# Dataset from proteomic analysis of human liver, lung, kidney and intestine microsomes

**DOI:** 10.1016/j.dib.2018.03.124

**Published:** 2018-03-30

**Authors:** Wei Song, Longjiang Yu, Zhihong Peng

**Affiliations:** a Hubei Province Key Laboratory of Biotechnology of Chinese Traditional Medicine, Hubei University, Wuhan 430062, China; b Department of Life Science, Hubei University, Wuhan 430062, China; c Institute of Resource Biology and Biotechnology, Department of Biotechnology, College of Life Science and Technology, Huazhong University of Science and Technology, Wuhan 430074, China

## Abstract

We provide detailed datasets from our analysis of proteins that are identified in human liver, lung, kidney and intestine microsomes by MS-based proteomics. Also included is a set of CYP450 enzymes and microsomal glutathione-*S*-transferase (MGSTs) activities in human liver microsomes. The data presented in this paper support the research article “Targeted label-free approach for quantification of epoxide hydrolase and glutathione transferases in microsomes” (Song et al., 2015) [1]. We expect that the data will contribute to the study of metabolism enzymes.

## Specifications Table

**Table:** 

Subject area	*Chemistry, Biology*
More specific subject area	*Proteomics, biology*
Type of data	*Text file, Table*
How data was acquired	*UPLC was used for enzyme activities assay, Nano-LC-MS/MS identification of proteins.*
Data format	*Filtered, Analyzed*
Experimental factors	*No sample pretreatment applied.*
Experimental features	a) *The difference of liver enzyme activities related age or gender*. b) *The different metabolism enzymes identified in human liver, lung, kidney and intestine microsomes*.
Data source location	*Wuhan, China*
Data accessibility	*With this article*

## Value of the data

• The proteins identified in human liver, lung, kidney and intestine microsomes with high sequence coverage.
• Data from the LC-ESI-MS/MS analysis will provide researchers with detailed information on metabolism enzymes in human liver, lung, kidney and intestine microsomes.
• Data from the enzyme activity analysis will enable researchers to observe the different activities of CYP450 enzymes and MGST enzyme in human liver microsomes.

## 1 Data

The data set shows the CYP450 enzymes and MGSTs activities in individual human liver microsomes (Table 1), the identified metabolism enzymes in human liver (Fig. S1), lung (Fig. S2), kidney (Fig. S3) and intestine (Fig. S4) microsomes are also reported.

**Table 1:** CYP450 enzymes, MEH, and MGSTs activities in human liver microsomes.

Enzymes	Individual human liver microsomes (*n* = 16)			Age < 60 (*n* = 10)	Age > 65 (*n* = 6)	Male (*n* = 8)	Female (*n* = 8)
	Max (pmol/mg protein/min)	Min (pmol/mg protein/min)	Max/min	pmol/mg protein/min	pmol/mg protein/min	pmol/mg protein/min	pmol/mg protein/min
CYP1A2	498	137	3.64	280 ± 130	259 ± 42.5	261 ± 121	285 ± 100
CYP2A6	1710	71.8	23.8	1188 ±535	472 ± 333	1254 ± 498	646 ± 520
CYP2B6	1330	76.3	17.4	570 ± 453	111 ± 35.0	498 ± 475	336 ± 399
CYP2C8	4370	629	6.95	2521 ± 956	1252 ± 486	2009 ± 674	2188 ± 1344
CYP2C9	3340	1710	1.95	2544 ± 780	2072 ± 591	2457 ± 635	2317 ± 899
CYP2C19	167	4.18	40.0	56.0 ± 64.4	25.8 ± 38.7	57.6 ± 67.9	39.6 ± 50.2
CYP2D6	651	120	5.43	397 ± 207	246 ± 93.6	260 ± 107	433 ± 219
CYP2E1	3770	730	5.16	2321 ± 1160	1750 ± 658	1908 ± 1006	2353 ± 1089
CYP3A4	5220	429	12.2	2779 ± 1403	1070 ± 448	1943 ± 457	2475 ± 2014
MEH							
MGSTs	0.774	0.509	1.52	0.638 ± 0.0768	0.646 ± 0.0566	0.659 ± 0.0679	0.622 ± 0.0694

## 2 Experimental design, materials and methods

### 2.1 Mass spectrometric analysis

All of the samples were prepared following the previous study [1]. A reversed phase Waters nanoACQUITY column (1.7 µm, BEH130 C18, 100 µm i.d. × 100 µm, Waters Corp.) coupled to a Thermo LTQ Velos Orbitrap tandem mass spectrometer (Thermo Fisher Scientific) was used to identify the proteins in human liver, lung, kidney and intestine microsomes. Samples were eluted at 1.2 µL/min. *t* = 0–5 min 99%*A*/1%*B*, *t* = 5.1 min 85%*A*/15%*B*, *t* = 50 min 40%*A*/60%*B*, *t* = 55 min 15%*A*/85%*B*, *t* = 55.1–65 min 99%*A*/1%*B* where *A* = 97% water/3% acetonitrile/0.1% formic acid and *B* = 0.1% formic acid in acetonitrile. Peptides were ionized via a nanoelectrospray ionization (ESI) source, and their mass spectra and collisionally induced dissociation (CID) fragmentation mass spectra were recorded. High resolution (60,000 resolving power), accurate mass spectra were recorded between *m*/*z* 395-2000 in ~ 1.2 s on the orbitrap mass analyzer. While the next high-resolution mass spectrum was being acquired on the orbitrap, the LTQ Velos linear ion trap independently recorded CID fragmentation mass spectra of the 8 most abundant-S2 ions present in the previous orbitrap mass spectrum. During the course of a 60-min nano-LC-MS/MS run, this approach typically generated ~ 3000 high-resolution mass spectra and between 12,000–15,000 CID MS/MS spectra. Thermo-Finnegan Proteome Discoverer 2.0 software (Thermo Fisher Scientific) was used to interface with the Mascot (Matrix Science) protein database search engine. MS/MS spectral information was used by Mascot to search the SwissProt Protein database, and a decoy search was employed to establish a false discovery rate. MS Data processed using Mascot (S1, S2, S3, S4) are presented in the Supplementary information tables.

### 2.2 Assessment of the activities of CYP450 enzymes and MGST in human liver microsomes

To assess the activities of CYP450 enzymes and MGST in human liver microsomes, known substrates specific for each enzyme were used. Briefly, liver microsomes (0.5 mg/mL), NADPH (1 mM) and known substrate were mixed with phosphate buffer (50 mM, pH 7.4) in a total volume of 200 μL. The CYP450 substrates used were phenacetin (20 μM) for CYP1A2-catalyzed phenacetin O-dealkylation, coumarin (4 μM) CYP2A6-catalyzed coumarin 7-hydroxylation, *S*-mephenytoin (10 μM) CYP2B6-catalyzed *S*-mephenytoin N-demethylation, amodiaquine (10 μM) CYP2C8-catalyzed amodiaquine N-dealkylation, tolbutamide (50 μM) for CYP2C9-catalyzed tolbutamide 4-methylhydroxylation, *S-*mephenytoin (50 μM) for CYP2C19-catalyzed *S-*mephenytoin 4'-hydroxylation, dextromethorphan (10 μM) for CYP2D6-catalyzed dextromethorphan O-demethylation, chlorzoxazone (10 μM) for CYP2E1-catalyzed chlorzoxazone 6-hydroxylation, and midazolam (10 μM) for CYP3A4-catalyzed 1′-Hydroxymidazolam. The substrate concentrations used were below their respective Km values. After preincubation at 37 °C for 5 min, 10 μL of NADPH (20 mM) was added to initiate the reaction. After incubation, 400 μL acetonitrile containing internal standard was added to stop the reaction. The samples were centrifuged at 10,000 g for 10 min and the supernatants were concentrated by drying *in vacuo*. The residue was re-dissolved in 100 µL acetonitrile/water (50/50, v/v) and analyzed following the previous study [2], [3].

Gutathione S-transferase activity was assayed following the method of Habig et al. [4] with 1 mM CDNB and 5 mM glutathione as the substrates.

## References

[1] Song W., Yu L., Peng Targeted Z. (2015). label-free approach for quantification of epoxide hydrolase and glutathione transferases in microsomes. Anal. Biochem..

[2] Alden P.G., Plumb R.S., Jones M.D., Rainville P.D., Shave D. (2010). A rapid ultra-performance liquid chromatography/tandem mass spectrometric methodology for the in vitro analysis of Pooled and Cocktail cytochrome P450 assays. Rapid Commun. Mass Spectrom..

[3] Spaggiari D., Geiser L., Daali Y., Rudaz S. (2014). Phenotyping of CYP450 in human liver microsomes using the cocktail approach. Anal. Bioanal. Chem..

[4] Habig W.H., Pabst M.J., Jakoby W.B. (1974). Glutathione S-transferases. The first enzymatic step in mercapturic acid formation. J. Biol. Chem..

