# Supplementary material for: Dataset from proteomic analysis of human liver, lung, kidney and intestine microsomes
Source: Data Brief. 2018 Mar 30;18:831–4. doi: 10.1016/j.dib.2018.03.124 (PMC5996730; doi:10.1016/j.dib.2018.03.124)
Supplement: Supplementary file 1 — Supplementary material [file mmc1.pdf]

Conflict of Interest statement

We confirm that the manuscript has been read and approved by all of the authors and there is no other person who satisfied the criteria for authorship but are not listed. We further confirm that the order of authors listed in the manuscript has been approved by all of us.

We understand that the corresponding author is the sole contact for the editorial process. He is responsible for communicating with the other authors about progress, submission of revisions and final approval of proofs.

The corresponding author should sign this declaration on behalf of all.

[illegible]

03-11-2018
