# Supplementary material for: Dataset from proteomic analysis of human liver, lung, kidney and intestine microsomes
Source: Data Brief. 2018 Mar 30;18:831–4. doi: 10.1016/j.dib.2018.03.124 (PMC5996730; doi:10.1016/j.dib.2018.03.124)
Supplement: Supplementary file 2 — Supplementary material [file mmc2.doc]

Mascot Search Results

**User	: zhihong**

**Email	: peng_zhihong@hotmail.com**

**Search title	: lung**

**MS data file	: lung-2.mgf**

**Database	: SwissProt 101509 (510076 sequences; 179409349 residues)**

**Taxonomy	: Homo sapiens (human) (20335 sequences)**

**Timestamp	: 8 May 2013 at 18:01:51 GMT**

**Warning	: Invalid value (10+) specified for the "CHARGE" parameter**

**Warning	: Invalid value () specified for the "CHARGE" parameter**

**Warning	: Invalid value (9+) specified for the "CHARGE" parameter**

**Warning	: Invalid value () specified for the "CHARGE" parameter**

**Warning	: Invalid value (9+) specified for the "CHARGE" parameter**

**Warning	: Invalid value () specified for the "CHARGE" parameter**

**Warning	: Invalid value (9+) specified for the "CHARGE" parameter**

**Warning	: Invalid value () specified for the "CHARGE" parameter**

**Warning	: Invalid value (9+) specified for the "CHARGE" parameter**

**Warning	: Invalid value () specified for the "CHARGE" parameter**

**Warning	: Error 61 has been detected 38 times and only the first 10 messages have been output**

**Enzyme	: Trypsin**

**Fixed modifications	: Carbamidomethyl (C)**

**Variable modifications : Oxidation (M)**

**Mass values	: Monoisotopic**

**Protein Mass	: Unrestricted**

**Peptide Mass Tolerance : ± 1.2 Da**

**Fragment Mass Tolerance: ± 0.6 Da**

**Max Missed Cleavages	: 1**

**Instrument type	: Default**

**Number of queries	: 31896**

| **Protein hits           :** | **P63261\|ACTG_HUMAN** | Actin, cytoplasmic 2 OS=Homo sapiens GN=ACTG1 PE=1 SV=1 |
| --- | --- | --- |
|  | **P60709\|ACTB_HUMAN** | Actin, cytoplasmic 1 OS=Homo sapiens GN=ACTB PE=1 SV=1 |
|  | **Q6S8J3\|POTEE_HUMAN** | POTE ankyrin domain family member E OS=Homo sapiens GN=POTEE PE=1 SV=3 |
|  | **P35579\|MYH9_HUMAN** | Myosin-9 OS=Homo sapiens GN=MYH9 PE=1 SV=4 |
|  | **Q00610\|CLH1_HUMAN** | Clathrin heavy chain 1 OS=Homo sapiens GN=CLTC PE=1 SV=5 |
|  | **P68032\|ACTC_HUMAN** | Actin, alpha cardiac muscle 1 OS=Homo sapiens GN=ACTC1 PE=1 SV=1 |
|  | **P62736\|ACTA_HUMAN** | Actin, aortic smooth muscle OS=Homo sapiens GN=ACTA2 PE=1 SV=1 |
|  | **P63267\|ACTH_HUMAN** | Actin, gamma-enteric smooth muscle OS=Homo sapiens GN=ACTG2 PE=1 SV=1 |
|  | **P68871\|HBB_HUMAN** | Hemoglobin subunit beta OS=Homo sapiens GN=HBB PE=1 SV=2 |
|  | **P07437\|TBB5_HUMAN** | Tubulin beta chain OS=Homo sapiens GN=TUBB PE=1 SV=2 |
|  | **P68133\|ACTS_HUMAN** | Actin, alpha skeletal muscle OS=Homo sapiens GN=ACTA1 PE=1 SV=1 |
|  | **P68371\|TBB2C_HUMAN** | Tubulin beta-2C chain OS=Homo sapiens GN=TUBB2C PE=1 SV=1 |
|  | **P02768\|ALBU_HUMAN** | Serum albumin OS=Homo sapiens GN=ALB PE=1 SV=2 |
|  | **Q14764\|MVP_HUMAN** | Major vault protein OS=Homo sapiens GN=MVP PE=1 SV=4 |
|  | **Q9BVA1\|TBB2B_HUMAN** | Tubulin beta-2B chain OS=Homo sapiens GN=TUBB2B PE=1 SV=1 |
|  | **Q8IWL1\|SFPA2_HUMAN** | Pulmonary surfactant-associated protein A2 OS=Homo sapiens GN=SFTPA2 PE=1 SV=1 |
|  | **P68363\|TBA1B_HUMAN** | Tubulin alpha-1B chain OS=Homo sapiens GN=TUBA1B PE=1 SV=1 |
|  | **P04350\|TBB4_HUMAN** | Tubulin beta-4 chain OS=Homo sapiens GN=TUBB4 PE=1 SV=2 |
|  | **Q71U36\|TBA1A_HUMAN** | Tubulin alpha-1A chain OS=Homo sapiens GN=TUBA1A PE=1 SV=1 |
|  | **P35580\|MYH10_HUMAN** | Myosin-10 OS=Homo sapiens GN=MYH10 PE=1 SV=3 |
|  | **Q13748\|TBA3C_HUMAN** | Tubulin alpha-3C/D chain OS=Homo sapiens GN=TUBA3C PE=1 SV=3 |
|  | **P35749\|MYH11_HUMAN** | Myosin-11 OS=Homo sapiens GN=MYH11 PE=1 SV=3 |
|  | **P07355\|ANXA2_HUMAN** | Annexin A2 OS=Homo sapiens GN=ANXA2 PE=1 SV=2 |
|  | **P05164\|PERM_HUMAN** | Myeloperoxidase OS=Homo sapiens GN=MPO PE=1 SV=1 |
|  | **P11021\|GRP78_HUMAN** | 78 kDa glucose-regulated protein OS=Homo sapiens GN=HSPA5 PE=1 SV=2 |
|  | **Q13509\|TBB3_HUMAN** | Tubulin beta-3 chain OS=Homo sapiens GN=TUBB3 PE=1 SV=2 |
|  | **Q6PEY2\|TBA3E_HUMAN** | Tubulin alpha-3E chain OS=Homo sapiens GN=TUBA3E PE=1 SV=2 |
|  | **P06396\|GELS_HUMAN** | Gelsolin OS=Homo sapiens GN=GSN PE=1 SV=1 |
|  | **P12111\|CO6A3_HUMAN** | Collagen alpha-3(VI) chain OS=Homo sapiens GN=COL6A3 PE=1 SV=4 |
|  | **P06576\|ATPB_HUMAN** | ATP synthase subunit beta, mitochondrial OS=Homo sapiens GN=ATP5B PE=1 SV=3 |
|  | **P02042\|HBD_HUMAN** | Hemoglobin subunit delta OS=Homo sapiens GN=HBD PE=1 SV=2 |
|  | **Q16853\|AOC3_HUMAN** | Membrane primary amine oxidase OS=Homo sapiens GN=AOC3 PE=1 SV=3 |
|  | **Q9UGT4\|SUSD2_HUMAN** | Sushi domain-containing protein 2 OS=Homo sapiens GN=SUSD2 PE=1 SV=1 |
|  | **P51659\|DHB4_HUMAN** | Peroxisomal multifunctional enzyme type 2 OS=Homo sapiens GN=HSD17B4 PE=1 SV=3 |
|  | **P02730\|B3AT_HUMAN** | Band 3 anion transport protein OS=Homo sapiens GN=SLC4A1 PE=1 SV=3 |
|  | **P14625\|ENPL_HUMAN** | Endoplasmin OS=Homo sapiens GN=HSP90B1 PE=1 SV=1 |
|  | **Q9NY65\|TBA8_HUMAN** | Tubulin alpha-8 chain OS=Homo sapiens GN=TUBA8 PE=1 SV=1 |
|  | **Q9BSJ8\|ESYT1_HUMAN** | Extended synaptotagmin-1 OS=Homo sapiens GN=ESYT1 PE=1 SV=1 |
|  | **P21333\|FLNA_HUMAN** | Filamin-A OS=Homo sapiens GN=FLNA PE=1 SV=4 |
|  | **A5A3E0\|POTEF_HUMAN** | POTE ankyrin domain family member F OS=Homo sapiens GN=POTEF PE=1 SV=2 |
|  | **P08670\|VIME_HUMAN** | Vimentin OS=Homo sapiens GN=VIM PE=1 SV=4 |
|  | **P69905\|HBA_HUMAN** | Hemoglobin subunit alpha OS=Homo sapiens GN=HBA1 PE=1 SV=2 |
|  | **Q562R1\|ACTBL_HUMAN** | Beta-actin-like protein 2 OS=Homo sapiens GN=ACTBL2 PE=1 SV=2 |
|  | **P25705\|ATPA_HUMAN** | ATP synthase subunit alpha, mitochondrial OS=Homo sapiens GN=ATP5A1 PE=1 SV=1 |
|  | **P30101\|PDIA3_HUMAN** | Protein disulfide-isomerase A3 OS=Homo sapiens GN=PDIA3 PE=1 SV=4 |
|  | **P02788\|TRFL_HUMAN** | Lactotransferrin OS=Homo sapiens GN=LTF PE=1 SV=6 |
|  | **P35555\|FBN1_HUMAN** | Fibrillin-1 OS=Homo sapiens GN=FBN1 PE=1 SV=2 |
|  | **Q01082\|SPTB2_HUMAN** | Spectrin beta chain, brain 1 OS=Homo sapiens GN=SPTBN1 PE=1 SV=2 |
|  | **Q99880\|H2B1L_HUMAN** | Histone H2B type 1-L OS=Homo sapiens GN=HIST1H2BL PE=1 SV=3 |
|  | **P58876\|H2B1D_HUMAN** | Histone H2B type 1-D OS=Homo sapiens GN=HIST1H2BD PE=1 SV=2 |
|  | **Q8N257\|H2B3B_HUMAN** | Histone H2B type 3-B OS=Homo sapiens GN=HIST3H2BB PE=1 SV=3 |
|  | **P53675\|CLH2_HUMAN** | Clathrin heavy chain 2 OS=Homo sapiens GN=CLTCL1 PE=1 SV=2 |
|  | **P12109\|CO6A1_HUMAN** | Collagen alpha-1(VI) chain OS=Homo sapiens GN=COL6A1 PE=1 SV=3 |
|  | **P27797\|CALR_HUMAN** | Calreticulin OS=Homo sapiens GN=CALR PE=1 SV=1 |
|  | **P05362\|ICAM1_HUMAN** | Intercellular adhesion molecule 1 OS=Homo sapiens GN=ICAM1 PE=1 SV=2 |
|  | **Q13813\|SPTA2_HUMAN** | Spectrin alpha chain, brain OS=Homo sapiens GN=SPTAN1 PE=1 SV=3 |
|  | **Q86VB7\|C163A_HUMAN** | Scavenger receptor cysteine-rich type 1 protein M130 OS=Homo sapiens GN=CD163 PE=1 SV=1 |
|  | **Q9BYX7\|ACTBM_HUMAN** | Beta-actin-like protein 3 OS=Homo sapiens GN=ACTBL3 PE=1 SV=1 |
|  | **P07237\|PDIA1_HUMAN** | Protein disulfide-isomerase OS=Homo sapiens GN=P4HB PE=1 SV=3 |
|  | **P45880\|VDAC2_HUMAN** | Voltage-dependent anion-selective channel protein 2 OS=Homo sapiens GN=VDAC2 PE=1 SV=2 |
|  | **P02549\|SPTA1_HUMAN** | Spectrin alpha chain, erythrocyte OS=Homo sapiens GN=SPTA1 PE=1 SV=4 |
|  | **P05556\|ITB1_HUMAN** | Integrin beta-1 OS=Homo sapiens GN=ITGB1 PE=1 SV=2 |
|  | **P05023\|AT1A1_HUMAN** | Sodium/potassium-transporting ATPase subunit alpha-1 OS=Homo sapiens GN=ATP1A1 PE=1 SV=1 |
|  | **P16157\|ANK1_HUMAN** | Ankyrin-1 OS=Homo sapiens GN=ANK1 PE=1 SV=3 |
|  | **P23141\|EST1_HUMAN** | Liver carboxylesterase 1 OS=Homo sapiens GN=CES1 PE=1 SV=2 |
|  | **Q5K4L6\|S27A3_HUMAN** | Long-chain fatty acid transport protein 3 OS=Homo sapiens GN=SLC27A3 PE=2 SV=3 |
|  | **P02751\|FINC_HUMAN** | Fibronectin OS=Homo sapiens GN=FN1 PE=1 SV=3 |
|  | **P05107\|ITB2_HUMAN** | Integrin beta-2 OS=Homo sapiens GN=ITGB2 PE=1 SV=2 |
|  | **P26038\|MOES_HUMAN** | Moesin OS=Homo sapiens GN=MSN PE=1 SV=3 |
|  | **P62873\|GBB1_HUMAN** | Guanine nucleotide-binding protein G(I)/G(S)/G(T) subunit beta-1 OS=Homo sapiens GN=GNB1 PE=1 SV=3 |
|  | **P11277\|SPTB1_HUMAN** | Spectrin beta chain, erythrocyte OS=Homo sapiens GN=SPTB PE=1 SV=5 |
|  | **P09382\|LEG1_HUMAN** | Galectin-1 OS=Homo sapiens GN=LGALS1 PE=1 SV=2 |
|  | **P07339\|CATD_HUMAN** | Cathepsin D OS=Homo sapiens GN=CTSD PE=1 SV=1 |
|  | **P16615\|AT2A2_HUMAN** | Sarcoplasmic/endoplasmic reticulum calcium ATPase 2 OS=Homo sapiens GN=ATP2A2 PE=1 SV=1 |
|  | **P21796\|VDAC1_HUMAN** | Voltage-dependent anion-selective channel protein 1 OS=Homo sapiens GN=VDAC1 PE=1 SV=2 |
|  | **P01834\|IGKC_HUMAN** | Ig kappa chain C region OS=Homo sapiens GN=IGKC PE=1 SV=1 |
|  | **P02545\|LMNA_HUMAN** | Lamin-A/C OS=Homo sapiens GN=LMNA PE=1 SV=1 |
|  | **P04040\|CATA_HUMAN** | Catalase OS=Homo sapiens GN=CAT PE=1 SV=3 |
|  | **Q6DD88\|ATLA3_HUMAN** | Atlastin-3 OS=Homo sapiens GN=ATL3 PE=1 SV=1 |
|  | **Q86WV6\|TM173_HUMAN** | Transmembrane protein 173 OS=Homo sapiens GN=TMEM173 PE=1 SV=1 |
|  | **P62879\|GBB2_HUMAN** | Guanine nucleotide-binding protein G(I)/G(S)/G(T) subunit beta-2 OS=Homo sapiens GN=GNB2 PE=1 SV=3 |
|  | **Q9BUF5\|TBB6_HUMAN** | Tubulin beta-6 chain OS=Homo sapiens GN=TUBB6 PE=1 SV=1 |
|  | **P14618\|KPYM_HUMAN** | Pyruvate kinase isozymes M1/M2 OS=Homo sapiens GN=PKM2 PE=1 SV=4 |
|  | **P62158\|CALM_HUMAN** | Calmodulin OS=Homo sapiens GN=CALM1 PE=1 SV=2 |
|  | **P11142\|HSP7C_HUMAN** | Heat shock cognate 71 kDa protein OS=Homo sapiens GN=HSPA8 PE=1 SV=1 |
|  | **P00387\|NB5R3_HUMAN** | NADH-cytochrome b5 reductase 3 OS=Homo sapiens GN=CYB5R3 PE=1 SV=3 |
|  | **P02787\|TRFE_HUMAN** | Serotransferrin OS=Homo sapiens GN=TF PE=1 SV=2 |
|  | **P10620\|MGST1_HUMAN** | Microsomal glutathione S-transferase 1 OS=Homo sapiens GN=MGST1 PE=1 SV=1 |
|  | **P04406\|G3P_HUMAN** | Glyceraldehyde-3-phosphate dehydrogenase OS=Homo sapiens GN=GAPDH PE=1 SV=3 |
|  | **P07099\|HYEP_HUMAN** | Epoxide hydrolase 1 OS=Homo sapiens GN=EPHX1 PE=1 SV=1 |
|  | **Q99536\|VAT1_HUMAN** | Synaptic vesicle membrane protein VAT-1 homolog OS=Homo sapiens GN=VAT1 PE=1 SV=2 |
|  | **P62820\|RAB1A_HUMAN** | Ras-related protein Rab-1A OS=Homo sapiens GN=RAB1A PE=1 SV=3 |
|  | **Q99758\|ABCA3_HUMAN** | ATP-binding cassette sub-family A member 3 OS=Homo sapiens GN=ABCA3 PE=1 SV=2 |
|  | **Q09666\|AHNK_HUMAN** | Neuroblast differentiation-associated protein AHNAK OS=Homo sapiens GN=AHNAK PE=1 SV=2 |
|  | **Q12797\|ASPH_HUMAN** | Aspartyl/asparaginyl beta-hydroxylase OS=Homo sapiens GN=ASPH PE=1 SV=3 |
|  | **P13010\|KU86_HUMAN** | ATP-dependent DNA helicase 2 subunit 2 OS=Homo sapiens GN=XRCC5 PE=1 SV=3 |
|  | **Q29963\|1C06_HUMAN** | HLA class I histocompatibility antigen, Cw-6 alpha chain OS=Homo sapiens GN=HLA-C PE=1 SV=2 |
|  | **P10321\|1C07_HUMAN** | HLA class I histocompatibility antigen, Cw-7 alpha chain OS=Homo sapiens GN=HLA-C PE=1 SV=3 |
|  | **Q8NF37\|PCAT1_HUMAN** | Lysophosphatidylcholine acyltransferase 1 OS=Homo sapiens GN=LPCAT1 PE=1 SV=2 |
|  | **Q07065\|CKAP4_HUMAN** | Cytoskeleton-associated protein 4 OS=Homo sapiens GN=CKAP4 PE=1 SV=2 |
|  | **Q9NZM1\|MYOF_HUMAN** | Myoferlin OS=Homo sapiens GN=MYOF PE=1 SV=1 |
|  | **P08758\|ANXA5_HUMAN** | Annexin A5 OS=Homo sapiens GN=ANXA5 PE=1 SV=2 |
|  | **P02679\|FIBG_HUMAN** | Fibrinogen gamma chain OS=Homo sapiens GN=FGG PE=1 SV=3 |
|  | **P02675\|FIBB_HUMAN** | Fibrinogen beta chain OS=Homo sapiens GN=FGB PE=1 SV=2 |
|  | **P55072\|TERA_HUMAN** | Transitional endoplasmic reticulum ATPase OS=Homo sapiens GN=VCP PE=1 SV=4 |
|  | **P60660\|MYL6_HUMAN** | Myosin light polypeptide 6 OS=Homo sapiens GN=MYL6 PE=1 SV=2 |
|  | **Q6NZI2\|PTRF_HUMAN** | Polymerase I and transcript release factor OS=Homo sapiens GN=PTRF PE=1 SV=1 |
|  | **O43707\|ACTN4_HUMAN** | Alpha-actinin-4 OS=Homo sapiens GN=ACTN4 PE=1 SV=2 |
|  | **P04899\|GNAI2_HUMAN** | Guanine nucleotide-binding protein G(i), alpha-2 subunit OS=Homo sapiens GN=GNAI2 PE=1 SV=3 |
|  | **P40939\|ECHA_HUMAN** | Trifunctional enzyme subunit alpha, mitochondrial OS=Homo sapiens GN=HADHA PE=1 SV=2 |
|  | **P00738\|HPT_HUMAN** | Haptoglobin OS=Homo sapiens GN=HP PE=1 SV=1 |
|  | **P06702\|S10A9_HUMAN** | Protein S100-A9 OS=Homo sapiens GN=S100A9 PE=1 SV=1 |
|  | **O75396\|SC22B_HUMAN** | Vesicle-trafficking protein SEC22b OS=Homo sapiens GN=SEC22B PE=1 SV=3 |
|  | **P30480\|1B42_HUMAN** | HLA class I histocompatibility antigen, B-42 alpha chain OS=Homo sapiens GN=HLA-B PE=1 SV=1 |
|  | **P01893\|HLAH_HUMAN** | Putative HLA class I histocompatibility antigen, alpha chain H OS=Homo sapiens GN=HLA-H PE=5 SV=2 |
|  | **P63104\|1433Z_HUMAN** | 14-3-3 protein zeta/delta OS=Homo sapiens GN=YWHAZ PE=1 SV=1 |
|  | **Q13510\|ASAH1_HUMAN** | Acid ceramidase OS=Homo sapiens GN=ASAH1 PE=1 SV=5 |
|  | **P30460\|1B08_HUMAN** | HLA class I histocompatibility antigen, B-8 alpha chain OS=Homo sapiens GN=HLA-B PE=1 SV=1 |
|  | **Q7Z406\|MYH14_HUMAN** | Myosin-14 OS=Homo sapiens GN=MYH14 PE=1 SV=1 |
|  | **P14780\|MMP9_HUMAN** | Matrix metalloproteinase-9 OS=Homo sapiens GN=MMP9 PE=1 SV=2 |
|  | **P04844\|RPN2_HUMAN** | Dolichyl-diphosphooligosaccharide--protein glycosyltransferase subunit 2 OS=Homo sapiens GN=RPN2 PE=1 SV=3 |
|  | **P16435\|NCPR_HUMAN** | NADPH--cytochrome P450 reductase OS=Homo sapiens GN=POR PE=1 SV=2 |
|  | **P0C0S8\|H2A1_HUMAN** | Histone H2A type 1 OS=Homo sapiens GN=HIST1H2AG PE=1 SV=2 |
|  | **P11215\|ITAM_HUMAN** | Integrin alpha-M OS=Homo sapiens GN=ITGAM PE=1 SV=2 |
|  | **Q07000\|1C15_HUMAN** | HLA class I histocompatibility antigen, Cw-15 alpha chain OS=Homo sapiens GN=HLA-C PE=1 SV=1 |
|  | **Q3ZCM7\|TBB8_HUMAN** | Tubulin beta-8 chain OS=Homo sapiens GN=TUBB8 PE=1 SV=2 |
|  | **P04839\|CY24B_HUMAN** | Cytochrome b-245 heavy chain OS=Homo sapiens GN=CYBB PE=1 SV=2 |
|  | **A6NNZ2\|TBB8B_HUMAN** | Tubulin beta-8 chain B OS=Homo sapiens PE=1 SV=1 |
|  | **P09601\|HMOX1_HUMAN** | Heme oxygenase 1 OS=Homo sapiens GN=HMOX1 PE=1 SV=1 |
|  | **P67936\|TPM4_HUMAN** | Tropomyosin alpha-4 chain OS=Homo sapiens GN=TPM4 PE=1 SV=3 |
|  | **P01889\|1B07_HUMAN** | HLA class I histocompatibility antigen, B-7 alpha chain OS=Homo sapiens GN=HLA-B PE=1 SV=3 |
|  | **P10316\|1A69_HUMAN** | HLA class I histocompatibility antigen, A-69 alpha chain OS=Homo sapiens GN=HLA-A PE=1 SV=2 |
|  | **P63027\|VAMP2_HUMAN** | Vesicle-associated membrane protein 2 OS=Homo sapiens GN=VAMP2 PE=1 SV=2 |
|  | **Q9NQC3\|RTN4_HUMAN** | Reticulon-4 OS=Homo sapiens GN=RTN4 PE=1 SV=2 |
|  | **P01892\|1A02_HUMAN** | HLA class I histocompatibility antigen, A-2 alpha chain OS=Homo sapiens GN=HLA-A PE=1 SV=1 |
|  | **A1L0T0\|ILVBL_HUMAN** | Acetolactate synthase-like protein OS=Homo sapiens GN=ILVBL PE=1 SV=2 |
|  | **P50993\|AT1A2_HUMAN** | Sodium/potassium-transporting ATPase subunit alpha-2 OS=Homo sapiens GN=ATP1A2 PE=1 SV=1 |
|  | **P13637\|AT1A3_HUMAN** | Sodium/potassium-transporting ATPase subunit alpha-3 OS=Homo sapiens GN=ATP1A3 PE=1 SV=3 |
|  | **Q9H0U4\|RAB1B_HUMAN** | Ras-related protein Rab-1B OS=Homo sapiens GN=RAB1B PE=1 SV=1 |
|  | **Q29836\|1B67_HUMAN** | HLA class I histocompatibility antigen, B-67 alpha chain OS=Homo sapiens GN=HLA-B PE=1 SV=1 |
|  | **Q6NUK1\|SCMC1_HUMAN** | Calcium-binding mitochondrial carrier protein SCaMC-1 OS=Homo sapiens GN=SLC25A24 PE=1 SV=2 |
|  | **P18463\|1B37_HUMAN** | HLA class I histocompatibility antigen, B-37 alpha chain OS=Homo sapiens GN=HLA-B PE=2 SV=1 |
|  | **P16188\|1A30_HUMAN** | HLA class I histocompatibility antigen, A-30 alpha chain OS=Homo sapiens GN=HLA-A PE=1 SV=2 |
|  | **P23284\|PPIB_HUMAN** | Peptidyl-prolyl cis-trans isomerase B OS=Homo sapiens GN=PPIB PE=1 SV=2 |
|  | **Q10567\|AP1B1_HUMAN** | AP-1 complex subunit beta-1 OS=Homo sapiens GN=AP1B1 PE=1 SV=1 |
|  | **Q95365\|1B38_HUMAN** | HLA class I histocompatibility antigen, B-38 alpha chain OS=Homo sapiens GN=HLA-B PE=2 SV=1 |
|  | **P61586\|RHOA_HUMAN** | Transforming protein RhoA OS=Homo sapiens GN=RHOA PE=1 SV=1 |
|  | **P07988\|PSPB_HUMAN** | Pulmonary surfactant-associated protein B OS=Homo sapiens GN=SFTPB PE=1 SV=3 |
|  | **P07858\|CATB_HUMAN** | Cathepsin B OS=Homo sapiens GN=CTSB PE=1 SV=3 |
|  | **P56199\|ITA1_HUMAN** | Integrin alpha-1 OS=Homo sapiens GN=ITGA1 PE=1 SV=2 |
|  | **Q53GQ0\|DHB12_HUMAN** | Estradiol 17-beta-dehydrogenase 12 OS=Homo sapiens GN=HSD17B12 PE=1 SV=2 |
|  | **P10809\|CH60_HUMAN** | 60 kDa heat shock protein, mitochondrial OS=Homo sapiens GN=HSPD1 PE=1 SV=2 |
|  | **Q15084\|PDIA6_HUMAN** | Protein disulfide-isomerase A6 OS=Homo sapiens GN=PDIA6 PE=1 SV=1 |
|  | **P30462\|1B14_HUMAN** | HLA class I histocompatibility antigen, B-14 alpha chain OS=Homo sapiens GN=HLA-B PE=1 SV=1 |
|  | **Q9ULC5\|ACSL5_HUMAN** | Long-chain-fatty-acid--CoA ligase 5 OS=Homo sapiens GN=ACSL5 PE=1 SV=1 |
|  | **Q9Y490\|TLN1_HUMAN** | Talin-1 OS=Homo sapiens GN=TLN1 PE=1 SV=3 |
|  | **P09110\|THIK_HUMAN** | 3-ketoacyl-CoA thiolase, peroxisomal OS=Homo sapiens GN=ACAA1 PE=1 SV=2 |
|  | **P18859\|ATP5J_HUMAN** | ATP synthase-coupling factor 6, mitochondrial OS=Homo sapiens GN=ATP5J PE=1 SV=1 |
|  | **Q29960\|1C16_HUMAN** | HLA class I histocompatibility antigen, Cw-16 alpha chain OS=Homo sapiens GN=HLA-C PE=2 SV=1 |
|  | **P60033\|CD81_HUMAN** | CD81 antigen OS=Homo sapiens GN=CD81 PE=1 SV=1 |
|  | **O00159\|MYO1C_HUMAN** | Myosin-Ic OS=Homo sapiens GN=MYO1C PE=1 SV=3 |
|  | **P05534\|1A24_HUMAN** | HLA class I histocompatibility antigen, A-24 alpha chain OS=Homo sapiens GN=HLA-A PE=1 SV=2 |
|  | **P30447\|1A23_HUMAN** | HLA class I histocompatibility antigen, A-23 alpha chain OS=Homo sapiens GN=HLA-A PE=1 SV=1 |
|  | **P06733\|ENOA_HUMAN** | Alpha-enolase OS=Homo sapiens GN=ENO1 PE=1 SV=2 |
|  | **Q29718\|1B82_HUMAN** | HLA class I histocompatibility antigen, B-82 alpha chain OS=Homo sapiens GN=HLA-B PE=2 SV=1 |
|  | **Q31612\|1B73_HUMAN** | HLA class I histocompatibility antigen, B-73 alpha chain OS=Homo sapiens GN=HLA-B PE=1 SV=1 |
|  | **P12814\|ACTN1_HUMAN** | Alpha-actinin-1 OS=Homo sapiens GN=ACTN1 PE=1 SV=2 |
|  | **P21397\|AOFA_HUMAN** | Amine oxidase [flavin-containing] A OS=Homo sapiens GN=MAOA PE=1 SV=1 |
|  | **Q99623\|PHB2_HUMAN** | Prohibitin-2 OS=Homo sapiens GN=PHB2 PE=1 SV=2 |
|  | **P07602\|SAP_HUMAN** | Proactivator polypeptide OS=Homo sapiens GN=PSAP PE=1 SV=2 |
|  | **Q99867\|TBB4Q_HUMAN** | Putative tubulin beta-4q chain OS=Homo sapiens GN=TUBB4Q PE=5 SV=1 |
|  | **P29972\|AQP1_HUMAN** | Aquaporin-1 OS=Homo sapiens GN=AQP1 PE=1 SV=3 |
|  | **P01842\|LAC_HUMAN** | Ig lambda chain C regions OS=Homo sapiens GN=IGLC1 PE=1 SV=1 |
|  | **P13746\|1A11_HUMAN** | HLA class I histocompatibility antigen, A-11 alpha chain OS=Homo sapiens GN=HLA-A PE=1 SV=1 |
|  | **P30443\|1A01_HUMAN** | HLA class I histocompatibility antigen, A-1 alpha chain OS=Homo sapiens GN=HLA-A PE=1 SV=1 |
|  | **P04439\|1A03_HUMAN** | HLA class I histocompatibility antigen, A-3 alpha chain OS=Homo sapiens GN=HLA-A PE=1 SV=2 |
|  | **Q15907\|RB11B_HUMAN** | Ras-related protein Rab-11B OS=Homo sapiens GN=RAB11B PE=1 SV=4 |
|  | **Q99805\|TM9S2_HUMAN** | Transmembrane 9 superfamily member 2 OS=Homo sapiens GN=TM9SF2 PE=1 SV=1 |
|  | **P31930\|QCR1_HUMAN** | Cytochrome b-c1 complex subunit 1, mitochondrial OS=Homo sapiens GN=UQCRC1 PE=1 SV=3 |
|  | **P61106\|RAB14_HUMAN** | Ras-related protein Rab-14 OS=Homo sapiens GN=RAB14 PE=1 SV=4 |
|  | **Q96A33\|CCD47_HUMAN** | Coiled-coil domain-containing protein 47 OS=Homo sapiens GN=CCDC47 PE=1 SV=1 |
|  | **Q03518\|TAP1_HUMAN** | Antigen peptide transporter 1 OS=Homo sapiens GN=TAP1 PE=1 SV=2 |
|  | **P30479\|1B41_HUMAN** | HLA class I histocompatibility antigen, B-41 alpha chain OS=Homo sapiens GN=HLA-B PE=1 SV=1 |
|  | **P28288\|ABCD3_HUMAN** | ATP-binding cassette sub-family D member 3 OS=Homo sapiens GN=ABCD3 PE=1 SV=1 |
|  | **Q31610\|1B81_HUMAN** | HLA class I histocompatibility antigen, B-81 alpha chain OS=Homo sapiens GN=HLA-B PE=2 SV=1 |
|  | **P63010\|AP2B1_HUMAN** | AP-2 complex subunit beta OS=Homo sapiens GN=AP2B1 PE=1 SV=1 |
|  | **P24557\|THAS_HUMAN** | Thromboxane-A synthase OS=Homo sapiens GN=TBXAS1 PE=1 SV=3 |
|  | **P20700\|LMNB1_HUMAN** | Lamin-B1 OS=Homo sapiens GN=LMNB1 PE=1 SV=2 |
|  | **Q9NP72\|RAB18_HUMAN** | Ras-related protein Rab-18 OS=Homo sapiens GN=RAB18 PE=1 SV=1 |
|  | **P30486\|1B48_HUMAN** | HLA class I histocompatibility antigen, B-48 alpha chain OS=Homo sapiens GN=HLA-B PE=2 SV=1 |
|  | **Q86Y82\|STX12_HUMAN** | Syntaxin-12 OS=Homo sapiens GN=STX12 PE=1 SV=1 |
|  | **Q5VTE0\|EF1A3_HUMAN** | Putative elongation factor 1-alpha-like 3 OS=Homo sapiens GN=EEF1AL3 PE=5 SV=1 |
|  | **P68104\|EF1A1_HUMAN** | Elongation factor 1-alpha 1 OS=Homo sapiens GN=EEF1A1 PE=1 SV=1 |
|  | **P13667\|PDIA4_HUMAN** | Protein disulfide-isomerase A4 OS=Homo sapiens GN=PDIA4 PE=1 SV=2 |
|  | **P30040\|ERP29_HUMAN** | Endoplasmic reticulum protein ERp29 OS=Homo sapiens GN=ERP29 PE=1 SV=4 |
|  | **P22695\|QCR2_HUMAN** | Cytochrome b-c1 complex subunit 2, mitochondrial OS=Homo sapiens GN=UQCRC2 PE=1 SV=3 |
|  | **P19105\|ML12A_HUMAN** | Myosin regulatory light chain 12A OS=Homo sapiens GN=MYL12A PE=1 SV=2 |
|  | **O95573\|ACSL3_HUMAN** | Long-chain-fatty-acid--CoA ligase 3 OS=Homo sapiens GN=ACSL3 PE=1 SV=3 |
|  | **P16070\|CD44_HUMAN** | CD44 antigen OS=Homo sapiens GN=CD44 PE=1 SV=2 |
|  | **P27105\|STOM_HUMAN** | Erythrocyte band 7 integral membrane protein OS=Homo sapiens GN=STOM PE=1 SV=3 |
|  | **Q9HCU5\|PREB_HUMAN** | Prolactin regulatory element-binding protein OS=Homo sapiens GN=PREB PE=1 SV=2 |
|  | **Q04826\|1B40_HUMAN** | HLA class I histocompatibility antigen, B-40 alpha chain OS=Homo sapiens GN=HLA-B PE=1 SV=1 |
|  | **Q95604\|1C17_HUMAN** | HLA class I histocompatibility antigen, Cw-17 alpha chain OS=Homo sapiens GN=HLA-C PE=1 SV=1 |
|  | **P28799\|GRN_HUMAN** | Granulins OS=Homo sapiens GN=GRN PE=1 SV=2 |
|  | **Q9NZN4\|EHD2_HUMAN** | EH domain-containing protein 2 OS=Homo sapiens GN=EHD2 PE=1 SV=2 |
|  | **P30493\|1B55_HUMAN** | HLA class I histocompatibility antigen, B-55 alpha chain OS=Homo sapiens GN=HLA-B PE=1 SV=1 |
|  | **P30492\|1B54_HUMAN** | HLA class I histocompatibility antigen, B-54 alpha chain OS=Homo sapiens GN=HLA-B PE=2 SV=1 |
|  | **P30049\|ATPD_HUMAN** | ATP synthase subunit delta, mitochondrial OS=Homo sapiens GN=ATP5D PE=1 SV=2 |
|  | **P08571\|CD14_HUMAN** | Monocyte differentiation antigen CD14 OS=Homo sapiens GN=CD14 PE=1 SV=2 |
|  | **P01023\|A2MG_HUMAN** | Alpha-2-macroglobulin OS=Homo sapiens GN=A2M PE=1 SV=1 |
|  | **P02786\|TFR1_HUMAN** | Transferrin receptor protein 1 OS=Homo sapiens GN=TFRC PE=1 SV=2 |
|  | **Q29940\|1B59_HUMAN** | HLA class I histocompatibility antigen, B-59 alpha chain OS=Homo sapiens GN=HLA-B PE=2 SV=1 |
|  | **P16452\|EPB42_HUMAN** | Erythrocyte membrane protein band 4.2 OS=Homo sapiens GN=EPB42 PE=1 SV=3 |
|  | **Q15005\|SPCS2_HUMAN** | Signal peptidase complex subunit 2 OS=Homo sapiens GN=SPCS2 PE=1 SV=3 |
|  | **P35232\|PHB_HUMAN** | Prohibitin OS=Homo sapiens GN=PHB PE=1 SV=1 |
|  | **Q29865\|1C18_HUMAN** | HLA class I histocompatibility antigen, Cw-18 alpha chain OS=Homo sapiens GN=HLA-C PE=2 SV=1 |
|  | **O14828\|SCAM3_HUMAN** | Secretory carrier-associated membrane protein 3 OS=Homo sapiens GN=SCAMP3 PE=1 SV=3 |
|  | **P24539\|AT5F1_HUMAN** | ATP synthase subunit b, mitochondrial OS=Homo sapiens GN=ATP5F1 PE=1 SV=2 |
|  | **O94973\|AP2A2_HUMAN** | AP-2 complex subunit alpha-2 OS=Homo sapiens GN=AP2A2 PE=1 SV=2 |
|  | **P80723\|BASP1_HUMAN** | Brain acid soluble protein 1 OS=Homo sapiens GN=BASP1 PE=1 SV=2 |
|  | **P24844\|MYL9_HUMAN** | Myosin regulatory light polypeptide 9 OS=Homo sapiens GN=MYL9 PE=1 SV=4 |
|  | **P30501\|1C02_HUMAN** | HLA class I histocompatibility antigen, Cw-2 alpha chain OS=Homo sapiens GN=HLA-C PE=1 SV=1 |
|  | **P84095\|RHOG_HUMAN** | Rho-related GTP-binding protein RhoG OS=Homo sapiens GN=RHOG PE=1 SV=1 |
|  | **P02735\|SAA_HUMAN** | Serum amyloid A protein OS=Homo sapiens GN=SAA1 PE=1 SV=2 |
|  | **P01891\|1A68_HUMAN** | HLA class I histocompatibility antigen, A-68 alpha chain OS=Homo sapiens GN=HLA-A PE=1 SV=4 |
|  | **P01911\|2B1E_HUMAN** | HLA class II histocompatibility antigen, DRB1-15 beta chain OS=Homo sapiens GN=HLA-DRB1 PE=1 SV=2 |
|  | **P54652\|HSP72_HUMAN** | Heat shock-related 70 kDa protein 2 OS=Homo sapiens GN=HSPA2 PE=1 SV=1 |
|  | **Q9NTJ5\|SAC1_HUMAN** | Phosphatidylinositide phosphatase SAC1 OS=Homo sapiens GN=SACM1L PE=1 SV=2 |
|  | **P21980\|TGM2_HUMAN** | Protein-glutamine gamma-glutamyltransferase 2 OS=Homo sapiens GN=TGM2 PE=1 SV=2 |
|  | **P08134\|RHOC_HUMAN** | Rho-related GTP-binding protein RhoC OS=Homo sapiens GN=RHOC PE=1 SV=1 |
|  | **P04229\|2B11_HUMAN** | HLA class II histocompatibility antigen, DRB1-1 beta chain OS=Homo sapiens GN=HLA-DRB1 PE=1 SV=2 |
|  | **Q30154\|2B51_HUMAN** | HLA class II histocompatibility antigen, DRB5 beta chain OS=Homo sapiens GN=HLA-DRB5 PE=1 SV=1 |
|  | **P61019\|RAB2A_HUMAN** | Ras-related protein Rab-2A OS=Homo sapiens GN=RAB2A PE=1 SV=1 |
|  | **Q14697\|GANAB_HUMAN** | Neutral alpha-glucosidase AB OS=Homo sapiens GN=GANAB PE=1 SV=3 |
|  | **Q9NR31\|SAR1A_HUMAN** | GTP-binding protein SAR1a OS=Homo sapiens GN=SAR1A PE=1 SV=1 |
|  | **P08133\|ANXA6_HUMAN** | Annexin A6 OS=Homo sapiens GN=ANXA6 PE=1 SV=3 |
|  | **P16284\|PECA1_HUMAN** | Platelet endothelial cell adhesion molecule OS=Homo sapiens GN=PECAM1 PE=1 SV=1 |
|  | **P14314\|GLU2B_HUMAN** | Glucosidase 2 subunit beta OS=Homo sapiens GN=PRKCSH PE=1 SV=2 |
|  | **P21281\|VATB2_HUMAN** | V-type proton ATPase subunit B, brain isoform OS=Homo sapiens GN=ATP6V1B2 PE=1 SV=3 |
|  | **P01912\|HB2B_HUMAN** | HLA class II histocompatibility antigen, DR-1 beta chain OS=Homo sapiens GN=HLA-DRB1 PE=1 SV=2 |
|  | **Q9UHQ9\|NB5R1_HUMAN** | NADH-cytochrome b5 reductase 1 OS=Homo sapiens GN=CYB5R1 PE=1 SV=1 |
|  | **P30453\|1A34_HUMAN** | HLA class I histocompatibility antigen, A-34 alpha chain OS=Homo sapiens GN=HLA-A PE=1 SV=1 |
|  | **O15127\|SCAM2_HUMAN** | Secretory carrier-associated membrane protein 2 OS=Homo sapiens GN=SCAMP2 PE=1 SV=2 |
|  | **P21926\|CD9_HUMAN** | CD9 antigen OS=Homo sapiens GN=CD9 PE=1 SV=4 |
|  | **P13796\|PLSL_HUMAN** | Plastin-2 OS=Homo sapiens GN=LCP1 PE=1 SV=5 |
|  | **P08246\|ELNE_HUMAN** | Neutrophil elastase OS=Homo sapiens GN=ELANE PE=1 SV=1 |
|  | **P00450\|CERU_HUMAN** | Ceruloplasmin OS=Homo sapiens GN=CP PE=1 SV=1 |
|  | **Q9Y6N5\|SQRD_HUMAN** | Sulfide:quinone oxidoreductase, mitochondrial OS=Homo sapiens GN=SQRDL PE=1 SV=1 |
|  | **Q13488\|VPP3_HUMAN** | V-type proton ATPase 116 kDa subunit a isoform 3 OS=Homo sapiens GN=TCIRG1 PE=1 SV=3 |
|  | **P00167\|CYB5_HUMAN** | Cytochrome b5 OS=Homo sapiens GN=CYB5A PE=1 SV=2 |
|  | **P30459\|1A74_HUMAN** | HLA class I histocompatibility antigen, A-74 alpha chain OS=Homo sapiens GN=HLA-A PE=1 SV=1 |
|  | **P10314\|1A32_HUMAN** | HLA class I histocompatibility antigen, A-32 alpha chain OS=Homo sapiens GN=HLA-A PE=2 SV=2 |
|  | **P30512\|1A29_HUMAN** | HLA class I histocompatibility antigen, A-29 alpha chain OS=Homo sapiens GN=HLA-A PE=2 SV=2 |
|  | **P16189\|1A31_HUMAN** | HLA class I histocompatibility antigen, A-31 alpha chain OS=Homo sapiens GN=HLA-A PE=2 SV=2 |
|  | **P01766\|HV305_HUMAN** | Ig heavy chain V-III region BRO OS=Homo sapiens PE=1 SV=1 |
|  | **P30499\|1C01_HUMAN** | HLA class I histocompatibility antigen, Cw-1 alpha chain OS=Homo sapiens GN=HLA-C PE=2 SV=1 |
|  | **P51572\|BAP31_HUMAN** | B-cell receptor-associated protein 31 OS=Homo sapiens GN=BCAP31 PE=1 SV=3 |
|  | **P61224\|RAP1B_HUMAN** | Ras-related protein Rap-1b OS=Homo sapiens GN=RAP1B PE=1 SV=1 |
|  | **P62834\|RAP1A_HUMAN** | Ras-related protein Rap-1A OS=Homo sapiens GN=RAP1A PE=1 SV=1 |
|  | **P16190\|1A33_HUMAN** | HLA class I histocompatibility antigen, A-33 alpha chain OS=Homo sapiens GN=HLA-A PE=1 SV=3 |
|  | **P02671\|FIBA_HUMAN** | Fibrinogen alpha chain OS=Homo sapiens GN=FGA PE=1 SV=2 |
|  | **P12236\|ADT3_HUMAN** | ADP/ATP translocase 3 OS=Homo sapiens GN=SLC25A6 PE=1 SV=4 |
|  | **P03989\|1B27_HUMAN** | HLA class I histocompatibility antigen, B-27 alpha chain OS=Homo sapiens GN=HLA-B PE=1 SV=2 |
|  | **P18465\|1B57_HUMAN** | HLA class I histocompatibility antigen, B-57 alpha chain OS=Homo sapiens GN=HLA-B PE=1 SV=1 |
|  | **P10319\|1B58_HUMAN** | HLA class I histocompatibility antigen, B-58 alpha chain OS=Homo sapiens GN=HLA-B PE=2 SV=1 |
|  | **P30464\|1B15_HUMAN** | HLA class I histocompatibility antigen, B-15 alpha chain OS=Homo sapiens GN=HLA-B PE=1 SV=2 |
|  | **P27338\|AOFB_HUMAN** | Amine oxidase [flavin-containing] B OS=Homo sapiens GN=MAOB PE=1 SV=3 |
|  | **Q5VSK2\|MRC1L_HUMAN** | Macrophage mannose receptor 1-like protein 1 OS=Homo sapiens GN=MRC1L1 PE=2 SV=1 |
|  | **P08107\|HSP71_HUMAN** | Heat shock 70 kDa protein 1 OS=Homo sapiens GN=HSPA1A PE=1 SV=5 |
|  | **P04217\|A1BG_HUMAN** | Alpha-1B-glycoprotein OS=Homo sapiens GN=A1BG PE=1 SV=3 |
|  | **P04083\|ANXA1_HUMAN** | Annexin A1 OS=Homo sapiens GN=ANXA1 PE=1 SV=2 |
|  | **P34931\|HS71L_HUMAN** | Heat shock 70 kDa protein 1L OS=Homo sapiens GN=HSPA1L PE=1 SV=2 |
|  | **Q96AG4\|LRC59_HUMAN** | Leucine-rich repeat-containing protein 59 OS=Homo sapiens GN=LRRC59 PE=1 SV=1 |
|  | **P62424\|RL7A_HUMAN** | 60S ribosomal protein L7a OS=Homo sapiens GN=RPL7A PE=1 SV=2 |
|  | **P01857\|IGHG1_HUMAN** | Ig gamma-1 chain C region OS=Homo sapiens GN=IGHG1 PE=1 SV=1 |
|  | **O75947\|ATP5H_HUMAN** | ATP synthase subunit d, mitochondrial OS=Homo sapiens GN=ATP5H PE=1 SV=3 |
|  | **P30487\|1B49_HUMAN** | HLA class I histocompatibility antigen, B-49 alpha chain OS=Homo sapiens GN=HLA-B PE=2 SV=2 |
|  | **P30461\|1B13_HUMAN** | HLA class I histocompatibility antigen, B-13 alpha chain OS=Homo sapiens GN=HLA-B PE=2 SV=1 |
|  | **P30485\|1B47_HUMAN** | HLA class I histocompatibility antigen, B-47 alpha chain OS=Homo sapiens GN=HLA-B PE=2 SV=1 |
|  | **P30481\|1B44_HUMAN** | HLA class I histocompatibility antigen, B-44 alpha chain OS=Homo sapiens GN=HLA-B PE=1 SV=1 |
|  | **P30483\|1B45_HUMAN** | HLA class I histocompatibility antigen, B-45 alpha chain OS=Homo sapiens GN=HLA-B PE=2 SV=1 |
|  | **P54920\|SNAA_HUMAN** | Alpha-soluble NSF attachment protein OS=Homo sapiens GN=NAPA PE=1 SV=3 |
|  | **P01903\|2DRA_HUMAN** | HLA class II histocompatibility antigen, DR alpha chain OS=Homo sapiens GN=HLA-DRA PE=1 SV=1 |
|  | **O43169\|CYB5B_HUMAN** | Cytochrome b5 type B OS=Homo sapiens GN=CYB5B PE=1 SV=2 |
|  | **Q15836\|VAMP3_HUMAN** | Vesicle-associated membrane protein 3 OS=Homo sapiens GN=VAMP3 PE=1 SV=3 |
|  | **P05387\|RLA2_HUMAN** | 60S acidic ribosomal protein P2 OS=Homo sapiens GN=RPLP2 PE=1 SV=1 |
|  | **O96009\|NAPSA_HUMAN** | Napsin-A OS=Homo sapiens GN=NAPSA PE=1 SV=1 |
|  | **O75915\|PRAF3_HUMAN** | PRA1 family protein 3 OS=Homo sapiens GN=ARL6IP5 PE=1 SV=1 |
|  | **P69891\|HBG1_HUMAN** | Hemoglobin subunit gamma-1 OS=Homo sapiens GN=HBG1 PE=1 SV=2 |
|  | **P02100\|HBE_HUMAN** | Hemoglobin subunit epsilon OS=Homo sapiens GN=HBE1 PE=1 SV=2 |
|  | **P30041\|PRDX6_HUMAN** | Peroxiredoxin-6 OS=Homo sapiens GN=PRDX6 PE=1 SV=3 |
|  | **P08238\|HS90B_HUMAN** | Heat shock protein HSP 90-beta OS=Homo sapiens GN=HSP90AB1 PE=1 SV=4 |
|  | **O75923\|DYSF_HUMAN** | Dysferlin OS=Homo sapiens GN=DYSF PE=1 SV=1 |
|  | **O15260\|SURF4_HUMAN** | Surfeit locus protein 4 OS=Homo sapiens GN=SURF4 PE=1 SV=3 |
|  | **P13284\|GILT_HUMAN** | Gamma-interferon-inducible lysosomal thiol reductase OS=Homo sapiens GN=IFI30 PE=1 SV=2 |
|  | **P49585\|PCY1A_HUMAN** | Choline-phosphate cytidylyltransferase A OS=Homo sapiens GN=PCYT1A PE=1 SV=2 |
|  | **P59665\|DEF1_HUMAN** | Neutrophil defensin 1 OS=Homo sapiens GN=DEFA1 PE=1 SV=1 |
|  | **P50897\|PPT1_HUMAN** | Palmitoyl-protein thioesterase 1 OS=Homo sapiens GN=PPT1 PE=1 SV=1 |
|  | **Q03252\|LMNB2_HUMAN** | Lamin-B2 OS=Homo sapiens GN=LMNB2 PE=1 SV=3 |
|  | **Q9BZQ8\|NIBAN_HUMAN** | Protein Niban OS=Homo sapiens GN=FAM129A PE=1 SV=1 |
|  | **P16671\|CD36_HUMAN** | Platelet glycoprotein 4 OS=Homo sapiens GN=CD36 PE=1 SV=2 |
|  | **P18464\|1B51_HUMAN** | HLA class I histocompatibility antigen, B-51 alpha chain OS=Homo sapiens GN=HLA-B PE=1 SV=1 |
|  | **Q13724\|MOGS_HUMAN** | Mannosyl-oligosaccharide glucosidase OS=Homo sapiens GN=MOGS PE=1 SV=5 |
|  | **Q93084\|AT2A3_HUMAN** | Sarcoplasmic/endoplasmic reticulum calcium ATPase 3 OS=Homo sapiens GN=ATP2A3 PE=1 SV=2 |
|  | **Q96QV6\|H2A1A_HUMAN** | Histone H2A type 1-A OS=Homo sapiens GN=HIST1H2AA PE=1 SV=3 |
|  | **P05388\|RLA0_HUMAN** | 60S acidic ribosomal protein P0 OS=Homo sapiens GN=RPLP0 PE=1 SV=1 |
|  | **Q00325\|MPCP_HUMAN** | Phosphate carrier protein, mitochondrial OS=Homo sapiens GN=SLC25A3 PE=1 SV=2 |
|  | **Q8NBQ5\|DHB11_HUMAN** | Estradiol 17-beta-dehydrogenase 11 OS=Homo sapiens GN=HSD17B11 PE=1 SV=2 |
|  | **P13760\|2B14_HUMAN** | HLA class II histocompatibility antigen, DRB1-4 beta chain OS=Homo sapiens GN=HLA-DRB1 PE=1 SV=1 |
|  | **P61204\|ARF3_HUMAN** | ADP-ribosylation factor 3 OS=Homo sapiens GN=ARF3 PE=1 SV=2 |
|  | **P08311\|CATG_HUMAN** | Cathepsin G OS=Homo sapiens GN=CTSG PE=1 SV=2 |
|  | **P11233\|RALA_HUMAN** | Ras-related protein Ral-A OS=Homo sapiens GN=RALA PE=1 SV=1 |
|  | **P20702\|ITAX_HUMAN** | Integrin alpha-X OS=Homo sapiens GN=ITGAX PE=1 SV=3 |
|  | **P18462\|1A25_HUMAN** | HLA class I histocompatibility antigen, A-25 alpha chain OS=Homo sapiens GN=HLA-A PE=2 SV=1 |
|  | **P01914\|HB2C_HUMAN** | HLA class II histocompatibility antigen, DR-1 beta chain OS=Homo sapiens PE=2 SV=2 |
|  | **P09104\|ENOG_HUMAN** | Gamma-enolase OS=Homo sapiens GN=ENO2 PE=1 SV=3 |
|  | **P13929\|ENOB_HUMAN** | Beta-enolase OS=Homo sapiens GN=ENO3 PE=1 SV=4 |
|  | **Q92597\|NDRG1_HUMAN** | Protein NDRG1 OS=Homo sapiens GN=NDRG1 PE=1 SV=1 |
|  | **P08195\|4F2_HUMAN** | 4F2 cell-surface antigen heavy chain OS=Homo sapiens GN=SLC3A2 PE=1 SV=3 |
|  | **P02649\|APOE_HUMAN** | Apolipoprotein E OS=Homo sapiens GN=APOE PE=1 SV=1 |
|  | **Q92928\|RAB1C_HUMAN** | Putative Ras-related protein Rab-1C OS=Homo sapiens GN=RAB1C PE=5 SV=2 |
|  | **Q09160\|1A80_HUMAN** | HLA class I histocompatibility antigen, A-80 alpha chain OS=Homo sapiens GN=HLA-A PE=2 SV=1 |
|  | **O95782\|AP2A1_HUMAN** | AP-2 complex subunit alpha-1 OS=Homo sapiens GN=AP2A1 PE=1 SV=2 |
|  | **O14653\|GOSR2_HUMAN** | Golgi SNAP receptor complex member 2 OS=Homo sapiens GN=GOSR2 PE=1 SV=2 |
|  | **Q9P0L0\|VAPA_HUMAN** | Vesicle-associated membrane protein-associated protein A OS=Homo sapiens GN=VAPA PE=1 SV=3 |
|  | **P30511\|HLAF_HUMAN** | HLA class I histocompatibility antigen, alpha chain F OS=Homo sapiens GN=HLA-F PE=2 SV=2 |
|  | **P21964\|COMT_HUMAN** | Catechol O-methyltransferase OS=Homo sapiens GN=COMT PE=1 SV=2 |
|  | **P17213\|BPI_HUMAN** | Bactericidal permeability-increasing protein OS=Homo sapiens GN=BPI PE=1 SV=4 |
|  | **P61006\|RAB8A_HUMAN** | Ras-related protein Rab-8A OS=Homo sapiens GN=RAB8A PE=1 SV=1 |
|  | **P31949\|S10AB_HUMAN** | Protein S100-A11 OS=Homo sapiens GN=S100A11 PE=1 SV=2 |
|  | **P33897\|ABCD1_HUMAN** | ATP-binding cassette sub-family D member 1 OS=Homo sapiens GN=ABCD1 PE=1 SV=2 |
|  | **P49755\|TMEDA_HUMAN** | Transmembrane emp24 domain-containing protein 10 OS=Homo sapiens GN=TMED10 PE=1 SV=2 |
|  | **P09211\|GSTP1_HUMAN** | Glutathione S-transferase P OS=Homo sapiens GN=GSTP1 PE=1 SV=2 |
|  | **P01876\|IGHA1_HUMAN** | Ig alpha-1 chain C region OS=Homo sapiens GN=IGHA1 PE=1 SV=2 |
|  | **Q9Y624\|JAM1_HUMAN** | Junctional adhesion molecule A OS=Homo sapiens GN=F11R PE=1 SV=1 |
|  | **P06756\|ITAV_HUMAN** | Integrin alpha-V OS=Homo sapiens GN=ITGAV PE=1 SV=2 |
|  | **Q9Y4L1\|HYOU1_HUMAN** | Hypoxia up-regulated protein 1 OS=Homo sapiens GN=HYOU1 PE=1 SV=1 |
|  | **O95994\|AGR2_HUMAN** | Anterior gradient protein 2 homolog OS=Homo sapiens GN=AGR2 PE=1 SV=1 |
|  | **P04792\|HSPB1_HUMAN** | Heat shock protein beta-1 OS=Homo sapiens GN=HSPB1 PE=1 SV=2 |
|  | **P55084\|ECHB_HUMAN** | Trifunctional enzyme subunit beta, mitochondrial OS=Homo sapiens GN=HADHB PE=1 SV=3 |
|  | **P04632\|CPNS1_HUMAN** | Calpain small subunit 1 OS=Homo sapiens GN=CAPNS1 PE=1 SV=1 |
|  | **P01871\|IGHM_HUMAN** | Ig mu chain C region OS=Homo sapiens GN=IGHM PE=1 SV=3 |
|  | **O00264\|PGRC1_HUMAN** | Membrane-associated progesterone receptor component 1 OS=Homo sapiens GN=PGRMC1 PE=1 SV=3 |
|  | **P18085\|ARF4_HUMAN** | ADP-ribosylation factor 4 OS=Homo sapiens GN=ARF4 PE=1 SV=3 |
|  | **O95292\|VAPB_HUMAN** | Vesicle-associated membrane protein-associated protein B/C OS=Homo sapiens GN=VAPB PE=1 SV=3 |
|  | **Q9H553\|ALG2_HUMAN** | Alpha-1,3-mannosyltransferase ALG2 OS=Homo sapiens GN=ALG2 PE=2 SV=1 |
|  | **P17066\|HSP76_HUMAN** | Heat shock 70 kDa protein 6 OS=Homo sapiens GN=HSPA6 PE=1 SV=2 |
|  | **P48741\|HSP77_HUMAN** | Putative heat shock 70 kDa protein 7 OS=Homo sapiens GN=HSPA7 PE=5 SV=2 |
|  | **Q9HAV0\|GBB4_HUMAN** | Guanine nucleotide-binding protein subunit beta-4 OS=Homo sapiens GN=GNB4 PE=1 SV=3 |
|  | **P00915\|CAH1_HUMAN** | Carbonic anhydrase 1 OS=Homo sapiens GN=CA1 PE=1 SV=2 |
|  | **Q8NBX0\|SCPDH_HUMAN** | Probable saccharopine dehydrogenase OS=Homo sapiens GN=SCCPDH PE=1 SV=1 |
|  | **P38606\|VATA_HUMAN** | V-type proton ATPase catalytic subunit A OS=Homo sapiens GN=ATP6V1A PE=1 SV=2 |
|  | **Q9Y3Z3\|SAMH1_HUMAN** | SAM domain and HD domain-containing protein 1 OS=Homo sapiens GN=SAMHD1 PE=1 SV=2 |
|  | **Q14573\|ITPR3_HUMAN** | Inositol 1,4,5-trisphosphate receptor type 3 OS=Homo sapiens GN=ITPR3 PE=1 SV=2 |
|  | **P39656\|OST48_HUMAN** | Dolichyl-diphosphooligosaccharide--protein glycosyltransferase 48 kDa subunit OS=Homo sapiens GN=DDOST PE=1 SV=4 |
|  | **P08729\|K2C7_HUMAN** | Keratin, type II cytoskeletal 7 OS=Homo sapiens GN=KRT7 PE=1 SV=3 |
|  | **Q12907\|LMAN2_HUMAN** | Vesicular integral-membrane protein VIP36 OS=Homo sapiens GN=LMAN2 PE=1 SV=1 |
|  | **P51148\|RAB5C_HUMAN** | Ras-related protein Rab-5C OS=Homo sapiens GN=RAB5C PE=1 SV=2 |
|  | **P30504\|1C04_HUMAN** | HLA class I histocompatibility antigen, Cw-4 alpha chain OS=Homo sapiens GN=HLA-C PE=1 SV=1 |
|  | **P09525\|ANXA4_HUMAN** | Annexin A4 OS=Homo sapiens GN=ANXA4 PE=1 SV=4 |
|  | **Q92959\|SO2A1_HUMAN** | Solute carrier organic anion transporter family member 2A1 OS=Homo sapiens GN=SLCO2A1 PE=1 SV=2 |
|  | **P20648\|ATP4A_HUMAN** | Potassium-transporting ATPase alpha chain 1 OS=Homo sapiens GN=ATP4A PE=2 SV=5 |
|  | **P51149\|RAB7A_HUMAN** | Ras-related protein Rab-7a OS=Homo sapiens GN=RAB7A PE=1 SV=1 |
|  | **P20674\|COX5A_HUMAN** | Cytochrome c oxidase subunit 5A, mitochondrial OS=Homo sapiens GN=COX5A PE=1 SV=2 |
|  | **O75381\|PEX14_HUMAN** | Peroxisomal membrane protein PEX14 OS=Homo sapiens GN=PEX14 PE=1 SV=1 |
|  | **P08575\|CD45_HUMAN** | Leukocyte common antigen OS=Homo sapiens GN=PTPRC PE=1 SV=2 |
|  | **Q07954\|LRP1_HUMAN** | Prolow-density lipoprotein receptor-related protein 1 OS=Homo sapiens GN=LRP1 PE=1 SV=1 |
|  | **P15144\|AMPN_HUMAN** | Aminopeptidase N OS=Homo sapiens GN=ANPEP PE=1 SV=4 |
|  | **P11908\|PRPS2_HUMAN** | Ribose-phosphate pyrophosphokinase 2 OS=Homo sapiens GN=PRPS2 PE=1 SV=2 |
|  | **Q9UKY3\|EST4_HUMAN** | Inactive carboxylesterase 4 OS=Homo sapiens GN=CES4 PE=2 SV=2 |
|  | **P51648\|AL3A2_HUMAN** | Fatty aldehyde dehydrogenase OS=Homo sapiens GN=ALDH3A2 PE=1 SV=1 |
|  | **P39210\|MPV17_HUMAN** | Protein Mpv17 OS=Homo sapiens GN=MPV17 PE=1 SV=1 |
|  | **Q9H853\|TBA4B_HUMAN** | Putative tubulin-like protein alpha-4B OS=Homo sapiens GN=TUBA4B PE=5 SV=2 |
|  | **Q08380\|LG3BP_HUMAN** | Galectin-3-binding protein OS=Homo sapiens GN=LGALS3BP PE=1 SV=1 |
|  | **P13747\|HLAE_HUMAN** | HLA class I histocompatibility antigen, alpha chain E OS=Homo sapiens GN=HLA-E PE=1 SV=3 |
|  | **P60891\|PRPS1_HUMAN** | Ribose-phosphate pyrophosphokinase 1 OS=Homo sapiens GN=PRPS1 PE=1 SV=2 |
|  | **P27449\|VATL_HUMAN** | V-type proton ATPase 16 kDa proteolipid subunit OS=Homo sapiens GN=ATP6V0C PE=1 SV=1 |
|  | **P63167\|DYL1_HUMAN** | Dynein light chain 1, cytoplasmic OS=Homo sapiens GN=DYNLL1 PE=1 SV=1 |
|  | **O43795\|MYO1B_HUMAN** | Myosin-Ib OS=Homo sapiens GN=MYO1B PE=1 SV=3 |
|  | **P12235\|ADT1_HUMAN** | ADP/ATP translocase 1 OS=Homo sapiens GN=SLC25A4 PE=1 SV=4 |
|  | **O75531\|BAF_HUMAN** | Barrier-to-autointegration factor OS=Homo sapiens GN=BANF1 PE=1 SV=1 |
|  | **P00395\|COX1_HUMAN** | Cytochrome c oxidase subunit 1 OS=Homo sapiens GN=MT-CO1 PE=1 SV=1 |
|  | **O14983\|AT2A1_HUMAN** | Sarcoplasmic/endoplasmic reticulum calcium ATPase 1 OS=Homo sapiens GN=ATP2A1 PE=1 SV=1 |
|  | **O95470\|SGPL1_HUMAN** | Sphingosine-1-phosphate lyase 1 OS=Homo sapiens GN=SGPL1 PE=1 SV=3 |
|  | **P36269\|GGT5_HUMAN** | Gamma-glutamyltransferase 5 OS=Homo sapiens GN=GGT5 PE=1 SV=2 |
|  | **P43490\|NAMPT_HUMAN** | Nicotinamide phosphoribosyltransferase OS=Homo sapiens GN=NAMPT PE=1 SV=1 |
|  | **Q03519\|TAP2_HUMAN** | Antigen peptide transporter 2 OS=Homo sapiens GN=TAP2 PE=1 SV=1 |
|  | **Q969Z3\|MOSC2_HUMAN** | MOSC domain-containing protein 2, mitochondrial OS=Homo sapiens GN=MOSC2 PE=1 SV=1 |
|  | **P27824\|CALX_HUMAN** | Calnexin OS=Homo sapiens GN=CANX PE=1 SV=2 |
|  | **P11234\|RALB_HUMAN** | Ras-related protein Ral-B OS=Homo sapiens GN=RALB PE=1 SV=1 |
|  | **Q8IY17\|PLPL6_HUMAN** | Neuropathy target esterase OS=Homo sapiens GN=PNPLA6 PE=1 SV=2 |
|  | **O75367\|H2AY_HUMAN** | Core histone macro-H2A.1 OS=Homo sapiens GN=H2AFY PE=1 SV=4 |
|  | **Q9P0M6\|H2AW_HUMAN** | Core histone macro-H2A.2 OS=Homo sapiens GN=H2AFY2 PE=1 SV=3 |
|  | **O60762\|DPM1_HUMAN** | Dolichol-phosphate mannosyltransferase OS=Homo sapiens GN=DPM1 PE=1 SV=1 |
|  | **P50454\|SERPH_HUMAN** | Serpin H1 OS=Homo sapiens GN=SERPINH1 PE=1 SV=2 |
|  | **P62745\|RHOB_HUMAN** | Rho-related GTP-binding protein RhoB OS=Homo sapiens GN=RHOB PE=1 SV=1 |
|  | **P00441\|SODC_HUMAN** | Superoxide dismutase [Cu-Zn] OS=Homo sapiens GN=SOD1 PE=1 SV=2 |
|  | **Q15067\|ACOX1_HUMAN** | Peroxisomal acyl-coenzyme A oxidase 1 OS=Homo sapiens GN=ACOX1 PE=1 SV=3 |
|  | **P61158\|ARP3_HUMAN** | Actin-related protein 3 OS=Homo sapiens GN=ACTR3 PE=1 SV=3 |
|  | **P41218\|MNDA_HUMAN** | Myeloid cell nuclear differentiation antigen OS=Homo sapiens GN=MNDA PE=1 SV=1 |
|  | **P48960\|CD97_HUMAN** | CD97 antigen OS=Homo sapiens GN=CD97 PE=1 SV=4 |
|  | **P80188\|NGAL_HUMAN** | Neutrophil gelatinase-associated lipocalin OS=Homo sapiens GN=LCN2 PE=1 SV=2 |
|  | **P12956\|KU70_HUMAN** | ATP-dependent DNA helicase 2 subunit 1 OS=Homo sapiens GN=XRCC6 PE=1 SV=2 |
|  | **P38646\|GRP75_HUMAN** | Stress-70 protein, mitochondrial OS=Homo sapiens GN=HSPA9 PE=1 SV=2 |
|  | **Q93050\|VPP1_HUMAN** | V-type proton ATPase 116 kDa subunit a isoform 1 OS=Homo sapiens GN=ATP6V0A1 PE=1 SV=3 |
|  | **Q9UBG0\|MRC2_HUMAN** | C-type mannose receptor 2 OS=Homo sapiens GN=MRC2 PE=1 SV=1 |
|  | **Q9H4G4\|GAPR1_HUMAN** | Golgi-associated plant pathogenesis-related protein 1 OS=Homo sapiens GN=GLIPR2 PE=1 SV=3 |
|  | **Q92575\|UBXN4_HUMAN** | UBX domain-containing protein 4 OS=Homo sapiens GN=UBXN4 PE=1 SV=2 |
|  | **O60716\|CTND1_HUMAN** | Catenin delta-1 OS=Homo sapiens GN=CTNND1 PE=1 SV=1 |
|  | **O15031\|PLXB2_HUMAN** | Plexin-B2 OS=Homo sapiens GN=PLXNB2 PE=1 SV=3 |
|  | **P02452\|CO1A1_HUMAN** | Collagen alpha-1(I) chain OS=Homo sapiens GN=COL1A1 PE=1 SV=4 |
|  | **P04003\|C4BPA_HUMAN** | C4b-binding protein alpha chain OS=Homo sapiens GN=C4BPA PE=1 SV=2 |
|  | **O00291\|HIP1_HUMAN** | Huntingtin-interacting protein 1 OS=Homo sapiens GN=HIP1 PE=1 SV=5 |
|  | **O00161\|SNP23_HUMAN** | Synaptosomal-associated protein 23 OS=Homo sapiens GN=SNAP23 PE=1 SV=1 |
|  | **P62937\|PPIA_HUMAN** | Peptidyl-prolyl cis-trans isomerase A OS=Homo sapiens GN=PPIA PE=1 SV=2 |
|  | **Q9Y673\|ALG5_HUMAN** | Dolichyl-phosphate beta-glucosyltransferase OS=Homo sapiens GN=ALG5 PE=1 SV=1 |
|  | **P31146\|COR1A_HUMAN** | Coronin-1A OS=Homo sapiens GN=CORO1A PE=1 SV=4 |
|  | **P08754\|GNAI3_HUMAN** | Guanine nucleotide-binding protein G(k) subunit alpha OS=Homo sapiens GN=GNAI3 PE=1 SV=3 |
|  | **Q8NFJ5\|RAI3_HUMAN** | Retinoic acid-induced protein 3 OS=Homo sapiens GN=GPRC5A PE=1 SV=2 |
|  | **P07910\|HNRPC_HUMAN** | Heterogeneous nuclear ribonucleoproteins C1/C2 OS=Homo sapiens GN=HNRNPC PE=1 SV=4 |
|  | **P00403\|COX2_HUMAN** | Cytochrome c oxidase subunit 2 OS=Homo sapiens GN=MT-CO2 PE=1 SV=1 |
|  | **Q15165\|PON2_HUMAN** | Serum paraoxonase/arylesterase 2 OS=Homo sapiens GN=PON2 PE=1 SV=2 |
|  | **Q15738\|NSDHL_HUMAN** | Sterol-4-alpha-carboxylate 3-dehydrogenase, decarboxylating OS=Homo sapiens GN=NSDHL PE=1 SV=2 |
|  | **P02656\|APOC3_HUMAN** | Apolipoprotein C-III OS=Homo sapiens GN=APOC3 PE=1 SV=1 |
|  | **P55290\|CAD13_HUMAN** | Cadherin-13 OS=Homo sapiens GN=CDH13 PE=1 SV=1 |
|  | **Q9Y5M8\|SRPRB_HUMAN** | Signal recognition particle receptor subunit beta OS=Homo sapiens GN=SRPRB PE=1 SV=3 |
|  | **P52597\|HNRPF_HUMAN** | Heterogeneous nuclear ribonucleoprotein F OS=Homo sapiens GN=HNRNPF PE=1 SV=3 |
|  | **Q9H8H3\|MET7A_HUMAN** | Methyltransferase-like protein 7A OS=Homo sapiens GN=METTL7A PE=1 SV=1 |
|  | **O00299\|CLIC1_HUMAN** | Chloride intracellular channel protein 1 OS=Homo sapiens GN=CLIC1 PE=1 SV=4 |
|  | **P61026\|RAB10_HUMAN** | Ras-related protein Rab-10 OS=Homo sapiens GN=RAB10 PE=1 SV=1 |
|  | **Q16718\|NDUA5_HUMAN** | NADH dehydrogenase [ubiquinone] 1 alpha subcomplex subunit 5 OS=Homo sapiens GN=NDUFA5 PE=1 SV=3 |
|  | **P33121\|ACSL1_HUMAN** | Long-chain-fatty-acid--CoA ligase 1 OS=Homo sapiens GN=ACSL1 PE=1 SV=1 |
|  | **Q96S66\|CLCC1_HUMAN** | Chloride channel CLIC-like protein 1 OS=Homo sapiens GN=CLCC1 PE=1 SV=1 |
|  | **Q8NHW5\|RLA0L_HUMAN** | 60S acidic ribosomal protein P0-like OS=Homo sapiens PE=2 SV=1 |
|  | **O15269\|SPTC1_HUMAN** | Serine palmitoyltransferase 1 OS=Homo sapiens GN=SPTLC1 PE=1 SV=1 |
|  | **Q32P51\|RA1L2_HUMAN** | Heterogeneous nuclear ribonucleoprotein A1-like protein 2 OS=Homo sapiens GN=HNRNPA1L2 PE=2 SV=2 |
|  | **P09651\|ROA1_HUMAN** | Heterogeneous nuclear ribonucleoprotein A1 OS=Homo sapiens GN=HNRNPA1 PE=1 SV=4 |
|  | **P0C7M2\|RA1L3_HUMAN** | Putative heterogeneous nuclear ribonucleoprotein A1-like protein 3 OS=Homo sapiens GN=HNRPA1L3 PE=5 SV=1 |
|  | **P02749\|APOH_HUMAN** | Beta-2-glycoprotein 1 OS=Homo sapiens GN=APOH PE=1 SV=3 |
|  | **P10909\|CLUS_HUMAN** | Clusterin OS=Homo sapiens GN=CLU PE=1 SV=1 |
|  | **P04206\|KV307_HUMAN** | Ig kappa chain V-III region GOL OS=Homo sapiens PE=1 SV=1 |
|  | **P01619\|KV301_HUMAN** | Ig kappa chain V-III region B6 OS=Homo sapiens PE=1 SV=1 |
|  | **Q15392\|DHC24_HUMAN** | 24-dehydrocholesterol reductase OS=Homo sapiens GN=DHCR24 PE=1 SV=2 |
|  | **P56134\|ATPK_HUMAN** | ATP synthase subunit f, mitochondrial OS=Homo sapiens GN=ATP5J2 PE=1 SV=3 |
|  | **Q8N3Y7\|RDHE2_HUMAN** | Epidermal retinal dehydrogenase 2 OS=Homo sapiens GN=SDR16C5 PE=2 SV=2 |
|  | **P13073\|COX41_HUMAN** | Cytochrome c oxidase subunit 4 isoform 1, mitochondrial OS=Homo sapiens GN=COX4I1 PE=1 SV=1 |
|  | **Q8WUD1\|RAB2B_HUMAN** | Ras-related protein Rab-2B OS=Homo sapiens GN=RAB2B PE=1 SV=1 |
|  | **P30050\|RL12_HUMAN** | 60S ribosomal protein L12 OS=Homo sapiens GN=RPL12 PE=1 SV=1 |
|  | **Q96S97\|MYADM_HUMAN** | Myeloid-associated differentiation marker OS=Homo sapiens GN=MYADM PE=1 SV=2 |
|  | **P15941\|MUC1_HUMAN** | Mucin-1 OS=Homo sapiens GN=MUC1 PE=1 SV=2 |
|  | **P06744\|G6PI_HUMAN** | Glucose-6-phosphate isomerase OS=Homo sapiens GN=GPI PE=1 SV=4 |
|  | **P20039\|2B1B_HUMAN** | HLA class II histocompatibility antigen, DRB1-11 beta chain OS=Homo sapiens GN=HLA-DRB1 PE=2 SV=1 |
|  | **Q14643\|ITPR1_HUMAN** | Inositol 1,4,5-trisphosphate receptor type 1 OS=Homo sapiens GN=ITPR1 PE=1 SV=2 |
|  | **P06753\|TPM3_HUMAN** | Tropomyosin alpha-3 chain OS=Homo sapiens GN=TPM3 PE=1 SV=1 |
|  | **Q16647\|PTGIS_HUMAN** | Prostacyclin synthase OS=Homo sapiens GN=PTGIS PE=1 SV=1 |
|  | **Q92930\|RAB8B_HUMAN** | Ras-related protein Rab-8B OS=Homo sapiens GN=RAB8B PE=1 SV=2 |
|  | **P20645\|MPRD_HUMAN** | Cation-dependent mannose-6-phosphate receptor OS=Homo sapiens GN=M6PR PE=1 SV=1 |
|  | **P15311\|EZRI_HUMAN** | Ezrin OS=Homo sapiens GN=EZR PE=1 SV=4 |
|  | **P05109\|S10A8_HUMAN** | Protein S100-A8 OS=Homo sapiens GN=S100A8 PE=1 SV=1 |
|  | **Q9NYU2\|UGGG1_HUMAN** | UDP-glucose:glycoprotein glucosyltransferase 1 OS=Homo sapiens GN=UGCGL1 PE=1 SV=3 |
|  | **O95716\|RAB3D_HUMAN** | Ras-related protein Rab-3D OS=Homo sapiens GN=RAB3D PE=1 SV=1 |
|  | **P20336\|RAB3A_HUMAN** | Ras-related protein Rab-3A OS=Homo sapiens GN=RAB3A PE=1 SV=1 |
|  | **Q13492\|PICAL_HUMAN** | Phosphatidylinositol-binding clathrin assembly protein OS=Homo sapiens GN=PICALM PE=1 SV=2 |
|  | **P04843\|RPN1_HUMAN** | Dolichyl-diphosphooligosaccharide--protein glycosyltransferase subunit 1 OS=Homo sapiens GN=RPN1 PE=1 SV=1 |
|  | **P04233\|HG2A_HUMAN** | HLA class II histocompatibility antigen gamma chain OS=Homo sapiens GN=CD74 PE=1 SV=3 |
|  | **P29401\|TKT_HUMAN** | Transketolase OS=Homo sapiens GN=TKT PE=1 SV=3 |
|  | **P40616\|ARL1_HUMAN** | ADP-ribosylation factor-like protein 1 OS=Homo sapiens GN=ARL1 PE=1 SV=1 |
|  | **Q9H4B7\|TBB1_HUMAN** | Tubulin beta-1 chain OS=Homo sapiens GN=TUBB1 PE=1 SV=1 |
|  | **P61421\|VA0D1_HUMAN** | V-type proton ATPase subunit d 1 OS=Homo sapiens GN=ATP6V0D1 PE=1 SV=1 |
|  | **Q6P4A8\|PLBL1_HUMAN** | Putative phospholipase B-like 1 OS=Homo sapiens GN=PLBD1 PE=1 SV=1 |
|  | **P08631\|HCK_HUMAN** | Tyrosine-protein kinase HCK OS=Homo sapiens GN=HCK PE=1 SV=5 |
|  | **O15173\|PGRC2_HUMAN** | Membrane-associated progesterone receptor component 2 OS=Homo sapiens GN=PGRMC2 PE=1 SV=1 |
|  | **P22748\|CAH4_HUMAN** | Carbonic anhydrase 4 OS=Homo sapiens GN=CA4 PE=1 SV=2 |
|  | **P02647\|APOA1_HUMAN** | Apolipoprotein A-I OS=Homo sapiens GN=APOA1 PE=1 SV=1 |
|  | **P07900\|HS90A_HUMAN** | Heat shock protein HSP 90-alpha OS=Homo sapiens GN=HSP90AA1 PE=1 SV=5 |
|  | **Q6UW68\|TM205_HUMAN** | Transmembrane protein 205 OS=Homo sapiens GN=TMEM205 PE=1 SV=1 |
|  | **P19823\|ITIH2_HUMAN** | Inter-alpha-trypsin inhibitor heavy chain H2 OS=Homo sapiens GN=ITIH2 PE=1 SV=2 |
|  | **P07951\|TPM2_HUMAN** | Tropomyosin beta chain OS=Homo sapiens GN=TPM2 PE=1 SV=1 |
|  | **P09493\|TPM1_HUMAN** | Tropomyosin alpha-1 chain OS=Homo sapiens GN=TPM1 PE=1 SV=2 |
|  | **Q6IAN0\|DRS7B_HUMAN** | Dehydrogenase/reductase SDR family member 7B OS=Homo sapiens GN=DHRS7B PE=1 SV=2 |
|  | **P31040\|DHSA_HUMAN** | Succinate dehydrogenase [ubiquinone] flavoprotein subunit, mitochondrial OS=Homo sapiens GN=SDHA PE=1 SV=2 |
|  | **P61981\|1433G_HUMAN** | 14-3-3 protein gamma OS=Homo sapiens GN=YWHAG PE=1 SV=2 |
|  | **P05186\|PPBT_HUMAN** | Alkaline phosphatase, tissue-nonspecific isozyme OS=Homo sapiens GN=ALPL PE=1 SV=4 |
|  | **Q9UBR2\|CATZ_HUMAN** | Cathepsin Z OS=Homo sapiens GN=CTSZ PE=1 SV=1 |
|  | **P61978\|HNRPK_HUMAN** | Heterogeneous nuclear ribonucleoprotein K OS=Homo sapiens GN=HNRNPK PE=1 SV=1 |
|  | **P12429\|ANXA3_HUMAN** | Annexin A3 OS=Homo sapiens GN=ANXA3 PE=1 SV=3 |
|  | **Q9BS26\|ERP44_HUMAN** | Endoplasmic reticulum resident protein ERp44 OS=Homo sapiens GN=ERP44 PE=1 SV=1 |
|  | **P31943\|HNRH1_HUMAN** | Heterogeneous nuclear ribonucleoprotein H OS=Homo sapiens GN=HNRNPH1 PE=1 SV=4 |
|  | **P55795\|HNRH2_HUMAN** | Heterogeneous nuclear ribonucleoprotein H2 OS=Homo sapiens GN=HNRNPH2 PE=1 SV=1 |
|  | **A6NMZ7\|CO6A6_HUMAN** | Collagen alpha-6(VI) chain OS=Homo sapiens GN=COL6A6 PE=1 SV=2 |
|  | **P60953\|CDC42_HUMAN** | Cell division control protein 42 homolog OS=Homo sapiens GN=CDC42 PE=1 SV=1 |
|  | **P60174\|TPIS_HUMAN** | Triosephosphate isomerase OS=Homo sapiens GN=TPI1 PE=1 SV=2 |
|  | **P40926\|MDHM_HUMAN** | Malate dehydrogenase, mitochondrial OS=Homo sapiens GN=MDH2 PE=1 SV=3 |
|  | **P07195\|LDHB_HUMAN** | L-lactate dehydrogenase B chain OS=Homo sapiens GN=LDHB PE=1 SV=2 |
|  | **P08727\|K1C19_HUMAN** | Keratin, type I cytoskeletal 19 OS=Homo sapiens GN=KRT19 PE=1 SV=3 |
|  | **O15400\|STX7_HUMAN** | Syntaxin-7 OS=Homo sapiens GN=STX7 PE=1 SV=4 |
|  | **Q9Y394\|DHRS7_HUMAN** | Dehydrogenase/reductase SDR family member 7 OS=Homo sapiens GN=DHRS7 PE=1 SV=1 |
|  | **P15328\|FOLR1_HUMAN** | Folate receptor alpha OS=Homo sapiens GN=FOLR1 PE=1 SV=3 |
|  | **Q12931\|TRAP1_HUMAN** | Heat shock protein 75 kDa, mitochondrial OS=Homo sapiens GN=TRAP1 PE=1 SV=3 |
|  | **Q92974\|ARHG2_HUMAN** | Rho guanine nucleotide exchange factor 2 OS=Homo sapiens GN=ARHGEF2 PE=1 SV=4 |
|  | **P14854\|CX6B1_HUMAN** | Cytochrome c oxidase subunit 6B1 OS=Homo sapiens GN=COX6B1 PE=1 SV=2 |
|  | **P02792\|FRIL_HUMAN** | Ferritin light chain OS=Homo sapiens GN=FTL PE=1 SV=2 |
|  | **P00558\|PGK1_HUMAN** | Phosphoglycerate kinase 1 OS=Homo sapiens GN=PGK1 PE=1 SV=3 |
|  | **P0C0S5\|H2AZ_HUMAN** | Histone H2A.Z OS=Homo sapiens GN=H2AFZ PE=1 SV=2 |
|  | **Q8NBS9\|TXND5_HUMAN** | Thioredoxin domain-containing protein 5 OS=Homo sapiens GN=TXNDC5 PE=1 SV=2 |
|  | **P62917\|RL8_HUMAN** | 60S ribosomal protein L8 OS=Homo sapiens GN=RPL8 PE=1 SV=2 |
|  | **O15126\|SCAM1_HUMAN** | Secretory carrier-associated membrane protein 1 OS=Homo sapiens GN=SCAMP1 PE=1 SV=2 |
|  | **P01861\|IGHG4_HUMAN** | Ig gamma-4 chain C region OS=Homo sapiens GN=IGHG4 PE=1 SV=1 |
|  | **P61916\|NPC2_HUMAN** | Epididymal secretory protein E1 OS=Homo sapiens GN=NPC2 PE=1 SV=1 |
|  | **Q9H1C4\|UN93B_HUMAN** | Protein unc-93 homolog B1 OS=Homo sapiens GN=UNC93B1 PE=1 SV=2 |
|  | **Q9GZP9\|DERL2_HUMAN** | Derlin-2 OS=Homo sapiens GN=DERL2 PE=1 SV=1 |
|  | **Q8TD06\|AGR3_HUMAN** | Anterior gradient protein 3 homolog OS=Homo sapiens GN=AGR3 PE=1 SV=1 |
|  | **P78527\|PRKDC_HUMAN** | DNA-dependent protein kinase catalytic subunit OS=Homo sapiens GN=PRKDC PE=1 SV=3 |
|  | **P01009\|A1AT_HUMAN** | Alpha-1-antitrypsin OS=Homo sapiens GN=SERPINA1 PE=1 SV=3 |
|  | **O95816\|BAG2_HUMAN** | BAG family molecular chaperone regulator 2 OS=Homo sapiens GN=BAG2 PE=1 SV=1 |
|  | **Q96GC9\|TMM49_HUMAN** | Transmembrane protein 49 OS=Homo sapiens GN=TMEM49 PE=2 SV=1 |
|  | **P62888\|RL30_HUMAN** | 60S ribosomal protein L30 OS=Homo sapiens GN=RPL30 PE=1 SV=2 |
|  | **Q99747\|SNAG_HUMAN** | Gamma-soluble NSF attachment protein OS=Homo sapiens GN=NAPG PE=1 SV=1 |
|  | **O75964\|ATP5L_HUMAN** | ATP synthase subunit g, mitochondrial OS=Homo sapiens GN=ATP5L PE=1 SV=3 |
|  | **Q15109\|RAGE_HUMAN** | Advanced glycosylation end product-specific receptor OS=Homo sapiens GN=AGER PE=1 SV=1 |
|  | **Q9P0I2\|TM111_HUMAN** | Transmembrane protein 111 OS=Homo sapiens GN=TMEM111 PE=1 SV=3 |
|  | **O75955\|FLOT1_HUMAN** | Flotillin-1 OS=Homo sapiens GN=FLOT1 PE=1 SV=3 |
|  | **P05141\|ADT2_HUMAN** | ADP/ATP translocase 2 OS=Homo sapiens GN=SLC25A5 PE=1 SV=6 |
|  | **P00739\|HPTR_HUMAN** | Haptoglobin-related protein OS=Homo sapiens GN=HPR PE=1 SV=1 |
|  | **P35613\|BASI_HUMAN** | Basigin OS=Homo sapiens GN=BSG PE=1 SV=2 |
|  | **Q9NS69\|TOM22_HUMAN** | Mitochondrial import receptor subunit TOM22 homolog OS=Homo sapiens GN=TOMM22 PE=1 SV=3 |
|  | **P40925\|MDHC_HUMAN** | Malate dehydrogenase, cytoplasmic OS=Homo sapiens GN=MDH1 PE=1 SV=4 |
|  | **P08473\|NEP_HUMAN** | Neprilysin OS=Homo sapiens GN=MME PE=1 SV=2 |
|  | **P17661\|DESM_HUMAN** | Desmin OS=Homo sapiens GN=DES PE=1 SV=3 |
|  | **Q6UW02\|CP20A_HUMAN** | Cytochrome P450 20A1 OS=Homo sapiens GN=CYP20A1 PE=1 SV=1 |
|  | **P05787\|K2C8_HUMAN** | Keratin, type II cytoskeletal 8 OS=Homo sapiens GN=KRT8 PE=1 SV=7 |
|  | **P22307\|NLTP_HUMAN** | Non-specific lipid-transfer protein OS=Homo sapiens GN=SCP2 PE=1 SV=2 |
|  | **Q92896\|GSLG1_HUMAN** | Golgi apparatus protein 1 OS=Homo sapiens GN=GLG1 PE=1 SV=2 |
|  | **P50895\|BCAM_HUMAN** | Basal cell adhesion molecule OS=Homo sapiens GN=BCAM PE=1 SV=2 |
|  | **Q15149\|PLEC1_HUMAN** | Plectin-1 OS=Homo sapiens GN=PLEC1 PE=1 SV=3 |
|  | **P29966\|MARCS_HUMAN** | Myristoylated alanine-rich C-kinase substrate OS=Homo sapiens GN=MARCKS PE=1 SV=4 |
|  | **P37802\|TAGL2_HUMAN** | Transgelin-2 OS=Homo sapiens GN=TAGLN2 PE=1 SV=3 |
|  | **P04062\|GLCM_HUMAN** | Glucosylceramidase OS=Homo sapiens GN=GBA PE=1 SV=3 |
|  | **Q9Y536\|PAL4B_HUMAN** | Peptidylprolyl cis-trans isomerase A-like 4B OS=Homo sapiens GN=PPIAL4B PE=1 SV=1 |
|  | **Q16891\|IMMT_HUMAN** | Mitochondrial inner membrane protein OS=Homo sapiens GN=IMMT PE=1 SV=1 |
|  | **Q06136\|KDSR_HUMAN** | 3-ketodihydrosphingosine reductase OS=Homo sapiens GN=KDSR PE=1 SV=1 |
|  | **Q9UKK3\|PARP4_HUMAN** | Poly [ADP-ribose] polymerase 4 OS=Homo sapiens GN=PARP4 PE=1 SV=2 |
|  | **P61626\|LYSC_HUMAN** | Lysozyme C OS=Homo sapiens GN=LYZ PE=1 SV=1 |
|  | **Q9UNL2\|SSRG_HUMAN** | Translocon-associated protein subunit gamma OS=Homo sapiens GN=SSR3 PE=1 SV=1 |
|  | **Q9Y277\|VDAC3_HUMAN** | Voltage-dependent anion-selective channel protein 3 OS=Homo sapiens GN=VDAC3 PE=1 SV=1 |
|  | **Q9NYL9\|TMOD3_HUMAN** | Tropomodulin-3 OS=Homo sapiens GN=TMOD3 PE=1 SV=1 |
|  | **Q13733\|AT1A4_HUMAN** | Sodium/potassium-transporting ATPase subunit alpha-4 OS=Homo sapiens GN=ATP1A4 PE=1 SV=3 |
|  | **P63208\|SKP1_HUMAN** | S-phase kinase-associated protein 1 OS=Homo sapiens GN=SKP1 PE=1 SV=2 |
|  | **P50151\|GBG10_HUMAN** | Guanine nucleotide-binding protein G(I)/G(S)/G(O) subunit gamma-10 OS=Homo sapiens GN=GNG10 PE=1 SV=1 |
|  | **P63096\|GNAI1_HUMAN** | Guanine nucleotide-binding protein G(i), alpha-1 subunit OS=Homo sapiens GN=GNAI1 PE=1 SV=2 |
|  | **Q16629\|SFRS7_HUMAN** | Splicing factor, arginine/serine-rich 7 OS=Homo sapiens GN=SFRS7 PE=1 SV=1 |
|  | **Q03135\|CAV1_HUMAN** | Caveolin-1 OS=Homo sapiens GN=CAV1 PE=1 SV=4 |
|  | **P20701\|ITAL_HUMAN** | Integrin alpha-L OS=Homo sapiens GN=ITGAL PE=1 SV=3 |
|  | **Q9UGP8\|SEC63_HUMAN** | Translocation protein SEC63 homolog OS=Homo sapiens GN=SEC63 PE=1 SV=2 |
|  | **P26006\|ITA3_HUMAN** | Integrin alpha-3 OS=Homo sapiens GN=ITGA3 PE=1 SV=3 |
|  | **P11686\|PSPC_HUMAN** | Pulmonary surfactant-associated protein C OS=Homo sapiens GN=SFTPC PE=1 SV=2 |
|  | **P01609\|KV117_HUMAN** | Ig kappa chain V-I region Scw OS=Homo sapiens PE=1 SV=1 |
|  | **P01599\|KV107_HUMAN** | Ig kappa chain V-I region Gal OS=Homo sapiens PE=1 SV=1 |
|  | **P01610\|KV118_HUMAN** | Ig kappa chain V-I region WEA OS=Homo sapiens PE=1 SV=1 |
|  | **P01597\|KV105_HUMAN** | Ig kappa chain V-I region DEE OS=Homo sapiens PE=1 SV=1 |
|  | **P08574\|CY1_HUMAN** | Cytochrome c1, heme protein, mitochondrial OS=Homo sapiens GN=CYC1 PE=1 SV=2 |
|  | **Q9H330\|CI005_HUMAN** | Transmembrane protein C9orf5 OS=Homo sapiens GN=C9orf5 PE=1 SV=2 |
|  | **P53007\|TXTP_HUMAN** | Tricarboxylate transport protein, mitochondrial OS=Homo sapiens GN=SLC25A1 PE=1 SV=2 |
|  | **P14384\|CBPM_HUMAN** | Carboxypeptidase M OS=Homo sapiens GN=CPM PE=1 SV=2 |
|  | **P05090\|APOD_HUMAN** | Apolipoprotein D OS=Homo sapiens GN=APOD PE=1 SV=1 |
|  | **P09668\|CATH_HUMAN** | Cathepsin H OS=Homo sapiens GN=CTSH PE=1 SV=3 |
|  | **Q9Y2Q3\|GSTK1_HUMAN** | Glutathione S-transferase kappa 1 OS=Homo sapiens GN=GSTK1 PE=1 SV=3 |
|  | **Q9Y2B0\|CNPY2_HUMAN** | Protein canopy homolog 2 OS=Homo sapiens GN=CNPY2 PE=1 SV=1 |
|  | **P08648\|ITA5_HUMAN** | Integrin alpha-5 OS=Homo sapiens GN=ITGA5 PE=1 SV=2 |
|  | **P84243\|H33_HUMAN** | Histone H3.3 OS=Homo sapiens GN=H3F3A PE=1 SV=2 |
|  | **P32969\|RL9_HUMAN** | 60S ribosomal protein L9 OS=Homo sapiens GN=RPL9 PE=1 SV=1 |
|  | **Q6RW13\|ATRAP_HUMAN** | Type-1 angiotensin II receptor-associated protein OS=Homo sapiens GN=AGTRAP PE=1 SV=1 |
|  | **P07204\|TRBM_HUMAN** | Thrombomodulin OS=Homo sapiens GN=THBD PE=1 SV=2 |
|  | **Q5JTV8\|TOIP1_HUMAN** | Torsin-1A-interacting protein 1 OS=Homo sapiens GN=TOR1AIP1 PE=1 SV=2 |
|  | **Q13423\|NNTM_HUMAN** | NAD(P) transhydrogenase, mitochondrial OS=Homo sapiens GN=NNT PE=1 SV=3 |
|  | **P01833\|PIGR_HUMAN** | Polymeric immunoglobulin receptor OS=Homo sapiens GN=PIGR PE=1 SV=4 |
|  | **P84085\|ARF5_HUMAN** | ADP-ribosylation factor 5 OS=Homo sapiens GN=ARF5 PE=1 SV=2 |
|  | **P11279\|LAMP1_HUMAN** | Lysosome-associated membrane glycoprotein 1 OS=Homo sapiens GN=LAMP1 PE=1 SV=3 |
|  | **O14773\|TPP1_HUMAN** | Tripeptidyl-peptidase 1 OS=Homo sapiens GN=TPP1 PE=1 SV=2 |
|  | **P18206\|VINC_HUMAN** | Vinculin OS=Homo sapiens GN=VCL PE=1 SV=4 |
|  | **Q00765\|REEP5_HUMAN** | Receptor expression-enhancing protein 5 OS=Homo sapiens GN=REEP5 PE=1 SV=3 |
|  | **O43504\|HBXIP_HUMAN** | Hepatitis B virus X-interacting protein OS=Homo sapiens GN=HBXIP PE=1 SV=1 |
|  | **O75380\|NDUS6_HUMAN** | NADH dehydrogenase [ubiquinone] iron-sulfur protein 6, mitochondrial OS=Homo sapiens GN=NDUFS6 PE=1 SV=1 |
|  | **O14817\|TSN4_HUMAN** | Tetraspanin-4 OS=Homo sapiens GN=TSPAN4 PE=1 SV=1 |
|  | **P13987\|CD59_HUMAN** | CD59 glycoprotein OS=Homo sapiens GN=CD59 PE=1 SV=1 |
|  | **P04220\|MUCB_HUMAN** | Ig mu heavy chain disease protein OS=Homo sapiens PE=1 SV=1 |
|  | **Q9Y639\|NPTN_HUMAN** | Neuroplastin OS=Homo sapiens GN=NPTN PE=1 SV=1 |
|  | **P15880\|RS2_HUMAN** | 40S ribosomal protein S2 OS=Homo sapiens GN=RPS2 PE=1 SV=2 |
|  | **P10606\|COX5B_HUMAN** | Cytochrome c oxidase subunit 5B, mitochondrial OS=Homo sapiens GN=COX5B PE=1 SV=2 |
|  | **P09758\|TACD2_HUMAN** | Tumor-associated calcium signal transducer 2 OS=Homo sapiens GN=TACSTD2 PE=1 SV=3 |
|  | **P63218\|GBG5_HUMAN** | Guanine nucleotide-binding protein G(I)/G(S)/G(O) subunit gamma-5 OS=Homo sapiens GN=GNG5 PE=1 SV=3 |
|  | **Q16555\|DPYL2_HUMAN** | Dihydropyrimidinase-related protein 2 OS=Homo sapiens GN=DPYSL2 PE=1 SV=1 |
|  | **P60903\|S10AA_HUMAN** | Protein S100-A10 OS=Homo sapiens GN=S100A10 PE=1 SV=2 |
|  | **O95674\|CDS2_HUMAN** | Phosphatidate cytidylyltransferase 2 OS=Homo sapiens GN=CDS2 PE=1 SV=1 |
|  | **P48047\|ATPO_HUMAN** | ATP synthase subunit O, mitochondrial OS=Homo sapiens GN=ATP5O PE=1 SV=1 |
|  | **P13498\|CY24A_HUMAN** | Cytochrome b-245 light chain OS=Homo sapiens GN=CYBA PE=1 SV=2 |
|  | **P62913\|RL11_HUMAN** | 60S ribosomal protein L11 OS=Homo sapiens GN=RPL11 PE=1 SV=2 |
|  | **P52943\|CRIP2_HUMAN** | Cysteine-rich protein 2 OS=Homo sapiens GN=CRIP2 PE=1 SV=1 |
|  | **O75881\|CP7B1_HUMAN** | 25-hydroxycholesterol 7-alpha-hydroxylase OS=Homo sapiens GN=CYP7B1 PE=1 SV=2 |
|  | **P55899\|FCGRN_HUMAN** | IgG receptor FcRn large subunit p51 OS=Homo sapiens GN=FCGRT PE=1 SV=1 |
|  | **P12110\|CO6A2_HUMAN** | Collagen alpha-2(VI) chain OS=Homo sapiens GN=COL6A2 PE=1 SV=4 |
|  | **P08174\|DAF_HUMAN** | Complement decay-accelerating factor OS=Homo sapiens GN=CD55 PE=1 SV=4 |
|  | **Q08431\|MFGM_HUMAN** | Lactadherin OS=Homo sapiens GN=MFGE8 PE=1 SV=2 |
|  | **Q02978\|M2OM_HUMAN** | Mitochondrial 2-oxoglutarate/malate carrier protein OS=Homo sapiens GN=SLC25A11 PE=1 SV=3 |
|  | **Q9HDC9\|APMAP_HUMAN** | Adipocyte plasma membrane-associated protein OS=Homo sapiens GN=APMAP PE=1 SV=2 |
|  | **P48509\|CD151_HUMAN** | CD151 antigen OS=Homo sapiens GN=CD151 PE=1 SV=3 |
|  | **Q86UE4\|LYRIC_HUMAN** | Protein LYRIC OS=Homo sapiens GN=MTDH PE=1 SV=2 |
|  | **Q9UEW3\|MARCO_HUMAN** | Macrophage receptor MARCO OS=Homo sapiens GN=MARCO PE=1 SV=1 |
|  | **P38919\|IF4A3_HUMAN** | Eukaryotic initiation factor 4A-III OS=Homo sapiens GN=EIF4A3 PE=1 SV=4 |
|  | **O14880\|MGST3_HUMAN** | Microsomal glutathione S-transferase 3 OS=Homo sapiens GN=MGST3 PE=1 SV=1 |
|  | **P61247\|RS3A_HUMAN** | 40S ribosomal protein S3a OS=Homo sapiens GN=RPS3A PE=1 SV=2 |
|  | **P50148\|GNAQ_HUMAN** | Guanine nucleotide-binding protein G(q) subunit alpha OS=Homo sapiens GN=GNAQ PE=1 SV=4 |
|  | **P62241\|RS8_HUMAN** | 40S ribosomal protein S8 OS=Homo sapiens GN=RPS8 PE=1 SV=2 |
|  | **O60256\|KPRB_HUMAN** | Phosphoribosyl pyrophosphate synthetase-associated protein 2 OS=Homo sapiens GN=PRPSAP2 PE=1 SV=1 |
|  | **Q92629\|SGCD_HUMAN** | Delta-sarcoglycan OS=Homo sapiens GN=SGCD PE=1 SV=2 |
|  | **P05386\|RLA1_HUMAN** | 60S acidic ribosomal protein P1 OS=Homo sapiens GN=RPLP1 PE=1 SV=1 |
|  | **P21810\|PGS1_HUMAN** | Biglycan OS=Homo sapiens GN=BGN PE=1 SV=2 |
|  | **Q8TBA6\|GOGA5_HUMAN** | Golgin subfamily A member 5 OS=Homo sapiens GN=GOLGA5 PE=1 SV=2 |
|  | **O00116\|ADAS_HUMAN** | Alkyldihydroxyacetonephosphate synthase, peroxisomal OS=Homo sapiens GN=AGPS PE=1 SV=1 |
|  | **P23634\|AT2B4_HUMAN** | Plasma membrane calcium-transporting ATPase 4 OS=Homo sapiens GN=ATP2B4 PE=1 SV=2 |
|  | **P52209\|6PGD_HUMAN** | 6-phosphogluconate dehydrogenase, decarboxylating OS=Homo sapiens GN=PGD PE=1 SV=3 |
|  | **P35610\|SOAT1_HUMAN** | Sterol O-acyltransferase 1 OS=Homo sapiens GN=SOAT1 PE=1 SV=3 |
|  | **P10301\|RRAS_HUMAN** | Ras-related protein R-Ras OS=Homo sapiens GN=RRAS PE=1 SV=1 |
|  | **P62070\|RRAS2_HUMAN** | Ras-related protein R-Ras2 OS=Homo sapiens GN=RRAS2 PE=1 SV=1 |
|  | **P62805\|H4_HUMAN** | Histone H4 OS=Homo sapiens GN=HIST1H4A PE=1 SV=2 |
|  | **P20340\|RAB6A_HUMAN** | Ras-related protein Rab-6A OS=Homo sapiens GN=RAB6A PE=1 SV=3 |
|  | **P17858\|K6PL_HUMAN** | 6-phosphofructokinase, liver type OS=Homo sapiens GN=PFKL PE=1 SV=6 |
|  | **Q96A08\|H2B1A_HUMAN** | Histone H2B type 1-A OS=Homo sapiens GN=HIST1H2BA PE=1 SV=3 |
|  | **O14786\|NRP1_HUMAN** | Neuropilin-1 OS=Homo sapiens GN=NRP1 PE=1 SV=3 |
|  | **O60831\|PRAF2_HUMAN** | PRA1 family protein 2 OS=Homo sapiens GN=PRAF2 PE=1 SV=1 |
|  | **P62491\|RB11A_HUMAN** | Ras-related protein Rab-11A OS=Homo sapiens GN=RAB11A PE=1 SV=3 |
|  | **Q16864\|VATF_HUMAN** | V-type proton ATPase subunit F OS=Homo sapiens GN=ATP6V1F PE=1 SV=2 |
|  | **Q9Y5L4\|TIM13_HUMAN** | Mitochondrial import inner membrane translocase subunit Tim13 OS=Homo sapiens GN=TIMM13 PE=1 SV=1 |
|  | **P46777\|RL5_HUMAN** | 60S ribosomal protein L5 OS=Homo sapiens GN=RPL5 PE=1 SV=3 |
|  | **O94826\|TOM70_HUMAN** | Mitochondrial import receptor subunit TOM70 OS=Homo sapiens GN=TOMM70A PE=1 SV=1 |
|  | **P26885\|FKBP2_HUMAN** | FK506-binding protein 2 OS=Homo sapiens GN=FKBP2 PE=1 SV=2 |
|  | **P30536\|TSPOA_HUMAN** | Translocator protein OS=Homo sapiens GN=TSPO PE=1 SV=2 |
|  | **Q7KYR7\|BT2A1_HUMAN** | Butyrophilin subfamily 2 member A1 OS=Homo sapiens GN=BTN2A1 PE=1 SV=3 |
|  | **Q9H0C2\|ADT4_HUMAN** | ADP/ATP translocase 4 OS=Homo sapiens GN=SLC25A31 PE=1 SV=1 |
|  | **P41219\|PERI_HUMAN** | Peripherin OS=Homo sapiens GN=PRPH PE=1 SV=2 |
|  | **Q15661\|TRYB1_HUMAN** | Tryptase beta-1 OS=Homo sapiens GN=TPSAB1 PE=1 SV=1 |
|  | **P15157\|TRYA1_HUMAN** | Tryptase alpha-1 OS=Homo sapiens GN=TPSAB1 PE=1 SV=3 |
|  | **Q07075\|AMPE_HUMAN** | Glutamyl aminopeptidase OS=Homo sapiens GN=ENPEP PE=1 SV=2 |
|  | **Q9NZ45\|CISD1_HUMAN** | CDGSH iron sulfur domain-containing protein 1 OS=Homo sapiens GN=CISD1 PE=1 SV=1 |
|  | **Q13011\|ECH1_HUMAN** | Delta(3,5)-Delta(2,4)-dienoyl-CoA isomerase, mitochondrial OS=Homo sapiens GN=ECH1 PE=1 SV=2 |
|  | **Q9UBI6\|GBG12_HUMAN** | Guanine nucleotide-binding protein G(I)/G(S)/G(O) subunit gamma-12 OS=Homo sapiens GN=GNG12 PE=1 SV=3 |
|  | **Q10589\|BST2_HUMAN** | Bone marrow stromal antigen 2 OS=Homo sapiens GN=BST2 PE=1 SV=1 |
|  | **P53621\|COPA_HUMAN** | Coatomer subunit alpha OS=Homo sapiens GN=COPA PE=1 SV=2 |
|  | **P01860\|IGHG3_HUMAN** | Ig gamma-3 chain C region OS=Homo sapiens GN=IGHG3 PE=1 SV=2 |
|  | **P02795\|MT2_HUMAN** | Metallothionein-2 OS=Homo sapiens GN=MT2A PE=1 SV=1 |
|  | **P80297\|MT1X_HUMAN** | Metallothionein-1X OS=Homo sapiens GN=MT1X PE=1 SV=1 |
|  | **Q687X5\|STEA4_HUMAN** | Metalloreductase STEAP4 OS=Homo sapiens GN=STEAP4 PE=1 SV=1 |
|  | **P55145\|MANF_HUMAN** | Mesencephalic astrocyte-derived neurotrophic factor OS=Homo sapiens GN=MANF PE=1 SV=2 |
|  | **P54707\|AT12A_HUMAN** | Potassium-transporting ATPase alpha chain 2 OS=Homo sapiens GN=ATP12A PE=2 SV=3 |
|  | **P09543\|CN37_HUMAN** | 2',3'-cyclic-nucleotide 3'-phosphodiesterase OS=Homo sapiens GN=CNP PE=1 SV=2 |
|  | **P00338\|LDHA_HUMAN** | L-lactate dehydrogenase A chain OS=Homo sapiens GN=LDHA PE=1 SV=2 |
|  | **P14174\|MIF_HUMAN** | Macrophage migration inhibitory factor OS=Homo sapiens GN=MIF PE=1 SV=4 |
|  | **O95837\|GNA14_HUMAN** | Guanine nucleotide-binding protein subunit alpha-14 OS=Homo sapiens GN=GNA14 PE=2 SV=1 |
|  | **Q9H8Y8\|GORS2_HUMAN** | Golgi reassembly-stacking protein 2 OS=Homo sapiens GN=GORASP2 PE=1 SV=3 |
|  | **P61225\|RAP2B_HUMAN** | Ras-related protein Rap-2b OS=Homo sapiens GN=RAP2B PE=1 SV=1 |
|  | **P26373\|RL13_HUMAN** | 60S ribosomal protein L13 OS=Homo sapiens GN=RPL13 PE=1 SV=4 |
|  | **P47712\|PA24A_HUMAN** | Cytosolic phospholipase A2 OS=Homo sapiens GN=PLA2G4A PE=1 SV=1 |
|  | **P62988\|UBIQ_HUMAN** | Ubiquitin OS=Homo sapiens GN=RPS27A PE=1 SV=1 |
|  | **Q9BTU6\|P4K2A_HUMAN** | Phosphatidylinositol 4-kinase type 2-alpha OS=Homo sapiens GN=PI4K2A PE=1 SV=1 |
|  | **Q9BTV4\|TMM43_HUMAN** | Transmembrane protein 43 OS=Homo sapiens GN=TMEM43 PE=1 SV=1 |
|  | **Q5T8D3\|ACBD5_HUMAN** | Acyl-CoA-binding domain-containing protein 5 OS=Homo sapiens GN=ACBD5 PE=1 SV=1 |
|  | **P08865\|RSSA_HUMAN** | 40S ribosomal protein SA OS=Homo sapiens GN=RPSA PE=1 SV=4 |
|  | **P05091\|ALDH2_HUMAN** | Aldehyde dehydrogenase, mitochondrial OS=Homo sapiens GN=ALDH2 PE=1 SV=2 |
|  | **P79483\|2B31_HUMAN** | HLA class II histocompatibility antigen, DRB3-1 beta chain OS=Homo sapiens GN=HLA-DRB3 PE=1 SV=1 |
|  | **Q9TQE0\|2B19_HUMAN** | HLA class II histocompatibility antigen, DRB1-9 beta chain OS=Homo sapiens GN=HLA-DRB1 PE=2 SV=1 |
|  | **Q8WWP7\|GIMA1_HUMAN** | GTPase IMAP family member 1 OS=Homo sapiens GN=GIMAP1 PE=2 SV=1 |
|  | **P29992\|GNA11_HUMAN** | Guanine nucleotide-binding protein subunit alpha-11 OS=Homo sapiens GN=GNA11 PE=1 SV=2 |
|  | **P51159\|RB27A_HUMAN** | Ras-related protein Rab-27A OS=Homo sapiens GN=RAB27A PE=1 SV=3 |
|  | **Q15006\|TTC35_HUMAN** | Tetratricopeptide repeat protein 35 OS=Homo sapiens GN=TTC35 PE=1 SV=1 |
|  | **O15511\|ARPC5_HUMAN** | Actin-related protein 2/3 complex subunit 5 OS=Homo sapiens GN=ARPC5 PE=1 SV=3 |
|  | **Q9BXP2\|S12A9_HUMAN** | Solute carrier family 12 member 9 OS=Homo sapiens GN=SLC12A9 PE=1 SV=1 |
|  | **O14672\|ADA10_HUMAN** | Disintegrin and metalloproteinase domain-containing protein 10 OS=Homo sapiens GN=ADAM10 PE=1 SV=1 |
|  | **P04075\|ALDOA_HUMAN** | Fructose-bisphosphate aldolase A OS=Homo sapiens GN=ALDOA PE=1 SV=2 |
|  | **O43747\|AP1G1_HUMAN** | AP-1 complex subunit gamma-1 OS=Homo sapiens GN=AP1G1 PE=1 SV=5 |
|  | **P55087\|AQP4_HUMAN** | Aquaporin-4 OS=Homo sapiens GN=AQP4 PE=1 SV=2 |
|  | **Q14108\|SCRB2_HUMAN** | Lysosome membrane protein 2 OS=Homo sapiens GN=SCARB2 PE=1 SV=2 |
|  | **Q9BVC6\|TM109_HUMAN** | Transmembrane protein 109 OS=Homo sapiens GN=TMEM109 PE=1 SV=1 |
|  | **Q16720\|AT2B3_HUMAN** | Plasma membrane calcium-transporting ATPase 3 OS=Homo sapiens GN=ATP2B3 PE=1 SV=3 |
|  | **P20020\|AT2B1_HUMAN** | Plasma membrane calcium-transporting ATPase 1 OS=Homo sapiens GN=ATP2B1 PE=1 SV=3 |
|  | **P62244\|RS15A_HUMAN** | 40S ribosomal protein S15a OS=Homo sapiens GN=RPS15A PE=1 SV=2 |
|  | **Q14651\|PLSI_HUMAN** | Plastin-1 OS=Homo sapiens GN=PLS1 PE=1 SV=2 |
|  | **P13797\|PLST_HUMAN** | Plastin-3 OS=Homo sapiens GN=PLS3 PE=1 SV=4 |
|  | **P31153\|METK2_HUMAN** | S-adenosylmethionine synthetase isoform type-2 OS=Homo sapiens GN=MAT2A PE=1 SV=1 |
|  | **P63000\|RAC1_HUMAN** | Ras-related C3 botulinum toxin substrate 1 OS=Homo sapiens GN=RAC1 PE=1 SV=1 |
|  | **P61313\|RL15_HUMAN** | 60S ribosomal protein L15 OS=Homo sapiens GN=RPL15 PE=1 SV=2 |
|  | **P48449\|ERG7_HUMAN** | Lanosterol synthase OS=Homo sapiens GN=LSS PE=1 SV=1 |
|  | **Q9NX63\|CHCH3_HUMAN** | Coiled-coil-helix-coiled-coil-helix domain-containing protein 3, mitochondrial OS=Homo sapiens GN=CHCHD3 PE=1 SV=1 |
|  | **O75165\|DJC13_HUMAN** | DnaJ homolog subfamily C member 13 OS=Homo sapiens GN=DNAJC13 PE=1 SV=4 |
|  | **P49913\|CAMP_HUMAN** | Cathelicidin antimicrobial peptide OS=Homo sapiens GN=CAMP PE=1 SV=1 |
|  | **P17693\|HLAG_HUMAN** | HLA class I histocompatibility antigen, alpha chain G OS=Homo sapiens GN=HLA-G PE=1 SV=1 |
|  | **Q86VR2\|F134C_HUMAN** | Protein FAM134C OS=Homo sapiens GN=FAM134C PE=1 SV=1 |
|  | **Q8N6Q3\|CD177_HUMAN** | CD177 antigen OS=Homo sapiens GN=CD177 PE=1 SV=2 |
|  | **P02763\|A1AG1_HUMAN** | Alpha-1-acid glycoprotein 1 OS=Homo sapiens GN=ORM1 PE=1 SV=1 |
|  | **P11717\|MPRI_HUMAN** | Cation-independent mannose-6-phosphate receptor OS=Homo sapiens GN=IGF2R PE=1 SV=2 |
|  | **P61604\|CH10_HUMAN** | 10 kDa heat shock protein, mitochondrial OS=Homo sapiens GN=HSPE1 PE=1 SV=2 |
|  | **Q9UHG3\|PCYOX_HUMAN** | Prenylcysteine oxidase 1 OS=Homo sapiens GN=PCYOX1 PE=1 SV=3 |
|  | **P35241\|RADI_HUMAN** | Radixin OS=Homo sapiens GN=RDX PE=1 SV=1 |
|  | **Q9H299\|SH3L3_HUMAN** | SH3 domain-binding glutamic acid-rich-like protein 3 OS=Homo sapiens GN=SH3BGRL3 PE=1 SV=1 |
|  | **P05026\|AT1B1_HUMAN** | Sodium/potassium-transporting ATPase subunit beta-1 OS=Homo sapiens GN=ATP1B1 PE=1 SV=1 |
|  | **O75390\|CISY_HUMAN** | Citrate synthase, mitochondrial OS=Homo sapiens GN=CS PE=1 SV=2 |
|  | **Q9BV40\|VAMP8_HUMAN** | Vesicle-associated membrane protein 8 OS=Homo sapiens GN=VAMP8 PE=1 SV=1 |
|  | **P51153\|RAB13_HUMAN** | Ras-related protein Rab-13 OS=Homo sapiens GN=RAB13 PE=1 SV=1 |
|  | **P98160\|PGBM_HUMAN** | Basement membrane-specific heparan sulfate proteoglycan core protein OS=Homo sapiens GN=HSPG2 PE=1 SV=3 |
|  | **Q07020\|RL18_HUMAN** | 60S ribosomal protein L18 OS=Homo sapiens GN=RPL18 PE=1 SV=2 |
|  | **P08590\|MYL3_HUMAN** | Myosin light chain 3 OS=Homo sapiens GN=MYL3 PE=1 SV=3 |
|  | **P05976\|MLE1_HUMAN** | Myosin light chain 1, skeletal muscle isoform OS=Homo sapiens GN=MYL1 PE=1 SV=3 |
|  | **Q9Y3D6\|FIS1_HUMAN** | Mitochondrial fission 1 protein OS=Homo sapiens GN=FIS1 PE=1 SV=2 |
|  | **Q969P0\|IGSF8_HUMAN** | Immunoglobulin superfamily member 8 OS=Homo sapiens GN=IGSF8 PE=1 SV=1 |
|  | **Q8N0U8\|VKORL_HUMAN** | Vitamin K epoxide reductase complex subunit 1-like protein 1 OS=Homo sapiens GN=VKORC1L1 PE=1 SV=2 |
|  | **Q9H115\|SNAB_HUMAN** | Beta-soluble NSF attachment protein OS=Homo sapiens GN=NAPB PE=1 SV=2 |
|  | **P62701\|RS4X_HUMAN** | 40S ribosomal protein S4, X isoform OS=Homo sapiens GN=RPS4X PE=1 SV=2 |
|  | **P46940\|IQGA1_HUMAN** | Ras GTPase-activating-like protein IQGAP1 OS=Homo sapiens GN=IQGAP1 PE=1 SV=1 |
|  | **O75954\|TSN9_HUMAN** | Tetraspanin-9 OS=Homo sapiens GN=TSPAN9 PE=1 SV=1 |
|  | **P11684\|UTER_HUMAN** | Uteroglobin OS=Homo sapiens GN=SCGB1A1 PE=1 SV=1 |
|  | **Q9H9B4\|SFXN1_HUMAN** | Sideroflexin-1 OS=Homo sapiens GN=SFXN1 PE=1 SV=4 |
|  | **P31946\|1433B_HUMAN** | 14-3-3 protein beta/alpha OS=Homo sapiens GN=YWHAB PE=1 SV=3 |
|  | **P28065\|PSB9_HUMAN** | Proteasome subunit beta type-9 OS=Homo sapiens GN=PSMB9 PE=1 SV=2 |
|  | **P59768\|GBG2_HUMAN** | Guanine nucleotide-binding protein G(I)/G(S)/G(O) subunit gamma-2 OS=Homo sapiens GN=GNG2 PE=1 SV=2 |
|  | **P40121\|CAPG_HUMAN** | Macrophage-capping protein OS=Homo sapiens GN=CAPG PE=1 SV=1 |
|  | **O00483\|NDUA4_HUMAN** | NADH dehydrogenase [ubiquinone] 1 alpha subcomplex subunit 4 OS=Homo sapiens GN=NDUFA4 PE=1 SV=1 |
|  | **P47756\|CAPZB_HUMAN** | F-actin-capping protein subunit beta OS=Homo sapiens GN=CAPZB PE=1 SV=4 |
|  | **Q96CX2\|KCD12_HUMAN** | BTB/POZ domain-containing protein KCTD12 OS=Homo sapiens GN=KCTD12 PE=1 SV=1 |
|  | **P11940\|PABP1_HUMAN** | Polyadenylate-binding protein 1 OS=Homo sapiens GN=PABPC1 PE=1 SV=2 |
|  | **Q13310\|PABP4_HUMAN** | Polyadenylate-binding protein 4 OS=Homo sapiens GN=PABPC4 PE=1 SV=1 |
|  | **Q9Y4P3\|TBL2_HUMAN** | Transducin beta-like protein 2 OS=Homo sapiens GN=TBL2 PE=1 SV=1 |
|  | **Q6ZMR3\|LDH6A_HUMAN** | L-lactate dehydrogenase A-like 6A OS=Homo sapiens GN=LDHAL6A PE=2 SV=1 |
|  | **P07864\|LDHC_HUMAN** | L-lactate dehydrogenase C chain OS=Homo sapiens GN=LDHC PE=2 SV=4 |
|  | **O00182\|LEG9_HUMAN** | Galectin-9 OS=Homo sapiens GN=LGALS9 PE=1 SV=2 |
|  | **P61769\|B2MG_HUMAN** | Beta-2-microglobulin OS=Homo sapiens GN=B2M PE=1 SV=1 |
|  | **P60468\|SC61B_HUMAN** | Protein transport protein Sec61 subunit beta OS=Homo sapiens GN=SEC61B PE=1 SV=2 |
|  | **Q16602\|CALRL_HUMAN** | Calcitonin gene-related peptide type 1 receptor OS=Homo sapiens GN=CALCRL PE=1 SV=2 |
|  | **Q14344\|GNA13_HUMAN** | Guanine nucleotide-binding protein subunit alpha-13 OS=Homo sapiens GN=GNA13 PE=1 SV=2 |
|  | **Q99653\|CHP1_HUMAN** | Calcium-binding protein p22 OS=Homo sapiens GN=CHP PE=1 SV=3 |
|  | **Q8N2K0\|ABD12_HUMAN** | Monoacylglycerol lipase ABHD12 OS=Homo sapiens GN=ABHD12 PE=2 SV=2 |
|  | **O15270\|SPTC2_HUMAN** | Serine palmitoyltransferase 2 OS=Homo sapiens GN=SPTLC2 PE=1 SV=1 |
|  | **Q9BWM7\|SFXN3_HUMAN** | Sideroflexin-3 OS=Homo sapiens GN=SFXN3 PE=2 SV=1 |
|  | **P01024\|CO3_HUMAN** | Complement C3 OS=Homo sapiens GN=C3 PE=1 SV=2 |
|  | **P80511\|S10AC_HUMAN** | Protein S100-A12 OS=Homo sapiens GN=S100A12 PE=1 SV=2 |
|  | **Q15286\|RAB35_HUMAN** | Ras-related protein Rab-35 OS=Homo sapiens GN=RAB35 PE=1 SV=1 |
|  | **P13693\|TCTP_HUMAN** | Translationally-controlled tumor protein OS=Homo sapiens GN=TPT1 PE=1 SV=1 |
|  | **Q9BXJ8\|T120A_HUMAN** | Transmembrane protein 120A OS=Homo sapiens GN=TMEM120A PE=2 SV=1 |
|  | **Q9NZ08\|ERAP1_HUMAN** | Endoplasmic reticulum aminopeptidase 1 OS=Homo sapiens GN=ERAP1 PE=1 SV=3 |
|  | **P08237\|K6PF_HUMAN** | 6-phosphofructokinase, muscle type OS=Homo sapiens GN=PFKM PE=1 SV=2 |
|  | **Q9UJU6\|DBNL_HUMAN** | Drebrin-like protein OS=Homo sapiens GN=DBNL PE=1 SV=1 |
|  | **O95831\|AIFM1_HUMAN** | Apoptosis-inducing factor 1, mitochondrial OS=Homo sapiens GN=AIFM1 PE=1 SV=1 |
|  | **Q14568\|HS902_HUMAN** | Putative heat shock protein HSP 90-alpha A2 OS=Homo sapiens GN=HSP90AA2 PE=1 SV=2 |
|  | **Q58FF8\|H90B2_HUMAN** | Putative heat shock protein HSP 90-beta 2 OS=Homo sapiens GN=HSP90AB2P PE=1 SV=2 |
|  | **P02655\|APOC2_HUMAN** | Apolipoprotein C-II OS=Homo sapiens GN=APOC2 PE=1 SV=1 |
|  | **Q96HE7\|ERO1A_HUMAN** | ERO1-like protein alpha OS=Homo sapiens GN=ERO1L PE=1 SV=2 |
|  | **P10253\|LYAG_HUMAN** | Lysosomal alpha-glucosidase OS=Homo sapiens GN=GAA PE=1 SV=3 |
|  | **Q9UBJ2\|ABCD2_HUMAN** | ATP-binding cassette sub-family D member 2 OS=Homo sapiens GN=ABCD2 PE=1 SV=1 |
|  | **Q13740\|CD166_HUMAN** | CD166 antigen OS=Homo sapiens GN=ALCAM PE=1 SV=2 |
|  | **Q96CW1\|AP2M1_HUMAN** | AP-2 complex subunit mu OS=Homo sapiens GN=AP2M1 PE=1 SV=2 |
|  | **P35221\|CTNA1_HUMAN** | Catenin alpha-1 OS=Homo sapiens GN=CTNNA1 PE=1 SV=1 |
|  | **Q8IUD2\|RB6I2_HUMAN** | ELKS/RAB6-interacting/CAST family member 1 OS=Homo sapiens GN=ERC1 PE=1 SV=1 |
|  | **O15083\|ERC2_HUMAN** | ERC protein 2 OS=Homo sapiens GN=ERC2 PE=1 SV=3 |
|  | **Q13501\|SQSTM_HUMAN** | Sequestosome-1 OS=Homo sapiens GN=SQSTM1 PE=1 SV=1 |
|  | **O60437\|PEPL_HUMAN** | Periplakin OS=Homo sapiens GN=PPL PE=1 SV=3 |
|  | **Q8N490\|PNKD_HUMAN** | Probable hydrolase PNKD OS=Homo sapiens GN=PNKD PE=1 SV=2 |
|  | **O75874\|IDHC_HUMAN** | Isocitrate dehydrogenase [NADP] cytoplasmic OS=Homo sapiens GN=IDH1 PE=1 SV=2 |
|  | **Q8N357\|CB018_HUMAN** | Transmembrane protein C2orf18 OS=Homo sapiens GN=C2orf18 PE=1 SV=1 |
|  | **Q969X0\|RIPL2_HUMAN** | RILP-like protein 2 OS=Homo sapiens GN=RILPL2 PE=1 SV=1 |
|  | **Q16534\|HLF_HUMAN** | Hepatic leukemia factor OS=Homo sapiens GN=HLF PE=2 SV=1 |
|  | **Q9NZ01\|TECR_HUMAN** | Trans-2,3-enoyl-CoA reductase OS=Homo sapiens GN=TECR PE=1 SV=1 |
|  | **Q9H223\|EHD4_HUMAN** | EH domain-containing protein 4 OS=Homo sapiens GN=EHD4 PE=1 SV=1 |
|  | **P01907\|HA25_HUMAN** | HLA class II histocompatibility antigen, DQ(5) alpha chain OS=Homo sapiens GN=HLA-DQA1 PE=1 SV=2 |
|  | **P20036\|HA2Q_HUMAN** | HLA class II histocompatibility antigen, DP alpha chain OS=Homo sapiens GN=HLA-DPA1 PE=1 SV=1 |
|  | **P05536\|HA27_HUMAN** | HLA class II histocompatibility antigen, DQ(W3) alpha chain OS=Homo sapiens GN=HLA-DQA1 PE=2 SV=1 |
|  | **P01909\|HA23_HUMAN** | HLA class II histocompatibility antigen, DQ(3) alpha chain OS=Homo sapiens GN=HLA-DQA1 PE=1 SV=1 |
|  | **Q6UWP7\|LCLT1_HUMAN** | Lysocardiolipin acyltransferase 1 OS=Homo sapiens GN=LCLAT1 PE=1 SV=1 |
|  | **Q8IWV7\|UBR1_HUMAN** | E3 ubiquitin-protein ligase UBR1 OS=Homo sapiens GN=UBR1 PE=1 SV=1 |
|  | **O94906\|PRP6_HUMAN** | Pre-mRNA-processing factor 6 OS=Homo sapiens GN=PRPF6 PE=1 SV=1 |
|  | **P09917\|LOX5_HUMAN** | Arachidonate 5-lipoxygenase OS=Homo sapiens GN=ALOX5 PE=1 SV=2 |
|  | **Q9C0E8\|LNP_HUMAN** | Protein lunapark OS=Homo sapiens GN=LNP PE=1 SV=2 |
|  | **Q04941\|PLP2_HUMAN** | Proteolipid protein 2 OS=Homo sapiens GN=PLP2 PE=1 SV=1 |
|  | **P98194\|AT2C1_HUMAN** | Calcium-transporting ATPase type 2C member 1 OS=Homo sapiens GN=ATP2C1 PE=1 SV=3 |
|  | **P49326\|FMO5_HUMAN** | Dimethylaniline monooxygenase [N-oxide-forming] 5 OS=Homo sapiens GN=FMO5 PE=2 SV=2 |
|  | **Q14677\|EPN4_HUMAN** | Clathrin interactor 1 OS=Homo sapiens GN=CLINT1 PE=1 SV=1 |
|  | **Q5VST9\|OBSCN_HUMAN** | Obscurin OS=Homo sapiens GN=OBSCN PE=1 SV=3 |
|  | **A0FGR8\|ESYT2_HUMAN** | Extended synaptotagmin-2 OS=Homo sapiens GN=ESYT2 PE=1 SV=1 |
|  | **Q9UQV4\|LAMP3_HUMAN** | Lysosome-associated membrane glycoprotein 3 OS=Homo sapiens GN=LAMP3 PE=1 SV=3 |
|  | **P17931\|LEG3_HUMAN** | Galectin-3 OS=Homo sapiens GN=LGALS3 PE=1 SV=5 |
|  | **P11169\|GTR3_HUMAN** | Solute carrier family 2, facilitated glucose transporter member 3 OS=Homo sapiens GN=SLC2A3 PE=1 SV=1 |
|  | **Q8TDB8\|GTR14_HUMAN** | Solute carrier family 2, facilitated glucose transporter member 14 OS=Homo sapiens GN=SLC2A14 PE=2 SV=1 |
|  | **P51571\|SSRD_HUMAN** | Translocon-associated protein subunit delta OS=Homo sapiens GN=SSR4 PE=1 SV=1 |
|  | **Q5JWF2\|GNAS1_HUMAN** | Guanine nucleotide-binding protein G(s) subunit alpha isoforms XLas OS=Homo sapiens GN=GNAS PE=1 SV=2 |
|  | **P01906\|HA26_HUMAN** | HLA class II histocompatibility antigen, DQ(6) alpha chain OS=Homo sapiens GN=HLA-DQA2 PE=2 SV=2 |
|  | **P01908\|HA21_HUMAN** | HLA class II histocompatibility antigen, DQ(1) alpha chain OS=Homo sapiens PE=2 SV=2 |
|  | **Q02543\|RL18A_HUMAN** | 60S ribosomal protein L18a OS=Homo sapiens GN=RPL18A PE=1 SV=2 |
|  | **Q9NV96\|CC50A_HUMAN** | Cell cycle control protein 50A OS=Homo sapiens GN=TMEM30A PE=1 SV=1 |
|  | **P30519\|HMOX2_HUMAN** | Heme oxygenase 2 OS=Homo sapiens GN=HMOX2 PE=1 SV=2 |
|  | **Q08722\|CD47_HUMAN** | Leukocyte surface antigen CD47 OS=Homo sapiens GN=CD47 PE=1 SV=1 |
|  | **Q969X5\|ERGI1_HUMAN** | Endoplasmic reticulum-Golgi intermediate compartment protein 1 OS=Homo sapiens GN=ERGIC1 PE=1 SV=1 |
|  | **O15254\|ACOX3_HUMAN** | Peroxisomal acyl-coenzyme A oxidase 3 OS=Homo sapiens GN=ACOX3 PE=2 SV=2 |
|  | **Q92820\|GGH_HUMAN** | Gamma-glutamyl hydrolase OS=Homo sapiens GN=GGH PE=1 SV=2 |
|  | **P24158\|PRTN3_HUMAN** | Myeloblastin OS=Homo sapiens GN=PRTN3 PE=1 SV=3 |
|  | **Q9UH99\|UN84B_HUMAN** | Protein unc-84 homolog B OS=Homo sapiens GN=UNC84B PE=1 SV=3 |
|  | **P59190\|RAB15_HUMAN** | Ras-related protein Rab-15 OS=Homo sapiens GN=RAB15 PE=1 SV=1 |
|  | **Q58FF3\|ENPLL_HUMAN** | Putative endoplasmin-like protein OS=Homo sapiens GN=HSP90B2P PE=5 SV=1 |
|  | **Q14571\|ITPR2_HUMAN** | Inositol 1,4,5-trisphosphate receptor type 2 OS=Homo sapiens GN=ITPR2 PE=1 SV=2 |
|  | **Q9NZR1\|TMOD2_HUMAN** | Tropomodulin-2 OS=Homo sapiens GN=TMOD2 PE=1 SV=1 |
|  | **P16422\|EPCAM_HUMAN** | Epithelial cell adhesion molecule OS=Homo sapiens GN=EPCAM PE=1 SV=2 |
|  | **Q14103\|HNRPD_HUMAN** | Heterogeneous nuclear ribonucleoprotein D0 OS=Homo sapiens GN=HNRNPD PE=1 SV=1 |
|  | **O43402\|CX4NB_HUMAN** | Neighbor of COX4 OS=Homo sapiens GN=COX4NB PE=1 SV=1 |
|  | **P18124\|RL7_HUMAN** | 60S ribosomal protein L7 OS=Homo sapiens GN=RPL7 PE=1 SV=1 |
|  | **P17813\|EGLN_HUMAN** | Endoglin OS=Homo sapiens GN=ENG PE=1 SV=2 |
|  | **Q8IVE3\|PKHH2_HUMAN** | Pleckstrin homology domain-containing family H member 2 OS=Homo sapiens GN=PLEKHH2 PE=2 SV=2 |
|  | **Q9BZW7\|TSG10_HUMAN** | Testis-specific gene 10 protein OS=Homo sapiens GN=TSGA10 PE=1 SV=1 |
|  | **O60488\|ACSL4_HUMAN** | Long-chain-fatty-acid--CoA ligase 4 OS=Homo sapiens GN=ACSL4 PE=1 SV=2 |
|  | **P10768\|ESTD_HUMAN** | S-formylglutathione hydrolase OS=Homo sapiens GN=ESD PE=1 SV=2 |
|  | **O95436\|NPT2B_HUMAN** | Sodium-dependent phosphate transport protein 2B OS=Homo sapiens GN=SLC34A2 PE=1 SV=2 |
|  | **P18031\|PTN1_HUMAN** | Tyrosine-protein phosphatase non-receptor type 1 OS=Homo sapiens GN=PTPN1 PE=1 SV=1 |
|  | **P18428\|LBP_HUMAN** | Lipopolysaccharide-binding protein OS=Homo sapiens GN=LBP PE=1 SV=3 |
|  | **P16520\|GBB3_HUMAN** | Guanine nucleotide-binding protein G(I)/G(S)/G(T) subunit beta-3 OS=Homo sapiens GN=GNB3 PE=1 SV=1 |
|  | **P60602\|ROMO1_HUMAN** | Reactive oxygen species modulator 1 OS=Homo sapiens GN=ROMO1 PE=1 SV=1 |
|  | **Q9NVJ2\|ARL8B_HUMAN** | ADP-ribosylation factor-like protein 8B OS=Homo sapiens GN=ARL8B PE=1 SV=1 |
|  | **Q96BM9\|ARL8A_HUMAN** | ADP-ribosylation factor-like protein 8A OS=Homo sapiens GN=ARL8A PE=1 SV=1 |
|  | **O95299\|NDUAA_HUMAN** | NADH dehydrogenase [ubiquinone] 1 alpha subcomplex subunit 10, mitochondrial OS=Homo sapiens GN=NDUFA10 PE=1 SV=1 |
|  | **O76024\|WFS1_HUMAN** | Wolframin OS=Homo sapiens GN=WFS1 PE=1 SV=1 |
|  | **Q01814\|AT2B2_HUMAN** | Plasma membrane calcium-transporting ATPase 2 OS=Homo sapiens GN=ATP2B2 PE=1 SV=2 |
|  | **P11488\|GNAT1_HUMAN** | Guanine nucleotide-binding protein G(t) subunit alpha-1 OS=Homo sapiens GN=GNAT1 PE=1 SV=5 |
|  | **P19087\|GNAT2_HUMAN** | Guanine nucleotide-binding protein G(t) subunit alpha-2 OS=Homo sapiens GN=GNAT2 PE=2 SV=4 |
|  | **A8MTJ3\|GNAT3_HUMAN** | Guanine nucleotide-binding protein G(t) subunit alpha-3 OS=Homo sapiens GN=GNAT3 PE=2 SV=2 |
|  | **Q6NUM9\|RETST_HUMAN** | All-trans-retinol 13,14-reductase OS=Homo sapiens GN=RETSAT PE=2 SV=2 |
|  | **Q6NUQ4\|TM214_HUMAN** | Transmembrane protein 214 OS=Homo sapiens GN=TMEM214 PE=1 SV=2 |
|  | **P84103\|SFRS3_HUMAN** | Splicing factor, arginine/serine-rich 3 OS=Homo sapiens GN=SFRS3 PE=1 SV=1 |
|  | **Q9BY67\|CADM1_HUMAN** | Cell adhesion molecule 1 OS=Homo sapiens GN=CADM1 PE=1 SV=2 |
|  | **P51608\|MECP2_HUMAN** | Methyl-CpG-binding protein 2 OS=Homo sapiens GN=MECP2 PE=1 SV=1 |
|  | **Q9BUL8\|PDC10_HUMAN** | Programmed cell death protein 10 OS=Homo sapiens GN=PDCD10 PE=1 SV=1 |
|  | **P50995\|ANX11_HUMAN** | Annexin A11 OS=Homo sapiens GN=ANXA11 PE=1 SV=1 |
|  | **Q02878\|RL6_HUMAN** | 60S ribosomal protein L6 OS=Homo sapiens GN=RPL6 PE=1 SV=3 |
|  | **Q9H4M9\|EHD1_HUMAN** | EH domain-containing protein 1 OS=Homo sapiens GN=EHD1 PE=1 SV=2 |
|  | **O43760\|SNG2_HUMAN** | Synaptogyrin-2 OS=Homo sapiens GN=SYNGR2 PE=1 SV=1 |
|  | **Q9UBC5\|MYO1A_HUMAN** | Myosin-Ia OS=Homo sapiens GN=MYO1A PE=1 SV=1 |
|  | **P53634\|CATC_HUMAN** | Dipeptidyl-peptidase 1 OS=Homo sapiens GN=CTSC PE=1 SV=1 |
|  | **Q12846\|STX4_HUMAN** | Syntaxin-4 OS=Homo sapiens GN=STX4 PE=1 SV=2 |
|  | **Q5VT99\|LRC38_HUMAN** | Leucine-rich repeat-containing protein 38 OS=Homo sapiens GN=LRRC38 PE=2 SV=1 |
|  | **O60613\|SEP15_HUMAN** | 15 kDa selenoprotein OS=Homo sapiens GN=SEP15 PE=1 SV=3 |
|  | **Q3B8N2\|LEG9B_HUMAN** | Galectin-9B OS=Homo sapiens GN=LGALS9B PE=2 SV=3 |
|  | **Q99442\|SEC62_HUMAN** | Translocation protein SEC62 OS=Homo sapiens GN=SEC62 PE=1 SV=1 |
|  | **Q9BX97\|PLVAP_HUMAN** | Plasmalemma vesicle-associated protein OS=Homo sapiens GN=PLVAP PE=2 SV=1 |
|  | **P22894\|MMP8_HUMAN** | Neutrophil collagenase OS=Homo sapiens GN=MMP8 PE=1 SV=1 |
|  | **P39023\|RL3_HUMAN** | 60S ribosomal protein L3 OS=Homo sapiens GN=RPL3 PE=1 SV=2 |
|  | **P12821\|ACE_HUMAN** | Angiotensin-converting enzyme OS=Homo sapiens GN=ACE PE=1 SV=1 |
|  | **P36543\|VATE1_HUMAN** | V-type proton ATPase subunit E 1 OS=Homo sapiens GN=ATP6V1E1 PE=1 SV=1 |
|  | **P03973\|SLPI_HUMAN** | Antileukoproteinase OS=Homo sapiens GN=SLPI PE=1 SV=2 |
|  | **P50416\|CPT1A_HUMAN** | Carnitine O-palmitoyltransferase 1, liver isoform OS=Homo sapiens GN=CPT1A PE=1 SV=2 |
|  | **P09669\|COX6C_HUMAN** | Cytochrome c oxidase subunit 6C OS=Homo sapiens GN=COX6C PE=1 SV=2 |
|  | **Q01518\|CAP1_HUMAN** | Adenylyl cyclase-associated protein 1 OS=Homo sapiens GN=CAP1 PE=1 SV=4 |
|  | **P13763\|HB2Q_HUMAN** | HLA class II histocompatibility antigen, DP(W2) beta chain OS=Homo sapiens GN=HLA-DPB1 PE=2 SV=1 |
|  | **P18669\|PGAM1_HUMAN** | Phosphoglycerate mutase 1 OS=Homo sapiens GN=PGAM1 PE=1 SV=2 |
|  | **Q08379\|GOGA2_HUMAN** | Golgin subfamily A member 2 OS=Homo sapiens GN=GOLGA2 PE=1 SV=2 |
|  | **Q9BVK6\|TMED9_HUMAN** | Transmembrane emp24 domain-containing protein 9 OS=Homo sapiens GN=TMED9 PE=1 SV=2 |
|  | **Q12840\|KIF5A_HUMAN** | Kinesin heavy chain isoform 5A OS=Homo sapiens GN=KIF5A PE=1 SV=2 |
|  | **O60282\|KIF5C_HUMAN** | Kinesin heavy chain isoform 5C OS=Homo sapiens GN=KIF5C PE=1 SV=1 |
|  | **P33176\|KINH_HUMAN** | Kinesin-1 heavy chain OS=Homo sapiens GN=KIF5B PE=1 SV=1 |
|  | **Q8NEW0\|ZNT7_HUMAN** | Zinc transporter 7 OS=Homo sapiens GN=SLC30A7 PE=1 SV=1 |

Top of Form

Select Summary Report

|  | Peptide Summary Select Summary (protein hits) Select Summary (unassigned) Export Search Results |  | Help |
| --- | --- | --- | --- |
|  | Significance threshold p< | Max. number of hits |  |
|  | Standard scoring  MudPIT scoring | Ions score or expect cut-off | Show sub-sets |
|  | Show pop-ups  Suppress pop-ups | Sort unassigned Decreasing Score Increasing query / Mr Decreasing Intensity | Require bold red |

Bottom of Form

Top of Form

| **1.** | P63261\|ACTG_HUMAN    **Mass:** 42108    **Score:** 8392   **Queries matched:** 351   **emPAI:** 49.67 |
| --- | --- |
|  | Actin, cytoplasmic 2 OS=Homo sapiens GN=ACTG1 PE=1 SV=1 |

|  | **Query** | **Observed** | **Mr(expt)** | **Mr(calc)** | **Delta** | **Miss** | **Score** | **Expect** | **Rank** | **Peptide** |
| --- | --- | --- | --- | --- | --- | --- | --- | --- | --- | --- |
|  | 1138 | **632.2894** | **631.2822** | **630.3701** | **0.9121** | **0** | **14** | **11** | **1** | **K.ILTER.G** 1129 1139 |
|  | 1169 | **644.3676** | **643.3603** | **643.3653** | **-0.0050** | **0** | **25** | **0.84** | **1** | **R.LDLAGR.D** |
|  | 1578 | **398.2373** | **794.4600** | **794.4650** | **-0.0050** | **0** | **22** | **0.87** | **1** | **K.IIAPPER.K** 1587 |
|  | 1635 | **400.7684** | **799.5222** | **799.5280** | **-0.0057** | **1** | **24** | **0.79** | **6** | **K.RGILTLK.Y** 1646 |
|  | 3056 | **453.2256** | **904.4366** | **904.4436** | **-0.0071** | **1** | **36** | **0.05** | **1** | **K.CDVDIRK.D** |
|  | 3326 | **462.2838** | **922.5531** | **922.5600** | **-0.0068** | **1** | **28** | **0.22** | **1** | **K.IIAPPERK.Y** 3327 3328 |
|  | 4062 | **488.7242** | **975.4339** | **975.4410** | **-0.0071** | **0** | **74** | **7.9e-006** | **1** | **K.AGFAGDDAPR.A** 4063 4064 4068 4075 |
|  | 4370 | **499.7436** | **997.4726** | **997.4790** | **-0.0064** | **0** | **44** | **0.0062** | **1** | **R.DLTDYLMK.I** 4366 4368 4369 4372 4401 |
|  | 4907 | **518.8259** | **1035.6373** | **1035.6440** | **-0.0067** | **1** | **25** | **0.45** | **1** | **K.IKIIAPPER.K** |
|  | 6245 | **566.7636** | **1131.5127** | **1131.5197** | **-0.0070** | **0** | **56** | **0.00041** | **1** | **R.GYSFTTTAER.E** 6242 6243 6244 |
|  | 6669 | **581.3090** | **1160.6034** | **1160.6111** | **-0.0077** | **0** | **44** | **0.006** | **1** | **K.EITALAPSTMK.I** 6667 6668 6670 6671 6685 |
|  | 6796 | **586.2847** | **1170.5549** | **1170.5638** | **-0.0089** | **0** | **55** | **0.00046** | **1** | **R.HQGVMVGMGQK.D** 6797 6798 6799 6800 |
|  | 6883 | **589.3063** | **1176.5981** | **1176.6060** | **-0.0079** | **0** | **(42)** | **0.011** | **1** | **K.EITALAPSTMK.I** 6885 |
|  | 7046 | **593.7484** | **1185.4823** | **1186.5587** | **-1.0764** | **0** | **(42)** | **0.0095** | **1** | **R.HQGVMVGMGQK.D** 7072 7074 7076 |
|  | 7069 | **594.2817** | **1186.5489** | **1186.5587** | **-0.0098** | **0** | **(41)** | **0.014** | **1** | **R.HQGVMVGMGQK.D** 7057 7068 7070 7071 7075 |
|  | 7313 | **599.7607** | **1197.5068** | **1197.5150** | **-0.0081** | **0** | **44** | **0.0055** | **1** | **K.DSYVGDEAQSK.R** 7289 7296 7311 |
|  | 7330 | **599.8522** | **1197.6899** | **1197.6982** | **-0.0083** | **0** | **58** | **0.00026** | **1** | **R.AVFPSIVGRPR.H** 7327 7328 7329 7331 7332 7333 7336 7342 |
|  | 7433 | **602.2789** | **1202.5433** | **1202.5536** | **-0.0103** | **0** | **(26)** | **0.42** | **1** | **R.HQGVMVGMGQK.D** 7436 7437 |
|  | 10424 | **677.8104** | **1353.6062** | **1353.6161** | **-0.0099** | **1** | **51** | **0.0011** | **1** | **K.DSYVGDEAQSKR.G** 10395 10415 10416 10417 10418 10419 10420 10421 10422 10423 10425 10426 10427 10429 10430 10445 10448 10450 |
|  | 13245 | **758.3734** | **1514.7323** | **1514.7419** | **-0.0096** | **0** | **45** | **0.0039** | **1** | **K.IWHHTFYNELR.V** 13243 13244 13248 13267 |
|  | 13260 | **758.8502** | **1515.6858** | **1515.6954** | **-0.0096** | **0** | **50** | **0.0014** | **1** | **K.QEYDESGPSIVHR.K** 13259 13261 |
|  | 13795 | **774.9045** | **1547.7944** | **1547.8051** | **-0.0107** | **1** | **16** | **3** | **1** | **R.MQKEITALAPSTMK.I** |
|  | 14290 | **395.7159** | **1578.8347** | **1579.7949** | **-0.9603** | **1** | **(4)** | **50** | **8** | **R.MQKEITALAPSTMK.I** |
|  | 15010 | **812.4193** | **1622.8239** | **1622.8338** | **-0.0098** | **1** | **27** | **0.24** | **1** | **R.LDLAGRDLTDYLMK.I** 15009 |
|  | 15307 | **548.9338** | **1643.7795** | **1643.7903** | **-0.0108** | **1** | **25** | **0.34** | **1** | **K.QEYDESGPSIVHRK.C** 15309 |
|  | 16932 | **872.9017** | **1743.7889** | **1743.8792** | **-0.0902** | **1** | **4** | **43** | **5** | **K.ILTERGYSFTTTAER.E** 16894 16929 |
|  | 17562 | **895.9443** | **1789.8740** | **1789.8846** | **-0.0106** | **0** | **72** | **7.6e-006** | **1** | **K.SYELPDGQVITIGNER.F** 17551 17553 17560 17561 17563 17564 17565 17566 17568 17569 17571 17572 17573 17574 17576 17577 17578 17579 17580 17581 17582 17583 17584 17585 17586 17587 17588 17591 17592 17593 17595 17596 17609 17610 17612 17613 17614 17616 17617 |
|  | 18513 | **618.3110** | **1851.9113** | **1850.8390** | **1.0723** | **0** | **0** | **93** | **8** | **M.EEEIAALVIDNGSGMCK.A** |
|  | 19713 | **973.9910** | **1945.9675** | **1945.8880** | **0.0795** | **0** | **0** | **96** | **2** | **K.YPIEHGIVTNWDDMEK.I** |
|  | 19788 | **977.5285** | **1953.0424** | **1953.0571** | **-0.0147** | **0** | **75** | **3.3e-006** | **1** | **R.VAPEEHPVLLTEAPLNPK.A** 19789 19791 19792 19795 19796 19797 19798 |
|  | 19968 | **655.9768** | **1964.9084** | **1965.8846** | **-0.9761** | **0** | **(3)** | **48** | **8** | **-.MEEEIAALVIDNGSGMCK.A** |
|  | 20131 | **661.6841** | **1982.0304** | **1981.8795** | **0.1509** | **0** | **7** | **17** | **1** | **-.MEEEIAALVIDNGSGMCK.A** |
|  | 20340 | **667.3346** | **1998.9819** | **1997.8744** | **1.1076** | **0** | **(2)** | **67** | **9** | **-.MEEEIAALVIDNGSGMCK.A** |
|  | 22479 | **1108.0314** | **2214.0482** | **2214.0627** | **-0.0145** | **0** | **101** | **6.9e-009** | **1** | **K.DLYANTVLSGGTTMYPGIADR.M** 22477 22478 22480 22481 22482 22483 22484 22485 22486 22487 22488 22489 22493 22495 22496 22497 22498 22501 22502 22509 22510 22511 22513 22514 22515 22516 22517 22520 22521 22541 22545 |
|  | 22682 | **1116.0297** | **2230.0448** | **2230.0576** | **-0.0128** | **0** | **(94)** | **3.4e-008** | **1** | **K.DLYANTVLSGGTTMYPGIADR.M** 22681 |
|  | 23560 | **1172.0783** | **2342.1419** | **2342.1576** | **-0.0157** | **1** | **102** | **4.6e-009** | **1** | **R.KDLYANTVLSGGTTMYPGIADR.M** 23559 23561 23562 23563 23568 |
|  | 23686 | **787.0530** | **2358.1371** | **2358.1526** | **-0.0154** | **1** | **(33)** | **0.039** | **1** | **R.KDLYANTVLSGGTTMYPGIADR.M** |
|  | 25087 | **1275.5856** | **2549.1566** | **2549.1665** | **-0.0099** | **0** | **137** | **1.1e-012** | **1** | **K.LCYVALDFEQEMATAASSSSLEK.S** 24992 24993 24994 24995 24997 24998 24999 25000 25001 25003 25004 25006 25007 25008 25009 25010 25012 25013 25014 25015 25016 25017 25018 25019 25020 25021 25022 25023 25024 25025 25026 25027 25028 25031 25032 25033 25035 25036 25038 25039 25040 25041 25042 25044 25045 25046 25047 25048 25049 25050 25051 25052 25053 25054 25055 25059 25061 25062 25063 25064 25065 25068 25069 25071 25072 25073 25075 25076 25077 25078 25079 25080 25082 25083 25084 25085 25086 25088 25089 25090 25091 25092 25093 25095 25096 25099 25100 25101 25103 25104 25106 25112 25113 25114 25115 |
|  | 25196 | **856.0536** | **2565.1391** | **2565.1614** | **-0.0223** | **0** | **(2)** | **37** | **2** | **K.LCYVALDFEQEMATAASSSSLEK.S** |
|  | 25421 | **868.1128** | **2601.3165** | **2601.3301** | **-0.0136** | **0** | **5** | **23** | **1** | **K.YSVWIGGSILASLSTFQQMWISK.Q** 25417 |
|  | 26609 | **1404.1532** | **2806.2918** | **2806.3041** | **-0.0122** | **1** | **117** | **1.2e-010** | **1** | **K.EKLCYVALDFEQEMATAASSSSLEK.S** 26602 26607 26608 26635 |
|  | 28409 | **1061.8699** | **3182.5878** | **3182.6071** | **-0.0193** | **0** | **67** | **8.6e-006** | **1** | **R.TTGIVMDSGDGVTHTVPIYEGYALPHAILR.L** 28404 28405 28406 28407 28408 28410 28411 28412 28413 28414 28415 28416 28417 28418 28419 28427 |
|  | 28488 | **1067.2017** | **3198.5832** | **3198.6020** | **-0.0188** | **0** | **(42)** | **0.0029** | **1** | **R.TTGIVMDSGDGVTHTVPIYEGYALPHAILR.L** 28489 |
|  | 28644 | **1077.8195** | **3230.4366** | **3230.4545** | **-0.0179** | **0** | **59** | **5.3e-005** | **1** | **R.CPEALFQPSFLGMESCGIHETTFNSIMK.C** 28639 28646 28647 28648 28650 28651 28652 28653 28658 28659 28661 28665 28666 |
|  | 28699 | **1083.1504** | **3246.4293** | **3246.4494** | **-0.0201** | **0** | **(35)** | **0.014** | **1** | **R.CPEALFQPSFLGMESCGIHETTFNSIMK.C** |
|  | 28700 | **1083.1504** | **3246.4293** | **3246.4494** | **-0.0201** | **0** | **(32)** | **0.027** | **1** | **R.CPEALFQPSFLGMESCGIHETTFNSIMK.C** |
|  | 29550 | **1178.8748** | **3533.6025** | **3533.6240** | **-0.0216** | **1** | **72** | **2.1e-006** | **1** | **R.FRCPEALFQPSFLGMESCGIHETTFNSIMK.C** 29549 29551 29552 29553 29554 29555 29556 29558 29563 29565 29566 |
|  | 29595 | **1184.2079** | **3549.6018** | **3549.6189** | **-0.0171** | **1** | **(65)** | **1e-005** | **1** | **R.FRCPEALFQPSFLGMESCGIHETTFNSIMK.C** 29592 29599 |

| **2.** | P60709\|ACTB_HUMAN    **Mass:** 42052    **Score:** 8392   **Queries matched:** 349   **emPAI:** 49.67 |
| --- | --- |
|  | Actin, cytoplasmic 1 OS=Homo sapiens GN=ACTB PE=1 SV=1 |

|  | **Query** | **Observed** | **Mr(expt)** | **Mr(calc)** | **Delta** | **Miss** | **Score** | **Expect** | **Rank** | **Peptide** |
| --- | --- | --- | --- | --- | --- | --- | --- | --- | --- | --- |
|  | 1138 | 632.2894 | 631.2822 | 630.3701 | 0.9121 | 0 | 14 | 11 | 1 | K.ILTER.G 1129 1139 |
|  | 1169 | 644.3676 | 643.3603 | 643.3653 | -0.0050 | 0 | 25 | 0.84 | 1 | R.LDLAGR.D |
|  | 1578 | 398.2373 | 794.4600 | 794.4650 | -0.0050 | 0 | 22 | 0.87 | 1 | K.IIAPPER.K 1587 |
|  | 1635 | 400.7684 | 799.5222 | 799.5280 | -0.0057 | 1 | 24 | 0.79 | 6 | K.RGILTLK.Y 1646 |
|  | 3056 | 453.2256 | 904.4366 | 904.4436 | -0.0071 | 1 | 36 | 0.05 | 1 | K.CDVDIRK.D |
|  | 3326 | 462.2838 | 922.5531 | 922.5600 | -0.0068 | 1 | 28 | 0.22 | 1 | K.IIAPPERK.Y 3327 3328 |
|  | 4062 | 488.7242 | 975.4339 | 975.4410 | -0.0071 | 0 | 74 | 7.9e-006 | 1 | K.AGFAGDDAPR.A 4063 4064 4068 4075 |
|  | 4370 | 499.7436 | 997.4726 | 997.4790 | -0.0064 | 0 | 44 | 0.0062 | 1 | R.DLTDYLMK.I 4366 4368 4369 4372 4401 |
|  | 4907 | 518.8259 | 1035.6373 | 1035.6440 | -0.0067 | 1 | 25 | 0.45 | 1 | K.IKIIAPPER.K |
|  | 6245 | 566.7636 | 1131.5127 | 1131.5197 | -0.0070 | 0 | 56 | 0.00041 | 1 | R.GYSFTTTAER.E 6242 6243 6244 |
|  | 6669 | 581.3090 | 1160.6034 | 1160.6111 | -0.0077 | 0 | 44 | 0.006 | 1 | K.EITALAPSTMK.I 6667 6668 6670 6671 6685 |
|  | 6796 | 586.2847 | 1170.5549 | 1170.5638 | -0.0089 | 0 | 55 | 0.00046 | 1 | R.HQGVMVGMGQK.D 6797 6798 6799 6800 |
|  | 6883 | 589.3063 | 1176.5981 | 1176.6060 | -0.0079 | 0 | (42) | 0.011 | 1 | K.EITALAPSTMK.I 6885 |
|  | 7046 | 593.7484 | 1185.4823 | 1186.5587 | -1.0764 | 0 | (42) | 0.0095 | 1 | R.HQGVMVGMGQK.D 7072 7074 7076 |
|  | 7069 | 594.2817 | 1186.5489 | 1186.5587 | -0.0098 | 0 | (41) | 0.014 | 1 | R.HQGVMVGMGQK.D 7057 7068 7070 7071 7075 |
|  | 7313 | 599.7607 | 1197.5068 | 1197.5150 | -0.0081 | 0 | 44 | 0.0055 | 1 | K.DSYVGDEAQSK.R 7289 7296 7311 |
|  | 7330 | 599.8522 | 1197.6899 | 1197.6982 | -0.0083 | 0 | 58 | 0.00026 | 1 | R.AVFPSIVGRPR.H 7327 7328 7329 7331 7332 7333 7336 7342 |
|  | 7433 | 602.2789 | 1202.5433 | 1202.5536 | -0.0103 | 0 | (26) | 0.42 | 1 | R.HQGVMVGMGQK.D 7436 7437 |
|  | 10424 | 677.8104 | 1353.6062 | 1353.6161 | -0.0099 | 1 | 51 | 0.0011 | 1 | K.DSYVGDEAQSKR.G 10395 10415 10416 10417 10418 10419 10420 10421 10422 10423 10425 10426 10427 10429 10430 10445 10448 10450 |
|  | 13245 | 758.3734 | 1514.7323 | 1514.7419 | -0.0096 | 0 | 45 | 0.0039 | 1 | K.IWHHTFYNELR.V 13243 13244 13248 13267 |
|  | 13260 | 758.8502 | 1515.6858 | 1515.6954 | -0.0096 | 0 | 50 | 0.0014 | 1 | K.QEYDESGPSIVHR.K 13259 13261 |
|  | 13795 | 774.9045 | 1547.7944 | 1547.8051 | -0.0107 | 1 | 16 | 3 | 1 | R.MQKEITALAPSTMK.I |
|  | 14290 | 395.7159 | 1578.8347 | 1579.7949 | -0.9603 | 1 | (4) | 50 | 8 | R.MQKEITALAPSTMK.I |
|  | 15010 | 812.4193 | 1622.8239 | 1622.8338 | -0.0098 | 1 | 27 | 0.24 | 1 | R.LDLAGRDLTDYLMK.I 15009 |
|  | 15307 | 548.9338 | 1643.7795 | 1643.7903 | -0.0108 | 1 | 25 | 0.34 | 1 | K.QEYDESGPSIVHRK.C 15309 |
|  | 16932 | 872.9017 | 1743.7889 | 1743.8792 | -0.0902 | 1 | 4 | 43 | 5 | K.ILTERGYSFTTTAER.E 16894 16929 |
|  | 17562 | 895.9443 | 1789.8740 | 1789.8846 | -0.0106 | 0 | 72 | 7.6e-006 | 1 | K.SYELPDGQVITIGNER.F 17551 17553 17560 17561 17563 17564 17565 17566 17568 17569 17571 17572 17573 17574 17576 17577 17578 17579 17580 17581 17582 17583 17584 17585 17586 17587 17588 17591 17592 17593 17595 17596 17609 17610 17612 17613 17614 17616 17617 |
|  | 19630 | **648.3676** | **1942.0810** | **1941.8118** | **0.2692** | **0** | **1** | **74** | **8** | **-.MDDDIAALVVDNGSGMCK.A** |
|  | 19713 | 973.9910 | 1945.9675 | 1945.8880 | 0.0795 | 0 | 0 | 96 | 2 | K.YPIEHGIVTNWDDMEK.I |
|  | 19788 | 977.5285 | 1953.0424 | 1953.0571 | -0.0147 | 0 | 75 | 3.3e-006 | 1 | R.VAPEEHPVLLTEAPLNPK.A 19789 19791 19792 19795 19796 19797 19798 |
|  | 22479 | 1108.0314 | 2214.0482 | 2214.0627 | -0.0145 | 0 | 101 | 6.9e-009 | 1 | K.DLYANTVLSGGTTMYPGIADR.M 22477 22478 22480 22481 22482 22483 22484 22485 22486 22487 22488 22489 22493 22495 22496 22497 22498 22501 22502 22509 22510 22511 22513 22514 22515 22516 22517 22520 22521 22541 22545 |
|  | 22682 | 1116.0297 | 2230.0448 | 2230.0576 | -0.0128 | 0 | (94) | 3.4e-008 | 1 | K.DLYANTVLSGGTTMYPGIADR.M 22681 |
|  | 23560 | 1172.0783 | 2342.1419 | 2342.1576 | -0.0157 | 1 | 102 | 4.6e-009 | 1 | R.KDLYANTVLSGGTTMYPGIADR.M 23559 23561 23562 23563 23568 |
|  | 23686 | 787.0530 | 2358.1371 | 2358.1526 | -0.0154 | 1 | (33) | 0.039 | 1 | R.KDLYANTVLSGGTTMYPGIADR.M |
|  | 25087 | 1275.5856 | 2549.1566 | 2549.1665 | -0.0099 | 0 | 137 | 1.1e-012 | 1 | K.LCYVALDFEQEMATAASSSSLEK.S 24992 24993 24994 24995 24997 24998 24999 25000 25001 25003 25004 25006 25007 25008 25009 25010 25012 25013 25014 25015 25016 25017 25018 25019 25020 25021 25022 25023 25024 25025 25026 25027 25028 25031 25032 25033 25035 25036 25038 25039 25040 25041 25042 25044 25045 25046 25047 25048 25049 25050 25051 25052 25053 25054 25055 25059 25061 25062 25063 25064 25065 25068 25069 25071 25072 25073 25075 25076 25077 25078 25079 25080 25082 25083 25084 25085 25086 25088 25089 25090 25091 25092 25093 25095 25096 25099 25100 25101 25103 25104 25106 25112 25113 25114 25115 |
|  | 25196 | 856.0536 | 2565.1391 | 2565.1614 | -0.0223 | 0 | (2) | 37 | 2 | K.LCYVALDFEQEMATAASSSSLEK.S |
|  | 25421 | 868.1128 | 2601.3165 | 2601.3301 | -0.0136 | 0 | 5 | 23 | 1 | K.YSVWIGGSILASLSTFQQMWISK.Q 25417 |
|  | 26310 | **918.4094** | **2752.2064** | **2752.2069** | **-0.0004** | **1** | **2** | **35** | **4** | **M.DDDIAALVVDNGSGMCKAGFAGDDAPR.A** |
|  | 26609 | 1404.1532 | 2806.2918 | 2806.3041 | -0.0122 | 1 | 117 | 1.2e-010 | 1 | K.EKLCYVALDFEQEMATAASSSSLEK.S 26602 26607 26608 26635 |
|  | 28409 | 1061.8699 | 3182.5878 | 3182.6071 | -0.0193 | 0 | 67 | 8.6e-006 | 1 | R.TTGIVMDSGDGVTHTVPIYEGYALPHAILR.L 28404 28405 28406 28407 28408 28410 28411 28412 28413 28414 28415 28416 28417 28418 28419 28427 |
|  | 28488 | 1067.2017 | 3198.5832 | 3198.6020 | -0.0188 | 0 | (42) | 0.0029 | 1 | R.TTGIVMDSGDGVTHTVPIYEGYALPHAILR.L 28489 |
|  | 28644 | 1077.8195 | 3230.4366 | 3230.4545 | -0.0179 | 0 | 59 | 5.3e-005 | 1 | R.CPEALFQPSFLGMESCGIHETTFNSIMK.C 28639 28646 28647 28648 28650 28651 28652 28653 28658 28659 28661 28665 28666 |
|  | 28699 | 1083.1504 | 3246.4293 | 3246.4494 | -0.0201 | 0 | (35) | 0.014 | 1 | R.CPEALFQPSFLGMESCGIHETTFNSIMK.C |
|  | 28700 | 1083.1504 | 3246.4293 | 3246.4494 | -0.0201 | 0 | (32) | 0.027 | 1 | R.CPEALFQPSFLGMESCGIHETTFNSIMK.C |
|  | 29550 | 1178.8748 | 3533.6025 | 3533.6240 | -0.0216 | 1 | 72 | 2.1e-006 | 1 | R.FRCPEALFQPSFLGMESCGIHETTFNSIMK.C 29549 29551 29552 29553 29554 29555 29556 29558 29563 29565 29566 |
|  | 29595 | 1184.2079 | 3549.6018 | 3549.6189 | -0.0171 | 1 | (65) | 1e-005 | 1 | R.FRCPEALFQPSFLGMESCGIHETTFNSIMK.C 29592 29599 |

| **3.** | Q6S8J3\|POTEE_HUMAN    **Mass:** 122882   **Score:** 5738   **Queries matched:** 206   **emPAI:** 0.51 |
| --- | --- |
|  | POTE ankyrin domain family member E OS=Homo sapiens GN=POTEE PE=1 SV=3 |

|  | **Query** | **Observed** | **Mr(expt)** | **Mr(calc)** | **Delta** | **Miss** | **Score** | **Expect** | **Rank** | **Peptide** |
| --- | --- | --- | --- | --- | --- | --- | --- | --- | --- | --- |
|  | 1138 | 632.2894 | 631.2822 | 630.3701 | 0.9121 | 0 | 14 | 11 | 1 | K.ILTER.G 1129 1139 |
|  | 1169 | 644.3676 | 643.3603 | 643.3653 | -0.0050 | 0 | 25 | 0.84 | 1 | R.LDLAGR.E |
|  | 1635 | 400.7684 | 799.5222 | 799.5280 | -0.0057 | 1 | 24 | 0.79 | 6 | K.RGILTLK.Y 1646 |
|  | 2841 | **443.7376** | **885.4606** | **885.4668** | **-0.0062** | **0** | **21** | **1.5** | **1** | **K.ANLNALDR.Y** |
|  | 4039 | **487.9703** | **973.9261** | **974.4305** | **-0.5044** | **0** | **2** | **89** | **5** | **K.GSENSQPEK.M** |
|  | 4062 | 488.7242 | 975.4339 | 975.4410 | -0.0071 | 0 | 74 | 7.9e-006 | 1 | K.AGFAGDDAPR.A 4063 4064 4068 4075 |
|  | 4601 | **507.7411** | **1013.4676** | **1013.5618** | **-0.0942** | **1** | **2** | **1.2e+002** | **3** | **K.KANLNALDR.Y** |
|  | 4738 | **513.2864** | **1024.5583** | **1023.4947** | **1.0637** | **0** | **3** | **79** | **8** | **R.ELPDYLMK.I** |
|  | 5921 | **554.8283** | **1107.6419** | **1106.5277** | **1.1142** | **0** | **8** | **27** | **5** | **K.MSQELEINK.D** |
|  | 6156 | **563.2864** | **1124.5583** | **1123.6039** | **0.9544** | **1** | **5** | **43** | **8** | **K.LHRAAWWGK.V** |
|  | 6339 | **571.0724** | **1140.1302** | **1139.5532** | **0.5770** | **0** | **0** | **95** | **8** | **R.ICELLSDYK.E** |
|  | 6779 | **585.3008** | **1168.5871** | **1167.5264** | **1.0608** | **0** | **2** | **83** | **7** | **K.MNSELSLSCK.K** |
|  | 7209 | **398.7393** | **1193.1960** | **1192.5831** | **0.6129** | **0** | **22** | **0.68** | **1** | **K.EIAALAPSMMK.I** |
|  | 7330 | 599.8522 | 1197.6899 | 1197.6982 | -0.0083 | 0 | 58 | 0.00026 | 1 | R.AVFPSIVGRPR.Q 7327 7328 7329 7331 7332 7333 7336 7342 |
|  | 8677 | **632.8616** | **1263.7087** | **1263.5159** | **0.1929** | **0** | **1** | **1.1e+002** | **6** | **R.QQGMMGGMHQK.E** |
|  | 10375 | **676.8123** | **1351.6101** | **1351.5562** | **0.0539** | **1** | **4** | **50** | **7** | **K.DGDREVEEEMK.K** |
|  | 11774 | **477.2324** | **1428.6754** | **1429.7785** | **-1.1030** | **1** | **7** | **27** | **2** | **K.EIAALAPSMMKIR.I** 11807 |
|  | 12809 | **497.2980** | **1488.8722** | **1489.6103** | **-0.7382** | **0** | **10** | **13** | **2** | **K.SNVGASGDHDDSAMK.T** 12805 12810 |
|  | 13245 | 758.3734 | 1514.7323 | 1514.7419 | -0.0096 | 0 | 45 | 0.0039 | 1 | K.IWHHTFYNELR.V 13243 13244 13248 13267 |
|  | 13260 | 758.8502 | 1515.6858 | 1515.6954 | -0.0096 | 0 | 50 | 0.0014 | 1 | K.QEYDESGPSIVHR.K 13259 13261 |
|  | 13553 | **767.3226** | **1532.6306** | **1533.7093** | **-1.0787** | **1** | **4** | **44** | **8** | **K.MSQELEINKDGDR.E** |
|  | 14076 | **783.3349** | **1564.6552** | **1564.7477** | **-0.0924** | **0** | **12** | **7.7** | **2** | **-.MVVEVDSMPAASSVK.K** 14064 14077 |
|  | 14289 | **395.7139** | **1578.8264** | **1579.7772** | **-0.9508** | **1** | **11** | **8.8** | **3** | **R.MQKEIAALAPSMMK.I** |
|  | 15077 | **543.3950** | **1627.1632** | **1626.7633** | **0.4000** | **1** | **9** | **11** | **1** | **R.ELENFMAIEEMKK.H** 15070 |
|  | 15307 | 548.9338 | 1643.7795 | 1643.7903 | -0.0108 | 1 | 25 | 0.34 | 1 | K.QEYDESGPSIVHRK.C 15309 |
|  | 15395 | **825.3951** | **1648.7756** | **1648.8494** | **-0.0738** | **1** | **6** | **27** | **2** | **R.LDLAGRELPDYLMK.I** 15386 15409 |
|  | 17562 | 895.9443 | 1789.8740 | 1789.8846 | -0.0106 | 0 | 72 | 7.6e-006 | 1 | K.SYELPDGQVITIGNER.F 17551 17553 17560 17561 17563 17564 17565 17566 17568 17569 17571 17572 17573 17574 17576 17577 17578 17579 17580 17581 17582 17583 17584 17585 17586 17587 17588 17591 17592 17593 17595 17596 17609 17610 17612 17613 17614 17616 17617 |
|  | 19251 | **956.4669** | **1910.9192** | **1910.8437** | **0.0754** | **1** | **1** | **82** | **3** | **R.QQGMMGGMHQKESYVGK.E** |
|  | 19426 | **643.0295** | **1926.0668** | **1926.8386** | **-0.7718** | **1** | **(1)** | **80** | **6** | **R.QQGMMGGMHQKESYVGK.E** |
|  | 19873 | **653.6609** | **1957.9608** | **1957.8397** | **0.1211** | **1** | **7** | **16** | **5** | **K.DGDRELENFMAIEEMK.K** |
|  | 20238 | **664.6633** | **1990.9682** | **1989.9677** | **1.0005** | **1** | **3** | **47** | **9** | **K.QMLKISSENSNPEQELK.L** 20235 |
|  | 20265 | **665.3200** | **1992.9382** | **1993.9051** | **-0.9669** | **1** | **2** | **55** | **4** | **K.QMPKYSSENSNPEQDLK.L** |
|  | 21056 | **688.4301** | **2062.2685** | **2062.9477** | **-0.6792** | **1** | **1** | **58** | **2** | **K.GSENSQPEKMSQELEINK.D** |
|  | 25087 | 1275.5856 | 2549.1566 | 2549.1665 | -0.0099 | 0 | 137 | 1.1e-012 | 1 | K.LCYVALDFEQEMATAASSSSLEK.S 24992 24993 24994 24995 24997 24998 24999 25000 25001 25003 25004 25006 25007 25008 25009 25010 25012 25013 25014 25015 25016 25017 25018 25019 25020 25021 25022 25023 25024 25025 25026 25027 25028 25031 25032 25033 25035 25036 25038 25039 25040 25041 25042 25044 25045 25046 25047 25048 25049 25050 25051 25052 25053 25054 25055 25059 25061 25062 25063 25064 25065 25068 25069 25071 25072 25073 25075 25076 25077 25078 25079 25080 25082 25083 25084 25085 25086 25088 25089 25090 25091 25092 25093 25095 25096 25099 25100 25101 25103 25104 25106 25112 25113 25114 25115 |
|  | 25196 | 856.0536 | 2565.1391 | 2565.1614 | -0.0223 | 0 | (2) | 37 | 2 | K.LCYVALDFEQEMATAASSSSLEK.S |
|  | 26609 | 1404.1532 | 2806.2918 | 2806.3041 | -0.0122 | 1 | 117 | 1.2e-010 | 1 | K.EKLCYVALDFEQEMATAASSSSLEK.S 26602 26607 26608 26635 |
|  | 27096 | **968.1269** | **2901.3590** | **2900.3802** | **0.9788** | **0** | **3** | **31** | **1** | **K.HGSTHVGFPENLTNGATAGNGDDGLIPPR.K** |

| **4.** | P35579\|MYH9_HUMAN    **Mass:** 227646   **Score:** 3887   **Queries matched:** 263   **emPAI:** 3.03 |
| --- | --- |
|  | Myosin-9 OS=Homo sapiens GN=MYH9 PE=1 SV=4 |

|  | **Query** | **Observed** | **Mr(expt)** | **Mr(calc)** | **Delta** | **Miss** | **Score** | **Expect** | **Rank** | **Peptide** |
| --- | --- | --- | --- | --- | --- | --- | --- | --- | --- | --- |
|  | 834 | **518.2890** | **517.2817** | **517.2608** | **0.0209** | **0** | **6** | **1e+002** | **9** | **R.ANASR.R** |
|  | 884 | **533.1866** | **532.1793** | **531.3017** | **0.8777** | **0** | **9** | **41** | **1** | **R.IGQSK.V** 880 882 |
|  | 988 | **572.1176** | **571.1103** | **571.3806** | **-0.2703** | **1** | **10** | **15** | **5** | **R.IKVGR.D** |
|  | 1026 | **586.2965** | **585.2892** | **585.2944** | **-0.0053** | **0** | **15** | **6.2** | **7** | **K.MNPPK.F** |
|  | 1213 | **662.3661** | **661.3588** | **661.3646** | **-0.0058** | **0** | **20** | **2.5** | **1** | **R.EVSSLK.N** |
|  | 1318 | **705.3721** | **704.3648** | **704.3639** | **0.0009** | **0** | **10** | **20** | **7** | **R.SMAVAAR.K** 1314 |
|  | 1458 | **767.3107** | **766.3035** | **766.3102** | **-0.0067** | **0** | **10** | **15** | **2** | **R.SMMQDR.E** |
|  | 1746 | **404.7289** | **807.4431** | **807.4491** | **-0.0059** | **0** | **23** | **0.97** | **1** | **R.TVGQLYK.E** 1763 |
|  | 2024 | **830.4562** | **829.4490** | **829.4545** | **-0.0055** | **0** | **30** | **0.21** | **1** | **K.GALALEEK.R** |
|  | 2184 | **841.1036** | **840.0963** | **840.4341** | **-0.3378** | **0** | **35** | **0.032** | **1** | **K.SVHELEK.S** |
|  | 2279 | **423.7447** | **845.4748** | **845.4793** | **-0.0045** | **1** | **19** | **2.6** | **3** | **R.KLQAQMK.D** 2280 |
|  | 2513 | **432.2372** | **862.4599** | **861.4742** | **0.9857** | **1** | **(17)** | **3.8** | **4** | **R.KLQAQMK.D** |
|  | 2881 | **445.7792** | **889.5439** | **888.5644** | **0.9795** | **1** | **4** | **89** | **7** | **K.LSLSTKLK.Q** |
|  | 2892 | **446.7125** | **891.4105** | **891.4160** | **-0.0055** | **0** | **16** | **4.8** | **1** | **K.ADEWLMK.N** |
|  | 3032 | **452.2575** | **902.5005** | **902.5073** | **-0.0067** | **0** | **11** | **17** | **1** | **K.ASITALEAK.I** |
|  | 3095 | **454.7447** | **907.4748** | **907.4803** | **-0.0056** | **0** | **16** | **3.9** | **1** | **K.FVSELWK.D** |
|  | 3242 | **459.2352** | **916.4558** | **916.4614** | **-0.0056** | **0** | **4** | **74** | **4** | **K.DLEGLSQR.H** |
|  | 3334 | **462.7474** | **923.4803** | **923.4865** | **-0.0062** | **0** | **35** | **0.044** | **1** | **R.VVFQEFR.Q** |
|  | 3422 | **466.2599** | **930.5052** | **930.5134** | **-0.0083** | **1** | **20** | **2** | **3** | **R.VRTELADK.V** 3423 |
|  | 3644 | **947.4919** | **946.4847** | **946.4906** | **-0.0059** | **0** | **31** | **0.14** | **1** | **R.QQQLTAMK.V** 3641 3643 |
|  | 3817 | **480.2577** | **958.5009** | **958.5083** | **-0.0074** | **1** | **33** | **0.1** | **1** | **K.VNKDDIQK.M** 3822 |
|  | 3855 | **481.2429** | **960.4712** | **960.4777** | **-0.0066** | **0** | **40** | **0.018** | **1** | **R.NTNPNFVR.C** |
|  | 4189 | **493.7816** | **985.5486** | **985.5556** | **-0.0070** | **1** | **14** | **7.4** | **1** | **K.GALALEEKR.R** |
|  | 4193 | **494.2553** | **986.4961** | **987.5237** | **-1.0276** | **1** | **7** | **33** | **9** | **K.LKQVEDEK.N** |
|  | 4391 | **500.2754** | **998.5362** | **998.5549** | **-0.0187** | **0** | **1** | **1.4e+002** | **10** | **R.GDLPFVVPR.R** |
|  | 4647 | **509.2570** | **1016.4994** | **1016.5073** | **-0.0079** | **0** | **32** | **0.13** | **1** | **R.CNGVLEGIR.I** |
|  | 4711 | **512.2675** | **1022.5205** | **1023.4734** | **-0.9529** | **1** | **9** | **19** | **1** | **K.NDNSSRFGK.F** 4724 |
|  | 4722 | **512.7650** | **1023.5153** | **1023.5237** | **-0.0083** | **1** | **23** | **0.77** | **1** | **K.ATDKSFVEK.V** 4732 4742 |
|  | 4782 | **514.8020** | **1027.5894** | **1026.3998** | **1.1897** | **0** | **2** | **1e+002** | **5** | **R.QEEEMMAK.E** |
|  | 4877 | **517.7595** | **1033.5045** | **1033.5114** | **-0.0069** | **0** | **16** | **4.7** | **1** | **K.LQEMEGTVK.S** |
|  | 5043 | **523.2861** | **1044.5576** | **1044.5637** | **-0.0061** | **0** | **25** | **0.61** | **1** | **R.LEVNLQAMK.A** 5042 |
|  | 5085 | **524.7491** | **1047.4837** | **1047.4906** | **-0.0069** | **0** | **45** | **0.005** | **1** | **R.ALEEAMEQK.A** |
|  | 5375 | **536.2752** | **1070.5359** | **1070.6124** | **-0.0766** | **1** | **5** | **53** | **7** | **K.KLVWVPSDK.S** 5377 |
|  | 5651 | **545.3001** | **1088.5857** | **1087.5621** | **1.0235** | **1** | **9** | **24** | **2** | **K.AKQTLENER.G** |
|  | 5670 | **546.2700** | **1090.5254** | **1091.5499** | **-1.0245** | **0** | **28** | **0.25** | **3** | **K.FDQLLAEEK.T** |
|  | 5854 | **552.2991** | **1102.5836** | **1102.5917** | **-0.0081** | **1** | **31** | **0.13** | **1** | **K.RQQQLTAMK.V** 5856 |
|  | 6070 | **560.2959** | **1118.5772** | **1118.5866** | **-0.0094** | **1** | **(22)** | **1.2** | **1** | **K.RQQQLTAMK.V** |
|  | 6193 | **564.8488** | **1127.6831** | **1128.5775** | **-0.8944** | **0** | **9** | **21** | **7** | **K.EEELQAALAR.V** |
|  | 6314 | **569.8013** | **1137.5880** | **1137.5964** | **-0.0084** | **1** | **25** | **0.48** | **1** | **R.CIIPNHEKK.A** |
|  | 6348 | **571.2879** | **1140.5612** | **1140.5710** | **-0.0097** | **1** | **6** | **35** | **1** | **R.CQHLQAEKK.K** |
|  | 6561 | **577.7795** | **1153.5445** | **1153.5550** | **-0.0105** | **0** | **17** | **3** | **1** | **K.VMQEQGTHPK.F** 6560 6562 6563 6565 6566 6569 6570 |
|  | 6581 | **578.3303** | **1154.6461** | **1154.6560** | **-0.0099** | **1** | **30** | **0.15** | **1** | **R.RGDLPFVVPR.R** |
|  | 6790 | **585.7502** | **1169.4858** | **1169.4944** | **-0.0086** | **0** | **62** | **9e-005** | **1** | **R.TEMEDLMSSK.D** |
|  | 7383 | **601.1215** | **1200.2284** | **1200.5809** | **-0.3525** | **1** | **5** | **30** | **4** | **K.DDIQKMNPPK.F** |
|  | 7616 | **606.7919** | **1211.5693** | **1211.5782** | **-0.0089** | **0** | **32** | **0.08** | **1** | **K.DLEAHIDSANK.N** 7611 7618 |
|  | 7687 | **608.3343** | **1214.6540** | **1214.6619** | **-0.0078** | **1** | **53** | **0.00089** | **1** | **R.ASREEILAQAK.E** 7677 7688 |
|  | 7794 | **610.8259** | **1219.6372** | **1219.6448** | **-0.0076** | **1** | **48** | **0.0025** | **1** | **K.KFDQLLAEEK.T** 7793 |
|  | 7855 | **408.8608** | **1223.5607** | **1222.6306** | **0.9301** | **0** | **7** | **26** | **4** | **R.AGVLAHLEEER.D** |
|  | 8202 | **621.7940** | **1241.5735** | **1241.5822** | **-0.0088** | **0** | **38** | **0.022** | **1** | **K.THEAQIQEMR.Q** 8200 |
|  | 8553 | **420.2319** | **1257.6739** | **1257.5772** | **0.0967** | **0** | **(9)** | **18** | **2** | **K.THEAQIQEMR.Q** |
|  | 8645 | **421.5636** | **1261.6691** | **1260.6748** | **0.9943** | **1** | **10** | **16** | **1** | **K.VKLQEMEGTVK.S** 8629 |
|  | 8714 | **423.0517** | **1266.1334** | **1265.5081** | **0.6252** | **0** | **2** | **57** | **8** | **R.EMEAELEDER.K** |
|  | 8879 | **637.8494** | **1273.6842** | **1273.6918** | **-0.0076** | **0** | **29** | **0.16** | **1** | **R.YEILTPNSIPK.G** 8862 8880 |
|  | 9477 | **653.3340** | **1304.6534** | **1304.6612** | **-0.0078** | **0** | **53** | **0.00072** | **1** | **K.EQADFAIEALAK.A** 9461 9485 |
|  | 9516 | **655.2991** | **1308.5837** | **1308.5946** | **-0.0109** | **1** | **44** | **0.0051** | **1** | **R.NAEQYKDQADK.A** |
|  | 9546 | **655.8514** | **1309.6882** | **1309.6990** | **-0.0108** | **1** | **40** | **0.015** | **1** | **K.VLLQGKGDSEHK.R** |
|  | 9578 | **656.8738** | **1311.7330** | **1311.5733** | **0.1597** | **1** | **12** | **8.9** | **2** | **K.LQAQMKDCMR.E** |
|  | 9744 | **440.2517** | **1317.7333** | **1317.7405** | **-0.0072** | **0** | **45** | **0.0048** | **1** | **K.LDPHLVLDQLR.C** 9743 |
|  | 10010 | **666.3024** | **1330.5903** | **1330.6000** | **-0.0097** | **0** | **19** | **1.8** | **1** | **R.QLEEAEEEAQR.A** |
|  | 10318 | **675.3514** | **1348.6882** | **1347.6154** | **1.0728** | **0** | **11** | **12** | **1** | **R.DELADEIANSSGK.G** |
|  | 10478 | **679.3525** | **1356.6904** | **1356.6997** | **-0.0093** | **1** | **25** | **0.43** | **1** | **K.DVLLQVDDERR.N** |
|  | 10878 | **460.1686** | **1377.4840** | **1377.6082** | **-0.1242** | **1** | **6** | **28** | **5** | **R.EMEAELEDERK.Q** |
|  | 11208 | **699.3379** | **1396.6612** | **1396.6697** | **-0.0084** | **1** | **55** | **0.00043** | **1** | **K.VDYKADEWLMK.N** 11207 |
|  | 11278 | **701.3161** | **1400.6176** | **1400.7147** | **-0.0970** | **1** | **4** | **56** | **10** | **R.EEILAQAKENEK.K** |
|  | 11478 | **706.9045** | **1411.7945** | **1411.8035** | **-0.0089** | **1** | **64** | **4.6e-005** | **1** | **K.KVEAQLQELQVK.F** 11452 11454 11463 |
|  | 11706 | **713.3742** | **1424.7339** | **1424.7446** | **-0.0107** | **0** | **50** | **0.0015** | **1** | **K.QIATLHAQVADMK.K** 11707 |
|  | 11837 | **717.3225** | **1432.6303** | **1432.6430** | **-0.0126** | **1** | **32** | **0.086** | **1** | **R.DLQGRDEQSEEK.K** |
|  | 11954 | **720.8527** | **1439.6908** | **1439.7004** | **-0.0097** | **1** | **15** | **4.3** | **1** | **K.DLEGLSQRHEEK.V** 11937 |
|  | 12608 | **739.8986** | **1477.7827** | **1477.7929** | **-0.0102** | **0** | **49** | **0.0017** | **1** | **K.VIQYLAYVASSHK.S** 12595 12609 |
|  | 12749 | **743.9499** | **1485.8852** | **1485.8919** | **-0.0067** | **0** | **69** | **1.7e-005** | **1** | **R.VISGVLQLGNIVFK.K** 12734 12747 |
|  | 12759 | **744.3530** | **1486.6915** | **1486.7011** | **-0.0096** | **1** | **55** | **0.00038** | **1** | **K.RQLEEAEEEAQR.A** |
|  | 13399 | **508.9368** | **1523.7885** | **1523.7984** | **-0.0099** | **1** | **17** | **2.7** | **1** | **K.TDLLLEPYNKYR.F** |
|  | 13516 | **765.8803** | **1529.7459** | **1529.7573** | **-0.0113** | **0** | **74** | **4.8e-006** | **1** | **K.IAQLEEQLDNETK.E** 13515 |
|  | 13618 | **513.2499** | **1536.7280** | **1536.7395** | **-0.0115** | **1** | **13** | **5.4** | **2** | **K.DKADFCIIHYAGK.V** |
|  | 13849 | **776.3460** | **1550.6775** | **1550.6882** | **-0.0108** | **0** | **18** | **1.8** | **1** | **R.EDQSILCTGESGAGK.T** |
|  | 13875 | **776.8790** | **1551.7435** | **1551.7538** | **-0.0103** | **0** | **50** | **0.0014** | **1** | **K.ITDVIIGFQACCR.G** 13856 |
|  | 13904 | **777.8485** | **1553.6825** | **1552.8395** | **0.8429** | **1** | **10** | **13** | **1** | **K.QIATLHAQVADMKK.K** |
|  | 14076 | 783.3349 | 1564.6552 | 1564.6675 | -0.0122 | 0 | 76 | 2.5e-006 | 1 | R.ELEDATETADAMNR.E 14077 |
|  | 14174 | **786.4255** | **1570.8364** | **1570.8468** | **-0.0103** | **0** | **37** | **0.026** | **1** | **K.VSHLLGINVTDFTR.G** 14175 |
|  | 14213 | **787.9492** | **1573.8839** | **1573.8940** | **-0.0101** | **1** | **46** | **0.0032** | **1** | **K.AGKLDPHLVLDQLR.C** 14209 |
|  | 14288 | **790.4199** | **1578.8253** | **1578.8365** | **-0.0112** | **1** | **45** | **0.0041** | **1** | **R.AGVLAHLEEERDLK.I** 14289 |
|  | 14375 | **793.3793** | **1584.7441** | **1584.7566** | **-0.0125** | **1** | **55** | **0.00036** | **1** | **K.NKHEAMITDLEER.L** 14374 |
|  | 14480 | **796.3496** | **1590.6845** | **1590.6944** | **-0.0098** | **0** | **(30)** | **0.12** | **1** | **R.NTDQASMPDNTAAQK.V** 14479 14481 |
|  | 14725 | **804.3456** | **1606.6767** | **1606.6893** | **-0.0126** | **0** | **43** | **0.0059** | **1** | **R.NTDQASMPDNTAAQK.V** |
|  | 14845 | **807.9951** | **1613.9756** | **1613.9869** | **-0.0113** | **1** | **35** | **0.037** | **1** | **R.VISGVLQLGNIVFKK.E** 14846 |
|  | 14859 | **808.4080** | **1614.8015** | **1614.8109** | **-0.0094** | **0** | **52** | **0.00076** | **1** | **R.IMGIPEEEQMGLLR.V** 14860 14862 |
|  | 14994 | **541.7350** | **1622.1831** | **1622.8318** | **-0.6486** | **1** | **2** | **46** | **4** | **R.QGFPNRVVFQEFR.Q** |
|  | 15327 | **823.9004** | **1645.7862** | **1645.7981** | **-0.0118** | **1** | **65** | **3.4e-005** | **1** | **R.ALEEAMEQKAELER.L** |
|  | 15352 | **549.9503** | **1646.8291** | **1646.8008** | **0.0284** | **0** | **(4)** | **55** | **6** | **R.IMGIPEEEQMGLLR.V** |
|  | 15466 | **827.3912** | **1652.7679** | **1652.7781** | **-0.0101** | **0** | **61** | **9.1e-005** | **1** | **R.IAEFTTNLTEEEEK.S** 15459 15464 |
|  | 15573 | **830.4460** | **1658.8775** | **1658.8661** | **0.0114** | **1** | **3** | **57** | **3** | **K.ALSLARALEEAMEQK.A** 15571 |
|  | 15615 | **554.7940** | **1661.3602** | **1661.8671** | **-0.5069** | **1** | **2** | **48** | **5** | **K.LMATLRNTNPNFVR.C** |
|  | 15784 | **836.9210** | **1671.8275** | **1671.8369** | **-0.0094** | **0** | **97** | **2.1e-008** | **1** | **K.NFINNPLAQADWAAK.K** 15770 |
|  | 15953 | **842.8677** | **1683.7209** | **1683.7331** | **-0.0122** | **1** | **62** | **6.8e-005** | **1** | **R.TEMEDLMSSKDDVGK.S** 15954 15981 |
|  | 16633 | **863.9731** | **1725.9316** | **1725.9413** | **-0.0097** | **0** | **83** | **5.2e-007** | **1** | **R.QLLQANPILEAFGNAK.T** 16634 16646 16647 |
|  | 16641 | **864.4268** | **1726.8391** | **1726.8487** | **-0.0096** | **0** | **67** | **2.3e-005** | **1** | **K.NLPIYSEEIVEMYK.G** 16643 |
|  | 16679 | **865.4333** | **1728.8520** | **1728.8642** | **-0.0122** | **1** | **29** | **0.15** | **1** | **K.QTLENERGELANEVK.V** 16707 |
|  | 17001 | **876.4255** | **1750.8365** | **1750.8481** | **-0.0116** | **0** | **78** | **1.8e-006** | **1** | **R.LTEMETLQSQLMAEK.L** 17003 17004 |
|  | 17133 | **881.4193** | **1760.8239** | **1759.8298** | **0.9942** | **0** | **53** | **0.00053** | **1** | **K.LEEEQIILEDQNCK.L** |
|  | 17196 | **884.4230** | **1766.8314** | **1766.8430** | **-0.0116** | **0** | **(49)** | **0.0014** | **1** | **R.LTEMETLQSQLMAEK.L** |
|  | 17220 | **590.6169** | **1768.8288** | **1768.8414** | **-0.0125** | **1** | **53** | **0.00056** | **1** | **K.KQELEEICHDLEAR.V** |
|  | 17963 | **908.4511** | **1814.8875** | **1814.9010** | **-0.0134** | **1** | **76** | **2.4e-006** | **1** | **K.IAQLEEQLDNETKER.Q** |
|  | 18511 | **618.2813** | **1851.8221** | **1851.8342** | **-0.0121** | **1** | **23** | **0.5** | **1** | **K.MEDSVGCLETAEEVKR.K** |
|  | 18710 | **934.9526** | **1867.8906** | **1867.9051** | **-0.0145** | **1** | **97** | **2e-008** | **1** | **R.IAEFTTNLTEEEEKSK.S** 18711 |
|  | 18721 | **935.4814** | **1868.9482** | **1868.9592** | **-0.0110** | **0** | **92** | **6e-008** | **1** | **K.ANLQIDQINTDLNLER.S** 18720 18738 |
|  | 18817 | **626.3577** | **1876.0512** | **1875.8381** | **0.2131** | **1** | **19** | **1.1** | **1** | **K.ERNTDQASMPDNTAAQK.V** |
|  | 18970 | **944.9629** | **1887.9112** | **1887.9247** | **-0.0135** | **1** | **113** | **4.9e-010** | **1** | **K.KLEEEQIILEDQNCK.L** 18969 18971 19000 |
|  | 19263 | **638.6226** | **1912.8460** | **1912.8585** | **-0.0124** | **1** | **6** | **23** | **3** | **R.VEEEEERCQHLQAEK.K** |
|  | 19487 | **966.0827** | **1930.1508** | **1929.9043** | **0.2465** | **0** | **2** | **59** | **6** | **R.HEMPPHIYAITDTAYR.S** |
|  | 19672 | **973.5026** | **1944.9907** | **1945.0004** | **-0.0097** | **0** | **83** | **4.7e-007** | **1** | **K.LQVELDNVTGLLSQSDSK.S** 19676 19679 19680 19716 19727 |
|  | 19752 | **650.6651** | **1948.9735** | **1948.9854** | **-0.0119** | **0** | **18** | **1.6** | **1** | **R.LQQELDDLLVDLDHQR.Q** |
|  | 19879 | **980.0175** | **1958.0204** | **1956.9462** | **1.0742** | **1** | **0** | **86** | **9** | **K.LEMDLKDLEAHIDSANK.N** 19878 |
|  | 19916 | **981.4566** | **1960.8986** | **1960.9113** | **-0.0126** | **0** | **110** | **9.5e-010** | **1** | **K.TQLEELEDELQATEDAK.L** 19915 |
|  | 20284 | **666.0028** | **1994.9866** | **1995.0021** | **-0.0155** | **1** | **65** | **3e-005** | **1** | **K.HSQAVEELAEQLEQTKR.V** 20285 20286 |
|  | 20306 | **999.5273** | **1997.0401** | **1997.0541** | **-0.0140** | **1** | **97** | **1.8e-008** | **1** | **K.KANLQIDQINTDLNLER.S** |
|  | 20530 | **1009.5308** | **2017.0470** | **2017.0554** | **-0.0084** | **0** | **71** | **8.1e-006** | **1** | **R.IIGLDQVAGMSETALPGAFK.T** 20503 20528 20529 |
|  | 20783 | **1021.9873** | **2041.9600** | **2041.9738** | **-0.0138** | **1** | **90** | **9.4e-008** | **1** | **K.TLEEEAKTHEAQIQEMR.Q** 20782 |
|  | 20866 | **1024.9794** | **2047.9442** | **2047.9554** | **-0.0112** | **0** | **67** | **2e-005** | **1** | **K.SMEAEMIQLQEELAAAER.A** |
|  | 21313 | **1044.9868** | **2087.9591** | **2087.9719** | **-0.0128** | **1** | **57** | **0.00016** | **1** | **R.QAQQERDELADEIANSSGK.G** 21312 |
|  | 22828 | **750.4149** | **2248.2228** | **2247.0874** | **1.1353** | **1** | **3** | **40** | **2** | **K.SMEAEMIQLQEELAAAERAK.R** |
|  | 23101 | **1143.5518** | **2285.0890** | **2285.1031** | **-0.0142** | **0** | **100** | **7.2e-009** | **1** | **K.VEDMAELTCLNEASVLHNLK.E** 23100 23102 |
|  | 23223 | **1150.4934** | **2298.9723** | **2298.9879** | **-0.0156** | **1** | **82** | **4.4e-007** | **1** | **R.SMMQDREDQSILCTGESGAGK.T** 23224 |
|  | 23249 | **1152.0840** | **2302.1534** | **2302.1288** | **0.0246** | **1** | **52** | **0.0005** | **1** | **K.IRELESQISELQEDLESER.A** 23246 23247 |
|  | 23479 | **1167.0245** | **2332.0345** | **2332.0488** | **-0.0143** | **0** | **135** | **2.2e-012** | **1** | **K.MQQNIQELEEQLEEEESAR.Q** 23478 |
|  | 23583 | **783.4247** | **2347.2524** | **2347.1139** | **0.1385** | **1** | **0** | **69** | **6** | **R.ELESQISELQEDLESERASR.N** |
|  | 23878 | **1193.6080** | **2385.2015** | **2384.2263** | **0.9752** | **0** | **67** | **1.5e-005** | **1** | **R.INFDVNGYIVGANIETYLLEK.S** 23875 23880 |
|  | 24335 | **818.7486** | **2453.2240** | **2453.2379** | **-0.0139** | **1** | **46** | **0.0017** | **1** | **K.YLYVDKNFINNPLAQADWAAK.K** |
|  | 24378 | **1231.0723** | **2460.1300** | **2460.1438** | **-0.0138** | **1** | **93** | **4e-008** | **1** | **K.KMQQNIQELEEQLEEEESAR.Q** 24381 |
|  | 24420 | **823.0679** | **2466.1820** | **2466.2002** | **-0.0182** | **0** | **6** | **19** | **2** | **K.LQQLFNHTMFILEQEEYQR.E** |
|  | 24459 | **1236.5830** | **2471.1515** | **2471.1663** | **-0.0148** | **0** | **103** | **3.1e-009** | **1** | **R.IAQLEEELEEEQGNTELINDR.L** 24457 24458 24460 24461 |
|  | 24625 | **1247.0846** | **2492.1546** | **2492.1667** | **-0.0120** | **0** | **80** | **7.9e-007** | **1** | **K.DFSALESQLQDTQELLQEENR.Q** 24624 24626 |
|  | 25009 | 850.7247 | 2549.1522 | 2549.2465 | -0.0943 | 1 | 3 | 32 | 2 | K.QLAAENRLTEMETLQSQLMAEK.L |
|  | 25224 | **643.5651** | **2570.2311** | **2570.2468** | **-0.0157** | **1** | **30** | **0.061** | **1** | **K.VEDMAELTCLNEASVLHNLKER.Y** |
|  | 25638 | **883.4349** | **2647.2830** | **2647.2985** | **-0.0155** | **1** | **81** | **4.7e-007** | **1** | **K.FSKVEDMAELTCLNEASVLHNLK.E** 25639 25641 |
|  | 26782 | **945.7994** | **2834.3765** | **2834.3934** | **-0.0169** | **1** | **69** | **7.4e-006** | **1** | **K.LTKDFSALESQLQDTQELLQEENR.Q** |
|  | 27596 | **1000.8221** | **2999.4444** | **3000.3523** | **-0.9079** | **1** | **5** | **18** | **3** | **K.DMFQETMEAMRIMGIPEEEQMGLLR.V** |
|  | 27609 | **751.3694** | **3001.4484** | **3000.3523** | **1.0961** | **1** | **(3)** | **25** | **7** | **K.DMFQETMEAMRIMGIPEEEQMGLLR.V** |
|  | 27637 | **1004.4554** | **3010.3443** | **3009.3783** | **0.9660** | **1** | **34** | **0.019** | **1** | **R.FLSNGHVTIPGQQDKDMFQETMEAMR.I** 27629 27636 |
|  | 27663 | **1005.8283** | **3014.4631** | **3014.4807** | **-0.0176** | **1** | **33** | **0.023** | **1** | **K.NMDPLNDNIATLLHQSSDKFVSELWK.D** 27664 27665 |
|  | 27677 | **1509.2357** | **3016.4569** | **3016.4724** | **-0.0155** | **1** | **100** | **5.5e-009** | **1** | **R.DLGEELEALKTELEDTLDSTAAQQELR.S** 27675 27676 27679 27680 27685 |
|  | 27744 | **758.6226** | **3030.4614** | **3030.4757** | **-0.0143** | **1** | **(21)** | **0.43** | **1** | **K.NMDPLNDNIATLLHQSSDKFVSELWK.D** 27745 27761 |
|  | 27768 | **759.1410** | **3032.5351** | **3032.3421** | **0.1930** | **1** | **(4)** | **19** | **1** | **K.DMFQETMEAMRIMGIPEEEQMGLLR.V** |
|  | 28246 | **1574.2373** | **3146.4600** | **3146.4812** | **-0.0212** | **1** | **86** | **1.1e-007** | **1** | **R.ALEQQVEEMKTQLEELEDELQATEDAK.L** 28247 28250 |
|  | 28309 | **1055.4946** | **3163.4621** | **3162.4761** | **0.9859** | **1** | **(3)** | **23** | **1** | **R.ALEQQVEEMKTQLEELEDELQATEDAK.L** |

| **5.** | Q00610\|CLH1_HUMAN    **Mass:** 193260   **Score:** 3344   **Queries matched:** 170   **emPAI:** 2.67 |
| --- | --- |
|  | Clathrin heavy chain 1 OS=Homo sapiens GN=CLTC PE=1 SV=5 |

|  | **Query** | **Observed** | **Mr(expt)** | **Mr(calc)** | **Delta** | **Miss** | **Score** | **Expect** | **Rank** | **Peptide** |
| --- | --- | --- | --- | --- | --- | --- | --- | --- | --- | --- |
|  | 1160 | **642.3525** | **641.3453** | **641.3497** | **-0.0044** | **0** | **11** | **11** | **2** | **R.ANVPNK.V** |
|  | 1293 | **698.3120** | **697.3047** | **697.3105** | **-0.0058** | **0** | **22** | **0.77** | **1** | **K.MYDAAK.L** 1300 |
|  | 2360 | **853.4170** | **852.4097** | **852.4164** | **-0.0066** | **0** | **24** | **0.65** | **1** | **K.YIQAACK.T** |
|  | 2509 | **432.2154** | **862.4162** | **862.4218** | **-0.0056** | **0** | **23** | **1.1** | **1** | **K.QSVELCK.K** |
|  | 2831 | **443.2058** | **884.3971** | **884.4028** | **-0.0057** | **0** | **36** | **0.042** | **1** | **R.AYEFAER.C** |
|  | 3337 | **462.8865** | **923.7585** | **923.4535** | **0.3051** | **0** | **7** | **18** | **3** | **R.VMEYINR.L** |
|  | 3611 | **472.7524** | **943.4903** | **942.4923** | **0.9980** | **0** | **4** | **78** | **2** | **K.HELIEFR.R** |
|  | 5340 | **535.2728** | **1068.5310** | **1068.5386** | **-0.0076** | **0** | **23** | **0.79** | **1** | **R.AHIAQLCEK.A** 5351 |
|  | 5391 | **536.3099** | **1070.6052** | **1070.6124** | **-0.0072** | **0** | **28** | **0.29** | **1** | **K.HDVVFLITK.Y** |
|  | 5953 | **555.8338** | **1109.6530** | **1109.6597** | **-0.0066** | **0** | **31** | **0.11** | **1** | **K.LLLPWLEAR.I** |
|  | 6756 | **584.7970** | **1167.5794** | **1168.4455** | **-0.8660** | **0** | **14** | **5.9** | **1** | **R.ESNCYDPER.V** |
|  | 6913 | **590.3400** | **1178.6655** | **1177.6343** | **1.0312** | **1** | **2** | **91** | **2** | **K.EGQFKDIITK.V** |
|  | 7914 | **614.0780** | **1226.1414** | **1225.7547** | **0.3868** | **0** | **1** | **76** | **7** | **K.QLPLVKPYLR.S** |
|  | 8030 | **617.2869** | **1232.5592** | **1231.6408** | **0.9184** | **1** | **7** | **29** | **3** | **K.VDKLDASESLR.K** |
|  | 8098 | **618.8328** | **1235.6510** | **1235.6584** | **-0.0074** | **0** | **58** | **0.00022** | **1** | **K.TLQIFNIEMK.S** 8097 |
|  | 9296 | **648.8338** | **1295.6530** | **1295.6622** | **-0.0092** | **0** | **58** | **0.00019** | **1** | **K.LLYNNVSNFGR.L** 9271 9315 |
|  | 9448 | **652.8292** | **1303.6439** | **1303.6520** | **-0.0081** | **0** | **51** | **0.0012** | **1** | **R.NNLAGAEELFAR.K** 9431 |
|  | 9997 | **444.2090** | **1329.6053** | **1329.6136** | **-0.0082** | **0** | **17** | **3.2** | **2** | **R.DPHLACVAYER.G** |
|  | 10062 | **667.8153** | **1333.6161** | **1333.6262** | **-0.0101** | **0** | **51** | **0.0012** | **1** | **K.IYIDSNNNPER.F** |
|  | 10110 | **669.3435** | **1336.6725** | **1336.6809** | **-0.0085** | **0** | **81** | **1.2e-006** | **1** | **R.VVGAMQLYSVDR.K** |
|  | 10408 | **677.4234** | **1352.8322** | **1352.8391** | **-0.0068** | **0** | **78** | **1.8e-006** | **1** | **R.NLQNLLILTAIK.A** 10441 |
|  | 10696 | **684.8477** | **1367.6809** | **1367.6906** | **-0.0097** | **0** | **49** | **0.0017** | **1** | **K.NNRPSEGPLQTR.L** 10697 10698 |
|  | 11302 | **701.8379** | **1401.6613** | **1401.6711** | **-0.0097** | **0** | **39** | **0.018** | **1** | **R.CNEPAVWSQLAK.A** |
|  | 11521 | **708.3626** | **1414.7105** | **1414.7205** | **-0.0099** | **0** | **62** | **9e-005** | **1** | **K.IVLDNSVFSEHR.N** |
|  | 11849 | **717.4002** | **1432.7857** | **1432.7926** | **-0.0068** | **0** | **63** | **7e-005** | **1** | **K.SVDPTLALSVYLR.A** 11833 11848 11850 |
|  | 12279 | **730.3465** | **1458.6784** | **1458.6891** | **-0.0107** | **1** | **15** | **3.9** | **1** | **R.FLRENPYYDSR.V** |
|  | 12373 | **732.8674** | **1463.7203** | **1463.7296** | **-0.0093** | **0** | **34** | **0.047** | **1** | **R.ALEHFTDLYDIK.R** 12374 12375 |
|  | 12389 | **733.3902** | **1464.7658** | **1464.7759** | **-0.0100** | **1** | **51** | **0.00096** | **1** | **R.VVGAMQLYSVDRK.V** |
|  | 12734 | 743.8596 | 1485.7046 | 1485.7147 | -0.0101 | 1 | 21 | 1.1 | 1 | K.RDPHLACVAYER.G |
|  | 12984 | **750.4152** | **1498.8159** | **1498.8256** | **-0.0097** | **0** | **47** | **0.0023** | **1** | **K.WLLLTGISAQQNR.V** 12985 |
|  | 13047 | **501.9188** | **1502.7345** | **1502.7452** | **-0.0108** | **1** | **20** | **1.2** | **1** | **K.MREHLELFWSR.V** |
|  | 13111 | **754.3767** | **1506.7389** | **1506.7501** | **-0.0112** | **0** | **74** | **4.8e-006** | **1** | **K.VIQCFAETGQVQK.I** |
|  | 13302 | **759.8725** | **1517.7304** | **1517.7409** | **-0.0104** | **0** | **47** | **0.0026** | **1** | **R.HSSLAGCQIINYR.T** 13301 13303 |
|  | 13856 | 776.3754 | 1550.7362 | 1550.7464 | -0.0102 | 0 | 64 | 5.2e-005 | 1 | R.GQFSTDELVAEVEK.R 13857 |
|  | 13932 | **778.8951** | **1555.7757** | **1556.7980** | **-1.0223** | **0** | **(2)** | **73** | **3** | **R.RPISADSAIMNPASK.V** 13948 |
|  | 14202 | **787.7786** | **1573.5426** | **1572.7930** | **0.7496** | **0** | **3** | **51** | **5** | **R.RPISADSAIMNPASK.V** |
|  | 14739 | **804.4467** | **1606.8788** | **1606.8871** | **-0.0084** | **1** | **80** | **1.2e-006** | **1** | **K.KVGYTPDWIFLLR.N** 14737 14738 |
|  | 14804 | **1612.8370** | **1611.8298** | **1611.8396** | **-0.0098** | **0** | **34** | **0.044** | **1** | **R.ESYVETELIFALAK.T** 14806 14807 |
|  | 14965 | **540.9476** | **1619.8209** | **1619.8307** | **-0.0099** | **1** | **43** | **0.006** | **1** | **R.ALEHFTDLYDIKR.A** 14964 |
|  | 15111 | **815.8914** | **1629.7683** | **1629.7787** | **-0.0104** | **0** | **44** | **0.0053** | **1** | **K.FNALFAQGNYSEAAK.V** |
|  | 16143 | **566.2503** | **1695.7291** | **1695.0043** | **0.7248** | **1** | **7** | **23** | **3** | **R.NLQNLLILTAIKADR.T** |
|  | 16319 | **854.4251** | **1706.8355** | **1706.8475** | **-0.0120** | **1** | **93** | **5.3e-008** | **1** | **R.GQFSTDELVAEVEKR.N** |
|  | 16470 | **858.8923** | **1715.7701** | **1715.8631** | **-0.0930** | **0** | **1** | **96** | **9** | **K.VSQPIEGHAASFAQFK.M** |
|  | 17009 | **584.6625** | **1750.9658** | **1749.8798** | **1.0860** | **1** | **4** | **45** | **5** | **K.IYIDSNNNPERFLR.E** |
|  | 17106 | **879.9382** | **1757.8618** | **1757.8736** | **-0.0119** | **1** | **91** | **8.1e-008** | **1** | **R.KFNALFAQGNYSEAAK.V** 17105 |
|  | 17317 | **888.9150** | **1775.8155** | **1775.8260** | **-0.0105** | **0** | **79** | **1.3e-006** | **1** | **R.IHEGCEEPATHNALAK.I** 17312 17314 17315 17316 17318 |
|  | 18386 | **922.9484** | **1843.8823** | **1843.9581** | **-0.0758** | **1** | **50** | **0.0011** | **1** | **R.KVSQPIEGHAASFAQFK.M** 18387 18388 |
|  | 18432 | **924.4681** | **1846.9216** | **1846.9359** | **-0.0143** | **0** | **44** | **0.0042** | **1** | **K.YESLELCRPVLQQGR.K** |
|  | 18624 | **930.5456** | **1859.0766** | **1859.8182** | **-0.7416** | **0** | **3** | **48** | **4** | **K.MEGNAEESTLFCFAVR.G** 18605 18607 |
|  | 19619 | **971.9538** | **1941.8930** | **1941.9068** | **-0.0138** | **0** | **101** | **7.5e-009** | **1** | **R.TSIDAYDNFDNISLAQR.L** 19620 |
|  | 19649 | **973.0247** | **1944.0349** | **1944.0469** | **-0.0120** | **0** | **69** | **1.2e-005** | **1** | **K.LHIIEVGTPPTGNQPFPK.K** 19650 |
|  | 19720 | **974.0068** | **1945.9991** | **1946.0070** | **-0.0079** | **0** | **84** | **3.8e-007** | **1** | **K.AFMTADLPNELIELLEK.I** 19717 19718 19719 |
|  | 20008 | **986.0126** | **1970.0107** | **1970.0221** | **-0.0114** | **0** | **75** | **2.7e-006** | **1** | **R.LASTLVHLGEYQAAVDGAR.K** |
|  | 20148 | **992.9735** | **1983.9325** | **1983.9409** | **-0.0084** | **0** | **56** | **0.00022** | **1** | **R.HNIMDFAMPYFIQVMK.E** 20159 |
|  | 20762 | **1021.0419** | **2040.0693** | **2040.0813** | **-0.0120** | **0** | **84** | **3.4e-007** | **1** | **R.LPVVIGGLLDVDCSEDVIK.N** |
|  | 21259 | **695.6557** | **2083.9453** | **2082.9727** | **0.9725** | **1** | **2** | **56** | **1** | **K.MFDRHSSLAGCQIINYR.T** |
|  | 21412 | **1049.5426** | **2097.0706** | **2097.1616** | **-0.0909** | **1** | **2** | **52** | **3** | **R.RPISADSAIMNPASKVIALK.A** 21413 |
|  | 21649 | **707.4123** | **2119.2152** | **2120.0313** | **-0.8161** | **0** | **2** | **49** | **5** | **K.DTELAEELLQWFLQEEK.R** |
|  | 21917 | **1076.9976** | **2151.9806** | **2151.9929** | **-0.0123** | **0** | **74** | **3.5e-006** | **1** | **R.GQCDLELINVCNENSLFK.S** 21915 21916 |
|  | 22280 | **1098.0525** | **2194.0904** | **2194.1052** | **-0.0148** | **0** | **83** | **4.4e-007** | **1** | **K.VGEQAQVVIIDMNDPSNPIR.R** 22279 |
|  | 22915 | **754.3733** | **2260.0982** | **2259.1827** | **0.9155** | **1** | **2** | **51** | **5** | **R.AMLSANIRQNLQICVQVASK.Y** |
|  | 23632 | **1177.1217** | **2352.2288** | **2352.2438** | **-0.0149** | **0** | **98** | **1.2e-008** | **1** | **R.ISGETIFVTAPHEATAGIIGVNR.K** 23631 23633 |
|  | 23647 | **1178.0700** | **2354.1253** | **2354.1390** | **-0.0136** | **0** | **114** | **3e-010** | **1** | **K.SVNESLNNLFITEEDYQALR.T** 23646 23648 23649 23650 |
|  | 23763 | **790.0874** | **2367.2404** | **2367.2547** | **-0.0143** | **1** | **45** | **0.0022** | **1** | **R.KFDVNTSAVQVLIEHIGNLDR.A** 23765 23766 |
|  | 23847 | **794.6973** | **2381.0702** | **2381.0820** | **-0.0119** | **0** | **20** | **0.73** | **1** | **K.YGYIHLYDLETGTCIYMNR.I** |
|  | 24299 | **1226.1097** | **2450.2049** | **2451.2428** | **-1.0378** | **1** | **20** | **0.79** | **1** | **R.EKVGEQAQVVIIDMNDPSNPIR.R** |
|  | 24395 | **1232.6060** | **2463.1974** | **2463.2142** | **-0.0169** | **0** | **91** | **5.9e-008** | **1** | **R.LAELEEFINGPNNAHIQQVGDR.C** 24394 24396 24404 24405 24407 24411 |
|  | 24499 | **827.4940** | **2479.4602** | **2480.3387** | **-0.8785** | **1** | **5** | **18** | **2** | **R.ISGETIFVTAPHEATAGIIGVNRK.G** |
|  | 25371 | **865.4624** | **2593.3654** | **2593.3786** | **-0.0132** | **1** | **34** | **0.027** | **1** | **K.VNPSRLPVVIGGLLDVDCSEDVIK.N** |
|  | 26090 | **903.1301** | **2706.3684** | **2706.3363** | **0.0320** | **1** | **36** | **0.015** | **1** | **K.KAVDVFFPPEAQNDFPVAMQISEK.H** |
|  | 26250 | **1370.6152** | **2739.2159** | **2739.2334** | **-0.0175** | **0** | **93** | **2.7e-008** | **1** | **K.ADDPSSYMEVVQAANTSGNWEELVK.Y** 26249 26251 26259 |
|  | 27002 | **1441.2531** | **2880.4915** | **2880.5080** | **-0.0165** | **0** | **76** | **1.4e-006** | **1** | **R.RPLIDQVVQTALSETQDPEEVSVTVK.A** 27001 27003 |
|  | 27930 | **1536.2485** | **3070.4825** | **3070.4923** | **-0.0098** | **0** | **96** | **1.2e-008** | **1** | **R.LDNYDAPDIANIAISNELFEEAFAIFR.K** 27929 |
|  | 28004 | **1031.1497** | **3090.4272** | **3090.4402** | **-0.0130** | **0** | **5** | **14** | **2** | **R.ECFGACLFTCYDLLRPDVVLETAWR.H** |
|  | 28158 | **1043.5027** | **3127.4862** | **3127.5041** | **-0.0179** | **0** | **48** | **0.00073** | **1** | **R.LLEMNLMHAPQVADAILGNQMFTHYDR.A** 28157 28161 28162 |
|  | 28230 | **786.8777** | **3143.4819** | **3143.4991** | **-0.0172** | **0** | **(22)** | **0.3** | **1** | **R.LLEMNLMHAPQVADAILGNQMFTHYDR.A** 28231 28237 |
|  | 28232 | **786.8787** | **3143.4858** | **3143.4991** | **-0.0133** | **0** | **(7)** | **10** | **1** | **R.LLEMNLMHAPQVADAILGNQMFTHYDR.A** |
|  | 28358 | **1058.8523** | **3173.5350** | **3173.5492** | **-0.0141** | **0** | **51** | **0.00036** | **1** | **K.WISLNTVALVTDNAVYHWSMEGESQPVK.M** 28361 28365 28370 28372 |
|  | 28443 | **1594.3339** | **3186.6532** | **3186.6714** | **-0.0182** | **0** | **79** | **5.4e-007** | **1** | **R.FQSVPAQPGQTSPLLQYFGILLDQGQLNK.Y** 28442 |
|  | 28451 | **798.4153** | **3189.6323** | **3189.5441** | **0.0882** | **0** | **(1)** | **39** | **1** | **K.WISLNTVALVTDNAVYHWSMEGESQPVK.M** |
|  | 28656 | **1077.8348** | **3230.4827** | **3230.4965** | **-0.0138** | **1** | **33** | **0.024** | **1** | **K.DAMQYASESKDTELAEELLQWFLQEEK.R** |
|  | 29304 | **1142.2709** | **3423.7908** | **3423.8072** | **-0.0164** | **1** | **31** | **0.028** | **1** | **R.KGQVLSVCVEEENIIPYITNVLQNPDLALR.M** 29299 29300 29301 29302 |
|  | 29481 | **1166.5847** | **3496.7323** | **3496.7449** | **-0.0126** | **0** | **11** | **3.1** | **1** | **R.AVVHTHLLNPEWLVNYFGSLSVEDSLECLR.A** |
|  | 29847 | **1220.5670** | **3658.6792** | **3658.6984** | **-0.0192** | **1** | **92** | **2e-008** | **1** | **K.EAIDSYIKADDPSSYMEVVQAANTSGNWEELVK.Y** 29846 |
|  | 30016 | **1245.6371** | **3733.8894** | **3732.8927** | **0.9967** | **1** | **22** | **0.24** | **1** | **K.VGEQAQVVIIDMNDPSNPIRRPISADSAIMNPASK.V** |
|  | 30021 | **1246.9878** | **3737.9415** | **3738.7811** | **-0.8395** | **1** | **2** | **22** | **10** | **K.WISLNTVALVTDNAVYHWSMEGESQPVKMFDR.H** |
|  | 30037 | **625.8077** | **3748.8028** | **3748.8876** | **-0.0848** | **1** | **(1)** | **28** | **1** | **K.VGEQAQVVIIDMNDPSNPIRRPISADSAIMNPASK.V** |
|  | 30215 | **1278.9763** | **3833.9071** | **3833.9233** | **-0.0161** | **1** | **58** | **5e-005** | **1** | **R.FQEHLQLQNLGINPANIGFSTLTMESDKFICIR.E** |
|  | 31288 | **1255.1552** | **5016.5915** | **5015.5967** | **0.9948** | **1** | **11** | **1.1** | **1** | **R.FQSVPAQPGQTSPLLQYFGILLDQGQLNKYESLELCRPVLQQGR.K** 31289 |
|  | 31764 | **1181.3336** | **5901.6317** | **5901.7859** | **-0.1542** | **1** | **1** | **6.5** | **2** | **R.KEEEQATETQPIVYGQPQLMLTAGPSVAVPPQAPFGYGYTAPPYGQPQPGFGYSM.-** |

| **6.** | P68032\|ACTC_HUMAN    **Mass:** 42334    **Score:** 2050   **Queries matched:** 173   **emPAI:** 16.93 |
| --- | --- |
|  | Actin, alpha cardiac muscle 1 OS=Homo sapiens GN=ACTC1 PE=1 SV=1 |

|  | **Query** | **Observed** | **Mr(expt)** | **Mr(calc)** | **Delta** | **Miss** | **Score** | **Expect** | **Rank** | **Peptide** |
| --- | --- | --- | --- | --- | --- | --- | --- | --- | --- | --- |
|  | 1138 | 632.2894 | 631.2822 | 630.3701 | 0.9121 | 0 | 14 | 11 | 1 | K.ILTER.G 1129 1139 |
|  | 1169 | 644.3676 | 643.3603 | 643.3653 | -0.0050 | 0 | 25 | 0.84 | 1 | R.LDLAGR.D |
|  | 1578 | 398.2373 | 794.4600 | 794.4650 | -0.0050 | 0 | 22 | 0.87 | 1 | K.IIAPPER.K 1587 |
|  | 1635 | 400.7684 | 799.5222 | 799.5280 | -0.0057 | 1 | 24 | 0.79 | 6 | K.RGILTLK.Y 1646 |
|  | 3263 | **460.2336** | **918.4526** | **918.4593** | **-0.0067** | **1** | **19** | **2.5** | **1** | **K.CDIDIRK.D** |
|  | 3326 | 462.2838 | 922.5531 | 922.5600 | -0.0068 | 1 | 28 | 0.22 | 1 | K.IIAPPERK.Y 3327 3328 |
|  | 4062 | 488.7242 | 975.4339 | 975.4410 | -0.0071 | 0 | 74 | 7.9e-006 | 1 | K.AGFAGDDAPR.A 4063 4064 4068 4075 |
|  | 4370 | 499.7436 | 997.4726 | 997.4790 | -0.0064 | 0 | 44 | 0.0062 | 1 | R.DLTDYLMK.I 4366 4368 4369 4372 4401 |
|  | 4907 | 518.8259 | 1035.6373 | 1035.6440 | -0.0067 | 1 | 25 | 0.45 | 1 | K.IKIIAPPER.K |
|  | 6669 | 581.3090 | 1160.6034 | 1160.6111 | -0.0077 | 0 | 44 | 0.006 | 1 | K.EITALAPSTMK.I 6667 6668 6670 6671 6685 |
|  | 6796 | 586.2847 | 1170.5549 | 1170.5638 | -0.0089 | 0 | 55 | 0.00046 | 1 | R.HQGVMVGMGQK.D 6797 6798 6799 6800 |
|  | 6883 | 589.3063 | 1176.5981 | 1176.6060 | -0.0079 | 0 | (42) | 0.011 | 1 | K.EITALAPSTMK.I 6885 |
|  | 7046 | 593.7484 | 1185.4823 | 1186.5587 | -1.0764 | 0 | (42) | 0.0095 | 1 | R.HQGVMVGMGQK.D 7072 7074 7076 |
|  | 7069 | 594.2817 | 1186.5489 | 1186.5587 | -0.0098 | 0 | (41) | 0.014 | 1 | R.HQGVMVGMGQK.D 7057 7068 7070 7071 7075 |
|  | 7313 | 599.7607 | 1197.5068 | 1197.5150 | -0.0081 | 0 | 44 | 0.0055 | 1 | K.DSYVGDEAQSK.R 7289 7296 7311 |
|  | 7330 | 599.8522 | 1197.6899 | 1197.6982 | -0.0083 | 0 | 58 | 0.00026 | 1 | R.AVFPSIVGRPR.H 7327 7328 7329 7331 7332 7333 7336 7342 |
|  | 7433 | 602.2789 | 1202.5433 | 1202.5536 | -0.0103 | 0 | (26) | 0.42 | 1 | R.HQGVMVGMGQK.D 7436 7437 |
|  | 10424 | 677.8104 | 1353.6062 | 1353.6161 | -0.0099 | 1 | 51 | 0.0011 | 1 | K.DSYVGDEAQSKR.G 10395 10415 10416 10417 10418 10419 10420 10421 10422 10423 10425 10426 10427 10429 10430 10445 10448 10450 |
|  | 12997 | **750.8526** | **1499.6906** | **1499.7005** | **-0.0098** | **0** | **36** | **0.029** | **1** | **K.QEYDEAGPSIVHR.K** 12977 12996 |
|  | 13245 | 758.3734 | 1514.7323 | 1514.7419 | -0.0096 | 0 | 45 | 0.0039 | 1 | K.IWHHTFYNELR.V 13243 13244 13248 13267 |
|  | 13795 | 774.9045 | 1547.7944 | 1547.8051 | -0.0107 | 1 | 16 | 3 | 1 | R.MQKEITALAPSTMK.I |
|  | 14290 | 395.7159 | 1578.8347 | 1579.7949 | -0.9603 | 1 | (4) | 50 | 8 | R.MQKEITALAPSTMK.I |
|  | 15010 | 812.4193 | 1622.8239 | 1622.8338 | -0.0098 | 1 | 27 | 0.24 | 1 | R.LDLAGRDLTDYLMK.I 15009 |
|  | 15068 | **814.3831** | **1626.7516** | **1627.7954** | **-1.0438** | **1** | **5** | **34** | **4** | **K.QEYDEAGPSIVHRK.C** |
|  | 17562 | 895.9443 | 1789.8740 | 1789.8846 | -0.0106 | 0 | 72 | 7.6e-006 | 1 | K.SYELPDGQVITIGNER.F 17551 17553 17560 17561 17563 17564 17565 17566 17568 17569 17571 17572 17573 17574 17576 17577 17578 17579 17580 17581 17582 17583 17584 17585 17586 17587 17588 17591 17592 17593 17595 17596 17609 17610 17612 17613 17614 17616 17617 |
|  | 19826 | **978.5183** | **1955.0221** | **1955.0364** | **-0.0143** | **0** | **55** | **0.00032** | **1** | **R.VAPEEHPTLLTEAPLNPK.A** 19828 19829 19831 |
|  | 19898 | **980.9523** | **1959.8900** | **1959.9036** | **-0.0137** | **0** | **53** | **0.00045** | **1** | **K.YPIEHGIITNWDDMEK.I** 19896 19897 19899 19900 19901 19912 |
|  | 22647 | **1114.5292** | **2227.0438** | **2227.0579** | **-0.0141** | **0** | **(86)** | **1.9e-007** | **1** | **K.DLYANNVLSGGTTMYPGIADR.M** 22648 22663 |
|  | 22804 | **1122.5265** | **2243.0384** | **2243.0528** | **-0.0144** | **0** | **104** | **3.1e-009** | **1** | **K.DLYANNVLSGGTTMYPGIADR.M** |
|  | 23659 | **1178.5748** | **2355.1351** | **2355.1529** | **-0.0178** | **1** | **91** | **5.5e-008** | **1** | **R.KDLYANNVLSGGTTMYPGIADR.M** 23660 23661 |
|  | 23781 | **791.0848** | **2370.2325** | **2371.1478** | **-0.9153** | **1** | **(2)** | **49** | **10** | **R.KDLYANNVLSGGTTMYPGIADR.M** |
|  | 24892 | **1268.5756** | **2535.1366** | **2535.1509** | **-0.0143** | **0** | **127** | **1.3e-011** | **1** | **K.LCYVALDFENEMATAASSSSLEK.S** 24893 24894 |
|  | 25114 | 1276.0881 | 2550.1617 | 2551.1458 | -0.9841 | 0 | (32) | 0.042 | 2 | K.LCYVALDFENEMATAASSSSLEK.S 25112 25113 |
|  | 25421 | 868.1128 | 2601.3165 | 2601.3301 | -0.0136 | 0 | 5 | 23 | 1 | K.YSVWIGGSILASLSTFQQMWISK.Q 25417 |
|  | 26526 | **931.7641** | **2792.2705** | **2792.2884** | **-0.0179** | **1** | **23** | **0.3** | **1** | **K.EKLCYVALDFENEMATAASSSSLEK.S** |
|  | 26641 | **937.3879** | **2809.1420** | **2808.2833** | **0.8587** | **1** | **(13)** | **2.4** | **1** | **K.EKLCYVALDFENEMATAASSSSLEK.S** |
|  | 28446 | **1063.4899** | **3187.4478** | **3187.4664** | **-0.0186** | **0** | **72** | **2.9e-006** | **1** | **R.CPETLFQPSFIGMESAGIHETTYNSIMK.C** |
|  | 28473 | **1066.2025** | **3195.5857** | **3195.6023** | **-0.0166** | **0** | **75** | **1.6e-006** | **1** | **R.TTGIVLDSGDGVTHNVPIYEGYALPHAIMR.L** 28469 28470 |
|  | 29467 | **1164.8806** | **3491.6200** | **3490.6360** | **0.9841** | **1** | **55** | **0.00012** | **1** | **R.FRCPETLFQPSFIGMESAGIHETTYNSIMK.C** 29461 29462 |
|  | 30413 | **994.7351** | **3974.9111** | **3975.8151** | **-0.9040** | **1** | **2** | **19** | **6** | **R.CPETLFQPSFIGMESAGIHETTYNSIMKCDIDIR.K** 30416 |

| **7.** | P62736\|ACTA_HUMAN    **Mass:** 42381    **Score:** 2022   **Queries matched:** 169   **emPAI:** 14.02 |
| --- | --- |
|  | Actin, aortic smooth muscle OS=Homo sapiens GN=ACTA2 PE=1 SV=1 |

|  | **Query** | **Observed** | **Mr(expt)** | **Mr(calc)** | **Delta** | **Miss** | **Score** | **Expect** | **Rank** | **Peptide** |
| --- | --- | --- | --- | --- | --- | --- | --- | --- | --- | --- |
|  | 1138 | 632.2894 | 631.2822 | 630.3701 | 0.9121 | 0 | 14 | 11 | 1 | K.ILTER.G 1129 1139 |
|  | 1169 | 644.3676 | 643.3603 | 643.3653 | -0.0050 | 0 | 25 | 0.84 | 1 | R.LDLAGR.D |
|  | 1578 | 398.2373 | 794.4600 | 794.4650 | -0.0050 | 0 | 22 | 0.87 | 1 | K.IIAPPER.K 1587 |
|  | 1635 | 400.7684 | 799.5222 | 799.5280 | -0.0057 | 1 | 24 | 0.79 | 6 | K.RGILTLK.Y 1646 |
|  | 3263 | 460.2336 | 918.4526 | 918.4593 | -0.0067 | 1 | 19 | 2.5 | 1 | K.CDIDIRK.D |
|  | 3326 | 462.2838 | 922.5531 | 922.5600 | -0.0068 | 1 | 28 | 0.22 | 1 | K.IIAPPERK.Y 3327 3328 |
|  | 4062 | 488.7242 | 975.4339 | 975.4410 | -0.0071 | 0 | 74 | 7.9e-006 | 1 | K.AGFAGDDAPR.A 4063 4064 4068 4075 |
|  | 4370 | 499.7436 | 997.4726 | 997.4790 | -0.0064 | 0 | 44 | 0.0062 | 1 | R.DLTDYLMK.I 4366 4368 4369 4372 4401 |
|  | 4907 | 518.8259 | 1035.6373 | 1035.6440 | -0.0067 | 1 | 25 | 0.45 | 1 | K.IKIIAPPER.K |
|  | 6669 | 581.3090 | 1160.6034 | 1160.6111 | -0.0077 | 0 | 44 | 0.006 | 1 | K.EITALAPSTMK.I 6667 6668 6670 6671 6685 |
|  | 6796 | 586.2847 | 1170.5549 | 1170.5638 | -0.0089 | 0 | 55 | 0.00046 | 1 | R.HQGVMVGMGQK.D 6797 6798 6799 6800 |
|  | 6883 | 589.3063 | 1176.5981 | 1176.6060 | -0.0079 | 0 | (42) | 0.011 | 1 | K.EITALAPSTMK.I 6885 |
|  | 7046 | 593.7484 | 1185.4823 | 1186.5587 | -1.0764 | 0 | (42) | 0.0095 | 1 | R.HQGVMVGMGQK.D 7072 7074 7076 |
|  | 7069 | 594.2817 | 1186.5489 | 1186.5587 | -0.0098 | 0 | (41) | 0.014 | 1 | R.HQGVMVGMGQK.D 7057 7068 7070 7071 7075 |
|  | 7313 | 599.7607 | 1197.5068 | 1197.5150 | -0.0081 | 0 | 44 | 0.0055 | 1 | K.DSYVGDEAQSK.R 7289 7296 7311 |
|  | 7330 | 599.8522 | 1197.6899 | 1197.6982 | -0.0083 | 0 | 58 | 0.00026 | 1 | R.AVFPSIVGRPR.H 7327 7328 7329 7331 7332 7333 7336 7342 |
|  | 7433 | 602.2789 | 1202.5433 | 1202.5536 | -0.0103 | 0 | (26) | 0.42 | 1 | R.HQGVMVGMGQK.D 7436 7437 |
|  | 10424 | 677.8104 | 1353.6062 | 1353.6161 | -0.0099 | 1 | 51 | 0.0011 | 1 | K.DSYVGDEAQSKR.G 10395 10415 10416 10417 10418 10419 10420 10421 10422 10423 10425 10426 10427 10429 10430 10445 10448 10450 |
|  | 12997 | 750.8526 | 1499.6906 | 1499.7005 | -0.0098 | 0 | 36 | 0.029 | 1 | K.QEYDEAGPSIVHR.K 12977 12996 |
|  | 13011 | **501.2465** | **1500.7176** | **1500.7262** | **-0.0087** | **0** | **15** | **4.4** | **1** | **K.IWHHSFYNELR.V** |
|  | 13795 | 774.9045 | 1547.7944 | 1547.8051 | -0.0107 | 1 | 16 | 3 | 1 | R.MQKEITALAPSTMK.I |
|  | 14290 | 395.7159 | 1578.8347 | 1579.7949 | -0.9603 | 1 | (4) | 50 | 8 | R.MQKEITALAPSTMK.I |
|  | 15010 | 812.4193 | 1622.8239 | 1622.8338 | -0.0098 | 1 | 27 | 0.24 | 1 | R.LDLAGRDLTDYLMK.I 15009 |
|  | 15068 | 814.3831 | 1626.7516 | 1627.7954 | -1.0438 | 1 | 5 | 34 | 4 | K.QEYDEAGPSIVHRK.C |
|  | 17562 | 895.9443 | 1789.8740 | 1789.8846 | -0.0106 | 0 | 72 | 7.6e-006 | 1 | K.SYELPDGQVITIGNER.F 17551 17553 17560 17561 17563 17564 17565 17566 17568 17569 17571 17572 17573 17574 17576 17577 17578 17579 17580 17581 17582 17583 17584 17585 17586 17587 17588 17591 17592 17593 17595 17596 17609 17610 17612 17613 17614 17616 17617 |
|  | 19826 | 978.5183 | 1955.0221 | 1955.0364 | -0.0143 | 0 | 55 | 0.00032 | 1 | R.VAPEEHPTLLTEAPLNPK.A 19828 19829 19831 |
|  | 19898 | 980.9523 | 1959.8900 | 1959.9036 | -0.0137 | 0 | 53 | 0.00045 | 1 | K.YPIEHGIITNWDDMEK.I 19896 19897 19899 19900 19901 19912 |
|  | 22647 | 1114.5292 | 2227.0438 | 2227.0579 | -0.0141 | 0 | (86) | 1.9e-007 | 1 | K.DLYANNVLSGGTTMYPGIADR.M 22648 22663 |
|  | 22804 | 1122.5265 | 2243.0384 | 2243.0528 | -0.0144 | 0 | 104 | 3.1e-009 | 1 | K.DLYANNVLSGGTTMYPGIADR.M |
|  | 23659 | 1178.5748 | 2355.1351 | 2355.1529 | -0.0178 | 1 | 91 | 5.5e-008 | 1 | R.KDLYANNVLSGGTTMYPGIADR.M 23660 23661 |
|  | 23781 | 791.0848 | 2370.2325 | 2371.1478 | -0.9153 | 1 | (2) | 49 | 10 | R.KDLYANNVLSGGTTMYPGIADR.M |
|  | 24892 | 1268.5756 | 2535.1366 | 2535.1509 | -0.0143 | 0 | 127 | 1.3e-011 | 1 | K.LCYVALDFENEMATAASSSSLEK.S 24893 24894 |
|  | 25114 | 1276.0881 | 2550.1617 | 2551.1458 | -0.9841 | 0 | (32) | 0.042 | 2 | K.LCYVALDFENEMATAASSSSLEK.S 25112 25113 |
|  | 25421 | 868.1128 | 2601.3165 | 2601.3301 | -0.0136 | 0 | 5 | 23 | 1 | K.YSVWIGGSILASLSTFQQMWISK.Q 25417 |
|  | 26526 | 931.7641 | 2792.2705 | 2792.2884 | -0.0179 | 1 | 23 | 0.3 | 1 | K.EKLCYVALDFENEMATAASSSSLEK.S |
|  | 26641 | 937.3879 | 2809.1420 | 2808.2833 | 0.8587 | 1 | (13) | 2.4 | 1 | K.EKLCYVALDFENEMATAASSSSLEK.S |
|  | 28446 | 1063.4899 | 3187.4478 | 3187.4664 | -0.0186 | 0 | 72 | 2.9e-006 | 1 | R.CPETLFQPSFIGMESAGIHETTYNSIMK.C |
|  | 28473 | 1066.2025 | 3195.5857 | 3195.6023 | -0.0166 | 0 | 75 | 1.6e-006 | 1 | R.TTGIVLDSGDGVTHNVPIYEGYALPHAIMR.L 28469 28470 |
|  | 29467 | 1164.8806 | 3491.6200 | 3490.6360 | 0.9841 | 1 | 55 | 0.00012 | 1 | R.FRCPETLFQPSFIGMESAGIHETTYNSIMK.C 29461 29462 |
|  | 30413 | 994.7351 | 3974.9111 | 3975.8151 | -0.9040 | 1 | 2 | 19 | 6 | R.CPETLFQPSFIGMESAGIHETTYNSIMKCDIDIR.K 30416 |

| **8.** | P63267\|ACTH_HUMAN    **Mass:** 42249    **Score:** 1996   **Queries matched:** 164   **emPAI:** 12.90 |
| --- | --- |
|  | Actin, gamma-enteric smooth muscle OS=Homo sapiens GN=ACTG2 PE=1 SV=1 |

|  | **Query** | **Observed** | **Mr(expt)** | **Mr(calc)** | **Delta** | **Miss** | **Score** | **Expect** | **Rank** | **Peptide** |
| --- | --- | --- | --- | --- | --- | --- | --- | --- | --- | --- |
|  | 1138 | 632.2894 | 631.2822 | 630.3701 | 0.9121 | 0 | 14 | 11 | 1 | K.ILTER.G 1129 1139 |
|  | 1169 | 644.3676 | 643.3603 | 643.3653 | -0.0050 | 0 | 25 | 0.84 | 1 | R.LDLAGR.D |
|  | 1578 | 398.2373 | 794.4600 | 794.4650 | -0.0050 | 0 | 22 | 0.87 | 1 | K.IIAPPER.K 1587 |
|  | 1635 | 400.7684 | 799.5222 | 799.5280 | -0.0057 | 1 | 24 | 0.79 | 6 | K.RGILTLK.Y 1646 |
|  | 3263 | 460.2336 | 918.4526 | 918.4593 | -0.0067 | 1 | 19 | 2.5 | 1 | K.CDIDIRK.D |
|  | 3326 | 462.2838 | 922.5531 | 922.5600 | -0.0068 | 1 | 28 | 0.22 | 1 | K.IIAPPERK.Y 3327 3328 |
|  | 4062 | 488.7242 | 975.4339 | 975.4410 | -0.0071 | 0 | 74 | 7.9e-006 | 1 | K.AGFAGDDAPR.A 4063 4064 4068 4075 |
|  | 4370 | 499.7436 | 997.4726 | 997.4790 | -0.0064 | 0 | 44 | 0.0062 | 1 | R.DLTDYLMK.I 4366 4368 4369 4372 4401 |
|  | 4907 | 518.8259 | 1035.6373 | 1035.6440 | -0.0067 | 1 | 25 | 0.45 | 1 | K.IKIIAPPER.K |
|  | 6669 | 581.3090 | 1160.6034 | 1160.6111 | -0.0077 | 0 | 44 | 0.006 | 1 | K.EITALAPSTMK.I 6667 6668 6670 6671 6685 |
|  | 6796 | 586.2847 | 1170.5549 | 1170.5638 | -0.0089 | 0 | 55 | 0.00046 | 1 | R.HQGVMVGMGQK.D 6797 6798 6799 6800 |
|  | 6883 | 589.3063 | 1176.5981 | 1176.6060 | -0.0079 | 0 | (42) | 0.011 | 1 | K.EITALAPSTMK.I 6885 |
|  | 7046 | 593.7484 | 1185.4823 | 1186.5587 | -1.0764 | 0 | (42) | 0.0095 | 1 | R.HQGVMVGMGQK.D 7072 7074 7076 |
|  | 7069 | 594.2817 | 1186.5489 | 1186.5587 | -0.0098 | 0 | (41) | 0.014 | 1 | R.HQGVMVGMGQK.D 7057 7068 7070 7071 7075 |
|  | 7313 | 599.7607 | 1197.5068 | 1197.5150 | -0.0081 | 0 | 44 | 0.0055 | 1 | K.DSYVGDEAQSK.R 7289 7296 7311 |
|  | 7330 | 599.8522 | 1197.6899 | 1197.6982 | -0.0083 | 0 | 58 | 0.00026 | 1 | R.AVFPSIVGRPR.H 7327 7328 7329 7331 7332 7333 7336 7342 |
|  | 7433 | 602.2789 | 1202.5433 | 1202.5536 | -0.0103 | 0 | (26) | 0.42 | 1 | R.HQGVMVGMGQK.D 7436 7437 |
|  | 10424 | 677.8104 | 1353.6062 | 1353.6161 | -0.0099 | 1 | 51 | 0.0011 | 1 | K.DSYVGDEAQSKR.G 10395 10415 10416 10417 10418 10419 10420 10421 10422 10423 10425 10426 10427 10429 10430 10445 10448 10450 |
|  | 13011 | 501.2465 | 1500.7176 | 1500.7262 | -0.0087 | 0 | 15 | 4.4 | 1 | K.IWHHSFYNELR.V |
|  | 13795 | 774.9045 | 1547.7944 | 1547.8051 | -0.0107 | 1 | 16 | 3 | 1 | R.MQKEITALAPSTMK.I |
|  | 14290 | 395.7159 | 1578.8347 | 1579.7949 | -0.9603 | 1 | (4) | 50 | 8 | R.MQKEITALAPSTMK.I |
|  | 15010 | 812.4193 | 1622.8239 | 1622.8338 | -0.0098 | 1 | 27 | 0.24 | 1 | R.LDLAGRDLTDYLMK.I 15009 |
|  | 17562 | 895.9443 | 1789.8740 | 1789.8846 | -0.0106 | 0 | 72 | 7.6e-006 | 1 | K.SYELPDGQVITIGNER.F 17551 17553 17560 17561 17563 17564 17565 17566 17568 17569 17571 17572 17573 17574 17576 17577 17578 17579 17580 17581 17582 17583 17584 17585 17586 17587 17588 17591 17592 17593 17595 17596 17609 17610 17612 17613 17614 17616 17617 |
|  | 19826 | 978.5183 | 1955.0221 | 1955.0364 | -0.0143 | 0 | 55 | 0.00032 | 1 | R.VAPEEHPTLLTEAPLNPK.A 19828 19829 19831 |
|  | 19898 | 980.9523 | 1959.8900 | 1959.9036 | -0.0137 | 0 | 53 | 0.00045 | 1 | K.YPIEHGIITNWDDMEK.I 19896 19897 19899 19900 19901 19912 |
|  | 20788 | **681.8486** | **2042.5241** | **2041.8391** | **0.6850** | **0** | **0** | **58** | **10** | **M.CEEETTALVCDNGSGLCK.A** |
|  | 22647 | 1114.5292 | 2227.0438 | 2227.0579 | -0.0141 | 0 | (86) | 1.9e-007 | 1 | K.DLYANNVLSGGTTMYPGIADR.M 22648 22663 |
|  | 22804 | 1122.5265 | 2243.0384 | 2243.0528 | -0.0144 | 0 | 104 | 3.1e-009 | 1 | K.DLYANNVLSGGTTMYPGIADR.M |
|  | 23659 | 1178.5748 | 2355.1351 | 2355.1529 | -0.0178 | 1 | 91 | 5.5e-008 | 1 | R.KDLYANNVLSGGTTMYPGIADR.M 23660 23661 |
|  | 23781 | 791.0848 | 2370.2325 | 2371.1478 | -0.9153 | 1 | (2) | 49 | 10 | R.KDLYANNVLSGGTTMYPGIADR.M |
|  | 24892 | 1268.5756 | 2535.1366 | 2535.1509 | -0.0143 | 0 | 127 | 1.3e-011 | 1 | K.LCYVALDFENEMATAASSSSLEK.S 24893 24894 |
|  | 25114 | 1276.0881 | 2550.1617 | 2551.1458 | -0.9841 | 0 | (32) | 0.042 | 2 | K.LCYVALDFENEMATAASSSSLEK.S 25112 25113 |
|  | 26526 | 931.7641 | 2792.2705 | 2792.2884 | -0.0179 | 1 | 23 | 0.3 | 1 | K.EKLCYVALDFENEMATAASSSSLEK.S |
|  | 26641 | 937.3879 | 2809.1420 | 2808.2833 | 0.8587 | 1 | (13) | 2.4 | 1 | K.EKLCYVALDFENEMATAASSSSLEK.S |
|  | 28446 | 1063.4899 | 3187.4478 | 3187.4664 | -0.0186 | 0 | 72 | 2.9e-006 | 1 | R.CPETLFQPSFIGMESAGIHETTYNSIMK.C |
|  | 28473 | 1066.2025 | 3195.5857 | 3195.6023 | -0.0166 | 0 | 75 | 1.6e-006 | 1 | R.TTGIVLDSGDGVTHNVPIYEGYALPHAIMR.L 28469 28470 |
|  | 29467 | 1164.8806 | 3491.6200 | 3490.6360 | 0.9841 | 1 | 55 | 0.00012 | 1 | R.FRCPETLFQPSFIGMESAGIHETTYNSIMK.C 29461 29462 |
|  | 30413 | 994.7351 | 3974.9111 | 3975.8151 | -0.9040 | 1 | 2 | 19 | 6 | R.CPETLFQPSFIGMESAGIHETTYNSIMKCDIDIR.K 30416 |

| **9.** | P68871\|HBB_HUMAN    **Mass:** 16102    **Score:** 1850   **Queries matched:** 106   **emPAI:** 38.59 |
| --- | --- |
|  | Hemoglobin subunit beta OS=Homo sapiens GN=HBB PE=1 SV=2 |

|  | **Query** | **Observed** | **Mr(expt)** | **Mr(calc)** | **Delta** | **Miss** | **Score** | **Expect** | **Rank** | **Peptide** |
| --- | --- | --- | --- | --- | --- | --- | --- | --- | --- | --- |
|  | 3443 | **466.7609** | **931.5073** | **931.5127** | **-0.0054** | **0** | **54** | **0.00074** | **1** | **K.SAVTALWGK.V** 3441 3442 |
|  | 3728 | **476.7553** | **951.4960** | **951.5025** | **-0.0065** | **0** | **22** | **1** | **1** | **M.VHLTPEEK.S** 3724 3726 |
|  | 6170 | **563.7820** | **1125.5495** | **1125.5567** | **-0.0072** | **0** | **19** | **1.8** | **1** | **K.LHVDPENFR.L** 6169 |
|  | 6479 | **575.3363** | **1148.6580** | **1148.6666** | **-0.0085** | **0** | **79** | **1.8e-006** | **1** | **K.VVAGVANALAHK.Y** 6477 6478 6482 6486 6487 |
|  | 8890 | **637.8627** | **1273.7108** | **1273.7183** | **-0.0075** | **0** | **56** | **0.00034** | **1** | **R.LLVVYPWTQR.F** 8883 8885 8886 8888 8891 8892 8893 8894 8896 8897 8898 8899 8900 8901 8903 8904 8912 |
|  | 9623 | **657.8317** | **1313.6489** | **1313.6575** | **-0.0086** | **0** | **74** | **5.7e-006** | **1** | **K.VNVDEVGGEALGR.L** 9616 9618 9619 9620 9622 9625 9626 9627 9628 9630 9631 9632 9633 9652 9676 |
|  | 10886 | **689.8488** | **1377.6829** | **1377.6929** | **-0.0099** | **0** | **54** | **0.00054** | **1** | **K.EFTPPVQAAYQK.V** 10884 10887 10888 |
|  | 12132 | **725.3964** | **1448.7782** | **1448.7888** | **-0.0107** | **1** | **60** | **0.00013** | **1** | **K.VVAGVANALAHKYH.-** 12131 12134 12136 12138 |
|  | 12601 | **739.8455** | **1477.6764** | **1477.6871** | **-0.0108** | **0** | **58** | **0.00023** | **1** | **K.GTFATLSELHCDK.L** 12600 |
|  | 15742 | **835.4432** | **1668.8718** | **1668.8835** | **-0.0117** | **0** | **105** | **3.9e-009** | **1** | **K.VLGAFSDGLAHLDNLK.G** 15726 15743 15748 15749 |
|  | 17326 | **445.0012** | **1775.9757** | **1775.9869** | **-0.0112** | **0** | **35** | **0.036** | **1** | **R.LLGNVLVCVLAHHFGK.E** 17327 17328 17352 |
|  | 17700 | **599.9964** | **1796.9674** | **1796.9785** | **-0.0111** | **1** | **63** | **5.4e-005** | **1** | **K.KVLGAFSDGLAHLDNLK.G** 17696 17698 17713 |
|  | 20986 | **1029.9712** | **2057.9278** | **2057.9405** | **-0.0126** | **0** | **116** | **2.3e-010** | **1** | **R.FFESFGDLSTPDAVMGNPK.V** 20981 20982 20983 20984 20985 20987 20988 20991 20992 20995 20996 20997 20998 21000 21001 21002 21003 21006 21007 21008 21011 21012 21013 21016 21018 |
|  | 21171 | **1037.9679** | **2073.9212** | **2073.9354** | **-0.0141** | **0** | **(62)** | **5.7e-005** | **1** | **R.FFESFGDLSTPDAVMGNPK.V** 21172 21173 |
|  | 23100 | 1143.5518 | 2285.0890 | 2285.1038 | -0.0149 | 1 | 3 | 37 | 2 | R.FFESFGDLSTPDAVMGNPKVK.A |
|  | 25322 | **862.4314** | **2584.2724** | **2585.2333** | **-0.9609** | **1** | **43** | **0.003** | **1** | **K.GTFATLSELHCDKLHVDPENFR.L** 25327 25329 25330 |

| **10.** | P07437\|TBB5_HUMAN    **Mass:** 50095    **Score:** 1747   **Queries matched:** 104   **emPAI:** 8.30 |
| --- | --- |
|  | Tubulin beta chain OS=Homo sapiens GN=TUBB PE=1 SV=2 |

|  | **Query** | **Observed** | **Mr(expt)** | **Mr(calc)** | **Delta** | **Miss** | **Score** | **Expect** | **Rank** | **Peptide** |
| --- | --- | --- | --- | --- | --- | --- | --- | --- | --- | --- |
|  | 1748 | **404.7345** | **807.4544** | **807.3399** | **0.1146** | **0** | **2** | **1e+002** | **6** | **R.EEYPDR.I** |
|  | 4775 | **514.7600** | **1027.5055** | **1027.5121** | **-0.0066** | **0** | **40** | **0.016** | **1** | **K.TAVCDIPPR.G** 4766 4769 4770 4772 4773 4776 |
|  | 4947 | **520.2974** | **1038.5802** | **1038.5862** | **-0.0060** | **0** | **60** | **0.00017** | **1** | **R.YLTVAAVFR.G** |
|  | 5302 | **533.2137** | **1064.4128** | **1064.4201** | **-0.0073** | **0** | **41** | **0.013** | **1** | **K.NMMAACDPR.H** 5301 |
|  | 5458 | **538.7742** | **1075.5338** | **1076.5250** | **-0.9913** | **1** | **21** | **1.4** | **1** | **K.IREEYPDR.I** 5480 |
|  | 5533 | **541.2109** | **1080.4073** | **1080.4151** | **-0.0077** | **0** | **(35)** | **0.047** | **1** | **K.NMMAACDPR.H** 5532 |
|  | 5550 | **541.7350** | **1081.4554** | **1080.4151** | **1.0404** | **0** | **(8)** | **23** | **2** | **K.NMMAACDPR.H** 5554 |
|  | 6379 | **572.3181** | **1142.6217** | **1142.6270** | **-0.0053** | **0** | **65** | **5.8e-005** | **1** | **K.LAVNMVPFPR.L** 6378 |
|  | 7957 | **615.2987** | **1228.5829** | **1228.5910** | **-0.0082** | **0** | **53** | **0.00081** | **1** | **R.ISEQFTAMFR.R** |
|  | 8582 | **630.3885** | **1258.7624** | **1257.6830** | **1.0795** | **1** | **9** | **17** | **3** | **R.FPGQLNADLRK.L** 8578 |
|  | 9375 | **651.3182** | **1300.6219** | **1300.6299** | **-0.0080** | **0** | **29** | **0.16** | **1** | **R.ISVYYNEATGGK.Y** |
|  | 9784 | **660.8520** | **1319.6894** | **1318.6955** | **0.9939** | **0** | **82** | **1e-006** | **1** | **R.IMNTFSVVPSPK.V** 9757 9774 |
|  | 12061 | **723.8434** | **1445.6722** | **1445.6820** | **-0.0098** | **0** | **62** | **8.4e-005** | **1** | **K.EVDEQMLNVQNK.N** 12062 12093 |
|  | 12344 | **488.2414** | **1461.7023** | **1461.6769** | **0.0253** | **0** | **(12)** | **8.5** | **2** | **K.EVDEQMLNVQNK.N** 12328 12329 12330 12336 12355 12361 |
|  | 14864 | **808.4167** | **1614.8188** | **1614.8287** | **-0.0099** | **0** | **80** | **1.1e-006** | **1** | **R.AILVDLEPGTMDSVR.S** 14863 14865 14883 |
|  | 14961 | **810.9169** | **1619.8193** | **1619.8283** | **-0.0090** | **0** | **24** | **0.53** | **1** | **R.LHFFMPGFAPLTSR.G** |
|  | 15133 | **816.4135** | **1630.8123** | **1630.8236** | **-0.0113** | **0** | **(62)** | **7.3e-005** | **1** | **R.AILVDLEPGTMDSVR.S** |
|  | 15571 | 830.4457 | 1658.8769 | 1658.8879 | -0.0110 | 0 | 43 | 0.005 | 1 | R.ALTVPELTQQVFDAK.N 15572 15573 15575 |
|  | 16151 | **848.9155** | **1695.8164** | **1695.8257** | **-0.0093** | **0** | **52** | **0.00079** | **1** | **K.NSSYFVEWIPNNVK.T** 16137 16148 16149 16165 16166 |
|  | 17961 | **605.9691** | **1814.8854** | **1815.9155** | **-1.0302** | **1** | **2** | **70** | **5** | **R.ISVYYNEATGGKYVPR.A** |
|  | 18073 | **911.9592** | **1821.9038** | **1821.9156** | **-0.0118** | **0** | **69** | **1.5e-005** | **1** | **R.EIVHIQAGQCGNQIGAK.F** 18072 18074 |
|  | 18725 | **935.4875** | **1868.9604** | **1868.9706** | **-0.0102** | **0** | **91** | **7.2e-008** | **1** | **K.MAVTFIGNSTAIQELFK.R** 18724 18746 |
|  | 19594 | **485.7540** | **1938.9868** | **1938.8849** | **0.1019** | **1** | **2** | **54** | **6** | **R.MSMKEVDEQMLNVQNK.N** |
|  | 19874 | **979.9879** | **1957.9613** | **1957.9745** | **-0.0132** | **0** | **110** | **8.4e-010** | **1** | **K.GHYTEGAELVDSVLDVVR.K** 19872 19873 |
|  | 20618 | **1013.5383** | **2025.0620** | **2025.0717** | **-0.0097** | **1** | **7** | **18** | **1** | **K.MAVTFIGNSTAIQELFKR.I** |
|  | 21275 | **1044.0358** | **2086.0570** | **2086.0695** | **-0.0125** | **1** | **118** | **1.6e-010** | **1** | **K.GHYTEGAELVDSVLDVVRK.E** 21274 21276 21277 21279 |
|  | 21524 | **528.0024** | **2107.9804** | **2108.0248** | **-0.0444** | **1** | **3** | **49** | **6** | **R.EEYPDRIMNTFSVVPSPK.V** |
|  | 21535 | **1055.5286** | **2109.0426** | **2109.0571** | **-0.0146** | **1** | **79** | **1.1e-006** | **1** | **-.MREIVHIQAGQCGNQIGAK.F** 21536 |
|  | 21696 | **532.5209** | **2126.0547** | **2125.0521** | **1.0026** | **1** | **(1)** | **72** | **10** | **-.MREIVHIQAGQCGNQIGAK.F** |
|  | 26095 | **1354.6655** | **2707.3165** | **2707.3310** | **-0.0145** | **0** | **90** | **5.5e-008** | **1** | **K.LTTPTYGDLNHLVSATMSGVTTCLR.F** 26091 26092 26093 26094 26096 |
|  | 26560 | **933.4475** | **2797.3205** | **2797.3361** | **-0.0156** | **0** | **80** | **6.4e-007** | **1** | **R.SGPFGQIFRPDNFVFGQSGAGNNWAK.G** 26559 26561 26562 26572 26573 26574 26575 26576 |
|  | 28036 | **1034.8008** | **3101.3805** | **3101.4003** | **-0.0198** | **0** | **52** | **0.0003** | **1** | **K.FWEVISDEHGIDPTGTYHGDSDLQLDR.I** 28038 |
|  | 28909 | **1104.5103** | **3310.5089** | **3310.5268** | **-0.0179** | **0** | **62** | **2.5e-005** | **1** | **K.EAESCDCLQGFQLTHSLGGGTGSGMGTLLISK.I** 28908 28910 28911 28912 28913 28914 |
|  | 28978 | **1109.8413** | **3326.5021** | **3326.5218** | **-0.0197** | **0** | **(27)** | **0.091** | **1** | **K.EAESCDCLQGFQLTHSLGGGTGSGMGTLLISK.I** |
|  | 29339 | **1147.5436** | **3439.6089** | **3438.6218** | **0.9871** | **1** | **52** | **0.00023** | **1** | **R.KEAESCDCLQGFQLTHSLGGGTGSGMGTLLISK.I** 29336 |
|  | 29368 | **1152.8380** | **3455.4922** | **3454.6167** | **0.8755** | **1** | **(2)** | **22** | **3** | **R.KEAESCDCLQGFQLTHSLGGGTGSGMGTLLISK.I** |
|  | 31044 | **1531.6991** | **4592.0755** | **4592.0999** | **-0.0245** | **0** | **49** | **0.0002** | **1** | **K.VSDTVVEPYNATLSVHQLVENTDETYCIDNEALYDICFR.T** 31043 31047 |

| **11.** | P68133\|ACTS_HUMAN    **Mass:** 42366    **Score:** 1676   **Queries matched:** 164   **emPAI:** 11.68 |
| --- | --- |
|  | Actin, alpha skeletal muscle OS=Homo sapiens GN=ACTA1 PE=1 SV=1 |

|  | **Query** | **Observed** | **Mr(expt)** | **Mr(calc)** | **Delta** | **Miss** | **Score** | **Expect** | **Rank** | **Peptide** |
| --- | --- | --- | --- | --- | --- | --- | --- | --- | --- | --- |
|  | 1138 | 632.2894 | 631.2822 | 630.3701 | 0.9121 | 0 | 14 | 11 | 1 | K.ILTER.G 1129 1139 |
|  | 1169 | 644.3676 | 643.3603 | 643.3653 | -0.0050 | 0 | 25 | 0.84 | 1 | R.LDLAGR.D |
|  | 1578 | 398.2373 | 794.4600 | 794.4650 | -0.0050 | 0 | 22 | 0.87 | 1 | K.IIAPPER.K 1587 |
|  | 1635 | 400.7684 | 799.5222 | 799.5280 | -0.0057 | 1 | 24 | 0.79 | 6 | K.RGILTLK.Y 1646 |
|  | 3263 | 460.2336 | 918.4526 | 918.4593 | -0.0067 | 1 | 19 | 2.5 | 1 | K.CDIDIRK.D |
|  | 3326 | 462.2838 | 922.5531 | 922.5600 | -0.0068 | 1 | 28 | 0.22 | 1 | K.IIAPPERK.Y 3327 3328 |
|  | 4062 | 488.7242 | 975.4339 | 975.4410 | -0.0071 | 0 | 74 | 7.9e-006 | 1 | K.AGFAGDDAPR.A 4063 4064 4068 4075 |
|  | 4370 | 499.7436 | 997.4726 | 997.4790 | -0.0064 | 0 | 44 | 0.0062 | 1 | R.DLTDYLMK.I 4366 4368 4369 4372 4401 |
|  | 4907 | 518.8259 | 1035.6373 | 1035.6440 | -0.0067 | 1 | 25 | 0.45 | 1 | K.IKIIAPPER.K |
|  | 6669 | 581.3090 | 1160.6034 | 1160.6111 | -0.0077 | 0 | 44 | 0.006 | 1 | K.EITALAPSTMK.I 6667 6668 6670 6671 6685 |
|  | 6796 | 586.2847 | 1170.5549 | 1170.5638 | -0.0089 | 0 | 55 | 0.00046 | 1 | R.HQGVMVGMGQK.D 6797 6798 6799 6800 |
|  | 6883 | 589.3063 | 1176.5981 | 1176.6060 | -0.0079 | 0 | (42) | 0.011 | 1 | K.EITALAPSTMK.I 6885 |
|  | 7046 | 593.7484 | 1185.4823 | 1186.5587 | -1.0764 | 0 | (42) | 0.0095 | 1 | R.HQGVMVGMGQK.D 7072 7074 7076 |
|  | 7069 | 594.2817 | 1186.5489 | 1186.5587 | -0.0098 | 0 | (41) | 0.014 | 1 | R.HQGVMVGMGQK.D 7057 7068 7070 7071 7075 |
|  | 7313 | 599.7607 | 1197.5068 | 1197.5150 | -0.0081 | 0 | 44 | 0.0055 | 1 | K.DSYVGDEAQSK.R 7289 7296 7311 |
|  | 7330 | 599.8522 | 1197.6899 | 1197.6982 | -0.0083 | 0 | 58 | 0.00026 | 1 | R.AVFPSIVGRPR.H 7327 7328 7329 7331 7332 7333 7336 7342 |
|  | 7433 | 602.2789 | 1202.5433 | 1202.5536 | -0.0103 | 0 | (26) | 0.42 | 1 | R.HQGVMVGMGQK.D 7436 7437 |
|  | 10424 | 677.8104 | 1353.6062 | 1353.6161 | -0.0099 | 1 | 51 | 0.0011 | 1 | K.DSYVGDEAQSKR.G 10395 10415 10416 10417 10418 10419 10420 10421 10422 10423 10425 10426 10427 10429 10430 10445 10448 10450 |
|  | 12997 | 750.8526 | 1499.6906 | 1499.7005 | -0.0098 | 0 | 36 | 0.029 | 1 | K.QEYDEAGPSIVHR.K 12977 12996 |
|  | 13245 | 758.3734 | 1514.7323 | 1514.7419 | -0.0096 | 0 | 45 | 0.0039 | 1 | K.IWHHTFYNELR.V 13243 13244 13248 13267 |
|  | 13795 | 774.9045 | 1547.7944 | 1547.8051 | -0.0107 | 1 | 16 | 3 | 1 | R.MQKEITALAPSTMK.I |
|  | 14290 | 395.7159 | 1578.8347 | 1579.7949 | -0.9603 | 1 | (4) | 50 | 8 | R.MQKEITALAPSTMK.I |
|  | 15010 | 812.4193 | 1622.8239 | 1622.8338 | -0.0098 | 1 | 27 | 0.24 | 1 | R.LDLAGRDLTDYLMK.I 15009 |
|  | 15068 | 814.3831 | 1626.7516 | 1627.7954 | -1.0438 | 1 | 5 | 34 | 4 | K.QEYDEAGPSIVHRK.C |
|  | 17562 | 895.9443 | 1789.8740 | 1789.8846 | -0.0106 | 0 | 72 | 7.6e-006 | 1 | K.SYELPDGQVITIGNER.F 17551 17553 17560 17561 17563 17564 17565 17566 17568 17569 17571 17572 17573 17574 17576 17577 17578 17579 17580 17581 17582 17583 17584 17585 17586 17587 17588 17591 17592 17593 17595 17596 17609 17610 17612 17613 17614 17616 17617 |
|  | 19826 | 978.5183 | 1955.0221 | 1955.0364 | -0.0143 | 0 | 55 | 0.00032 | 1 | R.VAPEEHPTLLTEAPLNPK.A 19828 19829 19831 |
|  | 19898 | 980.9523 | 1959.8900 | 1959.9036 | -0.0137 | 0 | 53 | 0.00045 | 1 | K.YPIEHGIITNWDDMEK.I 19896 19897 19899 19900 19901 19912 |
|  | 22677 | **744.1885** | **2229.5438** | **2228.9236** | **0.6202** | **0** | **0** | **55** | **2** | **-.MCDEDETTALVCDNGSGLVK.A** |
|  | 24892 | 1268.5756 | 2535.1366 | 2535.1509 | -0.0143 | 0 | 127 | 1.3e-011 | 1 | K.LCYVALDFENEMATAASSSSLEK.S 24893 24894 |
|  | 25114 | 1276.0881 | 2550.1617 | 2551.1458 | -0.9841 | 0 | (32) | 0.042 | 2 | K.LCYVALDFENEMATAASSSSLEK.S 25112 25113 |
|  | 26526 | 931.7641 | 2792.2705 | 2792.2884 | -0.0179 | 1 | 23 | 0.3 | 1 | K.EKLCYVALDFENEMATAASSSSLEK.S |
|  | 26641 | 937.3879 | 2809.1420 | 2808.2833 | 0.8587 | 1 | (13) | 2.4 | 1 | K.EKLCYVALDFENEMATAASSSSLEK.S |
|  | 28446 | 1063.4899 | 3187.4478 | 3187.4664 | -0.0186 | 0 | 72 | 2.9e-006 | 1 | R.CPETLFQPSFIGMESAGIHETTYNSIMK.C |
|  | 28473 | 1066.2025 | 3195.5857 | 3195.6023 | -0.0166 | 0 | 75 | 1.6e-006 | 1 | R.TTGIVLDSGDGVTHNVPIYEGYALPHAIMR.L 28469 28470 |
|  | 29467 | 1164.8806 | 3491.6200 | 3490.6360 | 0.9841 | 1 | 55 | 0.00012 | 1 | R.FRCPETLFQPSFIGMESAGIHETTYNSIMK.C 29461 29462 |
|  | 30413 | 994.7351 | 3974.9111 | 3975.8151 | -0.9040 | 1 | 2 | 19 | 6 | R.CPETLFQPSFIGMESAGIHETTYNSIMKCDIDIR.K 30416 |

| **12.** | P68371\|TBB2C_HUMAN    **Mass:** 50255    **Score:** 1582   **Queries matched:** 101   **emPAI:** 8.21 |
| --- | --- |
|  | Tubulin beta-2C chain OS=Homo sapiens GN=TUBB2C PE=1 SV=1 |

|  | **Query** | **Observed** | **Mr(expt)** | **Mr(calc)** | **Delta** | **Miss** | **Score** | **Expect** | **Rank** | **Peptide** |
| --- | --- | --- | --- | --- | --- | --- | --- | --- | --- | --- |
|  | 1748 | 404.7345 | 807.4544 | 807.3399 | 0.1146 | 0 | 2 | 1e+002 | 6 | R.EEYPDR.I |
|  | 4775 | 514.7600 | 1027.5055 | 1027.5121 | -0.0066 | 0 | 40 | 0.016 | 1 | K.TAVCDIPPR.G 4766 4769 4770 4772 4773 4776 |
|  | 4947 | 520.2974 | 1038.5802 | 1038.5862 | -0.0060 | 0 | 60 | 0.00017 | 1 | R.YLTVAAVFR.G |
|  | 5302 | 533.2137 | 1064.4128 | 1064.4201 | -0.0073 | 0 | 41 | 0.013 | 1 | K.NMMAACDPR.H 5301 |
|  | 5458 | 538.7742 | 1075.5338 | 1076.5250 | -0.9913 | 1 | 21 | 1.4 | 1 | K.IREEYPDR.I 5480 |
|  | 5533 | 541.2109 | 1080.4073 | 1080.4151 | -0.0077 | 0 | (35) | 0.047 | 1 | K.NMMAACDPR.H 5532 |
|  | 5550 | 541.7350 | 1081.4554 | 1080.4151 | 1.0404 | 0 | (8) | 23 | 2 | K.NMMAACDPR.H 5554 |
|  | 6379 | 572.3181 | 1142.6217 | 1142.6270 | -0.0053 | 0 | 65 | 5.8e-005 | 1 | K.LAVNMVPFPR.L 6378 |
|  | 7957 | 615.2987 | 1228.5829 | 1228.5910 | -0.0082 | 0 | 53 | 0.00081 | 1 | R.ISEQFTAMFR.R |
|  | 8582 | 630.3885 | 1258.7624 | 1257.6830 | 1.0795 | 1 | 9 | 17 | 3 | R.FPGQLNADLRK.L 8578 |
|  | 9784 | 660.8520 | 1319.6894 | 1318.6955 | 0.9939 | 0 | 82 | 1e-006 | 1 | R.IMNTFSVVPSPK.V 9757 9774 |
|  | 9972 | **664.8230** | **1327.6314** | **1327.6408** | **-0.0094** | **0** | **51** | **0.0012** | **1** | **R.INVYYNEATGGK.Y** |
|  | 12061 | 723.8434 | 1445.6722 | 1445.6820 | -0.0098 | 0 | 62 | 8.4e-005 | 1 | K.EVDEQMLNVQNK.N 12062 12093 |
|  | 12344 | 488.2414 | 1461.7023 | 1461.6769 | 0.0253 | 0 | (12) | 8.5 | 2 | K.EVDEQMLNVQNK.N 12328 12329 12330 12336 12355 12361 |
|  | 14633 | **801.4081** | **1600.8017** | **1600.8131** | **-0.0113** | **0** | **30** | **0.12** | **1** | **R.AVLVDLEPGTMDSVR.S** 14631 14634 |
|  | 14961 | 810.9169 | 1619.8193 | 1619.8283 | -0.0090 | 0 | 24 | 0.53 | 1 | R.LHFFMPGFAPLTSR.G |
|  | 16082 | **846.4330** | **1690.8514** | **1690.8600** | **-0.0086** | **0** | **53** | **0.00051** | **1** | **R.ALTVPELTQQMFDAK.N** 16080 16081 |
|  | 16151 | 848.9155 | 1695.8164 | 1695.8257 | -0.0093 | 0 | 52 | 0.00079 | 1 | K.NSSYFVEWIPNNVK.T 16137 16148 16149 16165 16166 |
|  | 18073 | 911.9592 | 1821.9038 | 1821.9156 | -0.0118 | 0 | 69 | 1.5e-005 | 1 | R.EIVHLQAGQCGNQIGAK.F 18072 18074 |
|  | 18581 | **929.4688** | **1856.9229** | **1856.9342** | **-0.0113** | **0** | **70** | **1e-005** | **2** | **K.MSATFIGNSTAIQELFK.R** 18585 18592 |
|  | 19594 | 485.7540 | 1938.9868 | 1938.8849 | 0.1019 | 1 | 2 | 54 | 6 | R.MSMKEVDEQMLNVQNK.N |
|  | 19874 | 979.9879 | 1957.9613 | 1957.9745 | -0.0132 | 0 | 110 | 8.4e-010 | 1 | K.GHYTEGAELVDSVLDVVR.K 19872 19873 |
|  | 20468 | **1007.5194** | **2013.0243** | **2013.0353** | **-0.0111** | **1** | **23** | **0.47** | **2** | **K.MSATFIGNSTAIQELFKR.I** 20467 |
|  | 21275 | 1044.0358 | 2086.0570 | 2086.0695 | -0.0125 | 1 | 118 | 1.6e-010 | 1 | K.GHYTEGAELVDSVLDVVRK.E 21274 21276 21277 21279 |
|  | 21524 | 528.0024 | 2107.9804 | 2108.0248 | -0.0444 | 1 | 3 | 49 | 6 | R.EEYPDRIMNTFSVVPSPK.V |
|  | 21535 | 1055.5286 | 2109.0426 | 2109.0571 | -0.0146 | 1 | 79 | 1.1e-006 | 1 | -.MREIVHLQAGQCGNQIGAK.F 21536 |
|  | 21610 | **529.7468** | **2114.9582** | **2116.0987** | **-1.1405** | **1** | **5** | **28** | **6** | **K.YVPRAVLVDLEPGTMDSVR.S** |
|  | 26095 | 1354.6655 | 2707.3165 | 2707.3310 | -0.0145 | 0 | 90 | 5.5e-008 | 1 | K.LTTPTYGDLNHLVSATMSGVTTCLR.F 26091 26092 26093 26094 26096 |
|  | 26560 | 933.4475 | 2797.3205 | 2797.3361 | -0.0156 | 0 | 80 | 6.4e-007 | 1 | R.SGPFGQIFRPDNFVFGQSGAGNNWAK.G 26559 26561 26562 26572 26573 26574 26575 26576 |
|  | 28092 | **1039.4719** | **3115.3939** | **3115.4159** | **-0.0220** | **0** | **47** | **0.00092** | **1** | **K.FWEVISDEHGIDPTGTYHGDSDLQLER.I** 28093 |
|  | 28909 | 1104.5103 | 3310.5089 | 3310.5268 | -0.0179 | 0 | 62 | 2.5e-005 | 1 | K.EAESCDCLQGFQLTHSLGGGTGSGMGTLLISK.I 28908 28910 28911 28912 28913 28914 |
|  | 28978 | 1109.8413 | 3326.5021 | 3326.5218 | -0.0197 | 0 | (27) | 0.091 | 1 | K.EAESCDCLQGFQLTHSLGGGTGSGMGTLLISK.I |
|  | 29339 | 1147.5436 | 3439.6089 | 3438.6218 | 0.9871 | 1 | 52 | 0.00023 | 1 | R.KEAESCDCLQGFQLTHSLGGGTGSGMGTLLISK.I 29336 |
|  | 29368 | 1152.8380 | 3455.4922 | 3454.6167 | 0.8755 | 1 | (2) | 22 | 3 | R.KEAESCDCLQGFQLTHSLGGGTGSGMGTLLISK.I |
|  | 31044 | 1531.6991 | 4592.0755 | 4592.0999 | -0.0245 | 0 | 49 | 0.0002 | 1 | K.VSDTVVEPYNATLSVHQLVENTDETYCIDNEALYDICFR.T 31043 31047 |

| **13.** | P02768\|ALBU_HUMAN    **Mass:** 71317    **Score:** 1577   **Queries matched:** 107   **emPAI:** 8.82 |
| --- | --- |
|  | Serum albumin OS=Homo sapiens GN=ALB PE=1 SV=2 |

|  | **Query** | **Observed** | **Mr(expt)** | **Mr(calc)** | **Delta** | **Miss** | **Score** | **Expect** | **Rank** | **Peptide** |
| --- | --- | --- | --- | --- | --- | --- | --- | --- | --- | --- |
|  | 1283 | **696.1799** | **695.1726** | **694.3286** | **0.8440** | **0** | **8** | **17** | **2** | **K.NYAEAK.D** |
|  | 2664 | **438.2555** | **874.4964** | **874.5025** | **-0.0061** | **1** | **14** | **9.1** | **4** | **R.LSQRFPK.A** |
|  | 2740 | **440.7213** | **879.4281** | **879.4338** | **-0.0057** | **0** | **36** | **0.044** | **1** | **K.AEFAEVSK.L** 2749 |
|  | 3368 | **464.2474** | **926.4803** | **926.4861** | **-0.0058** | **0** | **38** | **0.027** | **1** | **K.YLYEIAR.R** 3367 |
|  | 3463 | **467.2601** | **932.5056** | **932.5113** | **-0.0057** | **0** | **31** | **0.16** | **1** | **K.LCTVATLR.E** |
|  | 3537 | **470.7242** | **939.4339** | **939.4410** | **-0.0071** | **0** | **25** | **0.47** | **1** | **K.DDNPNLPR.L** |
|  | 3839 | **480.7814** | **959.5483** | **959.5552** | **-0.0069** | **0** | **45** | **0.0066** | **1** | **K.FQNALLVR.Y** |
|  | 4163 | **492.7447** | **983.4748** | **983.4811** | **-0.0063** | **0** | **30** | **0.15** | **1** | **K.TYETTLEK.C** 4161 4162 |
|  | 4421 | **500.8024** | **999.5902** | **999.5964** | **-0.0062** | **0** | **54** | **0.00062** | **1** | **K.QTALVELVK.H** 4418 |
|  | 4595 | **1013.5925** | **1012.5852** | **1012.5917** | **-0.0065** | **0** | **55** | **0.00048** | **1** | **K.LVAASQAALGL.-** 4596 |
|  | 5430 | **537.7708** | **1073.5269** | **1073.5353** | **-0.0083** | **1** | **38** | **0.027** | **1** | **K.LDELRDEGK.A** |
|  | 6193 | 564.8488 | 1127.6831 | 1127.6914 | -0.0083 | 1 | 42 | 0.011 | 1 | K.KQTALVELVK.H 6179 |
|  | 6329 | **570.3208** | **1138.6270** | **1137.4907** | **1.1364** | **0** | **8** | **21** | **2** | **K.CCTESLVNR.R** |
|  | 6456 | **574.9901** | **1147.9657** | **1148.5686** | **-0.6030** | **1** | **27** | **0.2** | **1** | **R.DAHKSEVAHR.F** |
|  | 6472 | **575.3072** | **1148.5998** | **1148.6077** | **-0.0079** | **0** | **50** | **0.0016** | **1** | **K.LVNEVTEFAK.T** 6471 6482 6490 |
|  | 7268 | **598.7994** | **1195.5843** | **1196.5495** | **-0.9652** | **1** | **6** | **39** | **2** | **K.DVCKNYAEAK.D** 7311 |
|  | 7898 | **409.5374** | **1225.5904** | **1225.5979** | **-0.0075** | **1** | **29** | **0.18** | **1** | **R.FKDLGEENFK.A** |
|  | 10194 | **671.8194** | **1341.6242** | **1341.6275** | **-0.0032** | **0** | **61** | **0.0001** | **1** | **K.AVMDDFAAFVEK.C** 10188 10189 10190 10193 |
|  | 10379 | **676.8425** | **1351.6705** | **1351.7612** | **-0.0907** | **1** | **3** | **63** | **4** | **K.FQNALLVRYTK.K** |
|  | 10750 | **686.2826** | **1370.5507** | **1370.5595** | **-0.0087** | **0** | **17** | **2.6** | **1** | **K.AAFTECCQAADK.A** 10765 |
|  | 11862 | **717.7654** | **1433.5163** | **1433.5261** | **-0.0098** | **0** | **34** | **0.048** | **1** | **R.ETYGEMADCCAK.Q** 11863 |
|  | 12003 | **722.3195** | **1442.6245** | **1442.6347** | **-0.0102** | **0** | **53** | **0.00063** | **1** | **K.YICENQDSISSK.L** 12004 12005 12022 12023 |
|  | 12427 | **489.9497** | **1466.8274** | **1466.8358** | **-0.0084** | **1** | **52** | **0.00083** | **1** | **R.RHPDYSVVLLLR.L** 12426 12428 |
|  | 12813 | **497.2995** | **1488.8766** | **1489.8980** | **-1.0214** | **1** | **3** | **65** | **8** | **K.QTALVELVKHKPK.A** |
|  | 12956 | **749.7871** | **1497.5597** | **1497.5712** | **-0.0115** | **0** | **84** | **4.5e-007** | **1** | **K.TCVADESAENCDK.S** 12955 12957 |
|  | 13760 | **773.8969** | **1545.7793** | **1545.7894** | **-0.0102** | **1** | **42** | **0.0071** | **1** | **K.LKECCEKPLLEK.S** 13758 13761 |
|  | 13866 | **776.7971** | **1551.5797** | **1551.5905** | **-0.0108** | **0** | **51** | **0.00079** | **1** | **K.CCAAADPHECYAK.V** 13865 13867 13884 |
|  | 15020 | **812.8950** | **1623.7754** | **1622.7803** | **0.9951** | **0** | **(36)** | **0.033** | **1** | **K.DVFLGMFLYEYAR.R** 14987 |
|  | 15239 | **820.3907** | **1638.7668** | **1638.7752** | **-0.0084** | **0** | **38** | **0.02** | **1** | **K.DVFLGMFLYEYAR.R** 15240 15246 |
|  | 15252 | **820.4670** | **1638.9194** | **1638.9305** | **-0.0111** | **1** | **65** | **3.6e-005** | **1** | **K.KVPQVSTPTLVEVSR.N** 15253 |
|  | 15626 | **831.8607** | **1661.7068** | **1661.7178** | **-0.0110** | **1** | **53** | **0.0005** | **1** | **R.YKAAFTECCQAADK.A** |
|  | 19237 | **955.9636** | **1909.9127** | **1909.9244** | **-0.0117** | **0** | **30** | **0.11** | **1** | **R.RPCFSALEVDETYVPK.E** |
|  | 19506 | **966.5169** | **1931.0193** | **1931.0299** | **-0.0106** | **1** | **48** | **0.0016** | **1** | **K.SLHTLFGDKLCTVATLR.E** 19505 |
|  | 20818 | **1023.0455** | **2044.0764** | **2044.0881** | **-0.0117** | **0** | **44** | **0.0035** | **1** | **K.VFDEFKPLVEEPQNLIK.Q** 20816 20817 20819 20820 20823 |
|  | 21269 | **696.2794** | **2085.8164** | **2085.8303** | **-0.0138** | **0** | **35** | **0.025** | **1** | **K.VHTECCHGDLLECADDR.A** |
|  | 22907 | **1130.5085** | **2259.0025** | **2259.0154** | **-0.0129** | **0** | **74** | **2.9e-006** | **1** | **K.EFNAETFTFHADICTLSEK.E** 22906 |
|  | 24586 | **1245.6395** | **2489.2645** | **2489.2777** | **-0.0132** | **0** | **94** | **2.8e-008** | **1** | **K.ALVLIAFAQYLQQCPFEDHVK.L** 24587 24590 |
|  | 24785 | **840.0726** | **2517.1961** | **2517.2066** | **-0.0105** | **0** | **69** | **8.8e-006** | **1** | **R.MPCAEDYLSVVLNQLCVLHEK.T** |
|  | 25318 | **517.8255** | **2584.0911** | **2584.1105** | **-0.0193** | **1** | **19** | **0.74** | **1** | **K.VHTECCHGDLLECADDRADLAK.Y** 25319 |
|  | 25396 | **867.0997** | **2598.2774** | **2598.2900** | **-0.0127** | **1** | **79** | **8.4e-007** | **1** | **K.QNCELFEQLGEYKFQNALLVR.Y** 25395 |
|  | 25647 | **884.0872** | **2649.2398** | **2649.2567** | **-0.0169** | **0** | **31** | **0.054** | **1** | **R.LVRPEVDVMCTAFHDNEETFLK.K** 25648 |
|  | 25835 | **669.3312** | **2673.2956** | **2673.3077** | **-0.0121** | **1** | **(44)** | **0.0022** | **1** | **K.RMPCAEDYLSVVLNQLCVLHEK.T** 25836 25837 25838 |
|  | 25935 | **897.7730** | **2690.2970** | **2689.3026** | **0.9944** | **1** | **56** | **0.00016** | **1** | **K.RMPCAEDYLSVVLNQLCVLHEK.T** |
|  | 26440 | **926.7847** | **2777.3324** | **2777.3517** | **-0.0193** | **1** | **37** | **0.013** | **1** | **R.LVRPEVDVMCTAFHDNEETFLKK.Y** 26439 26441 26442 26443 |
|  | 26541 | **699.3391** | **2793.3271** | **2793.3466** | **-0.0195** | **1** | **(11)** | **5.3** | **1** | **R.LVRPEVDVMCTAFHDNEETFLKK.Y** |
|  | 27431 | **1487.6670** | **2973.3194** | **2973.3372** | **-0.0177** | **0** | **101** | **4e-009** | **1** | **K.SHCIAEVENDEMPADLPSLAADFVESK.D** 27429 27430 27432 27433 27434 27439 |
|  | 29419 | **869.8902** | **3475.5317** | **3475.5582** | **-0.0265** | **1** | **6** | **9.6** | **1** | **K.SHCIAEVENDEMPADLPSLAADFVESKDVCK.N** |
|  | 30680 | **1038.7347** | **4150.9099** | **4150.9292** | **-0.0194** | **1** | **58** | **4.2e-005** | **1** | **R.RPCFSALEVDETYVPKEFNAETFTFHADICTLSEK.E** |

| **14.** | Q14764\|MVP_HUMAN    **Mass:** 99551    **Score:** 1450   **Queries matched:** 75   **emPAI:** 2.10 |
| --- | --- |
|  | Major vault protein OS=Homo sapiens GN=MVP PE=1 SV=4 |

|  | **Query** | **Observed** | **Mr(expt)** | **Mr(calc)** | **Delta** | **Miss** | **Score** | **Expect** | **Rank** | **Peptide** |
| --- | --- | --- | --- | --- | --- | --- | --- | --- | --- | --- |
|  | 1551 | **793.4394** | **792.4322** | **791.4627** | **0.9695** | **1** | **7** | **34** | **3** | **K.RPHARR.A** |
|  | 2296 | **424.6599** | **847.3053** | **848.4578** | **-1.1526** | **0** | **5** | **59** | **10** | **R.VLFAPMR.M** |
|  | 2365 | **427.2271** | **852.4397** | **852.4453** | **-0.0056** | **0** | **30** | **0.15** | **1** | **R.HADLEIR.L** 2363 |
|  | 2759 | **441.2609** | **880.5073** | **880.5130** | **-0.0057** | **0** | **30** | **0.14** | **1** | **K.SLQPLAPR.N** 2739 |
|  | 4617 | **508.2632** | **1014.5119** | **1015.5549** | **-1.0430** | **0** | **62** | **0.00011** | **1** | **R.AQLELEVSK.A** 4632 4633 4634 |
|  | 4834 | **516.7607** | **1031.5069** | **1031.5135** | **-0.0065** | **0** | **18** | **3** | **1** | **K.ILDQSEAEK.A** 4847 |
|  | 6661 | **581.2738** | **1160.5330** | **1159.5581** | **0.9749** | **1** | **5** | **50** | **6** | **K.AEAESRAEAAR.I** |
|  | 7214 | **398.8573** | **1193.5500** | **1193.5789** | **-0.0289** | **1** | **15** | **4.8** | **1** | **K.TYIRQDNER.V** |
|  | 8209 | **621.8514** | **1241.6882** | **1241.6979** | **-0.0097** | **1** | **45** | **0.0044** | **1** | **K.AQQLAEVEVKK.F** |
|  | 8346 | **624.3542** | **1246.6939** | **1246.7033** | **-0.0094** | **1** | **33** | **0.073** | **1** | **K.VRELELVYAR.A** |
|  | 8528 | **629.3250** | **1256.6355** | **1256.6435** | **-0.0080** | **0** | **52** | **0.0011** | **1** | **R.DLAVAGPEMQVK.L** |
|  | 8577 | **630.3547** | **1258.6949** | **1258.6517** | **0.0432** | **1** | **1** | **1.2e+002** | **10** | **K.ILDQSEAEKAR.K** |
|  | 8825 | **636.8087** | **1271.6029** | **1272.6384** | **-1.0355** | **0** | **(2)** | **86** | **8** | **R.DLAVAGPEMQVK.L** |
|  | 9416 | **652.3099** | **1302.6052** | **1302.6139** | **-0.0087** | **0** | **51** | **0.001** | **1** | **R.HYCTVANPVSR.D** |
|  | 9427 | **652.3442** | **1302.6738** | **1302.6602** | **0.0136** | **0** | **35** | **0.045** | **1** | **K.QMTEAIGPSTIR.D** |
|  | 11141 | **697.3363** | **1392.6580** | **1392.6674** | **-0.0094** | **0** | **42** | **0.0093** | **1** | **R.GAVASVTFDDFHK.N** |
|  | 12195 | **727.8490** | **1453.6834** | **1453.6912** | **-0.0077** | **0** | **53** | **0.00072** | **1** | **K.LFSVPDFVGDACK.A** 12194 |
|  | 12743 | **743.9038** | **1485.7931** | **1485.6834** | **0.1096** | **0** | **9** | **15** | **2** | **R.ALQPLEEGEDEEK.V** |
|  | 14800 | **538.2746** | **1611.8020** | **1610.9243** | **0.8777** | **0** | **5** | **41** | **6** | **K.EVEVVEIIQATIIR.Q** |
|  | 14933 | **809.9738** | **1617.9331** | **1616.8522** | **1.0808** | **0** | **61** | **9.2e-005** | **1** | **R.DAQGLVLFDVTGQVR.L** 14900 14905 |
|  | 16838 | **870.5122** | **1739.0099** | **1739.0193** | **-0.0094** | **1** | **102** | **6.5e-009** | **1** | **R.KEVEVVEIIQATIIR.Q** 16837 |
|  | 17463 | **892.4935** | **1782.9725** | **1782.9839** | **-0.0114** | **1** | **103** | **5.2e-009** | **1** | **K.LKAQALAIETEAELQR.V** 17462 |
|  | 17966 | **908.4750** | **1814.9355** | **1814.9454** | **-0.0099** | **0** | **47** | **0.002** | **1** | **R.LAQDPFPLYPGEVLEK.D** |
|  | 18753 | **936.5469** | **1871.0793** | **1871.0880** | **-0.0087** | **0** | **11** | **7.2** | **1** | **K.DITPLQVVLPNTALHLK.A** |
|  | 18784 | **937.4910** | **1872.9674** | **1872.9792** | **-0.0118** | **0** | **69** | **1.2e-005** | **1** | **R.SVQLAIEITTNSQEAAAK.H** 18758 |
|  | 19480 | **965.9847** | **1929.9548** | **1929.9684** | **-0.0135** | **0** | **58** | **0.00015** | **1** | **R.QAIPLDENEGIYVQDVK.T** |
|  | 20242 | **996.5081** | **1991.0017** | **1991.0132** | **-0.0115** | **0** | **36** | **0.024** | **1** | **K.ELLELEALSMAVESTGTAK.A** 20243 |
|  | 20429 | **670.3469** | **2008.0188** | **2007.0081** | **1.0106** | **0** | **(2)** | **62** | **5** | **K.ELLELEALSMAVESTGTAK.A** |
|  | 21250 | **1042.0199** | **2082.0252** | **2082.0343** | **-0.0091** | **0** | **72** | **5.7e-006** | **1** | **R.AVIGSTYMLTQDEVLWEK.E** 21248 |
|  | 21909 | **1076.5442** | **2151.0738** | **2151.0861** | **-0.0123** | **0** | **92** | **5.9e-008** | **1** | **R.IPPYHYIHVLDQNSNVSR.V** 21907 21908 21910 |
|  | 26659 | **938.1362** | **2811.3867** | **2811.4039** | **-0.0173** | **0** | **13** | **3.4** | **1** | **R.DQAVFPQNGLVVSSVDVQSVEPVDQR.T** |
|  | 26894 | **953.1846** | **2856.5321** | **2855.5433** | **0.9888** | **0** | **19** | **0.69** | **1** | **R.VVFGPELVSLGPEEQFTVLSLSAGRPK.R** |
|  | 27020 | **962.7988** | **2885.3745** | **2885.3944** | **-0.0199** | **1** | **27** | **0.11** | **1** | **R.ALQPLEEGEDEEKVSHQAGDHWLIR.G** |
|  | 28105 | **1040.5271** | **3118.5595** | **3118.5796** | **-0.0201** | **0** | **94** | **2e-008** | **1** | **R.VASGPSPGEGISPQSAQAPQAPGDNHVVPVLR.-** 28104 28106 |
|  | 28796 | **1637.8353** | **3273.6561** | **3273.6704** | **-0.0143** | **0** | **90** | **4.9e-008** | **1** | **K.STLITDGSTPINLFNTAFGLLGMGPEGQPLGR.R** 28794 28795 28797 |
|  | 28803 | **819.6724** | **3274.6603** | **3274.6807** | **-0.0204** | **1** | **29** | **0.06** | **1** | **R.RVASGPSPGEGISPQSAQAPQAPGDNHVVPVLR.-** |
|  | 28841 | **1097.5574** | **3289.6503** | **3289.6653** | **-0.0150** | **0** | **(3)** | **25** | **1** | **K.STLITDGSTPINLFNTAFGLLGMGPEGQPLGR.R** |
|  | 29012 | **1667.8628** | **3333.7110** | **3333.7245** | **-0.0135** | **0** | **76** | **1.1e-006** | **1** | **K.SFFLQPGEQLEQGIQDVYVLSEQQGLLLR.A** 29009 29010 29011 |
|  | 29243 | **1134.5832** | **3400.7279** | **3400.7476** | **-0.0197** | **1** | **55** | **0.00012** | **1** | **R.AVIGSTYMLTQDEVLWEKELPPGVEELLNK.G** 29242 |
|  | 29866 | **1223.6744** | **3668.0015** | **3668.0229** | **-0.0214** | **1** | **41** | **0.0025** | **1** | **R.LAQDPFPLYPGEVLEKDITPLQVVLPNTALHLK.A** 29865 29867 29869 |
|  | 29973 | **744.6002** | **3717.9647** | **3717.9645** | **0.0002** | **0** | **1** | **25** | **1** | **R.VTGEEWLVTTVGAYLPAVFEEVLDLVDAVILTEK.T** |
|  | 31439 | **1333.9224** | **5331.6603** | **5330.6446** | **1.0157** | **0** | **31** | **0.0094** | **1** | **R.TGEEWLVTVQDTEAHVPDVHEEVLGVVPITTLGPHNYCVILDPVGPDGK.N** 31437 31438 |
|  | 31493 | **1098.3484** | **5486.7056** | **5486.7457** | **-0.0402** | **1** | **33** | **0.0054** | **1** | **R.RTGEEWLVTVQDTEAHVPDVHEEVLGVVPITTLGPHNYCVILDPVGPDGK.N** 31494 |

| **15.** | Q9BVA1\|TBB2B_HUMAN    **Mass:** 50377    **Score:** 1334   **Queries matched:** 88   **emPAI:** 4.97 |
| --- | --- |
|  | Tubulin beta-2B chain OS=Homo sapiens GN=TUBB2B PE=1 SV=1 |

|  | **Query** | **Observed** | **Mr(expt)** | **Mr(calc)** | **Delta** | **Miss** | **Score** | **Expect** | **Rank** | **Peptide** |
| --- | --- | --- | --- | --- | --- | --- | --- | --- | --- | --- |
|  | 1748 | 404.7345 | 807.4544 | 807.3399 | 0.1146 | 0 | 2 | 1e+002 | 6 | R.EEYPDR.I |
|  | 4775 | 514.7600 | 1027.5055 | 1027.5121 | -0.0066 | 0 | 40 | 0.016 | 1 | K.TAVCDIPPR.G 4766 4769 4770 4772 4773 4776 |
|  | 5302 | 533.2137 | 1064.4128 | 1064.4201 | -0.0073 | 0 | 41 | 0.013 | 1 | K.NMMAACDPR.H 5301 |
|  | 5458 | 538.7742 | 1075.5338 | 1076.5250 | -0.9913 | 1 | 21 | 1.4 | 1 | K.IREEYPDR.I 5480 |
|  | 5533 | 541.2109 | 1080.4073 | 1080.4151 | -0.0077 | 0 | (35) | 0.047 | 1 | K.NMMAACDPR.H 5532 |
|  | 5550 | 541.7350 | 1081.4554 | 1080.4151 | 1.0404 | 0 | (8) | 23 | 2 | K.NMMAACDPR.H 5554 |
|  | 6379 | 572.3181 | 1142.6217 | 1142.6270 | -0.0053 | 0 | 65 | 5.8e-005 | 1 | K.LAVNMVPFPR.L 6378 |
|  | 7957 | 615.2987 | 1228.5829 | 1228.5910 | -0.0082 | 0 | 53 | 0.00081 | 1 | R.ISEQFTAMFR.R |
|  | 8582 | 630.3885 | 1258.7624 | 1257.6830 | 1.0795 | 1 | 9 | 17 | 3 | R.FPGQLNADLRK.L 8578 |
|  | 10345 | **676.3226** | **1350.6306** | **1350.6676** | **-0.0370** | **0** | **9** | **16** | **8** | **R.IMNTFSVMPSPK.V** |
|  | 10693 | **456.8826** | **1367.6260** | **1366.6625** | **0.9635** | **0** | **(5)** | **40** | **5** | **R.IMNTFSVMPSPK.V** |
|  | 10983 | **692.3545** | **1382.6944** | **1382.6574** | **0.0370** | **0** | **(2)** | **95** | **8** | **R.IMNTFSVMPSPK.V** |
|  | 12061 | 723.8434 | 1445.6722 | 1445.6820 | -0.0098 | 0 | 62 | 8.4e-005 | 1 | K.EVDEQMLNVQNK.N 12062 12093 |
|  | 12344 | 488.2414 | 1461.7023 | 1461.6769 | 0.0253 | 0 | (12) | 8.5 | 2 | K.EVDEQMLNVQNK.N 12328 12329 12330 12336 12355 12361 |
|  | 14864 | 808.4167 | 1614.8188 | 1614.8287 | -0.0099 | 0 | 80 | 1.1e-006 | 1 | R.AILVDLEPGTMDSVR.S 14863 14865 14883 |
|  | 14961 | 810.9169 | 1619.8193 | 1619.8283 | -0.0090 | 0 | 24 | 0.53 | 1 | R.LHFFMPGFAPLTSR.G |
|  | 15133 | 816.4135 | 1630.8123 | 1630.8236 | -0.0113 | 0 | (62) | 7.3e-005 | 1 | R.AILVDLEPGTMDSVR.S |
|  | 16151 | 848.9155 | 1695.8164 | 1695.8257 | -0.0093 | 0 | 52 | 0.00079 | 1 | K.NSSYFVEWIPNNVK.T 16137 16148 16149 16165 16166 |
|  | 16587 | **575.2979** | **1722.8717** | **1722.8498** | **0.0219** | **0** | **4** | **45** | **5** | **R.ALTVPELTQQMFDSK.N** |
|  | 18073 | 911.9592 | 1821.9038 | 1821.9156 | -0.0118 | 0 | 69 | 1.5e-005 | 1 | R.EIVHIQAGQCGNQIGAK.F 18072 18074 |
|  | 18581 | 929.4688 | 1856.9229 | 1856.9342 | -0.0113 | 0 | 70 | 1e-005 | 2 | K.MSATFIGNSTAIQELFK.R 18585 18592 |
|  | 19098 | **634.3061** | **1899.8964** | **1899.9479** | **-0.0514** | **1** | **9** | **14** | **1** | **R.INVYYNEATGNKYVPR.A** |
|  | 19594 | 485.7540 | 1938.9868 | 1938.8849 | 0.1019 | 1 | 2 | 54 | 6 | R.MSMKEVDEQMLNVQNK.N |
|  | 19874 | 979.9879 | 1957.9613 | 1957.9745 | -0.0132 | 0 | 110 | 8.4e-010 | 1 | K.GHYTEGAELVDSVLDVVR.K 19872 19873 |
|  | 20468 | 1007.5194 | 2013.0243 | 2013.0353 | -0.0111 | 1 | 23 | 0.47 | 2 | K.MSATFIGNSTAIQELFKR.I 20467 |
|  | 21275 | 1044.0358 | 2086.0570 | 2086.0695 | -0.0125 | 1 | 118 | 1.6e-010 | 1 | K.GHYTEGAELVDSVLDVVRK.E 21274 21276 21277 21279 |
|  | 21535 | 1055.5286 | 2109.0426 | 2109.0571 | -0.0146 | 1 | 79 | 1.1e-006 | 1 | -.MREIVHIQAGQCGNQIGAK.F 21536 |
|  | 21696 | 532.5209 | 2126.0547 | 2125.0521 | 1.0026 | 1 | (1) | 72 | 10 | -.MREIVHIQAGQCGNQIGAK.F |
|  | 26095 | 1354.6655 | 2707.3165 | 2707.3310 | -0.0145 | 0 | 90 | 5.5e-008 | 1 | K.LTTPTYGDLNHLVSATMSGVTTCLR.F 26091 26092 26093 26094 26096 |
|  | 26560 | 933.4475 | 2797.3205 | 2797.3361 | -0.0156 | 0 | 80 | 6.4e-007 | 1 | R.SGPFGQIFRPDNFVFGQSGAGNNWAK.G 26559 26561 26562 26572 26573 26574 26575 26576 |
|  | 28036 | 1034.8008 | 3101.3805 | 3101.4003 | -0.0198 | 0 | 33 | 0.023 | 2 | K.FWEVISDEHGIDPTGSYHGDSDLQLER.I |
|  | 29365 | **1152.4441** | **3454.3104** | **3454.6167** | **-0.3063** | **1** | **1** | **22** | **3** | **R.KESESCDCLQGFQLTHSLGGGTGSGMGTLLISK.I** |
|  | 31044 | 1531.6991 | 4592.0755 | 4592.0999 | -0.0245 | 0 | 49 | 0.0002 | 1 | K.VSDTVVEPYNATLSVHQLVENTDETYCIDNEALYDICFR.T 31043 31047 |

|  | **Proteins matching a subset of these peptides:** |
| --- | --- |

|  | Q13885\|TBB2A_HUMAN    **Mass:** 50274    **Score:** 1286   **Queries matched:** 84 |
| --- | --- |
|  | Tubulin beta-2A chain OS=Homo sapiens GN=TUBB2A PE=1 SV=1 |

| **16.** | Q8IWL1\|SFPA2_HUMAN    **Mass:** 26622    **Score:** 1303   **Queries matched:** 57   **emPAI:** 5.46 |
| --- | --- |
|  | Pulmonary surfactant-associated protein A2 OS=Homo sapiens GN=SFTPA2 PE=1 SV=1 |

|  | **Query** | **Observed** | **Mr(expt)** | **Mr(calc)** | **Delta** | **Miss** | **Score** | **Expect** | **Rank** | **Peptide** |
| --- | --- | --- | --- | --- | --- | --- | --- | --- | --- | --- |
|  | 1794 | **812.3660** | **811.3587** | **811.3647** | **-0.0060** | **0** | **24** | **0.53** | **1** | **R.NCLYSR.L** 1806 1807 1814 |
|  | 2921 | **448.2556** | **894.4967** | **894.5035** | **-0.0069** | **0** | **34** | **0.059** | **1** | **R.HQILQTR.G** 2919 2920 2922 2923 2924 2937 2939 |
|  | 2948 | **449.2441** | **896.4736** | **895.5352** | **0.9384** | **1** | **12** | **9.6** | **2** | **R.AGGRIAVPR.N** 2942 |
|  | 12089 | **724.3518** | **1446.6891** | **1446.6990** | **-0.0100** | **0** | **74** | **5.3e-006** | **1** | **R.NPEENEAIASFVK.K** 12088 |
|  | 12822 | **745.8924** | **1489.7702** | **1489.7810** | **-0.0108** | **0** | **66** | **3.3e-005** | **1** | **R.GALSLQGSIMTVGEK.V** 12797 12798 12820 12821 12823 12826 |
|  | 13093 | **753.8899** | **1505.7652** | **1505.7759** | **-0.0107** | **0** | **(43)** | **0.0058** | **1** | **R.GALSLQGSIMTVGEK.V** 13092 |
|  | 14223 | **788.3986** | **1574.7827** | **1574.7940** | **-0.0113** | **1** | **59** | **0.00014** | **1** | **R.NPEENEAIASFVKK.Y** |
|  | 19473 | **965.8807** | **1929.7468** | **1929.7622** | **-0.0154** | **0** | **23** | **0.42** | **1** | **K.EQCVEMYTDGQWNDR.N** |
|  | 20537 | **505.2804** | **2017.0926** | **2015.9848** | **1.1079** | **0** | **1** | **67** | **7** | **K.DVCVGSPGIPGTPGSHGLPGR.D** |
|  | 21494 | **1053.9861** | **2105.9576** | **2105.9694** | **-0.0118** | **0** | **108** | **1.4e-009** | **1** | **K.YNTYAYVGLTEGPSPGDFR.Y** 21493 21495 21497 21498 21506 |
|  | 21607 | **1058.4390** | **2114.8634** | **2114.8786** | **-0.0152** | **1** | **89** | **9.5e-008** | **1** | **R.GKEQCVEMYTDGQWNDR.N** 21606 21608 |
|  | 22335 | **1101.5032** | **2200.9918** | **2200.0219** | **0.9699** | **0** | **98** | **1.2e-008** | **1** | **K.VFSSNGQSITFDAIQEACAR.A** 22315 22317 22329 22330 22331 22333 22334 22336 22338 22339 22340 22341 22342 |
|  | 22721 | **1118.0315** | **2234.0484** | **2234.0644** | **-0.0160** | **1** | **107** | **1.5e-009** | **1** | **K.KYNTYAYVGLTEGPSPGDFR.Y** 22720 22732 |
|  | 25188 | **641.7995** | **2563.1689** | **2563.2670** | **-0.0981** | **0** | **3** | **32** | **8** | **-.MWLCPLALNLILMAASGAACEVK.D** 25195 |
|  | 26170 | **908.7066** | **2723.0980** | **2723.1163** | **-0.0183** | **1** | **21** | **0.38** | **1** | **K.EQCVEMYTDGQWNDRNCLYSR.L** |

|  | **Proteins matching a subset of these peptides:** |
| --- | --- |

|  | Q8IWL2\|SFTA1_HUMAN    **Mass:** 26739    **Score:** 1303   **Queries matched:** 55 |
| --- | --- |
|  | Pulmonary surfactant-associated protein A1 OS=Homo sapiens GN=SFTPA1 PE=1 SV=2 |

| **17.** | P68363\|TBA1B_HUMAN    **Mass:** 50804    **Score:** 1288   **Queries matched:** 58   **emPAI:** 3.75 |
| --- | --- |
|  | Tubulin alpha-1B chain OS=Homo sapiens GN=TUBA1B PE=1 SV=1 |

|  | **Query** | **Observed** | **Mr(expt)** | **Mr(calc)** | **Delta** | **Miss** | **Score** | **Expect** | **Rank** | **Peptide** |
| --- | --- | --- | --- | --- | --- | --- | --- | --- | --- | --- |
|  | 3129 | **455.7370** | **909.4595** | **908.4967** | **0.9628** | **1** | **14** | **5.3** | **1** | **R.LSVDYGKK.S** |
|  | 4625 | **508.2894** | **1014.5642** | **1014.5709** | **-0.0067** | **0** | **36** | **0.043** | **1** | **K.DVNAAIATIK.T** |
|  | 4720 | **512.7546** | **1023.4947** | **1022.4417** | **1.0530** | **0** | **1** | **1.3e+002** | **5** | **K.EDAANNYAR.G** |
|  | 4988 | **521.7651** | **1041.5156** | **1042.5270** | **-1.0114** | **1** | **7** | **34** | **10** | **K.FDLMYAKR.A** |
|  | 8367 | **625.2756** | **1248.5366** | **1248.5453** | **-0.0087** | **0** | **9** | **18** | **1** | **K.YMACCLLYR.G** 8371 |
|  | 10920 | **690.8480** | **1379.6814** | **1379.6907** | **-0.0094** | **1** | **25** | **0.5** | **1** | **R.LDHKFDLMYAK.R** 10921 |
|  | 12767 | **744.4402** | **1486.8658** | **1486.8719** | **-0.0060** | **0** | **72** | **8.7e-006** | **1** | **R.LISQIVSSITASLR.F** 12765 12766 |
|  | 14362 | **792.8755** | **1583.7364** | **1583.7443** | **-0.0078** | **0** | **25** | **0.4** | **1** | **R.SIQFVDWCPTGFK.V** |
|  | 16241 | **851.4521** | **1700.8896** | **1700.8985** | **-0.0089** | **0** | **76** | **2.6e-006** | **1** | **R.AVFVDLEPTVIDEVR.T** 16242 16243 |
|  | 16504 | **859.9384** | **1717.8623** | **1717.8747** | **-0.0124** | **0** | **44** | **0.0044** | **1** | **R.NLDIERPTYTNLNR.L** 16503 16505 |
|  | 17071 | **878.9804** | **1755.9463** | **1755.9559** | **-0.0097** | **0** | **65** | **3.1e-005** | **1** | **R.IHFPLATYAPVISAEK.A** 17060 17069 17070 17072 17074 |
|  | 18100 | **912.9898** | **1823.9651** | **1823.9782** | **-0.0131** | **0** | **57** | **0.00019** | **1** | **K.VGINYQPPTVVPGGDLAK.V** 18098 18099 18101 |
|  | 18338 | **461.2563** | **1840.9959** | **1841.0047** | **-0.0087** | **1** | **7** | **22** | **9** | **R.GHYTIGKEIIDLVLDR.I** |
|  | 18674 | **932.9498** | **1863.8851** | **1863.8971** | **-0.0120** | **0** | **84** | **3.8e-007** | **1** | **R.AVCMLSNTTAIAEAWAR.L** 18675 |
|  | 20417 | **1004.4446** | **2006.8746** | **2006.8858** | **-0.0112** | **0** | **69** | **1.1e-005** | **1** | **K.TIGGGDDSFNTFFSETGAGK.H** |
|  | 23458 | **777.3395** | **2328.9966** | **2329.0110** | **-0.0144** | **0** | **40** | **0.0071** | **1** | **R.AFVHWYVGEGMEEGEFSEAR.E** |
|  | 24021 | **1205.1011** | **2408.1876** | **2408.2012** | **-0.0136** | **0** | **116** | **1.8e-010** | **1** | **R.FDGALNVDLTEFQTNLVPYPR.I** 24022 24023 24024 24025 24026 |
|  | 24065 | **1208.0977** | **2414.1808** | **2414.1978** | **-0.0171** | **1** | **71** | **6.6e-006** | **1** | **R.QLFHPEQLITGKEDAANNYAR.G** 24063 24064 |
|  | 26296 | **1375.6395** | **2749.2645** | **2749.2840** | **-0.0194** | **0** | **84** | **2.1e-007** | **1** | **K.AYHEQLSVAEITNACFEPANQMVK.C** 26295 26298 26299 |
|  | 26370 | **922.7617** | **2765.2632** | **2765.2789** | **-0.0157** | **0** | **(32)** | **0.034** | **1** | **K.AYHEQLSVAEITNACFEPANQMVK.C** |
|  | 28599 | **805.1068** | **3216.3979** | **3216.4168** | **-0.0189** | **1** | **22** | **0.28** | **1** | **R.AFVHWYVGEGMEEGEFSEAREDMAALEK.D** |
|  | 29206 | **1130.8745** | **3389.6017** | **3389.6173** | **-0.0156** | **0** | **35** | **0.013** | **1** | **K.LADQCTGLQGFLVFHSFGGGTGSGFTSLLMER.L** |
|  | 29521 | **1173.5731** | **3517.6975** | **3517.7123** | **-0.0148** | **1** | **61** | **3.4e-005** | **1** | **R.KLADQCTGLQGFLVFHSFGGGTGSGFTSLLMER.L** 29522 |
|  | 30798 | **1074.9877** | **4295.9216** | **4295.9497** | **-0.0281** | **0** | **3** | **10** | **1** | **R.ECISIHVGQAGVQIGNACWELYCLEHGIQPDGQMPSDK.T** |
|  | 31036 | **1146.7736** | **4583.0651** | **4583.0913** | **-0.0261** | **1** | **57** | **3.5e-005** | **1** | **-.MRECISIHVGQAGVQIGNACWELYCLEHGIQPDGQMPSDK.T** 31035 31037 |
|  | 31716 | **1135.5408** | **5672.6675** | **5672.6902** | **-0.0227** | **1** | **12** | **0.58** | **1** | **K.LEFSIYPAPQVSTAVVEPYNSILTTHTTLEHSDCAFMVDNEAIYDICRR.N** |
|  | 31729 | **1147.5480** | **5732.7035** | **5731.7161** | **0.9874** | **1** | **3** | **4.8** | **1** | **K.SKLEFSIYPAPQVSTAVVEPYNSILTTHTTLEHSDCAFMVDNEAIYDICR.R** 31728 |

|  | **Proteins matching a subset of these peptides:** |
| --- | --- |

|  | P68366\|TBA4A_HUMAN    **Mass:** 50634    **Score:** 1072   **Queries matched:** 45 |
| --- | --- |
|  | Tubulin alpha-4A chain OS=Homo sapiens GN=TUBA4A PE=1 SV=1 |

| **18.** | P04350\|TBB4_HUMAN    **Mass:** 50010    **Score:** 1256   **Queries matched:** 78   **emPAI:** 5.49 |
| --- | --- |
|  | Tubulin beta-4 chain OS=Homo sapiens GN=TUBB4 PE=1 SV=2 |

|  | **Query** | **Observed** | **Mr(expt)** | **Mr(calc)** | **Delta** | **Miss** | **Score** | **Expect** | **Rank** | **Peptide** |
| --- | --- | --- | --- | --- | --- | --- | --- | --- | --- | --- |
|  | 4775 | 514.7600 | 1027.5055 | 1027.5121 | -0.0066 | 0 | 40 | 0.016 | 1 | K.TAVCDIPPR.G 4766 4769 4770 4772 4773 4776 |
|  | 4947 | 520.2974 | 1038.5802 | 1038.5862 | -0.0060 | 0 | 60 | 0.00017 | 1 | R.YLTVAAVFR.G |
|  | 5302 | 533.2137 | 1064.4128 | 1064.4201 | -0.0073 | 0 | 41 | 0.013 | 1 | K.NMMAACDPR.H 5301 |
|  | 5533 | 541.2109 | 1080.4073 | 1080.4151 | -0.0077 | 0 | (35) | 0.047 | 1 | K.NMMAACDPR.H 5532 |
|  | 5550 | 541.7350 | 1081.4554 | 1080.4151 | 1.0404 | 0 | (8) | 23 | 2 | K.NMMAACDPR.H 5554 |
|  | 6379 | 572.3181 | 1142.6217 | 1142.6270 | -0.0053 | 0 | 65 | 5.8e-005 | 1 | K.LAVNMVPFPR.L 6378 |
|  | 7957 | 615.2987 | 1228.5829 | 1228.5910 | -0.0082 | 0 | 53 | 0.00081 | 1 | R.ISEQFTAMFR.R |
|  | 8582 | 630.3885 | 1258.7624 | 1257.6830 | 1.0795 | 1 | 9 | 17 | 3 | R.FPGQLNADLRK.L 8578 |
|  | 9784 | 660.8520 | 1319.6894 | 1318.6955 | 0.9939 | 0 | 82 | 1e-006 | 1 | R.IMNTFSVVPSPK.V 9757 9774 |
|  | 14633 | 801.4081 | 1600.8017 | 1600.8131 | -0.0113 | 0 | 30 | 0.12 | 1 | R.AVLVDLEPGTMDSVR.S 14631 14634 |
|  | 14961 | 810.9169 | 1619.8193 | 1619.8283 | -0.0090 | 0 | 24 | 0.53 | 1 | R.LHFFMPGFAPLTSR.G |
|  | 16082 | 846.4330 | 1690.8514 | 1690.8600 | -0.0086 | 0 | 53 | 0.00051 | 1 | R.ALTVPELTQQMFDAK.N 16080 16081 |
|  | 16151 | 848.9155 | 1695.8164 | 1695.8257 | -0.0093 | 0 | 52 | 0.00079 | 1 | K.NSSYFVEWIPNNVK.T 16137 16148 16149 16165 16166 |
|  | 18073 | 911.9592 | 1821.9038 | 1821.9156 | -0.0118 | 0 | 69 | 1.5e-005 | 1 | R.EIVHLQAGQCGNQIGAK.F 18072 18074 |
|  | 18581 | 929.4688 | 1856.9229 | 1856.9342 | -0.0113 | 0 | 72 | 5.9e-006 | 1 | K.MAATFIGNSTAIQELFK.R 18585 18592 |
|  | 18735 | **624.2347** | **1869.6822** | **1868.8682** | **0.8140** | **1** | **3** | **40** | **1** | **R.MSMKEVDEQMLSVQSK.N** |
|  | 19626 | **971.9928** | **1941.9710** | **1941.9796** | **-0.0086** | **0** | **93** | **4.6e-008** | **1** | **K.GHYTEGAELVDAVLDVVR.K** |
|  | 20468 | 1007.5194 | 2013.0243 | 2013.0353 | -0.0111 | 1 | 24 | 0.38 | 1 | K.MAATFIGNSTAIQELFKR.I 20467 |
|  | 21535 | 1055.5286 | 2109.0426 | 2109.0571 | -0.0146 | 1 | 79 | 1.1e-006 | 1 | -.MREIVHLQAGQCGNQIGAK.F 21536 |
|  | 26095 | 1354.6655 | 2707.3165 | 2707.3310 | -0.0145 | 0 | 90 | 5.5e-008 | 1 | K.LTTPTYGDLNHLVSATMSGVTTCLR.F 26091 26092 26093 26094 26096 |
|  | 26560 | 933.4475 | 2797.3205 | 2797.3361 | -0.0156 | 0 | 80 | 6.4e-007 | 1 | R.SGPFGQIFRPDNFVFGQSGAGNNWAK.G 26559 26561 26562 26572 26573 26574 26575 26576 |
|  | 28092 | 1039.4719 | 3115.3939 | 3115.4159 | -0.0220 | 0 | 47 | 0.00092 | 1 | K.FWEVISDEHGIDPTGTYHGDSDLQLER.I 28093 |
|  | 28909 | 1104.5103 | 3310.5089 | 3310.5268 | -0.0179 | 0 | 62 | 2.5e-005 | 1 | K.EAESCDCLQGFQLTHSLGGGTGSGMGTLLISK.I 28908 28910 28911 28912 28913 28914 |
|  | 28978 | 1109.8413 | 3326.5021 | 3326.5218 | -0.0197 | 0 | (27) | 0.091 | 1 | K.EAESCDCLQGFQLTHSLGGGTGSGMGTLLISK.I |
|  | 29339 | 1147.5436 | 3439.6089 | 3438.6218 | 0.9871 | 1 | 52 | 0.00023 | 1 | R.KEAESCDCLQGFQLTHSLGGGTGSGMGTLLISK.I 29336 |
|  | 29368 | 1152.8380 | 3455.4922 | 3454.6167 | 0.8755 | 1 | (2) | 22 | 3 | R.KEAESCDCLQGFQLTHSLGGGTGSGMGTLLISK.I |
|  | 31044 | 1531.6991 | 4592.0755 | 4592.0999 | -0.0245 | 0 | 49 | 0.0002 | 1 | K.VSDTVVEPYNATLSVHQLVENTDETYCIDNEALYDICFR.T 31043 31047 |

| **19.** | Q71U36\|TBA1A_HUMAN    **Mass:** 50788    **Score:** 1248   **Queries matched:** 59   **emPAI:** 3.12 |
| --- | --- |
|  | Tubulin alpha-1A chain OS=Homo sapiens GN=TUBA1A PE=1 SV=1 |

|  | **Query** | **Observed** | **Mr(expt)** | **Mr(calc)** | **Delta** | **Miss** | **Score** | **Expect** | **Rank** | **Peptide** |
| --- | --- | --- | --- | --- | --- | --- | --- | --- | --- | --- |
|  | 3129 | 455.7370 | 909.4595 | 908.4967 | 0.9628 | 1 | 14 | 5.3 | 1 | R.LSVDYGKK.S |
|  | 4625 | 508.2894 | 1014.5642 | 1014.5709 | -0.0067 | 0 | 36 | 0.043 | 1 | K.DVNAAIATIK.T |
|  | 4720 | 512.7546 | 1023.4947 | 1022.4417 | 1.0530 | 0 | 1 | 1.3e+002 | 5 | K.EDAANNYAR.G |
|  | 4988 | 521.7651 | 1041.5156 | 1042.5270 | -1.0114 | 1 | 7 | 34 | 10 | K.FDLMYAKR.A |
|  | 8367 | 625.2756 | 1248.5366 | 1248.5453 | -0.0087 | 0 | 9 | 18 | 1 | K.YMACCLLYR.G 8371 |
|  | 10920 | 690.8480 | 1379.6814 | 1379.6907 | -0.0094 | 1 | 25 | 0.5 | 1 | R.LDHKFDLMYAK.R 10921 |
|  | 12255 | **729.4348** | **1456.8549** | **1456.8613** | **-0.0064** | **0** | **78** | **2e-006** | **1** | **R.LIGQIVSSITASLR.F** 12252 12253 12265 |
|  | 16241 | 851.4521 | 1700.8896 | 1700.8985 | -0.0089 | 0 | 76 | 2.6e-006 | 1 | R.AVFVDLEPTVIDEVR.T 16242 16243 |
|  | 16504 | 859.9384 | 1717.8623 | 1717.8747 | -0.0124 | 0 | 44 | 0.0044 | 1 | R.NLDIERPTYTNLNR.L 16503 16505 |
|  | 17071 | 878.9804 | 1755.9463 | 1755.9559 | -0.0097 | 0 | 65 | 3.1e-005 | 1 | R.IHFPLATYAPVISAEK.A 17060 17069 17070 17072 17074 |
|  | 18100 | 912.9898 | 1823.9651 | 1823.9782 | -0.0131 | 0 | 57 | 0.00019 | 1 | K.VGINYQPPTVVPGGDLAK.V 18098 18099 18101 |
|  | 18338 | 461.2563 | 1840.9959 | 1841.0047 | -0.0087 | 1 | 7 | 22 | 9 | R.GHYTIGKEIIDLVLDR.I |
|  | 18674 | 932.9498 | 1863.8851 | 1863.8971 | -0.0120 | 0 | 84 | 3.8e-007 | 1 | R.AVCMLSNTTAIAEAWAR.L 18675 |
|  | 20417 | 1004.4446 | 2006.8746 | 2006.8858 | -0.0112 | 0 | 69 | 1.1e-005 | 1 | K.TIGGGDDSFNTFFSETGAGK.H |
|  | 23458 | 777.3395 | 2328.9966 | 2329.0110 | -0.0144 | 0 | 40 | 0.0071 | 1 | R.AFVHWYVGEGMEEGEFSEAR.E |
|  | 24021 | 1205.1011 | 2408.1876 | 2408.2012 | -0.0136 | 0 | 116 | 1.8e-010 | 1 | R.FDGALNVDLTEFQTNLVPYPR.I 24022 24023 24024 24025 24026 |
|  | 24065 | 1208.0977 | 2414.1808 | 2414.1978 | -0.0171 | 1 | 71 | 6.6e-006 | 1 | R.QLFHPEQLITGKEDAANNYAR.G 24063 24064 |
|  | 26296 | 1375.6395 | 2749.2645 | 2749.2840 | -0.0194 | 0 | 84 | 2.1e-007 | 1 | K.AYHEQLSVAEITNACFEPANQMVK.C 26295 26298 26299 |
|  | 26370 | 922.7617 | 2765.2632 | 2765.2789 | -0.0157 | 0 | (32) | 0.034 | 1 | K.AYHEQLSVAEITNACFEPANQMVK.C |
|  | 28289 | **790.0373** | **3156.1201** | **3156.7255** | **-0.6054** | **1** | **1** | **27** | **1** | **R.NLDIERPTYTNLNRLIGQIVSSITASLR.F** |
|  | 28599 | 805.1068 | 3216.3979 | 3216.4168 | -0.0189 | 1 | 22 | 0.28 | 1 | R.AFVHWYVGEGMEEGEFSEAREDMAALEK.D |
|  | 29206 | 1130.8745 | 3389.6017 | 3389.6173 | -0.0156 | 0 | 35 | 0.013 | 1 | K.LADQCTGLQGFLVFHSFGGGTGSGFTSLLMER.L |
|  | 29521 | 1173.5731 | 3517.6975 | 3517.7123 | -0.0148 | 1 | 61 | 3.4e-005 | 1 | R.KLADQCTGLQGFLVFHSFGGGTGSGFTSLLMER.L 29522 |
|  | 30798 | 1074.9877 | 4295.9216 | 4295.9497 | -0.0281 | 0 | 3 | 10 | 1 | R.ECISIHVGQAGVQIGNACWELYCLEHGIQPDGQMPSDK.T |
|  | 31036 | 1146.7736 | 4583.0651 | 4583.0913 | -0.0261 | 1 | 57 | 3.5e-005 | 1 | -.MRECISIHVGQAGVQIGNACWELYCLEHGIQPDGQMPSDK.T 31035 31037 |
|  | 31716 | 1135.5408 | 5672.6675 | 5672.6902 | -0.0227 | 1 | 12 | 0.58 | 1 | K.LEFSIYPAPQVSTAVVEPYNSILTTHTTLEHSDCAFMVDNEAIYDICRR.N |
|  | 31729 | 1147.5480 | 5732.7035 | 5731.7161 | 0.9874 | 1 | 3 | 4.8 | 1 | K.SKLEFSIYPAPQVSTAVVEPYNSILTTHTTLEHSDCAFMVDNEAIYDICR.R 31728 |

| **20.** | P35580\|MYH10_HUMAN    **Mass:** 229827   **Score:** 1135   **Queries matched:** 117   **emPAI:** 0.56 |
| --- | --- |
|  | Myosin-10 OS=Homo sapiens GN=MYH10 PE=1 SV=3 |

|  | **Query** | **Observed** | **Mr(expt)** | **Mr(calc)** | **Delta** | **Miss** | **Score** | **Expect** | **Rank** | **Peptide** |
| --- | --- | --- | --- | --- | --- | --- | --- | --- | --- | --- |
|  | 834 | 518.2890 | 517.2817 | 517.2608 | 0.0209 | 0 | 6 | 1e+002 | 9 | R.ANASR.R |
|  | 884 | 533.1866 | 532.1793 | 531.3017 | 0.8777 | 0 | 9 | 41 | 1 | R.IGQSK.I 880 882 |
|  | 885 | **533.1890** | **532.1818** | **532.2969** | **-0.1151** | **0** | **10** | **26** | **1** | **K.SISAR.Y** 886 |
|  | 988 | 572.1176 | 571.1103 | 571.3806 | -0.2703 | 1 | 10 | 15 | 5 | R.IKVGR.D |
|  | 1026 | 586.2965 | 585.2892 | 585.2944 | -0.0053 | 0 | 15 | 6.2 | 7 | K.MNPPK.F |
|  | 1746 | 404.7289 | 807.4431 | 807.4491 | -0.0059 | 0 | 23 | 0.97 | 1 | R.TVGQLYK.E 1763 |
|  | 1795 | **406.6971** | **811.3797** | **811.4228** | **-0.0431** | **0** | **11** | **10** | **2** | **R.YLFVDR.A** 1802 |
|  | 1841 | **408.7010** | **815.3874** | **815.4501** | **-0.0626** | **1** | **6** | **53** | **9** | **K.ANLEKNK.Q** 1838 |
|  | 2279 | 423.7447 | 845.4748 | 845.4793 | -0.0045 | 1 | 19 | 2.6 | 3 | R.KLQAQMK.D 2280 |
|  | 2513 | 432.2372 | 862.4599 | 861.4742 | 0.9857 | 1 | (17) | 3.8 | 4 | R.KLQAQMK.D |
|  | 2892 | 446.7125 | 891.4105 | 891.4160 | -0.0055 | 0 | 16 | 4.8 | 1 | K.ADEWLMK.N |
|  | 3421 | **466.2501** | **930.4857** | **930.5134** | **-0.0277** | **1** | **22** | **1.3** | **1** | **K.SALLDEKR.R** |
|  | 3505 | **469.2765** | **936.5384** | **937.5022** | **-0.9637** | **0** | **13** | **7.2** | **1** | **R.IVFQEFR.Q** 3507 |
|  | 3855 | 481.2429 | 960.4712 | 960.4777 | -0.0066 | 0 | 40 | 0.018 | 1 | R.NTNPNFVR.C |
|  | 4647 | 509.2570 | 1016.4994 | 1016.5073 | -0.0079 | 0 | 32 | 0.13 | 1 | R.CNGVLEGIR.I |
|  | 4711 | 512.2675 | 1022.5205 | 1023.4734 | -0.9529 | 1 | 9 | 19 | 1 | K.NDNSSRFGK.F 4724 |
|  | 4725 | **512.7710** | **1023.5274** | **1023.5349** | **-0.0074** | **0** | **22** | **0.93** | **2** | **K.HQQLLEEK.N** |
|  | 5110 | **525.7916** | **1049.5687** | **1049.5757** | **-0.0070** | **1** | **24** | **0.61** | **1** | **K.ALAYDKLEK.T** |
|  | 5429 | **537.7565** | **1073.4985** | **1072.5764** | **0.9221** | **1** | **9** | **23** | **9** | **R.EQEVAELKK.A** 5409 |
|  | 5508 | **540.2557** | **1078.4969** | **1078.5012** | **-0.0042** | **1** | **7** | **35** | **2** | **K.QACERMIR.A** |
|  | 5670 | 546.2700 | 1090.5254 | 1091.5499 | -1.0245 | 0 | 28 | 0.25 | 3 | K.FDQLLAEEK.S |
|  | 5706 | **547.2819** | **1092.5492** | **1091.5029** | **1.0463** | **1** | **5** | **48** | **3** | **K.EQMEKANAR.M** |
|  | 6013 | **558.7814** | **1115.5482** | **1114.5618** | **0.9864** | **0** | **7** | **30** | **2** | **K.EEELQGALAR.G** |
|  | 6182 | **564.3276** | **1126.6406** | **1126.6499** | **-0.0092** | **1** | **7** | **29** | **2** | **K.KLVWIPSER.H** |
|  | 6523 | **576.8171** | **1151.6197** | **1151.6298** | **-0.0101** | **1** | **28** | **0.22** | **2** | **R.KHQQLLEEK.N** |
|  | 6948 | **591.7996** | **1181.5847** | **1180.5836** | **1.0011** | **1** | **11** | **11** | **5** | **K.SISARYAEER.D** |
|  | 7383 | 601.1215 | 1200.2284 | 1200.5809 | -0.3525 | 1 | 5 | 30 | 4 | K.DDIQKMNPPK.F |
|  | 7443 | **602.3182** | **1202.6218** | **1202.6295** | **-0.0077** | **0** | **7** | **30** | **4** | **R.ALELDPNLYR.I** |
|  | 7794 | 610.8259 | 1219.6372 | 1219.6448 | -0.0076 | 1 | 48 | 0.0025 | 1 | K.KFDQLLAEEK.S 7793 |
|  | 7855 | 408.8608 | 1223.5607 | 1222.6306 | 0.9301 | 0 | 7 | 26 | 4 | R.AGVLAHLEEER.D |
|  | 8013 | **616.7965** | **1231.5783** | **1231.5867** | **-0.0083** | **0** | **21** | **1.3** | **1** | **R.ALEQQVEEMR.T** |
|  | 8212 | **414.9101** | **1241.7086** | **1242.6568** | **-0.9482** | **1** | **1** | **1.2e+002** | **3** | **K.KEEELQGALAR.G** |
|  | 9271 | 648.3187 | 1294.6228 | 1293.6426 | 0.9802 | 1 | 1 | 1.1e+002 | 9 | R.GGPISFSSSRSGR.R |
|  | 9308 | **433.2030** | **1296.5873** | **1297.5543** | **-0.9671** | **1** | **10** | **14** | **2** | **K.GFMDGKQACER.M** |
|  | 9744 | 440.2517 | 1317.7333 | 1317.7405 | -0.0072 | 0 | 45 | 0.0048 | 1 | K.LDPHLVLDQLR.C 9743 |
|  | 10286 | **449.9686** | **1346.8841** | **1347.7002** | **-0.8162** | **1** | **6** | **29** | **2** | **K.LRLEVNMQAMK.A** |
|  | 11208 | 699.3379 | 1396.6612 | 1396.6697 | -0.0084 | 1 | 55 | 0.00043 | 1 | K.VDYKADEWLMK.N 11207 |
|  | 11301 | **468.2273** | **1401.6602** | **1401.6670** | **-0.0069** | **1** | **8** | **22** | **1** | **-.MAQRTGLEDPER.Y** |
|  | 11501 | **707.8937** | **1413.7728** | **1413.7827** | **-0.0099** | **0** | **5** | **38** | **1** | **K.QVLALQSQLADTK.K** |
|  | 11775 | **715.3574** | **1428.7002** | **1427.7368** | **0.9634** | **1** | **5** | **42** | **6** | **K.DLEAQIEAANKAR.D** |
|  | 12169 | **484.9333** | **1451.7780** | **1451.7885** | **-0.0104** | **0** | **4** | **45** | **2** | **K.VIQYLAHVASSHK.G** |
|  | 13060 | **502.2469** | **1503.7188** | **1503.6801** | **0.0387** | **1** | **2** | **77** | **3** | **R.DLQTRDEQNEEK.K** 13083 |
|  | 13223 | **757.8382** | **1513.6618** | **1513.7624** | **-0.1005** | **0** | **9** | **15** | **1** | **K.IGQLEEQLEQEAK.E** |
|  | 13334 | **760.7272** | **1519.4399** | **1518.6798** | **0.7601** | **0** | **8** | **14** | **1** | **R.ELDDATEANEGLSR.E** |
|  | 13618 | 513.2499 | 1536.7280 | 1536.7395 | -0.0115 | 1 | 13 | 5.4 | 2 | K.DKADFCIIHYAGK.V |
|  | 13688 | **771.4152** | **1540.8159** | **1541.8565** | **-1.0406** | **1** | **9** | **16** | **2** | **R.QRYEILTPNAIPK.G** 13687 |
|  | 13849 | 776.3460 | 1550.6775 | 1550.6882 | -0.0108 | 0 | 18 | 1.8 | 1 | R.EDQSILCTGESGAGK.T |
|  | 14213 | 787.9492 | 1573.8839 | 1573.8940 | -0.0101 | 1 | 46 | 0.0032 | 1 | R.AGKLDPHLVLDQLR.C 14209 |
|  | 14288 | 790.4199 | 1578.8253 | 1578.8365 | -0.0112 | 1 | 45 | 0.0041 | 1 | R.AGVLAHLEEERDLK.I 14289 |
|  | 14354 | **792.4155** | **1582.8164** | **1583.8882** | **-1.0718** | **1** | **3** | **53** | **7** | **K.ALSLARALEEALEAK.E** |
|  | 14475 | **795.8916** | **1589.7686** | **1589.7719** | **-0.0032** | **1** | **21** | **0.94** | **1** | **R.NKQEVMISDLEER.L** 14474 |
|  | 14564 | **532.9061** | **1595.6966** | **1594.8541** | **0.8424** | **0** | **14** | **4.1** | **2** | **K.ITDIIIFFQAVCR.G** |
|  | 14708 | **536.2587** | **1605.7542** | **1605.7668** | **-0.0126** | **1** | **(4)** | **42** | **3** | **R.NKQEVMISDLEER.L** |
|  | 14984 | **541.3418** | **1621.0036** | **1621.7141** | **-0.7105** | **0** | **1** | **74** | **10** | **R.IAECSSQLAEEEEK.A** |
|  | 15143 | **817.3729** | **1632.7313** | **1632.7413** | **-0.0100** | **0** | **71** | **1e-005** | **1** | **R.NTDQASMPENTVAQK.L** 15144 |
|  | 15615 | 554.7940 | 1661.3602 | 1661.8671 | -0.5069 | 1 | 2 | 48 | 5 | K.LMATLRNTNPNFVR.C |
|  | 15777 | **558.2557** | **1671.7452** | **1671.6968** | **0.0485** | **1** | **3** | **63** | **2** | **R.ADMEDLMSSKDDVGK.N** |
|  | 15824 | **838.3630** | **1674.7114** | **1675.8570** | **-1.1456** | **0** | **2** | **68** | **9** | **R.FLSNGYIPIPGQQDK.D** |
|  | 16058 | **845.9077** | **1689.8009** | **1689.8131** | **-0.0122** | **0** | **70** | **1.1e-005** | **1** | **K.MEEEILLLEDQNSK.F** 16083 |
|  | 16241 | 851.4521 | 1700.8896 | 1700.8468 | 0.0428 | 1 | 5 | 36 | 9 | R.QEEELQAKDEELLK.V |
|  | 16936 | **872.9517** | **1743.8888** | **1743.9003** | **-0.0115** | **0** | **50** | **0.001** | **1** | **K.TTLQVDTLNAELAAER.S** |
|  | 17048 | **877.9377** | **1753.8608** | **1753.8709** | **-0.0100** | **0** | **30** | **0.1** | **1** | **K.NLPIYSENIIEMYR.G** 17049 |
|  | 17716 | **900.3731** | **1798.7315** | **1798.9061** | **-0.1745** | **1** | **1** | **76** | **2** | **K.IGQLEEQLEQEAKER.A** |
|  | 18239 | **917.5032** | **1832.9919** | **1833.9029** | **-0.9110** | **1** | **3** | **48** | **1** | **K.KMEEEILLLEDQNSK.F** 18211 |
|  | 19511 | **966.9362** | **1931.8578** | **1932.9276** | **-1.0698** | **0** | **1** | **77** | **9** | **K.TELEDTLDTTAAQQELR.T** |
|  | 19645 | **972.9864** | **1943.9583** | **1943.9687** | **-0.0104** | **0** | **102** | **5.4e-009** | **1** | **K.LQNELDNVSTLLEEAEK.K** 19644 |
|  | 19779 | **651.6684** | **1951.9834** | **1951.9963** | **-0.0129** | **1** | **10** | **9.2** | **1** | **R.HATALEELSEQLEQAKR.F** 19781 |
|  | 19916 | 981.4566 | 1960.8986 | 1960.9113 | -0.0126 | 0 | 110 | 9.5e-010 | 1 | R.TQLEELEDELQATEDAK.L 19915 |
|  | 20410 | **1003.9751** | **2005.9356** | **2005.9480** | **-0.0123** | **0** | **17** | **1.8** | **2** | **R.ELQAQIAELQEDFESEK.A** |
|  | 20766 | **681.3201** | **2040.9386** | **2040.9534** | **-0.0149** | **1** | **30** | **0.1** | **1** | **K.ALEEETKNHEAQIQDMR.Q** |
|  | 21133 | **1036.4175** | **2070.8204** | **2071.9897** | **-1.1693** | **1** | **2** | **44** | **1** | **K.RHEMPPHIYAISESAYR.C** |
|  | 21270 | **696.3225** | **2085.9455** | **2085.9702** | **-0.0247** | **0** | **2** | **56** | **4** | **R.QLHLEGASLELSDDDTESK.T** |
|  | 21292 | **1044.5143** | **2087.0140** | **2087.0245** | **-0.0105** | **0** | **82** | **5.5e-007** | **1** | **R.IVGLDQVTGMTETAFGSAYK.T** |
|  | 21961 | **1079.0531** | **2156.0916** | **2155.1612** | **0.9304** | **1** | **2** | **50** | **1** | **K.ITDIIIFFQAVCRGYLAR.K** |
|  | 22310 | **734.0035** | **2198.9888** | **2199.0039** | **-0.0151** | **1** | **55** | **0.00027** | **1** | **R.HAEQERDELADEITNSASGK.S** |
|  | 22403 | **736.3840** | **2206.1301** | **2205.0648** | **1.0653** | **1** | **2** | **49** | **5** | **R.DELADEITNSASGKSALLDEK.R** |
|  | 22778 | **747.6699** | **2239.9878** | **2240.0015** | **-0.0137** | **0** | **47** | **0.0016** | **1** | **K.MQAHIQDLEEQLDEEEGAR.Q** |
|  | 23101 | 1143.5518 | 2285.0890 | 2285.1031 | -0.0142 | 0 | 100 | 7.2e-009 | 1 | K.VEDMAELTCLNEASVLHNLK.D 23100 23102 |
|  | 23786 | **1186.6175** | **2371.2205** | **2371.2311** | **-0.0106** | **0** | **42** | **0.0045** | **1** | **R.INFDVTGYIVGANIETYLLEK.S** |
|  | 23981 | **1202.5707** | **2403.1268** | **2403.1401** | **-0.0133** | **0** | **120** | **6.9e-011** | **1** | **K.DAASLESQLQDTQELLQEETR.Q** 23982 23983 |
|  | 24192 | **1218.5866** | **2435.1585** | **2434.1685** | **0.9900** | **0** | **100** | **6.1e-009** | **1** | **K.NILAEQLQAETELFAEAEEMR.A** 24180 |
|  | 24420 | 823.0679 | 2466.1820 | 2466.2002 | -0.0182 | 0 | 6 | 19 | 2 | K.LQQLFNHTMFILEQEEYQR.E |
|  | 25241 | **858.4396** | **2572.2969** | **2572.2479** | **0.0490** | **1** | **1** | **52** | **4** | **K.DVDRIVGLDQVTGMTETAFGSAYK.T** |
|  | 25638 | 883.4349 | 2647.2830 | 2647.2985 | -0.0155 | 1 | 81 | 4.7e-007 | 1 | K.FSKVEDMAELTCLNEASVLHNLK.D 25639 25641 |
|  | 28370 | 1059.1654 | 3174.4744 | 3174.4874 | -0.0130 | 1 | 34 | 0.02 | 1 | R.ALEQQVEEMRTQLEELEDELQATEDAK.L |

| **21.** | Q13748\|TBA3C_HUMAN    **Mass:** 50612    **Score:** 1116   **Queries matched:** 47   **emPAI:** 2.12 |
| --- | --- |
|  | Tubulin alpha-3C/D chain OS=Homo sapiens GN=TUBA3C PE=1 SV=3 |

|  | **Query** | **Observed** | **Mr(expt)** | **Mr(calc)** | **Delta** | **Miss** | **Score** | **Expect** | **Rank** | **Peptide** |
| --- | --- | --- | --- | --- | --- | --- | --- | --- | --- | --- |
|  | 3129 | 455.7370 | 909.4595 | 908.4967 | 0.9628 | 1 | 14 | 5.3 | 1 | R.LSVDYGKK.S |
|  | 4625 | 508.2894 | 1014.5642 | 1014.5709 | -0.0067 | 0 | 36 | 0.043 | 1 | K.DVNAAIATIK.T |
|  | 4720 | 512.7546 | 1023.4947 | 1022.4417 | 1.0530 | 0 | 1 | 1.3e+002 | 5 | K.EDAANNYAR.G |
|  | 4988 | 521.7651 | 1041.5156 | 1042.5270 | -1.0114 | 1 | 7 | 34 | 10 | K.FDLMYAKR.A |
|  | 10920 | 690.8480 | 1379.6814 | 1379.6907 | -0.0094 | 1 | 25 | 0.5 | 1 | R.LDHKFDLMYAK.R 10921 |
|  | 12255 | 729.4348 | 1456.8549 | 1456.8613 | -0.0064 | 0 | 78 | 2e-006 | 1 | R.LIGQIVSSITASLR.F 12252 12253 12265 |
|  | 16504 | 859.9384 | 1717.8623 | 1717.8747 | -0.0124 | 0 | 44 | 0.0044 | 1 | R.NLDIERPTYTNLNR.L 16503 16505 |
|  | 17071 | 878.9804 | 1755.9463 | 1755.9559 | -0.0097 | 0 | 65 | 3.1e-005 | 1 | R.IHFPLATYAPVISAEK.A 17060 17069 17070 17072 17074 |
|  | 18100 | 912.9898 | 1823.9651 | 1823.9782 | -0.0131 | 0 | 57 | 0.00019 | 1 | K.VGINYQPPTVVPGGDLAK.V 18098 18099 18101 |
|  | 18674 | 932.9498 | 1863.8851 | 1863.8971 | -0.0120 | 0 | 84 | 3.8e-007 | 1 | R.AVCMLSNTTAIAEAWAR.L 18675 |
|  | 20417 | 1004.4446 | 2006.8746 | 2006.8858 | -0.0112 | 0 | 69 | 1.1e-005 | 1 | K.TIGGGDDSFNTFFSETGAGK.H |
|  | 23458 | 777.3395 | 2328.9966 | 2329.0110 | -0.0144 | 0 | 40 | 0.0071 | 1 | R.AFVHWYVGEGMEEGEFSEAR.E |
|  | 24021 | 1205.1011 | 2408.1876 | 2408.2012 | -0.0136 | 0 | 116 | 1.8e-010 | 1 | R.FDGALNVDLTEFQTNLVPYPR.I 24022 24023 24024 24025 24026 |
|  | 24065 | 1208.0977 | 2414.1808 | 2414.1978 | -0.0171 | 1 | 71 | 6.6e-006 | 1 | R.QLFHPEQLITGKEDAANNYAR.G 24063 24064 |
|  | 26296 | 1375.6395 | 2749.2645 | 2749.2840 | -0.0194 | 0 | 84 | 2.1e-007 | 1 | K.AYHEQLSVAEITNACFEPANQMVK.C 26295 26298 26299 |
|  | 26370 | 922.7617 | 2765.2632 | 2765.2789 | -0.0157 | 0 | (32) | 0.034 | 1 | K.AYHEQLSVAEITNACFEPANQMVK.C |
|  | 28289 | 790.0373 | 3156.1201 | 3156.7255 | -0.6054 | 1 | 1 | 27 | 1 | R.NLDIERPTYTNLNRLIGQIVSSITASLR.F |
|  | 30798 | 1074.9877 | 4295.9216 | 4295.9497 | -0.0281 | 0 | 3 | 10 | 1 | R.ECISIHVGQAGVQIGNACWELYCLEHGIQPDGQMPSDK.T |
|  | 31036 | 1146.7736 | 4583.0651 | 4583.0913 | -0.0261 | 1 | 57 | 3.5e-005 | 1 | -.MRECISIHVGQAGVQIGNACWELYCLEHGIQPDGQMPSDK.T 31035 31037 |
|  | 31727 | **1147.3433** | **5731.6799** | **5731.7161** | **-0.0361** | **1** | **10** | **0.96** | **1** | **K.SKLEFAIYPAPQVSTAVVEPYNSILTTHTTLEHSDCAFMVDNEAIYDICR.R** |

| **22.** | P35749\|MYH11_HUMAN    **Mass:** 228054   **Score:** 1091   **Queries matched:** 121   **emPAI:** 0.62 |
| --- | --- |
|  | Myosin-11 OS=Homo sapiens GN=MYH11 PE=1 SV=3 |

|  | **Query** | **Observed** | **Mr(expt)** | **Mr(calc)** | **Delta** | **Miss** | **Score** | **Expect** | **Rank** | **Peptide** |
| --- | --- | --- | --- | --- | --- | --- | --- | --- | --- | --- |
|  | 884 | 533.1866 | 532.1793 | 531.3017 | 0.8777 | 0 | 9 | 41 | 1 | R.IGQSK.I 880 882 |
|  | 988 | 572.1176 | 571.1103 | 571.3806 | -0.2703 | 1 | 10 | 15 | 5 | R.IKVGR.D |
|  | 1026 | 586.2965 | 585.2892 | 585.2944 | -0.0053 | 0 | 15 | 6.2 | 7 | K.MNPPK.F |
|  | 1212 | **661.3826** | **660.3754** | **660.3806** | **-0.0053** | **0** | **29** | **0.33** | **1** | **K.LNVSTK.L** |
|  | 1746 | 404.7289 | 807.4431 | 807.4491 | -0.0059 | 0 | 23 | 0.97 | 1 | R.TVGQLYK.E 1763 |
|  | 2279 | 423.7447 | 845.4748 | 845.4793 | -0.0045 | 1 | 19 | 2.6 | 3 | R.KLQAQMK.D 2280 |
|  | 2513 | 432.2372 | 862.4599 | 861.4742 | 0.9857 | 1 | (17) | 3.8 | 4 | R.KLQAQMK.D |
|  | 3249 | **459.7278** | **917.4411** | **918.3930** | **-0.9519** | **0** | **13** | **9** | **5** | **R.LEEEEDR.G** 3271 |
|  | 3409 | **465.7571** | **929.4997** | **929.5182** | **-0.0184** | **0** | **4** | **68** | **10** | **R.QLVSNLEK.K** |
|  | 3505 | 469.2765 | 936.5384 | 937.5022 | -0.9637 | 0 | 13 | 7.2 | 1 | R.IVFQEFR.Q 3507 |
|  | 3644 | 947.4919 | 946.4847 | 946.4906 | -0.0059 | 0 | 31 | 0.14 | 1 | R.QQQLTAMK.V 3641 3643 |
|  | 3684 | **475.2343** | **948.4541** | **947.4825** | **0.9716** | **0** | **10** | **18** | **6** | **R.NTTPNFVR.C** |
|  | 4032 | **487.7671** | **973.5196** | **973.5266** | **-0.0071** | **0** | **36** | **0.045** | **1** | **R.IVGLDQMAK.M** |
|  | 4268 | **496.3571** | **990.6996** | **989.5216** | **1.1781** | **0** | **(1)** | **1.1e+002** | **2** | **R.IVGLDQMAK.M** |
|  | 4647 | 509.2570 | 1016.4994 | 1016.5073 | -0.0079 | 0 | 32 | 0.13 | 1 | R.CNGVLEGIR.I |
|  | 4711 | 512.2675 | 1022.5205 | 1023.4734 | -0.9529 | 1 | 9 | 19 | 1 | K.NDNSSRFGK.F 4724 |
|  | 4722 | 512.7650 | 1023.5153 | 1023.5237 | -0.0083 | 1 | 23 | 0.77 | 1 | K.ATDKSFVEK.L 4732 4742 |
|  | 5043 | 523.2861 | 1044.5576 | 1044.5637 | -0.0061 | 0 | 14 | 6.9 | 2 | R.LEVNMQALK.G 5042 |
|  | 5429 | 537.7565 | 1073.4985 | 1072.6128 | 0.8857 | 1 | 9 | 23 | 9 | R.EQEVTVLKK.A |
|  | 5670 | 546.2700 | 1090.5254 | 1091.5499 | -1.0245 | 0 | 28 | 0.25 | 3 | K.FDQLLAEEK.N |
|  | 5854 | 552.2991 | 1102.5836 | 1102.5917 | -0.0081 | 1 | 31 | 0.13 | 1 | K.RQQQLTAMK.V 5856 |
|  | 5987 | **557.3205** | **1112.6264** | **1112.6342** | **-0.0078** | **1** | **9** | **19** | **4** | **K.RLVWVPSEK.Q** |
|  | 6070 | 560.2959 | 1118.5772 | 1118.5866 | -0.0094 | 1 | (22) | 1.2 | 1 | K.RQQQLTAMK.V |
|  | 6193 | 564.8488 | 1127.6831 | 1128.5775 | -0.8944 | 0 | 9 | 21 | 7 | K.EEELQAALAR.L |
|  | 6539 | **577.2689** | **1152.5232** | **1152.5332** | **-0.0101** | **0** | **3** | **74** | **10** | **K.MTESSLPSASK.T** |
|  | 7383 | 601.1215 | 1200.2284 | 1200.5809 | -0.3525 | 1 | 5 | 30 | 4 | K.DDIQKMNPPK.F |
|  | 7409 | **401.2843** | **1200.8311** | **1201.6190** | **-0.7879** | **0** | **5** | **37** | **10** | **K.DLELQADSAIK.G** |
|  | 7443 | 602.3182 | 1202.6218 | 1202.6295 | -0.0077 | 0 | 7 | 30 | 4 | K.ALELDPNLYR.I |
|  | 7552 | **403.5449** | **1207.6128** | **1207.6197** | **-0.0068** | **1** | **14** | **5.3** | **1** | **R.ASRDEIFATAK.E** |
|  | 7665 | **607.7773** | **1213.5401** | **1213.5509** | **-0.0108** | **0** | **31** | **0.13** | **1** | **R.SHEAQVQEMR.Q** 7658 |
|  | 7794 | 610.8259 | 1219.6372 | 1219.6448 | -0.0076 | 1 | 48 | 0.0025 | 1 | R.KFDQLLAEEK.N 7793 |
|  | 8237 | **415.2272** | **1242.6598** | **1243.6230** | **-0.9633** | **1** | **3** | **71** | **10** | **K.LHEMEGAVKSK.F** |
|  | 8258 | **622.8114** | **1243.6082** | **1243.6230** | **-0.0148** | **1** | **9** | **20** | **3** | **R.SKLHEMEGAVK.S** |
|  | 8832 | **636.8386** | **1271.6626** | **1271.6721** | **-0.0095** | **0** | **20** | **1.3** | **1** | **K.LEAQVQELQSK.C** |
|  | 9709 | **658.8789** | **1315.7433** | **1315.7500** | **-0.0067** | **0** | **16** | **4.1** | **1** | **K.LDAFLVLEQLR.C** |
|  | 9971 | **664.8211** | **1327.6277** | **1328.6394** | **-1.0118** | **0** | **7** | **30** | **4** | **K.HESMISELEVR.L** 10000 |
|  | 10006 | **665.8409** | **1329.6672** | **1329.6776** | **-0.0103** | **1** | **49** | **0.002** | **1** | **K.AENELKELEQK.H** |
|  | 10430 | 677.8120 | 1353.6095 | 1353.6234 | -0.0139 | 1 | 25 | 0.49 | 1 | K.MAEQYKEQAEK.G |
|  | 10585 | **682.3154** | **1362.6162** | **1362.7507** | **-0.1345** | **1** | **8** | **22** | **3** | **R.TVGQLYKEQLGK.L** |
|  | 10955 | **691.3463** | **1380.6779** | **1380.6377** | **0.0403** | **1** | **12** | **8.1** | **3** | **K.RALETQMEEMK.T** |
|  | 11273 | **700.8856** | **1399.7566** | **1399.7671** | **-0.0105** | **1** | **94** | **5e-008** | **1** | **K.KLEAQVQELQSK.C** 11280 11292 |
|  | 12918 | **748.8350** | **1495.6555** | **1495.7262** | **-0.0707** | **1** | **3** | **69** | **10** | **K.MLKAEMEDLVSSK.D** |
|  | 13385 | **762.3592** | **1522.7038** | **1522.7151** | **-0.0112** | **0** | **46** | **0.0034** | **1** | **K.EIENLTQQYEEK.A** |
|  | 13719 | **515.3096** | **1542.9071** | **1541.7685** | **1.1386** | **0** | **4** | **49** | **5** | **K.IAQLEEQVEQEAR.E** |
|  | 13843 | **775.9301** | **1549.8455** | **1550.6518** | **-0.8063** | **0** | **11** | **10** | **2** | **R.ELDEATESNEAMGR.E** |
|  | 13849 | 776.3460 | 1550.6775 | 1550.6882 | -0.0108 | 0 | 18 | 1.8 | 1 | R.EDQSILCTGESGAGK.T |
|  | 14116 | **523.3077** | **1566.9012** | **1566.6467** | **0.2545** | **0** | **(5)** | **39** | **4** | **R.ELDEATESNEAMGR.E** |
|  | 14171 | **524.6159** | **1570.8259** | **1570.7306** | **0.0953** | **0** | **(5)** | **45** | **3** | **K.ITDVIMAFQAMCR.G** |
|  | 14354 | 792.4155 | 1582.8164 | 1583.8882 | -1.0718 | 1 | 3 | 53 | 7 | K.ALSLARALEEALEAK.E |
|  | 14356 | **528.6263** | **1582.8572** | **1582.8678** | **-0.0107** | **1** | **23** | **0.66** | **1** | **R.HISTLNIQLSDSKK.K** |
|  | 14415 | **529.7856** | **1586.3351** | **1586.7255** | **-0.3904** | **0** | **5** | **28** | **3** | **K.ITDVIMAFQAMCR.G** |
|  | 14480 | 796.3496 | 1590.6845 | 1590.6944 | -0.0098 | 0 | (30) | 0.12 | 1 | R.NTDQASMPDNTAAQK.V 14479 14481 |
|  | 14725 | 804.3456 | 1606.6767 | 1606.6893 | -0.0126 | 0 | 43 | 0.0059 | 1 | R.NTDQASMPDNTAAQK.V |
|  | 14755 | **805.3480** | **1608.6815** | **1608.8471** | **-0.1656** | **1** | **23** | **0.58** | **1** | **R.TGVLAHLEEERDLK.I** |
|  | 14982 | **541.3065** | **1620.8977** | **1621.7505** | **-0.8528** | **1** | **7** | **23** | **1** | **K.AEMEDLVSSKDDVGK.N** |
|  | 15102 | **815.4149** | **1628.8152** | **1628.8257** | **-0.0105** | **1** | **33** | **0.058** | **1** | **R.ALEEALEAKEELER.T** 15108 |
|  | 15191 | **818.9031** | **1635.7917** | **1635.7992** | **-0.0074** | **0** | **18** | **2** | **2** | **K.LQDFASTVEALEEGK.K** |
|  | 15392 | **825.3638** | **1648.7130** | **1648.7250** | **-0.0120** | **0** | **29** | **0.14** | **1** | **R.NSLQDQLDEEMEAK.Q** |
|  | 15630 | **554.9370** | **1661.7892** | **1661.8018** | **-0.0126** | **0** | **10** | **10** | **1** | **K.VCHLMGINVTDFTR.S** |
|  | 15632 | **831.9434** | **1661.8722** | **1661.8559** | **0.0163** | **1** | **12** | **6.6** | **3** | **R.LEVNMQALKGQFER.D** |
|  | 16633 | 863.9731 | 1725.9316 | 1725.9413 | -0.0097 | 0 | 83 | 5.2e-007 | 1 | K.QLLQANPILEAFGNAK.T 16634 16646 16647 |
|  | 16988 | **584.2689** | **1749.7849** | **1750.8964** | **-1.1114** | **1** | **3** | **60** | **5** | **K.HLPIYSEKIVDMYK.G** |
|  | 17046 | **585.6263** | **1753.8570** | **1753.8668** | **-0.0098** | **1** | **45** | **0.0033** | **1** | **K.KQELEEILHEMEAR.L** |
|  | 17220 | 590.6169 | 1768.8288 | 1769.8617 | -1.0329 | 1 | (17) | 2.1 | 2 | K.KQELEEILHEMEAR.L |
|  | 17597 | **895.9482** | **1789.8819** | **1789.8945** | **-0.0126** | **1** | **46** | **0.0028** | **1** | **R.ISDLTTNLAEEEEKAK.N** 17554 17598 |
|  | 17784 | **601.9619** | **1802.8637** | **1802.8720** | **-0.0083** | **1** | **1** | **87** | **6** | **K.KLEDEILVMDDQNNK.L** |
|  | 18166 | **915.4291** | **1828.8437** | **1828.8551** | **-0.0114** | **1** | **56** | **0.00029** | **1** | **R.LEEEEDRGQQLQAER.K** 18165 18167 |
|  | 18817 | 626.3577 | 1876.0512 | 1875.8381 | 0.2131 | 1 | 19 | 1.1 | 1 | K.ERNTDQASMPDNTAAQK.V |
|  | 19114 | **634.3879** | **1900.1420** | **1899.8937** | **0.2483** | **0** | **0** | **76** | **5** | **R.HEMPPHIYAIADTAYR.S** |
|  | 19257 | **956.9792** | **1911.9439** | **1911.9538** | **-0.0098** | **0** | **39** | **0.012** | **1** | **R.LQQELDDLVVDLDNQR.Q** |
|  | 19302 | **958.9811** | **1915.9476** | **1915.9599** | **-0.0123** | **1** | **151** | **8.1e-014** | **1** | **R.KATQQAEQLSNELATER.S** |
|  | 19419 | **642.9819** | **1925.9238** | **1925.9370** | **-0.0132** | **1** | **13** | **4.8** | **1** | **R.FQKEIENLTQQYEEK.A** |
|  | 19732 | **974.4639** | **1946.9132** | **1946.9255** | **-0.0123** | **0** | **107** | **2.1e-009** | **1** | **K.LQNEVESVTGMLNEAEGK.A** |
|  | 19916 | 981.4566 | 1960.8986 | 1960.9113 | -0.0126 | 0 | 110 | 9.5e-010 | 1 | K.TQLEELEDELQATEDAK.L 19915 |
|  | 20373 | **1001.9860** | **2001.9575** | **2001.9677** | **-0.0102** | **0** | **43** | **0.0052** | **1** | **K.SLEADLMQLQEDLAAAER.A** |
|  | 20646 | **676.9799** | **2027.9179** | **2027.9330** | **-0.0151** | **1** | **36** | **0.023** | **1** | **K.ALDEETRSHEAQVQEMR.Q** |
|  | 21061 | **688.8884** | **2063.6435** | **2062.9477** | **0.6958** | **0** | **5** | **18** | **4** | **K.NMDPLNDNVTSLLNASSDK.F** |
|  | 22386 | **736.0346** | **2205.0820** | **2205.0219** | **0.0601** | **1** | **1** | **61** | **4** | **R.ELDEATESNEAMGREVNALK.S** |
|  | 22854 | **752.0309** | **2253.0710** | **2253.0873** | **-0.0163** | **1** | **41** | **0.0063** | **1** | **K.IRELEGHISDLQEDLDSER.A** |
|  | 22929 | **1132.0064** | **2261.9981** | **2262.0143** | **-0.0162** | **0** | **94** | **3.1e-008** | **1** | **K.MAQQMLDLEEQLEEEEAAR.Q** 22930 |
|  | 23052 | **1140.4762** | **2278.9378** | **2278.0093** | **0.9286** | **0** | **(6)** | **17** | **1** | **K.MAQQMLDLEEQLEEEEAAR.Q** 23053 |
|  | 23786 | 1186.6175 | 2371.2205 | 2371.2311 | -0.0106 | 0 | 42 | 0.0045 | 1 | R.INFDVTGYIVGANIETYLLEK.S |
|  | 23899 | **1195.5818** | **2389.1490** | **2389.1609** | **-0.0118** | **0** | **58** | **0.00012** | **1** | **K.DVASLSSQLQDTQELLQEETR.Q** 23897 |
|  | 24120 | **808.4114** | **2422.2123** | **2422.0991** | **0.1132** | **1** | **2** | **44** | **1** | **K.KMAQQMLDLEEQLEEEEAAR.Q** |
|  | 24295 | **817.6879** | **2450.0420** | **2450.0577** | **-0.0157** | **0** | **30** | **0.069** | **1** | **R.IAQLEEELEEEQGNMEAMSDR.V** |
|  | 24420 | 823.0679 | 2466.1820 | 2466.2002 | -0.0182 | 0 | 6 | 19 | 2 | K.LQQLFNHTMFILEQEEYQR.E |
|  | 24720 | **1254.5938** | **2507.1729** | **2507.1849** | **-0.0120** | **0** | **77** | **1.4e-006** | **1** | **K.NLLQEQLQAETELYAEAEEMR.V** |
|  | 27837 | **1016.4963** | **3046.4672** | **3046.4830** | **-0.0158** | **1** | **42** | **0.0035** | **1** | **R.DLGEELEALKTELEDTLDSTATQQELR.A** 27838 |

| **23.** | P07355\|ANXA2_HUMAN    **Mass:** 38808    **Score:** 1077   **Queries matched:** 51   **emPAI:** 4.77 |
| --- | --- |
|  | Annexin A2 OS=Homo sapiens GN=ANXA2 PE=1 SV=2 |

|  | **Query** | **Observed** | **Mr(expt)** | **Mr(calc)** | **Delta** | **Miss** | **Score** | **Expect** | **Rank** | **Peptide** |
| --- | --- | --- | --- | --- | --- | --- | --- | --- | --- | --- |
|  | 1757 | **405.2063** | **808.3980** | **807.4603** | **0.9378** | **0** | **10** | **17** | **1** | **R.SVPHLQK.V** |
|  | 2649 | **437.2801** | **872.5456** | **872.5517** | **-0.0061** | **1** | **32** | **0.15** | **1** | **R.KLMVALAK.G** 2650 |
|  | 2739 | 880.4348 | 879.4275 | 879.4338 | -0.0063 | 0 | 34 | 0.064 | 1 | R.DLYDAGVK.R |
|  | 3824 | **480.2758** | **958.5371** | **957.5793** | **0.9578** | **1** | **9** | **22** | **10** | **K.LMVALAKGR.R** |
|  | 3833 | **480.7430** | **959.4715** | **959.4786** | **-0.0071** | **1** | **26** | **0.47** | **1** | **R.VYKEMYK.T** 3835 |
|  | 4889 | **518.2647** | **1034.5149** | **1034.5219** | **-0.0070** | **0** | **22** | **1.1** | **1** | **K.WISIMTER.S** 4890 |
|  | 7824 | **611.7963** | **1221.5780** | **1221.5877** | **-0.0097** | **0** | **29** | **0.19** | **1** | **K.TPAQYDASELK.A** 7835 7840 |
|  | 8258 | 622.8114 | 1243.6082 | 1243.6156 | -0.0074 | 0 | 21 | 1.1 | 1 | R.TNQELQEINR.V |
|  | 11660 | **474.9740** | **1421.9003** | **1422.7211** | **-0.8208** | **1** | **7** | **22** | **2** | **R.IMVSRSEVDMLK.I** 11664 11689 |
|  | 12308 | **730.8361** | **1459.6577** | **1459.6653** | **-0.0076** | **0** | **39** | **0.015** | **1** | **K.SYSPYDMLESIR.K** |
|  | 13685 | **771.3978** | **1540.7810** | **1541.8413** | **-1.0603** | **0** | **55** | **0.00038** | **1** | **K.GVDEVTIVNILTNR.S** 13702 |
|  | 14437 | **794.8827** | **1587.7509** | **1587.7603** | **-0.0093** | **1** | **53** | **0.00071** | **1** | **K.SYSPYDMLESIRK.E** 14436 |
|  | 15424 | **825.9897** | **1649.9649** | **1649.9716** | **-0.0066** | **0** | **90** | **1.1e-007** | **1** | **K.SALSGHLETVILGLLK.T** 15420 15422 15423 |
|  | 17261 | **886.9569** | **1771.8991** | **1770.9840** | **0.9152** | **1** | **31** | **0.091** | **1** | **K.TKGVDEVTIVNILTNR.S** 17260 |
|  | 17371 | **889.9323** | **1777.8501** | **1776.8564** | **0.9937** | **0** | **34** | **0.047** | **1** | **K.GLGTDEDSLIEIICSR.T** |
|  | 18387 | 922.9486 | 1843.8827 | 1843.8952 | -0.0125 | 0 | 73 | 5.6e-006 | 1 | K.LSLEGDHSTPPSAYGSVK.A 18386 18388 |
|  | 19206 | **954.9388** | **1907.8630** | **1907.8748** | **-0.0118** | **0** | **95** | **2.8e-008** | **1** | **R.AEDGSVIDYELIDQDAR.D** |
|  | 20454 | **1006.9775** | **2011.9404** | **2011.9527** | **-0.0123** | **1** | **68** | **1.6e-005** | **1** | **K.SLYYYIQQDTKGDYQK.A** 20453 20455 |
|  | 21070 | **1032.9893** | **2063.9640** | **2063.9760** | **-0.0120** | **1** | **92** | **5e-008** | **1** | **R.RAEDGSVIDYELIDQDAR.D** 21069 21085 |
|  | 21936 | **1078.0294** | **2154.0443** | **2154.0593** | **-0.0150** | **1** | **62** | **5.7e-005** | **1** | **K.AYTNFDAERDALNIETAIK.T** 21937 21938 |
|  | 26382 | **924.1008** | **2769.2807** | **2769.2981** | **-0.0174** | **1** | **42** | **0.0041** | **1** | **R.AEDGSVIDYELIDQDARDLYDAGVK.R** |
|  | 26801 | **1419.7100** | **2837.4054** | **2837.4170** | **-0.0117** | **0** | **86** | **1.3e-007** | **1** | **K.GDLENAFLNLVQCIQNKPLYFADR.L** 26799 26800 26802 26803 26804 26805 |
|  | 28463 | **1065.5442** | **3193.6107** | **3193.6230** | **-0.0122** | **1** | **75** | **1.4e-006** | **1** | **K.EVKGDLENAFLNLVQCIQNKPLYFADR.L** 28462 |

| **24.** | P05164\|PERM_HUMAN    **Mass:** 84784    **Score:** 1017   **Queries matched:** 83   **emPAI:** 1.56 |
| --- | --- |
|  | Myeloperoxidase OS=Homo sapiens GN=MPO PE=1 SV=1 |

|  | **Query** | **Observed** | **Mr(expt)** | **Mr(calc)** | **Delta** | **Miss** | **Score** | **Expect** | **Rank** | **Peptide** |
| --- | --- | --- | --- | --- | --- | --- | --- | --- | --- | --- |
|  | 1764 | **405.2986** | **808.5826** | **807.4239** | **1.1587** | **0** | **7** | **28** | **8** | **K.IPPNDPR.I** 1754 |
|  | 2849 | **444.2475** | **886.4804** | **886.4872** | **-0.0068** | **0** | **25** | **0.65** | **1** | **R.AVSNEIVR.F** |
|  | 3151 | **456.7371** | **911.4596** | **911.4654** | **-0.0058** | **0** | **16** | **3.3** | **1** | **R.VFFASWR.V** |
|  | 3513 | **469.7384** | **937.4623** | **937.4691** | **-0.0068** | **0** | **33** | **0.072** | **1** | **R.LFEQVMR.I** 3512 |
|  | 3541 | **470.7629** | **939.5113** | **939.5178** | **-0.0065** | **1** | **28** | **0.24** | **1** | **R.KYLPTYR.S** |
|  | 3613 | **472.7663** | **943.5181** | **943.5239** | **-0.0058** | **0** | **12** | **13** | **2** | **R.NGFPVALAR.A** |
|  | 5132 | **527.1538** | **1052.2931** | **1051.4570** | **0.8360** | **0** | **1** | **86** | **7** | **R.SYNDSVDPR.I** |
|  | 5201 | **529.7839** | **1057.5532** | **1057.5628** | **-0.0096** | **1** | **45** | **0.0058** | **1** | **R.RSPTLGASNR.A** 5187 5202 5203 5204 5205 5206 5207 5221 |
|  | 5313 | **533.7386** | **1065.4626** | **1065.4695** | **-0.0069** | **0** | **(15)** | **5.3** | **1** | **R.TITGMCNNR.R** |
|  | 5549 | **541.7348** | **1081.4550** | **1081.4645** | **-0.0094** | **0** | **52** | **0.00093** | **1** | **R.TITGMCNNR.R** 5538 5541 5548 5551 5560 |
|  | 5796 | **550.3364** | **1098.6583** | **1099.6250** | **-0.9667** | **1** | **4** | **67** | **5** | **K.RNGFPVALAR.A** |
|  | 6436 | **574.2600** | **1146.5053** | **1146.5128** | **-0.0074** | **0** | **16** | **4** | **2** | **R.YQPMEPNPR.V** |
|  | 6788 | **585.3675** | **1168.7204** | **1168.6676** | **0.0528** | **1** | **2** | **82** | **7** | **K.QPVAATRTAVR.A** |
|  | 7048 | **593.7826** | **1185.5506** | **1186.6492** | **-1.0986** | **1** | **5** | **54** | **6** | **R.GLMATPAKLNR.Q** |
|  | 7862 | **408.8881** | **1223.6424** | **1222.6128** | **1.0296** | **1** | **4** | **59** | **10** | **R.ERLFEQVMR.I** |
|  | 8132 | **619.7853** | **1237.5560** | **1237.5656** | **-0.0096** | **1** | **11** | **9.6** | **2** | **R.TITGMCNNRR.S** 8133 |
|  | 8988 | **640.8785** | **1279.7424** | **1279.7500** | **-0.0076** | **0** | **66** | **3.6e-005** | **1** | **R.VVLEGGIDPILR.G** |
|  | 10158 | **670.8674** | **1339.7203** | **1339.7282** | **-0.0079** | **0** | **64** | **5.4e-005** | **1** | **R.IGLDLPALNMQR.S** 10159 |
|  | 10494 | **679.8065** | **1357.5984** | **1357.6085** | **-0.0101** | **0** | **58** | **0.00024** | **1** | **K.NNIFMSNSYPR.D** 10495 |
|  | 10891 | **689.8510** | **1377.6875** | **1378.7643** | **-1.0768** | **0** | **4** | **53** | **2** | **K.IVGAMVQIITYR.D** |
|  | 11628 | **711.3643** | **1420.7140** | **1420.7232** | **-0.0092** | **0** | **83** | **7e-007** | **1** | **R.IICDNTGITTVSK.N** 11627 |
|  | 12353 | **488.2793** | **1461.8162** | **1460.7697** | **1.0464** | **0** | **9** | **18** | **3** | **R.DYLPLVLGPTAMR.K** 12352 |
|  | 12374 | 488.9141 | 1463.7204 | 1463.8038 | -0.0834 | 1 | 1 | 98 | 10 | R.VPLSRVFFASWR.V |
|  | 12459 | **735.8820** | **1469.7494** | **1469.7586** | **-0.0093** | **1** | **28** | **0.23** | **1** | **R.QNQIAVDEIRER.L** 12448 12458 |
|  | 13116 | **503.2863** | **1506.8370** | **1507.7606** | **-0.9235** | **1** | **7** | **25** | **8** | **R.IKNQADCIPFFR.S** |
|  | 13461 | **764.8684** | **1527.7223** | **1527.7331** | **-0.0108** | **1** | **23** | **0.68** | **1** | **R.SRDHGLPGYNAWR.R** 13460 13462 |
|  | 13737 | **772.9308** | **1543.8470** | **1543.8545** | **-0.0074** | **0** | **51** | **0.00092** | **1** | **R.VGPLLACIIGTQFR.K** 13738 |
|  | 14111 | **523.2590** | **1566.7553** | **1567.8430** | **-1.0878** | **1** | **6** | **29** | **2** | **K.LNRQNQIAVDEIR.E** |
|  | 14199 | **525.2728** | **1572.7967** | **1572.8042** | **-0.0075** | **0** | **6** | **32** | **4** | **R.NMSNQLGLLAVNQR.F** |
|  | 14592 | **533.6198** | **1597.8376** | **1598.8562** | **-1.0186** | **1** | **9** | **15** | **2** | **R.IGLDLPALNMQRSR.D** |
|  | 17770 | **601.3879** | **1801.1420** | **1800.9080** | **0.2340** | **1** | **1** | **61** | **7** | **R.LRSGSASPMELLSYFK.Q** |
|  | 18391 | **922.9609** | **1843.9073** | **1843.9172** | **-0.0098** | **0** | **86** | **2.8e-007** | **1** | **R.SSEMPELTSMHTLLLR.E** 18390 |
|  | 18605 | 930.4061 | 1858.7976 | 1858.8097 | -0.0121 | 0 | 80 | 1.1e-006 | 1 | R.FWWENEGVFSMQQR.Q 18607 |
|  | 19946 | **982.4829** | **1962.9513** | **1962.9622** | **-0.0109** | **0** | **51** | **0.00081** | **1** | **R.DFVNCSTLPALNLASWR.E** |
|  | 20020 | **986.5133** | **1971.0121** | **1971.0248** | **-0.0127** | **0** | **91** | **8.5e-008** | **1** | **R.FCGLPQPETVGQLGTVLR.N** 20021 |
|  | 20449 | **671.3714** | **2011.0924** | **2010.8672** | **0.2252** | **0** | **1** | **76** | **5** | **R.CMVDLGPCWAGGLTAEMK.L** |
|  | 21567 | **705.0204** | **2112.0395** | **2111.1739** | **0.8656** | **0** | **3** | **44** | **3** | **R.RPFNVTDVLTPAQLNVLSK.S** |
|  | 21711 | **710.0447** | **2127.1122** | **2127.1259** | **-0.0137** | **1** | **45** | **0.0029** | **1** | **R.RFCGLPQPETVGQLGTVLR.N** 21713 |
|  | 23247 | 768.3790 | 2302.1153 | 2301.9862 | 0.1291 | 1 | 6 | 20 | 2 | R.DGDRFWWENEGVFSMQQR.Q |
|  | 23337 | **772.3876** | **2314.1409** | **2314.1553** | **-0.0145** | **1** | **18** | **1.2** | **1** | **R.AVSNEIVRFPTDQLTPDQER.S** 23336 |
|  | 23946 | **599.8078** | **2395.2021** | **2396.1576** | **-0.9555** | **1** | **4** | **32** | **5** | **R.SSEMPELTSMHTLLLREHNR.L** |
|  | 24045 | **1206.5747** | **2411.1349** | **2411.1474** | **-0.0125** | **0** | **54** | **0.00027** | **1** | **R.WLPAEYEDGFSLPYGWTPGVK.R** 24042 24043 24044 |
|  | 25206 | **856.7527** | **2567.2364** | **2567.2485** | **-0.0121** | **1** | **2** | **46** | **3** | **R.WLPAEYEDGFSLPYGWTPGVKR.N** |
|  | 25467 | **1306.6299** | **2611.2452** | **2611.2588** | **-0.0136** | **0** | **113** | **3e-010** | **1** | **R.NQINALTSFVDASMVYGSEEPLAR.N** |
|  | 26157 | **907.4459** | **2719.3159** | **2719.3349** | **-0.0190** | **1** | **30** | **0.057** | **1** | **R.KLMEQYGTPNNIDIWMGGVSEPLK.R** |
|  | 26273 | **915.4282** | **2743.2628** | **2744.3262** | **-1.0633** | **1** | **1** | **46** | **6** | **R.IICDNTGITTVSKNNIFMSNSYPR.D** |
|  | 26285 | **916.7824** | **2747.3254** | **2747.3411** | **-0.0157** | **1** | **13** | **3.4** | **1** | **K.LMEQYGTPNNIDIWMGGVSEPLKR.K** |
|  | 26689 | **1409.6655** | **2817.3165** | **2817.3255** | **-0.0089** | **0** | **99** | **6.9e-009** | **1** | **R.SLMFMQWGQLLDHDLDFTPEPAAR.A** 26687 26688 |

| **25.** | P11021\|GRP78_HUMAN    **Mass:** 72402    **Score:** 1015   **Queries matched:** 75   **emPAI:** 3.26 |
| --- | --- |
|  | 78 kDa glucose-regulated protein OS=Homo sapiens GN=HSPA5 PE=1 SV=2 |

|  | **Query** | **Observed** | **Mr(expt)** | **Mr(calc)** | **Delta** | **Miss** | **Score** | **Expect** | **Rank** | **Peptide** |
| --- | --- | --- | --- | --- | --- | --- | --- | --- | --- | --- |
|  | 3030 | **452.2382** | **902.4618** | **902.4684** | **-0.0066** | **0** | **19** | **2.6** | **1** | **R.VMEHFIK.L** |
|  | 3251 | **459.7398** | **917.4649** | **917.4706** | **-0.0056** | **0** | **9** | **28** | **1** | **K.VLEDSDLK.K** |
|  | 4361 | **499.2587** | **996.5028** | **996.5101** | **-0.0072** | **0** | **31** | **0.13** | **1** | **R.ALSSQHQAR.I** 4358 4360 4375 |
|  | 5057 | **523.7863** | **1045.5581** | **1045.5655** | **-0.0074** | **1** | **17** | **3.4** | **1** | **K.VLEDSDLKK.S** |
|  | 5432 | **537.8115** | **1073.6085** | **1073.5465** | **0.0620** | **0** | **7** | **40** | **2** | **K.ITITNDQNR.L** |
|  | 6088 | **560.8666** | **1119.7187** | **1118.5568** | **1.1620** | **1** | **18** | **2.5** | **1** | **R.VTAEDKGTGNK.N** 6066 6089 |
|  | 7169 | **596.3179** | **1190.6213** | **1190.6295** | **-0.0082** | **0** | **56** | **0.00041** | **1** | **K.VYEGERPLTK.D** 7170 |
|  | 7581 | **605.7921** | **1209.5697** | **1209.5778** | **-0.0082** | **1** | **16** | **3.2** | **1** | **K.EFFNGKEPSR.G** |
|  | 7737 | **609.3154** | **1216.6162** | **1216.6234** | **-0.0072** | **0** | **56** | **0.00042** | **1** | **K.DAGTIAGLNVMR.I** |
|  | 7943 | **614.8138** | **1227.6130** | **1227.6207** | **-0.0077** | **0** | **58** | **0.00024** | **1** | **R.VEIIANDQGNR.I** 7942 |
|  | 8046 | **617.4311** | **1232.8476** | **1232.6183** | **0.2293** | **0** | **(2)** | **85** | **3** | **K.DAGTIAGLNVMR.I** |
|  | 8169 | **620.8120** | **1239.6093** | **1239.6169** | **-0.0075** | **1** | **33** | **0.072** | **1** | **K.MKETAEAYLGK.K** |
|  | 8472 | **628.2999** | **1254.5852** | **1255.6118** | **-1.0266** | **1** | **(9)** | **18** | **1** | **K.MKETAEAYLGK.K** |
|  | 9584 | **657.3099** | **1312.6052** | **1312.6122** | **-0.0069** | **0** | **37** | **0.03** | **1** | **K.FEELNMDLFR.S** |
|  | 9694 | **658.8174** | **1315.6203** | **1315.6295** | **-0.0092** | **0** | **38** | **0.024** | **1** | **R.NELESYAYSLK.N** |
|  | 9700 | **658.8447** | **1315.6749** | **1315.6844** | **-0.0095** | **1** | **42** | **0.01** | **1** | **K.NKITITNDQNR.L** |
|  | 9871 | **662.8501** | **1323.6856** | **1322.7057** | **0.9800** | **1** | **1** | **1.2e+002** | **8** | **R.VMEHFIKLYK.K** |
|  | 11211 | **699.3937** | **1396.7728** | **1396.7813** | **-0.0085** | **0** | **61** | **0.0001** | **1** | **K.ELEEIVQPIISK.L** |
|  | 11791 | **715.8441** | **1429.6736** | **1429.6838** | **-0.0102** | **0** | **66** | **3.4e-005** | **1** | **R.TWNDPSVQQDIK.F** 11790 |
|  | 13200 | **756.8751** | **1511.7356** | **1511.7442** | **-0.0086** | **1** | **61** | **0.00011** | **1** | **R.AKFEELNMDLFR.S** 13201 |
|  | 13410 | **763.3025** | **1524.5904** | **1524.6766** | **-0.0862** | **1** | **27** | **0.22** | **1** | **R.MVNDAEKFAEEDK.K** |
|  | 13437 | **763.9127** | **1525.8108** | **1524.8763** | **0.9345** | **1** | **9** | **15** | **5** | **K.KELEEIVQPIISK.L** |
|  | 13607 | **768.8987** | **1535.7828** | **1535.7905** | **-0.0077** | **0** | **23** | **0.59** | **1** | **K.TFAPEEISAMVLTK.M** 13606 |
|  | 13921 | **778.3951** | **1554.7756** | **1554.7862** | **-0.0106** | **1** | **44** | **0.0054** | **1** | **K.NGRVEIIANDQGNR.I** 13920 13929 13930 |
|  | 14098 | **783.8884** | **1565.7622** | **1565.7726** | **-0.0104** | **0** | **39** | **0.016** | **1** | **R.ITPSYVAFTPEGER.L** |
|  | 14441 | **530.2862** | **1587.8367** | **1587.8468** | **-0.0101** | **1** | **3** | **60** | **2** | **K.KSDIDEIVLVGGSTR.I** |
|  | 14673 | **802.9303** | **1603.8460** | **1603.8570** | **-0.0109** | **0** | **72** | **6.8e-006** | **1** | **K.TKPYIQVDIGGGQTK.T** 14672 14674 |
|  | 15576 | **830.4467** | **1658.8788** | **1658.8879** | **-0.0091** | **0** | **59** | **0.00013** | **1** | **R.IINEPTAAAIAYGLDK.R** 15592 |
|  | 15849 | **838.8917** | **1675.7689** | **1676.8006** | **-1.0317** | **0** | **69** | **1.4e-005** | **1** | **K.NQLTSNPENTVFDAK.R** 15862 15863 |
|  | 17735 | **450.9624** | **1799.8204** | **1800.0729** | **-0.2525** | **1** | **4** | **41** | **8** | **-.MKLSLVAAMLLLLSAAR.A** |
|  | 17756 | **901.4465** | **1800.8785** | **1800.8893** | **-0.0108** | **1** | **40** | **0.0099** | **1** | **R.IDTRNELESYAYSLK.N** 17757 |
|  | 17969 | **605.9999** | **1814.9780** | **1814.9890** | **-0.0110** | **1** | **25** | **0.34** | **1** | **R.IINEPTAAAIAYGLDKR.E** 17952 |
|  | 18199 | **916.8996** | **1831.7846** | **1832.0627** | **-0.2781** | **1** | **(4)** | **40** | **1** | **-.MKLSLVAAMLLLLSAAR.A** 18187 |
|  | 18231 | **917.4521** | **1832.8897** | **1832.9017** | **-0.0119** | **1** | **81** | **9.4e-007** | **1** | **K.NQLTSNPENTVFDAKR.L** |
|  | 18271 | **918.9644** | **1835.9142** | **1835.9265** | **-0.0123** | **0** | **80** | **1.1e-006** | **1** | **K.SQIFSTASDNQPTVTIK.V** |
|  | 18954 | **629.9907** | **1886.9502** | **1886.9639** | **-0.0138** | **0** | **20** | **1** | **1** | **K.VTHAVVTVPAYFNDAQR.Q** 18955 |
|  | 19519 | **967.0357** | **1932.0569** | **1933.0058** | **-0.9489** | **0** | **6** | **28** | **2** | **K.DNHLLGTFDLTGIPPAPR.G** |
|  | 19951 | **655.4091** | **1963.2055** | **1964.0215** | **-0.8159** | **1** | **4** | **30** | **3** | **K.KSQIFSTASDNQPTVTIK.V** |
|  | 20344 | **667.3652** | **1999.0737** | **1998.0786** | **0.9951** | **0** | **5** | **30** | **2** | **R.GVPQIEVTFEIDVNGILR.V** |
|  | 22036 | **1082.9939** | **2163.9732** | **2163.9848** | **-0.0115** | **0** | **113** | **4e-010** | **1** | **R.IEIESFYEGEDFSETLTR.A** 22035 |
|  | 22123 | **1087.5419** | **2173.0692** | **2173.0327** | **0.0365** | **1** | **36** | **0.022** | **1** | **K.IEWLESHQDADIEDFKAK.K** |
|  | 22145 | **1088.4932** | **2174.9718** | **2174.9855** | **-0.0138** | **1** | **95** | **2.4e-008** | **1** | **K.LYGSAGPPPTGEEDTAEKDEL.-** 22141 22143 22144 22146 |
|  | 22664 | **743.7079** | **2228.1018** | **2228.0969** | **0.0049** | **1** | **2** | **59** | **1** | **K.FEELNMDLFRSTMKPVQK.V** |
|  | 28053 | **1036.1991** | **3105.5755** | **3105.6247** | **-0.0493** | **1** | **1** | **35** | **1** | **K.VYEGERPLTKDNHLLGTFDLTGIPPAPR.G** |
|  | 31219 | **1225.6158** | **4898.4343** | **4898.4570** | **-0.0227** | **0** | **59** | **2.4e-005** | **1** | **R.GINPDEAVAYGAAVQAGVLSGDQDTGDLVLLDVCPLTLGIETVGGVMTK.L** 31218 31222 31225 |

| **26.** | Q13509\|TBB3_HUMAN    **Mass:** 50856    **Score:** 994    **Queries matched:** 47   **emPAI:** 2.11 |
| --- | --- |
|  | Tubulin beta-3 chain OS=Homo sapiens GN=TUBB3 PE=1 SV=2 |

|  | **Query** | **Observed** | **Mr(expt)** | **Mr(calc)** | **Delta** | **Miss** | **Score** | **Expect** | **Rank** | **Peptide** |
| --- | --- | --- | --- | --- | --- | --- | --- | --- | --- | --- |
|  | 1748 | 404.7345 | 807.4544 | 807.3399 | 0.1146 | 0 | 2 | 1e+002 | 6 | R.EEYPDR.I |
|  | 5302 | 533.2137 | 1064.4128 | 1064.4201 | -0.0073 | 0 | 41 | 0.013 | 1 | K.NMMAACDPR.H 5301 |
|  | 5347 | **535.3016** | **1068.5886** | **1068.5968** | **-0.0082** | **0** | **18** | **2.4** | **3** | **R.YLTVATVFR.G** |
|  | 5533 | 541.2109 | 1080.4073 | 1080.4151 | -0.0077 | 0 | (35) | 0.047 | 1 | K.NMMAACDPR.H 5532 |
|  | 5550 | 541.7350 | 1081.4554 | 1080.4151 | 1.0404 | 0 | (8) | 23 | 2 | K.NMMAACDPR.H 5554 |
|  | 6379 | 572.3181 | 1142.6217 | 1142.6270 | -0.0053 | 0 | 65 | 5.8e-005 | 1 | K.LAVNMVPFPR.L 6378 |
|  | 7957 | 615.2987 | 1228.5829 | 1228.5910 | -0.0082 | 0 | 53 | 0.00081 | 1 | R.ISEQFTAMFR.R |
|  | 8582 | 630.3885 | 1258.7624 | 1257.6830 | 1.0795 | 1 | 9 | 17 | 3 | R.FPGQLNADLRK.L 8578 |
|  | 9784 | 660.8520 | 1319.6894 | 1318.6955 | 0.9939 | 0 | 82 | 1e-006 | 1 | R.IMNTFSVVPSPK.V 9757 9774 |
|  | 11100 | **695.8426** | **1389.6706** | **1389.6810** | **-0.0103** | **0** | **0** | **1.2e+002** | **9** | **K.EVDEQMLAIQSK.N** |
|  | 14864 | 808.4167 | 1614.8188 | 1614.8287 | -0.0099 | 0 | 80 | 1.1e-006 | 1 | R.AILVDLEPGTMDSVR.S 14863 14865 14883 |
|  | 14961 | 810.9169 | 1619.8193 | 1619.8283 | -0.0090 | 0 | 6 | 28 | 2 | R.LHFFMPGFAPLTAR.G |
|  | 15133 | 816.4135 | 1630.8123 | 1630.8236 | -0.0113 | 0 | (62) | 7.3e-005 | 1 | R.AILVDLEPGTMDSVR.S |
|  | 16082 | 846.4330 | 1690.8514 | 1690.8600 | -0.0086 | 0 | 53 | 0.00051 | 1 | R.ALTVPELTQQMFDAK.N 16080 16081 |
|  | 16151 | 848.9155 | 1695.8164 | 1695.8257 | -0.0093 | 0 | 52 | 0.00079 | 1 | K.NSSYFVEWIPNNVK.V 16137 16148 16149 16165 16166 |
|  | 18073 | 911.9592 | 1821.9038 | 1821.9156 | -0.0118 | 0 | 69 | 1.5e-005 | 1 | R.EIVHIQAGQCGNQIGAK.F 18072 18074 |
|  | 19874 | 979.9879 | 1957.9613 | 1957.9745 | -0.0132 | 0 | 110 | 8.4e-010 | 1 | K.GHYTEGAELVDSVLDVVR.K 19872 19873 |
|  | 21275 | 1044.0358 | 2086.0570 | 2086.0695 | -0.0125 | 1 | 118 | 1.6e-010 | 1 | K.GHYTEGAELVDSVLDVVRK.E 21274 21276 21277 21279 |
|  | 21524 | 528.0024 | 2107.9804 | 2108.0248 | -0.0444 | 1 | 3 | 49 | 6 | R.EEYPDRIMNTFSVVPSPK.V |
|  | 21535 | 1055.5286 | 2109.0426 | 2109.0571 | -0.0146 | 1 | 79 | 1.1e-006 | 1 | -.MREIVHIQAGQCGNQIGAK.F 21536 |
|  | 21696 | 532.5209 | 2126.0547 | 2125.0521 | 1.0026 | 1 | (1) | 72 | 10 | -.MREIVHIQAGQCGNQIGAK.F |

| **27.** | Q6PEY2\|TBA3E_HUMAN    **Mass:** 50568    **Score:** 985    **Queries matched:** 39   **emPAI:** 1.52 |
| --- | --- |
|  | Tubulin alpha-3E chain OS=Homo sapiens GN=TUBA3E PE=1 SV=2 |

|  | **Query** | **Observed** | **Mr(expt)** | **Mr(calc)** | **Delta** | **Miss** | **Score** | **Expect** | **Rank** | **Peptide** |
| --- | --- | --- | --- | --- | --- | --- | --- | --- | --- | --- |
|  | 4625 | 508.2894 | 1014.5642 | 1014.5709 | -0.0067 | 0 | 36 | 0.043 | 1 | K.DVNAAIATIK.T |
|  | 10920 | 690.8480 | 1379.6814 | 1379.7271 | -0.0458 | 1 | 7 | 25 | 5 | R.LVHKFDLMYAK.W |
|  | 12255 | 729.4348 | 1456.8549 | 1456.8613 | -0.0064 | 0 | 78 | 2e-006 | 1 | R.LIGQIVSSITASLR.F 12252 12253 12265 |
|  | 16504 | 859.9384 | 1717.8623 | 1717.8747 | -0.0124 | 0 | 44 | 0.0044 | 1 | R.NLDIERPTYTNLNR.L 16503 16505 |
|  | 17071 | 878.9804 | 1755.9463 | 1755.9559 | -0.0097 | 0 | 65 | 3.1e-005 | 1 | R.IHFPLATYAPVISAEK.A 17060 17069 17070 17072 17074 |
|  | 18100 | 912.9898 | 1823.9651 | 1823.9782 | -0.0131 | 0 | 57 | 0.00019 | 1 | K.VGINYQPPTVVPGGDLAK.V 18098 18099 18101 |
|  | 18674 | 932.9498 | 1863.8851 | 1863.8971 | -0.0120 | 0 | 84 | 3.8e-007 | 1 | R.AVCMLSNTTAIAEAWAR.L 18675 |
|  | 20417 | 1004.4446 | 2006.8746 | 2006.8858 | -0.0112 | 0 | 69 | 1.1e-005 | 1 | K.TIGGGDDSFNTFFSETGAGK.H |
|  | 24021 | 1205.1011 | 2408.1876 | 2408.2012 | -0.0136 | 0 | 116 | 1.8e-010 | 1 | R.FDGALNVDLTEFQTNLVPYPR.I 24022 24023 24024 24025 24026 |
|  | 26296 | 1375.6395 | 2749.2645 | 2749.2840 | -0.0194 | 0 | 84 | 2.1e-007 | 1 | K.AYHEQLSVAEITNACFEPANQMVK.C 26295 26298 26299 |
|  | 26370 | 922.7617 | 2765.2632 | 2765.2789 | -0.0157 | 0 | (32) | 0.034 | 1 | K.AYHEQLSVAEITNACFEPANQMVK.C |
|  | 28289 | 790.0373 | 3156.1201 | 3156.7255 | -0.6054 | 1 | 1 | 27 | 1 | R.NLDIERPTYTNLNRLIGQIVSSITASLR.F |
|  | 30798 | 1074.9877 | 4295.9216 | 4295.9497 | -0.0281 | 0 | 3 | 10 | 1 | R.ECISIHVGQAGVQIGNACWELYCLEHGIQPDGQMPSDK.T |
|  | 31036 | 1146.7736 | 4583.0651 | 4583.0913 | -0.0261 | 1 | 57 | 3.5e-005 | 1 | -.MRECISIHVGQAGVQIGNACWELYCLEHGIQPDGQMPSDK.T 31035 31037 |
|  | 31727 | 1147.3433 | 5731.6799 | 5731.7161 | -0.0361 | 1 | 10 | 0.96 | 1 | K.SKLEFAIYPAPQVSTAVVEPYNSILTTHTTLEHSDCAFMVDNEAIYDICR.R |

| **28.** | P06396\|GELS_HUMAN    **Mass:** 86043    **Score:** 949    **Queries matched:** 40   **emPAI:** 0.88 |
| --- | --- |
|  | Gelsolin OS=Homo sapiens GN=GSN PE=1 SV=1 |

|  | **Query** | **Observed** | **Mr(expt)** | **Mr(calc)** | **Delta** | **Miss** | **Score** | **Expect** | **Rank** | **Peptide** |
| --- | --- | --- | --- | --- | --- | --- | --- | --- | --- | --- |
|  | 1273 | **692.9460** | **691.9387** | **691.4057** | **0.5330** | **0** | **8** | **19** | **1** | **K.IFVWK.G** |
|  | 2659 | **437.7681** | **873.5216** | **873.5284** | **-0.0068** | **1** | **17** | **4.6** | **4** | **R.LKATQVSK.G** |
|  | 2856 | **444.7130** | **887.4115** | **886.4872** | **0.9243** | **0** | **33** | **0.1** | **1** | **K.TGAQELLR.V** |
|  | 3174 | **457.2921** | **912.5695** | **912.5757** | **-0.0061** | **1** | **18** | **2.3** | **2** | **R.RTPITVVK.Q** |
|  | 5425 | **537.3213** | **1072.6280** | **1073.5101** | **-0.8821** | **0** | **22** | **1.2** | **1** | **R.EGGQTAPASTR.L** |
|  | 5487 | **539.7582** | **1077.5019** | **1077.5091** | **-0.0071** | **0** | **15** | **5.4** | **1** | **R.YIETDPANR.D** |
|  | 8058 | **412.2037** | **1233.5892** | **1233.6102** | **-0.0210** | **1** | **13** | **6.9** | **1** | **K.RYIETDPANR.D** |
|  | 8922 | **638.3576** | **1274.7006** | **1274.7095** | **-0.0089** | **0** | **42** | **0.0092** | **1** | **K.HVVPNEVVVQR.L** 8921 8923 |
|  | 9754 | **660.3469** | **1318.6793** | **1318.6881** | **-0.0088** | **0** | **69** | **1.7e-005** | **1** | **K.AGALNSNDAFVLK.T** 9755 9780 |
|  | 10289 | **450.1734** | **1347.4985** | **1348.6371** | **-1.1387** | **1** | **10** | **14** | **3** | **R.YIETDPANRDR.R** |
|  | 14949 | **540.9115** | **1619.7127** | **1619.7250** | **-0.0123** | **1** | **6** | **30** | **3** | **K.SEDCFILDHGKDGK.I** |
|  | 16563 | **861.9176** | **1721.8206** | **1721.8301** | **-0.0094** | **0** | **101** | **8.9e-009** | **1** | **R.EVQGFESATFLGYFK.S** 16586 |
|  | 18171 | **915.4806** | **1828.9466** | **1828.9571** | **-0.0105** | **0** | **64** | **4.3e-005** | **1** | **K.QTQVSVLPEGGETPLFK.Q** 18170 |
|  | 18279 | **919.4448** | **1836.8751** | **1836.8894** | **-0.0143** | **0** | **79** | **1.4e-006** | **1** | **K.TPSAAYLWVGTGASEAEK.T** 18280 18281 |
|  | 20852 | **683.3560** | **2047.0461** | **2047.0634** | **-0.0174** | **0** | **1** | **80** | **4** | **K.EPAHLMSLFGGKPMIIYK.G** |
|  | 22984 | **1136.5398** | **2271.0650** | **2271.0808** | **-0.0158** | **0** | **99** | **1.1e-008** | **1** | **R.AQPVQVAEGSEPDGFWEALGGK.A** 22985 |
|  | 23617 | **588.8139** | **2351.2265** | **2350.2103** | **1.0162** | **1** | **5** | **22** | **5** | **R.VPEARPNSMVVEHPEFLKAGK.E** |
|  | 24393 | **1232.5979** | **2463.1812** | **2462.2111** | **0.9701** | **0** | **124** | **2.9e-011** | **1** | **K.VSNGAGTMSVSLVADENPFAQGALK.S** 24391 |
|  | 26073 | **1352.6915** | **2703.3685** | **2703.3836** | **-0.0151** | **0** | **55** | **0.00024** | **1** | **K.FDLVPVPTNLYGDFFTGDAYVILK.T** |
|  | 26084 | **902.7902** | **2705.3487** | **2705.3660** | **-0.0174** | **1** | **68** | **1e-005** | **1** | **K.TPSAAYLWVGTGASEAEKTGAQELLR.V** |
|  | 26830 | **948.4561** | **2842.3465** | **2842.3634** | **-0.0169** | **1** | **67** | **1.3e-005** | **1** | **K.NWRDPDQTDGLGLSYLSSHIANVER.V** |
|  | 26962 | **958.4262** | **2872.2566** | **2872.2756** | **-0.0190** | **0** | **49** | **0.00068** | **1** | **R.VPFDAATLHTSTAMAAQHGMDDDGTGQK.Q** 26961 |
|  | 27885 | **1020.8663** | **3059.5772** | **3059.5896** | **-0.0124** | **1** | **55** | **0.00018** | **1** | **R.VEKFDLVPVPTNLYGDFFTGDAYVILK.T** |
|  | 27938 | **1025.4983** | **3073.4730** | **3074.5356** | **-1.0625** | **1** | **2** | **32** | **4** | **R.GASQAGAPQGRVPEARPNSMVVEHPEFLK.A** |
|  | 29148 | **843.6557** | **3370.5937** | **3370.8152** | **-0.2215** | **1** | **3** | **19** | **3** | **-.MAPHRPAPALLCALSLALCALSLPVRAATASR.G** |
|  | 29238 | **1133.8829** | **3398.6270** | **3398.6459** | **-0.0189** | **1** | **33** | **0.019** | **1** | **R.IEGSNKVPVDPATYGQFYGGDSYIILYNYR.H** |
|  | 30388 | **990.2358** | **3956.9140** | **3956.9425** | **-0.0285** | **1** | **26** | **0.084** | **1** | **R.VHVSEEGTEPEAMLQVLGPKPALPAGTEDTAKEDAANR.K** 30389 |
|  | 30486 | **1346.5951** | **4036.7634** | **4036.7857** | **-0.0223** | **0** | **71** | **2e-006** | **1** | **R.ATEVPVSWESFNNGDCFILDLGNNIHQWCGSNSNR.Y** |

| **29.** | P12111\|CO6A3_HUMAN    **Mass:** 345163   **Score:** 908    **Queries matched:** 103   **emPAI:** 0.32 |
| --- | --- |
|  | Collagen alpha-3(VI) chain OS=Homo sapiens GN=COL6A3 PE=1 SV=4 |

|  | **Query** | **Observed** | **Mr(expt)** | **Mr(calc)** | **Delta** | **Miss** | **Score** | **Expect** | **Rank** | **Peptide** |
| --- | --- | --- | --- | --- | --- | --- | --- | --- | --- | --- |
|  | 994 | **572.9940** | **571.9867** | **572.3031** | **-0.3164** | **0** | **11** | **16** | **3** | **K.GGIGNR.G** |
|  | 1157 | **637.3701** | **636.3628** | **635.3642** | **0.9985** | **0** | **27** | **0.3** | **1** | **K.VYLNK.F** |
|  | 1233 | **674.4033** | **673.3960** | **673.4010** | **-0.0050** | **0** | **24** | **1** | **1** | **K.SVLLDK.I** |
|  | 1355 | **718.3106** | **717.3033** | **716.4181** | **0.8852** | **0** | **14** | **9.7** | **3** | **R.VTQLTR.E** |
|  | 1665 | **401.7399** | **801.4653** | **801.4960** | **-0.0307** | **1** | **14** | **9.9** | **6** | **R.KSVLLDK.I** |
|  | 1959 | **413.2635** | **824.5125** | **824.4756** | **0.0369** | **0** | **7** | **26** | **10** | **R.GPIGSIGPK.G** 1964 |
|  | 2760 | **441.2663** | **880.5180** | **880.4589** | **0.0591** | **1** | **9** | **22** | **6** | **R.NGFLMRK.V** |
|  | 2951 | **449.3254** | **896.6362** | **895.4552** | **1.1810** | **0** | **5** | **42** | **9** | **K.TNFPYVR.D** |
|  | 3634 | **473.7739** | **945.5332** | **945.4880** | **0.0453** | **0** | **7** | **34** | **1** | **K.QNLTVTDR.V** |
|  | 3698 | **476.1920** | **950.3694** | **950.3772** | **-0.0079** | **0** | **13** | **7.7** | **1** | **K.ACCGVPCK.C** |
|  | 3813 | **480.2571** | **958.4996** | **957.4992** | **1.0004** | **1** | **7** | **36** | **8** | **K.GERGDVGIR.G** |
|  | 4380 | **499.9897** | **997.9648** | **998.5032** | **-0.5384** | **0** | **2** | **65** | **5** | **R.ELPNIEER.I** |
|  | 4664 | **509.8855** | **1017.7564** | **1017.5066** | **0.2499** | **0** | **2** | **98** | **6** | **R.LMHLEFGR.G** |
|  | 4902 | **518.5977** | **1035.1809** | **1035.5349** | **-0.3540** | **0** | **21** | **0.81** | **1** | **R.VAVVQYSDR.T** |
|  | 5087 | **524.7706** | **1047.5267** | **1047.5349** | **-0.0082** | **0** | **34** | **0.07** | **1** | **R.DQNVFVAQK.G** 5086 |
|  | 5981 | **557.2476** | **1112.4806** | **1112.6077** | **-0.1271** | **0** | **8** | **22** | **1** | **K.DGLALPSAELK.S** 5987 |
|  | 6568 | **577.7880** | **1153.5614** | **1153.5702** | **-0.0089** | **0** | **25** | **0.48** | **1** | **R.GFMYDRPLR.L** |
|  | 6572 | **577.8079** | **1153.6013** | **1152.6251** | **0.9762** | **1** | **7** | **31** | **10** | **R.GDRGPIGSIGPK.G** |
|  | 7110 | **397.2024** | **1188.5853** | **1189.5000** | **-0.9147** | **0** | **5** | **50** | **2** | **R.GYPGDEGGPGER.G** |
|  | 7708 | **608.8082** | **1215.6018** | **1215.6095** | **-0.0078** | **0** | **59** | **0.00018** | **1** | **R.VVESLDVGQDR.V** 7681 |
|  | 7886 | **409.2363** | **1224.6870** | **1224.6938** | **-0.0069** | **1** | **3** | **78** | **9** | **R.SQAPVLDAIRR.L** 7876 |
|  | 8247 | **415.2444** | **1242.7115** | **1243.6561** | **-0.9445** | **0** | **16** | **3.9** | **2** | **K.VEFLLNAHSSK.D** |
|  | 8565 | **630.3052** | **1258.5959** | **1257.6425** | **0.9534** | **1** | **3** | **70** | **1** | **R.ASENLRQEGVR.A** |
|  | 9270 | **648.3014** | **1294.5882** | **1293.6863** | **0.9019** | **1** | **6** | **33** | **4** | **K.RSSIMAFAIGNK.G** |
|  | 9541 | **655.8157** | **1309.6169** | **1309.6812** | **-0.0643** | **1** | **(0)** | **1.3e+002** | **8** | **K.RSSIMAFAIGNK.G** |
|  | 10071 | **445.5988** | **1333.7745** | **1333.6183** | **0.1562** | **0** | **4** | **55** | **7** | **K.STELNEEPLMR.F** |
|  | 10683 | **456.6109** | **1366.8108** | **1365.7405** | **1.0703** | **1** | **10** | **13** | **1** | **R.KVAVFFSNTPTR.A** |
|  | 10761 | **457.9094** | **1370.7063** | **1370.7154** | **-0.0091** | **1** | **42** | **0.008** | **1** | **R.SGDDVRNPSVVVK.R** 10760 10774 |
|  | 11338 | **469.0948** | **1404.2626** | **1404.7249** | **-0.4623** | **0** | **16** | **2.4** | **1** | **K.DVVFLLDGSEGVR.S** 11336 11337 11340 |
|  | 12262 | **486.8632** | **1457.5677** | **1456.7409** | **0.8268** | **0** | **4** | **51** | **9** | **K.SLDEISQPAQELK.R** |
|  | 12287 | **730.4312** | **1458.8477** | **1458.8558** | **-0.0081** | **0** | **30** | **0.12** | **1** | **K.IGDLHPQIVNLLK.S** 12288 |
|  | 12406 | **733.9041** | **1465.7937** | **1466.7517** | **-0.9581** | **0** | **11** | **10** | **1** | **K.ALNLGYALDYAQR.Y** |
|  | 12694 | **742.4044** | **1482.7942** | **1482.8042** | **-0.0100** | **0** | **7** | **23** | **1** | **K.IIDELNVKPEGTR.I** |
|  | 13225 | **757.8774** | **1513.7403** | **1512.7460** | **0.9943** | **0** | **75** | **4.3e-006** | **1** | **R.LVDYLDVGFDTTR.V** |
|  | 13234 | **758.2174** | **1514.4203** | **1514.8068** | **-0.3865** | **1** | **1** | **63** | **4** | **K.CKGYFFVVLGIGR.K** |
|  | 13517 | **510.9277** | **1529.7612** | **1529.7726** | **-0.0114** | **0** | **6** | **34** | **4** | **R.SQHPYVLTEDTLK.V** |
|  | 14300 | **790.8826** | **1579.7507** | **1578.8406** | **0.9101** | **0** | **4** | **48** | **9** | **K.ADIVFLLDGSINFR.R** |
|  | 14410 | **793.9615** | **1585.9085** | **1585.9192** | **-0.0106** | **0** | **63** | **5.8e-005** | **1** | **R.LQPVLQPLPSPGVGGK.R** |
|  | 14570 | **533.1865** | **1596.5376** | **1595.8056** | **0.7319** | **0** | **5** | **28** | **2** | **K.QASVFSFGLGAQAASR.A** |
|  | 14827 | **807.4211** | **1612.8276** | **1612.8420** | **-0.0144** | **1** | **42** | **0.0076** | **1** | **K.SLDEISQPAQELKR.S** 14830 |
|  | 14906 | **809.4314** | **1616.8482** | **1616.8886** | **-0.0403** | **1** | **7** | **24** | **3** | **K.RDIIFLLDGSANVGK.T** 14900 |
|  | 14912 | **809.4755** | **1616.9364** | **1616.9436** | **-0.0072** | **0** | **65** | **3.4e-005** | **1** | **R.AAPLQGMLPGLLAPLR.T** |
|  | 15131 | **816.4088** | **1630.8029** | **1630.8162** | **-0.0133** | **0** | **14** | **4.6** | **1** | **K.SVEDAQDVSLALTQR.G** 15111 |
|  | 15150 | **817.4399** | **1632.8653** | **1632.9385** | **-0.0732** | **0** | **(0)** | **1.1e+002** | **5** | **R.AAPLQGMLPGLLAPLR.T** |
|  | 15801 | **837.4778** | **1672.9411** | **1672.9512** | **-0.0101** | **0** | **32** | **0.074** | **1** | **R.IEDGVPQHLVLVLGGK.S** 15802 |
|  | 16417 | **857.3653** | **1712.7160** | **1713.9158** | **-1.1997** | **0** | **0** | **1e+002** | **2** | **K.VCAPVLAKPGVISVMGT.-** |
|  | 16549 | **574.6426** | **1720.9061** | **1720.8857** | **0.0204** | **1** | **4** | **47** | **2** | **K.GNPGEPGLNGTTGPKGIR.G** |
|  | 16571 | **575.2664** | **1722.7774** | **1723.8754** | **-1.0980** | **1** | **5** | **32** | **5** | **R.VNHFVPEAGSRLDQR.V** |
|  | 16724 | **866.4468** | **1730.8790** | **1730.8879** | **-0.0089** | **0** | **80** | **1.1e-006** | **1** | **K.ISLSPEYVFSVSTFR.E** 16702 |
|  | 16873 | **871.5104** | **1741.0062** | **1741.0138** | **-0.0076** | **0** | **102** | **5.8e-009** | **1** | **R.IEDGVLQFLVLLVAGR.S** |
|  | 17284 | **887.8353** | **1773.6561** | **1772.8978** | **0.7583** | **1** | **5** | **27** | **2** | **K.LVDKSTELNEEPLMR.F** |
|  | 17500 | **893.4516** | **1784.8886** | **1784.9017** | **-0.0130** | **1** | **2** | **71** | **2** | **R.NIDRTELQTITNDPR.L** |
|  | 17575 | **597.6325** | **1789.8757** | **1789.8880** | **-0.0123** | **1** | **4** | **40** | **3** | **R.GDSIDQCALIQSIKDK.C** |
|  | 17678 | **449.9915** | **1795.9369** | **1795.9468** | **-0.0100** | **0** | **11** | **8.1** | **2** | **R.FDEHQSKPEILNLVK.R** 17680 17681 |
|  | 18348 | **614.9557** | **1841.8452** | **1842.8683** | **-1.0230** | **1** | **3** | **57** | **5** | **R.QLDMSPDPKASQHFAR.V** |
|  | 18800 | **625.7985** | **1874.3737** | **1874.9487** | **-0.5749** | **1** | **3** | **36** | **3** | **K.EFVQRVVESLDVGQDR.V** |
|  | 19051 | **632.3566** | **1894.0479** | **1894.9537** | **-0.9058** | **1** | **1** | **76** | **7** | **R.NNLFTSSAGYRAAEGIPK.L** |
|  | 21799 | **1070.6029** | **2139.1913** | **2140.1025** | **-0.9113** | **0** | **3** | **38** | **10** | **R.GAQGPAGPAGPPGLIGEQGISGPR.G** |
|  | 22070 | **1084.6014** | **2167.1883** | **2167.2001** | **-0.0118** | **0** | **60** | **8.8e-005** | **1** | **R.QLTLLGGPTPNTGAALEFVLR.N** |
|  | 22636 | **1114.0486** | **2226.0826** | **2226.0957** | **-0.0131** | **0** | **63** | **4.1e-005** | **1** | **R.DVVFLIDGSQSAGPEFQYVR.T** |
|  | 23473 | **1166.5941** | **2331.1737** | **2331.1845** | **-0.0108** | **0** | **136** | **1.9e-012** | **1** | **R.LNLLDLDYELAEQLDNIAEK.A** 23474 23476 |
|  | 23972 | **801.4065** | **2401.1976** | **2401.2094** | **-0.0117** | **1** | **15** | **2.5** | **1** | **R.MRDVVLSIVNDLTIAESNCPR.G** |
|  | 25576 | **1318.7065** | **2635.3985** | **2635.4108** | **-0.0123** | **0** | **67** | **1.2e-005** | **1** | **K.NADPAELEQIVLSPAFILAAESLPK.I** 25563 25577 |
|  | 25667 | **1327.6473** | **2653.2801** | **2653.2911** | **-0.0110** | **0** | **73** | **3.3e-006** | **1** | **K.GADQAELEEIAFDSSLVFIPAEFR.A** 25665 25666 |
|  | 27586 | **1499.6971** | **2997.3797** | **2997.3954** | **-0.0156** | **0** | **66** | **1.2e-005** | **1** | **R.VSVVANTPSGPVEAFDFDEYQPEMLEK.F** 27582 27584 27585 |
|  | 28167 | **1043.5289** | **3127.5650** | **3127.5813** | **-0.0163** | **1** | **35** | **0.017** | **1** | **R.IEEGVPQFLVLISSGKSDDEVDDPAVELK.Q** 28165 28166 |
|  | 28416 | 796.6552 | 3182.5915 | 3182.5381 | 0.0534 | 0 | 10 | 4.7 | 2 | R.QLQLQGGSGLNTGSALSYVYANHFTEAGGSR.I 28406 28418 28427 |
|  | 29340 | **860.9294** | **3439.6887** | **3439.7048** | **-0.0162** | **0** | **47** | **0.00085** | **1** | **R.YPPPAVESDAADIVFLIDSSEGVRPDGFAHIR.D** |
|  | 29633 | **1188.5151** | **3562.5236** | **3562.8096** | **-0.2860** | **0** | **0** | **32** | **5** | **R.LEIGQDLIQVAVAQYADTVRPEFYFNTHPTK.R** |
|  | 29730 | **1196.9611** | **3587.8614** | **3587.8698** | **-0.0085** | **0** | **2** | **23** | **1** | **R.GGAVPIGIGIGNADITEMQTISFIPDFAVAIPTFR.Q** |
|  | 30188 | **955.9603** | **3819.8120** | **3819.8295** | **-0.0175** | **0** | **21** | **0.25** | **1** | **R.VGNVQELSELSEQVLETLHDAMHETLCPGVTDAAK.A** |

| **30.** | P06576\|ATPB_HUMAN    **Mass:** 56525    **Score:** 884    **Queries matched:** 68   **emPAI:** 1.96 |
| --- | --- |
|  | ATP synthase subunit beta, mitochondrial OS=Homo sapiens GN=ATP5B PE=1 SV=3 |

|  | **Query** | **Observed** | **Mr(expt)** | **Mr(calc)** | **Delta** | **Miss** | **Score** | **Expect** | **Rank** | **Peptide** |
| --- | --- | --- | --- | --- | --- | --- | --- | --- | --- | --- |
|  | 1585 | **398.7108** | **795.4070** | **794.4109** | **0.9961** | **0** | **16** | **4.1** | **1** | **-.MLGFVGR.V** |
|  | 1683 | **402.2878** | **802.5610** | **801.4821** | **1.0789** | **1** | **13** | **11** | **2** | **K.LTVSRAR.K** |
|  | 4054 | **488.2813** | **974.5479** | **974.5549** | **-0.0070** | **0** | **37** | **0.039** | **1** | **K.IGLFGGAGVGK.T** |
|  | 4925 | **519.6145** | **1037.2144** | **1037.5869** | **-0.3725** | **0** | **1** | **85** | **6** | **K.IPVGPETLGR.I** |
|  | 4960 | **520.8357** | **1039.6569** | **1039.6641** | **-0.0072** | **1** | **10** | **13** | **2** | **K.LVPLKETIK.G** 4942 |
|  | 5611 | **543.7526** | **1085.4906** | **1085.4989** | **-0.0083** | **1** | **27** | **0.34** | **1** | **K.ADKLAEEHSS.-** |
|  | 10003 | **444.2188** | **1329.6345** | **1329.7656** | **-0.1311** | **1** | **7** | **32** | **5** | **K.VVDLLAPYAKGGK.I** |
|  | 11037 | **462.9216** | **1385.7429** | **1384.7020** | **1.0409** | **0** | **5** | **42** | **6** | **R.IMNVIGEPIDER.G** |
|  | 11287 | **701.3676** | **1400.7205** | **1400.6969** | **0.0236** | **0** | **(5)** | **43** | **4** | **R.IMNVIGEPIDER.G** 11306 |
|  | 11373 | **469.5618** | **1405.6636** | **1405.6739** | **-0.0103** | **0** | **15** | **3.9** | **1** | **K.AHGGYSVFAGVGER.T** 11342 |
|  | 11880 | **718.3755** | **1434.7365** | **1434.7467** | **-0.0101** | **0** | **63** | **6.5e-005** | **1** | **R.FTQAGSEVSALLGR.I** |
|  | 12246 | **729.4199** | **1456.8252** | **1456.8323** | **-0.0071** | **0** | **77** | **2.4e-006** | **1** | **K.TVLIMELINNVAK.A** 12245 12247 |
|  | 12496 | **737.3639** | **1472.7132** | **1472.8272** | **-0.1140** | **0** | **(3)** | **69** | **2** | **K.TVLIMELINNVAK.A** |
|  | 14631 | 801.4036 | 1600.7927 | 1600.8031 | -0.0104 | 0 | 58 | 0.00019 | 1 | K.VALVYGQMNEPPGAR.A 14634 |
|  | 15417 | **825.9570** | **1649.8995** | **1649.9101** | **-0.0106** | **0** | **44** | **0.0042** | **1** | **R.LVLEVAQHLGESTVR.T** 15416 15418 |
|  | 16920 | **582.0122** | **1743.0148** | **1742.9614** | **0.0534** | **1** | **(3)** | **53** | **10** | **-.MLGFVGRVAAAPASGALR.R** |
|  | 17107 | **587.0676** | **1758.1809** | **1758.9563** | **-0.7754** | **1** | **5** | **22** | **7** | **-.MLGFVGRVAAAPASGALR.R** |
|  | 17418 | **890.9801** | **1779.9456** | **1779.9553** | **-0.0096** | **1** | **58** | **0.00015** | **1** | **R.IMNVIGEPIDERGPIK.T** 17416 17417 |
|  | 17956 | **908.4322** | **1814.8498** | **1814.8621** | **-0.0123** | **0** | **65** | **3.1e-005** | **1** | **R.IMDPNIVGSEHYDVAR.G** 17957 17958 |
|  | 18185 | **611.2886** | **1830.8439** | **1830.8570** | **-0.0132** | **0** | **(10)** | **11** | **2** | **R.IMDPNIVGSEHYDVAR.G** |
|  | 19324 | **640.3671** | **1918.0796** | **1918.0888** | **-0.0092** | **1** | **75** | **2.9e-006** | **1** | **K.VLDSGAPIKIPVGPETLGR.I** 19322 19323 19325 19326 |
|  | 20183 | **994.5146** | **1987.0146** | **1987.0262** | **-0.0116** | **0** | **8** | **16** | **2** | **R.AIAELGIYPAVDPLDSTSR.I** 20181 20182 |
|  | 20576 | **675.0040** | **2021.9903** | **2022.0033** | **-0.0131** | **0** | **17** | **1.8** | **1** | **R.FLSQPFQVAEVFTGHMGK.L** |
|  | 20743 | **1019.9870** | **2037.9594** | **2037.9983** | **-0.0388** | **0** | **(4)** | **37** | **4** | **R.FLSQPFQVAEVFTGHMGK.L** |
|  | 21456 | **1052.0739** | **2102.1331** | **2103.0419** | **-0.9087** | **1** | **2** | **49** | **5** | **K.DATSKVALVYGQMNEPPGAR.A** 21457 |
|  | 22951 | **1133.5393** | **2265.0641** | **2265.0770** | **-0.0129** | **0** | **77** | **1.7e-006** | **1** | **R.IPSAVGYQPTLATDMGTMQER.I** 22949 22950 |
|  | 23351 | **773.3818** | **2317.1235** | **2317.1372** | **-0.0137** | **1** | **43** | **0.0037** | **1** | **R.TREGNDLYHEMIESGVINLK.D** |
|  | 25841 | **1338.1868** | **2674.3590** | **2674.3735** | **-0.0145** | **1** | **98** | **9.4e-009** | **1** | **K.SLQDIIAILGMDELSEEDKLTVSR.A** 25839 25847 25848 25851 25852 25855 25859 25861 |
|  | 25936 | **897.7929** | **2690.3569** | **2690.3684** | **-0.0115** | **1** | **(2)** | **35** | **1** | **K.SLQDIIAILGMDELSEEDKLTVSR.A** 25927 |
|  | 27747 | **1011.2278** | **3030.6615** | **3030.6754** | **-0.0139** | **0** | **13** | **2.2** | **1** | **R.IVAVIGAVVDVQFDEGLPPILNALEVQGR.E** |
|  | 27826 | **1015.5037** | **3043.4892** | **3043.5034** | **-0.0143** | **0** | **40** | **0.0045** | **1** | **K.QFAPIHAEAPEFMEMSVEQEILVTGIK.V** 27825 |
|  | 29083 | **1117.5475** | **3349.6206** | **3349.6329** | **-0.0122** | **0** | **52** | **0.0003** | **1** | **K.GFQQILAGEYDHLPEQAFYMVGPIEEAVAK.A** 29080 29084 29085 |
|  | 30223 | **1281.6578** | **3841.9517** | **3841.9738** | **-0.0221** | **1** | **89** | **4.4e-008** | **1** | **K.KGSITSVQAIYVPADDLTDPAPATTFAHLDATTVLSR.A** 30224 |

| **31.** | P02042\|HBD_HUMAN    **Mass:** 16159    **Score:** 877    **Queries matched:** 57   **emPAI:** 15.36 |
| --- | --- |
|  | Hemoglobin subunit delta OS=Homo sapiens GN=HBD PE=1 SV=2 |

|  | **Query** | **Observed** | **Mr(expt)** | **Mr(calc)** | **Delta** | **Miss** | **Score** | **Expect** | **Rank** | **Peptide** |
| --- | --- | --- | --- | --- | --- | --- | --- | --- | --- | --- |
|  | 3728 | 476.7553 | 951.4960 | 951.5025 | -0.0065 | 0 | 22 | 1 | 1 | M.VHLTPEEK.T 3724 3726 |
|  | 6170 | 563.7820 | 1125.5495 | 1125.5567 | -0.0072 | 0 | 19 | 1.8 | 1 | K.LHVDPENFR.L 6169 |
|  | 6479 | 575.3363 | 1148.6580 | 1148.6666 | -0.0085 | 0 | 79 | 1.8e-006 | 1 | K.VVAGVANALAHK.Y 6477 6478 6482 6486 6487 |
|  | 8514 | **628.8292** | **1255.6439** | **1255.6521** | **-0.0082** | **0** | **74** | **5.7e-006** | **1** | **K.VNVDAVGGEALGR.L** 8477 |
|  | 8890 | 637.8627 | 1273.7108 | 1273.7183 | -0.0075 | 0 | 56 | 0.00034 | 1 | R.LLVVYPWTQR.F 8883 8885 8886 8888 8891 8892 8893 8894 8896 8897 8898 8899 8900 8901 8903 8904 8912 |
|  | 9911 | **663.8968** | **1325.7791** | **1325.7853** | **-0.0062** | **0** | **56** | **0.00032** | **1** | **R.LLGNVLVCVLAR.N** 9910 |
|  | 11968 | **721.3381** | **1440.6617** | **1440.6707** | **-0.0090** | **0** | **35** | **0.039** | **1** | **K.EFTPQMQAAYQK.V** |
|  | 12132 | 725.3964 | 1448.7782 | 1448.7888 | -0.0107 | 1 | 60 | 0.00013 | 1 | K.VVAGVANALAHKYH.- 12131 12134 12136 12138 |
|  | 15742 | 835.4432 | 1668.8718 | 1668.8835 | -0.0117 | 0 | 105 | 3.9e-009 | 1 | K.VLGAFSDGLAHLDNLK.G 15726 15743 15748 15749 |
|  | 17276 | **887.4957** | **1772.9768** | **1772.0131** | **0.9637** | **1** | **3** | **53** | **5** | **R.LLGNVLVCVLARNFGK.E** |
|  | 17700 | 599.9964 | 1796.9674 | 1796.9785 | -0.0111 | 1 | 63 | 5.4e-005 | 1 | K.KVLGAFSDGLAHLDNLK.G 17696 17698 17713 |
|  | 20803 | **1022.9633** | **2043.9120** | **2043.9248** | **-0.0128** | **0** | **94** | **3.4e-008** | **1** | **R.FFESFGDLSSPDAVMGNPK.V** 20804 20806 20817 20818 |
|  | 22293 | **1099.0909** | **2196.1673** | **2196.1651** | **0.0022** | **1** | **4** | **27** | **3** | **K.TAVNALWGKVNVDAVGGEALGR.L** |
|  | 23116 | **572.5228** | **2286.0622** | **2287.0831** | **-1.0209** | **1** | **1** | **68** | **9** | **R.FFESFGDLSSPDAVMGNPKVK.A** |
|  | 25539 | **877.0815** | **2628.2228** | **2628.2391** | **-0.0163** | **1** | **30** | **0.062** | **1** | **K.GTFSQLSELHCDKLHVDPENFR.L** |

| **32.** | Q16853\|AOC3_HUMAN    **Mass:** 85138    **Score:** 870    **Queries matched:** 76   **emPAI:** 1.25 |
| --- | --- |
|  | Membrane primary amine oxidase OS=Homo sapiens GN=AOC3 PE=1 SV=3 |

|  | **Query** | **Observed** | **Mr(expt)** | **Mr(calc)** | **Delta** | **Miss** | **Score** | **Expect** | **Rank** | **Peptide** |
| --- | --- | --- | --- | --- | --- | --- | --- | --- | --- | --- |
|  | 1164 | **643.3550** | **642.3478** | **641.3497** | **0.9981** | **0** | **14** | **8.5** | **1** | **K.ALDPAR.W** |
|  | 1462 | **769.3942** | **768.3870** | **768.3919** | **-0.0049** | **0** | **16** | **3.6** | **1** | **K.VFYQGR.Y** |
|  | 2336 | **426.2320** | **850.4494** | **850.4549** | **-0.0055** | **0** | **13** | **8.4** | **1** | **K.YTTPLTR.G** |
|  | 3503 | **469.2611** | **936.5077** | **936.5141** | **-0.0064** | **0** | **24** | **0.64** | **1** | **K.AAALAHLDR.G** |
|  | 3661 | **474.7417** | **947.4688** | **947.4746** | **-0.0057** | **0** | **27** | **0.4** | **1** | **R.EELTAVMR.F** |
|  | 4087 | **489.7684** | **977.5222** | **977.5294** | **-0.0072** | **0** | **46** | **0.0045** | **1** | **R.YQLAVTQR.K** 4088 4105 |
|  | 4803 | **515.7332** | **1029.4519** | **1029.4590** | **-0.0071** | **0** | **32** | **0.1** | **1** | **R.YVDGGFGMGK.Y** 4802 4813 |
|  | 4855 | **517.2660** | **1032.5175** | **1032.5253** | **-0.0078** | **0** | **15** | **5.2** | **2** | **R.HGGPLPYHR.R** 4833 4857 |
|  | 5051 | **523.7308** | **1045.4470** | **1045.4539** | **-0.0069** | **0** | **(11)** | **14** | **5** | **R.YVDGGFGMGK.Y** |
|  | 5851 | **552.2940** | **1102.5735** | **1102.5805** | **-0.0070** | **0** | **45** | **0.0051** | **1** | **R.NLVTMTTAPR.G** 5850 |
|  | 6085 | **560.7779** | **1119.5412** | **1118.5754** | **0.9658** | **0** | **(4)** | **66** | **5** | **R.NLVTMTTAPR.G** |
|  | 6118 | **561.8162** | **1121.6178** | **1121.6233** | **-0.0056** | **0** | **2** | **94** | **7** | **R.EALAIVFFGR.Q** |
|  | 9705 | **658.8549** | **1315.6952** | **1315.7030** | **-0.0079** | **1** | **3** | **78** | **2** | **R.GRNLVTMTTAPR.G** |
|  | 11699 | **713.3018** | **1424.5891** | **1424.7565** | **-0.1674** | **1** | **2** | **83** | **6** | **R.WTIQKVFYQGR.Y** |
|  | 17463 | 892.4935 | 1782.9725 | 1783.9733 | -1.0008 | 1 | 4 | 42 | 3 | R.GSPPPAREALAIVFFGR.Q |
|  | 17652 | **897.9315** | **1793.8484** | **1792.8567** | **0.9917** | **0** | **39** | **0.014** | **1** | **R.DAFCVFEQNQGLPLR.R** |
|  | 18845 | **939.9705** | **1877.9265** | **1877.8982** | **0.0283** | **1** | **1** | **81** | **4** | **R.YVDGGFGMGKYTTPLTR.G** |
|  | 19121 | **951.4755** | **1900.9364** | **1899.9407** | **0.9957** | **0** | **31** | **0.079** | **1** | **R.FYATGYISSAFLFGATGK.Y** 19118 19120 |
|  | 19751 | **975.4813** | **1948.9480** | **1948.9578** | **-0.0098** | **1** | **23** | **0.49** | **1** | **R.DAFCVFEQNQGLPLRR.H** 19750 |
|  | 19882 | **980.4565** | **1958.8984** | **1958.9131** | **-0.0147** | **0** | **78** | **1.4e-006** | **1** | **R.ELPQASGLLHHCCFYK.H** |
|  | 19933 | **982.0015** | **1961.9885** | **1960.9928** | **0.9957** | **0** | **37** | **0.018** | **1** | **K.LLEMEEQAAFLVGSATPR.Y** 19923 |
|  | 20159 | 993.4738 | 1984.9331 | 1986.0000 | -1.0669 | 0 | 28 | 0.16 | 2 | R.VASSLWTFSFGLGAFSGPR.I |
|  | 20364 | **1001.5236** | **2001.0326** | **2001.0472** | **-0.0147** | **0** | **45** | **0.0032** | **1** | **K.SPVPPGPAPPLQFYPQGPR.F** 20348 20365 20367 |
|  | 21326 | **1045.5448** | **2089.0750** | **2089.0877** | **-0.0127** | **1** | **99** | **1.2e-008** | **1** | **R.KLLEMEEQAAFLVGSATPR.Y** 21328 21329 |
|  | 23215 | **766.7546** | **2297.2421** | **2296.1674** | **1.0747** | **0** | **35** | **0.025** | **1** | **R.RPVLFQEYLDIDQMIFNR.E** |
|  | 23594 | **470.8294** | **2349.1105** | **2349.1251** | **-0.0145** | **0** | **21** | **0.65** | **1** | **K.YGNQVSEHTLGTVHTHSAHFK.V** 23593 |
|  | 25153 | **852.8412** | **2555.5019** | **2555.3033** | **0.1986** | **1** | **0** | **51** | **3** | **R.RHHSDLYSHYFGGLAETVLVVR.S** |
|  | 25770 | **890.1224** | **2667.3455** | **2667.3578** | **-0.0123** | **0** | **45** | **0.002** | **1** | **R.LVYEISLQEALAIYGGNSPAAMTTR.Y** 25756 25760 25761 25762 25765 25767 25769 25775 25778 25780 25782 25783 25784 25787 25789 25796 25797 25798 |
|  | 26111 | **1355.1477** | **2708.2809** | **2708.2945** | **-0.0136** | **0** | **68** | **1.1e-005** | **1** | **R.GVDCPYLATYVDWHFLLESQAPK.T** 26107 26110 26112 |
|  | 27469 | **993.5124** | **2977.5153** | **2977.5331** | **-0.0178** | **0** | **47** | **0.00096** | **1** | **R.LGPGLVDAAQARPSDNCVFSVELQLPPK.A** 27465 27467 27468 27470 |
|  | 30197 | **1274.8874** | **3821.6405** | **3821.6621** | **-0.0216** | **0** | **149** | **3.4e-014** | **1** | **R.GDQDAGACEVNPLACLPQAAACAPDLPAFSHGGFSHN.-** 30196 30199 |
|  | 30396 | **1321.9722** | **3962.8947** | **3962.9124** | **-0.0177** | **0** | **76** | **7.3e-007** | **1** | **K.VDLDVAGLENWVWAEDMVFVPMAVPWSPEHQLQR.L** |

| **33.** | Q9UGT4\|SUSD2_HUMAN    **Mass:** 91746    **Score:** 825    **Queries matched:** 46   **emPAI:** 1.12 |
| --- | --- |
|  | Sushi domain-containing protein 2 OS=Homo sapiens GN=SUSD2 PE=1 SV=1 |

|  | **Query** | **Observed** | **Mr(expt)** | **Mr(calc)** | **Delta** | **Miss** | **Score** | **Expect** | **Rank** | **Peptide** |
| --- | --- | --- | --- | --- | --- | --- | --- | --- | --- | --- |
|  | 790 | **504.3048** | **503.2975** | **504.2292** | **-0.9317** | **0** | **1** | **3.1e+002** | **5** | **R.ADSGR.F** |
|  | 1250 | **681.3737** | **680.3664** | **679.3289** | **1.0375** | **0** | **4** | **66** | **1** | **K.NYAGQK.D** |
|  | 1367 | **724.4049** | **723.3976** | **723.3295** | **0.0681** | **0** | **19** | **1.6** | **1** | **K.VSMMEK.S** |
|  | 1436 | **760.4258** | **759.4185** | **759.4239** | **-0.0054** | **0** | **20** | **2.2** | **1** | **R.SVQASLR.Y** |
|  | 5411 | **536.8128** | **1071.6111** | **1070.6448** | **0.9663** | **1** | **3** | **1.1e+002** | **10** | **R.VGALRIIDSK.N** |
|  | 9851 | **662.3003** | **1322.5861** | **1322.5959** | **-0.0097** | **0** | **48** | **0.0022** | **1** | **K.MSSPTDASVICR.F** |
|  | 10616 | **455.7241** | **1364.1504** | **1364.5990** | **-0.4486** | **0** | **6** | **23** | **2** | **R.AQPGTMSNGTETR.G** |
|  | 14040 | **781.9207** | **1561.8269** | **1561.8351** | **-0.0083** | **0** | **52** | **0.00072** | **1** | **R.GEYVLLEAALTDLR.V** |
|  | 16010 | **563.2864** | **1686.8375** | **1685.8526** | **0.9849** | **0** | **3** | **59** | **5** | **R.IPFTVSLDNGHSFPR.A** |
|  | 20352 | **1001.0095** | **2000.0045** | **2000.0175** | **-0.0130** | **0** | **91** | **7.2e-008** | **1** | **R.GTGLTAVAVQEGNSDVVEVR.L** 20339 20343 20350 20353 20363 |
|  | 23186 | **1148.0604** | **2294.1063** | **2293.1348** | **0.9715** | **0** | **31** | **0.063** | **1** | **R.MQSLQPVVSCGWLAPPPNGQK.E** 23185 |
|  | 24299 | 1226.1097 | 2450.2049 | 2449.2359 | 0.9691 | 1 | 8 | 11 | 2 | R.RMQSLQPVVSCGWLAPPPNGQK.E |
|  | 24811 | **841.0649** | **2520.1730** | **2520.1851** | **-0.0121** | **1** | **14** | **2.9** | **1** | **R.DFCLEILPYSGSMMGGKDFVVR.H** |
|  | 25125 | **1276.9971** | **2551.9796** | **2552.1750** | **-0.1954** | **1** | **(0)** | **44** | **3** | **R.DFCLEILPYSGSMMGGKDFVVR.H** 25119 |
|  | 26238 | **913.1268** | **2736.3585** | **2736.3680** | **-0.0095** | **0** | **74** | **2.9e-006** | **1** | **R.TGGLEVLLNQEVLSFTEQSWMDLK.G** 26237 26242 |
|  | 26255 | **1370.6692** | **2739.3238** | **2739.3392** | **-0.0153** | **0** | **50** | **0.00063** | **1** | **K.HDPTFEPLFPSETTLNPSLAQEAAK.L** 26253 26254 |
|  | 26293 | **917.0913** | **2748.2521** | **2749.3428** | **-1.0907** | **1** | **0** | **56** | **4** | **R.MQSLQPVVSCGWLAPPPNGQKEGNR.Y** |
|  | 26896 | **953.3732** | **2857.0979** | **2857.1169** | **-0.0191** | **0** | **29** | **0.048** | **1** | **R.CGALDGPCSCHPTCSGLGTCCLDFR.D** |
|  | 26987 | **960.4684** | **2878.3833** | **2878.3999** | **-0.0165** | **0** | **65** | **1.7e-005** | **1** | **K.FLTHTHGLLGTLNNDPTDDFTLHSGR.V** 26985 |
|  | 28014 | **1031.7550** | **3092.2432** | **3092.2674** | **-0.0243** | **0** | **68** | **6.7e-006** | **1** | **R.FFTDYGCDMEQGSVCTYHPGAVHCVR.S** 28015 28017 |
|  | 28597 | **1072.8566** | **3215.5479** | **3215.5670** | **-0.0191** | **1** | **76** | **1.2e-006** | **1** | **R.FKDSIQTLGHVDSSGQVHCVSPLLYESGR.I** 28598 |
|  | 28718 | **1085.2249** | **3252.6527** | **3253.5792** | **-0.9265** | **1** | **3** | **25** | **6** | **K.SELVNETRWQYYGTANTSGNLSLTWHVK.S** |
|  | 28741 | **1087.1830** | **3258.5271** | **3258.5431** | **-0.0160** | **0** | **10** | **4.3** | **1** | **K.SLPTQTITIELWGYEETGMPYSQEWTAK.W** |
|  | 29325 | **1145.5424** | **3433.6053** | **3433.6228** | **-0.0176** | **1** | **76** | **9.6e-007** | **1** | **K.DVQALWTNDHALAWHLSDDFREDPVAWAR.T** 29324 |
|  | 30136 | **1896.3450** | **3790.6754** | **3790.6913** | **-0.0159** | **0** | **48** | **0.00046** | **1** | **R.TQCQAWEELEDQLPNFLEELPDCPCTLTQAR.A** 30137 |
|  | 30460 | **1338.5819** | **4012.7239** | **4012.7520** | **-0.0281** | **0** | **77** | **5.7e-007** | **1** | **R.YLAGSTIYFHCDNGYSLAGAETSTCQADGTWSSPTPK.C** 30459 30461 |
|  | 30695 | **1044.2001** | **4172.7712** | **4172.7978** | **-0.0266** | **1** | **71** | **1.7e-006** | **1** | **R.YGSGQQCCYTADGTQLLTADSSGGSTPDRGHDWGAPPFR.T** |

| **34.** | P51659\|DHB4_HUMAN    **Mass:** 80092    **Score:** 795    **Queries matched:** 65   **emPAI:** 1.36 |
| --- | --- |
|  | Peroxisomal multifunctional enzyme type 2 OS=Homo sapiens GN=HSD17B4 PE=1 SV=3 |

|  | **Query** | **Observed** | **Mr(expt)** | **Mr(calc)** | **Delta** | **Miss** | **Score** | **Expect** | **Rank** | **Peptide** |
| --- | --- | --- | --- | --- | --- | --- | --- | --- | --- | --- |
|  | 1444 | **762.4080** | **761.4007** | **761.4072** | **-0.0064** | **0** | **24** | **0.85** | **1** | **K.VYQGPAK.G** 1442 |
|  | 1563 | **794.4104** | **793.4031** | **793.4083** | **-0.0051** | **0** | **14** | **6.6** | **1** | **R.GSFQVTR.A** |
|  | 2877 | **445.7387** | **889.4629** | **889.4691** | **-0.0062** | **0** | **28** | **0.36** | **1** | **R.GNIMLSQK.L** 2878 |
|  | 4501 | **503.2898** | **1004.5651** | **1003.5199** | **1.0452** | **1** | **7** | **33** | **8** | **M.GSPLRFDGR.V** 4479 |
|  | 6127 | **562.2783** | **1122.5421** | **1122.5492** | **-0.0071** | **0** | **7** | **34** | **1** | **K.NHPMTPEAVK.A** |
|  | 6773 | **585.2730** | **1168.5315** | **1168.6928** | **-0.1614** | **0** | **0** | **1.4e+002** | **9** | **R.VVLVTGAGAGLGR.A** |
|  | 7391 | **401.2118** | **1200.6135** | **1199.6775** | **0.9360** | **1** | **5** | **44** | **7** | **R.WERTLGAIVR.Q** |
|  | 8151 | **620.2996** | **1238.5847** | **1237.6740** | **0.9107** | **1** | **5** | **44** | **3** | **K.LQMILKDYAK.L** |
|  | 9025 | **641.8734** | **1281.7323** | **1281.7405** | **-0.0082** | **0** | **26** | **0.33** | **1** | **R.IDVVVNNAGILR.D** 9016 |
|  | 10208 | **448.5723** | **1342.6952** | **1342.7034** | **-0.0082** | **0** | **28** | **0.23** | **1** | **K.VNAVFEWHITK.G** |
|  | 10882 | **460.2308** | **1377.6707** | **1378.7027** | **-1.0320** | **1** | **2** | **81** | **7** | **R.QKNHPMTPEAVK.A** |
|  | 11036 | **693.8779** | **1385.7412** | **1385.7514** | **-0.0102** | **1** | **47** | **0.0024** | **1** | **K.GSLAADKVVEEIR.R** |
|  | 11226 | **466.8900** | **1397.6483** | **1397.6575** | **-0.0092** | **0** | **27** | **0.28** | **1** | **R.ISDEDWDIIHR.V** |
|  | 11771 | **715.3314** | **1428.6482** | **1428.6593** | **-0.0112** | **0** | **77** | **2.4e-006** | **1** | **K.IDSEGGVSANHTSR.A** 11759 11760 11772 |
|  | 12422 | **734.3703** | **1466.7260** | **1466.7365** | **-0.0105** | **0** | **57** | **0.00026** | **1** | **R.ATSTATSGFAGAIGQK.L** 12401 12407 12408 |
|  | 12926 | **748.9394** | **1495.8642** | **1495.8722** | **-0.0080** | **0** | **66** | **3.2e-005** | **1** | **K.LGLLGLANSLAIEGR.K** 12945 12948 |
|  | 13779 | **516.5991** | **1546.7755** | **1546.7852** | **-0.0097** | **1** | **40** | **0.013** | **1** | **R.RVLQQFADNDVSR.F** |
|  | 14318 | **791.4157** | **1580.8167** | **1580.8311** | **-0.0143** | **1** | **1** | **83** | **8** | **K.LQSTFVFEEIGRR.L** |
|  | 14878 | **808.8915** | **1615.7684** | **1615.9153** | **-0.1469** | **1** | **9** | **14** | **10** | **R.GNIMLSQKLQMILK.D** 14879 |
|  | 14989 | **811.8912** | **1621.7679** | **1621.8035** | **-0.0355** | **1** | **6** | **28** | **7** | **K.NHPMTPEAVKANWK.K** |
|  | 15030 | **542.3261** | **1623.9565** | **1623.9671** | **-0.0106** | **1** | **20** | **1.1** | **1** | **K.LGLLGLANSLAIEGRK.S** 15038 |
|  | 15317 | **549.1863** | **1644.5370** | **1643.9108** | **0.6262** | **1** | **2** | **55** | **4** | **R.FDGRVVLVTGAGAGLGR.A** |
|  | 16792 | **868.9327** | **1735.8508** | **1735.8628** | **-0.0120** | **1** | **61** | **9.4e-005** | **1** | **K.AVANYDSVEEGEKVVK.T** |
|  | 16832 | **870.4289** | **1738.8432** | **1738.8533** | **-0.0100** | **1** | **65** | **3.2e-005** | **1** | **R.KSNIHCNTIAPNAGSR.M** 16831 16847 |
|  | 16939 | **582.3213** | **1743.9420** | **1743.9520** | **-0.0099** | **1** | **27** | **0.24** | **1** | **R.GALVVVNDLGGDFKGVGK.G** |
|  | 19822 | **489.5212** | **1954.0556** | **1954.0676** | **-0.0120** | **0** | **17** | **1.8** | **1** | **K.VLHGEQYLELYKPLPR.A** |
|  | 20589 | **1012.5124** | **2023.0103** | **2023.0237** | **-0.0134** | **0** | **56** | **0.00025** | **1** | **R.FAKPVYPGQTLQTEMWK.E** 20590 20591 20605 |
|  | 22609 | **1112.5752** | **2223.1358** | **2223.0981** | **0.0378** | **0** | **11** | **6** | **1** | **K.GAADTTIILSDEDFMEVVLGK.L** |
|  | 22841 | **751.3938** | **2251.1596** | **2250.1619** | **0.9976** | **1** | **0** | **79** | **6** | **K.ARFAKPVYPGQTLQTEMWK.E** |
|  | 23605 | **1176.0636** | **2350.1126** | **2350.1263** | **-0.0137** | **0** | **96** | **2e-008** | **1** | **R.IIMTSSASGIYGNFGQANYSAAK.L** 23603 23604 |
|  | 24409 | **1233.1001** | **2464.1856** | **2464.1985** | **-0.0128** | **0** | **95** | **2.2e-008** | **1** | **K.FIYEGSSDFSCLPTFGVIIGQK.S** 24408 24410 24411 |
|  | 24899 | **1268.6333** | **2535.2520** | **2535.2704** | **-0.0184** | **0** | **51** | **0.00055** | **1** | **K.VQETGDIVISNAYVDLAPTSGTSAK.T** 24898 |
|  | 26598 | **935.8094** | **2804.4063** | **2804.4154** | **-0.0090** | **1** | **21** | **0.51** | **1** | **K.GAADTTIILSDEDFMEVVLGKLDPQK.A** |
|  | 26932 | **956.1800** | **2865.5181** | **2865.5348** | **-0.0167** | **0** | **17** | **1.1** | **1** | **K.VAVAIPNRPPDAVLTDTTSLNQAALYR.L** |
|  | 26990 | **1440.7096** | **2879.4046** | **2879.4222** | **-0.0176** | **0** | **38** | **0.01** | **1** | **K.ICDFENASKPQSIQESTGSIIEVLSK.I** 26989 |
|  | 27624 | **752.8820** | **3007.4990** | **3007.5172** | **-0.0182** | **1** | **2** | **33** | **3** | **K.KICDFENASKPQSIQESTGSIIEVLSK.I** |
|  | 27830 | **1015.5237** | **3043.5492** | **3043.5616** | **-0.0124** | **1** | **30** | **0.046** | **1** | **K.LPPFSYAYTELEAIMYALGVGASIKDPK.D** |
|  | 30404 | **993.4941** | **3969.9472** | **3969.9625** | **-0.0153** | **0** | **52** | **0.00019** | **1** | **R.LSGDWNPLHIDPNFASLAGFDKPILHGLCTFGFSAR.R** 30405 |

| **35.** | P02730\|B3AT_HUMAN    **Mass:** 102013   **Score:** 791    **Queries matched:** 42   **emPAI:** 0.97 |
| --- | --- |
|  | Band 3 anion transport protein OS=Homo sapiens GN=SLC4A1 PE=1 SV=3 |

|  | **Query** | **Observed** | **Mr(expt)** | **Mr(calc)** | **Delta** | **Miss** | **Score** | **Expect** | **Rank** | **Peptide** |
| --- | --- | --- | --- | --- | --- | --- | --- | --- | --- | --- |
|  | 2492 | **862.5181** | **861.5109** | **861.4596** | **0.0512** | **0** | **3** | **1.1e+002** | **2** | **K.LSVPDGFK.V** |
|  | 5146 | **527.7497** | **1053.4848** | **1053.4913** | **-0.0065** | **0** | **59** | **0.00017** | **1** | **R.IDAYMAQSR.G** |
|  | 5326 | **534.3110** | **1066.6075** | **1067.7107** | **-1.1031** | **0** | **0** | **1.4e+002** | **10** | **R.ILLLFKPPK.Y** |
|  | 6439 | **574.2895** | **1146.5645** | **1146.6662** | **-0.1017** | **0** | **6** | **36** | **3** | **R.GWVIHPLGLR.S** |
|  | 9975 | **664.8482** | **1327.6819** | **1327.6918** | **-0.0100** | **0** | **40** | **0.014** | **1** | **R.SVTHANALTVMGK.A** |
|  | 10258 | **673.3621** | **1344.7097** | **1343.6867** | **1.0230** | **0** | **(39)** | **0.018** | **1** | **R.SVTHANALTVMGK.A** |
|  | 10740 | **685.8942** | **1369.7738** | **1369.7201** | **0.0537** | **0** | **5** | **44** | **4** | **K.ASTPGAAAQIQEVK.E** |
|  | 12407 | 733.9094 | 1465.8043 | 1465.8140 | -0.0098 | 0 | 90 | 1.4e-007 | 1 | K.IPPDSEATLVLVGR.A 12384 12408 |
|  | 12828 | **745.8991** | **1489.7837** | **1489.7929** | **-0.0093** | **0** | **44** | **0.0053** | **1** | **R.ADFLEQPVLGFVR.L** 12794 |
|  | 14041 | **521.7286** | **1562.1641** | **1562.8053** | **-0.6412** | **1** | **0** | **80** | **5** | **K.LSVPDGFKVSNSSAR.G** |
|  | 14665 | **802.8784** | **1603.7423** | **1603.7518** | **-0.0095** | **0** | **46** | **0.0032** | **1** | **R.YQSSPAKPDSSFYK.G** 14664 |
|  | 18813 | **939.0184** | **1876.0223** | **1876.0305** | **-0.0082** | **0** | **70** | **8.9e-006** | **1** | **R.LQEAAELEAVELPVPIR.F** 18811 |
|  | 20777 | **1021.5537** | **2041.0929** | **2041.1069** | **-0.0140** | **0** | **94** | **3.3e-008** | **1** | **K.HSHAGELEALGGVKPAVLTR.S** 20778 20779 20785 |
|  | 22326 | **1101.4562** | **2200.8978** | **2200.9106** | **-0.0128** | **1** | **45** | **0.0023** | **1** | **K.ATFDEEEGRDEYDEVAMPV.-** |
|  | 22740 | **1118.5045** | **2234.9945** | **2234.1252** | **0.8692** | **1** | **88** | **1.1e-007** | **1** | **K.VYVELQELVMDEKNQELR.W** 22725 22739 |
|  | 23108 | **1143.6105** | **2285.2064** | **2285.2208** | **-0.0145** | **0** | **69** | **9.3e-006** | **1** | **R.FLFVLLGPEAPHIDYTQLGR.A** 23106 23107 23110 |
|  | 23432 | **1163.6283** | **2325.2420** | **2325.2540** | **-0.0119** | **0** | **81** | **6.2e-007** | **1** | **K.GTVLLDLQETSLAGVANQLLDR.F** 23433 23435 |
|  | 24425 | **1234.6174** | **2467.2203** | **2466.2503** | **0.9700** | **0** | **59** | **9.4e-005** | **1** | **K.GLDLNGGPDDPLQQTGQLFGGLVR.D** 24424 24426 |
|  | 28815 | **1093.4619** | **3277.3639** | **3277.9148** | **-0.5509** | **1** | **2** | **21** | **3** | **R.VWIGFWLILLVVLVVAFEGSFLVRFISR.Y** |
|  | 30315 | **1301.0126** | **3900.0159** | **3900.0343** | **-0.0184** | **0** | **61** | **2.2e-005** | **1** | **R.GELLHSLEGFLDCSLVLPPTDAPSEQALLSLVPVQR.E** 30316 |
|  | 30634 | **1373.9700** | **4118.8881** | **4118.9128** | **-0.0247** | **0** | **30** | **0.025** | **1** | **R.SGDPSQPLLPQHSSLETQLFCEQGDGGTEGHSPSGILEK.I** 30632 30633 |
|  | 31872 | **1169.3440** | **8178.3570** | **8177.4024** | **0.9546** | **1** | **1** | **1.8** | **2** | **-.MEELQDDYEDMMEENLEQEEYEDPDIPESQMEEPAAHDTEATATDYHTTSHPGTHKVYVELQELVMDEK.N** |

| **36.** | P14625\|ENPL_HUMAN    **Mass:** 92696    **Score:** 776    **Queries matched:** 55   **emPAI:** 1.27 |
| --- | --- |
|  | Endoplasmin OS=Homo sapiens GN=HSP90B1 PE=1 SV=1 |

|  | **Query** | **Observed** | **Mr(expt)** | **Mr(calc)** | **Delta** | **Miss** | **Score** | **Expect** | **Rank** | **Peptide** |
| --- | --- | --- | --- | --- | --- | --- | --- | --- | --- | --- |
|  | 1180 | **646.4075** | **645.4003** | **645.4061** | **-0.0059** | **0** | **3** | **1.4e+002** | **6** | **K.VIVTSK.H** |
|  | 1333 | **707.3767** | **706.3694** | **705.3810** | **0.9885** | **0** | **19** | **2.3** | **2** | **R.FQNVAK.E** |
|  | 1752 | **405.1950** | **808.3754** | **807.4127** | **0.9627** | **0** | **6** | **41** | **7** | **K.EFGTNIK.L** |
|  | 2671 | **438.7295** | **875.4445** | **875.4501** | **-0.0056** | **0** | **22** | **1.4** | **1** | **K.TFEINPR.H** |
|  | 3249 | 459.7278 | 917.4411 | 918.5287 | -1.0875 | 1 | 22 | 1.3 | 1 | K.NKEIFLR.E 3271 |
|  | 3631 | **473.2929** | **944.5712** | **944.5906** | **-0.0194** | **0** | **4** | **83** | **10** | **R.GTTITLVLK.E** |
|  | 3882 | **482.2940** | **962.5735** | **962.5800** | **-0.0066** | **0** | **18** | **2.5** | **1** | **K.LIINSLYK.N** |
|  | 4294 | **993.5187** | **992.5114** | **992.5179** | **-0.0064** | **0** | **39** | **0.019** | **1** | **R.SGYLLPDTK.A** 4293 |
|  | 4812 | **1031.4902** | **1030.4830** | **1030.4906** | **-0.0076** | **0** | **21** | **1.4** | **1** | **K.IYFMAGSSR.K** |
|  | 6324 | **570.2897** | **1138.5649** | **1138.5731** | **-0.0082** | **0** | **35** | **0.041** | **1** | **K.LGVIEDHSNR.T** |
|  | 6399 | **572.8155** | **1143.6164** | **1142.5608** | **1.0557** | **1** | **26** | **0.38** | **1** | **R.GLFDEYGSKK.S** |
|  | 7082 | **594.3389** | **1186.6632** | **1186.6710** | **-0.0078** | **0** | **35** | **0.049** | **1** | **K.SILFVPTSAPR.G** |
|  | 10713 | **457.2364** | **1368.6875** | **1368.7509** | **-0.0633** | **1** | **11** | **11** | **1** | **R.MMKLIINSLYK.N** |
|  | 11555 | **709.3465** | **1416.6784** | **1416.6885** | **-0.0100** | **1** | **29** | **0.18** | **1** | **K.DISTNYYASQKK.T** |
|  | 12720 | **743.3762** | **1484.7378** | **1484.7471** | **-0.0093** | **0** | **51** | **0.0011** | **1** | **K.GVVDSDDLPLNVSR.E** |
|  | 12847 | **498.0203** | **1491.0390** | **1491.8310** | **-0.7921** | **1** | **14** | **3.2** | **3** | **K.TFEINPRHPLIR.D** |
|  | 13008 | **501.0761** | **1500.2066** | **1500.7970** | **-0.5904** | **1** | **4** | **37** | **10** | **R.MLRLSLNIDPDAK.V** |
|  | 13068 | **502.3202** | **1503.9387** | **1503.7933** | **0.1454** | **1** | **6** | **30** | **5** | **K.EAESSPFVERLLK.K** |
|  | 13215 | **757.3886** | **1512.7625** | **1512.7719** | **-0.0093** | **0** | **44** | **0.0046** | **1** | **K.NLLHVTDTGVGMTR.E** |
|  | 13238 | **758.3544** | **1514.6942** | **1514.7041** | **-0.0100** | **1** | **55** | **0.00042** | **1** | **K.IADDKYNDTFWK.E** 13263 |
|  | 13702 | 771.9242 | 1541.8338 | 1542.9093 | -1.0755 | 1 | 9 | 15 | 9 | R.GNTLGRGTTITLVLK.E |
|  | 14567 | **798.8780** | **1595.7414** | **1594.7160** | **1.0254** | **0** | **34** | **0.048** | **1** | **R.VFITDDFHDMMPK.Y** |
|  | 14773 | **805.8886** | **1609.7625** | **1610.7109** | **-0.9484** | **0** | **(5)** | **38** | **2** | **R.VFITDDFHDMMPK.Y** |
|  | 15054 | **542.9637** | **1625.8692** | **1626.7058** | **-0.8366** | **0** | **(3)** | **60** | **7** | **R.VFITDDFHDMMPK.Y** |
|  | 16999 | **584.6090** | **1750.8050** | **1750.8171** | **-0.0121** | **1** | **32** | **0.065** | **1** | **R.RVFITDDFHDMMPK.Y** |
|  | 17815 | **602.3264** | **1803.9572** | **1804.7851** | **-0.8278** | **0** | **2** | **64** | **6** | **R.ADDEVDVDGTVEEDLGK.S** |
|  | 18652 | **931.9656** | **1861.9167** | **1861.9284** | **-0.0116** | **1** | **75** | **3.4e-006** | **1** | **R.EAVEKEFEPLLNWMK.D** 18664 |
|  | 18845 | 939.9705 | 1877.9265 | 1877.9352 | -0.0087 | 0 | 54 | 0.00042 | 1 | K.YSQFINFPIYVWSSK.T |
|  | 19520 | **967.0357** | **1932.0569** | **1933.0156** | **-0.9587** | **1** | **2** | **69** | **2** | **K.EIFLRELISNASDALDK.I** |
|  | 20832 | **1023.5276** | **2045.0406** | **2045.0528** | **-0.0122** | **0** | **96** | **2.3e-008** | **1** | **R.LISLTDENALSGNEELTVK.I** |
|  | 21560 | **1056.5529** | **2111.0912** | **2111.1045** | **-0.0133** | **1** | **72** | **4.8e-006** | **1** | **K.NLLHVTDTGVGMTREELVK.N** 21559 21561 |
|  | 22910 | **1130.5279** | **2259.0413** | **2259.0556** | **-0.0143** | **0** | **64** | **3.1e-005** | **1** | **R.FQSSHHPTDITSLDQYVER.M** 22908 22909 22911 |
|  | 23654 | **1178.5308** | **2355.0470** | **2355.0623** | **-0.0154** | **0** | **93** | **3.7e-008** | **1** | **R.LTESPCALVASQYGWSGNMER.I** 23653 23655 |
|  | 24327 | **818.4381** | **2452.2925** | **2451.2996** | **0.9929** | **1** | **34** | **0.025** | **1** | **R.GTTITLVLKEEASDYLELDTIK.N** 24307 24318 24319 |
|  | 24925 | **848.0975** | **2541.2708** | **2541.2838** | **-0.0131** | **0** | **22** | **0.42** | **1** | **K.TVWDWELMNDIKPIWQRPSK.E** |
|  | 28049 | **1035.4952** | **3103.4639** | **3103.4848** | **-0.0209** | **1** | **9** | **5.9** | **1** | **K.GYEVIYLTEPVDEYCIQALPEFDGKR.F** 28048 |
|  | 30375 | **1318.6526** | **3952.9359** | **3951.9452** | **0.9907** | **1** | **70** | **2.6e-006** | **1** | **K.MTEAQEDGQSTSELIGQFGVGFYSAFLVADKVIVTSK.H** 30376 30377 30378 |

| **37.** | Q9NY65\|TBA8_HUMAN    **Mass:** 50746    **Score:** 770    **Queries matched:** 31   **emPAI:** 0.89 |
| --- | --- |
|  | Tubulin alpha-8 chain OS=Homo sapiens GN=TUBA8 PE=1 SV=1 |

|  | **Query** | **Observed** | **Mr(expt)** | **Mr(calc)** | **Delta** | **Miss** | **Score** | **Expect** | **Rank** | **Peptide** |
| --- | --- | --- | --- | --- | --- | --- | --- | --- | --- | --- |
|  | 4720 | 512.7546 | 1023.4947 | 1022.4417 | 1.0530 | 0 | 1 | 1.3e+002 | 5 | K.EDAANNYAR.G |
|  | 4988 | 521.7651 | 1041.5156 | 1042.5270 | -1.0114 | 1 | 7 | 34 | 10 | K.FDLMYAKR.A |
|  | 10920 | 690.8480 | 1379.6814 | 1379.6907 | -0.0094 | 1 | 25 | 0.5 | 1 | R.LDHKFDLMYAK.R 10921 |
|  | 12767 | 744.4402 | 1486.8658 | 1486.8719 | -0.0060 | 0 | 72 | 8.7e-006 | 1 | R.LISQIVSSITASLR.F 12765 12766 |
|  | 16241 | 851.4521 | 1700.8896 | 1700.8655 | 0.0241 | 0 | 13 | 5 | 2 | R.AVMIDLEPTVVDEVR.A 16242 16243 |
|  | 16504 | 859.9384 | 1717.8623 | 1717.8747 | -0.0124 | 0 | 44 | 0.0044 | 1 | R.NLDIERPTYTNLNR.L 16503 16505 |
|  | 18100 | 912.9898 | 1823.9651 | 1823.9782 | -0.0131 | 0 | 57 | 0.00019 | 1 | K.VGINYQPPTVVPGGDLAK.V 18098 18099 18101 |
|  | 18674 | 932.9498 | 1863.8851 | 1863.8971 | -0.0120 | 0 | 84 | 3.8e-007 | 1 | R.AVCMLSNTTAIAEAWAR.L 18675 |
|  | 19880 | **653.8388** | **1958.4946** | **1958.0374** | **0.4572** | **1** | **4** | **22** | **1** | **R.AGTYRQLFHPEQLITGK.E** |
|  | 23458 | 777.3395 | 2328.9966 | 2329.0110 | -0.0144 | 0 | 40 | 0.0071 | 1 | R.AFVHWYVGEGMEEGEFSEAR.E |
|  | 24021 | 1205.1011 | 2408.1876 | 2408.2012 | -0.0136 | 0 | 116 | 1.8e-010 | 1 | R.FDGALNVDLTEFQTNLVPYPR.I 24022 24023 24024 24025 24026 |
|  | 24065 | 1208.0977 | 2414.1808 | 2414.1978 | -0.0171 | 1 | 71 | 6.6e-006 | 1 | R.QLFHPEQLITGKEDAANNYAR.G 24063 24064 |
|  | 31727 | 1147.3433 | 5731.6799 | 5731.7161 | -0.0361 | 1 | 10 | 0.96 | 1 | K.SKLEFAIYPAPQVSTAVVEPYNSILTTHTTLEHSDCAFMVDNEAIYDICR.R |

| **38.** | Q9BSJ8\|ESYT1_HUMAN    **Mass:** 123293   **Score:** 769    **Queries matched:** 44   **emPAI:** 0.60 |
| --- | --- |
|  | Extended synaptotagmin-1 OS=Homo sapiens GN=ESYT1 PE=1 SV=1 |

|  | **Query** | **Observed** | **Mr(expt)** | **Mr(calc)** | **Delta** | **Miss** | **Score** | **Expect** | **Rank** | **Peptide** |
| --- | --- | --- | --- | --- | --- | --- | --- | --- | --- | --- |
|  | 1710 | **403.2271** | **804.4396** | **805.4368** | **-0.9972** | **1** | **2** | **1.2e+002** | **1** | **R.MKLDVGK.V** |
|  | 3276 | **460.7423** | **919.4701** | **919.4763** | **-0.0063** | **0** | **23** | **0.9** | **1** | **K.SDPYALVR.L** |
|  | 4369 | 499.7435 | 997.4725 | 996.4699 | 1.0026 | 1 | 13 | 6.9 | 7 | K.DDFLGRMK.L |
|  | 4484 | **502.7945** | **1003.5744** | **1003.5815** | **-0.0070** | **1** | **17** | **3.2** | **1** | **K.DRFLGGLVK.G** |
|  | 4959 | **520.8054** | **1039.5962** | **1039.6026** | **-0.0064** | **0** | **19** | **1.7** | **1** | **R.VELGEKPLR.I** |
|  | 5569 | **542.7340** | **1083.4533** | **1084.5773** | **-1.1239** | **1** | **11** | **11** | **4** | **R.LYMKLVMR.I** |
|  | 5708 | **547.2880** | **1092.5614** | **1091.5247** | **1.0366** | **0** | **9** | **20** | **6** | **R.TLSPEFNER.F** 5675 |
|  | 6331 | **570.3393** | **1138.6640** | **1138.6710** | **-0.0070** | **0** | **22** | **0.89** | **1** | **K.LLAETVAPAVR.G** |
|  | 6763 | **584.8303** | **1167.6461** | **1167.6546** | **-0.0086** | **1** | **53** | **0.00075** | **1** | **R.KLVSIVHGCR.S** |
|  | 8677 | 632.8616 | 1263.7087 | 1264.7615 | -1.0528 | 1 | 3 | 75 | 4 | R.IHLLAARGLSSK.D |
|  | 9536 | **655.4103** | **1308.8061** | **1308.8129** | **-0.0068** | **0** | **28** | **0.19** | **1** | **R.ALTLGALTLPLAR.L** |
|  | 10231 | **448.9001** | **1343.6783** | **1342.5863** | **1.0920** | **1** | **7** | **31** | **2** | **R.WYDLMDNKDK.G** |
|  | 10327 | **450.5949** | **1348.7629** | **1348.7714** | **-0.0085** | **0** | **46** | **0.0039** | **1** | **R.IHVLEAQDLIAK.D** |
|  | 10974 | **461.5789** | **1381.7148** | **1380.7660** | **0.9489** | **1** | **5** | **40** | **2** | **K.AGVKGMQLHGVLR.V** |
|  | 13079 | **502.5847** | **1504.7323** | **1504.7423** | **-0.0100** | **0** | **18** | **1.8** | **1** | **R.GSNPHLQTFTFTR.V** |
|  | 14190 | **787.3511** | **1572.6877** | **1571.7614** | **0.9264** | **1** | **4** | **56** | **4** | **K.LDVSVKSNSSFMSR.E** 14201 |
|  | 14517 | **796.9304** | **1591.8463** | **1590.8518** | **0.9945** | **0** | **65** | **3.4e-005** | **1** | **R.WFTLSSGQGQVLLR.A** 14497 |
|  | 14980 | **541.3035** | **1620.8886** | **1619.8995** | **0.9891** | **1** | **11** | **9.3** | **1** | **R.IHVLEAQDLIAKDR.F** |
|  | 16028 | **563.6137** | **1687.8193** | **1687.8318** | **-0.0125** | **1** | **14** | **4.9** | **1** | **R.FEWELPLDEAQRR.K** |
|  | 16439 | **857.9469** | **1713.8792** | **1713.8897** | **-0.0105** | **0** | **107** | **2.3e-009** | **1** | **K.VQLDLAETDLSQGVAR.W** |
|  | 17539 | **895.0286** | **1788.0426** | **1788.0509** | **-0.0084** | **0** | **46** | **0.0025** | **1** | **R.LLVPLVPDLQDVAQLR.S** |
|  | 17866 | **904.9687** | **1807.9228** | **1807.9331** | **-0.0103** | **0** | **65** | **3.6e-005** | **1** | **K.IVAQVWPFLGQYMEK.L** 17865 |
|  | 18152 | **914.9140** | **1827.8134** | **1827.8250** | **-0.0116** | **0** | **80** | **9.5e-007** | **1** | **K.AVYSTNCPVWEEAFR.F** 18153 |
|  | 19344 | **960.9828** | **1919.9510** | **1919.9629** | **-0.0119** | **0** | **61** | **8.1e-005** | **1** | **R.FFLQDPQSQELDVQVK.D** |
|  | 21508 | **1054.5304** | **2107.0462** | **2107.0586** | **-0.0123** | **0** | **81** | **6.9e-007** | **1** | **K.TISQTSAPVWDESASFLIR.K** |
|  | 22894 | **1129.5709** | **2257.1273** | **2257.1419** | **-0.0146** | **1** | **65** | **2.3e-005** | **1** | **R.ELPAWVSFPDVEKAEWLNK.I** 22895 22896 |
|  | 23933 | **798.7183** | **2393.1331** | **2393.1499** | **-0.0168** | **1** | **34** | **0.032** | **1** | **R.FFLQDPQSQELDVQVKDDSR.A** |
|  | 25178 | **1281.6522** | **2561.2899** | **2561.3013** | **-0.0115** | **0** | **109** | **7.8e-010** | **1** | **R.LTTVLNSGFLDEWLTLEDVPSGR.L** 25177 25179 |
|  | 25233 | **1286.6676** | **2571.3206** | **2571.3333** | **-0.0126** | **0** | **78** | **1.1e-006** | **1** | **R.LLTAPELILDQWFQLSSSGPNSR.L** 25234 |
|  | 25442 | **869.4163** | **2605.2271** | **2605.2448** | **-0.0177** | **1** | **9** | **9.2** | **1** | **R.TLSPEFNERFEWELPLDEAQR.R** |
|  | 25879 | **893.4910** | **2677.4513** | **2677.4650** | **-0.0138** | **0** | **35** | **0.016** | **1** | **R.LTPRPTAAELEEVLQVNSLIQTQK.S** |
|  | 26548 | **932.4341** | **2794.2804** | **2795.3436** | **-1.0632** | **1** | **0** | **59** | **3** | **K.VQLDLAETDLSQGVARWYDLMDNK.D** |
|  | 26660 | **938.1584** | **2811.4533** | **2811.4688** | **-0.0155** | **0** | **35** | **0.02** | **1** | **R.GEGTGVLGSLSLPLSELLVADQLCLDR.W** |
|  | 29290 | **1141.5209** | **3421.5408** | **3421.5595** | **-0.0187** | **0** | **8** | **5.9** | **2** | **R.ILYLDSSEICFPTVPGCPGAWDVDSENPQR.G** |

| **39.** | P21333\|FLNA_HUMAN    **Mass:** 283301   **Score:** 762    **Queries matched:** 90   **emPAI:** 0.36 |
| --- | --- |
|  | Filamin-A OS=Homo sapiens GN=FLNA PE=1 SV=4 |

|  | **Query** | **Observed** | **Mr(expt)** | **Mr(calc)** | **Delta** | **Miss** | **Score** | **Expect** | **Rank** | **Peptide** |
| --- | --- | --- | --- | --- | --- | --- | --- | --- | --- | --- |
|  | 1506 | **395.2247** | **788.4348** | **789.4385** | **-1.0036** | **0** | **6** | **54** | **3** | **K.VYGPGVAK.T** |
|  | 2117 | **418.7994** | **835.5843** | **834.4824** | **1.1019** | **1** | **7** | **31** | **1** | **K.HGGKAPLR.V** 2099 |
|  | 2790 | **441.7708** | **881.5269** | **881.5334** | **-0.0065** | **0** | **29** | **0.17** | **1** | **K.AGVAPLQVK.V** |
|  | 2944 | **448.9064** | **895.7983** | **895.3706** | **0.4277** | **0** | **4** | **36** | **3** | **R.VTGDDSMR.M** |
|  | 3906 | **483.2362** | **964.4578** | **965.4454** | **-0.9876** | **0** | **2** | **1.1e+002** | **5** | **K.AEISFEDR.K** |
|  | 3979 | **486.2841** | **970.5537** | **970.5600** | **-0.0063** | **0** | **28** | **0.24** | **1** | **R.LLGWIQNK.L** |
|  | 4206 | **494.7703** | **987.5260** | **987.5349** | **-0.0089** | **1** | **11** | **15** | **2** | **K.KGEITGEVR.M** |
|  | 4239 | **495.7453** | **989.4760** | **990.4328** | **-0.9568** | **0** | **11** | **16** | **4** | **R.DAEMPATEK.D** |
|  | 4593 | **507.2862** | **1012.5579** | **1012.5665** | **-0.0086** | **1** | **17** | **3.4** | **1** | **K.VKAEGPGLSR.T** |
|  | 4649 | **509.2767** | **1016.5389** | **1016.6019** | **-0.0630** | **1** | **5** | **57** | **2** | **K.VKVYGPGVAK.T** |
|  | 4820 | **516.2860** | **1030.5575** | **1029.4437** | **1.1137** | **0** | **12** | **12** | **2** | **K.SSFTVDCSK.A** |
|  | 4840 | **516.7974** | **1031.5803** | **1030.5155** | **1.0647** | **1** | **7** | **39** | **8** | **K.VTANNDKNR.T** |
|  | 5305 | **533.2732** | **1064.5318** | **1064.5325** | **-0.0006** | **0** | **5** | **46** | **4** | **R.LSPFMADIR.D** |
|  | 5509 | **540.2783** | **1078.5420** | **1078.5520** | **-0.0100** | **0** | **45** | **0.005** | **1** | **R.VNVGAGSHPNK.V** |
|  | 5903 | **554.2836** | **1106.5526** | **1106.5972** | **-0.0446** | **0** | **8** | **25** | **9** | **K.LDVQFSGLTK.G** |
|  | 5917 | **554.8054** | **1107.5962** | **1107.6037** | **-0.0075** | **0** | **19** | **1.9** | **1** | **R.ALTQTGGPHVK.A** |
|  | 6284 | **568.3108** | **1134.6070** | **1134.6145** | **-0.0075** | **1** | **18** | **2.4** | **1** | **K.KIQQNTFTR.W** |
|  | 6409 | **573.3323** | **1144.6500** | **1144.6452** | **0.0048** | **1** | **1** | **1.2e+002** | **6** | **K.GAGSGELKVTVK.G** |
|  | 7270 | **598.8124** | **1195.6103** | **1195.6197** | **-0.0094** | **1** | **43** | **0.0079** | **1** | **R.VKVEPSHDASK.V** |
|  | 7904 | **613.8257** | **1225.6368** | **1225.6455** | **-0.0087** | **0** | **11** | **13** | **1** | **R.AWGPGLEGGVVGK.S** |
|  | 8464 | **418.9054** | **1253.6943** | **1254.7197** | **-1.0254** | **1** | **16** | **3.3** | **5** | **K.QRLLGWIQNK.L** |
|  | 8619 | **631.2521** | **1260.4897** | **1260.6925** | **-0.2028** | **0** | **8** | **23** | **6** | **R.LTVSSLQESGLK.V** |
|  | 9238 | **647.3611** | **1292.7076** | **1291.7136** | **0.9940** | **1** | **30** | **0.15** | **1** | **K.GKLDVQFSGLTK.G** |
|  | 9491 | **653.8593** | **1305.7041** | **1305.7153** | **-0.0113** | **1** | **29** | **0.17** | **1** | **R.VNVGAGSHPNKVK.V** |
|  | 10609 | **455.5706** | **1363.6899** | **1363.6984** | **-0.0084** | **1** | **20** | **1.4** | **1** | **K.VDVGKDQEFTVK.S** |
|  | 10887 | 689.8490 | 1377.6834 | 1378.6670 | -0.9835 | 0 | 13 | 7.4 | 2 | K.YGGPYHIGGSPFK.A |
|  | 11262 | **700.8236** | **1399.6327** | **1399.6408** | **-0.0082** | **0** | **11** | **11** | **1** | **K.YGGDEIPFSPYR.V** |
|  | 11523 | **708.3732** | **1414.7319** | **1414.7416** | **-0.0097** | **0** | **47** | **0.0026** | **1** | **R.IANLQTDLSDGLR.L** |
|  | 11540 | **708.8729** | **1415.7312** | **1416.7936** | **-1.0625** | **1** | **0** | **1.3e+002** | **4** | **R.RLTVSSLQESGLK.V** |
|  | 11775 | 715.3574 | 1428.7002 | 1428.7110 | -0.0108 | 0 | 66 | 3.6e-005 | 1 | K.AFGPGLQGGSAGSPAR.F |
|  | 11844 | **717.3528** | **1432.6910** | **1432.7020** | **-0.0110** | **0** | **34** | **0.048** | **1** | **R.AYGPGIEPTGNMVK.K** |
|  | 11866 | **717.8598** | **1433.7050** | **1433.7151** | **-0.0100** | **0** | **68** | **2.1e-005** | **1** | **R.ANLPQSFQVDTSK.A** |
|  | 12022 | 722.7633 | 1443.5121 | 1443.5217 | -0.0096 | 0 | 38 | 0.015 | 1 | K.MDCQECPEGYR.V |
|  | 12217 | **486.2467** | **1455.7184** | **1456.7497** | **-1.0313** | **1** | **2** | **85** | **4** | **K.AHVVPCFDASKVK.C** |
|  | 12297 | **487.3206** | **1458.9399** | **1459.7276** | **-0.7876** | **1** | **3** | **55** | **3** | **K.VKCSGPGLSPGMVR.A** |
|  | 13805 | **775.3636** | **1548.7126** | **1549.7624** | **-1.0498** | **0** | **2** | **79** | **2** | **R.DAGYGGLSLSIEGPSK.V** |
|  | 14138 | **523.9279** | **1568.7618** | **1569.8416** | **-1.0798** | **1** | **4** | **47** | **4** | **K.FNGTHIPGSPFKIR.V** |
|  | 14156 | **785.9020** | **1569.7894** | **1569.7999** | **-0.0104** | **0** | **58** | **0.00019** | **1** | **R.GAGTGGLGLAVEGPSEAK.M** |
|  | 14446 | **530.3017** | **1587.8833** | **1587.7351** | **0.1482** | **0** | **7** | **27** | **2** | **R.YAPSEAGLHEMDIR.Y** |
|  | 14643 | **534.9232** | **1601.7478** | **1601.7587** | **-0.0108** | **0** | **25** | **0.41** | **1** | **K.YNEQHVPGSPFTAR.V** 14647 |
|  | 14671 | **535.6140** | **1603.8200** | **1604.8522** | **-1.0322** | **1** | **16** | **2.9** | **1** | **K.LDVQFSGLTKGDAVR.D** |
|  | 15190 | **818.8436** | **1635.6726** | **1635.7046** | **-0.0320** | **0** | **2** | **62** | **3** | **K.VDINTEDLEDGTCR.V** |
|  | 15337 | **823.9427** | **1645.8708** | **1645.8675** | **0.0033** | **0** | **10** | **13** | **1** | **K.TGVAVNKPAEFTVDAK.H** |
|  | 15457 | **826.9282** | **1651.8419** | **1651.8529** | **-0.0110** | **0** | **45** | **0.004** | **1** | **K.VTAQGPGLEPSGNIANK.T** |
|  | 15602 | **831.3994** | **1660.7841** | **1659.8403** | **0.9439** | **1** | **2** | **70** | **3** | **K.ARAYGPGIEPTGNMVK.K** |
|  | 15908 | **560.9396** | **1679.7969** | **1678.7985** | **0.9984** | **1** | **10** | **10** | **2** | **R.SPFEVKVGTECGNQK.V** |
|  | 16190 | **566.9730** | **1697.8972** | **1697.9101** | **-0.0128** | **0** | **22** | **0.78** | **1** | **R.TGVELGKPTHFTVNAK.A** 16189 16192 |
|  | 16849 | **870.9606** | **1739.9066** | **1739.9168** | **-0.0102** | **0** | **30** | **0.11** | **1** | **R.VTYTPMAPGSYLISIK.Y** 16850 |
|  | 17378 | **593.8215** | **1778.4428** | **1777.8595** | **0.5833** | **1** | **0** | **68** | **6** | **K.GTVEPQLEARGDSTYR.C** |
|  | 17479 | **892.9400** | **1783.8654** | **1783.8775** | **-0.0120** | **0** | **34** | **0.042** | **1** | **R.VQVQDNEGCPVEALVK.D** 17487 |
|  | 17544 | **895.4117** | **1788.8089** | **1788.8213** | **-0.0124** | **0** | **71** | **7.7e-006** | **1** | **K.ATCAPQHGAPGPGPADASK.V** 17543 17545 |
|  | 18115 | **913.4808** | **1824.9471** | **1824.9846** | **-0.0375** | **1** | **5** | **33** | **2** | **K.VEPGLGADNSVVRFLPR.E** |
|  | 20208 | **995.5223** | **1989.0300** | **1989.9868** | **-0.9568** | **1** | **5** | **31** | **1** | **K.ARVANPSGNLTETYVQDR.G** |
|  | 20374 | **668.3267** | **2001.9583** | **2001.0088** | **0.9495** | **0** | **4** | **40** | **2** | **K.IPEISIQDMTAQVTSPSGK.T** |
|  | 20471 | **672.0267** | **2013.0584** | **2013.9830** | **-0.9246** | **0** | **1** | **66** | **5** | **K.CAPGVVGPAEADIDFDIIR.N** |
|  | 22534 | **739.3522** | **2215.0347** | **2214.0196** | **1.0151** | **1** | **(1)** | **59** | **2** | **-.MSSSHSRAGQSAAGAAPGGGVDTR.D** |
|  | 22685 | **558.5623** | **2230.2202** | **2230.0145** | **0.2057** | **1** | **4** | **31** | **2** | **-.MSSSHSRAGQSAAGAAPGGGVDTR.D** 22674 |
|  | 22889 | **1129.5096** | **2257.0047** | **2257.0209** | **-0.0162** | **0** | **38** | **0.013** | **1** | **K.DGSCGVAYVVQEPGDYEVSVK.F** 22888 |
|  | 23105 | **1143.5844** | **2285.1541** | **2285.1692** | **-0.0151** | **0** | **74** | **2.8e-006** | **1** | **K.YTPVQQGPVGVNVTYGGDPIPK.S** 23104 |
|  | 23312 | **1156.5336** | **2311.0526** | **2312.0921** | **-1.0395** | **0** | **2** | **46** | **2** | **R.SAGQGEVLVYVEDPAGHQEEAK.V** 23310 23311 |
|  | 23550 | **1171.6091** | **2341.2037** | **2341.2166** | **-0.0128** | **0** | **72** | **5.3e-006** | **1** | **K.ASGPGLNTTGVPASLPVEFTIDAK.D** |
|  | 23599 | **784.0725** | **2349.1955** | **2348.2046** | **0.9910** | **1** | **5** | **25** | **1** | **R.QMQLENVSVALEFLDRESIK.L** |
|  | 23878 | 1193.6080 | 2385.2015 | 2385.1159 | 0.0856 | 1 | 10 | 6.9 | 2 | R.KDGSCGVAYVVQEPGDYEVSVK.F 23880 |
|  | 24328 | **818.5800** | **2452.7182** | **2452.1032** | **0.6150** | **1** | **2** | **33** | **9** | **R.GAGTGGLGLAVEGPSEAKMSCMDNK.D** |
|  | 24501 | **827.7166** | **2480.1280** | **2479.1187** | **1.0094** | **1** | **3** | **37** | **1** | **K.YNEQHVPGSPFTARVTGDDSMR.M** |
|  | 24948 | **1272.6246** | **2543.2347** | **2543.2504** | **-0.0157** | **0** | **100** | **6.4e-009** | **1** | **K.GLVEPVDVVDNADGTQTVNYVPSR.E** 24931 24950 24951 |
|  | 28634 | **1077.1652** | **3228.4737** | **3228.4968** | **-0.0232** | **0** | **75** | **1.5e-006** | **1** | **R.ALGALVDSCAPGLCPDWDSWDASKPVTNAR.E** 28635 |
|  | 29442 | **1162.5753** | **3484.7041** | **3484.7290** | **-0.0249** | **0** | **54** | **0.00017** | **1** | **R.EEGPYEVEVTYDGVPVPGSPFPLEAVAPTKPSK.V** 29444 |
|  | 30450 | **1000.5107** | **3998.0136** | **3998.0353** | **-0.0217** | **1** | **6** | **8** | **4** | **R.FLPREEGPYEVEVTYDGVPVPGSPFPLEAVAPTKPSK.V** |
|  | 31248 | **1237.8053** | **4947.1921** | **4947.2168** | **-0.0247** | **1** | **25** | **0.042** | **1** | **R.GDGMYKVEYTPYEEGLHSVDVTYDGSPVPSSPFQVPVTEGCDPSR.V** |
|  | 31319 | **1270.8395** | **5079.3288** | **5078.4484** | **0.8804** | **0** | **3** | **6** | **1** | **R.YDNMHIPGSPLQFYVDYVNCGHVTAYGPGLTHGVVNKPATFTVNTK.D** |

| **40.** | A5A3E0\|POTEF_HUMAN    **Mass:** 123020   **Score:** 753    **Queries matched:** 103   **emPAI:** 0.34 |
| --- | --- |
|  | POTE ankyrin domain family member F OS=Homo sapiens GN=POTEF PE=1 SV=2 |

|  | **Query** | **Observed** | **Mr(expt)** | **Mr(calc)** | **Delta** | **Miss** | **Score** | **Expect** | **Rank** | **Peptide** |
| --- | --- | --- | --- | --- | --- | --- | --- | --- | --- | --- |
|  | 1169 | 644.3676 | 643.3603 | 643.3653 | -0.0050 | 0 | 25 | 0.84 | 1 | R.LDLAGR.E |
|  | 1635 | 400.7684 | 799.5222 | 799.5280 | -0.0057 | 1 | 24 | 0.79 | 6 | K.RGILTLK.Y 1646 |
|  | 2841 | 443.7376 | 885.4606 | 885.4668 | -0.0062 | 0 | 21 | 1.5 | 1 | K.ANLNALDR.Y |
|  | 4039 | 487.9703 | 973.9261 | 974.4305 | -0.5044 | 0 | 2 | 89 | 5 | K.GSENSQPEK.M |
|  | 4062 | 488.7242 | 975.4339 | 975.4410 | -0.0071 | 0 | 74 | 7.9e-006 | 1 | K.AGFAGDDAPR.A 4063 4064 4068 4075 |
|  | 4200 | **494.5775** | **987.1405** | **987.5138** | **-0.3732** | **0** | **12** | **7.4** | **2** | **K.ILTEHGYR.F** |
|  | 4601 | 507.7411 | 1013.4676 | 1013.5618 | -0.0942 | 1 | 2 | 1.2e+002 | 3 | K.KANLNALDR.Y |
|  | 4738 | 513.2864 | 1024.5583 | 1023.4947 | 1.0637 | 0 | 3 | 79 | 8 | R.ELPDYLMK.I |
|  | 6156 | 563.2864 | 1124.5583 | 1123.6039 | 0.9544 | 1 | 5 | 43 | 8 | K.LHRAAWWGK.V |
|  | 6339 | 571.0724 | 1140.1302 | 1139.5532 | 0.5770 | 0 | 0 | 95 | 8 | R.ICELLSDYK.E |
|  | 6779 | 585.3008 | 1168.5871 | 1167.5264 | 1.0608 | 0 | 2 | 83 | 7 | K.MNSELSLSCK.K |
|  | 7209 | 398.7393 | 1193.1960 | 1192.5831 | 0.6129 | 0 | 22 | 0.68 | 1 | K.EIAALAPSMMK.I |
|  | 7317 | **400.2046** | **1197.5919** | **1196.6149** | **0.9769** | **0** | **9** | **20** | **6** | **K.DILHENSTLR.E** |
|  | 7330 | 599.8522 | 1197.6899 | 1197.6982 | -0.0083 | 0 | 58 | 0.00026 | 1 | R.AVFPSIVGRPR.Q 7327 7328 7329 7331 7332 7333 7336 7342 |
|  | 8677 | 632.8616 | 1263.7087 | 1263.5159 | 0.1929 | 0 | 1 | 1.1e+002 | 6 | R.QQGMMGGMHQK.E |
|  | 10375 | 676.8123 | 1351.6101 | 1351.5562 | 0.0539 | 1 | 4 | 50 | 7 | K.DGDREVEEEMK.K |
|  | 11774 | 477.2324 | 1428.6754 | 1429.7785 | -1.1030 | 1 | 7 | 27 | 2 | K.EIAALAPSMMKIR.I 11807 |
|  | 12176 | **485.2370** | **1452.6893** | **1453.7525** | **-1.0632** | **1** | **5** | **38** | **7** | **K.EKDILHENSTLR.E** |
|  | 12809 | 497.2980 | 1488.8722 | 1489.6103 | -0.7382 | 0 | 10 | 13 | 2 | K.SNVGASGDHDDSAMK.T 12805 12810 |
|  | 13245 | 758.3734 | 1514.7323 | 1514.7419 | -0.0096 | 0 | 45 | 0.0039 | 1 | K.IWHHTFYNELR.V 13243 13244 13248 13267 |
|  | 13260 | 758.8502 | 1515.6858 | 1515.6954 | -0.0096 | 0 | 50 | 0.0014 | 1 | K.QEYDESGPSIVHR.K 13259 13261 |
|  | 14076 | 783.3349 | 1564.6552 | 1564.7477 | -0.0924 | 0 | 12 | 7.7 | 2 | -.MVVEVDSMPAASSVK.K 14064 14077 |
|  | 14289 | 395.7139 | 1578.8264 | 1579.7772 | -0.9508 | 1 | 11 | 8.8 | 3 | R.MQKEIAALAPSMMK.I |
|  | 15077 | 543.3950 | 1627.1632 | 1626.7633 | 0.4000 | 1 | 9 | 11 | 1 | R.ELENFMAIEEMKK.H 15070 |
|  | 15307 | 548.9338 | 1643.7795 | 1643.7903 | -0.0108 | 1 | 25 | 0.34 | 1 | K.QEYDESGPSIVHRK.C 15309 |
|  | 15395 | 825.3951 | 1648.7756 | 1648.8494 | -0.0738 | 1 | 6 | 27 | 2 | R.LDLAGRELPDYLMK.I 15386 15409 |
|  | 17562 | 895.9443 | 1789.8740 | 1789.8846 | -0.0106 | 0 | 72 | 7.6e-006 | 1 | K.SYELPDGQVITIGNER.F 17551 17553 17560 17561 17563 17564 17565 17566 17568 17569 17571 17572 17573 17574 17576 17577 17578 17579 17580 17581 17582 17583 17584 17585 17586 17587 17588 17591 17592 17593 17595 17596 17609 17610 17612 17613 17614 17616 17617 |
|  | 19251 | 956.4669 | 1910.9192 | 1910.8437 | 0.0754 | 1 | 1 | 82 | 3 | R.QQGMMGGMHQKESYVGK.E |
|  | 19426 | 643.0295 | 1926.0668 | 1926.8386 | -0.7718 | 1 | (1) | 80 | 6 | R.QQGMMGGMHQKESYVGK.E |
|  | 19873 | 653.6609 | 1957.9608 | 1957.8397 | 0.1211 | 1 | 7 | 16 | 5 | K.DGDRELENFMAIEEMK.K |
|  | 19899 | 980.9525 | 1959.8903 | 1959.9571 | -0.0668 | 1 | 7 | 18 | 3 | K.QMLKISSENSNPEQDLK.L 19898 |
|  | 20079 | **659.4740** | **1975.4002** | **1975.9520** | **-0.5519** | **1** | **(3)** | **33** | **6** | **K.QMLKISSENSNPEQDLK.L** |
|  | 20265 | 665.3200 | 1992.9382 | 1993.9051 | -0.9669 | 1 | 2 | 55 | 4 | K.QMPKYSSENSNPEQDLK.L |
|  | 27598 | **1000.9531** | **2999.8374** | **2999.4598** | **0.3775** | **1** | **3** | **19** | **10** | **K.HRSTHVGFPENLTNGATAGNGDDGLIPPR.K** 27603 |

| **41.** | P08670\|VIME_HUMAN    **Mass:** 53676    **Score:** 750    **Queries matched:** 53   **emPAI:** 3.09 |
| --- | --- |
|  | Vimentin OS=Homo sapiens GN=VIM PE=1 SV=4 |

|  | **Query** | **Observed** | **Mr(expt)** | **Mr(calc)** | **Delta** | **Miss** | **Score** | **Expect** | **Rank** | **Peptide** |
| --- | --- | --- | --- | --- | --- | --- | --- | --- | --- | --- |
|  | 2597 | **435.7184** | **869.4222** | **869.4283** | **-0.0061** | **0** | **28** | **0.26** | **1** | **R.FANYIDK.V** |
|  | 3084 | **454.2368** | **906.4590** | **905.4607** | **0.9984** | **0** | **6** | **41** | **9** | **R.FLEQQNK.I** |
|  | 4725 | 512.7710 | 1023.5274 | 1022.5033 | 1.0242 | 0 | 10 | 16 | 8 | R.QQYESVAAK.N |
|  | 5452 | **538.7526** | **1075.4907** | **1075.4968** | **-0.0061** | **0** | **10** | **19** | **1** | **R.DNLAEDIMR.L** |
|  | 5653 | **545.3002** | **1088.5859** | **1087.5258** | **1.0601** | **0** | **8** | **28** | **5** | **R.QDVDNASLAR.L** |
|  | 5699 | **547.2634** | **1092.5123** | **1092.5200** | **-0.0076** | **0** | **48** | **0.0023** | **1** | **K.FADLSEAANR.N** 5700 |
|  | 7714 | **608.8133** | **1215.6120** | **1215.6208** | **-0.0087** | **1** | **37** | **0.032** | **1** | **R.RQVDQLTNDK.A** 7713 7720 |
|  | 9113 | **644.3315** | **1286.6485** | **1286.6579** | **-0.0093** | **1** | **29** | **0.17** | **1** | **R.QVDQLTNDKAR.V** |
|  | 9272 | **648.3331** | **1294.6516** | **1294.6591** | **-0.0075** | **0** | **30** | **0.15** | **1** | **K.MALDIEIATYR.K** |
|  | 9423 | **652.3311** | **1302.6475** | **1302.6601** | **-0.0126** | **1** | **10** | **16** | **4** | **R.EKLQEEMLQR.E** |
|  | 9518 | **655.3018** | **1308.5891** | **1308.5986** | **-0.0095** | **0** | **45** | **0.0043** | **1** | **K.NLQEAEEWYK.S** 9517 |
|  | 9853 | **662.3082** | **1322.6019** | **1322.6102** | **-0.0083** | **0** | **3** | **63** | **3** | **R.EEAENTLQSFR.Q** |
|  | 10258 | 673.3621 | 1344.7097 | 1344.6819 | 0.0277 | 1 | 3 | 74 | 8 | R.DNLAEDIMRLR.E |
|  | 11365 | **469.3273** | **1404.9601** | **1404.7500** | **0.2101** | **0** | **8** | **16** | **3** | **K.VESLQEEIAFLK.K** |
|  | 11682 | **475.2555** | **1422.7447** | **1422.7540** | **-0.0093** | **1** | **22** | **0.86** | **1** | **K.MALDIEIATYRK.L** |
|  | 12936 | **749.3483** | **1496.6820** | **1496.6929** | **-0.0109** | **1** | **43** | **0.0065** | **1** | **K.SRLGDLYEEEMR.E** 12934 |
|  | 13454 | **509.9442** | **1526.8109** | **1526.8205** | **-0.0096** | **1** | **10** | **12** | **7** | **R.HLREYQDLLNVK.M** |
|  | 13564 | **767.4254** | **1532.8363** | **1532.8450** | **-0.0087** | **1** | **77** | **2.4e-006** | **1** | **R.KVESLQEEIAFLK.K** 13565 |
|  | 13658 | **770.4540** | **1538.8935** | **1538.9032** | **-0.0096** | **1** | **59** | **0.00017** | **1** | **K.ILLAELEQLKGQGK.S** 13649 13672 |
|  | 14425 | **794.3968** | **1586.7791** | **1586.7900** | **-0.0108** | **1** | **52** | **0.0009** | **1** | **R.TNEKVELQELNDR.F** 14426 14427 |
|  | 15421 | **550.9955** | **1649.9648** | **1649.8056** | **0.1592** | **1** | **3** | **58** | **2** | **R.RMFGGPGTASRPSSSR.S** |
|  | 15706 | **834.4334** | **1666.8521** | **1667.8366** | **-0.9845** | **0** | **2** | **68** | **3** | **R.ETNLDSLPLVDTHSK.R** |
|  | 16027 | **844.9114** | **1687.8083** | **1687.8199** | **-0.0116** | **1** | **40** | **0.013** | **1** | **R.VEVERDNLAEDIMR.L** |
|  | 16444 | **572.3555** | **1714.0446** | **1713.8645** | **0.1800** | **1** | **6** | **24** | **2** | **R.QDVDNASLARLDLER.K** |
|  | 16767 | **867.9054** | **1733.7962** | **1733.8076** | **-0.0114** | **1** | **61** | **8.3e-005** | **1** | **R.LQDEIQNMKEEMAR.H** 16787 |
|  | 17322 | **592.9548** | **1775.8427** | **1775.8550** | **-0.0124** | **1** | **14** | **4.4** | **2** | **K.FADLSEAANRNNDALR.Q** 17323 |
|  | 18263 | **918.8966** | **1835.7787** | **1835.7922** | **-0.0135** | **0** | **52** | **0.00072** | **1** | **R.DGQVINETSQHHDDLE.-** |
|  | 22218 | **1093.9791** | **2185.9437** | **2185.9586** | **-0.0149** | **0** | **119** | **9.1e-011** | **1** | **R.EMEENFAVEAANYQDTIGR.L** 22219 22220 22221 22222 |
|  | 23602 | **1176.0612** | **2350.1078** | **2350.1223** | **-0.0145** | **1** | **57** | **0.00014** | **1** | **K.LQEEMLQREEAENTLQSFR.Q** 23601 23614 |
|  | 23815 | **1189.0796** | **2376.1446** | **2376.1591** | **-0.0145** | **1** | **69** | **9.8e-006** | **1** | **R.QVQSLTCEVDALKGTNESLER.Q** 23814 |
|  | 23932 | **798.7106** | **2393.1101** | **2392.1255** | **0.9846** | **1** | **10** | **6.6** | **1** | **R.EEAENTLQSFRQDVDNASLAR.L** |
|  | 30336 | **981.5017** | **3921.9777** | **3922.0072** | **-0.0295** | **0** | **80** | **2.4e-007** | **1** | **K.LHEEEIQELQAQIQEQHVQIDVDVSKPDLTAALR.D** 30337 |
|  | 30508 | **811.0206** | **4050.0665** | **4050.1021** | **-0.0357** | **1** | **15** | **0.8** | **1** | **K.KLHEEEIQELQAQIQEQHVQIDVDVSKPDLTAALR.D** |

| **42.** | P69905\|HBA_HUMAN    **Mass:** 15305    **Score:** 707    **Queries matched:** 69   **emPAI:** 46.26 |
| --- | --- |
|  | Hemoglobin subunit alpha OS=Homo sapiens GN=HBA1 PE=1 SV=2 |

|  | **Query** | **Observed** | **Mr(expt)** | **Mr(calc)** | **Delta** | **Miss** | **Score** | **Expect** | **Rank** | **Peptide** |
| --- | --- | --- | --- | --- | --- | --- | --- | --- | --- | --- |
|  | 878 | **532.2830** | **531.2757** | **531.2805** | **-0.0048** | **0** | **18** | **5.7** | **1** | **K.AAWGK.V** |
|  | 1374 | **729.4081** | **728.4009** | **728.4068** | **-0.0060** | **0** | **11** | **17** | **2** | **M.VLSPADK.T** |
|  | 1872 | **409.7210** | **817.4275** | **817.4334** | **-0.0059** | **0** | **21** | **1.6** | **2** | **R.VDPVNFK.L** |
|  | 5380 | **536.2781** | **1070.5417** | **1070.5471** | **-0.0053** | **0** | **31** | **0.14** | **1** | **R.MFLSFPTTK.T** 5363 5365 5377 5378 5382 5385 |
|  | 5618 | **544.2750** | **1086.5355** | **1086.5420** | **-0.0065** | **0** | **(22)** | **1.1** | **1** | **R.MFLSFPTTK.T** |
|  | 5626 | **544.3126** | **1086.6106** | **1086.6186** | **-0.0080** | **1** | **29** | **0.22** | **1** | **K.LRVDPVNFK.L** |
|  | 6809 | **586.3337** | **1170.6529** | **1170.6608** | **-0.0079** | **1** | **36** | **0.037** | **1** | **M.VLSPADKTNVK.A** 6807 6811 6813 |
|  | 8435 | **626.8570** | **1251.6994** | **1251.7075** | **-0.0080** | **0** | **63** | **6.8e-005** | **1** | **K.FLASVSTVLTSK.Y** 8434 |
|  | 9409 | **651.8529** | **1301.6913** | **1301.7013** | **-0.0100** | **1** | **25** | **0.48** | **1** | **-.MVLSPADKTNVK.A** |
|  | 13486 | **765.3655** | **1528.7164** | **1528.7270** | **-0.0106** | **0** | **69** | **1.4e-005** | **1** | **K.VGAHAGEYGAEALER.M** 13458 13470 13476 13478 13480 13481 13482 13483 13484 13485 13487 13488 13495 13497 13498 |
|  | 18212 | **917.4434** | **1832.8723** | **1832.8846** | **-0.0123** | **0** | **64** | **4.3e-005** | **1** | **K.TYFPHFDLSHGSAQVK.G** 18203 18204 18209 18210 18211 18213 18215 18217 18218 18220 |
|  | 20779 | 511.2808 | 2041.0941 | 2041.9969 | -0.9029 | 1 | 5 | 30 | 6 | K.AAWGKVGAHAGEYGAEALER.M |
|  | 27552 | **999.4946** | **2995.4621** | **2995.4821** | **-0.0201** | **0** | **76** | **1.3e-006** | **1** | **K.VADALTNAVAHVDDMPNALSALSDLHAHK.L** 27551 27554 27555 27556 27557 27559 27560 27565 27574 27575 |
|  | 27642 | **753.8718** | **3011.4582** | **3011.4771** | **-0.0188** | **0** | **(5)** | **17** | **2** | **K.VADALTNAVAHVDDMPNALSALSDLHAHK.L** |
|  | 27721 | **756.9099** | **3023.6105** | **3023.6266** | **-0.0161** | **0** | **11** | **3.8** | **1** | **K.LLSHCLLVTLAAHLPAEFTPAVHASLDK.F** |
|  | 28132 | **1042.1942** | **3123.5608** | **3123.5771** | **-0.0163** | **1** | **80** | **5.3e-007** | **1** | **K.KVADALTNAVAHVDDMPNALSALSDLHAHK.L** 28125 28126 28127 28128 28129 28131 |
|  | 30774 | **852.4687** | **4257.3071** | **4257.3235** | **-0.0165** | **1** | **27** | **0.04** | **1** | **K.LLSHCLLVTLAAHLPAEFTPAVHASLDKFLASVSTVLTSK.Y** 30775 |

| **43.** | Q562R1\|ACTBL_HUMAN    **Mass:** 42318    **Score:** 692    **Queries matched:** 91   **emPAI:** 1.34 |
| --- | --- |
|  | Beta-actin-like protein 2 OS=Homo sapiens GN=ACTBL2 PE=1 SV=2 |

|  | **Query** | **Observed** | **Mr(expt)** | **Mr(calc)** | **Delta** | **Miss** | **Score** | **Expect** | **Rank** | **Peptide** |
| --- | --- | --- | --- | --- | --- | --- | --- | --- | --- | --- |
|  | 1138 | 632.2894 | 631.2822 | 630.3701 | 0.9121 | 0 | 14 | 11 | 1 | K.ILTER.G 1129 1139 |
|  | 1169 | 644.3676 | 643.3603 | 643.3653 | -0.0050 | 0 | 25 | 0.84 | 1 | R.LDLAGR.D |
|  | 1578 | 398.2373 | 794.4600 | 794.4650 | -0.0050 | 0 | 22 | 0.87 | 1 | K.IIAPPER.K 1587 |
|  | 3056 | 453.2256 | 904.4366 | 904.4436 | -0.0071 | 1 | 36 | 0.05 | 1 | K.CDVDIRK.D |
|  | 3326 | 462.2838 | 922.5531 | 922.5600 | -0.0068 | 1 | 28 | 0.22 | 1 | K.IIAPPERK.Y 3327 3328 |
|  | 4370 | 499.7436 | 997.4726 | 997.4790 | -0.0064 | 0 | 44 | 0.0062 | 1 | R.DLTDYLMK.I 4366 4368 4369 4372 4401 |
|  | 4907 | 518.8259 | 1035.6373 | 1035.6440 | -0.0067 | 1 | 25 | 0.45 | 1 | K.IKIIAPPER.K |
|  | 6796 | 586.2847 | 1170.5549 | 1170.5638 | -0.0089 | 0 | 55 | 0.00046 | 1 | R.HQGVMVGMGQK.D 6797 6798 6799 6800 |
|  | 7046 | 593.7484 | 1185.4823 | 1186.5587 | -1.0764 | 0 | (42) | 0.0095 | 1 | R.HQGVMVGMGQK.D 7072 7074 7076 |
|  | 7069 | 594.2817 | 1186.5489 | 1186.5587 | -0.0098 | 0 | (41) | 0.014 | 1 | R.HQGVMVGMGQK.D 7057 7068 7070 7071 7075 |
|  | 7433 | 602.2789 | 1202.5433 | 1202.5536 | -0.0103 | 0 | (26) | 0.42 | 1 | R.HQGVMVGMGQK.D 7436 7437 |
|  | 15010 | 812.4193 | 1622.8239 | 1622.8338 | -0.0098 | 1 | 27 | 0.24 | 1 | R.LDLAGRDLTDYLMK.I 15009 |
|  | 17256 | **886.9160** | **1771.8175** | **1771.8273** | **-0.0098** | **0** | **9** | **13** | **2** | **K.LCYVALDFEQEMVR.A** |
|  | 17562 | 895.9443 | 1789.8740 | 1789.8846 | -0.0106 | 0 | 72 | 7.6e-006 | 1 | R.SYELPDGQVITIGNER.F 17551 17553 17560 17561 17563 17564 17565 17566 17568 17569 17571 17572 17573 17574 17576 17577 17578 17579 17580 17581 17582 17583 17584 17585 17586 17587 17588 17591 17592 17593 17595 17596 17609 17610 17612 17613 17614 17616 17617 |
|  | 19788 | 977.5285 | 1953.0424 | 1953.0571 | -0.0147 | 0 | 25 | 0.32 | 2 | R.VAPDEHPILLTEAPLNPK.I 19784 19789 19791 19792 19796 19797 19798 |
|  | 22555 | **1109.5150** | **2217.0155** | **2216.0419** | **0.9735** | **0** | **4** | **32** | **1** | **K.DLYANTVLSGGSTMYPGIADR.M** 22541 |
|  | 23568 | 782.0491 | 2343.1256 | 2344.1369 | -1.0113 | 1 | 4 | 31 | 2 | R.KDLYANTVLSGGSTMYPGIADR.M |
|  | 25421 | 868.1128 | 2601.3165 | 2601.3301 | -0.0136 | 0 | 5 | 23 | 1 | K.YSVWIGGSILASLSTFQQMWISK.Q 25417 |

| **44.** | P25705\|ATPA_HUMAN    **Mass:** 59828    **Score:** 680    **Queries matched:** 49   **emPAI:** 1.33 |
| --- | --- |
|  | ATP synthase subunit alpha, mitochondrial OS=Homo sapiens GN=ATP5A1 PE=1 SV=1 |

|  | **Query** | **Observed** | **Mr(expt)** | **Mr(calc)** | **Delta** | **Miss** | **Score** | **Expect** | **Rank** | **Peptide** |
| --- | --- | --- | --- | --- | --- | --- | --- | --- | --- | --- |
|  | 1500 | **395.2058** | **788.3969** | **788.4141** | **-0.0171** | **0** | **5** | **75** | **8** | **R.VGSAAQTR.A** |
|  | 2269 | **423.2554** | **844.4962** | **844.5018** | **-0.0056** | **0** | **27** | **0.4** | **1** | **R.STVAQLVK.R** |
|  | 2519 | **864.4074** | **863.4001** | **863.4059** | **-0.0057** | **0** | **33** | **0.084** | **1** | **R.LTDADAMK.Y** |
|  | 2697 | **439.2315** | **876.4485** | **876.4189** | **0.0296** | **0** | **4** | **77** | **9** | **K.ISEQSDAK.L** |
|  | 4421 | 500.8024 | 999.5902 | 999.5713 | 0.0189 | 0 | 21 | 1.5 | 7 | R.VLSIGDGIAR.V 4437 |
|  | 4684 | **510.7573** | **1019.5001** | **1019.5070** | **-0.0069** | **1** | **20** | **1.6** | **2** | **K.RLTDADAMK.Y** |
|  | 4779 | **514.7999** | **1027.5853** | **1027.6390** | **-0.0537** | **1** | **8** | **27** | **2** | **R.GVRLTELLK.Q** |
|  | 4920 | **519.2681** | **1036.5217** | **1035.5019** | **1.0198** | **1** | **(5)** | **45** | **5** | **K.RLTDADAMK.Y** |
|  | 6802 | **586.3155** | **1170.6164** | **1170.6245** | **-0.0080** | **0** | **65** | **4.9e-005** | **1** | **R.VVDALGNAIDGK.G** |
|  | 7967 | **615.3464** | **1228.6783** | **1228.6775** | **0.0008** | **1** | **9** | **22** | **1** | **R.ELIIGDRQTGK.T** 7951 |
|  | 8817 | **424.6957** | **1271.0651** | **1270.7543** | **0.3108** | **1** | **(6)** | **26** | **3** | **-.MLSVRVAAAVVR.A** |
|  | 9130 | **644.8244** | **1287.6342** | **1286.7493** | **0.8850** | **1** | **7** | **27** | **4** | **-.MLSVRVAAAVVR.A** |
|  | 9708 | **658.8701** | **1315.7257** | **1315.7347** | **-0.0090** | **0** | **80** | **1.4e-006** | **1** | **K.TSIAIDTIINQK.R** 9707 |
|  | 11670 | **712.3355** | **1422.6565** | **1422.6660** | **-0.0096** | **0** | **64** | **5.3e-005** | **1** | **K.TGTAEMSSILEER.I** 11671 |
|  | 11933 | **719.9230** | **1437.8314** | **1437.8416** | **-0.0102** | **0** | **21** | **1.1** | **1** | **K.GIRPAINVGLSVSR.V** 11923 |
|  | 11938 | **720.3510** | **1438.6875** | **1438.6610** | **0.0265** | **0** | **(2)** | **72** | **9** | **K.TGTAEMSSILEER.I** |
|  | 11948 | **720.6953** | **1439.3761** | **1438.8191** | **0.5570** | **1** | **1** | **64** | **4** | **R.QMSLLLRRPPGR.E** |
|  | 12701 | **742.5339** | **1483.0533** | **1482.8017** | **0.2516** | **1** | **0** | **84** | **8** | **K.LYCIYVAIGQKR.S** |
|  | 14063 | **521.9846** | **1562.9319** | **1561.9053** | **1.0266** | **1** | **4** | **48** | **9** | **R.VLSIGDGIARVHGLR.N** |
|  | 14218 | **788.3912** | **1574.7678** | **1574.7788** | **-0.0110** | **0** | **89** | **1.5e-007** | **1** | **R.ILGADTSVDLEETGR.V** 14204 14217 14219 |
|  | 14745 | **536.7878** | **1607.3417** | **1606.8501** | **0.4916** | **1** | **5** | **26** | **2** | **K.QVAGTMKLELAQYR.E** |
|  | 15025 | **812.9437** | **1623.8729** | **1623.8832** | **-0.0103** | **0** | **30** | **0.12** | **1** | **R.TGAIVDVPVGEELLGR.V** |
|  | 15720 | **834.9141** | **1667.8137** | **1666.7872** | **1.0265** | **0** | **17** | **2.4** | **2** | **R.NVQAEEMVEFSSGLK.G** |
|  | 16360 | **855.4714** | **1708.9282** | **1709.9312** | **-1.0030** | **1** | **42** | **0.0064** | **1** | **R.VVDALGNAIDGKGPIGSK.T** |
|  | 17422 | **891.4079** | **1780.8012** | **1779.9843** | **0.8169** | **1** | **16** | **2.6** | **1** | **K.RTGAIVDVPVGEELLGR.V** 17399 |
|  | 21464 | **1052.5167** | **2103.0189** | **2103.0307** | **-0.0118** | **0** | **62** | **5.6e-005** | **1** | **K.GMSLNLEPDNVGVVVFGNDK.L** 21463 |
|  | 22811 | **749.0263** | **2244.0569** | **2245.1161** | **-1.0592** | **1** | **0** | **78** | **5** | **R.VHGLRNVQAEEMVEFSSGLK.G** |
|  | 23290 | **1155.0784** | **2308.1422** | **2308.1521** | **-0.0100** | **0** | **102** | **5.7e-009** | **1** | **K.QGQYSPMAIEEQVAVIYAGVR.G** 23291 |
|  | 23520 | **1169.5815** | **2337.1485** | **2337.1601** | **-0.0116** | **0** | **87** | **1.7e-007** | **1** | **R.EVAAFAQFGSDLDAATQQLLSR.G** 23521 |
|  | 23756 | **789.9003** | **2366.6790** | **2366.2495** | **0.4295** | **0** | **37** | **0.0099** | **1** | **K.FENAFLSHVVSQHQALLGTIR.A** 23751 23752 |
|  | 26119 | **542.6994** | **2708.4606** | **2708.4762** | **-0.0156** | **1** | **6** | **15** | **2** | **K.ITKFENAFLSHVVSQHQALLGTIR.A** |
|  | 27016 | **962.4655** | **2884.3747** | **2884.4429** | **-0.0682** | **1** | **2** | **35** | **2** | **K.QGQYSPMAIEEQVAVIYAGVRGYLDK.L** |
|  | 27619 | **602.3058** | **3006.4926** | **3005.5896** | **0.9030** | **1** | **6** | **15** | **4** | **R.LTELLKQGQYSPMAIEEQVAVIYAGVR.G** |
|  | 27714 | **605.3041** | **3021.4843** | **3021.5845** | **-0.1002** | **1** | **(0)** | **46** | **7** | **R.LTELLKQGQYSPMAIEEQVAVIYAGVR.G** |
|  | 29197 | **1694.7900** | **3387.5655** | **3387.5791** | **-0.0136** | **0** | **42** | **0.0025** | **1** | **K.YTIVVSATASDAAPLQYLAPYSGCSMGEYFR.D** |

| **45.** | P30101\|PDIA3_HUMAN    **Mass:** 57146    **Score:** 676    **Queries matched:** 55   **emPAI:** 2.32 |
| --- | --- |
|  | Protein disulfide-isomerase A3 OS=Homo sapiens GN=PDIA3 PE=1 SV=4 |

|  | **Query** | **Observed** | **Mr(expt)** | **Mr(calc)** | **Delta** | **Miss** | **Score** | **Expect** | **Rank** | **Peptide** |
| --- | --- | --- | --- | --- | --- | --- | --- | --- | --- | --- |
|  | 1056 | **601.0431** | **600.0359** | **599.3279** | **0.7080** | **0** | **5** | **42** | **3** | **K.NLEPK.Y** |
|  | 1245 | **678.3631** | **677.3558** | **677.3604** | **-0.0046** | **0** | **35** | **0.059** | **1** | **R.VMMVAK.K** |
|  | 2550 | **866.4542** | **865.4469** | **865.4545** | **-0.0076** | **1** | **24** | **0.67** | **1** | **K.YKELGEK.L** |
|  | 2700 | **439.2451** | **876.4756** | **876.4817** | **-0.0062** | **0** | **25** | **0.63** | **1** | **K.LNFAVASR.K** |
|  | 3498 | **469.2292** | **936.4439** | **937.6324** | **-1.1885** | **1** | **14** | **5.8** | **2** | **R.LKGIVPLAK.V** |
|  | 4967 | **521.2661** | **1040.5177** | **1039.5662** | **0.9515** | **0** | **22** | **0.96** | **2** | **R.TADGIVSHLK.K** |
|  | 5141 | **527.2961** | **1052.5777** | **1051.4948** | **1.0830** | **1** | **14** | **5.8** | **1** | **K.GSNYWRNR.V** 5142 |
|  | 5575 | **542.7835** | **1083.5523** | **1083.5601** | **-0.0077** | **0** | **31** | **0.13** | **1** | **K.YGVSGYPTLK.I** 5576 |
|  | 6764 | **584.8341** | **1167.6535** | **1167.6612** | **-0.0076** | **1** | **52** | **0.001** | **1** | **R.TADGIVSHLKK.Q** |
|  | 6817 | **586.7695** | **1171.5244** | **1171.5332** | **-0.0088** | **0** | **27** | **0.34** | **1** | **K.FVMQEEFSR.D** |
|  | 7164 | **596.3002** | **1190.5858** | **1190.5931** | **-0.0073** | **0** | **64** | **6e-005** | **1** | **R.LAPEYEAAATR.L** 7161 7163 |
|  | 7302 | **599.3591** | **1196.7037** | **1196.7128** | **-0.0091** | **1** | **42** | **0.0088** | **1** | **K.LSKDPNIVIAK.M** 7303 |
|  | 10281 | **674.3497** | **1346.6848** | **1346.6942** | **-0.0094** | **1** | **56** | **0.00036** | **1** | **K.RLAPEYEAAATR.L** 10299 |
|  | 10695 | **684.8307** | **1367.6468** | **1367.6569** | **-0.0100** | **0** | **18** | **1.9** | **1** | **K.SEPIPESNDGPVK.V** |
|  | 10734 | **685.8473** | **1369.6800** | **1369.6878** | **-0.0077** | **0** | **37** | **0.027** | **1** | **R.ELSDFISYLQR.E** 10735 10755 |
|  | 11201 | **699.2877** | **1396.5608** | **1396.5711** | **-0.0104** | **0** | **83** | **5.7e-007** | **1** | **K.VDCTANTNTCNK.Y** 11196 11197 11198 11199 11200 11202 11203 11204 11208 11223 |
|  | 12266 | **729.8533** | **1457.6920** | **1457.7038** | **-0.0118** | **1** | **51** | **0.0011** | **1** | **K.FEDKTVAYTEQK.M** |
|  | 12505 | **737.4145** | **1472.8145** | **1471.6766** | **1.1380** | **1** | **1** | **93** | **10** | **K.FVMQEEFSRDGK.A** |
|  | 13251 | **758.3785** | **1514.7425** | **1514.7518** | **-0.0092** | **1** | **28** | **0.18** | **1** | **R.FLQDYFDGNLKR.Y** 13250 |
|  | 14941 | **810.3905** | **1618.7664** | **1618.7766** | **-0.0102** | **0** | **56** | **0.00032** | **1** | **K.DLLIAYYDVDYEK.N** |
|  | 15321 | **823.4100** | **1644.8055** | **1644.8736** | **-0.0681** | **1** | **16** | **2.6** | **2** | **K.FLDAGHKLNFAVASR.K** 15320 |
|  | 15452 | **826.8813** | **1651.7480** | **1651.7590** | **-0.0110** | **1** | **64** | **4.5e-005** | **1** | **K.IFRDGEEAGAYDGPR.T** 15451 15453 15454 |
|  | 15660 | **832.8768** | **1663.7391** | **1663.7512** | **-0.0121** | **0** | **18** | **1.9** | **1** | **K.MDATANDVPSPYEVR.G** 15676 |
|  | 17271 | **887.4100** | **1772.8055** | **1771.8992** | **0.9063** | **1** | **18** | **1.8** | **1** | **R.YLKSEPIPESNDGPVK.V** |
|  | 25252 | **1288.1480** | **2574.2813** | **2574.2966** | **-0.0152** | **0** | **81** | **5.8e-007** | **1** | **K.TFSHELSDFGLESTAGEIPVVAIR.T** 25251 25253 |
|  | 26065 | **901.7997** | **2702.3772** | **2702.3915** | **-0.0143** | **1** | **41** | **0.0055** | **1** | **R.KTFSHELSDFGLESTAGEIPVVAIR.T** |
|  | 26847 | **949.7927** | **2846.3563** | **2846.3731** | **-0.0168** | **1** | **59** | **7.1e-005** | **1** | **K.FIQENIFGICPHMTEDNKDLIQGK.D** 26846 |
|  | 27263 | **980.1251** | **2937.3534** | **2937.3709** | **-0.0175** | **1** | **68** | **8.9e-006** | **1** | **K.FISDKDASIVGFFDDSFSEAHSEFLK.A** |

| **46.** | P02788\|TRFL_HUMAN    **Mass:** 80014    **Score:** 673    **Queries matched:** 61   **emPAI:** 2.10 |
| --- | --- |
|  | Lactotransferrin OS=Homo sapiens GN=LTF PE=1 SV=6 |

|  | **Query** | **Observed** | **Mr(expt)** | **Mr(calc)** | **Delta** | **Miss** | **Score** | **Expect** | **Rank** | **Peptide** |
| --- | --- | --- | --- | --- | --- | --- | --- | --- | --- | --- |
|  | 2342 | **426.7114** | **851.4082** | **851.4137** | **-0.0056** | **0** | **31** | **0.13** | **1** | **K.DSAIGFSR.V** |
|  | 3620 | **473.2101** | **944.4057** | **944.4134** | **-0.0078** | **0** | **13** | **9** | **2** | **K.SCHTAVDR.T** |
|  | 3683 | **475.2199** | **948.4253** | **948.4335** | **-0.0082** | **0** | **21** | **1.3** | **3** | **R.LCAGTGENK.C** 3681 |
|  | 3783 | **478.7818** | **955.5491** | **955.5563** | **-0.0072** | **1** | **20** | **1.6** | **1** | **K.RKPVTEAR.S** 3782 |
|  | 4693 | **511.2577** | **1020.5008** | **1019.5036** | **0.9972** | **0** | **57** | **0.00034** | **1** | **R.DGAGDVAFIR.E** 4683 4691 |
|  | 5970 | **556.8124** | **1111.6102** | **1111.6172** | **-0.0070** | **1** | **32** | **0.099** | **1** | **K.VRGPPVSCIK.R** |
|  | 6486 | 575.8118 | 1149.6090 | 1149.6182 | -0.0093 | 0 | 25 | 0.45 | 1 | R.THYYAVAVVK.K 6478 |
|  | 7241 | **598.3053** | **1194.5960** | **1194.6033** | **-0.0073** | **0** | **38** | **0.023** | **1** | **K.FQLFGSPSGQK.D** 7240 |
|  | 9075 | **643.2938** | **1284.5730** | **1284.5809** | **-0.0079** | **0** | **16** | **3.3** | **1** | **R.FFSASCVPGADK.G** |
|  | 9483 | **435.9355** | **1304.7846** | **1304.7928** | **-0.0082** | **1** | **14** | **5.2** | **2** | **R.LKQVLLHQQAK.F** |
|  | 10087 | **668.3491** | **1334.6837** | **1334.6904** | **-0.0067** | **0** | **58** | **0.0002** | **1** | **K.LADFALLCLDGK.R** 10086 |
|  | 11148 | **697.3729** | **1392.7312** | **1392.6707** | **0.0604** | **0** | **5** | **39** | **1** | **R.CLAENAGDVAFVK.D** |
|  | 11161 | **697.4554** | **1392.8962** | **1392.6997** | **0.1965** | **1** | **4** | **44** | **2** | **R.RSDTSLTWNSVK.G** |
|  | 12043 | **723.3590** | **1444.7035** | **1444.7133** | **-0.0098** | **0** | **51** | **0.0011** | **1** | **R.VVWCAVGEQELR.K** |
|  | 12313 | **730.8914** | **1459.7683** | **1459.7783** | **-0.0100** | **0** | **7** | **26** | **1** | **K.LRPVAAEVYGTER.Q** 12314 |
|  | 13308 | **759.9119** | **1517.8092** | **1517.8202** | **-0.0110** | **1** | **89** | **1.8e-007** | **1** | **K.KGGSFQLNELQGLK.S** 13309 |
|  | 14191 | **787.3570** | **1572.6994** | **1572.8082** | **-0.1088** | **1** | **34** | **0.049** | **1** | **R.VVWCAVGEQELRK.C** |
|  | 14277 | **790.3754** | **1578.7363** | **1578.7460** | **-0.0097** | **0** | **27** | **0.25** | **1** | **K.NLLFNDNTECLAR.L** 14301 |
|  | 14843 | **807.9287** | **1613.8429** | **1613.8525** | **-0.0097** | **1** | **65** | **4.1e-005** | **1** | **R.SVNGKEDAIWNLLR.Q** |
|  | 15069 | **543.2579** | **1626.7520** | **1625.7057** | **1.0463** | **0** | **2** | **70** | **4** | **R.ESTVFEDLSDEAER.D** |
|  | 15398 | **413.2027** | **1648.7818** | **1648.7926** | **-0.0108** | **0** | **35** | **0.04** | **1** | **R.SCHLAMAPNHAVVSR.M** 15381 15410 |
|  | 15669 | **832.9669** | **1663.9193** | **1663.9297** | **-0.0104** | **1** | **75** | **3.7e-006** | **1** | **K.YLGPQYVAGITNLKK.C** 15670 |
|  | 15675 | **417.2017** | **1664.7778** | **1664.7875** | **-0.0097** | **0** | **(18)** | **1.7** | **1** | **R.SCHLAMAPNHAVVSR.M** |
|  | 15932 | **841.8919** | **1681.7693** | **1681.7804** | **-0.0111** | **0** | **29** | **0.13** | **1** | **K.CSTSPLLEACEFLR.K** |
|  | 17217 | **885.4147** | **1768.8149** | **1769.8842** | **-1.0693** | **1** | **7** | **22** | **2** | **K.RDSPIQCIQAIAENR.A** |
|  | 17796 | **902.9013** | **1803.7881** | **1803.7985** | **-0.0105** | **0** | **35** | **0.032** | **2** | **K.GEADAMSLDGGYVYTAGK.C** 17802 17808 |
|  | 17847 | **904.3947** | **1806.7749** | **1806.7876** | **-0.0128** | **0** | **82** | **6.4e-007** | **1** | **R.SNLCALCIGDEQGENK.C** 17846 |
|  | 18356 | **615.0619** | **1842.1638** | **1840.9908** | **1.1731** | **1** | **12** | **5** | **1** | **K.LRPVAAEVYGTERQPR.T** |
|  | 18647 | **931.8799** | **1861.7453** | **1861.7577** | **-0.0124** | **0** | **50** | **0.00091** | **1** | **K.FDEYFSQSCAPGSDPR.S** |
|  | 20192 | **994.9615** | **1987.9084** | **1987.9235** | **-0.0151** | **0** | **54** | **0.00037** | **1** | **K.DVTVLQNTDGNNNEAWAK.D** 20190 |
|  | 20496 | **672.6468** | **2014.9187** | **2014.0054** | **0.9133** | **1** | **1** | **79** | **3** | **K.NLLFNDNTECLARLHGK.T** |
|  | 20824 | **682.3677** | **2044.0812** | **2044.2271** | **-0.1459** | **0** | **1** | **81** | **2** | **K.LVFLVLLFLGALGLCLAGR.R** 20827 |
|  | 21122 | **1036.0175** | **2070.0204** | **2070.0310** | **-0.0106** | **0** | **7** | **16** | **1** | **R.ADAVTLDGGFIYEAGLAPYK.L** |
|  | 21296 | **1044.5350** | **2087.0555** | **2087.0687** | **-0.0132** | **0** | **55** | **0.0003** | **1** | **R.IDSGLYLGSGYFTAIQNLR.K** 21297 21298 21299 |
|  | 22890 | **753.3492** | **2257.0259** | **2257.0409** | **-0.0150** | **1** | **28** | **0.12** | **1** | **R.FFSASCVPGADKGQFPNLCR.L** |
|  | 26363 | **922.0676** | **2763.1809** | **2763.2010** | **-0.0202** | **1** | **40** | **0.0051** | **1** | **R.SNLCALCIGDEQGENKCVPNSNER.Y** |
|  | 26898 | **953.4569** | **2857.3487** | **2857.3665** | **-0.0178** | **0** | **50** | **0.00051** | **1** | **K.SQQSSDPDPNCVDRPVEGYLAVAVVR.R** 26900 |
|  | 28186 | **1044.7854** | **3131.3344** | **3131.3513** | **-0.0169** | **1** | **21** | **0.31** | **1** | **R.ESTVFEDLSDEAERDEYELLCPDNTR.K** |
|  | 29121 | **1121.8610** | **3362.5611** | **3362.5837** | **-0.0227** | **1** | **26** | **0.099** | **1** | **R.CLAENAGDVAFVKDVTVLQNTDGNNNEAWAK.D** |

| **47.** | P35555\|FBN1_HUMAN    **Mass:** 332667   **Score:** 658    **Queries matched:** 95   **emPAI:** 0.37 |
| --- | --- |
|  | Fibrillin-1 OS=Homo sapiens GN=FBN1 PE=1 SV=2 |

|  | **Query** | **Observed** | **Mr(expt)** | **Mr(calc)** | **Delta** | **Miss** | **Score** | **Expect** | **Rank** | **Peptide** |
| --- | --- | --- | --- | --- | --- | --- | --- | --- | --- | --- |
|  | 878 | 532.2830 | 531.2757 | 531.3129 | -0.0372 | 1 | 12 | 21 | 6 | R.ASRAK.R |
|  | 1412 | **746.5228** | **745.5155** | **746.3269** | **-0.8114** | **0** | **4** | **1e+002** | **2** | **K.EPDVCK.H** |
|  | 1520 | **395.4237** | **788.8329** | **788.3123** | **0.5206** | **0** | **0** | **1.4e+002** | **3** | **K.HDCTEK.Q** |
|  | 2254 | **422.8719** | **843.7292** | **843.3545** | **0.3747** | **0** | **8** | **23** | **1** | **K.NTMGSYR.C** |
|  | 2953 | **449.7274** | **897.4402** | **897.4491** | **-0.0089** | **0** | **12** | **9.3** | **1** | **K.VIHDVCR.N** |
|  | 3308 | **461.6902** | **921.3659** | **921.3724** | **-0.0065** | **0** | **8** | **28** | **1** | **R.MSYCYAK.F** |
|  | 3518 | **469.7476** | **937.4806** | **937.4691** | **0.0114** | **0** | **12** | **9.3** | **3** | **R.LETCFLR.Y** |
|  | 3530 | **470.2448** | **938.4751** | **937.3674** | **1.1078** | **0** | **(7)** | **27** | **9** | **R.MSYCYAK.F** |
|  | 3998 | **486.7872** | **971.5598** | **971.5665** | **-0.0066** | **1** | **16** | **4.9** | **6** | **R.RGFIPNIR.T** |
|  | 4202 | **494.6028** | **987.1909** | **987.4873** | **-0.2963** | **0** | **4** | **48** | **6** | **K.ELNQLEDK.Y** |
|  | 4557 | **506.2415** | **1010.4684** | **1009.3706** | **1.0979** | **0** | **4** | **55** | **3** | **R.DACGNGTCR.N** |
|  | 4641 | **509.2302** | **1016.4458** | **1016.4532** | **-0.0074** | **0** | **15** | **6** | **1** | **R.VCVDTHMR.S** |
|  | 4718 | **512.7158** | **1023.4171** | **1023.4266** | **-0.0095** | **0** | **18** | **2.5** | **1** | **R.GSYHCICK.T** |
|  | 4759 | **514.2628** | **1026.5111** | **1026.5029** | **0.0082** | **0** | **18** | **2.4** | **1** | **R.SIQHCNIR.C** 4757 |
|  | 4913 | **519.2432** | **1036.4719** | **1036.4794** | **-0.0075** | **0** | **33** | **0.077** | **1** | **K.TLCCATVGR.A** 4925 |
|  | 4983 | **521.7047** | **1041.3949** | **1041.4042** | **-0.0093** | **0** | **37** | **0.031** | **1** | **K.ASCCCSLGK.A** 4984 |
|  | 5108 | **525.7790** | **1049.5434** | **1049.5142** | **0.0293** | **0** | **2** | **1.1e+002** | **5** | **K.GYILQEDGR.S** |
|  | 5668 | **546.2459** | **1090.4773** | **1090.4866** | **-0.0093** | **1** | **20** | **1.8** | **1** | **R.STCYGGYKR.G** |
|  | 6371 | **572.2826** | **1142.5506** | **1142.5576** | **-0.0070** | **0** | **16** | **4** | **1** | **K.MIPSLCTHGK.C** |
|  | 6586 | **578.7836** | **1155.5527** | **1156.3882** | **-0.8355** | **0** | **6** | **40** | **3** | **K.MQCCCDAGR.C** |
|  | 6648 | **580.3230** | **1158.6314** | **1158.5526** | **0.0789** | **0** | **(1)** | **1.3e+002** | **3** | **K.MIPSLCTHGK.C** |
|  | 6838 | **587.7941** | **1173.5737** | **1172.3831** | **1.1906** | **0** | **(2)** | **1.1e+002** | **2** | **K.MQCCCDAGR.C** |
|  | 7249 | **399.2321** | **1194.6744** | **1193.6081** | **1.0663** | **0** | **7** | **30** | **8** | **K.EGISYLHFTK.K** |
|  | 8918 | **638.3173** | **1274.6200** | **1274.6289** | **-0.0089** | **0** | **23** | **0.8** | **1** | **R.CSNQLPQSITK.M** |
|  | 9210 | **646.8024** | **1291.5903** | **1291.5979** | **-0.0076** | **0** | **35** | **0.041** | **1** | **K.GTCWQTVIDGR.C** |
|  | 9734 | **659.7661** | **1317.5177** | **1317.5271** | **-0.0094** | **0** | **19** | **1.6** | **2** | **R.YNAYCCPGWK.T** |
|  | 9771 | **660.7468** | **1319.4791** | **1319.4879** | **-0.0088** | **0** | **43** | **0.0057** | **1** | **K.MCCCSYNIGR.A** 9793 |
|  | 9841 | **661.8148** | **1321.6151** | **1321.7030** | **-0.0879** | **1** | **0** | **1.3e+002** | **9** | **K.EGISYLHFTKK.K** |
|  | 9902 | **663.7818** | **1325.5490** | **1325.5591** | **-0.0101** | **1** | **32** | **0.089** | **1** | **R.SCKDLDECATK.Q** |
|  | 10343 | **676.3148** | **1350.6150** | **1349.6510** | **0.9640** | **0** | **24** | **0.57** | **1** | **R.SGNCYLDIRPR.G** |
|  | 10377 | **676.8321** | **1351.6497** | **1351.6667** | **-0.0169** | **1** | **6** | **36** | **3** | **K.NGLCVNTRGSFK.C** |
|  | 11580 | **473.5958** | **1417.7655** | **1417.7752** | **-0.0097** | **0** | **26** | **0.31** | **1** | **R.GQCIKPLFGAVTK.S** 11581 11597 |
|  | 11999 | **722.2925** | **1442.5704** | **1442.5806** | **-0.0102** | **0** | **48** | **0.0018** | **1** | **R.CECEIGYELDR.S** |
|  | 12022 | 722.7633 | 1443.5121 | 1443.5065 | 0.0056 | 0 | 1 | 77 | 8 | K.NCEDMDECSIR.N |
|  | 12375 | 488.9142 | 1463.7208 | 1463.5778 | 0.1430 | 1 | 4 | 48 | 6 | K.KMCCCSYNIGR.A 12374 |
|  | 13599 | **768.8146** | **1535.6146** | **1534.6221** | **0.9925** | **0** | **54** | **0.0005** | **1** | **R.CECPVGFFYNDK.L** 13584 |
|  | 13711 | **772.3812** | **1542.7479** | **1541.7256** | **1.0223** | **1** | **5** | **40** | **2** | **R.AGYQSTLTRTECR.D** |
|  | 13833 | **775.8540** | **1549.6934** | **1549.7018** | **-0.0083** | **0** | **52** | **0.00073** | **1** | **R.CECFPGLAVGLDGR.V** |
|  | 13871 | **776.8453** | **1551.6761** | **1551.6875** | **-0.0114** | **0** | **60** | **0.00012** | **1** | **R.YEDEECTLPIAGR.H** |
|  | 13894 | **777.3234** | **1552.6322** | **1552.6433** | **-0.0111** | **0** | **41** | **0.0097** | **1** | **K.CQCPSGMTLDATGR.I** |
|  | 13928 | **778.8403** | **1555.6660** | **1555.6759** | **-0.0099** | **0** | **20** | **1.3** | **1** | **K.CLCPEGFSLSSSGR.R** |
|  | 14005 | **781.3304** | **1560.6462** | **1561.6878** | **-1.0416** | **0** | **5** | **39** | **3** | **R.CQHGCQNIIGGYR.C** |
|  | 14364 | **792.8976** | **1583.7806** | **1583.7912** | **-0.0106** | **0** | **9** | **14** | **1** | **K.TLPGGNQCIVPICR.H** |
|  | 14454 | **795.3271** | **1588.6397** | **1588.6498** | **-0.0100** | **0** | **34** | **0.048** | **1** | **K.CICNSGYEVDSTGK.N** |
|  | 14751 | **804.8796** | **1607.7446** | **1607.7548** | **-0.0102** | **0** | **33** | **0.057** | **1** | **K.CICKPGFQLASDGR.Y** 14750 |
|  | 14918 | **809.8762** | **1617.7379** | **1617.7491** | **-0.0112** | **0** | **42** | **0.0083** | **1** | **R.TCVDINECLLEPR.K** |
|  | 16619 | **863.8262** | **1725.6379** | **1725.6507** | **-0.0128** | **0** | **32** | **0.057** | **1** | **R.CLCYDGFMASEDMK.T** |
|  | 16630 | **576.3022** | **1725.8847** | **1725.7563** | **0.1285** | **1** | **9** | **13** | **1** | **R.KCAPGTCQNLDGSYR.C** |
|  | 17189 | **883.8546** | **1765.6947** | **1764.7229** | **0.9717** | **0** | **6** | **26** | **1** | **K.DINECETPGICMNGR.C** |
|  | 17406 | **890.8807** | **1779.7469** | **1778.7741** | **0.9728** | **0** | **35** | **0.034** | **1** | **R.GDNGDTACSNEIGVGVSK.A** |
|  | 18104 | **913.3669** | **1824.7192** | **1824.7308** | **-0.0116** | **0** | **64** | **3.2e-005** | **1** | **K.YQCACNPGYHSTPDR.L** 18103 |
|  | 18791 | **937.9617** | **1873.9089** | **1874.8478** | **-0.9389** | **0** | **2** | **67** | **6** | **R.CWSPGVTVAPEMCPIR.A** |
|  | 20707 | **1018.4105** | **2034.8065** | **2034.8194** | **-0.0129** | **0** | **102** | **5.4e-009** | **1** | **K.TGCTDINECEIGAHNCGK.H** 20706 |
|  | 21237 | **694.4192** | **2080.2357** | **2080.1568** | **0.0789** | **1** | **1** | **68** | **4** | **K.KKPVAGTYSLQISSTPLYK.K** |
|  | 21339 | **697.6187** | **2089.8341** | **2089.8492** | **-0.0150** | **0** | **39** | **0.0099** | **1** | **R.AWGHPCEMCPAQPHPCR.R** 21338 |
|  | 21378 | **1047.9377** | **2093.8609** | **2093.8751** | **-0.0142** | **1** | **13** | **3.5** | **2** | **K.NCMDIDECQRDPLLCR.G** 21376 21377 |
|  | 21814 | **1071.6339** | **2141.2533** | **2141.8704** | **-0.6172** | **0** | **4** | **28** | **1** | **K.GTQCEDIDECEVFPGVCK.N** |
|  | 22378 | **1103.4756** | **2204.9366** | **2203.9660** | **0.9706** | **0** | **18** | **1.3** | **1** | **K.LLVCEDIDECQNGPVCQR.N** 22377 |
|  | 22931 | **755.0472** | **2262.1199** | **2261.9452** | **0.1747** | **1** | **0** | **79** | **2** | **R.AWGHPCEMCPAQPHPCRR.G** |
|  | 23538 | **780.9261** | **2339.7564** | **2339.0491** | **0.7074** | **1** | **0** | **49** | **8** | **K.CQCPSGMTLDATGRICLDIR.L** |
|  | 23860 | **795.3519** | **2383.0338** | **2383.0494** | **-0.0157** | **1** | **42** | **0.0047** | **1** | **R.IKGTQCEDIDECEVFPGVCK.N** |
|  | 25905 | **896.0820** | **2685.2243** | **2685.2383** | **-0.0141** | **0** | **19** | **0.83** | **1** | **R.TGPCFTVISNQMCQGQLSGIVCTK.T** 25898 |
|  | 26429 | **695.0428** | **2776.1421** | **2776.1599** | **-0.0179** | **0** | **38** | **0.0072** | **1** | **R.RPDGEGCVDENECQTKPGICENGR.C** 26436 26437 |
|  | 26661 | **938.3923** | **2812.1550** | **2812.1772** | **-0.0222** | **0** | **27** | **0.1** | **1** | **K.TCVDVNECDLNPNICLSGTCENTK.G** |
|  | 27078 | **967.0765** | **2898.2078** | **2898.2228** | **-0.0150** | **0** | **9** | **5.3** | **1** | **K.SQCCSSLGAAWGSPCTLCQVDPICGK.G** |
|  | 27337 | **739.3515** | **2953.3769** | **2953.0461** | **0.3308** | **1** | **3** | **28** | **3** | **K.CDEGYESGFMMMKNCMDIDECQR.D** |
|  | 28153 | **1042.7926** | **3125.3560** | **3124.3129** | **1.0430** | **1** | **0** | **39** | **9** | **K.TLCCATVGRAWGHPCEMCPAQPHPCR.R** |
|  | 29102 | **1119.1523** | **3354.4352** | **3353.4560** | **0.9792** | **1** | **67** | **6.6e-006** | **1** | **R.QICPYGSGIIVGPDDSAVDMDECKEPDVCK.H** |
|  | 29382 | **1154.1741** | **3459.5004** | **3459.5204** | **-0.0200** | **0** | **37** | **0.0083** | **1** | **K.TGYTPDITGTSCVDLNECNQAPKPCNFICK.N** |
|  | 29582 | **1772.7952** | **3543.5758** | **3544.4786** | **-0.9028** | **0** | **1** | **29** | **5** | **R.TGACQDVDECQAIPGLCQGGNCINTVGSFECK.C** |
|  | 30168 | **1269.5945** | **3805.7616** | **3804.6535** | **1.1081** | **0** | **0** | **27** | **2** | **R.CECPPGHQLSPNISACIDINECELSAHLCPNGR.C** |
|  | 30282 | **1294.6277** | **3880.8612** | **3880.8798** | **-0.0185** | **0** | **44** | **0.0012** | **1** | **K.ILCPGGEGFRPNPITVILEDIDECQELPGLCQGGK.C** 30278 30279 30280 30281 |
|  | 31330 | **1278.8401** | **5111.3313** | **5111.3562** | **-0.0250** | **0** | **25** | **0.04** | **1** | **R.AWNKPCEQCPIPSTDEFATLCGSQRPGFVIDIYTGLPVDIDECR.E** |

| **48.** | Q01082\|SPTB2_HUMAN    **Mass:** 275237   **Score:** 647    **Queries matched:** 88   **emPAI:** 0.25 |
| --- | --- |
|  | Spectrin beta chain, brain 1 OS=Homo sapiens GN=SPTBN1 PE=1 SV=2 |

|  | **Query** | **Observed** | **Mr(expt)** | **Mr(calc)** | **Delta** | **Miss** | **Score** | **Expect** | **Rank** | **Peptide** |
| --- | --- | --- | --- | --- | --- | --- | --- | --- | --- | --- |
|  | 1264 | **689.4144** | **688.4071** | **688.4119** | **-0.0048** | **0** | **7** | **53** | **2** | **R.LTVQTK.F** |
|  | 1480 | **782.9713** | **781.9640** | **781.4446** | **0.5194** | **0** | **10** | **12** | **1** | **K.HNLLASK.E** 1483 |
|  | 1739 | **404.6921** | **807.3697** | **806.4399** | **0.9298** | **1** | **8** | **28** | **2** | **R.LFERSR.I** |
|  | 1948 | **413.2135** | **824.4124** | **824.4293** | **-0.0169** | **1** | **16** | **3.8** | **9** | **R.FREFAR.D** |
|  | 2146 | **419.3137** | **836.6129** | **836.4062** | **0.2067** | **0** | **2** | **79** | **7** | **K.DLTSVMR.L** |
|  | 2434 | **429.7475** | **857.4804** | **857.4858** | **-0.0054** | **0** | **15** | **6.5** | **2** | **R.DLVAIEAK.L** |
|  | 2776 | **441.2962** | **880.5778** | **879.3822** | **1.1956** | **0** | **4** | **59** | **5** | **K.STDEVDSK.R** |
|  | 3153 | **456.7820** | **911.5494** | **911.5553** | **-0.0058** | **0** | **44** | **0.0055** | **1** | **R.VQAVVAVAR.E** 3158 |
|  | 3419 | **466.2379** | **930.4613** | **929.4566** | **1.0047** | **0** | **3** | **91** | **8** | **R.QLQEDAAR.L** |
|  | 3892 | **482.7482** | **963.4818** | **963.4886** | **-0.0068** | **0** | **29** | **0.2** | **1** | **K.EIQGHQPR.I** 3895 |
|  | 4138 | **491.7609** | **981.5073** | **981.5144** | **-0.0071** | **0** | **12** | **8.9** | **1** | **K.WVNSHLAR.V** 4135 |
|  | 5041 | **523.2858** | **1044.5571** | **1044.5637** | **-0.0066** | **0** | **15** | **5.6** | **1** | **R.LEMNLGLQK.I** |
|  | 5268 | **531.3847** | **1060.7548** | **1060.5587** | **0.1962** | **0** | **(3)** | **70** | **3** | **R.LEMNLGLQK.I** |
|  | 5398 | **536.7685** | **1071.5224** | **1072.5625** | **-1.0401** | **1** | **22** | **1.2** | **1** | **K.AQQDKLNTR.W** |
|  | 5735 | **548.3156** | **1094.6167** | **1094.5720** | **0.0447** | **0** | **6** | **36** | **1** | **R.RPPSPEPSTK.V** |
|  | 5737 | **1095.6332** | **1094.6259** | **1094.6084** | **0.0175** | **0** | **3** | **80** | **7** | **K.LLTQHENIK.N** |
|  | 5791 | **550.2944** | **1098.5743** | **1097.5829** | **0.9914** | **0** | **7** | **32** | **3** | **R.QIEAQEKPR.D** |
|  | 7428 | **602.2477** | **1202.4809** | **1202.5891** | **-0.1082** | **0** | **11** | **13** | **6** | **R.DQNTVETLQR.M** |
|  | 7773 | **407.2117** | **1218.6132** | **1219.6131** | **-1.0000** | **1** | **8** | **25** | **6** | **R.ANNQKVYMPR.E** |
|  | 7885 | **409.2277** | **1224.6612** | **1225.7142** | **-1.0531** | **1** | **9** | **17** | **3** | **R.ELALRNELIR.Q** |
|  | 8010 | **616.7667** | **1231.5188** | **1232.6037** | **-1.0849** | **1** | **11** | **11** | **1** | **K.HYASEEIKEK.L** |
|  | 8021 | **616.8495** | **1231.6844** | **1231.5932** | **0.0912** | **0** | **1** | **1.2e+002** | **9** | **R.EIGQSVDEVEK.L** |
|  | 8677 | 632.8616 | 1263.7087 | 1263.7187 | -0.0100 | 0 | 40 | 0.014 | 1 | K.HLLGVEDLLQK.H |
|  | 8737 | **634.3881** | **1266.7617** | **1265.5823** | **1.1794** | **0** | **5** | **47** | **6** | **R.QLMHSGHPSEK.E** 8736 |
|  | 9502 | **654.4252** | **1306.8358** | **1305.6598** | **1.1760** | **1** | **39** | **0.017** | **1** | **K.AKDEQSAVSMLK.K** |
|  | 9541 | 655.8157 | 1309.6169 | 1309.6262 | -0.0093 | 0 | 45 | 0.0039 | 1 | R.ALVADSHPESER.I |
|  | 10949 | **691.3182** | **1380.6219** | **1380.6310** | **-0.0091** | **0** | **23** | **0.65** | **1** | **R.DLDDFQSWLSR.T** |
|  | 11637 | **474.7235** | **1421.1486** | **1420.6439** | **0.5048** | **0** | **10** | **7.8** | **1** | **K.QQMLENQMEVR.K** |
|  | 12165 | **726.8510** | **1451.6875** | **1451.6966** | **-0.0092** | **0** | **12** | **8.2** | **1** | **K.MWEVLESTTQTK.A** |
|  | 12286 | **487.2860** | **1458.8363** | **1458.7732** | **0.0631** | **1** | **3** | **75** | **6** | **K.TFTKWVNSHLAR.V** |
|  | 12351 | **731.9072** | **1461.7999** | **1460.8239** | **0.9760** | **0** | **1** | **1.1e+002** | **3** | **K.GNLEVLLFTIQSK.M** |
|  | 12946 | **499.9865** | **1496.9378** | **1496.6817** | **0.2561** | **0** | **7** | **21** | **5** | **K.AELFTQSCADLDK.W** |
|  | 12999 | **750.8580** | **1499.7014** | **1499.8745** | **-0.1731** | **1** | **9** | **17** | **5** | **R.MLIKLLEVLSGER.L** |
|  | 13421 | **763.3878** | **1524.7610** | **1523.7732** | **0.9877** | **0** | **40** | **0.014** | **1** | **R.LQALDTGWNELHK.M** |
|  | 13806 | **775.3702** | **1548.7259** | **1548.7388** | **-0.0129** | **1** | **12** | **8.6** | **3** | **K.QQMLENQMEVRK.K** |
|  | 13857 | 776.3755 | 1550.7365 | 1550.8053 | -0.0687 | 0 | 5 | 37 | 3 | K.HTLVEADIGIQAER.V |
|  | 13940 | **778.9232** | **1555.8318** | **1556.7947** | **-0.9629** | **0** | **10** | **14** | **2** | **R.EQWANLEQLSAIR.K** 13941 |
|  | 14081 | **783.3735** | **1564.7324** | **1564.7337** | **-0.0013** | **1** | **5** | **37** | **1** | **K.KQQMLENQMEVR.K** 14080 |
|  | 14211 | **787.9294** | **1573.8442** | **1573.8980** | **-0.0538** | **1** | **10** | **13** | **3** | **R.LWEYLLELLRAR.R** 14199 |
|  | 14849 | **539.2483** | **1614.7230** | **1613.8777** | **0.8454** | **1** | **1** | **89** | **4** | **K.QLWGLLIEETEKR.H** |
|  | 15133 | 816.4135 | 1630.8123 | 1631.8440 | -1.0316 | 1 | 3 | 59 | 7 | K.VLDNAIETEKMIEK.Y |
|  | 15837 | **559.3032** | **1674.8878** | **1674.8060** | **0.0818** | **1** | **11** | **8.1** | **1** | **R.TSSKESSPIPSPTSDR.K** |
|  | 16332 | **854.8961** | **1707.7777** | **1707.7886** | **-0.0109** | **0** | **50** | **0.0011** | **1** | **R.TQETPSAQMEGFLNR.K** 16333 |
|  | 16587 | 575.2979 | 1722.8717 | 1723.7835 | -0.9118 | 0 | (3) | 60 | 7 | R.TQETPSAQMEGFLNR.K |
|  | 16672 | **865.3831** | **1728.7516** | **1728.7606** | **-0.0090** | **0** | **12** | **7.2** | **1** | **K.FFWEMAEEEGWIR.E** |
|  | 17355 | **889.4436** | **1776.8726** | **1776.8853** | **-0.0127** | **0** | **104** | **4.3e-009** | **1** | **R.SQNIVTDSSSLSAEAIR.Q** 17356 |
|  | 17452 | **892.4485** | **1782.8825** | **1782.8934** | **-0.0109** | **0** | **17** | **1.9** | **1** | **R.DVSSVELLMNNHQGIK.A** |
|  | 17732 | **900.8925** | **1799.7704** | **1798.8771** | **0.8933** | **1** | **45** | **0.0033** | **1** | **K.ILSSDDYGKDLTSVMR.L** 17718 |
|  | 17846 | 904.3947 | 1806.7749 | 1806.8934 | -0.1185 | 1 | (0) | 1.1e+002 | 9 | K.MWEVLESTTQTKAQR.L |
|  | 18083 | **608.6329** | **1822.8770** | **1822.8883** | **-0.0114** | **1** | **1** | **94** | **5** | **K.MWEVLESTTQTKAQR.L** |
|  | 20570 | **1011.5067** | **2020.9989** | **2021.0106** | **-0.0117** | **0** | **32** | **0.061** | **1** | **R.LVSQDNFGFDLPAVEAATK.K** |
|  | 20876 | **1025.4790** | **2048.9434** | **2048.9572** | **-0.0137** | **0** | **85** | **3e-007** | **1** | **R.TQTAIASEDMPNTLTEAEK.L** 20877 |
|  | 21188 | **693.2362** | **2076.6868** | **2076.0925** | **0.5943** | **1** | **6** | **16** | **1** | **R.LTVQTKFMELLEPLNER.K** |
|  | 21693 | **1064.0283** | **2126.0421** | **2124.9568** | **1.0853** | **1** | **2** | **53** | **2** | **K.MLTAQDMSYDEARNLHSK.W** |
|  | 21729 | **1065.5420** | **2129.0694** | **2129.0787** | **-0.0092** | **0** | **65** | **2.9e-005** | **1** | **K.VIESTQDLGNDLAGVMALQR.K** 21730 21738 |
|  | 21890 | **717.6889** | **2150.0449** | **2150.0603** | **-0.0154** | **1** | **19** | **1.1** | **1** | **K.EAEKLESEHPDQAQAILSR.L** 21891 |
|  | 21941 | **1078.0498** | **2154.0850** | **2153.9182** | **0.1669** | **0** | **2** | **55** | **3** | **K.IFQEMLYIMDWMDEMK.V** |
|  | 22530 | **554.5350** | **2214.1110** | **2215.2113** | **-1.1003** | **1** | **1** | **61** | **10** | **R.IIYIREQWANLEQLSAIR.K** |
|  | 22577 | **1110.9834** | **2219.9522** | **2219.9650** | **-0.0127** | **0** | **85** | **2.4e-007** | **1** | **R.DMGEMVTQGQTDAQYMFLR.Q** |
|  | 22756 | **746.5587** | **2236.6543** | **2235.9599** | **0.6944** | **0** | **(4)** | **21** | **1** | **R.DMGEMVTQGQTDAQYMFLR.Q** |
|  | 22759 | **560.2806** | **2237.0935** | **2235.9599** | **1.1336** | **0** | **(0)** | **79** | **7** | **R.DMGEMVTQGQTDAQYMFLR.Q** |
|  | 23519 | **780.0568** | **2337.1485** | **2337.1601** | **-0.0116** | **0** | **61** | **5.5e-005** | **1** | **K.HQILEQAVEDYAETVHQLSK.T** 23518 |
|  | 23690 | **787.0767** | **2358.2082** | **2358.2213** | **-0.0131** | **1** | **1** | **58** | **2** | **K.TKVIESTQDLGNDLAGVMALQR.K** |
|  | 23890 | **796.9033** | **2387.6881** | **2387.0581** | **0.6301** | **1** | **0** | **45** | **2** | **R.MAETVDTSEMVNGATEQRTSSK.E** |
|  | 24431 | **823.7296** | **2468.1668** | **2468.2271** | **-0.0602** | **1** | **2** | **50** | **6** | **R.DGMAFNALIHKHRPDLIDFDK.L** |
|  | 24835 | **843.0612** | **2526.1618** | **2526.1809** | **-0.0191** | **0** | **26** | **0.17** | **1** | **R.EVVAGSHELGQDYEHVTMLQER.F** |
|  | 25572 | **879.4153** | **2635.2242** | **2635.2401** | **-0.0159** | **1** | **8** | **9.7** | **1** | **K.LLTQHENIKNEIDNYEEDYQK.M** |
|  | 26110 | 903.7671 | 2708.2794 | 2707.3024 | 0.9770 | 1 | 2 | 41 | 4 | R.GVNASAQKFATDGEGYKPCDPQVIR.D |
|  | 26351 | **921.3449** | **2761.0129** | **2760.4697** | **0.5432** | **1** | **2** | **27** | **2** | **K.YESLASDLLEWIEQTIIILNNRK.F** |
|  | 26601 | **936.1220** | **2805.3442** | **2805.3569** | **-0.0127** | **0** | **17** | **1.2** | **1** | **R.VDTVNHLADELINSGHSDAATIAEWK.D** |
|  | 27596 | 1000.8221 | 2999.4444 | 2999.4586 | -0.0142 | 0 | 46 | 0.0013 | 1 | R.LEEASLLHQFQADADDIDAWMLDILK.I |
|  | 28840 | **1097.2015** | **3288.5828** | **3288.5972** | **-0.0144** | **0** | **74** | **1.9e-006** | **1** | **K.QAEAFLNNQEYVLAHTEMPTTLEGAEAAIK.K** |

| **49.** | Q99880\|H2B1L_HUMAN    **Mass:** 13944    **Score:** 638    **Queries matched:** 63   **emPAI:** 1.69 |
| --- | --- |
|  | Histone H2B type 1-L OS=Homo sapiens GN=HIST1H2BL PE=1 SV=3 |

|  | **Query** | **Observed** | **Mr(expt)** | **Mr(calc)** | **Delta** | **Miss** | **Score** | **Expect** | **Rank** | **Peptide** |
| --- | --- | --- | --- | --- | --- | --- | --- | --- | --- | --- |
|  | 1843 | **408.7292** | **815.4438** | **815.4501** | **-0.0063** | **0** | **26** | **0.56** | **1** | **R.EIQTAVR.L** 1844 1845 |
|  | 1896 | **410.7329** | **819.4513** | **819.4563** | **-0.0050** | **1** | **17** | **3.9** | **3** | **K.RSTITSR.E** |
|  | 1994 | **414.7114** | **827.4082** | **827.4137** | **-0.0055** | **0** | **31** | **0.13** | **1** | **K.HAVSEGTK.A** 1990 1991 1992 1993 2000 2001 2004 2005 2006 2007 2008 2009 2010 |
|  | 2993 | **451.2501** | **900.4857** | **900.4929** | **-0.0073** | **1** | **32** | **0.13** | **1** | **R.LAHYNKR.S** 2989 2991 2992 3001 |
|  | 3744 | **477.3026** | **952.5906** | **952.5957** | **-0.0051** | **0** | **38** | **0.024** | **1** | **R.LLLPGELAK.H** 3740 3741 3742 |
|  | 5936 | **555.2867** | **1108.5588** | **1107.6288** | **0.9300** | **1** | **3** | **71** | **7** | **M.PELAKSAPAPK.K** |
|  | 6757 | **584.7971** | **1167.5797** | **1167.5884** | **-0.0087** | **0** | **24** | **0.59** | **1** | **K.QVHPDTGISSK.A** 6753 6755 6788 |
|  | 12311 | **730.8779** | **1459.7412** | **1458.7579** | **0.9833** | **1** | **6** | **34** | **1** | **R.IASEASRLAHYNK.R** |
|  | 13148 | **503.9497** | **1508.8273** | **1507.8358** | **0.9915** | **1** | **11** | **9.6** | **1** | **K.VLKQVHPDTGISSK.A** |
|  | 16889 | **872.4083** | **1742.8021** | **1742.8120** | **-0.0099** | **0** | **106** | **2.7e-009** | **1** | **K.AMGIMNSFVNDIFER.I** 16885 16886 16887 16888 16890 16891 16893 16894 16895 16896 16897 16898 16899 16901 16902 16903 16904 16925 16927 16929 16931 16932 |
|  | 17112 | **880.4065** | **1758.7984** | **1758.8069** | **-0.0085** | **0** | **(80)** | **9.8e-007** | **1** | **K.AMGIMNSFVNDIFER.I** 17113 |
|  | 17114 | **880.4066** | **1758.7987** | **1758.8069** | **-0.0082** | **0** | **(103)** | **5.1e-009** | **1** | **K.AMGIMNSFVNDIFER.I** 17111 17125 17126 |

| **50.** | P58876\|H2B1D_HUMAN    **Mass:** 13928    **Score:** 638    **Queries matched:** 64   **emPAI:** 1.71 |
| --- | --- |
|  | Histone H2B type 1-D OS=Homo sapiens GN=HIST1H2BD PE=1 SV=2 |

|  | **Query** | **Observed** | **Mr(expt)** | **Mr(calc)** | **Delta** | **Miss** | **Score** | **Expect** | **Rank** | **Peptide** |
| --- | --- | --- | --- | --- | --- | --- | --- | --- | --- | --- |
|  | 1843 | 408.7292 | 815.4438 | 815.4501 | -0.0063 | 0 | 26 | 0.56 | 1 | R.EIQTAVR.L 1844 1845 |
|  | 1896 | 410.7329 | 819.4513 | 819.4563 | -0.0050 | 1 | 17 | 3.9 | 3 | K.RSTITSR.E |
|  | 1994 | 414.7114 | 827.4082 | 827.4137 | -0.0055 | 0 | 31 | 0.13 | 1 | K.HAVSEGTK.A 1990 1991 1992 1993 2000 2001 2004 2005 2006 2007 2008 2009 2010 |
|  | 2993 | 451.2501 | 900.4857 | 900.4929 | -0.0073 | 1 | 32 | 0.13 | 1 | R.LAHYNKR.S 2989 2991 2992 3001 |
|  | 3744 | 477.3026 | 952.5906 | 952.5957 | -0.0051 | 0 | 38 | 0.024 | 1 | R.LLLPGELAK.H 3740 3741 3742 |
|  | 6757 | 584.7971 | 1167.5797 | 1167.5884 | -0.0087 | 0 | 24 | 0.59 | 1 | K.QVHPDTGISSK.A 6753 6755 6788 |
|  | 8749 | **423.5754** | **1267.7043** | **1268.6434** | **-0.9391** | **1** | **6** | **34** | **5** | **-.MPEPTKSAPAPK.K** 8760 |
|  | 13148 | 503.9497 | 1508.8273 | 1507.8358 | 0.9915 | 1 | 11 | 9.6 | 1 | K.VLKQVHPDTGISSK.A |
|  | 16889 | 872.4083 | 1742.8021 | 1742.8120 | -0.0099 | 0 | 106 | 2.7e-009 | 1 | K.AMGIMNSFVNDIFER.I 16885 16886 16887 16888 16890 16891 16893 16894 16895 16896 16897 16898 16899 16901 16902 16903 16904 16925 16927 16929 16931 16932 |
|  | 17112 | 880.4065 | 1758.7984 | 1758.8069 | -0.0085 | 0 | (80) | 9.8e-007 | 1 | K.AMGIMNSFVNDIFER.I 17113 |
|  | 17114 | 880.4066 | 1758.7987 | 1758.8069 | -0.0082 | 0 | (103) | 5.1e-009 | 1 | K.AMGIMNSFVNDIFER.I 17111 17125 17126 |
|  | 24252 | **815.3892** | **2443.1457** | **2443.1624** | **-0.0167** | **1** | **0** | **71** | **1** | **K.AMGIMNSFVNDIFERIAGEASR.L** |

|  | **Proteins matching a subset of these peptides:** |
| --- | --- |

|  | Q5QNW6\|H2B2F_HUMAN    **Mass:** 13912    **Score:** 638    **Queries matched:** 62 |
| --- | --- |
|  | Histone H2B type 2-F OS=Homo sapiens GN=HIST2H2BF PE=1 SV=3 |

|  | P06899\|H2B1J_HUMAN    **Mass:** 13896    **Score:** 638    **Queries matched:** 62 |
| --- | --- |
|  | Histone H2B type 1-J OS=Homo sapiens GN=HIST1H2BJ PE=1 SV=3 |

|  | Q16778\|H2B2E_HUMAN    **Mass:** 13912    **Score:** 638    **Queries matched:** 62 |
| --- | --- |
|  | Histone H2B type 2-E OS=Homo sapiens GN=HIST2H2BE PE=1 SV=3 |

|  | P23527\|H2B1O_HUMAN    **Mass:** 13898    **Score:** 638    **Queries matched:** 62 |
| --- | --- |
|  | Histone H2B type 1-O OS=Homo sapiens GN=HIST1H2BO PE=1 SV=3 |

|  | P33778\|H2B1B_HUMAN    **Mass:** 13942    **Score:** 638    **Queries matched:** 62 |
| --- | --- |
|  | Histone H2B type 1-B OS=Homo sapiens GN=HIST1H2BB PE=1 SV=2 |

|  | O60814\|H2B1K_HUMAN    **Mass:** 13882    **Score:** 638    **Queries matched:** 62 |
| --- | --- |
|  | Histone H2B type 1-K OS=Homo sapiens GN=HIST1H2BK PE=1 SV=3 |

|  | P62807\|H2B1C_HUMAN    **Mass:** 13811    **Score:** 638    **Queries matched:** 62 |
| --- | --- |
|  | Histone H2B type 1-C/E/F/G/I OS=Homo sapiens GN=HIST1H2BC PE=1 SV=3 |

|  | Q93079\|H2B1H_HUMAN    **Mass:** 13884    **Score:** 638    **Queries matched:** 62 |
| --- | --- |
|  | Histone H2B type 1-H OS=Homo sapiens GN=HIST1H2BH PE=1 SV=3 |

|  | Q99877\|H2B1N_HUMAN    **Mass:** 13914    **Score:** 638    **Queries matched:** 62 |
| --- | --- |
|  | Histone H2B type 1-N OS=Homo sapiens GN=HIST1H2BN PE=1 SV=3 |

|  | Q99879\|H2B1M_HUMAN    **Mass:** 13981    **Score:** 638    **Queries matched:** 62 |
| --- | --- |
|  | Histone H2B type 1-M OS=Homo sapiens GN=HIST1H2BM PE=1 SV=3 |

|  | P57053\|H2BFS_HUMAN    **Mass:** 13936    **Score:** 638    **Queries matched:** 57 |
| --- | --- |
|  | Histone H2B type F-S OS=Homo sapiens GN=H2BFS PE=1 SV=2 |

| **51.** | Q8N257\|H2B3B_HUMAN    **Mass:** 13900    **Score:** 638    **Queries matched:** 61   **emPAI:** 1.71 |
| --- | --- |
|  | Histone H2B type 3-B OS=Homo sapiens GN=HIST3H2BB PE=1 SV=3 |

|  | **Query** | **Observed** | **Mr(expt)** | **Mr(calc)** | **Delta** | **Miss** | **Score** | **Expect** | **Rank** | **Peptide** |
| --- | --- | --- | --- | --- | --- | --- | --- | --- | --- | --- |
|  | 1682 | **402.2437** | **802.4729** | **801.4345** | **1.0384** | **0** | **10** | **25** | **10** | **R.EVQTAVR.L** |
|  | 1896 | 410.7329 | 819.4513 | 819.4563 | -0.0050 | 1 | 17 | 3.9 | 3 | K.RSTITSR.E |
|  | 1994 | 414.7114 | 827.4082 | 827.4137 | -0.0055 | 0 | 31 | 0.13 | 1 | K.HAVSEGTK.A 1990 1991 1992 1993 2000 2001 2004 2005 2006 2007 2008 2009 2010 |
|  | 2993 | 451.2501 | 900.4857 | 900.4929 | -0.0073 | 1 | 32 | 0.13 | 1 | R.LAHYNKR.S 2989 2991 2992 3001 |
|  | 3744 | 477.3026 | 952.5906 | 952.5957 | -0.0051 | 0 | 38 | 0.024 | 1 | R.LLLPGELAK.H 3740 3741 3742 |
|  | 6757 | 584.7971 | 1167.5797 | 1167.5884 | -0.0087 | 0 | 24 | 0.59 | 1 | K.QVHPDTGISSK.A 6753 6755 6788 |
|  | 7865 | **408.9092** | **1223.7059** | **1224.6172** | **-0.9113** | **1** | **4** | **57** | **6** | **-.MPDPSKSAPAPK.K** |
|  | 12311 | 730.8779 | 1459.7412 | 1458.7579 | 0.9833 | 1 | 6 | 34 | 1 | R.IASEASRLAHYNK.R |
|  | 13148 | 503.9497 | 1508.8273 | 1507.8358 | 0.9915 | 1 | 11 | 9.6 | 1 | K.VLKQVHPDTGISSK.A |
|  | 16889 | 872.4083 | 1742.8021 | 1742.8120 | -0.0099 | 0 | 106 | 2.7e-009 | 1 | K.AMGIMNSFVNDIFER.I 16885 16886 16887 16888 16890 16891 16893 16894 16895 16896 16897 16898 16899 16901 16902 16903 16904 16925 16927 16929 16931 16932 |
|  | 17112 | 880.4065 | 1758.7984 | 1758.8069 | -0.0085 | 0 | (80) | 9.8e-007 | 1 | K.AMGIMNSFVNDIFER.I 17113 |
|  | 17114 | 880.4066 | 1758.7987 | 1758.8069 | -0.0082 | 0 | (103) | 5.1e-009 | 1 | K.AMGIMNSFVNDIFER.I 17111 17125 17126 |

| **52.** | P53675\|CLH2_HUMAN    **Mass:** 189020   **Score:** 637    **Queries matched:** 42   **emPAI:** 0.31 |
| --- | --- |
|  | Clathrin heavy chain 2 OS=Homo sapiens GN=CLTCL1 PE=1 SV=2 |

|  | **Query** | **Observed** | **Mr(expt)** | **Mr(calc)** | **Delta** | **Miss** | **Score** | **Expect** | **Rank** | **Peptide** |
| --- | --- | --- | --- | --- | --- | --- | --- | --- | --- | --- |
|  | 2360 | 853.4170 | 852.4097 | 852.4164 | -0.0066 | 0 | 24 | 0.65 | 1 | K.YIQAACK.T |
|  | 2831 | 443.2058 | 884.3971 | 884.4028 | -0.0057 | 0 | 36 | 0.042 | 1 | R.AYEFAER.C |
|  | 5340 | 535.2728 | 1068.5310 | 1068.5386 | -0.0076 | 0 | 23 | 0.79 | 1 | R.AHIAQLCEK.A 5351 |
|  | 6913 | 590.3400 | 1178.6655 | 1177.6343 | 1.0312 | 1 | 2 | 91 | 2 | K.EGQFKDIITK.V |
|  | 8098 | 618.8328 | 1235.6510 | 1235.6584 | -0.0074 | 0 | 58 | 0.00022 | 1 | K.TLQIFNIEMK.S 8097 |
|  | 9476 | **653.3339** | **1304.6533** | **1303.7248** | **0.9285** | **1** | **0** | **1.3e+002** | **10** | **R.SNLAGAEKLFVR.K** |
|  | 9997 | 444.2090 | 1329.6053 | 1329.6136 | -0.0082 | 0 | 17 | 3.2 | 2 | R.DPHLACVAYER.G |
|  | 10110 | 669.3435 | 1336.6725 | 1336.6809 | -0.0085 | 0 | 81 | 1.2e-006 | 1 | R.VVGAMQLYSVDR.K |
|  | 10408 | 677.4234 | 1352.8322 | 1352.8391 | -0.0068 | 0 | 78 | 1.8e-006 | 1 | R.NLQNLLILTAIK.A 10441 |
|  | 10538 | **454.2352** | **1359.6838** | **1358.6102** | **1.0735** | **0** | **2** | **1e+002** | **9** | **R.ENAYYDSSVVGR.Y** |
|  | 11521 | 708.3626 | 1414.7105 | 1414.7205 | -0.0099 | 0 | 62 | 9e-005 | 1 | K.IVLDNSVFSEHR.N |
|  | 11624 | **711.3532** | **1420.6919** | **1419.7721** | **0.9197** | **1** | **15** | **4.3** | **2** | **K.DLVKEAINSYIR.G** |
|  | 12389 | 733.3902 | 1464.7658 | 1464.7759 | -0.0100 | 1 | 51 | 0.00096 | 1 | R.VVGAMQLYSVDRK.V |
|  | 12734 | 743.8596 | 1485.7046 | 1485.7147 | -0.0101 | 1 | 21 | 1.1 | 1 | K.RDPHLACVAYER.G |
|  | 13856 | 776.3754 | 1550.7362 | 1550.7464 | -0.0102 | 0 | 64 | 5.2e-005 | 1 | R.GQFSTDELVAEVEK.R 13857 |
|  | 13913 | **519.1365** | **1554.3878** | **1554.7501** | **-0.3623** | **0** | **4** | **29** | **3** | **K.VIQCFAETGQFQK.I** |
|  | 14159 | **524.2736** | **1569.7990** | **1570.8137** | **-1.0146** | **0** | **1** | **1e+002** | **5** | **R.RPISAESAIMNPASK.V** |
|  | 14498 | **531.3005** | **1590.8798** | **1590.7249** | **0.1549** | **0** | **1** | **89** | **7** | **K.GNNWWAQSVELCK.K** |
|  | 14739 | 804.4467 | 1606.8788 | 1606.8871 | -0.0084 | 1 | 80 | 1.2e-006 | 1 | K.KVGYTPDWIFLLR.G 14737 14738 |
|  | 16143 | 566.2503 | 1695.7291 | 1695.0043 | 0.7248 | 1 | 7 | 23 | 3 | R.NLQNLLILTAIKADR.T |
|  | 16201 | **567.2478** | **1698.7216** | **1699.9105** | **-1.1889** | **0** | **8** | **16** | **1** | **R.QLIDQVVQTALSETR.D** |
|  | 16319 | 854.4251 | 1706.8355 | 1706.8475 | -0.0120 | 1 | 93 | 5.3e-008 | 1 | R.GQFSTDELVAEVEKR.N |
|  | 16625 | **576.2828** | **1725.8267** | **1726.7984** | **-0.9718** | **0** | **2** | **64** | **5** | **K.IYIDSNNSPECFLR.E** 16624 |
|  | 18158 | **610.2988** | **1827.8745** | **1826.8808** | **0.9937** | **0** | **2** | **62** | **3** | **K.MEGNAKPATLFCFAVR.N** |
|  | 18951 | **629.9689** | **1886.8850** | **1885.8588** | **1.0262** | **1** | **3** | **54** | **4** | **K.DAMQHAAESRDAELAQK.L** |
|  | 19720 | 974.0068 | 1945.9991 | 1946.0070 | -0.0079 | 0 | 84 | 3.8e-007 | 1 | K.AFMTADLPNELIELLEK.I 19717 19718 19719 |
|  | 19859 | **653.3289** | **1956.9649** | **1957.9542** | **-0.9893** | **1** | **0** | **82** | **6** | **K.HQLMEFRCIAAYLYK.G** |
|  | 20474 | **672.3094** | **2013.9065** | **2014.0293** | **-0.1227** | **0** | **1** | **68** | **10** | **R.TPAVIGGLLDVDCSEEVIK.H** |
|  | 22932 | **755.0948** | **2262.2625** | **2261.1804** | **1.0821** | **1** | **3** | **38** | **6** | **R.TDEYQKWLLLVGISAQQNR.V** |
|  | 25567 | **528.0476** | **2635.2014** | **2634.3152** | **0.8862** | **1** | **1** | **55** | **10** | **K.KAVDVFFPPEAQNDFPVAMQIGAK.H** |
|  | 30092 | **629.6799** | **3772.0356** | **3770.8641** | **1.1715** | **1** | **2** | **20** | **2** | **K.VGEQAQVTIIDMSDPMAPIRRPISAESAIMNPASK.V** |
|  | 31715 | **1135.5403** | **5672.6650** | **5672.8694** | **-0.2044** | **1** | **3** | **5.1** | **1** | **R.AVVHTHLLNPEWLVNFFGSLSVEDSVECLHAMLSANIRQNLQLCVQVASK.Y** |

| **53.** | P12109\|CO6A1_HUMAN    **Mass:** 109602   **Score:** 624    **Queries matched:** 48   **emPAI:** 0.54 |
| --- | --- |
|  | Collagen alpha-1(VI) chain OS=Homo sapiens GN=COL6A1 PE=1 SV=3 |

|  | **Query** | **Observed** | **Mr(expt)** | **Mr(calc)** | **Delta** | **Miss** | **Score** | **Expect** | **Rank** | **Peptide** |
| --- | --- | --- | --- | --- | --- | --- | --- | --- | --- | --- |
|  | 879 | **533.1862** | **532.1789** | **532.2428** | **-0.0639** | **0** | **6** | **78** | **5** | **R.MPGGR.D** 880 883 |
|  | 1318 | 705.3721 | 704.3648 | 704.3705 | -0.0057 | 0 | 14 | 8.4 | 3 | K.SSVDAVK.Y 1314 |
|  | 1382 | **731.3697** | **730.3624** | **729.4021** | **0.9603** | **0** | **22** | **1.7** | **1** | **R.NVQELK.E** |
|  | 3571 | **472.2629** | **942.5112** | **943.4909** | **-0.9797** | **1** | **2** | **1.1e+002** | **5** | **R.MPGGRDALK.S** |
|  | 3803 | **479.2869** | **956.5592** | **956.5655** | **-0.0063** | **0** | **5** | **57** | **6** | **R.IALVITDGR.S** |
|  | 3827 | **480.2774** | **958.5402** | **958.5447** | **-0.0045** | **1** | **13** | **10** | **1** | **R.LSRDELVK.F** 3826 3830 |
|  | 3890 | **482.7321** | **963.4497** | **962.4094** | **1.0404** | **0** | **5** | **56** | **2** | **R.GDPGFEGER.G** |
|  | 4637 | **508.7995** | **1015.5844** | **1014.5458** | **1.0386** | **1** | **13** | **8.8** | **3** | **K.RGIDGVDGVK.G** |
|  | 4767 | **514.7359** | **1027.4572** | **1027.4644** | **-0.0072** | **0** | **5** | **53** | **3** | **K.GTYTDCAIK.K** |
|  | 5848 | **552.2617** | **1102.5088** | **1102.6386** | **-0.1299** | **0** | **9** | **24** | **10** | **R.LKPYGALVDK.V** |
|  | 6613 | **579.3049** | **1156.5952** | **1155.5594** | **1.0358** | **1** | **13** | **7.9** | **7** | **K.GTYTDCAIKK.G** 6592 |
|  | 8417 | **626.3288** | **1250.6430** | **1249.6415** | **1.0016** | **0** | **4** | **56** | **5** | **R.DQLLPPSPNNR.I** |
|  | 10343 | 676.3148 | 1350.6150 | 1349.7667 | 0.8483 | 0 | 10 | 15 | 2 | K.GLEQLLVGGSHLK.E |
|  | 12778 | **744.8646** | **1487.7147** | **1487.6562** | **0.0585** | **0** | **1** | **96** | **8** | **K.EPCGGLEDAVNEAK.H** |
|  | 13070 | **502.3394** | **1503.9963** | **1503.6702** | **0.3261** | **1** | **2** | **63** | **4** | **K.GYRGDEGPPGSEGAR.G** |
|  | 14305 | **527.6158** | **1579.8257** | **1579.8358** | **-0.0102** | **0** | **33** | **0.063** | **1** | **K.VFSVAITPDHLEPR.L** |
|  | 15361 | **550.2128** | **1647.6167** | **1647.7675** | **-0.1509** | **0** | **5** | **32** | **5** | **R.GAPGPAGPPGDPGLMGER.G** |
|  | 15921 | **841.4118** | **1680.8090** | **1680.6976** | **0.1115** | **0** | **1** | **80** | **5** | **K.GEAGDEGNPGPDGAPGER.G** |
|  | 17087 | **879.4041** | **1756.7937** | **1756.8056** | **-0.0120** | **0** | **72** | **6.2e-006** | **1** | **K.TAEYDVAYGESHLFR.V** 17088 17089 |
|  | 17293 | **592.3072** | **1773.8997** | **1773.9122** | **-0.0125** | **0** | **52** | **0.00071** | **1** | **R.VAVVQYSGTGQQRPER.A** 17294 |
|  | 19503 | **966.5009** | **1930.9871** | **1931.0000** | **-0.0128** | **0** | **65** | **3.2e-005** | **1** | **R.LLLFSDGNSQGATPAAIEK.A** 19502 19505 |
|  | 20235 | 996.4906 | 1990.9666 | 1990.9769 | -0.0102 | 0 | 96 | 2.6e-008 | 1 | R.DAEEAISQTIDTIVDMIK.N 20236 20237 20238 |
|  | 20302 | **999.4091** | **1996.8036** | **1996.8190** | **-0.0154** | **0** | **50** | **0.00082** | **1** | **K.NNVEQVCCSFECQPAR.G** |
|  | 21479 | **527.0283** | **2104.0842** | **2104.9922** | **-0.9080** | **1** | **3** | **42** | **8** | **R.GIDGVDGVKGEMGYPGLPGCK.G** |
|  | 21620 | **1059.0034** | **2115.9923** | **2116.0048** | **-0.0125** | **0** | **102** | **5.8e-009** | **1** | **K.SLQWMAGGTFTGEALQYTR.D** 21631 |
|  | 22294 | **1099.0916** | **2196.1685** | **2196.1824** | **-0.0139** | **0** | **66** | **1.7e-005** | **1** | **R.DTTPLNVLCSPGIQVVSVGIK.D** 22293 |
|  | 24849 | **632.8398** | **2527.3303** | **2528.1051** | **-0.7749** | **1** | **2** | **45** | **1** | **K.GYPGLKGDEGEAGDPGDDNNDIAPR.G** |
|  | 25830 | **891.7750** | **2672.3031** | **2672.2363** | **0.0667** | **1** | **2** | **36** | **3** | **K.GEMGYPGLPGCKGSPGFDGIQGPPGPK.G** |
|  | 28352 | **1058.5149** | **3172.5228** | **3172.5387** | **-0.0158** | **1** | **61** | **3.4e-005** | **1** | **K.YLIVVTDGHPLEGYKEPCGGLEDAVNEAK.H** |
|  | 29347 | **862.9139** | **3447.6267** | **3447.6517** | **-0.0251** | **1** | **9** | **5.1** | **1** | **R.DELVKFEPGQSYAGVVQYSHSQMQEHVSLR.S** |
|  | 29515 | **1172.2661** | **3513.7765** | **3513.7926** | **-0.0161** | **0** | **60** | **4.2e-005** | **1** | **K.DVFDFIPGSDQLNVISCQGLAPSQGRPGLSLVK.E** |
|  | 31737 | **1438.1550** | **5748.5910** | **5748.8118** | **-0.2208** | **1** | **1** | **6.8** | **8** | **R.VAVVQYSGTGQQRPERASLQFLQNYTALASAVDAMDFINDATDVNDALGYVTR.F** |

| **54.** | P27797\|CALR_HUMAN    **Mass:** 48283    **Score:** 606    **Queries matched:** 36   **emPAI:** 1.84 |
| --- | --- |
|  | Calreticulin OS=Homo sapiens GN=CALR PE=1 SV=1 |

|  | **Query** | **Observed** | **Mr(expt)** | **Mr(calc)** | **Delta** | **Miss** | **Score** | **Expect** | **Rank** | **Peptide** |
| --- | --- | --- | --- | --- | --- | --- | --- | --- | --- | --- |
|  | 1708 | **403.2270** | **804.4394** | **803.3409** | **1.0984** | **0** | **9** | **28** | **3** | **K.QDEEQR.L** |
|  | 4273 | **496.7345** | **991.4544** | **991.4611** | **-0.0066** | **0** | **12** | **10** | **3** | **R.QIDNPDYK.G** |
|  | 6427 | **573.7903** | **1145.5660** | **1146.6550** | **-1.0889** | **1** | **10** | **14** | **3** | **K.KVHVIFNYK.G** |
|  | 7748 | **609.8021** | **1217.5896** | **1217.6000** | **-0.0105** | **1** | **21** | **1.1** | **1** | **K.DKGLQTSQDAR.F** |
|  | 11433 | **705.8132** | **1409.6119** | **1409.6212** | **-0.0093** | **0** | **50** | **0.0013** | **1** | **K.EQFLDGDGWTSR.W** |
|  | 12551 | **738.8251** | **1475.6356** | **1475.6463** | **-0.0107** | **0** | **36** | **0.035** | **1** | **K.HEQNIDCGGGYVK.L** |
|  | 14727 | **804.3859** | **1606.7572** | **1606.7667** | **-0.0096** | **0** | **93** | **5.6e-008** | **1** | **R.FYALSASFEPFSNK.G** 14728 |
|  | 16345 | **570.3040** | **1707.8902** | **1708.6435** | **-0.7532** | **1** | **4** | **48** | **8** | **K.EEEEAEDKEDDEDK.D** |
|  | 16952 | **873.7910** | **1745.5675** | **1744.8533** | **0.7142** | **1** | **0** | **70** | **5** | **K.GEWKPRQIDNPDYK.G** |
|  | 17733 | **900.9169** | **1799.8193** | **1799.8326** | **-0.0133** | **1** | **69** | **1.3e-005** | **1** | **K.IKDPDASKPEDWDER.A** 17734 |
|  | 24794 | **1260.0929** | **2518.1712** | **2518.1864** | **-0.0151** | **1** | **59** | **8.2e-005** | **1** | **K.IDNSQVESGSLEDDWDFLPPKK.I** 24792 24793 24795 24805 |
|  | 26886 | **714.8480** | **2855.3627** | **2855.3800** | **-0.0173** | **1** | **32** | **0.033** | **1** | **R.CKDDEFTHLYTLIVRPDNTYEVK.I** |
|  | 27361 | **987.1309** | **2958.3708** | **2958.3883** | **-0.0175** | **1** | **43** | **0.003** | **1** | **R.AKIDDPTDSKPEDWDKPEHIPDPDAK.K** 27360 |
|  | 27365 | **987.4304** | **2959.2694** | **2959.2858** | **-0.0164** | **0** | **46** | **0.0011** | **1** | **K.KPEDWDEEMDGEWEPPVIQNPEYK.G** 27363 27364 |
|  | 28762 | **1634.7432** | **3267.4718** | **3267.4884** | **-0.0167** | **0** | **61** | **3.4e-005** | **1** | **K.SGTIFDNFLITNDEAYAEEFGNETWGVTK.A** 28763 28764 28766 |
|  | 29369 | **1152.8383** | **3455.4930** | **3455.5108** | **-0.0179** | **0** | **43** | **0.0016** | **1** | **K.LFPNSLDQTDMHGDSEYNIMFGPDICGPGTK.K** 29366 29368 29372 |
|  | 30673 | **1383.6632** | **4147.9678** | **4147.9844** | **-0.0166** | **0** | **58** | **4e-005** | **1** | **K.GTWIHPEIDNPEYSPDPSIYAYDNFGVLGLDLWQVK.S** 30670 30672 30674 |
|  | 31244 | **1639.0830** | **4914.2272** | **4913.1466** | **1.0806** | **1** | **6** | **4** | **1** | **K.HEQNIDCGGGYVKLFPNSLDQTDMHGDSEYNIMFGPDICGPGTK.K** |

| **55.** | P05362\|ICAM1_HUMAN    **Mass:** 58587    **Score:** 600    **Queries matched:** 32   **emPAI:** 1.09 |
| --- | --- |
|  | Intercellular adhesion molecule 1 OS=Homo sapiens GN=ICAM1 PE=1 SV=2 |

|  | **Query** | **Observed** | **Mr(expt)** | **Mr(calc)** | **Delta** | **Miss** | **Score** | **Expect** | **Rank** | **Peptide** |
| --- | --- | --- | --- | --- | --- | --- | --- | --- | --- | --- |
|  | 1316 | **704.4043** | **703.3970** | **703.4017** | **-0.0047** | **0** | **9** | **33** | **4** | **R.VLYGPR.L** |
|  | 4007 | **487.2253** | **972.4359** | **972.4447** | **-0.0088** | **0** | **25** | **0.62** | **1** | **R.CQVEGGAPR.A** 4012 |
|  | 4161 | 492.7443 | 983.4740 | 983.5764 | -0.1024 | 0 | 4 | 64 | 10 | K.VTVNVLSPR.Y |
|  | 4727 | **512.7834** | **1023.5522** | **1024.5665** | **-1.0143** | **0** | **6** | **38** | **6** | **K.ELLLPGNNR.K** |
|  | 5523 | **540.8333** | **1079.6519** | **1079.6590** | **-0.0071** | **0** | **58** | **0.00025** | **1** | **K.LLGIETPLPK.K** 5525 |
|  | 10144 | **670.3327** | **1338.6508** | **1338.6602** | **-0.0093** | **0** | **24** | **0.58** | **1** | **K.GTPMKPNTQATPP.-** |
|  | 11473 | **706.8597** | **1411.7049** | **1411.7136** | **-0.0087** | **0** | **45** | **0.004** | **1** | **K.TFLTVYWTPER.V** 11474 |
|  | 11588 | **473.9091** | **1418.7054** | **1417.8042** | **0.9012** | **0** | **10** | **13** | **1** | **K.VTLNGVPAQPLGPR.A** |
|  | 13347 | **760.9231** | **1519.8316** | **1519.8399** | **-0.0082** | **0** | **70** | **1.2e-005** | **1** | **R.VELAPLPSWQPVGK.N** 13348 13364 |
|  | 13418 | **509.2560** | **1524.7463** | **1524.8260** | **-0.0797** | **1** | **2** | **83** | **1** | **R.AQLLLKATPEDNGR.S** |
|  | 14158 | **785.9066** | **1569.7987** | **1569.8046** | **-0.0059** | **1** | **3** | **70** | **2** | **K.NLTLRCQVEGGAPR.A** |
|  | 14795 | **806.8914** | **1611.7683** | **1612.7733** | **-1.0050** | **0** | **4** | **49** | **6** | **R.LNPTVTYGNDSFSAK.A** |
|  | 14930 | **809.9619** | **1617.9091** | **1616.9362** | **0.9729** | **1** | **36** | **0.032** | **1** | **R.AKVTLNGVPAQPLGPR.A** |
|  | 16035 | **844.9420** | **1687.8694** | **1687.8781** | **-0.0088** | **0** | **62** | **7.2e-005** | **1** | **K.DGTFPLPIGESVTVTR.D** 16036 |
|  | 16123 | **848.3811** | **1694.7476** | **1694.7604** | **-0.0128** | **0** | **65** | **3.3e-005** | **1** | **R.GGSVLVTCSTSCDQPK.L** 16144 |
|  | 19382 | **962.5222** | **1923.0298** | **1923.0425** | **-0.0128** | **1** | **83** | **5.2e-007** | **1** | **K.REPAVGEPAEVTTTVLVR.R** 19381 19383 19384 |
|  | 20897 | **684.3404** | **2049.9993** | **2051.0470** | **-1.0476** | **1** | **3** | **44** | **8** | **R.LQQAQKGTPMKPNTQATPP.-** |
|  | 27161 | **1457.8306** | **2913.6466** | **2913.5429** | **0.1037** | **1** | **6** | **11** | **1** | **K.TFLTVYWTPERVELAPLPSWQPVGK.N** |
|  | 28057 | **1554.1454** | **3106.2762** | **3106.2954** | **-0.0192** | **0** | **81** | **2.9e-007** | **1** | **K.VYELSNVQEDSQPMCYSNCPDGQSTAK.T** 28055 28056 |
|  | 28673 | **1079.1292** | **3234.3656** | **3234.3904** | **-0.0247** | **1** | **53** | **0.00018** | **1** | **R.KVYELSNVQEDSQPMCYSNCPDGQSTAK.T** |

| **56.** | Q13813\|SPTA2_HUMAN    **Mass:** 285163   **Score:** 594    **Queries matched:** 79   **emPAI:** 0.33 |
| --- | --- |
|  | Spectrin alpha chain, brain OS=Homo sapiens GN=SPTAN1 PE=1 SV=3 |

|  | **Query** | **Observed** | **Mr(expt)** | **Mr(calc)** | **Delta** | **Miss** | **Score** | **Expect** | **Rank** | **Peptide** |
| --- | --- | --- | --- | --- | --- | --- | --- | --- | --- | --- |
|  | 1233 | 674.4033 | 673.3960 | 673.3759 | 0.0201 | 0 | 14 | 10 | 3 | R.DALLSR.R |
|  | 2646 | **437.2705** | **872.5264** | **871.4399** | **1.0865** | **0** | **2** | **1.3e+002** | **9** | **K.DPTNLQGK.L** |
|  | 2729 | **440.2218** | **878.4291** | **877.3712** | **1.0579** | **0** | **3** | **88** | **2** | **R.EQAQSCR.Q** |
|  | 2970 | **450.2784** | **898.5423** | **897.5760** | **0.9664** | **1** | **9** | **20** | **7** | **R.LKGLALQR.Q** |
|  | 3199 | **458.2517** | **914.4889** | **913.5345** | **0.9544** | **1** | **18** | **2.7** | **6** | **K.KQEALVAR.Y** 3190 3195 3207 3210 |
|  | 3362 | **464.2291** | **926.4437** | **926.4498** | **-0.0061** | **1** | **26** | **0.44** | **2** | **K.KFDDFQK.D** |
|  | 3962 | **485.7686** | **969.5227** | **969.5468** | **-0.0240** | **1** | **14** | **6.6** | **2** | **K.QLAAARGQR.L** |
|  | 4243 | **495.7684** | **989.5222** | **989.5294** | **-0.0072** | **0** | **19** | **2.6** | **1** | **K.LFGAAEVQR.F** |
|  | 4617 | 508.2632 | 1014.5119 | 1014.5458 | -0.0339 | 0 | 9 | 21 | 5 | R.DLTGVQNLR.K |
|  | 5305 | 533.2732 | 1064.5318 | 1065.4978 | -0.9660 | 0 | 3 | 84 | 10 | R.EAFLNTEDK.G |
|  | 5403 | **536.7878** | **1071.5611** | **1071.5672** | **-0.0061** | **1** | **(8)** | **30** | **1** | **K.KLDPAQSASR.E** 5425 |
|  | 5760 | **549.4134** | **1096.8122** | **1096.5560** | **0.2563** | **1** | **6** | **24** | **7** | **R.NALHERAMR.R** |
|  | 5822 | **551.3074** | **1100.6002** | **1100.6077** | **-0.0075** | **1** | **11** | **13** | **1** | **R.DLAALEDKVK.A** |
|  | 6073 | **560.3048** | **1118.5951** | **1118.5794** | **0.0157** | **0** | **5** | **52** | **1** | **R.MNEVISLWK.K** |
|  | 6649 | **580.3381** | **1158.6617** | **1159.6561** | **-0.9944** | **1** | **1** | **1.3e+002** | **2** | **R.ITKEAGSVSLR.M** |
|  | 7717 | **608.8216** | **1215.6286** | **1215.5632** | **0.0654** | **0** | **3** | **73** | **10** | **K.QDEVNAAWQR.L** |
|  | 7946 | **615.0615** | **1228.1084** | **1227.6724** | **0.4360** | **1** | **6** | **29** | **8** | **K.KLLEAQSHFR.K** |
|  | 8632 | **631.3717** | **1260.7288** | **1261.5799** | **-0.8511** | **0** | **7** | **27** | **2** | **R.EANQQQQFNR.N** |
|  | 8975 | **640.3495** | **1278.6844** | **1278.6819** | **0.0025** | **0** | **13** | **7.4** | **4** | **R.ALSSEGKPYVTK.E** |
|  | 9406 | **651.8259** | **1301.6372** | **1301.6463** | **-0.0091** | **0** | **62** | **8.4e-005** | **1** | **K.VLETAEDIQER.R** |
|  | 9548 | **655.8680** | **1309.7214** | **1309.7282** | **-0.0068** | **1** | **9** | **19** | **1** | **R.KVEDLFLTFAK.K** |
|  | 9856 | **662.3348** | **1322.6550** | **1321.6700** | **0.9850** | **1** | **8** | **21** | **6** | **R.YEALKEPMVAR.K** 9797 |
|  | 9869 | **442.2342** | **1323.6809** | **1323.6895** | **-0.0086** | **0** | **19** | **1.8** | **1** | **R.SQLLGSAHEVQR.F** |
|  | 10572 | **454.9035** | **1361.6888** | **1360.7173** | **0.9715** | **1** | **8** | **24** | **4** | **R.QWELLLEKMR.E** |
|  | 12030 | **482.2711** | **1443.7914** | **1444.7059** | **-0.9145** | **1** | **6** | **34** | **6** | **K.TKQDEVNAAWQR.L** |
|  | 12262 | 486.8632 | 1457.5677 | 1456.7422 | 0.8255 | 1 | 5 | 36 | 3 | K.QDEVNAAWQRLK.G |
|  | 12903 | **748.3705** | **1494.7265** | **1494.7361** | **-0.0096** | **0** | **45** | **0.004** | **1** | **R.MQHNLEQQIQAR.N** |
|  | 13010 | **751.3626** | **1500.7107** | **1500.7208** | **-0.0102** | **0** | **44** | **0.0052** | **1** | **R.EANELQQWINEK.E** 13042 |
|  | 13533 | **766.3851** | **1530.7557** | **1530.7651** | **-0.0094** | **1** | **70** | **1.1e-005** | **1** | **K.LREANQQQQFNR.N** |
|  | 13931 | **519.5986** | **1555.7741** | **1555.7855** | **-0.0114** | **0** | **37** | **0.024** | **1** | **K.HQALQAEIAGHEPR.I** |
|  | 14419 | **794.3712** | **1586.7279** | **1586.7437** | **-0.0158** | **0** | **15** | **3.9** | **1** | **K.HQAFEAELSANQSR.I** |
|  | 14690 | **803.4780** | **1604.9415** | **1604.8409** | **0.1006** | **0** | **7** | **24** | **8** | **K.LIQEQHPEEELIK.T** |
|  | 14990 | **541.6070** | **1621.7991** | **1621.8100** | **-0.0109** | **1** | **22** | **0.69** | **1** | **R.FNRDVDETISWIK.E** |
|  | 15164 | **545.6002** | **1633.7787** | **1632.7856** | **0.9931** | **0** | **50** | **0.0013** | **1** | **K.HQLLEADISAHEDR.L** |
|  | 15870 | **839.4625** | **1676.9104** | **1675.7689** | **1.1415** | **1** | **10** | **11** | **1** | **K.HEDFEKSLSAQEEK.I** |
|  | 15968 | **421.9744** | **1683.8685** | **1683.8805** | **-0.0120** | **1** | **11** | **9.6** | **3** | **K.KHQALQAEIAGHEPR.I** 15970 |
|  | 16258 | **852.3828** | **1702.7511** | **1702.7621** | **-0.0110** | **0** | **51** | **0.00084** | **1** | **K.GNAMVEEGHFAAEDVK.A** |
|  | 16581 | **575.2842** | **1722.8307** | **1722.8424** | **-0.0117** | **0** | **30** | **0.12** | **1** | **R.LIQSHPESAEDLQEK.C** |
|  | 16942 | **873.4305** | **1744.8464** | **1744.8591** | **-0.0127** | **1** | **43** | **0.0057** | **1** | **K.LSDDNTIGKEEIQQR.L** 16941 |
|  | 16950 | **582.8298** | **1745.4675** | **1745.8367** | **-0.3692** | **1** | **3** | **35** | **8** | **K.DRVNDVCTNGQDLIK.K** |
|  | 17610 | 896.4360 | 1790.8574 | 1789.9509 | 0.9065 | 1 | 1 | 84 | 7 | K.SARLMVHTVATFNSIK.E |
|  | 18525 | **618.3607** | **1852.0602** | **1852.9869** | **-0.9267** | **1** | **8** | **17** | **2** | **R.LMELHRQWELLLEK.M** |
|  | 18595 | **929.9736** | **1857.9326** | **1857.9432** | **-0.0106** | **1** | **67** | **2.3e-005** | **1** | **R.DLAALGDKVNSLGETAER.L** 18594 |
|  | 19307 | **639.9464** | **1916.8174** | **1917.8891** | **-1.0717** | **1** | **2** | **57** | **7** | **K.GNAMVEEGHFAAEDVKAK.L** |
|  | 19767 | **976.4661** | **1950.9177** | **1950.9283** | **-0.0106** | **0** | **112** | **5.9e-010** | **1** | **R.SSLSSAQADFNQLAELDR.Q** |
|  | 20514 | **1009.0467** | **2016.0788** | **2015.9834** | **0.0955** | **1** | **5** | **29** | **4** | **-.MDPSGVKVLETAEDIQER.R** |
|  | 20601 | **675.6585** | **2023.9537** | **2023.9698** | **-0.0161** | **1** | **28** | **0.17** | **1** | **K.LQTASDESYKDPTNIQSK.H** 20602 |
|  | 21049 | **688.3290** | **2061.9651** | **2061.9751** | **-0.0100** | **1** | **5** | **26** | **2** | **R.NTTGVTEEALKEFSMMFK.H** |
|  | 21389 | **1048.5225** | **2095.0304** | **2094.9469** | **0.0835** | **1** | **2** | **61** | **7** | **R.LHQFFRDMDDEESWIK.E** |
|  | 21618 | **706.3287** | **2115.9642** | **2117.0939** | **-1.1297** | **1** | **2** | **51** | **2** | **R.LMVHTVATFNSIKELNER.W** |
|  | 21698 | **1064.0452** | **2126.0758** | **2126.0855** | **-0.0097** | **0** | **41** | **0.0059** | **1** | **K.ALINADELASDVAGAEALLDR.H** |
|  | 23179 | **765.3810** | **2293.1213** | **2293.1325** | **-0.0112** | **1** | **9** | **10** | **2** | **R.EAFLNTEDKGDSLDSVEALIK.K** |
|  | 23211 | **1149.5127** | **2297.0108** | **2297.0236** | **-0.0128** | **0** | **51** | **0.00059** | **1** | **K.ASAFNSWFENAEEDLTDPVR.C** |
|  | 23472 | **1166.5894** | **2331.1642** | **2330.0677** | **1.0964** | **0** | **40** | **0.0074** | **1** | **R.VASNPYTWFTMEALEETWR.N** |
|  | 23597 | **588.3026** | **2349.1811** | **2350.1587** | **-0.9776** | **1** | **10** | **7.1** | **1** | **K.EQLMASDDFGRDLASVQALLR.K** |
|  | 24140 | **809.4175** | **2425.2306** | **2425.2489** | **-0.0182** | **1** | **37** | **0.014** | **1** | **R.ALSSEGKPYVTKEELYQNLTR.E** |
|  | 24348 | **819.1068** | **2454.2984** | **2455.2204** | **-0.9219** | **1** | **1** | **59** | **3** | **K.NQALNTDNYGHDLASVQALQRK.H** |
|  | 24703 | **835.4614** | **2503.3623** | **2503.1384** | **0.2238** | **1** | **17** | **1.2** | **1** | **K.DLNSQADSLMTSSAFDTSQVKDK.R** |
|  | 25326 | **647.3106** | **2585.2133** | **2586.2827** | **-1.0693** | **1** | **2** | **43** | **5** | **R.IDGITIQARQFQDAGHFDAENIK.K** 25325 |
|  | 25869 | **670.0861** | **2676.3154** | **2676.2146** | **0.1008** | **0** | **11** | **5.1** | **1** | **R.TYLLDGSCMVEESGTLESQLEATK.R** |
|  | 26314 | **918.4498** | **2752.3275** | **2752.3443** | **-0.0168** | **1** | **18** | **0.87** | **1** | **R.QQVAPTDDETGKELVLALYDYQEK.S** |
|  | 27600 | **1001.1124** | **3000.3155** | **3000.3308** | **-0.0153** | **1** | **68** | **7.6e-006** | **1** | **R.RQDLEDSLQAQQYFADANEAESWMR.E** 27599 |
|  | 28038 | 776.3527 | 3101.3818 | 3101.3672 | 0.0146 | 1 | 2 | 29 | 10 | R.QDLEDSLQAQQYFADANEAESWMREK.E |
|  | 30024 | **749.1156** | **3740.5416** | **3739.8428** | **0.6988** | **1** | **3** | **15** | **4** | **R.EANELQQWINEKEAALTSEEVGADLEQVEVLQK.K** |
|  | 30231 | **1283.2867** | **3846.8384** | **3846.8588** | **-0.0204** | **1** | **83** | **1.7e-007** | **1** | **R.NVEDIELWLYEVEGHLASDDYGKDLTNVQNLQK.K** 30232 |

| **57.** | Q86VB7\|C163A_HUMAN    **Mass:** 129575   **Score:** 589    **Queries matched:** 41   **emPAI:** 0.48 |
| --- | --- |
|  | Scavenger receptor cysteine-rich type 1 protein M130 OS=Homo sapiens GN=CD163 PE=1 SV=1 |

|  | **Query** | **Observed** | **Mr(expt)** | **Mr(calc)** | **Delta** | **Miss** | **Score** | **Expect** | **Rank** | **Peptide** |
| --- | --- | --- | --- | --- | --- | --- | --- | --- | --- | --- |
|  | 1384 | **731.3995** | **730.3923** | **730.3973** | **-0.0051** | **0** | **15** | **7.5** | **1** | **K.GADLSLR.L** |
|  | 2311 | **425.2055** | **848.3964** | **847.3858** | **1.0106** | **0** | **12** | **14** | **1** | **K.EADVVCR.Q** 2294 |
|  | 2401 | **428.9195** | **855.8244** | **856.4654** | **-0.6410** | **0** | **13** | **6.5** | **6** | **K.INPASLDK.A** |
|  | 3456 | **467.2396** | **932.4647** | **933.4702** | **-1.0055** | **1** | **11** | **14** | **1** | **K.CSGRVEVK.V** |
|  | 3465 | **467.2887** | **932.5628** | **932.4749** | **0.0878** | **0** | **11** | **15** | **4** | **R.QLGCGSALK.T** |
|  | 4929 | **519.8588** | **1037.7030** | **1036.5263** | **1.1767** | **0** | **2** | **77** | **2** | **K.FLPISGMEK.E** |
|  | 5005 | **522.2740** | **1042.5335** | **1041.5818** | **0.9517** | **1** | **7** | **34** | **7** | **K.GKINPASLDK.A** |
|  | 7152 | **596.2704** | **1190.5263** | **1190.5350** | **-0.0086** | **0** | **46** | **0.0042** | **1** | **R.LQEGPTSCSGR.V** 7151 7153 7154 |
|  | 7177 | **596.7811** | **1191.5477** | **1191.5554** | **-0.0077** | **0** | **48** | **0.0023** | **1** | **R.LVDGVTECSGR.L** 7157 |
|  | 7429 | **602.2694** | **1202.5243** | **1202.5325** | **-0.0082** | **0** | **10** | **17** | **1** | **R.IWMDHVSCR.G** |
|  | 7878 | **409.2167** | **1224.6284** | **1223.5499** | **1.0785** | **1** | **7** | **31** | **5** | **R.LTRGGNMCSGR.I** |
|  | 7897 | **613.8018** | **1225.5891** | **1225.5986** | **-0.0095** | **1** | **9** | **17** | **3** | **K.ITCSAHREPR.L** 7901 |
|  | 10207 | **672.3541** | **1342.6936** | **1342.7027** | **-0.0092** | **0** | **55** | **0.00046** | **1** | **K.QLGCPTAVTAIGR.V** |
|  | 10472 | **678.8497** | **1355.6849** | **1355.6946** | **-0.0097** | **0** | **51** | **0.0012** | **1** | **R.GENLVHQIQYR.E** |
|  | 11467 | **706.8237** | **1411.6328** | **1411.6436** | **-0.0108** | **0** | **65** | **3.9e-005** | **1** | **K.SSMSETTVGVVCR.Q** |
|  | 12284 | **487.2831** | **1458.8273** | **1459.7202** | **-0.8928** | **1** | **12** | **8.3** | **2** | **K.IRLQEGPTSCSGR.V** 12286 12334 |
|  | 13197 | **504.8067** | **1511.3983** | **1511.7515** | **-0.3532** | **1** | **4** | **36** | **7** | **R.EPRLVGGDIPCSGR.V** |
|  | 13803 | **517.2366** | **1548.6881** | **1548.7090** | **-0.0209** | **0** | **2** | **84** | **7** | **K.EDAAVNCTDISVQK.T** |
|  | 16402 | **856.8534** | **1711.6923** | **1711.7043** | **-0.0119** | **0** | **89** | **1.2e-007** | **1** | **K.HNCDHAEDAGVICSK.G** 16401 16403 16404 |
|  | 16572 | **862.4045** | **1722.7945** | **1723.8563** | **-1.0618** | **1** | **2** | **71** | **3** | **K.LRMVLLEDSGSADFR.R** |
|  | 18792 | **937.9617** | **1873.9089** | **1873.9210** | **-0.0121** | **0** | **60** | **9.1e-005** | **1** | **K.EAEFGQGTGPIWLNEVK.C** 18791 18796 |
|  | 19786 | **651.9453** | **1952.8141** | **1952.8271** | **-0.0130** | **0** | **31** | **0.073** | **1** | **R.IWQCHSHGWGQQNCR.H** |
|  | 24985 | **850.3463** | **2548.0169** | **2548.0343** | **-0.0174** | **0** | **101** | **4.4e-009** | **1** | **K.HYCNHNEDAGVTCSDGSDLELR.L** |
|  | 25974 | **900.0757** | **2697.2052** | **2697.2217** | **-0.0165** | **0** | **24** | **0.25** | **1** | **K.GFGHIWLDSVSCQGHEPAVWQCK.H** |
|  | 27796 | **1013.4735** | **3037.3987** | **3037.4175** | **-0.0188** | **0** | **44** | **0.0019** | **1** | **K.TLGAWGSLCNSHWDIEDAHVLCQQLK.C** |
|  | 28525 | **1069.4630** | **3205.3672** | **3205.3870** | **-0.0198** | **0** | **46** | **0.001** | **1** | **R.VEIYHEGSWGTICDDSWDLSDAHVVCR.Q** |
|  | 30525 | **1353.6010** | **4057.7810** | **4057.8034** | **-0.0224** | **0** | **72** | **1.8e-006** | **1** | **R.VEIWHGGSWGTVCDDSWDLDDAQVVCQQLGCGPALK.A** 30524 |

| **58.** | Q9BYX7\|ACTBM_HUMAN    **Mass:** 42331    **Score:** 584    **Queries matched:** 59   **emPAI:** 0.97 |
| --- | --- |
|  | Beta-actin-like protein 3 OS=Homo sapiens GN=ACTBL3 PE=1 SV=1 |

|  | **Query** | **Observed** | **Mr(expt)** | **Mr(calc)** | **Delta** | **Miss** | **Score** | **Expect** | **Rank** | **Peptide** |
| --- | --- | --- | --- | --- | --- | --- | --- | --- | --- | --- |
|  | 1138 | 632.2894 | 631.2822 | 630.3701 | 0.9121 | 0 | 14 | 11 | 1 | K.ILTER.G 1129 1139 |
|  | 1169 | 644.3676 | 643.3603 | 643.3653 | -0.0050 | 0 | 25 | 0.84 | 1 | R.LDLAGR.E |
|  | 2744 | **440.7445** | **879.4745** | **878.5953** | **0.8792** | **1** | **5** | **49** | **8** | **K.IKIIAPPK.R** 2748 |
|  | 9981 | **443.7373** | **1328.1900** | **1328.5424** | **-0.3524** | **0** | **5** | **34** | **6** | **R.HQGMMEGMHQK.E** |
|  | 13245 | 758.3734 | 1514.7323 | 1514.7419 | -0.0096 | 0 | 45 | 0.0039 | 1 | K.IWHHTFYNELR.V 13243 13244 13248 13267 |
|  | 13260 | 758.8502 | 1515.6858 | 1515.6954 | -0.0096 | 0 | 50 | 0.0014 | 1 | K.QEYDESGPSIVHR.K 13259 13261 |
|  | 15307 | 548.9338 | 1643.7795 | 1643.7903 | -0.0108 | 1 | 25 | 0.34 | 1 | K.QEYDESGPSIVHRK.C 15309 |
|  | 17562 | 895.9443 | 1789.8740 | 1789.8846 | -0.0106 | 0 | 72 | 7.6e-006 | 1 | K.SYELPDGQVITIGNER.F 17551 17553 17560 17561 17563 17564 17565 17566 17568 17569 17571 17572 17573 17574 17576 17577 17578 17579 17580 17581 17582 17583 17584 17585 17586 17587 17588 17591 17592 17593 17595 17596 17609 17610 17612 17613 17614 17616 17617 |
|  | 24032 | **804.0716** | **2409.1929** | **2410.1951** | **-1.0022** | **1** | **1** | **60** | **1** | **R.KDLYTNTVLSGGTTMYPGIAHR.M** |
|  | 29364 | **1152.1793** | **3453.5161** | **3453.6129** | **-0.0968** | **1** | **2** | **24** | **3** | **K.AGFAGDDAPQAVFPSIVGRPRHQGMMEGMHQK.E** |

| **59.** | P07237\|PDIA1_HUMAN    **Mass:** 57480    **Score:** 583    **Queries matched:** 45   **emPAI:** 3.23 |
| --- | --- |
|  | Protein disulfide-isomerase OS=Homo sapiens GN=P4HB PE=1 SV=3 |

|  | **Query** | **Observed** | **Mr(expt)** | **Mr(calc)** | **Delta** | **Miss** | **Score** | **Expect** | **Rank** | **Peptide** |
| --- | --- | --- | --- | --- | --- | --- | --- | --- | --- | --- |
|  | 1424 | **753.3718** | **752.3646** | **752.3705** | **-0.0059** | **0** | **30** | **0.16** | **1** | **K.TAAESFK.G** |
|  | 2487 | **431.7343** | **861.4541** | **861.4596** | **-0.0055** | **0** | **9** | **26** | **2** | **K.ALAPEYAK.A** |
|  | 3379 | **928.5187** | **927.5114** | **927.5178** | **-0.0064** | **0** | **23** | **0.76** | **1** | **K.VHSFPTLK.F** 3378 |
|  | 3517 | **469.7464** | **937.4783** | **937.4869** | **-0.0086** | **1** | **23** | **0.68** | **1** | **K.TAAESFKGK.I** |
|  | 3864 | **481.7262** | **961.4379** | **961.4440** | **-0.0061** | **0** | **33** | **0.1** | **1** | **R.ITEFCHR.F** |
|  | 3962 | 485.7686 | 969.5227 | 969.5284 | -0.0056 | 0 | 33 | 0.07 | 1 | K.QLAPIWDK.L |
|  | 4203 | **494.7431** | **987.4716** | **987.4807** | **-0.0091** | **1** | **12** | **12** | **2** | **K.KEECPAVR.L** |
|  | 5315 | **533.7586** | **1065.5027** | **1065.5091** | **-0.0064** | **0** | **34** | **0.065** | **1** | **R.TVIDYNGER.T** |
|  | 5542 | **541.3384** | **1080.6622** | **1080.6695** | **-0.0073** | **0** | **8** | **23** | **1** | **K.THILLFLPK.S** |
|  | 7417 | **601.8023** | **1201.5901** | **1201.5979** | **-0.0078** | **0** | **30** | **0.15** | **1** | **R.EADDIVNWLK.K** |
|  | 9096 | **429.5638** | **1285.6694** | **1285.6778** | **-0.0084** | **1** | **37** | **0.03** | **1** | **R.KSNFAEALAAHK.Y** 9102 |
|  | 9227 | **647.2993** | **1292.5840** | **1292.5918** | **-0.0078** | **0** | **34** | **0.055** | **1** | **K.MDSTANEVEAVK.V** |
|  | 10187 | **448.2133** | **1341.6182** | **1340.6248** | **0.9934** | **1** | **3** | **63** | **8** | **K.NFEDVAFDEKK.N** |
|  | 11696 | **712.8881** | **1423.7617** | **1423.7711** | **-0.0094** | **1** | **1** | **97** | **4** | **K.YQLDKDGVVLFK.K** |
|  | 12155 | **726.3496** | **1450.6847** | **1450.6939** | **-0.0093** | **0** | **26** | **0.29** | **1** | **K.YKPESEELTAER.I** 12151 12154 |
|  | 16682 | **865.4530** | **1728.8914** | **1728.9046** | **-0.0132** | **1** | **44** | **0.0044** | **1** | **K.LGETYKDHENIVIAK.M** 16683 16684 |
|  | 17411 | **890.9153** | **1779.8161** | **1779.8275** | **-0.0114** | **0** | **65** | **3.2e-005** | **1** | **K.VDATEESDLAQQYGVR.G** 17410 |
|  | 18115 | 913.4808 | 1824.9471 | 1825.0866 | -0.1395 | 1 | 3 | 49 | 7 | K.IFGGEIKTHILLFLPK.S |
|  | 18234 | **917.4551** | **1832.8956** | **1832.9057** | **-0.0101** | **0** | **68** | **1.9e-005** | **1** | **K.ILFIFIDSDHTDNQR.I** 18232 18233 |
|  | 18861 | **940.5453** | **1879.0761** | **1878.9112** | **0.1650** | **1** | **12** | **6.6** | **1** | **K.EYTAGREADDIVNWLK.K** |
|  | 19955 | **983.0201** | **1964.0256** | **1964.0367** | **-0.0111** | **0** | **61** | **6.8e-005** | **1** | **K.HNQLPLVIEFTEQTAPK.I** 19954 19957 |
|  | 22354 | **735.3690** | **2203.0852** | **2202.0991** | **0.9862** | **1** | **30** | **0.079** | **1** | **K.MDSTANEVEAVKVHSFPTLK.F** |
|  | 24105 | **605.3134** | **2417.2246** | **2417.2413** | **-0.0167** | **1** | **39** | **0.0099** | **1** | **K.IKPHLMSQELPEDWDKQPVK.V** 24104 |
|  | 26135 | **1357.1636** | **2712.3126** | **2712.3283** | **-0.0157** | **0** | **77** | **1.3e-006** | **1** | **K.QFLQAAEAIDDIPFGITSNSDVFSK.Y** 26136 |
|  | 27243 | **1468.2432** | **2934.4718** | **2934.4862** | **-0.0145** | **0** | **72** | **3.3e-006** | **1** | **R.TGPAATTLPDGAAAESLVESSEVAVIGFFK.D** 27236 27244 |
|  | 27960 | **771.1433** | **3080.5441** | **3081.4629** | **-0.9188** | **1** | **4** | **18** | **3** | **K.SNFAEALAAHKYLLVEFYAPWCGHCK.A** |
|  | 30076 | **1256.2849** | **3765.8329** | **3765.8473** | **-0.0144** | **1** | **54** | **0.00015** | **1** | **R.TGPAATTLPDGAAAESLVESSEVAVIGFFKDVESDSAK.Q** 30074 30075 30077 |

| **60.** | P45880\|VDAC2_HUMAN    **Mass:** 32060    **Score:** 574    **Queries matched:** 27   **emPAI:** 2.04 |
| --- | --- |
|  | Voltage-dependent anion-selective channel protein 2 OS=Homo sapiens GN=VDAC2 PE=1 SV=2 |

|  | **Query** | **Observed** | **Mr(expt)** | **Mr(calc)** | **Delta** | **Miss** | **Score** | **Expect** | **Rank** | **Peptide** |
| --- | --- | --- | --- | --- | --- | --- | --- | --- | --- | --- |
|  | 2304 | **424.7368** | **847.4590** | **847.4651** | **-0.0061** | **0** | **24** | **0.85** | **1** | **K.VTGTLETK.Y** 2301 2302 2310 |
|  | 3538 | **470.7320** | **939.4494** | **939.4563** | **-0.0068** | **0** | **19** | **1.8** | **1** | **R.NNFAVGYR.T** 3526 |
|  | 4656 | **509.3012** | **1016.5879** | **1015.5914** | **0.9965** | **0** | **59** | **0.00023** | **1** | **K.LTLSALVDGK.S** 4637 |
|  | 8963 | **427.0746** | **1278.2019** | **1278.7336** | **-0.5317** | **1** | **1** | **74** | **9** | **K.GFGFGLVKLDVK.T** |
|  | 11751 | **714.8492** | **1427.6838** | **1427.6933** | **-0.0095** | **0** | **41** | **0.011** | **1** | **K.LTFDTTFSPNTGK.K** |
|  | 13933 | **778.8956** | **1555.7766** | **1555.7882** | **-0.0117** | **1** | **24** | **0.48** | **1** | **K.LTFDTTFSPNTGKK.S** 13919 13934 |
|  | 19189 | **954.3937** | **1906.7728** | **1906.7851** | **-0.0123** | **0** | **101** | **6.9e-009** | **1** | **K.SCSGVEFSTSGSSNTDTGK.V** 19188 |
|  | 21457 | 1052.0740 | 2102.1334 | 2102.1484 | -0.0150 | 0 | 97 | 1.8e-008 | 1 | K.VNNSSLIGVGYTQTLRPGVK.L 21456 21458 21459 21460 |
|  | 24798 | **1260.0999** | **2518.1851** | **2518.2010** | **-0.0158** | **0** | **103** | **3.2e-009** | **1** | **K.WNTDNTLGTEIAIEDQICQGLK.L** 24799 24800 |
|  | 24844 | **1264.5806** | **2527.1466** | **2527.1616** | **-0.0150** | **0** | **80** | **6.4e-007** | **1** | **R.TGDFQLHTNVNDGTEFGGSIYQK.V** 24843 |
|  | 26195 | **910.7560** | **2729.2463** | **2728.2996** | **0.9467** | **1** | **0** | **60** | **3** | **M.ATHGQTCARPMCIPPSYADLGKAAR.D** |
|  | 26234 | **913.0809** | **2736.2210** | **2736.2396** | **-0.0187** | **1** | **36** | **0.016** | **1** | **K.SCSGVEFSTSGSSNTDTGKVTGTLETK.Y** |

| **61.** | P02549\|SPTA1_HUMAN    **Mass:** 281023   **Score:** 541    **Queries matched:** 87   **emPAI:** 0.42 |
| --- | --- |
|  | Spectrin alpha chain, erythrocyte OS=Homo sapiens GN=SPTA1 PE=1 SV=4 |

|  | **Query** | **Observed** | **Mr(expt)** | **Mr(calc)** | **Delta** | **Miss** | **Score** | **Expect** | **Rank** | **Peptide** |
| --- | --- | --- | --- | --- | --- | --- | --- | --- | --- | --- |
|  | 1848 | **408.7371** | **815.4597** | **815.4654** | **-0.0056** | **1** | **4** | **92** | **9** | **R.VLDRWK.A** |
|  | 1861 | **409.2279** | **816.4412** | **815.4388** | **1.0023** | **0** | **5** | **72** | **9** | **K.IEALNEK.T** |
|  | 2262 | **423.2150** | **844.4155** | **843.4450** | **0.9705** | **0** | **3** | **1.1e+002** | **3** | **K.AQLIDER.T** |
|  | 2568 | **434.7632** | **867.5118** | **866.4610** | **1.0508** | **0** | **11** | **12** | **1** | **K.AHIEELR.H** 2566 |
|  | 2862 | **445.0335** | **888.0523** | **887.4712** | **0.5811** | **0** | **12** | **9.5** | **2** | **R.IQEITER.G** |
|  | 2898 | **446.7746** | **891.5346** | **890.4280** | **1.1066** | **0** | **7** | **42** | **9** | **R.QESLVCR.F** |
|  | 3325 | **462.2711** | **922.5277** | **922.5348** | **-0.0071** | **1** | **15** | **4.6** | **1** | **K.KHQLLER.E** |
|  | 4833 | 516.7601 | 1031.5056 | 1031.5135 | -0.0079 | 0 | 8 | 29 | 5 | K.ETVVESSGPK.V 4868 |
|  | 5184 | **529.2873** | **1056.5601** | **1056.5676** | **-0.0074** | **0** | **16** | **4.6** | **1** | **K.ALSNAANLQR.F** |
|  | 6061 | **560.2421** | **1118.4697** | **1117.5662** | **0.9035** | **1** | **26** | **0.38** | **1** | **R.ARQESLVCR.F** 6050 |
|  | 6131 | **562.3108** | **1122.6071** | **1122.6145** | **-0.0074** | **0** | **40** | **0.016** | **1** | **K.HGLLESAVAAR.Q** 6135 |
|  | 6157 | **563.2993** | **1124.5841** | **1124.5938** | **-0.0097** | **0** | **20** | **1.5** | **2** | **K.LISAQHNQSK.A** |
|  | 6172 | **563.8072** | **1125.5999** | **1125.6070** | **-0.0070** | **0** | **21** | **1.1** | **1** | **R.AYFLDGSLLK.E** |
|  | 7068 | 594.2813 | 1186.5479 | 1186.6306 | -0.0826 | 1 | 6 | 38 | 3 | R.IQEITERGNK.M |
|  | 7158 | **596.2889** | **1190.5632** | **1190.5713** | **-0.0081** | **0** | **30** | **0.14** | **1** | **K.SLNQNMESLR.A** 7159 |
|  | 7724 | **608.9744** | **1215.9343** | **1215.5632** | **0.3711** | **0** | **3** | **55** | **4** | **K.QNEVNAAWER.L** |
|  | 8409 | **417.7297** | **1250.1672** | **1250.7095** | **-0.5423** | **1** | **6** | **24** | **7** | **R.KHGLLESAVAAR.Q** |
|  | 8563 | **630.2789** | **1258.5433** | **1258.7133** | **-0.1699** | **0** | **0** | **1.4e+002** | **3** | **K.GDVLTLLSSINK.D** |
|  | 8575 | **630.3417** | **1258.6689** | **1258.6591** | **0.0098** | **0** | **7** | **32** | **9** | **K.CLLELDQQIK.A** |
|  | 8771 | **635.3206** | **1268.6266** | **1268.6361** | **-0.0095** | **0** | **41** | **0.01** | **1** | **K.HQSLEAEVQTK.S** 8772 |
|  | 8861 | **425.2592** | **1272.7558** | **1272.6098** | **0.1460** | **0** | **1** | **1e+002** | **7** | **K.DHEDFLASLAR.A** |
|  | 8881 | **637.8542** | **1273.6939** | **1273.7030** | **-0.0091** | **1** | **46** | **0.0034** | **1** | **R.FEALKEPLATR.K** |
|  | 9312 | **649.3045** | **1296.5944** | **1295.6007** | **0.9937** | **0** | **33** | **0.061** | **1** | **K.HQTFAHEVDGR.S** 9281 |
|  | 9472 | **435.8849** | **1304.6329** | **1304.6401** | **-0.0072** | **0** | **9** | **18** | **9** | **K.AEDLFVEFAHK.A** |
|  | 9583 | **438.3406** | **1311.9998** | **1312.7211** | **-0.7213** | **1** | **5** | **27** | **5** | **R.QKALSNAANLQR.F** |
|  | 9698 | **658.8339** | **1315.6533** | **1315.6619** | **-0.0086** | **0** | **8** | **25** | **2** | **K.VLETAEEIQER.R** |
|  | 10279 | **674.3420** | **1346.6694** | **1347.6816** | **-1.0122** | **1** | **1** | **1.3e+002** | **10** | **K.GLERNLAVMSDK.V** |
|  | 10425 | 452.2093 | 1353.6062 | 1354.5459 | -0.9397 | 0 | 4 | 52 | 9 | R.DSEQVDSWMSR.Q |
|  | 10513 | **453.7809** | **1358.3208** | **1357.7354** | **0.5854** | **1** | **7** | **21** | **7** | **K.RDVTEAIQWIK.E** 10515 |
|  | 11587 | **710.3465** | **1418.6784** | **1418.6889** | **-0.0104** | **0** | **36** | **0.036** | **1** | **K.ETGTLESQLEANK.R** |
|  | 12421 | **489.9142** | **1466.7206** | **1466.7300** | **-0.0093** | **0** | **15** | **4** | **1** | **R.MQHNLEQQIQAK.D** |
|  | 12800 | **497.2688** | **1488.7847** | **1488.8010** | **-0.0163** | **1** | **3** | **62** | **8** | **K.WIMEKVNILTDK.S** |
|  | 14138 | 523.9279 | 1568.7618 | 1568.7769 | -0.0152 | 1 | 3 | 70 | 9 | R.VMALYDFQARSPR.E |
|  | 14422 | **794.3848** | **1586.7550** | **1586.7722** | **-0.0173** | **0** | **37** | **0.029** | **1** | **R.VQNVCAQGEDILNK.V** 14424 |
|  | 14542 | **532.2830** | **1593.8272** | **1593.8375** | **-0.0103** | **1** | **21** | **0.96** | **1** | **K.RWEQLLEASAVHR.Q** |
|  | 14754 | **537.0322** | **1608.0749** | **1608.8195** | **-0.7446** | **1** | **(4)** | **33** | **2** | **R.RLAHDEFPMLPQR.R** |
|  | 14920 | **809.9112** | **1617.8078** | **1617.8209** | **-0.0131** | **1** | **50** | **0.0013** | **1** | **K.VLQEESQNKEISSK.I** 14921 |
|  | 15029 | **542.3198** | **1623.9376** | **1624.8144** | **-0.8767** | **1** | **5** | **33** | **2** | **R.RLAHDEFPMLPQR.R** |
|  | 15225 | **819.8934** | **1637.7723** | **1637.7818** | **-0.0095** | **1** | **26** | **0.3** | **1** | **R.SDDKSSLDSLEALMK.K** |
|  | 15344 | **824.3696** | **1646.7247** | **1646.7359** | **-0.0111** | **0** | **68** | **1.8e-005** | **1** | **K.MVEEGHFAAEDVASR.V** |
|  | 15908 | 560.9396 | 1679.7969 | 1678.8050 | 0.9920 | 1 | 14 | 4.6 | 1 | K.LADDEDYKDIQNLK.S |
|  | 16035 | 844.9420 | 1687.8694 | 1687.8529 | 0.0164 | 1 | 4 | 45 | 9 | K.YQKHQSLEAEVQTK.S |
|  | 17025 | **438.9914** | **1751.9365** | **1751.8115** | **0.1250** | **1** | **7** | **20** | **8** | **K.FDEFQKDLNTNEPR.L** |
|  | 17238 | **590.9721** | **1769.8945** | **1769.9060** | **-0.0115** | **1** | **27** | **0.24** | **1** | **R.QLQKDHEDFLASLAR.A** |
|  | 17800 | **602.2812** | **1803.8217** | **1803.7846** | **0.0372** | **1** | **1** | **92** | **5** | **R.EERFTMGHSAHEETK.A** |
|  | 18077 | **608.3353** | **1821.9842** | **1822.9359** | **-0.9518** | **1** | **2** | **79** | **5** | **R.MQHNLEQQIQAKDIK.G** |
|  | 18242 | **612.2925** | **1833.8556** | **1834.8519** | **-0.9963** | **1** | **9** | **15** | **2** | **K.MELNEAWEDLQGRTK.D** 18241 |
|  | 19660 | **973.4465** | **1944.8785** | **1944.8894** | **-0.0109** | **0** | **29** | **0.11** | **1** | **K.LQLEDDYAFQEFNWK.A** |
|  | 19778 | **651.6563** | **1951.9469** | **1950.9509** | **0.9960** | **1** | **28** | **0.15** | **1** | **K.KHEAFLLDLNSFGDSMK.A** |
|  | 19940 | **655.0240** | **1962.0503** | **1961.8901** | **0.1602** | **1** | **0** | **88** | **3** | **R.GNKMVEEGHFAAEDVASR.V** |
|  | 20133 | **992.4459** | **1982.8772** | **1982.8898** | **-0.0126** | **0** | **8** | **13** | **1** | **R.WLEDVEWQVTSEDYGK.G** |
|  | 20457 | **671.8174** | **2012.4305** | **2012.0731** | **0.3574** | **1** | **0** | **56** | **4** | **K.QLPLQKAEDLFVEFAHK.A** |
|  | 20967 | **1029.0431** | **2056.0716** | **2056.0841** | **-0.0124** | **0** | **19** | **1** | **1** | **K.VADDLLFEGLLTPEGAQIR.Q** |
|  | 21082 | **1033.4579** | **2064.9012** | **2064.9171** | **-0.0159** | **0** | **63** | **4.1e-005** | **1** | **K.TGQEMIEGGHYASDNVTTR.L** 21083 |
|  | 21624 | **1059.0627** | **2116.1109** | **2116.1205** | **-0.0095** | **0** | **2** | **54** | **2** | **K.ALGVPSSPYTWLTVEVLER.T** |
|  | 22782 | **1121.0686** | **2240.1226** | **2239.1332** | **0.9895** | **0** | **85** | **2.5e-007** | **1** | **K.TAAINADELPTDVAGGEVLLDR.H** 22771 |
|  | 22851 | **1127.0811** | **2252.1475** | **2252.1590** | **-0.0114** | **0** | **24** | **0.36** | **1** | **K.YSTIGLAQQWDQLYQLGLR.M** 22849 |
|  | 23535 | **780.7050** | **2339.0932** | **2338.2995** | **0.7937** | **1** | **9** | **10** | **1** | **R.QLTKIVDLGDNLEDALILDIK.Y** |
|  | 23637 | **1177.5408** | **2353.0670** | **2353.0829** | **-0.0159** | **0** | **59** | **9e-005** | **1** | **R.NQANACQQQQAAPVEGVAGEQR.V** 23638 |
|  | 24382 | **821.3875** | **2461.1407** | **2461.1511** | **-0.0104** | **0** | **23** | **0.41** | **1** | **R.YNEFLLAYEAGDMLEWIQEK.K** |
|  | 24805 | 840.7304 | 2519.1692 | 2519.3119 | -0.1427 | 1 | 0 | 63 | 2 | K.DLNTLAEDLLSSGTFNVDQIVKK.K 24793 |
|  | 24838 | **632.5602** | **2526.2116** | **2526.3329** | **-0.1213** | **1** | **1** | **51** | **4** | **R.DINKVADDLLFEGLLTPEGAQIR.Q** |
|  | 25308 | **861.7387** | **2582.1943** | **2582.2109** | **-0.0166** | **0** | **4** | **25** | **2** | **K.GTQLHEANQQLQFENNAEDLQR.W** |
|  | 17227 | **885.7828** | **2654.3267** | **2653.1723** | **1.1544** | **0** | **3** | **77** | **1** | **K.QALTPEQVSFCATHMQQYMDPR.G** |
|  | 27071 | **966.1243** | **2895.3512** | **2895.3708** | **-0.0197** | **0** | **46** | **0.0015** | **1** | **R.VVEVNQYANECAEENHPDLPLIQSK.Q** |
|  | 27592 | **750.5955** | **2998.3527** | **2999.4876** | **-1.1348** | **1** | **0** | **46** | **3** | **K.AITAQEGKITDLEHFAESLIADEHYAK.E** |
|  | 27763 | **1011.4847** | **3031.4322** | **3030.4471** | **0.9851** | **0** | **9** | **6.4** | **1** | **R.QNDLEANVQFQQYLADLHEAETWIR.E** |
|  | 28242 | **787.3918** | **3145.5380** | **3145.5502** | **-0.0122** | **1** | **17** | **0.87** | **1** | **K.LTLSHPSDAPQIQEMKEDLVSSWEHIR.A** |
|  | 28780 | **1090.8536** | **3269.5391** | **3269.5437** | **-0.0046** | **1** | **43** | **0.0021** | **1** | **R.GLKLEESLEYLQFMQNAEEEEAWINEK.N** |
|  | 29720 | **897.1599** | **3584.6105** | **3584.6253** | **-0.0147** | **0** | **8** | **5.2** | **2** | **R.ADMEAEAPTFQALEDFSAELIDSGHHASPEIEK.K** |
|  | 30686 | **1042.4813** | **4165.8962** | **4165.9239** | **-0.0278** | **1** | **17** | **0.49** | **1** | **R.QEAFLENEDLGNSLGSAEALLQKHEDFEEAFTAQEEK.I** |

| **62.** | P05556\|ITB1_HUMAN    **Mass:** 91664    **Score:** 507    **Queries matched:** 44   **emPAI:** 0.74 |
| --- | --- |
|  | Integrin beta-1 OS=Homo sapiens GN=ITGB1 PE=1 SV=2 |

|  | **Query** | **Observed** | **Mr(expt)** | **Mr(calc)** | **Delta** | **Miss** | **Score** | **Expect** | **Rank** | **Peptide** |
| --- | --- | --- | --- | --- | --- | --- | --- | --- | --- | --- |
|  | 1546 | **397.2108** | **792.4071** | **792.4130** | **-0.0058** | **1** | **9** | **23** | **5** | **R.AFNKGEK.K** |
|  | 2278 | **423.7446** | **845.4746** | **844.4515** | **1.0231** | **1** | **7** | **47** | **6** | **K.NKNVTNR.S** |
|  | 2905 | **447.2278** | **892.4410** | **893.4719** | **-1.0309** | **1** | **5** | **62** | **8** | **R.RITSDFR.I** |
|  | 4150 | **492.2595** | **982.5045** | **982.5124** | **-0.0078** | **0** | **32** | **0.1** | **1** | **R.IGFGSFVEK.T** |
|  | 5547 | **541.7209** | **1081.4272** | **1081.4355** | **-0.0083** | **0** | **16** | **3.2** | **1** | **R.GICECGVCK.C** |
|  | 5670 | 546.2700 | 1090.5254 | 1090.5328 | -0.0075 | 1 | 32 | 0.11 | 1 | R.CDDLEALKK.K |
|  | 5903 | 554.2836 | 1106.5526 | 1106.5608 | -0.0082 | 0 | 25 | 0.52 | 1 | R.SGEPQTFTLK.F 5902 |
|  | 8703 | **633.7903** | **1265.5660** | **1265.5744** | **-0.0084** | **0** | **56** | **0.00039** | **1** | **K.SLGTDLMNEMR.R** |
|  | 8749 | 423.5754 | 1267.7043 | 1268.5455 | -0.8412 | 0 | 8 | 22 | 4 | K.GCPPDDIENPR.G |
|  | 9282 | **648.8077** | **1295.6008** | **1295.6106** | **-0.0098** | **1** | **35** | **0.044** | **1** | **K.RDNTNEIYSGK.F** |
|  | 10064 | **667.8259** | **1333.6373** | **1334.6071** | **-0.9698** | **0** | **8** | **22** | **2** | **R.SNGLICGGNGVCK.C** |
|  | 11182 | **698.7486** | **1395.4826** | **1396.6405** | **-1.1579** | **1** | **22** | **0.61** | **1** | **K.KGCPPDDIENPR.G** 11206 |
|  | 12207 | **485.9094** | **1454.7063** | **1453.6653** | **1.0410** | **1** | **11** | **10** | **4** | **K.SLGTDLMNEMRR.I** 12206 |
|  | 13596 | **768.7628** | **1535.5111** | **1535.5228** | **-0.0117** | **0** | **59** | **0.00011** | **1** | **K.FCECDNFNCDR.S** 13584 13597 |
|  | 15919 | **561.2480** | **1680.7221** | **1681.7770** | **-1.0548** | **1** | **3** | **52** | **5** | **K.MNAKWDTGENPIYK.S** |
|  | 18392 | **615.6446** | **1843.9121** | **1843.9251** | **-0.0130** | **1** | **36** | **0.027** | **1** | **R.DKLPQPVQPDPVSHCK.E** 18382 |
|  | 18598 | **620.3407** | **1858.0003** | **1857.9407** | **0.0596** | **1** | **4** | **44** | **8** | **K.LPQPVQPDPVSHCKEK.D** |
|  | 19907 | **981.0329** | **1960.0512** | **1960.0629** | **-0.0117** | **1** | **102** | **6.6e-009** | **1** | **K.NVLSLTNKGEVFNELVGK.Q** 19908 19909 |
|  | 20546 | **673.7203** | **2018.1392** | **2018.1524** | **-0.0132** | **0** | **48** | **0.0012** | **1** | **K.LKPEDITQIQPQQLVLR.L** 20545 20553 |
|  | 22567 | **740.4249** | **2218.2530** | **2218.1270** | **0.1260** | **1** | **0** | **69** | **10** | **K.WDTGENPIYKSAVTTVVNPK.Y** |
|  | 23820 | **1189.4174** | **2376.8202** | **2376.8351** | **-0.0150** | **0** | **74** | **2.3e-006** | **1** | **R.HCECSTDEVNSEDMDAYCR.K** 23819 |
|  | 24246 | **815.3295** | **2442.9668** | **2442.9848** | **-0.0180** | **0** | **24** | **0.22** | **1** | **K.FQGQTCEMCQTCLGVCAEHK.E** |
|  | 24442 | **1235.6224** | **2469.2303** | **2469.2427** | **-0.0124** | **0** | **97** | **1.5e-008** | **1** | **K.LSENNIQTIFAVTEEFQPVYK.E** 24443 24444 24446 |
|  | 24792 | 840.3970 | 2518.1693 | 2517.1443 | 1.0249 | 1 | 0 | 65 | 4 | K.RAEDYPIDLYYLMDLSYSMK.D |
|  | 28024 | **775.3815** | **3097.4968** | **3097.4824** | **0.0144** | **0** | **0** | **47** | **7** | **-.MNLQPIFWIGLISSVCCVFAQTDENR.C** |
|  | 28225 | **1048.4834** | **3142.4284** | **3142.4402** | **-0.0118** | **1** | **9** | **5.5** | **1** | **R.AEDYPIDLYYLMDLSYSMKDDLENVK.S** |
|  | 28807 | **819.8305** | **3275.2929** | **3275.3168** | **-0.0238** | **1** | **40** | **0.0033** | **1** | **K.FQGQTCEMCQTCLGVCAEHKECVQCR.A** 28808 |
|  | 30697 | **1045.2383** | **4176.9240** | **4176.9496** | **-0.0256** | **0** | **4** | **9.8** | **1** | **K.LGGIVLPNDGQCHLENNMYTMSHYYDYPSIAHLVQK.L** 30699 |

| **63.** | P05023\|AT1A1_HUMAN    **Mass:** 114135   **Score:** 483    **Queries matched:** 39   **emPAI:** 0.51 |
| --- | --- |
|  | Sodium/potassium-transporting ATPase subunit alpha-1 OS=Homo sapiens GN=ATP1A1 PE=1 SV=1 |

|  | **Query** | **Observed** | **Mr(expt)** | **Mr(calc)** | **Delta** | **Miss** | **Score** | **Expect** | **Rank** | **Peptide** |
| --- | --- | --- | --- | --- | --- | --- | --- | --- | --- | --- |
|  | 875 | **530.2573** | **529.2500** | **528.2656** | **0.9844** | **0** | **11** | **24** | **8** | **K.GAPER.I** |
|  | 1783 | **811.3888** | **810.3815** | **810.3872** | **-0.0057** | **0** | **19** | **1.7** | **1** | **K.YGTDLSR.G** |
|  | 2508 | **863.4226** | **862.4153** | **861.4378** | **0.9775** | **0** | **6** | **53** | **9** | **R.IISANGCK.V** 2501 |
|  | 4105 | 490.2605 | 978.5065 | 978.5135 | -0.0069 | 0 | 12 | 10 | 1 | R.GIVVYTGDR.T |
|  | 4608 | **507.7695** | **1013.5244** | **1013.5328** | **-0.0084** | **0** | **19** | **2.2** | **1** | **R.CSSILLHGK.E** 4586 |
|  | 4924 | **519.5986** | **1037.1827** | **1037.4964** | **-0.3137** | **0** | **6** | **24** | **6** | **R.NSVFQQGMK.N** |
|  | 5596 | **543.2701** | **1084.5257** | **1084.5335** | **-0.0078** | **0** | **25** | **0.46** | **1** | **K.ACVVHGSDLK.D** |
|  | 7993 | **616.3256** | **1230.6367** | **1230.6456** | **-0.0089** | **0** | **66** | **3.7e-005** | **1** | **R.AVAGDASESALLK.C** |
|  | 8100 | **618.8520** | **1235.6895** | **1235.6986** | **-0.0091** | **0** | **29** | **0.18** | **1** | **R.LNIPVSQVNPR.D** 8072 |
|  | 8627 | **631.3384** | **1260.6622** | **1260.6714** | **-0.0092** | **0** | **55** | **0.00043** | **1** | **K.IVEIPFNSTNK.Y** |
|  | 8748 | **634.8565** | **1267.6983** | **1267.7071** | **-0.0087** | **0** | **27** | **0.26** | **3** | **K.NMVPQQALVIR.N** |
|  | 10962 | **691.3739** | **1380.7332** | **1380.7435** | **-0.0103** | **0** | **47** | **0.0027** | **1** | **K.VIMVTGDHPITAK.A** 10963 10964 |
|  | 13091 | **502.9265** | **1505.7576** | **1505.7396** | **0.0180** | **0** | **1** | **87** | **9** | **K.ADIGVAMGIAGSDVSK.Q** |
|  | 14383 | **793.4395** | **1584.8645** | **1583.8671** | **0.9974** | **0** | **41** | **0.011** | **1** | **R.AVFQANQENLPILK.R** |
|  | 14939 | **810.3740** | **1618.7335** | **1618.7435** | **-0.0100** | **0** | **68** | **2e-005** | **1** | **K.VDNSSLTGESEPQTR.S** 14925 14938 |
|  | 17400 | **593.9977** | **1778.9714** | **1779.9917** | **-1.0203** | **1** | **1** | **80** | **3** | **K.VIMVTGDHPITAKAIAK.G** |
|  | 17912 | **906.4724** | **1810.9303** | **1810.9425** | **-0.0122** | **0** | **83** | **5.3e-007** | **1** | **R.QGAIVAVTGDGVNDSPALK.K** |
|  | 18169 | **915.4591** | **1828.9037** | **1828.9167** | **-0.0130** | **0** | **86** | **2.9e-007** | **1** | **K.GVGIISEGNETVEDIAAR.L** |
|  | 18181 | **915.9738** | **1829.9329** | **1829.9445** | **-0.0115** | **0** | **81** | **7.8e-007** | **1** | **K.MSINAEEVVVGDLVEVK.G** 18188 18189 |
|  | 19391 | **962.9550** | **1923.8954** | **1923.9095** | **-0.0142** | **0** | **1** | **82** | **1** | **K.NLEAVETLGSTSTICSDK.T** |
|  | 22534 | 739.3522 | 2215.0347 | 2215.1154 | -0.0808 | 1 | 10 | 8.1 | 1 | K.MSINAEEVVVGDLVEVKGGDR.I 22550 |
|  | 24392 | **822.0676** | **2463.1811** | **2463.1951** | **-0.0141** | **0** | **38** | **0.012** | **1** | **K.QAADMILLDDNFASIVTGVEEGR.L** |
|  | 24493 | **826.7657** | **2477.2752** | **2476.2896** | **0.9856** | **1** | **33** | **0.041** | **1** | **K.YQLSIHKNPNTSEPQHLLVMK.G** 24488 |
|  | 26769 | **1417.6893** | **2833.3641** | **2833.3770** | **-0.0129** | **1** | **73** | **2.6e-006** | **1** | **K.EQPLDEELKDAFQNAYLELGGLGER.V** 26770 |
|  | 28677 | **1080.1383** | **3237.3931** | **3237.4132** | **-0.0201** | **0** | **48** | **0.0007** | **1** | **R.MTVAHMWFDNQIHEADTTENQSGVSFDK.T** |
|  | 28900 | **1103.4862** | **3307.4368** | **3306.6806** | **0.7562** | **1** | **1** | **29** | **1** | **K.QAADMILLDDNFASIVTGVEEGRLIFDNLK.K** |
|  | 31322 | **1018.2991** | **5086.4590** | **5085.6289** | **0.8301** | **1** | **0** | **12** | **3** | **R.LISMAYGQIGMIQALGGFFTYFVILAENGFLPIHLLGLRVDWDDR.W** |

| **64.** | P16157\|ANK1_HUMAN    **Mass:** 207334   **Score:** 482    **Queries matched:** 68   **emPAI:** 0.30 |
| --- | --- |
|  | Ankyrin-1 OS=Homo sapiens GN=ANK1 PE=1 SV=3 |

|  | **Query** | **Observed** | **Mr(expt)** | **Mr(calc)** | **Delta** | **Miss** | **Score** | **Expect** | **Rank** | **Peptide** |
| --- | --- | --- | --- | --- | --- | --- | --- | --- | --- | --- |
|  | 1232 | **674.2064** | **673.1991** | **673.3833** | **-0.1842** | **0** | **1** | **2.2e+002** | **3** | **R.LAMPVK.V** |
|  | 1690 | **402.7208** | **803.4271** | **803.4324** | **-0.0053** | **0** | **12** | **15** | **1** | **R.GNVIMVR.L** |
|  | 1966 | **413.7093** | **825.4040** | **824.4181** | **0.9859** | **0** | **13** | **7.1** | **2** | **M.PYSVGFR.E** 1968 |
|  | 2965 | **450.2375** | **898.4604** | **898.4695** | **-0.0091** | **0** | **8** | **26** | **4** | **R.IGHTNMVK.L** |
|  | 3817 | 480.2577 | 958.5009 | 959.5328 | -1.0318 | 0 | 17 | 4.2 | 7 | K.ELLDFVPK.L 3858 |
|  | 3855 | 481.2429 | 960.4712 | 959.5335 | 0.9377 | 1 | 12 | 13 | 8 | R.RGNVIMVR.L |
|  | 3978 | **486.2642** | **970.5138** | **971.4535** | **-0.9397** | **0** | **18** | **2.8** | **1** | **-.MPYSVGFR.E** 3976 |
|  | 4324 | **498.2686** | **994.5227** | **994.5825** | **-0.0598** | **0** | **8** | **25** | **6** | **K.FHRPIGLR.I** |
|  | 5281 | **532.2832** | **1062.5518** | **1061.5506** | **1.0013** | **0** | **13** | **9** | **2** | **R.EPGGSLSFLR.K** 5266 5270 5278 5279 |
|  | 5535 | **541.2676** | **1080.5207** | **1079.5247** | **0.9960** | **0** | **41** | **0.012** | **1** | **R.EADAATSFLR.A** 5519 |
|  | 5856 | 552.2993 | 1102.5840 | 1101.5237 | 1.0603 | 0 | 18 | 2.6 | 2 | K.HGVMVDATTR.M |
|  | 6870 | **588.8016** | **1175.5887** | **1174.6809** | **0.9079** | **1** | **5** | **47** | **5** | **K.EIILETTTKK.G** 6869 |
|  | 7717 | 608.8216 | 1215.6286 | 1215.7564 | -0.1278 | 1 | 4 | 57 | 5 | K.VRLPALHIAAR.N |
|  | 10442 | **452.2912** | **1353.8519** | **1352.6911** | **1.1608** | **1** | **6** | **31** | **3** | **K.FVIFAKMNDPR.E** |
|  | 10986 | **692.3676** | **1382.7207** | **1382.7306** | **-0.0100** | **0** | **56** | **0.00035** | **1** | **K.FLLQHQADVNAK.T** |
|  | 11491 | **707.4150** | **1412.8154** | **1413.8126** | **-0.9972** | **1** | **5** | **41** | **6** | **R.GNVIMVRLLLDR.G** |
|  | 12181 | **727.3677** | **1452.7209** | **1453.7525** | **-1.0316** | **0** | **5** | **40** | **1** | **R.ITHSPTVSQVTER.S** 12211 |
|  | 12673 | **494.9051** | **1481.6935** | **1481.7045** | **-0.0110** | **1** | **29** | **0.14** | **1** | **K.AKDDQTPLHCAAR.I** 12672 |
|  | 13097 | **503.1030** | **1506.2873** | **1506.7427** | **-0.4554** | **1** | **9** | **10** | **3** | **R.DSSREPGGSLSFLR.K** 13098 13099 |
|  | 13617 | **769.3522** | **1536.6898** | **1537.7876** | **-1.0978** | **0** | **25** | **0.41** | **1** | **K.VVTDETSFVLVSDK.H** |
|  | 14040 | 781.9207 | 1561.8269 | 1561.8325 | -0.0056 | 1 | 9 | 15 | 7 | R.LPALHIAARNDDTR.T |
|  | 14177 | **786.4305** | **1570.8464** | **1570.7740** | **0.0725** | **0** | **21** | **0.91** | **2** | **K.TLEQHENFVEVAR.S** 14178 |
|  | 14675 | **535.6350** | **1603.8830** | **1603.8933** | **-0.0103** | **0** | **55** | **0.00034** | **1** | **R.ISEILLDHGAPIQAK.T** |
|  | 14764 | **537.2809** | **1608.8208** | **1608.7910** | **0.0299** | **1** | **4** | **52** | **3** | **K.AAQQRSFHFQSFR.E** |
|  | 14873 | **539.4131** | **1615.2174** | **1614.8729** | **0.3445** | **1** | **0** | **76** | **9** | **R.ELVVLRSENGSVWK.E** |
|  | 14950 | **405.9355** | **1619.7127** | **1620.9087** | **-1.1959** | **0** | **4** | **42** | **7** | **K.LALQAQPVPDELVTK.L** |
|  | 15175 | **545.7600** | **1634.2580** | **1633.8060** | **0.4521** | **0** | **3** | **39** | **2** | **R.ELVNYGANVNAQSQK.G** |
|  | 15689 | **833.9302** | **1665.8458** | **1665.8573** | **-0.0115** | **0** | **25** | **0.35** | **1** | **K.LSTPPPLAEEEGLASR.I** 15690 |
|  | 15827 | **838.3903** | **1674.7660** | **1674.8352** | **-0.0693** | **1** | **5** | **39** | **3** | **R.DVDEEKELLDFVPK.L** 15828 |
|  | 16635 | **863.9855** | **1725.9565** | **1725.9665** | **-0.0100** | **0** | **48** | **0.0015** | **1** | **K.LVPLVQATFPENAVTK.R** |
|  | 17018 | **876.9026** | **1751.7906** | **1751.8036** | **-0.0130** | **0** | **83** | **5.9e-007** | **1** | **R.LCQDYDTIGPEGGSLK.S** 17027 |
|  | 17661 | **897.9780** | **1793.9415** | **1793.9523** | **-0.0108** | **0** | **22** | **0.64** | **1** | **R.TAAVLLQNDPNPDVLSK.T** |
|  | 17950 | **605.8107** | **1814.4102** | **1813.9071** | **0.5031** | **1** | **0** | **66** | **6** | **K.TLEQHENFVEVARSR.D** |
|  | 17968 | **908.4862** | **1814.9579** | **1814.9641** | **-0.0062** | **0** | **50** | **0.00093** | **1** | **R.IITTDFPLYFVIMSR.L** |
|  | 18235 | **611.9727** | **1832.8962** | **1833.9697** | **-1.0735** | **0** | **2** | **74** | **3** | **K.GNTALHIAALAGQDEVVR.E** |
|  | 19466 | **965.4677** | **1928.9209** | **1927.9487** | **0.9722** | **0** | **53** | **0.00044** | **1** | **K.NGASPNEVSSDGTTPLAIAK.R** 19467 |
|  | 19538 | **967.9565** | **1933.8985** | **1932.9840** | **0.9146** | **1** | **2** | **61** | **3** | **K.VETPLHMAARAGHTEVAK.Y** |
|  | 20832 | 1023.5276 | 2045.0406 | 2046.0244 | -0.9838 | 0 | 8 | 13 | 2 | K.GFTPLYMAAQENHLEVVK.F |
|  | 21046 | **688.3206** | **2061.9400** | **2062.0193** | **-0.0793** | **0** | **(0)** | **91** | **10** | **K.GFTPLYMAAQENHLEVVK.F** |
|  | 23936 | **798.7863** | **2393.3371** | **2392.3478** | **0.9893** | **0** | **0** | **65** | **8** | **K.SGLTPLHLVAQEGHVPVADVLIK.H** |
|  | 26739 | **707.6426** | **2826.5412** | **2826.5545** | **-0.0133** | **0** | **60** | **5.2e-005** | **1** | **R.IIALGPTGAQFLSPVIVEIPHFASHGR.G** 26740 |
|  | 27310 | **983.5180** | **2947.5322** | **2947.5477** | **-0.0155** | **0** | **42** | **0.0032** | **1** | **K.TGASIDAVTESGLTPLHVASFMGHLPIVK.N** |
|  | 28326 | **634.5303** | **3167.6150** | **3166.4652** | **1.1497** | **1** | **1** | **41** | **2** | **K.HRMSFPETVDEILDVSEDEGEELISFK.A** |
|  | 29256 | **1137.2014** | **3408.5824** | **3408.6038** | **-0.0213** | **1** | **116** | **1e-010** | **1** | **R.EGQNANMENLYTALQSIDRGEIVNMLEGSGR.Q** |
|  | 29728 | **1196.9420** | **3587.8042** | **3587.9134** | **-0.1092** | **1** | **2** | **23** | **5** | **K.TGASIDAVTESGLTPLHVASFMGHLPIVKNLLQR.G** |
|  | 30299 | **973.2536** | **3888.9853** | **3889.0043** | **-0.0191** | **0** | **12** | **2** | **1** | **R.SLLQYGGSANAESVQGVTPLHLAAQEGHAEMVALLLSK.Q** |
|  | 30910 | **1099.0580** | **4392.2028** | **4392.2139** | **-0.0110** | **0** | **47** | **0.00042** | **1** | **K.FLLENGANQNVATEDGFTPLAVALQQGHENVVAHLINYGTK.G** |
|  | 31031 | **1524.7255** | **4571.1546** | **4570.3125** | **0.8420** | **1** | **1** | **13** | **8** | **R.VENPNSLLEQSVALLNLWVIREGQNANMENLYTALQSIDR.G** |
|  | 31160 | **1600.7554** | **4799.2443** | **4800.2171** | **-0.9728** | **1** | **1** | **12** | **3** | **K.EYDEDSLIPSSPATETSDNISPVASPVHTGFLVSFMVDARGGSMR.G** |

| **65.** | P23141\|EST1_HUMAN    **Mass:** 62766    **Score:** 481    **Queries matched:** 39   **emPAI:** 0.59 |
| --- | --- |
|  | Liver carboxylesterase 1 OS=Homo sapiens GN=CES1 PE=1 SV=2 |

|  | **Query** | **Observed** | **Mr(expt)** | **Mr(calc)** | **Delta** | **Miss** | **Score** | **Expect** | **Rank** | **Peptide** |
| --- | --- | --- | --- | --- | --- | --- | --- | --- | --- | --- |
|  | 2208 | **421.5331** | **841.0516** | **840.5069** | **0.5447** | **1** | **11** | **8.4** | **2** | **R.KENIPLK.L** |
|  | 3140 | **456.2269** | **910.4393** | **910.4450** | **-0.0057** | **0** | **15** | **4.1** | **2** | **K.FWANFAR.N** |
|  | 5109 | **525.7829** | **1049.5512** | **1049.5579** | **-0.0067** | **0** | **12** | **11** | **1** | **K.SYPLVCIAK.E** |
|  | 8594 | **630.8284** | **1259.6422** | **1259.6510** | **-0.0088** | **0** | **38** | **0.022** | **1** | **K.FLSLDLQGDPR.E** 8595 |
|  | 10275 | **674.2061** | **1346.3977** | **1346.6677** | **-0.2700** | **1** | **3** | **50** | **5** | **K.EGASEEEIRLSK.M** |
|  | 10300 | **674.8608** | **1347.7071** | **1347.7146** | **-0.0075** | **0** | **35** | **0.046** | **1** | **K.AGQLLSELFTNR.K** |
|  | 11902 | **479.5814** | **1435.7224** | **1435.7320** | **-0.0096** | **0** | **6** | **34** | **1** | **R.GNWGHLDQVAALR.W** |
|  | 13315 | **507.2344** | **1518.6815** | **1518.7864** | **-0.1049** | **1** | **5** | **40** | **8** | **K.MKFLSLDLQGDPR.E** |
|  | 13325 | **507.2783** | **1518.8132** | **1518.7653** | **0.0479** | **0** | **5** | **43** | **9** | **R.NFHTVPYMVGINK.Q** |
|  | 13612 | **512.9687** | **1535.8842** | **1534.7814** | **1.1029** | **1** | **(0)** | **1.1e+002** | **5** | **K.MKFLSLDLQGDPR.E** |
|  | 14485 | **796.4014** | **1590.7882** | **1590.8002** | **-0.0120** | **0** | **71** | **9.6e-006** | **1** | **K.EGYLQIGANTQAAQK.L** |
|  | 14928 | **809.9384** | **1617.8621** | **1617.8726** | **-0.0104** | **1** | **11** | **8.9** | **1** | **K.KAVEKPPQTEHIEL.-** |
|  | 18128 | **914.0145** | **1826.0145** | **1826.0223** | **-0.0078** | **0** | **65** | **2.9e-005** | **1** | **R.ESQPLLGTVIDGMLLLK.T** 18129 |
|  | 18230 | **459.2271** | **1832.8792** | **1831.9792** | **0.9000** | **1** | **10** | **9.9** | **1** | **K.EGYLQIGANTQAAQKLK.D** |
|  | 18375 | **615.3405** | **1842.9997** | **1842.0172** | **0.9825** | **0** | **(8)** | **18** | **1** | **R.ESQPLLGTVIDGMLLLK.T** |
|  | 21717 | **1065.0212** | **2128.0279** | **2128.0398** | **-0.0119** | **0** | **72** | **4.8e-006** | **1** | **K.LSEDCLYLNIYTPADLTK.K** 21718 |
|  | 21872 | **1075.0469** | **2148.0792** | **2148.0892** | **-0.0100** | **0** | **18** | **1.4** | **1** | **K.TVIGDHGDELFSVFGAPFLK.E** 21871 |
|  | 26676 | **939.4213** | **2815.2420** | **2815.2622** | **-0.0202** | **0** | **29** | **0.061** | **1** | **R.DAGAPTYMYEFQYRPSFSSDMKPK.T** |
|  | 27488 | **994.5674** | **2980.6803** | **2980.6943** | **-0.0139** | **0** | **46** | **0.0012** | **1** | **K.FVSLEGFAQPVAIFLGIPFAKPPLGPLR.F** |
|  | 28260 | **788.1373** | **3148.5202** | **3148.5353** | **-0.0151** | **1** | **2** | **31** | **8** | **K.TVIGDHGDELFSVFGAPFLKEGASEEEIR.L** |
|  | 30121 | **1263.9773** | **3788.9100** | **3788.9261** | **-0.0161** | **0** | **60** | **3.2e-005** | **1** | **R.WVQDNIASFGGNPGSVTIFGESAGGESVSVLVLSPLAK.N** 30109 30112 30113 30114 30115 30116 30118 30120 30122 30124 30125 30126 30127 30134 |

| **66.** | Q5K4L6\|S27A3_HUMAN    **Mass:** 79393    **Score:** 481    **Queries matched:** 36   **emPAI:** 0.26 |
| --- | --- |
|  | Long-chain fatty acid transport protein 3 OS=Homo sapiens GN=SLC27A3 PE=2 SV=3 |

|  | **Query** | **Observed** | **Mr(expt)** | **Mr(calc)** | **Delta** | **Miss** | **Score** | **Expect** | **Rank** | **Peptide** |
| --- | --- | --- | --- | --- | --- | --- | --- | --- | --- | --- |
|  | 643 | **459.0106** | **458.0033** | **458.2601** | **-0.2568** | **0** | **4** | **69** | **1** | **R.GAVGR.A** 640 647 663 |
|  | 1680 | **402.2432** | **802.4719** | **802.4045** | **0.0674** | **1** | **12** | **13** | **9** | **R.ESNRAAR.A** |
|  | 3377 | **464.7610** | **927.5074** | **927.5137** | **-0.0063** | **0** | **10** | **17** | **5** | **R.LAELAQQR.A** |
|  | 4055 | **488.2816** | **974.5486** | **974.5549** | **-0.0063** | **0** | **12** | **13** | **2** | **R.TAFVPTALR.R** |
|  | 4204 | **494.7531** | **987.4917** | **987.4297** | **0.0620** | **0** | **13** | **10** | **1** | **R.FSYSEAER.E** 4223 |
|  | 5040 | **523.2839** | **1044.5533** | **1044.5604** | **-0.0071** | **0** | **22** | **1** | **1** | **R.YLVNQPPSK.A** |
|  | 5256 | **531.2894** | **1060.5643** | **1061.6134** | **-1.0491** | **0** | **14** | **7** | **2** | **K.LHLWPQLR.W** 5253 5258 5259 5286 |
|  | 5489 | **539.7666** | **1077.5186** | **1076.5978** | **0.9208** | **0** | **6** | **41** | **2** | **R.YSALLAGNLR.I** 5479 |
|  | 6113 | **561.7703** | **1121.5261** | **1120.6288** | **0.8973** | **1** | **5** | **53** | **10** | **R.RGPLLHCLR.S** |
|  | 6392 | **572.7687** | **1143.5228** | **1143.5309** | **-0.0080** | **1** | **12** | **10** | **5** | **R.RFSYSEAER.E** |
|  | 12533 | **738.3809** | **1474.7473** | **1473.6484** | **1.0989** | **1** | **0** | **1.2e+002** | **1** | **R.FSYSEAERESNR.A** |
|  | 13936 | **778.9077** | **1555.8009** | **1555.8107** | **-0.0098** | **0** | **17** | **2.7** | **2** | **R.LAVGSGLRPDTWER.F** |
|  | 16993 | **584.3019** | **1749.8838** | **1750.9101** | **-1.0263** | **1** | **3** | **61** | **2** | **R.LQESLATTETFKQQK.V** |
|  | 20059 | **987.9419** | **1973.8692** | **1973.8827** | **-0.0135** | **0** | **121** | **7.9e-011** | **1** | **R.AAPGAGDAAAGSGAEFAGGDGAAR.G** 20048 20058 20078 |
|  | 21114 | **1035.4823** | **2068.9500** | **2068.9636** | **-0.0136** | **0** | **90** | **8.1e-008** | **1** | **R.ALAAAAADPEGPEGGCSLAWR.L** 21115 |
|  | 22270 | **1097.1065** | **2192.1983** | **2192.2092** | **-0.0109** | **0** | **26** | **0.19** | **1** | **R.ALVLAPEFLESLEPDLPALR.A** 22271 22272 |
|  | 22918 | **1131.4623** | **2260.9100** | **2260.9258** | **-0.0157** | **0** | **119** | **9e-011** | **1** | **R.ALGWDWGPDGGDSGEGSAGEGER.A** 22917 |
|  | 26303 | **688.3568** | **2749.3979** | **2748.2164** | **1.1814** | **1** | **4** | **24** | **1** | **R.AFLRALGWDWGPDGGDSGEGSAGEGER.A** |
|  | 29306 | **1142.5633** | **3424.6682** | **3424.6861** | **-0.0178** | **0** | **24** | **0.16** | **1** | **R.MANEGFDPSTLSDPLYVLDQAVGAYLPLTTAR.Y** |
|  | 31364 | **1295.8749** | **5179.4704** | **5179.5020** | **-0.0315** | **1** | **9** | **1.6** | **1** | **R.YDVTTGEPIRDPQGHCMATSPGEPGLLVAPVSQQSPFLGYAGGPELAQGK.L** |

| **67.** | P02751\|FINC_HUMAN    **Mass:** 266034   **Score:** 478    **Queries matched:** 38   **emPAI:** 0.13 |
| --- | --- |
|  | Fibronectin OS=Homo sapiens GN=FN1 PE=1 SV=3 |

|  | **Query** | **Observed** | **Mr(expt)** | **Mr(calc)** | **Delta** | **Miss** | **Score** | **Expect** | **Rank** | **Peptide** |
| --- | --- | --- | --- | --- | --- | --- | --- | --- | --- | --- |
|  | 1513 | **395.4232** | **788.8318** | **789.4055** | **-0.5737** | **1** | **5** | **52** | **2** | **K.EKTGPMK.E** |
|  | 1731 | **404.2018** | **806.3891** | **807.4239** | **-1.0348** | **0** | **7** | **35** | **5** | **R.AQITGYR.L** 1765 |
|  | 3473 | **467.7395** | **933.4644** | **933.4702** | **-0.0057** | **0** | **28** | **0.27** | **1** | **R.ISCTIANR.C** |
|  | 4343 | **498.7556** | **995.4965** | **994.4944** | **1.0021** | **1** | **6** | **36** | **3** | **R.EDRVPHSR.N** |
|  | 4812 | 1031.4902 | 1030.4830 | 1029.5243 | 0.9586 | 0 | 1 | 1.5e+002 | 9 | K.YEKPGSPPR.E |
|  | 5292 | **532.7679** | **1063.5213** | **1063.5298** | **-0.0085** | **0** | **25** | **0.52** | **1** | **R.VGDTYERPK.D** 5291 |
|  | 5774 | **550.2128** | **1098.4110** | **1098.6046** | **-0.1937** | **1** | **5** | **46** | **9** | **R.WRPKNSVGR.W** |
|  | 8872 | **425.5480** | **1273.6223** | **1274.6044** | **-0.9821** | **0** | **9** | **19** | **8** | **K.TYHVGEQWQK.E** 8914 8922 |
|  | 9860 | **662.3558** | **1322.6970** | **1322.7055** | **-0.0085** | **0** | **12** | **8.2** | **1** | **K.LGVRPSQGGEAPR.E** |
|  | 10017 | **666.3559** | **1330.6972** | **1330.7092** | **-0.0120** | **0** | **11** | **11** | **1** | **R.ESKPLTAQQTTK.L** |
|  | 11281 | **701.3311** | **1400.6475** | **1400.6585** | **-0.0110** | **0** | **15** | **4.1** | **1** | **K.HYQINQQWER.T** |
|  | 12325 | **487.9050** | **1460.6931** | **1460.7835** | **-0.0904** | **0** | **8** | **23** | **7** | **R.VPGTSTSATLTGLTR.G** |
|  | 12352 | 488.2792 | 1461.8158 | 1461.6817 | 0.1341 | 0 | 5 | 38 | 6 | R.GNLLQCICTGNGR.G |
|  | 13711 | 772.3812 | 1542.7479 | 1542.7566 | -0.0087 | 0 | 15 | 3.5 | 1 | R.SYTITGLQPGTDYK.I |
|  | 13790 | **774.8552** | **1547.6958** | **1547.8784** | **-0.1826** | **1** | **17** | **2.6** | **2** | **R.AQITGYRLTVGLTR.R** |
|  | 14393 | **793.8259** | **1585.6372** | **1585.6467** | **-0.0095** | **0** | **76** | **2.7e-006** | **1** | **K.WCGTTQNYDADQK.F** 14392 |
|  | 14536 | **797.9105** | **1593.8065** | **1592.8046** | **1.0019** | **0** | **3** | **57** | **1** | **R.VTDATETTITISWR.T** |
|  | 15094 | **815.3892** | **1628.7638** | **1628.8635** | **-0.0997** | **0** | **6** | **29** | **1** | **R.VDVIPVNLPGEHGQR.L** |
|  | 15520 | **553.0558** | **1656.1455** | **1656.6695** | **-0.5239** | **0** | **3** | **43** | **9** | **K.DSMIWDCTCIGAGR.G** |
|  | 16736 | **866.9722** | **1731.9299** | **1731.9407** | **-0.0108** | **0** | **50** | **0.00096** | **1** | **R.NLQPASEYTVSLVAIK.G** |
|  | 17208 | **590.3047** | **1767.8922** | **1767.9744** | **-0.0822** | **1** | **4** | **43** | **2** | **R.IGFKLGVRPSQGGEAPR.E** |
|  | 19261 | **956.9960** | **1911.9774** | **1911.9902** | **-0.0128** | **0** | **71** | **8.2e-006** | **1** | **R.SSPVVIDASTAIDAPSNLR.F** 19260 |
|  | 22066 | **1084.5226** | **2167.0306** | **2167.0433** | **-0.0127** | **0** | **68** | **1.5e-005** | **1** | **R.ITYGETGGNSPVQEFTVPGSK.S** 22067 |
|  | 24823 | **1262.6765** | **2523.3385** | **2523.3585** | **-0.0200** | **0** | **101** | **5e-009** | **1** | **R.DLEVVAATPTSLLISWDAPAVTVR.Y** |
|  | 25505 | **874.0355** | **2619.0847** | **2619.1015** | **-0.0167** | **0** | **13** | **2.6** | **1** | **K.FGFCPMAAHEEICTTNEGVMYR.I** |
|  | 27103 | **968.4049** | **2902.1927** | **2902.2174** | **-0.0247** | **1** | **50** | **0.00043** | **1** | **R.GFNCESKPEAEETCFDKYTGNTYR.V** |
|  | 28387 | **1060.4432** | **3178.3079** | **3178.3285** | **-0.0206** | **1** | **73** | **1.9e-006** | **1** | **R.WKCDPVDQCQDSETGTFYQIGDSWEK.Y** |
|  | 31349 | **1290.6421** | **5158.5393** | **5157.5452** | **0.9940** | **0** | **46** | **0.00033** | **1** | **K.TAGPDQTEMTIEGLQPTVEYVVSVYAQNPSGESQPLVQTAVTNIDRPK.G** 31348 |
|  | 31491 | **1366.1779** | **5460.6823** | **5460.7100** | **-0.0277** | **1** | **24** | **0.045** | **1** | **R.YSPVKNEEDVAELSISPSDNAVVLTNLLPGTEYVVSVSSVYEQHESTPLR.G** 31490 |

| **68.** | P05107\|ITB2_HUMAN    **Mass:** 87976    **Score:** 476    **Queries matched:** 36   **emPAI:** 0.71 |
| --- | --- |
|  | Integrin beta-2 OS=Homo sapiens GN=ITGB2 PE=1 SV=2 |

|  | **Query** | **Observed** | **Mr(expt)** | **Mr(calc)** | **Delta** | **Miss** | **Score** | **Expect** | **Rank** | **Peptide** |
| --- | --- | --- | --- | --- | --- | --- | --- | --- | --- | --- |
|  | 2221 | **421.7574** | **841.5002** | **840.3622** | **1.1380** | **0** | **1** | **1.3e+002** | **8** | **R.GLCFCGK.C** |
|  | 2505 | **432.2090** | **862.4033** | **862.4079** | **-0.0046** | **1** | **10** | **20** | **1** | **R.RVECSGR.G** |
|  | 3130 | **455.7376** | **909.4607** | **908.4756** | **0.9851** | **1** | **12** | **9.5** | **2** | **K.FEKGPFGK.N** 3098 3123 |
|  | 3162 | **457.2048** | **912.3950** | **912.4011** | **-0.0061** | **0** | **38** | **0.022** | **1** | **R.CDTGYIGK.N** |
|  | 3174 | 457.2921 | 912.5695 | 913.4579 | -0.8883 | 1 | 11 | 11 | 7 | R.MVKTYEK.L |
|  | 5067 | **524.2445** | **1046.4743** | **1046.4815** | **-0.0071** | **0** | **31** | **0.16** | **1** | **R.TTEGCLNPR.R** |
|  | 5644 | **545.2769** | **1088.5393** | **1088.5462** | **-0.0069** | **0** | **37** | **0.035** | **1** | **R.ALNEITESGR.I** |
|  | 6511 | **576.7461** | **1151.4777** | **1151.4877** | **-0.0099** | **0** | **38** | **0.022** | **1** | **R.SSQELEGSCR.K** |
|  | 6532 | **577.1214** | **1152.2282** | **1151.4448** | **0.7835** | **0** | **17** | **2** | **1** | **K.NCECQTQGR.S** 6531 |
|  | 6709 | **583.2614** | **1164.5083** | **1163.5142** | **0.9941** | **0** | **43** | **0.0071** | **1** | **R.YNGQVCGGPGR.G** 6708 |
|  | 12676 | **741.8953** | **1481.7761** | **1481.7878** | **-0.0117** | **0** | **20** | **1.4** | **1** | **R.VFLDHNALPDTLK.V** 12677 |
|  | 12690 | **742.3441** | **1482.6736** | **1482.7024** | **-0.0289** | **1** | **8** | **21** | **5** | **K.SATTTVMNPKFAES.-** |
|  | 14300 | 790.8826 | 1579.7507 | 1578.7638 | 0.9869 | 0 | 4 | 41 | 7 | K.LTNNSNQFQTEVGK.Q |
|  | 15789 | **836.9492** | **1671.8838** | **1671.8944** | **-0.0106** | **0** | **56** | **0.00026** | **1** | **K.LAENNIQPIFAVTSR.M** |
|  | 16073 | **846.4003** | **1690.7860** | **1690.7951** | **-0.0091** | **0** | **34** | **0.041** | **1** | **R.SNEFDYPSVGQLAHK.L** |
|  | 16107 | **847.8217** | **1693.6289** | **1693.6396** | **-0.0107** | **0** | **31** | **0.075** | **1** | **R.CHPGFEGSACQCER.T** |
|  | 16372 | **856.3410** | **1710.6674** | **1710.6800** | **-0.0126** | **0** | **35** | **0.03** | **1** | **R.ECIESGPGCTWCQK.L** |
|  | 17414 | **890.9425** | **1779.8704** | **1779.8825** | **-0.0121** | **0** | **43** | **0.0054** | **1** | **K.VTATECIQEQSFVIR.A** |
|  | 17879 | **905.4394** | **1808.8642** | **1808.8734** | **-0.0092** | **0** | **65** | **3.8e-005** | **1** | **R.LLVFATDDGFHFAGDGK.L** 17878 17901 |
|  | 18437 | **616.7888** | **1847.3446** | **1846.8962** | **0.4484** | **1** | **0** | **64** | **2** | **K.RSNEFDYPSVGQLAHK.L** |
|  | 19004 | **945.9701** | **1889.9256** | **1888.9353** | **0.9903** | **0** | **60** | **0.00011** | **1** | **R.GDCDGVQINVPITFQVK.V** 18967 18993 |
|  | 21375 | **1047.9342** | **2093.8538** | **2092.8652** | **0.9886** | **0** | **95** | **2.2e-008** | **1** | **K.LIYGQYCECDTINCER.Y** 21369 |
|  | 22385 | **1103.5474** | **2205.0802** | **2205.0922** | **-0.0121** | **0** | **58** | **0.00012** | **1** | **R.ALGFTDIVTVQVLPQCECR.C** 22384 |
|  | 28696 | **812.1727** | **3244.6618** | **3243.4998** | **1.1620** | **0** | **5** | **14** | **2** | **K.QLISGNLDAPEGGLDAMMQVAACPEEIGWR.N** |
|  | 29048 | **835.9078** | **3339.6023** | **3340.4936** | **-0.8913** | **1** | **2** | **25** | **2** | **R.GCAADDIMDPTSLAETQEDHNGGQKQLSPQK.V** |

| **69.** | P26038\|MOES_HUMAN    **Mass:** 67892    **Score:** 473    **Queries matched:** 46   **emPAI:** 0.61 |
| --- | --- |
|  | Moesin OS=Homo sapiens GN=MSN PE=1 SV=3 |

|  | **Query** | **Observed** | **Mr(expt)** | **Mr(calc)** | **Delta** | **Miss** | **Score** | **Expect** | **Rank** | **Peptide** |
| --- | --- | --- | --- | --- | --- | --- | --- | --- | --- | --- |
|  | 1487 | **788.4194** | **787.4121** | **787.4188** | **-0.0067** | **0** | **14** | **8.3** | **1** | **K.VTAQDVR.K** 1491 |
|  | 1790 | **406.2345** | **810.4545** | **809.3919** | **1.0626** | **0** | **21** | **1.2** | **1** | **K.SGYLAGDK.L** |
|  | 2103 | **418.2001** | **834.3857** | **833.3953** | **0.9904** | **0** | **12** | **12** | **3** | **R.AMLENEK.K** |
|  | 2840 | **443.7373** | **885.4600** | **886.4872** | **-1.0272** | **0** | **9** | **24** | **6** | **K.EALLQASR.D** |
|  | 3642 | **474.2491** | **946.4835** | **946.4906** | **-0.0070** | **0** | **32** | **0.12** | **1** | **R.ISQLEMAR.Q** |
|  | 3908 | **483.2527** | **964.4909** | **964.4978** | **-0.0068** | **1** | **12** | **11** | **3** | **R.NISFNDKK.F** |
|  | 4194 | **494.2559** | **986.4972** | **986.5032** | **-0.0061** | **0** | **24** | **0.73** | **2** | **R.ALELEQER.K** 4195 |
|  | 5055 | **523.7709** | **1045.5273** | **1044.5563** | **0.9710** | **0** | **4** | **68** | **5** | **K.ALTSELANAR.D** |
|  | 5918 | **554.8221** | **1107.6297** | **1107.6400** | **-0.0103** | **1** | **13** | **7.6** | **1** | **R.VLEQHKLNK.D** |
|  | 6023 | **558.8121** | **1115.6096** | **1115.6186** | **-0.0090** | **1** | **21** | **1.3** | **1** | **R.LKQIEEQTK.K** |
|  | 7444 | **602.3182** | **1202.6219** | **1202.6441** | **-0.0222** | **1** | **(7)** | **32** | **7** | **R.ISQLEMARQK.K** |
|  | 7502 | **603.8315** | **1205.6484** | **1205.5598** | **0.0886** | **0** | **12** | **10** | **1** | **K.AQMVQEDLEK.T** |
|  | 7780 | **407.2234** | **1218.6484** | **1218.6390** | **0.0094** | **1** | **7** | **28** | **2** | **R.ISQLEMARQK.K** |
|  | 8030 | 617.2869 | 1232.5592 | 1232.5673 | -0.0081 | 0 | 32 | 0.1 | 1 | K.ESEAVEWQQK.A |
|  | 9717 | **439.8649** | **1316.5729** | **1315.6806** | **0.8923** | **0** | **3** | **79** | **7** | **R.KPDTIEVQQMK.A** |
|  | 10508 | **453.7401** | **1358.1986** | **1358.6790** | **-0.4804** | **1** | **3** | **49** | **9** | **K.KAQQELEEQTR.R** |
|  | 10557 | **454.5838** | **1360.7295** | **1359.7986** | **0.9308** | **1** | **3** | **74** | **8** | **K.LLPQRVLEQHK.L** |
|  | 11054 | **694.3447** | **1386.6749** | **1386.6851** | **-0.0102** | **1** | **11** | **12** | **2** | **K.AQQELEEQTRR.A** 11055 |
|  | 11578 | **709.8644** | **1417.7143** | **1417.6078** | **0.1065** | **0** | **4** | **51** | **6** | **K.TANDMIHAENMR.L** |
|  | 12487 | **736.8934** | **1471.7722** | **1471.7817** | **-0.0095** | **1** | **45** | **0.0037** | **1** | **R.RKPDTIEVQQMK.A** 12492 |
|  | 13061 | **752.8780** | **1503.7414** | **1503.7529** | **-0.0114** | **1** | **45** | **0.0042** | **1** | **K.ALTSELANARDESK.K** 13085 |
|  | 13509 | **510.9062** | **1529.6968** | **1529.7078** | **-0.0110** | **1** | **16** | **3** | **1** | **K.KTANDMIHAENMR.L** |
|  | 13609 | **768.9038** | **1535.7931** | **1535.8017** | **-0.0087** | **1** | **24** | **0.53** | **1** | **R.GMLREDAVLEYLK.I** 13610 |
|  | 15587 | **830.8997** | **1659.7848** | **1659.7933** | **-0.0086** | **0** | **44** | **0.0044** | **1** | **R.EVWFFGLQYQDTK.G** 15603 |
|  | 16262 | **852.4295** | **1702.8444** | **1702.8559** | **-0.0115** | **0** | **103** | **6.3e-009** | **1** | **K.TQEQLALEMAELTAR.I** |
|  | 16670 | **577.1319** | **1728.3739** | **1727.8195** | **0.5544** | **1** | **5** | **24** | **4** | **K.TANDMIHAENMRLGR.D** |
|  | 16931 | 872.9007 | 1743.7868 | 1743.8144 | -0.0276 | 1 | (3) | 55 | 6 | K.TANDMIHAENMRLGR.D |
|  | 18190 | **916.4762** | **1830.9378** | **1830.9509** | **-0.0130** | **1** | **113** | **5e-010** | **1** | **K.KTQEQLALEMAELTAR.I** 18207 |
|  | 19184 | **636.3672** | **1906.0797** | **1905.9182** | **0.1615** | **0** | **1** | **83** | **4** | **K.IAQDLEMYGVNYFSIK.N** |
|  | 19378 | **962.4416** | **1922.8687** | **1921.9686** | **0.9001** | **1** | **4** | **37** | **2** | **K.IGFPWSEIRNISFNDK.K** |
|  | 21202 | **693.3671** | **2077.0794** | **2075.9562** | **1.1232** | **1** | **2** | **60** | **9** | **K.DQWEERIQVWHEEHR.G** |
|  | 21241 | **1041.4994** | **2080.9842** | **2080.9953** | **-0.0111** | **0** | **5** | **27** | **1** | **K.FYPEDVSEELIQDITQR.L** |
|  | 23066 | **761.0159** | **2280.0258** | **2280.1274** | **-0.1016** | **1** | **6** | **21** | **4** | **R.AKFYPEDVSEELIQDITQR.L** |
|  | 23709 | **1181.5818** | **2361.1490** | **2361.1601** | **-0.0110** | **0** | **104** | **2.7e-009** | **1** | **K.GSELWLGVDALGLNIYEQNDR.L** 23706 |
|  | 26939 | **1434.2082** | **2866.4019** | **2865.4106** | **0.9913** | **0** | **62** | **3.2e-005** | **1** | **K.EGILNDDIYCPPETAVLLASYAVQSK.Y** 26930 |
|  | 28917 | **1104.7610** | **3311.2611** | **3311.4120** | **-0.1508** | **0** | **0** | **31** | **8** | **K.TAMSTPHVAEPAENEQDEQDENGAEASADLR.A** |

| **70.** | P62873\|GBB1_HUMAN    **Mass:** 38151    **Score:** 472    **Queries matched:** 30   **emPAI:** 1.33 |
| --- | --- |
|  | Guanine nucleotide-binding protein G(I)/G(S)/G(T) subunit beta-1 OS=Homo sapiens GN=GNB1 PE=1 SV=3 |

|  | **Query** | **Observed** | **Mr(expt)** | **Mr(calc)** | **Delta** | **Miss** | **Score** | **Expect** | **Rank** | **Peptide** |
| --- | --- | --- | --- | --- | --- | --- | --- | --- | --- | --- |
|  | 1725 | **403.7397** | **805.4649** | **804.4970** | **0.9679** | **0** | **7** | **36** | **5** | **K.VHAIPLR.S** 1701 |
|  | 2050 | **416.2589** | **830.5033** | **830.4359** | **0.0675** | **1** | **10** | **22** | **9** | **K.TREGNVR.V** |
|  | 4530 | **504.7602** | **1007.5059** | **1006.4753** | **1.0306** | **0** | **6** | **37** | **6** | **-.MSELDQLR.Q** |
|  | 4540 | **505.2582** | **1008.5018** | **1008.5101** | **-0.0083** | **0** | **55** | **0.00047** | **1** | **R.AGVLAGHDNR.V** 4532 4533 4535 4538 |
|  | 4651 | **509.2787** | **1016.5428** | **1016.5502** | **-0.0074** | **0** | **36** | **0.051** | **1** | **R.LLVSASQDGK.L** 4637 4650 |
|  | 9805 | **661.3829** | **1320.7513** | **1320.6067** | **0.1446** | **1** | **1** | **97** | **5** | **K.LWDVREGMCR.Q** |
|  | 10093 | **668.8040** | **1335.5934** | **1335.6030** | **-0.0096** | **0** | **48** | **0.0021** | **1** | **K.IYAMHWGTDSR.L** |
|  | 10401 | **677.3509** | **1352.6872** | **1352.6976** | **-0.0104** | **0** | **3** | **75** | **8** | **K.LIIWDSYTTNK.V** |
|  | 13382 | **762.3356** | **1522.6566** | **1522.6657** | **-0.0091** | **0** | **50** | **0.0012** | **1** | **R.ELAGHTGYLSCCR.F** 13381 |
|  | 16006 | **844.4204** | **1686.8263** | **1685.8584** | **0.9679** | **1** | **1** | **1e+002** | **10** | **M.SELDQLRQEAEQLK.N** |
|  | 21689 | **1064.0088** | **2126.0030** | **2126.0143** | **-0.0112** | **0** | **73** | **3.8e-006** | **1** | **R.LLLAGYDDFNCNVWDALK.A** |
|  | 21835 | **715.3586** | **2143.0541** | **2143.0692** | **-0.0151** | **1** | **7** | **18** | **1** | **R.KACADATLSQITNNIDPVGR.I** |
|  | 24070 | **1208.5540** | **2415.0934** | **2415.1087** | **-0.0153** | **0** | **124** | **2.9e-011** | **1** | **R.VSCLGVTDDGMAVATGSWDSFLK.I** 24071 24072 24073 |
|  | 24431 | 823.7296 | 2468.1668 | 2468.1794 | -0.0126 | 1 | 36 | 0.017 | 1 | R.LLLAGYDDFNCNVWDALKADR.A |
|  | 26144 | **906.4201** | **2716.2385** | **2715.2520** | **0.9865** | **0** | **4** | **22** | **2** | **R.ADQELMTYSHDNIICGITSVSFSK.S** |
|  | 29115 | **1120.9196** | **3359.7369** | **3359.6166** | **0.1202** | **1** | **1** | **30** | **2** | **R.LFDLRADQELMTYSHDNIICGITSVSFSK.S** |
|  | 29411 | **1157.8485** | **3470.5237** | **3470.5403** | **-0.0166** | **0** | **46** | **0.00088** | **1** | **R.SSWVMTCAYAPSGNYVACGGLDNICSIYNLK.T** 29410 |
|  | 29531 | **1174.8307** | **3521.4702** | **3520.5049** | **0.9654** | **0** | **22** | **0.17** | **1** | **R.QTFTGHESDINAICFFPNGNAFATGSDDATCR.L** |

| **71.** | P11277\|SPTB1_HUMAN    **Mass:** 247171   **Score:** 468    **Queries matched:** 73   **emPAI:** 0.32 |
| --- | --- |
|  | Spectrin beta chain, erythrocyte OS=Homo sapiens GN=SPTB PE=1 SV=5 |

|  | **Query** | **Observed** | **Mr(expt)** | **Mr(calc)** | **Delta** | **Miss** | **Score** | **Expect** | **Rank** | **Peptide** |
| --- | --- | --- | --- | --- | --- | --- | --- | --- | --- | --- |
|  | 1488 | **788.4826** | **787.4753** | **787.4803** | **-0.0050** | **0** | **6** | **63** | **1** | **K.AITAATLK.F** |
|  | 1739 | 404.6921 | 807.3697 | 806.4399 | 0.9298 | 1 | 8 | 28 | 2 | R.LFERSR.I |
|  | 2263 | **845.4396** | **844.4324** | **845.4243** | **-0.9919** | **1** | **5** | **63** | **5** | **K.DVAEERK.R** |
|  | 2628 | **437.2136** | **872.4127** | **871.4763** | **0.9364** | **0** | **3** | **1e+002** | **4** | **K.VQLIEDR.H** |
|  | 3732 | **476.7793** | **951.5440** | **951.4848** | **0.0593** | **1** | **1** | **1.3e+002** | **3** | **K.DLAAFCKK.N** |
|  | 3809 | **479.7836** | **957.5526** | **957.4880** | **0.0647** | **0** | **17** | **3.6** | **1** | **R.VEDQVNVR.K** 3815 |
|  | 3847 | **481.1504** | **960.2863** | **959.4672** | **0.8191** | **0** | **6** | **39** | **10** | **R.ETGAIGQER.V** |
|  | 3975 | **486.2630** | **970.5114** | **970.5308** | **-0.0194** | **1** | **11** | **13** | **3** | **R.EAAAGRLQR.L** |
|  | 4138 | 491.7609 | 981.5073 | 981.5144 | -0.0071 | 0 | 12 | 8.9 | 1 | K.WVNSHLAR.V 4135 |
|  | 4190 | **493.7821** | **985.5496** | **985.5556** | **-0.0060** | **0** | **31** | **0.14** | **1** | **K.AQEASVLLR.D** |
|  | 4267 | **496.3375** | **990.6604** | **989.5579** | **1.1025** | **1** | **8** | **27** | **2** | **K.MKVLAVEGK.R** |
|  | 4618 | **508.2709** | **1014.5272** | **1014.5346** | **-0.0074** | **0** | **14** | **6.4** | **1** | **K.VLSPVDSGNK.L** |
|  | 4850 | **517.2532** | **1032.4919** | **1032.5498** | **-0.0579** | **1** | **8** | **27** | **2** | **K.LQQVMSRR.K** 4851 |
|  | 5349 | **535.3189** | **1068.6231** | **1068.6291** | **-0.0060** | **0** | **39** | **0.019** | **1** | **R.DLAGIIAIQR.K** |
|  | 5412 | **537.0322** | **1072.0499** | **1071.5713** | **0.4786** | **0** | **3** | **61** | **4** | **K.AHLLSAEFGK.H** |
|  | 6571 | **577.8016** | **1153.5886** | **1153.5979** | **-0.0093** | **0** | **14** | **6.1** | **1** | **K.VIDHAIETEK.M** |
|  | 6868 | **588.8002** | **1175.5859** | **1175.5935** | **-0.0076** | **0** | **37** | **0.036** | **1** | **R.VDNVNAFIER.L** |
|  | 7430 | **401.8488** | **1202.5245** | **1203.6135** | **-1.0890** | **0** | **14** | **6.9** | **2** | **R.LWDELQATTK.E** |
|  | 7885 | 409.2277 | 1224.6612 | 1225.7142 | -1.0531 | 1 | 9 | 17 | 3 | R.ELALRNELIR.Q |
|  | 8526 | **419.8727** | **1256.5961** | **1257.6565** | **-1.0604** | **1** | **4** | **54** | **9** | **R.DKVLSPVDSGNK.L** 8555 |
|  | 9236 | **647.3572** | **1292.6999** | **1292.6725** | **0.0275** | **1** | **3** | **78** | **2** | **R.TQLVDTADKFR.F** |
|  | 10099 | **668.8736** | **1335.7326** | **1335.7398** | **-0.0071** | **0** | **26** | **0.32** | **1** | **K.HLLEVEDLLQK.H** 10077 |
|  | 10574 | **681.8541** | **1361.6937** | **1361.7013** | **-0.0076** | **0** | **50** | **0.0014** | **1** | **R.DLLSWMESIIR.Q** |
|  | 11312 | **702.3368** | **1402.6590** | **1402.6688** | **-0.0098** | **1** | **39** | **0.015** | **1** | **R.SDDPGKDLTSVNR.M** 11313 |
|  | 11899 | **479.5690** | **1435.6853** | **1435.6956** | **-0.0104** | **0** | **47** | **0.0023** | **1** | **R.HNLEHAFNVAER.Q** 11898 |
|  | 12110 | **483.5546** | **1447.6419** | **1448.7008** | **-1.0589** | **1** | **9** | **18** | **2** | **R.DFARETGAIGQER.V** |
|  | 12286 | 487.2860 | 1458.8363 | 1458.7732 | 0.0631 | 1 | 3 | 75 | 6 | K.TFTKWVNSHLAR.V |
|  | 12316 | **487.6135** | **1459.8186** | **1459.8286** | **-0.0100** | **0** | **10** | **13** | **1** | **R.FAALEKPTTLELK.E** |
|  | 12803 | **745.4184** | **1488.8222** | **1488.8300** | **-0.0078** | **0** | **10** | **12** | **1** | **K.GNLEVLLFTIQSR.M** |
|  | 12923 | **748.8598** | **1495.7050** | **1495.7154** | **-0.0104** | **0** | **63** | **6.1e-005** | **1** | **K.LLTSQDVSYDEAR.N** 12899 12935 |
|  | 13149 | **503.9558** | **1508.8457** | **1509.8627** | **-1.0170** | **1** | **2** | **76** | **6** | **K.DLGRDLAGIIAIQR.K** |
|  | 13349 | **760.9247** | **1519.8349** | **1519.7896** | **0.0454** | **1** | **1** | **1.1e+002** | **10** | **R.WQAFQTLVSERR.E** |
|  | 13499 | **765.3847** | **1528.7548** | **1529.7586** | **-1.0038** | **1** | **2** | **75** | **10** | **R.EVKQYQDHLNTR.W** 13491 |
|  | 13640 | **769.8597** | **1537.7048** | **1537.7161** | **-0.0113** | **1** | **39** | **0.014** | **1** | **R.AWESLEEAEYRR.E** 13641 13642 |
|  | 13754 | **773.8655** | **1545.7164** | **1545.7271** | **-0.0107** | **0** | **84** | **5.3e-007** | **1** | **R.LLSGEDVGQDEGATR.A** |
|  | 13783 | **774.4458** | **1546.8770** | **1545.8072** | **1.0698** | **1** | **4** | **44** | **5** | **K.LMEADIAIQGDKVK.A** |
|  | 14060 | **521.9462** | **1562.8169** | **1562.8279** | **-0.0110** | **1** | **14** | **4.9** | **1** | **R.KLENMYHLFQLK.R** |
|  | 14565 | **798.8579** | **1595.7013** | **1596.8220** | **-1.1207** | **1** | **8** | **19** | **1** | **R.SSDLRLQTHADLNK.W** |
|  | 14948 | **810.8413** | **1619.6681** | **1619.8380** | **-0.1699** | **0** | **3** | **58** | **6** | **K.NQTLQNEILGHTPR.V** |
|  | 15489 | **552.3166** | **1653.9279** | **1653.9375** | **-0.0096** | **0** | **21** | **0.82** | **1** | **K.LLEVLSGEMLPKPTK.G** 15495 |
|  | 16486 | **859.3773** | **1716.7400** | **1716.7494** | **-0.0094** | **0** | **49** | **0.0013** | **1** | **K.FFWEMDEAESWIK.E** |
|  | 17303 | **888.4401** | **1774.8657** | **1774.9326** | **-0.0669** | **0** | **28** | **0.17** | **1** | **R.AQGLLSAGHPEGEQIIR.L** |
|  | 18172 | **915.4851** | **1828.9557** | **1828.9683** | **-0.0126** | **0** | **89** | **1.4e-007** | **1** | **K.VIQGQTDPEYLLLGQR.L** 18173 |
|  | 19046 | **947.9739** | **1893.9332** | **1893.9472** | **-0.0140** | **0** | **103** | **5.2e-009** | **1** | **R.LVAQDNFGYDLAAVEAAK.K** 19045 |
|  | 19370 | **641.6901** | **1922.0484** | **1923.0214** | **-0.9730** | **1** | **3** | **48** | **4** | **K.LEALHRLWDELQATTK.E** |
|  | 20637 | **507.7520** | **2026.9788** | **2026.9054** | **0.0734** | **0** | **4** | **37** | **5** | **-.MTSATEFENVGNQPPYSR.I** |
|  | 21658 | **531.2650** | **2121.0309** | **2121.0425** | **-0.0116** | **0** | **22** | **0.59** | **1** | **R.GLDAHLEQIFQEAHGMVAR.K** |
|  | 22484 | 1108.0317 | 2214.0489 | 2213.9865 | 0.0624 | 0 | 22 | 0.47 | 2 | K.NLQDAENFFQFQGDADDLK.A |
|  | 22579 | **741.0294** | **2220.0663** | **2219.0463** | **1.0200** | **1** | **0** | **80** | **5** | **R.ISHLEQCFEELSNMAAGRK.A** |
|  | 22861 | **752.3448** | **2254.0127** | **2254.0284** | **-0.0157** | **1** | **31** | **0.068** | **1** | **R.ESQQLMDSHPEQKEDIGQR.Q** |
|  | 24232 | **814.7169** | **2441.1289** | **2440.1403** | **0.9887** | **0** | **29** | **0.092** | **1** | **K.WLAEMEMPDTLEDLEVVQHR.F** |
|  | 24498 | **827.4360** | **2479.2863** | **2479.2958** | **-0.0095** | **0** | **28** | **0.11** | **1** | **K.YSGLASDLLTWIEQTITVLNSR.K** |
|  | 27798 | **760.6019** | **3038.3784** | **3038.3941** | **-0.0157** | **1** | **7** | **9.5** | **1** | **R.GVMEHLEQQAQGFPEEFRDSPDVTHR.L** |
|  | 27941 | **769.3800** | **3073.4909** | **3073.5041** | **-0.0132** | **1** | **16** | **1.3** | **1** | **K.LFQDMLHSIDWMDEIKAHLLSAEFGK.H** 27940 |
|  | 28414 | 796.6549 | 3182.5903 | 3182.5318 | 0.0585 | 1 | 1 | 38 | 9 | K.DALLLWCQMKTAGYPHVNVTNFTSSWK.D |
|  | 28675 | **1079.5334** | **3235.5785** | **3235.5996** | **-0.0211** | **0** | **28** | **0.08** | **1** | **K.QAEAILSNQEYTLAHLEPPDSLEAAEAGIR.K** |
|  | 29576 | **886.4014** | **3541.5766** | **3541.7545** | **-0.1779** | **1** | **0** | **35** | **4** | **R.FDILDQEMKTLMTQIDGVNLAANSLVESGHPR.S** |

| **72.** | P09382\|LEG1_HUMAN    **Mass:** 15048    **Score:** 463    **Queries matched:** 30   **emPAI:** 4.00 |
| --- | --- |
|  | Galectin-1 OS=Homo sapiens GN=LGALS1 PE=1 SV=2 |

|  | **Query** | **Observed** | **Mr(expt)** | **Mr(calc)** | **Delta** | **Miss** | **Score** | **Expect** | **Rank** | **Peptide** |
| --- | --- | --- | --- | --- | --- | --- | --- | --- | --- | --- |
|  | 2704 | **439.2581** | **876.5017** | **876.5069** | **-0.0052** | **0** | **38** | **0.029** | **1** | **K.SFVLNLGK.D** 2703 |
|  | 3957 | **485.2878** | **968.5611** | **967.4651** | **1.0960** | **0** | **8** | **24** | **2** | **K.LPDGYEFK.F** |
|  | 4972 | **521.2833** | **1040.5520** | **1040.5614** | **-0.0095** | **1** | **45** | **0.0046** | **1** | **R.VRGEVAPDAK.S** 4955 4956 4958 4971 4973 4974 4975 4976 4979 |
|  | 5451 | **538.7379** | **1075.4613** | **1075.4683** | **-0.0070** | **0** | **42** | **0.0097** | **1** | **K.DGGAWGTEQR.E** 5459 |
|  | 12732 | **743.8410** | **1485.6674** | **1485.6783** | **-0.0108** | **0** | **56** | **0.00036** | **1** | **K.DSNNLCLHFNPR.F** 12733 |
|  | 15346 | **824.3747** | **1646.7348** | **1646.7471** | **-0.0123** | **0** | **57** | **0.00024** | **1** | **R.FNAHGDANTIVCNSK.D** 15345 |
|  | 17472 | **892.9247** | **1783.8348** | **1783.8450** | **-0.0102** | **0** | **107** | **1.9e-009** | **1** | **R.LNLEAINYMAADGDFK.I** 17469 17471 17473 17483 17493 |
|  | 26944 | **1434.2118** | **2866.4090** | **2866.4212** | **-0.0121** | **0** | **82** | **3.6e-007** | **1** | **R.EAVFPFQPGSVAEVCITFDQANLTVK.L** 26930 26939 26942 26943 |

| **73.** | P07339\|CATD_HUMAN    **Mass:** 45037    **Score:** 456    **Queries matched:** 25   **emPAI:** 1.22 |
| --- | --- |
|  | Cathepsin D OS=Homo sapiens GN=CTSD PE=1 SV=1 |

|  | **Query** | **Observed** | **Mr(expt)** | **Mr(calc)** | **Delta** | **Miss** | **Score** | **Expect** | **Rank** | **Peptide** |
| --- | --- | --- | --- | --- | --- | --- | --- | --- | --- | --- |
|  | 3443 | 466.7609 | 931.5073 | 932.5080 | -1.0006 | 1 | 15 | 5.4 | 3 | R.VGFAEAARL.- |
|  | 4992 | **521.8253** | **1041.6361** | **1041.6434** | **-0.0073** | **0** | **11** | **12** | **1** | **K.VSTLPAITLK.L** |
|  | 8153 | **620.3098** | **1238.6051** | **1238.6118** | **-0.0067** | **0** | **65** | **4.1e-005** | **1** | **K.FDGILGMAYPR.I** 8136 |
|  | 8499 | **419.3133** | **1254.9181** | **1254.6067** | **0.3114** | **0** | **(7)** | **20** | **5** | **K.FDGILGMAYPR.I** |
|  | 8644 | **631.8375** | **1261.6604** | **1262.6619** | **-1.0015** | **1** | **4** | **60** | **1** | **K.VERQVFGEATK.Q** |
|  | 12339 | **731.8343** | **1461.6541** | **1461.6637** | **-0.0095** | **1** | **20** | **1.3** | **1** | **R.YYTVFDRDNNR.V** 12338 |
|  | 13589 | **768.3793** | **1534.7440** | **1534.7549** | **-0.0109** | **0** | **15** | **3.9** | **1** | **R.TMSEVGGSVEDLIAK.G** |
|  | 14635 | **801.4152** | **1600.8159** | **1600.8249** | **-0.0090** | **0** | **69** | **1.3e-005** | **1** | **K.LVDQNIFSFYLSR.D** |
|  | 17521 | **894.4033** | **1786.7921** | **1786.8044** | **-0.0123** | **0** | **55** | **0.0003** | **1** | **R.DPDAQPGGELMLGGTDSK.Y** 17520 |
|  | 19879 | 980.0175 | 1958.0204 | 1958.0295 | -0.0092 | 0 | 50 | 0.00087 | 1 | R.ISVNNVLPVFDNLMQQK.L 19878 |
|  | 20177 | **994.5059** | **1986.9972** | **1988.0111** | **-1.0139** | **0** | **68** | **1.4e-005** | **1** | **K.AIGAVPLIQGEYMIPCEK.V** 20193 20194 |
|  | 20393 | **1003.0048** | **2003.9951** | **2004.0060** | **-0.0109** | **0** | **(50)** | **0.00089** | **1** | **K.AIGAVPLIQGEYMIPCEK.V** 20392 |
|  | 23348 | **773.0554** | **2316.1443** | **2316.1573** | **-0.0130** | **0** | **9** | **9.4** | **1** | **K.AYWQVHLDQVEVASGLTLCK.E** |
|  | 23483 | **1167.5443** | **2333.0741** | **2333.0879** | **-0.0139** | **0** | **66** | **2e-005** | **1** | **K.EGCEAIVDTGTSLMVGPVDEVR.E** 23482 |
|  | 24263 | **815.7535** | **2444.2386** | **2444.2522** | **-0.0136** | **1** | **31** | **0.06** | **1** | **R.KAYWQVHLDQVEVASGLTLCK.E** |
|  | 28468 | **799.9027** | **3195.5817** | **3194.6257** | **0.9561** | **1** | **2** | **31** | **4** | **K.FDGILGMAYPRISVNNVLPVFDNLMQQK.L** |
|  | 29143 | **1124.2085** | **3369.6037** | **3369.6187** | **-0.0151** | **1** | **30** | **0.039** | **1** | **K.LVDQNIFSFYLSRDPDAQPGGELMLGGTDSK.Y** |

| **74.** | P16615\|AT2A2_HUMAN    **Mass:** 116336   **Score:** 455    **Queries matched:** 49   **emPAI:** 0.65 |
| --- | --- |
|  | Sarcoplasmic/endoplasmic reticulum calcium ATPase 2 OS=Homo sapiens GN=ATP2A2 PE=1 SV=1 |

|  | **Query** | **Observed** | **Mr(expt)** | **Mr(calc)** | **Delta** | **Miss** | **Score** | **Expect** | **Rank** | **Peptide** |
| --- | --- | --- | --- | --- | --- | --- | --- | --- | --- | --- |
|  | 1881 | **819.3711** | **818.3639** | **818.3705** | **-0.0066** | **0** | **22** | **1.3** | **1** | **R.DACLNAR.C** |
|  | 2149 | **419.3141** | **836.6136** | **835.4300** | **1.1836** | **1** | **3** | **70** | **6** | **K.VYRQDR.K** |
|  | 4082 | **489.2997** | **976.5848** | **975.4807** | **1.1041** | **0** | **11** | **15** | **3** | **R.ANACNSVIK.Q** |
|  | 4850 | 517.2532 | 1032.4919 | 1032.4989 | -0.0070 | 0 | 5 | 62 | 7 | K.HTDPVPDPR.A |
|  | 5752 | **548.9382** | **1095.8619** | **1095.5270** | **0.3349** | **0** | **32** | **0.063** | **1** | **K.MNVFDTELK.G** |
|  | 5897 | **554.2530** | **1106.4914** | **1106.4993** | **-0.0078** | **0** | **35** | **0.057** | **1** | **R.EWGSGSDTLR.C** |
|  | 9176 | **430.9050** | **1289.6933** | **1289.7013** | **-0.0081** | **1** | **15** | **4.8** | **1** | **R.VGSTKVPMTSGVK.Q** 9175 |
|  | 9463 | **653.3022** | **1304.5899** | **1304.5997** | **-0.0097** | **0** | **55** | **0.00043** | **1** | **R.EFDELNPSAQR.D** |
|  | 9498 | **654.3335** | **1306.6524** | **1305.6963** | **0.9562** | **1** | **(3)** | **61** | **3** | **R.VGSTKVPMTSGVK.Q** |
|  | 9856 | 662.3348 | 1322.6550 | 1322.6653 | -0.0102 | 0 | 52 | 0.00093 | 1 | K.NMLFSGTNIAAGK.A |
|  | 10951 | **691.3199** | **1380.6253** | **1379.6980** | **0.9274** | **0** | **9** | **18** | **4** | **R.CLALATHDNPLR.R** 10905 |
|  | 11121 | **464.7628** | **1391.2666** | **1390.7126** | **0.5540** | **0** | **1** | **65** | **8** | **K.AEIGIAMGSGTAVAK.T** |
|  | 11405 | **470.2500** | **1407.7283** | **1407.6882** | **0.0401** | **0** | **4** | **57** | **7** | **R.IGIFGQDEDVTSK.A** |
|  | 11467 | 706.8237 | 1411.6328 | 1412.7234 | -1.0906 | 1 | 3 | 59 | 8 | R.AIYNNMKQFIR.Y |
|  | 12431 | **734.8424** | **1467.6703** | **1467.6810** | **-0.0107** | **0** | **62** | **7.9e-005** | **1** | **K.TGTLTTNQMSVCR.M** |
|  | 12558 | **738.8549** | **1475.6952** | **1475.7038** | **-0.0086** | **1** | **64** | **5.3e-005** | **1** | **K.IRDEMVATEQER.T** |
|  | 12655 | **494.5906** | **1480.7499** | **1480.7596** | **-0.0096** | **1** | **16** | **3.1** | **1** | **K.MNVFDTELKGLSK.I** |
|  | 13049 | **752.4084** | **1502.8022** | **1502.8126** | **-0.0104** | **1** | **36** | **0.031** | **1** | **K.KAEIGIAMGSGTAVAK.T** 13040 |
|  | 13297 | **506.9037** | **1517.6892** | **1518.8076** | **-1.1183** | **1** | **(4)** | **52** | **8** | **K.KAEIGIAMGSGTAVAK.T** 13343 |
|  | 13433 | **763.8533** | **1525.6920** | **1525.7017** | **-0.0097** | **0** | **38** | **0.019** | **1** | **K.SMSVYCTPNKPSR.T** |
|  | 13537 | **511.2841** | **1530.8304** | **1530.8188** | **0.0116** | **1** | **12** | **7** | **1** | **R.QAGIRVIMITGDNK.G** |
|  | 13608 | **512.9362** | **1535.7867** | **1535.7991** | **-0.0124** | **1** | **10** | **13** | **1** | **R.CLALATHDNPLRR.E** |
|  | 13706 | **515.0280** | **1542.0620** | **1541.6966** | **0.3654** | **0** | **(0)** | **83** | **8** | **K.SMSVYCTPNKPSR.T** |
|  | 13747 | **773.4134** | **1544.8122** | **1544.8232** | **-0.0110** | **0** | **69** | **1.6e-005** | **1** | **K.AMGVVVATGVNTEIGK.I** |
|  | 14057 | **521.9318** | **1562.7736** | **1561.7082** | **1.0654** | **0** | **10** | **13** | **1** | **R.EEMHLEDSANFIK.Y** |
|  | 14068 | **782.8964** | **1563.7783** | **1563.7893** | **-0.0110** | **1** | **48** | **0.0019** | **1** | **R.RIGIFGQDEDVTSK.A** 14067 |
|  | 14211 | 787.9294 | 1573.8442 | 1573.8563 | -0.0121 | 0 | 63 | 6e-005 | 1 | R.VDQSILTGESVSVIK.H 14191 14195 |
|  | 14266 | **526.9374** | **1577.7903** | **1577.7031** | **0.0872** | **0** | **(5)** | **34** | **10** | **R.EEMHLEDSANFIK.Y** |
|  | 14958 | **810.9049** | **1619.7953** | **1619.8076** | **-0.0124** | **0** | **91** | **1e-007** | **1** | **K.VGEATETALTCLVEK.M** 14977 |
|  | 16019 | **844.4675** | **1686.9205** | **1687.8278** | **-0.9073** | **1** | **3** | **55** | **5** | **K.HTDPVPDPRAVNQDK.K** |
|  | 16746 | **867.4238** | **1732.8331** | **1732.8454** | **-0.0123** | **1** | **7** | **22** | **1** | **R.NYLEPGKECVQPATK.S** 16745 |
|  | 19776 | **976.9628** | **1951.9110** | **1951.9231** | **-0.0121** | **0** | **35** | **0.031** | **1** | **R.SLPSVETLGCTSVICSDK.T** |
|  | 22251 | **1096.0955** | **2190.1764** | **2190.1896** | **-0.0132** | **1** | **71** | **7.2e-006** | **1** | **K.DIVPGDIVEIAVGDKVPADIR.L** |
|  | 24462 | **824.7540** | **2471.2403** | **2471.2544** | **-0.0141** | **0** | **6** | **18** | **1** | **K.TVEEVLGHFGVNESTGLSLEQVK.K** |
|  | 25407 | **867.4521** | **2599.3344** | **2599.3494** | **-0.0149** | **1** | **66** | **1.6e-005** | **1** | **K.TVEEVLGHFGVNESTGLSLEQVKK.L** 25406 |
|  | 26943 | 1434.2118 | 2866.4090 | 2865.4729 | 0.9362 | 1 | 0 | 53 | 5 | K.NMLFSGTNIAAGKAMGVVVATGVNTEIGK.I |
|  | 27142 | **970.8201** | **2909.4386** | **2908.4528** | **0.9857** | **1** | **24** | **0.22** | **1** | **K.IVEFLQSFDEITAMTGDGVNDAPALKK.A** 27131 |
|  | 28872 | **1100.9034** | **3299.6885** | **3298.6140** | **1.0745** | **1** | **2** | **28** | **2** | **-.MENAHTKTVEEVLGHFGVNESTGLSLEQVK.K** |

| **75.** | P21796\|VDAC1_HUMAN    **Mass:** 30868    **Score:** 445    **Queries matched:** 26   **emPAI:** 1.25 |
| --- | --- |
|  | Voltage-dependent anion-selective channel protein 1 OS=Homo sapiens GN=VDAC1 PE=1 SV=2 |

|  | **Query** | **Observed** | **Mr(expt)** | **Mr(calc)** | **Delta** | **Miss** | **Score** | **Expect** | **Rank** | **Peptide** |
| --- | --- | --- | --- | --- | --- | --- | --- | --- | --- | --- |
|  | 1077 | **609.3193** | **608.3121** | **608.3170** | **-0.0049** | **0** | **15** | **4.2** | **1** | **R.DVFTK.G** |
|  | 2375 | **427.7398** | **853.4649** | **853.4698** | **-0.0048** | **0** | **12** | **9.2** | **3** | **K.GYGFGLIK.L** |
|  | 4805 | **515.7590** | **1029.5035** | **1029.6070** | **-0.1035** | **0** | **2** | **1e+002** | **8** | **K.LTLSALLDGK.N** |
|  | 6526 | **576.8680** | **1151.7215** | **1152.6139** | **-0.8923** | **1** | **9** | **19** | **5** | **K.VTGSLETKYR.W** |
|  | 10820 | **687.8276** | **1373.6407** | **1373.6503** | **-0.0096** | **0** | **40** | **0.013** | **1** | **R.WTEYGLTFTEK.W** 10821 |
|  | 11493 | **707.8140** | **1413.6134** | **1413.6235** | **-0.0101** | **0** | **50** | **0.0014** | **1** | **K.YQIDPDACFSAK.V** |
|  | 13466 | **764.8804** | **1527.7463** | **1527.7569** | **-0.0106** | **1** | **39** | **0.014** | **1** | **K.LTFDSSFSPNTGKK.N** 13467 |
|  | 19669 | **973.5015** | **1944.9885** | **1945.0017** | **-0.0132** | **1** | **94** | **3.5e-008** | **1** | **K.KLETAVNLAWTAGNSNTR.F** 19668 |
|  | 19888 | **980.5000** | **1958.9854** | **1958.8705** | **0.1149** | **0** | **3** | **47** | **5** | **K.SENGLEFTSSGSANTETTK.V** |
|  | 21460 | 1052.0869 | 2102.1593 | 2102.1735 | -0.0143 | 0 | 52 | 0.00048 | 1 | K.VNNSSLIGLGYTQTLKPGIK.L 21456 21457 21458 21459 |
|  | 21981 | **720.3626** | **2158.0658** | **2158.9776** | **-0.9117** | **0** | **11** | **7** | **1** | **R.EHINLGCDMDFDIAGPSIR.G** |
|  | 22148 | **1088.5221** | **2175.0296** | **2175.0444** | **-0.0148** | **0** | **109** | **1.2e-009** | **1** | **K.WNTDNTLGTEITVEDQLAR.G** 22149 |
|  | 22229 | **1095.0055** | **2187.9964** | **2188.0131** | **-0.0167** | **1** | **115** | **2.9e-010** | **1** | **K.TKSENGLEFTSSGSANTETTK.V** 22228 22235 |
|  | 24094 | **1209.5851** | **2417.1556** | **2417.1725** | **-0.0169** | **0** | **0** | **65** | **1** | **R.GALVLGYEGWLAGYQMNFETAK.S** |
|  | 25403 | **867.3952** | **2599.1638** | **2599.1827** | **-0.0189** | **0** | **19** | **0.75** | **1** | **K.TDEFQLHTNVNDGTEFGGSIYQK.V** |
|  | 26413 | **925.4710** | **2773.3912** | **2774.3094** | **-0.9182** | **1** | **2** | **34** | **2** | **K.SENGLEFTSSGSANTETTKVTGSLETK.Y** |

| **76.** | P01834\|IGKC_HUMAN    **Mass:** 11773    **Score:** 430    **Queries matched:** 18   **emPAI:** 9.24 |
| --- | --- |
|  | Ig kappa chain C region OS=Homo sapiens GN=IGKC PE=1 SV=1 |

|  | **Query** | **Observed** | **Mr(expt)** | **Mr(calc)** | **Delta** | **Miss** | **Score** | **Expect** | **Rank** | **Peptide** |
| --- | --- | --- | --- | --- | --- | --- | --- | --- | --- | --- |
|  | 1112 | **625.3140** | **624.3067** | **624.2755** | **0.0312** | **0** | **8** | **18** | **8** | **K.ADYEK.H** |
|  | 13042 | 751.8775 | 1501.7404 | 1501.7512 | -0.0107 | 0 | 36 | 0.034 | 1 | K.DSTYSLSSTLTLSK.A |
|  | 18804 | **938.4605** | **1874.9063** | **1874.9197** | **-0.0133** | **0** | **68** | **1.5e-005** | **1** | **K.VYACEVTHQGLSSPVTK.S** 18801 18802 18803 |
|  | 19679 | 973.5120 | 1945.0095 | 1945.0197 | -0.0102 | 0 | 33 | 0.044 | 1 | -.TVAAPSVFIFPPSDEQLK.S 19675 19676 19680 19722 |
|  | 21771 | **1068.4811** | **2134.9476** | **2134.9614** | **-0.0138** | **0** | **102** | **5e-009** | **1** | **K.VDNALQSGNSQESVTEQDSK.D** 21770 |
|  | 21803 | **714.3602** | **2140.0589** | **2140.0735** | **-0.0147** | **1** | **63** | **4.6e-005** | **1** | **K.HKVYACEVTHQGLSSPVTK.S** 21802 |
|  | 29784 | **1207.2333** | **3618.6780** | **3618.7020** | **-0.0240** | **1** | **64** | **1.5e-005** | **1** | **K.VDNALQSGNSQESVTEQDSKDSTYSLSSTLTLSK.A** 29782 29783 |

| **77.** | P02545\|LMNA_HUMAN    **Mass:** 74380    **Score:** 399    **Queries matched:** 58   **emPAI:** 1.08 |
| --- | --- |
|  | Lamin-A/C OS=Homo sapiens GN=LMNA PE=1 SV=1 |

|  | **Query** | **Observed** | **Mr(expt)** | **Mr(calc)** | **Delta** | **Miss** | **Score** | **Expect** | **Rank** | **Peptide** |
| --- | --- | --- | --- | --- | --- | --- | --- | --- | --- | --- |
|  | 1000 | **573.3556** | **572.3484** | **572.3533** | **-0.0050** | **0** | **22** | **2** | **1** | **R.LLAEK.E** |
|  | 1032 | **588.9893** | **587.9820** | **587.3027** | **0.6793** | **0** | **11** | **19** | **7** | **K.LDNAR.Q** 1031 |
|  | 1391 | **733.4037** | **732.3964** | **732.4018** | **-0.0053** | **0** | **23** | **1** | **1** | **K.TLDSVAK.E** |
|  | 2027 | **415.7501** | **829.4856** | **829.4909** | **-0.0053** | **0** | **20** | **2** | **1** | **R.LQLELSK.V** |
|  | 2435 | **429.7521** | **857.4896** | **857.4970** | **-0.0074** | **1** | **14** | **7.5** | **10** | **R.LLAEKER.E** |
|  | 2480 | **431.2522** | **860.4898** | **860.4967** | **-0.0069** | **1** | **17** | **3.8** | **1** | **R.KTLDSVAK.E** 2471 |
|  | 3242 | 459.2352 | 916.4558 | 917.4454 | -0.9896 | 0 | 6 | 47 | 1 | R.DLEDSLAR.E 3248 |
|  | 3993 | **486.7557** | **971.4969** | **971.5036** | **-0.0067** | **0** | **23** | **0.97** | **1** | **R.LSPSPTSQR.S** |
|  | 4046 | **488.2414** | **974.4682** | **973.4716** | **0.9966** | **0** | **20** | **1.9** | **1** | **K.LLEGEEER.L** 4028 |
|  | 4673 | **510.2860** | **1018.5573** | **1017.5455** | **1.0119** | **1** | **2** | **1.2e+002** | **3** | **K.TLDSVAKER.A** |
|  | 5004 | **1043.5399** | **1042.5326** | **1042.5407** | **-0.0081** | **0** | **45** | **0.0054** | **1** | **K.EGDLIAAQAR.L** 5005 |
|  | 5120 | **526.2940** | **1050.5735** | **1050.4586** | **0.1149** | **1** | **7** | **32** | **6** | **K.EREMAEMR.A** |
|  | 5700 | 547.2637 | 1092.5128 | 1092.5564 | -0.0436 | 0 | 6 | 36 | 10 | R.SYLLGNSSPR.T 5686 |
|  | 6087 | **560.8181** | **1119.6217** | **1118.5819** | **1.0398** | **0** | **4** | **65** | **4** | **K.EAALSTALSEK.R** |
|  | 7079 | **594.3184** | **1186.6223** | **1186.6306** | **-0.0083** | **1** | **53** | **0.00073** | **1** | **K.LRDLEDSLAR.E** |
|  | 8246 | **622.3624** | **1242.7103** | **1242.7183** | **-0.0080** | **1** | **36** | **0.038** | **1** | **R.LKDLEALLNSK.E** 8247 |
|  | 10503 | **679.8616** | **1357.7087** | **1358.6790** | **-0.9703** | **0** | **1** | **1.1e+002** | **5** | **R.SGAQASSTPLSPTR.I** |
|  | 10514 | **453.7819** | **1358.3238** | **1357.7313** | **0.5924** | **1** | **7** | **19** | **6** | **R.SLETENAGLRLR.I** |
|  | 10584 | **682.3070** | **1362.5993** | **1362.6099** | **-0.0105** | **0** | **49** | **0.0019** | **1** | **K.AQNTWGCGNSLR.T** |
|  | 10955 | 691.3463 | 1380.6779 | 1380.6885 | -0.0105 | 1 | 31 | 0.11 | 1 | K.NIYSEELRETK.R 10956 |
|  | 11565 | **473.2862** | **1416.8367** | **1416.7572** | **0.0795** | **1** | **7** | **26** | **4** | **R.LRITESEEVVSR.E** 11541 |
|  | 11799 | **715.8912** | **1429.7678** | **1429.7776** | **-0.0098** | **0** | **40** | **0.012** | **1** | **R.IDSLSAQLSQLQK.Q** |
|  | 12839 | **746.3723** | **1490.7301** | **1490.7399** | **-0.0098** | **0** | **66** | **3.4e-005** | **1** | **R.TALINSTGEEVAMR.K** 12850 |
|  | 13036 | **751.8586** | **1501.7026** | **1501.7161** | **-0.0135** | **1** | **59** | **0.00016** | **1** | **R.AQHEDQVEQYKK.E** 13035 13037 13039 |
|  | 13109 | **503.2528** | **1506.7366** | **1506.7348** | **0.0018** | **0** | **(5)** | **38** | **5** | **R.TALINSTGEEVAMR.K** |
|  | 13143 | **755.3799** | **1508.7452** | **1508.7545** | **-0.0092** | **1** | **53** | **0.00058** | **1** | **R.LQTMKEELDFQK.N** 13142 |
|  | 13298 | **506.9056** | **1517.6950** | **1517.7070** | **-0.0120** | **0** | **5** | **44** | **2** | **R.ASSHSSQTQGGGSVTK.K** 13328 |
|  | 13600 | **768.8354** | **1535.6562** | **1535.6674** | **-0.0112** | **0** | **55** | **0.00038** | **1** | **K.SNEDQSMGNWQIK.R** |
|  | 14092 | **522.7712** | **1565.2917** | **1565.7434** | **-0.4517** | **0** | **2** | **53** | **10** | **R.SVGGSGGGSFGDNLVTR.S** 14097 |
|  | 14717 | **536.2837** | **1605.8292** | **1604.8046** | **1.0246** | **1** | **4** | **45** | **8** | **R.VAVEEVDEEGKFVR.L** |
|  | 15096 | **815.4023** | **1628.7901** | **1628.8005** | **-0.0104** | **1** | **49** | **0.0015** | **1** | **R.LQEKEDLQELNDR.L** |
|  | 15679 | **555.9651** | **1664.8734** | **1664.8846** | **-0.0111** | **1** | **7** | **25** | **5** | **R.TLEGELHDLRGQVAK.L** |
|  | 16020 | **563.3247** | **1686.9521** | **1686.8649** | **0.0872** | **1** | **7** | **24** | **1** | **R.ATRSGAQASSTPLSPTR.I** |
|  | 16223 | **850.9222** | **1699.8299** | **1698.9628** | **0.8671** | **1** | **13** | **6** | **1** | **R.IRIDSLSAQLSQLQK.Q** 16201 |
|  | 17005 | **876.4543** | **1750.8940** | **1751.8550** | **-0.9610** | **0** | **72** | **6.3e-006** | **1** | **R.NSNLVGAAHEELQQSR.I** 17021 17022 |
|  | 17128 | **880.9388** | **1759.8631** | **1760.9156** | **-1.0525** | **1** | **2** | **67** | **3** | **R.ITESEEVVSREVSGIK.A** |
|  | 17350 | **593.2785** | **1776.8137** | **1777.8846** | **-1.0709** | **1** | **5** | **32** | **1** | **R.EVSGIKAAYEAELGDAR.K** |
|  | 19035 | **947.4605** | **1892.9063** | **1892.9189** | **-0.0126** | **0** | **58** | **0.00017** | **1** | **R.MQQQLDEYQELLDIK.L** |
|  | 24876 | **845.1068** | **2532.2984** | **2532.3125** | **-0.0141** | **0** | **9** | **7.6** | **1** | **K.AGQVVTIWAAGAGATHSPPTDLVWK.A** |

| **78.** | P04040\|CATA_HUMAN    **Mass:** 59947    **Score:** 398    **Queries matched:** 25   **emPAI:** 0.94 |
| --- | --- |
|  | Catalase OS=Homo sapiens GN=CAT PE=1 SV=3 |

|  | **Query** | **Observed** | **Mr(expt)** | **Mr(calc)** | **Delta** | **Miss** | **Score** | **Expect** | **Rank** | **Peptide** |
| --- | --- | --- | --- | --- | --- | --- | --- | --- | --- | --- |
|  | 3796 | **479.2759** | **956.5373** | **956.5443** | **-0.0071** | **1** | **6** | **43** | **8** | **K.VFEHIGKK.T** |
|  | 3857 | **481.2443** | **960.4740** | **961.5233** | **-1.0492** | **0** | **21** | **1.5** | **1** | **K.DAQIFIQK.K** |
|  | 4047 | **488.2459** | **974.4773** | **973.4828** | **0.9944** | **0** | **8** | **32** | **8** | **K.NLSVEDAAR.L** |
|  | 6065 | **560.2789** | **1118.5433** | **1118.5509** | **-0.0076** | **0** | **18** | **2.4** | **1** | **R.LFAYPDTHR.H** |
|  | 9212 | **646.8050** | **1291.5954** | **1291.6044** | **-0.0090** | **0** | **20** | **1.4** | **1** | **R.LSQEDPDYGIR.D** |
|  | 12651 | **741.3682** | **1480.7219** | **1480.7310** | **-0.0091** | **0** | **71** | **9.5e-006** | **1** | **R.AFYVNVLNEEQR.K** 12653 |
|  | 15396 | **825.3978** | **1648.7810** | **1648.7917** | **-0.0108** | **1** | **53** | **0.00057** | **1** | **R.RFNTANDDNVTQVR.A** 15393 |
|  | 16341 | **854.9418** | **1707.8691** | **1707.8805** | **-0.0114** | **0** | **29** | **0.14** | **1** | **K.NAIHTFVQSGSHLAAR.E** 16342 |
|  | 17938 | **605.3086** | **1812.9039** | **1811.9141** | **0.9898** | **0** | **8** | **17** | **1** | **R.LGPNYLHIPVNCPYR.A** |
|  | 18497 | **617.9613** | **1850.8621** | **1850.8759** | **-0.0138** | **1** | **25** | **0.35** | **1** | **R.FSTVAGESGSADTVRDPR.G** 18498 18499 |
|  | 23725 | **1182.6394** | **2363.2642** | **2363.2809** | **-0.0167** | **1** | **100** | **7.1e-009** | **1** | **K.ADVLTTGAGNPVGDKLNVITVGPR.G** 23728 |
|  | 24784 | **1259.6001** | **2517.1856** | **2517.1965** | **-0.0108** | **0** | **71** | **4.9e-006** | **1** | **K.FYTEDGNWDLVGNNTPIFFIR.D** |
|  | 27655 | **754.3756** | **3013.4733** | **3013.4933** | **-0.0200** | **1** | **28** | **0.084** | **1** | **K.NFTEVHPDYGSHIQALLDKYNAEKPK.N** 27654 27656 |
|  | 30088 | **1257.9548** | **3770.8427** | **3770.8614** | **-0.0187** | **1** | **58** | **6e-005** | **1** | **K.LVLNRNPVNYFAEVEQIAFDPSNMPPGIEASPDK.M** |
|  | 30475 | **1007.7527** | **4026.9817** | **4026.9945** | **-0.0128** | **1** | **25** | **0.091** | **1** | **K.FYTEDGNWDLVGNNTPIFFIRDPILFPSFIHSQK.R** |
|  | 31064 | **1542.9974** | **4625.9705** | **4625.9911** | **-0.0206** | **0** | **54** | **5.3e-005** | **1** | **R.DGPMCMQDNQGGAPNYYPNSFGAPEQQPSALEHSIQYSGEVR.R** |
|  | 31151 | **1196.5225** | **4782.0607** | **4782.0922** | **-0.0314** | **1** | **10** | **1.6** | **1** | **R.DGPMCMQDNQGGAPNYYPNSFGAPEQQPSALEHSIQYSGEVRR.F** |

| **79.** | Q6DD88\|ATLA3_HUMAN    **Mass:** 60960    **Score:** 396    **Queries matched:** 28   **emPAI:** 0.61 |
| --- | --- |
|  | Atlastin-3 OS=Homo sapiens GN=ATL3 PE=1 SV=1 |

|  | **Query** | **Observed** | **Mr(expt)** | **Mr(calc)** | **Delta** | **Miss** | **Score** | **Expect** | **Rank** | **Peptide** |
| --- | --- | --- | --- | --- | --- | --- | --- | --- | --- | --- |
|  | 3962 | 485.7686 | 969.5227 | 970.5236 | -1.0009 | 0 | 11 | 11 | 8 | K.QLALDHFK.K |
|  | 6861 | **588.3669** | **1174.7192** | **1173.5812** | **1.1380** | **0** | **2** | **1.1e+002** | **7** | **R.DAVVGRPSMDK.K** |
|  | 9407 | **651.8409** | **1301.6673** | **1301.6762** | **-0.0088** | **1** | **18** | **2.3** | **1** | **R.DAVVGRPSMDKK.A** 9408 |
|  | 9895 | **663.3769** | **1324.7392** | **1325.7166** | **-0.9773** | **1** | **5** | **38** | **5** | **K.GKSFILDFMLR.Y** |
|  | 10328 | **450.5984** | **1348.7734** | **1348.7827** | **-0.0092** | **0** | **27** | **0.32** | **1** | **K.ALASILLQDHIR.D** 10294 |
|  | 11575 | **709.8370** | **1417.6594** | **1417.6698** | **-0.0104** | **0** | **25** | **0.48** | **1** | **K.EHQHEEIQNVR.N** 11574 |
|  | 16410 | **856.9780** | **1711.9414** | **1711.9508** | **-0.0094** | **0** | **46** | **0.0025** | **1** | **K.EQLQALIPYVLNPSK.L** 16400 |
|  | 18206 | **916.9570** | **1831.8995** | **1831.9097** | **-0.0102** | **0** | **118** | **1.6e-010** | **1** | **K.SMLQATAEANNLAAAASAK.D** 18205 |
|  | 19889 | **654.0026** | **1958.9859** | **1959.9214** | **-0.9355** | **1** | **1** | **77** | **9** | **K.DFSFRYQQELEEEIK.E** |
|  | 21940 | **1078.0497** | **2154.0848** | **2154.0991** | **-0.0143** | **0** | **64** | **3.2e-005** | **1** | **R.GADDAMESSKPGPVQVVLVQK.D** 21941 21942 |
|  | 22666 | **558.0503** | **2228.1721** | **2229.2078** | **-1.0358** | **1** | **3** | **43** | **6** | **K.EQLQALIPYVLNPSKLMEK.E** |
|  | 23438 | **776.4111** | **2326.2116** | **2326.2218** | **-0.0102** | **0** | **27** | **0.17** | **1** | **R.LAMDEIFQKPFQTLMFLVR.D** |
|  | 23919 | **798.0343** | **2391.0811** | **2391.0940** | **-0.0129** | **1** | **3** | **34** | **2** | **R.YQQELEEEIKELYENFCK.H** |
|  | 23962 | **801.0283** | **2400.0630** | **2400.0771** | **-0.0141** | **0** | **42** | **0.0041** | **1** | **K.ESGHSNWLGDPEEPLTGFSWR.G** 23963 23964 |
|  | 25501 | **873.7650** | **2618.2730** | **2618.2864** | **-0.0134** | **0** | **9** | **8.3** | **1** | **R.GGSDPETTGIQIWSEVFTVEKPGGK.K** |
|  | 25544 | **877.3993** | **2629.1760** | **2629.1947** | **-0.0187** | **1** | **8** | **9.8** | **1** | **R.DWSFPYEYSYGLQGGMAFLDKR.L** |
|  | 28050 | **1035.8008** | **3104.3805** | **3104.3994** | **-0.0189** | **0** | **32** | **0.025** | **1** | **K.DIYYNNMEEVCGGEKPYLSPDILEEK.H** 28051 |
|  | 29479 | **874.9106** | **3495.6132** | **3495.6340** | **-0.0208** | **0** | **41** | **0.0032** | **1** | **R.NHIHSCFSDVTCFLLPHPGLQVATSPDFDGK.L** |

| **80.** | Q86WV6\|TM173_HUMAN    **Mass:** 42736    **Score:** 389    **Queries matched:** 16   **emPAI:** 0.40 |
| --- | --- |
|  | Transmembrane protein 173 OS=Homo sapiens GN=TMEM173 PE=1 SV=1 |

|  | **Query** | **Observed** | **Mr(expt)** | **Mr(calc)** | **Delta** | **Miss** | **Score** | **Expect** | **Rank** | **Peptide** |
| --- | --- | --- | --- | --- | --- | --- | --- | --- | --- | --- |
|  | 3528 | **470.2447** | **938.4748** | **938.4933** | **-0.0186** | **1** | **9** | **18** | **7** | **R.HLRQEEK.E** |
|  | 5813 | **551.2770** | **1100.5394** | **1101.5535** | **-1.0141** | **1** | **4** | **72** | **10** | **R.ACLGCPLRR.G** |
|  | 10207 | 672.3541 | 1342.6936 | 1343.6755 | -0.9819 | 0 | 6 | 37 | 7 | K.GLAPAEISAVCEK.G |
|  | 11878 | **479.2375** | **1434.6907** | **1434.7004** | **-0.0096** | **0** | **5** | **39** | **4** | **R.TYNQHYNNLLR.G** |
|  | 14870 | **539.3032** | **1614.8877** | **1614.7759** | **0.1118** | **0** | **5** | **35** | **5** | **-.MPHSSLHPSIPCPR.G** 14868 14872 |
|  | 17445 | **595.2778** | **1782.8117** | **1783.8740** | **-1.0624** | **0** | **2** | **71** | **7** | **R.VYSNSIYELLENGQR.A** |
|  | 19656 | **973.4439** | **1944.8731** | **1944.8847** | **-0.0115** | **0** | **76** | **2.6e-006** | **1** | **R.TLEDILADAPESQNNCR.L** 19655 |
|  | 23759 | **1184.5791** | **2367.1436** | **2367.1594** | **-0.0157** | **0** | **104** | **3e-009** | **1** | **R.LIAYQEPADDSSFSLSQEVLR.H** 23758 |
|  | 25398 | **1300.1600** | **2598.3055** | **2598.3186** | **-0.0131** | **0** | **80** | **6.8e-007** | **1** | **R.LYILLPLDCGVPDNLSMADPNIR.F** 25397 25399 25400 |

| **81.** | P62879\|GBB2_HUMAN    **Mass:** 38048    **Score:** 386    **Queries matched:** 25   **emPAI:** 0.93 |
| --- | --- |
|  | Guanine nucleotide-binding protein G(I)/G(S)/G(T) subunit beta-2 OS=Homo sapiens GN=GNB2 PE=1 SV=3 |

|  | **Query** | **Observed** | **Mr(expt)** | **Mr(calc)** | **Delta** | **Miss** | **Score** | **Expect** | **Rank** | **Peptide** |
| --- | --- | --- | --- | --- | --- | --- | --- | --- | --- | --- |
|  | 1725 | 403.7397 | 805.4649 | 804.4970 | 0.9679 | 0 | 7 | 36 | 5 | K.VHAIPLR.S 1701 |
|  | 2050 | 416.2589 | 830.5033 | 830.4359 | 0.0675 | 1 | 10 | 22 | 9 | K.TREGNVR.V |
|  | 4540 | 505.2582 | 1008.5018 | 1008.5101 | -0.0083 | 0 | 55 | 0.00047 | 1 | R.AGVLAGHDNR.V 4532 4533 4535 4538 |
|  | 4651 | 509.2787 | 1016.5428 | 1016.5502 | -0.0074 | 0 | 36 | 0.051 | 1 | R.LLVSASQDGK.L 4637 4650 |
|  | 8512 | **628.8063** | **1255.5980** | **1254.5914** | **1.0065** | **0** | **18** | **2.2** | **1** | **R.TFVSGACDASIK.L** |
|  | 10093 | 668.8040 | 1335.5934 | 1335.6030 | -0.0096 | 0 | 48 | 0.0021 | 1 | K.IYAMHWGTDSR.L |
|  | 10123 | **669.8093** | **1337.6040** | **1336.6596** | **0.9444** | **1** | **1** | **1.1e+002** | **3** | **K.GDRAGVLAGHDNR.V** |
|  | 10401 | 677.3509 | 1352.6872 | 1352.6976 | -0.0104 | 0 | 3 | 75 | 8 | K.LIIWDSYTTNK.V |
|  | 13802 | **775.3424** | **1548.6703** | **1548.6813** | **-0.0111** | **0** | **47** | **0.0025** | **1** | **R.ELPGHTGYLSCCR.F** |
|  | 18809 | **938.4981** | **1874.9817** | **1874.9156** | **0.0661** | **1** | **6** | **28** | **4** | **-.MSELEQLRQEAEQLR.N** |
|  | 19499 | **966.4728** | **1930.9310** | **1930.9419** | **-0.0109** | **0** | **19** | **1.3** | **1** | **K.ACGDSTLTQITAGLDPVGR.I** |
|  | 21977 | **1079.9928** | **2157.9710** | **2157.9863** | **-0.0153** | **0** | **61** | **7.1e-005** | **1** | **R.LLLAGYDDFNCNIWDAMK.G** 21979 |
|  | 24070 | 1208.5540 | 2415.0934 | 2415.1087 | -0.0153 | 0 | 124 | 2.9e-011 | 1 | R.VSCLGVTDDGMAVATGSWDSFLK.I 24071 24072 24073 |
|  | 26252 | **914.1025** | **2739.2856** | **2739.2996** | **-0.0140** | **0** | **5** | **21** | **1** | **R.ADQELLMYSHDNIICGITSVAFSR.S** |

| **82.** | Q9BUF5\|TBB6_HUMAN    **Mass:** 50281    **Score:** 376    **Queries matched:** 40   **emPAI:** 1.20 |
| --- | --- |
|  | Tubulin beta-6 chain OS=Homo sapiens GN=TUBB6 PE=1 SV=1 |

|  | **Query** | **Observed** | **Mr(expt)** | **Mr(calc)** | **Delta** | **Miss** | **Score** | **Expect** | **Rank** | **Peptide** |
| --- | --- | --- | --- | --- | --- | --- | --- | --- | --- | --- |
|  | 1252 | **682.3712** | **681.3639** | **681.2826** | **0.0814** | **0** | **3** | **59** | **1** | **R.GPMSMK.E** |
|  | 5302 | 533.2137 | 1064.4128 | 1064.4201 | -0.0073 | 0 | 41 | 0.013 | 1 | R.NMMAACDPR.H 5301 |
|  | 5347 | 535.3016 | 1068.5886 | 1068.5968 | -0.0082 | 0 | 18 | 2.4 | 3 | R.YLTVATVFR.G |
|  | 5533 | 541.2109 | 1080.4073 | 1080.4151 | -0.0077 | 0 | (35) | 0.047 | 1 | R.NMMAACDPR.H 5532 |
|  | 5550 | 541.7350 | 1081.4554 | 1080.4151 | 1.0404 | 0 | (8) | 23 | 2 | R.NMMAACDPR.H 5554 |
|  | 6379 | 572.3181 | 1142.6217 | 1142.6270 | -0.0053 | 0 | 65 | 5.8e-005 | 1 | K.LAVNMVPFPR.L 6378 |
|  | 8020 | **616.8351** | **1231.6557** | **1230.5703** | **1.0854** | **0** | **7** | **35** | **4** | **R.ISEQFSAMFR.R** |
|  | 8582 | 630.3885 | 1258.7624 | 1257.6830 | 1.0795 | 1 | 9 | 17 | 3 | R.FPGQLNADLRK.L 8578 |
|  | 10345 | 676.3226 | 1350.6306 | 1350.6676 | -0.0370 | 0 | 9 | 16 | 8 | R.IMNTFSVMPSPK.V |
|  | 10693 | 456.8826 | 1367.6260 | 1366.6625 | 0.9635 | 0 | (5) | 40 | 5 | R.IMNTFSVMPSPK.V |
|  | 10735 | 685.8475 | 1369.6804 | 1370.6765 | -0.9961 | 1 | 1 | 1e+002 | 9 | K.RISEQFSAMFR.R |
|  | 10983 | 692.3545 | 1382.6944 | 1382.6574 | 0.0370 | 0 | (2) | 95 | 8 | R.IMNTFSVMPSPK.V |
|  | 11100 | 695.8426 | 1389.6706 | 1389.6810 | -0.0103 | 0 | 0 | 1.2e+002 | 9 | K.EVDEQMLAIQSK.N |
|  | 11821 | **716.8492** | **1431.6839** | **1430.6677** | **1.0162** | **0** | **15** | **4.1** | **1** | **R.INVYYNESSSQK.Y** |
|  | 14961 | 810.9169 | 1619.8193 | 1619.8283 | -0.0090 | 0 | 24 | 0.53 | 1 | R.LHFFMPGFAPLTSR.G |
|  | 16151 | 848.9155 | 1695.8164 | 1695.8257 | -0.0093 | 0 | 52 | 0.00079 | 1 | K.NSSYFVEWIPNNVK.V 16137 16148 16149 16165 16166 |
|  | 16518 | **860.4363** | **1718.8580** | **1718.8662** | **-0.0081** | **0** | **45** | **0.004** | **1** | **R.ALTVPELTQQMFDAR.N** |
|  | 18581 | 929.4688 | 1856.9229 | 1856.9342 | -0.0113 | 0 | 70 | 1e-005 | 2 | K.MASTFIGNSTAIQELFK.R 18585 18592 |
|  | 19626 | 971.9928 | 1941.9710 | 1941.9796 | -0.0086 | 0 | 93 | 4.6e-008 | 1 | K.GHYTEGAELVDAVLDVVR.K |
|  | 20468 | 1007.5194 | 2013.0243 | 2013.0353 | -0.0111 | 1 | 23 | 0.47 | 2 | K.MASTFIGNSTAIQELFKR.I 20467 |
|  | 20672 | **508.4994** | **2029.9683** | **2029.0302** | **0.9381** | **1** | **(2)** | **66** | **3** | **K.MASTFIGNSTAIQELFKR.I** |
|  | 25564 | **879.1141** | **2634.3206** | **2634.3323** | **-0.0117** | **0** | **11** | **4.6** | **1** | **K.LTTPTYGDLNHLVSATMSGVTTSLR.F** |
|  | 26728 | **942.7913** | **2825.3521** | **2825.3674** | **-0.0153** | **0** | **51** | **0.00045** | **1** | **R.SGPFGQLFRPDNFIFGQTGAGNNWAK.G** 26727 |
|  | 31044 | 1531.6991 | 4592.0755 | 4592.0999 | -0.0245 | 0 | 49 | 0.0002 | 1 | K.VSDTVVEPYNATLSVHQLVENTDETYCIDNEALYDICFR.T 31043 31047 |

| **83.** | P14618\|KPYM_HUMAN    **Mass:** 58470    **Score:** 376    **Queries matched:** 19   **emPAI:** 0.36 |
| --- | --- |
|  | Pyruvate kinase isozymes M1/M2 OS=Homo sapiens GN=PKM2 PE=1 SV=4 |

|  | **Query** | **Observed** | **Mr(expt)** | **Mr(calc)** | **Delta** | **Miss** | **Score** | **Expect** | **Rank** | **Peptide** |
| --- | --- | --- | --- | --- | --- | --- | --- | --- | --- | --- |
|  | 1393 | **734.3554** | **733.3481** | **733.3541** | **-0.0060** | **0** | **20** | **2** | **1** | **K.SGMNVAR.L** |
|  | 3147 | **456.3136** | **910.6126** | **911.4461** | **-0.8334** | **0** | **10** | **14** | **6** | **K.ASDVHEVR.K** |
|  | 3751 | **477.7271** | **953.4395** | **952.4726** | **0.9669** | **0** | **5** | **45** | **10** | **K.IENHEGVR.R** 3755 |
|  | 7285 | **599.2906** | **1196.5666** | **1196.5747** | **-0.0081** | **0** | **28** | **0.23** | **1** | **K.ITLDNAYMEK.C** |
|  | 7311 | 599.7598 | 1197.5051 | 1196.6401 | 0.8650 | 0 | 10 | 16 | 2 | R.LDIDSPPITAR.N 7270 7310 |
|  | 7644 | **607.3098** | **1212.6049** | **1212.5696** | **0.0353** | **0** | **(4)** | **64** | **7** | **K.ITLDNAYMEK.C** |
|  | 8014 | **616.8002** | **1231.5858** | **1231.6595** | **-0.0737** | **1** | **2** | **94** | **4** | **K.NICKVVEVGSK.I** |
|  | 9524 | **437.2274** | **1308.6604** | **1308.7046** | **-0.0442** | **1** | **9** | **18** | **1** | **K.VFLAQKMMIGR.C** |
|  | 17413 | **890.9274** | **1779.8403** | **1778.8687** | **0.9716** | **0** | **11** | **8.7** | **1** | **K.GADFLVTEVENGGSLGSK.K** |
|  | 18611 | **930.4504** | **1858.8863** | **1858.8924** | **-0.0061** | **0** | **120** | **1.1e-010** | **1** | **K.FGVEQDVDMVFASFIR.K** |
|  | 22129 | **1088.0561** | **2174.0977** | **2174.1107** | **-0.0129** | **0** | **101** | **6.8e-009** | **1** | **R.LAPITSDPTEATAVGAVEASFK.C** 22130 |
|  | 23464 | **777.7212** | **2330.1417** | **2330.2118** | **-0.0700** | **1** | **4** | **29** | **1** | **R.RLAPITSDPTEATAVGAVEASFK.C** |
|  | 24414 | **822.4301** | **2464.2685** | **2464.2849** | **-0.0164** | **0** | **30** | **0.078** | **1** | **R.TATESFASDPILYRPVAVALDTK.G** |
|  | 29474 | **1165.5458** | **3493.6155** | **3493.6341** | **-0.0186** | **1** | **114** | **1.6e-010** | **1** | **R.AEGSDVANAVLDGADCIMLSGETAKGDYPLEAVR.M** 29473 |

| **84.** | P62158\|CALM_HUMAN    **Mass:** 16827    **Score:** 375    **Queries matched:** 25   **emPAI:** 1.81 |
| --- | --- |
|  | Calmodulin OS=Homo sapiens GN=CALM1 PE=1 SV=2 |

|  | **Query** | **Observed** | **Mr(expt)** | **Mr(calc)** | **Delta** | **Miss** | **Score** | **Expect** | **Rank** | **Peptide** |
| --- | --- | --- | --- | --- | --- | --- | --- | --- | --- | --- |
|  | 1707 | **403.2131** | **804.4116** | **804.4164** | **-0.0048** | **0** | **17** | **3.9** | **1** | **K.ELGTVMR.S** 1705 |
|  | 1910 | **411.2250** | **820.4355** | **820.4113** | **0.0242** | **0** | **(2)** | **1e+002** | **8** | **K.ELGTVMR.S** |
|  | 14564 | 532.9061 | 1595.6966 | 1595.7063 | -0.0098 | 1 | 17 | 2.3 | 1 | K.DTDSEEEIREAFR.V |
|  | 15728 | **557.0919** | **1668.2539** | **1667.7712** | **0.4827** | **0** | **2** | **54** | **5** | **-.MADQLTEEQIAEFK.E** |
|  | 17044 | **877.9332** | **1753.8519** | **1753.8635** | **-0.0116** | **1** | **85** | **3.6e-007** | **1** | **R.VFDKDGNGYISAAELR.H** 17031 17053 17055 17056 17057 17058 |
|  | 18384 | **922.9433** | **1843.8720** | **1843.8840** | **-0.0119** | **1** | **51** | **0.00088** | **1** | **K.EAFSLFDKDGDGTITTK.E** 18385 |
|  | 24582 | **1245.5374** | **2489.0601** | **2489.0726** | **-0.0125** | **0** | **91** | **4.8e-008** | **1** | **R.EADIDGDGQVNYEEFVQMMTAK.-** 24580 24581 24583 |
|  | 30553 | **1357.6116** | **4069.8129** | **4068.8391** | **0.9738** | **0** | **(30)** | **0.027** | **1** | **R.SLGQNPTEAELQDMINEVDADGNGTIDFPEFLTMMAR.K** 30552 |
|  | 30577 | **1362.9398** | **4085.7976** | **4084.8340** | **0.9636** | **0** | **(21)** | **0.17** | **1** | **R.SLGQNPTEAELQDMINEVDADGNGTIDFPEFLTMMAR.K** |
|  | 30579 | **1362.9431** | **4085.8075** | **4084.8340** | **0.9735** | **0** | **52** | **0.00015** | **1** | **R.SLGQNPTEAELQDMINEVDADGNGTIDFPEFLTMMAR.K** 30578 |
|  | 30630 | **1372.9581** | **4115.8526** | **4116.8238** | **-0.9713** | **0** | **(1)** | **18** | **1** | **R.SLGQNPTEAELQDMINEVDADGNGTIDFPEFLTMMAR.K** |
|  | 30713 | **1400.3691** | **4198.0856** | **4196.9340** | **1.1516** | **1** | **3** | **11** | **2** | **R.SLGQNPTEAELQDMINEVDADGNGTIDFPEFLTMMARK.M** |

| **85.** | P11142\|HSP7C_HUMAN    **Mass:** 71082    **Score:** 373    **Queries matched:** 49   **emPAI:** 0.94 |
| --- | --- |
|  | Heat shock cognate 71 kDa protein OS=Homo sapiens GN=HSPA8 PE=1 SV=1 |

|  | **Query** | **Observed** | **Mr(expt)** | **Mr(calc)** | **Delta** | **Miss** | **Score** | **Expect** | **Rank** | **Peptide** |
| --- | --- | --- | --- | --- | --- | --- | --- | --- | --- | --- |
|  | 1691 | **804.4410** | **803.4337** | **803.4389** | **-0.0052** | **0** | **27** | **0.48** | **1** | **K.ITITNDK.G** |
|  | 2453 | **430.7156** | **859.4167** | **860.4352** | **-1.0184** | **1** | **14** | **8.7** | **9** | **K.DISENKR.A** |
|  | 3424 | **466.2736** | **930.5326** | **929.5294** | **1.0031** | **1** | **15** | **5.7** | **1** | **K.RNTTIPTK.Q** |
|  | 3607 | **472.7241** | **943.4337** | **943.5161** | **-0.0824** | **0** | **13** | **9.2** | **3** | **K.VCNPIITK.L** |
|  | 4228 | **495.2636** | **988.5125** | **988.5189** | **-0.0063** | **1** | **9** | **23** | **1** | **R.LSKEDIER.M** |
|  | 4652 | **509.2835** | **1016.5525** | **1016.5614** | **-0.0089** | **1** | **27** | **0.36** | **1** | **K.ITITNDKGR.L** |
|  | 6153 | **563.2797** | **1124.5449** | **1124.5536** | **-0.0087** | **1** | **23** | **0.8** | **1** | **R.MVQEAEKYK.A** |
|  | 6921 | **590.8102** | **1179.6059** | **1179.6135** | **-0.0076** | **1** | **5** | **49** | **5** | **K.VQVEYKGETK.S** |
|  | 7943 | 614.8138 | 1227.6130 | 1227.6207 | -0.0077 | 0 | 58 | 0.00024 | 1 | K.VEIIANDQGNR.T 7942 |
|  | 8075 | **618.3116** | **1234.6087** | **1234.6169** | **-0.0081** | **0** | **48** | **0.0026** | **1** | **R.MVNHFIAEFK.R** 8076 |
|  | 8453 | **627.7834** | **1253.5522** | **1253.5598** | **-0.0076** | **0** | **41** | **0.012** | **1** | **R.FDDAVVQSDMK.H** 8441 |
|  | 8748 | 634.8565 | 1267.6983 | 1267.6482 | 0.0502 | 1 | 14 | 5.4 | 8 | K.MKEIAEAYLGK.T |
|  | 8794 | **424.2204** | **1269.6394** | **1269.5547** | **0.0846** | **0** | **(5)** | **40** | **4** | **R.FDDAVVQSDMK.H** 8787 8803 |
|  | 9399 | **434.8811** | **1301.6214** | **1302.5914** | **-0.9700** | **0** | **6** | **37** | **3** | **K.NSLESYAFNMK.A** |
|  | 11437 | **705.8328** | **1409.6510** | **1409.6609** | **-0.0100** | **1** | **49** | **0.0015** | **1** | **R.RFDDAVVQSDMK.H** 11434 11435 |
|  | 12658 | **741.4022** | **1480.7899** | **1480.7998** | **-0.0099** | **0** | **53** | **0.00059** | **1** | **K.SQIHDIVLVGGSTR.I** 12659 |
|  | 12758 | **744.3493** | **1486.6840** | **1486.6940** | **-0.0100** | **0** | **33** | **0.064** | **1** | **R.TTPSYVAFTDTER.L** |
|  | 14076 | 783.3349 | 1564.6552 | 1564.8249 | -0.1697 | 1 | 2 | 75 | 9 | K.LLQDFFNGKELNK.S |
|  | 14882 | **808.8950** | **1615.7755** | **1615.7804** | **-0.0049** | **0** | **64** | **4.3e-005** | **1** | **K.SFYPEEVSSMVLTK.M** 14851 14880 14883 |
|  | 15395 | 825.3951 | 1648.7756 | 1648.7879 | -0.0123 | 0 | 61 | 8.9e-005 | 1 | K.NQVAMNPTNTVFDAK.R 15370 |
|  | 15468 | **551.9452** | **1652.8139** | **1652.8246** | **-0.0106** | **0** | **24** | **0.52** | **1** | **K.HWPFMVVNDAGRPK.V** |
|  | 15576 | 830.4467 | 1658.8788 | 1658.8879 | -0.0091 | 0 | 59 | 0.00013 | 1 | R.IINEPTAAAIAYGLDK.K 15592 |
|  | 15679 | 555.9651 | 1664.8734 | 1664.7828 | 0.0906 | 0 | (8) | 17 | 2 | K.NQVAMNPTNTVFDAK.R |
|  | 15746 | **557.2985** | **1668.8736** | **1668.8195** | **0.0541** | **0** | **(2)** | **65** | **4** | **K.HWPFMVVNDAGRPK.V** 15724 |
|  | 16070 | **846.3608** | **1690.7071** | **1690.7183** | **-0.0112** | **0** | **51** | **0.00079** | **1** | **K.STAGDTHLGGEDFDNR.M** 16069 |
|  | 16291 | **569.3043** | **1704.8910** | **1703.8188** | **1.0721** | **1** | **4** | **45** | **7** | **K.VSSKNSLESYAFNMK.A** |
|  | 17258 | **591.6353** | **1771.8841** | **1772.9131** | **-1.0290** | **1** | **3** | **57** | **6** | **K.ILDKCNEIINWLDK.N** |
|  | 17528 | **894.4936** | **1786.9726** | **1786.9828** | **-0.0102** | **1** | **56** | **0.00028** | **1** | **R.IINEPTAAAIAYGLDKK.V** |
|  | 17830 | **602.8280** | **1805.4622** | **1804.8890** | **0.5732** | **1** | **4** | **32** | **4** | **K.NQVAMNPTNTVFDAKR.L** |
|  | 18290 | **613.3387** | **1836.9942** | **1837.0058** | **-0.0116** | **1** | **13** | **5.4** | **1** | **K.LDKSQIHDIVLVGGSTR.I** |
|  | 20116 | **991.4958** | **1980.9770** | **1980.9905** | **-0.0135** | **0** | **7** | **18** | **1** | **K.TVTNAVVTVPAYFNDSQR.Q** |
|  | 23490 | **778.8956** | **2333.6649** | **2333.1685** | **0.4963** | **1** | **2** | **33** | **2** | **R.LIGDAAKNQVAMNPTNTVFDAK.R** |
|  | 26409 | **925.4422** | **2773.3048** | **2773.3195** | **-0.0147** | **0** | **31** | **0.045** | **1** | **K.QTQTFTTYSDNQPGVLIQVYEGER.A** |
|  | 30793 | **1072.6027** | **4286.3815** | **4286.9923** | **-0.6108** | **1** | **3** | **10** | **2** | **K.VCNPIITKLYQSAGGMPGGMPGGFPGGGAPPSGGASSGPTIEEVD.-** |

| **86.** | P00387\|NB5R3_HUMAN    **Mass:** 34441    **Score:** 371    **Queries matched:** 28   **emPAI:** 1.07 |
| --- | --- |
|  | NADH-cytochrome b5 reductase 3 OS=Homo sapiens GN=CYB5R3 PE=1 SV=3 |

|  | **Query** | **Observed** | **Mr(expt)** | **Mr(calc)** | **Delta** | **Miss** | **Score** | **Expect** | **Rank** | **Peptide** |
| --- | --- | --- | --- | --- | --- | --- | --- | --- | --- | --- |
|  | 3935 | **484.2804** | **966.5463** | **965.4971** | **1.0493** | **0** | **22** | **0.98** | **1** | **K.LWYTLDR.A** |
|  | 4766 | 514.6375 | 1027.2605 | 1026.6186 | 0.6419 | 1 | 1 | 92 | 6 | K.SNPIIRTVK.S |
|  | 4835 | **516.7863** | **1031.5579** | **1030.5923** | **0.9656** | **1** | **1** | **1.3e+002** | **5** | **K.GKFAIRPDK.K** |
|  | 6275 | **567.7966** | **1133.5786** | **1133.5869** | **-0.0084** | **1** | **15** | **4.7** | **1** | **K.VYFKDTHPK.F** |
|  | 10742 | **457.5987** | **1369.7742** | **1370.7293** | **-0.9551** | **0** | **7** | **28** | **5** | **R.STPAITLESPDIK.Y** 10740 10756 |
|  | 12425 | **734.3946** | **1466.7746** | **1466.7841** | **-0.0095** | **1** | **11** | **9.1** | **1** | **R.LIDREIISHDTR.R** |
|  | 12912 | **748.4227** | **1494.8309** | **1494.8405** | **-0.0096** | **0** | **9** | **17** | **4** | **K.DILLRPELEELR.N** |
|  | 19106 | **951.0158** | **1900.0169** | **1900.0274** | **-0.0105** | **0** | **76** | **2.4e-006** | **1** | **K.SVGMIAGGTGITPMLQVIR.A** 19103 19116 |
|  | 19110 | **951.0167** | **1900.0189** | **1900.0305** | **-0.0117** | **1** | **70** | **1.1e-005** | **1** | **R.STPAITLESPDIKYPLR.L** |
|  | 21555 | **1056.4727** | **2110.9308** | **2110.9418** | **-0.0111** | **0** | **83** | **3.9e-007** | **1** | **R.APEAWDYGQGFVNEEMIR.D** 21554 |
|  | 22812 | **562.2512** | **2244.9755** | **2244.2334** | **0.7421** | **1** | **10** | **8.3** | **1** | **R.TVKSVGMIAGGTGITPMLQVIR.A** |
|  | 23689 | **590.5487** | **2358.1657** | **2359.2790** | **-1.1132** | **1** | **3** | **35** | **6** | **K.SVGMIAGGTGITPMLQVIRAIMK.D** |
|  | 24760 | **838.7992** | **2513.3757** | **2513.3907** | **-0.0149** | **0** | **9** | **8.2** | **1** | **R.FALPSPQHILGLPVGQHIYLSAR.I** |
|  | 25309 | **861.7433** | **2582.2080** | **2582.2257** | **-0.0177** | **1** | **39** | **0.0074** | **1** | **R.AIMKDPDDHTVCHLLFANQTEK.D** |
|  | 25510 | **1310.9006** | **2619.7867** | **2620.2301** | **-0.4434** | **1** | **1** | **35** | **1** | **K.FPAGGKMSQYLESMQIGDTIEFR.G** |
|  | 27449 | **992.5239** | **2974.5500** | **2974.5652** | **-0.0152** | **1** | **53** | **0.00028** | **1** | **R.IDGNLVVRPYTPISSDDDKGFVDLVIK.V** 27446 27447 27448 27450 |
|  | 27879 | **1020.4775** | **3058.4106** | **3058.4283** | **-0.0177** | **1** | **64** | **2.1e-005** | **1** | **K.LWYTLDRAPEAWDYGQGFVNEEMIR.D** 27880 |
|  | 30859 | **1089.7669** | **4355.0383** | **4355.0636** | **-0.0253** | **0** | **3** | **9.4** | **2** | **R.DHLPPPEEEPLVLMCGPPPMIQYACLPNLDHVGHPTER.C** |

| **87.** | P02787\|TRFE_HUMAN    **Mass:** 79280    **Score:** 369    **Queries matched:** 43   **emPAI:** 0.65 |
| --- | --- |
|  | Serotransferrin OS=Homo sapiens GN=TF PE=1 SV=2 |

|  | **Query** | **Observed** | **Mr(expt)** | **Mr(calc)** | **Delta** | **Miss** | **Score** | **Expect** | **Rank** | **Peptide** |
| --- | --- | --- | --- | --- | --- | --- | --- | --- | --- | --- |
|  | 734 | **479.3557** | **478.3484** | **478.2210** | **0.1274** | **0** | **1** | **1.3e+002** | **1** | **R.SMGGK.E** |
|  | 2141 | **419.3130** | **836.6115** | **836.4062** | **0.2054** | **1** | **9** | **18** | **5** | **K.KDSSLCK.L** |
|  | 2687 | **439.2233** | **876.4320** | **877.4545** | **-1.0225** | **0** | **2** | **1.2e+002** | **9** | **R.KPVDEYK.D** |
|  | 6165 | **563.3363** | **1124.6580** | **1124.5648** | **0.0932** | **1** | **3** | **81** | **3** | **K.KASYLDCIR.A** |
|  | 6728 | **583.7951** | **1165.5756** | **1165.5840** | **-0.0084** | **0** | **37** | **0.028** | **1** | **K.HQTVPQNTGGK.N** 6723 6724 6725 6726 6727 6729 6730 6731 6733 |
|  | 7232 | **598.2708** | **1194.5271** | **1194.5352** | **-0.0082** | **0** | **20** | **1.4** | **1** | **K.WCALSHHER.L** 7267 |
|  | 8371 | 625.3026 | 1248.5907 | 1248.5986 | -0.0080 | 0 | 34 | 0.054 | 1 | K.SASDLTWDNLK.G |
|  | 8904 | 637.8639 | 1273.7132 | 1272.6462 | 1.0670 | 0 | 4 | 56 | 8 | K.HSTIFENLANK.A 8898 |
|  | 9716 | **659.2931** | **1316.5717** | **1316.5819** | **-0.0102** | **0** | **50** | **0.0013** | **1** | **R.WCAVSEHEATK.C** 9691 |
|  | 10635 | **683.3560** | **1364.6974** | **1363.7170** | **0.9804** | **1** | **2** | **88** | **5** | **R.CLVEKGDVAFVK.H** |
|  | 11520 | **708.3591** | **1414.7037** | **1414.7126** | **-0.0089** | **0** | **7** | **29** | **1** | **K.SVIPSDGPSVACVK.K** |
|  | 12840 | **497.9205** | **1490.7397** | **1490.7518** | **-0.0121** | **1** | **7** | **26** | **1** | **K.SKEFQLFSSPHGK.D** |
|  | 13358 | **761.3671** | **1520.7197** | **1520.7293** | **-0.0096** | **1** | **52** | **0.00085** | **1** | **R.LKCDEWSVNSVGK.I** |
|  | 13521 | **766.0736** | **1530.1327** | **1530.6807** | **-0.5480** | **0** | **0** | **78** | **3** | **K.CSTSSLLEACTFR.R** |
|  | 13724 | **515.3261** | **1542.9563** | **1542.8076** | **0.1487** | **1** | **4** | **42** | **9** | **K.SVIPSDGPSVACVKK.A** |
|  | 14087 | **783.3992** | **1564.7838** | **1564.7919** | **-0.0081** | **1** | **39** | **0.016** | **1** | **K.DLLFRDDTVCLAK.L** 14069 14085 |
|  | 16550 | **574.6526** | **1720.9361** | **1721.7535** | **-0.8174** | **0** | **2** | **77** | **9** | **K.LCMGSGLNLCEPNNK.E** |
|  | 16613 | **863.3820** | **1724.7494** | **1724.7597** | **-0.0103** | **0** | **17** | **2** | **1** | **K.IECVSAETTEDCIAK.I** |
|  | 17983 | **909.4003** | **1816.7860** | **1816.7972** | **-0.0112** | **0** | **58** | **0.00017** | **1** | **R.EGTCPEAPTDECKPVK.W** 17982 |
|  | 19787 | **489.2448** | **1952.9499** | **1951.9309** | **1.0190** | **1** | **2** | **64** | **7** | **K.NLNEKDYELLCLDGTR.K** |
|  | 20064 | **658.9929** | **1973.9568** | **1973.9708** | **-0.0140** | **1** | **20** | **0.92** | **1** | **K.HQTVPQNTGGKNPDPWAK.N** |
|  | 22094 | **1086.0475** | **2170.0804** | **2170.0881** | **-0.0077** | **0** | **90** | **8.4e-008** | **1** | **R.SAGWNIPIGLLYCDLPEPR.K** 22093 |
|  | 23306 | **1156.5112** | **2311.0079** | **2312.1437** | **-1.1358** | **1** | **1** | **61** | **3** | **K.EDLIWELLNQAQEHFGKDK.S** 23305 |
|  | 24860 | **844.0807** | **2529.2202** | **2529.2322** | **-0.0119** | **1** | **57** | **0.00015** | **1** | **R.SMGGKEDLIWELLNQAQEHFGK.D** |
|  | 31273 | **1248.0232** | **4988.0637** | **4987.0904** | **0.9732** | **0** | **37** | **0.002** | **1** | **K.AVANFFSGSCAPCADGTDFPQLCQLCPGCGCSTLNQYFGYSGAFK.C** 31272 |

| **88.** | P10620\|MGST1_HUMAN    **Mass:** 17644    **Score:** 368    **Queries matched:** 25   **emPAI:** 3.00 |
| --- | --- |
|  | Microsomal glutathione S-transferase 1 OS=Homo sapiens GN=MGST1 PE=1 SV=1 |

|  | **Query** | **Observed** | **Mr(expt)** | **Mr(calc)** | **Delta** | **Miss** | **Score** | **Expect** | **Rank** | **Peptide** |
| --- | --- | --- | --- | --- | --- | --- | --- | --- | --- | --- |
|  | 11645 | **711.8285** | **1421.6425** | **1421.6505** | **-0.0080** | **0** | **84** | **5.5e-007** | **1** | **K.MMLMSTATAFYR.L** 11673 |
|  | 12172 | **727.3378** | **1452.6610** | **1452.6708** | **-0.0098** | **0** | **38** | **0.018** | **1** | **K.VFANPEDCVAFGK.G** |
|  | 14314 | **791.3848** | **1580.7551** | **1580.7657** | **-0.0106** | **1** | **63** | **6.3e-005** | **1** | **R.KVFANPEDCVAFGK.G** 14313 |
|  | 17848 | **603.2692** | **1806.7859** | **1807.8783** | **-1.0924** | **1** | **7** | **22** | **1** | **K.MMLMSTATAFYRLTR.K** |
|  | 18874 | **941.3316** | **1880.6486** | **1880.9495** | **-0.3008** | **0** | **2** | **45** | **6** | **R.ALSFFVGYGVTLSMAYR.L** 18873 |
|  | 19774 | **651.6387** | **1951.8942** | **1951.9098** | **-0.0156** | **1** | **18** | **1.5** | **1** | **K.VFANPEDCVAFGKGENAK.K** |
|  | 26619 | **1404.1914** | **2806.3683** | **2806.3809** | **-0.0126** | **0** | **78** | **9.8e-007** | **1** | **M.VDLTQVMDDEVFMAFASYATIILSK.M** 26612 26615 26616 26618 26620 26622 26623 26628 26629 26630 26631 26632 26633 |
|  | 26712 | **941.7948** | **2822.3626** | **2822.3758** | **-0.0133** | **0** | **(59)** | **7e-005** | **1** | **M.VDLTQVMDDEVFMAFASYATIILSK.M** 26713 |

| **89.** | P04406\|G3P_HUMAN    **Mass:** 36201    **Score:** 360    **Queries matched:** 24   **emPAI:** 1.21 |
| --- | --- |
|  | Glyceraldehyde-3-phosphate dehydrogenase OS=Homo sapiens GN=GAPDH PE=1 SV=3 |

|  | **Query** | **Observed** | **Mr(expt)** | **Mr(calc)** | **Delta** | **Miss** | **Score** | **Expect** | **Rank** | **Peptide** |
| --- | --- | --- | --- | --- | --- | --- | --- | --- | --- | --- |
|  | 1399 | **739.3559** | **738.3486** | **738.3548** | **-0.0062** | **0** | **20** | **1.4** | **1** | **K.YDNSLK.I** |
|  | 2599 | **435.7475** | **869.4804** | **868.5018** | **0.9786** | **0** | **6** | **38** | **2** | **K.VIPELNGK.L** |
|  | 3119 | **455.5956** | **909.1765** | **908.4828** | **0.6937** | **0** | **3** | **51** | **8** | **K.AGAHLQGGAK.R** |
|  | 4830 | **516.5991** | **1031.1837** | **1031.5876** | **-0.4039** | **1** | **3** | **65** | **4** | **K.VKVGVNGFGR.I** |
|  | 7399 | **401.2420** | **1200.7043** | **1200.5995** | **0.1048** | **0** | **10** | **16** | **2** | **R.VVDLMAHMASK.E** |
|  | 10002 | **665.8240** | **1329.6335** | **1329.6421** | **-0.0085** | **1** | **50** | **0.0013** | **1** | **R.VVDLMAHMASKE.-** 10001 10003 10008 |
|  | 11459 | **706.3941** | **1410.7736** | **1410.7831** | **-0.0094** | **0** | **11** | **9.1** | **2** | **R.GALQNIIPASTGAAK.A** |
|  | 13493 | **510.5810** | **1528.7212** | **1529.7872** | **-1.0660** | **0** | **6** | **29** | **4** | **R.VPTANVSVVDLTCR.L** |
|  | 17159 | **882.3998** | **1762.7850** | **1762.7951** | **-0.0101** | **0** | **43** | **0.0051** | **1** | **K.LISWYDNEFGYSNR.V** 17158 |
|  | 18237 | **611.9746** | **1832.9018** | **1832.9124** | **-0.0106** | **0** | **4** | **48** | **1** | **K.IISNASCTTNCLAPLAK.V** |
|  | 21570 | **1057.0282** | **2112.0418** | **2112.1327** | **-0.0909** | **1** | **1** | **73** | **5** | **K.AENGKLVINGNPITIFQER.D** |
|  | 22461 | **1107.0524** | **2212.0902** | **2212.1020** | **-0.0118** | **0** | **36** | **0.023** | **1** | **R.VIISAPSADAPMFVMGVNHEK.Y** 22460 |
|  | 23022 | **1139.0155** | **2276.0164** | **2276.0307** | **-0.0143** | **0** | **112** | **5.2e-010** | **1** | **K.WGDAGAEYVVESTGVFTTMEK.A** 23023 23024 |
|  | 25374 | **865.7868** | **2594.3386** | **2594.3527** | **-0.0141** | **0** | **73** | **3.5e-006** | **1** | **K.VIHDNFGIVEGLMTTVHAITATQK.T** |
|  | 28902 | **1103.5219** | **3307.5437** | **3307.5570** | **-0.0133** | **0** | **41** | **0.0038** | **1** | **K.VDIVAINDPFIDLNYMVYMFQYDSTHGK.F** |
|  | 30482 | **1009.9738** | **4035.8659** | **4035.8875** | **-0.0216** | **0** | **37** | **0.0054** | **1** | **K.GILGYTEHQVVSSDFNSDTHSSTFDAGAGIALNDHFVK.L** 30483 |

| **90.** | P07099\|HYEP_HUMAN    **Mass:** 53143    **Score:** 360    **Queries matched:** 36   **emPAI:** 1.76 |
| --- | --- |
|  | Epoxide hydrolase 1 OS=Homo sapiens GN=EPHX1 PE=1 SV=1 |

|  | **Query** | **Observed** | **Mr(expt)** | **Mr(calc)** | **Delta** | **Miss** | **Score** | **Expect** | **Rank** | **Peptide** |
| --- | --- | --- | --- | --- | --- | --- | --- | --- | --- | --- |
|  | 1930 | **412.2242** | **822.4339** | **822.4388** | **-0.0049** | **0** | **6** | **42** | **8** | **K.VISYWR.N** |
|  | 2108 | **418.2346** | **834.4547** | **834.4599** | **-0.0052** | **0** | **19** | **2.3** | **1** | **R.FLGLTER.D** 2113 |
|  | 2159 | **838.8300** | **837.8227** | **837.3657** | **0.4570** | **0** | **7** | **18** | **1** | **R.NEFDWK.K** 2160 2162 |
|  | 2514 | **432.2500** | **862.4854** | **862.4912** | **-0.0059** | **0** | **37** | **0.042** | **1** | **K.FLSVLER.Q** |
|  | 2608 | **436.2502** | **870.4859** | **870.4923** | **-0.0064** | **0** | **17** | **3.7** | **2** | **K.QVEILNR.Y** |
|  | 3198 | **458.2384** | **914.4622** | **914.4684** | **-0.0062** | **0** | **35** | **0.063** | **1** | **K.VFYSLMR.E** |
|  | 3914 | **483.7345** | **965.4545** | **965.4607** | **-0.0062** | **1** | **7** | **35** | **4** | **R.NEFDWKK.Q** |
|  | 4261 | **496.2790** | **990.5435** | **990.5498** | **-0.0063** | **1** | **33** | **0.1** | **1** | **K.FLSVLERQ.-** |
|  | 4398 | **500.2973** | **998.5800** | **998.5873** | **-0.0072** | **1** | **17** | **3.7** | **2** | **K.KQVEILNR.Y** |
|  | 4544 | **505.3151** | **1008.6156** | **1008.6219** | **-0.0063** | **0** | **20** | **1.5** | **1** | **K.IIPLLTDPK.N** |
|  | 5113 | **526.2529** | **1050.4913** | **1050.4982** | **-0.0069** | **0** | **22** | **1.1** | **1** | **R.YLEDGGLER.K** |
|  | 5117 | **526.2863** | **1050.5579** | **1049.5618** | **0.9962** | **1** | **28** | **0.26** | **1** | **K.KGFNSVATAR.I** |
|  | 6434 | **574.2473** | **1146.4799** | **1146.5743** | **-0.0944** | **0** | **8** | **24** | **1** | **K.LISYSYMVR.G** |
|  | 9187 | **646.3039** | **1290.5932** | **1290.6027** | **-0.0094** | **0** | **33** | **0.072** | **1** | **K.ENLGQGWMTQK.H** |
|  | 9504 | **436.7244** | **1307.1514** | **1306.5976** | **0.5538** | **0** | **(8)** | **15** | **1** | **K.ENLGQGWMTQK.H** |
|  | 16676 | **865.4160** | **1728.8175** | **1728.8294** | **-0.0119** | **1** | **48** | **0.0016** | **1** | **R.FYKENLGQGWMTQK.H** 16677 |
|  | 16678 | **577.2820** | **1728.8241** | **1728.8002** | **0.0239** | **1** | **3** | **51** | **5** | **K.ENLGQGWMTQKHER.M** |
|  | 20334 | **1000.4900** | **1998.9654** | **1998.9799** | **-0.0145** | **0** | **62** | **5.8e-005** | **1** | **R.GGHFAAFEEPELLAQDIR.K** |
|  | 21700 | **709.7069** | **2126.0989** | **2127.0749** | **-0.9760** | **1** | **30** | **0.081** | **1** | **R.GGHFAAFEEPELLAQDIRK.F** |
|  | 23041 | **1140.0851** | **2278.1556** | **2278.1674** | **-0.0118** | **0** | **15** | **2.5** | **1** | **K.VYVPTGFSAFPFELLHTPEK.W** |
|  | 24190 | **1218.5448** | **2435.0750** | **2435.0892** | **-0.0142** | **0** | **27** | **0.13** | **1** | **R.FTPPLEDSCFHYGFNSNYLK.K** 24191 |
|  | 26412 | **1387.7025** | **2773.3905** | **2773.3997** | **-0.0092** | **0** | **106** | **1.6e-009** | **1** | **K.FSLDDLLTNVMLYWTTGTIISSQR.F** 26413 |
|  | 26549 | **699.5802** | **2794.2917** | **2794.3158** | **-0.0241** | **1** | **20** | **0.65** | **1** | **R.EDDSIRPFKVETSDEEIHDLHQR.I** 26550 26551 |
|  | 27098 | **968.1689** | **2901.4848** | **2901.4946** | **-0.0098** | **1** | **24** | **0.23** | **1** | **R.KFSLDDLLTNVMLYWTTGTIISSQR.F** |
|  | 27902 | **766.6048** | **3062.3901** | **3062.4080** | **-0.0179** | **0** | **29** | **0.062** | **1** | **K.NHGLSDEHVFEVICPSIPGYGFSEASSK.K** 27900 27901 |
|  | 29637 | **1188.9108** | **3563.7105** | **3563.7276** | **-0.0171** | **0** | **70** | **4.1e-006** | **1** | **R.ESGYMHIQCTKPDTVGSALNDSPVGLAAYILEK.F** |

| **91.** | Q99536\|VAT1_HUMAN    **Mass:** 42122    **Score:** 359    **Queries matched:** 26   **emPAI:** 1.16 |
| --- | --- |
|  | Synaptic vesicle membrane protein VAT-1 homolog OS=Homo sapiens GN=VAT1 PE=1 SV=2 |

|  | **Query** | **Observed** | **Mr(expt)** | **Mr(calc)** | **Delta** | **Miss** | **Score** | **Expect** | **Rank** | **Peptide** |
| --- | --- | --- | --- | --- | --- | --- | --- | --- | --- | --- |
|  | 3629 | **473.2790** | **944.5434** | **943.5385** | **1.0049** | **1** | **11** | **17** | **10** | **K.RNLMALAR.T** |
|  | 6105 | **561.3102** | **1120.6059** | **1119.6110** | **0.9949** | **0** | **39** | **0.02** | **1** | **K.GYNLLKPMGK.V** 6110 |
|  | 7222 | **597.8363** | **1193.6580** | **1193.6656** | **-0.0075** | **1** | **28** | **0.22** | **1** | **K.VLLVPGPEKEN.-** |
|  | 10125 | **669.8147** | **1337.6148** | **1337.6220** | **-0.0072** | **0** | **86** | **3.4e-007** | **1** | **R.ACGLNFADLMAR.Q** |
|  | 10292 | **450.2122** | **1347.6147** | **1348.8078** | **-1.1931** | **1** | **3** | **84** | **8** | **K.NVGKVLLVPGPEK.E** |
|  | 13155 | **755.8855** | **1509.7564** | **1509.7675** | **-0.0111** | **0** | **35** | **0.039** | **1** | **R.TVENVTVFGTASASK.H** |
|  | 14208 | **787.8846** | **1573.7547** | **1573.7658** | **-0.0111** | **0** | **89** | **1.6e-007** | **1** | **K.GVDIVMDPLGGSDTAK.G** 14207 |
|  | 14454 | 795.3271 | 1588.6397 | 1589.7607 | -1.1210 | 0 | (7) | 21 | 2 | K.GVDIVMDPLGGSDTAK.G 14491 14502 |
|  | 14918 | 809.8762 | 1617.7379 | 1618.8865 | -1.1486 | 1 | 6 | 30 | 3 | K.VVTYGMANLLTGPKR.N 14947 14957 |
|  | 19425 | **964.0403** | **1926.0661** | **1926.0799** | **-0.0138** | **0** | **65** | **2.6e-005** | **1** | **K.LQSRPAAPPAPGPGQLTLR.L** 19426 19427 19428 19429 |
|  | 21333 | **697.4195** | **2089.2366** | **2088.0851** | **1.1515** | **1** | **3** | **41** | **3** | **R.TVENVTVFGTASASKHEALK.E** |
|  | 25386 | **650.0530** | **2596.1828** | **2596.3757** | **-0.1929** | **1** | **5** | **21** | **1** | **K.GYNLLKPMGKVVTYGMANLLTGPK.R** 25387 |
|  | 25625 | **882.4778** | **2644.4115** | **2644.4258** | **-0.0143** | **1** | **37** | **0.01** | **1** | **R.LPPLPVTPGMEGAGVVIAVGEGVSDRK.A** |
|  | 28707 | **1083.8973** | **3248.6702** | **3248.6864** | **-0.0162** | **1** | **74** | **2e-006** | **1** | **R.QGLYDRLPPLPVTPGMEGAGVVIAVGEGVSDR.K** 28706 |

| **92.** | P62820\|RAB1A_HUMAN    **Mass:** 22891    **Score:** 356    **Queries matched:** 35   **emPAI:** 4.47 |
| --- | --- |
|  | Ras-related protein Rab-1A OS=Homo sapiens GN=RAB1A PE=1 SV=3 |

|  | **Query** | **Observed** | **Mr(expt)** | **Mr(calc)** | **Delta** | **Miss** | **Score** | **Expect** | **Rank** | **Peptide** |
| --- | --- | --- | --- | --- | --- | --- | --- | --- | --- | --- |
|  | 1162 | **643.2711** | **642.2638** | **642.4064** | **-0.1427** | **0** | **27** | **0.41** | **1** | **K.LLVGNK.C** |
|  | 1467 | **772.4502** | **771.4429** | **771.4491** | **-0.0061** | **0** | **21** | **1.6** | **2** | **K.IQSTPVK.Q** |
|  | 3332 | **462.7205** | **923.4265** | **923.4348** | **-0.0083** | **0** | **13** | **6.7** | **1** | **R.YASENVNK.L** |
|  | 4239 | 495.7453 | 989.4760 | 989.4818 | -0.0058 | 0 | 33 | 0.1 | 1 | R.TITSSYYR.G |
|  | 4728 | **512.7835** | **1023.5523** | **1023.5601** | **-0.0077** | **1** | **36** | **0.041** | **1** | **K.KVVDYTTAK.E** 4708 4727 |
|  | 5052 | **523.7465** | **1045.4784** | **1045.4862** | **-0.0079** | **0** | **42** | **0.012** | **1** | **R.MGPGATAGGAEK.S** 5036 5066 5078 5081 |
|  | 5272 | **531.8570** | **1061.6994** | **1061.4812** | **0.2183** | **0** | **(2)** | **1e+002** | **9** | **R.MGPGATAGGAEK.S** |
|  | 5394 | **536.3203** | **1070.6261** | **1070.6336** | **-0.0075** | **0** | **45** | **0.006** | **1** | **K.LLLIGDSGVGK.S** 5392 |
|  | 5639 | **544.8228** | **1087.6309** | **1086.5458** | **1.0852** | **0** | **10** | **19** | **9** | **K.QWLQEIDR.Y** |
|  | 7446 | **602.3568** | **1202.6989** | **1201.5873** | **1.1116** | **1** | **54** | **0.00057** | **1** | **K.RMGPGATAGGAEK.S** |
|  | 7742 | **406.5653** | **1216.6740** | **1217.5823** | **-0.9082** | **1** | **(8)** | **28** | **4** | **K.RMGPGATAGGAEK.S** |
|  | 9695 | **658.8284** | **1315.6422** | **1315.6521** | **-0.0099** | **0** | **18** | **2.6** | **1** | **K.LQIWDTAGQER.F** 9685 |
|  | 12794 | 745.3779 | 1488.7413 | 1489.7195 | -0.9782 | 1 | 13 | 6.1 | 3 | R.MGPGATAGGAEKSNVK.I 12828 |
|  | 12857 | **498.2625** | **1491.7655** | **1492.6544** | **-0.8889** | **0** | **10** | **15** | **3** | **M.SSMNPEYDYLFK.L** 12866 |
|  | 16601 | **862.9358** | **1723.8570** | **1723.8669** | **-0.0098** | **0** | **35** | **0.033** | **1** | **K.EFADSLGIPFLETSAK.N** 16602 |
|  | 18924 | **943.4422** | **1884.8698** | **1883.8757** | **0.9941** | **0** | **101** | **8.4e-009** | **1** | **K.NATNVEQSFMTMAAEIK.K** 18914 18915 |
|  | 19106 | 951.0158 | 1900.0169 | 1899.8706 | 0.1463 | 0 | (1) | 75 | 7 | K.NATNVEQSFMTMAAEIK.K |
|  | 20253 | **996.9864** | **1991.9583** | **1991.9701** | **-0.0117** | **1** | **46** | **0.0024** | **1** | **K.QWLQEIDRYASENVNK.L** 20252 |
|  | 22107 | **1086.4988** | **2170.9830** | **2170.9947** | **-0.0117** | **0** | **72** | **5.5e-006** | **1** | **R.FADDTYTESYISTIGVDFK.I** 22106 |
|  | 23254 | **769.0485** | **2304.1237** | **2304.1386** | **-0.0149** | **0** | **5** | **24** | **1** | **R.GAHGIIVVYDVTDQESFNNVK.Q** |

| **93.** | Q99758\|ABCA3_HUMAN    **Mass:** 192836   **Score:** 355    **Queries matched:** 48   **emPAI:** 0.16 |
| --- | --- |
|  | ATP-binding cassette sub-family A member 3 OS=Homo sapiens GN=ABCA3 PE=1 SV=2 |

|  | **Query** | **Observed** | **Mr(expt)** | **Mr(calc)** | **Delta** | **Miss** | **Score** | **Expect** | **Rank** | **Peptide** |
| --- | --- | --- | --- | --- | --- | --- | --- | --- | --- | --- |
|  | 869 | **528.2405** | **527.2333** | **526.3479** | **0.8854** | **0** | **6** | **33** | **9** | **R.IPAVK.L** |
|  | 1318 | 705.3721 | 704.3648 | 704.3705 | -0.0057 | 0 | 16 | 5.8 | 1 | R.ISSDVGK.V |
|  | 1425 | **754.3671** | **753.3599** | **752.3605** | **0.9993** | **0** | **11** | **12** | **6** | **K.AAYSWR.E** |
|  | 1717 | **403.2294** | **804.4442** | **804.4341** | **0.0100** | **0** | **14** | **8.9** | **4** | **K.TVTETVR.R** |
|  | 2545 | **433.5917** | **865.1688** | **864.3726** | **0.7962** | **0** | **8** | **19** | **5** | **K.DQFNEGR.K** |
|  | 2786 | **441.7235** | **881.4324** | **881.4355** | **-0.0031** | **1** | **3** | **76** | **5** | **R.TYSGGNKR.K** |
|  | 3862 | **481.2705** | **960.5265** | **960.5352** | **-0.0088** | **1** | **11** | **15** | **4** | **K.TVTETVRR.A** |
|  | 3997 | **486.7794** | **971.5442** | **971.5698** | **-0.0257** | **1** | **9** | **23** | **10** | **R.RALVINMR.V** |
|  | 4561 | **506.2566** | **1010.4986** | **1009.5127** | **0.9858** | **1** | **7** | **31** | **4** | **R.SRFLSGGMR.R** |
|  | 4753 | **513.9840** | **1025.9535** | **1025.5076** | **0.4458** | **1** | **(1)** | **85** | **7** | **R.SRFLSGGMR.R** |
|  | 4939 | **520.2498** | **1038.4851** | **1038.5280** | **-0.0430** | **0** | **3** | **75** | **3** | **K.CLGSPQHLK.S** |
|  | 5048 | **523.3311** | **1044.6475** | **1043.4780** | **1.1696** | **0** | **3** | **82** | **2** | **R.EMLVMYAR.L** |
|  | 5383 | **536.2787** | **1070.5429** | **1070.6382** | **-0.0953** | **1** | **7** | **32** | **8** | **R.ALVINMRVR.G** |
|  | 5621 | **544.2840** | **1086.5534** | **1086.6332** | **-0.0797** | **1** | **(4)** | **70** | **6** | **R.ALVINMRVR.G** |
|  | 6130 | **562.3049** | **1122.5953** | **1121.5101** | **1.0852** | **0** | **3** | **81** | **10** | **R.ASVEGGGFNER.C** |
|  | 6386 | **572.3725** | **1142.7304** | **1141.7070** | **1.0234** | **0** | **1** | **1.2e+002** | **4** | **K.LSIGIALIAGSK.V** |
|  | 7646 | **607.3157** | **1212.6169** | **1212.5735** | **0.0435** | **0** | **4** | **52** | **6** | **K.DALQAEGQEPR.E** 7655 |
|  | 8495 | **419.3127** | **1254.9164** | **1254.6067** | **0.3097** | **0** | **1** | **76** | **8** | **K.YGAGYHMTLVK.E** |
|  | 8769 | **635.3176** | **1268.6206** | **1268.6295** | **-0.0090** | **0** | **18** | **2.5** | **1** | **R.HIGACVENTLR.G** 8770 |
|  | 8807 | **636.3146** | **1270.6146** | **1269.8020** | **0.8126** | **1** | **5** | **45** | **8** | **R.KLSIGIALIAGSK.V** |
|  | 9611 | **438.7606** | **1313.2599** | **1312.6631** | **0.5968** | **1** | **4** | **40** | **4** | **R.EMLVMYARLR.G** |
|  | 10293 | **674.8149** | **1347.6153** | **1347.6241** | **-0.0088** | **0** | **42** | **0.01** | **1** | **R.AIMEYHADAATR.Q** 10292 |
|  | 11632 | **711.3883** | **1420.7621** | **1420.7099** | **0.0522** | **1** | **1** | **1e+002** | **9** | **R.ESTHRFEGLFAK.L** |
|  | 12496 | 737.3639 | 1472.7132 | 1473.6736 | -0.9604 | 0 | 8 | 21 | 1 | R.EPTSPDGGEPGYIR.E |
|  | 14284 | **790.4180** | **1578.8215** | **1578.8293** | **-0.0078** | **0** | **40** | **0.011** | **1** | **R.EVLGDLEEFLIFR.A** 14285 |
|  | 14818 | **807.3719** | **1612.7292** | **1612.7403** | **-0.0111** | **0** | **84** | **4.6e-007** | **1** | **R.MPVLPEDQDVADER.T** 14817 |
|  | 14995 | **541.7775** | **1622.3106** | **1621.7599** | **0.5507** | **0** | **0** | **69** | **4** | **R.NYMWTQTGSFFLK.E** |
|  | 16475 | **858.9398** | **1715.8651** | **1715.8764** | **-0.0113** | **0** | **70** | **1.1e-005** | **1** | **K.VLILDEPTSGMDAISR.R** 16476 |
|  | 17397 | **890.4639** | **1778.9132** | **1778.9237** | **-0.0105** | **0** | **18** | **1.7** | **1** | **K.TTTLSMLTGLFPPTSGR.A** |
|  | 18080 | **912.4325** | **1822.8504** | **1822.8625** | **-0.0120** | **0** | **49** | **0.0015** | **1** | **R.NEYFEAEPEDLVAGIK.I** |
|  | 18546 | **619.2819** | **1854.8239** | **1854.9410** | **-0.1171** | **1** | **0** | **95** | **8** | **K.QMLHIIGLEDKWNSR.S** |
|  | 20244 | **664.6747** | **1991.0022** | **1991.0411** | **-0.0389** | **0** | **0** | **84** | **3** | **K.LLCGPHASIVVSNFPQPR.S** |
|  | 20561 | **674.3497** | **2020.0272** | **2019.9948** | **0.0323** | **1** | **0** | **83** | **10** | **R.AIMEYHADAATRQLFQR.L** |
|  | 23077 | **761.7075** | **2282.1006** | **2282.1120** | **-0.0115** | **0** | **14** | **3.3** | **1** | **K.ETEGWHTTSLFPLFPNPGPR.E** |
|  | 25143 | **852.4303** | **2554.2691** | **2554.2849** | **-0.0159** | **0** | **18** | **1** | **1** | **K.LVDSSMDIQAIQLPALQYQHER.R** |
|  | 25821 | **891.4088** | **2671.2046** | **2670.2972** | **0.9074** | **1** | **1** | **45** | **5** | **R.EGFLAVQHAVDRAIMEYHADAATR.Q** |
|  | 27219 | **977.4424** | **2929.3053** | **2929.5445** | **-0.2392** | **0** | **1** | **42** | **2** | **R.FVASMAASGCAYLILLFLIETNLLQR.L** |
|  | 27742 | **1010.8697** | **3029.5872** | **3029.5974** | **-0.0102** | **0** | **45** | **0.0014** | **1** | **R.TVVNALFNNQAYHSPATALAVVDNLLFK.L** 27746 |
|  | 29219 | **1131.8789** | **3392.6149** | **3392.5500** | **0.0649** | **1** | **2** | **25** | **1** | **R.ASDWAVDSNLCGAMDPSDGIGALIEEERTAVK.L** |
|  | 29690 | **1194.9124** | **3581.7152** | **3581.7314** | **-0.0162** | **0** | **76** | **8.9e-007** | **1** | **K.YGVDDYSVSQISLEQVFLSFAHLQPPTAEEGR.-** 29689 |

| **94.** | Q09666\|AHNK_HUMAN    **Mass:** 629213   **Score:** 351    **Queries matched:** 201   **emPAI:** 0.10 |
| --- | --- |
|  | Neuroblast differentiation-associated protein AHNAK OS=Homo sapiens GN=AHNAK PE=1 SV=2 |

|  | **Query** | **Observed** | **Mr(expt)** | **Mr(calc)** | **Delta** | **Miss** | **Score** | **Expect** | **Rank** | **Peptide** |
| --- | --- | --- | --- | --- | --- | --- | --- | --- | --- | --- |
|  | 987 | **571.3319** | **570.3246** | **569.3537** | **0.9709** | **0** | **14** | **6** | **1** | **K.LNVPK.M** |
|  | 1192 | **650.0526** | **649.0453** | **649.3291** | **-0.2838** | **1** | **12** | **10** | **1** | **K.MPKMK.M** 1191 1195 1198 |
|  | 1994 | 414.7114 | 827.4082 | 828.4891 | -1.0809 | 1 | 4 | 73 | 8 | K.LNVPKMK.V |
|  | 2799 | **442.5624** | **883.1103** | **882.4633** | **0.6470** | **0** | **2** | **67** | **3** | **K.IPEMHLK.A** |
|  | 3002 | **451.2679** | **900.5213** | **900.4553** | **0.0660** | **0** | **6** | **53** | **10** | **K.ADVDISGPK.I** |
|  | 3056 | 453.2256 | 904.4366 | 905.4277 | -0.9911 | 0 | 22 | 1.1 | 3 | K.MQVGGDGVK.V 3084 |
|  | 3112 | **455.2117** | **908.4088** | **908.4967** | **-0.0879** | **0** | **6** | **42** | **8** | **K.AEAPLPSPK.L** |
|  | 3152 | **456.7453** | **911.4761** | **912.5280** | **-1.0519** | **0** | **23** | **0.71** | **2** | **K.GDVVVSLPK.V** |
|  | 3398 | **465.2720** | **928.5295** | **928.4865** | **0.0430** | **0** | **17** | **3.6** | **1** | **K.ADIEISGPK.V** 3373 3392 3395 |
|  | 3408 | **465.7541** | **929.4937** | **928.4866** | **1.0071** | **0** | **35** | **0.055** | **1** | **K.GDVDVSLPK.L** |
|  | 3868 | **481.7764** | **961.5383** | **960.4586** | **1.0797** | **0** | **4** | **72** | **8** | **K.GDMDVSLPK.V** |
|  | 4272 | **496.5989** | **991.1831** | **990.4658** | **0.7173** | **0** | **4** | **48** | **8** | **R.LDFEGPDAK.L** |
|  | 4417 | **500.7920** | **999.5695** | **1000.5302** | **-0.9607** | **0** | **8** | **28** | **8** | **K.VGGSGVNVNAK.G** |
|  | 4554 | **506.2310** | **1010.4474** | **1010.5396** | **-0.0922** | **1** | **3** | **76** | **1** | **K.GPEADIKGPK.V** |
|  | 4589 | **507.2345** | **1012.4545** | **1013.5393** | **-1.0848** | **0** | **4** | **63** | **6** | **K.VQTPEVDVK.G** |
|  | 4947 | 520.2974 | 1038.5802 | 1038.4651 | 0.1150 | 1 | 14 | 6.5 | 9 | -.MEKEETTR.E |
|  | 5037 | **523.2590** | **1044.5035** | **1045.5768** | **-1.0733** | **0** | **14** | **7.8** | **3** | **K.LQGSGVSLASK.K** |
|  | 5737 | 1095.6332 | 1094.6259 | 1094.6084 | 0.0175 | 1 | 5 | 43 | 3 | K.VPDVDIRGPK.V |
|  | 5750 | **548.8361** | **1095.6576** | **1094.6084** | **1.0492** | **1** | **10** | **13** | **1** | **K.VPEVDVRGPK.V** |
|  | 5813 | 551.2770 | 1100.5394 | 1099.5584 | 0.9811 | 0 | 8 | 27 | 3 | K.MPDVDISVPK.I |
|  | 6022 | **558.7985** | **1115.5825** | **1115.5533** | **0.0292** | **0** | **(7)** | **30** | **5** | **K.MPDVDISVPK.I** |
|  | 6067 | **560.2850** | **1118.5555** | **1118.6158** | **-0.0603** | **1** | **6** | **41** | **8** | **K.FKMPEINIK.A** |
|  | 6101 | **561.2814** | **1120.5483** | **1121.5791** | **-1.0308** | **0** | **1** | **1.4e+002** | **5** | **K.MPFLSISSPK.V** |
|  | 6231 | **566.2930** | **1130.5715** | **1130.5827** | **-0.0112** | **1** | **(5)** | **57** | **1** | **K.MPEMSIKAPK.I** 6220 |
|  | 6345 | **571.2792** | **1140.5439** | **1140.5784** | **-0.0344** | **1** | **10** | **17** | **10** | **K.MPKIHMSGPK.I** |
|  | 6360 | **571.7579** | **1141.5013** | **1140.5784** | **0.9230** | **1** | **(4)** | **58** | **8** | **K.MPKIHMSGPK.I** |
|  | 6366 | **571.7991** | **1141.5837** | **1141.5511** | **0.0326** | **1** | **1** | **1.2e+002** | **7** | **K.FKMPEMSIK.A** |
|  | 6422 | **573.7815** | **1145.5484** | **1146.5777** | **-1.0292** | **1** | **7** | **29** | **5** | **K.MPEMSIKAPK.I** 6437 |
|  | 6594 | **578.8281** | **1155.6417** | **1156.5733** | **-0.9316** | **1** | **(8)** | **22** | **4** | **K.MPKIHMSGPK.I** |
|  | 6600 | **579.0106** | **1156.0067** | **1155.6321** | **0.3745** | **1** | **2** | **60** | **5** | **K.MPEINIKAPK.I** |
|  | 6659 | **581.2735** | **1160.5324** | **1159.5729** | **0.9595** | **1** | **10** | **15** | **5** | **K.MPDMNIKAPK.I** 6642 |
|  | 6723 | 583.7938 | 1165.5731 | 1166.5828 | -1.0097 | 1 | 2 | 82 | 5 | K.MPKISMPGFK.G 6729 |
|  | 6792 | **585.8414** | **1169.6682** | **1169.6656** | **0.0026** | **1** | **3** | **81** | **1** | **K.VKGDVDVTLPK.V** |
|  | 6937 | **591.3317** | **1180.6488** | **1180.5984** | **0.0504** | **1** | **3** | **76** | **9** | **K.MPKFSMPSLK.G** |
|  | 6974 | **592.3015** | **1182.5885** | **1183.6635** | **-1.0750** | **0** | **5** | **50** | **3** | **K.TPEMIIQKPK.I** |
|  | 7065 | **594.2525** | **1186.4904** | **1185.6250** | **0.8655** | **1** | **3** | **71** | **6** | **K.MPEMNIKVPK.I** |
|  | 7075 | 396.5243 | 1186.5510 | 1187.6584 | -1.1074 | 1 | 12 | 9.5 | 4 | K.MKGDVVVSLPK.V |
|  | 7139 | **397.4028** | **1189.1865** | **1189.6013** | **-0.4148** | **1** | **5** | **35** | **1** | **K.VKGDMDVSVPK.V** 7125 |
|  | 7169 | 596.3179 | 1190.6213 | 1191.5780 | -0.9567 | 1 | 3 | 74 | 8 | K.LKMPEMHFK.A 7201 |
|  | 7373 | **600.8315** | **1199.6484** | **1198.5297** | **1.1187** | **0** | **16** | **3.9** | **1** | **K.MPDMHVNMPK.I** 7322 |
|  | 7429 | 602.2694 | 1202.5243 | 1201.6376 | 0.8866 | 1 | 9 | 21 | 2 | K.IKGDMDISVPK.L |
|  | 7430 | 401.8488 | 1202.5245 | 1201.6376 | 0.8869 | 0 | 13 | 7.8 | 4 | K.ISMPDVGLNLK.A 7417 |
|  | 7438 | **602.2905** | **1202.5665** | **1201.5584** | **1.0082** | **0** | **0** | **1.6e+002** | **10** | **K.MPSMNIQTHK.I** |
|  | 7445 | **602.3564** | **1202.6982** | **1203.5991** | **-0.9009** | **0** | **2** | **90** | **7** | **K.MPEMSIKPQK.I** |
|  | 7590 | **606.0446** | **1210.0746** | **1210.6710** | **-0.5965** | **1** | **4** | **36** | **9** | **K.LKFGTFGGLGSK.S** |
|  | 7594 | **606.2866** | **1210.5586** | **1210.6194** | **-0.0608** | **1** | **11** | **11** | **2** | **K.GEGPDGDVKLPK.A** 7595 7596 7597 7599 |
|  | 7628 | **607.2072** | **1212.3999** | **1211.6034** | **0.7965** | **0** | **7** | **21** | **3** | **K.GEGPDVDVSLPK.A** |
|  | 7637 | **405.2060** | **1212.5961** | **1211.5467** | **1.0494** | **1** | **10** | **16** | **5** | **K.FKMPDMHFK.A** |
|  | 7643 | **405.2064** | **1212.5973** | **1211.5757** | **1.0215** | **1** | **11** | **12** | **2** | **K.FSMPGFKGEGR.E** |
|  | 7691 | **608.3362** | **1214.6578** | **1213.6918** | **0.9660** | **1** | **5** | **46** | **5** | **K.LKTDVDVSLPK.V** |
|  | 7729 | **609.2986** | **1216.5826** | **1216.5733** | **0.0093** | **1** | **15** | **4.7** | **1** | **K.MPDMHFKAPK.I** 7743 |
|  | 7744 | **406.6971** | **1217.0696** | **1217.6326** | **-0.5630** | **1** | **10** | **10** | **9** | **K.VKGDMDISLPK.V** |
|  | 7748 | 609.8021 | 1217.5896 | 1216.5733 | 1.0163 | 1 | (3) | 78 | 7 | K.MPDMHFKAPK.I |
|  | 7785 | **610.3678** | **1218.7210** | **1217.6326** | **1.0885** | **1** | **(3)** | **72** | **4** | **K.IKGDMDISVPK.L** |
|  | 7914 | 614.0780 | 1226.1414 | 1225.6190 | 0.5224 | 0 | 2 | 64 | 6 | K.GEGPDVDVTLPK.A |
|  | 8151 | 620.2996 | 1238.5847 | 1237.5736 | 1.0111 | 1 | 4 | 54 | 6 | K.FKMPEMHFR.A |
|  | 8167 | **620.8095** | **1239.6045** | **1238.6870** | **0.9174** | **1** | **5** | **46** | **5** | **K.LPKADIDVSGPK.V** |
|  | 8186 | **414.5581** | **1240.6526** | **1239.6347** | **1.0179** | **0** | **1** | **1.1e+002** | **7** | **K.GEGPEVDVTLPK.A** |
|  | 8287 | **416.0273** | **1245.0602** | **1244.6322** | **0.4280** | **0** | **5** | **34** | **5** | **K.MSLPDVDLDLK.G** |
|  | 8296 | **416.2181** | **1245.6325** | **1245.6275** | **0.0050** | **0** | **11** | **13** | **2** | **K.VSMPDVDLNLK.G** |
|  | 8724 | **634.3209** | **1266.6272** | **1265.6326** | **0.9946** | **1** | **(4)** | **51** | **4** | **K.VKGEYDMTVPK.L** |
|  | 8726 | **634.3257** | **1266.6369** | **1266.6456** | **-0.0086** | **0** | **48** | **0.0022** | **1** | **K.AEGPEVDVNLPK.A** |
|  | 8731 | **634.3361** | **1266.6577** | **1266.6642** | **-0.0065** | **0** | **2** | **98** | **9** | **K.ISMPDVDLHLK.G** |
|  | 8749 | 423.5754 | 1267.7043 | 1267.7136 | -0.0093 | 1 | 47 | 0.0025 | 1 | K.GKKPDIDITGPK.V |
|  | 8838 | **636.8536** | **1271.6927** | **1271.6795** | **0.0132** | **0** | **5** | **46** | **8** | **K.ISMPEIDLNLK.G** |
|  | 9023 | **428.2283** | **1281.6631** | **1281.6275** | **0.0356** | **1** | **6** | **36** | **10** | **K.VKGEYDMTVPK.L** 9036 |
|  | 9140 | **430.2996** | **1287.8769** | **1287.6744** | **0.2024** | **0** | **(4)** | **44** | **10** | **K.ISMPEIDLNLK.G** |
|  | 9173 | **645.8397** | **1289.6648** | **1288.7213** | **0.9434** | **1** | **4** | **53** | **7** | **K.FSMPKIGIPGVK.M** |
|  | 9314 | **649.3160** | **1296.6174** | **1295.6180** | **0.9994** | **0** | **10** | **14** | **2** | **K.GEGPDVHMTLPK.G** |
|  | 9355 | **434.2050** | **1299.5931** | **1300.6412** | **-1.0481** | **1** | **4** | **58** | **8** | **K.GPRLDFEGPDAK.L** |
|  | 9375 | 651.3182 | 1300.6219 | 1300.6155 | 0.0064 | 0 | 4 | 54 | 2 | K.ISMPDVDLHMK.G |
|  | 9688 | **658.6285** | **1315.2424** | **1314.6642** | **0.5782** | **0** | **4** | **40** | **2** | **K.ISMPDFDLHLK.G** |
|  | 9976 | **443.5682** | **1327.6829** | **1326.7217** | **0.9612** | **1** | **(6)** | **32** | **8** | **K.VKMPDVDISVPK.I** |
|  | 10237 | **448.9059** | **1343.6960** | **1342.7166** | **0.9793** | **1** | **8** | **21** | **9** | **K.VKMPDVDISVPK.I** |
|  | 10593 | **682.3494** | **1362.6843** | **1362.6892** | **-0.0049** | **1** | **1** | **1.1e+002** | **6** | **K.FGVSTGREGQTPK.A** |
|  | 10761 | 457.9094 | 1370.7063 | 1369.7089 | 0.9974 | 1 | 8 | 21 | 7 | K.VEGDLKGPEADIK.G 10758 |
|  | 10982 | **692.3544** | **1382.6942** | **1382.7042** | **-0.0100** | **0** | **45** | **0.0048** | **1** | **K.VDINAPDVEVQGK.V** 10986 |
|  | 11233 | **467.0576** | **1398.1509** | **1397.7766** | **0.3743** | **1** | **1** | **77** | **10** | **K.LEGDIKAPSLDIK.G** |
|  | 11726 | **476.2777** | **1425.8112** | **1426.8217** | **-1.0105** | **1** | **3** | **62** | **6** | **K.VKTPEMIIQKPK.I** |
|  | 12091 | **724.3649** | **1446.7152** | **1445.7072** | **1.0080** | **1** | **1** | **97** | **5** | **K.MEGDLKGPEVDIK.G** |
|  | 12258 | **486.7551** | **1457.2434** | **1457.7436** | **-0.5002** | **1** | **5** | **26** | **9** | **K.VEGEMKVPDVDIK.G** 12262 |
|  | 12291 | **487.2949** | **1458.8628** | **1459.7229** | **-0.8600** | **1** | **5** | **39** | **7** | **K.VEGDMKVPDVDIK.G** |
|  | 12363 | **732.3857** | **1462.7568** | **1462.7676** | **-0.0108** | **1** | **(7)** | **27** | **9** | **K.FKMPEMSIKPQK.I** |
|  | 12422 | 734.3703 | 1466.7260 | 1466.7617 | -0.0356 | 1 | 2 | 68 | 10 | K.GPEVDIEGPEGKLK.G |
|  | 12465 | **736.3173** | **1470.6201** | **1471.6985** | **-1.0785** | **1** | **(1)** | **1e+002** | **2** | **K.LKMPDMHVNMPK.I** |
|  | 12501 | **491.9287** | **1472.7643** | **1471.6985** | **1.0658** | **1** | **3** | **66** | **8** | **K.LKMPDMHVNMPK.I** |
|  | 12517 | **737.8763** | **1473.7380** | **1472.7545** | **0.9836** | **0** | **27** | **0.29** | **1** | **R.ISMSEVDLNVAAPK.V** |
|  | 12620 | **740.3712** | **1478.7279** | **1478.7625** | **-0.0346** | **1** | **12** | **7.4** | **2** | **K.FKMPEMSIKPQK.I** |
|  | 12807 | **497.2972** | **1488.8699** | **1488.7494** | **0.1205** | **0** | **(7)** | **23** | **8** | **R.ISMSEVDLNVAAPK.V** 12816 |
|  | 12822 | 745.8924 | 1489.7702 | 1488.7494 | 1.0209 | 0 | 7 | 25 | 3 | K.ISMQDVDLSLGSPK.L |
|  | 12887 | **498.9128** | **1493.7167** | **1493.7838** | **-0.0671** | **1** | **12** | **7.9** | **2** | **K.GPKVDINAPDVDVR.G** |
|  | 13243 | 505.9178 | 1514.7317 | 1515.7239 | -0.9922 | 0 | 6 | 29 | 3 | K.IEGEMQVPDVDIR.G 13244 13266 |
|  | 13289 | **759.3256** | **1516.6366** | **1517.7686** | **-1.1320** | **1** | **2** | **72** | **3** | **K.GKGGVTGSPEASISGSK.G** |
|  | 13394 | **762.8483** | **1523.6820** | **1522.8606** | **0.8213** | **1** | **2** | **82** | **10** | **K.APKISIPDVDLDLK.G** |
|  | 13688 | 771.4152 | 1540.8159 | 1541.8123 | -0.9964 | 1 | 10 | 12 | 1 | K.ISMPDVDLNLKGPK.L 13687 |
|  | 13918 | **519.2574** | **1554.7503** | **1555.8280** | **-1.0776** | **1** | **2** | **76** | **7** | **K.APKISMPDVDLNLK.G** |
|  | 14162 | **524.2842** | **1569.8307** | **1569.7787** | **0.0520** | **0** | **3** | **69** | **10** | **K.GSEVGFHGAAPDISVK.G** |
|  | 14354 | 792.4155 | 1582.8164 | 1582.7847 | 0.0317 | 1 | 4 | 51 | 6 | K.ISMPDVDLHMKGPK.V |
|  | 14417 | **529.9048** | **1586.6925** | **1587.8330** | **-1.1405** | **1** | **0** | **1.2e+002** | **10** | **K.APKISMPDIDFNLK.G** |
|  | 14536 | 797.9105 | 1593.8065 | 1592.8596 | 0.9469 | 1 | 1 | 1e+002 | 2 | K.APKISMPDLDLHLK.S |
|  | 14592 | 533.6198 | 1597.8376 | 1596.8334 | 1.0042 | 1 | 7 | 22 | 6 | K.ISMPDFDLHLKGPK.V 14564 |
|  | 14707 | **536.2450** | **1605.7132** | **1606.8541** | **-1.1410** | **1** | **4** | **48** | **7** | **K.IPMPDFDLHLKGPK.V** |
|  | 14754 | 537.0322 | 1608.0749 | 1606.8753 | 1.1996 | 1 | 4 | 36 | 4 | K.VPKISMPDVDLHLK.G |
|  | 14764 | 537.2809 | 1608.8208 | 1608.7744 | 0.0464 | 0 | 1 | 1e+002 | 9 | R.AVEVQGPSLESGDHGK.I |
|  | 14786 | **806.4040** | **1610.7934** | **1609.8675** | **0.9259** | **1** | **7** | **25** | **3** | **K.VDINAPDVEVQGKVK.G** |
|  | 15193 | **546.2812** | **1635.8217** | **1636.8169** | **-0.9951** | **0** | **2** | **70** | **10** | **R.AGAISASGPELQGAGHSK.L** |
|  | 15203 | **819.4165** | **1636.8184** | **1636.8308** | **-0.0124** | **0** | **31** | **0.083** | **1** | **K.VGVEVPDVNIEGPEGK.L** 15204 |
|  | 15214 | **410.2192** | **1636.8475** | **1636.8647** | **-0.0172** | **1** | **3** | **59** | **9** | **K.APKIPMPDFDLHLK.G** |
|  | 15702 | **834.4040** | **1666.7934** | **1666.8050** | **-0.0116** | **0** | **47** | **0.0022** | **1** | **K.VDIEGPDVNIEGPEGK.L** |
|  | 15720 | 834.9141 | 1667.8137 | 1667.8254 | -0.0117 | 0 | 36 | 0.03 | 1 | K.VDVEVPDVSLEGPEGK.L 15721 |
|  | 15921 | 841.4118 | 1680.8090 | 1680.8207 | -0.0116 | 0 | 27 | 0.2 | 1 | K.VDVDVPDVNIEGPDAK.L |
|  | 16000 | **844.3898** | **1686.7650** | **1686.7771** | **-0.0121** | **0** | **63** | **5.3e-005** | **1** | **K.MDVNVGDIDIEGPEGK.L** |
|  | 16185 | **849.9194** | **1697.8242** | **1697.8360** | **-0.0118** | **0** | **39** | **0.013** | **1** | **K.IDVTAPDVSIEEPEGK.L** 16186 |
|  | 16203 | **850.3898** | **1698.7651** | **1698.7771** | **-0.0119** | **0** | **25** | **0.32** | **1** | **K.MDAEVPDVNIEGPDAK.L** 16202 |
|  | 16385 | **856.4405** | **1710.8664** | **1710.8788** | **-0.0124** | **0** | **26** | **0.27** | **1** | **R.VDIETPNLEGTLTGPR.L** |
|  | 16428 | **572.2664** | **1713.7773** | **1714.7720** | **-0.9947** | **0** | **(1)** | **85** | **2** | **K.MDAEVPDVNIEGPDAK.L** |
|  | 16469 | **858.8922** | **1715.7699** | **1715.9128** | **-0.1429** | **1** | **12** | **6.3** | **1** | **R.ISMSEVDLNVAAPKVK.G** 16470 |
|  | 16480 | **572.9941** | **1715.9606** | **1714.8276** | **1.1330** | **1** | **6** | **25** | **6** | **K.MYFPDVEFDIKSPK.F** |
|  | 17046 | 585.6263 | 1753.8570 | 1753.9648 | -0.1078 | 1 | 4 | 49 | 8 | K.ISMPDIDLNLTGPKIK.G |
|  | 17092 | **879.4533** | **1756.8921** | **1755.8713** | **1.0208** | **1** | **9** | **14** | **5** | **K.VEGDMQVPDLDIKGPK.V** |
|  | 17378 | 593.8215 | 1778.4428 | 1777.9397 | 0.5031 | 1 | 2 | 46 | 3 | K.ISMPDVDLHLKGPNVK.G 17384 |
|  | 17539 | 895.0286 | 1788.0426 | 1787.9088 | 0.1338 | 1 | 3 | 51 | 9 | K.MQVGGDGVKVPGIDATTK.L 17549 |
|  | 18013 | **607.3141** | **1818.9204** | **1818.9033** | **0.0171** | **1** | **3** | **61** | **3** | **K.MKGEVDVSLANVEGDLK.G** |
|  | 18095 | **608.9744** | **1823.9015** | **1823.9451** | **-0.0437** | **1** | **4** | **38** | **6** | **K.APNISMPDVDLNLKGPK.I** |
|  | 18203 | 916.9377 | 1831.8608 | 1831.8701 | -0.0093 | 0 | 94 | 4.4e-008 | 1 | R.DDGVFVQEVTQNSPAAR.T |
|  | 18286 | **919.4811** | **1836.9476** | **1836.9581** | **-0.0105** | **0** | **29** | **0.14** | **1** | **K.VQANLGAPDINIEGLDAK.V** |
|  | 19486 | **966.0825** | **1930.1504** | **1931.0000** | **-0.8496** | **1** | **0** | **85** | **3** | **K.IDVDAPDIDIHGPDAKLK.G** |
|  | 19587 | **647.3043** | **1938.8910** | **1938.9357** | **-0.0447** | **1** | **4** | **37** | **1** | **K.GPKLDADMPEVAVEGPNGK.W** |
|  | 19855 | **653.2852** | **1956.8337** | **1955.9510** | **0.8827** | **1** | **0** | **84** | **6** | **K.MDAEVPDVNIEGPDAKLK.G** |
|  | 20134 | **496.7382** | **1982.9237** | **1984.0776** | **-1.1539** | **1** | **3** | **46** | **9** | **K.LQVTMPGIKVGGSGVNVNAK.G** |
|  | 20137 | **496.7448** | **1982.9500** | **1982.9158** | **0.0342** | **1** | **1** | **80** | **5** | **K.TMFGKMYFPDVEFDIK.S** |
|  | 20332 | **1000.4692** | **1998.9239** | **1998.9357** | **-0.0118** | **0** | **77** | **1.8e-006** | **1** | **K.VDVSAPDVEMQGPDWNLK.M** |
|  | 20553 | 1010.5787 | 2019.1428 | 2019.9976 | -0.8548 | 1 | 1 | 71 | 7 | K.FSMPGFKGEGPDVDVTLPK.A |
|  | 20632 | **676.5798** | **2026.7177** | **2025.9909** | **0.7268** | **0** | **8** | **9.6** | **1** | **K.VDINAPDVDVHGPDWHLK.M** |
|  | 20694 | **678.6672** | **2032.9797** | **2032.9928** | **-0.0131** | **1** | **18** | **1.5** | **1** | **K.FSMPGFKGEGPDVDVNLPK.A** |
|  | 20711 | **679.3612** | **2035.0618** | **2036.1266** | **-1.0648** | **1** | **9** | **12** | **1** | **K.VPGIDATTKLNVGAPDVTLR.G** |
|  | 20755 | **680.7997** | **2039.3772** | **2040.0065** | **-0.6293** | **0** | **1** | **53** | **8** | **K.VDINAPDVEVHGPDWHLK.M** |
|  | 20811 | **682.3486** | **2044.0239** | **2043.0347** | **0.9892** | **1** | **8** | **13** | **2** | **K.FSMPSLKGEGPEVDVNLPK.A** 20787 |
|  | 20841 | **683.0053** | **2045.9941** | **2046.0059** | **-0.0118** | **0** | **16** | **2.6** | **2** | **K.VDIDVPDVDVQGPDWHLK.M** |
|  | 20965 | **515.0041** | **2055.9875** | **2056.0014** | **-0.0139** | **0** | **4** | **31** | **1** | **K.VDINTPDVDVHGPDWHLK.M** |
|  | 21067 | **688.9906** | **2063.9500** | **2063.9623** | **-0.0123** | **0** | **2** | **58** | **4** | **K.MDIDAPDVEVQGPDWHLK.M** 21084 |
|  | 21214 | **1040.9609** | **2079.9073** | **2078.9805** | **0.9268** | **1** | **1** | **77** | **1** | **K.FSMPGFKGEGPEVDMNLPK.A** |
|  | 21361 | **698.3330** | **2091.9772** | **2091.9936** | **-0.0164** | **0** | **8** | **15** | **2** | **K.MDIDVPDVEVQGPDWHLK.M** |
|  | 21516 | **703.6510** | **2107.9312** | **2109.0412** | **-1.1100** | **1** | **0** | **82** | **7** | **K.GEGPEVDMNLPKADLGVSGPK.V** |
|  | 21629 | **706.3941** | **2116.1605** | **2117.0389** | **-0.8784** | **1** | **15** | **2.7** | **1** | **K.GSGGEWKGPQVSSALNLDTSK.F** |
|  | 22159 | **726.3820** | **2176.1241** | **2176.1389** | **-0.0148** | **0** | **0** | **80** | **5** | **R.ELLLPNWQGSGSHGLTIAQR.D** |
|  | 22165 | **545.2803** | **2177.0922** | **2176.0623** | **1.0299** | **1** | **9** | **11** | **3** | **K.GPAFNMASPESDFGINLKGPK.I** |
|  | 23376 | **774.0673** | **2319.1801** | **2319.1958** | **-0.0157** | **1** | **11** | **5.4** | **1** | **K.IKGDVPSVGLEGPDVDLQGPEAK.I** |
|  | 23719 | **788.4282** | **2362.2628** | **2362.1264** | **0.1365** | **1** | **0** | **67** | **8** | **K.GPKMDIDAPDVEVQGPDWHLK.M** |
|  | 23794 | **594.0680** | **2372.2429** | **2373.1787** | **-0.9359** | **1** | **2** | **41** | **4** | **K.VDINAPDVDVQGPDWHLKMPK.I** |
|  | 23821 | **1189.4946** | **2376.9747** | **2378.1577** | **-1.1829** | **1** | **0** | **63** | **9** | **K.SAKMDIDVPDVEVQGPDWHLK.M** |
|  | 24080 | **806.3704** | **2416.0894** | **2417.2299** | **-1.1405** | **0** | **0** | **68** | **7** | **K.GGQIGLQAPGLSVSGPQGHLESGSGK.V** |
|  | 24199 | **813.0273** | **2436.0602** | **2436.1454** | **-0.0852** | **1** | **2** | **43** | **10** | **K.MDIDAPDVEVQGPDWHLKMPK.M** |
|  | 24746 | **1256.6603** | **2511.3060** | **2511.1501** | **0.1560** | **0** | **40** | **0.0063** | **2** | **K.ADIDVSGPSVDTDAPDLDIEGPEGK.L** |
|  | 25191 | **855.4223** | **2563.2451** | **2563.2654** | **-0.0203** | **1** | **15** | **1.9** | **1** | **K.ADIDVSGPKVDVDVPDVNIEGPDAK.L** 25189 25190 |
|  | 25444 | **869.4706** | **2605.3901** | **2604.3694** | **1.0207** | **1** | **0** | **64** | **10** | **R.AGAISASGPELQGAGHSKLQVTMPGIK.V** |
|  | 25905 | 896.0820 | 2685.2243 | 2686.3921 | -1.1679 | 1 | 0 | 63 | 2 | K.TPEMIIQKPKISMQDVDLSLGSPK.L |
|  | 26072 | **902.0859** | **2703.2358** | **2704.3443** | **-1.1085** | **1** | **2** | **47** | **5** | **K.GPEIDIKGPSLDIDTPDVNIEGPEGK.L** |
|  | 27620 | **1003.1769** | **3006.5088** | **3007.5601** | **-1.0513** | **1** | **3** | **25** | **5** | **K.ADIKSPSLDVTVPEAELNLETPEISVGGK.G** |
|  | 28743 | **1087.5419** | **3259.6038** | **3260.6385** | **-1.0348** | **1** | **3** | **25** | **9** | **R.LPSGSGAASPTGSAVDIRAGAISASGPELQGAGHSK.L** 28746 |

| **95.** | Q12797\|ASPH_HUMAN    **Mass:** 86266    **Score:** 348    **Queries matched:** 16   **emPAI:** 0.09 |
| --- | --- |
|  | Aspartyl/asparaginyl beta-hydroxylase OS=Homo sapiens GN=ASPH PE=1 SV=3 |

|  | **Query** | **Observed** | **Mr(expt)** | **Mr(calc)** | **Delta** | **Miss** | **Score** | **Expect** | **Rank** | **Peptide** |
| --- | --- | --- | --- | --- | --- | --- | --- | --- | --- | --- |
|  | 3380 | **464.9011** | **927.7877** | **926.5913** | **1.1964** | **1** | **8** | **21** | **2** | **K.VLLGLKER.S** |
|  | 4073 | **489.0251** | **976.0356** | **976.4535** | **-0.4179** | **0** | **(3)** | **65** | **3** | **R.DEGLAVMDK.A** |
|  | 4307 | **497.7228** | **993.4310** | **992.4485** | **0.9826** | **0** | **8** | **27** | **5** | **R.DEGLAVMDK.A** |
|  | 7105 | **595.2916** | **1188.5686** | **1187.6397** | **0.9288** | **1** | **2** | **96** | **4** | **K.TIKAELDAAEK.L** |
|  | 8918 | 638.3173 | 1274.6200 | 1275.7849 | -1.1649 | 1 | 5 | 49 | 6 | R.LRMHLGLVIPK.E |
|  | 10845 | **688.4019** | **1374.7893** | **1373.7555** | **1.0338** | **0** | **6** | **32** | **4** | **R.LVQLFPNDTSLK.N** |
|  | 11276 | **467.7553** | **1400.2440** | **1399.6343** | **0.6097** | **0** | **2** | **51** | **9** | **R.FYFHLGDAMQR.V** |
|  | 11330 | **468.9214** | **1403.7423** | **1403.5801** | **0.1622** | **0** | **1** | **1.1e+002** | **10** | **K.EGIESGDPGTDDGR.F** |
|  | 13019 | **501.2624** | **1500.7653** | **1500.7936** | **-0.0283** | **1** | **8** | **19** | **6** | **K.AKGLFLPEDENLR.E** |
|  | 17550 | **597.3337** | **1788.9792** | **1789.8846** | **-0.9054** | **0** | **6** | **26** | **7** | **K.NDLGVGYLLIGDNDNAK.K** |
|  | 18905 | **628.6581** | **1882.9524** | **1884.0152** | **-1.0628** | **1** | **1** | **75** | **5** | **R.QQFLGHMRGSLLTLQR.L** |
|  | 19095 | **950.9030** | **1899.7915** | **1899.8011** | **-0.0096** | **0** | **85** | **2.6e-007** | **1** | **K.LGIYDADGDGDFDVDDAK.V** 19094 |
|  | 21454 | **1051.9365** | **2101.8585** | **2101.8744** | **-0.0159** | **0** | **151** | **6.6e-014** | **1** | **K.SSGNSSSSGSGSGSTSAGSSSPGAR.R** 21453 |
|  | 24110 | **807.2112** | **2418.6119** | **2418.1550** | **0.4568** | **1** | **9** | **5.1** | **1** | **K.IAESIPYLKEGIESGDPGTDDGR.F** |

| **96.** | P13010\|KU86_HUMAN    **Mass:** 83222    **Score:** 345    **Queries matched:** 27   **emPAI:** 0.30 |
| --- | --- |
|  | ATP-dependent DNA helicase 2 subunit 2 OS=Homo sapiens GN=XRCC5 PE=1 SV=3 |

|  | **Query** | **Observed** | **Mr(expt)** | **Mr(calc)** | **Delta** | **Miss** | **Score** | **Expect** | **Rank** | **Peptide** |
| --- | --- | --- | --- | --- | --- | --- | --- | --- | --- | --- |
|  | 982 | **565.2680** | **564.2607** | **564.3271** | **-0.0664** | **0** | **11** | **14** | **9** | **R.IAAYK.S** |
|  | 5075 | **524.2829** | **1046.5512** | **1046.5760** | **-0.0248** | **1** | **7** | **36** | **9** | **K.KTWTVVDAK.T** 5077 5079 |
|  | 5419 | **537.2897** | **1072.5648** | **1072.6240** | **-0.0593** | **0** | **7** | **35** | **6** | **R.LTIGSNLSIR.I** |
|  | 5562 | **542.2838** | **1082.5531** | **1082.5583** | **-0.0052** | **0** | **2** | **95** | **1** | **R.FFMGNQVLK.V** |
|  | 5613 | **543.8207** | **1085.6268** | **1085.5804** | **0.0464** | **0** | **35** | **0.055** | **1** | **R.LFQCLLHR.A** |
|  | 5949 | **555.7625** | **1109.5105** | **1108.6029** | **0.9075** | **0** | **18** | **2.4** | **6** | **R.LGGHGPSFPLK.G** 5948 |
|  | 6630 | **579.8179** | **1157.6213** | **1158.7012** | **-1.0799** | **1** | **5** | **54** | **1** | **K.TLFPLIEAKK.K** |
|  | 10621 | **683.2406** | **1364.4666** | **1363.5753** | **0.8914** | **1** | **3** | **50** | **2** | **K.EDGSGDRGDGPFR.L** |
|  | 10857 | **459.7333** | **1376.1779** | **1376.7565** | **-0.5785** | **0** | **2** | **58** | **7** | **R.ANPQVGVAFPHIK.H** |
|  | 14177 | 786.4305 | 1570.8464 | 1570.8566 | -0.0102 | 1 | 38 | 0.022 | 1 | K.GITEQQKEGLEIVK.M |
|  | 16809 | **869.5057** | **1736.9969** | **1737.8951** | **-0.8982** | **1** | **1** | **80** | **1** | **R.GDGPFRLGGHGPSFPLK.G** |
|  | 23383 | **1161.0812** | **2320.1478** | **2320.1620** | **-0.0142** | **0** | **96** | **1.8e-008** | **1** | **K.YAPTEAQLNAVDALIDSMSLAK.K** 23385 |
|  | 24285 | **817.0888** | **2448.2444** | **2448.2570** | **-0.0126** | **1** | **2** | **44** | **1** | **K.KYAPTEAQLNAVDALIDSMSLAK.K** |
|  | 24734 | **1255.5647** | **2509.1148** | **2508.1214** | **0.9934** | **0** | **71** | **4.6e-006** | **1** | **K.DKPSGDTAAVFEEGGDVDDLLDMI.-** 24725 |
|  | 25660 | **885.1268** | **2652.3585** | **2652.3734** | **-0.0149** | **0** | **5** | **19** | **1** | **R.EPLPPIQQHIWNMLNPPAEVTTK.S** |
|  | 26166 | **1362.1923** | **2722.3700** | **2722.3847** | **-0.0148** | **0** | **110** | **6.7e-010** | **1** | **R.DDEAAAVALSSLIHALDDLDMVAIVR.Y** 26167 26168 |
|  | 27912 | **1022.7632** | **3065.2677** | **3064.4587** | **0.8090** | **1** | **1** | **30** | **3** | **K.FLAPKDKPSGDTAAVFEEGGDVDDLLDMI.-** |
|  | 28143 | **521.7643** | **3124.5424** | **3125.5236** | **-0.9812** | **1** | **2** | **29** | **1** | **K.AAVVLCMDVGFTMSNSIPGIESPFEQAKK.V** |
|  | 28397 | **796.1661** | **3180.6352** | **3180.6489** | **-0.0137** | **1** | **12** | **3** | **1** | **K.IQPGSQQADFLDALIVSMDVIQHETIGKK.F** |
|  | 29199 | **1130.5264** | **3388.5573** | **3387.6465** | **0.9108** | **1** | **0** | **42** | **1** | **K.EGLEIVKMVMISLEGEDGLDEIYSFSESLR.K** |

| **97.** | Q29963\|1C06_HUMAN    **Mass:** 41399    **Score:** 343    **Queries matched:** 29   **emPAI:** 1.18 |
| --- | --- |
|  | HLA class I histocompatibility antigen, Cw-6 alpha chain OS=Homo sapiens GN=HLA-C PE=1 SV=2 |

|  | **Query** | **Observed** | **Mr(expt)** | **Mr(calc)** | **Delta** | **Miss** | **Score** | **Expect** | **Rank** | **Peptide** |
| --- | --- | --- | --- | --- | --- | --- | --- | --- | --- | --- |
|  | 3888 | **482.7234** | **963.4322** | **964.4250** | **-0.9929** | **0** | **33** | **0.088** | **1** | **R.FDSDAASPR.G** 3900 3901 3907 |
|  | 10335 | **675.8494** | **1349.6843** | **1349.6939** | **-0.0096** | **1** | **38** | **0.024** | **1** | **R.YLENGKETLQR.A** 10311 10351 |
|  | 11313 | 468.5608 | 1402.6605 | 1403.6429 | -0.9825 | 1 | 8 | 22 | 2 | R.FDSDAASPRGEPR.A |
|  | 11736 | **714.3626** | **1426.7105** | **1426.7205** | **-0.0099** | **0** | **46** | **0.0031** | **1** | **K.WAAVVVPSGEEQR.Y** 11721 11735 11751 |
|  | 12921 | **748.8587** | **1495.7028** | **1495.7129** | **-0.0101** | **0** | **57** | **0.00026** | **1** | **R.AYLEGTCVEWLR.R** |
|  | 13321 | **760.3871** | **1518.7596** | **1518.7427** | **0.0169** | **0** | **62** | **7.5e-005** | **1** | **R.SWTAADTAAQITQR.K** |
|  | 15320 | 823.4096 | 1644.8047 | 1644.8148 | -0.0101 | 0 | 69 | 1.4e-005 | 1 | R.FISVGYVDDTQFVR.F 15321 |
|  | 18283 | **613.3167** | **1836.9283** | **1835.9027** | **1.0256** | **0** | **15** | **3.7** | **1** | **K.THVTHHPVSDHEATLR.C** |
|  | 20871 | **410.6170** | **2048.0485** | **2049.0102** | **-0.9617** | **0** | **9** | **11** | **2** | **R.YTCHVQHEGLPEPLTLR.W** 20872 20886 20897 |
|  | 21557 | **1056.5175** | **2111.0204** | **2111.0299** | **-0.0095** | **0** | **86** | **2.1e-007** | **1** | **R.CWALGFYPAEITLTWQR.D** 21558 |
|  | 25366 | **865.0411** | **2592.1016** | **2591.2293** | **0.8723** | **1** | **0** | **56** | **6** | **R.FISVGYVDDTQFVRFDSDAASPR.G** |
|  | 25578 | **1319.0889** | **2636.1632** | **2636.1838** | **-0.0206** | **0** | **57** | **0.00013** | **1** | **R.DGEDQTQDTELVETRPAGDGTFQK.W** 25579 25580 25581 |
|  | 30193 | **1274.3292** | **3819.9658** | **3819.0865** | **0.8793** | **0** | **2** | **22** | **4** | **R.WEPSSQPTIPIVGIVAGLAVLAVLAVLGAVMAVVMCR.R** |

| **98.** | P10321\|1C07_HUMAN    **Mass:** 41136    **Score:** 342    **Queries matched:** 26   **emPAI:** 1.19 |
| --- | --- |
|  | HLA class I histocompatibility antigen, Cw-7 alpha chain OS=Homo sapiens GN=HLA-C PE=1 SV=3 |

|  | **Query** | **Observed** | **Mr(expt)** | **Mr(calc)** | **Delta** | **Miss** | **Score** | **Expect** | **Rank** | **Peptide** |
| --- | --- | --- | --- | --- | --- | --- | --- | --- | --- | --- |
|  | 965 | **559.2914** | **558.2842** | **558.3125** | **-0.0284** | **0** | **15** | **8.3** | **10** | **K.LEAAR.A** |
|  | 3888 | 482.7234 | 963.4322 | 964.4250 | -0.9929 | 0 | 33 | 0.088 | 1 | R.FDSDAASPR.G 3900 3901 3907 |
|  | 6691 | **582.2626** | **1162.5107** | **1163.4700** | **-0.9592** | **0** | **7** | **29** | **3** | **R.MSGCDLGPDGR.L** |
|  | 10335 | 675.8494 | 1349.6843 | 1349.6939 | -0.0096 | 1 | 38 | 0.024 | 1 | R.YLENGKETLQR.A 10311 10351 |
|  | 11313 | 468.5608 | 1402.6605 | 1403.6429 | -0.9825 | 1 | 8 | 22 | 2 | R.FDSDAASPRGEPR.A |
|  | 11721 | 713.8703 | 1425.7260 | 1425.7365 | -0.0104 | 0 | 45 | 0.0043 | 1 | K.WAAVVVPSGQEQR.Y 11735 11736 |
|  | 12921 | 748.8587 | 1495.7028 | 1495.7129 | -0.0101 | 0 | 57 | 0.00026 | 1 | R.AYLEGTCVEWLR.R |
|  | 13321 | 760.3871 | 1518.7596 | 1518.7427 | 0.0169 | 0 | 62 | 7.5e-005 | 1 | R.SWTAADTAAQITQR.K |
|  | 15320 | 823.4096 | 1644.8047 | 1644.8148 | -0.0101 | 0 | 69 | 1.4e-005 | 1 | R.FISVGYVDDTQFVR.F 15321 |
|  | 18486 | **463.4837** | **1849.9057** | **1849.9183** | **-0.0127** | **0** | **16** | **2.9** | **1** | **K.THVTHHPLSDHEATLR.C** 18485 |
|  | 21557 | 1056.5175 | 2111.0204 | 2111.0299 | -0.0095 | 0 | 86 | 2.1e-007 | 1 | R.CWALGFYPAEITLTWQR.D 21558 |
|  | 25366 | 865.0411 | 2592.1016 | 2591.2293 | 0.8723 | 1 | 0 | 56 | 6 | R.FISVGYVDDTQFVRFDSDAASPR.G |
|  | 25578 | 1319.0889 | 2636.1632 | 2636.1838 | -0.0206 | 0 | 57 | 0.00013 | 1 | R.DGEDQTQDTELVETRPAGDGTFQK.W 25579 25580 25581 |

| **99.** | Q8NF37\|PCAT1_HUMAN    **Mass:** 59741    **Score:** 341    **Queries matched:** 20   **emPAI:** 0.27 |
| --- | --- |
|  | Lysophosphatidylcholine acyltransferase 1 OS=Homo sapiens GN=LPCAT1 PE=1 SV=2 |

|  | **Query** | **Observed** | **Mr(expt)** | **Mr(calc)** | **Delta** | **Miss** | **Score** | **Expect** | **Rank** | **Peptide** |
| --- | --- | --- | --- | --- | --- | --- | --- | --- | --- | --- |
|  | 5134 | **527.2477** | **1052.4808** | **1052.4887** | **-0.0079** | **1** | **11** | **12** | **1** | **K.DLDRYSER.A** |
|  | 5886 | **553.3418** | **1104.6690** | **1103.5723** | **1.0967** | **0** | **12** | **10** | **1** | **R.NPALYASNVR.R** |
|  | 6223 | **566.2682** | **1130.5218** | **1130.5316** | **-0.0098** | **0** | **67** | **2.9e-005** | **1** | **R.AAPASSAGASDAR.L** 6227 6228 6229 |
|  | 6475 | **575.3252** | **1148.6358** | **1148.6441** | **-0.0083** | **0** | **5** | **46** | **3** | **R.TLDTIQLAFK.M** |
|  | 7901 | 409.5513 | 1225.6322 | 1224.5499 | 1.0823 | 0 | 9 | 20 | 6 | R.TMWFAGGFHR.V |
|  | 8589 | **420.8481** | **1259.5223** | **1259.6734** | **-0.1511** | **1** | **8** | **25** | **3** | **K.RNPALYASNVR.R** 8590 8611 |
|  | 8621 | **631.2831** | **1260.5516** | **1259.6734** | **0.8782** | **1** | **11** | **12** | **3** | **R.NPALYASNVRR.V** |
|  | 9836 | **441.3191** | **1320.9355** | **1319.7635** | **1.1720** | **1** | **3** | **44** | **5** | **K.VVDFLLKAIMR.T** |
|  | 13993 | **520.8357** | **1559.4854** | **1558.8905** | **0.5949** | **0** | **3** | **40** | **8** | **K.AQVALMTLTLFPVR.L** |
|  | 14687 | **803.4424** | **1604.8702** | **1604.8774** | **-0.0072** | **0** | **69** | **1.5e-005** | **1** | **K.TALGVAELTVTDLFR.A** |
|  | 21715 | **1064.9738** | **2127.9329** | **2127.9453** | **-0.0123** | **0** | **121** | **6.9e-011** | **1** | **K.MYGAQEDGSVGEGDLSCILK.T** 21714 |
|  | 24509 | **827.9594** | **2480.8564** | **2480.3978** | **0.4586** | **0** | **0** | **49** | **1** | **R.TCLITFKPGAFIPGAPVQPVVLR.Y** |
|  | 29808 | **1212.2841** | **3633.8304** | **3633.8496** | **-0.0193** | **0** | **49** | **0.00044** | **1** | **R.QALPTEAAILTLAPHSSYFDAIPVTMTMSSIVMK.A** |
|  | 30064 | **752.7905** | **3758.9163** | **3758.0303** | **0.8860** | **1** | **1** | **25** | **7** | **R.LLVAAAMMLLAWPLALVASLGSAEKEPEQPPALWR.K** |

| **100.** | Q07065\|CKAP4_HUMAN    **Mass:** 66097    **Score:** 339    **Queries matched:** 31   **emPAI:** 0.55 |
| --- | --- |
|  | Cytoskeleton-associated protein 4 OS=Homo sapiens GN=CKAP4 PE=1 SV=2 |

|  | **Query** | **Observed** | **Mr(expt)** | **Mr(calc)** | **Delta** | **Miss** | **Score** | **Expect** | **Rank** | **Peptide** |
| --- | --- | --- | --- | --- | --- | --- | --- | --- | --- | --- |
|  | 942 | **550.2479** | **549.2406** | **548.2628** | **0.9778** | **0** | **2** | **1.3e+002** | **9** | **-.MPSAK.Q** |
|  | 1532 | **396.2047** | **790.3947** | **790.3457** | **0.0491** | **0** | **6** | **58** | **7** | **K.EAADTER.L** |
|  | 2493 | **431.7628** | **861.5110** | **861.5324** | **-0.0214** | **1** | **11** | **16** | **3** | **R.LFVKVEK.I** |
|  | 2871 | **445.2596** | **888.5046** | **888.4916** | **0.0130** | **0** | **10** | **20** | **6** | **R.LSSLDNLK.A** |
|  | 5684 | **546.7659** | **1091.5173** | **1091.5281** | **-0.0108** | **1** | **13** | **7.7** | **1** | **R.SQKEINDMK.A** |
|  | 6411 | **573.4047** | **1144.7948** | **1145.5928** | **-0.7980** | **0** | **1** | **1.2e+002** | **5** | **K.ASVSQVEADLK.M** |
|  | 8775 | **635.3473** | **1268.6800** | **1268.6109** | **0.0691** | **1** | **4** | **50** | **4** | **R.GSKGGHGAASPSEK.G** |
|  | 9108 | **429.8797** | **1286.6172** | **1286.6255** | **-0.0083** | **0** | **17** | **3** | **2** | **R.HSEAFEALQQK.S** |
|  | 9209 | **646.8021** | **1291.5897** | **1291.5979** | **-0.0082** | **1** | **32** | **0.084** | **1** | **K.SREWDMEALR.S** |
|  | 10534 | **454.2072** | **1359.5998** | **1360.6834** | **-1.0836** | **1** | **12** | **9.8** | **1** | **K.AKVASLEESEGNK.Q** 10538 |
|  | 10571 | **681.8488** | **1361.6831** | **1360.6470** | **1.0361** | **0** | **3** | **79** | **2** | **K.IETNENNLESAK.G** 10577 |
|  | 11069 | **463.5404** | **1387.5993** | **1387.6117** | **-0.0124** | **1** | **25** | **0.39** | **1** | **K.SDSHGPKEDGGFR.H** 11056 11082 |
|  | 11491 | 707.4150 | 1412.8154 | 1413.7212 | -0.9058 | 1 | 13 | 6.9 | 1 | K.GLLDDLRNDLDR.L |
|  | 11676 | **475.2373** | **1422.6900** | **1423.7419** | **-1.0520** | **1** | **8** | **20** | **3** | **K.DLSDGIHVVKDAR.E** 11707 |
|  | 12500 | **737.3887** | **1472.7629** | **1472.7722** | **-0.0093** | **0** | **31** | **0.1** | **1** | **R.SVGELPSTVESLQK.V** |
|  | 15083 | **814.9467** | **1627.8789** | **1628.8733** | **-0.9944** | **1** | **4** | **45** | **8** | **K.RSVGELPSTVESLQK.V** |
|  | 15331 | **823.9098** | **1645.8050** | **1645.8159** | **-0.0108** | **1** | **82** | **8.5e-007** | **1** | **K.VASLEESEGNKQDLK.A** |
|  | 15729 | **557.0920** | **1668.2541** | **1667.8916** | **0.3625** | **1** | **9** | **11** | **1** | **K.MLRTAVDSLVAYSVK.I** |
|  | 16008 | **563.2854** | **1686.8344** | **1687.8741** | **-1.0397** | **1** | **5** | **34** | **5** | **K.QGESEVSRISEVLQK.L** |
|  | 16161 | **566.6040** | **1696.7902** | **1697.8696** | **-1.0795** | **1** | **3** | **60** | **4** | **R.QREELGQGLQGVEQK.V** |
|  | 17256 | 886.9160 | 1771.8175 | 1771.8298 | -0.0123 | 0 | 68 | 1.8e-005 | 1 | R.STLQTMESDIYTEVR.E 17255 |
|  | 17694 | **899.4765** | **1796.9384** | **1795.9469** | **0.9916** | **0** | **64** | **3.7e-005** | **1** | **K.VQSLQATFGTFESILR.S** |
|  | 19154 | **952.9559** | **1903.8972** | **1903.9123** | **-0.0151** | **0** | **81** | **8.2e-007** | **1** | **R.LEGLGSSEADQDGLASTVR.S** |
|  | 20572 | **1011.5147** | **2021.0147** | **2021.0290** | **-0.0143** | **0** | **110** | **9.4e-010** | **1** | **K.VQEQVHTLLSQDQAQAAR.L** |
|  | 26769 | 1417.6893 | 2833.3641 | 2834.2738 | -0.9097 | 1 | 2 | 36 | 4 | R.EWDMEALRSTLQTMESDIYTEVR.E |

| **101.** | Q9NZM1\|MYOF_HUMAN    **Mass:** 236100   **Score:** 337    **Queries matched:** 90   **emPAI:** 0.38 |
| --- | --- |
|  | Myoferlin OS=Homo sapiens GN=MYOF PE=1 SV=1 |

|  | **Query** | **Observed** | **Mr(expt)** | **Mr(calc)** | **Delta** | **Miss** | **Score** | **Expect** | **Rank** | **Peptide** |
| --- | --- | --- | --- | --- | --- | --- | --- | --- | --- | --- |
|  | 848 | **520.2706** | **519.2633** | **520.2679** | **-1.0046** | **0** | **3** | **1.5e+002** | **1** | **R.VMAGK.V** |
|  | 2931 | **448.7145** | **895.4143** | **895.4222** | **-0.0078** | **0** | **22** | **0.9** | **1** | **K.CGQYIQK.G** |
|  | 3402 | **465.2840** | **928.5534** | **928.5593** | **-0.0059** | **0** | **4** | **69** | **8** | **K.LISLLNEK.G** |
|  | 3434 | **466.7360** | **931.4574** | **931.4876** | **-0.0302** | **0** | **20** | **1.7** | **1** | **K.WGTSGLVGR.H** 3415 3431 3433 3435 3436 3448 3449 3451 3452 3455 |
|  | 3878 | **482.2711** | **962.5276** | **962.5338** | **-0.0062** | **0** | **15** | **5.9** | **1** | **R.VYIYHLR.C** |
|  | 3912 | **483.3000** | **964.5854** | **963.4886** | **1.0968** | **1** | **4** | **61** | **10** | **K.GEGVAYRGR.I** |
|  | 4661 | **509.7653** | **1017.5160** | **1017.5244** | **-0.0084** | **1** | **33** | **0.097** | **1** | **R.HKFSDVTGK.I** 4671 |
|  | 5488 | **539.7637** | **1077.5129** | **1077.5455** | **-0.0325** | **1** | **13** | **7.4** | **7** | **K.EIFGGNADKK.N** 5476 |
|  | 5701 | **547.2655** | **1092.5164** | **1092.5247** | **-0.0083** | **0** | **21** | **1.3** | **1** | **K.VHVCGQTHR.T** |
|  | 5879 | **553.2515** | **1104.4885** | **1103.6009** | **0.8876** | **1** | **14** | **7.1** | **1** | **K.KVCTNIIEK.N** |
|  | 6430 | **573.7997** | **1145.5849** | **1146.5591** | **-0.9741** | **0** | **3** | **89** | **8** | **K.LNSEMDITPK.L** |
|  | 6484 | **575.7849** | **1149.5553** | **1149.5626** | **-0.0073** | **0** | **32** | **0.1** | **1** | **K.DLTQTASSTAR.A** 6499 |
|  | 6558 | **577.7543** | **1153.4940** | **1153.5040** | **-0.0100** | **0** | **34** | **0.063** | **1** | **K.FYASSGEHEK.C** |
|  | 6594 | 578.8281 | 1155.6417 | 1155.6499 | -0.0082 | 0 | 45 | 0.0043 | 1 | R.VIVESASNIPK.T |
|  | 6606 | **579.2670** | **1156.5194** | **1156.5295** | **-0.0101** | **1** | **6** | **44** | **10** | **K.GRDEPNMNPK.L** |
|  | 7395 | **401.2190** | **1200.6352** | **1201.6190** | **-0.9838** | **0** | **6** | **42** | **8** | **K.LIDEVIEDTR.Y** |
|  | 7964 | **615.3414** | **1228.6682** | **1228.6776** | **-0.0094** | **0** | **65** | **5.1e-005** | **1** | **K.ANVTVLDTQIR.K** |
|  | 8583 | **630.3890** | **1258.7635** | **1257.6565** | **1.1071** | **0** | **5** | **47** | **5** | **K.VGETIIDLENR.F** |
|  | 8640 | **631.8302** | **1261.6458** | **1261.6529** | **-0.0071** | **0** | **14** | **6** | **1** | **K.LQMWVDVFPK.S** |
|  | 8903 | 637.8638 | 1273.7130 | 1274.7486 | -1.0356 | 0 | 7 | 28 | 7 | R.ILVELATFLEK.T 8885 8891 8892 |
|  | 8954 | **426.7168** | **1277.1287** | **1277.6575** | **-0.5289** | **1** | **3** | **52** | **10** | **K.KDLTQTASSTAR.A** |
|  | 9744 | 440.2517 | 1317.7333 | 1317.6850 | 0.0483 | 0 | 15 | 4.5 | 3 | K.VEMTLEILNEK.E |
|  | 10634 | **683.3547** | **1364.6949** | **1364.7048** | **-0.0099** | **0** | **29** | **0.16** | **1** | **R.TQGLVPEHVETR.T** |
|  | 10899 | **690.3226** | **1378.6306** | **1378.6405** | **-0.0099** | **0** | **29** | **0.16** | **1** | **K.ISVYDYDTFTR.D** |
|  | 11242 | **700.3468** | **1398.6790** | **1398.6891** | **-0.0101** | **1** | **4** | **52** | **6** | **K.GWIPGNEENKQK.T** |
|  | 11248 | **467.2498** | **1398.7277** | **1398.7368** | **-0.0090** | **0** | **51** | **0.0012** | **1** | **K.ILHQHLGAPEER.L** |
|  | 11522 | **708.3630** | **1414.7114** | **1415.7442** | **-1.0328** | **0** | **0** | **1.2e+002** | **9** | **R.LDAVNTLLAMAER.L** |
|  | 11793 | **715.8536** | **1429.6926** | **1429.7024** | **-0.0098** | **0** | **51** | **0.0011** | **1** | **K.MAPSETHGAAAIFK.L** |
|  | 12067 | **723.8779** | **1445.7412** | **1445.7515** | **-0.0103** | **0** | **15** | **4.3** | **1** | **K.NDVVGTTYLHLSK.I** 12068 |
|  | 12086 | **724.3309** | **1446.6472** | **1445.6973** | **0.9499** | **0** | **(6)** | **36** | **1** | **K.MAPSETHGAAAIFK.L** 12095 |
|  | 12130 | **725.3912** | **1448.7678** | **1448.7776** | **-0.0098** | **0** | **32** | **0.087** | **1** | **K.SLGPPGPPFNITPR.K** |
|  | 12470 | **736.3484** | **1470.6822** | **1470.6912** | **-0.0090** | **0** | **3** | **72** | **4** | **K.SITGEEMSDIYVK.G** |
|  | 12530 | **738.3769** | **1474.7393** | **1475.7555** | **-1.0161** | **0** | **10** | **14** | **3** | **R.MLSNKPQDFQIR.V** |
|  | 12936 | 749.3483 | 1496.6820 | 1496.7881 | -0.1062 | 1 | 8 | 21 | 6 | R.SLSQIHEAAVRMR.S |
|  | 13166 | **504.3062** | **1509.8967** | **1510.7854** | **-0.8887** | **1** | **4** | **49** | **4** | **K.KTPNFPSSVLFMK.V** |
|  | 13975 | **520.3456** | **1558.0149** | **1557.7973** | **0.2176** | **1** | **2** | **64** | **4** | **R.KMAPSETHGAAAIFK.L** |
|  | 14006 | **781.3462** | **1560.6778** | **1559.7000** | **0.9779** | **0** | **4** | **50** | **2** | **R.MYELSCYLPQEK.D** |
|  | 14355 | **792.4252** | **1582.8359** | **1582.8454** | **-0.0095** | **0** | **28** | **0.17** | **1** | **K.LEPISNDDLLVVEK.Y** 14328 |
|  | 14523 | **797.3739** | **1592.7332** | **1592.8562** | **-0.1230** | **1** | **4** | **51** | **4** | **K.DEFLGRSIFSPVVK.L** |
|  | 14844 | **538.9550** | **1613.8431** | **1613.8521** | **-0.0090** | **1** | **5** | **38** | **4** | **R.LDMIPDLKAMNPLK.A** |
|  | 15107 | **544.1848** | **1629.5324** | **1629.8210** | **-0.2885** | **1** | **3** | **46** | **5** | **R.DEKVGETIIDLENR.F** |
|  | 15115 | **544.2840** | **1629.8302** | **1629.8470** | **-0.0168** | **1** | **(1)** | **92** | **9** | **R.LDMIPDLKAMNPLK.A** |
|  | 15169 | **817.9293** | **1633.8440** | **1632.8512** | **0.9928** | **0** | **4** | **49** | **2** | **R.IYPLPDDPSVPAPPR.Q** |
|  | 15368 | **824.8568** | **1647.6989** | **1647.8515** | **-0.1525** | **1** | **2** | **71** | **7** | **R.RMLSNKPQDFQIR.V** |
|  | 15443 | **551.3093** | **1650.9060** | **1650.9165** | **-0.0106** | **0** | **43** | **0.0055** | **1** | **R.DQLRPTQLLQNVAR.F** 15442 |
|  | 15601 | **831.3994** | **1660.7841** | **1660.7944** | **-0.0103** | **0** | **41** | **0.0099** | **1** | **K.WLLLNDPEDTSSGSK.G** |
|  | 16379 | **571.2784** | **1710.8133** | **1710.9403** | **-0.1270** | **1** | **2** | **72** | **4** | **K.KLEPISNDDLLVVEK.Y** |
|  | 16550 | 574.6526 | 1720.9361 | 1719.9268 | 1.0093 | 1 | 2 | 78 | 10 | R.GPGPKGPVGTVSEAQLAR.R |
|  | 17522 | **596.6048** | **1786.7926** | **1786.7951** | **-0.0025** | **0** | **2** | **72** | **10** | **K.GWEWEGEWIVDPER.S** |
|  | 17602 | **597.8958** | **1790.6656** | **1790.8727** | **-0.2070** | **1** | **2** | **51** | **1** | **K.LTEKEEEIVDWWSK.F** |
|  | 17762 | **901.4794** | **1800.9443** | **1800.9543** | **-0.0100** | **0** | **71** | **8.8e-006** | **1** | **R.DIVIEMEDTKPLLASK.L** |
|  | 17980 | **606.3255** | **1815.9547** | **1816.9492** | **-0.9945** | **0** | **(8)** | **15** | **1** | **R.DIVIEMEDTKPLLASK.L** |
|  | 18013 | 607.3141 | 1818.9204 | 1817.9927 | 0.9277 | 1 | 5 | 35 | 2 | K.FGKPDPIVSVIFKDEK.K |
|  | 18579 | **929.4569** | **1856.8991** | **1857.9221** | **-1.0229** | **0** | **7** | **23** | **2** | **R.IPAHQVLYSTSGENASGK.Y** |
|  | 21163 | **1037.4935** | **2072.9725** | **2072.9837** | **-0.0112** | **0** | **42** | **0.0051** | **1** | **R.GLELQPQDNNGLCDPYIK.I** 21162 |
|  | 22649 | **743.3566** | **2227.0481** | **2226.0627** | **0.9854** | **1** | **37** | **0.015** | **1** | **K.IYRAEDIPQMDDAFSQTVK.E** |
|  | 22862 | **752.3851** | **2254.1336** | **2254.1495** | **-0.0159** | **1** | **29** | **0.095** | **1** | **K.VIEDRDHYIPNTLNPVFGR.M** |
|  | 23565 | **1172.0958** | **2342.1771** | **2342.1906** | **-0.0135** | **1** | **75** | **2.5e-006** | **1** | **K.KVDNELNPVWNEILEFDLR.G** 23566 |
|  | 23643 | **785.6757** | **2354.0052** | **2354.0209** | **-0.0157** | **0** | **13** | **3.7** | **1** | **R.FGSHCGIPEEYCVSGVNTWR.D** |
|  | 24179 | **812.0873** | **2433.2400** | **2432.2448** | **0.9952** | **1** | **1** | **60** | **6** | **R.LAYARIPAHQVLYSTSGENASGK.Y** |
|  | 25136 | **852.0767** | **2553.2083** | **2554.1978** | **-0.9895** | **1** | **6** | **17** | **2** | **R.NMKNFQMASITSPSLVVECGGER.V** |
|  | 25231 | **858.0583** | **2571.1530** | **2570.1927** | **0.9603** | **1** | **(2)** | **37** | **7** | **R.NMKNFQMASITSPSLVVECGGER.V** |
|  | 25273 | **860.0562** | **2577.1468** | **2577.1660** | **-0.0192** | **1** | **5** | **21** | **3** | **R.EYTGFPDPYDELNTGKGEGVAYR.G** |
|  | 27109 | **968.7880** | **2903.3422** | **2903.4890** | **-0.1468** | **1** | **3** | **28** | **6** | **R.TQGLVPEHVETRTLHSTFQPNISQGK.L** |
|  | 27251 | **979.4949** | **2935.4630** | **2935.4783** | **-0.0154** | **1** | **18** | **0.89** | **1** | **K.NFQMASITSPSLVVECGGERVESVVIK.N** |
|  | 29396 | **1156.6256** | **3466.8550** | **3467.6054** | **-0.7504** | **0** | **0** | **35** | **3** | **R.GNNPFFDELFFYNVNMTPSELMDEIISIR.V** |
|  | 30785 | **1423.0432** | **4266.1078** | **4266.1372** | **-0.0294** | **0** | **23** | **0.11** | **1** | **K.TTEIIHSTLNPTWDQTIIFDEVEIYGEPQTVLQNPPK.V** |
|  | 31413 | **1326.8412** | **5303.3357** | **5303.3709** | **-0.0353** | **1** | **13** | **0.63** | **1** | **K.GQDTGATIDLVIGYDPPSAPHPNDLSGPSVPGMGGDGEEDEGDEDRLDNAVR.G** |

| **102.** | P08758\|ANXA5_HUMAN    **Mass:** 35971    **Score:** 332    **Queries matched:** 20   **emPAI:** 1.01 |
| --- | --- |
|  | Annexin A5 OS=Homo sapiens GN=ANXA5 PE=1 SV=2 |

|  | **Query** | **Observed** | **Mr(expt)** | **Mr(calc)** | **Delta** | **Miss** | **Score** | **Expect** | **Rank** | **Peptide** |
| --- | --- | --- | --- | --- | --- | --- | --- | --- | --- | --- |
|  | 1152 | **636.3047** | **635.2974** | **635.3643** | **-0.0668** | **1** | **9** | **24** | **7** | **R.KVFDK.Y** |
|  | 2212 | **421.7047** | **841.3948** | **841.3454** | **0.0494** | **0** | **8** | **27** | **2** | **K.GDTSGDYK.K** |
|  | 3031 | **452.2441** | **902.4737** | **902.4821** | **-0.0084** | **1** | **15** | **7** | **1** | **R.ADAETLRK.A** 3019 |
|  | 3761 | **478.2446** | **954.4747** | **953.5335** | **0.9412** | **0** | **5** | **53** | **5** | **K.FITIFGTR.S** |
|  | 4417 | 500.7920 | 999.5695 | 1000.5917 | -1.0222 | 0 | 51 | 0.0013 | 1 | K.VLTEIIASR.T 4426 |
|  | 4622 | **508.2835** | **1014.5524** | **1013.5069** | **1.0455** | **0** | **9** | **24** | **8** | **R.LYDAYELK.H** |
|  | 5904 | **554.2937** | **1106.5728** | **1105.5768** | **0.9961** | **0** | **13** | **8.1** | **1** | **R.SEIDLFNIR.K** |
|  | 6158 | **563.2994** | **1124.5842** | **1124.5938** | **-0.0096** | **1** | **17** | **2.7** | **1** | **K.HALKGAGTNEK.V** |
|  | 10156 | **670.8048** | **1339.5951** | **1339.6045** | **-0.0094** | **0** | **31** | **0.1** | **1** | **R.GTVTDFPGFDER.A** |
|  | 13795 | 774.9045 | 1547.7944 | 1546.8905 | 0.9039 | 1 | 13 | 6 | 2 | K.FEKLIVALMKPSR.L |
|  | 16276 | **852.9497** | **1703.8849** | **1703.8941** | **-0.0093** | **0** | **77** | **2.2e-006** | **1** | **K.GLGTDEESILTLLTSR.S** |
|  | 17720 | **900.4469** | **1798.8792** | **1798.8890** | **-0.0098** | **1** | **70** | **9.2e-006** | **1** | **K.WGTDEEKFITIFGTR.S** 17719 |
|  | 20907 | **684.6671** | **2050.9793** | **2050.0616** | **0.9177** | **1** | **4** | **36** | **2** | **K.AMKGLGTDEESILTLLTSR.S** |
|  | 29233 | **1133.2523** | **3396.7351** | **3396.7486** | **-0.0135** | **1** | **91** | **3.6e-008** | **1** | **K.YMTISGFQIEETIDRETSGNLEQLLLAVVK.S** 29234 |
|  | 30148 | **1265.9636** | **3794.8690** | **3794.9149** | **-0.0458** | **1** | **78** | **5.6e-007** | **1** | **R.MLVVLLQANRDPDAGIDEAQVEQDAQALFQAGELK.W** 30151 |

| **103.** | P02679\|FIBG_HUMAN    **Mass:** 52106    **Score:** 328    **Queries matched:** 20   **emPAI:** 0.51 |
| --- | --- |
|  | Fibrinogen gamma chain OS=Homo sapiens GN=FGG PE=1 SV=3 |

|  | **Query** | **Observed** | **Mr(expt)** | **Mr(calc)** | **Delta** | **Miss** | **Score** | **Expect** | **Rank** | **Peptide** |
| --- | --- | --- | --- | --- | --- | --- | --- | --- | --- | --- |
|  | 2253 | **422.7633** | **843.5121** | **842.5225** | **0.9896** | **1** | **10** | **21** | **7** | **K.IVNLKEK.V** 2241 |
|  | 2902 | **447.2241** | **892.4336** | **892.4398** | **-0.0062** | **0** | **14** | **7.2** | **1** | **K.MLEEIMK.Y** |
|  | 3121 | **455.7154** | **909.4163** | **908.4347** | **0.9816** | **0** | **(6)** | **39** | **5** | **K.MLEEIMK.Y** |
|  | 4772 | 514.7596 | 1027.5046 | 1028.4862 | -0.9816 | 0 | 9 | 20 | 2 | -.MSWSLHPR.N |
|  | 4879 | **517.7628** | **1033.5111** | **1033.5192** | **-0.0082** | **1** | **29** | **0.24** | **1** | **K.VGPEADKYR.L** |
|  | 7789 | **610.7106** | **1219.4067** | **1218.5663** | **0.8404** | **1** | **5** | **38** | **8** | **K.DCQDIANKGAK.Q** |
|  | 12838 | **497.9166** | **1490.7280** | **1490.7365** | **-0.0085** | **0** | **9** | **16** | **1** | **K.YEASILTHDSSIR.Y** |
|  | 13210 | **757.3624** | **1512.7103** | **1512.7208** | **-0.0105** | **0** | **64** | **5.4e-005** | **1** | **R.YLQEIYNSNNQK.I** 13209 |
|  | 17984 | **909.4277** | **1816.8408** | **1816.8447** | **-0.0040** | **1** | **1** | **93** | **4** | **K.EKVAQLEAQCQEPCK.D** |
|  | 20441 | **671.3044** | **2010.8915** | **2010.9007** | **-0.0092** | **0** | **5** | **28** | **1** | **K.CHAGHLNGVYYQGGTYSK.A** |
|  | 20474 | 672.3094 | 2013.9065 | 2014.7938 | -0.8873 | 0 | 2 | 66 | 9 | K.FEGNCAEQDGSGWWMNK.C |
|  | 22398 | **1104.0177** | **2206.0208** | **2206.0331** | **-0.0123** | **0** | **40** | **0.0088** | **1** | **K.EGFGHLSPTGTTEFWLGNEK.I** |
|  | 24077 | **1209.0356** | **2416.0567** | **2416.0716** | **-0.0148** | **0** | **82** | **4.3e-007** | **1** | **R.FGSYCPTTCGIADFLSTYQTK.V** 24078 |
|  | 24806 | **1260.6263** | **2519.2381** | **2519.2577** | **-0.0196** | **0** | **79** | **8.9e-007** | **1** | **K.AIQLTYNPDESSKPNMIDAATLK.S** 24807 |
|  | 26378 | **923.4436** | **2767.3090** | **2767.3269** | **-0.0179** | **1** | **52** | **0.00038** | **1** | **K.VAQLEAQCQEPCKDTVQIHDITGK.D** 26379 |

| **104.** | P02675\|FIBB_HUMAN    **Mass:** 56577    **Score:** 326    **Queries matched:** 23   **emPAI:** 0.56 |
| --- | --- |
|  | Fibrinogen beta chain OS=Homo sapiens GN=FGB PE=1 SV=2 |

|  | **Query** | **Observed** | **Mr(expt)** | **Mr(calc)** | **Delta** | **Miss** | **Score** | **Expect** | **Rank** | **Peptide** |
| --- | --- | --- | --- | --- | --- | --- | --- | --- | --- | --- |
|  | 1355 | 718.3106 | 717.3033 | 716.4181 | 0.8852 | 0 | 14 | 9.7 | 3 | K.ISQLTR.M |
|  | 2337 | **426.2321** | **850.4496** | **850.4548** | **-0.0053** | **0** | **12** | **9.5** | **1** | **K.YQISVNK.Y** |
|  | 8148 | **620.2581** | **1238.5017** | **1238.5105** | **-0.0088** | **0** | **3** | **58** | **2** | **K.EDGGGWWYNR.C** |
|  | 8632 | 631.3717 | 1260.7288 | 1261.6641 | -0.9353 | 1 | 3 | 72 | 7 | R.MVSWSFHKLK.T |
|  | 9508 | **654.8080** | **1307.6015** | **1307.6106** | **-0.0091** | **0** | **71** | **1.1e-005** | **1** | **K.QGFGNVATNTDGK.N** |
|  | 14399 | **793.8762** | **1585.7378** | **1586.6705** | **-0.9327** | **0** | **5** | **42** | **1** | **K.LESDVSAQMEYCR.T** |
|  | 14915 | **809.8637** | **1617.7127** | **1617.7855** | **-0.0727** | **0** | **18** | **2** | **1** | **R.TPCTVSCNIPVVSGK.E** |
|  | 15717 | **834.8568** | **1667.6989** | **1667.7079** | **-0.0089** | **0** | **21** | **0.91** | **1** | **R.YYWGGQYTWDMAK.H** |
|  | 16075 | **846.4020** | **1690.7894** | **1690.7946** | **-0.0052** | **0** | **59** | **0.00013** | **1** | **R.MGPTELLIEMEDWK.G** |
|  | 17490 | **893.3940** | **1784.7735** | **1784.7828** | **-0.0093** | **0** | **69** | **1.3e-005** | **1** | **K.NYCGLPGEYWLGNDK.I** 17489 |
|  | 20234 | **664.6487** | **1990.9244** | **1990.9380** | **-0.0136** | **1** | **17** | **1.9** | **1** | **R.MGPTELLIEMEDWKGDK.V** |
|  | 21953 | **1078.9713** | **2155.9281** | **2155.9415** | **-0.0135** | **0** | **93** | **4.1e-008** | **1** | **R.VYCDMNTENGGWTVIQNR.Q** 21963 21964 |
|  | 22874 | **753.0261** | **2256.0564** | **2256.0732** | **-0.0169** | **0** | **41** | **0.0072** | **1** | **K.GGETSEMYLIQPDSSVKPYR.V** 22875 |
|  | 23768 | **790.3893** | **2368.1460** | **2367.1607** | **0.9853** | **1** | **47** | **0.0014** | **1** | **K.AHYGGFTVQNEANKYQISVNK.Y** |
|  | 23873 | **795.7253** | **2384.1542** | **2384.1682** | **-0.0140** | **1** | **23** | **0.42** | **1** | **R.KGGETSEMYLIQPDSSVKPYR.V** |
|  | 24467 | **825.1325** | **2472.3757** | **2473.3189** | **-0.9432** | **1** | **6** | **14** | **2** | **R.EEAPSLRPAPPPISGGGYRARPAK.A** |
|  | 28321 | **1056.4943** | **3166.4610** | **3166.4764** | **-0.0155** | **0** | **14** | **2.1** | **1** | **R.NSVDELNNNVEAVSQTSSSSFQYMYLLK.D** |
|  | 30567 | **1021.7540** | **4082.9868** | **4083.0088** | **-0.0221** | **1** | **42** | **0.0016** | **1** | **R.KAPDAGGCLHADPDLGVLCPTGCQLQEALLQQERPIR.N** 30568 |

| **105.** | P55072\|TERA_HUMAN    **Mass:** 89950    **Score:** 325    **Queries matched:** 45   **emPAI:** 0.38 |
| --- | --- |
|  | Transitional endoplasmic reticulum ATPase OS=Homo sapiens GN=VCP PE=1 SV=4 |

|  | **Query** | **Observed** | **Mr(expt)** | **Mr(calc)** | **Delta** | **Miss** | **Score** | **Expect** | **Rank** | **Peptide** |
| --- | --- | --- | --- | --- | --- | --- | --- | --- | --- | --- |
|  | 1977 | **414.2008** | **826.3870** | **826.3933** | **-0.0063** | **0** | **9** | **18** | **1** | **K.THGEVER.R** |
|  | 3669 | **474.7858** | **947.5570** | **946.5600** | **0.9970** | **1** | **12** | **12** | **2** | **R.KGDIFLVR.G** 3640 |
|  | 4187 | **493.7669** | **985.5193** | **985.5266** | **-0.0073** | **0** | **13** | **9.3** | **1** | **K.EMVELPLR.H** |
|  | 4872 | **517.7179** | **1033.4212** | **1033.4287** | **-0.0075** | **0** | **10** | **16** | **1** | **R.DHFEEAMR.F** |
|  | 6039 | **559.3067** | **1116.5988** | **1115.4956** | **1.1033** | **0** | **9** | **23** | **1** | **R.GGNIGDGGGAADR.V** 6038 |
|  | 6823 | **586.8337** | **1171.6528** | **1171.6601** | **-0.0073** | **0** | **31** | **0.14** | **1** | **R.GILLYGPPGTGK.T** |
|  | 7536 | **403.2272** | **1206.6599** | **1205.5247** | **1.1351** | **1** | **8** | **24** | **4** | **R.RDHFEEAMR.F** |
|  | 8413 | **626.3076** | **1250.6006** | **1250.6118** | **-0.0112** | **0** | **6** | **33** | **2** | **K.GVLFYGPPGCGK.T** |
|  | 9139 | **430.2988** | **1287.8745** | **1287.7034** | **0.1711** | **1** | **13** | **6** | **1** | **K.GDDLSTAILKQK.N** |
|  | 9987 | **665.3446** | **1328.6746** | **1328.6837** | **-0.0090** | **0** | **54** | **0.00058** | **1** | **R.WALSQSNPSALR.E** 9990 |
|  | 11802 | **715.9145** | **1429.8144** | **1429.8214** | **-0.0070** | **0** | **82** | **9e-007** | **1** | **R.IVSQLLTLMDGLK.Q** 11801 |
|  | 12086 | 724.3309 | 1446.6472 | 1445.8163 | 0.8308 | 0 | (5) | 48 | 4 | R.IVSQLLTLMDGLK.Q 12085 |
|  | 13942 | **778.9271** | **1555.8396** | **1555.8497** | **-0.0102** | **0** | **61** | **0.0001** | **1** | **R.LDQLIYIPLPDEK.S** |
|  | 14629 | **534.6032** | **1600.7878** | **1601.9174** | **-1.1296** | **1** | **2** | **70** | **8** | **R.RIVSQLLTLMDGLK.Q** |
|  | 15095 | **815.4008** | **1628.7871** | **1628.7981** | **-0.0110** | **1** | **66** | **2.8e-005** | **1** | **R.KYEMFAQTLQQSR.G** |
|  | 15349 | **549.9402** | **1646.7989** | **1647.8315** | **-1.0326** | **1** | **7** | **24** | **2** | **M.ASGADSKGDDLSTAILK.Q** |
|  | 15872 | **839.8618** | **1677.7090** | **1678.8270** | **-1.1180** | **0** | **5** | **33** | **1** | **R.VINQILTEMDGMSTK.K** |
|  | 16684 | 577.3048 | 1728.8924 | 1729.9760 | -1.0836 | 1 | 5 | 34 | 2 | R.IVSQLLTLMDGLKQR.A 16673 |
|  | 17654 | **449.4719** | **1793.8585** | **1794.8669** | **-1.0085** | **1** | **1** | **96** | **10** | **-.MASGADSKGDDLSTAILK.Q** 17675 |
|  | 17732 | 900.8925 | 1799.7704 | 1798.7978 | 0.9725 | 0 | 15 | 3.2 | 2 | K.MTNGFSGADLTEICQR.A |
|  | 17849 | **603.2900** | **1806.8481** | **1806.9219** | **-0.0738** | **1** | **1** | **81** | **1** | **R.VINQILTEMDGMSTKK.N** |
|  | 17899 | **905.9972** | **1809.9798** | **1809.9876** | **-0.0078** | **0** | **53** | **0.0005** | **1** | **K.NAPAIIFIDELDAIAPK.R** |
|  | 21903 | **717.8289** | **2150.4649** | **2150.0830** | **0.3819** | **1** | **4** | **24** | **1** | **K.ARQAAPCVLFFDELDSIAK.A** |
|  | 22085 | **1085.5591** | **2169.1036** | **2169.1165** | **-0.0129** | **0** | **65** | **2.5e-005** | **1** | **R.LIVDEAINEDNSVVSLSQPK.M** 22083 |
|  | 22456 | **738.0555** | **2211.1446** | **2211.1575** | **-0.0129** | **1** | **9** | **11** | **1** | **R.ELQELVQYPVEHPDKFLK.F** |
|  | 22869 | **752.7130** | **2255.1172** | **2255.1296** | **-0.0124** | **0** | **57** | **0.00017** | **1** | **R.AVANETGAFFFLINGPEIMSK.L** 22868 |
|  | 23234 | **1151.3561** | **2300.6976** | **2301.2488** | **-0.5512** | **1** | **7** | **10** | **2** | **R.AHVIVMAATNRPNSIDPALRR.F** 23233 |
|  | 23308 | **771.3551** | **2311.0435** | **2311.0750** | **-0.0315** | **1** | **4** | **29** | **3** | **R.QTNPSAMEVEEDDPVPEIRR.D** |
|  | 23326 | **1157.0502** | **2312.0858** | **2312.9703** | **-0.8845** | **1** | **3** | **37** | **3** | **R.EDEEESLNEVGYDDIGGCRK.Q** |
|  | 24788 | **840.1410** | **2517.4011** | **2517.4180** | **-0.0168** | **0** | **11** | **5.2** | **1** | **K.NVFIIGATNRPDIIDPAILRPGR.L** |
|  | 26637 | **702.8552** | **2807.3918** | **2808.3018** | **-0.9101** | **1** | **9** | **7.5** | **1** | **R.GGNIGDGGGAADRVINQILTEMDGMSTK.K** |
|  | 28094 | **1039.4984** | **3115.4734** | **3116.3876** | **-0.9142** | **0** | **1** | **42** | **5** | **K.MDLIDLEDETIDAEVMNSLAVTMDDFR.W** |
|  | 29876 | **918.9485** | **3671.7649** | **3671.7849** | **-0.0201** | **0** | **2** | **23** | **1** | **K.LADDVDLEQVANETHGHVGADLAALCSEAALQAIR.K** |
|  | 30538 | **581.1558** | **4061.0394** | **4060.9582** | **0.0812** | **1** | **5** | **9** | **1** | **K.NMKLADDVDLEQVANETHGHVGADLAALCSEAALQAIR.K** |
|  | 30927 | **1471.5968** | **4411.7686** | **4411.0658** | **0.7028** | **1** | **9** | **1.9** | **1** | **K.MDLIDLEDETIDAEVMNSLAVTMDDFRWALSQSNPSALR.E** |

| **106.** | P60660\|MYL6_HUMAN    **Mass:** 17090    **Score:** 324    **Queries matched:** 28   **emPAI:** 5.29 |
| --- | --- |
|  | Myosin light polypeptide 6 OS=Homo sapiens GN=MYL6 PE=1 SV=2 |

|  | **Query** | **Observed** | **Mr(expt)** | **Mr(calc)** | **Delta** | **Miss** | **Score** | **Expect** | **Rank** | **Peptide** |
| --- | --- | --- | --- | --- | --- | --- | --- | --- | --- | --- |
|  | 863 | **527.0978** | **526.0905** | **525.2911** | **0.7994** | **0** | **7** | **15** | **1** | **R.HILSG.-** |
|  | 1114 | **626.7722** | **625.7649** | **626.3752** | **-0.6102** | **0** | **21** | **0.6** | **1** | **K.VLGNPK.S** 1115 |
|  | 1917 | **822.3594** | **821.3521** | **821.3589** | **-0.0068** | **0** | **39** | **0.022** | **1** | **K.SDEMNVK.V** |
|  | 2166 | **420.2213** | **838.4280** | **837.3538** | **1.0742** | **0** | **(10)** | **16** | **3** | **K.SDEMNVK.V** |
|  | 4325 | **498.2942** | **994.5739** | **994.5811** | **-0.0072** | **0** | **52** | **0.00097** | **1** | **R.HVLVTLGEK.M** 4309 4326 4339 |
|  | 4733 | **513.2528** | **1024.4911** | **1024.4978** | **-0.0067** | **0** | **5** | **44** | **3** | **K.EAFQLFDR.T** |
|  | 10437 | **677.8653** | **1353.7160** | **1353.7252** | **-0.0092** | **0** | **45** | **0.0042** | **1** | **R.ALGQNPTNAEVLK.V** 10438 10458 |
|  | 13732 | **772.8422** | **1543.6699** | **1543.6791** | **-0.0092** | **0** | **64** | **4.7e-005** | **1** | **K.DQGTYEDYVEGLR.V** |
|  | 16564 | **861.9216** | **1721.8286** | **1721.8407** | **-0.0121** | **1** | **44** | **0.004** | **1** | **R.VFDKEGNGTVMGAEIR.H** 16575 16576 16577 16578 16579 |
|  | 16812 | **869.8542** | **1737.6938** | **1737.8356** | **-0.1418** | **1** | **(1)** | **74** | **5** | **R.VFDKEGNGTVMGAEIR.H** |
|  | 17508 | **893.9097** | **1785.8048** | **1785.8169** | **-0.0122** | **1** | **92** | **6.8e-008** | **1** | **K.NKDQGTYEDYVEGLR.V** 17509 |
|  | 18960 | **630.0033** | **1886.9881** | **1886.9964** | **-0.0084** | **0** | **51** | **0.00073** | **1** | **K.VLDFEHFLPMLQTVAK.N** 18957 18958 18959 |
|  | 19152 | **952.9285** | **1903.8425** | **1902.9914** | **0.8511** | **0** | **(5)** | **27** | **1** | **K.VLDFEHFLPMLQTVAK.N** |

| **107.** | Q6NZI2\|PTRF_HUMAN    **Mass:** 43450    **Score:** 319    **Queries matched:** 26   **emPAI:** 0.64 |
| --- | --- |
|  | Polymerase I and transcript release factor OS=Homo sapiens GN=PTRF PE=1 SV=1 |

|  | **Query** | **Observed** | **Mr(expt)** | **Mr(calc)** | **Delta** | **Miss** | **Score** | **Expect** | **Rank** | **Peptide** |
| --- | --- | --- | --- | --- | --- | --- | --- | --- | --- | --- |
|  | 1067 | **604.9559** | **603.9486** | **603.3704** | **0.5782** | **1** | **9** | **24** | **3** | **K.LKTSR.D** |
|  | 1176 | **645.3880** | **644.3807** | **644.3857** | **-0.0050** | **0** | **19** | **3.3** | **1** | **K.VSVNVK.T** |
|  | 1352 | **716.3771** | **715.3699** | **716.3453** | **-0.9754** | **0** | **7** | **47** | **1** | **R.GEAGDLR.R** |
|  | 6014 | **558.7816** | **1115.5486** | **1115.5571** | **-0.0085** | **0** | **47** | **0.0036** | **1** | **K.AHATTSNTVSK.L** 6009 6010 6011 6012 6013 6015 6016 6017 6018 6019 6026 |
|  | 13464 | **764.8786** | **1527.7426** | **1528.7596** | **-1.0169** | **1** | **9** | **15** | **5** | **R.NFKVMIYQDEVK.L** |
|  | 14611 | **800.4476** | **1598.8807** | **1598.8879** | **-0.0072** | **0** | **61** | **9e-005** | **1** | **K.SDQVNGVLVLSLLDK.I** 14610 14618 |
|  | 17804 | **902.9348** | **1803.8551** | **1803.8672** | **-0.0122** | **0** | **80** | **1.1e-006** | **1** | **R.QAEMEGAVQSIQGELSK.L** |
|  | 20607 | **1013.0469** | **2024.0792** | **2024.0902** | **-0.0110** | **0** | **106** | **2.4e-009** | **1** | **K.IIGAVDQIQLTQAQLEER.Q** 20606 20608 |
|  | 24987 | **850.3680** | **2548.0821** | **2548.0983** | **-0.0162** | **1** | **17** | **1.2** | **1** | **K.ATEMVEVGADDDEGGAERGEAGDLR.R** |
|  | 26293 | 917.0913 | 2748.2521 | 2748.2396 | 0.0125 | 1 | 2 | 40 | 1 | R.EGQVEVLKATEMVEVGADDDEGGAER.G |
|  | 31161 | **803.0233** | **4812.0963** | **4813.1948** | **-1.0985** | **1** | **2** | **8.5** | **3** | **K.ESEALPEKEGEELGEGERPEEDAAALELSSDEAVEVEEVIEESR.A** |

| **108.** | O43707\|ACTN4_HUMAN    **Mass:** 105245   **Score:** 314    **Queries matched:** 30   **emPAI:** 0.36 |
| --- | --- |
|  | Alpha-actinin-4 OS=Homo sapiens GN=ACTN4 PE=1 SV=2 |

|  | **Query** | **Observed** | **Mr(expt)** | **Mr(calc)** | **Delta** | **Miss** | **Score** | **Expect** | **Rank** | **Peptide** |
| --- | --- | --- | --- | --- | --- | --- | --- | --- | --- | --- |
|  | 2523 | **432.7420** | **863.4695** | **863.4752** | **-0.0057** | **0** | **28** | **0.33** | **1** | **K.ALDFIASK.G** |
|  | 2635 | **437.2473** | **872.4799** | **873.4192** | **-0.9393** | **0** | **11** | **16** | **2** | **K.STLPDADR.E** |
|  | 6853 | **588.3041** | **1174.5937** | **1173.5965** | **0.9973** | **0** | **7** | **32** | **1** | **K.EGLLLWCQR.K** |
|  | 8335 | **624.3159** | **1246.6172** | **1247.6622** | **-1.0450** | **1** | **2** | **88** | **5** | **K.NFITAEELRR.E** |
|  | 8455 | **627.7901** | **1253.5656** | **1254.5979** | **-1.0323** | **0** | **3** | **67** | **2** | **R.DYETATLSDIK.A** |
|  | 9373 | **434.5373** | **1300.5900** | **1300.5982** | **-0.0082** | **0** | **23** | **0.66** | **1** | **K.HTNYTMEHIR.V** 9374 |
|  | 9890 | **663.3394** | **1324.6642** | **1324.6735** | **-0.0093** | **1** | **30** | **0.13** | **1** | **K.RDHALLEEQSK.Q** |
|  | 10375 | 676.8123 | 1351.6101 | 1351.6190 | -0.0089 | 0 | 38 | 0.021 | 1 | K.GISQEQMQEFR.A |
|  | 11021 | **693.3895** | **1384.7645** | **1385.7667** | **-1.0022** | **0** | **74** | **5.6e-006** | **1** | **R.VGWEQLLTTIAR.T** 11042 |
|  | 11779 | **715.3815** | **1428.7485** | **1428.7572** | **-0.0087** | **0** | **31** | **0.12** | **1** | **R.TINEVENQILTR.D** |
|  | 13600 | 768.8354 | 1535.6562 | 1536.7671 | -1.1109 | 0 | 7 | 23 | 6 | R.FAIQDISVEETSAK.E |
|  | 14147 | **523.9534** | **1568.8385** | **1567.8470** | **0.9914** | **1** | **19** | **1.6** | **1** | **R.HRPELIEYDKLR.K** |
|  | 15024 | **542.2936** | **1623.8589** | **1623.7563** | **0.1027** | **0** | **0** | **1.1e+002** | **4** | **K.ACLISLGYDVENDR.Q** |
|  | 16354 | **570.6419** | **1708.9039** | **1707.9043** | **0.9996** | **1** | **1** | **80** | **6** | **R.DYETATLSDIKALIR.K** |
|  | 17254 | **886.9034** | **1771.7923** | **1772.9210** | **-1.1286** | **0** | **4** | **47** | **8** | **R.QFASQANVVGPWIQTK.M** |
|  | 17311 | **592.8822** | **1775.6248** | **1774.8373** | **0.7874** | **0** | **2** | **55** | **4** | **K.DDPVTNLNNAFEVAEK.Y** |
|  | 17625 | **896.9267** | **1791.8388** | **1791.8502** | **-0.0113** | **0** | **73** | **6e-006** | **1** | **R.MAPYQGPDAVPGALDYK.S** 17615 |
|  | 17986 | **606.6270** | **1816.8592** | **1815.8672** | **0.9920** | **0** | **16** | **2.4** | **1** | **K.MLDAEDIVNTARPDEK.A** |
|  | 18206 | 916.9570 | 1831.8995 | 1831.8622 | 0.0373 | 0 | (3) | 48 | 9 | K.MLDAEDIVNTARPDEK.A 18205 |
|  | 18848 | **627.0032** | **1877.9877** | **1876.9829** | **1.0048** | **1** | **5** | **33** | **6** | **K.CQLEINFNTLQTKLR.L** |
|  | 19331 | **960.5015** | **1918.9884** | **1919.0000** | **-0.0116** | **0** | **100** | **1.1e-008** | **1** | **K.LSGSNPYTTVTPQIINSK.W** |
|  | 21035 | **687.6529** | **2059.9368** | **2059.9520** | **-0.0152** | **0** | **8** | **13** | **1** | **K.VLAVNQENEHLMEDYEK.L** |
|  | 22346 | **735.0065** | **2201.9976** | **2202.0130** | **-0.0154** | **1** | **25** | **0.27** | **1** | **R.ASFNHFDKDHGGALGPEEFK.A** |
|  | 25170 | **854.4144** | **2560.2215** | **2561.2683** | **-1.0468** | **1** | **3** | **35** | **4** | **K.YLDIPKMLDAEDIVNTARPDEK.A** |
|  | 28963 | **1108.9146** | **3323.7218** | **3323.7401** | **-0.0183** | **0** | **63** | **2.2e-005** | **1** | **R.SIVDYKPNLDLLEQQHQLIQEALIFDNK.H** 28964 |

| **109.** | P04899\|GNAI2_HUMAN    **Mass:** 40995    **Score:** 314    **Queries matched:** 26   **emPAI:** 2.12 |
| --- | --- |
|  | Guanine nucleotide-binding protein G(i), alpha-2 subunit OS=Homo sapiens GN=GNAI2 PE=1 SV=3 |

|  | **Query** | **Observed** | **Mr(expt)** | **Mr(calc)** | **Delta** | **Miss** | **Score** | **Expect** | **Rank** | **Peptide** |
| --- | --- | --- | --- | --- | --- | --- | --- | --- | --- | --- |
|  | 3113 | **455.2127** | **908.4109** | **908.4175** | **-0.0066** | **0** | **33** | **0.081** | **1** | **K.MFDVGGQR.S** |
|  | 3832 | **480.7394** | **959.4641** | **959.4672** | **-0.0030** | **1** | **5** | **56** | **6** | **K.NLREDGEK.A** 3850 |
|  | 5181 | **529.2853** | **1056.5560** | **1056.6179** | **-0.0619** | **0** | **28** | **0.31** | **1** | **K.LLLLGAGESGK.S** |
|  | 5949 | 555.7625 | 1109.5105 | 1109.5175 | -0.0071 | 0 | 58 | 0.0002 | 1 | K.LFDSICNNK.W |
|  | 8882 | **637.8601** | **1273.7055** | **1273.5826** | **0.1229** | **0** | **3** | **73** | **10** | **K.YDEAASYIQSK.F** |
|  | 10924 | **690.8571** | **1379.6997** | **1379.7085** | **-0.0089** | **0** | **15** | **4.8** | **1** | **K.TTGIVETHFTFK.D** |
|  | 13262 | **758.8539** | **1515.6933** | **1515.7041** | **-0.0108** | **0** | **42** | **0.0088** | **1** | **R.LWADHGVQACFGR.S** |
|  | 13569 | **767.8644** | **1533.7142** | **1533.7246** | **-0.0104** | **0** | **41** | **0.011** | **1** | **K.AMGNLQIDFADPSR.A** 13570 |
|  | 13800 | **517.2173** | **1548.6300** | **1549.7195** | **-1.0894** | **0** | **(9)** | **15** | **4** | **K.AMGNLQIDFADPSR.A** |
|  | 14390 | **529.3709** | **1585.0909** | **1584.9450** | **0.1459** | **1** | **1** | **67** | **4** | **K.LLLLGAGESGKSTIVK.Q** |
|  | 15190 | 818.8436 | 1635.6726 | 1635.6835 | -0.0109 | 0 | 40 | 0.012 | 1 | K.IIHEDGYSEEECR.Q |
|  | 15780 | **558.2720** | **1671.7941** | **1671.8052** | **-0.0112** | **1** | **47** | **0.0023** | **1** | **R.RLWADHGVQACFGR.S** |
|  | 16346 | **854.9661** | **1707.9176** | **1706.9243** | **0.9932** | **0** | **29** | **0.14** | **1** | **K.NVQFVFDAVTDVIIK.N** |
|  | 16796 | **868.9796** | **1735.9445** | **1735.9542** | **-0.0097** | **0** | **61** | **9.4e-005** | **1** | **R.AVVYSNTIQSIMAIVK.A** |
|  | 16955 | **873.9488** | **1745.8831** | **1745.8948** | **-0.0117** | **0** | **38** | **0.016** | **1** | **R.IAQSDYIPTQQDVLR.T** |
|  | 18931 | **629.3328** | **1884.9765** | **1884.8168** | **0.1596** | **1** | **4** | **43** | **1** | **R.MHESMKLFDSICNNK.W** |
|  | 21046 | 688.3206 | 2061.9400 | 2061.9538 | -0.0137 | 1 | 17 | 1.8 | 1 | K.AMGNLQIDFADPSRADDAR.Q |
|  | 21185 | **1038.9725** | **2075.9305** | **2075.9436** | **-0.0131** | **0** | **2** | **59** | **1** | **R.EYQLNDSAAYYLNDLER.I** |
|  | 22311 | **550.7828** | **2199.1020** | **2199.1721** | **-0.0701** | **1** | **7** | **16** | **2** | **R.QYRAVVYSNTIQSIMAIVK.A** |
|  | 23367 | **1160.5376** | **2319.0606** | **2319.0767** | **-0.0160** | **1** | **87** | **1.5e-007** | **1** | **R.SREYQLNDSAAYYLNDLER.I** 23368 |
|  | 26411 | **925.4703** | **2773.3892** | **2773.4069** | **-0.0177** | **1** | **31** | **0.043** | **1** | **R.QLFALSCTAEEQGVLPDDLSGVIRR.L** |
|  | 28884 | **826.8962** | **3303.5559** | **3303.5758** | **-0.0199** | **1** | **34** | **0.017** | **1** | **K.ITHSPLTICFPEYTGANKYDEAASYIQSK.F** 28885 |

| **110.** | P40939\|ECHA_HUMAN    **Mass:** 83688    **Score:** 307    **Queries matched:** 39   **emPAI:** 0.41 |
| --- | --- |
|  | Trifunctional enzyme subunit alpha, mitochondrial OS=Homo sapiens GN=HADHA PE=1 SV=2 |

|  | **Query** | **Observed** | **Mr(expt)** | **Mr(calc)** | **Delta** | **Miss** | **Score** | **Expect** | **Rank** | **Peptide** |
| --- | --- | --- | --- | --- | --- | --- | --- | --- | --- | --- |
|  | 1348 | **714.9851** | **713.9778** | **714.4025** | **-0.4246** | **0** | **1** | **1.1e+002** | **1** | **K.GDVAVVR.I** |
|  | 2111 | **418.2632** | **834.5119** | **833.4396** | **1.0723** | **0** | **7** | **37** | **4** | **R.GQQQVFK.G** |
|  | 2443 | **859.1540** | **858.1468** | **857.5222** | **0.6246** | **1** | **1** | **1.3e+002** | **5** | **R.IVEKLEK.S** |
|  | 2564 | **434.7324** | **867.4502** | **867.4450** | **0.0052** | **0** | **3** | **73** | **7** | **K.HVAEDLGK.V** |
|  | 3388 | **465.2357** | **928.4568** | **928.4654** | **-0.0086** | **1** | **27** | **0.4** | **1** | **K.KYEAAYGK.Q** 3387 |
|  | 3421 | 466.2501 | 930.4857 | 930.4923 | -0.0066 | 0 | 11 | 17 | 8 | R.THINYGVK.G |
|  | 3690 | **475.7298** | **949.4451** | **950.5185** | **-1.0734** | **1** | **8** | **25** | **6** | **K.KALTSFER.D** |
|  | 4034 | **487.7715** | **973.5285** | **974.5033** | **-0.9748** | **0** | **4** | **85** | **8** | **K.DATLTALDR.G** |
|  | 6054 | **1118.5980** | **1117.5907** | **1117.5979** | **-0.0072** | **0** | **26** | **0.38** | **1** | **K.DTSASAVAVGLK.Q** |
|  | 7444 | 602.3182 | 1202.6219 | 1202.5972 | 0.0248 | 0 | 15 | 5.3 | 2 | K.GFYIYQEGVK.R |
|  | 9087 | **643.4093** | **1284.8040** | **1283.6431** | **1.1609** | **1** | **4** | **58** | **3** | **K.FGELVMTKESK.A** |
|  | 9822 | **441.2963** | **1320.8672** | **1321.6192** | **-0.7521** | **0** | **4** | **45** | **9** | **R.CLAPMMSEVIR.I** |
|  | 10120 | **446.7746** | **1337.3019** | **1336.7615** | **0.5403** | **1** | **3** | **51** | **6** | **R.AIGILSRFSAFR.I** |
|  | 11247 | **467.2449** | **1398.7130** | **1397.7377** | **0.9753** | **0** | **2** | **94** | **8** | **K.LTAYAMTIPFVR.Q** |
|  | 11461 | **471.2893** | **1410.8460** | **1410.6263** | **0.2197** | **0** | **0** | **1.1e+002** | **4** | **K.SEVSSDEDIQFR.L** |
|  | 11885 | **479.2625** | **1434.7655** | **1433.7726** | **0.9930** | **1** | **20** | **1.2** | **1** | **K.TSKDTSASAVAVGLK.Q** |
|  | 12815 | **745.4584** | **1488.9023** | **1488.7581** | **0.1442** | **0** | **0** | **1.2e+002** | **10** | **K.ALMGLYHGQVLCK.K** |
|  | 13359 | **761.3731** | **1520.7315** | **1520.7392** | **-0.0076** | **0** | **31** | **0.11** | **1** | **K.DLNSDMDSILASLK.L** |
|  | 13410 | 763.3025 | 1524.5904 | 1524.9279 | -0.3375 | 1 | 6 | 29 | 5 | K.GLYPAPLKIIDVVK.T |
|  | 14016 | **781.3950** | **1560.7754** | **1560.7826** | **-0.0073** | **0** | **1** | **1e+002** | **5** | **K.MVGVPAALDMMLTGR.S** |
|  | 14248 | **789.3991** | **1576.7837** | **1576.7919** | **-0.0083** | **0** | **4** | **48** | **1** | **R.FGGGNPELLTQMVSK.G** |
|  | 14929 | **540.2952** | **1617.8639** | **1616.8531** | **1.0108** | **1** | **4** | **47** | **7** | **K.ALMGLYHGQVLCKK.N** |
|  | 15114 | **815.9183** | **1629.8221** | **1629.8322** | **-0.0101** | **0** | **42** | **0.0078** | **1** | **K.TLQEVTQLSQEAQR.I** |
|  | 15894 | **840.4288** | **1678.8431** | **1678.8488** | **-0.0056** | **0** | **33** | **0.052** | **1** | **K.ADMVIEAVFEDLSLK.H** |
|  | 18344 | **921.9067** | **1841.7988** | **1841.8102** | **-0.0113** | **0** | **57** | **0.00021** | **1** | **K.TGIEQGSDAGYLCESQK.F** |
|  | 19406 | **642.6772** | **1925.0097** | **1924.0492** | **0.9605** | **1** | **2** | **66** | **7** | **K.GLVEKLTAYAMTIPFVR.Q** |
|  | 19518 | **645.0203** | **1932.0390** | **1931.0042** | **1.0347** | **1** | **1** | **81** | **8** | **R.LPKMVGVPAALDMMLTGR.S** |
|  | 19609 | **970.9124** | **1939.8103** | **1939.9210** | **-0.1108** | **0** | **5** | **25** | **4** | **K.QFTPCQLLADHANSPNK.K** |
|  | 19743 | **650.3462** | **1948.0167** | **1946.9992** | **1.0176** | **1** | **(0)** | **93** | **10** | **R.LPKMVGVPAALDMMLTGR.S** |
|  | 20563 | **674.3747** | **2020.1022** | **2020.1139** | **-0.0117** | **0** | **4** | **37** | **2** | **K.HLAILGAGLMGAGIAQVSVDK.G** |
|  | 21042 | **1031.4868** | **2060.9591** | **2060.9691** | **-0.0100** | **0** | **77** | **1.8e-006** | **1** | **R.DSIFSNLTGQLDYQGFEK.A** 21048 |
|  | 25370 | **865.4367** | **2593.2883** | **2593.3066** | **-0.0183** | **0** | **13** | **3.1** | **1** | **R.SAVLISSKPGCFIAGADINMLAACK.T** |
|  | 26791 | **1418.7478** | **2835.4810** | **2835.4906** | **-0.0096** | **1** | **80** | **5.5e-007** | **1** | **K.KLDSLTTSFGFPVGAATLVDEVGVDVAK.H** 26790 |
|  | 27922 | **1023.5195** | **3067.5366** | **3067.5536** | **-0.0170** | **0** | **13** | **2.8** | **1** | **K.EVEAVIPDHCIFASNTSALPISEIAAVSK.R** |
|  | 29254 | **852.9528** | **3407.7819** | **3407.8010** | **-0.0191** | **1** | **2** | **23** | **1** | **R.VLKEVEAVIPDHCIFASNTSALPISEIAAVSK.R** |
|  | 31283 | **1671.5214** | **5011.5423** | **5010.4024** | **1.1399** | **1** | **5** | **3.7** | **5** | **K.ELHSEFSEVMNEIWASDQIRSAVLISSKPGCFIAGADINMLAACK.T** |

| **111.** | P00738\|HPT_HUMAN    **Mass:** 45861    **Score:** 304    **Queries matched:** 24   **emPAI:** 0.87 |
| --- | --- |
|  | Haptoglobin OS=Homo sapiens GN=HP PE=1 SV=1 |

|  | **Query** | **Observed** | **Mr(expt)** | **Mr(calc)** | **Delta** | **Miss** | **Score** | **Expect** | **Rank** | **Peptide** |
| --- | --- | --- | --- | --- | --- | --- | --- | --- | --- | --- |
|  | 2424 | **429.2731** | **856.5317** | **857.4858** | **-0.9541** | **0** | **34** | **0.085** | **2** | **K.QLVEIEK.V** 2423 |
|  | 6439 | 574.2895 | 1146.5645 | 1145.5353 | 1.0293 | 0 | 6 | 42 | 4 | R.HYEGSTVPEK.K |
|  | 7444 | 602.3182 | 1202.6219 | 1202.6295 | -0.0076 | 0 | 50 | 0.0016 | 1 | K.VTSIQDWVQK.T |
|  | 8867 | **637.8170** | **1273.6194** | **1273.6302** | **-0.0109** | **1** | **39** | **0.019** | **1** | **R.HYEGSTVPEKK.T** 8868 8869 8873 8874 |
|  | 10247 | **673.3211** | **1344.6277** | **1344.6384** | **-0.0107** | **0** | **40** | **0.014** | **1** | **K.SCAVAEYGVYVK.V** |
|  | 11583 | **709.9116** | **1417.8087** | **1417.8181** | **-0.0094** | **1** | **23** | **0.66** | **1** | **K.DIAPTLTLYVGKK.Q** |
|  | 14317 | **791.4034** | **1580.7923** | **1579.7842** | **1.0081** | **1** | **36** | **0.026** | **1** | **K.LRTEGDGVYTLNDK.K** |
|  | 16316 | **854.4075** | **1706.8004** | **1706.8120** | **-0.0116** | **0** | **8** | **19** | **2** | **K.YVMLPVADQDQCIR.H** |
|  | 16338 | **854.9229** | **1707.8313** | **1707.8428** | **-0.0115** | **1** | **63** | **5.7e-005** | **1** | **K.LRTEGDGVYTLNNEK.Q** |
|  | 17745 | **601.0431** | **1800.1074** | **1798.9802** | **1.1272** | **1** | **2** | **50** | **4** | **K.NPANPVQRILGGHLDAK.G** |
|  | 18248 | **917.9573** | **1833.9001** | **1833.9117** | **-0.0116** | **1** | **49** | **0.0013** | **1** | **R.VMPICLPSKDYAEVGR.V** |
|  | 18579 | 929.4569 | 1856.8991 | 1856.9124 | -0.0133 | 1 | 22 | 0.64 | 1 | K.AVGDKLPECEAVCGKPK.N 18578 |
|  | 22108 | **724.6857** | **2171.0354** | **2171.0504** | **-0.0150** | **0** | **13** | **3.9** | **1** | **K.SPVGVQPILNEHTFCAGMSK.Y** |
|  | 24409 | 1233.1001 | 2464.1856 | 2464.1879 | -0.0023 | 1 | 1 | 59 | 4 | K.FTDHLKYVMLPVADQDQCIR.H 24408 |
|  | 30271 | **1292.2213** | **3873.6421** | **3873.6628** | **-0.0207** | **0** | **94** | **1.1e-008** | **1** | **K.YQEDTCYGDAGSAFAVHDLEEDTWYATGILSFDK.S** 30269 30270 |

| **112.** | P06702\|S10A9_HUMAN    **Mass:** 13291    **Score:** 299    **Queries matched:** 17   **emPAI:** 5.12 |
| --- | --- |
|  | Protein S100-A9 OS=Homo sapiens GN=S100A9 PE=1 SV=1 |

|  | **Query** | **Observed** | **Mr(expt)** | **Mr(calc)** | **Delta** | **Miss** | **Score** | **Expect** | **Rank** | **Peptide** |
| --- | --- | --- | --- | --- | --- | --- | --- | --- | --- | --- |
|  | 3969 | **486.2470** | **970.4793** | **970.4872** | **-0.0079** | **0** | **9** | **22** | **4** | **R.LTWASHEK.M** |
|  | 12207 | 485.9094 | 1454.7063 | 1454.7154 | -0.0091 | 0 | 38 | 0.02 | 1 | K.LGHPDTLNQGEFK.E 12206 |
|  | 14839 | **807.9019** | **1613.7892** | **1613.7945** | **-0.0054** | **0** | **33** | **0.057** | **1** | **K.QLSFEEFIMLMAR.L** 14834 |
|  | 16877 | **871.9117** | **1741.8088** | **1741.8192** | **-0.0104** | **0** | **71** | **8e-006** | **1** | **K.VIEHIMEDLDTNADK.Q** 16876 |
|  | 17838 | **903.9675** | **1805.9205** | **1805.9312** | **-0.0107** | **0** | **53** | **0.00059** | **1** | **R.NIETIINTFHQYSVK.L** 17835 17836 17837 17842 |
|  | 22132 | **725.9865** | **2174.9377** | **2174.9552** | **-0.0175** | **0** | **25** | **0.26** | **1** | **K.MHEGDEGPGHHHKPGLGEGTP.-** 22137 |
|  | 29024 | **1113.1735** | **3336.4986** | **3337.6032** | **-1.1047** | **1** | **88** | **6.9e-008** | **1** | **K.VIEHIMEDLDTNADKQLSFEEFIMLMAR.L** 29025 29026 |

| **113.** | O75396\|SC22B_HUMAN    **Mass:** 24896    **Score:** 296    **Queries matched:** 21   **emPAI:** 1.71 |
| --- | --- |
|  | Vesicle-trafficking protein SEC22b OS=Homo sapiens GN=SEC22B PE=1 SV=3 |

|  | **Query** | **Observed** | **Mr(expt)** | **Mr(calc)** | **Delta** | **Miss** | **Score** | **Expect** | **Rank** | **Peptide** |
| --- | --- | --- | --- | --- | --- | --- | --- | --- | --- | --- |
|  | 3220 | **458.7830** | **915.5515** | **915.5575** | **-0.0061** | **0** | **31** | **0.15** | **1** | **M.VLLTMIAR.V** |
|  | 3458 | **467.2503** | **932.4861** | **932.4927** | **-0.0065** | **0** | **20** | **1.7** | **1** | **K.ANNLSSLSK.K** |
|  | 4258 | **496.2562** | **990.4978** | **989.5029** | **0.9949** | **0** | **8** | **30** | **2** | **R.GEALSALDSK.A** |
|  | 5229 | **530.7545** | **1059.4944** | **1060.5876** | **-1.0933** | **1** | **11** | **15** | **2** | **K.ANNLSSLSKK.Y** 5228 |
|  | 5401 | **536.7861** | **1071.5576** | **1071.5672** | **-0.0097** | **1** | **52** | **0.0012** | **1** | **R.KLNEQSPTR.C** 5402 5403 5404 5425 |
|  | 5508 | 540.2557 | 1078.4969 | 1078.5879 | -0.0909 | 0 | 6 | 38 | 3 | -.MVLLTMIAR.V |
|  | 7545 | **604.7940** | **1207.5735** | **1207.5833** | **-0.0098** | **0** | **46** | **0.0034** | **1** | **R.DLQQYQSQAK.Q** |
|  | 11499 | **707.8848** | **1413.7550** | **1413.7650** | **-0.0100** | **0** | **68** | **2.3e-005** | **1** | **R.IMVANIEEVLQR.G** |
|  | 14403 | **793.9047** | **1585.7948** | **1585.8060** | **-0.0112** | **0** | **73** | **6.5e-006** | **1** | **R.NLGSINTELQDVQR.I** 14367 |
|  | 16873 | 871.5104 | 1741.0062 | 1741.9071 | -0.9009 | 1 | 6 | 23 | 8 | R.RNLGSINTELQDVQR.I |
|  | 20043 | **987.4589** | **1972.9033** | **1972.9160** | **-0.0127** | **0** | **75** | **2.7e-006** | **1** | **R.VADGLPLAASMQEDEQSGR.D** 20041 |
|  | 23881 | **796.0899** | **2385.2479** | **2385.2573** | **-0.0094** | **1** | **57** | **0.00015** | **1** | **R.IMVANIEEVLQRGEALSALDSK.A** |
|  | 23921 | **798.0860** | **2391.2362** | **2390.3436** | **0.8926** | **1** | **4** | **27** | **1** | **R.STYAKLAAVAVFFIMLIVYVR.F** |
|  | 28306 | **1055.1622** | **3162.4649** | **3162.4887** | **-0.0239** | **1** | **27** | **0.097** | **1** | **R.VADGLPLAASMQEDEQSGRDLQQYQSQAK.Q** |

| **114.** | P30480\|1B42_HUMAN    **Mass:** 40650    **Score:** 295    **Queries matched:** 34   **emPAI:** 1.42 |
| --- | --- |
|  | HLA class I histocompatibility antigen, B-42 alpha chain OS=Homo sapiens GN=HLA-B PE=1 SV=1 |

|  | **Query** | **Observed** | **Mr(expt)** | **Mr(calc)** | **Delta** | **Miss** | **Score** | **Expect** | **Rank** | **Peptide** |
| --- | --- | --- | --- | --- | --- | --- | --- | --- | --- | --- |
|  | 1309 | **702.8578** | **701.8505** | **701.3894** | **0.4611** | **0** | **10** | **18** | **2** | **M.LVMAPR.T** 1307 1311 |
|  | 1455 | **766.4029** | **765.3956** | **765.4021** | **-0.0065** | **0** | **37** | **0.038** | **1** | **R.NTQIYK.A** |
|  | 3888 | 482.7234 | 963.4322 | 964.4250 | -0.9929 | 0 | 33 | 0.088 | 1 | R.FDSDAASPR.E 3900 3901 3907 |
|  | 10029 | **666.8338** | **1331.6530** | **1331.6622** | **-0.0092** | **0** | **17** | **3.1** | **1** | **R.YFYTSVSRPGR.G** |
|  | 11736 | 714.3626 | 1426.7105 | 1426.7205 | -0.0099 | 0 | 46 | 0.0031 | 1 | K.WAAVVVPSGEEQR.Y 11721 11735 11751 |
|  | 12552 | **492.8911** | **1475.6516** | **1475.6641** | **-0.0125** | **1** | **13** | **6.8** | **1** | **R.FDSDAASPREEPR.A** 12553 |
|  | 12921 | 748.8587 | 1495.7028 | 1495.7129 | -0.0101 | 0 | 57 | 0.00026 | 1 | R.AYLEGTCVEWLR.R |
|  | 13321 | 760.3871 | 1518.7596 | 1518.7427 | 0.0169 | 0 | 62 | 7.5e-005 | 1 | R.SWTAADTAAQITQR.K |
|  | 15320 | 823.4096 | 1644.8047 | 1644.8148 | -0.0101 | 0 | 69 | 1.4e-005 | 1 | R.FISVGYVDDTQFVR.F 15321 |
|  | 18486 | 463.4837 | 1849.9057 | 1849.9183 | -0.0127 | 0 | 16 | 2.9 | 1 | K.THVTHHPISDHEATLR.C 18485 |
|  | 20872 | 513.0196 | 2048.0493 | 2048.0626 | -0.0133 | 0 | 16 | 2.1 | 1 | R.YTCHVQHEGLPKPLTLR.W 20848 20850 20871 |
|  | 21557 | 1056.5175 | 2111.0204 | 2111.0299 | -0.0095 | 0 | 86 | 2.1e-007 | 1 | R.CWALGFYPAEITLTWQR.D 21558 |
|  | 22689 | **744.6669** | **2230.9790** | **2230.9938** | **-0.0149** | **0** | **44** | **0.0032** | **1** | **R.DGEDQTQDTELVETRPAGDR.T** 22688 |
|  | 23097 | **1143.5082** | **2285.0018** | **2286.0341** | **-1.0323** | **1** | **11** | **6.4** | **1** | **R.EEPRAPWIEQEGPEYWDR.N** |
|  | 23645 | **785.7004** | **2354.0793** | **2354.0927** | **-0.0134** | **1** | **36** | **0.02** | **1** | **R.GHNQYAYDGKDYIALNEDLR.S** |
|  | 25366 | 865.0411 | 2592.1016 | 2591.2293 | 0.8723 | 1 | 0 | 56 | 6 | R.FISVGYVDDTQFVRFDSDAASPR.E |
|  | 28130 | **521.6007** | **3123.5606** | **3123.6936** | **-0.1330** | **1** | **13** | **2.7** | **1** | **M.LVMAPRTVLLLLSAALALTETWAGSHSMR.Y** |
|  | 30136 | 1896.3450 | 3790.6754 | 3790.1002 | 0.5752 | 1 | 2 | 20 | 5 | R.WEPSSQSTVPIVGIVAGLAVLAVVVIGAVVAAVMCRR.K |

| **115.** | P01893\|HLAH_HUMAN    **Mass:** 41052    **Score:** 294    **Queries matched:** 29   **emPAI:** 1.01 |
| --- | --- |
|  | Putative HLA class I histocompatibility antigen, alpha chain H OS=Homo sapiens GN=HLA-H PE=5 SV=2 |

|  | **Query** | **Observed** | **Mr(expt)** | **Mr(calc)** | **Delta** | **Miss** | **Score** | **Expect** | **Rank** | **Peptide** |
| --- | --- | --- | --- | --- | --- | --- | --- | --- | --- | --- |
|  | 1446 | **763.1124** | **762.1051** | **762.3694** | **-0.2643** | **0** | **2** | **96** | **3** | **R.NTQICK.A** |
|  | 4176 | **493.2762** | **984.5379** | **983.5876** | **0.9503** | **1** | **10** | **18** | **10** | **R.ENLRIALR.Y** 4164 |
|  | 4546 | **505.7235** | **1009.4323** | **1008.4149** | **1.0175** | **0** | **8** | **22** | **4** | **R.FDSDDASPR.E** |
|  | 6565 | 577.7809 | 1153.5473 | 1153.6091 | -0.0618 | 1 | 3 | 67 | 9 | K.ETLQRADPPK.T |
|  | 10335 | 675.8494 | 1349.6843 | 1349.6939 | -0.0096 | 1 | 38 | 0.024 | 1 | R.YLENGKETLQR.A 10311 10351 |
|  | 11736 | 714.3626 | 1426.7105 | 1426.7205 | -0.0099 | 0 | 46 | 0.0031 | 1 | K.WAAVVVPSGEEQR.Y 11721 11735 11751 |
|  | 13806 | 775.3702 | 1548.7259 | 1548.7718 | -0.0459 | 1 | 44 | 0.0053 | 1 | R.SWTAADMAAQITKR.K |
|  | 14092 | 522.7712 | 1565.2917 | 1564.7668 | 0.5250 | 1 | (8) | 12 | 3 | R.SWTAADMAAQITKR.K |
|  | 15320 | 823.4096 | 1644.8047 | 1644.8148 | -0.0101 | 0 | 69 | 1.4e-005 | 1 | R.FISVGYVDDTQFVR.F 15321 |
|  | 18889 | **628.3002** | **1881.8787** | **1881.8904** | **-0.0117** | **0** | **24** | **0.42** | **1** | **K.THMTHHPISDHEATLR.C** |
|  | 20871 | 410.6170 | 2048.0485 | 2049.0102 | -0.9617 | 0 | 9 | 11 | 2 | R.YTCHVQHEGLPEPLTLR.W 20872 20886 20897 |
|  | 21557 | 1056.5175 | 2111.0204 | 2111.0299 | -0.0095 | 0 | 86 | 2.1e-007 | 1 | R.CWALGFYPAEITLTWQR.D 21558 |
|  | 25378 | **866.1122** | **2595.3149** | **2595.2639** | **0.0510** | **1** | **1** | **48** | **1** | **K.DYIALNEDLRSWTAADMAAQITK.R** |
|  | 25578 | 1319.0889 | 2636.1632 | 2636.1838 | -0.0206 | 0 | 57 | 0.00013 | 1 | R.DGEDQTQDTELVETRPAGDGTFQK.W 25579 25580 25581 |
|  | 29808 | 1212.2841 | 3633.8304 | 3633.6439 | 0.1864 | 1 | 1 | 29 | 4 | R.IALRYYNQSEGGSHTMQVMYGCDVGPDGPFLR.G |

| **116.** | P63104\|1433Z_HUMAN    **Mass:** 27899    **Score:** 293    **Queries matched:** 17   **emPAI:** 0.67 |
| --- | --- |
|  | 14-3-3 protein zeta/delta OS=Homo sapiens GN=YWHAZ PE=1 SV=1 |

|  | **Query** | **Observed** | **Mr(expt)** | **Mr(calc)** | **Delta** | **Miss** | **Score** | **Expect** | **Rank** | **Peptide** |
| --- | --- | --- | --- | --- | --- | --- | --- | --- | --- | --- |
|  | 1097 | **616.1310** | **615.1238** | **614.3500** | **0.7738** | **0** | **1** | **1.9e+002** | **2** | **K.NVVGAR.R** |
|  | 1190 | **649.2011** | **648.1938** | **648.3013** | **-0.1075** | **0** | **8** | **42** | **1** | **K.QQMAR.E** |
|  | 5854 | 552.2991 | 1102.5836 | 1103.4086 | -0.8250 | 0 | 10 | 17 | 7 | R.YDDMAACMK.S |
|  | 6485 | **575.7877** | **1149.5608** | **1150.5506** | **-0.9898** | **0** | **8** | **23** | **3** | **R.YLAEVAAGDDK.K** 6505 |
|  | 7120 | **397.2190** | **1188.6351** | **1188.6536** | **-0.0186** | **0** | **11** | **12** | **1** | **K.DSTLIMQLLR.D** |
|  | 11325 | **702.8574** | **1403.7003** | **1404.7361** | **-1.0358** | **1** | **4** | **58** | **3** | **R.SSWRVVSSIEQK.T** |
|  | 13790 | 774.8552 | 1547.6958 | 1547.7063 | -0.0105 | 0 | 57 | 0.00023 | 1 | K.SVTEQGAELSNEER.N 13791 |
|  | 20758 | **1020.9908** | **2039.9671** | **2039.9800** | **-0.0129** | **0** | **116** | **2.3e-010** | **1** | **K.GIVDQSQQAYQEAFEISK.K** 20754 20767 |
|  | 24012 | **803.4449** | **2407.3128** | **2407.9888** | **-0.6759** | **0** | **0** | **64** | **3** | **R.DNLTLWTSDTQGDEAEAGEGGEN.-** |
|  | 28878 | **1101.5436** | **3301.6089** | **3301.6275** | **-0.0186** | **1** | **79** | **6.1e-007** | **1** | **K.TAFDEAIAELDTLSEESYKDSTLIMQLLR.D** 28879 28880 28881 |

| **117.** | Q13510\|ASAH1_HUMAN    **Mass:** 45087    **Score:** 291    **Queries matched:** 17   **emPAI:** 0.27 |
| --- | --- |
|  | Acid ceramidase OS=Homo sapiens GN=ASAH1 PE=1 SV=5 |

|  | **Query** | **Observed** | **Mr(expt)** | **Mr(calc)** | **Delta** | **Miss** | **Score** | **Expect** | **Rank** | **Peptide** |
| --- | --- | --- | --- | --- | --- | --- | --- | --- | --- | --- |
|  | 1507 | **395.2248** | **788.4350** | **788.4406** | **-0.0055** | **0** | **21** | **2** | **1** | **K.GHLIHGR.N** |
|  | 3241 | **459.2262** | **916.4378** | **916.5355** | **-0.0977** | **1** | **1** | **1.6e+002** | **7** | **K.KGHLIHGR.N** |
|  | 3866 | **481.7549** | **961.4953** | **960.4950** | **1.0003** | **0** | **9** | **24** | **2** | **K.IMQVVDEK.L** |
|  | 4590 | **507.2528** | **1012.4910** | **1012.4978** | **-0.0068** | **0** | **21** | **1.2** | **2** | **K.GQFETYLR.D** |
|  | 7426 | **401.7402** | **1202.1987** | **1201.5992** | **0.5994** | **1** | **7** | **20** | **7** | **K.HPFFLDDRR.T** |
|  | 7872 | **613.2918** | **1224.5691** | **1224.5775** | **-0.0084** | **0** | **10** | **14** | **1** | **K.STYPPSGPTYR.G** |
|  | 8133 | 619.7855 | 1237.5564 | 1236.6325 | 0.9238 | 0 | 8 | 20 | 3 | K.DVMWIGFLTR.T |
|  | 8998 | **641.3104** | **1280.6062** | **1279.8227** | **0.7834** | **1** | **5** | **41** | **10** | **K.APVLKVIVNSLK.N** |
|  | 10627 | **683.3278** | **1364.6411** | **1364.7275** | **-0.0864** | **1** | **4** | **51** | **4** | **K.KDVMWIGFLTR.T** |
|  | 18793 | **937.9667** | **1873.9188** | **1873.9284** | **-0.0096** | **0** | **101** | **8.6e-009** | **1** | **K.LPGLLGNFPGPFEEEMK.G** 18794 |
|  | 19909 | 654.3577 | 1960.0514 | 1960.0816 | -0.0302 | 1 | 1 | 73 | 7 | K.VIVNSLKNMINTFVPSGK.I |
|  | 20753 | **680.3931** | **2038.1574** | **2039.0422** | **-0.8848** | **1** | **1** | **58** | **3** | **R.TVLENSTSYEEAKNLLTK.T** |
|  | 25618 | **1322.6545** | **2643.2945** | **2643.3102** | **-0.0156** | **0** | **92** | **3.8e-008** | **1** | **R.TSQENISFETMYDVLSTKPVLNK.L** 25617 |
|  | 26683 | **939.8065** | **2816.3976** | **2816.4129** | **-0.0153** | **1** | **58** | **9.2e-005** | **1** | **K.IMQVVDEKLPGLLGNFPGPFEEEMK.G** 26684 |

| **118.** | P30460\|1B08_HUMAN    **Mass:** 40648    **Score:** 289    **Queries matched:** 35   **emPAI:** 1.21 |
| --- | --- |
|  | HLA class I histocompatibility antigen, B-8 alpha chain OS=Homo sapiens GN=HLA-B PE=1 SV=1 |

|  | **Query** | **Observed** | **Mr(expt)** | **Mr(calc)** | **Delta** | **Miss** | **Score** | **Expect** | **Rank** | **Peptide** |
| --- | --- | --- | --- | --- | --- | --- | --- | --- | --- | --- |
|  | 1309 | 702.8578 | 701.8505 | 701.3894 | 0.4611 | 0 | 10 | 18 | 2 | M.LVMAPR.T 1307 1311 |
|  | 3888 | 482.7234 | 963.4322 | 964.4250 | -0.9929 | 0 | 33 | 0.088 | 1 | R.FDSDAASPR.E 3900 3901 3907 |
|  | 9355 | 434.2050 | 1299.5931 | 1299.6030 | -0.0099 | 0 | 5 | 43 | 5 | R.YFDTAMSRPGR.G |
|  | 11736 | 714.3626 | 1426.7105 | 1426.7205 | -0.0099 | 0 | 46 | 0.0031 | 1 | K.WAAVVVPSGEEQR.Y 11721 11735 11751 |
|  | 12552 | 492.8911 | 1475.6516 | 1475.6641 | -0.0125 | 1 | 13 | 6.8 | 1 | R.FDSDAASPREEPR.A 12553 |
|  | 12921 | 748.8587 | 1495.7028 | 1495.7129 | -0.0101 | 0 | 57 | 0.00026 | 1 | R.AYLEGTCVEWLR.R |
|  | 13321 | 760.3871 | 1518.7596 | 1518.7427 | 0.0169 | 0 | 62 | 7.5e-005 | 1 | R.SWTAADTAAQITQR.K |
|  | 15320 | 823.4096 | 1644.8047 | 1644.8148 | -0.0101 | 0 | 69 | 1.4e-005 | 1 | R.FISVGYVDDTQFVR.F 15321 |
|  | 16845 | **580.9057** | **1739.6953** | **1738.8209** | **0.8743** | **1** | **6** | **25** | **6** | **R.YFDTAMSRPGRGEPR.F** 16824 |
|  | 18486 | 463.4837 | 1849.9057 | 1849.9183 | -0.0127 | 0 | 16 | 2.9 | 1 | K.THVTHHPISDHEATLR.C 18485 |
|  | 20872 | 513.0196 | 2048.0493 | 2048.0626 | -0.0133 | 0 | 16 | 2.1 | 1 | R.YTCHVQHEGLPKPLTLR.W 20848 20850 20871 |
|  | 21557 | 1056.5175 | 2111.0204 | 2111.0299 | -0.0095 | 0 | 86 | 2.1e-007 | 1 | R.CWALGFYPAEITLTWQR.D 21558 |
|  | 22689 | 744.6669 | 2230.9790 | 2230.9938 | -0.0149 | 0 | 44 | 0.0032 | 1 | R.DGEDQTQDTELVETRPAGDR.T 22688 |
|  | 23097 | 1143.5082 | 2285.0018 | 2286.0341 | -1.0323 | 1 | 11 | 6.4 | 1 | R.EEPRAPWIEQEGPEYWDR.N |
|  | 23645 | 785.7004 | 2354.0793 | 2354.0927 | -0.0134 | 1 | 36 | 0.02 | 1 | R.GHNQYAYDGKDYIALNEDLR.S |
|  | 25366 | 865.0411 | 2592.1016 | 2591.2293 | 0.8723 | 1 | 0 | 56 | 6 | R.FISVGYVDDTQFVRFDSDAASPR.E |
|  | 28130 | 521.6007 | 3123.5606 | 3123.6936 | -0.1330 | 1 | 13 | 2.7 | 1 | M.LVMAPRTVLLLLSAALALTETWAGSHSMR.Y |
|  | 30136 | 1896.3450 | 3790.6754 | 3790.1002 | 0.5752 | 1 | 2 | 20 | 5 | R.WEPSSQSTVPIVGIVAGLAVLAVVVIGAVVAAVMCRR.K |

| **119.** | Q7Z406\|MYH14_HUMAN    **Mass:** 228889   **Score:** 289    **Queries matched:** 88   **emPAI:** 0.19 |
| --- | --- |
|  | Myosin-14 OS=Homo sapiens GN=MYH14 PE=1 SV=1 |

|  | **Query** | **Observed** | **Mr(expt)** | **Mr(calc)** | **Delta** | **Miss** | **Score** | **Expect** | **Rank** | **Peptide** |
| --- | --- | --- | --- | --- | --- | --- | --- | --- | --- | --- |
|  | 988 | 572.1176 | 571.1103 | 571.3806 | -0.2703 | 1 | 10 | 15 | 5 | R.IKVGR.D |
|  | 1026 | 586.2965 | 585.2892 | 585.2944 | -0.0053 | 0 | 15 | 6.2 | 7 | R.MNPPK.F |
|  | 1746 | 404.7289 | 807.4431 | 807.4491 | -0.0059 | 0 | 23 | 0.97 | 1 | R.TVGQLYK.E 1763 |
|  | 2399 | **428.7632** | **855.5117** | **855.5178** | **-0.0060** | **0** | **28** | **0.24** | **2** | **R.LVLDQLR.C** |
|  | 2892 | 446.7125 | 891.4105 | 890.4320 | 0.9785 | 0 | 10 | 19 | 8 | K.ANEWLMK.N |
|  | 3216 | **458.7556** | **915.4965** | **916.4250** | **-0.9285** | **0** | **19** | **2.7** | **2** | **R.LGEEDAGAR.A** 3243 3251 |
|  | 3393 | **465.2702** | **928.5258** | **928.5341** | **-0.0083** | **1** | **7** | **35** | **4** | **K.AAILEEKR.Q** |
|  | 3971 | **486.2522** | **970.4898** | **971.5400** | **-1.0501** | **1** | **4** | **59** | **8** | **K.EEAVKQLR.K** |
|  | 4176 | 493.2762 | 984.5379 | 984.5828 | -0.0450 | 1 | 11 | 14 | 3 | K.QRTLAVAAR.K 4164 |
|  | 4478 | **502.7088** | **1003.4030** | **1003.5121** | **-0.1091** | **0** | **1** | **1.3e+002** | **7** | **M.AAVTMSVPGR.K** |
|  | 4647 | 509.2570 | 1016.4994 | 1016.5073 | -0.0079 | 0 | 32 | 0.13 | 1 | R.CNGVLEGIR.I |
|  | 4711 | 512.2675 | 1022.5205 | 1023.4734 | -0.9529 | 1 | 9 | 19 | 1 | K.NDNSSRFGK.F 4724 |
|  | 4722 | 512.7650 | 1023.5153 | 1023.5237 | -0.0083 | 1 | 23 | 0.77 | 1 | K.ATDKSFVEK.V 4732 4742 |
|  | 4951 | **520.3456** | **1038.6766** | **1038.5094** | **0.1672** | **1** | **3** | **80** | **7** | **R.SFSAKAESGR.Q** |
|  | 5590 | **543.2252** | **1084.4358** | **1085.5465** | **-1.1107** | **0** | **26** | **0.39** | **1** | **R.VAEQAANDLR.A** |
|  | 5670 | 546.2700 | 1090.5254 | 1091.5499 | -1.0245 | 0 | 28 | 0.25 | 3 | K.FDQLLAEEK.A |
|  | 5729 | **548.2722** | **1094.5299** | **1093.4346** | **1.0953** | **0** | **5** | **48** | **10** | **R.VGEEEECSR.Q** |
|  | 5837 | **551.8051** | **1101.5957** | **1102.5870** | **-0.9913** | **1** | **6** | **44** | **9** | **R.EQEVTELKK.T** |
|  | 5902 | 554.2835 | 1106.5525 | 1105.5363 | 1.0161 | 1 | 8 | 29 | 8 | R.EVEETRTSR.E |
|  | 6193 | 564.8488 | 1127.6831 | 1128.5775 | -0.8944 | 0 | 9 | 21 | 7 | K.EEELQAALAR.A |
|  | 6400 | **572.9938** | **1143.9731** | **1143.5771** | **0.3960** | **0** | **8** | **16** | **4** | **R.DLGEELEALR.G** 6387 |
|  | 6511 | 576.7461 | 1151.4777 | 1151.5869 | -0.1092 | 1 | 12 | 10 | 4 | R.CIVPNHEKR.A |
|  | 6715 | **583.3160** | **1164.6174** | **1164.6251** | **-0.0077** | **0** | **23** | **0.69** | **1** | **R.IHEAAVQELR.Q** |
|  | 7051 | **593.8081** | **1185.6017** | **1185.6102** | **-0.0085** | **0** | **41** | **0.013** | **1** | **K.VQELQQQSAR.E** 7050 |
|  | 7794 | 610.8259 | 1219.6372 | 1219.6448 | -0.0076 | 1 | 48 | 0.0025 | 1 | R.KFDQLLAEEK.A 7793 |
|  | 7914 | 614.0780 | 1226.1414 | 1225.6237 | 0.5177 | 1 | 4 | 34 | 1 | R.DQIQRMNPPK.F |
|  | 8196 | **414.7112** | **1241.1119** | **1241.6186** | **-0.5067** | **1** | **(4)** | **36** | **7** | **R.DQIQRMNPPK.F** |
|  | 8322 | **624.2343** | **1246.4541** | **1246.6526** | **-0.1985** | **1** | **1** | **1.1e+002** | **3** | **-.MAAVTMSVPGRK.A** |
|  | 8791 | **635.8025** | **1269.5904** | **1269.5482** | **0.0422** | **1** | **0** | **1.3e+002** | **10** | **K.GFMDGKQACEK.M** |
|  | 8875 | **425.5497** | **1273.6272** | **1272.6608** | **0.9664** | **1** | **4** | **62** | **4** | **R.VRAMEAEAAGLR.E** |
|  | 9477 | 653.3340 | 1304.6534 | 1304.6612 | -0.0078 | 0 | 53 | 0.00072 | 1 | K.EQADFALEALAK.A 9461 9485 |
|  | 9793 | 661.2485 | 1320.4825 | 1319.6478 | 0.8347 | 1 | 1 | 89 | 8 | K.MQAQMKELWR.E |
|  | 10654 | **456.2248** | **1365.6525** | **1365.6637** | **-0.0112** | **1** | **16** | **3.1** | **2** | **R.TSREEIFSQNR.E** |
|  | 10715 | **457.2405** | **1368.6998** | **1368.6642** | **0.0356** | **1** | **4** | **55** | **7** | **R.VMQRNCAAYLK.L** |
|  | 10834 | **459.1706** | **1374.4899** | **1373.6171** | **0.8728** | **1** | **2** | **69** | **8** | **R.DLQGRDEAGEER.R** |
|  | 11965 | **481.1504** | **1440.4295** | **1439.7844** | **0.6451** | **1** | **6** | **20** | **10** | **R.QQLERQIQELR.G** |
|  | 12455 | **735.8251** | **1469.6357** | **1469.8089** | **-0.1732** | **1** | **3** | **69** | **5** | **R.LKEVVLQVEEER.R** |
|  | 12589 | **739.3726** | **1476.7306** | **1477.6354** | **-0.9049** | **0** | **0** | **1.2e+002** | **5** | **R.AEAELCAEAEETR.G** |
|  | 13078 | **753.3717** | **1504.7288** | **1503.7351** | **0.9937** | **1** | **7** | **23** | **4** | **K.AQMASAGQGKEEAVK.Q** |
|  | 13359 | 761.3731 | 1520.7315 | 1519.7300 | 1.0015 | 1 | (4) | 48 | 3 | K.AQMASAGQGKEEAVK.Q 13330 |
|  | 13673 | **514.3074** | **1539.9005** | **1539.8773** | **0.0232** | **1** | **3** | **57** | **10** | **K.KVIQYLAHVASSPK.G** 13658 |
|  | 13688 | 771.4152 | 1540.8159 | 1541.8565 | -1.0406 | 1 | 9 | 16 | 2 | R.QRYEILTPNAIPK.G 13687 |
|  | 13750 | **773.4462** | **1544.8778** | **1543.7478** | **1.1300** | **0** | **11** | **9.8** | **1** | **K.LAQAEEQLEQETR.E** 13722 |
|  | 13823 | **517.2972** | **1548.8699** | **1549.7923** | **-0.9224** | **0** | **3** | **62** | **8** | **R.LMATLSNTNPSFVR.C** |
|  | 13849 | 776.3460 | 1550.6775 | 1550.6882 | -0.0108 | 0 | 18 | 1.8 | 1 | R.EDQSILCTGESGAGK.T |
|  | 13980 | **780.3900** | **1558.7655** | **1558.7739** | **-0.0085** | **0** | **37** | **0.027** | **1** | **R.ELQTAQAQLSEWR.R** |
|  | 14217 | 788.3901 | 1574.7656 | 1573.8199 | 0.9457 | 1 | 5 | 35 | 8 | R.AELEALLSSKDDVGK.S |
|  | 14679 | **803.3572** | **1604.6998** | **1604.7100** | **-0.0102** | **0** | **7** | **25** | **1** | **R.NTDQATMPDNTAAQK.L** 14665 |
|  | 14755 | 805.3480 | 1608.6815 | 1608.6937 | -0.0122 | 0 | 4 | 43 | 4 | R.ELEDVTESAESMNR.E |
|  | 14976 | **811.4011** | **1620.7876** | **1620.7050** | **0.0826** | **0** | **(3)** | **63** | **7** | **R.NTDQATMPDNTAAQK.L** |
|  | 15490 | **552.3167** | **1653.9283** | **1654.8638** | **-0.9355** | **1** | **3** | **53** | **1** | **R.QDEVLQARAQELQK.V** 15485 |
|  | 16633 | 863.9731 | 1725.9316 | 1725.9413 | -0.0097 | 0 | 83 | 5.2e-007 | 1 | R.QLLQANPILEAFGNAK.T 16634 16646 16647 |
|  | 16882 | **581.6765** | **1742.0075** | **1740.8756** | **1.1319** | **0** | **5** | **28** | **1** | **K.QLPIYTEAIVEMYR.G** 16854 |
|  | 17769 | **601.3876** | **1801.1409** | **1801.8918** | **-0.7509** | **1** | **2** | **57** | **7** | **R.AELSSLQTARQEGEQR.R** |
|  | 18609 | **930.4293** | **1858.8441** | **1858.8544** | **-0.0103** | **0** | **78** | **1.6e-006** | **1** | **R.EAQAALAEAQEDLESER.V** |
|  | 19814 | **652.3251** | **1953.9536** | **1954.9133** | **-0.9598** | **0** | **4** | **34** | **1** | **R.GPSAGGGPGSGTSPQVEWTAR.R** |
|  | 20385 | **501.7471** | **2002.9592** | **2003.9833** | **-1.0241** | **1** | **8** | **17** | **4** | **K.LEGELEELKAQMASAGQGK.E** |
|  | 21842 | **715.6746** | **2144.0020** | **2144.0167** | **-0.0147** | **1** | **37** | **0.018** | **1** | **R.AMEAEAAGLREQLEEEAAAR.E** 21834 |
|  | 22223 | **729.6846** | **2186.0319** | **2185.0610** | **0.9708** | **1** | **2** | **59** | **4** | **R.EAQAALAEAQEDLESERVAR.T** |
|  | 22712 | **745.4080** | **2233.2021** | **2233.9869** | **-0.7849** | **1** | **1** | **57** | **8** | **R.QAQQDRDEMADEVANGNLSK.A** |
|  | 24143 | **809.7209** | **2426.1408** | **2426.1561** | **-0.0152** | **0** | **24** | **0.27** | **1** | **K.ELSSTEAQLHDAQELLQEETR.A** |
|  | 27486 | **1491.2794** | **2980.5443** | **2980.4712** | **0.0731** | **1** | **0** | **48** | **1** | **K.NMDPLNDNVAALLHQSTDRLTAEIWK.D** |
|  | 27824 | **1015.4893** | **3043.4460** | **3043.4581** | **-0.0122** | **1** | **45** | **0.0017** | **1** | **R.DLGEELEALRGELEDTLDSTNAQQELR.S** |
|  | 28908 | 828.6339 | 3310.5063 | 3310.4385 | 0.0678 | 0 | 5 | 13 | 2 | R.LEEGVASDEEAEEAQPGSGPSPEPEGSPPAHPQ.- |
|  | 31417 | **1062.9253** | **5309.5901** | **5309.6457** | **-0.0557** | **1** | **0** | **12** | **2** | **R.EGIPWTFLDFGLDLQPCIDLIERPANPPGLLALLDEECWFPKATDK.S** |

| **120.** | P14780\|MMP9_HUMAN    **Mass:** 79433    **Score:** 283    **Queries matched:** 19   **emPAI:** 0.38 |
| --- | --- |
|  | Matrix metalloproteinase-9 OS=Homo sapiens GN=MMP9 PE=1 SV=2 |

|  | **Query** | **Observed** | **Mr(expt)** | **Mr(calc)** | **Delta** | **Miss** | **Score** | **Expect** | **Rank** | **Peptide** |
| --- | --- | --- | --- | --- | --- | --- | --- | --- | --- | --- |
|  | 4066 | **488.7446** | **975.4746** | **976.4978** | **-1.0232** | **0** | **0** | **1.8e+002** | **7** | **R.AVIDDAFAR.A** |
|  | 4725 | 512.7710 | 1023.5274 | 1024.5342 | -1.0067 | 0 | 9 | 20 | 10 | R.FTEGPPLHK.D |
|  | 6028 | **559.2275** | **1116.4405** | **1116.4506** | **-0.0101** | **0** | **26** | **0.41** | **1** | **R.SYSACTTDGR.S** |
|  | 6526 | 576.8680 | 1151.7215 | 1151.7278 | -0.0062 | 0 | 39 | 0.018 | 1 | K.SLGPALLLLQK.Q |
|  | 11110 | **696.3701** | **1390.7256** | **1390.7344** | **-0.0088** | **1** | **36** | **0.037** | **1** | **R.KLDSVFEEPLSK.K** |
|  | 11958 | **720.9083** | **1439.8021** | **1439.8096** | **-0.0075** | **0** | **5** | **44** | **2** | **K.LGLGADVAQVTGALR.S** |
|  | 12994 | **750.8250** | **1499.6355** | **1499.6463** | **-0.0108** | **1** | **55** | **0.00043** | **1** | **R.WCATTANYDRDK.L** |
|  | 15910 | **840.9544** | **1679.8943** | **1679.9035** | **-0.0093** | **0** | **5** | **34** | **1** | **R.AFALWSAVTPLTFTR.V** |
|  | 16031 | **563.6261** | **1687.8565** | **1687.9158** | **-0.0594** | **1** | **4** | **51** | **6** | **R.QVWVYTGASVLGPRR.L** |
|  | 16239 | **851.4428** | **1700.8709** | **1700.8832** | **-0.0123** | **0** | **26** | **0.28** | **1** | **K.QLSLPETGELDSATLK.A** |
|  | 17670 | **449.7261** | **1794.8755** | **1793.9061** | **0.9694** | **1** | **5** | **33** | **3** | **R.FTEGPPLHKDDVNGIR.H** 17655 |
|  | 17684 | **599.7883** | **1796.3432** | **1796.0156** | **0.3276** | **1** | **2** | **45** | **3** | **R.LDKLGLGADVAQVTGALR.S** |
|  | 20835 | **682.7005** | **2045.0797** | **2045.0906** | **-0.0109** | **1** | **9** | **10** | **1** | **R.QSTLVLFPGDLRTNLTDR.Q** |
|  | 24706 | **1253.0859** | **2504.1573** | **2504.1716** | **-0.0143** | **0** | **92** | **4.1e-008** | **1** | **R.ADSTVMGGNSAGELCVFPFTFLGK.E** 24707 |
|  | 24933 | **848.4460** | **2542.3163** | **2542.3626** | **-0.0463** | **0** | **1** | **60** | **4** | **-.MSLWQPLVLVLLVLGCCFAAPR.Q** |
|  | 27866 | **1018.7534** | **3053.2383** | **3053.2556** | **-0.0174** | **1** | **50** | **0.00037** | **1** | **R.SDGLPWCSTTANYDTDDRFGFCPSER.L** |
|  | 29797 | **1210.5310** | **3628.5712** | **3628.5988** | **-0.0276** | **0** | **75** | **1.1e-006** | **1** | **R.LYTQDGNADGKPCQFPFIFQGQSYSACTTDGR.S** |

| **121.** | P04844\|RPN2_HUMAN    **Mass:** 69355    **Score:** 283    **Queries matched:** 23   **emPAI:** 0.37 |
| --- | --- |
|  | Dolichyl-diphosphooligosaccharide--protein glycosyltransferase subunit 2 OS=Homo sapiens GN=RPN2 PE=1 SV=3 |

|  | **Query** | **Observed** | **Mr(expt)** | **Mr(calc)** | **Delta** | **Miss** | **Score** | **Expect** | **Rank** | **Peptide** |
| --- | --- | --- | --- | --- | --- | --- | --- | --- | --- | --- |
|  | 1208 | **659.4036** | **658.3964** | **658.4014** | **-0.0050** | **0** | **15** | **7.7** | **1** | **R.ATVLQK.T** |
|  | 5260 | **531.3126** | **1060.6106** | **1059.5859** | **1.0247** | **1** | **3** | **81** | **5** | **R.MLAQQAVKR.T** |
|  | 6011 | 558.7811 | 1115.5477 | 1115.5935 | -0.0458 | 1 | 4 | 59 | 6 | K.DQSIAPKTTR.V |
|  | 6635 | **580.2920** | **1158.5694** | **1158.6721** | **-0.1026** | **1** | **16** | **4.5** | **3** | **K.SVASRATVLQK.T** 6632 |
|  | 9485 | 653.7911 | 1305.5676 | 1304.7452 | 0.8224 | 1 | 3 | 63 | 7 | R.YIANTVELRVK.I |
|  | 10766 | **686.6925** | **1371.3704** | **1371.7245** | **-0.3541** | **0** | **25** | **0.34** | **1** | **R.SIVEEIEDLVAR.L** |
|  | 18306 | **920.5248** | **1839.0350** | **1839.0466** | **-0.0116** | **0** | **92** | **6e-008** | **1** | **R.LQVTNVLSQPLTQATVK.L** 18307 |
|  | 20807 | **1023.0006** | **2043.9865** | **2043.9976** | **-0.0110** | **0** | **21** | **0.77** | **1** | **K.TSFTPVGDVFELNFMNVK.F** 20819 20820 20823 |
|  | 21028 | **515.7853** | **2059.1120** | **2059.9925** | **-0.8805** | **0** | **(11)** | **6.9** | **1** | **K.TSFTPVGDVFELNFMNVK.F** |
|  | 23265 | **769.7134** | **2306.1185** | **2306.1291** | **-0.0106** | **0** | **5** | **23** | **1** | **K.NFESLSEAFSVASAAAVLSHNR.Y** |
|  | 24194 | **812.7459** | **2435.2159** | **2434.2240** | **0.9919** | **1** | **71** | **5.9e-006** | **1** | **K.KNFESLSEAFSVASAAAVLSHNR.Y** |
|  | 24863 | **844.1075** | **2529.3008** | **2529.3174** | **-0.0166** | **1** | **12** | **3.8** | **1** | **K.ISTEVGITNVDLSTVDKDQSIAPK.T** |
|  | 27261 | **979.8549** | **2936.5429** | **2936.5567** | **-0.0138** | **1** | **49** | **0.00061** | **1** | **R.LSKEETVLATVQALQTASHLSQQADLR.S** 27260 |
|  | 28718 | 1085.2249 | 3252.6527 | 3252.6666 | -0.0139 | 0 | 45 | 0.0015 | 1 | K.ASLDRPFTNLESAFYSIVGLSSLGAQVPDAK.K 28719 |
|  | 29177 | **846.1937** | **3380.7458** | **3380.7616** | **-0.0158** | **1** | **2** | **26** | **1** | **K.ASLDRPFTNLESAFYSIVGLSSLGAQVPDAKK.A** |
|  | 29737 | **1199.3018** | **3594.8835** | **3594.8974** | **-0.0139** | **1** | **7** | **7.6** | **1** | **K.NPILWNVADVVIKFPEEEAPSTVLSQNLFTPK.Q** |

| **122.** | P16435\|NCPR_HUMAN    **Mass:** 77097    **Score:** 276    **Queries matched:** 32   **emPAI:** 0.60 |
| --- | --- |
|  | NADPH--cytochrome P450 reductase OS=Homo sapiens GN=POR PE=1 SV=2 |

|  | **Query** | **Observed** | **Mr(expt)** | **Mr(calc)** | **Delta** | **Miss** | **Score** | **Expect** | **Rank** | **Peptide** |
| --- | --- | --- | --- | --- | --- | --- | --- | --- | --- | --- |
|  | 1963 | **413.2647** | **824.5148** | **825.4457** | **-0.9309** | **1** | **3** | **77** | **5** | **R.LSKDAHR.Y** |
|  | 2832 | **443.2146** | **884.4146** | **885.3940** | **-0.9794** | **0** | **3** | **82** | **10** | **K.EPAGENGGR.A** |
|  | 3185 | **457.7534** | **913.4922** | **913.4981** | **-0.0059** | **0** | **31** | **0.15** | **1** | **R.LEQLGAQR.I** |
|  | 3215 | **458.7509** | **915.4871** | **915.4926** | **-0.0055** | **0** | **8** | **34** | **4** | **K.GVATNWLR.A** |
|  | 3550 | **471.2372** | **940.4599** | **941.4099** | **-0.9500** | **0** | **14** | **5.6** | **1** | **K.VYMGEMGR.L** |
|  | 3620 | 473.2101 | 944.4057 | 944.5039 | -0.0983 | 1 | 12 | 12 | 6 | R.KLNQGTER.H 3634 |
|  | 4399 | **500.2999** | **998.5852** | **998.5913** | **-0.0061** | **0** | **15** | **5.8** | **1** | **K.VYVQHLLK.Q** |
|  | 4483 | **502.7871** | **1003.5596** | **1003.5662** | **-0.0066** | **0** | **27** | **0.35** | **1** | **K.IQTLTSSVR.E** |
|  | 7281 | **599.2676** | **1196.5207** | **1196.5284** | **-0.0077** | **0** | **48** | **0.0023** | **1** | **K.TYEHFNAMGK.Y** |
|  | 7421 | **601.9619** | **1201.9091** | **1202.6408** | **-0.7316** | **0** | **36** | **0.03** | **1** | **K.NPFLAAVTTNR.K** |
|  | 7617 | **606.7940** | **1211.5735** | **1212.5233** | **-0.9499** | **0** | **(6)** | **36** | **6** | **K.TYEHFNAMGK.Y** |
|  | 11672 | **712.3411** | **1422.6676** | **1422.6779** | **-0.0103** | **0** | **41** | **0.011** | **1** | **K.SYENQKPPFDAK.N** |
|  | 13621 | **769.3813** | **1536.7480** | **1536.7606** | **-0.0126** | **0** | **39** | **0.014** | **1** | **R.HLMHLELDISDSK.I** 13620 13624 13626 |
|  | 14632 | **534.6072** | **1600.7997** | **1600.8097** | **-0.0100** | **0** | **14** | **4.4** | **1** | **R.QYELVVHTDIDAAK.V** 14627 |
|  | 14866 | **539.2805** | **1614.8196** | **1614.7824** | **0.0371** | **1** | **2** | **70** | **10** | **K.EVGETLLYYGCRR.S** |
|  | 21119 | **690.9841** | **2069.9304** | **2069.9442** | **-0.0139** | **1** | **6** | **21** | **1** | **R.SDEDYLYREELAQFHR.D** |
|  | 21442 | **701.3213** | **2100.9420** | **2102.0149** | **-1.0729** | **1** | **0** | **86** | **2** | **K.LIEGGAHIYVCGDARNMAR.D** |
|  | 24084 | **806.4073** | **2416.2002** | **2416.2155** | **-0.0153** | **1** | **13** | **4.2** | **1** | **K.ILGADLDVVMSLNNLDEESNKK.H** |
|  | 24272 | **816.3942** | **2446.1609** | **2446.1765** | **-0.0156** | **0** | **13** | **4.1** | **1** | **R.YESGDHVAVYPANDSALVNQLGK.I** |
|  | 24331 | **1227.5698** | **2453.1251** | **2453.1387** | **-0.0136** | **0** | **114** | **2.5e-010** | **1** | **R.IFELGLGDDDGNLEEDFITWR.E** 24332 |
|  | 24366 | **1229.6552** | **2457.2957** | **2457.3090** | **-0.0133** | **0** | **27** | **0.15** | **1** | **K.ATTPVIMVGPGTGVAPFIGFIQER.A** |
|  | 25739 | **889.0644** | **2664.1715** | **2664.1915** | **-0.0199** | **0** | **4** | **24** | **1** | **R.EQFWPAVCEHFGVEATGEESSIR.Q** |
|  | 26146 | **906.4564** | **2716.3474** | **2715.3616** | **0.9858** | **1** | **48** | **0.001** | **1** | **K.IRYESGDHVAVYPANDSALVNQLGK.I** |
|  | 27269 | **980.8033** | **2939.3882** | **2939.4011** | **-0.0129** | **0** | **53** | **0.00026** | **1** | **R.DVQNTFYDIVAELGAMEHAQAVDYIK.K** |
|  | 27456 | **744.8998** | **2975.5700** | **2975.5837** | **-0.0137** | **0** | **19** | **0.7** | **1** | **R.HILAILQDCPSLRPPIDHLCELLPR.L** |
|  | 27919 | **767.8779** | **3067.4826** | **3067.4961** | **-0.0134** | **1** | **15** | **1.6** | **1** | **R.DVQNTFYDIVAELGAMEHAQAVDYIKK.L** |

| **123.** | P0C0S8\|H2A1_HUMAN    **Mass:** 14083    **Score:** 276    **Queries matched:** 25   **emPAI:** 6.12 |
| --- | --- |
|  | Histone H2A type 1 OS=Homo sapiens GN=HIST1H2AG PE=1 SV=2 |

|  | **Query** | **Observed** | **Mr(expt)** | **Mr(calc)** | **Delta** | **Miss** | **Score** | **Expect** | **Rank** | **Peptide** |
| --- | --- | --- | --- | --- | --- | --- | --- | --- | --- | --- |
|  | 1100 | **617.3092** | **616.3019** | **615.3452** | **0.9567** | **1** | **19** | **4.4** | **4** | **K.QGGKAR.A** |
|  | 2130 | **419.2117** | **836.4088** | **836.4140** | **-0.0052** | **1** | **22** | **1.1** | **1** | **R.KGNYAER.V** 2129 |
|  | 2328 | **425.7633** | **849.5121** | **849.5184** | **-0.0064** | **0** | **32** | **0.11** | **1** | **R.HLQLAIR.N** 2329 2330 2339 |
|  | 2469 | **431.2187** | **860.4229** | **860.3875** | **0.0353** | **0** | **14** | **7.7** | **4** | **R.NDEELNK.L** |
|  | 3614 | **472.7664** | **943.5183** | **943.5240** | **-0.0057** | **0** | **45** | **0.0057** | **1** | **R.AGLQFPVGR.V** 3604 3613 3617 |
|  | 8835 | **636.8390** | **1271.6634** | **1271.6721** | **-0.0087** | **1** | **49** | **0.0017** | **1** | **R.NDEELNKLLGK.V** 8833 8834 8856 |
|  | 8870 | **637.8182** | **1273.6218** | **1273.6891** | **-0.0673** | **1** | **8** | **21** | **3** | **R.SSRAGLQFPVGR.V** |
|  | 16093 | **846.9494** | **1691.8842** | **1691.8954** | **-0.0112** | **1** | **75** | **3.6e-006** | **1** | **R.HLQLAIRNDEELNK.L** 16091 16092 16094 |
|  | 19487 | 966.0827 | 1930.1508 | 1930.1615 | -0.0107 | 0 | 69 | 1.2e-005 | 1 | K.VTIAQGGVLPNIQAVLLPK.K 19486 19488 19490 |

|  | **Proteins matching the same set of peptides:** |
| --- | --- |

|  | Q6FI13\|H2A2A_HUMAN    **Mass:** 14087    **Score:** 276    **Queries matched:** 25 |
| --- | --- |
|  | Histone H2A type 2-A OS=Homo sapiens GN=HIST2H2AA3 PE=1 SV=3 |

|  | Q96KK5\|H2A1H_HUMAN    **Mass:** 13898    **Score:** 276    **Queries matched:** 25 |
| --- | --- |
|  | Histone H2A type 1-H OS=Homo sapiens GN=HIST1H2AH PE=1 SV=3 |

|  | Q16777\|H2A2C_HUMAN    **Mass:** 13980    **Score:** 276    **Queries matched:** 25 |
| --- | --- |
|  | Histone H2A type 2-C OS=Homo sapiens GN=HIST2H2AC PE=1 SV=4 |

|  | Q99878\|H2A1J_HUMAN    **Mass:** 13928    **Score:** 276    **Queries matched:** 25 |
| --- | --- |
|  | Histone H2A type 1-J OS=Homo sapiens GN=HIST1H2AJ PE=1 SV=3 |

|  | **Proteins matching a subset of these peptides:** |
| --- | --- |

|  | Q9BTM1\|H2AJ_HUMAN    **Mass:** 14011    **Score:** 276    **Queries matched:** 24 |
| --- | --- |
|  | Histone H2A.J OS=Homo sapiens GN=H2AFJ PE=1 SV=1 |

|  | P20671\|H2A1D_HUMAN    **Mass:** 14099    **Score:** 276    **Queries matched:** 23 |
| --- | --- |
|  | Histone H2A type 1-D OS=Homo sapiens GN=HIST1H2AD PE=1 SV=2 |

|  | Q93077\|H2A1C_HUMAN    **Mass:** 14097    **Score:** 242    **Queries matched:** 21 |
| --- | --- |
|  | Histone H2A type 1-C OS=Homo sapiens GN=HIST1H2AC PE=1 SV=3 |

|  | Q7L7L0\|H2A3_HUMAN    **Mass:** 14113    **Score:** 242    **Queries matched:** 19 |
| --- | --- |
|  | Histone H2A type 3 OS=Homo sapiens GN=HIST3H2A PE=1 SV=3 |

|  | P04908\|H2A1B_HUMAN    **Mass:** 14127    **Score:** 242    **Queries matched:** 19 |
| --- | --- |
|  | Histone H2A type 1-B/E OS=Homo sapiens GN=HIST1H2AB PE=1 SV=2 |

| **124.** | P11215\|ITAM_HUMAN    **Mass:** 128410   **Score:** 276    **Queries matched:** 29   **emPAI:** 0.25 |
| --- | --- |
|  | Integrin alpha-M OS=Homo sapiens GN=ITGAM PE=1 SV=2 |

|  | **Query** | **Observed** | **Mr(expt)** | **Mr(calc)** | **Delta** | **Miss** | **Score** | **Expect** | **Rank** | **Peptide** |
| --- | --- | --- | --- | --- | --- | --- | --- | --- | --- | --- |
|  | 2619 | **436.7427** | **871.4709** | **871.4875** | **-0.0166** | **0** | **8** | **35** | **8** | **K.TIQNQLR.E** |
|  | 4950 | **520.3132** | **1038.6119** | **1038.6186** | **-0.0067** | **0** | **9** | **19** | **1** | **R.VQSLVLGAPR.Y** |
|  | 6262 | **567.2868** | **1132.5590** | **1133.5829** | **-1.0239** | **0** | **2** | **93** | **8** | **R.ELFNITNGAR.K** |
|  | 6763 | 584.8303 | 1167.6461 | 1168.6023 | -0.9562 | 0 | 6 | 40 | 5 | R.GGQVSVCPLPR.G 6760 |
|  | 7607 | **404.6921** | **1211.0545** | **1211.6590** | **-0.6045** | **0** | **6** | **22** | **1** | **R.LFTALFPFEK.N** 7631 |
|  | 8219 | **622.1002** | **1242.1859** | **1241.6081** | **0.5778** | **0** | **1** | **85** | **8** | **K.GNLSFDWYIK.T** |
|  | 10575 | **681.8546** | **1361.6945** | **1361.7052** | **-0.0106** | **0** | **16** | **3.5** | **1** | **R.GFGQSVVQLQGSR.V** 10593 |
|  | 11868 | **478.9265** | **1433.7576** | **1433.5439** | **0.2137** | **0** | **8** | **23** | **7** | **K.DMMSEGGPPGAEPQ.-** 11869 |
|  | 11946 | **720.4497** | **1438.8847** | **1438.8912** | **-0.0064** | **0** | **44** | **0.0051** | **1** | **R.SLPISLVFLVPVR.L** 11924 11932 |
|  | 13843 | 775.9301 | 1549.8455 | 1549.8576 | -0.0121 | 0 | 36 | 0.028 | 1 | R.VVVGAPQEIVAANQR.G |
|  | 14014 | **781.3943** | **1560.7740** | **1559.7732** | **1.0008** | **0** | **5** | **39** | **1** | **R.DHVFQVNNFEALK.T** |
|  | 14405 | **397.4617** | **1585.8177** | **1584.7891** | **1.0286** | **1** | **9** | **16** | **5** | **R.MKEFVSTVMEQLK.K** |
|  | 14757 | **805.3853** | **1608.7561** | **1608.7665** | **-0.0104** | **0** | **42** | **0.0084** | **1** | **R.LACESASSTEVSGALK.S** |
|  | 16002 | **844.4142** | **1686.8138** | **1686.8260** | **-0.0122** | **0** | **71** | **8.9e-006** | **1** | **R.VMQHQYQVSNLGQR.S** |
|  | 16520 | **860.4448** | **1718.8750** | **1718.8873** | **-0.0123** | **0** | **56** | **0.00028** | **1** | **R.QTQVLGLTQTCETLK.L** |
|  | 17629 | **896.9432** | **1791.8719** | **1791.8315** | **0.0404** | **0** | **5** | **34** | **3** | **K.FGDPLGYEDVIPEADR.E** 17611 |
|  | 20251 | **996.9181** | **1991.8216** | **1991.8353** | **-0.0137** | **0** | **94** | **3e-008** | **1** | **R.GSLYQCDYSTGSCEPIR.L** |
|  | 21843 | **715.6829** | **2144.0269** | **2144.0399** | **-0.0130** | **0** | **40** | **0.0083** | **1** | **R.GAVYLFHGTSGSGISPSHSQR.I** |
|  | 23578 | **783.0523** | **2346.1351** | **2346.1492** | **-0.0141** | **1** | **48** | **0.0013** | **1** | **K.FGDPLGYEDVIPEADREGVIR.Y** |
|  | 23790 | **791.4603** | **2371.3592** | **2370.2994** | **1.0597** | **1** | **1** | **45** | **2** | **R.VQSLVLGAPRYQHIGLVAMFR.Q** |
|  | 24113 | **807.3972** | **2419.1698** | **2420.3288** | **-1.1590** | **1** | **1** | **58** | **7** | **K.EFQNNPNPRSLVKPITQLLGR.T** |
|  | 29912 | **924.5129** | **3694.0227** | **3694.8269** | **-0.8043** | **1** | **1** | **23** | **5** | **-.MALRVLLLTALTLCHGFNLDTENAMTFQENAR.G** |

| **125.** | Q07000\|1C15_HUMAN    **Mass:** 41293    **Score:** 274    **Queries matched:** 29   **emPAI:** 1.00 |
| --- | --- |
|  | HLA class I histocompatibility antigen, Cw-15 alpha chain OS=Homo sapiens GN=HLA-C PE=1 SV=1 |

|  | **Query** | **Observed** | **Mr(expt)** | **Mr(calc)** | **Delta** | **Miss** | **Score** | **Expect** | **Rank** | **Peptide** |
| --- | --- | --- | --- | --- | --- | --- | --- | --- | --- | --- |
|  | 1409 | **746.1623** | **745.1551** | **744.3766** | **0.7785** | **0** | **12** | **10** | **6** | **R.EAEQLR.A** |
|  | 1478 | **781.3572** | **780.3499** | **781.3606** | **-1.0107** | **0** | **7** | **40** | **6** | **R.ETQNYK.R** |
|  | 3888 | 482.7234 | 963.4322 | 964.4250 | -0.9929 | 0 | 33 | 0.088 | 1 | R.FDSDAASPR.G 3900 3901 3907 |
|  | 9722 | **659.3238** | **1316.6331** | **1315.6673** | **0.9658** | **0** | **5** | **45** | **8** | **R.YFYTAVSRPGR.G** |
|  | 10335 | 675.8494 | 1349.6843 | 1349.6939 | -0.0096 | 1 | 38 | 0.024 | 1 | R.YLENGKETLQR.A 10311 10351 |
|  | 11313 | 468.5608 | 1402.6605 | 1403.6429 | -0.9825 | 1 | 8 | 22 | 2 | R.FDSDAASPRGEPR.A |
|  | 11736 | 714.3626 | 1426.7105 | 1426.7205 | -0.0099 | 0 | 46 | 0.0031 | 1 | K.WAAVVVPSGEEQR.Y 11721 11735 11751 |
|  | 12921 | 748.8587 | 1495.7028 | 1495.7129 | -0.0101 | 0 | 57 | 0.00026 | 1 | R.AYLEGTCVEWLR.R |
|  | 13321 | 760.3871 | 1518.7596 | 1518.7427 | 0.0169 | 0 | 62 | 7.5e-005 | 1 | R.SWTAADTAAQITQR.K |
|  | 18283 | 613.3167 | 1836.9283 | 1835.9027 | 1.0256 | 0 | 15 | 3.7 | 1 | K.THVTHHPVSDHEATLR.C |
|  | 20871 | 410.6170 | 2048.0485 | 2049.0102 | -0.9617 | 0 | 9 | 11 | 2 | R.YTCHVQHEGLPEPLTLR.W 20872 20886 20897 |
|  | 21557 | 1056.5175 | 2111.0204 | 2111.0299 | -0.0095 | 0 | 86 | 2.1e-007 | 1 | R.CWALGFYPAEITLTWQR.D 21558 |
|  | 25578 | 1319.0889 | 2636.1632 | 2636.1838 | -0.0206 | 0 | 57 | 0.00013 | 1 | R.DGEDQTQDTELVETRPAGDGTFQK.W 25579 25580 25581 |
|  | 30193 | 1274.3292 | 3819.9658 | 3819.0865 | 0.8793 | 0 | 2 | 22 | 4 | R.WEPSSQPTIPIVGIVAGLAVLAVLAVLGAVMAVVMCR.R |

|  | **Proteins matching a subset of these peptides:** |
| --- | --- |

|  | P30508\|1C12_HUMAN    **Mass:** 41316    **Score:** 274    **Queries matched:** 27 |
| --- | --- |
|  | HLA class I histocompatibility antigen, Cw-12 alpha chain OS=Homo sapiens GN=HLA-C PE=1 SV=2 |

|  | P30505\|1C08_HUMAN    **Mass:** 41203    **Score:** 262    **Queries matched:** 18 |
| --- | --- |
|  | HLA class I histocompatibility antigen, Cw-8 alpha chain OS=Homo sapiens GN=HLA-C PE=2 SV=1 |

|  | Q9TNN7\|1C05_HUMAN    **Mass:** 41342    **Score:** 226    **Queries matched:** 17 |
| --- | --- |
|  | HLA class I histocompatibility antigen, Cw-5 alpha chain OS=Homo sapiens GN=HLA-C PE=2 SV=1 |

| **126.** | Q3ZCM7\|TBB8_HUMAN    **Mass:** 50257    **Score:** 271    **Queries matched:** 43   **emPAI:** 0.91 |
| --- | --- |
|  | Tubulin beta-8 chain OS=Homo sapiens GN=TUBB8 PE=1 SV=2 |

|  | **Query** | **Observed** | **Mr(expt)** | **Mr(calc)** | **Delta** | **Miss** | **Score** | **Expect** | **Rank** | **Peptide** |
| --- | --- | --- | --- | --- | --- | --- | --- | --- | --- | --- |
|  | 1748 | 404.7345 | 807.4544 | 807.3399 | 0.1146 | 0 | 2 | 1e+002 | 6 | R.EEYPDR.I |
|  | 4775 | 514.7600 | 1027.5055 | 1027.5121 | -0.0066 | 0 | 40 | 0.016 | 1 | K.TAVCDIPPR.G 4766 4769 4770 4772 4773 4776 |
|  | 5302 | 533.2137 | 1064.4128 | 1064.4201 | -0.0073 | 0 | 41 | 0.013 | 1 | K.NMMAACDPR.H 5301 |
|  | 5458 | 538.7742 | 1075.5338 | 1076.5250 | -0.9913 | 1 | 21 | 1.4 | 1 | K.IREEYPDR.I 5480 |
|  | 5533 | 541.2109 | 1080.4073 | 1080.4151 | -0.0077 | 0 | (35) | 0.047 | 1 | K.NMMAACDPR.H 5532 |
|  | 5550 | 541.7350 | 1081.4554 | 1080.4151 | 1.0404 | 0 | (8) | 23 | 2 | K.NMMAACDPR.H 5554 |
|  | 6379 | 572.3181 | 1142.6217 | 1142.6270 | -0.0053 | 0 | 65 | 5.8e-005 | 1 | K.LAVNMVPFPR.L 6378 |
|  | 7696 | **406.0252** | **1215.0539** | **1214.5754** | **0.4785** | **0** | **3** | **54** | **5** | **R.VSEQFTAMFR.R** |
|  | 8582 | 630.3885 | 1258.7624 | 1257.6830 | 1.0795 | 1 | 9 | 17 | 3 | R.FPGQLNADLRK.L 8578 |
|  | 10169 | **671.3342** | **1340.6538** | **1341.6313** | **-0.9775** | **0** | **7** | **26** | **5** | **R.INVYYNEASGGR.Y** 10215 |
|  | 10735 | 685.8475 | 1369.6804 | 1370.6765 | -0.9961 | 1 | 1 | 1.1e+002 | 10 | K.RVSEQFTAMFR.R |
|  | 14633 | 801.4081 | 1600.8017 | 1600.8131 | -0.0113 | 0 | 30 | 0.12 | 1 | R.AVLVDLEPGTMDSVR.S 14631 14634 |
|  | 14961 | 810.9169 | 1619.8193 | 1619.8283 | -0.0090 | 0 | 24 | 0.53 | 1 | R.LHFFMPGFAPLTSR.G |
|  | 20742 | **680.2991** | **2037.8756** | **2037.9136** | **-0.0380** | **0** | **1** | **66** | **6** | **K.GHYTEGAELMESVMDVVR.K** |
|  | 20791 | **1022.5081** | **2043.0017** | **2041.8907** | **1.1110** | **1** | **3** | **46** | **2** | **R.MPMREVDEQMFNIQDK.N** |
|  | 21610 | 529.7468 | 2114.9582 | 2116.0987 | -1.1405 | 1 | 5 | 28 | 6 | R.YVPRAVLVDLEPGTMDSVR.S |
|  | 22059 | **542.5454** | **2166.1525** | **2166.0085** | **0.1440** | **1** | **1** | **63** | **8** | **K.GHYTEGAELMESVMDVVRK.E** |
|  | 28909 | 1104.5103 | 3310.5089 | 3310.5268 | -0.0179 | 0 | 62 | 2.5e-005 | 1 | K.EAESCDCLQGFQLTHSLGGGTGSGMGTLLLSK.I 28908 28910 28911 28912 28913 28914 |
|  | 28978 | 1109.8413 | 3326.5021 | 3326.5218 | -0.0197 | 0 | (27) | 0.091 | 1 | K.EAESCDCLQGFQLTHSLGGGTGSGMGTLLLSK.I |
|  | 29339 | 1147.5436 | 3439.6089 | 3438.6218 | 0.9871 | 1 | 52 | 0.00023 | 1 | R.KEAESCDCLQGFQLTHSLGGGTGSGMGTLLLSK.I 29336 |
|  | 29368 | 1152.8380 | 3455.4922 | 3454.6167 | 0.8755 | 1 | (2) | 22 | 3 | R.KEAESCDCLQGFQLTHSLGGGTGSGMGTLLLSK.I |

| **127.** | P04839\|CY24B_HUMAN    **Mass:** 66206    **Score:** 270    **Queries matched:** 31   **emPAI:** 0.39 |
| --- | --- |
|  | Cytochrome b-245 heavy chain OS=Homo sapiens GN=CYBB PE=1 SV=2 |

|  | **Query** | **Observed** | **Mr(expt)** | **Mr(calc)** | **Delta** | **Miss** | **Score** | **Expect** | **Rank** | **Peptide** |
| --- | --- | --- | --- | --- | --- | --- | --- | --- | --- | --- |
|  | 1983 | **414.2393** | **826.4640** | **826.4701** | **-0.0061** | **0** | **24** | **0.67** | **1** | **K.VVTHPFK.T** 1982 |
|  | 3860 | **481.2631** | **960.5116** | **960.5181** | **-0.0066** | **0** | **12** | **14** | **1** | **R.GVHFIFNK.E** |
|  | 5972 | **556.8577** | **1111.7009** | **1111.7077** | **-0.0068** | **1** | **28** | **0.23** | **1** | **R.KLLGSALALAR.A** |
|  | 6142 | **562.7891** | **1123.5636** | **1123.5734** | **-0.0098** | **0** | **56** | **0.00036** | **1** | **K.TIASQHPNTR.I** 6122 6143 6144 6145 6146 6149 6159 |
|  | 6660 | **581.2738** | **1160.5330** | **1160.5422** | **-0.0091** | **0** | **42** | **0.011** | **1** | **K.QSISNSESGPR.G** 6658 6664 |
|  | 7645 | **607.3141** | **1212.6136** | **1212.6213** | **-0.0076** | **0** | **26** | **0.33** | **1** | **K.MEVGQYIFVK.C** |
|  | 10378 | **676.8405** | **1351.6663** | **1350.6721** | **0.9943** | **1** | **23** | **0.61** | **1** | **R.GVHFIFNKENF.-** |
|  | 14831 | **538.7742** | **1613.3007** | **1613.7946** | **-0.4939** | **1** | **0** | **71** | **7** | **K.MEVGQYIFVKCPK.V** |
|  | 15241 | **820.3911** | **1638.7677** | **1638.7790** | **-0.0114** | **0** | **17** | **2.4** | **1** | **K.TLYGRPNWDNEFK.T** 15242 |
|  | 15955 | **562.2513** | **1683.7320** | **1682.8313** | **0.9007** | **0** | **2** | **68** | **7** | **K.WIVGPMFLYLCER.L** |
|  | 17811 | **902.9744** | **1803.9343** | **1803.9441** | **-0.0098** | **0** | **72** | **7.4e-006** | **1** | **R.IGVFLCGPEALAETLSK.Q** 17812 |
|  | 18157 | **610.2952** | **1827.8639** | **1827.8720** | **-0.0081** | **1** | **1** | **85** | **3** | **R.NLLSFLRGSSACCSTR.V** |
|  | 18259 | **918.4733** | **1834.9320** | **1834.8697** | **0.0623** | **0** | **9** | **14** | **2** | **R.VNNSDPYSVALSELGDR.Q** |
|  | 25826 | **891.4383** | **2671.2930** | **2671.3071** | **-0.0140** | **0** | **32** | **0.041** | **1** | **K.LEWHPFTLTSAPEEDFFSIHIR.I** |
|  | 27428 | **992.1033** | **2973.2880** | **2973.3062** | **-0.0182** | **1** | **80** | **4.6e-007** | **1** | **R.IVGDWTEGLFNACGCDKQEFQDAWK.L** 27427 |
|  | 29649 | **714.6976** | **3568.4515** | **3568.6804** | **-0.2289** | **1** | **1** | **24** | **4** | **K.ECPIPQFAGNPPMTWKWIVGPMFLYLCER.L** |
|  | 30016 | 1245.6371 | 3733.8894 | 3733.9523 | -0.0629 | 0 | 2 | 20 | 5 | -.MGNWAVNEGLSIFVILVWLGLNVFLFVWYYR.V |
|  | 30839 | **867.4131** | **4332.0294** | **4332.0512** | **-0.0218** | **1** | **1** | **18** | **1** | **R.NNAGFLSYNIYLTGWDESQANHFAVHHDEEKDVITGLK.Q** |

| **128.** | A6NNZ2\|TBB8B_HUMAN    **Mass:** 50168    **Score:** 270    **Queries matched:** 38   **emPAI:** 0.78 |
| --- | --- |
|  | Tubulin beta-8 chain B OS=Homo sapiens PE=1 SV=1 |

|  | **Query** | **Observed** | **Mr(expt)** | **Mr(calc)** | **Delta** | **Miss** | **Score** | **Expect** | **Rank** | **Peptide** |
| --- | --- | --- | --- | --- | --- | --- | --- | --- | --- | --- |
|  | 1748 | 404.7345 | 807.4544 | 807.3399 | 0.1146 | 0 | 2 | 1e+002 | 6 | R.EEYPDR.I |
|  | 4775 | 514.7600 | 1027.5055 | 1027.5121 | -0.0066 | 0 | 40 | 0.016 | 1 | K.TAVCDIPPR.G 4766 4769 4770 4772 4773 4776 |
|  | 5302 | 533.2137 | 1064.4128 | 1064.4201 | -0.0073 | 0 | 41 | 0.013 | 1 | K.NMMAACDPR.H 5301 |
|  | 5458 | 538.7742 | 1075.5338 | 1076.5250 | -0.9913 | 1 | 21 | 1.4 | 1 | K.IREEYPDR.I 5480 |
|  | 5533 | 541.2109 | 1080.4073 | 1080.4151 | -0.0077 | 0 | (35) | 0.047 | 1 | K.NMMAACDPR.H 5532 |
|  | 5550 | 541.7350 | 1081.4554 | 1080.4151 | 1.0404 | 0 | (8) | 23 | 2 | K.NMMAACDPR.H 5554 |
|  | 6379 | 572.3181 | 1142.6217 | 1142.6270 | -0.0053 | 0 | 65 | 5.8e-005 | 1 | K.LAVNMVPFPR.L 6378 |
|  | 8582 | 630.3885 | 1258.7624 | 1257.6830 | 1.0795 | 1 | 9 | 17 | 3 | R.FPGQLNADLRK.L 8578 |
|  | 14950 | 405.9355 | 1619.7127 | 1619.8355 | -0.1227 | 1 | 4 | 52 | 9 | R.HGCYLTVAAIFRGR.M |
|  | 14961 | 810.9169 | 1619.8193 | 1619.8283 | -0.0090 | 0 | 24 | 0.53 | 1 | R.LHFFMPGFAPLTSR.G |
|  | 16061 | **564.2816** | **1689.8230** | **1688.7471** | **1.0759** | **0** | **2** | **75** | **10** | **K.NSSYFADWFPDNVK.T** |
|  | 17723 | **600.6609** | **1798.9610** | **1797.8455** | **1.1155** | **0** | **0** | **90** | **2** | **R.YTEGAELTESVMDVVR.K** |
|  | 18139 | **914.4130** | **1826.8115** | **1827.9241** | **-1.1126** | **1** | **5** | **29** | **1** | **R.INVHHHEASGGRYVPR.A** |
|  | 20791 | 1022.5081 | 2043.0017 | 2041.8907 | 1.1110 | 1 | 3 | 46 | 2 | R.MPMREVDEQMFNIQDK.N |
|  | 28599 | 805.1068 | 3216.3979 | 3215.4726 | 0.9253 | 0 | 1 | 37 | 5 | K.MSATFIGNNAAIQELFTCVSEQFTAMFR.R |
|  | 28909 | 1104.5103 | 3310.5089 | 3310.5268 | -0.0179 | 0 | 62 | 2.5e-005 | 1 | K.EAESCDCLQGFQLTHSLGGGTGSGMGTLLISK.I 28908 28910 28911 28912 28913 28914 |
|  | 28978 | 1109.8413 | 3326.5021 | 3326.5218 | -0.0197 | 0 | (27) | 0.091 | 1 | K.EAESCDCLQGFQLTHSLGGGTGSGMGTLLISK.I |
|  | 29339 | 1147.5436 | 3439.6089 | 3438.6218 | 0.9871 | 1 | 52 | 0.00023 | 1 | R.KEAESCDCLQGFQLTHSLGGGTGSGMGTLLISK.I 29336 |
|  | 29368 | 1152.8380 | 3455.4922 | 3454.6167 | 0.8755 | 1 | (2) | 22 | 3 | R.KEAESCDCLQGFQLTHSLGGGTGSGMGTLLISK.I |

| **129.** | P09601\|HMOX1_HUMAN    **Mass:** 32798    **Score:** 269    **Queries matched:** 20   **emPAI:** 0.92 |
| --- | --- |
|  | Heme oxygenase 1 OS=Homo sapiens GN=HMOX1 PE=1 SV=1 |

|  | **Query** | **Observed** | **Mr(expt)** | **Mr(calc)** | **Delta** | **Miss** | **Score** | **Expect** | **Rank** | **Peptide** |
| --- | --- | --- | --- | --- | --- | --- | --- | --- | --- | --- |
|  | 1029 | **587.3468** | **586.3395** | **586.3802** | **-0.0407** | **1** | **10** | **23** | **6** | **K.KIAQK.A** |
|  | 8393 | **417.2431** | **1248.7076** | **1247.6002** | **1.1074** | **0** | **7** | **31** | **8** | **R.MNSLEMTPAVR.Q** |
|  | 8669 | **632.8101** | **1263.6056** | **1263.5951** | **0.0104** | **0** | **(0)** | **1.3e+002** | **7** | **R.MNSLEMTPAVR.Q** |
|  | 10869 | **459.9262** | **1376.7569** | **1376.7664** | **-0.0095** | **1** | **9** | **16** | **2** | **R.YLGDLSGGQVLKK.I** 10888 |
|  | 12825 | **745.8946** | **1489.7746** | **1490.7333** | **-0.9587** | **1** | **3** | **67** | **1** | **R.SRMNSLEMTPAVR.Q** |
|  | 14032 | **781.8525** | **1561.6905** | **1560.6991** | **0.9915** | **0** | **34** | **0.045** | **1** | **K.EVHTQAENAEFMR.N** 14007 |
|  | 14589 | **799.9052** | **1597.7957** | **1597.8060** | **-0.0102** | **1** | **41** | **0.0098** | **1** | **R.ASNKVQDSAPVETPR.G** 14586 |
|  | 14919 | **809.9004** | **1617.7863** | **1617.7973** | **-0.0110** | **0** | **27** | **0.27** | **1** | **R.WQEVIPYTPAMQR.Y** |
|  | 15192 | **818.9044** | **1635.7942** | **1635.8045** | **-0.0104** | **0** | **40** | **0.012** | **1** | **K.AALEQDLAFWYGPR.W** |
|  | 15354 | **549.9829** | **1646.9269** | **1646.9580** | **-0.0311** | **1** | **4** | **50** | **10** | **R.GKPPLNTRSQAPLLR.W** |
|  | 21380 | **1047.9520** | **2093.8895** | **2093.9589** | **-0.0694** | **1** | **5** | **29** | **4** | **K.EVHTQAENAEFMRNFQK.G** |
|  | 21983 | **720.3649** | **2158.0730** | **2158.0847** | **-0.0118** | **1** | **40** | **0.0094** | **1** | **R.NKESPVFAPVYFPEELHR.K** |
|  | 24336 | **1227.6235** | **2453.2325** | **2453.2478** | **-0.0153** | **0** | **101** | **5.5e-009** | **1** | **K.ALDLPSSGEGLAFFTFPNIASATK.F** 24337 24338 24339 24340 |

| **130.** | P67936\|TPM4_HUMAN    **Mass:** 28619    **Score:** 268    **Queries matched:** 27   **emPAI:** 1.39 |
| --- | --- |
|  | Tropomyosin alpha-4 chain OS=Homo sapiens GN=TPM4 PE=1 SV=3 |

|  | **Query** | **Observed** | **Mr(expt)** | **Mr(calc)** | **Delta** | **Miss** | **Score** | **Expect** | **Rank** | **Peptide** |
| --- | --- | --- | --- | --- | --- | --- | --- | --- | --- | --- |
|  | 1361 | **722.3414** | **721.3341** | **721.3395** | **-0.0054** | **0** | **13** | **7.8** | **1** | **R.AEFAER.T** |
|  | 2293 | **424.3009** | **846.5872** | **845.4606** | **1.1266** | **1** | **3** | **1.1e+002** | **9** | **K.LKEAETR.A** 2284 |
|  | 3859 | **481.2626** | **960.5106** | **959.5222** | **0.9884** | **1** | **2** | **1.2e+002** | **6** | **K.VIENRAMK.D** |
|  | 4074 | **489.2248** | **976.4350** | **975.5171** | **0.9178** | **1** | **(2)** | **1.3e+002** | **4** | **K.VIENRAMK.D** |
|  | 4426 | 501.0761 | 1000.1377 | 1000.5553 | -0.4176 | 0 | 9 | 14 | 1 | M.AGLNSLEAVK.R |
|  | 6462 | **575.2893** | **1148.5639** | **1147.5907** | **0.9733** | **0** | **3** | **78** | **1** | **-.MAGLNSLEAVK.R** |
|  | 6818 | **586.8067** | **1171.5988** | **1170.6105** | **0.9883** | **1** | **2** | **92** | **5** | **K.AEGDVAALNRR.I** |
|  | 8233 | **622.3256** | **1242.6366** | **1242.6456** | **-0.0090** | **0** | **34** | **0.056** | **1** | **R.IQLVEEELDR.A** |
|  | 8744 | **634.8156** | **1267.6165** | **1267.6156** | **0.0009** | **1** | **7** | **27** | **9** | **K.EAKHIAEEADR.K** |
|  | 9334 | **649.8831** | **1297.7516** | **1297.7605** | **-0.0089** | **1** | **28** | **0.21** | **1** | **R.KLVILEGELER.A** 9317 9335 |
|  | 10171 | **671.3363** | **1340.6580** | **1340.6459** | **0.0121** | **1** | **8** | **21** | **3** | **K.SLEAASEKYSEK.E** |
|  | 12015 | **722.4007** | **1442.7868** | **1442.7980** | **-0.0112** | **1** | **21** | **1** | **1** | **R.LATALQKLEEAEK.A** 12016 |
|  | 12554 | **738.8392** | **1475.6639** | **1475.6739** | **-0.0100** | **1** | **63** | **6.9e-005** | **1** | **K.LEEAEKAADESER.G** 12555 12567 |
|  | 14834 | 807.8834 | 1613.7523 | 1613.7645 | -0.0122 | 0 | 82 | 8.3e-007 | 1 | K.IQALQQQADEAEDR.A |
|  | 15935 | **841.9561** | **1681.8977** | **1681.7538** | **0.1438** | **1** | **2** | **64** | **8** | **K.DEEKMEIQEMQLK.E** |
|  | 16644 | **864.4434** | **1726.8722** | **1726.8849** | **-0.0128** | **1** | **34** | **0.039** | **1** | **R.IQLVEEELDRAQER.L** |
|  | 16881 | **871.9314** | **1741.8482** | **1741.8595** | **-0.0112** | **1** | **66** | **2.6e-005** | **1** | **R.KIQALQQQADEAEDR.A** |
|  | 22965 | **756.7108** | **2267.1106** | **2267.1254** | **-0.0148** | **1** | **47** | **0.0017** | **1** | **K.IQALQQQADEAEDRAQGLQR.E** |
|  | 23534 | **1170.5532** | **2339.0919** | **2339.1063** | **-0.0144** | **0** | **54** | **0.00031** | **1** | **K.EENVGLHQTLDQTLNELNCI.-** 23533 |
|  | 26865 | **951.1417** | **2850.4033** | **2850.4181** | **-0.0148** | **1** | **23** | **0.26** | **1** | **K.LAQAKEENVGLHQTLDQTLNELNCI.-** |

| **131.** | P01889\|1B07_HUMAN    **Mass:** 40777    **Score:** 268    **Queries matched:** 34   **emPAI:** 1.21 |
| --- | --- |
|  | HLA class I histocompatibility antigen, B-7 alpha chain OS=Homo sapiens GN=HLA-B PE=1 SV=3 |

|  | **Query** | **Observed** | **Mr(expt)** | **Mr(calc)** | **Delta** | **Miss** | **Score** | **Expect** | **Rank** | **Peptide** |
| --- | --- | --- | --- | --- | --- | --- | --- | --- | --- | --- |
|  | 1309 | 702.8578 | 701.8505 | 701.3894 | 0.4611 | 0 | 10 | 18 | 2 | M.LVMAPR.T 1307 1311 |
|  | 1455 | 766.4029 | 765.3956 | 765.4021 | -0.0065 | 0 | 37 | 0.038 | 1 | R.NTQIYK.A |
|  | 3888 | 482.7234 | 963.4322 | 964.4250 | -0.9929 | 0 | 33 | 0.088 | 1 | R.FDSDAASPR.E 3900 3901 3907 |
|  | 10029 | 666.8338 | 1331.6530 | 1331.6622 | -0.0092 | 0 | 17 | 3.1 | 1 | R.YFYTSVSRPGR.G |
|  | 11736 | 714.3626 | 1426.7105 | 1426.7205 | -0.0099 | 0 | 46 | 0.0031 | 1 | K.WAAVVVPSGEEQR.Y 11721 11735 11751 |
|  | 12552 | 492.8911 | 1475.6516 | 1475.6641 | -0.0125 | 1 | 13 | 6.8 | 1 | R.FDSDAASPREEPR.A 12553 |
|  | 13321 | 760.3871 | 1518.7596 | 1518.7427 | 0.0169 | 0 | 62 | 7.5e-005 | 1 | R.SWTAADTAAQITQR.K |
|  | 15320 | 823.4096 | 1644.8047 | 1644.8148 | -0.0101 | 0 | 69 | 1.4e-005 | 1 | R.FISVGYVDDTQFVR.F 15321 |
|  | 18486 | 463.4837 | 1849.9057 | 1849.9183 | -0.0127 | 0 | 16 | 2.9 | 1 | K.THVTHHPISDHEATLR.C 18485 |
|  | 20872 | 513.0196 | 2048.0493 | 2048.0626 | -0.0133 | 0 | 16 | 2.1 | 1 | R.YTCHVQHEGLPKPLTLR.W 20848 20850 20871 |
|  | 21557 | 1056.5175 | 2111.0204 | 2111.0299 | -0.0095 | 0 | 86 | 2.1e-007 | 1 | R.CWALGFYPAEITLTWQR.D 21558 |
|  | 22689 | 744.6669 | 2230.9790 | 2230.9938 | -0.0149 | 0 | 44 | 0.0032 | 1 | R.DGEDQTQDTELVETRPAGDR.T 22688 |
|  | 23097 | 1143.5082 | 2285.0018 | 2286.0341 | -1.0323 | 1 | 11 | 6.4 | 1 | R.EEPRAPWIEQEGPEYWDR.N |
|  | 23645 | 785.7004 | 2354.0793 | 2355.0767 | -0.9974 | 1 | 32 | 0.05 | 2 | R.GHDQYAYDGKDYIALNEDLR.S 23656 |
|  | 25366 | 865.0411 | 2592.1016 | 2591.2293 | 0.8723 | 1 | 0 | 56 | 6 | R.FISVGYVDDTQFVRFDSDAASPR.E |
|  | 28130 | 521.6007 | 3123.5606 | 3123.6936 | -0.1330 | 1 | 13 | 2.7 | 1 | M.LVMAPRTVLLLLSAALALTETWAGSHSMR.Y |
|  | 30136 | 1896.3450 | 3790.6754 | 3790.1002 | 0.5752 | 1 | 2 | 20 | 5 | R.WEPSSQSTVPIVGIVAGLAVLAVVVIGAVVAAVMCRR.K |

| **132.** | P10316\|1A69_HUMAN    **Mass:** 41236    **Score:** 268    **Queries matched:** 33   **emPAI:** 1.85 |
| --- | --- |
|  | HLA class I histocompatibility antigen, A-69 alpha chain OS=Homo sapiens GN=HLA-A PE=1 SV=2 |

|  | **Query** | **Observed** | **Mr(expt)** | **Mr(calc)** | **Delta** | **Miss** | **Score** | **Expect** | **Rank** | **Peptide** |
| --- | --- | --- | --- | --- | --- | --- | --- | --- | --- | --- |
|  | 4359 | **499.2580** | **996.5014** | **995.4308** | **1.0706** | **0** | **8** | **25** | **1** | **R.FDSDAASQR.M** |
|  | 7402 | **601.3879** | **1200.7613** | **1200.5200** | **0.2414** | **0** | **11** | **12** | **1** | **R.GYHQYAYDGK.D** |
|  | 10029 | 666.8338 | 1331.6530 | 1331.6622 | -0.0092 | 0 | 17 | 3.1 | 1 | R.YFYTSVSRPGR.G |
|  | 10245 | **673.2651** | **1344.5156** | **1344.5227** | **-0.0071** | **0** | **35** | **0.041** | **1** | **R.MYGCDVGSDWR.F** 10244 |
|  | 10335 | 675.8494 | 1349.6843 | 1349.6939 | -0.0096 | 1 | 38 | 0.024 | 1 | R.YLENGKETLQR.T 10311 10351 |
|  | 10951 | 691.3199 | 1380.6253 | 1380.6344 | -0.0090 | 0 | 54 | 0.00056 | 1 | R.SWTAADMAAQTTK.H |
|  | 11721 | 713.8703 | 1425.7260 | 1425.7365 | -0.0104 | 0 | 45 | 0.0043 | 1 | K.WAAVVVPSGQEQR.Y 11735 11736 |
|  | 12921 | 748.8587 | 1495.7028 | 1495.7129 | -0.0101 | 0 | 57 | 0.00026 | 1 | R.AYLEGTCVEWLR.R |
|  | 13404 | **508.9781** | **1523.9124** | **1524.6627** | **-0.7502** | **1** | **4** | **52** | **6** | **R.FDSDAASQRMEPR.A** |
|  | 14210 | **787.9075** | **1573.8005** | **1573.8113** | **-0.0108** | **1** | **59** | **0.00016** | **1** | **K.HKWEAAHVAEQLR.A** 14209 |
|  | 18347 | **461.4684** | **1841.8447** | **1841.8591** | **-0.0145** | **0** | **28** | **0.18** | **1** | **K.THMTHHAVSDHEATLR.C** 18348 |
|  | 19153 | **635.6392** | **1903.8958** | **1903.9104** | **-0.0146** | **1** | **29** | **0.12** | **1** | **R.GYHQYAYDGKDYIALK.E** |
|  | 20872 | 513.0196 | 2048.0493 | 2048.0626 | -0.0133 | 0 | 16 | 2.1 | 1 | R.YTCHVQHEGLPKPLTLR.W 20848 20850 20871 |
|  | 23132 | **763.4412** | **2287.3018** | **2288.0320** | **-0.7302** | **1** | **26** | **0.17** | **1** | **R.MEPRAPWIEQEGPEYWDR.N** |
|  | 24297 | **613.5440** | **2450.1469** | **2450.1938** | **-0.0469** | **1** | **4** | **29** | **2** | **R.VDLGTLRGYYNQSEAGSHTVQR.M** |
|  | 25578 | 1319.0889 | 2636.1632 | 2636.1838 | -0.0206 | 0 | 57 | 0.00013 | 1 | R.DGEDQTQDTELVETRPAGDGTFQK.W 25579 25580 25581 |
|  | 27799 | **760.6081** | **3038.4033** | **3038.2672** | **0.1361** | **1** | **2** | **29** | **2** | **R.GYYNQSEAGSHTVQRMYGCDVGSDWR.F** |
|  | 28413 | 796.6549 | 3182.5903 | 3181.6926 | 0.8977 | 1 | (2) | 31 | 6 | -.MAVMAPRTLVLLLSGALALTQTWAGSHSMR.Y |
|  | 28476 | **533.7578** | **3196.5032** | **3197.6875** | **-1.1843** | **1** | **(0)** | **46** | **8** | **-.MAVMAPRTLVLLLSGALALTQTWAGSHSMR.Y** |
|  | 28578 | **804.6039** | **3214.3867** | **3213.6824** | **0.7042** | **1** | **4** | **16** | **2** | **-.MAVMAPRTLVLLLSGALALTQTWAGSHSMR.Y** |

| **133.** | P63027\|VAMP2_HUMAN    **Mass:** 12698    **Score:** 267    **Queries matched:** 9   **emPAI:** 1.96 |
| --- | --- |
|  | Vesicle-associated membrane protein 2 OS=Homo sapiens GN=VAMP2 PE=1 SV=2 |

|  | **Query** | **Observed** | **Mr(expt)** | **Mr(calc)** | **Delta** | **Miss** | **Score** | **Expect** | **Rank** | **Peptide** |
| --- | --- | --- | --- | --- | --- | --- | --- | --- | --- | --- |
|  | 5389 | **536.3080** | **1070.6015** | **1070.6084** | **-0.0069** | **1** | **27** | **0.38** | **1** | **R.VNVDKVLER.D** |
|  | 15676 | 833.4023 | 1664.7900 | 1664.8006 | -0.0105 | 0 | 43 | 0.0063 | 1 | R.ADALQAGASQFETSAAK.L |
|  | 18762 | **936.9722** | **1871.9299** | **1871.9411** | **-0.0112** | **0** | **109** | **1.2e-009** | **1** | **R.LQQTQAQVDEVVDIMR.V** 18763 |
|  | 18967 | 630.3052 | 1887.8939 | 1887.9360 | -0.0421 | 0 | (1) | 86 | 9 | R.LQQTQAQVDEVVDIMR.V |
|  | 24635 | **1247.5978** | **2493.1810** | **2493.1983** | **-0.0173** | **1** | **63** | **3.9e-005** | **1** | **K.LSELDDRADALQAGASQFETSAAK.L** 24636 |
|  | 27005 | **961.4518** | **2881.3337** | **2881.6498** | **-0.3161** | **1** | **1** | **46** | **1** | **K.NLKMMIILGVICAIILIIIIVYFSS.-** |
|  | 27209 | **586.1216** | **2925.5718** | **2924.4563** | **1.1156** | **1** | **2** | **36** | **5** | **-.MSATAATAPPAAPAGEGGPPAPPPNLTSNRR.L** |

| **134.** | Q9NQC3\|RTN4_HUMAN    **Mass:** 130250   **Score:** 267    **Queries matched:** 33   **emPAI:** 0.15 |
| --- | --- |
|  | Reticulon-4 OS=Homo sapiens GN=RTN4 PE=1 SV=2 |

|  | **Query** | **Observed** | **Mr(expt)** | **Mr(calc)** | **Delta** | **Miss** | **Score** | **Expect** | **Rank** | **Peptide** |
| --- | --- | --- | --- | --- | --- | --- | --- | --- | --- | --- |
|  | 869 | 528.2405 | 527.2333 | 526.3479 | 0.8854 | 0 | 6 | 33 | 9 | K.IPGLK.R |
|  | 1494 | **395.0779** | **788.1411** | **788.3916** | **-0.2504** | **0** | **10** | **16** | **1** | **K.LPSDTEK.E** 1507 1510 |
|  | 2395 | **428.7448** | **855.4750** | **855.5178** | **-0.0428** | **0** | **5** | **48** | **5** | **K.GVIQAIQK.S** |
|  | 2689 | **439.2267** | **876.4388** | **875.4535** | **0.9853** | **1** | **9** | **24** | **6** | **K.NVKDAMAK.I** 2690 |
|  | 2885 | **446.2256** | **890.4366** | **891.4484** | **-1.0118** | **1** | **(7)** | **40** | **2** | **K.NVKDAMAK.I** |
|  | 3605 | **472.7105** | **943.4064** | **943.4148** | **-0.0084** | **0** | **54** | **0.0008** | **1** | **K.SDEGHPFR.A** |
|  | 4571 | **506.2935** | **1010.5725** | **1009.4386** | **1.1339** | **0** | **6** | **37** | **6** | **R.SSAENMDLK.E** 4560 4563 |
|  | 5699 | 547.2634 | 1092.5123 | 1092.4757 | 0.0366 | 0 | 7 | 32 | 9 | K.EDSDMLAAGGK.I |
|  | 8098 | 618.8328 | 1235.6510 | 1235.6397 | 0.0112 | 0 | 16 | 3.2 | 4 | R.SPSAIFSAELSK.T |
|  | 8812 | **424.5786** | **1270.7141** | **1270.7034** | **0.0107** | **0** | **6** | **34** | **4** | **R.GPLPAAPPVAPER.Q** |
|  | 9754 | 660.3469 | 1318.6793 | 1318.7092 | -0.0299 | 1 | 6 | 40 | 5 | K.AQIVTEKNTSTK.T 9785 |
|  | 10848 | **459.2899** | **1374.8479** | **1375.7599** | **-0.9120** | **0** | **7** | **28** | **4** | **R.LFLVDDLVDSLK.F** |
|  | 11682 | 475.2555 | 1422.7447 | 1422.6297 | 0.1151 | 1 | 12 | 7.7 | 3 | K.DSKEDSDMLAAGGK.I |
|  | 14053 | **782.3776** | **1562.7406** | **1562.7511** | **-0.0105** | **0** | **69** | **1.5e-005** | **1** | **K.YSNSALGHVNCTIK.E** 14050 14051 14052 14066 |
|  | 14735 | **804.4122** | **1606.8099** | **1606.8216** | **-0.0117** | **0** | **64** | **5.3e-005** | **1** | **R.HQAQIDHYLGLANK.N** |
|  | 15205 | **819.4198** | **1636.8250** | **1635.8104** | **1.0147** | **1** | **1** | **88** | **10** | **K.EDRSPSAIFSAELSK.T** |
|  | 17854 | **904.4647** | **1806.9149** | **1806.9251** | **-0.0102** | **0** | **53** | **0.00053** | **1** | **R.AYLESEVAISEELVQK.Y** |
|  | 18218 | 459.2255 | 1832.8729 | 1831.9720 | 0.9009 | 0 | 4 | 40 | 7 | K.LSALPPEGGKPYLESFK.L |
|  | 18275 | **919.3950** | **1836.7755** | **1837.0309** | **-0.2554** | **1** | **0** | **92** | **8** | **K.AESAVIVANPREEIIVK.N** |
|  | 24652 | **625.0255** | **2496.0729** | **2496.2132** | **-0.1403** | **0** | **1** | **52** | **5** | **M.EDLDQSPLVSSSDSPPRPQPAFK.Y** |
|  | 26275 | **687.3196** | **2745.2495** | **2746.3814** | **-1.1319** | **0** | **7** | **13** | **2** | **R.QPSWDPSPVSSTVPAPSPLSAAAVSPSK.L** |
|  | 30704 | **1396.2396** | **4185.6970** | **4185.7192** | **-0.0222** | **1** | **94** | **8.5e-009** | **1** | **K.YQFVREPEDEEEEEEEEEEDEDEDLEELEVLER.K** |
|  | 31842 | **1445.1322** | **7220.6246** | **7219.6459** | **0.9788** | **1** | **7** | **0.74** | **1** | **R.QPSWDPSPVSSTVPAPSPLSAAAVSPSKLPEDDEPPARPPPPPPASVSPQAEPVWTPPAPAPAAPPSTPAAPK.R** |

| **135.** | P01892\|1A02_HUMAN    **Mass:** 41181    **Score:** 261    **Queries matched:** 32   **emPAI:** 1.61 |
| --- | --- |
|  | HLA class I histocompatibility antigen, A-2 alpha chain OS=Homo sapiens GN=HLA-A PE=1 SV=1 |

|  | **Query** | **Observed** | **Mr(expt)** | **Mr(calc)** | **Delta** | **Miss** | **Score** | **Expect** | **Rank** | **Peptide** |
| --- | --- | --- | --- | --- | --- | --- | --- | --- | --- | --- |
|  | 4359 | 499.2580 | 996.5014 | 995.4308 | 1.0706 | 0 | 8 | 25 | 1 | R.FDSDAASQR.M |
|  | 7402 | 601.3879 | 1200.7613 | 1200.5200 | 0.2414 | 0 | 11 | 12 | 1 | R.GYHQYAYDGK.D |
|  | 9699 | **658.8362** | **1315.6579** | **1315.6673** | **-0.0094** | **0** | **14** | **6.2** | **2** | **R.YFFTSVSRPGR.G** |
|  | 10245 | 673.2651 | 1344.5156 | 1344.5227 | -0.0071 | 0 | 35 | 0.041 | 1 | R.MYGCDVGSDWR.F 10244 |
|  | 10335 | 675.8494 | 1349.6843 | 1349.6939 | -0.0096 | 1 | 38 | 0.024 | 1 | R.YLENGKETLQR.T 10311 10351 |
|  | 10951 | 691.3199 | 1380.6253 | 1380.6344 | -0.0090 | 0 | 54 | 0.00056 | 1 | R.SWTAADMAAQTTK.H |
|  | 11721 | 713.8703 | 1425.7260 | 1425.7365 | -0.0104 | 0 | 45 | 0.0043 | 1 | K.WAAVVVPSGQEQR.Y 11735 11736 |
|  | 12921 | 748.8587 | 1495.7028 | 1495.7129 | -0.0101 | 0 | 57 | 0.00026 | 1 | R.AYLEGTCVEWLR.R |
|  | 13404 | 508.9781 | 1523.9124 | 1524.6627 | -0.7502 | 1 | 4 | 52 | 6 | R.FDSDAASQRMEPR.A |
|  | 14210 | 787.9075 | 1573.8005 | 1573.8113 | -0.0108 | 1 | 59 | 0.00016 | 1 | K.HKWEAAHVAEQLR.A 14209 |
|  | 18347 | 461.4684 | 1841.8447 | 1841.8591 | -0.0145 | 0 | 28 | 0.18 | 1 | K.THMTHHAVSDHEATLR.C 18348 |
|  | 19153 | 635.6392 | 1903.8958 | 1903.9104 | -0.0146 | 1 | 29 | 0.12 | 1 | R.GYHQYAYDGKDYIALK.E |
|  | 20872 | 513.0196 | 2048.0493 | 2048.0626 | -0.0133 | 0 | 16 | 2.1 | 1 | R.YTCHVQHEGLPKPLTLR.W 20848 20850 20871 |
|  | 24297 | 613.5440 | 2450.1469 | 2450.1938 | -0.0469 | 1 | 4 | 29 | 2 | R.VDLGTLRGYYNQSEAGSHTVQR.M |
|  | 25578 | 1319.0889 | 2636.1632 | 2636.1838 | -0.0206 | 0 | 57 | 0.00013 | 1 | R.DGEDQTQDTELVETRPAGDGTFQK.W 25579 25580 25581 |
|  | 27799 | 760.6081 | 3038.4033 | 3038.2672 | 0.1361 | 1 | 2 | 29 | 2 | R.GYYNQSEAGSHTVQRMYGCDVGSDWR.F |
|  | 28413 | 796.6549 | 3182.5903 | 3181.6926 | 0.8977 | 1 | (2) | 31 | 6 | -.MAVMAPRTLVLLLSGALALTQTWAGSHSMR.Y |
|  | 28476 | 533.7578 | 3196.5032 | 3197.6875 | -1.1843 | 1 | (0) | 46 | 8 | -.MAVMAPRTLVLLLSGALALTQTWAGSHSMR.Y |
|  | 28578 | 804.6039 | 3214.3867 | 3213.6824 | 0.7042 | 1 | 4 | 16 | 2 | -.MAVMAPRTLVLLLSGALALTQTWAGSHSMR.Y |

| **136.** | A1L0T0\|ILVBL_HUMAN    **Mass:** 68452    **Score:** 259    **Queries matched:** 25   **emPAI:** 0.37 |
| --- | --- |
|  | Acetolactate synthase-like protein OS=Homo sapiens GN=ILVBL PE=1 SV=2 |

|  | **Query** | **Observed** | **Mr(expt)** | **Mr(calc)** | **Delta** | **Miss** | **Score** | **Expect** | **Rank** | **Peptide** |
| --- | --- | --- | --- | --- | --- | --- | --- | --- | --- | --- |
|  | 1823 | **407.7058** | **813.3971** | **812.4756** | **0.9215** | **0** | **7** | **32** | **1** | **R.DIVPTLR.A** 1811 |
|  | 2705 | **877.5140** | **876.5067** | **875.4535** | **1.0532** | **0** | **2** | **1.2e+002** | **7** | **R.CVEILSR.A** |
|  | 2868 | **445.2184** | **888.4223** | **887.4824** | **0.9398** | **1** | **4** | **85** | **7** | **R.ENRSAALK.K** |
|  | 3823 | **480.2755** | **958.5365** | **959.4560** | **-0.9194** | **0** | **11** | **14** | **5** | **R.ENEDQVVK.V** 3848 |
|  | 4571 | 506.2935 | 1010.5725 | 1011.5423 | -0.9697 | 0 | 5 | 45 | 8 | K.EMVPAKPPK.G |
|  | 8458 | **627.7992** | **1253.5838** | **1253.5935** | **-0.0097** | **0** | **37** | **0.027** | **1** | **K.VLHDAQQQCR.D** 8457 |
|  | 11076 | **694.8995** | **1387.7845** | **1387.7936** | **-0.0091** | **0** | **42** | **0.0079** | **1** | **R.DGHPVVVNILIGR.T** 11088 |
|  | 20385 | 501.7471 | 2002.9592 | 2004.0469 | -1.0877 | 0 | 11 | 8 | 2 | R.WLDPGAFGTLGVGAGFALGAK.L |
|  | 22200 | **1092.6082** | **2183.2017** | **2183.2162** | **-0.0144** | **0** | **70** | **8.3e-006** | **1** | **R.LSGTVGVAAVTAGPGLTNTVTAVK.N** 22201 |
|  | 22460 | 738.3698 | 2212.0876 | 2212.2249 | -0.1373 | 1 | 1 | 61 | 10 | R.AKRPLMVLGSQALLTPTSADK.L |
|  | 22638 | **1114.0785** | **2226.1424** | **2225.1117** | **1.0307** | **0** | **8** | **12** | **2** | **K.LVEGLQGQTWAPDWVEELR.E** |
|  | 23083 | **1142.5674** | **2283.1202** | **2282.2780** | **0.8422** | **1** | **0** | **72** | **3** | **K.RPLMVLGSQALLTPTSADKLR.A** |
|  | 23754 | **1184.1390** | **2366.2635** | **2366.2740** | **-0.0104** | **0** | **54** | **0.00032** | **1** | **K.NAQMAQSPILLLGGAASTLLQNR.G** 23755 |
|  | 25970 | **899.7753** | **2696.3042** | **2696.3194** | **-0.0153** | **1** | **35** | **0.021** | **1** | **K.LVEGLQGQTWAPDWVEELREADR.Q** |
|  | 29427 | **870.9356** | **3479.7133** | **3480.8261** | **-1.1128** | **0** | **0** | **38** | **1** | **-.METPAAAAPAGSLFPSFLLLACGTLVAALLGAAHR.L** |
|  | 29866 | 1223.6744 | 3668.0015 | 3668.9196 | -0.9181 | 1 | 1 | 22 | 3 | R.HEVTAVFAADAMARLSGTVGVAAVTAGPGLTNTVTAVK.N |
|  | 31187 | **1215.3893** | **4857.5280** | **4857.5488** | **-0.0208** | **0** | **84** | **6e-008** | **1** | **K.AAMPVAQHLNPVQVLQLVEETLPDNSILVVDGGDFVGTAAHLVQPR.G** 31186 |
|  | 31201 | **1219.5609** | **4874.2145** | **4873.5437** | **0.6708** | **0** | **(3)** | **7** | **1** | **K.AAMPVAQHLNPVQVLQLVEETLPDNSILVVDGGDFVGTAAHLVQPR.G** |

| **137.** | P50993\|AT1A2_HUMAN    **Mass:** 113505   **Score:** 259    **Queries matched:** 37   **emPAI:** 0.21 |
| --- | --- |
|  | Sodium/potassium-transporting ATPase subunit alpha-2 OS=Homo sapiens GN=ATP1A2 PE=1 SV=1 |

|  | **Query** | **Observed** | **Mr(expt)** | **Mr(calc)** | **Delta** | **Miss** | **Score** | **Expect** | **Rank** | **Peptide** |
| --- | --- | --- | --- | --- | --- | --- | --- | --- | --- | --- |
|  | 875 | 530.2573 | 529.2500 | 528.2656 | 0.9844 | 0 | 11 | 24 | 8 | K.GAPER.I |
|  | 2981 | **451.2256** | **900.4367** | **901.4869** | **-1.0501** | **0** | **9** | **26** | **6** | **K.LSLDELGR.K** |
|  | 4924 | 519.5986 | 1037.1827 | 1037.4964 | -0.3137 | 0 | 6 | 24 | 6 | R.NSVFQQGMK.N |
|  | 5596 | 543.2701 | 1084.5257 | 1084.5335 | -0.0078 | 0 | 25 | 0.46 | 1 | K.ACVVHGSDLK.D |
|  | 6863 | **588.3676** | **1174.7205** | **1173.6254** | **1.0951** | **1** | **4** | **60** | **5** | **K.RSPTWTALSR.I** 6839 |
|  | 7117 | **595.3146** | **1188.6147** | **1187.6258** | **0.9889** | **1** | **10** | **16** | **1** | **K.AGQENISVSKR.D** |
|  | 8748 | 634.8565 | 1267.6983 | 1267.7071 | -0.0087 | 0 | 27 | 0.26 | 3 | K.NMVPQQALVIR.E |
|  | 9070 | **429.0871** | **1284.2394** | **1283.6656** | **0.5738** | **0** | **5** | **26** | **6** | **R.LNIPMSQVNPR.E** |
|  | 10962 | 691.3739 | 1380.7332 | 1380.7435 | -0.0103 | 0 | 47 | 0.0027 | 1 | K.VIMVTGDHPITAK.A 10963 10964 |
|  | 11503 | **472.2731** | **1413.7976** | **1412.7082** | **1.0894** | **1** | **1** | **95** | **4** | **R.EAKACVVHGSDLK.D** |
|  | 12207 | 485.9094 | 1454.7063 | 1454.6565 | 0.0498 | 0 | 10 | 15 | 6 | K.FDTDELNFPTEK.L 12206 |
|  | 12576 | **493.2352** | **1476.6839** | **1476.7936** | **-0.1098** | **1** | **3** | **61** | **7** | **R.AVFKAGQENISVSK.R** |
|  | 14939 | 810.3740 | 1618.7335 | 1618.7435 | -0.0100 | 0 | 68 | 2e-005 | 1 | K.VDNSSLTGESEPQTR.S 14925 14938 |
|  | 15437 | **551.2950** | **1650.8631** | **1650.7932** | **0.0699** | **0** | **(3)** | **64** | **6** | **K.LCFVGLMSMIDPPR.A** |
|  | 15692 | **556.3090** | **1665.9051** | **1666.7881** | **-0.8831** | **0** | **4** | **44** | **9** | **K.LCFVGLMSMIDPPR.A** 15689 |
|  | 16234 | **567.7708** | **1700.2906** | **1699.9178** | **0.3728** | **1** | **7** | **13** | **2** | **R.CSTILVQGKEIPLDK.E** |
|  | 16961 | **874.4321** | **1746.8497** | **1745.9458** | **0.9039** | **1** | **5** | **32** | **2** | **R.TVMGRIATLASGLEVGR.T** |
|  | 17400 | 593.9977 | 1778.9714 | 1779.9917 | -1.0203 | 1 | 1 | 80 | 3 | K.VIMVTGDHPITAKAIAK.G |
|  | 17912 | 906.4724 | 1810.9303 | 1810.9425 | -0.0122 | 0 | 83 | 5.3e-007 | 1 | R.QGAIVAVTGDGVNDSPALK.K |
|  | 18169 | 915.4591 | 1828.9037 | 1828.9167 | -0.0130 | 0 | 86 | 2.9e-007 | 1 | K.GVGIISEGNETVEDIAAR.L |
|  | 18848 | 627.0032 | 1877.9877 | 1878.9258 | -0.9381 | 1 | 6 | 26 | 2 | R.EDSPQSHVLVMKGAPER.I |
|  | 19391 | 962.9550 | 1923.8954 | 1923.9095 | -0.0142 | 0 | 1 | 82 | 1 | K.NLEAVETLGSTSTICSDK.T |
|  | 22891 | **753.3717** | **2257.0933** | **2256.1420** | **0.9513** | **1** | **5** | **26** | **4** | **K.MQINAEEVVVGDLVEVKGGDR.V** |
|  | 22993 | **758.0573** | **2271.1501** | **2272.1369** | **-0.9868** | **1** | **(1)** | **61** | **8** | **K.MQINAEEVVVGDLVEVKGGDR.V** |
|  | 23458 | 777.3395 | 2328.9966 | 2330.1675 | -1.1709 | 1 | 3 | 37 | 3 | R.EGEKMQINAEEVVVGDLVEVK.G |
|  | 24392 | 822.0676 | 2463.1811 | 2463.1951 | -0.0141 | 0 | 38 | 0.012 | 1 | K.QAADMILLDDNFASIVTGVEEGR.L |
|  | 26883 | **714.3783** | **2853.4841** | **2853.3313** | **0.1528** | **1** | **3** | **26** | **4** | **K.EIPLDKEMQDAFQNAYMELGGLGER.V** |
|  | 28297 | **1053.1774** | **3156.5103** | **3156.4596** | **0.0507** | **1** | **0** | **43** | **5** | **K.VDNSSLTGESEPQTRSPEFTHENPLETR.N** |
|  | 28900 | 1103.4862 | 3307.4368 | 3306.6806 | 0.7562 | 1 | 1 | 29 | 1 | K.QAADMILLDDNFASIVTGVEEGRLIFDNLK.K |
|  | 29236 | **1133.5529** | **3397.6368** | **3396.4776** | **1.1592** | **1** | **2** | **25** | **1** | **R.MTVAHMWFDNQIHEADTTEDQSGATFDKR.S** |

| **138.** | P13637\|AT1A3_HUMAN    **Mass:** 113102   **Score:** 259    **Queries matched:** 24   **emPAI:** 0.21 |
| --- | --- |
|  | Sodium/potassium-transporting ATPase subunit alpha-3 OS=Homo sapiens GN=ATP1A3 PE=1 SV=3 |

|  | **Query** | **Observed** | **Mr(expt)** | **Mr(calc)** | **Delta** | **Miss** | **Score** | **Expect** | **Rank** | **Peptide** |
| --- | --- | --- | --- | --- | --- | --- | --- | --- | --- | --- |
|  | 875 | 530.2573 | 529.2500 | 528.2656 | 0.9844 | 0 | 11 | 24 | 8 | K.GAPER.I |
|  | 4924 | 519.5986 | 1037.1827 | 1037.4964 | -0.3137 | 0 | 6 | 24 | 6 | R.NSVFQQGMK.N |
|  | 6287 | **568.3408** | **1134.6670** | **1133.4910** | **1.1759** | **0** | **3** | **81** | **2** | **K.EQPLDEEMK.E** |
|  | 8100 | 618.8520 | 1235.6895 | 1235.6986 | -0.0091 | 0 | 29 | 0.18 | 1 | R.LNIPVSQVNPR.D 8072 |
|  | 8748 | 634.8565 | 1267.6983 | 1267.7071 | -0.0087 | 0 | 27 | 0.26 | 3 | K.NMVPQQALVIR.E |
|  | 10962 | 691.3739 | 1380.7332 | 1380.7435 | -0.0103 | 0 | 47 | 0.0027 | 1 | K.VIMVTGDHPITAK.A 10963 10964 |
|  | 11845 | **717.3739** | **1432.7332** | **1432.7457** | **-0.0124** | **1** | **5** | **45** | **1** | **R.GVVVATGDRTVMGR.I** |
|  | 13091 | 502.9265 | 1505.7576 | 1505.7396 | 0.0180 | 0 | 1 | 87 | 9 | K.ADIGVAMGIAGSDVSK.Q |
|  | 14939 | 810.3740 | 1618.7335 | 1618.7435 | -0.0100 | 0 | 68 | 2e-005 | 1 | K.VDNSSLTGESEPQTR.S 14925 14938 |
|  | 16523 | **430.7463** | **1718.9560** | **1717.9397** | **1.0164** | **1** | **12** | **6.9** | **2** | **R.TVMGRIATLASGLEVGK.T** |
|  | 17400 | 593.9977 | 1778.9714 | 1779.9917 | -1.0203 | 1 | 1 | 80 | 3 | K.VIMVTGDHPITAKAIAK.G |
|  | 17912 | 906.4724 | 1810.9303 | 1810.9425 | -0.0122 | 0 | 83 | 5.3e-007 | 1 | R.QGAIVAVTGDGVNDSPALK.K |
|  | 18169 | 915.4591 | 1828.9037 | 1828.9167 | -0.0130 | 0 | 86 | 2.9e-007 | 1 | K.GVGIISEGNETVEDIAAR.L |
|  | 18761 | **936.9641** | **1871.9135** | **1870.9710** | **0.9425** | **0** | **1** | **85** | **5** | **K.MQVNAEEVVVGDLVEIK.G** |
|  | 19391 | 962.9550 | 1923.8954 | 1923.9095 | -0.0142 | 0 | 1 | 82 | 1 | K.NLEAVETLGSTSTICSDK.T |
|  | 22891 | 753.3717 | 2257.0933 | 2256.1420 | 0.9513 | 1 | 4 | 33 | 5 | K.MQVNAEEVVVGDLVEIKGGDR.V |
|  | 24392 | 822.0676 | 2463.1811 | 2463.1951 | -0.0141 | 0 | 38 | 0.012 | 1 | K.QAADMILLDDNFASIVTGVEEGR.L |
|  | 28900 | 1103.4862 | 3307.4368 | 3306.6806 | 0.7562 | 1 | 1 | 29 | 1 | K.QAADMILLDDNFASIVTGVEEGRLIFDNLK.K |
|  | 30626 | **1371.3502** | **4111.0288** | **4111.8277** | **-0.7988** | **1** | **2** | **16** | **7** | **K.TGTLTQNRMTVAHMWFDNQIHEADTTEDQSGTSFDK.S** |

| **139.** | Q9H0U4\|RAB1B_HUMAN    **Mass:** 22328    **Score:** 259    **Queries matched:** 24   **emPAI:** 3.15 |
| --- | --- |
|  | Ras-related protein Rab-1B OS=Homo sapiens GN=RAB1B PE=1 SV=1 |

|  | **Query** | **Observed** | **Mr(expt)** | **Mr(calc)** | **Delta** | **Miss** | **Score** | **Expect** | **Rank** | **Peptide** |
| --- | --- | --- | --- | --- | --- | --- | --- | --- | --- | --- |
|  | 1162 | 643.2711 | 642.2638 | 642.4064 | -0.1427 | 0 | 27 | 0.41 | 1 | K.LLVGNK.S |
|  | 3332 | 462.7205 | 923.4265 | 923.4348 | -0.0083 | 0 | 13 | 6.7 | 1 | R.YASENVNK.L |
|  | 4239 | 495.7453 | 989.4760 | 989.4818 | -0.0058 | 0 | 33 | 0.1 | 1 | R.TITSSYYR.G |
|  | 5394 | 536.3203 | 1070.6261 | 1070.6336 | -0.0075 | 0 | 45 | 0.006 | 1 | K.LLLIGDSGVGK.S 5392 |
|  | 5639 | 544.8228 | 1087.6309 | 1086.5458 | 1.0852 | 0 | 10 | 19 | 9 | K.QWLQEIDR.Y |
|  | 9139 | 430.2988 | 1287.8745 | 1287.7398 | 0.1347 | 1 | 2 | 68 | 10 | K.LLVGNKSDLTTK.K |
|  | 9695 | 658.8284 | 1315.6422 | 1315.6521 | -0.0099 | 0 | 18 | 2.6 | 1 | K.LQIWDTAGQER.F 9685 |
|  | 11972 | **721.3595** | **1440.7044** | **1440.7143** | **-0.0099** | **0** | **32** | **0.081** | **1** | **R.MGPGAASGGERPNLK.I** 11961 11973 |
|  | 14585 | **799.7875** | **1597.5604** | **1596.8154** | **0.7450** | **1** | **5** | **29** | **8** | **K.RMGPGAASGGERPNLK.I** |
|  | 16601 | 862.9358 | 1723.8570 | 1723.8669 | -0.0098 | 0 | 35 | 0.033 | 1 | K.EFADSLGIPFLETSAK.N 16602 |
|  | 18924 | 943.4422 | 1884.8698 | 1883.8757 | 0.9941 | 0 | 74 | 4e-006 | 2 | K.NATNVEQAFMTMAAEIK.K 18915 |
|  | 19106 | 951.0158 | 1900.0169 | 1899.8706 | 0.1463 | 0 | (3) | 52 | 4 | K.NATNVEQAFMTMAAEIK.K 19117 |
|  | 20253 | 996.9864 | 1991.9583 | 1991.9701 | -0.0117 | 1 | 46 | 0.0024 | 1 | K.QWLQEIDRYASENVNK.L 20252 |
|  | 22107 | 1086.4988 | 2170.9830 | 2170.9947 | -0.0117 | 0 | 72 | 5.5e-006 | 1 | R.FADDTYTESYISTIGVDFK.I 22106 |
|  | 23034 | **1139.5297** | **2277.0448** | **2277.1277** | **-0.0830** | **0** | **6** | **21** | **1** | **R.GAHGIIVVYDVTDQESYANVK.Q** |

| **140.** | Q29836\|1B67_HUMAN    **Mass:** 40602    **Score:** 259    **Queries matched:** 38   **emPAI:** 1.42 |
| --- | --- |
|  | HLA class I histocompatibility antigen, B-67 alpha chain OS=Homo sapiens GN=HLA-B PE=1 SV=1 |

|  | **Query** | **Observed** | **Mr(expt)** | **Mr(calc)** | **Delta** | **Miss** | **Score** | **Expect** | **Rank** | **Peptide** |
| --- | --- | --- | --- | --- | --- | --- | --- | --- | --- | --- |
|  | 1309 | 702.8578 | 701.8505 | 701.3894 | 0.4611 | 0 | 10 | 18 | 2 | M.LVMAPR.T 1307 1311 |
|  | 1455 | 766.4029 | 765.3956 | 765.4021 | -0.0065 | 0 | 37 | 0.038 | 1 | R.NTQIYK.A |
|  | 3888 | 482.7234 | 963.4322 | 964.4250 | -0.9929 | 0 | 33 | 0.088 | 1 | R.FDSDAASPR.E 3900 3901 3907 |
|  | 6565 | 577.7809 | 1153.5473 | 1153.6091 | -0.0618 | 1 | 3 | 67 | 9 | K.ETLQRADPPK.T |
|  | 10029 | 666.8338 | 1331.6530 | 1331.6622 | -0.0092 | 0 | 17 | 3.1 | 1 | R.YFYTSVSRPGR.G |
|  | 10335 | 675.8494 | 1349.6843 | 1349.6939 | -0.0096 | 1 | 38 | 0.024 | 1 | R.YLENGKETLQR.A 10311 10351 |
|  | 11736 | 714.3626 | 1426.7105 | 1426.7205 | -0.0099 | 0 | 46 | 0.0031 | 1 | K.WAAVVVPSGEEQR.Y 11721 11735 11751 |
|  | 12552 | 492.8911 | 1475.6516 | 1475.6641 | -0.0125 | 1 | 13 | 6.8 | 1 | R.FDSDAASPREEPR.A 12553 |
|  | 13435 | **763.8641** | **1525.7136** | **1525.7235** | **-0.0099** | **0** | **36** | **0.032** | **1** | **R.TYLEGTCVEWLR.R** |
|  | 15320 | 823.4096 | 1644.8047 | 1644.8148 | -0.0101 | 0 | 69 | 1.4e-005 | 1 | R.FISVGYVDDTQFVR.F 15321 |
|  | 18486 | 463.4837 | 1849.9057 | 1849.9183 | -0.0127 | 0 | 16 | 2.9 | 1 | K.THVTHHPISDHEATLR.C 18485 |
|  | 20872 | 513.0196 | 2048.0493 | 2048.0626 | -0.0133 | 0 | 16 | 2.1 | 1 | R.YTCHVQHEGLPKPLTLR.W 20848 20850 20871 |
|  | 21557 | 1056.5175 | 2111.0204 | 2111.0299 | -0.0095 | 0 | 86 | 2.1e-007 | 1 | R.CWALGFYPAEITLTWQR.D 21558 |
|  | 22689 | 744.6669 | 2230.9790 | 2230.9938 | -0.0149 | 0 | 44 | 0.0032 | 1 | R.DGEDQTQDTELVETRPAGDR.T 22688 |
|  | 23097 | 1143.5082 | 2285.0018 | 2286.0341 | -1.0323 | 1 | 11 | 6.4 | 1 | R.EEPRAPWIEQEGPEYWDR.N |
|  | 25366 | 865.0411 | 2592.1016 | 2591.2293 | 0.8723 | 1 | 0 | 56 | 6 | R.FISVGYVDDTQFVRFDSDAASPR.E |
|  | 28130 | 521.6007 | 3123.5606 | 3123.6936 | -0.1330 | 1 | 13 | 2.7 | 1 | M.LVMAPRTVLLLLSAALALTETWAGSHSMR.Y |
|  | 30083 | **943.4384** | **3769.7246** | **3769.7608** | **-0.0362** | **1** | **29** | **0.038** | **1** | **R.GHNQFAYDGKDYIALNEDLSSWTAADTAAQITQR.K** 30082 |
|  | 30136 | 1896.3450 | 3790.6754 | 3790.1002 | 0.5752 | 1 | 2 | 20 | 5 | R.WEPSSQSTVPIVGIVAGLAVLAVVVIGAVVAAVMCRR.K |

| **141.** | Q6NUK1\|SCMC1_HUMAN    **Mass:** 53548    **Score:** 258    **Queries matched:** 25   **emPAI:** 0.50 |
| --- | --- |
|  | Calcium-binding mitochondrial carrier protein SCaMC-1 OS=Homo sapiens GN=SLC25A24 PE=1 SV=2 |

|  | **Query** | **Observed** | **Mr(expt)** | **Mr(calc)** | **Delta** | **Miss** | **Score** | **Expect** | **Rank** | **Peptide** |
| --- | --- | --- | --- | --- | --- | --- | --- | --- | --- | --- |
|  | 877 | **530.9559** | **529.9487** | **530.2813** | **-0.3326** | **0** | **12** | **14** | **2** | **K.EGGIR.S** |
|  | 3227 | **458.8808** | **915.7470** | **916.4865** | **-0.7395** | **0** | **1** | **1.2e+002** | **5** | **K.LLTEEGQK.I** |
|  | 4805 | 515.7590 | 1029.5035 | 1029.5099 | -0.0064 | 0 | 21 | 1.3 | 1 | K.IMMQVHGSK.S |
|  | 7606 | **606.3569** | **1210.6992** | **1211.6986** | **-0.9994** | **0** | **5** | **43** | **5** | **R.QLLAGGIAGAVSR.T** 7636 |
|  | 8832 | 636.8386 | 1271.6626 | 1270.6890 | 0.9736 | 1 | (4) | 62 | 8 | R.LKIMMQVHGSK.S |
|  | 9129 | **644.8184** | **1287.6223** | **1286.6839** | **0.9384** | **1** | **(2)** | **1e+002** | **9** | **R.LKIMMQVHGSK.S** |
|  | 9439 | **435.2688** | **1302.7845** | **1302.6788** | **0.1057** | **1** | **4** | **56** | **9** | **R.LKIMMQVHGSK.S** |
|  | 10563 | **681.7982** | **1361.5819** | **1361.5922** | **-0.0103** | **0** | **36** | **0.034** | **1** | **K.TGQYSGIYDCAK.K** 10579 |
|  | 10983 | 692.3545 | 1382.6944 | 1382.7041 | -0.0097 | 0 | 30 | 0.14 | 1 | R.NLGIPLGQDAEEK.I |
|  | 15763 | **835.9157** | **1669.8167** | **1669.8271** | **-0.0104** | **0** | **58** | **0.00019** | **1** | **R.NGDGVVDIGELQEGLR.N** 15770 |
|  | 17428 | **891.4727** | **1780.9309** | **1780.9433** | **-0.0124** | **0** | **50** | **0.001** | **1** | **K.VLPAVGISYVVYENMK.Q** |
|  | 19321 | **640.3495** | **1918.0266** | **1917.8891** | **0.1376** | **0** | **0** | **1e+002** | **10** | **R.DFVLPTAACQDAEQPTR.Y** |
|  | 20443 | **671.3097** | **2010.9072** | **2012.0704** | **-1.1632** | **1** | **0** | **88** | **6** | **K.SGQWWRQLLAGGIAGAVSR.T** |
|  | 22580 | **1111.0436** | **2220.0726** | **2220.0853** | **-0.0127** | **0** | **112** | **4.8e-010** | **1** | **R.MQAQAMLEGSPQLNMVGLFR.R** 22581 22582 |
|  | 23946 | 599.8078 | 2395.2021 | 2395.1262 | 0.0759 | 0 | 5 | 25 | 3 | R.FISGSMAGATAQTFIYPMEVMK.T |
|  | 24049 | **603.8195** | **2411.2487** | **2411.1211** | **0.1276** | **0** | **(0)** | **69** | **7** | **R.FISGSMAGATAQTFIYPMEVMK.T** |
|  | 24642 | **832.2368** | **2493.6885** | **2493.2290** | **0.4594** | **1** | **3** | **26** | **1** | **R.TRMQAQAMLEGSPQLNMVGLFR.R** |
|  | 24968 | **849.7360** | **2546.1862** | **2546.2024** | **-0.0162** | **1** | **12** | **4.8** | **1** | **K.HSTGIDIGDSLTIPDEFTEDEKK.S** 24967 |
|  | 25806 | **890.7380** | **2669.1923** | **2668.2699** | **0.9224** | **1** | **3** | **35** | **3** | **R.FISGSMAGATAQTFIYPMEVMKTR.L** |

| **142.** | P18463\|1B37_HUMAN    **Mass:** 40716    **Score:** 258    **Queries matched:** 29   **emPAI:** 1.02 |
| --- | --- |
|  | HLA class I histocompatibility antigen, B-37 alpha chain OS=Homo sapiens GN=HLA-B PE=2 SV=1 |

|  | **Query** | **Observed** | **Mr(expt)** | **Mr(calc)** | **Delta** | **Miss** | **Score** | **Expect** | **Rank** | **Peptide** |
| --- | --- | --- | --- | --- | --- | --- | --- | --- | --- | --- |
|  | 3888 | 482.7234 | 963.4322 | 964.4250 | -0.9929 | 0 | 33 | 0.088 | 1 | R.FDSDAASPR.T 3900 3901 3907 |
|  | 6565 | 577.7809 | 1153.5473 | 1153.6091 | -0.0618 | 1 | 3 | 67 | 9 | K.ETLQRADPPK.T |
|  | 6736 | **584.2381** | **1166.4616** | **1165.4492** | **1.0124** | **0** | **6** | **34** | **1** | **R.MSGCDVGPDGR.L** 6729 6740 |
|  | 10335 | 675.8494 | 1349.6843 | 1349.6939 | -0.0096 | 1 | 38 | 0.024 | 1 | R.YLENGKETLQR.A 10311 10351 |
|  | 11736 | 714.3626 | 1426.7105 | 1426.7205 | -0.0099 | 0 | 46 | 0.0031 | 1 | K.WAAVVVPSGEEQR.Y 11721 11735 11751 |
|  | 12921 | 748.8587 | 1495.7028 | 1495.7129 | -0.0101 | 0 | 57 | 0.00026 | 1 | R.AYLEGTCVEWLR.R |
|  | 15320 | 823.4096 | 1644.8047 | 1644.8148 | -0.0101 | 0 | 69 | 1.4e-005 | 1 | R.FISVGYVDDTQFVR.F 15321 |
|  | 18486 | 463.4837 | 1849.9057 | 1849.9183 | -0.0127 | 0 | 16 | 2.9 | 1 | K.THVTHHPISDHEATLR.C 18485 |
|  | 20872 | 513.0196 | 2048.0493 | 2048.0626 | -0.0133 | 0 | 16 | 2.1 | 1 | R.YTCHVQHEGLPKPLTLR.W 20848 20850 20871 |
|  | 21557 | 1056.5175 | 2111.0204 | 2111.0299 | -0.0095 | 0 | 86 | 2.1e-007 | 1 | R.CWALGFYPAEITLTWQR.D 21558 |
|  | 22689 | 744.6669 | 2230.9790 | 2230.9938 | -0.0149 | 0 | 44 | 0.0032 | 1 | R.DGEDQTQDTELVETRPAGDR.T 22688 |
|  | 25366 | 865.0411 | 2592.1016 | 2591.2293 | 0.8723 | 1 | 0 | 56 | 6 | R.FISVGYVDDTQFVRFDSDAASPR.T |

| **143.** | P16188\|1A30_HUMAN    **Mass:** 41164    **Score:** 255    **Queries matched:** 23   **emPAI:** 1.01 |
| --- | --- |
|  | HLA class I histocompatibility antigen, A-30 alpha chain OS=Homo sapiens GN=HLA-A PE=1 SV=2 |

|  | **Query** | **Observed** | **Mr(expt)** | **Mr(calc)** | **Delta** | **Miss** | **Score** | **Expect** | **Rank** | **Peptide** |
| --- | --- | --- | --- | --- | --- | --- | --- | --- | --- | --- |
|  | 4359 | 499.2580 | 996.5014 | 995.4308 | 1.0706 | 0 | 8 | 25 | 1 | R.FDSDAASQR.M |
|  | 10335 | 675.8494 | 1349.6843 | 1349.6939 | -0.0096 | 1 | 38 | 0.024 | 1 | R.YLENGKETLQR.T 10311 10351 |
|  | 11736 | 714.3626 | 1426.7105 | 1426.7205 | -0.0099 | 0 | 46 | 0.0031 | 1 | K.WAAVVVPSGEEQR.Y 11721 11735 11751 |
|  | 12921 | 748.8587 | 1495.7028 | 1495.7129 | -0.0101 | 0 | 57 | 0.00026 | 1 | R.AYLEGTCVEWLR.R |
|  | 13404 | 508.9781 | 1523.9124 | 1524.6627 | -0.7502 | 1 | 4 | 52 | 6 | R.FDSDAASQRMEPR.A |
|  | 13806 | 775.3702 | 1548.7259 | 1548.7355 | -0.0095 | 0 | 44 | 0.0053 | 1 | R.SWTAADMAAQITQR.K |
|  | 14092 | 522.7712 | 1565.2917 | 1564.7304 | 0.5613 | 0 | (8) | 12 | 3 | R.SWTAADMAAQITQR.K |
|  | 18889 | 628.3002 | 1881.8787 | 1881.8904 | -0.0117 | 0 | 24 | 0.42 | 1 | K.THMTHHPISDHEATLR.C |
|  | 20872 | 513.0196 | 2048.0493 | 2048.0626 | -0.0133 | 0 | 16 | 2.1 | 1 | R.YTCHVQHEGLPKPLTLR.W 20848 20850 20871 |
|  | 21557 | 1056.5175 | 2111.0204 | 2111.0299 | -0.0095 | 0 | 86 | 2.1e-007 | 1 | R.CWALGFYPAEITLTWQR.D 21558 |
|  | 25578 | 1319.0889 | 2636.1632 | 2636.1838 | -0.0206 | 0 | 57 | 0.00013 | 1 | R.DGEDQTQDTELVETRPAGDGTFQK.W 25579 25580 25581 |

| **144.** | P23284\|PPIB_HUMAN    **Mass:** 23785    **Score:** 255    **Queries matched:** 22   **emPAI:** 2.29 |
| --- | --- |
|  | Peptidyl-prolyl cis-trans isomerase B OS=Homo sapiens GN=PPIB PE=1 SV=2 |

|  | **Query** | **Observed** | **Mr(expt)** | **Mr(calc)** | **Delta** | **Miss** | **Score** | **Expect** | **Rank** | **Peptide** |
| --- | --- | --- | --- | --- | --- | --- | --- | --- | --- | --- |
|  | 1258 | **687.3368** | **686.3295** | **685.3912** | **0.9384** | **0** | **13** | **12** | **1** | **K.HVVFGK.V** |
|  | 2751 | **440.9221** | **879.8296** | **879.5218** | **0.3077** | **0** | **1** | **76** | **1** | **R.VIFGLFGK.T** |
|  | 2930 | **448.7050** | **895.3954** | **895.4076** | **-0.0122** | **0** | **8** | **23** | **8** | **R.FPDENFK.L** |
|  | 4819 | **516.2778** | **1030.5410** | **1030.5481** | **-0.0071** | **0** | **57** | **0.00037** | **1** | **K.VLEGMEVVR.K** 4818 |
|  | 6857 | **588.3216** | **1174.6286** | **1174.6380** | **-0.0094** | **1** | **5** | **54** | **6** | **K.VLEGMEVVRK.V** |
|  | 9388 | **434.7631** | **1301.2674** | **1301.5711** | **-0.3036** | **0** | **7** | **18** | **3** | **K.DFMIQGGDFTR.G** 9379 |
|  | 10608 | **682.8520** | **1363.6894** | **1363.6983** | **-0.0089** | **0** | **84** | **5.5e-007** | **1** | **K.TVDNFVALATGEK.G** 10591 |
|  | 10775 | **686.8470** | **1371.6794** | **1372.7602** | **-1.0808** | **1** | **8** | **23** | **9** | **K.IEVEKPFAIAKE.-** |
|  | 12260 | **729.6846** | **1457.3546** | **1456.7198** | **0.6348** | **0** | **12** | **6.4** | **1** | **K.DTNGSQFFITTVK.T** |
|  | 12511 | **737.8431** | **1473.6717** | **1473.6823** | **-0.0106** | **0** | **47** | **0.0028** | **1** | **K.HYGPGWVSMANAGK.D** |
|  | 14173 | **786.4213** | **1570.8281** | **1570.8389** | **-0.0108** | **1** | **75** | **4.4e-006** | **1** | **R.DKPLKDVIIADCGK.I** 14154 14172 |
|  | 14627 | 801.3777 | 1600.7408 | 1600.7522 | -0.0113 | 1 | 49 | 0.0015 | 1 | K.SIYGERFPDENFK.L 14628 |
|  | 15041 | **813.4197** | **1624.8248** | **1625.8236** | **-0.9988** | **1** | **47** | **0.0023** | **1** | **R.VIKDFMIQGGDFTR.G** 15051 |
|  | 15294 | **548.5715** | **1642.6926** | **1641.8185** | **0.8741** | **1** | **(2)** | **71** | **4** | **R.VIKDFMIQGGDFTR.G** |
|  | 18790 | **625.6313** | **1873.8720** | **1873.8265** | **0.0455** | **1** | **1** | **81** | **7** | **K.DFMIQGGDFTRGDGTGGK.S** |

| **145.** | Q10567\|AP1B1_HUMAN    **Mass:** 105452   **Score:** 254    **Queries matched:** 33   **emPAI:** 0.19 |
| --- | --- |
|  | AP-1 complex subunit beta-1 OS=Homo sapiens GN=AP1B1 PE=1 SV=1 |

|  | **Query** | **Observed** | **Mr(expt)** | **Mr(calc)** | **Delta** | **Miss** | **Score** | **Expect** | **Rank** | **Peptide** |
| --- | --- | --- | --- | --- | --- | --- | --- | --- | --- | --- |
|  | 1887 | **819.4338** | **818.4266** | **818.4320** | **-0.0055** | **0** | **13** | **9.8** | **1** | **K.TAAVCVAK.L** |
|  | 2909 | **447.3449** | **892.6753** | **892.3927** | **0.2827** | **0** | **4** | **51** | **2** | **K.DEDPYVR.K** 2901 |
|  | 3646 | **474.2669** | **946.5192** | **946.5270** | **-0.0078** | **1** | **10** | **17** | **7** | **R.KTAAVCVAK.L** |
|  | 4282 | **993.0422** | **992.0350** | **991.4393** | **0.5957** | **0** | **8** | **18** | **2** | **R.EAQSICER.V** |
|  | 4562 | **506.2573** | **1010.5001** | **1010.5073** | **-0.0071** | **0** | **17** | **3.1** | **1** | **K.YNDPIYVK.L** |
|  | 7166 | **596.3094** | **1190.6043** | **1190.5819** | **0.0224** | **1** | **3** | **74** | **3** | **M.TDSKYFTTTK.K** |
|  | 7937 | **614.7806** | **1227.5467** | **1227.5554** | **-0.0086** | **0** | **20** | **1.5** | **1** | **K.DCEDPNPLIR.A** 7938 |
|  | 8339 | **416.5507** | **1246.6304** | **1246.6638** | **-0.0334** | **1** | **10** | **16** | **6** | **R.ALAVRTMGCIR.V** |
|  | 8620 | **631.2825** | **1260.5504** | **1261.5608** | **-1.0104** | **0** | **6** | **35** | **6** | **R.DCPLNAEAASSK.L** |
|  | 9242 | **647.5536** | **1293.0927** | **1293.6023** | **-0.5096** | **1** | **0** | **88** | **10** | **K.CLKDEDPYVR.K** |
|  | 10483 | **453.2467** | **1356.7184** | **1355.7015** | **1.0169** | **1** | **11** | **12** | **4** | **K.VLMKFMEMLSK.D** |
|  | 10783 | **458.2710** | **1371.7913** | **1370.7228** | **1.0685** | **0** | **6** | **34** | **3** | **K.MEPLNNLQVAVK.N** |
|  | 11083 | **463.7531** | **1388.2374** | **1387.6913** | **0.5461** | **1** | **(9)** | **12** | **1** | **K.VLMKFMEMLSK.D** |
|  | 11102 | **695.8710** | **1389.7275** | **1389.7366** | **-0.0091** | **0** | **25** | **0.47** | **1** | **K.LVYLYLMNYAK.S** |
|  | 11657 | **474.9176** | **1421.7310** | **1420.7384** | **0.9926** | **1** | **6** | **30** | **3** | **K.ITEYLCEPLRK.C** 11629 |
|  | 13396 | **762.8666** | **1523.7186** | **1523.7290** | **-0.0104** | **0** | **58** | **0.0002** | **1** | **R.NVEGQDMLYQSLK.L** |
|  | 13580 | **767.9166** | **1533.8187** | **1532.7868** | **1.0319** | **0** | **3** | **70** | **3** | **R.QVGSISMDLQLTNK.A** |
|  | 13689 | **771.4247** | **1540.8348** | **1539.7239** | **1.1109** | **0** | **(2)** | **77** | **6** | **R.NVEGQDMLYQSLK.L** |
|  | 15686 | **556.2821** | **1665.8245** | **1666.8059** | **-0.9814** | **0** | **2** | **75** | **9** | **K.SQPDMAIMAVNTFVK.D** |
|  | 16148 | 848.9149 | 1695.8152 | 1695.8250 | -0.0098 | 1 | 1 | 87 | 6 | K.RNVEGQDMLYQSLK.L |
|  | 18885 | **628.1918** | **1881.5537** | **1882.0710** | **-0.5173** | **1** | **2** | **42** | **2** | **R.LSHANSAVVLSAVKVLMK.F** 18889 |
|  | 27119 | **969.8306** | **2906.4701** | **2905.5007** | **0.9693** | **1** | **2** | **29** | **1** | **R.NVEGQDMLYQSLKLTNGIWVLAELR.I** |
|  | 27537 | **998.1878** | **2991.5416** | **2991.5553** | **-0.0137** | **1** | **94** | **2.3e-008** | **1** | **R.LASQANIAQVLAELKEYATEVDVDFVR.K** 27538 |
|  | 28909 | 1104.5103 | 3310.5089 | 3309.6295 | 0.8794 | 1 | 1 | 38 | 4 | K.VIASMTVGKDVSALFPDVVNCMQTDNLELK.K |
|  | 29778 | **1205.9476** | **3614.8211** | **3614.8356** | **-0.0145** | **0** | **87** | **6.2e-008** | **1** | **R.IDNADELLESFLEGFHDESTQVQLQLLTAIVK.L** 29777 |
|  | 30087 | **1257.6708** | **3769.9905** | **3770.0077** | **-0.0172** | **0** | **52** | **0.00019** | **1** | **R.NSFGLAPAAPLQVHAPLSPNQTVEISLPLSTVGSVMK.M** 30085 |

| **146.** | Q95365\|1B38_HUMAN    **Mass:** 40733    **Score:** 253    **Queries matched:** 40   **emPAI:** 1.21 |
| --- | --- |
|  | HLA class I histocompatibility antigen, B-38 alpha chain OS=Homo sapiens GN=HLA-B PE=2 SV=1 |

|  | **Query** | **Observed** | **Mr(expt)** | **Mr(calc)** | **Delta** | **Miss** | **Score** | **Expect** | **Rank** | **Peptide** |
| --- | --- | --- | --- | --- | --- | --- | --- | --- | --- | --- |
|  | 1309 | 702.8578 | 701.8505 | 701.3894 | 0.4611 | 0 | 10 | 18 | 2 | M.LVMAPR.T 1307 1311 |
|  | 1446 | 763.1124 | 762.1051 | 762.3694 | -0.2643 | 0 | 2 | 96 | 3 | R.NTQICK.T |
|  | 3888 | 482.7234 | 963.4322 | 964.4250 | -0.9929 | 0 | 33 | 0.088 | 1 | R.FDSDAASPR.E 3900 3901 3907 |
|  | 4176 | 493.2762 | 984.5379 | 983.5876 | 0.9503 | 1 | 10 | 18 | 10 | R.ENLRIALR.Y 4164 |
|  | 6565 | 577.7809 | 1153.5473 | 1153.6091 | -0.0618 | 1 | 3 | 67 | 9 | K.ETLQRADPPK.T |
|  | 10029 | 666.8338 | 1331.6530 | 1331.6622 | -0.0092 | 0 | 17 | 3.1 | 1 | R.YFYTSVSRPGR.G |
|  | 10335 | 675.8494 | 1349.6843 | 1349.6939 | -0.0096 | 1 | 38 | 0.024 | 1 | R.YLENGKETLQR.A 10311 10351 |
|  | 11736 | 714.3626 | 1426.7105 | 1426.7205 | -0.0099 | 0 | 46 | 0.0031 | 1 | K.WAAVVVPSGEEQR.Y 11721 11735 11751 |
|  | 12552 | 492.8911 | 1475.6516 | 1475.6641 | -0.0125 | 1 | 13 | 6.8 | 1 | R.FDSDAASPREEPR.A 12553 |
|  | 13435 | 763.8641 | 1525.7136 | 1525.7235 | -0.0099 | 0 | 36 | 0.032 | 1 | R.TYLEGTCVEWLR.R |
|  | 15320 | 823.4096 | 1644.8047 | 1644.8148 | -0.0101 | 0 | 69 | 1.4e-005 | 1 | R.FISVGYVDDTQFVR.F 15321 |
|  | 18486 | 463.4837 | 1849.9057 | 1849.9183 | -0.0127 | 0 | 16 | 2.9 | 1 | K.THVTHHPISDHEATLR.C 18485 |
|  | 20872 | 513.0196 | 2048.0493 | 2048.0626 | -0.0133 | 0 | 16 | 2.1 | 1 | R.YTCHVQHEGLPKPLTLR.W 20848 20850 20871 |
|  | 21557 | 1056.5175 | 2111.0204 | 2111.0299 | -0.0095 | 0 | 86 | 2.1e-007 | 1 | R.CWALGFYPAEITLTWQR.D 21558 |
|  | 22689 | 744.6669 | 2230.9790 | 2230.9938 | -0.0149 | 0 | 44 | 0.0032 | 1 | R.DGEDQTQDTELVETRPAGDR.T 22688 |
|  | 23097 | 1143.5082 | 2285.0018 | 2286.0341 | -1.0323 | 1 | 11 | 6.4 | 1 | R.EEPRAPWIEQEGPEYWDR.N |
|  | 25366 | 865.0411 | 2592.1016 | 2591.2293 | 0.8723 | 1 | 0 | 56 | 6 | R.FISVGYVDDTQFVRFDSDAASPR.E |
|  | 28130 | 521.6007 | 3123.5606 | 3123.6936 | -0.1330 | 1 | 13 | 2.7 | 1 | M.LVMAPRTVLLLLSAALALTETWAGSHSMR.Y |
|  | 30083 | 943.4384 | 3769.7246 | 3769.7608 | -0.0362 | 1 | 29 | 0.038 | 1 | R.GHNQFAYDGKDYIALNEDLSSWTAADTAAQITQR.K 30082 |
|  | 30136 | 1896.3450 | 3790.6754 | 3790.1002 | 0.5752 | 1 | 2 | 20 | 5 | R.WEPSSQSTVPIVGIVAGLAVLAVVVIGAVVAAVMCRR.K |

|  | **Proteins matching a subset of these peptides:** |
| --- | --- |

|  | P30475\|1B39_HUMAN    **Mass:** 40645    **Score:** 253    **Queries matched:** 38 |
| --- | --- |
|  | HLA class I histocompatibility antigen, B-39 alpha chain OS=Homo sapiens GN=HLA-B PE=2 SV=1 |

| **147.** | P61586\|RHOA_HUMAN    **Mass:** 22096    **Score:** 253    **Queries matched:** 16   **emPAI:** 1.22 |
| --- | --- |
|  | Transforming protein RhoA OS=Homo sapiens GN=RHOA PE=1 SV=1 |

|  | **Query** | **Observed** | **Mr(expt)** | **Mr(calc)** | **Delta** | **Miss** | **Score** | **Expect** | **Rank** | **Peptide** |
| --- | --- | --- | --- | --- | --- | --- | --- | --- | --- | --- |
|  | 4129 | **491.2532** | **980.4918** | **981.4590** | **-0.9672** | **0** | **(1)** | **1.3e+002** | **6** | **R.EVFEMATR.A** |
|  | 4401 | 500.3102 | 998.6059 | 997.4539 | 1.1521 | 0 | 8 | 26 | 9 | R.EVFEMATR.A |
|  | 5636 | **544.7886** | **1087.5626** | **1087.5696** | **-0.0070** | **0** | **19** | **2.2** | **1** | **K.LVIVGDGACGK.T** 5635 |
|  | 10051 | **667.2955** | **1332.5765** | **1332.5842** | **-0.0077** | **0** | **46** | **0.0039** | **1** | **R.IGAFGYMECSAK.T** |
|  | 12010 | **481.9096** | **1442.7068** | **1442.7187** | **-0.0119** | **1** | **15** | **4.3** | **2** | **K.MKQEPVKPEEGR.D** 11993 |
|  | 14269 | **527.1537** | **1578.4394** | **1577.7218** | **0.7176** | **1** | **2** | **55** | **10** | **R.IGAFGYMECSAKTK.D** |
|  | 14513 | **796.8949** | **1591.7752** | **1591.8140** | **-0.0388** | **1** | **0** | **1.2e+002** | **4** | **R.EVFEMATRAALQAR.R** |
|  | 16786 | **579.3232** | **1734.9479** | **1734.9603** | **-0.0124** | **1** | **50** | **0.0012** | **1** | **K.HFCPNVPIILVGNKK.D** |
|  | 17081 | **586.3284** | **1755.9633** | **1754.8369** | **1.1263** | **1** | **4** | **45** | **8** | **K.QEPVKPEEGRDMANR.I** |
|  | 20422 | **1004.9587** | **2007.9028** | **2007.9174** | **-0.0146** | **0** | **63** | **4.7e-005** | **1** | **K.QVELALWDTAGQEDYDR.L** |
|  | 26400 | **1387.1586** | **2772.3026** | **2772.3171** | **-0.0145** | **0** | **62** | **3.5e-005** | **1** | **K.DQFPEVYVPTVFENYVADIEVDGK.Q** 26401 |
|  | 29393 | **1155.5691** | **3463.6854** | **3463.6891** | **-0.0036** | **0** | **75** | **1.1e-006** | **1** | **R.LRPLSYPDTDVILMCFSIDSPDSLENIPEK.W** 29394 |

| **148.** | P07988\|PSPB_HUMAN    **Mass:** 43514    **Score:** 253    **Queries matched:** 18   **emPAI:** 0.94 |
| --- | --- |
|  | Pulmonary surfactant-associated protein B OS=Homo sapiens GN=SFTPB PE=1 SV=3 |

|  | **Query** | **Observed** | **Mr(expt)** | **Mr(calc)** | **Delta** | **Miss** | **Score** | **Expect** | **Rank** | **Peptide** |
| --- | --- | --- | --- | --- | --- | --- | --- | --- | --- | --- |
|  | 3967 | **486.2457** | **970.4768** | **971.5586** | **-1.0817** | **1** | **7** | **36** | **1** | **K.RIQAMIPK.G** |
|  | 4629 | **508.7693** | **1015.5241** | **1015.5307** | **-0.0066** | **0** | **8** | **27** | **3** | **R.MLPQLVCR.L** |
|  | 5726 | **548.2087** | **1094.4029** | **1094.4121** | **-0.0092** | **0** | **26** | **0.37** | **1** | **R.CSMDDSAGPR.S** |
|  | 7668 | **607.8336** | **1213.6527** | **1213.6601** | **-0.0074** | **0** | **32** | **0.087** | **1** | **K.GALAVAVAQVCR.V** |
|  | 10577 | 681.9003 | 1361.7861 | 1361.7918 | -0.0057 | 0 | 92 | 8.9e-008 | 1 | R.YSVILLDTLLGR.M |
|  | 12798 | 745.3846 | 1488.7546 | 1488.7646 | -0.0100 | 0 | 36 | 0.036 | 1 | K.FLEQECNVLPLK.L 12797 |
|  | 14492 | **531.2846** | **1590.8320** | **1591.7447** | **-0.9127** | **1** | **9** | **14** | **1** | **R.LVLRCSMDDSAGPR.S** |
|  | 14907 | **809.4314** | **1616.8482** | **1616.8596** | **-0.0113** | **1** | **53** | **0.00056** | **1** | **R.KFLEQECNVLPLK.L** 14906 |
|  | 16862 | **871.4525** | **1740.8903** | **1740.9015** | **-0.0112** | **0** | **54** | **0.00044** | **1** | **R.VVPLVAGGICQCLAER.Y** 16861 16863 |
|  | 28485 | **1067.1338** | **3198.3795** | **3198.3992** | **-0.0196** | **0** | **18** | **0.62** | **1** | **R.GWDAHTTCQALGVCGTMSSPLQCIHSPDL.-** |
|  | 29774 | **904.6710** | **3614.6547** | **3614.6705** | **-0.0157** | **0** | **48** | **0.00051** | **1** | **R.ALGHCLQEVWGHVGADDLCQECEDIVHILNK.M** 29773 |
|  | 31043 | 1531.6987 | 4592.0744 | 4591.0991 | 0.9753 | 0 | 2 | 11 | 5 | K.LLMPQCNQVLDDYFPLVIDYFQNQTDSNGICMHLGLCK.S 31044 |

| **149.** | P07858\|CATB_HUMAN    **Mass:** 38766    **Score:** 252    **Queries matched:** 15   **emPAI:** 0.74 |
| --- | --- |
|  | Cathepsin B OS=Homo sapiens GN=CTSB PE=1 SV=3 |

|  | **Query** | **Observed** | **Mr(expt)** | **Mr(calc)** | **Delta** | **Miss** | **Score** | **Expect** | **Rank** | **Peptide** |
| --- | --- | --- | --- | --- | --- | --- | --- | --- | --- | --- |
|  | 4145 | **491.9287** | **981.8429** | **981.4841** | **0.3588** | **0** | **6** | **23** | **7** | **K.DIMAEIYK.N** |
|  | 9613 | **657.8010** | **1313.5874** | **1313.5962** | **-0.0088** | **0** | **37** | **0.03** | **1** | **K.ICEPGYSPTYK.Q** 9612 |
|  | 15982 | **843.3897** | **1684.7649** | **1684.7767** | **-0.0118** | **1** | **29** | **0.13** | **1** | **K.ICEPGYSPTYKQDK.H** |
|  | 18068 | **911.9208** | **1821.8270** | **1822.8632** | **-1.0362** | **0** | **14** | **4.7** | **1** | **R.GQDHCGIESEVVAGIPR.T** 18081 |
|  | 19659 | **649.3000** | **1944.8781** | **1944.8935** | **-0.0153** | **0** | **50** | **0.00097** | **1** | **K.SGVYQHVTGEMMGGHAIR.I** 19658 |
|  | 19914 | **654.6323** | **1960.8750** | **1960.8884** | **-0.0134** | **0** | **(2)** | **61** | **2** | **K.SGVYQHVTGEMMGGHAIR.I** |
|  | 21439 | **701.3161** | **2100.9265** | **2101.0592** | **-0.1328** | **0** | **3** | **41** | **2** | **R.SRPSFHPLSDELVNYVNK.R** |
|  | 22115 | **1086.9507** | **2171.8868** | **2171.9001** | **-0.0133** | **0** | **108** | **1.1e-009** | **1** | **R.DQGSCGSCWAFGAVEAISDR.I** |
|  | 22403 | 736.3840 | 2206.1301 | 2205.1324 | 0.9977 | 1 | 12 | 5 | 1 | K.ILRGQDHCGIESEVVAGIPR.T 22374 |
|  | 25222 | **857.7117** | **2570.1132** | **2570.1278** | **-0.0146** | **1** | **48** | **0.00097** | **1** | **K.EIRDQGSCGSCWAFGAVEAISDR.I** |
|  | 25392 | **866.7264** | **2597.1575** | **2597.1744** | **-0.0169** | **1** | **81** | **4.7e-007** | **1** | **K.HYGYNSYSVSNSEKDIMAEIYK.N** |

| **150.** | P56199\|ITA1_HUMAN    **Mass:** 132304   **Score:** 252    **Queries matched:** 28   **emPAI:** 0.15 |
| --- | --- |
|  | Integrin alpha-1 OS=Homo sapiens GN=ITGA1 PE=1 SV=2 |

|  | **Query** | **Observed** | **Mr(expt)** | **Mr(calc)** | **Delta** | **Miss** | **Score** | **Expect** | **Rank** | **Peptide** |
| --- | --- | --- | --- | --- | --- | --- | --- | --- | --- | --- |
|  | 1770 | **405.7243** | **809.4340** | **810.3946** | **-0.9605** | **0** | **1** | **1.1e+002** | **9** | **R.GEMVTFK.I** |
|  | 2339 | 426.2394 | 850.4643 | 850.4701 | -0.0058 | 0 | 23 | 0.94 | 1 | K.VGYPFLR.R |
|  | 2702 | **877.5079** | **876.5006** | **876.5069** | **-0.0063** | **0** | **14** | **8.2** | **3** | **R.FGTAIAAVK.D** |
|  | 3095 | 454.7447 | 907.4748 | 906.5175 | 0.9573 | 0 | 13 | 8.6 | 3 | K.FNVSLTVK.N 3091 |
|  | 7109 | **595.2982** | **1188.5818** | **1188.5921** | **-0.0103** | **1** | **(3)** | **75** | **10** | **K.MTTSTDHLKR.G** |
|  | 7471 | **402.2431** | **1203.7076** | **1204.5870** | **-0.8795** | **1** | **11** | **12** | **1** | **K.MTTSTDHLKR.G** |
|  | 9249 | **432.0402** | **1293.0987** | **1292.6394** | **0.4593** | **0** | **6** | **24** | **6** | **R.QTMTALGIDTAR.K** |
|  | 11632 | 711.3883 | 1420.7621 | 1420.7344 | 0.0277 | 1 | 9 | 15 | 3 | R.QTMTALGIDTARK.E |
|  | 12350 | **488.2591** | **1461.7554** | **1462.7263** | **-0.9710** | **0** | **2** | **75** | **7** | **R.SENASLVLSSSNQK.R** |
|  | 12889 | **747.8704** | **1493.7262** | **1492.7773** | **0.9489** | **1** | **5** | **36** | **7** | **R.GNLSTEKFVEEIK.S** |
|  | 13229 | **505.7325** | **1514.1757** | **1514.6889** | **-0.5132** | **0** | **5** | **25** | **3** | **K.EDTIYEADLQYR.V** |
|  | 13296 | **759.8412** | **1517.6679** | **1517.6780** | **-0.0101** | **0** | **36** | **0.034** | **1** | **K.VIQDCEDENIQR.F** |
|  | 16316 | 854.4075 | 1706.8004 | 1705.8774 | 0.9230 | 1 | 13 | 5.8 | 1 | K.FVEEIKSIASEPTEK.H |
|  | 16325 | **569.9788** | **1706.9145** | **1705.8709** | **1.0435** | **1** | **5** | **38** | **1** | **R.DVAVVKVTMNFEPNK.V** |
|  | 16704 | **865.9104** | **1729.8062** | **1729.8159** | **-0.0096** | **1** | **63** | **5.5e-005** | **1** | **K.SKEDTIYEADLQYR.V** 16703 |
|  | 16811 | **869.8537** | **1737.6928** | **1737.9513** | **-0.2584** | **0** | **5** | **28** | **2** | **K.LDLPVNTSIPNVTEVK.E** |
|  | 18184 | **611.2456** | **1830.7150** | **1829.9193** | **0.7957** | **1** | **4** | **33** | **8** | **K.EKCISDLSLHVATTEK.D** |
|  | 19968 | 655.9768 | 1964.9084 | 1964.9738 | -0.0654 | 1 | 5 | 29 | 6 | K.VMVIVTDGESHDNHRLK.K |
|  | 27398 | **989.4649** | **2965.3729** | **2965.3739** | **-0.0010** | **0** | **3** | **28** | **9** | **K.ENMTFGSTLVTNPNGGFLACGPLYAYR.C** |
|  | 28687 | **1081.5415** | **3241.6027** | **3241.6255** | **-0.0228** | **1** | **74** | **1.8e-006** | **1** | **K.KNEPLASYLGYTVNSATASSGDVLYIAGQPR.Y** 28688 |
|  | 29151 | **843.6564** | **3370.5964** | **3370.6106** | **-0.0142** | **0** | **7** | **7.6** | **1** | **K.DLNLDGFNDIVIGAPLEDDHGGAVYIYHGSGK.T** |
|  | 30865 | **1454.6851** | **4361.0334** | **4361.0661** | **-0.0327** | **1** | **90** | **2.4e-008** | **1** | **K.NSMTFSGPVEDMFGYTVQQYENEEGKWVLIGSPLVGQPK.N** 30868 |
|  | 31694 | **932.9766** | **5591.8157** | **5591.5095** | **0.3062** | **0** | **0** | **9.7** | **2** | **R.IFALEATADQSAASFEMEMSQTGFSAHYSQDWVMLGAVGAYDWNGTVVMQK.A** |
|  | 31710 | **1411.6395** | **5642.5290** | **5641.7623** | **0.7667** | **1** | **0** | **8.8** | **4** | **R.GEMVTFKILFQFNTSYLMENVTIYLSATSDSEEPPETLSDNVVNISIPVK.Y** |

| **151.** | Q53GQ0\|DHB12_HUMAN    **Mass:** 34416    **Score:** 251    **Queries matched:** 24   **emPAI:** 0.87 |
| --- | --- |
|  | Estradiol 17-beta-dehydrogenase 12 OS=Homo sapiens GN=HSD17B12 PE=1 SV=2 |

|  | **Query** | **Observed** | **Mr(expt)** | **Mr(calc)** | **Delta** | **Miss** | **Score** | **Expect** | **Rank** | **Peptide** |
| --- | --- | --- | --- | --- | --- | --- | --- | --- | --- | --- |
|  | 1432 | **759.3942** | **758.3870** | **759.4239** | **-1.0370** | **0** | **13** | **11** | **1** | **K.TVGLQSR.T** |
|  | 2325 | **425.7166** | **849.4187** | **848.4248** | **0.9939** | **0** | **17** | **3.3** | **1** | **K.IVMNMNK.S** |
|  | 2768 | **441.2950** | **880.5755** | **880.4146** | **0.1608** | **0** | **(2)** | **85** | **10** | **K.IVMNMNK.S** |
|  | 6318 | **570.2564** | **1138.4983** | **1138.6645** | **-0.1662** | **1** | **(2)** | **83** | **3** | **K.HGMKVVLISR.S** |
|  | 6576 | **578.2687** | **1154.5229** | **1154.6594** | **-0.1365** | **1** | **10** | **17** | **3** | **K.HGMKVVLISR.S** |
|  | 7550 | **604.8043** | **1207.5940** | **1208.6005** | **-1.0066** | **1** | **1** | **1.1e+002** | **7** | **K.IVMNMNKSTR.A** |
|  | 10805 | **687.3635** | **1372.7125** | **1372.7207** | **-0.0082** | **0** | **44** | **0.0054** | **1** | **K.MTQLVLPGMVER.S** 10772 10804 |
|  | 11344 | **469.2223** | **1404.6451** | **1404.7105** | **-0.0654** | **0** | **(8)** | **19** | **3** | **K.MTQLVLPGMVER.S** |
|  | 14400 | **793.8763** | **1585.7380** | **1585.7512** | **-0.0132** | **0** | **18** | **2** | **1** | **R.TIAVDFASEDIYDK.I** |
|  | 15506 | **828.4582** | **1654.9018** | **1653.9131** | **0.9888** | **0** | **9** | **13** | **1** | **K.GVFVQSVLPYFVATK.L** 15488 |
|  | 18146 | **914.4820** | **1826.9494** | **1826.9302** | **0.0193** | **1** | **47** | **0.0022** | **1** | **R.TIAVDFASEDIYDKIK.T** 18145 |
|  | 18557 | **464.7681** | **1855.0432** | **1855.0567** | **-0.0135** | **1** | **21** | **0.85** | **1** | **K.IRKPTLDKPSPETFVK.S** 18558 |
|  | 24696 | **1252.1909** | **2502.3673** | **2502.3767** | **-0.0094** | **0** | **108** | **9.7e-010** | **1** | **K.GAILNISSGSGMLPVPLLTIYSATK.T** 24695 |
|  | 26151 | **906.8370** | **2717.4891** | **2717.5037** | **-0.0146** | **1** | **19** | **0.68** | **1** | **R.SKGAILNISSGSGMLPVPLLTIYSATK.T** 26152 |
|  | 29597 | **888.4337** | **3549.7058** | **3549.8549** | **-0.1492** | **1** | **3** | **18** | **3** | **R.TNGYLIHALMGSIISNLPSWIYLKIVMNMNK.S** |
|  | 29847 | 1220.5670 | 3658.6792 | 3659.8108 | -1.1315 | 1 | 0 | 33 | 9 | R.VWGVGNEAGVGPGLGEWAVVTGSTDGIGKSYAEELAK.H |
|  | 30598 | **1367.0310** | **4098.0712** | **4098.0911** | **-0.0199** | **1** | **76** | **6.3e-007** | **1** | **K.TGLAGLEIGILVNNVGMSYEYPEYFLDVPDLDNVIKK.M** |

| **152.** | P10809\|CH60_HUMAN    **Mass:** 61187    **Score:** 250    **Queries matched:** 37   **emPAI:** 0.43 |
| --- | --- |
|  | 60 kDa heat shock protein, mitochondrial OS=Homo sapiens GN=HSPD1 PE=1 SV=2 |

|  | **Query** | **Observed** | **Mr(expt)** | **Mr(calc)** | **Delta** | **Miss** | **Score** | **Expect** | **Rank** | **Peptide** |
| --- | --- | --- | --- | --- | --- | --- | --- | --- | --- | --- |
|  | 778 | **503.1039** | **502.0966** | **501.2911** | **0.8055** | **0** | **3** | **2.1e+002** | **6** | **K.NIGAK.L** |
|  | 1561 | **397.4029** | **792.7912** | **792.4494** | **0.3418** | **1** | **6** | **30** | **1** | **K.YKNIGAK.L** |
|  | 2074 | **417.2359** | **832.4572** | **832.3828** | **0.0745** | **0** | **7** | **37** | **3** | **K.APGFGDNR.K** |
|  | 2257 | **423.0518** | **844.0890** | **843.4888** | **0.6002** | **0** | **5** | **42** | **4** | **K.IPAMTIAK.N** |
|  | 2391 | **428.6752** | **855.3358** | **854.4610** | **0.8748** | **0** | **3** | **87** | **8** | **K.GANPVEIR.R** |
|  | 2975 | **450.7583** | **899.5020** | **900.5280** | **-1.0260** | **0** | **6** | **40** | **8** | **K.LSDGVAVLK.V** |
|  | 3163 | **457.2163** | **912.4179** | **911.5804** | **0.8375** | **0** | **53** | **0.0008** | **1** | **K.VGLQVVAVK.A** 3153 |
|  | 3205 | **458.2942** | **914.5739** | **915.5389** | **-0.9651** | **1** | **12** | **10** | **8** | **K.GVITVKDGK.T** |
|  | 3564 | **471.5981** | **941.1817** | **940.6069** | **0.5748** | **1** | **1** | **90** | **9** | **K.IGIEIIKR.T** |
|  | 3839 | 480.7814 | 959.5483 | 959.5036 | 0.0447 | 0 | 19 | 2.6 | 2 | R.VTDALNATR.A |
|  | 4639 | **508.9778** | **1015.9411** | **1016.5866** | **-0.6455** | **1** | **3** | **59** | **4** | **K.VTKDGVTVAK.S** |
|  | 6979 | **395.2063** | **1182.5972** | **1182.6720** | **-0.0749** | **1** | **9** | **20** | **3** | **K.ISKGANPVEIR.R** |
|  | 7146 | **595.8184** | **1189.6223** | **1189.6012** | **0.0210** | **0** | **6** | **40** | **8** | **K.EIGNIISDAMK.K** |
|  | 7433 | 602.2789 | 1202.5433 | 1201.7104 | 0.8329 | 1 | 7 | 31 | 5 | R.TLKIPAMTIAK.N |
|  | 7698 | **406.0259** | **1215.0559** | **1214.6507** | **0.4053** | **0** | **6** | **30** | **2** | **K.NAGVEGSLIVEK.I** |
|  | 13080 | **753.3795** | **1504.7444** | **1503.7490** | **0.9953** | **0** | **51** | **0.00099** | **1** | **K.TLNDELEIIEGMK.F** |
|  | 13943 | **778.9536** | **1555.8927** | **1555.9007** | **-0.0081** | **1** | **25** | **0.42** | **1** | **R.GVMLAVDAVIAELKK.Q** |
|  | 14184 | **786.9418** | **1571.8690** | **1571.8956** | **-0.0267** | **1** | **(4)** | **54** | **3** | **R.GVMLAVDAVIAELKK.Q** 14178 |
|  | 15970 | 842.9498 | 1683.8850 | 1683.8978 | -0.0128 | 0 | 32 | 0.063 | 1 | R.AAVEEGIVLGGGCALLR.C |
|  | 16309 | **569.7489** | **1706.2249** | **1705.6027** | **0.6221** | **0** | **7** | **16** | **1** | **K.DPGMGAMGGMGGGMGGGMF.-** |
|  | 16557 | **574.9318** | **1721.7735** | **1721.5977** | **0.1758** | **0** | **(5)** | **35** | **4** | **K.DPGMGAMGGMGGGMGGGMF.-** |
|  | 16570 | **575.1019** | **1722.2838** | **1721.5977** | **0.6861** | **0** | **(2)** | **46** | **4** | **K.DPGMGAMGGMGGGMGGGMF.-** |
|  | 16629 | **576.2997** | **1725.8774** | **1725.9526** | **-0.0752** | **1** | **2** | **69** | **5** | **K.VGLQVVAVKAPGFGDNR.K** 16630 |
|  | 16804 | **869.4706** | **1736.9267** | **1737.5926** | **-0.6658** | **0** | **(4)** | **45** | **2** | **K.DPGMGAMGGMGGGMGGGMF.-** |
|  | 16818 | **580.3048** | **1737.8926** | **1737.5926** | **0.3000** | **0** | **(3)** | **56** | **3** | **K.DPGMGAMGGMGGGMGGGMF.-** |
|  | 17337 | **593.1526** | **1776.4361** | **1775.9941** | **0.4420** | **1** | **6** | **19** | **2** | **R.QMRPVSRVLAPHLTR.A** |
|  | 19322 | 960.0452 | 1918.0759 | 1918.0636 | 0.0123 | 0 | 3 | 48 | 2 | K.ISSIQSIVPALEIANAHR.K |
|  | 21362 | **698.3461** | **2092.0164** | **2091.7829** | **0.2335** | **1** | **2** | **56** | **4** | **K.EEKDPGMGAMGGMGGGMGGGMF.-** |
|  | 21575 | **705.0482** | **2112.1228** | **2112.1323** | **-0.0094** | **0** | **24** | **0.38** | **1** | **R.ALMLQGVDLLADAVAVTMGPK.G** |
|  | 23742 | **592.3132** | **2365.2236** | **2364.3264** | **0.8972** | **0** | **2** | **48** | **4** | **R.KPLVIIAEDVDGEALSTLVLNR.L** |
|  | 25150 | **852.7430** | **2555.2073** | **2555.0866** | **0.1207** | **0** | **0** | **68** | **2** | **K.IMQSSSEVGYDAMAGDFVNMVEK.G** |
|  | 25166 | **1280.6201** | **2559.2257** | **2559.2413** | **-0.0156** | **0** | **121** | **5.4e-011** | **1** | **K.LVQDVANNTNEEAGDGTTTATVLAR.S** 25164 25167 |

| **153.** | Q15084\|PDIA6_HUMAN    **Mass:** 48490    **Score:** 250    **Queries matched:** 24   **emPAI:** 0.68 |
| --- | --- |
|  | Protein disulfide-isomerase A6 OS=Homo sapiens GN=PDIA6 PE=1 SV=1 |

|  | **Query** | **Observed** | **Mr(expt)** | **Mr(calc)** | **Delta** | **Miss** | **Score** | **Expect** | **Rank** | **Peptide** |
| --- | --- | --- | --- | --- | --- | --- | --- | --- | --- | --- |
|  | 1217 | **665.3567** | **664.3494** | **664.3544** | **-0.0050** | **0** | **28** | **0.31** | **1** | **K.IFGSNK.N** |
|  | 3460 | **467.2546** | **932.4946** | **932.5039** | **-0.0093** | **1** | **31** | **0.14** | **1** | **R.TRSDIVSR.A** |
|  | 4626 | **508.3077** | **1014.6009** | **1014.6073** | **-0.0065** | **1** | **18** | **2.6** | **2** | **K.AATALKDVVK.V** 4606 |
|  | 5474 | **539.2805** | **1076.5464** | **1077.6070** | **-1.0606** | **0** | **26** | **0.43** | **1** | **R.NSYLEVLLK.L** |
|  | 6492 | **575.8831** | **1149.7516** | **1150.4891** | **-0.7375** | **0** | **1** | **1.1e+002** | **7** | **K.GESPVDYDGGR.T** |
|  | 7700 | **406.1897** | **1215.5473** | **1214.6507** | **0.8966** | **1** | **7** | **35** | **2** | **K.DVVKVGAVDADK.H** 7679 7683 |
|  | 11038 | **693.8793** | **1385.7440** | **1385.7514** | **-0.0074** | **0** | **77** | **2.9e-006** | **1** | **R.TGEAIVDAALSALR.Q** 11040 |
|  | 11411 | **470.5636** | **1408.6689** | **1407.6379** | **1.0310** | **1** | **7** | **24** | **1** | **K.GESPVDYDGGRTR.S** |
|  | 13455 | **764.4238** | **1526.8330** | **1526.8416** | **-0.0087** | **0** | **23** | **0.64** | **1** | **K.LAAVDATVNQVLASR.Y** |
|  | 15700 | **834.3983** | **1666.7821** | **1666.7951** | **-0.0130** | **1** | **42** | **0.007** | **1** | **K.IFQKGESPVDYDGGR.T** |
|  | 20353 | 1001.0119 | 2000.0092 | 1999.9850 | 0.0242 | 1 | 27 | 0.2 | 2 | K.NLEPEWAAAASEVKEQTK.G 20349 |
|  | 23962 | 801.0283 | 2400.0630 | 2400.1068 | -0.0438 | 0 | 1 | 56 | 4 | R.DGELPVEDDIDLSDVELDDLGK.D |
|  | 25294 | **861.1031** | **2580.2874** | **2580.3085** | **-0.0211** | **1** | **58** | **0.00011** | **1** | **K.VGAVDADKHHSLGGQYGVQGFPTIK.I** 25295 |
|  | 25584 | **1319.1786** | **2636.3426** | **2636.3585** | **-0.0158** | **0** | **61** | **5.2e-005** | **1** | **R.ALDLFSDNAPPPELLEIINEDIAK.R** |
|  | 25629 | **882.7707** | **2645.2902** | **2645.3054** | **-0.0152** | **0** | **52** | **0.00041** | **1** | **R.TCEEHQLCVVAVLPHILDTGAAGR.N** |
|  | 26341 | **920.4621** | **2758.3645** | **2757.2604** | **1.1041** | **1** | **3** | **29** | **2** | **R.DGELPVEDDIDLSDVELDDLGKDEL.-** |
|  | 26535 | **931.8232** | **2792.4477** | **2792.4596** | **-0.0119** | **1** | **49** | **0.00084** | **1** | **R.ALDLFSDNAPPPELLEIINEDIAKR.T** 26536 |

| **154.** | P30462\|1B14_HUMAN    **Mass:** 40675    **Score:** 248    **Queries matched:** 34   **emPAI:** 0.86 |
| --- | --- |
|  | HLA class I histocompatibility antigen, B-14 alpha chain OS=Homo sapiens GN=HLA-B PE=1 SV=1 |

|  | **Query** | **Observed** | **Mr(expt)** | **Mr(calc)** | **Delta** | **Miss** | **Score** | **Expect** | **Rank** | **Peptide** |
| --- | --- | --- | --- | --- | --- | --- | --- | --- | --- | --- |
|  | 1309 | 702.8578 | 701.8505 | 701.3894 | 0.4611 | 0 | 10 | 18 | 2 | M.LVMAPR.T 1307 1311 |
|  | 1409 | 746.1623 | 745.1551 | 744.3766 | 0.7785 | 0 | 12 | 10 | 6 | R.EAEQLR.A |
|  | 1446 | 763.1124 | 762.1051 | 762.3694 | -0.2643 | 0 | 2 | 96 | 3 | R.NTQICK.T |
|  | 3888 | 482.7234 | 963.4322 | 964.4250 | -0.9929 | 0 | 33 | 0.088 | 1 | R.FDSDAASPR.E 3900 3901 3907 |
|  | 6565 | 577.7809 | 1153.5473 | 1153.6091 | -0.0618 | 1 | 3 | 67 | 9 | K.ETLQRADPPK.T |
|  | 10029 | 666.8338 | 1331.6530 | 1331.6622 | -0.0092 | 0 | 17 | 3.1 | 1 | R.YFYTSVSRPGR.G |
|  | 11736 | 714.3626 | 1426.7105 | 1426.7205 | -0.0099 | 0 | 46 | 0.0031 | 1 | K.WAAVVVPSGEEQR.Y 11721 11735 11751 |
|  | 12552 | 492.8911 | 1475.6516 | 1475.6641 | -0.0125 | 1 | 13 | 6.8 | 1 | R.FDSDAASPREEPR.A 12553 |
|  | 12921 | 748.8587 | 1495.7028 | 1495.7129 | -0.0101 | 0 | 57 | 0.00026 | 1 | R.AYLEGTCVEWLR.R |
|  | 15320 | 823.4096 | 1644.8047 | 1644.8148 | -0.0101 | 0 | 69 | 1.4e-005 | 1 | R.FISVGYVDDTQFVR.F 15321 |
|  | 18486 | 463.4837 | 1849.9057 | 1849.9183 | -0.0127 | 0 | 16 | 2.9 | 1 | K.THVTHHPISDHEATLR.C 18485 |
|  | 20872 | 513.0196 | 2048.0493 | 2048.0626 | -0.0133 | 0 | 16 | 2.1 | 1 | R.YTCHVQHEGLPKPLTLR.W 20848 20850 20871 |
|  | 21557 | 1056.5175 | 2111.0204 | 2111.0299 | -0.0095 | 0 | 86 | 2.1e-007 | 1 | R.CWALGFYPAEITLTWQR.D 21558 |
|  | 22689 | 744.6669 | 2230.9790 | 2230.9938 | -0.0149 | 0 | 44 | 0.0032 | 1 | R.DGEDQTQDTELVETRPAGDR.T 22688 |
|  | 23097 | 1143.5082 | 2285.0018 | 2286.0341 | -1.0323 | 1 | 11 | 6.4 | 1 | R.EEPRAPWIEQEGPEYWDR.N |
|  | 25366 | 865.0411 | 2592.1016 | 2591.2293 | 0.8723 | 1 | 0 | 56 | 6 | R.FISVGYVDDTQFVRFDSDAASPR.E |
|  | 28130 | 521.6007 | 3123.5606 | 3123.6936 | -0.1330 | 1 | 13 | 2.7 | 1 | M.LVMAPRTVLLLLSAALALTETWAGSHSMR.Y |
|  | 30136 | 1896.3450 | 3790.6754 | 3790.1002 | 0.5752 | 1 | 2 | 20 | 5 | R.WEPSSQSTVPIVGIVAGLAVLAVVVIGAVVAAVMCRR.K |

| **155.** | Q9ULC5\|ACSL5_HUMAN    **Mass:** 76855    **Score:** 246    **Queries matched:** 23   **emPAI:** 0.33 |
| --- | --- |
|  | Long-chain-fatty-acid--CoA ligase 5 OS=Homo sapiens GN=ACSL5 PE=1 SV=1 |

|  | **Query** | **Observed** | **Mr(expt)** | **Mr(calc)** | **Delta** | **Miss** | **Score** | **Expect** | **Rank** | **Peptide** |
| --- | --- | --- | --- | --- | --- | --- | --- | --- | --- | --- |
|  | 1172 | **644.9523** | **643.9450** | **643.4017** | **0.5433** | **1** | **0** | **1.7e+002** | **2** | **K.IIDRK.K** 1174 |
|  | 1218 | **665.3569** | **664.3496** | **664.3544** | **-0.0048** | **0** | **24** | **0.83** | **1** | **K.GTNVFK.G** |
|  | 2264 | **423.2250** | **844.4355** | **844.4403** | **-0.0048** | **0** | **3** | **1e+002** | **4** | **K.IQDSLGGR.V** |
|  | 2721 | **440.0291** | **878.0437** | **878.5225** | **-0.4789** | **1** | **3** | **62** | **6** | **K.LAVSSKFK.E** |
|  | 4377 | **499.8001** | **997.5856** | **998.5185** | **-0.9329** | **1** | **10** | **14** | **4** | **R.GELSKYFR.T** |
|  | 7528 | **604.3140** | **1206.6135** | **1206.6244** | **-0.0109** | **1** | **20** | **1.3** | **1** | **R.IYDKVQNEAK.T** 7551 |
|  | 12304 | **730.7925** | **1459.5704** | **1460.7003** | **-1.1299** | **0** | **(2)** | **81** | **8** | **K.ADIAMVICDTPQK.A** |
|  | 12585 | **739.3595** | **1476.7044** | **1476.6953** | **0.0092** | **0** | **4** | **51** | **1** | **K.ADIAMVICDTPQK.A** |
|  | 12603 | **739.8516** | **1477.6887** | **1477.6984** | **-0.0097** | **0** | **50** | **0.0013** | **1** | **R.GLAVSDNGPCLGYR.K** 12602 |
|  | 13091 | 502.9265 | 1505.7576 | 1505.7667 | -0.0091 | 1 | 2 | 74 | 6 | R.HDSFWDKLIFAK.I |
|  | 13523 | **766.3057** | **1530.5969** | **1530.6079** | **-0.0110** | **0** | **38** | **0.016** | **1** | **K.NNDLTSCCFSDAK.T** |
|  | 14080 | 783.3669 | 1564.7192 | 1564.7304 | -0.0112 | 0 | 50 | 0.0012 | 1 | K.GSFEELCQNQVVR.E |
|  | 16547 | **861.4467** | **1720.8789** | **1720.8883** | **-0.0094** | **0** | **102** | **6.9e-009** | **1** | **K.SGIEILSLYDAENLGK.E** |
|  | 18932 | **943.4955** | **1884.9765** | **1884.9880** | **-0.0114** | **0** | **75** | **3e-006** | **1** | **K.GAMITHQNIVSNAAAFLK.C** 18940 |
|  | 19485 | **644.3907** | **1930.1502** | **1929.9693** | **0.1810** | **0** | **1** | **72** | **5** | **R.VIVTGAAPMSTSVMTFFR.A** |
|  | 20354 | **1001.0429** | **2000.0713** | **2000.0830** | **-0.0118** | **0** | **24** | **0.38** | **1** | **R.SSLVGVVVPDTDVLPSFAAK.L** |
|  | 22314 | **734.3204** | **2199.9395** | **2201.1337** | **-1.1943** | **1** | **2** | **56** | **7** | **R.VRVIVTGAAPMSTSVMTFFR.A** |
|  | 24845 | **843.4222** | **2527.2447** | **2528.2469** | **-1.0022** | **0** | **11** | **5** | **1** | **R.KPVPPSPEDLSVICFTSGTTGDPK.G** |
|  | 28024 | 775.3815 | 3097.4968 | 3097.5179 | -0.0211 | 1 | 2 | 35 | 2 | K.EHFRKPVPPSPEDLSVICFTSGTTGDPK.G |

| **156.** | Q9Y490\|TLN1_HUMAN    **Mass:** 271766   **Score:** 244    **Queries matched:** 58   **emPAI:** 0.06 |
| --- | --- |
|  | Talin-1 OS=Homo sapiens GN=TLN1 PE=1 SV=3 |

|  | **Query** | **Observed** | **Mr(expt)** | **Mr(calc)** | **Delta** | **Miss** | **Score** | **Expect** | **Rank** | **Peptide** |
| --- | --- | --- | --- | --- | --- | --- | --- | --- | --- | --- |
|  | 1171 | **644.9520** | **643.9447** | **643.4268** | **0.5178** | **0** | **0** | **1.7e+002** | **1** | **R.LLGITK.E** |
|  | 1436 | 760.4258 | 759.4185 | 758.4399 | 0.9786 | 1 | 3 | 1.2e+002 | 8 | K.AKSVAQR.T |
|  | 1538 | **792.4239** | **791.4167** | **791.4211** | **-0.0044** | **0** | **12** | **11** | **2** | **K.GITMATAK.A** |
|  | 1672 | **402.2142** | **802.4138** | **801.3981** | **1.0157** | **0** | **14** | **10** | **4** | **R.DVDNALR.A** |
|  | 1689 | **402.7194** | **803.4242** | **804.4164** | **-0.9922** | **0** | **7** | **42** | **4** | **R.AIADMLR.A** |
|  | 1749 | **404.7345** | **807.4544** | **806.4286** | **1.0258** | **0** | **9** | **24** | **9** | **R.QFVQSAK.E** |
|  | 2016 | **415.2451** | **828.4757** | **828.5069** | **-0.0312** | **0** | **10** | **20** | **2** | **K.ISIGNVVK.T** |
|  | 3093 | **454.7279** | **907.4413** | **907.4321** | **0.0092** | **0** | **11** | **12** | **1** | **K.TIMVDDSK.T** |
|  | 3555 | **471.2531** | **940.4916** | **939.4774** | **1.0142** | **0** | **1** | **1.1e+002** | **8** | **R.AEASQLGHK.V** |
|  | 3882 | 482.2940 | 962.5735 | 962.5219 | 0.0516 | 1 | 8 | 25 | 6 | K.LITSMRDK.A 3897 |
|  | 3912 | 483.3000 | 964.5854 | 964.4839 | 0.1015 | 0 | 8 | 24 | 3 | K.AHATGAGPAGR.Y |
|  | 4761 | **514.2951** | **1026.5756** | **1026.5934** | **-0.0178** | **1** | **18** | **2.7** | **1** | **K.RLQAAGNAVK.R** |
|  | 5212 | **529.8182** | **1057.6218** | **1056.5927** | **1.0291** | **0** | **22** | **1.1** | **1** | **K.NLGTALAELR.T** 5190 |
|  | 5303 | **533.2609** | **1064.5073** | **1064.5138** | **-0.0065** | **0** | **7** | **31** | **4** | **K.TSTPEDFIR.M** |
|  | 5589 | **543.2067** | **1084.3988** | **1084.6240** | **-0.2253** | **0** | **1** | **1.3e+002** | **9** | **K.GLAGAVSELLR.S** |
|  | 6116 | **561.7769** | **1121.5393** | **1122.4975** | **-0.9583** | **0** | **9** | **21** | **7** | **K.CTQDLGNSTK.A** |
|  | 6997 | **395.2346** | **1182.6819** | **1183.5941** | **-0.9121** | **1** | **10** | **13** | **9** | **R.MTKGITMATAK.A** 6976 |
|  | 7477 | **603.2562** | **1204.4979** | **1204.5077** | **-0.0098** | **0** | **10** | **17** | **1** | **K.HTSALCNSCR.L** |
|  | 7814 | **407.8886** | **1220.6439** | **1221.5626** | **-0.9186** | **0** | **6** | **40** | **4** | **K.ALDGAFTEENR.A** |
|  | 8183 | **414.5554** | **1240.6443** | **1241.6040** | **-0.9597** | **1** | **1** | **1.1e+002** | **10** | **K.FLPSELRDEH.-** |
|  | 8882 | 637.8601 | 1273.7055 | 1274.7095 | -1.0039 | 1 | 4 | 55 | 2 | K.LAQIRQQQYK.F |
|  | 9463 | 653.3022 | 1304.5899 | 1304.7010 | -0.1110 | 0 | 7 | 26 | 7 | K.AIAVTVQEMVTK.S |
|  | 9764 | **660.3881** | **1318.7616** | **1318.7101** | **0.0515** | **1** | **5** | **48** | **2** | **R.LLGITKECVMR.V** |
|  | 11005 | **462.2840** | **1383.8302** | **1384.8289** | **-0.9987** | **1** | **10** | **12** | **2** | **K.LLSAAKILADATAK.M** 11003 11006 11035 |
|  | 11577 | **709.8633** | **1417.7120** | **1417.7235** | **-0.0115** | **0** | **12** | **7.9** | **1** | **K.VLGEAMTGISQNAK.N** |
|  | 11639 | **474.7422** | **1421.2048** | **1420.7959** | **0.4089** | **1** | **6** | **20** | **1** | **K.GISMSSSKLLLAAK.A** |
|  | 11809 | **716.3525** | **1430.6904** | **1430.7803** | **-0.0898** | **1** | **2** | **88** | **4** | **K.ILADATAKMVEAAK.G** |
|  | 12435 | **490.2406** | **1467.7000** | **1467.6664** | **0.0336** | **0** | **5** | **36** | **6** | **K.AGALQCSPSDAYTK.K** |
|  | 13594 | **512.7667** | **1535.2783** | **1535.8096** | **-0.5313** | **1** | **1** | **67** | **7** | **R.QQQYKFLPSELR.D** |
|  | 13640 | 769.8597 | 1537.7048 | 1536.7024 | 1.0024 | 1 | 16 | 2.8 | 2 | K.NCGQMSEIEAKVR.Y 13641 |
|  | 13892 | **518.3221** | **1551.9444** | **1551.7893** | **0.1552** | **0** | **2** | **67** | **3** | **R.EQGVEEHETLLLR.R** |
|  | 15444 | **413.7348** | **1650.9102** | **1651.8161** | **-0.9059** | **1** | **7** | **21** | **5** | **R.MLDGTVKTIMVDDSK.T** |
|  | 17461 | **595.3314** | **1782.9723** | **1781.9272** | **1.0451** | **0** | **5** | **30** | **4** | **R.DLDQASLAAVSQQLAPR.E** |
|  | 17848 | 603.2692 | 1806.7859 | 1806.0614 | 0.7244 | 1 | 6 | 26 | 4 | K.LLLAAKALSTDPAAPNLK.S |
|  | 18391 | 922.9609 | 1843.9073 | 1844.9488 | -1.0415 | 0 | 7 | 20 | 8 | K.MVGGIAQIIAAQEEMLR.K |
|  | 19470 | **644.0005** | **1928.9796** | **1928.9737** | **0.0059** | **1** | **4** | **41** | **3** | **R.EAAEGLRMATNAAAQNAIK.K** |
|  | 19556 | **968.9703** | **1935.9261** | **1934.9109** | **1.0153** | **0** | **1** | **81** | **2** | **K.EADESLNFEEQILEAAK.S** |
|  | 19667 | **649.3256** | **1944.9550** | **1944.9687** | **-0.0136** | **1** | **(2)** | **54** | **6** | **R.EAAEGLRMATNAAAQNAIK.K** |
|  | 20626 | **1014.0232** | **2026.0318** | **2026.0443** | **-0.0125** | **0** | **111** | **7.5e-010** | **1** | **R.LNEAAAGLNQAATELVQASR.G** |
|  | 21271 | **1043.9804** | **2085.9461** | **2086.0914** | **-0.1453** | **1** | **4** | **39** | **1** | **K.EKMVGGIAQIIAAQEEMLR.K** |
|  | 21346 | **697.7205** | **2090.1397** | **2090.1484** | **-0.0087** | **0** | **21** | **0.64** | **1** | **R.GVGAAATAVTQALNELLQHVK.A** |
|  | 21643 | **707.3480** | **2119.0221** | **2118.0813** | **0.9408** | **1** | **(1)** | **73** | **10** | **K.EKMVGGIAQIIAAQEEMLR.K** |
|  | 22210 | **729.3879** | **2185.1420** | **2184.1685** | **0.9735** | **1** | **1** | **65** | **3** | **K.TMLESAGGLIQTARALAVNPR.D** |
|  | 22728 | **745.7197** | **2234.1372** | **2234.1542** | **-0.0170** | **0** | **13** | **3.6** | **1** | **R.LASEAKPAAVAAENEEIGSHIK.H** |
|  | 27775 | **759.3673** | **3033.4401** | **3034.5836** | **-1.1434** | **1** | **1** | **42** | **6** | **R.ELVAQGKVGAIPANALDDGQWSQGLISAAR.M** |
|  | 27796 | 1013.4735 | 3037.3987 | 3036.6217 | 0.7770 | 1 | 2 | 28 | 3 | R.GVGAAATAVTQALNELLQHVKAHATGAGPAGR.Y |
|  | 28173 | **626.7164** | **3128.5458** | **3128.4140** | **0.1317** | **0** | **0** | **44** | **2** | **R.ELLENPVQPINDMSYFGCLDSVMENSK.V** |
|  | 28446 | 1063.4899 | 3187.4478 | 3187.5965 | -0.1487 | 1 | 2 | 27 | 3 | K.ASVPTIQDQASAMQLSQCAKNLGTALAELR.T |
|  | 29006 | **834.4010** | **3333.5749** | **3334.5446** | **-0.9697** | **1** | **0** | **42** | **8** | **R.YDQATDTILTVTENIFSSMGDAGEMVRQAR.I** |
|  | 30652 | **1379.3424** | **4135.0054** | **4135.0280** | **-0.0226** | **0** | **123** | **1.4e-011** | **1** | **R.QNLLQAAGNVGQASGELLQQIGESDTDPHFQDALMQLAK.A** 30653 |

| **157.** | P09110\|THIK_HUMAN    **Mass:** 44834    **Score:** 243    **Queries matched:** 23   **emPAI:** 0.90 |
| --- | --- |
|  | 3-ketoacyl-CoA thiolase, peroxisomal OS=Homo sapiens GN=ACAA1 PE=1 SV=2 |

|  | **Query** | **Observed** | **Mr(expt)** | **Mr(calc)** | **Delta** | **Miss** | **Score** | **Expect** | **Rank** | **Peptide** |
| --- | --- | --- | --- | --- | --- | --- | --- | --- | --- | --- |
|  | 1556 | **397.4023** | **792.7900** | **792.4051** | **0.3849** | **1** | **7** | **23** | **6** | **R.LMEKEK.A** |
|  | 3201 | **458.2710** | **914.5274** | **914.4570** | **0.0705** | **0** | **3** | **85** | **1** | **R.GNPGNITSR.L** 3181 |
|  | 10911 | **690.4128** | **1378.8110** | **1378.8184** | **-0.0074** | **0** | **6** | **35** | **1** | **K.AEELGLPILGVLR.S** 10894 |
|  | 12370 | **488.8959** | **1463.6660** | **1464.8347** | **-1.1687** | **1** | **2** | **73** | **8** | **-.MQRLQVVLGHLR.G** |
|  | 12874 | **747.3784** | **1492.7423** | **1492.7522** | **-0.0099** | **1** | **37** | **0.026** | **1** | **R.EKQDTFALASQQK.A** 12875 |
|  | 20679 | **677.9987** | **2030.9742** | **2030.9877** | **-0.0136** | **1** | **24** | **0.39** | **1** | **K.ARDCLIPMGITSENVAER.F** 20686 |
|  | 21427 | **1050.5651** | **2099.1156** | **2099.1263** | **-0.0107** | **0** | **46** | **0.002** | **1** | **R.IAQFLSDIPETVPLSTVNR.Q** |
|  | 22004 | **1081.5310** | **2161.0475** | **2161.0611** | **-0.0136** | **0** | **109** | **9.6e-010** | **1** | **K.DGSTTAGNSSQVSDGAAAILLAR.R** |
|  | 22268 | **731.7174** | **2192.1304** | **2192.1399** | **-0.0095** | **1** | **48** | **0.0014** | **1** | **R.GGFKDTTPDELLSAVMTAVLK.D** |
|  | 22707 | **1117.5560** | **2233.0975** | **2233.1260** | **-0.0285** | **0** | **26** | **0.2** | **1** | **R.SITVTQDEGIRPSTTMEGLAK.L** 22699 |
|  | 23146 | **764.0737** | **2289.1994** | **2289.1561** | **0.0433** | **1** | **7** | **14** | **2** | **K.KDGSTTAGNSSQVSDGAAAILLAR.R** 23153 |
|  | 23844 | **477.0346** | **2380.1367** | **2380.1515** | **-0.0148** | **1** | **2** | **45** | **6** | **R.DCLIPMGITSENVAERFGISR.E** |
|  | 25454 | **870.3801** | **2608.1186** | **2609.1713** | **-1.0527** | **1** | **1** | **51** | **2** | **K.RAYGVVSMCIGTGMGAAAVFEYPGN.-** |
|  | 25531 | **1313.7139** | **2625.4132** | **2625.4240** | **-0.0108** | **0** | **68** | **1e-005** | **1** | **R.SYAVVGVPPDIMGIGPAYAIPVALQK.A** |
|  | 27305 | **983.4666** | **2947.3780** | **2946.3957** | **0.9823** | **0** | **8** | **8.6** | **1** | **K.AGLTVSDVDIFEINEAFASQAAYCVEK.L** |
|  | 27387 | **988.5053** | **2962.4939** | **2962.5117** | **-0.0178** | **0** | **33** | **0.029** | **1** | **K.DVNLRPEQLGDICVGNVLQPGAGAIMAR.I** |
|  | 29000 | **833.8968** | **3331.5581** | **3330.5986** | **0.9594** | **1** | **2** | **30** | **6** | **R.GPADSGWMPQAAPCLSGAPQASAADVVVVHGRR.T** |

| **158.** | P18859\|ATP5J_HUMAN    **Mass:** 12580    **Score:** 240    **Queries matched:** 9   **emPAI:** 1.99 |
| --- | --- |
|  | ATP synthase-coupling factor 6, mitochondrial OS=Homo sapiens GN=ATP5J PE=1 SV=1 |

|  | **Query** | **Observed** | **Mr(expt)** | **Mr(calc)** | **Delta** | **Miss** | **Score** | **Expect** | **Rank** | **Peptide** |
| --- | --- | --- | --- | --- | --- | --- | --- | --- | --- | --- |
|  | 2576 | **434.8812** | **867.7478** | **867.4926** | **0.2551** | **0** | **4** | **38** | **6** | **R.SAVSVHLR.R** |
|  | 5239 | **531.0914** | **1060.1683** | **1059.5600** | **0.6083** | **0** | **1** | **93** | **3** | **K.FEVIEKPQA.-** |
|  | 9174 | **430.8984** | **1289.6734** | **1288.7252** | **0.9482** | **1** | **6** | **40** | **3** | **R.RNIGVTAVAFNK.E** |
|  | 15868 | **839.4267** | **1676.8388** | **1675.8457** | **0.9931** | **1** | **28** | **0.17** | **1** | **K.FEDPKFEVIEKPQA.-** 15825 |
|  | 16975 | **874.8872** | **1747.7597** | **1747.7698** | **-0.0101** | **0** | **31** | **0.077** | **1** | **K.QMFGNADMNTFPTFK.F** |
|  | 21217 | **1040.9675** | **2079.9205** | **2079.9345** | **-0.0140** | **0** | **116** | **2e-010** | **1** | **R.QTSGGPVDASSEYQQELER.E** 21218 |
|  | 22744 | **746.3477** | **2236.0212** | **2236.0356** | **-0.0144** | **1** | **47** | **0.0015** | **1** | **K.RQTSGGPVDASSEYQQELER.E** |

| **159.** | Q29960\|1C16_HUMAN    **Mass:** 41240    **Score:** 239    **Queries matched:** 28   **emPAI:** 0.84 |
| --- | --- |
|  | HLA class I histocompatibility antigen, Cw-16 alpha chain OS=Homo sapiens GN=HLA-C PE=2 SV=1 |

|  | **Query** | **Observed** | **Mr(expt)** | **Mr(calc)** | **Delta** | **Miss** | **Score** | **Expect** | **Rank** | **Peptide** |
| --- | --- | --- | --- | --- | --- | --- | --- | --- | --- | --- |
|  | 3888 | 482.7234 | 963.4322 | 964.4250 | -0.9929 | 0 | 33 | 0.088 | 1 | R.FDSDAASPR.G 3900 3901 3907 |
|  | 8237 | 415.2272 | 1242.6598 | 1242.6357 | 0.0241 | 0 | 11 | 12 | 1 | K.DYIALNEHLR.S 8251 |
|  | 9722 | 659.3238 | 1316.6331 | 1315.6673 | 0.9658 | 0 | 5 | 45 | 8 | R.YFYTAVSRPGR.G |
|  | 10335 | 675.8494 | 1349.6843 | 1349.6939 | -0.0096 | 1 | 38 | 0.024 | 1 | R.YLENGKETLQR.A 10311 10351 |
|  | 11313 | 468.5608 | 1402.6605 | 1403.6429 | -0.9825 | 1 | 8 | 22 | 2 | R.FDSDAASPRGEPR.A |
|  | 11736 | 714.3626 | 1426.7105 | 1426.7205 | -0.0099 | 0 | 46 | 0.0031 | 1 | K.WAAVVVPSGEEQR.Y 11721 11735 11751 |
|  | 12880 | **498.7321** | **1493.1744** | **1492.6940** | **0.4804** | **0** | **1** | **60** | **8** | **R.SCTAADTAAQITQR.K** |
|  | 12921 | 748.8587 | 1495.7028 | 1495.7129 | -0.0101 | 0 | 57 | 0.00026 | 1 | R.AYLEGTCVEWLR.R |
|  | 14969 | **541.1163** | **1620.3270** | **1620.7890** | **-0.4620** | **1** | **6** | **21** | **3** | **R.SCTAADTAAQITQRK.W** |
|  | 20871 | 410.6170 | 2048.0485 | 2049.0102 | -0.9617 | 0 | 9 | 11 | 2 | R.YTCHVQHEGLPEPLTLR.W 20872 20886 20897 |
|  | 21557 | 1056.5175 | 2111.0204 | 2111.0299 | -0.0095 | 0 | 86 | 2.1e-007 | 1 | R.CWALGFYPAEITLTWQR.D 21558 |
|  | 25578 | 1319.0889 | 2636.1632 | 2636.1838 | -0.0206 | 0 | 57 | 0.00013 | 1 | R.DGEDQTQDTELVETRPAGDGTFQK.W 25579 25580 25581 |

| **160.** | P60033\|CD81_HUMAN    **Mass:** 26476    **Score:** 238    **Queries matched:** 6   **emPAI:** 0.49 |
| --- | --- |
|  | CD81 antigen OS=Homo sapiens GN=CD81 PE=1 SV=1 |

|  | **Query** | **Observed** | **Mr(expt)** | **Mr(calc)** | **Delta** | **Miss** | **Score** | **Expect** | **Rank** | **Peptide** |
| --- | --- | --- | --- | --- | --- | --- | --- | --- | --- | --- |
|  | 17354 | **889.4417** | **1776.8689** | **1776.8828** | **-0.0140** | **0** | **31** | **0.094** | **1** | **K.NNLCPSGSNIISNLFK.E** |
|  | 22846 | **1127.0153** | **2252.0160** | **2252.0345** | **-0.0186** | **0** | **132** | **5.5e-012** | **1** | **K.QFYDQALQQAVVDDDANNAK.A** 22845 |
|  | 24919 | **1271.6040** | **2541.1934** | **2541.2091** | **-0.0157** | **0** | **54** | **0.0003** | **1** | **K.TFHETLDCCGSSTLTALTTSVLK.N** 24920 24921 |

| **161.** | O00159\|MYO1C_HUMAN    **Mass:** 122503   **Score:** 238    **Queries matched:** 45   **emPAI:** 0.39 |
| --- | --- |
|  | Myosin-Ic OS=Homo sapiens GN=MYO1C PE=1 SV=3 |

|  | **Query** | **Observed** | **Mr(expt)** | **Mr(calc)** | **Delta** | **Miss** | **Score** | **Expect** | **Rank** | **Peptide** |
| --- | --- | --- | --- | --- | --- | --- | --- | --- | --- | --- |
|  | 639 | **458.2336** | **457.2263** | **458.2601** | **-1.0338** | **0** | **8** | **39** | **4** | **R.GGAVR.D** 641 |
|  | 2522 | **432.7419** | **863.4693** | **863.4752** | **-0.0060** | **0** | **3** | **95** | **3** | **K.AVASEIFK.G** |
|  | 4053 | **488.2793** | **974.5441** | **974.4458** | **0.0984** | **0** | **10** | **19** | **3** | **K.DVESPSWR.S** 4043 |
|  | 4205 | **494.7566** | **987.4987** | **986.5033** | **0.9954** | **0** | **27** | **0.38** | **1** | **R.LGTDEISPR.V** |
|  | 4442 | **501.5708** | **1001.1270** | **1000.5566** | **0.5704** | **1** | **11** | **11** | **2** | **K.WAAQTIRR.L** |
|  | 4711 | 512.2675 | 1022.5205 | 1023.4734 | -0.9529 | 1 | 9 | 19 | 1 | R.NDNSSRFGK.Y 4724 |
|  | 5149 | **527.7838** | **1053.5531** | **1053.5607** | **-0.0076** | **1** | **11** | **13** | **1** | **R.KYEAFLQR.Y** |
|  | 5428 | **537.7393** | **1073.4640** | **1073.6081** | **-0.1441** | **0** | **5** | **59** | **9** | **R.LLSVEGSTLR.E** |
|  | 5491 | **539.7785** | **1077.5424** | **1077.5488** | **-0.0064** | **0** | **52** | **0.001** | **1** | **R.VTMESALTAR.D** |
|  | 5666 | **545.8132** | **1089.6119** | **1089.6182** | **-0.0063** | **0** | **45** | **0.0056** | **1** | **K.YLGLLENLR.V** |
|  | 5716 | **547.7754** | **1093.5363** | **1093.5437** | **-0.0074** | **0** | **(8)** | **24** | **4** | **R.VTMESALTAR.D** |
|  | 6055 | **559.8342** | **1117.6538** | **1117.5689** | **0.0849** | **0** | **3** | **92** | **10** | **K.IICDLVEEK.F** |
|  | 7150 | **596.2355** | **1190.4565** | **1191.5271** | **-1.0705** | **0** | **1** | **1.1e+002** | **7** | **K.YMDVQFDFK.G** |
|  | 7284 | **599.2794** | **1196.5442** | **1196.5529** | **-0.0087** | **1** | **34** | **0.064** | **1** | **R.NLKETMCSSK.N** 7283 |
|  | 8335 | 624.3159 | 1246.6172 | 1245.6829 | 0.9342 | 1 | 2 | 96 | 6 | K.NNDLLFRNLK.E |
|  | 8661 | **632.3178** | **1262.6209** | **1262.6295** | **-0.0086** | **0** | **31** | **0.12** | **1** | **R.HLGYKPEEYK.M** |
|  | 8957 | **639.8155** | **1277.6164** | **1277.6252** | **-0.0087** | **1** | **29** | **0.19** | **1** | **K.VSSINDKSDWK.V** |
|  | 9452 | **652.8645** | **1303.7144** | **1303.7248** | **-0.0104** | **1** | **21** | **1.2** | **1** | **K.KRPETVATQFK.M** |
|  | 10231 | 448.9001 | 1343.6783 | 1344.7877 | -1.1094 | 1 | 3 | 63 | 9 | K.YLGLLENLRVR.R |
|  | 10320 | **450.5858** | **1348.7354** | **1348.6769** | **0.0585** | **1** | **(1)** | **1.2e+002** | **8** | **R.VTMESALTARDR.V** |
|  | 10334 | **675.8352** | **1349.6559** | **1348.6293** | **1.0266** | **0** | **7** | **32** | **4** | **R.DQAVMISGESGAGK.T** |
|  | 10636 | **683.3655** | **1364.7165** | **1364.6242** | **0.0923** | **0** | **(1)** | **1.1e+002** | **1** | **R.DQAVMISGESGAGK.T** |
|  | 10649 | **456.2016** | **1365.5829** | **1364.6718** | **0.9111** | **1** | **9** | **18** | **4** | **R.VTMESALTARDR.V** |
|  | 10992 | **462.1445** | **1383.4116** | **1382.7088** | **0.7027** | **1** | **2** | **58** | **4** | **R.CIKPNDAKQPGR.F** |
|  | 12353 | 488.2793 | 1461.8162 | 1460.7259 | 1.0902 | 1 | 9 | 18 | 2 | R.SLASKDVESPSWR.S |
|  | 13026 | **751.4334** | **1500.8523** | **1500.8585** | **-0.0063** | **0** | **54** | **0.00047** | **1** | **K.MSLLQLVEILQSK.E** |
|  | 13632 | **513.2758** | **1536.8056** | **1536.8875** | **-0.0819** | **0** | **3** | **61** | **5** | **M.ALQVELVPTGEIIR.V** |
|  | 14616 | **800.9340** | **1599.8534** | **1599.8620** | **-0.0087** | **0** | **58** | **0.00018** | **1** | **R.LLQSNPVLEAFGNAK.T** |
|  | 14997 | **812.3030** | **1622.5914** | **1622.7875** | **-0.1961** | **1** | **1** | **83** | **9** | **R.HLGYKPEEYKMGR.T** |
|  | 15564 | **553.9438** | **1658.8095** | **1659.8402** | **-1.0307** | **1** | **12** | **7.1** | **1** | **K.EPAYVRCIKPNDAK.Q** |
|  | 15664 | **832.8964** | **1663.7783** | **1662.8730** | **0.9053** | **0** | **3** | **56** | **2** | **R.NVLDTSWPTPPPALR.E** |
|  | 15701 | **556.6014** | **1666.7825** | **1667.9280** | **-1.1455** | **0** | **(2)** | **75** | **7** | **-.MALQVELVPTGEIIR.V** |
|  | 15974 | **842.9838** | **1683.9530** | **1683.9229** | **0.0300** | **0** | **4** | **41** | **9** | **-.MALQVELVPTGEIIR.V** |
|  | 16115 | **847.9087** | **1693.8028** | **1693.8134** | **-0.0105** | **0** | **63** | **4.9e-005** | **1** | **R.LLQFYAETCPAPER.G** |
|  | 16290 | **569.3038** | **1704.8895** | **1703.9206** | **0.9689** | **1** | **2** | **73** | **9** | **R.LFISTRLGTDEISPR.V** |
|  | 16304 | **853.9409** | **1705.8673** | **1705.8774** | **-0.0102** | **0** | **22** | **0.67** | **1** | **R.DGTIDFTPGSELLITK.A** 16305 |
|  | 18781 | **937.4810** | **1872.9474** | **1872.9581** | **-0.0107** | **0** | **76** | **2.3e-006** | **1** | **K.GEELLSPLNLEQAAYAR.D** |
|  | 23597 | 588.3026 | 2349.1811 | 2350.1706 | -0.9895 | 1 | 3 | 39 | 6 | R.NFHIFYQLLEGGEEETLRR.L |
|  | 25373 | **865.7720** | **2594.2941** | **2594.3064** | **-0.0123** | **0** | **5** | **19** | **1** | **K.SLCPETWPTWAGRPQDGVAVLVR.H** |
|  | 26155 | **907.1284** | **2718.3634** | **2718.3786** | **-0.0151** | **0** | **9** | **7.8** | **1** | **K.GIISILDEECLRPGEATDLTFLEK.L** |

| **162.** | P05534\|1A24_HUMAN    **Mass:** 40948    **Score:** 236    **Queries matched:** 30   **emPAI:** 1.02 |
| --- | --- |
|  | HLA class I histocompatibility antigen, A-24 alpha chain OS=Homo sapiens GN=HLA-A PE=1 SV=2 |

|  | **Query** | **Observed** | **Mr(expt)** | **Mr(calc)** | **Delta** | **Miss** | **Score** | **Expect** | **Rank** | **Peptide** |
| --- | --- | --- | --- | --- | --- | --- | --- | --- | --- | --- |
|  | 1838 | 408.2467 | 814.4788 | 813.3729 | 1.1059 | 0 | 4 | 80 | 3 | K.AHSQTDR.E |
|  | 4176 | 493.2762 | 984.5379 | 983.5876 | 0.9503 | 1 | 10 | 18 | 10 | R.ENLRIALR.Y 4164 |
|  | 4359 | 499.2580 | 996.5014 | 995.4308 | 1.0706 | 0 | 8 | 25 | 1 | R.FDSDAASQR.M |
|  | 7402 | 601.3879 | 1200.7613 | 1200.5200 | 0.2414 | 0 | 11 | 12 | 1 | R.GYHQYAYDGK.D |
|  | 10335 | 675.8494 | 1349.6843 | 1349.6939 | -0.0096 | 1 | 38 | 0.024 | 1 | R.YLENGKETLQR.T 10311 10351 |
|  | 11736 | 714.3626 | 1426.7105 | 1426.7205 | -0.0099 | 0 | 46 | 0.0031 | 1 | K.WAAVVVPSGEEQR.Y 11721 11735 11751 |
|  | 13404 | 508.9781 | 1523.9124 | 1524.6627 | -0.7502 | 1 | 4 | 52 | 6 | R.FDSDAASQRMEPR.A |
|  | 13806 | 775.3702 | 1548.7259 | 1548.7718 | -0.0459 | 1 | 44 | 0.0053 | 1 | R.SWTAADMAAQITKR.K |
|  | 14092 | 522.7712 | 1565.2917 | 1564.7668 | 0.5250 | 1 | (8) | 12 | 3 | R.SWTAADMAAQITKR.K |
|  | 18889 | 628.3002 | 1881.8787 | 1881.8904 | -0.0117 | 0 | 24 | 0.42 | 1 | K.THMTHHPISDHEATLR.C |
|  | 19153 | 635.6392 | 1903.8958 | 1903.9104 | -0.0146 | 1 | 29 | 0.12 | 1 | R.GYHQYAYDGKDYIALK.E |
|  | 20872 | 513.0196 | 2048.0493 | 2048.0626 | -0.0133 | 0 | 16 | 2.1 | 1 | R.YTCHVQHEGLPKPLTLR.W 20848 20850 20871 |
|  | 21557 | 1056.5175 | 2111.0204 | 2111.0299 | -0.0095 | 0 | 86 | 2.1e-007 | 1 | R.CWALGFYPAEITLTWQR.D 21558 |
|  | 25578 | 1319.0889 | 2636.1632 | 2636.1838 | -0.0206 | 0 | 57 | 0.00013 | 1 | R.DGEDQTQDTELVETRPAGDGTFQK.W 25579 25580 25581 |
|  | 28413 | 796.6549 | 3182.5903 | 3181.6926 | 0.8977 | 1 | (2) | 31 | 6 | -.MAVMAPRTLVLLLSGALALTQTWAGSHSMR.Y |
|  | 28476 | 533.7578 | 3196.5032 | 3197.6875 | -1.1843 | 1 | (0) | 46 | 8 | -.MAVMAPRTLVLLLSGALALTQTWAGSHSMR.Y |
|  | 28578 | 804.6039 | 3214.3867 | 3213.6824 | 0.7042 | 1 | 4 | 16 | 2 | -.MAVMAPRTLVLLLSGALALTQTWAGSHSMR.Y |

| **163.** | P30447\|1A23_HUMAN    **Mass:** 40992    **Score:** 236    **Queries matched:** 31   **emPAI:** 1.02 |
| --- | --- |
|  | HLA class I histocompatibility antigen, A-23 alpha chain OS=Homo sapiens GN=HLA-A PE=1 SV=1 |

|  | **Query** | **Observed** | **Mr(expt)** | **Mr(calc)** | **Delta** | **Miss** | **Score** | **Expect** | **Rank** | **Peptide** |
| --- | --- | --- | --- | --- | --- | --- | --- | --- | --- | --- |
|  | 1838 | 408.2467 | 814.4788 | 813.3729 | 1.1059 | 0 | 4 | 80 | 3 | K.AHSQTDR.E |
|  | 4176 | 493.2762 | 984.5379 | 983.5876 | 0.9503 | 1 | 10 | 18 | 10 | R.ENLRIALR.Y 4164 |
|  | 4359 | 499.2580 | 996.5014 | 995.4308 | 1.0706 | 0 | 8 | 25 | 1 | R.FDSDAASQR.M |
|  | 7402 | 601.3879 | 1200.7613 | 1200.5200 | 0.2414 | 0 | 11 | 12 | 1 | R.GYHQYAYDGK.D |
|  | 10335 | 675.8494 | 1349.6843 | 1349.6939 | -0.0096 | 1 | 38 | 0.024 | 1 | R.YLENGKETLQR.T 10311 10351 |
|  | 11736 | 714.3626 | 1426.7105 | 1426.7205 | -0.0099 | 0 | 46 | 0.0031 | 1 | K.WAAVVVPSGEEQR.Y 11721 11735 11751 |
|  | 13404 | 508.9781 | 1523.9124 | 1524.6627 | -0.7502 | 1 | 4 | 52 | 6 | R.FDSDAASQRMEPR.A |
|  | 13806 | 775.3702 | 1548.7259 | 1548.7355 | -0.0095 | 0 | 44 | 0.0053 | 1 | R.SWTAADMAAQITQR.K |
|  | 14092 | 522.7712 | 1565.2917 | 1564.7304 | 0.5613 | 0 | (8) | 12 | 3 | R.SWTAADMAAQITQR.K |
|  | 18889 | 628.3002 | 1881.8787 | 1881.8904 | -0.0117 | 0 | 24 | 0.42 | 1 | K.THMTHHPISDHEATLR.C |
|  | 19153 | 635.6392 | 1903.8958 | 1903.9104 | -0.0146 | 1 | 29 | 0.12 | 1 | R.GYHQYAYDGKDYIALK.E |
|  | 20872 | 513.0196 | 2048.0493 | 2048.0626 | -0.0133 | 0 | 16 | 2.1 | 1 | R.YTCHVQHEGLPKPLTLR.W 20848 20850 20871 |
|  | 20884 | **684.1162** | **2049.3268** | **2049.0313** | **0.2955** | **1** | **1** | **55** | **10** | **R.VAEQLRAYLEGTCVDGLR.R** |
|  | 21557 | 1056.5175 | 2111.0204 | 2111.0299 | -0.0095 | 0 | 86 | 2.1e-007 | 1 | R.CWALGFYPAEITLTWQR.D 21558 |
|  | 25578 | 1319.0889 | 2636.1632 | 2636.1838 | -0.0206 | 0 | 57 | 0.00013 | 1 | R.DGEDQTQDTELVETRPAGDGTFQK.W 25579 25580 25581 |
|  | 28413 | 796.6549 | 3182.5903 | 3181.6926 | 0.8977 | 1 | (2) | 31 | 6 | -.MAVMAPRTLVLLLSGALALTQTWAGSHSMR.Y |
|  | 28476 | 533.7578 | 3196.5032 | 3197.6875 | -1.1843 | 1 | (0) | 46 | 8 | -.MAVMAPRTLVLLLSGALALTQTWAGSHSMR.Y |
|  | 28578 | 804.6039 | 3214.3867 | 3213.6824 | 0.7042 | 1 | 4 | 16 | 2 | -.MAVMAPRTLVLLLSGALALTQTWAGSHSMR.Y |

| **164.** | P06733\|ENOA_HUMAN    **Mass:** 47481    **Score:** 234    **Queries matched:** 20   **emPAI:** 0.58 |
| --- | --- |
|  | Alpha-enolase OS=Homo sapiens GN=ENO1 PE=1 SV=2 |

|  | **Query** | **Observed** | **Mr(expt)** | **Mr(calc)** | **Delta** | **Miss** | **Score** | **Expect** | **Rank** | **Peptide** |
| --- | --- | --- | --- | --- | --- | --- | --- | --- | --- | --- |
|  | 1705 | 805.4183 | 804.4111 | 805.4446 | -1.0336 | 0 | 6 | 50 | 10 | K.YNQLLR.I |
|  | 2623 | **436.8877** | **871.7608** | **871.5127** | **0.2482** | **1** | **5** | **43** | **1** | **R.IAKAVNEK.S** |
|  | 3859 | 481.2626 | 960.5106 | 959.4924 | 1.0183 | 0 | 2 | 1.2e+002 | 8 | K.LNVTEQEK.I |
|  | 4511 | **504.2507** | **1006.4869** | **1006.4940** | **-0.0070** | **0** | **15** | **5.5** | **1** | **K.SCNCLLLK.V** |
|  | 5421 | **537.2977** | **1072.5809** | **1071.5237** | **1.0572** | **1** | **12** | **12** | **4** | **R.SGKYDLDFK.S** |
|  | 6946 | **591.7815** | **1181.5484** | **1180.6564** | **0.8920** | **1** | **3** | **75** | **9** | **K.GVSKAVEHINK.T** |
|  | 8984 | **640.7922** | **1279.5699** | **1279.5788** | **-0.0089** | **0** | **10** | **14** | **2** | **K.LMIEMDGTENK.S** |
|  | 11704 | **713.3620** | **1424.7094** | **1424.7187** | **-0.0093** | **0** | **55** | **0.00037** | **1** | **R.YISPDQLADLYK.S** |
|  | 13686 | **771.4091** | **1540.8036** | **1539.7756** | **1.0280** | **0** | **30** | **0.14** | **1** | **K.VVIGMDVAASEFFR.S** |
|  | 13782 | **774.4282** | **1546.8418** | **1547.7800** | **-0.9382** | **1** | **2** | **75** | **9** | **K.AVNEKSCNCLLLK.V** |
|  | 17806 | **902.9704** | **1803.9262** | **1803.9366** | **-0.0104** | **0** | **88** | **1.9e-007** | **1** | **R.AAVPSGASTGIYEALELR.D** |
|  | 19110 | 951.0167 | 1900.0189 | 1899.9982 | 0.0207 | 1 | 1 | 85 | 3 | R.YISPDQLADLYKSFIK.D |
|  | 20687 | **1017.0247** | **2032.0348** | **2032.0477** | **-0.0129** | **0** | **40** | **0.009** | **1** | **K.FTASAGIQVVGDDLTVTNPK.R** |
|  | 22237 | **730.7196** | **2189.1370** | **2188.1488** | **0.9882** | **1** | **5** | **27** | **3** | **K.FTASAGIQVVGDDLTVTNPKR.I** |
|  | 23295 | **770.7010** | **2309.0811** | **2308.0971** | **0.9840** | **1** | **3** | **41** | **1** | **K.YGKDATNVGDEGGFAPNILENK.E** |
|  | 23624 | **1177.0768** | **2352.1390** | **2352.1519** | **-0.0129** | **0** | **77** | **1.6e-006** | **1** | **R.SGETEDTFIADLVVGLCTGQIK.T** 23627 |
|  | 24039 | **804.4561** | **2410.3465** | **2410.1959** | **0.1506** | **1** | **3** | **32** | **5** | **K.LAMQEFMILPVGAANFREAMR.I** |
|  | 24733 | **1255.5547** | **2509.0948** | **2509.1074** | **-0.0126** | **0** | **67** | **1.3e-005** | **1** | **K.DYPVVSIEDPFDQDDWGAWQK.F** |
|  | 28290 | **1053.0474** | **3156.1203** | **3155.5604** | **0.5598** | **1** | **1** | **25** | **4** | **K.VNQIGSVTESLQACKLAQANGWGVMVSHR.S** |

| **165.** | Q29718\|1B82_HUMAN    **Mass:** 40681    **Score:** 233    **Queries matched:** 32   **emPAI:** 1.03 |
| --- | --- |
|  | HLA class I histocompatibility antigen, B-82 alpha chain OS=Homo sapiens GN=HLA-B PE=2 SV=1 |

|  | **Query** | **Observed** | **Mr(expt)** | **Mr(calc)** | **Delta** | **Miss** | **Score** | **Expect** | **Rank** | **Peptide** |
| --- | --- | --- | --- | --- | --- | --- | --- | --- | --- | --- |
|  | 1455 | 766.4029 | 765.3956 | 765.4021 | -0.0065 | 0 | 37 | 0.038 | 1 | R.NTQIYK.A |
|  | 3888 | 482.7234 | 963.4322 | 964.4250 | -0.9929 | 0 | 33 | 0.088 | 1 | R.FDSDAASPR.E 3900 3901 3907 |
|  | 6565 | 577.7809 | 1153.5473 | 1153.6091 | -0.0618 | 1 | 3 | 67 | 9 | K.ETLQRADPPK.T |
|  | 10335 | 675.8494 | 1349.6843 | 1349.6939 | -0.0096 | 1 | 38 | 0.024 | 1 | R.YLENGKETLQR.A 10311 10351 |
|  | 10618 | **455.7262** | **1364.1569** | **1363.6343** | **0.5226** | **0** | **3** | **50** | **7** | **R.YFYTAMSRPGR.G** |
|  | 11736 | 714.3626 | 1426.7105 | 1426.7205 | -0.0099 | 0 | 46 | 0.0031 | 1 | K.WAAVVVPSGEEQR.Y 11721 11735 11751 |
|  | 12552 | 492.8911 | 1475.6516 | 1475.6641 | -0.0125 | 1 | 13 | 6.8 | 1 | R.FDSDAASPREEPR.A 12553 |
|  | 14680 | **803.3827** | **1604.7508** | **1605.7756** | **-1.0247** | **1** | **2** | **71** | **6** | **R.MFGCDLGPDGRLLR.G** |
|  | 15320 | 823.4096 | 1644.8047 | 1644.8148 | -0.0101 | 0 | 69 | 1.4e-005 | 1 | R.FISVGYVDDTQFVR.F 15321 |
|  | 17808 | 902.9733 | 1803.9321 | 1802.8522 | 1.0799 | 1 | 3 | 60 | 8 | R.YFYTAMSRPGRGEPR.F |
|  | 18486 | 463.4837 | 1849.9057 | 1849.9183 | -0.0127 | 0 | 16 | 2.9 | 1 | K.THVTHHPISDHEATLR.C 18485 |
|  | 20872 | 513.0196 | 2048.0493 | 2048.0626 | -0.0133 | 0 | 16 | 2.1 | 1 | R.YTCHVQHEGLPKPLTLR.W 20848 20850 20871 |
|  | 21557 | 1056.5175 | 2111.0204 | 2111.0299 | -0.0095 | 0 | 86 | 2.1e-007 | 1 | R.CWALGFYPAEITLTWQR.D 21558 |
|  | 22689 | 744.6669 | 2230.9790 | 2230.9938 | -0.0149 | 0 | 44 | 0.0032 | 1 | R.DGEDQTQDTELVETRPAGDR.T 22688 |
|  | 23097 | 1143.5082 | 2285.0018 | 2286.0341 | -1.0323 | 1 | 11 | 6.4 | 1 | R.EEPRAPWIEQEGPEYWDR.N |
|  | 25366 | 865.0411 | 2592.1016 | 2591.2293 | 0.8723 | 1 | 0 | 56 | 6 | R.FISVGYVDDTQFVRFDSDAASPR.E |

| **166.** | Q31612\|1B73_HUMAN    **Mass:** 40809    **Score:** 233    **Queries matched:** 25   **emPAI:** 0.69 |
| --- | --- |
|  | HLA class I histocompatibility antigen, B-73 alpha chain OS=Homo sapiens GN=HLA-B PE=1 SV=1 |

|  | **Query** | **Observed** | **Mr(expt)** | **Mr(calc)** | **Delta** | **Miss** | **Score** | **Expect** | **Rank** | **Peptide** |
| --- | --- | --- | --- | --- | --- | --- | --- | --- | --- | --- |
|  | 1309 | 702.8578 | 701.8505 | 701.3894 | 0.4611 | 0 | 10 | 18 | 2 | M.LVMAPR.T 1307 1311 |
|  | 1446 | 763.1124 | 762.1051 | 762.3694 | -0.2643 | 0 | 2 | 96 | 3 | R.NTQICK.A |
|  | 3888 | 482.7234 | 963.4322 | 964.4250 | -0.9929 | 0 | 33 | 0.088 | 1 | R.FDSDAASPR.E 3900 3901 3907 |
|  | 6565 | 577.7809 | 1153.5473 | 1153.6091 | -0.0618 | 1 | 3 | 67 | 9 | K.ETLQRADPPK.T |
|  | 11721 | 713.8703 | 1425.7260 | 1425.7365 | -0.0104 | 0 | 45 | 0.0043 | 1 | K.WAAVVVPSGQEQR.Y 11735 11736 |
|  | 12552 | 492.8911 | 1475.6516 | 1475.6641 | -0.0125 | 1 | 13 | 6.8 | 1 | R.FDSDAASPREEPR.A 12553 |
|  | 13321 | 760.3871 | 1518.7596 | 1518.7427 | 0.0169 | 0 | 62 | 7.5e-005 | 1 | R.SWTAADTAAQITQR.K |
|  | 18486 | 463.4837 | 1849.9057 | 1849.9183 | -0.0127 | 0 | 16 | 2.9 | 1 | K.THVTHHPISDHEATLR.C 18485 |
|  | 21557 | 1056.5175 | 2111.0204 | 2111.0299 | -0.0095 | 0 | 86 | 2.1e-007 | 1 | R.CWALGFYPAEITLTWQR.D 21558 |
|  | 23097 | 1143.5082 | 2285.0018 | 2286.0341 | -1.0323 | 1 | 11 | 6.4 | 1 | R.EEPRAPWIEQEGPEYWDR.N |
|  | 25578 | 1319.0889 | 2636.1632 | 2636.1838 | -0.0206 | 0 | 57 | 0.00013 | 1 | R.DGEDQTQDTELVETRPAGDGTFQK.W 25579 25580 25581 |
|  | 28130 | 521.6007 | 3123.5606 | 3123.6936 | -0.1330 | 1 | 13 | 2.7 | 1 | M.LVMAPRTVLLLLSAALALTETWAGSHSMR.Y |

| **167.** | P12814\|ACTN1_HUMAN    **Mass:** 103563   **Score:** 233    **Queries matched:** 15   **emPAI:** 0.19 |
| --- | --- |
|  | Alpha-actinin-1 OS=Homo sapiens GN=ACTN1 PE=1 SV=2 |

|  | **Query** | **Observed** | **Mr(expt)** | **Mr(calc)** | **Delta** | **Miss** | **Score** | **Expect** | **Rank** | **Peptide** |
| --- | --- | --- | --- | --- | --- | --- | --- | --- | --- | --- |
|  | 2523 | 432.7420 | 863.4695 | 863.4752 | -0.0057 | 0 | 28 | 0.33 | 1 | K.ALDFIASK.G |
|  | 6853 | 588.3041 | 1174.5937 | 1173.5965 | 0.9973 | 0 | 7 | 32 | 1 | K.EGLLLWCQR.K |
|  | 9373 | 434.5373 | 1300.5900 | 1300.5982 | -0.0082 | 0 | 23 | 0.66 | 1 | K.HTNYTMEHIR.V 9374 |
|  | 11021 | 693.3895 | 1384.7645 | 1385.7667 | -1.0022 | 0 | 74 | 5.6e-006 | 1 | R.VGWEQLLTTIAR.T 11042 |
|  | 11779 | 715.3815 | 1428.7485 | 1428.7572 | -0.0087 | 0 | 31 | 0.12 | 1 | R.TINEVENQILTR.D |
|  | 13600 | 768.8354 | 1535.6562 | 1536.7671 | -1.1109 | 0 | 7 | 23 | 6 | R.FAIQDISVEETSAK.E |
|  | 16386 | **571.2988** | **1710.8747** | **1710.9080** | **-0.0333** | **0** | **24** | **0.41** | **1** | **K.LLETIDQLYLEYAK.R** 16388 16400 |
|  | 18848 | 627.0032 | 1877.9877 | 1876.9829 | 1.0048 | 1 | 5 | 33 | 6 | K.CQLEINFNTLQTKLR.L |
|  | 20905 | **1026.4766** | **2050.9386** | **2050.9517** | **-0.0131** | **0** | **92** | **6.2e-008** | **1** | **K.VLAVNQENEQLMEDYEK.L** |
|  | 26394 | **924.8205** | **2771.4397** | **2771.4139** | **0.0258** | **1** | **1** | **48** | **3** | **R.IMSIVDPNRLGVVTFQAFIDFMSR.E** |
|  | 28702 | **1083.5371** | **3247.5895** | **3247.6070** | **-0.0175** | **1** | **67** | **9.4e-006** | **1** | **K.VLAVNQENEQLMEDYEKLASDLLEWIR.R** |

| **168.** | P21397\|AOFA_HUMAN    **Mass:** 60157    **Score:** 232    **Queries matched:** 33   **emPAI:** 0.43 |
| --- | --- |
|  | Amine oxidase [flavin-containing] A OS=Homo sapiens GN=MAOA PE=1 SV=1 |

|  | **Query** | **Observed** | **Mr(expt)** | **Mr(calc)** | **Delta** | **Miss** | **Score** | **Expect** | **Rank** | **Peptide** |
| --- | --- | --- | --- | --- | --- | --- | --- | --- | --- | --- |
|  | 1186 | **647.3828** | **646.3755** | **646.2922** | **0.0833** | **0** | **14** | **12** | **4** | **M.ENQEK.A** |
|  | 1415 | **748.3610** | **747.3537** | **748.4483** | **-1.0946** | **0** | **10** | **20** | **1** | **R.LVQYVK.G** |
|  | 1477 | **778.3701** | **777.3628** | **777.3691** | **-0.0063** | **0** | **18** | **3.5** | **1** | **R.TIDNMGK.E** |
|  | 1517 | **395.4235** | **788.8324** | **788.4908** | **0.3415** | **1** | **6** | **42** | **2** | **K.YKLLPR.S** 1518 1519 |
|  | 2269 | 423.2554 | 844.4962 | 843.4888 | 1.0074 | 0 | 9 | 31 | 4 | R.LPMGAVIK.C 2256 |
|  | 3150 | **456.7370** | **911.4594** | **910.3387** | **1.1207** | **0** | **1** | **1.3e+002** | **4** | **K.CMMYYK.E** |
|  | 3777 | **478.7629** | **955.5113** | **954.4303** | **1.0810** | **1** | **4** | **68** | **3** | **K.WDKMTMK.E** |
|  | 6115 | **561.7767** | **1121.5389** | **1121.5466** | **-0.0076** | **0** | **62** | **0.00011** | **1** | **K.FVGGSGQVSER.I** 6116 |
|  | 6141 | **562.7696** | **1123.5246** | **1123.5617** | **-0.0371** | **1** | **8** | **23** | **1** | **K.MTMKELIDK.I** |
|  | 7039 | **593.3316** | **1184.6486** | **1184.6077** | **0.0409** | **0** | **5** | **56** | **4** | **R.IFFAGTETATK.W** |
|  | 8402 | **625.8235** | **1249.6325** | **1249.6415** | **-0.0090** | **1** | **42** | **0.0087** | **1** | **R.KFVGGSGQVSER.I** 8381 8420 |
|  | 9118 | **644.3682** | **1286.7218** | **1285.7242** | **0.9976** | **1** | **4** | **56** | **6** | **R.EVLNGLGKVTEK.D** |
|  | 11272 | **700.8711** | **1399.7276** | **1399.8075** | **-0.0799** | **0** | **14** | **4.8** | **1** | **K.YVINAIPPTLTAK.I** |
|  | 13676 | **771.3332** | **1540.6518** | **1540.6616** | **-0.0098** | **0** | **37** | **0.023** | **1** | **K.WSGYMEGAVEAGER.A** |
|  | 15197 | **546.2928** | **1635.8565** | **1635.8468** | **0.0098** | **1** | **6** | **28** | **1** | **K.ELGIETYKVNVSER.L** |
|  | 15603 | 831.4009 | 1660.7873 | 1660.9400 | -0.1526 | 0 | 12 | 7.9 | 1 | K.LLTEYGVSVLVLEAR.D 15587 15608 |
|  | 16009 | **844.4246** | **1686.8347** | **1686.8465** | **-0.0118** | **1** | **60** | **0.00012** | **1** | **K.VTEKDIWVQEPESK.D** 16007 16008 |
|  | 16207 | **850.4203** | **1698.8261** | **1698.8366** | **-0.0104** | **0** | **65** | **3.5e-005** | **1** | **K.DVPAVEITHTFWER.N** 16206 |
|  | 16537 | **860.9603** | **1719.9061** | **1719.8220** | **0.0841** | **1** | **0** | **1e+002** | **2** | **R.LPMGAVIKCMMYYK.E** |
|  | 18257 | **612.6432** | **1834.9077** | **1834.9213** | **-0.0136** | **0** | **6** | **30** | **2** | **K.VLGSQEALHPVHYEEK.N** |
|  | 18298 | **613.8869** | **1838.6389** | **1838.8369** | **-0.1981** | **1** | **6** | **20** | **3** | **K.WSGYMEGAVEAGERAAR.E** |
|  | 22886 | **565.2549** | **2256.9904** | **2256.1936** | **0.7968** | **0** | **1** | **68** | **5** | **K.ASIAGHMFDVVVIGGGISGLSAAK.L** |

| **169.** | Q99623\|PHB2_HUMAN    **Mass:** 33276    **Score:** 232    **Queries matched:** 16   **emPAI:** 0.71 |
| --- | --- |
|  | Prohibitin-2 OS=Homo sapiens GN=PHB2 PE=1 SV=2 |

|  | **Query** | **Observed** | **Mr(expt)** | **Mr(calc)** | **Delta** | **Miss** | **Score** | **Expect** | **Rank** | **Peptide** |
| --- | --- | --- | --- | --- | --- | --- | --- | --- | --- | --- |
|  | 782 | **503.1056** | **502.0983** | **502.3115** | **-0.2132** | **0** | **1** | **3.3e+002** | **3** | **K.SVVAK.F** |
|  | 1244 | **677.3590** | **676.3517** | **676.3578** | **-0.0061** | **0** | **14** | **8.7** | **3** | **R.GMGTALK.L** |
|  | 1358 | **719.3890** | **718.3817** | **718.3861** | **-0.0044** | **0** | **19** | **3** | **2** | **R.GSDSLIK.G** |
|  | 3030 | 452.2382 | 902.4618 | 903.5025 | -1.0408 | 1 | 13 | 9.6 | 2 | R.GSDSLIKGK.K |
|  | 4127 | **491.2447** | **980.4749** | **980.4814** | **-0.0066** | **0** | **21** | **1.3** | **1** | **R.EYTAAVEAK.Q** |
|  | 5190 | 529.3060 | 1056.5974 | 1056.5312 | 0.0662 | 0 | 3 | 92 | 5 | K.QVAQQEAQR.A |
|  | 7589 | **605.8703** | **1209.7260** | **1209.7333** | **-0.0072** | **0** | **1** | **1e+002** | **10** | **R.VLPSIVNEVLK.S** |
|  | 7682 | **608.3102** | **1214.6058** | **1214.6142** | **-0.0084** | **0** | **54** | **0.00057** | **1** | **K.IVQAEGEAEAAK.M** 7668 |
|  | 7728 | **609.2948** | **1216.5750** | **1216.5837** | **-0.0086** | **0** | **31** | **0.13** | **1** | **R.ESVFTVEGGHR.A** |
|  | 8544 | **420.2052** | **1257.5937** | **1258.7397** | **-1.1461** | **0** | **3** | **75** | **8** | **K.LLLGAGAVAYGVR.E** |
|  | 18540 | **618.9977** | **1853.9714** | **1852.9796** | **0.9918** | **0** | **43** | **0.0047** | **1** | **R.IGGVQQDTILAEGLHFR.I** |
|  | 18978 | **944.9816** | **1887.9486** | **1887.9625** | **-0.0139** | **0** | **50** | **0.00096** | **1** | **R.VLSRPNAQELPSMYQR.L** |
|  | 22619 | **1113.0697** | **2224.1248** | **2224.1375** | **-0.0127** | **0** | **107** | **1.9e-009** | **1** | **R.IYLTADNLVLNLQDESFTR.G** 22617 22618 |

| **170.** | P07602\|SAP_HUMAN    **Mass:** 59899    **Score:** 231    **Queries matched:** 28   **emPAI:** 0.35 |
| --- | --- |
|  | Proactivator polypeptide OS=Homo sapiens GN=PSAP PE=1 SV=2 |

|  | **Query** | **Observed** | **Mr(expt)** | **Mr(calc)** | **Delta** | **Miss** | **Score** | **Expect** | **Rank** | **Peptide** |
| --- | --- | --- | --- | --- | --- | --- | --- | --- | --- | --- |
|  | 2107 | **418.2346** | **834.4546** | **834.4599** | **-0.0054** | **0** | **3** | **94** | **6** | **K.LVGYLDR.N** |
|  | 2734 | **440.2951** | **878.5756** | **878.4354** | **0.1403** | **1** | **6** | **49** | **4** | **K.MCSKLPK.S** |
|  | 3895 | 482.7833 | 963.5520 | 962.5549 | 0.9971 | 1 | 24 | 0.72 | 1 | K.KLVGYLDR.N 3880 |
|  | 5240 | **531.2559** | **1060.4973** | **1060.5045** | **-0.0072** | **0** | **50** | **0.0018** | **1** | **R.LGPGMADICK.N** 5241 |
|  | 6270 | **567.7695** | **1133.5244** | **1134.5591** | **-1.0347** | **0** | **7** | **36** | **1** | **K.DVVTAAGDMLK.D** |
|  | 7346 | **600.2587** | **1198.5029** | **1198.5111** | **-0.0082** | **1** | **22** | **0.98** | **1** | **K.DGGFCEVCKK.L** 7359 |
|  | 9559 | **656.3015** | **1310.5885** | **1310.5965** | **-0.0081** | **0** | **25** | **0.4** | **1** | **K.GCSFLPDPYQK.Q** 9558 |
|  | 14184 | 786.9418 | 1571.8690 | 1571.8308 | 0.0382 | 0 | 3 | 59 | 4 | R.TNSTFVQALVEHVK.E |
|  | 14776 | **537.6168** | **1609.8286** | **1609.8399** | **-0.0113** | **0** | **8** | **19** | **1** | **K.HCLQTVWNKPTVK.S** 14775 |
|  | 16693 | **865.4993** | **1728.9840** | **1728.9913** | **-0.0073** | **0** | **59** | **0.00013** | **1** | **K.EIVDSYLPVILDIIK.G** 16694 16695 |
|  | 21519 | **703.6511** | **2107.9315** | **2107.9952** | **-0.0637** | **1** | **3** | **48** | **2** | **K.SLPCDICKDVVTAAGDMLK.D** 21517 21520 |
|  | 24235 | **1222.0591** | **2442.1036** | **2442.1204** | **-0.0168** | **0** | **91** | **6.4e-008** | **1** | **K.NYISQYSEIAIQMMMHMQPK.E** 24236 24239 24240 24249 |
|  | 24371 | **820.4052** | **2458.1938** | **2458.1153** | **0.0785** | **0** | **(1)** | **65** | **2** | **K.NYISQYSEIAIQMMMHMQPK.E** |
|  | 26919 | **955.4451** | **2863.3136** | **2863.5703** | **-0.2568** | **1** | **1** | **48** | **10** | **-.MYALFLLASLLGAALAGPVLGLKECTR.G** |
|  | 29528 | **1174.2523** | **3519.7351** | **3520.7656** | **-1.0304** | **1** | **0** | **37** | **8** | **K.TCDWLPKPNMSASCKEIVDSYLPVILDIIK.G** |

| **171.** | Q99867\|TBB4Q_HUMAN    **Mass:** 48917    **Score:** 228    **Queries matched:** 28   **emPAI:** 0.44 |
| --- | --- |
|  | Putative tubulin beta-4q chain OS=Homo sapiens GN=TUBB4Q PE=5 SV=1 |

|  | **Query** | **Observed** | **Mr(expt)** | **Mr(calc)** | **Delta** | **Miss** | **Score** | **Expect** | **Rank** | **Peptide** |
| --- | --- | --- | --- | --- | --- | --- | --- | --- | --- | --- |
|  | 1748 | 404.7345 | 807.4544 | 807.3399 | 0.1146 | 0 | 2 | 1e+002 | 6 | R.EEYPDR.I |
|  | 5458 | 538.7742 | 1075.5338 | 1076.5250 | -0.9913 | 1 | 21 | 1.4 | 1 | K.IREEYPDR.I 5480 |
|  | 5703 | **547.2711** | **1092.5277** | **1092.4804** | **0.0473** | **1** | **2** | **92** | **8** | **K.NMMAARDPR.H** |
|  | 6379 | 572.3181 | 1142.6217 | 1142.6270 | -0.0053 | 0 | 65 | 5.8e-005 | 1 | K.LAVNMVPFPR.L 6378 |
|  | 8582 | 630.3885 | 1258.7624 | 1257.6830 | 1.0795 | 1 | 9 | 17 | 3 | R.FPGQLNADLRK.L 8578 |
|  | 14495 | **531.2855** | **1590.8347** | **1591.8042** | **-0.9694** | **1** | **2** | **68** | **4** | **R.HGCYLTAAAIFRGR.M** |
|  | 14633 | 801.4081 | 1600.8017 | 1600.8131 | -0.0113 | 0 | 30 | 0.12 | 1 | R.AVLVDLEPGTMDSVR.S 14631 14634 |
|  | 18139 | 914.4130 | 1826.8115 | 1827.9241 | -1.1126 | 1 | 5 | 29 | 1 | R.INVHHHEASGGRYVPR.A |
|  | 18375 | 615.3405 | 1842.9997 | 1843.8332 | -0.8335 | 0 | 7 | 22 | 2 | R.YTEGAELMESVMDVVR.K |
|  | 18377 | **615.3776** | **1843.1110** | **1843.8332** | **-0.7222** | **0** | **(2)** | **58** | **8** | **R.YTEGAELMESVMDVVR.K** |
|  | 20791 | 1022.5081 | 2043.0017 | 2041.8907 | 1.1110 | 1 | 3 | 46 | 2 | R.MPMREVDEQMFNIQDK.N |
|  | 21610 | 529.7468 | 2114.9582 | 2116.0987 | -1.1405 | 1 | 5 | 28 | 6 | R.YVPRAVLVDLEPGTMDSVR.S |
|  | 28909 | 1104.5103 | 3310.5089 | 3310.5268 | -0.0179 | 0 | 62 | 2.5e-005 | 1 | K.EAESCDCLQGFQLTHSLGGGTGSGMGTLLLSK.I 28908 28910 28911 28912 28913 28914 |
|  | 28978 | 1109.8413 | 3326.5021 | 3326.5218 | -0.0197 | 0 | (27) | 0.091 | 1 | K.EAESCDCLQGFQLTHSLGGGTGSGMGTLLLSK.I |
|  | 29339 | 1147.5436 | 3439.6089 | 3438.6218 | 0.9871 | 1 | 52 | 0.00023 | 1 | R.KEAESCDCLQGFQLTHSLGGGTGSGMGTLLLSK.I 29336 |
|  | 29368 | 1152.8380 | 3455.4922 | 3454.6167 | 0.8755 | 1 | (2) | 22 | 3 | R.KEAESCDCLQGFQLTHSLGGGTGSGMGTLLLSK.I |

| **172.** | P29972\|AQP1_HUMAN    **Mass:** 28736    **Score:** 227    **Queries matched:** 7   **emPAI:** 0.28 |
| --- | --- |
|  | Aquaporin-1 OS=Homo sapiens GN=AQP1 PE=1 SV=3 |

|  | **Query** | **Observed** | **Mr(expt)** | **Mr(calc)** | **Delta** | **Miss** | **Score** | **Expect** | **Rank** | **Peptide** |
| --- | --- | --- | --- | --- | --- | --- | --- | --- | --- | --- |
|  | 1386 | **731.4064** | **730.3992** | **730.4047** | **-0.0056** | **0** | **22** | **1.7** | **1** | **R.VEMKPK.-** |
|  | 15327 | 823.9004 | 1645.7862 | 1645.8060 | -0.0198 | 0 | 3 | 59 | 4 | K.YPVGNNQTAVQDNVK.V |
|  | 23305 | 1156.5112 | 2311.0079 | 2311.0240 | -0.0161 | 0 | 125 | 2.6e-011 | 1 | K.VWTSGQVEEYDLDADDINSR.V 23306 |
|  | 24911 | **847.0646** | **2538.1719** | **2538.1874** | **-0.0155** | **1** | **15** | **2.3** | **1** | **R.VKVWTSGQVEEYDLDADDINSR.V** |
|  | 27720 | **756.8588** | **3023.4062** | **3023.4182** | **-0.0120** | **1** | **12** | **3.1** | **1** | **K.VWTSGQVEEYDLDADDINSRVEMKPK.-** |
|  | 28746 | 1088.2419 | 3261.7040 | 3261.7180 | -0.0140 | 0 | 39 | 0.0052 | 1 | R.DLGGSAPLAIGLSVALGHLLAIDYTGCGINPAR.S |

| **173.** | P01842\|LAC_HUMAN    **Mass:** 11401    **Score:** 227    **Queries matched:** 9   **emPAI:** 2.30 |
| --- | --- |
|  | Ig lambda chain C regions OS=Homo sapiens GN=IGLC1 PE=1 SV=1 |

|  | **Query** | **Observed** | **Mr(expt)** | **Mr(calc)** | **Delta** | **Miss** | **Score** | **Expect** | **Rank** | **Peptide** |
| --- | --- | --- | --- | --- | --- | --- | --- | --- | --- | --- |
|  | 2537 | **865.0821** | **864.0748** | **863.3695** | **0.7053** | **0** | **33** | **0.066** | **1** | **K.TVAPTECS.-** |
|  | 4242 | **495.7549** | **989.4953** | **989.5029** | **-0.0076** | **0** | **63** | **8.5e-005** | **1** | **K.AGVETTTPSK.Q** 4240 4241 |
|  | 16375 | **856.3773** | **1710.7400** | **1710.7519** | **-0.0120** | **0** | **78** | **1.7e-006** | **1** | **R.SYSCQVTHEGSTVEK.T** 16374 |
|  | 16909 | **872.4266** | **1742.8387** | **1742.8515** | **-0.0128** | **0** | **33** | **0.048** | **1** | **K.YAASSYLSLTPEQWK.S** |
|  | 20164 | **993.5060** | **1984.9975** | **1985.0105** | **-0.0130** | **0** | **15** | **3** | **1** | **K.AAPSVTLFPPSSEELQANK.A** 20162 |

| **174.** | P13746\|1A11_HUMAN    **Mass:** 41196    **Score:** 225    **Queries matched:** 30   **emPAI:** 0.84 |
| --- | --- |
|  | HLA class I histocompatibility antigen, A-11 alpha chain OS=Homo sapiens GN=HLA-A PE=1 SV=1 |

|  | **Query** | **Observed** | **Mr(expt)** | **Mr(calc)** | **Delta** | **Miss** | **Score** | **Expect** | **Rank** | **Peptide** |
| --- | --- | --- | --- | --- | --- | --- | --- | --- | --- | --- |
|  | 1793 | **406.5443** | **811.0741** | **810.4500** | **0.6241** | **1** | **3** | **49** | **10** | **R.FLRGYR.Q** |
|  | 4359 | 499.2580 | 996.5014 | 995.4308 | 1.0706 | 0 | 8 | 25 | 1 | R.FDSDAASQR.M |
|  | 9280 | **648.8030** | **1295.5915** | **1295.6007** | **-0.0091** | **0** | **13** | **7** | **1** | **K.WEAAHAAEQQR.A** |
|  | 10029 | 666.8338 | 1331.6530 | 1331.6622 | -0.0092 | 0 | 17 | 3.1 | 1 | R.YFYTSVSRPGR.G |
|  | 10335 | 675.8494 | 1349.6843 | 1349.6939 | -0.0096 | 1 | 38 | 0.024 | 1 | R.YLENGKETLQR.T 10311 10351 |
|  | 11693 | **475.5686** | **1423.6841** | **1423.6956** | **-0.0116** | **1** | **21** | **0.99** | **1** | **R.KWEAAHAAEQQR.A** |
|  | 11736 | 714.3626 | 1426.7105 | 1426.7205 | -0.0099 | 0 | 46 | 0.0031 | 1 | K.WAAVVVPSGEEQR.Y 11721 11735 11751 |
|  | 13404 | 508.9781 | 1523.9124 | 1524.6627 | -0.7502 | 1 | 4 | 52 | 6 | R.FDSDAASQRMEPR.A |
|  | 13806 | 775.3702 | 1548.7259 | 1548.7718 | -0.0459 | 1 | 44 | 0.0053 | 1 | R.SWTAADMAAQITKR.K |
|  | 14092 | 522.7712 | 1565.2917 | 1564.7668 | 0.5250 | 1 | (8) | 12 | 3 | R.SWTAADMAAQITKR.K |
|  | 18889 | 628.3002 | 1881.8787 | 1881.8904 | -0.0117 | 0 | 24 | 0.42 | 1 | K.THMTHHPISDHEATLR.C |
|  | 20872 | 513.0196 | 2048.0493 | 2048.0626 | -0.0133 | 0 | 16 | 2.1 | 1 | R.YTCHVQHEGLPKPLTLR.W 20848 20850 20871 |
|  | 21557 | 1056.5175 | 2111.0204 | 2111.0299 | -0.0095 | 0 | 86 | 2.1e-007 | 1 | R.CWALGFYPAEITLTWQR.D 21558 |
|  | 25378 | 866.1122 | 2595.3149 | 2595.2639 | 0.0510 | 1 | 1 | 48 | 1 | K.DYIALNEDLRSWTAADMAAQITK.R |
|  | 25578 | 1319.0889 | 2636.1632 | 2636.1838 | -0.0206 | 0 | 57 | 0.00013 | 1 | R.DGEDQTQDTELVETRPAGDGTFQK.W 25579 25580 25581 |
|  | 25731 | **888.4573** | **2662.3502** | **2662.1758** | **0.1744** | **1** | **2** | **41** | **3** | **R.MEPRAPWIEQEGPEYWDQETR.N** |
|  | 27956 | **1027.8076** | **3080.4010** | **3080.6627** | **-0.2617** | **1** | **3** | **24** | **1** | **M.AVMAPRTLLLLLSGALALTQTWAGSHSMR.Y** |
|  | 28626 | **1076.5287** | **3226.5642** | **3227.6981** | **-1.1338** | **1** | **1** | **34** | **3** | **-.MAVMAPRTLLLLLSGALALTQTWAGSHSMR.Y** |

| **175.** | P30443\|1A01_HUMAN    **Mass:** 41105    **Score:** 225    **Queries matched:** 31   **emPAI:** 0.84 |
| --- | --- |
|  | HLA class I histocompatibility antigen, A-1 alpha chain OS=Homo sapiens GN=HLA-A PE=1 SV=1 |

|  | **Query** | **Observed** | **Mr(expt)** | **Mr(calc)** | **Delta** | **Miss** | **Score** | **Expect** | **Rank** | **Peptide** |
| --- | --- | --- | --- | --- | --- | --- | --- | --- | --- | --- |
|  | 1793 | 406.5443 | 811.0741 | 810.4500 | 0.6241 | 1 | 3 | 49 | 10 | R.FLRGYR.Q |
|  | 1838 | 408.2467 | 814.4788 | 813.3729 | 1.1059 | 0 | 4 | 80 | 3 | K.AHSQTDR.A |
|  | 3955 | **485.2459** | **968.4772** | **967.4247** | **1.0525** | **0** | **7** | **30** | **4** | **R.FDSDAASQK.M** |
|  | 7264 | **399.5143** | **1195.5211** | **1195.5734** | **-0.0523** | **0** | **12** | **9.5** | **6** | **K.WEAVHAAEQR.R** 7273 |
|  | 9699 | 658.8362 | 1315.6579 | 1315.6673 | -0.0094 | 0 | 14 | 6.2 | 2 | R.YFFTSVSRPGR.G |
|  | 10335 | 675.8494 | 1349.6843 | 1349.6939 | -0.0096 | 1 | 38 | 0.024 | 1 | R.YLENGKETLQR.T 10311 10351 |
|  | 10404 | **677.3697** | **1352.7248** | **1351.6745** | **1.0503** | **1** | **2** | **89** | **7** | **K.WEAVHAAEQRR.V** |
|  | 11736 | 714.3626 | 1426.7105 | 1426.7205 | -0.0099 | 0 | 46 | 0.0031 | 1 | K.WAAVVVPSGEEQR.Y 11721 11735 11751 |
|  | 13806 | 775.3702 | 1548.7259 | 1548.7718 | -0.0459 | 1 | 44 | 0.0053 | 1 | R.SWTAADMAAQITKR.K |
|  | 14092 | 522.7712 | 1565.2917 | 1564.7668 | 0.5250 | 1 | (8) | 12 | 3 | R.SWTAADMAAQITKR.K |
|  | 18889 | 628.3002 | 1881.8787 | 1881.8904 | -0.0117 | 0 | 24 | 0.42 | 1 | K.THMTHHPISDHEATLR.C |
|  | 20872 | 513.0196 | 2048.0493 | 2048.0626 | -0.0133 | 0 | 16 | 2.1 | 1 | R.YTCHVQHEGLPKPLTLR.W 20848 20850 20871 |
|  | 21557 | 1056.5175 | 2111.0204 | 2111.0299 | -0.0095 | 0 | 86 | 2.1e-007 | 1 | R.CWALGFYPAEITLTWQR.D 21558 |
|  | 25378 | 866.1122 | 2595.3149 | 2595.2639 | 0.0510 | 1 | 1 | 48 | 1 | K.DYIALNEDLRSWTAADMAAQITK.R |
|  | 25578 | 1319.0889 | 2636.1632 | 2636.1838 | -0.0206 | 0 | 57 | 0.00013 | 1 | R.DGEDQTQDTELVETRPAGDGTFQK.W 25579 25580 25581 |
|  | 25731 | 888.4573 | 2662.3502 | 2662.1758 | 0.1744 | 1 | 2 | 41 | 3 | K.MEPRAPWIEQEGPEYWDQETR.N |
|  | 27956 | 1027.8076 | 3080.4010 | 3080.6627 | -0.2617 | 1 | 3 | 24 | 1 | M.AVMAPRTLLLLLSGALALTQTWAGSHSMR.Y |
|  | 28626 | 1076.5287 | 3226.5642 | 3227.6981 | -1.1338 | 1 | 1 | 34 | 3 | -.MAVMAPRTLLLLLSGALALTQTWAGSHSMR.Y |

|  | **Proteins matching the same set of peptides:** |
| --- | --- |

|  | P30455\|1A36_HUMAN    **Mass:** 41193    **Score:** 225    **Queries matched:** 31 |
| --- | --- |
|  | HLA class I histocompatibility antigen, A-36 alpha chain OS=Homo sapiens GN=HLA-A PE=1 SV=1 |

| **176.** | P04439\|1A03_HUMAN    **Mass:** 41100    **Score:** 225    **Queries matched:** 28   **emPAI:** 0.84 |
| --- | --- |
|  | HLA class I histocompatibility antigen, A-3 alpha chain OS=Homo sapiens GN=HLA-A PE=1 SV=2 |

|  | **Query** | **Observed** | **Mr(expt)** | **Mr(calc)** | **Delta** | **Miss** | **Score** | **Expect** | **Rank** | **Peptide** |
| --- | --- | --- | --- | --- | --- | --- | --- | --- | --- | --- |
|  | 1793 | 406.5443 | 811.0741 | 810.4500 | 0.6241 | 1 | 3 | 49 | 10 | R.FLRGYR.Q |
|  | 4359 | 499.2580 | 996.5014 | 995.4308 | 1.0706 | 0 | 8 | 25 | 1 | R.FDSDAASQR.M |
|  | 9699 | 658.8362 | 1315.6579 | 1315.6673 | -0.0094 | 0 | 14 | 6.2 | 2 | R.YFFTSVSRPGR.G |
|  | 10335 | 675.8494 | 1349.6843 | 1349.6939 | -0.0096 | 1 | 38 | 0.024 | 1 | R.YLENGKETLQR.T 10311 10351 |
|  | 11736 | 714.3626 | 1426.7105 | 1426.7205 | -0.0099 | 0 | 46 | 0.0031 | 1 | K.WAAVVVPSGEEQR.Y 11721 11735 11751 |
|  | 13404 | 508.9781 | 1523.9124 | 1524.6627 | -0.7502 | 1 | 4 | 52 | 6 | R.FDSDAASQRMEPR.A |
|  | 13806 | 775.3702 | 1548.7259 | 1548.7718 | -0.0459 | 1 | 44 | 0.0053 | 1 | R.SWTAADMAAQITKR.K |
|  | 14092 | 522.7712 | 1565.2917 | 1564.7668 | 0.5250 | 1 | (8) | 12 | 3 | R.SWTAADMAAQITKR.K |
|  | 18889 | 628.3002 | 1881.8787 | 1881.8904 | -0.0117 | 0 | 24 | 0.42 | 1 | K.THMTHHPISDHEATLR.C |
|  | 20872 | 513.0196 | 2048.0493 | 2048.0626 | -0.0133 | 0 | 16 | 2.1 | 1 | R.YTCHVQHEGLPKPLTLR.W 20848 20850 20871 |
|  | 21557 | 1056.5175 | 2111.0204 | 2111.0299 | -0.0095 | 0 | 86 | 2.1e-007 | 1 | R.CWALGFYPAEITLTWQR.D 21558 |
|  | 25378 | 866.1122 | 2595.3149 | 2595.2639 | 0.0510 | 1 | 1 | 48 | 1 | K.DYIALNEDLRSWTAADMAAQITK.R |
|  | 25578 | 1319.0889 | 2636.1632 | 2636.1838 | -0.0206 | 0 | 57 | 0.00013 | 1 | R.DGEDQTQDTELVETRPAGDGTFQK.W 25579 25580 25581 |
|  | 25731 | 888.4573 | 2662.3502 | 2662.1758 | 0.1744 | 1 | 2 | 41 | 3 | R.MEPRAPWIEQEGPEYWDQETR.N |
|  | 27956 | 1027.8076 | 3080.4010 | 3080.6627 | -0.2617 | 1 | 3 | 24 | 1 | M.AVMAPRTLLLLLSGALALTQTWAGSHSMR.Y |
|  | 28626 | 1076.5287 | 3226.5642 | 3227.6981 | -1.1338 | 1 | 1 | 34 | 3 | -.MAVMAPRTLLLLLSGALALTQTWAGSHSMR.Y |

| **177.** | Q15907\|RB11B_HUMAN    **Mass:** 24588    **Score:** 225    **Queries matched:** 11   **emPAI:** 1.05 |
| --- | --- |
|  | Ras-related protein Rab-11B OS=Homo sapiens GN=RAB11B PE=1 SV=4 |

|  | **Query** | **Observed** | **Mr(expt)** | **Mr(calc)** | **Delta** | **Miss** | **Score** | **Expect** | **Rank** | **Peptide** |
| --- | --- | --- | --- | --- | --- | --- | --- | --- | --- | --- |
|  | 1007 | **575.3103** | **574.3030** | **573.3486** | **0.9544** | **0** | **7** | **62** | **3** | **R.IVSQK.Q** |
|  | 3610 | **944.4777** | **943.4704** | **943.4763** | **-0.0059** | **0** | **6** | **47** | **1** | **R.AITSAYYR.G** |
|  | 5011 | **522.3045** | **1042.5944** | **1042.6023** | **-0.0078** | **0** | **45** | **0.0051** | **1** | **K.VVLIGDSGVGK.S** |
|  | 8677 | 632.8616 | 1263.7087 | 1263.6684 | 0.0403 | 1 | 6 | 39 | 3 | R.HLRAVPTDEAR.A |
|  | 8865 | **637.8054** | **1273.5962** | **1273.6051** | **-0.0089** | **0** | **39** | **0.018** | **1** | **K.AQIWDTAGQER.Y** |
|  | 15039 | **542.6121** | **1624.8144** | **1624.8243** | **-0.0099** | **0** | **29** | **0.15** | **1** | **R.DHADSNIVIMLVGNK.S** 15040 |
|  | 15454 | 551.5901 | 1651.7486 | 1651.7188 | 0.0298 | 1 | 4 | 46 | 2 | -.MGTRDDEYDYLFK.V |
|  | 23449 | **1165.0542** | **2328.0938** | **2328.1121** | **-0.0183** | **0** | **111** | **6.1e-010** | **1** | **K.NNLSFIETSALDSTNVEEAFK.N** 23450 |
|  | 28994 | **1111.2225** | **3330.6458** | **3330.6619** | **-0.0161** | **1** | **77** | **7.8e-007** | **1** | **K.NNLSFIETSALDSTNVEEAFKNILTEIYR.I** |

| **178.** | Q99805\|TM9S2_HUMAN    **Mass:** 76809    **Score:** 224    **Queries matched:** 7   **emPAI:** 0.10 |
| --- | --- |
|  | Transmembrane 9 superfamily member 2 OS=Homo sapiens GN=TM9SF2 PE=1 SV=1 |

|  | **Query** | **Observed** | **Mr(expt)** | **Mr(calc)** | **Delta** | **Miss** | **Score** | **Expect** | **Rank** | **Peptide** |
| --- | --- | --- | --- | --- | --- | --- | --- | --- | --- | --- |
|  | 3481 | **468.2528** | **934.4911** | **934.4984** | **-0.0073** | **0** | **13** | **9.3** | **2** | **K.NAIEHPVR.T** |
|  | 7192 | **596.8977** | **1191.7809** | **1191.7088** | **0.0721** | **1** | **6** | **34** | **7** | **M.SARLPVLSPPR.W** |
|  | 17534 | **894.9023** | **1787.7900** | **1787.8011** | **-0.0111** | **0** | **44** | **0.0043** | **1** | **R.FCNPGFPIGCYITDK.G** 17535 |
|  | 21189 | **1039.4187** | **2076.8228** | **2076.8371** | **-0.0142** | **0** | **114** | **3e-010** | **1** | **R.YNQMDSTEDAQEEFGWK.L** 21190 |
|  | 27287 | **589.6857** | **2943.3923** | **2942.5551** | **0.8372** | **1** | **2** | **33** | **5** | **R.GALMTCAVVLWVLLGTPAGYVAARFYK.S** |

| **179.** | P31930\|QCR1_HUMAN    **Mass:** 53297    **Score:** 224    **Queries matched:** 27   **emPAI:** 0.31 |
| --- | --- |
|  | Cytochrome b-c1 complex subunit 1, mitochondrial OS=Homo sapiens GN=UQCRC1 PE=1 SV=3 |

|  | **Query** | **Observed** | **Mr(expt)** | **Mr(calc)** | **Delta** | **Miss** | **Score** | **Expect** | **Rank** | **Peptide** |
| --- | --- | --- | --- | --- | --- | --- | --- | --- | --- | --- |
|  | 1772 | **405.7424** | **809.4703** | **808.4443** | **1.0260** | **0** | **6** | **38** | **5** | **R.SLLTYGR.R** 1765 |
|  | 5209 | **529.7921** | **1057.5697** | **1057.5768** | **-0.0071** | **0** | **45** | **0.0052** | **1** | **R.IAEVDASVVR.E** |
|  | 5352 | **535.7466** | **1069.4786** | **1069.6244** | **-0.1458** | **0** | **8** | **28** | **9** | **R.AATAGAQVLLR.A** |
|  | 8228 | **415.2132** | **1242.6178** | **1241.4652** | **1.1525** | **0** | **12** | **9.4** | **1** | **R.EMQENDASMR.D** 8211 8216 |
|  | 8515 | **628.8373** | **1255.6600** | **1255.6673** | **-0.0073** | **1** | **25** | **0.49** | **1** | **R.RIPLAEWESR.I** 8480 8516 |
|  | 9325 | **433.2634** | **1296.7684** | **1296.7626** | **0.0058** | **1** | **4** | **49** | **8** | **R.AATAGAQVLLRAR.R** |
|  | 11970 | **481.2365** | **1440.6876** | **1439.6932** | **0.9944** | **0** | **16** | **3.2** | **1** | **R.ADLTEYLSTHYK.A** |
|  | 15645 | **832.3817** | **1662.7487** | **1663.7624** | **-1.0136** | **0** | **(1)** | **97** | **10** | **K.EVESMGAHLNAYSTR.E** |
|  | 15921 | 841.4118 | 1680.8090 | 1679.7573 | 1.0517 | 0 | 1 | 80 | 4 | K.EVESMGAHLNAYSTR.E |
|  | 17673 | **599.3460** | **1795.0162** | **1795.9104** | **-0.8942** | **1** | **1** | **82** | **9** | **K.LSRADLTEYLSTHYK.A** |
|  | 17756 | 901.4465 | 1800.8785 | 1800.7997 | 0.0788 | 0 | 4 | 46 | 6 | K.IDDMMFVLQGQWMR.L |
|  | 17941 | **605.3223** | **1812.9450** | **1812.9992** | **-0.0542** | **1** | **4** | **41** | **1** | **M.AASVVCRAATAGAQVLLR.A** |
|  | 18294 | **613.6711** | **1837.9916** | **1837.8298** | **0.1618** | **1** | **3** | **54** | **3** | **R.DVILREMQENDASMR.D** |
|  | 20648 | **676.9857** | **2027.9352** | **2027.9453** | **-0.0102** | **1** | **3** | **44** | **5** | **R.MKIDDMMFVLQGQWMR.L** 20646 |
|  | 20928 | **1027.4952** | **2052.9759** | **2052.9899** | **-0.0139** | **0** | **87** | **1.9e-007** | **1** | **R.NALVSHLDGTTPVCEDIGR.S** 20927 |
|  | 21357 | **698.0408** | **2091.1007** | **2091.1146** | **-0.0140** | **0** | **27** | **0.17** | **1** | **R.MVLAAAGGVEHQQLLDLAQK.H** |
|  | 25487 | **655.0704** | **2616.2526** | **2616.2714** | **-0.0188** | **1** | **16** | **1.7** | **1** | **K.NRPGSALEKEVESMGAHLNAYSTR.E** 25491 |
|  | 26052 | **1351.6228** | **2701.2310** | **2701.2482** | **-0.0172** | **0** | **89** | **7.6e-008** | **1** | **K.YIYDQCPAVAGYGPIEQLPDYNR.I** 26053 |

| **180.** | P61106\|RAB14_HUMAN    **Mass:** 24110    **Score:** 223    **Queries matched:** 22   **emPAI:** 1.80 |
| --- | --- |
|  | Ras-related protein Rab-14 OS=Homo sapiens GN=RAB14 PE=1 SV=4 |

|  | **Query** | **Observed** | **Mr(expt)** | **Mr(calc)** | **Delta** | **Miss** | **Score** | **Expect** | **Rank** | **Peptide** |
| --- | --- | --- | --- | --- | --- | --- | --- | --- | --- | --- |
|  | 2660 | **437.7725** | **873.5305** | **872.4967** | **1.0338** | **0** | **17** | **4.2** | **6** | **R.IIEVSGQK.I** 2658 |
|  | 9695 | 658.8284 | 1315.6422 | 1315.6521 | -0.0099 | 0 | 18 | 2.6 | 1 | K.LQIWDTAGQER.F 9685 |
|  | 11116 | **696.4263** | **1390.8380** | **1389.7075** | **1.1305** | **1** | **2** | **88** | **1** | **K.SCLLHQFTEKK.F** |
|  | 12876 | **498.5979** | **1492.7719** | **1492.7820** | **-0.0101** | **1** | **15** | **3.9** | **1** | **R.GAAGALMVYDITRR.S** |
|  | 13145 | **503.9282** | **1508.7627** | **1508.7769** | **-0.0143** | **1** | **(3)** | **65** | **7** | **R.GAAGALMVYDITRR.S** |
|  | 13956 | **779.4173** | **1556.8200** | **1556.8311** | **-0.0110** | **1** | **32** | **0.081** | **1** | **K.IKLQIWDTAGQER.F** 13955 13957 |
|  | 14976 | 811.4011 | 1620.7876 | 1620.7995 | -0.0119 | 1 | 50 | 0.0011 | 1 | K.TGENVEDAFLEAAKK.I 14953 |
|  | 15014 | **812.4702** | **1622.9259** | **1622.9355** | **-0.0097** | **0** | **75** | **3.2e-006** | **1** | **R.NLTNPNTVIILIGNK.A** 15023 |
|  | 15392 | 825.3638 | 1648.7130 | 1649.7798 | -1.0668 | 0 | 19 | 1.3 | 2 | R.STYNHLSSWLTDAR.N |
|  | 17852 | **603.2980** | **1806.8721** | **1805.8809** | **0.9912** | **1** | **8** | **16** | **1** | **R.RSTYNHLSSWLTDAR.N** 17839 |
|  | 18293 | **613.6134** | **1837.8184** | **1836.8288** | **0.9896** | **0** | **11** | **7.9** | **1** | **K.FMADCPHTIGVEFGTR.I** |
|  | 19968 | 655.9768 | 1964.9084 | 1964.9237 | -0.0153 | 1 | 33 | 0.049 | 1 | K.KFMADCPHTIGVEFGTR.I |
|  | 28266 | **1050.8481** | **3149.5226** | **3149.5490** | **-0.0264** | **0** | **60** | **4.7e-005** | **1** | **K.IYQNIQDGSLDLNAAESGVQHKPSAPQGGR.L** 28269 |
|  | 28821 | **820.4123** | **3277.6201** | **3277.6439** | **-0.0239** | **1** | **90** | **4.6e-008** | **1** | **K.KIYQNIQDGSLDLNAAESGVQHKPSAPQGGR.L** |

| **181.** | Q96A33\|CCD47_HUMAN    **Mass:** 56123    **Score:** 223    **Queries matched:** 20   **emPAI:** 0.38 |
| --- | --- |
|  | Coiled-coil domain-containing protein 47 OS=Homo sapiens GN=CCDC47 PE=1 SV=1 |

|  | **Query** | **Observed** | **Mr(expt)** | **Mr(calc)** | **Delta** | **Miss** | **Score** | **Expect** | **Rank** | **Peptide** |
| --- | --- | --- | --- | --- | --- | --- | --- | --- | --- | --- |
|  | 3795 | **479.2744** | **956.5341** | **956.5403** | **-0.0061** | **1** | **30** | **0.19** | **1** | **R.RLEEAALR.R** |
|  | 6092 | **560.9396** | **1119.8646** | **1119.4754** | **0.3892** | **0** | **9** | **14** | **3** | **R.IMNEEDPEK.Q** |
|  | 7343 | **400.2387** | **1197.6944** | **1196.6989** | **0.9955** | **1** | **8** | **21** | **6** | **K.RQDLLNVLAR.M** |
|  | 11346 | **469.2292** | **1404.6658** | **1404.6191** | **0.0468** | **1** | **1** | **1e+002** | **9** | **K.ERIMNEEDPEK.Q** |
|  | 11830 | **716.8721** | **1431.7297** | **1430.7374** | **0.9923** | **0** | **27** | **0.24** | **1** | **R.MMRPVSDQVQIK.V** 11828 |
|  | 14255 | **526.6314** | **1576.8722** | **1576.7522** | **0.1200** | **0** | **10** | **11** | **2** | **K.IESVHFSDQFSGPK.I** |
|  | 18552 | **928.4783** | **1854.9421** | **1854.9516** | **-0.0095** | **0** | **42** | **0.0059** | **1** | **R.TLLFTFNVPGSGNTYPK.D** 18551 |
|  | 20683 | **678.3096** | **2031.9071** | **2031.9221** | **-0.0150** | **0** | **19** | **1** | **1** | **K.LNQENEHIYNLWCSGR.V** |
|  | 21388 | **1048.5225** | **2095.0304** | **2095.0403** | **-0.0099** | **0** | **59** | **0.00012** | **1** | **K.DMEALLPLMNMVIYSIDK.A** 21389 21391 |
|  | 21549 | **704.4074** | **2110.2004** | **2111.0352** | **-0.8348** | **0** | **(4)** | **28** | **2** | **K.DMEALLPLMNMVIYSIDK.A** |
|  | 23301 | **578.5483** | **2310.1643** | **2310.1673** | **-0.0030** | **1** | **2** | **51** | **10** | **K.DMEALLPLMNMVIYSIDKAK.K** |
|  | 25239 | **1287.0906** | **2572.1666** | **2572.1747** | **-0.0081** | **0** | **90** | **6e-008** | **1** | **K.YGLPDSLAILSEMGEVTDGMMDTK.M** 25238 |
|  | 29466 | **873.9063** | **3491.5961** | **3491.6194** | **-0.0232** | **1** | **5** | **11** | **1** | **R.MMRPVSDQVQIKVTMNDEDMDTYVFAVGTR.K** |
|  | 29534 | **881.6829** | **3522.7026** | **3523.6092** | **-0.9066** | **1** | **(1)** | **30** | **2** | **R.MMRPVSDQVQIKVTMNDEDMDTYVFAVGTR.K** |
|  | 30364 | **1316.9180** | **3947.7321** | **3946.8137** | **0.9183** | **1** | **10** | **2.5** | **1** | **K.YGLPDSLAILSEMGEVTDGMMDTKMVHFLTHYADK.I** |

| **182.** | Q03518\|TAP1_HUMAN    **Mass:** 87733    **Score:** 220    **Queries matched:** 15   **emPAI:** 0.18 |
| --- | --- |
|  | Antigen peptide transporter 1 OS=Homo sapiens GN=TAP1 PE=1 SV=2 |

|  | **Query** | **Observed** | **Mr(expt)** | **Mr(calc)** | **Delta** | **Miss** | **Score** | **Expect** | **Rank** | **Peptide** |
| --- | --- | --- | --- | --- | --- | --- | --- | --- | --- | --- |
|  | 3370 | **464.2817** | **926.5487** | **925.4730** | **1.0758** | **0** | **0** | **1.5e+002** | **6** | **R.GAESPRPGR.D** |
|  | 8607 | **421.0562** | **1260.1468** | **1260.6608** | **-0.5140** | **1** | **4** | **42** | **8** | **R.RLLGCLGSETR.R** |
|  | 8647 | **421.5653** | **1261.6741** | **1260.6608** | **1.0133** | **1** | **2** | **1e+002** | **10** | **R.LLGCLGSETRR.L** |
|  | 11024 | **462.7268** | **1385.1586** | **1384.6769** | **0.4817** | **1** | **1** | **74** | **9** | **R.EGGTHQQLMEKK.G** |
|  | 13031 | **501.3377** | **1500.9912** | **1499.8071** | **1.1841** | **0** | **3** | **49** | **3** | **R.WAVLWLGACGVLR.A** |
|  | 15833 | **559.2760** | **1674.8062** | **1674.8611** | **-0.0549** | **0** | **3** | **56** | **9** | **K.SSQVAIEALSAMPTVR.S** |
|  | 18292 | **919.8867** | **1837.7588** | **1837.8847** | **-0.1259** | **0** | **16** | **2.4** | **1** | **R.LTDWILQDGSADTFTR.N** |
|  | 21954 | **719.6915** | **2156.0526** | **2156.0823** | **-0.0298** | **0** | **4** | **32** | **2** | **K.EAVAYAVNSWTTSISGMLLK.V** |
|  | 26086 | **902.7997** | **2705.3774** | **2705.3945** | **-0.0171** | **0** | **48** | **0.00091** | **1** | **R.SLQENIAYGLTQKPTMEEITAAAVK.S** |
|  | 27849 | **1017.4895** | **3049.4467** | **3049.4014** | **0.0453** | **0** | **42** | **0.0031** | **1** | **K.SGAHSFISGLPQGYDTEVDEAGSQLSGGQR.Q** |
|  | 29284 | **1140.9403** | **3419.7991** | **3419.8201** | **-0.0210** | **0** | **107** | **6.7e-010** | **1** | **K.STVAALLQNLYQPTGGQLLLDGKPLPQYEHR.Y** 29283 |
|  | 29775 | **1205.9395** | **3614.7965** | **3614.8138** | **-0.0172** | **0** | **34** | **0.014** | **1** | **R.KPCVLILDDATSALDANSQLQVEQLLYESPER.Y** 29776 |
|  | 30062 | **1253.9089** | **3758.7050** | **3758.8248** | **-0.1199** | **1** | **1** | **23** | **4** | **K.SGAHSFISGLPQGYDTEVDEAGSQLSGGQRQAVALAR.A** |

| **183.** | P30479\|1B41_HUMAN    **Mass:** 40856    **Score:** 218    **Queries matched:** 22   **emPAI:** 0.85 |
| --- | --- |
|  | HLA class I histocompatibility antigen, B-41 alpha chain OS=Homo sapiens GN=HLA-B PE=1 SV=1 |

|  | **Query** | **Observed** | **Mr(expt)** | **Mr(calc)** | **Delta** | **Miss** | **Score** | **Expect** | **Rank** | **Peptide** |
| --- | --- | --- | --- | --- | --- | --- | --- | --- | --- | --- |
|  | 4343 | 498.7556 | 995.4965 | 994.4356 | 1.0610 | 0 | 8 | 23 | 2 | R.FDSDATSPR.K 4329 |
|  | 6145 | 562.7893 | 1123.5639 | 1122.5305 | 1.0334 | 1 | 4 | 54 | 3 | R.FDSDATSPRK.E 6130 |
|  | 11736 | 714.3626 | 1426.7105 | 1426.7205 | -0.0099 | 0 | 46 | 0.0031 | 1 | K.WAAVVVPSGEEQR.Y 11721 11735 11751 |
|  | 12921 | 748.8587 | 1495.7028 | 1495.7129 | -0.0101 | 0 | 57 | 0.00026 | 1 | R.AYLEGTCVEWLR.R |
|  | 13321 | 760.3871 | 1518.7596 | 1518.7427 | 0.0169 | 0 | 62 | 7.5e-005 | 1 | R.SWTAADTAAQITQR.K |
|  | 18486 | 463.4837 | 1849.9057 | 1849.9183 | -0.0127 | 0 | 16 | 2.9 | 1 | K.THVTHHPISDHEATLR.C 18485 |
|  | 20872 | 513.0196 | 2048.0493 | 2048.0626 | -0.0133 | 0 | 16 | 2.1 | 1 | R.YTCHVQHEGLPKPLTLR.W 20848 20850 20871 |
|  | 21557 | 1056.5175 | 2111.0204 | 2111.0299 | -0.0095 | 0 | 86 | 2.1e-007 | 1 | R.CWALGFYPAEITLTWQR.D 21558 |
|  | 22689 | 744.6669 | 2230.9790 | 2230.9938 | -0.0149 | 0 | 44 | 0.0032 | 1 | R.DGEDQTQDTELVETRPAGDR.T 22688 |
|  | 23645 | 785.7004 | 2354.0793 | 2354.0927 | -0.0134 | 1 | 36 | 0.02 | 1 | R.GHNQYAYDGKDYIALNEDLR.S |
|  | 30136 | 1896.3450 | 3790.6754 | 3790.1002 | 0.5752 | 1 | 2 | 20 | 5 | R.WEPSSQSTVPIVGIVAGLAVLAVVVIGAVVAAVMCRR.K |

| **184.** | P28288\|ABCD3_HUMAN    **Mass:** 75941    **Score:** 216    **Queries matched:** 31   **emPAI:** 0.27 |
| --- | --- |
|  | ATP-binding cassette sub-family D member 3 OS=Homo sapiens GN=ABCD3 PE=1 SV=1 |

|  | **Query** | **Observed** | **Mr(expt)** | **Mr(calc)** | **Delta** | **Miss** | **Score** | **Expect** | **Rank** | **Peptide** |
| --- | --- | --- | --- | --- | --- | --- | --- | --- | --- | --- |
|  | 1429 | **758.3306** | **757.3234** | **756.3442** | **0.9791** | **0** | **2** | **1.3e+002** | **8** | **R.GNYEFK.Q** |
|  | 1891 | **410.7172** | **819.4199** | **818.3705** | **1.0495** | **0** | **6** | **53** | **9** | **K.MGNLDNR.I** |
|  | 2895 | **446.7271** | **891.4396** | **891.4450** | **-0.0054** | **0** | **24** | **0.74** | **1** | **R.DLNFEVR.S** |
|  | 4007 | 487.2253 | 972.4359 | 971.6015 | 0.8344 | 1 | 13 | 9.1 | 6 | R.KGISDLVLK.E |
|  | 5281 | 532.2832 | 1062.5518 | 1061.5142 | 1.0377 | 0 | 11 | 15 | 6 | R.DQVIYPDGR.E |
|  | 5432 | 537.8115 | 1073.6085 | 1073.6154 | -0.0070 | 0 | 2 | 1.2e+002 | 5 | R.ITELMQVLK.D |
|  | 6680 | **581.3213** | **1160.6280** | **1160.6336** | **-0.0055** | **1** | **6** | **38** | **8** | **R.IVLAGREMTR.L** |
|  | 7603 | **606.3166** | **1210.6186** | **1210.6306** | **-0.0119** | **0** | **11** | **11** | **1** | **K.SGKPPLQNNEK.E** |
|  | 8434 | 626.8568 | 1251.6991 | 1251.6394 | 0.0597 | 1 | 4 | 57 | 7 | R.EMTRLAGFTAR.I |
|  | 8920 | **425.8990** | **1274.6753** | **1274.6951** | **-0.0198** | **1** | **(3)** | **68** | **10** | **R.MLLRMSQALGR.I** |
|  | 9164 | **645.8213** | **1289.6280** | **1290.6900** | **-1.0620** | **1** | **(4)** | **56** | **3** | **R.MLLRMSQALGR.I** |
|  | 9179 | **430.9111** | **1289.7115** | **1290.6900** | **-0.9785** | **1** | **6** | **36** | **8** | **R.MLLRMSQALGR.I** |
|  | 9957 | **664.3571** | **1326.6996** | **1327.6846** | **-0.9850** | **0** | **(5)** | **46** | **2** | **R.FSMGFIDSIIAK.Y** |
|  | 10138 | **670.3170** | **1338.6195** | **1338.7255** | **-0.1060** | **1** | **6** | **34** | **5** | **K.KSGKPPLQNNEK.E** |
|  | 10214 | **672.3733** | **1342.7320** | **1342.7398** | **-0.0077** | **0** | **5** | **41** | **2** | **R.VLGELWPLFGGR.L** |
|  | 10267 | **449.2767** | **1344.8082** | **1343.6795** | **1.1287** | **0** | **9** | **20** | **1** | **R.FSMGFIDSIIAK.Y** |
|  | 11053 | **694.3446** | **1386.6746** | **1386.7765** | **-0.1019** | **1** | **4** | **53** | **8** | **R.MSQALGRIVLAGR.E** |
|  | 12515 | **737.8740** | **1473.7335** | **1472.6255** | **1.1080** | **0** | **1** | **98** | **3** | **K.HHEYYLHMDGR.G** |
|  | 14018 | **781.4044** | **1560.7943** | **1561.7372** | **-0.9430** | **1** | **14** | **4.6** | **1** | **R.DQVIYPDGREDQK.R** |
|  | 16187 | **849.9388** | **1697.8630** | **1697.8737** | **-0.0106** | **0** | **68** | **1.9e-005** | **1** | **K.EYLDNVQLGHILER.E** |
|  | 20020 | 986.5133 | 1971.0121 | 1970.9210 | 0.0911 | 1 | 1 | 73 | 7 | K.SLWKHHEYYLHMDGR.G 20021 |
|  | 20193 | 995.0066 | 1987.9986 | 1986.9159 | 1.0828 | 1 | (0) | 84 | 4 | K.SLWKHHEYYLHMDGR.G 20177 20194 |
|  | 21380 | 1047.9520 | 2093.8895 | 2093.9001 | -0.0105 | 0 | 121 | 5.9e-011 | 1 | R.EGGWDSVQDWMDVLSGGEK.Q |
|  | 24825 | **842.1257** | **2523.3554** | **2523.3697** | **-0.0143** | **1** | **46** | **0.0014** | **1** | **K.GISDLVLKEYLDNVQLGHILER.E** |
|  | 24928 | **1271.7401** | **2541.4657** | **2541.4781** | **-0.0125** | **0** | **43** | **0.0032** | **1** | **K.GIEGVQVIPLIPGAGEIIIADNIIK.F** 24929 24930 |
|  | 30793 | 1072.6027 | 4286.3815 | 4286.4042 | -0.0227 | 1 | 5 | 6 | 1 | K.GIEGVQVIPLIPGAGEIIIADNIIKFDHVPLATPNGDVLIR.D |

| **185.** | Q31610\|1B81_HUMAN    **Mass:** 40717    **Score:** 216    **Queries matched:** 29   **emPAI:** 0.85 |
| --- | --- |
|  | HLA class I histocompatibility antigen, B-81 alpha chain OS=Homo sapiens GN=HLA-B PE=2 SV=1 |

|  | **Query** | **Observed** | **Mr(expt)** | **Mr(calc)** | **Delta** | **Miss** | **Score** | **Expect** | **Rank** | **Peptide** |
| --- | --- | --- | --- | --- | --- | --- | --- | --- | --- | --- |
|  | 965 | 559.2914 | 558.2842 | 558.3125 | -0.0284 | 0 | 15 | 8.3 | 10 | K.LEAAR.V |
|  | 1309 | 702.8578 | 701.8505 | 701.3894 | 0.4611 | 0 | 10 | 18 | 2 | M.LVMAPR.T 1307 1311 |
|  | 1455 | 766.4029 | 765.3956 | 765.4021 | -0.0065 | 0 | 37 | 0.038 | 1 | R.NTQIYK.A |
|  | 3888 | 482.7234 | 963.4322 | 964.4250 | -0.9929 | 0 | 33 | 0.088 | 1 | R.FDSDAASPR.E 3900 3901 3907 |
|  | 10029 | 666.8338 | 1331.6530 | 1331.6622 | -0.0092 | 0 | 17 | 3.1 | 1 | R.YFYTSVSRPGR.G |
|  | 12552 | 492.8911 | 1475.6516 | 1475.6641 | -0.0125 | 1 | 13 | 6.8 | 1 | R.FDSDAASPREEPR.A 12553 |
|  | 15320 | 823.4096 | 1644.8047 | 1644.8148 | -0.0101 | 0 | 69 | 1.4e-005 | 1 | R.FISVGYVDDTQFVR.F 15321 |
|  | 18486 | 463.4837 | 1849.9057 | 1849.9183 | -0.0127 | 0 | 16 | 2.9 | 1 | K.THVTHHPISDHEATLR.C 18485 |
|  | 20872 | 513.0196 | 2048.0493 | 2048.0626 | -0.0133 | 0 | 16 | 2.1 | 1 | R.YTCHVQHEGLPKPLTLR.W 20848 20850 20871 |
|  | 21557 | 1056.5175 | 2111.0204 | 2111.0299 | -0.0095 | 0 | 86 | 2.1e-007 | 1 | R.CWALGFYPAEITLTWQR.D 21558 |
|  | 22689 | 744.6669 | 2230.9790 | 2230.9938 | -0.0149 | 0 | 44 | 0.0032 | 1 | R.DGEDQTQDTELVETRPAGDR.T 22688 |
|  | 23097 | 1143.5082 | 2285.0018 | 2286.0341 | -1.0323 | 1 | 11 | 6.4 | 1 | R.EEPRAPWIEQEGPEYWDR.N |
|  | 23645 | 785.7004 | 2354.0793 | 2354.0927 | -0.0134 | 1 | 36 | 0.02 | 1 | R.GHNQYAYDGKDYIALNEDLR.S |
|  | 25366 | 865.0411 | 2592.1016 | 2591.2293 | 0.8723 | 1 | 0 | 56 | 6 | R.FISVGYVDDTQFVRFDSDAASPR.E |
|  | 28470 | 799.9033 | 3195.5839 | 3194.7097 | 0.8743 | 1 | 3 | 25 | 4 | M.LVMAPRTVLLLLWGAVALTETWAGSHSMR.Y |
|  | 30136 | 1896.3450 | 3790.6754 | 3790.1002 | 0.5752 | 1 | 2 | 20 | 5 | R.WEPSSQSTVPIVGIVAGLAVLAVVVIGAVVAAVMCRR.K |

| **186.** | P63010\|AP2B1_HUMAN    **Mass:** 105398   **Score:** 215    **Queries matched:** 37   **emPAI:** 0.23 |
| --- | --- |
|  | AP-2 complex subunit beta OS=Homo sapiens GN=AP2B1 PE=1 SV=1 |

|  | **Query** | **Observed** | **Mr(expt)** | **Mr(calc)** | **Delta** | **Miss** | **Score** | **Expect** | **Rank** | **Peptide** |
| --- | --- | --- | --- | --- | --- | --- | --- | --- | --- | --- |
|  | 1887 | 819.4338 | 818.4266 | 818.4320 | -0.0055 | 0 | 13 | 9.8 | 1 | K.TAAVCVAK.L |
|  | 2909 | 447.3449 | 892.6753 | 892.3927 | 0.2827 | 0 | 4 | 51 | 2 | K.DEDPYVR.K 2901 |
|  | 3646 | 474.2669 | 946.5192 | 946.5270 | -0.0078 | 1 | 10 | 17 | 7 | R.KTAAVCVAK.L |
|  | 4276 | **496.7641** | **991.5136** | **991.5491** | **-0.0355** | **0** | **11** | **13** | **2** | **R.QVFLATWK.D** |
|  | 4282 | 993.0422 | 992.0350 | 991.4393 | 0.5957 | 0 | 8 | 18 | 2 | R.EAQSICER.V |
|  | 4562 | 506.2573 | 1010.5001 | 1010.5073 | -0.0071 | 0 | 17 | 3.1 | 1 | K.YNDPIYVK.L |
|  | 7937 | 614.7806 | 1227.5467 | 1227.5554 | -0.0086 | 0 | 20 | 1.5 | 1 | K.DCEDPNPLIR.A 7938 |
|  | 8339 | 416.5507 | 1246.6304 | 1246.6638 | -0.0334 | 1 | 10 | 16 | 6 | R.ALAVRTMGCIR.V |
|  | 8943 | **639.3419** | **1276.6693** | **1276.6775** | **-0.0082** | **0** | **24** | **0.55** | **1** | **K.LQNNNVYTIAK.R** 8959 |
|  | 9242 | 647.5536 | 1293.0927 | 1293.6023 | -0.5096 | 1 | 0 | 88 | 10 | K.CLKDEDPYVR.K |
|  | 10529 | **680.3931** | **1358.7716** | **1359.6089** | **-0.8373** | **0** | **9** | **17** | **1** | **K.ECHLNADTVSSK.L** |
|  | 10783 | 458.2710 | 1371.7913 | 1370.7228 | 1.0685 | 0 | 6 | 34 | 3 | K.MEPLNNLQVAVK.N |
|  | 11102 | 695.8710 | 1389.7275 | 1389.7366 | -0.0091 | 0 | 25 | 0.47 | 1 | K.LVYLYLMNYAK.S |
|  | 11251 | **700.3967** | **1398.7789** | **1397.8031** | **0.9759** | **0** | **20** | **1.2** | **3** | **K.LTNGIWILAELR.I** |
|  | 11657 | 474.9176 | 1421.7310 | 1420.7384 | 0.9926 | 1 | 6 | 30 | 3 | K.ITEYLCEPLRK.C 11629 |
|  | 12028 | **722.8884** | **1443.7623** | **1443.7722** | **-0.0098** | **0** | **18** | **2.1** | **1** | **R.IQPGNPNYTLSLK.C** |
|  | 13396 | 762.8666 | 1523.7186 | 1523.7290 | -0.0104 | 0 | 58 | 0.0002 | 1 | R.NVEGQDMLYQSLK.L |
|  | 13689 | 771.4247 | 1540.8348 | 1539.7239 | 1.1109 | 0 | (2) | 77 | 6 | R.NVEGQDMLYQSLK.L |
|  | 14428 | **794.3985** | **1586.7824** | **1586.7940** | **-0.0116** | **0** | **11** | **11** | **4** | **K.DIPNENELQFQIK.E** |
|  | 15098 | **543.9428** | **1628.8064** | **1627.7123** | **1.0941** | **0** | **1** | **87** | **2** | **R.QGHIYMEMNFTNK.A** |
|  | 15191 | 818.9031 | 1635.7917 | 1636.7953 | -1.0036 | 0 | 27 | 0.23 | 1 | K.SQPDMAIMAVNSFVK.D |
|  | 15746 | 557.2985 | 1668.8736 | 1668.7851 | 0.0884 | 0 | (4) | 50 | 2 | K.SQPDMAIMAVNSFVK.D |
|  | 16148 | 848.9149 | 1695.8152 | 1695.8250 | -0.0098 | 1 | 1 | 87 | 6 | K.RNVEGQDMLYQSLK.L |
|  | 18885 | 628.1918 | 1881.5537 | 1882.0710 | -0.5173 | 1 | 2 | 42 | 2 | R.LSHANSAVVLSAVKVLMK.F 18889 |
|  | 27537 | 998.1878 | 2991.5416 | 2991.5553 | -0.0137 | 1 | 94 | 2.3e-008 | 1 | R.LASQANIAQVLAELKEYATEVDVDFVR.K 27538 |
|  | 27682 | **755.3418** | **3017.3381** | **3016.4746** | **0.8635** | **1** | **0** | **49** | **2** | **K.TAAVCVAKLHDINAQMVEDQGFLDSLR.D** |
|  | 28400 | **796.3995** | **3181.5688** | **3181.5851** | **-0.0163** | **1** | **4** | **20** | **1** | **K.KPSETQELVQQVLSLATQDSDNPDLRDR.G** |
|  | 29728 | 1196.9420 | 3587.8042 | 3587.8247 | -0.0204 | 0 | 75 | 1.2e-006 | 1 | R.IDNADELLESFLEGFHDESTQVQLTLLTAIVK.L 29729 |
|  | 30313 | **1300.3584** | **3898.0534** | **3898.0737** | **-0.0203** | **0** | **32** | **0.016** | **1** | **K.NSFGVIPSTPLAIHTPLMPNQSIDVSLPLNTLGPVMK.M** 30314 |

| **187.** | P24557\|THAS_HUMAN    **Mass:** 61163    **Score:** 214    **Queries matched:** 19   **emPAI:** 0.34 |
| --- | --- |
|  | Thromboxane-A synthase OS=Homo sapiens GN=TBXAS1 PE=1 SV=3 |

|  | **Query** | **Observed** | **Mr(expt)** | **Mr(calc)** | **Delta** | **Miss** | **Score** | **Expect** | **Rank** | **Peptide** |
| --- | --- | --- | --- | --- | --- | --- | --- | --- | --- | --- |
|  | 2365 | 427.2271 | 852.4397 | 853.5134 | -1.0736 | 1 | 11 | 11 | 4 | R.ILPNKNR.D 2374 |
|  | 2767 | **441.2950** | **880.5754** | **881.4317** | **-0.8563** | **0** | **10** | **14** | **6** | **R.MASGLEFK.S** 2773 2788 |
|  | 6305 | **569.3038** | **1136.5930** | **1136.5536** | **0.0394** | **0** | **5** | **44** | **2** | **R.GALMSAFSPEK.L** |
|  | 10838 | **688.3131** | **1374.6117** | **1374.6198** | **-0.0081** | **0** | **36** | **0.033** | **1** | **R.EAAQDCEVLGQR.I** |
|  | 10865 | **459.9142** | **1376.7209** | **1375.8452** | **0.8757** | **1** | **14** | **5.5** | **1** | **K.LTLLHVLHKFR.F** |
|  | 11717 | **476.1968** | **1425.5686** | **1425.6347** | **-0.0661** | **0** | **5** | **39** | **6** | **R.QGFWESQMELR.K** |
|  | 13854 | **776.3596** | **1550.7046** | **1550.7148** | **-0.0102** | **0** | **69** | **1.6e-005** | **1** | **R.DVFSSTGCKPNPSR.Q** 13852 13853 |
|  | 14124 | **784.8934** | **1567.7722** | **1566.7791** | **0.9931** | **0** | **48** | **0.0022** | **1** | **K.QVLVENFSNFTNR.M** |
|  | 14543 | **532.2832** | **1593.8278** | **1593.8259** | **0.0019** | **1** | **4** | **45** | **9** | **R.RMFIVISEPDMIK.Q** |
|  | 14802 | **806.9175** | **1611.8205** | **1611.8328** | **-0.0123** | **1** | **17** | **2.6** | **1** | **R.NVIALRDQQAAEER.R** |
|  | 15545 | **829.8966** | **1657.7785** | **1657.7883** | **-0.0097** | **0** | **84** | **4.9e-007** | **1** | **R.HSASPMGVQDFDIVR.D** |
|  | 18805 | **938.4652** | **1874.9159** | **1873.9244** | **0.9915** | **0** | **46** | **0.0027** | **1** | **R.FQACPETQVPLQLESK.S** |
|  | 22064 | **542.7549** | **2166.9904** | **2166.2697** | **0.7207** | **0** | **3** | **40** | **2** | **K.LEVNGPMVTVALSVALLALLK.W** |
|  | 27261 | 979.8549 | 2936.5429 | 2936.3758 | 0.1671 | 0 | 1 | 45 | 4 | K.HMAPEFCSLEEGLPYLDMVIAETLR.M |

| **188.** | P20700\|LMNB1_HUMAN    **Mass:** 66653    **Score:** 214    **Queries matched:** 34   **emPAI:** 0.63 |
| --- | --- |
|  | Lamin-B1 OS=Homo sapiens GN=LMNB1 PE=1 SV=2 |

|  | **Query** | **Observed** | **Mr(expt)** | **Mr(calc)** | **Delta** | **Miss** | **Score** | **Expect** | **Rank** | **Peptide** |
| --- | --- | --- | --- | --- | --- | --- | --- | --- | --- | --- |
|  | 2911 | **447.7343** | **893.4541** | **892.3960** | **1.0581** | **0** | **13** | **8.8** | **4** | **R.EELMESR.M** |
|  | 3112 | 455.2117 | 908.4088 | 908.5080 | -0.0991 | 0 | 6 | 39 | 6 | M.ATATPVPPR.M 3111 |
|  | 3952 | **485.2438** | **968.4731** | **968.4815** | **-0.0083** | **0** | **15** | **4.6** | **1** | **K.IGDTSVSYK.Y** |
|  | 4046 | 488.2414 | 974.4682 | 973.4716 | 0.9966 | 0 | 20 | 1.9 | 1 | K.LLEGEEER.L 4028 |
|  | 4143 | **491.7788** | **981.5429** | **981.4628** | **0.0801** | **0** | **6** | **35** | **4** | **R.EQHDAQVR.L** |
|  | 4386 | **500.2547** | **998.4949** | **998.6124** | **-0.1175** | **1** | **21** | **1.4** | **1** | **R.AKLQIELGK.C** |
|  | 5039 | **523.2837** | **1044.5528** | **1043.5611** | **0.9917** | **1** | **6** | **48** | **7** | **R.LQEKEELR.E** |
|  | 5102 | **525.2725** | **1048.5304** | **1048.5625** | **-0.0321** | **1** | **5** | **60** | **2** | **R.VTVSRASSSR.S** |
|  | 5390 | **536.3082** | **1070.6019** | **1070.6084** | **-0.0065** | **1** | **5** | **59** | **3** | **R.LKLSPSPSSR.V** |
|  | 6543 | **577.2835** | **1152.5523** | **1153.6091** | **-1.0568** | **0** | **5** | **53** | **10** | **R.AGGPTTPLSPTR.L** |
|  | 9233 | **647.3390** | **1292.6634** | **1292.6724** | **-0.0090** | **1** | **45** | **0.0046** | **1** | **K.LREYEAALNSK.D** |
|  | 9318 | **649.3438** | **1296.6729** | **1296.6384** | **0.0346** | **0** | **1** | **1e+002** | **3** | **K.LALDMEISAYR.K** |
|  | 10060 | **667.7971** | **1333.5797** | **1333.5899** | **-0.0102** | **0** | **21** | **1.2** | **1** | **K.NQNSWGTGEDVK.V** 10051 10078 |
|  | 11260 | **700.7983** | **1399.5820** | **1399.5925** | **-0.0105** | **0** | **17** | **2.9** | **1** | **K.SMYEEEINETR.R** |
|  | 12542 | **492.6281** | **1474.8626** | **1475.7256** | **-0.8630** | **1** | **0** | **1.2e+002** | **9** | **K.IGDTSVSYKYTSR.Y** |
|  | 12920 | **748.8484** | **1495.6823** | **1495.6936** | **-0.0113** | **0** | **93** | **6.5e-008** | **1** | **R.LSSEMNTSTVNSAR.E** |
|  | 13178 | **756.3547** | **1510.6948** | **1511.6886** | **-0.9938** | **0** | **(41)** | **0.011** | **1** | **R.LSSEMNTSTVNSAR.E** |
|  | 13417 | **509.2453** | **1524.7142** | **1524.7242** | **-0.0101** | **1** | **5** | **43** | **1** | **R.CQSLTEDLEFRK.S** |
|  | 13499 | 765.3847 | 1528.7548 | 1527.6875 | 1.0674 | 1 | 7 | 23 | 2 | R.KSMYEEEINETR.R |
|  | 13942 | 778.9271 | 1555.8396 | 1555.8569 | -0.0174 | 1 | 9 | 16 | 5 | K.DQIAQLEASLAAAKK.Q 13915 |
|  | 15433 | **826.4137** | **1650.8128** | **1650.8253** | **-0.0124** | **1** | **38** | **0.017** | **1** | **R.LYKEELEQTYHAK.L** 15432 |
|  | 16750 | **867.4595** | **1732.9045** | **1732.9141** | **-0.0096** | **1** | **66** | **3e-005** | **1** | **R.MRIESLSSQLSNLQK.E** |
|  | 19236 | **637.6447** | **1909.9123** | **1909.7935** | **0.1188** | **0** | **2** | **67** | **4** | **K.NTSEQDQPMGGWEMIR.K** |
|  | 21663 | **708.3971** | **2122.1694** | **2122.9841** | **-0.8146** | **0** | **3** | **44** | **8** | **R.DQMQQQLNDYEQLLDVK.L** |
|  | 21909 | 1076.5442 | 2151.0738 | 2150.9725 | 0.1014 | 1 | 0 | 85 | 8 | R.LKNTSEQDQPMGGWEMIR.K |
|  | 24847 | **632.8300** | **2527.2907** | **2527.3017** | **-0.0109** | **1** | **42** | **0.0042** | **1** | **K.SLEGDLEDLKDQIAQLEASLAAAK.K** 24848 |
|  | 29258 | **1137.8551** | **3410.5435** | **3410.5637** | **-0.0203** | **0** | **31** | **0.033** | **1** | **K.TTIPEEEEEEEEAAGVVVEEELFHQQGTPR.A** |

| **189.** | Q9NP72\|RAB18_HUMAN    **Mass:** 23248    **Score:** 213    **Queries matched:** 12   **emPAI:** 0.84 |
| --- | --- |
|  | Ras-related protein Rab-18 OS=Homo sapiens GN=RAB18 PE=1 SV=1 |

|  | **Query** | **Observed** | **Mr(expt)** | **Mr(calc)** | **Delta** | **Miss** | **Score** | **Expect** | **Rank** | **Peptide** |
| --- | --- | --- | --- | --- | --- | --- | --- | --- | --- | --- |
|  | 5245 | **531.2725** | **1060.5305** | **1059.5924** | **0.9381** | **1** | **5** | **62** | **4** | **K.VKTISVDGNK.A** |
|  | 5605 | **543.3285** | **1084.6424** | **1084.6492** | **-0.0068** | **0** | **13** | **7.7** | **7** | **K.ILIIGESGVGK.S** |
|  | 8568 | **630.3182** | **1258.6219** | **1258.6306** | **-0.0087** | **0** | **54** | **0.00054** | **1** | **K.LAIWDTAGQER.F** |
|  | 17067 | **878.9408** | **1755.8670** | **1755.8791** | **-0.0121** | **0** | **56** | **0.00029** | **1** | **K.IIQTPGLWESENQNK.G** 17068 |
|  | 17468 | **892.8892** | **1783.7639** | **1783.7757** | **-0.0118** | **0** | **66** | **2.6e-005** | **1** | **K.TCDGVQCAFEELVEK.I** 17452 |
|  | 18134 | **609.8073** | **1826.4001** | **1825.8305** | **0.5696** | **0** | **5** | **21** | **3** | **K.LDNWLNELETYCTR.N** |
|  | 21358 | **1046.9159** | **2091.8172** | **2092.9055** | **-1.0883** | **1** | **2** | **46** | **5** | **K.LSHREEGQGGGACGGYCSVL.-** |
|  | 21446 | **1051.4960** | **2100.9774** | **2100.9892** | **-0.0118** | **0** | **91** | **7.4e-008** | **1** | **R.FTDDTFDPELAATIGVDFK.V** 21445 |
|  | 27668 | **754.8504** | **3015.3725** | **3014.3824** | **0.9901** | **1** | **1** | **38** | **9** | **K.HSMLFIEASAKTCDGVQCAFEELVEK.I** |

| **190.** | P30486\|1B48_HUMAN    **Mass:** 40679    **Score:** 210    **Queries matched:** 28   **emPAI:** 0.70 |
| --- | --- |
|  | HLA class I histocompatibility antigen, B-48 alpha chain OS=Homo sapiens GN=HLA-B PE=2 SV=1 |

|  | **Query** | **Observed** | **Mr(expt)** | **Mr(calc)** | **Delta** | **Miss** | **Score** | **Expect** | **Rank** | **Peptide** |
| --- | --- | --- | --- | --- | --- | --- | --- | --- | --- | --- |
|  | 965 | 559.2914 | 558.2842 | 558.3125 | -0.0284 | 0 | 15 | 8.3 | 10 | K.LEAAR.V |
|  | 1309 | 702.8578 | 701.8505 | 701.3894 | 0.4611 | 0 | 10 | 18 | 2 | M.LVMAPR.T 1307 1311 |
|  | 3888 | 482.7234 | 963.4322 | 964.4250 | -0.9929 | 0 | 33 | 0.088 | 1 | R.FDSDAASPR.E 3900 3901 3907 |
|  | 10029 | 666.8338 | 1331.6530 | 1331.6622 | -0.0092 | 0 | 17 | 3.1 | 1 | R.YFYTSVSRPGR.G |
|  | 12552 | 492.8911 | 1475.6516 | 1475.6641 | -0.0125 | 1 | 13 | 6.8 | 1 | R.FDSDAASPREEPR.A 12553 |
|  | 15320 | 823.4096 | 1644.8047 | 1644.8148 | -0.0101 | 0 | 69 | 1.4e-005 | 1 | R.FISVGYVDDTQFVR.F 15321 |
|  | 18486 | 463.4837 | 1849.9057 | 1849.9183 | -0.0127 | 0 | 16 | 2.9 | 1 | K.THVTHHPISDHEATLR.C 18485 |
|  | 20872 | 513.0196 | 2048.0493 | 2048.0626 | -0.0133 | 0 | 16 | 2.1 | 1 | R.YTCHVQHEGLPKPLTLR.W 20848 20850 20871 |
|  | 21557 | 1056.5175 | 2111.0204 | 2111.0299 | -0.0095 | 0 | 86 | 2.1e-007 | 1 | R.CWALGFYPAEITLTWQR.D 21558 |
|  | 22689 | 744.6669 | 2230.9790 | 2230.9938 | -0.0149 | 0 | 44 | 0.0032 | 1 | R.DGEDQTQDTELVETRPAGDR.T 22688 |
|  | 23097 | 1143.5082 | 2285.0018 | 2286.0341 | -1.0323 | 1 | 11 | 6.4 | 1 | R.EEPRAPWIEQEGPEYWDR.E |
|  | 23645 | 785.7004 | 2354.0793 | 2354.0927 | -0.0134 | 1 | 36 | 0.02 | 1 | R.GHNQYAYDGKDYIALNEDLR.S |
|  | 25366 | 865.0411 | 2592.1016 | 2591.2293 | 0.8723 | 1 | 0 | 56 | 6 | R.FISVGYVDDTQFVRFDSDAASPR.E |
|  | 28130 | 521.6007 | 3123.5606 | 3123.6936 | -0.1330 | 1 | 13 | 2.7 | 1 | M.LVMAPRTVLLLLSAALALTETWAGSHSMR.Y |
|  | 30136 | 1896.3450 | 3790.6754 | 3790.1002 | 0.5752 | 1 | 2 | 20 | 5 | R.WEPSSQSTVPIVGIVAGLAVLAVVVIGAVVAAVMCRR.K |

| **191.** | Q86Y82\|STX12_HUMAN    **Mass:** 31736    **Score:** 210    **Queries matched:** 13   **emPAI:** 0.75 |
| --- | --- |
|  | Syntaxin-12 OS=Homo sapiens GN=STX12 PE=1 SV=1 |

|  | **Query** | **Observed** | **Mr(expt)** | **Mr(calc)** | **Delta** | **Miss** | **Score** | **Expect** | **Rank** | **Peptide** |
| --- | --- | --- | --- | --- | --- | --- | --- | --- | --- | --- |
|  | 2263 | 845.4396 | 844.4324 | 844.4403 | -0.0079 | 0 | 11 | 16 | 1 | R.ATEQLQR.A |
|  | 5457 | **1076.5260** | **1075.5187** | **1074.5417** | **0.9770** | **1** | **13** | **7.9** | **2** | **R.AGSRLSAEER.Q** |
|  | 6028 | 559.2275 | 1116.4405 | 1116.4910 | -0.0505 | 0 | 8 | 29 | 5 | M.SYGPLDMYR.N |
|  | 15053 | **813.9340** | **1625.8534** | **1625.8624** | **-0.0091** | **0** | **24** | **0.51** | **1** | **K.ELGSLPLPLSTSEQR.Q** 15068 |
|  | 15335 | **823.9409** | **1645.8673** | **1644.8723** | **0.9950** | **0** | **64** | **4.5e-005** | **1** | **R.QLEADILDVNQIFK.D** |
|  | 19511 | 966.9362 | 1931.8578 | 1931.0510 | 0.8068 | 1 | 2 | 72 | 8 | R.ISQATAQIKNLMSQLGTK.Q |
|  | 19577 | **969.9725** | **1937.9305** | **1937.9418** | **-0.0112** | **0** | **99** | **1.2e-008** | **1** | **R.LMNDFSAALNNFQAVQR.R** 19576 |
|  | 20257 | **997.0201** | **1992.0257** | **1992.0388** | **-0.0131** | **0** | **60** | **0.0001** | **1** | **K.LQENLQQLQHSTNQLAK.E** 20256 |
|  | 22781 | **561.0361** | **2240.1152** | **2239.0804** | **1.0348** | **1** | **2** | **50** | **5** | **K.ERLMNDFSAALNNFQAVQR.R** |
|  | 28711 | **1084.8364** | **3251.4875** | **3251.5074** | **-0.0200** | **0** | **13** | **2.1** | **1** | **K.DLAMMIHDQGDLIDSIEANVESSEVHVER.A** |

| **192.** | Q5VTE0\|EF1A3_HUMAN    **Mass:** 50495    **Score:** 209    **Queries matched:** 21   **emPAI:** 0.43 |
| --- | --- |
|  | Putative elongation factor 1-alpha-like 3 OS=Homo sapiens GN=EEF1AL3 PE=5 SV=1 |

|  | **Query** | **Observed** | **Mr(expt)** | **Mr(calc)** | **Delta** | **Miss** | **Score** | **Expect** | **Rank** | **Peptide** |
| --- | --- | --- | --- | --- | --- | --- | --- | --- | --- | --- |
|  | 2601 | **435.7712** | **869.5278** | **869.5334** | **-0.0056** | **0** | **33** | **0.079** | **2** | **K.QLIVGVNK.M** |
|  | 3176 | **457.3114** | **912.6082** | **913.5597** | **-0.9515** | **0** | **1** | **1.1e+002** | **8** | **R.QTVAVGVIK.A** |
|  | 3934 | **484.2733** | **966.5319** | **966.5386** | **-0.0066** | **1** | **22** | **1** | **1** | **K.EVSTYIKK.I** |
|  | 9335 | 433.5917 | 1297.7532 | 1297.5496 | 0.2036 | 0 | 11 | 10 | 1 | K.MDSTEPPYSQK.R 9320 |
|  | 9586 | **657.3124** | **1312.6102** | **1313.7343** | **-1.1241** | **0** | **28** | **0.23** | **1** | **R.EHALLAYTLGVK.Q** 9585 |
|  | 9977 | **664.8523** | **1327.6900** | **1326.7871** | **0.9029** | **1** | **2** | **97** | **10** | **R.QTVAVGVIKAVDK.K** |
|  | 24766 | **1258.1876** | **2514.3607** | **2514.3768** | **-0.0161** | **0** | **62** | **3.8e-005** | **1** | **R.VETGVLKPGMVVTFAPVNVTTEVK.S** 24765 24775 |
|  | 25939 | **897.8292** | **2690.4658** | **2689.4552** | **1.0107** | **1** | **2** | **39** | **4** | **K.THINIVVIGHVDSGKSTTTGHLIYK.C** |
|  | 28261 | **630.7337** | **3148.6321** | **3148.6550** | **-0.0229** | **1** | **6** | **10** | **4** | **R.KDGNASGTTLLEALDCILPPTRPTDKPLR.L** |
|  | 29644 | **1784.3322** | **3566.6497** | **3566.6639** | **-0.0141** | **0** | **41** | **0.0028** | **1** | **K.IGYNPDTVAFVPISGWNGDNMLEPSANMPWFK.G** 29642 29646 |
|  | 29697 | **1195.5667** | **3583.6781** | **3582.6588** | **1.0193** | **0** | **(9)** | **4.9** | **1** | **K.IGYNPDTVAFVPISGWNGDNMLEPSANMPWFK.G** 29696 |
|  | 29914 | **1232.5858** | **3694.7356** | **3694.7589** | **-0.0232** | **1** | **64** | **1.4e-005** | **1** | **K.KIGYNPDTVAFVPISGWNGDNMLEPSANMPWFK.G** 29913 29916 |

| **193.** | P68104\|EF1A1_HUMAN    **Mass:** 50451    **Score:** 209    **Queries matched:** 22   **emPAI:** 0.43 |
| --- | --- |
|  | Elongation factor 1-alpha 1 OS=Homo sapiens GN=EEF1A1 PE=1 SV=1 |

|  | **Query** | **Observed** | **Mr(expt)** | **Mr(calc)** | **Delta** | **Miss** | **Score** | **Expect** | **Rank** | **Peptide** |
| --- | --- | --- | --- | --- | --- | --- | --- | --- | --- | --- |
|  | 2601 | 435.7712 | 869.5278 | 869.5334 | -0.0056 | 0 | 33 | 0.079 | 2 | K.QLIVGVNK.M |
|  | 3176 | 457.3114 | 912.6082 | 913.5597 | -0.9515 | 0 | 1 | 1.1e+002 | 8 | R.QTVAVGVIK.A |
|  | 3934 | 484.2733 | 966.5319 | 966.5386 | -0.0066 | 1 | 22 | 1 | 1 | K.EVSTYIKK.I |
|  | 9335 | 433.5917 | 1297.7532 | 1297.5496 | 0.2036 | 0 | 11 | 10 | 1 | K.MDSTEPPYSQK.R 9320 |
|  | 9586 | 657.3124 | 1312.6102 | 1313.7343 | -1.1241 | 0 | 28 | 0.23 | 1 | R.EHALLAYTLGVK.Q 9585 |
|  | 9977 | 664.8523 | 1327.6900 | 1326.7871 | 0.9029 | 1 | 2 | 97 | 10 | R.QTVAVGVIKAVDK.K |
|  | 24766 | 1258.1876 | 2514.3607 | 2514.3768 | -0.0161 | 0 | 62 | 3.8e-005 | 1 | R.VETGVLKPGMVVTFAPVNVTTEVK.S 24765 24775 |
|  | 25939 | 897.8292 | 2690.4658 | 2689.4552 | 1.0107 | 1 | 2 | 39 | 4 | K.THINIVVIGHVDSGKSTTTGHLIYK.C |
|  | 27712 | **756.3781** | **3021.4833** | **3022.4818** | **-0.9985** | **1** | **1** | **37** | **3** | **K.SVEMHHEALSEALPGDNVGFNVKNVSVK.D** |
|  | 28261 | 630.7337 | 3148.6321 | 3148.6550 | -0.0229 | 1 | 6 | 10 | 4 | R.KDGNASGTTLLEALDCILPPTRPTDKPLR.L |
|  | 29644 | 1784.3322 | 3566.6497 | 3566.6639 | -0.0141 | 0 | 41 | 0.0028 | 1 | K.IGYNPDTVAFVPISGWNGDNMLEPSANMPWFK.G 29642 29646 |
|  | 29697 | 1195.5667 | 3583.6781 | 3582.6588 | 1.0193 | 0 | (9) | 4.9 | 1 | K.IGYNPDTVAFVPISGWNGDNMLEPSANMPWFK.G 29696 |
|  | 29914 | 1232.5858 | 3694.7356 | 3694.7589 | -0.0232 | 1 | 64 | 1.4e-005 | 1 | K.KIGYNPDTVAFVPISGWNGDNMLEPSANMPWFK.G 29913 29916 |

| **194.** | P13667\|PDIA4_HUMAN    **Mass:** 73229    **Score:** 209    **Queries matched:** 30   **emPAI:** 0.35 |
| --- | --- |
|  | Protein disulfide-isomerase A4 OS=Homo sapiens GN=PDIA4 PE=1 SV=2 |

|  | **Query** | **Observed** | **Mr(expt)** | **Mr(calc)** | **Delta** | **Miss** | **Score** | **Expect** | **Rank** | **Peptide** |
| --- | --- | --- | --- | --- | --- | --- | --- | --- | --- | --- |
|  | 2782 | **441.3191** | **880.6237** | **881.5699** | **-0.9462** | **1** | **1** | **1e+002** | **6** | **K.GPVKVVVGK.T** |
|  | 4029 | **487.7470** | **973.4793** | **973.4869** | **-0.0075** | **0** | **26** | **0.46** | **1** | **K.FIEEHATK.L** |
|  | 4065 | **488.7441** | **975.4735** | **976.5229** | **-1.0494** | **1** | **8** | **29** | **10** | **K.KLAPEYEK.A** |
|  | 4091 | **489.8052** | **977.5957** | **977.6022** | **-0.0064** | **1** | **22** | **1** | **1** | **K.RSPPIPLAK.V** |
|  | 4558 | **506.2508** | **1010.4871** | **1010.5509** | **-0.0638** | **1** | **6** | **41** | **5** | **K.SQPVPKNNK.G** |
|  | 6048 | **559.3817** | **1116.7489** | **1117.5516** | **-0.8028** | **1** | **1** | **1.2e+002** | **10** | **K.NPVKFEGGDR.D** |
|  | 6184 | **564.3558** | **1126.6970** | **1125.5706** | **1.1264** | **0** | **7** | **30** | **3** | **R.FDVSGYPTIK.I** |
|  | 6542 | **577.2820** | **1152.5494** | **1151.5533** | **0.9961** | **0** | **8** | **21** | **2** | **K.TFDSIVMDPK.K** |
|  | 6754 | **584.7967** | **1167.5789** | **1167.5482** | **0.0308** | **0** | **(7)** | **32** | **7** | **K.TFDSIVMDPK.K** |
|  | 7146 | 595.8184 | 1189.6223 | 1189.6302 | -0.0080 | 0 | 58 | 0.00024 | 1 | K.IDATSASVLASR.F |
|  | 7566 | **605.3196** | **1208.6246** | **1208.5786** | **0.0461** | **1** | **23** | **0.74** | **1** | **K.KGQAVDYEGSR.T** 7560 |
|  | 9152 | **645.3342** | **1288.6538** | **1288.6623** | **-0.0085** | **1** | **29** | **0.17** | **1** | **K.VDATAETDLAKR.F** 9158 |
|  | 9185 | **646.2778** | **1290.5411** | **1290.5510** | **-0.0099** | **0** | **54** | **0.0006** | **1** | **K.MDATANDVPSDR.Y** |
|  | 9733 | **440.0297** | **1317.0673** | **1316.6513** | **0.4160** | **0** | **4** | **35** | **4** | **K.FHHTFSTEIAK.F** |
|  | 11990 | **721.8818** | **1441.7491** | **1441.7599** | **-0.0108** | **0** | **41** | **0.01** | **1** | **K.VSQGQLVVMQPEK.F** |
|  | 13042 | 751.8775 | 1501.7404 | 1501.7161 | 0.0243 | 1 | 5 | 46 | 3 | K.FEGGDRDLEHLSK.F |
|  | 15670 | 555.6471 | 1663.9194 | 1662.8981 | 1.0214 | 1 | 5 | 36 | 4 | K.QFAPEYEKIANILK.D |
|  | 17475 | **892.9276** | **1783.8405** | **1782.8498** | **0.9907** | **0** | **78** | **1.6e-006** | **1** | **K.YGIVDYMIEQSGPPSK.E** 17447 |
|  | 18997 | **630.6707** | **1888.9901** | **1887.9360** | **1.0541** | **1** | **5** | **31** | **4** | **K.GLVIAKMDATANDVPSDR.Y** 18995 |
|  | 20758 | 1020.9908 | 2039.9671 | 2039.9874 | -0.0202 | 1 | 5 | 28 | 4 | R.EKYGIVDYMIEQSGPPSK.E 20767 |
|  | 21773 | **1068.5576** | **2135.1007** | **2135.1151** | **-0.0144** | **0** | **18** | **1.3** | **1** | **R.EVSQPDWTPPPEVTLVLTK.E** |
|  | 22030 | **722.0579** | **2163.1519** | **2162.1623** | **0.9896** | **1** | **28** | **0.15** | **1** | **K.QVQEFLKDGDDVIIIGVFK.G** |
|  | 22820 | **749.6993** | **2246.0760** | **2246.0914** | **-0.0154** | **1** | **17** | **1.7** | **1** | **K.DLGLSESGEDVNAAILDESGKK.F** |
|  | 23049 | **760.4150** | **2278.2231** | **2279.0311** | **-0.8079** | **1** | **0** | **76** | **2** | **K.YEPRSHMMDVQGSTQDSAIK.D** |
|  | 23509 | **779.7070** | **2336.0991** | **2336.1141** | **-0.0150** | **1** | **29** | **0.11** | **1** | **R.SHMMDVQGSTQDSAIKDFVLK.Y** |

| **195.** | P30040\|ERP29_HUMAN    **Mass:** 29032    **Score:** 208    **Queries matched:** 14   **emPAI:** 0.63 |
| --- | --- |
|  | Endoplasmic reticulum protein ERp29 OS=Homo sapiens GN=ERP29 PE=1 SV=4 |

|  | **Query** | **Observed** | **Mr(expt)** | **Mr(calc)** | **Delta** | **Miss** | **Score** | **Expect** | **Rank** | **Peptide** |
| --- | --- | --- | --- | --- | --- | --- | --- | --- | --- | --- |
|  | 2224 | **843.0986** | **842.0913** | **841.5385** | **0.5528** | **1** | **11** | **9.4** | **5** | **R.IARLIEK.N** |
|  | 8808 | **636.3494** | **1270.6842** | **1269.6274** | **1.0567** | **1** | **1** | **1e+002** | **6** | **K.LNMELSEKYK.L** |
|  | 14749 | **804.8745** | **1607.7345** | **1607.7468** | **-0.0123** | **0** | **44** | **0.0052** | **1** | **R.DGDFENPVPYTGAVK.V** |
|  | 15077 | 543.3950 | 1627.1632 | 1627.8893 | -0.7261 | 1 | 6 | 19 | 2 | R.QALLKQGQDNLSSVK.E |
|  | 16331 | **854.8902** | **1707.7658** | **1707.7774** | **-0.0115** | **0** | **55** | **0.00037** | **1** | **K.ILDQGEDFPASEMTR.I** 16330 |
|  | 16581 | 575.2842 | 1722.8307 | 1723.7723 | -0.9416 | 0 | (10) | 11 | 2 | K.ILDQGEDFPASEMTR.I 16616 |
|  | 21937 | 1078.0304 | 2154.0462 | 2153.0133 | 1.0330 | 1 | 3 | 47 | 4 | K.IMGKILDQGEDFPASEMTR.I |
|  | 22689 | 744.6669 | 2230.9790 | 2232.1175 | -1.1385 | 1 | 0 | 74 | 6 | R.DGDFENPVPYTGAVKVGAIQR.W |
|  | 24429 | **1234.6865** | **2467.3585** | **2467.1602** | **0.1983** | **0** | **6** | **16** | **1** | **R.LAENSASSDDLLVAEVGISDYGDK.L** |
|  | 25310 | **1292.1335** | **2582.2525** | **2582.2662** | **-0.0136** | **0** | **68** | **9.4e-006** | **1** | **K.GQGVYLGMPGCLPVYDALAGEFIR.A** 25311 |
|  | 29259 | **1138.2092** | **3411.6059** | **3411.6239** | **-0.0180** | **1** | **73** | **1.9e-006** | **1** | **R.LAENSASSDDLLVAEVGISDYGDKLNMELSEK.Y** |

| **196.** | P22695\|QCR2_HUMAN    **Mass:** 48584    **Score:** 208    **Queries matched:** 27   **emPAI:** 0.95 |
| --- | --- |
|  | Cytochrome b-c1 complex subunit 2, mitochondrial OS=Homo sapiens GN=UQCRC2 PE=1 SV=3 |

|  | **Query** | **Observed** | **Mr(expt)** | **Mr(calc)** | **Delta** | **Miss** | **Score** | **Expect** | **Rank** | **Peptide** |
| --- | --- | --- | --- | --- | --- | --- | --- | --- | --- | --- |
|  | 877 | 530.9559 | 529.9487 | 530.2813 | -0.3326 | 0 | 11 | 20 | 6 | R.GGEIR.E |
|  | 1437 | **760.4339** | **759.4266** | **758.4286** | **0.9980** | **0** | **27** | **0.45** | **1** | **R.GGLGLSGAK.A** |
|  | 5240 | 531.2559 | 1060.4973 | 1060.6393 | -0.1420 | 1 | 4 | 70 | 9 | R.IGLFIKAGSR.Y |
|  | 8099 | **412.8921** | **1235.6544** | **1234.6128** | **1.0416** | **0** | **(3)** | **70** | **7** | **K.QVAEQFLNMR.G** |
|  | 8428 | **418.2222** | **1251.6447** | **1250.6077** | **1.0369** | **0** | **4** | **62** | **4** | **K.QVAEQFLNMR.G** |
|  | 11745 | **476.7599** | **1427.2579** | **1426.7668** | **0.4912** | **1** | **7** | **18** | **2** | **R.LTSSLTTKGASSFK.I** |
|  | 12144 | **725.8674** | **1449.7203** | **1449.7324** | **-0.0121** | **0** | **63** | **7.5e-005** | **1** | **R.GSNTTSHLHQAVAK.A** |
|  | 12679 | **741.9023** | **1481.7900** | **1481.7990** | **-0.0090** | **1** | **37** | **0.024** | **1** | **R.RWEVADLQPQLK.I** 12692 |
|  | 14100 | **522.9426** | **1565.8061** | **1565.7296** | **0.0764** | **0** | **4** | **51** | **2** | **R.NALANPLYCPDYR.I** |
|  | 14714 | **803.9175** | **1605.8205** | **1605.8335** | **-0.0130** | **1** | **62** | **7.1e-005** | **1** | **K.RGSNTTSHLHQAVAK.A** 14694 14712 |
|  | 15113 | **815.9183** | **1629.8220** | **1629.8322** | **-0.0102** | **0** | **71** | **1e-005** | **1** | **K.TIAQGNLSNTDVQAAK.N** 15104 |
|  | 15677 | **555.9424** | **1664.8053** | **1664.8158** | **-0.0105** | **0** | **38** | **0.016** | **1** | **R.YEDFSNLGTTHLLR.L** |
|  | 17146 | **588.2947** | **1761.8622** | **1760.8040** | **1.0583** | **0** | **4** | **43** | **5** | **K.SMAASGNLGHTPFVDEL.-** |
|  | 21736 | **710.9629** | **2129.8668** | **2129.0245** | **0.8423** | **1** | **1** | **56** | **8** | **K.LSVTATRENMAYTVECLR.G** |
|  | 22041 | **722.6020** | **2164.7843** | **2164.0338** | **0.7506** | **0** | **5** | **18** | **1** | **K.VTSEELHYFVQNHFTSAR.M** |
|  | 23057 | **760.7203** | **2279.1390** | **2279.1508** | **-0.0118** | **0** | **4** | **35** | **1** | **R.GDVDILMEFLLNVTTAPEFR.R** |
|  | 23190 | **765.7109** | **2294.1108** | **2295.1457** | **-1.0349** | **0** | **(1)** | **55** | **7** | **R.GDVDILMEFLLNVTTAPEFR.R** |
|  | 23596 | **784.0673** | **2349.1800** | **2349.1978** | **-0.0178** | **0** | **27** | **0.13** | **1** | **K.AVAFQNPQTHVIENLHAAAYR.N** 23598 |
|  | 24199 | 813.0273 | 2436.0602 | 2435.2519 | 0.8083 | 1 | 3 | 29 | 8 | R.GDVDILMEFLLNVTTAPEFRR.W |
|  | 25748 | **534.0580** | **2665.2535** | **2666.4400** | **-1.1865** | **1** | **1** | **53** | **6** | **R.MALIGLGVSHPVLKQVAEQFLNMR.G** |
|  | 30193 | 1274.3292 | 3819.9658 | 3818.9843 | 0.9816 | 1 | 38 | 0.0045 | 1 | K.ATAAPAGAPPQPQDLEFTKLPNGLVIASLENYSPVSR.I |
|  | 30219 | **1280.9517** | **3839.8332** | **3839.8530** | **-0.0199** | **0** | **39** | **0.004** | **1** | **K.ATQQPFDVSAFNASYSDSGLFGIYTISQATAAGDVIK.A** |

| **197.** | P19105\|ML12A_HUMAN    **Mass:** 19839    **Score:** 207    **Queries matched:** 15   **emPAI:** 1.89 |
| --- | --- |
|  | Myosin regulatory light chain 12A OS=Homo sapiens GN=MYL12A PE=1 SV=2 |

|  | **Query** | **Observed** | **Mr(expt)** | **Mr(calc)** | **Delta** | **Miss** | **Score** | **Expect** | **Rank** | **Peptide** |
| --- | --- | --- | --- | --- | --- | --- | --- | --- | --- | --- |
|  | 4887 | **518.2572** | **1034.4998** | **1034.5066** | **-0.0068** | **0** | **34** | **0.068** | **1** | **R.ELLTTMGDR.F** 4888 |
|  | 7958 | **615.3007** | **1228.5868** | **1227.6095** | **0.9772** | **0** | **27** | **0.3** | **1** | **K.LNGTDPEDVIR.N** 7954 7964 |
|  | 8103 | **619.2805** | **1236.5464** | **1236.5557** | **-0.0093** | **0** | **55** | **0.0004** | **1** | **K.EAFNMIDQNR.D** |
|  | 8592 | **630.8001** | **1259.5857** | **1259.5935** | **-0.0078** | **0** | **53** | **0.00073** | **1** | **K.GNFNYIEFTR.I** 8570 |
|  | 11091 | **695.3731** | **1388.7317** | **1387.6884** | **1.0432** | **1** | **4** | **54** | **3** | **K.KGNFNYIEFTR.I** |
|  | 11519 | **708.3155** | **1414.6164** | **1414.6252** | **-0.0088** | **0** | **61** | **9.3e-005** | **1** | **R.FTDEEVDELYR.E** |
|  | 21341 | **1045.9894** | **2089.9642** | **2089.9779** | **-0.0137** | **0** | **105** | **2.5e-009** | **1** | **R.ATSNVFAMFDQSQIQEFK.E** 21342 |
|  | 24172 | **1216.5618** | **2431.1090** | **2431.1213** | **-0.0123** | **1** | **48** | **0.0013** | **1** | **R.ELLTTMGDRFTDEEVDELYR.E** 24171 |
|  | 31158 | **960.2320** | **4796.1236** | **4797.1304** | **-1.0069** | **1** | **1** | **12** | **4** | **K.EDLHDMLASLGKNPTDEYLDAMMNEAPGPINFTMFLTMFGEK.L** |

|  | **Proteins matching a subset of these peptides:** |
| --- | --- |

|  | O14950\|ML12B_HUMAN    **Mass:** 19824    **Score:** 207    **Queries matched:** 14 |
| --- | --- |
|  | Myosin regulatory light chain 12B OS=Homo sapiens GN=MYL12B PE=1 SV=2 |

| **198.** | O95573\|ACSL3_HUMAN    **Mass:** 81338    **Score:** 206    **Queries matched:** 33   **emPAI:** 0.43 |
| --- | --- |
|  | Long-chain-fatty-acid--CoA ligase 3 OS=Homo sapiens GN=ACSL3 PE=1 SV=3 |

|  | **Query** | **Observed** | **Mr(expt)** | **Mr(calc)** | **Delta** | **Miss** | **Score** | **Expect** | **Rank** | **Peptide** |
| --- | --- | --- | --- | --- | --- | --- | --- | --- | --- | --- |
|  | 1172 | 644.9523 | 643.9450 | 643.4017 | 0.5433 | 1 | 0 | 1.7e+002 | 2 | K.IIDRK.K 1174 |
|  | 2026 | **415.7376** | **829.4607** | **828.4817** | **0.9790** | **0** | **7** | **39** | **9** | **R.SLLGGNIR.L** |
|  | 2054 | **416.2842** | **830.5539** | **830.4498** | **0.1042** | **0** | **22** | **1.3** | **3** | **K.ELTELAR.K** |
|  | 5352 | 535.7466 | 1069.4786 | 1069.4862 | -0.0076 | 0 | 29 | 0.21 | 1 | K.VSEMSSFQR.N 5386 |
|  | 6258 | **567.2706** | **1132.5267** | **1131.5309** | **0.9958** | **0** | **18** | **2.6** | **1** | **K.THYQADIER.M** |
|  | 9972 | 664.8230 | 1327.6314 | 1328.6507 | -1.0192 | 0 | 3 | 78 | 9 | M.NNHVSSKPSTMK.L |
|  | 11832 | **716.8748** | **1431.7350** | **1431.7470** | **-0.0120** | **0** | **16** | **3.6** | **1** | **K.AKPVNSKPDSAYR.S** 11831 |
|  | 12552 | 492.8911 | 1475.6516 | 1475.6861 | -0.0345 | 0 | 3 | 66 | 7 | -.MNNHVSSKPSTMK.L 12532 |
|  | 13958 | **779.4187** | **1556.8228** | **1556.8345** | **-0.0116** | **0** | **28** | **0.22** | **1** | **R.LLLCGGAPLSATTQR.F** |
|  | 14315 | **791.3856** | **1580.7567** | **1580.7682** | **-0.0115** | **0** | **54** | **0.00047** | **1** | **R.IGYSSPQTLADQSSK.I** |
|  | 15509 | **828.7673** | **1655.5200** | **1655.7759** | **-0.2560** | **1** | **2** | **49** | **1** | **K.NVMNKVSEMSSFQR.N** |
|  | 16002 | 844.4142 | 1686.8138 | 1687.7658 | -0.9520 | 1 | (2) | 81 | 9 | K.NVMNKVSEMSSFQR.N |
|  | 16454 | **858.4318** | **1714.8491** | **1714.8600** | **-0.0109** | **0** | **20** | **1.1** | **1** | **R.GEILIGGQSVTMGYYK.N** |
|  | 19781 | 651.6771 | 1952.0095 | 1951.0204 | 0.9892 | 0 | 9 | 11 | 1 | R.HIITVDGKPPTWSEFPK.G 19764 |
|  | 20291 | **998.9370** | **1995.8595** | **1995.8707** | **-0.0112** | **0** | **53** | **0.00047** | **1** | **R.WLCTGDIGEFEPDGCLK.I** |
|  | 21955 | **540.0230** | **2156.0627** | **2157.0776** | **-1.0149** | **1** | **1** | **77** | **5** | **R.GEILIGGQSVTMGYYKNEAK.T** |
|  | 22151 | **726.0283** | **2175.0631** | **2175.0738** | **-0.0106** | **0** | **25** | **0.26** | **1** | **K.GDTSMLKPTLMAAVPEIMDR.I** |
|  | 22225 | **547.7667** | **2187.0378** | **2187.0497** | **-0.0120** | **1** | **18** | **1.4** | **1** | **K.LKNWEEGGYFNTDKPHPR.G** |
|  | 22604 | **1112.5363** | **2223.0579** | **2223.0729** | **-0.0150** | **0** | **32** | **0.058** | **1** | **R.SVNSLDGLASVLYPGCDTLDK.V** 22605 |
|  | 22645 | **1114.4625** | **2226.9105** | **2226.9231** | **-0.0126** | **0** | **96** | **1.6e-008** | **1** | **K.GTWEELCNSCEMENEVLK.V** |
|  | 23430 | **1163.5962** | **2325.1778** | **2325.1463** | **0.0315** | **1** | **2** | **51** | **2** | **K.VSEMSSFQRNLFILAYNYK.M** 23431 23440 |
|  | 24270 | **816.3733** | **2446.0982** | **2447.2222** | **-1.1240** | **1** | **1** | **62** | **7** | **K.GSKGDTSMLKPTLMAAVPEIMDR.I** |
|  | 28341 | **635.1435** | **3170.6811** | **3171.5580** | **-0.8769** | **0** | **0** | **46** | **3** | **K.ASMENQPHSKPLPSDIAVIMYTSGSTGLPK.G** |
|  | 29845 | **915.4623** | **3657.8203** | **3658.7151** | **-0.8948** | **1** | **1** | **27** | **5** | **K.NWEEGGYFNTDKPHPRGEILIGGQSVTMGYYK.N** |
|  | 30329 | **1306.5676** | **3916.6811** | **3916.6954** | **-0.0143** | **0** | **21** | **0.2** | **1** | **R.FMNICFCCPVGQGYGLTESAGAGTISEVWDYNTGR.V** |

| **199.** | P16070\|CD44_HUMAN    **Mass:** 82017    **Score:** 204    **Queries matched:** 23   **emPAI:** 0.36 |
| --- | --- |
|  | CD44 antigen OS=Homo sapiens GN=CD44 PE=1 SV=2 |

|  | **Query** | **Observed** | **Mr(expt)** | **Mr(calc)** | **Delta** | **Miss** | **Score** | **Expect** | **Rank** | **Peptide** |
| --- | --- | --- | --- | --- | --- | --- | --- | --- | --- | --- |
|  | 1357 | **719.3677** | **718.3605** | **717.3406** | **1.0199** | **0** | **1** | **1.7e+002** | **8** | **R.NDVTGGR.R** |
|  | 2628 | 437.2136 | 872.4127 | 873.4417 | -1.0290 | 1 | 4 | 91 | 2 | R.NDVTGGRR.D |
|  | 3083 | **454.2097** | **906.4049** | **906.4117** | **-0.0068** | **0** | **34** | **0.079** | **1** | **R.TEAADLCK.A** |
|  | 3852 | **481.2391** | **960.4637** | **960.4698** | **-0.0061** | **0** | **33** | **0.11** | **1** | **R.NLQNVDMK.I** |
|  | 4988 | 521.7651 | 1041.5156 | 1041.5243 | -0.0087 | 1 | 27 | 0.31 | 1 | R.YVQKGEYR.T 4987 |
|  | 6543 | 577.2835 | 1152.5523 | 1152.5597 | -0.0074 | 0 | 47 | 0.0028 | 1 | K.ALSIGFETCR.Y 6542 |
|  | 8332 | **624.3126** | **1246.6106** | **1245.6387** | **0.9718** | **1** | **1** | **1.1e+002** | **10** | **R.NLQNVDMKIGV.-** |
|  | 10229 | **672.8365** | **1343.6584** | **1342.6841** | **0.9743** | **0** | **6** | **34** | **2** | **K.LVINSGNGAVEDR.K** |
|  | 11033 | **693.8757** | **1385.7369** | **1385.7456** | **-0.0087** | **0** | **54** | **0.00055** | **1** | **R.YGFIEGHVVIPR.I** 11031 11034 11035 11067 |
|  | 11530 | **472.7524** | **1415.2354** | **1415.6641** | **-0.4287** | **0** | **1** | **75** | **4** | **K.DHPTTSTLTSSNR.N** |
|  | 12465 | 736.3173 | 1470.6201 | 1470.7790 | -0.1589 | 1 | 0 | 1.2e+002 | 8 | K.KLVINSGNGAVEDR.K |
|  | 14349 | **792.3863** | **1582.7581** | **1583.7323** | **-0.9742** | **0** | **0** | **1.1e+002** | **4** | **K.AFNSTLPTMAQMEK.A** |
|  | 18357 | **922.3805** | **1842.7465** | **1842.7578** | **-0.0112** | **0** | **52** | **0.00064** | **1** | **K.ESSETPDQFMTADETR.N** |
|  | 27627 | **1003.7849** | **3008.3327** | **3008.3492** | **-0.0164** | **1** | **64** | **1.8e-005** | **1** | **K.SQEMVHLVNKESSETPDQFMTADETR.N** 27625 27626 |
|  | 29544 | **707.1279** | **3530.6030** | **3529.7461** | **0.8568** | **1** | **1** | **25** | **4** | **-.MDKFWWHAAWGLCLVPLSLAQIDLNITCR.F** |

| **200.** | P27105\|STOM_HUMAN    **Mass:** 31882    **Score:** 203    **Queries matched:** 22   **emPAI:** 0.96 |
| --- | --- |
|  | Erythrocyte band 7 integral membrane protein OS=Homo sapiens GN=STOM PE=1 SV=3 |

|  | **Query** | **Observed** | **Mr(expt)** | **Mr(calc)** | **Delta** | **Miss** | **Score** | **Expect** | **Rank** | **Peptide** |
| --- | --- | --- | --- | --- | --- | --- | --- | --- | --- | --- |
|  | 888 | **533.3637** | **532.3564** | **532.2493** | **0.1072** | **0** | **9** | **36** | **6** | **K.DSPSK.G** |
|  | 1140 | **632.3311** | **631.3238** | **630.3701** | **0.9537** | **0** | **17** | **5.3** | **2** | **R.NVLGTK.N** |
|  | 1256 | **686.4136** | **685.4063** | **685.4123** | **-0.0060** | **0** | **19** | **2.4** | **1** | **R.ILQGGAK.G** |
|  | 1328 | **707.2164** | **706.2091** | **705.3697** | **0.8394** | **0** | **4** | **68** | **9** | **R.LPDSFK.D** |
|  | 3203 | **458.2819** | **914.5492** | **914.5549** | **-0.0057** | **0** | **38** | **0.03** | **1** | **R.LLAQTTLR.N** |
|  | 4508 | **503.9497** | **1005.8849** | **1005.4549** | **0.4300** | **0** | **5** | **38** | **10** | **R.AMAAEAEASR.E** |
|  | 7267 | 399.5266 | 1195.5579 | 1195.6349 | -0.0770 | 1 | 8 | 25 | 7 | K.EYERAIIFR.L |
|  | 8351 | **624.8001** | **1247.5857** | **1246.5975** | **0.9881** | **0** | **30** | **0.13** | **2** | **K.VIAAEGEMNASR.A** |
|  | 10352 | **451.2305** | **1350.6696** | **1350.7395** | **-0.0699** | **0** | **7** | **26** | **5** | **R.YLQTLTTIAAEK.N** |
|  | 10587 | **682.3253** | **1362.6361** | **1361.6357** | **1.0004** | **1** | **4** | **50** | **3** | **R.AMAAEAEASREAR.A** |
|  | 12335 | **487.9706** | **1460.8899** | **1461.7245** | **-0.8346** | **1** | **3** | **66** | **9** | **R.AKVIAAEGEMNASR.A** |
|  | 14217 | 788.3901 | 1574.7656 | 1574.8086 | -0.0430 | 1 | 7 | 23 | 5 | K.VIAAEGEMNASRALK.E |
|  | 14636 | **801.4382** | **1600.8619** | **1600.8712** | **-0.0093** | **0** | **10** | **11** | **1** | **R.TISFDIPPQEILTK.D** |
|  | 16457 | **858.4482** | **1714.8819** | **1714.8923** | **-0.0104** | **0** | **5** | **32** | **3** | **K.EASMVITESPAALQLR.Y** |
|  | 20649 | **1015.0569** | **2028.0992** | **2027.1085** | **0.9907** | **1** | **68** | **1.4e-005** | **1** | **R.ALKEASMVITESPAALQLR.Y** 20639 |
|  | 21712 | **1064.5950** | **2127.1754** | **2126.1809** | **0.9944** | **0** | **51** | **0.00066** | **1** | **K.NSTIVFPLPIDMLQGIIGAK.H** 21701 21713 |
|  | 29146 | **1124.5381** | **3370.5924** | **3370.6099** | **-0.0175** | **1** | **59** | **4.9e-005** | **1** | **K.NLSQILSDREEIAHNMQSTLDDATDAWGIK.V** 29147 29148 |

| **201.** | Q9HCU5\|PREB_HUMAN    **Mass:** 46010    **Score:** 200    **Queries matched:** 21   **emPAI:** 0.48 |
| --- | --- |
|  | Prolactin regulatory element-binding protein OS=Homo sapiens GN=PREB PE=1 SV=2 |

|  | **Query** | **Observed** | **Mr(expt)** | **Mr(calc)** | **Delta** | **Miss** | **Score** | **Expect** | **Rank** | **Peptide** |
| --- | --- | --- | --- | --- | --- | --- | --- | --- | --- | --- |
|  | 3394 | **465.2703** | **928.5261** | **928.4726** | **0.0535** | **1** | **24** | **0.73** | **1** | **K.AGSKEQGPR.Q** 3387 3391 3393 |
|  | 7036 | **593.3227** | **1184.6308** | **1184.5687** | **0.0622** | **0** | **5** | **55** | **4** | **R.FQAHQQQGNK.A** 7031 7039 |
|  | 8960 | **426.9100** | **1277.7081** | **1278.6979** | **-0.9898** | **1** | **4** | **57** | **3** | **R.CQLHLLPSRR.S** |
|  | 9085 | **643.3839** | **1284.7533** | **1283.6622** | **1.0910** | **0** | **25** | **0.43** | **1** | **R.FGQVPDQPAGLR.L** 9058 |
|  | 9127 | **644.4914** | **1286.9682** | **1287.7438** | **-0.7756** | **1** | **0** | **91** | **7** | **K.VPSLEKVLEFK.A** |
|  | 12986 | **500.6127** | **1498.8164** | **1498.6834** | **0.1329** | **0** | **6** | **30** | **4** | **K.CGAETQHEGLELR.V** |
|  | 13211 | **757.3725** | **1512.7304** | **1512.7433** | **-0.0129** | **1** | **35** | **0.036** | **1** | **R.FQAHQQQGNKAEK.A** 13223 |
|  | 14751 | 804.8796 | 1607.7446 | 1608.8736 | -1.1290 | 0 | 2 | 77 | 10 | K.NGVHFLQLELINGR.L |
|  | 15412 | **825.8964** | **1649.7783** | **1649.7897** | **-0.0114** | **0** | **67** | **2.4e-005** | **1** | **K.AHEGEIEDLALGPDGK.L** |
|  | 20541 | **1009.9974** | **2017.9803** | **2017.9957** | **-0.0153** | **0** | **84** | **3.5e-007** | **1** | **R.VENLQAVQTDFSSDPLQK.V** 20542 |
|  | 24688 | **834.7592** | **2501.2558** | **2500.2685** | **0.9873** | **1** | **24** | **0.26** | **1** | **R.LRQPPPCYLTAWDGSNFLPLR.T** |
|  | 24821 | **841.7601** | **2522.2586** | **2522.2733** | **-0.0148** | **0** | **5** | **21** | **1** | **R.ATMNLALAGDILAAGQDAHCQLLR.F** |
|  | 25439 | **868.7490** | **2603.2252** | **2603.2405** | **-0.0152** | **0** | **26** | **0.17** | **1** | **K.DQLVTQLHWQENGPTFSSTPYR.Y** |

| **202.** | Q04826\|1B40_HUMAN    **Mass:** 40822    **Score:** 197    **Queries matched:** 25   **emPAI:** 0.85 |
| --- | --- |
|  | HLA class I histocompatibility antigen, B-40 alpha chain OS=Homo sapiens GN=HLA-B PE=1 SV=1 |

|  | **Query** | **Observed** | **Mr(expt)** | **Mr(calc)** | **Delta** | **Miss** | **Score** | **Expect** | **Rank** | **Peptide** |
| --- | --- | --- | --- | --- | --- | --- | --- | --- | --- | --- |
|  | 4343 | 498.7556 | 995.4965 | 994.4356 | 1.0610 | 0 | 8 | 23 | 2 | R.FDSDATSPR.K 4329 |
|  | 6145 | 562.7893 | 1123.5639 | 1122.5305 | 1.0334 | 1 | 4 | 54 | 3 | R.FDSDATSPRK.E 6130 |
|  | 6565 | 577.7809 | 1153.5473 | 1153.6091 | -0.0618 | 1 | 3 | 67 | 9 | K.ETLQRADPPK.T |
|  | 10335 | 675.8494 | 1349.6843 | 1349.6939 | -0.0096 | 1 | 38 | 0.024 | 1 | R.YLENGKETLQR.A 10311 10351 |
|  | 11736 | 714.3626 | 1426.7105 | 1426.7205 | -0.0099 | 0 | 46 | 0.0031 | 1 | K.WAAVVVPSGEEQR.Y 11721 11735 11751 |
|  | 13321 | 760.3871 | 1518.7596 | 1518.7427 | 0.0169 | 0 | 62 | 7.5e-005 | 1 | R.SWTAADTAAQITQR.K |
|  | 18486 | 463.4837 | 1849.9057 | 1849.9183 | -0.0127 | 0 | 16 | 2.9 | 1 | K.THVTHHPISDHEATLR.C 18485 |
|  | 20872 | 513.0196 | 2048.0493 | 2048.0626 | -0.0133 | 0 | 16 | 2.1 | 1 | R.YTCHVQHEGLPKPLTLR.W 20848 20850 20871 |
|  | 21557 | 1056.5175 | 2111.0204 | 2111.0299 | -0.0095 | 0 | 86 | 2.1e-007 | 1 | R.CWALGFYPAEITLTWQR.D 21558 |
|  | 22689 | 744.6669 | 2230.9790 | 2230.9938 | -0.0149 | 0 | 44 | 0.0032 | 1 | R.DGEDQTQDTELVETRPAGDR.T 22688 |
|  | 23645 | 785.7004 | 2354.0793 | 2354.0927 | -0.0134 | 1 | 36 | 0.02 | 1 | R.GHNQYAYDGKDYIALNEDLR.S |
|  | 30136 | 1896.3450 | 3790.6754 | 3790.1002 | 0.5752 | 1 | 2 | 20 | 5 | R.WEPSSQSTVPIVGIVAGLAVLAVVVIGAVVAAVMCRR.K |

| **203.** | Q95604\|1C17_HUMAN    **Mass:** 41612    **Score:** 197    **Queries matched:** 19   **emPAI:** 0.54 |
| --- | --- |
|  | HLA class I histocompatibility antigen, Cw-17 alpha chain OS=Homo sapiens GN=HLA-C PE=1 SV=1 |

|  | **Query** | **Observed** | **Mr(expt)** | **Mr(calc)** | **Delta** | **Miss** | **Score** | **Expect** | **Rank** | **Peptide** |
| --- | --- | --- | --- | --- | --- | --- | --- | --- | --- | --- |
|  | 965 | 559.2914 | 558.2842 | 558.3125 | -0.0284 | 0 | 15 | 8.3 | 10 | K.LEAAR.E |
|  | 1409 | 746.1623 | 745.1551 | 744.3766 | 0.7785 | 0 | 12 | 10 | 6 | R.EAEQLR.A |
|  | 3888 | 482.7234 | 963.4322 | 964.4250 | -0.9929 | 0 | 33 | 0.088 | 1 | R.FDSDAASPR.G 3900 3901 3907 |
|  | 9722 | 659.3238 | 1316.6331 | 1315.6673 | 0.9658 | 0 | 5 | 45 | 8 | R.YFYTAVSRPGR.G |
|  | 11313 | 468.5608 | 1402.6605 | 1403.6429 | -0.9825 | 1 | 8 | 22 | 2 | R.FDSDAASPRGEPR.A |
|  | 11721 | 713.8703 | 1425.7260 | 1425.7365 | -0.0104 | 0 | 45 | 0.0043 | 1 | K.WAAVVVPSGQEQR.Y 11735 11736 |
|  | 18283 | 613.3167 | 1836.9283 | 1835.9027 | 1.0256 | 0 | 15 | 3.7 | 1 | K.THVTHHPVSDHEATLR.C |
|  | 21557 | 1056.5175 | 2111.0204 | 2111.0299 | -0.0095 | 0 | 86 | 2.1e-007 | 1 | R.CWALGFYPAEITLTWQR.D 21558 |
|  | 25578 | 1319.0889 | 2636.1632 | 2636.1838 | -0.0206 | 0 | 57 | 0.00013 | 1 | R.DGEDQTQDTELVETRPAGDGTFQK.W 25579 25580 25581 |
|  | 27401 | **742.4013** | **2965.5759** | **2964.5929** | **0.9830** | **0** | **9** | **6.7** | **1** | **R.VMAPQALLLLLSGALALIETWAGSHSMR.Y** |

| **204.** | P28799\|GRN_HUMAN    **Mass:** 68518    **Score:** 196    **Queries matched:** 24   **emPAI:** 0.30 |
| --- | --- |
|  | Granulins OS=Homo sapiens GN=GRN PE=1 SV=2 |

|  | **Query** | **Observed** | **Mr(expt)** | **Mr(calc)** | **Delta** | **Miss** | **Score** | **Expect** | **Rank** | **Peptide** |
| --- | --- | --- | --- | --- | --- | --- | --- | --- | --- | --- |
|  | 1366 | **723.4456** | **722.4383** | **722.4075** | **0.0308** | **0** | **15** | **4.7** | **5** | **R.SPHVGVK.D** |
|  | 4461 | **502.2469** | **1002.4792** | **1001.5393** | **0.9399** | **0** | **9** | **23** | **8** | **R.GSEIVAGLEK.M** |
|  | 4480 | **502.7088** | **1003.4030** | **1003.4117** | **-0.0086** | **0** | **22** | **1.2** | **1** | **R.HCCPAGFR.C** 4478 4479 4481 4488 4489 4495 4501 |
|  | 6096 | **561.2463** | **1120.4781** | **1120.4866** | **-0.0085** | **1** | **12** | **10** | **5** | **R.QGVCCADRR.H** |
|  | 6650 | **580.7587** | **1159.5029** | **1159.5128** | **-0.0098** | **1** | **10** | **15** | **1** | **R.RHCCPAGFR.C** |
|  | 7309 | **599.7476** | **1197.4807** | **1196.4855** | **0.9952** | **0** | **28** | **0.21** | **1** | **R.QGWACCPYR.Q** |
|  | 9531 | **437.2584** | **1308.7533** | **1308.6938** | **0.0594** | **1** | **9** | **18** | **7** | **R.WDAPLRDPALR.Q** |
|  | 12349 | **488.2577** | **1461.7512** | **1462.6908** | **-0.9396** | **0** | **4** | **53** | **7** | **R.AVALSSSVMCPDAR.S** |
|  | 14530 | **532.2198** | **1593.6377** | **1593.6487** | **-0.0109** | **0** | **24** | **0.4** | **1** | **R.QHCCPAGYTCNVK.A** 14531 |
|  | 17885 | **905.8738** | **1809.7330** | **1809.7444** | **-0.0114** | **1** | **57** | **0.00017** | **1** | **R.SRCPDGSTCCELPSGK.Y** |
|  | 19223 | **955.3286** | **1908.6427** | **1908.6569** | **-0.0143** | **0** | **74** | **2.8e-006** | **1** | **K.CDMEVSCPDGYTCCR.L** 19209 19224 |
|  | 19404 | **963.3264** | **1924.6381** | **1924.6519** | **-0.0137** | **0** | **(31)** | **0.058** | **1** | **K.CDMEVSCPDGYTCCR.L** |
|  | 23062 | **760.9420** | **2279.8041** | **2279.8201** | **-0.0161** | **0** | **10** | **5.6** | **1** | **K.DVECGEGHFCHDNQTCCR.D** |
|  | 30522 | **1353.5374** | **4057.5902** | **4057.6192** | **-0.0290** | **0** | **57** | **4.3e-005** | **1** | **R.LQSGAWGCCPFTQAVCCEDHIHCCPAGFTCDTQK.G** |

| **205.** | Q9NZN4\|EHD2_HUMAN    **Mass:** 61294    **Score:** 195    **Queries matched:** 22   **emPAI:** 0.34 |
| --- | --- |
|  | EH domain-containing protein 2 OS=Homo sapiens GN=EHD2 PE=1 SV=2 |

|  | **Query** | **Observed** | **Mr(expt)** | **Mr(calc)** | **Delta** | **Miss** | **Score** | **Expect** | **Rank** | **Peptide** |
| --- | --- | --- | --- | --- | --- | --- | --- | --- | --- | --- |
|  | 815 | **515.3254** | **514.3182** | **515.2928** | **-0.9747** | **1** | **15** | **6.6** | **4** | **K.RGGAR.G** 810 812 |
|  | 1604 | **399.3458** | **796.6770** | **795.4967** | **1.1803** | **1** | **3** | **47** | **1** | **R.RLVPPSK.R** |
|  | 2415 | **429.1953** | **856.3760** | **855.4967** | **0.8793** | **0** | **16** | **5** | **3** | **K.FHSLKPK.L** |
|  | 2926 | **448.2560** | **894.4975** | **893.4317** | **1.0658** | **0** | **7** | **34** | **8** | **K.EMPSVFGK.E** |
|  | 8996 | **641.3063** | **1280.5980** | **1280.6071** | **-0.0091** | **1** | **0** | **1.2e+002** | **4** | **K.EMPSVFGKENK.K** |
|  | 9796 | **661.3117** | **1320.6089** | **1320.6166** | **-0.0077** | **0** | **34** | **0.052** | **1** | **K.ADMVETQQLMR.V** 9825 |
|  | 10409 | **451.9515** | **1352.8325** | **1352.6064** | **0.2261** | **0** | **(8)** | **19** | **1** | **K.ADMVETQQLMR.V** 10431 |
|  | 11228 | **466.9263** | **1397.7572** | **1397.7667** | **-0.0095** | **1** | **8** | **23** | **1** | **R.TKLLPLEEHYR.F** |
|  | 16387 | **856.4456** | **1710.8767** | **1710.8862** | **-0.0095** | **0** | **30** | **0.1** | **1** | **K.LLEALDEMLTHDIAK.L** 16386 16408 |
|  | 20224 | **996.0168** | **1990.0191** | **1990.0313** | **-0.0121** | **0** | **40** | **0.0088** | **1** | **R.VYIGSFWSQPLLVPDNR.R** 20225 |
|  | 26489 | **558.6740** | **2788.3337** | **2788.3225** | **0.0112** | **1** | **0** | **56** | **9** | **K.LSDVDRDGMLDDEEFALASHLIEAK.L** |
|  | 27099 | **1451.7499** | **2901.4852** | **2901.4980** | **-0.0128** | **0** | **81** | **4.3e-007** | **1** | **R.FMCAQLPNQVLESISIIDTPGILSGAK.Q** 27100 |
|  | 28283 | **1052.5040** | **3154.4903** | **3154.5070** | **-0.0167** | **0** | **48** | **0.00082** | **1** | **R.FGAFHSPALEDADFDGKPMVLVAGQYSTGK.T** |
|  | 28332 | **1057.5121** | **3169.5144** | **3170.5019** | **-0.9875** | **0** | **(1)** | **39** | **9** | **R.FGAFHSPALEDADFDGKPMVLVAGQYSTGK.T** |

| **206.** | P30493\|1B55_HUMAN    **Mass:** 40756    **Score:** 195    **Queries matched:** 30   **emPAI:** 1.02 |
| --- | --- |
|  | HLA class I histocompatibility antigen, B-55 alpha chain OS=Homo sapiens GN=HLA-B PE=1 SV=1 |

|  | **Query** | **Observed** | **Mr(expt)** | **Mr(calc)** | **Delta** | **Miss** | **Score** | **Expect** | **Rank** | **Peptide** |
| --- | --- | --- | --- | --- | --- | --- | --- | --- | --- | --- |
|  | 1409 | 746.1623 | 745.1551 | 744.3766 | 0.7785 | 0 | 12 | 10 | 6 | R.EAEQLR.A |
|  | 1455 | 766.4029 | 765.3956 | 765.4021 | -0.0065 | 0 | 37 | 0.038 | 1 | R.NTQIYK.A |
|  | 3888 | 482.7234 | 963.4322 | 964.4250 | -0.9929 | 0 | 33 | 0.088 | 1 | R.FDSDAASPR.E 3900 3901 3907 |
|  | 6565 | 577.7809 | 1153.5473 | 1153.6091 | -0.0618 | 1 | 3 | 67 | 9 | K.ETLQRADPPK.T |
|  | 10335 | 675.8494 | 1349.6843 | 1349.6939 | -0.0096 | 1 | 38 | 0.024 | 1 | R.YLENGKETLQR.A 10311 10351 |
|  | 10618 | 455.7262 | 1364.1569 | 1363.6343 | 0.5226 | 0 | 3 | 50 | 7 | R.YFYTAMSRPGR.G |
|  | 11736 | 714.3626 | 1426.7105 | 1426.7205 | -0.0099 | 0 | 46 | 0.0031 | 1 | K.WAAVVVPSGEEQR.Y 11721 11735 11751 |
|  | 12552 | 492.8911 | 1475.6516 | 1475.6641 | -0.0125 | 1 | 13 | 6.8 | 1 | R.FDSDAASPREEPR.A 12553 |
|  | 12921 | 748.8587 | 1495.7028 | 1495.7129 | -0.0101 | 0 | 57 | 0.00026 | 1 | R.AYLEGTCVEWLR.R |
|  | 17808 | 902.9733 | 1803.9321 | 1802.8522 | 1.0799 | 1 | 3 | 60 | 8 | R.YFYTAMSRPGRGEPR.F |
|  | 18486 | 463.4837 | 1849.9057 | 1849.9183 | -0.0127 | 0 | 16 | 2.9 | 1 | K.THVTHHPISDHEATLR.C 18485 |
|  | 20872 | 513.0196 | 2048.0493 | 2048.0626 | -0.0133 | 0 | 16 | 2.1 | 1 | R.YTCHVQHEGLPKPLTLR.W 20848 20850 20871 |
|  | 21557 | 1056.5175 | 2111.0204 | 2111.0299 | -0.0095 | 0 | 86 | 2.1e-007 | 1 | R.CWALGFYPAEITLTWQR.D 21558 |
|  | 22689 | 744.6669 | 2230.9790 | 2230.9938 | -0.0149 | 0 | 44 | 0.0032 | 1 | R.DGEDQTQDTELVETRPAGDR.T 22688 |
|  | 23097 | 1143.5082 | 2285.0018 | 2286.0341 | -1.0323 | 1 | 11 | 6.4 | 1 | R.EEPRAPWIEQEGPEYWDR.N |

| **207.** | P30492\|1B54_HUMAN    **Mass:** 40640    **Score:** 195    **Queries matched:** 27   **emPAI:** 1.03 |
| --- | --- |
|  | HLA class I histocompatibility antigen, B-54 alpha chain OS=Homo sapiens GN=HLA-B PE=2 SV=1 |

|  | **Query** | **Observed** | **Mr(expt)** | **Mr(calc)** | **Delta** | **Miss** | **Score** | **Expect** | **Rank** | **Peptide** |
| --- | --- | --- | --- | --- | --- | --- | --- | --- | --- | --- |
|  | 1455 | 766.4029 | 765.3956 | 765.4021 | -0.0065 | 0 | 37 | 0.038 | 1 | R.NTQIYK.A |
|  | 3888 | 482.7234 | 963.4322 | 964.4250 | -0.9929 | 0 | 33 | 0.088 | 1 | R.FDSDAASPR.G 3900 3901 3907 |
|  | 6565 | 577.7809 | 1153.5473 | 1153.6091 | -0.0618 | 1 | 3 | 67 | 9 | K.ETLQRADPPK.T |
|  | 10335 | 675.8494 | 1349.6843 | 1349.6939 | -0.0096 | 1 | 38 | 0.024 | 1 | R.YLENGKETLQR.A 10311 10351 |
|  | 10618 | 455.7262 | 1364.1569 | 1363.6343 | 0.5226 | 0 | 3 | 50 | 7 | R.YFYTAMSRPGR.G |
|  | 11313 | 468.5608 | 1402.6605 | 1403.6429 | -0.9825 | 1 | 8 | 22 | 2 | R.FDSDAASPRGEPR.A |
|  | 11736 | 714.3626 | 1426.7105 | 1426.7205 | -0.0099 | 0 | 46 | 0.0031 | 1 | K.WAAVVVPSGEEQR.Y 11721 11735 11751 |
|  | 12921 | 748.8587 | 1495.7028 | 1495.7129 | -0.0101 | 0 | 57 | 0.00026 | 1 | R.AYLEGTCVEWLR.R |
|  | 17808 | 902.9733 | 1803.9321 | 1802.8522 | 1.0799 | 1 | 3 | 60 | 8 | R.YFYTAMSRPGRGEPR.F |
|  | 18486 | 463.4837 | 1849.9057 | 1849.9183 | -0.0127 | 0 | 16 | 2.9 | 1 | K.THVTHHPISDHEATLR.C 18485 |
|  | 20872 | 513.0196 | 2048.0493 | 2048.0626 | -0.0133 | 0 | 16 | 2.1 | 1 | R.YTCHVQHEGLPKPLTLR.W 20848 20850 20871 |
|  | 21557 | 1056.5175 | 2111.0204 | 2111.0299 | -0.0095 | 0 | 86 | 2.1e-007 | 1 | R.CWALGFYPAEITLTWQR.D 21558 |
|  | 22689 | 744.6669 | 2230.9790 | 2230.9938 | -0.0149 | 0 | 44 | 0.0032 | 1 | R.DGEDQTQDTELVETRPAGDR.T 22688 |

| **208.** | P30049\|ATPD_HUMAN    **Mass:** 17479    **Score:** 193    **Queries matched:** 17   **emPAI:** 0.49 |
| --- | --- |
|  | ATP synthase subunit delta, mitochondrial OS=Homo sapiens GN=ATP5D PE=1 SV=2 |

|  | **Query** | **Observed** | **Mr(expt)** | **Mr(calc)** | **Delta** | **Miss** | **Score** | **Expect** | **Rank** | **Peptide** |
| --- | --- | --- | --- | --- | --- | --- | --- | --- | --- | --- |
|  | 4171 | **493.2191** | **984.4236** | **985.5444** | **-1.1208** | **0** | **19** | **1.9** | **1** | **R.IEANEALVK.A** |
|  | 11809 | 716.3525 | 1430.6904 | 1430.7001 | -0.0097 | 0 | 68 | 2e-005 | 1 | K.AQAELVGTADEATR.A |
|  | 13364 | 761.4247 | 1520.8349 | 1519.9133 | 0.9216 | 1 | 7 | 27 | 3 | -.MLPAALLRRPGLGR.L |
|  | 21817 | **714.9844** | **2141.9315** | **2141.1076** | **0.8239** | **1** | **3** | **43** | **2** | **K.AQAELVGTADEATRAEIQIR.I** |
|  | 29707 | **1195.6008** | **3583.7807** | **3583.7967** | **-0.0161** | **0** | **45** | **0.0011** | **1** | **K.YFVSSGSIAVNADSSVQLLAEEAVTLDMLDLGAAK.A** 29698 29700 29701 29704 29705 29708 29710 29712 29713 29714 29715 29717 |

| **209.** | P08571\|CD14_HUMAN    **Mass:** 40678    **Score:** 192    **Queries matched:** 13   **emPAI:** 0.56 |
| --- | --- |
|  | Monocyte differentiation antigen CD14 OS=Homo sapiens GN=CD14 PE=1 SV=2 |

|  | **Query** | **Observed** | **Mr(expt)** | **Mr(calc)** | **Delta** | **Miss** | **Score** | **Expect** | **Rank** | **Peptide** |
| --- | --- | --- | --- | --- | --- | --- | --- | --- | --- | --- |
|  | 3152 | 456.7453 | 911.4761 | 911.4825 | -0.0064 | 0 | 47 | 0.0032 | 1 | R.ATVNPSAPR.C |
|  | 3858 | 481.2480 | 960.4815 | 959.5175 | 0.9640 | 0 | 7 | 40 | 10 | K.ELTLEDLK.I |
|  | 6341 | **571.2756** | **1140.5366** | **1141.6608** | **-1.1242** | **0** | **11** | **12** | **7** | **K.FPAIQNLALR.N** |
|  | 17331 | **889.0324** | **1776.0503** | **1776.0621** | **-0.0119** | **0** | **39** | **0.012** | **1** | **R.LTVGAAQVPAQLLVGALR.V** 17355 |
|  | 17681 | 899.0027 | 1795.9909 | 1795.9985 | -0.0075 | 0 | 72 | 6.3e-006 | 1 | R.SWLAELQQWLKPGLK.V 17680 |
|  | 19537 | **645.3925** | **1933.1557** | **1932.1633** | **0.9925** | **1** | **11** | **7.5** | **1** | **R.RLTVGAAQVPAQLLVGALR.V** |
|  | 20340 | 667.3346 | 1998.9819 | 1998.9945 | -0.0126 | 0 | 50 | 0.00093 | 1 | K.VLSIAQAHSPAFSCEQVR.A 20338 20339 |
|  | 21780 | **1069.5920** | **2137.1695** | **2137.1817** | **-0.0121** | **0** | **21** | **0.58** | **1** | **K.ITGTMPPLPLEATGLALSSLR.L** 21782 |

| **210.** | P01023\|A2MG_HUMAN    **Mass:** 164600   **Score:** 191    **Queries matched:** 33   **emPAI:** 0.14 |
| --- | --- |
|  | Alpha-2-macroglobulin OS=Homo sapiens GN=A2M PE=1 SV=1 |

|  | **Query** | **Observed** | **Mr(expt)** | **Mr(calc)** | **Delta** | **Miss** | **Score** | **Expect** | **Rank** | **Peptide** |
| --- | --- | --- | --- | --- | --- | --- | --- | --- | --- | --- |
|  | 751 | **488.2478** | **487.2405** | **488.2231** | **-0.9826** | **0** | **5** | **1.2e+002** | **9** | **K.DLGNA.-** |
|  | 1280 | **694.3460** | **693.3387** | **693.3731** | **-0.0344** | **0** | **20** | **1.5** | **1** | **R.TTVMVK.N** |
|  | 2225 | **843.0993** | **842.0920** | **842.3844** | **-0.2923** | **1** | **3** | **62** | **5** | **R.KEYEMK.L** |
|  | 3483 | **468.3268** | **934.6391** | **933.4920** | **1.1471** | **1** | **3** | **79** | **4** | **K.GPTQEFKK.R** |
|  | 5040 | 523.2839 | 1044.5533 | 1045.5808 | -1.0275 | 0 | 9 | 20 | 4 | K.FEVQVTVPK.I 5058 |
|  | 7891 | **613.6134** | **1225.2122** | **1224.5445** | **0.6678** | **0** | **0** | **92** | **1** | **K.YDVENCLANK.V** |
|  | 8479 | **628.3209** | **1254.6273** | **1254.6357** | **-0.0084** | **0** | **38** | **0.023** | **1** | **K.AIGYLNTGYQR.Q** 8478 |
|  | 10494 | 679.8065 | 1357.5984 | 1357.7453 | -0.1469 | 1 | 7 | 28 | 4 | K.EVLKSLNEEAVK.K 10495 |
|  | 11572 | **709.7975** | **1417.5804** | **1417.5899** | **-0.0094** | **0** | **37** | **0.028** | **1** | **K.HYDGSYSTFGER.Y** 11590 |
|  | 12373 | 732.8674 | 1463.7203 | 1463.6312 | 0.0891 | 0 | 3 | 66 | 6 | K.DMYSFLEDMGLK.A |
|  | 12830 | **745.9003** | **1489.7860** | **1490.7882** | **-1.0022** | **0** | **3** | **67** | **1** | **R.NQGNTWLTAFVLK.T** |
|  | 13922 | **519.2676** | **1554.7809** | **1555.8028** | **-1.0219** | **1** | **4** | **49** | **2** | **R.TGTHGLLVKQEDMK.G** 13945 |
|  | 17699 | **599.9961** | **1796.9666** | **1796.9268** | **0.0398** | **1** | **4** | **44** | **10** | **R.TGKAAQVTIQSSGTFSSK.F** |
|  | 18401 | **923.0177** | **1844.0208** | **1844.0295** | **-0.0087** | **0** | **14** | **4.3** | **1** | **R.LLIYAVLPTGDVIGDSAK.Y** |
|  | 18909 | **942.5215** | **1883.0284** | **1883.0404** | **-0.0120** | **0** | **52** | **0.00069** | **1** | **R.VSVQLEASPAFLAVPVEK.E** 18908 |
|  | 19050 | **474.5119** | **1894.0186** | **1893.0472** | **0.9714** | **1** | **12** | **5.7** | **3** | **K.GHFSISIPVKSDIAPVAR.L** |
|  | 19667 | 649.3256 | 1944.9550 | 1945.0893 | -0.1342 | 1 | (1) | 79 | 10 | K.MVSGFIPLKPTVKMLER.S |
|  | 19937 | **491.5196** | **1962.0494** | **1961.0842** | **0.9652** | **1** | **2** | **53** | **6** | **K.MVSGFIPLKPTVKMLER.S** |
|  | 20822 | **1023.0475** | **2044.0804** | **2044.0914** | **-0.0110** | **0** | **60** | **8.8e-005** | **1** | **R.LLLQQVSLPELPGEYSMK.V** |
|  | 22705 | **745.2916** | **2232.8529** | **2232.8688** | **-0.0160** | **1** | **42** | **0.0043** | **1** | **R.KYSDASDCHGEDSQAFCEK.F** |
|  | 24042 | 804.7184 | 2411.1335 | 2411.2406 | -0.1071 | 1 | 6 | 20 | 2 | K.VTGEGCVYLQTSLKYNILPEK.E |
|  | 28078 | **779.1556** | **3112.5932** | **3112.6056** | **-0.0123** | **1** | **6** | **12** | **1** | **K.FRVVSMDENFHPLNELIPLVYIQDPK.G** 28075 |
|  | 28307 | **1055.1876** | **3162.5410** | **3162.5583** | **-0.0173** | **1** | **13** | **2.2** | **1** | **K.YNILPEKEEFPFALGVQTLPQTCDEPK.A** 28308 |
|  | 29063 | **837.4099** | **3345.6105** | **3345.4675** | **0.1430** | **1** | **1** | **35** | **5** | **K.MCPQLQQYEMHGPEGLRVGFYESDVMGR.G** |
|  | 31237 | **1227.6036** | **4906.3855** | **4906.4263** | **-0.0408** | **0** | **41** | **0.0012** | **1** | **K.APVGHFYEPQAPSAEVEMTSYVLLAYLTAQPAPTSEDLTSATNIVK.W** |

| **211.** | P02786\|TFR1_HUMAN    **Mass:** 85274    **Score:** 191    **Queries matched:** 26   **emPAI:** 0.24 |
| --- | --- |
|  | Transferrin receptor protein 1 OS=Homo sapiens GN=TFRC PE=1 SV=2 |

|  | **Query** | **Observed** | **Mr(expt)** | **Mr(calc)** | **Delta** | **Miss** | **Score** | **Expect** | **Rank** | **Peptide** |
| --- | --- | --- | --- | --- | --- | --- | --- | --- | --- | --- |
|  | 3023 | **902.9807** | **901.9734** | **902.4756** | **-0.5022** | **1** | **8** | **21** | **1** | **K.LNDRVMR.V** |
|  | 3810 | **479.7911** | **957.5677** | **957.5859** | **-0.0181** | **0** | **14** | **7.6** | **2** | **K.SGVGTALLLK.L** |
|  | 7269 | **399.5360** | **1195.5860** | **1196.5925** | **-1.0065** | **0** | **8** | **20** | **5** | **K.LDSTDFTGTIK.L** |
|  | 7457 | **602.8155** | **1203.6164** | **1203.6248** | **-0.0083** | **0** | **56** | **0.00041** | **1** | **K.LLNENSYVPR.E** |
|  | 7673 | **405.7243** | **1214.1511** | **1213.6356** | **0.5154** | **1** | **4** | **46** | **3** | **K.VWRDQHFVK.I** |
|  | 8256 | **622.7920** | **1243.5694** | **1242.7547** | **0.8147** | **1** | **0** | **1.4e+002** | **6** | **K.LTVSNVLKEIK.I** |
|  | 9621 | **657.8317** | **1313.6489** | **1313.6359** | **0.0130** | **0** | **5** | **49** | **2** | **K.LAQMFSDMVLK.D** 9660 |
|  | 11837 | 717.3225 | 1432.6303 | 1432.8177 | -0.1874 | 0 | 11 | 9.9 | 4 | K.VSASPLLYTLIEK.T 11854 |
|  | 12434 | **734.8528** | **1467.6910** | **1467.8045** | **-0.1135** | **0** | **8** | **19** | **3** | **R.SSGLPNIPVQTISR.A** 12420 |
|  | 13896 | **777.4051** | **1552.7956** | **1552.7708** | **0.0249** | **0** | **2** | **77** | **2** | **K.EMGLSLQWLYSAR.G** |
|  | 14017 | **781.3975** | **1560.7805** | **1560.6903** | **0.0902** | **0** | **31** | **0.098** | **1** | **K.LAVDEEENADNNTK.A** |
|  | 14069 | 522.2869 | 1563.8390 | 1564.7926 | -0.9536 | 0 | 30 | 0.13 | 1 | R.VEYHFLSPYVSPK.E 14087 14099 |
|  | 15492 | **828.3405** | **1654.6665** | **1654.6756** | **-0.0091** | **0** | **50** | **0.0011** | **1** | **K.LFGNMEGDCPSDWK.T** 15491 |
|  | 15782 | **836.9202** | **1671.8258** | **1671.8369** | **-0.0111** | **0** | **64** | **5e-005** | **1** | **K.GFVEPDHYVVVGAQR.D** 15779 15783 |
|  | 17247 | **886.4615** | **1770.9085** | **1770.9192** | **-0.0107** | **0** | **4** | **48** | **2** | **R.LVYLVENPGGYVAYSK.A** |
|  | 20285 | 499.7544 | 1994.9886 | 1996.0088 | -1.0201 | 1 | 5 | 31 | 4 | R.ADIKEMGLSLQWLYSAR.G |
|  | 21488 | **527.2809** | **2105.0947** | **2105.1058** | **-0.0111** | **0** | **23** | **0.43** | **1** | **R.HVFWGSGSHTLPALLENLK.L** |
|  | 22590 | **741.2894** | **2220.8463** | **2221.2214** | **-0.3751** | **1** | **2** | **36** | **5** | **K.SGVGTALLLKLAQMFSDMVLK.D** |

| **212.** | Q29940\|1B59_HUMAN    **Mass:** 40844    **Score:** 189    **Queries matched:** 30   **emPAI:** 0.85 |
| --- | --- |
|  | HLA class I histocompatibility antigen, B-59 alpha chain OS=Homo sapiens GN=HLA-B PE=2 SV=1 |

|  | **Query** | **Observed** | **Mr(expt)** | **Mr(calc)** | **Delta** | **Miss** | **Score** | **Expect** | **Rank** | **Peptide** |
| --- | --- | --- | --- | --- | --- | --- | --- | --- | --- | --- |
|  | 3888 | 482.7234 | 963.4322 | 964.4250 | -0.9929 | 0 | 33 | 0.088 | 1 | R.FDSDAASPR.E 3900 3901 3907 |
|  | 4176 | 493.2762 | 984.5379 | 983.5876 | 0.9503 | 1 | 10 | 18 | 10 | R.ENLRIALR.Y 4164 |
|  | 6565 | 577.7809 | 1153.5473 | 1153.6091 | -0.0618 | 1 | 3 | 67 | 9 | K.ETLQRADPPK.T |
|  | 10335 | 675.8494 | 1349.6843 | 1349.6939 | -0.0096 | 1 | 38 | 0.024 | 1 | R.YLENGKETLQR.A 10311 10351 |
|  | 10618 | 455.7262 | 1364.1569 | 1363.6343 | 0.5226 | 0 | 3 | 50 | 7 | R.YFYTAMSRPGR.G |
|  | 11736 | 714.3626 | 1426.7105 | 1426.7205 | -0.0099 | 0 | 46 | 0.0031 | 1 | K.WAAVVVPSGEEQR.Y 11721 11735 11751 |
|  | 12552 | 492.8911 | 1475.6516 | 1475.6641 | -0.0125 | 1 | 13 | 6.8 | 1 | R.FDSDAASPREEPR.A 12553 |
|  | 12921 | 748.8587 | 1495.7028 | 1495.7129 | -0.0101 | 0 | 57 | 0.00026 | 1 | R.AYLEGTCVEWLR.R |
|  | 17808 | 902.9733 | 1803.9321 | 1802.8522 | 1.0799 | 1 | 3 | 60 | 8 | R.YFYTAMSRPGRGEPR.F |
|  | 18486 | 463.4837 | 1849.9057 | 1849.9183 | -0.0127 | 0 | 16 | 2.9 | 1 | K.THVTHHPISDHEATLR.C 18485 |
|  | 20872 | 513.0196 | 2048.0493 | 2048.0626 | -0.0133 | 0 | 16 | 2.1 | 1 | R.YTCHVQHEGLPKPLTLR.W 20848 20850 20871 |
|  | 21557 | 1056.5175 | 2111.0204 | 2111.0299 | -0.0095 | 0 | 86 | 2.1e-007 | 1 | R.CWALGFYPAEITLTWQR.D 21558 |
|  | 22689 | 744.6669 | 2230.9790 | 2230.9938 | -0.0149 | 0 | 44 | 0.0032 | 1 | R.DGEDQTQDTELVETRPAGDR.T 22688 |
|  | 23097 | 1143.5082 | 2285.0018 | 2286.0341 | -1.0323 | 1 | 11 | 6.4 | 1 | R.EEPRAPWIEQEGPEYWDR.N |

| **213.** | P16452\|EPB42_HUMAN    **Mass:** 77816    **Score:** 189    **Queries matched:** 14   **emPAI:** 0.15 |
| --- | --- |
|  | Erythrocyte membrane protein band 4.2 OS=Homo sapiens GN=EPB42 PE=1 SV=3 |

|  | **Query** | **Observed** | **Mr(expt)** | **Mr(calc)** | **Delta** | **Miss** | **Score** | **Expect** | **Rank** | **Peptide** |
| --- | --- | --- | --- | --- | --- | --- | --- | --- | --- | --- |
|  | 5936 | 555.2867 | 1108.5588 | 1109.6961 | -1.1373 | 0 | 5 | 46 | 3 | R.VLGALLHFLK.E |
|  | 9153 | **645.3342** | **1288.6539** | **1288.6564** | **-0.0025** | **1** | **9** | **18** | **4** | **R.KWWSAVVEER.D** |
|  | 9988 | **665.3489** | **1328.6833** | **1328.6936** | **-0.0103** | **0** | **38** | **0.021** | **1** | **K.VALTAQTGEQPSK.I** 9990 |
|  | 10639 | **683.4304** | **1364.8463** | **1365.7980** | **-0.9517** | **1** | **1** | **90** | **4** | **K.KLHLTLSANLEK.I** |
|  | 13162 | **504.2931** | **1509.8575** | **1509.8456** | **0.0119** | **1** | **4** | **52** | **8** | **R.RGQPFTIILYFR.A** |
|  | 15275 | **821.4006** | **1640.7866** | **1640.7439** | **0.0427** | **1** | **3** | **63** | **4** | **R.FRSVWPENTMCAK.F** |
|  | 19216 | **955.0164** | **1908.0182** | **1908.0316** | **-0.0135** | **0** | **76** | **2.7e-006** | **1** | **R.VLPTPQTQATQEGALLNK.R** 19215 |
|  | 20573 | **674.8416** | **2021.5030** | **2021.1422** | **0.3608** | **0** | **1** | **50** | **1** | **K.AVQLAIGVQAVHYNGVLAAK.L** |
|  | 24351 | **819.1284** | **2454.3633** | **2453.3529** | **1.0103** | **1** | **4** | **23** | **2** | **R.EKDNGIRPPSLETASPLYLLLK.A** |
|  | 27826 | 1015.5037 | 3043.4892 | 3042.5974 | 0.8917 | 1 | 0 | 46 | 6 | R.QWLTGRGRPVYDGQAWVLAAVACTVLR.C |
|  | 30302 | **1297.6414** | **3889.9023** | **3889.8900** | **0.0123** | **1** | **50** | **0.00033** | **1** | **K.MPEKAEQYQPLTASVSLQNSLDAPMEDCVISILGR.G** 30301 |

| **214.** | Q15005\|SPCS2_HUMAN    **Mass:** 25272    **Score:** 188    **Queries matched:** 8   **emPAI:** 0.32 |
| --- | --- |
|  | Signal peptidase complex subunit 2 OS=Homo sapiens GN=SPCS2 PE=1 SV=3 |

|  | **Query** | **Observed** | **Mr(expt)** | **Mr(calc)** | **Delta** | **Miss** | **Score** | **Expect** | **Rank** | **Peptide** |
| --- | --- | --- | --- | --- | --- | --- | --- | --- | --- | --- |
|  | 3638 | **474.2296** | **946.4447** | **946.4654** | **-0.0208** | **0** | **9** | **21** | **5** | **-.MAAAAVQGGR.S** |
|  | 4803 | 515.7332 | 1029.4519 | 1028.5179 | 0.9340 | 1 | 7 | 38 | 3 | R.FDDKYTLK.L |
|  | 7355 | **400.5566** | **1198.6480** | **1198.6822** | **-0.0342** | **1** | **17** | **2.9** | **1** | **K.EKSIFLVAHR.K** |
|  | 9039 | **642.2831** | **1282.5517** | **1281.6353** | **0.9164** | **0** | **4** | **51** | **7** | **K.YVENFGLIDGR.L** |
|  | 14509 | **796.8070** | **1591.5995** | **1591.7478** | **-0.1483** | **1** | **1** | **88** | **8** | **K.WDGSAVKNSLDDSAK.K** |
|  | 18572 | **929.3568** | **1856.6991** | **1856.7126** | **-0.0136** | **0** | **84** | **3.6e-007** | **1** | **R.SGGSGGCSGAGGASNCGTGSGR.S** |
|  | 20389 | **1002.9586** | **2003.9026** | **2003.9146** | **-0.0121** | **0** | **81** | **7.1e-007** | **1** | **K.DPTGMDPDDIWQLSSSLK.R** 20390 |

| **215.** | P35232\|PHB_HUMAN    **Mass:** 29843    **Score:** 188    **Queries matched:** 12   **emPAI:** 1.05 |
| --- | --- |
|  | Prohibitin OS=Homo sapiens GN=PHB PE=1 SV=1 |

|  | **Query** | **Observed** | **Mr(expt)** | **Mr(calc)** | **Delta** | **Miss** | **Score** | **Expect** | **Rank** | **Peptide** |
| --- | --- | --- | --- | --- | --- | --- | --- | --- | --- | --- |
|  | 1103 | **620.3097** | **619.3024** | **620.3534** | **-1.0509** | **0** | **10** | **20** | **1** | **R.FVVEK.A** |
|  | 6466 | **575.2947** | **1148.5749** | **1148.5826** | **-0.0077** | **0** | **68** | **2.7e-005** | **1** | **R.FDAGELITQR.E** |
|  | 7106 | **397.1993** | **1188.5761** | **1188.6350** | **-0.0589** | **1** | **2** | **99** | **10** | **K.KAAIISAEGDSK.A** |
|  | 7486 | **603.3251** | **1204.6357** | **1204.6452** | **-0.0095** | **1** | **8** | **28** | **3** | **R.FVVEKAEQQK.K** |
|  | 12038 | **723.3297** | **1444.6449** | **1443.6518** | **0.9931** | **0** | **21** | **1.1** | **1** | **R.IFTSIGEDYDER.V** |
|  | 14715 | **803.9201** | **1605.8256** | **1605.8362** | **-0.0105** | **1** | **90** | **1.2e-007** | **1** | **R.KLEAAEDIAYQLSR.S** |
|  | 18544 | **928.0150** | **1854.0153** | **1854.0251** | **-0.0097** | **0** | **13** | **5.2** | **1** | **R.NITYLPAGQSVLLQLPQ.-** |
|  | 20308 | **999.5411** | **1997.0677** | **1997.0793** | **-0.0115** | **0** | **42** | **0.0064** | **1** | **K.AAELIANSLATAGDGLIELR.K** 20310 |
|  | 23781 | 791.0848 | 2370.2325 | 2370.2444 | -0.0119 | 0 | 39 | 0.0097 | 1 | K.FGLALAVAGGVVNSALYNVDAGHR.A |
|  | 28123 | **1041.8816** | **3122.6229** | **3122.6376** | **-0.0146** | **0** | **47** | **0.001** | **1** | **R.GVQDIVVGEGTHFLIPWVQKPIIFDCR.S** 28122 |

| **216.** | Q29865\|1C18_HUMAN    **Mass:** 41363    **Score:** 186    **Queries matched:** 23   **emPAI:** 0.68 |
| --- | --- |
|  | HLA class I histocompatibility antigen, Cw-18 alpha chain OS=Homo sapiens GN=HLA-C PE=2 SV=1 |

|  | **Query** | **Observed** | **Mr(expt)** | **Mr(calc)** | **Delta** | **Miss** | **Score** | **Expect** | **Rank** | **Peptide** |
| --- | --- | --- | --- | --- | --- | --- | --- | --- | --- | --- |
|  | 3888 | 482.7234 | 963.4322 | 964.4250 | -0.9929 | 0 | 33 | 0.088 | 1 | R.FDSDAASPR.G 3900 3901 3907 |
|  | 10335 | 675.8494 | 1349.6843 | 1349.6939 | -0.0096 | 1 | 38 | 0.024 | 1 | R.YLENGKETLQR.A 10311 10351 |
|  | 11313 | 468.5608 | 1402.6605 | 1403.6429 | -0.9825 | 1 | 8 | 22 | 2 | R.FDSDAASPRGEPR.A |
|  | 11736 | 714.3626 | 1426.7105 | 1426.7205 | -0.0099 | 0 | 46 | 0.0031 | 1 | K.WAAVVVPSGEEQR.Y 11721 11735 11751 |
|  | 12921 | 748.8587 | 1495.7028 | 1495.7129 | -0.0101 | 0 | 57 | 0.00026 | 1 | R.AYLEGTCVEWLR.R |
|  | 13321 | 760.3871 | 1518.7596 | 1518.7427 | 0.0169 | 0 | 62 | 7.5e-005 | 1 | R.SWTAADTAAQITQR.K |
|  | 14680 | 803.3827 | 1604.7508 | 1605.7756 | -1.0247 | 1 | 2 | 71 | 6 | R.MFGCDLGPDGRLLR.G |
|  | 15320 | 823.4096 | 1644.8047 | 1644.8148 | -0.0101 | 0 | 69 | 1.4e-005 | 1 | R.FISVGYVDDTQFVR.F 15321 |
|  | 18283 | 613.3167 | 1836.9283 | 1835.9027 | 1.0256 | 0 | 15 | 3.7 | 1 | K.THVTHHPVSDHEATLR.C |
|  | 20871 | 410.6170 | 2048.0485 | 2049.0102 | -0.9617 | 0 | 9 | 11 | 2 | R.YTCHVQHEGLPEPLTLR.W 20872 20886 20897 |
|  | 25366 | 865.0411 | 2592.1016 | 2591.2293 | 0.8723 | 1 | 0 | 56 | 6 | R.FISVGYVDDTQFVRFDSDAASPR.G |

| **217.** | O14828\|SCAM3_HUMAN    **Mass:** 38661    **Score:** 185    **Queries matched:** 7   **emPAI:** 0.45 |
| --- | --- |
|  | Secretory carrier-associated membrane protein 3 OS=Homo sapiens GN=SCAMP3 PE=1 SV=3 |

|  | **Query** | **Observed** | **Mr(expt)** | **Mr(calc)** | **Delta** | **Miss** | **Score** | **Expect** | **Rank** | **Peptide** |
| --- | --- | --- | --- | --- | --- | --- | --- | --- | --- | --- |
|  | 9867 | **662.8475** | **1323.6804** | **1323.6895** | **-0.0091** | **0** | **60** | **0.00014** | **1** | **R.ELQHAALGGTATR.Q** |
|  | 12581 | **739.3588** | **1476.7030** | **1475.7117** | **0.9913** | **0** | **63** | **7.6e-005** | **1** | **R.TAAANAAAGAAENAFR.A** 12538 12541 |
|  | 14767 | **537.2977** | **1608.8714** | **1608.8332** | **0.0382** | **1** | **4** | **46** | **4** | **R.ERELQHAALGGTATR.Q** |
|  | 16078 | **846.4233** | **1690.8320** | **1690.8427** | **-0.0107** | **0** | **37** | **0.02** | **1** | **K.AQQEFAAGVFSNPAVR.T** |
|  | 20505 | **1008.9910** | **2015.9674** | **2015.9799** | **-0.0126** | **0** | **96** | **2.5e-008** | **1** | **K.NYGSYSTQASAAAATAELLK.K** |

| **218.** | P24539\|AT5F1_HUMAN    **Mass:** 28947    **Score:** 184    **Queries matched:** 11   **emPAI:** 0.45 |
| --- | --- |
|  | ATP synthase subunit b, mitochondrial OS=Homo sapiens GN=ATP5F1 PE=1 SV=2 |

|  | **Query** | **Observed** | **Mr(expt)** | **Mr(calc)** | **Delta** | **Miss** | **Score** | **Expect** | **Rank** | **Peptide** |
| --- | --- | --- | --- | --- | --- | --- | --- | --- | --- | --- |
|  | 5187 | 529.3044 | 1056.5943 | 1056.6040 | -0.0096 | 1 | 24 | 0.75 | 1 | K.SQQALVQKR.H |
|  | 5472 | **539.2740** | **1076.5334** | **1076.5403** | **-0.0069** | **0** | **19** | **2.4** | **1** | **R.HYLFDVQR.N** |
|  | 6418 | **573.4057** | **1144.7968** | **1143.6685** | **1.1283** | **1** | **3** | **74** | **6** | **K.CIADLKLLAK.K** |
|  | 11001 | **692.8619** | **1383.7092** | **1382.7154** | **0.9938** | **0** | **34** | **0.058** | **1** | **K.HVVQSISTQQEK.E** |
|  | 12266 | 729.8533 | 1457.6920 | 1457.6609 | 0.0311 | 0 | 3 | 61 | 5 | K.EQEHMINWVEK.H |
|  | 13076 | **502.5835** | **1504.7286** | **1505.7119** | **-0.9833** | **0** | **7** | **25** | **8** | **R.LDYHISVQNMMR.R** |
|  | 16126 | **848.4309** | **1694.8473** | **1694.8587** | **-0.0115** | **0** | **10** | **10** | **1** | **K.QASIQHIQNAIDTEK.S** |
|  | 19408 | **963.5123** | **1925.0101** | **1925.0218** | **-0.0117** | **1** | **94** | **4.1e-008** | **1** | **K.HVVQSISTQQEKETIAK.C** 19405 19406 19407 |

| **219.** | O94973\|AP2A2_HUMAN    **Mass:** 104807   **Score:** 184    **Queries matched:** 27   **emPAI:** 0.23 |
| --- | --- |
|  | AP-2 complex subunit alpha-2 OS=Homo sapiens GN=AP2A2 PE=1 SV=2 |

|  | **Query** | **Observed** | **Mr(expt)** | **Mr(calc)** | **Delta** | **Miss** | **Score** | **Expect** | **Rank** | **Peptide** |
| --- | --- | --- | --- | --- | --- | --- | --- | --- | --- | --- |
|  | 2819 | **443.0392** | **884.0637** | **883.4763** | **0.5874** | **1** | **22** | **0.67** | **1** | **K.AQEPPKSK.K** 2807 |
|  | 3754 | **477.7527** | **953.4909** | **953.6022** | **-0.1113** | **0** | **6** | **33** | **7** | **R.VIQIVINR.D** |
|  | 4646 | **509.2560** | **1016.4975** | **1016.5073** | **-0.0098** | **1** | **27** | **0.37** | **1** | **M.PAVSKGDGMR.G** |
|  | 4700 | **511.3149** | **1020.6153** | **1020.6219** | **-0.0066** | **0** | **14** | **7.4** | **1** | **R.ALLLSTYIK.F** |
|  | 5262 | **531.3209** | **1060.6272** | **1060.5699** | **0.0573** | **0** | **4** | **72** | **8** | **K.TTQIGCLLR.L** |
|  | 5371 | **536.2676** | **1070.5206** | **1069.6243** | **0.8963** | **1** | **23** | **0.85** | **1** | **R.INKELANIR.S** 5386 |
|  | 5423 | **537.3097** | **1072.6048** | **1071.5309** | **1.0740** | **0** | **29** | **0.24** | **1** | **R.NADVELQQR.A** 5409 |
|  | 6457 | **575.1019** | **1148.1892** | **1147.5478** | **0.6414** | **1** | **1** | **74** | **10** | **-.MPAVSKGDGMR.G** |
|  | 6717 | **583.3302** | **1164.6458** | **1163.5427** | **1.1031** | **1** | **(1)** | **1.2e+002** | **6** | **-.MPAVSKGDGMR.G** |
|  | 8375 | **625.3159** | **1248.6173** | **1249.6224** | **-1.0051** | **0** | **2** | **91** | **2** | **K.VLVAGDTMDSVK.Q** |
|  | 10192 | **671.8174** | **1341.6203** | **1341.6057** | **0.0146** | **0** | **3** | **75** | **1** | **R.AVDLLYAMCDR.S** |
|  | 12473 | **491.2423** | **1470.7051** | **1470.7150** | **-0.0099** | **0** | **26** | **0.31** | **1** | **R.ACNQLGQFLQHR.E** |
|  | 13763 | **516.2960** | **1545.8661** | **1545.8185** | **0.0476** | **1** | **9** | **16** | **3** | **R.GRLTECLETILNK.A** |
|  | 14996 | **541.8044** | **1622.3913** | **1621.8246** | **0.5667** | **1** | **2** | **51** | **2** | **K.GDGMRGLAVFISDIR.N** |
|  | 15001 | **812.3603** | **1622.7060** | **1623.8944** | **-1.1884** | **1** | **10** | **11** | **2** | **K.THIETVINALKTER.D** 15027 |
|  | 15073 | **543.3282** | **1626.9627** | **1625.7654** | **1.1974** | **1** | **3** | **51** | **9** | **R.QRAVDLLYAMCDR.S** |
|  | 15787 | **836.9313** | **1671.8481** | **1671.8580** | **-0.0099** | **0** | **73** | **6e-006** | **1** | **K.QLSNPQQEVQNIFK.A** |
|  | 17815 | 602.3264 | 1803.9572 | 1802.9275 | 1.0297 | 1 | 5 | 37 | 3 | R.NADVELQQRAVEYLR.L |
|  | 18839 | **939.9220** | **1877.8294** | **1877.8407** | **-0.0112** | **0** | **70** | **9.4e-006** | **1** | **K.FFQPTEMASQDFFQR.W** |
|  | 20094 | **990.0273** | **1978.0400** | **1978.0524** | **-0.0124** | **0** | **66** | **2.2e-005** | **1** | **R.YGGTFQNVSVQLPITLNK.F** |
|  | 22930 | 1132.0070 | 2261.9994 | 2262.1712 | -0.1718 | 1 | 5 | 22 | 3 | K.VLVAGDTMDSVKQSAALCLLR.L |
|  | 26988 | **960.5085** | **2878.5038** | **2878.5229** | **-0.0191** | **0** | **43** | **0.0031** | **1** | **K.IIGFGSALLEEVDPNPANFVGAGIIHTK.T** |
|  | 28695 | **649.9312** | **3244.6197** | **3245.7012** | **-1.0816** | **1** | **0** | **44** | **5** | **R.IVTSASTDLQDYTYYFVPAPWLSVKLLR.L** |

| **220.** | P80723\|BASP1_HUMAN    **Mass:** 22680    **Score:** 183    **Queries matched:** 15   **emPAI:** 1.18 |
| --- | --- |
|  | Brain acid soluble protein 1 OS=Homo sapiens GN=BASP1 PE=1 SV=2 |

|  | **Query** | **Observed** | **Mr(expt)** | **Mr(calc)** | **Delta** | **Miss** | **Score** | **Expect** | **Rank** | **Peptide** |
| --- | --- | --- | --- | --- | --- | --- | --- | --- | --- | --- |
|  | 9177 | **430.9050** | **1289.6933** | **1288.5783** | **1.1150** | **0** | **10** | **16** | **1** | **K.AEGAATEEEGTPK.E** 9142 |
|  | 10000 | 665.8217 | 1329.6289 | 1329.6412 | -0.0123 | 1 | 2 | 89 | 3 | K.AEPEKTEGAAEAK.A |
|  | 11028 | **693.8356** | **1385.6566** | **1385.6674** | **-0.0108** | **0** | **55** | **0.00043** | **1** | **K.ETPAATEAPSSTPK.A** |
|  | 11475 | **706.8672** | **1411.7198** | **1411.7307** | **-0.0108** | **1** | **75** | **3.9e-006** | **1** | **K.KTEAPAAPAAQETK.S** |
|  | 16860 | **581.2963** | **1740.8670** | **1740.8166** | **0.0504** | **1** | **10** | **10** | **1** | **K.ESEPQAAAEPAEAKEGK.E** |
|  | 17320 | **444.9630** | **1775.8228** | **1774.8486** | **0.9743** | **0** | **7** | **22** | **3** | **K.APEQEQAAPGPAAGGEAPK.A** |
|  | 19511 | 966.9362 | 1931.8578 | 1931.8708 | -0.0131 | 0 | 57 | 0.00019 | 1 | K.SDGAPASDSKPGSSEAAPSSK.E 19513 19521 |
|  | 19972 | **656.0112** | **1965.0117** | **1965.9279** | **-0.9162** | **0** | **3** | **49** | **3** | **K.AAEAAAAPAESAAPAAGEEPSK.E** |
|  | 23212 | **766.7120** | **2297.1141** | **2297.1287** | **-0.0146** | **1** | **50** | **0.00074** | **1** | **K.AEPPKAPEQEQAAPGPAAGGEAPK.A** |
|  | 25570 | **879.4090** | **2635.2052** | **2635.2248** | **-0.0197** | **1** | **17** | **1.3** | **1** | **K.AAEAAAAPAESAAPAAGEEPSKEEGEPK.K** |
|  | 27053 | **964.8056** | **2891.3950** | **2891.4148** | **-0.0199** | **1** | **56** | **0.00015** | **1** | **K.AQGPAASAEEPKPVEAPAANSDQTVTVKE.-** |
|  | 29982 | **1241.6078** | **3721.8015** | **3722.7659** | **-0.9643** | **1** | **4** | **15** | **1** | **K.APEQEQAAPGPAAGGEAPKAAEAAAAPAESAAPAAGEEPSK.E** |

| **221.** | P24844\|MYL9_HUMAN    **Mass:** 19871    **Score:** 183    **Queries matched:** 13   **emPAI:** 1.89 |
| --- | --- |
|  | Myosin regulatory light polypeptide 9 OS=Homo sapiens GN=MYL9 PE=1 SV=4 |

|  | **Query** | **Observed** | **Mr(expt)** | **Mr(calc)** | **Delta** | **Miss** | **Score** | **Expect** | **Rank** | **Peptide** |
| --- | --- | --- | --- | --- | --- | --- | --- | --- | --- | --- |
|  | 4887 | 518.2572 | 1034.4998 | 1034.5066 | -0.0068 | 0 | 34 | 0.068 | 1 | R.ELLTTMGDR.F 4888 |
|  | 7958 | 615.3007 | 1228.5868 | 1227.6095 | 0.9772 | 0 | 27 | 0.3 | 1 | K.LNGTDPEDVIR.N 7954 7964 |
|  | 8103 | 619.2805 | 1236.5464 | 1236.5557 | -0.0093 | 0 | 55 | 0.0004 | 1 | K.EAFNMIDQNR.D |
|  | 8293 | **623.7921** | **1245.5695** | **1245.5778** | **-0.0083** | **0** | **41** | **0.011** | **1** | **K.GNFNYVEFTR.I** |
|  | 11868 | 478.9265 | 1433.7576 | 1432.5817 | 1.1760 | 0 | 6 | 34 | 10 | R.FTDEEVDEMYR.E |
|  | 21270 | 696.3225 | 2085.9455 | 2085.9201 | 0.0254 | 1 | 2 | 54 | 3 | R.FTDEEVDEMYREAPIDK.K |
|  | 21341 | 1045.9894 | 2089.9642 | 2089.9779 | -0.0137 | 0 | 105 | 2.5e-009 | 1 | R.ATSNVFAMFDQSQIQEFK.E 21342 |
|  | 24288 | **1225.5381** | **2449.0616** | **2449.0777** | **-0.0161** | **1** | **47** | **0.0013** | **1** | **R.ELLTTMGDRFTDEEVDEMYR.E** 24289 |

| **222.** | P30501\|1C02_HUMAN    **Mass:** 41468    **Score:** 182    **Queries matched:** 23   **emPAI:** 0.68 |
| --- | --- |
|  | HLA class I histocompatibility antigen, Cw-2 alpha chain OS=Homo sapiens GN=HLA-C PE=1 SV=1 |

|  | **Query** | **Observed** | **Mr(expt)** | **Mr(calc)** | **Delta** | **Miss** | **Score** | **Expect** | **Rank** | **Peptide** |
| --- | --- | --- | --- | --- | --- | --- | --- | --- | --- | --- |
|  | 3888 | 482.7234 | 963.4322 | 964.4250 | -0.9929 | 0 | 33 | 0.088 | 1 | R.FDSDAASPR.G 3900 3901 3907 |
|  | 11313 | 468.5608 | 1402.6605 | 1403.6429 | -0.9825 | 1 | 8 | 22 | 2 | R.FDSDAASPRGEPR.G |
|  | 11736 | 714.3626 | 1426.7105 | 1426.7205 | -0.0099 | 0 | 46 | 0.0031 | 1 | K.WAAVVVPSGEEQR.Y 11721 11735 11751 |
|  | 13321 | 760.3871 | 1518.7596 | 1518.7427 | 0.0169 | 0 | 62 | 7.5e-005 | 1 | R.SWTAADTAAQITQR.K |
|  | 14519 | **797.3466** | **1592.6787** | **1592.6896** | **-0.0109** | **0** | **50** | **0.0012** | **1** | **R.WVEQEGPEYWDR.E** |
|  | 18283 | 613.3167 | 1836.9283 | 1835.9027 | 1.0256 | 0 | 15 | 3.7 | 1 | K.THVTHHPVSDHEATLR.C |
|  | 20871 | 410.6170 | 2048.0485 | 2049.0102 | -0.9617 | 0 | 9 | 11 | 2 | R.YTCHVQHEGLPEPLTLR.W 20872 20886 20897 |
|  | 21209 | **693.9805** | **2078.9196** | **2078.9334** | **-0.0138** | **1** | **21** | **0.75** | **1** | **R.WVEQEGPEYWDRETQK.Y** |
|  | 23768 | 790.3893 | 2368.1460 | 2369.0401 | -0.8941 | 1 | 3 | 39 | 2 | K.GGSCSQAASSNSAQGSDESLIASKA.- |
|  | 25578 | 1319.0889 | 2636.1632 | 2636.1838 | -0.0206 | 0 | 57 | 0.00013 | 1 | R.DGEDQTQDTELVETRPAGDGTFQK.W 25579 25580 25581 |
|  | 28443 | 1594.3339 | 3186.6532 | 3187.6192 | -0.9660 | 1 | 1 | 39 | 7 | R.VMEPRTLILLLSGALALTETWACSHSMR.Y |

| **223.** | P84095\|RHOG_HUMAN    **Mass:** 21751    **Score:** 182    **Queries matched:** 8   **emPAI:** 0.91 |
| --- | --- |
|  | Rho-related GTP-binding protein RhoG OS=Homo sapiens GN=RHOG PE=1 SV=1 |

|  | **Query** | **Observed** | **Mr(expt)** | **Mr(calc)** | **Delta** | **Miss** | **Score** | **Expect** | **Rank** | **Peptide** |
| --- | --- | --- | --- | --- | --- | --- | --- | --- | --- | --- |
|  | 3778 | **478.7632** | **955.5117** | **955.5199** | **-0.0082** | **1** | **20** | **1.8** | **1** | **R.AQPDTLRR.L** |
|  | 13184 | **756.3738** | **1510.7331** | **1509.7133** | **1.0198** | **0** | **44** | **0.0046** | **1** | **R.YLECSALQQDGVK.E** |
|  | 15328 | **823.9026** | **1645.7907** | **1646.8484** | **-1.0577** | **1** | **6** | **33** | **4** | **-.MQSIKCVVVGDGAVGK.T** |
|  | 20407 | **1003.5481** | **2005.0816** | **2005.0956** | **-0.0140** | **1** | **71** | **6.6e-006** | **1** | **R.LKEQGQAPITPQQGQALAK.Q** 20406 20408 |
|  | 21743 | **1066.4929** | **2130.9713** | **2130.9858** | **-0.0145** | **0** | **62** | **5.5e-005** | **1** | **K.EYIPTVFDNYSAQSAVDGR.T** 21742 |

| **224.** | P02735\|SAA_HUMAN    **Mass:** 13581    **Score:** 182    **Queries matched:** 8   **emPAI:** 1.15 |
| --- | --- |
|  | Serum amyloid A protein OS=Homo sapiens GN=SAA1 PE=1 SV=2 |

|  | **Query** | **Observed** | **Mr(expt)** | **Mr(calc)** | **Delta** | **Miss** | **Score** | **Expect** | **Rank** | **Peptide** |
| --- | --- | --- | --- | --- | --- | --- | --- | --- | --- | --- |
|  | 4318 | **498.2099** | **994.4052** | **995.4560** | **-1.0507** | **0** | **5** | **45** | **7** | **R.EANYIGSDK.Y** 4334 |
|  | 13834 | **775.8631** | **1549.7116** | **1549.7202** | **-0.0085** | **0** | **39** | **0.016** | **1** | **R.SFFSFLGEAFDGAR.D** |
|  | 14799 | **806.9076** | **1611.8006** | **1611.8117** | **-0.0111** | **1** | **18** | **2** | **1** | **K.RGPGGVWAAEAISDAR.E** |
|  | 22163 | **1089.4781** | **2176.9417** | **2176.9562** | **-0.0145** | **0** | **101** | **5.7e-009** | **1** | **R.FFGHGAEDSLADQAANEWGR.S** 22157 22162 22164 |

| **225.** | P01891\|1A68_HUMAN    **Mass:** 41168    **Score:** 182    **Queries matched:** 23   **emPAI:** 0.84 |
| --- | --- |
|  | HLA class I histocompatibility antigen, A-68 alpha chain OS=Homo sapiens GN=HLA-A PE=1 SV=4 |

|  | **Query** | **Observed** | **Mr(expt)** | **Mr(calc)** | **Delta** | **Miss** | **Score** | **Expect** | **Rank** | **Peptide** |
| --- | --- | --- | --- | --- | --- | --- | --- | --- | --- | --- |
|  | 1793 | 406.5443 | 811.0741 | 810.4500 | 0.6241 | 1 | 3 | 49 | 10 | R.FLRGYR.Q |
|  | 4359 | 499.2580 | 996.5014 | 995.4308 | 1.0706 | 0 | 8 | 25 | 1 | R.FDSDAASQR.M |
|  | 10029 | 666.8338 | 1331.6530 | 1331.6622 | -0.0092 | 0 | 17 | 3.1 | 1 | R.YFYTSVSRPGR.G |
|  | 10335 | 675.8494 | 1349.6843 | 1349.6939 | -0.0096 | 1 | 38 | 0.024 | 1 | R.YLENGKETLQR.T 10311 10351 |
|  | 10951 | 691.3199 | 1380.6253 | 1380.6344 | -0.0090 | 0 | 54 | 0.00056 | 1 | R.SWTAADMAAQTTK.H |
|  | 12921 | 748.8587 | 1495.7028 | 1495.7129 | -0.0101 | 0 | 57 | 0.00026 | 1 | R.AYLEGTCVEWLR.R |
|  | 13404 | 508.9781 | 1523.9124 | 1524.6627 | -0.7502 | 1 | 4 | 52 | 6 | R.FDSDAASQRMEPR.A |
|  | 18347 | 461.4684 | 1841.8447 | 1841.8591 | -0.0145 | 0 | 28 | 0.18 | 1 | K.THMTHHAVSDHEATLR.C 18348 |
|  | 20872 | 513.0196 | 2048.0493 | 2048.0626 | -0.0133 | 0 | 16 | 2.1 | 1 | R.YTCHVQHEGLPKPLTLR.W 20848 20850 20871 |
|  | 23132 | 763.4412 | 2287.3018 | 2288.0320 | -0.7302 | 1 | 26 | 0.17 | 1 | R.MEPRAPWIEQEGPEYWDR.N |
|  | 25578 | 1319.0889 | 2636.1632 | 2636.1838 | -0.0206 | 0 | 57 | 0.00013 | 1 | R.DGEDQTQDTELVETRPAGDGTFQK.W 25579 25580 25581 |
|  | 28413 | 796.6549 | 3182.5903 | 3181.6926 | 0.8977 | 1 | (2) | 31 | 6 | -.MAVMAPRTLVLLLSGALALTQTWAGSHSMR.Y |
|  | 28476 | 533.7578 | 3196.5032 | 3197.6875 | -1.1843 | 1 | (0) | 46 | 8 | -.MAVMAPRTLVLLLSGALALTQTWAGSHSMR.Y |
|  | 28578 | 804.6039 | 3214.3867 | 3213.6824 | 0.7042 | 1 | 4 | 16 | 2 | -.MAVMAPRTLVLLLSGALALTQTWAGSHSMR.Y |

| **226.** | P01911\|2B1E_HUMAN    **Mass:** 30289    **Score:** 181    **Queries matched:** 16   **emPAI:** 1.02 |
| --- | --- |
|  | HLA class II histocompatibility antigen, DRB1-15 beta chain OS=Homo sapiens GN=HLA-DRB1 PE=1 SV=2 |

|  | **Query** | **Observed** | **Mr(expt)** | **Mr(calc)** | **Delta** | **Miss** | **Score** | **Expect** | **Rank** | **Peptide** |
| --- | --- | --- | --- | --- | --- | --- | --- | --- | --- | --- |
|  | 1551 | 793.4394 | 792.4322 | 792.4382 | -0.0060 | 0 | 16 | 4.3 | 1 | K.VTVYPSK.T |
|  | 3775 | **478.7490** | **955.4834** | **954.4229** | **1.0605** | **0** | **16** | **4** | **1** | **R.AAVDTYCR.H** |
|  | 8307 | **416.2588** | **1245.7546** | **1244.7129** | **1.0418** | **1** | **3** | **66** | **10** | **R.VQPKVTVYPSK.T** |
|  | 10061 | **667.7997** | **1333.5849** | **1333.5939** | **-0.0089** | **0** | **19** | **1.7** | **1** | **R.YFYNQEESVR.F** |
|  | 13588 | **768.3789** | **1534.7431** | **1534.7529** | **-0.0097** | **0** | **44** | **0.0045** | **1** | **R.HNYGVVESFTVQR.R** |
|  | 13843 | 775.9301 | 1549.8455 | 1550.7048 | -0.8593 | 1 | 5 | 43 | 8 | R.ECHFFNGTERVR.F |
|  | 19944 | **982.4727** | **1962.9308** | **1962.9435** | **-0.0128** | **0** | **84** | **3.5e-007** | **1** | **R.AVTELGRPDAEYWNSQK.D** 19943 |
|  | 25716 | **1331.1263** | **2660.2381** | **2660.2541** | **-0.0160** | **0** | **64** | **2.4e-005** | **1** | **R.SGEVYTCQVEHPSVTSPLTVEWR.A** 25715 |
|  | 26513 | **930.7947** | **2789.3624** | **2788.3780** | **0.9844** | **1** | **24** | **0.22** | **1** | **R.AVTELGRPDAEYWNSQKDILEQAR.A** |
|  | 27392 | **989.1784** | **2964.5134** | **2963.4885** | **1.0249** | **0** | **20** | **0.56** | **1** | **K.AGMVSTGLIQNGDWTFQTLVMLETVPR.S** |
|  | 30317 | **976.0123** | **3900.0202** | **3900.0464** | **-0.0262** | **1** | **3** | **14** | **2** | **K.LPGGSCMTALTVTLMVLSSPLALSGDTRPRFLWQPK.R** |
|  | 30621 | **1371.0184** | **4110.0335** | **4111.0183** | **-0.9849** | **1** | **8** | **3.9** | **2** | **R.WFLNGQEEKAGMVSTGLIQNGDWTFQTLVMLETVPR.S** |
|  | 30626 | 1371.3502 | 4111.0288 | 4111.0183 | 0.0105 | 1 | (6) | 7.2 | 2 | R.WFLNGQEEKAGMVSTGLIQNGDWTFQTLVMLETVPR.S 30623 |

| **227.** | P54652\|HSP72_HUMAN    **Mass:** 70263    **Score:** 181    **Queries matched:** 24   **emPAI:** 0.29 |
| --- | --- |
|  | Heat shock-related 70 kDa protein 2 OS=Homo sapiens GN=HSPA2 PE=1 SV=1 |

|  | **Query** | **Observed** | **Mr(expt)** | **Mr(calc)** | **Delta** | **Miss** | **Score** | **Expect** | **Rank** | **Peptide** |
| --- | --- | --- | --- | --- | --- | --- | --- | --- | --- | --- |
|  | 1463 | **771.4301** | **770.4228** | **770.4286** | **-0.0058** | **1** | **14** | **6.9** | **5** | **K.KDIGPNK.R** |
|  | 1691 | 804.4410 | 803.4337 | 803.4389 | -0.0052 | 0 | 27 | 0.48 | 1 | K.ITITNDK.G |
|  | 3002 | 451.2679 | 900.5213 | 899.5076 | 1.0136 | 0 | 12 | 13 | 3 | R.VVSEGGKPK.V |
|  | 3424 | 466.2736 | 930.5326 | 929.5294 | 1.0031 | 1 | 15 | 5.7 | 1 | K.RNTTIPTK.Q |
|  | 4652 | 509.2835 | 1016.5525 | 1016.5614 | -0.0089 | 1 | 27 | 0.36 | 1 | K.ITITNDKGR.L |
|  | 6511 | 576.7461 | 1151.4777 | 1152.5597 | -1.0820 | 1 | 10 | 17 | 10 | R.MVQEAERYK.S |
|  | 6921 | 590.8102 | 1179.6059 | 1179.6135 | -0.0076 | 1 | 5 | 49 | 5 | K.VQVEYKGETK.T |
|  | 7496 | **402.7194** | **1205.1363** | **1205.5750** | **-0.4388** | **0** | **8** | **16** | **5** | **R.MVSHLAEEFK.R** |
|  | 7943 | 614.8138 | 1227.6130 | 1227.6207 | -0.0077 | 0 | 58 | 0.00024 | 1 | K.VEIIANDQGNR.T 7942 |
|  | 7958 | 615.3007 | 1228.5868 | 1228.6776 | -0.0908 | 0 | 8 | 23 | 6 | K.DAGTITGLNVLR.I 7956 7971 |
|  | 11242 | 700.3468 | 1398.6790 | 1397.6497 | 1.0294 | 1 | (3) | 61 | 7 | R.KFEDATVQSDMK.H |
|  | 11493 | 707.8140 | 1413.6134 | 1413.6446 | -0.0312 | 1 | 8 | 19 | 3 | R.KFEDATVQSDMK.H |
|  | 12222 | **486.2630** | **1455.7671** | **1455.8046** | **-0.0375** | **0** | **9** | **16** | **2** | **K.GQIQEIVLVGGSTR.I** |
|  | 12758 | 744.3493 | 1486.6840 | 1486.6940 | -0.0100 | 0 | 33 | 0.064 | 1 | R.TTPSYVAFTDTER.L |
|  | 14076 | 783.3349 | 1564.6552 | 1564.8249 | -0.1697 | 1 | 2 | 75 | 9 | K.LLQDFFNGKELNK.S |
|  | 15576 | 830.4467 | 1658.8788 | 1658.8879 | -0.0091 | 0 | 59 | 0.00013 | 1 | R.IINEPTAAAIAYGLDK.K 15592 |
|  | 16070 | 846.3608 | 1690.7071 | 1690.7183 | -0.0112 | 0 | 51 | 0.00079 | 1 | K.STAGDTHLGGEDFDNR.M 16069 |
|  | 17528 | 894.4936 | 1786.9726 | 1786.9828 | -0.0102 | 1 | 56 | 0.00028 | 1 | R.IINEPTAAAIAYGLDKK.G |
|  | 20880 | **1025.4983** | **2048.9820** | **2048.9408** | **0.0413** | **1** | **3** | **51** | **3** | **K.CQEVINWLDRNQMAEK.D** |

| **228.** | Q9NTJ5\|SAC1_HUMAN    **Mass:** 67437    **Score:** 178    **Queries matched:** 27   **emPAI:** 0.62 |
| --- | --- |
|  | Phosphatidylinositide phosphatase SAC1 OS=Homo sapiens GN=SACM1L PE=1 SV=2 |

|  | **Query** | **Observed** | **Mr(expt)** | **Mr(calc)** | **Delta** | **Miss** | **Score** | **Expect** | **Rank** | **Peptide** |
| --- | --- | --- | --- | --- | --- | --- | --- | --- | --- | --- |
|  | 1881 | 819.3711 | 818.3639 | 818.3923 | -0.0284 | 0 | 9 | 26 | 7 | K.DFVDAPR.L |
|  | 2135 | **837.4772** | **836.4700** | **836.4756** | **-0.0056** | **0** | **38** | **0.024** | **1** | **K.LHITPEK.F** |
|  | 3615 | **472.7684** | **943.5223** | **942.5247** | **0.9977** | **0** | **18** | **3.2** | **2** | **R.SLQAQLQR.L** |
|  | 3695 | **475.7895** | **949.5645** | **949.5709** | **-0.0064** | **0** | **23** | **0.84** | **1** | **R.LGVLHVGQK.L** |
|  | 5066 | 524.2424 | 1046.4702 | 1045.6019 | 0.8683 | 0 | 9 | 23 | 4 | R.VSTEVTLAVK.K |
|  | 5783 | **550.2598** | **1098.5050** | **1098.6258** | **-0.1208** | **1** | **12** | **9.4** | **3** | **R.RSLQAQLQR.L** |
|  | 6704 | **582.8727** | **1163.7309** | **1164.5887** | **-0.8578** | **0** | **6** | **33** | **10** | **R.ELSAQPEVHR.F** |
|  | 6789 | **585.7167** | **1169.4188** | **1169.4264** | **-0.0076** | **0** | **46** | **0.0029** | **1** | **R.SNCMDCLDR.T** |
|  | 6941 | **591.6409** | **1181.2673** | **1181.7132** | **-0.4459** | **0** | **1** | **74** | **8** | **K.QVIINLINQK.G** |
|  | 9123 | **429.9565** | **1286.8475** | **1285.6554** | **1.1921** | **1** | **3** | **62** | **10** | **K.ATDFDVLSYKK.T** |
|  | 9461 | 653.2813 | 1304.5481 | 1304.5567 | -0.0087 | 0 | 79 | 1.9e-006 | 1 | K.NAWADNANACAK.Q |
|  | 9489 | **653.8388** | **1305.6631** | **1305.6717** | **-0.0087** | **0** | **42** | **0.0083** | **1** | **R.HFDSQVIIYGK.Q** |
|  | 14173 | 786.4213 | 1570.8281 | 1569.7562 | 1.0719 | 1 | 2 | 73 | 6 | K.LEEQDEFEKIYK.N |
|  | 15082 | **814.9430** | **1627.8714** | **1627.8835** | **-0.0120** | **0** | **43** | **0.0058** | **1** | **R.GSIPVFWSQRPNLK.Y** |
|  | 16910 | **581.9573** | **1742.8502** | **1742.8596** | **-0.0094** | **0** | **43** | **0.0056** | **1** | **R.THLGLIMDGWNSMIR.Y** 16911 |
|  | 18866 | **940.9512** | **1879.8878** | **1879.8985** | **-0.0108** | **0** | **39** | **0.014** | **1** | **R.LSNTSPEFQEMSLLER.A** |
|  | 19070 | **633.0252** | **1896.0536** | **1895.8935** | **0.1602** | **0** | **(1)** | **70** | **2** | **R.LSNTSPEFQEMSLLER.A** |
|  | 19492 | **644.4299** | **1930.2678** | **1930.9506** | **-0.6828** | **1** | **2** | **41** | **3** | **K.RTHLGLIMDGWNSMIR.Y** 19494 |
|  | 21409 | **700.0272** | **2097.0597** | **2097.0742** | **-0.0146** | **1** | **14** | **3.2** | **1** | **R.LGVLHVGQKLEEQDEFEK.I** |
|  | 22804 | 1122.5265 | 2243.0384 | 2244.1473 | -1.1089 | 1 | 8 | 13 | 2 | K.YKPLPQISKVANHMDGFQR.H |
|  | 24491 | **620.0926** | **2476.3413** | **2475.1444** | **1.1969** | **0** | **8** | **12** | **3** | **K.GSEKPLEQTFATMVSSLGSGMMR.Y** |
|  | 25433 | **868.3992** | **2602.1759** | **2601.2095** | **0.9663** | **0** | **4** | **27** | **1** | **R.GIDSEGHAANFVETEQIVHYNGSK.A** |
|  | 30287 | **1295.3114** | **3882.9124** | **3882.9350** | **-0.0226** | **0** | **18** | **0.43** | **1** | **R.LSILLDQVAEMQDELSYFLVDSAGQVVANQEGVFR.S** 30288 |
|  | 30315 | 1301.0126 | 3900.0159 | 3898.9299 | 1.0860 | 0 | (2) | 20 | 9 | R.LSILLDQVAEMQDELSYFLVDSAGQVVANQEGVFR.S |

| **229.** | P21980\|TGM2_HUMAN    **Mass:** 78420    **Score:** 176    **Queries matched:** 28   **emPAI:** 0.26 |
| --- | --- |
|  | Protein-glutamine gamma-glutamyltransferase 2 OS=Homo sapiens GN=TGM2 PE=1 SV=2 |

|  | **Query** | **Observed** | **Mr(expt)** | **Mr(calc)** | **Delta** | **Miss** | **Score** | **Expect** | **Rank** | **Peptide** |
| --- | --- | --- | --- | --- | --- | --- | --- | --- | --- | --- |
|  | 1879 | **409.8863** | **817.7580** | **817.4042** | **0.3538** | **1** | **12** | **9.3** | **3** | **K.SVGRDER.E** |
|  | 2527 | **432.8830** | **863.7514** | **863.4501** | **0.3013** | **0** | **2** | **69** | **6** | **R.SSPVYVGR.V** |
|  | 3340 | **463.2142** | **924.4138** | **923.3841** | **1.0298** | **0** | **12** | **9.7** | **1** | **K.EETGMAMR.I** |
|  | 3780 | **478.7769** | **955.5392** | **955.3739** | **0.1653** | **0** | **(10)** | **15** | **3** | **K.EETGMAMR.I** |
|  | 5234 | **530.7998** | **1059.5850** | **1060.5764** | **-0.9914** | **1** | **9** | **24** | **7** | **R.AIKEGDLSTK.Y** |
|  | 5872 | **553.0171** | **1104.0196** | **1104.5485** | **-0.5288** | **0** | **0** | **1e+002** | **10** | **-.MAEELVLER.C** |
|  | 7561 | **605.2972** | **1208.5798** | **1208.5641** | **0.0157** | **1** | **(4)** | **58** | **5** | **K.EETGMAMRIR.V** |
|  | 7577 | **404.0297** | **1209.0673** | **1208.5641** | **0.5032** | **1** | **4** | **34** | **9** | **K.EETGMAMRIR.V** 7576 |
|  | 8037 | **617.3178** | **1232.6211** | **1232.6288** | **-0.0078** | **0** | **42** | **0.0096** | **1** | **R.DLYLENPEIK.I** |
|  | 10945 | **691.2612** | **1380.5078** | **1380.6377** | **-0.1299** | **1** | **2** | **76** | **4** | **K.LAEKEETGMAMR.I** |
|  | 12648 | **741.3244** | **1480.6342** | **1480.6439** | **-0.0097** | **0** | **44** | **0.0048** | **1** | **K.SEGTYCCGPVPVR.A** |
|  | 13184 | 756.3738 | 1510.7331 | 1510.7449 | -0.0118 | 1 | 29 | 0.15 | 2 | K.YRDCLTESNLIK.V |
|  | 14304 | **790.9186** | **1579.8226** | **1578.8293** | **0.9933** | **0** | **49** | **0.0014** | **1** | **K.YLLNLNLEPFSEK.S** 14283 |
|  | 14566 | **798.8773** | **1595.7401** | **1594.7661** | **0.9740** | **0** | **11** | **8.8** | **1** | **R.TVSYNGILGPECGTK.Y** |
|  | 14787 | **806.4040** | **1610.7934** | **1610.8039** | **-0.0105** | **0** | **22** | **0.75** | **1** | **K.TVEIPDPVEAGEEVK.V** 14786 |
|  | 17719 | 600.6335 | 1798.8788 | 1799.0192 | -0.1404 | 0 | 10 | 11 | 4 | R.ALLVEPVINSYLLAER.D 17727 |
|  | 18691 | **622.9952** | **1865.9637** | **1865.9735** | **-0.0097** | **1** | **1** | **81** | **7** | **K.TVEIPDPVEAGEEVKVR.M** |
|  | 19515 | **966.9599** | **1931.9052** | **1931.9194** | **-0.0141** | **0** | **60** | **0.00012** | **1** | **R.VVSGMVNCNDDQGVLLGR.W** 19516 |
|  | 23397 | **581.5372** | **2322.1196** | **2321.1872** | **0.9324** | **1** | **3** | **40** | **5** | **R.LLLCARTVSYNGILGPECGTK.Y** |
|  | 24321 | **818.3189** | **2451.9347** | **2451.2654** | **0.6693** | **1** | **0** | **51** | **6** | **R.MDLLPLHMGLHKLVVNFESDK.L** |
|  | 24500 | **1241.0706** | **2480.1266** | **2480.1431** | **-0.0165** | **0** | **12** | **3.9** | **1** | **R.WDNNYGDGVSPMSWIGSVDILR.R** |
|  | 28285 | **789.9017** | **3155.5778** | **3155.5849** | **-0.0071** | **1** | **31** | **0.04** | **1** | **R.TVSYNGILGPECGTKYLLNLNLEPFSEK.S** |
|  | 29320 | **687.3195** | **3431.5612** | **3430.7999** | **0.7613** | **1** | **3** | **19** | **3** | **K.NIPWNFGQFEDGILDICLILLDVNPKFLK.N** |

| **230.** | P08134\|RHOC_HUMAN    **Mass:** 22334    **Score:** 176    **Queries matched:** 11   **emPAI:** 0.61 |
| --- | --- |
|  | Rho-related GTP-binding protein RhoC OS=Homo sapiens GN=RHOC PE=1 SV=1 |

|  | **Query** | **Observed** | **Mr(expt)** | **Mr(calc)** | **Delta** | **Miss** | **Score** | **Expect** | **Rank** | **Peptide** |
| --- | --- | --- | --- | --- | --- | --- | --- | --- | --- | --- |
|  | 1122 | **629.1870** | **628.1797** | **627.3340** | **0.8457** | **0** | **2** | **1.2e+002** | **2** | **K.QEPVR.S** |
|  | 1168 | **644.3676** | **643.3603** | **642.3813** | **0.9790** | **0** | **9** | **32** | **8** | **R.AGLQVR.K** |
|  | 4129 | 491.2532 | 980.4918 | 981.4590 | -0.9672 | 0 | (1) | 1.3e+002 | 6 | R.EVFEMATR.A |
|  | 4401 | 500.3102 | 998.6059 | 997.4539 | 1.1521 | 0 | 8 | 26 | 9 | R.EVFEMATR.A |
|  | 5636 | 544.7886 | 1087.5626 | 1087.5696 | -0.0070 | 0 | 19 | 2.2 | 1 | K.LVIVGDGACGK.T 5635 |
|  | 6770 | **1168.9121** | **1167.9048** | **1168.5585** | **-0.6537** | **1** | **1** | **85** | **8** | **K.DLRQDEHTR.R** |
|  | 16786 | 579.3232 | 1734.9479 | 1734.9603 | -0.0124 | 1 | 50 | 0.0012 | 1 | K.HFCPNVPIILVGNKK.D |
|  | 20422 | 1004.9587 | 2007.9028 | 2007.9174 | -0.0146 | 0 | 63 | 4.7e-005 | 1 | K.QVELALWDTAGQEDYDR.L |
|  | 29393 | 1155.5691 | 3463.6854 | 3463.6891 | -0.0036 | 0 | 75 | 1.1e-006 | 1 | R.LRPLSYPDTDVILMCFSIDSPDSLENIPEK.W 29394 |

| **231.** | P04229\|2B11_HUMAN    **Mass:** 30294    **Score:** 176    **Queries matched:** 13   **emPAI:** 0.80 |
| --- | --- |
|  | HLA class II histocompatibility antigen, DRB1-1 beta chain OS=Homo sapiens GN=HLA-DRB1 PE=1 SV=2 |

|  | **Query** | **Observed** | **Mr(expt)** | **Mr(calc)** | **Delta** | **Miss** | **Score** | **Expect** | **Rank** | **Peptide** |
| --- | --- | --- | --- | --- | --- | --- | --- | --- | --- | --- |
|  | 1551 | 793.4394 | 792.4322 | 792.4382 | -0.0060 | 0 | 16 | 4.3 | 1 | K.VTVYPSK.T |
|  | 3775 | 478.7490 | 955.4834 | 954.4229 | 1.0605 | 0 | 16 | 4 | 1 | R.AAVDTYCR.H |
|  | 5634 | **544.7355** | **1087.4564** | **1086.4618** | **0.9946** | **0** | **21** | **1.4** | **1** | **R.FDSDVGEYR.A** 5635 |
|  | 7210 | **597.6307** | **1193.2468** | **1192.5625** | **0.6843** | **1** | **1** | **88** | **3** | **R.WFRNGQEEK.A** |
|  | 12023 | 722.8113 | 1443.6081 | 1442.6037 | 1.0044 | 0 | 9 | 15 | 3 | K.FECHFFNGTER.V 12005 |
|  | 12872 | **747.3553** | **1492.6960** | **1492.7059** | **-0.0099** | **0** | **50** | **0.0013** | **1** | **R.HNYGVGESFTVQR.R** |
|  | 19944 | 982.4727 | 1962.9308 | 1962.9435 | -0.0128 | 0 | 84 | 3.5e-007 | 1 | R.AVTELGRPDAEYWNSQK.D 19943 |
|  | 25716 | 1331.1263 | 2660.2381 | 2660.2541 | -0.0160 | 0 | 64 | 2.4e-005 | 1 | R.SGEVYTCQVEHPSVTSPLTVEWR.A 25715 |
|  | 30317 | 976.0123 | 3900.0202 | 3900.0828 | -0.0626 | 1 | 3 | 14 | 1 | K.LPGGSCMTALTVTLMVLSSPLALAGDTRPRFLWQLK.F |

| **232.** | Q30154\|2B51_HUMAN    **Mass:** 30322    **Score:** 176    **Queries matched:** 15   **emPAI:** 0.80 |
| --- | --- |
|  | HLA class II histocompatibility antigen, DRB5 beta chain OS=Homo sapiens GN=HLA-DRB5 PE=1 SV=1 |

|  | **Query** | **Observed** | **Mr(expt)** | **Mr(calc)** | **Delta** | **Miss** | **Score** | **Expect** | **Rank** | **Peptide** |
| --- | --- | --- | --- | --- | --- | --- | --- | --- | --- | --- |
|  | 3775 | 478.7490 | 955.4834 | 954.4229 | 1.0605 | 0 | 16 | 4 | 1 | R.AAVDTYCR.H |
|  | 5634 | 544.7355 | 1087.4564 | 1086.4618 | 0.9946 | 0 | 21 | 1.4 | 1 | R.FDSDVGEYR.A 5635 |
|  | 12872 | 747.3553 | 1492.6960 | 1492.7059 | -0.0099 | 0 | 50 | 0.0013 | 1 | R.HNYGVGESFTVQR.R |
|  | 14145 | **523.9473** | **1568.8202** | **1569.7717** | **-0.9516** | **1** | **(1)** | **90** | **10** | **-.MVCLKLPGGSYMAK.L** |
|  | 14162 | 524.2842 | 1569.8307 | 1569.7717 | 0.0590 | 1 | (4) | 54 | 7 | -.MVCLKLPGGSYMAK.L |
|  | 14384 | **529.3041** | **1584.8904** | **1585.7666** | **-0.8762** | **1** | **4** | **43** | **10** | **-.MVCLKLPGGSYMAK.L** |
|  | 16422 | **571.9651** | **1712.8736** | **1713.7682** | **-0.8946** | **1** | **2** | **77** | **6** | **K.YECHFFNGTERVR.F** |
|  | 19944 | 982.4727 | 1962.9308 | 1962.9435 | -0.0128 | 0 | 84 | 3.5e-007 | 1 | R.AVTELGRPDAEYWNSQK.D 19943 |
|  | 22562 | **740.3012** | **2217.8816** | **2217.9902** | **-0.1086** | **1** | **11** | **5.5** | **2** | **R.FLQQDKYECHFFNGTER.V** |
|  | 25716 | 1331.1263 | 2660.2381 | 2660.2541 | -0.0160 | 0 | 64 | 2.4e-005 | 1 | R.SGEVYTCQVEHPSVTSPLTVEWR.A 25715 |
|  | 28187 | **783.8524** | **3131.3803** | **3130.6882** | **0.6921** | **1** | **7** | **8.4** | **1** | **K.LPGGSYMAKLTVTLMVLSSPLALAGDTRPR.F** |
|  | 28253 | **1050.1315** | **3147.3726** | **3146.6832** | **0.6894** | **1** | **(1)** | **35** | **4** | **K.LPGGSYMAKLTVTLMVLSSPLALAGDTRPR.F** |

| **233.** | P61019\|RAB2A_HUMAN    **Mass:** 23702    **Score:** 176    **Queries matched:** 10   **emPAI:** 0.81 |
| --- | --- |
|  | Ras-related protein Rab-2A OS=Homo sapiens GN=RAB2A PE=1 SV=1 |

|  | **Query** | **Observed** | **Mr(expt)** | **Mr(calc)** | **Delta** | **Miss** | **Score** | **Expect** | **Rank** | **Peptide** |
| --- | --- | --- | --- | --- | --- | --- | --- | --- | --- | --- |
|  | 4897 | **518.2892** | **1034.5638** | **1035.4985** | **-0.9347** | **1** | **3** | **78** | **7** | **K.KEEGEAFAR.E** |
|  | 10575 | 681.8546 | 1361.6945 | 1361.6649 | 0.0296 | 0 | 6 | 34 | 5 | R.EHGLIFMETSAK.T |
|  | 12889 | 747.8704 | 1493.7262 | 1493.7362 | -0.0100 | 0 | 63 | 6.5e-005 | 1 | K.TASNVEEAFINTAK.E |
|  | 13838 | **775.8782** | **1549.7419** | **1549.7525** | **-0.0106** | **0** | **81** | **1.1e-006** | **1** | **K.LQIWDTAGQESFR.S** 13837 |
|  | 16502 | **859.9042** | **1717.7938** | **1717.8060** | **-0.0122** | **0** | **38** | **0.017** | **1** | **R.DTFNHLTTWLEDAR.Q** 16515 |
|  | 17128 | 880.9388 | 1759.8631 | 1759.8741 | -0.0109 | 0 | 53 | 0.00062 | 1 | K.IQEGVFDINNEANGIK.I |
|  | 23989 | **602.0718** | **2404.2580** | **2404.1475** | **0.1106** | **1** | **6** | **20** | **1** | **R.QHSNSNMVIMLIGNKSDLESR.R** |
|  | 24811 | 841.0649 | 2520.1730 | 2519.0779 | 1.0951 | 0 | 1 | 54 | 7 | K.IGPQHAATNATHAGNQGGQQAGGGCC.- |

| **234.** | Q14697\|GANAB_HUMAN    **Mass:** 107263   **Score:** 175    **Queries matched:** 26   **emPAI:** 0.18 |
| --- | --- |
|  | Neutral alpha-glucosidase AB OS=Homo sapiens GN=GANAB PE=1 SV=3 |

|  | **Query** | **Observed** | **Mr(expt)** | **Mr(calc)** | **Delta** | **Miss** | **Score** | **Expect** | **Rank** | **Peptide** |
| --- | --- | --- | --- | --- | --- | --- | --- | --- | --- | --- |
|  | 2637 | **437.2529** | **872.4912** | **872.4603** | **0.0309** | **0** | **9** | **28** | **5** | **R.LDLLEDR.S** 2628 |
|  | 3775 | 478.7490 | 955.4834 | 954.5723 | 0.9111 | 1 | 5 | 49 | 3 | M.AAVAAVAARR.R |
|  | 3999 | **486.7926** | **971.5706** | **971.5764** | **-0.0057** | **0** | **14** | **6.5** | **1** | **R.SLLLSVNAR.G** |
|  | 5605 | 543.3285 | 1084.6424 | 1085.6709 | -1.0285 | 0 | 15 | 5.5 | 3 | K.IILTARPFR.L |
|  | 9411 | **652.2681** | **1302.5217** | **1302.5333** | **-0.0116** | **1** | **26** | **0.39** | **1** | **K.TCEESSFCKR.Q** |
|  | 10385 | **676.9255** | **1351.8364** | **1351.8439** | **-0.0075** | **0** | **3** | **57** | **8** | **K.VLLVLELQGLQK.N** |
|  | 12293 | **487.3033** | **1458.8880** | **1458.7215** | **0.1665** | **1** | **6** | **32** | **6** | **K.VDSGYRVHEELR.N** |
|  | 14140 | **523.9348** | **1568.7826** | **1567.6750** | **1.1076** | **0** | **12** | **8.5** | **1** | **K.DPAEGDGAQPEETPR.D** |
|  | 14146 | **785.4177** | **1568.8208** | **1568.8674** | **-0.0467** | **1** | **2** | **82** | **8** | **R.VHEELRNLGLYVK.T** 14144 |
|  | 15653 | **555.3508** | **1663.0307** | **1663.0396** | **-0.0090** | **0** | **14** | **3.7** | **1** | **R.VVIIGAGKPAAVVLQTK.G** |
|  | 15903 | **560.8671** | **1679.5794** | **1679.9359** | **-0.3565** | **1** | **3** | **46** | **5** | **R.YRVPDVLVADPPIAR.L** |
|  | 16852 | **870.9623** | **1739.9101** | **1739.9206** | **-0.0105** | **0** | **35** | **0.03** | **1** | **R.LSFQHDPETSVLVLR.K** |
|  | 18768 | **625.0344** | **1872.0814** | **1872.9958** | **-0.9144** | **1** | **0** | **83** | **10** | **R.REPWLLPSQHNDIIR.D** |
|  | 19432 | **964.4241** | **1926.8336** | **1926.8452** | **-0.0116** | **0** | **84** | **3.7e-007** | **1** | **K.MMDYLQGSGETPQTDVR.W** 19433 |
|  | 19707 | **649.6349** | **1945.8828** | **1945.8959** | **-0.0130** | **0** | **8** | **14** | **2** | **R.FGAVWTGDNTAEWDHLK.I** |
|  | 23683 | **786.9418** | **2357.8035** | **2357.2240** | **0.5795** | **1** | **5** | **18** | **3** | **R.EPWLLPSQHNDIIRDALGQR.Y** 23673 |
|  | 26643 | **937.4586** | **2809.3539** | **2810.3201** | **-0.9663** | **1** | **6** | **15** | **2** | **R.AFFAGSQRFGAVWTGDNTAEWDHLK.I** |
|  | 27631 | **1004.1393** | **3009.3962** | **3009.4145** | **-0.0183** | **0** | **56** | **0.00012** | **1** | **R.FSFSGNTLVSSSADPEGHFETPIWIER.V** 27630 |
|  | 28119 | **1041.7660** | **3122.2761** | **3122.2923** | **-0.0162** | **1** | **27** | **0.087** | **1** | **K.TRDGSDYEGWCWPGSAGYPDFTNPTMR.A** |
|  | 30374 | **791.5734** | **3952.8304** | **3952.8513** | **-0.0208** | **0** | **38** | **0.0042** | **1** | **K.THSDSKPYGPMSVGLDFSLPGMEHVYGIPEHADNLR.L** |
|  | 30403 | **794.9966** | **3969.9465** | **3968.8462** | **1.1003** | **0** | **(1)** | **22** | **8** | **K.THSDSKPYGPMSVGLDFSLPGMEHVYGIPEHADNLR.L** |

| **235.** | Q9NR31\|SAR1A_HUMAN    **Mass:** 22467    **Score:** 175    **Queries matched:** 3   **emPAI:** 0.17 |
| --- | --- |
|  | GTP-binding protein SAR1a OS=Homo sapiens GN=SAR1A PE=1 SV=1 |

|  | **Query** | **Observed** | **Mr(expt)** | **Mr(calc)** | **Delta** | **Miss** | **Score** | **Expect** | **Rank** | **Peptide** |
| --- | --- | --- | --- | --- | --- | --- | --- | --- | --- | --- |
|  | 24655 | **1249.1744** | **2496.3343** | **2496.3509** | **-0.0166** | **0** | **102** | **4.1e-009** | **1** | **K.VELNALMTDETISNVPILILGNK.I** 24654 |
|  | 25122 | **638.8647** | **2551.4297** | **2551.2961** | **0.1336** | **1** | **1** | **48** | **7** | **K.GNVTLKELNARPMEVFMCSVLK.R** |

| **236.** | P08133\|ANXA6_HUMAN    **Mass:** 76168    **Score:** 175    **Queries matched:** 32   **emPAI:** 0.27 |
| --- | --- |
|  | Annexin A6 OS=Homo sapiens GN=ANXA6 PE=1 SV=3 |

|  | **Query** | **Observed** | **Mr(expt)** | **Mr(calc)** | **Delta** | **Miss** | **Score** | **Expect** | **Rank** | **Peptide** |
| --- | --- | --- | --- | --- | --- | --- | --- | --- | --- | --- |
|  | 1331 | **707.3439** | **706.3366** | **705.3657** | **0.9709** | **0** | **8** | **31** | **1** | **K.TSLETR.F** |
|  | 1424 | 753.3718 | 752.3646 | 752.4255 | -0.0609 | 1 | 11 | 11 | 2 | R.LFKAMK.G |
|  | 1522 | **395.7057** | **789.3968** | **788.4830** | **0.9138** | **0** | **6** | **57** | **3** | **K.LMLAVVK.C** |
|  | 1556 | 397.4023 | 792.7900 | 792.4494 | 0.3406 | 1 | 7 | 22 | 3 | R.EIFRTK.Y |
|  | 1561 | 397.4029 | 792.7912 | 792.3436 | 0.4476 | 0 | 5 | 39 | 4 | K.EMSGDVR.D |
|  | 3219 | **458.7816** | **915.5487** | **916.4800** | **-0.9313** | **0** | **13** | **9.5** | **1** | **-.MAKPAQGAK.Y** |
|  | 4401 | 500.3102 | 998.6059 | 998.6124 | -0.0065 | 0 | 25 | 0.5 | 2 | K.ALIEILATR.T |
|  | 4762 | **514.3063** | **1026.5981** | **1025.5433** | **1.0548** | **0** | **2** | **1e+002** | **3** | **R.LVFDEYLK.T** |
|  | 4926 | **519.7069** | **1037.3993** | **1038.4652** | **-1.0659** | **1** | **6** | **42** | **10** | **K.SMKGAGTDEK.T** 4929 |
|  | 5523 | 540.8333 | 1079.6519 | 1079.6451 | 0.0068 | 0 | 12 | 8.6 | 2 | R.ILISLATGHR.E 5525 |
|  | 6402 | **572.9941** | **1143.9737** | **1144.6638** | **-0.6901** | **0** | **12** | **7.1** | **3** | **K.MLVVLLQGTR.E** |
|  | 6834 | **587.3101** | **1172.6056** | **1171.6561** | **0.9495** | **0** | **5** | **49** | **2** | **K.TTGKPIEASIR.G** |
|  | 8091 | **618.8053** | **1235.5960** | **1235.7462** | **-0.1502** | **1** | **1** | **1.1e+002** | **8** | **R.RILISLATGHR.E** |
|  | 8092 | **412.8738** | **1235.5995** | **1235.6444** | **-0.0449** | **1** | **4** | **53** | **5** | **-.MAKPAQGAKYR.G** |
|  | 9885 | **663.3108** | **1324.6071** | **1324.6194** | **-0.0122** | **1** | **9** | **17** | **2** | **R.QRQEVCQSYK.S** |
|  | 10647 | **683.4712** | **1364.9278** | **1365.6235** | **-0.6956** | **0** | **1** | **76** | **2** | **K.MTNYDVEHTIK.K** |
|  | 12560 | **738.8618** | **1475.7090** | **1474.7515** | **0.9575** | **1** | **15** | **4.3** | **2** | **K.EIKDAISGIGTDEK.C** 12511 12561 |
|  | 16001 | **563.2692** | **1686.7857** | **1686.7962** | **-0.0105** | **0** | **5** | **39** | **2** | **K.GTVRPANDFNPDADAK.A** |
|  | 16489 | **859.4181** | **1716.8216** | **1716.8319** | **-0.0103** | **0** | **60** | **0.0001** | **1** | **K.SLHQAIEGDTSGDFLK.A** |
|  | 17000 | **438.7086** | **1750.8055** | **1750.8083** | **-0.0028** | **1** | **2** | **73** | **8** | **K.SLYSMIKNDTSGEYK.K** |
|  | 17334 | **593.1525** | **1776.4358** | **1776.8391** | **-0.4033** | **1** | **0** | **72** | **10** | **K.SLEDALSSDTSGHFRR.I** |
|  | 17438 | **891.9414** | **1781.8681** | **1781.8737** | **-0.0055** | **0** | **60** | **0.00012** | **1** | **K.WGTDEAQFIYILGNR.S** |
|  | 20931 | **685.3710** | **2053.0913** | **2054.1056** | **-1.0144** | **1** | **4** | **31** | **3** | **R.LILGLMMPPAHYDAKQLK.K** |
|  | 23174 | **1147.4551** | **2292.8956** | **2293.0247** | **-0.1291** | **1** | **1** | **53** | **4** | **K.EDYHKSLEDALSSDTSGHFR.R** 23173 |
|  | 23838 | **794.3686** | **2380.0839** | **2379.0979** | **0.9861** | **1** | **31** | **0.05** | **1** | **K.DAYERDLEADIIGDTSGHFQK.M** |
|  | 24822 | **842.0481** | **2523.1225** | **2523.1376** | **-0.0152** | **0** | **36** | **0.017** | **1** | **R.GSIHDFPGFDPNQDAEALYTAMK.G** |
|  | 30414 | **1325.9902** | **3974.9489** | **3974.9671** | **-0.0182** | **1** | **62** | **2e-005** | **1** | **K.MLVVLLQGTREEDDVVSEDLVQQDVQDLYEAGELK.W** |

| **237.** | P16284\|PECA1_HUMAN    **Mass:** 83396    **Score:** 175    **Queries matched:** 22   **emPAI:** 0.14 |
| --- | --- |
|  | Platelet endothelial cell adhesion molecule OS=Homo sapiens GN=PECAM1 PE=1 SV=1 |

|  | **Query** | **Observed** | **Mr(expt)** | **Mr(calc)** | **Delta** | **Miss** | **Score** | **Expect** | **Rank** | **Peptide** |
| --- | --- | --- | --- | --- | --- | --- | --- | --- | --- | --- |
|  | 1979 | **414.2126** | **826.4106** | **825.5112** | **0.8994** | **0** | **10** | **15** | **1** | **R.VILAPWK.K** 1972 |
|  | 2292 | **424.2362** | **846.4579** | **846.4633** | **-0.0054** | **0** | **(16)** | **5.7** | **1** | **K.MLSEVLR.V** |
|  | 2514 | 432.2500 | 862.4854 | 862.4582 | 0.0272 | 0 | 17 | 4.4 | 2 | K.MLSEVLR.V |
|  | 3603 | **472.5019** | **942.9892** | **942.4771** | **0.5121** | **0** | **4** | **49** | **10** | **K.AVPDAVESR.Y** |
|  | 6542 | 577.2820 | 1152.5494 | 1152.5999 | -0.0505 | 1 | 6 | 37 | 5 | R.ANHASSVPRSK.I |
|  | 7684 | **608.3279** | **1214.6413** | **1215.6585** | **-1.0171** | **1** | **5** | **51** | **2** | **K.AIVAHNRHGNK.A** |
|  | 9314 | 649.3160 | 1296.6174 | 1296.6244 | -0.0070 | 0 | 4 | 56 | 6 | R.NHAMKPINDNK.E |
|  | 13398 | **762.8887** | **1523.7628** | **1523.7719** | **-0.0091** | **0** | **47** | **0.0026** | **1** | **K.SELVTVTESFSTPK.F** 13399 |
|  | 18941 | **629.6190** | **1885.8352** | **1885.9091** | **-0.0739** | **1** | **2** | **68** | **8** | **K.IASKSDSGTYICTAGIDK.V** |
|  | 19236 | 637.6447 | 1909.9123 | 1909.9633 | -0.0510 | 1 | 1 | 85 | 8 | K.EDTIVSQTQDFTKIASK.S |
|  | 22356 | **551.7893** | **2203.1281** | **2203.8647** | **-0.7366** | **0** | **0** | **79** | **4** | **K.MSDPNMEANSHYGHNDDVR.N** |
|  | 22867 | **1128.5608** | **2255.1070** | **2254.0794** | **1.0277** | **1** | **7** | **17** | **1** | **K.STESYFIPEVRIYDSGTYK.C** 22866 |
|  | 24527 | **828.8638** | **2483.5697** | **2484.2464** | **-0.6768** | **1** | **6** | **13** | **1** | **K.AKQMPVEMSRPAVPLLNSNNEK.M** |
|  | 25190 | 1282.6250 | 2563.2354 | 2563.2476 | -0.0121 | 0 | 89 | 7.9e-008 | 1 | K.VVESGEDIVLQCAVNEGSGPITYK.F 25189 25191 |
|  | 25286 | **860.7706** | **2579.2901** | **2579.3020** | **-0.0119** | **1** | **27** | **0.14** | **1** | **R.DQNFVILEFPVEEQDRVLSFR.C** |
|  | 25324 | **862.6060** | **2584.7962** | **2585.1353** | **-0.3391** | **1** | **0** | **44** | **1** | **K.EQEGEYYCTAFNRANHASSVPR.S** |
|  | 28929 | **830.3663** | **3317.4362** | **3316.4547** | **0.9815** | **1** | **5** | **12** | **9** | **K.DNPTEDVEYQCVADNCHSHAKMLSEVLR.V** |

| **238.** | P14314\|GLU2B_HUMAN    **Mass:** 60357    **Score:** 175    **Queries matched:** 22   **emPAI:** 0.27 |
| --- | --- |
|  | Glucosidase 2 subunit beta OS=Homo sapiens GN=PRKCSH PE=1 SV=2 |

|  | **Query** | **Observed** | **Mr(expt)** | **Mr(calc)** | **Delta** | **Miss** | **Score** | **Expect** | **Rank** | **Peptide** |
| --- | --- | --- | --- | --- | --- | --- | --- | --- | --- | --- |
|  | 4351 | **499.2080** | **996.4015** | **996.4988** | **-0.0973** | **1** | **9** | **16** | **7** | **R.EAKEQHQK.L** |
|  | 4394 | **500.2793** | **998.5441** | **998.6124** | **-0.0683** | **1** | **9** | **23** | **8** | **K.KLIELQAGK.K** |
|  | 4400 | **500.3098** | **998.6049** | **998.6124** | **-0.0074** | **1** | **15** | **5.5** | **4** | **K.LIELQAGKK.S** 4413 |
|  | 4702 | **511.7444** | **1021.4742** | **1021.4828** | **-0.0086** | **1** | **16** | **4.3** | **1** | **R.NKFEEAER.S** |
|  | 6607 | **579.2735** | **1156.5324** | **1157.6080** | **-1.0756** | **0** | **56** | **0.00045** | **1** | **K.LWEEQLAAAK.A** 6627 6628 |
|  | 7771 | **610.2991** | **1218.5837** | **1218.5914** | **-0.0077** | **0** | **5** | **48** | **2** | **K.SLEDQVEMLR.T** |
|  | 9191 | **646.3146** | **1290.6146** | **1290.6238** | **-0.0092** | **0** | **32** | **0.081** | **1** | **R.ESLQQMAEVTR.E** 9190 |
|  | 9668 | **658.3421** | **1314.6696** | **1314.6779** | **-0.0083** | **1** | **32** | **0.1** | **1** | **R.TVKEEAEKPER.E** |
|  | 10149 | **447.2372** | **1338.6898** | **1337.6133** | **1.0765** | **0** | **(3)** | **70** | **4** | **K.ETMVTSTTEPSR.C** |
|  | 10396 | **451.8996** | **1352.6770** | **1353.6082** | **-0.9312** | **0** | **7** | **30** | **4** | **K.ETMVTSTTEPSR.C** |
|  | 10584 | 682.3070 | 1362.5993 | 1362.6813 | -0.0819 | 1 | 8 | 21 | 3 | K.KSLEDQVEMLR.T |
|  | 12125 | **725.3675** | **1448.7204** | **1447.6943** | **1.0261** | **0** | **2** | **88** | **4** | **K.AQQEQELAADAFK.E** |
|  | 22181 | **727.4567** | **2179.3484** | **2179.9844** | **-0.6360** | **0** | **(1)** | **50** | **10** | **K.MPPYDEQTQAFIDAAQEAR.N** |
|  | 22289 | **1098.7446** | **2195.4747** | **2195.9793** | **-0.5046** | **0** | **2** | **36** | **1** | **K.MPPYDEQTQAFIDAAQEAR.N** |
|  | 26210 | **683.8298** | **2731.2902** | **2731.3065** | **-0.0162** | **1** | **23** | **0.28** | **1** | **K.LGGSPTSLGTWGSWIGPDHDKFSAMK.Y** |
|  | 27322 | **983.8074** | **2948.4003** | **2947.5192** | **0.8811** | **1** | **0** | **50** | **1** | **K.LVSQKPKLGGSPTSLGTWGSWIGPDHDK.F** |
|  | 27807 | **1521.0590** | **3040.1034** | **3040.1250** | **-0.0216** | **0** | **85** | **1.2e-007** | **1** | **R.VNDGVCDCCDGTDEYNSGVICENTCK.E** |
|  | 31625 | **927.8134** | **5560.8369** | **5560.4232** | **0.4137** | **0** | **0** | **9.6** | **2** | **K.ELDDDMDGTVSVTELQTHPELDTDGDGALSEAEAQALLSGDTQTDATSFYDR.V** |

| **239.** | P21281\|VATB2_HUMAN    **Mass:** 56807    **Score:** 173    **Queries matched:** 24   **emPAI:** 0.29 |
| --- | --- |
|  | V-type proton ATPase subunit B, brain isoform OS=Homo sapiens GN=ATP6V1B2 PE=1 SV=3 |

|  | **Query** | **Observed** | **Mr(expt)** | **Mr(calc)** | **Delta** | **Miss** | **Score** | **Expect** | **Rank** | **Peptide** |
| --- | --- | --- | --- | --- | --- | --- | --- | --- | --- | --- |
|  | 1328 | 707.2164 | 706.2091 | 706.3320 | -0.1229 | 0 | 5 | 54 | 6 | K.DVQAMK.A |
|  | 2627 | **437.2111** | **872.4077** | **872.4716** | **-0.0639** | **0** | **19** | **2.8** | **1** | **R.EQALAVSR.N** |
|  | 2715 | **439.7368** | **877.4590** | **876.4453** | **1.0137** | **0** | **8** | **29** | **2** | **R.NYLSQPR.L** |
|  | 3490 | **469.0947** | **936.1748** | **936.4335** | **-0.2586** | **0** | **8** | **18** | **2** | **K.SAIGEGMTR.K** |
|  | 8320 | **624.2341** | **1246.4537** | **1245.6677** | **0.7860** | **1** | **5** | **39** | **4** | **K.RSGQVLEVSGSK.A** 8289 |
|  | 9511 | **436.8877** | **1307.6413** | **1308.6530** | **-1.0117** | **1** | **5** | **42** | **5** | **R.LMKSAIGEGMTR.K** |
|  | 9526 | **437.2473** | **1308.7199** | **1307.6258** | **1.0941** | **0** | **11** | **12** | **3** | **R.NFIAQGPYENR.T** |
|  | 13344 | **760.8970** | **1519.7794** | **1519.7882** | **-0.0089** | **0** | **82** | **8.9e-007** | **1** | **K.AVVQVFEGTSGIDAK.K** |
|  | 14522 | **797.3732** | **1592.7319** | **1592.8311** | **-0.0992** | **1** | **3** | **55** | **5** | **K.RIPQSTLSEFYPR.D** |
|  | 16065 | **845.9575** | **1689.9005** | **1689.9090** | **-0.0085** | **0** | **46** | **0.003** | **1** | **R.TVFETLDIGWQLLR.I** |
|  | 19381 | 962.5222 | 1923.0298 | 1923.8785 | -0.8487 | 0 | 3 | 47 | 3 | K.DHADVSNQLYACYAIGK.D |
|  | 19537 | 645.3925 | 1933.1557 | 1932.9979 | 0.1579 | 0 | 3 | 44 | 5 | K.HVLVILTDMSSYAEALR.E |
|  | 20342 | **667.3522** | **1999.0347** | **1999.0851** | **-0.0504** | **0** | **0** | **89** | **2** | **R.GIVNGAAPELPVPTGGPAVGAR.E** |
|  | 24018 | **1205.0848** | **2408.1551** | **2408.1715** | **-0.0164** | **0** | **74** | **2.8e-006** | **1** | **R.IYPEEMIQTGISAIDGMNSIAR.G** |
|  | 24432 | **823.7450** | **2468.2132** | **2468.2192** | **-0.0060** | **0** | **9** | **8.6** | **1** | **R.GPVVLAEDFLDIMGQPINPQCR.I** |
|  | 26442 | 695.3413 | 2777.3361 | 2777.2677 | 0.0685 | 0 | (2) | 41 | 7 | K.DVVDYSEENFAIVFAAMGVNMETAR.F |
|  | 26643 | 937.4586 | 2809.3539 | 2809.2575 | 0.0964 | 0 | 2 | 33 | 10 | K.DVVDYSEENFAIVFAAMGVNMETAR.F |
|  | 27538 | 748.8931 | 2991.5432 | 2992.3946 | -0.8515 | 1 | 3 | 24 | 3 | K.SKDVVDYSEENFAIVFAAMGVNMETAR.F |
|  | 28581 | **1072.4723** | **3214.3950** | **3215.4944** | **-1.0993** | **1** | **3** | **23** | **5** | **K.DVVDYSEENFAIVFAAMGVNMETARFFK.S** |
|  | 30623 | 1371.0223 | 4110.0452 | 4110.0620 | -0.0168 | 0 | 36 | 0.0069 | 1 | R.NGSITQIPILTMPNDDITHPIPDLTGYITEGQIYVDR.Q 30621 30626 30627 |

| **240.** | P01912\|HB2B_HUMAN    **Mass:** 30443    **Score:** 172    **Queries matched:** 12   **emPAI:** 0.60 |
| --- | --- |
|  | HLA class II histocompatibility antigen, DR-1 beta chain OS=Homo sapiens GN=HLA-DRB1 PE=1 SV=2 |

|  | **Query** | **Observed** | **Mr(expt)** | **Mr(calc)** | **Delta** | **Miss** | **Score** | **Expect** | **Rank** | **Peptide** |
| --- | --- | --- | --- | --- | --- | --- | --- | --- | --- | --- |
|  | 1551 | 793.4394 | 792.4322 | 792.4382 | -0.0060 | 0 | 16 | 4.3 | 1 | K.VTVYPSK.T |
|  | 1964 | 413.2647 | 824.5149 | 825.3439 | -0.8290 | 0 | 7 | 28 | 4 | R.VDNYCR.H |
|  | 2994 | **451.2558** | **900.4970** | **900.5029** | **-0.0058** | **1** | **17** | **3.7** | **1** | **K.DLLEQKR.G** |
|  | 7210 | 597.6307 | 1193.2468 | 1192.5625 | 0.6843 | 1 | 1 | 88 | 3 | R.WFRNGQEEK.T |
|  | 7827 | **611.8204** | **1221.6263** | **1220.6374** | **0.9889** | **1** | **4** | **59** | **6** | **R.NQKGHSGLQPR.G** |
|  | 8495 | 419.3127 | 1254.9164 | 1254.6469 | 0.2695 | 1 | 3 | 52 | 4 | K.GHSGLQPRGFLS.- |
|  | 13588 | 768.3789 | 1534.7431 | 1534.7529 | -0.0097 | 0 | 44 | 0.0045 | 1 | R.HNYGVVESFTVQR.R |
|  | 19944 | 982.4727 | 1962.9308 | 1962.9435 | -0.0128 | 0 | 84 | 3.5e-007 | 1 | R.AVTELGRPDAEYWNSQK.D 19943 |
|  | 25716 | 1331.1263 | 2660.2381 | 2660.2541 | -0.0160 | 0 | 64 | 2.4e-005 | 1 | R.SGEVYTCQVEHPSVTSPLTVEWR.A 25715 |
|  | 31376 | **1735.1232** | **5202.3477** | **5203.5239** | **-1.1763** | **1** | **1** | **11** | **7** | **R.LPGGSCMAVLTVTLMVLSSPLALAGDTRPRFLEYSTSECHFFNGTER.V** |

| **241.** | Q9UHQ9\|NB5R1_HUMAN    **Mass:** 34244    **Score:** 172    **Queries matched:** 17   **emPAI:** 0.52 |
| --- | --- |
|  | NADH-cytochrome b5 reductase 1 OS=Homo sapiens GN=CYB5R1 PE=1 SV=1 |

|  | **Query** | **Observed** | **Mr(expt)** | **Mr(calc)** | **Delta** | **Miss** | **Score** | **Expect** | **Rank** | **Peptide** |
| --- | --- | --- | --- | --- | --- | --- | --- | --- | --- | --- |
|  | 3301 | **461.2396** | **920.4647** | **919.4763** | **0.9884** | **0** | **27** | **0.32** | **1** | **K.VGDVVEFR.G** 3267 3268 |
|  | 4106 | **490.2625** | **978.5104** | **979.5451** | **-1.0347** | **1** | **7** | **36** | **2** | **K.SPPEPRVAK.K** |
|  | 5575 | 542.7835 | 1083.5523 | 1083.5270 | 0.0253 | 0 | 6 | 40 | 4 | K.MSQYLDSLK.V |
|  | 6954 | **591.8182** | **1181.6219** | **1181.6305** | **-0.0086** | **1** | **27** | **0.31** | **1** | **K.GHFNIQPNKK.S** |
|  | 15457 | 826.9282 | 1651.8419 | 1651.9005 | -0.0587 | 1 | 0 | 1e+002 | 7 | R.SRRPQVTLLDPNEK.Y |
|  | 16470 | 858.8923 | 1715.7701 | 1714.8236 | 0.9465 | 1 | 3 | 59 | 5 | K.FPEGGKMSQYLDSLK.V |
|  | 17104 | **586.9414** | **1757.8022** | **1758.8399** | **-1.0377** | **1** | **1** | **84** | **8** | **K.DWAYSKGFVTADMIR.E** |
|  | 18339 | **921.5162** | **1841.0179** | **1841.0267** | **-0.0088** | **0** | **80** | **9.3e-007** | **1** | **K.LGMIAGGTGITPMLQLIR.A** 18340 |
|  | 18779 | **625.3168** | **1872.9287** | **1873.0165** | **-0.0879** | **0** | **(1)** | **71** | **7** | **K.LGMIAGGTGITPMLQLIR.A** |
|  | 20354 | 1001.0429 | 2000.0713 | 2000.9877 | -0.9165 | 1 | 1 | 80 | 2 | K.MSQYLDSLKVGDVVEFR.G |
|  | 21769 | **1068.4442** | **2134.8739** | **2136.0198** | **-1.1459** | **0** | **0** | **72** | **1** | **K.VPEDPTQCFLLFANQTEK.D** 21768 |
|  | 27536 | **998.1838** | **2991.5297** | **2991.5441** | **-0.0144** | **0** | **62** | **3.4e-005** | **1** | **R.IDGSLVIRPYTPVTSDEDQGYVDLVIK.V** 27537 |

| **242.** | P30453\|1A34_HUMAN    **Mass:** 41314    **Score:** 172    **Queries matched:** 23   **emPAI:** 0.84 |
| --- | --- |
|  | HLA class I histocompatibility antigen, A-34 alpha chain OS=Homo sapiens GN=HLA-A PE=1 SV=1 |

|  | **Query** | **Observed** | **Mr(expt)** | **Mr(calc)** | **Delta** | **Miss** | **Score** | **Expect** | **Rank** | **Peptide** |
| --- | --- | --- | --- | --- | --- | --- | --- | --- | --- | --- |
|  | 4359 | 499.2580 | 996.5014 | 995.4308 | 1.0706 | 0 | 8 | 25 | 1 | R.FDSDAASQR.M |
|  | 10029 | 666.8338 | 1331.6530 | 1331.6622 | -0.0092 | 0 | 17 | 3.1 | 1 | R.YFYTSVSRPGR.G |
|  | 10335 | 675.8494 | 1349.6843 | 1349.6939 | -0.0096 | 1 | 38 | 0.024 | 1 | R.YLENGKETLQR.T 10311 10351 |
|  | 12921 | 748.8587 | 1495.7028 | 1495.7129 | -0.0101 | 0 | 57 | 0.00026 | 1 | R.AYLEGTCVEWLR.R |
|  | 13404 | 508.9781 | 1523.9124 | 1524.6627 | -0.7502 | 1 | 4 | 52 | 6 | R.FDSDAASQRMEPR.A |
|  | 13806 | 775.3702 | 1548.7259 | 1548.7355 | -0.0095 | 0 | 44 | 0.0053 | 1 | R.SWTAADMAAQITQR.K |
|  | 14092 | 522.7712 | 1565.2917 | 1564.7304 | 0.5613 | 0 | (8) | 12 | 3 | R.SWTAADMAAQITQR.K |
|  | 18347 | 461.4684 | 1841.8447 | 1841.8591 | -0.0145 | 0 | 28 | 0.18 | 1 | K.THMTHHAVSDHEATLR.C 18348 |
|  | 20872 | 513.0196 | 2048.0493 | 2048.0626 | -0.0133 | 0 | 16 | 2.1 | 1 | R.YTCHVQHEGLPKPLTLR.W 20848 20850 20871 |
|  | 23132 | 763.4412 | 2287.3018 | 2288.0320 | -0.7302 | 1 | 26 | 0.17 | 1 | R.MEPRAPWIEQEGPEYWDR.N |
|  | 23842 | **477.0307** | **2380.1170** | **2380.9747** | **-0.8578** | **0** | **10** | **6.5** | **3** | **K.GGSYSQAASSDSAQGSDMSLTACK.V** |
|  | 25578 | 1319.0889 | 2636.1632 | 2636.1838 | -0.0206 | 0 | 57 | 0.00013 | 1 | R.DGEDQTQDTELVETRPAGDGTFQK.W 25579 25580 25581 |
|  | 27956 | 1027.8076 | 3080.4010 | 3080.6627 | -0.2617 | 1 | 1 | 36 | 2 | M.AIMAPRTLVLLLSGALALTQTWAGSHSMR.Y |
|  | 28626 | 1076.5287 | 3226.5642 | 3227.6981 | -1.1338 | 1 | 1 | 38 | 5 | -.MAIMAPRTLVLLLSGALALTQTWAGSHSMR.Y |

| **243.** | O15127\|SCAM2_HUMAN    **Mass:** 37082    **Score:** 169    **Queries matched:** 5   **emPAI:** 0.34 |
| --- | --- |
|  | Secretory carrier-associated membrane protein 2 OS=Homo sapiens GN=SCAMP2 PE=1 SV=2 |

|  | **Query** | **Observed** | **Mr(expt)** | **Mr(calc)** | **Delta** | **Miss** | **Score** | **Expect** | **Rank** | **Peptide** |
| --- | --- | --- | --- | --- | --- | --- | --- | --- | --- | --- |
|  | 11150 | **697.3763** | **1392.7380** | **1392.7473** | **-0.0093** | **0** | **56** | **0.00034** | **1** | **R.ELQNTVANLHVR.Q** |
|  | 15883 | **560.3003** | **1677.8790** | **1677.8910** | **-0.0120** | **1** | **28** | **0.19** | **1** | **K.ERELQNTVANLHVR.Q** |
|  | 22359 | **1103.0085** | **2204.0025** | **2204.0134** | **-0.0109** | **0** | **126** | **1.9e-011** | **1** | **R.TGASFQQAQEEFSQGIFSSR.T** 22358 22360 |

| **244.** | P21926\|CD9_HUMAN    **Mass:** 25969    **Score:** 168    **Queries matched:** 13   **emPAI:** 0.51 |
| --- | --- |
|  | CD9 antigen OS=Homo sapiens GN=CD9 PE=1 SV=4 |

|  | **Query** | **Observed** | **Mr(expt)** | **Mr(calc)** | **Delta** | **Miss** | **Score** | **Expect** | **Rank** | **Peptide** |
| --- | --- | --- | --- | --- | --- | --- | --- | --- | --- | --- |
|  | 3554 | **471.2415** | **940.4684** | **941.4494** | **-0.9810** | **0** | **3** | **72** | **10** | **K.EVQEFYK.D** |
|  | 6911 | **590.3310** | **1178.6474** | **1178.6547** | **-0.0073** | **1** | **43** | **0.0076** | **1** | **K.KDVLETFTVK.S** 6912 |
|  | 13434 | **763.8549** | **1525.6953** | **1525.7664** | **-0.0711** | **1** | **4** | **49** | **2** | **K.DEVIKEVQEFYK.D** |
|  | 26527 | **1397.1544** | **2792.2943** | **2792.3084** | **-0.0141** | **0** | **87** | **1.2e-007** | **1** | **K.AIHYALNCCGLAGGVEQFISDICPK.K** 26528 26529 26530 26531 26532 26539 26540 |
|  | 29432 | **871.1172** | **3480.4397** | **3480.8457** | **-0.4060** | **1** | **2** | **20** | **2** | **K.FHIIGAVGIGIAVVMIFGMIFSMILCCAIRR.N** |

| **245.** | P13796\|PLSL_HUMAN    **Mass:** 70815    **Score:** 167    **Queries matched:** 36   **emPAI:** 0.36 |
| --- | --- |
|  | Plastin-2 OS=Homo sapiens GN=LCP1 PE=1 SV=5 |

|  | **Query** | **Observed** | **Mr(expt)** | **Mr(calc)** | **Delta** | **Miss** | **Score** | **Expect** | **Rank** | **Peptide** |
| --- | --- | --- | --- | --- | --- | --- | --- | --- | --- | --- |
|  | 923 | **545.2996** | **544.2923** | **543.3493** | **0.9430** | **1** | **9** | **43** | **1** | **R.KIGAR.V** |
|  | 3005 | **451.7209** | **901.4272** | **902.4457** | **-1.0186** | **1** | **15** | **5.9** | **5** | **R.EKDDIQR.A** 3031 |
|  | 4585 | **507.2258** | **1012.4371** | **1011.6076** | **0.8295** | **0** | **7** | **33** | **8** | **R.NEALIALLR.E** |
|  | 6183 | **564.3340** | **1126.6534** | **1125.6295** | **1.0239** | **1** | **4** | **57** | **1** | **K.IKVPVDWNR.V** |
|  | 8093 | **618.8155** | **1235.6164** | **1234.5434** | **1.0730** | **0** | **28** | **0.23** | **2** | **R.AECMLQQAER.L** |
|  | 9439 | 435.2688 | 1302.7845 | 1302.6139 | 0.1706 | 0 | 10 | 14 | 1 | R.NWMNSLGVNPR.V 9416 |
|  | 9675 | **439.2352** | **1314.6837** | **1314.7296** | **-0.0459** | **1** | **12** | **9.4** | **1** | **K.IFHGLKSTDVAK.T** 9659 |
|  | 9883 | **663.3048** | **1324.5951** | **1323.6670** | **0.9280** | **1** | **28** | **0.21** | **1** | **K.IGNFSTDIKDSK.A** |
|  | 10694 | **456.8828** | **1367.6266** | **1366.7027** | **0.9239** | **1** | **5** | **45** | **5** | **K.LNNAKYAISMAR.K** 10653 10661 10691 10693 |
|  | 11232 | **467.0434** | **1398.1083** | **1397.5803** | **0.5280** | **0** | **2** | **51** | **5** | **R.GSVSDEEMMELR.E** |
|  | 11357 | **703.3975** | **1404.7805** | **1404.7877** | **-0.0072** | **0** | **45** | **0.004** | **1** | **K.LNLAFIANLFNR.Y** 11358 |
|  | 11611 | **474.2787** | **1419.8142** | **1418.6430** | **1.1712** | **0** | **8** | **22** | **3** | **K.MVMTVFACLMGK.G** |
|  | 13041 | **751.8748** | **1501.7351** | **1501.7446** | **-0.0095** | **0** | **3** | **67** | **4** | **K.MINLSVPDTIDER.T** |
|  | 13623 | **769.3820** | **1536.7494** | **1536.7606** | **-0.0112** | **1** | **47** | **0.0024** | **1** | **K.KLENCNYAVELGK.N** |
|  | 15054 | 542.9637 | 1625.8692 | 1624.7185 | 1.1507 | 1 | 5 | 37 | 5 | M.ARGSVSDEEMMELR.E |
|  | 15834 | **838.4175** | **1674.8205** | **1674.8326** | **-0.0120** | **0** | **50** | **0.0012** | **1** | **K.FSLVGIGGQDLNEGNR.T** |
|  | 16823 | **580.3160** | **1737.9261** | **1736.7756** | **1.1505** | **1** | **6** | **27** | **6** | **R.AECMLQQAERLGCR.Q** 16814 |
|  | 16911 | 581.9573 | 1742.8502 | 1742.8509 | -0.0007 | 0 | 10 | 11 | 4 | K.GDEEGVPAVVIDMSGLR.E |
|  | 17081 | 586.3284 | 1755.9633 | 1755.7590 | 0.2043 | 1 | 7 | 21 | 2 | -.MARGSVSDEEMMELR.E |
|  | 17097 | **440.2437** | **1756.9459** | **1755.7590** | **1.1869** | **1** | **(4)** | **40** | **3** | **-.MARGSVSDEEMMELR.E** |
|  | 17269 | **591.8420** | **1772.5041** | **1771.7539** | **0.7502** | **1** | **(4)** | **28** | **2** | **-.MARGSVSDEEMMELR.E** |
|  | 17716 | 900.3731 | 1798.7315 | 1798.9214 | -0.1898 | 0 | 4 | 37 | 1 | K.VNDDIIVNWVNETLR.E |
|  | 20514 | 1009.0467 | 2016.0788 | 2015.9834 | 0.0955 | 1 | 2 | 51 | 10 | K.GDEEGVPAVVIDMSGLREK.D 20533 |
|  | 22538 | **739.3581** | **2215.0524** | **2216.1246** | **-1.0721** | **1** | **3** | **37** | **2** | **R.EGESLEDLMKLSPEELLLR.W** |
|  | 25977 | **1349.7433** | **2697.4720** | **2697.4840** | **-0.0120** | **0** | **48** | **0.00083** | **1** | **K.ISTSLPVLDLIDAIQPGSINYDLLK.T** |
|  | 28286 | **1052.8668** | **3155.5786** | **3155.5961** | **-0.0175** | **1** | **45** | **0.0016** | **1** | **K.AYYHLLEQVAPKGDEEGVPAVVIDMSGLR.E** |

| **246.** | P08246\|ELNE_HUMAN    **Mass:** 29127    **Score:** 166    **Queries matched:** 10   **emPAI:** 0.63 |
| --- | --- |
|  | Neutrophil elastase OS=Homo sapiens GN=ELANE PE=1 SV=1 |

|  | **Query** | **Observed** | **Mr(expt)** | **Mr(calc)** | **Delta** | **Miss** | **Score** | **Expect** | **Rank** | **Peptide** |
| --- | --- | --- | --- | --- | --- | --- | --- | --- | --- | --- |
|  | 3687 | **475.2838** | **948.5530** | **947.4859** | **1.0672** | **0** | **8** | **28** | **1** | **R.SNVCTLVR.G** |
|  | 5309 | **533.3082** | **1064.6018** | **1064.6091** | **-0.0073** | **0** | **45** | **0.0055** | **1** | **R.VVLGAHNLSR.R** |
|  | 17762 | 901.4794 | 1800.9443 | 1800.9127 | 0.0316 | 0 | 0 | 96 | 10 | R.LGNGVQCLAMGWGLLGR.N |
|  | 19877 | **653.6691** | **1957.9856** | **1957.0138** | **0.9717** | **1** | **12** | **5.7** | **1** | **R.RLGNGVQCLAMGWGLLGR.N** |
|  | 26690 | **940.1257** | **2817.3554** | **2817.3691** | **-0.0137** | **0** | **45** | **0.002** | **1** | **R.QAGVCFGDSGSPLVCNGLIHGIASFVR.G** 26694 |
|  | 27860 | **1526.7472** | **3051.4798** | **3051.4913** | **-0.0115** | **0** | **64** | **1.8e-005** | **1** | **R.GGCASGLYPDAFAPVAQFVNWIDSIIQR.S** 27857 27858 27859 |

| **247.** | P00450\|CERU_HUMAN    **Mass:** 122983   **Score:** 166    **Queries matched:** 28   **emPAI:** 0.13 |
| --- | --- |
|  | Ceruloplasmin OS=Homo sapiens GN=CP PE=1 SV=1 |

|  | **Query** | **Observed** | **Mr(expt)** | **Mr(calc)** | **Delta** | **Miss** | **Score** | **Expect** | **Rank** | **Peptide** |
| --- | --- | --- | --- | --- | --- | --- | --- | --- | --- | --- |
|  | 1618 | **400.0322** | **798.0499** | **797.3966** | **0.6533** | **0** | **6** | **27** | **5** | **K.MHAINGR.M** |
|  | 1797 | **406.7158** | **811.4171** | **810.4096** | **1.0075** | **0** | **9** | **17** | **10** | **K.GSLHANGR.Q** |
|  | 2103 | 418.2001 | 834.3857 | 833.4130 | 0.9727 | 1 | 12 | 12 | 3 | K.DSLDKEK.E |
|  | 3536 | **470.7153** | **939.4161** | **939.4232** | **-0.0071** | **0** | **31** | **0.11** | **1** | **K.YTVNQCR.R** 3527 |
|  | 6919 | **590.8047** | **1179.5948** | **1179.6036** | **-0.0088** | **0** | **21** | **1.1** | **1** | **R.IYHSHIDAPK.D** 6907 |
|  | 7156 | **596.2827** | **1190.5509** | **1189.5325** | **1.0184** | **0** | **6** | **39** | **5** | **K.MYYSAVDPTK.D** |
|  | 10764 | **686.3809** | **1370.7472** | **1370.7558** | **-0.0086** | **0** | **41** | **0.012** | **1** | **K.GAYPLSIEPIGVR.F** 10763 |
|  | 12456 | **735.8611** | **1469.7076** | **1469.7184** | **-0.0108** | **0** | **50** | **0.0012** | **1** | **K.EVGPTNADPVCLAK.M** |
|  | 19134 | **952.4070** | **1902.7995** | **1902.8132** | **-0.0137** | **0** | **46** | **0.0024** | **1** | **K.NNEGTYYSPNYNPQSR.S** 19135 |
|  | 22743 | **559.7965** | **2235.1567** | **2234.9040** | **0.2527** | **0** | **6** | **22** | **4** | **K.MHSMNGFMYGNQPGLTMCK.G** 22742 |
|  | 22847 | **564.0182** | **2252.0437** | **2250.8989** | **1.1448** | **0** | **(4)** | **33** | **3** | **K.MHSMNGFMYGNQPGLTMCK.G** |
|  | 23165 | **574.0211** | **2292.0554** | **2292.0195** | **0.0358** | **1** | **0** | **70** | **10** | **R.FNKNNEGTYYSPNYNPQSR.S** |
|  | 23828 | **793.7423** | **2378.2051** | **2378.1538** | **0.0513** | **1** | **0** | **65** | **6** | **K.MYYSAVDPTKDIFTGLIGPMK.I** 23821 |
|  | 24468 | **825.3546** | **2473.0418** | **2473.0591** | **-0.0173** | **1** | **5** | **18** | **1** | **R.MFTTAPDQVDKEDEDFQESNK.M** |
|  | 25793 | **1335.1265** | **2668.2384** | **2668.2520** | **-0.0136** | **0** | **56** | **0.00017** | **1** | **R.GVYSSDVFDIFPGTYQTLEMFPR.T** 25792 25794 |
|  | 26179 | **909.4526** | **2725.3361** | **2725.2622** | **0.0739** | **0** | **5** | **18** | **1** | **R.EFVVMFSVVDENFSWYLEDNIK.T** |
|  | 27604 | **751.1470** | **3000.5588** | **2999.3971** | **1.1617** | **1** | **6** | **14** | **1** | **K.IPERSGAGTEDSACIPWAYYSTVDQVK.D** |
|  | 30299 | 973.2536 | 3888.9853 | 3887.9153 | 1.0700 | 0 | 1 | 22 | 3 | K.NMATRPYSIHAHGVQTESSTVTPTLPGETLTYVWK.I |
|  | 30834 | **1082.9923** | **4327.9401** | **4327.9501** | **-0.0100** | **0** | **(3)** | **12** | **2** | **R.DTANLFPQTSLTLHMWPDTEGTFNVECLTTDHYTGGMK.Q** |
|  | 30863 | **1091.2623** | **4361.0202** | **4359.9399** | **1.0803** | **0** | **4** | **7.9** | **2** | **R.DTANLFPQTSLTLHMWPDTEGTFNVECLTTDHYTGGMK.Q** |

| **248.** | Q9Y6N5\|SQRD_HUMAN    **Mass:** 50214    **Score:** 166    **Queries matched:** 22   **emPAI:** 0.54 |
| --- | --- |
|  | Sulfide:quinone oxidoreductase, mitochondrial OS=Homo sapiens GN=SQRDL PE=1 SV=1 |

|  | **Query** | **Observed** | **Mr(expt)** | **Mr(calc)** | **Delta** | **Miss** | **Score** | **Expect** | **Rank** | **Peptide** |
| --- | --- | --- | --- | --- | --- | --- | --- | --- | --- | --- |
|  | 7398 | **401.2366** | **1200.6881** | **1201.5835** | **-0.8954** | **1** | **15** | **5.4** | **2** | **K.ERLSMYLMK.A** |
|  | 7853 | **612.7823** | **1223.5500** | **1223.7060** | **-0.1560** | **0** | **1** | **1.1e+002** | **5** | **-.MVPLVAVVSGPR.A** |
|  | 9208 | **431.5370** | **1291.5891** | **1291.6271** | **-0.0380** | **0** | **10** | **15** | **1** | **K.IMYLSEAYFR.K** |
|  | 10211 | **672.3633** | **1342.7120** | **1342.7205** | **-0.0085** | **0** | **66** | **3.1e-005** | **1** | **K.TAAAVAAQSGILDR.T** 10210 |
|  | 12113 | **724.8680** | **1447.7215** | **1447.7307** | **-0.0091** | **0** | **10** | **14** | **1** | **K.YADALQEIIQER.N** |
|  | 12444 | **735.3782** | **1468.7418** | **1468.7522** | **-0.0104** | **0** | **36** | **0.03** | **1** | **K.VGAENVAIVEPSER.H** |
|  | 12531 | **492.5872** | **1474.7397** | **1475.8018** | **-1.0621** | **1** | **1** | **96** | **9** | **R.TISVIMKNQTPTK.K** |
|  | 14581 | **533.2857** | **1596.8353** | **1596.8471** | **-0.0118** | **1** | **27** | **0.23** | **1** | **R.KVGAENVAIVEPSER.H** |
|  | 15423 | 825.9897 | 1649.9648 | 1650.9232 | -0.9584 | 0 | 7 | 23 | 5 | R.YLIIALGIQLDYEK.I 15432 |
|  | 15650 | **555.3048** | **1662.8924** | **1663.9297** | **-1.0373** | **0** | **0** | **1.1e+002** | **8** | **K.ANIIFNTSLGAIFGVK.K** |
|  | 16013 | **844.4465** | **1686.8784** | **1686.8882** | **-0.0098** | **0** | **64** | **4.9e-005** | **1** | **R.HFYQPIWTLVGAGAK.Q** 16012 |
|  | 20743 | 1019.9870 | 2037.9594 | 2037.9942 | -0.0348 | 1 | 35 | 0.026 | 2 | R.RYPNVFGIGDCTNLPTSK.T 20744 |
|  | 22307 | **733.7260** | **2198.1562** | **2198.1695** | **-0.0133** | **0** | **20** | **0.72** | **1** | **K.QLSSSGRPTASVIPSGVEWIK.A** |
|  | 23190 | 765.7109 | 2294.1108 | 2294.1291 | -0.0183 | 1 | 3 | 39 | 6 | K.TSPVADAAGWVDVDKETLQHR.R |
|  | 25346 | **863.0994** | **2586.2763** | **2586.2894** | **-0.0131** | **1** | **10** | **6.3** | **1** | **R.VILAEFDYKAEPLETFPFDQSK.E** 25345 |
|  | 27259 | **979.8098** | **2936.4074** | **2936.3964** | **0.0110** | **1** | **1** | **46** | **1** | **K.ADLMPFLYWNMMLRGYWGGPAFLR.K** |
|  | 30762 | **1064.0277** | **4252.0817** | **4252.1072** | **-0.0254** | **1** | **35** | **0.0066** | **1** | **R.ADKQEAVFENLDKPGETQVISYEMLHVTPPMSPPDVLK.T** |

| **249.** | Q13488\|VPP3_HUMAN    **Mass:** 93650    **Score:** 166    **Queries matched:** 26   **emPAI:** 0.08 |
| --- | --- |
|  | V-type proton ATPase 116 kDa subunit a isoform 3 OS=Homo sapiens GN=TCIRG1 PE=1 SV=3 |

|  | **Query** | **Observed** | **Mr(expt)** | **Mr(calc)** | **Delta** | **Miss** | **Score** | **Expect** | **Rank** | **Peptide** |
| --- | --- | --- | --- | --- | --- | --- | --- | --- | --- | --- |
|  | 1203 | **656.3315** | **655.3242** | **655.3653** | **-0.0411** | **0** | **13** | **6.6** | **1** | **K.APALER.L** |
|  | 2885 | 446.2256 | 890.4366 | 890.5589 | -0.1223 | 0 | 12 | 13 | 1 | R.AGLVLPPPK.G 2898 |
|  | 3127 | **455.7262** | **909.4379** | **909.5072** | **-0.0693** | **0** | **13** | **8.1** | **1** | **R.GFLIASFR.E** 3126 |
|  | 3277 | **460.7552** | **919.4958** | **918.5287** | **0.9671** | **0** | **5** | **57** | **9** | **R.FLSQVLGR.V** |
|  | 5859 | **552.5961** | **1103.1777** | **1103.6815** | **-0.5038** | **1** | **5** | **37** | **8** | **R.AGLVLPPPKGR.L** |
|  | 6264 | **567.2972** | **1132.5798** | **1131.6288** | **0.9510** | **0** | **4** | **72** | **7** | **R.LGELGLVEFR.D** |
|  | 11718 | **713.8199** | **1425.6252** | **1424.7412** | **0.8840** | **1** | **1** | **1.1e+002** | **9** | **K.TFTFLQEEVRR.A** |
|  | 12439 | **735.3156** | **1468.6165** | **1467.7690** | **0.8475** | **1** | **3** | **57** | **1** | **R.IPCRDMPPTLIR.T** |
|  | 12509 | **737.8277** | **1473.6408** | **1473.6518** | **-0.0109** | **0** | **10** | **14** | **1** | **R.DSSMEEGVSAVAHR.I** |
|  | 12813 | 497.2995 | 1488.8766 | 1489.6467 | -0.7701 | 0 | (4) | 51 | 6 | R.DSSMEEGVSAVAHR.I |
|  | 13908 | **518.9713** | **1553.8921** | **1554.9246** | **-1.0325** | **0** | **2** | **83** | **8** | **R.VLQLLPPGQVQVHK.M** |
|  | 14575 | **799.3568** | **1596.6991** | **1596.9212** | **-0.2222** | **0** | **1** | **98** | **6** | **R.AQLHQLQLHAAVLR.Q** |
|  | 14612 | **800.8879** | **1599.7613** | **1598.8529** | **0.9084** | **0** | **16** | **2.7** | **1** | **R.TPLLQAPGGPHQDLR.V** |
|  | 14861 | **539.2747** | **1614.8023** | **1613.8373** | **0.9651** | **1** | **0** | **1.1e+002** | **6** | **R.IQEETERLAQELR.D** |
|  | 18183 | **611.1550** | **1830.4433** | **1830.0550** | **0.3883** | **1** | **2** | **48** | **2** | **R.VLQLLPPGQVQVHKMK.A** |
|  | 18788 | **625.6249** | **1873.8528** | **1873.8667** | **-0.0139** | **0** | **21** | **0.75** | **1** | **R.QGHEPQLAAAHTDGASER.T** |
|  | 19159 | **635.6790** | **1904.0152** | **1904.0268** | **-0.0116** | **1** | **21** | **0.75** | **1** | **R.VNFVAGAVEPHKAPALER.L** |
|  | 21270 | 696.3225 | 2085.9455 | 2086.9524 | -1.0069 | 1 | 6 | 24 | 2 | K.FYSGTGYKLSPFTFAATDD.- |
|  | 24131 | **1213.1169** | **2424.2193** | **2424.2359** | **-0.0166** | **0** | **3** | **37** | **1** | **R.SEEVALVQLFLPTAAAYTCVSR.L** |
|  | 24232 | 814.7169 | 2441.1289 | 2440.2759 | 0.8530 | 0 | 1 | 64 | 10 | R.LWALSLAHAQLSEVLWAMVMR.I |
|  | 25523 | **1313.1713** | **2624.3280** | **2624.3405** | **-0.0126** | **0** | **100** | **6.2e-009** | **1** | **R.LGALQQLQQQSQELQEVLGETER.F** 25522 25524 |
|  | 25680 | **886.4324** | **2656.2753** | **2656.3584** | **-0.0831** | **0** | **4** | **21** | **3** | **R.AASAPSILIHFINMFLFSHSPSNR.L** |

| **250.** | P00167\|CYB5_HUMAN    **Mass:** 15321    **Score:** 165    **Queries matched:** 14   **emPAI:** 3.89 |
| --- | --- |
|  | Cytochrome b5 OS=Homo sapiens GN=CYB5A PE=1 SV=2 |

|  | **Query** | **Observed** | **Mr(expt)** | **Mr(calc)** | **Delta** | **Miss** | **Score** | **Expect** | **Rank** | **Peptide** |
| --- | --- | --- | --- | --- | --- | --- | --- | --- | --- | --- |
|  | 6276 | **567.8209** | **1133.6273** | **1133.6346** | **-0.0073** | **0** | **29** | **0.22** | **1** | **K.STWLILHHK.V** |
|  | 7057 | 593.8384 | 1185.6623 | 1185.5917 | 0.0706 | 0 | 8 | 26 | 5 | K.YYTLEEIQK.H |
|  | 13182 | **756.3724** | **1510.7303** | **1510.7416** | **-0.0113** | **0** | **43** | **0.0058** | **1** | **K.FLEEHPGGEEVLR.E** 13183 |
|  | 15213 | **819.4308** | **1636.8470** | **1636.8573** | **-0.0103** | **0** | **34** | **0.049** | **1** | **K.TFIIGELHPDDRPK.L** 15210 15212 15214 |
|  | 22376 | **1103.4602** | **2204.9059** | **2204.9207** | **-0.0148** | **0** | **84** | **2.9e-007** | **1** | **R.EQAGGDATENFEDVGHSTDAR.E** 22375 |
|  | 29919 | **1233.5496** | **3697.6269** | **3697.6517** | **-0.0248** | **1** | **52** | **0.00018** | **1** | **K.FLEEHPGGEEVLREQAGGDATENFEDVGHSTDAR.E** 29917 29920 |
|  | 31737 | 1438.1550 | 5748.5910 | 5749.0018 | -0.4108 | 1 | 1 | 6.2 | 5 | K.TFIIGELHPDDRPKLNKPPETLITTIDSSSSWWTNWVIPAISAVAVALMYR.L |

| **251.** | P30459\|1A74_HUMAN    **Mass:** 41150    **Score:** 165    **Queries matched:** 24   **emPAI:** 0.69 |
| --- | --- |
|  | HLA class I histocompatibility antigen, A-74 alpha chain OS=Homo sapiens GN=HLA-A PE=1 SV=1 |

|  | **Query** | **Observed** | **Mr(expt)** | **Mr(calc)** | **Delta** | **Miss** | **Score** | **Expect** | **Rank** | **Peptide** |
| --- | --- | --- | --- | --- | --- | --- | --- | --- | --- | --- |
|  | 1838 | 408.2467 | 814.4788 | 813.3729 | 1.1059 | 0 | 4 | 80 | 3 | K.AHSQTDR.V |
|  | 4359 | 499.2580 | 996.5014 | 995.4308 | 1.0706 | 0 | 8 | 25 | 1 | R.FDSDAASQR.M |
|  | 9699 | 658.8362 | 1315.6579 | 1315.6673 | -0.0094 | 0 | 14 | 6.2 | 2 | R.YFFTSVSRPGR.G |
|  | 10335 | 675.8494 | 1349.6843 | 1349.6939 | -0.0096 | 1 | 38 | 0.024 | 1 | R.YLENGKETLQR.T 10311 10351 |
|  | 12921 | 748.8587 | 1495.7028 | 1495.7129 | -0.0101 | 0 | 57 | 0.00026 | 1 | R.AYLEGTCVEWLR.R |
|  | 13404 | 508.9781 | 1523.9124 | 1524.6627 | -0.7502 | 1 | 4 | 52 | 6 | R.FDSDAASQRMEPR.A |
|  | 13806 | 775.3702 | 1548.7259 | 1548.7355 | -0.0095 | 0 | 44 | 0.0053 | 1 | R.SWTAADMAAQITQR.K |
|  | 14092 | 522.7712 | 1565.2917 | 1564.7304 | 0.5613 | 0 | (8) | 12 | 3 | R.SWTAADMAAQITQR.K |
|  | 14114 | **523.2885** | **1566.8435** | **1567.8067** | **-0.9631** | **1** | **12** | **7.4** | **1** | **K.AHSQTDRVDLGTLR.G** |
|  | 18347 | 461.4684 | 1841.8447 | 1841.8591 | -0.0145 | 0 | 28 | 0.18 | 1 | K.THMTHHAVSDHEATLR.C 18348 |
|  | 20872 | 513.0196 | 2048.0493 | 2048.0626 | -0.0133 | 0 | 16 | 2.1 | 1 | R.YTCHVQHEGLPKPLTLR.W 20848 20850 20871 |
|  | 23506 | **779.4187** | **2335.2343** | **2334.4185** | **0.8158** | **1** | **3** | **34** | **2** | **M.AVMAPRTLLLLLLGALALTQTR.A** |
|  | 23842 | 477.0307 | 2380.1170 | 2380.9747 | -0.8578 | 0 | 10 | 6.5 | 3 | K.GGSYSQAASSDSAQGSDMSLTACK.V |
|  | 25578 | 1319.0889 | 2636.1632 | 2636.1838 | -0.0206 | 0 | 57 | 0.00013 | 1 | R.DGEDQTQDTELVETRPAGDGTFQK.W 25579 25580 25581 |
|  | 25731 | 888.4573 | 2662.3502 | 2662.1758 | 0.1744 | 1 | 2 | 41 | 3 | R.MEPRAPWIEQEGPEYWDQETR.N |

| **252.** | P10314\|1A32_HUMAN    **Mass:** 41307    **Score:** 165    **Queries matched:** 24   **emPAI:** 0.68 |
| --- | --- |
|  | HLA class I histocompatibility antigen, A-32 alpha chain OS=Homo sapiens GN=HLA-A PE=2 SV=2 |

|  | **Query** | **Observed** | **Mr(expt)** | **Mr(calc)** | **Delta** | **Miss** | **Score** | **Expect** | **Rank** | **Peptide** |
| --- | --- | --- | --- | --- | --- | --- | --- | --- | --- | --- |
|  | 1838 | 408.2467 | 814.4788 | 813.3729 | 1.1059 | 0 | 4 | 80 | 3 | K.AHSQTDR.E |
|  | 4359 | 499.2580 | 996.5014 | 995.4308 | 1.0706 | 0 | 8 | 25 | 1 | R.FDSDAASQR.M |
|  | 9699 | 658.8362 | 1315.6579 | 1315.6673 | -0.0094 | 0 | 14 | 6.2 | 2 | R.YFFTSVSRPGR.G |
|  | 10335 | 675.8494 | 1349.6843 | 1349.6939 | -0.0096 | 1 | 38 | 0.024 | 1 | R.YLENGKETLQR.T 10311 10351 |
|  | 12921 | 748.8587 | 1495.7028 | 1495.7129 | -0.0101 | 0 | 57 | 0.00026 | 1 | R.AYLEGTCVEWLR.R |
|  | 13404 | 508.9781 | 1523.9124 | 1524.6627 | -0.7502 | 1 | 4 | 52 | 6 | R.FDSDAASQRMEPR.A |
|  | 13806 | 775.3702 | 1548.7259 | 1548.7355 | -0.0095 | 0 | 44 | 0.0053 | 1 | R.SWTAADMAAQITQR.K |
|  | 14092 | 522.7712 | 1565.2917 | 1564.7304 | 0.5613 | 0 | (8) | 12 | 3 | R.SWTAADMAAQITQR.K |
|  | 18347 | 461.4684 | 1841.8447 | 1841.8591 | -0.0145 | 0 | 28 | 0.18 | 1 | K.THMTHHAVSDHEATLR.C 18348 |
|  | 20872 | 513.0196 | 2048.0493 | 2048.0626 | -0.0133 | 0 | 16 | 2.1 | 1 | R.YTCHVQHEGLPKPLTLR.W 20848 20850 20871 |
|  | 23842 | 477.0307 | 2380.1170 | 2380.9747 | -0.8578 | 0 | 10 | 6.5 | 3 | K.GGSYSQAASSDSAQGSDMSLTACK.V |
|  | 25578 | 1319.0889 | 2636.1632 | 2636.1838 | -0.0206 | 0 | 57 | 0.00013 | 1 | R.DGEDQTQDTELVETRPAGDGTFQK.W 25579 25580 25581 |
|  | 25731 | 888.4573 | 2662.3502 | 2662.1758 | 0.1744 | 1 | 2 | 41 | 3 | R.MEPRAPWIEQEGPEYWDQETR.N |
|  | 28612 | **1075.3120** | **3222.9142** | **3221.7603** | **1.1539** | **1** | **7** | **6.2** | **1** | **-.MAVMAPRTLLLLLLGALALTQTWAGSHSMR.Y** 28610 |

| **253.** | P30512\|1A29_HUMAN    **Mass:** 41122    **Score:** 165    **Queries matched:** 23   **emPAI:** 0.69 |
| --- | --- |
|  | HLA class I histocompatibility antigen, A-29 alpha chain OS=Homo sapiens GN=HLA-A PE=2 SV=2 |

|  | **Query** | **Observed** | **Mr(expt)** | **Mr(calc)** | **Delta** | **Miss** | **Score** | **Expect** | **Rank** | **Peptide** |
| --- | --- | --- | --- | --- | --- | --- | --- | --- | --- | --- |
|  | 1793 | 406.5443 | 811.0741 | 810.4500 | 0.6241 | 1 | 3 | 49 | 10 | R.FLRGYR.Q |
|  | 4359 | 499.2580 | 996.5014 | 995.4308 | 1.0706 | 0 | 8 | 25 | 1 | R.FDSDAASQR.M |
|  | 8805 | **636.0600** | **1270.1054** | **1269.6466** | **0.4589** | **0** | **1** | **75** | **2** | **R.YFTTSVSRPGR.G** |
|  | 10335 | 675.8494 | 1349.6843 | 1349.6939 | -0.0096 | 1 | 38 | 0.024 | 1 | R.YLENGKETLQR.T 10311 10351 |
|  | 12921 | 748.8587 | 1495.7028 | 1495.7129 | -0.0101 | 0 | 57 | 0.00026 | 1 | R.AYLEGTCVEWLR.R |
|  | 13404 | 508.9781 | 1523.9124 | 1524.6627 | -0.7502 | 1 | 4 | 52 | 6 | R.FDSDAASQRMEPR.A |
|  | 13806 | 775.3702 | 1548.7259 | 1548.7355 | -0.0095 | 0 | 44 | 0.0053 | 1 | R.SWTAADMAAQITQR.K |
|  | 14092 | 522.7712 | 1565.2917 | 1564.7304 | 0.5613 | 0 | (8) | 12 | 3 | R.SWTAADMAAQITQR.K |
|  | 18347 | 461.4684 | 1841.8447 | 1841.8591 | -0.0145 | 0 | 28 | 0.18 | 1 | K.THMTHHAVSDHEATLR.C 18348 |
|  | 20872 | 513.0196 | 2048.0493 | 2048.0626 | -0.0133 | 0 | 16 | 2.1 | 1 | R.YTCHVQHEGLPKPLTLR.W 20848 20850 20871 |
|  | 23842 | 477.0307 | 2380.1170 | 2380.9747 | -0.8578 | 0 | 10 | 6.5 | 3 | K.GGSYSQAASSDSAQGSDMSLTACK.V |
|  | 25578 | 1319.0889 | 2636.1632 | 2636.1838 | -0.0206 | 0 | 57 | 0.00013 | 1 | R.DGEDQTQDTELVETRPAGDGTFQK.W 25579 25580 25581 |
|  | 28612 | 1075.3120 | 3222.9142 | 3221.7603 | 1.1539 | 1 | 7 | 6.2 | 1 | -.MAVMAPRTLLLLLLGALALTQTWAGSHSMR.Y 28610 |

| **254.** | P16189\|1A31_HUMAN    **Mass:** 41263    **Score:** 165    **Queries matched:** 23   **emPAI:** 0.68 |
| --- | --- |
|  | HLA class I histocompatibility antigen, A-31 alpha chain OS=Homo sapiens GN=HLA-A PE=2 SV=2 |

|  | **Query** | **Observed** | **Mr(expt)** | **Mr(calc)** | **Delta** | **Miss** | **Score** | **Expect** | **Rank** | **Peptide** |
| --- | --- | --- | --- | --- | --- | --- | --- | --- | --- | --- |
|  | 4359 | 499.2580 | 996.5014 | 995.4308 | 1.0706 | 0 | 8 | 25 | 1 | R.FDSDAASQR.M |
|  | 8805 | 636.0600 | 1270.1054 | 1269.6466 | 0.4589 | 0 | 1 | 75 | 2 | R.YFTTSVSRPGR.G |
|  | 10335 | 675.8494 | 1349.6843 | 1349.6939 | -0.0096 | 1 | 38 | 0.024 | 1 | R.YLENGKETLQR.T 10311 10351 |
|  | 12921 | 748.8587 | 1495.7028 | 1495.7129 | -0.0101 | 0 | 57 | 0.00026 | 1 | R.AYLEGTCVEWLR.R |
|  | 13404 | 508.9781 | 1523.9124 | 1524.6627 | -0.7502 | 1 | 4 | 52 | 6 | R.FDSDAASQRMEPR.A |
|  | 13806 | 775.3702 | 1548.7259 | 1548.7355 | -0.0095 | 0 | 44 | 0.0053 | 1 | R.SWTAADMAAQITQR.K |
|  | 14092 | 522.7712 | 1565.2917 | 1564.7304 | 0.5613 | 0 | (8) | 12 | 3 | R.SWTAADMAAQITQR.K |
|  | 14315 | 791.3856 | 1580.7567 | 1579.8430 | 0.9136 | 1 | 1 | 1e+002 | 10 | K.AHSQIDRVDLGTLR.G |
|  | 18347 | 461.4684 | 1841.8447 | 1841.8591 | -0.0145 | 0 | 28 | 0.18 | 1 | K.THMTHHAVSDHEATLR.C 18348 |
|  | 20872 | 513.0196 | 2048.0493 | 2048.0626 | -0.0133 | 0 | 16 | 2.1 | 1 | R.YTCHVQHEGLPKPLTLR.W 20848 20850 20871 |
|  | 23842 | 477.0307 | 2380.1170 | 2380.9747 | -0.8578 | 0 | 10 | 6.5 | 3 | K.GGSYSQAASSDSAQGSDMSLTACK.V |
|  | 25578 | 1319.0889 | 2636.1632 | 2636.1838 | -0.0206 | 0 | 57 | 0.00013 | 1 | R.DGEDQTQDTELVETRPAGDGTFQK.W 25579 25580 25581 |
|  | 28612 | 1075.3120 | 3222.9142 | 3221.7603 | 1.1539 | 1 | 7 | 6.2 | 1 | -.MAVMAPRTLLLLLLGALALTQTWAGSHSMR.Y 28610 |

| **255.** | P01766\|HV305_HUMAN    **Mass:** 13332    **Score:** 164    **Queries matched:** 4   **emPAI:** 0.30 |
| --- | --- |
|  | Ig heavy chain V-III region BRO OS=Homo sapiens PE=1 SV=1 |

|  | **Query** | **Observed** | **Mr(expt)** | **Mr(calc)** | **Delta** | **Miss** | **Score** | **Expect** | **Rank** | **Peptide** |
| --- | --- | --- | --- | --- | --- | --- | --- | --- | --- | --- |
|  | 887 | **533.3010** | **532.2938** | **531.3017** | **0.9921** | **0** | **11** | **25** | **8** | **R.QVTGK.G** |
|  | 10406 | **677.3821** | **1352.7496** | **1353.6711** | **-0.9214** | **0** | **7** | **29** | **2** | **K.NTLYLNMNSLR.A** |
|  | 18882 | **941.4990** | **1880.9834** | **1880.9956** | **-0.0122** | **0** | **104** | **3.7e-009** | **1** | **-.EVQLVESGGGLVQPGGSLR.L** 18883 |

|  | **Proteins matching a subset of these peptides:** |
| --- | --- |

|  | P01777\|HV316_HUMAN    **Mass:** 12908    **Score:** 164    **Queries matched:** 2 |
| --- | --- |
|  | Ig heavy chain V-III region TEI OS=Homo sapiens PE=1 SV=1 |

| **256.** | P30499\|1C01_HUMAN    **Mass:** 41395    **Score:** 164    **Queries matched:** 19   **emPAI:** 0.41 |
| --- | --- |
|  | HLA class I histocompatibility antigen, Cw-1 alpha chain OS=Homo sapiens GN=HLA-C PE=2 SV=1 |

|  | **Query** | **Observed** | **Mr(expt)** | **Mr(calc)** | **Delta** | **Miss** | **Score** | **Expect** | **Rank** | **Peptide** |
| --- | --- | --- | --- | --- | --- | --- | --- | --- | --- | --- |
|  | 3888 | 482.7234 | 963.4322 | 964.4250 | -0.9929 | 0 | 33 | 0.088 | 1 | R.FDSDAASPR.G 3900 3901 3907 |
|  | 9699 | 658.8362 | 1315.6579 | 1315.6673 | -0.0094 | 0 | 14 | 6.2 | 2 | K.YFFTSVSRPGR.G |
|  | 11313 | 468.5608 | 1402.6605 | 1403.6429 | -0.9825 | 1 | 8 | 22 | 2 | R.FDSDAASPRGEPR.A |
|  | 12538 | 492.6275 | 1474.8607 | 1474.6875 | 0.1732 | 0 | 3 | 70 | 7 | K.WAAVMVPSGEEQR.Y 12532 |
|  | 12921 | 748.8587 | 1495.7028 | 1495.7129 | -0.0101 | 0 | 57 | 0.00026 | 1 | R.AYLEGTCVEWLR.R |
|  | 13321 | 760.3871 | 1518.7596 | 1518.7427 | 0.0169 | 0 | 62 | 7.5e-005 | 1 | R.SWTAADTAAQITQR.K |
|  | 15320 | 823.4096 | 1644.8047 | 1644.8148 | -0.0101 | 0 | 69 | 1.4e-005 | 1 | R.FISVGYVDDTQFVR.F 15321 |
|  | 18283 | 613.3167 | 1836.9283 | 1835.9027 | 1.0256 | 0 | 15 | 3.7 | 1 | K.THVTHHPVSDHEATLR.C |
|  | 20871 | 410.6170 | 2048.0485 | 2049.0102 | -0.9617 | 0 | 9 | 11 | 2 | R.YTCHVQHEGLPEPLTLR.W 20872 20886 20897 |
|  | 23768 | 790.3893 | 2368.1460 | 2369.0401 | -0.8941 | 1 | 3 | 39 | 2 | K.GGSCSQAASSNSAQGSDESLIASKA.- |
|  | 25366 | 865.0411 | 2592.1016 | 2591.2293 | 0.8723 | 1 | 0 | 56 | 6 | R.FISVGYVDDTQFVRFDSDAASPR.G |

| **257.** | P51572\|BAP31_HUMAN    **Mass:** 28031    **Score:** 163    **Queries matched:** 29   **emPAI:** 1.14 |
| --- | --- |
|  | B-cell receptor-associated protein 31 OS=Homo sapiens GN=BCAP31 PE=1 SV=3 |

|  | **Query** | **Observed** | **Mr(expt)** | **Mr(calc)** | **Delta** | **Miss** | **Score** | **Expect** | **Rank** | **Peptide** |
| --- | --- | --- | --- | --- | --- | --- | --- | --- | --- | --- |
|  | 2169 | **839.4554** | **838.4482** | **838.4548** | **-0.0067** | **0** | **25** | **0.46** | **1** | **R.LLEEHAK.L** |
|  | 2305 | **424.8756** | **847.7367** | **848.4908** | **-0.7541** | **1** | **3** | **66** | **5** | **R.WQKIFK.S** |
|  | 2983 | **451.2271** | **900.4397** | **901.5233** | **-1.0835** | **1** | **31** | **0.16** | **1** | **R.SLKADLQK.L** 3017 |
|  | 4246 | **495.7739** | **989.5332** | **990.4618** | **-0.9285** | **0** | **15** | **6.3** | **1** | **K.QAESASEAAK.K** |
|  | 4461 | 502.2469 | 1002.4792 | 1003.5549 | -1.0758 | 1 | 23 | 0.83 | 1 | K.LKDELASTK.Q 4500 |
|  | 4816 | **516.2645** | **1030.5145** | **1030.5229** | **-0.0084** | **0** | **48** | **0.0032** | **1** | **K.AENQVLAMR.K** 4808 4815 |
|  | 6051 | **559.7921** | **1117.5695** | **1118.5567** | **-0.9872** | **1** | **10** | **18** | **2** | **K.KQAESASEAAK.K** |
|  | 6067 | 560.2850 | 1118.5555 | 1118.5567 | -0.0012 | 1 | 16 | 4 | 1 | K.QAESASEAAKK.Y 6066 6090 |
|  | 6394 | **572.7826** | **1143.5506** | **1143.5594** | **-0.0088** | **0** | **35** | **0.051** | **1** | **K.LQAAVDGPMDK.K** |
|  | 6671 | 1161.6117 | 1160.6044 | 1159.5543 | 1.0501 | 0 | (3) | 87 | 5 | K.LQAAVDGPMDK.K |
|  | 6771 | **585.2567** | **1168.4989** | **1168.5070** | **-0.0081** | **0** | **30** | **0.15** | **1** | **K.YMEENDQLK.K** |
|  | 9310 | **649.3036** | **1296.5926** | **1296.6020** | **-0.0093** | **1** | **32** | **0.079** | **1** | **K.KYMEENDQLK.K** 9305 |
|  | 9311 | **649.3039** | **1296.5932** | **1296.6020** | **-0.0087** | **1** | **48** | **0.0023** | **1** | **K.YMEENDQLKK.G** 9279 |
|  | 9614 | **438.8709** | **1313.5907** | **1312.5969** | **0.9938** | **1** | **(14)** | **6.3** | **3** | **K.YMEENDQLKK.G** |
|  | 9624 | **438.8903** | **1313.6491** | **1312.5969** | **1.0523** | **1** | **(4)** | **57** | **6** | **K.KYMEENDQLK.K** |
|  | 11577 | 709.8633 | 1417.7120 | 1416.7395 | 0.9726 | 1 | 4 | 57 | 2 | K.LEKAENQVLAMR.K |
|  | 14600 | **533.7891** | **1598.3454** | **1598.8264** | **-0.4810** | **1** | **6** | **19** | **1** | **K.GAAVDGGKLDVGNAEVK.L** |
|  | 16433 | **857.9280** | **1713.8415** | **1713.8533** | **-0.0118** | **1** | **73** | **5.7e-006** | **1** | **K.LDVGNAEVKLEEENR.S** 16434 |
|  | 20121 | **661.3378** | **1980.9917** | **1979.9986** | **0.9931** | **1** | **1** | **85** | **3** | **R.LLEEHAKLQAAVDGPMDK.K** |
|  | 22125 | **725.4090** | **2173.2052** | **2174.2310** | **-1.0259** | **1** | **0** | **71** | **3** | **R.LVTLISQQATLLASNEAFKK.Q** |

| **258.** | P61224\|RAP1B_HUMAN    **Mass:** 21040    **Score:** 162    **Queries matched:** 12   **emPAI:** 0.65 |
| --- | --- |
|  | Ras-related protein Rap-1b OS=Homo sapiens GN=RAP1B PE=1 SV=1 |

|  | **Query** | **Observed** | **Mr(expt)** | **Mr(calc)** | **Delta** | **Miss** | **Score** | **Expect** | **Rank** | **Peptide** |
| --- | --- | --- | --- | --- | --- | --- | --- | --- | --- | --- |
|  | 4180 | **493.3026** | **984.5907** | **984.5968** | **-0.0061** | **0** | **36** | **0.037** | **1** | **K.LVVLGSGGVGK.S** 4189 |
|  | 9333 | **649.8571** | **1297.6997** | **1297.7102** | **-0.0105** | **1** | **19** | **1.6** | **1** | **R.VVGKEQGQNLAR.Q** |
|  | 11027 | **462.8865** | **1385.6378** | **1385.6463** | **-0.0085** | **1** | **3** | **63** | **3** | **K.YDPTIEDSYRK.Q** |
|  | 11545 | **473.0665** | **1416.1777** | **1415.6966** | **0.4810** | **0** | **3** | **47** | **1** | **K.DTDDVPMILVGNK.C** |
|  | 12893 | **747.8976** | **1493.7807** | **1493.7878** | **-0.0071** | **0** | **77** | **2.6e-006** | **1** | **K.INVNEIFYDLVR.Q** 12894 |
|  | 13904 | 777.8485 | 1553.6825 | 1553.6933 | -0.0108 | 0 | 8 | 18 | 2 | R.QWNNCAFLESSAK.S |
|  | 15296 | **822.3887** | **1642.7628** | **1642.8600** | **-0.0972** | **1** | **14** | **4.3** | **1** | **R.VKDTDDVPMILVGNK.C** |
|  | 28856 | **1099.8944** | **3296.6614** | **3296.6789** | **-0.0175** | **1** | **78** | **7.4e-007** | **1** | **K.NGQGFALVYSITAQSTFNDLQDLREQILR.V** |
|  | 29836 | **1217.5470** | **3649.6192** | **3649.6772** | **-0.0581** | **1** | **(1)** | **28** | **1** | **K.QVEVDAQQCMLEILDTAGTEQFTAMRDLYMK.N** |
|  | 29856 | **1222.5659** | **3664.6759** | **3665.6721** | **-0.9962** | **1** | **4** | **13** | **1** | **K.QVEVDAQQCMLEILDTAGTEQFTAMRDLYMK.N** |

|  | **Proteins matching a subset of these peptides:** |
| --- | --- |

|  | A6NIZ1\|RP1BL_HUMAN    **Mass:** 21140    **Score:** 154    **Queries matched:** 7 |
| --- | --- |
|  | Ras-related protein Rap-1b-like protein OS=Homo sapiens PE=2 SV=1 |

| **259.** | P62834\|RAP1A_HUMAN    **Mass:** 21316    **Score:** 162    **Queries matched:** 10   **emPAI:** 0.64 |
| --- | --- |
|  | Ras-related protein Rap-1A OS=Homo sapiens GN=RAP1A PE=1 SV=1 |

|  | **Query** | **Observed** | **Mr(expt)** | **Mr(calc)** | **Delta** | **Miss** | **Score** | **Expect** | **Rank** | **Peptide** |
| --- | --- | --- | --- | --- | --- | --- | --- | --- | --- | --- |
|  | 4180 | 493.3026 | 984.5907 | 984.5968 | -0.0061 | 0 | 36 | 0.037 | 1 | K.LVVLGSGGVGK.S 4189 |
|  | 9333 | 649.8571 | 1297.6997 | 1297.7102 | -0.0105 | 1 | 19 | 1.6 | 1 | R.VVGKEQGQNLAR.Q |
|  | 11027 | 462.8865 | 1385.6378 | 1385.6463 | -0.0085 | 1 | 3 | 63 | 3 | K.YDPTIEDSYRK.Q |
|  | 12893 | 747.8976 | 1493.7807 | 1493.7878 | -0.0071 | 0 | 77 | 2.6e-006 | 1 | K.INVNEIFYDLVR.Q 12894 |
|  | 15547 | **829.9042** | **1657.7938** | **1656.8757** | **0.9181** | **1** | **15** | **3.6** | **1** | **R.VKDTEDVPMILVGNK.C** 15519 |
|  | 23718 | **788.4243** | **2362.2510** | **2363.0621** | **-0.8111** | **1** | **2** | **45** | **3** | **K.DTEDVPMILVGNKCDLEDER.V** |
|  | 28856 | 1099.8944 | 3296.6614 | 3296.6789 | -0.0175 | 1 | 78 | 7.4e-007 | 1 | K.NGQGFALVYSITAQSTFNDLQDLREQILR.V |

| **260.** | P16190\|1A33_HUMAN    **Mass:** 41151    **Score:** 162    **Queries matched:** 21   **emPAI:** 0.69 |
| --- | --- |
|  | HLA class I histocompatibility antigen, A-33 alpha chain OS=Homo sapiens GN=HLA-A PE=1 SV=3 |

|  | **Query** | **Observed** | **Mr(expt)** | **Mr(calc)** | **Delta** | **Miss** | **Score** | **Expect** | **Rank** | **Peptide** |
| --- | --- | --- | --- | --- | --- | --- | --- | --- | --- | --- |
|  | 4359 | 499.2580 | 996.5014 | 995.4308 | 1.0706 | 0 | 8 | 25 | 1 | R.FDSDAASQR.M |
|  | 8805 | 636.0600 | 1270.1054 | 1269.6466 | 0.4589 | 0 | 1 | 75 | 2 | R.YFTTSVSRPGR.G |
|  | 12921 | 748.8587 | 1495.7028 | 1495.7129 | -0.0101 | 0 | 57 | 0.00026 | 1 | R.AYLEGTCVEWLR.R |
|  | 13404 | 508.9781 | 1523.9124 | 1524.6627 | -0.7502 | 1 | 4 | 52 | 6 | R.FDSDAASQRMEPR.A |
|  | 13806 | 775.3702 | 1548.7259 | 1548.7355 | -0.0095 | 0 | 44 | 0.0053 | 1 | R.SWTAADMAAQITQR.K |
|  | 14092 | 522.7712 | 1565.2917 | 1564.7304 | 0.5613 | 0 | (8) | 12 | 3 | R.SWTAADMAAQITQR.K |
|  | 14315 | 791.3856 | 1580.7567 | 1579.8430 | 0.9136 | 1 | 1 | 1e+002 | 10 | K.AHSQIDRVDLGTLR.G |
|  | 18347 | 461.4684 | 1841.8447 | 1841.8591 | -0.0145 | 0 | 28 | 0.18 | 1 | K.THMTHHAVSDHEATLR.C 18348 |
|  | 20872 | 513.0196 | 2048.0493 | 2048.0626 | -0.0133 | 0 | 16 | 2.1 | 1 | R.YTCHVQHEGLPKPLTLR.W 20848 20850 20871 |
|  | 23132 | 763.4412 | 2287.3018 | 2288.0320 | -0.7302 | 1 | 26 | 0.17 | 1 | R.MEPRAPWIEQEGPEYWDR.N |
|  | 23842 | 477.0307 | 2380.1170 | 2380.9747 | -0.8578 | 0 | 10 | 6.5 | 3 | K.GGSYSQAASSDSAQGSDMSLTACK.V |
|  | 25578 | 1319.0889 | 2636.1632 | 2636.1838 | -0.0206 | 0 | 57 | 0.00013 | 1 | R.DGEDQTQDTELVETRPAGDGTFQK.W 25579 25580 25581 |
|  | 28612 | 1075.3120 | 3222.9142 | 3221.7603 | 1.1539 | 1 | 7 | 6.2 | 1 | -.MAVMAPRTLLLLLLGALALTQTWAGSHSMR.Y 28610 |

| **261.** | P02671\|FIBA_HUMAN    **Mass:** 95656    **Score:** 160    **Queries matched:** 33   **emPAI:** 0.26 |
| --- | --- |
|  | Fibrinogen alpha chain OS=Homo sapiens GN=FGA PE=1 SV=2 |

|  | **Query** | **Observed** | **Mr(expt)** | **Mr(calc)** | **Delta** | **Miss** | **Score** | **Expect** | **Rank** | **Peptide** |
| --- | --- | --- | --- | --- | --- | --- | --- | --- | --- | --- |
|  | 936 | **548.2909** | **547.2837** | **546.3377** | **0.9460** | **0** | **6** | **64** | **10** | **K.LVTSK.G** |
|  | 1033 | **589.2972** | **588.2900** | **587.3279** | **0.9621** | **0** | **6** | **86** | **1** | **K.LPGSSK.I** |
|  | 1357 | 719.3677 | 718.3605 | 717.3657 | 0.9948 | 0 | 5 | 73 | 3 | R.VSEDLR.S |
|  | 3140 | 456.2269 | 910.4393 | 909.4590 | 0.9803 | 1 | 8 | 25 | 7 | R.SCSKTVTK.T |
|  | 3319 | **462.2364** | **922.4583** | **922.4872** | **-0.0289** | **0** | **3** | **83** | **1** | **K.TVIGPDGHK.E** |
|  | 3926 | **484.2318** | **966.4490** | **966.4155** | **0.0335** | **0** | **10** | **15** | **3** | **R.GDFSSANNR.D** |
|  | 4549 | **505.7556** | **1009.4965** | **1009.5040** | **-0.0075** | **0** | **34** | **0.057** | **1** | **K.VTSGSTTTTR.R** |
|  | 5242 | **531.2624** | **1060.5102** | **1061.4998** | **-0.9895** | **0** | **9** | **21** | **1** | **K.ALTDMPQMR.M** 5243 |
|  | 5920 | **554.8235** | **1107.6324** | **1106.5832** | **1.0492** | **1** | **4** | **57** | **8** | **R.GADYSLRAVR.M** |
|  | 6335 | **570.7763** | **1139.5379** | **1139.5459** | **-0.0079** | **0** | **22** | **0.88** | **1** | **R.GSESGIFTNTK.E** |
|  | 7124 | **397.2296** | **1188.6668** | **1188.6285** | **0.0383** | **1** | **6** | **36** | **4** | **K.NVRAQLVDMK.R** |
|  | 8796 | **424.2299** | **1269.6678** | **1268.6765** | **0.9913** | **1** | **8** | **24** | **2** | **K.LKNSLFEYQK.N** |
|  | 10928 | **690.8730** | **1379.7314** | **1379.7409** | **-0.0095** | **1** | **49** | **0.0018** | **1** | **K.TVIGPDGHKEVTK.E** |
|  | 10986 | 692.3676 | 1382.7207 | 1383.6782 | -0.9576 | 1 | 10 | 12 | 4 | K.NSLFEYQKNNK.D |
|  | 13013 | **751.3701** | **1500.7256** | **1500.7354** | **-0.0099** | **0** | **24** | **0.58** | **1** | **R.MELERPGGNEITR.G** 13012 13014 13027 |
|  | 13123 | **754.8350** | **1507.6555** | **1508.6994** | **-1.0440** | **1** | **3** | **64** | **2** | **R.EVDLKDYEDQQK.Q** |
|  | 13341 | **760.8654** | **1519.7163** | **1519.7267** | **-0.0104** | **0** | **54** | **0.00048** | **1** | **K.GLIDEVNQDFTNR.I** 13340 |
|  | 14182 | **786.8372** | **1571.6599** | **1571.6700** | **-0.0101** | **0** | **46** | **0.0033** | **1** | **R.GGSTSYGTGSETESPR.N** |
|  | 16701 | **865.8712** | **1729.7279** | **1729.7404** | **-0.0125** | **1** | **26** | **0.23** | **1** | **R.GDFSSANNRDNTYNR.V** 16700 |
|  | 19014 | **631.2831** | **1890.8274** | **1889.8366** | **0.9908** | **1** | **1** | **86** | **3** | **R.MDGSLNFNRTWQDYK.R** |
|  | 19953 | **982.8705** | **1963.7264** | **1962.7367** | **0.9897** | **0** | **53** | **0.00033** | **1** | **K.DSDWPFCSDEDWNYK.C** 19941 |
|  | 20160 | **662.6697** | **1984.9872** | **1985.0000** | **-0.0128** | **1** | **(2)** | **53** | **3** | **K.NNKDSHSLTTNIMEILR.G** |
|  | 20349 | 667.6650 | 1999.9733 | 2000.9949 | -1.0216 | 1 | 4 | 39 | 3 | K.NNKDSHSLTTNIMEILR.G |
|  | 27929 | 1536.2485 | 3070.4825 | 3070.3898 | 0.0928 | 1 | 7 | 11 | 2 | R.MELERPGGNEITRGGSTSYGTGSETESPR.N |
|  | 28169 | **626.6649** | **3128.2882** | **3127.3962** | **0.8920** | **0** | **7** | **8.4** | **2** | **K.EVVTSEDGSDCPEAMDLGTLSGIGTLDGFR.H** |
|  | 29938 | **1234.9054** | **3701.6944** | **3702.7845** | **-1.0901** | **1** | **3** | **15** | **7** | **-.MFSMRIVCLVLSVVGTAWTADSGEGDFLAEGGGVR.G** |

| **262.** | P12236\|ADT3_HUMAN    **Mass:** 33073    **Score:** 160    **Queries matched:** 21   **emPAI:** 1.13 |
| --- | --- |
|  | ADP/ATP translocase 3 OS=Homo sapiens GN=SLC25A6 PE=1 SV=4 |

|  | **Query** | **Observed** | **Mr(expt)** | **Mr(calc)** | **Delta** | **Miss** | **Score** | **Expect** | **Rank** | **Peptide** |
| --- | --- | --- | --- | --- | --- | --- | --- | --- | --- | --- |
|  | 2175 | **420.6770** | **839.3394** | **839.3452** | **-0.0058** | **0** | **(11)** | **11** | **5** | **R.MMMQSGR.K** 2188 |
|  | 2406 | **429.0863** | **856.1580** | **855.3401** | **0.8179** | **0** | **(10)** | **12** | **8** | **R.MMMQSGR.K** |
|  | 2408 | **429.0867** | **856.1589** | **855.3401** | **0.8188** | **0** | **16** | **3.3** | **1** | **R.MMMQSGR.K** 2394 |
|  | 2425 | **429.3168** | **856.6190** | **855.4814** | **1.1376** | **0** | **10** | **18** | **1** | **K.TAVAPIER.V** 2392 |
|  | 2606 | **436.2230** | **870.4314** | **871.3350** | **-0.9037** | **0** | **(3)** | **87** | **6** | **R.MMMQSGR.K** |
|  | 3011 | **451.7430** | **901.4713** | **901.4770** | **-0.0056** | **0** | **40** | **0.02** | **1** | **K.GAWSNVLR.G** 3009 |
|  | 3284 | **461.0064** | **919.9982** | **920.4716** | **-0.4734** | **1** | **17** | **2.2** | **1** | **K.IFRDEGGK.A** |
|  | 4577 | **506.6468** | **1011.2790** | **1011.4412** | **-0.1622** | **1** | **(0)** | **1.1e+002** | **8** | **R.RMMMQSGR.K** |
|  | 5032 | **523.0566** | **1044.0987** | **1043.4310** | **0.6677** | **1** | **0** | **1.1e+002** | **9** | **R.RMMMQSGR.K** |
|  | 6295 | **568.8387** | **1135.6628** | **1135.6713** | **-0.0085** | **0** | **31** | **0.14** | **1** | **K.LLLQVQHASK.Q** |
|  | 8040 | **617.3419** | **1232.6693** | **1232.6765** | **-0.0072** | **0** | **84** | **5.5e-007** | **1** | **K.DFLAGGIAAAISK.T** 8055 |
|  | 12063 | **723.8700** | **1445.7254** | **1445.7343** | **-0.0089** | **0** | **54** | **0.00053** | **1** | **R.YFPTQALNFAFK.D** |
|  | 15304 | **822.8554** | **1643.6961** | **1643.7072** | **-0.0111** | **0** | **35** | **0.032** | **1** | **K.GADIMYTGTVDCWR.K** |
|  | 17252 | **886.9025** | **1771.7904** | **1771.8022** | **-0.0118** | **1** | **51** | **0.00092** | **1** | **R.KGADIMYTGTVDCWR.K** |
|  | 21138 | **691.3179** | **2070.9320** | **2070.1473** | **0.7847** | **1** | **1** | **60** | **7** | **K.DFLAGGIAAAISKTAVAPIER.V** |
|  | 26555 | **1398.6686** | **2795.3226** | **2795.3377** | **-0.0151** | **0** | **37** | **0.013** | **1** | **R.YFAGNLASGGAAGATSLCFVYPLDFAR.T** |

| **263.** | P03989\|1B27_HUMAN    **Mass:** 40802    **Score:** 159    **Queries matched:** 27   **emPAI:** 0.69 |
| --- | --- |
|  | HLA class I histocompatibility antigen, B-27 alpha chain OS=Homo sapiens GN=HLA-B PE=1 SV=2 |

|  | **Query** | **Observed** | **Mr(expt)** | **Mr(calc)** | **Delta** | **Miss** | **Score** | **Expect** | **Rank** | **Peptide** |
| --- | --- | --- | --- | --- | --- | --- | --- | --- | --- | --- |
|  | 3888 | 482.7234 | 963.4322 | 964.4250 | -0.9929 | 0 | 33 | 0.088 | 1 | R.FDSDAASPR.E 3900 3901 3907 |
|  | 4068 | 488.7570 | 975.4994 | 976.5011 | -1.0018 | 1 | 12 | 13 | 10 | R.ETQICKAK.A |
|  | 6565 | 577.7809 | 1153.5473 | 1153.6091 | -0.0618 | 1 | 3 | 67 | 9 | K.ETLQRADPPK.T |
|  | 10335 | 675.8494 | 1349.6843 | 1349.6939 | -0.0096 | 1 | 38 | 0.024 | 1 | R.YLENGKETLQR.A 10311 10351 |
|  | 11736 | 714.3626 | 1426.7105 | 1426.7205 | -0.0099 | 0 | 46 | 0.0031 | 1 | K.WAAVVVPSGEEQR.Y 11721 11735 11751 |
|  | 12552 | 492.8911 | 1475.6516 | 1475.6641 | -0.0125 | 1 | 13 | 6.8 | 1 | R.FDSDAASPREEPR.A 12553 |
|  | 18486 | 463.4837 | 1849.9057 | 1849.9183 | -0.0127 | 0 | 16 | 2.9 | 1 | K.THVTHHPISDHEATLR.C 18485 |
|  | 20872 | 513.0196 | 2048.0493 | 2048.0626 | -0.0133 | 0 | 16 | 2.1 | 1 | R.YTCHVQHEGLPKPLTLR.W 20848 20850 20871 |
|  | 21557 | 1056.5175 | 2111.0204 | 2111.0299 | -0.0095 | 0 | 86 | 2.1e-007 | 1 | R.CWALGFYPAEITLTWQR.D 21558 |
|  | 22689 | 744.6669 | 2230.9790 | 2230.9938 | -0.0149 | 0 | 44 | 0.0032 | 1 | R.DGEDQTQDTELVETRPAGDR.T 22688 |
|  | 23097 | 1143.5082 | 2285.0018 | 2286.0341 | -1.0323 | 1 | 11 | 6.4 | 1 | R.EEPRAPWIEQEGPEYWDR.E |
|  | 30136 | 1896.3450 | 3790.6754 | 3790.1002 | 0.5752 | 1 | 2 | 20 | 5 | R.WEPSSQSTVPIVGIVAGLAVLAVVVIGAVVAAVMCRR.K |

| **264.** | P18465\|1B57_HUMAN    **Mass:** 40541    **Score:** 159    **Queries matched:** 29   **emPAI:** 0.70 |
| --- | --- |
|  | HLA class I histocompatibility antigen, B-57 alpha chain OS=Homo sapiens GN=HLA-B PE=1 SV=1 |

|  | **Query** | **Observed** | **Mr(expt)** | **Mr(calc)** | **Delta** | **Miss** | **Score** | **Expect** | **Rank** | **Peptide** |
| --- | --- | --- | --- | --- | --- | --- | --- | --- | --- | --- |
|  | 3888 | 482.7234 | 963.4322 | 964.4250 | -0.9929 | 0 | 33 | 0.088 | 1 | R.FDSDAASPR.M 3900 3901 3907 |
|  | 4176 | 493.2762 | 984.5379 | 983.5876 | 0.9503 | 1 | 10 | 18 | 10 | R.ENLRIALR.Y 4164 |
|  | 6565 | 577.7809 | 1153.5473 | 1153.6091 | -0.0618 | 1 | 3 | 67 | 9 | K.ETLQRADPPK.T |
|  | 6759 | **584.7974** | **1167.5802** | **1168.5659** | **-0.9857** | **1** | **3** | **72** | **10** | **R.NMKASAQTYR.E** |
|  | 10335 | 675.8494 | 1349.6843 | 1349.6939 | -0.0096 | 1 | 38 | 0.024 | 1 | R.YLENGKETLQR.A 10311 10351 |
|  | 10618 | 455.7262 | 1364.1569 | 1363.6343 | 0.5226 | 0 | 3 | 50 | 7 | R.YFYTAMSRPGR.G |
|  | 11600 | **710.4257** | **1418.8368** | **1419.6565** | **-0.8197** | **1** | **4** | **48** | **6** | **R.FDSDAASPRMAPR.A** |
|  | 11736 | 714.3626 | 1426.7105 | 1426.7205 | -0.0099 | 0 | 46 | 0.0031 | 1 | K.WAAVVVPSGEEQR.Y 11721 11735 11751 |
|  | 17808 | 902.9733 | 1803.9321 | 1802.8522 | 1.0799 | 1 | 3 | 60 | 8 | R.YFYTAMSRPGRGEPR.F |
|  | 18486 | 463.4837 | 1849.9057 | 1849.9183 | -0.0127 | 0 | 16 | 2.9 | 1 | K.THVTHHPISDHEATLR.C 18485 |
|  | 20872 | 513.0196 | 2048.0493 | 2048.0626 | -0.0133 | 0 | 16 | 2.1 | 1 | R.YTCHVQHEGLPKPLTLR.W 20848 20850 20871 |
|  | 21557 | 1056.5175 | 2111.0204 | 2111.0299 | -0.0095 | 0 | 86 | 2.1e-007 | 1 | R.CWALGFYPAEITLTWQR.D 21558 |
|  | 22689 | 744.6669 | 2230.9790 | 2230.9938 | -0.0149 | 0 | 44 | 0.0032 | 1 | R.DGEDQTQDTELVETRPAGDR.T 22688 |
|  | 30136 | 1896.3450 | 3790.6754 | 3790.1002 | 0.5752 | 1 | 2 | 20 | 5 | R.WEPSSQSTVPIVGIVAGLAVLAVVVIGAVVAAVMCRR.K |

| **265.** | P10319\|1B58_HUMAN    **Mass:** 40597    **Score:** 159    **Queries matched:** 26   **emPAI:** 0.70 |
| --- | --- |
|  | HLA class I histocompatibility antigen, B-58 alpha chain OS=Homo sapiens GN=HLA-B PE=2 SV=1 |

|  | **Query** | **Observed** | **Mr(expt)** | **Mr(calc)** | **Delta** | **Miss** | **Score** | **Expect** | **Rank** | **Peptide** |
| --- | --- | --- | --- | --- | --- | --- | --- | --- | --- | --- |
|  | 3888 | 482.7234 | 963.4322 | 964.4250 | -0.9929 | 0 | 33 | 0.088 | 1 | R.FDSDAASPR.T 3900 3901 3907 |
|  | 4176 | 493.2762 | 984.5379 | 983.5876 | 0.9503 | 1 | 10 | 18 | 10 | R.ENLRIALR.Y 4164 |
|  | 6565 | 577.7809 | 1153.5473 | 1153.6091 | -0.0618 | 1 | 3 | 67 | 9 | K.ETLQRADPPK.T |
|  | 6759 | 584.7974 | 1167.5802 | 1168.5659 | -0.9857 | 1 | 3 | 72 | 10 | R.NMKASAQTYR.E |
|  | 10335 | 675.8494 | 1349.6843 | 1349.6939 | -0.0096 | 1 | 38 | 0.024 | 1 | R.YLENGKETLQR.A 10311 10351 |
|  | 10618 | 455.7262 | 1364.1569 | 1363.6343 | 0.5226 | 0 | 3 | 50 | 7 | R.YFYTAMSRPGR.G |
|  | 11736 | 714.3626 | 1426.7105 | 1426.7205 | -0.0099 | 0 | 46 | 0.0031 | 1 | K.WAAVVVPSGEEQR.Y 11721 11735 11751 |
|  | 17808 | 902.9733 | 1803.9321 | 1802.8522 | 1.0799 | 1 | 3 | 60 | 8 | R.YFYTAMSRPGRGEPR.F |
|  | 18283 | 613.3167 | 1836.9283 | 1835.9027 | 1.0256 | 0 | 15 | 3.7 | 1 | K.THVTHHPVSDHEATLR.C |
|  | 20872 | 513.0196 | 2048.0493 | 2048.0626 | -0.0133 | 0 | 16 | 2.1 | 1 | R.YTCHVQHEGLPKPLTLR.W 20848 20850 20871 |
|  | 21557 | 1056.5175 | 2111.0204 | 2111.0299 | -0.0095 | 0 | 86 | 2.1e-007 | 1 | R.CWALGFYPAEITLTWQR.D 21558 |
|  | 22689 | 744.6669 | 2230.9790 | 2230.9938 | -0.0149 | 0 | 44 | 0.0032 | 1 | R.DGEDQTQDTELVETRPAGDR.T 22688 |

|  | **Proteins matching a subset of these peptides:** |
| --- | --- |

|  | P30491\|1B53_HUMAN    **Mass:** 40755    **Score:** 159    **Queries matched:** 25 |
| --- | --- |
|  | HLA class I histocompatibility antigen, B-53 alpha chain OS=Homo sapiens GN=HLA-B PE=1 SV=1 |

|  | P30685\|1B35_HUMAN    **Mass:** 40715    **Score:** 159    **Queries matched:** 23 |
| --- | --- |
|  | HLA class I histocompatibility antigen, B-35 alpha chain OS=Homo sapiens GN=HLA-B PE=1 SV=1 |

| **266.** | P30464\|1B15_HUMAN    **Mass:** 40648    **Score:** 159    **Queries matched:** 26   **emPAI:** 0.70 |
| --- | --- |
|  | HLA class I histocompatibility antigen, B-15 alpha chain OS=Homo sapiens GN=HLA-B PE=1 SV=2 |

|  | **Query** | **Observed** | **Mr(expt)** | **Mr(calc)** | **Delta** | **Miss** | **Score** | **Expect** | **Rank** | **Peptide** |
| --- | --- | --- | --- | --- | --- | --- | --- | --- | --- | --- |
|  | 3888 | 482.7234 | 963.4322 | 964.4250 | -0.9929 | 0 | 33 | 0.088 | 1 | R.FDSDAASPR.M 3900 3901 3907 |
|  | 6565 | 577.7809 | 1153.5473 | 1153.6091 | -0.0618 | 1 | 3 | 67 | 9 | K.ETLQRADPPK.T |
|  | 10335 | 675.8494 | 1349.6843 | 1349.6939 | -0.0096 | 1 | 38 | 0.024 | 1 | R.YLENGKETLQR.A 10311 10351 |
|  | 10618 | 455.7262 | 1364.1569 | 1363.6343 | 0.5226 | 0 | 3 | 50 | 7 | R.YFYTAMSRPGR.G |
|  | 11600 | 710.4257 | 1418.8368 | 1419.6565 | -0.8197 | 1 | 4 | 48 | 6 | R.FDSDAASPRMAPR.A |
|  | 11736 | 714.3626 | 1426.7105 | 1426.7205 | -0.0099 | 0 | 46 | 0.0031 | 1 | K.WAAVVVPSGEEQR.Y 11721 11735 11751 |
|  | 17808 | 902.9733 | 1803.9321 | 1802.8522 | 1.0799 | 1 | 3 | 60 | 8 | R.YFYTAMSRPGRGEPR.F |
|  | 18486 | 463.4837 | 1849.9057 | 1849.9183 | -0.0127 | 0 | 16 | 2.9 | 1 | K.THVTHHPISDHEATLR.C 18485 |
|  | 20872 | 513.0196 | 2048.0493 | 2048.0626 | -0.0133 | 0 | 16 | 2.1 | 1 | R.YTCHVQHEGLPKPLTLR.W 20848 20850 20871 |
|  | 21557 | 1056.5175 | 2111.0204 | 2111.0299 | -0.0095 | 0 | 86 | 2.1e-007 | 1 | R.CWALGFYPAEITLTWQR.D 21558 |
|  | 22689 | 744.6669 | 2230.9790 | 2230.9938 | -0.0149 | 0 | 44 | 0.0032 | 1 | R.DGEDQTQDTELVETRPAGDR.T 22688 |
|  | 27405 | **742.6110** | **2966.4147** | **2966.6012** | **-0.1864** | **1** | **2** | **37** | **1** | **R.VTAPRTVLLLLSGALALTETWAGSHSMR.Y** |

|  | **Proteins matching the same set of peptides:** |
| --- | --- |

|  | P30484\|1B46_HUMAN    **Mass:** 40700    **Score:** 159    **Queries matched:** 26 |
| --- | --- |
|  | HLA class I histocompatibility antigen, B-46 alpha chain OS=Homo sapiens GN=HLA-B PE=2 SV=1 |

| **267.** | P27338\|AOFB_HUMAN    **Mass:** 59238    **Score:** 159    **Queries matched:** 26   **emPAI:** 0.20 |
| --- | --- |
|  | Amine oxidase [flavin-containing] B OS=Homo sapiens GN=MAOB PE=1 SV=3 |

|  | **Query** | **Observed** | **Mr(expt)** | **Mr(calc)** | **Delta** | **Miss** | **Score** | **Expect** | **Rank** | **Peptide** |
| --- | --- | --- | --- | --- | --- | --- | --- | --- | --- | --- |
|  | 2210 | **421.5636** | **841.1127** | **840.3106** | **0.8021** | **0** | **9** | **14** | **4** | **R.TMDDMGR.E** 2178 2204 |
|  | 6115 | 561.7767 | 1121.5389 | 1121.5466 | -0.0076 | 0 | 62 | 0.00011 | 1 | K.FVGGSGQVSER.I 6116 |
|  | 6867 | **588.7920** | **1175.5694** | **1174.5942** | **0.9752** | **0** | **17** | **3.2** | **1** | **R.IISTTNGGQER.K** |
|  | 8402 | 625.8235 | 1249.6325 | 1249.6415 | -0.0090 | 1 | 42 | 0.0087 | 1 | R.KFVGGSGQVSER.I 8381 8420 |
|  | 10381 | **451.5677** | **1351.6814** | **1351.6893** | **-0.0079** | **0** | **16** | **3.8** | **1** | **K.IHFNPPLPMMR.N** 10364 |
|  | 10995 | **462.2278** | **1383.6616** | **1383.6791** | **-0.0175** | **0** | **(4)** | **57** | **10** | **K.IHFNPPLPMMR.N** |
|  | 11331 | **468.9816** | **1403.9231** | **1404.7687** | **-0.8456** | **0** | **3** | **52** | **5** | **K.YVISAIPPTLGMK.I** 11373 |
|  | 14714 | 803.9175 | 1605.8205 | 1605.7855 | 0.0350 | 0 | 5 | 35 | 9 | K.CDVVVVGGGISGMAAAK.L 14703 |
|  | 15037 | **813.3938** | **1624.7730** | **1624.7845** | **-0.0115** | **0** | **70** | **1.1e-005** | **1** | **K.YVDLGGSYVGPTQNR.I** 15036 |
|  | 15169 | 817.9293 | 1633.8440 | 1633.9151 | -0.0712 | 0 | 6 | 28 | 1 | K.LLHDSGLNVVVLEAR.D |
|  | 15229 | **819.9407** | **1637.8669** | **1637.9215** | **-0.0546** | **1** | **3** | **61** | **1** | **R.VPLGSVIKCIVYYK.E** |
|  | 15871 | **560.2421** | **1677.7046** | **1677.8573** | **-0.1528** | **1** | **4** | **48** | **3** | **K.ELGLETYKVNEVER.L** |
|  | 18658 | **621.9190** | **1862.7350** | **1863.8131** | **-1.0781** | **1** | **9** | **10** | **1** | **R.TMDDMGREIPSDAPWK.A** |
|  | 21549 | 704.4074 | 2110.2004 | 2111.0591 | -0.8587 | 1 | 5 | 23 | 1 | K.IHFNPPLPMMRNQMITR.V |
|  | 26300 | **917.4301** | **2749.2683** | **2748.2126** | **1.0557** | **0** | **0** | **54** | **3** | **R.IYFAGTETATHWSGYMEGAVEAGER.A** |
|  | 28982 | **555.6068** | **3327.5968** | **3327.5255** | **0.0714** | **1** | **3** | **21** | **2** | **R.QPVDRIYFAGTETATHWSGYMEGAVEAGER.A** |
|  | 31247 | **989.0356** | **4940.1418** | **4940.2738** | **-0.1320** | **1** | **1** | **9.2** | **2** | **K.VLGSLEALEPVHYEEKNWCEEQYSGGCYTTYFPPGILTQYGR.V** |

| **268.** | Q5VSK2\|MRC1L_HUMAN    **Mass:** 168904   **Score:** 159    **Queries matched:** 28   **emPAI:** 0.14 |
| --- | --- |
|  | Macrophage mannose receptor 1-like protein 1 OS=Homo sapiens GN=MRC1L1 PE=2 SV=1 |

|  | **Query** | **Observed** | **Mr(expt)** | **Mr(calc)** | **Delta** | **Miss** | **Score** | **Expect** | **Rank** | **Peptide** |
| --- | --- | --- | --- | --- | --- | --- | --- | --- | --- | --- |
|  | 1246 | **678.3873** | **677.3801** | **678.4064** | **-1.0264** | **1** | **7** | **40** | **3** | **K.LYAKGK.H** |
|  | 1478 | 781.3572 | 780.3499 | 780.4204 | -0.0705 | 0 | 7 | 36 | 2 | K.NIMLYK.G |
|  | 2650 | 437.2801 | 872.5456 | 872.4967 | 0.0488 | 0 | 20 | 2.4 | 10 | K.NDTLLGIK.G |
|  | 3477 | **468.1966** | **934.3786** | **933.4524** | **0.9262** | **0** | **5** | **61** | **5** | **R.KPGCVAMR.T** |
|  | 3844 | **481.1499** | **960.2853** | **960.4699** | **-0.1846** | **0** | **11** | **13** | **2** | **K.SCVSLNPGK.N** |
|  | 5141 | 527.2961 | 1052.5777 | 1052.5185 | 0.0592 | 1 | 10 | 15 | 6 | K.RGYICQTR.S |
|  | 5951 | **555.7842** | **1109.5539** | **1108.4819** | **1.0721** | **1** | **1** | **1.1e+002** | **9** | **K.EKETMDNAR.A** |
|  | 6402 | 572.9941 | 1143.9737 | 1143.4985 | 0.4752 | 0 | 9 | 15 | 8 | K.EGWNFYSNK.C |
|  | 6822 | **586.8334** | **1171.6522** | **1171.6197** | **0.0325** | **0** | **1** | **1.3e+002** | **7** | **R.ALGGDLASINNK.E** |
|  | 7045 | **593.7468** | **1185.4791** | **1185.4873** | **-0.0082** | **0** | **40** | **0.014** | **1** | **K.WYADCTSAGR.S** 7081 |
|  | 7207 | **398.6926** | **1193.0560** | **1192.5580** | **0.4979** | **1** | **7** | **22** | **8** | **R.QEDCVVMKGK.D** |
|  | 7213 | **597.7712** | **1193.5278** | **1193.5353** | **-0.0075** | **0** | **15** | **4.5** | **1** | **R.YTNWAADEPK.L** |
|  | 7479 | **603.2806** | **1204.5466** | **1204.5546** | **-0.0080** | **0** | **23** | **0.82** | **1** | **K.WENLECVQK.L** 7480 |
|  | 9344 | **650.2987** | **1298.5829** | **1298.5925** | **-0.0096** | **0** | **44** | **0.0059** | **1** | **K.IYGTTDNLCSR.G** |
|  | 10277 | **674.2936** | **1346.5726** | **1346.5826** | **-0.0100** | **0** | **8** | **26** | **1** | **R.FTHWNSDMPGR.K** |
|  | 12341 | **731.8379** | **1461.6613** | **1461.7364** | **-0.0751** | **1** | **2** | **90** | **7** | **R.QFLIYNEDHKR.C** |
|  | 13185 | **756.3776** | **1510.7406** | **1510.7490** | **-0.0084** | **0** | **56** | **0.00031** | **1** | **R.WVSESQIMSVAFK.L** 13166 |
|  | 14703 | 536.1638 | 1605.4695 | 1605.7998 | -0.3304 | 0 | 1 | 61 | 3 | R.SDEIPATEPPQLPGR.C |
|  | 14857 | **808.4044** | **1614.7943** | **1614.8042** | **-0.0099** | **0** | **16** | **3.3** | **1** | **R.YLNWLPGSPSAEPGK.S** 14856 |
|  | 15281 | **821.7719** | **1641.5293** | **1640.7513** | **0.7780** | **1** | **8** | **13** | **1** | **R.GCEWPLGYICKMK.S** |
|  | 21544 | **704.0554** | **2109.1444** | **2109.0643** | **0.0801** | **0** | **0** | **84** | **7** | **K.HWAEGVTHPPKPTTTPEPK.C** |
|  | 22799 | **748.3804** | **2242.1195** | **2242.1342** | **-0.0147** | **1** | **29** | **0.11** | **1** | **R.ALGGDLASINNKEEQQTIWR.L** |
|  | 27589 | **1000.4385** | **2998.2936** | **2998.3154** | **-0.0218** | **1** | **27** | **0.093** | **1** | **R.SDGWLWCGTTTDYDTDKLFGYCPLK.F** |
|  | 28606 | **1074.1908** | **3219.5506** | **3219.5658** | **-0.0153** | **0** | **79** | **5.4e-007** | **1** | **K.LHNSLIASILDPYSNAFAWLQMETSNER.V** |

|  | **Proteins matching the same set of peptides:** |
| --- | --- |

|  | P22897\|MRC1_HUMAN    **Mass:** 168870   **Score:** 159    **Queries matched:** 28 |
| --- | --- |
|  | Macrophage mannose receptor 1 OS=Homo sapiens GN=MRC1 PE=1 SV=1 |

| **269.** | P08107\|HSP71_HUMAN    **Mass:** 70294    **Score:** 158    **Queries matched:** 23   **emPAI:** 0.36 |
| --- | --- |
|  | Heat shock 70 kDa protein 1 OS=Homo sapiens GN=HSPA1A PE=1 SV=5 |

|  | **Query** | **Observed** | **Mr(expt)** | **Mr(calc)** | **Delta** | **Miss** | **Score** | **Expect** | **Rank** | **Peptide** |
| --- | --- | --- | --- | --- | --- | --- | --- | --- | --- | --- |
|  | 1691 | 804.4410 | 803.4337 | 803.4389 | -0.0052 | 0 | 27 | 0.48 | 1 | K.ITITNDK.G |
|  | 4652 | 509.2835 | 1016.5525 | 1016.5614 | -0.0089 | 1 | 27 | 0.36 | 1 | K.ITITNDKGR.L |
|  | 6153 | 563.2797 | 1124.5449 | 1124.5536 | -0.0087 | 1 | 23 | 0.8 | 1 | R.MVQEAEKYK.A |
|  | 6301 | **569.2761** | **1136.5377** | **1136.5462** | **-0.0085** | **1** | **16** | **3.9** | **1** | **K.YKAEDEVQR.E** |
|  | 6315 | **569.8052** | **1137.5958** | **1137.6030** | **-0.0072** | **1** | **14** | **6.1** | **2** | **K.VQVSYKGETK.A** |
|  | 7828 | **408.2211** | **1221.6416** | **1221.5700** | **0.0716** | **0** | **9** | **19** | **4** | **K.FGDPVVQSDMK.H** |
|  | 7943 | 614.8138 | 1227.6130 | 1227.6207 | -0.0077 | 0 | 58 | 0.00024 | 1 | K.VEIIANDQGNR.T 7942 |
|  | 8157 | **620.3475** | **1238.6804** | **1237.5649** | **1.1155** | **0** | **(3)** | **72** | **2** | **K.FGDPVVQSDMK.H** |
|  | 8627 | 631.3384 | 1260.6622 | 1260.6503 | 0.0119 | 0 | 2 | 92 | 7 | R.LVNHFVEEFK.R |
|  | 10334 | 675.8352 | 1349.6559 | 1349.6649 | -0.0091 | 1 | 21 | 1.1 | 1 | R.KFGDPVVQSDMK.H |
|  | 11579 | **473.5890** | **1417.7452** | **1416.7514** | **0.9939** | **1** | **29** | **0.19** | **1** | **R.LVNHFVEEFKR.K** |
|  | 12758 | 744.3493 | 1486.6840 | 1486.6940 | -0.0100 | 0 | 33 | 0.064 | 1 | R.TTPSYVAFTDTER.L |
|  | 14838 | **807.9019** | **1613.7892** | **1613.8011** | **-0.0119** | **0** | **2** | **68** | **3** | **K.AFYPEEISSMVLTK.M** |
|  | 15037 | 813.3938 | 1624.7730 | 1624.9260 | -0.1530 | 1 | 15 | 3.9 | 2 | R.QATKDAGVIAGLNVLR.I 15036 |
|  | 15549 | **829.9229** | **1657.8313** | **1657.8424** | **-0.0111** | **0** | **61** | **9.2e-005** | **1** | **K.NQVALNPQNTVFDAK.R** |
|  | 15824 | 838.3630 | 1674.7114 | 1674.7234 | -0.0120 | 0 | 66 | 2.6e-005 | 1 | K.ATAGDTHLGGEDFDNR.L 15835 |
|  | 15909 | **560.9501** | **1679.8284** | **1679.8420** | **-0.0136** | **0** | **18** | **2** | **1** | **K.HWPFQVINDGDKPK.V** 15927 |
|  | 22943 | **755.7114** | **2264.1125** | **2264.1260** | **-0.0135** | **0** | **15** | **2.5** | **1** | **K.AAAIGIDLGTTYSCVGVFQHGK.V** |
|  | 28744 | **1087.5436** | **3259.6089** | **3259.6223** | **-0.0134** | **1** | **5** | **14** | **1** | **K.MKEIAEAYLGYPVTNAVITVPAYFNDSQR.Q** |

| **270.** | P04217\|A1BG_HUMAN    **Mass:** 54809    **Score:** 158    **Queries matched:** 13   **emPAI:** 0.39 |
| --- | --- |
|  | Alpha-1B-glycoprotein OS=Homo sapiens GN=A1BG PE=1 SV=3 |

|  | **Query** | **Observed** | **Mr(expt)** | **Mr(calc)** | **Delta** | **Miss** | **Score** | **Expect** | **Rank** | **Peptide** |
| --- | --- | --- | --- | --- | --- | --- | --- | --- | --- | --- |
|  | 2581 | **435.2266** | **868.4386** | **869.5222** | **-1.0836** | **0** | **15** | **5** | **2** | **K.LLELTGPK.S** |
|  | 4981 | **521.3148** | **1040.6150** | **1039.6026** | **1.0124** | **1** | **2** | **87** | **6** | **R.GEKELLVPR.S** |
|  | 7973 | **410.7327** | **1229.1763** | **1229.7608** | **-0.5846** | **1** | **9** | **14** | **10** | **R.GVLRGVTFLLR.R** |
|  | 8672 | **632.8264** | **1263.6383** | **1263.6459** | **-0.0076** | **0** | **49** | **0.0019** | **1** | **R.SGLSTGWTQLSK.L** |
|  | 15322 | **823.4122** | **1644.8098** | **1644.8181** | **-0.0084** | **0** | **41** | **0.0097** | **1** | **R.CEGPIPDVTFELLR.E** 15334 |
|  | 18808 | **938.4981** | **1874.9817** | **1874.9924** | **-0.0108** | **0** | **58** | **0.00018** | **1** | **K.VTLTCVAPLSGVDFQLR.R** 18809 |
|  | 21875 | **538.0497** | **2148.1699** | **2147.0371** | **1.1328** | **0** | **9** | **9.5** | **1** | **R.IFFHLNAVALGDGGHYTCR.Y** |
|  | 21898 | **1076.0809** | **2150.1473** | **2150.1598** | **-0.0125** | **0** | **55** | **0.00026** | **1** | **K.SLPAPWLSMAPVSWITPGLK.T** 21896 |
|  | 24448 | **1236.0945** | **2470.1744** | **2470.1904** | **-0.0160** | **0** | **7** | **14** | **1** | **R.SWVPHTFESELSDPVELLVAES.-** |
|  | 30442 | **1331.2805** | **3990.8197** | **3989.8555** | **0.9642** | **0** | **16** | **0.69** | **1** | **R.LHDNQNGWSGDSAPVELILSDETLPAPEFSPEPESGR.A** |

| **271.** | P04083\|ANXA1_HUMAN    **Mass:** 38918    **Score:** 158    **Queries matched:** 16   **emPAI:** 0.74 |
| --- | --- |
|  | Annexin A1 OS=Homo sapiens GN=ANXA1 PE=1 SV=2 |

|  | **Query** | **Observed** | **Mr(expt)** | **Mr(calc)** | **Delta** | **Miss** | **Score** | **Expect** | **Rank** | **Peptide** |
| --- | --- | --- | --- | --- | --- | --- | --- | --- | --- | --- |
|  | 3151 | 456.7371 | 911.4596 | 912.5069 | -1.0473 | 1 | 12 | 8.3 | 2 | R.VFQKYTK.Y |
|  | 4942 | 520.2661 | 1038.5177 | 1037.4600 | 1.0577 | 1 | 6 | 39 | 7 | K.YSKHDMNK.V |
|  | 5290 | **532.7463** | **1063.4781** | **1063.4856** | **-0.0074** | **0** | **28** | **0.27** | **1** | **R.SEIDMNDIK.A** |
|  | 5384 | **536.2788** | **1070.5431** | **1070.5140** | **0.0290** | **0** | **3** | **99** | **2** | **-.MAMVSEFLK.Q** |
|  | 7666 | **607.7892** | **1213.5638** | **1212.6397** | **0.9241** | **1** | **6** | **35** | **1** | **K.LHQAMKGVGTR.H** 7657 |
|  | 10773 | **458.2321** | **1371.6744** | **1370.7657** | **0.9088** | **1** | **6** | **35** | **9** | **K.VLDLELKGDIEK.C** |
|  | 11063 | **694.3839** | **1386.7533** | **1386.7606** | **-0.0073** | **0** | **77** | **2.6e-006** | **1** | **K.GVDEATIIDILTK.R** |
|  | 13842 | **775.9081** | **1549.8017** | **1549.8100** | **-0.0083** | **0** | **29** | **0.14** | **1** | **K.GTDVNVFNTILTTR.S** |
|  | 14690 | 803.4780 | 1604.9415 | 1604.9501 | -0.0086 | 0 | 67 | 2.1e-005 | 1 | K.ALTGHLEEVVLALLK.T |
|  | 15641 | **555.1686** | **1662.4839** | **1662.8035** | **-0.3196** | **1** | **8** | **14** | **2** | **K.TPAQFDADELRAAMK.G** |
|  | 16840 | **580.6774** | **1739.0103** | **1738.7282** | **0.2821** | **0** | **2** | **65** | **3** | **R.SEDFGVNEDLADSDAR.A** |
|  | 19158 | **635.6782** | **1904.0128** | **1904.0255** | **-0.0126** | **1** | **17** | **2.1** | **1** | **K.AAYLQETGKPLDETLKK.A** |
|  | 19728 | **974.0311** | **1946.0477** | **1945.0806** | **0.9672** | **1** | **2** | **55** | **4** | **K.AIMVKGVDEATIIDILTK.R** |
|  | 23485 | **778.7006** | **2333.0800** | **2333.0919** | **-0.0119** | **1** | **31** | **0.057** | **1** | **K.MYGISLCQAILDETKGDYEK.I** |
|  | 23658 | **786.0517** | **2355.1333** | **2355.1495** | **-0.0163** | **0** | **53** | **0.0004** | **1** | **K.GGPGSAVSPYPTFNPSSDVAALHK.A** |

| **272.** | P34931\|HS71L_HUMAN    **Mass:** 70730    **Score:** 158    **Queries matched:** 20   **emPAI:** 0.23 |
| --- | --- |
|  | Heat shock 70 kDa protein 1L OS=Homo sapiens GN=HSPA1L PE=1 SV=2 |

|  | **Query** | **Observed** | **Mr(expt)** | **Mr(calc)** | **Delta** | **Miss** | **Score** | **Expect** | **Rank** | **Peptide** |
| --- | --- | --- | --- | --- | --- | --- | --- | --- | --- | --- |
|  | 1404 | **741.1929** | **740.1857** | **739.4704** | **0.7152** | **1** | **7** | **20** | **1** | **R.IPKVQR.L** |
|  | 1691 | 804.4410 | 803.4337 | 803.4389 | -0.0052 | 0 | 27 | 0.48 | 1 | K.ITITNDK.G |
|  | 4652 | 509.2835 | 1016.5525 | 1016.5614 | -0.0089 | 1 | 27 | 0.36 | 1 | K.ITITNDKGR.L |
|  | 6301 | 569.2761 | 1136.5377 | 1136.5462 | -0.0085 | 1 | 16 | 3.9 | 1 | K.YKAEDEVQR.E |
|  | 7943 | 614.8138 | 1227.6130 | 1227.6207 | -0.0077 | 0 | 58 | 0.00024 | 1 | K.VEIIANDQGNR.T 7942 |
|  | 12758 | 744.3493 | 1486.6840 | 1486.6940 | -0.0100 | 0 | 33 | 0.064 | 1 | R.TTPSYVAFTDTER.L |
|  | 14838 | 807.9019 | 1613.7892 | 1613.8011 | -0.0119 | 0 | 2 | 68 | 3 | K.AFYPEEISSMVLTK.L |
|  | 15037 | 813.3938 | 1624.7730 | 1624.9260 | -0.1530 | 1 | 15 | 3.9 | 2 | R.QATKDAGVIAGLNVLR.I 15036 |
|  | 15576 | 830.4467 | 1658.8788 | 1658.8879 | -0.0091 | 0 | 59 | 0.00013 | 1 | R.IINEPTAAAIAYGLDK.G 15592 |
|  | 15824 | 838.3630 | 1674.7114 | 1674.7234 | -0.0120 | 0 | 66 | 2.6e-005 | 1 | K.ATAGDTHLGGEDFDNR.L 15835 |
|  | 15849 | 838.8917 | 1675.7689 | 1675.7988 | -0.0299 | 0 | 8 | 20 | 3 | K.NQVAMNPQNTVFDAK.R 15862 |
|  | 19689 | **649.3548** | **1945.0426** | **1944.9615** | **0.0811** | **0** | **5** | **32** | **3** | **K.CNELLSWLEVNQLAEK.D** 19707 |
|  | 20979 | **686.8098** | **2057.4074** | **2057.9979** | **-0.5905** | **1** | **1** | **47** | **6** | **K.GENKAFYPEEISSMVLTK.L** |
|  | 22793 | **748.3554** | **2242.0444** | **2243.1546** | **-1.1102** | **1** | **3** | **42** | **3** | **R.IINEPTAAAIAYGLDKGGQGER.H** |

| **273.** | Q96AG4\|LRC59_HUMAN    **Mass:** 35308    **Score:** 158    **Queries matched:** 15   **emPAI:** 0.50 |
| --- | --- |
|  | Leucine-rich repeat-containing protein 59 OS=Homo sapiens GN=LRRC59 PE=1 SV=1 |

|  | **Query** | **Observed** | **Mr(expt)** | **Mr(calc)** | **Delta** | **Miss** | **Score** | **Expect** | **Rank** | **Peptide** |
| --- | --- | --- | --- | --- | --- | --- | --- | --- | --- | --- |
|  | 832 | **517.3303** | **516.3230** | **515.2816** | **1.0415** | **0** | **6** | **1e+002** | **7** | **K.GGNLR.D** |
|  | 1208 | 659.4036 | 658.3964 | 658.3511 | 0.0453 | 0 | 12 | 18 | 7 | K.SGSRPR.K |
|  | 2454 | **430.7371** | **859.4596** | **858.4671** | **0.9924** | **1** | **10** | **24** | **3** | **K.AGSKGGNLR.D** 2442 |
|  | 2634 | **437.2406** | **872.4666** | **873.4780** | **-1.0115** | **1** | **4** | **91** | **2** | **K.SKSGSRPR.K** |
|  | 6274 | **567.7908** | **1133.5670** | **1133.5750** | **-0.0081** | **0** | **2** | **94** | **2** | **K.ATILDLSCNK.L** |
|  | 6395 | **572.8053** | **1143.5960** | **1143.6037** | **-0.0076** | **0** | **21** | **1.2** | **1** | **K.LQQLPADFGR.L** |
|  | 13570 | 767.8647 | 1533.7149 | 1532.8674 | 0.8475 | 0 | 48 | 0.002 | 1 | R.LVNLQHLDLLNNK.L 13566 13569 |
|  | 16794 | **579.6543** | **1735.9411** | **1735.9509** | **-0.0098** | **1** | **28** | **0.18** | **1** | **K.WLDLKDNPLDPVLAK.V** 16786 |
|  | 18869 | **940.9783** | **1879.9420** | **1879.9541** | **-0.0121** | **1** | **98** | **1.6e-008** | **1** | **R.RHEILQWVLQTDSQQ.-** 18870 18877 |

| **274.** | P62424\|RL7A_HUMAN    **Mass:** 30148    **Score:** 157    **Queries matched:** 11   **emPAI:** 0.42 |
| --- | --- |
|  | 60S ribosomal protein L7a OS=Homo sapiens GN=RPL7A PE=1 SV=2 |

|  | **Query** | **Observed** | **Mr(expt)** | **Mr(calc)** | **Delta** | **Miss** | **Score** | **Expect** | **Rank** | **Peptide** |
| --- | --- | --- | --- | --- | --- | --- | --- | --- | --- | --- |
|  | 643 | 459.0106 | 458.0033 | 458.2853 | -0.2820 | 0 | 2 | 1.2e+002 | 7 | K.GALAK.L |
|  | 5273 | **532.2487** | **1062.4829** | **1063.5563** | **-1.0734** | **0** | **21** | **1.4** | **1** | **R.HWGGNVLGPK.S** 5293 |
|  | 7685 | **608.3280** | **1214.6414** | **1215.6248** | **-0.9833** | **0** | **4** | **64** | **6** | **K.NFGIGQDIQPK.R** |
|  | 9926 | **443.0383** | **1326.0932** | **1325.7819** | **0.3112** | **1** | **3** | **43** | **9** | **K.VVNPLFEKRPK.N** |
|  | 10262 | **673.3652** | **1344.7158** | **1344.7249** | **-0.0091** | **0** | **48** | **0.0023** | **1** | **R.AGVNTVTTLVENK.K** 10261 |
|  | 14144 | 785.4171 | 1568.8197 | 1568.8311 | -0.0114 | 0 | 83 | 6.1e-007 | 1 | K.VPPAINQFTQALDR.Q 14146 |
|  | 17901 | 604.3405 | 1809.9997 | 1810.0101 | -0.0104 | 1 | 2 | 59 | 3 | R.LKVPPAINQFTQALDR.Q |
|  | 26676 | 939.4213 | 2815.2420 | 2814.5830 | 0.6590 | 1 | 2 | 33 | 4 | K.AQLVVIAHDVDPIELVVFLPALCRK.M |

| **275.** | P01857\|IGHG1_HUMAN    **Mass:** 36596    **Score:** 157    **Queries matched:** 19   **emPAI:** 0.63 |
| --- | --- |
|  | Ig gamma-1 chain C region OS=Homo sapiens GN=IGHG1 PE=1 SV=1 |

|  | **Query** | **Observed** | **Mr(expt)** | **Mr(calc)** | **Delta** | **Miss** | **Score** | **Expect** | **Rank** | **Peptide** |
| --- | --- | --- | --- | --- | --- | --- | --- | --- | --- | --- |
|  | 1491 | 395.0771 | 788.1396 | 787.4440 | 0.6957 | 0 | 12 | 11 | 4 | K.SLSLSPGK.- 1494 |
|  | 2156 | **419.7523** | **837.4901** | **837.4960** | **-0.0058** | **0** | **2** | **93** | **9** | **K.ALPAPIEK.T** |
|  | 6657 | **580.9736** | **1159.9327** | **1160.6223** | **-0.6896** | **0** | **3** | **62** | **8** | **K.NQVSLTCLVK.G** |
|  | 7043 | **593.5035** | **1184.9925** | **1185.6394** | **-0.6468** | **0** | **2** | **74** | **5** | **K.GPSVFPLAPSSK.S** |
|  | 9103 | **429.7351** | **1286.1833** | **1285.6666** | **0.5167** | **0** | **14** | **3.9** | **4** | **R.EPQVYTLPPSR.D** |
|  | 15859 | **839.3992** | **1676.7839** | **1676.7947** | **-0.0108** | **0** | **61** | **9.7e-005** | **1** | **K.FNWYVDGVEVHNAK.T** 15860 |
|  | 17858 | **904.5013** | **1806.9881** | **1806.9992** | **-0.0111** | **0** | **46** | **0.0025** | **1** | **R.VVSVLTVLHQDWLNGK.E** 17868 |
|  | 21789 | **713.6762** | **2138.0068** | **2138.0202** | **-0.0134** | **0** | **30** | **0.076** | **1** | **R.TPEVTCVVVDVSHEDPEVK.F** |
|  | 24591 | **830.7993** | **2489.3761** | **2488.2996** | **1.0766** | **1** | **0** | **59** | **3** | **K.GPSVFPLAPSSKSTSGGTAALGCLVK.D** |
|  | 24943 | **1272.5602** | **2543.1058** | **2543.1241** | **-0.0183** | **0** | **38** | **0.01** | **1** | **K.GFYPSDIAVEWESNGQPENNYK.T** 24942 24944 24958 24960 |
|  | 26676 | 939.4213 | 2815.2420 | 2816.2548 | -1.0128 | 0 | 1 | 41 | 7 | R.WQQGNVFSCSVMHEALHNHYTQK.S |
|  | 31806 | **1343.4609** | **6712.2683** | **6712.3072** | **-0.0389** | **0** | **23** | **0.026** | **1** | **K.DYFPEPVTVSWNSGALTSGVHTFPAVLQSSGLYSLSSVVTVPSSSLGTQTYICNVNHKPSNTK.V** |

| **276.** | O75947\|ATP5H_HUMAN    **Mass:** 18537    **Score:** 157    **Queries matched:** 8   **emPAI:** 0.21 |
| --- | --- |
|  | ATP synthase subunit d, mitochondrial OS=Homo sapiens GN=ATP5H PE=1 SV=3 |

|  | **Query** | **Observed** | **Mr(expt)** | **Mr(calc)** | **Delta** | **Miss** | **Score** | **Expect** | **Rank** | **Peptide** |
| --- | --- | --- | --- | --- | --- | --- | --- | --- | --- | --- |
|  | 1265 | **689.4147** | **688.4075** | **688.4119** | **-0.0045** | **0** | **20** | **2.6** | **1** | **K.AIASSLK.S** |
|  | 9314 | 649.3160 | 1296.6174 | 1296.6271 | -0.0097 | 1 | 19 | 1.9 | 1 | R.IVEYEKEMEK.M |
|  | 15965 | **562.2839** | **1683.8300** | **1683.8409** | **-0.0109** | **1** | **14** | **3.9** | **1** | **K.KYPYWPHQPIENL.-** 15951 15964 |
|  | 24402 | **1233.0931** | **2464.1717** | **2464.1832** | **-0.0114** | **0** | **82** | **4.9e-007** | **1** | **K.NLIPFDQMTIEDLNEAFPETK.L** 24401 24415 |

| **277.** | P30487\|1B49_HUMAN    **Mass:** 40841    **Score:** 156    **Queries matched:** 26   **emPAI:** 0.55 |
| --- | --- |
|  | HLA class I histocompatibility antigen, B-49 alpha chain OS=Homo sapiens GN=HLA-B PE=2 SV=2 |

|  | **Query** | **Observed** | **Mr(expt)** | **Mr(calc)** | **Delta** | **Miss** | **Score** | **Expect** | **Rank** | **Peptide** |
| --- | --- | --- | --- | --- | --- | --- | --- | --- | --- | --- |
|  | 1409 | 746.1623 | 745.1551 | 744.3766 | 0.7785 | 0 | 12 | 10 | 6 | R.EAEQLR.A |
|  | 4176 | 493.2762 | 984.5379 | 983.5876 | 0.9503 | 1 | 10 | 18 | 10 | R.ENLRIALR.Y 4164 |
|  | 4343 | 498.7556 | 995.4965 | 994.4356 | 1.0610 | 0 | 8 | 23 | 2 | R.FDSDATSPR.K 4329 |
|  | 6145 | 562.7893 | 1123.5639 | 1122.5305 | 1.0334 | 1 | 4 | 54 | 3 | R.FDSDATSPRK.E 6130 |
|  | 6565 | 577.7809 | 1153.5473 | 1153.6091 | -0.0618 | 1 | 3 | 67 | 9 | K.ETLQRADPPK.T |
|  | 10335 | 675.8494 | 1349.6843 | 1349.6939 | -0.0096 | 1 | 38 | 0.024 | 1 | R.YLENGKETLQR.A 10311 10351 |
|  | 11736 | 714.3626 | 1426.7105 | 1426.7205 | -0.0099 | 0 | 46 | 0.0031 | 1 | K.WAAVVVPSGEEQR.Y 11721 11735 11751 |
|  | 18486 | 463.4837 | 1849.9057 | 1849.9183 | -0.0127 | 0 | 16 | 2.9 | 1 | K.THVTHHPISDHEATLR.C 18485 |
|  | 20872 | 513.0196 | 2048.0493 | 2048.0626 | -0.0133 | 0 | 16 | 2.1 | 1 | R.YTCHVQHEGLPKPLTLR.W 20848 20850 20871 |
|  | 21557 | 1056.5175 | 2111.0204 | 2111.0299 | -0.0095 | 0 | 86 | 2.1e-007 | 1 | R.CWALGFYPAEITLTWQR.D 21558 |
|  | 22181 | 727.4567 | 2179.3484 | 2179.0559 | 0.2925 | 1 | 2 | 41 | 3 | R.IALRYYNQSEAGSHTWQR.M |
|  | 22689 | 744.6669 | 2230.9790 | 2230.9938 | -0.0149 | 0 | 44 | 0.0032 | 1 | R.DGEDQTQDTELVETRPAGDR.T 22688 |

|  | **Proteins matching a subset of these peptides:** |
| --- | --- |

|  | P30488\|1B50_HUMAN    **Mass:** 40801    **Score:** 156    **Queries matched:** 23 |
| --- | --- |
|  | HLA class I histocompatibility antigen, B-50 alpha chain OS=Homo sapiens GN=HLA-B PE=2 SV=1 |

| **278.** | P30461\|1B13_HUMAN    **Mass:** 40791    **Score:** 156    **Queries matched:** 25   **emPAI:** 0.55 |
| --- | --- |
|  | HLA class I histocompatibility antigen, B-13 alpha chain OS=Homo sapiens GN=HLA-B PE=2 SV=1 |

|  | **Query** | **Observed** | **Mr(expt)** | **Mr(calc)** | **Delta** | **Miss** | **Score** | **Expect** | **Rank** | **Peptide** |
| --- | --- | --- | --- | --- | --- | --- | --- | --- | --- | --- |
|  | 3992 | **486.7554** | **971.4963** | **971.5512** | **-0.0549** | **1** | **11** | **16** | **9** | **R.ENLRTALR.Y** |
|  | 4343 | 498.7556 | 995.4965 | 994.4356 | 1.0610 | 0 | 8 | 23 | 2 | R.FDSDATSPR.M 4329 |
|  | 6565 | 577.7809 | 1153.5473 | 1153.6091 | -0.0618 | 1 | 3 | 67 | 9 | K.ETLQRADPPK.T |
|  | 10335 | 675.8494 | 1349.6843 | 1349.6939 | -0.0096 | 1 | 38 | 0.024 | 1 | R.YLENGKETLQR.A 10311 10351 |
|  | 10618 | 455.7262 | 1364.1569 | 1363.6343 | 0.5226 | 0 | 3 | 50 | 7 | R.YFYTAMSRPGR.G |
|  | 11736 | 714.3626 | 1426.7105 | 1426.7205 | -0.0099 | 0 | 46 | 0.0031 | 1 | K.WAAVVVPSGEEQR.Y 11721 11735 11751 |
|  | 17808 | 902.9733 | 1803.9321 | 1802.8522 | 1.0799 | 1 | 3 | 60 | 8 | R.YFYTAMSRPGRGEPR.F |
|  | 18486 | 463.4837 | 1849.9057 | 1849.9183 | -0.0127 | 0 | 16 | 2.9 | 1 | K.THVTHHPISDHEATLR.C 18485 |
|  | 20872 | 513.0196 | 2048.0493 | 2048.0626 | -0.0133 | 0 | 16 | 2.1 | 1 | R.YTCHVQHEGLPKPLTLR.W 20848 20850 20871 |
|  | 21557 | 1056.5175 | 2111.0204 | 2111.0299 | -0.0095 | 0 | 86 | 2.1e-007 | 1 | R.CWALGFYPAEITLTWQR.D 21558 |
|  | 22689 | 744.6669 | 2230.9790 | 2230.9938 | -0.0149 | 0 | 44 | 0.0032 | 1 | R.DGEDQTQDTELVETRPAGDR.T 22688 |
|  | 30136 | 1896.3450 | 3790.6754 | 3790.1002 | 0.5752 | 1 | 2 | 20 | 5 | R.WEPSSQSTVPIVGIVAGLAVLAVVVIGAVVAAVMCRR.K |
|  | 30295 | **972.7078** | **3886.8022** | **3886.9651** | **-0.1629** | **1** | **5** | **11** | **1** | **R.TLLLLLWGAVALTETWAGSHSMRYFYTAMSRPGR.G** |

| **279.** | P30485\|1B47_HUMAN    **Mass:** 40888    **Score:** 156    **Queries matched:** 30   **emPAI:** 0.55 |
| --- | --- |
|  | HLA class I histocompatibility antigen, B-47 alpha chain OS=Homo sapiens GN=HLA-B PE=2 SV=1 |

|  | **Query** | **Observed** | **Mr(expt)** | **Mr(calc)** | **Delta** | **Miss** | **Score** | **Expect** | **Rank** | **Peptide** |
| --- | --- | --- | --- | --- | --- | --- | --- | --- | --- | --- |
|  | 4343 | 498.7556 | 995.4965 | 994.4356 | 1.0610 | 0 | 8 | 23 | 2 | R.FDSDATSPR.K 4329 |
|  | 6145 | 562.7893 | 1123.5639 | 1122.5305 | 1.0334 | 1 | 4 | 54 | 3 | R.FDSDATSPRK.E 6130 |
|  | 6565 | 577.7809 | 1153.5473 | 1153.6091 | -0.0618 | 1 | 3 | 67 | 9 | K.ETLQRADPPK.T |
|  | 10335 | 675.8494 | 1349.6843 | 1349.6939 | -0.0096 | 1 | 38 | 0.024 | 1 | R.YLENGKETLQR.A 10311 10351 |
|  | 10618 | 455.7262 | 1364.1569 | 1363.6343 | 0.5226 | 0 | 3 | 50 | 7 | R.YFYTAMSRPGR.G |
|  | 11736 | 714.3626 | 1426.7105 | 1426.7205 | -0.0099 | 0 | 46 | 0.0031 | 1 | K.WAAVVVPSGEEQR.Y 11721 11735 11751 |
|  | 17808 | 902.9733 | 1803.9321 | 1802.8522 | 1.0799 | 1 | 3 | 60 | 8 | R.YFYTAMSRPGRGEPR.F |
|  | 18486 | 463.4837 | 1849.9057 | 1849.9183 | -0.0127 | 0 | 16 | 2.9 | 1 | K.THVTHHPISDHEATLR.C 18485 |
|  | 20872 | 513.0196 | 2048.0493 | 2048.0626 | -0.0133 | 0 | 16 | 2.1 | 1 | R.YTCHVQHEGLPKPLTLR.W 20848 20850 20871 |
|  | 21557 | 1056.5175 | 2111.0204 | 2111.0299 | -0.0095 | 0 | 86 | 2.1e-007 | 1 | R.CWALGFYPAEITLTWQR.D 21558 |
|  | 22689 | 744.6669 | 2230.9790 | 2230.9938 | -0.0149 | 0 | 44 | 0.0032 | 1 | R.DGEDQTQDTELVETRPAGDR.T 22688 |
|  | 30034 | **749.5159** | **3742.5430** | **3742.1332** | **0.4098** | **1** | **5** | **8.5** | **1** | **R.WEPSSQSTVPIVGIVAGLAVLAVVVIGAVVAAVVCRR.K** 30029 30030 30031 30035 |
|  | 30295 | 972.7078 | 3886.8022 | 3886.9651 | -0.1629 | 1 | 5 | 11 | 1 | R.TLLLLLWGAVALTETWAGSHSMRYFYTAMSRPGR.G |

| **280.** | P30481\|1B44_HUMAN    **Mass:** 40798    **Score:** 156    **Queries matched:** 26   **emPAI:** 0.55 |
| --- | --- |
|  | HLA class I histocompatibility antigen, B-44 alpha chain OS=Homo sapiens GN=HLA-B PE=1 SV=1 |

|  | **Query** | **Observed** | **Mr(expt)** | **Mr(calc)** | **Delta** | **Miss** | **Score** | **Expect** | **Rank** | **Peptide** |
| --- | --- | --- | --- | --- | --- | --- | --- | --- | --- | --- |
|  | 3992 | 486.7554 | 971.4963 | 971.5512 | -0.0549 | 1 | 11 | 16 | 9 | R.ENLRTALR.Y |
|  | 4343 | 498.7556 | 995.4965 | 994.4356 | 1.0610 | 0 | 8 | 23 | 2 | R.FDSDATSPR.K 4329 |
|  | 6145 | 562.7893 | 1123.5639 | 1122.5305 | 1.0334 | 1 | 4 | 54 | 3 | R.FDSDATSPRK.E 6130 |
|  | 6565 | 577.7809 | 1153.5473 | 1153.6091 | -0.0618 | 1 | 3 | 67 | 9 | K.ETLQRADPPK.T |
|  | 10335 | 675.8494 | 1349.6843 | 1349.6939 | -0.0096 | 1 | 38 | 0.024 | 1 | R.YLENGKETLQR.A 10311 10351 |
|  | 10618 | 455.7262 | 1364.1569 | 1363.6343 | 0.5226 | 0 | 3 | 50 | 7 | R.YFYTAMSRPGR.G |
|  | 11736 | 714.3626 | 1426.7105 | 1426.7205 | -0.0099 | 0 | 46 | 0.0031 | 1 | K.WAAVVVPSGEEQR.Y 11721 11735 11751 |
|  | 17808 | 902.9733 | 1803.9321 | 1802.8522 | 1.0799 | 1 | 3 | 60 | 8 | R.YFYTAMSRPGRGEPR.F |
|  | 20872 | 513.0196 | 2048.0493 | 2048.0626 | -0.0133 | 0 | 16 | 2.1 | 1 | R.YTCHVQHEGLPKPLTLR.W 20848 20850 20871 |
|  | 21506 | 1054.4789 | 2106.9432 | 2107.0368 | -0.0936 | 1 | 1 | 70 | 2 | R.VAEQDRAYLEGLCVESLR.R |
|  | 21557 | 1056.5175 | 2111.0204 | 2111.0299 | -0.0095 | 0 | 86 | 2.1e-007 | 1 | R.CWALGFYPAEITLTWQR.D 21558 |
|  | 22689 | 744.6669 | 2230.9790 | 2230.9938 | -0.0149 | 0 | 44 | 0.0032 | 1 | R.DGEDQTQDTELVETRPAGDR.T 22688 |
|  | 30136 | 1896.3450 | 3790.6754 | 3790.1002 | 0.5752 | 1 | 2 | 20 | 5 | R.WEPSSQSTVPIVGIVAGLAVLAVVVIGAVVAAVMCRR.K |
|  | 30295 | 972.7078 | 3886.8022 | 3886.9651 | -0.1629 | 1 | 5 | 11 | 1 | R.TLLLLLWGAVALTETWAGSHSMRYFYTAMSRPGR.G |

| **281.** | P30483\|1B45_HUMAN    **Mass:** 40674    **Score:** 156    **Queries matched:** 23   **emPAI:** 0.56 |
| --- | --- |
|  | HLA class I histocompatibility antigen, B-45 alpha chain OS=Homo sapiens GN=HLA-B PE=2 SV=1 |

|  | **Query** | **Observed** | **Mr(expt)** | **Mr(calc)** | **Delta** | **Miss** | **Score** | **Expect** | **Rank** | **Peptide** |
| --- | --- | --- | --- | --- | --- | --- | --- | --- | --- | --- |
|  | 4343 | 498.7556 | 995.4965 | 994.4356 | 1.0610 | 0 | 8 | 23 | 2 | R.FDSDATSPR.K 4329 |
|  | 6145 | 562.7893 | 1123.5639 | 1122.5305 | 1.0334 | 1 | 4 | 54 | 3 | R.FDSDATSPRK.E 6130 |
|  | 6565 | 577.7809 | 1153.5473 | 1153.6091 | -0.0618 | 1 | 3 | 67 | 9 | K.ETLQRADPPK.T |
|  | 10335 | 675.8494 | 1349.6843 | 1349.6939 | -0.0096 | 1 | 38 | 0.024 | 1 | R.YLENGKETLQR.A 10311 10351 |
|  | 11736 | 714.3626 | 1426.7105 | 1426.7205 | -0.0099 | 0 | 46 | 0.0031 | 1 | K.WAAVVVPSGEEQR.Y 11721 11735 11751 |
|  | 18486 | 463.4837 | 1849.9057 | 1849.9183 | -0.0127 | 0 | 16 | 2.9 | 1 | K.THVTHHPISDHEATLR.C 18485 |
|  | 20872 | 513.0196 | 2048.0493 | 2048.0626 | -0.0133 | 0 | 16 | 2.1 | 1 | R.YTCHVQHEGLPKPLTLR.W 20848 20850 20871 |
|  | 21506 | 1054.4789 | 2106.9432 | 2107.0368 | -0.0936 | 1 | 1 | 70 | 2 | R.VAEQDRAYLEGLCVESLR.R |
|  | 21557 | 1056.5175 | 2111.0204 | 2111.0299 | -0.0095 | 0 | 86 | 2.1e-007 | 1 | R.CWALGFYPAEITLTWQR.D 21558 |
|  | 22689 | 744.6669 | 2230.9790 | 2230.9938 | -0.0149 | 0 | 44 | 0.0032 | 1 | R.DGEDQTQDTELVETRPAGDR.T 22688 |

| **282.** | P54920\|SNAA_HUMAN    **Mass:** 33667    **Score:** 156    **Queries matched:** 12   **emPAI:** 0.53 |
| --- | --- |
|  | Alpha-soluble NSF attachment protein OS=Homo sapiens GN=NAPA PE=1 SV=3 |

|  | **Query** | **Observed** | **Mr(expt)** | **Mr(calc)** | **Delta** | **Miss** | **Score** | **Expect** | **Rank** | **Peptide** |
| --- | --- | --- | --- | --- | --- | --- | --- | --- | --- | --- |
|  | 8439 | **627.2905** | **1252.5664** | **1252.5757** | **-0.0094** | **0** | **51** | **0.0011** | **1** | **K.IEEACEIYAR.A** |
|  | 11552 | **709.3277** | **1416.6408** | **1416.6489** | **-0.0081** | **0** | **11** | **12** | **1** | **K.ADPQEAINCLMR.A** 11551 |
|  | 12305 | **730.8338** | **1459.6530** | **1459.6619** | **-0.0089** | **0** | **19** | **1.6** | **1** | **K.YEELFPAFSDSR.E** |
|  | 13805 | 775.3636 | 1548.7126 | 1548.7209 | -0.0083 | 0 | 3 | 58 | 1 | K.NSQSFFSGLFGGSSK.I |
|  | 14023 | **521.3036** | **1560.8891** | **1560.7388** | **0.1503** | **1** | **0** | **1.2e+002** | **8** | **K.KADPQEAINCLMR.A** |
|  | 15001 | 812.3603 | 1622.7060 | 1622.7148 | -0.0087 | 0 | 61 | 9.8e-005 | 1 | K.HDAATCFVDAGNAFK.K 15000 |
|  | 21790 | **535.5266** | **2138.0773** | **2138.0895** | **-0.0122** | **0** | **51** | **0.00073** | **1** | **K.HHISIAEIYETELVDIEK.A** |
|  | 22874 | 753.0261 | 2256.0564 | 2255.1256 | 0.9308 | 1 | 2 | 47 | 7 | K.EYDSISRLDQWLTTMLLR.I |
|  | 25175 | **854.7119** | **2561.1139** | **2561.1306** | **-0.0167** | **1** | **44** | **0.0026** | **1** | **K.AIAHYEQSADYYKGEESNSSANK.C** |
|  | 26494 | **930.4628** | **2788.3665** | **2788.3537** | **0.0128** | **1** | **6** | **16** | **3** | **K.MAKNWSAAGNAFCQAAQLHLQLQSK.H** |

| **283.** | P01903\|2DRA_HUMAN    **Mass:** 28760    **Score:** 156    **Queries matched:** 10   **emPAI:** 0.86 |
| --- | --- |
|  | HLA class II histocompatibility antigen, DR alpha chain OS=Homo sapiens GN=HLA-DRA PE=1 SV=1 |

|  | **Query** | **Observed** | **Mr(expt)** | **Mr(calc)** | **Delta** | **Miss** | **Score** | **Expect** | **Rank** | **Peptide** |
| --- | --- | --- | --- | --- | --- | --- | --- | --- | --- | --- |
|  | 11882 | **479.2542** | **1434.7408** | **1434.7507** | **-0.0099** | **0** | **38** | **0.021** | **1** | **R.VEHWGLDEPLLK.H** 11868 11869 |
|  | 17776 | **601.6565** | **1801.9476** | **1800.9734** | **0.9742** | **0** | **12** | **6.4** | **1** | **R.NGKPVTTGVSETVFLPR.E** 17763 |
|  | 21976 | **720.3306** | **2157.9701** | **2157.9830** | **-0.0129** | **0** | **44** | **0.0039** | **1** | **K.FHYLPFLPSTEDVYDCR.V** 21972 |
|  | 23117 | **763.0289** | **2286.0648** | **2286.0779** | **-0.0132** | **1** | **42** | **0.0052** | **1** | **R.KFHYLPFLPSTEDVYDCR.V** |
|  | 25654 | **1326.6815** | **2651.3485** | **2651.3628** | **-0.0144** | **1** | **76** | **1.5e-006** | **1** | **R.FASFEAQGALANIAVDKANLEIMTK.R** 25655 |

| **284.** | O43169\|CYB5B_HUMAN    **Mass:** 16436    **Score:** 155    **Queries matched:** 6   **emPAI:** 1.34 |
| --- | --- |
|  | Cytochrome b5 type B OS=Homo sapiens GN=CYB5B PE=1 SV=2 |

|  | **Query** | **Observed** | **Mr(expt)** | **Mr(calc)** | **Delta** | **Miss** | **Score** | **Expect** | **Rank** | **Peptide** |
| --- | --- | --- | --- | --- | --- | --- | --- | --- | --- | --- |
|  | 6322 | **570.2752** | **1138.5359** | **1139.4765** | **-0.9406** | **0** | **35** | **0.041** | **2** | **-.MATAEASGSDGK.G** 6320 |
|  | 14072 | **522.3002** | **1563.8789** | **1563.8885** | **-0.0097** | **1** | **57** | **0.00026** | **1** | **R.NSLKELWLVIHGR.V** |
|  | 29794 | **1209.8811** | **3626.6215** | **3626.6397** | **-0.0183** | **0** | **85** | **1.1e-007** | **1** | **R.FLNEHPGGEEVLLEQAGVDASESFEDVGHSSDAR.E** 29793 29795 |

| **285.** | Q15836\|VAMP3_HUMAN    **Mass:** 11359    **Score:** 153    **Queries matched:** 7   **emPAI:** 3.51 |
| --- | --- |
|  | Vesicle-associated membrane protein 3 OS=Homo sapiens GN=VAMP3 PE=1 SV=3 |

|  | **Query** | **Observed** | **Mr(expt)** | **Mr(calc)** | **Delta** | **Miss** | **Score** | **Expect** | **Rank** | **Peptide** |
| --- | --- | --- | --- | --- | --- | --- | --- | --- | --- | --- |
|  | 5389 | 536.3080 | 1070.6015 | 1070.6084 | -0.0069 | 1 | 27 | 0.38 | 1 | R.VNVDKVLER.D |
|  | 11657 | 474.9176 | 1421.7310 | 1421.6681 | 0.0629 | 1 | 3 | 67 | 10 | -.MSTGPTAATGSNRR.L |
|  | 15676 | 833.4023 | 1664.7900 | 1664.8006 | -0.0105 | 0 | 43 | 0.0063 | 1 | R.ADALQAGASQFETSAAK.L |
|  | 19292 | **958.4738** | **1914.9331** | **1914.9469** | **-0.0138** | **0** | **52** | **0.00068** | **1** | **R.LQQTQNQVDEVVDIMR.V** |
|  | 21157 | **691.6865** | **2072.0376** | **2071.0480** | **0.9895** | **1** | **26** | **0.2** | **1** | **R.RLQQTQNQVDEVVDIMR.V** |
|  | 24635 | 1247.5978 | 2493.1810 | 2493.1983 | -0.0173 | 1 | 63 | 3.9e-005 | 1 | K.LSELDDRADALQAGASQFETSAAK.L 24636 |

| **286.** | P05387\|RLA2_HUMAN    **Mass:** 11658    **Score:** 153    **Queries matched:** 10   **emPAI:** 3.34 |
| --- | --- |
|  | 60S acidic ribosomal protein P2 OS=Homo sapiens GN=RPLP2 PE=1 SV=1 |

|  | **Query** | **Observed** | **Mr(expt)** | **Mr(calc)** | **Delta** | **Miss** | **Score** | **Expect** | **Rank** | **Peptide** |
| --- | --- | --- | --- | --- | --- | --- | --- | --- | --- | --- |
|  | 8518 | **628.8424** | **1255.6701** | **1255.6772** | **-0.0071** | **0** | **47** | **0.0032** | **1** | **K.NIEDVIAQGIGK.L** |
|  | 17257 | **886.9493** | **1771.8840** | **1771.8952** | **-0.0112** | **1** | **52** | **0.00067** | **1** | **K.ILDSVGIEADDDRLNK.V** 17258 |
|  | 18719 | **935.4784** | **1868.9423** | **1867.9679** | **0.9744** | **0** | **27** | **0.21** | **1** | **R.YVASYLLAALGGNSSPSAK.D** 18715 |
|  | 21416 | **700.0474** | **2097.1204** | **2096.1477** | **0.9727** | **1** | **6** | **21** | **1** | **K.VISELNGKNIEDVIAQGIGK.L** |
|  | 26415 | **1387.7106** | **2773.4066** | **2773.4246** | **-0.0180** | **0** | **62** | **3.4e-005** | **1** | **K.LASVPAGGAVAVSAAPGSAAPAAGSAPAAAEEK.K** 26416 26419 26427 |

| **287.** | O96009\|NAPSA_HUMAN    **Mass:** 45700    **Score:** 152    **Queries matched:** 10   **emPAI:** 0.27 |
| --- | --- |
|  | Napsin-A OS=Homo sapiens GN=NAPSA PE=1 SV=1 |

|  | **Query** | **Observed** | **Mr(expt)** | **Mr(calc)** | **Delta** | **Miss** | **Score** | **Expect** | **Rank** | **Peptide** |
| --- | --- | --- | --- | --- | --- | --- | --- | --- | --- | --- |
|  | 3207 | 458.3048 | 914.5951 | 915.5250 | -0.9299 | 1 | 6 | 47 | 9 | K.SSARVGLAR.A |
|  | 4057 | **488.3037** | **974.5929** | **974.4920** | **0.1008** | **0** | **7** | **40** | **9** | **R.VDGILSEDK.L** |
|  | 4615 | **508.2348** | **1014.4551** | **1014.5532** | **-0.0981** | **0** | **4** | **66** | **8** | **K.VGPGLTLCAK.G** |
|  | 6954 | 591.8182 | 1181.6219 | 1181.6193 | 0.0027 | 1 | 3 | 68 | 10 | R.GWREPAELPK.L 6972 |
|  | 15537 | **553.3132** | **1656.9179** | **1656.9298** | **-0.0119** | **1** | **45** | **0.0037** | **1** | **R.VDGILSEDKLTIGGIK.G** 15538 |
|  | 22127 | **1088.0447** | **2174.0748** | **2174.0889** | **-0.0141** | **0** | **77** | **1.6e-006** | **1** | **K.GCAAILDTGTSLITGPTEEIR.A** 22128 |
|  | 27714 | 605.3041 | 3021.4843 | 3020.7711 | 0.7132 | 0 | 4 | 20 | 2 | -.MSPPPLLQPLLLLLPLLNVEPSGATLIR.I |

| **288.** | O75915\|PRAF3_HUMAN    **Mass:** 21600    **Score:** 152    **Queries matched:** 9   **emPAI:** 0.63 |
| --- | --- |
|  | PRA1 family protein 3 OS=Homo sapiens GN=ARL6IP5 PE=1 SV=1 |

|  | **Query** | **Observed** | **Mr(expt)** | **Mr(calc)** | **Delta** | **Miss** | **Score** | **Expect** | **Rank** | **Peptide** |
| --- | --- | --- | --- | --- | --- | --- | --- | --- | --- | --- |
|  | 2945 | **449.1122** | **896.2099** | **896.5080** | **-0.2980** | **0** | **0** | **1e+002** | **5** | **M.DVNIAPLR.A** |
|  | 3043 | **452.7564** | **903.4982** | **902.5007** | **0.9975** | **1** | **15** | **6** | **1** | **K.MEGIGLKR.T** |
|  | 4852 | **517.2557** | **1032.4968** | **1031.5148** | **0.9820** | **1** | **5** | **54** | **8** | **R.DISKWNNR.V** |
|  | 8011 | **616.7811** | **1231.5476** | **1230.6642** | **0.8834** | **1** | **6** | **40** | **1** | **K.LENKMEGIGLK.R** |
|  | 9568 | **656.7794** | **1311.5443** | **1311.5520** | **-0.0077** | **0** | **47** | **0.0026** | **1** | **R.AWDDFFPGSDR.F** |
|  | 21570 | 1057.0282 | 2112.0418 | 2112.0521 | -0.0102 | 0 | 90 | 9.7e-008 | 1 | R.TPMGIVLDALEQQEEGINR.L |
|  | 22250 | **548.5344** | **2190.1086** | **2190.0494** | **0.0592** | **1** | **3** | **46** | **4** | **M.DVNIAPLRAWDDFFPGSDR.F** |
|  | 22966 | **756.7187** | **2267.1342** | **2268.1532** | **-1.0190** | **1** | **47** | **0.0016** | **1** | **K.RTPMGIVLDALEQQEEGINR.L** 22971 |

| **289.** | P69891\|HBG1_HUMAN    **Mass:** 16187    **Score:** 152    **Queries matched:** 28   **emPAI:** 0.24 |
| --- | --- |
|  | Hemoglobin subunit gamma-1 OS=Homo sapiens GN=HBG1 PE=1 SV=2 |

|  | **Query** | **Observed** | **Mr(expt)** | **Mr(calc)** | **Delta** | **Miss** | **Score** | **Expect** | **Rank** | **Peptide** |
| --- | --- | --- | --- | --- | --- | --- | --- | --- | --- | --- |
|  | 4629 | 508.7693 | 1015.5241 | 1015.5914 | -0.0673 | 0 | 3 | 86 | 10 | K.VLTSLGDAIK.H |
|  | 6400 | 572.9938 | 1143.9731 | 1143.6863 | 0.2868 | 1 | 8 | 16 | 3 | K.KVLTSLGDAIK.H 6405 |
|  | 7556 | **403.7084** | **1208.1035** | **1207.6231** | **0.4804** | **0** | **8** | **16** | **9** | **K.MVTAVASALSSR.Y** |
|  | 8890 | 637.8627 | 1273.7108 | 1273.7183 | -0.0075 | 0 | 56 | 0.00034 | 1 | R.LLVVYPWTQR.F 8883 8885 8886 8888 8891 8892 8893 8894 8896 8897 8898 8899 8900 8901 8903 8904 8912 |
|  | 9686 | **439.3737** | **1315.0994** | **1315.6368** | **-0.5374** | **0** | **4** | **43** | **10** | **K.VNVEDAGGETLGR.L** 9681 |
|  | 13105 | **503.2262** | **1506.6567** | **1507.7453** | **-1.0886** | **1** | **2** | **84** | **2** | **K.MVTAVASALSSRYH.-** |
|  | 20205 | **995.4670** | **1988.9194** | **1988.9302** | **-0.0108** | **0** | **3** | **45** | **1** | **R.FFDSFGNLSSASAIMGNPK.V** |
|  | 22655 | **743.4082** | **2227.2028** | **2226.0739** | **1.1289** | **1** | **3** | **38** | **1** | **K.HLDDLKGTFAQLSELHCDK.L** |
|  | 28696 | 812.1727 | 3244.6618 | 3244.6379 | 0.0239 | 1 | 1 | 32 | 5 | R.LLVVYPWTQRFFDSFGNLSSASAIMGNPK.V |

|  | **Proteins matching a subset of these peptides:** |
| --- | --- |

|  | P69892\|HBG2_HUMAN    **Mass:** 16173    **Score:** 152    **Queries matched:** 26 |
| --- | --- |
|  | Hemoglobin subunit gamma-2 OS=Homo sapiens GN=HBG2 PE=1 SV=2 |

| **290.** | P02100\|HBE_HUMAN    **Mass:** 16249    **Score:** 152    **Queries matched:** 20   **emPAI:** 0.24 |
| --- | --- |
|  | Hemoglobin subunit epsilon OS=Homo sapiens GN=HBE1 PE=1 SV=2 |

|  | **Query** | **Observed** | **Mr(expt)** | **Mr(calc)** | **Delta** | **Miss** | **Score** | **Expect** | **Rank** | **Peptide** |
| --- | --- | --- | --- | --- | --- | --- | --- | --- | --- | --- |
|  | 5919 | **554.8225** | **1107.6305** | **1106.5066** | **1.1238** | **0** | **6** | **35** | **4** | **-.MVHFTAEEK.A** |
|  | 8890 | 637.8627 | 1273.7108 | 1273.7183 | -0.0075 | 0 | 56 | 0.00034 | 1 | R.LLVVYPWTQR.F 8883 8885 8886 8888 8891 8892 8893 8894 8896 8897 8898 8899 8900 8901 8903 8904 8912 |
|  | 20898 | **684.3415** | **2050.0028** | **2050.0193** | **-0.0165** | **1** | **3** | **49** | **8** | **-.MVHFTAEEKAAVTSLWSK.M** |

| **291.** | P30041\|PRDX6_HUMAN    **Mass:** 25133    **Score:** 152    **Queries matched:** 7   **emPAI:** 0.15 |
| --- | --- |
|  | Peroxiredoxin-6 OS=Homo sapiens GN=PRDX6 PE=1 SV=3 |

|  | **Query** | **Observed** | **Mr(expt)** | **Mr(calc)** | **Delta** | **Miss** | **Score** | **Expect** | **Rank** | **Peptide** |
| --- | --- | --- | --- | --- | --- | --- | --- | --- | --- | --- |
|  | 6080 | **560.7415** | **1119.4685** | **1118.5390** | **0.9295** | **1** | **8** | **27** | **5** | **K.DEKGMPVTAR.V** 6073 |
|  | 10466 | **452.7407** | **1355.2004** | **1354.7357** | **0.4647** | **1** | **7** | **19** | **2** | **K.LPFPIIDDRNR.E** |
|  | 16515 | 573.9402 | 1718.7987 | 1718.9178 | -0.1191 | 1 | 3 | 53 | 5 | K.GMPVTARVVFVFGPDK.K |
|  | 19069 | **633.0036** | **1895.9890** | **1895.9993** | **-0.0103** | **0** | **15** | **3.3** | **1** | **K.LIALSIDSVEDHLAWSK.D** |
|  | 21413 | 1049.5432 | 2097.0719 | 2097.0855 | -0.0136 | 0 | 93 | 4.5e-008 | 1 | M.PGGLLLGDVAPNFEANTTVGR.I 21412 |

| **292.** | P08238\|HS90B_HUMAN    **Mass:** 83554    **Score:** 151    **Queries matched:** 30   **emPAI:** 0.19 |
| --- | --- |
|  | Heat shock protein HSP 90-beta OS=Homo sapiens GN=HSP90AB1 PE=1 SV=4 |

|  | **Query** | **Observed** | **Mr(expt)** | **Mr(calc)** | **Delta** | **Miss** | **Score** | **Expect** | **Rank** | **Peptide** |
| --- | --- | --- | --- | --- | --- | --- | --- | --- | --- | --- |
|  | 1100 | 617.3092 | 616.3019 | 615.3704 | 0.9315 | 1 | 22 | 1.9 | 1 | K.KNNIK.L |
|  | 2507 | **863.4213** | **862.4140** | **862.4218** | **-0.0078** | **0** | **20** | **1.8** | **1** | **R.EMLQQSK.I** |
|  | 4184 | **986.5083** | **985.5010** | **984.6232** | **0.8778** | **1** | **15** | **5.8** | **4** | **R.ALLFIPRR.A** |
|  | 4529 | **504.7289** | **1007.4431** | **1007.6491** | **-0.2060** | **1** | **8** | **28** | **6** | **R.GTKVILHLK.E** |
|  | 6668 | 581.3089 | 1160.6032 | 1159.5761 | 1.0272 | 0 | 1 | 1.4e+002 | 10 | K.SIYYITGESK.E |
|  | 8036 | **411.8792** | **1232.6159** | **1232.6798** | **-0.0639** | **1** | **3** | **71** | **8** | **R.EMLQQSKILK.V** 8066 |
|  | 8102 | **618.9977** | **1235.9809** | **1235.6299** | **0.3511** | **1** | **2** | **53** | **1** | **R.RAPFDLFENK.K** |
|  | 8140 | **619.9091** | **1237.8037** | **1236.6211** | **1.1826** | **1** | **0** | **1.1e+002** | **1** | **R.ADHGEPIGRGTK.V** |
|  | 8244 | **622.3537** | **1242.6928** | **1241.6979** | **0.9949** | **0** | **41** | **0.012** | **1** | **K.ADLINNLGTIAK.S** 8227 |
|  | 8320 | 624.2341 | 1246.4537 | 1247.4984 | -1.0447 | 0 | 4 | 56 | 8 | R.DNSTMGYMMAK.K |
|  | 8335 | 624.3159 | 1246.6172 | 1245.6393 | 0.9778 | 1 | 2 | 96 | 9 | K.FYEAFSKNLK.L |
|  | 9299 | **432.8983** | **1295.6730** | **1295.4832** | **0.1898** | **0** | **(3)** | **66** | **8** | **R.DNSTMGYMMAK.K** |
|  | 10305 | **450.2784** | **1347.8135** | **1347.6572** | **0.1563** | **0** | **4** | **65** | **8** | **K.HFSVEGQLEFR.A** |
|  | 10869 | 459.9262 | 1376.7569 | 1375.5934 | 1.1635 | 1 | 5 | 44 | 10 | R.DNSTMGYMMAKK.H |
|  | 12818 | **745.8749** | **1489.7353** | **1489.6420** | **0.0934** | **1** | **8** | **21** | **3** | **K.EEEDKDDEEKPK.I** |
|  | 13217 | **757.3913** | **1512.7680** | **1512.7784** | **-0.0103** | **0** | **78** | **2.2e-006** | **1** | **R.GVVDSEDLPLNISR.E** |
|  | 13450 | **509.9157** | **1526.7252** | **1526.7365** | **-0.0113** | **0** | **20** | **1.1** | **1** | **K.SLTNDWEDHLAVK.H** |
|  | 14317 | 791.4034 | 1580.7923 | 1580.7756 | 0.0168 | 1 | 3 | 61 | 7 | K.KCLELFSELAEDK.E |
|  | 15321 | 823.4100 | 1644.8055 | 1645.8199 | -1.0144 | 1 | 3 | 58 | 6 | K.ETQKSIYYITGESK.E 15320 |
|  | 17813 | **902.9799** | **1803.9452** | **1802.8113** | **1.1338** | **1** | **3** | **58** | **2** | **K.AQALRDNSTMGYMMAK.K** |
|  | 18430 | **924.3949** | **1846.7752** | **1846.7897** | **-0.0145** | **0** | **93** | **5.3e-008** | **1** | **R.NPDDITQEEYGEFYK.S** |
|  | 19520 | 967.0357 | 1932.0569 | 1933.0156 | -0.9587 | 1 | 2 | 69 | 2 | K.EIFLRELISNASDALDK.I |
|  | 20489 | **672.4362** | **2014.2867** | **2014.0371** | **0.2496** | **1** | **19** | **0.81** | **1** | **K.VILHLKEDQTEYLEER.R** 20481 |
|  | 24200 | **610.0450** | **2436.1508** | **2435.0733** | **1.0775** | **1** | **4** | **27** | **5** | **R.YHTSQSGDEMTSLSEYVSRMK.E** |
|  | 28043 | **776.3547** | **3101.3899** | **3101.4733** | **-0.0834** | **1** | **1** | **43** | **1** | **K.VTISNRLVSSPCCIVTSTYGWTANMER.I** |
|  | 29877 | **1225.1727** | **3672.4964** | **3672.7844** | **-0.2880** | **1** | **1** | **22** | **6** | **K.SGTKAFMEALQAGADISMIGQFGVGFYSAYLVAEK.V** |

| **293.** | O75923\|DYSF_HUMAN    **Mass:** 239254   **Score:** 151    **Queries matched:** 42   **emPAI:** 0.05 |
| --- | --- |
|  | Dysferlin OS=Homo sapiens GN=DYSF PE=1 SV=1 |

|  | **Query** | **Observed** | **Mr(expt)** | **Mr(calc)** | **Delta** | **Miss** | **Score** | **Expect** | **Rank** | **Peptide** |
| --- | --- | --- | --- | --- | --- | --- | --- | --- | --- | --- |
|  | 1408 | **745.4407** | **744.4335** | **745.3680** | **-0.9345** | **0** | **12** | **15** | **4** | **R.MEPLEK.T** |
|  | 2501 | 863.0857 | 862.0784 | 861.5324 | 0.5460 | 0 | 5 | 42 | 2 | K.LQTIFLK.Y |
|  | 2547 | **433.7268** | **865.4390** | **865.4446** | **-0.0056** | **0** | **31** | **0.14** | **1** | **K.HWVPAEK.M** |
|  | 3354 | **463.7399** | **925.4653** | **924.4236** | **1.0417** | **1** | **8** | **21** | **5** | **R.RGANYCGK.N** |
|  | 3414 | **466.2109** | **930.4073** | **930.5247** | **-0.1173** | **1** | **15** | **6.4** | **2** | **K.VTAAGQTKR.T** |
|  | 3912 | 483.3000 | 964.5854 | 963.4886 | 1.0968 | 1 | 4 | 61 | 10 | K.GEGVAYRGR.L |
|  | 5987 | 557.3205 | 1112.6264 | 1112.5172 | 0.1093 | 1 | 6 | 39 | 9 | R.EKCGSYLEK.D |
|  | 7157 | 596.2887 | 1190.5628 | 1189.6125 | 0.9504 | 1 | 3 | 70 | 9 | R.RDLSQMEALK.R |
|  | 7492 | **603.3777** | **1204.7408** | **1205.6074** | **-0.8666** | **1** | **(2)** | **88** | **3** | **R.RDLSQMEALK.R** |
|  | 8188 | **621.3358** | **1240.6571** | **1241.7132** | **-1.0561** | **0** | **4** | **61** | **5** | **R.TAIEILAWGLR.N** |
|  | 10228 | **672.8213** | **1343.6280** | **1342.7020** | **0.9260** | **0** | **0** | **1.3e+002** | **3** | **K.ITLYDYDLLSK.D** |
|  | 11600 | 710.4257 | 1418.8368 | 1419.8085 | -0.9718 | 0 | 2 | 75 | 9 | R.LEAGLEQVHLALK.A |
|  | 13143 | 755.3799 | 1508.7452 | 1509.8265 | -1.0813 | 1 | 15 | 4.2 | 2 | K.LQTIFLKYPMEK.V |
|  | 13516 | 765.8803 | 1529.7459 | 1529.7249 | 0.0210 | 0 | 6 | 29 | 6 | K.LSVFAETYENETK.L |
|  | 13927 | **778.4302** | **1554.8459** | **1553.7144** | **1.1316** | **1** | **(1)** | **88** | **4** | **R.DEPNMNPKLEDPR.R** |
|  | 14005 | 781.3304 | 1560.6462 | 1561.7163 | -1.0701 | 1 | 5 | 39 | 2 | R.CICQPSLERMPR.L |
|  | 14171 | 524.6159 | 1570.8259 | 1569.7093 | 1.1166 | 1 | 3 | 60 | 5 | R.DEPNMNPKLEDPR.R |
|  | 14410 | 793.9615 | 1585.9085 | 1585.9093 | -0.0007 | 1 | 21 | 0.96 | 2 | R.KLPSRPPPHYPGIK.R |
|  | 14435 | **530.2576** | **1587.7509** | **1586.8780** | **0.8729** | **1** | **3** | **60** | **1** | **R.KLLSDKPQDFQIR.V** |
|  | 14866 | 539.2805 | 1614.8196 | 1615.8053 | -0.9858 | 1 | 3 | 64 | 9 | K.DEKIGETVVDLENR.L |
|  | 15401 | **550.6251** | **1648.8535** | **1647.9321** | **0.9214** | **1** | **2** | **77** | **3** | **K.ALGRPGPPFNITPRR.A** |
|  | 15607 | **831.4273** | **1660.8401** | **1661.8049** | **-0.9649** | **0** | **3** | **53** | **1** | **K.WLLLSDPDDFSAGAR.G** |
|  | 16182 | **849.9092** | **1697.8039** | **1698.9080** | **-1.1040** | **1** | **0** | **1e+002** | **10** | **K.DLKITLYDYDLLSK.D** |
|  | 16421 | **857.4216** | **1712.8286** | **1712.8403** | **-0.0117** | **0** | **14** | **4.4** | **1** | **K.TSLCVLGPGDEAPLER.K** |
|  | 17315 | 444.9609 | 1775.8147 | 1774.8349 | 0.9798 | 1 | 5 | 34 | 6 | K.TVKGWWPCVAEEGEK.K |
|  | 17554 | 895.8943 | 1789.7740 | 1789.7862 | -0.0122 | 0 | 15 | 3.7 | 1 | R.AEDLPQMDDAVMDNVK.Q 17598 |
|  | 17829 | **602.8215** | **1805.4426** | **1805.7812** | **-0.3386** | **0** | **(6)** | **19** | **1** | **R.AEDLPQMDDAVMDNVK.Q** |
|  | 18189 | 916.4759 | 1830.9372 | 1830.9476 | -0.0103 | 0 | 70 | 9.6e-006 | 1 | R.GSQPSGELLASFELIQR.E 18181 18188 |
|  | 20143 | **496.7788** | **1983.0860** | **1984.0564** | **-0.9704** | **0** | **5** | **27** | **8** | **R.EANIYMVPQNIKPALQR.T** |
|  | 21982 | **1080.0405** | **2158.0665** | **2158.0809** | **-0.0144** | **0** | **49** | **0.001** | **1** | **R.FIFPFDYLPAEQVCTIAK.K** |
|  | 22050 | **722.7070** | **2165.0993** | **2166.1547** | **-1.0554** | **1** | **1** | **65** | **6** | **K.GLFKIYPLPEDPAIPMPPR.Q** |
|  | 22076 | **1085.0054** | **2167.9962** | **2168.0096** | **-0.0134** | **0** | **3** | **45** | **1** | **K.SVSDQDNYIPCTLEPVFGK.M** |
|  | 22623 | **742.5345** | **2224.5818** | **2224.0843** | **0.4975** | **1** | **1** | **48** | **10** | **R.KNPNFDICTLFMEVMLPR.E** |
|  | 23601 | 1176.0612 | 2350.1078 | 2350.0767 | 0.0311 | 1 | 2 | 46 | 2 | R.HRQAEAEGEGWEYASLFGWK.F 23602 |
|  | 28612 | 1075.3120 | 3222.9142 | 3223.7539 | -0.8397 | 1 | 0 | 32 | 10 | R.EANIYMVPQNIKPALQRTAIEILAWGLR.N |
|  | 29974 | **930.4993** | **3717.9682** | **3717.9842** | **-0.0160** | **0** | **14** | **1.2** | **1** | **R.LALHVLQQQGLVPEHVESRPLYSPLQPDIEQGK.L** |
|  | 30050 | **751.9674** | **3754.8007** | **3755.8927** | **-1.0921** | **1** | **3** | **17** | **1** | **R.LTHNDIVATTYLSMSKISAPGGEIEEEPAGAVKPSK.A** |
|  | 31398 | **1315.3723** | **5257.4601** | **5257.4820** | **-0.0218** | **0** | **18** | **0.2** | **1** | **K.AQCSTEDVDSLVAQLTDELIAGCSQPLGDIHETPSATHLDQYLYQLR.T** |

| **294.** | O15260\|SURF4_HUMAN    **Mass:** 30602    **Score:** 151    **Queries matched:** 11   **emPAI:** 0.12 |
| --- | --- |
|  | Surfeit locus protein 4 OS=Homo sapiens GN=SURF4 PE=1 SV=3 |

|  | **Query** | **Observed** | **Mr(expt)** | **Mr(calc)** | **Delta** | **Miss** | **Score** | **Expect** | **Rank** | **Peptide** |
| --- | --- | --- | --- | --- | --- | --- | --- | --- | --- | --- |
|  | 4137 | **491.7527** | **981.4908** | **982.5348** | **-1.0441** | **0** | **8** | **25** | **3** | **K.QYLPHVAR.L** 4159 |
|  | 5744 | **548.7639** | **1095.5131** | **1095.5205** | **-0.0074** | **0** | **8** | **22** | **1** | **K.SMFAGVPTMR.E** 5759 |
|  | 13214 | **505.2596** | **1512.7570** | **1512.7065** | **0.0505** | **1** | **7** | **25** | **3** | **R.SEGKSMFAGVPTMR.E** |
|  | 15518 | **553.0173** | **1656.0302** | **1655.7647** | **0.2654** | **1** | **3** | **50** | **4** | **K.SMFAGVPTMRESSPK.Q** |
|  | 20635 | **1014.4559** | **2026.8972** | **2026.9055** | **-0.0083** | **0** | **86** | **2.1e-007** | **1** | **M.GQNDLMGTAEDFADQFLR.V** 20645 |
|  | 22242 | **731.0263** | **2190.0571** | **2189.9358** | **0.1213** | **0** | **8** | **12** | **2** | **-.MGQNDLMGTAEDFADQFLR.V** |
|  | 24667 | **834.0725** | **2499.1957** | **2500.3260** | **-1.1303** | **1** | **0** | **62** | **8** | **K.QYLPHVARLCLISTFLEDGIR.M** |
|  | 24796 | **840.4005** | **2518.1797** | **2518.1468** | **0.0329** | **1** | **1** | **53** | **4** | **-.MGQNDLMGTAEDFADQFLRVTK.Q** |

| **295.** | P13284\|GILT_HUMAN    **Mass:** 29757    **Score:** 151    **Queries matched:** 11   **emPAI:** 0.43 |
| --- | --- |
|  | Gamma-interferon-inducible lysosomal thiol reductase OS=Homo sapiens GN=IFI30 PE=1 SV=2 |

|  | **Query** | **Observed** | **Mr(expt)** | **Mr(calc)** | **Delta** | **Miss** | **Score** | **Expect** | **Rank** | **Peptide** |
| --- | --- | --- | --- | --- | --- | --- | --- | --- | --- | --- |
|  | 2105 | **418.2025** | **834.3903** | **835.4552** | **-1.0648** | **0** | **7** | **42** | **7** | **K.TGNLYLR.G** |
|  | 8511 | **628.7988** | **1255.5831** | **1255.5914** | **-0.0083** | **0** | **63** | **8.1e-005** | **1** | **R.GMQLMHANAQR.T** |
|  | 20931 | 685.3710 | 2053.0913 | 2053.9925 | -0.9012 | 1 | 3 | 42 | 6 | K.KPDVCPSSTSSLRSVCFK.- |
|  | 27124 | **1454.6843** | **2907.3541** | **2907.3639** | **-0.0098** | **0** | **51** | **0.00047** | **1** | **R.SLPLCLQLYAPGLSPDTIMECAMGDR.G** 27121 27122 |
|  | 30888 | **1460.4039** | **4378.1900** | **4378.2308** | **-0.0408** | **0** | **33** | **0.012** | **1** | **R.TDALQPPHEYVPWVTVNGKPLEDQTQLLTLVCQLYQGK.K** 30889 30891 30893 30894 |

| **296.** | P49585\|PCY1A_HUMAN    **Mass:** 42047    **Score:** 150    **Queries matched:** 13   **emPAI:** 0.19 |
| --- | --- |
|  | Choline-phosphate cytidylyltransferase A OS=Homo sapiens GN=PCYT1A PE=1 SV=2 |

|  | **Query** | **Observed** | **Mr(expt)** | **Mr(calc)** | **Delta** | **Miss** | **Score** | **Expect** | **Rank** | **Peptide** |
| --- | --- | --- | --- | --- | --- | --- | --- | --- | --- | --- |
|  | 3140 | 456.2269 | 910.4393 | 909.3684 | 1.0709 | 0 | 11 | 10 | 5 | -.MDAQCSAK.V |
|  | 5084 | **524.7307** | **1047.4469** | **1047.4556** | **-0.0087** | **0** | **22** | **1.2** | **1** | **R.YDAVQHCR.Y** |
|  | 5190 | 529.3060 | 1056.5974 | 1057.5815 | -0.9841 | 1 | 6 | 41 | 1 | K.VQRCAVGLR.Q |
|  | 5814 | **551.2789** | **1100.5432** | **1099.6172** | **0.9260** | **1** | **(1)** | **1.3e+002** | **1** | **K.GRMLQAISPK.Q** |
|  | 5919 | 554.8225 | 1107.6305 | 1106.5179 | 1.1126 | 0 | 5 | 45 | 9 | K.EAGMFAPTQR.T |
|  | 6011 | 558.7811 | 1115.5477 | 1115.6121 | -0.0644 | 1 | 6 | 44 | 4 | K.GRMLQAISPK.Q |
|  | 6130 | 562.3049 | 1122.5953 | 1122.5128 | 0.0825 | 0 | (5) | 51 | 3 | K.EAGMFAPTQR.T |
|  | 15479 | **827.8846** | **1653.7547** | **1653.7482** | **0.0065** | **0** | **4** | **48** | **4** | **K.EAPGPNGATEEDGVPSK.V** 15480 |
|  | 18761 | 936.9641 | 1871.9135 | 1870.9175 | 0.9960 | 0 | 93 | 5.6e-008 | 1 | R.EFIGSFLEMFGPEGALK.H 18749 |
|  | 21598 | **705.6766** | **2114.0081** | **2114.0506** | **-0.0426** | **1** | **2** | **62** | **6** | **K.SREFIGSFLEMFGPEGALK.H** |
|  | 23838 | 794.3686 | 2380.0839 | 2380.1693 | -0.0853 | 1 | 2 | 48 | 7 | K.EAGMFAPTQRTEGISTSDIITR.I |

| **297.** | P59665\|DEF1_HUMAN    **Mass:** 10536    **Score:** 150    **Queries matched:** 11   **emPAI:** 2.63 |
| --- | --- |
|  | Neutrophil defensin 1 OS=Homo sapiens GN=DEFA1 PE=1 SV=1 |

|  | **Query** | **Observed** | **Mr(expt)** | **Mr(calc)** | **Delta** | **Miss** | **Score** | **Expect** | **Rank** | **Peptide** |
| --- | --- | --- | --- | --- | --- | --- | --- | --- | --- | --- |
|  | 4194 | 494.2559 | 986.4972 | 985.5015 | 0.9957 | 0 | 27 | 0.34 | 1 | R.IPACIAGER.R 4195 |
|  | 5828 | **551.7285** | **1101.4425** | **1101.4518** | **-0.0093** | **1** | **(4)** | **68** | **8** | **R.KNMACYCR.I** |
|  | 6029 | **559.2543** | **1116.4941** | **1116.5022** | **-0.0081** | **0** | **42** | **0.012** | **1** | **R.YGTCIYQGR.L** 6030 6031 6036 |
|  | 6063 | **560.2682** | **1118.5218** | **1117.4467** | **1.0751** | **1** | **5** | **48** | **3** | **R.KNMACYCR.I** |
|  | 8851 | **637.3045** | **1272.5944** | **1272.6033** | **-0.0089** | **1** | **25** | **0.43** | **1** | **R.RYGTCIYQGR.L** |
|  | 28519 | **1068.8772** | **3203.6098** | **3203.6237** | **-0.0140** | **0** | **71** | **4e-006** | **1** | **R.ADEVAAAPEQIAADIPEVVVSLAWDESLAPK.H** 28518 |

|  | **Proteins matching a subset of these peptides:** |
| --- | --- |

|  | P59666\|DEF3_HUMAN    **Mass:** 10580    **Score:** 150    **Queries matched:** 9 |
| --- | --- |
|  | Neutrophil defensin 3 OS=Homo sapiens GN=DEFA3 PE=1 SV=1 |

| **298.** | P50897\|PPT1_HUMAN    **Mass:** 34627    **Score:** 150    **Queries matched:** 6   **emPAI:** 0.23 |
| --- | --- |
|  | Palmitoyl-protein thioesterase 1 OS=Homo sapiens GN=PPT1 PE=1 SV=1 |

|  | **Query** | **Observed** | **Mr(expt)** | **Mr(calc)** | **Delta** | **Miss** | **Score** | **Expect** | **Rank** | **Peptide** |
| --- | --- | --- | --- | --- | --- | --- | --- | --- | --- | --- |
|  | 5126 | **526.7859** | **1051.5573** | **1051.5662** | **-0.0088** | **1** | **19** | **1.9** | **1** | **R.KTLNAGAYSK.V** |
|  | 13594 | 512.7667 | 1535.2783 | 1534.8103 | 0.4680 | 1 | 3 | 47 | 4 | K.TLNAGAYSKVVQER.L |
|  | 17640 | **897.4449** | **1792.8753** | **1792.8843** | **-0.0089** | **0** | **61** | **8e-005** | **1** | **K.ETIPLQETSLYTQDR.L** |
|  | 20363 | 1001.4770 | 2000.9394 | 2000.9527 | -0.0133 | 0 | 111 | 7.3e-010 | 1 | K.LQQGYNAMGFSQGGQFLR.A |
|  | 20546 | 673.7203 | 2018.1392 | 2016.9476 | 1.1916 | 0 | (5) | 25 | 7 | K.LQQGYNAMGFSQGGQFLR.A 20545 |

| **299.** | Q03252\|LMNB2_HUMAN    **Mass:** 67762    **Score:** 150    **Queries matched:** 26   **emPAI:** 0.24 |
| --- | --- |
|  | Lamin-B2 OS=Homo sapiens GN=LMNB2 PE=1 SV=3 |

|  | **Query** | **Observed** | **Mr(expt)** | **Mr(calc)** | **Delta** | **Miss** | **Score** | **Expect** | **Rank** | **Peptide** |
| --- | --- | --- | --- | --- | --- | --- | --- | --- | --- | --- |
|  | 4046 | 488.2414 | 974.4682 | 973.4716 | 0.9966 | 0 | 20 | 1.9 | 1 | K.LLEGEEER.L 4028 |
|  | 5039 | 523.2837 | 1044.5528 | 1043.5611 | 0.9917 | 1 | 6 | 48 | 7 | R.LQEKEELR.E |
|  | 5390 | 536.3082 | 1070.6019 | 1070.6084 | -0.0065 | 1 | 5 | 59 | 3 | R.LKLSPSPSSR.V |
|  | 7167 | **596.3099** | **1190.6053** | **1190.6143** | **-0.0089** | **1** | **25** | **0.49** | **1** | **K.ISEKEEVTTR.E** |
|  | 7657 | 405.2986 | 1212.8739 | 1213.6415 | -0.7676 | 1 | 6 | 31 | 4 | R.VRALELENDR.L |
|  | 7821 | **611.7589** | **1221.5031** | **1221.6241** | **-0.1209** | **0** | **17** | **2.8** | **2** | **K.LELEQTYQAK.L** |
|  | 8742 | **634.8027** | **1267.5909** | **1267.6004** | **-0.0095** | **0** | **43** | **0.0074** | **1** | **R.ATSSSSGSLSATGR.L** 8732 8761 8767 |
|  | 10817 | **458.8807** | **1373.6201** | **1374.6925** | **-1.0724** | **1** | **4** | **57** | **4** | **K.ETLMRVDLENR.C** 10813 |
|  | 10937 | **461.0072** | **1379.9999** | **1379.6681** | **0.3318** | **1** | **6** | **21** | **6** | **K.SVFEEEVRETR.R** |
|  | 11147 | **465.2374** | **1392.6903** | **1392.6997** | **-0.0094** | **1** | **0** | **1.3e+002** | **9** | **K.ALYESELADARR.V** |
|  | 11617 | **711.0309** | **1420.0472** | **1419.6841** | **0.3631** | **1** | **3** | **39** | **2** | **K.LDSAKLSSDQNDK.A** |
|  | 11851 | **717.4081** | **1432.8017** | **1432.6906** | **0.1111** | **1** | **50** | **0.0015** | **1** | **K.LSSDQNDKAASAAR.E** |
|  | 13045 | **752.3721** | **1502.7297** | **1502.7399** | **-0.0102** | **0** | **71** | **1.1e-005** | **1** | **R.TVLVNADGEEVAMR.T** |
|  | 13304 | **506.9265** | **1517.7578** | **1518.7348** | **-0.9770** | **0** | **(3)** | **62** | **8** | **R.TVLVNADGEEVAMR.T** 13298 |
|  | 13558 | **767.3508** | **1532.6870** | **1532.6968** | **-0.0098** | **1** | **47** | **0.0024** | **1** | **K.NNSDKDQSLGNWR.I** 13557 |
|  | 14608 | **800.4081** | **1598.8016** | **1598.8627** | **-0.0611** | **1** | **1** | **99** | **9** | **R.GLESDVAELRAQLAK.A** |
|  | 15303 | **548.8361** | **1643.4864** | **1642.7000** | **0.7863** | **1** | **6** | **21** | **4** | **K.MLDAKEQEMTEMR.D** |
|  | 19706 | **649.6210** | **1945.8411** | **1947.0360** | **-1.1949** | **1** | **3** | **44** | **2** | **-.MATPLPGRAGGPATPLSPTR.L** |
|  | 24905 | **1269.5945** | **2537.1744** | **2536.2897** | **0.8847** | **0** | **1** | **57** | **1** | **R.AGQMVTVWAAGAGVAHSPPSTLVWK.G** |

| **300.** | Q9BZQ8\|NIBAN_HUMAN    **Mass:** 104039   **Score:** 150    **Queries matched:** 15   **emPAI:** 0.07 |
| --- | --- |
|  | Protein Niban OS=Homo sapiens GN=FAM129A PE=1 SV=1 |

|  | **Query** | **Observed** | **Mr(expt)** | **Mr(calc)** | **Delta** | **Miss** | **Score** | **Expect** | **Rank** | **Peptide** |
| --- | --- | --- | --- | --- | --- | --- | --- | --- | --- | --- |
|  | 484 | **443.0389** | **442.0316** | **442.2540** | **-0.2224** | **0** | **4** | **14** | **2** | **R.GAAPK.C** 475 |
|  | 1385 | **731.4045** | **730.3972** | **730.4225** | **-0.0253** | **0** | **15** | **7.6** | **2** | **K.TAVAIEK.V** 1387 |
|  | 6295 | 568.8387 | 1135.6628 | 1135.5179 | 0.1449 | 0 | (13) | 8.1 | 3 | R.SDMDQIVNSK.N |
|  | 6500 | **576.2841** | **1150.5537** | **1151.5128** | **-0.9592** | **0** | **14** | **6.3** | **1** | **R.SDMDQIVNSK.N** |
|  | 15324 | **823.4228** | **1644.8310** | **1645.8100** | **-0.9790** | **1** | **6** | **26** | **4** | **K.TEAAIKNFSPYYSR.Q** |
|  | 15467 | **827.3970** | **1652.7794** | **1652.7893** | **-0.0099** | **0** | **70** | **1.2e-005** | **1** | **K.VLTSEDEYNLLSDR.H** |
|  | 17798 | **902.9075** | **1803.8004** | **1804.8600** | **-1.0596** | **1** | **7** | **21** | **2** | **K.MEPCYTKVNLLHER.L** |
|  | 18843 | **939.9409** | **1877.8673** | **1877.9153** | **-0.0480** | **1** | **10** | **10** | **10** | **K.GLEGTIRSDMDQIVNSK.N** |
|  | 27269 | 980.8033 | 2939.3882 | 2940.5709 | -1.1827 | 1 | 1 | 40 | 6 | K.RTWLGLLEEAYTLVQHQVSEGLSALK.E |
|  | 29436 | **1161.8839** | **3482.6299** | **3482.6486** | **-0.0187** | **0** | **91** | **3.2e-008** | **1** | **R.TQNYMQELMENAVFTFEQLLSPHLQGEASK.T** 29437 |
|  | 30622 | **1028.5175** | **4110.0407** | **4109.0159** | **1.0248** | **1** | **3** | **13** | **1** | **K.AMVAQPAEKSCLESVQPFLASILEELMGPVSSGFSEVR.V** |
|  | 30627 | 1371.3508 | 4111.0307 | 4109.8746 | 1.1560 | 1 | 1 | 22 | 8 | R.KLLTASVEVPVDSAPVMEEDTNGESHVPQENEEEEEK.E |

| **301.** | P16671\|CD36_HUMAN    **Mass:** 53589    **Score:** 149    **Queries matched:** 9   **emPAI:** 0.31 |
| --- | --- |
|  | Platelet glycoprotein 4 OS=Homo sapiens GN=CD36 PE=1 SV=2 |

|  | **Query** | **Observed** | **Mr(expt)** | **Mr(calc)** | **Delta** | **Miss** | **Score** | **Expect** | **Rank** | **Peptide** |
| --- | --- | --- | --- | --- | --- | --- | --- | --- | --- | --- |
|  | 3507 | 469.3271 | 936.6396 | 935.4865 | 1.1531 | 0 | 6 | 34 | 10 | R.ELLWGYR.D |
|  | 4954 | **520.7630** | **1039.5114** | **1039.5199** | **-0.0085** | **1** | **39** | **0.017** | **1** | **K.QRGPYTYR.V** |
|  | 14401 | **793.8810** | **1585.7475** | **1585.7559** | **-0.0084** | **0** | **80** | **1.2e-006** | **1** | **K.SQVLQFFSSDICR.S** |
|  | 18628 | **620.8032** | **1859.3878** | **1860.0145** | **-0.6267** | **1** | **3** | **36** | **3** | **K.QVVLEEGTIAFKNWVK.T** |
|  | 19751 | 975.4813 | 1948.9480 | 1948.2085 | 0.7395 | 1 | 2 | 60 | 5 | R.LQVNLLVKPSEKIQVLK.N |
|  | 19850 | **979.0149** | **1956.0152** | **1956.0244** | **-0.0092** | **0** | **28** | **0.16** | **1** | **R.TYLDIEPITGFTLQFAK.R** |
|  | 21881 | **1075.5509** | **2149.0872** | **2147.8928** | **1.1944** | **0** | **43** | **0.0041** | **1** | **K.AFASPVENPDNYCFCTEK.I** 21870 |
|  | 30275 | **969.9845** | **3875.9089** | **3875.8754** | **0.0335** | **0** | **8** | **5.2** | **1** | **K.EGRPVYISLPHFLYASPDVSEPIDGLNPNEEEHR.T** |

| **302.** | P18464\|1B51_HUMAN    **Mass:** 40826    **Score:** 149    **Queries matched:** 23   **emPAI:** 0.55 |
| --- | --- |
|  | HLA class I histocompatibility antigen, B-51 alpha chain OS=Homo sapiens GN=HLA-B PE=1 SV=1 |

|  | **Query** | **Observed** | **Mr(expt)** | **Mr(calc)** | **Delta** | **Miss** | **Score** | **Expect** | **Rank** | **Peptide** |
| --- | --- | --- | --- | --- | --- | --- | --- | --- | --- | --- |
|  | 1409 | 746.1623 | 745.1551 | 744.3766 | 0.7785 | 0 | 12 | 10 | 6 | R.EAEQLR.A |
|  | 3888 | 482.7234 | 963.4322 | 964.4250 | -0.9929 | 0 | 33 | 0.088 | 1 | R.FDSDAASPR.T 3900 3901 3907 |
|  | 4176 | 493.2762 | 984.5379 | 983.5876 | 0.9503 | 1 | 10 | 18 | 10 | R.ENLRIALR.Y 4164 |
|  | 6565 | 577.7809 | 1153.5473 | 1153.6091 | -0.0618 | 1 | 3 | 67 | 9 | K.ETLQRADPPK.T |
|  | 10618 | 455.7262 | 1364.1569 | 1363.6343 | 0.5226 | 0 | 3 | 50 | 7 | R.YFYTAMSRPGR.G |
|  | 11736 | 714.3626 | 1426.7105 | 1426.7205 | -0.0099 | 0 | 46 | 0.0031 | 1 | K.WAAVVVPSGEEQR.Y 11721 11735 11751 |
|  | 17808 | 902.9733 | 1803.9321 | 1802.8522 | 1.0799 | 1 | 3 | 60 | 8 | R.YFYTAMSRPGRGEPR.F |
|  | 18283 | 613.3167 | 1836.9283 | 1835.9027 | 1.0256 | 0 | 15 | 3.7 | 1 | K.THVTHHPVSDHEATLR.C |
|  | 20872 | 513.0196 | 2048.0493 | 2048.0626 | -0.0133 | 0 | 16 | 2.1 | 1 | R.YTCHVQHEGLPKPLTLR.W 20848 20850 20871 |
|  | 21557 | 1056.5175 | 2111.0204 | 2111.0299 | -0.0095 | 0 | 86 | 2.1e-007 | 1 | R.CWALGFYPAEITLTWQR.D 21558 |
|  | 22689 | 744.6669 | 2230.9790 | 2230.9938 | -0.0149 | 0 | 44 | 0.0032 | 1 | R.DGEDQTQDTELVETRPAGDR.T 22688 |

|  | **Proteins matching the same set of peptides:** |
| --- | --- |

|  | P30490\|1B52_HUMAN    **Mass:** 40781    **Score:** 149    **Queries matched:** 23 |
| --- | --- |
|  | HLA class I histocompatibility antigen, B-52 alpha chain OS=Homo sapiens GN=HLA-B PE=2 SV=1 |

|  | **Proteins matching a subset of these peptides:** |
| --- | --- |

|  | P30498\|1B78_HUMAN    **Mass:** 40738    **Score:** 149    **Queries matched:** 21 |
| --- | --- |
|  | HLA class I histocompatibility antigen, B-78 alpha chain OS=Homo sapiens GN=HLA-B PE=2 SV=1 |

| **303.** | Q13724\|MOGS_HUMAN    **Mass:** 92032    **Score:** 149    **Queries matched:** 23   **emPAI:** 0.08 |
| --- | --- |
|  | Mannosyl-oligosaccharide glucosidase OS=Homo sapiens GN=MOGS PE=1 SV=5 |

|  | **Query** | **Observed** | **Mr(expt)** | **Mr(calc)** | **Delta** | **Miss** | **Score** | **Expect** | **Rank** | **Peptide** |
| --- | --- | --- | --- | --- | --- | --- | --- | --- | --- | --- |
|  | 437 | **442.2744** | **441.2671** | **442.2288** | **-0.9618** | **0** | **10** | **7.4** | **1** | **R.GGGPR.S** 470 477 |
|  | 472 | **443.0381** | **442.0308** | **442.2288** | **-0.1980** | **0** | **6** | **10** | **1** | **R.GGPGR.R** 464 468 503 |
|  | 1624 | **400.2350** | **798.4555** | **797.4395** | **1.0159** | **0** | **5** | **57** | **10** | **R.AVPAEGVR.T** |
|  | 4590 | 507.2528 | 1012.4910 | 1013.5407 | -1.0497 | 0 | 28 | 0.24 | 1 | R.ANVVGNVWR.Q |
|  | 5602 | **543.3282** | **1084.6418** | **1083.6077** | **1.0341** | **0** | **3** | **86** | **4** | **R.VPPEFLVQR.A** |
|  | 8024 | **411.6841** | **1232.0303** | **1232.6037** | **-0.5734** | **0** | **5** | **34** | **2** | **K.TLPSGLDDYPR.A** |
|  | 8851 | 637.3045 | 1272.5944 | 1272.6098 | -0.0154 | 0 | 5 | 47 | 6 | R.SLAASSSFYGQR.N 8877 |
|  | 11102 | 695.8710 | 1389.7275 | 1390.7932 | -1.0657 | 1 | 6 | 36 | 3 | R.GRDPALPTLLNPK.T 11101 11132 |
|  | 18989 | **630.3885** | **1888.1436** | **1887.9492** | **0.1944** | **0** | **4** | **32** | **3** | **R.LNSWFQHRPPGAPPER.Y** |
|  | 24770 | **1258.6244** | **2515.2342** | **2515.2457** | **-0.0115** | **0** | **100** | **7.3e-009** | **1** | **K.YGSYNVFWTSNPGLPLLTEMVK.S** 24771 |
|  | 25199 | **856.3926** | **2566.1559** | **2565.3737** | **0.7822** | **1** | **1** | **47** | **8** | **K.TRSPKPLLTGLMWAQQGTTPGTPK.L** |
|  | 25913 | **896.4455** | **2686.3147** | **2685.3452** | **0.9694** | **1** | **0** | **61** | **10** | **R.GFLWDEGFHQLVVQRWDPSLTR.E** |
|  | 30322 | **1302.3617** | **3904.0632** | **3904.0762** | **-0.0129** | **1** | **37** | **0.0061** | **1** | **R.VTVEPQDSGTSALPLVSLFFYVVTDGKEVLLPEVGAK.G** 30321 |

| **304.** | Q93084\|AT2A3_HUMAN    **Mass:** 115444   **Score:** 148    **Queries matched:** 22   **emPAI:** 0.13 |
| --- | --- |
|  | Sarcoplasmic/endoplasmic reticulum calcium ATPase 3 OS=Homo sapiens GN=ATP2A3 PE=1 SV=2 |

|  | **Query** | **Observed** | **Mr(expt)** | **Mr(calc)** | **Delta** | **Miss** | **Score** | **Expect** | **Rank** | **Peptide** |
| --- | --- | --- | --- | --- | --- | --- | --- | --- | --- | --- |
|  | 2759 | 441.2609 | 880.5073 | 880.4337 | 0.0736 | 1 | 9 | 18 | 6 | R.TARCFAR.V |
|  | 3473 | 467.7395 | 933.4644 | 932.4749 | 0.9895 | 0 | 13 | 10 | 5 | R.AGACNTVIK.Q |
|  | 4275 | **496.7523** | **991.4901** | **991.5008** | **-0.0108** | **0** | **10** | **17** | **3** | **R.VVMITGDNK.G** |
|  | 6834 | 587.3101 | 1172.6056 | 1172.7128 | -0.1073 | 1 | 1 | 1.2e+002 | 7 | R.LIEIKSTTLR.V |
|  | 8686 | **633.3200** | **1264.6255** | **1265.7707** | **-1.1452** | **1** | **0** | **1.3e+002** | **6** | **K.VPADLRLIEIK.S** |
|  | 10140 | **447.2191** | **1338.6354** | **1339.7030** | **-1.0676** | **1** | **4** | **61** | **9** | **R.CLALATRDAPPR.K** |
|  | 10587 | 682.3253 | 1362.6361 | 1361.6688 | 0.9673 | 1 | 4 | 51 | 4 | K.IRDWGSGSDTLR.C |
|  | 11121 | 464.7628 | 1391.2666 | 1390.7126 | 0.5540 | 0 | 1 | 65 | 8 | K.AEIGIAMGSGTAVAK.S |
|  | 12431 | 734.8424 | 1467.6703 | 1467.6810 | -0.0107 | 0 | 62 | 7.9e-005 | 1 | K.TGTLTTNQMSVCR.M |
|  | 13049 | 752.4084 | 1502.8022 | 1502.8126 | -0.0104 | 1 | 36 | 0.031 | 1 | K.KAEIGIAMGSGTAVAK.S 13040 |
|  | 13297 | 506.9037 | 1517.6892 | 1518.8076 | -1.1183 | 1 | (4) | 52 | 8 | K.KAEIGIAMGSGTAVAK.S 13343 |
|  | 13361 | **761.3756** | **1520.7367** | **1521.7973** | **-1.0607** | **0** | **10** | **14** | **2** | **-.MEAAHLLPAADVLR.H** |
|  | 13427 | **509.3012** | **1524.8818** | **1524.7242** | **0.1576** | **0** | **7** | **23** | **4** | **K.MNVFDTDLQALSR.V** |
|  | 14328 | 528.2849 | 1581.8329 | 1580.7704 | 1.0625 | 0 | 1 | 93 | 10 | R.NHMHACLYPGLLR.T |
|  | 14958 | 810.9049 | 1619.7953 | 1619.8076 | -0.0124 | 0 | 91 | 1e-007 | 1 | K.VGEATETALTCLVEK.M 14977 |
|  | 17816 | **602.3564** | **1804.0473** | **1803.9335** | **0.1138** | **1** | **1** | **75** | **10** | **R.VVMITGDNKGTAVAICR.R** |
|  | 18933 | **943.5040** | **1884.9934** | **1883.9490** | **1.0444** | **0** | **4** | **36** | **1** | **R.HFSVTAEGGLSPAQVTGAR.E** |
|  | 19776 | 976.9628 | 1951.9110 | 1951.9231 | -0.0121 | 0 | 35 | 0.031 | 1 | R.SLPSVETLGCTSVICSDK.T |
|  | 26366 | **922.1540** | **2763.4401** | **2762.3433** | **1.0969** | **0** | **1** | **51** | **8** | **R.IVENLQSFNEITAMTGDGVNDAPALK.K** |

| **305.** | Q96QV6\|H2A1A_HUMAN    **Mass:** 14225    **Score:** 148    **Queries matched:** 19   **emPAI:** 3.31 |
| --- | --- |
|  | Histone H2A type 1-A OS=Homo sapiens GN=HIST1H2AA PE=1 SV=3 |

|  | **Query** | **Observed** | **Mr(expt)** | **Mr(calc)** | **Delta** | **Miss** | **Score** | **Expect** | **Rank** | **Peptide** |
| --- | --- | --- | --- | --- | --- | --- | --- | --- | --- | --- |
|  | 1100 | 617.3092 | 616.3019 | 615.3452 | 0.9567 | 1 | 19 | 4.4 | 4 | K.QGGKAR.A |
|  | 2130 | 419.2117 | 836.4088 | 836.4140 | -0.0052 | 1 | 22 | 1.1 | 1 | R.KGNYAER.I 2129 |
|  | 2328 | 425.7633 | 849.5121 | 849.5184 | -0.0064 | 0 | 32 | 0.11 | 1 | R.HLQLAIR.N 2329 2330 2339 |
|  | 2469 | 431.2187 | 860.4229 | 860.3875 | 0.0353 | 0 | 14 | 7.7 | 4 | R.NDEELNK.L |
|  | 3614 | 472.7664 | 943.5183 | 943.5240 | -0.0057 | 0 | 45 | 0.0057 | 1 | R.AGLQFPVGR.I 3604 3613 3617 |
|  | 8870 | 637.8182 | 1273.6218 | 1273.6891 | -0.0673 | 1 | 8 | 21 | 3 | R.SSRAGLQFPVGR.I |
|  | 16093 | 846.9494 | 1691.8842 | 1691.8954 | -0.0112 | 1 | 75 | 3.6e-006 | 1 | R.HLQLAIRNDEELNK.L 16091 16092 16094 |
|  | 22977 | **1136.1876** | **2270.3607** | **2270.3726** | **-0.0119** | **0** | **34** | **0.025** | **1** | **K.LLGGVTIAQGGVLPNIQAVLLPK.K** 22976 |

|  | **Proteins matching a subset of these peptides:** |
| --- | --- |

|  | P16104\|H2AX_HUMAN    **Mass:** 15135    **Score:** 148    **Queries matched:** 16 |
| --- | --- |
|  | Histone H2A.x OS=Homo sapiens GN=H2AFX PE=1 SV=2 |

| **306.** | P05388\|RLA0_HUMAN    **Mass:** 34423    **Score:** 147    **Queries matched:** 10   **emPAI:** 0.37 |
| --- | --- |
|  | 60S acidic ribosomal protein P0 OS=Homo sapiens GN=RPLP0 PE=1 SV=1 |

|  | **Query** | **Observed** | **Mr(expt)** | **Mr(calc)** | **Delta** | **Miss** | **Score** | **Expect** | **Rank** | **Peptide** |
| --- | --- | --- | --- | --- | --- | --- | --- | --- | --- | --- |
|  | 1674 | **402.2424** | **802.4703** | **802.4119** | **0.0584** | **0** | **24** | **1** | **3** | **K.QMQQIR.M** |
|  | 17476 | **595.6254** | **1783.8545** | **1784.0407** | **-0.1863** | **1** | **0** | **1e+002** | **4** | **K.ISRGTIEILSDVQLIK.T** |
|  | 19059 | **948.4975** | **1894.9804** | **1894.9928** | **-0.0123** | **0** | **70** | **9.2e-006** | **1** | **R.VLALSVETDYTFPLAEK.V** 19060 |
|  | 19750 | 975.4804 | 1948.9461 | 1950.0799 | -1.1337 | 1 | 7 | 22 | 3 | R.GHLENNPALEKLLPHIR.G |
|  | 20899 | **684.3521** | **2050.0345** | **2051.0939** | **-1.0594** | **1** | **1** | **72** | **8** | **K.RVLALSVETDYTFPLAEK.V** |
|  | 22180 | **1090.5470** | **2179.0794** | **2179.0943** | **-0.0149** | **0** | **63** | **4.2e-005** | **1** | **R.AGAIAPCEVTVPAQNTGLGPEK.T** 22179 |
|  | 26308 | **918.1542** | **2751.4407** | **2751.4595** | **-0.0188** | **0** | **19** | **0.83** | **1** | **K.AFLADPSAFVAAAPVAAATTAAPAAAAAPAK.V** 26309 |

| **307.** | Q00325\|MPCP_HUMAN    **Mass:** 40525    **Score:** 146    **Queries matched:** 14   **emPAI:** 0.30 |
| --- | --- |
|  | Phosphate carrier protein, mitochondrial OS=Homo sapiens GN=SLC25A3 PE=1 SV=2 |

|  | **Query** | **Observed** | **Mr(expt)** | **Mr(calc)** | **Delta** | **Miss** | **Score** | **Expect** | **Rank** | **Peptide** |
| --- | --- | --- | --- | --- | --- | --- | --- | --- | --- | --- |
|  | 1931 | **412.2291** | **822.4436** | **822.4487** | **-0.0051** | **0** | **18** | **2.4** | **1** | **R.TVEALYK.F** |
|  | 2025 | **415.7318** | **829.4491** | **828.3589** | **1.0902** | **0** | **9** | **28** | **2** | **K.FACFER.T** |
|  | 2261 | **423.2098** | **844.4051** | **844.4113** | **-0.0062** | **0** | **23** | **1.1** | **3** | **R.MQVDPQK.Y** 2275 |
|  | 6279 | **567.9222** | **1133.8299** | **1133.5652** | **0.2648** | **0** | **0** | **1.1e+002** | **7** | **-.MFSSVAHLAR.A** |
|  | 6292 | **568.7883** | **1135.5620** | **1135.5696** | **-0.0076** | **1** | **17** | **3.3** | **1** | **R.MQVDPQKYK.G** |
|  | 10554 | **681.3577** | **1360.7009** | **1360.7099** | **-0.0090** | **0** | **46** | **0.0035** | **1** | **R.IQTQPGYANTLR.D** 10533 |
|  | 16803 | **869.4097** | **1736.8048** | **1737.9050** | **-1.1002** | **1** | **32** | **0.063** | **1** | **K.GIFNGFSVTLKEDGVR.G** |
|  | 17955 | **908.4249** | **1814.8353** | **1814.8484** | **-0.0131** | **0** | **67** | **1.9e-005** | **1** | **K.GWAPTFLGYSMQGLCK.F** 17954 |
|  | 20183 | 994.5146 | 1987.0146 | 1986.9509 | 0.0637 | 0 | 11 | 7.9 | 1 | K.VLYSNMLGEENTYLWR.T |
|  | 28397 | 796.1661 | 3180.6352 | 3181.6534 | -1.0181 | 1 | 4 | 22 | 5 | M.FSSVAHLARANPFNTPHLQLVHDGLGDLR.S |
|  | 31066 | **926.2326** | **4626.1266** | **4627.2654** | **-1.1388** | **1** | **1** | **14** | **9** | **K.VLYSNMLGEENTYLWRTSLYLAASASAEFFADIALAPMEAAK.V** |

| **308.** | Q8NBQ5\|DHB11_HUMAN    **Mass:** 33285    **Score:** 146    **Queries matched:** 10   **emPAI:** 0.54 |
| --- | --- |
|  | Estradiol 17-beta-dehydrogenase 11 OS=Homo sapiens GN=HSD17B11 PE=1 SV=2 |

|  | **Query** | **Observed** | **Mr(expt)** | **Mr(calc)** | **Delta** | **Miss** | **Score** | **Expect** | **Rank** | **Peptide** |
| --- | --- | --- | --- | --- | --- | --- | --- | --- | --- | --- |
|  | 7068 | 594.2813 | 1186.5479 | 1186.6056 | -0.0577 | 1 | 4 | 68 | 8 | K.FDAVIGYKMK.A |
|  | 8254 | **622.4181** | **1242.8216** | **1242.6026** | **0.2190** | **1** | **0** | **1.2e+002** | **4** | **K.HGLEETAAKCK.G** |
|  | 14184 | 786.9418 | 1571.8690 | 1571.8770 | -0.0080 | 0 | 41 | 0.0096 | 1 | K.TLTDELAALQITGVK.T 14197 |
|  | 16088 | **564.9598** | **1691.8577** | **1691.8672** | **-0.0095** | **0** | **21** | **0.89** | **1** | **K.TFEVNVLAHFWTTK.A** |
|  | 17243 | **886.4230** | **1770.8314** | **1770.8433** | **-0.0120** | **0** | **42** | **0.0079** | **1** | **K.TTCLCPNFVNTGFIK.N** 17242 |
|  | 19560 | **646.3510** | **1936.0312** | **1936.0418** | **-0.0106** | **1** | **20** | **0.94** | **1** | **K.LVLWDINKHGLEETAAK.C** |
|  | 19579 | **969.9851** | **1937.9557** | **1937.9694** | **-0.0138** | **0** | **64** | **3.8e-005** | **1** | **K.NPSTSLGPTLEPEEVVNR.L** 19580 |

| **309.** | P13760\|2B14_HUMAN    **Mass:** 30435    **Score:** 145    **Queries matched:** 10   **emPAI:** 0.60 |
| --- | --- |
|  | HLA class II histocompatibility antigen, DRB1-4 beta chain OS=Homo sapiens GN=HLA-DRB1 PE=1 SV=1 |

|  | **Query** | **Observed** | **Mr(expt)** | **Mr(calc)** | **Delta** | **Miss** | **Score** | **Expect** | **Rank** | **Peptide** |
| --- | --- | --- | --- | --- | --- | --- | --- | --- | --- | --- |
|  | 2994 | 451.2558 | 900.4970 | 900.5029 | -0.0058 | 1 | 17 | 3.7 | 1 | K.DLLEQKR.A |
|  | 3775 | 478.7490 | 955.4834 | 954.4229 | 1.0605 | 0 | 16 | 4 | 1 | R.AAVDTYCR.H |
|  | 5634 | 544.7355 | 1087.4564 | 1086.4618 | 0.9946 | 0 | 21 | 1.4 | 1 | R.FDSDVGEYR.A 5635 |
|  | 7210 | 597.6307 | 1193.2468 | 1192.5625 | 0.6843 | 1 | 1 | 88 | 3 | R.WFRNGQEEK.T |
|  | 8690 | **633.3376** | **1264.6606** | **1264.6703** | **-0.0097** | **0** | **18** | **2.2** | **1** | **R.VYPEVTVYPAK.T** |
|  | 11632 | 711.3883 | 1420.7621 | 1420.7714 | -0.0094 | 1 | 30 | 0.14 | 1 | R.RVYPEVTVYPAK.T |
|  | 12872 | 747.3553 | 1492.6960 | 1492.7059 | -0.0099 | 0 | 50 | 0.0013 | 1 | R.HNYGVGESFTVQR.R |
|  | 19944 | 982.4727 | 1962.9308 | 1962.9435 | -0.0128 | 0 | 84 | 3.5e-007 | 1 | R.AVTELGRPDAEYWNSQK.D 19943 |

| **310.** | P61204\|ARF3_HUMAN    **Mass:** 20645    **Score:** 144    **Queries matched:** 17   **emPAI:** 2.30 |
| --- | --- |
|  | ADP-ribosylation factor 3 OS=Homo sapiens GN=ARF3 PE=1 SV=2 |

|  | **Query** | **Observed** | **Mr(expt)** | **Mr(calc)** | **Delta** | **Miss** | **Score** | **Expect** | **Rank** | **Peptide** |
| --- | --- | --- | --- | --- | --- | --- | --- | --- | --- | --- |
|  | 1584 | **398.7023** | **795.3900** | **794.4763** | **0.9138** | **0** | **15** | **4.3** | **1** | **K.LGLHSLR.H** |
|  | 2204 | 421.2071 | 840.3996 | 839.5130 | 0.8866 | 0 | 14 | 6 | 1 | K.IRPLWR.H |
|  | 4071 | **488.8959** | **975.7773** | **975.4695** | **0.3078** | **0** | **(0)** | **1.2e+002** | **4** | **R.MLAEDELR.D** |
|  | 4295 | **497.2657** | **992.5168** | **991.4644** | **1.0524** | **0** | **15** | **5.8** | **2** | **R.MLAEDELR.D** |
|  | 8331 | **624.3115** | **1246.6085** | **1245.6135** | **0.9950** | **1** | **26** | **0.41** | **1** | **R.VNEAREELMR.M** |
|  | 14083 | **783.3784** | **1564.7422** | **1564.7522** | **-0.0100** | **0** | **50** | **0.0011** | **1** | **K.NISFTVWDVGGQDK.I** |
|  | 15110 | **815.8854** | **1629.7563** | **1629.7668** | **-0.0105** | **0** | **57** | **0.00024** | **1** | **K.QDLPNAMNAAEITDK.L** |
|  | 15343 | **549.8127** | **1646.4162** | **1645.7617** | **0.6545** | **0** | **(2)** | **53** | **7** | **K.QDLPNAMNAAEITDK.L** |
|  | 15428 | **826.3663** | **1650.7181** | **1649.7752** | **0.9429** | **1** | **7** | **21** | **1** | **R.EELMRMLAEDELR.D** |
|  | 20842 | **683.0316** | **2046.0730** | **2046.0819** | **-0.0089** | **1** | **38** | **0.013** | **1** | **R.MLAEDELRDAVLLVFANK.Q** 20849 20853 |
|  | 23401 | **1162.1196** | **2322.2247** | **2322.2359** | **-0.0112** | **0** | **24** | **0.29** | **1** | **K.LGEIVTTIPTIGFNVETVEYK.N** 23402 |
|  | 23879 | **1193.6084** | **2385.2022** | **2386.2546** | **-1.0524** | **1** | **0** | **74** | **7** | **K.NISFTVWDVGGQDKIRPLWR.H** |
|  | 24211 | **813.3935** | **2437.1586** | **2437.1775** | **-0.0188** | **1** | **49** | **0.0009** | **1** | **R.HYFQNTQGLIFVVDSNDRER.V** |
|  | 25193 | **855.4740** | **2563.4002** | **2563.4149** | **-0.0147** | **1** | **27** | **0.13** | **1** | **K.LKLGEIVTTIPTIGFNVETVEYK.N** |

|  | **Proteins matching the same set of peptides:** |
| --- | --- |

|  | P84077\|ARF1_HUMAN    **Mass:** 20741    **Score:** 144    **Queries matched:** 17 |
| --- | --- |
|  | ADP-ribosylation factor 1 OS=Homo sapiens GN=ARF1 PE=1 SV=2 |

| **311.** | P08311\|CATG_HUMAN    **Mass:** 29161    **Score:** 142    **Queries matched:** 20   **emPAI:** 1.35 |
| --- | --- |
|  | Cathepsin G OS=Homo sapiens GN=CTSG PE=1 SV=2 |

|  | **Query** | **Observed** | **Mr(expt)** | **Mr(calc)** | **Delta** | **Miss** | **Score** | **Expect** | **Rank** | **Peptide** |
| --- | --- | --- | --- | --- | --- | --- | --- | --- | --- | --- |
|  | 1762 | **405.2268** | **808.4391** | **807.4062** | **1.0330** | **0** | **2** | **1.1e+002** | **10** | **R.CGGFLVR.E** |
|  | 1863 | **409.2428** | **816.4710** | **817.4406** | **-0.9696** | **1** | **8** | **32** | **8** | **R.RGTDTLR.E** |
|  | 4110 | **490.2845** | **978.5544** | **978.5611** | **-0.0066** | **0** | **27** | **0.34** | **1** | **R.NVNPVALPR.A** 4114 |
|  | 7262 | **598.7664** | **1195.5183** | **1196.5898** | **-1.0715** | **0** | **38** | **0.025** | **1** | **R.ENTQQHITAR.R** 7288 7289 |
|  | 9021 | **428.2268** | **1281.6586** | **1281.6690** | **-0.0104** | **1** | **26** | **0.3** | **1** | **R.AIRHPQYNQR.T** |
|  | 10398 | **677.3477** | **1352.6808** | **1352.6909** | **-0.0101** | **1** | **46** | **0.0036** | **1** | **R.RENTQQHITAR.R** 10396 10397 10399 10400 10413 |
|  | 13748 | **773.4239** | **1544.8333** | **1543.8392** | **0.9942** | **0** | **19** | **1.4** | **1** | **R.TIQNDIMLLQLSR.R** |
|  | 19451 | **964.9851** | **1927.9555** | **1927.9687** | **-0.0131** | **0** | **55** | **0.00031** | **1** | **R.AQEGLRPGTLCTVAGWGR.V** 19452 |
|  | 21598 | 705.6766 | 2114.0081 | 2114.0215 | -0.0134 | 0 | 35 | 0.025 | 1 | K.GDSGGPLLCNNVAHGIVSYGK.S 21597 |
|  | 25367 | **865.0419** | **2592.1040** | **2592.4713** | **-0.3673** | **0** | **0** | **53** | **3** | **-.MQPLLLLLAFLLPTGAEAGEIIGGR.E** |

| **312.** | P11233\|RALA_HUMAN    **Mass:** 23723    **Score:** 142    **Queries matched:** 14   **emPAI:** 1.11 |
| --- | --- |
|  | Ras-related protein Ral-A OS=Homo sapiens GN=RALA PE=1 SV=1 |

|  | **Query** | **Observed** | **Mr(expt)** | **Mr(calc)** | **Delta** | **Miss** | **Score** | **Expect** | **Rank** | **Peptide** |
| --- | --- | --- | --- | --- | --- | --- | --- | --- | --- | --- |
|  | 2506 | **432.2138** | **862.4130** | **861.4192** | **0.9938** | **1** | **6** | **48** | **9** | **K.SDLEDKR.Q** |
|  | 3365 | **464.2388** | **926.4631** | **926.4684** | **-0.0053** | **0** | **34** | **0.063** | **1** | **K.VFFDLMR.E** |
|  | 3932 | **484.2661** | **966.5177** | **966.5247** | **-0.0069** | **0** | **29** | **0.19** | **1** | **K.GQNSLALHK.V** |
|  | 12196 | **485.5840** | **1453.7301** | **1453.7388** | **-0.0087** | **1** | **30** | **0.12** | **1** | **R.ANVDKVFFDLMR.E** |
|  | 12468 | **736.3428** | **1470.6710** | **1469.7337** | **0.9373** | **1** | **(0)** | **1.2e+002** | **4** | **R.ANVDKVFFDLMR.E** |
|  | 15213 | 819.4308 | 1636.8470 | 1637.7685 | -0.9215 | 0 | 25 | 0.35 | 2 | R.AEQWNVNYVETSAK.T 15210 15241 |
|  | 19209 | 954.9565 | 1907.8985 | 1907.9126 | -0.0140 | 1 | 81 | 8.2e-007 | 1 | K.NRAEQWNVNYVETSAK.T 19207 19208 19224 |
|  | 24136 | **1213.5502** | **2425.0858** | **2425.1035** | **-0.0177** | **0** | **2** | **41** | **1** | **K.SALTLQFMYDEFVEDYEPTK.A** |
|  | 27683 | **1006.7897** | **3017.3474** | **3017.3640** | **-0.0167** | **1** | **23** | **0.24** | **1** | **K.SALTLQFMYDEFVEDYEPTKADSYR.K** |

| **313.** | P20702\|ITAX_HUMAN    **Mass:** 128946   **Score:** 141    **Queries matched:** 21   **emPAI:** 0.12 |
| --- | --- |
|  | Integrin alpha-X OS=Homo sapiens GN=ITGAX PE=1 SV=3 |

|  | **Query** | **Observed** | **Mr(expt)** | **Mr(calc)** | **Delta** | **Miss** | **Score** | **Expect** | **Rank** | **Peptide** |
| --- | --- | --- | --- | --- | --- | --- | --- | --- | --- | --- |
|  | 4244 | **495.7733** | **989.5319** | **989.5393** | **-0.0074** | **0** | **17** | **3.8** | **1** | **R.AQTTTVLEK.Y** |
|  | 4695 | **511.2610** | **1020.5074** | **1020.5141** | **-0.0067** | **0** | **14** | **6.8** | **1** | **R.LFHASYGAR.R** |
|  | 4906 | **518.7839** | **1035.5532** | **1034.5298** | **1.0234** | **0** | **3** | **77** | **8** | **K.GNLSFGWVR.Q** |
|  | 5672 | **546.2800** | **1090.5454** | **1090.5519** | **-0.0066** | **0** | **48** | **0.0029** | **1** | **R.YQVNNLGQR.D** |
|  | 6763 | 584.8303 | 1167.6461 | 1168.6023 | -0.9562 | 0 | 6 | 40 | 5 | R.GGQVSVCPLPR.G 6760 |
|  | 8313 | **416.2949** | **1245.8629** | **1245.5634** | **0.2995** | **0** | **6** | **32** | **5** | **R.NFATMMNFVR.A** |
|  | 10459 | **678.3594** | **1354.7043** | **1354.7139** | **-0.0096** | **0** | **41** | **0.01** | **1** | **R.NLRPMLAADAQR.Y** |
|  | 10478 | 679.3525 | 1356.6904 | 1356.7071 | -0.0167 | 0 | 6 | 35 | 9 | K.DVIPMADAAGIIR.Y |
|  | 15442 | 826.4599 | 1650.9052 | 1650.8617 | 0.0435 | 0 | 3 | 55 | 9 | R.GGAQITFLATFDVSPK.A |
|  | 16222 | **567.6016** | **1699.7829** | **1698.8788** | **0.9040** | **0** | **3** | **54** | **3** | **R.DLQSSVTLDLALDPGR.L** |
|  | 16770 | **434.4991** | **1733.9675** | **1733.9213** | **0.0462** | **1** | **3** | **55** | **6** | **R.YQHTGKAVIFTQVSR.Q** |
|  | 19651 | **649.0206** | **1944.0399** | **1944.0276** | **0.0123** | **1** | **0** | **88** | **6** | **R.LLLTANVSSENNTPRTSK.T** |
|  | 22827 | **750.3980** | **2248.1720** | **2248.1045** | **0.0675** | **1** | **0** | **70** | **1** | **K.EGDSLDYKDVIPMADAAGIIR.Y** |
|  | 23202 | **766.0892** | **2295.2457** | **2295.2562** | **-0.0105** | **0** | **5** | **21** | **1** | **R.TRPVLWVGVSMQFIPAEIPR.S** |
|  | 24296 | **817.7094** | **2450.1064** | **2449.1155** | **0.9909** | **0** | **15** | **2.4** | **1** | **K.NCGADHICQDNLGISFSFPGLK.S** |
|  | 25150 | 852.7430 | 2555.2073 | 2554.3002 | 0.9071 | 1 | 1 | 57 | 1 | R.NLRPMLAADAQRYFTASLPFEK.N |
|  | 26524 | **1396.7057** | **2791.3968** | **2791.4141** | **-0.0173** | **0** | **86** | **1.7e-007** | **1** | **R.LQYFGQALSGGQDLTQDGLVDLAVGAR.G** 26523 |
|  | 26544 | **932.1707** | **2793.4901** | **2793.4670** | **0.0231** | **1** | **0** | **55** | **1** | **R.NMYLTGLCFLLGPTQLTQRLPVSR.Q** |
|  | 30390 | **792.7909** | **3958.9184** | **3959.9516** | **-1.0333** | **0** | **0** | **30** | **6** | **R.DLPVSINFWVPVELNQEAVWMDVEVSHPQNPSLR.C** |

| **314.** | P18462\|1A25_HUMAN    **Mass:** 41477    **Score:** 141    **Queries matched:** 24   **emPAI:** 0.68 |
| --- | --- |
|  | HLA class I histocompatibility antigen, A-25 alpha chain OS=Homo sapiens GN=HLA-A PE=2 SV=1 |

|  | **Query** | **Observed** | **Mr(expt)** | **Mr(calc)** | **Delta** | **Miss** | **Score** | **Expect** | **Rank** | **Peptide** |
| --- | --- | --- | --- | --- | --- | --- | --- | --- | --- | --- |
|  | 1838 | 408.2467 | 814.4788 | 813.3729 | 1.1059 | 0 | 4 | 80 | 3 | K.AHSQTDR.E |
|  | 4359 | 499.2580 | 996.5014 | 995.4308 | 1.0706 | 0 | 8 | 25 | 1 | R.FDSDAASQR.M |
|  | 10029 | 666.8338 | 1331.6530 | 1331.6622 | -0.0092 | 0 | 17 | 3.1 | 1 | R.YFYTSVSRPGR.G |
|  | 10335 | 675.8494 | 1349.6843 | 1349.6939 | -0.0096 | 1 | 38 | 0.024 | 1 | R.YLENGKETLQR.T 10311 10351 |
|  | 13404 | 508.9781 | 1523.9124 | 1524.6627 | -0.7502 | 1 | 4 | 52 | 6 | R.FDSDAASQRMEPR.A |
|  | 13806 | 775.3702 | 1548.7259 | 1548.7355 | -0.0095 | 0 | 44 | 0.0053 | 1 | R.SWTAADMAAQITQR.K |
|  | 14092 | 522.7712 | 1565.2917 | 1564.7304 | 0.5613 | 0 | (8) | 12 | 3 | R.SWTAADMAAQITQR.K |
|  | 18347 | 461.4684 | 1841.8447 | 1841.8591 | -0.0145 | 0 | 28 | 0.18 | 1 | K.THMTHHAVSDHEATLR.C 18348 |
|  | 20872 | 513.0196 | 2048.0493 | 2048.0626 | -0.0133 | 0 | 16 | 2.1 | 1 | R.YTCHVQHEGLPKPLTLR.W 20848 20850 20871 |
|  | 23132 | 763.4412 | 2287.3018 | 2288.0320 | -0.7302 | 1 | 26 | 0.17 | 1 | R.MEPRAPWIEQEGPEYWDR.N |
|  | 23842 | 477.0307 | 2380.1170 | 2380.9747 | -0.8578 | 0 | 10 | 6.5 | 3 | K.GGSYSQAASSDSAQGSDMSLTACK.V |
|  | 25578 | 1319.0889 | 2636.1632 | 2636.1838 | -0.0206 | 0 | 57 | 0.00013 | 1 | R.DGEDQTQDTELVETRPAGDGTFQK.W 25579 25580 25581 |
|  | 28413 | 796.6549 | 3182.5903 | 3181.6926 | 0.8977 | 1 | (2) | 31 | 6 | -.MAVMAPRTLVLLLSGALALTQTWAGSHSMR.Y |
|  | 28476 | 533.7578 | 3196.5032 | 3197.6875 | -1.1843 | 1 | (0) | 46 | 8 | -.MAVMAPRTLVLLLSGALALTQTWAGSHSMR.Y |
|  | 28578 | 804.6039 | 3214.3867 | 3213.6824 | 0.7042 | 1 | 4 | 16 | 2 | -.MAVMAPRTLVLLLSGALALTQTWAGSHSMR.Y |

|  | **Proteins matching the same set of peptides:** |
| --- | --- |

|  | P30450\|1A26_HUMAN    **Mass:** 41321    **Score:** 141    **Queries matched:** 24 |
| --- | --- |
|  | HLA class I histocompatibility antigen, A-26 alpha chain OS=Homo sapiens GN=HLA-A PE=1 SV=2 |

|  | **Proteins matching a subset of these peptides:** |
| --- | --- |

|  | P30457\|1A66_HUMAN    **Mass:** 41341    **Score:** 141    **Queries matched:** 23 |
| --- | --- |
|  | HLA class I histocompatibility antigen, A-66 alpha chain OS=Homo sapiens GN=HLA-A PE=1 SV=1 |

|  | P30456\|1A43_HUMAN    **Mass:** 41292    **Score:** 134    **Queries matched:** 23 |
| --- | --- |
|  | HLA class I histocompatibility antigen, A-43 alpha chain OS=Homo sapiens GN=HLA-A PE=2 SV=1 |

| **315.** | P01914\|HB2C_HUMAN    **Mass:** 30325    **Score:** 140    **Queries matched:** 9   **emPAI:** 0.27 |
| --- | --- |
|  | HLA class II histocompatibility antigen, DR-1 beta chain OS=Homo sapiens PE=2 SV=2 |

|  | **Query** | **Observed** | **Mr(expt)** | **Mr(calc)** | **Delta** | **Miss** | **Score** | **Expect** | **Rank** | **Peptide** |
| --- | --- | --- | --- | --- | --- | --- | --- | --- | --- | --- |
|  | 1551 | 793.4394 | 792.4322 | 792.4382 | -0.0060 | 0 | 16 | 4.3 | 1 | K.VTVYPSK.T |
|  | 3775 | 478.7490 | 955.4834 | 954.4229 | 1.0605 | 0 | 16 | 4 | 1 | R.AAVDTYCR.H |
|  | 7210 | 597.6307 | 1193.2468 | 1192.5625 | 0.6843 | 1 | 1 | 88 | 3 | R.WFRNGQEEK.T |
|  | 8307 | 416.2588 | 1245.7546 | 1244.7129 | 1.0418 | 1 | 3 | 66 | 10 | R.VQPKVTVYPSK.T |
|  | 12023 | 722.8113 | 1443.6081 | 1442.6037 | 1.0044 | 0 | 9 | 15 | 3 | K.FECHFFNGTER.V 12005 |
|  | 12872 | 747.3553 | 1492.6960 | 1492.7059 | -0.0099 | 0 | 50 | 0.0013 | 1 | R.HNYGVGESFTVQR.R |
|  | 19944 | 982.4727 | 1962.9308 | 1962.9435 | -0.0128 | 0 | 84 | 3.5e-007 | 1 | R.AVTELGRPDAEYWNSQK.D 19943 |

|  | **Proteins matching the same set of peptides:** |
| --- | --- |

|  | Q30167\|2B1A_HUMAN    **Mass:** 30397    **Score:** 140    **Queries matched:** 9 |
| --- | --- |
|  | HLA class II histocompatibility antigen, DRB1-10 beta chain OS=Homo sapiens GN=HLA-DRB1 PE=2 SV=1 |

| **316.** | P09104\|ENOG_HUMAN    **Mass:** 47581    **Score:** 140    **Queries matched:** 9   **emPAI:** 0.16 |
| --- | --- |
|  | Gamma-enolase OS=Homo sapiens GN=ENO2 PE=1 SV=3 |

|  | **Query** | **Observed** | **Mr(expt)** | **Mr(calc)** | **Delta** | **Miss** | **Score** | **Expect** | **Rank** | **Peptide** |
| --- | --- | --- | --- | --- | --- | --- | --- | --- | --- | --- |
|  | 2297 | **424.6954** | **847.3763** | **847.4089** | **-0.0326** | **0** | **11** | **15** | **5** | **R.FAGHNFR.N** 2298 2299 |
|  | 14388 | **529.3411** | **1585.0014** | **1585.7810** | **-0.7797** | **0** | **1** | **75** | **9** | **K.IVIGMDVAASEFYR.D** |
|  | 17806 | 902.9704 | 1803.9262 | 1803.9366 | -0.0104 | 0 | 88 | 1.9e-007 | 1 | R.AAVPSGASTGIYEALELR.D |
|  | 19570 | **646.8156** | **1937.4250** | **1937.9743** | **-0.5493** | **0** | **1** | **55** | **5** | **K.LAMQEFMILPVGAESFR.D** |
|  | 23624 | 1177.0768 | 2352.1390 | 2352.1519 | -0.0129 | 0 | 77 | 1.6e-006 | 1 | R.SGETEDTFIADLVVGLCTGQIK.T 23627 |
|  | 24039 | 804.4561 | 2410.3465 | 2411.1799 | -0.8334 | 1 | 3 | 31 | 2 | K.LAMQEFMILPVGAESFRDAMR.L |

| **317.** | P13929\|ENOB_HUMAN    **Mass:** 47244    **Score:** 140    **Queries matched:** 6   **emPAI:** 0.16 |
| --- | --- |
|  | Beta-enolase OS=Homo sapiens GN=ENO3 PE=1 SV=4 |

|  | **Query** | **Observed** | **Mr(expt)** | **Mr(calc)** | **Delta** | **Miss** | **Score** | **Expect** | **Rank** | **Peptide** |
| --- | --- | --- | --- | --- | --- | --- | --- | --- | --- | --- |
|  | 12791 | **745.3743** | **1488.7340** | **1488.7936** | **-0.0596** | **1** | **5** | **38** | **2** | **R.IEEALGDKAIFAGR.K** |
|  | 17806 | 902.9704 | 1803.9262 | 1803.9366 | -0.0104 | 0 | 88 | 1.9e-007 | 1 | R.AAVPSGASTGIYEALELR.D |
|  | 18760 | **468.9846** | **1871.9091** | **1870.9247** | **0.9844** | **1** | **0** | **90** | **6** | **K.VVIGMDVAASEFYRNGK.Y** |
|  | 23624 | 1177.0768 | 2352.1390 | 2352.1519 | -0.0129 | 0 | 77 | 1.6e-006 | 1 | R.SGETEDTFIADLVVGLCTGQIK.T 23627 |
|  | 30243 | **965.2549** | **3856.9904** | **3857.8959** | **-0.9055** | **1** | **8** | **5.4** | **2** | **K.DATNVGDEGGFAPNILENNEALELLKTAIQAAGYPDK.V** |

| **318.** | Q92597\|NDRG1_HUMAN    **Mass:** 43264    **Score:** 138    **Queries matched:** 9   **emPAI:** 0.09 |
| --- | --- |
|  | Protein NDRG1 OS=Homo sapiens GN=NDRG1 PE=1 SV=1 |

|  | **Query** | **Observed** | **Mr(expt)** | **Mr(calc)** | **Delta** | **Miss** | **Score** | **Expect** | **Rank** | **Peptide** |
| --- | --- | --- | --- | --- | --- | --- | --- | --- | --- | --- |
|  | 14188 | **525.1024** | **1572.2853** | **1571.7436** | **0.5417** | **0** | **3** | **48** | **8** | **K.MADCGGLPQISQPAK.L** |
|  | 16540 | **574.3301** | **1719.9684** | **1719.7886** | **0.1798** | **0** | **9** | **14** | **2** | **K.EEMQSNVEVVHTYR.Q** |
|  | 18105 | **913.4008** | **1824.7870** | **1824.7997** | **-0.0128** | **0** | **82** | **5.9e-007** | **1** | **K.YFVQGMGYMPSASMTR.L** 18106 |
|  | 18319 | **614.3257** | **1839.9552** | **1840.7946** | **-0.8394** | **0** | **(3)** | **54** | **3** | **K.YFVQGMGYMPSASMTR.L** |
|  | 22712 | 745.4080 | 2233.2021 | 2232.1130 | 1.0891 | 1 | 1 | 57 | 6 | -.MSREMQDVDLAEVKPLVEK.G |
|  | 22886 | 565.2549 | 2256.9904 | 2257.0152 | -0.0248 | 1 | 1 | 59 | 4 | K.YFVQGMGYMPSASMTRLMR.S |
|  | 29461 | 873.6622 | 3490.6198 | 3490.6359 | -0.0161 | 0 | 5 | 13 | 3 | R.FALNNPEMVEGLVLINVNPCAEGWMDWAASK.I 29462 |

| **319.** | P08195\|4F2_HUMAN    **Mass:** 68180    **Score:** 136    **Queries matched:** 15   **emPAI:** 0.30 |
| --- | --- |
|  | 4F2 cell-surface antigen heavy chain OS=Homo sapiens GN=SLC3A2 PE=1 SV=3 |

|  | **Query** | **Observed** | **Mr(expt)** | **Mr(calc)** | **Delta** | **Miss** | **Score** | **Expect** | **Rank** | **Peptide** |
| --- | --- | --- | --- | --- | --- | --- | --- | --- | --- | --- |
|  | 8675 | **632.8571** | **1263.6997** | **1263.7074** | **-0.0077** | **1** | **14** | **5.8** | **1** | **K.FTGLSKEELLK.V** |
|  | 8998 | 641.3104 | 1280.6062 | 1279.5768 | 1.0294 | 0 | 26 | 0.37 | 1 | R.WCSWSLSQAR.L 8983 |
|  | 11565 | 473.2862 | 1416.8367 | 1416.8452 | -0.0085 | 1 | 39 | 0.018 | 1 | R.LKLEPHEGLLLR.F 11584 |
|  | 12740 | **743.8855** | **1485.7564** | **1485.7674** | **-0.0110** | **1** | **61** | **9.5e-005** | **1** | **K.IKVAEDEAEAAAAAK.F** 12730 |
|  | 15615 | 554.7940 | 1661.3602 | 1660.8533 | 0.5069 | 1 | 2 | 46 | 4 | K.GQSEDPGSLLSLFRR.L |
|  | 16521 | **573.9684** | **1718.8833** | **1719.7920** | **-0.9086** | **0** | **(7)** | **23** | **1** | **K.QPMNAASGAAMSLAGAEK.N** |
|  | 16551 | **431.2414** | **1720.9365** | **1719.7920** | **1.1445** | **0** | **12** | **7.4** | **1** | **K.QPMNAASGAAMSLAGAEK.N** |
|  | 17217 | 885.4147 | 1768.8149 | 1768.8268 | -0.0119 | 0 | 57 | 0.0002 | 1 | R.GENSWFSTQVDTVATK.V |
|  | 22166 | **726.7127** | **2177.1161** | **2177.1270** | **-0.0108** | **1** | **48** | **0.0014** | **1** | **K.VKDALEFWLQAGVDGFQVR.D** |
|  | 22685 | 558.5623 | 2230.2202 | 2231.1038 | -0.8836 | 1 | 1 | 67 | 9 | K.QPMNAASGAAMSLAGAEKNGLVK.I |
|  | 28468 | 799.9027 | 3195.5817 | 3196.4275 | -0.8458 | 1 | 0 | 43 | 10 | K.NAEVTGTMSQDTEVDMKEVELNELEPEK.Q 28481 |

| **320.** | P02649\|APOE_HUMAN    **Mass:** 36246    **Score:** 135    **Queries matched:** 16   **emPAI:** 0.64 |
| --- | --- |
|  | Apolipoprotein E OS=Homo sapiens GN=APOE PE=1 SV=1 |

|  | **Query** | **Observed** | **Mr(expt)** | **Mr(calc)** | **Delta** | **Miss** | **Score** | **Expect** | **Rank** | **Peptide** |
| --- | --- | --- | --- | --- | --- | --- | --- | --- | --- | --- |
|  | 5781 | **550.2506** | **1098.4867** | **1097.4594** | **1.0273** | **1** | **12** | **9** | **3** | **R.ARMEEMGSR.T** 5769 |
|  | 7058 | **396.2281** | **1185.6624** | **1185.6353** | **0.0271** | **1** | **11** | **13** | **5** | **R.LLRDADDLQK.R** 7057 |
|  | 7821 | 611.7589 | 1221.5031 | 1221.5118 | -0.0087 | 0 | 33 | 0.072 | 1 | R.LGADMEDVCGR.L |
|  | 9591 | **657.3574** | **1312.7003** | **1312.7099** | **-0.0096** | **1** | **61** | **0.00012** | **1** | **R.AKLEEQAQQIR.L** 9593 |
|  | 12942 | **749.3995** | **1496.7845** | **1496.7947** | **-0.0102** | **0** | **45** | **0.0037** | **1** | **R.AATVGSLAGQPLQER.A** |
|  | 14956 | **810.8978** | **1619.7811** | **1619.7904** | **-0.0093** | **0** | **50** | **0.0013** | **1** | **K.VQAAVGTSAAPVPSDNH.-** |
|  | 15347 | **824.3986** | **1646.7827** | **1646.7934** | **-0.0107** | **0** | **46** | **0.0029** | **1** | **R.GEVQAMLGQSTEELR.V** |
|  | 15641 | 555.1686 | 1662.4839 | 1662.7883 | -0.3044 | 0 | (7) | 17 | 6 | R.GEVQAMLGQSTEELR.V |
|  | 16705 | **865.9329** | **1729.8512** | **1729.8370** | **0.0142** | **0** | **20** | **1.1** | **2** | **K.SELEEQLTPVAEETR.A** 16706 |
|  | 19073 | **475.2195** | **1896.8488** | **1896.8822** | **-0.0334** | **1** | **8** | **15** | **1** | **R.LGADMEDVCGRLVQYR.G** |
|  | 21490 | **527.2919** | **2105.1384** | **2104.9630** | **0.1754** | **1** | **4** | **34** | **7** | **K.ELQAAQARLGADMEDVCGR.L** |
|  | 26204 | **910.7959** | **2729.3659** | **2729.3872** | **-0.0213** | **0** | **16** | **1.5** | **1** | **R.WVQTLSEQVQEELLSSQVTQELR.A** |

| **321.** | Q92928\|RAB1C_HUMAN    **Mass:** 22231    **Score:** 134    **Queries matched:** 16   **emPAI:** 0.89 |
| --- | --- |
|  | Putative Ras-related protein Rab-1C OS=Homo sapiens GN=RAB1C PE=5 SV=2 |

|  | **Query** | **Observed** | **Mr(expt)** | **Mr(calc)** | **Delta** | **Miss** | **Score** | **Expect** | **Rank** | **Peptide** |
| --- | --- | --- | --- | --- | --- | --- | --- | --- | --- | --- |
|  | 1162 | 643.2711 | 642.2638 | 642.4064 | -0.1427 | 0 | 27 | 0.41 | 1 | K.LLVGNK.S |
|  | 5394 | 536.3203 | 1070.6261 | 1070.6336 | -0.0075 | 0 | 45 | 0.006 | 1 | K.LLLIGDSGVGK.S 5392 |
|  | 5639 | 544.8228 | 1087.6309 | 1086.5458 | 1.0852 | 0 | 10 | 19 | 9 | K.QWLQEIDR.H |
|  | 9139 | 430.2988 | 1287.8745 | 1287.7398 | 0.1347 | 1 | 2 | 68 | 10 | K.LLVGNKSDLTTK.K |
|  | 9695 | 658.8284 | 1315.6422 | 1315.6521 | -0.0099 | 0 | 18 | 2.6 | 1 | K.LQIWDTAGQER.F 9685 |
|  | 10505 | **679.8719** | **1357.7293** | **1357.7817** | **-0.0523** | **1** | **1** | **1e+002** | **6** | **K.IQTIELDGKTIK.L** |
|  | 14383 | 793.4395 | 1584.8645 | 1584.7678 | 0.0966 | 0 | 5 | 41 | 8 | K.QMGPGAASGGERPNLK.I |
|  | 16601 | 862.9358 | 1723.8570 | 1723.8669 | -0.0098 | 0 | 35 | 0.033 | 1 | K.EFADSLGIPFLETSAK.N 16602 |
|  | 18924 | 943.4422 | 1884.8698 | 1883.8757 | 0.9941 | 0 | 74 | 4e-006 | 2 | K.NATNVEQAFMTMAAEIK.K 18915 |
|  | 19106 | 951.0158 | 1900.0169 | 1899.8706 | 0.1463 | 0 | (3) | 52 | 4 | K.NATNVEQAFMTMAAEIK.K 19117 |
|  | 28320 | **792.4089** | **3165.6064** | **3164.5441** | **1.0622** | **1** | **2** | **29** | **2** | **R.FADDPYTESYISTIGVDFKIQTIELDGK.T** |

| **322.** | Q09160\|1A80_HUMAN    **Mass:** 41051    **Score:** 131    **Queries matched:** 19   **emPAI:** 0.55 |
| --- | --- |
|  | HLA class I histocompatibility antigen, A-80 alpha chain OS=Homo sapiens GN=HLA-A PE=2 SV=1 |

|  | **Query** | **Observed** | **Mr(expt)** | **Mr(calc)** | **Delta** | **Miss** | **Score** | **Expect** | **Rank** | **Peptide** |
| --- | --- | --- | --- | --- | --- | --- | --- | --- | --- | --- |
|  | 1793 | 406.5443 | 811.0741 | 810.4500 | 0.6241 | 1 | 3 | 49 | 10 | R.FLRGYR.Q |
|  | 1878 | **409.7938** | **817.5730** | **816.3986** | **1.1744** | **0** | **5** | **69** | **1** | **-.MAVMPPR.T** |
|  | 9699 | 658.8362 | 1315.6579 | 1315.6673 | -0.0094 | 0 | 14 | 6.2 | 2 | R.YFFTSVSRPGR.G |
|  | 10335 | 675.8494 | 1349.6843 | 1349.6939 | -0.0096 | 1 | 38 | 0.024 | 1 | R.YLENGKETLQR.T 10311 10351 |
|  | 13637 | **513.3106** | **1536.9098** | **1536.7355** | **0.1744** | **1** | **2** | **76** | **3** | **R.AYLEGECVDGLRR.Y** |
|  | 13806 | 775.3702 | 1548.7259 | 1548.7718 | -0.0459 | 1 | 44 | 0.0053 | 1 | R.SWTAADMAAQITKR.K |
|  | 14092 | 522.7712 | 1565.2917 | 1564.7668 | 0.5250 | 1 | (8) | 12 | 3 | R.SWTAADMAAQITKR.K |
|  | 18889 | 628.3002 | 1881.8787 | 1881.8904 | -0.0117 | 0 | 24 | 0.42 | 1 | K.THMTHHPISDHEATLR.C |
|  | 20871 | 410.6170 | 2048.0485 | 2049.0102 | -0.9617 | 0 | 9 | 11 | 2 | R.YTCHVQHEGLPEPLTLR.W 20872 20886 20897 |
|  | 25378 | 866.1122 | 2595.3149 | 2595.2639 | 0.0510 | 1 | 1 | 48 | 1 | K.DYIALNEDLRSWTAADMAAQITK.R |
|  | 25578 | 1319.0889 | 2636.1632 | 2636.1838 | -0.0206 | 0 | 57 | 0.00013 | 1 | R.DGEDQTQDTELVETRPAGDGTFQK.W 25579 25580 25581 |

| **323.** | O95782\|AP2A1_HUMAN    **Mass:** 108570   **Score:** 131    **Queries matched:** 33   **emPAI:** 0.22 |
| --- | --- |
|  | AP-2 complex subunit alpha-1 OS=Homo sapiens GN=AP2A1 PE=1 SV=2 |

|  | **Query** | **Observed** | **Mr(expt)** | **Mr(calc)** | **Delta** | **Miss** | **Score** | **Expect** | **Rank** | **Peptide** |
| --- | --- | --- | --- | --- | --- | --- | --- | --- | --- | --- |
|  | 2819 | 443.0392 | 884.0637 | 883.4763 | 0.5874 | 1 | 22 | 0.67 | 1 | K.AQEPPKSK.K 2807 |
|  | 4646 | 509.2560 | 1016.4975 | 1016.5073 | -0.0098 | 1 | 27 | 0.37 | 1 | M.PAVSKGDGMR.G |
|  | 4700 | 511.3149 | 1020.6153 | 1020.6219 | -0.0066 | 0 | 14 | 7.4 | 1 | R.ALLLSTYIK.F |
|  | 5371 | 536.2676 | 1070.5206 | 1069.6243 | 0.8963 | 1 | 23 | 0.85 | 1 | R.INKELANIR.S 5386 |
|  | 5423 | 537.3097 | 1072.6048 | 1071.5309 | 1.0740 | 0 | 29 | 0.24 | 1 | R.NADVELQQR.A 5409 |
|  | 6457 | 575.1019 | 1148.1892 | 1147.5478 | 0.6414 | 1 | 1 | 74 | 10 | -.MPAVSKGDGMR.G |
|  | 6717 | 583.3302 | 1164.6458 | 1163.5427 | 1.1031 | 1 | (1) | 1.2e+002 | 6 | -.MPAVSKGDGMR.G |
|  | 7365 | **400.7322** | **1199.1749** | **1199.5895** | **-0.4146** | **1** | **7** | **19** | **3** | **K.KGPGAGSALDDGR.R** |
|  | 7633 | **405.1925** | **1212.5558** | **1211.5605** | **0.9953** | **0** | **8** | **24** | **3** | **K.ANHPMDAEVTK.A** |
|  | 8061 | **617.8168** | **1233.6190** | **1233.6275** | **-0.0085** | **0** | **9** | **20** | **1** | **R.ILVAGDSMDSVK.Q** |
|  | 9495 | **654.2715** | **1306.5284** | **1305.6023** | **0.9261** | **0** | **22** | **0.84** | **1** | **R.EMGEAFAADIPR.I** |
|  | 9838 | **661.7555** | **1321.4964** | **1321.5972** | **-0.1008** | **0** | **(2)** | **68** | **4** | **R.EMGEAFAADIPR.I** |
|  | 11631 | **711.3857** | **1420.7568** | **1420.7827** | **-0.0259** | **1** | **9** | **16** | **4** | **R.FRYGGAPQALTLK.L** |
|  | 11712 | **476.1944** | **1425.5613** | **1425.7616** | **-0.2003** | **0** | **7** | **26** | **2** | **R.SSPPVQFSLLHSK.F** |
|  | 12473 | 491.2423 | 1470.7051 | 1470.7150 | -0.0099 | 0 | 26 | 0.31 | 1 | R.ACNQLGQFLQHR.E |
|  | 13766 | **516.3099** | **1545.9078** | **1544.7293** | **1.1785** | **0** | **5** | **35** | **4** | **K.ASPDLVPMGEWTAR.V** |
|  | 14347 | **792.3510** | **1582.6875** | **1582.8467** | **-0.1592** | **1** | **2** | **66** | **8** | **R.WKQLSLPQQEAQK.I** |
|  | 14996 | 541.8044 | 1622.3913 | 1621.8246 | 0.5667 | 1 | 2 | 51 | 2 | K.GDGMRGLAVFISDIR.N |
|  | 18650 | **931.9250** | **1861.8354** | **1861.8458** | **-0.0103** | **0** | **(40)** | **0.01** | **1** | **K.FFQPTEMAAQDFFQR.W** |
|  | 18839 | 939.9220 | 1877.8294 | 1877.8407 | -0.0112 | 0 | 48 | 0.0016 | 2 | K.FFQPTEMAAQDFFQR.W |
|  | 18997 | 630.6707 | 1888.9901 | 1889.0007 | -0.0105 | 1 | 18 | 1.6 | 1 | R.VLQIVTNRDDVQGYAAK.T |
|  | 22930 | 1132.0070 | 2261.9994 | 2262.1712 | -0.1718 | 1 | 5 | 22 | 3 | R.ILVAGDSMDSVKQSAALCLLR.L |
|  | 27081 | **1450.2545** | **2898.4945** | **2898.5127** | **-0.0182** | **0** | **73** | **2.5e-006** | **1** | **K.LLGFGSALLDNVDPNPENFVGAGIIQTK.A** |
|  | 30124 | 1263.9780 | 3788.9123 | 3788.8654 | 0.0468 | 1 | 3 | 16 | 3 | K.TVFEALQAPACHENMVKVGGYILGEFGNLIAGDPR.S 30113 30118 30120 30121 30126 |
|  | 30306 | **1298.2665** | **3891.7776** | **3892.0067** | **-0.2291** | **1** | **1** | **24** | **5** | **R.AVEYLTLSSVASTDVLATVLEEMPPFPERESSILAK.L** |

| **324.** | O14653\|GOSR2_HUMAN    **Mass:** 24874    **Score:** 130    **Queries matched:** 10   **emPAI:** 0.33 |
| --- | --- |
|  | Golgi SNAP receptor complex member 2 OS=Homo sapiens GN=GOSR2 PE=1 SV=2 |

|  | **Query** | **Observed** | **Mr(expt)** | **Mr(calc)** | **Delta** | **Miss** | **Score** | **Expect** | **Rank** | **Peptide** |
| --- | --- | --- | --- | --- | --- | --- | --- | --- | --- | --- |
|  | 2871 | 445.2596 | 888.5046 | 887.5440 | 0.9606 | 1 | 4 | 76 | 8 | R.LTLKGTQK.K |
|  | 5975 | **557.0919** | **1112.1693** | **1112.5615** | **-0.3922** | **0** | **4** | **42** | **3** | **M.DPLFQQTHK.Q** |
|  | 8615 | **421.0573** | **1260.1500** | **1259.5969** | **0.5531** | **0** | **2** | **68** | **10** | **-.MDPLFQQTHK.Q** |
|  | 17130 | **880.9691** | **1759.9237** | **1759.9324** | **-0.0087** | **0** | **93** | **5.3e-008** | **1** | **K.ILDIANMLGLSNTVMR.L** 17129 |
|  | 17361 | **593.3218** | **1776.9437** | **1775.9274** | **1.0163** | **0** | **(2)** | **64** | **6** | **K.ILDIANMLGLSNTVMR.L** |
|  | 17640 | 897.4449 | 1792.8753 | 1791.9223 | 0.9531 | 0 | (2) | 71 | 6 | K.ILDIANMLGLSNTVMR.L |
|  | 22533 | **739.3519** | **2215.0338** | **2216.1008** | **-1.0670** | **0** | **3** | **36** | **2** | **K.VHNGMDDLILDGHNILDGLR.T** |
|  | 25323 | **1293.1492** | **2584.2838** | **2585.1937** | **-0.9100** | **1** | **0** | **65** | **6** | **-.MDPLFQQTHKQVHEIQSCMGR.L** |
|  | 27412 | **743.3805** | **2969.4931** | **2969.5094** | **-0.0163** | **1** | **18** | **0.78** | **1** | **R.LETADKQSVHIVENEIQASIDQIFSR.L** |

| **325.** | Q9P0L0\|VAPA_HUMAN    **Mass:** 28103    **Score:** 129    **Queries matched:** 35   **emPAI:** 1.14 |
| --- | --- |
|  | Vesicle-associated membrane protein-associated protein A OS=Homo sapiens GN=VAPA PE=1 SV=3 |

|  | **Query** | **Observed** | **Mr(expt)** | **Mr(calc)** | **Delta** | **Miss** | **Score** | **Expect** | **Rank** | **Peptide** |
| --- | --- | --- | --- | --- | --- | --- | --- | --- | --- | --- |
|  | 1034 | **589.3373** | **588.3300** | **588.2867** | **0.0433** | **0** | **2** | **2.1e+002** | **2** | **R.DEGLR.L** |
|  | 1623 | **799.4621** | **798.4548** | **798.4599** | **-0.0051** | **0** | **15** | **5.4** | **1** | **K.AVPLNASK.Q** |
|  | 2115 | **418.7009** | **835.3873** | **835.3932** | **-0.0059** | **0** | **16** | **4.8** | **2** | **R.LQGEMMK.L** 2114 |
|  | 2418 | **429.2300** | **856.4454** | **856.4515** | **-0.0061** | **1** | **13** | **10** | **5** | **K.LRNPSDR.K** |
|  | 2592 | **435.2686** | **868.5226** | **867.3830** | **1.1396** | **0** | **(9)** | **19** | **1** | **R.LQGEMMK.L** |
|  | 3317 | **462.2278** | **922.4411** | **923.4204** | **-0.9794** | **0** | **(5)** | **55** | **5** | **-.MASASGAMAK.H** |
|  | 3545 | **471.2341** | **940.4536** | **939.4154** | **1.0382** | **0** | **10** | **15** | **10** | **-.MASASGAMAK.H** |
|  | 4274 | **496.7513** | **991.4880** | **991.4943** | **-0.0063** | **1** | **18** | **2.4** | **1** | **K.RLQGEMMK.L** |
|  | 4323 | **498.2684** | **994.5222** | **994.5308** | **-0.0086** | **1** | **30** | **0.14** | **1** | **R.HLRDEGLR.L** 4324 |
|  | 6546 | **577.2976** | **1152.5805** | **1152.5999** | **-0.0194** | **1** | **7** | **29** | **3** | **K.LSEENRHLR.D** |
|  | 8635 | **631.7956** | **1261.5766** | **1261.5860** | **-0.0094** | **0** | **12** | **9** | **1** | **K.EAKPDELMDSK.L** 8620 |
|  | 10969 | **691.7844** | **1381.5542** | **1381.5642** | **-0.0101** | **0** | **20** | **1.5** | **1** | **R.CVFEMPNENDK.L** |
|  | 11230 | **699.9178** | **1397.8210** | **1397.5591** | **0.2619** | **0** | **(2)** | **90** | **4** | **R.CVFEMPNENDK.L** |
|  | 13531 | **511.2577** | **1530.7512** | **1530.7711** | **-0.0200** | **1** | **7** | **27** | **4** | **K.EAKPDELMDSKLR.C** 13518 13543 |
|  | 13768 | **516.3293** | **1545.9662** | **1546.7661** | **-0.7998** | **1** | **(6)** | **24** | **5** | **K.EAKPDELMDSKLR.C** |
|  | 14101 | **783.9244** | **1565.8343** | **1565.8454** | **-0.0110** | **1** | **32** | **0.078** | **1** | **K.FKGPFTDVVTTNLK.L** |
|  | 14908 | **809.4402** | **1616.8658** | **1616.8774** | **-0.0115** | **0** | **44** | **0.0044** | **1** | **K.HEQILVLDPPTDLK.F** 14909 14910 |
|  | 16443 | **572.3476** | **1714.0210** | **1712.8767** | **1.1443** | **1** | **1** | **77** | **8** | **K.LNDMEPSKAVPLNASK.Q** |
|  | 17304 | **592.6351** | **1774.8834** | **1774.8962** | **-0.0128** | **1** | **48** | **0.0017** | **1** | **R.KVAHSDKPGSTSTASFR.D** |
|  | 21524 | 528.0024 | 2107.9804 | 2107.9957 | -0.0153 | 0 | 12 | 5.9 | 1 | K.QDGPMPKPHSVSLNDTETR.K |
|  | 23315 | **1156.5515** | **2311.0885** | **2311.1017** | **-0.0132** | **0** | **38** | **0.013** | **1** | **K.FMVQTIFAPPNTSDMEAVWK.E** 23314 23316 23317 |
|  | 23318 | **771.3713** | **2311.0920** | **2311.9759** | **-0.8839** | **1** | **5** | **27** | **1** | **R.CVFEMPNENDKLNDMEPSK.A** |
|  | 29729 | 897.9603 | 3587.8120 | 3586.6670 | 1.1450 | 1 | (2) | 25 | 3 | K.FMVQTIFAPPNTSDMEAVWKEAKPDELMDSK.L |
|  | 29741 | **1200.2382** | **3597.6927** | **3597.7120** | **-0.0193** | **0** | **15** | **1.1** | **1** | **R.YCVRPNSGIIDPGSTVTVSVMLQPFDYDPNEK.S** |
|  | 29744 | **901.6545** | **3602.5891** | **3602.6619** | **-0.0728** | **1** | **3** | **15** | **8** | **K.FMVQTIFAPPNTSDMEAVWKEAKPDELMDSK.L** |

| **326.** | P30511\|HLAF_HUMAN    **Mass:** 39255    **Score:** 127    **Queries matched:** 11   **emPAI:** 0.31 |
| --- | --- |
|  | HLA class I histocompatibility antigen, alpha chain F OS=Homo sapiens GN=HLA-F PE=2 SV=2 |

|  | **Query** | **Observed** | **Mr(expt)** | **Mr(calc)** | **Delta** | **Miss** | **Score** | **Expect** | **Rank** | **Peptide** |
| --- | --- | --- | --- | --- | --- | --- | --- | --- | --- | --- |
|  | 6565 | 577.7809 | 1153.5473 | 1153.6091 | -0.0618 | 1 | 3 | 67 | 9 | K.ETLQRADPPK.A |
|  | 10335 | 675.8494 | 1349.6843 | 1349.6939 | -0.0096 | 1 | 38 | 0.024 | 1 | R.YLENGKETLQR.A 10311 10351 |
|  | 11736 | 714.3626 | 1426.7105 | 1426.7205 | -0.0099 | 0 | 46 | 0.0031 | 1 | K.WAAVVVPSGEEQR.Y 11721 11735 11751 |
|  | 21557 | 1056.5175 | 2111.0204 | 2111.0299 | -0.0095 | 0 | 86 | 2.1e-007 | 1 | R.CWALGFYPAEITLTWQR.D 21558 |
|  | 25651 | **663.6119** | **2650.4184** | **2650.1995** | **0.2190** | **0** | **1** | **45** | **10** | **R.DGEEQTQDTELVETRPAGDGTFQK.W** |

| **327.** | P21964\|COMT_HUMAN    **Mass:** 30474    **Score:** 127    **Queries matched:** 18   **emPAI:** 0.60 |
| --- | --- |
|  | Catechol O-methyltransferase OS=Homo sapiens GN=COMT PE=1 SV=2 |

|  | **Query** | **Observed** | **Mr(expt)** | **Mr(calc)** | **Delta** | **Miss** | **Score** | **Expect** | **Rank** | **Peptide** |
| --- | --- | --- | --- | --- | --- | --- | --- | --- | --- | --- |
|  | 5615 | **543.9191** | **1085.8237** | **1086.5968** | **-0.7731** | **1** | **2** | **76** | **5** | **R.MARLLSPGAR.L** |
|  | 5931 | **555.1694** | **1108.3243** | **1108.5587** | **-0.2344** | **1** | **5** | **32** | **2** | **R.MVDFAGVKDK.V** |
|  | 6425 | **573.7891** | **1145.5637** | **1145.5717** | **-0.0080** | **1** | **31** | **0.12** | **1** | **K.AIYKGPGSEAGP.-** 6424 |
|  | 6880 | **589.2829** | **1176.5512** | **1176.5597** | **-0.0085** | **1** | **12** | **10** | **1** | **K.EWAMNVGDKK.G** |
|  | 15914 | **840.9932** | **1679.9718** | **1679.9822** | **-0.0104** | **0** | **20** | **0.98** | **1** | **K.VTLVVGASQDIIPQLK.K** 15896 |
|  | 17808 | 902.9733 | 1803.9321 | 1803.9440 | -0.0119 | 0 | 60 | 0.00011 | 1 | R.YLPDTLLLEECGLLR.K 17809 |
|  | 18146 | 914.4820 | 1826.9494 | 1826.9560 | -0.0066 | 0 | 38 | 0.017 | 2 | R.LITIEINPDCAAITQR.M 18144 18145 |
|  | 19378 | 962.4416 | 1922.8687 | 1923.1041 | -0.2353 | 1 | 1 | 73 | 8 | K.DKVTLVVGASQDIIPQLK.K |
|  | 21187 | **693.0281** | **2076.0626** | **2075.0721** | **0.9905** | **1** | **16** | **2.2** | **2** | **K.DRYLPDTLLLEECGLLR.K** |
|  | 22105 | **724.6330** | **2170.8772** | **2169.8884** | **0.9887** | **0** | **6** | **18** | **1** | **R.GSSCFECTHYQSFLEYR.E** |
|  | 23496 | **779.0744** | **2334.2014** | **2334.2155** | **-0.0141** | **0** | **0** | **72** | **2** | **K.GTVLLADNVICPGAPDFLAHVR.G** |
|  | 28381 | **795.1412** | **3176.5359** | **3176.5560** | **-0.0202** | **0** | **10** | **4.5** | **1** | **R.ILNHVLQHAEPGNAQSVLEAIDTYCEQK.E** |
|  | 30731 | **1053.0050** | **4207.9909** | **4207.0102** | **0.9807** | **1** | **31** | **0.02** | **1** | **R.ILNHVLQHAEPGNAQSVLEAIDTYCEQKEWAMNVGDK.K** |

| **328.** | P17213\|BPI_HUMAN    **Mass:** 54093    **Score:** 126    **Queries matched:** 16   **emPAI:** 0.22 |
| --- | --- |
|  | Bactericidal permeability-increasing protein OS=Homo sapiens GN=BPI PE=1 SV=4 |

|  | **Query** | **Observed** | **Mr(expt)** | **Mr(calc)** | **Delta** | **Miss** | **Score** | **Expect** | **Rank** | **Peptide** |
| --- | --- | --- | --- | --- | --- | --- | --- | --- | --- | --- |
|  | 4276 | 496.7641 | 991.5136 | 992.5291 | -1.0154 | 0 | 15 | 5.9 | 1 | K.FSISNANIK.I 4296 |
|  | 4320 | **498.2138** | **994.4131** | **994.4212** | **-0.0081** | **0** | **17** | **2.8** | **1** | **K.MNSQVCEK.V** |
|  | 8066 | 412.2242 | 1233.6509 | 1234.6050 | -0.9541 | 1 | 9 | 18 | 1 | K.MTLRDDMIPK.E |
|  | 8517 | **628.8375** | **1255.6605** | **1254.6649** | **0.9956** | **0** | **29** | **0.19** | **1** | **K.FFGTFLPEVAK.K** |
|  | 9878 | **442.2443** | **1323.7111** | **1323.7663** | **-0.0552** | **1** | **6** | **31** | **9** | **K.LQKGFPLPTPAR.V** 9860 |
|  | 10776 | **686.8500** | **1371.6855** | **1371.6136** | **0.0720** | **1** | **4** | **55** | **8** | **R.ENMARGPCNAPR.W** |
|  | 13840 | **775.8882** | **1549.7618** | **1549.7736** | **-0.0118** | **0** | **18** | **2.2** | **1** | **K.GLDYASQQGTAALQK.E** |
|  | 23874 | **1193.0974** | **2384.1803** | **2384.1934** | **-0.0131** | **0** | **64** | **3.2e-005** | **1** | **K.VTNSVSSELQPYFQTLPVMTK.I** |
|  | 26414 | **1387.7101** | **2773.4056** | **2773.4208** | **-0.0152** | **0** | **54** | **0.00025** | **1** | **K.IDSVAGINYGLVAPPATTAETLDVQMK.G** 26417 |
|  | 26567 | **933.4714** | **2797.3923** | **2797.4037** | **-0.0114** | **0** | **44** | **0.0026** | **1** | **R.MVYLGLSDYFFNTAGLVYQEAGVLK.M** |
|  | 27387 | 988.5053 | 2962.4939 | 2963.5426 | -1.0487 | 1 | 2 | 38 | 5 | R.EFQLPSSQISMVPNVGLKFSISNANIK.I |
|  | 31377 | **1735.1232** | **5202.3477** | **5203.4683** | **-1.1206** | **1** | **3** | **6.5** | **1** | **K.MSGNFDLSIEGMSISADLKLGSNPTSGKPTITCSSCSSHINSVHVHISK.S** 31376 |

| **329.** | P61006\|RAB8A_HUMAN    **Mass:** 23824    **Score:** 126    **Queries matched:** 19   **emPAI:** 1.43 |
| --- | --- |
|  | Ras-related protein Rab-8A OS=Homo sapiens GN=RAB8A PE=1 SV=1 |

|  | **Query** | **Observed** | **Mr(expt)** | **Mr(calc)** | **Delta** | **Miss** | **Score** | **Expect** | **Rank** | **Peptide** |
| --- | --- | --- | --- | --- | --- | --- | --- | --- | --- | --- |
|  | 2003 | **415.2057** | **828.3968** | **828.3687** | **0.0280** | **0** | **11** | **17** | **1** | **K.FMETSAK.A** 2001 2004 |
|  | 5394 | 536.3203 | 1070.6261 | 1070.6336 | -0.0075 | 0 | 45 | 0.006 | 1 | K.LLLIGDSGVGK.T 5392 |
|  | 9253 | **432.2090** | **1293.6050** | **1294.6267** | **-1.0217** | **1** | **3** | **68** | **5** | **-.MAKTYDYLFK.L** |
|  | 9695 | 658.8284 | 1315.6422 | 1315.6521 | -0.0099 | 0 | 18 | 2.6 | 1 | K.LQIWDTAGQER.F 9685 |
|  | 10160 | **670.8881** | **1339.7617** | **1340.6208** | **-0.8591** | **0** | **2** | **92** | **3** | **R.NIEEHASADVEK.M** |
|  | 11495 | **707.8443** | **1413.6740** | **1413.6848** | **-0.0108** | **0** | **58** | **0.00019** | **1** | **K.LEGNSPQGSNQGVK.I** |
|  | 13705 | **514.9953** | **1541.9641** | **1541.7798** | **0.1843** | **1** | **4** | **42** | **4** | **K.KLEGNSPQGSNQGVK.I** |
|  | 13956 | 779.4173 | 1556.8200 | 1556.8311 | -0.0110 | 1 | 32 | 0.081 | 1 | R.IKLQIWDTAGQER.F 13955 13957 |
|  | 14281 | **790.4106** | **1578.8066** | **1578.8154** | **-0.0088** | **0** | **28** | **0.16** | **1** | **K.ANINVENAFFTLAR.D** |
|  | 15483 | **827.9073** | **1653.8001** | **1653.8106** | **-0.0104** | **0** | **75** | **3.7e-006** | **1** | **R.GAMGIMLVYDITNEK.S** 15501 |
|  | 20487 | **672.3746** | **2014.1019** | **2012.9837** | **1.1182** | **1** | **6** | **21** | **2** | **R.NIEEHASADVEKMILGNK.C** |
|  | 23895 | **598.0458** | **2388.1540** | **2389.1736** | **-1.0196** | **1** | **1** | **53** | **1** | **K.FMETSAKANINVENAFFTLAR.D** |

| **330.** | P31949\|S10AB_HUMAN    **Mass:** 11847    **Score:** 126    **Queries matched:** 5   **emPAI:** 1.37 |
| --- | --- |
|  | Protein S100-A11 OS=Homo sapiens GN=S100A11 PE=1 SV=2 |

|  | **Query** | **Observed** | **Mr(expt)** | **Mr(calc)** | **Delta** | **Miss** | **Score** | **Expect** | **Rank** | **Peptide** |
| --- | --- | --- | --- | --- | --- | --- | --- | --- | --- | --- |
|  | 18465 | **925.4764** | **1848.9383** | **1848.8968** | **0.0416** | **0** | **60** | **0.00011** | **1** | **K.TEFLSFMNTELAAFTK.N** 18471 |
|  | 29310 | **1143.5381** | **3427.5924** | **3427.6064** | **-0.0140** | **0** | **61** | **3.1e-005** | **1** | **K.LDTNSDGQLDFSEFLNLIGGLAMACHDSFLK.A** |
|  | 29618 | **889.9294** | **3555.6884** | **3555.7014** | **-0.0130** | **1** | **42** | **0.0024** | **1** | **K.KLDTNSDGQLDFSEFLNLIGGLAMACHDSFLK.A** 29617 |

| **331.** | P33897\|ABCD1_HUMAN    **Mass:** 83398    **Score:** 126    **Queries matched:** 15   **emPAI:** 0.04 |
| --- | --- |
|  | ATP-binding cassette sub-family D member 1 OS=Homo sapiens GN=ABCD1 PE=1 SV=2 |

|  | **Query** | **Observed** | **Mr(expt)** | **Mr(calc)** | **Delta** | **Miss** | **Score** | **Expect** | **Rank** | **Peptide** |
| --- | --- | --- | --- | --- | --- | --- | --- | --- | --- | --- |
|  | 3157 | **456.8823** | **911.7500** | **910.5600** | **1.1900** | **1** | **0** | **93** | **7** | **R.VEGPLKIR.G** |
|  | 4916 | **519.2632** | **1036.5118** | **1035.5349** | **0.9769** | **1** | **3** | **70** | **9** | **K.FEKLDSAAR.L** |
|  | 5372 | **536.2705** | **1070.5265** | **1071.5937** | **-1.0673** | **1** | **6** | **49** | **1** | **R.SRLVAHAYR.L** |
|  | 11311 | **702.3367** | **1402.6588** | **1402.6688** | **-0.0100** | **0** | **19** | **1.7** | **1** | **R.ELEDAQAGSGTIGR.S** |
|  | 12903 | 748.3705 | 1494.7265 | 1495.8147 | -1.0882 | 1 | 9 | 15 | 7 | R.TFLSVYVARLDGR.L |
|  | 13622 | **769.3817** | **1536.7489** | **1536.8624** | **-0.1135** | **0** | **5** | **37** | **4** | **R.ETGLLALHSAALVSR.T** |
|  | 15396 | 825.3978 | 1648.7810 | 1649.8413 | -1.0603 | 1 | 9 | 15 | 5 | R.AFSPKFGELVAEEAR.R 15416 |
|  | 17513 | **893.9513** | **1785.8880** | **1786.8427** | **-0.9547** | **0** | **1** | **92** | **4** | **K.YHTHLLQFDGEGGWK.F** |
|  | 17919 | **604.9623** | **1811.8652** | **1810.8706** | **0.9946** | **0** | **1** | **72** | **3** | **R.VEEGMHLLITGPNGCGK.S** |
|  | 18516 | **926.9671** | **1851.9196** | **1851.9326** | **-0.0130** | **0** | **82** | **6.8e-007** | **1** | **R.GLQAPAGEPTQEASGVAAAK.A** 18515 18522 |
|  | 25641 | 883.4700 | 2647.3883 | 2648.3340 | -0.9458 | 1 | 1 | 49 | 2 | R.QCLAPARGLQAPAGEPTQEASGVAAAK.A |
|  | 27296 | **589.8774** | **2944.3505** | **2944.4099** | **-0.0594** | **1** | **5** | **18** | **1** | **R.MFYHRPKYALLDECTSAVSIDVEGK.I** |

| **332.** | P49755\|TMEDA_HUMAN    **Mass:** 25131    **Score:** 125    **Queries matched:** 10   **emPAI:** 0.15 |
| --- | --- |
|  | Transmembrane emp24 domain-containing protein 10 OS=Homo sapiens GN=TMED10 PE=1 SV=2 |

|  | **Query** | **Observed** | **Mr(expt)** | **Mr(calc)** | **Delta** | **Miss** | **Score** | **Expect** | **Rank** | **Peptide** |
| --- | --- | --- | --- | --- | --- | --- | --- | --- | --- | --- |
|  | 4217 | **495.0602** | **988.1059** | **987.4808** | **0.6252** | **0** | **1** | **1e+002** | **9** | **-.MSGLSGPPAR.R** |
|  | 6384 | **572.3725** | **1142.7304** | **1143.5819** | **-0.8514** | **1** | **6** | **40** | **7** | **-.MSGLSGPPARR.G** |
|  | 9423 | 652.3311 | 1302.6475 | 1303.6772 | -1.0297 | 0 | 27 | 0.31 | 1 | K.ITDSAGHILYSK.E |
|  | 16624 | 576.2828 | 1725.8265 | 1726.7064 | -0.8799 | 1 | 2 | 66 | 6 | R.EEEMRDTNESTNTR.V |
|  | 19031 | **631.8248** | **1892.4527** | **1892.1764** | **0.2763** | **1** | **1** | **57** | **2** | **R.RGPFPLALLLLFLLGPR.L** |
|  | 20353 | 1001.0119 | 2000.0092 | 2000.9765 | -0.9672 | 1 | (0) | 91 | 7 | R.LEDLSESIVNDFAYMKK.R |
|  | 20505 | 1008.9910 | 2015.9674 | 2016.9714 | -1.0040 | 1 | 7 | 18 | 2 | R.LEDLSESIVNDFAYMKK.R |
|  | 20662 | **1015.4937** | **2028.9727** | **2028.9826** | **-0.0099** | **1** | **90** | **8.6e-008** | **1** | **R.RLEDLSESIVNDFAYMK.K** 20661 |
|  | 20836 | **682.8336** | **2045.4789** | **2044.9775** | **0.5013** | **1** | **(4)** | **28** | **5** | **R.RLEDLSESIVNDFAYMK.K** |

| **333.** | P09211\|GSTP1_HUMAN    **Mass:** 23569    **Score:** 125    **Queries matched:** 8   **emPAI:** 0.16 |
| --- | --- |
|  | Glutathione S-transferase P OS=Homo sapiens GN=GSTP1 PE=1 SV=2 |

|  | **Query** | **Observed** | **Mr(expt)** | **Mr(calc)** | **Delta** | **Miss** | **Score** | **Expect** | **Rank** | **Peptide** |
| --- | --- | --- | --- | --- | --- | --- | --- | --- | --- | --- |
|  | 6291 | **568.7880** | **1135.5615** | **1135.5696** | **-0.0081** | **0** | **17** | **2.9** | **1** | **K.ASCLYGQLPK.F** |
|  | 16730 | **433.9638** | **1731.8263** | **1732.8519** | **-1.0257** | **0** | **7** | **22** | **7** | **K.EEVVTVETWQEGSLK.A** |
|  | 18904 | **942.4725** | **1882.9304** | **1882.9425** | **-0.0121** | **0** | **125** | **3.2e-011** | **1** | **K.FQDGDLTLYQSNTILR.H** |
|  | 20674 | **677.8180** | **2030.4323** | **2031.0425** | **-0.6102** | **1** | **1** | **48** | **1** | **K.AFLASPEYVNLPINGNGKQ.-** |
|  | 21683 | **1063.5776** | **2125.1407** | **2125.1531** | **-0.0124** | **0** | **5** | **26** | **1** | **K.ALPGQLKPFETLLSQNQGGK.T** |
|  | 26857 | **950.4662** | **2848.3769** | **2848.3913** | **-0.0144** | **1** | **7** | **11** | **1** | **R.TLGLYGKDQQEAALVDMVNDGVEDLR.C** 26858 |
|  | 27533 | **997.8251** | **2990.4534** | **2990.4695** | **-0.0161** | **1** | **16** | **1.3** | **1** | **R.MLLADQGQSWKEEVVTVETWQEGSLK.A** |

| **334.** | P01876\|IGHA1_HUMAN    **Mass:** 38486    **Score:** 125    **Queries matched:** 10   **emPAI:** 0.45 |
| --- | --- |
|  | Ig alpha-1 chain C region OS=Homo sapiens GN=IGHA1 PE=1 SV=2 |

|  | **Query** | **Observed** | **Mr(expt)** | **Mr(calc)** | **Delta** | **Miss** | **Score** | **Expect** | **Rank** | **Peptide** |
| --- | --- | --- | --- | --- | --- | --- | --- | --- | --- | --- |
|  | 2934 | **448.7318** | **895.4490** | **895.4552** | **-0.0062** | **0** | **8** | **25** | **1** | **K.YLTWASR.Q** 2940 |
|  | 3430 | **466.7297** | **931.4448** | **930.5386** | **0.9062** | **0** | **9** | **23** | **3** | **K.TPLTATLSK.S** |
|  | 7646 | 607.3157 | 1212.6169 | 1212.6251 | -0.0082 | 0 | 14 | 5.6 | 1 | R.WLQGSQELPR.E |
|  | 10847 | **688.4301** | **1374.8457** | **1374.6126** | **0.2331** | **0** | **31** | **0.095** | **1** | **K.TFTCTAAYPESK.T** |
|  | 12448 | 490.6162 | 1468.8269 | 1469.7626 | -0.9358 | 1 | 6 | 31 | 1 | R.WLQGSQELPREK.Y |
|  | 18259 | 918.4733 | 1834.9320 | 1834.9425 | -0.0105 | 0 | 109 | 1.3e-009 | 1 | R.QEPSQGTTTFAVTSILR.V |
|  | 23517 | **780.0471** | **2337.1194** | **2336.1293** | **0.9900** | **1** | **23** | **0.37** | **1** | **K.KGDTFSCMVGHEALPLAFTQK.T** |
|  | 25367 | 865.0419 | 2592.1040 | 2592.1196 | -0.0156 | 0 | 3 | 32 | 1 | R.DLCGCYSVSSVLPGCAEPWNHGK.T |
|  | 29666 | **1191.9596** | **3572.8569** | **3572.8661** | **-0.0091** | **0** | **20** | **0.41** | **1** | **K.SGNTFRPEVHLLPPPSEELALNELVTLTCLAR.G** |

|  | **Proteins matching a subset of these peptides:** |
| --- | --- |

|  | P01877\|IGHA2_HUMAN    **Mass:** 37301    **Score:** 114    **Queries matched:** 7 |
| --- | --- |
|  | Ig alpha-2 chain C region OS=Homo sapiens GN=IGHA2 PE=1 SV=3 |

| **335.** | Q9Y624\|JAM1_HUMAN    **Mass:** 32962    **Score:** 125    **Queries matched:** 14   **emPAI:** 0.38 |
| --- | --- |
|  | Junctional adhesion molecule A OS=Homo sapiens GN=F11R PE=1 SV=1 |

|  | **Query** | **Observed** | **Mr(expt)** | **Mr(calc)** | **Delta** | **Miss** | **Score** | **Expect** | **Rank** | **Peptide** |
| --- | --- | --- | --- | --- | --- | --- | --- | --- | --- | --- |
|  | 3138 | **456.2247** | **910.4348** | **909.4378** | **0.9970** | **0** | **14** | **5.7** | **2** | **R.LVCYNNK.I** |
|  | 3524 | **470.2184** | **938.4222** | **938.4094** | **0.0128** | **0** | **8** | **24** | **3** | **K.FDQGDTTR.L** 3536 |
|  | 4669 | **510.2455** | **1018.4763** | **1018.5229** | **-0.0466** | **1** | **5** | **55** | **5** | **-.MGTKAQVER.K** |
|  | 5399 | **536.7768** | **1071.5390** | **1070.5430** | **0.9960** | **0** | **37** | **0.038** | **1** | **K.DGIVMPTNPK.S** 5374 |
|  | 7832 | **408.2467** | **1221.7182** | **1222.6962** | **-0.9780** | **0** | **7** | **27** | **2** | **R.VTFLPTGITFK.S** |
|  | 10672 | **684.3165** | **1366.6184** | **1366.6299** | **-0.0116** | **0** | **9** | **18** | **2** | **R.NGYGTPMTSNAVR.M** 10688 |
|  | 23236 | **1151.5123** | **2301.0101** | **2301.0260** | **-0.0159** | **0** | **71** | **5.4e-006** | **1** | **R.AVLTCSEQDGSPPSEYTWFK.D** 23237 |
|  | 23797 | **791.7936** | **2372.3591** | **2372.3791** | **-0.0200** | **0** | **3** | **29** | **1** | **K.LIVLVPPSKPTVNIPSSATIGNR.A** |
|  | 29097 | **1118.8531** | **3353.5376** | **3353.5584** | **-0.0208** | **1** | **46** | **0.0011** | **1** | **R.AVLTCSEQDGSPPSEYTWFKDGIVMPTNPK.S** |
|  | 30475 | 1007.7527 | 4026.9817 | 4027.8382 | -0.8565 | 1 | 6 | 6.9 | 3 | K.STRAFSNSSYVLNPTTGELVFDPLSASDTGEYSCEAR.N |

| **336.** | P06756\|ITAV_HUMAN    **Mass:** 117048   **Score:** 125    **Queries matched:** 33   **emPAI:** 0.10 |
| --- | --- |
|  | Integrin alpha-V OS=Homo sapiens GN=ITGAV PE=1 SV=2 |

|  | **Query** | **Observed** | **Mr(expt)** | **Mr(calc)** | **Delta** | **Miss** | **Score** | **Expect** | **Rank** | **Peptide** |
| --- | --- | --- | --- | --- | --- | --- | --- | --- | --- | --- |
|  | 1031 | 588.9891 | 587.9818 | 587.3391 | 0.6427 | 1 | 9 | 26 | 4 | R.LDRGK.S |
|  | 1357 | 719.3677 | 718.3605 | 717.3632 | 0.9973 | 0 | 5 | 77 | 6 | -.MAFPPR.R |
|  | 2647 | **437.2705** | **872.5265** | **873.4643** | **-0.9378** | **1** | **17** | **4.2** | **1** | **-.MAFPPRR.R** 2635 2645 2653 |
|  | 2789 | **441.7292** | **881.4438** | **880.4225** | **1.0213** | **0** | **3** | **67** | **1** | **K.VSCFNVR.F** |
|  | 3152 | 456.7453 | 911.4761 | 911.5189 | -0.0428 | 1 | 10 | 13 | 8 | K.ADGKGVLPR.K 3167 |
|  | 3503 | 469.2611 | 936.5077 | 936.4335 | 0.0742 | 1 | 5 | 50 | 9 | R.TEMKQER.E |
|  | 4103 | **490.2482** | **978.4819** | **978.4883** | **-0.0064** | **0** | **9** | **20** | **2** | **K.YNNQLATR.T** |
|  | 4340 | **498.7326** | **995.4507** | **994.4542** | **0.9965** | **0** | **23** | **0.67** | **1** | **K.TVEYAPCR.S** |
|  | 4421 | 500.8024 | 999.5902 | 998.5873 | 1.0029 | 0 | 22 | 1.2 | 6 | K.AGTQLLAGLR.F |
|  | 5083 | **524.2969** | **1046.5792** | **1046.5430** | **0.0362** | **0** | **5** | **59** | **7** | **K.TCSLPGTALK.V** |
|  | 8338 | **624.3223** | **1246.6301** | **1246.6405** | **-0.0104** | **1** | **13** | **7.7** | **1** | **K.LEVSVDSDQKK.I** |
|  | 8341 | **624.3303** | **1246.6461** | **1246.7132** | **-0.0672** | **1** | **5** | **53** | **10** | **R.IKISSLQTTEK.N** |
|  | 8570 | 630.3245 | 1258.6344 | 1259.6002 | -0.9659 | 0 | 8 | 24 | 3 | R.QVVCDLGNPMK.A |
|  | 8946 | **639.4044** | **1276.7943** | **1275.5952** | **1.1991** | **0** | **(5)** | **48** | **1** | **R.QVVCDLGNPMK.A** |
|  | 11271 | **467.5780** | **1399.7122** | **1398.7217** | **0.9905** | **0** | **2** | **80** | **7** | **K.LTPITIFMEYR.L** |
|  | 11993 | 721.9053 | 1441.7961 | 1441.7889 | 0.0072 | 0 | 14 | 4.9 | 1 | K.LQEVGQVSVSLQR.A |
|  | 15205 | 819.4198 | 1636.8250 | 1637.7356 | -0.9105 | 0 | 2 | 81 | 8 | R.FSVHQQSEMDTSVK.F |
|  | 18663 | **621.9772** | **1862.9097** | **1863.8785** | **-0.9688** | **1** | **4** | **44** | **9** | **K.QEREPVGTCFLQDGTK.T** |
|  | 19938 | **491.5198** | **1962.0500** | **1962.8703** | **-0.8203** | **1** | **2** | **54** | **2** | **R.SMPPSFGYSMKGATDIDK.N** |
|  | 20276 | **998.4167** | **1994.8189** | **1995.9506** | **-1.1317** | **0** | **9** | **11** | **2** | **R.QAHILLDCGEDNVCKPK.L** |
|  | 20305 | **999.5180** | **1997.0215** | **1997.0330** | **-0.0116** | **0** | **35** | **0.03** | **1** | **R.STGLNAVPSQILEGQWAAR.S** |
|  | 21991 | **720.3879** | **2158.1420** | **2159.1336** | **-0.9916** | **1** | **1** | **77** | **8** | **K.SAILYVKSLLWTETFMNK.E** |
|  | 23163 | **573.7914** | **2291.1364** | **2292.1386** | **-1.0022** | **1** | **4** | **29** | **4** | **K.GATDIDKNGYPDLIVGAFGVDR.A** |
|  | 26516 | **1396.2367** | **2790.4588** | **2790.4592** | **-0.0004** | **0** | **86** | **1.4e-007** | **1** | **R.VLLGGPGSFYWQGQLISDQVAEIVSK.Y** 26514 26515 |
|  | 28315 | **1055.8441** | **3164.5105** | **3164.5302** | **-0.0197** | **1** | **6** | **11** | **1** | **R.FGSAIAPLGDLDQDGFNDIAIAAPYGGEDKK.G** |
|  | 28686 | **541.2668** | **3241.5574** | **3241.5536** | **0.0038** | **1** | **0** | **43** | **9** | **R.STGLNAVPSQILEGQWAARSMPPSFGYSMK.G** |
|  | 28902 | 1103.5219 | 3307.5437 | 3307.7717 | -0.2280 | 1 | 5 | 14 | 5 | K.VDLAVLAAVEIRGVSSPDHVFLPIPNWEHK.E |

| **337.** | Q9Y4L1\|HYOU1_HUMAN    **Mass:** 111494   **Score:** 124    **Queries matched:** 34   **emPAI:** 0.10 |
| --- | --- |
|  | Hypoxia up-regulated protein 1 OS=Homo sapiens GN=HYOU1 PE=1 SV=1 |

|  | **Query** | **Observed** | **Mr(expt)** | **Mr(calc)** | **Delta** | **Miss** | **Score** | **Expect** | **Rank** | **Peptide** |
| --- | --- | --- | --- | --- | --- | --- | --- | --- | --- | --- |
|  | 1419 | **752.3884** | **751.3812** | **751.3357** | **0.0455** | **0** | **10** | **18** | **8** | **K.MMALDR.E** |
|  | 2800 | **442.7428** | **883.4710** | **883.5352** | **-0.0642** | **1** | **7** | **32** | **3** | **R.RAVLQAAR.M** |
|  | 3410 | **465.7621** | **929.5097** | **929.5182** | **-0.0085** | **1** | **24** | **0.77** | **1** | **K.AVGKEELGK.N** |
|  | 6371 | 572.2826 | 1142.5506 | 1142.5455 | 0.0051 | 0 | 8 | 30 | 8 | K.DEPGEQVELK.E |
|  | 7927 | **614.3460** | **1226.6773** | **1227.7121** | **-1.0348** | **1** | **11** | **11** | **1** | **R.AVLQAARMAGLK.V** |
|  | 8224 | **415.2129** | **1242.6170** | **1243.5907** | **-0.9737** | **0** | **1** | **1.1e+002** | **10** | **R.FFGDSAASMAIK.N** |
|  | 8798 | **424.2362** | **1269.6868** | **1270.6993** | **-1.0125** | **1** | **6** | **32** | **6** | **K.EQSTGQKRPLK.N** 8833 |
|  | 8807 | 636.3146 | 1270.6146 | 1269.6499 | 0.9647 | 0 | 9 | 17 | 2 | K.EAGMQPQLQIR.G |
|  | 9890 | 663.3394 | 1324.6642 | 1324.8091 | -0.1450 | 1 | 1 | 1e+002 | 9 | K.AKFTKPRPRPK.D |
|  | 11838 | **478.5515** | **1432.6328** | **1433.8242** | **-1.1914** | **1** | **5** | **40** | **6** | **R.VFGSQNLTTVKLK.G** |
|  | 12673 | 494.9051 | 1481.6935 | 1481.7375 | -0.0440 | 0 | 2 | 76 | 6 | K.QADNPHVALYQAR.F |
|  | 13124 | **503.5597** | **1507.6572** | **1507.7154** | **-0.0583** | **0** | **5** | **40** | **5** | **K.AEAGPEGVAPAPEGEK.K** |
|  | 13228 | **505.6842** | **1514.0307** | **1514.7875** | **-0.7568** | **1** | **1** | **70** | **5** | **K.TKEAGMQPQLQIR.G** |
|  | 14797 | **403.9496** | **1611.7694** | **1611.9308** | **-0.1614** | **1** | **11** | **9.3** | **7** | **K.LAQSVQKLQDLTLR.D** |
|  | 15190 | 818.8436 | 1635.6726 | 1635.8104 | -0.1378 | 1 | 6 | 25 | 2 | K.AEAGPEGVAPAPEGEKK.Q |
|  | 16952 | 873.7910 | 1745.5675 | 1744.8744 | 0.6931 | 1 | 5 | 24 | 4 | R.EVEEEPGIHSLKHNK.R |
|  | 17766 | **601.3417** | **1801.0034** | **1801.9396** | **-0.9362** | **0** | **(4)** | **41** | **3** | **R.LSALDNLLNHSSMFLK.G** |
|  | 17996 | **606.6576** | **1816.9511** | **1817.9345** | **-0.9834** | **0** | **7** | **23** | **3** | **R.LSALDNLLNHSSMFLK.G** 17993 |
|  | 18640 | **621.3199** | **1860.9378** | **1860.9846** | **-0.0468** | **1** | **1** | **76** | **9** | **K.DAVITVPVFFNQAERR.A** |
|  | 21382 | **1048.0570** | **2094.0995** | **2094.1109** | **-0.0115** | **0** | **73** | **4.7e-006** | **1** | **K.VLQLINDNTATALSYGVFR.R** |
|  | 24203 | **813.0751** | **2436.2036** | **2435.2254** | **0.9782** | **1** | **3** | **34** | **1** | **K.LSAASTWLEDEGVGATTVMLKEK.L** |
|  | 24804 | **840.7244** | **2519.1515** | **2519.1672** | **-0.0158** | **0** | **16** | **1.6** | **1** | **K.TVLSANADHMAQIEGLMDDVDFK.A** |
|  | 24917 | **848.0638** | **2541.1695** | **2541.1870** | **-0.0175** | **1** | **12** | **3.9** | **1** | **K.LYQPEYQEVSTEEQREEISGK.L** |
|  | 24997 | 1275.5824 | 2549.1502 | 2550.2709 | -1.1207 | 1 | 3 | 31 | 3 | K.GARLIPEMDQIFTEVEMTTLEK.V 25020 |
|  | 25122 | 638.8647 | 2551.4297 | 2551.1571 | 0.2726 | 0 | (3) | 32 | 3 | K.TVLSANADHMAQIEGLMDDVDFK.A |
|  | 25562 | **878.8090** | **2633.4051** | **2634.2959** | **-0.8908** | **1** | **4** | **24** | **3** | **K.EELGKNINADEAAAMGAVYQAAALSK.A** |
|  | 28197 | **1568.3167** | **3134.6187** | **3134.6282** | **-0.0094** | **0** | **77** | **1e-006** | **1** | **R.VPGPVQQALQSAEMSLDEIEQVILVGGATR.V** |
|  | 29330 | **1146.5676** | **3436.6811** | **3435.5343** | **1.1468** | **0** | **3** | **23** | **6** | **K.DINTTAQNIMFYDMGSGSTVCTIVTYQMVK.T** |
|  | 29505 | **877.6786** | **3506.6853** | **3505.8122** | **0.8731** | **0** | **4** | **16** | **1** | **R.VCWALVAVLLADLLALSDTLAVMSVDLGSESMK.V** |
|  | 29506 | **877.6804** | **3506.6926** | **3505.8122** | **0.8804** | **0** | **(1)** | **32** | **1** | **R.VCWALVAVLLADLLALSDTLAVMSVDLGSESMK.V** |
|  | 29858 | **917.4238** | **3665.6662** | **3664.6769** | **0.9893** | **1** | **3** | **16** | **1** | **K.DINTTAQNIMFYDMGSGSTVCTIVTYQMVKTK.E** |

| **338.** | O95994\|AGR2_HUMAN    **Mass:** 20024    **Score:** 124    **Queries matched:** 10   **emPAI:** 1.02 |
| --- | --- |
|  | Anterior gradient protein 2 homolog OS=Homo sapiens GN=AGR2 PE=1 SV=1 |

|  | **Query** | **Observed** | **Mr(expt)** | **Mr(calc)** | **Delta** | **Miss** | **Score** | **Expect** | **Rank** | **Peptide** |
| --- | --- | --- | --- | --- | --- | --- | --- | --- | --- | --- |
|  | 3652 | **474.3254** | **946.6362** | **945.4879** | **1.1483** | **1** | **8** | **31** | **2** | **K.DTKDSRPK.L** |
|  | 7486 | 603.3251 | 1204.6357 | 1204.6452 | -0.0094 | 1 | 25 | 0.51 | 1 | K.VFAENKEIQK.L |
|  | 11197 | 699.2870 | 1396.5594 | 1396.7786 | -0.2192 | 1 | 6 | 32 | 5 | K.DSRPKLPQTLSR.G |
|  | 19615 | **971.4828** | **1940.9510** | **1939.9601** | **0.9910** | **0** | **50** | **0.0009** | **1** | **R.LYAYEPADTALLLDNMK.K** 19601 |
|  | 20290 | **998.5449** | **1995.0752** | **1995.0564** | **0.0187** | **0** | **5** | **27** | **1** | **K.LAEQFVLLNLVYETTDK.H** |
|  | 21107 | **690.3546** | **2068.0418** | **2068.0550** | **-0.0132** | **1** | **7** | **17** | **1** | **R.LYAYEPADTALLLDNMKK.A** |
|  | 22318 | **1101.0250** | **2200.0355** | **2200.0477** | **-0.0122** | **0** | **47** | **0.0016** | **1** | **R.GWGDQLIWTQTYEEALYK.S** |
|  | 28697 | **812.1728** | **3244.6621** | **3244.6768** | **-0.0148** | **1** | **37** | **0.0088** | **1** | **K.LAEQFVLLNLVYETTDKHLSPDGQYVPR.I** 28696 |

| **339.** | P04792\|HSPB1_HUMAN    **Mass:** 22826    **Score:** 124    **Queries matched:** 7   **emPAI:** 1.17 |
| --- | --- |
|  | Heat shock protein beta-1 OS=Homo sapiens GN=HSPB1 PE=1 SV=2 |

|  | **Query** | **Observed** | **Mr(expt)** | **Mr(calc)** | **Delta** | **Miss** | **Score** | **Expect** | **Rank** | **Peptide** |
| --- | --- | --- | --- | --- | --- | --- | --- | --- | --- | --- |
|  | 3548 | **471.2368** | **940.4590** | **940.4978** | **-0.0388** | **0** | **11** | **11** | **9** | **R.AQLGGPEAAK.S** |
|  | 5445 | **538.2871** | **1074.5597** | **1074.5669** | **-0.0073** | **0** | **29** | **0.2** | **1** | **R.QLSSGVSEIR.H** |
|  | 6700 | **582.3110** | **1162.6074** | **1162.6135** | **-0.0061** | **0** | **35** | **0.053** | **1** | **R.LFDQAFGLPR.L** |
|  | 15494 | **552.5843** | **1654.7312** | **1654.7448** | **-0.0135** | **1** | **18** | **1.8** | **1** | **K.HEERQDEHGYISR.C** |
|  | 17454 | **892.4591** | **1782.9037** | **1782.9152** | **-0.0116** | **0** | **35** | **0.032** | **1** | **R.VSLDVNHFAPDELTVK.T** |
|  | 28624 | **1076.2170** | **3225.6293** | **3225.6479** | **-0.0186** | **1** | **40** | **0.0041** | **1** | **R.KYTLPPGVDPTQVSSSLSPEGTLTVEAPMPK.L** |
|  | 30594 | **1365.0176** | **4092.0309** | **4092.0533** | **-0.0224** | **0** | **67** | **5.5e-006** | **1** | **R.LPEEWSQWLGGSSWPGYVRPLPPAAIESPAVAAPAYSR.A** |

| **340.** | P55084\|ECHB_HUMAN    **Mass:** 51547    **Score:** 123    **Queries matched:** 17   **emPAI:** 0.42 |
| --- | --- |
|  | Trifunctional enzyme subunit beta, mitochondrial OS=Homo sapiens GN=HADHB PE=1 SV=3 |

|  | **Query** | **Observed** | **Mr(expt)** | **Mr(calc)** | **Delta** | **Miss** | **Score** | **Expect** | **Rank** | **Peptide** |
| --- | --- | --- | --- | --- | --- | --- | --- | --- | --- | --- |
|  | 2267 | **423.2273** | **844.4400** | **844.4589** | **-0.0188** | **0** | **9** | **29** | **7** | **R.LVMAAANR.L** |
|  | 4036 | **487.7855** | **973.5564** | **973.5630** | **-0.0066** | **1** | **29** | **0.26** | **1** | **R.KLMLDLNK.A** 4029 4035 4056 |
|  | 4262 | **496.2841** | **990.5536** | **989.5579** | **0.9957** | **1** | **(12)** | **12** | **5** | **R.KLMLDLNK.A** |
|  | 6290 | **568.7763** | **1135.5379** | **1135.5509** | **-0.0130** | **0** | **37** | **0.033** | **1** | **R.LEQDEYALR.S** |
|  | 7639 | **405.2061** | **1212.5964** | **1212.6390** | **-0.0426** | **0** | **10** | **14** | **4** | **R.TPFLLSGTSYK.D** |
|  | 8533 | **629.3573** | **1256.7000** | **1255.7037** | **0.9964** | **1** | **4** | **58** | **7** | **K.NLPTASKWALR.F** 8527 |
|  | 16085 | **846.8364** | **1691.6582** | **1691.6708** | **-0.0126** | **0** | **22** | **0.66** | **1** | **K.AMDSDWFAENYMGR.K** |
|  | 18523 | **927.0058** | **1851.9970** | **1851.0030** | **0.9941** | **0** | **75** | **3.3e-006** | **1** | **K.EVVDYIIFGTVIQEVK.T** |
|  | 18920 | **629.1860** | **1884.5361** | **1885.0560** | **-0.5200** | **1** | **2** | **42** | **2** | **K.DQLLLGPTYATPKVLEK.A** |
|  | 21754 | **712.3295** | **2133.9668** | **2133.9803** | **-0.0136** | **0** | **44** | **0.0037** | **1** | **K.FNNWGGSLSLGHPFGATGCR.L** |
|  | 23735 | **789.1034** | **2364.2883** | **2363.2988** | **0.9895** | **1** | **4** | **25** | **1** | **R.TSVPKEVVDYIIFGTVIQEVK.T** |
|  | 26078 | **902.4262** | **2704.2568** | **2704.2737** | **-0.0169** | **0** | **34** | **0.025** | **1** | **K.EGGQYGLVAACAAGGQGHAMIVEAYPK.-** |
|  | 29660 | **893.9611** | **3571.8151** | **3571.8306** | **-0.0154** | **0** | **0** | **36** | **1** | **K.LKPAFIKPYGTVTAANSSFLTDGASAMLIMAEEK.A** |

| **341.** | P04632\|CPNS1_HUMAN    **Mass:** 28469    **Score:** 123    **Queries matched:** 9   **emPAI:** 0.13 |
| --- | --- |
|  | Calpain small subunit 1 OS=Homo sapiens GN=CAPNS1 PE=1 SV=1 |

|  | **Query** | **Observed** | **Mr(expt)** | **Mr(calc)** | **Delta** | **Miss** | **Score** | **Expect** | **Rank** | **Peptide** |
| --- | --- | --- | --- | --- | --- | --- | --- | --- | --- | --- |
|  | 1766 | **405.2987** | **808.5829** | **807.4279** | **1.1550** | **0** | **4** | **62** | **6** | **R.WQAIYK.Q** 1765 |
|  | 2596 | **435.5555** | **869.0963** | **868.4331** | **0.6633** | **0** | **4** | **38** | **9** | **K.LGFEEFK.Y** |
|  | 17718 | 600.6293 | 1798.8662 | 1799.9246 | -1.0585 | 1 | 3 | 50 | 8 | K.LGFEEFKYLWNNIK.R |
|  | 22414 | **736.8245** | **2207.4517** | **2207.0126** | **0.4391** | **1** | **4** | **20** | **1** | **R.SMVAVMDSDTTGKLGFEEFK.Y** |
|  | 24819 | **1262.1211** | **2522.2276** | **2522.2396** | **-0.0120** | **0** | **123** | **3.2e-011** | **1** | **R.LFAQLAGDDMEVSATELMNILNK.V** |
|  | 25969 | **899.4765** | **2695.4077** | **2694.3356** | **1.0720** | **1** | **1** | **43** | **1** | **R.RLFAQLAGDDMEVSATELMNILNK.V** |
|  | 30996 | **1133.3387** | **4529.3259** | **4529.1968** | **0.1291** | **1** | **5** | **6** | **1** | **M.FLVNSFLKGGGGGGGGGGGLGGGLGNVLGGLISGAGGGGGGGGGGGGGGGGGGGGTAMR.I** 31000 |

| **342.** | P01871\|IGHM_HUMAN    **Mass:** 49960    **Score:** 123    **Queries matched:** 21   **emPAI:** 0.24 |
| --- | --- |
|  | Ig mu chain C region OS=Homo sapiens GN=IGHM PE=1 SV=3 |

|  | **Query** | **Observed** | **Mr(expt)** | **Mr(calc)** | **Delta** | **Miss** | **Score** | **Expect** | **Rank** | **Peptide** |
| --- | --- | --- | --- | --- | --- | --- | --- | --- | --- | --- |
|  | 1476 | **776.4440** | **775.4367** | **774.4388** | **0.9979** | **0** | **19** | **2.7** | **1** | **R.GFPSVLR.G** |
|  | 2491 | **431.7627** | **861.5108** | **861.5171** | **-0.0063** | **0** | **10** | **20** | **4** | **K.VTSTLTIK.E** |
|  | 3002 | 451.2679 | 900.5213 | 899.5229 | 0.9984 | 0 | 22 | 1.3 | 1 | K.VSVFVPPR.D |
|  | 3112 | 455.2117 | 908.4088 | 908.4141 | -0.0052 | 0 | 29 | 0.21 | 1 | R.DGFFGNPR.K 3111 |
|  | 3638 | 474.2296 | 946.4447 | 946.5084 | -0.0637 | 1 | 12 | 10 | 3 | R.VTERTVDK.S |
|  | 4795 | **515.3259** | **1028.6373** | **1028.5767** | **0.0606** | **0** | **6** | **40** | **4** | **R.QIQVSWLR.E** 4768 |
|  | 11065 | **694.4192** | **1386.8238** | **1385.8282** | **0.9956** | **0** | **5** | **44** | **2** | **K.NVPLPVIAELPPK.V** |
|  | 14612 | 800.8879 | 1599.7613 | 1599.7715 | -0.0102 | 0 | 16 | 3.2 | 2 | K.YVTSAPMPEPQAPGR.Y |
|  | 14872 | 539.3262 | 1614.9569 | 1615.7664 | -0.8096 | 0 | (1) | 90 | 9 | K.YVTSAPMPEPQAPGR.Y |
|  | 14903 | **809.4022** | **1616.7899** | **1616.8006** | **-0.0107** | **0** | **62** | **7.2e-005** | **1** | **K.QVGSGVTTDQVQAEAK.E** 14902 14932 |
|  | 15220 | **819.4654** | **1636.9162** | **1636.9229** | **-0.0066** | **0** | **22** | **0.69** | **1** | **R.VFAIPPSFASIFLTK.S** 15217 15219 15230 |
|  | 16474 | **858.9295** | **1715.8444** | **1715.8553** | **-0.0108** | **0** | **59** | **0.00014** | **1** | **R.FTCTVTHTDLPSPLK.Q** |
|  | 17277 | **592.0045** | **1772.9917** | **1773.0050** | **-0.0132** | **0** | **18** | **1.7** | **1** | **K.GVALHRPDVYLLPPAR.E** |
|  | 19509 | **644.8262** | **1931.4569** | **1930.9596** | **0.4973** | **1** | **1** | **51** | **2** | **R.EGKQVGSGVTTDQVQAEAK.E** |

| **343.** | O00264\|PGRC1_HUMAN    **Mass:** 21772    **Score:** 123    **Queries matched:** 7   **emPAI:** 0.63 |
| --- | --- |
|  | Membrane-associated progesterone receptor component 1 OS=Homo sapiens GN=PGRMC1 PE=1 SV=3 |

|  | **Query** | **Observed** | **Mr(expt)** | **Mr(calc)** | **Delta** | **Miss** | **Score** | **Expect** | **Rank** | **Peptide** |
| --- | --- | --- | --- | --- | --- | --- | --- | --- | --- | --- |
|  | 3659 | **474.7401** | **947.4657** | **947.4712** | **-0.0055** | **0** | **11** | **13** | **1** | **R.DFTPAELR.R** |
|  | 5697 | **547.2581** | **1092.5017** | **1093.6396** | **-1.1379** | **1** | **3** | **78** | **6** | **K.YHHVGKLLK.E** |
|  | 5877 | **553.2507** | **1104.4868** | **1103.5723** | **0.9144** | **1** | **5** | **60** | **8** | **R.RDFTPAELR.R** |
|  | 13264 | **758.8592** | **1515.7039** | **1515.7147** | **-0.0107** | **0** | **47** | **0.0026** | **1** | **K.FYGPEGPYGVFAGR.D** |
|  | 15314 | **822.9068** | **1643.7990** | **1643.8096** | **-0.0106** | **1** | **54** | **0.00043** | **1** | **R.KFYGPEGPYGVFAGR.D** 15315 15316 |

| **344.** | P18085\|ARF4_HUMAN    **Mass:** 20612    **Score:** 123    **Queries matched:** 10   **emPAI:** 0.98 |
| --- | --- |
|  | ADP-ribosylation factor 4 OS=Homo sapiens GN=ARF4 PE=1 SV=3 |

|  | **Query** | **Observed** | **Mr(expt)** | **Mr(calc)** | **Delta** | **Miss** | **Score** | **Expect** | **Rank** | **Peptide** |
| --- | --- | --- | --- | --- | --- | --- | --- | --- | --- | --- |
|  | 4219 | **495.2240** | **988.4334** | **987.5423** | **0.8912** | **0** | **5** | **58** | **2** | **K.MLLVDELR.D** |
|  | 15645 | 832.3817 | 1662.7487 | 1662.7593 | -0.0105 | 0 | 92 | 6.7e-008 | 1 | K.QDLPNAMAISEMTDK.L |
|  | 15904 | **560.9003** | **1679.6792** | **1678.7542** | **0.9250** | **0** | **(2)** | **63** | **6** | **K.QDLPNAMAISEMTDK.L** |
|  | 16109 | **847.8941** | **1693.7736** | **1694.7491** | **-0.9755** | **0** | **(12)** | **7.1** | **1** | **K.QDLPNAMAISEMTDK.L** 16110 |
|  | 21324 | **697.3478** | **2089.0217** | **2088.1653** | **0.8564** | **1** | **1** | **66** | **1** | **K.MLLVDELRDAVLLLFANK.Q** |
|  | 23401 | 1162.1196 | 2322.2247 | 2322.2359 | -0.0112 | 0 | 24 | 0.29 | 1 | K.LGEIVTTIPTIGFNVETVEYK.N 23402 |
|  | 24211 | 813.3935 | 2437.1586 | 2437.1775 | -0.0188 | 1 | 49 | 0.0009 | 1 | K.HYFQNTQGLIFVVDSNDRER.I |
|  | 25193 | 855.4740 | 2563.4002 | 2563.4149 | -0.0147 | 1 | 27 | 0.13 | 1 | K.LKLGEIVTTIPTIGFNVETVEYK.N |

| **345.** | O95292\|VAPB_HUMAN    **Mass:** 27439    **Score:** 122    **Queries matched:** 16   **emPAI:** 0.48 |
| --- | --- |
|  | Vesicle-associated membrane protein-associated protein B/C OS=Homo sapiens GN=VAPB PE=1 SV=3 |

|  | **Query** | **Observed** | **Mr(expt)** | **Mr(calc)** | **Delta** | **Miss** | **Score** | **Expect** | **Rank** | **Peptide** |
| --- | --- | --- | --- | --- | --- | --- | --- | --- | --- | --- |
|  | 1465 | **772.3889** | **771.3816** | **771.3875** | **-0.0059** | **0** | **10** | **20** | **1** | **K.LGNPTDR.N** |
|  | 3718 | **476.2793** | **950.5440** | **950.4314** | **0.1126** | **1** | **8** | **27** | **1** | **K.VMEECKR.L** |
|  | 8620 | 631.2825 | 1260.5504 | 1261.5860 | -1.0356 | 0 | 6 | 39 | 9 | K.EAKPEDLMDSK.L |
|  | 13531 | 511.2577 | 1530.7512 | 1530.7711 | -0.0200 | 1 | 7 | 27 | 4 | K.EAKPEDLMDSKLR.C 13518 13539 13543 |
|  | 13768 | 516.3293 | 1545.9662 | 1546.7661 | -0.7998 | 1 | (6) | 24 | 5 | K.EAKPEDLMDSKLR.C |
|  | 15387 | **824.9429** | **1647.8712** | **1647.8832** | **-0.0120** | **0** | **51** | **0.001** | **1** | **K.VEQVLSLEPQHELK.F** 15366 15386 |
|  | 23381 | **1161.0127** | **2320.0108** | **2320.0214** | **-0.0106** | **0** | **57** | **0.00013** | **1** | **K.FMVQSMFAPTDTSDMEAVWK.E** 23379 23380 |
|  | 25416 | **651.3056** | **2601.1933** | **2601.1702** | **0.0231** | **1** | **9** | **8.2** | **2** | **K.HKFMVQSMFAPTDTSDMEAVWK.E** |
|  | 29795 | 907.6627 | 3626.6215 | 3627.5765 | -0.9550 | 1 | 0 | 31 | 7 | K.FMVQSMFAPTDTSDMEAVWKEAKPEDLMDSK.L |

| **346.** | Q9H553\|ALG2_HUMAN    **Mass:** 47746    **Score:** 122    **Queries matched:** 12   **emPAI:** 0.08 |
| --- | --- |
|  | Alpha-1,3-mannosyltransferase ALG2 OS=Homo sapiens GN=ALG2 PE=2 SV=1 |

|  | **Query** | **Observed** | **Mr(expt)** | **Mr(calc)** | **Delta** | **Miss** | **Score** | **Expect** | **Rank** | **Peptide** |
| --- | --- | --- | --- | --- | --- | --- | --- | --- | --- | --- |
|  | 1552 | **397.2237** | **792.4328** | **791.3960** | **1.0369** | **0** | **6** | **44** | **1** | **K.ATMGLAGR.A** 1536 |
|  | 4243 | 495.7684 | 989.5222 | 988.5705 | 0.9516 | 1 | 11 | 16 | 7 | K.FIREPSLK.A |
|  | 10002 | 665.8240 | 1329.6335 | 1329.7074 | -0.0739 | 1 | 5 | 44 | 4 | R.EPSLKATMGLAGR.A |
|  | 14142 | **785.4085** | **1568.8025** | **1567.7929** | **1.0095** | **1** | **0** | **1.2e+002** | **1** | **R.ELPVRCAGDWLPR.G** |
|  | 17480 | **892.9488** | **1783.8830** | **1783.8927** | **-0.0097** | **0** | **79** | **1.2e-006** | **1** | **K.MVQQSDLGQYVTFLR.S** 17461 17481 |
|  | 19465 | **483.2285** | **1928.8850** | **1927.9826** | **0.9024** | **1** | **5** | **28** | **1** | **K.KMVQQSDLGQYVTFLR.S** 19438 |
|  | 20090 | **660.3342** | **1977.9807** | **1977.9836** | **-0.0029** | **1** | **1** | **86** | **7** | **K.FSPEAFTEQLYRYVTK.L** |
|  | 23146 | 764.0737 | 2289.1994 | 2289.2117 | -0.0124 | 0 | 16 | 2 | 1 | R.DSVPKPSVLFLHPDLGVGGAER.L |

| **347.** | P17066\|HSP76_HUMAN    **Mass:** 71440    **Score:** 122    **Queries matched:** 14   **emPAI:** 0.16 |
| --- | --- |
|  | Heat shock 70 kDa protein 6 OS=Homo sapiens GN=HSPA6 PE=1 SV=2 |

|  | **Query** | **Observed** | **Mr(expt)** | **Mr(calc)** | **Delta** | **Miss** | **Score** | **Expect** | **Rank** | **Peptide** |
| --- | --- | --- | --- | --- | --- | --- | --- | --- | --- | --- |
|  | 1691 | 804.4410 | 803.4337 | 803.4389 | -0.0052 | 0 | 27 | 0.48 | 1 | K.ITITNDK.G |
|  | 3002 | 451.2679 | 900.5213 | 899.5076 | 1.0136 | 0 | 12 | 13 | 3 | R.VVSEGGKPK.V |
|  | 4652 | 509.2835 | 1016.5525 | 1016.5614 | -0.0089 | 1 | 27 | 0.36 | 1 | K.ITITNDKGR.L |
|  | 7943 | 614.8138 | 1227.6130 | 1227.6207 | -0.0077 | 0 | 58 | 0.00024 | 1 | R.VEILANDQGNR.T 7942 |
|  | 12758 | 744.3493 | 1486.6840 | 1486.6940 | -0.0100 | 0 | 33 | 0.064 | 1 | R.TTPSYVAFTDTER.L |
|  | 14076 | 783.3349 | 1564.6552 | 1564.8249 | -0.1697 | 1 | 2 | 75 | 9 | K.LLQDFFNGKELNK.S |
|  | 15824 | 838.3630 | 1674.7114 | 1674.7234 | -0.0120 | 0 | 66 | 2.6e-005 | 1 | K.ATAGDTHLGGEDFDNR.L 15835 |
|  | 17857 | **603.3363** | **1806.9871** | **1806.9952** | **-0.0081** | **1** | **3** | **56** | **2** | **K.LDKAQIHDVVLVGGSTR.I** |
|  | 19361 | **641.3984** | **1921.1735** | **1920.9213** | **0.2522** | **1** | **2** | **51** | **4** | **K.TFYPEEISSMVLSKMK.E** |
|  | 20272 | **998.0218** | **1994.0290** | **1994.9996** | **-0.9706** | **1** | **2** | **55** | **3** | **K.CREVLAWLEHNQLAEK.E** |
|  | 27858 | 1018.1669 | 3051.4790 | 3051.6890 | -0.2100 | 0 | 4 | 19 | 2 | K.VQDLLLLDVAPLSLGLETAGGVMTTLIQR.N 27857 |

| **348.** | P48741\|HSP77_HUMAN    **Mass:** 40448    **Score:** 122    **Queries matched:** 8   **emPAI:** 0.31 |
| --- | --- |
|  | Putative heat shock 70 kDa protein 7 OS=Homo sapiens GN=HSPA7 PE=5 SV=2 |

|  | **Query** | **Observed** | **Mr(expt)** | **Mr(calc)** | **Delta** | **Miss** | **Score** | **Expect** | **Rank** | **Peptide** |
| --- | --- | --- | --- | --- | --- | --- | --- | --- | --- | --- |
|  | 7943 | 614.8138 | 1227.6130 | 1227.6207 | -0.0077 | 0 | 58 | 0.00024 | 1 | R.VEILANDQGNR.T 7942 |
|  | 8248 | **415.2451** | **1242.7135** | **1242.6932** | **0.0204** | **1** | **12** | **9.6** | **1** | **R.QATKDAGAIAGLK.V** |
|  | 12758 | 744.3493 | 1486.6840 | 1486.6940 | -0.0100 | 0 | 33 | 0.064 | 1 | R.TTPSYVAFTDTER.L |
|  | 15824 | 838.3630 | 1674.7114 | 1674.7234 | -0.0120 | 0 | 66 | 2.6e-005 | 1 | K.ATAGDTHLGGEDFDNR.L 15835 |
|  | 19361 | 641.3984 | 1921.1735 | 1920.9213 | 0.2522 | 1 | 2 | 51 | 4 | K.TFYPEEISSMVLSKMK.E |
|  | 20009 | **493.5208** | **1970.0541** | **1970.0836** | **-0.0295** | **0** | **2** | **55** | **7** | **K.VLPIINEATAAAIAYGLDR.R** |

| **349.** | Q9HAV0\|GBB4_HUMAN    **Mass:** 38284    **Score:** 122    **Queries matched:** 17   **emPAI:** 0.45 |
| --- | --- |
|  | Guanine nucleotide-binding protein subunit beta-4 OS=Homo sapiens GN=GNB4 PE=1 SV=3 |

|  | **Query** | **Observed** | **Mr(expt)** | **Mr(calc)** | **Delta** | **Miss** | **Score** | **Expect** | **Rank** | **Peptide** |
| --- | --- | --- | --- | --- | --- | --- | --- | --- | --- | --- |
|  | 2050 | 416.2589 | 830.5033 | 830.4359 | 0.0675 | 1 | 10 | 22 | 9 | K.TREGNVR.V |
|  | 4540 | 505.2582 | 1008.5018 | 1008.5101 | -0.0083 | 0 | 55 | 0.00047 | 1 | R.AGVLAGHDNR.V 4532 4533 4535 4538 |
|  | 4651 | 509.2787 | 1016.5428 | 1016.5502 | -0.0074 | 0 | 36 | 0.051 | 1 | R.LLVSASQDGK.L 4637 4650 |
|  | 7939 | **614.7857** | **1227.5569** | **1228.5394** | **-0.9825** | **0** | **17** | **3** | **2** | **R.TFVSGACDASSK.L** |
|  | 10123 | 669.8093 | 1337.6040 | 1336.6596 | 0.9444 | 1 | 1 | 1.1e+002 | 3 | K.GDRAGVLAGHDNR.V |
|  | 10401 | 677.3509 | 1352.6872 | 1352.6976 | -0.0104 | 0 | 3 | 75 | 8 | K.LIIWDSYTTNK.M |
|  | 13802 | 775.3424 | 1548.6703 | 1548.6813 | -0.0111 | 0 | 47 | 0.0025 | 1 | R.ELPGHTGYLSCCR.F |
|  | 18809 | 938.4981 | 1874.9817 | 1874.9156 | 0.0661 | 1 | 6 | 28 | 4 | -.MSELEQLRQEAEQLR.N |
|  | 29337 | **1147.4795** | **3439.4166** | **3438.5212** | **0.8955** | **0** | **2** | **21** | **2** | **R.QSFTGHVSDINAVSFFPNGYAFATGSDDATCR.L** |
|  | 29411 | 1157.8485 | 3470.5237 | 3470.5403 | -0.0166 | 0 | 46 | 0.00088 | 1 | R.SSWVMTCAYAPSGNYVACGGLDNICSIYNLK.T 29410 |

| **350.** | P00915\|CAH1_HUMAN    **Mass:** 28909    **Score:** 122    **Queries matched:** 15   **emPAI:** 0.45 |
| --- | --- |
|  | Carbonic anhydrase 1 OS=Homo sapiens GN=CA1 PE=1 SV=2 |

|  | **Query** | **Observed** | **Mr(expt)** | **Mr(calc)** | **Delta** | **Miss** | **Score** | **Expect** | **Rank** | **Peptide** |
| --- | --- | --- | --- | --- | --- | --- | --- | --- | --- | --- |
|  | 3979 | 486.2841 | 970.5537 | 969.5859 | 0.9678 | 0 | 8 | 27 | 10 | K.VLDALQAIK.T |
|  | 7060 | **593.8439** | **1185.6733** | **1185.6791** | **-0.0058** | **0** | **87** | **3.2e-007** | **1** | **K.ADGLAVIGVLMK.V** |
|  | 7414 | **401.2857** | **1200.8352** | **1201.6740** | **-0.8388** | **0** | **(14)** | **4.9** | **4** | **K.ADGLAVIGVLMK.V** |
|  | 14312 | **791.3820** | **1580.7494** | **1579.7842** | **0.9652** | **0** | **21** | **0.98** | **1** | **K.ESISVSSEQLAQFR.S** 14282 |
|  | 14795 | 806.8914 | 1611.7683 | 1611.7794 | -0.0111 | 0 | 39 | 0.016 | 1 | K.YSAELHVAHWNSAK.Y |
|  | 16914 | **872.4426** | **1742.8707** | **1741.8999** | **0.9708** | **0** | **20** | **1** | **2** | **K.LYPIANGNNQSPVDIK.T** 16913 |
|  | 18883 | 941.4990 | 1880.9834 | 1881.0394 | -0.0560 | 1 | 7 | 19 | 7 | K.ADGLAVIGVLMKVGEANPK.L |
|  | 22275 | **732.0114** | **2193.0124** | **2193.1715** | **-0.1591** | **1** | **1** | **72** | **3** | **K.YSSLAEAASKADGLAVIGVLMK.V** |
|  | 22864 | **752.6808** | **2255.0207** | **2255.0356** | **-0.0148** | **0** | **15** | **2.7** | **1** | **K.EIINVGHSFHVNFEDNDNR.S** |
|  | 24478 | **619.5695** | **2474.2487** | **2474.2653** | **-0.0165** | **1** | **9** | **9.4** | **1** | **K.TSETKHDTSLKPISVSYNPATAK.E** |
|  | 25758 | **890.0529** | **2667.1369** | **2668.3245** | **-1.1876** | **1** | **2** | **41** | **2** | **K.NGPEQWSKLYPIANGNNQSPVDIK.T** |
|  | 26341 | 920.4621 | 2758.3645 | 2758.3820 | -0.0176 | 0 | 44 | 0.0025 | 1 | R.SLLSNVEGDNAVPMQHNNRPTQPLK.G 26340 |

| **351.** | Q8NBX0\|SCPDH_HUMAN    **Mass:** 47464    **Score:** 121    **Queries matched:** 15   **emPAI:** 0.46 |
| --- | --- |
|  | Probable saccharopine dehydrogenase OS=Homo sapiens GN=SCCPDH PE=1 SV=1 |

|  | **Query** | **Observed** | **Mr(expt)** | **Mr(calc)** | **Delta** | **Miss** | **Score** | **Expect** | **Rank** | **Peptide** |
| --- | --- | --- | --- | --- | --- | --- | --- | --- | --- | --- |
|  | 1416 | **748.3962** | **747.3889** | **747.3949** | **-0.0060** | **0** | **15** | **7** | **3** | **K.ICTQVK.G** |
|  | 11732 | **714.3451** | **1426.6757** | **1426.6841** | **-0.0083** | **0** | **39** | **0.018** | **1** | **K.SAIYGFGDQSNLR.K** 11733 |
|  | 12544 | **492.6334** | **1474.8783** | **1474.8871** | **-0.0088** | **0** | **11** | **11** | **1** | **R.NVSNLKPVPLIGPK.L** 12566 |
|  | 12563 | **738.8805** | **1475.7464** | **1475.7555** | **-0.0091** | **0** | **30** | **0.14** | **1** | **K.QATVVLNCVGPYR.F** |
|  | 13944 | **519.6383** | **1555.8931** | **1554.7790** | **1.1141** | **1** | **9** | **17** | **1** | **K.SAIYGFGDQSNLRK.L** |
|  | 15098 | 543.9428 | 1628.8064 | 1628.7869 | 0.0196 | 0 | 0 | 1.1e+002 | 4 | K.GYSIPFMGSDVSVVR.R |
|  | 16491 | **573.2949** | **1716.8628** | **1716.0661** | **0.7966** | **1** | **1** | **83** | **9** | **R.NVSNLKPVPLIGPKLK.R** |
|  | 20348 | 1000.9531 | 1999.8916 | 1999.0085 | 0.8831 | 1 | 3 | 45 | 3 | R.ELKGYSIPFMGSDVSVVR.R |
|  | 26476 | **929.4819** | **2785.4238** | **2784.4334** | **0.9904** | **1** | **27** | **0.13** | **1** | **K.AADKGVYIIGSSGFDSIPADLGVIYTR.N** |
|  | 26813 | **568.8630** | **2839.2785** | **2838.3238** | **0.9547** | **0** | **0** | **51** | **8** | **K.ACIENGASCIDISGEPQFLELMQLK.Y** |
|  | 27267 | **980.5035** | **2938.4888** | **2938.4932** | **-0.0044** | **0** | **58** | **7.7e-005** | **1** | **K.GPEAGYVATPIAMVQAAMTLLSDASHLPK.A** |
|  | 28027 | **775.6298** | **3098.4899** | **3097.5179** | **0.9720** | **0** | **34** | **0.02** | **1** | **K.MNGTLTAVESFLTIHSGPEGLSIHDGTWK.S** |
|  | 30057 | **752.3171** | **3756.5493** | **3755.8249** | **0.7245** | **1** | **0** | **25** | **6** | **R.FYGEPVIKACIENGASCIDISGEPQFLELMQLK.Y** |

| **352.** | P38606\|VATA_HUMAN    **Mass:** 68660    **Score:** 121    **Queries matched:** 18   **emPAI:** 0.30 |
| --- | --- |
|  | V-type proton ATPase catalytic subunit A OS=Homo sapiens GN=ATP6V1A PE=1 SV=2 |

|  | **Query** | **Observed** | **Mr(expt)** | **Mr(calc)** | **Delta** | **Miss** | **Score** | **Expect** | **Rank** | **Peptide** |
| --- | --- | --- | --- | --- | --- | --- | --- | --- | --- | --- |
|  | 6700 | 582.3110 | 1162.6074 | 1162.5466 | 0.0608 | 0 | 5 | 55 | 4 | R.AVETTAQSDNK.I |
|  | 8583 | 630.3890 | 1258.7635 | 1257.7041 | 1.0594 | 1 | 8 | 23 | 2 | R.GVNVSALSRDIK.W |
|  | 9520 | **655.3170** | **1308.6194** | **1307.7197** | **0.8996** | **0** | **37** | **0.03** | **1** | **R.VGHSELVGEIIR.L** |
|  | 9748 | **440.3561** | **1318.0464** | **1318.6477** | **-0.6013** | **1** | **5** | **32** | **2** | **R.RAVETTAQSDNK.I** 9777 |
|  | 16700 | 577.5832 | 1729.7277 | 1730.7934 | -1.0656 | 0 | 7 | 22 | 3 | K.YSNSDVIIYVGCGER.G |
|  | 16713 | **577.7795** | **1730.3168** | **1730.7536** | **-0.4368** | **0** | **1** | **55** | **6** | **K.DDFLQQNGYTPYDR.F** |
|  | 17750 | **901.3907** | **1800.7668** | **1799.8148** | **0.9520** | **0** | **47** | **0.0021** | **1** | **K.SDYAQLLEDMQNAFR.S** |
|  | 18259 | 918.4733 | 1834.9320 | 1834.8416 | 0.0904 | 0 | 5 | 37 | 4 | K.TVGMLSNMIAFYDMAR.R |
|  | 18523 | 927.0058 | 1851.9970 | 1850.8365 | 1.1605 | 0 | (1) | 85 | 10 | K.TVGMLSNMIAFYDMAR.R |
|  | 20585 | **675.3259** | **2022.9560** | **2022.9325** | **0.0234** | **1** | **0** | **88** | **7** | **K.TVGMLSNMIAFYDMARR.A** |
|  | 22858 | **752.0743** | **2253.2010** | **2253.2103** | **-0.0093** | **1** | **35** | **0.026** | **1** | **K.AKEILQEEEDLAEIVQLVGK.A** |
|  | 24188 | **812.6985** | **2435.0738** | **2435.1978** | **-0.1240** | **0** | **2** | **44** | **7** | **R.VLDALFPCVQGGTTAIPGAFGCGK.T** |
|  | 25952 | **539.4844** | **2692.3855** | **2693.2150** | **-0.8295** | **1** | **1** | **50** | **5** | **R.FCPFYKTVGMLSNMIAFYDMAR.R** |
|  | 28333 | **1057.5288** | **3169.5646** | **3169.5707** | **-0.0061** | **1** | **1** | **44** | **1** | **R.GTVTYIAPPGNYDTSDVVLELEFEGVKEK.F** |
|  | 28699 | 1083.1504 | 3246.4293 | 3245.6390 | 0.7903 | 1 | 1 | 35 | 4 | R.TALVANTSNMPVAAREASIYTGITLSEYFR.D |
|  | 30181 | **1272.9873** | **3815.9401** | **3815.9622** | **-0.0222** | **1** | **40** | **0.0034** | **1** | **R.EGSVSIVGAVSPPGGDFSDPVTSATLGIVQVFWGLDKK.L** |
|  | 30663 | **1381.3991** | **4141.1753** | **4141.1882** | **-0.0128** | **0** | **45** | **0.00069** | **1** | **R.TGKPLSVELGPGIMGAIFDGIQRPLSDISSQTQSIYIPR.G** |

| **353.** | Q9Y3Z3\|SAMH1_HUMAN    **Mass:** 72896    **Score:** 120    **Queries matched:** 13   **emPAI:** 0.10 |
| --- | --- |
|  | SAM domain and HD domain-containing protein 1 OS=Homo sapiens GN=SAMHD1 PE=1 SV=2 |

|  | **Query** | **Observed** | **Mr(expt)** | **Mr(calc)** | **Delta** | **Miss** | **Score** | **Expect** | **Rank** | **Peptide** |
| --- | --- | --- | --- | --- | --- | --- | --- | --- | --- | --- |
|  | 1445 | **763.1114** | **762.1042** | **761.3854** | **0.7188** | **1** | **3** | **69** | **3** | **R.ICARDK.E** |
|  | 2017 | **415.2452** | **828.4759** | **828.4705** | **0.0054** | **0** | **13** | **11** | **6** | **K.EVASAKPK.V** |
|  | 6402 | 572.9941 | 1143.9737 | 1144.5472 | -0.5735 | 1 | 7 | 21 | 10 | M.QRADSEQPSK.R |
|  | 7182 | **596.8097** | **1191.6048** | **1190.6659** | **0.9389** | **1** | **7** | **32** | **1** | **R.EILKQIEYR.N** |
|  | 7361 | **600.3783** | **1198.7420** | **1198.6380** | **0.1040** | **0** | **10** | **13** | **1** | **R.LVQIHVDTMK.V** |
|  | 9224 | **647.2817** | **1292.5489** | **1291.5826** | **0.9663** | **1** | **5** | **48** | **2** | **-.MQRADSEQPSK.R** |
|  | 9782 | **660.8388** | **1319.6629** | **1319.6721** | **-0.0092** | **0** | **12** | **8.4** | **1** | **K.YVGETQPTGQIK.I** |
|  | 11881 | **718.3764** | **1434.7382** | **1434.7467** | **-0.0084** | **1** | **4** | **52** | **3** | **R.FENLGVSSLGERK.K** |
|  | 12234 | **729.3396** | **1456.6646** | **1456.6755** | **-0.0109** | **0** | **68** | **2e-005** | **1** | **R.ISTAIDDMEAYTK.L** |
|  | 12943 | **499.9373** | **1496.7900** | **1495.8974** | **0.8927** | **1** | **5** | **37** | **4** | **K.EVASAKPKVLLDVK.L** |
|  | 15439 | **826.4396** | **1650.8647** | **1649.8698** | **0.9949** | **0** | **81** | **9.1e-007** | **1** | **K.VGNIIDTMITDAFLK.A** |
|  | 17784 | 601.9619 | 1802.8637 | 1801.8707 | 0.9930 | 1 | 1 | 74 | 3 | K.EWNDSTSVQNPTRLR.E |
|  | 22430 | **737.3279** | **2208.9618** | **2209.2107** | **-0.2489** | **1** | **5** | **26** | **1** | **R.NFTKPQDGDVIAPLITPQKK.E** |

| **354.** | Q14573\|ITPR3_HUMAN    **Mass:** 306820   **Score:** 119    **Queries matched:** 50   **emPAI:** 0.06 |
| --- | --- |
|  | Inositol 1,4,5-trisphosphate receptor type 3 OS=Homo sapiens GN=ITPR3 PE=1 SV=2 |

|  | **Query** | **Observed** | **Mr(expt)** | **Mr(calc)** | **Delta** | **Miss** | **Score** | **Expect** | **Rank** | **Peptide** |
| --- | --- | --- | --- | --- | --- | --- | --- | --- | --- | --- |
|  | 1186 | 647.3828 | 646.3755 | 646.3286 | 0.0470 | 1 | 13 | 12 | 7 | K.QDKEK.I 1187 |
|  | 1391 | 733.4037 | 732.3964 | 732.4204 | -0.0240 | 0 | 13 | 12 | 2 | R.TLAMVAK.G |
|  | 2035 | **416.2475** | **830.4804** | **829.4657** | **1.0147** | **0** | **21** | **1.6** | **5** | **K.NSDILIR.T** 2020 |
|  | 2953 | 449.7274 | 897.4402 | 897.4854 | -0.0453 | 1 | 7 | 30 | 4 | K.VLHDRMK.R |
|  | 3477 | 468.1966 | 934.3786 | 933.4556 | 0.9230 | 0 | 6 | 41 | 3 | K.DESLFPAR.V |
|  | 3874 | **963.2475** | **962.2402** | **961.5379** | **0.7024** | **1** | **10** | **14** | **1** | **R.TLAMVAKGR.A** |
|  | 3945 | **484.7477** | **967.4809** | **968.4788** | **-0.9978** | **1** | **0** | **1.4e+002** | **10** | **R.RGHEVSER.V** |
|  | 5060 | **523.8058** | **1045.5971** | **1044.6001** | **0.9970** | **0** | **42** | **0.012** | **1** | **K.LLLALMESR.H** |
|  | 5336 | **534.9232** | **1067.8319** | **1067.5586** | **0.2733** | **0** | **7** | **22** | **1** | **K.INLFMQFR.D** |
|  | 5547 | 541.7209 | 1081.4272 | 1082.4307 | -1.0035 | 0 | 3 | 67 | 4 | K.MCGVGEQMR.K |
|  | 5794 | **550.3282** | **1098.6418** | **1098.5669** | **0.0749** | **1** | **4** | **58** | **5** | **R.EPVDPTTKGR.V** |
|  | 6451 | **574.7893** | **1147.5641** | **1147.5516** | **0.0124** | **1** | **1** | **1.2e+002** | **7** | **K.AAGMGAQGRTGR.R** |
|  | 6733 | 583.8053 | 1165.5960 | 1164.5775 | 1.0186 | 1 | 10 | 13 | 2 | K.KAYLQEEER.E |
|  | 6969 | **592.2934** | **1182.5722** | **1182.5815** | **-0.0093** | **0** | **5** | **49** | **3** | **K.LQHAAQMEQK.Q** |
|  | 7109 | 595.2982 | 1188.5818 | 1188.6040 | -0.0222 | 1 | 4 | 64 | 5 | K.NKNLDWFPR.M |
|  | 7253 | **399.2413** | **1194.7021** | **1194.5308** | **0.1713** | **1** | **7** | **29** | **4** | **K.MCGVGEQMRK.K** |
|  | 7609 | **404.7052** | **1211.0937** | **1210.5257** | **0.5680** | **1** | **(7)** | **20** | **7** | **K.MCGVGEQMRK.K** |
|  | 7908 | **613.8795** | **1225.7445** | **1226.5206** | **-0.7761** | **1** | **(1)** | **1.2e+002** | **6** | **K.MCGVGEQMRK.K** |
|  | 8174 | **414.2374** | **1239.6904** | **1240.5216** | **-0.8313** | **0** | **8** | **24** | **1** | **K.SFHNLMMSDK.K** |
|  | 11168 | **697.7999** | **1393.5852** | **1394.5984** | **-1.0132** | **0** | **2** | **90** | **6** | **K.TSSMLEVDDEGGR.M** |
|  | 11211 | 699.3937 | 1396.7728 | 1396.7787 | -0.0058 | 1 | 6 | 30 | 6 | R.NGGGVGDILRKPSK.D |
|  | 11605 | **710.8451** | **1419.6757** | **1419.7656** | **-0.0899** | **1** | **3** | **67** | **9** | **R.KMLLQNYLQNR.K** |
|  | 11627 | 711.3633 | 1420.7120 | 1421.6577 | -0.9457 | 1 | 6 | 31 | 10 | R.LNKMCGVGEQMR.K |
|  | 12690 | 742.3441 | 1482.6736 | 1482.6838 | -0.0103 | 0 | 50 | 0.0012 | 1 | R.LFTTTEQDEQGSK.V |
|  | 12812 | **497.2987** | **1488.8743** | **1487.6787** | **1.1956** | **1** | **6** | **31** | **1** | **K.GDASDPKAAGMGAQGR.T** 12789 |
|  | 12834 | **497.7432** | **1490.2077** | **1489.7810** | **0.4266** | **1** | **2** | **52** | **8** | **R.MDLVLQLKDNASK.L** |
|  | 13053 | **501.9715** | **1502.8928** | **1503.6736** | **-0.7808** | **1** | **(5)** | **38** | **3** | **K.GDASDPKAAGMGAQGR.T** |
|  | 14231 | **526.2740** | **1575.8001** | **1575.8290** | **-0.0289** | **1** | **1** | **1.1e+002** | **5** | **K.DNASKLLLALMESR.H** |
|  | 17935 | **605.2907** | **1812.8503** | **1812.8451** | **0.0052** | **1** | **3** | **54** | **3** | **R.SKFEENEDIVVMETK.L** |
|  | 18242 | 612.2925 | 1833.8556 | 1833.9546 | -0.0990 | 1 | 3 | 58 | 4 | K.VMLDLLQIPYDKGDAK.M 18214 |
|  | 18649 | **931.9240** | **1861.8335** | **1861.8476** | **-0.0141** | **0** | **57** | **0.00019** | **1** | **R.AMSLVSNEGEGEQNEIR.I** |
|  | 18832 | **626.8443** | **1877.5111** | **1876.9321** | **0.5789** | **1** | **7** | **15** | **1** | **R.SIQGVGHMMSTMVLSRK.Q** |
|  | 19902 | **654.3060** | **1959.8961** | **1959.9248** | **-0.0287** | **1** | **4** | **42** | **1** | **K.TDYTGPESYVAQMIKNK.N** |
|  | 20170 | **662.8571** | **1985.5495** | **1984.8909** | **0.6587** | **0** | **3** | **30** | **1** | **K.STVAVNMNDLGSQPHEDR.E** |
|  | 20235 | 996.4906 | 1990.9666 | 1990.9517 | 0.0150 | 0 | 1 | 69 | 6 | R.IQEEEAEGISSMLSLNNK.Q |
|  | 20326 | **667.1732** | **1998.4978** | **1998.9792** | **-0.4814** | **1** | **2** | **38** | **3** | **K.LLLALMESRHDSENAER.I** |
|  | 21773 | 1068.5576 | 2135.1007 | 2134.9996 | 0.1011 | 0 | 5 | 26 | 2 | R.ACDTLLMCIVTVMNHGLR.N 21775 |
|  | 22049 | **722.6736** | **2164.9989** | **2164.9841** | **0.0148** | **1** | **2** | **55** | **5** | **R.MRAMSLVSNEGEGEQNEIR.I** |
|  | 23761 | **790.0761** | **2367.2063** | **2367.2216** | **-0.0153** | **1** | **37** | **0.015** | **1** | **K.LVSHLTAQLNELKEQMTEQR.K** |
|  | 25956 | **898.7368** | **2693.1885** | **2693.1659** | **0.0225** | **0** | **6** | **13** | **1** | **K.CQDMIMTELTNAGDDVVVFYNDK.A** |
|  | 26792 | **946.4428** | **2836.3064** | **2836.3298** | **-0.0233** | **1** | **26** | **0.16** | **1** | **K.STVAVNMNDLGSQPHEDREPVDPTTK.G** |
|  | 28280 | **1052.1213** | **3153.3422** | **3153.5223** | **-0.1801** | **1** | **2** | **22** | **4** | **K.AARDGVEDHSPLMYHISLVDLLAACAEGK.N** |
|  | 28468 | 799.9027 | 3195.5817 | 3195.6215 | -0.0398 | 1 | 3 | 21 | 3 | K.TVSFEEHIKLEHNMWNYLYFIVLVR.V 28470 |
|  | 29000 | 833.8968 | 3331.5581 | 3332.4602 | -0.9021 | 0 | 4 | 18 | 1 | K.EFVEVFPMQDSGADGTAPAFDSTTANMNLDR.I |

| **355.** | P39656\|OST48_HUMAN    **Mass:** 50940    **Score:** 119    **Queries matched:** 6   **emPAI:** 0.24 |
| --- | --- |
|  | Dolichyl-diphosphooligosaccharide--protein glycosyltransferase 48 kDa subunit OS=Homo sapiens GN=DDOST PE=1 SV=4 |

|  | **Query** | **Observed** | **Mr(expt)** | **Mr(calc)** | **Delta** | **Miss** | **Score** | **Expect** | **Rank** | **Peptide** |
| --- | --- | --- | --- | --- | --- | --- | --- | --- | --- | --- |
|  | 8762 | **635.2881** | **1268.5617** | **1268.7452** | **-0.1835** | **0** | **0** | **1.3e+002** | **9** | **R.TLVLLDNLNVR.E** |
|  | 15020 | 812.8950 | 1623.7754 | 1623.9168 | -0.1415 | 1 | 4 | 50 | 3 | K.NTLLIAGLQARNNAR.V |
|  | 30845 | **1084.2915** | **4333.1369** | **4333.1617** | **-0.0248** | **0** | **(45)** | **0.00067** | **1** | **R.GVGMVADPDNPLVLDILTGSSTSYSFFPDKPITQYPHAVGK.N** 30846 30848 |
|  | 30855 | **1088.2914** | **4349.1364** | **4349.1566** | **-0.0202** | **0** | **46** | **0.00051** | **1** | **R.GVGMVADPDNPLVLDILTGSSTSYSFFPDKPITQYPHAVGK.N** |

| **356.** | P08729\|K2C7_HUMAN    **Mass:** 51443    **Score:** 119    **Queries matched:** 20   **emPAI:** 0.23 |
| --- | --- |
|  | Keratin, type II cytoskeletal 7 OS=Homo sapiens GN=KRT7 PE=1 SV=3 |

|  | **Query** | **Observed** | **Mr(expt)** | **Mr(calc)** | **Delta** | **Miss** | **Score** | **Expect** | **Rank** | **Peptide** |
| --- | --- | --- | --- | --- | --- | --- | --- | --- | --- | --- |
|  | 3084 | 454.2368 | 906.4590 | 905.4607 | 0.9984 | 0 | 6 | 41 | 9 | R.FLEQQNK.L |
|  | 4014 | **487.2860** | **972.5575** | **971.5400** | **1.0176** | **1** | **3** | **88** | **10** | **R.GELALKDAR.A** |
|  | 4648 | **509.2752** | **1016.5359** | **1016.5185** | **0.0174** | **1** | **8** | **27** | **7** | **K.QDMARQLR.E** |
|  | 7495 | **402.7117** | **1205.1132** | **1205.6265** | **-0.5133** | **1** | **9** | **12** | **6** | **R.SAAFSGRGAQVR.L** |
|  | 8838 | 636.8536 | 1271.6927 | 1271.6973 | -0.0045 | 0 | 77 | 2.9e-006 | 1 | R.SLDLDGIIAEVK.A |
|  | 10304 | **450.2506** | **1347.7300** | **1347.7398** | **-0.0098** | **1** | **3** | **74** | **9** | **R.TAAENEFVVLKK.D** |
|  | 11018 | **693.3680** | **1384.7215** | **1384.7310** | **-0.0095** | **1** | **44** | **0.0056** | **1** | **R.AKQEELEAALQR.A** 11017 11019 |
|  | 11201 | 699.2877 | 1396.5608 | 1396.7350 | -0.1743 | 1 | 8 | 20 | 4 | K.TLNNKFASFIDK.V 11202 |
|  | 11578 | 709.8644 | 1417.7143 | 1417.7201 | -0.0058 | 0 | 55 | 0.0004 | 1 | K.VDALNDEINFLR.T |
|  | 12802 | **497.2805** | **1488.8197** | **1489.8140** | **-0.9943** | **1** | **6** | **33** | **5** | **R.FLEQQNKLLETK.W** 12793 12833 |
|  | 15122 | **544.3161** | **1629.9265** | **1628.9137** | **1.0127** | **1** | **6** | **30** | **6** | **K.LLETKWTLLQEQK.S** |
|  | 17945 | **454.2688** | **1813.0461** | **1811.9628** | **1.0833** | **1** | **6** | **24** | **1** | **K.LEAAIAEAEERGELALK.D** |
|  | 22813 | **749.3325** | **2244.9756** | **2245.1927** | **-0.2172** | **0** | **8** | **13** | **1** | **R.LSSARPGGLGSSSLYGLGASRPR.V** 22814 |
|  | 31369 | **1298.6235** | **5190.4651** | **5189.5609** | **0.9041** | **1** | **0** | **13** | **3** | **R.LAGDGVGAVNISVMNSTGGSSSGGGIGLTLGGTMGSNALSFSSSAGPGLLKAYSIR.T** |

| **357.** | Q12907\|LMAN2_HUMAN    **Mass:** 40545    **Score:** 119    **Queries matched:** 9   **emPAI:** 0.30 |
| --- | --- |
|  | Vesicular integral-membrane protein VIP36 OS=Homo sapiens GN=LMAN2 PE=1 SV=1 |

|  | **Query** | **Observed** | **Mr(expt)** | **Mr(calc)** | **Delta** | **Miss** | **Score** | **Expect** | **Rank** | **Peptide** |
| --- | --- | --- | --- | --- | --- | --- | --- | --- | --- | --- |
|  | 5070 | **524.2625** | **1046.5105** | **1046.5179** | **-0.0074** | **0** | **6** | **41** | **2** | **K.NCIDITGVR.L** |
|  | 11718 | 713.8199 | 1425.6252 | 1425.6347 | -0.0095 | 0 | 46 | 0.0035 | 1 | R.WTELAGCTADFR.N |
|  | 16533 | **574.2848** | **1719.8325** | **1720.8342** | **-1.0016** | **1** | **6** | **26** | **2** | **R.LTVMTDLEDKNEWK.N** |
|  | 23350 | **773.3672** | **2317.0797** | **2317.0936** | **-0.0139** | **0** | **20** | **0.82** | **1** | **K.LFQLMVEHTPDEESIDWTK.I** |
|  | 24421 | **823.3783** | **2467.1131** | **2467.1292** | **-0.0161** | **0** | **13** | **3.7** | **1** | **K.DNFHGLAIFLDTYPNDETTER.V** |
|  | 25985 | **900.9923** | **2699.9551** | **2700.5839** | **-0.6288** | **0** | **1** | **33** | **3** | **R.VFLLLLCALLGIVVCAVVGAVVFQK.R** |
|  | 26195 | 910.7560 | 2729.2463 | 2729.2643 | -0.0180 | 0 | 92 | 4.3e-008 | 1 | R.LPTGYYFGASAGTGDLSDNHDIISMK.L |
|  | 29058 | **837.1686** | **3344.6452** | **3344.6639** | **-0.0186** | **1** | **13** | **2.4** | **1** | **K.LFQLMVEHTPDEESIDWTKIEPSVNFLK.S** |
|  | 30315 | 1301.0126 | 3900.0159 | 3900.8927 | -0.8768 | 1 | 2 | 20 | 10 | K.REHSLIKPYQGVGSSSMPLWDFQGSTMLTSQYVR.L |

| **358.** | P51148\|RAB5C_HUMAN    **Mass:** 23696    **Score:** 119    **Queries matched:** 24   **emPAI:** 0.35 |
| --- | --- |
|  | Ras-related protein Rab-5C OS=Homo sapiens GN=RAB5C PE=1 SV=2 |

|  | **Query** | **Observed** | **Mr(expt)** | **Mr(calc)** | **Delta** | **Miss** | **Score** | **Expect** | **Rank** | **Peptide** |
| --- | --- | --- | --- | --- | --- | --- | --- | --- | --- | --- |
|  | 5409 | 536.8001 | 1071.5855 | 1071.5825 | 0.0030 | 1 | 12 | 11 | 2 | K.NWVKELQR.Q 5423 |
|  | 7594 | 606.2866 | 1210.5586 | 1210.5690 | -0.0105 | 0 | 29 | 0.16 | 1 | K.NEPQNATGAPGR.N 7597 7599 7616 7617 |
|  | 9358 | **650.8064** | **1299.5982** | **1299.6070** | **-0.0087** | **0** | **29** | **0.16** | **1** | **R.YHSLAPMYYR.G** 9356 |
|  | 13880 | **776.8928** | **1551.7711** | **1551.7789** | **-0.0078** | **0** | **64** | **5.5e-005** | **1** | **K.TAMNVNEIFMAIAK.K** 13866 13878 13879 |
|  | 14152 | **524.2541** | **1569.7404** | **1568.7655** | **0.9749** | **1** | **5** | **42** | **2** | **R.NRGVDLQENNPASR.S** |
|  | 15441 | **551.3081** | **1650.9025** | **1651.8325** | **-0.9300** | **1** | **(0)** | **1.1e+002** | **6** | **-.MAGRGGAARPNGPAAGNK.I** |
|  | 15710 | **834.4656** | **1666.9166** | **1667.8274** | **-0.9108** | **1** | **8** | **16** | **1** | **-.MAGRGGAARPNGPAAGNK.I** 15700 15739 15749 15750 |
|  | 16855 | **871.0092** | **1740.0038** | **1740.0509** | **-0.0471** | **1** | **0** | **96** | **7** | **K.LVLLGESAVGKSSLVLR.F** |
|  | 19348 | **641.2129** | **1920.6170** | **1921.0745** | **-0.4574** | **1** | **2** | **47** | **10** | **K.ELQRQASPNIVIALAGNK.A** |
|  | 28946 | **665.3643** | **3321.7849** | **3322.6081** | **-0.8232** | **1** | **1** | **28** | **8** | **R.YHSLAPMYYRGAQAAIVVYDITNTDTFAR.A** |
|  | 30780 | **1065.9747** | **4259.8698** | **4258.9748** | **0.8950** | **1** | **0** | **20** | **1** | **R.AVEFQEAQAYADDNSLLFMETSAKTAMNVNEIFMAIAK.K** |

| **359.** | P30504\|1C04_HUMAN    **Mass:** 41368    **Score:** 118    **Queries matched:** 21   **emPAI:** 0.54 |
| --- | --- |
|  | HLA class I histocompatibility antigen, Cw-4 alpha chain OS=Homo sapiens GN=HLA-C PE=1 SV=1 |

|  | **Query** | **Observed** | **Mr(expt)** | **Mr(calc)** | **Delta** | **Miss** | **Score** | **Expect** | **Rank** | **Peptide** |
| --- | --- | --- | --- | --- | --- | --- | --- | --- | --- | --- |
|  | 3888 | 482.7234 | 963.4322 | 964.4250 | -0.9929 | 0 | 33 | 0.088 | 1 | R.FDSDAASPR.G 3900 3901 3907 |
|  | 10335 | 675.8494 | 1349.6843 | 1349.6939 | -0.0096 | 1 | 38 | 0.024 | 1 | R.YLENGKETLQR.A 10311 10351 |
|  | 11313 | 468.5608 | 1402.6605 | 1403.6429 | -0.9825 | 1 | 8 | 22 | 2 | R.FDSDAASPRGEPR.E |
|  | 11736 | 714.3626 | 1426.7105 | 1426.7205 | -0.0099 | 0 | 46 | 0.0031 | 1 | K.WAAVVVPSGEEQR.Y 11721 11735 11751 |
|  | 12921 | 748.8587 | 1495.7028 | 1495.7129 | -0.0101 | 0 | 57 | 0.00026 | 1 | R.AYLEGTCVEWLR.R |
|  | 13321 | 760.3871 | 1518.7596 | 1518.7427 | 0.0169 | 0 | 62 | 7.5e-005 | 1 | R.SWTAADTAAQITQR.K |
|  | 14680 | 803.3827 | 1604.7508 | 1605.7756 | -1.0247 | 1 | 2 | 71 | 6 | R.MFGCDLGPDGRLLR.G |
|  | 18283 | 613.3167 | 1836.9283 | 1835.9027 | 1.0256 | 0 | 15 | 3.7 | 1 | K.THVTHHPVSDHEATLR.C |
|  | 20871 | 410.6170 | 2048.0485 | 2049.0102 | -0.9617 | 0 | 9 | 11 | 2 | R.YTCHVQHEGLPEPLTLR.W 20872 20886 20897 |
|  | 29986 | **1242.2772** | **3723.8098** | **3723.9083** | **-0.0985** | **1** | **1** | **29** | **3** | **R.TLILLLSGALALTETWAGSHSMRYFSTSVSWPGR.G** |

| **360.** | P09525\|ANXA4_HUMAN    **Mass:** 36088    **Score:** 118    **Queries matched:** 12   **emPAI:** 0.64 |
| --- | --- |
|  | Annexin A4 OS=Homo sapiens GN=ANXA4 PE=1 SV=4 |

|  | **Query** | **Observed** | **Mr(expt)** | **Mr(calc)** | **Delta** | **Miss** | **Score** | **Expect** | **Rank** | **Peptide** |
| --- | --- | --- | --- | --- | --- | --- | --- | --- | --- | --- |
|  | 2604 | **435.8868** | **869.7591** | **869.3515** | **0.4076** | **0** | **9** | **15** | **7** | **K.GDTSGDYR.K** |
|  | 5440 | **538.2725** | **1074.5304** | **1074.5379** | **-0.0076** | **0** | **41** | **0.013** | **1** | **R.AEIDMLDIR.A** 5461 |
|  | 6859 | **588.3276** | **1174.6406** | **1173.5990** | **1.0416** | **0** | **4** | **61** | **4** | **K.GLGTDDNTLIR.V** 6860 |
|  | 9146 | **645.3015** | **1288.5885** | **1287.7034** | **0.8851** | **1** | **11** | **11** | **1** | **R.ISQKDIEQSIK.S** |
|  | 10757 | **686.3313** | **1370.6480** | **1370.6579** | **-0.0098** | **0** | **43** | **0.0065** | **1** | **R.ISQTYQQQYGR.S** |
|  | 15606 | **831.4073** | **1660.8001** | **1660.8090** | **-0.0089** | **0** | **78** | **1.8e-006** | **1** | **K.GAGTDEGCLIEILASR.T** |
|  | 15646 | **555.2731** | **1662.7974** | **1662.8433** | **-0.0459** | **1** | **0** | **1.1e+002** | **7** | **R.VMVSRAEIDMLDIR.A** |
|  | 15688 | **833.9260** | **1665.8374** | **1665.8461** | **-0.0087** | **0** | **23** | **0.52** | **1** | **K.SETSGSFEDALLAIVK.C** |
|  | 23365 | **773.7309** | **2318.1709** | **2318.1866** | **-0.0158** | **1** | **34** | **0.029** | **1** | **R.VLVSLSAGGRDEGNYLDDALVR.Q** |
|  | 30284 | **1294.9709** | **3881.8910** | **3882.9271** | **-1.0361** | **1** | **0** | **30** | **9** | **R.DLIDDLKSELSGNFEQVIVGMMTPTVLYDVQELR.R** |

| **361.** | Q92959\|SO2A1_HUMAN    **Mass:** 71367    **Score:** 117    **Queries matched:** 9   **emPAI:** 0.05 |
| --- | --- |
|  | Solute carrier organic anion transporter family member 2A1 OS=Homo sapiens GN=SLCO2A1 PE=1 SV=2 |

|  | **Query** | **Observed** | **Mr(expt)** | **Mr(calc)** | **Delta** | **Miss** | **Score** | **Expect** | **Rank** | **Peptide** |
| --- | --- | --- | --- | --- | --- | --- | --- | --- | --- | --- |
|  | 2270 | **423.2587** | **844.5029** | **844.4290** | **0.0739** | **0** | **3** | **1.2e+002** | **6** | **R.VVNQEEK.S** 2264 |
|  | 5211 | **529.8102** | **1057.6059** | **1056.5312** | **1.0747** | **1** | **13** | **8.3** | **2** | **K.RAPATADEAR.K** |
|  | 8705 | **633.7940** | **1265.5735** | **1265.5848** | **-0.0113** | **0** | **78** | **2.2e-006** | **1** | **K.LGASQGSDTSTSR.A** 8704 |
|  | 19345 | **960.9830** | **1919.9515** | **1920.9575** | **-1.0060** | **1** | **1** | **73** | **1** | **-.MGLLPKLGASQGSDTSTSR.A** 19364 |
|  | 19470 | 644.0005 | 1928.9796 | 1927.9132 | 1.0665 | 0 | 1 | 75 | 7 | K.QLIYLNCSCVTGGSASAK.T |
|  | 25638 | 883.4349 | 2647.2830 | 2648.3681 | -1.0851 | 1 | 4 | 29 | 6 | R.YLGLQMGYKALGMLLLCFISWR.V |

| **362.** | P20648\|ATP4A_HUMAN    **Mass:** 115756   **Score:** 117    **Queries matched:** 26   **emPAI:** 0.10 |
| --- | --- |
|  | Potassium-transporting ATPase alpha chain 1 OS=Homo sapiens GN=ATP4A PE=2 SV=5 |

|  | **Query** | **Observed** | **Mr(expt)** | **Mr(calc)** | **Delta** | **Miss** | **Score** | **Expect** | **Rank** | **Peptide** |
| --- | --- | --- | --- | --- | --- | --- | --- | --- | --- | --- |
|  | 875 | 530.2573 | 529.2500 | 528.2656 | 0.9844 | 0 | 11 | 24 | 8 | K.GAPER.V |
|  | 2073 | **417.2333** | **832.4520** | **832.4337** | **0.0183** | **1** | **21** | **1.8** | **2** | **K.CRTAGIR.V** |
|  | 3152 | 456.7453 | 911.4761 | 912.5280 | -1.0519 | 0 | 17 | 3.2 | 3 | R.ATVPDAVLK.C |
|  | 4108 | **490.2739** | **978.5332** | **979.5338** | **-1.0007** | **0** | **9** | **21** | **7** | **K.STNIIASFK.N** |
|  | 4720 | 512.7546 | 1023.4947 | 1023.5964 | -0.1017 | 1 | 1 | 1.3e+002 | 6 | K.KSIAYTLTK.N |
|  | 6278 | **567.8857** | **1133.7569** | **1132.5733** | **1.1837** | **0** | **8** | **25** | **1** | **R.ACVINGMQLK.D** |
|  | 7392 | **401.2121** | **1200.6146** | **1199.6087** | **1.0058** | **0** | **11** | **13** | **3** | **R.LSAFQQGFFR.N** |
|  | 10826 | **459.0107** | **1374.0101** | **1374.7224** | **-0.7123** | **1** | **6** | **22** | **2** | **R.ALCRVLTLCNR.A** |
|  | 10962 | 691.3739 | 1380.7332 | 1380.7435 | -0.0103 | 0 | 47 | 0.0027 | 1 | R.VIMVTGDHPITAK.A 10963 10964 |
|  | 11104 | **696.1816** | **1390.3486** | **1389.6446** | **0.7040** | **0** | **1** | **73** | **10** | **K.DMDPSELVEALR.T** |
|  | 12199 | **485.7471** | **1454.2194** | **1454.7518** | **-0.5324** | **0** | **3** | **40** | **9** | **K.FQLSIHTLEDPR.D** |
|  | 12535 | **492.6123** | **1474.8150** | **1474.7384** | **0.0765** | **1** | **0** | **1.3e+002** | **9** | **K.DARACVINGMQLK.D** |
|  | 13169 | **756.0540** | **1510.0934** | **1510.8290** | **-0.7356** | **1** | **3** | **39** | **2** | **R.VPADIRILAAQGCK.V** |
|  | 14200 | **787.4094** | **1572.8042** | **1573.8134** | **-1.0092** | **1** | **2** | **91** | **5** | **K.KADIGVAMGIAGSDAAK.N** |
|  | 14939 | 810.3740 | 1618.7335 | 1618.7435 | -0.0100 | 0 | 68 | 2e-005 | 1 | K.VDNSSLTGESEPQTR.S 14925 14938 |
|  | 15625 | **554.9095** | **1661.7066** | **1662.8624** | **-1.1558** | **1** | **4** | **45** | **2** | **K.AESDIMHLRPRNPK.R** |
|  | 19381 | 962.5222 | 1923.0298 | 1924.0629 | -1.0332 | 1 | 4 | 35 | 2 | R.LGAIVAVTGDGVNDSPALKK.A |
|  | 15557 | **830.3532** | **2488.0378** | **2488.2124** | **-0.1746** | **1** | **(0)** | **1.5e+002** | **3** | **R.ACVINGMQLKDMDPSELVEALR.T** |
|  | 24816 | **631.3106** | **2521.2131** | **2520.2022** | **1.0109** | **1** | **1** | **61** | **8** | **R.ACVINGMQLKDMDPSELVEALR.T** |
|  | 25539 | 877.0815 | 2628.2228 | 2629.2040 | -0.9812 | 1 | 0 | 53 | 4 | K.AENYELYSVELGPGPGGDMAAKMSK.K |
|  | 26604 | **562.2648** | **2806.2875** | **2807.3283** | **-1.0408** | **1** | **0** | **54** | **5** | **K.EMEINDHQLSVAELEQKYQTSATK.G** 26613 |

| **363.** | P51149\|RAB7A_HUMAN    **Mass:** 23760    **Score:** 117    **Queries matched:** 12   **emPAI:** 1.11 |
| --- | --- |
|  | Ras-related protein Rab-7a OS=Homo sapiens GN=RAB7A PE=1 SV=1 |

|  | **Query** | **Observed** | **Mr(expt)** | **Mr(calc)** | **Delta** | **Miss** | **Score** | **Expect** | **Rank** | **Peptide** |
| --- | --- | --- | --- | --- | --- | --- | --- | --- | --- | --- |
|  | 2501 | 863.0857 | 862.0784 | 862.3855 | -0.3071 | 0 | 3 | 72 | 8 | K.EVMVDDR.L |
|  | 4906 | 518.7839 | 1035.5532 | 1035.5601 | -0.0069 | 0 | 38 | 0.025 | 1 | K.ATIGADFLTK.E |
|  | 5192 | **529.3130** | **1056.6114** | **1056.6179** | **-0.0065** | **0** | **38** | **0.029** | **1** | **K.VIILGDSGVGK.T** |
|  | 7078 | **594.3106** | **1186.6065** | **1186.6135** | **-0.0069** | **0** | **36** | **0.041** | **1** | **R.FQSLGVAFYR.G** |
|  | 7645 | 607.3141 | 1212.6136 | 1212.5809 | 0.0328 | 0 | 4 | 55 | 8 | K.TSLMNQYVNK.K |
|  | 14460 | **795.4128** | **1588.8110** | **1588.8209** | **-0.0099** | **0** | **64** | **5.5e-005** | **1** | **K.EAINVEQAFQTIAR.N** 14459 |
|  | 15389 | **412.9833** | **1647.9039** | **1646.8086** | **1.0953** | **0** | **9** | **15** | **3** | **R.LVTMQIWDTAGQER.F** |
|  | 15632 | 831.9434 | 1661.8722 | 1662.8036 | -0.9314 | 0 | (9) | 15 | 6 | R.LVTMQIWDTAGQER.F 15648 |
|  | 19528 | **967.4823** | **1932.9500** | **1932.9694** | **-0.0193** | **1** | **25** | **0.3** | **1** | **K.TLDSWRDEFLIQASPR.D** |
|  | 28401 | **796.6171** | **3182.4394** | **3181.5366** | **0.9029** | **1** | **1** | **33** | **1** | **R.FQSLGVAFYRGADCCVLVFDVTAPNTFK.T** |

| **364.** | P20674\|COX5A_HUMAN    **Mass:** 16923    **Score:** 117    **Queries matched:** 15   **emPAI:** 2.46 |
| --- | --- |
|  | Cytochrome c oxidase subunit 5A, mitochondrial OS=Homo sapiens GN=COX5A PE=1 SV=2 |

|  | **Query** | **Observed** | **Mr(expt)** | **Mr(calc)** | **Delta** | **Miss** | **Score** | **Expect** | **Rank** | **Peptide** |
| --- | --- | --- | --- | --- | --- | --- | --- | --- | --- | --- |
|  | 1464 | **771.4673** | **770.4600** | **770.4650** | **-0.0050** | **0** | **35** | **0.05** | **1** | **K.IIDAALR.A** |
|  | 4493 | **1005.4136** | **1004.4064** | **1004.5185** | **-0.1122** | **1** | **10** | **16** | **6** | **R.RCAVAATTR.A** |
|  | 4704 | **511.7634** | **1021.5122** | **1021.5193** | **-0.0070** | **0** | **52** | **0.001** | **1** | **R.LNDFASTVR.I** 4712 |
|  | 6610 | **579.2920** | **1156.5694** | **1157.6339** | **-1.0645** | **1** | **10** | **18** | **1** | **K.IIDAALRACR.R** |
|  | 7168 | **397.8761** | **1190.6065** | **1191.6472** | **-1.0408** | **1** | **7** | **33** | **5** | **R.ADPRGLLHSAR.T** |
|  | 15851 | **838.9265** | **1675.8385** | **1675.8491** | **-0.0106** | **0** | **47** | **0.0024** | **1** | **K.GINTLVTYDMVPEPK.I** 15826 |
|  | 20917 | **684.7358** | **2051.1855** | **2052.0105** | **-0.8250** | **0** | **1** | **64** | **2** | **R.WVTYFNKPDIDAWELR.K** |
|  | 22188 | **727.7047** | **2180.0921** | **2180.1055** | **-0.0133** | **1** | **28** | **0.13** | **1** | **R.WVTYFNKPDIDAWELRK.G** |
|  | 24150 | **810.1216** | **2427.3431** | **2428.3036** | **-0.9605** | **1** | **1** | **57** | **8** | **K.GINTLVTYDMVPEPKIIDAALR.A** |
|  | 29311 | **1143.6054** | **3427.7942** | **3427.8126** | **-0.0184** | **1** | **30** | **0.032** | **1** | **K.EIYPYVIQELRPTLNELGISTPEELGLDKV.-** 29313 29314 29316 |

| **365.** | O75381\|PEX14_HUMAN    **Mass:** 41269    **Score:** 115    **Queries matched:** 10   **emPAI:** 0.30 |
| --- | --- |
|  | Peroxisomal membrane protein PEX14 OS=Homo sapiens GN=PEX14 PE=1 SV=1 |

|  | **Query** | **Observed** | **Mr(expt)** | **Mr(calc)** | **Delta** | **Miss** | **Score** | **Expect** | **Rank** | **Peptide** |
| --- | --- | --- | --- | --- | --- | --- | --- | --- | --- | --- |
|  | 4658 | **509.7493** | **1017.4840** | **1018.5672** | **-1.0832** | **1** | **2** | **1e+002** | **8** | **K.FLQNSRVR.Q** |
|  | 8199 | **414.7479** | **1241.2219** | **1241.7860** | **-0.5640** | **1** | **7** | **19** | **5** | **K.KYLLPLILGGR.E** |
|  | 9236 | 647.3572 | 1292.6999 | 1292.7088 | -0.0089 | 0 | 15 | 5.1 | 1 | K.IQELAHELAAAK.A |
|  | 25539 | 877.0815 | 2628.2228 | 2629.1926 | -0.9698 | 0 | 2 | 37 | 3 | -.MASSEQAEQPSQPSSTPGSENVLPR.E |
|  | 26763 | **945.1526** | **2832.4359** | **2832.4505** | **-0.0145** | **1** | **31** | **0.045** | **1** | **K.ATTSTNWILESQNINELKSEINSLK.G** 26760 |
|  | 30242 | **1286.6704** | **3856.9894** | **3857.0092** | **-0.0198** | **0** | **58** | **4.7e-005** | **1** | **R.MEAGLSELSGSVAQTVTQLQTTLASVQELLIQQQQK.I** 30240 30241 30243 |

| **366.** | P08575\|CD45_HUMAN    **Mass:** 148644   **Score:** 115    **Queries matched:** 26   **emPAI:** 0.08 |
| --- | --- |
|  | Leukocyte common antigen OS=Homo sapiens GN=PTPRC PE=1 SV=2 |

|  | **Query** | **Observed** | **Mr(expt)** | **Mr(calc)** | **Delta** | **Miss** | **Score** | **Expect** | **Rank** | **Peptide** |
| --- | --- | --- | --- | --- | --- | --- | --- | --- | --- | --- |
|  | 4486 | **502.8076** | **1003.6006** | **1004.5689** | **-0.9683** | **0** | **8** | **29** | **4** | **K.ATVIVMVTR.C** |
|  | 7205 | **597.4044** | **1192.7943** | **1191.6070** | **1.1873** | **1** | **12** | **7.4** | **1** | **K.RCPDYIIQK.L** |
|  | 9110 | **644.3243** | **1286.6341** | **1285.6150** | **1.0191** | **1** | **1** | **1.1e+002** | **8** | **K.DTDKSSTYTLR.V** |
|  | 9329 | **649.8125** | **1297.6104** | **1296.6058** | **1.0046** | **0** | **27** | **0.25** | **1** | **R.TQHIGNQEENK.S** |
|  | 10431 | 677.8180 | 1353.6215 | 1353.6313 | -0.0098 | 0 | 7 | 27 | 1 | R.NSNVIPYDYNR.V 10419 |
|  | 10794 | **687.2976** | **1372.5807** | **1372.5904** | **-0.0097** | **0** | **42** | **0.0079** | **1** | **R.FQCGNMIFDNK.E** 10790 |
|  | 10985 | **692.3659** | **1382.7173** | **1381.7241** | **0.9931** | **0** | **20** | **1.3** | **1** | **K.YDLQNLKPYTK.Y** 10974 |
|  | 11102 | 695.8710 | 1389.7275 | 1389.8020 | -0.0745 | 0 | 11 | 12 | 2 | K.YVLSLHAYIIAK.V |
|  | 11324 | **702.8277** | **1403.6408** | **1403.6504** | **-0.0095** | **0** | **12** | **8.5** | **1** | **K.FQLHDCTQVEK.A** |
|  | 13008 | 501.0761 | 1500.2066 | 1499.7732 | 0.4334 | 0 | 7 | 16 | 4 | R.YHLEVEAGNTLVR.N |
|  | 14729 | **804.3959** | **1606.7772** | **1606.7879** | **-0.0107** | **1** | **37** | **0.022** | **1** | **R.VKDLQYSTDYTFK.A** |
|  | 16573 | **862.4045** | **1722.7945** | **1722.8036** | **-0.0090** | **0** | **15** | **3.3** | **1** | **K.TDFGSPGEPQIIFCR.S** 16572 |
|  | 17945 | 454.2688 | 1813.0461 | 1813.8821 | -0.8360 | 1 | 2 | 57 | 9 | K.ETIGDFWQMIFQRK.V |
|  | 18492 | **617.7871** | **1850.3393** | **1850.0301** | **0.3092** | **1** | **0** | **66** | **2** | **K.NLIKYDLQNLKPYTK.Y** |
|  | 19928 | **654.8262** | **1961.4567** | **1961.0370** | **0.4196** | **1** | **1** | **49** | **4** | **K.IADEGRLFLAEFQSIPR.V** |
|  | 24253 | **1222.5829** | **2443.1512** | **2443.1696** | **-0.0183** | **0** | **91** | **5.5e-008** | **1** | **R.TVYQYQYTNWSVEQLPAEPK.E** |
|  | 24985 | 850.3463 | 2548.0169 | 2549.1349 | -1.1180 | 0 | 5 | 16 | 4 | K.SAPPSQVWNMTVSMTSDNSMHVK.C |
|  | 26497 | **698.1039** | **2788.3867** | **2788.3378** | **0.0488** | **0** | **1** | **42** | **2** | **K.MPSVPLSSDPLPTHTTAFSPASTFER.E** |
|  | 26553 | **699.6058** | **2794.3942** | **2794.4079** | **-0.0137** | **0** | **18** | **1.1** | **1** | **R.EVTHIQFTSWPDHGVPEDPHLLLK.L** |
|  | 27901 | 1021.8038 | 3062.3895 | 3062.5158 | -0.1263 | 1 | 1 | 41 | 2 | R.TGTYIGIDAMLEGLEAENKVDVYGYVVK.L |
|  | 28796 | 1637.8353 | 3273.6561 | 3272.9920 | 0.6641 | 1 | 0 | 43 | 7 | K.ALIAFLAFLIIVTSIALLVVLYKIYDLHK.K |
|  | 30320 | **976.5136** | **3902.0253** | **3902.6791** | **-0.6537** | **1** | **0** | **30** | **1** | **R.NGSAAMCHFTTKSAPPSQVWNMTVSMTSDNSMHVK.C** |

| **367.** | Q07954\|LRP1_HUMAN    **Mass:** 523119   **Score:** 114    **Queries matched:** 76   **emPAI:** 0.04 |
| --- | --- |
|  | Prolow-density lipoprotein receptor-related protein 1 OS=Homo sapiens GN=LRP1 PE=1 SV=1 |

|  | **Query** | **Observed** | **Mr(expt)** | **Mr(calc)** | **Delta** | **Miss** | **Score** | **Expect** | **Rank** | **Peptide** |
| --- | --- | --- | --- | --- | --- | --- | --- | --- | --- | --- |
|  | 1028 | **587.3462** | **586.3389** | **586.3438** | **-0.0049** | **0** | **14** | **10** | **8** | **R.ELLGR.G** |
|  | 1829 | **407.8882** | **813.7619** | **814.4913** | **-0.7293** | **0** | **8** | **19** | **2** | **R.TGIGVQLK.D** |
|  | 1976 | **414.1979** | **826.3813** | **826.4297** | **-0.0484** | **0** | **11** | **11** | **3** | **R.THANGSIK.R** |
|  | 2391 | 428.6752 | 855.3358 | 856.3498 | -1.0139 | 0 | 3 | 78 | 7 | R.GGDPHSCK.A |
|  | 2888 | **446.2529** | **890.4913** | **890.4974** | **-0.0061** | **1** | **10** | **19** | **9** | **K.DIKVFNR.D** 2876 |
|  | 3321 | **462.2424** | **922.4703** | **923.4501** | **-0.9798** | **0** | **14** | **6.2** | **1** | **R.VYWSDVR.T** |
|  | 5584 | **542.8183** | **1083.6220** | **1083.5607** | **0.0613** | **1** | **12** | **10** | **3** | **R.DCPGVKRPR.C** |
|  | 5824 | **551.3922** | **1100.7697** | **1100.5397** | **0.2301** | **1** | **(3)** | **69** | **9** | **R.ANKHVGSNMK.L** 5826 |
|  | 5872 | 553.0171 | 1104.0196 | 1104.5386 | -0.5190 | 0 | 3 | 63 | 5 | K.GVLFQPCER.T |
|  | 6260 | **567.2805** | **1132.5464** | **1132.5547** | **-0.0083** | **0** | **32** | **0.097** | **1** | **R.ILQDDLTCR.A** |
|  | 6446 | **574.7630** | **1147.5114** | **1148.5318** | **-1.0204** | **0** | **29** | **0.19** | **1** | **R.CLEGACVVNK.Q** |
|  | 6804 | **586.3257** | **1170.6368** | **1170.6972** | **-0.0604** | **1** | **2** | **92** | **6** | **R.TGIGVQLKDIK.V** |
|  | 7207 | 398.6926 | 1193.0560 | 1192.5758 | 0.4802 | 0 | 7 | 20 | 5 | K.IETAAMDGTLR.E 7191 |
|  | 7665 | 607.7773 | 1213.5401 | 1214.5350 | -0.9949 | 0 | 10 | 17 | 2 | K.QPEVTNPCDR.K |
|  | 7805 | **611.3051** | **1220.5957** | **1220.5931** | **0.0025** | **1** | **7** | **34** | **1** | **R.IEAASMSGAGRR.T** |
|  | 8113 | **619.3877** | **1236.7608** | **1236.7190** | **0.0418** | **0** | **4** | **48** | **1** | **R.GVGGAPPTVTLLR.S** |
|  | 8251 | 415.2455 | 1242.7148 | 1242.6720 | 0.0427 | 0 | 6 | 39 | 10 | R.SERPPIFEIR.M |
|  | 9151 | **430.5543** | **1288.6410** | **1288.6499** | **-0.0089** | **0** | **6** | **36** | **5** | **R.FVCPPNRPFR.C** |
|  | 9855 | **441.8848** | **1322.6325** | **1321.6990** | **0.9335** | **0** | **3** | **64** | **10** | **R.TTLLAGDIEHPR.A** |
|  | 9982 | **665.2363** | **1328.4581** | **1328.6031** | **-0.1450** | **1** | **4** | **43** | **8** | **R.SGFSLGSDGKSCK.K** |
|  | 10304 | 450.2506 | 1347.7300 | 1346.6295 | 1.1005 | 0 | 4 | 55 | 2 | R.LYWVDAFYDR.I |
|  | 10783 | 458.2710 | 1371.7913 | 1372.6306 | -0.8393 | 1 | 3 | 65 | 8 | R.LERAWMDGSHR.D |
|  | 10800 | **687.3436** | **1372.6727** | **1373.7667** | **-1.0940** | **0** | **15** | **4.5** | **1** | **R.YVVISQGLDKPR.A** 10822 |
|  | 11939 | **720.3676** | **1438.7207** | **1438.7755** | **-0.0548** | **1** | **5** | **37** | **8** | **K.FLLYARQMEIR.G** |
|  | 12249 | **729.4205** | **1456.8265** | **1455.7681** | **1.0584** | **1** | **4** | **46** | **1** | **R.LNGTDPIVAADSKR.G** |
|  | 12866 | 498.3366 | 1491.9880 | 1492.7899 | -0.8019 | 1 | 3 | 46 | 7 | R.VDKGGALHIYHQR.R |
|  | 12983 | **750.4149** | **1498.8152** | **1497.6630** | **1.1522** | **1** | **4** | **55** | **3** | **K.SDEKPSYCNSRR.C** |
|  | 13023 | **751.4296** | **1500.8447** | **1499.7507** | **1.0940** | **1** | **2** | **83** | **8** | **R.AEKLYFSDATLDK.I** |
|  | 13042 | 751.8775 | 1501.7404 | 1501.8075 | -0.0671 | 1 | 1 | 1.1e+002 | 7 | R.LGVKGVLFQPCER.T |
|  | 13151 | **755.8463** | **1509.6781** | **1509.6882** | **-0.0101** | **0** | **78** | **1.9e-006** | **1** | **R.MYDAQQQQVGTNK.C** |
|  | 13328 | 507.3002 | 1518.8787 | 1519.7678 | -0.8891 | 0 | 1 | 1e+002 | 7 | R.MHLNGSNVQVLHR.T |
|  | 13659 | **513.9840** | **1538.9302** | **1537.8399** | **1.0903** | **1** | **8** | **18** | **1** | **R.GVTHLNISGLKMPR.G** |
|  | 14103 | **522.9686** | **1565.8839** | **1565.8930** | **-0.0091** | **0** | **31** | **0.096** | **1** | **K.IVFPHGITLDLVSR.L** |
|  | 14302 | **527.5957** | **1579.7653** | **1579.5151** | **0.2501** | **0** | **1** | **83** | **2** | **K.CDGDHDCADGSDEK.D** |
|  | 14372 | **529.2308** | **1584.6707** | **1584.6838** | **-0.0131** | **1** | **10** | **12** | **1** | **K.DKSDEKPSYCNSR.R** |
|  | 14762 | **805.4125** | **1608.8105** | **1608.8485** | **-0.0380** | **1** | **5** | **43** | **5** | **K.ANKWTGHNVTVVQR.T** |
|  | 15024 | 542.2936 | 1623.8589 | 1622.6600 | 1.1990 | 0 | 5 | 41 | 2 | R.NGGTCAASPSGMPTCR.C |
|  | 15045 | **542.7728** | **1625.2967** | **1624.5478** | **0.7488** | **0** | **1** | **67** | **10** | **R.CNGQDNCGDGEDER.D** |
|  | 15243 | **547.2634** | **1638.7685** | **1639.7658** | **-0.9973** | **1** | **8** | **17** | **3** | **K.MGTCSKADGSGSVVLR.N** |
|  | 15398 | 413.2027 | 1648.7818 | 1649.9213 | -1.1395 | 1 | 13 | 5.6 | 2 | R.QIDRGVTHLNISGLK.M |
|  | 15480 | 827.8846 | 1653.7547 | 1654.5359 | -0.7812 | 0 | 9 | 13 | 1 | K.CDGEDDCGDGSDEPK.E 15479 15491 |
|  | 15674 | **555.9327** | **1664.7762** | **1664.7498** | **0.0264** | **0** | **4** | **49** | **6** | **R.CNLDGSGLEVIDAMR.S** |
|  | 16077 | **423.7147** | **1690.8298** | **1690.9406** | **-0.1108** | **1** | **6** | **25** | **5** | **R.LYWADAKLSVIGSIR.L** |
|  | 16158 | **849.3812** | **1696.7479** | **1696.8211** | **-0.0732** | **1** | **1** | **81** | **2** | **R.TVSCACPHLMKLHK.D** |
|  | 16992 | **584.2997** | **1749.8774** | **1748.9209** | **0.9565** | **1** | **6** | **25** | **1** | **R.ILWIDARSDAIYSAR.Y** |
|  | 17065 | **586.2495** | **1755.7267** | **1754.7967** | **0.9300** | **0** | **5** | **31** | **3** | **R.MTNGAMNVEIGNPTYK.M** |
|  | 17230 | **590.9188** | **1769.7345** | **1770.7917** | **-1.0572** | **0** | **(0)** | **96** | **10** | **R.MTNGAMNVEIGNPTYK.M** |
|  | 17373 | **889.9428** | **1777.8711** | **1776.9046** | **0.9664** | **0** | **23** | **0.54** | **1** | **K.ITWPNGLTLDYVTER.I** 17328 17354 |
|  | 18584 | **929.4699** | **1856.9253** | **1855.8444** | **1.0808** | **1** | **1** | **85** | **1** | **K.LSGCSQDCEDLKIGFK.C** |
|  | 18905 | 628.6581 | 1882.9524 | 1883.0112 | -0.0588 | 1 | 5 | 35 | 1 | K.DNATDSVPLRTGIGVQLK.D |
|  | 19354 | **641.3100** | **1920.9082** | **1919.9233** | **0.9848** | **0** | **4** | **42** | **1** | **K.VPDEHMIPIENLMNPR.A** |
|  | 19583 | **647.2817** | **1938.8234** | **1939.8993** | **-1.0759** | **0** | **0** | **85** | **7** | **K.VNNGGCSNLCLLSPGGGHK.C** |
|  | 19957 | 655.6827 | 1964.0262 | 1963.9561 | 0.0701 | 0 | 2 | 65 | 6 | R.NGDTCVTLLDLELYNPK.G |
|  | 21052 | **688.3510** | **2062.0312** | **2062.1436** | **-0.1124** | **0** | **1** | **67** | **10** | **K.NAVVQGLEQPHGLVVHPLR.G** |
|  | 21200 | **693.3599** | **2077.0579** | **2078.0069** | **-0.9489** | **1** | **1** | **73** | **8** | **R.ETGSGGWPNGLTVDYLEKR.I** |
|  | 21700 | 709.7069 | 2126.0989 | 2125.1743 | 0.9246 | 1 | 1 | 72 | 10 | K.LSVIGSIRLNGTDPIVAADSK.R |
|  | 22254 | **548.7408** | **2190.9340** | **2190.8946** | **0.0394** | **0** | **3** | **36** | **4** | **K.LYWTDGDNISMANMDGSNR.T** |
|  | 22981 | **758.0070** | **2270.9992** | **2271.0161** | **-0.0169** | **0** | **35** | **0.024** | **1** | **R.SGFHTVPGQPGCQDINECLR.F** 22982 |
|  | 23264 | **1154.0536** | **2306.0926** | **2305.7828** | **0.3098** | **0** | **0** | **74** | **2** | **R.CDADADCMDGSDEEACGTGVR.T** |
|  | 25608 | **661.3241** | **2641.2673** | **2642.1961** | **-0.9288** | **1** | **2** | **37** | **5** | **R.GMDMGAKVPDEHMIPIENLMNPR.A** 25609 |
|  | 27419 | **1486.7773** | **2971.5401** | **2971.6178** | **-0.0777** | **1** | **0** | **45** | **1** | **K.TTGTNKTLLISTLHRPMDLHVFHALR.Q** |
|  | 27907 | **1022.4689** | **3064.3850** | **3065.3859** | **-1.0009** | **1** | **0** | **47** | **8** | **K.LYWTDGDNISMANMDGSNRTLLFSGQK.G** |
|  | 29363 | **1152.1202** | **3453.3389** | **3453.3643** | **-0.0254** | **1** | **28** | **0.044** | **1** | **R.TSLCYAPSWVCDGANDCGDYSDERDCPGVK.R** |
|  | 30196 | 1274.8864 | 3821.6372 | 3820.9312 | 0.7060 | 1 | 6 | 7.2 | 3 | K.GDYSVLVPGLRNTIALDFHLSQSALYWTDVVEDK.I 30197 |
|  | 31060 | **770.8435** | **4619.0174** | **4620.0719** | **-1.0545** | **1** | **1** | **14** | **7** | **R.ETVITMSGDDHPRAFVLDECQNLMFWTNWNEQHPSIMR.A** |

| **368.** | P15144\|AMPN_HUMAN    **Mass:** 109870   **Score:** 114    **Queries matched:** 30   **emPAI:** 0.10 |
| --- | --- |
|  | Aminopeptidase N OS=Homo sapiens GN=ANPEP PE=1 SV=4 |

|  | **Query** | **Observed** | **Mr(expt)** | **Mr(calc)** | **Delta** | **Miss** | **Score** | **Expect** | **Rank** | **Peptide** |
| --- | --- | --- | --- | --- | --- | --- | --- | --- | --- | --- |
|  | 2429 | **429.7095** | **857.4044** | **857.5123** | **-0.1079** | **1** | **6** | **47** | **6** | **K.ANIKWVK.E** |
|  | 3139 | **456.2248** | **910.4350** | **909.4266** | **1.0084** | **0** | **2** | **82** | **10** | **R.SEVYGPMK.N** |
|  | 4614 | **507.8025** | **1013.5904** | **1013.5982** | **-0.0077** | **1** | **24** | **0.65** | **1** | **R.KIQTQLQR.D** |
|  | 7096 | **594.9755** | **1187.9364** | **1187.6047** | **0.3317** | **0** | **1** | **79** | **1** | **K.LNYTLSQGHR.V** 7097 |
|  | 7765 | **610.2800** | **1218.5455** | **1217.6326** | **0.9130** | **0** | **14** | **6.6** | **1** | **K.DLTALSNMLPK.G** |
|  | 8460 | **627.8149** | **1253.6152** | **1252.6122** | **1.0030** | **0** | **1** | **1.2e+002** | **7** | **K.DLMVLNDVYR.V** |
|  | 8792 | **635.8185** | **1269.6224** | **1268.5269** | **1.0955** | **0** | **3** | **69** | **1** | **K.DNEETGFGSGTR.A** 8775 |
|  | 9397 | **434.8808** | **1301.6206** | **1302.6932** | **-1.0726** | **0** | **13** | **6.9** | **1** | **R.LPNTLKPDSYR.V** 9396 9398 9399 9400 9401 9402 |
|  | 10551 | **681.3413** | **1360.6681** | **1360.6769** | **-0.0088** | **0** | **36** | **0.035** | **1** | **K.VVATTQMQAADAR.K** |
|  | 12030 | 482.2711 | 1443.7914 | 1443.7834 | 0.0080 | 0 | 5 | 40 | 8 | R.VTLRPYLTPNDR.G |
|  | 12424 | **734.3906** | **1466.7667** | **1466.7769** | **-0.0102** | **0** | **18** | **1.9** | **1** | **R.YLSYTLNPDLIR.K** 12426 12427 12428 |
|  | 12819 | **497.5952** | **1489.7637** | **1488.7719** | **0.9919** | **1** | **30** | **0.13** | **1** | **R.KVVATTQMQAADAR.K** 12792 |
|  | 13073 | **502.3714** | **1504.0925** | **1504.7668** | **-0.6743** | **1** | **2** | **55** | **6** | **K.VVATTQMQAADARK.S** 13072 |
|  | 14674 | 535.6227 | 1603.8462 | 1603.7374 | 0.1088 | 1 | 15 | 3.8 | 2 | K.LMFDRSEVYGPMK.N |
|  | 28173 | 626.7164 | 3128.5458 | 3128.7883 | -0.2425 | 1 | 3 | 25 | 1 | K.SLGILGILLGVAAVCTIIALSVVYSQEKNK.N |
|  | 30252 | **1287.6400** | **3859.8982** | **3859.9189** | **-0.0207** | **0** | **75** | **9.8e-007** | **1** | **R.VMAVDALASSHPLSTPASEINTPAQISELFDAISYSK.G** 30253 |

| **369.** | P11908\|PRPS2_HUMAN    **Mass:** 35146    **Score:** 113    **Queries matched:** 7   **emPAI:** 0.11 |
| --- | --- |
|  | Ribose-phosphate pyrophosphokinase 2 OS=Homo sapiens GN=PRPS2 PE=1 SV=2 |

|  | **Query** | **Observed** | **Mr(expt)** | **Mr(calc)** | **Delta** | **Miss** | **Score** | **Expect** | **Rank** | **Peptide** |
| --- | --- | --- | --- | --- | --- | --- | --- | --- | --- | --- |
|  | 2017 | 415.2452 | 828.4759 | 828.4817 | -0.0058 | 1 | 19 | 2.8 | 2 | K.SRAPISAK.L |
|  | 2696 | **439.2315** | **876.4483** | **875.4787** | **0.9697** | **0** | **3** | **92** | **5** | **R.MVLVGDVK.D** |
|  | 6426 | **573.7902** | **1145.5659** | **1146.6067** | **-1.0408** | **1** | **11** | **13** | **3** | **R.MVLVGDVKDR.V** |
|  | 15974 | 842.9838 | 1683.9530 | 1683.9593 | -0.0063 | 0 | 113 | 5.8e-010 | 1 | K.IQVIDISMILAEAIR.R |
|  | 16215 | **567.4080** | **1699.2021** | **1699.9542** | **-0.7522** | **0** | **(2)** | **45** | **3** | **K.IQVIDISMILAEAIR.R** |
|  | 18324 | **614.3940** | **1840.1603** | **1840.0604** | **0.0999** | **1** | **1** | **59** | **10** | **K.IQVIDISMILAEAIRR.T** |
|  | 23016 | **759.3444** | **2275.0113** | **2275.0283** | **-0.0171** | **0** | **4** | **31** | **2** | **R.VAILVDDMADTCGTICHAADK.L** |

| **370.** | Q9UKY3\|EST4_HUMAN    **Mass:** 30773    **Score:** 112    **Queries matched:** 7   **emPAI:** 0.26 |
| --- | --- |
|  | Inactive carboxylesterase 4 OS=Homo sapiens GN=CES4 PE=2 SV=2 |

|  | **Query** | **Observed** | **Mr(expt)** | **Mr(calc)** | **Delta** | **Miss** | **Score** | **Expect** | **Rank** | **Peptide** |
| --- | --- | --- | --- | --- | --- | --- | --- | --- | --- | --- |
|  | 2208 | 421.5331 | 841.0516 | 840.5069 | 0.5447 | 1 | 11 | 8.4 | 2 | R.KENIPLK.L |
|  | 10300 | 674.8608 | 1347.7071 | 1347.7146 | -0.0075 | 0 | 35 | 0.046 | 1 | K.AGQLISELFTNR.K |
|  | 10924 | 690.8571 | 1379.6997 | 1380.8089 | -1.1092 | 1 | 3 | 79 | 5 | K.KGDVKPLAEVGLR.L |
|  | 21717 | 1065.0212 | 2128.0279 | 2128.0398 | -0.0119 | 0 | 72 | 4.8e-006 | 1 | K.LSEDCLYLNIYTPADLTK.K 21718 |
|  | 31063 | **1541.5437** | **4621.6093** | **4621.5294** | **0.0799** | **1** | **3** | **8.2** | **3** | **K.FVSLEGFAQPVAVFLGIPFAKPPLGPLRFTLPQPAEPWNFVK.N** |
|  | 31821 | **1358.6365** | **6788.1460** | **6788.3729** | **-0.2269** | **0** | **0** | **5.4** | **3** | **R.LGIWGFFSTGDEHSPGNWGHLDQLAALHWVQDNIASFGGNPGSVTIFGGSVGGESVSVLVLSPLAK.N** |

| **371.** | P51648\|AL3A2_HUMAN    **Mass:** 55269    **Score:** 112    **Queries matched:** 23   **emPAI:** 0.30 |
| --- | --- |
|  | Fatty aldehyde dehydrogenase OS=Homo sapiens GN=ALDH3A2 PE=1 SV=1 |

|  | **Query** | **Observed** | **Mr(expt)** | **Mr(calc)** | **Delta** | **Miss** | **Score** | **Expect** | **Rank** | **Peptide** |
| --- | --- | --- | --- | --- | --- | --- | --- | --- | --- | --- |
|  | 1235 | **675.1210** | **674.1138** | **673.3507** | **0.7630** | **1** | **6** | **50** | **2** | **K.REGANK.L** |
|  | 1640 | **801.4147** | **800.4075** | **800.4504** | **-0.0430** | **1** | **7** | **48** | **6** | **M.ELEVRR.V** |
|  | 2072 | **833.4576** | **832.4504** | **831.4524** | **0.9980** | **0** | **24** | **0.89** | **3** | **K.IVMEAAAK.H** 2065 |
|  | 3289 | **461.0075** | **920.0003** | **919.4399** | **0.5604** | **0** | **9** | **14** | **1** | **R.YPPNSQSK.V** |
|  | 3964 | **485.7842** | **969.5539** | **969.5607** | **-0.0068** | **0** | **22** | **1** | **2** | **R.LQQLEALR.R** |
|  | 4419 | **500.8023** | **999.5899** | **999.5964** | **-0.0065** | **0** | **15** | **6** | **1** | **R.ILSLLEGQK.I** |
|  | 6173 | **563.8337** | **1125.6529** | **1125.6618** | **-0.0089** | **1** | **19** | **2** | **1** | **R.LQQLEALRR.M** |
|  | 8677 | 632.8616 | 1263.7087 | 1263.7187 | -0.0100 | 0 | 3 | 78 | 5 | K.HLTPVTLELGGK.S |
|  | 8865 | 637.8054 | 1273.5962 | 1272.7302 | 0.8659 | 1 | 5 | 49 | 7 | R.FRLQQLEALR.R |
|  | 11811 | **716.3849** | **1430.7552** | **1430.7657** | **-0.0105** | **0** | **11** | **12** | **1** | **R.YIAPTVLTDVDPK.T** |
|  | 11842 | **717.3491** | **1432.6837** | **1432.6946** | **-0.0109** | **0** | **37** | **0.029** | **1** | **K.NVDEAINFINER.E** |
|  | 14000 | **520.9456** | **1559.8149** | **1559.8229** | **-0.0080** | **1** | **42** | **0.008** | **1** | **R.EKDILTAIAADLCK.S** 14019 |
|  | 15943 | **842.4031** | **1682.7917** | **1681.8940** | **0.8977** | **0** | **8** | **18** | **1** | **R.EKPLALYVFSHNHK.L** |
|  | 18776 | **625.3051** | **1872.8935** | **1871.9101** | **0.9834** | **0** | **1** | **80** | **3** | **K.HSFDTFSHQRPCLLK.S** |
|  | 21207 | **693.6892** | **2078.0458** | **2077.1605** | **0.8853** | **1** | **2** | **65** | **7** | **K.IVMEAAAKHLTPVTLELGGK.S** |
|  | 21532 | **1055.4791** | **2108.9437** | **2109.3541** | **-0.4104** | **1** | **3** | **41** | **1** | **K.EKLGLLLLTFLGIVAAVLVK.A** 21547 |
|  | 27461 | **993.1924** | **2976.5555** | **2975.5743** | **0.9812** | **0** | **0** | **50** | **2** | **K.LLPQYLDQDLYIVINGGVEETTELLK.Q** |
|  | 28950 | **1108.2931** | **3321.8574** | **3322.7635** | **-0.9061** | **1** | **48** | **0.00059** | **1** | **K.VMQEEIFGPILPIVPVKNVDEAINFINER.E** |
|  | 30535 | **1016.2114** | **4060.8163** | **4060.8394** | **-0.0231** | **0** | **45** | **0.00072** | **1** | **R.MIDETSSGGVTGNDVIMHFTLNSFPFGGVGSSGMGAYHGK.H** 30534 |

| **372.** | P39210\|MPV17_HUMAN    **Mass:** 19948    **Score:** 112    **Queries matched:** 5   **emPAI:** 0.19 |
| --- | --- |
|  | Protein Mpv17 OS=Homo sapiens GN=MPV17 PE=1 SV=1 |

|  | **Query** | **Observed** | **Mr(expt)** | **Mr(calc)** | **Delta** | **Miss** | **Score** | **Expect** | **Rank** | **Peptide** |
| --- | --- | --- | --- | --- | --- | --- | --- | --- | --- | --- |
|  | 7213 | 597.7712 | 1193.5278 | 1193.6127 | -0.0849 | 1 | 2 | 84 | 10 | -.MALWRAYQR.A |
|  | 8347 | **416.5725** | **1246.6957** | **1245.7081** | **0.9876** | **1** | **7** | **28** | **5** | **K.VLDRFIPGTTK.V** 8292 |
|  | 24145 | **1214.1615** | **2426.3084** | **2426.3203** | **-0.0118** | **0** | **83** | **3.4e-007** | **1** | **K.VQVLTAGSLMGLGDIISQQLVER.R** 24146 |

| **373.** | Q9H853\|TBA4B_HUMAN    **Mass:** 27819    **Score:** 112    **Queries matched:** 5   **emPAI:** 0.29 |
| --- | --- |
|  | Putative tubulin-like protein alpha-4B OS=Homo sapiens GN=TUBA4B PE=5 SV=2 |

|  | **Query** | **Observed** | **Mr(expt)** | **Mr(calc)** | **Delta** | **Miss** | **Score** | **Expect** | **Rank** | **Peptide** |
| --- | --- | --- | --- | --- | --- | --- | --- | --- | --- | --- |
|  | 4778 | **514.7999** | **1027.5853** | **1026.5094** | **1.0759** | **0** | **8** | **23** | **10** | **R.QDPSQPLSR.Q** |
|  | 5810 | **551.2314** | **1100.4482** | **1100.5145** | **-0.0663** | **1** | **8** | **27** | **1** | **-.MRHQQTER.Q** |
|  | 12767 | 744.4402 | 1486.8658 | 1486.8719 | -0.0060 | 0 | 72 | 8.7e-006 | 1 | R.LISQIVSSITASLR.F 12765 12766 |

| **374.** | Q08380\|LG3BP_HUMAN    **Mass:** 66202    **Score:** 111    **Queries matched:** 7   **emPAI:** 0.06 |
| --- | --- |
|  | Galectin-3-binding protein OS=Homo sapiens GN=LGALS3BP PE=1 SV=1 |

|  | **Query** | **Observed** | **Mr(expt)** | **Mr(calc)** | **Delta** | **Miss** | **Score** | **Expect** | **Rank** | **Peptide** |
| --- | --- | --- | --- | --- | --- | --- | --- | --- | --- | --- |
|  | 1756 | **405.2062** | **808.3979** | **807.4239** | **0.9741** | **0** | **8** | **26** | **7** | **K.LASAYGAR.Q** |
|  | 6239 | **566.7563** | **1131.4980** | **1130.6659** | **0.8321** | **1** | **21** | **1.3** | **1** | **R.RIDITLSSVK.C** |
|  | 10464 | **678.3886** | **1354.7627** | **1354.7708** | **-0.0081** | **0** | **10** | **13** | **1** | **R.SDLAVPSELALLK.A** |
|  | 14513 | 796.8949 | 1591.7752 | 1591.7842 | -0.0089 | 0 | 9 | 16 | 1 | R.ELSEALGQIFDSQR.G |
|  | 24305 | **1226.5496** | **2451.0846** | **2451.0948** | **-0.0102** | **0** | **111** | **5.1e-010** | **1** | **R.GQWGTVCDNLWDLTDASVVCR.A** |
|  | 27154 | **971.7678** | **2912.2816** | **2912.2991** | **-0.0174** | **0** | **24** | **0.22** | **1** | **R.AAFGQGSGPIMLDEVQCTGTEASLADCK.S** |
|  | 28996 | **1111.5129** | **3331.5170** | **3332.6361** | **-1.1191** | **1** | **10** | **4.1** | **1** | **R.LFWVWLLVAGTQGVNDGDMRLADGGATNQGR.V** |

| **375.** | P13747\|HLAE_HUMAN    **Mass:** 40360    **Score:** 111    **Queries matched:** 14   **emPAI:** 0.19 |
| --- | --- |
|  | HLA class I histocompatibility antigen, alpha chain E OS=Homo sapiens GN=HLA-E PE=1 SV=3 |

|  | **Query** | **Observed** | **Mr(expt)** | **Mr(calc)** | **Delta** | **Miss** | **Score** | **Expect** | **Rank** | **Peptide** |
| --- | --- | --- | --- | --- | --- | --- | --- | --- | --- | --- |
|  | 2609 | **436.2505** | **870.4864** | **870.5399** | **-0.0535** | **1** | **15** | **5.7** | **7** | **R.VNLRTLR.G** |
|  | 8427 | **418.2025** | **1251.5855** | **1250.6143** | **0.9713** | **0** | **7** | **29** | **4** | **K.DYLTLNEDLR.S** |
|  | 10549 | **681.3309** | **1360.6473** | **1360.7714** | **-0.1241** | **1** | **3** | **69** | **3** | **K.GKETLLHLEPPK.T** |
|  | 11736 | 714.3626 | 1426.7105 | 1426.7205 | -0.0099 | 0 | 46 | 0.0031 | 1 | K.WAAVVVPSGEEQR.Y 11721 11735 11751 |
|  | 15181 | **818.4025** | **1634.7904** | **1633.7948** | **0.9956** | **0** | **5** | **36** | **1** | **R.SWTAVDTAAQISEQK.S** |
|  | 15320 | 823.4096 | 1644.8047 | 1644.8148 | -0.0101 | 0 | 69 | 1.4e-005 | 1 | R.FISVGYVDDTQFVR.F 15321 |
|  | 15625 | 554.9095 | 1661.7066 | 1662.7712 | -1.0646 | 0 | 3 | 53 | 3 | R.AYLEDTCVEWLHK.Y |
|  | 18486 | 463.4837 | 1849.9057 | 1849.9183 | -0.0127 | 0 | 16 | 2.9 | 1 | K.THVTHHPISDHEATLR.C 18485 |
|  | 23083 | 1142.5674 | 2283.1202 | 2281.9884 | 1.1318 | 1 | 0 | 72 | 3 | R.MVPRAPWMEQEGSEYWDR.E |

| **376.** | P60891\|PRPS1_HUMAN    **Mass:** 35325    **Score:** 111    **Queries matched:** 12   **emPAI:** 0.22 |
| --- | --- |
|  | Ribose-phosphate pyrophosphokinase 1 OS=Homo sapiens GN=PRPS1 PE=1 SV=2 |

|  | **Query** | **Observed** | **Mr(expt)** | **Mr(calc)** | **Delta** | **Miss** | **Score** | **Expect** | **Rank** | **Peptide** |
| --- | --- | --- | --- | --- | --- | --- | --- | --- | --- | --- |
|  | 1066 | **604.9557** | **603.9485** | **603.3340** | **0.6145** | **0** | **9** | **22** | **5** | **K.IASASR.V** 1064 1067 |
|  | 2017 | 415.2452 | 828.4759 | 828.4817 | -0.0058 | 1 | 19 | 2.8 | 2 | K.SRAPISAK.L |
|  | 2696 | 439.2315 | 876.4483 | 875.4787 | 0.9697 | 0 | 3 | 92 | 5 | R.MVLVGDVK.D |
|  | 6426 | 573.7902 | 1145.5659 | 1146.6067 | -1.0408 | 1 | 11 | 13 | 3 | R.MVLVGDVKDR.V |
|  | 11865 | **478.8888** | **1433.6446** | **1432.6947** | **0.9500** | **0** | **6** | **30** | **3** | **K.IFSGSSHQDLSQK.I** |
|  | 15974 | 842.9838 | 1683.9530 | 1683.9593 | -0.0063 | 0 | 113 | 5.8e-010 | 1 | K.IQVIDISMILAEAIR.R |
|  | 16215 | 567.4080 | 1699.2021 | 1699.9542 | -0.7522 | 0 | (2) | 45 | 3 | K.IQVIDISMILAEAIR.R |
|  | 18324 | 614.3940 | 1840.1603 | 1840.0604 | 0.0999 | 1 | 1 | 59 | 10 | K.IQVIDISMILAEAIRR.T |
|  | 23016 | 759.3444 | 2275.0113 | 2275.0283 | -0.0171 | 0 | 4 | 31 | 2 | R.VAILVDDMADTCGTICHAADK.L |
|  | 31081 | **1160.0950** | **4636.3508** | **4636.3709** | **-0.0202** | **0** | **21** | **0.15** | **1** | **K.LVANMLSVAGADHIITMDLHASQIQGFFDIPVDNLYAEPAVLK.W** |

| **377.** | P27449\|VATL_HUMAN    **Mass:** 15725    **Score:** 110    **Queries matched:** 6   **emPAI:** 0.25 |
| --- | --- |
|  | V-type proton ATPase 16 kDa proteolipid subunit OS=Homo sapiens GN=ATP6V0C PE=1 SV=1 |

|  | **Query** | **Observed** | **Mr(expt)** | **Mr(calc)** | **Delta** | **Miss** | **Score** | **Expect** | **Rank** | **Peptide** |
| --- | --- | --- | --- | --- | --- | --- | --- | --- | --- | --- |
|  | 19586 | **647.2993** | **1938.8760** | **1937.9373** | **0.9387** | **0** | **3** | **53** | **2** | **K.SGTGIAAMSVMRPEQIMK.S** |
|  | 27739 | **1515.7156** | **3029.4166** | **3029.4303** | **-0.0137** | **0** | **110** | **5.5e-010** | **1** | **K.SGPEYASFFAVMGASAAMVFSALGAAYGTAK.S** |
|  | 29471 | **1165.2455** | **3492.7146** | **3492.6217** | **0.0929** | **1** | **3** | **20** | **2** | **M.SESKSGPEYASFFAVMGASAAMVFSALGAAYGTAK.S** |
|  | 31262 | **997.5112** | **4982.5198** | **4981.3468** | **1.1729** | **1** | **1** | **11** | **4** | **K.SGPEYASFFAVMGASAAMVFSALGAAYGTAKSGTGIAAMSVMRPEQIMK.S** |
|  | 31490 | 1366.1752 | 5460.6716 | 5460.9512 | -0.2796 | 1 | (1) | 9.4 | 3 | K.SGTGIAAMSVMRPEQIMKSIIPVVMAGIIAIYGLVVAVLIANSLNDDISLYK.S |
|  | 31491 | 1366.1779 | 5460.6823 | 5460.9512 | -0.2688 | 1 | 6 | 2.6 | 2 | K.SGTGIAAMSVMRPEQIMKSIIPVVMAGIIAIYGLVVAVLIANSLNDDISLYK.S |

| **378.** | P63167\|DYL1_HUMAN    **Mass:** 10530    **Score:** 109    **Queries matched:** 2   **emPAI:** 0.38 |
| --- | --- |
|  | Dynein light chain 1, cytoplasmic OS=Homo sapiens GN=DYNLL1 PE=1 SV=1 |

|  | **Query** | **Observed** | **Mr(expt)** | **Mr(calc)** | **Delta** | **Miss** | **Score** | **Expect** | **Rank** | **Peptide** |
| --- | --- | --- | --- | --- | --- | --- | --- | --- | --- | --- |
|  | 24756 | **1257.5154** | **2513.0162** | **2513.0356** | **-0.0194** | **0** | **109** | **6.8e-010** | **1** | **K.NADMSEEMQQDSVECATQALEK.Y** 24757 |

| **379.** | O43795\|MYO1B_HUMAN    **Mass:** 132928   **Score:** 109    **Queries matched:** 22   **emPAI:** 0.12 |
| --- | --- |
|  | Myosin-Ib OS=Homo sapiens GN=MYO1B PE=1 SV=3 |

|  | **Query** | **Observed** | **Mr(expt)** | **Mr(calc)** | **Delta** | **Miss** | **Score** | **Expect** | **Rank** | **Peptide** |
| --- | --- | --- | --- | --- | --- | --- | --- | --- | --- | --- |
|  | 1313 | **703.3932** | **702.3860** | **702.3912** | **-0.0052** | **0** | **28** | **0.42** | **4** | **R.INESIK.A** |
|  | 3141 | **456.2296** | **910.4445** | **909.4821** | **0.9625** | **1** | **4** | **60** | **6** | **K.FFRANAGK.K** |
|  | 4378 | **499.8126** | **997.6106** | **997.6172** | **-0.0066** | **0** | **22** | **0.95** | **1** | **K.IIIAEVVNK.I** |
|  | 4711 | 512.2675 | 1022.5205 | 1023.4734 | -0.9529 | 1 | 9 | 19 | 1 | R.NDNSSRFGK.Y 4724 |
|  | 4895 | **518.2889** | **1034.5633** | **1033.5709** | **0.9924** | **0** | **6** | **46** | **5** | **R.LFSWLVNR.I** |
|  | 5461 | 538.8049 | 1075.5953 | 1075.6026 | -0.0073 | 0 | 18 | 2.6 | 1 | R.YLGLLENVR.V |
|  | 5637 | **544.7921** | **1087.5695** | **1087.5774** | **-0.0079** | **0** | **31** | **0.15** | **1** | **K.RPPTAGSQFK.A** |
|  | 9963 | **664.3732** | **1326.7319** | **1327.6157** | **-0.8838** | **1** | **6** | **34** | **5** | **K.YRDQFTDQQK.L** |
|  | 12121 | **483.7765** | **1448.3078** | **1447.6977** | **0.6101** | **0** | **6** | **21** | **1** | **K.DQCILITGESGAGK.T** |
|  | 16622 | **432.4543** | **1725.7880** | **1725.8005** | **-0.0125** | **0** | **2** | **70** | **7** | **K.LNQVCATHQHFESR.M** |
|  | 16691 | **865.4911** | **1728.9676** | **1728.9774** | **-0.0098** | **0** | **46** | **0.0028** | **1** | **R.IFLLTNNNLLLADQK.S** 16690 |
|  | 17440 | **594.9766** | **1781.9080** | **1781.8407** | **0.0673** | **0** | **5** | **38** | **1** | **K.VSMSSQNDGFFAVHLK.E** |
|  | 18586 | **929.4856** | **1856.9566** | **1856.9632** | **-0.0065** | **0** | **35** | **0.033** | **1** | **K.EQLLQSNPVLEAFGNAK.T** 18593 |
|  | 24052 | **1207.0944** | **2412.1742** | **2412.1849** | **-0.0107** | **0** | **46** | **0.002** | **1** | **R.SGVEVLFNELEIPVEEYSFGR.S** |
|  | 24813 | **841.1020** | **2520.2843** | **2520.3012** | **-0.0169** | **1** | **2** | **45** | **1** | **K.ALYPSSVGQPFQGAYLEINKNPK.Y** |
|  | 25611 | **881.4578** | **2641.3515** | **2641.3608** | **-0.0093** | **0** | **26** | **0.17** | **1** | **R.NAMQIVGFMDHEAESVLAVVAAVLK.L** |
|  | 26444 | **556.4755** | **2777.3409** | **2778.3462** | **-1.0053** | **1** | **0** | **55** | **6** | **K.YMDIEFDFKGDPLGGVISNYLLEK.S** |
|  | 28703 | **1083.8326** | **3248.4761** | **3247.6356** | **0.8405** | **0** | **1** | **35** | **1** | **K.TSLLDNMIGVGDMVLLEPLNEETFINNLK.K** |
|  | 29165 | **1126.5587** | **3376.6543** | **3375.7305** | **0.9238** | **1** | **2** | **28** | **1** | **K.TSLLDNMIGVGDMVLLEPLNEETFINNLKK.R** |

| **380.** | P12235\|ADT1_HUMAN    **Mass:** 33271    **Score:** 109    **Queries matched:** 24   **emPAI:** 0.90 |
| --- | --- |
|  | ADP/ATP translocase 1 OS=Homo sapiens GN=SLC25A4 PE=1 SV=4 |

|  | **Query** | **Observed** | **Mr(expt)** | **Mr(calc)** | **Delta** | **Miss** | **Score** | **Expect** | **Rank** | **Peptide** |
| --- | --- | --- | --- | --- | --- | --- | --- | --- | --- | --- |
|  | 2175 | 420.6770 | 839.3394 | 839.3452 | -0.0058 | 0 | (11) | 11 | 5 | R.MMMQSGR.K 2188 |
|  | 2406 | 429.0863 | 856.1580 | 855.3401 | 0.8179 | 0 | (10) | 12 | 8 | R.MMMQSGR.K |
|  | 2408 | 429.0867 | 856.1589 | 855.3401 | 0.8188 | 0 | 16 | 3.3 | 1 | R.MMMQSGR.K 2394 |
|  | 2425 | 429.3168 | 856.6190 | 855.4814 | 1.1376 | 0 | 10 | 18 | 1 | K.TAVAPIER.V 2392 |
|  | 2606 | 436.2230 | 870.4314 | 871.3350 | -0.9037 | 0 | (3) | 87 | 6 | R.MMMQSGR.K |
|  | 3011 | 451.7430 | 901.4713 | 901.4770 | -0.0056 | 0 | 40 | 0.02 | 1 | K.GAWSNVLR.G 3009 |
|  | 3480 | **468.2245** | **934.4344** | **934.5236** | **-0.0892** | **1** | **5** | **55** | **1** | **K.IFKSDGLR.G** |
|  | 4501 | 503.2898 | 1004.5651 | 1003.5451 | 1.0200 | 0 | 11 | 14 | 3 | K.QLFLGGVDR.H 4481 4495 |
|  | 4577 | 506.6468 | 1011.2790 | 1011.4412 | -0.1622 | 1 | (0) | 1.1e+002 | 8 | R.RMMMQSGR.K |
|  | 5032 | 523.0566 | 1044.0987 | 1043.4310 | 0.6677 | 1 | 0 | 1.1e+002 | 9 | R.RMMMQSGR.K |
|  | 6295 | 568.8387 | 1135.6628 | 1135.6713 | -0.0085 | 0 | 31 | 0.14 | 1 | K.LLLQVQHASK.Q |
|  | 9153 | 645.3342 | 1288.6539 | 1287.6282 | 1.0257 | 0 | 10 | 16 | 2 | R.EFHGLGDCIIK.I |
|  | 12063 | 723.8700 | 1445.7254 | 1445.7343 | -0.0089 | 0 | 54 | 0.00053 | 1 | R.YFPTQALNFAFK.D |
|  | 15304 | 822.8554 | 1643.6961 | 1643.7072 | -0.0111 | 0 | 35 | 0.032 | 1 | K.GADIMYTGTVDCWR.K |
|  | 17252 | 886.9025 | 1771.7904 | 1771.8022 | -0.0118 | 1 | 51 | 0.00092 | 1 | R.KGADIMYTGTVDCWR.K |
|  | 19427 | 643.0295 | 1926.0668 | 1926.0363 | 0.0305 | 0 | 2 | 54 | 6 | R.GLYQGFNVSVQGIIIYR.A 19429 |
|  | 26555 | 1398.6686 | 2795.3226 | 2795.3377 | -0.0151 | 0 | 37 | 0.013 | 1 | R.YFAGNLASGGAAGATSLCFVYPLDFAR.T |

| **381.** | O75531\|BAF_HUMAN    **Mass:** 10280    **Score:** 109    **Queries matched:** 2   **emPAI:** 0.93 |
| --- | --- |
|  | Barrier-to-autointegration factor OS=Homo sapiens GN=BANF1 PE=1 SV=1 |

|  | **Query** | **Observed** | **Mr(expt)** | **Mr(calc)** | **Delta** | **Miss** | **Score** | **Expect** | **Rank** | **Peptide** |
| --- | --- | --- | --- | --- | --- | --- | --- | --- | --- | --- |
|  | 10329 | **675.4097** | **1348.8049** | **1348.8119** | **-0.0070** | **0** | **82** | **8.6e-007** | **1** | **K.AYVVLGQFLVLK.K** |
|  | 23959 | **1200.6204** | **2399.2262** | **2399.2406** | **-0.0145** | **0** | **55** | **0.00024** | **1** | **R.DFVAEPMGEKPVGSLAGIGEVLGK.K** |

| **382.** | P00395\|COX1_HUMAN    **Mass:** 57060    **Score:** 108    **Queries matched:** 2   **emPAI:** 0.07 |
| --- | --- |
|  | Cytochrome c oxidase subunit 1 OS=Homo sapiens GN=MT-CO1 PE=1 SV=1 |

|  | **Query** | **Observed** | **Mr(expt)** | **Mr(calc)** | **Delta** | **Miss** | **Score** | **Expect** | **Rank** | **Peptide** |
| --- | --- | --- | --- | --- | --- | --- | --- | --- | --- | --- |
|  | 30103 | **1262.5774** | **3784.7103** | **3784.7325** | **-0.0222** | **0** | **84** | **1.3e-007** | **1** | **K.VLMVEEPSMNLEWLYGCPPPYHTFEEPVYMK.S** 30104 |

| **383.** | O14983\|AT2A1_HUMAN    **Mass:** 111550   **Score:** 108    **Queries matched:** 28   **emPAI:** 0.14 |
| --- | --- |
|  | Sarcoplasmic/endoplasmic reticulum calcium ATPase 1 OS=Homo sapiens GN=ATP2A1 PE=1 SV=1 |

|  | **Query** | **Observed** | **Mr(expt)** | **Mr(calc)** | **Delta** | **Miss** | **Score** | **Expect** | **Rank** | **Peptide** |
| --- | --- | --- | --- | --- | --- | --- | --- | --- | --- | --- |
|  | 877 | 530.9559 | 529.9487 | 530.2813 | -0.3326 | 0 | 15 | 7.1 | 1 | R.DAGIR.V |
|  | 4658 | 509.7493 | 1017.4840 | 1016.5549 | 0.9291 | 1 | 5 | 57 | 4 | K.GTAIAICRR.I |
|  | 6698 | **582.3026** | **1162.5907** | **1163.5128** | **-0.9222** | **0** | **9** | **19** | **6** | **R.EEMVLDDSAR.F** |
|  | 8913 | **637.8908** | **1273.7669** | **1272.5842** | **1.1827** | **1** | **5** | **47** | **2** | **R.KSMSVYCSPAK.S** 8882 |
|  | 9856 | 662.3348 | 1322.6550 | 1322.6653 | -0.0102 | 0 | 52 | 0.00093 | 1 | K.NMLFSGTNIAAGK.A |
|  | 10200 | **448.2736** | **1341.7989** | **1341.7075** | **0.0915** | **1** | **5** | **43** | **9** | **R.CLALATRDTPPK.R** |
|  | 11121 | 464.7628 | 1391.2666 | 1390.7126 | 0.5540 | 0 | 1 | 65 | 8 | K.AEIGIAMGSGTAVAK.T |
|  | 11467 | 706.8237 | 1411.6328 | 1412.7234 | -1.0906 | 1 | 3 | 59 | 8 | R.AIYNNMKQFIR.Y |
|  | 11629 | 711.3708 | 1420.7270 | 1420.6616 | 0.0654 | 1 | 1 | 93 | 3 | K.IRDQMAATEQDK.T |
|  | 12087 | **483.2362** | **1446.6867** | **1447.6943** | **-1.0076** | **0** | **1** | **1.1e+002** | **9** | **R.IGIFGENEEVADR.A** |
|  | 13049 | 752.4084 | 1502.8022 | 1502.8126 | -0.0104 | 1 | 36 | 0.031 | 1 | K.KAEIGIAMGSGTAVAK.T 13040 |
|  | 13297 | 506.9037 | 1517.6892 | 1518.8076 | -1.1183 | 1 | (4) | 52 | 8 | K.KAEIGIAMGSGTAVAK.T 13343 |
|  | 14011 | **521.2601** | **1560.7586** | **1560.8247** | **-0.0661** | **0** | **2** | **71** | **7** | **K.VGEATETALTTLVEK.M** |
|  | 14211 | 787.9294 | 1573.8442 | 1573.8563 | -0.0121 | 0 | 63 | 6e-005 | 1 | R.VDQSILTGESVSVIK.H 14191 14195 |
|  | 14693 | **536.1608** | **1605.4605** | **1604.7729** | **0.6876** | **1** | **3** | **35** | **4** | **K.GAPEGVIDRCNYVR.V** 14694 |
|  | 18203 | 916.9377 | 1831.8608 | 1831.9648 | -0.1040 | 1 | 1 | 75 | 6 | R.VIMITGDNKGTAIAICR.R |
|  | 18902 | **628.4963** | **1882.4672** | **1882.1292** | **0.3380** | **1** | **10** | **6.6** | **1** | **K.ISLPVIGLDEILKFVAR.N** |
|  | 19776 | 976.9628 | 1951.9110 | 1951.9231 | -0.0121 | 0 | 35 | 0.031 | 1 | R.SLPSVETLGCTSVICSDK.T |
|  | 22353 | **735.3623** | **2203.0651** | **2203.0687** | **-0.0036** | **1** | **1** | **69** | **6** | **K.TGTLTTNQMSVCKMFIIDK.V** |
|  | 29160 | **1125.5485** | **3373.6236** | **3373.5874** | **0.0362** | **1** | **1** | **31** | **1** | **R.SLPSVETLGCTSVICSDKTGTLTTNQMSVCK.M** |
|  | 30532 | **812.8143** | **4059.0353** | **4059.1302** | **-0.0950** | **0** | **9** | **3.7** | **1** | **R.MPPWVNIWLLGSICLSMSLHFLILYVDPLPMIFK.L** |
|  | 30865 | 1454.6851 | 4361.0334 | 4360.3052 | 0.7281 | 1 | 1 | 16 | 6 | R.MPPWVNIWLLGSICLSMSLHFLILYVDPLPMIFKLR.A |

| **384.** | O95470\|SGPL1_HUMAN    **Mass:** 64053    **Score:** 108    **Queries matched:** 22   **emPAI:** 0.12 |
| --- | --- |
|  | Sphingosine-1-phosphate lyase 1 OS=Homo sapiens GN=SGPL1 PE=1 SV=3 |

|  | **Query** | **Observed** | **Mr(expt)** | **Mr(calc)** | **Delta** | **Miss** | **Score** | **Expect** | **Rank** | **Peptide** |
| --- | --- | --- | --- | --- | --- | --- | --- | --- | --- | --- |
|  | 1294 | **698.4140** | **697.4067** | **697.3435** | **0.0632** | **0** | **1** | **1.1e+002** | **1** | **K.YGYAPK.G** |
|  | 4838 | **516.7900** | **1031.5655** | **1032.5525** | **-0.9870** | **0** | **2** | **1.1e+002** | **5** | **M.PSTDLLMLK.A** |
|  | 5993 | **557.7811** | **1113.5476** | **1114.5619** | **-1.0143** | **0** | **20** | **1.8** | **1** | **K.GVTSISADTHK.Y** |
|  | 7406 | **401.2841** | **1200.8305** | **1201.7183** | **-0.8877** | **1** | **9** | **16** | **7** | **R.VAIQFLKDIR.E** 7415 |
|  | 8437 | **418.2632** | **1251.7678** | **1252.5726** | **-0.8048** | **1** | **(9)** | **18** | **6** | **K.MMEVDVRAMR.R** |
|  | 9077 | **643.3159** | **1284.6173** | **1284.5625** | **0.0548** | **1** | **13** | **7.4** | **1** | **K.MMEVDVRAMR.R** 9066 |
|  | 9293 | **432.8842** | **1295.6308** | **1296.6384** | **-1.0075** | **1** | **5** | **41** | **4** | **K.DDISKNMSFLK.V** |
|  | 9582 | **656.9977** | **1311.9809** | **1312.6333** | **-0.6524** | **1** | **(3)** | **43** | **3** | **K.DDISKNMSFLK.V** |
|  | 10198 | **448.2559** | **1341.7460** | **1341.7252** | **0.0208** | **1** | **0** | **1.2e+002** | **8** | **R.VKGVTSISADTHK.Y** |
|  | 12743 | 743.9038 | 1485.7931 | 1485.8038 | -0.0108 | 0 | 61 | 9.7e-005 | 1 | K.ALPSQGLSSSAVLEK.L |
|  | 14665 | 802.8784 | 1603.7423 | 1604.6565 | -0.9143 | 0 | 5 | 36 | 3 | K.EYSSMDAFWQEGR.A 14664 |
|  | 17269 | 591.8420 | 1772.5041 | 1771.8233 | 0.6808 | 0 | 1 | 52 | 7 | K.TTGMGAIYGMAQTTVDR.N |
|  | 17642 | **598.7664** | **1793.2774** | **1793.8948** | **-0.6174** | **1** | **3** | **39** | **5** | **K.GVTSISADTHKYGYAPK.G** |
|  | 18797 | **938.0219** | **1874.0293** | **1874.0414** | **-0.0122** | **0** | **69** | **1.3e-005** | **1** | **K.GIFVFGNPQLSVIALGSR.D** |
|  | 20093 | **660.3520** | **1978.0341** | **1978.0636** | **-0.0294** | **1** | **2** | **62** | **8** | **K.GIKTPEIVAPQSAHAAFNK.A** |
|  | 20176 | **663.3311** | **1986.9715** | **1986.9503** | **0.0212** | **1** | **3** | **49** | **4** | **K.AKTTGMGAIYGMAQTTVDR.N** |
|  | 20402 | **669.3231** | **2004.9474** | **2005.0731** | **-0.1258** | **1** | **5** | **30** | **5** | **K.EYVKALPSQGLSSSAVLEK.L** |
|  | 31444 | **1336.6230** | **5342.4631** | **5341.4524** | **1.0107** | **1** | **1** | **8.1** | **2** | **K.TTGMGAIYGMAQTTVDRNMVAELSSVFLDSLYSTDTVTQGSQMNGSPKPH.-** |
|  | 31711 | **1882.8058** | **5645.3955** | **5645.6198** | **-0.2242** | **1** | **3** | **3.8** | **2** | **K.YRNYQFFVDTDWQGGIYASPTIAGSRPGGISAACWAALMHFGENGYVEATK.Q** |

| **385.** | P36269\|GGT5_HUMAN    **Mass:** 62736    **Score:** 108    **Queries matched:** 7   **emPAI:** 0.12 |
| --- | --- |
|  | Gamma-glutamyltransferase 5 OS=Homo sapiens GN=GGT5 PE=1 SV=2 |

|  | **Query** | **Observed** | **Mr(expt)** | **Mr(calc)** | **Delta** | **Miss** | **Score** | **Expect** | **Rank** | **Peptide** |
| --- | --- | --- | --- | --- | --- | --- | --- | --- | --- | --- |
|  | 9413 | **652.3007** | **1302.5868** | **1303.7612** | **-1.1744** | **0** | **3** | **67** | **3** | **R.AAIAAPILHVNSK.G** |
|  | 10180 | **671.3867** | **1340.7588** | **1340.7663** | **-0.0076** | **0** | **7** | **25** | **1** | **R.DLLGETLAQLIR.Q** 10178 |
|  | 12160 | **484.7607** | **1451.2604** | **1450.7932** | **0.4671** | **0** | **9** | **9.9** | **1** | **R.VNVYHHLVETLK.F** |
|  | 18332 | **921.4034** | **1840.7923** | **1840.8050** | **-0.0127** | **0** | **50** | **0.00099** | **1** | **K.GCVEYEPNFSQEVQR.G** |
|  | 29819 | **1213.9512** | **3638.8317** | **3638.8515** | **-0.0198** | **0** | **63** | **2e-005** | **1** | **R.ETVPASHAPSLLDQCAQALPLGTGAQWIGVPGELR.G** 29818 |

| **386.** | P43490\|NAMPT_HUMAN    **Mass:** 55772    **Score:** 107    **Queries matched:** 7   **emPAI:** 0.14 |
| --- | --- |
|  | Nicotinamide phosphoribosyltransferase OS=Homo sapiens GN=NAMPT PE=1 SV=1 |

|  | **Query** | **Observed** | **Mr(expt)** | **Mr(calc)** | **Delta** | **Miss** | **Score** | **Expect** | **Rank** | **Peptide** |
| --- | --- | --- | --- | --- | --- | --- | --- | --- | --- | --- |
|  | 2858 | **444.7294** | **887.4443** | **887.4501** | **-0.0058** | **0** | **5** | **61** | **8** | **K.IWGEDLR.H** |
|  | 4696 | **511.2699** | **1020.5253** | **1021.5233** | **-0.9979** | **0** | **0** | **1.6e+002** | **6** | **K.GWNYILEK.Y** |
|  | 16457 | 858.4482 | 1714.8819 | 1713.8461 | 1.0358 | 0 | 11 | 8.8 | 2 | K.YLLETSGNLDGLEYK.L |
|  | 21567 | 705.0204 | 2112.0395 | 2113.0037 | -0.9642 | 0 | 0 | 87 | 7 | -.MNPAAEAEFNILLATDSYK.V |
|  | 21968 | **1079.5512** | **2157.0877** | **2157.0987** | **-0.0110** | **0** | **87** | **1.8e-007** | **1** | **R.VIQGDGVDINTLQEIVEGMK.Q** 21967 |
|  | 22731 | **745.7450** | **2234.2132** | **2234.2270** | **-0.0139** | **0** | **17** | **1.4** | **1** | **R.STQAPLIIRPDSGNPLDTVLK.V** |

| **387.** | Q03519\|TAP2_HUMAN    **Mass:** 76186    **Score:** 106    **Queries matched:** 12   **emPAI:** 0.10 |
| --- | --- |
|  | Antigen peptide transporter 2 OS=Homo sapiens GN=TAP2 PE=1 SV=1 |

|  | **Query** | **Observed** | **Mr(expt)** | **Mr(calc)** | **Delta** | **Miss** | **Score** | **Expect** | **Rank** | **Peptide** |
| --- | --- | --- | --- | --- | --- | --- | --- | --- | --- | --- |
|  | 3014 | **451.7518** | **901.4891** | **900.4665** | **1.0226** | **0** | **5** | **72** | **7** | **R.EIQDAVAR.A** |
|  | 4794 | **515.3256** | **1028.6366** | **1028.5615** | **0.0751** | **0** | **7** | **32** | **9** | **R.EAVGGLQTVR.S** |
|  | 7232 | 598.2708 | 1194.5271 | 1195.5655 | -1.0385 | 1 | 13 | 8 | 2 | R.YKEALEQCR.Q 7243 7267 |
|  | 8086 | **412.6955** | **1235.0648** | **1235.7099** | **-0.6451** | **1** | **4** | **38** | **2** | **R.GDRTVLVIAHR.L** |
|  | 9772 | **660.7813** | **1319.5481** | **1319.5564** | **-0.0084** | **0** | **56** | **0.00032** | **1** | **R.SFGAEEHEVCR.Y** |
|  | 14654 | **535.2943** | **1602.8609** | **1603.9409** | **-1.0800** | **1** | **4** | **47** | **6** | **R.AHQILVLQEGKLQK.L** 14657 |
|  | 19923 | 654.6682 | 1960.9828 | 1960.1218 | 0.8610 | 1 | 8 | 16 | 2 | R.LQTVQRAHQILVLQEGK.L |
|  | 28504 | **1068.1799** | **3201.5180** | **3201.5360** | **-0.0181** | **1** | **75** | **1.7e-006** | **1** | **R.VLILDEATSALDVQCEQALQDWNSRGDR.T** |
|  | 31093 | **1164.7688** | **4655.0461** | **4654.0686** | **0.9775** | **1** | **4** | **6.9** | **1** | **R.NNIAYGLQSCEDDKVMAAAQAAHADDFIQEMEHGIYTDVGEK.G** |

| **388.** | Q969Z3\|MOSC2_HUMAN    **Mass:** 38569    **Score:** 106    **Queries matched:** 9   **emPAI:** 0.10 |
| --- | --- |
|  | MOSC domain-containing protein 2, mitochondrial OS=Homo sapiens GN=MOSC2 PE=1 SV=1 |

|  | **Query** | **Observed** | **Mr(expt)** | **Mr(calc)** | **Delta** | **Miss** | **Score** | **Expect** | **Rank** | **Peptide** |
| --- | --- | --- | --- | --- | --- | --- | --- | --- | --- | --- |
|  | 1719 | **403.2419** | **804.4691** | **804.4745** | **-0.0054** | **0** | **21** | **1.8** | **1** | **R.IFGLDIK.G** |
|  | 1846 | **408.7297** | **815.4449** | **816.4202** | **-0.9753** | **1** | **13** | **11** | **8** | **R.SGNLRDR.F** |
|  | 2700 | 439.2451 | 876.4756 | 876.4310 | 0.0446 | 1 | 5 | 59 | 9 | K.KVMACPR.C 2711 |
|  | 4670 | **510.2471** | **1018.4796** | **1017.5971** | **0.8825** | **1** | **8** | **29** | **6** | **R.IFGLDIKGR.D** |
|  | 5144 | **527.3307** | **1052.6468** | **1052.4920** | **0.1548** | **0** | **2** | **1e+002** | **7** | **-.MGASSSSALAR.L** |
|  | 9988 | 665.3489 | 1328.6833 | 1328.6646 | 0.0187 | 0 | 7 | 27 | 4 | R.APDMDQLVLPSK.Q |
|  | 30355 | **1313.3359** | **3936.9860** | **3937.0005** | **-0.0145** | **1** | **64** | **1.1e-005** | **1** | **R.KLLPTLDQNFQVAYPDYCPLLIMTDASLVDLNTR.M** 30354 |

| **389.** | P27824\|CALX_HUMAN    **Mass:** 67982    **Score:** 106    **Queries matched:** 21   **emPAI:** 0.17 |
| --- | --- |
|  | Calnexin OS=Homo sapiens GN=CANX PE=1 SV=2 |

|  | **Query** | **Observed** | **Mr(expt)** | **Mr(calc)** | **Delta** | **Miss** | **Score** | **Expect** | **Rank** | **Peptide** |
| --- | --- | --- | --- | --- | --- | --- | --- | --- | --- | --- |
|  | 3007 | **451.7310** | **901.4475** | **901.4545** | **-0.0070** | **1** | **16** | **4.8** | **2** | **K.TYFTDKK.T** 3006 |
|  | 3867 | **481.7759** | **961.5373** | **961.5457** | **-0.0084** | **1** | **22** | **1.3** | **1** | **R.AKHHAISAK.L** |
|  | 5623 | **544.2867** | **1086.5588** | **1086.5015** | **0.0573** | **1** | **13** | **8.6** | **2** | **K.QTSGMEYKK.T** |
|  | 9391 | **651.6684** | **1301.3222** | **1300.7173** | **0.6049** | **1** | **4** | **38** | **2** | **K.LPGDKGLVLMSR.A** 9393 |
|  | 9539 | **655.7674** | **1309.5202** | **1309.5860** | **-0.0657** | **1** | **1** | **1e+002** | **5** | **K.WEVEEMKESK.L** |
|  | 12777 | **744.8549** | **1487.6953** | **1487.7045** | **-0.0092** | **0** | **64** | **4.6e-005** | **1** | **R.IVDDWANDGWGLK.K** |
|  | 14885 | **808.9016** | **1615.7887** | **1615.7995** | **-0.0108** | **1** | **53** | **0.00056** | **1** | **R.IVDDWANDGWGLKK.A** 14859 14884 14886 |
|  | 18663 | 621.9772 | 1862.9097 | 1862.9203 | -0.0106 | 1 | 23 | 0.48 | 1 | R.KIPNPDFFEDLEPFR.M |
|  | 22463 | **554.0352** | **2212.1115** | **2212.1251** | **-0.0136** | **0** | **8** | **13** | **2** | **K.WKPPMIDNPSYQGIWKPR.K** 22464 |
|  | 22657 | **743.4256** | **2227.2550** | **2228.1201** | **-0.8651** | **0** | **(2)** | **44** | **2** | **K.WKPPMIDNPSYQGIWKPR.K** |
|  | 23080 | **762.0185** | **2283.0336** | **2282.0451** | **0.9886** | **1** | **15** | **2.4** | **1** | **R.EIEDPEDRKPEDWDERPK.I** |
|  | 24279 | **1224.5587** | **2447.1029** | **2446.1158** | **0.9871** | **0** | **9** | **9.1** | **1** | **R.CESAPGCGVWQRPVIDNPNYK.G** |
|  | 29703 | **1195.6001** | **3583.7785** | **3584.6778** | **-0.8994** | **1** | **4** | **14** | **1** | **R.MTPFSAIGLELWSMTSDIFFDNFIICADRR.I** |
|  | 29934 | **926.2264** | **3700.8767** | **3700.8963** | **-0.0197** | **0** | **25** | **0.1** | **1** | **K.LNKPFLFDTKPLIVQYEVNFQNGIECGGAYVK.L** 29939 |

| **390.** | P11234\|RALB_HUMAN    **Mass:** 23508    **Score:** 105    **Queries matched:** 9   **emPAI:** 0.82 |
| --- | --- |
|  | Ras-related protein Ral-B OS=Homo sapiens GN=RALB PE=1 SV=1 |

|  | **Query** | **Observed** | **Mr(expt)** | **Mr(calc)** | **Delta** | **Miss** | **Score** | **Expect** | **Rank** | **Peptide** |
| --- | --- | --- | --- | --- | --- | --- | --- | --- | --- | --- |
|  | 3365 | 464.2388 | 926.4631 | 926.4684 | -0.0053 | 0 | 34 | 0.063 | 1 | K.VFFDLMR.E |
|  | 12196 | 485.5840 | 1453.7301 | 1453.7388 | -0.0087 | 1 | 30 | 0.12 | 1 | R.ANVDKVFFDLMR.E |
|  | 12468 | 736.3428 | 1470.6710 | 1469.7337 | 0.9373 | 1 | (0) | 1.2e+002 | 4 | R.ANVDKVFFDLMR.E |
|  | 15927 | 841.4610 | 1680.9074 | 1680.9410 | -0.0336 | 1 | 0 | 1e+002 | 10 | K.IPLLVVGNKSDLEER.R |
|  | 17907 | **906.4385** | **1810.8625** | **1810.8737** | **-0.0112** | **1** | **74** | **4.2e-006** | **1** | **R.SKAEEWGVQYVETSAK.T** 17904 17906 |
|  | 24136 | 1213.5502 | 2425.0858 | 2425.1035 | -0.0177 | 0 | 2 | 41 | 1 | K.SALTLQFMYDEFVEDYEPTK.A |
|  | 27683 | 1006.7897 | 3017.3474 | 3017.3640 | -0.0167 | 1 | 23 | 0.24 | 1 | K.SALTLQFMYDEFVEDYEPTKADSYR.K |

| **391.** | Q8IY17\|PLPL6_HUMAN    **Mass:** 151383   **Score:** 105    **Queries matched:** 25   **emPAI:** 0.07 |
| --- | --- |
|  | Neuropathy target esterase OS=Homo sapiens GN=PNPLA6 PE=1 SV=2 |

|  | **Query** | **Observed** | **Mr(expt)** | **Mr(calc)** | **Delta** | **Miss** | **Score** | **Expect** | **Rank** | **Peptide** |
| --- | --- | --- | --- | --- | --- | --- | --- | --- | --- | --- |
|  | 1982 | 414.2393 | 826.4640 | 827.4137 | -0.9497 | 0 | 12 | 10 | 3 | R.TPTQEPR.E 1985 |
|  | 2042 | **416.2586** | **830.5027** | **830.4722** | **0.0304** | **1** | **22** | **1.4** | **1** | **R.TVSARAAR.D** 2050 |
|  | 2617 | **436.7244** | **871.4342** | **870.5399** | **0.8943** | **1** | **6** | **49** | **8** | **R.LRSVIQR.G** |
|  | 5098 | **525.2643** | **1048.5141** | **1047.5171** | **0.9970** | **0** | **1** | **1.3e+002** | **10** | **R.TVEWLNMR.S** |
|  | 6662 | **581.2741** | **1160.5337** | **1160.5496** | **-0.0159** | **0** | **3** | **79** | **4** | **K.VPDMAEIQSR.L** |
|  | 7780 | 407.2234 | 1218.6484 | 1219.7037 | -1.0553 | 1 | 6 | 40 | 3 | K.LPEGTLGHIKR.R |
|  | 10310 | **675.2675** | **1348.5204** | **1348.6405** | **-0.1201** | **1** | **2** | **96** | **7** | **K.RMVSTSATDEPR.E** |
|  | 10727 | **457.2921** | **1368.8544** | **1369.8228** | **-0.9684** | **1** | **3** | **55** | **3** | **R.VVQIIMVRLQR.V** |
|  | 10834 | 459.1706 | 1374.4899 | 1373.6535 | 0.8364 | 0 | 5 | 39 | 4 | M.GTSSHGLATNSSGAK.V |
|  | 11376 | **703.8624** | **1405.7102** | **1405.7201** | **-0.0099** | **0** | **49** | **0.0017** | **1** | **R.LGASALDSIQEFR.L** 11364 |
|  | 14127 | **784.9547** | **1567.8947** | **1567.9046** | **-0.0099** | **0** | **72** | **8.2e-006** | **1** | **R.VLTGNTIALVLGGGGAR.G** |
|  | 14246 | **789.3895** | **1576.7644** | **1576.8685** | **-0.1041** | **0** | **3** | **57** | **7** | **R.AQPSVVLSAAHTVAAR.M** |
|  | 15164 | 545.6002 | 1633.7787 | 1632.8583 | 0.9203 | 1 | 7 | 24 | 2 | R.ARLGASALDSIQEFR.L |
|  | 16351 | **855.3148** | **1708.6150** | **1708.7879** | **-0.1729** | **0** | **25** | **0.3** | **1** | **R.QMDFAIDWTAVEAGR.A** |
|  | 17651 | **598.9493** | **1793.8260** | **1794.0516** | **-0.2256** | **1** | **3** | **53** | **4** | **R.YPQVVTRLIHLLSQK.I** 17644 |
|  | 18654 | **931.9981** | **1861.9817** | **1861.9938** | **-0.0121** | **0** | **2** | **69** | **1** | **R.IVLYQTDASLTPWTVR.C** |
|  | 19199 | **636.6832** | **1907.0279** | **1908.1269** | **-1.0990** | **1** | **3** | **45** | **10** | **R.LARVLTGNTIALVLGGGGAR.G** |
|  | 19887 | **490.7532** | **1958.9836** | **1959.9432** | **-0.9596** | **1** | **3** | **43** | **10** | **-.MGTSSHGLATNSSGAKVAER.D** |
|  | 29935 | **1234.6865** | **3701.0377** | **3701.9265** | **-0.8887** | **0** | **4** | **11** | **2** | **R.ETPGRPPDPTGAPLPGPTGDPVKPTSLETPSAPLLSR.C** |
|  | 30243 | 965.2549 | 3856.9904 | 3858.0311 | -1.0407 | 1 | 2 | 18 | 3 | R.MIDKAEDVCLFVAQPGELVGQLAVLTGEPLIFTLR.A |
|  | 30315 | 1301.0126 | 3900.0159 | 3900.8848 | -0.8690 | 1 | 3 | 15 | 3 | R.ASMTLSGYLPPLCDPKDGHLLMDGGYINNLPADIAR.S |

| **392.** | O75367\|H2AY_HUMAN    **Mass:** 39764    **Score:** 104    **Queries matched:** 6   **emPAI:** 0.09 |
| --- | --- |
|  | Core histone macro-H2A.1 OS=Homo sapiens GN=H2AFY PE=1 SV=4 |

|  | **Query** | **Observed** | **Mr(expt)** | **Mr(calc)** | **Delta** | **Miss** | **Score** | **Expect** | **Rank** | **Peptide** |
| --- | --- | --- | --- | --- | --- | --- | --- | --- | --- | --- |
|  | 4471 | **502.3194** | **1002.6242** | **1003.4822** | **-0.8579** | **0** | **6** | **43** | **1** | **K.DDLGNTLEK.K** |
|  | 6436 | 574.2600 | 1146.5053 | 1146.5703 | -0.0649 | 1 | 8 | 26 | 9 | K.NCLALADDKK.L |
|  | 7488 | **603.3407** | **1204.6668** | **1203.6499** | **1.0169** | **0** | **6** | **40** | **10** | **K.EFVEAVLELR.K** |
|  | 19520 | 967.0357 | 1932.0569 | 1932.0680 | -0.0111 | 0 | 64 | 4.6e-005 | 1 | R.HILLAVANDEELNQLLK.G 19519 |
|  | 21595 | **705.4249** | **2113.2528** | **2113.2259** | **0.0269** | **1** | **4** | **28** | **2** | **K.GVTIASGGVLPNIHPELLAKK.R** |

| **393.** | Q9P0M6\|H2AW_HUMAN    **Mass:** 40261    **Score:** 104    **Queries matched:** 7   **emPAI:** 0.09 |
| --- | --- |
|  | Core histone macro-H2A.2 OS=Homo sapiens GN=H2AFY2 PE=1 SV=3 |

|  | **Query** | **Observed** | **Mr(expt)** | **Mr(calc)** | **Delta** | **Miss** | **Score** | **Expect** | **Rank** | **Peptide** |
| --- | --- | --- | --- | --- | --- | --- | --- | --- | --- | --- |
|  | 1039 | **590.4236** | **589.4163** | **590.3136** | **-0.8973** | **1** | **12** | **20** | **1** | **M.SGRSGK.K** |
|  | 3292 | **921.1241** | **920.1168** | **919.5491** | **0.5677** | **0** | **1** | **85** | **3** | **R.IHPELLAK.K** |
|  | 6678 | **581.3192** | **1160.6239** | **1160.5979** | **0.0260** | **0** | **7** | **36** | **3** | **K.SVAFPPFPSGR.N** |
|  | 10393 | **677.3225** | **1352.6303** | **1352.7299** | **-0.0996** | **1** | **2** | **85** | **10** | **K.SETILSPPPEKR.G** |
|  | 16592 | **575.3350** | **1722.9832** | **1722.8301** | **0.1532** | **0** | **3** | **50** | **6** | **K.FVIHCHIPQWGSDK.C** |
|  | 19520 | 967.0357 | 1932.0569 | 1932.0680 | -0.0111 | 0 | 64 | 4.6e-005 | 1 | R.HILLAVANDEELNQLLK.G 19519 |

| **394.** | O60762\|DPM1_HUMAN    **Mass:** 29673    **Score:** 104    **Queries matched:** 9   **emPAI:** 0.27 |
| --- | --- |
|  | Dolichol-phosphate mannosyltransferase OS=Homo sapiens GN=DPM1 PE=1 SV=1 |

|  | **Query** | **Observed** | **Mr(expt)** | **Mr(calc)** | **Delta** | **Miss** | **Score** | **Expect** | **Rank** | **Peptide** |
| --- | --- | --- | --- | --- | --- | --- | --- | --- | --- | --- |
|  | 1337 | **711.0902** | **710.0829** | **709.3395** | **0.7434** | **0** | **10** | **8.8** | **1** | **K.IYGSDR.I** |
|  | 1408 | 745.4407 | 744.4335 | 744.4381 | -0.0047 | 1 | 11 | 20 | 7 | R.KEVLEK.L |
|  | 1660 | **401.7167** | **801.4188** | **800.4504** | **0.9684** | **1** | **7** | **41** | **8** | **R.RELEVR.S** |
|  | 6877 | **588.8315** | **1175.6485** | **1175.6550** | **-0.0065** | **0** | **67** | **3.1e-005** | **1** | **K.LGGNEIVSFLK.G** |
|  | 10430 | 677.8120 | 1353.6095 | 1353.6928 | -0.0834 | 0 | 3 | 71 | 9 | K.YSVLLPTYNER.E |
|  | 11331 | 468.9816 | 1403.9231 | 1403.7595 | 0.1636 | 1 | 4 | 43 | 4 | K.KLGLGTAYIHGMK.H |
|  | 16602 | 862.9359 | 1723.8573 | 1723.8893 | -0.0320 | 1 | 0 | 1e+002 | 5 | R.QNKYSVLLPTYNER.E |
|  | 18854 | **470.7428** | **1878.9422** | **1877.8838** | **1.0584** | **1** | **5** | **35** | **10** | **K.CVSKGYVFQMEMIVR.A** |
|  | 29232 | **1133.2069** | **3396.5989** | **3396.6209** | **-0.0220** | **1** | **60** | **4e-005** | **1** | **K.SFSESGINYEIIIIDDGSPDGTRDVAEQLEK.I** |

| **395.** | P50454\|SERPH_HUMAN    **Mass:** 46525    **Score:** 104    **Queries matched:** 17   **emPAI:** 0.36 |
| --- | --- |
|  | Serpin H1 OS=Homo sapiens GN=SERPINH1 PE=1 SV=2 |

|  | **Query** | **Observed** | **Mr(expt)** | **Mr(calc)** | **Delta** | **Miss** | **Score** | **Expect** | **Rank** | **Peptide** |
| --- | --- | --- | --- | --- | --- | --- | --- | --- | --- | --- |
|  | 3142 | **456.2518** | **910.4890** | **911.5552** | **-1.0662** | **1** | **4** | **54** | **9** | **R.LVRPKGDK.M** |
|  | 6463 | **575.2893** | **1148.5641** | **1147.6059** | **0.9581** | **1** | **6** | **42** | **5** | **K.EQLKIWMGK.M** |
|  | 6995 | **395.2264** | **1182.6573** | **1181.6404** | **1.0169** | **0** | **9** | **18** | **10** | **K.KPAAAAAPGTAEK.L** |
|  | 7861 | **612.8281** | **1223.6416** | **1223.6510** | **-0.0094** | **0** | **47** | **0.0027** | **1** | **K.GVVEVTHDLQK.H** 7860 |
|  | 8959 | 639.8608 | 1277.7070 | 1277.7166 | -0.0095 | 0 | 25 | 0.42 | 1 | K.LQIVEMPLAHK.L |
|  | 9896 | **663.3858** | **1324.7570** | **1324.6380** | **0.1190** | **1** | **(3)** | **62** | **3** | **K.MVDNRGFMVTR.S** |
|  | 10171 | 671.3363 | 1340.6580 | 1340.6329 | 0.0251 | 1 | 5 | 45 | 8 | K.MVDNRGFMVTR.S |
|  | 13847 | **776.2288** | **1550.4430** | **1550.8490** | **-0.4060** | **1** | **2** | **50** | **2** | **K.EKLQIVEMPLAHK.L** |
|  | 14293 | **527.2945** | **1578.8616** | **1578.8729** | **-0.0113** | **1** | **47** | **0.0025** | **1** | **K.HLAGLGLTEAIDKNK.A** |
|  | 14749 | 804.8745 | 1607.7345 | 1606.9042 | 0.8303 | 1 | 6 | 31 | 7 | K.KPAAAAAPGTAEKLSPK.A |
|  | 15563 | **830.3994** | **1658.7843** | **1658.7941** | **-0.0098** | **0** | **43** | **0.0054** | **1** | **R.LYGPSSVSFADDFVR.S** 15562 |
|  | 18941 | 629.6190 | 1885.8352 | 1886.0738 | -0.2385 | 1 | 12 | 6.9 | 3 | R.DTQSGSLLFIGRLVRPK.G |
|  | 20143 | 496.7788 | 1983.0860 | 1983.0975 | -0.0115 | 0 | 22 | 0.56 | 1 | K.LSSLIILMPHHVEPLER.L |
|  | 20388 | **668.8656** | **2003.5750** | **2003.1415** | **0.4334** | **1** | **1** | **53** | **4** | **K.AVAISLPKGVVEVTHDLQK.H** |
|  | 24547 | **829.7572** | **2486.2498** | **2486.2653** | **-0.0155** | **1** | **18** | **1.1** | **1** | **R.SALQSINEWAAQTTDGKLPEVTK.D** |

| **396.** | P62745\|RHOB_HUMAN    **Mass:** 22565    **Score:** 104    **Queries matched:** 8   **emPAI:** 0.37 |
| --- | --- |
|  | Rho-related GTP-binding protein RhoB OS=Homo sapiens GN=RHOB PE=1 SV=1 |

|  | **Query** | **Observed** | **Mr(expt)** | **Mr(calc)** | **Delta** | **Miss** | **Score** | **Expect** | **Rank** | **Peptide** |
| --- | --- | --- | --- | --- | --- | --- | --- | --- | --- | --- |
|  | 1122 | 629.1870 | 628.1797 | 627.3340 | 0.8457 | 0 | 2 | 1.2e+002 | 2 | K.QEPVR.T |
|  | 3734 | **477.1937** | **952.3729** | **951.4662** | **0.9068** | **0** | **10** | **15** | **6** | **R.EVFETATR.A** |
|  | 5902 | 554.2835 | 1106.5525 | 1106.5139 | 0.0386 | 1 | 11 | 13 | 2 | R.TDDGRAMAVR.I |
|  | 11133 | **464.9422** | **1391.8049** | **1392.6997** | **-0.8949** | **1** | **4** | **50** | **8** | **K.EGVREVFETATR.A** |
|  | 20422 | 1004.9587 | 2007.9028 | 2007.9174 | -0.0146 | 0 | 63 | 4.7e-005 | 1 | K.QVELALWDTAGQEDYDR.L |
|  | 26400 | 1387.1586 | 2772.3026 | 2773.3011 | -0.9985 | 0 | 54 | 0.00024 | 2 | K.DEFPEVYVPTVFENYVADIEVDGK.Q 26401 |
|  | 29394 | 1155.8984 | 3464.6735 | 3465.6684 | -0.9949 | 0 | 27 | 0.073 | 2 | R.LRPLSYPDTDVILMCFSVDSPDSLENIPEK.W |

| **397.** | P00441\|SODC_HUMAN    **Mass:** 16154    **Score:** 103    **Queries matched:** 6   **emPAI:** 0.91 |
| --- | --- |
|  | Superoxide dismutase [Cu-Zn] OS=Homo sapiens GN=SOD1 PE=1 SV=2 |

|  | **Query** | **Observed** | **Mr(expt)** | **Mr(calc)** | **Delta** | **Miss** | **Score** | **Expect** | **Rank** | **Peptide** |
| --- | --- | --- | --- | --- | --- | --- | --- | --- | --- | --- |
|  | 13016 | **751.3813** | **1500.7480** | **1500.7573** | **-0.0092** | **0** | **50** | **0.0013** | **1** | **K.GDGPVQGIINFEQK.E** |
|  | 22470 | **738.6684** | **2212.9834** | **2212.1124** | **0.8710** | **1** | **0** | **70** | **3** | **K.GDGPVQGIINFEQKESNGPVK.V** |
|  | 29526 | **880.9050** | **3519.5908** | **3518.6175** | **0.9733** | **0** | **21** | **0.31** | **1** | **K.GLTEGLHGFHVHEFGDNTAGCTSAGPHFNPLSR.K** 29523 |
|  | 29975 | **930.9521** | **3719.7793** | **3719.8061** | **-0.0269** | **1** | **47** | **0.00073** | **1** | **R.HVGDLGNVTADKDGVADVSIEDSVISLSGDHCIIGR.T** 29976 |

| **398.** | Q15067\|ACOX1_HUMAN    **Mass:** 74889    **Score:** 103    **Queries matched:** 19   **emPAI:** 0.10 |
| --- | --- |
|  | Peroxisomal acyl-coenzyme A oxidase 1 OS=Homo sapiens GN=ACOX1 PE=1 SV=3 |

|  | **Query** | **Observed** | **Mr(expt)** | **Mr(calc)** | **Delta** | **Miss** | **Score** | **Expect** | **Rank** | **Peptide** |
| --- | --- | --- | --- | --- | --- | --- | --- | --- | --- | --- |
|  | 1706 | **403.2130** | **804.4114** | **803.4323** | **0.9791** | **0** | **9** | **26** | **9** | **K.ACTIAIR.Y** |
|  | 2987 | **451.2419** | **900.4693** | **900.4599** | **0.0093** | **1** | **9** | **26** | **8** | **-.MNPDLRR.E** |
|  | 3693 | **475.7477** | **949.4809** | **948.5029** | **0.9781** | **0** | **7** | **33** | **2** | **R.SFLVGEAAR.A** |
|  | 4962 | **521.2543** | **1040.4940** | **1040.6342** | **-0.1402** | **1** | **8** | **25** | **5** | **R.AARLVEIAAK.N** 4955 4956 4958 |
|  | 5594 | **543.2562** | **1084.4978** | **1083.7016** | **0.7962** | **1** | **8** | **25** | **2** | **R.VKELLTLIR.S** |
|  | 7421 | 601.9619 | 1201.9091 | 1202.6805 | -0.7713 | 1 | 4 | 39 | 5 | R.ALSKACTIAIR.Y |
|  | 7427 | **401.7968** | **1202.3686** | **1201.6165** | **0.7521** | **0** | **6** | **31** | **5** | **K.LTYGTMVFVR.S** |
|  | 10179 | **671.3854** | **1340.7563** | **1339.6884** | **1.0679** | **1** | **1** | **1e+002** | **7** | **K.AEVHESYKHLK.S** |
|  | 13107 | **754.3733** | **1506.7321** | **1507.8722** | **-1.1401** | **0** | **1** | **1e+002** | **1** | **K.TSNHAIVLAQLITK.G** |
|  | 16977 | **874.8984** | **1747.7823** | **1746.7889** | **0.9934** | **0** | **32** | **0.068** | **1** | **R.YDGNVYENLFEWAK.N** |
|  | 18126 | **609.6530** | **1825.9370** | **1824.8756** | **1.0614** | **1** | **5** | **32** | **1** | **R.EFGIADPDEIMWFKK.L** |
|  | 19178 | **953.9547** | **1905.8949** | **1907.0152** | **-1.1204** | **0** | **0** | **95** | **9** | **K.YAQVKPDGTYVKPLSNK.L** |
|  | 21463 | 702.0134 | 2103.0185 | 2101.9163 | 1.1021 | 1 | 2 | 55 | 6 | K.FGYDEIDNGYLKMDNHR.I |
|  | 23940 | **1198.1045** | **2394.1944** | **2394.2067** | **-0.0123** | **0** | **90** | **7e-008** | **1** | **R.SDAVALVDAFDFQDVTLGSVLGR.Y** |
|  | 26900 | 1429.6823 | 2857.3499 | 2858.3657 | -1.0158 | 1 | 4 | 22 | 4 | R.REIENMILNDPDFQHEDLNFLTR.S |
|  | 30394 | **1321.2263** | **3960.6571** | **3959.9397** | **0.7174** | **0** | **1** | **18** | **4** | **R.SLCLLYSLYGISQNAGDFLQGSIMTEPQITQVNQR.V** |

| **399.** | P61158\|ARP3_HUMAN    **Mass:** 47797    **Score:** 102    **Queries matched:** 15   **emPAI:** 0.16 |
| --- | --- |
|  | Actin-related protein 3 OS=Homo sapiens GN=ACTR3 PE=1 SV=3 |

|  | **Query** | **Observed** | **Mr(expt)** | **Mr(calc)** | **Delta** | **Miss** | **Score** | **Expect** | **Rank** | **Peptide** |
| --- | --- | --- | --- | --- | --- | --- | --- | --- | --- | --- |
|  | 1030 | **588.8319** | **587.8246** | **587.3027** | **0.5219** | **0** | **10** | **22** | **5** | **R.QVGER.T** |
|  | 3638 | 474.2296 | 946.4447 | 946.4720 | -0.0273 | 0 | 16 | 4.6 | 2 | K.LSEELSGGR.L 3668 |
|  | 4968 | **521.2723** | **1040.5300** | **1040.5365** | **-0.0065** | **0** | **33** | **0.086** | **1** | **R.FMEQVIFK.Y** |
|  | 5712 | **547.6155** | **1093.2165** | **1093.5768** | **-0.3602** | **0** | **1** | **82** | **8** | **K.QYTGINAISK.K** |
|  | 9287 | **432.8830** | **1295.6271** | **1296.6496** | **-1.0225** | **0** | **3** | **63** | **6** | **K.NIVLSGGSTMFR.D** |
|  | 9442 | **652.7922** | **1303.5699** | **1302.5728** | **0.9971** | **1** | **17** | **2.7** | **1** | **K.EFNKYDTDGSK.W** |
|  | 12418 | **734.3516** | **1466.6887** | **1465.6871** | **1.0016** | **1** | **10** | **11** | **1** | **K.KDYEEIGPSICR.H** |
|  | 12964 | **500.2607** | **1497.7601** | **1498.6875** | **-0.9273** | **0** | **7** | **27** | **7** | **R.HGIVEDWDLMER.F** |
|  | 17215 | **885.3763** | **1768.7380** | **1767.9003** | **0.8377** | **1** | **8** | **17** | **2** | **R.DREVGIPPEQSLETAK.A** |
|  | 20755 | 680.7997 | 2039.3772 | 2039.1462 | 0.2310 | 0 | 2 | 38 | 3 | R.LKPKPIDVQVITHHMQR.Y |
|  | 22260 | **1096.5682** | **2191.1219** | **2191.1347** | **-0.0128** | **0** | **61** | **6.2e-005** | **1** | **K.LGYAGNTEPQFIIPSCIAIK.E** 22259 |
|  | 27560 | 600.1009 | 2995.4681 | 2995.4967 | -0.0287 | 1 | 0 | 52 | 6 | K.GVDDLDFFIGDEAIEKPTYATKWPIR.H |
|  | 27878 | **1020.1898** | **3057.5475** | **3057.5693** | **-0.0219** | **0** | **14** | **1.9** | **1** | **R.TLTGTVIDSGDGVTHVIPVAEGYVIGSCIK.H** |

| **400.** | P41218\|MNDA_HUMAN    **Mass:** 46092    **Score:** 102    **Queries matched:** 12   **emPAI:** 0.17 |
| --- | --- |
|  | Myeloid cell nuclear differentiation antigen OS=Homo sapiens GN=MNDA PE=1 SV=1 |

|  | **Query** | **Observed** | **Mr(expt)** | **Mr(calc)** | **Delta** | **Miss** | **Score** | **Expect** | **Rank** | **Peptide** |
| --- | --- | --- | --- | --- | --- | --- | --- | --- | --- | --- |
|  | 1784 | **406.2053** | **810.3961** | **810.5076** | **-0.1115** | **1** | **7** | **25** | **4** | **R.IPVAQKR.K** |
|  | 2322 | **425.5480** | **849.0815** | **848.4313** | **0.6502** | **0** | **1** | **90** | **7** | **K.ITDLMEK.K** |
|  | 10817 | 458.8807 | 1373.6201 | 1372.7635 | 0.8566 | 1 | 8 | 21 | 2 | K.LIELAKDMPSLK.N 10789 |
|  | 12756 | **496.5585** | **1486.6538** | **1486.8330** | **-0.1792** | **1** | **1** | **1.1e+002** | **6** | **K.LVCGSHSFIKVIK.A** |
|  | 13623 | 769.3820 | 1536.7494 | 1537.7412 | -0.9919 | 1 | 7 | 25 | 4 | K.ATAPFKYESPENGK.S |
|  | 15806 | **558.7833** | **1673.3282** | **1672.8317** | **0.4965** | **0** | **(4)** | **32** | **8** | **K.QASGTMVYGLFMLQK.K** |
|  | 16280 | **569.2352** | **1704.6837** | **1704.8215** | **-0.1378** | **0** | **11** | **8.2** | **1** | **K.QASGTMVYGLFMLQK.K** |
|  | 17170 | **882.8194** | **1763.6242** | **1763.9530** | **-0.3287** | **1** | **1** | **70** | **1** | **K.KINQEEVGLAAPAPTAR.N** |
|  | 17995 | **455.2450** | **1816.9510** | **1816.9216** | **0.0295** | **1** | **4** | **39** | **3** | **K.QASGTMVYGLFMLQKK.S** |
|  | 19584 | **970.4357** | **1938.8569** | **1938.8708** | **-0.0139** | **0** | **58** | **0.00015** | **1** | **K.EASSVSDFNQNFEVPNR.I** |
|  | 21705 | **1064.4905** | **2126.9664** | **2126.9790** | **-0.0126** | **0** | **69** | **9.7e-006** | **1** | **K.NTIYEIQDNTGSMDVVGSGK.W** |

| **401.** | P48960\|CD97_HUMAN    **Mass:** 94603    **Score:** 101    **Queries matched:** 9   **emPAI:** 0.04 |
| --- | --- |
|  | CD97 antigen OS=Homo sapiens GN=CD97 PE=1 SV=4 |

|  | **Query** | **Observed** | **Mr(expt)** | **Mr(calc)** | **Delta** | **Miss** | **Score** | **Expect** | **Rank** | **Peptide** |
| --- | --- | --- | --- | --- | --- | --- | --- | --- | --- | --- |
|  | 5301 | 533.2137 | 1064.4128 | 1065.4873 | -1.0745 | 0 | 5 | 53 | 8 | K.NVTMGQSSAR.M |
|  | 7862 | 408.8881 | 1223.6424 | 1223.6299 | 0.0126 | 1 | 6 | 38 | 2 | R.FFDKVQDLGR.D |
|  | 18692 | **934.0048** | **1865.9950** | **1866.0033** | **-0.0083** | **0** | **101** | **7.2e-009** | **1** | **R.HLIATQLLSNLEDIMR.I** |
|  | 18787 | **625.6113** | **1873.8120** | **1872.8239** | **0.9881** | **0** | **14** | **4.3** | **1** | **K.YSEFTSTTSGTGHNQTR.A** |
|  | 22495 | 1108.0325 | 2214.0504 | 2213.0461 | 1.0043 | 1 | 1 | 68 | 2 | K.YSEFTSTTSGTGHNQTRALR.A 22485 22489 |
|  | 30018 | **935.4380** | **3737.7229** | **3736.7363** | **0.9866** | **1** | **0** | **31** | **4** | **R.HGIPNNQKDTVCEDMTFSTWTPPPGVHSQTLSR.F** |
|  | 30725 | **841.8332** | **4204.1296** | **4203.1820** | **0.9476** | **1** | **2** | **14** | **1** | **R.MKLNWAVAAGAEDPGPAVAGILSIQNMTTLLANASLNLHSK.K** |

| **402.** | P80188\|NGAL_HUMAN    **Mass:** 22745    **Score:** 101    **Queries matched:** 11   **emPAI:** 0.36 |
| --- | --- |
|  | Neutrophil gelatinase-associated lipocalin OS=Homo sapiens GN=LCN2 PE=1 SV=2 |

|  | **Query** | **Observed** | **Mr(expt)** | **Mr(calc)** | **Delta** | **Miss** | **Score** | **Expect** | **Rank** | **Peptide** |
| --- | --- | --- | --- | --- | --- | --- | --- | --- | --- | --- |
|  | 3698 | 476.1920 | 950.3694 | 950.5549 | -0.1855 | 1 | 7 | 34 | 8 | K.ITLYGRTK.E |
|  | 7054 | **593.8235** | **1185.6324** | **1184.6190** | **1.0135** | **0** | **1** | **1.3e+002** | **6** | **K.SYNVTSVLFR.K** |
|  | 8479 | 628.3209 | 1254.6273 | 1254.6608 | -0.0335 | 0 | 11 | 12 | 4 | K.SYPGLTSYLVR.V 8478 8482 |
|  | 9585 | 657.3121 | 1312.6097 | 1312.7139 | -0.1042 | 1 | 11 | 11 | 2 | K.SYNVTSVLFRK.K |
|  | 11829 | **716.8715** | **1431.7284** | **1430.8034** | **0.9250** | **0** | **17** | **2.8** | **1** | **K.WYVVGLAGNAILR.E** |
|  | 17738 | **600.9641** | **1799.8703** | **1799.8665** | **0.0038** | **0** | **1** | **92** | **7** | **R.VVSTNYNQHAMVFFK.K** |
|  | 18677 | **932.9607** | **1863.9068** | **1863.9189** | **-0.0121** | **0** | **45** | **0.0037** | **1** | **R.TFVPGCQPGEFTLGNIK.S** |
|  | 23373 | **1160.5795** | **2319.1444** | **2319.1569** | **-0.0125** | **0** | **49** | **0.001** | **1** | **K.SLGLPENHIVFPVPIDQCIDG.-** 23382 |

| **403.** | P12956\|KU70_HUMAN    **Mass:** 70084    **Score:** 101    **Queries matched:** 22   **emPAI:** 0.23 |
| --- | --- |
|  | ATP-dependent DNA helicase 2 subunit 1 OS=Homo sapiens GN=XRCC6 PE=1 SV=2 |

|  | **Query** | **Observed** | **Mr(expt)** | **Mr(calc)** | **Delta** | **Miss** | **Score** | **Expect** | **Rank** | **Peptide** |
| --- | --- | --- | --- | --- | --- | --- | --- | --- | --- | --- |
|  | 5418 | **537.2832** | **1072.5518** | **1072.5587** | **-0.0068** | **0** | **(13)** | **9.4** | **1** | **K.IMATPEQVGK.M** |
|  | 5645 | **545.2805** | **1088.5465** | **1088.5536** | **-0.0071** | **0** | **22** | **1.1** | **1** | **K.IMATPEQVGK.M** |
|  | 6826 | **586.8439** | **1171.6733** | **1171.6812** | **-0.0079** | **1** | **59** | **0.00022** | **1** | **K.KQELLEALTK.H** 6825 |
|  | 7512 | **403.0458** | **1206.1157** | **1206.6496** | **-0.5339** | **0** | **5** | **35** | **8** | **R.DSLIFLVDASK.A** |
|  | 10316 | **675.3313** | **1348.6480** | **1347.6890** | **0.9590** | **1** | **(0)** | **1.4e+002** | **5** | **K.IMATPEQVGKMK.A** |
|  | 10326 | **450.5901** | **1348.7485** | **1347.6890** | **1.0595** | **1** | **6** | **41** | **7** | **K.IMATPEQVGKMK.A** |
|  | 10379 | 676.8425 | 1351.6705 | 1350.6788 | 0.9917 | 1 | 1 | 1e+002 | 7 | K.FTVPMLKEACR.A |
|  | 11271 | 467.5780 | 1399.7122 | 1398.7296 | 0.9826 | 0 | 3 | 72 | 6 | K.KPGGFDISLFYR.D |
|  | 11306 | 701.8704 | 1401.7263 | 1401.7326 | -0.0063 | 0 | 14 | 5.1 | 1 | R.DTGIFLDLMHLK.K |
|  | 11574 | 473.5604 | 1417.6593 | 1417.7275 | -0.0683 | 0 | (4) | 61 | 9 | R.DTGIFLDLMHLK.K |
|  | 13337 | **760.8619** | **1519.7092** | **1519.7195** | **-0.0102** | **0** | **11** | **9.9** | **2** | **K.ELVYPPDYNPEGK.V** |
|  | 14200 | 787.4094 | 1572.8042 | 1572.8147 | -0.0106 | 0 | 55 | 0.00038 | 1 | K.NIYVLQELDNPGAK.R |
|  | 16259 | **852.4049** | **1702.7951** | **1702.8063** | **-0.0112** | **0** | **38** | **0.02** | **1** | **R.SDSFENPVLQQHFR.N** 16260 |
|  | 17855 | **603.3189** | **1806.9347** | **1806.9476** | **-0.0129** | **1** | **7** | **24** | **2** | **R.TFNTSTGGLLLPSDTKR.S** |
|  | 19275 | **638.9728** | **1913.8967** | **1914.0033** | **-0.1066** | **1** | **0** | **94** | **6** | **K.AGDLRDTGIFLDLMHLK.K** |
|  | 20698 | **1017.5568** | **2033.0990** | **2033.1094** | **-0.0104** | **0** | **23** | **0.48** | **1** | **R.FDDPGLMLMGFKPLVLLK.K** |
|  | 20902 | **684.3987** | **2050.1742** | **2049.1043** | **1.0699** | **0** | **(0)** | **74** | **3** | **R.FDDPGLMLMGFKPLVLLK.K** |
|  | 21744 | **711.3497** | **2131.0274** | **2131.1354** | **-0.1080** | **0** | **3** | **43** | **2** | **K.IQVTPPGFQLVFLPFADDK.R** |
|  | 22009 | **541.3055** | **2161.1931** | **2161.2043** | **-0.0113** | **1** | **5** | **28** | **3** | **R.FDDPGLMLMGFKPLVLLKK.H** |
|  | 30471 | **1342.5330** | **4024.5771** | **4024.7893** | **-0.2122** | **1** | **4** | **9.1** | **1** | **R.FQDMMGHGSDYSLSEVLWVCANLFSDVQFKMSHK.R** |

| **404.** | P38646\|GRP75_HUMAN    **Mass:** 73920    **Score:** 101    **Queries matched:** 34   **emPAI:** 0.10 |
| --- | --- |
|  | Stress-70 protein, mitochondrial OS=Homo sapiens GN=HSPA9 PE=1 SV=2 |

|  | **Query** | **Observed** | **Mr(expt)** | **Mr(calc)** | **Delta** | **Miss** | **Score** | **Expect** | **Rank** | **Peptide** |
| --- | --- | --- | --- | --- | --- | --- | --- | --- | --- | --- |
|  | 888 | 533.3637 | 532.3564 | 532.2969 | 0.0595 | 0 | 11 | 24 | 1 | M.ISASR.A |
|  | 2603 | **435.7762** | **869.5379** | **870.5109** | **-0.9731** | **1** | **7** | **30** | **9** | **R.LVGMPAKR.Q** |
|  | 3031 | 452.2441 | 902.4737 | 903.4960 | -1.0223 | 1 | 12 | 13 | 3 | K.MRELLAR.K 3054 |
|  | 3843 | **481.1499** | **960.2852** | **959.5440** | **0.7412** | **1** | **12** | **9.1** | **3** | **K.DIKNVPFK.I** 3835 |
|  | 4011 | **487.2735** | **972.5325** | **971.5916** | **0.9409** | **1** | **4** | **76** | **7** | **K.NVPFKIVR.A** |
|  | 4016 | **487.2949** | **972.5752** | **972.5175** | **0.0578** | **1** | **8** | **29** | **6** | **R.RTIAPCQK.A** |
|  | 4089 | **489.7725** | **977.5305** | **978.4328** | **-0.9023** | **0** | **18** | **3** | **2** | **K.DDIENMVK.N** |
|  | 6104 | **561.2953** | **1120.5760** | **1119.5818** | **0.9942** | **1** | **3** | **77** | **8** | **-.MISASRAAAAR.L** |
|  | 7853 | 612.7823 | 1223.5500 | 1223.5418 | 0.0082 | 1 | 2 | 97 | 3 | K.NAEKYAEEDR.R |
|  | 9173 | 645.8397 | 1289.6648 | 1289.6728 | -0.0081 | 0 | 23 | 0.65 | 1 | K.VQQTVQDLFGR.A |
|  | 9330 | **649.8176** | **1297.6206** | **1296.7262** | **0.8944** | **1** | **13** | **6.3** | **1** | **R.LVGAAASRGPTAAR.H** |
|  | 10556 | **681.3706** | **1360.7267** | **1360.7351** | **-0.0084** | **0** | **39** | **0.02** | **1** | **R.AQFEGIVTDLIR.R** 10538 |
|  | 11642 | **711.8259** | **1421.6372** | **1420.6504** | **0.9868** | **1** | **4** | **53** | **2** | **K.DDIENMVKNAEK.Y** |
|  | 12349 | 488.2577 | 1461.7512 | 1461.7497 | 0.0015 | 0 | 8 | 21 | 2 | K.SDIGEVILVGGMTR.M 12343 12352 12354 |
|  | 15746 | 557.2985 | 1668.8736 | 1669.9111 | -1.0376 | 1 | 1 | 94 | 7 | R.QATKDAGQISGLNVLR.V |
|  | 16065 | 845.9575 | 1689.9005 | 1689.8356 | 0.0649 | 1 | 5 | 38 | 4 | R.ETGVDLTKDNMALQR.V |
|  | 16124 | **848.4265** | **1694.8383** | **1693.8424** | **0.9960** | **0** | **12** | **7.4** | **1** | **K.NAVITVPAYFNDSQR.Q** 16099 |
|  | 16176 | **566.7687** | **1697.2844** | **1696.7322** | **0.5522** | **1** | **7** | **16** | **3** | **K.MASEREGSGSSGTGEQK.E** |
|  | 16409 | **571.6436** | **1711.9088** | **1712.7271** | **-0.8183** | **1** | **(5)** | **39** | **4** | **K.MASEREGSGSSGTGEQK.E** 16415 16418 |
|  | 16596 | **575.4100** | **1723.2081** | **1723.8642** | **-0.6561** | **1** | **6** | **18** | **1** | **R.QAVTNPNNTFYATKR.L** |
|  | 17627 | **598.2946** | **1791.8620** | **1791.8495** | **0.0125** | **1** | **2** | **77** | **10** | **R.TIAPCQKAMQDAEVSK.S** |
|  | 21197 | **1039.5114** | **2077.0081** | **2077.0184** | **-0.0103** | **0** | **81** | **7.3e-007** | **1** | **K.GAVVGIDLGTTNSCVAVMEGK.Q** |
|  | 21863 | **716.5123** | **2146.5152** | **2146.1093** | **0.4059** | **1** | **1** | **45** | **8** | **R.TTPSVVAFTADGERLVGMPAK.R** |
|  | 23998 | **802.7326** | **2405.1760** | **2405.1930** | **-0.0171** | **1** | **6** | **19** | **1** | **K.AMQDAEVSKSDIGEVILVGGMTR.M** |
|  | 24103 | **806.7384** | **2417.1934** | **2417.2108** | **-0.0174** | **1** | **0** | **71** | **2** | **R.EQQIVIQSSGGLSKDDIENMVK.N** |

| **405.** | Q93050\|VPP1_HUMAN    **Mass:** 97148    **Score:** 99     **Queries matched:** 19   **emPAI:** 0.21 |
| --- | --- |
|  | V-type proton ATPase 116 kDa subunit a isoform 1 OS=Homo sapiens GN=ATP6V0A1 PE=1 SV=3 |

|  | **Query** | **Observed** | **Mr(expt)** | **Mr(calc)** | **Delta** | **Miss** | **Score** | **Expect** | **Rank** | **Peptide** |
| --- | --- | --- | --- | --- | --- | --- | --- | --- | --- | --- |
|  | 1478 | 781.3572 | 780.3499 | 780.3589 | -0.0090 | 0 | 7 | 40 | 5 | K.ICEGFR.A |
|  | 3944 | **484.7474** | **967.4802** | **968.5331** | **-1.0529** | **0** | **8** | **21** | **4** | **K.LTFLNSFK.M** |
|  | 5047 | **523.3077** | **1044.6008** | **1044.6080** | **-0.0072** | **0** | **45** | **0.0055** | **1** | **R.LGFVAGVINR.E** |
|  | 5565 | **542.3198** | **1082.6251** | **1082.6560** | **-0.0309** | **1** | **2** | **1e+002** | **3** | **R.VLQAAAKNIR.V** |
|  | 5685 | **546.7722** | **1091.5299** | **1091.5393** | **-0.0094** | **1** | **35** | **0.056** | **1** | **R.KEMASGVNTR.I** 5670 5694 5695 |
|  | 7034 | **395.8780** | **1184.6121** | **1183.6462** | **0.9659** | **0** | **7** | **36** | **8** | **K.HLGTLNFGGIR.V** |
|  | 9204 | **431.2716** | **1290.7929** | **1291.6713** | **-0.8784** | **0** | **7** | **28** | **5** | **K.FLPFSFEHIR.E** |
|  | 11647 | **711.8325** | **1421.6505** | **1421.6609** | **-0.0104** | **0** | **48** | **0.0019** | **1** | **R.MQTNQTPPTYNK.T** 11640 11646 |
|  | 13773 | **774.3552** | **1546.6959** | **1546.7086** | **-0.0127** | **0** | **12** | **7.9** | **1** | **R.ASLYPCPETPQER.K** |
|  | 13903 | **777.8465** | **1553.6784** | **1553.7798** | **-0.1013** | **0** | **6** | **29** | **2** | **R.GTEHSGSTVPSILNR.M** |
|  | 16366 | **570.9631** | **1709.8674** | **1709.8809** | **-0.0135** | **1** | **24** | **0.48** | **1** | **R.RGTEHSGSTVPSILNR.M** |
|  | 18123 | **609.6244** | **1825.8513** | **1825.8629** | **-0.0115** | **0** | **37** | **0.021** | **1** | **R.IDDLQMVLNQTEDHR.Q** |
|  | 28987 | **1110.5048** | **3328.4925** | **3328.5115** | **-0.0191** | **1** | **37** | **0.0077** | **1** | **R.KTQQFFDEMADPDLLEESSSLLEPSEMGR.G** |
|  | 30325 | **783.5927** | **3912.9269** | **3911.8089** | **1.1179** | **1** | **1** | **22** | **2** | **-.MGELFRSEEMTLAQLFLQSEAAYCCVSELGELGK.V** |

| **406.** | Q9UBG0\|MRC2_HUMAN    **Mass:** 169742   **Score:** 99     **Queries matched:** 18   **emPAI:** 0.07 |
| --- | --- |
|  | C-type mannose receptor 2 OS=Homo sapiens GN=MRC2 PE=1 SV=1 |

|  | **Query** | **Observed** | **Mr(expt)** | **Mr(calc)** | **Delta** | **Miss** | **Score** | **Expect** | **Rank** | **Peptide** |
| --- | --- | --- | --- | --- | --- | --- | --- | --- | --- | --- |
|  | 3070 | **453.7797** | **905.5448** | **904.3675** | **1.1773** | **0** | **10** | **18** | **4** | **R.EDWGDQR.C** |
|  | 3189 | **458.2312** | **914.4478** | **913.4181** | **1.0297** | **0** | **7** | **39** | **9** | **R.FQEAEYK.F** 3193 |
|  | 6589 | **578.7938** | **1155.5730** | **1155.5269** | **0.0461** | **1** | **7** | **34** | **10** | **R.EPDDSPQGRR.E** |
|  | 8379 | **625.3289** | **1248.6432** | **1249.5914** | **-0.9482** | **0** | **3** | **79** | **3** | **R.GAWLGMNFNPK.G** |
|  | 8574 | **630.3406** | **1258.6666** | **1257.6731** | **0.9935** | **0** | **4** | **60** | **10** | **M.GPGRPAPAPWPR.H** |
|  | 9388 | 434.7631 | 1301.2674 | 1301.6939 | -0.4265 | 0 | 3 | 43 | 9 | R.TSNISKPGTLER.G |
|  | 9797 | 441.2136 | 1320.6191 | 1319.6582 | 0.9609 | 1 | 10 | 13 | 1 | R.QSIERGAFEGAR.Y 9768 |
|  | 11373 | 469.5618 | 1405.6636 | 1404.7085 | 0.9551 | 0 | 5 | 43 | 2 | -.MGPGRPAPAPWPR.H |
|  | 13473 | **510.2879** | **1527.8418** | **1527.6914** | **0.1504** | **1** | **9** | **14** | **1** | **R.GTDVREPDDSPQGR.R** |
|  | 15978 | **843.0734** | **1684.1322** | **1684.7223** | **-0.5902** | **0** | **35** | **0.024** | **1** | **K.AGQLSQGAAEEDHGCR.K** 15980 15994 |
|  | 19297 | **639.3493** | **1915.0261** | **1915.8709** | **-0.8448** | **0** | **0** | **87** | **5** | **R.EHCYSFHMELLLGHK.E** |
|  | 19510 | **644.9520** | **1931.8341** | **1932.8822** | **-1.0481** | **1** | **1** | **84** | **8** | **R.WNDSPCNQSLPSICKK.A** |
|  | 28753 | **1088.8533** | **3263.5380** | **3263.5557** | **-0.0177** | **0** | **42** | **0.0033** | **1** | **R.AGGAVLSILDEMENVFVWEHLQSYEGQSR.G** |
|  | 30194 | **1274.6085** | **3820.8037** | **3820.8188** | **-0.0151** | **0** | **59** | **3.7e-005** | **1** | **K.SWVQAQGACQELGAQLLSLASYEEEHFVANMLNK.I** |

| **407.** | Q9H4G4\|GAPR1_HUMAN    **Mass:** 17322    **Score:** 98     **Queries matched:** 8   **emPAI:** 0.50 |
| --- | --- |
|  | Golgi-associated plant pathogenesis-related protein 1 OS=Homo sapiens GN=GLIPR2 PE=1 SV=3 |

|  | **Query** | **Observed** | **Mr(expt)** | **Mr(calc)** | **Delta** | **Miss** | **Score** | **Expect** | **Rank** | **Peptide** |
| --- | --- | --- | --- | --- | --- | --- | --- | --- | --- | --- |
|  | 1966 | 413.7093 | 825.4040 | 824.4068 | 0.9972 | 0 | 16 | 3.7 | 1 | R.WYSEIK.N |
|  | 6519 | **576.8077** | **1151.6009** | **1152.6251** | **-1.0242** | **1** | **3** | **83** | **1** | **R.ILKHSPESSR.G** |
|  | 8440 | **627.3055** | **1252.5965** | **1252.6048** | **-0.0083** | **0** | **10** | **14** | **3** | **K.ASASDGSSFVVAR.Y** |
|  | 12174 | **727.3447** | **1452.6748** | **1452.6844** | **-0.0097** | **0** | **21** | **0.94** | **1** | **R.EAQQYSEALASTR.I** |
|  | 18912 | **942.9064** | **1883.7983** | **1883.8108** | **-0.0125** | **0** | **90** | **8.8e-008** | **1** | **R.GQCGENLAWASYDQTGK.E** |
|  | 24345 | **819.0303** | **2454.0690** | **2454.0870** | **-0.0180** | **1** | **27** | **0.12** | **1** | **R.GQCGENLAWASYDQTGKEVADR.W** |
|  | 24782 | **840.0532** | **2517.1378** | **2517.1536** | **-0.0158** | **0** | **8** | **11** | **1** | **K.NYNFQQPGFTSGTGHFTAMVWK.N** 24783 |

| **408.** | Q92575\|UBXN4_HUMAN    **Mass:** 57028    **Score:** 98     **Queries matched:** 9   **emPAI:** 0.21 |
| --- | --- |
|  | UBX domain-containing protein 4 OS=Homo sapiens GN=UBXN4 PE=1 SV=2 |

|  | **Query** | **Observed** | **Mr(expt)** | **Mr(calc)** | **Delta** | **Miss** | **Score** | **Expect** | **Rank** | **Peptide** |
| --- | --- | --- | --- | --- | --- | --- | --- | --- | --- | --- |
|  | 886 | 533.1894 | 532.1821 | 532.2969 | -0.1148 | 0 | 2 | 1.9e+002 | 4 | R.STVAR.I |
|  | 1828 | **407.8619** | **813.7093** | **813.3578** | **0.3514** | **0** | **8** | **22** | **3** | **K.EMLDYK.R** |
|  | 11051 | **694.3335** | **1386.6524** | **1386.6627** | **-0.0102** | **0** | **34** | **0.049** | **1** | **R.VTSSEPPNPASSSK.S** 11050 |
|  | 12757 | **496.5664** | **1486.6773** | **1487.8017** | **-1.1244** | **1** | **1** | **1.1e+002** | **9** | **K.AAALLAKQAEMEVK.R** |
|  | 16037 | **844.9591** | **1687.9037** | **1687.9120** | **-0.0083** | **0** | **22** | **0.68** | **1** | **-.MLWFQGAIPAAIATAK.R** |
|  | 18412 | **616.0738** | **1845.1997** | **1844.0131** | **1.1867** | **1** | **1** | **59** | **4** | **-.MLWFQGAIPAAIATAKR.S** |
|  | 23032 | **1139.5281** | **2277.0416** | **2277.0549** | **-0.0133** | **0** | **88** | **1.3e-007** | **1** | **R.LPDGSSFTNQFPSDAPLEEAR.Q** |
|  | 31301 | **1009.8733** | **5044.3301** | **5043.2330** | **1.0971** | **1** | **4** | **6.1** | **1** | **R.NAELCEIPPTSDTKSDTATGGESAGHATSSQEPSGCSDQRPAEDLNIR.V** |

| **409.** | O60716\|CTND1_HUMAN    **Mass:** 108674   **Score:** 97     **Queries matched:** 19   **emPAI:** 0.03 |
| --- | --- |
|  | Catenin delta-1 OS=Homo sapiens GN=CTNND1 PE=1 SV=1 |

|  | **Query** | **Observed** | **Mr(expt)** | **Mr(calc)** | **Delta** | **Miss** | **Score** | **Expect** | **Rank** | **Peptide** |
| --- | --- | --- | --- | --- | --- | --- | --- | --- | --- | --- |
|  | 4184 | 986.5083 | 985.5010 | 985.4941 | 0.0069 | 1 | 15 | 5.7 | 3 | R.TGTPSDPRR.R 4185 |
|  | 4560 | 506.2550 | 1010.4955 | 1011.6076 | -1.1121 | 1 | 3 | 70 | 10 | K.ELRKPLEK.E 4598 |
|  | 4749 | **513.7970** | **1025.5794** | **1025.5254** | **0.0540** | **0** | **3** | **68** | **7** | **R.VGGSSVDLHR.F** |
|  | 5887 | **553.6002** | **1105.1858** | **1104.5829** | **0.6029** | **1** | **3** | **57** | **6** | **R.KGGPPPPNWR.Q** |
|  | 6380 | **572.3476** | **1142.6806** | **1143.4978** | **-0.8172** | **0** | **1** | **1.3e+002** | **9** | **R.EPNEDCKPR.H** |
|  | 10613 | **455.5956** | **1363.7648** | **1363.6480** | **0.1168** | **0** | **8** | **24** | **3** | **K.SDFQVNLNNASR.S** 10593 10608 |
|  | 11807 | 477.7720 | 1430.2942 | 1430.8609 | -0.5667 | 1 | 4 | 36 | 8 | K.ELIGKHAIPNLVK.N |
|  | 14848 | **539.2473** | **1614.7199** | **1614.8439** | **-0.1240** | **0** | **3** | **60** | **8** | **R.QPELPEVIAMLGFR.L** 14849 |
|  | 15327 | 823.9004 | 1645.7862 | 1645.7981 | -0.0119 | 1 | 4 | 46 | 2 | R.TLDRSGDLGDMEPLK.G |
|  | 19828 | 652.6816 | 1955.0231 | 1955.9847 | -0.9616 | 1 | 1 | 72 | 9 | R.VSPQDANPLMANGTLTRR.H |
|  | 24477 | **825.7383** | **2474.1930** | **2474.1999** | **-0.0069** | **0** | **9** | **9.4** | **1** | **R.DMDLTEVITGTLWNLSSHDSIK.M** |
|  | 29629 | **1187.8738** | **3560.5995** | **3559.7941** | **0.8054** | **1** | **4** | **14** | **2** | **K.TVTTRTVQPVAMGPDGLPVDASSVSNNYIQTLGR.D** 29628 |
|  | 30665 | **1382.0247** | **4143.0522** | **4143.0760** | **-0.0238** | **1** | **97** | **4.7e-009** | **1** | **K.NLPGGQQNSSWNFSEDTVISILNTINEVIAENLEAAKK.L** |

| **410.** | O15031\|PLXB2_HUMAN    **Mass:** 207734   **Score:** 97     **Queries matched:** 32   **emPAI:** 0.04 |
| --- | --- |
|  | Plexin-B2 OS=Homo sapiens GN=PLXNB2 PE=1 SV=3 |

|  | **Query** | **Observed** | **Mr(expt)** | **Mr(calc)** | **Delta** | **Miss** | **Score** | **Expect** | **Rank** | **Peptide** |
| --- | --- | --- | --- | --- | --- | --- | --- | --- | --- | --- |
|  | 1011 | **577.1249** | **576.1176** | **575.2915** | **0.8261** | **0** | **0** | **1.8e+002** | **4** | **K.NIDSK.L** |
|  | 1261 | **688.9907** | **687.9834** | **687.4214** | **0.5621** | **1** | **1** | **1.5e+002** | **9** | **K.LMLRR.S** |
|  | 2951 | 449.3254 | 896.6362 | 896.3480 | 0.2882 | 0 | 11 | 11 | 3 | R.SDCSLCR.A |
|  | 3754 | 477.7527 | 953.4909 | 953.5334 | -0.0426 | 0 | 6 | 33 | 7 | R.VLYAVFSR.D |
|  | 4061 | **488.5781** | **975.1416** | **974.5145** | **0.6271** | **1** | **11** | **9.4** | **4** | **R.LLDRTDSR.E** 4039 |
|  | 4185 | 493.7580 | 985.5014 | 986.5219 | -1.0205 | 0 | 9 | 23 | 9 | R.VTLNGVPCK.V |
|  | 5364 | **536.1632** | **1070.3119** | **1070.5291** | **-0.2172** | **1** | **1** | **98** | **10** | **K.VHAKMEANR.N** |
|  | 7048 | 593.7826 | 1185.5506 | 1184.6626 | 0.8881 | 1 | 4 | 63 | 7 | R.DGLRGTAVLQR.G |
|  | 8910 | **637.8643** | **1273.7140** | **1272.6172** | **1.0967** | **1** | **6** | **34** | **3** | **K.MVEDYYKGIR.Q** |
|  | 9689 | **658.6415** | **1315.2685** | **1314.7330** | **0.5356** | **1** | **7** | **21** | **3** | **R.VTLNGVPCKVTK.F** 9616 |
|  | 11159 | **465.2784** | **1392.8133** | **1393.7102** | **-0.8969** | **1** | **6** | **31** | **2** | **R.AFEPLRSFASGGR.S** |
|  | 12382 | **489.2261** | **1464.6564** | **1465.7136** | **-1.0572** | **0** | **2** | **83** | **7** | **K.LHVTLYNCSFGR.S** |
|  | 13316 | **507.2345** | **1518.6818** | **1517.8606** | **0.8212** | **0** | **2** | **84** | **6** | **K.VYFASLLTVALHGK.L** |
|  | 13566 | 511.9600 | 1532.8581 | 1532.7868 | 0.0713 | 1 | 11 | 9.7 | 2 | K.VLNCDTISQVKEK.I |
|  | 13717 | **515.2927** | **1542.8563** | **1542.8664** | **-0.0101** | **1** | **0** | **1.2e+002** | **8** | **K.GICALRALSNISLR.L** |
|  | 14989 | 811.8912 | 1621.7679 | 1621.7770 | -0.0091 | 0 | 82 | 8e-007 | 1 | K.AMTLQEAEAFVGAER.C 14987 14988 |
|  | 16551 | 431.2414 | 1720.9365 | 1721.8957 | -0.9592 | 1 | 5 | 37 | 6 | R.LVECGSLFKGICALR.A |
|  | 17408 | **890.8970** | **1779.7795** | **1778.8952** | **0.8844** | **0** | **1** | **89** | **2** | **R.VWHLVRPTDEVDEGK.S** |
|  | 17632 | **598.3516** | **1792.0330** | **1790.8516** | **1.1815** | **1** | **2** | **72** | **9** | **K.SCVAVTSAQPQNMSRR.A** |
|  | 18825 | **939.4371** | **1876.8597** | **1876.8488** | **0.0109** | **0** | **45** | **0.0031** | **1** | **K.FMEPVTMQESGTFAFR.T** |
|  | 19228 | **955.4108** | **1908.8070** | **1908.8386** | **-0.0316** | **0** | **(6)** | **22** | **2** | **K.FMEPVTMQESGTFAFR.T** |
|  | 19535 | **645.3733** | **1933.0980** | **1932.9364** | **0.1617** | **1** | **4** | **35** | **6** | **R.DSRSSGGPGAGLCLFPLDK.V** |
|  | 19613 | **647.8611** | **1940.5614** | **1940.9401** | **-0.3787** | **0** | **2** | **39** | **5** | **R.DLVLSGDLGSLYAMTQDK.V** |
|  | 23316 | 1156.5519 | 2311.0892 | 2311.1015 | -0.0123 | 0 | 1 | 58 | 3 | R.DPDIHAAAFGTCLAASVAAPGSGR.V 23315 |
|  | 23378 | **774.3397** | **2319.9972** | **2321.1916** | **-1.1945** | **1** | **4** | **29** | **5** | **K.SFLINFIHTLENQREFSAR.A** |
|  | 27976 | **1029.4951** | **3085.4635** | **3086.4842** | **-1.0206** | **1** | **3** | **21** | **1** | **K.GSSLHVGSDLLKFMEPVTMQESGTFAFR.T** |
|  | 29898 | **922.4084** | **3685.6045** | **3685.8298** | **-0.2253** | **0** | **3** | **14** | **3** | **R.AQGEVQLTVSPLPALSEEDELLCLFGESPPHPAR.V** |

| **411.** | P02452\|CO1A1_HUMAN    **Mass:** 139853   **Score:** 97     **Queries matched:** 37   **emPAI:** 0.03 |
| --- | --- |
|  | Collagen alpha-1(I) chain OS=Homo sapiens GN=COL1A1 PE=1 SV=4 |

|  | **Query** | **Observed** | **Mr(expt)** | **Mr(calc)** | **Delta** | **Miss** | **Score** | **Expect** | **Rank** | **Peptide** |
| --- | --- | --- | --- | --- | --- | --- | --- | --- | --- | --- |
|  | 2128 | **419.1952** | **836.3759** | **835.4301** | **0.9458** | **0** | **5** | **49** | **4** | **R.GPPGPQGAR.G** |
|  | 3171 | **457.2555** | **912.4964** | **911.4573** | **1.0390** | **1** | **12** | **8.9** | **10** | **K.GDTGPRGPR.G** |
|  | 4859 | **517.2763** | **1032.5379** | **1032.5339** | **0.0040** | **0** | **8** | **27** | **9** | **R.DLEVDTTLK.S** |
|  | 5391 | 536.3099 | 1070.6052 | 1071.5349 | -0.9297 | 0 | 1 | 1.5e+002 | 10 | R.GFPGADGVAGPK.G |
|  | 5591 | **543.2338** | **1084.4531** | **1083.4945** | **0.9586** | **0** | **4** | **66** | **3** | **R.EGAPGAEGSPGR.D** |
|  | 5697 | 547.2581 | 1092.5017 | 1093.5053 | -1.0036 | 1 | 3 | 78 | 6 | K.AHDGGRYYR.A |
|  | 6686 | **581.7878** | **1161.5610** | **1160.5649** | **0.9962** | **0** | **24** | **0.57** | **1** | **R.GQAGVMGFPGPK.G** |
|  | 7048 | 593.7826 | 1185.5506 | 1186.6306 | -1.0799 | 0 | 4 | 68 | 9 | K.SLSQQIENIR.S |
|  | 9286 | **432.8828** | **1295.6266** | **1296.6058** | **-0.9792** | **0** | **4** | **56** | **10** | **K.GESGPSGPAGPTGAR.G** |
|  | 12112 | **483.5688** | **1447.6845** | **1447.7718** | **-0.0873** | **1** | **6** | **35** | **4** | **R.GLPGTAGLPGMKGHR.G** |
|  | 12380 | **489.0251** | **1464.0534** | **1463.7667** | **0.2867** | **1** | **(5)** | **24** | **2** | **R.GLPGTAGLPGMKGHR.G** |
|  | 13750 | 773.4462 | 1544.8778 | 1545.7900 | -0.9122 | 0 | 4 | 53 | 8 | R.GETGPAGPAGPVGPVGAR.G |
|  | 13972 | **520.2704** | **1557.7893** | **1557.8151** | **-0.0258** | **0** | **4** | **54** | **1** | **R.GLTGPIGPPGPAGAPGDK.G** |
|  | 14406 | **529.6142** | **1585.8208** | **1585.7332** | **0.0875** | **1** | **7** | **23** | **2** | **K.GNSGEPGAPGSKGDTGAK.G** |
|  | 14707 | 536.2450 | 1605.7132 | 1606.8063 | -1.0931 | 0 | 2 | 71 | 10 | K.GSPGEAGRPGEAGLPGAK.G 14710 |
|  | 15210 | 819.4298 | 1636.8451 | 1637.8162 | -0.9711 | 1 | 0 | 1.1e+002 | 4 | R.GERGFPGLPGPSGEPGK.Q |
|  | 15232 | **547.0836** | **1638.2290** | **1638.8114** | **-0.5824** | **1** | **1** | **59** | **8** | **R.GFPGADGVAGPKGPAGER.G** 15245 |
|  | 16734 | **578.3133** | **1731.9180** | **1731.8805** | **0.0375** | **1** | **2** | **71** | **6** | **R.GFPGERGVQGPPGPAGPR.G** |
|  | 16772 | **579.0114** | **1734.0124** | **1734.8398** | **-0.8274** | **1** | **1** | **75** | **10** | **K.GEAGPQGPRGSEGPQGVR.G** |
|  | 18175 | **915.4922** | **1828.9698** | **1827.8938** | **1.0760** | **1** | **0** | **1e+002** | **7** | **R.GQAGVMGFPGPKGAAGEPGK.A** |
|  | 19918 | **981.4858** | **1960.9571** | **1960.9715** | **-0.0144** | **1** | **66** | **2.6e-005** | **1** | **K.SGDRGETGPAGPAGPVGPVGAR.G** 19895 19919 |
|  | 21649 | 707.4123 | 2119.2152 | 2120.0651 | -0.8499 | 0 | 2 | 44 | 2 | R.GETGPAGPPGAPGAPGAPGPVGPAGK.S |
|  | 21910 | 538.7760 | 2151.0749 | 2149.9811 | 1.0938 | 0 | 1 | 69 | 5 | K.GDAGAPGAPGSQGAPGLQGMPGER.G |
|  | 22592 | **741.3472** | **2221.0197** | **2222.0716** | **-1.0519** | **1** | **3** | **44** | **5** | **R.GFPGLPGPSGEPGKQGPSGASGER.G** |
|  | 22715 | **745.4404** | **2233.2995** | **2232.1288** | **1.1707** | **0** | **5** | **21** | **1** | **K.GDAGPPGPAGPAGPPGPIGNVGAPGAK.G** |
|  | 23658 | 786.0517 | 2355.1333 | 2355.1931 | -0.0598 | 1 | 1 | 64 | 10 | R.GSPGPAGPKGSPGEAGRPGEAGLPGAK.G |
|  | 26612 | 936.4615 | 2806.3626 | 2806.3273 | 0.0354 | 1 | 4 | 22 | 2 | R.FTYSVTVDGCTSHTGAWGKTVIEYK.T |
|  | 27597 | **1000.9499** | **2999.8278** | **2999.4991** | **0.3287** | **0** | **2** | **24** | **2** | **R.LPIIDVAPLDVGAPDQEFGFDVGPVCFL.-** |
|  | 28107 | **780.9173** | **3119.6401** | **3118.5520** | **1.0881** | **1** | **3** | **25** | **2** | **K.TGPPGPAGQDGRPGPPGPPGARGQAGVMGFPGPK.G** |
|  | 28230 | 786.8777 | 3143.4819 | 3143.4439 | 0.0380 | 1 | 1 | 41 | 5 | R.GANGAPGNDGAKGDAGAPGAPGSQGAPGLQGMPGER.G |
|  | 29553 | 1178.8755 | 3533.6046 | 3532.7084 | 0.8962 | 1 | 0 | 33 | 4 | R.GFSGLQGPPGPPGSPGEQGPSGASGPAGPRGPPGSAGAPGK.D |
|  | 31009 | **908.6199** | **4538.0633** | **4538.1860** | **-0.1228** | **1** | **3** | **9.7** | **3** | **K.STGGISVPGPMGPSGPRGLPGPPGAPGPQGFQGPPGEPGEPGASGPMGPR.G** |
|  | 31063 | 1541.5437 | 4621.6093 | 4622.0499 | -0.4406 | 1 | 1 | 12 | 5 | K.SGEYWIDPNQGCNLDAIKVFCNMETGETCVYPTQPSVAQK.N |

| **412.** | P04003\|C4BPA_HUMAN    **Mass:** 69042    **Score:** 97     **Queries matched:** 12   **emPAI:** 0.17 |
| --- | --- |
|  | C4b-binding protein alpha chain OS=Homo sapiens GN=C4BPA PE=1 SV=2 |

|  | **Query** | **Observed** | **Mr(expt)** | **Mr(calc)** | **Delta** | **Miss** | **Score** | **Expect** | **Rank** | **Peptide** |
| --- | --- | --- | --- | --- | --- | --- | --- | --- | --- | --- |
|  | 8359 | **416.9043** | **1247.6910** | **1248.6714** | **-0.9804** | **0** | **6** | **38** | **8** | **K.EDVYVVGTVLR.Y** |
|  | 9290 | **432.8839** | **1295.6300** | **1296.7051** | **-1.0751** | **1** | **6** | **37** | **2** | **M.HPPKTPSGALHR.K** 9316 |
|  | 11774 | 477.2324 | 1428.6754 | 1427.7456 | 0.9299 | 1 | 6 | 32 | 3 | -.MHPPKTPSGALHR.K 11767 |
|  | 12049 | **482.6137** | **1444.8193** | **1443.7405** | **1.0788** | **1** | **(2)** | **82** | **7** | **-.MHPPKTPSGALHR.K** |
|  | 14311 | **791.3544** | **1580.6943** | **1580.7042** | **-0.0099** | **0** | **30** | **0.12** | **1** | **R.FSAICQGDGTWSPR.T** |
|  | 16303 | **569.6238** | **1705.8495** | **1705.8610** | **-0.0115** | **0** | **28** | **0.18** | **1** | **R.GVGWSHPLPQCEIVK.C** |
|  | 18954 | 629.9907 | 1886.9502 | 1885.9111 | 1.0390 | 1 | 6 | 26 | 4 | K.IAHGHYKQSSSYSFFK.E |
|  | 20276 | 998.4167 | 1994.8189 | 1994.8325 | -0.0135 | 0 | 54 | 0.00031 | 1 | R.WTPYQGCEALCCPEPK.L 20277 |
|  | 31283 | 1671.5214 | 5011.5423 | 5010.6313 | 0.9110 | 1 | 5 | 4 | 7 | R.LWKVSDPILFQMTLIAALLPAVLGNCGPPPTLSFAAPMDITLTETR.F |

| **413.** | O00291\|HIP1_HUMAN    **Mass:** 117232   **Score:** 97     **Queries matched:** 30   **emPAI:** 0.03 |
| --- | --- |
|  | Huntingtin-interacting protein 1 OS=Homo sapiens GN=HIP1 PE=1 SV=5 |

|  | **Query** | **Observed** | **Mr(expt)** | **Mr(calc)** | **Delta** | **Miss** | **Score** | **Expect** | **Rank** | **Peptide** |
| --- | --- | --- | --- | --- | --- | --- | --- | --- | --- | --- |
|  | 968 | **560.8668** | **559.8595** | **559.2966** | **0.5630** | **0** | **4** | **86** | **5** | **R.GTASPK.E** |
|  | 2909 | 447.3449 | 892.6753 | 891.5178 | 1.1575 | 0 | 4 | 50 | 1 | K.QVPNPLPK.V |
|  | 3545 | 471.2341 | 940.4536 | 940.4106 | 0.0430 | 1 | 14 | 6.3 | 1 | M.DRMASSMK.Q 3564 |
|  | 3929 | **484.2398** | **966.4650** | **966.4076** | **0.0574** | **0** | **9** | **18** | **5** | **R.NELSDMSR.M** |
|  | 4033 | **487.7714** | **973.5283** | **973.5080** | **0.0203** | **0** | **13** | **11** | **5** | **K.ELQDTQLK.L** |
|  | 4361 | 499.2587 | 996.5028 | 996.5604 | -0.0575 | 0 | 13 | 8.1 | 2 | K.AIGEELLPR.G 4348 4358 4360 |
|  | 4537 | **505.2522** | **1008.4899** | **1007.4342** | **1.0557** | **0** | **8** | **23** | **5** | **R.QEMDSQVR.V** |
|  | 4804 | **515.7517** | **1029.4889** | **1028.5251** | **0.9638** | **0** | **10** | **18** | **2** | **R.GVGAGLEAAER.E** |
|  | 5408 | **536.7999** | **1071.5853** | **1071.5924** | **-0.0071** | **0** | **12** | **11** | **8** | **K.AINTQEVAVK.E** |
|  | 6448 | **574.7726** | **1147.5306** | **1147.5404** | **-0.0098** | **1** | **22** | **1.1** | **1** | **K.RQEMDSQVR.V** |
|  | 7028 | **593.1561** | **1184.2977** | **1184.6262** | **-0.3285** | **1** | **5** | **34** | **2** | **R.RGVGAGLEAAER.E** |
|  | 7232 | 598.2708 | 1194.5271 | 1194.5815 | -0.0545 | 0 | 11 | 11 | 3 | R.TCILGTHHEK.G |
|  | 7420 | **401.5683** | **1201.6831** | **1200.5597** | **1.1233** | **1** | **5** | **49** | **9** | **R.DRFMEQFTK.L** |
|  | 8145 | **413.7272** | **1238.1598** | **1237.7394** | **0.4204** | **1** | **12** | **5.3** | **2** | **K.IKAIGEELLPR.G** |
|  | 9361 | **650.8364** | **1299.6583** | **1300.6735** | **-1.0152** | **1** | **5** | **42** | **10** | **K.DLQREIVESGR.G** |
|  | 10305 | 450.2784 | 1347.8135 | 1348.6769 | -0.8634 | 1 | 5 | 51 | 5 | K.NAEVTKQVSMAR.Q |
|  | 11059 | **463.2399** | **1386.6979** | **1386.7329** | **-0.0351** | **0** | **6** | **32** | **1** | **R.LPLSSNAVLCWK.F** |
|  | 12693 | **495.2550** | **1482.7431** | **1482.6694** | **0.0737** | **0** | **4** | **50** | **5** | **K.LASTEESMCQLAK.D** |
|  | 13153 | **504.2507** | **1509.7304** | **1508.7140** | **1.0163** | **0** | **4** | **50** | **7** | **K.EMAATSAAIETATAR.I** 13129 |
|  | 13986 | **780.4180** | **1558.8214** | **1558.8315** | **-0.0101** | **0** | **97** | **2.5e-008** | **1** | **R.GVNQATAGVVASTISGK.S** |
|  | 13991 | **780.7042** | **1559.3939** | **1558.7847** | **0.6092** | **1** | **0** | **80** | **1** | **R.MASSMKQVPNPLPK.V** |
|  | 21488 | 527.2809 | 2105.0947 | 2105.1204 | -0.0258 | 1 | 11 | 7.7 | 3 | K.LLFKLHSCLPADTLQGHR.D |
|  | 21987 | **540.5336** | **2158.1054** | **2157.0023** | **1.1031** | **0** | **6** | **20** | **3** | **R.MWGHLSEGYGQLCSIYLK.L** |
|  | 22543 | **739.3591** | **2215.0556** | **2215.1420** | **-0.0864** | **1** | **1** | **66** | **10** | **K.AVGWGATVMVDAADLVVQGRGK.F** |
|  | 24209 | **610.2930** | **2437.1430** | **2438.1669** | **-1.0238** | **1** | **1** | **60** | **9** | **K.ELQDTQLKLASTEESMCQLAK.D** |

| **414.** | O00161\|SNP23_HUMAN    **Mass:** 23682    **Score:** 97     **Queries matched:** 6   **emPAI:** 0.35 |
| --- | --- |
|  | Synaptosomal-associated protein 23 OS=Homo sapiens GN=SNAP23 PE=1 SV=1 |

|  | **Query** | **Observed** | **Mr(expt)** | **Mr(calc)** | **Delta** | **Miss** | **Score** | **Expect** | **Rank** | **Peptide** |
| --- | --- | --- | --- | --- | --- | --- | --- | --- | --- | --- |
|  | 4876 | **517.7451** | **1033.4757** | **1032.5200** | **0.9557** | **1** | **6** | **45** | **4** | **R.ITDKADTNR.D** |
|  | 9760 | **440.5828** | **1318.7265** | **1317.6160** | **1.1104** | **0** | **8** | **23** | **2** | **M.DNLSSEEIQQR.A** |
|  | 14324 | **528.2590** | **1581.7553** | **1582.9042** | **-1.1490** | **1** | **5** | **40** | **3** | **R.RILGLAIESQDAGIK.T** |
|  | 17845 | **904.3938** | **1806.7730** | **1806.7843** | **-0.0113** | **0** | **69** | **1.2e-005** | **1** | **K.TTWGDGGENSPCNVVSK.Q** |
|  | 22080 | **1085.5256** | **2169.0367** | **2168.9797** | **0.0570** | **1** | **0** | **79** | **5** | **K.AYKTTWGDGGENSPCNVVSK.Q** |
|  | 26491 | **930.4537** | **2788.3394** | **2788.3549** | **-0.0155** | **1** | **53** | **0.00026** | **1** | **R.ITNDAREDEMEENLTQVGSILGNLK.D** |

| **415.** | P62937\|PPIA_HUMAN    **Mass:** 18229    **Score:** 96     **Queries matched:** 16   **emPAI:** 0.47 |
| --- | --- |
|  | Peptidyl-prolyl cis-trans isomerase A OS=Homo sapiens GN=PPIA PE=1 SV=2 |

|  | **Query** | **Observed** | **Mr(expt)** | **Mr(calc)** | **Delta** | **Miss** | **Score** | **Expect** | **Rank** | **Peptide** |
| --- | --- | --- | --- | --- | --- | --- | --- | --- | --- | --- |
|  | 1258 | 687.3368 | 686.3295 | 685.3912 | 0.9384 | 0 | 13 | 12 | 1 | K.HVVFGK.V |
|  | 1397 | **737.3513** | **736.3440** | **736.3504** | **-0.0063** | **0** | **14** | **6** | **1** | **K.TAENFR.A** |
|  | 6567 | **577.7863** | **1153.5581** | **1153.5655** | **-0.0074** | **0** | **26** | **0.37** | **1** | **K.FEDENFILK.H** |
|  | 9557 | **437.7726** | **1310.2961** | **1309.5642** | **0.7319** | **0** | **10** | **9.5** | **1** | **K.EGMNIVEAMER.F** |
|  | 10907 | **460.5877** | **1378.7411** | **1378.7496** | **-0.0085** | **1** | **12** | **8.8** | **3** | **R.VSFELFADKVPK.T** |
|  | 13077 | **753.3716** | **1504.7286** | **1504.7377** | **-0.0091** | **1** | **55** | **0.00035** | **1** | **K.VKEGMNIVEAMER.F** 13075 |
|  | 13362 | **507.9317** | **1520.7732** | **1520.7327** | **0.0405** | **1** | **(3)** | **70** | **4** | **K.VKEGMNIVEAMER.F** |
|  | 14588 | **799.8714** | **1597.7282** | **1597.7381** | **-0.0099** | **0** | **47** | **0.0021** | **1** | **R.IIPGFMCQGGDFTR.H** 14587 |
|  | 14835 | **538.9323** | **1613.7751** | **1613.7331** | **0.0420** | **0** | **(3)** | **59** | **2** | **R.IIPGFMCQGGDFTR.H** |
|  | 18190 | 916.4762 | 1830.9378 | 1830.9039 | 0.0339 | 1 | 1 | 79 | 6 | K.SIYGEKFEDENFILK.H |
|  | 22947 | **756.0196** | **2265.0369** | **2265.0419** | **-0.0050** | **1** | **1** | **61** | **6** | **R.IIPGFMCQGGDFTRHNGTGGK.S** |
|  | 26519 | **931.4379** | **2791.2920** | **2790.3218** | **0.9702** | **0** | **27** | **0.11** | **1** | **K.HTGPGILSMANAGPNTNGSQFFICTAK.T** 26518 |
|  | 26614 | **936.4621** | **2806.3645** | **2806.3167** | **0.0478** | **0** | **(1)** | **44** | **1** | **K.HTGPGILSMANAGPNTNGSQFFICTAK.T** |

| **416.** | Q9Y673\|ALG5_HUMAN    **Mass:** 37150    **Score:** 96     **Queries matched:** 9   **emPAI:** 0.10 |
| --- | --- |
|  | Dolichyl-phosphate beta-glucosyltransferase OS=Homo sapiens GN=ALG5 PE=1 SV=1 |

|  | **Query** | **Observed** | **Mr(expt)** | **Mr(calc)** | **Delta** | **Miss** | **Score** | **Expect** | **Rank** | **Peptide** |
| --- | --- | --- | --- | --- | --- | --- | --- | --- | --- | --- |
|  | 8436 | **418.2585** | **1251.7536** | **1250.6554** | **1.0982** | **1** | **6** | **30** | **4** | **K.GGAIRMGIFSSR.G** |
|  | 11614 | **474.2997** | **1419.8774** | **1418.7075** | **1.1699** | **1** | **12** | **7.3** | **2** | **R.GEKILMADADGATK.F** |
|  | 14438 | **530.2705** | **1587.7897** | **1588.7957** | **-1.0060** | **1** | **2** | **79** | **9** | **R.EAASRTFSSLHVER.W** |
|  | 15986 | **843.4283** | **1684.8421** | **1685.8624** | **-1.0203** | **1** | **11** | **8.1** | **1** | **K.GQKETLPSIWDSPTK.Q** |
|  | 17650 | **897.9052** | **1793.7959** | **1793.9055** | **-0.1097** | **1** | **3** | **56** | **4** | **R.LPVMMDEALSYLEKR.Q** |
|  | 17656 | **598.9727** | **1793.8962** | **1793.9055** | **-0.0094** | **1** | **5** | **39** | **2** | **K.RLPVMMDEALSYLEK.R** |
|  | 21707 | **1064.4998** | **2126.9850** | **2126.9990** | **-0.0140** | **0** | **67** | **1.6e-005** | **1** | **K.GLNDLQPWPNQMAIACGSR.A** 21706 |
|  | 26077 | **902.4176** | **2704.2310** | **2705.3166** | **-1.0856** | **1** | **2** | **38** | **1** | **K.GLNDLQPWPNQMAIACGSRAHLEK.E** |

| **417.** | P31146\|COR1A_HUMAN    **Mass:** 51678    **Score:** 96     **Queries matched:** 6   **emPAI:** 0.15 |
| --- | --- |
|  | Coronin-1A OS=Homo sapiens GN=CORO1A PE=1 SV=4 |

|  | **Query** | **Observed** | **Mr(expt)** | **Mr(calc)** | **Delta** | **Miss** | **Score** | **Expect** | **Rank** | **Peptide** |
| --- | --- | --- | --- | --- | --- | --- | --- | --- | --- | --- |
|  | 3819 | **480.2582** | **958.5019** | **959.5076** | **-1.0057** | **0** | **8** | **27** | **10** | **R.QVALWDTK.H** |
|  | 6316 | **569.9208** | **1137.8270** | **1137.4907** | **0.3363** | **0** | **3** | **57** | **6** | **R.DGGLICTSCR.D** |
|  | 9987 | 665.3446 | 1328.6746 | 1328.6693 | 0.0054 | 1 | 10 | 15 | 6 | R.RCEPIAMTVPR.K |
|  | 13033 | **751.8520** | **1501.6894** | **1501.7009** | **-0.0114** | **0** | **48** | **0.0019** | **1** | **R.AAPEASGTPSSDAVSR.L** |
|  | 30230 | **1282.9697** | **3845.8874** | **3845.9074** | **-0.0200** | **0** | **58** | **5.3e-005** | **1** | **K.HLEEPLSLQELDTSSGVLLPFFDPDTNIVYLCGK.G** 30229 |

| **418.** | P08754\|GNAI3_HUMAN    **Mass:** 41076    **Score:** 96     **Queries matched:** 17   **emPAI:** 0.55 |
| --- | --- |
|  | Guanine nucleotide-binding protein G(k) subunit alpha OS=Homo sapiens GN=GNAI3 PE=1 SV=3 |

|  | **Query** | **Observed** | **Mr(expt)** | **Mr(calc)** | **Delta** | **Miss** | **Score** | **Expect** | **Rank** | **Peptide** |
| --- | --- | --- | --- | --- | --- | --- | --- | --- | --- | --- |
|  | 3113 | 455.2127 | 908.4109 | 908.4175 | -0.0066 | 0 | 33 | 0.081 | 1 | K.MFDVGGQR.S |
|  | 3832 | 480.7394 | 959.4641 | 959.4672 | -0.0030 | 1 | 5 | 56 | 6 | R.NLREDGEK.A 3850 |
|  | 5181 | 529.2853 | 1056.5560 | 1056.6179 | -0.0619 | 0 | 28 | 0.31 | 1 | K.LLLLGAGESGK.S |
|  | 5743 | **548.7582** | **1095.5019** | **1095.4768** | **0.0252** | **0** | **0** | **1.4e+002** | **8** | **R.DGGVQACFSR.S** |
|  | 5949 | 555.7625 | 1109.5105 | 1109.5175 | -0.0071 | 0 | 58 | 0.0002 | 1 | K.LFDSICNNK.W |
|  | 10924 | 690.8571 | 1379.6997 | 1379.7085 | -0.0089 | 0 | 15 | 4.8 | 1 | K.TTGIVETHFTFK.D |
|  | 14390 | 529.3709 | 1585.0909 | 1584.9450 | 0.1459 | 1 | 1 | 67 | 4 | K.LLLLGAGESGKSTIVK.Q |
|  | 14514 | **796.9033** | **1591.7921** | **1590.7501** | **1.0420** | **1** | **4** | **43** | **1** | **K.DLYFKMFDVGGQR.S** |
|  | 15226 | **546.9317** | **1637.7733** | **1636.7549** | **1.0184** | **1** | **10** | **10** | **2** | **-.MGCTLSAEDKAAVER.S** |
|  | 16346 | 854.9661 | 1707.9176 | 1706.9243 | 0.9932 | 0 | 29 | 0.14 | 1 | K.NVQFVFDAVTDVIIK.N |
|  | 17136 | **881.4542** | **1760.8938** | **1760.9057** | **-0.0119** | **0** | **6** | **27** | **2** | **R.ISQSNYIPTQQDVLR.T** |
|  | 18931 | 629.3328 | 1884.9765 | 1884.8168 | 0.1596 | 1 | 4 | 43 | 1 | R.MHESMKLFDSICNNK.W |
|  | 20458 | **671.9625** | **2012.8657** | **2012.8785** | **-0.0128** | **1** | **14** | **3.4** | **1** | **K.IIHEDGYSEDECKQYK.V** |
|  | 23684 | **786.9432** | **2357.8077** | **2358.2505** | **-0.4428** | **0** | **3** | **28** | **3** | **R.QLFVLAGSAEEGVMTPELAGVIK.R** |
|  | 27102 | **726.3900** | **2901.5307** | **2902.4746** | **-0.9439** | **1** | **1** | **44** | **5** | **R.ADDARQLFVLAGSAEEGVMTPELAGVIK.R** |
|  | 30162 | **1268.5618** | **3802.6635** | **3802.6767** | **-0.0132** | **0** | **46** | **0.00069** | **1** | **R.SPLTICYPEYTGSNTYEEAAAYIQCQFEDLNR.R** |

| **419.** | Q8NFJ5\|RAI3_HUMAN    **Mass:** 40624    **Score:** 95     **Queries matched:** 6   **emPAI:** 0.09 |
| --- | --- |
|  | Retinoic acid-induced protein 3 OS=Homo sapiens GN=GPRC5A PE=1 SV=2 |

|  | **Query** | **Observed** | **Mr(expt)** | **Mr(calc)** | **Delta** | **Miss** | **Score** | **Expect** | **Rank** | **Peptide** |
| --- | --- | --- | --- | --- | --- | --- | --- | --- | --- | --- |
|  | 3725 | **476.7415** | **951.4685** | **951.4774** | **-0.0089** | **1** | **16** | **3.9** | **3** | **K.KSYGVENR.A** |
|  | 11858 | **478.7423** | **1433.2051** | **1432.7310** | **0.4741** | **0** | **3** | **40** | **3** | **R.TNVNVFSELSAPR.R** |
|  | 16059 | **564.2753** | **1689.8040** | **1689.8151** | **-0.0111** | **1** | **5** | **33** | **1** | **R.AHAWPSPYKDYEVK.K** |
|  | 29162 | **844.6289** | **3374.4865** | **3374.8234** | **-0.3368** | **0** | **1** | **30** | **5** | **K.AEAWGIVLETVATAGVVTSVAFMLTLPILVCK.V** |
|  | 29922 | **1233.5953** | **3697.7642** | **3696.9677** | **0.7965** | **1** | **2** | **21** | **2** | **K.RHGAHIYLTMLLSIAIWVAWITLLMLPDFDR.R** |
|  | 30501 | **1349.2878** | **4044.8417** | **4044.8654** | **-0.0237** | **0** | **95** | **7.6e-009** | **1** | **R.AYSQEEITQGFEETGDTLYAPYSTHFQLQNQPPQK.E** |

| **420.** | P07910\|HNRPC_HUMAN    **Mass:** 33707    **Score:** 95     **Queries matched:** 15   **emPAI:** 0.11 |
| --- | --- |
|  | Heterogeneous nuclear ribonucleoproteins C1/C2 OS=Homo sapiens GN=HNRNPC PE=1 SV=4 |

|  | **Query** | **Observed** | **Mr(expt)** | **Mr(calc)** | **Delta** | **Miss** | **Score** | **Expect** | **Rank** | **Peptide** |
| --- | --- | --- | --- | --- | --- | --- | --- | --- | --- | --- |
|  | 2953 | 449.7274 | 897.4402 | 898.4695 | -1.0293 | 0 | 7 | 28 | 3 | K.IVGCSVHK.G |
|  | 3105 | **908.9982** | **907.9910** | **908.2995** | **-0.3086** | **0** | **3** | **56** | **1** | **R.DSANGEDDS.-** |
|  | 3608 | **472.7242** | **943.4339** | **942.5651** | **0.8688** | **0** | **15** | **6.4** | **4** | **R.VPPPPPIAR.A** 3577 3590 3598 3606 3607 |
|  | 7190 | **596.8565** | **1191.6985** | **1192.5758** | **-0.8773** | **1** | **7** | **28** | **3** | **K.EQSKQAVEMK.N** |
|  | 7445 | 602.3564 | 1202.6982 | 1201.6051 | 1.0931 | 1 | 1 | 1.1e+002 | 9 | M.ASNVTNKTDPR.S |
|  | 9704 | **439.5715** | **1315.6928** | **1315.7864** | **-0.0936** | **0** | **7** | **30** | **8** | **R.VFIGNLNTLVVK.K** |
|  | 10312 | **675.3098** | **1348.6051** | **1348.6405** | **-0.0354** | **1** | **5** | **44** | **4** | **-.MASNVTNKTDPR.S** |
|  | 15935 | 841.9561 | 1681.8977 | 1681.9073 | -0.0096 | 0 | 95 | 3.3e-008 | 1 | R.MIAGQVLDINLAAEPK.V |
|  | 19183 | **636.3502** | **1906.0288** | **1907.0299** | **-1.0010** | **1** | **1** | **83** | **8** | **R.SMNSRVFIGNLNTLVVK.K** |
|  | 29347 | 862.9139 | 3447.6267 | 3448.5405 | -0.9139 | 0 | 1 | 28 | 3 | R.SAAEMYGSVTEHPSPSPLLSSSFDLDYDFQR.D |

| **421.** | P00403\|COX2_HUMAN    **Mass:** 25719    **Score:** 95     **Queries matched:** 7   **emPAI:** 0.32 |
| --- | --- |
|  | Cytochrome c oxidase subunit 2 OS=Homo sapiens GN=MT-CO2 PE=1 SV=1 |

|  | **Query** | **Observed** | **Mr(expt)** | **Mr(calc)** | **Delta** | **Miss** | **Score** | **Expect** | **Rank** | **Peptide** |
| --- | --- | --- | --- | --- | --- | --- | --- | --- | --- | --- |
|  | 2259 | **423.0525** | **844.0905** | **843.4450** | **0.6455** | **0** | **11** | **11** | **2** | **R.LLDVDNR.V** 2265 2267 |
|  | 18511 | 618.2813 | 1851.8221 | 1850.9335 | 0.8886 | 0 | 4 | 35 | 10 | R.ILYMTDEVNDPSLTIK.S |
|  | 22638 | 1114.0785 | 2226.1424 | 2226.1541 | -0.0117 | 0 | 78 | 1.2e-006 | 1 | R.MMITSQDVLHSWAVPTLGLK.T |
|  | 22800 | **748.3862** | **2242.1369** | **2242.1490** | **-0.0121** | **0** | **(40)** | **0.0076** | **1** | **R.MMITSQDVLHSWAVPTLGLK.T** |
|  | 22890 | 753.3492 | 2257.0259 | 2258.1439 | -1.1180 | 0 | (0) | 72 | 8 | R.MMITSQDVLHSWAVPTLGLK.T |

| **422.** | Q15165\|PON2_HUMAN    **Mass:** 39658    **Score:** 95     **Queries matched:** 7   **emPAI:** 0.09 |
| --- | --- |
|  | Serum paraoxonase/arylesterase 2 OS=Homo sapiens GN=PON2 PE=1 SV=2 |

|  | **Query** | **Observed** | **Mr(expt)** | **Mr(calc)** | **Delta** | **Miss** | **Score** | **Expect** | **Rank** | **Peptide** |
| --- | --- | --- | --- | --- | --- | --- | --- | --- | --- | --- |
|  | 2322 | 425.5480 | 849.0815 | 849.4596 | -0.3781 | 0 | 3 | 58 | 4 | K.NTVEIFK.F |
|  | 5599 | **543.3096** | **1084.6046** | **1084.6393** | **-0.0347** | **0** | **7** | **32** | **3** | **K.LLIGTLYHR.A** |
|  | 18420 | **923.9648** | **1845.9150** | **1845.9261** | **-0.0111** | **0** | **70** | **9.6e-006** | **1** | **K.LFVYDPNNPPSSEVLR.I** 18419 |
|  | 24387 | **821.7628** | **2462.2666** | **2461.2740** | **0.9926** | **0** | **2** | **53** | **3** | **K.GIEAGSEDIDILPNGLAFFSVGLK.F** |
|  | 30377 | 1318.6537 | 3952.9392 | 3954.0051 | -1.0658 | 1 | 5 | 8.4 | 2 | K.VVAEGFDSANGINISPDDKYIYVADILAHEIHVLEK.H 30382 |

| **423.** | Q15738\|NSDHL_HUMAN    **Mass:** 42159    **Score:** 95     **Queries matched:** 9   **emPAI:** 0.19 |
| --- | --- |
|  | Sterol-4-alpha-carboxylate 3-dehydrogenase, decarboxylating OS=Homo sapiens GN=NSDHL PE=1 SV=2 |

|  | **Query** | **Observed** | **Mr(expt)** | **Mr(calc)** | **Delta** | **Miss** | **Score** | **Expect** | **Rank** | **Peptide** |
| --- | --- | --- | --- | --- | --- | --- | --- | --- | --- | --- |
|  | 4597 | **507.3266** | **1012.6387** | **1012.5189** | **0.1198** | **0** | **2** | **94** | **1** | **R.AVLGANDPEK.N** 4598 |
|  | 4964 | **521.2601** | **1040.5057** | **1040.5138** | **-0.0081** | **0** | **4** | **67** | **6** | **R.THLTEDTPK.V** |
|  | 6383 | **572.3723** | **1142.7301** | **1142.6309** | **0.0992** | **1** | **4** | **65** | **6** | **R.TVQSFRHLR.R** |
|  | 6905 | **590.2849** | **1178.5551** | **1177.5107** | **1.0444** | **0** | **5** | **55** | **8** | **-.MEPAVSEPMR.D** |
|  | 15239 | 820.3907 | 1638.7668 | 1637.8447 | 0.9221 | 1 | 6 | 29 | 3 | R.VNYIGTKNVIETCK.E |
|  | 17683 | **599.7476** | **1796.2210** | **1795.9846** | **0.2365** | **0** | **3** | **33** | **10** | **K.NFLTTAIRPHGIFGPR.D** |
|  | 18138 | **914.4020** | **1826.7894** | **1826.8001** | **-0.0107** | **0** | **36** | **0.027** | **1** | **K.AMGYQPLVTMDDAMER.T** |
|  | 22056 | **1084.0247** | **2166.0348** | **2166.0494** | **-0.0147** | **0** | **89** | **1.1e-007** | **1** | **R.GYAVNVFDIQQGFDNPQVR.F** |

| **424.** | P02656\|APOC3_HUMAN    **Mass:** 10846    **Score:** 94     **Queries matched:** 5   **emPAI:** 0.87 |
| --- | --- |
|  | Apolipoprotein C-III OS=Homo sapiens GN=APOC3 PE=1 SV=1 |

|  | **Query** | **Observed** | **Mr(expt)** | **Mr(calc)** | **Delta** | **Miss** | **Score** | **Expect** | **Rank** | **Peptide** |
| --- | --- | --- | --- | --- | --- | --- | --- | --- | --- | --- |
|  | 16471 | **858.9226** | **1715.8305** | **1715.8438** | **-0.0133** | **0** | **72** | **6.8e-006** | **1** | **K.DALSSVQESQVAQQAR.G** 16472 |
|  | 20251 | 996.9181 | 1991.8216 | 1992.8808 | -1.0592 | 0 | 2 | 50 | 2 | R.ASEAEDASLLSFMQGYMK.H |
|  | 20519 | **673.0344** | **2016.0814** | **2016.0236** | **0.0579** | **1** | **5** | **27** | **5** | **K.TAKDALSSVQESQVAQQAR.G** 20516 |

| **425.** | P55290\|CAD13_HUMAN    **Mass:** 78694    **Score:** 94     **Queries matched:** 18   **emPAI:** 0.05 |
| --- | --- |
|  | Cadherin-13 OS=Homo sapiens GN=CDH13 PE=1 SV=1 |

|  | **Query** | **Observed** | **Mr(expt)** | **Mr(calc)** | **Delta** | **Miss** | **Score** | **Expect** | **Rank** | **Peptide** |
| --- | --- | --- | --- | --- | --- | --- | --- | --- | --- | --- |
|  | 5398 | 536.7685 | 1071.5224 | 1071.5309 | -0.0085 | 0 | 19 | 2.5 | 2 | K.VVDSDRPER.S |
|  | 6053 | **559.7936** | **1117.5727** | **1118.5284** | **-0.9557** | **0** | **1** | **1.4e+002** | **2** | **R.YEVSSPYFK.V** |
|  | 6804 | 586.3257 | 1170.6368 | 1170.6245 | 0.0124 | 1 | 2 | 1e+002 | 8 | R.LTGKGVDQEPK.G |
|  | 7711 | **406.2096** | **1215.6069** | **1214.6870** | **0.9199** | **0** | **6** | **36** | **10** | **K.NLSVVILGASDK.D** 7697 |
|  | 8644 | 631.8375 | 1261.6604 | 1262.6507 | -0.9903 | 0 | 1 | 1.3e+002 | 3 | K.DIQGSLQDIFK.F |
|  | 8926 | **638.8236** | **1275.6327** | **1275.6419** | **-0.0092** | **0** | **72** | **9.7e-006** | **1** | **R.INENTGSVSVTR.T** 8922 8941 |
|  | 13413 | **509.2330** | **1524.6771** | **1525.8729** | **-1.1958** | **1** | **1** | **1e+002** | **10** | **R.NITAVGKTLFVHAR.T** |
|  | 14048 | **782.3763** | **1562.7380** | **1563.8984** | **-1.1604** | **0** | **10** | **12** | **3** | **R.SIVVSPILIPENQR.Q** |
|  | 20611 | **1013.4147** | **2024.8148** | **2024.0466** | **0.7682** | **0** | **0** | **73** | **1** | **R.EVIAVYQLFVETTDVNGK.T** |
|  | 25562 | 878.8090 | 2633.4051 | 2633.4177 | -0.0126 | 0 | 14 | 2.5 | 1 | K.TLEGPVPLEVIVIDQNDNRPIFR.E |
|  | 25942 | **898.1239** | **2691.3499** | **2692.3200** | **-0.9702** | **0** | **2** | **39** | **2** | **K.ANYNLPIMVTDSGKPPMTNITDLR.V** |
|  | 28416 | 796.6552 | 3182.5915 | 3183.5567 | -0.9652 | 0 | 7 | 9.6 | 3 | K.YELIIEAQDMAGLDVGLTGTATATIMIDDK.N 28417 28418 28422 |

| **426.** | Q9Y5M8\|SRPRB_HUMAN    **Mass:** 29912    **Score:** 94     **Queries matched:** 11   **emPAI:** 0.82 |
| --- | --- |
|  | Signal recognition particle receptor subunit beta OS=Homo sapiens GN=SRPRB PE=1 SV=3 |

|  | **Query** | **Observed** | **Mr(expt)** | **Mr(calc)** | **Delta** | **Miss** | **Score** | **Expect** | **Rank** | **Peptide** |
| --- | --- | --- | --- | --- | --- | --- | --- | --- | --- | --- |
|  | 4397 | **500.2923** | **998.5700** | **998.5760** | **-0.0060** | **0** | **34** | **0.06** | **1** | **K.LIQQQLEK.E** |
|  | 10268 | **673.8200** | **1345.6255** | **1345.6361** | **-0.0107** | **0** | **22** | **1** | **1** | **R.GDVGSADIQDLEK.W** |
|  | 14071 | **522.2997** | **1563.8774** | **1563.9501** | **-0.0727** | **1** | **5** | **37** | **4** | **K.TLLFVRLLTGLYR.D** |
|  | 14878 | 808.8915 | 1615.7684 | 1615.7802 | -0.0118 | 1 | 57 | 0.00026 | 1 | K.GGRGDVGSADIQDLEK.W 14879 |
|  | 17536 | **894.9459** | **1787.8772** | **1787.8901** | **-0.0129** | **0** | **44** | **0.0047** | **1** | **R.SAAPSTLDSSSTAPAQLGK.K** |
|  | 19605 | **647.5552** | **1939.6439** | **1939.9965** | **-0.3526** | **0** | **12** | **4.8** | **1** | **K.DVAEFLYQVLIDSMGLK.N** 19602 19603 19606 |
|  | 23209 | **766.4048** | **2296.1925** | **2296.2025** | **-0.0099** | **1** | **41** | **0.0069** | **1** | **R.EVKDVAEFLYQVLIDSMGLK.N** |

| **427.** | P52597\|HNRPF_HUMAN    **Mass:** 45985    **Score:** 94     **Queries matched:** 8   **emPAI:** 0.08 |
| --- | --- |
|  | Heterogeneous nuclear ribonucleoprotein F OS=Homo sapiens GN=HNRNPF PE=1 SV=3 |

|  | **Query** | **Observed** | **Mr(expt)** | **Mr(calc)** | **Delta** | **Miss** | **Score** | **Expect** | **Rank** | **Peptide** |
| --- | --- | --- | --- | --- | --- | --- | --- | --- | --- | --- |
|  | 4201 | **494.5906** | **987.1666** | **986.4352** | **0.7314** | **1** | **2** | **87** | **6** | **K.DRESMGHR.Y** |
|  | 12386 | **733.3450** | **1464.6755** | **1465.6945** | **-1.0190** | **0** | **5** | **38** | **4** | **-.MMLGPEGGEGFVVK.L** |
|  | 13161 | **504.2783** | **1509.8132** | **1510.7238** | **-0.9106** | **1** | **1** | **94** | **10** | **R.ESMGHRYIEVFK.S** |
|  | 14650 | **535.2728** | **1602.7965** | **1603.8392** | **-1.0427** | **1** | **6** | **34** | **1** | **M.MLGPEGGEGFVVKLR.G** 14648 |
|  | 15097 | **543.9376** | **1628.7910** | **1629.7132** | **-0.9221** | **0** | **1** | **1e+002** | **3** | **K.HSGPNSADSANDGFVR.L** |
|  | 18698 | **934.4710** | **1866.9273** | **1866.9363** | **-0.0090** | **0** | **94** | **3.8e-008** | **1** | **K.ITGEAFVQFASQELAEK.A** |
|  | 22429 | **553.0385** | **2208.1247** | **2207.0052** | **1.1195** | **0** | **1** | **70** | **7** | **R.VTGEADVEFATHEEAVAAMSK.D** |

| **428.** | Q9H8H3\|MET7A_HUMAN    **Mass:** 28814    **Score:** 94     **Queries matched:** 8   **emPAI:** 0.45 |
| --- | --- |
|  | Methyltransferase-like protein 7A OS=Homo sapiens GN=METTL7A PE=1 SV=1 |

|  | **Query** | **Observed** | **Mr(expt)** | **Mr(calc)** | **Delta** | **Miss** | **Score** | **Expect** | **Rank** | **Peptide** |
| --- | --- | --- | --- | --- | --- | --- | --- | --- | --- | --- |
|  | 4501 | 503.2898 | 1004.5651 | 1003.6066 | 0.9585 | 0 | 7 | 34 | 9 | M.ELTIFILR.L |
|  | 11840 | **717.3355** | **1432.6565** | **1432.6657** | **-0.0092** | **0** | **57** | **0.00027** | **1** | **R.VTCIDPNPNFEK.F** 11839 |
|  | 14086 | **783.3983** | **1564.7821** | **1564.7919** | **-0.0098** | **0** | **52** | **0.00074** | **1** | **K.LSLLEVGCGTGANFK.F** |
|  | 15005 | **812.3988** | **1622.7830** | **1622.7940** | **-0.0110** | **0** | **39** | **0.014** | **1** | **R.ELFSNLQEFAGPSGK.L** |
|  | 26149 | **680.3737** | **2717.4658** | **2717.4805** | **-0.0148** | **0** | **13** | **2.7** | **1** | **K.LQHIQAPLSWELVRPHIYGYAVK.-** 26148 26152 |

| **429.** | O00299\|CLIC1_HUMAN    **Mass:** 27248    **Score:** 94     **Queries matched:** 7   **emPAI:** 0.14 |
| --- | --- |
|  | Chloride intracellular channel protein 1 OS=Homo sapiens GN=CLIC1 PE=1 SV=4 |

|  | **Query** | **Observed** | **Mr(expt)** | **Mr(calc)** | **Delta** | **Miss** | **Score** | **Expect** | **Rank** | **Peptide** |
| --- | --- | --- | --- | --- | --- | --- | --- | --- | --- | --- |
|  | 5307 | **533.2857** | **1064.5569** | **1064.6093** | **-0.0524** | **0** | **4** | **61** | **9** | **R.LFMVLWLK.G** |
|  | 5486 | **539.7552** | **1077.4958** | **1077.5026** | **-0.0067** | **0** | **18** | **2.7** | **1** | **K.IGNCPFSQR.L** 5514 |
|  | 9971 | 664.8211 | 1327.6277 | 1327.6368 | -0.0091 | 0 | 17 | 2.6 | 1 | K.NSNPALNDNLEK.G |
|  | 11390 | **469.9109** | **1406.7108** | **1405.7215** | **0.9894** | **1** | **7** | **24** | **8** | **R.GVHRYLSNAYAR.E** |
|  | 18398 | **922.9855** | **1843.9565** | **1843.9679** | **-0.0114** | **0** | **71** | **7.7e-006** | **1** | **K.LAALNPESNTAGLDIFAK.F** 18399 |

| **430.** | P61026\|RAB10_HUMAN    **Mass:** 22755    **Score:** 93     **Queries matched:** 16   **emPAI:** 1.17 |
| --- | --- |
|  | Ras-related protein Rab-10 OS=Homo sapiens GN=RAB10 PE=1 SV=1 |

|  | **Query** | **Observed** | **Mr(expt)** | **Mr(calc)** | **Delta** | **Miss** | **Score** | **Expect** | **Rank** | **Peptide** |
| --- | --- | --- | --- | --- | --- | --- | --- | --- | --- | --- |
|  | 992 | **572.4080** | **571.4007** | **571.3693** | **0.0314** | **1** | **22** | **1.4** | **1** | **R.KTPVK.E** |
|  | 2003 | 415.2057 | 828.3968 | 828.4018 | -0.0050 | 0 | 11 | 17 | 1 | R.FFETSAK.A 2001 2004 |
|  | 2983 | 451.2271 | 900.4397 | 901.5233 | -1.0835 | 1 | 17 | 4.2 | 6 | K.TVELQGKK.I |
|  | 5394 | 536.3203 | 1070.6261 | 1070.6336 | -0.0075 | 0 | 45 | 0.006 | 1 | K.LLLIGDSGVGK.T 5392 |
|  | 8630 | **631.3578** | **1260.7011** | **1260.7078** | **-0.0066** | **0** | **51** | **0.0012** | **1** | **K.AFLTLAEDILR.K** |
|  | 9695 | 658.8284 | 1315.6422 | 1315.6521 | -0.0099 | 0 | 18 | 2.6 | 1 | K.LQIWDTAGQER.F 9685 |
|  | 11950 | **720.8157** | **1439.6168** | **1439.6277** | **-0.0109** | **0** | **55** | **0.00043** | **1** | **R.NIDEHANEDVER.M** |
|  | 12195 | 727.8490 | 1453.6834 | 1454.6204 | -0.9369 | 1 | 11 | 11 | 7 | R.MLLGNKCDMDDK.R 12194 |
|  | 13956 | 779.4173 | 1556.8200 | 1556.8311 | -0.0110 | 1 | 32 | 0.081 | 1 | K.IKLQIWDTAGQER.F 13955 13957 |

| **431.** | Q16718\|NDUA5_HUMAN    **Mass:** 13507    **Score:** 93     **Queries matched:** 3   **emPAI:** 0.29 |
| --- | --- |
|  | NADH dehydrogenase [ubiquinone] 1 alpha subcomplex subunit 5 OS=Homo sapiens GN=NDUFA5 PE=1 SV=3 |

|  | **Query** | **Observed** | **Mr(expt)** | **Mr(calc)** | **Delta** | **Miss** | **Score** | **Expect** | **Rank** | **Peptide** |
| --- | --- | --- | --- | --- | --- | --- | --- | --- | --- | --- |
|  | 750 | **486.8631** | **485.8558** | **486.3166** | **-0.4607** | **0** | **4** | **66** | **6** | **M.AGVLK.K** |
|  | 18519 | **618.3243** | **1851.9512** | **1851.9625** | **-0.0113** | **1** | **8** | **18** | **1** | **K.KTTGLVGLAVCNTPHER.L** |
|  | 27426 | **991.8558** | **2972.5455** | **2972.5567** | **-0.0112** | **1** | **93** | **2.3e-008** | **1** | **K.KLEDQLQGGQLEEVILQAEHELNLAR.K** |

| **432.** | P33121\|ACSL1_HUMAN    **Mass:** 78919    **Score:** 93     **Queries matched:** 30   **emPAI:** 0.32 |
| --- | --- |
|  | Long-chain-fatty-acid--CoA ligase 1 OS=Homo sapiens GN=ACSL1 PE=1 SV=1 |

|  | **Query** | **Observed** | **Mr(expt)** | **Mr(calc)** | **Delta** | **Miss** | **Score** | **Expect** | **Rank** | **Peptide** |
| --- | --- | --- | --- | --- | --- | --- | --- | --- | --- | --- |
|  | 1172 | 644.9523 | 643.9450 | 643.4017 | 0.5433 | 1 | 0 | 1.7e+002 | 2 | K.IIDRK.K 1174 |
|  | 1407 | **745.3782** | **744.3709** | **744.4130** | **-0.0421** | **1** | **5** | **79** | **4** | **R.KEAELR.S** |
|  | 1531 | **791.3663** | **790.3591** | **790.3643** | **-0.0053** | **0** | **21** | **1.5** | **1** | **K.AMEDLGR.A** 1538 |
|  | 1673 | **803.4301** | **802.4228** | **802.4297** | **-0.0069** | **0** | **39** | **0.031** | **1** | **K.VQSSLGGR.V** |
|  | 3047 | **905.1038** | **904.0965** | **903.4199** | **0.6767** | **0** | **0** | **1.2e+002** | **3** | **R.NNSLWDR.L** |
|  | 3880 | 482.2814 | 962.5483 | 961.4902 | 1.0581 | 0 | 8 | 27 | 8 | K.AILEDMVR.L |
|  | 4547 | **505.7325** | **1009.4505** | **1009.4573** | **-0.0068** | **0** | **42** | **0.01** | **1** | **R.CGVEVTSMK.A** |
|  | 5440 | 538.2725 | 1074.5304 | 1073.5903 | 0.9401 | 1 | 6 | 40 | 6 | K.KAILEDMVR.L |
|  | 5525 | 540.9420 | 1079.8695 | 1079.5546 | 0.3149 | 1 | 3 | 56 | 5 | R.LLNRMFDR.I |
|  | 8166 | **620.8032** | **1239.5919** | **1238.5965** | **0.9954** | **0** | **31** | **0.097** | **1** | **R.NIVSDCSAFVK.A** 8148 |
|  | 8341 | 624.3303 | 1246.6461 | 1246.6557 | -0.0097 | 0 | 31 | 0.13 | 1 | K.DSGLKPFEQVK.G 8342 |
|  | 8358 | **624.8525** | **1247.6905** | **1247.6986** | **-0.0081** | **1** | **42** | **0.0091** | **1** | **R.IFGQANTTLKR.W** 8357 |
|  | 10354 | **451.2379** | **1350.6918** | **1350.6384** | **0.0534** | **1** | **9** | **18** | **3** | **R.GQRCGVEVTSMK.A** |
|  | 11292 | 467.9274 | 1400.7605 | 1401.6567 | -0.8962 | 0 | 4 | 49 | 7 | R.VVECVMLCHGAK.I |
|  | 16924 | **582.2632** | **1743.7677** | **1744.9029** | **-1.1352** | **0** | **8** | **19** | **5** | **K.QVAELSECIGSALIQK.G** 16922 |
|  | 17438 | 891.9414 | 1781.8681 | 1781.8110 | 0.0571 | 1 | (3) | 56 | 3 | R.CGVEVTSMKAMEDLGR.A |
|  | 17719 | 600.6335 | 1798.8788 | 1797.8059 | 1.0729 | 1 | 4 | 37 | 7 | R.CGVEVTSMKAMEDLGR.A |
|  | 17937 | **605.3024** | **1812.8855** | **1813.8009** | **-0.9154** | **1** | **(1)** | **80** | **10** | **R.CGVEVTSMKAMEDLGR.A** |
|  | 18865 | **940.9260** | **1879.8375** | **1879.9866** | **-0.1491** | **1** | **1** | **75** | **8** | **K.IGFFQGDIRLLMDDLK.V** |
|  | 19420 | **642.9861** | **1925.9366** | **1925.9483** | **-0.0117** | **1** | **48** | **0.0013** | **1** | **K.TAEALDKDGWLHTGDIGK.W** 19432 19433 |
|  | 23792 | **1187.0266** | **2372.0387** | **2371.0382** | **1.0005** | **0** | **20** | **0.61** | **1** | **K.LVDVEEMNYMAAEGEGEVCVK.G** |
|  | 24297 | 613.5440 | 2450.1469 | 2451.1862 | -1.0392 | 1 | 2 | 45 | 6 | R.VVECVMLCHGAKIGFFQGDIR.L |

| **433.** | Q96S66\|CLCC1_HUMAN    **Mass:** 62667    **Score:** 93     **Queries matched:** 7   **emPAI:** 0.19 |
| --- | --- |
|  | Chloride channel CLIC-like protein 1 OS=Homo sapiens GN=CLCC1 PE=1 SV=1 |

|  | **Query** | **Observed** | **Mr(expt)** | **Mr(calc)** | **Delta** | **Miss** | **Score** | **Expect** | **Rank** | **Peptide** |
| --- | --- | --- | --- | --- | --- | --- | --- | --- | --- | --- |
|  | 6636 | **580.2986** | **1158.5827** | **1157.5928** | **0.9899** | **0** | **4** | **68** | **5** | **K.ETGGILGEGTPK.E** |
|  | 10585 | 682.3154 | 1362.6162 | 1362.6238 | -0.0076 | 0 | 23 | 0.75 | 1 | R.GQMGPTEQGPYAK.T |
|  | 13216 | **757.3900** | **1512.7655** | **1513.6507** | **-0.8853** | **1** | **4** | **46** | **6** | **R.SSWTYKDDPCQK.Y** |
|  | 15905 | **840.8712** | **1679.7279** | **1679.7387** | **-0.0108** | **0** | **42** | **0.0074** | **1** | **K.SEAAGSPDQGSTYSPAR.G** |
|  | 27335 | **985.1033** | **2952.2880** | **2952.3068** | **-0.0188** | **0** | **73** | **2.3e-006** | **1** | **K.ESSTESSQSAKPVSGQDTSGNTEGSPAAEK.A** 27338 |
|  | 31086 | **1550.7063** | **4649.0971** | **4650.0555** | **-0.9584** | **0** | **1** | **11** | **10** | **-.MLCSLLLCECLLLVAGYAHDDDWIDPTDMLNYDAASGTMR.K** |

| **434.** | Q8NHW5\|RLA0L_HUMAN    **Mass:** 34514    **Score:** 93     **Queries matched:** 6   **emPAI:** 0.11 |
| --- | --- |
|  | 60S acidic ribosomal protein P0-like OS=Homo sapiens PE=2 SV=1 |

|  | **Query** | **Observed** | **Mr(expt)** | **Mr(calc)** | **Delta** | **Miss** | **Score** | **Expect** | **Rank** | **Peptide** |
| --- | --- | --- | --- | --- | --- | --- | --- | --- | --- | --- |
|  | 1674 | 402.2424 | 802.4703 | 802.4119 | 0.0584 | 0 | 24 | 1 | 3 | K.QMQQIR.M |
|  | 11165 | **465.4647** | **1393.3723** | **1393.7244** | **-0.3520** | **1** | **6** | **24** | **3** | **K.VVVLMGKNTMMR.K** 11146 |
|  | 17476 | 595.6254 | 1783.8545 | 1784.0407 | -0.1863 | 1 | 0 | 1e+002 | 4 | K.ISRGTIEILSDVQLIK.T |
|  | 22180 | 1090.5470 | 2179.0794 | 2179.0943 | -0.0149 | 0 | 63 | 4.2e-005 | 1 | R.AGAIAPCEVTVPAQNTGLGPEK.T 22179 |

| **435.** | O15269\|SPTC1_HUMAN    **Mass:** 53281    **Score:** 92     **Queries matched:** 14   **emPAI:** 0.14 |
| --- | --- |
|  | Serine palmitoyltransferase 1 OS=Homo sapiens GN=SPTLC1 PE=1 SV=1 |

|  | **Query** | **Observed** | **Mr(expt)** | **Mr(calc)** | **Delta** | **Miss** | **Score** | **Expect** | **Rank** | **Peptide** |
| --- | --- | --- | --- | --- | --- | --- | --- | --- | --- | --- |
|  | 1424 | 753.3718 | 752.3646 | 752.4255 | -0.0609 | 1 | 8 | 20 | 5 | R.LAKFMK.T |
|  | 3926 | 484.2318 | 966.4490 | 965.4389 | 1.0101 | 0 | 11 | 13 | 2 | K.YGVGTCGPR.G |
|  | 4432 | **501.2579** | **1000.5013** | **1000.5090** | **-0.0077** | **0** | **2** | **1.1e+002** | **7** | **R.SFVIDHQR.L** |
|  | 4676 | **510.2887** | **1018.5628** | **1017.4614** | **1.1013** | **0** | **11** | **16** | **10** | **K.EQEIEDQK.N** |
|  | 5726 | 548.2087 | 1094.4029 | 1093.5339 | 0.8691 | 1 | 5 | 46 | 5 | K.KYGVGTCGPR.G |
|  | 5783 | 550.2598 | 1098.5050 | 1099.4716 | -0.9666 | 0 | 11 | 14 | 6 | K.HNDMADLER.L |
|  | 7112 | **595.3017** | **1188.5888** | **1187.6510** | **0.9379** | **1** | **6** | **40** | **9** | **K.LQERSDLTVK.E** |
|  | 13058 | **502.2465** | **1503.7176** | **1503.7140** | **0.0036** | **1** | **1** | **1e+002** | **9** | **K.LFKHNDMADLER.L** |
|  | 13300 | **759.8692** | **1517.7238** | **1517.7330** | **-0.0092** | **0** | **46** | **0.0032** | **1** | **R.LLQEIVDQCMNR.S** |
|  | 15565 | **830.4194** | **1658.8242** | **1658.8363** | **-0.0121** | **0** | **68** | **1.8e-005** | **1** | **R.VVVTVEQTEEELER.A** |
|  | 19004 | 945.9701 | 1889.9256 | 1888.9683 | 0.9573 | 0 | 0 | 98 | 9 | R.IFLEESLSFGVLGEHGR.G |
|  | 21616 | **706.2950** | **2115.8631** | **2116.1065** | **-0.2434** | **1** | **3** | **38** | **4** | **K.ARIFLEESLSFGVLGEHGR.G** |
|  | 21690 | **1064.0142** | **2126.0138** | **2126.0255** | **-0.0117** | **0** | **1** | **61** | **1** | **K.ECINFASFNFLGLLDNPR.V** |
|  | 22798 | **748.3766** | **2242.1079** | **2242.1230** | **-0.0150** | **0** | **6** | **22** | **1** | **K.VVGESLSPAFHLQLEESTGSR.E** |

| **436.** | Q32P51\|RA1L2_HUMAN    **Mass:** 34375    **Score:** 92     **Queries matched:** 12   **emPAI:** 0.11 |
| --- | --- |
|  | Heterogeneous nuclear ribonucleoprotein A1-like protein 2 OS=Homo sapiens GN=HNRNPA1L2 PE=2 SV=2 |

|  | **Query** | **Observed** | **Mr(expt)** | **Mr(calc)** | **Delta** | **Miss** | **Score** | **Expect** | **Rank** | **Peptide** |
| --- | --- | --- | --- | --- | --- | --- | --- | --- | --- | --- |
|  | 1279 | **694.3460** | **693.3387** | **693.3194** | **0.0192** | **0** | **8** | **23** | **9** | **R.GGNFSGR.G** |
|  | 1744 | **404.7116** | **807.4087** | **807.3624** | **0.0463** | **0** | **15** | **5.2** | **1** | **R.GSGNFGGGR.G** |
|  | 2485 | **431.5669** | **861.1193** | **860.5484** | **0.5709** | **1** | **14** | **5.1** | **3** | **K.KIFVGGIK.E** 2490 |
|  | 4391 | 500.2754 | 998.5362 | 997.5556 | 0.9806 | 1 | 3 | 84 | 5 | K.VDGRVVEPK.R |
|  | 7267 | 399.5266 | 1195.5579 | 1196.5091 | -0.9512 | 0 | 8 | 25 | 9 | K.QEMASASSSQR.G 7278 7335 |
|  | 7605 | **404.5566** | **1210.6481** | **1211.4956** | **-0.8475** | **0** | **4** | **54** | **10** | **R.GDGFGGNDNFGR.G** |
|  | 11433 | 705.8132 | 1409.6119 | 1409.6317 | -0.0198 | 1 | 9 | 15 | 3 | K.QEMASASSSQRGR.R |
|  | 11909 | **479.9163** | **1436.7270** | **1436.7372** | **-0.0101** | **0** | **9** | **17** | **1** | **R.EDSQRPGAHLTVK.K** |
|  | 17483 | 892.9523 | 1783.8900 | 1783.8992 | -0.0092 | 0 | 92 | 6.7e-008 | 1 | K.LFIGGLSFETTDESLR.S |

| **437.** | P09651\|ROA1_HUMAN    **Mass:** 38936    **Score:** 92     **Queries matched:** 12   **emPAI:** 0.10 |
| --- | --- |
|  | Heterogeneous nuclear ribonucleoprotein A1 OS=Homo sapiens GN=HNRNPA1 PE=1 SV=4 |

|  | **Query** | **Observed** | **Mr(expt)** | **Mr(calc)** | **Delta** | **Miss** | **Score** | **Expect** | **Rank** | **Peptide** |
| --- | --- | --- | --- | --- | --- | --- | --- | --- | --- | --- |
|  | 1153 | **637.0497** | **636.0424** | **636.2980** | **-0.2556** | **0** | **0** | **1.1e+002** | **6** | **R.GGFGGSR.G** |
|  | 1279 | 694.3460 | 693.3387 | 693.3194 | 0.0192 | 0 | 8 | 23 | 9 | R.GGNFSGR.G |
|  | 2485 | 431.5669 | 861.1193 | 860.5484 | 0.5709 | 1 | 14 | 5.1 | 3 | K.KIFVGGIK.E 2490 |
|  | 4391 | 500.2754 | 998.5362 | 997.5556 | 0.9806 | 1 | 3 | 84 | 5 | K.VDGRVVEPK.R |
|  | 7267 | 399.5266 | 1195.5579 | 1196.5091 | -0.9512 | 0 | 8 | 25 | 9 | K.QEMASASSSQR.G 7278 7335 |
|  | 11433 | 705.8132 | 1409.6119 | 1409.6317 | -0.0198 | 1 | 9 | 15 | 3 | K.QEMASASSSQRGR.S |
|  | 11909 | 479.9163 | 1436.7270 | 1436.7372 | -0.0101 | 0 | 9 | 17 | 1 | R.EDSQRPGAHLTVK.K |
|  | 16095 | **565.2449** | **1692.7128** | **1693.6928** | **-0.9800** | **0** | **6** | **26** | **3** | **R.NQGGYGGSSSSSSYGSGR.R** |
|  | 17483 | 892.9523 | 1783.8900 | 1783.8992 | -0.0092 | 0 | 92 | 6.7e-008 | 1 | K.LFIGGLSFETTDESLR.S |

| **438.** | P0C7M2\|RA1L3_HUMAN    **Mass:** 34373    **Score:** 92     **Queries matched:** 12   **emPAI:** 0.11 |
| --- | --- |
|  | Putative heterogeneous nuclear ribonucleoprotein A1-like protein 3 OS=Homo sapiens GN=HNRPA1L3 PE=5 SV=1 |

|  | **Query** | **Observed** | **Mr(expt)** | **Mr(calc)** | **Delta** | **Miss** | **Score** | **Expect** | **Rank** | **Peptide** |
| --- | --- | --- | --- | --- | --- | --- | --- | --- | --- | --- |
|  | 1153 | 637.0497 | 636.0424 | 636.2980 | -0.2556 | 0 | 0 | 1.1e+002 | 6 | R.GGFGGSR.A |
|  | 2485 | 431.5669 | 861.1193 | 860.5484 | 0.5709 | 1 | 14 | 5.1 | 3 | K.KIFVGGIK.E 2490 |
|  | 4093 | **489.9110** | **977.8073** | **977.4791** | **0.3282** | **1** | **5** | **32** | **4** | **R.GGFGGSRAGGR.Y** |
|  | 4391 | 500.2754 | 998.5362 | 997.5556 | 0.9806 | 1 | 3 | 84 | 5 | K.VDGRVVEPK.R |
|  | 7267 | 399.5266 | 1195.5579 | 1196.5091 | -0.9512 | 0 | 8 | 25 | 9 | K.QEMASASSSQR.G 7278 7335 |
|  | 11433 | 705.8132 | 1409.6119 | 1409.6317 | -0.0198 | 1 | 9 | 15 | 3 | K.QEMASASSSQRGR.S |
|  | 11909 | 479.9163 | 1436.7270 | 1436.7372 | -0.0101 | 0 | 9 | 17 | 1 | R.EDSQRPGAHLTVK.K |
|  | 15480 | 827.8846 | 1653.7547 | 1654.7852 | -1.0305 | 0 | 7 | 25 | 3 | R.NSGPYGGGGQYFAKPR.N |
|  | 17483 | 892.9523 | 1783.8900 | 1783.8992 | -0.0092 | 0 | 92 | 6.7e-008 | 1 | K.LFIGGLSFETTDESLR.S |

| **439.** | P02749\|APOH_HUMAN    **Mass:** 39584    **Score:** 92     **Queries matched:** 16   **emPAI:** 0.44 |
| --- | --- |
|  | Beta-2-glycoprotein 1 OS=Homo sapiens GN=APOH PE=1 SV=3 |

|  | **Query** | **Observed** | **Mr(expt)** | **Mr(calc)** | **Delta** | **Miss** | **Score** | **Expect** | **Rank** | **Peptide** |
| --- | --- | --- | --- | --- | --- | --- | --- | --- | --- | --- |
|  | 4254 | **496.2115** | **990.4085** | **991.4281** | **-1.0195** | **0** | **23** | **1** | **1** | **K.TDASDVKPC.-** 4253 |
|  | 4715 | **512.2790** | **1022.5435** | **1021.5193** | **1.0243** | **0** | **1** | **1.3e+002** | **3** | **K.ATVVYQGER.V** |
|  | 5866 | **552.7737** | **1103.5329** | **1103.5400** | **-0.0070** | **0** | **13** | **8.5** | **3** | **K.EHSSLAFWK.T** |
|  | 8720 | **634.3061** | **1266.5976** | **1265.5533** | **1.0444** | **0** | **7** | **28** | **6** | **K.LGNWSAMPSCK.A** |
|  | 22470 | 738.6684 | 2212.9834 | 2212.9980 | -0.0147 | 1 | 16 | 2 | 1 | K.KCSYTEDAQCIDGTIEVPK.C |
|  | 23854 | **795.0290** | **2382.0653** | **2382.0838** | **-0.0185** | **0** | **18** | **1.1** | **1** | **K.TFYEPGEEITYSCKPGYVSR.G** 23855 |
|  | 23876 | **795.9984** | **2384.9734** | **2384.9889** | **-0.0155** | **0** | **34** | **0.027** | **1** | **K.ATFGCHDGYSLDGPEEIECTK.L** 23879 |
|  | 25538 | **877.0393** | **2628.0959** | **2628.1108** | **-0.0149** | **1** | **46** | **0.0013** | **1** | **K.DKATFGCHDGYSLDGPEEIECTK.L** |
|  | 26699 | **1410.2267** | **2818.4388** | **2818.4550** | **-0.0162** | **0** | **31** | **0.047** | **1** | **K.WSPELPVCAPIICPPPSIPTFATLR.V** 26696 26697 26698 |
|  | 29535 | **1175.2445** | **3522.7117** | **3522.7350** | **-0.0233** | **1** | **14** | **1.6** | **1** | **K.CTEEGKWSPELPVCAPIICPPPSIPTFATLR.V** |

| **440.** | P10909\|CLUS_HUMAN    **Mass:** 53031    **Score:** 92     **Queries matched:** 16   **emPAI:** 0.15 |
| --- | --- |
|  | Clusterin OS=Homo sapiens GN=CLU PE=1 SV=1 |

|  | **Query** | **Observed** | **Mr(expt)** | **Mr(calc)** | **Delta** | **Miss** | **Score** | **Expect** | **Rank** | **Peptide** |
| --- | --- | --- | --- | --- | --- | --- | --- | --- | --- | --- |
|  | 3543 | **471.0326** | **940.0507** | **939.3790** | **0.6717** | **1** | **1** | **75** | **2** | **R.MKDQCDK.C** |
|  | 7788 | **407.4496** | **1219.3270** | **1219.5478** | **-0.2208** | **1** | **7** | **19** | **9** | **K.QTCMKFYAR.V** 7757 |
|  | 9129 | 644.8184 | 1287.6223 | 1287.6306 | -0.0083 | 0 | 20 | 1.4 | 1 | R.ELDESLQVAER.L |
|  | 12000 | **722.3110** | **1442.6075** | **1443.7317** | **-1.1242** | **1** | **13** | **6.2** | **7** | **R.RELDESLQVAER.L** |
|  | 16712 | **577.7791** | **1730.3155** | **1729.8345** | **0.4810** | **1** | **12** | **4.5** | **1** | **K.FMETVAEKALQEYR.K** 16713 16714 16715 16716 |
|  | 17143 | **881.9114** | **1761.8082** | **1761.8203** | **-0.0121** | **0** | **21** | **0.97** | **1** | **R.EILSVDCSTNNPSQAK.L** |
|  | 23331 | **1157.5846** | **2313.1546** | **2313.1700** | **-0.0154** | **0** | **58** | **0.00012** | **1** | **R.VTTVASHTSDSDVPSGVTEVVVK.L** 23332 |
|  | 27364 | 987.4294 | 2959.2663 | 2959.3553 | -0.0889 | 1 | 3 | 22 | 5 | R.IDSLLENDRQQTHMLDVMQDHFSR.A 27365 |
|  | 30544 | **1017.4780** | **4065.8828** | **4065.0253** | **0.8575** | **0** | **1** | **22** | **3** | **K.TLLLFVGLLLTWESGQVLGDQTVSDNELQEMSNQGSK.Y** |

| **441.** | P04206\|KV307_HUMAN    **Mass:** 11937    **Score:** 91     **Queries matched:** 6   **emPAI:** 0.33 |
| --- | --- |
|  | Ig kappa chain V-III region GOL OS=Homo sapiens PE=1 SV=1 |

|  | **Query** | **Observed** | **Mr(expt)** | **Mr(calc)** | **Delta** | **Miss** | **Score** | **Expect** | **Rank** | **Peptide** |
| --- | --- | --- | --- | --- | --- | --- | --- | --- | --- | --- |
|  | 7194 | **597.2576** | **1192.5006** | **1192.6452** | **-0.1446** | **1** | **9** | **20** | **1** | **R.SFGQGTKVEIK.R** |
|  | 11339 | **469.0963** | **1404.2670** | **1404.7507** | **-0.4837** | **1** | **8** | **15** | **1** | **R.ATLSCRAALLSSR.G** |
|  | 16571 | 575.2664 | 1722.7774 | 1722.8723 | -0.0949 | 1 | 7 | 21 | 3 | R.LLMYGASSRATGIPDR.F 16570 16591 |
|  | 18907 | **942.5009** | **1882.9873** | **1883.0000** | **-0.0127** | **0** | **91** | **7.2e-008** | **1** | **-.EIVLTQSPGTLSLSPGER.A** |

|  | **Proteins matching a subset of these peptides:** |
| --- | --- |

|  | P01620\|KV302_HUMAN    **Mass:** 11882    **Score:** 91     **Queries matched:** 1 |
| --- | --- |
|  | Ig kappa chain V-III region SIE OS=Homo sapiens PE=1 SV=1 |

|  | P01622\|KV304_HUMAN    **Mass:** 11895    **Score:** 91     **Queries matched:** 1 |
| --- | --- |
|  | Ig kappa chain V-III region Ti OS=Homo sapiens PE=1 SV=1 |

|  | P01623\|KV305_HUMAN    **Mass:** 11853    **Score:** 91     **Queries matched:** 1 |
| --- | --- |
|  | Ig kappa chain V-III region WOL OS=Homo sapiens PE=1 SV=1 |

| **442.** | P01619\|KV301_HUMAN    **Score:** 91     **Queries matched:** 2 |
| --- | --- |
|  | Ig kappa chain V-III region B6 OS=Homo sapiens PE=1 SV=1 |

|  | **Query** | **Observed** | **Mr(expt)** | **Mr(calc)** | **Delta** | **Miss** | **Score** | **Expect** | **Rank** | **Peptide** |
| --- | --- | --- | --- | --- | --- | --- | --- | --- | --- | --- |
|  | 18900 | **628.3727** | **1882.0962** | **1883.0000** | **-0.9038** | **0** | **5** | **28** | **6** | **-.QIVLTESPGTLSLSPGER.A** |
|  | 18907 | 942.5009 | 1882.9873 | 1883.0000 | -0.0127 | 0 | 91 | 7.2e-008 | 1 | -.EIVLTQSPGTLSLSPGER.A |

| **443.** | Q15392\|DHC24_HUMAN    **Mass:** 60803    **Score:** 91     **Queries matched:** 15   **emPAI:** 0.06 |
| --- | --- |
|  | 24-dehydrocholesterol reductase OS=Homo sapiens GN=DHCR24 PE=1 SV=2 |

|  | **Query** | **Observed** | **Mr(expt)** | **Mr(calc)** | **Delta** | **Miss** | **Score** | **Expect** | **Rank** | **Peptide** |
| --- | --- | --- | --- | --- | --- | --- | --- | --- | --- | --- |
|  | 1132 | **631.0332** | **630.0259** | **629.3497** | **0.6763** | **0** | **13** | **11** | **10** | **K.LSSAPR.L** |
|  | 3205 | 458.2942 | 914.5739 | 915.5290 | -0.9551 | 1 | 17 | 3.9 | 1 | K.LRFEPVR.G |
|  | 7575 | **404.0295** | **1209.0665** | **1208.5179** | **0.5486** | **1** | **6** | **26** | **5** | **K.HFEARSCMR.Q** |
|  | 8864 | **425.4343** | **1273.2810** | **1272.6575** | **0.6235** | **1** | **8** | **15** | **6** | **K.HVENYLKTNR.E** |
|  | 12470 | 736.3484 | 1470.6822 | 1470.8770 | -0.1947 | 1 | 7 | 25 | 1 | K.ISLLKLTQGETLR.K |
|  | 13678 | **771.3455** | **1540.6765** | **1540.6868** | **-0.0103** | **0** | **60** | **0.00012** | **1** | **K.LGCQDAFPEVYDK.I** 13677 |
|  | 18692 | 934.0048 | 1865.9950 | 1865.8795 | 0.1155 | 0 | 4 | 39 | 3 | K.GNEAELYIDIGAYGEPR.V |
|  | 18993 | 945.4688 | 1888.9231 | 1889.0002 | -0.0771 | 1 | (1) | 77 | 6 | K.NIMINLMDILEVDTKK.Q |
|  | 19180 | **636.3409** | **1906.0008** | **1904.9951** | **1.0057** | **1** | **3** | **51** | **2** | **K.NIMINLMDILEVDTKK.Q** |
|  | 21548 | **704.4044** | **2110.1913** | **2110.0703** | **0.1209** | **1** | **3** | **37** | **1** | **R.KLYEQHHVVQDMLVPMK.C** 21553 |
|  | 21994 | **720.6953** | **2159.0641** | **2159.0966** | **-0.0325** | **1** | **1** | **64** | **2** | **K.THKNIMINLMDILEVDTK.K** |
|  | 24017 | **603.0319** | **2408.0983** | **2409.0817** | **-0.9833** | **1** | **4** | **29** | **6** | **K.FVRSVHGFQMLYADCYMNR.E** 24015 |

| **444.** | P56134\|ATPK_HUMAN    **Mass:** 11025    **Score:** 91     **Queries matched:** 4   **emPAI:** 0.36 |
| --- | --- |
|  | ATP synthase subunit f, mitochondrial OS=Homo sapiens GN=ATP5J2 PE=1 SV=3 |

|  | **Query** | **Observed** | **Mr(expt)** | **Mr(calc)** | **Delta** | **Miss** | **Score** | **Expect** | **Rank** | **Peptide** |
| --- | --- | --- | --- | --- | --- | --- | --- | --- | --- | --- |
|  | 9644 | **657.8619** | **1313.7092** | **1313.7166** | **-0.0073** | **0** | **73** | **7.3e-006** | **1** | **K.LGELPSWILMR.D** 9643 |
|  | 9990 | 665.3822 | 1328.7498 | 1329.7115 | -0.9616 | 0 | (9) | 17 | 1 | K.LGELPSWILMR.D |
|  | 11750 | **714.8450** | **1427.6755** | **1427.6834** | **-0.0079** | **0** | **8** | **23** | **8** | **R.DFSPSGIFGAFQR.G** |

| **445.** | Q8N3Y7\|RDHE2_HUMAN    **Mass:** 34586    **Score:** 91     **Queries matched:** 9   **emPAI:** 0.23 |
| --- | --- |
|  | Epidermal retinal dehydrogenase 2 OS=Homo sapiens GN=SDR16C5 PE=2 SV=2 |

|  | **Query** | **Observed** | **Mr(expt)** | **Mr(calc)** | **Delta** | **Miss** | **Score** | **Expect** | **Rank** | **Peptide** |
| --- | --- | --- | --- | --- | --- | --- | --- | --- | --- | --- |
|  | 6753 | 584.7967 | 1167.5788 | 1168.5910 | -1.0122 | 1 | 6 | 35 | 7 | -.MSFNLQSSKK.L |
|  | 10687 | **684.7811** | **1367.5477** | **1367.5598** | **-0.0121** | **0** | **38** | **0.018** | **1** | **R.VHAYTCDCSQK.E** |
|  | 20157 | **662.3940** | **1984.1601** | **1984.0413** | **0.1188** | **1** | **8** | **12** | **1** | **K.IVEAILQEKMYLYMPK.L** |
|  | 20831 | **682.6786** | **2045.0139** | **2045.1747** | **-0.1608** | **0** | **1** | **83** | **8** | **K.SLFSLLEAMIFALLPKPR.K** |
|  | 22291 | **732.8897** | **2195.6473** | **2195.0579** | **0.5894** | **1** | **0** | **47** | **5** | **K.MYLYMPKLLYFMMFLK.S** |
|  | 23273 | **1154.5470** | **2307.0794** | **2307.0949** | **-0.0155** | **0** | **67** | **1.5e-005** | **1** | **K.TGMFEGCTTGCPSLLPILEPK.Y** 23272 |
|  | 23900 | **598.2990** | **2389.1669** | **2389.2352** | **-0.0682** | **0** | **(1)** | **55** | **5** | **K.TGLLIADYLGILHAMDGFVDQK.K** |
|  | 24001 | **803.0591** | **2406.1556** | **2405.2301** | **0.9255** | **0** | **2** | **41** | **3** | **K.TGLLIADYLGILHAMDGFVDQK.K** |

| **446.** | P13073\|COX41_HUMAN    **Mass:** 19621    **Score:** 90     **Queries matched:** 9   **emPAI:** 0.71 |
| --- | --- |
|  | Cytochrome c oxidase subunit 4 isoform 1, mitochondrial OS=Homo sapiens GN=COX4I1 PE=1 SV=1 |

|  | **Query** | **Observed** | **Mr(expt)** | **Mr(calc)** | **Delta** | **Miss** | **Score** | **Expect** | **Rank** | **Peptide** |
| --- | --- | --- | --- | --- | --- | --- | --- | --- | --- | --- |
|  | 4151 | **492.2776** | **982.5407** | **982.4178** | **0.1229** | **0** | **6** | **37** | **2** | **K.ESFAEMNR.G** |
|  | 4749 | 513.7970 | 1025.5794 | 1025.5869 | -0.0075 | 0 | 39 | 0.017 | 1 | K.VNPIQGLASK.W 4750 |
|  | 8185 | **414.5566** | **1240.6480** | **1240.6564** | **-0.0084** | **0** | **18** | **2.3** | **1** | **R.DHPLPEVAHVK.H** |
|  | 8567 | **630.3150** | **1258.6154** | **1257.5812** | **1.0343** | **1** | **1** | **1.3e+002** | **8** | **K.FKESFAEMNR.G** |
|  | 11782 | **715.8117** | **1429.6089** | **1429.6184** | **-0.0095** | **0** | **22** | **0.84** | **1** | **K.SEDFSLPAYMDR.R** |
|  | 12935 | 749.3437 | 1496.6728 | 1496.6817 | -0.0088 | 1 | 13 | 6.6 | 1 | K.EKASWSSLSMDEK.V |
|  | 14396 | **793.8610** | **1585.7075** | **1585.7195** | **-0.0120** | **1** | **24** | **0.5** | **1** | **K.SEDFSLPAYMDRR.D** |
|  | 19097 | **950.9521** | **1899.8897** | **1899.9036** | **-0.0139** | **1** | **66** | **2.7e-005** | **1** | **K.ASWSSLSMDEKVELYR.I** |

| **447.** | Q8WUD1\|RAB2B_HUMAN    **Mass:** 24427    **Score:** 90     **Queries matched:** 10   **emPAI:** 0.16 |
| --- | --- |
|  | Ras-related protein Rab-2B OS=Homo sapiens GN=RAB2B PE=1 SV=1 |

|  | **Query** | **Observed** | **Mr(expt)** | **Mr(calc)** | **Delta** | **Miss** | **Score** | **Expect** | **Rank** | **Peptide** |
| --- | --- | --- | --- | --- | --- | --- | --- | --- | --- | --- |
|  | 5308 | **533.2887** | **1064.5628** | **1063.5046** | **1.0582** | **1** | **3** | **85** | **10** | **K.REEGEAFAR.E** |
|  | 9960 | **664.3617** | **1326.7088** | **1325.5088** | **1.2000** | **1** | **2** | **97** | **4** | **R.NSRDIGSNSGCC.-** |
|  | 10575 | 681.8546 | 1361.6945 | 1361.6649 | 0.0296 | 0 | 6 | 34 | 5 | R.EHGLIFMETSAK.T |
|  | 13838 | 775.8782 | 1549.7419 | 1549.7525 | -0.0106 | 0 | 81 | 1.1e-006 | 1 | K.LQIWDTAGQESFR.S 13837 |
|  | 16502 | 859.9042 | 1717.7938 | 1717.8060 | -0.0122 | 0 | 6 | 30 | 2 | R.ETFNHLTSWLEDAR.Q 16515 |
|  | 21906 | **1076.5287** | **2151.0428** | **2152.1278** | **-1.0850** | **1** | **0** | **83** | **5** | **-.MTYAYLFKYIIIGDTGVGK.S** |
|  | 21970 | **540.3052** | **2157.1916** | **2156.0934** | **1.0982** | **1** | **1** | **62** | **8** | **K.IGPQQSISTSVGPSASQRNSR.D** |
|  | 29160 | 1125.5485 | 3373.6236 | 3373.6183 | 0.0052 | 1 | 0 | 42 | 3 | R.ETFNHLTSWLEDARQHSSSNMVIMLIGNK.S |

| **448.** | P30050\|RL12_HUMAN    **Mass:** 17979    **Score:** 90     **Queries matched:** 5   **emPAI:** 0.21 |
| --- | --- |
|  | 60S ribosomal protein L12 OS=Homo sapiens GN=RPL12 PE=1 SV=1 |

|  | **Query** | **Observed** | **Mr(expt)** | **Mr(calc)** | **Delta** | **Miss** | **Score** | **Expect** | **Rank** | **Peptide** |
| --- | --- | --- | --- | --- | --- | --- | --- | --- | --- | --- |
|  | 1772 | 405.7424 | 809.4703 | 809.4759 | -0.0056 | 1 | 8 | 26 | 4 | K.ALKEPPR.D |
|  | 9665 | **658.3357** | **1314.6569** | **1314.6642** | **-0.0072** | **1** | **7** | **26** | **5** | **-.MPPKFDPNEIK.V** |
|  | 15831 | **838.4013** | **1674.7879** | **1674.7995** | **-0.0116** | **0** | **58** | **0.0002** | **1** | **K.EILGTAQSVGCNVDGR.H** 15830 |
|  | 23065 | **760.9908** | **2279.9507** | **2280.2148** | **-0.2641** | **1** | **4** | **27** | **2** | **R.CTGGEVGATSALAPKIGPLGLSPK.K** |

| **449.** | Q96S97\|MYADM_HUMAN    **Mass:** 36049    **Score:** 89     **Queries matched:** 7   **emPAI:** 0.10 |
| --- | --- |
|  | Myeloid-associated differentiation marker OS=Homo sapiens GN=MYADM PE=1 SV=2 |

|  | **Query** | **Observed** | **Mr(expt)** | **Mr(calc)** | **Delta** | **Miss** | **Score** | **Expect** | **Rank** | **Peptide** |
| --- | --- | --- | --- | --- | --- | --- | --- | --- | --- | --- |
|  | 22836 | **1126.5681** | **2251.1217** | **2251.1366** | **-0.0149** | **0** | **73** | **3.9e-006** | **1** | **R.TTITTTTTSSSGLGSPMIVGSPR.A** 22837 22838 |
|  | 27447 | 744.6445 | 2974.5490 | 2973.7419 | 0.8071 | 0 | 2 | 36 | 2 | R.LAVAILTAINLLAYVADLVHSAHLVFVK.V |
|  | 27865 | **1018.5563** | **3052.6472** | **3051.5581** | **1.0891** | **1** | **(2)** | **32** | **1** | **-.MPVTVTRTTITTTTTSSSGLGSPMIVGSPR.A** |
|  | 27919 | 767.8779 | 3067.4826 | 3067.5530 | -0.0704 | 1 | 2 | 31 | 4 | -.MPVTVTRTTITTTTTSSSGLGSPMIVGSPR.A |
|  | 27940 | 769.3799 | 3073.4907 | 3073.4538 | 0.0368 | 1 | 1 | 42 | 5 | R.SRDHAIAATFFSCIACVAYATEVAWTR.A |

| **450.** | P15941\|MUC1_HUMAN    **Mass:** 122170   **Score:** 89     **Queries matched:** 7   **emPAI:** 0.06 |
| --- | --- |
|  | Mucin-1 OS=Homo sapiens GN=MUC1 PE=1 SV=2 |

|  | **Query** | **Observed** | **Mr(expt)** | **Mr(calc)** | **Delta** | **Miss** | **Score** | **Expect** | **Rank** | **Peptide** |
| --- | --- | --- | --- | --- | --- | --- | --- | --- | --- | --- |
|  | 6265 | **567.3151** | **1132.6156** | **1132.6241** | **-0.0085** | **0** | **53** | **0.00087** | **1** | **K.QGGFLGLSNIK.F** 6262 6263 |
|  | 9249 | 432.0402 | 1293.0987 | 1292.6513 | 0.4474 | 0 | 7 | 20 | 5 | K.NYGQLDIFPAR.D |
|  | 11282 | **701.3320** | **1400.6495** | **1401.6850** | **-1.0355** | **0** | **6** | **38** | **1** | **R.DISEMFLQIYK.Q** |
|  | 11621 | **474.5597** | **1420.6573** | **1420.7463** | **-0.0889** | **1** | **3** | **66** | **7** | **R.KNYGQLDIFPAR.D** |
|  | 20568 | **1011.4760** | **2020.9373** | **2020.9490** | **-0.0117** | **0** | **63** | **4.4e-005** | **1** | **R.EGTINVHDVETQFNQYK.T** |

| **451.** | P06744\|G6PI_HUMAN    **Mass:** 63335    **Score:** 89     **Queries matched:** 9   **emPAI:** 0.06 |
| --- | --- |
|  | Glucose-6-phosphate isomerase OS=Homo sapiens GN=GPI PE=1 SV=4 |

|  | **Query** | **Observed** | **Mr(expt)** | **Mr(calc)** | **Delta** | **Miss** | **Score** | **Expect** | **Rank** | **Peptide** |
| --- | --- | --- | --- | --- | --- | --- | --- | --- | --- | --- |
|  | 1029 | 587.3468 | 586.3395 | 586.3802 | -0.0407 | 1 | 10 | 23 | 6 | K.QLAKK.I |
|  | 5444 | **538.2836** | **1074.5526** | **1075.5332** | **-0.9806** | **0** | **9** | **23** | **6** | **K.NLVTEDVMR.M** |
|  | 7784 | **610.3524** | **1218.6903** | **1217.6404** | **1.0498** | **0** | **2** | **98** | **7** | **K.HFVALSTNTTK.V** |
|  | 16267 | **852.4769** | **1702.9392** | **1702.9440** | **-0.0048** | **0** | **89** | **1.5e-007** | **1** | **K.ILLANFLAQTEALMR.G** |
|  | 16312 | **854.3817** | **1706.7489** | **1707.8944** | **-1.1456** | **0** | **1** | **83** | **3** | **K.VFEGNRPTNSIVFTK.L** |
|  | 18195 | **916.5055** | **1830.9965** | **1831.0091** | **-0.0125** | **0** | **7** | **21** | **2** | **K.TLAQLNPESSLFIIASK.T** |
|  | 20023 | **986.5359** | **1971.0573** | **1970.9983** | **0.0591** | **1** | **0** | **86** | **1** | **R.SNTPILVDGKDVMPEVNK.V** |
|  | 25542 | **877.1048** | **2628.2926** | **2628.3071** | **-0.0146** | **1** | **3** | **31** | **1** | **K.TFTTQETITNAETAKEWFLQAAK.D** |
|  | 30501 | 1349.2878 | 4044.8417 | 4044.9734 | -0.1317 | 0 | 6 | 6.4 | 3 | R.YSLWSAIGLSIALHVGFDNFEQLLSGAHWMDQHFR.T |

| **452.** | P20039\|2B1B_HUMAN    **Mass:** 30483    **Score:** 88     **Queries matched:** 9   **emPAI:** 0.42 |
| --- | --- |
|  | HLA class II histocompatibility antigen, DRB1-11 beta chain OS=Homo sapiens GN=HLA-DRB1 PE=2 SV=1 |

|  | **Query** | **Observed** | **Mr(expt)** | **Mr(calc)** | **Delta** | **Miss** | **Score** | **Expect** | **Rank** | **Peptide** |
| --- | --- | --- | --- | --- | --- | --- | --- | --- | --- | --- |
|  | 1551 | 793.4394 | 792.4322 | 792.4382 | -0.0060 | 0 | 16 | 4.3 | 1 | K.VTVYPSK.T |
|  | 3775 | 478.7490 | 955.4834 | 954.4229 | 1.0605 | 0 | 16 | 4 | 1 | R.AAVDTYCR.H |
|  | 7210 | 597.6307 | 1193.2468 | 1192.5625 | 0.6843 | 1 | 1 | 88 | 3 | R.WFRNGQEEK.T |
|  | 7827 | 611.8204 | 1221.6263 | 1220.6374 | 0.9889 | 1 | 4 | 59 | 6 | R.NQKGHSGLQPR.G |
|  | 8495 | 419.3127 | 1254.9164 | 1254.6469 | 0.2695 | 1 | 3 | 52 | 4 | K.GHSGLQPRGFLS.- |
|  | 12872 | 747.3553 | 1492.6960 | 1492.7059 | -0.0099 | 0 | 50 | 0.0013 | 1 | R.HNYGVGESFTVQR.R |
|  | 25716 | 1331.1263 | 2660.2381 | 2660.2541 | -0.0160 | 0 | 64 | 2.4e-005 | 1 | R.SGEVYTCQVEHPSVTSPLTVEWR.A 25715 |
|  | 31376 | 1735.1232 | 5202.3477 | 5203.5239 | -1.1763 | 1 | 1 | 11 | 7 | R.LPGGSCMAVLTVTLMVLSSPLALAGDTRPRFLEYSTSECHFFNGTER.V |

| **453.** | Q14643\|ITPR1_HUMAN    **Mass:** 317167   **Score:** 88     **Queries matched:** 58   **emPAI:** 0.02 |
| --- | --- |
|  | Inositol 1,4,5-trisphosphate receptor type 1 OS=Homo sapiens GN=ITPR1 PE=1 SV=2 |

|  | **Query** | **Observed** | **Mr(expt)** | **Mr(calc)** | **Delta** | **Miss** | **Score** | **Expect** | **Rank** | **Peptide** |
| --- | --- | --- | --- | --- | --- | --- | --- | --- | --- | --- |
|  | 926 | **546.2509** | **545.2436** | **544.2605** | **0.9831** | **0** | **13** | **21** | **4** | **R.DAPSR.K** 927 |
|  | 1387 | 731.4252 | 730.4179 | 730.4225 | -0.0046 | 0 | 14 | 10 | 1 | R.VLSDVAK.S |
|  | 1718 | **403.2294** | **804.4443** | **805.3575** | **-0.9132** | **0** | **10** | **20** | **10** | **K.LCPMNR.Y** |
|  | 1959 | 413.2635 | 824.5125 | 823.3824 | 1.1301 | 1 | 8 | 22 | 7 | K.DRGYGEK.L 1961 |
|  | 2426 | **429.5389** | **857.0633** | **857.4831** | **-0.4198** | **1** | **17** | **2.8** | **2** | **R.LSARNAAR.R** 2425 2428 |
|  | 2596 | 435.5555 | 869.0963 | 869.4759 | -0.3796 | 1 | 13 | 4.4 | 1 | R.KAFTTFR.R |
|  | 3081 | **454.2072** | **906.3999** | **906.3865** | **0.0133** | **0** | **6** | **40** | **2** | **K.DQMTEQR.K** |
|  | 3801 | **479.2868** | **956.5590** | **956.6018** | **-0.0428** | **0** | **1** | **1.3e+002** | **5** | **R.ALIASTILR.L** |
|  | 3857 | 481.2443 | 960.4740 | 959.5361 | 0.9379 | 0 | (5) | 55 | 10 | R.MDLVLELK.N |
|  | 4082 | 489.2997 | 976.5848 | 975.5311 | 1.0538 | 0 | 11 | 16 | 5 | R.MDLVLELK.N |
|  | 5060 | 523.8058 | 1045.5971 | 1044.6001 | 0.9970 | 0 | 42 | 0.012 | 1 | K.LLLAIMESR.H |
|  | 5118 | **526.2913** | **1050.5681** | **1049.5692** | **0.9989** | **0** | **0** | **1.5e+002** | **10** | **R.ILYNMRPK.E** |
|  | 5320 | **533.8739** | **1065.7332** | **1065.5931** | **0.1402** | **0** | **8** | **19** | **3** | **K.HTAQIEIVR.L** |
|  | 5508 | 540.2557 | 1078.4969 | 1077.5567 | 0.9403 | 1 | 4 | 69 | 10 | R.DYRNIIER.L |
|  | 6863 | 588.3676 | 1174.7205 | 1173.5989 | 1.1216 | 0 | 6 | 37 | 2 | R.DQLLEASAATR.K |
|  | 7740 | **609.3201** | **1216.6257** | **1215.6095** | **1.0162** | **1** | **17** | **3.4** | **2** | **R.ELAQDAKEGQK.E** 7701 |
|  | 8834 | 636.8387 | 1271.6628 | 1271.6469 | 0.0159 | 1 | 16 | 3.5 | 2 | K.KTEEGNNKPQK.H 8835 |
|  | 8922 | 638.3576 | 1274.7006 | 1275.6605 | -0.9599 | 1 | 1 | 1.1e+002 | 6 | K.LCVQESASVRK.S |
|  | 9862 | **662.3624** | **1322.7102** | **1321.6621** | **1.0480** | **1** | **6** | **30** | **3** | **K.EDIMVMDTKLK.I** 9857 |
|  | 10040 | **445.0337** | **1332.0792** | **1331.5953** | **0.4839** | **1** | **2** | **66** | **8** | **K.GEENKGNNDVEK.L** |
|  | 10786 | **458.3048** | **1371.8927** | **1371.7510** | **0.1417** | **1** | **4** | **53** | **7** | **R.KPSKEEPLFAAR.V** |
|  | 10992 | 462.1445 | 1383.4116 | 1382.6170 | 0.7946 | 1 | 6 | 23 | 3 | K.AEDTKMQEIMR.L |
|  | 13959 | **779.4241** | **1556.8337** | **1555.6613** | **1.1724** | **0** | **4** | **55** | **3** | **R.SEDLFNEMNWQK.K** |
|  | 15157 | **545.3002** | **1632.8789** | **1631.7283** | **1.1505** | **0** | **3** | **58** | **7** | **R.GEMSLAEVQCHLDK.E** |
|  | 15734 | **557.2476** | **1668.7209** | **1669.8999** | **-1.1790** | **0** | **4** | **47** | **1** | **K.AAKPGANSTTDAVLLNK.L** |
|  | 15883 | 560.3003 | 1677.8790 | 1677.7708 | 0.1082 | 0 | 2 | 74 | 7 | R.FAQTMEFVEEYLR.D 15860 |
|  | 16825 | **580.3178** | **1737.9314** | **1736.8304** | **1.1010** | **0** | **1** | **76** | **7** | **K.ALWEVEVVQHDPCR.G** |
|  | 17376 | **593.7484** | **1778.2234** | **1778.8066** | **-0.5832** | **1** | **1** | **63** | **4** | **K.QAEPEKEDIMVMDTK.L** |
|  | 17475 | 892.9276 | 1783.8405 | 1783.9753 | -0.1348 | 1 | 5 | 36 | 4 | K.EEEKPVMLKIGTSPVK.E 17447 |
|  | 17679 | **599.6701** | **1795.9885** | **1794.8934** | **1.0950** | **1** | **6** | **24** | **1** | **R.CVVQPETGDLNNPPKK.F** |
|  | 17835 | 602.9806 | 1805.9201 | 1805.8441 | 0.0761 | 0 | 5 | 39 | 3 | K.LLQAPFTDCGDGPMLR.L |
|  | 18371 | **922.4866** | **1842.9586** | **1844.0593** | **-1.1007** | **1** | **3** | **53** | **2** | **R.ILYNMRPKELVEVIK.K** 18370 |
|  | 18496 | **926.4169** | **1850.8193** | **1850.8316** | **-0.0123** | **0** | **75** | **3.1e-006** | **1** | **R.AMSLVSSDSEGEQNELR.N** |
|  | 18586 | 929.4856 | 1856.9566 | 1857.7435 | -0.7869 | 0 | 1 | 76 | 3 | K.GQGPDETMDGASGENEHK.K |
|  | 18830 | **470.2359** | **1876.9145** | **1875.8859** | **1.0286** | **0** | **3** | **46** | **10** | **R.GGGFLPMTPMAAAPEGNVK.Q** |
|  | 19349 | **641.2758** | **1920.8055** | **1919.9258** | **0.8796** | **0** | **5** | **29** | **1** | **K.EGASNLVIDLIMNASSDR.V** |
|  | 19559 | **485.0150** | **1936.0308** | **1934.9785** | **1.0523** | **0** | **4** | **36** | **6** | **R.IGLLGHPPHMNVNPQQPA.-** |
|  | 19905 | **490.9995** | **1959.9689** | **1960.0009** | **-0.0320** | **0** | **7** | **17** | **4** | **K.DDLEMSAVITIMQPILR.F** |
|  | 20326 | 667.1732 | 1998.4978 | 1998.9792 | -0.4814 | 1 | 2 | 38 | 3 | K.LLLAIMESRHDSENAER.I |
|  | 21454 | 1051.9365 | 2101.8585 | 2101.9812 | -0.1228 | 1 | 4 | 33 | 6 | R.EPRFLDYLSDLCVSMNK.S |
|  | 21492 | **702.8574** | **2105.5504** | **2106.1242** | **-0.5738** | **1** | **1** | **46** | **2** | **K.LESTMKLVTNLSGQLSELK.D** |
|  | 21890 | 717.6889 | 2150.0449 | 2148.9027 | 1.1422 | 1 | 0 | 79 | 4 | R.CMSDENLPYDLRASFCR.L |
|  | 24515 | **828.4087** | **2482.2042** | **2482.4055** | **-0.2013** | **1** | **2** | **41** | **3** | **K.ILLAILDCVHVTTIFPISKMAK.G** |
|  | 24822 | 842.0481 | 2523.1225 | 2524.2155 | -1.0931 | 0 | 0 | 57 | 4 | K.ELQSMLKPGGQVDGDEALEFYAK.H |
|  | 24936 | **848.5294** | **2542.5664** | **2542.2196** | **0.3469** | **1** | **1** | **44** | **2** | **R.GGGFLPMTPMAAAPEGNVKQAEPEK.E** |
|  | 28508 | **1068.5576** | **3202.6510** | **3201.6071** | **1.0439** | **0** | **0** | **48** | **5** | **R.NMSFWSSISFNLAVLMNLLVAFFYPFK.G** |
|  | 29565 | 884.6543 | 3534.5881 | 3533.6436 | 0.9445 | 1 | 1 | 26 | 4 | R.GEMSLAEVQCHLDKEGASNLVIDLIMNASSDR.V |
|  | 31252 | **1240.6456** | **4958.5534** | **4958.5059** | **0.0476** | **1** | **2** | **8.6** | **1** | **K.EGASNLVIDLIMNASSDRVFHESILLAIALLEGGNTTIQHSFFCR.L** |

| **454.** | P06753\|TPM3_HUMAN    **Mass:** 32856    **Score:** 88     **Queries matched:** 20   **emPAI:** 0.54 |
| --- | --- |
|  | Tropomyosin alpha-3 chain OS=Homo sapiens GN=TPM3 PE=1 SV=1 |

|  | **Query** | **Observed** | **Mr(expt)** | **Mr(calc)** | **Delta** | **Miss** | **Score** | **Expect** | **Rank** | **Peptide** |
| --- | --- | --- | --- | --- | --- | --- | --- | --- | --- | --- |
|  | 1352 | 716.3771 | 715.3699 | 716.3453 | -0.9754 | 0 | 7 | 48 | 4 | K.ENALDR.A |
|  | 1361 | 722.3414 | 721.3341 | 721.3395 | -0.0054 | 0 | 13 | 7.8 | 1 | R.AEFAER.S |
|  | 1544 | **397.2009** | **792.3873** | **793.4190** | **-1.0317** | **1** | **3** | **1e+002** | **7** | **K.KMQMLK.L** |
|  | 2293 | 424.3009 | 846.5872 | 845.4606 | 1.1266 | 1 | 3 | 1.1e+002 | 9 | K.LKEAETR.A 2284 |
|  | 8233 | 622.3256 | 1242.6366 | 1242.6456 | -0.0090 | 0 | 34 | 0.056 | 1 | R.IQLVEEELDR.A |
|  | 8744 | 634.8156 | 1267.6165 | 1267.6156 | 0.0009 | 1 | 7 | 27 | 9 | K.EAKHIAEEADR.K |
|  | 9064 | **428.9196** | **1283.7371** | **1283.7449** | **-0.0078** | **1** | **33** | **0.063** | **1** | **R.KLVIIEGDLER.T** 9066 9068 |
|  | 9714 | **439.7368** | **1316.1885** | **1315.6368** | **0.5518** | **0** | **10** | **9.8** | **1** | **K.AADAEAEVASLNR.R** |
|  | 11324 | 702.8277 | 1403.6408 | 1402.7126 | 0.9283 | 1 | 6 | 35 | 3 | K.QLEDELAAMQKK.L |
|  | 12015 | 722.4007 | 1442.7868 | 1442.7980 | -0.0112 | 1 | 21 | 1 | 1 | R.LATALQKLEEAEK.A 12016 |
|  | 12030 | 482.2711 | 1443.7914 | 1443.7317 | 0.0596 | 1 | 5 | 37 | 7 | K.KAADAEAEVASLNR.R |
|  | 12554 | 738.8392 | 1475.6639 | 1475.6739 | -0.0100 | 1 | 63 | 6.9e-005 | 1 | K.LEEAEKAADESER.G 12555 12567 |
|  | 16253 | **568.3276** | **1701.9611** | **1700.7965** | **1.1646** | **1** | **10** | **12** | **4** | **K.ENALDRAEQAEAEQK.Q** |
|  | 16644 | 864.4434 | 1726.8722 | 1726.8849 | -0.0128 | 1 | 34 | 0.039 | 1 | R.IQLVEEELDRAQER.L |

| **455.** | Q16647\|PTGIS_HUMAN    **Mass:** 57182    **Score:** 88     **Queries matched:** 13   **emPAI:** 0.13 |
| --- | --- |
|  | Prostacyclin synthase OS=Homo sapiens GN=PTGIS PE=1 SV=1 |

|  | **Query** | **Observed** | **Mr(expt)** | **Mr(calc)** | **Delta** | **Miss** | **Score** | **Expect** | **Rank** | **Peptide** |
| --- | --- | --- | --- | --- | --- | --- | --- | --- | --- | --- |
|  | 1445 | 763.1114 | 762.1042 | 761.3919 | 0.7122 | 0 | 9 | 17 | 1 | R.GSLSVGDK.D |
|  | 4235 | **495.2898** | **988.5651** | **988.5706** | **-0.0054** | **0** | **18** | **3.3** | **1** | **R.LTAAPFITR.E** |
|  | 6272 | **567.7725** | **1133.5305** | **1133.5717** | **-0.0412** | **1** | **9** | **21** | **2** | **R.FLNPDGSEKK.D** |
|  | 13322 | **507.2615** | **1518.7627** | **1517.7218** | **1.0409** | **0** | **4** | **50** | **6** | **R.EVVVDLAMPMADGR.E** |
|  | 15177 | **545.7731** | **1634.2976** | **1634.7393** | **-0.4417** | **1** | **6** | **18** | **1** | **R.GSLSVGDKDHMCSVK.S** 15170 15182 |
|  | 18175 | 915.4922 | 1828.9698 | 1828.9782 | -0.0084 | 0 | 81 | 8.9e-007 | 1 | K.VLDSTPVLDSVLSESLR.L |
|  | 21060 | **688.6569** | **2062.9488** | **2063.0047** | **-0.0560** | **1** | **0** | **82** | **10** | **R.YGFGLMQPEHDVPVRYR.I** |
|  | 21465 | **1052.5366** | **2103.0587** | **2103.3547** | **-0.2960** | **0** | **3** | **47** | **7** | **M.AWAALLGLLAALLLLLLLSR.R** 21466 |
|  | 22916 | **566.0480** | **2260.1630** | **2259.4558** | **0.7072** | **1** | **3** | **41** | **7** | **M.AWAALLGLLAALLLLLLLSRR.R** |
|  | 26366 | 922.1540 | 2763.4401 | 2763.4523 | -0.0122 | 1 | 28 | 0.099 | 1 | R.LLLFPFLSPQRDPEIYTDPEVFK.Y |

| **456.** | Q92930\|RAB8B_HUMAN    **Mass:** 23740    **Score:** 87     **Queries matched:** 16   **emPAI:** 0.81 |
| --- | --- |
|  | Ras-related protein Rab-8B OS=Homo sapiens GN=RAB8B PE=1 SV=2 |

|  | **Query** | **Observed** | **Mr(expt)** | **Mr(calc)** | **Delta** | **Miss** | **Score** | **Expect** | **Rank** | **Peptide** |
| --- | --- | --- | --- | --- | --- | --- | --- | --- | --- | --- |
|  | 1585 | 398.7108 | 795.4070 | 794.4174 | 0.9896 | 0 | 9 | 17 | 8 | K.FLETSAK.S |
|  | 3768 | **478.2791** | **954.5436** | **953.3695** | **1.1741** | **1** | **0** | **1.4e+002** | **9** | **K.CDMNDKR.Q** |
|  | 5394 | 536.3203 | 1070.6261 | 1070.6336 | -0.0075 | 0 | 45 | 0.006 | 1 | K.LLLIGDSGVGK.T 5392 |
|  | 9253 | 432.2090 | 1293.6050 | 1294.6267 | -1.0217 | 1 | 3 | 68 | 5 | -.MAKTYDYLFK.L |
|  | 9695 | 658.8284 | 1315.6422 | 1315.6521 | -0.0099 | 0 | 18 | 2.6 | 1 | K.LQIWDTAGQER.F 9685 |
|  | 9757 | 660.3506 | 1318.6867 | 1319.5776 | -0.8908 | 0 | 3 | 80 | 6 | K.MNDSNSAGAGGPVK.I |
|  | 11850 | 717.4002 | 1432.7859 | 1431.6776 | 1.1083 | 1 | 3 | 66 | 5 | R.KMNDSNSAGAGGPVK.I |
|  | 13956 | 779.4173 | 1556.8200 | 1556.8311 | -0.0110 | 1 | 32 | 0.081 | 1 | K.IKLQIWDTAGQER.F 13955 13957 |
|  | 15483 | 827.9073 | 1653.8001 | 1653.8106 | -0.0104 | 0 | 75 | 3.7e-006 | 1 | R.GAMGIMLVYDITNEK.S 15501 |
|  | 21723 | **710.3903** | **2128.1491** | **2129.0463** | **-0.8971** | **1** | **7** | **16** | **2** | **K.SSANVEEAFFTLARDIMTK.L** |
|  | 21849 | **716.0195** | **2145.0366** | **2145.0412** | **-0.0046** | **1** | **(2)** | **50** | **4** | **K.SSANVEEAFFTLARDIMTK.L** |

| **457.** | P20645\|MPRD_HUMAN    **Mass:** 31487    **Score:** 87     **Queries matched:** 3   **emPAI:** 0.12 |
| --- | --- |
|  | Cation-dependent mannose-6-phosphate receptor OS=Homo sapiens GN=M6PR PE=1 SV=1 |

|  | **Query** | **Observed** | **Mr(expt)** | **Mr(calc)** | **Delta** | **Miss** | **Score** | **Expect** | **Rank** | **Peptide** |
| --- | --- | --- | --- | --- | --- | --- | --- | --- | --- | --- |
|  | 5067 | 524.2445 | 1046.4743 | 1045.5516 | 0.9227 | 1 | 15 | 6.1 | 6 | K.SNGKETVVGR.L |
|  | 28398 | **1061.4779** | **3181.4119** | **3181.4209** | **-0.0090** | **0** | **87** | **9.1e-008** | **1** | **K.GMEQFPHLAFWQDLGNLVADGCDFVCR.S** |
|  | 29337 | 1147.4795 | 3439.4166 | 3440.5554 | -1.1387 | 1 | 1 | 24 | 5 | R.LNETHIFNGSNWIMLIYKGGDEYDNHCGK.E |

| **458.** | P15311\|EZRI_HUMAN    **Mass:** 69484    **Score:** 87     **Queries matched:** 21   **emPAI:** 0.17 |
| --- | --- |
|  | Ezrin OS=Homo sapiens GN=EZR PE=1 SV=4 |

|  | **Query** | **Observed** | **Mr(expt)** | **Mr(calc)** | **Delta** | **Miss** | **Score** | **Expect** | **Rank** | **Peptide** |
| --- | --- | --- | --- | --- | --- | --- | --- | --- | --- | --- |
|  | 1727 | **404.0297** | **806.0449** | **805.4004** | **0.6445** | **0** | **3** | **69** | **9** | **K.EELMLR.L** |
|  | 2663 | **438.2500** | **874.4854** | **874.4548** | **0.0306** | **0** | **15** | **6.7** | **8** | **K.ENPLQFK.F** |
|  | 3908 | 483.2527 | 964.4909 | 964.4978 | -0.0068 | 1 | 12 | 11 | 3 | R.NISFNDKK.F |
|  | 3982 | **486.3357** | **970.6568** | **969.5430** | **1.1138** | **0** | **7** | **28** | **8** | **-.MPKPINVR.V** |
|  | 4446 | **501.7610** | **1001.5075** | **1001.5141** | **-0.0067** | **0** | **12** | **11** | **1** | **R.ELSEQIQR.A** 4470 |
|  | 4465 | **502.2789** | **1002.5432** | **1002.5498** | **-0.0066** | **1** | **25** | **0.61** | **1** | **R.KENPLQFK.F** |
|  | 6021 | **558.7940** | **1115.5733** | **1115.5822** | **-0.0089** | **1** | **18** | **2.4** | **1** | **R.AKEAQDDLVK.T** |
|  | 9717 | 439.8649 | 1316.5729 | 1315.6806 | 0.8923 | 0 | 3 | 79 | 7 | R.KPDTIEVQQMK.A |
|  | 11541 | 472.9216 | 1415.7430 | 1415.6640 | 0.0789 | 1 | 2 | 83 | 6 | R.AQEEAERLEADR.M |
|  | 12487 | 736.8934 | 1471.7722 | 1471.7817 | -0.0095 | 1 | 45 | 0.0037 | 1 | R.RKPDTIEVQQMK.A 12492 |
|  | 13092 | 753.8885 | 1505.7624 | 1504.7998 | 0.9627 | 1 | 3 | 61 | 10 | K.SGYLSSERLIPQR.V |
|  | 15431 | **826.4067** | **1650.7988** | **1650.8100** | **-0.0112** | **0** | **59** | **0.00014** | **1** | **K.SQEQLAAELAEYTAK.I** 15411 |
|  | 17327 | 592.9996 | 1775.9771 | 1776.8473 | -0.8703 | 0 | 3 | 57 | 10 | R.ILQLCMGNHELYMR.R |
|  | 19378 | 962.4416 | 1922.8687 | 1921.9686 | 0.9001 | 1 | 4 | 37 | 2 | K.IGFPWSEIRNISFNDK.K |
|  | 20738 | **1019.5007** | **2036.9868** | **2036.9942** | **-0.0074** | **0** | **0** | **81** | **1** | **K.FYPEDVAEELIQDITQK.L** |
|  | 23506 | 779.4187 | 2335.2343 | 2334.1743 | 1.0599 | 1 | 0 | 67 | 9 | K.GTDLWLGVDALGLNIYEKDDK.L |
|  | 26714 | **941.8041** | **2822.3906** | **2822.4048** | **-0.0142** | **0** | **24** | **0.26** | **1** | **K.EGILSDEIYCPPETAVLLGSYAVQAK.F** |
|  | 31546 | **1112.5709** | **5557.8182** | **5557.6875** | **0.1307** | **1** | **0** | **8.8** | **1** | **K.TKEELHLVMTAPPPPPPPVYEPVSYHVQESLQDEGAEPTGYSAELSSEGIR.D** |

| **459.** | P05109\|S10A8_HUMAN    **Mass:** 10885    **Score:** 87     **Queries matched:** 10   **emPAI:** 0.87 |
| --- | --- |
|  | Protein S100-A8 OS=Homo sapiens GN=S100A8 PE=1 SV=1 |

|  | **Query** | **Observed** | **Mr(expt)** | **Mr(calc)** | **Delta** | **Miss** | **Score** | **Expect** | **Rank** | **Peptide** |
| --- | --- | --- | --- | --- | --- | --- | --- | --- | --- | --- |
|  | 2198 | **421.0565** | **840.0984** | **840.4640** | **-0.3655** | **1** | **34** | **0.035** | **1** | **K.MGVAAHKK.S** 2182 2195 |
|  | 2420 | **429.2425** | **856.4704** | **856.4589** | **0.0115** | **1** | **(4)** | **79** | **3** | **K.MGVAAHKK.S** |
|  | 8837 | **424.9004** | **1271.6793** | **1271.6874** | **-0.0080** | **0** | **31** | **0.12** | **1** | **K.ALNSIIDVYHK.Y** |
|  | 11624 | 711.3532 | 1420.6919 | 1420.7020 | -0.0102 | 0 | 85 | 4.4e-007 | 1 | K.LLETECPQYIR.K 11625 |
|  | 13813 | **775.4002** | **1548.7859** | **1548.7970** | **-0.0111** | **1** | **18** | **2.1** | **1** | **K.KLLETECPQYIR.K** |
|  | 23916 | **797.7617** | **2390.2633** | **2390.2733** | **-0.0100** | **0** | **11** | **5.4** | **1** | **K.ELDINTDGAVNFQEFLILVIK.M** 23924 |

| **460.** | Q9NYU2\|UGGG1_HUMAN    **Mass:** 177819   **Score:** 86     **Queries matched:** 24   **emPAI:** 0.04 |
| --- | --- |
|  | UDP-glucose:glycoprotein glucosyltransferase 1 OS=Homo sapiens GN=UGCGL1 PE=1 SV=3 |

|  | **Query** | **Observed** | **Mr(expt)** | **Mr(calc)** | **Delta** | **Miss** | **Score** | **Expect** | **Rank** | **Peptide** |
| --- | --- | --- | --- | --- | --- | --- | --- | --- | --- | --- |
|  | 1586 | **398.7222** | **795.4298** | **794.4109** | **1.0190** | **0** | **13** | **7.6** | **3** | **K.FCLSLR.S** |
|  | 2427 | **858.1300** | **857.1227** | **856.4225** | **0.7002** | **0** | **0** | **1.3e+002** | **4** | **R.VMEGLHR.L** |
|  | 3640 | 474.2401 | 946.4655 | 945.4821 | 0.9835 | 0 | 22 | 1.1 | 1 | R.HYIFNPR.K |
|  | 4185 | 493.7580 | 985.5014 | 986.5219 | -1.0205 | 0 | 15 | 4.9 | 1 | R.ISMINNPAK.E |
|  | 6113 | 561.7703 | 1121.5261 | 1121.6669 | -0.1408 | 1 | 5 | 50 | 9 | K.IKSHIQQLR.V |
|  | 8728 | **634.3310** | **1266.6474** | **1265.7356** | **0.9118** | **1** | **2** | **90** | **6** | **K.ANPGAWILRLR.K** |
|  | 8932 | **426.2314** | **1275.6724** | **1274.7194** | **0.9530** | **1** | **4** | **56** | **9** | **R.AITKTAVSSELR.T** |
|  | 9927 | **443.0384** | **1326.0934** | **1326.6462** | **-0.5528** | **1** | **6** | **26** | **9** | **R.NEARVMEGLHR.L** |
|  | 10089 | **668.3685** | **1334.7224** | **1333.6435** | **1.0789** | **0** | **2** | **90** | **2** | **R.VEEDVASDLVMK.V** |
|  | 10329 | 675.4097 | 1348.8049 | 1349.7927 | -0.9878 | 1 | 20 | 1.5 | 2 | R.FLRIMMLSVLK.N |
|  | 15655 | **555.3522** | **1663.0347** | **1661.8526** | **1.1821** | **0** | **4** | **43** | **6** | **K.VWQLQDLSFQTAAR.I** |
|  | 17691 | **599.9667** | **1796.8782** | **1797.8455** | **-0.9673** | **0** | **2** | **67** | **4** | **R.EQLDPDELETITMHK.I** |
|  | 23378 | 774.3397 | 2319.9972 | 2319.1495 | 0.8476 | 1 | 3 | 34 | 8 | K.QDKDDIINIFSVASGHLYER.F |
|  | 23679 | **786.7407** | **2357.2002** | **2356.2196** | **0.9806** | **1** | **6** | **17** | **1** | **R.VEEDVASDLVMKVDALLSAQPK.G** 23675 |
|  | 24099 | **1209.6021** | **2417.1895** | **2416.0644** | **1.1252** | **1** | **7** | **15** | **1** | **-.MGCKGDASGACAAGALPVTGVCYK.M** |
|  | 25627 | **882.7583** | **2645.2531** | **2644.2657** | **0.9874** | **0** | **16** | **1.4** | **1** | **R.IYSHDGTDSPPDADEVVIVLNNFK.S** |
|  | 27280 | **981.7696** | **2942.2869** | **2941.4433** | **0.8437** | **1** | **6** | **12** | **1** | **K.EARGYYEQTGVGPLPVVLFNGMPFER.E** |
|  | 27546 | **999.1649** | **2994.4728** | **2994.4909** | **-0.0182** | **0** | **8** | **8.8** | **1** | **R.AVYLGELPHDQDVVEYIMNQPNVVPR.I** |
|  | 28069 | **778.6527** | **3110.5817** | **3109.6522** | **0.9295** | **0** | **0** | **43** | **8** | **R.GLQFTLGTSANPVIVDTIVMANLGYFQLK.A** |
|  | 28154 | **626.1204** | **3125.5657** | **3125.7311** | **-0.1654** | **1** | **3** | **26** | **3** | **K.VWQLQDLSFQTAARILASPVELALVVMK.D** |
|  | 28157 | 782.8787 | 3127.4855 | 3127.4775 | 0.0081 | 1 | 2 | 31 | 4 | R.DYLDLTASNNFFVDDYARFTILDSQGK.T |
|  | 29391 | **1155.2300** | **3462.6681** | **3462.6838** | **-0.0157** | **0** | **89** | **5.5e-008** | **1** | **R.GQYQGLSQDPNSLSNLDQDLPNNMIHQVPIK.S** |
|  | 30565 | **817.1792** | **4080.8596** | **4080.9859** | **-0.1263** | **1** | **2** | **14** | **1** | **R.KNLHNMVFIVDPAHETTAELMNTAEMFLSNHIPLR.I** |

| **461.** | O95716\|RAB3D_HUMAN    **Mass:** 24480    **Score:** 86     **Queries matched:** 14   **emPAI:** 0.54 |
| --- | --- |
|  | Ras-related protein Rab-3D OS=Homo sapiens GN=RAB3D PE=1 SV=1 |

|  | **Query** | **Observed** | **Mr(expt)** | **Mr(calc)** | **Delta** | **Miss** | **Score** | **Expect** | **Rank** | **Peptide** |
| --- | --- | --- | --- | --- | --- | --- | --- | --- | --- | --- |
|  | 1246 | 678.3873 | 677.3801 | 677.3497 | 0.0304 | 0 | 4 | 71 | 7 | K.QVFER.L |
|  | 4051 | **488.2591** | **974.5036** | **974.5107** | **-0.0071** | **0** | **17** | **3.7** | **1** | **R.LVDVICEK.M** |
|  | 5825 | **551.3923** | **1100.7701** | **1099.6601** | **1.1100** | **0** | **3** | **69** | **6** | **K.LLLIGNSSVGK.T** |
|  | 6664 | 581.2745 | 1160.5344 | 1160.5244 | 0.0100 | 0 | 5 | 48 | 10 | -.MASAGDTQAGPR.D |
|  | 9695 | 658.8284 | 1315.6422 | 1315.6521 | -0.0099 | 0 | 18 | 2.6 | 1 | K.LQIWDTAGQER.Y 9685 |
|  | 13956 | 779.4173 | 1556.8200 | 1556.8311 | -0.0110 | 1 | 32 | 0.081 | 1 | R.IKLQIWDTAGQER.Y 13955 13957 |
|  | 14756 | **805.3773** | **1608.7401** | **1609.6890** | **-0.9489** | **0** | **9** | **14** | **1** | **K.MNESLEPSSSSGSNGK.G** |
|  | 18507 | **618.0749** | **1851.2028** | **1851.0618** | **0.1410** | **1** | **3** | **39** | **2** | **K.LLLIGNSSVGKTSFLFR.Y** |
|  | 21211 | **1040.4945** | **2078.9745** | **2078.9837** | **-0.0093** | **0** | **77** | **1.8e-006** | **1** | **R.YADDSFTPAFVSTVGIDFK.V** 21212 |
|  | 31166 | **965.4336** | **4822.1316** | **4822.3411** | **-0.2095** | **1** | **0** | **12** | **3** | **R.GAMGFLLMYDIANQESFAAVQDWATQIKTYSWDNAQVILVGNK.C** |

| **462.** | P20336\|RAB3A_HUMAN    **Mass:** 25196    **Score:** 86     **Queries matched:** 13   **emPAI:** 0.53 |
| --- | --- |
|  | Ras-related protein Rab-3A OS=Homo sapiens GN=RAB3A PE=1 SV=1 |

|  | **Query** | **Observed** | **Mr(expt)** | **Mr(calc)** | **Delta** | **Miss** | **Score** | **Expect** | **Rank** | **Peptide** |
| --- | --- | --- | --- | --- | --- | --- | --- | --- | --- | --- |
|  | 2364 | **427.2214** | **852.4282** | **853.3600** | **-0.9318** | **0** | **5** | **45** | **6** | **-.MASATDSR.Y** |
|  | 4051 | 488.2591 | 974.5036 | 974.5107 | -0.0071 | 0 | 17 | 3.7 | 1 | R.LVDVICEK.M |
|  | 5825 | 551.3923 | 1100.7701 | 1099.6601 | 1.1100 | 0 | 3 | 69 | 6 | K.ILIIGNSSVGK.T |
|  | 9695 | 658.8284 | 1315.6422 | 1315.6521 | -0.0099 | 0 | 18 | 2.6 | 1 | K.LQIWDTAGQER.Y 9685 |
|  | 10570 | **681.8486** | **1361.6827** | **1362.6892** | **-1.0065** | **1** | **8** | **25** | **1** | **K.DNINVKQTFER.L** |
|  | 13956 | 779.4173 | 1556.8200 | 1556.8311 | -0.0110 | 1 | 32 | 0.081 | 1 | R.IKLQIWDTAGQER.Y 13955 13957 |
|  | 18507 | 618.0749 | 1851.2028 | 1851.0618 | 0.1410 | 1 | 3 | 39 | 2 | K.ILIIGNSSVGKTSFLFR.Y |
|  | 21211 | 1040.4945 | 2078.9745 | 2078.9837 | -0.0093 | 0 | 77 | 1.8e-006 | 1 | R.YADDSFTPAFVSTVGIDFK.V 21212 |
|  | 25188 | 641.7995 | 2563.1689 | 2564.2350 | -1.0661 | 1 | 3 | 32 | 10 | R.LVDVICEKMSESLDTADPAVTGAK.Q |

| **463.** | Q13492\|PICAL_HUMAN    **Mass:** 70881    **Score:** 86     **Queries matched:** 17   **emPAI:** 0.11 |
| --- | --- |
|  | Phosphatidylinositol-binding clathrin assembly protein OS=Homo sapiens GN=PICALM PE=1 SV=2 |

|  | **Query** | **Observed** | **Mr(expt)** | **Mr(calc)** | **Delta** | **Miss** | **Score** | **Expect** | **Rank** | **Peptide** |
| --- | --- | --- | --- | --- | --- | --- | --- | --- | --- | --- |
|  | 4371 | **499.7440** | **997.4735** | **996.5392** | **0.9342** | **0** | **10** | **16** | **2** | **R.FIQYLASR.N** |
|  | 4565 | **506.2627** | **1010.5109** | **1009.4499** | **1.0610** | **0** | **9** | **18** | **1** | **-.MSGQSLTDR.I** 4561 |
|  | 5697 | 547.2581 | 1092.5017 | 1092.5849 | -0.0832 | 1 | 3 | 76 | 4 | R.TMNTEKLLK.T |
|  | 11305 | **468.2492** | **1401.7258** | **1400.6895** | **1.0363** | **1** | **8** | **24** | **1** | **R.EKQAALEEEQAR.L** |
|  | 11968 | 721.3381 | 1440.6617 | 1440.6337 | 0.0280 | 1 | 2 | 86 | 9 | R.GADGVMRTMNTEK.L |
|  | 19647 | **649.0177** | **1944.0313** | **1944.0099** | **0.0214** | **1** | **0** | **87** | **10** | **R.ITAAQHSVTGSAVSKTVCK.A** |
|  | 24270 | 816.3733 | 2446.0982 | 2447.2075 | -1.1092 | 1 | 4 | 28 | 1 | -.MSGQSLTDRITAAQHSVTGSAVSK.T |
|  | 28184 | **1044.7765** | **3131.3076** | **3132.5008** | **-1.1932** | **0** | **3** | **21** | **3** | **K.HLDYLIQCTNEMNVNIPQLADSLFER.T** |
|  | 29137 | **1123.5867** | **3367.7382** | **3367.7624** | **-0.0242** | **0** | **64** | **1.7e-005** | **1** | **K.STNVIVDSGGFDELGGLLKPTVASQNQNLPVAK.L** |
|  | 30029 | 749.3171 | 3741.5490 | 3740.7934 | 0.7556 | 0 | 4 | 11 | 1 | R.TPTHEMFVGFTPSPVAQPHPSAGLNVDFESVFGNK.S 30024 |
|  | 30090 | **943.7361** | **3770.9152** | **3770.9326** | **-0.0174** | **1** | **36** | **0.0082** | **1** | **K.VAEQVGIDRGDIPDLSQAPSSLLDALEQHLASLEGK.K** 30088 30091 |
|  | 30911 | **1099.3029** | **4393.1823** | **4393.3065** | **-0.1242** | **1** | **1** | **17** | **3** | **K.LLKTVPIIQNQMDALLDFNVNSNELTNGVINAAFMLLFK.D** |
|  | 31848 | **1249.6067** | **7491.5965** | **7491.6324** | **-0.0359** | **0** | **0** | **3.7** | **1** | **K.VAPTTAWNAATMAPPVMAYPATTPTGMIGYGIPPQMGSVPVMTQPTLIYSQPVMRPPNPFGPVSGAQIQFM.-** |

| **464.** | P04843\|RPN1_HUMAN    **Mass:** 68641    **Score:** 86     **Queries matched:** 37   **emPAI:** 0.30 |
| --- | --- |
|  | Dolichyl-diphosphooligosaccharide--protein glycosyltransferase subunit 1 OS=Homo sapiens GN=RPN1 PE=1 SV=1 |

|  | **Query** | **Observed** | **Mr(expt)** | **Mr(calc)** | **Delta** | **Miss** | **Score** | **Expect** | **Rank** | **Peptide** |
| --- | --- | --- | --- | --- | --- | --- | --- | --- | --- | --- |
|  | 750 | 486.8631 | 485.8558 | 486.3166 | -0.4607 | 0 | 7 | 30 | 4 | R.LVAGK.L |
|  | 2176 | **840.4030** | **839.3957** | **839.4025** | **-0.0068** | **0** | **18** | **2.2** | **1** | **R.NVESYTK.L** 2164 2196 |
|  | 2179 | **420.7602** | **839.5059** | **839.5116** | **-0.0057** | **0** | **13** | **7.2** | **4** | **K.IILPEGAK.N** 2180 2183 |
|  | 2643 | **437.2589** | **872.5033** | **872.5080** | **-0.0047** | **1** | **17** | **4.2** | **5** | **K.RQELVTK.I** |
|  | 3118 | **455.2526** | **908.4906** | **908.4967** | **-0.0061** | **0** | **24** | **0.62** | **1** | **K.IDHILDAL.-** |
|  | 3559 | **471.2898** | **940.5650** | **940.5131** | **0.0519** | **1** | **6** | **38** | **6** | **K.SGRFFTVK.L** 3552 |
|  | 4644 | **509.2370** | **1016.4594** | **1016.4676** | **-0.0081** | **0** | **22** | **1.3** | **1** | **R.HFDETVNR.Y** 4642 4643 4645 |
|  | 5353 | **535.7468** | **1069.4790** | **1069.5768** | **-0.0978** | **0** | **6** | **42** | **8** | **R.TVDLSSHLAK.V** |
|  | 6320 | 1139.5430 | 1138.5357 | 1138.5506 | -0.0149 | 1 | 5 | 42 | 5 | K.KDTYIENEK.L |
|  | 7913 | **614.0779** | **1226.1412** | **1225.6779** | **0.4633** | **1** | **1** | **76** | **9** | **K.RTVDLSSHLAK.V** |
|  | 8464 | 418.9054 | 1253.6943 | 1254.7547 | -1.0605 | 1 | 16 | 3.5 | 6 | K.LDAQVKELVLK.S |
|  | 9230 | **431.8722** | **1292.5948** | **1292.6031** | **-0.0082** | **1** | **30** | **0.16** | **1** | **R.LKTEGSDLCDR.V** 9221 9228 9229 9231 |
|  | 9380 | **651.3703** | **1300.7260** | **1300.7350** | **-0.0090** | **0** | **33** | **0.075** | **1** | **K.ALTSEIALLQSR.L** 9381 |
|  | 9492 | **436.2505** | **1305.7297** | **1304.6684** | **1.0613** | **1** | **6** | **35** | **3** | **K.QSRDISTLNSGK.K** |
|  | 9560 | **656.3098** | **1310.6049** | **1310.6143** | **-0.0093** | **0** | **28** | **0.21** | **1** | **R.SEDLLDYGPFR.D** |
|  | 11838 | 478.5515 | 1432.6328 | 1431.6113 | 1.0214 | 0 | 5 | 41 | 7 | K.GEDEEENNLEVR.E |
|  | 11876 | **718.3517** | **1434.6888** | **1434.6990** | **-0.0102** | **0** | **15** | **4.5** | **2** | **K.NIEIDSPYEISR.A** |
|  | 14425 | 794.3968 | 1586.7791 | 1586.8702 | -0.0910 | 0 | 9 | 16 | 6 | K.VACITEQVLTLVNK.R |
|  | 15470 | **827.4447** | **1652.8748** | **1652.8846** | **-0.0098** | **0** | **34** | **0.044** | **1** | **K.VTAEVVLAHLGGGSTSR.A** 15457 |
|  | 15577 | **830.4470** | **1658.8795** | **1658.8879** | **-0.0084** | **0** | **8** | **19** | **1** | **R.ATSFLLALEPELEAR.L** |
|  | 17487 | 893.3784 | 1784.7422 | 1783.8489 | 0.8933 | 1 | 8 | 18 | 1 | R.YDYQRQPDSGISSIR.S |
|  | 19752 | 650.6651 | 1948.9735 | 1948.9643 | 0.0092 | 1 | 2 | 61 | 4 | K.LGNPTRSEDLLDYGPFR.D |
|  | 20222 | **996.0041** | **1989.9936** | **1990.0048** | **-0.0112** | **0** | **67** | **1.8e-005** | **1** | **R.FVDHVFDEQVIDSLTVK.I** |

| **465.** | P04233\|HG2A_HUMAN    **Mass:** 33950    **Score:** 86     **Queries matched:** 11   **emPAI:** 0.11 |
| --- | --- |
|  | HLA class II histocompatibility antigen gamma chain OS=Homo sapiens GN=CD74 PE=1 SV=3 |

|  | **Query** | **Observed** | **Mr(expt)** | **Mr(calc)** | **Delta** | **Miss** | **Score** | **Expect** | **Rank** | **Peptide** |
| --- | --- | --- | --- | --- | --- | --- | --- | --- | --- | --- |
|  | 9310 | 649.3036 | 1296.5926 | 1296.6938 | -0.1012 | 1 | 6 | 36 | 9 | K.GSFPENLRHLK.N |
|  | 10617 | **455.7253** | **1364.1540** | **1364.8213** | **-0.6673** | **1** | **5** | **27** | **1** | **R.MKLPKPPKPVSK.M** |
|  | 14607 | **800.4059** | **1598.7972** | **1598.8086** | **-0.0114** | **0** | **16** | **2.9** | **1** | **R.DLISNNEQLPMLGR.R** |
|  | 14897 | **539.8238** | **1616.4495** | **1615.8722** | **0.5773** | **1** | **4** | **35** | **2** | **K.VYPPLKGSFPENLR.H** |
|  | 15083 | 814.9467 | 1627.8789 | 1627.8893 | -0.0104 | 0 | 72 | 6.9e-006 | 1 | K.LTVTSQNLQLENLR.M 15084 |
|  | 23424 | **1163.4725** | **2324.9305** | **2325.0882** | **-0.1577** | **0** | **2** | **37** | **1** | **K.YGNMTEDHVMHLLQNADPLK.V** 23425 |
|  | 27364 | 987.4294 | 2959.2663 | 2960.4414 | -1.1751 | 1 | 3 | 25 | 6 | K.MRMATPLLMQALPMGALPQGPMQNATK.Y 27365 |
|  | 27368 | **592.8847** | **2959.3871** | **2960.4414** | **-1.0543** | **1** | **(2)** | **32** | **4** | **K.MRMATPLLMQALPMGALPQGPMQNATK.Y** |

| **466.** | P29401\|TKT_HUMAN    **Mass:** 68519    **Score:** 85     **Queries matched:** 21   **emPAI:** 0.17 |
| --- | --- |
|  | Transketolase OS=Homo sapiens GN=TKT PE=1 SV=3 |

|  | **Query** | **Observed** | **Mr(expt)** | **Mr(calc)** | **Delta** | **Miss** | **Score** | **Expect** | **Rank** | **Peptide** |
| --- | --- | --- | --- | --- | --- | --- | --- | --- | --- | --- |
|  | 3192 | **458.2316** | **914.4487** | **914.5549** | **-0.1062** | **1** | **11** | **15** | **4** | **R.KLILDSAR.A** 3190 3193 3198 3206 |
|  | 3216 | 458.7556 | 915.4965 | 915.5025 | -0.0060 | 0 | 33 | 0.093 | 1 | K.AVELAANTK.G |
|  | 4090 | **489.7865** | **977.5585** | **977.5658** | **-0.0073** | **0** | **15** | **5.7** | **1** | **K.HQPTAIIAK.T** |
|  | 10073 | **445.6937** | **1334.0594** | **1334.6076** | **-0.5482** | **1** | **2** | **63** | **8** | **K.SQDPRNPHNDR.F** |
|  | 10702 | **684.8920** | **1367.7694** | **1367.7772** | **-0.0078** | **0** | **14** | **5.2** | **1** | **K.LDNLVAILDINR.L** |
|  | 15885 | **560.3108** | **1677.9105** | **1676.8920** | **1.0186** | **1** | **8** | **19** | **1** | **R.SGKPAELLKMFGIDR.D** |
|  | 18927 | **943.4600** | **1884.9055** | **1883.9153** | **0.9902** | **0** | **28** | **0.16** | **1** | **R.SVPTSTVFYPSDGVATEK.A** 18916 |
|  | 19888 | 980.5000 | 1958.9854 | 1958.9884 | -0.0029 | 1 | 5 | 30 | 2 | -.MESYHKPDQQKLQALK.D |
|  | 20551 | **1010.5331** | **2019.0517** | **2019.0636** | **-0.0119** | **0** | **66** | **2.5e-005** | **1** | **K.ILATPPQEDAPSVDIANIR.M** |
|  | 20583 | **675.2819** | **2022.8238** | **2022.0091** | **0.8146** | **0** | **6** | **22** | **4** | **K.NMAEQIIQEIYSQIQSK.K** 20577 20579 |
|  | 21862 | **716.4250** | **2146.2531** | **2147.1586** | **-0.9055** | **1** | **1** | **51** | **8** | **K.KILATPPQEDAPSVDIANIR.M** |
|  | 24721 | **836.7359** | **2507.1859** | **2507.2040** | **-0.0182** | **0** | **2** | **45** | **2** | **R.TSRPENAIIYNNNEDFQVGQAK.V** |
|  | 26464 | **1392.2154** | **2782.4163** | **2781.4073** | **1.0091** | **1** | **1** | **50** | **8** | **R.SVPTSTVFYPSDGVATEKAVELAANTK.G** |
|  | 31109 | **1175.2716** | **4697.0573** | **4697.1441** | **-0.0867** | **1** | **0** | **14** | **2** | **R.AFDQIRMAAISESNINLCGSHCGVSIGEDGPSQMALEDLAMFR.S** |

| **467.** | P40616\|ARL1_HUMAN    **Mass:** 20518    **Score:** 85     **Queries matched:** 8   **emPAI:** 0.19 |
| --- | --- |
|  | ADP-ribosylation factor-like protein 1 OS=Homo sapiens GN=ARL1 PE=1 SV=1 |

|  | **Query** | **Observed** | **Mr(expt)** | **Mr(calc)** | **Delta** | **Miss** | **Score** | **Expect** | **Rank** | **Peptide** |
| --- | --- | --- | --- | --- | --- | --- | --- | --- | --- | --- |
|  | 13771 | **774.3397** | **1546.6648** | **1546.7548** | **-0.0901** | **0** | **2** | **76** | **10** | **K.SELVAMLEEEELR.K** 13764 |
|  | 17076 | **586.3241** | **1755.9505** | **1755.8291** | **0.1214** | **0** | **2** | **66** | **4** | **-.MGGFFSSIFSSLFGTR.E** |
|  | 17629 | 896.9432 | 1791.8719 | 1790.8760 | 0.9959 | 0 | 59 | 0.00015 | 1 | K.GTGLDEAMEWLVETLK.S 17611 |
|  | 17843 | **602.9961** | **1805.9666** | **1806.8710** | **-0.9043** | **0** | **(4)** | **44** | **7** | **K.GTGLDEAMEWLVETLK.S** |
|  | 20837 | **682.8387** | **2045.4942** | **2045.0714** | **0.4228** | **1** | **5** | **21** | **2** | **R.IGISKSELVAMLEEEELR.K** |
|  | 21517 | 703.6511 | 2107.9314 | 2108.0592 | -0.1279 | 0 | 1 | 71 | 7 | K.FQVWDLGGQTSIRPYWR.C |

| **468.** | Q9H4B7\|TBB1_HUMAN    **Mass:** 50865    **Score:** 84     **Queries matched:** 10   **emPAI:** 0.07 |
| --- | --- |
|  | Tubulin beta-1 chain OS=Homo sapiens GN=TUBB1 PE=1 SV=1 |

|  | **Query** | **Observed** | **Mr(expt)** | **Mr(calc)** | **Delta** | **Miss** | **Score** | **Expect** | **Rank** | **Peptide** |
| --- | --- | --- | --- | --- | --- | --- | --- | --- | --- | --- |
|  | 1748 | 404.7345 | 807.4544 | 807.3399 | 0.1146 | 0 | 2 | 1e+002 | 6 | R.EEYPDR.I |
|  | 5458 | 538.7742 | 1075.5338 | 1076.5250 | -0.9913 | 1 | 21 | 1.4 | 1 | K.IREEYPDR.I 5480 |
|  | 6379 | 572.3181 | 1142.6217 | 1142.6270 | -0.0053 | 0 | 65 | 5.8e-005 | 1 | K.LAVNMVPFPR.L 6378 |
|  | 8582 | 630.3885 | 1258.7624 | 1257.6830 | 1.0795 | 1 | 9 | 17 | 3 | R.FPGQLNADLRK.L 8578 |
|  | 10444 | **452.3924** | **1354.1554** | **1354.7180** | **-0.5626** | **1** | **8** | **14** | **1** | **R.GRYLTVACIFR.G** |
|  | 16156 | **566.3198** | **1695.9375** | **1694.8298** | **1.1077** | **0** | **2** | **63** | **8** | **R.ALSVAELTQQMFDAR.N** |
|  | 30178 | **952.9835** | **3807.9047** | **3807.7437** | **0.1610** | **1** | **5** | **9.7** | **2** | **R.HESESCDCLQGFQIVHSLGGGTGSGMGTLLMNKIR.E** |

| **469.** | P61421\|VA0D1_HUMAN    **Mass:** 40759    **Score:** 84     **Queries matched:** 9   **emPAI:** 0.30 |
| --- | --- |
|  | V-type proton ATPase subunit d 1 OS=Homo sapiens GN=ATP6V0D1 PE=1 SV=1 |

|  | **Query** | **Observed** | **Mr(expt)** | **Mr(calc)** | **Delta** | **Miss** | **Score** | **Expect** | **Rank** | **Peptide** |
| --- | --- | --- | --- | --- | --- | --- | --- | --- | --- | --- |
|  | 2840 | 443.7373 | 885.4600 | 885.4279 | 0.0320 | 0 | 8 | 33 | 9 | K.LFPHCGR.L |
|  | 3933 | **484.2686** | **966.5226** | **966.4294** | **0.0932** | **0** | **4** | **57** | **5** | **R.ADDYEQVK.N** |
|  | 4682 | **510.7526** | **1019.4906** | **1019.4964** | **-0.0057** | **0** | **12** | **12** | **1** | **K.AYLESFYK.F** |
|  | 8086 | 412.6955 | 1235.0648 | 1235.5903 | -0.5256 | 1 | 2 | 60 | 5 | K.MVVEFRHMR.N |
|  | 9443 | **652.8234** | **1303.6323** | **1303.6408** | **-0.0085** | **0** | **55** | **0.00045** | **1** | **K.LLFEGAGSNPGDK.T** |
|  | 15258 | **820.8748** | **1639.7350** | **1639.8821** | **-0.1471** | **0** | **20** | **1.2** | **1** | **R.AFIITINSFGTELSK.E** |
|  | 17184 | **589.3264** | **1764.9574** | **1764.9206** | **0.0369** | **1** | **3** | **53** | **9** | **R.NIVWIAECIAQRHR.A** |
|  | 26273 | 915.4282 | 2743.2628 | 2743.2768 | -0.0140 | 1 | 42 | 0.0034 | 1 | K.FCTLLGGTTADAMCPILEFEADRR.A |
|  | 31483 | **1359.6741** | **5434.6672** | **5434.6879** | **-0.0207** | **1** | **34** | **0.0043** | **1** | **K.AGVLSQADYLNLVQCETLEDLKLHLQSTDYGNFLANEASPLTVSVIDDR.L** |

| **470.** | Q6P4A8\|PLBL1_HUMAN    **Mass:** 63374    **Score:** 84     **Queries matched:** 12   **emPAI:** 0.06 |
| --- | --- |
|  | Putative phospholipase B-like 1 OS=Homo sapiens GN=PLBD1 PE=1 SV=1 |

|  | **Query** | **Observed** | **Mr(expt)** | **Mr(calc)** | **Delta** | **Miss** | **Score** | **Expect** | **Rank** | **Peptide** |
| --- | --- | --- | --- | --- | --- | --- | --- | --- | --- | --- |
|  | 2351 | **853.0877** | **852.0804** | **851.4059** | **0.6746** | **0** | **13** | **5.4** | **1** | **K.VTDTASMK.Y** 2344 |
|  | 4942 | 520.2661 | 1038.5177 | 1038.4474 | 0.0703 | 0 | 6 | 39 | 6 | R.VANMMADSGK.R |
|  | 7624 | **404.8874** | **1211.6403** | **1210.5434** | **1.0969** | **1** | **4** | **56** | **6** | **R.VANMMADSGKR.W** |
|  | 8446 | **627.3456** | **1252.6766** | **1251.4795** | **1.1971** | **0** | **15** | **4.7** | **1** | **R.GDPCNTICCR.E** |
|  | 8979 | **427.3379** | **1278.9918** | **1279.6078** | **-0.6161** | **1** | **4** | **37** | **2** | **R.DQGKVTDTASMK.Y** 8977 8980 |
|  | 10973 | **691.8463** | **1381.6781** | **1381.6878** | **-0.0097** | **0** | **84** | **5.5e-007** | **1** | **K.LGLDYSYDLAPR.A** |
|  | 11797 | **477.5865** | **1429.7376** | **1430.6898** | **-0.9522** | **1** | **9** | **18** | **4** | **K.VTDTASMKYIMR.Y** |
|  | 14304 | 790.9186 | 1579.8226 | 1579.8398 | -0.0172 | 0 | 2 | 70 | 9 | K.IYNWSGYPLLVQK.L |
|  | 20898 | 684.3415 | 2050.0028 | 2050.9054 | -0.9026 | 1 | 7 | 19 | 4 | K.NVMDKNGDAYGFYNNSVK.T |

| **471.** | P08631\|HCK_HUMAN    **Mass:** 60075    **Score:** 84     **Queries matched:** 20   **emPAI:** 0.06 |
| --- | --- |
|  | Tyrosine-protein kinase HCK OS=Homo sapiens GN=HCK PE=1 SV=5 |

|  | **Query** | **Observed** | **Mr(expt)** | **Mr(calc)** | **Delta** | **Miss** | **Score** | **Expect** | **Rank** | **Peptide** |
| --- | --- | --- | --- | --- | --- | --- | --- | --- | --- | --- |
|  | 2475 | **431.2207** | **860.4268** | **861.4708** | **-1.0440** | **0** | **17** | **4.3** | **4** | **K.IADFGLAR.V** 2476 2487 2489 2511 |
|  | 2523 | 432.7420 | 863.4695 | 863.4613 | 0.0082 | 1 | 6 | 47 | 10 | R.ALERGYR.M |
|  | 4666 | **509.9442** | **1017.8739** | **1018.4866** | **-0.6126** | **1** | **9** | **14** | **4** | **K.KGNDGLCQK.L** |
|  | 7558 | **605.2869** | **1208.5593** | **1208.5673** | **-0.0080** | **0** | **17** | **2.6** | **1** | **R.VIEDNEYTAR.E** |
|  | 8748 | 634.8565 | 1267.6983 | 1267.6197 | 0.0787 | 0 | 28 | 0.23 | 1 | K.EGYIPSNYVAR.V 8747 |
|  | 12759 | 744.3530 | 1486.6915 | 1487.7442 | -1.0527 | 0 | 6 | 35 | 7 | R.IPYPGMSNPEVIR.A |
|  | 14310 | **791.2982** | **1580.5819** | **1580.6130** | **-0.0311** | **1** | **1** | **81** | **3** | **-.MGGRSSCEDPGCPR.D** |
|  | 15904 | 560.9003 | 1679.6792 | 1678.8535 | 0.8257 | 0 | 2 | 72 | 7 | R.QLLAPGNMLGSFMIR.D |
|  | 22110 | **724.7224** | **2171.1454** | **2172.0265** | **-0.8811** | **0** | **(2)** | **54** | **2** | **K.TMKPGSMSVEAFLAEANVMK.T** |
|  | 22115 | 1086.9507 | 2171.8868 | 2172.0265 | -0.1397 | 0 | 9 | 9.3 | 2 | K.TMKPGSMSVEAFLAEANVMK.T |
|  | 22354 | 735.3690 | 2203.0852 | 2202.2122 | 0.8730 | 1 | 0 | 85 | 10 | K.LHAVVTKEPIYIITEFMAK.G |
|  | 25211 | **857.1094** | **2568.3063** | **2569.2954** | **-0.9891** | **1** | **6** | **16** | **1** | **K.VAVKTMKPGSMSVEAFLAEANVMK.T** |
|  | 26981 | **960.1438** | **2877.4096** | **2878.4027** | **-0.9931** | **1** | **1** | **44** | **4** | **K.TMKPGSMSVEAFLAEANVMKTLQHDK.L** 26983 |
|  | 30099 | **1260.5783** | **3778.7129** | **3778.7387** | **-0.0258** | **0** | **84** | **1.3e-007** | **1** | **K.NRPEERPTFEYIQSVLDDFYTATESQYQQQP.-** |

| **472.** | O15173\|PGRC2_HUMAN    **Mass:** 23861    **Score:** 84     **Queries matched:** 8   **emPAI:** 0.35 |
| --- | --- |
|  | Membrane-associated progesterone receptor component 2 OS=Homo sapiens GN=PGRMC2 PE=1 SV=1 |

|  | **Query** | **Observed** | **Mr(expt)** | **Mr(calc)** | **Delta** | **Miss** | **Score** | **Expect** | **Rank** | **Peptide** |
| --- | --- | --- | --- | --- | --- | --- | --- | --- | --- | --- |
|  | 2510 | **432.2273** | **862.4401** | **862.3855** | **0.0546** | **0** | **2** | **1.3e+002** | **5** | **-.MAAGDGDVK.L** |
|  | 4353 | **499.2225** | **996.4305** | **996.4375** | **-0.0070** | **0** | **15** | **4.4** | **2** | **R.EWEMQFK.E** |
|  | 4530 | 504.7602 | 1007.5059 | 1006.5083 | 0.9976 | 0 | 37 | 0.035 | 1 | R.DFSLEQLR.Q |
|  | 4804 | 515.7517 | 1029.4889 | 1028.4927 | 0.9962 | 1 | 9 | 20 | 4 | K.EKYDYVGR.L |
|  | 12484 | **736.8644** | **1471.7143** | **1471.7248** | **-0.0105** | **0** | **49** | **0.0018** | **1** | **K.FYGPAGPYGIFAGR.D** 12485 12493 |
|  | 18540 | 618.9977 | 1853.9714 | 1852.9391 | 1.0323 | 1 | 11 | 8.4 | 2 | R.RGLGAGAGAGEESPATSLPR.M |

| **473.** | P22748\|CAH4_HUMAN    **Mass:** 35295    **Score:** 84     **Queries matched:** 10   **emPAI:** 0.50 |
| --- | --- |
|  | Carbonic anhydrase 4 OS=Homo sapiens GN=CA4 PE=1 SV=2 |

|  | **Query** | **Observed** | **Mr(expt)** | **Mr(calc)** | **Delta** | **Miss** | **Score** | **Expect** | **Rank** | **Peptide** |
| --- | --- | --- | --- | --- | --- | --- | --- | --- | --- | --- |
|  | 5283 | **532.2890** | **1062.5634** | **1062.5709** | **-0.0075** | **0** | **18** | **2.5** | **1** | **R.EQILAFSQK.L** |
|  | 6067 | 560.2850 | 1118.5555 | 1119.4880 | -0.9325 | 1 | 7 | 34 | 6 | K.WGGNCQKDR.Q |
|  | 10777 | **458.2360** | **1371.6861** | **1370.7518** | **0.9343** | **1** | **12** | **9.6** | **1** | **K.DRQSPINIVTTK.A** |
|  | 11540 | 708.8729 | 1415.7312 | 1415.7409 | -0.0097 | 0 | 18 | 2.3 | 1 | K.ASISGGGLPAPYQAK.Q 11566 |
|  | 11686 | **712.3870** | **1422.7594** | **1422.7692** | **-0.0098** | **0** | **44** | **0.0057** | **1** | **K.DNVRPLQQLGQR.T** 11685 |
|  | 12703 | **742.8453** | **1483.6761** | **1483.6865** | **-0.0103** | **0** | **47** | **0.0028** | **1** | **R.YLGSLTTPTCDEK.V** |
|  | 13059 | **752.8664** | **1503.7182** | **1503.7279** | **-0.0097** | **1** | **55** | **0.00043** | **1** | **K.LYYDKEQTVSMK.D** |
|  | 24189 | **812.6988** | **2435.0747** | **2435.0634** | **0.0113** | **0** | **3** | **32** | **4** | **K.GSEHSLDGEHFAMEMHIVHEK.E** |

| **474.** | P02647\|APOA1_HUMAN    **Mass:** 30759    **Score:** 83     **Queries matched:** 15   **emPAI:** 0.26 |
| --- | --- |
|  | Apolipoprotein A-I OS=Homo sapiens GN=APOA1 PE=1 SV=1 |

|  | **Query** | **Observed** | **Mr(expt)** | **Mr(calc)** | **Delta** | **Miss** | **Score** | **Expect** | **Rank** | **Peptide** |
| --- | --- | --- | --- | --- | --- | --- | --- | --- | --- | --- |
|  | 1098 | **616.3615** | **615.3542** | **614.3751** | **0.9791** | **0** | **9** | **37** | **2** | **K.QLNLK.L** |
|  | 1351 | **715.3513** | **714.3440** | **713.4296** | **0.9144** | **1** | **7** | **37** | **9** | **R.QRLAAR.L** |
|  | 2932 | **448.7231** | **895.4315** | **895.4763** | **-0.0448** | **0** | **14** | **5.9** | **1** | **K.LHELQEK.L** 2950 |
|  | 4579 | **506.7371** | **1011.4596** | **1011.5713** | **-0.1116** | **0** | **5** | **43** | **5** | **K.AKPALEDLR.Q** |
|  | 6511 | 576.7461 | 1151.4777 | 1151.6298 | -0.1521 | 1 | 12 | 9.4 | 3 | R.QKLHELQEK.L |
|  | 9360 | **434.2189** | **1299.6349** | **1298.5601** | **1.0748** | **0** | **12** | **9.2** | **1** | **K.WQEEMELYR.Q** |
|  | 9405 | **651.8232** | **1301.6319** | **1300.6411** | **0.9908** | **0** | **12** | **8.7** | **1** | **R.THLAPYSDELR.Q** |
|  | 11032 | **693.8570** | **1385.6994** | **1385.7078** | **-0.0084** | **0** | **41** | **0.011** | **1** | **K.VSFLSALEEYTK.K** 11058 |
|  | 11505 | **472.2783** | **1413.8131** | **1414.6953** | **-0.8821** | **1** | **5** | **40** | **6** | **K.ENGGARLAEYHAK.A** |
|  | 12412 | **489.7444** | **1466.2115** | **1466.7841** | **-0.5726** | **1** | **2** | **48** | **3** | **K.VEPLRAELQEGAR.Q** |
|  | 14796 | **806.8916** | **1611.7686** | **1611.7781** | **-0.0094** | **0** | **66** | **3.3e-005** | **1** | **K.LLDNWDSVTSTFSK.L** |
|  | 16689 | **577.3220** | **1728.9441** | **1730.0090** | **-1.0650** | **0** | **1** | **88** | **5** | **K.AAVLTLAVLFLTGSQAR.H** |
|  | 22189 | **546.0309** | **2180.0944** | **2179.0235** | **1.0709** | **1** | **4** | **32** | **4** | **R.HFWQQDEPPQSPWDRVK.D** |

| **475.** | P07900\|HS90A_HUMAN    **Mass:** 85006    **Score:** 83     **Queries matched:** 20   **emPAI:** 0.14 |
| --- | --- |
|  | Heat shock protein HSP 90-alpha OS=Homo sapiens GN=HSP90AA1 PE=1 SV=5 |

|  | **Query** | **Observed** | **Mr(expt)** | **Mr(calc)** | **Delta** | **Miss** | **Score** | **Expect** | **Rank** | **Peptide** |
| --- | --- | --- | --- | --- | --- | --- | --- | --- | --- | --- |
|  | 1100 | 617.3092 | 616.3019 | 615.3704 | 0.9315 | 1 | 22 | 1.9 | 1 | K.KNNIK.L |
|  | 2507 | 863.4213 | 862.4140 | 862.4218 | -0.0078 | 0 | 20 | 1.8 | 1 | R.EMLQQSK.I |
|  | 4498 | **503.2768** | **1004.5390** | **1004.5576** | **-0.0186** | **1** | **5** | **51** | **5** | **K.IMKDILEK.K** |
|  | 4529 | 504.7289 | 1007.4431 | 1007.6491 | -0.2060 | 1 | 8 | 28 | 6 | R.GTKVILHLK.E |
|  | 6757 | 584.7971 | 1167.5797 | 1167.5632 | 0.0165 | 0 | 10 | 15 | 5 | K.LGIHEDSQNR.K |
|  | 7785 | 610.3678 | 1218.7210 | 1219.4849 | -0.7639 | 0 | 1 | 1.1e+002 | 7 | R.DNSTMGYMAAK.K |
|  | 8036 | 411.8792 | 1232.6159 | 1232.6798 | -0.0639 | 1 | 3 | 71 | 8 | R.EMLQQSKILK.V 8066 |
|  | 8244 | 622.3537 | 1242.6928 | 1241.6979 | 0.9949 | 0 | 41 | 0.012 | 1 | K.ADLINNLGTIAK.S 8227 |
|  | 10305 | 450.2784 | 1347.8135 | 1347.6572 | 0.1563 | 0 | 4 | 65 | 8 | K.HFSVEGQLEFR.A |
|  | 13217 | 757.3913 | 1512.7680 | 1512.7784 | -0.0103 | 0 | 78 | 2.2e-006 | 1 | R.GVVDSEDLPLNISR.E |
|  | 13450 | 509.9157 | 1526.7252 | 1526.7365 | -0.0113 | 0 | 20 | 1.1 | 1 | K.SLTNDWEDHLAVK.H |
|  | 13986 | 780.4180 | 1558.8214 | 1559.8155 | -0.9941 | 1 | 2 | 75 | 2 | R.ELISNSSDALDKIR.Y |
|  | 20489 | 672.4362 | 2014.2867 | 2014.0371 | 0.2496 | 1 | 19 | 0.81 | 1 | K.VILHLKEDQTEYLEER.R 20481 |
|  | 25328 | **647.3115** | **2585.2170** | **2586.2610** | **-1.0441** | **1** | **5** | **23** | **2** | **R.RVFIMDNCEELIPEYLNFIR.G** |
|  | 29877 | 1225.1727 | 3672.4964 | 3672.7844 | -0.2880 | 1 | 1 | 22 | 6 | K.SGTKAFMEALQAGADISMIGQFGVGFYSAYLVAEK.V |
|  | 30702 | **1046.4877** | **4181.9216** | **4181.7424** | **0.1792** | **1** | **3** | **13** | **3** | **K.LGLGIDEDDPTADDTSAAVTEEMPPLEGDDDTSRMEEVD.-** |
|  | 31177 | **969.2288** | **4841.1077** | **4841.1921** | **-0.0844** | **0** | **3** | **6.9** | **1** | **-.MPEETQTQDQPMEEEEVETFAFQAEIAQLMSLIINTFYSNK.E** |

| **476.** | Q6UW68\|TM205_HUMAN    **Mass:** 21469    **Score:** 83     **Queries matched:** 8   **emPAI:** 0.64 |
| --- | --- |
|  | Transmembrane protein 205 OS=Homo sapiens GN=TMEM205 PE=1 SV=1 |

|  | **Query** | **Observed** | **Mr(expt)** | **Mr(calc)** | **Delta** | **Miss** | **Score** | **Expect** | **Rank** | **Peptide** |
| --- | --- | --- | --- | --- | --- | --- | --- | --- | --- | --- |
|  | 5609 | **543.3824** | **1084.7503** | **1085.5717** | **-0.8213** | **0** | **8** | **21** | **2** | **M.EEGGNLGGLIK.M** |
|  | 12126 | **725.3699** | **1448.7252** | **1448.7333** | **-0.0081** | **0** | **26** | **0.38** | **1** | **R.TTAAMWALQTVEK.E** |
|  | 12391 | **489.2724** | **1464.7955** | **1464.7283** | **0.0672** | **0** | **(2)** | **75** | **8** | **R.TTAAMWALQTVEK.E** |
|  | 16562 | **861.9073** | **1721.8001** | **1721.8122** | **-0.0120** | **0** | **68** | **1.9e-005** | **1** | **R.GLGGEVPGSHQGPDPYR.Q** 16559 16560 16561 |
|  | 28130 | 521.6007 | 3123.5606 | 3124.6217 | -1.0611 | 0 | 1 | 40 | 3 | K.MVHLLVLSGAWGMQMWVTFVSGFLLFR.S |

| **477.** | P19823\|ITIH2_HUMAN    **Mass:** 106853   **Score:** 82     **Queries matched:** 35   **emPAI:** 0.07 |
| --- | --- |
|  | Inter-alpha-trypsin inhibitor heavy chain H2 OS=Homo sapiens GN=ITIH2 PE=1 SV=2 |

|  | **Query** | **Observed** | **Mr(expt)** | **Mr(calc)** | **Delta** | **Miss** | **Score** | **Expect** | **Rank** | **Peptide** |
| --- | --- | --- | --- | --- | --- | --- | --- | --- | --- | --- |
|  | 1649 | **401.2841** | **800.5536** | **800.4868** | **0.0668** | **1** | **13** | **11** | **9** | **K.GKTAGLVR.S** |
|  | 2476 | 431.2256 | 860.4367 | 860.4140 | 0.0226 | 0 | 8 | 35 | 10 | K.LGSYEHR.I 2455 |
|  | 2491 | 431.7627 | 861.5108 | 860.4603 | 1.0505 | 0 | 11 | 16 | 2 | R.NDLISATK.T |
|  | 4754 | **514.2169** | **1026.4192** | **1025.4885** | **0.9306** | **0** | **7** | **30** | **4** | **R.MATTMIQSK.V** |
|  | 6257 | **567.2573** | **1132.5001** | **1131.6149** | **0.8852** | **1** | **0** | **1.5e+002** | **6** | **K.FQLVAENRR.Y** |
|  | 6759 | 584.7974 | 1167.5802 | 1168.5183 | -0.9381 | 0 | 5 | 47 | 6 | R.SSALDMENFR.T |
|  | 7760 | **407.1331** | **1218.3774** | **1217.6227** | **0.7547** | **0** | **11** | **9.8** | **3** | **K.EMSFSVLLHR.V** 7745 7759 7762 7783 |
|  | 8135 | **619.8101** | **1237.6057** | **1237.6163** | **-0.0106** | **0** | **42** | **0.0082** | **1** | **R.LSNENHGIAQR.I** 8137 |
|  | 10125 | 669.8147 | 1337.6148 | 1336.7139 | 0.9009 | 0 | 7 | 26 | 10 | K.FYNQVSTPLLR.N |
|  | 10829 | **459.0112** | **1374.0117** | **1374.6337** | **-0.6220** | **0** | **5** | **29** | **5** | **K.DPEKPEASMEVK.G** 10845 |
|  | 14329 | **791.9252** | **1581.8359** | **1581.8474** | **-0.0115** | **0** | **50** | **0.0013** | **1** | **K.IQPSGGTNINEALLR.A** 14318 14330 |
|  | 14384 | 529.3041 | 1584.8904 | 1583.8348 | 1.0556 | 0 | 9 | 16 | 2 | R.VENDPHFIIYLPK.S |
|  | 15382 | **550.2944** | **1647.8613** | **1647.8502** | **0.0111** | **1** | **4** | **49** | **2** | **K.QTVEAMKTILDDLR.A** |
|  | 16006 | 844.4204 | 1686.8263 | 1687.8087 | -0.9824 | 1 | 10 | 11 | 3 | K.DPEKPEASMEVKGQK.L 16032 |
|  | 17434 | **594.8461** | **1781.5164** | **1781.8730** | **-0.3566** | **1** | **1** | **59** | **3** | **K.TAGLVRSSALDMENFR.T** |
|  | 17595 | 895.9478 | 1789.8811 | 1788.9523 | 0.9288 | 0 | 1 | 96 | 8 | K.HLEVDVWVIEPQGLR.F |
|  | 21664 | **708.4086** | **2122.2041** | **2121.1219** | **1.0822** | **0** | **1** | **61** | **3** | **K.VVNNSPQPQNVVFDVQIPK.G** |
|  | 22789 | **561.2785** | **2241.0849** | **2240.1266** | **0.9582** | **0** | **5** | **29** | **4** | **R.FLHVPDTFEGHFDGVPVISK.G** |
|  | 27643 | **603.2995** | **3011.4611** | **3010.3576** | **1.1036** | **1** | **4** | **21** | **4** | **R.YQRSLPGESEEMMEEVDQVTLYSYK.V** |
|  | 28100 | **780.1487** | **3116.5659** | **3116.5812** | **-0.0153** | **0** | **6** | **14** | **1** | **R.SILQMSLDHHIVTPLTSLVIENEAGDER.M** |
|  | 29677 | **1193.0712** | **3576.1917** | **3575.8083** | **0.3834** | **1** | **0** | **27** | **1** | **K.VVNNSPQPQNVVFDVQIPKGAFISNFSMTVDGK.T** |
|  | 29889 | **921.6879** | **3682.7224** | **3682.7441** | **-0.0217** | **0** | **9** | **4.2** | **1** | **R.NVQFNYPHTSVTDVTQNNFHNYFGGSEIVVAGK.F** |
|  | 30044 | **1251.2909** | **3750.8508** | **3749.8921** | **0.9588** | **0** | **0** | **31** | **10** | **K.LDQIESVITATSANTQLVLETLAQMDDLQDFLSK.D** |
|  | 30472 | **1342.5341** | **4024.5804** | **4025.0027** | **-0.4223** | **0** | **0** | **18** | **1** | **K.VVPDSTPSWANPSPTPVISMLAQGSQVLESTPPPHVMR.V** |
|  | 30595 | **1024.0190** | **4092.0471** | **4091.2405** | **0.8065** | **1** | **7** | **5.6** | **1** | **R.AIFILNEANNLGLLDPNSVSLIILVSDGDPTVGELKLSK.I** |

| **478.** | P07951\|TPM2_HUMAN    **Mass:** 32945    **Score:** 82     **Queries matched:** 26   **emPAI:** 0.38 |
| --- | --- |
|  | Tropomyosin beta chain OS=Homo sapiens GN=TPM2 PE=1 SV=1 |

|  | **Query** | **Observed** | **Mr(expt)** | **Mr(calc)** | **Delta** | **Miss** | **Score** | **Expect** | **Rank** | **Peptide** |
| --- | --- | --- | --- | --- | --- | --- | --- | --- | --- | --- |
|  | 1006 | **574.9904** | **573.9831** | **573.3486** | **0.6346** | **1** | **11** | **17** | **2** | **M.DAIKK.K** |
|  | 1129 | 630.3877 | 629.3804 | 630.3588 | -0.9784 | 0 | 10 | 24 | 6 | K.LLEEK.L |
|  | 1352 | 716.3771 | 715.3699 | 716.3453 | -0.9754 | 0 | 7 | 48 | 4 | K.ENAIDR.A |
|  | 1361 | 722.3414 | 721.3341 | 721.3395 | -0.0054 | 0 | 13 | 7.8 | 1 | R.AEFAER.S |
|  | 1544 | 397.2009 | 792.3873 | 793.4190 | -1.0317 | 1 | 3 | 1e+002 | 7 | K.KMQMLK.L |
|  | 2293 | 424.3009 | 846.5872 | 845.4606 | 1.1266 | 1 | 3 | 1.1e+002 | 9 | K.LKEAETR.A 2284 |
|  | 3859 | 481.2626 | 960.5106 | 959.5222 | 0.9884 | 1 | 2 | 1.2e+002 | 6 | K.VIENRAMK.D |
|  | 4074 | 489.2248 | 976.4350 | 975.5171 | 0.9178 | 1 | (2) | 1.3e+002 | 4 | K.VIENRAMK.D |
|  | 8083 | **618.3342** | **1234.6538** | **1233.5837** | **1.0701** | **1** | **6** | **38** | **1** | **R.SEERAEVAESK.C** |
|  | 8233 | 622.3256 | 1242.6366 | 1242.6456 | -0.0090 | 0 | 34 | 0.056 | 1 | R.IQLVEEELDR.A |
|  | 9334 | 649.8831 | 1297.7516 | 1297.7605 | -0.0089 | 1 | 28 | 0.21 | 1 | R.KLVILEGELER.S 9317 9335 |
|  | 12015 | 722.4007 | 1442.7868 | 1442.7980 | -0.0112 | 1 | 21 | 1 | 1 | R.LATALQKLEEAEK.A 12016 |
|  | 12333 | **487.9703** | **1460.8892** | **1459.7267** | **1.1625** | **1** | **6** | **34** | **1** | **K.KATDAEADVASLNR.R** 12282 |
|  | 12554 | 738.8392 | 1475.6639 | 1475.6739 | -0.0100 | 1 | 63 | 6.9e-005 | 1 | K.LEEAEKAADESER.G 12555 12567 |
|  | 12795 | **745.3789** | **1488.7433** | **1487.7328** | **1.0105** | **1** | **2** | **77** | **4** | **K.ATDAEADVASLNRR.I** |
|  | 15793 | **558.3575** | **1672.0506** | **1670.8839** | **1.1667** | **1** | **11** | **7** | **2** | **K.LVILEGELERSEER.A** |
|  | 15935 | 841.9561 | 1681.8977 | 1681.7538 | 0.1438 | 1 | 2 | 64 | 8 | K.DEEKMELQEMQLK.E |
|  | 16644 | 864.4434 | 1726.8722 | 1726.8849 | -0.0128 | 1 | 34 | 0.039 | 1 | R.IQLVEEELDRAQER.L |
|  | 19222 | **637.0802** | **1908.2188** | **1907.9364** | **0.2824** | **1** | **3** | **36** | **9** | **K.LEKTIDDLEDEVYAQK.M** |

| **479.** | P09493\|TPM1_HUMAN    **Mass:** 32746    **Score:** 82     **Queries matched:** 16   **emPAI:** 0.39 |
| --- | --- |
|  | Tropomyosin alpha-1 chain OS=Homo sapiens GN=TPM1 PE=1 SV=2 |

|  | **Query** | **Observed** | **Mr(expt)** | **Mr(calc)** | **Delta** | **Miss** | **Score** | **Expect** | **Rank** | **Peptide** |
| --- | --- | --- | --- | --- | --- | --- | --- | --- | --- | --- |
|  | 1006 | 574.9904 | 573.9831 | 573.3486 | 0.6346 | 1 | 11 | 17 | 2 | M.DAIKK.K |
|  | 1352 | 716.3771 | 715.3699 | 716.3453 | -0.9754 | 0 | 7 | 48 | 4 | K.ENALDR.A |
|  | 1361 | 722.3414 | 721.3341 | 721.3395 | -0.0054 | 0 | 13 | 7.8 | 1 | R.AEFAER.S |
|  | 1544 | 397.2009 | 792.3873 | 793.4190 | -1.0317 | 1 | 3 | 1e+002 | 7 | K.KMQMLK.L |
|  | 2293 | 424.3009 | 846.5872 | 845.4606 | 1.1266 | 1 | 3 | 1.1e+002 | 9 | K.LKEAETR.A 2284 |
|  | 8233 | 622.3256 | 1242.6366 | 1242.6456 | -0.0090 | 0 | 34 | 0.056 | 1 | R.IQLVEEELDR.A |
|  | 12015 | 722.4007 | 1442.7868 | 1442.7980 | -0.0112 | 1 | 21 | 1 | 1 | R.LATALQKLEEAEK.A 12016 |
|  | 12333 | 487.9703 | 1460.8892 | 1459.7267 | 1.1625 | 1 | 6 | 34 | 1 | K.KATDAEADVASLNR.R 12282 |
|  | 12554 | 738.8392 | 1475.6639 | 1475.6739 | -0.0100 | 1 | 63 | 6.9e-005 | 1 | K.LEEAEKAADESER.G 12555 12567 |
|  | 12795 | 745.3789 | 1488.7433 | 1487.7328 | 1.0105 | 1 | 2 | 77 | 4 | K.ATDAEADVASLNRR.I |
|  | 16644 | 864.4434 | 1726.8722 | 1726.8849 | -0.0128 | 1 | 34 | 0.039 | 1 | R.IQLVEEELDRAQER.L |

| **480.** | Q6IAN0\|DRS7B_HUMAN    **Mass:** 35382    **Score:** 82     **Queries matched:** 9   **emPAI:** 0.22 |
| --- | --- |
|  | Dehydrogenase/reductase SDR family member 7B OS=Homo sapiens GN=DHRS7B PE=1 SV=2 |

|  | **Query** | **Observed** | **Mr(expt)** | **Mr(calc)** | **Delta** | **Miss** | **Score** | **Expect** | **Rank** | **Peptide** |
| --- | --- | --- | --- | --- | --- | --- | --- | --- | --- | --- |
|  | 990 | **572.3035** | **571.2963** | **571.3693** | **-0.0731** | **1** | **7** | **42** | **2** | **R.KSLPK.V** 989 |
|  | 4397 | 500.2923 | 998.5700 | 998.6025 | -0.0325 | 1 | 10 | 16 | 10 | R.LLQWVRGK.A |
|  | 9343 | **650.2985** | **1298.5825** | **1298.5925** | **-0.0100** | **0** | **41** | **0.012** | **1** | **R.YGVMDTTTAQGR.S** |
|  | 9534 | **437.2705** | **1308.7896** | **1309.6072** | **-0.8176** | **0** | **7** | **29** | **4** | **R.GTIMDTTVDVDK.R** |
|  | 9606 | **438.6988** | **1313.0747** | **1313.6438** | **-0.5691** | **1** | **1** | **70** | **10** | **K.ECAKVFYAAGAK.L** |
|  | 9676 | 658.3496 | 1314.6847 | 1314.5874 | 0.0972 | 0 | (7) | 31 | 7 | R.YGVMDTTTAQGR.S |
|  | 12687 | **495.2266** | **1482.6580** | **1481.8090** | **0.8491** | **0** | **1** | **86** | **3** | **R.SPVEVAQDVLAAVGK.K** |
|  | 14139 | **785.3974** | **1568.7802** | **1568.7909** | **-0.0106** | **0** | **75** | **3.8e-006** | **1** | **R.VMETNYFGPVALTK.A** |

| **481.** | P31040\|DHSA_HUMAN    **Mass:** 73672    **Score:** 82     **Queries matched:** 12   **emPAI:** 0.16 |
| --- | --- |
|  | Succinate dehydrogenase [ubiquinone] flavoprotein subunit, mitochondrial OS=Homo sapiens GN=SDHA PE=1 SV=2 |

|  | **Query** | **Observed** | **Mr(expt)** | **Mr(calc)** | **Delta** | **Miss** | **Score** | **Expect** | **Rank** | **Peptide** |
| --- | --- | --- | --- | --- | --- | --- | --- | --- | --- | --- |
|  | 1188 | **648.3890** | **647.3818** | **647.3642** | **0.0175** | **0** | **9** | **28** | **2** | **R.YAPVAK.D** |
|  | 2793 | **442.2351** | **882.4556** | **882.4420** | **0.0136** | **1** | **11** | **12** | **2** | **K.ESRGAHAR.E** |
|  | 3878 | 482.2711 | 962.5276 | 961.5127 | 1.0148 | 1 | 10 | 17 | 7 | -.MSGVRGLSR.L |
|  | 4105 | 490.2605 | 978.5065 | 977.5076 | 0.9989 | 1 | (6) | 42 | 8 | -.MSGVRGLSR.L 4109 |
|  | 4855 | 517.2660 | 1032.5175 | 1033.5669 | -1.0493 | 1 | 10 | 20 | 5 | K.LRFADGSIR.T |
|  | 9231 | 647.3100 | 1292.6054 | 1292.6837 | -0.0783 | 1 | 9 | 19 | 9 | R.AKNTVVATGGYGR.T |
|  | 11329 | **468.9181** | **1403.7325** | **1403.7409** | **-0.0084** | **1** | **4** | **48** | **3** | **R.KHTLSYVDVGTGK.V** |
|  | 12506 | **737.4219** | **1472.8292** | **1472.8351** | **-0.0059** | **0** | **31** | **0.091** | **1** | **R.LGANSLLDLVVFGR.A** |
|  | 17919 | 604.9623 | 1811.8652 | 1810.8737 | 0.9915 | 0 | 36 | 0.026 | 1 | R.IDEYDYSKPIQGQQK.K |
|  | 29133 | **1122.8573** | **3365.5501** | **3365.5632** | **-0.0131** | **0** | **44** | **0.0015** | **1** | **R.AGLPCQDLEFVQFHPTGIYGAGCLITEGCR.G** 29132 |

| **482.** | P61981\|1433G_HUMAN    **Mass:** 28456    **Score:** 82     **Queries matched:** 14   **emPAI:** 0.13 |
| --- | --- |
|  | 14-3-3 protein gamma OS=Homo sapiens GN=YWHAG PE=1 SV=2 |

|  | **Query** | **Observed** | **Mr(expt)** | **Mr(calc)** | **Delta** | **Miss** | **Score** | **Expect** | **Rank** | **Peptide** |
| --- | --- | --- | --- | --- | --- | --- | --- | --- | --- | --- |
|  | 1097 | 616.1310 | 615.1238 | 614.3500 | 0.7738 | 0 | 1 | 1.9e+002 | 2 | K.NVVGAR.R |
|  | 1181 | **646.7200** | **645.7127** | **645.3697** | **0.3430** | **1** | **8** | **32** | **6** | **R.EKIEK.E** |
|  | 3045 | **452.7598** | **903.5050** | **902.5073** | **0.9978** | **0** | **11** | **14** | **5** | **R.VISSIEQK.T** |
|  | 6271 | **567.7708** | **1133.5271** | **1133.5353** | **-0.0082** | **0** | **18** | **2.8** | **2** | **K.AYSEAHEISK.E** |
|  | 7120 | 397.2190 | 1188.6351 | 1188.6536 | -0.0186 | 0 | 11 | 12 | 1 | K.DSTLIMQLLR.D |
|  | 15292 | **548.3200** | **1641.9382** | **1642.7798** | **-0.8416** | **0** | **7** | **21** | **1** | **K.NVTELNEPLSNEER.N** |
|  | 28874 | **1101.2040** | **3300.5901** | **3300.6071** | **-0.0170** | **1** | **71** | **3.6e-006** | **1** | **K.TAFDDAIAELDTLNEDSYKDSTLIMQLLR.D** 28873 28875 28876 28880 28881 |
|  | 28925 | **829.8880** | **3315.5229** | **3316.6020** | **-1.0791** | **1** | **(3)** | **24** | **2** | **K.TAFDDAIAELDTLNEDSYKDSTLIMQLLR.D** |
|  | 29171 | **1690.2941** | **3378.5736** | **3379.5110** | **-0.9374** | **1** | **3** | **20** | **6** | **K.DSTLIMQLLRDNLTLWTSDQQDDDGGEGNN.-** |

| **483.** | P05186\|PPBT_HUMAN    **Mass:** 57611    **Score:** 81     **Queries matched:** 21   **emPAI:** 0.13 |
| --- | --- |
|  | Alkaline phosphatase, tissue-nonspecific isozyme OS=Homo sapiens GN=ALPL PE=1 SV=4 |

|  | **Query** | **Observed** | **Mr(expt)** | **Mr(calc)** | **Delta** | **Miss** | **Score** | **Expect** | **Rank** | **Peptide** |
| --- | --- | --- | --- | --- | --- | --- | --- | --- | --- | --- |
|  | 3464 | **933.5302** | **932.5229** | **932.5291** | **-0.0062** | **0** | **11** | **16** | **1** | **K.SVGIVTTTR.V** 3439 |
|  | 4836 | **516.7867** | **1031.5588** | **1031.5070** | **0.0518** | **0** | **13** | **9.8** | **1** | **R.DIDVIMGGGR.K** |
|  | 6849 | **588.2947** | **1174.5749** | **1175.5969** | **-1.0219** | **1** | **1** | **1.4e+002** | **6** | **R.DIDVIMGGGRK.Y** |
|  | 7943 | 614.8138 | 1227.6130 | 1227.6109 | 0.0022 | 1 | 9 | 21 | 5 | R.IDHGHHEGKAK.Q 7968 |
|  | 8876 | **637.8312** | **1273.6478** | **1273.6554** | **-0.0076** | **0** | **6** | **38** | **2** | **R.LDGLDLVDTWK.S** |
|  | 9482 | **653.3677** | **1304.7209** | **1303.7096** | **1.0113** | **1** | **1** | **98** | **4** | **K.DAGKSVGIVTTTR.V** |
|  | 10549 | 681.3309 | 1360.6473 | 1360.6583 | -0.0110 | 0 | 48 | 0.0023 | 1 | K.ANEGTVGVSAATER.S 10538 |
|  | 10841 | **688.3510** | **1374.6875** | **1373.6436** | **1.0439** | **0** | **1** | **1.1e+002** | **10** | **K.GQLHHNPGEETR.L** |
|  | 11976 | **721.3630** | **1440.7114** | **1440.6368** | **0.0746** | **1** | **7** | **25** | **2** | **K.TDVEYESDEKAR.G** |
|  | 14508 | **796.8069** | **1591.5992** | **1591.7624** | **-0.1632** | **0** | **6** | **27** | **2** | **R.CNTTQGNEVTSILR.W** |
|  | 15699 | **556.5998** | **1666.7775** | **1666.7812** | **-0.0036** | **0** | **1** | **85** | **5** | **R.VNHATPSAAYAHSADR.D** |
|  | 15912 | **420.9818** | **1679.8981** | **1680.8029** | **-0.9048** | **0** | **1** | **85** | **9** | **R.GNSIFGLAPMLSDTDK.K** |
|  | 17335 | **593.1525** | **1776.4358** | **1776.9257** | **-0.4900** | **1** | **8** | **11** | **1** | **R.DQAQETLKYALELQK.L** |
|  | 18259 | 918.4733 | 1834.9320 | 1834.8956 | 0.0364 | 1 | 8 | 18 | 3 | R.SRCNTTQGNEVTSILR.W |
|  | 19818 | **652.3442** | **1954.0107** | **1953.9652** | **0.0455** | **0** | **5** | **33** | **4** | **K.NVIMFLGDGMGVSTVTAAR.I** |
|  | 23970 | **1201.5604** | **2401.1063** | **2401.1220** | **-0.0157** | **0** | **53** | **0.00038** | **1** | **K.TYNTNAQVPDSAGTATAYLCGVK.A** |
|  | 25878 | **893.4751** | **2677.4035** | **2678.3884** | **-0.9849** | **1** | **0** | **54** | **8** | **K.LNTNVAKNVIMFLGDGMGVSTVTAAR.I** |
|  | 29533 | **881.4299** | **3521.6906** | **3521.7063** | **-0.0157** | **0** | **5** | **13** | **1** | **R.AIGQAGSLTSSEDTLTVVTADHSHVFTFGGYTPR.G** |

| **484.** | Q9UBR2\|CATZ_HUMAN    **Mass:** 34530    **Score:** 81     **Queries matched:** 6   **emPAI:** 0.23 |
| --- | --- |
|  | Cathepsin Z OS=Homo sapiens GN=CTSZ PE=1 SV=1 |

|  | **Query** | **Observed** | **Mr(expt)** | **Mr(calc)** | **Delta** | **Miss** | **Score** | **Expect** | **Rank** | **Peptide** |
| --- | --- | --- | --- | --- | --- | --- | --- | --- | --- | --- |
|  | 4548 | **505.7451** | **1009.4756** | **1009.4829** | **-0.0073** | **0** | **76** | **3.5e-006** | **1** | **R.VGDYGSLSGR.E** |
|  | 18384 | 922.9433 | 1843.8720 | 1842.9013 | 0.9708 | 1 | 10 | 11 | 2 | R.NYTLWRVGDYGSLSGR.E 18385 |
|  | 18825 | 939.4371 | 1876.8597 | 1876.8665 | -0.0068 | 0 | 35 | 0.031 | 2 | R.YNLAIEEHCTFGDPIV.- |
|  | 24902 | **846.4656** | **2536.3751** | **2537.4635** | **-1.0884** | **0** | **2** | **44** | **3** | **R.GPGWRPLLLLVLLAGAAQGGLYFR.R** |
|  | 31500 | **1377.1041** | **5504.3874** | **5504.4157** | **-0.0283** | **0** | **4** | **3.9** | **1** | **K.GAWPSTLLSVQNVIDCGNAGSCEGGNDLSVWDYAHQHGIPDETCNNYQAK.D** |

| **485.** | P61978\|HNRPK_HUMAN    **Mass:** 51230    **Score:** 81     **Queries matched:** 22   **emPAI:** 0.23 |
| --- | --- |
|  | Heterogeneous nuclear ribonucleoprotein K OS=Homo sapiens GN=HNRNPK PE=1 SV=1 |

|  | **Query** | **Observed** | **Mr(expt)** | **Mr(calc)** | **Delta** | **Miss** | **Score** | **Expect** | **Rank** | **Peptide** |
| --- | --- | --- | --- | --- | --- | --- | --- | --- | --- | --- |
|  | 2611 | **436.2687** | **870.5228** | **870.4712** | **0.0516** | **0** | **11** | **15** | **1** | **R.GPPPPPPGR.G** |
|  | 2637 | 437.2529 | 872.4912 | 872.4967 | -0.0055 | 0 | 8 | 33 | 7 | K.DLAGSIIGK.G |
|  | 4390 | **500.2688** | **998.5231** | **997.3811** | **1.1420** | **0** | **7** | **36** | **5** | **R.DYDDMSPR.R** 4384 4385 |
|  | 4758 | **514.2583** | **1026.5020** | **1026.5723** | **-0.0702** | **1** | **9** | **20** | **2** | **R.RGPPPPPPGR.G** 4749 4767 |
|  | 4818 | 516.2776 | 1030.5406 | 1031.5335 | -0.9929 | 0 | 8 | 31 | 10 | R.GRPVGFPMR.G 4857 |
|  | 7892 | **409.4391** | **1225.2956** | **1224.6575** | **0.6382** | **1** | **11** | **7.8** | **1** | **K.QIRHESGASIK.I** |
|  | 8586 | **420.7054** | **1259.0944** | **1258.5677** | **0.5267** | **0** | **8** | **14** | **1** | **K.IDEPLEGSEDR.I** |
|  | 8816 | **424.6599** | **1270.9579** | **1270.6993** | **0.2586** | **1** | **14** | **3.6** | **1** | **K.DLAGSIIGKGGQR.I** |
|  | 10161 | **670.9020** | **1339.7894** | **1339.7962** | **-0.0068** | **0** | **65** | **4.5e-005** | **1** | **K.IILDLISESPIK.G** |
|  | 11610 | **474.2669** | **1419.7788** | **1420.8303** | **-1.0515** | **1** | **3** | **65** | **9** | **R.ARNLPLPPPPPPR.G** |
|  | 13309 | 506.9440 | 1517.8102 | 1517.9293 | -0.1192 | 0 | 6 | 32 | 9 | R.LLIHQSLAGGIIGVK.G |
|  | 13811 | **775.3882** | **1548.7618** | **1548.6450** | **0.1169** | **0** | **2** | **74** | **7** | **K.LFQECCPHSTDR.V** |
|  | 14531 | 797.8264 | 1593.6383 | 1594.6933 | -1.0550 | 0 | 3 | 60 | 8 | K.RPAEDMEEEQAFK.R |
|  | 17407 | **890.8966** | **1779.7787** | **1779.7911** | **-0.0125** | **0** | **7** | **23** | **1** | **R.TDYNASVSVPDSSGPER.I** |
|  | 19305 | **959.0138** | **1916.0130** | **1916.0255** | **-0.0125** | **0** | **31** | **0.074** | **1** | **R.GSYGDLGGPIITTQVTIPK.D** 19289 |
|  | 25352 | **863.7971** | **2588.3695** | **2588.3810** | **-0.0114** | **0** | **25** | **0.2** | **1** | **R.IITITGTQDQIQNAQYLLQNSVK.Q** |

| **486.** | P12429\|ANXA3_HUMAN    **Mass:** 36524    **Score:** 81     **Queries matched:** 14   **emPAI:** 0.34 |
| --- | --- |
|  | Annexin A3 OS=Homo sapiens GN=ANXA3 PE=1 SV=3 |

|  | **Query** | **Observed** | **Mr(expt)** | **Mr(calc)** | **Delta** | **Miss** | **Score** | **Expect** | **Rank** | **Peptide** |
| --- | --- | --- | --- | --- | --- | --- | --- | --- | --- | --- |
|  | 4091 | 489.8052 | 977.5957 | 977.5182 | 0.0776 | 0 | 17 | 3.3 | 2 | K.QDAQILYK.A |
|  | 5417 | **537.2708** | **1072.5271** | **1071.5284** | **0.9987** | **0** | **31** | **0.16** | **1** | **-.MASIWVGHR.G** 5415 5416 |
|  | 5420 | **537.2919** | **1072.5693** | **1072.5764** | **-0.0071** | **0** | **28** | **0.3** | **1** | **R.SEIDLLDIR.T** |
|  | 5448 | **538.3099** | **1074.6052** | **1074.6107** | **-0.0055** | **0** | **51** | **0.0015** | **1** | **K.MLISILTER.S** |
|  | 5688 | **546.7898** | **1091.5650** | **1090.6056** | **0.9594** | **0** | **(8)** | **25** | **5** | **K.MLISILTER.S** |
|  | 10121 | **446.7750** | **1337.3031** | **1337.7317** | **-0.4286** | **1** | **6** | **24** | **2** | **M.ASIWVGHRGTVR.D** 10120 |
|  | 10321 | **675.3780** | **1348.7414** | **1349.6503** | **-0.9089** | **0** | **2** | **89** | **1** | **K.DISQAYYTVYK.K** |
|  | 11971 | **721.3524** | **1440.6903** | **1440.6984** | **-0.0081** | **0** | **2** | **90** | **2** | **K.SDTSGDYEITLLK.I** |
|  | 17436 | **891.9108** | **1781.8070** | **1780.8156** | **0.9914** | **0** | **49** | **0.0015** | **1** | **R.DYPDFSPSVDAEAIQK.A** 17423 |
|  | 27151 | **728.8818** | **2911.4983** | **2911.5113** | **-0.0131** | **1** | **4** | **24** | **1** | **K.DIVDSIKGELSGHFEDLLLAIVNCVR.N** |

| **487.** | Q9BS26\|ERP44_HUMAN    **Mass:** 47341    **Score:** 80     **Queries matched:** 9   **emPAI:** 0.08 |
| --- | --- |
|  | Endoplasmic reticulum resident protein ERp44 OS=Homo sapiens GN=ERP44 PE=1 SV=1 |

|  | **Query** | **Observed** | **Mr(expt)** | **Mr(calc)** | **Delta** | **Miss** | **Score** | **Expect** | **Rank** | **Peptide** |
| --- | --- | --- | --- | --- | --- | --- | --- | --- | --- | --- |
|  | 2967 | **450.2432** | **898.4718** | **898.3823** | **0.0895** | **1** | **(9)** | **19** | **3** | **R.NGMMMKR.E** |
|  | 2970 | 450.2784 | 898.5423 | 898.3823 | 0.1600 | 1 | 12 | 10 | 3 | R.NGMMMKR.E |
|  | 6648 | 580.3230 | 1158.6314 | 1158.5348 | 0.0967 | 1 | 2 | 1e+002 | 1 | K.LFRNGMMMK.R |
|  | 12000 | 722.3110 | 1442.6075 | 1442.6209 | -0.0133 | 0 | 61 | 9.1e-005 | 1 | R.VDCDQHSDIAQR.Y 12001 12002 |
|  | 26782 | 945.7994 | 2834.3765 | 2835.4041 | -1.0276 | 0 | 2 | 34 | 3 | R.EITFENGEELTEEGLPFLILFHMK.E |
|  | 29596 | **1184.2412** | **3549.7018** | **3549.7238** | **-0.0220** | **1** | **4** | **14** | **1** | **R.FSQMLHPIFEEASDVIKEEFPNENQVVFAR.V** |
|  | 30751 | **848.4221** | **4237.0739** | **4235.9609** | **1.1131** | **0** | **0** | **24** | **2** | **R.YSGDNIIYKPPGHSAPDMVYLGAMTNFDVTYNWIQDK.C** |

| **488.** | P31943\|HNRH1_HUMAN    **Mass:** 49484    **Score:** 80     **Queries matched:** 11   **emPAI:** 0.08 |
| --- | --- |
|  | Heterogeneous nuclear ribonucleoprotein H OS=Homo sapiens GN=HNRNPH1 PE=1 SV=4 |

|  | **Query** | **Observed** | **Mr(expt)** | **Mr(calc)** | **Delta** | **Miss** | **Score** | **Expect** | **Rank** | **Peptide** |
| --- | --- | --- | --- | --- | --- | --- | --- | --- | --- | --- |
|  | 1721 | **403.2472** | **804.4798** | **803.4249** | **1.0549** | **1** | **10** | **20** | **2** | **K.SSRAEVR.T** |
|  | 10157 | **670.8404** | **1339.6662** | **1338.6490** | **1.0173** | **0** | **3** | **68** | **4** | **M.MLGTEGGEGFVVK.V** |
|  | 10977 | **461.6902** | **1382.0488** | **1382.6691** | **-0.6203** | **1** | **5** | **29** | **5** | **R.GYNSIGRGAGFER.M** |
|  | 16834 | **580.6434** | **1738.9083** | **1739.8413** | **-0.9331** | **1** | **4** | **45** | **3** | **R.FFSDCKIQNGAQGIR.F** |
|  | 16865 | **581.3109** | **1740.9109** | **1740.8539** | **0.0570** | **1** | **3** | **53** | **4** | **-.MMLGTEGGEGFVVKVR.G** |
|  | 18333 | **921.4440** | **1840.8735** | **1840.8843** | **-0.0108** | **0** | **65** | **3.3e-005** | **1** | **R.STGEAFVQFASQEIAEK.A** 18327 18334 |
|  | 18969 | 630.3107 | 1887.9104 | 1887.9196 | -0.0092 | 0 | 5 | 34 | 6 | K.LMAMQRPGPYDRPGAGR.G |
|  | 22166 | 726.7127 | 2177.1161 | 2176.9947 | 0.1215 | 0 | 2 | 54 | 3 | R.VTGEADVEFATHEDAVAAMSK.D |
|  | 26484 | **697.8585** | **2787.4050** | **2786.3399** | **1.0651** | **1** | **3** | **25** | **5** | **R.FIYTREGRPSGEAFVELESEDEVK.L** |

| **489.** | P55795\|HNRH2_HUMAN    **Mass:** 49517    **Score:** 80     **Queries matched:** 9   **emPAI:** 0.08 |
| --- | --- |
|  | Heterogeneous nuclear ribonucleoprotein H2 OS=Homo sapiens GN=HNRNPH2 PE=1 SV=1 |

|  | **Query** | **Observed** | **Mr(expt)** | **Mr(calc)** | **Delta** | **Miss** | **Score** | **Expect** | **Rank** | **Peptide** |
| --- | --- | --- | --- | --- | --- | --- | --- | --- | --- | --- |
|  | 1721 | 403.2472 | 804.4798 | 803.4249 | 1.0549 | 1 | 10 | 20 | 2 | K.SSRAEVR.T |
|  | 10977 | 461.6902 | 1382.0488 | 1382.6691 | -0.6203 | 1 | 5 | 29 | 5 | R.GYNSIGRGAGFER.M |
|  | 18333 | 921.4440 | 1840.8735 | 1840.8843 | -0.0108 | 0 | 65 | 3.3e-005 | 1 | R.STGEAFVQFASQEIAEK.A 18327 18334 |
|  | 18969 | 630.3107 | 1887.9104 | 1887.9196 | -0.0092 | 0 | 5 | 34 | 6 | K.LMAMQRPGPYDRPGAGR.G |
|  | 21251 | **695.0684** | **2082.1833** | **2082.8636** | **-0.6804** | **0** | **7** | **16** | **1** | **R.YGDGGSSFQSTTGHCVHMR.G** |
|  | 22010 | **721.4191** | **2161.2354** | **2160.9997** | **0.2356** | **0** | **0** | **69** | **2** | **R.VTGEADVEFATHEDAVAAMAK.D** |
|  | 29924 | **925.4545** | **3697.7890** | **3697.4828** | **0.3062** | **1** | **1** | **29** | **10** | **R.DLNYCFSGMSDHRYGDGGSSFQSTTGHCVHMR.G** |

| **490.** | A6NMZ7\|CO6A6_HUMAN    **Mass:** 248672   **Score:** 80     **Queries matched:** 58   **emPAI:** 0.01 |
| --- | --- |
|  | Collagen alpha-6(VI) chain OS=Homo sapiens GN=COL6A6 PE=1 SV=2 |

|  | **Query** | **Observed** | **Mr(expt)** | **Mr(calc)** | **Delta** | **Miss** | **Score** | **Expect** | **Rank** | **Peptide** |
| --- | --- | --- | --- | --- | --- | --- | --- | --- | --- | --- |
|  | 969 | **560.8668** | **559.8595** | **559.3329** | **0.5266** | **0** | **7** | **47** | **3** | **K.NLVSK.S** |
|  | 1004 | **574.9901** | **573.9828** | **574.3326** | **-0.3498** | **0** | **14** | **9.1** | **4** | **K.DITVK.G** |
|  | 1473 | **775.4331** | **774.4258** | **774.3945** | **0.0313** | **0** | **13** | **11** | **8** | **R.MIESAPK.Q** |
|  | 1879 | 409.8863 | 817.7580 | 817.4294 | 0.3286 | 1 | 11 | 12 | 4 | K.GEKGGVGSK.G |
|  | 2070 | **417.2050** | **832.3954** | **831.4385** | **0.9570** | **1** | **18** | **3.2** | **5** | **R.EIGRAMR.F** |
|  | 2340 | **426.2398** | **850.4650** | **851.4613** | **-0.9963** | **0** | **2** | **1.1e+002** | **10** | **R.TLPGAHTR.K** |
|  | 2946 | **449.2250** | **896.4355** | **895.4698** | **0.9657** | **1** | **2** | **87** | **10** | **R.AMRFISR.N** |
|  | 3420 | **466.2478** | **930.4810** | **930.4770** | **0.0040** | **1** | **12** | **13** | **9** | **R.EIAGEEKR.V** |
|  | 3731 | **476.7652** | **951.5159** | **951.5250** | **-0.0091** | **1** | **18** | **2.5** | **2** | **R.LREEHIR.V** |
|  | 3772 | **478.7167** | **955.4188** | **956.5767** | **-1.1579** | **1** | **2** | **1.1e+002** | **5** | **K.LNATAKALR.D** |
|  | 4132 | **491.2792** | **980.5438** | **979.5563** | **0.9875** | **1** | **4** | **62** | **2** | **R.TLPGAHTRK.I** |
|  | 4845 | **1032.9374** | **1031.9301** | **1031.5876** | **0.3425** | **1** | **10** | **12** | **3** | **K.NQVRFGALK.Y** 4853 |
|  | 5133 | **527.1563** | **1052.2979** | **1051.5298** | **0.7681** | **1** | **2** | **72** | **4** | **K.YSNKQDLGK.A** 5131 5132 |
|  | 5613 | 543.8207 | 1085.6268 | 1084.6644 | 0.9624 | 1 | 16 | 4.3 | 7 | K.YPPINLKIK.C |
|  | 5991 | **557.7581** | **1113.5016** | **1113.5455** | **-0.0439** | **0** | **3** | **86** | **5** | **R.AEFNLTTYR.S** |
|  | 6023 | 558.8121 | 1115.6096 | 1116.5775 | -0.9679 | 1 | 13 | 8 | 5 | K.LEQKSDELR.K |
|  | 7128 | **595.4182** | **1188.8219** | **1188.5411** | **0.2807** | **0** | **0** | **1.3e+002** | **9** | **K.GQEGFPGESGPK.G** 7132 |
|  | 7166 | 596.3094 | 1190.6043 | 1190.6329 | -0.0285 | 0 | 4 | 61 | 1 | K.VINSLSMGINK.S 7181 |
|  | 7285 | 599.2906 | 1196.5666 | 1195.6244 | 0.9422 | 1 | 7 | 29 | 6 | K.GRSPMLNHLR.K |
|  | 7788 | 407.4496 | 1219.3270 | 1218.6166 | 0.7105 | 0 | 12 | 6.9 | 5 | K.MISSLPIEADK.Y |
|  | 9114 | **429.8901** | **1286.6486** | **1286.6185** | **0.0301** | **1** | **3** | **66** | **10** | **R.MKEMMAFLVR.D** |
|  | 9124 | **644.4312** | **1286.8479** | **1286.6185** | **0.2294** | **1** | **(0)** | **1.2e+002** | **5** | **R.MKEMMAFLVR.D** |
|  | 9647 | **438.9161** | **1313.7266** | **1312.7099** | **1.0167** | **1** | **3** | **66** | **6** | **K.NLVSKSQIGPDR.V** |
|  | 9778 | **440.8850** | **1319.6332** | **1320.6687** | **-1.0355** | **1** | **8** | **22** | **4** | **R.GWPGPPGTPGSRR.K** |
|  | 10267 | 449.2767 | 1344.8082 | 1344.6826 | 0.1255 | 0 | 4 | 53 | 7 | K.LHSEFHLSTFK.G 10243 |
|  | 12076 | **482.9862** | **1445.9366** | **1445.7878** | **0.1488** | **0** | **6** | **28** | **4** | **R.VQIGVVQFSDINK.E** 12099 |
|  | 12109 | **724.8282** | **1447.6418** | **1446.7613** | **0.8806** | **1** | **7** | **30** | **10** | **R.TQLSIGMRELGSR.L** |
|  | 13280 | **506.2780** | **1515.8121** | **1514.6494** | **1.1627** | **0** | **3** | **60** | **5** | **K.CIGGDGTMGDPGPPGK.R** 13234 |
|  | 13539 | 511.3128 | 1530.9166 | 1530.7638 | 0.1528 | 1 | 8 | 20 | 2 | R.DSEDNVTKAAVNLR.R |
|  | 13922 | 519.2676 | 1554.7809 | 1553.8058 | 0.9751 | 0 | 1 | 90 | 4 | K.VPCHLVVLTNGMSK.D |
|  | 14845 | 807.9951 | 1613.9756 | 1613.8988 | 0.0768 | 1 | 6 | 28 | 2 | K.IISVGVQKASEENLK.A |
|  | 15364 | **550.2140** | **1647.6201** | **1647.7965** | **-0.1764** | **1** | **1** | **82** | **10** | **R.GLQGNDGSPGYGSVGRK.G** |
|  | 15721 | 834.9253 | 1667.8360 | 1666.8275 | 1.0086 | 1 | 3 | 62 | 2 | K.GEIGDPGGPGETGLKGAR.G |
|  | 16021 | **563.3261** | **1686.9563** | **1686.7454** | **0.2109** | **1** | **3** | **57** | **1** | **K.CIGGDGTMGDPGPPGKR.G** |
|  | 16594 | **575.3737** | **1723.0992** | **1721.9729** | **1.1262** | **1** | **1** | **69** | **5** | **K.QPPRPFRSFVPGPLK.A** |
|  | 17016 | **876.9019** | **1751.7892** | **1752.8530** | **-1.0639** | **0** | **2** | **74** | **1** | **R.GDPGAPGVDSSIEGPTGLK.G** |
|  | 18300 | **920.4428** | **1838.8709** | **1838.9522** | **-0.0812** | **1** | **0** | **1e+002** | **5** | **K.MFITKMISSLPIEADK.Y** |
|  | 18514 | **618.3116** | **1851.9131** | **1852.9652** | **-1.0520** | **1** | **4** | **40** | **3** | **R.GNKVPCHLVVLTNGMSK.D** |
|  | 18980 | **630.3402** | **1887.9988** | **1887.9340** | **0.0648** | **1** | **4** | **42** | **3** | **K.ALQEAHRTYFSAPANGR.D** |
|  | 20240 | **498.7552** | **1990.9919** | **1992.0164** | **-1.0245** | **0** | **4** | **36** | **4** | **K.GVPQVLIVITDGESHDADK.L** |
|  | 22278 | **732.3486** | **2194.0241** | **2193.0636** | **0.9604** | **1** | **1** | **69** | **1** | **K.ASEENLKAMATSQFHFNLR.T** |
|  | 23510 | **585.0369** | **2336.1184** | **2336.2410** | **-0.1226** | **1** | **1** | **68** | **4** | **R.VGLVAYSNETKVINSLSMGINK.S** |
|  | 23911 | **1196.0961** | **2390.1776** | **2390.1940** | **-0.0164** | **0** | **80** | **7.3e-007** | **1** | **K.GQTLLEGQPWMETYLQDILR.A** |
|  | 24109 | **605.5606** | **2418.2131** | **2419.2456** | **-1.0325** | **1** | **4** | **27** | **2** | **K.NQGVPQIAVLVTHRDSEDNVTK.A** |
|  | 24288 | 1225.5381 | 2449.0616 | 2450.2237 | -1.1621 | 1 | 0 | 64 | 4 | K.VRENSCPVGAHIAILSYNSHAR.H |
|  | 24737 | **628.3417** | **2509.3379** | **2509.3462** | **-0.0083** | **0** | **(1)** | **54** | **4** | **K.GILVLAVGIDGANPVELLAMAGSSDK.Y** |
|  | 24838 | 632.5602 | 2526.2116 | 2525.3411 | 0.8705 | 0 | 4 | 30 | 1 | K.GILVLAVGIDGANPVELLAMAGSSDK.Y |
|  | 25145 | **852.4446** | **2554.3121** | **2553.1983** | **1.1137** | **1** | **0** | **63** | **9** | **K.GQEGFPGESGPKGEIGDPGGPGETGLK.G** |
|  | 25487 | 655.0704 | 2616.2526 | 2615.3330 | 0.9196 | 1 | 3 | 31 | 4 | K.KQFPPILVVLASSESEDNVEEASK.A |
|  | 28389 | **795.6529** | **3178.5825** | **3178.4624** | **0.1201** | **1** | **2** | **28** | **5** | **K.GNRGLNGQEGEVGENGIDGLNGEQGDNGLPGR.K** |

| **491.** | P60953\|CDC42_HUMAN    **Mass:** 21696    **Score:** 80     **Queries matched:** 8   **emPAI:** 0.39 |
| --- | --- |
|  | Cell division control protein 42 homolog OS=Homo sapiens GN=CDC42 PE=1 SV=1 |

|  | **Query** | **Observed** | **Mr(expt)** | **Mr(calc)** | **Delta** | **Miss** | **Score** | **Expect** | **Rank** | **Peptide** |
| --- | --- | --- | --- | --- | --- | --- | --- | --- | --- | --- |
|  | 15599 | **831.3904** | **1660.7662** | **1660.8641** | **-0.0979** | **1** | **4** | **44** | **6** | **-.MQTIKCVVVGDGAVGK.T** 15606 |
|  | 23636 | **785.3524** | **2353.0353** | **2354.1974** | **-1.1622** | **1** | **2** | **45** | **4** | **K.CVVVGDGAVGKTCLLISYTTNK.F** |
|  | 23680 | **1179.6237** | **2357.2328** | **2357.2478** | **-0.0151** | **1** | **42** | **0.0043** | **1** | **K.TPFLLVGTQIDLRDDPSTIEK.L** 23681 23682 23683 |
|  | 26896 | 953.3732 | 2857.0979 | 2856.5109 | 0.5870 | 1 | 1 | 31 | 8 | K.WVPEITHHCPKTPFLLVGTQIDLR.D |

| **492.** | P60174\|TPIS_HUMAN    **Mass:** 26938    **Score:** 80     **Queries matched:** 5   **emPAI:** 0.30 |
| --- | --- |
|  | Triosephosphate isomerase OS=Homo sapiens GN=TPI1 PE=1 SV=2 |

|  | **Query** | **Observed** | **Mr(expt)** | **Mr(calc)** | **Delta** | **Miss** | **Score** | **Expect** | **Rank** | **Peptide** |
| --- | --- | --- | --- | --- | --- | --- | --- | --- | --- | --- |
|  | 9906 | **663.8351** | **1325.6557** | **1325.6649** | **-0.0092** | **0** | **59** | **0.00019** | **1** | **R.IIYGGSVTGATCK.E** |
|  | 12401 | 733.8601 | 1465.7057 | 1465.7161 | -0.0104 | 0 | 50 | 0.0012 | 1 | K.TATPQQAQEVHEK.L |
|  | 14398 | **529.5809** | **1585.7210** | **1585.7307** | **-0.0098** | **0** | **9** | **14** | **1** | **K.DCGATWVVLGHSER.R** 14397 |
|  | 14841 | **538.9415** | **1613.8028** | **1613.8162** | **-0.0134** | **1** | **10** | **12** | **1** | **R.RHVFGESDELIGQK.V** |

| **493.** | P40926\|MDHM_HUMAN    **Mass:** 35937    **Score:** 79     **Queries matched:** 11   **emPAI:** 0.35 |
| --- | --- |
|  | Malate dehydrogenase, mitochondrial OS=Homo sapiens GN=MDH2 PE=1 SV=3 |

|  | **Query** | **Observed** | **Mr(expt)** | **Mr(calc)** | **Delta** | **Miss** | **Score** | **Expect** | **Rank** | **Peptide** |
| --- | --- | --- | --- | --- | --- | --- | --- | --- | --- | --- |
|  | 1280 | 694.3460 | 693.3387 | 693.3334 | 0.0054 | 0 | 9 | 21 | 6 | K.GEDFVK.T |
|  | 1929 | **412.2240** | **822.4334** | **821.4283** | **1.0051** | **1** | **13** | **7.8** | **2** | **K.KGEDFVK.T** |
|  | 3363 | **464.2315** | **926.4485** | **927.4563** | **-1.0078** | **0** | **17** | **3.3** | **1** | **K.HGVYNPNK.I** |
|  | 4801 | **515.3557** | **1028.6969** | **1027.6026** | **1.0943** | **1** | **3** | **71** | **9** | **K.GIEKNLGIGK.V** |
|  | 6022 | 558.7985 | 1115.5825 | 1115.5896 | -0.0071 | 0 | 32 | 0.094 | 1 | K.MISDAIPELK.A |
|  | 6548 | **577.3048** | **1152.5951** | **1152.5485** | **0.0465** | **0** | **10** | **14** | **4** | **K.EGVVECSFVK.S** |
|  | 9309 | **433.2047** | **1296.5924** | **1295.7673** | **0.8251** | **0** | **4** | **57** | **3** | **M.LSALARPASAALR.R** |
|  | 9530 | **437.2530** | **1308.7371** | **1309.6375** | **-0.9003** | **1** | **8** | **24** | **7** | **R.RSFSTSAQNNAK.V** |
|  | 12817 | **745.8674** | **1489.7202** | **1488.7283** | **0.9919** | **0** | **18** | **2** | **1** | **K.GYLGPEQLPDCLK.G** |
|  | 17634 | **897.0441** | **1792.0737** | **1792.0822** | **-0.0085** | **0** | **72** | **5.8e-006** | **1** | **K.VAVLGASGGIGQPLSLLLK.N** |
|  | 21829 | **1072.3926** | **2142.7706** | **2142.1586** | **0.6120** | **1** | **4** | **28** | **2** | **K.HGVYNPNKIFGVTTLDIVR.A** |

| **494.** | P07195\|LDHB_HUMAN    **Mass:** 36900    **Score:** 79     **Queries matched:** 9   **emPAI:** 0.21 |
| --- | --- |
|  | L-lactate dehydrogenase B chain OS=Homo sapiens GN=LDHB PE=1 SV=2 |

|  | **Query** | **Observed** | **Mr(expt)** | **Mr(calc)** | **Delta** | **Miss** | **Score** | **Expect** | **Rank** | **Peptide** |
| --- | --- | --- | --- | --- | --- | --- | --- | --- | --- | --- |
|  | 1890 | **410.7000** | **819.3855** | **819.4524** | **-0.0669** | **1** | **8** | **28** | **8** | **-.MATLKEK.L** |
|  | 8351 | 624.8001 | 1247.5857 | 1247.5928 | -0.0072 | 0 | 43 | 0.0078 | 1 | R.VIGSGCNLDSAR.F |
|  | 8727 | **634.3292** | **1266.6438** | **1266.6530** | **-0.0092** | **0** | **8** | **23** | **1** | **K.MVVESAYEVIK.L** |
|  | 13156 | **504.2595** | **1509.7566** | **1509.7675** | **-0.0108** | **1** | **19** | **1.6** | **1** | **K.IVADKDYSVTANSK.I** |
|  | 16107 | 847.8217 | 1693.6289 | 1693.8886 | -0.2597 | 0 | 15 | 3 | 2 | K.LIAPVAEEEATVPNNK.I 16116 |
|  | 17331 | 889.0324 | 1776.0503 | 1775.9128 | 0.1375 | 1 | 2 | 66 | 6 | K.EVHKMVVESAYEVIK.L |
|  | 20209 | **664.0251** | **1989.0534** | **1988.0799** | **0.9736** | **0** | **0** | **88** | **4** | **K.ITVVGVGQVGMACAISILGK.S** |
|  | 23197 | **1148.5719** | **2295.1292** | **2295.1391** | **-0.0099** | **0** | **64** | **3e-005** | **1** | **K.GMYGIENEVFLSLPCILNAR.G** |

| **495.** | P08727\|K1C19_HUMAN    **Mass:** 44065    **Score:** 79     **Queries matched:** 17   **emPAI:** 0.50 |
| --- | --- |
|  | Keratin, type I cytoskeletal 19 OS=Homo sapiens GN=KRT19 PE=1 SV=3 |

|  | **Query** | **Observed** | **Mr(expt)** | **Mr(calc)** | **Delta** | **Miss** | **Score** | **Expect** | **Rank** | **Peptide** |
| --- | --- | --- | --- | --- | --- | --- | --- | --- | --- | --- |
|  | 3912 | 483.3000 | 964.5854 | 964.4362 | 0.1491 | 0 | 14 | 6 | 1 | R.QNQEYQR.L 3910 |
|  | 4306 | **497.5984** | **993.1822** | **992.4927** | **0.6895** | **0** | **1** | **85** | **8** | **K.FETEQALR.M** |
|  | 4537 | 505.2522 | 1008.4899 | 1007.5188 | 0.9711 | 1 | 9 | 20 | 2 | K.IRDWYQK.Q |
|  | 4978 | **521.3021** | **1040.5897** | **1040.5978** | **-0.0081** | **0** | **29** | **0.2** | **1** | **R.IVLQIDNAR.L** |
|  | 5421 | 537.2977 | 1072.5809 | 1072.5876 | -0.0067 | 0 | 27 | 0.37 | 1 | K.ILGATIENSR.I |
|  | 7801 | **611.2713** | **1220.5280** | **1221.6353** | **-1.1073** | **1** | **19** | **2.1** | **1** | **R.TKFETEQALR.M** |
|  | 8259 | **622.8180** | **1243.6214** | **1242.6455** | **0.9759** | **0** | **18** | **2.6** | **1** | **R.ALEAANGELEVK.I** |
|  | 10535 | **454.2078** | **1359.6015** | **1359.6929** | **-0.0914** | **1** | **13** | **7.7** | **4** | **R.MSVEADINGLRR.V** |
|  | 10603 | **682.8336** | **1363.6526** | **1364.7048** | **-1.0522** | **1** | **29** | **0.18** | **1** | **K.SRLEQEIATYR.S** |
|  | 11100 | 695.8426 | 1389.6706 | 1388.6783 | 0.9923 | 0 | 54 | 0.00058 | 1 | K.AALEDTLAETEAR.F |
|  | 12671 | **741.8489** | **1481.6832** | **1481.6932** | **-0.0100** | **1** | **36** | **0.03** | **1** | **R.SQYEVMAEQNRK.D** |
|  | 12718 | **743.3541** | **1484.6936** | **1484.7042** | **-0.0106** | **0** | **18** | **2.3** | **1** | **R.EVAGHTEQLQMSR.S** |
|  | 22650 | **743.3573** | **2227.0501** | **2227.0651** | **-0.0150** | **1** | **32** | **0.05** | **1** | **R.TEELNREVAGHTEQLQMSR.S** |
|  | 23128 | **572.7921** | **2287.1393** | **2286.2141** | **0.9253** | **1** | **6** | **20** | **4** | **R.VLDELTLARTDLEMQIEGLK.E** |
|  | 23876 | 795.9984 | 2384.9734 | 2385.1826 | -0.2092 | 1 | 2 | 38 | 3 | R.QSSATSSFGGLGGGSVRFGPGVAFR.A |
|  | 28509 | **1068.8485** | **3203.5237** | **3203.6231** | **-0.0994** | **1** | **14** | **2** | **1** | **R.TLQGLEIELQSQLSMKAALEDTLAETEAR.F** |

| **496.** | O15400\|STX7_HUMAN    **Mass:** 29911    **Score:** 79     **Queries matched:** 9   **emPAI:** 0.13 |
| --- | --- |
|  | Syntaxin-7 OS=Homo sapiens GN=STX7 PE=1 SV=4 |

|  | **Query** | **Observed** | **Mr(expt)** | **Mr(calc)** | **Delta** | **Miss** | **Score** | **Expect** | **Rank** | **Peptide** |
| --- | --- | --- | --- | --- | --- | --- | --- | --- | --- | --- |
|  | 5707 | **547.2856** | **1092.5566** | **1092.5564** | **0.0003** | **0** | **15** | **5.2** | **1** | **K.QQYTNQLAK.E** |
|  | 6336 | **570.7820** | **1139.5494** | **1138.5142** | **1.0352** | **0** | **22** | **0.82** | **1** | **R.VSGSFPEDSSK.E** |
|  | 11670 | 712.3355 | 1422.6565 | 1423.6579 | -1.0015 | 1 | 8 | 21 | 9 | R.VSGSFPEDSSKER.N |
|  | 14590 | **799.9200** | **1597.8255** | **1597.8352** | **-0.0096** | **0** | **79** | **1.6e-006** | **1** | **R.LVAEFTTSLTNFQK.V** |
|  | 15721 | 834.9253 | 1667.8360 | 1667.8479 | -0.0118 | 0 | 14 | 4.9 | 1 | R.TLNQLGTPQDSPELR.Q |
|  | 17156 | **882.3635** | **1762.7124** | **1762.8308** | **-0.1185** | **0** | **11** | **7.7** | **1** | **-.MSYTPGVGGDPAQLAQR.I** |
|  | 24796 | 840.4005 | 2518.1797 | 2517.2646 | 0.9151 | 1 | 0 | 61 | 10 | -.MSYTPGVGGDPAQLAQRISSNIQK.I |
|  | 24886 | **845.7663** | **2534.2771** | **2533.2595** | **1.0176** | **1** | **(0)** | **66** | **4** | **-.MSYTPGVGGDPAQLAQRISSNIQK.I** |
|  | 27974 | **1029.4841** | **3085.4306** | **3085.4476** | **-0.0171** | **0** | **7** | **8.6** | **1** | **R.NLVSWESQTQPQVQVQDEEITEDDLR.L** |

| **497.** | Q9Y394\|DHRS7_HUMAN    **Mass:** 38673    **Score:** 78     **Queries matched:** 17   **emPAI:** 0.32 |
| --- | --- |
|  | Dehydrogenase/reductase SDR family member 7 OS=Homo sapiens GN=DHRS7 PE=1 SV=1 |

|  | **Query** | **Observed** | **Mr(expt)** | **Mr(calc)** | **Delta** | **Miss** | **Score** | **Expect** | **Rank** | **Peptide** |
| --- | --- | --- | --- | --- | --- | --- | --- | --- | --- | --- |
|  | 1732 | **404.2046** | **806.3947** | **805.4446** | **0.9501** | **1** | **9** | **24** | **5** | **K.RIENFK.S** |
|  | 3267 | 460.2504 | 918.4862 | 918.4923 | -0.0061 | 0 | 45 | 0.0052 | 1 | K.AVLQEFGR.I |
|  | 4603 | **507.7521** | **1013.4897** | **1013.6233** | **-0.1337** | **0** | **19** | **2.4** | **1** | **K.LGVSLVLSAR.R** |
|  | 6855 | **588.3068** | **1174.5990** | **1174.5142** | **0.0847** | **0** | **0** | **1.6e+002** | **6** | **K.SGVDADSSYFK.I** |
|  | 11521 | 708.3626 | 1414.7105 | 1415.7191 | -1.0085 | 0 | 22 | 0.83 | 2 | R.IDILVNNGGMSQR.S |
|  | 14048 | 782.3763 | 1562.7380 | 1562.7617 | -0.0237 | 1 | 1 | 99 | 10 | K.SGVDADSSYFKIFK.T |
|  | 14061 | **782.4490** | **1562.8835** | **1562.8919** | **-0.0084** | **0** | **11** | **8.8** | **1** | **K.LIELNYLGTVSLTK.C** |
|  | 14420 | **794.3734** | **1586.7323** | **1586.7433** | **-0.0110** | **1** | **58** | **0.00019** | **1** | **R.SLCMDTSLDVYRK.L** |
|  | 15878 | **560.2828** | **1677.8265** | **1678.8568** | **-1.0303** | **1** | **(3)** | **59** | **5** | **R.CVRLMLISMANDLK.E** |
|  | 15895 | **560.6449** | **1678.9129** | **1678.8568** | **0.0560** | **1** | **3** | **51** | **1** | **R.CVRLMLISMANDLK.E** |
|  | 17149 | **588.3041** | **1761.8906** | **1761.8064** | **0.0842** | **1** | **3** | **53** | **1** | **K.TIGNNGDQSHKMTTSR.C** |
|  | 21508 | 1054.5304 | 2107.0462 | 2108.1001 | -1.0539 | 0 | 12 | 5.5 | 2 | K.DILVLPLDLTDTGSHEAATK.A 21529 |
|  | 23743 | **789.4156** | **2365.2249** | **2365.2377** | **-0.0127** | **1** | **28** | **0.12** | **1** | **K.EKDILVLPLDLTDTGSHEAATK.A** |
|  | 23986 | **802.1189** | **2403.3349** | **2403.3447** | **-0.0098** | **0** | **25** | **0.24** | **1** | **K.IVTVNSILGIISVPLSIGYCASK.H** |
|  | 29654 | **893.4609** | **3569.8144** | **3569.8287** | **-0.0143** | **0** | **17** | **0.75** | **1** | **R.TELATYPGIIVSNICPGPVQSNIVENSLAGEVTK.T** 29652 |

| **498.** | P15328\|FOLR1_HUMAN    **Mass:** 30712    **Score:** 78     **Queries matched:** 9   **emPAI:** 0.12 |
| --- | --- |
|  | Folate receptor alpha OS=Homo sapiens GN=FOLR1 PE=1 SV=3 |

|  | **Query** | **Observed** | **Mr(expt)** | **Mr(calc)** | **Delta** | **Miss** | **Score** | **Expect** | **Rank** | **Peptide** |
| --- | --- | --- | --- | --- | --- | --- | --- | --- | --- | --- |
|  | 3202 | **458.2710** | **914.5275** | **914.4498** | **0.0777** | **0** | **5** | **61** | **4** | **K.DVSYLYR.F** |
|  | 9000 | **427.8777** | **1280.6114** | **1280.6196** | **-0.0082** | **0** | **11** | **11** | **1** | **K.LHEQCRPWR.K** |
|  | 9391 | 651.6684 | 1301.3222 | 1300.6524 | 0.6699 | 1 | 5 | 32 | 1 | K.HHKEKPGPEDK.L |
|  | 11464 | **471.3299** | **1410.9678** | **1410.6350** | **0.3327** | **1** | **2** | **52** | **6** | **R.TSYTCKSNWHK.G** |
|  | 18820 | **626.6005** | **1876.7797** | **1876.7919** | **-0.0122** | **1** | **9** | **13** | **1** | **R.FNWNHCGEMAPACKR.H** |
|  | 22921 | **1131.5007** | **2260.9869** | **2260.9994** | **-0.0125** | **0** | **54** | **0.00032** | **1** | **R.CIQMWFDPAQGNPNEEVAR.F** 22920 |
|  | 24889 | **846.0357** | **2535.0853** | **2535.0981** | **-0.0128** | **1** | **6** | **15** | **1** | **R.VLNVPLCKEDCEQWWEDCR.T** |
|  | 29164 | **1126.5145** | **3376.5218** | **3376.5394** | **-0.0176** | **0** | **1** | **31** | **1** | **R.HFIQDTCLYECSPNLGPWIQQVDQSWR.K** |

| **499.** | Q12931\|TRAP1_HUMAN    **Mass:** 80345    **Score:** 78     **Queries matched:** 23   **emPAI:** 0.05 |
| --- | --- |
|  | Heat shock protein 75 kDa, mitochondrial OS=Homo sapiens GN=TRAP1 PE=1 SV=3 |

|  | **Query** | **Observed** | **Mr(expt)** | **Mr(calc)** | **Delta** | **Miss** | **Score** | **Expect** | **Rank** | **Peptide** |
| --- | --- | --- | --- | --- | --- | --- | --- | --- | --- | --- |
|  | 2967 | 450.2432 | 898.4718 | 898.4872 | -0.0154 | 0 | 7 | 33 | 5 | K.TDAPLNIR.S |
|  | 4382 | **500.2098** | **998.4051** | **998.5621** | **-0.1570** | **1** | **2** | **97** | **9** | **R.TTAQLGPRR.N** |
|  | 5520 | **540.7842** | **1079.5539** | **1079.5360** | **0.0180** | **1** | **5** | **50** | **6** | **K.EFSSEARVR.D** |
|  | 9132 | **644.8262** | **1287.6379** | **1288.6510** | **-1.0131** | **0** | **3** | **69** | **3** | **R.ELISNASDALEK.L** |
|  | 9394 | **434.8799** | **1301.6180** | **1302.6667** | **-1.0487** | **0** | **2** | **86** | **10** | **R.EGIVTATEQEVK.E** |
|  | 9531 | 437.2584 | 1308.7533 | 1308.7037 | 0.0495 | 1 | 11 | 12 | 3 | R.ELGSSVALYSRK.V |
|  | 10877 | **689.5555** | **1377.0965** | **1376.6718** | **0.4247** | **1** | **2** | **59** | **3** | **R.MQQLAKTQEER.A** |
|  | 13217 | 757.3913 | 1512.7680 | 1512.7784 | -0.0103 | 0 | 78 | 2.2e-006 | 1 | R.GVVDSEDIPLNLSR.E |
|  | 13961 | **520.1339** | **1557.3799** | **1557.8362** | **-0.4563** | **1** | **1** | **72** | **10** | **R.ELISNASDALEKLR.H** |
|  | 14481 | 796.3497 | 1590.6848 | 1591.7842 | -1.0994 | 0 | 2 | 77 | 5 | K.AFLDALQNQAEASSK.I |
|  | 16251 | **568.2975** | **1701.8706** | **1702.8137** | **-0.9430** | **1** | **10** | **12** | **1** | **R.HLAEHSPYYEAMKK.K** |
|  | 16274 | **568.9247** | **1703.7524** | **1703.8995** | **-0.1471** | **1** | **4** | **43** | **1** | **R.YTLHYKTDAPLNIR.S** |
|  | 18155 | **914.9384** | **1827.8621** | **1826.9019** | **0.9603** | **0** | **1** | **83** | **10** | **R.LDTHPAMVTVLEMGAAR.H** 18156 |
|  | 18187 | 611.3106 | 1830.9098 | 1831.9369 | -1.0271 | 1 | 2 | 72 | 7 | K.YSNFVSFPLYLNGRR.M |
|  | 19736 | **974.8051** | **1947.5955** | **1947.9369** | **-0.3413** | **1** | **1** | **56** | **2** | **R.MNTLQAIWMMDPKDVR.E** |
|  | 20936 | **685.4557** | **2053.3454** | **2054.2000** | **-0.8546** | **1** | **2** | **41** | **7** | **R.AQLLQPTLEINPRHALIK.K** |
|  | 21629 | 706.3941 | 2116.1605 | 2115.0331 | 1.1273 | 0 | 2 | 55 | 2 | K.EEPLHSIISSTESVQGSTSK.H |
|  | 23061 | **760.7282** | **2279.1626** | **2280.2082** | **-1.0456** | **1** | **(1)** | **61** | **2** | **K.VTLRLDTHPAMVTVLEMGAAR.H** |
|  | 23206 | **766.3721** | **2296.0946** | **2296.2032** | **-0.1086** | **1** | **8** | **13** | **1** | **K.VTLRLDTHPAMVTVLEMGAAR.H** |
|  | 23845 | 596.0415 | 2380.1369 | 2380.2144 | -0.0775 | 1 | 3 | 32 | 2 | R.LDTHPAMVTVLEMGAARHFLR.M 23851 |
|  | 29689 | 1194.9117 | 3581.7134 | 3582.8174 | -1.1040 | 1 | 1 | 30 | 2 | R.ASEPGLAQLLVDQIYENAMIAAGLVDDPRAMVGR.L |

| **500.** | Q92974\|ARHG2_HUMAN    **Mass:** 112386   **Score:** 78     **Queries matched:** 27   **emPAI:** 0.03 |
| --- | --- |
|  | Rho guanine nucleotide exchange factor 2 OS=Homo sapiens GN=ARHGEF2 PE=1 SV=4 |

|  | **Query** | **Observed** | **Mr(expt)** | **Mr(calc)** | **Delta** | **Miss** | **Score** | **Expect** | **Rank** | **Peptide** |
| --- | --- | --- | --- | --- | --- | --- | --- | --- | --- | --- |
|  | 1148 | **635.3127** | **634.3054** | **633.3520** | **0.9534** | **1** | **19** | **3** | **1** | **K.KEVMK.Q** |
|  | 1611 | **399.7481** | **797.4816** | **796.4443** | **1.0373** | **0** | **7** | **33** | **3** | **R.AQTPVPGK.G** |
|  | 2019 | **415.2453** | **828.4761** | **827.4865** | **0.9896** | **0** | **6** | **54** | **7** | **R.LLQDAIR.E** |
|  | 2724 | **440.0295** | **878.0445** | **877.5055** | **0.5390** | **1** | **5** | **42** | **3** | **R.TLKIMTR.L** |
|  | 3138 | 456.2247 | 910.4348 | 909.4920 | 0.9429 | 0 | 14 | 5.6 | 1 | R.AGAAPVAPEK.Q 3111 3112 3137 |
|  | 5176 | **529.2239** | **1056.4333** | **1056.5200** | **-0.0866** | **0** | **8** | **25** | **6** | **R.SPQEEALQR.L** 5210 |
|  | 8294 | **623.8116** | **1245.6087** | **1245.5949** | **0.0138** | **1** | **1** | **1.3e+002** | **10** | **R.SESLESPRGER.L** |
|  | 9387 | **434.7327** | **1301.1763** | **1300.6735** | **0.5028** | **1** | **8** | **15** | **1** | **R.ESEQARALLER.E** 9364 |
|  | 9595 | **657.3601** | **1312.7055** | **1311.6605** | **1.0450** | **1** | **5** | **49** | **2** | **R.MDPRAQTPVPGK.G** |
|  | 10360 | **676.3720** | **1350.7295** | **1349.5769** | **1.1526** | **0** | **2** | **81** | **4** | **R.MQDIPEETESR.D** |
|  | 13592 | **512.6769** | **1535.0090** | **1535.7726** | **-0.7636** | **1** | **1** | **84** | **4** | **R.RILSQSTDSLNMR.N** |
|  | 13895 | **518.5977** | **1552.7713** | **1551.8192** | **0.9522** | **0** | **5** | **41** | **4** | **R.HGVQECILLVTQR.I** |
|  | 14778 | **537.7393** | **1610.1960** | **1610.7689** | **-0.5729** | **0** | **3** | **40** | **6** | **R.ERPSSAIYPSDSFR.Q** |
|  | 16834 | 580.6434 | 1738.9083 | 1738.8710 | 0.0373 | 1 | 0 | 1e+002 | 8 | R.NGNQLRSPQEEALQR.L |
|  | 21598 | 705.6766 | 2114.0081 | 2114.0392 | -0.0312 | 0 | 4 | 32 | 2 | K.SVSTTNIAGHFNDESPLGLR.R 21593 |
|  | 22135 | **544.7424** | **2174.9404** | **2174.1596** | **0.7808** | **1** | **6** | **23** | **2** | **R.RQLAALGQTEPLPAEAPWAR.R** 22134 |
|  | 26165 | **908.4603** | **2722.3590** | **2723.4428** | **-1.0839** | **0** | **59** | **7e-005** | **1** | **R.LVNLYGLLHGLQAAVAQQDTLMEAR.F** 26175 |
|  | 26843 | **1424.1150** | **2846.2154** | **2846.4207** | **-0.2053** | **1** | **3** | **27** | **2** | **R.NFVIHRLGDLLISQFSGPSAEQMCK.T** |
|  | 27591 | **1000.4479** | **2998.3220** | **2997.3242** | **0.9978** | **1** | **0** | **46** | **9** | **K.DARYTNGHLFTTISVSGMTMCYACNK.S** |

| **501.** | P14854\|CX6B1_HUMAN    **Mass:** 10414    **Score:** 77     **Queries matched:** 7   **emPAI:** 2.67 |
| --- | --- |
|  | Cytochrome c oxidase subunit 6B1 OS=Homo sapiens GN=COX6B1 PE=1 SV=2 |

|  | **Query** | **Observed** | **Mr(expt)** | **Mr(calc)** | **Delta** | **Miss** | **Score** | **Expect** | **Rank** | **Peptide** |
| --- | --- | --- | --- | --- | --- | --- | --- | --- | --- | --- |
|  | 3733 | **476.9329** | **951.8513** | **952.4470** | **-0.5957** | **1** | **1** | **77** | **3** | **R.CQKAMTAK.G** |
|  | 3746 | **477.5526** | **953.0906** | **953.3834** | **-0.2928** | **0** | **3** | **46** | **8** | **-.MAEDMETK.I** |
|  | 7612 | **404.7289** | **1211.1648** | **1210.5573** | **0.6075** | **1** | **4** | **40** | **10** | **-.MAEDMETKIK.N** |
|  | 12440 | **735.3228** | **1468.6311** | **1468.6405** | **-0.0095** | **0** | **31** | **0.091** | **1** | **K.GGDISVCEWYQR.V** |
|  | 13869 | **776.8355** | **1551.6565** | **1551.6677** | **-0.0113** | **0** | **45** | **0.0042** | **1** | **R.NCWQNYLDFHR.C** 13870 |
|  | 27876 | **1019.8014** | **3056.3823** | **3056.3975** | **-0.0151** | **1** | **31** | **0.044** | **1** | **R.VYQSLCPTSWVTDWDEQRAEGTFPGK.I** |

| **502.** | P02792\|FRIL_HUMAN    **Mass:** 20064    **Score:** 77     **Queries matched:** 3   **emPAI:** 0.19 |
| --- | --- |
|  | Ferritin light chain OS=Homo sapiens GN=FTL PE=1 SV=2 |

|  | **Query** | **Observed** | **Mr(expt)** | **Mr(calc)** | **Delta** | **Miss** | **Score** | **Expect** | **Rank** | **Peptide** |
| --- | --- | --- | --- | --- | --- | --- | --- | --- | --- | --- |
|  | 11128 | **696.8814** | **1391.7483** | **1391.6788** | **0.0694** | **1** | **3** | **62** | **3** | **K.TPDAMKAAMALEK.K** |
|  | 11401 | **470.2340** | **1407.6803** | **1407.6738** | **0.0065** | **1** | **(1)** | **1e+002** | **10** | **K.TPDAMKAAMALEK.K** |
|  | 14731 | **804.4020** | **1606.7894** | **1606.7991** | **-0.0097** | **0** | **77** | **2.2e-006** | **1** | **R.LGGPEAGLGEYLFER.L** |

| **503.** | P00558\|PGK1_HUMAN    **Mass:** 44985    **Score:** 77     **Queries matched:** 18   **emPAI:** 0.27 |
| --- | --- |
|  | Phosphoglycerate kinase 1 OS=Homo sapiens GN=PGK1 PE=1 SV=3 |

|  | **Query** | **Observed** | **Mr(expt)** | **Mr(calc)** | **Delta** | **Miss** | **Score** | **Expect** | **Rank** | **Peptide** |
| --- | --- | --- | --- | --- | --- | --- | --- | --- | --- | --- |
|  | 3344 | **463.2302** | **924.4458** | **925.5960** | **-1.1502** | **1** | **1** | **1.1e+002** | **8** | **R.IKAAVPSIK.F** |
|  | 3516 | **469.7421** | **937.4696** | **936.4586** | **1.0110** | **1** | **6** | **37** | **4** | **K.DLMSKAEK.N** |
|  | 8372 | **625.3051** | **1248.5957** | **1248.6747** | **-0.0791** | **1** | **1** | **1.1e+002** | **5** | **-.MSLSNKLTLDK.L** |
|  | 9763 | **440.5876** | **1318.7410** | **1318.6915** | **0.0496** | **1** | **5** | **48** | **8** | **K.ALMDEVVKATSR.G** |
|  | 9764 | 660.3881 | 1318.7616 | 1317.7180 | 1.0435 | 0 | 4 | 54 | 6 | K.ITLPVDFVTADK.F |
|  | 12133 | **483.9336** | **1448.7789** | **1449.7472** | **-0.9683** | **1** | **(1)** | **97** | **7** | **R.VVMRVDFNVPMK.N** |
|  | 12134 | 483.9338 | 1448.7796 | 1449.7472 | -0.9676 | 1 | (7) | 29 | 5 | R.VVMRVDFNVPMK.N 12136 12146 |
|  | 12405 | **489.6034** | **1465.7883** | **1465.7422** | **0.0462** | **1** | **9** | **14** | **2** | **R.VVMRVDFNVPMK.N** 12424 |
|  | 16918 | **581.9947** | **1742.9624** | **1742.8736** | **0.0889** | **0** | **7** | **20** | **3** | **K.VNEMIIGGGMAFTFLK.V** |
|  | 17041 | **877.8912** | **1753.7679** | **1753.7798** | **-0.0118** | **0** | **48** | **0.0017** | **1** | **R.GCITIIGGGDTATCCAK.W** 17042 |
|  | 17122 | **587.3070** | **1758.8990** | **1758.8685** | **0.0306** | **0** | **(2)** | **66** | **4** | **K.VNEMIIGGGMAFTFLK.V** |
|  | 17209 | **884.9668** | **1767.9190** | **1767.9301** | **-0.0111** | **0** | **45** | **0.0033** | **1** | **K.ACANPAAGSVILLENLR.F** |
|  | 25444 | 869.4706 | 2605.3901 | 2604.2312 | 1.1589 | 1 | 1 | 51 | 6 | K.TGQATVASGIPAGWMGLDCGPESSKK.Y |
|  | 28750 | **816.6794** | **3262.6887** | **3262.7094** | **-0.0207** | **1** | **20** | **0.43** | **1** | **K.SVVLMSHLGRPDGVPMPDKYSLEPVAVELK.S** |

| **504.** | P0C0S5\|H2AZ_HUMAN    **Mass:** 13545    **Score:** 77     **Queries matched:** 14   **emPAI:** 1.77 |
| --- | --- |
|  | Histone H2A.Z OS=Homo sapiens GN=H2AFZ PE=1 SV=2 |

|  | **Query** | **Observed** | **Mr(expt)** | **Mr(calc)** | **Delta** | **Miss** | **Score** | **Expect** | **Rank** | **Peptide** |
| --- | --- | --- | --- | --- | --- | --- | --- | --- | --- | --- |
|  | 734 | 479.3557 | 478.3484 | 478.2210 | 0.1274 | 0 | 1 | 1.3e+002 | 1 | -.MAGGK.A |
|  | 1356 | **719.3671** | **718.3599** | **718.3796** | **-0.0197** | **1** | **5** | **73** | **9** | **-.MAGGKAGK.D** |
|  | 1680 | 402.2432 | 802.4719 | 802.4409 | 0.0310 | 1 | 15 | 8 | 8 | K.AVSRSQR.A |
|  | 2328 | 425.7633 | 849.5121 | 849.5184 | -0.0064 | 0 | 32 | 0.11 | 1 | R.HLQLAIR.G 2329 2330 2339 |
|  | 3614 | 472.7664 | 943.5183 | 943.5240 | -0.0057 | 0 | 45 | 0.0057 | 1 | R.AGLQFPVGR.I 3604 3613 3617 |
|  | 9699 | 658.8362 | 1315.6579 | 1314.7157 | 0.9423 | 1 | 4 | 54 | 8 | R.SQRAGLQFPVGR.I |
|  | 10740 | 685.8942 | 1369.7738 | 1369.7830 | -0.0092 | 0 | 49 | 0.0016 | 1 | K.ATIAGGGVIPHIHK.S 10741 |

|  | **Proteins matching the same set of peptides:** |
| --- | --- |

|  | Q71UI9\|H2AV_HUMAN    **Mass:** 13501    **Score:** 77     **Queries matched:** 14 |
| --- | --- |
|  | Histone H2A.V OS=Homo sapiens GN=H2AFV PE=1 SV=3 |

| **505.** | Q8NBS9\|TXND5_HUMAN    **Mass:** 48283    **Score:** 77     **Queries matched:** 8   **emPAI:** 0.16 |
| --- | --- |
|  | Thioredoxin domain-containing protein 5 OS=Homo sapiens GN=TXNDC5 PE=1 SV=2 |

|  | **Query** | **Observed** | **Mr(expt)** | **Mr(calc)** | **Delta** | **Miss** | **Score** | **Expect** | **Rank** | **Peptide** |
| --- | --- | --- | --- | --- | --- | --- | --- | --- | --- | --- |
|  | 1023 | **580.3036** | **579.2964** | **578.3428** | **0.9536** | **0** | **13** | **8.7** | **1** | **K.VYVAK.V** |
|  | 2019 | 415.2453 | 828.4761 | 827.3886 | 1.0875 | 0 | 6 | 56 | 9 | K.VSEHSGGR.D |
|  | 6473 | **575.3075** | **1148.6004** | **1148.6077** | **-0.0073** | **1** | **36** | **0.041** | **1** | **R.FVLSQAKDEL.-** |
|  | 6539 | 577.2689 | 1152.5232 | 1151.6128 | 0.9104 | 0 | 7 | 29 | 2 | R.GYPTLLWFR.D |
|  | 9796 | 661.3117 | 1320.6089 | 1320.7554 | -0.1465 | 1 | 4 | 50 | 6 | R.GYPTLLLFRGGK.K |
|  | 11292 | 467.9274 | 1400.7605 | 1401.7504 | -0.9899 | 1 | 6 | 35 | 2 | K.TLAPTWEELSKK.E |
|  | 17132 | **881.3793** | **1760.7440** | **1760.7570** | **-0.0131** | **0** | **68** | **1.6e-005** | **1** | **K.VDCTAHSDVCSAQGVR.G** |
|  | 19962 | **655.9514** | **1964.8324** | **1964.8469** | **-0.0145** | **0** | **14** | **3.3** | **1** | **K.VDCTQHYELCSGNQVR.G** |

| **506.** | P62917\|RL8_HUMAN    **Mass:** 28235    **Score:** 77     **Queries matched:** 9   **emPAI:** 0.29 |
| --- | --- |
|  | 60S ribosomal protein L8 OS=Homo sapiens GN=RPL8 PE=1 SV=2 |

|  | **Query** | **Observed** | **Mr(expt)** | **Mr(calc)** | **Delta** | **Miss** | **Score** | **Expect** | **Rank** | **Peptide** |
| --- | --- | --- | --- | --- | --- | --- | --- | --- | --- | --- |
|  | 1897 | **410.7334** | **819.4522** | **820.4555** | **-1.0033** | **1** | **9** | **24** | **10** | **R.KGAGSVFR.A** 1910 1920 |
|  | 2653 | 437.7215 | 873.4285 | 873.5032 | -0.0747 | 1 | 7 | 39 | 9 | K.KVISSANR.A |
|  | 3564 | 471.5981 | 941.1817 | 940.5454 | 0.6363 | 0 | 5 | 32 | 3 | R.AVVGVVAGGGR.I |
|  | 5193 | **529.3411** | **1056.6676** | **1056.6444** | **0.0232** | **1** | **7** | **38** | **8** | **R.GAPLAKVVFR.D** |
|  | 9760 | 440.5828 | 1318.7265 | 1318.7357 | -0.0093 | 1 | 20 | 1.3 | 1 | K.GIVKDIIHDPGR.G |
|  | 16025 | **844.9095** | **1687.8044** | **1687.8165** | **-0.0121** | **0** | **55** | **0.00035** | **1** | **R.ASGNYATVISHNPETK.K** |
|  | 28760 | **1089.8700** | **3266.5882** | **3266.6098** | **-0.0216** | **0** | **50** | **0.00043** | **1** | **K.AQLNIGNVLPVGTMPEGTIVCCLEEKPGDR.G** |

| **507.** | O15126\|SCAM1_HUMAN    **Mass:** 38295    **Score:** 77     **Queries matched:** 6   **emPAI:** 0.10 |
| --- | --- |
|  | Secretory carrier-associated membrane protein 1 OS=Homo sapiens GN=SCAMP1 PE=1 SV=2 |

|  | **Query** | **Observed** | **Mr(expt)** | **Mr(calc)** | **Delta** | **Miss** | **Score** | **Expect** | **Rank** | **Peptide** |
| --- | --- | --- | --- | --- | --- | --- | --- | --- | --- | --- |
|  | 2191 | **841.1047** | **840.0975** | **839.4025** | **0.6950** | **0** | **19** | **1.2** | **1** | **R.TTGASFEK.A** |
|  | 2628 | 437.2136 | 872.4127 | 871.5028 | 0.9099 | 1 | 2 | 1.2e+002 | 8 | K.KVHGLYR.T |
|  | 7671 | **405.6917** | **1214.0534** | **1214.5462** | **-0.4928** | **0** | **2** | **66** | **10** | **R.EMQNLSQHGR.K** |
|  | 11436 | **705.8326** | **1409.6506** | **1409.6609** | **-0.0103** | **0** | **77** | **2.7e-006** | **1** | **K.AQQEFATGVMSNK.T** |
|  | 25188 | 641.7995 | 2563.1689 | 2563.2626 | -0.0937 | 1 | 5 | 21 | 3 | K.TVQTAAANAASTAASSAAQNAFKGNQI.- |
|  | 29582 | 1772.7952 | 3543.5758 | 3542.7059 | 0.8699 | 1 | 1 | 29 | 6 | K.AQQEFATGVMSNKTVQTAAANAASTAASSAAQNAFK.G |

| **508.** | P01861\|IGHG4_HUMAN    **Mass:** 36431    **Score:** 76     **Queries matched:** 13   **emPAI:** 0.34 |
| --- | --- |
|  | Ig gamma-4 chain C region OS=Homo sapiens GN=IGHG4 PE=1 SV=1 |

|  | **Query** | **Observed** | **Mr(expt)** | **Mr(calc)** | **Delta** | **Miss** | **Score** | **Expect** | **Rank** | **Peptide** |
| --- | --- | --- | --- | --- | --- | --- | --- | --- | --- | --- |
|  | 6657 | 580.9736 | 1159.9327 | 1160.6223 | -0.6896 | 0 | 3 | 62 | 8 | K.NQVSLTCLVK.G |
|  | 15670 | 555.6471 | 1663.9194 | 1663.7624 | 0.1570 | 0 | 5 | 34 | 3 | K.TYTCNVDHKPSNTK.V 15665 |
|  | 17858 | 904.5013 | 1806.9881 | 1806.9992 | -0.0111 | 0 | 46 | 0.0025 | 1 | R.VVSVLTVLHQDWLNGK.E 17868 |
|  | 19021 | **631.3734** | **1891.0984** | **1891.8873** | **-0.7889** | **0** | **1** | **75** | **7** | **R.EPQVYTLPPSQEEMTK.N** |
|  | 24943 | 1272.5602 | 2543.1058 | 2543.1241 | -0.0183 | 0 | 38 | 0.01 | 1 | K.GFYPSDIAVEWESNGQPENNYK.T 24942 24944 24958 24960 |
|  | 27281 | **981.8173** | **2942.4300** | **2942.4500** | **-0.0200** | **0** | **32** | **0.032** | **1** | **K.YGPPCPSCPAPEFLGGPSVFLFPPKPK.D** |
|  | 31805 | **1341.4612** | **6702.2695** | **6701.2913** | **0.9783** | **1** | **16** | **0.11** | **1** | **K.DYFPEPVTVSWNSGALTSGVHTFPAVLQSSGLYSLSSVVTVPSSSLGTKTYTCNVDHKPSNTK.V** |

| **509.** | P61916\|NPC2_HUMAN    **Mass:** 16902    **Score:** 76     **Queries matched:** 8   **emPAI:** 0.51 |
| --- | --- |
|  | Epididymal secretory protein E1 OS=Homo sapiens GN=NPC2 PE=1 SV=1 |

|  | **Query** | **Observed** | **Mr(expt)** | **Mr(calc)** | **Delta** | **Miss** | **Score** | **Expect** | **Rank** | **Peptide** |
| --- | --- | --- | --- | --- | --- | --- | --- | --- | --- | --- |
|  | 5094 | **525.2463** | **1048.4781** | **1048.4859** | **-0.0078** | **0** | **29** | **0.2** | **1** | **K.DCGSVDGVIK.E** |
|  | 6214 | **565.7890** | **1129.5634** | **1130.5608** | **-0.9973** | **1** | **11** | **14** | **3** | **K.DKTYSYLNK.L** |
|  | 8610 | **421.0565** | **1260.1476** | **1259.7125** | **0.4351** | **1** | **9** | **14** | **6** | **K.LPVKSEYPSIK.L** |
|  | 18363 | **922.4313** | **1842.8481** | **1842.8604** | **-0.0123** | **0** | **45** | **0.0031** | **1** | **K.EVNVSPCPTQPCQLSK.G** 18362 |
|  | 18598 | 620.3407 | 1858.0003 | 1858.9061 | -0.9058 | 0 | 4 | 39 | 7 | K.GQSYSVNVTFTSNIQSK.S |
|  | 24151 | **486.4813** | **2427.3702** | **2428.2647** | **-0.8945** | **0** | **(3)** | **33** | **6** | **K.AVVHGILMGVPVPFPIPEPDGCK.S** |
|  | 24265 | **815.7564** | **2444.2472** | **2444.2596** | **-0.0124** | **0** | **4** | **31** | **1** | **K.AVVHGILMGVPVPFPIPEPDGCK.S** |

| **510.** | Q9H1C4\|UN93B_HUMAN    **Mass:** 66930    **Score:** 76     **Queries matched:** 6   **emPAI:** 0.06 |
| --- | --- |
|  | Protein unc-93 homolog B1 OS=Homo sapiens GN=UNC93B1 PE=1 SV=2 |

|  | **Query** | **Observed** | **Mr(expt)** | **Mr(calc)** | **Delta** | **Miss** | **Score** | **Expect** | **Rank** | **Peptide** |
| --- | --- | --- | --- | --- | --- | --- | --- | --- | --- | --- |
|  | 1674 | 402.2424 | 802.4703 | 803.4323 | -0.9620 | 1 | 24 | 1 | 3 | R.MEQKLR.R |
|  | 12858 | **746.8978** | **1491.7810** | **1491.7874** | **-0.0065** | **0** | **76** | **3.3e-006** | **1** | **R.SVGWGNIFQLPFK.H** |
|  | 14346 | **792.3489** | **1582.6832** | **1583.7191** | **-1.0358** | **1** | **2** | **71** | **10** | **R.MAQKYHEYSHYK.E** |
|  | 23464 | 777.7212 | 2330.1417 | 2330.3106 | -0.1688 | 0 | 0 | 68 | 7 | K.MLMGINVTPIAALLYTPVLIR.F |
|  | 28545 | **1070.8915** | **3209.6526** | **3208.5555** | **1.0971** | **1** | **0** | **38** | **6** | **R.FFGTKWMMFLAVGIYALFVSTNYWER.Y** |
|  | 29942 | **926.6768** | **3702.6782** | **3701.8493** | **0.8288** | **1** | **0** | **29** | **5** | **K.ERQDFIFTIYHWWQAVAIFTVYLGSSLHMK.A** |

| **511.** | Q9GZP9\|DERL2_HUMAN    **Mass:** 27663    **Score:** 75     **Queries matched:** 4   **emPAI:** 0.14 |
| --- | --- |
|  | Derlin-2 OS=Homo sapiens GN=DERL2 PE=1 SV=1 |

|  | **Query** | **Observed** | **Mr(expt)** | **Mr(calc)** | **Delta** | **Miss** | **Score** | **Expect** | **Rank** | **Peptide** |
| --- | --- | --- | --- | --- | --- | --- | --- | --- | --- | --- |
|  | 1690 | 402.7208 | 803.4271 | 803.4402 | -0.0131 | 1 | 10 | 19 | 3 | R.RNPYVR.M |
|  | 7269 | 399.5360 | 1195.5860 | 1196.5608 | -0.9748 | 1 | 8 | 24 | 8 | R.MLEEGSFRGR.T |
|  | 29156 | **1125.5094** | **3373.5064** | **3373.5276** | **-0.0212** | **0** | **75** | **1.3e-006** | **1** | **K.AIFDTPDEDPNYNPLPEERPGGFAWGEGQR.L** 29157 |

| **512.** | Q8TD06\|AGR3_HUMAN    **Mass:** 19273    **Score:** 75     **Queries matched:** 5   **emPAI:** 0.20 |
| --- | --- |
|  | Anterior gradient protein 3 homolog OS=Homo sapiens GN=AGR3 PE=1 SV=1 |

|  | **Query** | **Observed** | **Mr(expt)** | **Mr(calc)** | **Delta** | **Miss** | **Score** | **Expect** | **Rank** | **Peptide** |
| --- | --- | --- | --- | --- | --- | --- | --- | --- | --- | --- |
|  | 7582 | **404.2095** | **1209.6068** | **1210.6782** | **-1.0714** | **1** | **7** | **29** | **5** | **K.EKRPPQTLSR.G** |
|  | 14728 | 804.3862 | 1606.7579 | 1607.7687 | -1.0108 | 0 | 4 | 52 | 3 | K.FIMLNLMHETTDK.N 14752 |
|  | 17369 | **889.9165** | **1777.8184** | **1777.8305** | **-0.0120** | **0** | **75** | **3.4e-006** | **1** | **K.VFAQNEEIQEMAQNK.F** |
|  | 23424 | 1163.4725 | 2324.9305 | 2325.1609 | -0.2304 | 0 | 0 | 63 | 5 | K.KPLMVIHHLEDCQYSQALK.K |

| **513.** | P78527\|PRKDC_HUMAN    **Mass:** 473749   **Score:** 75     **Queries matched:** 97   **emPAI:** 0.03 |
| --- | --- |
|  | DNA-dependent protein kinase catalytic subunit OS=Homo sapiens GN=PRKDC PE=1 SV=3 |

|  | **Query** | **Observed** | **Mr(expt)** | **Mr(calc)** | **Delta** | **Miss** | **Score** | **Expect** | **Rank** | **Peptide** |
| --- | --- | --- | --- | --- | --- | --- | --- | --- | --- | --- |
|  | 363 | **432.2363** | **431.2291** | **430.2288** | **1.0002** | **0** | **8** | **70** | **1** | **K.GAAGR.T** 343 364 |
|  | 1255 | **684.2012** | **683.1940** | **683.3239** | **-0.1299** | **0** | **4** | **46** | **2** | **K.DDVHAK.E** |
|  | 1693 | **402.7331** | **803.4516** | **802.3933** | **1.0583** | **0** | **7** | **44** | **2** | **K.LNSNEAR.L** |
|  | 1719 | 403.2419 | 804.4691 | 804.3912 | 0.0779 | 0 | 13 | 11 | 7 | -.MAGSGAGVR.C |
|  | 1766 | 405.2987 | 808.5829 | 808.4807 | 0.1022 | 0 | 3 | 66 | 10 | R.LPPDVLR.W |
|  | 1898 | **410.7394** | **819.4643** | **820.3861** | **-0.9219** | **0** | **(8)** | **29** | **4** | **-.MAGSGAGVR.C** 1919 |
|  | 2072 | 833.4576 | 832.4504 | 831.5218 | 0.9286 | 0 | 26 | 0.54 | 2 | K.VFLALAAK.G |
|  | 2355 | **427.1396** | **852.2647** | **852.4527** | **-0.1880** | **0** | **3** | **64** | **4** | **K.LSLMYAR.K** |
|  | 2374 | 427.7374 | 853.4602 | 853.5385 | -0.0783 | 0 | 6 | 33 | 1 | K.LKPVELR.E 2358 |
|  | 2558 | **434.2407** | **866.4669** | **865.5021** | **0.9648** | **0** | **12** | **9.4** | **1** | **R.HNLEIIK.T** |
|  | 2698 | **439.2352** | **876.4558** | **876.5069** | **-0.0511** | **1** | **10** | **18** | **2** | **K.FGKEVAVK.M** |
|  | 2699 | **439.2398** | **876.4651** | **875.4535** | **1.0117** | **0** | **3** | **93** | **5** | **K.QVSNMVAK.N** |
|  | 2907 | **447.2372** | **892.4599** | **891.4484** | **1.0115** | **0** | **(3)** | **94** | **6** | **K.QVSNMVAK.N** |
|  | 3157 | 456.8823 | 911.7500 | 910.5600 | 1.1900 | 1 | 0 | 93 | 7 | K.VPADLLKR.A |
|  | 3616 | **944.9847** | **943.9775** | **944.5728** | **-0.5954** | **0** | **0** | **1.3e+002** | **7** | **K.MAVLALLAK.I** |
|  | 3919 | **483.7765** | **965.5385** | **965.4245** | **0.1140** | **0** | **10** | **15** | **3** | **K.MQLVCCR.A** |
|  | 4423 | **500.9520** | **999.8894** | **1000.6182** | **-0.7287** | **1** | **6** | **33** | **2** | **K.RTFPVLLR.L** |
|  | 4555 | **506.2367** | **1010.4589** | **1009.4400** | **1.0189** | **0** | **24** | **0.56** | **1** | **K.CFGTGAAGNR.T** 4546 4547 |
|  | 4568 | **506.2715** | **1010.5283** | **1011.5059** | **-0.9776** | **0** | **8** | **26** | **1** | **K.ILDVMYSR.L** |
|  | 4804 | 515.7517 | 1029.4889 | 1028.5727 | 0.9162 | 1 | 10 | 16 | 1 | K.GAAGRTDLLR.L |
|  | 5413 | **537.2463** | **1072.4781** | **1072.4859** | **-0.0078** | **0** | **16** | **4.4** | **1** | **K.NTCTSVYTK.D** |
|  | 5980 | **557.2041** | **1112.3936** | **1111.5332** | **0.8605** | **1** | **1** | **99** | **5** | **R.SSRLMDEFK.I** |
|  | 6152 | **563.2562** | **1124.4979** | **1124.5648** | **-0.0669** | **1** | **0** | **1.4e+002** | **7** | **K.SKDFVQVMR.H** |
|  | 6674 | **581.3109** | **1160.6073** | **1160.6336** | **-0.0263** | **1** | **4** | **71** | **8** | **R.SRLAAVVSACK.Q** |
|  | 7242 | **598.3259** | **1194.6372** | **1194.4823** | **0.1549** | **0** | **4** | **59** | **9** | **K.SMEEDPQTSR.E** |
|  | 7742 | 406.5653 | 1216.6740 | 1217.7019 | -1.0279 | 0 | 8 | 26 | 3 | R.LLALNSLYSPK.I 7752 |
|  | 8053 | **617.4319** | **1232.8492** | **1233.6613** | **-0.8121** | **0** | **(0)** | **1.2e+002** | **9** | **R.QFINLMLPMK.E** |
|  | 8383 | **417.2426** | **1248.7059** | **1249.6563** | **-0.9504** | **0** | **10** | **13** | **3** | **R.QFINLMLPMK.E** |
|  | 8420 | 626.3577 | 1250.7008 | 1249.6563 | 1.0445 | 0 | (1) | 1.2e+002 | 5 | R.QFINLMLPMK.E |
|  | 8447 | **418.5665** | **1252.6776** | **1253.6656** | **-0.9880** | **1** | **6** | **35** | **8** | **K.YFEGVSPKSLK.H** 8477 |
|  | 8845 | **425.0476** | **1272.1208** | **1272.7336** | **-0.6128** | **1** | **6** | **27** | **8** | **R.VTVMASLRRPK.R** |
|  | 9870 | **442.2351** | **1323.6834** | **1323.7584** | **-0.0750** | **1** | **5** | **41** | **5** | **R.EPKLPVLAGCLK.G** |
|  | 9969 | **664.7797** | **1327.5448** | **1327.6918** | **-0.1470** | **1** | **12** | **9.6** | **1** | **K.MNKDSKPPGNLK.E** |
|  | 10350 | **451.2287** | **1350.6643** | **1349.7125** | **0.9517** | **1** | **2** | **92** | **7** | **K.MKQDAQVVLYR.S** |
|  | 10425 | 452.2093 | 1353.6062 | 1354.6994 | -1.0931 | 1 | 4 | 55 | 10 | R.LSFTRQSFVDR.S |
|  | 10529 | 680.3931 | 1358.7716 | 1358.6871 | 0.0845 | 1 | 0 | 1.4e+002 | 6 | K.FIQTFGKEFDK.H |
|  | 10985 | 692.3659 | 1382.7173 | 1382.7405 | -0.0233 | 1 | 1 | 99 | 7 | R.LGLPGDEVDNKVK.G |
|  | 11001 | 692.8619 | 1383.7092 | 1384.6843 | -0.9751 | 0 | 3 | 68 | 4 | R.HECMAPLTALVK.H |
|  | 11161 | 697.4554 | 1392.8962 | 1392.7040 | 0.1922 | 1 | 2 | 65 | 6 | K.MQLVCCRAIVK.V |
|  | 11435 | 470.8908 | 1409.6506 | 1409.6721 | -0.0215 | 1 | 6 | 29 | 6 | K.SLNSIEFRECR.E |
|  | 11811 | 716.3849 | 1430.7552 | 1429.7201 | 1.0351 | 0 | 7 | 25 | 3 | K.GGSWIQEINVAEK.N |
|  | 11908 | **479.7911** | **1436.3516** | **1435.6864** | **0.6652** | **0** | **7** | **18** | **5** | **K.DLLLNTMSQEEK.A** |
|  | 12361 | 488.5781 | 1462.7124 | 1462.7854 | -0.0730 | 1 | 8 | 21 | 5 | K.MGQKIAPYSVEIK.N |
|  | 12382 | 489.2261 | 1464.6564 | 1465.7428 | -1.0864 | 1 | 2 | 79 | 5 | K.YSCFALFVKFGK.E |
|  | 13242 | **505.9137** | **1514.7193** | **1514.7147** | **0.0046** | **1** | **2** | **79** | **8** | **K.QLKQHQNTMEDK.F** |
|  | 13284 | **506.2939** | **1515.8599** | **1516.7807** | **-0.9208** | **0** | **3** | **58** | **6** | **K.NILEESLCELVAK.Q** |
|  | 13467 | 510.2563 | 1527.7471 | 1526.7439 | 1.0032 | 0 | 0 | 1.1e+002 | 10 | K.DVDFMYVELIQR.C |
|  | 13501 | **765.4282** | **1528.8419** | **1528.8514** | **-0.0095** | **1** | **9** | **17** | **5** | **K.RLYSLALHPNAFK.R** |
|  | 13685 | 771.3978 | 1540.7810 | 1541.7656 | -0.9846 | 1 | 7 | 26 | 6 | K.VCLDIIYKMMPK.L |
|  | 13730 | **772.8130** | **1543.6114** | **1542.6621** | **0.9494** | **0** | **1** | **97** | **3** | **K.QMFLTQTDTGDDR.V** |
|  | 14214 | **525.6355** | **1573.8847** | **1573.7947** | **0.0900** | **0** | **11** | **9.2** | **2** | **K.NLSSNEAISLEEIR.I** |
|  | 14240 | **526.2945** | **1575.8616** | **1575.7061** | **0.1555** | **1** | **7** | **26** | **7** | **K.MYERMYAALGDPK.A** |
|  | 14752 | 804.8847 | 1607.7548 | 1607.7654 | -0.0105 | 0 | 26 | 0.32 | 1 | R.YNFPVEVEVPMER.K |
|  | 14900 | 809.3615 | 1616.7085 | 1615.8644 | 0.8441 | 0 | 4 | 51 | 2 | R.GFPPSASLCLLDLVK.W |
|  | 14939 | 810.3740 | 1618.7335 | 1618.8031 | -0.0696 | 0 | 11 | 8.9 | 3 | K.FYQGFLFSEKPEK.N 14938 |
|  | 15086 | **814.9634** | **1627.9122** | **1627.9185** | **-0.0063** | **0** | **70** | **1.1e-005** | **1** | **R.LGLIEWLENTVTLK.D** |
|  | 15195 | **546.2866** | **1635.8380** | **1635.8178** | **0.0202** | **0** | **2** | **69** | **5** | **R.SDPGLLTNTMDVFVK.E** |
|  | 15756 | **557.3003** | **1668.8792** | **1669.7844** | **-0.9052** | **1** | **1** | **88** | **6** | **K.MGYYKILDVMYSR.L** |
|  | 15908 | 560.9396 | 1679.7969 | 1679.8665 | -0.0696 | 1 | 4 | 44 | 9 | R.IAGFDERVTVMASLR.R |
|  | 16340 | **570.2968** | **1707.8686** | **1706.8490** | **1.0196** | **0** | **4** | **41** | **2** | **K.LQYFMEQFYGIIR.N** |
|  | 16468 | **572.9139** | **1715.7200** | **1714.9539** | **0.7661** | **1** | **10** | **11** | **1** | **K.DSPLETKMAVLALLAK.I** |
|  | 16689 | 577.3220 | 1728.9441 | 1727.9716 | 0.9724 | 1 | 4 | 44 | 2 | R.IRVVQMLGSLGGQINK.N 16657 16674 |
|  | 17373 | 889.9428 | 1777.8711 | 1777.7837 | 0.0873 | 1 | 2 | 76 | 10 | K.MSGKHDVGAYMLMYK.G |
|  | 17532 | **596.8995** | **1787.6768** | **1787.0570** | **0.6198** | **1** | **3** | **44** | **3** | **K.FLHGKLGNPIVPLNIR.L** |
|  | 17805 | **602.3060** | **1803.8963** | **1802.9373** | **0.9589** | **1** | **4** | **45** | **7** | **K.TKNLSSNEAISLEEIR.I** |
|  | 17956 | 908.4322 | 1814.8498 | 1814.0149 | 0.8349 | 0 | 4 | 41 | 9 | R.TVGALQVLGTEAQSSLLK.A |
|  | 18525 | 618.3607 | 1852.0602 | 1850.9713 | 1.0889 | 1 | 3 | 52 | 4 | K.ELSIAIRGYGLFAGPCK.V 18513 |
|  | 19138 | **635.3002** | **1902.8789** | **1903.0302** | **-0.1514** | **0** | **0** | **93** | **10** | **K.LLLQGEADQSLLTFIDK.A** |
|  | 19316 | **640.3111** | **1917.9115** | **1916.8977** | **1.0138** | **0** | **0** | **1e+002** | **3** | **R.ATQQQHDFTLTQTADGR.S** |
|  | 19487 | 966.0827 | 1930.1508 | 1929.9488 | 0.2021 | 1 | 2 | 58 | 4 | R.HECMAPLTALVKHMHR.S 19488 |
|  | 21151 | **691.4603** | **2071.3592** | **2070.9292** | **0.4300** | **1** | **1** | **50** | **2** | **R.EFPPGTPRFNNYVDCMK.K** |
|  | 21647 | **707.3926** | **2119.1561** | **2118.2048** | **0.9513** | **0** | **2** | **58** | **3** | **K.DVLIQGLIDENPGLQLIIR.N** |
|  | 22418 | **737.0007** | **2207.9802** | **2209.1758** | **-1.1956** | **1** | **3** | **44** | **1** | **K.SFPPLADRFMNAVFFLLPK.F** |
|  | 22674 | 744.0658 | 2229.1756 | 2230.0875 | -0.9119 | 1 | 1 | 60 | 6 | R.RGSCVTQVGLLESVYEMFR.K |
|  | 22863 | **752.4097** | **2254.2074** | **2254.0511** | **0.1563** | **1** | **1** | **57** | **9** | **K.ATQMPEGGQGAPPMYQLYKR.T** |
|  | 23190 | 765.7109 | 2294.1108 | 2293.1260 | 0.9849 | 1 | 3 | 37 | 5 | K.DLLLNTMSQEEKAAYLSDPR.A |
|  | 23424 | 1163.4725 | 2324.9305 | 2325.1353 | -0.2048 | 1 | 1 | 48 | 3 | R.QTKVAACELLHSMVMFMLGK.A |
|  | 24513 | **828.3624** | **2482.0655** | **2482.3067** | **-0.2413** | **1** | **0** | **62** | **1** | **K.TVSLLDENNVSSYLSKNILAFR.D** |
|  | 26117 | **903.7920** | **2708.3541** | **2707.3634** | **0.9907** | **0** | **22** | **0.4** | **1** | **K.FLDALELSQSPMLLELMTEVLCR.E** |
|  | 26893 | **953.1534** | **2856.4383** | **2856.4480** | **-0.0097** | **0** | **26** | **0.15** | **1** | **K.VYELLGLLGEVHPSEMINNAENLFR.A** |
|  | 27134 | **970.4825** | **2908.4258** | **2908.4708** | **-0.0450** | **1** | **1** | **46** | **2** | **R.VVQMLGSLGGQINKNLLTVTSSDEMMK.S** |
|  | 27528 | **997.4750** | **2989.4033** | **2990.5066** | **-1.1033** | **1** | **3** | **28** | **2** | **R.VEQLFQVMNGILAQDSACSQRALQLR.T** 27533 |
|  | 30463 | **1005.2268** | **4016.8781** | **4015.8837** | **0.9945** | **1** | **0** | **27** | **1** | **K.VAACELLHSMVMFMLGKATQMPEGGQGAPPMYQLYK.R** |

| **514.** | P01009\|A1AT_HUMAN    **Mass:** 46878    **Score:** 75     **Queries matched:** 10   **emPAI:** 0.17 |
| --- | --- |
|  | Alpha-1-antitrypsin OS=Homo sapiens GN=SERPINA1 PE=1 SV=3 |

|  | **Query** | **Observed** | **Mr(expt)** | **Mr(calc)** | **Delta** | **Miss** | **Score** | **Expect** | **Rank** | **Peptide** |
| --- | --- | --- | --- | --- | --- | --- | --- | --- | --- | --- |
|  | 2356 | **427.1657** | **852.3169** | **851.4865** | **0.8304** | **0** | **11** | **12** | **2** | **R.SASLHLPK.L** |
|  | 4636 | **508.7994** | **1015.5841** | **1014.6073** | **0.9768** | **0** | **4** | **80** | **4** | **K.SVLGQLGITK.V** |
|  | 5488 | 539.7637 | 1077.5129 | 1077.5203 | -0.0074 | 1 | 19 | 2.2 | 2 | K.FLENEDRR.S |
|  | 6296 | **568.8658** | **1135.7171** | **1135.5873** | **0.1298** | **1** | **5** | **53** | **4** | **K.KQINDYVEK.G** |
|  | 7465 | **602.8562** | **1203.6978** | **1203.7049** | **-0.0071** | **1** | **56** | **0.00043** | **1** | **K.KLSSWVLLMK.Y** |
|  | 12621 | **493.9225** | **1478.7456** | **1478.7365** | **0.0091** | **1** | **10** | **14** | **6** | **K.QINDYVEKGTQGK.I** |
|  | 15292 | 548.3200 | 1641.9382 | 1640.8562 | 1.0820 | 0 | 3 | 57 | 10 | K.ITPNLAEFAFSLYR.Q 15285 |
|  | 17381 | **593.9229** | **1778.7467** | **1778.7609** | **-0.0141** | **0** | **21** | **0.83** | **1** | **K.TDTSHHDQDHPTFNK.I** |
|  | 21330 | **697.3673** | **2089.0799** | **2089.0884** | **-0.0085** | **1** | **47** | **0.0016** | **1** | **K.ELDRDTVFALVNYIFFK.G** |

| **515.** | O95816\|BAG2_HUMAN    **Mass:** 23928    **Score:** 74     **Queries matched:** 13   **emPAI:** 0.34 |
| --- | --- |
|  | BAG family molecular chaperone regulator 2 OS=Homo sapiens GN=BAG2 PE=1 SV=1 |

|  | **Query** | **Observed** | **Mr(expt)** | **Mr(calc)** | **Delta** | **Miss** | **Score** | **Expect** | **Rank** | **Peptide** |
| --- | --- | --- | --- | --- | --- | --- | --- | --- | --- | --- |
|  | 4025 | **487.3289** | **972.6432** | **971.5148** | **1.1284** | **1** | **4** | **72** | **2** | **K.INAKANEGR.F** |
|  | 4047 | 488.2459 | 974.4773 | 973.5378 | 0.9394 | 1 | 8 | 33 | 9 | -.MAQAKINAK.A |
|  | 4291 | **497.2544** | **992.4942** | **991.4975** | **0.9967** | **0** | **16** | **4.7** | **1** | **K.FLDDLGNAK.S** 4271 4275 4287 |
|  | 6157 | 563.2993 | 1124.5841 | 1125.6142 | -1.0301 | 1 | 9 | 19 | 7 | K.LLEHSKGAGSK.T |
|  | 12081 | **483.1478** | **1446.4217** | **1445.7222** | **0.6994** | **1** | **3** | **42** | **5** | **K.GAGSKTLQQNAESR.F** |
|  | 13535 | **766.3994** | **1530.7841** | **1531.7776** | **-0.9935** | **1** | **1** | **1.1e+002** | **4** | **R.EELNLTANRLMGR.T** |
|  | 14737 | 536.6329 | 1606.8768 | 1606.8025 | 0.0743 | 0 | 6 | 28 | 6 | K.FQSIVIGCALEDQK.K 14740 |
|  | 28075 | 1038.5070 | 3112.4991 | 3112.5168 | -0.0178 | 1 | 70 | 4.5e-006 | 1 | R.EAATAVEQEKEILLEMIHSIQNSQDMR.Q 28076 |

| **516.** | Q96GC9\|TMM49_HUMAN    **Mass:** 46607    **Score:** 74     **Queries matched:** 8   **emPAI:** 0.08 |
| --- | --- |
|  | Transmembrane protein 49 OS=Homo sapiens GN=TMEM49 PE=2 SV=1 |

|  | **Query** | **Observed** | **Mr(expt)** | **Mr(calc)** | **Delta** | **Miss** | **Score** | **Expect** | **Rank** | **Peptide** |
| --- | --- | --- | --- | --- | --- | --- | --- | --- | --- | --- |
|  | 1203 | 656.3315 | 655.3242 | 655.3475 | -0.0233 | 0 | 7 | 26 | 6 | K.MHIQK.I |
|  | 7158 | 596.2889 | 1190.5632 | 1190.5098 | 0.0534 | 1 | 11 | 13 | 2 | M.AENGKNCDQR.R 7159 |
|  | 8261 | **622.8588** | **1243.7030** | **1243.6520** | **0.0509** | **1** | **13** | **7.3** | **5** | **R.IQQRLNSEEK.T** |
|  | 10139 | **670.3232** | **1338.6319** | **1337.5452** | **1.0867** | **1** | **2** | **79** | **8** | **-.MAENGKNCDQR.R** |
|  | 16525 | **573.9963** | **1718.9670** | **1719.9746** | **-1.0076** | **1** | **1** | **90** | **5** | **K.MHIQKIFVIITFSK.H** |
|  | 29379 | **1153.8133** | **3458.4182** | **3458.4368** | **-0.0186** | **0** | **52** | **0.0002** | **1** | **R.LSGAEPDDEEYQEFEEMLEHAESAQDFASR.A** 29378 |

| **517.** | P62888\|RL30_HUMAN    **Mass:** 12947    **Score:** 74     **Queries matched:** 6   **emPAI:** 0.30 |
| --- | --- |
|  | 60S ribosomal protein L30 OS=Homo sapiens GN=RPL30 PE=1 SV=2 |

|  | **Query** | **Observed** | **Mr(expt)** | **Mr(calc)** | **Delta** | **Miss** | **Score** | **Expect** | **Rank** | **Peptide** |
| --- | --- | --- | --- | --- | --- | --- | --- | --- | --- | --- |
|  | 4716 | **512.2832** | **1022.5518** | **1021.4386** | **1.1132** | **0** | **8** | **27** | **3** | **R.SMPEQTGEK.-** |
|  | 4860 | **517.2815** | **1032.5484** | **1032.5563** | **-0.0079** | **1** | **35** | **0.057** | **1** | **K.KSLESINSR.L** |
|  | 9699 | 658.8362 | 1315.6579 | 1316.6322 | -0.9743 | 0 | 7 | 28 | 6 | K.SEIEYYAMLAK.T |
|  | 17092 | 879.4533 | 1756.8921 | 1756.9029 | -0.0109 | 0 | 66 | 2.8e-005 | 1 | R.VCTLAIIDPGDSDIIR.S 17093 |
|  | 20493 | **672.5223** | **2014.5452** | **2013.9327** | **0.6125** | **0** | **2** | **42** | **5** | **K.TGVHHYSGNNIELGTACGK.Y** |

| **518.** | Q99747\|SNAG_HUMAN    **Mass:** 35066    **Score:** 74     **Queries matched:** 11   **emPAI:** 0.11 |
| --- | --- |
|  | Gamma-soluble NSF attachment protein OS=Homo sapiens GN=NAPG PE=1 SV=1 |

|  | **Query** | **Observed** | **Mr(expt)** | **Mr(calc)** | **Delta** | **Miss** | **Score** | **Expect** | **Rank** | **Peptide** |
| --- | --- | --- | --- | --- | --- | --- | --- | --- | --- | --- |
|  | 5178 | **529.2551** | **1056.4957** | **1055.5499** | **0.9458** | **0** | **7** | **33** | **1** | **K.LIENVDPEK.A** |
|  | 6113 | 561.7703 | 1121.5261 | 1120.5764 | 0.9497 | 0 | 7 | 32 | 2 | R.FDEAALSIQK.E |
|  | 7574 | **605.3686** | **1208.7226** | **1208.7493** | **-0.0266** | **0** | **6** | **35** | **3** | **K.LGLSLVVPGGGIK.K** |
|  | 9603 | **657.3998** | **1312.7850** | **1311.7034** | **1.0816** | **1** | **3** | **68** | **5** | **R.AGKLIENVDPEK.A** |
|  | 10626 | **455.8856** | **1364.6349** | **1364.6507** | **-0.0158** | **1** | **5** | **42** | **5** | **K.QFEQAKDACLR.E** |
|  | 15729 | 557.0920 | 1668.2541 | 1667.8664 | 0.3877 | 1 | 0 | 73 | 7 | -.MAAQKINEGLEHLAK.A |
|  | 15792 | **558.3198** | **1671.9375** | **1670.8913** | **1.0462** | **1** | **1** | **86** | **10** | **K.EMQKLPEAVQLIEK.A** |
|  | 20744 | 1019.9870 | 2037.9594 | 2037.9756 | -0.0161 | 0 | 54 | 0.00031 | 1 | K.AVQLYQQTANVFENEER.L 20743 20754 |
|  | 24242 | **815.0646** | **2442.1721** | **2443.1181** | **-0.9460** | **1** | **0** | **71** | **4** | **K.ASMMYLENGTPDTAAMALERAGK.L** |

| **519.** | O75964\|ATP5L_HUMAN    **Mass:** 11421    **Score:** 74     **Queries matched:** 3   **emPAI:** 0.82 |
| --- | --- |
|  | ATP synthase subunit g, mitochondrial OS=Homo sapiens GN=ATP5L PE=1 SV=3 |

|  | **Query** | **Observed** | **Mr(expt)** | **Mr(calc)** | **Delta** | **Miss** | **Score** | **Expect** | **Rank** | **Peptide** |
| --- | --- | --- | --- | --- | --- | --- | --- | --- | --- | --- |
|  | 6507 | **576.3022** | **1150.5898** | **1150.5982** | **-0.0084** | **0** | **41** | **0.013** | **1** | **K.IVNSAQTGSFK.Q** |
|  | 8975 | 640.3495 | 1278.6844 | 1278.6932 | -0.0088 | 1 | 59 | 0.00016 | 1 | K.KIVNSAQTGSFK.Q |
|  | 10342 | **676.3120** | **1350.6095** | **1349.7125** | **0.8969** | **1** | **4** | **54** | **1** | **-.MAQFVRNLVEK.T** |

| **520.** | Q15109\|RAGE_HUMAN    **Mass:** 43118    **Score:** 73     **Queries matched:** 14   **emPAI:** 0.52 |
| --- | --- |
|  | Advanced glycosylation end product-specific receptor OS=Homo sapiens GN=AGER PE=1 SV=1 |

|  | **Query** | **Observed** | **Mr(expt)** | **Mr(calc)** | **Delta** | **Miss** | **Score** | **Expect** | **Rank** | **Peptide** |
| --- | --- | --- | --- | --- | --- | --- | --- | --- | --- | --- |
|  | 970 | **560.8671** | **559.8599** | **559.3078** | **0.5521** | **0** | **8** | **36** | **5** | **K.LNTGR.T** 969 |
|  | 13421 | 763.3878 | 1524.7610 | 1524.7685 | -0.0075 | 0 | 22 | 0.72 | 2 | K.VLSPQGGGPWDSVAR.V |
|  | 24746 | 1256.6603 | 2511.3060 | 2511.3221 | -0.0160 | 0 | 48 | 0.001 | 1 | R.VYQIPGKPEIVDSASELTAGVPNK.V 24747 |
|  | 28024 | 775.3815 | 3097.4968 | 3097.5179 | -0.0211 | 0 | 26 | 0.11 | 1 | K.VGTCVSEGSYPAGTLSWHLDGKPLVPNEK.G |
|  | 30009 | **933.8853** | **3731.5119** | **3731.0960** | **0.4159** | **1** | **5** | **8.1** | **1** | **-.MAAGTAVGAWVLVLSLWGAVVGAQNITARIGEPLVLK.C** |
|  | 30843 | **1084.0388** | **4332.1262** | **4332.1485** | **-0.0223** | **0** | **20** | **0.2** | **1** | **K.DGVPLPLPPSPVLILPEIGPQDQGTYSCVATHSSHGPQESR.A** 30840 30842 30844 |
|  | 31191 | **1216.1138** | **4860.4260** | **4860.4507** | **-0.0247** | **0** | **30** | **0.015** | **1** | **R.VWEPVPLEEVQLVVEPEGGAVAPGGTVTLTCEVPAQPSPQIHWMK.D** 31192 |
|  | 31694 | 932.9766 | 5591.8157 | 5590.8294 | 0.9863 | 1 | 11 | 0.79 | 1 | R.VYQIPGKPEIVDSASELTAGVPNKVGTCVSEGSYPAGTLSWHLDGKPLVPNEK.G |

| **521.** | Q9P0I2\|TM111_HUMAN    **Mass:** 29932    **Score:** 73     **Queries matched:** 12   **emPAI:** 0.13 |
| --- | --- |
|  | Transmembrane protein 111 OS=Homo sapiens GN=TMEM111 PE=1 SV=3 |

|  | **Query** | **Observed** | **Mr(expt)** | **Mr(calc)** | **Delta** | **Miss** | **Score** | **Expect** | **Rank** | **Peptide** |
| --- | --- | --- | --- | --- | --- | --- | --- | --- | --- | --- |
|  | 9426 | **652.3373** | **1302.6600** | **1301.6979** | **0.9621** | **0** | **4** | **53** | **1** | **R.HYVSILLQSDK.K** |
|  | 12013 | **481.9246** | **1442.7519** | **1443.7391** | **-0.9872** | **0** | **9** | **16** | **6** | **-.MAGPELLLDSNIR.L** 12025 |
|  | 14889 | **539.6174** | **1615.8303** | **1614.8577** | **0.9726** | **0** | **8** | **20** | **10** | **K.LTQEQVSDSQVLIR.S** |
|  | 18496 | 926.4169 | 1850.8193 | 1849.9170 | 0.9023 | 0 | 4 | 42 | 9 | R.SIYSLILGQDNAADQSR.M |
|  | 20957 | **1028.9287** | **2055.8429** | **2055.8556** | **-0.0127** | **0** | **73** | **3.6e-006** | **1** | **R.MMQEQMTGAAMAMPADTNK.A** |
|  | 21130 | **691.2429** | **2070.7068** | **2071.8505** | **-1.1437** | **0** | **(2)** | **35** | **3** | **R.MMQEQMTGAAMAMPADTNK.A** |
|  | 21322 | **697.3218** | **2088.9435** | **2087.8454** | **1.0981** | **0** | **(2)** | **53** | **2** | **R.MMQEQMTGAAMAMPADTNK.A** |
|  | 21882 | **717.3739** | **2149.0999** | **2150.0132** | **-0.9133** | **1** | **(1)** | **69** | **1** | **R.KVVPPSPMTDPTMLTDMMK.G** |
|  | 22060 | **723.1801** | **2166.5183** | **2166.0081** | **0.5102** | **1** | **2** | **39** | **4** | **R.KVVPPSPMTDPTMLTDMMK.G** |
|  | 24183 | **609.5472** | **2434.1599** | **2434.0459** | **0.1140** | **1** | **3** | **33** | **3** | **R.MMQEQMTGAAMAMPADTNKAFK.T** |
|  | 31244 | 1639.0830 | 4914.2272 | 4913.4260 | 0.8012 | 1 | 0 | 14 | 3 | K.VVPPSPMTDPTMLTDMMKGNVTNVLPMILIGGWINMTFSGFVTTK.V |

| **522.** | O75955\|FLOT1_HUMAN    **Mass:** 47554    **Score:** 73     **Queries matched:** 18   **emPAI:** 0.26 |
| --- | --- |
|  | Flotillin-1 OS=Homo sapiens GN=FLOT1 PE=1 SV=3 |

|  | **Query** | **Observed** | **Mr(expt)** | **Mr(calc)** | **Delta** | **Miss** | **Score** | **Expect** | **Rank** | **Peptide** |
| --- | --- | --- | --- | --- | --- | --- | --- | --- | --- | --- |
|  | 877 | 530.9559 | 529.9487 | 530.2813 | -0.3326 | 0 | 15 | 7.1 | 1 | R.DAGIR.E |
|  | 2611 | 436.2687 | 870.5228 | 871.4875 | -0.9647 | 1 | 2 | 1e+002 | 8 | K.ELEARVR.K |
|  | 2904 | **447.2260** | **892.4375** | **892.3960** | **0.0415** | **0** | **6** | **49** | **8** | **R.AEAEQMAK.K** |
|  | 3960 | **485.7575** | **969.5005** | **969.5066** | **-0.0061** | **0** | **30** | **0.16** | **1** | **R.SPPVMVAGGR.V** 3982 |
|  | 5156 | **528.2849** | **1054.5553** | **1054.5883** | **-0.0330** | **1** | **11** | **12** | **1** | **R.VRKPAEAER.Y** |
|  | 5863 | **552.7719** | **1103.5293** | **1103.5393** | **-0.0100** | **1** | **32** | **0.12** | **1** | **R.ARAEAEQMAK.K** 5850 |
|  | 10893 | **460.2561** | **1377.7465** | **1377.6823** | **0.0642** | **1** | **10** | **15** | **2** | **R.MRGEAEAFAIGAR.A** |
|  | 11174 | **698.3185** | **1394.6224** | **1394.7075** | **-0.0851** | **0** | **2** | **86** | **7** | **K.ITLVSSGSGTMGAAK.V** |
|  | 12434 | 734.8528 | 1467.6910 | 1467.7279 | -0.0369 | 0 | 15 | 4.2 | 1 | K.VSAQYLSEIEMAK.A |
|  | 13966 | **520.2512** | **1557.7318** | **1558.8930** | **-1.1612** | **1** | **4** | **49** | **4** | **R.ISLNTLTLNVKSEK.V** |
|  | 14649 | **802.4036** | **1602.7926** | **1602.8035** | **-0.0109** | **0** | **60** | **0.00012** | **1** | **K.SQLIMQAEAEAASVR.M** 14675 |
|  | 14918 | 809.8762 | 1617.7379 | 1618.7984 | -1.0605 | 0 | (3) | 64 | 6 | K.SQLIMQAEAEAASVR.M |
|  | 25256 | **859.1539** | **2574.4400** | **2575.3891** | **-0.9492** | **1** | **3** | **32** | **1** | **K.ITLVSSGSGTMGAAKVTGEVLDILTR.L** |
|  | 29599 | 888.6584 | 3550.6045 | 3549.7595 | 0.8449 | 1 | 3 | 19 | 6 | K.TEAEIAHIALETLEGHQRAIMAHMTVEEIYK.D 29592 |

| **523.** | P05141\|ADT2_HUMAN    **Mass:** 33102    **Score:** 73     **Queries matched:** 25   **emPAI:** 0.72 |
| --- | --- |
|  | ADP/ATP translocase 2 OS=Homo sapiens GN=SLC25A5 PE=1 SV=6 |

|  | **Query** | **Observed** | **Mr(expt)** | **Mr(calc)** | **Delta** | **Miss** | **Score** | **Expect** | **Rank** | **Peptide** |
| --- | --- | --- | --- | --- | --- | --- | --- | --- | --- | --- |
|  | 2175 | 420.6770 | 839.3394 | 839.3452 | -0.0058 | 0 | (11) | 11 | 5 | R.MMMQSGR.K 2188 |
|  | 2406 | 429.0863 | 856.1580 | 855.3401 | 0.8179 | 0 | (10) | 12 | 8 | R.MMMQSGR.K |
|  | 2408 | 429.0867 | 856.1589 | 855.3401 | 0.8188 | 0 | 16 | 3.3 | 1 | R.MMMQSGR.K 2394 |
|  | 2425 | 429.3168 | 856.6190 | 855.4814 | 1.1376 | 0 | 10 | 18 | 1 | K.TAVAPIER.V 2392 |
|  | 2606 | 436.2230 | 870.4314 | 871.3350 | -0.9037 | 0 | (3) | 87 | 6 | R.MMMQSGR.K |
|  | 3011 | 451.7430 | 901.4713 | 901.4770 | -0.0056 | 0 | 40 | 0.02 | 1 | K.GAWSNVLR.G 3009 |
|  | 3324 | **462.2595** | **922.5045** | **922.5124** | **-0.0078** | **1** | **11** | **13** | **4** | **K.IYKSDGIK.G** |
|  | 4577 | 506.6468 | 1011.2790 | 1011.4412 | -0.1622 | 1 | (0) | 1.1e+002 | 8 | R.RMMMQSGR.K |
|  | 5032 | 523.0566 | 1044.0987 | 1043.4310 | 0.6677 | 1 | 0 | 1.1e+002 | 9 | R.RMMMQSGR.K |
|  | 5177 | **529.2255** | **1056.4365** | **1055.4957** | **0.9407** | **0** | **3** | **90** | **1** | **-.MTDAAVSFAK.D** 5153 |
|  | 6249 | **566.8231** | **1131.6316** | **1131.6400** | **-0.0085** | **1** | **23** | **0.84** | **1** | **K.QIFLGGVDKR.T** |
|  | 6295 | 568.8387 | 1135.6628 | 1135.6713 | -0.0085 | 0 | 31 | 0.14 | 1 | K.LLLQVQHASK.Q |
|  | 7770 | **610.2991** | **1218.5837** | **1218.5921** | **-0.0084** | **0** | **30** | **0.15** | **1** | **R.AAYFGIYDTAK.G** |
|  | 7783 | 407.2260 | 1218.6561 | 1218.6608 | -0.0047 | 0 | 3 | 74 | 3 | K.DFLAGGVAAAISK.T 7781 |
|  | 12063 | 723.8700 | 1445.7254 | 1445.7343 | -0.0089 | 0 | 54 | 0.00053 | 1 | R.YFPTQALNFAFK.D |
|  | 19427 | 643.0295 | 1926.0668 | 1926.0363 | 0.0305 | 0 | 2 | 54 | 6 | K.GLYQGFNVSVQGIIIYR.A 19429 |
|  | 22789 | 561.2785 | 2241.0849 | 2240.1511 | 0.9338 | 1 | 7 | 18 | 1 | -.MTDAAVSFAKDFLAGGVAAAISK.T |
|  | 26555 | 1398.6686 | 2795.3226 | 2795.3377 | -0.0151 | 0 | 37 | 0.013 | 1 | R.YFAGNLASGGAAGATSLCFVYPLDFAR.T |

| **524.** | P00739\|HPTR_HUMAN    **Mass:** 39496    **Score:** 73     **Queries matched:** 11   **emPAI:** 0.44 |
| --- | --- |
|  | Haptoglobin-related protein OS=Homo sapiens GN=HPR PE=1 SV=1 |

|  | **Query** | **Observed** | **Mr(expt)** | **Mr(calc)** | **Delta** | **Miss** | **Score** | **Expect** | **Rank** | **Peptide** |
| --- | --- | --- | --- | --- | --- | --- | --- | --- | --- | --- |
|  | 2424 | 429.2731 | 856.5317 | 857.4858 | -0.9541 | 0 | 34 | 0.085 | 2 | K.QLVEIEK.V 2423 |
|  | 7444 | 602.3182 | 1202.6219 | 1202.6295 | -0.0076 | 0 | 50 | 0.0016 | 1 | K.VTSIQDWVQK.T |
|  | 10247 | 673.3211 | 1344.6277 | 1344.6384 | -0.0107 | 0 | 40 | 0.014 | 1 | K.SCAVAEYGVYVK.V |
|  | 11583 | 709.9116 | 1417.8087 | 1417.8181 | -0.0094 | 1 | 23 | 0.66 | 1 | K.DIAPTLTLYVGKK.Q |
|  | 14317 | 791.4034 | 1580.7923 | 1579.7842 | 1.0081 | 1 | 36 | 0.026 | 1 | R.LRTEGDGVYTLNDK.K |
|  | 15356 | **412.7470** | **1646.9588** | **1645.8862** | **1.0727** | **0** | **8** | **19** | **4** | **-.MSDLGAVISLLLWGR.Q** 15354 |
|  | 17745 | 601.0431 | 1800.1074 | 1798.9802 | 1.1272 | 1 | 2 | 50 | 4 | K.NPANPVQRILGGHLDAK.G |
|  | 18579 | 929.4569 | 1856.8991 | 1856.9124 | -0.0133 | 1 | 22 | 0.64 | 1 | K.AVGDKLPECEAVCGKPK.N 18578 |

| **525.** | P35613\|BASI_HUMAN    **Mass:** 42573    **Score:** 73     **Queries matched:** 10   **emPAI:** 0.52 |
| --- | --- |
|  | Basigin OS=Homo sapiens GN=BSG PE=1 SV=2 |

|  | **Query** | **Observed** | **Mr(expt)** | **Mr(calc)** | **Delta** | **Miss** | **Score** | **Expect** | **Rank** | **Peptide** |
| --- | --- | --- | --- | --- | --- | --- | --- | --- | --- | --- |
|  | 4131 | **491.2782** | **980.5419** | **979.4393** | **1.1026** | **0** | **4** | **57** | **6** | **K.ALMNGSESR.F** |
|  | 5431 | **537.7941** | **1073.5737** | **1072.5877** | **0.9860** | **0** | **2** | **1.1e+002** | **4** | **K.GSDQAIITLR.V** |
|  | 9976 | 443.5682 | 1327.6829 | 1327.7572 | -0.0743 | 1 | 8 | 25 | 5 | K.GSDQAIITLRVR.S |
|  | 11172 | **465.7571** | **1394.2496** | **1394.6651** | **-0.4155** | **1** | **1** | **63** | **10** | **R.ASNDPDRNHLTR.A** |
|  | 11441 | **470.9332** | **1409.7779** | **1409.7878** | **-0.0100** | **1** | **23** | **0.68** | **1** | **K.GGVVLKEDALPGQK.T** 11440 |
|  | 15663 | **832.8964** | **1663.7783** | **1663.7882** | **-0.0099** | **0** | **46** | **0.0028** | **1** | **K.SESVPPVTDWAWYK.I** |
|  | 17798 | 902.9075 | 1803.8004 | 1803.8131 | -0.0127 | 0 | 39 | 0.013 | 1 | K.SSEHINEGETAMLVCK.S 17821 |
|  | 29516 | **1172.5365** | **3514.5877** | **3514.5922** | **-0.0045** | **0** | **25** | **0.12** | **1** | **K.VDSDDQWGEYSCVFLPEPMGTANIQLHGPPR.V** |

| **526.** | Q9NS69\|TOM22_HUMAN    **Mass:** 15512    **Score:** 73     **Queries matched:** 1   **emPAI:** 0.25 |
| --- | --- |
|  | Mitochondrial import receptor subunit TOM22 homolog OS=Homo sapiens GN=TOMM22 PE=1 SV=3 |

|  | **Query** | **Observed** | **Mr(expt)** | **Mr(calc)** | **Delta** | **Miss** | **Score** | **Expect** | **Rank** | **Peptide** |
| --- | --- | --- | --- | --- | --- | --- | --- | --- | --- | --- |
|  | 13950 | **779.3884** | **1556.7622** | **1556.7729** | **-0.0107** | **0** | **73** | **6.9e-006** | **1** | **K.LQMEQQQQLQQR.Q** |

| **527.** | P40925\|MDHC_HUMAN    **Mass:** 36631    **Score:** 72     **Queries matched:** 11   **emPAI:** 0.22 |
| --- | --- |
|  | Malate dehydrogenase, cytoplasmic OS=Homo sapiens GN=MDH1 PE=1 SV=4 |

|  | **Query** | **Observed** | **Mr(expt)** | **Mr(calc)** | **Delta** | **Miss** | **Score** | **Expect** | **Rank** | **Peptide** |
| --- | --- | --- | --- | --- | --- | --- | --- | --- | --- | --- |
|  | 1537 | **396.7133** | **791.4120** | **792.3548** | **-0.9428** | **1** | **5** | **66** | **3** | **R.REGMER.K** |
|  | 3205 | 458.2942 | 914.5739 | 915.5025 | -0.9287 | 0 | 13 | 9.2 | 7 | K.LGVTANDVK.N |
|  | 4297 | **497.2693** | **992.5240** | **992.5324** | **-0.0084** | **1** | **24** | **0.75** | **1** | **R.KLSSAMSAAK.A** 4273 |
|  | 6710 | **583.2732** | **1164.5319** | **1163.5935** | **0.9384** | **0** | **12** | **10** | **1** | **K.GEFVTTVQQR.G** |
|  | 11820 | **716.8487** | **1431.6828** | **1432.7926** | **-1.1097** | **1** | **2** | **80** | **9** | **K.LQGKEVGVYEALK.D** |
|  | 12128 | **725.3862** | **1448.7579** | **1449.7351** | **-0.9772** | **1** | **3** | **67** | **1** | **K.DVIATDKEDVAFK.D** |
|  | 16456 | **572.6317** | **1714.8733** | **1715.8447** | **-0.9714** | **1** | **39** | **0.015** | **1** | **K.LSSAMSAAKAICDHVR.D** |
|  | 17094 | **879.4590** | **1756.9034** | **1756.9142** | **-0.0108** | **0** | **59** | **0.00015** | **1** | **K.VIVVGNPANTNCLTASK.S** |
|  | 21171 | 1037.9679 | 2073.9212 | 2074.0769 | -0.1556 | 1 | 8 | 13 | 2 | K.EDVAFKDLDVAILVGSMPR.R 21172 |

| **528.** | P08473\|NEP_HUMAN    **Mass:** 86144    **Score:** 72     **Queries matched:** 12   **emPAI:** 0.04 |
| --- | --- |
|  | Neprilysin OS=Homo sapiens GN=MME PE=1 SV=2 |

|  | **Query** | **Observed** | **Mr(expt)** | **Mr(calc)** | **Delta** | **Miss** | **Score** | **Expect** | **Rank** | **Peptide** |
| --- | --- | --- | --- | --- | --- | --- | --- | --- | --- | --- |
|  | 4579 | 506.7371 | 1011.4596 | 1011.4661 | -0.0065 | 0 | 28 | 0.22 | 1 | K.YGASWTAEK.A |
|  | 8730 | **634.3361** | **1266.6577** | **1266.6642** | **-0.0065** | **0** | **72** | **8.9e-006** | **1** | **R.FIMDLVSSLSR.T** |
|  | 9051 | **428.7283** | **1283.1631** | **1283.6397** | **-0.4767** | **0** | **3** | **46** | **10** | **R.LYVEAAFAGESK.H** |
|  | 9657 | **658.2791** | **1314.5437** | **1313.6575** | **0.8862** | **0** | **1** | **1.1e+002** | **8** | **K.EIANATAKPEDR.N** |
|  | 12236 | **729.3680** | **1456.7214** | **1455.6994** | **1.0220** | **0** | **0** | **1.2e+002** | **10** | **K.ALYGTTSETATWR.R** 12228 |
|  | 15310 | **548.9370** | **1643.7890** | **1642.8236** | **0.9654** | **0** | **3** | **58** | **2** | **R.LPIDENQLALEMNK.V** |
|  | 15956 | **562.2526** | **1683.7359** | **1683.7093** | **0.0265** | **0** | **1** | **92** | **9** | **R.CANYVNGNMENAVGR.L** |
|  | 16965 | **583.3062** | **1746.8968** | **1747.7909** | **-0.8941** | **0** | **4** | **49** | **5** | **K.EACTAYVDFMISVAR.L** |
|  | 19687 | **649.3546** | **1945.0420** | **1944.9575** | **0.0845** | **1** | **2** | **65** | **2** | **R.SCINESAIDSRGGEPLLK.L** |
|  | 22990 | **568.7889** | **2271.1267** | **2270.0968** | **1.0299** | **1** | **4** | **30** | **1** | **K.VDKDEWISGAAVVNAFYSSGR.N** |
|  | 23790 | 791.4603 | 2371.3592 | 2372.1967 | -0.8375 | 1 | 0 | 57 | 7 | R.LPIDENQLALEMNKVMELEK.E |

| **529.** | P17661\|DESM_HUMAN    **Mass:** 53560    **Score:** 72     **Queries matched:** 17   **emPAI:** 0.14 |
| --- | --- |
|  | Desmin OS=Homo sapiens GN=DES PE=1 SV=3 |

|  | **Query** | **Observed** | **Mr(expt)** | **Mr(calc)** | **Delta** | **Miss** | **Score** | **Expect** | **Rank** | **Peptide** |
| --- | --- | --- | --- | --- | --- | --- | --- | --- | --- | --- |
|  | 6636 | 580.2986 | 1158.5827 | 1159.4928 | -0.9100 | 0 | 4 | 72 | 6 | -.MSQAYSSSQR.V |
|  | 7506 | **403.0455** | **1206.1146** | **1206.6642** | **-0.5496** | **1** | **9** | **13** | **5** | **K.TVMIKTIETR.D** |
|  | 8631 | **631.3582** | **1260.7017** | **1260.6674** | **0.0344** | **1** | **9** | **21** | **1** | **K.SKVSDLTQAANK.N** |
|  | 10904 | **460.5713** | **1378.6920** | **1377.7252** | **0.9669** | **1** | **1** | **1e+002** | **5** | **R.DIRAQYETIAAK.N** |
|  | 11365 | 469.3273 | 1404.9601 | 1404.7500 | 0.2101 | 0 | 8 | 16 | 3 | R.IESLNEEIAFLK.K |
|  | 13454 | 509.9442 | 1526.8109 | 1526.8205 | -0.0096 | 1 | 10 | 12 | 7 | R.HLREYQDLLNVK.M |
|  | 13565 | 511.9530 | 1532.8371 | 1532.8450 | -0.0079 | 1 | 10 | 13 | 3 | R.IESLNEEIAFLKK.V |
|  | 14425 | 794.3968 | 1586.7791 | 1586.7900 | -0.0108 | 1 | 52 | 0.0009 | 1 | R.TNEKVELQELNDR.F 14426 14427 |
|  | 15064 | **543.2474** | **1626.7205** | **1627.8529** | **-1.1324** | **1** | **4** | **49** | **8** | **R.ADVDAATLARIDLER.R** |
|  | 15411 | 550.9299 | 1649.7680 | 1648.8937 | 0.8743 | 0 | 4 | 40 | 4 | R.INLPIQTYSALNFR.E |
|  | 16672 | 865.3831 | 1728.7516 | 1728.8754 | -0.1239 | 1 | 2 | 64 | 2 | K.VSDLTQAANKNNDALR.Q |
|  | 17167 | **588.8160** | **1763.4261** | **1763.9279** | **-0.5017** | **1** | **6** | **20** | **1** | **R.VYQVSRTSGGAGGLGSLR.A** |
|  | 17204 | **884.9106** | **1767.8066** | **1767.8275** | **-0.0209** | **0** | **1** | **81** | **2** | **R.DGEVVSEATQQQHEVL.-** |
|  | 22810 | **1122.9883** | **2243.9620** | **2244.1386** | **-0.1765** | **1** | **0** | **73** | **3** | **K.LQEEIQLKEEAENNLAAFR.A** |
|  | 29484 | **700.5436** | **3497.6818** | **3498.7446** | **-1.0628** | **1** | **7** | **7.8** | **1** | **R.ELQAQLQEQQVQVEMDMSKPDLTAALRDIR.A** |

| **530.** | Q6UW02\|CP20A_HUMAN    **Mass:** 52741    **Score:** 71     **Queries matched:** 7   **emPAI:** 0.07 |
| --- | --- |
|  | Cytochrome P450 20A1 OS=Homo sapiens GN=CYP20A1 PE=1 SV=1 |

|  | **Query** | **Observed** | **Mr(expt)** | **Mr(calc)** | **Delta** | **Miss** | **Score** | **Expect** | **Rank** | **Peptide** |
| --- | --- | --- | --- | --- | --- | --- | --- | --- | --- | --- |
|  | 4338 | **498.7307** | **995.4469** | **995.4634** | **-0.0165** | **0** | **5** | **44** | **9** | **R.FDDELVMK.T** |
|  | 8154 | **620.3114** | **1238.6082** | **1237.6190** | **0.9892** | **0** | **7** | **25** | **4** | **K.LYENGVTDSLK.S** |
|  | 12384 | 489.2264 | 1464.6575 | 1463.6650 | 0.9925 | 0 | 2 | 72 | 3 | R.YCQHVLCETVR.T |
|  | 13121 | **754.8256** | **1507.6366** | **1507.6474** | **-0.0108** | **0** | **71** | **9e-006** | **1** | **R.YQSGGGSVSENHMR.K** 13136 |
|  | 19298 | **639.3500** | **1915.0281** | **1914.8782** | **0.1499** | **0** | **1** | **75** | **3** | **K.TFSSLGFSGTQECPELR.F** |
|  | 27130 | **970.4640** | **2908.3701** | **2908.3259** | **0.0442** | **1** | **2** | **37** | **2** | **R.FDDELVMKTFSSLGFSGTQECPELR.F** |

| **531.** | P05787\|K2C8_HUMAN    **Mass:** 53671    **Score:** 71     **Queries matched:** 21   **emPAI:** 0.22 |
| --- | --- |
|  | Keratin, type II cytoskeletal 8 OS=Homo sapiens GN=KRT8 PE=1 SV=7 |

|  | **Query** | **Observed** | **Mr(expt)** | **Mr(calc)** | **Delta** | **Miss** | **Score** | **Expect** | **Rank** | **Peptide** |
| --- | --- | --- | --- | --- | --- | --- | --- | --- | --- | --- |
|  | 983 | **566.8838** | **565.8765** | **566.2813** | **-0.4047** | **0** | **3** | **37** | **5** | **R.AFSSR.S** |
|  | 3084 | 454.2368 | 906.4590 | 905.4607 | 0.9984 | 0 | 6 | 41 | 9 | R.FLEQQNK.M |
|  | 3145 | **456.2680** | **910.5215** | **910.4145** | **0.1071** | **0** | **1** | **1.2e+002** | **6** | **R.SYTSGPGSR.I** |
|  | 4416 | **500.7841** | **999.5536** | **999.5600** | **-0.0064** | **0** | **4** | **64** | **3** | **R.LQAEIEGLK.G** |
|  | 4453 | **501.9941** | **1001.9737** | **1002.5345** | **-0.5608** | **1** | **3** | **61** | **5** | **R.TQEKEQIK.T** |
|  | 4648 | 509.2752 | 1016.5359 | 1016.5185 | 0.0174 | 1 | 8 | 27 | 7 | K.QDMARQLR.E |
|  | 5508 | 540.2557 | 1078.4969 | 1078.5043 | -0.0074 | 0 | 26 | 0.39 | 1 | K.AQYEDIANR.S |
|  | 6204 | **565.3102** | **1128.6058** | **1128.6138** | **-0.0080** | **0** | **54** | **0.00073** | **1** | **K.LSELEAALQR.A** 6203 |
|  | 11201 | 699.2877 | 1396.5608 | 1396.7350 | -0.1743 | 1 | 8 | 20 | 4 | K.TLNNKFASFIDK.V 11202 |
|  | 11470 | **706.8409** | **1411.6672** | **1411.6765** | **-0.0093** | **1** | **33** | **0.059** | **1** | **R.SRAEAESMYQIK.Y** |
|  | 11593 | **710.3737** | **1418.7329** | **1418.7405** | **-0.0076** | **0** | **12** | **8.2** | **1** | **R.LEGLTDEINFLR.Q** 11578 11594 |
|  | 12840 | 497.9205 | 1490.7397 | 1490.6857 | 0.0539 | 0 | (4) | 47 | 2 | R.LESGMQNMSIHTK.T |
|  | 13120 | **503.5108** | **1507.5105** | **1506.6806** | **0.8298** | **0** | **5** | **34** | **1** | **R.LESGMQNMSIHTK.T** |
|  | 13145 | 503.9282 | 1508.7627 | 1507.7704 | 0.9922 | 1 | 2 | 75 | 9 | R.FLEQQNKMLETK.W |
|  | 19844 | **653.0009** | **1955.9807** | **1955.0323** | **0.9484** | **1** | **1** | **70** | **4** | **R.ASLEAAIADAEQRGELAIK.D** |
|  | 20555 | **674.2061** | **2019.5965** | **2018.8938** | **0.7027** | **1** | **1** | **53** | **3** | **R.SNMDNMFESYINNLRR.Q** |
|  | 21669 | **708.9681** | **2123.8824** | **2124.0005** | **-0.1181** | **0** | **1** | **56** | **8** | **R.ELQSQISDTSVVLSMDNSR.S** |

| **532.** | P22307\|NLTP_HUMAN    **Mass:** 59640    **Score:** 71     **Queries matched:** 27   **emPAI:** 0.20 |
| --- | --- |
|  | Non-specific lipid-transfer protein OS=Homo sapiens GN=SCP2 PE=1 SV=2 |

|  | **Query** | **Observed** | **Mr(expt)** | **Mr(calc)** | **Delta** | **Miss** | **Score** | **Expect** | **Rank** | **Peptide** |
| --- | --- | --- | --- | --- | --- | --- | --- | --- | --- | --- |
|  | 679 | **462.1423** | **461.1350** | **460.2281** | **0.9068** | **0** | **6** | **65** | **5** | **R.GEAGK.R** 678 680 |
|  | 3232 | **459.0111** | **916.0076** | **915.4662** | **0.5415** | **0** | **7** | **28** | **3** | **K.GSVLPNSDK.K** |
|  | 3397 | **465.2715** | **928.5283** | **929.3987** | **-0.8703** | **0** | **12** | **12** | **3** | **K.MVGFDMSK.E** 3396 |
|  | 4892 | **518.2665** | **1034.5184** | **1034.5253** | **-0.0068** | **0** | **34** | **0.068** | **1** | **K.ITGNMGLAMK.L** 4891 |
|  | 7330 | 599.8522 | 1197.6899 | 1198.5441 | -0.8542 | 0 | 5 | 44 | 8 | K.MGFPEAASSFR.T |
|  | 7561 | 605.2972 | 1208.5798 | 1207.6747 | 0.9051 | 1 | 4 | 60 | 7 | R.RVFVVGVGMTK.F |
|  | 8456 | **627.7961** | **1253.5776** | **1253.5863** | **-0.0087** | **0** | **6** | **35** | **2** | **K.MNPQSAFFQGK.L** |
|  | 8942 | **639.3302** | **1276.6458** | **1275.7043** | **0.9416** | **1** | **7** | **30** | **1** | **K.LKITGNMGLAMK.L** |
|  | 9533 | **437.2589** | **1308.7549** | **1307.6941** | **1.0608** | **1** | **(4)** | **51** | **9** | **K.LKITGNMGLAMK.L** 9513 |
|  | 10081 | **668.3381** | **1334.6617** | **1334.6718** | **-0.0101** | **1** | **61** | **0.0001** | **1** | **K.KLEEEGEQFVK.K** |
|  | 10502 | **679.8505** | **1357.6865** | **1356.6166** | **1.0699** | **1** | **(0)** | **1.3e+002** | **7** | **K.MVGFDMSKEAAR.K** |
|  | 10801 | **687.3462** | **1372.6778** | **1372.6115** | **0.0663** | **1** | **1** | **1.3e+002** | **4** | **K.MVGFDMSKEAAR.K** |
|  | 11021 | 693.3895 | 1384.7645 | 1385.7051 | -0.9406 | 1 | 9 | 16 | 3 | M.SSSPWEPATLRR.V 11042 |
|  | 11903 | **718.9094** | **1435.8042** | **1435.8147** | **-0.0105** | **1** | **23** | **0.6** | **1** | **K.LQNLQLQPGNAKL.-** |
|  | 13173 | **756.3480** | **1510.6815** | **1510.7602** | **-0.0787** | **1** | **(10)** | **13** | **6** | **K.MNPQSAFFQGKLK.I** 13175 13184 |
|  | 21048 | 1031.9884 | 2061.9622 | 2062.0484 | -0.0861 | 1 | 1 | 78 | 4 | R.GDNTYGGKWVINPSGGLISK.G |
|  | 22369 | **1103.1041** | **2204.1937** | **2204.0163** | **0.1774** | **1** | **1** | **71** | **1** | **K.KADCTITMADSDFLALMTGK.M** |
|  | 25884 | **894.1592** | **2679.4559** | **2679.3010** | **0.1549** | **1** | **4** | **21** | **4** | **K.GHPLGATGLAQCAELCWQLRGEAGK.R** |
|  | 29057 | **1115.4473** | **3343.3200** | **3343.4869** | **-0.1670** | **1** | **1** | **25** | **9** | **K.ADCTITMADSDFLALMTGKMNPQSAFFQGK.L** |

| **533.** | Q92896\|GSLG1_HUMAN    **Mass:** 138341   **Score:** 71     **Queries matched:** 27   **emPAI:** 0.05 |
| --- | --- |
|  | Golgi apparatus protein 1 OS=Homo sapiens GN=GLG1 PE=1 SV=2 |

|  | **Query** | **Observed** | **Mr(expt)** | **Mr(calc)** | **Delta** | **Miss** | **Score** | **Expect** | **Rank** | **Peptide** |
| --- | --- | --- | --- | --- | --- | --- | --- | --- | --- | --- |
|  | 1059 | **603.3407** | **602.3334** | **602.3275** | **0.0059** | **0** | **18** | **5.3** | **3** | **K.EDVLK.L** |
|  | 1850 | **408.7420** | **815.4694** | **816.3879** | **-0.9184** | **0** | **6** | **52** | **6** | **R.GGAGAGGGWK.L** |
|  | 1966 | 413.7093 | 825.4040 | 826.5640 | -1.1600 | 1 | 10 | 14 | 5 | K.VNLLKIK.T |
|  | 3252 | **459.7607** | **917.5069** | **917.4454** | **0.0615** | **0** | **2** | **1.2e+002** | **3** | **R.NDTLQEAK.E** |
|  | 3276 | 460.7423 | 919.4701 | 919.4480 | 0.0221 | 1 | 23 | 0.9 | 2 | -.MAACGRVR.R |
|  | 4035 | 487.7755 | 973.5365 | 973.5630 | -0.0265 | 1 | 14 | 8.4 | 7 | K.KEVLNMLK.E |
|  | 4819 | 516.2778 | 1030.5410 | 1029.5454 | 0.9955 | 1 | 10 | 19 | 5 | K.GLVKEAEER.E |
|  | 6609 | **579.2897** | **1156.5648** | **1156.5771** | **-0.0123** | **1** | **4** | **69** | **9** | **K.QLSTRCHQK.V** |
|  | 8381 | 417.2252 | 1248.6537 | 1248.7051 | -0.0513 | 1 | 8 | 25 | 5 | R.AEVQRILHQR.A |
|  | 8765 | **635.3023** | **1268.5901** | **1267.5251** | **1.0649** | **0** | **39** | **0.02** | **1** | **R.FCENTQAGEGR.V** 8759 |
|  | 9005 | **641.3604** | **1280.7063** | **1280.7128** | **-0.0066** | **0** | **3** | **72** | **4** | **R.LLELQYFISR.D** |
|  | 9266 | **432.2776** | **1293.8110** | **1293.6425** | **0.1685** | **1** | **6** | **35** | **3** | **K.RQITQNTDYR.L** |
|  | 9423 | 652.3311 | 1302.6475 | 1302.6238 | 0.0238 | 1 | 8 | 25 | 5 | K.QNKNSELMDPK.C |
|  | 9425 | **435.2266** | **1302.6579** | **1303.6925** | **-1.0345** | **1** | **5** | **41** | **10** | **R.DWKLDPVLYR.K** |
|  | 9780 | 660.8386 | 1319.6626 | 1319.7085 | -0.0459 | 0 | 13 | 6.8 | 2 | R.IIIQESALDYR.L |
|  | 10046 | **445.1185** | **1332.3336** | **1331.6629** | **0.6707** | **1** | **6** | **23** | **4** | **K.HHCAAITPGRGR.Q** |
|  | 11318 | **702.3759** | **1402.7371** | **1401.7398** | **0.9973** | **1** | **10** | **12** | **3** | **K.NDINILKCGSIR.L** |
|  | 12179 | **485.2441** | **1452.7103** | **1453.6251** | **-0.9148** | **0** | **4** | **45** | **7** | **R.QMSCLMEALEDK.R** |
|  | 13146 | **503.9382** | **1508.7928** | **1507.7163** | **1.0765** | **1** | **2** | **71** | **6** | **K.CLIDLGKWCSEK.T** |
|  | 17328 | 445.0019 | 1775.9785 | 1775.8723 | 0.1061 | 1 | 5 | 36 | 9 | R.QLRVEELEMTEDIR.L 17332 |
|  | 21735 | **710.8423** | **2129.5050** | **2129.9319** | **-0.4269** | **0** | **1** | **45** | **5** | **K.LQETEMMDPELDYTLMR.V** |
|  | 24348 | 819.1068 | 2454.2984 | 2455.1044 | -0.8060 | 1 | 0 | 62 | 4 | K.MTAIIFSDYRLICGFMDDCK.N |
|  | 25183 | **854.9661** | **2561.8764** | **2561.2076** | **0.6687** | **1** | **5** | **17** | **1** | **K.HIRSGDPMILSCLMEHLYTEK.M** |
|  | 28174 | **783.1677** | **3128.6418** | **3129.4713** | **-0.8295** | **1** | **2** | **30** | **2** | **R.EPENEISSDCNHLLWNYKLNLTTDPK.F** |
|  | 28376 | **1059.5234** | **3175.5485** | **3176.4979** | **-0.9494** | **1** | **52** | **0.00029** | **1** | **K.GNLGMNCQQALQTLIQETDPGADYRIDR.A** |

| **534.** | P50895\|BCAM_HUMAN    **Mass:** 68161    **Score:** 71     **Queries matched:** 17   **emPAI:** 0.11 |
| --- | --- |
|  | Basal cell adhesion molecule OS=Homo sapiens GN=BCAM PE=1 SV=2 |

|  | **Query** | **Observed** | **Mr(expt)** | **Mr(calc)** | **Delta** | **Miss** | **Score** | **Expect** | **Rank** | **Peptide** |
| --- | --- | --- | --- | --- | --- | --- | --- | --- | --- | --- |
|  | 1328 | 707.2164 | 706.2091 | 705.2687 | 0.9404 | 0 | 9 | 24 | 1 | K.GGPCCR.Q |
|  | 5054 | **523.7602** | **1045.5058** | **1045.5152** | **-0.0094** | **0** | **35** | **0.06** | **1** | **R.AGAAGTAEATAR.L** 5036 5053 5080 5081 |
|  | 7287 | **599.2960** | **1196.5775** | **1197.5448** | **-0.9673** | **0** | **5** | **47** | **5** | **-.MEPPDAPAQAR.G** |
|  | 11537 | **472.8985** | **1415.6737** | **1415.7310** | **-0.0573** | **1** | **1** | **1.1e+002** | **6** | **R.NGNPAPKITWYR.N** |
|  | 14063 | 521.9846 | 1562.9319 | 1562.7623 | 0.1695 | 1 | 3 | 58 | 10 | -.MEPPDAPAQARGAPR.L |
|  | 14308 | **527.6293** | **1579.8660** | **1578.7572** | **1.1087** | **1** | **(2)** | **76** | **1** | **-.MEPPDAPAQARGAPR.L** 14286 |
|  | 15268 | **821.3663** | **1640.7181** | **1640.7325** | **-0.0144** | **1** | **65** | **3.7e-005** | **1** | **R.DGISCEASNPHGNKR.H** 15269 |
|  | 17006 | **876.4655** | **1750.9165** | **1750.9002** | **0.0163** | **0** | **26** | **0.26** | **1** | **K.LSWSQLGGSPAEPIPGR.Q** 17007 |
|  | 18485 | 463.4835 | 1849.9049 | 1850.8542 | -0.9494 | 0 | 10 | 12 | 4 | R.LEVPVEMNPEGYMTSR.T |
|  | 21462 | **701.9933** | **2102.9582** | **2102.9725** | **-0.0143** | **0** | **9** | **11** | **1** | **R.LASAEMQGSELQVTMHDTR.G** |

| **535.** | Q15149\|PLEC1_HUMAN    **Mass:** 533462   **Score:** 70     **Queries matched:** 125   **emPAI:** 0.01 |
| --- | --- |
|  | Plectin-1 OS=Homo sapiens GN=PLEC1 PE=1 SV=3 |

|  | **Query** | **Observed** | **Mr(expt)** | **Mr(calc)** | **Delta** | **Miss** | **Score** | **Expect** | **Rank** | **Peptide** |
| --- | --- | --- | --- | --- | --- | --- | --- | --- | --- | --- |
|  | 1219 | **667.1705** | **666.1633** | **666.3925** | **-0.2293** | **1** | **1** | **98** | **10** | **R.HRQVK.L** |
|  | 2465 | **431.2135** | **860.4125** | **861.5185** | **-1.1060** | **1** | **8** | **31** | **7** | **K.LTFRGLR.K** |
|  | 2476 | 431.2256 | 860.4367 | 859.4035 | 1.0332 | 0 | 11 | 16 | 3 | R.QVEEAER.L |
|  | 2654 | **437.7415** | **873.4684** | **872.4352** | **1.0332** | **0** | **2** | **1.5e+002** | **10** | **K.QVAEEAAR.L** |
|  | 2855 | **444.7130** | **887.4115** | **886.4508** | **0.9607** | **0** | **5** | **60** | **3** | **R.AQAQAAAEK.L** |
|  | 2983 | 451.2271 | 900.4397 | 901.4505 | -1.0108 | 0 | 21 | 1.5 | 5 | R.EAEGQLQK.L 3017 |
|  | 3136 | **455.9019** | **909.7892** | **909.5760** | **0.2132** | **0** | **9** | **12** | **1** | **K.AVVQLKPR.H** |
|  | 3218 | **458.7635** | **915.5124** | **914.5185** | **0.9939** | **0** | **4** | **86** | **9** | **K.GLLSAEVAR.L** |
|  | 3795 | 479.2744 | 956.5341 | 956.5039 | 0.0302 | 0 | 14 | 6.8 | 7 | R.AQAEQAALR.Q |
|  | 3957 | 485.2878 | 968.5611 | 967.6066 | 0.9545 | 1 | 3 | 67 | 6 | R.LLPLKGAEK.A |
|  | 3961 | **485.7594** | **969.5042** | **968.6131** | **0.8911** | **1** | **10** | **14** | **2** | **R.QVKLVNIR.N** 3965 |
|  | 3970 | **486.2500** | **970.4855** | **971.4382** | **-0.9527** | **0** | **9** | **22** | **4** | **K.ESYSALMR.E** 3971 |
|  | 4034 | 487.7715 | 973.5285 | 974.5033 | -0.9748 | 0 | 3 | 87 | 9 | K.DLSELGSVR.T |
|  | 4119 | **490.7767** | **979.5389** | **978.4692** | **1.0697** | **0** | **3** | **77** | **4** | **R.VQMEELSK.L** |
|  | 4215 | **495.0591** | **988.1037** | **988.4574** | **-0.3537** | **0** | **4** | **46** | **6** | **R.QVQDESQR.K** |
|  | 4291 | 497.2544 | 992.4942 | 991.5338 | 0.9604 | 1 | 9 | 20 | 6 | R.FIEQEKAK.L 4275 |
|  | 4390 | 500.2688 | 998.5231 | 998.5298 | -0.0066 | 0 | 8 | 27 | 2 | K.AHAFAVQQK.E |
|  | 4447 | **501.7610** | **1001.5075** | **1000.5189** | **0.9886** | **0** | **14** | **7.6** | **2** | **K.AQAEVEGLGK.G** |
|  | 4563 | 506.2607 | 1010.5067 | 1009.5266 | 0.9801 | 1 | 1 | 1.2e+002 | 7 | K.YLTCPKTK.L |
|  | 4794 | 515.3256 | 1028.6366 | 1028.5363 | 0.1003 | 1 | 9 | 19 | 1 | K.QRLEAEAGR.F 4806 |
|  | 4820 | 516.2860 | 1030.5575 | 1030.5658 | -0.0084 | 0 | 19 | 2.1 | 1 | R.LLEAAAQSTK.G |
|  | 4913 | 519.2432 | 1036.4719 | 1036.4868 | -0.0149 | 0 | 6 | 36 | 5 | -.MVAGMLMPR.D |
|  | 5066 | 524.2424 | 1046.4702 | 1045.5338 | 0.9363 | 1 | 8 | 30 | 5 | R.MQEEVVRR.E |
|  | 5095 | **525.2527** | **1048.4908** | **1047.5461** | **0.9447** | **1** | **3** | **84** | **8** | **R.LEAEAGRFR.E** |
|  | 5145 | **527.3386** | **1052.6626** | **1051.5121** | **1.1505** | **0** | **3** | **80** | **2** | **R.MVEGYQGLR.C** |
|  | 5222 | **530.2887** | **1058.5628** | **1058.5720** | **-0.0091** | **1** | **20** | **1.9** | **1** | **R.LKAEATEAAR.Q** |
|  | 5234 | 530.7998 | 1059.5850 | 1059.5084 | 0.0766 | 0 | 8 | 29 | 9 | R.VLADPSDDTK.G |
|  | 5329 | **534.3660** | **1066.7174** | **1065.5892** | **1.1282** | **0** | **0** | **1.3e+002** | **6** | **K.LISLFQAMK.K** |
|  | 5417 | 537.2708 | 1072.5271 | 1072.6029 | -0.0758 | 0 | 5 | 58 | 8 | K.SLLAWQSLR.R |
|  | 5486 | 539.7552 | 1077.4958 | 1077.5091 | -0.0132 | 0 | 14 | 7.2 | 4 | R.LAEDEAFQR.R 5469 |
|  | 5568 | **542.6312** | **1083.2479** | **1082.5794** | **0.6685** | **1** | **4** | **43** | **2** | **R.LSVYQAMKK.G** |
|  | 5770 | **549.9402** | **1097.8659** | **1097.4600** | **0.4059** | **0** | **0** | **94** | **7** | **K.AFCGFEDPR.T** |
|  | 5823 | **551.3133** | **1100.6120** | **1100.5826** | **0.0295** | **1** | **2** | **1.1e+002** | **9** | **R.QVEEAERLK.Q** |
|  | 5828 | 551.7285 | 1101.4425 | 1100.5574 | 0.8851 | 0 | 8 | 29 | 4 | R.QLAEGTAQQR.L |
|  | 6088 | 560.8666 | 1119.7187 | 1119.5520 | 0.1667 | 1 | 18 | 2.5 | 1 | R.SNAEDTLRSK.E 6066 |
|  | 6243 | 566.7634 | 1131.5123 | 1131.4655 | 0.0468 | 0 | 13 | 7.7 | 2 | R.GYFSEEMNR.V 6240 6242 6244 6245 |
|  | 6277 | **567.8259** | **1133.6373** | **1133.5750** | **0.0623** | **0** | **5** | **51** | **3** | **K.QITMEELVR.S** |
|  | 6336 | 570.7820 | 1139.5494 | 1139.5757 | -0.0263 | 1 | 2 | 95 | 9 | K.VSIYEAMRR.G |
|  | 6361 | **571.7731** | **1141.5316** | **1142.6052** | **-1.0737** | **0** | **(3)** | **73** | **1** | **R.RPVAMVMPAR.R** |
|  | 6366 | 571.7991 | 1141.5837 | 1142.6052 | -1.0215 | 0 | (1) | 1.3e+002 | 10 | R.RPVAMVMPAR.R |
|  | 6369 | **572.2406** | **1142.4666** | **1142.5931** | **-0.1265** | **0** | **6** | **40** | **1** | **R.LQAEEVAQQK.S** |
|  | 6375 | **572.3037** | **1142.5929** | **1141.6819** | **0.9110** | **1** | **13** | **8.6** | **1** | **K.LAAIGEATRLK.T** |
|  | 6456 | 574.9901 | 1147.9657 | 1148.6778 | -0.7121 | 1 | 8 | 18 | 2 | K.GLIVREHGIR.L |
|  | 6638 | **580.3044** | **1158.5943** | **1158.5993** | **-0.0050** | **1** | **17** | **3.6** | **1** | **R.DVAEVDTVRR.A** |
|  | 6644 | **580.3159** | **1158.6172** | **1158.6002** | **0.0170** | **0** | **11** | **13** | **5** | **R.RPVAMVMPAR.R** |
|  | 6977 | **395.2058** | **1182.5954** | **1181.6153** | **0.9802** | **1** | **3** | **73** | **3** | **K.RGPLPTEEQR.V** |
|  | 7350 | **600.3087** | **1198.6029** | **1198.6669** | **-0.0640** | **1** | **4** | **62** | **7** | **R.LREAIAELER.E** |
|  | 7575 | 404.0295 | 1209.0665 | 1209.5850 | -0.5185 | 1 | 8 | 16 | 1 | R.RQHEAEEGVR.R |
|  | 7788 | 407.4496 | 1219.3270 | 1219.6673 | -0.3403 | 1 | 8 | 18 | 8 | R.KGLVGPELHDR.L |
|  | 7858 | **612.8232** | **1223.6319** | **1223.6696** | **-0.0377** | **0** | **1** | **1.2e+002** | **5** | **R.HKPLLIDMNK.V** |
|  | 7907 | **409.5725** | **1225.6956** | **1224.5847** | **1.1109** | **1** | **8** | **20** | **4** | **R.HGERDVEVER.W** |
|  | 7943 | 614.8138 | 1227.6130 | 1226.6619 | 0.9511 | 0 | 7 | 29 | 8 | R.QLQLAQEAAQK.R |
|  | 8346 | 624.3542 | 1246.6939 | 1247.5816 | -0.8876 | 1 | 8 | 23 | 2 | R.QKQAADAEMEK.H |
|  | 8434 | 626.8568 | 1251.6991 | 1251.6169 | 0.0822 | 1 | 3 | 65 | 10 | R.FLQEEAEKMK.Q |
|  | 8665 | **632.3756** | **1262.7367** | **1263.7438** | **-1.0071** | **1** | **5** | **45** | **4** | **R.SLSAIYLEKLK.T** |
|  | 8692 | **633.3418** | **1264.6690** | **1263.5765** | **1.0926** | **1** | **(4)** | **59** | **9** | **R.QKQAADAEMEK.H** |
|  | 8861 | 425.2592 | 1272.7558 | 1273.6812 | -0.9254 | 1 | 11 | 10 | 1 | K.MQAVQEATRLK.A |
|  | 9054 | **428.7576** | **1283.2511** | **1283.7309** | **-0.4799** | **1** | **18** | **1.4** | **1** | **R.LSVAAQEAARLR.Q** 9061 |
|  | 9072 | **429.1604** | **1284.4595** | **1285.6374** | **-1.1780** | **1** | **11** | **9.3** | **5** | **R.QLAEEDAARQR.A** 9075 |
|  | 9201 | **431.2529** | **1290.7368** | **1289.6761** | **1.0606** | **1** | **(5)** | **46** | **8** | **K.MQAVQEATRLK.A** 9161 |
|  | 9349 | **433.8929** | **1298.6568** | **1298.7063** | **-0.0495** | **1** | **9** | **19** | **5** | **R.RPVAMVMPARR.T** |
|  | 9373 | 434.5373 | 1300.5900 | 1299.6419 | 0.9482 | 0 | 8 | 21 | 6 | R.QLAEEDLAQQR.A |
|  | 9572 | **656.8387** | **1311.6628** | **1312.7099** | **-1.0471** | **1** | **0** | **1.4e+002** | **1** | **K.ADIEERLAQLR.K** |
|  | 9647 | 438.9161 | 1313.7266 | 1314.7013 | -0.9747 | 1 | (3) | 68 | 8 | R.RPVAMVMPARR.T |
|  | 9739 | **440.2262** | **1317.6569** | **1316.6044** | **1.0525** | **1** | **4** | **57** | **6** | **R.HPQGEQMYRR.V** |
|  | 9936 | **443.0392** | **1326.0958** | **1325.7415** | **0.3543** | **1** | **4** | **36** | **2** | **K.QRALQALEELR.L** |
|  | 10466 | 452.7407 | 1355.2004 | 1355.7408 | -0.5404 | 1 | 4 | 38 | 6 | R.LAEVEAALEKQR.Q |
|  | 10480 | **679.3572** | **1356.6998** | **1357.6586** | **-0.9588** | **1** | **1** | **1.2e+002** | **8** | **R.DELRGAQEVGER.L** |
|  | 10501 | **679.8490** | **1357.6834** | **1357.6949** | **-0.0115** | **1** | **73** | **7e-006** | **1** | **R.LKQSAEEQAQAR.A** |
|  | 10539 | **454.2368** | **1359.6885** | **1360.7384** | **-1.0499** | **1** | **6** | **35** | **9** | **K.VRAEMEVLLASK.A** |
|  | 10925 | **460.9073** | **1379.7000** | **1379.7092** | **-0.0092** | **1** | **12** | **8.4** | **2** | **K.SHRVPLDVACAR.G** 10936 |
|  | 11007 | **693.0281** | **1384.0417** | **1383.7218** | **0.3199** | **1** | **1** | **74** | **4** | **R.QRGGAEGELQALR.A** |
|  | 11038 | 693.8793 | 1385.7440 | 1385.7271 | 0.0168 | 1 | 14 | 4.9 | 5 | M.VAGMLMPRDQLR.A 11032 |
|  | 11605 | 710.8451 | 1419.6757 | 1419.7027 | -0.0270 | 1 | 10 | 13 | 2 | R.QLEMSAEAERLK.L |
|  | 12966 | **500.2754** | **1497.8043** | **1496.8310** | **0.9733** | **1** | **2** | **81** | **8** | **R.QLLEEELARLQR.E** |
|  | 13058 | 502.2465 | 1503.7176 | 1502.7511 | 0.9665 | 1 | 3 | 56 | 6 | K.DIRLQLEACETR.T |
|  | 13319 | **760.3837** | **1518.7529** | **1517.8202** | **0.9327** | **1** | **1** | **1.1e+002** | **2** | **R.LLFNDVQTLKDGR.H** |
|  | 13538 | **766.4410** | **1530.8674** | **1530.8770** | **-0.0095** | **0** | **23** | **0.65** | **1** | **K.VLALPEPSPAAPTLR.S** |
|  | 13558 | 767.3508 | 1532.6870 | 1532.7625 | -0.0756 | 1 | 8 | 21 | 3 | -.MVAGMLMPRDQLR.A |
|  | 13857 | 776.3755 | 1550.7365 | 1550.7875 | -0.0510 | 1 | 1 | 1.1e+002 | 5 | R.AQLMADFQAGRVTK.E |
|  | 13938 | **778.9120** | **1555.8094** | **1555.8205** | **-0.0111** | **0** | **30** | **0.12** | **1** | **R.LQEAGILSAEELQR.L** |
|  | 13984 | **520.6134** | **1558.8184** | **1557.7383** | **1.0801** | **1** | **2** | **77** | **9** | **R.AAEEAEEARVQAER.E** |
|  | 14019 | 521.2734 | 1560.7985 | 1559.8420 | 0.9565 | 1 | 9 | 16 | 4 | R.VIDRELYQQLQR.G |
|  | 14828 | **538.6174** | **1612.8303** | **1612.8672** | **-0.0369** | **0** | **12** | **7.3** | **1** | **R.LLDAQLSTGGIVDPSK.S** |
|  | 14963 | **540.9473** | **1619.8200** | **1620.8332** | **-1.0132** | **1** | **9** | **16** | **1** | **R.QLAEAHAQAKAQAER.E** |
|  | 15253 | 547.3138 | 1638.9195 | 1638.8689 | 0.0506 | 1 | 5 | 33 | 10 | R.VPLDEALQRGTVDAR.T |
|  | 15500 | **552.6062** | **1654.7968** | **1654.9518** | **-0.1551** | **1** | **3** | **58** | **5** | **R.HYLQGRSSIAGLLLK.A** 15508 |
|  | 15882 | **560.2999** | **1677.8780** | **1676.8482** | **1.0298** | **0** | **4** | **43** | **8** | **R.TLARPGPEPAPATDER.D** |
|  | 15980 | 562.5768 | 1684.7087 | 1685.8736 | -1.1649 | 1 | 3 | 50 | 9 | K.EQLNEYKGHLSGLAK.R |
|  | 16034 | **844.9414** | **1687.8683** | **1687.8992** | **-0.0309** | **1** | **0** | **1.1e+002** | **3** | **R.EGLTSIEEVTKNLQK.F** |
|  | 16752 | **578.6432** | **1732.9077** | **1732.9247** | **-0.0169** | **1** | **0** | **99** | **6** | **R.QVEEEILALKASFEK.A** |
|  | 17436 | 891.9108 | 1781.8070 | 1780.9359 | 0.8710 | 1 | 1 | 86 | 8 | R.DPYTGQSVSLFQALKK.G |
|  | 17693 | **450.2394** | **1796.9284** | **1798.0240** | **-1.0956** | **0** | **7** | **19** | **1** | **R.LLLEAQAATGFLLDPVK.G** |
|  | 17769 | 601.3876 | 1801.1409 | 1799.9465 | 1.1944 | 0 | 2 | 56 | 5 | R.SLHPHVPGVTNLQVMR.A |
|  | 17866 | 904.9687 | 1807.9228 | 1807.9064 | 0.0164 | 1 | 3 | 52 | 3 | R.QEQALLEEIERHGEK.V 17865 |
|  | 18462 | **925.4478** | **1848.8809** | **1849.9363** | **-1.0553** | **1** | **8** | **19** | **1** | **K.VPPGYHPLDVEKEWGK.L** |
|  | 18892 | **471.5038** | **1881.9861** | **1882.8796** | **-0.8935** | **0** | **4** | **43** | **3** | **K.AEVVETTQVYTEEETR.R** |
|  | 19053 | **632.3756** | **1894.1050** | **1893.9544** | **0.1506** | **1** | **5** | **26** | **1** | **R.GTQGAEEVLRAHEEQLK.E** |
|  | 19298 | 639.3500 | 1915.0281 | 1914.9397 | 0.0884 | 1 | 2 | 66 | 2 | R.DPYTEQTISLFQAMKK.E |
|  | 19861 | **979.4940** | **1956.9734** | **1955.9847** | **0.9887** | **1** | **5** | **30** | **4** | **R.RQEQIQAMPLADSQAVR.E** |
|  | 21317 | **697.0363** | **2088.0869** | **2086.9919** | **1.0950** | **1** | **6** | **24** | **2** | **R.GYLNKDTHDQLSEPSEVR.S** |
|  | 21419 | **700.4077** | **2098.2013** | **2099.0358** | **-0.8344** | **1** | **1** | **57** | **4** | **K.FLEGTSCIAGVFVDATKER.L** |
|  | 21982 | 1080.0405 | 2158.0665 | 2159.1408 | -1.0743 | 1 | 1 | 71 | 4 | R.LCFEGLRSLVPAAELLESR.V |
|  | 22080 | 1085.5256 | 2169.0367 | 2169.0960 | -0.0593 | 1 | 5 | 25 | 2 | K.VSITEAMHRNLVDNITGQR.L |
|  | 23085 | **762.0491** | **2283.1254** | **2282.1100** | **1.0154** | **1** | **2** | **45** | **10** | **R.SQVEEELFSVRVQMEELSK.L** |
|  | 27895 | **766.4007** | **3061.5737** | **3060.5914** | **0.9823** | **1** | **2** | **31** | **3** | **K.KDLLPSDMAVALLEAQAGTGHIIDPATSAR.L** |
|  | 30579 | 1362.9431 | 4085.8075 | 4086.9051 | -1.0976 | 1 | 1 | 20 | 2 | R.RAFEETQIDIPGGGSHGGSTMSLWEVMQSDLIPEEQR.A |

| **536.** | P29966\|MARCS_HUMAN    **Mass:** 31707    **Score:** 70     **Queries matched:** 10   **emPAI:** 0.57 |
| --- | --- |
|  | Myristoylated alanine-rich C-kinase substrate OS=Homo sapiens GN=MARCKS PE=1 SV=4 |

|  | **Query** | **Observed** | **Mr(expt)** | **Mr(calc)** | **Delta** | **Miss** | **Score** | **Expect** | **Rank** | **Peptide** |
| --- | --- | --- | --- | --- | --- | --- | --- | --- | --- | --- |
|  | 6322 | 570.2752 | 1138.5359 | 1138.5805 | -0.0446 | 1 | 11 | 11 | 8 | -.MGAQFSKTAAK.G |
|  | 12995 | **750.8398** | **1499.6650** | **1499.6740** | **-0.0090** | **0** | **22** | **0.79** | **1** | **K.AEDGATPSPSNETPK.K** |
|  | 15084 | 814.9469 | 1627.8792 | 1627.7689 | 0.1103 | 1 | 13 | 6.2 | 2 | K.AEDGATPSPSNETPKK.K 15083 |
|  | 17477 | **892.9358** | **1783.8571** | **1783.8700** | **-0.0129** | **0** | **56** | **0.00026** | **1** | **K.GEAAAERPGEAAVASSPSK.A** 17457 17476 17498 |
|  | 21948 | **719.3641** | **2155.0704** | **2155.0869** | **-0.0165** | **1** | **33** | **0.042** | **1** | **K.TAAKGEAAAERPGEAAVASSPSK.A** |
|  | 26182 | **909.4661** | **2725.3764** | **2725.2790** | **0.0973** | **1** | **4** | **21** | **1** | **K.VNGDASPAAAESGAKEELQANGSAPAADK.E** |

| **537.** | P37802\|TAGL2_HUMAN    **Mass:** 22548    **Score:** 69     **Queries matched:** 15   **emPAI:** 0.60 |
| --- | --- |
|  | Transgelin-2 OS=Homo sapiens GN=TAGLN2 PE=1 SV=3 |

|  | **Query** | **Observed** | **Mr(expt)** | **Mr(calc)** | **Delta** | **Miss** | **Score** | **Expect** | **Rank** | **Peptide** |
| --- | --- | --- | --- | --- | --- | --- | --- | --- | --- | --- |
|  | 1896 | 410.7329 | 819.4513 | 819.4239 | 0.0274 | 0 | 14 | 7.9 | 9 | R.GPAYGLSR.E |
|  | 4130 | **491.2602** | **980.5058** | **980.5152** | **-0.0094** | **0** | **15** | **4.8** | **1** | **K.DVGRPQPGR.E** |
|  | 7687 | 608.3343 | 1214.6540 | 1214.6805 | -0.0265 | 0 | 18 | 2.5 | 5 | R.TLMNLGGLAVAR.D 7688 7717 |
|  | 8970 | **640.2952** | **1278.5759** | **1278.5840** | **-0.0081** | **0** | **51** | **0.0012** | **1** | **R.NFSDNQLQEGK.N** 8985 |
|  | 10971 | **691.7932** | **1381.5719** | **1382.6071** | **-1.0352** | **0** | **(9)** | **17** | **2** | **R.GASQAGMTGYGMPR.Q** |
|  | 11239 | **467.1001** | **1398.2785** | **1398.6020** | **-0.3235** | **0** | **(1)** | **78** | **9** | **R.GASQAGMTGYGMPR.Q** |
|  | 11532 | **472.7663** | **1415.2772** | **1414.5969** | **0.6802** | **0** | **9** | **10** | **1** | **R.GASQAGMTGYGMPR.Q** |
|  | 11824 | **716.8537** | **1431.6928** | **1431.7470** | **-0.0542** | **1** | **6** | **35** | **5** | **R.GPAYGLSREVQQK.I** |
|  | 14532 | **797.8578** | **1593.7011** | **1593.7100** | **-0.0088** | **0** | **48** | **0.0018** | **1** | **R.DDGLFSGDPNWFPK.K** |
|  | 21502 | **703.0331** | **2106.0774** | **2106.0496** | **0.0278** | **1** | **12** | **5.1** | **2** | **K.NMACVQRTLMNLGGLAVAR.D** |
|  | 23935 | **1197.5911** | **2393.1676** | **2393.1685** | **-0.0010** | **0** | **15** | **2.3** | **1** | **K.QYDADLEQILIQWITTQCR.K** |
|  | 24067 | **805.7537** | **2414.2392** | **2414.2515** | **-0.0124** | **1** | **21** | **0.67** | **1** | **K.DGTVLCELINALYPEGQAPVKK.I** |

| **538.** | P04062\|GLCM_HUMAN    **Mass:** 60134    **Score:** 69     **Queries matched:** 9   **emPAI:** 0.06 |
| --- | --- |
|  | Glucosylceramidase OS=Homo sapiens GN=GBA PE=1 SV=3 |

|  | **Query** | **Observed** | **Mr(expt)** | **Mr(calc)** | **Delta** | **Miss** | **Score** | **Expect** | **Rank** | **Peptide** |
| --- | --- | --- | --- | --- | --- | --- | --- | --- | --- | --- |
|  | 4471 | 502.3194 | 1002.6242 | 1002.5168 | 0.1074 | 0 | 1 | 1.3e+002 | 9 | R.LLMLDDQR.L |
|  | 4985 | **521.7286** | **1041.4427** | **1041.5706** | **-0.1279** | **0** | **8** | **27** | **2** | **K.VVLTDPEAAK.Y** 4973 |
|  | 6409 | 573.3323 | 1144.6500 | 1144.6200 | 0.0300 | 1 | 2 | 1e+002 | 4 | K.TNGAVNGKGSLK.G |
|  | 18960 | 630.0033 | 1886.9881 | 1886.8907 | 0.0974 | 0 | 8 | 14 | 6 | R.LFPNTMLFASEACVGSK.F |
|  | 27213 | **586.4871** | **2927.3992** | **2926.3629** | **1.0363** | **1** | **4** | **22** | **8** | **K.SYFSEEGIGYNIIRVPMASCDFSIR.T** |
|  | 27474 | **993.7375** | **2978.1906** | **2978.5317** | **-0.3411** | **1** | **2** | **27** | **9** | **R.RMELSMGPIQANHTGTGLLLTLQPEQK.F** |
|  | 30040 | **1250.9324** | **3749.7753** | **3749.7937** | **-0.0184** | **0** | **69** | **4e-006** | **1** | **K.LQFWAVTAENEPSAGLLSGYPFQCLGFTPEHQR.D** |
|  | 31695 | **1866.1035** | **5595.2887** | **5594.8085** | **0.4802** | **1** | **1** | **6** | **1** | **R.GMQYSHSIITNLLYHVVGWTDWNLALNPEGGPNWVRNFVDSPIIVDITK.D** |

| **539.** | Q9Y536\|PAL4B_HUMAN    **Mass:** 18398    **Score:** 69     **Queries matched:** 5   **emPAI:** 0.21 |
| --- | --- |
|  | Peptidylprolyl cis-trans isomerase A-like 4B OS=Homo sapiens GN=PPIAL4B PE=1 SV=1 |

|  | **Query** | **Observed** | **Mr(expt)** | **Mr(calc)** | **Delta** | **Miss** | **Score** | **Expect** | **Rank** | **Peptide** |
| --- | --- | --- | --- | --- | --- | --- | --- | --- | --- | --- |
|  | 1397 | 737.3513 | 736.3440 | 736.3504 | -0.0063 | 0 | 14 | 6 | 1 | K.TAENFR.A |
|  | 4692 | **511.2468** | **1020.4791** | **1020.4876** | **-0.0085** | **0** | **9** | **19** | **7** | **K.FDDENLIR.K** |
|  | 14588 | 799.8714 | 1597.7282 | 1597.7381 | -0.0099 | 0 | 47 | 0.0021 | 1 | R.IIPGFMCQGGDFTR.H 14587 |
|  | 14835 | 538.9323 | 1613.7751 | 1613.7331 | 0.0420 | 0 | (3) | 59 | 2 | R.IIPGFMCQGGDFTR.H |

| **540.** | Q16891\|IMMT_HUMAN    **Mass:** 84026    **Score:** 69     **Queries matched:** 30   **emPAI:** 0.19 |
| --- | --- |
|  | Mitochondrial inner membrane protein OS=Homo sapiens GN=IMMT PE=1 SV=1 |

|  | **Query** | **Observed** | **Mr(expt)** | **Mr(calc)** | **Delta** | **Miss** | **Score** | **Expect** | **Rank** | **Peptide** |
| --- | --- | --- | --- | --- | --- | --- | --- | --- | --- | --- |
|  | 1430 | **758.4463** | **757.4390** | **757.4082** | **0.0308** | **0** | **10** | **21** | **3** | **R.IDQLNR.E** |
|  | 3695 | 475.7895 | 949.5645 | 949.4539 | 0.1106 | 0 | 6 | 45 | 7 | R.VAMIDETR.N |
|  | 3800 | **479.2864** | **956.5583** | **956.5655** | **-0.0071** | **1** | **6** | **41** | **9** | **K.KSIQSGPLK.I** |
|  | 4344 | **498.7592** | **995.5038** | **994.5005** | **1.0033** | **0** | **8** | **25** | **3** | **K.ISSVSEVMK.E** |
|  | 4623 | **508.2843** | **1014.5540** | **1014.5611** | **-0.0070** | **1** | **18** | **2.9** | **2** | **R.RVAQDWLK.E** |
|  | 4874 | **517.7378** | **1033.4610** | **1034.5430** | **-1.0820** | **1** | **10** | **18** | **3** | **K.MKSVIENAK.K** |
|  | 5136 | **527.2605** | **1052.5064** | **1052.5138** | **-0.0074** | **0** | **39** | **0.018** | **1** | **R.GVYSEETLR.A** |
|  | 5223 | **530.2888** | **1058.5631** | **1058.5720** | **-0.0089** | **1** | **24** | **0.73** | **1** | **K.KVQAAQSEAK.V** |
|  | 5726 | 548.2087 | 1094.4029 | 1093.5437 | 0.8592 | 1 | 4 | 58 | 7 | K.EARMTLETK.Q |
|  | 5739 | **548.3202** | **1094.6259** | **1094.6236** | **0.0023** | **1** | **1** | **1.3e+002** | **7** | **R.FYAVQKLAR.R** |
|  | 6090 | 560.8671 | 1119.7196 | 1118.5581 | 1.1615 | 0 | 5 | 46 | 6 | R.QAAAHTDHLR.D |
|  | 6548 | 577.3048 | 1152.5951 | 1152.5887 | 0.0064 | 0 | 20 | 1.6 | 1 | R.ERPPEEVAAR.L |
|  | 7428 | 602.2477 | 1202.4809 | 1202.5891 | -0.1082 | 1 | 10 | 16 | 10 | R.SEIQAEQDRK.I |
|  | 8240 | **622.3438** | **1242.6729** | **1242.6819** | **-0.0090** | **1** | **9** | **18** | **1** | **R.KAVDEAADALLK.A** |
|  | 12230 | **486.3485** | **1456.0237** | **1455.7681** | **0.2556** | **1** | **7** | **18** | **3** | **R.IDQLNRELAEQK.A** |
|  | 13147 | **755.4146** | **1508.8147** | **1508.8021** | **0.0126** | **0** | **21** | **0.93** | **1** | **K.LHNMIVDLDNVVK.K** |
|  | 13449 | **764.3673** | **1526.7199** | **1526.8205** | **-0.1006** | **0** | **6** | **34** | **7** | **K.VVSQYHELVVQAR.D** 13448 |
|  | 14354 | 792.4155 | 1582.8164 | 1582.8243 | -0.0079 | 0 | 16 | 3.3 | 1 | R.ELDSITPEVLPGWK.G |
|  | 14650 | 535.2728 | 1602.7965 | 1601.8386 | 0.9579 | 1 | 0 | 1.2e+002 | 10 | R.QAAAHTDHLRDVLR.V |
|  | 15643 | **555.1708** | **1662.4905** | **1661.8485** | **0.6420** | **0** | **4** | **32** | **3** | **K.LSTDDLNSLIAHAHR.R** |
|  | 16820 | **580.3155** | **1737.9246** | **1738.9254** | **-1.0007** | **1** | **5** | **31** | **7** | **K.RELDSITPEVLPGWK.G** |
|  | 18750 | **936.4969** | **1870.9792** | **1870.9888** | **-0.0095** | **0** | **44** | **0.0039** | **1** | **K.TSSAETPTIPLGSAVEAIK.A** 18738 |
|  | 19347 | **641.0345** | **1920.0816** | **1918.9975** | **1.0841** | **1** | **6** | **23** | **3** | **K.AHQLWLSVEALKYSMK.T** |
|  | 19729 | **649.7935** | **1946.3586** | **1947.1193** | **-0.7608** | **0** | **0** | **65** | **3** | **K.IAGAGLLFVGGGIGGTILYAK.W** |
|  | 21071 | **689.0026** | **2063.9860** | **2064.0011** | **-0.0151** | **1** | **28** | **0.13** | **1** | **R.VQEQELKSEFEQNLSEK.L** |
|  | 23217 | **767.0064** | **2297.9974** | **2297.0749** | **0.9225** | **1** | **4** | **29** | **1** | **-.MLRACQLSGVTAAAQSCLCGK.F** |
|  | 25732 | **888.4723** | **2662.3950** | **2662.4080** | **-0.0130** | **1** | **15** | **1.8** | **1** | **K.TIPYSDKLFEMVLGPAAYNVPLPK.K** |
|  | 25750 | **534.0673** | **2665.3002** | **2665.3130** | **-0.0127** | **1** | **18** | **0.97** | **1** | **K.GMSVSDLADKLSTDDLNSLIAHAHR.R** |

| **541.** | Q06136\|KDSR_HUMAN    **Mass:** 36563    **Score:** 69     **Queries matched:** 9   **emPAI:** 0.22 |
| --- | --- |
|  | 3-ketodihydrosphingosine reductase OS=Homo sapiens GN=KDSR PE=1 SV=1 |

|  | **Query** | **Observed** | **Mr(expt)** | **Mr(calc)** | **Delta** | **Miss** | **Score** | **Expect** | **Rank** | **Peptide** |
| --- | --- | --- | --- | --- | --- | --- | --- | --- | --- | --- |
|  | 2256 | 423.0517 | 844.0889 | 843.4814 | 0.6075 | 0 | 7 | 30 | 4 | R.TKPLETR.L 2268 |
|  | 3285 | **461.0066** | **919.9986** | **919.4247** | **0.5739** | **1** | **11** | **10** | **4** | **R.EKSENADK.T** |
|  | 5443 | **538.2793** | **1074.5440** | **1074.6186** | **-0.0745** | **0** | **3** | **97** | **2** | **K.QGAFITLVAR.N** |
|  | 14608 | 800.4081 | 1598.8016 | 1598.8126 | -0.0110 | 0 | 39 | 0.015 | 1 | R.LMSINYLGSVYPSR.A |
|  | 14844 | 538.9550 | 1613.8431 | 1614.8076 | -0.9645 | 0 | (6) | 30 | 3 | R.LMSINYLGSVYPSR.A 14848 14855 |
|  | 17548 | **895.4661** | **1788.9177** | **1788.9291** | **-0.0114** | **0** | **52** | **0.00062** | **1** | **R.LISETTSVCKPEQVAK.Q** |

| **542.** | Q9UKK3\|PARP4_HUMAN    **Mass:** 194633   **Score:** 69     **Queries matched:** 24   **emPAI:** 0.04 |
| --- | --- |
|  | Poly [ADP-ribose] polymerase 4 OS=Homo sapiens GN=PARP4 PE=1 SV=2 |

|  | **Query** | **Observed** | **Mr(expt)** | **Mr(calc)** | **Delta** | **Miss** | **Score** | **Expect** | **Rank** | **Peptide** |
| --- | --- | --- | --- | --- | --- | --- | --- | --- | --- | --- |
|  | 3496 | **469.2215** | **936.4284** | **935.4018** | **1.0265** | **0** | **7** | **28** | **2** | **K.MDDPSISR.N** 3497 |
|  | 6311 | **569.7836** | **1137.5527** | **1136.6376** | **0.9151** | **1** | **3** | **79** | **2** | **R.LIPHKGTMPK.E** |
|  | 7061 | **396.2357** | **1185.6852** | **1186.6095** | **-0.9243** | **0** | **1** | **1.3e+002** | **10** | **R.QVASFGSAAPPR.Q** 7064 |
|  | 8730 | 634.3361 | 1266.6577 | 1265.6187 | 1.0390 | 0 | 16 | 3.2 | 4 | R.LFACGIGSTANR.H |
|  | 10295 | **674.8180** | **1347.6214** | **1347.6306** | **-0.0092** | **0** | **35** | **0.053** | **1** | **R.LELGNDWDSATK.Q** |
|  | 10800 | 687.3436 | 1372.6727 | 1372.6834 | -0.0107 | 0 | 10 | 13 | 2 | K.ALNENLQDTVEK.I 10822 |
|  | 10929 | **460.9178** | **1379.7315** | **1378.6584** | **1.0731** | **1** | **12** | **9.6** | **2** | **K.SLMKMDDPSISR.N** |
|  | 15104 | 815.4563 | 1628.8980 | 1628.8158 | 0.0823 | 0 | 4 | 48 | 6 | R.NQSLLASSEWPELR.L 15102 |
|  | 16527 | **860.5780** | **1719.1414** | **1718.8017** | **0.3398** | **0** | **2** | **48** | **3** | **-.MVMGIFANCIFCLK.V** |
|  | 19481 | **644.3275** | **1929.9605** | **1929.9701** | **-0.0096** | **1** | **1** | **84** | **4** | **-.MVMGIFANCIFCLKVK.Y** |
|  | 19728 | 974.0311 | 1946.0477 | 1946.0275 | 0.0202 | 1 | 1 | 80 | 6 | K.NHVHIANPDFIWKSIR.E |
|  | 24050 | **604.0391** | **2412.1271** | **2411.0740** | **1.0532** | **0** | **7** | **15** | **1** | **K.EDVDFLPYMSWQGEPQEAVR.N** |
|  | 25192 | **641.8314** | **2563.2963** | **2562.4533** | **0.8430** | **1** | **2** | **43** | **5** | **K.LTPELGLILNLNTNGLHSFLKQK.G** |
|  | 25251 | 1288.1480 | 2574.2813 | 2575.3183 | -1.0370 | 0 | 23 | 0.38 | 2 | K.WQQLNPDAPEALQAPAQVPSLFR.N 25252 25253 |
|  | 28128 | 625.7194 | 3123.5604 | 3123.4821 | 0.0783 | 0 | 0 | 47 | 6 | K.QSFSLTMSIEMPYVIEFIFSDTHELK.Q |
|  | 28685 | **649.3180** | **3241.5539** | **3240.6781** | **0.8758** | **0** | **0** | **41** | **10** | **K.ITYITELSILGTVGVFFMPATVAPWQQDK.A** |
|  | 29471 | 1165.2455 | 3492.7146 | 3492.7347 | -0.0201 | 0 | 54 | 0.00014 | 1 | K.FSFSLNPQCTHIILDNADVLSQYQLNSIQK.N |
|  | 29872 | **918.4329** | **3669.7023** | **3670.8011** | **-1.0988** | **1** | **6** | **8.7** | **1** | **K.AVISTMEGSSLDSSGFSLHIGLSAAYLPRMWVEK.H** |

| **543.** | P61626\|LYSC_HUMAN    **Mass:** 16982    **Score:** 69     **Queries matched:** 13   **emPAI:** 0.51 |
| --- | --- |
|  | Lysozyme C OS=Homo sapiens GN=LYZ PE=1 SV=1 |

|  | **Query** | **Observed** | **Mr(expt)** | **Mr(calc)** | **Delta** | **Miss** | **Score** | **Expect** | **Rank** | **Peptide** |
| --- | --- | --- | --- | --- | --- | --- | --- | --- | --- | --- |
|  | 1781 | **406.1897** | **810.3648** | **810.3694** | **-0.0046** | **0** | **21** | **1.2** | **1** | **R.LGMDGYR.G** 1780 |
|  | 1987 | **414.5554** | **827.0962** | **826.3643** | **0.7319** | **0** | **(13)** | **5.4** | **1** | **R.LGMDGYR.G** |
|  | 2294 | 424.5369 | 847.0593 | 846.3878 | 0.6715 | 1 | 6 | 35 | 9 | R.NRCQNR.D |
|  | 3929 | 484.2398 | 966.4650 | 966.4705 | -0.0055 | 1 | 14 | 6.3 | 1 | K.RLGMDGYR.G |
|  | 4124 | **491.2193** | **980.4240** | **980.4312** | **-0.0071** | **0** | **40** | **0.015** | **1** | **R.ATNYNAGDR.S** 4125 |
|  | 4578 | **506.7243** | **1011.4341** | **1011.4410** | **-0.0069** | **0** | **18** | **2.6** | **1** | **K.WESGYNTR.A** 4585 |
|  | 11267 | **700.8395** | **1399.6645** | **1399.6732** | **-0.0087** | **0** | **56** | **0.00033** | **1** | **R.STDYGIFQINSR.Y** 11250 |
|  | 20119 | **661.3372** | **1980.9897** | **1981.2373** | **-0.2477** | **1** | **12** | **5.9** | **1** | **-.MKALIVLGLVLLSVTVQGK.V** |
|  | 20315 | **667.0290** | **1998.0651** | **1997.2323** | **0.8329** | **1** | **(5)** | **32** | **1** | **-.MKALIVLGLVLLSVTVQGK.V** |

| **544.** | Q9UNL2\|SSRG_HUMAN    **Mass:** 21067    **Score:** 68     **Queries matched:** 4   **emPAI:** 0.18 |
| --- | --- |
|  | Translocon-associated protein subunit gamma OS=Homo sapiens GN=SSR3 PE=1 SV=1 |

|  | **Query** | **Observed** | **Mr(expt)** | **Mr(calc)** | **Delta** | **Miss** | **Score** | **Expect** | **Rank** | **Peptide** |
| --- | --- | --- | --- | --- | --- | --- | --- | --- | --- | --- |
|  | 1686 | **402.6978** | **803.3810** | **803.3773** | **0.0037** | **0** | **10** | **23** | **8** | **K.LSEADNR.K** |
|  | 6275 | 567.7966 | 1133.5786 | 1132.5724 | 1.0062 | 1 | 5 | 51 | 4 | R.EDAVSKEVTR.K |
|  | 16315 | **854.4069** | **1706.7993** | **1706.8111** | **-0.0118** | **0** | **46** | **0.0029** | **1** | **K.QQSEEDLLLQDFSR.N** 16314 |

| **545.** | Q9Y277\|VDAC3_HUMAN    **Mass:** 30981    **Score:** 68     **Queries matched:** 7   **emPAI:** 0.26 |
| --- | --- |
|  | Voltage-dependent anion-selective channel protein 3 OS=Homo sapiens GN=VDAC3 PE=1 SV=1 |

|  | **Query** | **Observed** | **Mr(expt)** | **Mr(calc)** | **Delta** | **Miss** | **Score** | **Expect** | **Rank** | **Peptide** |
| --- | --- | --- | --- | --- | --- | --- | --- | --- | --- | --- |
|  | 1883 | **410.1973** | **818.3801** | **818.4134** | **-0.0333** | **0** | **2** | **1.4e+002** | **4** | **K.ASGNLETK.Y** |
|  | 4805 | 515.7590 | 1029.5035 | 1029.6070 | -0.1035 | 0 | 2 | 1e+002 | 8 | K.LTLSALIDGK.N |
|  | 8461 | **627.8238** | **1253.6330** | **1253.6404** | **-0.0074** | **0** | **48** | **0.0026** | **1** | **K.LSQNNFALGYK.A** |
|  | 10223 | **448.7318** | **1343.1735** | **1342.6955** | **0.4780** | **1** | **2** | **61** | **3** | **K.GYGFGMVKIDLK.T** |
|  | 13763 | 516.2960 | 1545.8661 | 1545.8766 | -0.0106 | 1 | 22 | 0.73 | 1 | K.LTLDTIFVPNTGKK.S |
|  | 24798 | 1260.0999 | 2518.1851 | 2518.2339 | -0.0488 | 1 | 46 | 0.0017 | 2 | K.WNTDNTLGTEISWENKLAEGLK.L 24800 |

| **546.** | Q9NYL9\|TMOD3_HUMAN    **Mass:** 39741    **Score:** 68     **Queries matched:** 7   **emPAI:** 0.31 |
| --- | --- |
|  | Tropomodulin-3 OS=Homo sapiens GN=TMOD3 PE=1 SV=1 |

|  | **Query** | **Observed** | **Mr(expt)** | **Mr(calc)** | **Delta** | **Miss** | **Score** | **Expect** | **Rank** | **Peptide** |
| --- | --- | --- | --- | --- | --- | --- | --- | --- | --- | --- |
|  | 2414 | **429.1604** | **856.3063** | **857.4719** | **-1.1656** | **1** | **7** | **39** | **10** | **K.NNDLVRK.R** 2449 |
|  | 9984 | **665.3200** | **1328.6255** | **1327.6310** | **0.9945** | **0** | **37** | **0.027** | **1** | **K.FGYQFTQQGPR.T** |
|  | 21780 | 1069.5920 | 2137.1695 | 2137.0943 | 0.0752 | 0 | 0 | 74 | 5 | K.ILPVFDEPPNPTNVEESLK.R 21782 |
|  | 23429 | **1163.5962** | **2325.1778** | **2325.1852** | **-0.0074** | **0** | **27** | **0.16** | **1** | **K.QLETVLDDLDPENALLPAGFR.Q** |
|  | 30290 | **1295.6382** | **3883.8927** | **3883.9071** | **-0.0143** | **0** | **53** | **0.00016** | **1** | **K.VSLDPELEEALTSASDTELCDLAAILGMHNLITNTK.F** |

| **547.** | Q13733\|AT1A4_HUMAN    **Mass:** 115119   **Score:** 68     **Queries matched:** 18   **emPAI:** 0.10 |
| --- | --- |
|  | Sodium/potassium-transporting ATPase subunit alpha-4 OS=Homo sapiens GN=ATP1A4 PE=1 SV=3 |

|  | **Query** | **Observed** | **Mr(expt)** | **Mr(calc)** | **Delta** | **Miss** | **Score** | **Expect** | **Rank** | **Peptide** |
| --- | --- | --- | --- | --- | --- | --- | --- | --- | --- | --- |
|  | 875 | 530.2573 | 529.2500 | 528.2656 | 0.9844 | 0 | 11 | 24 | 8 | K.GAPER.I |
|  | 8748 | 634.8565 | 1267.6983 | 1267.7071 | -0.0087 | 0 | 27 | 0.26 | 3 | K.NMVPQQALVIR.G |
|  | 10962 | 691.3739 | 1380.7332 | 1380.7435 | -0.0103 | 0 | 47 | 0.0027 | 1 | K.VIMVTGDHPITAK.A 10963 10964 |
|  | 14038 | **521.6078** | **1561.8017** | **1561.7082** | **0.0935** | **0** | **2** | **77** | **7** | **K.FIEQSYSSVAEMR.E** |
|  | 16324 | **569.9765** | **1706.9077** | **1705.8134** | **1.0943** | **1** | **7** | **25** | **1** | **K.TFTKSSDTWFMLAR.I** 16306 |
|  | 17400 | 593.9977 | 1778.9714 | 1779.9917 | -1.0203 | 1 | 1 | 80 | 3 | K.VIMVTGDHPITAKAIAK.G |
|  | 17442 | **595.1495** | **1782.4266** | **1781.9523** | **0.4743** | **0** | **1** | **62** | **1** | **R.LGAVVAVTGDGVNDSPALK.K** |
|  | 19391 | 962.9550 | 1923.8954 | 1923.9095 | -0.0142 | 0 | 1 | 82 | 1 | K.NLEAVETLGSTSTICSDK.T |
|  | 19414 | **642.8575** | **1925.5506** | **1926.0496** | **-0.4990** | **0** | **(2)** | **43** | **5** | **K.MQINVQEVVLGDLVEIK.G** 19425 |
|  | 19626 | 971.9928 | 1941.9710 | 1942.0445 | -0.0735 | 0 | 7 | 20 | 3 | K.MQINVQEVVLGDLVEIK.G |
|  | 23327 | **1157.0502** | **2312.0858** | **2313.2250** | **-1.1392** | **1** | **3** | **43** | **1** | **R.GGEKMQINVQEVVLGDLVEIK.G** |
|  | 24392 | 822.0676 | 2463.1811 | 2463.1951 | -0.0141 | 0 | 38 | 0.012 | 1 | K.QAADMILLDDNFASIVTGVEEGR.L |
|  | 25898 | 537.8540 | 2684.2336 | 2684.1808 | 0.0528 | 0 | 1 | 57 | 7 | R.ILEFCSTFLLNGQEYSMNDEMK.E |
|  | 28900 | 1103.4862 | 3307.4368 | 3306.6806 | 0.7562 | 1 | 1 | 29 | 1 | K.QAADMILLDDNFASIVTGVEEGRLIFDNLK.K |

| **548.** | P63208\|SKP1_HUMAN    **Mass:** 18817    **Score:** 68     **Queries matched:** 5   **emPAI:** 0.20 |
| --- | --- |
|  | S-phase kinase-associated protein 1 OS=Homo sapiens GN=SKP1 PE=1 SV=2 |

|  | **Query** | **Observed** | **Mr(expt)** | **Mr(calc)** | **Delta** | **Miss** | **Score** | **Expect** | **Rank** | **Peptide** |
| --- | --- | --- | --- | --- | --- | --- | --- | --- | --- | --- |
|  | 21769 | 1068.4442 | 2134.8739 | 2135.1514 | -0.2775 | 0 | 0 | 73 | 2 | K.VDQGTLFELILAANYLDIK.G |
|  | 28142 | **1042.4984** | **3124.4734** | **3124.4944** | **-0.0210** | **1** | **68** | **7.5e-006** | **1** | **K.TMLEDLGMDDEGDDDPVPLPNVNAAILKK.V** |
|  | 29897 | **922.4080** | **3685.6027** | **3684.7750** | **0.8277** | **1** | **2** | **19** | **3** | **K.QSVTIKTMLEDLGMDDEGDDDPVPLPNVNAAILK.K** 29896 29898 |

| **549.** | P50151\|GBG10_HUMAN    **Mass:** 7372     **Score:** 68     **Queries matched:** 6   **emPAI:** 0.57 |
| --- | --- |
|  | Guanine nucleotide-binding protein G(I)/G(S)/G(O) subunit gamma-10 OS=Homo sapiens GN=GNG10 PE=1 SV=1 |

|  | **Query** | **Observed** | **Mr(expt)** | **Mr(calc)** | **Delta** | **Miss** | **Score** | **Expect** | **Rank** | **Peptide** |
| --- | --- | --- | --- | --- | --- | --- | --- | --- | --- | --- |
|  | 4885 | **518.2338** | **1034.4530** | **1033.5152** | **0.9378** | **0** | **14** | **6.5** | **6** | **M.SSGASASALQR.L** |
|  | 6716 | **583.3262** | **1164.6378** | **1164.5557** | **0.0821** | **0** | **9** | **17** | **1** | **-.MSSGASASALQR.L** 6711 |
|  | 19040 | **947.9095** | **1893.8044** | **1894.0061** | **-0.2017** | **1** | **7** | **18** | **1** | **K.DALLVGVPAGSNPFREPR.S** |
|  | 21423 | **1050.4620** | **2098.9095** | **2098.9234** | **-0.0139** | **0** | **68** | **1.3e-005** | **1** | **K.VSQAAAELQQYCMQNACK.D** |
|  | 23542 | **781.0356** | **2340.0851** | **2340.1024** | **-0.0173** | **1** | **14** | **3.3** | **1** | **R.IKVSQAAAELQQYCMQNACK.D** |

| **550.** | P63096\|GNAI1_HUMAN    **Mass:** 40905    **Score:** 68     **Queries matched:** 14   **emPAI:** 0.42 |
| --- | --- |
|  | Guanine nucleotide-binding protein G(i), alpha-1 subunit OS=Homo sapiens GN=GNAI1 PE=1 SV=2 |

|  | **Query** | **Observed** | **Mr(expt)** | **Mr(calc)** | **Delta** | **Miss** | **Score** | **Expect** | **Rank** | **Peptide** |
| --- | --- | --- | --- | --- | --- | --- | --- | --- | --- | --- |
|  | 3113 | 455.2127 | 908.4109 | 908.4175 | -0.0066 | 0 | 33 | 0.081 | 1 | K.MFDVGGQR.S |
|  | 3832 | 480.7394 | 959.4641 | 959.4672 | -0.0030 | 1 | 5 | 56 | 6 | R.NLREDGEK.A 3850 |
|  | 5181 | 529.2853 | 1056.5560 | 1056.6179 | -0.0619 | 0 | 28 | 0.31 | 1 | K.LLLLGAGESGK.S |
|  | 5949 | 555.7625 | 1109.5105 | 1109.5175 | -0.0071 | 0 | 58 | 0.0002 | 1 | K.LFDSICNNK.W |
|  | 6111 | **561.5390** | **1121.0634** | **1120.5877** | **0.4758** | **1** | **1** | **91** | **4** | **R.LKIDFGDSAR.A** |
|  | 10924 | 690.8571 | 1379.6997 | 1379.7085 | -0.0089 | 0 | 15 | 4.8 | 1 | K.TTGIVETHFTFK.D |
|  | 11191 | **466.2379** | **1395.6919** | **1395.6313** | **0.0606** | **1** | **6** | **34** | **10** | **K.DSGVQACFNRSR.E** 11190 |
|  | 11411 | 470.5636 | 1408.6689 | 1407.6379 | 1.0310 | 1 | 6 | 29 | 2 | K.IDFGDSARADDAR.Q |
|  | 14390 | 529.3709 | 1585.0909 | 1584.9450 | 0.1459 | 1 | 1 | 67 | 4 | K.LLLLGAGESGKSTIVK.Q |
|  | 15226 | 546.9317 | 1637.7733 | 1636.7549 | 1.0184 | 1 | 10 | 10 | 2 | -.MGCTLSAEDKAAVER.S |
|  | 16346 | 854.9661 | 1707.9176 | 1706.9243 | 0.9932 | 0 | 29 | 0.14 | 1 | K.NVQFVFDAVTDVIIK.N |
|  | 18931 | 629.3328 | 1884.9765 | 1884.8168 | 0.1596 | 1 | 4 | 43 | 1 | R.MHESMKLFDSICNNK.W |

| **551.** | Q16629\|SFRS7_HUMAN    **Mass:** 27578    **Score:** 68     **Queries matched:** 13   **emPAI:** 0.14 |
| --- | --- |
|  | Splicing factor, arginine/serine-rich 7 OS=Homo sapiens GN=SFRS7 PE=1 SV=1 |

|  | **Query** | **Observed** | **Mr(expt)** | **Mr(calc)** | **Delta** | **Miss** | **Score** | **Expect** | **Rank** | **Peptide** |
| --- | --- | --- | --- | --- | --- | --- | --- | --- | --- | --- |
|  | 833 | **518.2852** | **517.2779** | **516.2656** | **1.0123** | **0** | **2** | **2.8e+002** | **4** | **R.SASPR.R** |
|  | 885 | 533.1890 | 532.1818 | 532.2969 | -0.1151 | 0 | 6 | 72 | 8 | R.SASLR.R |
|  | 1652 | **401.2843** | **800.5540** | **801.4093** | **-0.8553** | **1** | **10** | **23** | **9** | **R.RSASPER.M** |
|  | 5046 | **523.3047** | **1044.5948** | **1044.5788** | **0.0160** | **1** | **9** | **23** | **4** | **R.SISRPRSSR.S** 5021 5038 |
|  | 5417 | 537.2708 | 1072.5271 | 1072.5342 | -0.0071 | 0 | 27 | 0.41 | 2 | R.AFSYYGPLR.T 5416 |
|  | 7028 | 593.1561 | 1184.2977 | 1183.5734 | 0.7243 | 1 | 3 | 59 | 8 | K.GSRYFQSPSR.S 7018 |
|  | 8584 | **420.6770** | **1259.0091** | **1259.6656** | **-0.6565** | **1** | **2** | **57** | **10** | **R.VRVELSTGMPR.R** 8612 |
|  | 16535 | **860.9513** | **1719.8880** | **1718.8951** | **0.9929** | **1** | **68** | **1.9e-005** | **1** | **K.VYVGNLGTGAGKGELER.A** |

| **552.** | Q03135\|CAV1_HUMAN    **Mass:** 20630    **Score:** 67     **Queries matched:** 11   **emPAI:** 0.41 |
| --- | --- |
|  | Caveolin-1 OS=Homo sapiens GN=CAV1 PE=1 SV=4 |

|  | **Query** | **Observed** | **Mr(expt)** | **Mr(calc)** | **Delta** | **Miss** | **Score** | **Expect** | **Rank** | **Peptide** |
| --- | --- | --- | --- | --- | --- | --- | --- | --- | --- | --- |
|  | 734 | 479.3557 | 478.3484 | 478.2210 | 0.1274 | 0 | 1 | 1.3e+002 | 1 | -.MSGGK.Y |
|  | 3528 | 470.2447 | 938.4748 | 938.4821 | -0.0074 | 0 | 37 | 0.029 | 1 | K.HLNDDVVK.I 3529 |
|  | 4286 | **497.2282** | **992.4419** | **992.4484** | **-0.0065** | **0** | **23** | **0.79** | **1** | **K.AMADELSEK.Q** 4292 |
|  | 7320 | **599.8211** | **1197.6277** | **1197.6353** | **-0.0077** | **1** | **24** | **0.67** | **1** | **K.EIDLVNRDPK.H** |
|  | 9445 | **652.8280** | **1303.6414** | **1303.6520** | **-0.0106** | **0** | **25** | **0.46** | **1** | **R.EQGNIYKPNNK.A** |
|  | 15378 | **824.9139** | **1647.8133** | **1647.8257** | **-0.0123** | **0** | **59** | **0.00016** | **1** | **K.YVDSEGHLYTVPIR.E** 15387 |
|  | 19779 | 651.6684 | 1951.9834 | 1950.8993 | 1.0841 | 1 | 3 | 49 | 4 | K.AMADELSEKQVYDAHTK.E |
|  | 21539 | **704.0266** | **2109.0580** | **2108.0361** | **1.0219** | **1** | **0** | **83** | **2** | **-.MSGGKYVDSEGHLYTVPIR.E** |

| **553.** | P20701\|ITAL_HUMAN    **Mass:** 129942   **Score:** 67     **Queries matched:** 13   **emPAI:** 0.09 |
| --- | --- |
|  | Integrin alpha-L OS=Homo sapiens GN=ITGAL PE=1 SV=3 |

|  | **Query** | **Observed** | **Mr(expt)** | **Mr(calc)** | **Delta** | **Miss** | **Score** | **Expect** | **Rank** | **Peptide** |
| --- | --- | --- | --- | --- | --- | --- | --- | --- | --- | --- |
|  | 3911 | **965.5462** | **964.5389** | **964.5454** | **-0.0065** | **0** | **63** | **8.3e-005** | **1** | **R.GHAVVGAVGAK.D** 3910 |
|  | 6699 | **582.3055** | **1162.5965** | **1162.5843** | **0.0122** | **0** | **33** | **0.083** | **1** | **R.HGGLSPQPSQR.I** |
|  | 9813 | **441.2951** | **1320.8635** | **1321.6700** | **-0.8065** | **0** | **2** | **70** | **9** | **R.ALSCNVSSPIFK.A** |
|  | 14353 | **792.4108** | **1582.8071** | **1582.8144** | **-0.0073** | **0** | **20** | **1.1** | **1** | **R.AGYLGYTVTWLPSR.Q** |
|  | 14796 | 806.8916 | 1611.7686 | 1611.8005 | -0.0319 | 1 | 1 | 90 | 10 | K.HFQTKESQETLHK.F |
|  | 16063 | **845.9444** | **1689.8742** | **1689.8839** | **-0.0096** | **0** | **16** | **2.5** | **1** | **R.IEGTQVLSGIQWFGR.S** |
|  | 21733 | **710.7026** | **2129.0861** | **2129.0277** | **0.0584** | **0** | **1** | **65** | **3** | **K.ADLQDDTFIGNEPLTPEVR.A** 21732 |
|  | 22253 | **731.0671** | **2190.1794** | **2190.0593** | **0.1201** | **0** | **4** | **32** | **6** | **R.QLGFEEVSELQGDPGYPLGR.F** |
|  | 23303 | **771.3276** | **2310.9609** | **2311.2437** | **-0.2828** | **1** | **4** | **28** | **1** | **R.IEGTQVLSGIQWFGRSIHGVK.D** |
|  | 28698 | **1082.8002** | **3245.3787** | **3245.5236** | **-0.1449** | **0** | **1** | **28** | **3** | **K.DSCITVMAMALLSGFFFFAPASSYNLDVR.G** |
|  | 29679 | **895.2165** | **3576.8371** | **3576.8399** | **-0.0028** | **1** | **17** | **0.69** | **1** | **K.HMLLLTNTFGAINYVATEVFREELGARPDATK.V** |

| **554.** | Q9UGP8\|SEC63_HUMAN    **Mass:** 88341    **Score:** 67     **Queries matched:** 18   **emPAI:** 0.04 |
| --- | --- |
|  | Translocation protein SEC63 homolog OS=Homo sapiens GN=SEC63 PE=1 SV=2 |

|  | **Query** | **Observed** | **Mr(expt)** | **Mr(calc)** | **Delta** | **Miss** | **Score** | **Expect** | **Rank** | **Peptide** |
| --- | --- | --- | --- | --- | --- | --- | --- | --- | --- | --- |
|  | 1753 | **405.2060** | **808.3974** | **809.3524** | **-0.9549** | **1** | **11** | **12** | **7** | **R.NMDMKR.L** 1758 |
|  | 3376 | **464.7558** | **927.4971** | **926.3950** | **1.1021** | **1** | **3** | **81** | **8** | **K.TRNMDMK.R** |
|  | 3858 | 481.2480 | 960.4815 | 961.4604 | -0.9789 | 0 | 10 | 21 | 3 | K.DTEEVELK.F |
|  | 4438 | **501.3110** | **1000.6074** | **1000.5917** | **0.0158** | **1** | **1** | **1.5e+002** | **4** | **R.EIGSINLKK.N** |
|  | 4676 | 510.2887 | 1018.5628 | 1017.4727 | 1.0901 | 1 | 12 | 12 | 6 | R.EQDEKQNK.D |
|  | 8514 | 628.8292 | 1255.6439 | 1255.6521 | -0.0082 | 0 | 7 | 30 | 9 | K.QANGVVGNEAAVK.E |
|  | 10320 | 450.5858 | 1348.7354 | 1349.6319 | -0.8965 | 0 | 1 | 1.1e+002 | 6 | R.APTLASLENCMK.L |
|  | 10691 | 456.8821 | 1367.6246 | 1368.6530 | -1.0284 | 1 | (1) | 1.1e+002 | 9 | K.GGDEVMFMRIAK.A |
|  | 11037 | 462.9216 | 1385.7429 | 1384.6479 | 1.0950 | 1 | 7 | 29 | 2 | K.GGDEVMFMRIAK.A |
|  | 12591 | **739.3905** | **1476.7664** | **1476.7759** | **-0.0094** | **0** | **67** | **2.5e-005** | **1** | **K.LSQMAVQGLQQFK.S** |
|  | 13751 | **773.4467** | **1544.8789** | **1545.7861** | **-0.9072** | **1** | **4** | **50** | **2** | **K.KNEPPLTCPYSLK.A** |
|  | 14465 | **530.6224** | **1588.8453** | **1588.0076** | **0.8377** | **1** | **5** | **40** | **5** | **R.LLKPQPNIIPTVKK.I** |
|  | 14530 | 532.2198 | 1593.6377 | 1593.8072 | -0.1695 | 0 | 3 | 48 | 9 | R.SDSYMGLDQIKPLK.L |
|  | 16147 | **566.2748** | **1695.8027** | **1696.7338** | **-0.9311** | **1** | **1** | **91** | **3** | **K.YHPDKGGDEVMFMR.I** |
|  | 20373 | 1001.9860 | 2001.9575 | 2001.9392 | 0.0183 | 1 | 1 | 81 | 10 | K.DDEAEWQELQQSIQRK.E |
|  | 20897 | 684.3404 | 2049.9993 | 2051.0510 | -1.0516 | 1 | 5 | 26 | 5 | K.RLIMVLAGASEFDPQYNK.D |
|  | 26673 | **939.1335** | **2814.3786** | **2814.4975** | **-0.1189** | **1** | **1** | **47** | **2** | **K.SQVLDDEDSNNITVGSLVTVLVKLTR.Q** |

| **555.** | P26006\|ITA3_HUMAN    **Mass:** 119820   **Score:** 67     **Queries matched:** 17   **emPAI:** 0.10 |
| --- | --- |
|  | Integrin alpha-3 OS=Homo sapiens GN=ITGA3 PE=1 SV=3 |

|  | **Query** | **Observed** | **Mr(expt)** | **Mr(calc)** | **Delta** | **Miss** | **Score** | **Expect** | **Rank** | **Peptide** |
| --- | --- | --- | --- | --- | --- | --- | --- | --- | --- | --- |
|  | 4790 | **515.2967** | **1028.5788** | **1027.4943** | **1.0845** | **1** | **10** | **16** | **2** | **R.MVGKCYVR.G** |
|  | 7215 | **597.7886** | **1193.5626** | **1193.5710** | **-0.0085** | **0** | **44** | **0.0058** | **1** | **K.SETVLTCATGR.A** |
|  | 7972 | **410.7327** | **1229.1763** | **1228.6849** | **0.4914** | **0** | **8** | **15** | **6** | **K.LELLLMDNLR.D** 7967 |
|  | 8300 | **416.2553** | **1245.7441** | **1244.6798** | **1.0643** | **0** | **(7)** | **29** | **6** | **K.LELLLMDNLR.D** |
|  | 10772 | 458.2317 | 1371.6733 | 1370.8245 | 0.8488 | 1 | 13 | 7.6 | 1 | R.KLLLSINVTNTR.T |
|  | 14108 | **784.3242** | **1566.6338** | **1567.7969** | **-1.1632** | **1** | **7** | **23** | **2** | **R.YYQIMPKYHAVR.I** |
|  | 15826 | 838.3896 | 1674.7646 | 1674.7746 | -0.0100 | 0 | 53 | 0.00063 | 1 | R.LQSFFGGTVMGESGMK.T |
|  | 17276 | 887.4957 | 1772.9768 | 1773.8798 | -0.9030 | 0 | 21 | 0.77 | 1 | K.EAGNPGSLFGYSVALHR.Q |
|  | 19128 | **951.5152** | **1901.0158** | **1901.0371** | **-0.0212** | **1** | **0** | **91** | **7** | **R.QLDPGGGQGPPPVTLAAAKK.A** |
|  | 19467 | 965.4677 | 1928.9209 | 1929.0432 | -0.1223 | 1 | 1 | 85 | 8 | R.RQLDPGGGQGPPPVTLAAAK.K |
|  | 21485 | **527.2453** | **2104.9521** | **2104.9670** | **-0.0149** | **1** | **7** | **18** | **1** | **R.TGAVYLCPLTAHKDDCER.M** |
|  | 21546 | **704.3102** | **2109.9089** | **2109.0538** | **0.8551** | **1** | **4** | **36** | **2** | **R.HRHMGAVFLLSQEAGGDLR.R** |
|  | 25301 | **861.3626** | **2581.0658** | **2581.1189** | **-0.0530** | **1** | **2** | **38** | **2** | **R.FAGSESAVFHGFFSMPEMRCQK.L** |
|  | 28475 | **640.2986** | **3196.4569** | **3195.6676** | **0.7893** | **1** | **3** | **24** | **4** | **R.LGLRSLDAYPILNQAQALENHTEVQFQK.E** |
|  | 28725 | **1086.1627** | **3255.4663** | **3254.7052** | **0.7611** | **1** | **3** | **23** | **2** | **R.LMLCALALMVAAGGCVVSAFNLDTRFLVVK.E** |
|  | 31318 | **1270.8387** | **5079.3259** | **5078.5573** | **0.7686** | **1** | **2** | **6.2** | **2** | **K.WLLYPTEITVHGNGSWPCRPPGDLINPLNLTLSDPGDRPSSPQRR.R** |

| **556.** | P11686\|PSPC_HUMAN    **Mass:** 21381    **Score:** 67     **Queries matched:** 4   **emPAI:** 0.18 |
| --- | --- |
|  | Pulmonary surfactant-associated protein C OS=Homo sapiens GN=SFTPC PE=1 SV=2 |

|  | **Query** | **Observed** | **Mr(expt)** | **Mr(calc)** | **Delta** | **Miss** | **Score** | **Expect** | **Rank** | **Peptide** |
| --- | --- | --- | --- | --- | --- | --- | --- | --- | --- | --- |
|  | 13147 | 755.4146 | 1508.8147 | 1508.8198 | -0.0051 | 0 | 6 | 29 | 2 | K.IAPESIPSLEALNR.K |
|  | 15598 | **831.3896** | **1660.7647** | **1660.7766** | **-0.0119** | **0** | **51** | **0.00083** | **1** | **K.EVLMESPPDYSAAPR.G** 15599 |
|  | 20619 | **676.0297** | **2025.0672** | **2025.9612** | **-0.8940** | **0** | **3** | **49** | **3** | **K.HTEMVLEMSIGAPEAQQR.L** |

| **557.** | P01609\|KV117_HUMAN    **Mass:** 11871    **Score:** 67     **Queries matched:** 5   **emPAI:** 0.33 |
| --- | --- |
|  | Ig kappa chain V-I region Scw OS=Homo sapiens PE=1 SV=1 |

|  | **Query** | **Observed** | **Mr(expt)** | **Mr(calc)** | **Delta** | **Miss** | **Score** | **Expect** | **Rank** | **Peptide** |
| --- | --- | --- | --- | --- | --- | --- | --- | --- | --- | --- |
|  | 13296 | 759.8412 | 1517.6679 | 1518.7824 | -1.1145 | 1 | 11 | 9.4 | 3 | R.VTITCQASQDIRK.H |
|  | 16347 | **570.3172** | **1707.9298** | **1708.8797** | **-0.9500** | **1** | **2** | **65** | **2** | **K.HLNWYDQKPGKAPR.L** |
|  | 18843 | 939.9409 | 1877.8673 | 1877.8789 | -0.0116 | 0 | 67 | 2.1e-005 | 1 | -.DIQMTQSPSSLSASVGDR.V |
|  | 28709 | **813.3790** | **3249.4868** | **3250.5558** | **-1.0690** | **1** | **5** | **13** | **1** | **-.DIQMTQSPSSLSASVGDRVTITCQASQDIR.K** |
|  | 28759 | **1089.7064** | **3266.0974** | **3266.5507** | **-0.4533** | **1** | **(2)** | **21** | **2** | **-.DIQMTQSPSSLSASVGDRVTITCQASQDIR.K** |

|  | **Proteins matching a subset of these peptides:** |
| --- | --- |

|  | P01593\|KV101_HUMAN    **Mass:** 12099    **Score:** 67     **Queries matched:** 1 |
| --- | --- |
|  | Ig kappa chain V-I region AG OS=Homo sapiens PE=1 SV=1 |

|  | P01594\|KV102_HUMAN    **Mass:** 12046    **Score:** 67     **Queries matched:** 1 |
| --- | --- |
|  | Ig kappa chain V-I region AU OS=Homo sapiens PE=1 SV=1 |

|  | P01600\|KV108_HUMAN    **Mass:** 11778    **Score:** 67     **Queries matched:** 1 |
| --- | --- |
|  | Ig kappa chain V-I region Hau OS=Homo sapiens PE=1 SV=1 |

|  | P01607\|KV115_HUMAN    **Mass:** 12009    **Score:** 67     **Queries matched:** 1 |
| --- | --- |
|  | Ig kappa chain V-I region Rei OS=Homo sapiens PE=1 SV=1 |

|  | P01608\|KV116_HUMAN    **Mass:** 11889    **Score:** 67     **Queries matched:** 1 |
| --- | --- |
|  | Ig kappa chain V-I region Roy OS=Homo sapiens PE=1 SV=1 |

|  | P80362\|KV125_HUMAN    **Mass:** 11844    **Score:** 67     **Queries matched:** 1 |
| --- | --- |
|  | Ig kappa chain V-I region WAT OS=Homo sapiens PE=1 SV=1 |

|  | P01606\|KV114_HUMAN    **Score:** 67     **Queries matched:** 1 |
| --- | --- |

| **558.** | P01599\|KV107_HUMAN    **Mass:** 11921    **Score:** 67     **Queries matched:** 3   **emPAI:** 0.33 |
| --- | --- |
|  | Ig kappa chain V-I region Gal OS=Homo sapiens PE=1 SV=1 |

|  | **Query** | **Observed** | **Mr(expt)** | **Mr(calc)** | **Delta** | **Miss** | **Score** | **Expect** | **Rank** | **Peptide** |
| --- | --- | --- | --- | --- | --- | --- | --- | --- | --- | --- |
|  | 7194 | 597.2576 | 1192.5006 | 1192.6452 | -0.1446 | 1 | 9 | 20 | 1 | R.SFGQGTKVEIK.R |
|  | 16266 | **568.6332** | **1702.8777** | **1703.8842** | **-1.0065** | **0** | **1** | **98** | **7** | **K.ELIYAASNLQSGVPSR.F** |
|  | 18843 | 939.9409 | 1877.8673 | 1877.8789 | -0.0116 | 0 | 67 | 2.1e-005 | 1 | -.DIQMTQSPSSLSASVGDR.V |

| **559.** | P01610\|KV118_HUMAN    **Mass:** 11947    **Score:** 67     **Queries matched:** 4   **emPAI:** 0.33 |
| --- | --- |
|  | Ig kappa chain V-I region WEA OS=Homo sapiens PE=1 SV=1 |

|  | **Query** | **Observed** | **Mr(expt)** | **Mr(calc)** | **Delta** | **Miss** | **Score** | **Expect** | **Rank** | **Peptide** |
| --- | --- | --- | --- | --- | --- | --- | --- | --- | --- | --- |
|  | 13779 | 516.5991 | 1546.7755 | 1547.8308 | -1.0552 | 0 | 4 | 54 | 5 | R.LIYGATSLQSGVPSR.F |
|  | 16266 | 568.6332 | 1702.8777 | 1703.9319 | -1.0541 | 1 | 4 | 40 | 2 | K.RLIYGATSLQSGVPSR.F 16291 |
|  | 18843 | 939.9409 | 1877.8673 | 1877.8789 | -0.0116 | 0 | 67 | 2.1e-005 | 1 | -.DIQMTQSPSSLSASVGDR.V |

| **560.** | P01597\|KV105_HUMAN    **Score:** 67     **Queries matched:** 2 |
| --- | --- |
|  | Ig kappa chain V-I region DEE OS=Homo sapiens PE=1 SV=1 |

|  | **Query** | **Observed** | **Mr(expt)** | **Mr(calc)** | **Delta** | **Miss** | **Score** | **Expect** | **Rank** | **Peptide** |
| --- | --- | --- | --- | --- | --- | --- | --- | --- | --- | --- |
|  | 18843 | 939.9409 | 1877.8673 | 1877.8789 | -0.0116 | 0 | 67 | 2.1e-005 | 1 | -.DIQMTQSPSSLSASVGDR.V |
|  | 18854 | 470.7428 | 1878.9422 | 1878.8629 | 0.0793 | 0 | 6 | 26 | 7 | -.DIEMTQSPSSLSASVGDR.V |

| **561.** | P08574\|CY1_HUMAN    **Mass:** 35709    **Score:** 67     **Queries matched:** 9   **emPAI:** 0.22 |
| --- | --- |
|  | Cytochrome c1, heme protein, mitochondrial OS=Homo sapiens GN=CYC1 PE=1 SV=2 |

|  | **Query** | **Observed** | **Mr(expt)** | **Mr(calc)** | **Delta** | **Miss** | **Score** | **Expect** | **Rank** | **Peptide** |
| --- | --- | --- | --- | --- | --- | --- | --- | --- | --- | --- |
|  | 3993 | 486.7557 | 971.4969 | 971.5916 | -0.0947 | 1 | 15 | 6.1 | 8 | R.KLAYRPPK.- 3996 |
|  | 12183 | **485.2527** | **1452.7362** | **1453.8001** | **-1.0639** | **1** | **4** | **48** | **2** | **R.GLLSSLDHTSIRR.G** |
|  | 15738 | **418.2195** | **1668.8488** | **1669.8787** | **-1.0299** | **0** | **7** | **25** | **8** | **R.AANNGALPPDLSYIVR.A** |
|  | 19078 | **633.3249** | **1896.9528** | **1898.0153** | **-1.0625** | **0** | **1** | **74** | **6** | **K.MLMMMALLVPLVYTIK.R** |
|  | 19278 | **957.9670** | **1913.9195** | **1913.9312** | **-0.0116** | **0** | **28** | **0.15** | **1** | **K.LFDYFPKPYPNSEAAR.A** |
|  | 19297 | 639.3493 | 1915.0261 | 1914.0102 | 1.0158 | 0 | (0) | 95 | 7 | K.MLMMMALLVPLVYTIK.R |
|  | 20943 | **685.8473** | **2054.5200** | **2054.1164** | **0.4036** | **1** | **1** | **43** | **5** | **K.MLMMMALLVPLVYTIKR.H** |
|  | 30677 | **1038.2207** | **4148.8537** | **4148.8765** | **-0.0228** | **1** | **56** | **5.6e-005** | **1** | **R.HLVGVCYTEDEAKELAAEVEVQDGPNEDGEMFMRPGK.L** |

| **562.** | Q9H330\|CI005_HUMAN    **Mass:** 101508   **Score:** 67     **Queries matched:** 11   **emPAI:** 0.04 |
| --- | --- |
|  | Transmembrane protein C9orf5 OS=Homo sapiens GN=C9orf5 PE=1 SV=2 |

|  | **Query** | **Observed** | **Mr(expt)** | **Mr(calc)** | **Delta** | **Miss** | **Score** | **Expect** | **Rank** | **Peptide** |
| --- | --- | --- | --- | --- | --- | --- | --- | --- | --- | --- |
|  | 926 | 546.2509 | 545.2436 | 544.3221 | 0.9216 | 0 | 11 | 31 | 7 | K.ILGDK.V |
|  | 6345 | 571.2792 | 1140.5439 | 1140.5524 | -0.0084 | 0 | 67 | 3.3e-005 | 1 | R.AVGPSGGGGETPR.T 6344 6346 |
|  | 8237 | 415.2272 | 1242.6598 | 1243.7149 | -1.0551 | 1 | 3 | 69 | 7 | R.RRPEIGTFLR.K |
|  | 9286 | 432.8828 | 1295.6266 | 1294.6418 | 0.9848 | 1 | 8 | 20 | 4 | K.WNASTERYLR.A |
|  | 11869 | 478.9267 | 1433.7584 | 1434.7614 | -1.0031 | 1 | 8 | 21 | 7 | K.MIIWLEKMLDK.I |
|  | 12852 | **498.2093** | **1491.6060** | **1492.7747** | **-1.1686** | **1** | **5** | **36** | **9** | **R.VPRAVGPSGGGGETPR.T** |
|  | 20277 | 998.4167 | 1994.8189 | 1995.1041 | -0.2851 | 1 | 3 | 44 | 4 | R.SEIFSKVDVVSSAVIFLR.K |
|  | 20621 | **676.2929** | **2025.8567** | **2026.0847** | **-0.2280** | **1** | **1** | **65** | **5** | **K.VNNTAVIEKQVLELWDR.L** |
|  | 31063 | 1541.5437 | 4621.6093 | 4622.6511 | -1.0418 | 1 | 3 | 8 | 2 | K.TSDIYFVSLVWAIVVMQIWLNLWIVQLLPVPIAVWILKK.L |

| **563.** | P53007\|TXTP_HUMAN    **Mass:** 34333    **Score:** 67     **Queries matched:** 10   **emPAI:** 0.37 |
| --- | --- |
|  | Tricarboxylate transport protein, mitochondrial OS=Homo sapiens GN=SLC25A1 PE=1 SV=2 |

|  | **Query** | **Observed** | **Mr(expt)** | **Mr(calc)** | **Delta** | **Miss** | **Score** | **Expect** | **Rank** | **Peptide** |
| --- | --- | --- | --- | --- | --- | --- | --- | --- | --- | --- |
|  | 1451 | **765.1744** | **764.1671** | **763.4453** | **0.7218** | **1** | **4** | **57** | **5** | **M.PAPRAPR.A** |
|  | 4914 | **519.2557** | **1036.4968** | **1037.5658** | **-1.0690** | **1** | **9** | **18** | **2** | **K.AFYKGTVPR.L** |
|  | 8069 | **617.8523** | **1233.6900** | **1233.6969** | **-0.0068** | **0** | **41** | **0.011** | **1** | **R.GLSSLLYGSIPK.A** |
|  | 9041 | **642.3179** | **1282.6212** | **1282.6306** | **-0.0094** | **0** | **15** | **4.3** | **1** | **K.FIHDQTSPNPK.Y** 9056 |
|  | 13270 | **758.8837** | **1515.7529** | **1514.6799** | **1.0730** | **0** | **41** | **0.01** | **1** | **R.FGMFEFLSNHMR.D** 13237 |
|  | 20786 | **681.8206** | **2042.4400** | **2041.9251** | **0.5150** | **1** | **0** | **60** | **5** | **R.FGMFEFLSNHMRDAQGR.L** |
|  | 29477 | **1165.9432** | **3494.8079** | **3494.8232** | **-0.0153** | **0** | **16** | **1** | **1** | **R.GDNPNKPMNPLITGVFGAIAGAASVFGNTPLDVIK.T** 29476 |

| **564.** | P14384\|CBPM_HUMAN    **Mass:** 50938    **Score:** 67     **Queries matched:** 6   **emPAI:** 0.15 |
| --- | --- |
|  | Carboxypeptidase M OS=Homo sapiens GN=CPM PE=1 SV=2 |

|  | **Query** | **Observed** | **Mr(expt)** | **Mr(calc)** | **Delta** | **Miss** | **Score** | **Expect** | **Rank** | **Peptide** |
| --- | --- | --- | --- | --- | --- | --- | --- | --- | --- | --- |
|  | 8738 | **423.4547** | **1267.3423** | **1267.7513** | **-0.4090** | **1** | **2** | **55** | **8** | **K.GRNLWVLVVGR.F** 8732 |
|  | 12109 | 724.8282 | 1447.6418 | 1447.6514 | -0.0096 | 0 | 55 | 0.00044 | 1 | K.YVANMHGDETVGR.E 12110 |
|  | 22303 | **733.6790** | **2198.0151** | **2198.0280** | **-0.0130** | **0** | **8** | **12** | **1** | **R.SLTPDDDVFQYLAHTYASR.N** |
|  | 27927 | **1535.7846** | **3069.5545** | **3069.5667** | **-0.0122** | **0** | **40** | **0.0048** | **1** | **K.DILLPFQGQLDSIPVSNPSCPMIPLYR.N** |

| **565.** | P05090\|APOD_HUMAN    **Mass:** 21547    **Score:** 66     **Queries matched:** 6   **emPAI:** 0.39 |
| --- | --- |
|  | Apolipoprotein D OS=Homo sapiens GN=APOD PE=1 SV=1 |

|  | **Query** | **Observed** | **Mr(expt)** | **Mr(calc)** | **Delta** | **Miss** | **Score** | **Expect** | **Rank** | **Peptide** |
| --- | --- | --- | --- | --- | --- | --- | --- | --- | --- | --- |
|  | 2609 | 436.2505 | 870.4864 | 870.4923 | -0.0059 | 0 | 39 | 0.02 | 1 | K.VLNQELR.A |
|  | 5968 | **556.7678** | **1111.5211** | **1111.6713** | **-0.1502** | **1** | **6** | **38** | **1** | **K.IKVLNQELR.A** |
|  | 11605 | 710.8451 | 1419.6757 | 1419.6850 | -0.0093 | 1 | 58 | 0.00022 | 1 | K.KMTVTDQVNCPK.L |
|  | 13104 | **503.2259** | **1506.6558** | **1507.7011** | **-1.0452** | **1** | **0** | **1.1e+002** | **10** | **K.MTVTDQVNCPKLS.-** |
|  | 17498 | 893.4500 | 1784.8855 | 1783.8597 | 1.0258 | 1 | 1 | 88 | 7 | R.CIQANYSLMENGKIK.V |
|  | 26170 | 908.7066 | 2723.0980 | 2722.4025 | 0.6955 | 1 | 1 | 38 | 2 | R.ADGTVNQIEGEATPVNLTEPAKLEVK.F |

| **566.** | P09668\|CATH_HUMAN    **Mass:** 37980    **Score:** 66     **Queries matched:** 4   **emPAI:** 0.21 |
| --- | --- |
|  | Cathepsin H OS=Homo sapiens GN=CTSH PE=1 SV=3 |

|  | **Query** | **Observed** | **Mr(expt)** | **Mr(calc)** | **Delta** | **Miss** | **Score** | **Expect** | **Rank** | **Peptide** |
| --- | --- | --- | --- | --- | --- | --- | --- | --- | --- | --- |
|  | 1166 | **643.3841** | **642.3768** | **641.2479** | **1.1289** | **0** | **8** | **36** | **1** | **K.DGYCK.F** |
|  | 8161 | **620.7799** | **1239.5453** | **1239.5554** | **-0.0101** | **0** | **35** | **0.039** | **1** | **R.TGIYSSTSCHK.T** |
|  | 12264 | **729.8275** | **1457.6405** | **1457.6497** | **-0.0092** | **0** | **55** | **0.00046** | **1** | **K.GIMGEDTYPYQGK.D** |
|  | 17692 | **599.9832** | **1796.9278** | **1796.9421** | **-0.0143** | **1** | **7** | **20** | **1** | **K.TPDKVNHAVLAVGYGEK.N** |

| **567.** | Q9Y2Q3\|GSTK1_HUMAN    **Mass:** 25594    **Score:** 66     **Queries matched:** 9   **emPAI:** 0.51 |
| --- | --- |
|  | Glutathione S-transferase kappa 1 OS=Homo sapiens GN=GSTK1 PE=1 SV=3 |

|  | **Query** | **Observed** | **Mr(expt)** | **Mr(calc)** | **Delta** | **Miss** | **Score** | **Expect** | **Rank** | **Peptide** |
| --- | --- | --- | --- | --- | --- | --- | --- | --- | --- | --- |
|  | 3491 | **469.0948** | **936.1750** | **936.3971** | **-0.2221** | **0** | **7** | **20** | **7** | **K.ETTEAACR.Y** 3494 |
|  | 6535 | **577.1239** | **1152.2332** | **1151.6008** | **0.6324** | **1** | **4** | **45** | **9** | **R.KGLYMANDLK.L** |
|  | 8451 | **627.4652** | **1252.9159** | **1252.6849** | **0.2310** | **0** | **0** | **98** | **10** | **R.MELLAHLLGEK.W** |
|  | 11600 | 710.4257 | 1418.8368 | 1419.6776 | -0.8408 | 1 | 21 | 1.1 | 1 | K.NQLKETTEAACR.Y |
|  | 11824 | 716.8537 | 1431.6928 | 1431.7027 | -0.0099 | 0 | 67 | 2.7e-005 | 1 | K.AGMSAEQAQGLLEK.I |
|  | 16660 | **864.9302** | **1727.8458** | **1727.8577** | **-0.0119** | **0** | **24** | **0.42** | **1** | **R.NEDITEPQSILAAAEK.A** 16659 |
|  | 17222 | **590.6605** | **1768.9597** | **1769.9022** | **-0.9424** | **0** | **1** | **89** | **3** | **R.FLTAVNLEHPEMLEK.A** |

| **568.** | Q9Y2B0\|CNPY2_HUMAN    **Mass:** 20981    **Score:** 66     **Queries matched:** 7   **emPAI:** 0.65 |
| --- | --- |
|  | Protein canopy homolog 2 OS=Homo sapiens GN=CNPY2 PE=1 SV=1 |

|  | **Query** | **Observed** | **Mr(expt)** | **Mr(calc)** | **Delta** | **Miss** | **Score** | **Expect** | **Rank** | **Peptide** |
| --- | --- | --- | --- | --- | --- | --- | --- | --- | --- | --- |
|  | 7428 | 602.2477 | 1202.4809 | 1202.4921 | -0.0112 | 0 | 36 | 0.039 | 1 | R.SQDLHCGACR.A |
|  | 11559 | **473.2505** | **1416.7296** | **1416.6845** | **0.0452** | **0** | **8** | **22** | **2** | **R.NGESSELDLQGIR.I** |
|  | 16705 | 865.9329 | 1729.8512 | 1729.8635 | -0.0123 | 0 | 32 | 0.067 | 1 | R.INPDGSQSVVEVPYAR.S 16706 |
|  | 18538 | **927.9737** | **1853.9328** | **1853.9411** | **-0.0082** | **0** | **59** | **0.00014** | **1** | **R.ALVDELEWEIAQVDPK.K** |
|  | 23226 | **767.3711** | **2299.0914** | **2299.2663** | **-0.1749** | **1** | **4** | **31** | **3** | **-.MKGWGWLALLLGALLGTAWAR.R** |
|  | 24270 | 816.3733 | 2446.0982 | 2446.2187 | -0.1205 | 1 | 4 | 29 | 2 | R.NGESSELDLQGIRIDSDISGTLK.F |

| **569.** | P08648\|ITA5_HUMAN    **Mass:** 115605   **Score:** 65     **Queries matched:** 22   **emPAI:** 0.03 |
| --- | --- |
|  | Integrin alpha-5 OS=Homo sapiens GN=ITGA5 PE=1 SV=2 |

|  | **Query** | **Observed** | **Mr(expt)** | **Mr(calc)** | **Delta** | **Miss** | **Score** | **Expect** | **Rank** | **Peptide** |
| --- | --- | --- | --- | --- | --- | --- | --- | --- | --- | --- |
|  | 2587 | **435.2550** | **868.4954** | **868.4919** | **0.0035** | **0** | **9** | **17** | **1** | **R.FTVPHLR.D** 2567 2588 2594 |
|  | 4704 | 511.7634 | 1021.5122 | 1020.5062 | 1.0060 | 0 | 13 | 8.5 | 4 | R.ILEYAPCR.S |
|  | 8570 | 630.3245 | 1258.6344 | 1258.6414 | -0.0070 | 0 | 24 | 0.59 | 1 | R.LLVCDLGNPMK.A |
|  | 8733 | **634.3466** | **1266.6787** | **1267.6230** | **-0.9444** | **1** | **7** | **26** | **2** | **K.RSLPYGTAMEK.A** |
|  | 8896 | 637.8632 | 1273.7118 | 1274.6363 | -0.9245 | 0 | (11) | 11 | 2 | R.LLVCDLGNPMK.A 8892 8903 |
|  | 10150 | **670.3632** | **1338.7118** | **1338.7184** | **-0.0066** | **0** | **4** | **58** | **1** | **K.TIQFDFQILSK.N** |
|  | 12429 | **734.4871** | **1466.9597** | **1466.8133** | **0.1464** | **1** | **1** | **74** | **2** | **K.KTIQFDFQILSK.N** |
|  | 12782 | **744.8838** | **1487.7530** | **1487.7620** | **-0.0090** | **0** | **24** | **0.49** | **1** | **R.VTAPPEAEYSGLVR.H** |
|  | 15676 | 833.4023 | 1664.7900 | 1664.8304 | -0.0404 | 0 | 2 | 69 | 7 | R.VTGLNCTTNHPINPK.G |
|  | 17355 | 889.4436 | 1776.8726 | 1777.9257 | -1.0531 | 1 | 6 | 27 | 6 | -.MGSRTPESPLHAVQLR.W 17356 17368 |
|  | 20128 | **991.9680** | **1981.9215** | **1981.9368** | **-0.0153** | **0** | **65** | **2.8e-005** | **1** | **R.LLESSLSSSEGEEPVEYK.S** |
|  | 22113 | **724.8841** | **2171.6304** | **2170.9841** | **0.6463** | **0** | **3** | **27** | **3** | **K.EPLSDPVGTCYLSTDNFTR.I** |
|  | 22138 | **1088.4797** | **2174.9449** | **2175.0881** | **-0.1432** | **1** | **0** | **77** | **1** | **R.SLPYGTAMEKAQLKPPATSDA.-** |
|  | 25208 | **856.9780** | **2567.9121** | **2568.2860** | **-0.3740** | **1** | **0** | **46** | **4** | **R.DLDGNGYPDLIVGSFGVDKAVVYR.G** |
|  | 27638 | **753.6324** | **3010.5005** | **3010.5189** | **-0.0184** | **0** | **9** | **6.7** | **1** | **R.VYVYLQHPAGIEPTPTLTLTGHDEFGR.F** |

| **570.** | P84243\|H33_HUMAN    **Mass:** 15376    **Score:** 65     **Queries matched:** 6   **emPAI:** 0.57 |
| --- | --- |
|  | Histone H3.3 OS=Homo sapiens GN=H3F3A PE=1 SV=2 |

|  | **Query** | **Observed** | **Mr(expt)** | **Mr(calc)** | **Delta** | **Miss** | **Score** | **Expect** | **Rank** | **Peptide** |
| --- | --- | --- | --- | --- | --- | --- | --- | --- | --- | --- |
|  | 2035 | 416.2475 | 830.4804 | 830.4861 | -0.0057 | 0 | 53 | 0.0011 | 1 | K.STELLIR.K 2037 |
|  | 2252 | **422.7546** | **843.4947** | **843.5000** | **-0.0053** | **1** | **15** | **6** | **3** | **K.RVTIMPK.D** |
|  | 3417 | **466.2368** | **930.4590** | **930.5134** | **-0.0545** | **1** | **15** | **5.4** | **3** | **R.KSAPSTGGVK.K** |
|  | 9778 | 440.8850 | 1319.6332 | 1320.7262 | -1.0930 | 1 | 8 | 22 | 6 | K.SAPSTGGVKKPHR.Y |
|  | 29330 | 1146.5676 | 3436.6811 | 3436.6973 | -0.0162 | 0 | 40 | 0.0039 | 1 | R.FQSAAIGALQEASEAYLVGLFEDTNLCAIHAK.R |

|  | **Proteins matching a subset of these peptides:** |
| --- | --- |

|  | Q6NXT2\|H3L_HUMAN    **Mass:** 15318    **Score:** 53     **Queries matched:** 3 |
| --- | --- |
|  | Histone H3-like OS=Homo sapiens PE=1 SV=3 |

|  | Q71DI3\|H32_HUMAN    **Mass:** 15436    **Score:** 53     **Queries matched:** 3 |
| --- | --- |
|  | Histone H3.2 OS=Homo sapiens GN=HIST2H3A PE=1 SV=3 |

|  | Q16695\|H31T_HUMAN    **Mass:** 15613    **Score:** 53     **Queries matched:** 3 |
| --- | --- |
|  | Histone H3.1t OS=Homo sapiens GN=HIST3H3 PE=1 SV=3 |

|  | P68431\|H31_HUMAN    **Mass:** 15509    **Score:** 53     **Queries matched:** 3 |
| --- | --- |
|  | Histone H3.1 OS=Homo sapiens GN=HIST1H3A PE=1 SV=2 |

| **571.** | P32969\|RL9_HUMAN    **Mass:** 21964    **Score:** 65     **Queries matched:** 14   **emPAI:** 0.38 |
| --- | --- |
|  | 60S ribosomal protein L9 OS=Homo sapiens GN=RPL9 PE=1 SV=1 |

|  | **Query** | **Observed** | **Mr(expt)** | **Mr(calc)** | **Delta** | **Miss** | **Score** | **Expect** | **Rank** | **Peptide** |
| --- | --- | --- | --- | --- | --- | --- | --- | --- | --- | --- |
|  | 1843 | 408.7292 | 815.4438 | 815.4865 | -0.0427 | 1 | 15 | 6.7 | 4 | R.KELATVR.T 1844 1845 |
|  | 6791 | **585.8016** | **1169.5886** | **1169.5968** | **-0.0082** | **0** | **17** | **3.2** | **1** | **K.FLDGIYVSEK.G** |
|  | 10005 | **665.8297** | **1329.6447** | **1329.6533** | **-0.0086** | **0** | **28** | **0.25** | **1** | **R.TICSHVQNMIK.G** 10004 |
|  | 13299 | **759.8600** | **1517.7054** | **1517.7443** | **-0.0388** | **0** | **9** | **14** | **2** | **R.MRPGVACSVSQAQK.D** |
|  | 17290 | **592.3013** | **1773.8822** | **1772.9138** | **0.9684** | **1** | **2** | **71** | **7** | **R.VRMRPGVACSVSQAQK.D** |
|  | 20323 | **667.0429** | **1998.1069** | **1999.0343** | **-0.9274** | **1** | **2** | **52** | **3** | **K.ELATVRTICSHVQNMIK.G** |
|  | 21574 | **1057.0665** | **2112.1185** | **2112.1314** | **-0.0129** | **0** | **58** | **0.00014** | **1** | **K.TILSNQTVDIPENVDITLK.G** 21572 |
|  | 24537 | **829.1041** | **2484.2906** | **2483.3172** | **0.9733** | **0** | **22** | **0.39** | **1** | **R.SVYAHFPINVVIQENGSLVEIR.N** 24515 24538 |

| **572.** | Q6RW13\|ATRAP_HUMAN    **Mass:** 17579    **Score:** 65     **Queries matched:** 4   **emPAI:** 0.22 |
| --- | --- |
|  | Type-1 angiotensin II receptor-associated protein OS=Homo sapiens GN=AGTRAP PE=1 SV=1 |

|  | **Query** | **Observed** | **Mr(expt)** | **Mr(calc)** | **Delta** | **Miss** | **Score** | **Expect** | **Rank** | **Peptide** |
| --- | --- | --- | --- | --- | --- | --- | --- | --- | --- | --- |
|  | 23158 | **1146.5360** | **2291.0575** | **2291.0706** | **-0.0131** | **0** | **65** | **2.6e-005** | **1** | **R.SAYQTIDSAEAPADPFAVPEGR.S** 23159 23166 |
|  | 30501 | 1349.2878 | 4044.8417 | 4044.9454 | -0.1037 | 1 | 5 | 8.8 | 4 | R.GGELLVHTGFLGSSQDRSAYQTIDSAEAPADPFAVPEGR.S |

| **573.** | P07204\|TRBM_HUMAN    **Mass:** 63083    **Score:** 65     **Queries matched:** 4   **emPAI:** 0.06 |
| --- | --- |
|  | Thrombomodulin OS=Homo sapiens GN=THBD PE=1 SV=2 |

|  | **Query** | **Observed** | **Mr(expt)** | **Mr(calc)** | **Delta** | **Miss** | **Score** | **Expect** | **Rank** | **Peptide** |
| --- | --- | --- | --- | --- | --- | --- | --- | --- | --- | --- |
|  | 959 | **556.3165** | **555.3092** | **554.3540** | **0.9552** | **0** | **8** | **20** | **8** | **R.LGPLR.G** 956 |
|  | 17834 | **602.9631** | **1805.8674** | **1805.9611** | **-0.0937** | **1** | **2** | **66** | **2** | **R.RLWIGLQLPPGCGDPK.R** |
|  | 29933 | **1234.6226** | **3700.8459** | **3700.8607** | **-0.0148** | **0** | **65** | **1.2e-005** | **1** | **R.GADFQALPVGSSAAVAPLGLQLMCTAPPGAVQGHWAR.E** |

| **574.** | Q5JTV8\|TOIP1_HUMAN    **Mass:** 66379    **Score:** 65     **Queries matched:** 10   **emPAI:** 0.06 |
| --- | --- |
|  | Torsin-1A-interacting protein 1 OS=Homo sapiens GN=TOR1AIP1 PE=1 SV=2 |

|  | **Query** | **Observed** | **Mr(expt)** | **Mr(calc)** | **Delta** | **Miss** | **Score** | **Expect** | **Rank** | **Peptide** |
| --- | --- | --- | --- | --- | --- | --- | --- | --- | --- | --- |
|  | 711 | **474.9764** | **473.9691** | **474.2187** | **-0.2495** | **0** | **6** | **65** | **3** | **M.AGDGR.R** |
|  | 921 | **544.3155** | **543.3082** | **544.2969** | **-0.9887** | **0** | **14** | **10** | **1** | **R.AEAVR.E** |
|  | 4543 | **505.2947** | **1008.5749** | **1007.5400** | **1.0349** | **1** | **4** | **63** | **10** | **K.RSQTFLEK.H** |
|  | 7501 | **402.8850** | **1205.6330** | **1204.6234** | **1.0096** | **1** | **5** | **51** | **8** | **R.TRMQNDSILK.S** |
|  | 7815 | **611.3371** | **1220.6596** | **1220.6183** | **0.0413** | **1** | **(3)** | **70** | **1** | **R.TRMQNDSILK.S** |
|  | 9056 | 428.8827 | 1283.6263 | 1283.6258 | 0.0005 | 0 | 12 | 9.5 | 4 | K.TPQEWAPQTAR.I |
|  | 10313 | **675.3140** | **1348.6134** | **1348.6219** | **-0.0085** | **0** | **65** | **5.4e-005** | **1** | **K.SELGNQSPSTSSR.Q** 10289 |
|  | 18095 | 608.9744 | 1823.9015 | 1824.0105 | -0.1090 | 1 | 10 | 11 | 2 | R.SIQEAPVSEDLVIRLR.R |
|  | 22414 | 736.8245 | 2207.4517 | 2207.9906 | -0.5388 | 1 | 1 | 48 | 9 | K.VKFTNSNTPNSYNHMDPDK.L |

| **575.** | Q13423\|NNTM_HUMAN    **Mass:** 114564   **Score:** 64     **Queries matched:** 23   **emPAI:** 0.07 |
| --- | --- |
|  | NAD(P) transhydrogenase, mitochondrial OS=Homo sapiens GN=NNT PE=1 SV=3 |

|  | **Query** | **Observed** | **Mr(expt)** | **Mr(calc)** | **Delta** | **Miss** | **Score** | **Expect** | **Rank** | **Peptide** |
| --- | --- | --- | --- | --- | --- | --- | --- | --- | --- | --- |
|  | 2176 | 840.4030 | 839.3957 | 840.5069 | -1.1112 | 0 | 4 | 55 | 7 | K.QLTVGVPK.E |
|  | 3329 | **462.4843** | **922.9541** | **922.5236** | **0.4305** | **0** | **10** | **10** | **1** | **K.NIPQGAPVK.Q** |
|  | 3527 | 470.2397 | 938.4649 | 938.3882 | 0.0766 | 0 | 19 | 1.7 | 1 | K.FSDDHYR.V |
|  | 5411 | 536.8128 | 1071.6111 | 1071.6176 | -0.0065 | 0 | 19 | 2.4 | 1 | K.EVLASDLVVK.V |
|  | 5563 | **542.3001** | **1082.5855** | **1081.4676** | **1.1179** | **0** | **7** | **32** | **2** | **K.ESGEGQGGYAK.E** |
|  | 6909 | **590.3214** | **1178.6281** | **1178.6771** | **-0.0490** | **1** | **3** | **82** | **4** | **K.NIPQGAPVKQK.T** |
|  | 8282 | **623.3471** | **1244.6797** | **1244.6612** | **0.0185** | **1** | **5** | **51** | **9** | **K.QKTVAELEAEK.A** |
|  | 10478 | 679.3525 | 1356.6904 | 1357.7388 | -1.0484 | 1 | 21 | 1 | 2 | R.KTTVLAMDQVPR.V 10522 |
|  | 13402 | **762.9547** | **1523.8947** | **1523.9035** | **-0.0088** | **0** | **5** | **39** | **2** | **K.ILIVGGGVAGLASAGAAK.S** 13394 |
|  | 14270 | **527.1538** | **1578.4396** | **1577.7686** | **0.6710** | **0** | **3** | **44** | **10** | **K.QGFNVVVESGAGEASK.F** |
|  | 15575 | 553.9665 | 1658.8778 | 1657.7981 | 1.0797 | 0 | 2 | 77 | 9 | R.MATQASTLYSNNITK.L |
|  | 16666 | **577.1227** | **1728.3464** | **1728.9410** | **-0.5946** | **1** | **7** | **16** | **1** | **K.QLTVGVPKEIFQNEK.R** 16667 16681 |
|  | 21021 | **687.3462** | **2059.0167** | **2059.1248** | **-0.1081** | **1** | **9** | **12** | **1** | **K.VRAPMVNPTLGVHEADLLK.T** |
|  | 21155 | **1037.0138** | **2072.0130** | **2072.0248** | **-0.0118** | **0** | **58** | **0.00014** | **1** | **R.VTIAQGYDALSSMANIAGYK.A** 21146 |
|  | 22388 | **1103.5852** | **2205.1559** | **2205.1681** | **-0.0122** | **0** | **11** | **6.6** | **1** | **K.TSGTLISFIYPAQNPELLNK.L** |
|  | 26719 | **942.4836** | **2824.4289** | **2824.4469** | **-0.0180** | **0** | **23** | **0.32** | **1** | **R.SLGVGYAAVDNPIFYKPNTAMLLGDAK.K** |
|  | 28612 | 1075.3120 | 3222.9142 | 3223.6448 | -0.7306 | 1 | 0 | 30 | 7 | R.VTIAQGYDALSSMANIAGYKAVVLAANHFGR.F |
|  | 30145 | **949.4458** | **3793.7541** | **3794.8383** | **-1.0842** | **1** | **4** | **11** | **1** | **K.EMIESMKEGSVVVDLAAEAGGNFETTKPGELYIHK.G** |

| **576.** | P01833\|PIGR_HUMAN    **Mass:** 84429    **Score:** 64     **Queries matched:** 14   **emPAI:** 0.09 |
| --- | --- |
|  | Polymeric immunoglobulin receptor OS=Homo sapiens GN=PIGR PE=1 SV=4 |

|  | **Query** | **Observed** | **Mr(expt)** | **Mr(calc)** | **Delta** | **Miss** | **Score** | **Expect** | **Rank** | **Peptide** |
| --- | --- | --- | --- | --- | --- | --- | --- | --- | --- | --- |
|  | 1562 | **397.5615** | **793.1085** | **792.4130** | **0.6955** | **0** | **4** | **44** | **1** | **K.VLDSGFR.E** |
|  | 8393 | 417.2431 | 1248.7076 | 1249.6667 | -0.9591 | 0 | 12 | 10 | 2 | K.DGSFSVVITGLR.K 8382 8388 8407 |
|  | 11194 | **466.2736** | **1395.7989** | **1396.7926** | **-0.9937** | **0** | **8** | **22** | **3** | **K.VPGNVTAVLGETLK.V** |
|  | 11779 | 715.3815 | 1428.7485 | 1428.7096 | 0.0389 | 1 | 4 | 52 | 5 | R.DVSLAKADAAPDEK.V |
|  | 13321 | 760.3871 | 1518.7596 | 1518.7613 | -0.0017 | 0 | 8 | 19 | 3 | K.GVAGGSVAVLCPYNR.K |
|  | 15514 | **828.9202** | **1655.8259** | **1656.8029** | **-0.9770** | **0** | **3** | **63** | **8** | **R.GGCITLISSEGYVSSK.Y** |
|  | 22087 | **724.3218** | **2169.9435** | **2169.9597** | **-0.0161** | **1** | **36** | **0.02** | **1** | **R.LSDAGQYLCQAGDDSNSNKK.N** |
|  | 29800 | **1210.9069** | **3629.6988** | **3629.7209** | **-0.0221** | **0** | **52** | **0.00023** | **1** | **R.YLCGAHSDGQLQEGSPIQAWQLFVNEESTIPR.S** 29801 |
|  | 30077 | 942.4658 | 3765.8342 | 3766.7003 | -0.8662 | 1 | 4 | 15 | 4 | R.EFGANDNMGASSITQETSLGGKEEFVATTESTTETK.E |
|  | 31480 | **1087.7373** | **5433.6501** | **5432.7387** | **0.9115** | **1** | **1** | **10** | **1** | **-.MLLFVLTCLLAVFPAISTKSPIFGPEEVNSVEGNSVSITCYYPPTSVNR.H** |

| **577.** | P84085\|ARF5_HUMAN    **Mass:** 20631    **Score:** 64     **Queries matched:** 8   **emPAI:** 0.98 |
| --- | --- |
|  | ADP-ribosylation factor 5 OS=Homo sapiens GN=ARF5 PE=1 SV=2 |

|  | **Query** | **Observed** | **Mr(expt)** | **Mr(calc)** | **Delta** | **Miss** | **Score** | **Expect** | **Rank** | **Peptide** |
| --- | --- | --- | --- | --- | --- | --- | --- | --- | --- | --- |
|  | 2107 | 418.2346 | 834.4546 | 835.5028 | -1.0482 | 0 | 3 | 98 | 8 | K.LGLQHLR.S |
|  | 2204 | 421.2071 | 840.3996 | 839.5130 | 0.8866 | 0 | 14 | 6 | 1 | K.IRPLWR.H |
|  | 6422 | 573.7815 | 1145.5484 | 1145.5564 | -0.0080 | 0 | 33 | 0.085 | 1 | R.VQESADELQK.M |
|  | 23401 | 1162.1196 | 2322.2247 | 2322.2359 | -0.0112 | 0 | 24 | 0.29 | 1 | K.LGEIVTTIPTIGFNVETVEYK.N 23402 |
|  | 24211 | 813.3935 | 2437.1586 | 2437.1775 | -0.0188 | 1 | 49 | 0.0009 | 1 | R.HYFQNTQGLIFVVDSNDRER.V |
|  | 25193 | 855.4740 | 2563.4002 | 2563.4149 | -0.0147 | 1 | 27 | 0.13 | 1 | K.LKLGEIVTTIPTIGFNVETVEYK.N |
|  | 26442 | 695.3413 | 2777.3361 | 2777.3616 | -0.0254 | 1 | 5 | 21 | 3 | R.DAVLLVFANKQDMPNAMPVSELTDK.L |

| **578.** | P11279\|LAMP1_HUMAN    **Mass:** 45367    **Score:** 64     **Queries matched:** 13   **emPAI:** 0.17 |
| --- | --- |
|  | Lysosome-associated membrane glycoprotein 1 OS=Homo sapiens GN=LAMP1 PE=1 SV=3 |

|  | **Query** | **Observed** | **Mr(expt)** | **Mr(calc)** | **Delta** | **Miss** | **Score** | **Expect** | **Rank** | **Peptide** |
| --- | --- | --- | --- | --- | --- | --- | --- | --- | --- | --- |
|  | 4600 | **507.7209** | **1013.4272** | **1013.4349** | **-0.0077** | **0** | **13** | **8.5** | **2** | **K.CNAEEHVR.V** |
|  | 4859 | 517.2763 | 1032.5379 | 1032.5451 | -0.0072 | 0 | 32 | 0.1 | 1 | K.TVESITDIR.A |
|  | 6508 | **576.3026** | **1150.5905** | **1150.5982** | **-0.0077** | **0** | **53** | **0.00083** | **1** | **R.ALQATVGNSYK.C** 6487 |
|  | 8427 | 418.2025 | 1251.5855 | 1252.7180 | -1.1324 | 1 | 10 | 14 | 2 | R.VTKAFSVNIFK.V 8438 |
|  | 17627 | 598.2946 | 1791.8620 | 1791.8825 | -0.0205 | 0 | 2 | 66 | 8 | K.NMTFDLPSDATVVLNR.S |
|  | 18410 | **923.5112** | **1845.0078** | **1845.0149** | **-0.0071** | **0** | **40** | **0.0092** | **1** | **R.FFLQGIQLNTILPDAR.D** 18393 18394 |
|  | 23985 | **802.1023** | **2403.2850** | **2403.2951** | **-0.0100** | **1** | **18** | **1** | **1** | **R.FFLQGIQLNTILPDARDPAFK.A** |
|  | 29957 | **1237.6555** | **3709.9447** | **3710.0457** | **-0.1010** | **1** | **(2)** | **20** | **1** | **-.MAAPGSARRPLLLLLLLLLLGLMHCASAAMFMVK.N** |
|  | 29958 | **928.6798** | **3710.6899** | **3710.0457** | **0.6442** | **1** | **3** | **17** | **2** | **-.MAAPGSARRPLLLLLLLLLLGLMHCASAAMFMVK.N** |

| **579.** | O14773\|TPP1_HUMAN    **Mass:** 61723    **Score:** 63     **Queries matched:** 9   **emPAI:** 0.12 |
| --- | --- |
|  | Tripeptidyl-peptidase 1 OS=Homo sapiens GN=TPP1 PE=1 SV=2 |

|  | **Query** | **Observed** | **Mr(expt)** | **Mr(calc)** | **Delta** | **Miss** | **Score** | **Expect** | **Rank** | **Peptide** |
| --- | --- | --- | --- | --- | --- | --- | --- | --- | --- | --- |
|  | 7758 | **407.1323** | **1218.3751** | **1218.6390** | **-0.2639** | **1** | **6** | **29** | **7** | **R.VNTELMKAAAR.G** |
|  | 12516 | **492.2527** | **1473.7362** | **1473.7477** | **-0.0115** | **0** | **4** | **49** | **7** | **R.LFGGNFAHQASVAR.V** |
|  | 13611 | **512.9678** | **1535.8817** | **1535.8936** | **-0.0120** | **0** | **6** | **27** | **2** | **R.ILSGRPPLGFLNPR.L** 13589 |
|  | 14668 | **802.9072** | **1603.7998** | **1603.8107** | **-0.0109** | **0** | **59** | **0.00014** | **1** | **R.LYQQHGAGLFDVTR.G** 14669 |
|  | 19336 | **960.9506** | **1919.8866** | **1920.0576** | **-0.1711** | **0** | **1** | **87** | **5** | **-.MGLQACLLGLFALILSGK.C** |
|  | 29283 | 1140.9383 | 3419.7932 | 3420.7844 | -0.9912 | 1 | 1 | 28 | 5 | R.SPHPYQLPQALAPHVDFVGGLHRFPPTSSLR.Q |
|  | 30708 | **1048.4582** | **4189.8039** | **4189.8245** | **-0.0206** | **0** | **5** | **6.5** | **1** | **R.GCHESCLDEEVEGQGFCSGPGWDPVTGWGTPNFPALLK.T** |

| **580.** | P18206\|VINC_HUMAN    **Mass:** 124292   **Score:** 63     **Queries matched:** 30   **emPAI:** 0.03 |
| --- | --- |
|  | Vinculin OS=Homo sapiens GN=VCL PE=1 SV=4 |

|  | **Query** | **Observed** | **Mr(expt)** | **Mr(calc)** | **Delta** | **Miss** | **Score** | **Expect** | **Rank** | **Peptide** |
| --- | --- | --- | --- | --- | --- | --- | --- | --- | --- | --- |
|  | 1335 | **709.3397** | **708.3324** | **709.2952** | **-0.9628** | **0** | **7** | **32** | **6** | **K.DTEAMK.R** |
|  | 1464 | 771.4673 | 770.4600 | 770.4399 | 0.0201 | 0 | 19 | 2.1 | 4 | R.GVGQAAIR.G |
|  | 2649 | 437.2801 | 872.5456 | 872.4603 | 0.0852 | 0 | 19 | 2.4 | 6 | R.QILDEAGK.V |
|  | 2750 | **440.9004** | **879.7862** | **879.4450** | **0.3412** | **0** | **5** | **35** | **1** | **R.TDAGFTLR.W** |
|  | 3410 | 465.7621 | 929.5097 | 929.4203 | 0.0894 | 0 | 8 | 27 | 8 | R.GEGESPQAR.A |
|  | 5032 | 523.0566 | 1044.0987 | 1043.4996 | 0.5992 | 1 | 3 | 58 | 5 | R.QGKGDSPEAR.A |
|  | 5656 | **545.3362** | **1088.6578** | **1088.5284** | **0.1294** | **0** | **6** | **48** | **2** | **R.GQGSSPVAMQK.A** |
|  | 6060 | **559.9462** | **1117.8778** | **1117.5550** | **0.3228** | **1** | **0** | **1.1e+002** | **5** | **K.VGELCAGKER.R** |
|  | 6464 | **575.2895** | **1148.5645** | **1148.6302** | **-0.0657** | **1** | **5** | **53** | **2** | **K.IRTDAGFTLR.W** |
|  | 6779 | 585.3008 | 1168.5871 | 1167.5450 | 1.0421 | 0 | (2) | 88 | 9 | R.MALLMAEMSR.L |
|  | 6981 | **395.2072** | **1182.5997** | **1183.5399** | **-0.9402** | **0** | **10** | **15** | **2** | **R.MALLMAEMSR.L** |
|  | 7471 | 402.2431 | 1203.7076 | 1203.6095 | 0.0980 | 1 | 10 | 17 | 6 | K.DIAKASDEVTR.L |
|  | 7644 | 607.3098 | 1212.6049 | 1212.7151 | -0.1102 | 0 | 5 | 42 | 4 | K.ELLPVLISAMK.I |
|  | 7980 | **615.8109** | **1229.6073** | **1228.7101** | **0.8972** | **0** | **(3)** | **76** | **3** | **K.ELLPVLISAMK.I** |
|  | 8418 | **626.3566** | **1250.6987** | **1250.6363** | **0.0624** | **0** | **0** | **1.4e+002** | **7** | **R.VMLVNSMNTVK.E** |
|  | 9044 | **642.3528** | **1282.6911** | **1283.7058** | **-1.0147** | **1** | **5** | **38** | **3** | **K.TNRAVANSRPAK.A** |
|  | 9886 | **663.3110** | **1324.6074** | **1323.6461** | **0.9613** | **1** | **8** | **21** | **2** | **K.RMALLMAEMSR.L** |
|  | 10485 | **453.2532** | **1356.7378** | **1355.6359** | **1.1018** | **1** | **(3)** | **67** | **2** | **K.RMALLMAEMSR.L** |
|  | 11284 | **701.3328** | **1400.6510** | **1400.7558** | **-0.1048** | **1** | **3** | **63** | **10** | **R.IRTNLLQVCER.I** |
|  | 12083 | **483.1866** | **1446.5378** | **1445.7838** | **0.7540** | **1** | **4** | **48** | **7** | **K.STVEGIQASVKTAR.E** |
|  | 12706 | **742.8630** | **1483.7114** | **1483.7994** | **-0.0880** | **0** | **2** | **88** | **3** | **R.VDQLTAQLADLAAR.G** |
|  | 13299 | 759.8600 | 1517.7054 | 1517.7152 | -0.0098 | 0 | 63 | 7e-005 | 1 | K.AGEVINQPMMMAAR.Q |
|  | 16892 | **581.9419** | **1742.8038** | **1743.8540** | **-1.0501** | **1** | **1** | **92** | **4** | **R.AANFENHSGKLGATAEK.A** |
|  | 18495 | **926.4080** | **1850.8015** | **1850.9382** | **-0.1368** | **1** | **4** | **34** | **1** | **R.LANVMMGPYRQDLLAK.C** |
|  | 19230 | **637.3315** | **1908.9728** | **1908.9615** | **0.0113** | **1** | **4** | **39** | **8** | **R.NFTVEKMSAEINEIIR.V** 19220 |
|  | 19995 | **657.3258** | **1968.9556** | **1969.9978** | **-1.0422** | **1** | **(1)** | **74** | **3** | **R.GLVAEGHRLANVMMGPYR.Q** |
|  | 20383 | **668.6597** | **2002.9572** | **2001.9877** | **0.9695** | **1** | **1** | **75** | **9** | **R.GLVAEGHRLANVMMGPYR.Q** |
|  | 24902 | 846.4656 | 2536.3751 | 2536.1396 | 0.2355 | 1 | 2 | 43 | 2 | K.DEEFPEQKAGEVINQPMMMAAR.Q |
|  | 25145 | 852.4446 | 2554.3121 | 2553.2428 | 1.0693 | 1 | 0 | 65 | 10 | R.QQELTHQEHRVMLVNSMNTVK.E |

| **581.** | Q00765\|REEP5_HUMAN    **Mass:** 21707    **Score:** 63     **Queries matched:** 11   **emPAI:** 0.38 |
| --- | --- |
|  | Receptor expression-enhancing protein 5 OS=Homo sapiens GN=REEP5 PE=1 SV=3 |

|  | **Query** | **Observed** | **Mr(expt)** | **Mr(calc)** | **Delta** | **Miss** | **Score** | **Expect** | **Rank** | **Peptide** |
| --- | --- | --- | --- | --- | --- | --- | --- | --- | --- | --- |
|  | 4135 | 491.7077 | 981.4008 | 982.4324 | -1.0316 | 1 | 11 | 11 | 2 | -.MSAAMRER.F |
|  | 5075 | 524.2829 | 1046.5512 | 1046.5608 | -0.0095 | 1 | 41 | 0.015 | 1 | K.AKETADAITK.E 5053 5076 5077 5078 5079 5080 5081 |
|  | 6847 | **588.2889** | **1174.5632** | **1174.5288** | **0.0344** | **0** | **19** | **2** | **1** | **K.HESQMDSVVK.D** |
|  | 7393 | **401.2123** | **1200.6150** | **1200.6714** | **-0.0564** | **1** | **10** | **15** | **7** | **K.KATVNLLGEEK.K** |

| **582.** | O43504\|HBXIP_HUMAN    **Mass:** 9779     **Score:** 62     **Queries matched:** 2   **emPAI:** 0.41 |
| --- | --- |
|  | Hepatitis B virus X-interacting protein OS=Homo sapiens GN=HBXIP PE=1 SV=1 |

|  | **Query** | **Observed** | **Mr(expt)** | **Mr(calc)** | **Delta** | **Miss** | **Score** | **Expect** | **Rank** | **Peptide** |
| --- | --- | --- | --- | --- | --- | --- | --- | --- | --- | --- |
|  | 20272 | 998.0218 | 1994.0290 | 1994.0433 | -0.0142 | 0 | 62 | 5.5e-005 | 1 | R.GTLSDEHAGVISVLAQQAAK.L 20271 |

| **583.** | O75380\|NDUS6_HUMAN    **Mass:** 14045    **Score:** 62     **Queries matched:** 2   **emPAI:** 0.28 |
| --- | --- |
|  | NADH dehydrogenase [ubiquinone] iron-sulfur protein 6, mitochondrial OS=Homo sapiens GN=NDUFS6 PE=1 SV=1 |

|  | **Query** | **Observed** | **Mr(expt)** | **Mr(calc)** | **Delta** | **Miss** | **Score** | **Expect** | **Rank** | **Peptide** |
| --- | --- | --- | --- | --- | --- | --- | --- | --- | --- | --- |
|  | 11419 | **705.4249** | **1408.8352** | **1407.6929** | **1.1423** | **0** | **42** | **0.0076** | **1** | **R.VIACDGGGGALGHPK.V** 11402 |

| **584.** | O14817\|TSN4_HUMAN    **Mass:** 26841    **Score:** 62     **Queries matched:** 1   **emPAI:** 0.14 |
| --- | --- |
|  | Tetraspanin-4 OS=Homo sapiens GN=TSPAN4 PE=1 SV=1 |

|  | **Query** | **Observed** | **Mr(expt)** | **Mr(calc)** | **Delta** | **Miss** | **Score** | **Expect** | **Rank** | **Peptide** |
| --- | --- | --- | --- | --- | --- | --- | --- | --- | --- | --- |
|  | 26348 | **921.1339** | **2760.3797** | **2760.3984** | **-0.0187** | **0** | **62** | **3.4e-005** | **1** | **K.GLHLYGTQGNVGLTNAWSIIQTDFR.C** |

| **585.** | P13987\|CD59_HUMAN    **Mass:** 14795    **Score:** 62     **Queries matched:** 9   **emPAI:** 1.02 |
| --- | --- |
|  | CD59 glycoprotein OS=Homo sapiens GN=CD59 PE=1 SV=1 |

|  | **Query** | **Observed** | **Mr(expt)** | **Mr(calc)** | **Delta** | **Miss** | **Score** | **Expect** | **Rank** | **Peptide** |
| --- | --- | --- | --- | --- | --- | --- | --- | --- | --- | --- |
|  | 2886 | **446.2406** | **890.4666** | **891.4814** | **-1.0148** | **0** | **45** | **0.0064** | **1** | **K.AGLQVYNK.C** 2900 |
|  | 10898 | **690.2792** | **1378.5439** | **1378.5533** | **-0.0094** | **0** | **20** | **1.3** | **1** | **R.ENELTYYCCK.K** |
|  | 13662 | **514.2169** | **1539.6288** | **1538.6572** | **0.9715** | **0** | **30** | **0.13** | **1** | **K.FEHCNFNDVTTR.L** 13651 13663 |
|  | 15371 | **824.8705** | **1647.7265** | **1647.7385** | **-0.0120** | **1** | **40** | **0.011** | **1** | **R.LRENELTYYCCK.K** 15370 15372 |

| **586.** | P04220\|MUCB_HUMAN    **Mass:** 43543    **Score:** 62     **Queries matched:** 16   **emPAI:** 0.18 |
| --- | --- |
|  | Ig mu heavy chain disease protein OS=Homo sapiens PE=1 SV=1 |

|  | **Query** | **Observed** | **Mr(expt)** | **Mr(calc)** | **Delta** | **Miss** | **Score** | **Expect** | **Rank** | **Peptide** |
| --- | --- | --- | --- | --- | --- | --- | --- | --- | --- | --- |
|  | 2491 | 431.7627 | 861.5108 | 861.5171 | -0.0063 | 0 | 10 | 20 | 4 | K.VTSTLTIK.E |
|  | 3002 | 451.2679 | 900.5213 | 899.5229 | 0.9984 | 0 | 22 | 1.3 | 1 | K.VSVFVPPR.D |
|  | 3112 | 455.2117 | 908.4088 | 908.4141 | -0.0052 | 0 | 29 | 0.21 | 1 | R.DGFFGNPR.K 3111 |
|  | 3638 | 474.2296 | 946.4447 | 946.5084 | -0.0637 | 1 | 12 | 10 | 3 | R.VTERTVDK.S |
|  | 14612 | 800.8879 | 1599.7613 | 1599.7715 | -0.0102 | 0 | 16 | 3.2 | 2 | K.YVTSAPMPEPQAPGR.Y |
|  | 14726 | **804.3552** | **1606.6958** | **1607.8155** | **-1.1197** | **0** | **11** | **10** | **1** | **-.DSPLEQSGHEVGILK.E** |
|  | 14872 | 539.3262 | 1614.9569 | 1615.7664 | -0.8096 | 0 | (1) | 90 | 9 | K.YVTSAPMPEPQAPGR.Y |
|  | 14932 | 540.3129 | 1617.9168 | 1618.7686 | -0.8518 | 0 | 3 | 63 | 4 | K.QVGSGVTTDEVEAEAK.E |
|  | 15220 | 819.4654 | 1636.9162 | 1636.9229 | -0.0066 | 0 | 22 | 0.69 | 1 | R.VFAIPPSFASIFLTK.S 15217 15219 15230 |
|  | 16474 | 858.9295 | 1715.8444 | 1715.8553 | -0.0108 | 0 | 59 | 0.00014 | 1 | R.FTCTVTHTDLPSPLK.Q |
|  | 17277 | 592.0045 | 1772.9917 | 1773.0050 | -0.0132 | 0 | 18 | 1.7 | 1 | K.GVALHRPDVYLLPPAR.E |
|  | 22041 | 722.6020 | 2164.7843 | 2165.1514 | -0.3671 | 1 | 2 | 36 | 5 | R.LSGRDMQVTSQPVIAELPPK.V |

| **587.** | Q9Y639\|NPTN_HUMAN    **Mass:** 31500    **Score:** 62     **Queries matched:** 6   **emPAI:** 0.25 |
| --- | --- |
|  | Neuroplastin OS=Homo sapiens GN=NPTN PE=1 SV=1 |

|  | **Query** | **Observed** | **Mr(expt)** | **Mr(calc)** | **Delta** | **Miss** | **Score** | **Expect** | **Rank** | **Peptide** |
| --- | --- | --- | --- | --- | --- | --- | --- | --- | --- | --- |
|  | 6611 | **579.2973** | **1156.5800** | **1157.6656** | **-1.0855** | **0** | **1** | **1.4e+002** | **2** | **R.IVTSEEVIIR.D** |
|  | 13894 | 777.3234 | 1552.6322 | 1551.6657 | 0.9664 | 0 | 6 | 30 | 3 | K.ENGMPMDIVNTSGR.F |
|  | 14518 | **531.7704** | **1592.2893** | **1591.7888** | **0.5005** | **1** | **4** | **29** | **10** | **K.NASNMEYRINKPR.A** |
|  | 15675 | 417.2017 | 1664.7778 | 1663.7658 | 1.0120 | 1 | 11 | 8.2 | 3 | K.KENGMPMDIVNTSGR.F |
|  | 17285 | **887.8649** | **1773.7153** | **1773.7298** | **-0.0145** | **1** | **60** | **9.6e-005** | **1** | **R.SENKNEGQDATMYCK.S** |
|  | 21385 | **699.3045** | **2094.8917** | **2094.9105** | **-0.0189** | **0** | **24** | **0.35** | **1** | **R.AEDSGEYHCVYHFVSAPK.A** |

| **588.** | P15880\|RS2_HUMAN    **Mass:** 31590    **Score:** 62     **Queries matched:** 22   **emPAI:** 0.25 |
| --- | --- |
|  | 40S ribosomal protein S2 OS=Homo sapiens GN=RPS2 PE=1 SV=2 |

|  | **Query** | **Observed** | **Mr(expt)** | **Mr(calc)** | **Delta** | **Miss** | **Score** | **Expect** | **Rank** | **Peptide** |
| --- | --- | --- | --- | --- | --- | --- | --- | --- | --- | --- |
|  | 812 | 515.3253 | 514.3180 | 515.2928 | -0.9748 | 1 | 6 | 59 | 8 | R.GRGAR.G |
|  | 1433 | **759.4307** | **758.4234** | **758.4286** | **-0.0053** | **0** | **12** | **15** | **1** | **K.EVATAIR.G** |
|  | 1533 | **396.2167** | **790.4189** | **789.4167** | **1.0022** | **0** | **10** | **23** | **3** | **R.CGSVLVR.L** |
|  | 2344 | 852.4405 | 851.4332 | 851.4389 | -0.0057 | 0 | 13 | 7.5 | 2 | K.ATFDAISK.T |
|  | 4740 | **513.2993** | **1024.5840** | **1024.5917** | **-0.0077** | **0** | **16** | **4.1** | **1** | **R.GTGIVSAPVPK.K** 4724 4739 4751 |
|  | 5820 | **551.3072** | **1100.5998** | **1099.6172** | **0.9826** | **1** | **8** | **27** | **2** | **K.IMPVQKQTR.A** |
|  | 6322 | 570.2752 | 1138.5359 | 1138.5441 | -0.0082 | 0 | 50 | 0.0015 | 1 | R.GCTATLGNFAK.A 6320 |
|  | 9359 | **434.2152** | **1299.6239** | **1298.7268** | **0.8971** | **1** | **2** | **89** | **4** | **K.DEVLKIMPVQK.Q** |
|  | 10035 | **445.0025** | **1331.9858** | **1332.6384** | **-0.6526** | **1** | **7** | **19** | **8** | **K.AEDKEWMPVTK.L** |
|  | 12135 | **483.9348** | **1448.7826** | **1448.9442** | **-0.1616** | **1** | **5** | **44** | **9** | **R.GAIILAKLSIVPVR.R** |
|  | 15788 | **836.9454** | **1671.8763** | **1672.0036** | **-0.1273** | **1** | **1** | **97** | **3** | **R.LIPAPRGTGIVSAPVPK.K** |
|  | 16472 | 572.9515 | 1715.8326 | 1715.8995 | -0.0669 | 0 | 6 | 25 | 4 | K.AFVAIGDYNGHVGLGVK.C |
|  | 17050 | **877.9517** | **1753.8889** | **1753.7438** | **0.1451** | **0** | **3** | **54** | **2** | **M.ADDAGAAGGPGGPGGPGMGNR.G** |
|  | 18405 | **615.8333** | **1844.4779** | **1843.8630** | **0.6149** | **1** | **1** | **56** | **2** | **K.KLLMMAGIDDCYTSAR.G** |
|  | 18718 | **623.9792** | **1868.9157** | **1868.7894** | **0.1263** | **0** | **(1)** | **72** | **6** | **-.MADDAGAAGGPGGPGGPGMGNR.G** |
|  | 19098 | 634.3061 | 1899.8964 | 1900.7792 | -0.8828 | 0 | 2 | 62 | 9 | -.MADDAGAAGGPGGPGGPGMGNR.G |
|  | 23108 | 1143.6105 | 2285.2064 | 2286.0019 | -0.7955 | 1 | 1 | 56 | 6 | -.MADDAGAAGGPGGPGGPGMGNRGGFR.G 23111 |

| **589.** | P10606\|COX5B_HUMAN    **Mass:** 13915    **Score:** 62     **Queries matched:** 7   **emPAI:** 0.28 |
| --- | --- |
|  | Cytochrome c oxidase subunit 5B, mitochondrial OS=Homo sapiens GN=COX5B PE=1 SV=2 |

|  | **Query** | **Observed** | **Mr(expt)** | **Mr(calc)** | **Delta** | **Miss** | **Score** | **Expect** | **Rank** | **Peptide** |
| --- | --- | --- | --- | --- | --- | --- | --- | --- | --- | --- |
|  | 1473 | 775.4331 | 774.4258 | 774.4309 | -0.0051 | 0 | 25 | 0.69 | 1 | R.EIMLAAK.K |
|  | 2095 | **417.7297** | **833.4448** | **832.3861** | **1.0587** | **0** | **10** | **18** | **6** | **R.GPSGAAAMR.S** |
|  | 3079 | **453.8193** | **905.6240** | **904.5130** | **1.1109** | **0** | **1** | **1.5e+002** | **10** | **K.LVPQQLAH.-** |
|  | 7060 | 593.8439 | 1185.6733 | 1185.6394 | 0.0339 | 0 | 11 | 12 | 6 | K.GLDPYNVLAPK.G |
|  | 9645 | **657.8701** | **1313.7257** | **1313.7343** | **-0.0086** | **1** | **49** | **0.0019** | **1** | **K.KGLDPYNVLAPK.G** 9587 9646 |

| **590.** | P09758\|TACD2_HUMAN    **Mass:** 36371    **Score:** 61     **Queries matched:** 5   **emPAI:** 0.22 |
| --- | --- |
|  | Tumor-associated calcium signal transducer 2 OS=Homo sapiens GN=TACSTD2 PE=1 SV=3 |

|  | **Query** | **Observed** | **Mr(expt)** | **Mr(calc)** | **Delta** | **Miss** | **Score** | **Expect** | **Rank** | **Peptide** |
| --- | --- | --- | --- | --- | --- | --- | --- | --- | --- | --- |
|  | 2662 | **438.2147** | **874.4149** | **873.4490** | **0.9659** | **1** | **8** | **35** | **8** | **R.MSAPKNAR.T** |
|  | 8027 | **617.2672** | **1232.5199** | **1232.5278** | **-0.0079** | **0** | **31** | **0.13** | **1** | **K.MTVCSPDGPGGR.C** |
|  | 14578 | **799.3755** | **1596.7364** | **1596.7487** | **-0.0123** | **0** | **52** | **0.00068** | **1** | **R.ALGSGMAVDCSTLTSK.C** |
|  | 22562 | 740.3012 | 2217.8816 | 2216.9647 | 0.9170 | 1 | 3 | 34 | 4 | R.CQCRALGSGMAVDCSTLTSK.C |
|  | 24091 | **806.7150** | **2417.1233** | **2418.1088** | **-0.9855** | **1** | **3** | **35** | **5** | **R.QNTSQKAAGDVDIGDAAYYFER.D** |

| **591.** | P63218\|GBG5_HUMAN    **Mass:** 7428     **Score:** 61     **Queries matched:** 6   **emPAI:** 1.43 |
| --- | --- |
|  | Guanine nucleotide-binding protein G(I)/G(S)/G(O) subunit gamma-5 OS=Homo sapiens GN=GNG5 PE=1 SV=3 |

|  | **Query** | **Observed** | **Mr(expt)** | **Mr(calc)** | **Delta** | **Miss** | **Score** | **Expect** | **Rank** | **Peptide** |
| --- | --- | --- | --- | --- | --- | --- | --- | --- | --- | --- |
|  | 2603 | 435.7762 | 869.5379 | 869.5447 | -0.0068 | 1 | 8 | 26 | 4 | K.KVVQQLR.L |
|  | 3012 | **451.7477** | **901.4808** | **901.4869** | **-0.0061** | **0** | **53** | **0.001** | **1** | **K.VSQAAADLK.Q** 3013 3017 |
|  | 12896 | **499.0518** | **1494.1335** | **1494.8630** | **-0.7296** | **1** | **4** | **35** | **4** | **K.VVQQLRLEAGLNR.V** |
|  | 30366 | **988.9908** | **3951.9340** | **3952.9854** | **-1.0514** | **1** | **3** | **15** | **1** | **K.VSQAAADLKQFCLQNAQHDPLLTGVSSSTNPFRPQK.V** |

| **592.** | Q16555\|DPYL2_HUMAN    **Mass:** 62711    **Score:** 60     **Queries matched:** 17   **emPAI:** 0.12 |
| --- | --- |
|  | Dihydropyrimidinase-related protein 2 OS=Homo sapiens GN=DPYSL2 PE=1 SV=1 |

|  | **Query** | **Observed** | **Mr(expt)** | **Mr(calc)** | **Delta** | **Miss** | **Score** | **Expect** | **Rank** | **Peptide** |
| --- | --- | --- | --- | --- | --- | --- | --- | --- | --- | --- |
|  | 1115 | 627.3774 | 626.3702 | 626.3864 | -0.0162 | 1 | 7 | 25 | 3 | K.KNIPR.I |
|  | 1584 | 398.7023 | 795.3900 | 794.4399 | 0.9502 | 0 | 6 | 36 | 6 | K.QQAPPVR.N |
|  | 4569 | **506.2780** | **1010.5414** | **1009.6032** | **0.9382** | **1** | **1** | **1.1e+002** | **6** | **R.LAELRGVPR.G** |
|  | 5371 | 536.2676 | 1070.5206 | 1070.5832 | -0.0626 | 1 | 7 | 35 | 4 | K.NIPRITSDR.L |
|  | 5563 | 542.3001 | 1082.5855 | 1083.6288 | -1.0433 | 0 | 6 | 39 | 3 | R.GSPLVVISQGK.I 5582 |
|  | 14955 | **810.8952** | **1619.7758** | **1619.7865** | **-0.0107** | **0** | **50** | **0.0011** | **1** | **R.GLYDGPVCEVSVTPK.T** |
|  | 15303 | 548.8361 | 1643.4864 | 1642.7814 | 0.7050 | 0 | 1 | 60 | 9 | K.DHGVNSFLVYMAFK.D |
|  | 16614 | **863.4028** | **1724.7911** | **1724.8039** | **-0.0128** | **0** | **7** | **21** | **2** | **K.MDENQFVAVTSTNAAK.V** |
|  | 18032 | **910.9763** | **1819.9381** | **1819.9502** | **-0.0121** | **0** | **27** | **0.25** | **1** | **R.AITIANQTNCPLYITK.V** 18033 |
|  | 20148 | 992.9735 | 1983.9325 | 1983.0135 | 0.9189 | 0 | 6 | 21 | 2 | R.FQLTDCQIYEVLSVIR.D |
|  | 22078 | **723.8182** | **2168.4329** | **2168.0610** | **0.3719** | **0** | **4** | **23** | **3** | **R.NLHQSGFSLSGAQIDDNIPR.R** |
|  | 23589 | **1175.5514** | **2349.0882** | **2348.0882** | **1.0000** | **0** | **10** | **6.7** | **2** | **K.IVNDDQSFYADIYMEDGLIK.Q** 23591 |
|  | 27085 | **967.5034** | **2899.4884** | **2899.5039** | **-0.0155** | **0** | **31** | **0.044** | **1** | **R.ILDLGITGPEGHVLSRPEEVEAEAVNR.A** 27084 |

| **593.** | P60903\|S10AA_HUMAN    **Mass:** 11310    **Score:** 60     **Queries matched:** 10   **emPAI:** 0.83 |
| --- | --- |
|  | Protein S100-A10 OS=Homo sapiens GN=S100A10 PE=1 SV=2 |

|  | **Query** | **Observed** | **Mr(expt)** | **Mr(calc)** | **Delta** | **Miss** | **Score** | **Expect** | **Rank** | **Peptide** |
| --- | --- | --- | --- | --- | --- | --- | --- | --- | --- | --- |
|  | 5797 | **550.5945** | **1099.1744** | **1098.5710** | **0.6035** | **1** | **16** | **2.8** | **2** | **K.FAGDKGYLTK.E** |
|  | 6436 | 574.2600 | 1146.5053 | 1147.5907 | -1.0853 | 1 | 10 | 17 | 6 | K.EDLRVLMEK.E 6473 |
|  | 19712 | **973.9909** | **1945.9673** | **1945.9785** | **-0.0113** | **1** | **42** | **0.0066** | **1** | **K.EFPGFLENQKDPLAVDK.I** 19683 19713 19714 |
|  | 21244 | **521.4752** | **2081.8718** | **2081.8831** | **-0.0114** | **0** | **41** | **0.0064** | **1** | **M.PSQMEHAMETMMFTFHK.F** |
|  | 22929 | 1132.0064 | 2261.9981 | 2260.9084 | 1.0898 | 0 | 2 | 50 | 5 | -.MPSQMEHAMETMMFTFHK.F 22930 |

| **594.** | O95674\|CDS2_HUMAN    **Mass:** 51954    **Score:** 60     **Queries matched:** 3   **emPAI:** 0.07 |
| --- | --- |
|  | Phosphatidate cytidylyltransferase 2 OS=Homo sapiens GN=CDS2 PE=1 SV=1 |

|  | **Query** | **Observed** | **Mr(expt)** | **Mr(calc)** | **Delta** | **Miss** | **Score** | **Expect** | **Rank** | **Peptide** |
| --- | --- | --- | --- | --- | --- | --- | --- | --- | --- | --- |
|  | 18338 | 461.2563 | 1840.9959 | 1840.9254 | 0.0705 | 1 | 9 | 12 | 4 | K.IKDFANTIPGHGGIMDR.F 18335 |
|  | 20108 | **990.9903** | **1979.9660** | **1979.9800** | **-0.0139** | **0** | **60** | **9.8e-005** | **1** | **R.AESAPLPVSADDTPEVLNR.A** |

| **595.** | P48047\|ATPO_HUMAN    **Mass:** 23377    **Score:** 60     **Queries matched:** 9   **emPAI:** 0.16 |
| --- | --- |
|  | ATP synthase subunit O, mitochondrial OS=Homo sapiens GN=ATP5O PE=1 SV=1 |

|  | **Query** | **Observed** | **Mr(expt)** | **Mr(calc)** | **Delta** | **Miss** | **Score** | **Expect** | **Rank** | **Peptide** |
| --- | --- | --- | --- | --- | --- | --- | --- | --- | --- | --- |
|  | 2487 | 431.7343 | 861.4541 | 860.4603 | 0.9937 | 0 | 5 | 66 | 7 | K.SLNDITAK.E |
|  | 4744 | **513.3179** | **1024.6212** | **1024.6281** | **-0.0069** | **1** | **14** | **6.3** | **1** | **R.VAQILKEPK.V** |
|  | 5220 | **530.2839** | **1058.5532** | **1058.5542** | **-0.0011** | **0** | **(1)** | **1.6e+002** | **9** | **-.MAAPAVSGLSR.Q** |
|  | 5452 | 538.7526 | 1075.4907 | 1074.5492 | 0.9415 | 0 | 2 | 1.1e+002 | 10 | -.MAAPAVSGLSR.Q |
|  | 8516 | 628.8373 | 1255.6600 | 1255.7136 | -0.0536 | 1 | 3 | 68 | 5 | K.LEQVEKELLR.V |
|  | 13222 | **757.8058** | **1513.5970** | **1514.7729** | **-1.1758** | **1** | **3** | **51** | **6** | **R.YATALYSAASKQNK.L** |
|  | 21252 | **695.0690** | **2082.1853** | **2082.9827** | **-0.7974** | **0** | **1** | **67** | **6** | **R.LSNTQGVVSAFSTMMSVHR.G** |
|  | 23353 | **1159.5693** | **2317.1241** | **2317.1359** | **-0.0118** | **0** | **60** | **7.6e-005** | **1** | **R.GEVPCTVTSASPLEEATLSELK.T** |
|  | 26336 | **1379.9210** | **2757.8275** | **2758.4310** | **-0.6035** | **1** | **5** | **15** | **5** | **R.GEVPCTVTSASPLEEATLSELKTVLK.S** |

| **596.** | P13498\|CY24A_HUMAN    **Mass:** 21059    **Score:** 59     **Queries matched:** 8   **emPAI:** 0.40 |
| --- | --- |
|  | Cytochrome b-245 light chain OS=Homo sapiens GN=CYBA PE=1 SV=2 |

|  | **Query** | **Observed** | **Mr(expt)** | **Mr(calc)** | **Delta** | **Miss** | **Score** | **Expect** | **Rank** | **Peptide** |
| --- | --- | --- | --- | --- | --- | --- | --- | --- | --- | --- |
|  | 1949 | **413.2135** | **824.4124** | **823.3858** | **1.0267** | **1** | **4** | **54** | **3** | **K.KGSTMER.W** |
|  | 5768 | **549.8132** | **1097.6118** | **1097.6193** | **-0.0075** | **0** | **36** | **0.034** | **1** | **R.ERPQIGGTIK.Q** |
|  | 9349 | 433.8929 | 1298.6568 | 1299.6758 | -1.0190 | 1 | 9 | 18 | 2 | R.WGQKHMTAVVK.L |
|  | 11924 | 480.2581 | 1437.7524 | 1436.7412 | 1.0112 | 0 | 5 | 42 | 3 | R.GEQWTPIEPKPR.E |
|  | 14957 | 540.9348 | 1619.7824 | 1618.8654 | 0.9170 | 1 | 0 | 1.1e+002 | 9 | K.HMTAVVKLFGPFTR.N |
|  | 27639 | **1004.5089** | **3010.5047** | **3010.5247** | **-0.0200** | **1** | **47** | **0.0012** | **1** | **R.KKPSEEEAAAAAGGPPGGPQVNPIPVTDEVV.-** 27635 |
|  | 28554 | **643.5023** | **3212.4752** | **3212.6652** | **-0.1900** | **0** | **2** | **32** | **3** | **M.GQIEWAMWANEQALASGLILITGGIVATAGR.F** |

| **597.** | P62913\|RL11_HUMAN    **Mass:** 20468    **Score:** 59     **Queries matched:** 6   **emPAI:** 0.19 |
| --- | --- |
|  | 60S ribosomal protein L11 OS=Homo sapiens GN=RPL11 PE=1 SV=2 |

|  | **Query** | **Observed** | **Mr(expt)** | **Mr(calc)** | **Delta** | **Miss** | **Score** | **Expect** | **Rank** | **Peptide** |
| --- | --- | --- | --- | --- | --- | --- | --- | --- | --- | --- |
|  | 3763 | **478.2568** | **954.4991** | **954.5069** | **-0.0079** | **0** | **5** | **44** | **9** | **K.IAVHCTVR.G** |
|  | 5247 | **531.2833** | **1060.5520** | **1059.5131** | **1.0389** | **1** | **15** | **6.3** | **5** | **K.ENPMRELR.I** 5249 5260 |
|  | 13762 | **773.9227** | **1545.8309** | **1545.8403** | **-0.0094** | **0** | **59** | **0.00014** | **1** | **K.VLEQLTGQTPVFSK.A** |
|  | 13824 | **517.3050** | **1548.8931** | **1548.6660** | **0.2271** | **1** | **3** | **66** | **5** | **-.MAQDQGEKENPMR.E** |

| **598.** | P52943\|CRIP2_HUMAN    **Mass:** 23276    **Score:** 59     **Queries matched:** 1   **emPAI:** 0.16 |
| --- | --- |
|  | Cysteine-rich protein 2 OS=Homo sapiens GN=CRIP2 PE=1 SV=1 |

|  | **Query** | **Observed** | **Mr(expt)** | **Mr(calc)** | **Delta** | **Miss** | **Score** | **Expect** | **Rank** | **Peptide** |
| --- | --- | --- | --- | --- | --- | --- | --- | --- | --- | --- |
|  | 28544 | **1070.8899** | **3209.6478** | **3209.6721** | **-0.0242** | **0** | **59** | **4.9e-005** | **1** | **K.GVNIGGAGSYIYEKPLAEGPQVTGPIEVPAAR.A** |

| **599.** | O75881\|CP7B1_HUMAN    **Mass:** 58732    **Score:** 59     **Queries matched:** 14   **emPAI:** 0.06 |
| --- | --- |
|  | 25-hydroxycholesterol 7-alpha-hydroxylase OS=Homo sapiens GN=CYP7B1 PE=1 SV=2 |

|  | **Query** | **Observed** | **Mr(expt)** | **Mr(calc)** | **Delta** | **Miss** | **Score** | **Expect** | **Rank** | **Peptide** |
| --- | --- | --- | --- | --- | --- | --- | --- | --- | --- | --- |
|  | 4360 | 499.2582 | 996.5019 | 996.4811 | 0.0208 | 0 | 10 | 15 | 3 | R.HPEAMAAVR.D 4348 |
|  | 4775 | 514.7600 | 1027.5055 | 1026.5346 | 0.9709 | 0 | 19 | 1.8 | 2 | R.LSSYSTTIR.F 4759 |
|  | 5095 | 525.2527 | 1048.4908 | 1048.4971 | -0.0063 | 0 | 5 | 52 | 3 | -.MAGEVSAATGR.F |
|  | 5811 | **551.2681** | **1100.5217** | **1100.6230** | **-0.1013** | **0** | **2** | **98** | **4** | **K.QVFEPQLLK.T** |
|  | 10450 | 678.3154 | 1354.6162 | 1353.6136 | 1.0026 | 0 | 4 | 57 | 2 | K.MQGWSEVFQSR.Q |
|  | 10987 | **461.9147** | **1382.7222** | **1381.7242** | **0.9980** | **1** | **8** | **22** | **6** | **K.FISELRDDFLK.F** |
|  | 13069 | **502.3202** | **1503.9388** | **1503.8660** | **0.0728** | **1** | **10** | **11** | **1** | **K.LLEKAFSISQLQK.N** 13066 |
|  | 15167 | **817.9249** | **1633.8352** | **1633.8419** | **-0.0067** | **0** | **22** | **0.71** | **1** | **K.SLDILLESMMQNLK.Q** |
|  | 16187 | 849.9388 | 1697.8630 | 1696.8203 | 1.0428 | 1 | 7 | 25 | 3 | -.MAGEVSAATGRFSLER.L |
|  | 17441 | **891.9703** | **1781.9261** | **1781.9352** | **-0.0091** | **0** | **59** | **0.00013** | **1** | **R.LLFGIQYPDSDVLFR.Y** |
|  | 22919 | **754.6648** | **2260.9725** | **2260.9994** | **-0.0268** | **0** | **0** | **69** | **9** | **K.NHDMNDELHLCYQFLQGK.S** |

| **600.** | P55899\|FCGRN_HUMAN    **Mass:** 40060    **Score:** 59     **Queries matched:** 9   **emPAI:** 0.09 |
| --- | --- |
|  | IgG receptor FcRn large subunit p51 OS=Homo sapiens GN=FCGRT PE=1 SV=1 |

|  | **Query** | **Observed** | **Mr(expt)** | **Mr(calc)** | **Delta** | **Miss** | **Score** | **Expect** | **Rank** | **Peptide** |
| --- | --- | --- | --- | --- | --- | --- | --- | --- | --- | --- |
|  | 2922 | 448.2557 | 894.4967 | 895.4624 | -0.9656 | 1 | 8 | 27 | 10 | R.EHLERGR.G 2946 |
|  | 3731 | 476.7652 | 951.5159 | 951.5250 | -0.0091 | 1 | 18 | 2.4 | 1 | R.LREHLER.G |
|  | 7258 | **399.3456** | **1195.0148** | **1195.6713** | **-0.6565** | **0** | **8** | **17** | **1** | **R.SGLPAPWISLR.G** |
|  | 9256 | **432.2138** | **1293.6195** | **1292.7492** | **0.8703** | **1** | **9** | **19** | **6** | **K.LFLEAFKALGGK.G** |
|  | 12672 | 494.9049 | 1481.6930 | 1482.8129 | -1.1199 | 1 | 13 | 6.1 | 1 | R.MRSGLPAPWISLR.G |
|  | 15824 | 838.3630 | 1674.7114 | 1673.7759 | 0.9355 | 0 | 5 | 38 | 3 | K.FALNGEEFMNFDLK.Q |
|  | 18748 | **936.4493** | **1870.8841** | **1870.8962** | **-0.0121** | **0** | **59** | **0.00012** | **1** | **K.QGTWGGDWPEALAISQR.W** |
|  | 28472 | **640.1242** | **3195.5847** | **3194.5381** | **1.0465** | **1** | **6** | **11** | **4** | **R.FLRNGLAAGTGQGDFGPNSDGSFHASSSLTVK.S** |

| **601.** | P12110\|CO6A2_HUMAN    **Mass:** 109709   **Score:** 59     **Queries matched:** 24   **emPAI:** 0.07 |
| --- | --- |
|  | Collagen alpha-2(VI) chain OS=Homo sapiens GN=COL6A2 PE=1 SV=4 |

|  | **Query** | **Observed** | **Mr(expt)** | **Mr(calc)** | **Delta** | **Miss** | **Score** | **Expect** | **Rank** | **Peptide** |
| --- | --- | --- | --- | --- | --- | --- | --- | --- | --- | --- |
|  | 832 | 517.3303 | 516.3230 | 515.2928 | 1.0302 | 1 | 6 | 1e+002 | 7 | R.GGARR.H |
|  | 1087 | **612.3412** | **611.3339** | **611.3027** | **0.0312** | **0** | **8** | **19** | **5** | **K.GEPGPR.G** |
|  | 1595 | **399.2320** | **796.4494** | **795.4351** | **1.0142** | **1** | **6** | **34** | **8** | **K.GAKGGPGPR.G** |
|  | 1761 | **405.2261** | **808.4377** | **807.3511** | **1.0866** | **0** | **7** | **35** | **5** | **K.GEFGADGR.K** |
|  | 2193 | **421.0562** | **840.0979** | **840.3726** | **-0.2747** | **0** | **5** | **29** | **4** | **R.GDPGDAGPR.G** |
|  | 2783 | **441.4719** | **880.9293** | **880.4039** | **0.5254** | **0** | **2** | **61** | **8** | **K.GDPGPEGPR.G** |
|  | 3990 | **486.7453** | **971.4760** | **971.4825** | **-0.0065** | **0** | **38** | **0.027** | **1** | **R.LGEQNFHK.A** |
|  | 4256 | **496.2473** | **990.4801** | **989.4778** | **1.0023** | **1** | **11** | **14** | **3** | **K.DPKSETGTR.V** |
|  | 4499 | **503.2844** | **1004.5543** | **1003.5563** | **0.9980** | **1** | **26** | **0.46** | **1** | **R.RFVEQVAR.R** |
|  | 4697 | **511.2715** | **1020.5285** | **1020.5352** | **-0.0068** | **0** | **15** | **5.1** | **1** | **K.NLQGISSFR.R** |
|  | 5370 | **536.2587** | **1070.5028** | **1070.5105** | **-0.0077** | **1** | **5** | **62** | **2** | **R.RDDDPLNAR.V** |
|  | 5424 | **537.3162** | **1072.6179** | **1073.5982** | **-0.9803** | **0** | **22** | **1.2** | **1** | **K.NFVINVVNR.L** |
|  | 5779 | **550.2147** | **1098.4149** | **1099.5986** | **-1.1837** | **1** | **4** | **61** | **5** | **R.GLAGEVGNKGAK.G** |
|  | 10626 | 455.8856 | 1364.6349 | 1364.6433 | -0.0084 | 1 | 4 | 58 | 8 | R.HDPRDDDLNLR.A |
|  | 10715 | 457.2405 | 1368.6998 | 1369.6950 | -0.9952 | 1 | 4 | 60 | 9 | K.GAPGLAGKNGTDGQK.G |
|  | 11506 | **472.2783** | **1413.8132** | **1412.6467** | **1.1666** | **1** | **6** | **36** | **9** | **K.GNMGEPGEPGQKGR.Q** 11477 |
|  | 15370 | 550.2492 | 1647.7258 | 1646.8013 | 0.9245 | 1 | 8 | 20 | 2 | K.GSKGYQGNSGAPGSPGVK.G 15363 |
|  | 16463 | **572.7687** | **1715.2842** | **1715.8665** | **-0.5823** | **1** | **3** | **39** | **5** | **K.VSCLEIPGPSGPKGYR.G** |
|  | 16937 | **872.9529** | **1743.8913** | **1742.8992** | **0.9922** | **0** | **17** | **2.2** | **1** | **K.NLEWIAGGTWTPSALK.F** |
|  | 17686 | **599.9489** | **1796.8249** | **1797.9737** | **-1.1488** | **1** | **3** | **49** | **9** | **R.EEGIRLFAVAPNQNLK.E** |
|  | 26746 | **943.4912** | **2827.4518** | **2827.4638** | **-0.0120** | **0** | **52** | **0.00034** | **1** | **K.QNVVPTVLALGSDVDMDVLTTLSLGDR.A** 26740 |

| **602.** | P08174\|DAF_HUMAN    **Mass:** 42400    **Score:** 59     **Queries matched:** 13   **emPAI:** 0.18 |
| --- | --- |
|  | Complement decay-accelerating factor OS=Homo sapiens GN=CD55 PE=1 SV=4 |

|  | **Query** | **Observed** | **Mr(expt)** | **Mr(calc)** | **Delta** | **Miss** | **Score** | **Expect** | **Rank** | **Peptide** |
| --- | --- | --- | --- | --- | --- | --- | --- | --- | --- | --- |
|  | 2301 | 848.3865 | 847.3792 | 847.3858 | -0.0066 | 0 | 15 | 6.1 | 1 | R.SCEVPTR.L 2302 2304 |
|  | 3170 | **457.2553** | **912.4961** | **912.5029** | **-0.0067** | **1** | **33** | **0.082** | **1** | **R.REPSLSPK.L** 3171 |
|  | 4247 | **495.7743** | **989.5340** | **988.5375** | **0.9964** | **0** | **27** | **0.4** | **1** | **K.LTCLQNLK.W** |
|  | 4786 | **515.2391** | **1028.4637** | **1028.4709** | **-0.0072** | **0** | **7** | **35** | **4** | **K.SCPNPGEIR.N** 4787 |
|  | 11269 | **700.8455** | **1399.6765** | **1399.6871** | **-0.0106** | **0** | **9** | **16** | **1** | **R.TSFPEDTVITYK.C** |
|  | 12857 | 498.2625 | 1491.7655 | 1490.7689 | 0.9966 | 1 | 5 | 38 | 10 | K.TTTKTTTPNAQATR.S |
|  | 14008 | **781.3580** | **1560.7015** | **1560.7930** | **-0.0915** | **1** | **1** | **1.1e+002** | **6** | **R.SCEVPTRLNSASLK.Q** |
|  | 22080 | 1085.5256 | 2169.0367 | 2169.0524 | -0.0157 | 0 | 51 | 0.0006 | 1 | R.EIYCPAPPQIDNGIIQGER.D |
|  | 29044 | **1114.1362** | **3339.3869** | **3338.4067** | **0.9802** | **0** | **25** | **0.1** | **1** | **K.GFTMIGEHSIYCTVNNDEGEWSGPPPECR.G** |

| **603.** | Q08431\|MFGM_HUMAN    **Mass:** 43894    **Score:** 59     **Queries matched:** 9   **emPAI:** 0.09 |
| --- | --- |
|  | Lactadherin OS=Homo sapiens GN=MFGE8 PE=1 SV=2 |

|  | **Query** | **Observed** | **Mr(expt)** | **Mr(calc)** | **Delta** | **Miss** | **Score** | **Expect** | **Rank** | **Peptide** |
| --- | --- | --- | --- | --- | --- | --- | --- | --- | --- | --- |
|  | 4307 | 497.7228 | 993.4310 | 992.4716 | 0.9594 | 0 | 9 | 18 | 1 | K.EFVGNWNK.N |
|  | 6374 | **572.3032** | **1142.5919** | **1143.6248** | **-1.0329** | **0** | **2** | **97** | **2** | **K.EVTGIITQGAR.N** |
|  | 6835 | **587.3317** | **1172.6488** | **1172.6553** | **-0.0065** | **0** | **11** | **13** | **3** | **K.NLFETPILAR.Y** 6816 |
|  | 13792 | **774.8672** | **1547.7198** | **1546.6796** | **1.0402** | **0** | **5** | **40** | **2** | **R.GDVFPSYTCTCLK.G** 13772 |
|  | 14286 | 527.2817 | 1578.8232 | 1578.7283 | 0.0949 | 0 | 1 | 96 | 8 | R.LYPTSCHTACTLR.F |
|  | 17022 | 876.9287 | 1751.8429 | 1751.8690 | -0.0261 | 1 | 3 | 49 | 8 | K.NNSIPDKQITASSSYK.T |
|  | 25979 | **1350.1491** | **2698.2835** | **2697.2969** | **0.9866** | **0** | **59** | **8e-005** | **1** | **R.AGMVNAWTPSSNDDNPWIQVNLLR.R** |

| **604.** | Q02978\|M2OM_HUMAN    **Mass:** 34211    **Score:** 59     **Queries matched:** 9   **emPAI:** 0.23 |
| --- | --- |
|  | Mitochondrial 2-oxoglutarate/malate carrier protein OS=Homo sapiens GN=SLC25A11 PE=1 SV=3 |

|  | **Query** | **Observed** | **Mr(expt)** | **Mr(calc)** | **Delta** | **Miss** | **Score** | **Expect** | **Rank** | **Peptide** |
| --- | --- | --- | --- | --- | --- | --- | --- | --- | --- | --- |
|  | 3303 | **461.2580** | **920.5015** | **919.4433** | **1.0582** | **0** | **5** | **53** | **7** | **R.MQLSGEGAK.T** |
|  | 3622 | **473.2505** | **944.4864** | **945.5396** | **-1.0532** | **0** | **6** | **53** | **9** | **K.NVFNALIR.I** |
|  | 7160 | **596.2907** | **1190.5669** | **1189.5873** | **0.9795** | **1** | **5** | **51** | **3** | **K.NRMQLSGEGAK.T** |
|  | 7205 | 597.4044 | 1192.7943 | 1192.5870 | 0.2073 | 1 | 2 | 85 | 8 | R.MQLSGEGAKTR.E |
|  | 11248 | 467.2498 | 1398.7277 | 1398.7215 | 0.0062 | 0 | 10 | 15 | 5 | M.AATASAGAGGIDGKPR.T 11246 |
|  | 12488 | **491.6088** | **1471.8047** | **1471.8147** | **-0.0100** | **1** | **22** | **0.83** | **1** | **R.ITREEGVLTLWR.G** |
|  | 16967 | **583.3160** | **1746.9261** | **1745.9788** | **0.9473** | **1** | **7** | **24** | **4** | **K.AEGLRGIYTGLSAGLLR.Q** |
|  | 23005 | **1137.1128** | **2272.2110** | **2272.2249** | **-0.0139** | **0** | **53** | **0.00038** | **1** | **K.AVIGMTAGATGAFVGTPAEVALIR.M** |

| **605.** | Q9HDC9\|APMAP_HUMAN    **Mass:** 46622    **Score:** 59     **Queries matched:** 13   **emPAI:** 0.36 |
| --- | --- |
|  | Adipocyte plasma membrane-associated protein OS=Homo sapiens GN=APMAP PE=1 SV=2 |

|  | **Query** | **Observed** | **Mr(expt)** | **Mr(calc)** | **Delta** | **Miss** | **Score** | **Expect** | **Rank** | **Peptide** |
| --- | --- | --- | --- | --- | --- | --- | --- | --- | --- | --- |
|  | 2399 | 428.7632 | 855.5117 | 855.5178 | -0.0060 | 0 | 32 | 0.093 | 1 | K.VLLDQLR.F |
|  | 6899 | **589.8016** | **1177.5886** | **1177.5509** | **0.0376** | **1** | **2** | **93** | **5** | **-.MSEADGLRQR.R** |
|  | 7660 | **607.4551** | **1212.8957** | **1213.5840** | **-0.6883** | **1** | **2** | **70** | **1** | **K.DGSSFSGRVFR.V** |
|  | 9099 | **643.8588** | **1285.7031** | **1285.7129** | **-0.0098** | **0** | **39** | **0.018** | **1** | **K.LLLSSETPIEGK.N** 9110 |
|  | 11684 | **712.3815** | **1422.7484** | **1422.7143** | **0.0341** | **0** | **9** | **16** | **3** | **R.AGPNGTLFVADAYK.G** 11668 |
|  | 14572 | **799.3218** | **1596.6291** | **1596.8334** | **-0.2043** | **1** | **0** | **98** | **4** | **K.LFSQETVMKFVPR.Y** |
|  | 16150 | **566.2792** | **1695.8159** | **1695.7887** | **0.0272** | **0** | **2** | **79** | **7** | **K.NMSFVNDLTVTQDGR.K** |
|  | 21355 | **698.0254** | **2091.0545** | **2091.0709** | **-0.0164** | **0** | **31** | **0.07** | **1** | **R.RPLRPQVVTDDDGQAPEAK.D** 21356 |
|  | 23809 | **594.8107** | **2375.2138** | **2375.2306** | **-0.0167** | **1** | **22** | **0.42** | **1** | **R.QRRPLRPQVVTDDDGQAPEAK.D** |
|  | 26966 | **958.8032** | **2873.3877** | **2873.4018** | **-0.0142** | **0** | **31** | **0.039** | **1** | **R.LFENQLVGPESIAHIGDVMFTGTADGR.V** |

| **606.** | P48509\|CD151_HUMAN    **Mass:** 29132    **Score:** 58     **Queries matched:** 7   **emPAI:** 0.28 |
| --- | --- |
|  | CD151 antigen OS=Homo sapiens GN=CD151 PE=1 SV=3 |

|  | **Query** | **Observed** | **Mr(expt)** | **Mr(calc)** | **Delta** | **Miss** | **Score** | **Expect** | **Rank** | **Peptide** |
| --- | --- | --- | --- | --- | --- | --- | --- | --- | --- | --- |
|  | 3650 | **474.2988** | **946.5830** | **946.4508** | **0.1322** | **0** | **10** | **20** | **4** | **R.DHASNIYK.V** |
|  | 3886 | **964.4146** | **963.4073** | **963.4154** | **-0.0081** | **0** | **43** | **0.0089** | **1** | **R.VVPDSCCK.T** |
|  | 4448 | **501.7612** | **1001.5078** | **1002.5281** | **-1.0202** | **0** | **44** | **0.0066** | **1** | **K.TVVALCGQR.D** 4463 |
|  | 12834 | 497.7432 | 1490.2077 | 1489.6909 | 0.5167 | 1 | 4 | 36 | 5 | R.DSEWIRSQEAGGR.V |
|  | 28253 | 1050.1315 | 3147.3726 | 3147.6106 | -0.2380 | 1 | 1 | 33 | 3 | R.VIGAVGIGIACVQVFGMIFTCCLYRSLK.L |
|  | 30604 | **1026.0083** | **4100.0041** | **4100.0924** | **-0.0883** | **0** | **1** | **21** | **1** | **K.SDYISLLASGTYLATAYILVVAGTVVMVTGVLGCCATFK.E** |

| **607.** | Q86UE4\|LYRIC_HUMAN    **Mass:** 63856    **Score:** 58     **Queries matched:** 14   **emPAI:** 0.06 |
| --- | --- |
|  | Protein LYRIC OS=Homo sapiens GN=MTDH PE=1 SV=2 |

|  | **Query** | **Observed** | **Mr(expt)** | **Mr(calc)** | **Delta** | **Miss** | **Score** | **Expect** | **Rank** | **Peptide** |
| --- | --- | --- | --- | --- | --- | --- | --- | --- | --- | --- |
|  | 2017 | 415.2452 | 828.4759 | 828.4341 | 0.0418 | 0 | 11 | 16 | 10 | K.GEGALPTGK.S |
|  | 3689 | **475.4319** | **948.8492** | **949.5233** | **-0.6740** | **1** | **2** | **77** | **9** | **K.QEKAFSLK.T** |
|  | 4451 | **501.9188** | **1001.8230** | **1001.5029** | **0.3200** | **0** | **5** | **42** | **1** | **R.EDLPVNTSK.T** |
|  | 7333 | 400.2373 | 1197.6901 | 1197.5850 | 0.1050 | 1 | 9 | 17 | 8 | K.AVQNSSRHDGK.E |
|  | 11249 | **700.3820** | **1398.7495** | **1399.7307** | **-0.9812** | **1** | **2** | **78** | **3** | **K.EIREDLPVNTSK.T** |
|  | 11594 | 710.3737 | 1418.7329 | 1418.7041 | 0.0288 | 1 | 2 | 92 | 8 | K.SETSWESPKQIK.K 11593 |
|  | 11976 | 721.3630 | 1440.7114 | 1439.7984 | 0.9130 | 1 | 4 | 49 | 4 | R.TELGLDLGLEPKR.Y |
|  | 13097 | 503.1030 | 1506.2873 | 1505.8388 | 0.4485 | 1 | 6 | 22 | 7 | R.LREMLSVGLGFLR.T 13099 |
|  | 13461 | 764.8684 | 1527.7223 | 1527.7780 | -0.0558 | 0 | 2 | 79 | 10 | K.SSSQVPPILQETDK.S |
|  | 17796 | 902.9013 | 1803.7881 | 1803.8023 | -0.0143 | 0 | 58 | 0.00016 | 1 | R.SWQDELAQQAEEGSAR.L |
|  | 19364 | 961.7358 | 1921.4571 | 1920.8548 | 0.6023 | 1 | 4 | 29 | 1 | K.KQGEDNSTAQDTEELEK.E |
|  | 22115 | 1086.9507 | 2171.8868 | 2172.1161 | -0.2293 | 1 | 2 | 48 | 9 | K.TLPPATSTEPSVILSKSDSDK.S |

| **608.** | Q9UEW3\|MARCO_HUMAN    **Mass:** 52968    **Score:** 58     **Queries matched:** 11   **emPAI:** 0.07 |
| --- | --- |
|  | Macrophage receptor MARCO OS=Homo sapiens GN=MARCO PE=1 SV=1 |

|  | **Query** | **Observed** | **Mr(expt)** | **Mr(calc)** | **Delta** | **Miss** | **Score** | **Expect** | **Rank** | **Peptide** |
| --- | --- | --- | --- | --- | --- | --- | --- | --- | --- | --- |
|  | 3128 | **455.7317** | **909.4488** | **910.4695** | **-1.0206** | **1** | **5** | **49** | **3** | **R.MLGYSKGR.A** |
|  | 3994 | **486.7593** | **971.5040** | **970.4468** | **1.0572** | **0** | **4** | **74** | **9** | **K.GEPGSAGSPGR.A** |
|  | 7932 | **614.5691** | **1227.1236** | **1226.5891** | **0.5345** | **0** | **2** | **74** | **6** | **K.QGATGTPGPQGEK.G** |
|  | 10232 | **448.9043** | **1343.6910** | **1343.6140** | **0.0770** | **0** | **8** | **20** | **2** | **K.GDAGVMGPPGAQGSK.G** |
|  | 12194 | 727.8489 | 1453.6832 | 1453.8405 | -0.1573 | 0 | 8 | 20 | 7 | R.LQVLQAQLTWVR.V |
|  | 15564 | 553.9438 | 1658.8095 | 1659.7709 | -0.9613 | 1 | 6 | 25 | 5 | R.GMKGDAGVMGPPGAQGSK.G |
|  | 15854 | **559.7936** | **1676.3591** | **1675.7658** | **0.5933** | **1** | **(5)** | **23** | **3** | **R.GMKGDAGVMGPPGAQGSK.G** |
|  | 16176 | 566.7687 | 1697.2844 | 1696.8686 | 0.4158 | 0 | 6 | 21 | 4 | K.GDFGRPGPPGLAGFPGAK.G |
|  | 17272 | **887.4332** | **1772.8518** | **1772.8628** | **-0.0110** | **0** | **58** | **0.00018** | **1** | **K.VGAGTGQIWLDNVQCR.G** |
|  | 19234 | **637.5612** | **1909.6617** | **1908.9112** | **0.7504** | **1** | **3** | **32** | **2** | **K.GAMGRDGATGPSGPQGPPGVK.G** |
|  | 26444 | 556.4755 | 2777.3409 | 2777.3378 | 0.0032 | 1 | 3 | 30 | 2 | K.GEQGAPGLQGHKGAMGMPGAPGPPGPPAEK.G |

| **609.** | P38919\|IF4A3_HUMAN    **Mass:** 47126    **Score:** 58     **Queries matched:** 19   **emPAI:** 0.08 |
| --- | --- |
|  | Eukaryotic initiation factor 4A-III OS=Homo sapiens GN=EIF4A3 PE=1 SV=4 |

|  | **Query** | **Observed** | **Mr(expt)** | **Mr(calc)** | **Delta** | **Miss** | **Score** | **Expect** | **Rank** | **Peptide** |
| --- | --- | --- | --- | --- | --- | --- | --- | --- | --- | --- |
|  | 3485 | **468.7117** | **935.4088** | **935.5011** | **-0.0923** | **1** | **14** | **6.2** | **4** | **R.VFDMIRR.R** |
|  | 7877 | **613.3167** | **1224.6189** | **1224.5768** | **0.0420** | **0** | **6** | **36** | **2** | **M.ATTATMATSGSAR.K** |
|  | 10451 | **678.3231** | **1354.6317** | **1355.6173** | **-0.9856** | **0** | **(6)** | **33** | **6** | **-.MATTATMATSGSAR.K** |
|  | 10777 | 458.2360 | 1371.6861 | 1371.6122 | 0.0738 | 0 | 10 | 15 | 3 | -.MATTATMATSGSAR.K 10768 10769 10770 10771 |
|  | 11316 | **702.3607** | **1402.7068** | **1402.7165** | **-0.0097** | **1** | **58** | **0.00023** | **1** | **K.GRDVIAQSQSGTGK.T** |
|  | 14120 | **523.4695** | **1567.3866** | **1566.7831** | **0.6035** | **1** | **1** | **63** | **5** | **K.QFFVAVEREEWK.F** |
|  | 14580 | **533.2825** | **1596.8256** | **1596.8373** | **-0.0117** | **1** | **2** | **72** | **1** | **R.KLDYGQHVVAGTPGR.V** |
|  | 14916 | **540.2471** | **1617.7196** | **1616.8634** | **0.8561** | **1** | **6** | **31** | **3** | **R.SGASRVLISTDVWAR.G** 14893 14896 14898 14924 |
|  | 17881 | **603.9935** | **1808.9586** | **1809.7662** | **-0.8076** | **0** | **2** | **67** | **7** | **R.EANFTVSSMHGDMPQK.E** |
|  | 21385 | 699.3045 | 2094.8917 | 2094.9099 | -0.0182 | 1 | 1 | 68 | 5 | R.EANFTVSSMHGDMPQKER.E |
|  | 24093 | **484.4371** | **2417.1491** | **2417.0945** | **0.0547** | **0** | **5** | **21** | **6** | **K.VEFETSEEVDVTPTFDTMGLR.E** |

| **610.** | O14880\|MGST3_HUMAN    **Mass:** 16734    **Score:** 58     **Queries matched:** 8   **emPAI:** 0.87 |
| --- | --- |
|  | Microsomal glutathione S-transferase 3 OS=Homo sapiens GN=MGST3 PE=1 SV=1 |

|  | **Query** | **Observed** | **Mr(expt)** | **Mr(calc)** | **Delta** | **Miss** | **Score** | **Expect** | **Rank** | **Peptide** |
| --- | --- | --- | --- | --- | --- | --- | --- | --- | --- | --- |
|  | 1306 | **702.3721** | **701.3648** | **701.3708** | **-0.0060** | **0** | **38** | **0.045** | **1** | **K.SGLGSGPK.C** 1312 |
|  | 27436 | **992.1276** | **2973.3609** | **2973.3789** | **-0.0181** | **1** | **42** | **0.0029** | **1** | **K.YKVEYPIMYSTDPENGHIFNCIQR.A** 27435 27440 27441 27442 |
|  | 27588 | **750.4102** | **2997.6118** | **2997.6223** | **-0.0105** | **1** | **20** | **0.54** | **1** | **R.SRGALGSIALLGLVGTTVCSAFQHLGWVK.S** |

| **611.** | P61247\|RS3A_HUMAN    **Mass:** 30154    **Score:** 57     **Queries matched:** 18   **emPAI:** 0.13 |
| --- | --- |
|  | 40S ribosomal protein S3a OS=Homo sapiens GN=RPS3A PE=1 SV=2 |

|  | **Query** | **Observed** | **Mr(expt)** | **Mr(calc)** | **Delta** | **Miss** | **Score** | **Expect** | **Rank** | **Peptide** |
| --- | --- | --- | --- | --- | --- | --- | --- | --- | --- | --- |
|  | 1632 | **400.7376** | **799.4607** | **799.4413** | **0.0195** | **1** | **19** | **2.2** | **5** | **K.RNNQIR.K** 1631 1637 1648 |
|  | 2243 | **422.2529** | **842.4913** | **841.4909** | **1.0004** | **0** | **7** | **40** | **10** | **K.LIPDSIGK.D** |
|  | 3228 | **916.8997** | **915.8924** | **915.5138** | **0.3786** | **1** | **0** | **1.2e+002** | **6** | **K.IASDGLKGR.V** |
|  | 3377 | 464.7610 | 927.5074 | 926.4024 | 1.1051 | 0 | 8 | 29 | 10 | K.MMEIMTR.E |
|  | 3518 | 469.7476 | 937.4806 | 937.4869 | -0.0063 | 0 | 13 | 7.8 | 2 | K.TTDGYLLR.L 3517 |
|  | 5152 | **528.2471** | **1054.4796** | **1054.4973** | **-0.0177** | **1** | **7** | **28** | **2** | **K.KMMEIMTR.E** |
|  | 5387 | **536.2842** | **1070.5538** | **1070.4922** | **0.0616** | **1** | **(4)** | **73** | **3** | **K.KMMEIMTR.E** |
|  | 7730 | **609.2994** | **1216.5843** | **1216.5949** | **-0.0106** | **0** | **57** | **0.00029** | **1** | **K.TSYAQHQQVR.Q** |
|  | 9913 | **663.9348** | **1325.8549** | **1326.7395** | **-0.8845** | **1** | **1** | **1e+002** | **1** | **K.LIPDSIGKDIEK.A** |
|  | 10252 | **449.2340** | **1344.6803** | **1344.6898** | **-0.0095** | **1** | **25** | **0.49** | **1** | **R.KTSYAQHQQVR.Q** 10236 |
|  | 14537 | **532.2793** | **1593.8161** | **1593.7028** | **0.1133** | **0** | **6** | **33** | **1** | **K.NCLTNFHGMDLTR.D** |
|  | 16275 | **568.9525** | **1703.8355** | **1703.8454** | **-0.0098** | **0** | **4** | **46** | **2** | **K.ACQSIYPLHDVFVR.K** |
|  | 19008 | **631.0326** | **1890.0761** | **1889.9710** | **0.1051** | **1** | **1** | **75** | **10** | **K.TTDGYLLRLFCVGFTK.K** |

| **612.** | P50148\|GNAQ_HUMAN    **Mass:** 42400    **Score:** 57     **Queries matched:** 12   **emPAI:** 0.18 |
| --- | --- |
|  | Guanine nucleotide-binding protein G(q) subunit alpha OS=Homo sapiens GN=GNAQ PE=1 SV=4 |

|  | **Query** | **Observed** | **Mr(expt)** | **Mr(calc)** | **Delta** | **Miss** | **Score** | **Expect** | **Rank** | **Peptide** |
| --- | --- | --- | --- | --- | --- | --- | --- | --- | --- | --- |
|  | 1077 | 609.3193 | 608.3121 | 607.3442 | 0.9679 | 1 | 7 | 28 | 9 | K.RGFTK.L |
|  | 5019 | **522.7712** | **1043.5278** | **1043.5359** | **-0.0081** | **1** | **18** | **2.5** | **2** | **R.RINDEIER.Q** |
|  | 6171 | **563.7968** | **1125.5791** | **1125.5488** | **0.0303** | **1** | **8** | **23** | **6** | **R.MEESKALFR.T** |
|  | 8386 | **417.2427** | **1248.7062** | **1248.5881** | **0.1181** | **1** | **8** | **25** | **10** | **R.MVDVGGQRSER.R** |
|  | 9079 | **643.3355** | **1284.6563** | **1284.6786** | **-0.0222** | **1** | **3** | **72** | **7** | **R.INDEIERQLR.R** |
|  | 9789 | **440.9221** | **1319.7444** | **1319.5994** | **0.1450** | **0** | **12** | **8.4** | **2** | **R.IIHGSGYSDEDK.R** |
|  | 12171 | **727.3048** | **1452.5951** | **1451.7296** | **0.8654** | **0** | **7** | **24** | **1** | **K.VSAFENPYVDAIK.S** |
|  | 12238 | **486.5818** | **1456.7235** | **1456.8501** | **-0.1266** | **1** | **1** | **1.1e+002** | **6** | **R.ELKLLLLGTGESGK.S** |
|  | 12587 | **739.3609** | **1476.7072** | **1475.7005** | **1.0068** | **1** | **40** | **0.013** | **1** | **R.IIHGSGYSDEDKR.G** 12575 |
|  | 24293 | **817.4488** | **2449.3245** | **2449.3369** | **-0.0124** | **1** | **38** | **0.011** | **1** | **R.VRVPTTGIIEYPFDLQSVIFR.M** |
|  | 27867 | **1018.8331** | **3053.4774** | **3052.5692** | **0.9082** | **1** | **2** | **35** | **1** | **R.VPTTGIIEYPFDLQSVIFRMVDVGGQR.S** |

| **613.** | P62241\|RS8_HUMAN    **Mass:** 24475    **Score:** 57     **Queries matched:** 7   **emPAI:** 0.34 |
| --- | --- |
|  | 40S ribosomal protein S8 OS=Homo sapiens GN=RPS8 PE=1 SV=2 |

|  | **Query** | **Observed** | **Mr(expt)** | **Mr(calc)** | **Delta** | **Miss** | **Score** | **Expect** | **Rank** | **Peptide** |
| --- | --- | --- | --- | --- | --- | --- | --- | --- | --- | --- |
|  | 783 | **503.1058** | **502.0985** | **502.2863** | **-0.1878** | **1** | **2** | **2.7e+002** | **8** | **R.GGNKK.Y** |
|  | 2777 | **441.2963** | **880.5780** | **879.5403** | **1.0378** | **1** | **3** | **72** | **6** | **R.IHTVRVR.G** |
|  | 3716 | **476.2777** | **950.5408** | **950.4709** | **0.0699** | **0** | **6** | **39** | **5** | **R.ADGYVLEGK.E** |
|  | 13092 | 753.8885 | 1505.7624 | 1505.7726 | -0.0101 | 0 | 58 | 0.0002 | 1 | K.ISSLLEEQFQQGK.L |
|  | 13969 | **520.2661** | **1557.7765** | **1557.7868** | **-0.0103** | **0** | **30** | **0.12** | **1** | **K.LLACIASRPGQCGR.A** 13970 |
|  | 15295 | **411.6975** | **1642.7611** | **1641.8951** | **0.8660** | **1** | **7** | **21** | **2** | **K.YELGRPAANTKIGPR.R** |

| **614.** | O60256\|KPRB_HUMAN    **Mass:** 41299    **Score:** 57     **Queries matched:** 13   **emPAI:** 0.19 |
| --- | --- |
|  | Phosphoribosyl pyrophosphate synthetase-associated protein 2 OS=Homo sapiens GN=PRPSAP2 PE=1 SV=1 |

|  | **Query** | **Observed** | **Mr(expt)** | **Mr(calc)** | **Delta** | **Miss** | **Score** | **Expect** | **Rank** | **Peptide** |
| --- | --- | --- | --- | --- | --- | --- | --- | --- | --- | --- |
|  | 1351 | 715.3513 | 714.3440 | 713.4436 | 0.9005 | 0 | 22 | 1.4 | 1 | R.NAVIVAK.S |
|  | 3809 | 479.7836 | 957.5526 | 957.5243 | 0.0283 | 0 | 14 | 6.9 | 5 | R.VQIQESVR.G |
|  | 4165 | **492.8800** | **983.7454** | **984.4442** | **-0.6988** | **0** | **9** | **16** | **2** | **K.LLASMMCK.A** |
|  | 12348 | **488.2576** | **1461.7511** | **1462.7701** | **-1.0190** | **0** | **4** | **51** | **9** | **K.TVDISMILSEAIR.R** |
|  | 14521 | **531.9179** | **1592.7317** | **1592.8345** | **-0.1027** | **0** | **4** | **48** | **1** | **K.AGLTHLITMDLHQK.E** |
|  | 16006 | 844.4204 | 1686.8263 | 1687.9542 | -1.1279 | 1 | 12 | 7.7 | 1 | K.IKTVDISMILSEAIR.R |
|  | 18740 | **624.3126** | **1869.9159** | **1869.9883** | **-0.0725** | **1** | **2** | **65** | **10** | **K.IFVMATHGLLSSDAPRR.I** 18745 |
|  | 21389 | 1048.5225 | 2095.0304 | 2095.9633 | -0.9329 | 1 | 3 | 48 | 4 | R.IHNGESMSYLFRNIGLDD.- |
|  | 24375 | **1230.6545** | **2459.2945** | **2459.3047** | **-0.0101** | **0** | **55** | **0.0002** | **1** | **R.IAIIVDDIIDDVDSFLAAAETLK.E** 24376 |
|  | 24754 | **838.4001** | **2512.1784** | **2511.2834** | **0.8951** | **1** | **(1)** | **61** | **5** | **K.LLASMMCKAGLTHLITMDLHQK.E** |
|  | 24933 | 848.4460 | 2542.3163 | 2543.2732 | -0.9569 | 1 | 1 | 50 | 2 | K.LLASMMCKAGLTHLITMDLHQK.E |

| **615.** | Q92629\|SGCD_HUMAN    **Mass:** 32393    **Score:** 57     **Queries matched:** 6   **emPAI:** 0.25 |
| --- | --- |
|  | Delta-sarcoglycan OS=Homo sapiens GN=SGCD PE=1 SV=2 |

|  | **Query** | **Observed** | **Mr(expt)** | **Mr(calc)** | **Delta** | **Miss** | **Score** | **Expect** | **Rank** | **Peptide** |
| --- | --- | --- | --- | --- | --- | --- | --- | --- | --- | --- |
|  | 2339 | 426.2394 | 850.4643 | 849.4497 | 1.0146 | 0 | 7 | 36 | 2 | K.VGIYGWR.K |
|  | 5350 | **535.3312** | **1068.6479** | **1068.6543** | **-0.0064** | **0** | **37** | **0.032** | **1** | **K.VLTQLITGPK.A** |
|  | 12698 | **495.2886** | **1482.8441** | **1482.6959** | **0.1482** | **0** | **2** | **81** | **10** | **K.VMNFTIDGMGNLR.I** |
|  | 16733 | **866.9408** | **1731.8670** | **1731.8792** | **-0.0121** | **0** | **45** | **0.0032** | **1** | **K.LLFSADNNEVVVGAER.L** |
|  | 22737 | **745.9861** | **2234.9364** | **2236.0212** | **-1.0848** | **1** | **2** | **42** | **3** | **K.GVEINAEAGNMEATCRTELR.L** |
|  | 30987 | **904.2649** | **4516.2881** | **4517.4553** | **-1.1672** | **1** | **0** | **18** | **4** | **R.CLYFFVLLLMILILVNLAMTIWILKVMNFTIDGMGNLR.I** |

| **616.** | P05386\|RLA1_HUMAN    **Mass:** 11621    **Score:** 57     **Queries matched:** 1   **emPAI:** 0.34 |
| --- | --- |
|  | 60S acidic ribosomal protein P1 OS=Homo sapiens GN=RPLP1 PE=1 SV=1 |

|  | **Query** | **Observed** | **Mr(expt)** | **Mr(calc)** | **Delta** | **Miss** | **Score** | **Expect** | **Rank** | **Peptide** |
| --- | --- | --- | --- | --- | --- | --- | --- | --- | --- | --- |
|  | 30178 | 952.9835 | 3807.9047 | 3807.9213 | -0.0166 | 0 | 57 | 6.4e-005 | 1 | K.ALANVNIGSLICNVGAGGPAPAAGAAPAGGPAPSTAAAPAEEK.K |

| **617.** | P21810\|PGS1_HUMAN    **Mass:** 42027    **Score:** 57     **Queries matched:** 7   **emPAI:** 0.09 |
| --- | --- |
|  | Biglycan OS=Homo sapiens GN=BGN PE=1 SV=2 |

|  | **Query** | **Observed** | **Mr(expt)** | **Mr(calc)** | **Delta** | **Miss** | **Score** | **Expect** | **Rank** | **Peptide** |
| --- | --- | --- | --- | --- | --- | --- | --- | --- | --- | --- |
|  | 1119 | **628.8543** | **627.8470** | **627.3956** | **0.4515** | **0** | **1** | **98** | **3** | **K.LTGIPK.D** |
|  | 6054 | 1118.5980 | 1117.5907 | 1117.5802 | 0.0106 | 0 | 4 | 66 | 4 | R.VVQCSDLGLK.S |
|  | 9578 | 656.8738 | 1311.7330 | 1311.7398 | -0.0068 | 0 | 24 | 0.62 | 1 | K.IQAIELEDLLR.Y |
|  | 13434 | 763.8549 | 1525.6953 | 1525.7058 | -0.0105 | 0 | 57 | 0.00025 | 1 | K.VGVNDFCPMGFGVK.R |
|  | 13696 | **514.7589** | **1541.2549** | **1541.7007** | **-0.4458** | **0** | **(2)** | **51** | **9** | **K.VGVNDFCPMGFGVK.R** |
|  | 14246 | 789.3895 | 1576.7644 | 1576.8283 | -0.0639 | 0 | 3 | 60 | 9 | R.MIENGSLSFLPTLR.E |
|  | 24927 | **424.5591** | **2541.3106** | **2540.3057** | **1.0050** | **1** | **16** | **1.9** | **1** | **R.MIENGSLSFLPTLRELHLDNNK.L** |

| **618.** | Q8TBA6\|GOGA5_HUMAN    **Mass:** 82997    **Score:** 57     **Queries matched:** 20   **emPAI:** 0.09 |
| --- | --- |
|  | Golgin subfamily A member 5 OS=Homo sapiens GN=GOLGA5 PE=1 SV=2 |

|  | **Query** | **Observed** | **Mr(expt)** | **Mr(calc)** | **Delta** | **Miss** | **Score** | **Expect** | **Rank** | **Peptide** |
| --- | --- | --- | --- | --- | --- | --- | --- | --- | --- | --- |
|  | 3646 | 474.2669 | 946.5192 | 946.5196 | -0.0004 | 1 | 11 | 13 | 5 | R.TKNTLQSR.I |
|  | 5628 | **544.3161** | **1086.6176** | **1087.5622** | **-0.9445** | **0** | **6** | **40** | **4** | **R.VDQGAATALSR.K** |
|  | 7102 | **397.1854** | **1188.5343** | **1189.5761** | **-1.0417** | **1** | **7** | **31** | **4** | **K.EMQREEIQK.L** |
|  | 7724 | 608.9744 | 1215.9343 | 1215.6571 | 0.2772 | 1 | 12 | 7.3 | 1 | R.VDQGAATALSRK.D |
|  | 7746 | **609.6530** | **1217.2913** | **1216.6047** | **0.6866** | **1** | **1** | **78** | **9** | **K.ETQEELNKAR.A** |
|  | 7750 | **609.8102** | **1217.6059** | **1217.6139** | **-0.0080** | **0** | **24** | **0.58** | **1** | **R.TEALEALQSEK.S** |
|  | 9844 | **441.6406** | **1321.8999** | **1321.6990** | **0.2010** | **1** | **5** | **34** | **1** | **R.KAASSIDQFSIR.L** 9846 |
|  | 11053 | 694.3446 | 1386.6746 | 1386.7354 | -0.0608 | 1 | 5 | 49 | 3 | K.QELETELERLK.Q |
|  | 12311 | 730.8779 | 1459.7412 | 1460.7470 | -1.0059 | 1 | 3 | 64 | 6 | R.TEALEALQSEKSR.I |
|  | 12371 | **732.8452** | **1463.6759** | **1463.6852** | **-0.0093** | **0** | **58** | **0.0002** | **1** | **K.TLSNSSQSELENR.L** |
|  | 13469 | **510.2668** | **1527.7786** | **1527.8369** | **-0.0583** | **1** | **6** | **32** | **6** | **K.VRLQEADQLLSTR.T** |
|  | 16818 | 580.3048 | 1737.8926 | 1737.9196 | -0.0270 | 1 | 3 | 52 | 2 | R.EEIQKLMGQIHQLR.S |
|  | 18198 | **611.4221** | **1831.2445** | **1830.8206** | **0.4239** | **1** | **(1)** | **58** | **1** | **R.EQESYKQMQSEFAAR.L** |
|  | 18427 | **616.3981** | **1846.1726** | **1846.8155** | **-0.6430** | **1** | **4** | **31** | **2** | **R.EQESYKQMQSEFAAR.L** |
|  | 18749 | 936.4617 | 1870.9089 | 1869.8897 | 1.0192 | 1 | 2 | 69 | 5 | K.QEFHYIEEDLYRTK.N |
|  | 20888 | **684.1995** | **2049.5766** | **2049.8909** | **-0.3143** | **0** | **2** | **41** | **6** | **R.SELQDMEAQQVNEAESAR.E** |
|  | 27749 | **1011.2279** | **3030.6619** | **3030.2891** | **0.3728** | **0** | **3** | **24** | **1** | **R.LEQQMNSASGSSSNGSSINMSGIDNGEGTR.L** |
|  | 28873 | 1101.2040 | 3300.5901 | 3299.4742 | 1.1159 | 1 | 4 | 20 | 3 | R.LEQQMNSASGSSSNGSSINMSGIDNGEGTRLR.N 28874 |

| **619.** | O00116\|ADAS_HUMAN    **Mass:** 73664    **Score:** 57     **Queries matched:** 15   **emPAI:** 0.05 |
| --- | --- |
|  | Alkyldihydroxyacetonephosphate synthase, peroxisomal OS=Homo sapiens GN=AGPS PE=1 SV=1 |

|  | **Query** | **Observed** | **Mr(expt)** | **Mr(calc)** | **Delta** | **Miss** | **Score** | **Expect** | **Rank** | **Peptide** |
| --- | --- | --- | --- | --- | --- | --- | --- | --- | --- | --- |
|  | 1148 | 635.3127 | 634.3054 | 633.3156 | 0.9898 | 0 | 19 | 3 | 1 | R.QEVMK.W |
|  | 1153 | 637.0497 | 636.0424 | 635.2948 | 0.7476 | 0 | 5 | 34 | 3 | R.LMDNK.Q 1154 1156 |
|  | 9916 | **663.9356** | **1325.8565** | **1325.6690** | **0.1876** | **0** | **0** | **1.1e+002** | **2** | **R.YPLSGMGLPTFK.E** |
|  | 12523 | **492.2890** | **1473.8451** | **1472.9052** | **0.9399** | **1** | **4** | **51** | **7** | **R.LRVLSGHLLGRPR.E** |
|  | 12697 | **495.2884** | **1482.8434** | **1481.7701** | **1.0734** | **1** | **6** | **30** | **4** | **K.RYPLSGMGLPTFK.E** |
|  | 14799 | 806.9076 | 1611.8006 | 1611.8178 | -0.0172 | 1 | 0 | 1.1e+002 | 10 | K.ESISDVGFGMLKSVK.E 14804 |
|  | 15054 | 542.9637 | 1625.8692 | 1625.8777 | -0.0085 | 0 | 6 | 27 | 2 | K.NIYGNIEDLVVHIK.M |
|  | 15702 | 834.4040 | 1666.7934 | 1665.8475 | 0.9460 | 0 | 6 | 27 | 3 | K.EWIQNTLGVNVEHK.T |
|  | 18337 | **614.6726** | **1840.9958** | **1840.9180** | **0.0778** | **0** | **5** | **33** | **1** | **R.EEILANGGSLSHHHGVGK.L** |
|  | 22178 | **727.3668** | **2179.0785** | **2179.0658** | **0.0127** | **1** | **0** | **79** | **7** | **K.QVYDIAAKFGGLAAGEDNGQR.G** |
|  | 22767 | **747.0555** | **2238.1446** | **2238.1604** | **-0.0158** | **1** | **57** | **0.00018** | **1** | **R.RAASAATAAPTATPAAQESGTIPK.K** |
|  | 27442 | 992.4552 | 2974.3438 | 2973.5059 | 0.8379 | 1 | 0 | 46 | 4 | R.YPLSGMGLPTFKEWIQNTLGVNVEHK.T |

| **620.** | P23634\|AT2B4_HUMAN    **Mass:** 139030   **Score:** 56     **Queries matched:** 50   **emPAI:** 0.14 |
| --- | --- |
|  | Plasma membrane calcium-transporting ATPase 4 OS=Homo sapiens GN=ATP2B4 PE=1 SV=2 |

|  | **Query** | **Observed** | **Mr(expt)** | **Mr(calc)** | **Delta** | **Miss** | **Score** | **Expect** | **Rank** | **Peptide** |
| --- | --- | --- | --- | --- | --- | --- | --- | --- | --- | --- |
|  | 1172 | 644.9523 | 643.9450 | 643.4017 | 0.5433 | 1 | 0 | 1.7e+002 | 2 | R.ILDRK.G 1174 |
|  | 1557 | **397.4026** | **792.7906** | **792.4164** | **0.3743** | **0** | **12** | **6.9** | **4** | **K.SMSTVIR.N** |
|  | 1738 | **404.6886** | **807.3626** | **808.4113** | **-1.0487** | **0** | **(8)** | **31** | **8** | **K.SMSTVIR.N** 1750 |
|  | 2663 | 438.2500 | 874.4854 | 874.4912 | -0.0058 | 1 | 25 | 0.7 | 1 | R.KGEAVPFK.N |
|  | 3280 | **460.7609** | **919.5072** | **919.4909** | **0.0163** | **1** | **7** | **33** | **6** | **K.LMELRSR.D** |
|  | 4822 | **516.3041** | **1030.5936** | **1031.5546** | **-0.9610** | **1** | **1** | **1.3e+002** | **7** | **K.CKQAGITVR.M** |
|  | 4964 | 521.2601 | 1040.5057 | 1040.5363 | -0.0306 | 1 | 4 | 59 | 3 | K.QGVPENRNK.A |
|  | 6975 | **592.3020** | **1182.5894** | **1182.5993** | **-0.0099** | **0** | **27** | **0.26** | **1** | **K.GIIDSTVGEHR.Q** |
|  | 7039 | 593.3316 | 1184.6486 | 1184.5608 | 0.0878 | 0 | 4 | 71 | 5 | R.QNMGQHLDVK.L |
|  | 7157 | 596.2887 | 1190.5628 | 1190.5714 | -0.0085 | 0 | 35 | 0.051 | 1 | R.MVTGDNINTAR.A 7177 |
|  | 7173 | **397.9013** | **1190.6822** | **1189.5761** | **1.1061** | **0** | **11** | **13** | **3** | **R.VLPANSMAESR.E** 7126 7128 7129 7131 7135 7138 |
|  | 7631 | 607.2716 | 1212.5287 | 1211.6510 | 0.8777 | 1 | 6 | 40 | 2 | R.SSPTDKHTLVK.G 7635 7648 |
|  | 8151 | 620.2996 | 1238.5847 | 1239.6863 | -1.1016 | 0 | 2 | 87 | 8 | R.QIPSPDVFLPK.V |
|  | 9747 | **440.2951** | **1317.8635** | **1318.7860** | **-0.9226** | **1** | **3** | **58** | **6** | **K.LVPSSSYIKVVK.A** |
|  | 9873 | **442.2376** | **1323.6910** | **1324.6670** | **-0.9760** | **1** | **14** | **4.9** | **1** | **R.RQNMGQHLDVK.L** 9891 |
|  | 10493 | **679.8007** | **1357.5868** | **1358.7571** | **-1.1704** | **1** | **2** | **91** | **2** | **R.GQILWFRGLNR.I** |
|  | 10999 | **462.2364** | **1383.6875** | **1382.7227** | **0.9648** | **1** | **3** | **66** | **7** | **R.MYSKGASEIILR.K** |
|  | 13242 | 505.9137 | 1514.7193 | 1515.7053 | -0.9860 | 0 | 5 | 39 | 4 | K.IDESSLTGESDHVK.K |
|  | 13303 | 506.9177 | 1517.7312 | 1518.7943 | -1.0631 | 1 | 11 | 11 | 5 | K.IHGEKNVFSGIYR.N |
|  | 13969 | 520.2661 | 1557.7765 | 1556.6963 | 1.0802 | 1 | 8 | 19 | 3 | R.EGDFGCTVMELRK.L |
|  | 14811 | **403.9776** | **1611.8813** | **1612.7944** | **-0.9131** | **0** | **7** | **24** | **7** | **K.TSPVEGLSGNPADLEK.R** |
|  | 15278 | **821.4200** | **1640.8255** | **1640.8370** | **-0.0115** | **0** | **26** | **0.28** | **1** | **R.QVVAVTGDGTNDGPALK.K** 15272 |
|  | 18411 | **616.0099** | **1845.0078** | **1843.8846** | **1.1232** | **1** | **1** | **89** | **8** | **M.TNPSDRVLPANSMAESR.E** |
|  | 18539 | **618.9940** | **1853.9602** | **1853.9734** | **-0.0132** | **1** | **24** | **0.39** | **1** | **R.LKTSPVEGLSGNPADLEK.R** |
|  | 18623 | **930.4847** | **1858.9549** | **1857.8050** | **1.1499** | **0** | **5** | **32** | **1** | **K.DAEGLDEIDHAEMELR.R** 18577 |
|  | 18868 | **940.9717** | **1879.9289** | **1879.9415** | **-0.0126** | **0** | **28** | **0.17** | **1** | **K.EASDIILTDDNFTSIVK.A** 18867 |
|  | 20226 | **664.3571** | **1990.0494** | **1990.9200** | **-0.8707** | **1** | **10** | **9.9** | **1** | **-.MTNPSDRVLPANSMAESR.E** |
|  | 20315 | 667.0290 | 1998.0651 | 1997.9112 | 0.1539 | 1 | 4 | 33 | 2 | K.DAEGLDEIDHAEMELRR.G |
|  | 22973 | **757.3769** | **2269.1090** | **2269.0837** | **0.0253** | **0** | **5** | **24** | **2** | **K.SIHSFMTHPEFAIEEELPR.T** |
|  | 25326 | 647.3106 | 2585.2133 | 2584.1720 | 1.0414 | 1 | 2 | 41 | 4 | R.VLPANSMAESREGDFGCTVMELR.K |
|  | 26944 | 1434.2118 | 2866.4090 | 2867.2670 | -0.8580 | 1 | 1 | 41 | 3 | R.HLDACETMGNATAICSDKTGTLTMNR.M 26943 |
|  | 29012 | 1667.8628 | 3333.7110 | 3333.7240 | -0.0130 | 0 | 2 | 31 | 3 | K.AVQMLWVNLIMDTFASLALATEPPTESLLK.R 29011 |
|  | 30599 | **1025.7557** | **4098.9939** | **4099.2569** | **-0.2630** | **1** | **6** | **7.7** | **1** | **R.NGQLIQLPVAEIVVGDIAQVKYGDLLPADGILIQGNDLK.I** |

| **621.** | P52209\|6PGD_HUMAN    **Mass:** 53619    **Score:** 56     **Queries matched:** 9   **emPAI:** 0.07 |
| --- | --- |
|  | 6-phosphogluconate dehydrogenase, decarboxylating OS=Homo sapiens GN=PGD PE=1 SV=3 |

|  | **Query** | **Observed** | **Mr(expt)** | **Mr(calc)** | **Delta** | **Miss** | **Score** | **Expect** | **Rank** | **Peptide** |
| --- | --- | --- | --- | --- | --- | --- | --- | --- | --- | --- |
|  | 3655 | **474.7230** | **947.4315** | **947.4672** | **-0.0357** | **1** | **5** | **63** | **4** | **R.DSAGQKGTGK.W** |
|  | 7190 | 596.8565 | 1191.6985 | 1191.5772 | 0.1213 | 0 | 3 | 71 | 10 | K.AGQAVDDFIEK.L |
|  | 10351 | 676.3420 | 1350.6694 | 1350.5623 | 0.1072 | 0 | 10 | 13 | 3 | K.SAVENCQDSWR.R |
|  | 14486 | **796.4026** | **1590.7907** | **1590.8002** | **-0.0095** | **0** | **56** | **0.00026** | **1** | **K.GILFVGSGVSGGEEGAR.Y** |
|  | 17886 | **604.2712** | **1809.7917** | **1808.9937** | **0.7980** | **1** | **9** | **13** | **1** | **K.EAWPHIKTIFQGIAAK.V** 17896 |
|  | 20106 | **660.9791** | **1979.9154** | **1980.9880** | **-1.0726** | **1** | **1** | **74** | **7** | **R.YGPSLMPGGNKEAWPHIK.T** |
|  | 23020 | **759.6566** | **2275.9480** | **2275.9627** | **-0.0147** | **0** | **5** | **23** | **1** | **K.VGTGEPCCDWVGDEGAGHFVK.M** 23019 |

| **622.** | P35610\|SOAT1_HUMAN    **Mass:** 65205    **Score:** 56     **Queries matched:** 15   **emPAI:** 0.06 |
| --- | --- |
|  | Sterol O-acyltransferase 1 OS=Homo sapiens GN=SOAT1 PE=1 SV=3 |

|  | **Query** | **Observed** | **Mr(expt)** | **Mr(calc)** | **Delta** | **Miss** | **Score** | **Expect** | **Rank** | **Peptide** |
| --- | --- | --- | --- | --- | --- | --- | --- | --- | --- | --- |
|  | 1058 | **602.8279** | **601.8206** | **601.3547** | **0.4659** | **1** | **24** | **0.78** | **1** | **R.AKDLR.A** |
|  | 1767 | **405.6210** | **809.2274** | **808.4555** | **0.7719** | **0** | **11** | **9.9** | **3** | **K.SSHPLIR.S** |
|  | 2591 | **435.2685** | **868.5225** | **869.4106** | **-0.8881** | **0** | **4** | **50** | **6** | **R.WGYVAMK.F** |
|  | 5286 | 532.2898 | 1062.5650 | 1063.5332 | -0.9681 | 1 | 13 | 8.4 | 4 | M.VGEEKMSLR.N 5278 5279 5284 5292 5295 |
|  | 5664 | **545.8127** | **1089.6108** | **1088.5331** | **1.0777** | **0** | **1** | **1.3e+002** | **10** | **K.DFLWFFSK.R** |
|  | 8115 | **413.2633** | **1236.7680** | **1236.6438** | **0.1243** | **1** | **6** | **32** | **4** | **R.FVMKAHSFVR.E** |
|  | 16229 | **567.6450** | **1699.9131** | **1698.9531** | **0.9599** | **1** | **0** | **1.1e+002** | **9** | **R.FIIIFEQIRFVMK.A** |
|  | 18480 | **617.4319** | **1849.2738** | **1848.8894** | **0.3844** | **0** | **1** | **65** | **4** | **K.EVGSHFDDFVTNLIEK.S** |
|  | 19050 | 474.5119 | 1894.0186 | 1894.0274 | -0.0087 | 1 | 56 | 0.00023 | 1 | K.IKLTAEAEELKPFFMK.E |
|  | 31617 | **795.2686** | **5559.8294** | **5559.7613** | **0.0681** | **0** | **4** | **4.6** | **1** | **K.SAAMLAVFAVSAVVHEYALAVCLSFFYPVLFVLFMFFGMAFNFIVNDSR.K** |

| **623.** | P10301\|RRAS_HUMAN    **Mass:** 23637    **Score:** 56     **Queries matched:** 13   **emPAI:** 0.16 |
| --- | --- |
|  | Ras-related protein R-Ras OS=Homo sapiens GN=RRAS PE=1 SV=1 |

|  | **Query** | **Observed** | **Mr(expt)** | **Mr(calc)** | **Delta** | **Miss** | **Score** | **Expect** | **Rank** | **Peptide** |
| --- | --- | --- | --- | --- | --- | --- | --- | --- | --- | --- |
|  | 1859 | **409.2274** | **816.4402** | **817.3930** | **-0.9527** | **0** | **10** | **22** | **3** | **K.ADLESQR.Q** |
|  | 3557 | **471.2893** | **940.5640** | **940.5706** | **-0.0066** | **0** | **9** | **18** | **1** | **K.LVVVGGGGVGK.S** |
|  | 4140 | **491.7653** | **981.5161** | **980.4345** | **1.0815** | **0** | **16** | **3.6** | **3** | **-.MSSGAASGTGR.G** 4131 |
|  | 4371 | 499.7440 | 997.4735 | 996.4295 | 1.0440 | 0 | (8) | 26 | 4 | -.MSSGAASGTGR.G |
|  | 5636 | 544.7886 | 1087.5626 | 1086.5492 | 1.0134 | 0 | 10 | 19 | 2 | K.ICSVDGIPAR.L |
|  | 9721 | **659.3236** | **1316.6327** | **1315.6705** | **0.9622** | **1** | **9** | **18** | **1** | **M.SSGAASGTGRGRPR.G** 9692 |
|  | 12757 | 496.5664 | 1486.6773 | 1485.6848 | 0.9925 | 0 | 1 | 1e+002 | 8 | R.GGGPGPGDPPPSETHK.L |
|  | 17178 | **883.4197** | **1764.8249** | **1764.8352** | **-0.0103** | **0** | **56** | **0.00025** | **1** | **R.LDILDTAGQEEFGAMR.E** 17180 |
|  | 24023 | 803.7367 | 2408.1882 | 2408.2449 | -0.0566 | 1 | 1 | 63 | 9 | R.GGGPGPGDPPPSETHKLVVVGGGGVGK.S 24022 |

| **624.** | P62070\|RRAS2_HUMAN    **Mass:** 23613    **Score:** 56     **Queries matched:** 7   **emPAI:** 0.16 |
| --- | --- |
|  | Ras-related protein R-Ras2 OS=Homo sapiens GN=RRAS2 PE=1 SV=1 |

|  | **Query** | **Observed** | **Mr(expt)** | **Mr(calc)** | **Delta** | **Miss** | **Score** | **Expect** | **Rank** | **Peptide** |
| --- | --- | --- | --- | --- | --- | --- | --- | --- | --- | --- |
|  | 3557 | 471.2893 | 940.5640 | 940.5706 | -0.0066 | 0 | 9 | 18 | 1 | R.LVVVGGGGVGK.S |
|  | 12348 | 488.2576 | 1461.7511 | 1462.6623 | -0.9112 | 1 | 7 | 29 | 3 | -.MAAAGWRDGSGQEK.Y |
|  | 14034 | **781.8884** | **1561.7622** | **1562.7763** | **-1.0141** | **1** | **1** | **94** | **9** | **K.DRDEFPMILIGNK.A** |
|  | 17078 | **586.3246** | **1755.9519** | **1754.9275** | **1.0244** | **1** | **0** | **1e+002** | **10** | **R.QVTQEEGQQLARQLK.V** |
|  | 17178 | 883.4197 | 1764.8249 | 1764.8352 | -0.0103 | 0 | 56 | 0.00025 | 1 | R.LDILDTAGQEEFGAMR.E 17180 |
|  | 21700 | 709.7069 | 2126.0989 | 2127.0419 | -0.9430 | 1 | 4 | 33 | 2 | R.DEFPMILIGNKADLDHQR.Q |

| **625.** | P62805\|H4_HUMAN    **Mass:** 11360    **Score:** 56     **Queries matched:** 10   **emPAI:** 0.83 |
| --- | --- |
|  | Histone H4 OS=Homo sapiens GN=HIST1H4A PE=1 SV=2 |

|  | **Query** | **Observed** | **Mr(expt)** | **Mr(calc)** | **Delta** | **Miss** | **Score** | **Expect** | **Rank** | **Peptide** |
| --- | --- | --- | --- | --- | --- | --- | --- | --- | --- | --- |
|  | 813 | **515.3253** | **514.3180** | **515.3180** | **-0.9999** | **1** | **12** | **15** | **10** | **R.GGVKR.I** 832 |
|  | 1341 | **714.8925** | **713.8852** | **713.3385** | **0.5467** | **0** | **15** | **4.6** | **1** | **R.TLYGFGG.-** |
|  | 4233 | **495.2897** | **988.5649** | **988.5706** | **-0.0056** | **0** | **39** | **0.025** | **1** | **K.VFLENVIR.D** 4249 |
|  | 6920 | **590.8098** | **1179.6051** | **1179.6135** | **-0.0085** | **0** | **46** | **0.0042** | **1** | **R.ISGLIYEETR.G** |
|  | 9894 | **442.5862** | **1324.7368** | **1324.7463** | **-0.0095** | **0** | **5** | **42** | **9** | **R.DNIQGITKPAIR.R** |
|  | 12405 | 489.6034 | 1465.7883 | 1465.7963 | -0.0079 | 1 | 18 | 1.8 | 1 | K.TVTAMDVVYALKR.Q |
|  | 14255 | 526.6314 | 1576.8722 | 1576.8824 | -0.0102 | 1 | 11 | 10 | 1 | R.ISGLIYEETRGVLK.V |
|  | 16101 | **565.3045** | **1692.8917** | **1692.9999** | **-0.1082** | **1** | **4** | **42** | **10** | **K.VLRDNIQGITKPAIR.R** |

| **626.** | P20340\|RAB6A_HUMAN    **Mass:** 23692    **Score:** 55     **Queries matched:** 9   **emPAI:** 0.35 |
| --- | --- |
|  | Ras-related protein Rab-6A OS=Homo sapiens GN=RAB6A PE=1 SV=3 |

|  | **Query** | **Observed** | **Mr(expt)** | **Mr(calc)** | **Delta** | **Miss** | **Score** | **Expect** | **Rank** | **Peptide** |
| --- | --- | --- | --- | --- | --- | --- | --- | --- | --- | --- |
|  | 7107 | **397.2007** | **1188.5801** | **1188.5887** | **-0.0086** | **1** | **6** | **39** | **9** | **K.WIDDVRTER.G** |
|  | 8411 | **417.7552** | **1250.2437** | **1250.5714** | **-0.3277** | **0** | **10** | **9.7** | **4** | **-.MSTGGDFGNPLR.K** |
|  | 8641 | **631.8304** | **1261.6463** | **1260.6748** | **0.9716** | **0** | **9** | **20** | **2** | **R.GSDVIIMLVGNK.T** |
|  | 9695 | 658.8284 | 1315.6422 | 1315.6521 | -0.0099 | 0 | 18 | 2.6 | 1 | R.LQLWDTAGQER.F 9685 |
|  | 12040 | **723.3511** | **1444.6876** | **1444.6980** | **-0.0104** | **0** | **9** | **15** | **1** | **R.VAAALPGMESTQDR.S** |
|  | 14630 | **801.4017** | **1600.7888** | **1600.7991** | **-0.0103** | **1** | **56** | **0.00027** | **1** | **R.RVAAALPGMESTQDR.S** |
|  | 15125 | **544.3312** | **1629.9717** | **1630.8712** | **-0.8995** | **1** | **3** | **58** | **10** | **R.TERGSDVIIMLVGNK.T** |
|  | 24594 | **623.5525** | **2490.1809** | **2491.1254** | **-0.9445** | **0** | **0** | **66** | **1** | **R.FMYDSFDNTYQATIGIDFLSK.T** |

| **627.** | P17858\|K6PL_HUMAN    **Mass:** 85762    **Score:** 55     **Queries matched:** 20   **emPAI:** 0.04 |
| --- | --- |
|  | 6-phosphofructokinase, liver type OS=Homo sapiens GN=PFKL PE=1 SV=6 |

|  | **Query** | **Observed** | **Mr(expt)** | **Mr(calc)** | **Delta** | **Miss** | **Score** | **Expect** | **Rank** | **Peptide** |
| --- | --- | --- | --- | --- | --- | --- | --- | --- | --- | --- |
|  | 936 | 548.2909 | 547.2837 | 546.3377 | 0.9460 | 0 | 7 | 62 | 7 | R.ILSSK.M |
|  | 1323 | **707.2159** | **706.2086** | **707.3602** | **-1.1516** | **0** | **2** | **1e+002** | **7** | **R.LGFDTR.V** |
|  | 1607 | **399.5150** | **797.0155** | **796.3901** | **0.6253** | **0** | **6** | **21** | **6** | **K.MLAQYR.I** |
|  | 2098 | **417.7552** | **833.4958** | **834.4712** | **-0.9754** | **1** | **15** | **5.9** | **6** | **K.LREVYR.K** |
|  | 2543 | **433.4150** | **864.8155** | **865.4327** | **-0.6173** | **1** | **4** | **41** | **5** | **R.RTLSMDK.G** |
|  | 2900 | 447.2101 | 892.4057 | 891.4371 | 0.9685 | 0 | 6 | 44 | 9 | -.MAAVDLEK.L |
|  | 3071 | **453.7805** | **905.5464** | **905.4753** | **0.0711** | **1** | **7** | **38** | **10** | **R.GGSMLGTKR.T** |
|  | 3261 | **460.1686** | **918.3227** | **918.3832** | **-0.0605** | **0** | **5** | **61** | **2** | **K.DTDFEHR.M** |
|  | 3304 | **461.2600** | **920.5055** | **921.4702** | **-0.9647** | **1** | **(1)** | **1.3e+002** | **4** | **R.GGSMLGTKR.T** |
|  | 4820 | 516.2860 | 1030.5575 | 1030.6287 | -0.0713 | 0 | 6 | 42 | 10 | K.LLAHQKPPK.E |
|  | 5515 | **540.3129** | **1078.6112** | **1078.4965** | **0.1147** | **1** | **5** | **53** | **10** | **K.EVQKAMDDK.R** |
|  | 6663 | **581.2742** | **1160.5339** | **1160.6223** | **-0.0884** | **1** | **13** | **7.3** | **2** | **-.MAAVDLEKLR.A** |
|  | 8990 | **427.7104** | **1280.1093** | **1280.6911** | **-0.5817** | **1** | **5** | **27** | **6** | **R.AVTRMGIYVGAK.V** 9008 |
|  | 9037 | **642.0643** | **1282.1141** | **1281.6937** | **0.4204** | **1** | **2** | **55** | **1** | **R.LMLKMLAQYR.I** |
|  | 15350 | **824.4168** | **1646.8191** | **1645.8199** | **0.9992** | **0** | **55** | **0.00035** | **1** | **R.SEWGSLLEELVAEGK.I** |
|  | 17399 | 445.7462 | 1778.9558 | 1777.8669 | 1.0889 | 0 | 4 | 38 | 7 | R.ISMAAYVSGELEHVTR.R |
|  | 17514 | **893.9520** | **1785.8894** | **1786.8996** | **-1.0102** | **0** | **4** | **41** | **1** | **K.AIGVLTSGGDAQGMNAAVR.A** |
|  | 19261 | 956.9960 | 1911.9774 | 1912.9677 | -0.9903 | 0 | 1 | 79 | 5 | R.IMEVIDAITTTAQSHQR.T 19260 |

| **628.** | Q96A08\|H2B1A_HUMAN    **Mass:** 14159    **Score:** 55     **Queries matched:** 33   **emPAI:** 0.63 |
| --- | --- |
|  | Histone H2B type 1-A OS=Homo sapiens GN=HIST1H2BA PE=1 SV=3 |

|  | **Query** | **Observed** | **Mr(expt)** | **Mr(calc)** | **Delta** | **Miss** | **Score** | **Expect** | **Rank** | **Peptide** |
| --- | --- | --- | --- | --- | --- | --- | --- | --- | --- | --- |
|  | 1843 | 408.7292 | 815.4438 | 815.4501 | -0.0063 | 0 | 26 | 0.56 | 1 | R.EIQTAVR.L 1844 1845 |
|  | 1994 | 414.7114 | 827.4082 | 827.4137 | -0.0055 | 0 | 31 | 0.13 | 1 | K.HAVSEGTK.A 1990 1991 1992 1993 2000 2001 2004 2005 2006 2007 2008 2009 2010 |
|  | 3744 | 477.3026 | 952.5906 | 952.5957 | -0.0051 | 0 | 38 | 0.024 | 1 | R.LLLPGELAK.H 3740 3741 3742 |
|  | 6757 | 584.7971 | 1167.5797 | 1167.5884 | -0.0087 | 0 | 24 | 0.59 | 1 | K.QVHPDTGISSK.A 6753 6755 6788 |
|  | 10353 | **676.3473** | **1350.6800** | **1349.6861** | **0.9940** | **1** | **3** | **72** | **7** | **-.MPEVSSKGATISK.K** 10360 |
|  | 13148 | 503.9497 | 1508.8273 | 1507.8358 | 0.9915 | 1 | 11 | 9.6 | 1 | K.VLKQVHPDTGISSK.A |
|  | 17111 | 880.4059 | 1758.7972 | 1759.8273 | -1.0301 | 0 | 12 | 7.1 | 3 | K.AMSIMNSFVTDIFER.I 17112 17129 17130 |
|  | 17323 | 888.9288 | 1775.8430 | 1775.8222 | 0.0208 | 0 | (5) | 39 | 2 | K.AMSIMNSFVTDIFER.I |

| **629.** | O14786\|NRP1_HUMAN    **Mass:** 104323   **Score:** 55     **Queries matched:** 20   **emPAI:** 0.04 |
| --- | --- |
|  | Neuropilin-1 OS=Homo sapiens GN=NRP1 PE=1 SV=3 |

|  | **Query** | **Observed** | **Mr(expt)** | **Mr(calc)** | **Delta** | **Miss** | **Score** | **Expect** | **Rank** | **Peptide** |
| --- | --- | --- | --- | --- | --- | --- | --- | --- | --- | --- |
|  | 1303 | **701.3879** | **700.3806** | **699.3955** | **0.9851** | **1** | **10** | **23** | **2** | **K.KYYVK.T** |
|  | 3522 | **469.9109** | **937.8072** | **936.6120** | **1.1952** | **1** | **0** | **97** | **4** | **R.VLLHKSLK.L** |
|  | 3707 | **951.4792** | **950.4720** | **950.5661** | **-0.0942** | **1** | **6** | **38** | **2** | **K.EGRVLLHK.S** 3691 |
|  | 4745 | **513.7283** | **1025.4420** | **1026.4474** | **-1.0054** | **1** | **9** | **20** | **8** | **K.MIMDDSKR.K** 4772 |
|  | 5235 | **530.8184** | **1059.6222** | **1058.4967** | **1.1254** | **0** | **9** | **22** | **6** | **R.NWMPENIR.L** |
|  | 13238 | 758.3544 | 1514.6942 | 1514.7154 | -0.0212 | 1 | 4 | 49 | 8 | K.FKIGYSNNGSDWK.M |
|  | 15510 | **828.8638** | **1655.7131** | **1654.7223** | **0.9908** | **0** | **47** | **0.0023** | **1** | **K.SFEGNNNYDTPELR.T** 15493 |
|  | 16584 | **575.2893** | **1722.8461** | **1722.9556** | **-0.1095** | **1** | **0** | **1.1e+002** | **10** | **K.SLKLYQVIFEGEIGK.G** |
|  | 17125 | 880.8978 | 1759.7810 | 1759.8352 | -0.0542 | 0 | 2 | 73 | 5 | R.IMINFNPHFDLEDR.D |
|  | 19203 | **636.8179** | **1907.4320** | **1907.0087** | **0.4232** | **1** | **6** | **18** | **1** | **R.FVRIKPATWETGISMR.F** |
|  | 21197 | 1039.5114 | 2077.0081 | 2075.8928 | 1.1153 | 1 | 1 | 68 | 2 | K.IGYSNNGSDWKMIMDDSK.R |
|  | 22785 | **449.0421** | **2240.1739** | **2241.0967** | **-0.9228** | **1** | **3** | **36** | **9** | **R.YDRLEIWDGFPDVGPHIGR.Y** |
|  | 24350 | **614.5967** | **2454.3576** | **2453.3651** | **0.9926** | **1** | **3** | **33** | **9** | **-.MERGLPLLCAVLALVLAPAGAFR.N** |
|  | 24885 | **845.7124** | **2534.1154** | **2534.1310** | **-0.0156** | **0** | **9** | **8.5** | **1** | **K.TGPIQDHTGDGNFIYSQADENQK.G** |
|  | 28263 | **788.3728** | **3149.4621** | **3149.7237** | **-0.2616** | **0** | **1** | **36** | **6** | **K.EGNKPVLFQGNTNPTDVVVAVFPKPLITR.F** |
|  | 29438 | **697.5596** | **3482.7615** | **3482.5959** | **0.1656** | **0** | **1** | **34** | **3** | **R.LVSPVVYSQNSAHCMTFWYHMSGSHVGTLR.V** |
|  | 31293 | **1676.4082** | **5026.2028** | **5025.6139** | **0.5889** | **1** | **2** | **8.2** | **6** | **R.KPGNVLKTLDPILITIIAMSALGVLLGAVCGVVLYCACWHNGMSER.N** |

| **630.** | O60831\|PRAF2_HUMAN    **Mass:** 19588    **Score:** 55     **Queries matched:** 2   **emPAI:** 0.20 |
| --- | --- |
|  | PRA1 family protein 2 OS=Homo sapiens GN=PRAF2 PE=1 SV=1 |

|  | **Query** | **Observed** | **Mr(expt)** | **Mr(calc)** | **Delta** | **Miss** | **Score** | **Expect** | **Rank** | **Peptide** |
| --- | --- | --- | --- | --- | --- | --- | --- | --- | --- | --- |
|  | 6698 | 582.3026 | 1162.5907 | 1162.5982 | -0.0076 | 0 | 55 | 0.00047 | 1 | R.ALDDFVLGSAR.L |
|  | 7267 | 399.5266 | 1195.5579 | 1196.6699 | -1.1120 | 1 | 8 | 25 | 8 | -.MSEVRLPPLR.A |

| **631.** | P62491\|RB11A_HUMAN    **Mass:** 24492    **Score:** 55     **Queries matched:** 10   **emPAI:** 0.54 |
| --- | --- |
|  | Ras-related protein Rab-11A OS=Homo sapiens GN=RAB11A PE=1 SV=3 |

|  | **Query** | **Observed** | **Mr(expt)** | **Mr(calc)** | **Delta** | **Miss** | **Score** | **Expect** | **Rank** | **Peptide** |
| --- | --- | --- | --- | --- | --- | --- | --- | --- | --- | --- |
|  | 1007 | 575.3103 | 574.3030 | 573.3486 | 0.9544 | 0 | 7 | 62 | 3 | R.IVSQK.Q |
|  | 3610 | 944.4777 | 943.4704 | 943.4763 | -0.0059 | 0 | 6 | 47 | 1 | R.AITSAYYR.G |
|  | 5011 | 522.3045 | 1042.5944 | 1042.6023 | -0.0078 | 0 | 45 | 0.0051 | 1 | K.VVLIGDSGVGK.S |
|  | 8677 | 632.8616 | 1263.7087 | 1263.6684 | 0.0403 | 1 | 6 | 39 | 3 | R.HLRAVPTDEAR.A |
|  | 8865 | 637.8054 | 1273.5962 | 1273.6051 | -0.0089 | 0 | 39 | 0.018 | 1 | K.AQIWDTAGQER.Y |
|  | 15039 | 542.6121 | 1624.8144 | 1624.8243 | -0.0099 | 0 | 29 | 0.15 | 1 | R.DHADSNIVIMLVGNK.S 15040 |
|  | 15454 | 551.5901 | 1651.7486 | 1651.7188 | 0.0298 | 1 | 4 | 46 | 2 | -.MGTRDDEYDYLFK.V |
|  | 28511 | **1068.8654** | **3203.5743** | **3202.6034** | **0.9709** | **0** | **18** | **0.76** | **1** | **K.NGLSFIETSALDSTNVEAAFQTILTEIYR.I** 28510 |

| **632.** | Q16864\|VATF_HUMAN    **Mass:** 13362    **Score:** 55     **Queries matched:** 2   **emPAI:** 0.30 |
| --- | --- |
|  | V-type proton ATPase subunit F OS=Homo sapiens GN=ATP6V1F PE=1 SV=2 |

|  | **Query** | **Observed** | **Mr(expt)** | **Mr(calc)** | **Delta** | **Miss** | **Score** | **Expect** | **Rank** | **Peptide** |
| --- | --- | --- | --- | --- | --- | --- | --- | --- | --- | --- |
|  | 1061 | **604.3153** | **603.3080** | **602.3388** | **0.9693** | **0** | **12** | **16** | **1** | **K.DSILR.R** |
|  | 24255 | **1222.6628** | **2443.3111** | **2443.3210** | **-0.0099** | **0** | **55** | **0.00022** | **1** | **K.LIAVIGDEDTVTGFLLGGIGELNK.N** |

| **633.** | Q9Y5L4\|TIM13_HUMAN    **Mass:** 10721    **Score:** 55     **Queries matched:** 2   **emPAI:** 0.37 |
| --- | --- |
|  | Mitochondrial import inner membrane translocase subunit Tim13 OS=Homo sapiens GN=TIMM13 PE=1 SV=1 |

|  | **Query** | **Observed** | **Mr(expt)** | **Mr(calc)** | **Delta** | **Miss** | **Score** | **Expect** | **Rank** | **Peptide** |
| --- | --- | --- | --- | --- | --- | --- | --- | --- | --- | --- |
|  | 8533 | 629.3573 | 1256.7000 | 1257.5448 | -0.8448 | 0 | 2 | 99 | 10 | R.YMDAWNTVSR.A |
|  | 13890 | **776.9392** | **1551.8637** | **1551.8733** | **-0.0095** | **0** | **55** | **0.0004** | **1** | **K.VQIAVANAQELLQR.M** |

| **634.** | P46777\|RL5_HUMAN    **Mass:** 34569    **Score:** 55     **Queries matched:** 21   **emPAI:** 0.23 |
| --- | --- |
|  | 60S ribosomal protein L5 OS=Homo sapiens GN=RPL5 PE=1 SV=3 |

|  | **Query** | **Observed** | **Mr(expt)** | **Mr(calc)** | **Delta** | **Miss** | **Score** | **Expect** | **Rank** | **Peptide** |
| --- | --- | --- | --- | --- | --- | --- | --- | --- | --- | --- |
|  | 4429 | **501.2406** | **1000.4666** | **1000.4726** | **-0.0060** | **0** | **11** | **16** | **5** | **K.EFNAEVHR.K** |
|  | 5942 | **555.3520** | **1108.6894** | **1108.5699** | **0.1195** | **1** | **5** | **48** | **10** | **R.LLNRFGMDK.I** |
|  | 7030 | **593.2761** | **1184.5377** | **1184.5462** | **-0.0085** | **1** | **22** | **1** | **1** | **K.RFPGYDSESK.E** 7029 |
|  | 10128 | **669.8492** | **1337.6838** | **1337.6939** | **-0.0101** | **0** | **53** | **0.00064** | **1** | **K.GAVDGGLSIPHSTK.R** 10129 |
|  | 11864 | **717.8289** | **1433.6433** | **1433.6544** | **-0.0111** | **0** | **29** | **0.15** | **1** | **K.HIMGQNVADYMR.Y** |
|  | 12132 | 725.3964 | 1448.7782 | 1449.6493 | -0.8711 | 0 | (7) | 27 | 2 | K.HIMGQNVADYMR.Y 12131 |
|  | 12151 | 484.3822 | 1450.1249 | 1449.6493 | 0.4756 | 0 | (0) | 79 | 6 | K.HIMGQNVADYMR.Y |
|  | 12893 | 747.8976 | 1493.7807 | 1493.7950 | -0.0143 | 1 | 8 | 18 | 6 | K.GAVDGGLSIPHSTKR.F 12894 |
|  | 13828 | **517.3689** | **1549.0849** | **1548.6653** | **0.4195** | **1** | **6** | **22** | **1** | **R.YLMEEDEDAYKK.Q** |
|  | 14038 | 521.6078 | 1561.8017 | 1561.7493 | 0.0524 | 1 | 6 | 30 | 3 | R.KHIMGQNVADYMR.Y |
|  | 14204 | 787.8253 | 1573.6361 | 1573.6462 | -0.0101 | 0 | 26 | 0.28 | 1 | K.NSVTPDMMEEMYK.K 14205 |
|  | 14286 | 527.2817 | 1578.8232 | 1577.7442 | 1.0790 | 1 | (3) | 60 | 2 | R.KHIMGQNVADYMR.Y |
|  | 14487 | **796.4210** | **1590.8274** | **1589.6411** | **1.1862** | **0** | **(2)** | **74** | **4** | **K.NSVTPDMMEEMYK.K** |
|  | 16246 | **851.8724** | **1701.7302** | **1701.7412** | **-0.0110** | **1** | **28** | **0.17** | **1** | **K.NSVTPDMMEEMYKK.A** |
|  | 16777 | **579.2735** | **1734.7987** | **1733.7310** | **1.0677** | **1** | **(5)** | **31** | **7** | **K.NSVTPDMMEEMYKK.A** |
|  | 19438 | 643.3420 | 1927.0043 | 1926.0033 | 1.0010 | 0 | 3 | 43 | 1 | K.VGLTNYAAAYCTGLLLAR.R |

| **635.** | O94826\|TOM70_HUMAN    **Mass:** 68096    **Score:** 55     **Queries matched:** 15   **emPAI:** 0.05 |
| --- | --- |
|  | Mitochondrial import receptor subunit TOM70 OS=Homo sapiens GN=TOMM70A PE=1 SV=1 |

|  | **Query** | **Observed** | **Mr(expt)** | **Mr(calc)** | **Delta** | **Miss** | **Score** | **Expect** | **Rank** | **Peptide** |
| --- | --- | --- | --- | --- | --- | --- | --- | --- | --- | --- |
|  | 2020 | 415.2455 | 828.4765 | 828.4817 | -0.0052 | 1 | 8 | 35 | 7 | K.EANVKLR.A |
|  | 6656 | **580.8573** | **1159.7000** | **1159.6601** | **0.0400** | **1** | **3** | **78** | **3** | **K.AVELNPKYVK.A** |
|  | 8683 | **633.3056** | **1264.5966** | **1263.5918** | **1.0049** | **1** | **0** | **1.4e+002** | **4** | **K.WKEVAQDCTK.A** |
|  | 9099 | 643.8588 | 1285.7031 | 1285.6740 | 0.0291 | 0 | 10 | 15 | 3 | R.EPLMPSPQFIK.S 9113 |
|  | 9418 | **652.3186** | **1302.6226** | **1301.6689** | **0.9537** | **0** | **(1)** | **1.2e+002** | **8** | **R.EPLMPSPQFIK.S** |
|  | 9563 | **656.3682** | **1310.7219** | **1310.7306** | **-0.0087** | **0** | **55** | **0.00045** | **1** | **R.LRPESALAQAQK.C** |
|  | 12382 | 489.2261 | 1464.6564 | 1463.7806 | 0.8758 | 1 | 2 | 83 | 7 | K.AIDMFNKAINLAK.S |
|  | 13930 | 778.8863 | 1555.7581 | 1555.8181 | -0.0599 | 1 | 6 | 30 | 5 | K.NREPLMPSPQFIK.S |
|  | 14185 | **524.9638** | **1571.8696** | **1571.8130** | **0.0566** | **1** | **(5)** | **38** | **3** | **K.NREPLMPSPQFIK.S** |
|  | 24767 | **629.5978** | **2514.3623** | **2513.2220** | **1.1403** | **1** | **(0)** | **61** | **5** | **R.QAYTGNNSSQIQAAMKGFEEVIK.K** |
|  | 24854 | **843.7911** | **2528.3516** | **2529.2169** | **-0.8653** | **1** | **4** | **26** | **6** | **R.QAYTGNNSSQIQAAMKGFEEVIK.K** 24863 |
|  | 27132 | **728.1002** | **2908.3715** | **2909.3475** | **-0.9759** | **1** | **3** | **28** | **1** | **K.TPEGRASPAPGSGHPEGPGAHLDMNSLDR.A** |
|  | 30399 | **1322.2898** | **3963.8475** | **3964.7567** | **-0.9092** | **0** | **4** | **12** | **1** | **R.GSMYMQQQQPLLSTQDFNMAADIDPQNADVYHHR.G** |

| **636.** | P26885\|FKBP2_HUMAN    **Mass:** 15810    **Score:** 54     **Queries matched:** 2   **emPAI:** 0.25 |
| --- | --- |
|  | FK506-binding protein 2 OS=Homo sapiens GN=FKBP2 PE=1 SV=2 |

|  | **Query** | **Observed** | **Mr(expt)** | **Mr(calc)** | **Delta** | **Miss** | **Score** | **Expect** | **Rank** | **Peptide** |
| --- | --- | --- | --- | --- | --- | --- | --- | --- | --- | --- |
|  | 11030 | **693.8474** | **1385.6803** | **1384.6922** | **0.9881** | **1** | **2** | **96** | **5** | **R.KGDVLHMHYTGK.L** |
|  | 14274 | **790.3427** | **1578.6707** | **1578.6807** | **-0.0099** | **0** | **54** | **0.0004** | **1** | **K.GWDQGLLGMCEGEK.R** |

| **637.** | P30536\|TSPOA_HUMAN    **Mass:** 18881    **Score:** 54     **Queries matched:** 7   **emPAI:** 0.45 |
| --- | --- |
|  | Translocator protein OS=Homo sapiens GN=TSPO PE=1 SV=2 |

|  | **Query** | **Observed** | **Mr(expt)** | **Mr(calc)** | **Delta** | **Miss** | **Score** | **Expect** | **Rank** | **Peptide** |
| --- | --- | --- | --- | --- | --- | --- | --- | --- | --- | --- |
|  | 3182 | **457.7423** | **913.4699** | **913.4770** | **-0.0071** | **0** | **10** | **18** | **2** | **R.FVHGEGLR.W** |
|  | 7178 | **398.1976** | **1191.5709** | **1190.5442** | **1.0267** | **1** | **10** | **15** | **1** | **R.DNHGWHGGRR.L** |
|  | 24102 | **806.7377** | **2417.1914** | **2417.2025** | **-0.0111** | **0** | **42** | **0.0045** | **1** | **M.APPWVPAMGFTLAPSLGCFVGSR.F** 24099 24100 24101 |
|  | 30842 | 1084.0383 | 4332.1242 | 4333.1400 | -1.0158 | 0 | 1 | 19 | 7 | R.WYAGLQKPSWHPPHWVLGPVWGTLYSAMGYGSYLVWK.E |

| **638.** | Q7KYR7\|BT2A1_HUMAN    **Mass:** 60393    **Score:** 54     **Queries matched:** 9   **emPAI:** 0.06 |
| --- | --- |
|  | Butyrophilin subfamily 2 member A1 OS=Homo sapiens GN=BTN2A1 PE=1 SV=3 |

|  | **Query** | **Observed** | **Mr(expt)** | **Mr(calc)** | **Delta** | **Miss** | **Score** | **Expect** | **Rank** | **Peptide** |
| --- | --- | --- | --- | --- | --- | --- | --- | --- | --- | --- |
|  | 1002 | **574.3513** | **573.3440** | **572.3533** | **0.9907** | **0** | **16** | **8.5** | **8** | **R.EIALK.E** |
|  | 1005 | **574.9903** | **573.9830** | **573.2983** | **0.6847** | **1** | **11** | **17** | **1** | **K.GGRER.T** |
|  | 1185 | **646.9983** | **645.9910** | **645.3697** | **0.6213** | **0** | **7** | **35** | **2** | **K.ILSGEK.E** |
|  | 2192 | **421.0562** | **840.0978** | **839.3886** | **0.7092** | **0** | **4** | **41** | **5** | **R.GHEDGGIR.L** |
|  | 7940 | **614.8077** | **1227.6009** | **1227.7009** | **-0.1000** | **1** | **0** | **1.6e+002** | **8** | **R.ILPLKESLCR.V** |
|  | 16584 | 575.2893 | 1722.8461 | 1722.8393 | 0.0068 | 1 | 1 | 79 | 4 | R.NMSCSINNTLLGQKK.E |
|  | 18165 | 610.6216 | 1828.8429 | 1829.8036 | -0.9607 | 1 | 6 | 25 | 6 | R.CHLSPEKNAEDMEVR.W |
|  | 24441 | **824.0830** | **2469.2272** | **2468.3461** | **0.8811** | **0** | **54** | **0.00028** | **1** | **R.SYDEAILHLVVAGLGSKPLISMR.G** |
|  | 24453 | **824.4818** | **2470.4234** | **2470.3161** | **0.1073** | **1** | **1** | **43** | **4** | **R.GWYPKPLTVWRDPYGGVAPALK.E** |

| **639.** | Q9H0C2\|ADT4_HUMAN    **Mass:** 35285    **Score:** 54     **Queries matched:** 14   **emPAI:** 0.11 |
| --- | --- |
|  | ADP/ATP translocase 4 OS=Homo sapiens GN=SLC25A31 PE=1 SV=1 |

|  | **Query** | **Observed** | **Mr(expt)** | **Mr(calc)** | **Delta** | **Miss** | **Score** | **Expect** | **Rank** | **Peptide** |
| --- | --- | --- | --- | --- | --- | --- | --- | --- | --- | --- |
|  | 2425 | 429.3168 | 856.6190 | 855.4814 | 1.1376 | 0 | 10 | 18 | 1 | K.TAVAPIER.V 2392 |
|  | 3126 | 455.7253 | 909.4360 | 908.4096 | 1.0264 | 0 | 1 | 1.1e+002 | 9 | K.GLGDCIMK.I |
|  | 5605 | 543.3285 | 1084.6424 | 1085.6444 | -1.0020 | 0 | 15 | 5.5 | 3 | K.LLLQVQASSK.Q |
|  | 7171 | **397.8887** | **1190.6443** | **1189.5880** | **1.0563** | **0** | **6** | **44** | **7** | **K.EFFHIDIGGR.-** |
|  | 7378 | **601.0430** | **1200.0715** | **1199.5097** | **0.5618** | **1** | **4** | **40** | **6** | **R.RMMMQSGEAK.R** 7361 |
|  | 8148 | 620.2581 | 1238.5017 | 1239.6104 | -1.1087 | 1 | 1 | 1e+002 | 3 | R.YKGMVDCLVR.I |
|  | 9607 | **438.7120** | **1313.1142** | **1313.6802** | **-0.5660** | **1** | **9** | **13** | **1** | **K.YKQLFMSGVNK.E** 9601 |
|  | 11791 | 715.8441 | 1429.6736 | 1430.7670 | -1.0935 | 1 | 15 | 4.2 | 3 | K.IKEFFHIDIGGR.- |
|  | 12063 | 723.8700 | 1445.7254 | 1445.7343 | -0.0089 | 0 | 54 | 0.00053 | 1 | R.YFPTQALNFAFK.D |
|  | 13748 | 773.4239 | 1544.8333 | 1545.9130 | -1.0797 | 1 | 8 | 20 | 3 | R.GTGGALVLVLYDKIK.E |
|  | 26729 | **707.3458** | **2825.3539** | **2826.4163** | **-1.0624** | **0** | **10** | **5.7** | **1** | **R.WFLANLASGGAAGATSLCVVYPLDFAR.T** |

| **640.** | P41219\|PERI_HUMAN    **Mass:** 53732    **Score:** 54     **Queries matched:** 11   **emPAI:** 0.14 |
| --- | --- |
|  | Peripherin OS=Homo sapiens GN=PRPH PE=1 SV=2 |

|  | **Query** | **Observed** | **Mr(expt)** | **Mr(calc)** | **Delta** | **Miss** | **Score** | **Expect** | **Rank** | **Peptide** |
| --- | --- | --- | --- | --- | --- | --- | --- | --- | --- | --- |
|  | 4915 | **519.2574** | **1036.5002** | **1036.4872** | **0.0130** | **0** | **3** | **80** | **8** | **-.MSHHPSGLR.A** |
|  | 5139 | **527.2890** | **1052.5634** | **1052.5614** | **0.0020** | **1** | **3** | **82** | **9** | **K.KLHEEELR.D** |
|  | 6279 | 567.9222 | 1133.8299 | 1134.5921 | -0.7621 | 0 | 0 | 1.1e+002 | 5 | R.EYQELLNVK.M |
|  | 7088 | **396.8585** | **1187.5537** | **1188.6251** | **-1.0714** | **0** | **10** | **15** | **2** | **R.FLEQQNAALR.G** |
|  | 8812 | 424.5786 | 1270.7141 | 1269.7153 | 0.9988 | 1 | 5 | 42 | 7 | R.GTNEALLRQLR.E |
|  | 9272 | 648.3331 | 1294.6516 | 1294.6591 | -0.0075 | 0 | 30 | 0.15 | 1 | K.MALDIEIATYR.K |
|  | 9518 | 655.3018 | 1308.5891 | 1308.5986 | -0.0095 | 0 | 45 | 0.0043 | 1 | K.NLQEAEEWYK.S 9517 |
|  | 11682 | 475.2555 | 1422.7447 | 1422.7540 | -0.0093 | 1 | 22 | 0.86 | 1 | K.MALDIEIATYRK.L |
|  | 12415 | **734.3204** | **1466.6263** | **1465.7374** | **0.8889** | **0** | **12** | **7.8** | **1** | **K.IESLMDEIEFLK.K** |
|  | 15108 | 815.8441 | 1629.6737 | 1630.8162 | -1.1425 | 1 | 4 | 51 | 6 | K.DVDDATLSRLELER.K |

| **641.** | Q15661\|TRYB1_HUMAN    **Mass:** 30952    **Score:** 54     **Queries matched:** 6   **emPAI:** 0.26 |
| --- | --- |
|  | Tryptase beta-1 OS=Homo sapiens GN=TPSAB1 PE=1 SV=1 |

|  | **Query** | **Observed** | **Mr(expt)** | **Mr(calc)** | **Delta** | **Miss** | **Score** | **Expect** | **Rank** | **Peptide** |
| --- | --- | --- | --- | --- | --- | --- | --- | --- | --- | --- |
|  | 9870 | 442.2351 | 1323.6834 | 1323.5660 | 0.1174 | 1 | 6 | 31 | 3 | R.DDMLCAGNTRR.D |
|  | 10651 | **683.8180** | **1365.6215** | **1365.6313** | **-0.0098** | **0** | **32** | **0.087** | **1** | **K.YHLGAYTGDDVR.I** |
|  | 12618 | **740.3088** | **1478.6030** | **1478.6130** | **-0.0100** | **0** | **45** | **0.004** | **1** | **R.DSCQGDSGGPLVCK.V** |
|  | 13077 | 753.3716 | 1504.7286 | 1504.9704 | -0.2418 | 0 | 9 | 17 | 3 | M.LNLLLLALPVLASR.A 13078 |
|  | 13338 | **760.8640** | **1519.7135** | **1519.7235** | **-0.0101** | **1** | **21** | **0.99** | **1** | **R.IVRDDMLCAGNTR.R** |

|  | **Proteins matching the same set of peptides:** |
| --- | --- |

|  | P20231\|TRYB2_HUMAN    **Mass:** 30952    **Score:** 54     **Queries matched:** 6 |
| --- | --- |
|  | Tryptase beta-2 OS=Homo sapiens GN=TPSB2 PE=1 SV=2 |

| **642.** | P15157\|TRYA1_HUMAN    **Mass:** 31209    **Score:** 54     **Queries matched:** 3   **emPAI:** 0.26 |
| --- | --- |
|  | Tryptase alpha-1 OS=Homo sapiens GN=TPSAB1 PE=1 SV=3 |

|  | **Query** | **Observed** | **Mr(expt)** | **Mr(calc)** | **Delta** | **Miss** | **Score** | **Expect** | **Rank** | **Peptide** |
| --- | --- | --- | --- | --- | --- | --- | --- | --- | --- | --- |
|  | 9870 | 442.2351 | 1323.6834 | 1323.5660 | 0.1174 | 1 | 6 | 31 | 3 | R.DDMLCAGNTRR.D |
|  | 10651 | 683.8180 | 1365.6215 | 1365.6313 | -0.0098 | 0 | 32 | 0.087 | 1 | K.YHLGAYTGDDVR.I |
|  | 12618 | 740.3088 | 1478.6030 | 1478.6494 | -0.0464 | 1 | 45 | 0.004 | 1 | R.DSCKGDSGGPLVCK.V |

| **643.** | Q07075\|AMPE_HUMAN    **Mass:** 109717   **Score:** 54     **Queries matched:** 15   **emPAI:** 0.03 |
| --- | --- |
|  | Glutamyl aminopeptidase OS=Homo sapiens GN=ENPEP PE=1 SV=2 |

|  | **Query** | **Observed** | **Mr(expt)** | **Mr(calc)** | **Delta** | **Miss** | **Score** | **Expect** | **Rank** | **Peptide** |
| --- | --- | --- | --- | --- | --- | --- | --- | --- | --- | --- |
|  | 3882 | 482.2940 | 962.5735 | 962.5800 | -0.0066 | 0 | 10 | 17 | 3 | K.LLYGLASVK.N |
|  | 4911 | **519.1362** | **1036.2578** | **1036.4937** | **-0.2360** | **1** | **7** | **24** | **4** | **M.NFAEREGSK.R** |
|  | 4921 | **519.2839** | **1036.5533** | **1036.4648** | **0.0885** | **0** | **8** | **26** | **7** | **K.EVMDTWTR.Q** |
|  | 7035 | **593.3218** | **1184.6291** | **1183.5291** | **1.1000** | **1** | **1** | **1.2e+002** | **8** | **-.MNFAEREGSK.R** |
|  | 8192 | **621.5304** | **1241.0462** | **1240.5764** | **0.4698** | **0** | **3** | **44** | **1** | **R.EWFFNLLESG.-** |
|  | 9263 | **432.2535** | **1293.7387** | **1294.6227** | **-0.8840** | **0** | **4** | **54** | **7** | **K.EYGALSNMPVAK.E** |
|  | 10336 | **450.9026** | **1349.6859** | **1349.7489** | **-0.0631** | **1** | **9** | **19** | **3** | **K.LLRSSVLGFACK.M** 10311 |
|  | 11451 | **471.2372** | **1410.6899** | **1410.6739** | **0.0160** | **0** | **2** | **74** | **8** | **K.SIVATDHEPTDAR.K** |
|  | 14770 | **805.7994** | **1609.5843** | **1609.7269** | **-0.1425** | **1** | **1** | **79** | **2** | **K.GCQMYLEKYQFK.N** |
|  | 17653 | **598.9595** | **1793.8568** | **1794.8536** | **-0.9969** | **1** | **6** | **25** | **3** | **K.NAKTSDFWAALEEASR.L** |
|  | 18186 | **611.3051** | **1830.8935** | **1829.9121** | **0.9814** | **0** | **12** | **7.2** | **1** | **R.VISAVTYIISMFEDDK.E** |
|  | 21254 | **1042.5193** | **2083.0240** | **2083.0374** | **-0.0134** | **0** | **54** | **0.0004** | **1** | **R.ANPSQPPSDLGYTWNIPVK.W** 21255 |
|  | 22692 | **744.7103** | **2231.1092** | **2231.1222** | **-0.0131** | **0** | **5** | **25** | **1** | **R.VNYEVATWDSIATALSLNHK.T** |

| **644.** | Q9NZ45\|CISD1_HUMAN    **Mass:** 12362    **Score:** 54     **Queries matched:** 5   **emPAI:** 0.32 |
| --- | --- |
|  | CDGSH iron sulfur domain-containing protein 1 OS=Homo sapiens GN=CISD1 PE=1 SV=1 |

|  | **Query** | **Observed** | **Mr(expt)** | **Mr(calc)** | **Delta** | **Miss** | **Score** | **Expect** | **Rank** | **Peptide** |
| --- | --- | --- | --- | --- | --- | --- | --- | --- | --- | --- |
|  | 5319 | **533.8018** | **1065.5891** | **1066.5957** | **-1.0066** | **0** | **14** | **5.6** | **2** | **K.AMINLHIQK.D** 5325 |
|  | 12785 | **497.2347** | **1488.6823** | **1488.6919** | **-0.0096** | **0** | **21** | **0.99** | **1** | **K.IVHAFDMEDLGDK.A** |
|  | 15182 | 818.4145 | 1634.8144 | 1634.8264 | -0.0120 | 0 | 54 | 0.00049 | 1 | K.HNEETGDNVGPLIIK.K |
|  | 19909 | 654.3577 | 1960.0514 | 1958.9044 | 1.1470 | 1 | 6 | 22 | 2 | K.DNPKIVHAFDMEDLGDK.A |

| **645.** | Q13011\|ECH1_HUMAN    **Mass:** 36136    **Score:** 54     **Queries matched:** 10   **emPAI:** 0.10 |
| --- | --- |
|  | Delta(3,5)-Delta(2,4)-dienoyl-CoA isomerase, mitochondrial OS=Homo sapiens GN=ECH1 PE=1 SV=2 |

|  | **Query** | **Observed** | **Mr(expt)** | **Mr(calc)** | **Delta** | **Miss** | **Score** | **Expect** | **Rank** | **Peptide** |
| --- | --- | --- | --- | --- | --- | --- | --- | --- | --- | --- |
|  | 3233 | **459.0112** | **916.0078** | **915.5025** | **0.5052** | **0** | **12** | **8.4** | **1** | **K.SPVAVQSTK.V** 3232 |
|  | 10852 | **688.8135** | **1375.6125** | **1375.6231** | **-0.0105** | **0** | **5** | **41** | **3** | **R.YCAQDAFFQVK.E** |
|  | 11580 | 473.5958 | 1417.7655 | 1417.6984 | 0.0671 | 1 | 6 | 38 | 4 | R.DADCRAVVISGAGK.M 11581 |
|  | 14068 | 782.8964 | 1563.7783 | 1563.7749 | 0.0034 | 1 | (1) | 88 | 8 | R.KMMADEALGSGLVSR.V |
|  | 14321 | **791.4603** | **1580.9061** | **1579.7698** | **1.1363** | **1** | **3** | **52** | **3** | **R.KMMADEALGSGLVSR.V** |
|  | 15159 | **545.3196** | **1632.9369** | **1632.8947** | **0.0422** | **1** | **4** | **44** | **6** | **R.RLTGSNYPGLSISLR.L** |
|  | 16725 | **866.4680** | **1730.9214** | **1730.9315** | **-0.0102** | **0** | **54** | **0.00048** | **1** | **K.VIGNQSLVNELAFTAR.K** |
|  | 20937 | **685.4705** | **2053.3896** | **2053.9813** | **-0.5917** | **1** | **2** | **39** | **4** | **K.MMADEALGSGLVSRVFPDK.E** |

| **646.** | Q9UBI6\|GBG12_HUMAN    **Mass:** 8115     **Score:** 53     **Queries matched:** 7   **emPAI:** 0.51 |
| --- | --- |
|  | Guanine nucleotide-binding protein G(I)/G(S)/G(O) subunit gamma-12 OS=Homo sapiens GN=GNG12 PE=1 SV=3 |

|  | **Query** | **Observed** | **Mr(expt)** | **Mr(calc)** | **Delta** | **Miss** | **Score** | **Expect** | **Rank** | **Peptide** |
| --- | --- | --- | --- | --- | --- | --- | --- | --- | --- | --- |
|  | 6428 | **573.7925** | **1145.5705** | **1145.5789** | **-0.0083** | **0** | **53** | **0.00081** | **1** | **K.TASTNNIAQAR.R** 6438 6444 |
|  | 13707 | **515.0947** | **1542.2622** | **1541.8525** | **0.4097** | **1** | **0** | **77** | **9** | **R.TVQQLRLEASIER.I** |
|  | 16645 | **864.4605** | **1726.9063** | **1726.9141** | **-0.0078** | **0** | **6** | **27** | **1** | **R.SDPLLIGIPTSENPFK.D** |
|  | 19993 | **657.3221** | **1968.9446** | **1968.8669** | **0.0777** | **1** | **4** | **34** | **1** | **K.VSKASADLMSYCEEHAR.S** 19995 |

| **647.** | Q10589\|BST2_HUMAN    **Mass:** 20041    **Score:** 53     **Queries matched:** 3   **emPAI:** 0.19 |
| --- | --- |
|  | Bone marrow stromal antigen 2 OS=Homo sapiens GN=BST2 PE=1 SV=1 |

|  | **Query** | **Observed** | **Mr(expt)** | **Mr(calc)** | **Delta** | **Miss** | **Score** | **Expect** | **Rank** | **Peptide** |
| --- | --- | --- | --- | --- | --- | --- | --- | --- | --- | --- |
|  | 16833 | **870.4563** | **1738.8980** | **1738.9101** | **-0.0120** | **1** | **53** | **0.00058** | **1** | **K.KVEELEGEITTLNHK.L** |
|  | 17001 | 876.4255 | 1750.8365 | 1750.9213 | -0.0848 | 0 | 5 | 32 | 3 | R.NVTHLLQQELTEAQK.G 17003 |

| **648.** | P53621\|COPA_HUMAN    **Mass:** 139797   **Score:** 53     **Queries matched:** 21   **emPAI:** 0.08 |
| --- | --- |
|  | Coatomer subunit alpha OS=Homo sapiens GN=COPA PE=1 SV=2 |

|  | **Query** | **Observed** | **Mr(expt)** | **Mr(calc)** | **Delta** | **Miss** | **Score** | **Expect** | **Rank** | **Peptide** |
| --- | --- | --- | --- | --- | --- | --- | --- | --- | --- | --- |
|  | 3641 | 474.2490 | 946.4835 | 946.4906 | -0.0071 | 0 | 17 | 3.8 | 1 | K.DVAVMQLR.S 3635 |
|  | 4139 | **491.7627** | **981.5108** | **981.5178** | **-0.0070** | **0** | **18** | **2.5** | **1** | **K.HAIVICNR.K** |
|  | 7142 | **595.7946** | **1189.5746** | **1189.6707** | **-0.0961** | **0** | **1** | **1.3e+002** | **1** | **R.TLDLPIYVTR.V** |
|  | 8231 | **415.2163** | **1242.6272** | **1242.6204** | **0.0068** | **0** | **10** | **14** | **2** | **K.NLSPGAVESDVR.G** 8253 8259 |
|  | 9495 | 654.2715 | 1306.5284 | 1305.6711 | 0.8573 | 1 | 1 | 93 | 3 | K.DVAVMQLRSGSK.F |
|  | 10152 | **670.3842** | **1338.7538** | **1337.7667** | **0.9871** | **1** | **0** | **1.3e+002** | **10** | **K.DAGLKNGVPAVGLK.L** |
|  | 14524 | **797.4036** | **1592.7926** | **1591.8458** | **0.9468** | **0** | **43** | **0.0059** | **1** | **R.GITGVDLFGTTDAVVK.H** 14525 |
|  | 14979 | **541.2819** | **1620.8239** | **1619.8494** | **0.9746** | **0** | **1** | **97** | **4** | **R.CLFTLLGHLDYIR.T** |
|  | 15535 | **553.2993** | **1656.8761** | **1656.7579** | **0.1182** | **0** | **39** | **0.015** | **1** | **R.GHYNNVSCAVFHPR.Q** |
|  | 16132 | **848.5294** | **1695.0443** | **1693.8675** | **1.1768** | **1** | **0** | **90** | **4** | **K.QQPLFVSGGDDYKIK.V** |
|  | 18917 | **471.9910** | **1883.9349** | **1883.9464** | **-0.0115** | **0** | **20** | **0.97** | **1** | **R.ERPAYAVHGNMLHYVK.D** |
|  | 20846 | **683.3459** | **2047.0158** | **2046.0972** | **0.9186** | **1** | **3** | **45** | **3** | **K.VKYVIWSADMSHVALLAK.H** 20856 |
|  | 21123 | **691.0283** | **2070.0631** | **2069.9113** | **0.1519** | **0** | **1** | **64** | **1** | **K.DMSGHYQNALYLGDVSER.V** |
|  | 24258 | **815.4557** | **2443.3454** | **2443.3549** | **-0.0095** | **0** | **11** | **5.5** | **1** | **K.LLQPPAPIMPLDTNWPLLTVSK.G** |
|  | 26410 | **925.4478** | **2773.3214** | **2774.3706** | **-1.0492** | **0** | **2** | **35** | **3** | **R.ICEMAAYFTHSNLQPVHMILVLR.T** |
|  | 30530 | **677.5122** | **4059.0296** | **4059.8700** | **-0.8404** | **0** | **0** | **28** | **5** | **R.TCVCVLTGHNHYVMCAQFHPTEDLVVSASLDQTVR.V** |

| **649.** | P01860\|IGHG3_HUMAN    **Mass:** 42287    **Score:** 53     **Queries matched:** 14   **emPAI:** 0.19 |
| --- | --- |
|  | Ig gamma-3 chain C region OS=Homo sapiens GN=IGHG3 PE=1 SV=2 |

|  | **Query** | **Observed** | **Mr(expt)** | **Mr(calc)** | **Delta** | **Miss** | **Score** | **Expect** | **Rank** | **Peptide** |
| --- | --- | --- | --- | --- | --- | --- | --- | --- | --- | --- |
|  | 1491 | 395.0771 | 788.1396 | 787.4440 | 0.6957 | 0 | 12 | 11 | 4 | K.SLSLSPGK.- 1494 |
|  | 2156 | 419.7523 | 837.4901 | 837.4960 | -0.0058 | 0 | 2 | 93 | 9 | K.ALPAPIEK.T |
|  | 6657 | 580.9736 | 1159.9327 | 1160.6223 | -0.6896 | 0 | 3 | 62 | 8 | K.NQVSLTCLVK.G |
|  | 7072 | 594.2824 | 1186.5503 | 1185.4907 | 1.0596 | 0 | 9 | 22 | 3 | K.SCDTPPPCPR.C 7046 7073 |
|  | 9103 | 429.7351 | 1286.1833 | 1285.6666 | 0.5167 | 0 | 14 | 3.9 | 4 | R.EPQVYTLPPSR.E |
|  | 11537 | 472.8985 | 1415.6737 | 1415.6834 | -0.0097 | 0 | 8 | 20 | 1 | K.WYVDGVEVHNAK.T 11557 |
|  | 17858 | 904.5013 | 1806.9881 | 1806.9992 | -0.0111 | 0 | 46 | 0.0025 | 1 | R.VVSVLTVLHQDWLNGK.E 17868 |
|  | 19341 | **960.9615** | **1919.9085** | **1919.9299** | **-0.0213** | **1** | **4** | **39** | **4** | **R.EPQVYTLPPSREEMTK.N** |
|  | 24057 | **805.3848** | **2413.1327** | **2413.1472** | **-0.0145** | **0** | **26** | **0.18** | **1** | **R.TPEVTCVVVDVSHEDPEVQFK.W** |

| **650.** | P02795\|MT2_HUMAN    **Mass:** 7178     **Score:** 53     **Queries matched:** 2   **emPAI:** 0.58 |
| --- | --- |
|  | Metallothionein-2 OS=Homo sapiens GN=MT2A PE=1 SV=1 |

|  | **Query** | **Observed** | **Mr(expt)** | **Mr(calc)** | **Delta** | **Miss** | **Score** | **Expect** | **Rank** | **Peptide** |
| --- | --- | --- | --- | --- | --- | --- | --- | --- | --- | --- |
|  | 12037 | **723.2525** | **1444.4904** | **1444.5026** | **-0.0122** | **0** | **53** | **0.00057** | **1** | **K.SCCSCCPVGCAK.C** |
|  | 24652 | 625.0255 | 2496.0729 | 2495.8725 | 0.2004 | 1 | 7 | 14 | 1 | -.MDPNCSCAAGDSCTCAGSCKCK.E |

|  | **Proteins matching a subset of these peptides:** |
| --- | --- |

|  | Q8N339\|MT1M_HUMAN    **Mass:** 7246     **Score:** 53     **Queries matched:** 1 |
| --- | --- |
|  | Metallothionein-1M OS=Homo sapiens GN=MT1M PE=2 SV=2 |

|  | P04732\|MT1E_HUMAN    **Mass:** 7150     **Score:** 53     **Queries matched:** 1 |
| --- | --- |
|  | Metallothionein-1E OS=Homo sapiens GN=MT1E PE=1 SV=1 |

|  | P13640\|MT1G_HUMAN    **Mass:** 7277     **Score:** 53     **Queries matched:** 1 |
| --- | --- |
|  | Metallothionein-1G OS=Homo sapiens GN=MT1G PE=1 SV=2 |

| **651.** | P80297\|MT1X_HUMAN    **Mass:** 7204     **Score:** 53     **Queries matched:** 2   **emPAI:** 0.58 |
| --- | --- |
|  | Metallothionein-1X OS=Homo sapiens GN=MT1X PE=1 SV=1 |

|  | **Query** | **Observed** | **Mr(expt)** | **Mr(calc)** | **Delta** | **Miss** | **Score** | **Expect** | **Rank** | **Peptide** |
| --- | --- | --- | --- | --- | --- | --- | --- | --- | --- | --- |
|  | 12037 | 723.2525 | 1444.4904 | 1444.5026 | -0.0122 | 0 | 53 | 0.00057 | 1 | K.SCCSCCPVGCAK.C |
|  | 22354 | 735.3690 | 2203.0852 | 2203.7884 | -0.7031 | 0 | 2 | 57 | 6 | -.MDPNCSCSPVGSCACAGSCK.C |

| **652.** | Q687X5\|STEA4_HUMAN    **Mass:** 52518    **Score:** 52     **Queries matched:** 2   **emPAI:** 0.07 |
| --- | --- |
|  | Metalloreductase STEAP4 OS=Homo sapiens GN=STEAP4 PE=1 SV=1 |

|  | **Query** | **Observed** | **Mr(expt)** | **Mr(calc)** | **Delta** | **Miss** | **Score** | **Expect** | **Rank** | **Peptide** |
| --- | --- | --- | --- | --- | --- | --- | --- | --- | --- | --- |
|  | 6514 | **576.7797** | **1151.5449** | **1152.5234** | **-0.9785** | **0** | **4** | **60** | **8** | **R.QVFVCGNDSK.A** |
|  | 15330 | **823.9098** | **1645.8050** | **1645.8167** | **-0.0117** | **0** | **52** | **0.0007** | **1** | **R.NLGLTPMDQGSLMAAK.E** |

| **653.** | P55145\|MANF_HUMAN    **Mass:** 20700    **Score:** 52     **Queries matched:** 7   **emPAI:** 0.19 |
| --- | --- |
|  | Mesencephalic astrocyte-derived neurotrophic factor OS=Homo sapiens GN=MANF PE=1 SV=2 |

|  | **Query** | **Observed** | **Mr(expt)** | **Mr(calc)** | **Delta** | **Miss** | **Score** | **Expect** | **Rank** | **Peptide** |
| --- | --- | --- | --- | --- | --- | --- | --- | --- | --- | --- |
|  | 3996 | 486.7778 | 971.5410 | 971.5473 | -0.0063 | 1 | 13 | 8.5 | 1 | R.KINELMPK.Y |
|  | 6101 | 561.2814 | 1120.5483 | 1119.5594 | 0.9889 | 1 | 4 | 65 | 3 | K.KDSQICELK.Y |
|  | 8581 | **630.3737** | **1258.7328** | **1258.7133** | **0.0195** | **1** | **6** | **38** | **1** | **K.QIDLSTVDLKK.L** |
|  | 13600 | 768.8354 | 1535.6562 | 1536.8035 | -1.1473 | 1 | 12 | 8.1 | 4 | K.YDKQIDLSTVDLK.K |
|  | 14008 | 781.3580 | 1560.7015 | 1560.7130 | -0.0115 | 0 | 52 | 0.00073 | 1 | R.LCYYIGATDDAATK.I |
|  | 15847 | **838.7866** | **1675.5586** | **1675.8669** | **-0.3083** | **0** | **2** | **47** | **1** | **R.DVTFSPATIENELIK.F** |
|  | 19708 | **649.6459** | **1945.9158** | **1946.9949** | **-1.0791** | **1** | **2** | **61** | **5** | **K.DRDVTFSPATIENELIK.F** |

| **654.** | P54707\|AT12A_HUMAN    **Mass:** 116292   **Score:** 52     **Queries matched:** 19   **emPAI:** 0.06 |
| --- | --- |
|  | Potassium-transporting ATPase alpha chain 2 OS=Homo sapiens GN=ATP12A PE=2 SV=3 |

|  | **Query** | **Observed** | **Mr(expt)** | **Mr(calc)** | **Delta** | **Miss** | **Score** | **Expect** | **Rank** | **Peptide** |
| --- | --- | --- | --- | --- | --- | --- | --- | --- | --- | --- |
|  | 875 | 530.2573 | 529.2500 | 528.2656 | 0.9844 | 0 | 11 | 24 | 8 | K.GAPER.I |
|  | 4735 | **513.2739** | **1024.5332** | **1024.5189** | **0.0142** | **0** | **5** | **52** | **9** | **R.DGPNSLTPPK.Q** |
|  | 7412 | **401.2851** | **1200.8334** | **1201.7070** | **-0.8737** | **1** | **11** | **9.1** | **7** | **K.QTPEIVKFLK.Q** 7442 |
|  | 7574 | 605.3686 | 1208.7226 | 1208.6302 | 0.0924 | 0 | 4 | 54 | 9 | R.NSIFQQGLFR.N |
|  | 9547 | **437.5808** | **1309.7204** | **1309.6523** | **0.0682** | **1** | **6** | **39** | **2** | **R.FLMVMKGAPER.I** |
|  | 10962 | 691.3739 | 1380.7332 | 1380.7435 | -0.0103 | 0 | 47 | 0.0027 | 1 | K.VIMVTGDHPITAK.A 10963 10964 |
|  | 11279 | **701.3162** | **1400.6179** | **1399.7823** | **0.8356** | **1** | **1** | **1.1e+002** | **8** | **R.TLINLRVEWEK.D** |
|  | 11792 | **715.8500** | **1429.6855** | **1428.6919** | **0.9936** | **0** | **1** | **1e+002** | **8** | **K.YGTDIIMGLSSTR.A** |
|  | 12307 | **730.8358** | **1459.6571** | **1459.7341** | **-0.0770** | **0** | **2** | **78** | **7** | **K.ADIGIAMGIAGSDAAK.N** |
|  | 12559 | **492.9094** | **1475.7065** | **1475.7290** | **-0.0225** | **0** | **(2)** | **85** | **9** | **K.ADIGIAMGIAGSDAAK.N** 12550 |
|  | 15172 | **545.6569** | **1633.9488** | **1634.8702** | **-0.9214** | **1** | **3** | **54** | **5** | **K.FSEVILGDVMEIRK.R** |
|  | 15744 | **557.2984** | **1668.8734** | **1668.7566** | **0.1168** | **0** | **1** | **88** | **7** | **K.FQLSIHEMDDPHGK.R** |
|  | 16177 | **566.8231** | **1697.4474** | **1696.7879** | **0.6594** | **0** | **4** | **33** | **4** | **K.TFHTAYMELGGLGER.V** |
|  | 17400 | 593.9977 | 1778.9714 | 1779.9917 | -1.0203 | 1 | 1 | 80 | 3 | K.VIMVTGDHPITAKAIAK.S |
|  | 30306 | 1298.2665 | 3891.7776 | 3892.8823 | -1.1047 | 1 | 0 | 28 | 6 | K.ADIGIAMGIAGSDAAKNAADMVLLDDNFASIVTGVEEGR.L |

| **655.** | P09543\|CN37_HUMAN    **Mass:** 47948    **Score:** 51     **Queries matched:** 15   **emPAI:** 0.16 |
| --- | --- |
|  | 2',3'-cyclic-nucleotide 3'-phosphodiesterase OS=Homo sapiens GN=CNP PE=1 SV=2 |

|  | **Query** | **Observed** | **Mr(expt)** | **Mr(calc)** | **Delta** | **Miss** | **Score** | **Expect** | **Rank** | **Peptide** |
| --- | --- | --- | --- | --- | --- | --- | --- | --- | --- | --- |
|  | 1507 | 395.2248 | 788.4350 | 788.3163 | 0.1187 | 0 | 8 | 41 | 4 | K.FCDYGK.A |
|  | 5040 | 523.2839 | 1044.5533 | 1043.5036 | 1.0497 | 0 | 8 | 27 | 7 | R.QFVPGDEPR.E 5013 |
|  | 6438 | 574.2848 | 1146.5550 | 1146.6067 | -0.0517 | 1 | 21 | 1.1 | 2 | R.KGGALQSCTII.- 6435 6440 |
|  | 7902 | **409.5515** | **1225.6327** | **1224.5445** | **1.0882** | **0** | **6** | **36** | **7** | **R.LDEDLAAYCR.R** |
|  | 9399 | 434.8811 | 1301.6214 | 1300.6412 | 0.9802 | 1 | 3 | 67 | 8 | R.QFVPGDEPREK.M |
|  | 13110 | **503.2528** | **1506.7366** | **1506.7687** | **-0.0321** | **1** | **7** | **25** | **7** | **R.WMLTLAKNMEVR.A** |
|  | 15408 | **825.4819** | **1648.9492** | **1647.8079** | **1.1413** | **1** | **(0)** | **98** | **2** | **K.NMEVRAIFTGYYGK.G** |
|  | 15647 | **555.2734** | **1662.7985** | **1663.8028** | **-1.0043** | **1** | **3** | **59** | **2** | **K.NMEVRAIFTGYYGK.G** |
|  | 20048 | 658.7424 | 1973.2055 | 1973.0874 | 0.1181 | 1 | 6 | 21 | 2 | K.SYSKAFTLTISALFVTPK.T |
|  | 25878 | 893.4751 | 2677.4035 | 2677.4109 | -0.0074 | 0 | 22 | 0.37 | 1 | R.AHITLGCAADVEAVQTGLDLLEILR.Q |
|  | 27620 | 1003.1769 | 3006.5088 | 3006.5298 | -0.0210 | 1 | 51 | 0.00045 | 1 | R.VELSEQQLQLWPSDVDKLSPTDNLPR.G |
|  | 27902 | 766.6048 | 3062.3901 | 3062.6070 | -0.2169 | 1 | 1 | 37 | 9 | R.AHITLGCAADVEAVQTGLDLLEILRQEK.G |

| **656.** | P00338\|LDHA_HUMAN    **Mass:** 36950    **Score:** 51     **Queries matched:** 10   **emPAI:** 0.21 |
| --- | --- |
|  | L-lactate dehydrogenase A chain OS=Homo sapiens GN=LDHA PE=1 SV=2 |

|  | **Query** | **Observed** | **Mr(expt)** | **Mr(calc)** | **Delta** | **Miss** | **Score** | **Expect** | **Rank** | **Peptide** |
| --- | --- | --- | --- | --- | --- | --- | --- | --- | --- | --- |
|  | 2018 | **415.2452** | **828.4759** | **828.5069** | **-0.0310** | **1** | **13** | **9.8** | **1** | **R.TPKIVSGK.D** |
|  | 3175 | **457.2921** | **912.5696** | **912.5756** | **-0.0060** | **0** | **7** | **34** | **3** | **K.LVIITAGAR.Q** |
|  | 4029 | 487.7470 | 973.4793 | 972.4512 | 1.0281 | 0 | 19 | 2.2 | 5 | K.EEQTPQNK.I |
|  | 8351 | 624.8001 | 1247.5857 | 1247.5928 | -0.0072 | 0 | 43 | 0.0078 | 1 | R.VIGSGCNLDSAR.F |
|  | 15194 | **546.2839** | **1635.8300** | **1634.8304** | **0.9996** | **1** | **4** | **49** | **9** | **K.SADTLWGIQKELQF.-** |
|  | 15633 | **416.5107** | **1662.0139** | **1662.9378** | **-0.9240** | **1** | **7** | **19** | **7** | **-.MATLKDQLIYNLLK.E** |
|  | 18795 | **469.4924** | **1873.9405** | **1873.9542** | **-0.0137** | **1** | **13** | **5.7** | **2** | **K.LKGEMMDLQHGSLFLR.T** |
|  | 20565 | **674.4662** | **2020.3769** | **2021.0723** | **-0.6954** | **0** | **0** | **61** | **3** | **K.ITVVGVGAVGMACAISILMK.D** |
|  | 21576 | **1057.0691** | **2112.1236** | **2111.0609** | **1.0628** | **0** | **33** | **0.042** | **1** | **K.GYTSWAIGLSVADLAESIMK.N** 21560 |

| **657.** | P14174\|MIF_HUMAN    **Mass:** 12639    **Score:** 51     **Queries matched:** 4   **emPAI:** 0.31 |
| --- | --- |
|  | Macrophage migration inhibitory factor OS=Homo sapiens GN=MIF PE=1 SV=4 |

|  | **Query** | **Observed** | **Mr(expt)** | **Mr(calc)** | **Delta** | **Miss** | **Score** | **Expect** | **Rank** | **Peptide** |
| --- | --- | --- | --- | --- | --- | --- | --- | --- | --- | --- |
|  | 5022 | **522.7963** | **1043.5780** | **1043.5797** | **-0.0018** | **0** | **22** | **1.2** | **1** | **K.LLCGLLAER.L** |
|  | 6929 | **590.9711** | **1179.9276** | **1179.5996** | **0.3280** | **1** | **2** | **66** | **2** | **K.IGGAQNRSYSK.L** |
|  | 9115 | **644.3436** | **1286.6726** | **1286.6805** | **-0.0079** | **0** | **51** | **0.0012** | **1** | **M.PMFIVNTNVPR.A** |
|  | 31744 | **1162.3766** | **5806.8466** | **5806.8797** | **-0.0332** | **0** | **3** | **3.6** | **2** | **R.ASVPDGFLSELTQQLAQATGKPPQYIAVHVVPDQLMAFGGSSEPCALCSLHSIGK.I** |

| **658.** | O95837\|GNA14_HUMAN    **Mass:** 42000    **Score:** 51     **Queries matched:** 9   **emPAI:** 0.19 |
| --- | --- |
|  | Guanine nucleotide-binding protein subunit alpha-14 OS=Homo sapiens GN=GNA14 PE=2 SV=1 |

|  | **Query** | **Observed** | **Mr(expt)** | **Mr(calc)** | **Delta** | **Miss** | **Score** | **Expect** | **Rank** | **Peptide** |
| --- | --- | --- | --- | --- | --- | --- | --- | --- | --- | --- |
|  | 8386 | 417.2427 | 1248.7062 | 1248.5881 | 0.1181 | 1 | 8 | 25 | 10 | R.MVDVGGQRSER.R |
|  | 9051 | 428.7283 | 1283.1631 | 1283.4944 | -0.3314 | 0 | 5 | 26 | 2 | M.AGCCCLSAEEK.E |
|  | 9720 | **659.3235** | **1316.6324** | **1316.6684** | **-0.0360** | **1** | **8** | **24** | **5** | **K.ESQRISAEIER.Q** 9692 |
|  | 12238 | 486.5818 | 1456.7235 | 1456.8501 | -0.1266 | 1 | 1 | 1.1e+002 | 6 | R.ELKLLLLGTGESGK.S |
|  | 12587 | 739.3609 | 1476.7072 | 1475.7005 | 1.0068 | 1 | 39 | 0.018 | 2 | R.IIHGSGYSDEDRK.G |
|  | 16029 | **844.9352** | **1687.8558** | **1686.9305** | **0.9254** | **1** | **2** | **76** | **1** | **K.DTILQLNLREFNLV.-** |
|  | 24600 | **831.4537** | **2491.3392** | **2491.3475** | **-0.0083** | **1** | **35** | **0.023** | **1** | **R.VRVPTTGIIEYPFDLENIIFR.M** |
|  | 24879 | **845.3620** | **2533.0642** | **2533.2491** | **-0.1849** | **1** | **2** | **39** | **2** | **K.LVYQNIFTAMQAMIRAMDTLR.I** |

| **659.** | Q9H8Y8\|GORS2_HUMAN    **Mass:** 47287    **Score:** 51     **Queries matched:** 4   **emPAI:** 0.08 |
| --- | --- |
|  | Golgi reassembly-stacking protein 2 OS=Homo sapiens GN=GORASP2 PE=1 SV=3 |

|  | **Query** | **Observed** | **Mr(expt)** | **Mr(calc)** | **Delta** | **Miss** | **Score** | **Expect** | **Rank** | **Peptide** |
| --- | --- | --- | --- | --- | --- | --- | --- | --- | --- | --- |
|  | 6852 | **588.3022** | **1174.5898** | **1173.6241** | **0.9657** | **1** | **5** | **52** | **4** | **K.DNDTLKDLLK.A** |
|  | 17006 | 876.4655 | 1750.9165 | 1751.0015 | -0.0850 | 1 | 15 | 3.1 | 3 | K.KISLPGQMAGTPITPLK.D 17007 |
|  | 22100 | **1086.0706** | **2170.1266** | **2170.1382** | **-0.0117** | **0** | **51** | **0.00072** | **1** | **R.ETSVTPSNLWGGQGLLGVSIR.F** |

| **660.** | P61225\|RAP2B_HUMAN    **Mass:** 20719    **Score:** 51     **Queries matched:** 3   **emPAI:** 0.18 |
| --- | --- |
|  | Ras-related protein Rap-2b OS=Homo sapiens GN=RAP2B PE=1 SV=1 |

|  | **Query** | **Observed** | **Mr(expt)** | **Mr(calc)** | **Delta** | **Miss** | **Score** | **Expect** | **Rank** | **Peptide** |
| --- | --- | --- | --- | --- | --- | --- | --- | --- | --- | --- |
|  | 17198 | **589.9877** | **1766.9414** | **1767.9553** | **-1.0139** | **1** | **1** | **90** | **8** | **R.VPMILVGNKVDLEGER.E** |
|  | 18567 | **928.9076** | **1855.8006** | **1855.8120** | **-0.0114** | **0** | **51** | **0.00086** | **1** | **K.ALAEEWSCPFMETSAK.N** |
|  | 26716 | **942.1160** | **2823.3263** | **2823.3484** | **-0.0221** | **0** | **25** | **0.18** | **1** | **K.EIEVDSSPSVLEILDTAGTEQFASMR.D** |

| **661.** | P26373\|RL13_HUMAN    **Mass:** 24304    **Score:** 50     **Queries matched:** 8   **emPAI:** 0.16 |
| --- | --- |
|  | 60S ribosomal protein L13 OS=Homo sapiens GN=RPL13 PE=1 SV=4 |

|  | **Query** | **Observed** | **Mr(expt)** | **Mr(calc)** | **Delta** | **Miss** | **Score** | **Expect** | **Rank** | **Peptide** |
| --- | --- | --- | --- | --- | --- | --- | --- | --- | --- | --- |
|  | 1114 | 626.7722 | 625.7649 | 626.3751 | -0.6102 | 0 | 5 | 22 | 3 | R.KPSAPK.K |
|  | 2279 | 423.7447 | 845.4748 | 846.4334 | -0.9586 | 0 | 15 | 7.3 | 8 | R.VITEEEK.N 2282 |
|  | 5990 | **557.6767** | **1113.3388** | **1112.6302** | **0.7086** | **1** | **6** | **31** | **3** | **R.TIGISVDPRR.R** |
|  | 7144 | **595.8082** | **1189.6019** | **1188.6040** | **0.9979** | **0** | **3** | **75** | **5** | **R.VATWFNQPAR.K** |
|  | 8015 | **616.8106** | **1231.6065** | **1231.6157** | **-0.0091** | **0** | **50** | **0.0014** | **1** | **K.STESLQANVQR.L** |
|  | 9844 | 441.6406 | 1321.8999 | 1322.6918 | -0.7918 | 0 | 2 | 59 | 6 | R.NGMVLKPHFHK.D |
|  | 17064 | **878.4735** | **1754.9325** | **1755.0744** | **-0.1419** | **1** | **0** | **1e+002** | **5** | **R.RIAPRPASGPIRPIVR.C** |

| **662.** | P47712\|PA24A_HUMAN    **Mass:** 85669    **Score:** 50     **Queries matched:** 16   **emPAI:** 0.04 |
| --- | --- |
|  | Cytosolic phospholipase A2 OS=Homo sapiens GN=PLA2G4A PE=1 SV=1 |

|  | **Query** | **Observed** | **Mr(expt)** | **Mr(calc)** | **Delta** | **Miss** | **Score** | **Expect** | **Rank** | **Peptide** |
| --- | --- | --- | --- | --- | --- | --- | --- | --- | --- | --- |
|  | 2096 | **417.7353** | **833.4561** | **832.5171** | **0.9391** | **0** | **15** | **5.6** | **4** | **K.FTVVVLR.A** |
|  | 2855 | 444.7130 | 887.4115 | 886.5025 | 0.9091 | 1 | 7 | 43 | 2 | K.YKAPGVPR.E |
|  | 3272 | **460.5713** | **919.1280** | **918.5174** | **0.6106** | **1** | **1** | **93** | **6** | **K.EFLSKPKA.-** |
|  | 3334 | 462.7474 | 923.4803 | 923.4752 | 0.0050 | 0 | 8 | 22 | 9 | R.YVESLWK.K |
|  | 3369 | **464.2543** | **926.4941** | **927.4888** | **-0.9947** | **0** | **(7)** | **32** | **3** | **K.FFMGTVVK.K** |
|  | 3591 | **472.2804** | **942.5462** | **943.4837** | **-0.9375** | **0** | **14** | **6.2** | **2** | **K.FFMGTVVK.K** 3582 3587 |
|  | 4215 | 495.0591 | 988.1037 | 988.5302 | -0.4265 | 0 | 4 | 52 | 8 | R.VLGVSGSQSR.G 4249 |
|  | 4552 | **505.7794** | **1009.5442** | **1009.5114** | **0.0328** | **0** | **4** | **66** | **2** | **R.MNTTLSSLK.E** |
|  | 4983 | 521.7047 | 1041.3949 | 1041.4987 | -0.1039 | 0 | 12 | 8.8 | 2 | R.AMVGFSGVMK.A |
|  | 4995 | **521.9318** | **1041.8491** | **1041.4987** | **0.3504** | **0** | **(7)** | **22** | **4** | **R.AMVGFSGVMK.A** |
|  | 7913 | 614.0779 | 1226.1412 | 1225.5649 | 0.5764 | 0 | 2 | 60 | 5 | K.EAMVESIEYR.R |
|  | 11215 | **466.7356** | **1397.1849** | **1397.6609** | **-0.4760** | **1** | **6** | **25** | **6** | **K.EAMVESIEYRR.Q** |
|  | 30561 | **1358.9276** | **4073.7610** | **4073.8007** | **-0.0397** | **1** | **50** | **0.00022** | **1** | **R.ETEEEKEIADFDIFDDPESPFSTFNFQYPNQAFK.R** |

| **663.** | P62988\|UBIQ_HUMAN    **Mass:** 8560     **Score:** 50     **Queries matched:** 3   **emPAI:** 0.48 |
| --- | --- |
|  | Ubiquitin OS=Homo sapiens GN=RPS27A PE=1 SV=1 |

|  | **Query** | **Observed** | **Mr(expt)** | **Mr(calc)** | **Delta** | **Miss** | **Score** | **Expect** | **Rank** | **Peptide** |
| --- | --- | --- | --- | --- | --- | --- | --- | --- | --- | --- |
|  | 10275 | 674.2061 | 1346.3977 | 1345.7354 | 0.6623 | 1 | 5 | 34 | 2 | R.LIFAGKQLEDGR.T |
|  | 17526 | **894.4610** | **1786.9074** | **1786.9200** | **-0.0126** | **0** | **50** | **0.0011** | **1** | **K.TITLEVEPSDTIENVK.A** 17525 |

| **664.** | Q9BTU6\|P4K2A_HUMAN    **Mass:** 54388    **Score:** 50     **Queries matched:** 15   **emPAI:** 0.22 |
| --- | --- |
|  | Phosphatidylinositol 4-kinase type 2-alpha OS=Homo sapiens GN=PI4K2A PE=1 SV=1 |

|  | **Query** | **Observed** | **Mr(expt)** | **Mr(calc)** | **Delta** | **Miss** | **Score** | **Expect** | **Rank** | **Peptide** |
| --- | --- | --- | --- | --- | --- | --- | --- | --- | --- | --- |
|  | 1027 | **586.4140** | **585.4067** | **584.3533** | **1.0534** | **0** | **9** | **23** | **5** | **K.EPVIK.V** |
|  | 1109 | **624.2950** | **623.2878** | **623.4006** | **-0.1129** | **0** | **6** | **33** | **5** | **R.IGLPPK.V** |
|  | 3203 | 458.2819 | 914.5492 | 914.5953 | -0.0461 | 0 | 19 | 2.1 | 4 | R.IIAVFKPK.N |
|  | 3741 | 477.3017 | 952.5888 | 952.5705 | 0.0182 | 0 | 11 | 11 | 10 | K.LELNIVPR.T 3749 |
|  | 9320 | 433.2336 | 1296.6791 | 1296.6098 | 0.0693 | 0 | 7 | 28 | 4 | K.NEEPYGHLNPK.W |
|  | 9638 | **657.8357** | **1313.6568** | **1313.7027** | **-0.0458** | **1** | **2** | **98** | **1** | **R.GQFHKQIAVMR.G** |
|  | 10081 | 668.3381 | 1334.6617 | 1334.6507 | 0.0110 | 0 | 7 | 30 | 8 | R.IYQGSSGSYFVK.D |
|  | 14592 | 533.6198 | 1597.8376 | 1597.9079 | -0.0703 | 1 | 7 | 25 | 8 | K.DLILPKISDPNFVK.D |
|  | 19274 | **479.2697** | **1913.0497** | **1912.0928** | **0.9570** | **1** | **4** | **35** | **9** | **K.QIAVMRGQILNLTQALK.D** |
|  | 22274 | **731.8814** | **2192.6222** | **2193.1946** | **-0.5724** | **1** | **1** | **47** | **5** | **R.IIAVFKPKNEEPYGHLNPK.W** |
|  | 23043 | **760.3944** | **2278.1612** | **2279.1104** | **-0.9491** | **0** | **4** | **29** | **2** | **R.DCLVLNQGYLSEAGASLVDQK.L** |
|  | 23169 | **765.0447** | **2292.1124** | **2292.1215** | **-0.0091** | **1** | **31** | **0.062** | **1** | **K.VGSFQLFVEGYKDADYWLR.R** |
|  | 23330 | **772.0515** | **2313.1325** | **2313.1416** | **-0.0091** | **1** | **26** | **0.19** | **1** | **K.ISDPNFVKDLEEDLYELFK.K** |
|  | 27960 | 771.1433 | 3080.5441 | 3080.5612 | -0.0171 | 0 | 35 | 0.016 | 1 | R.GAAAQGQTQTVAAQAQALAAQAAAAAHAAQAHR.E |

| **665.** | Q9BTV4\|TMM43_HUMAN    **Mass:** 44904    **Score:** 50     **Queries matched:** 11   **emPAI:** 0.17 |
| --- | --- |
|  | Transmembrane protein 43 OS=Homo sapiens GN=TMEM43 PE=1 SV=1 |

|  | **Query** | **Observed** | **Mr(expt)** | **Mr(calc)** | **Delta** | **Miss** | **Score** | **Expect** | **Rank** | **Peptide** |
| --- | --- | --- | --- | --- | --- | --- | --- | --- | --- | --- |
|  | 4259 | **496.2627** | **990.5109** | **990.6338** | **-0.1229** | **0** | **10** | **18** | **1** | **R.LVHIIGALR.T** |
|  | 8090 | **412.8691** | **1235.5853** | **1236.6714** | **-1.0861** | **1** | **8** | **20** | **4** | **K.VDNFKSLSLSK.L** |
|  | 18197 | **916.5489** | **1831.0832** | **1831.8242** | **-0.7409** | **0** | **1** | **72** | **1** | **R.AAGWMAMFMGLNLMTR.I** |
|  | 19020 | **631.3718** | **1891.0935** | **1891.0679** | **0.0255** | **1** | **0** | **90** | **7** | **K.LLSDPNYGVHLPAVKLR.R** |
|  | 19596 | **647.3484** | **1939.0235** | **1938.0574** | **0.9661** | **1** | **3** | **45** | **2** | **R.TSKLLSDPNYGVHLPAVK.L** |
|  | 22777 | **747.4301** | **2239.2683** | **2239.0766** | **0.1918** | **0** | **2** | **46** | **3** | **K.NPSAMAVESFMATAPFVQIGR.F** |
|  | 22971 | 757.0544 | 2268.1415 | 2269.0440 | -0.9025 | 1 | 4 | 34 | 6 | R.GDFFYHSENPKYPEVGDLR.V |
|  | 25613 | **881.7800** | **2642.3183** | **2642.3341** | **-0.0158** | **0** | **42** | **0.0041** | **1** | **R.VSFSYAGLSGDDPDLGPAHVVTVIAR.Q** |
|  | 26787 | **946.1428** | **2835.4066** | **2835.4250** | **-0.0184** | **0** | **25** | **0.18** | **1** | **K.TATSLAEGLSLVVSPDSIHSVAPENEGR.L** |
|  | 28093 | 779.8568 | 3115.3981 | 3115.5399 | -0.1417 | 0 | 3 | 22 | 4 | R.LSETSGGMFVGLMAFLLSFYLIFTNEGR.A |
|  | 30177 | **952.9830** | **3807.9028** | **3808.0483** | **-0.1455** | **1** | **0** | **28** | **8** | **K.TATSLAEGLSLVVSPDSIHSVAPENEGRLVHIIGALR.T** |

| **666.** | Q5T8D3\|ACBD5_HUMAN    **Mass:** 60510    **Score:** 50     **Queries matched:** 16   **emPAI:** 0.06 |
| --- | --- |
|  | Acyl-CoA-binding domain-containing protein 5 OS=Homo sapiens GN=ACBD5 PE=1 SV=1 |

|  | **Query** | **Observed** | **Mr(expt)** | **Mr(calc)** | **Delta** | **Miss** | **Score** | **Expect** | **Rank** | **Peptide** |
| --- | --- | --- | --- | --- | --- | --- | --- | --- | --- | --- |
|  | 2361 | **427.2128** | **852.4110** | **851.3998** | **1.0113** | **0** | **13** | **7.6** | **1** | **R.NNSGAPHR.E** 2345 2346 2353 2358 |
|  | 2377 | **427.9070** | **853.7995** | **853.4010** | **0.3985** | **0** | **7** | **18** | **8** | **K.FYSFYK.Q** |
|  | 3865 | **481.7288** | **961.4431** | **960.4261** | **1.0170** | **0** | **11** | **15** | **3** | **R.QVGSGGDGER.W** |
|  | 7590 | 606.0446 | 1210.0746 | 1209.5262 | 0.5484 | 0 | 8 | 14 | 4 | R.GGETDEFSNVR.R 7606 |
|  | 8070 | **412.2584** | **1233.7533** | **1233.6565** | **0.0969** | **1** | **9** | **17** | **5** | **R.SSDITSVRLEK.I** |
|  | 10382 | **676.8650** | **1351.7154** | **1351.6727** | **0.0427** | **1** | **1** | **1.2e+002** | **5** | **K.KIIETMPMTEK.V** |
|  | 10799 | **687.3409** | **1372.6672** | **1372.6769** | **-0.0097** | **0** | **50** | **0.0015** | **1** | **R.LQEDMQNVLQR.L** |
|  | 12259 | **486.7759** | **1457.3059** | **1457.6418** | **-0.3359** | **0** | **2** | **59** | **8** | **K.EEAMIAYVEEMK.K** |
|  | 16918 | 581.9947 | 1742.9624 | 1741.9145 | 1.0479 | 1 | 5 | 30 | 8 | R.LQEDMQNVLQRLQK.L |
|  | 22923 | **754.7358** | **2261.1855** | **2261.1548** | **0.0307** | **1** | **3** | **44** | **7** | **K.VIQSLPKNGSFQPTNEMMLK.F** |
|  | 23838 | 794.3686 | 2380.0839 | 2380.1693 | -0.0853 | 0 | 4 | 27 | 4 | R.EDIQVPPGNGNIGNMQVVAVEGK.G |

| **667.** | P08865\|RSSA_HUMAN    **Mass:** 32947    **Score:** 50     **Queries matched:** 11   **emPAI:** 0.11 |
| --- | --- |
|  | 40S ribosomal protein SA OS=Homo sapiens GN=RPSA PE=1 SV=4 |

|  | **Query** | **Observed** | **Mr(expt)** | **Mr(calc)** | **Delta** | **Miss** | **Score** | **Expect** | **Rank** | **Peptide** |
| --- | --- | --- | --- | --- | --- | --- | --- | --- | --- | --- |
|  | 7555 | **604.9623** | **1207.9101** | **1207.5941** | **0.3161** | **0** | **4** | **43** | **1** | **-.MSGALDVLQMK.E** |
|  | 8873 | 425.5484 | 1273.6233 | 1273.6514 | -0.0281 | 1 | 6 | 40 | 10 | K.EEQAAAEKAVTK.E 8871 |
|  | 14868 | 539.2896 | 1614.8470 | 1613.9253 | 0.9217 | 1 | 9 | 17 | 1 | R.AVLKFAAATGATPIAGR.F |
|  | 16210 | **850.4296** | **1698.8447** | **1697.8526** | **0.9921** | **0** | **50** | **0.0012** | **1** | **R.FTPGTFTNQIQAAFR.E** 16163 |
|  | 16826 | **870.3801** | **1738.7457** | **1739.9417** | **-1.1960** | **0** | **9** | **14** | **1** | **R.AIVAIENPADVSVISSR.N** |
|  | 19350 | **641.2820** | **1920.8241** | **1920.9536** | **-0.1295** | **1** | **2** | **63** | **7** | **-.MSGALDVLQMKEEDVLK.F** |
|  | 21700 | 709.7069 | 2126.0989 | 2127.0870 | -0.9881 | 1 | 1 | 72 | 9 | K.GAHSVGLMWWMLAREVLR.M |
|  | 27550 | **999.4908** | **2995.4507** | **2995.4709** | **-0.0202** | **0** | **22** | **0.33** | **1** | **R.ADHQPLTEASYVNLPTIALCNTDSPLR.Y** |
|  | 29065 | **837.4302** | **3345.6916** | **3345.6227** | **0.0689** | **1** | **1** | **33** | **2** | **K.EEDVLKFLAAGTHLGGTNLDFQMEQYIYK.R** |

| **668.** | P05091\|ALDH2_HUMAN    **Mass:** 56859    **Score:** 50     **Queries matched:** 9   **emPAI:** 0.07 |
| --- | --- |
|  | Aldehyde dehydrogenase, mitochondrial OS=Homo sapiens GN=ALDH2 PE=1 SV=2 |

|  | **Query** | **Observed** | **Mr(expt)** | **Mr(calc)** | **Delta** | **Miss** | **Score** | **Expect** | **Rank** | **Peptide** |
| --- | --- | --- | --- | --- | --- | --- | --- | --- | --- | --- |
|  | 1644 | **401.2190** | **800.4235** | **801.4610** | **-1.0375** | **1** | **12** | **14** | **3** | **R.FGPRLGR.R** |
|  | 3042 | **452.7407** | **903.4669** | **902.4709** | **0.9960** | **1** | **9** | **27** | **3** | **K.EDVDKAVK.A** |
|  | 7483 | **603.2916** | **1204.5686** | **1204.6200** | **-0.0515** | **1** | **3** | **79** | **3** | **K.SRVVGNPFDSK.T** |
|  | 10718 | **457.2558** | **1368.7456** | **1368.7799** | **-0.0343** | **0** | **7** | **27** | **2** | **K.LGPALATGNVVVMK.V** |
|  | 12278 | **730.3451** | **1458.6756** | **1457.6728** | **1.0029** | **1** | **2** | **89** | **6** | **R.YYAGWADKYHGK.T** |
|  | 13530 | **766.3697** | **1530.7249** | **1530.7355** | **-0.0105** | **0** | **16** | **3.3** | **1** | **K.TIPIDGDFFSYTR.H** |
|  | 18376 | **922.5251** | **1843.0356** | **1843.0455** | **-0.0098** | **0** | **50** | **0.0011** | **1** | **K.VAEQTPLTALYVANLIK.E** |
|  | 29653 | **1190.9441** | **3569.8104** | **3570.8143** | **-1.0038** | **1** | **4** | **14** | **1** | **R.GYFIQPTVFGDVQDGMTIAKEEIFGPVMQILK.F** |
|  | 31173 | **806.0139** | **4830.0399** | **4830.1757** | **-0.1359** | **1** | **0** | **12** | **2** | **R.VTLELGGKSPNIIMSDADMDWAVEQAHFALFFNQGQCCCAGSR.T** |

| **669.** | P79483\|2B31_HUMAN    **Mass:** 30228    **Score:** 50     **Queries matched:** 8   **emPAI:** 0.27 |
| --- | --- |
|  | HLA class II histocompatibility antigen, DRB3-1 beta chain OS=Homo sapiens GN=HLA-DRB3 PE=1 SV=1 |

|  | **Query** | **Observed** | **Mr(expt)** | **Mr(calc)** | **Delta** | **Miss** | **Score** | **Expect** | **Rank** | **Peptide** |
| --- | --- | --- | --- | --- | --- | --- | --- | --- | --- | --- |
|  | 1964 | 413.2647 | 824.5149 | 825.3439 | -0.8290 | 0 | 7 | 28 | 4 | R.VDNYCR.H |
|  | 2994 | 451.2558 | 900.4970 | 900.5029 | -0.0058 | 1 | 17 | 3.7 | 1 | K.DLLEQKR.G |
|  | 5634 | 544.7355 | 1087.4564 | 1086.4618 | 0.9946 | 0 | 21 | 1.4 | 1 | R.FDSDVGEYR.A 5635 |
|  | 7210 | 597.6307 | 1193.2468 | 1192.5625 | 0.6843 | 1 | 1 | 88 | 3 | R.WFRNGQEEK.A |
|  | 12872 | 747.3553 | 1492.6960 | 1492.7059 | -0.0099 | 0 | 50 | 0.0013 | 1 | R.HNYGVGESFTVQR.R |
|  | 25566 | **659.8074** | **2635.2006** | **2635.4111** | **-0.2105** | **1** | **2** | **40** | **3** | **-.MVCLKLPGGSSLAALTVTLMVLSSR.L** 25568 |

|  | **Proteins matching a subset of these peptides:** |
| --- | --- |

|  | P01913\|2B32_HUMAN    **Mass:** 30255    **Score:** 50     **Queries matched:** 7 |
| --- | --- |
|  | HLA class II histocompatibility antigen, DRB3-2 beta chain OS=Homo sapiens GN=HLA-DRB3 PE=2 SV=3 |

| **670.** | Q9TQE0\|2B19_HUMAN    **Mass:** 30149    **Score:** 50     **Queries matched:** 8   **emPAI:** 0.27 |
| --- | --- |
|  | HLA class II histocompatibility antigen, DRB1-9 beta chain OS=Homo sapiens GN=HLA-DRB1 PE=2 SV=1 |

|  | **Query** | **Observed** | **Mr(expt)** | **Mr(calc)** | **Delta** | **Miss** | **Score** | **Expect** | **Rank** | **Peptide** |
| --- | --- | --- | --- | --- | --- | --- | --- | --- | --- | --- |
|  | 5634 | 544.7355 | 1087.4564 | 1086.4618 | 0.9946 | 0 | 21 | 1.4 | 1 | R.FDSDVGEYR.A 5635 |
|  | 7210 | 597.6307 | 1193.2468 | 1192.5625 | 0.6843 | 1 | 1 | 88 | 3 | R.WFRNGQEEK.A |
|  | 7816 | **407.8961** | **1220.6664** | **1220.5785** | **0.0878** | **0** | **9** | **20** | **7** | **R.GIYNQEENVR.F** |
|  | 12023 | 722.8113 | 1443.6081 | 1442.6037 | 1.0044 | 0 | 9 | 15 | 3 | K.FECHFFNGTER.V 12005 |
|  | 12872 | 747.3553 | 1492.6960 | 1492.7059 | -0.0099 | 0 | 50 | 0.0013 | 1 | R.HNYGVGESFTVQR.R |
|  | 29320 | 687.3195 | 3431.5612 | 3430.8026 | 0.7586 | 1 | 2 | 23 | 7 | K.LPGGSCMAALTVTLMVLSSPLALAGDTQPRFLK.Q |

| **671.** | Q8WWP7\|GIMA1_HUMAN    **Mass:** 34690    **Score:** 50     **Queries matched:** 9   **emPAI:** 0.23 |
| --- | --- |
|  | GTPase IMAP family member 1 OS=Homo sapiens GN=GIMAP1 PE=2 SV=1 |

|  | **Query** | **Observed** | **Mr(expt)** | **Mr(calc)** | **Delta** | **Miss** | **Score** | **Expect** | **Rank** | **Peptide** |
| --- | --- | --- | --- | --- | --- | --- | --- | --- | --- | --- |
|  | 3890 | 482.7321 | 963.4497 | 962.3764 | 1.0734 | 0 | 4 | 70 | 6 | K.TDPGCEER.G |
|  | 5859 | 552.5961 | 1103.1777 | 1102.5731 | 0.6046 | 0 | 21 | 0.86 | 1 | K.SATGNSILGQR.R |
|  | 6697 | **582.2899** | **1162.5653** | **1162.5731** | **-0.0078** | **0** | **44** | **0.0065** | **1** | **R.FTAQDQQAVR.Q** |
|  | 8590 | 420.8488 | 1259.5245 | 1258.6742 | 0.8504 | 1 | 13 | 7.6 | 1 | K.SATGNSILGQRR.F 8557 |
|  | 11892 | **479.2870** | **1434.8391** | **1435.7130** | **-0.8738** | **1** | **4** | **47** | **8** | **R.QVRDMFGEDVLK.W** |
|  | 21475 | **702.3300** | **2103.9682** | **2103.9821** | **-0.0139** | **1** | **35** | **0.026** | **1** | **R.KEDLAGGSLHDYVSNTENR.A** |
|  | 23828 | 793.7423 | 2378.2051 | 2378.2151 | -0.0100 | 0 | 1 | 61 | 4 | R.EQEAQVEQLLGMVEGLVLEHK.G |
|  | 26364 | **922.1425** | **2763.4057** | **2763.4225** | **-0.0168** | **1** | **15** | **2** | **1** | **R.ATGREQEAQVEQLLGMVEGLVLEHK.G** |

| **672.** | P29992\|GNA11_HUMAN    **Mass:** 42382    **Score:** 50     **Queries matched:** 9   **emPAI:** 0.18 |
| --- | --- |
|  | Guanine nucleotide-binding protein subunit alpha-11 OS=Homo sapiens GN=GNA11 PE=1 SV=2 |

|  | **Query** | **Observed** | **Mr(expt)** | **Mr(calc)** | **Delta** | **Miss** | **Score** | **Expect** | **Rank** | **Peptide** |
| --- | --- | --- | --- | --- | --- | --- | --- | --- | --- | --- |
|  | 1077 | 609.3193 | 608.3121 | 607.3442 | 0.9679 | 1 | 7 | 28 | 9 | K.RGFTK.L |
|  | 2478 | **431.2299** | **860.4452** | **859.4651** | **0.9802** | **1** | **10** | **23** | **1** | **K.KDLLEDK.I** |
|  | 6171 | 563.7968 | 1125.5791 | 1125.5488 | 0.0303 | 1 | 8 | 23 | 6 | R.MEESKALFR.T |
|  | 8386 | 417.2427 | 1248.7062 | 1248.5881 | 0.1181 | 1 | 8 | 25 | 10 | R.MVDVGGQRSER.R |
|  | 11848 | 478.6021 | 1432.7845 | 1431.7834 | 1.0011 | 1 | 6 | 32 | 2 | K.YEQNKANALLIR.E |
|  | 12238 | 486.5818 | 1456.7235 | 1456.8501 | -0.1266 | 1 | 1 | 1.1e+002 | 6 | R.ELKLLLLGTGESGK.S |
|  | 16019 | 844.4675 | 1686.9205 | 1686.9305 | -0.0100 | 0 | 38 | 0.018 | 1 | R.IATLGYLPTQQDVLR.V 16031 |
|  | 24600 | 831.4537 | 2491.3392 | 2491.3475 | -0.0083 | 1 | 35 | 0.023 | 1 | R.VRVPTTGIIEYPFDLENIIFR.M |

| **673.** | P51159\|RB27A_HUMAN    **Mass:** 25137    **Score:** 50     **Queries matched:** 10   **emPAI:** 0.15 |
| --- | --- |
|  | Ras-related protein Rab-27A OS=Homo sapiens GN=RAB27A PE=1 SV=3 |

|  | **Query** | **Observed** | **Mr(expt)** | **Mr(calc)** | **Delta** | **Miss** | **Score** | **Expect** | **Rank** | **Peptide** |
| --- | --- | --- | --- | --- | --- | --- | --- | --- | --- | --- |
|  | 2506 | 432.2138 | 862.4130 | 861.3828 | 1.0302 | 0 | 6 | 48 | 9 | K.SDLEDQR.V |
|  | 4992 | 521.8253 | 1041.6361 | 1041.5607 | 0.0754 | 0 | 6 | 38 | 7 | K.SWIPEGVVR.S |
|  | 6762 | **584.8183** | **1167.6220** | **1167.6288** | **-0.0068** | **0** | **11** | **13** | **1** | **K.FITTVGIDFR.E** |
|  | 8283 | **623.3574** | **1244.7002** | **1244.6666** | **0.0336** | **1** | **1** | **1.2e+002** | **1** | **R.FRSLTTAFFR.D** |
|  | 11756 | **714.8955** | **1427.7765** | **1427.7871** | **-0.0106** | **1** | **50** | **0.0015** | **1** | **R.VVKEEEAIALAEK.Y** 11757 |
|  | 14099 | 522.9358 | 1565.7855 | 1565.7950 | -0.0095 | 0 | 5 | 38 | 5 | R.IHLQLWDTAGQER.F |
|  | 16991 | **875.9352** | **1749.8559** | **1749.8574** | **-0.0014** | **1** | **0** | **1.1e+002** | **7** | **K.TSVLYQYTDGKFNSK.F** |
|  | 17129 | 880.9688 | 1759.9231 | 1758.8020 | 1.1211 | 1 | 1 | 81 | 6 | R.SNGHASTDQLSEEKEK.G |
|  | 31391 | **1050.2363** | **5246.1453** | **5245.4770** | **0.6683** | **1** | **8** | **1.4** | **1** | **R.DAMGFLLLFDLTNEQSFLNVRNWISQLQMHAYCENPDIVLCGNK.S** |

| **674.** | Q15006\|TTC35_HUMAN    **Mass:** 34982    **Score:** 49     **Queries matched:** 8   **emPAI:** 0.11 |
| --- | --- |
|  | Tetratricopeptide repeat protein 35 OS=Homo sapiens GN=TTC35 PE=1 SV=1 |

|  | **Query** | **Observed** | **Mr(expt)** | **Mr(calc)** | **Delta** | **Miss** | **Score** | **Expect** | **Rank** | **Peptide** |
| --- | --- | --- | --- | --- | --- | --- | --- | --- | --- | --- |
|  | 5593 | **543.2474** | **1084.4803** | **1084.5989** | **-0.1185** | **1** | **3** | **80** | **5** | **K.AQGKNVEAIR.E** |
|  | 9973 | **664.8395** | **1327.6645** | **1327.6732** | **-0.0086** | **0** | **49** | **0.0017** | **1** | **R.ILQEDPTNTAAR.K** 9964 9979 |
|  | 15503 | **552.6229** | **1654.8469** | **1655.7501** | **-0.9032** | **0** | **5** | **37** | **3** | **K.VSELYDVTWEEMR.D** 15499 |
|  | 19112 | **634.3513** | **1900.0320** | **1898.8720** | **1.1599** | **1** | **13** | **5.4** | **1** | **K.VSELYDVTWEEMRDK.M** |
|  | 22181 | 727.4567 | 2179.3484 | 2179.1095 | 0.2388 | 1 | 1 | 48 | 7 | R.ALFGLYMSASHIASNPKASAK.T |

| **675.** | O15511\|ARPC5_HUMAN    **Mass:** 16367    **Score:** 49     **Queries matched:** 9   **emPAI:** 0.24 |
| --- | --- |
|  | Actin-related protein 2/3 complex subunit 5 OS=Homo sapiens GN=ARPC5 PE=1 SV=3 |

|  | **Query** | **Observed** | **Mr(expt)** | **Mr(calc)** | **Delta** | **Miss** | **Score** | **Expect** | **Rank** | **Peptide** |
| --- | --- | --- | --- | --- | --- | --- | --- | --- | --- | --- |
|  | 5355 | **535.8154** | **1069.6163** | **1069.6244** | **-0.0081** | **0** | **49** | **0.002** | **1** | **K.ALAAGGVGSIVR.V** |
|  | 5742 | **548.7565** | **1095.4985** | **1095.5342** | **-0.0357** | **1** | **3** | **71** | **10** | **-.MSKNTVSSAR.F** |
|  | 10040 | 445.0337 | 1332.0792 | 1331.6867 | 0.3925 | 0 | 3 | 55 | 6 | R.QGNMTAALQAALK.N |
|  | 10824 | **687.9674** | **1373.9203** | **1373.8646** | **0.0557** | **1** | **8** | **20** | **1** | **R.AGSIVLKVLISFK.A** 10818 10842 |
|  | 10867 | **689.3807** | **1376.7469** | **1375.7711** | **0.9758** | **1** | **4** | **55** | **3** | **K.VLISFKANDIEK.A** |
|  | 12204 | **728.3551** | **1454.6956** | **1455.7795** | **-1.0839** | **1** | **1** | **1.2e+002** | **6** | **K.NGVDLLMKYIYK.G** |
|  | 12465 | 736.3173 | 1470.6201 | 1471.7745 | -1.1544 | 1 | (0) | 1.2e+002 | 6 | K.NGVDLLMKYIYK.G |

| **676.** | Q9BXP2\|S12A9_HUMAN    **Mass:** 96733    **Score:** 49     **Queries matched:** 8   **emPAI:** 0.04 |
| --- | --- |
|  | Solute carrier family 12 member 9 OS=Homo sapiens GN=SLC12A9 PE=1 SV=1 |

|  | **Query** | **Observed** | **Mr(expt)** | **Mr(calc)** | **Delta** | **Miss** | **Score** | **Expect** | **Rank** | **Peptide** |
| --- | --- | --- | --- | --- | --- | --- | --- | --- | --- | --- |
|  | 1808 | **407.1990** | **812.3835** | **813.5072** | **-1.1237** | **1** | **5** | **48** | **10** | **R.LANQLKK.G** |
|  | 5058 | 523.7941 | 1045.5737 | 1046.6236 | -1.0499 | 1 | 3 | 98 | 5 | K.YLLRLDVR.K |
|  | 5344 | **535.2875** | **1068.5605** | **1068.6767** | **-0.1162** | **1** | **17** | **2.9** | **1** | **R.LRALLSQLR.I** 5330 |
|  | 13913 | 519.1365 | 1554.3878 | 1554.8042 | -0.4164 | 0 | 1 | 67 | 10 | R.EGSSPALSTLFPPPR.A |
|  | 19460 | **643.8417** | **1928.5034** | **1929.0544** | **-0.5510** | **1** | **7** | **14** | **1** | **R.DIRLTPRPGPNGSSLPPR.F** |
|  | 29120 | **1121.5614** | **3361.6624** | **3360.6739** | **0.9885** | **0** | **49** | **0.00056** | **1** | **R.GTGGGPGGPEGGDAEGPITALTFLYLPRPPADPAR.Y** |
|  | 31035 | 917.4439 | 4582.1832 | 4582.0437 | 0.1395 | 0 | 4 | 6.8 | 6 | K.DNLGAGYAEDYTTGAVMNFASVFAVLFNGCTGIMAGANMSGELK.D |

| **677.** | O14672\|ADA10_HUMAN    **Mass:** 86140    **Score:** 48     **Queries matched:** 22   **emPAI:** 0.09 |
| --- | --- |
|  | Disintegrin and metalloproteinase domain-containing protein 10 OS=Homo sapiens GN=ADAM10 PE=1 SV=1 |

|  | **Query** | **Observed** | **Mr(expt)** | **Mr(calc)** | **Delta** | **Miss** | **Score** | **Expect** | **Rank** | **Peptide** |
| --- | --- | --- | --- | --- | --- | --- | --- | --- | --- | --- |
|  | 2787 | **441.7257** | **881.4369** | **881.4429** | **-0.0060** | **0** | **33** | **0.073** | **1** | **K.FSLCSIR.N** |
|  | 4332 | **498.3366** | **994.6587** | **993.5430** | **1.1157** | **1** | **2** | **92** | **9** | **R.NISFMVKR.I** |
|  | 4764 | **514.3672** | **1026.7198** | **1025.5506** | **1.1693** | **0** | **1** | **1.1e+002** | **8** | **R.LVDADGPLAR.L** |
|  | 8984 | 640.7922 | 1279.5699 | 1280.7088 | -1.1389 | 0 | 18 | 2.3 | 1 | R.EAVIAQISSHVK.A |
|  | 9908 | **442.9084** | **1325.7033** | **1325.6398** | **0.0635** | **0** | **6** | **35** | **3** | **K.ICSVHTPSSNPK.L** 9934 |
|  | 10916 | **460.7609** | **1379.2608** | **1378.5581** | **0.7028** | **1** | **2** | **53** | **9** | **R.GYCDVFMRCR.L** |
|  | 13004 | **500.9520** | **1499.8342** | **1499.8433** | **-0.0091** | **1** | **8** | **20** | **5** | **R.RRPPQPIQQPQR.Q** |
|  | 14681 | **803.3831** | **1604.7517** | **1604.7617** | **-0.0100** | **0** | **35** | **0.039** | **1** | **R.TITLQPGSPCNDFR.G** 14680 14693 14694 14696 |
|  | 14773 | 805.8886 | 1609.7625 | 1609.6357 | 0.1268 | 1 | 4 | 46 | 4 | K.DDKELCHVCCMK.K |
|  | 17215 | 885.3763 | 1768.7380 | 1768.7509 | -0.0129 | 0 | 37 | 0.02 | 1 | K.MDPSTCASTGSVQWSR.H |
|  | 17821 | 602.6086 | 1804.8039 | 1804.8050 | -0.0011 | 1 | 7 | 23 | 2 | K.ENGNYIMYARATSGDK.L |
|  | 18431 | **616.6446** | **1846.9121** | **1845.8632** | **1.0489** | **1** | **0** | **1e+002** | **3** | **R.DTSLFSDEFKVETSNK.V** |
|  | 22353 | 735.3623 | 2203.0651 | 2203.0698 | -0.0048 | 1 | 0 | 85 | 8 | R.FEGFIQTRGGTFYVEPAER.Y |
|  | 23341 | **772.4629** | **2314.3668** | **2314.2507** | **0.1161** | **0** | **1** | **47** | **3** | **R.VLILLLSWAAGMGGQYGNPLNK.Y** |
|  | 26664 | **704.0917** | **2812.3376** | **2811.5833** | **0.7543** | **1** | **10** | **5.5** | **1** | **M.VLLRVLILLLSWAAGMGGQYGNPLNK.Y** 26663 |
|  | 27919 | 767.8779 | 3067.4826 | 3066.5080 | 0.9746 | 1 | 1 | 41 | 8 | R.KYQMTGVEEVTQIPQEEHAANGPELLR.K |

| **678.** | P04075\|ALDOA_HUMAN    **Mass:** 39851    **Score:** 48     **Queries matched:** 16   **emPAI:** 0.31 |
| --- | --- |
|  | Fructose-bisphosphate aldolase A OS=Homo sapiens GN=ALDOA PE=1 SV=2 |

|  | **Query** | **Observed** | **Mr(expt)** | **Mr(calc)** | **Delta** | **Miss** | **Score** | **Expect** | **Rank** | **Peptide** |
| --- | --- | --- | --- | --- | --- | --- | --- | --- | --- | --- |
|  | 2257 | 423.0518 | 844.0890 | 843.5178 | 0.5712 | 1 | 10 | 14 | 1 | K.SKGGVVGIK.V |
|  | 5343 | **535.2873** | **1068.5601** | **1067.5723** | **0.9878** | **1** | **3** | **73** | **6** | **K.KELSDIAHR.I** |
|  | 6247 | **566.7889** | **1131.5633** | **1131.5706** | **-0.0073** | **0** | **46** | **0.0044** | **1** | **R.ALANSLACQGK.Y** |
|  | 9128 | **430.2142** | **1287.6207** | **1287.6717** | **-0.0510** | **1** | **9** | **17** | **5** | **K.RALANSLACQGK.Y** 9138 |
|  | 10055 | **667.3522** | **1332.6898** | **1331.6932** | **0.9966** | **0** | **10** | **14** | **1** | **K.GILAADESTGSIAK.R** |
|  | 10211 | 672.3633 | 1342.7120 | 1341.7041 | 1.0079 | 0 | 13 | 6.7 | 2 | K.ADDGRPFPQVIK.S |
|  | 11877 | **718.3519** | **1434.6892** | **1433.7191** | **0.9701** | **0** | **23** | **0.62** | **1** | **M.PYQYPALTPEQK.K** 11864 |
|  | 14038 | 521.6078 | 1561.8017 | 1561.8140 | -0.0123 | 1 | 6 | 35 | 4 | M.PYQYPALTPEQKK.E |
|  | 15328 | 823.9026 | 1645.7907 | 1645.8019 | -0.0112 | 1 | 16 | 3.1 | 1 | R.LQSIGTENTEENRR.F 15330 |
|  | 18449 | **925.0004** | **1847.9862** | **1846.9359** | **1.0502** | **1** | **2** | **64** | **5** | **K.FSHEEIAMATVTALRR.T** |
|  | 21502 | 703.0331 | 2106.0774 | 2106.0891 | -0.0117 | 0 | 28 | 0.15 | 1 | K.IGEHTPSALAIMENANVLAR.Y |
|  | 21658 | 531.2650 | 2121.0309 | 2122.0840 | -1.0531 | 0 | (5) | 29 | 5 | K.IGEHTPSALAIMENANVLAR.Y |
|  | 25479 | **872.4380** | **2614.2923** | **2613.3246** | **0.9677** | **1** | **2** | **41** | **4** | **K.VDKGVVPLAGTNGETTTQGLDGLSER.C** |

| **679.** | O43747\|AP1G1_HUMAN    **Mass:** 92148    **Score:** 48     **Queries matched:** 16   **emPAI:** 0.04 |
| --- | --- |
|  | AP-1 complex subunit gamma-1 OS=Homo sapiens GN=AP1G1 PE=1 SV=5 |

|  | **Query** | **Observed** | **Mr(expt)** | **Mr(calc)** | **Delta** | **Miss** | **Score** | **Expect** | **Rank** | **Peptide** |
| --- | --- | --- | --- | --- | --- | --- | --- | --- | --- | --- |
|  | 1916 | **411.5931** | **821.1715** | **821.5235** | **-0.3520** | **1** | **1** | **1.1e+002** | **2** | **M.PAPIRLR.E** |
|  | 3615 | 472.7684 | 943.5223 | 944.5291 | -1.0068 | 1 | 16 | 4.7 | 4 | K.DLDVSIKR.R |
|  | 5146 | 527.7497 | 1053.4848 | 1053.5495 | -0.0646 | 0 | 5 | 47 | 6 | R.AVEYNALFK.K |
|  | 5323 | **534.2712** | **1066.5279** | **1065.5819** | **0.9461** | **0** | **10** | **14** | **2** | **R.VLTTAGSYVR.D** 5312 |
|  | 7252 | **598.3516** | **1194.6887** | **1193.6768** | **1.0119** | **1** | **5** | **43** | **2** | **K.LLKTSNSYLR.K** |
|  | 8253 | 415.2513 | 1242.7319 | 1242.6292 | 0.1028 | 1 | 11 | 13 | 1 | K.RWHIDTIMR.V |
|  | 8310 | **416.2591** | **1245.7554** | **1245.6829** | **0.0725** | **1** | **8** | **23** | **8** | **R.FLLNNDKNIR.Y** 8343 |
|  | 8744 | 634.8156 | 1267.6165 | 1267.6269 | -0.0104 | 0 | 48 | 0.0021 | 1 | K.TVQTDHNAVQR.H |
|  | 12763 | **496.6285** | **1486.8636** | **1486.7272** | **0.1364** | **0** | **5** | **42** | **3** | **R.QDVHLLMTNCIK.N** |
|  | 14917 | **540.2491** | **1617.7256** | **1618.8865** | **-1.1608** | **1** | **7** | **24** | **2** | **K.RIGYLGAMLLLDER.Q** |
|  | 17909 | **604.6312** | **1810.8719** | **1812.0621** | **-1.1903** | **1** | **2** | **64** | **5** | **R.VLAINILGRFLLNNDK.N** |
|  | 21378 | 1047.9377 | 2093.8609 | 2094.0350 | -0.1741 | 0 | 0 | 71 | 8 | K.NHGVLHTSVVLLTEMCER.S |
|  | 25318 | 517.8255 | 2584.0911 | 2584.1082 | -0.0171 | 0 | 3 | 29 | 5 | R.NDDDSSEAMNDILAQVATNTETSK.N |
|  | 27050 | **1446.2980** | **2890.5814** | **2889.5852** | **0.9962** | **0** | **10** | **4.6** | **1** | **K.TFQLQLLSPSSSIVPAFNTGTITQVIK.V** |

| **680.** | P55087\|AQP4_HUMAN    **Mass:** 35263    **Score:** 48     **Queries matched:** 3   **emPAI:** 0.11 |
| --- | --- |
|  | Aquaporin-4 OS=Homo sapiens GN=AQP4 PE=1 SV=2 |

|  | **Query** | **Observed** | **Mr(expt)** | **Mr(calc)** | **Delta** | **Miss** | **Score** | **Expect** | **Rank** | **Peptide** |
| --- | --- | --- | --- | --- | --- | --- | --- | --- | --- | --- |
|  | 2393 | **428.7283** | **855.4420** | **855.4490** | **-0.0070** | **1** | **23** | **0.82** | **2** | **R.FKEAFSK.A** |
|  | 2499 | **432.0402** | **862.0658** | **862.3790** | **-0.3131** | **0** | **5** | **41** | **7** | **K.CGPLCTR.E** |
|  | 27042 | **964.1707** | **2889.4903** | **2889.5084** | **-0.0180** | **1** | **48** | **0.00082** | **1** | **R.SQVETDDLILKPGVVHVIDVDRGEEK.K** |

| **681.** | Q14108\|SCRB2_HUMAN    **Mass:** 54712    **Score:** 48     **Queries matched:** 17   **emPAI:** 0.14 |
| --- | --- |
|  | Lysosome membrane protein 2 OS=Homo sapiens GN=SCARB2 PE=1 SV=2 |

|  | **Query** | **Observed** | **Mr(expt)** | **Mr(calc)** | **Delta** | **Miss** | **Score** | **Expect** | **Rank** | **Peptide** |
| --- | --- | --- | --- | --- | --- | --- | --- | --- | --- | --- |
|  | 2252 | 422.7546 | 843.4947 | 844.3927 | -0.8979 | 0 | 12 | 14 | 10 | R.DQSVGDPK.I 2251 |
|  | 2267 | 423.2273 | 844.4400 | 844.4443 | -0.0043 | 0 | 14 | 9.5 | 2 | K.IVEWNGK.T |
|  | 4640 | **508.9781** | **1015.9416** | **1016.5502** | **-0.6086** | **1** | **3** | **59** | **5** | **K.AVDQSIEKK.I** |
|  | 4726 | **512.7745** | **1023.5345** | **1023.5753** | **-0.0408** | **0** | **7** | **32** | **3** | **R.FQINIYVK.K** |
|  | 7618 | 606.7941 | 1211.5736 | 1211.5822 | -0.0087 | 0 | 24 | 0.56 | 1 | R.VEEVGPYTYR.E 7616 7617 |
|  | 10402 | **451.9049** | **1352.6928** | **1351.5310** | **1.1618** | **0** | **(2)** | **90** | **8** | **K.GQGSMDEGTADER.A** |
|  | 10692 | **456.8823** | **1367.6250** | **1367.5259** | **0.0991** | **0** | **3** | **61** | **2** | **K.GQGSMDEGTADER.A** |
|  | 14060 | 521.9462 | 1562.8169 | 1563.8330 | -1.0161 | 1 | 2 | 85 | 3 | R.EIIEAMLKAYQQK.L |
|  | 21370 | **1047.4847** | **2092.9549** | **2091.9836** | **0.9713** | **0** | **47** | **0.0019** | **1** | **K.NGAPIIMSFPHFYQADER.F** 21371 |
|  | 21526 | **528.0095** | **2108.0087** | **2107.9785** | **0.0302** | **0** | **(3)** | **48** | **3** | **K.NGAPIIMSFPHFYQADER.F** 21510 |
|  | 22944 | **755.7611** | **2264.2615** | **2264.2681** | **-0.0066** | **0** | **2** | **47** | **8** | **R.TLNIPVLTVIEWSQVHFLR.E** |
|  | 29123 | **841.9231** | **3363.6633** | **3363.6809** | **-0.0176** | **0** | **5** | **14** | **1** | **R.FVSAIEGMHPNQEDHETFVDINPLTGIILK.A** |

| **682.** | Q9BVC6\|TM109_HUMAN    **Mass:** 26194    **Score:** 48     **Queries matched:** 3   **emPAI:** 0.15 |
| --- | --- |
|  | Transmembrane protein 109 OS=Homo sapiens GN=TMEM109 PE=1 SV=1 |

|  | **Query** | **Observed** | **Mr(expt)** | **Mr(calc)** | **Delta** | **Miss** | **Score** | **Expect** | **Rank** | **Peptide** |
| --- | --- | --- | --- | --- | --- | --- | --- | --- | --- | --- |
|  | 9321 | **649.3554** | **1296.6961** | **1296.7038** | **-0.0076** | **0** | **48** | **0.0023** | **1** | **R.EAPVDVLTQIGR.S** |
|  | 11054 | 694.3447 | 1386.6749 | 1387.7419 | -1.0670 | 1 | 5 | 41 | 9 | R.LTGSRASGAQLEAK.V |
|  | 30234 | **963.4348** | **3849.7101** | **3849.2688** | **0.4413** | **0** | **0** | **27** | **4** | **K.LSPGQVQTFLLWGAGALVVYWLLSLLLGLVLALLGR.I** |

| **683.** | Q16720\|AT2B3_HUMAN    **Mass:** 135253   **Score:** 47     **Queries matched:** 32   **emPAI:** 0.08 |
| --- | --- |
|  | Plasma membrane calcium-transporting ATPase 3 OS=Homo sapiens GN=ATP2B3 PE=1 SV=3 |

|  | **Query** | **Observed** | **Mr(expt)** | **Mr(calc)** | **Delta** | **Miss** | **Score** | **Expect** | **Rank** | **Peptide** |
| --- | --- | --- | --- | --- | --- | --- | --- | --- | --- | --- |
|  | 1126 | **629.3937** | **628.3865** | **627.3704** | **1.0161** | **1** | **10** | **23** | **4** | **K.KANAPK.K** |
|  | 1557 | 397.4026 | 792.7906 | 792.4164 | 0.3743 | 0 | 12 | 6.9 | 4 | K.SMSTVIR.M |
|  | 1738 | 404.6886 | 807.3626 | 808.4113 | -1.0487 | 0 | (8) | 31 | 8 | K.SMSTVIR.M 1750 |
|  | 7157 | 596.2887 | 1190.5628 | 1190.5714 | -0.0085 | 0 | 35 | 0.051 | 1 | R.MVTGDNINTAR.A 7177 |
|  | 7343 | 400.2387 | 1197.6944 | 1197.6942 | 0.0002 | 1 | 15 | 4.5 | 1 | R.GLNRIQTQIR.V 7304 7332 7336 |
|  | 7631 | 607.2716 | 1212.5287 | 1211.6510 | 0.8777 | 1 | 6 | 40 | 2 | R.SSPTDKHTLVK.G 7635 7648 |
|  | 7991 | **411.2052** | **1230.5939** | **1230.5840** | **0.0099** | **1** | **7** | **30** | **6** | **R.NEKGEIEQER.L** 8000 |
|  | 9435 | **435.2682** | **1302.7827** | **1303.6925** | **-0.9097** | **0** | **6** | **36** | **8** | **R.QIYGQNFIPPK.Q** |
|  | 10346 | **451.2256** | **1350.6549** | **1350.6999** | **-0.0451** | **1** | **9** | **17** | **2** | **R.DKPLISRTMMK.N** |
|  | 10493 | 679.8007 | 1357.5868 | 1358.7571 | -1.1704 | 1 | 2 | 91 | 2 | R.GQILWFRGLNR.I |
|  | 11565 | 473.2862 | 1416.8367 | 1417.7057 | -0.8690 | 1 | 9 | 15 | 2 | R.KIIEPMACDGLR.T |
|  | 13394 | 762.8483 | 1523.6820 | 1523.6886 | -0.0066 | 1 | 2 | 76 | 8 | K.QQDGAMESSQTKAK.K |
|  | 14153 | **524.2572** | **1569.7498** | **1568.7439** | **1.0058** | **1** | **3** | **63** | **8** | **K.SMSTVIRMPDGGFR.L** |
|  | 15278 | 821.4200 | 1640.8255 | 1640.8370 | -0.0115 | 0 | 26 | 0.28 | 1 | R.QVVAVTGDGTNDGPALK.K 15272 |
|  | 15528 | **829.3869** | **1656.7592** | **1656.8988** | **-0.1395** | **1** | **4** | **48** | **7** | **R.QIYGQNFIPPKQPK.T** 15517 |
|  | 15591 | **415.9702** | **1659.8517** | **1660.8566** | **-1.0049** | **1** | **8** | **18** | **10** | **R.MVTGDNINTARAIAAK.C** |
|  | 16065 | 845.9575 | 1689.9005 | 1689.8145 | 0.0860 | 0 | 1 | 79 | 10 | R.DVPQAGGFGCTLAELR.T |
|  | 18245 | **917.9488** | **1833.8830** | **1834.8594** | **-0.9763** | **0** | **7** | **24** | **1** | **K.CGIIQPGEDFLCLEGK.E** |
|  | 18868 | 940.9717 | 1879.9289 | 1879.9415 | -0.0126 | 0 | 28 | 0.17 | 1 | K.EASDIILTDDNFTSIVK.A 18867 |
|  | 19679 | 973.5120 | 1945.0095 | 1944.9640 | 0.0455 | 1 | 2 | 55 | 3 | R.LKTSPTEGLADNTNDLEK.R |
|  | 30268 | **775.5860** | **3872.8936** | **3872.0409** | **0.8527** | **1** | **6** | **9.1** | **1** | **R.TMMKNILGHAVYQLAIIFTLLFVGELFFDIDSGR.N** |

| **684.** | P20020\|AT2B1_HUMAN    **Mass:** 139637   **Score:** 47     **Queries matched:** 27   **emPAI:** 0.08 |
| --- | --- |
|  | Plasma membrane calcium-transporting ATPase 1 OS=Homo sapiens GN=ATP2B1 PE=1 SV=3 |

|  | **Query** | **Observed** | **Mr(expt)** | **Mr(calc)** | **Delta** | **Miss** | **Score** | **Expect** | **Rank** | **Peptide** |
| --- | --- | --- | --- | --- | --- | --- | --- | --- | --- | --- |
|  | 1225 | **670.3827** | **669.3755** | **669.4173** | **-0.0419** | **1** | **12** | **7.9** | **2** | **K.KANLPK.K** |
|  | 1580 | **398.6880** | **795.3614** | **794.3559** | **1.0055** | **0** | **4** | **55** | **6** | **R.DYQDVR.N** |
|  | 7157 | 596.2887 | 1190.5628 | 1190.5714 | -0.0085 | 0 | 35 | 0.051 | 1 | R.MVTGDNINTAR.A 7177 |
|  | 7631 | 607.2716 | 1212.5287 | 1211.6510 | 0.8777 | 1 | 6 | 40 | 2 | R.SSPTDKHTLVK.G 7635 7648 |
|  | 7991 | 411.2052 | 1230.5939 | 1230.5840 | 0.0099 | 1 | 7 | 30 | 6 | R.NEKGEIEQER.I 8000 |
|  | 10493 | 679.8007 | 1357.5868 | 1358.7571 | -1.1704 | 1 | 2 | 91 | 2 | R.GQILWFRGLNR.I |
|  | 10840 | **688.3290** | **1374.6434** | **1374.7289** | **-0.0855** | **1** | **2** | **84** | **2** | **R.ALMELRSTDALR.K** |
|  | 13242 | 505.9137 | 1514.7193 | 1515.7053 | -0.9860 | 0 | 5 | 39 | 4 | K.IDESSLTGESDHVK.K |
|  | 13718 | **515.2967** | **1542.8682** | **1543.7300** | **-0.8618** | **1** | **3** | **56** | **3** | **K.SMSTVLKNSDGSYR.I** |
|  | 14224 | **788.4200** | **1574.8254** | **1574.6705** | **0.1549** | **0** | **2** | **80** | **1** | **-.MGDMANNSVAYSGVK.N** |
|  | 14835 | 538.9323 | 1613.7751 | 1613.9174 | -0.1423 | 1 | 3 | 61 | 4 | K.TECALLGLLLDLKR.D 14854 |
|  | 15278 | 821.4200 | 1640.8255 | 1640.8370 | -0.0115 | 0 | 26 | 0.28 | 1 | R.QVVAVTGDGTNDGPALK.K 15272 |
|  | 15736 | **557.2872** | **1668.8399** | **1667.8301** | **1.0098** | **0** | **3** | **60** | **2** | **K.DPLLLSGTHVMEGSGR.M** |
|  | 17926 | **605.2645** | **1812.7716** | **1811.8762** | **0.8954** | **1** | **1** | **78** | **8** | **K.TSPNEGLSGNPADLERR.E** |
|  | 18868 | 940.9717 | 1879.9289 | 1879.9415 | -0.0126 | 0 | 28 | 0.17 | 1 | K.EASDIILTDDNFTSIVK.A 18867 |
|  | 20377 | **668.3685** | **2002.0836** | **2000.9295** | **1.1540** | **1** | **1** | **74** | **6** | **-.MGDMANNSVAYSGVKNSLK.E** |
|  | 23590 | **588.2794** | **2349.0886** | **2349.1747** | **-0.0861** | **0** | **2** | **44** | **5** | **R.IQTQMDVVNAFQSGSSIQGALR.R** |
|  | 26944 | 1434.2118 | 2866.4090 | 2867.2670 | -0.8580 | 1 | 1 | 41 | 3 | R.HLDACETMGNATAICSDKTGTLTMNR.M 26943 |
|  | 27052 | **964.7676** | **2891.2811** | **2890.3879** | **0.8932** | **1** | **2** | **39** | **5** | **R.NSSPPPSPNKNNNAVDSGIHLTIEMNK.S** |

| **685.** | P62244\|RS15A_HUMAN    **Mass:** 14944    **Score:** 47     **Queries matched:** 9   **emPAI:** 0.26 |
| --- | --- |
|  | 40S ribosomal protein S15a OS=Homo sapiens GN=RPS15A PE=1 SV=2 |

|  | **Query** | **Observed** | **Mr(expt)** | **Mr(calc)** | **Delta** | **Miss** | **Score** | **Expect** | **Rank** | **Peptide** |
| --- | --- | --- | --- | --- | --- | --- | --- | --- | --- | --- |
|  | 4031 | **487.7669** | **973.5193** | **973.5266** | **-0.0073** | **0** | **47** | **0.0036** | **1** | **R.MNVLADALK.S** 4021 |
|  | 6379 | 572.3181 | 1142.6217 | 1142.6230 | -0.0013 | 1 | 7 | 37 | 9 | R.LNKCGVISPR.F |
|  | 7850 | **408.7371** | **1223.1896** | **1222.6499** | **0.5397** | **1** | **3** | **48** | **6** | **K.HTGGKILGFFF.-** |
|  | 8256 | 622.7920 | 1243.5694 | 1244.6911 | -1.1216 | 1 | 2 | 99 | 4 | M.VRMNVLADALK.S |
|  | 14126 | **523.6233** | **1567.8480** | **1566.9392** | **0.9088** | **1** | **1** | **1.1e+002** | **5** | **R.QVLIRPCSKVIVR.F** |
|  | 16222 | 567.6016 | 1699.7829 | 1699.7954 | -0.0126 | 0 | 2 | 71 | 5 | K.HGYIGEFEIIDDHR.A |
|  | 16688 | **577.3206** | **1728.9400** | **1729.9032** | **-0.9632** | **1** | **11** | **7.9** | **1** | **R.MNVLADALKSINNAEK.R** |
|  | 23936 | 798.7863 | 2393.3371 | 2393.1798 | 0.1574 | 1 | 1 | 52 | 5 | R.QFGFIVLTTSAGIMDHEEARR.K |

| **686.** | Q14651\|PLSI_HUMAN    **Mass:** 70608    **Score:** 47     **Queries matched:** 16   **emPAI:** 0.05 |
| --- | --- |
|  | Plastin-1 OS=Homo sapiens GN=PLS1 PE=1 SV=2 |

|  | **Query** | **Observed** | **Mr(expt)** | **Mr(calc)** | **Delta** | **Miss** | **Score** | **Expect** | **Rank** | **Peptide** |
| --- | --- | --- | --- | --- | --- | --- | --- | --- | --- | --- |
|  | 923 | 545.2996 | 544.2923 | 543.3493 | 0.9430 | 1 | 9 | 43 | 1 | R.KIGAR.I |
|  | 2546 | **433.7099** | **865.4052** | **865.4658** | **-0.0606** | **1** | **5** | **55** | **6** | **K.DISKTFR.K** |
|  | 2615 | **872.3856** | **871.3783** | **871.5127** | **-0.1344** | **1** | **10** | **22** | **7** | **K.VREIVEK.I** |
|  | 6584 | **578.3921** | **1154.7696** | **1154.5237** | **0.2459** | **0** | **1** | **1.1e+002** | **10** | **-.MENSTTTISR.E** |
|  | 9768 | 440.7276 | 1319.1611 | 1318.7357 | 0.4254 | 1 | 6 | 26 | 5 | K.LNNAKYAISVAR.K 9764 |
|  | 9985 | **665.3254** | **1328.6363** | **1328.7452** | **-0.1089** | **1** | **14** | **6.3** | **2** | **K.EASLPLPGYKVR.E** |
|  | 11611 | 474.2787 | 1419.8142 | 1418.6430 | 1.1712 | 0 | 8 | 22 | 3 | K.MVMTVFACLMGK.G |
|  | 12238 | 486.5818 | 1456.7235 | 1457.7515 | -1.0280 | 0 | 0 | 1.2e+002 | 8 | K.QFVTPADVVSGNPK.L |
|  | 13523 | 766.3057 | 1530.5969 | 1531.7188 | -1.1219 | 0 | 2 | 73 | 3 | K.MINLSEPDTIDER.A |
|  | 13564 | 767.4254 | 1532.8363 | 1532.7691 | 0.0672 | 1 | 7 | 23 | 5 | R.AGLMLQEADKLGCK.Q |
|  | 13608 | 512.9362 | 1535.7867 | 1536.7671 | -0.9805 | 0 | 3 | 60 | 6 | R.YTLNVLSDLGEGEK.V |
|  | 13623 | 769.3820 | 1536.7494 | 1536.7606 | -0.0112 | 1 | 47 | 0.0024 | 1 | K.KIENCNYAVELGK.N |
|  | 20474 | 672.3094 | 2013.9065 | 2014.9921 | -1.0856 | 1 | 2 | 65 | 8 | K.DGKISFEEFVSLMQELK.S |
|  | 26883 | 714.3783 | 2853.4841 | 2853.3264 | 0.1577 | 1 | 4 | 24 | 3 | K.REGITAIGGTSTISSEGTQHSYSEEEK.V |
|  | 28619 | **1075.8023** | **3224.3849** | **3223.6897** | **0.6953** | **1** | **1** | **32** | **5** | **R.NEALIALLNEGEELEELMKLSPEELLLR.W** |

| **687.** | P13797\|PLST_HUMAN    **Mass:** 71279    **Score:** 47     **Queries matched:** 14   **emPAI:** 0.05 |
| --- | --- |
|  | Plastin-3 OS=Homo sapiens GN=PLS3 PE=1 SV=4 |

|  | **Query** | **Observed** | **Mr(expt)** | **Mr(calc)** | **Delta** | **Miss** | **Score** | **Expect** | **Rank** | **Peptide** |
| --- | --- | --- | --- | --- | --- | --- | --- | --- | --- | --- |
|  | 1135 | **631.3724** | **630.3651** | **629.3748** | **0.9903** | **0** | **12** | **19** | **2** | **R.EIIQK.L** |
|  | 2108 | 418.2346 | 834.4547 | 834.3905 | 0.0642 | 0 | 12 | 13 | 5 | K.LMLDGDR.N 2100 |
|  | 4814 | **516.2596** | **1030.5047** | **1030.5481** | **-0.0434** | **0** | **10** | **19** | **4** | **K.AVGDGIVLCK.M** |
|  | 5373 | **536.2705** | **1070.5265** | **1070.6124** | **-0.0860** | **1** | **8** | **27** | **1** | **R.IKVPVDWSK.V** |
|  | 8762 | 635.2881 | 1268.5617 | 1269.5581 | -0.9963 | 0 | 5 | 46 | 5 | -.MDEMATTQISK.D 8797 |
|  | 11111 | **696.3881** | **1390.7616** | **1389.7074** | **1.0541** | **1** | **1** | **96** | **3** | **K.EANMPLPGYKVR.E** |
|  | 12057 | **482.7833** | **1445.3280** | **1445.7548** | **-0.4267** | **1** | **10** | **8.9** | **1** | **R.EIIQKLMLDGDR.N** |
|  | 12238 | 486.5818 | 1456.7235 | 1457.7515 | -1.0280 | 0 | 0 | 1.2e+002 | 8 | R.QFVTPADVVSGNPK.L |
|  | 13041 | 751.8748 | 1501.7351 | 1501.7446 | -0.0095 | 0 | 3 | 67 | 4 | K.MINLSVPDTIDER.A |
|  | 13623 | 769.3820 | 1536.7494 | 1536.7606 | -0.0112 | 1 | 47 | 0.0024 | 1 | K.KLENCNYAVELGK.H |
|  | 22689 | 744.6669 | 2230.9790 | 2230.1402 | 0.8387 | 1 | 0 | 80 | 7 | R.DGETLEELMKLSPEELLLR.W |
|  | 30066 | **1255.0620** | **3762.1642** | **3762.8873** | **-0.7231** | **1** | **0** | **26** | **3** | **K.TISSSLAVVDLIDAIQPGCINYDLVKSGNLTEDDK.H** |

| **688.** | P31153\|METK2_HUMAN    **Mass:** 43975    **Score:** 47     **Queries matched:** 7   **emPAI:** 0.09 |
| --- | --- |
|  | S-adenosylmethionine synthetase isoform type-2 OS=Homo sapiens GN=MAT2A PE=1 SV=1 |

|  | **Query** | **Observed** | **Mr(expt)** | **Mr(calc)** | **Delta** | **Miss** | **Score** | **Expect** | **Rank** | **Peptide** |
| --- | --- | --- | --- | --- | --- | --- | --- | --- | --- | --- |
|  | 976 | **563.0463** | **562.0391** | **561.2693** | **0.7697** | **0** | **11** | **15** | **1** | **K.GGLCR.R** 972 |
|  | 1315 | **704.3531** | **703.3458** | **702.3912** | **0.9546** | **1** | **8** | **40** | **6** | **R.DALKEK.V** |
|  | 4761 | 514.2951 | 1026.5756 | 1026.6185 | -0.0429 | 1 | 10 | 14 | 10 | K.LNAKLAELR.R |
|  | 10991 | **462.1427** | **1383.4063** | **1383.6453** | **-0.2390** | **0** | **1** | **87** | **7** | **K.TQVTVQYMQDR.G** |
|  | 12052 | **723.4352** | **1444.8558** | **1443.7471** | **1.1088** | **0** | **47** | **0.0026** | **1** | **R.FVIGGPQGDAGLTGR.K** |
|  | 28189 | **1044.8468** | **3131.5186** | **3130.5903** | **0.9282** | **1** | **3** | **25** | **2** | **R.GAVLPIRVHTIVISVQHDEEVCLDEMR.D** |

| **689.** | P63000\|RAC1_HUMAN    **Mass:** 21835    **Score:** 47     **Queries matched:** 7   **emPAI:** 0.38 |
| --- | --- |
|  | Ras-related C3 botulinum toxin substrate 1 OS=Homo sapiens GN=RAC1 PE=1 SV=1 |

|  | **Query** | **Observed** | **Mr(expt)** | **Mr(calc)** | **Delta** | **Miss** | **Score** | **Expect** | **Rank** | **Peptide** |
| --- | --- | --- | --- | --- | --- | --- | --- | --- | --- | --- |
|  | 8162 | **414.1979** | **1239.5720** | **1239.5918** | **-0.0198** | **0** | **7** | **28** | **4** | **K.YLECSALTQR.G** |
|  | 8340 | **624.3287** | **1246.6428** | **1247.6874** | **-1.0446** | **1** | **1** | **1.2e+002** | **2** | **R.GLKTVFDEAIR.A** |
|  | 13050 | **752.4097** | **1502.8049** | **1502.8167** | **-0.0118** | **0** | **30** | **0.12** | **1** | **K.LTPITYPQGLAMAK.E** |
|  | 14407 | **529.6168** | **1585.8285** | **1585.8399** | **-0.0114** | **0** | **12** | **7.2** | **1** | **R.HHCPNTPIILVGTK.L** |
|  | 15136 | **816.4564** | **1630.8982** | **1630.9116** | **-0.0135** | **1** | **44** | **0.0052** | **1** | **K.KLTPITYPQGLAMAK.E** 15112 |
|  | 15328 | 823.9026 | 1645.7907 | 1646.8484 | -1.0577 | 1 | 5 | 40 | 6 | -.MQAIKCVVVGDGAVGK.T |

| **690.** | P61313\|RL15_HUMAN    **Mass:** 24245    **Score:** 47     **Queries matched:** 9   **emPAI:** 0.16 |
| --- | --- |
|  | 60S ribosomal protein L15 OS=Homo sapiens GN=RPL15 PE=1 SV=2 |

|  | **Query** | **Observed** | **Mr(expt)** | **Mr(calc)** | **Delta** | **Miss** | **Score** | **Expect** | **Rank** | **Peptide** |
| --- | --- | --- | --- | --- | --- | --- | --- | --- | --- | --- |
|  | 2723 | **440.0291** | **878.0437** | **878.4280** | **-0.3843** | **1** | **9** | **17** | **3** | **K.KQSDVMR.F** 2717 2719 |
|  | 4660 | **509.7584** | **1017.5023** | **1017.5091** | **-0.0068** | **0** | **25** | **0.61** | **1** | **R.SLQSVAEER.A** |
|  | 9007 | **427.9234** | **1280.7484** | **1279.6707** | **1.0777** | **1** | **5** | **45** | **4** | **K.QSDVMRFLLR.V** 8971 |
|  | 11702 | **475.9031** | **1424.6873** | **1425.7404** | **-1.0531** | **1** | **7** | **25** | **1** | **M.GAYKYIQELWR.K** |
|  | 15586 | **830.8904** | **1659.7663** | **1659.7780** | **-0.0117** | **0** | **47** | **0.0024** | **1** | **R.VLNSYWVGEDSTYK.F** |
|  | 16293 | **569.3070** | **1704.8992** | **1704.9060** | **-0.0068** | **0** | **5** | **35** | **1** | **K.GATYGKPVHHGVNQLK.F** |

| **691.** | P48449\|ERG7_HUMAN    **Mass:** 84453    **Score:** 47     **Queries matched:** 18   **emPAI:** 0.04 |
| --- | --- |
|  | Lanosterol synthase OS=Homo sapiens GN=LSS PE=1 SV=1 |

|  | **Query** | **Observed** | **Mr(expt)** | **Mr(calc)** | **Delta** | **Miss** | **Score** | **Expect** | **Rank** | **Peptide** |
| --- | --- | --- | --- | --- | --- | --- | --- | --- | --- | --- |
|  | 2105 | 418.2025 | 834.3903 | 835.3858 | -0.9955 | 0 | 7 | 42 | 7 | M.TEGTCLR.R |
|  | 2979 | **451.1976** | **900.3806** | **900.5280** | **-0.1474** | **0** | **11** | **16** | **4** | **K.SISIGPISK.T** |
|  | 3837 | **480.7686** | **959.5226** | **958.4720** | **1.0507** | **0** | **10** | **20** | **7** | **K.TEPATDLGR.W** |
|  | 5501 | **539.8238** | **1077.6330** | **1076.5978** | **1.0352** | **1** | **4** | **71** | **10** | **R.EEIVRYLR.S** |
|  | 5710 | **547.3155** | **1092.6164** | **1093.5152** | **-0.8988** | **0** | **1** | **1.3e+002** | **7** | **R.HPDIEAQER.G** |
|  | 7062 | **593.9537** | **1185.8929** | **1185.6658** | **0.2271** | **0** | **2** | **73** | **2** | **R.NIFPIWALGR.F** 7080 |
|  | 7894 | **409.4391** | **1225.2956** | **1224.6363** | **0.6593** | **1** | **1** | **82** | **4** | **R.FPEHRAAEIR.E** |
|  | 10392 | **677.3223** | **1352.6300** | **1352.6394** | **-0.0095** | **0** | **5** | **44** | **1** | **R.ETLTQGLEFCR.R** |
|  | 13012 | 751.3687 | 1500.7227 | 1499.7256 | 0.9971 | 0 | 15 | 4.1 | 2 | R.LSQVPDNPPDYQK.Y 13013 |
|  | 13202 | **756.8944** | **1511.7743** | **1511.8460** | **-0.0717** | **1** | **7** | **28** | **2** | **R.IPLPAGYREEIVR.Y** |
|  | 13454 | 509.9442 | 1526.8109 | 1526.8093 | 0.0016 | 0 | 10 | 12 | 6 | K.STVFGTALNYVSLR.I |
|  | 14761 | **805.4058** | **1608.7970** | **1607.8042** | **0.9927** | **0** | **47** | **0.0026** | **1** | **R.EQTGLEAYALGLDTK.N** |
|  | 16690 | 865.4906 | 1728.9666 | 1727.9968 | 0.9699 | 1 | 5 | 33 | 4 | K.SISIGPISKTINMLVR.W |
|  | 19764 | 651.0179 | 1950.0320 | 1948.9068 | 1.1252 | 0 | 16 | 2.6 | 1 | R.WYVDGPASTAFQEHVSR.I |
|  | 26273 | 915.4282 | 2743.2628 | 2743.2339 | 0.0290 | 0 | 1 | 52 | 10 | R.YLQSAQSQIHNTCWAMMGLMAVR.H |
|  | 26940 | **956.4753** | **2866.4042** | **2867.3451** | **-0.9409** | **1** | **2** | **31** | **1** | **R.RYLQSAQSQIHNTCWAMMGLMAVR.H** |

| **692.** | Q9NX63\|CHCH3_HUMAN    **Mass:** 26421    **Score:** 47     **Queries matched:** 14   **emPAI:** 0.31 |
| --- | --- |
|  | Coiled-coil-helix-coiled-coil-helix domain-containing protein 3, mitochondrial OS=Homo sapiens GN=CHCHD3 PE=1 SV=1 |

|  | **Query** | **Observed** | **Mr(expt)** | **Mr(calc)** | **Delta** | **Miss** | **Score** | **Expect** | **Rank** | **Peptide** |
| --- | --- | --- | --- | --- | --- | --- | --- | --- | --- | --- |
|  | 1756 | 405.2062 | 808.3979 | 809.3702 | -0.9722 | 0 | 12 | 11 | 2 | -.MGGTTSTR.R 1761 |
|  | 2521 | **432.7351** | **863.4557** | **862.4032** | **1.0525** | **1** | **15** | **6.3** | **2** | **K.KESEDQK.R** |
|  | 2899 | **446.7750** | **891.5354** | **890.4093** | **1.1260** | **1** | **14** | **8.2** | **1** | **K.ESEDQKR.L** 2898 |
|  | 3622 | 473.2505 | 944.4864 | 944.4927 | -0.0063 | 0 | 13 | 10 | 1 | R.LSENVIDR.M |
|  | 3918 | **483.7578** | **965.5010** | **965.4713** | **0.0297** | **1** | **8** | **26** | **7** | **-.MGGTTSTRR.V** |
|  | 6460 | **575.2789** | **1148.5433** | **1148.5422** | **0.0011** | **1** | **7** | **31** | **5** | **K.ESSPSGSKSQR.Y** |
|  | 7772 | **407.2080** | **1218.6021** | **1219.6230** | **-1.0209** | **1** | **5** | **45** | **8** | **R.LSENVIDRMK.E** |
|  | 13291 | **759.3514** | **1516.6882** | **1516.6980** | **-0.0098** | **0** | **51** | **0.00098** | **1** | **R.YESHPVCADLQAK.I** 13290 |
|  | 14215 | **788.3568** | **1574.6991** | **1574.7100** | **-0.0109** | **0** | **13** | **6.4** | **1** | **R.YSGAYGASVSDEELK.R** |
|  | 19730 | **649.8424** | **1946.5054** | **1945.9017** | **0.6037** | **1** | **2** | **48** | **2** | **K.SQRYSGAYGASVSDEELK.R** 19720 |

| **693.** | O75165\|DJC13_HUMAN    **Mass:** 256549   **Score:** 46     **Queries matched:** 54   **emPAI:** 0.01 |
| --- | --- |
|  | DnaJ homolog subfamily C member 13 OS=Homo sapiens GN=DNAJC13 PE=1 SV=4 |

|  | **Query** | **Observed** | **Mr(expt)** | **Mr(calc)** | **Delta** | **Miss** | **Score** | **Expect** | **Rank** | **Peptide** |
| --- | --- | --- | --- | --- | --- | --- | --- | --- | --- | --- |
|  | 1216 | **664.3572** | **663.3500** | **662.3421** | **1.0078** | **0** | **9** | **27** | **1** | **R.MGIAQK.E** |
|  | 1405 | **742.3122** | **741.3049** | **741.3293** | **-0.0244** | **0** | **3** | **76** | **3** | **K.YSSTER.D** |
|  | 2483 | **431.2716** | **860.5286** | **860.5484** | **-0.0197** | **0** | **7** | **42** | **6** | **K.LIFSLLR.V** |
|  | 2496 | **431.8722** | **861.7299** | **862.4297** | **-0.6998** | **0** | **13** | **7.6** | **4** | **K.GQDIFQR.S** |
|  | 2965 | 450.2375 | 898.4604 | 898.4548 | 0.0055 | 0 | 7 | 28 | 7 | K.IGDYYLR.L |
|  | 3939 | **484.3202** | **966.6259** | **967.5022** | **-0.8762** | **0** | **16** | **3.9** | **1** | **K.VIQAMNHR.N** |
|  | 4286 | 497.2282 | 992.4419 | 993.5317 | -1.0898 | 0 | 12 | 12 | 5 | R.YIIGMLER.C |
|  | 5161 | **528.3102** | **1054.6058** | **1054.5342** | **0.0716** | **1** | **3** | **78** | **3** | **K.MTPTHKGQR.W** |
|  | 5520 | 540.7842 | 1079.5539 | 1079.4780 | 0.0760 | 0 | 6 | 37 | 5 | R.EMMLEHFK.N 5543 |
|  | 6046 | **559.3520** | **1116.6894** | **1117.6026** | **-0.9132** | **1** | **1** | **1.4e+002** | **6** | **R.MLTNRQLSR.H** |
|  | 6450 | **574.7847** | **1147.5549** | **1148.6302** | **-1.0753** | **1** | **4** | **57** | **8** | **K.ISPRHSEPVK.R** |
|  | 6619 | **579.3298** | **1156.6450** | **1156.5876** | **0.0573** | **1** | **2** | **96** | **3** | **R.HKEDLQPYK.Y** |
|  | 7195 | **597.2682** | **1192.5218** | **1191.6652** | **0.8566** | **0** | **1** | **1.2e+002** | **5** | **K.AGFLAFTQLPK.F** |
|  | 7430 | 401.8488 | 1202.5245 | 1202.6196 | -0.0951 | 1 | 10 | 16 | 8 | K.FNKVPEWQR.L |
|  | 8759 | 635.2524 | 1268.4902 | 1269.6540 | -1.1638 | 1 | 6 | 35 | 2 | K.DLCRVLYFGK.S 8807 |
|  | 8820 | **636.7953** | **1271.5760** | **1270.7860** | **0.7900** | **1** | **1** | **1.1e+002** | **6** | **R.KLAVASVTEILK.M** |
|  | 10016 | **666.3511** | **1330.6877** | **1330.6374** | **0.0504** | **1** | **1** | **1.1e+002** | **3** | **R.DVCVKMTPTHK.G** |
|  | 10327 | 450.5949 | 1348.7629 | 1347.7510 | 1.0119 | 1 | 11 | 12 | 5 | R.TELLTEALRFR.T |
|  | 10862 | **459.8888** | **1376.6445** | **1376.6718** | **-0.0273** | **1** | **6** | **35** | **7** | **K.NVKDLMDSNGIR.I** |
|  | 11523 | 708.3732 | 1414.7319 | 1413.7286 | 1.0033 | 0 | 25 | 0.45 | 2 | K.MQELALSEGALPR.H |
|  | 11648 | **474.8962** | **1421.6668** | **1421.7701** | **-0.1032** | **1** | **5** | **41** | **8** | **K.SLAGMLTPYVARK.L** |
|  | 11661 | **474.9746** | **1421.9018** | **1421.7701** | **0.1318** | **1** | **7** | **23** | **2** | **R.KSLAGMLTPYVAR.K** 11660 |
|  | 12997 | 750.8526 | 1499.6906 | 1500.8334 | -1.1427 | 1 | 7 | 27 | 2 | R.VEMALEALRNVIK.Y 12996 |
|  | 13649 | 513.7749 | 1538.3029 | 1538.7875 | -0.4846 | 0 | 3 | 45 | 4 | R.VIHALSENELCVR.A |
|  | 14510 | **796.8757** | **1591.7369** | **1590.8109** | **0.9260** | **1** | **2** | **73** | **4** | **K.ITEMPSIIKDLCR.V** 14503 |
|  | 14665 | 802.8784 | 1603.7423 | 1604.8569 | -1.1146 | 1 | 4 | 42 | 4 | K.VIQAMNHRNNAIPK.S |
|  | 14892 | **539.7582** | **1616.2529** | **1615.7651** | **0.4878** | **0** | **0** | **79** | **9** | **R.TITMETSDDLLFSK.E** |
|  | 15882 | 560.2999 | 1677.8780 | 1678.8092 | -0.9312 | 0 | 6 | 32 | 4 | R.AMASLETIGPLMNGMK.K |
|  | 16127 | **565.9707** | **1694.8903** | **1694.8041** | **0.0861** | **0** | **(3)** | **52** | **6** | **R.AMASLETIGPLMNGMK.K** |
|  | 16148 | 848.9149 | 1695.8152 | 1694.8041 | 1.0110 | 0 | (1) | 95 | 8 | R.AMASLETIGPLMNGMK.K |
|  | 16853 | **870.9670** | **1739.9194** | **1739.9305** | **-0.0111** | **0** | **46** | **0.0023** | **1** | **K.LLEGIGLENLDSPAATK.A** |
|  | 17701 | **600.2989** | **1797.8748** | **1798.9135** | **-1.0386** | **0** | **0** | **95** | **6** | **R.ATVPLQSNVIEAAPDMK.R** |
|  | 18073 | 911.9592 | 1821.9038 | 1822.8991 | -0.9953 | 1 | 5 | 33 | 4 | R.AMASLETIGPLMNGMKK.R 18074 |
|  | 18888 | **628.2706** | **1881.7899** | **1881.9625** | **-0.1726** | **1** | **4** | **38** | **2** | **K.EPLEFEQYLNLRFGK.Y** |
|  | 19297 | 639.3493 | 1915.0261 | 1915.9415 | -0.9154 | 0 | 2 | 65 | 3 | K.YSTDESITSLAEFVVQK.I |
|  | 19513 | 644.9609 | 1931.8610 | 1930.9719 | 0.8891 | 1 | 3 | 51 | 5 | R.ITLMKFLPSVFMDAMR.D |
|  | 19843 | **978.9974** | **1955.9802** | **1956.0350** | **-0.0548** | **1** | **18** | **1.3** | **1** | **K.EIATKMQELALSEGALPR.H** 19845 19861 |
|  | 19976 | **656.4045** | **1966.1916** | **1966.0418** | **0.1498** | **1** | **1** | **53** | **1** | **K.SAIRVIHALSENELCVR.A** |
|  | 21188 | 693.2362 | 2076.6868 | 2077.8687 | -1.1819 | 1 | 1 | 56 | 6 | K.GDCDKTYGSEFVYSDHAK.E |
|  | 21380 | 1047.9520 | 2093.8895 | 2093.9629 | -0.0734 | 1 | 8 | 12 | 3 | K.ERSGPYGFHEMQELWTK.G |
|  | 21797 | **1070.5590** | **2139.1034** | **2139.0055** | **0.0979** | **0** | **2** | **53** | **6** | **K.MLNSNTESPYLIWNNSTR.A** |
|  | 22301 | **1099.9832** | **2197.9517** | **2198.1695** | **-0.2177** | **1** | **1** | **60** | **4** | **R.EEIIKSAIDHAGNYIGISLR.I** |
|  | 22898 | **753.3926** | **2257.1561** | **2258.1048** | **-0.9487** | **1** | **3** | **39** | **1** | **R.HLHTAMFTISSDQRMLTNR.Q** |
|  | 23069 | **457.0238** | **2280.0825** | **2279.2736** | **0.8088** | **1** | **4** | **29** | **5** | **K.LLEGIGLENLDSPAATKAQIVK.A** |
|  | 27799 | 760.6081 | 3038.4033 | 3038.5066 | -0.1033 | 1 | 2 | 30 | 4 | K.MQELALSEGALPRHLHTAMFTISSDQR.M |
|  | 29394 | 1155.8984 | 3464.6735 | 3464.6744 | -0.0009 | 1 | 4 | 17 | 8 | K.GQDIFQRSILGHILPEAMVCYLENYEPEK.F |

| **694.** | P49913\|CAMP_HUMAN    **Mass:** 19517    **Score:** 46     **Queries matched:** 9   **emPAI:** 0.43 |
| --- | --- |
|  | Cathelicidin antimicrobial peptide OS=Homo sapiens GN=CAMP PE=1 SV=1 |

|  | **Query** | **Observed** | **Mr(expt)** | **Mr(calc)** | **Delta** | **Miss** | **Score** | **Expect** | **Rank** | **Peptide** |
| --- | --- | --- | --- | --- | --- | --- | --- | --- | --- | --- |
|  | 2840 | 443.7373 | 885.4600 | 885.4668 | -0.0068 | 0 | 14 | 7.4 | 1 | R.AIDGINQR.S |
|  | 3217 | **458.7573** | **915.5001** | **914.4821** | **1.0180** | **1** | **0** | **1.9e+002** | **3** | **R.NLVPRTES.-** |
|  | 3342 | **463.2193** | **924.4240** | **924.4301** | **-0.0060** | **0** | **47** | **0.0026** | **1** | **R.SSDANLYR.L** 3341 |
|  | 8410 | **417.7423** | **1250.2050** | **1249.5907** | **0.6143** | **0** | **11** | **7.6** | **4** | **R.CMGTVTLNQAR.G** |
|  | 11648 | 474.8962 | 1421.6668 | 1421.6868 | -0.0199 | 1 | 6 | 29 | 5 | K.RCMGTVTLNQAR.G 11667 |
|  | 14346 | 792.3489 | 1582.6832 | 1582.6933 | -0.0101 | 1 | 18 | 2 | 1 | R.TTQQSPEDCDFKK.D |
|  | 28608 | **806.1533** | **3220.5842** | **3220.9389** | **-0.3547** | **1** | **1** | **37** | **6** | **R.WSLVLLLLGLVMPLAIIAQVLSYKEAVLR.A** |

| **695.** | P17693\|HLAG_HUMAN    **Mass:** 38542    **Score:** 46     **Queries matched:** 6   **emPAI:** 0.10 |
| --- | --- |
|  | HLA class I histocompatibility antigen, alpha chain G OS=Homo sapiens GN=HLA-G PE=1 SV=1 |

|  | **Query** | **Observed** | **Mr(expt)** | **Mr(calc)** | **Delta** | **Miss** | **Score** | **Expect** | **Rank** | **Peptide** |
| --- | --- | --- | --- | --- | --- | --- | --- | --- | --- | --- |
|  | 1599 | **399.2450** | **796.4754** | **797.3780** | **-0.9026** | **0** | **6** | **35** | **6** | **K.AHAQTDR.M** 1600 |
|  | 11736 | 714.3626 | 1426.7105 | 1426.7205 | -0.0099 | 0 | 46 | 0.0031 | 1 | K.WAAVVVPSGEEQR.Y 11721 11735 11751 |

| **696.** | Q86VR2\|F134C_HUMAN    **Mass:** 51763    **Score:** 46     **Queries matched:** 5   **emPAI:** 0.07 |
| --- | --- |
|  | Protein FAM134C OS=Homo sapiens GN=FAM134C PE=1 SV=1 |

|  | **Query** | **Observed** | **Mr(expt)** | **Mr(calc)** | **Delta** | **Miss** | **Score** | **Expect** | **Rank** | **Peptide** |
| --- | --- | --- | --- | --- | --- | --- | --- | --- | --- | --- |
|  | 4831 | **516.6799** | **1031.3452** | **1031.4528** | **-0.1076** | **1** | **4** | **71** | **1** | **R.GYMMSKQR.E** |
|  | 12127 | **725.3861** | **1448.7577** | **1448.6792** | **0.0785** | **1** | **3** | **61** | **3** | **R.LDFSVRGYMMSK.Q** |
|  | 15507 | **828.4681** | **1654.9216** | **1654.9294** | **-0.0078** | **0** | **46** | **0.0026** | **1** | **R.ALVEVLGPYEPLLSR.V** 15503 |
|  | 27587 | **750.3790** | **2997.4868** | **2996.5730** | **0.9138** | **0** | **1** | **43** | **8** | **R.YVPGLLLSYLMLVTVMMWPLAVYHR.L** |

| **697.** | Q8N6Q3\|CD177_HUMAN    **Mass:** 47929    **Score:** 46     **Queries matched:** 5   **emPAI:** 0.08 |
| --- | --- |
|  | CD177 antigen OS=Homo sapiens GN=CD177 PE=1 SV=2 |

|  | **Query** | **Observed** | **Mr(expt)** | **Mr(calc)** | **Delta** | **Miss** | **Score** | **Expect** | **Rank** | **Peptide** |
| --- | --- | --- | --- | --- | --- | --- | --- | --- | --- | --- |
|  | 3292 | 921.1241 | 920.1168 | 919.4876 | 0.6292 | 0 | 1 | 1.1e+002 | 8 | R.GGGIFSNLR.V |
|  | 8109 | **619.3256** | **1236.6367** | **1235.5565** | **1.0802** | **0** | **4** | **56** | **8** | **K.GCSTVGAQNSQK.T** |
|  | 20232 | **996.4328** | **1990.8510** | **1990.8660** | **-0.0149** | **0** | **46** | **0.0021** | **1** | **R.QCPTCVQPLGTCSSGSPR.M** |
|  | 24538 | 829.1095 | 2484.3067 | 2485.0304 | -0.7237 | 0 | 0 | 63 | 5 | R.CPVCLSMEGCLEGTTEEICPK.G |
|  | 29417 | **869.3975** | **3473.5607** | **3474.5571** | **-0.9964** | **1** | **2** | **25** | **4** | **R.VQGCMPQPGCNLLNGTQEIGPVGMTENCNRK.D** |

| **698.** | P02763\|A1AG1_HUMAN    **Mass:** 23725    **Score:** 46     **Queries matched:** 3   **emPAI:** 0.35 |
| --- | --- |
|  | Alpha-1-acid glycoprotein 1 OS=Homo sapiens GN=ORM1 PE=1 SV=1 |

|  | **Query** | **Observed** | **Mr(expt)** | **Mr(calc)** | **Delta** | **Miss** | **Score** | **Expect** | **Rank** | **Peptide** |
| --- | --- | --- | --- | --- | --- | --- | --- | --- | --- | --- |
|  | 16874 | **871.9019** | **1741.7892** | **1741.7981** | **-0.0089** | **0** | **42** | **0.007** | **1** | **K.EQLGEFYEALDCLR.I** |
|  | 17038 | **585.3205** | **1752.9397** | **1751.9471** | **0.9926** | **0** | **24** | **0.45** | **1** | **R.YVGGQEHFAHLLILR.D** 17025 |

| **699.** | P11717\|MPRI_HUMAN    **Mass:** 281056   **Score:** 46     **Queries matched:** 31   **emPAI:** 0.01 |
| --- | --- |
|  | Cation-independent mannose-6-phosphate receptor OS=Homo sapiens GN=IGF2R PE=1 SV=2 |

|  | **Query** | **Observed** | **Mr(expt)** | **Mr(calc)** | **Delta** | **Miss** | **Score** | **Expect** | **Rank** | **Peptide** |
| --- | --- | --- | --- | --- | --- | --- | --- | --- | --- | --- |
|  | 1630 | **400.7360** | **799.4574** | **799.4552** | **0.0022** | **1** | **12** | **13** | **10** | **R.REGTIPK.L** |
|  | 1841 | 408.7010 | 815.3874 | 815.4865 | -0.0991 | 1 | 6 | 53 | 9 | R.ISVGKANK.R |
|  | 1983 | 414.2393 | 826.4640 | 826.4735 | -0.0095 | 1 | 8 | 22 | 10 | K.VVCPPKK.L 1982 |
|  | 2968 | **450.2446** | **898.4746** | **899.5188** | **-1.0443** | **1** | **12** | **8.7** | **3** | **R.VLQEGKAR.G** |
|  | 3683 | 475.2199 | 948.4253 | 948.5029 | -0.0776 | 0 | 13 | 8.5 | 5 | K.GTADAFIVR.F |
|  | 5096 | **525.2559** | **1048.4972** | **1048.4495** | **0.0476** | **0** | **8** | **28** | **3** | **R.VEGDNCEVK.D** |
|  | 6151 | **563.2279** | **1124.4413** | **1124.5145** | **-0.0733** | **1** | **2** | **95** | **3** | **K.CGNQRFSTR.I** |
|  | 6213 | **565.7770** | **1129.5395** | **1129.5476** | **-0.0080** | **0** | **46** | **0.0045** | **1** | **R.NAQSNALQER.E** 6232 |
|  | 6750 | **584.7966** | **1167.5786** | **1168.5910** | **-1.0124** | **1** | **11** | **13** | **3** | **R.LQSMKLDYR.H** 6746 6747 6749 6751 6753 6754 |
|  | 6844 | **588.2750** | **1174.5355** | **1173.6540** | **0.8815** | **1** | **7** | **30** | **8** | **R.GVSMGTPKLLR.T** |
|  | 7319 | **599.8210** | **1197.6275** | **1196.5972** | **1.0303** | **1** | **2** | **92** | **9** | **K.GASFGRLQSMK.L** |
|  | 7554 | **604.9549** | **1207.8952** | **1208.6223** | **-0.7271** | **0** | **1** | **71** | **5** | **R.VQSSIAFLCGK.T** |
|  | 7572 | **605.3365** | **1208.6585** | **1207.5391** | **1.1195** | **0** | **14** | **6** | **1** | **K.LSSDVCPTSDK.S** |
|  | 11880 | 718.3755 | 1434.7365 | 1434.6660 | 0.0705 | 1 | 7 | 29 | 6 | K.EKEDLLCGATDGK.K |
|  | 12725 | **743.4358** | **1484.8570** | **1484.7372** | **0.1198** | **0** | **2** | **80** | **4** | **K.FLHQDIDSGQGIR.N** |
|  | 12932 | **499.7605** | **1496.2597** | **1495.8147** | **0.4450** | **1** | **6** | **21** | **5** | **R.DGPQWRDGIIVLK.Y** |
|  | 13911 | **519.1347** | **1554.3823** | **1554.7024** | **-0.3202** | **0** | **0** | **72** | **8** | **K.EVPCYVFDEELR.K** |
|  | 14911 | **539.9860** | **1616.9361** | **1615.8365** | **1.0996** | **1** | **4** | **49** | **1** | **-.MGAAAGRSPHLGPAPAR.R** |
|  | 16783 | **579.3201** | **1734.9384** | **1734.8835** | **0.0548** | **1** | **4** | **45** | **5** | **R.QTTYTTRIHLVCSR.G** |
|  | 19094 | 950.9030 | 1899.7915 | 1899.8455 | -0.0540 | 1 | 2 | 54 | 5 | R.MSVINFECNKTAGNDGK.G |
|  | 23397 | 581.5372 | 2322.1196 | 2323.2933 | -1.1738 | 1 | 3 | 34 | 4 | K.IKTNITLVCKPGDLESAPVLR.T |
|  | 24056 | **805.3773** | **2413.1101** | **2412.0829** | **1.0272** | **1** | **17** | **1.6** | **1** | **R.HAEPEQNWEAVDGSQTETEKK.H** |
|  | 29961 | **743.3486** | **3711.7065** | **3712.8593** | **-1.1528** | **1** | **1** | **25** | **4** | **R.AACAVKPQEVQMVNGTITNPINGKSFSLGDIYFK.L** |

| **700.** | P61604\|CH10_HUMAN    **Mass:** 10925    **Score:** 46     **Queries matched:** 6   **emPAI:** 0.87 |
| --- | --- |
|  | 10 kDa heat shock protein, mitochondrial OS=Homo sapiens GN=HSPE1 PE=1 SV=2 |

|  | **Query** | **Observed** | **Mr(expt)** | **Mr(calc)** | **Delta** | **Miss** | **Score** | **Expect** | **Rank** | **Peptide** |
| --- | --- | --- | --- | --- | --- | --- | --- | --- | --- | --- |
|  | 1097 | 616.1310 | 615.1238 | 614.3752 | 0.7486 | 0 | 3 | 1.2e+002 | 1 | R.VLVER.S |
|  | 1582 | **398.6926** | **795.3706** | **795.3698** | **0.0009** | **0** | **8** | **26** | **8** | **-.MAGQAFR.K** |
|  | 5460 | **538.7996** | **1075.5847** | **1075.5914** | **-0.0067** | **0** | **14** | **7.4** | **1** | **K.VLLPEYGGTK.V** |
|  | 7324 | **599.8389** | **1197.6633** | **1197.6717** | **-0.0084** | **1** | **8** | **25** | **1** | **K.GKGGEIQPVSVK.V** |
|  | 13507 | **765.7634** | **1529.5122** | **1528.7926** | **0.7196** | **1** | **43** | **0.0049** | **1** | **K.VVLDDKDYFLFR.D** 13500 |

| **701.** | Q9UHG3\|PCYOX_HUMAN    **Mass:** 57003    **Score:** 45     **Queries matched:** 18   **emPAI:** 0.07 |
| --- | --- |
|  | Prenylcysteine oxidase 1 OS=Homo sapiens GN=PCYOX1 PE=1 SV=3 |

|  | **Query** | **Observed** | **Mr(expt)** | **Mr(calc)** | **Delta** | **Miss** | **Score** | **Expect** | **Rank** | **Peptide** |
| --- | --- | --- | --- | --- | --- | --- | --- | --- | --- | --- |
|  | 2806 | **443.0380** | **884.0614** | **884.4691** | **-0.4076** | **1** | **7** | **19** | **7** | **K.FMRIYR.Y** |
|  | 5317 | **533.7891** | **1065.5636** | **1065.5706** | **-0.0070** | **0** | **18** | **2.7** | **2** | **K.IFSQETLTK.A** 5307 |
|  | 6857 | 588.3216 | 1174.6286 | 1174.6380 | -0.0094 | 0 | 9 | 21 | 3 | K.LVCSGLLQASK.S |
|  | 7748 | 609.8021 | 1217.5896 | 1217.6332 | -0.0436 | 0 | 2 | 90 | 10 | K.LFLSYDYAVK.K |
|  | 11839 | 717.3354 | 1432.6562 | 1433.6319 | -0.9757 | 0 | 10 | 12 | 3 | R.MHMWVEDVLDK.F |
|  | 13995 | **780.8467** | **1559.6788** | **1559.6892** | **-0.0104** | **0** | **45** | **0.0034** | **1** | **R.YQSHDYAFSSVEK.L** |
|  | 14149 | **785.6016** | **1569.1887** | **1568.7756** | **0.4131** | **0** | **3** | **41** | **2** | **K.SNLISGSVMYIEEK.T** 14122 |
|  | 14923 | **540.2806** | **1617.8201** | **1618.9294** | **-1.1093** | **1** | **2** | **78** | **6** | **K.IFSQETLTKAQILK.L** |
|  | 16566 | **861.9524** | **1721.8902** | **1721.8988** | **-0.0086** | **0** | **10** | **12** | **1** | **R.SDFYDIVLVATPLNR.K** |
|  | 17717 | **600.5846** | **1798.7319** | **1797.9182** | **0.8137** | **1** | **4** | **37** | **5** | **K.SNLISGSVMYIEEKTK.T** |
|  | 18274 | **919.1107** | **1836.2068** | **1835.8521** | **0.3547** | **1** | **0** | **74** | **4** | **R.MHMWVEDVLDKFMR.I** |
|  | 23017 | **569.8289** | **2275.2863** | **2274.2372** | **1.0492** | **1** | **8** | **12** | **8** | **R.APPDKIAIIGAGIGGTSAAYYLR.Q** |
|  | 26262 | **914.4642** | **2740.3709** | **2741.3979** | **-1.0271** | **1** | **1** | **43** | **5** | **K.LVCSGLLQASKSNLISGSVMYIEEK.T** |
|  | 27818 | **1014.9570** | **3041.8493** | **3041.4885** | **0.3608** | **1** | **1** | **30** | **3** | **R.LATMMVQGQEYEAGGSVIHPLNLHMKR.F** |
|  | 29223 | **849.6598** | **3394.6101** | **3394.6260** | **-0.0159** | **0** | **18** | **0.7** | **1** | **R.LYYLNGIECAASAMEMSAIAAHNAALLAYHR.W** |
|  | 29480 | **1166.5200** | **3496.5382** | **3496.6901** | **-0.1519** | **1** | **1** | **27** | **5** | **R.EEVGGRLATMMVQGQEYEAGGSVIHPLNLHMK.R** |

| **702.** | P35241\|RADI_HUMAN    **Mass:** 68635    **Score:** 45     **Queries matched:** 17   **emPAI:** 0.05 |
| --- | --- |
|  | Radixin OS=Homo sapiens GN=RDX PE=1 SV=1 |

|  | **Query** | **Observed** | **Mr(expt)** | **Mr(calc)** | **Delta** | **Miss** | **Score** | **Expect** | **Rank** | **Peptide** |
| --- | --- | --- | --- | --- | --- | --- | --- | --- | --- | --- |
|  | 1410 | **746.3748** | **745.3675** | **744.4130** | **0.9545** | **1** | **6** | **61** | **2** | **K.REIAEK.E** |
|  | 1732 | 404.2046 | 806.3947 | 806.3592 | 0.0355 | 0 | 10 | 19 | 4 | K.QAADQMK.N |
|  | 2663 | 438.2500 | 874.4854 | 874.4548 | 0.0306 | 0 | 15 | 6.7 | 8 | K.ENPLQFK.F |
|  | 3908 | 483.2527 | 964.4909 | 964.4978 | -0.0068 | 1 | 12 | 11 | 3 | R.NISFNDKK.F |
|  | 3982 | 486.3357 | 970.6568 | 969.5430 | 1.1138 | 0 | 7 | 28 | 8 | -.MPKPINVR.V |
|  | 4465 | 502.2789 | 1002.5432 | 1002.5498 | -0.0066 | 1 | 25 | 0.61 | 1 | K.KENPLQFK.F |
|  | 9717 | 439.8649 | 1316.5729 | 1315.6806 | 0.8923 | 0 | 3 | 79 | 7 | R.KPDTIEVQQMK.A |
|  | 10557 | 454.5838 | 1360.7295 | 1359.7986 | 0.9308 | 1 | 3 | 74 | 8 | R.LLPQRVLEQHK.L |
|  | 10691 | 456.8821 | 1367.6246 | 1366.6841 | 0.9405 | 0 | 5 | 38 | 1 | K.TQNDVLHAENVK.A 10678 |
|  | 12487 | 736.8934 | 1471.7722 | 1471.7817 | -0.0095 | 1 | 45 | 0.0037 | 1 | R.RKPDTIEVQQMK.A 12492 |
|  | 13721 | **515.3255** | **1542.9547** | **1541.8525** | **1.1022** | **1** | **6** | **25** | **10** | **K.KQLQALSSELAQAR.D** 13727 |
|  | 14899 | **809.3208** | **1616.6270** | **1617.7200** | **-1.0930** | **1** | **(1)** | **90** | **4** | **R.GMLREDSMMEYLK.I** |
|  | 15172 | 545.6569 | 1633.9488 | 1633.7150 | 0.2338 | 1 | 1 | 87 | 10 | R.GMLREDSMMEYLK.I |
|  | 19378 | 962.4416 | 1922.8687 | 1921.9686 | 0.9001 | 1 | 4 | 37 | 2 | K.IGFPWSEIRNISFNDK.K |

| **703.** | Q9H299\|SH3L3_HUMAN    **Mass:** 10488    **Score:** 45     **Queries matched:** 6   **emPAI:** 0.92 |
| --- | --- |
|  | SH3 domain-binding glutamic acid-rich-like protein 3 OS=Homo sapiens GN=SH3BGRL3 PE=1 SV=1 |

|  | **Query** | **Observed** | **Mr(expt)** | **Mr(calc)** | **Delta** | **Miss** | **Score** | **Expect** | **Rank** | **Peptide** |
| --- | --- | --- | --- | --- | --- | --- | --- | --- | --- | --- |
|  | 1225 | 670.3827 | 669.3755 | 669.3810 | -0.0055 | 0 | 24 | 0.56 | 1 | R.ALAGNPK.A |
|  | 5186 | **529.2945** | **1056.5744** | **1055.5248** | **1.0497** | **0** | **46** | **0.0044** | **1** | **R.VYSTSVTGSR.E** 5179 5189 |
|  | 14882 | 808.8950 | 1615.7755 | 1615.7988 | -0.0233 | 1 | 2 | 77 | 9 | -.MSGLRVYSTSVTGSR.E |
|  | 23266 | **769.7138** | **2306.1194** | **2306.1325** | **-0.0130** | **1** | **23** | **0.42** | **1** | **R.IQYQLVDISQDNALRDEMR.A** |

| **704.** | P05026\|AT1B1_HUMAN    **Mass:** 35438    **Score:** 45     **Queries matched:** 10   **emPAI:** 0.22 |
| --- | --- |
|  | Sodium/potassium-transporting ATPase subunit beta-1 OS=Homo sapiens GN=ATP1B1 PE=1 SV=1 |

|  | **Query** | **Observed** | **Mr(expt)** | **Mr(calc)** | **Delta** | **Miss** | **Score** | **Expect** | **Rank** | **Peptide** |
| --- | --- | --- | --- | --- | --- | --- | --- | --- | --- | --- |
|  | 4085 | **489.7629** | **977.5112** | **976.5593** | **0.9519** | **1** | **6** | **42** | **6** | **R.FDVKIEVK.S** |
|  | 4325 | 498.2942 | 994.5739 | 995.5189 | -0.9450 | 1 | 6 | 39 | 6 | R.FQGRFDVK.I |
|  | 7979 | **615.7813** | **1229.5479** | **1229.5564** | **-0.0084** | **0** | **1** | **1.3e+002** | **3** | **K.AYGENIGYSEK.D** |
|  | 9422 | **435.2231** | **1302.6473** | **1302.6568** | **-0.0095** | **0** | **11** | **11** | **1** | **K.TEISFRPNDPK.S** |
|  | 9542 | **437.5535** | **1309.6387** | **1309.6224** | **0.0163** | **0** | **3** | **66** | **7** | **K.NESLETYPVMK.Y** |
|  | 12783 | **744.8889** | **1487.7633** | **1488.8664** | **-1.1031** | **0** | **30** | **0.12** | **1** | **R.VAPPGLTQIPQIQK.T** 12805 |
|  | 12791 | 745.3743 | 1488.7340 | 1488.7395 | -0.0055 | 0 | 7 | 25 | 1 | K.YNPNVLPVQCTGK.R |
|  | 15324 | 823.4228 | 1644.8310 | 1644.8406 | -0.0096 | 1 | 20 | 1 | 1 | K.YNPNVLPVQCTGKR.D |
|  | 26748 | **1415.1724** | **2828.3302** | **2828.3486** | **-0.0185** | **0** | **35** | **0.021** | **1** | **K.VGNVEYFGLGNSPGFPLQYYPYYGK.L** |

| **705.** | O75390\|CISY_HUMAN    **Mass:** 51908    **Score:** 45     **Queries matched:** 11   **emPAI:** 0.07 |
| --- | --- |
|  | Citrate synthase, mitochondrial OS=Homo sapiens GN=CS PE=1 SV=2 |

|  | **Query** | **Observed** | **Mr(expt)** | **Mr(calc)** | **Delta** | **Miss** | **Score** | **Expect** | **Rank** | **Peptide** |
| --- | --- | --- | --- | --- | --- | --- | --- | --- | --- | --- |
|  | 2536 | **432.8992** | **863.7838** | **864.4453** | **-0.6615** | **0** | **18** | **2** | **3** | **R.AYAQGISR.T** |
|  | 3229 | **459.0105** | **916.0064** | **916.5164** | **-0.5100** | **0** | **8** | **22** | **4** | **-.MALLTAAAR.L** |
|  | 10723 | **685.4193** | **1368.8241** | **1367.6656** | **1.1585** | **1** | **1** | **1e+002** | **3** | **R.FRGFSIPECQK.L** |
|  | 12064 | **723.8709** | **1445.7271** | **1444.8435** | **0.8836** | **1** | **1** | **1.2e+002** | **3** | **-.MALLTAAARLLGTK.N** |
|  | 14412 | **529.7041** | **1586.0905** | **1585.8974** | **0.1931** | **1** | **8** | **16** | **1** | **R.LLGTKNASCLVLAAR.H** |
|  | 14737 | 536.6329 | 1606.8768 | 1606.8216 | 0.0552 | 1 | 6 | 32 | 9 | K.LRDYIWNTLNSGR.V |
|  | 17148 | **881.9428** | **1761.8711** | **1761.8785** | **-0.0074** | **0** | **45** | **0.0037** | **1** | **K.GLVYETSVLDPDEGIR.F** |
|  | 21477 | **702.3420** | **2104.0043** | **2104.9778** | **-0.9735** | **1** | **(1)** | **63** | **5** | **K.TVVGQITVDMMYGGMRGMK.G** |
|  | 21500 | **703.0120** | **2106.0142** | **2104.9778** | **1.0364** | **1** | **5** | **30** | **2** | **K.TVVGQITVDMMYGGMRGMK.G** |
|  | 21788 | **535.5022** | **2137.9797** | **2136.9676** | **1.0121** | **1** | **(2)** | **50** | **3** | **K.TVVGQITVDMMYGGMRGMK.G** |
|  | 23861 | **596.7921** | **2383.1391** | **2383.1083** | **0.0308** | **1** | **3** | **44** | **7** | **R.QQHGKTVVGQITVDMMYGGMR.G** |

| **706.** | Q9BV40\|VAMP8_HUMAN    **Mass:** 11488    **Score:** 45     **Queries matched:** 9   **emPAI:** 0.35 |
| --- | --- |
|  | Vesicle-associated membrane protein 8 OS=Homo sapiens GN=VAMP8 PE=1 SV=1 |

|  | **Query** | **Observed** | **Mr(expt)** | **Mr(calc)** | **Delta** | **Miss** | **Score** | **Expect** | **Rank** | **Peptide** |
| --- | --- | --- | --- | --- | --- | --- | --- | --- | --- | --- |
|  | 5833 | **551.7863** | **1101.5579** | **1101.5666** | **-0.0086** | **0** | **23** | **0.94** | **1** | **R.NLQSEVEGVK.N** 5834 |
|  | 5873 | **553.0173** | **1104.0201** | **1103.5393** | **0.4808** | **0** | **3** | **59** | **9** | **K.NIMTQNVER.I** |
|  | 8419 | **626.3575** | **1250.7004** | **1250.4833** | **0.2171** | **0** | **6** | **35** | **8** | **-.MEEASEGGGNDR.V** |
|  | 8710 | **422.8981** | **1265.6725** | **1266.4782** | **-0.8058** | **0** | **(3)** | **71** | **7** | **-.MEEASEGGGNDR.V** 8739 8740 |
|  | 15374 | **550.2618** | **1647.7635** | **1647.7740** | **-0.0105** | **1** | **45** | **0.004** | **1** | **R.NKTEDLEATSEHFK.T** |
|  | 22225 | 547.7667 | 2187.0378 | 2187.0954 | -0.0576 | 1 | 2 | 58 | 10 | R.NLQSEVEGVKNIMTQNVER.I |

| **707.** | P51153\|RAB13_HUMAN    **Mass:** 22988    **Score:** 45     **Queries matched:** 13   **emPAI:** 0.17 |
| --- | --- |
|  | Ras-related protein Rab-13 OS=Homo sapiens GN=RAB13 PE=1 SV=1 |

|  | **Query** | **Observed** | **Mr(expt)** | **Mr(calc)** | **Delta** | **Miss** | **Score** | **Expect** | **Rank** | **Peptide** |
| --- | --- | --- | --- | --- | --- | --- | --- | --- | --- | --- |
|  | 2003 | 415.2057 | 828.3968 | 828.4018 | -0.0050 | 0 | 11 | 17 | 1 | R.FFETSAK.S 2001 2004 |
|  | 5394 | 536.3203 | 1070.6261 | 1070.6336 | -0.0075 | 0 | 45 | 0.006 | 1 | K.LLLIGDSGVGK.T 5392 |
|  | 8621 | 631.2831 | 1260.5516 | 1259.6469 | 0.9047 | 1 | 25 | 0.44 | 1 | K.SIKENASAGVER.L 8597 |
|  | 9374 | 651.3024 | 1300.5902 | 1301.6364 | -1.0462 | 0 | 1 | 98 | 7 | K.LQVWDTAGQER.F |
|  | 13494 | **510.5812** | **1528.7217** | **1528.6828** | **0.0390** | **0** | **0** | **1.1e+002** | **5** | **K.SSMNVDEAFSSLAR.D** |
|  | 15219 | 819.4651 | 1636.9156 | 1636.8382 | 0.0774 | 0 | (6) | 26 | 3 | R.GAMGIILVYDITDEK.S |
|  | 15483 | 827.9073 | 1653.8001 | 1652.8331 | 0.9670 | 0 | 8 | 17 | 3 | R.GAMGIILVYDITDEK.S |
|  | 19718 | 974.0065 | 1945.9984 | 1945.0673 | 0.9311 | 1 | 1 | 71 | 5 | K.AYDHLFKLLLIGDSGVGK.T |
|  | 23537 | **780.9259** | **2339.7559** | **2339.0740** | **0.6819** | **1** | **1** | **45** | **5** | **R.FFETSAKSSMNVDEAFSSLAR.D** |

| **708.** | P98160\|PGBM_HUMAN    **Mass:** 479221   **Score:** 45     **Queries matched:** 37   **emPAI:** 0.01 |
| --- | --- |
|  | Basement membrane-specific heparan sulfate proteoglycan core protein OS=Homo sapiens GN=HSPG2 PE=1 SV=3 |

|  | **Query** | **Observed** | **Mr(expt)** | **Mr(calc)** | **Delta** | **Miss** | **Score** | **Expect** | **Rank** | **Peptide** |
| --- | --- | --- | --- | --- | --- | --- | --- | --- | --- | --- |
|  | 935 | **547.3167** | **546.3094** | **546.2762** | **0.0333** | **0** | **11** | **23** | **1** | **K.DGSLR.V** |
|  | 966 | **559.3525** | **558.3452** | **558.3489** | **-0.0037** | **0** | **5** | **93** | **7** | **R.VTALR.E** |
|  | 1908 | **411.2208** | **820.4270** | **819.4100** | **1.0170** | **0** | **8** | **28** | **2** | **R.HQVHGSR.L** |
|  | 2103 | 418.2001 | 834.3857 | 833.4508 | 0.9349 | 1 | 12 | 12 | 3 | R.FRDQIR.L |
|  | 4113 | **490.7344** | **979.4543** | **978.5974** | **0.8568** | **0** | **16** | **3.7** | **3** | **R.LLAVTHGLR.A** |
|  | 4575 | **506.3045** | **1010.5944** | **1010.5145** | **0.0799** | **0** | **4** | **65** | **4** | **K.EGGSLPPQAR.S** |
|  | 4602 | **507.7470** | **1013.4795** | **1013.5658** | **-0.0863** | **0** | **13** | **8.5** | **3** | **R.LGTVPQFPR.A** |
|  | 4612 | **507.7883** | **1013.5621** | **1013.6233** | **-0.0612** | **1** | **6** | **41** | **10** | **R.GTLIIRDVK.E** |
|  | 5164 | **528.3723** | **1054.7299** | **1054.5454** | **0.1845** | **1** | **0** | **1.2e+002** | **8** | **R.CHLGRSGLR.C** |
|  | 5296 | **532.8153** | **1063.6161** | **1062.5319** | **1.0842** | **0** | **8** | **26** | **3** | **R.HQTHGSQLR.L** |
|  | 5969 | **556.7842** | **1111.5538** | **1110.5530** | **1.0008** | **0** | **6** | **36** | **2** | **R.AHSSAGQQVAR.A** |
|  | 6581 | 578.3303 | 1154.6461 | 1155.6261 | -0.9801 | 0 | 8 | 23 | 8 | R.AVLHVHGGGGPR.V 6590 |
|  | 6892 | **589.3516** | **1176.6886** | **1176.6503** | **0.0383** | **0** | **4** | **60** | **7** | **R.YELGSGLAVLR.S** 6875 6901 |
|  | 7230 | **597.8964** | **1193.7783** | **1192.6241** | **1.1542** | **0** | **0** | **1.2e+002** | **6** | **K.SPAYTLVWTR.L** |
|  | 7536 | 403.2272 | 1206.6599 | 1206.5629 | 0.0970 | 0 | 10 | 16 | 1 | R.ASYAQQPAESR.V |
|  | 9035 | **641.9224** | **1281.8303** | **1280.6949** | **1.1354** | **1** | **0** | **1e+002** | **10** | **K.RVPGSPTNLANR.Q** |
|  | 11081 | **463.7523** | **1388.2352** | **1387.8663** | **0.3689** | **0** | **6** | **24** | **2** | **R.AAGALLLALLLHGR.L** |
|  | 12540 | **492.6276** | **1474.8609** | **1474.6987** | **0.1622** | **0** | **4** | **51** | **3** | **K.RPEEVCGPTQFR.C** 12539 12547 |
|  | 13288 | **506.4505** | **1516.3296** | **1515.5719** | **0.7577** | **0** | **3** | **48** | **4** | **R.GPSCQDCDTGYTR.T** |
|  | 13383 | **762.3561** | **1522.6976** | **1521.7980** | **0.8996** | **0** | **3** | **57** | **2** | **R.LLSGPYFWSLPSR.F** |
|  | 13676 | 771.3332 | 1540.6518 | 1540.7886 | -0.1367 | 1 | 2 | 81 | 4 | R.FLGDKVTSYGGELR.F |
|  | 15702 | 834.4040 | 1666.7934 | 1665.7747 | 1.0187 | 0 | 2 | 67 | 7 | R.LVSEDPINDGEWHR.V |
|  | 15785 | **558.2841** | **1671.8304** | **1671.6399** | **0.1904** | **1** | **6** | **31** | **2** | **R.CDERGSMGTSGEACR.C** |
|  | 16062 | **845.9227** | **1689.8309** | **1689.8686** | **-0.0377** | **0** | **5** | **32** | **10** | **K.GSVYIGGAPDVATLTGGR.F** |
|  | 17896 | 604.3182 | 1809.9329 | 1809.8884 | 0.0445 | 0 | 0 | 94 | 8 | R.EVSEAVVDTLESEYLK.I |
|  | 18165 | 610.6216 | 1828.8429 | 1828.9530 | -0.1101 | 1 | 8 | 18 | 4 | R.IEPSSSRVAEGQTLDLK.C |
|  | 19778 | 651.6563 | 1951.9469 | 1951.0500 | 0.8969 | 1 | 5 | 30 | 3 | R.AVLHVHGGGGPRVQVSPER.T |
|  | 19874 | 979.9879 | 1957.9613 | 1958.0433 | -0.0820 | 0 | 10 | 10 | 4 | R.VVGSSGTQEASVLVTIQQR.L 19872 |
|  | 22284 | **732.4199** | **2194.2378** | **2195.0416** | **-0.8038** | **0** | **1** | **61** | **1** | **R.AYDGLSLPEDIETVTASQMR.W** |
|  | 24467 | 825.1325 | 2472.3757 | 2472.3839 | -0.0082 | 0 | 45 | 0.0022 | 1 | R.AELLQVLQSLEAVLIQTVYNTK.M |
|  | 28977 | **1109.8214** | **3326.4424** | **3325.5238** | **0.9186** | **1** | **1** | **35** | **3** | **K.MASVGLSDIAMDTTVTHATSHGRAHSVEECR.C** |

| **709.** | Q07020\|RL18_HUMAN    **Mass:** 21735    **Score:** 44     **Queries matched:** 7   **emPAI:** 0.18 |
| --- | --- |
|  | 60S ribosomal protein L18 OS=Homo sapiens GN=RPL18 PE=1 SV=2 |

|  | **Query** | **Observed** | **Mr(expt)** | **Mr(calc)** | **Delta** | **Miss** | **Score** | **Expect** | **Rank** | **Peptide** |
| --- | --- | --- | --- | --- | --- | --- | --- | --- | --- | --- |
|  | 1301 | **699.3976** | **698.3903** | **698.3963** | **-0.0060** | **0** | **15** | **4.5** | **3** | **R.VQEVPK.L** |
|  | 3560 | **471.3299** | **940.6452** | **939.5753** | **1.0699** | **1** | **5** | **43** | **2** | **R.VQEVPKLK.V** |
|  | 6020 | **558.7916** | **1115.5687** | **1115.5757** | **-0.0070** | **0** | **44** | **0.0059** | **1** | **K.GCGTVLLSGPR.K** |
|  | 6338 | **570.8238** | **1139.6330** | **1139.6411** | **-0.0081** | **0** | **21** | **1.3** | **1** | **R.TNRPPLSLSR.M** 6335 |
|  | 10263 | **673.3652** | **1344.7158** | **1344.7249** | **-0.0091** | **0** | **17** | **2.8** | **1** | **K.TAVVVGTITDDVR.V** |
|  | 11383 | **704.3102** | **1406.6059** | **1405.7678** | **0.8382** | **1** | **24** | **0.54** | **1** | **R.TNSTFNQVVLKR.L** |

| **710.** | P08590\|MYL3_HUMAN    **Mass:** 22089    **Score:** 44     **Queries matched:** 12   **emPAI:** 0.17 |
| --- | --- |
|  | Myosin light chain 3 OS=Homo sapiens GN=MYL3 PE=1 SV=3 |

|  | **Query** | **Observed** | **Mr(expt)** | **Mr(calc)** | **Delta** | **Miss** | **Score** | **Expect** | **Rank** | **Peptide** |
| --- | --- | --- | --- | --- | --- | --- | --- | --- | --- | --- |
|  | 3864 | 481.7262 | 961.4379 | 961.4604 | -0.0225 | 0 | 17 | 3.5 | 2 | R.LTEDEVEK.L |
|  | 4325 | 498.2942 | 994.5739 | 994.5560 | 0.0179 | 0 | 19 | 2 | 2 | R.HVLATLGER.L 4309 4326 |
|  | 16564 | 861.9216 | 1721.8286 | 1721.8407 | -0.0121 | 1 | 44 | 0.004 | 1 | R.VFDKEGNGTVMGAELR.H 16575 16576 16577 16578 16579 |
|  | 16812 | 869.8542 | 1737.6938 | 1737.8356 | -0.1418 | 1 | (1) | 74 | 5 | R.VFDKEGNGTVMGAELR.H |
|  | 18414 | **616.1291** | **1845.3654** | **1844.8219** | **0.5435** | **1** | **1** | **57** | **10** | **K.CEMKITYGQCGDVLR.A** |

| **711.** | P05976\|MLE1_HUMAN    **Mass:** 21189    **Score:** 44     **Queries matched:** 9   **emPAI:** 0.18 |
| --- | --- |
|  | Myosin light chain 1, skeletal muscle isoform OS=Homo sapiens GN=MYL1 PE=1 SV=3 |

|  | **Query** | **Observed** | **Mr(expt)** | **Mr(calc)** | **Delta** | **Miss** | **Score** | **Expect** | **Rank** | **Peptide** |
| --- | --- | --- | --- | --- | --- | --- | --- | --- | --- | --- |
|  | 16564 | 861.9216 | 1721.8286 | 1721.8407 | -0.0121 | 1 | 44 | 0.004 | 1 | R.VFDKEGNGTVMGAELR.H 16575 16576 16577 16578 16579 |
|  | 16812 | 869.8542 | 1737.6938 | 1737.8356 | -0.1418 | 1 | (1) | 74 | 5 | R.VFDKEGNGTVMGAELR.H |
|  | 25607 | **529.2601** | **2641.2643** | **2640.5115** | **0.7528** | **1** | **6** | **17** | **1** | **K.DVKKPVAAAAAAPAPAPAPAPAPAPAKPK.E** |
|  | 26756 | **708.8174** | **2831.2407** | **2831.2265** | **0.0141** | **0** | **3** | **29** | **7** | **K.EEEVEALMAGQEDSNGCINYEAFVK.H** |

|  | **Proteins matching a subset of these peptides:** |
| --- | --- |

|  | P06741\|MLE3_HUMAN    **Score:** 44     **Queries matched:** 8 |
| --- | --- |

| **712.** | Q9Y3D6\|FIS1_HUMAN    **Mass:** 16984    **Score:** 44     **Queries matched:** 5   **emPAI:** 0.23 |
| --- | --- |
|  | Mitochondrial fission 1 protein OS=Homo sapiens GN=FIS1 PE=1 SV=2 |

|  | **Query** | **Observed** | **Mr(expt)** | **Mr(calc)** | **Delta** | **Miss** | **Score** | **Expect** | **Rank** | **Peptide** |
| --- | --- | --- | --- | --- | --- | --- | --- | --- | --- | --- |
|  | 10356 | **451.2493** | **1350.7262** | **1350.8486** | **-0.1224** | **1** | **5** | **44** | **2** | **R.KGIVLLEELLPK.G** |
|  | 11955 | **720.8709** | **1439.7271** | **1439.7368** | **-0.0097** | **0** | **44** | **0.005** | **1** | **R.GLLQTEPQNNQAK.E** |
|  | 18588 | **465.2643** | **1857.0281** | **1857.9697** | **-0.9415** | **1** | **4** | **39** | **10** | **K.YVRGLLQTEPQNNQAK.E** |
|  | 25533 | **876.4543** | **2626.3410** | **2625.4598** | **0.8812** | **0** | **8** | **9.1** | **1** | **K.DGLVGMAIVGGMALGVAGLAGLIGLAVSK.S** |
|  | 26719 | 942.4836 | 2824.4289 | 2824.5918 | -0.1629 | 1 | 0 | 53 | 3 | K.DGLVGMAIVGGMALGVAGLAGLIGLAVSKSK.S |

| **713.** | Q969P0\|IGSF8_HUMAN    **Mass:** 65621    **Score:** 44     **Queries matched:** 8   **emPAI:** 0.06 |
| --- | --- |
|  | Immunoglobulin superfamily member 8 OS=Homo sapiens GN=IGSF8 PE=1 SV=1 |

|  | **Query** | **Observed** | **Mr(expt)** | **Mr(calc)** | **Delta** | **Miss** | **Score** | **Expect** | **Rank** | **Peptide** |
| --- | --- | --- | --- | --- | --- | --- | --- | --- | --- | --- |
|  | 2350 | **426.7662** | **851.5178** | **852.4454** | **-0.9276** | **0** | **6** | **34** | **8** | **R.QAPTSPPR.M** |
|  | 9930 | **443.0390** | **1326.0953** | **1326.7368** | **-0.6415** | **1** | **4** | **34** | **5** | **K.SRVVAGEVQVQR.L** 9926 9967 |
|  | 11608 | **474.2490** | **1419.7252** | **1418.7188** | **1.0065** | **0** | **9** | **17** | **4** | **R.GETASLLCNISVR.G** |
|  | 17717 | 600.5846 | 1798.7319 | 1799.8658 | -1.1339 | 0 | 4 | 38 | 6 | R.MTVHEGQELALGCLAR.T |
|  | 20526 | **505.2599** | **2017.0103** | **2016.0654** | **0.9450** | **1** | **2** | **58** | **9** | **K.HTHLAVSFGRSVPEAPVGR.S** |
|  | 31339 | **1284.6621** | **5134.6193** | **5134.6476** | **-0.0283** | **0** | **44** | **0.0005** | **1** | **R.LAASWWVERPEDGELSSVPAQLVGGVGQDGVAELGVRPGGGPVSVELVGPR.S** |

| **714.** | Q8N0U8\|VKORL_HUMAN    **Mass:** 20108    **Score:** 44     **Queries matched:** 2   **emPAI:** 0.19 |
| --- | --- |
|  | Vitamin K epoxide reductase complex subunit 1-like protein 1 OS=Homo sapiens GN=VKORC1L1 PE=1 SV=2 |

|  | **Query** | **Observed** | **Mr(expt)** | **Mr(calc)** | **Delta** | **Miss** | **Score** | **Expect** | **Rank** | **Peptide** |
| --- | --- | --- | --- | --- | --- | --- | --- | --- | --- | --- |
|  | 6637 | **580.3008** | **1158.5870** | **1157.5903** | **0.9967** | **0** | **44** | **0.0067** | **1** | **R.ALCDLGPWVK.C** |
|  | 9221 | 431.5669 | 1291.6790 | 1290.7084 | 0.9705 | 1 | 5 | 45 | 5 | K.RLVYLNEAWK.R |

| **715.** | Q9H115\|SNAB_HUMAN    **Mass:** 33878    **Score:** 44     **Queries matched:** 8   **emPAI:** 0.11 |
| --- | --- |
|  | Beta-soluble NSF attachment protein OS=Homo sapiens GN=NAPB PE=1 SV=2 |

|  | **Query** | **Observed** | **Mr(expt)** | **Mr(calc)** | **Delta** | **Miss** | **Score** | **Expect** | **Rank** | **Peptide** |
| --- | --- | --- | --- | --- | --- | --- | --- | --- | --- | --- |
|  | 1899 | **410.7395** | **819.4645** | **820.4192** | **-0.9547** | **0** | **5** | **57** | **4** | **R.GLFGGNTR.I** |
|  | 8067 | **412.2281** | **1233.6624** | **1233.5911** | **0.0714** | **0** | **3** | **68** | **9** | **R.EAVQLMAEAEK.R** |
|  | 11096 | **463.9505** | **1388.8297** | **1389.6922** | **-0.8625** | **1** | **3** | **67** | **6** | **R.EAVQLMAEAEKR.V** |
|  | 12693 | 495.2550 | 1482.7431 | 1481.7514 | 0.9917 | 0 | 5 | 36 | 2 | K.VAAYAAQLEQYQK.A |
|  | 12838 | 497.9166 | 1490.7280 | 1491.6340 | -0.9060 | 0 | 5 | 38 | 4 | K.YEEMFPAFTDSR.E |
|  | 14634 | 801.4093 | 1600.8040 | 1599.8079 | 0.9962 | 0 | 5 | 38 | 2 | K.AALCHFIVDELNAK.L |
|  | 21952 | **719.4397** | **2155.2973** | **2154.1507** | **1.1466** | **1** | **3** | **35** | **5** | **K.AALCHFIVDELNAKLALEK.Y** |
|  | 25175 | 854.7119 | 2561.1139 | 2561.1306 | -0.0167 | 1 | 44 | 0.0026 | 1 | K.AIAHYEQSADYYKGEESNSSANK.C |

| **716.** | P62701\|RS4X_HUMAN    **Mass:** 29807    **Score:** 44     **Queries matched:** 15   **emPAI:** 0.27 |
| --- | --- |
|  | 40S ribosomal protein S4, X isoform OS=Homo sapiens GN=RPS4X PE=1 SV=2 |

|  | **Query** | **Observed** | **Mr(expt)** | **Mr(calc)** | **Delta** | **Miss** | **Score** | **Expect** | **Rank** | **Peptide** |
| --- | --- | --- | --- | --- | --- | --- | --- | --- | --- | --- |
|  | 1543 | **397.1854** | **792.3562** | **791.4905** | **0.8657** | **1** | **10** | **20** | **5** | **R.KIFVGTK.G** |
|  | 2053 | **416.2592** | **830.5038** | **830.4498** | **0.0541** | **0** | **7** | **47** | **2** | **R.LTIAEER.D** |
|  | 3898 | **482.9862** | **963.9577** | **964.5342** | **-0.5764** | **1** | **4** | **47** | **10** | **R.LIYDTKGR.F** |
|  | 4249 | 495.7991 | 989.5836 | 989.5910 | -0.0074 | 0 | 22 | 1.2 | 1 | R.LSNIFVIGK.G |
|  | 4326 | 498.2944 | 994.5742 | 994.4971 | 0.0771 | 0 | 9 | 20 | 4 | K.YALTGDEVK.K 4325 |
|  | 5432 | 537.8115 | 1073.6085 | 1073.5717 | 0.0368 | 1 | 4 | 76 | 3 | R.LTIAEERDK.R |
|  | 17437 | **891.9108** | **1781.8071** | **1781.8189** | **-0.0118** | **0** | **37** | **0.02** | **1** | **K.FDTGNLCMVTGGANLGR.I** |
|  | 17697 | **450.2489** | **1796.9665** | **1797.8138** | **-0.8473** | **0** | **(4)** | **39** | **5** | **K.FDTGNLCMVTGGANLGR.I** |
|  | 18928 | **943.4603** | **1884.9060** | **1884.9179** | **-0.0119** | **0** | **25** | **0.33** | **1** | **R.TDITYPAGFMDVISIDK.T** 18926 |
|  | 19108 | **634.3466** | **1900.0179** | **1900.9128** | **-0.8950** | **0** | **(2)** | **66** | **5** | **R.TDITYPAGFMDVISIDK.T** |
|  | 21811 | **714.6825** | **2141.0257** | **2140.0874** | **0.9382** | **1** | **4** | **32** | **4** | **K.VRTDITYPAGFMDVISIDK.T** |
|  | 22863 | 752.4097 | 2254.2074 | 2254.0880 | 0.1194 | 1 | 1 | 59 | 10 | R.HPGSFDVVHVKDANGNSFATR.L |
|  | 23002 | **758.3785** | **2272.1138** | **2271.1998** | **0.9140** | **1** | **2** | **48** | **8** | **R.YPDPLIKVNDTIQIDLETGK.I** |

| **717.** | P46940\|IQGA1_HUMAN    **Mass:** 189761   **Score:** 44     **Queries matched:** 51   **emPAI:** 0.06 |
| --- | --- |
|  | Ras GTPase-activating-like protein IQGAP1 OS=Homo sapiens GN=IQGAP1 PE=1 SV=1 |

|  | **Query** | **Observed** | **Mr(expt)** | **Mr(calc)** | **Delta** | **Miss** | **Score** | **Expect** | **Rank** | **Peptide** |
| --- | --- | --- | --- | --- | --- | --- | --- | --- | --- | --- |
|  | 961 | **558.3563** | **557.3490** | **558.3377** | **-0.9886** | **0** | **4** | **95** | **4** | **K.AELVK.L** |
|  | 1108 | **624.2340** | **623.2267** | **622.3438** | **0.8829** | **1** | **5** | **40** | **1** | **R.GYKQK.K** |
|  | 1305 | **702.3479** | **701.3406** | **701.4072** | **-0.0665** | **0** | **15** | **8.9** | **1** | **R.NLGSIAK.M** |
|  | 1653 | **401.2843** | **800.5540** | **800.4253** | **0.1288** | **1** | **11** | **18** | **9** | **R.DIRNQR.R** |
|  | 2056 | **416.2951** | **830.5756** | **829.4657** | **1.1098** | **0** | **7** | **48** | **8** | **K.ELATLQR.Q** 2045 |
|  | 3122 | **455.7228** | **909.4309** | **910.4695** | **-1.0385** | **1** | **4** | **54** | **4** | **R.SRMNFLK.K** |
|  | 3368 | 464.2474 | 926.4803 | 926.4419 | 0.0384 | 0 | 11 | 12 | 9 | R.YLDELMK.L 3366 |
|  | 3724 | 952.3312 | 951.3240 | 950.4491 | 0.8749 | 1 | 0 | 1.3e+002 | 9 | K.QDKMTNAK.N |
|  | 4721 | **512.7549** | **1023.4953** | **1024.6029** | **-1.1076** | **0** | **8** | **22** | **2** | **R.ALQSPALGLR.G** 4751 |
|  | 5400 | **536.7813** | **1071.5481** | **1071.5812** | **-0.0331** | **0** | **3** | **1.1e+002** | **10** | **K.LTELGTVDPK.N** |
|  | 6269 | **567.7564** | **1133.4983** | **1133.5903** | **-0.0920** | **0** | **2** | **1.1e+002** | **6** | **K.LIFQMPQNK.S** |
|  | 6780 | **585.3049** | **1168.5952** | **1167.6209** | **0.9743** | **1** | **2** | **90** | **1** | **R.YLDELMKLK.A** |
|  | 7370 | **600.8284** | **1199.6423** | **1199.6761** | **-0.0338** | **1** | **9** | **18** | **2** | **K.KLTELGTVDPK.N** 7403 |
|  | 9462 | **653.2922** | **1304.5699** | **1304.6646** | **-0.0946** | **1** | **3** | **74** | **7** | **K.EIMDDKSLNIK.T** |
|  | 9624 | 438.8903 | 1313.6491 | 1313.7191 | -0.0699 | 1 | 6 | 33 | 4 | K.LTELGTVDPKNK.Y |
|  | 9750 | **440.5440** | **1318.6100** | **1317.6565** | **0.9535** | **0** | **12** | **9.2** | **2** | **R.ALESGDVNTVWK.Q** 9733 |
|  | 9936 | 443.0392 | 1326.0958 | 1325.6986 | 0.3972 | 1 | 2 | 60 | 5 | K.IQSLARMHQAR.K |
|  | 10007 | **665.8436** | **1329.6726** | **1329.7041** | **-0.0315** | **1** | **2** | **1e+002** | **2** | **K.LAVGDNNSKWVK.H** |
|  | 10771 | 458.2316 | 1371.6731 | 1370.5880 | 1.0851 | 0 | 6 | 34 | 8 | K.EQLSDMMMINK.Q |
|  | 11496 | **472.2450** | **1413.7133** | **1414.7820** | **-1.0687** | **0** | **2** | **82** | **10** | **K.LGLAPQIQDLYGK.V** |
|  | 12603 | 739.8516 | 1477.6887 | 1478.7154 | -1.0267 | 0 | 6 | 30 | 5 | R.GLQQQNSDWYLK.Q |
|  | 13467 | 510.2563 | 1527.7471 | 1526.9548 | 0.7923 | 1 | 1 | 1.1e+002 | 9 | K.VNVNLLIFLLNKK.F |
|  | 14834 | 807.8834 | 1613.7523 | 1613.8698 | -0.1175 | 1 | 6 | 29 | 4 | R.QILAPVVKEIMDDK.S |
|  | 15909 | 560.9501 | 1679.8284 | 1680.7413 | -0.9130 | 0 | 0 | 1.1e+002 | 5 | K.SWVNQMESQTGEASK.L |
|  | 16175 | **566.7563** | **1697.2470** | **1697.8882** | **-0.6412** | **1** | **3** | **41** | **3** | **R.NLGSIAKMLQHAASNK.M** |
|  | 16938 | **872.9731** | **1743.9316** | **1743.9407** | **-0.0091** | **0** | **37** | **0.02** | **1** | **K.GVLLEIEDLQVNQFK.N** |
|  | 17289 | **887.9293** | **1773.8441** | **1773.8567** | **-0.0126** | **0** | **32** | **0.065** | **1** | **R.SNQQLENDLNLMDIK.I** |
|  | 20938 | **685.6964** | **2054.0672** | **2054.0796** | **-0.0124** | **0** | **18** | **1.5** | **1** | **K.LEGVLAEVAQHYQDTLIR.A** 20947 |
|  | 21084 | 689.3234 | 2064.9483 | 2063.9508 | 0.9975 | 0 | 2 | 51 | 3 | K.EELQSGVDAANSAAQQYQR.R |
|  | 23530 | **780.5502** | **2338.6289** | **2338.2209** | **0.4080** | **1** | **7** | **11** | **1** | **K.FPDAGEDELLKIIGNLLYYR.Y** |
|  | 23736 | **592.2640** | **2365.0271** | **2364.1267** | **0.9003** | **1** | **1** | **61** | **10** | **K.FDVPGDENAEMDARTILLNTK.R** |
|  | 23958 | **480.8451** | **2399.1889** | **2400.1420** | **-0.9531** | **1** | **3** | **37** | **3** | **K.STKFMDSVIFTLYNYASNQR.E** |
|  | 25225 | **857.7514** | **2570.2324** | **2570.2475** | **-0.0151** | **0** | **21** | **0.55** | **1** | **K.MFLGDNAHLSIINEYLSQSYQK.F** |
|  | 25330 | 518.0508 | 2585.2178 | 2586.2424 | -1.0246 | 0 | (3) | 35 | 10 | K.MFLGDNAHLSIINEYLSQSYQK.F 25334 |
|  | 25500 | **873.7603** | **2618.2589** | **2617.2152** | **1.0437** | **1** | **1** | **55** | **9** | **K.RWMEACLGEDLPPTTELEEGLR.N** |
|  | 26089 | **903.0839** | **2706.2298** | **2706.2483** | **-0.0186** | **0** | **0** | **54** | **1** | **R.SPDVGLYGVIPECGETYHSDLAEAK.K** |
|  | 26273 | 915.4282 | 2743.2628 | 2743.4062 | -0.1433 | 1 | 1 | 51 | 9 | R.EEVITLIRSNQQLENDLNLMDIK.I |
|  | 26779 | **945.5555** | **2833.6448** | **2834.4160** | **-0.7712** | **0** | **3** | **22** | **1** | **K.NPNAMLVNLEEPLASTYQDILYQAK.Q** |
|  | 26821 | **947.7843** | **2840.3311** | **2840.3464** | **-0.0153** | **0** | **23** | **0.3** | **1** | **R.FQPGETLTEILETPATSEQEAEHQR.A** |
|  | 27353 | **986.8637** | **2957.5691** | **2957.5821** | **-0.0130** | **0** | **9** | **6.2** | **1** | **K.IGGILANELSVDEAALHAAVIAINEAIDR.R** |
|  | 28084 | **779.4248** | **3113.6701** | **3113.6832** | **-0.0131** | **1** | **2** | **34** | **4** | **K.IGGILANELSVDEAALHAAVIAINEAIDRR.I** |
|  | 28976 | **1109.7690** | **3326.2853** | **3326.5837** | **-0.2984** | **1** | **3** | **19** | **1** | **R.FQPGETLTEILETPATSEQEAEHQRAMQR.R** |
|  | 29389 | **1155.1747** | **3462.5022** | **3463.6525** | **-1.1503** | **1** | **1** | **26** | **2** | **R.ALESGDVNTVWKQLSSSVTGLTNIEEENCQR.Y** |
|  | 29685 | **1194.2566** | **3579.7479** | **3579.7274** | **0.0205** | **1** | **1** | **30** | **9** | **K.AKFMGVQMETFMLHYQDLLQLQYEGVAVMK.L** |

| **718.** | O75954\|TSN9_HUMAN    **Mass:** 27559    **Score:** 44     **Queries matched:** 3   **emPAI:** 0.14 |
| --- | --- |
|  | Tetraspanin-9 OS=Homo sapiens GN=TSPAN9 PE=1 SV=1 |

|  | **Query** | **Observed** | **Mr(expt)** | **Mr(calc)** | **Delta** | **Miss** | **Score** | **Expect** | **Rank** | **Peptide** |
| --- | --- | --- | --- | --- | --- | --- | --- | --- | --- | --- |
|  | 10491 | **679.7330** | **1357.4515** | **1357.4632** | **-0.0117** | **0** | **44** | **0.0046** | **1** | **R.CCMENSQGCGR.N** |
|  | 10827 | **459.0107** | **1374.0103** | **1373.4581** | **0.5522** | **0** | **(12)** | **5.6** | **2** | **R.CCMENSQGCGR.N** 10814 |

| **719.** | P11684\|UTER_HUMAN    **Mass:** 10215    **Score:** 43     **Queries matched:** 3   **emPAI:** 0.39 |
| --- | --- |
|  | Uteroglobin OS=Homo sapiens GN=SCGB1A1 PE=1 SV=1 |

|  | **Query** | **Observed** | **Mr(expt)** | **Mr(calc)** | **Delta** | **Miss** | **Score** | **Expect** | **Rank** | **Peptide** |
| --- | --- | --- | --- | --- | --- | --- | --- | --- | --- | --- |
|  | 2258 | **423.0522** | **844.0899** | **843.4814** | **0.6085** | **1** | **8** | **21** | **3** | **R.EAGAQLKK.L** |
|  | 9265 | **432.2632** | **1293.7678** | **1293.7769** | **-0.0091** | **1** | **24** | **0.57** | **1** | **K.KLVDTLPQKPR.E** |
|  | 27993 | **1545.2078** | **3088.4010** | **3088.4079** | **-0.0069** | **0** | **43** | **0.0021** | **1** | **R.VIETLLMDTPSSYEAAMELFSPDQDMR.E** |

| **720.** | Q9H9B4\|SFXN1_HUMAN    **Mass:** 35881    **Score:** 43     **Queries matched:** 11   **emPAI:** 0.10 |
| --- | --- |
|  | Sideroflexin-1 OS=Homo sapiens GN=SFXN1 PE=1 SV=4 |

|  | **Query** | **Observed** | **Mr(expt)** | **Mr(calc)** | **Delta** | **Miss** | **Score** | **Expect** | **Rank** | **Peptide** |
| --- | --- | --- | --- | --- | --- | --- | --- | --- | --- | --- |
|  | 3409 | 465.7571 | 929.4997 | 929.5083 | -0.0086 | 1 | 10 | 19 | 1 | R.KIVHDYR.Q 3413 3418 |
|  | 9479 | **435.9008** | **1304.6807** | **1303.6310** | **1.0498** | **0** | **3** | **68** | **10** | **R.ANHFFTVTDPR.N** |
|  | 13002 | **750.8997** | **1499.7849** | **1499.7943** | **-0.0094** | **0** | **43** | **0.0057** | **1** | **R.NILLTNEQLESAR.K** |
|  | 16106 | **565.5499** | **1693.6278** | **1693.8821** | **-0.2543** | **1** | **4** | **35** | **2** | **-.MSGELPPNINIKEPR.W** 16115 |
|  | 27917 | **767.8762** | **3067.4758** | **3068.4270** | **-0.9513** | **1** | **(3)** | **25** | **1** | **K.MILIGRMSAQVPMNMTITGCMMTFYR.T** |
|  | 27966 | **1028.7822** | **3083.3249** | **3084.4220** | **-1.0971** | **1** | **(11)** | **3.6** | **1** | **K.MILIGRMSAQVPMNMTITGCMMTFYR.T** |
|  | 27967 | **1028.7825** | **3083.3256** | **3084.4220** | **-1.0964** | **1** | **11** | **3.2** | **1** | **K.MILIGRMSAQVPMNMTITGCMMTFYR.T** |
|  | 28184 | 1044.7765 | 3131.3076 | 3132.4067 | -1.0991 | 1 | (3) | 21 | 4 | K.MILIGRMSAQVPMNMTITGCMMTFYR.T |

| **721.** | P31946\|1433B_HUMAN    **Mass:** 28179    **Score:** 43     **Queries matched:** 8   **emPAI:** 0.29 |
| --- | --- |
|  | 14-3-3 protein beta/alpha OS=Homo sapiens GN=YWHAB PE=1 SV=3 |

|  | **Query** | **Observed** | **Mr(expt)** | **Mr(calc)** | **Delta** | **Miss** | **Score** | **Expect** | **Rank** | **Peptide** |
| --- | --- | --- | --- | --- | --- | --- | --- | --- | --- | --- |
|  | 1097 | 616.1310 | 615.1238 | 614.3500 | 0.7738 | 0 | 1 | 1.9e+002 | 2 | K.NVVGAR.R |
|  | 3045 | 452.7598 | 903.5050 | 902.5073 | 0.9978 | 0 | 11 | 14 | 5 | R.VISSIEQK.T |
|  | 3426 | **466.2826** | **930.5507** | **931.4222** | **-0.8715** | **1** | **0** | **1.8e+002** | **6** | **K.MKGDYFR.Y** |
|  | 6946 | 591.7815 | 1181.5484 | 1181.5564 | -0.0080 | 0 | 37 | 0.029 | 1 | R.YLSEVASGDNK.Q |
|  | 7120 | 397.2190 | 1188.6351 | 1188.6536 | -0.0186 | 0 | 11 | 12 | 1 | K.DSTLIMQLLR.D |
|  | 9882 | **442.2986** | **1323.8739** | **1324.6367** | **-0.7627** | **1** | **7** | **23** | **5** | **-.MTMDKSELVQK.A** |
|  | 14586 | 533.5812 | 1597.7219 | 1597.7332 | -0.0113 | 0 | 20 | 1.1 | 1 | K.AVTEQGHELSNEER.N |
|  | 28988 | **1110.5482** | **3328.6228** | **3328.6384** | **-0.0156** | **1** | **30** | **0.043** | **1** | **K.TAFDEAIAELDTLNEESYKDSTLIMQLLR.D** |

| **722.** | P28065\|PSB9_HUMAN    **Mass:** 23364    **Score:** 43     **Queries matched:** 6   **emPAI:** 0.16 |
| --- | --- |
|  | Proteasome subunit beta type-9 OS=Homo sapiens GN=PSMB9 PE=1 SV=2 |

|  | **Query** | **Observed** | **Mr(expt)** | **Mr(calc)** | **Delta** | **Miss** | **Score** | **Expect** | **Rank** | **Peptide** |
| --- | --- | --- | --- | --- | --- | --- | --- | --- | --- | --- |
|  | 4442 | 501.5708 | 1001.1270 | 1000.5302 | 0.5969 | 0 | 43 | 0.0058 | 1 | R.VSAGEAVVNR.V |
|  | 9307 | **649.3008** | **1296.5871** | **1295.6544** | **0.9328** | **0** | **3** | **76** | **2** | **R.FTTDAIALAMSR.D** |
|  | 10449 | **452.5439** | **1354.6099** | **1353.7187** | **0.8912** | **1** | **7** | **27** | **9** | **-.MLRAGAPTGDLPR.A** |
|  | 10742 | 457.5987 | 1369.7742 | 1369.7136 | 0.0605 | 1 | (3) | 66 | 9 | -.MLRAGAPTGDLPR.A |
|  | 12420 | 489.9110 | 1466.7110 | 1467.7504 | -1.0394 | 1 | 5 | 34 | 7 | R.RFTTDAIALAMSR.D |
|  | 31215 | **1223.8722** | **4891.4597** | **4890.2728** | **1.1869** | **1** | **2** | **10** | **3** | **R.EGGQVYGTLGGMLTRQPFAIGGSGSTFIYGYVDAAYKPGMSPEECR.R** |

| **723.** | P59768\|GBG2_HUMAN    **Mass:** 7959     **Score:** 43     **Queries matched:** 6   **emPAI:** 0.51 |
| --- | --- |
|  | Guanine nucleotide-binding protein G(I)/G(S)/G(O) subunit gamma-2 OS=Homo sapiens GN=GNG2 PE=1 SV=2 |

|  | **Query** | **Observed** | **Mr(expt)** | **Mr(calc)** | **Delta** | **Miss** | **Score** | **Expect** | **Rank** | **Peptide** |
| --- | --- | --- | --- | --- | --- | --- | --- | --- | --- | --- |
|  | 2319 | **425.2592** | **848.5039** | **847.3858** | **1.1181** | **0** | **21** | **1.5** | **1** | **K.MEANIDR.I** |
|  | 2424 | 429.2731 | 856.5317 | 856.5382 | -0.0065 | 1 | 43 | 0.009 | 1 | R.KLVEQLK.M 2393 2423 |
|  | 2959 | **899.0863** | **898.0790** | **897.4782** | **0.6008** | **1** | **7** | **20** | **1** | **K.KFFCAIL.-** |
|  | 10361 | **451.2576** | **1350.7510** | **1349.6357** | **1.1152** | **0** | **5** | **48** | **5** | **-.MASNNTASIAQAR.K** |

| **724.** | P40121\|CAPG_HUMAN    **Mass:** 38779    **Score:** 43     **Queries matched:** 6   **emPAI:** 0.10 |
| --- | --- |
|  | Macrophage-capping protein OS=Homo sapiens GN=CAPG PE=1 SV=1 |

|  | **Query** | **Observed** | **Mr(expt)** | **Mr(calc)** | **Delta** | **Miss** | **Score** | **Expect** | **Rank** | **Peptide** |
| --- | --- | --- | --- | --- | --- | --- | --- | --- | --- | --- |
|  | 1464 | 771.4673 | 770.4600 | 770.4650 | -0.0050 | 0 | 18 | 2.9 | 8 | R.DLALAIR.D |
|  | 4373 | **499.7446** | **997.4747** | **997.6032** | **-0.1285** | **1** | **7** | **31** | **5** | **K.ARDLALAIR.D** |
|  | 18394 | 922.9643 | 1843.9140 | 1843.9250 | -0.0110 | 0 | 40 | 0.012 | 1 | R.MQYAPNTQVEILPQGR.E 18393 |
|  | 26352 | **921.4880** | **2761.4421** | **2761.4572** | **-0.0150** | **0** | **16** | **1.6** | **1** | **K.AQVEIVTDGEEPAEMIQVLGPKPALK.E** 26346 |

| **725.** | O00483\|NDUA4_HUMAN    **Mass:** 9421     **Score:** 43     **Queries matched:** 2   **emPAI:** 0.43 |
| --- | --- |
|  | NADH dehydrogenase [ubiquinone] 1 alpha subcomplex subunit 4 OS=Homo sapiens GN=NDUFA4 PE=1 SV=1 |

|  | **Query** | **Observed** | **Mr(expt)** | **Mr(calc)** | **Delta** | **Miss** | **Score** | **Expect** | **Rank** | **Peptide** |
| --- | --- | --- | --- | --- | --- | --- | --- | --- | --- | --- |
|  | 1526 | **395.7163** | **789.4181** | **790.3973** | **-0.9792** | **1** | **2** | **1.6e+002** | **7** | **K.KERPDF.-** |
|  | 3471 | **467.7314** | **933.4482** | **933.4556** | **-0.0074** | **0** | **43** | **0.0094** | **1** | **K.LGPNDQYK.F** |

| **726.** | P47756\|CAPZB_HUMAN    **Mass:** 31616    **Score:** 43     **Queries matched:** 10   **emPAI:** 0.25 |
| --- | --- |
|  | F-actin-capping protein subunit beta OS=Homo sapiens GN=CAPZB PE=1 SV=4 |

|  | **Query** | **Observed** | **Mr(expt)** | **Mr(calc)** | **Delta** | **Miss** | **Score** | **Expect** | **Rank** | **Peptide** |
| --- | --- | --- | --- | --- | --- | --- | --- | --- | --- | --- |
|  | 5407 | **536.7995** | **1071.5844** | **1072.5877** | **-1.0032** | **0** | **8** | **33** | **4** | **R.ELSQVLTQR.Q** |
|  | 8343 | 416.5564 | 1246.6473 | 1245.6387 | 1.0086 | 1 | 7 | 31 | 6 | R.LVEDMENKIR.S |
|  | 10106 | **669.3227** | **1336.6308** | **1336.6405** | **-0.0097** | **0** | **21** | **1** | **1** | **K.SGSGTMNLGGSLTR.Q** |
|  | 14294 | **527.3150** | **1578.9232** | **1579.6970** | **-0.7739** | **0** | **3** | **58** | **4** | **M.SDQQLDCALDLMR.R** |
|  | 15953 | 842.8677 | 1683.7209 | 1684.7879 | -1.0670 | 0 | 2 | 60 | 7 | K.GCWDSIHVVEVQEK.S |
|  | 16137 | 848.7376 | 1695.4605 | 1695.8216 | -0.3611 | 1 | 0 | 70 | 4 | R.KLEVEANNAFDQYR.D |
|  | 17516 | **596.3055** | **1785.8946** | **1786.8672** | **-0.9726** | **1** | **8** | **18** | **3** | **K.YDPPLEDGAMPSARLR.K** |
|  | 21883 | **717.4002** | **2149.1788** | **2150.1194** | **-0.9406** | **1** | **2** | **57** | **10** | **R.TAHYKLTSTVMLWLQTNK.S** |
|  | 23113 | **763.0141** | **2286.0205** | **2286.0369** | **-0.0164** | **1** | **10** | **7.7** | **1** | **R.QMEKDETVSDCSPHIANIGR.L** |
|  | 26464 | 1392.2154 | 2782.4163 | 2782.4310 | -0.0147 | 0 | 44 | 0.0023 | 1 | K.NLSDLIDLVPSLCEDLLSSVDQPLK.I |

| **727.** | Q96CX2\|KCD12_HUMAN    **Mass:** 35964    **Score:** 43     **Queries matched:** 9   **emPAI:** 0.10 |
| --- | --- |
|  | BTB/POZ domain-containing protein KCTD12 OS=Homo sapiens GN=KCTD12 PE=1 SV=1 |

|  | **Query** | **Observed** | **Mr(expt)** | **Mr(calc)** | **Delta** | **Miss** | **Score** | **Expect** | **Rank** | **Peptide** |
| --- | --- | --- | --- | --- | --- | --- | --- | --- | --- | --- |
|  | 12202 | **728.3078** | **1454.6010** | **1454.7630** | **-0.1620** | **0** | **7** | **25** | **1** | **R.LGAPQQPGPGPPPSR.R** |
|  | 12560 | 738.8618 | 1475.7090 | 1475.7191 | -0.0101 | 0 | 36 | 0.036 | 1 | R.MFTQQQPQELAR.D 12561 |
|  | 12862 | **498.3358** | **1491.9855** | **1491.7140** | **0.2715** | **0** | **(3)** | **47** | **7** | **R.MFTQQQPQELAR.D** |
|  | 14670 | **802.9155** | **1603.8164** | **1603.8246** | **-0.0082** | **0** | **5** | **36** | **1** | **R.DLQLVLPDYFPER.S** |
|  | 14814 | **538.3413** | **1612.0021** | **1610.8641** | **1.1380** | **1** | **0** | **95** | **6** | **R.LGAPQQPGPGPPPSRR.G** |
|  | 19814 | 652.3251 | 1953.9536 | 1953.9756 | -0.0220 | 0 | 3 | 50 | 5 | R.SPSGGAAGPLLTPSQSLDGSR.R |
|  | 21562 | **704.7148** | **2111.1227** | **2110.0767** | **1.0460** | **1** | **3** | **39** | **1** | **R.SPSGGAAGPLLTPSQSLDGSRR.S** 21551 |

| **728.** | P11940\|PABP1_HUMAN    **Mass:** 70854    **Score:** 43     **Queries matched:** 21   **emPAI:** 0.05 |
| --- | --- |
|  | Polyadenylate-binding protein 1 OS=Homo sapiens GN=PABPC1 PE=1 SV=2 |

|  | **Query** | **Observed** | **Mr(expt)** | **Mr(calc)** | **Delta** | **Miss** | **Score** | **Expect** | **Rank** | **Peptide** |
| --- | --- | --- | --- | --- | --- | --- | --- | --- | --- | --- |
|  | 930 | **547.1404** | **546.1331** | **545.2809** | **0.8522** | **0** | **6** | **79** | **4** | **K.EAAQK.A** |
|  | 1251 | **682.3351** | **681.3279** | **681.3156** | **0.0123** | **0** | **9** | **15** | **3** | **K.FEQMK.Q** |
|  | 1786 | **406.2089** | **810.4032** | **810.3364** | **0.0668** | **0** | **4** | **51** | **9** | **K.VMMEGGR.S** |
|  | 4738 | 513.2864 | 1024.5583 | 1025.4634 | -0.9051 | 1 | 6 | 40 | 1 | K.VMMEGGRSK.G 4753 |
|  | 4827 | **516.3293** | **1030.6441** | **1029.5203** | **1.1239** | **1** | **7** | **33** | **3** | **K.EREAELGAR.A** |
|  | 8605 | **421.0560** | **1260.1461** | **1259.6299** | **0.5162** | **1** | **11** | **7.3** | **1** | **K.SKGFGFVSFER.H** |
|  | 10879 | **460.2242** | **1377.6509** | **1377.7656** | **-0.1147** | **1** | **4** | **52** | **5** | **K.DLFGKFGPALSVK.V** |
|  | 11452 | 471.2377 | 1410.6912 | 1411.7347 | -1.0435 | 1 | 8 | 19 | 2 | R.KEFSPFGTITSAK.V |
|  | 12610 | **493.6081** | **1477.8024** | **1477.7889** | **0.0135** | **0** | **1** | **1.1e+002** | **10** | **K.VDEAVAVLQAHQAK.E** |
|  | 13315 | 507.2344 | 1518.6815 | 1519.7486 | -1.0672 | 1 | 8 | 23 | 4 | R.AIEKMNGMLLNDR.K |
|  | 17809 | 902.9735 | 1803.9325 | 1802.9713 | 0.9612 | 1 | 0 | 1.1e+002 | 9 | R.ALDTMNFDVIKGKPVR.I |
|  | 19518 | 645.0203 | 1932.0390 | 1932.9410 | -0.9021 | 1 | 2 | 65 | 6 | R.QAHLTNQYMQRMASVR.A |
|  | 20791 | 1022.5081 | 2043.0017 | 2043.9758 | -0.9741 | 1 | 5 | 28 | 1 | K.EFSPFGTITSAKVMMEGGR.S |
|  | 23861 | 596.7921 | 2383.1391 | 2382.2552 | 0.8839 | 1 | 4 | 35 | 2 | K.QMLGERLFPLIQAMHPTLAGK.I |
|  | 24085 | **806.4224** | **2416.2453** | **2416.3260** | **-0.0808** | **1** | **3** | **41** | **3** | **K.AVTEMNGRIVATKPLYVALAQR.K** |
|  | 26262 | 914.4642 | 2740.3709 | 2739.3822 | 0.9886 | 0 | 43 | 0.0031 | 1 | K.ITGMLLEIDNSELLHMLESPESLR.S |
|  | 29049 | **1114.4570** | **3340.3493** | **3341.5294** | **-1.1801** | **0** | **1** | **27** | **5** | **-.MNPSAPSYPMASLYVGDLHPDVTEAMLYEK.F** |
|  | 30846 | 1445.3864 | 4333.1372 | 4333.1439 | -0.0067 | 1 | 5 | 7.4 | 3 | M.NPSAPSYPMASLYVGDLHPDVTEAMLYEKFSPAGPILSIR.V 30840 |
|  | 31289 | 1255.1552 | 5016.5915 | 5015.5082 | 1.0833 | 1 | 1 | 11 | 10 | R.WTAQGARPHPFQNMPGAIRPAAPRPPFSTMRPASSQVPRVMSTQR.V |

| **729.** | Q13310\|PABP4_HUMAN    **Mass:** 71080    **Score:** 43     **Queries matched:** 10   **emPAI:** 0.05 |
| --- | --- |
|  | Polyadenylate-binding protein 4 OS=Homo sapiens GN=PABPC4 PE=1 SV=1 |

|  | **Query** | **Observed** | **Mr(expt)** | **Mr(calc)** | **Delta** | **Miss** | **Score** | **Expect** | **Rank** | **Peptide** |
| --- | --- | --- | --- | --- | --- | --- | --- | --- | --- | --- |
|  | 930 | 547.1404 | 546.1331 | 545.2809 | 0.8522 | 0 | 6 | 79 | 4 | K.EAAQK.V |
|  | 986 | **570.3567** | **569.3494** | **569.3649** | **-0.0155** | **0** | **6** | **27** | **8** | **K.GKPIR.I** |
|  | 4443 | **501.5751** | **1001.1357** | **1001.5141** | **-0.3784** | **1** | **0** | **1.1e+002** | **10** | **K.EREAELGAK.A** |
|  | 7766 | **610.2862** | **1218.5578** | **1217.7132** | **0.8446** | **1** | **3** | **80** | **3** | **K.EISGKIIFVGR.A** |
|  | 8782 | **424.0357** | **1269.0854** | **1269.6241** | **-0.5387** | **0** | **4** | **37** | **5** | **K.EFSPFGSITSAK.V** |
|  | 13315 | 507.2344 | 1518.6815 | 1519.7486 | -1.0672 | 1 | 8 | 23 | 4 | K.AIEKMNGMLLNDR.K |
|  | 15761 | **835.8978** | **1669.7810** | **1668.9021** | **0.8789** | **0** | **(0)** | **1e+002** | **1** | **R.LFPLIQTMHSNLAGK.I** |
|  | 15989 | **843.4710** | **1684.9275** | **1684.8970** | **0.0304** | **0** | **3** | **55** | **8** | **R.LFPLIQTMHSNLAGK.I** |
|  | 23889 | **597.8254** | **2387.2727** | **2388.2947** | **-1.0221** | **1** | **7** | **14** | **1** | **K.AVTEMNGRIVGSKPLYVALAQR.K** |
|  | 26262 | 914.4642 | 2740.3709 | 2739.3822 | 0.9886 | 0 | 43 | 0.0031 | 1 | K.ITGMLLEIDNSELLHMLESPESLR.S |

| **730.** | Q9Y4P3\|TBL2_HUMAN    **Mass:** 50393    **Score:** 43     **Queries matched:** 20   **emPAI:** 0.15 |
| --- | --- |
|  | Transducin beta-like protein 2 OS=Homo sapiens GN=TBL2 PE=1 SV=1 |

|  | **Query** | **Observed** | **Mr(expt)** | **Mr(calc)** | **Delta** | **Miss** | **Score** | **Expect** | **Rank** | **Peptide** |
| --- | --- | --- | --- | --- | --- | --- | --- | --- | --- | --- |
|  | 1461 | **769.1857** | **768.1785** | **768.4204** | **-0.2419** | **1** | **11** | **7.9** | **1** | **R.VFKMTK.R** |
|  | 3019 | 451.9015 | 901.7884 | 902.4392 | -0.6509 | 0 | 10 | 14 | 6 | R.SGRPACQK.A |
|  | 5467 | **539.2598** | **1076.5051** | **1077.5468** | **-1.0417** | **0** | **22** | **1.2** | **1** | **R.LFHNTPGHR.A** |
|  | 5571 | **542.7361** | **1083.4576** | **1083.4655** | **-0.0079** | **0** | **39** | **0.017** | **1** | **K.YLATCADDR.T** 5570 |
|  | 6512 | **576.7750** | **1151.5355** | **1151.5798** | **-0.0443** | **1** | **3** | **78** | **5** | **K.VWEVCFGKK.G** |
|  | 8755 | **423.7443** | **1268.2110** | **1267.7136** | **0.4974** | **0** | **1** | **74** | **5** | **K.APVIDIGIANTGK.F** |
|  | 9230 | 431.8722 | 1292.5948 | 1292.6877 | -0.0929 | 1 | 7 | 29 | 10 | R.IWSTKDFLQR.E |
|  | 9949 | **664.3164** | **1326.6181** | **1326.6279** | **-0.0097** | **0** | **32** | **0.08** | **1** | **R.FVASCGFTPDVK.V** 9950 |
|  | 11623 | **711.3497** | **1420.6849** | **1420.6960** | **-0.0111** | **0** | **17** | **2.3** | **1** | **K.EKPQQHNFTHR.L** |
|  | 13501 | 765.4282 | 1528.8419 | 1527.8257 | 1.0162 | 0 | 21 | 0.88 | 1 | R.LQQQLTQAQETLK.S 13470 |
|  | 16652 | **576.7803** | **1727.3190** | **1726.8784** | **0.4405** | **1** | **4** | **27** | **4** | **R.SMRANVELDHATLVR.F** |
|  | 21456 | 1052.0739 | 2102.1331 | 2101.1544 | 0.9787 | 1 | 4 | 30 | 3 | K.EKPQQHNFTHRLLAAALK.S 21457 |
|  | 24647 | **832.7859** | **2495.3358** | **2495.3496** | **-0.0137** | **0** | **15** | **2.1** | **1** | **R.LALSPNAQVLALASGSSIHLYNTR.R** |
|  | 27775 | 759.3673 | 3033.4401 | 3034.5938 | -1.1537 | 0 | 1 | 44 | 10 | -.MELSQMSELMGLSVLLGLLALMATAAVAR.G |
|  | 28384 | **1060.1405** | **3177.3997** | **3177.6995** | **-0.2999** | **1** | **0** | **39** | **1** | **K.APVIDIGIANTGKFIMTASSDTTVLIWSLK.G** |
|  | 29683 | **1194.2559** | **3579.7458** | **3578.8696** | **0.8761** | **1** | **4** | **14** | **1** | **-.MELSQMSELMGLSVLLGLLALMATAAVARGWLR.A** |

| **731.** | Q6ZMR3\|LDH6A_HUMAN    **Mass:** 36826    **Score:** 43     **Queries matched:** 4   **emPAI:** 0.10 |
| --- | --- |
|  | L-lactate dehydrogenase A-like 6A OS=Homo sapiens GN=LDHAL6A PE=2 SV=1 |

|  | **Query** | **Observed** | **Mr(expt)** | **Mr(calc)** | **Delta** | **Miss** | **Score** | **Expect** | **Rank** | **Peptide** |
| --- | --- | --- | --- | --- | --- | --- | --- | --- | --- | --- |
|  | 8351 | 624.8001 | 1247.5857 | 1247.5928 | -0.0072 | 0 | 43 | 0.0078 | 1 | R.VIGSGCNLDSAR.F |
|  | 8487 | **419.2409** | **1254.7009** | **1255.6482** | **-0.9473** | **1** | **5** | **42** | **7** | **K.KVISSGYEMVK.M** 8493 |
|  | 11784 | **715.8240** | **1429.6335** | **1430.8245** | **-1.1910** | **1** | **8** | **21** | **6** | **R.LDLVQRNVSIFK.L** |

| **732.** | P07864\|LDHC_HUMAN    **Mass:** 36630    **Score:** 43     **Queries matched:** 5   **emPAI:** 0.10 |
| --- | --- |
|  | L-lactate dehydrogenase C chain OS=Homo sapiens GN=LDHC PE=2 SV=4 |

|  | **Query** | **Observed** | **Mr(expt)** | **Mr(calc)** | **Delta** | **Miss** | **Score** | **Expect** | **Rank** | **Peptide** |
| --- | --- | --- | --- | --- | --- | --- | --- | --- | --- | --- |
|  | 8351 | 624.8001 | 1247.5857 | 1247.5928 | -0.0072 | 0 | 43 | 0.0078 | 1 | R.VIGSGCNLDSAR.F |
|  | 13435 | 763.8641 | 1525.7136 | 1526.8165 | -1.1029 | 1 | 2 | 72 | 4 | R.QQEGETRLALVQR.N |
|  | 13563 | **511.9468** | **1532.8187** | **1533.8038** | **-0.9851** | **1** | **7** | **27** | **6** | **K.INLNSEEEALFKK.S** |
|  | 19463 | **643.9551** | **1928.8434** | **1929.8601** | **-1.0167** | **0** | **1** | **80** | **4** | **K.GEMMDLQHGSLFFSTSK.I** |
|  | 24724 | **836.9492** | **2507.8257** | **2508.3410** | **-0.5154** | **1** | **0** | **45** | **3** | **K.GYTSWAIGLSVMDLVGSILKNLR.R** |

| **733.** | O00182\|LEG9_HUMAN    **Mass:** 39835    **Score:** 43     **Queries matched:** 6   **emPAI:** 0.20 |
| --- | --- |
|  | Galectin-9 OS=Homo sapiens GN=LGALS9 PE=1 SV=2 |

|  | **Query** | **Observed** | **Mr(expt)** | **Mr(calc)** | **Delta** | **Miss** | **Score** | **Expect** | **Rank** | **Peptide** |
| --- | --- | --- | --- | --- | --- | --- | --- | --- | --- | --- |
|  | 1189 | **649.2006** | **648.1933** | **648.3418** | **-0.1485** | **0** | **0** | **2.3e+002** | **9** | **K.MPFVR.G** |
|  | 4630 | **508.7725** | **1015.5305** | **1015.5273** | **0.0032** | **1** | **8** | **31** | **7** | **R.KTHMPFQK.G** |
|  | 13586 | **768.3338** | **1534.6530** | **1534.6648** | **-0.0117** | **0** | **25** | **0.35** | **1** | **R.NTQIDNSWGSEER.S** |
|  | 20369 | **668.0267** | **2001.0584** | **2002.0173** | **-0.9590** | **1** | **2** | **64** | **2** | **K.VAVDGQHLFEYYHRLR.N** |
|  | 23877 | **796.0455** | **2385.1148** | **2385.1291** | **-0.0143** | **0** | **42** | **0.0044** | **1** | **R.FAVNFQTGFSGNDIAFHFNPR.F** |
|  | 25304 | **1291.6307** | **2581.2469** | **2580.2440** | **1.0029** | **1** | **1** | **48** | **3** | **K.MPFVRGQSFSVWILCEAHCLK.V** |

| **734.** | P61769\|B2MG_HUMAN    **Mass:** 13820    **Score:** 42     **Queries matched:** 4   **emPAI:** 0.65 |
| --- | --- |
|  | Beta-2-microglobulin OS=Homo sapiens GN=B2M PE=1 SV=1 |

|  | **Query** | **Observed** | **Mr(expt)** | **Mr(calc)** | **Delta** | **Miss** | **Score** | **Expect** | **Rank** | **Peptide** |
| --- | --- | --- | --- | --- | --- | --- | --- | --- | --- | --- |
|  | 6116 | 561.7769 | 1121.5393 | 1121.6193 | -0.0800 | 0 | 15 | 4.6 | 2 | R.VNHVTLSQPK.I |
|  | 6461 | **575.2808** | **1148.5471** | **1147.5509** | **0.9961** | **0** | **20** | **1.6** | **1** | **K.VEHSDLSFSK.D** |
|  | 25136 | 852.0767 | 2553.2083 | 2553.2210 | -0.0126 | 0 | 31 | 0.058 | 1 | K.SNFLNCYVSGFHPSDIEVDLLK.N 25137 |

| **735.** | P60468\|SC61B_HUMAN    **Mass:** 10025    **Score:** 42     **Queries matched:** 4   **emPAI:** 0.40 |
| --- | --- |
|  | Protein transport protein Sec61 subunit beta OS=Homo sapiens GN=SEC61B PE=1 SV=2 |

|  | **Query** | **Observed** | **Mr(expt)** | **Mr(calc)** | **Delta** | **Miss** | **Score** | **Expect** | **Rank** | **Peptide** |
| --- | --- | --- | --- | --- | --- | --- | --- | --- | --- | --- |
|  | 6139 | **562.7566** | **1123.4986** | **1123.5081** | **-0.0094** | **0** | **36** | **0.037** | **1** | **R.TTSAGTGGMWR.F** 6140 |
|  | 6341 | 571.2756 | 1140.5366 | 1139.5030 | 1.0336 | 0 | (13) | 8.1 | 3 | R.TTSAGTGGMWR.F |
|  | 13017 | **501.2579** | **1500.7520** | **1500.6991** | **0.0529** | **0** | **3** | **72** | **6** | **-.MPGPTPSGTNVGSSGR.S** |

| **736.** | Q16602\|CALRL_HUMAN    **Mass:** 53864    **Score:** 42     **Queries matched:** 5   **emPAI:** 0.07 |
| --- | --- |
|  | Calcitonin gene-related peptide type 1 receptor OS=Homo sapiens GN=CALCRL PE=1 SV=2 |

|  | **Query** | **Observed** | **Mr(expt)** | **Mr(calc)** | **Delta** | **Miss** | **Score** | **Expect** | **Rank** | **Peptide** |
| --- | --- | --- | --- | --- | --- | --- | --- | --- | --- | --- |
|  | 1818 | **407.2550** | **812.4954** | **813.5688** | **-1.0734** | **1** | **5** | **50** | **5** | **R.VLITKLK.V** |
|  | 14137 | **785.3810** | **1568.7474** | **1568.7583** | **-0.0109** | **0** | **42** | **0.0079** | **1** | **K.IQFGNSFSNSEALR.S** |
|  | 15865 | **559.9448** | **1676.8126** | **1676.8556** | **-0.0429** | **1** | **8** | **19** | **2** | **K.LKVTHQAESNLYMK.A** 15863 |
|  | 31187 | 1215.3893 | 4857.5280 | 4857.2828 | 0.2452 | 1 | 1 | 14 | 5 | R.SASYTVSTISDGPGYSHDCPSEHLNGKSIHDIENVLLKPENLYN.- |

| **737.** | Q14344\|GNA13_HUMAN    **Mass:** 44364    **Score:** 42     **Queries matched:** 11   **emPAI:** 0.18 |
| --- | --- |
|  | Guanine nucleotide-binding protein subunit alpha-13 OS=Homo sapiens GN=GNA13 PE=1 SV=2 |

|  | **Query** | **Observed** | **Mr(expt)** | **Mr(calc)** | **Delta** | **Miss** | **Score** | **Expect** | **Rank** | **Peptide** |
| --- | --- | --- | --- | --- | --- | --- | --- | --- | --- | --- |
|  | 2845 | **444.2090** | **886.4035** | **886.3677** | **0.0358** | **0** | **14** | **8.1** | **1** | **K.MMSFDTR.A** |
|  | 3034 | **452.3924** | **902.7702** | **902.3626** | **0.4076** | **0** | **(0)** | **1.2e+002** | **5** | **K.MMSFDTR.A** |
|  | 4912 | **519.1365** | **1036.2585** | **1035.5237** | **0.7349** | **0** | **4** | **45** | **6** | **R.EFQLGESVK.Y** |
|  | 5181 | 529.2853 | 1056.5560 | 1056.6179 | -0.0619 | 0 | 28 | 0.31 | 1 | K.ILLLGAGESGK.S |
|  | 7775 | **610.3232** | **1218.6319** | **1218.7125** | **-0.0805** | **0** | **5** | **49** | **9** | **R.VFLQYLPAIR.A** |
|  | 8386 | 417.2427 | 1248.7062 | 1248.5881 | 0.1181 | 1 | 8 | 25 | 10 | K.MVDVGGQRSER.K |
|  | 9241 | **431.9937** | **1292.9591** | **1292.5853** | **0.3738** | **0** | **3** | **53** | **7** | **R.APMAAQGMVETR.V** |
|  | 11973 | 481.2421 | 1440.7045 | 1439.7231 | 0.9813 | 1 | 8 | 21 | 1 | R.DVQKFLVECFR.N |
|  | 14819 | **807.3815** | **1612.7484** | **1613.9240** | **-1.1756** | **1** | **2** | **77** | **7** | **K.TDLLEEKVQIVSIK.D** |
|  | 16176 | 566.7687 | 1697.2844 | 1696.8818 | 0.4026 | 1 | 10 | 7.8 | 1 | K.DTILHDNLKQLMLQ.- |
|  | 27255 | **979.5011** | **2935.4815** | **2935.4967** | **-0.0152** | **1** | **36** | **0.012** | **1** | **K.YFLDNLDKLGEPDYIPSQQDILLAR.R** |

| **738.** | Q99653\|CHP1_HUMAN    **Mass:** 22442    **Score:** 42     **Queries matched:** 9   **emPAI:** 0.17 |
| --- | --- |
|  | Calcium-binding protein p22 OS=Homo sapiens GN=CHP PE=1 SV=3 |

|  | **Query** | **Observed** | **Mr(expt)** | **Mr(calc)** | **Delta** | **Miss** | **Score** | **Expect** | **Rank** | **Peptide** |
| --- | --- | --- | --- | --- | --- | --- | --- | --- | --- | --- |
|  | 5668 | 546.2459 | 1090.4773 | 1090.5917 | -0.1144 | 1 | 7 | 36 | 8 | -.MGSRASTLLR.D |
|  | 7057 | 593.8384 | 1185.6623 | 1185.6605 | 0.0018 | 1 | 7 | 32 | 8 | K.VLEKVDVEQK.M |
|  | 14140 | 523.9348 | 1568.7826 | 1568.7947 | -0.0121 | 0 | 4 | 53 | 5 | R.TLAHFRPIEDNEK.S 14138 |
|  | 19056 | **948.4629** | **1894.9112** | **1894.9214** | **-0.0101** | **0** | **42** | **0.0064** | **1** | **R.IINAFFPEGEDQVNFR.G** |
|  | 20111 | **661.3111** | **1980.9115** | **1982.0221** | **-1.1106** | **1** | **1** | **68** | **9** | **R.EDFQRIPELAINPLGDR.I** |
|  | 22339 | 1101.5040 | 2200.9935 | 2200.0172 | 0.9763 | 0 | 4 | 37 | 2 | R.TIQEADQDGDSAISFTEFVK.V |
|  | 23886 | **796.5387** | **2386.5943** | **2386.1528** | **0.4414** | **1** | **2** | **33** | **4** | **R.IINAFFPEGEDQVNFRGFMR.T** |
|  | 28436 | **796.9063** | **3183.5959** | **3183.6353** | **-0.0394** | **1** | **1** | **39** | **3** | **R.IPELAINPLGDRIINAFFPEGEDQVNFR.G** |

| **739.** | Q8N2K0\|ABD12_HUMAN    **Mass:** 45524    **Score:** 42     **Queries matched:** 10   **emPAI:** 0.08 |
| --- | --- |
|  | Monoacylglycerol lipase ABHD12 OS=Homo sapiens GN=ABHD12 PE=2 SV=2 |

|  | **Query** | **Observed** | **Mr(expt)** | **Mr(calc)** | **Delta** | **Miss** | **Score** | **Expect** | **Rank** | **Peptide** |
| --- | --- | --- | --- | --- | --- | --- | --- | --- | --- | --- |
|  | 916 | **540.2403** | **539.2330** | **540.2405** | **-1.0074** | **0** | **3** | **51** | **1** | **R.GGDHR.V** |
|  | 2494 | **862.5748** | **861.5675** | **862.3569** | **-0.7894** | **0** | **1** | **1.7e+002** | **5** | **K.SEPEHQH.-** |
|  | 4141 | **491.7662** | **981.5178** | **981.5243** | **-0.0065** | **0** | **42** | **0.01** | **1** | **R.LTGPAAAEPR.C** 4151 4153 |
|  | 5659 | **545.6002** | **1089.1858** | **1088.6342** | **0.5516** | **1** | **5** | **34** | **8** | **R.KLYSIAAPAR.S** |
|  | 17506 | **893.9040** | **1785.7934** | **1785.8502** | **-0.0567** | **1** | **7** | **20** | **1** | **R.LTGPAAAEPRCAADAGMK.R** 17505 |
|  | 23775 | **593.2990** | **2369.1669** | **2369.2114** | **-0.0445** | **1** | **2** | **49** | **6** | **R.ETPPDALILESPFTNIREEAK.S** |
|  | 28280 | 1052.1213 | 3153.3422 | 3153.5124 | -0.1702 | 1 | 7 | 7.7 | 1 | R.YFPGFDWFFLDPITSSGIKFANDENVK.H |

| **740.** | O15270\|SPTC2_HUMAN    **Mass:** 63568    **Score:** 42     **Queries matched:** 20   **emPAI:** 0.06 |
| --- | --- |
|  | Serine palmitoyltransferase 2 OS=Homo sapiens GN=SPTLC2 PE=1 SV=1 |

|  | **Query** | **Observed** | **Mr(expt)** | **Mr(calc)** | **Delta** | **Miss** | **Score** | **Expect** | **Rank** | **Peptide** |
| --- | --- | --- | --- | --- | --- | --- | --- | --- | --- | --- |
|  | 3908 | 483.2527 | 964.4909 | 964.5276 | -0.0367 | 1 | 9 | 19 | 5 | R.NLYMRIR.D |
|  | 4113 | 490.7344 | 979.4543 | 980.5225 | -1.0683 | 1 | (9) | 19 | 5 | R.NLYMRIR.D |
|  | 5879 | 553.2515 | 1104.4885 | 1104.6655 | -0.1770 | 1 | 9 | 24 | 7 | R.LSGATIRIFK.H |
|  | 6429 | **573.7941** | **1145.5737** | **1145.5248** | **0.0490** | **0** | **6** | **40** | **3** | **R.ANGCVANGEVR.N** |
|  | 8367 | 625.2756 | 1248.5366 | 1247.4958 | 1.0408 | 0 | 7 | 32 | 3 | M.RPEPGGCCCR.R |
|  | 10800 | 687.3436 | 1372.6727 | 1371.7874 | 0.8853 | 1 | 5 | 51 | 6 | K.LLKDAIVYGQPR.T |
|  | 11171 | **465.6140** | **1393.8203** | **1394.5312** | **-0.7109** | **0** | **3** | **64** | **7** | **-.MRPEPGGCCCR.R** |
|  | 12795 | 745.3789 | 1488.7433 | 1487.7554 | 0.9878 | 1 | 1 | 98 | 8 | R.IFKHNNMQSLEK.L |
|  | 13605 | **512.9341** | **1535.7804** | **1534.6374** | **1.1430** | **1** | **3** | **57** | **8** | **-.MRPEPGGCCCRR.T** |
|  | 15272 | 821.3831 | 1640.7517 | 1640.7841 | -0.0325 | 0 | 11 | 9.3 | 4 | R.DNWNRPICSVPGAR.V 15271 |
|  | 20711 | 679.3612 | 2035.0618 | 2035.1387 | -0.0769 | 1 | 0 | 91 | 8 | K.KILILVEGIYSMEGSIVR.L |
|  | 20732 | **679.8947** | **2036.6623** | **2037.0677** | **-0.4054** | **0** | **25** | **0.18** | **1** | **K.GCLILSDELNHASLVLGAR.L** |
|  | 21386 | **699.3193** | **2094.9360** | **2094.1950** | **0.7410** | **1** | **3** | **42** | **3** | **R.NIGVVVVGFPATPIIESRAR.F** 21374 |
|  | 24281 | **1225.0632** | **2448.1119** | **2448.1229** | **-0.0110** | **0** | **42** | **0.005** | **1** | **R.GVVEYFGLDPEDVDVMMGTFTK.S** 24284 |
|  | 26201 | **910.7744** | **2729.3014** | **2729.3154** | **-0.0140** | **0** | **1** | **51** | **2** | **K.EMGFIIYGNEDSPVVPLMLYMPAK.I** |
|  | 27422 | **743.9155** | **2971.6330** | **2970.4945** | **1.1386** | **1** | **5** | **15** | **1** | **R.LKEMGFIIYGNEDSPVVPLMLYMPAK.I** |
|  | 29001 | **833.9615** | **3331.8171** | **3330.6491** | **1.1680** | **1** | **2** | **24** | **4** | **K.EMGFIIYGNEDSPVVPLMLYMPAKIGAFGR.E** |

| **741.** | Q9BWM7\|SFXN3_HUMAN    **Mass:** 35823    **Score:** 41     **Queries matched:** 11   **emPAI:** 0.10 |
| --- | --- |
|  | Sideroflexin-3 OS=Homo sapiens GN=SFXN3 PE=2 SV=1 |

|  | **Query** | **Observed** | **Mr(expt)** | **Mr(calc)** | **Delta** | **Miss** | **Score** | **Expect** | **Rank** | **Peptide** |
| --- | --- | --- | --- | --- | --- | --- | --- | --- | --- | --- |
|  | 2867 | **445.1185** | **888.2224** | **887.5341** | **0.6883** | **0** | **6** | **40** | **6** | **K.HLPPLVGR.F** |
|  | 3061 | **453.4645** | **904.9145** | **905.4719** | **-0.5574** | **0** | **5** | **40** | **4** | **R.NIVQNYR.A** |
|  | 3120 | **455.7051** | **909.3956** | **908.4967** | **0.8989** | **0** | **20** | **1.5** | **1** | **R.LGYSVTAAK.Q** |
|  | 12899 | 499.2080 | 1494.6023 | 1493.7838 | 0.8185 | 0 | 5 | 34 | 3 | K.SSIHISNLEPELR.A |
|  | 14303 | **395.9538** | **1579.7862** | **1580.8158** | **-1.0296** | **0** | **5** | **34** | **4** | **R.ELQVGIPVADEAGQR.L** |
|  | 15272 | 821.3831 | 1640.7517 | 1640.8522 | -0.1005 | 0 | 41 | 0.0081 | 1 | R.AGVVTPGITEDQLWR.A 15271 |
|  | 24741 | **837.8664** | **2510.5773** | **2511.1452** | **-0.5679** | **1** | **6** | **11** | **1** | **R.MSAQVPMNMTITGCMLTFYRK.T** |
|  | 24742 | **837.9181** | **2510.7324** | **2511.1452** | **-0.4128** | **1** | **(1)** | **34** | **2** | **R.MSAQVPMNMTITGCMLTFYRK.T** |
|  | 27712 | 756.3781 | 3021.4833 | 3020.4778 | 1.0055 | 1 | 2 | 33 | 1 | K.VVLIGRMSAQVPMNMTITGCMLTFYR.K |
|  | 29471 | 1165.2455 | 3492.7146 | 3492.7637 | -0.0491 | 1 | 0 | 35 | 5 | K.SSIHISNLEPELRAQIHEQNPSVEVVYYNK.G |

| **742.** | P01024\|CO3_HUMAN    **Mass:** 188569   **Score:** 41     **Queries matched:** 29   **emPAI:** 0.06 |
| --- | --- |
|  | Complement C3 OS=Homo sapiens GN=C3 PE=1 SV=2 |

|  | **Query** | **Observed** | **Mr(expt)** | **Mr(calc)** | **Delta** | **Miss** | **Score** | **Expect** | **Rank** | **Peptide** |
| --- | --- | --- | --- | --- | --- | --- | --- | --- | --- | --- |
|  | 1475 | **776.3657** | **775.3584** | **774.4487** | **0.9097** | **0** | **7** | **46** | **2** | **K.LVLSSEK.T** |
|  | 2762 | **441.2917** | **880.5688** | **881.5698** | **-1.0010** | **1** | **6** | **40** | **10** | **R.LKGPLLNK.F** |
|  | 3841 | **480.7816** | **959.5486** | **958.5335** | **1.0152** | **1** | **3** | **98** | **8** | **R.EALKLEEK.K** |
|  | 3848 | 481.1511 | 960.2876 | 959.5400 | 0.7476 | 1 | 13 | 8.5 | 4 | R.RSVQLTEK.R |
|  | 8109 | 619.3256 | 1236.6367 | 1237.7143 | -1.0776 | 1 | 14 | 5.5 | 1 | R.KVLLDGVQNPR.A |
|  | 8216 | 415.0344 | 1242.0813 | 1242.5880 | -0.5067 | 1 | 10 | 11 | 1 | K.YELDKAFSDR.N 8217 8225 8226 8230 |
|  | 8238 | **415.2273** | **1242.6600** | **1241.6438** | **1.0161** | **0** | **8** | **24** | **2** | **R.QPVPGQQMTLK.I** |
|  | 8468 | **419.1817** | **1254.5233** | **1255.6884** | **-1.1652** | **1** | **14** | **5.9** | **1** | **K.TVAVRTLDPER.L** |
|  | 8634 | **631.4468** | **1260.8791** | **1260.5946** | **0.2846** | **0** | **2** | **77** | **4** | **K.QELSEAEQATR.T** |
|  | 9452 | 652.8645 | 1303.7144 | 1303.7361 | -0.0216 | 1 | 5 | 42 | 5 | R.RHQQTVTIPPK.S |
|  | 9860 | 662.3558 | 1322.6970 | 1323.5812 | -0.8842 | 1 | 4 | 53 | 3 | R.ENPMRFSCQR.R |
|  | 11284 | 701.3328 | 1400.6510 | 1400.8279 | -0.1769 | 0 | 4 | 55 | 7 | K.SSLSVPYVIVPLK.T |
|  | 15258 | 820.8748 | 1639.7350 | 1638.8651 | 0.8699 | 0 | 3 | 60 | 5 | R.TVMVNIENPEGIPVK.Q |
|  | 15272 | 821.3831 | 1640.7517 | 1640.7617 | -0.0100 | 0 | 33 | 0.058 | 2 | K.AGDFLEANYMNLQR.S 15271 |
|  | 16321 | **854.4266** | **1706.8387** | **1705.8821** | **0.9566** | **1** | **5** | **40** | **1** | **K.GQGTLSVVTMYHAKAK.D** |
|  | 17177 | **589.2618** | **1764.7637** | **1764.9332** | **-0.1695** | **0** | **7** | **23** | **5** | **K.DMALTAFVLISLQEAK.D** |
|  | 17748 | **601.1215** | **1800.3426** | **1799.9240** | **0.4186** | **0** | **2** | **44** | **9** | **R.VELLHNPAFCSLATTK.R** |
|  | 19016 | **631.3281** | **1890.9626** | **1890.0687** | **0.8939** | **0** | **29** | **0.13** | **1** | **K.LSINTHPSQKPLSITVR.T** 19019 |
|  | 22044 | **1083.5051** | **2164.9957** | **2164.9953** | **0.0004** | **0** | **4** | **33** | **2** | **R.AYYENSPQQVFSTEFEVK.E** |
|  | 22980 | **757.9340** | **2270.7801** | **2270.1478** | **0.6323** | **0** | **1** | **47** | **2** | **K.TVLTPATNHMGNVTFTIPANR.E** |
|  | 23032 | 1139.5281 | 2277.0416 | 2278.1263 | -1.0847 | 0 | 0 | 72 | 7 | K.SLYVSATVILHSGSDMVQAER.S |
|  | 23729 | **788.7646** | **2363.2721** | **2364.1995** | **-0.9273** | **1** | **0** | **67** | **2** | **R.NNNEKDMALTAFVLISLQEAK.D** |
|  | 31234 | **1226.6263** | **4902.4763** | **4902.4936** | **-0.0173** | **1** | **29** | **0.022** | **1** | **R.TVMVNIENPEGIPVKQDSLSSQNQLGVLPLSWDIPELVNMGQWK.I** |

| **743.** | P80511\|S10AC_HUMAN    **Mass:** 10569    **Score:** 41     **Queries matched:** 6   **emPAI:** 0.90 |
| --- | --- |
|  | Protein S100-A12 OS=Homo sapiens GN=S100A12 PE=1 SV=2 |

|  | **Query** | **Observed** | **Mr(expt)** | **Mr(calc)** | **Delta** | **Miss** | **Score** | **Expect** | **Rank** | **Peptide** |
| --- | --- | --- | --- | --- | --- | --- | --- | --- | --- | --- |
|  | 1488 | 788.4826 | 787.4753 | 787.4439 | 0.0314 | 0 | 4 | 83 | 2 | K.ELANTIK.N |
|  | 3038 | **452.7268** | **903.4390** | **903.4450** | **-0.0060** | **0** | **28** | **0.28** | **1** | **K.GHFDTLSK.G** 3037 |
|  | 10014 | **666.3337** | **1330.6529** | **1330.6881** | **-0.0352** | **1** | **3** | **83** | **3** | **K.GHFDTLSKGELK.Q** |
|  | 10780 | **458.2517** | **1371.7334** | **1370.8133** | **0.9201** | **1** | **7** | **30** | **3** | **K.QLLTKELANTIK.N** |
|  | 24047 | **804.7554** | **2411.2445** | **2411.2597** | **-0.0153** | **1** | **42** | **0.0049** | **1** | **M.TKLEEHLEGIVNIFHQYSVR.K** |

| **744.** | Q15286\|RAB35_HUMAN    **Mass:** 23296    **Score:** 41     **Queries matched:** 12   **emPAI:** 0.58 |
| --- | --- |
|  | Ras-related protein Rab-35 OS=Homo sapiens GN=RAB35 PE=1 SV=1 |

|  | **Query** | **Observed** | **Mr(expt)** | **Mr(calc)** | **Delta** | **Miss** | **Score** | **Expect** | **Rank** | **Peptide** |
| --- | --- | --- | --- | --- | --- | --- | --- | --- | --- | --- |
|  | 1162 | 643.2711 | 642.2638 | 642.4064 | -0.1427 | 0 | 27 | 0.41 | 1 | R.ILVGNK.N |
|  | 4475 | **502.3713** | **1002.7281** | **1003.4975** | **-0.7694** | **0** | **10** | **15** | **1** | **R.TITSTYYR.G** |
|  | 5150 | **527.7943** | **1053.5739** | **1052.5026** | **1.0714** | **0** | **8** | **26** | **2** | **K.VVETEDAYK.F** |
|  | 5394 | 536.3203 | 1070.6261 | 1070.6336 | -0.0075 | 0 | 45 | 0.006 | 1 | K.LLIIGDSGVGK.S 5392 |
|  | 6633 | **580.2920** | **1158.5694** | **1157.6404** | **0.9290** | **1** | **1** | **1.3e+002** | **9** | **K.IRTVEINGEK.V** |
|  | 6936 | **591.3015** | **1180.5885** | **1180.5975** | **-0.0091** | **1** | **34** | **0.062** | **1** | **R.KVVETEDAYK.F** |
|  | 9695 | 658.8284 | 1315.6422 | 1315.6521 | -0.0099 | 0 | 18 | 2.6 | 1 | K.LQIWDTAGQER.F 9685 |
|  | 15073 | 543.3282 | 1626.9627 | 1626.8076 | 0.1552 | 0 | 5 | 37 | 6 | K.FAGQMGIQLFETSAK.E |
|  | 16851 | **580.9766** | **1739.9079** | **1740.0509** | **-0.1431** | **1** | **9** | **13** | **1** | **K.LLIIGDSGVGKSSLLLR.F** |
|  | 20471 | 672.0267 | 2013.0584 | 2013.8898 | -0.8314 | 1 | 1 | 71 | 8 | K.RWLHEINQNCDDVCR.I |

| **745.** | P13693\|TCTP_HUMAN    **Mass:** 19697    **Score:** 41     **Queries matched:** 7   **emPAI:** 0.20 |
| --- | --- |
|  | Translationally-controlled tumor protein OS=Homo sapiens GN=TPT1 PE=1 SV=1 |

|  | **Query** | **Observed** | **Mr(expt)** | **Mr(calc)** | **Delta** | **Miss** | **Score** | **Expect** | **Rank** | **Peptide** |
| --- | --- | --- | --- | --- | --- | --- | --- | --- | --- | --- |
|  | 2210 | 421.5636 | 841.1127 | 841.4810 | -0.3683 | 0 | 5 | 31 | 7 | K.HILANFK.N |
|  | 8182 | **621.3277** | **1240.6408** | **1240.6524** | **-0.0115** | **1** | **3** | **76** | **6** | **K.GKLEEQRPER.V** |
|  | 12098 | **483.2545** | **1446.7416** | **1445.6901** | **1.0515** | **0** | **8** | **23** | **8** | **R.EDGVTPYMIFFK.D** 12054 |
|  | 16641 | 864.4268 | 1726.8391 | 1727.7712 | -0.9322 | 0 | 1 | 84 | 8 | R.DLISHDEMFSDIYK.I |
|  | 20125 | **661.4480** | **1981.3222** | **1980.9615** | **0.3607** | **1** | **0** | **60** | **4** | **R.DLISHDEMFSDIYKIR.E** |
|  | 31184 | **1215.0776** | **4856.2815** | **4856.2571** | **0.0244** | **0** | **41** | **0.0012** | **1** | **R.TEGNIDDSLIGGNASAEGPEGEGTESTVITGVDIVMNHHLQETSFTK.E** |

| **746.** | Q9BXJ8\|T120A_HUMAN    **Mass:** 41040    **Score:** 41     **Queries matched:** 4   **emPAI:** 0.09 |
| --- | --- |
|  | Transmembrane protein 120A OS=Homo sapiens GN=TMEM120A PE=2 SV=1 |

|  | **Query** | **Observed** | **Mr(expt)** | **Mr(calc)** | **Delta** | **Miss** | **Score** | **Expect** | **Rank** | **Peptide** |
| --- | --- | --- | --- | --- | --- | --- | --- | --- | --- | --- |
|  | 9231 | 647.3100 | 1292.6054 | 1292.6143 | -0.0088 | 0 | 41 | 0.011 | 1 | K.LQNNCTSSITR.Q 9228 |
|  | 11330 | 468.9214 | 1403.7423 | 1402.7027 | 1.0395 | 0 | 1 | 99 | 7 | M.QPPPPGPLGDCLR.D |
|  | 13589 | 768.3793 | 1534.7440 | 1533.7432 | 1.0008 | 0 | 2 | 87 | 5 | -.MQPPPPGPLGDCLR.D |

| **747.** | Q9NZ08\|ERAP1_HUMAN    **Mass:** 107736   **Score:** 41     **Queries matched:** 19   **emPAI:** 0.03 |
| --- | --- |
|  | Endoplasmic reticulum aminopeptidase 1 OS=Homo sapiens GN=ERAP1 PE=1 SV=3 |

|  | **Query** | **Observed** | **Mr(expt)** | **Mr(calc)** | **Delta** | **Miss** | **Score** | **Expect** | **Rank** | **Peptide** |
| --- | --- | --- | --- | --- | --- | --- | --- | --- | --- | --- |
|  | 1692 | **402.7250** | **803.4355** | **802.3933** | **1.0422** | **0** | **10** | **20** | **3** | **K.ENGSQLR.C** |
|  | 2502 | **863.0859** | **862.0787** | **862.4986** | **-0.4200** | **0** | **7** | **26** | **1** | **-.MVFLPLK.W** 2494 |
|  | 6470 | **575.3070** | **1148.5993** | **1147.5298** | **1.0695** | **0** | **3** | **84** | **4** | **R.SDGTPFPWNK.I** |
|  | 7091 | **594.8003** | **1187.5860** | **1188.5663** | **-0.9802** | **0** | **5** | **50** | **5** | **K.YQFSLSSTEK.S** |
|  | 8151 | 620.2996 | 1238.5847 | 1237.6853 | 0.8994 | 0 | 4 | 48 | 4 | R.HLAISNMPLVK.S |
|  | 9451 | **652.8619** | **1303.7092** | **1303.6309** | **0.0783** | **1** | **16** | **3.3** | **1** | **K.RSDGTPFPWNK.I** |
|  | 11328 | **702.8735** | **1403.7324** | **1403.7409** | **-0.0085** | **0** | **41** | **0.01** | **1** | **R.ILASTQFEPTAAR.M** |
|  | 14675 | 535.6350 | 1603.8830 | 1604.9389 | -1.0558 | 1 | 8 | 17 | 3 | K.LSIEKALDLSLYLK.H |
|  | 18564 | **619.5244** | **1855.5512** | **1855.9138** | **-0.3626** | **1** | **3** | **39** | **3** | **R.DMNEVETQFKAFLIR.L** 18565 |
|  | 20566 | **674.6351** | **2020.8836** | **2020.8987** | **-0.0152** | **0** | **21** | **0.7** | **1** | **R.SQHSSSSSHWHQEGVDVK.T** |
|  | 20926 | **685.1999** | **2052.5780** | **2052.8769** | **-0.2988** | **0** | **2** | **33** | **7** | **K.NEDLWDSMASICPTDGVK.G** |
|  | 24113 | 807.3972 | 2419.1698 | 2420.1756 | -1.0058 | 0 | 1 | 54 | 4 | K.HETEIMPVFQGLNELIPMYK.L |
|  | 26836 | **711.8652** | **2843.4316** | **2844.3615** | **-0.9299** | **1** | **0** | **55** | **8** | **R.ILASTQFEPTAARMAFPCFDEPAFK.A** |
|  | 27021 | **578.0824** | **2885.3756** | **2884.4212** | **0.9545** | **1** | **4** | **22** | **8** | **K.LVQKFELGSSSIAHMVMGTTNQFSTR.T** |
|  | 27596 | 1000.8221 | 2999.4444 | 2999.3753 | 0.0691 | 0 | 0 | 52 | 9 | K.CFDAMEVDALNSSHPVSTPVENPAQIR.E |
|  | 29795 | 907.6627 | 3626.6215 | 3627.8205 | -1.1989 | 0 | 1 | 29 | 5 | K.WSLATMSFLLSSLLALLTVSTPSWCQSTEASPK.R |
|  | 30832 | **1082.1965** | **4324.7570** | **4325.2731** | **-0.5161** | **1** | **1** | **14** | **2** | **M.VFLPLKWSLATMSFLLSSLLALLTVSTPSWCQSTEASPK.R** |

| **748.** | P08237\|K6PF_HUMAN    **Mass:** 85984    **Score:** 41     **Queries matched:** 23   **emPAI:** 0.04 |
| --- | --- |
|  | 6-phosphofructokinase, muscle type OS=Homo sapiens GN=PFKM PE=1 SV=2 |

|  | **Query** | **Observed** | **Mr(expt)** | **Mr(calc)** | **Delta** | **Miss** | **Score** | **Expect** | **Rank** | **Peptide** |
| --- | --- | --- | --- | --- | --- | --- | --- | --- | --- | --- |
|  | 926 | 546.2509 | 545.2436 | 544.3333 | 0.9104 | 0 | 11 | 31 | 7 | R.ILGSR.M |
|  | 1854 | **409.1947** | **816.3748** | **817.4406** | **-1.0658** | **1** | **8** | **32** | **8** | **K.QSAAGTKR.R** |
|  | 1905 | **411.2088** | **820.4031** | **819.4603** | **0.9428** | **0** | **41** | **0.014** | **1** | **R.VGIFTGAR.V** 1897 1900 1906 |
|  | 7316 | **599.8028** | **1197.5910** | **1196.5682** | **1.0229** | **1** | **3** | **80** | **4** | **K.AMNWMSGKIK.E** |
|  | 8108 | **619.3182** | **1236.6219** | **1236.6350** | **-0.0131** | **0** | **0** | **1.3e+002** | **9** | **K.SFEQISANITK.F** |
|  | 10728 | **685.4557** | **1368.8969** | **1367.8024** | **1.0946** | **0** | **3** | **53** | **8** | **R.LNIIIVAEGAIDK.N** 10695 10707 10730 |
|  | 18311 | **614.2518** | **1839.7337** | **1839.8686** | **-0.1350** | **0** | **3** | **47** | **4** | **K.NVLGHMQQGGSPTPFDR.N** |
|  | 19261 | 956.9960 | 1911.9774 | 1912.9677 | -0.9903 | 0 | 1 | 79 | 5 | R.IMEIVDAITTTAQSHQR.T 19260 |
|  | 22305 | **550.5257** | **2198.0737** | **2199.0525** | **-0.9788** | **0** | **5** | **27** | **1** | **K.SGSHTVAVMNVGAPAAGMNAAVR.S** 22316 |
|  | 24109 | 605.5606 | 2418.2131 | 2417.1546 | 1.0584 | 1 | 0 | 63 | 10 | K.NVLGHMQQGGSPTPFDRNFATK.M |
|  | 25608 | 661.3241 | 2641.2673 | 2642.3017 | -1.0344 | 1 | 16 | 1.4 | 1 | K.SGSHTVAVMNVGAPAAGMNAAVRSTVR.I 25609 |
|  | 25899 | **672.0662** | **2684.2358** | **2683.3680** | **0.8678** | **0** | **0** | **59** | **7** | **K.FNIQGLVIIGGFEAYTGGLELMEGR.K** |
|  | 29123 | 841.9231 | 3363.6633 | 3364.7972 | -1.1339 | 1 | 0 | 37 | 3 | K.LLAHVRPPVSKSGSHTVAVMNVGAPAAGMNAAVR.S |
|  | 30904 | **1097.5127** | **4386.0217** | **4386.1766** | **-0.1549** | **1** | **3** | **11** | **1** | **R.MGVEAVMALLEGTPDTPACVVSLSGNQAVRLPLMECVQVTK.D** |

| **749.** | Q9UJU6\|DBNL_HUMAN    **Mass:** 48463    **Score:** 41     **Queries matched:** 11   **emPAI:** 0.08 |
| --- | --- |
|  | Drebrin-like protein OS=Homo sapiens GN=DBNL PE=1 SV=1 |

|  | **Query** | **Observed** | **Mr(expt)** | **Mr(calc)** | **Delta** | **Miss** | **Score** | **Expect** | **Rank** | **Peptide** |
| --- | --- | --- | --- | --- | --- | --- | --- | --- | --- | --- |
|  | 1137 | **631.8159** | **630.8086** | **630.3449** | **0.4637** | **0** | **5** | **64** | **1** | **M.AANLSR.N** |
|  | 4488 | 503.1030 | 1004.1915 | 1003.4570 | 0.7345 | 1 | 4 | 47 | 7 | K.AEKEEENR.R |
|  | 5333 | **534.7837** | **1067.5528** | **1066.5883** | **0.9645** | **0** | **5** | **47** | **6** | **R.EPAAAISRPR.A** |
|  | 9603 | 657.3998 | 1312.7850 | 1312.6524 | 0.1326 | 0 | 4 | 54 | 2 | K.QLTQPETHFGR.E |
|  | 10311 | 675.2819 | 1348.5492 | 1348.6007 | -0.0516 | 0 | 3 | 70 | 7 | R.YQEQGGEASPQR.T |
|  | 15842 | **559.3401** | **1674.9986** | **1675.8563** | **-0.8577** | **1** | **3** | **50** | **6** | **R.AMSTTSISSPQPGKLR.S** |
|  | 19036 | **631.9763** | **1892.9070** | **1891.8543** | **1.0527** | **1** | **1** | **87** | **6** | **R.AEEDVEPECIMEKVAK.A** |
|  | 20410 | 1003.9751 | 2005.9356 | 2004.9310 | 1.0047 | 0 | 41 | 0.0069 | 1 | R.VAGTGEGGLEEMVEELNSGK.V |
|  | 23719 | 788.4282 | 2362.2628 | 2361.2301 | 1.0327 | 1 | 0 | 67 | 8 | K.QLTQPETHFGREPAAAISRPR.A |
|  | 24850 | **843.4710** | **2527.3912** | **2526.2867** | **1.1045** | **1** | **6** | **15** | **2** | **K.DPNSGLPKFVLINWTGEGVNDVR.K** |
|  | 25604 | **881.4193** | **2641.2359** | **2641.2660** | **-0.0301** | **1** | **1** | **51** | **6** | **R.VVTEKSPTDWALFTYEGNSNDIR.V** |

| **750.** | O95831\|AIFM1_HUMAN    **Mass:** 67144    **Score:** 41     **Queries matched:** 17   **emPAI:** 0.18 |
| --- | --- |
|  | Apoptosis-inducing factor 1, mitochondrial OS=Homo sapiens GN=AIFM1 PE=1 SV=1 |

|  | **Query** | **Observed** | **Mr(expt)** | **Mr(calc)** | **Delta** | **Miss** | **Score** | **Expect** | **Rank** | **Peptide** |
| --- | --- | --- | --- | --- | --- | --- | --- | --- | --- | --- |
|  | 1027 | 586.4140 | 585.4067 | 586.3261 | -0.9194 | 0 | 8 | 29 | 7 | R.MPIAR.K |
|  | 2018 | 415.2452 | 828.4759 | 827.4865 | 0.9894 | 0 | 9 | 25 | 10 | K.VVQLDVR.D |
|  | 3734 | 477.1937 | 952.3729 | 951.6229 | 0.7500 | 1 | 12 | 9.5 | 3 | K.QKLVPLVR.T |
|  | 4336 | **498.7287** | **995.4428** | **995.4342** | **0.0086** | **0** | **14** | **5.4** | **2** | **R.QMASSGASGGK.I** 4337 |
|  | 8720 | 634.3061 | 1266.5976 | 1266.6092 | -0.0115 | 0 | 26 | 0.37 | 1 | K.LNDGSQITYEK.C |
|  | 9704 | 439.5715 | 1315.6928 | 1315.7095 | -0.0168 | 1 | 9 | 20 | 5 | R.SLSAIDRAGAEVK.S |
|  | 10678 | 684.3625 | 1366.7104 | 1366.6850 | 0.0255 | 1 | 3 | 70 | 4 | -.MFRCGGLAAGALK.Q |
|  | 11226 | 466.8900 | 1397.6483 | 1397.7878 | -0.1395 | 1 | 9 | 16 | 3 | R.ISGLGLTPEQKQK.K |
|  | 12469 | **736.3442** | **1470.6738** | **1469.6787** | **0.9951** | **0** | **32** | **0.077** | **1** | **K.TGGLEIDSDFGGFR.V** |
|  | 12969 | **500.2793** | **1497.8162** | **1497.7549** | **0.0612** | **1** | **4** | **49** | **8** | **R.RVEHHDHAVVSGR.L** |
|  | 14183 | **786.9186** | **1571.8226** | **1571.8341** | **-0.0115** | **0** | **26** | **0.35** | **1** | **K.VMPNAIVQSVGVSSGK.L** |
|  | 15252 | 820.4670 | 1638.9194 | 1638.7963 | 0.1231 | 0 | 7 | 23 | 6 | K.ILPEYLSNWTMEK.V |
|  | 18042 | **911.4396** | **1820.8646** | **1819.9105** | **0.9541** | **1** | **0** | **1.1e+002** | **4** | **K.ELWFSDDPNVTKTLR.F** |
|  | 18550 | **928.4490** | **1854.8835** | **1853.8829** | **1.0006** | **1** | **4** | **40** | **3** | **R.DNMVKLNDGSQITYEK.C** |
|  | 20248 | **664.8434** | **1991.5085** | **1991.0510** | **0.4575** | **0** | **2** | **38** | **5** | **K.SITIIGGGFLGSELACALGR.K** |
|  | 23885 | **796.4210** | **2386.2411** | **2386.1522** | **0.0889** | **1** | **2** | **48** | **1** | **R.WHVPLELQMTRQMASSGASGGK.I** |

| **751.** | Q14568\|HS902_HUMAN    **Mass:** 39454    **Score:** 41     **Queries matched:** 8   **emPAI:** 0.10 |
| --- | --- |
|  | Putative heat shock protein HSP 90-alpha A2 OS=Homo sapiens GN=HSP90AA2 PE=1 SV=2 |

|  | **Query** | **Observed** | **Mr(expt)** | **Mr(calc)** | **Delta** | **Miss** | **Score** | **Expect** | **Rank** | **Peptide** |
| --- | --- | --- | --- | --- | --- | --- | --- | --- | --- | --- |
|  | 4529 | 504.7289 | 1007.4431 | 1007.6491 | -0.2060 | 1 | 8 | 28 | 6 | R.GTKVILHLK.E |
|  | 4940 | **520.2509** | **1038.4873** | **1037.4560** | **1.0313** | **1** | **5** | **49** | **7** | **R.TDTGERMGR.G** |
|  | 8244 | 622.3537 | 1242.6928 | 1241.6979 | 0.9949 | 0 | 41 | 0.012 | 1 | K.ADLINNLGTIAK.S 8227 |
|  | 10305 | 450.2784 | 1347.8135 | 1347.6572 | 0.1563 | 0 | 4 | 65 | 8 | K.HFSVEGQLEFR.A |
|  | 21809 | **429.2114** | **2141.0206** | **2142.0957** | **-1.0750** | **1** | **6** | **20** | **4** | **K.VILHLKEDQTEYLEEQR.I** |
|  | 27054 | **723.8604** | **2891.4125** | **2891.5426** | **-0.1301** | **1** | **0** | **56** | **5** | **K.ELHINLIPNKQDQTLTIVDTGIGMTK.A** |
|  | 31177 | 969.2288 | 4841.1077 | 4841.1921 | -0.0844 | 0 | 3 | 6.9 | 1 | -.MPEETQTQDQPMEEEEVETFAFQAEIAQLMSLIINTFYSNK.E |

| **752.** | Q58FF8\|H90B2_HUMAN    **Mass:** 44492    **Score:** 41     **Queries matched:** 7   **emPAI:** 0.08 |
| --- | --- |
|  | Putative heat shock protein HSP 90-beta 2 OS=Homo sapiens GN=HSP90AB2P PE=1 SV=2 |

|  | **Query** | **Observed** | **Mr(expt)** | **Mr(calc)** | **Delta** | **Miss** | **Score** | **Expect** | **Rank** | **Peptide** |
| --- | --- | --- | --- | --- | --- | --- | --- | --- | --- | --- |
|  | 3857 | 481.2443 | 960.4740 | 961.4352 | -0.9612 | 1 | 8 | 32 | 6 | K.GEKEEENK.D |
|  | 4879 | 517.7628 | 1033.5111 | 1033.5226 | -0.0115 | 0 | 6 | 40 | 6 | K.VEMVTVSNR.L |
|  | 6668 | 581.3089 | 1160.6032 | 1159.5761 | 1.0272 | 0 | 1 | 1.4e+002 | 10 | K.SIYYITGESK.E |
|  | 7041 | **593.3355** | **1184.6563** | **1183.5543** | **1.1020** | **0** | **0** | **1.5e+002** | **5** | **-.MPEEVHLGEK.E** |
|  | 7731 | **406.5356** | **1216.5850** | **1215.5780** | **1.0070** | **1** | **2** | **96** | **6** | **K.FENLCKFMK.E** |
|  | 8244 | 622.3537 | 1242.6928 | 1241.6979 | 0.9949 | 0 | 41 | 0.012 | 1 | K.ADLINNLGTIAK.F 8227 |

| **753.** | P02655\|APOC2_HUMAN    **Mass:** 11277    **Score:** 41     **Queries matched:** 1   **emPAI:** 0.35 |
| --- | --- |
|  | Apolipoprotein C-II OS=Homo sapiens GN=APOC2 PE=1 SV=1 |

|  | **Query** | **Observed** | **Mr(expt)** | **Mr(calc)** | **Delta** | **Miss** | **Score** | **Expect** | **Rank** | **Peptide** |
| --- | --- | --- | --- | --- | --- | --- | --- | --- | --- | --- |
|  | 4928 | **519.7648** | **1037.5151** | **1036.5189** | **0.9962** | **0** | **41** | **0.013** | **1** | **K.TAAQNLYEK.T** |

| **754.** | Q96HE7\|ERO1A_HUMAN    **Mass:** 55213    **Score:** 41     **Queries matched:** 5   **emPAI:** 0.07 |
| --- | --- |
|  | ERO1-like protein alpha OS=Homo sapiens GN=ERO1L PE=1 SV=2 |

|  | **Query** | **Observed** | **Mr(expt)** | **Mr(calc)** | **Delta** | **Miss** | **Score** | **Expect** | **Rank** | **Peptide** |
| --- | --- | --- | --- | --- | --- | --- | --- | --- | --- | --- |
|  | 1144 | **634.3284** | **633.3212** | **633.3697** | **-0.0486** | **0** | **10** | **26** | **6** | **R.ISTSVK.E** |
|  | 9850 | **662.2886** | **1322.5626** | **1321.6918** | **0.8708** | **0** | **4** | **54** | **9** | **R.YLLQETWLEK.K** |
|  | 10904 | 460.5713 | 1378.6920 | 1379.7197 | -1.0277 | 0 | 0 | 1.3e+002 | 8 | R.QEIVSLFNAFGR.I |
|  | 13074 | **753.3646** | **1504.7147** | **1504.7257** | **-0.0110** | **0** | **41** | **0.01** | **1** | **R.LGAVDESLSEETQK.A** |
|  | 18414 | 616.1291 | 1845.3654 | 1846.0564 | -0.6909 | 1 | 2 | 49 | 6 | K.LQTQGLGTALKILFSEK.L |

| **755.** | P10253\|LYAG_HUMAN    **Mass:** 106107   **Score:** 40     **Queries matched:** 14   **emPAI:** 0.03 |
| --- | --- |
|  | Lysosomal alpha-glucosidase OS=Homo sapiens GN=GAA PE=1 SV=3 |

|  | **Query** | **Observed** | **Mr(expt)** | **Mr(calc)** | **Delta** | **Miss** | **Score** | **Expect** | **Rank** | **Peptide** |
| --- | --- | --- | --- | --- | --- | --- | --- | --- | --- | --- |
|  | 2087 | **417.2429** | **832.4713** | **831.3988** | **1.0725** | **0** | **18** | **3.6** | **1** | **R.STFAGHGR.Y** 2085 |
|  | 3517 | 469.7464 | 937.4783 | 937.4909 | -0.0126 | 0 | 6 | 33 | 7 | R.TTPTFFPK.D |
|  | 6358 | **571.5082** | **1141.0019** | **1140.5564** | **0.4456** | **0** | **5** | **37** | **2** | **R.WGYSSTAITR.Q** |
|  | 7399 | 401.2420 | 1200.7043 | 1201.5887 | -0.8844 | 1 | 6 | 43 | 10 | M.GVRHPPCSHR.L |
|  | 7478 | **603.2782** | **1204.5418** | **1204.5506** | **-0.0088** | **0** | **29** | **0.19** | **1** | **K.AITQEQCEAR.G** |
|  | 8771 | 635.3206 | 1268.6266 | 1269.7115 | -1.0849 | 0 | (1) | 1.1e+002 | 4 | R.QQPMALAVALTK.G |
|  | 8780 | **635.4979** | **1268.9812** | **1268.5377** | **0.4435** | **0** | **7** | **18** | **2** | **R.LDVMMETENR.L** |
|  | 9080 | **643.3420** | **1284.6695** | **1285.7064** | **-1.0369** | **0** | **7** | **29** | **1** | **R.QQPMALAVALTK.G** |
|  | 13265 | **758.8683** | **1515.7221** | **1515.7333** | **-0.0111** | **0** | **40** | **0.011** | **1** | **R.WTQLGAFYPFMR.N** |
|  | 13531 | 511.2577 | 1530.7512 | 1531.7282 | -0.9771 | 0 | (4) | 46 | 8 | R.WTQLGAFYPFMR.N |
|  | 18777 | **625.3151** | **1872.9235** | **1871.8935** | **1.0301** | **0** | **0** | **90** | **9** | **K.LENLSSSEMGYTATLTR.T** |
|  | 23719 | 788.4282 | 2362.2628 | 2363.1030 | -0.8401 | 1 | 0 | 67 | 8 | K.GGEARGELFWDDGESLEVLER.G |
|  | 29679 | 895.2165 | 3576.8371 | 3577.9238 | -1.0867 | 0 | 6 | 9.6 | 2 | R.YALLPHLYTLFHQAHVAGETVARPLFLEFPK.D |

| **756.** | Q9UBJ2\|ABCD2_HUMAN    **Mass:** 83750    **Score:** 40     **Queries matched:** 21   **emPAI:** 0.04 |
| --- | --- |
|  | ATP-binding cassette sub-family D member 2 OS=Homo sapiens GN=ABCD2 PE=1 SV=1 |

|  | **Query** | **Observed** | **Mr(expt)** | **Mr(calc)** | **Delta** | **Miss** | **Score** | **Expect** | **Rank** | **Peptide** |
| --- | --- | --- | --- | --- | --- | --- | --- | --- | --- | --- |
|  | 2538 | **433.2030** | **864.3915** | **863.4171** | **0.9744** | **0** | **12** | **11** | **3** | **K.QVMVSER.T** 2529 |
|  | 2760 | 441.2663 | 880.5180 | 880.4007 | 0.1173 | 1 | 11 | 11 | 3 | K.QRMGMAR.M |
|  | 3660 | **474.7415** | **947.4685** | **946.5712** | **0.8973** | **1** | **5** | **53** | **2** | **K.LALAFRTR.L** |
|  | 4466 | **502.2883** | **1002.5620** | **1001.6386** | **0.9235** | **1** | **14** | **6.9** | **4** | **K.KPRTFIIK.L** |
|  | 5147 | **527.7708** | **1053.5269** | **1054.6022** | **-1.0753** | **0** | **9** | **17** | **3** | **K.LESQLAGIPK.M** 5164 |
|  | 5759 | 549.3278 | 1096.6411 | 1095.4833 | 1.1578 | 0 | 4 | 56 | 4 | K.GYTDQDLER.I |
|  | 7988 | **616.1291** | **1230.2436** | **1229.5645** | **0.6791** | **0** | **6** | **27** | **3** | **-.MTHMLNAAADR.V** |
|  | 9641 | **438.8952** | **1313.6637** | **1314.6415** | **-0.9779** | **0** | **4** | **56** | **2** | **R.TAVIQESESHSK.N** |
|  | 9666 | **439.2267** | **1314.6582** | **1315.7169** | **-1.0587** | **0** | **20** | **1.5** | **1** | **K.ALADQMNLILSK.R** |
|  | 10885 | **689.8487** | **1377.6828** | **1376.7598** | **0.9230** | **1** | **1** | **1.2e+002** | **9** | **K.RAACLVAAAYALK.T** |
|  | 12787 | **497.2384** | **1488.6933** | **1487.8130** | **0.8803** | **1** | **3** | **59** | **7** | **K.ALADQMNLILSKR.L** 12796 |
|  | 14593 | **799.9409** | **1597.8672** | **1597.8610** | **0.0062** | **1** | **4** | **47** | **3** | **K.LESQLAGIPKMQQR.L** |
|  | 14819 | 807.3815 | 1612.7484 | 1613.8559 | -1.1075 | 1 | (3) | 66 | 5 | K.LESQLAGIPKMQQR.L |
|  | 14857 | 808.4044 | 1614.7943 | 1613.8349 | 0.9593 | 0 | 5 | 40 | 2 | R.LWYIMIEQFLMK.Y |
|  | 17919 | 604.9623 | 1811.8652 | 1810.8706 | 0.9946 | 0 | 1 | 72 | 3 | K.VEEGMHLLITGPNGCGK.S |
|  | 19590 | **485.7427** | **1938.9416** | **1937.9880** | **0.9536** | **1** | **7** | **19** | **6** | **R.NLLASGADAIERIMSSYK.E** |
|  | 21380 | 1047.9520 | 2093.8895 | 2093.9364 | -0.0469 | 1 | 40 | 0.0074 | 2 | R.EGGWDAVMDWKDVLSGGEK.Q |
|  | 27086 | **967.7754** | **2900.3045** | **2900.4088** | **-0.1043** | **1** | **3** | **31** | **7** | **R.MFYHKPKYALLDECTSAVSIDVEGK.I** |

| **757.** | Q13740\|CD166_HUMAN    **Mass:** 65745    **Score:** 40     **Queries matched:** 11   **emPAI:** 0.06 |
| --- | --- |
|  | CD166 antigen OS=Homo sapiens GN=ALCAM PE=1 SV=2 |

|  | **Query** | **Observed** | **Mr(expt)** | **Mr(calc)** | **Delta** | **Miss** | **Score** | **Expect** | **Rank** | **Peptide** |
| --- | --- | --- | --- | --- | --- | --- | --- | --- | --- | --- |
|  | 8642 | **631.8320** | **1261.6494** | **1260.6536** | **0.9957** | **0** | **6** | **37** | **6** | **R.LDVPQNLMFGK.W** |
|  | 8952 | **639.4445** | **1276.8744** | **1276.6486** | **0.2258** | **0** | **(5)** | **33** | **2** | **R.LDVPQNLMFGK.W** 8950 8951 |
|  | 9617 | **657.8313** | **1313.6480** | **1314.7143** | **-1.0662** | **1** | **1** | **1.3e+002** | **1** | **K.NAIKEGDNITLK.C** |
|  | 11471 | **706.8552** | **1411.6958** | **1411.7056** | **-0.0098** | **1** | **9** | **16** | **1** | **R.SSNTYTLTDVRR.N** |
|  | 13908 | 518.9713 | 1553.8921 | 1553.9028 | -0.0107 | 0 | 12 | 7.9 | 1 | R.ESLTLIVEGKPQIK.M |
|  | 15835 | 559.2837 | 1674.8292 | 1673.8407 | 0.9886 | 0 | 3 | 52 | 6 | R.QIGDALPVSCTISASR.N |
|  | 18253 | **612.3351** | **1833.9834** | **1833.0876** | **0.8958** | **1** | **3** | **61** | **1** | **R.NGKVLHPLEGAVVIIFK.K** |
|  | 28596 | **1072.8252** | **3215.4538** | **3215.4717** | **-0.0179** | **0** | **40** | **0.0042** | **1** | **R.SSPSFSSLHYQDAGNYVCETALQEVEGLK.K** |
|  | 29057 | 1115.4473 | 3343.3200 | 3343.9346 | -0.6146 | 1 | 11 | 2.9 | 1 | K.VNDQAKLIVGIVVGLLLAALVAGVVYWLYMK.K |

| **758.** | Q96CW1\|AP2M1_HUMAN    **Mass:** 49965    **Score:** 40     **Queries matched:** 24   **emPAI:** 0.07 |
| --- | --- |
|  | AP-2 complex subunit mu OS=Homo sapiens GN=AP2M1 PE=1 SV=2 |

|  | **Query** | **Observed** | **Mr(expt)** | **Mr(calc)** | **Delta** | **Miss** | **Score** | **Expect** | **Rank** | **Peptide** |
| --- | --- | --- | --- | --- | --- | --- | --- | --- | --- | --- |
|  | 1369 | **726.3353** | **725.3280** | **726.3007** | **-0.9727** | **0** | **4** | **53** | **7** | **K.FGMNDK.I** |
|  | 1537 | 396.7133 | 791.4120 | 791.3926 | 0.0194 | 0 | 6 | 54 | 1 | R.NAVDAFR.V |
|  | 1972 | 413.7707 | 825.5268 | 824.4028 | 1.1240 | 0 | 5 | 48 | 5 | R.SGIYETR.C |
|  | 2644 | **437.2604** | **872.5061** | **872.5120** | **-0.0058** | **0** | **12** | **14** | **2** | **K.DIILPFR.V** 2622 2628 2629 2648 |
|  | 3194 | **458.2321** | **914.4496** | **913.5596** | **0.8900** | **1** | **7** | **34** | **4** | **K.IVIEKQGK.G** |
|  | 4131 | 491.2782 | 980.5419 | 980.4927 | 0.0492 | 1 | 8 | 25 | 3 | R.LSKFDSER.S |
|  | 4292 | 497.2557 | 992.4969 | 992.5039 | -0.0070 | 1 | 11 | 14 | 3 | R.VYRDDIGR.N |
|  | 4647 | 509.2570 | 1016.4994 | 1016.5291 | -0.0297 | 0 | 19 | 2.4 | 3 | K.ASENAIVWK.I |
|  | 8542 | **629.8037** | **1257.5929** | **1257.7081** | **-0.1152** | **1** | **5** | **43** | **7** | **K.ASENAIVWKIK.R** |
|  | 9505 | **654.6323** | **1307.2500** | **1307.6696** | **-0.4196** | **0** | **2** | **59** | **1** | **-.MIGGLFIYNHK.G** |
|  | 9521 | **655.3258** | **1308.6371** | **1307.6873** | **0.9497** | **1** | **40** | **0.013** | **1** | **K.YKASENAIVWK.I** |
|  | 18858 | **627.3412** | **1879.0017** | **1878.7950** | **0.2067** | **1** | **3** | **47** | **7** | **K.SYLSGMPECKFGMNDK.I** 18845 |
|  | 23372 | **580.7869** | **2319.1184** | **2320.1290** | **-1.0106** | **1** | **5** | **23** | **6** | **R.MAGMKESQISAEIELLPTNDK.K** |
|  | 23514 | **1169.5306** | **2337.0467** | **2336.1239** | **0.9228** | **1** | **(1)** | **61** | **1** | **R.MAGMKESQISAEIELLPTNDK.K** 23515 |
|  | 23781 | 791.0848 | 2370.2325 | 2371.2511 | -1.0186 | 1 | 5 | 21 | 3 | K.KWARPPISMNFEVPFAPSGLK.V |
|  | 28324 | **1056.8275** | **3167.4607** | **3167.6285** | **-0.1678** | **0** | **1** | **34** | **5** | **R.NELFLDVLESVNLLMSPQGQVLSAHVSGR.V** |
|  | 29825 | **1214.8982** | **3641.6727** | **3640.8957** | **0.7771** | **1** | **3** | **18** | **1** | **R.NELFLDVLESVNLLMSPQGQVLSAHVSGRVVMK.S** 29824 |

| **759.** | P35221\|CTNA1_HUMAN    **Mass:** 100693   **Score:** 40     **Queries matched:** 23   **emPAI:** 0.04 |
| --- | --- |
|  | Catenin alpha-1 OS=Homo sapiens GN=CTNNA1 PE=1 SV=1 |

|  | **Query** | **Observed** | **Mr(expt)** | **Mr(calc)** | **Delta** | **Miss** | **Score** | **Expect** | **Rank** | **Peptide** |
| --- | --- | --- | --- | --- | --- | --- | --- | --- | --- | --- |
|  | 2057 | **416.2952** | **830.5759** | **829.5208** | **1.0552** | **1** | **3** | **1.2e+002** | **8** | **R.KAVLMIR.T** |
|  | 2100 | 418.0311 | 834.0477 | 834.4599 | -0.4122 | 0 | 14 | 4.7 | 1 | R.LVYDGIR.D 2107 |
|  | 4819 | 516.2778 | 1030.5410 | 1030.5229 | 0.0180 | 0 | 8 | 26 | 9 | R.IVAECNAVR.Q |
|  | 4873 | **517.7297** | **1033.4449** | **1032.5087** | **0.9362** | **0** | **9** | **20** | **4** | **R.SDALNSAIDK.M** |
|  | 5697 | 547.2581 | 1092.5017 | 1092.5233 | -0.0216 | 1 | 3 | 76 | 4 | K.IAEAGSRMDK.L |
|  | 8851 | 637.3045 | 1272.5944 | 1271.6543 | 0.9401 | 0 | 3 | 69 | 10 | R.AIMAQLPQEQK.A |
|  | 9666 | 439.2267 | 1314.6582 | 1315.6666 | -1.0084 | 1 | 9 | 19 | 10 | R.ERIVAECNAVR.Q |
|  | 9719 | **439.8766** | **1316.6079** | **1317.6499** | **-1.0420** | **0** | **9** | **20** | **3** | **-.MTAVHAGNINFK.W** 9716 |
|  | 10528 | **680.3570** | **1358.6994** | **1357.6950** | **1.0044** | **1** | **8** | **21** | **2** | **K.DVDGLDRTAGAIR.G** |
|  | 10881 | **689.8414** | **1377.6683** | **1377.6776** | **-0.0093** | **0** | **1** | **1.2e+002** | **10** | **K.IAEQVASFQEEK.S** |
|  | 11139 | **465.2126** | **1392.6158** | **1392.6918** | **-0.0760** | **1** | **1** | **1.1e+002** | **9** | **R.SDALNSAIDKMTK.K** 11165 |
|  | 16704 | 865.9104 | 1729.8062 | 1729.8669 | -0.0606 | 1 | 1 | 78 | 4 | K.CVIALQEKDVDGLDR.T |
|  | 17986 | 606.6270 | 1816.8592 | 1817.8755 | -1.0163 | 0 | 1 | 85 | 10 | R.TSVQTEDDQLIAGQSAR.A |
|  | 18375 | 615.3405 | 1842.9997 | 1843.9039 | -0.9042 | 1 | 4 | 39 | 4 | -.MTAVHAGNINFKWDPK.S 18373 |
|  | 18988 | **630.3607** | **1888.0602** | **1887.7004** | **0.3598** | **0** | **1** | **76** | **7** | **K.QMCMIMMEMTDFTR.G** |
|  | 20748 | **510.5053** | **2037.9923** | **2039.0721** | **-1.0799** | **1** | **10** | **9.5** | **1** | **K.NLMNAVVQTVKASYVASTK.Y** |
|  | 20980 | **686.8579** | **2057.5519** | **2056.8219** | **0.7300** | **1** | **10** | **6.2** | **1** | **K.QMCMIMMEMTDFTRGK.G** |
|  | 26672 | **938.5031** | **2812.4875** | **2812.5004** | **-0.0129** | **1** | **40** | **0.0056** | **1** | **K.VKAEVQNLGGELVVSGVDSAMSLIQAAK.N** |
|  | 28780 | 1090.8536 | 3269.5391 | 3268.6649 | 0.8742 | 1 | 2 | 30 | 2 | K.AVMDHVSDSFLETNVPLLVLIEAAKNGNEK.E |

| **760.** | Q8IUD2\|RB6I2_HUMAN    **Mass:** 128236   **Score:** 40     **Queries matched:** 29   **emPAI:** 0.03 |
| --- | --- |
|  | ELKS/RAB6-interacting/CAST family member 1 OS=Homo sapiens GN=ERC1 PE=1 SV=1 |

|  | **Query** | **Observed** | **Mr(expt)** | **Mr(calc)** | **Delta** | **Miss** | **Score** | **Expect** | **Rank** | **Peptide** |
| --- | --- | --- | --- | --- | --- | --- | --- | --- | --- | --- |
|  | 2761 | **441.2733** | **880.5320** | **879.4273** | **1.1047** | **1** | **16** | **3.5** | **1** | **R.SHSKFMK.N** |
|  | 3943 | **968.4445** | **967.4372** | **966.4883** | **0.9489** | **0** | **3** | **78** | **5** | **K.AHEAALEAR.A** |
|  | 4104 | **490.2589** | **978.5031** | **977.4454** | **1.0577** | **0** | **8** | **28** | **2** | **R.FENAPDSAK.T** |
|  | 4685 | **510.7678** | **1019.5210** | **1019.4957** | **0.0253** | **0** | **40** | **0.018** | **1** | **K.TLEEMELR.I** |
|  | 4692 | 511.2468 | 1020.4791 | 1020.4910 | -0.0119 | 1 | 7 | 31 | 10 | K.DMLDVKER.K |
|  | 4812 | 1031.4902 | 1030.4830 | 1031.5685 | -1.0855 | 0 | 4 | 74 | 4 | K.ALQTVIEMK.D |
|  | 4855 | 517.2660 | 1032.5175 | 1033.4862 | -0.9687 | 0 | 9 | 23 | 6 | K.SAQMLEEAR.R |
|  | 5228 | 530.7545 | 1059.4944 | 1060.5513 | -1.0569 | 1 | 7 | 32 | 8 | K.ESLTAKEQR.A |
|  | 6692 | **582.2628** | **1162.5111** | **1163.5856** | **-1.0745** | **1** | **8** | **23** | **10** | **R.KTLEEMELR.I** 6711 6712 |
|  | 6727 | 583.7945 | 1165.5744 | 1165.5615 | 0.0130 | 1 | 1 | 1.1e+002 | 8 | K.QEEIDNYKK.D |
|  | 7078 | 594.3106 | 1186.6065 | 1187.6398 | -1.0332 | 0 | 9 | 19 | 6 | K.VSLLQGDLSEK.E 7095 |
|  | 7120 | 397.2190 | 1188.6351 | 1189.5873 | -0.9523 | 1 | 10 | 16 | 3 | K.SAQMLEEARR.R |
|  | 8398 | **625.7427** | **1249.4709** | **1249.6224** | **-0.1514** | **1** | **6** | **31** | **2** | **R.LEEKETMLNK.K** |
|  | 8542 | 629.8037 | 1257.5929 | 1258.7132 | -1.1204 | 1 | 4 | 58 | 10 | R.KDTELLALQTK.L |
|  | 8807 | 636.3146 | 1270.6146 | 1269.7041 | 0.9106 | 1 | 9 | 19 | 4 | K.KIENLQEQLR.D |
|  | 9079 | 643.3355 | 1284.6563 | 1284.7653 | -0.1089 | 1 | 3 | 78 | 9 | R.LKTLEIALEQK.K |
|  | 9280 | 648.8030 | 1295.5915 | 1294.6041 | 0.9875 | 1 | 0 | 1.2e+002 | 7 | R.EKQEEIDNYK.K |
|  | 9797 | 441.2136 | 1320.6191 | 1319.6391 | 0.9800 | 1 | 8 | 24 | 5 | K.TKQIQDMAEEK.G |
|  | 11096 | 463.9505 | 1388.8297 | 1389.7463 | -0.9167 | 1 | 2 | 84 | 10 | K.AKLSSTQQSLAEK.E 11104 |
|  | 11516 | **472.4472** | **1414.3197** | **1413.7212** | **0.5985** | **1** | **2** | **51** | **6** | **R.SVGKVEPSSQSPGR.S** |
|  | 15230 | 819.9658 | 1637.9171 | 1638.7558 | -0.8387 | 1 | 2 | 69 | 7 | R.RTNSTGGSSGSSVGGGSGK.T |
|  | 17615 | 896.4455 | 1790.8764 | 1789.8992 | 0.9772 | 1 | 2 | 74 | 5 | R.QARDNTIMDLQTQLK.E |
|  | 17804 | 902.9348 | 1803.8551 | 1804.8625 | -1.0074 | 1 | 6 | 27 | 7 | R.EMVLAQEESARTNAEK.Q |
|  | 21725 | **710.4257** | **2128.2552** | **2129.0575** | **-0.8023** | **0** | **2** | **41** | **4** | **R.LAEAEMHVHHLESLLEQK.E** |
|  | 24759 | **838.7866** | **2513.3379** | **2513.3588** | **-0.0209** | **1** | **1** | **50** | **2** | **K.QEALLAAISEKDANIALLELSSSK.K** |

| **761.** | O15083\|ERC2_HUMAN    **Mass:** 110661   **Score:** 40     **Queries matched:** 30   **emPAI:** 0.03 |
| --- | --- |
|  | ERC protein 2 OS=Homo sapiens GN=ERC2 PE=1 SV=3 |

|  | **Query** | **Observed** | **Mr(expt)** | **Mr(calc)** | **Delta** | **Miss** | **Score** | **Expect** | **Rank** | **Peptide** |
| --- | --- | --- | --- | --- | --- | --- | --- | --- | --- | --- |
|  | 1410 | 746.3748 | 745.3675 | 744.4381 | 0.9294 | 1 | 6 | 66 | 3 | R.KELDIK.D |
|  | 2238 | **843.1906** | **842.1833** | **841.5385** | **0.6448** | **1** | **7** | **26** | **7** | **K.INVLQKK.I** |
|  | 2761 | 441.2733 | 880.5320 | 879.4273 | 1.1047 | 1 | 16 | 3.5 | 1 | K.SHSKFMK.T |
|  | 4685 | 510.7678 | 1019.5210 | 1019.4957 | 0.0253 | 0 | 40 | 0.018 | 1 | K.TLEEMELR.I |
|  | 4812 | 1031.4902 | 1030.4830 | 1031.5685 | -1.0855 | 0 | 4 | 74 | 4 | K.ALQTVIEMK.D |
|  | 4856 | **517.2684** | **1032.5223** | **1031.5611** | **0.9612** | **1** | **5** | **63** | **4** | **K.DTKIASLER.N** 4832 |
|  | 4902 | 518.5977 | 1035.1809 | 1034.5066 | 0.6743 | 1 | 2 | 67 | 5 | K.DMLEVKER.K |
|  | 5228 | 530.7545 | 1059.4944 | 1060.5513 | -1.0569 | 1 | 7 | 32 | 8 | K.ESLTAKEQR.A |
|  | 5288 | **532.3242** | **1062.6338** | **1063.5219** | **-0.8882** | **0** | **6** | **47** | **10** | **K.TQEEVMALK.R** |
|  | 6413 | **573.4052** | **1144.7957** | **1143.6499** | **1.1458** | **0** | **1** | **1.1e+002** | **2** | **K.IAELESLTLR.H** |
|  | 6489 | **575.8820** | **1149.7494** | **1148.5859** | **1.1634** | **1** | **4** | **57** | **5** | **K.EEAARMSVLK.E** |
|  | 6692 | 582.2628 | 1162.5111 | 1163.5856 | -1.0745 | 1 | 8 | 23 | 10 | R.KTLEEMELR.I 6711 6712 |
|  | 6845 | **588.2765** | **1174.5384** | **1173.5990** | **0.9395** | **0** | **6** | **44** | **2** | **R.TITNLEGSPSR.S** |
|  | 7218 | **597.8121** | **1193.6096** | **1194.6105** | **-1.0009** | **1** | **6** | **36** | **1** | **R.LQAEHDRQAK.E** |
|  | 8807 | 636.3146 | 1270.6146 | 1269.7041 | 0.9106 | 1 | 9 | 19 | 4 | K.KIENLQEQLR.D |
|  | 10075 | **445.7387** | **1334.1944** | **1333.6547** | **0.5397** | **1** | **0** | **86** | **7** | **K.EECSKLEAQLK.K** |
|  | 12009 | **481.9044** | **1442.6914** | **1442.8167** | **-0.1252** | **1** | **5** | **40** | **7** | **K.LLEMLQSKGLPSK.S** 12008 |
|  | 12859 | **498.3003** | **1491.8792** | **1491.8270** | **0.0522** | **1** | **8** | **20** | **8** | **R.LVHQLKQQTQNR.M** |
|  | 19103 | 634.3330 | 1899.9772 | 1899.9901 | -0.0129 | 1 | 3 | 44 | 1 | K.QLQDLTEEKGTLAGEIR.D |
|  | 19397 | **642.3784** | **1924.1133** | **1923.9764** | **0.1368** | **1** | **(3)** | **45** | **6** | **K.LGSSMNSIKTFWSPELK.K** 19387 |
|  | 19608 | **970.9124** | **1939.8103** | **1939.9713** | **-0.1611** | **1** | **5** | **26** | **8** | **K.LGSSMNSIKTFWSPELK.K** |
|  | 22866 | 1128.5601 | 2255.1056 | 2255.1216 | -0.0160 | 1 | 4 | 29 | 1 | K.LETLSNQNSDCKQHIEVLK.E 22867 |
|  | 25180 | **641.3396** | **2561.3293** | **2560.1421** | **1.1872** | **0** | **0** | **66** | **8** | **R.EDSMADNSQHLQIEELMNALEK.T** |
|  | 31481 | **1359.5964** | **5434.3567** | **5433.5869** | **0.7698** | **1** | **1** | **7** | **1** | **R.AVYGGRVTAMGSSPNIASAGLSHTDVLSYTDQHGGLTGSSHHHHHQVPSMLR.Q** |

| **762.** | Q13501\|SQSTM_HUMAN    **Mass:** 48455    **Score:** 40     **Queries matched:** 5   **emPAI:** 0.08 |
| --- | --- |
|  | Sequestosome-1 OS=Homo sapiens GN=SQSTM1 PE=1 SV=1 |

|  | **Query** | **Observed** | **Mr(expt)** | **Mr(calc)** | **Delta** | **Miss** | **Score** | **Expect** | **Rank** | **Peptide** |
| --- | --- | --- | --- | --- | --- | --- | --- | --- | --- | --- |
|  | 6950 | **591.8054** | **1181.5963** | **1181.6444** | **-0.0482** | **1** | **8** | **21** | **10** | **K.DDIFRIYIK.E** |
|  | 11437 | 705.8328 | 1409.6510 | 1409.7952 | -0.1442 | 1 | 3 | 57 | 9 | -.MASLTVKAYLLGK.E |
|  | 15814 | **558.9296** | **1673.7671** | **1674.8365** | **-1.0695** | **0** | **6** | **29** | **1** | **K.EAALYPHLPPEADPR.L** |
|  | 24468 | 825.3546 | 2473.0418 | 2472.1665 | 0.8753 | 0 | 4 | 27 | 2 | R.LIESLSQMLSMGFSDEGGWLTR.L |
|  | 25223 | **857.7434** | **2570.2084** | **2570.2249** | **-0.0165** | **0** | **40** | **0.0065** | **1** | **R.AGEARPGPTAESASGPSEDPSVNFLK.N** |

| **763.** | O60437\|PEPL_HUMAN    **Mass:** 205124   **Score:** 40     **Queries matched:** 41   **emPAI:** 0.04 |
| --- | --- |
|  | Periplakin OS=Homo sapiens GN=PPL PE=1 SV=3 |

|  | **Query** | **Observed** | **Mr(expt)** | **Mr(calc)** | **Delta** | **Miss** | **Score** | **Expect** | **Rank** | **Peptide** |
| --- | --- | --- | --- | --- | --- | --- | --- | --- | --- | --- |
|  | 965 | 559.2914 | 558.2842 | 558.3238 | -0.0396 | 1 | 15 | 8.2 | 5 | R.QKGAR.E |
|  | 1889 | **410.5484** | **819.0822** | **818.4134** | **0.6689** | **0** | **10** | **15** | **2** | **R.EVSDLTR.Q** |
|  | 2005 | 415.2130 | 828.4114 | 828.5181 | -0.1067 | 1 | 4 | 78 | 9 | K.RLGSVAVK.R |
|  | 2026 | 415.7376 | 829.4607 | 828.5181 | 0.9426 | 1 | 9 | 28 | 5 | R.LGSVAVKR.E |
|  | 2539 | **433.2303** | **864.4461** | **864.3535** | **0.0927** | **0** | **8** | **29** | **7** | **K.TDPEMEK.E** |
|  | 2854 | **444.7129** | **887.4112** | **887.4712** | **-0.0600** | **1** | **19** | **2.6** | **5** | **R.ELDDLKR.L** |
|  | 3247 | **459.2899** | **916.5653** | **915.4661** | **1.0992** | **0** | **21** | **1.5** | **1** | **R.QLEELER.E** 3205 |
|  | 4019 | **487.2954** | **972.5763** | **971.5400** | **1.0363** | **0** | **8** | **32** | **9** | **K.NITNELLR.I** |
|  | 4194 | 494.2559 | 986.4972 | 987.4985 | -1.0013 | 1 | 11 | 15 | 8 | R.NLEAKEER.I |
|  | 4203 | 494.7431 | 987.4716 | 988.5189 | -1.0472 | 1 | 12 | 12 | 2 | K.EEELSRVK.E |
|  | 4809 | **515.8074** | **1029.6003** | **1029.5454** | **0.0549** | **0** | **8** | **27** | **2** | **K.NLLDEIASR.E** |
|  | 5035 | **523.2582** | **1044.5019** | **1043.5360** | **0.9660** | **1** | **6** | **44** | **6** | **K.QVDLERER.A** |
|  | 5056 | **523.7725** | **1045.5305** | **1045.5404** | **-0.0099** | **1** | **11** | **14** | **1** | **R.KVDSDLNQK.Y** |
|  | 5184 | 529.2873 | 1056.5601 | 1055.5836 | 0.9766 | 1 | 6 | 44 | 6 | R.EVQRLQQR.L |
|  | 5415 | 537.2684 | 1072.5223 | 1072.5876 | -0.0653 | 1 | 4 | 80 | 3 | R.VAALAEEKSR.A |
|  | 6378 | 572.3179 | 1142.6212 | 1141.6091 | 1.0121 | 0 | 8 | 26 | 5 | R.AQADEVLQLR.E 6379 |
|  | 7215 | 597.7886 | 1193.5626 | 1192.6564 | 0.9062 | 0 | 12 | 8.7 | 2 | R.SIVVIHPDTGR.E |
|  | 7303 | 399.9089 | 1196.7049 | 1197.6717 | -0.9668 | 0 | 13 | 6.9 | 6 | R.EAEVLLLQQR.V 7292 |
|  | 8176 | **414.2557** | **1239.7454** | **1240.6524** | **-0.9070** | **1** | **3** | **73** | **8** | **R.NQGPQESVVRK.E** |
|  | 8699 | **422.7451** | **1265.2134** | **1264.6299** | **0.5836** | **1** | **3** | **47** | **8** | **K.DYELEAEKLR.S** |
|  | 8708 | **422.8719** | **1265.5938** | **1266.5953** | **-1.0014** | **0** | **3** | **75** | **4** | **K.GPNGESSVIHDR.K** |
|  | 9003 | **641.3450** | **1280.6754** | **1280.6837** | **-0.0083** | **0** | **34** | **0.059** | **1** | **K.VVLQQDPQQAR.E** 8988 |
|  | 9630 | 657.8333 | 1313.6519 | 1312.5619 | 1.0901 | 1 | 3 | 81 | 10 | K.GRMQYDWSDR.N |
|  | 9985 | 665.3254 | 1328.6363 | 1328.6572 | -0.0208 | 0 | 29 | 0.17 | 1 | R.LAALEQEEAEAR.E |
|  | 10846 | **459.2771** | **1374.8094** | **1373.7402** | **1.0692** | **1** | **14** | **5** | **1** | **R.DVTLQKVLDSEK.L** |
|  | 11049 | **463.2142** | **1386.6208** | **1386.6528** | **-0.0320** | **0** | **8** | **21** | **1** | **R.NRPDSGVEEAWK.I** 11066 |
|  | 12661 | **494.6195** | **1480.8367** | **1481.8314** | **-0.9947** | **1** | **8** | **18** | **8** | **K.GAREAEVLLLQQR.V** |
|  | 12847 | 498.0203 | 1491.0390 | 1490.7729 | 0.2660 | 1 | 17 | 1.8 | 2 | R.DGGQEYVVKEVLR.I |
|  | 13078 | 753.3717 | 1504.7288 | 1503.6372 | 1.0916 | 0 | 17 | 2.3 | 1 | R.NMTVADSGTNHDSR.L |
|  | 13141 | **755.3778** | **1508.7411** | **1508.6453** | **0.0958** | **0** | **0** | **1.1e+002** | **10** | **R.SQECDWEEISVK.G** |
|  | 14249 | **395.2062** | **1576.7959** | **1577.8388** | **-1.0430** | **1** | **10** | **13** | **4** | **R.AGLIDWNMFVKLR.S** |
|  | 14388 | 529.3411 | 1585.0014 | 1585.7947 | -0.7933 | 1 | 8 | 17 | 2 | R.LAALEQEEAEAREK.V |
|  | 15166 | **545.6187** | **1633.8343** | **1632.8393** | **0.9950** | **0** | **5** | **33** | **4** | **K.DMSIQELAVLVSGQK.-** |
|  | 18599 | **620.3461** | **1858.0166** | **1858.0047** | **0.0119** | **1** | **2** | **69** | **8** | **R.SISNKELSELIEQLQK.N** |
|  | 18649 | 931.9240 | 1861.8335 | 1862.9156 | -1.0821 | 1 | 9 | 13 | 2 | R.LQSEINMAATETRDLR.N |
|  | 24510 | **828.0527** | **2481.1362** | **2481.1520** | **-0.0158** | **0** | **7** | **14** | **2** | **K.LQNDPQLEAEYQQLQEDHQR.Q** |

| **764.** | Q8N490\|PNKD_HUMAN    **Mass:** 43248    **Score:** 40     **Queries matched:** 7   **emPAI:** 0.18 |
| --- | --- |
|  | Probable hydrolase PNKD OS=Homo sapiens GN=PNKD PE=1 SV=2 |

|  | **Query** | **Observed** | **Mr(expt)** | **Mr(calc)** | **Delta** | **Miss** | **Score** | **Expect** | **Rank** | **Peptide** |
| --- | --- | --- | --- | --- | --- | --- | --- | --- | --- | --- |
|  | 812 | 515.3253 | 514.3180 | 515.2928 | -0.9748 | 1 | 6 | 59 | 8 | K.GRGAR.N |
|  | 4254 | 496.2115 | 990.4085 | 990.5069 | -0.0984 | 1 | 14 | 7.7 | 7 | R.KMQWVQR.Q |
|  | 8914 | 637.9522 | 1273.8899 | 1273.7176 | 0.1722 | 1 | 1 | 81 | 4 | -.MAAVVAATALKGR.G |
|  | 10065 | **667.8262** | **1333.6379** | **1333.6296** | **0.0083** | **1** | **41** | **0.012** | **1** | **R.KGTCPSTLGEER.S** |
|  | 11973 | 481.2421 | 1440.7045 | 1439.7806 | 0.9238 | 0 | 7 | 28 | 4 | K.EGVTLVAILCTHK.H |
|  | 16911 | 581.9573 | 1742.8502 | 1742.9256 | -0.0755 | 1 | 4 | 39 | 8 | R.TWLGYLFYRQQLR.R |
|  | 24834 | **843.0507** | **2526.1302** | **2526.1445** | **-0.0144** | **0** | **24** | **0.3** | **1** | **R.THCLALQEALGPGPGPTGDDDYSR.A** |

| **765.** | O75874\|IDHC_HUMAN    **Mass:** 46915    **Score:** 40     **Queries matched:** 10   **emPAI:** 0.08 |
| --- | --- |
|  | Isocitrate dehydrogenase [NADP] cytoplasmic OS=Homo sapiens GN=IDH1 PE=1 SV=2 |

|  | **Query** | **Observed** | **Mr(expt)** | **Mr(calc)** | **Delta** | **Miss** | **Score** | **Expect** | **Rank** | **Peptide** |
| --- | --- | --- | --- | --- | --- | --- | --- | --- | --- | --- |
|  | 7123 | **397.2237** | **1188.6492** | **1189.5761** | **-0.9269** | **1** | **11** | **13** | **3** | **K.CATITPDEKR.V** |
|  | 7646 | 607.3157 | 1212.6169 | 1212.6648 | -0.0479 | 1 | 3 | 68 | 10 | R.EAIICKNIPR.L |
|  | 8398 | 625.7427 | 1249.4709 | 1249.6046 | -0.1337 | 0 | (1) | 1.1e+002 | 7 | R.LIDDMVAQAMK.S |
|  | 8725 | **634.3229** | **1266.6312** | **1265.5995** | **1.0317** | **0** | **12** | **9.4** | **3** | **R.LIDDMVAQAMK.S** |
|  | 10171 | 671.3363 | 1340.6580 | 1340.6684 | -0.0104 | 0 | 40 | 0.014 | 1 | K.TVEAEAAHGTVTR.H |
|  | 13428 | **509.3129** | **1524.9169** | **1524.6443** | **0.2726** | **0** | **5** | **39** | **2** | **R.SDYLNTFEFMDK.L** |
|  | 16234 | 567.7708 | 1700.2906 | 1700.9322 | -0.6416 | 1 | 3 | 34 | 5 | K.SPNGTIRNILGGTVFR.E |
|  | 16388 | 856.4562 | 1710.8979 | 1710.7553 | 0.1426 | 0 | 1 | 91 | 10 | K.ISGGSVVEMQGDEMTR.I |
|  | 19305 | 959.0138 | 1916.0130 | 1917.1676 | -1.1546 | 1 | 1 | 83 | 2 | K.NIPRLVSGWVKPIIIGR.H |
|  | 25728 | **666.5716** | **2662.2573** | **2662.3642** | **-0.1070** | **1** | **1** | **48** | **5** | **K.EKLIFPYVELDLHSYDLGIENR.D** |

| **766.** | Q8N357\|CB018_HUMAN    **Mass:** 40473    **Score:** 40     **Queries matched:** 4   **emPAI:** 0.09 |
| --- | --- |
|  | Transmembrane protein C2orf18 OS=Homo sapiens GN=C2orf18 PE=1 SV=1 |

|  | **Query** | **Observed** | **Mr(expt)** | **Mr(calc)** | **Delta** | **Miss** | **Score** | **Expect** | **Rank** | **Peptide** |
| --- | --- | --- | --- | --- | --- | --- | --- | --- | --- | --- |
|  | 11241 | **700.2995** | **1398.5844** | **1399.6691** | **-1.0847** | **0** | **40** | **0.013** | **1** | **R.GRPLAEESEQER.L** |
|  | 13733 | **515.5778** | **1543.7115** | **1544.6024** | **-0.8910** | **0** | **5** | **38** | **7** | **K.WADNFMAEGCGGSK.E** |
|  | 19368 | **641.6443** | **1921.9110** | **1923.1095** | **-1.1984** | **1** | **2** | **67** | **10** | **R.GAVIIFTGLFSVAFLGRR.L** |
|  | 27591 | 1000.4479 | 2998.3220 | 2997.6745 | 0.6475 | 0 | 0 | 46 | 8 | K.LSEVITGDLLIIMAQIIVAIQMVLEEK.F |

| **767.** | Q969X0\|RIPL2_HUMAN    **Mass:** 24028    **Score:** 40     **Queries matched:** 5   **emPAI:** 0.16 |
| --- | --- |
|  | RILP-like protein 2 OS=Homo sapiens GN=RILPL2 PE=1 SV=1 |

|  | **Query** | **Observed** | **Mr(expt)** | **Mr(calc)** | **Delta** | **Miss** | **Score** | **Expect** | **Rank** | **Peptide** |
| --- | --- | --- | --- | --- | --- | --- | --- | --- | --- | --- |
|  | 2033 | **416.2108** | **830.4069** | **829.4657** | **0.9412** | **1** | **40** | **0.024** | **1** | **R.KEVEGLR.R** |
|  | 2468 | **431.2187** | **860.4229** | **859.5014** | **0.9214** | **1** | **8** | **32** | **10** | **K.EEKTIIK.K** |
|  | 9220 | **431.5627** | **1291.6663** | **1291.6997** | **-0.0334** | **1** | **6** | **33** | **2** | **K.SGLIPPREGPGGR.R** |
|  | 22304 | **733.6948** | **2198.0626** | **2199.1708** | **-1.1081** | **0** | **2** | **54** | **5** | **R.VLEMLEALVNEGSLALEELK.M** |
|  | 27035 | **722.8475** | **2887.3610** | **2887.4610** | **-0.1000** | **1** | **0** | **52** | **5** | **R.QSPPASGEVNLGPNKMVVDLTDPNRPR.F** |

| **768.** | Q16534\|HLF_HUMAN    **Score:** 40     **Queries matched:** 2 |
| --- | --- |
|  | Hepatic leukemia factor OS=Homo sapiens GN=HLF PE=2 SV=1 |

|  | **Query** | **Observed** | **Mr(expt)** | **Mr(calc)** | **Delta** | **Miss** | **Score** | **Expect** | **Rank** | **Peptide** |
| --- | --- | --- | --- | --- | --- | --- | --- | --- | --- | --- |
|  | 1188 | 648.3890 | 647.3818 | 647.3061 | 0.0757 | 0 | 6 | 62 | 6 | K.NNMAAK.R |
|  | 2033 | 416.2108 | 830.4069 | 829.4294 | 0.9776 | 0 | 40 | 0.024 | 1 | R.QEVADLR.K |

| **769.** | Q9NZ01\|TECR_HUMAN    **Mass:** 36410    **Score:** 40     **Queries matched:** 9   **emPAI:** 0.34 |
| --- | --- |
|  | Trans-2,3-enoyl-CoA reductase OS=Homo sapiens GN=TECR PE=1 SV=1 |

|  | **Query** | **Observed** | **Mr(expt)** | **Mr(calc)** | **Delta** | **Miss** | **Score** | **Expect** | **Rank** | **Peptide** |
| --- | --- | --- | --- | --- | --- | --- | --- | --- | --- | --- |
|  | 3600 | **472.3210** | **942.6274** | **942.5612** | **0.0662** | **0** | **6** | **42** | **3** | **R.MPIIPFLL.-** |
|  | 6579 | **578.3181** | **1154.6217** | **1154.5621** | **0.0596** | **0** | **9** | **20** | **1** | **K.THPQWYPAR.Q** |
|  | 6861 | 588.3669 | 1174.7192 | 1173.6241 | 1.0951 | 1 | 4 | 70 | 4 | K.SLKDEDVLQK.L |
|  | 10236 | 448.9048 | 1343.6926 | 1343.7085 | -0.0158 | 1 | 8 | 21 | 4 | M.KHYEVEILDAK.T 10229 |
|  | 12530 | 738.3769 | 1474.7393 | 1474.7490 | -0.0096 | 1 | 37 | 0.024 | 1 | -.MKHYEVEILDAK.T 12531 |
|  | 20140 | **662.0192** | **1983.0359** | **1983.0499** | **-0.0140** | **1** | **28** | **0.16** | **1** | **K.LCFLDKVEPHATIAEIK.N** |
|  | 30910 | 1099.0580 | 4392.2028 | 4392.0601 | 0.1427 | 1 | 1 | 16 | 4 | R.NIFKNCTYYWGFAAWMAYYINHPLYTPPTYGAQQVK.L |

| **770.** | Q9H223\|EHD4_HUMAN    **Mass:** 61365    **Score:** 39     **Queries matched:** 12   **emPAI:** 0.06 |
| --- | --- |
|  | EH domain-containing protein 4 OS=Homo sapiens GN=EHD4 PE=1 SV=1 |

|  | **Query** | **Observed** | **Mr(expt)** | **Mr(calc)** | **Delta** | **Miss** | **Score** | **Expect** | **Rank** | **Peptide** |
| --- | --- | --- | --- | --- | --- | --- | --- | --- | --- | --- |
|  | 751 | 488.2478 | 487.2405 | 487.2503 | -0.0098 | 0 | 6 | 1.1e+002 | 7 | R.QAGGR.E |
|  | 990 | 572.3035 | 571.2963 | 571.3693 | -0.0731 | 1 | 7 | 42 | 2 | R.KSLPK.A 989 |
|  | 2415 | 429.1953 | 856.3760 | 855.4967 | 0.8793 | 0 | 16 | 5 | 3 | K.FHSLKPK.L |
|  | 2926 | 448.2560 | 894.4975 | 893.4317 | 1.0658 | 0 | 7 | 34 | 8 | K.EMPSVFGK.E |
|  | 7702 | **406.2053** | **1215.5941** | **1216.6815** | **-1.0874** | **1** | **6** | **40** | **10** | **R.KVLPLEEAYR.F** |
|  | 8732 | 634.3363 | 1266.6580 | 1267.5880 | -0.9300 | 1 | 0 | 1.3e+002 | 7 | M.FSWMGRQAGGR.E |
|  | 8996 | 641.3063 | 1280.5980 | 1280.6071 | -0.0091 | 1 | 0 | 1.2e+002 | 4 | K.EMPSVFGKENK.K |
|  | 10779 | **686.8579** | **1371.7013** | **1371.7107** | **-0.0094** | **0** | **39** | **0.016** | **1** | **R.AGGADAVQTVTGGLR.S** |
|  | 11238 | **467.0996** | **1398.2771** | **1398.6285** | **-0.3514** | **1** | **7** | **16** | **10** | **-.MFSWMGRQAGGR.E** |
|  | 11946 | 720.4497 | 1438.8847 | 1438.8296 | 0.0551 | 1 | 9 | 15 | 2 | R.VDRIILLFDAHK.L |
|  | 21421 | **700.4305** | **2098.2698** | **2098.0623** | **0.2075** | **0** | **4** | **26** | **1** | **K.DKPVYDELFYTLSPINGK.I** |

| **771.** | P01907\|HA25_HUMAN    **Mass:** 28267    **Score:** 39     **Queries matched:** 6   **emPAI:** 0.13 |
| --- | --- |
|  | HLA class II histocompatibility antigen, DQ(5) alpha chain OS=Homo sapiens GN=HLA-DQA1 PE=1 SV=2 |

|  | **Query** | **Observed** | **Mr(expt)** | **Mr(calc)** | **Delta** | **Miss** | **Score** | **Expect** | **Rank** | **Peptide** |
| --- | --- | --- | --- | --- | --- | --- | --- | --- | --- | --- |
|  | 4142 | **491.7752** | **981.5359** | **981.5429** | **-0.0070** | **0** | **26** | **0.41** | **1** | **K.HNLNIMIK.R** |
|  | 11869 | 478.9267 | 1433.7584 | 1433.7667 | -0.0083 | 0 | 39 | 0.015 | 1 | K.VEHWGLDQPLLK.H 11836 11868 11872 11882 |

| **772.** | P20036\|HA2Q_HUMAN    **Score:** 39     **Queries matched:** 9 |
| --- | --- |
|  | HLA class II histocompatibility antigen, DP alpha chain OS=Homo sapiens GN=HLA-DPA1 PE=1 SV=1 |

|  | **Query** | **Observed** | **Mr(expt)** | **Mr(calc)** | **Delta** | **Miss** | **Score** | **Expect** | **Rank** | **Peptide** |
| --- | --- | --- | --- | --- | --- | --- | --- | --- | --- | --- |
|  | 1661 | **401.7170** | **801.4194** | **802.3755** | **-0.9561** | **0** | **(9)** | **27** | **6** | **-.MRPEDR.M** |
|  | 1877 | **409.7369** | **817.4593** | **818.3705** | **-0.9111** | **0** | **10** | **21** | **5** | **-.MRPEDR.M** |
|  | 7462 | **602.8329** | **1203.6512** | **1202.7387** | **0.9126** | **0** | **3** | **84** | **4** | **R.ALSLAFLLSLR.G** |
|  | 11869 | 478.9267 | 1433.7584 | 1433.7667 | -0.0083 | 0 | 39 | 0.015 | 1 | R.VEHWGLDQPLLK.H 11836 11868 11872 11882 |
|  | 16245 | **567.9875** | **1700.9406** | **1700.0348** | **0.9058** | **1** | **4** | **45** | **4** | **R.ALSLAFLLSLRGAGAIK.A** |

| **773.** | P05536\|HA27_HUMAN    **Mass:** 28112    **Score:** 39     **Queries matched:** 9   **emPAI:** 0.13 |
| --- | --- |
|  | HLA class II histocompatibility antigen, DQ(W3) alpha chain OS=Homo sapiens GN=HLA-DQA1 PE=2 SV=1 |

|  | **Query** | **Observed** | **Mr(expt)** | **Mr(calc)** | **Delta** | **Miss** | **Score** | **Expect** | **Rank** | **Peptide** |
| --- | --- | --- | --- | --- | --- | --- | --- | --- | --- | --- |
|  | 3532 | **470.3055** | **938.5965** | **937.5345** | **1.0620** | **0** | **2** | **1e+002** | **7** | **K.HNLNSLIK.R** |
|  | 11869 | 478.9267 | 1433.7584 | 1433.8031 | -0.0447 | 0 | 39 | 0.015 | 1 | K.VEHWGLDKPLLK.H 11836 11868 11872 11882 |
|  | 14611 | 800.4476 | 1598.8807 | 1599.8409 | -0.9602 | 0 | 3 | 54 | 7 | R.FDPQFALTNIAVHK.H |
|  | 20172 | **663.3058** | **1986.8957** | **1987.9448** | **-1.0491** | **0** | **3** | **42** | **3** | **K.ISYLTLLPSAEESYDCK.V** |
|  | 30724 | **841.8199** | **4204.0631** | **4204.2316** | **-0.1686** | **0** | **3** | **13** | **5** | **K.HWEPEIPAPMSELTETVVCALGLSVGLVGIVVGTVFIIR.G** |

| **774.** | P01909\|HA23_HUMAN    **Mass:** 28072    **Score:** 39     **Queries matched:** 9   **emPAI:** 0.13 |
| --- | --- |
|  | HLA class II histocompatibility antigen, DQ(3) alpha chain OS=Homo sapiens GN=HLA-DQA1 PE=1 SV=1 |

|  | **Query** | **Observed** | **Mr(expt)** | **Mr(calc)** | **Delta** | **Miss** | **Score** | **Expect** | **Rank** | **Peptide** |
| --- | --- | --- | --- | --- | --- | --- | --- | --- | --- | --- |
|  | 3532 | 470.3055 | 938.5965 | 937.5345 | 1.0620 | 0 | 2 | 1e+002 | 7 | K.HNLNSLIK.R |
|  | 11869 | 478.9267 | 1433.7584 | 1433.8031 | -0.0447 | 0 | 39 | 0.015 | 1 | K.VEHWGLDKPLLK.H 11836 11868 11872 11882 |
|  | 14254 | **789.4358** | **1576.8571** | **1575.8661** | **0.9911** | **0** | **1** | **87** | **9** | **R.FDPQFALTNIAVLK.H** |
|  | 20172 | 663.3058 | 1986.8957 | 1987.9448 | -1.0491 | 0 | 3 | 42 | 3 | K.ISYLTLLPSAEESYDCK.V |
|  | 30724 | 841.8199 | 4204.0631 | 4204.2316 | -0.1686 | 0 | 3 | 13 | 5 | K.HWEPEIPAPMSELTETVVCALGLSVGLVGIVVGTVFIIR.G |

| **775.** | Q6UWP7\|LCLT1_HUMAN    **Mass:** 49344    **Score:** 39     **Queries matched:** 6   **emPAI:** 0.08 |
| --- | --- |
|  | Lysocardiolipin acyltransferase 1 OS=Homo sapiens GN=LCLAT1 PE=1 SV=1 |

|  | **Query** | **Observed** | **Mr(expt)** | **Mr(calc)** | **Delta** | **Miss** | **Score** | **Expect** | **Rank** | **Peptide** |
| --- | --- | --- | --- | --- | --- | --- | --- | --- | --- | --- |
|  | 5373 | 536.2705 | 1070.5265 | 1070.5760 | -0.0495 | 1 | 3 | 82 | 5 | R.YSYLRLEK.I 5372 |
|  | 6365 | **571.7917** | **1141.5689** | **1141.5768** | **-0.0079** | **0** | **39** | **0.019** | **1** | **R.TTGFTFVVDR.L** |
|  | 8244 | 622.3537 | 1242.6928 | 1241.6663 | 1.0266 | 1 | 6 | 36 | 6 | R.SVIIMNHRTR.M |
|  | 10812 | **458.7830** | **1373.3272** | **1372.7351** | **0.5922** | **0** | **11** | **7.3** | **1** | **K.VIITGDAFVPGER.S** |
|  | 21515 | **703.6509** | **2107.9308** | **2107.0462** | **0.8846** | **0** | **0** | **83** | **10** | **K.GVPGFGWAMQAAAYIFIHR.K** |

| **776.** | Q8IWV7\|UBR1_HUMAN    **Mass:** 203445   **Score:** 39     **Queries matched:** 38   **emPAI:** 0.02 |
| --- | --- |
|  | E3 ubiquitin-protein ligase UBR1 OS=Homo sapiens GN=UBR1 PE=1 SV=1 |

|  | **Query** | **Observed** | **Mr(expt)** | **Mr(calc)** | **Delta** | **Miss** | **Score** | **Expect** | **Rank** | **Peptide** |
| --- | --- | --- | --- | --- | --- | --- | --- | --- | --- | --- |
|  | 1909 | **411.2218** | **820.4291** | **821.4395** | **-1.0104** | **0** | **10** | **17** | **1** | **R.ISGYNIR.H** 1919 |
|  | 3439 | 466.7478 | 931.4810 | 930.5902 | 0.8908 | 1 | 5 | 58 | 8 | R.KIFPSVIK.Y |
|  | 3825 | **480.2765** | **958.5384** | **957.5719** | **0.9665** | **1** | **6** | **53** | **6** | **R.LSRLGAVSR.L** |
|  | 3983 | **486.3485** | **970.6825** | **970.4356** | **0.2469** | **0** | **2** | **87** | **4** | **K.NDEITHDK.E** |
|  | 5107 | **525.7571** | **1049.4997** | **1048.5890** | **0.9107** | **1** | **8** | **27** | **5** | **K.RGNPLHLSR.E** |
|  | 5256 | 531.2894 | 1060.5643 | 1059.4291 | 1.1352 | 0 | 7 | 35 | 5 | K.SGETTYSCR.D |
|  | 5511 | **540.2849** | **1078.5551** | **1079.5360** | **-0.9808** | **1** | **6** | **38** | **5** | **K.VPPDERDPR.V** |
|  | 5719 | **547.8203** | **1093.6261** | **1093.6131** | **0.0129** | **0** | **19** | **1.9** | **1** | **K.ELPPELQIR.E** 5705 5718 5720 5721 5723 5724 5732 |
|  | 6300 | **569.2352** | **1136.4558** | **1135.5729** | **0.8829** | **0** | **3** | **77** | **5** | **K.IMAQMSALQK.N** |
|  | 9009 | **641.3984** | **1280.7823** | **1279.6998** | **1.0825** | **1** | **3** | **63** | **5** | **R.LMLWDAKLYK.G** |
|  | 11567 | **473.3066** | **1416.8978** | **1416.5901** | **0.3077** | **0** | **6** | **34** | **2** | **K.LMYDNTSEMPGK.E** |
|  | 11724 | **476.2689** | **1425.7848** | **1426.7238** | **-0.9390** | **0** | **5** | **47** | **7** | **R.CPLNEEVIVQAR.K** |
|  | 12044 | **723.3599** | **1444.7053** | **1444.6980** | **0.0072** | **0** | **11** | **11** | **2** | **K.TVVQSCGHSLETK.S** |
|  | 12920 | 748.8484 | 1495.6823 | 1495.6833 | -0.0009 | 0 | 7 | 27 | 4 | K.ILTCMQGMEEIR.R |
|  | 13046 | **501.9179** | **1502.7318** | **1501.7751** | **0.9567** | **1** | **8** | **22** | **1** | **R.MQFLEGFRSFLK.I** |
|  | 13222 | 757.8058 | 1513.5970 | 1514.7399 | -1.1428 | 0 | 7 | 25 | 2 | R.MEISAELPQTPQR.L |
|  | 13944 | 519.6383 | 1555.8931 | 1554.8188 | 1.0743 | 1 | 1 | 1e+002 | 9 | R.CPLNEEVIVQARK.I |
|  | 15451 | 551.5899 | 1651.7479 | 1651.7844 | -0.0365 | 1 | (0) | 1e+002 | 10 | K.ILTCMQGMEEIRR.Q |
|  | 15463 | **414.1979** | **1652.7627** | **1651.7844** | **0.9783** | **1** | **1** | **98** | **10** | **K.ILTCMQGMEEIRR.Q** |
|  | 17058 | 585.9530 | 1754.8372 | 1755.8576 | -1.0204 | 0 | 2 | 63 | 5 | K.DMITWILQMFDTVK.R |
|  | 17301 | **592.5470** | **1774.6192** | **1773.9005** | **0.7187** | **0** | **3** | **44** | **2** | **K.DIIMLQIGASLMDPNK.F** |
|  | 17315 | 444.9609 | 1775.8147 | 1774.9685 | 0.8462 | 1 | 4 | 40 | 9 | R.LGSSAMNIQMLLEKLK.G 17305 |
|  | 17605 | **597.9549** | **1790.8429** | **1790.9634** | **-0.1205** | **1** | **(4)** | **40** | **1** | **R.LGSSAMNIQMLLEKLK.G** |
|  | 17668 | **599.2910** | **1794.8512** | **1793.8982** | **0.9530** | **1** | **8** | **18** | **3** | **R.YVPGVGNVTKEEVTMR.E** |
|  | 18866 | 940.9512 | 1879.8878 | 1879.9495 | -0.0617 | 1 | 2 | 64 | 9 | K.ASRLGSSAMNIQMLLEK.L |
|  | 19567 | **969.4775** | **1936.9405** | **1936.8811** | **0.0594** | **1** | **1** | **77** | **4** | **R.LGSWMNKIMSYSSDFR.Q** |
|  | 20008 | 986.0126 | 1970.0107 | 1970.0585 | -0.0478 | 1 | 39 | 0.011 | 2 | K.SYRVSEDLVSIHLPLSR.T |
|  | 20338 | 667.3329 | 1998.9768 | 1997.9728 | 1.0041 | 0 | 8 | 17 | 3 | R.ALDCELAEAQLHTTAIDK.E |
|  | 20776 | **681.3643** | **2041.0710** | **2039.9759** | **1.0950** | **0** | **2** | **59** | **10** | **K.NLPENENNETGLENVINK.V** |

| **777.** | O94906\|PRP6_HUMAN    **Mass:** 107656   **Score:** 39     **Queries matched:** 28   **emPAI:** 0.03 |
| --- | --- |
|  | Pre-mRNA-processing factor 6 OS=Homo sapiens GN=PRPF6 PE=1 SV=1 |

|  | **Query** | **Observed** | **Mr(expt)** | **Mr(calc)** | **Delta** | **Miss** | **Score** | **Expect** | **Rank** | **Peptide** |
| --- | --- | --- | --- | --- | --- | --- | --- | --- | --- | --- |
|  | 813 | 515.3253 | 514.3180 | 514.3227 | -0.0047 | 0 | 12 | 13 | 8 | R.LVAGR.I 808 |
|  | 924 | **545.3014** | **544.2941** | **543.3129** | **0.9812** | **0** | **3** | **1.7e+002** | **1** | **K.IGQAR.N** |
|  | 1835 | **408.2217** | **814.4287** | **813.5072** | **0.9215** | **1** | **6** | **55** | **8** | **K.KIGDILR.L** |
|  | 1999 | **414.7604** | **827.5062** | **827.5593** | **-0.0530** | **1** | **8** | **26** | **2** | **R.LLLKSVR.E** |
|  | 2149 | 419.3141 | 836.6136 | 835.4664 | 1.1472 | 0 | 3 | 67 | 4 | R.HLPQSVR.I |
|  | 2932 | 448.7231 | 895.4315 | 895.3892 | 0.0424 | 0 | 10 | 15 | 2 | R.NTLMDMR.L |
|  | 3528 | 470.2447 | 938.4748 | 938.5338 | -0.0590 | 0 | 13 | 7.4 | 3 | R.HIWITAAK.L 3529 |
|  | 3689 | 475.4319 | 948.8492 | 948.5392 | 0.3100 | 1 | 4 | 44 | 3 | R.LEYRAGLK.N |
|  | 3928 | **484.2362** | **966.4579** | **965.4818** | **0.9762** | **1** | **7** | **29** | **3** | **K.EEIEKYR.M** 3907 3940 |
|  | 4039 | 487.9703 | 973.9261 | 974.5219 | -0.5957 | 0 | 3 | 62 | 3 | K.NIANTLMAK.A |
|  | 6043 | **559.3400** | **1116.6655** | **1116.6001** | **0.0654** | **0** | **7** | **35** | **6** | **K.AEVLWLMGAK.S** |
|  | 6610 | 579.2920 | 1156.5694 | 1156.6928 | -0.1234 | 1 | 7 | 31 | 4 | K.IIDRAITSLR.A |
|  | 7055 | **396.2254** | **1185.6544** | **1185.5601** | **0.0943** | **0** | **0** | **1.5e+002** | **3** | **R.HGELWCAVSK.D** |
|  | 7831 | **611.8416** | **1221.6687** | **1220.6441** | **1.0245** | **0** | **2** | **93** | **6** | **K.LTPVPDSFFAK.H** |
|  | 10207 | 672.3541 | 1342.6936 | 1343.7595 | -1.0659 | 1 | 8 | 22 | 4 | R.AGLKNIANTLMAK.A |
|  | 10929 | 460.9178 | 1379.7315 | 1380.7990 | -1.0674 | 1 | 3 | 79 | 9 | R.HLPQSVRIYIR.A |
|  | 12359 | **732.3494** | **1462.6842** | **1461.6769** | **1.0073** | **0** | **3** | **72** | **3** | **K.LEEANGNTQMVEK.I** |
|  | 14040 | 781.9207 | 1561.8269 | 1561.8464 | -0.0195 | 1 | 39 | 0.015 | 2 | R.IYIRAAELETDIR.A |
|  | 15955 | 562.2513 | 1683.7320 | 1682.8277 | 0.9043 | 0 | 5 | 37 | 4 | R.ETNPHHPPAWIASAR.L |
|  | 18040 | **911.3987** | **1820.7828** | **1820.9567** | **-0.1739** | **1** | **2** | **73** | **3** | **R.ESLEALLQRAVAHCPK.A** |
|  | 19647 | 649.0177 | 1944.0313 | 1943.9622 | 0.0691 | 1 | 4 | 36 | 4 | K.AAVELEEPEDARIMLSR.A |
|  | 25723 | **1331.7179** | **2661.4212** | **2660.4261** | **0.9951** | **1** | **3** | **31** | **1** | **K.KPFLGMPAPLGYVPGLGRGATGFTTR.S** 25722 |
|  | 30598 | 1367.0310 | 4098.0712 | 4098.9126 | -0.8414 | 1 | 0 | 26 | 10 | R.QTQFGGLNTPYPGGLNTPYPGGMTPGLMTPGTGELDMRK.I |

| **778.** | P09917\|LOX5_HUMAN    **Mass:** 78675    **Score:** 39     **Queries matched:** 8   **emPAI:** 0.05 |
| --- | --- |
|  | Arachidonate 5-lipoxygenase OS=Homo sapiens GN=ALOX5 PE=1 SV=2 |

|  | **Query** | **Observed** | **Mr(expt)** | **Mr(calc)** | **Delta** | **Miss** | **Score** | **Expect** | **Rank** | **Peptide** |
| --- | --- | --- | --- | --- | --- | --- | --- | --- | --- | --- |
|  | 12678 | **494.9372** | **1481.7899** | **1481.7158** | **0.0741** | **0** | **3** | **60** | **6** | **K.ANATGGGGHVQMVQR.A** |
|  | 14843 | 807.9287 | 1613.8429 | 1613.8526 | -0.0097 | 1 | 6 | 31 | 6 | R.WITGDVEVVLRDGR.A |
|  | 15248 | **547.2833** | **1638.8282** | **1637.8236** | **1.0046** | **0** | **12** | **6.5** | **1** | **R.THLVSEVFGIAMYR.Q** 15234 |
|  | 17556 | **895.9271** | **1789.8397** | **1789.8498** | **-0.0101** | **0** | **39** | **0.014** | **1** | **R.WMEWNPGFPLSIDAK.C** |
|  | 18360 | **461.7180** | **1842.8429** | **1843.8781** | **-1.0353** | **1** | **8** | **18** | **1** | **K.ANATGGGGHVQMVQRAMK.D** |
|  | 22059 | 542.5454 | 2166.1525 | 2165.8975 | 0.2550 | 0 | 1 | 67 | 9 | R.FMHMFQSSWNDFADFEK.I |
|  | 22751 | **746.3738** | **2236.0995** | **2237.0728** | **-0.9733** | **1** | **4** | **32** | **1** | **K.QYRWMEWNPGFPLSIDAK.C** |

| **779.** | Q9C0E8\|LNP_HUMAN    **Mass:** 48052    **Score:** 39     **Queries matched:** 10   **emPAI:** 0.08 |
| --- | --- |
|  | Protein lunapark OS=Homo sapiens GN=LNP PE=1 SV=2 |

|  | **Query** | **Observed** | **Mr(expt)** | **Mr(calc)** | **Delta** | **Miss** | **Score** | **Expect** | **Rank** | **Peptide** |
| --- | --- | --- | --- | --- | --- | --- | --- | --- | --- | --- |
|  | 2185 | **841.1036** | **840.0964** | **839.4389** | **0.6575** | **1** | **7** | **20** | **5** | **K.ETYKTAK.L** |
|  | 3594 | **472.2843** | **942.5540** | **942.5862** | **-0.0322** | **1** | **4** | **67** | **7** | **K.TAKLILER.F** |
|  | 6284 | 568.3108 | 1134.6070 | 1133.6002 | 1.0069 | 1 | 8 | 28 | 6 | K.KILEEVMEK.E |
|  | 10053 | **445.2184** | **1332.6334** | **1331.7085** | **0.9249** | **1** | **5** | **50** | **5** | **K.LILERFDPDSK.K** |
|  | 10451 | 678.3231 | 1354.6317 | 1353.7616 | 0.8701 | 0 | 16 | 3.2 | 1 | R.TVTPALSSNVLPR.H |
|  | 13187 | **756.3818** | **1510.7491** | **1510.7588** | **-0.0097** | **1** | **39** | **0.015** | **1** | **K.ILEEVMEKETYK.T** |
|  | 21398 | **699.6735** | **2095.9986** | **2095.0116** | **0.9869** | **0** | **8** | **15** | **2** | **K.ECEPPSAGAAVTARPGQEIR.Q** |
|  | 28685 | 649.3180 | 3241.5539 | 3240.4831 | 1.0708 | 1 | 4 | 18 | 4 | R.YALICQQCFSHNGMALKEEFEYIAFR.C |
|  | 29481 | 1166.5847 | 3496.7323 | 3496.6690 | 0.0633 | 1 | 1 | 33 | 5 | R.IVEYLVGDGPQNRYALICQQCFSHNGMALK.E |
|  | 30683 | **1387.0847** | **4158.2323** | **4157.3048** | **0.9275** | **1** | **2** | **13** | **1** | **R.TVTPALSSNVLPRHLGSPATSVPGMGLHPPGPPLARPILPR.E** |

| **780.** | Q04941\|PLP2_HUMAN    **Mass:** 17022    **Score:** 39     **Queries matched:** 2   **emPAI:** 0.23 |
| --- | --- |
|  | Proteolipid protein 2 OS=Homo sapiens GN=PLP2 PE=1 SV=1 |

|  | **Query** | **Observed** | **Mr(expt)** | **Mr(calc)** | **Delta** | **Miss** | **Score** | **Expect** | **Rank** | **Peptide** |
| --- | --- | --- | --- | --- | --- | --- | --- | --- | --- | --- |
|  | 8350 | **624.7926** | **1247.5706** | **1247.5782** | **-0.0076** | **0** | **39** | **0.019** | **1** | **R.HTAAPTDPADGPV.-** 8368 |

| **781.** | P98194\|AT2C1_HUMAN    **Mass:** 101653   **Score:** 39     **Queries matched:** 16   **emPAI:** 0.07 |
| --- | --- |
|  | Calcium-transporting ATPase type 2C member 1 OS=Homo sapiens GN=ATP2C1 PE=1 SV=3 |

|  | **Query** | **Observed** | **Mr(expt)** | **Mr(calc)** | **Delta** | **Miss** | **Score** | **Expect** | **Rank** | **Peptide** |
| --- | --- | --- | --- | --- | --- | --- | --- | --- | --- | --- |
|  | 3254 | **459.8888** | **917.7630** | **917.4865** | **0.2765** | **1** | **12** | **7.5** | **1** | **K.ARMGSAGLR.V** 3242 3253 |
|  | 4110 | 490.2845 | 978.5544 | 979.5927 | -1.0383 | 1 | 5 | 46 | 4 | K.DVIRKPPR.N |
|  | 8857 | **637.3416** | **1272.6687** | **1272.6786** | **-0.0100** | **0** | **35** | **0.041** | **1** | **K.GQTLTLTQQQR.D** 8880 |
|  | 9996 | **665.7878** | **1329.5610** | **1329.7326** | **-0.1716** | **1** | **14** | **6.2** | **1** | **K.TPLQKSMDLLGK.Q** 10003 |
|  | 11400 | **704.8427** | **1407.6709** | **1407.6816** | **-0.0108** | **0** | **24** | **0.52** | **1** | **K.MGLDGLQQDYIR.K** |
|  | 14114 | 523.2885 | 1566.8435 | 1567.7954 | -0.9519 | 0 | 5 | 41 | 8 | K.VTAPQPAATNGDLASR.S 14109 |
|  | 17212 | **590.3310** | **1767.9711** | **1767.8680** | **0.1032** | **0** | **4** | **45** | **3** | **K.GVVIGTGENSEFGEVFK.M** |
|  | 20177 | 994.5059 | 1986.9972 | 1988.0434 | -1.0463 | 0 | 3 | 47 | 2 | R.NNTLMGKPTEGALIALAMK.M |
|  | 27480 | **746.1303** | **2980.4919** | **2981.3398** | **-0.8479** | **1** | **5** | **16** | **2** | **R.DTTMTFTCFVFFDMFNALSSRSQTK.S** |
|  | 29842 | **1218.8398** | **3653.4977** | **3654.0392** | **-0.5415** | **0** | **3** | **14** | **1** | **K.DILEMFTISVSLAVAAIPEGLPIVVTVTLALGVMR.M** |
|  | 29844 | **914.5029** | **3653.9826** | **3654.0392** | **-0.0566** | **0** | **(0)** | **30** | **3** | **K.DILEMFTISVSLAVAAIPEGLPIVVTVTLALGVMR.M** |

| **782.** | P49326\|FMO5_HUMAN    **Mass:** 60638    **Score:** 39     **Queries matched:** 18   **emPAI:** 0.06 |
| --- | --- |
|  | Dimethylaniline monooxygenase [N-oxide-forming] 5 OS=Homo sapiens GN=FMO5 PE=2 SV=2 |

|  | **Query** | **Observed** | **Mr(expt)** | **Mr(calc)** | **Delta** | **Miss** | **Score** | **Expect** | **Rank** | **Peptide** |
| --- | --- | --- | --- | --- | --- | --- | --- | --- | --- | --- |
|  | 2524 | **432.7481** | **863.4816** | **864.5069** | **-1.0253** | **1** | **10** | **19** | **1** | **K.NKISLYK.K** |
|  | 4176 | 493.2762 | 984.5379 | 984.5617 | -0.0238 | 1 | 10 | 15 | 5 | R.RGAWILNR.V |
|  | 4820 | 516.2860 | 1030.5575 | 1029.6117 | 0.9458 | 1 | 9 | 21 | 4 | R.IRKPLMTR.V |
|  | 4832 | 516.7557 | 1031.4968 | 1031.5036 | -0.0068 | 0 | 10 | 19 | 1 | R.TDDIGGLWR.F 4856 |
|  | 4861 | **517.2924** | **1032.5703** | **1031.5611** | **1.0092** | **1** | **15** | **5.4** | **2** | **R.KAILTTDDR.I** 4824 |
|  | 5348 | **535.3050** | **1068.5954** | **1068.5638** | **0.0316** | **1** | **4** | **63** | **7** | **R.FKTTVCSVK.K** |
|  | 7989 | **411.0957** | **1230.2653** | **1229.5632** | **0.7021** | **0** | **0** | **95** | **8** | **R.SSSMTSTMTIGK.F** |
|  | 8238 | 415.2273 | 1242.6600 | 1243.7071 | -1.0471 | 1 | 5 | 45 | 6 | R.KPLMTRVVER.S 8237 |
|  | 13855 | **776.3683** | **1550.7221** | **1550.7320** | **-0.0098** | **0** | **39** | **0.017** | **1** | **K.TLPSQSEMMAEISK.A** |
|  | 14098 | 783.8884 | 1565.7622 | 1566.7269 | -0.9647 | 0 | (9) | 17 | 3 | K.TLPSQSEMMAEISK.A |
|  | 16952 | 873.7910 | 1745.5675 | 1744.8335 | 0.7339 | 1 | 10 | 6.9 | 1 | R.VVERSSSMTSTMTIGK.F |
|  | 18899 | **628.3613** | **1882.0620** | **1880.9223** | **1.1397** | **1** | **4** | **34** | **7** | **K.GLKTLPSQSEMMAEISK.A** 18884 |
|  | 29875 | **918.7181** | **3670.8435** | **3670.8553** | **-0.0118** | **0** | **2** | **21** | **1** | **R.HTIQGDYIDTMEELADLVGVRPNLLSLAFTDPK.L** |
|  | 29878 | **1225.1733** | **3672.4982** | **3672.7164** | **-0.2182** | **1** | **1** | **21** | **2** | **K.EMNVFDGVMVCTGHHTNAHLPLESFPGIEKFK.G** |

| **783.** | Q14677\|EPN4_HUMAN    **Mass:** 68273    **Score:** 39     **Queries matched:** 7   **emPAI:** 0.11 |
| --- | --- |
|  | Clathrin interactor 1 OS=Homo sapiens GN=CLINT1 PE=1 SV=1 |

|  | **Query** | **Observed** | **Mr(expt)** | **Mr(calc)** | **Delta** | **Miss** | **Score** | **Expect** | **Rank** | **Peptide** |
| --- | --- | --- | --- | --- | --- | --- | --- | --- | --- | --- |
|  | 14227 | **788.4282** | **1574.8419** | **1573.7161** | **1.1258** | **0** | **4** | **43** | **5** | **R.SLENYHFVDEHGK.D** |
|  | 14641 | **801.4779** | **1600.9413** | **1599.7450** | **1.1963** | **0** | **0** | **1.1e+002** | **1** | **K.ATNVVMNYSEIESK.V** |
|  | 14762 | 805.4125 | 1608.8105 | 1608.8220 | -0.0115 | 0 | 33 | 0.055 | 1 | K.HIHITQATETTTTR.H 14763 |
|  | 15254 | **547.3155** | **1638.9246** | **1639.8093** | **-0.8847** | **1** | **3** | **57** | **8** | **K.SAFPFSDKLGELSDK.I** |
|  | 16488 | **573.2811** | **1716.8216** | **1716.9886** | **-0.1670** | **1** | **2** | **70** | **2** | **K.SLLLLAYLIRNGSER.V** |
|  | 23175 | **765.3496** | **2293.0270** | **2293.0370** | **-0.0100** | **0** | **12** | **4.8** | **1** | **K.ATFMYEQFPELMNMLWSR.M** |

| **784.** | Q5VST9\|OBSCN_HUMAN    **Mass:** 879630   **Score:** 38     **Queries matched:** 115   **emPAI:** 0.00 |
| --- | --- |
|  | Obscurin OS=Homo sapiens GN=OBSCN PE=1 SV=3 |

|  | **Query** | **Observed** | **Mr(expt)** | **Mr(calc)** | **Delta** | **Miss** | **Score** | **Expect** | **Rank** | **Peptide** |
| --- | --- | --- | --- | --- | --- | --- | --- | --- | --- | --- |
|  | 870 | **528.2734** | **527.2662** | **526.3227** | **0.9435** | **0** | **1** | **1.3e+002** | **6** | **R.ALPAR.F** |
|  | 874 | **529.7631** | **528.7558** | **529.2972** | **-0.5414** | **0** | **12** | **11** | **8** | **K.NAAVR.A** |
|  | 883 | 533.1865 | 532.1792 | 532.2605 | -0.0813 | 0 | 3 | 1.4e+002 | 6 | K.SSPSR.Q |
|  | 1215 | **664.3569** | **663.3496** | **663.3010** | **0.0486** | **0** | **5** | **60** | **3** | **R.CELSR.A** |
|  | 1261 | 688.9907 | 687.9834 | 687.4214 | 0.5621 | 1 | 1 | 1.5e+002 | 9 | R.LMLRR.T |
|  | 1567 | **397.8761** | **793.7376** | **793.4123** | **0.3254** | **0** | **6** | **28** | **3** | **K.AVQWYK.D** |
|  | 2135 | 837.4772 | 836.4700 | 836.4756 | -0.0056 | 0 | 38 | 0.024 | 1 | R.LHITEPK.M |
|  | 2242 | **422.2407** | **842.4668** | **841.5022** | **0.9647** | **0** | **9** | **24** | **1** | **R.LVVQQAGK.T** |
|  | 2246 | **422.7362** | **843.4579** | **843.5429** | **-0.0850** | **0** | **10** | **19** | **8** | **R.VLISATLK.N** |
|  | 2247 | **844.4819** | **843.4746** | **843.4926** | **-0.0180** | **1** | **4** | **88** | **10** | **K.ASGRALAAK.I** |
|  | 2798 | **442.2986** | **882.5826** | **881.3987** | **1.1839** | **0** | **9** | **17** | **1** | **R.VEMCISK.E** |
|  | 2822 | **443.0394** | **884.0642** | **883.5239** | **0.5402** | **0** | **8** | **15** | **7** | **R.AQLLVQGR.R** 2823 |
|  | 2890 | **446.2687** | **890.5229** | **890.4280** | **0.0949** | **0** | **6** | **47** | **10** | **R.VEAVGCTR.R** |
|  | 2964 | **450.2372** | **898.4598** | **899.4647** | **-1.0049** | **1** | **8** | **26** | **2** | **K.IAMEKHR.G** 2971 |
|  | 3051 | **453.2162** | **904.4178** | **903.4199** | **0.9980** | **0** | **6** | **53** | **6** | **K.EGATGQWR.L** |
|  | 3220 | 458.7830 | 915.5515 | 915.4661 | 0.0854 | 0 | 11 | 17 | 6 | R.VLEEEAAR.E 3229 |
|  | 3310 | **461.7378** | **921.4611** | **922.5236** | **-1.0625** | **1** | **5** | **58** | **5** | **K.LSFSSKVR.M** |
|  | 3344 | 463.2302 | 924.4458 | 924.4917 | -0.0458 | 0 | 3 | 81 | 4 | K.GSSITFSVK.V |
|  | 3407 | **465.4830** | **928.9515** | **928.5018** | **0.4497** | **0** | **2** | **71** | **8** | **R.TYTLIYR.R** |
|  | 3496 | 469.2215 | 936.4284 | 935.4712 | 0.9572 | 0 | 6 | 35 | 4 | R.FEALTEAR.Q |
|  | 4015 | **487.2922** | **972.5698** | **973.5821** | **-1.0123** | **1** | **5** | **67** | **1** | **K.AVIFRNVR.D** |
|  | 4125 | 491.2207 | 980.4268 | 980.4675 | -0.0407 | 1 | 5 | 53 | 7 | R.DGDRYSLR.Q |
|  | 4201 | 494.5906 | 987.1666 | 987.6229 | -0.4563 | 1 | 0 | 1.2e+002 | 8 | K.QLKFLLAR.S |
|  | 4351 | 499.2080 | 996.4015 | 995.5148 | 0.8867 | 1 | 9 | 18 | 8 | R.RLGEPDGPR.V |
|  | 4735 | 513.2739 | 1024.5332 | 1024.5705 | -0.0374 | 1 | 9 | 19 | 2 | K.IQAAFKGYK.V |
|  | 4890 | 518.2651 | 1034.5156 | 1035.5349 | -1.0193 | 0 | 12 | 11 | 3 | K.EGVELHAGPK.Y 4889 |
|  | 5194 | **529.3709** | **1056.7273** | **1057.5152** | **-0.7880** | **0** | **2** | **1e+002** | **8** | **R.NQEEVVAGGR.V** |
|  | 5316 | **533.7808** | **1065.5471** | **1064.5475** | **0.9996** | **1** | **11** | **12** | **2** | **K.TPSASPRHGR.S** 5305 5309 |
|  | 5446 | **538.2941** | **1074.5736** | **1073.5577** | **1.0158** | **1** | **5** | **52** | **4** | **R.LSRASGQEAR.W** |
|  | 5466 | **539.2597** | **1076.5048** | **1077.5818** | **-1.0770** | **1** | **4** | **61** | **7** | **R.EYEALKGLR.H** |
|  | 5718 | 547.8201 | 1093.6257 | 1094.5468 | -0.9211 | 0 | 8 | 26 | 9 | K.NVEIHAEGAR.H |
|  | 5785 | **550.2785** | **1098.5424** | **1099.5509** | **-1.0085** | **0** | **6** | **39** | **3** | **K.LEPQTVEER.S** 5774 5808 |
|  | 5836 | **551.8049** | **1101.5953** | **1101.5778** | **0.0175** | **1** | **8** | **26** | **6** | **K.KDGQLVTEGR.R** 5855 |
|  | 5939 | **555.3467** | **1108.6788** | **1107.5383** | **1.1405** | **0** | **6** | **39** | **1** | **R.LMPADAGVYR.C** |
|  | 6079 | **560.7188** | **1119.4231** | **1118.5567** | **0.8663** | **0** | **8** | **26** | **4** | **K.EATEGATATLR.C** |
|  | 6117 | **561.7927** | **1121.5709** | **1121.6193** | **-0.0484** | **0** | **1** | **1.4e+002** | **10** | **R.LPSAPSGGAPIR.D** |
|  | 6392 | 572.7687 | 1143.5228 | 1143.6975 | -0.1747 | 1 | 13 | 8 | 4 | R.GLTSKASLIVR.E 6400 |
|  | 6414 | **573.4053** | **1144.7960** | **1143.6400** | **1.1560** | **0** | **9** | **17** | **1** | **K.GGYIAGALPGLR.E** 6411 |
|  | 6450 | 574.7847 | 1147.5549 | 1148.5648 | -1.0099 | 0 | 4 | 68 | 10 | R.FQMVAEGPVR.S |
|  | 7015 | **395.7125** | **1184.1157** | **1184.6626** | **-0.5468** | **1** | **17** | **2.1** | **1** | **K.SIQRADAGIVR.A** |
|  | 7190 | 596.8565 | 1191.6985 | 1192.6815 | -0.9831 | 0 | 9 | 17 | 1 | R.ELLIHQLEAK.D |
|  | 7969 | **615.3776** | **1228.7407** | **1227.6459** | **1.0948** | **1** | **4** | **58** | **8** | **K.KLEPQTVEER.S** 7960 |
|  | 8343 | 416.5564 | 1246.6473 | 1245.7445 | 0.9028 | 1 | 7 | 31 | 6 | R.YQALLKELIR.N |
|  | 8629 | 631.3401 | 1260.6657 | 1259.6544 | 1.0114 | 0 | 1 | 1.3e+002 | 8 | R.QEGAMLELVVR.N |
|  | 8696 | **633.3911** | **1264.7677** | **1263.6645** | **1.1031** | **1** | **1** | **1e+002** | **3** | **R.AMPSKFIEGLR.N** |
|  | 8966 | **427.1657** | **1278.4754** | **1279.5900** | **-1.1146** | **0** | **6** | **34** | **3** | **K.MSLEGQMAELR.I** |
|  | 9204 | 431.2716 | 1290.7929 | 1291.6521 | -0.8592 | 1 | 7 | 25 | 4 | R.VGEDSVDFRLR.V |
|  | 9479 | 435.9008 | 1304.6807 | 1304.6659 | 0.0148 | 1 | 4 | 58 | 7 | K.RFQMVAEGPVR.S |
|  | 9849 | **662.2820** | **1322.5494** | **1322.6752** | **-0.1257** | **0** | **8** | **19** | **1** | **K.LTSQITEMVSAK.I** |
|  | 10527 | **680.3569** | **1358.6993** | **1358.6943** | **0.0050** | **1** | **3** | **83** | **10** | **R.VEVKEGATGQWR.L** |
|  | 10558 | **454.7279** | **1361.1619** | **1361.6497** | **-0.4878** | **1** | **7** | **20** | **4** | **K.DLGDPSMDKAAVK.I** |
|  | 10769 | 458.2314 | 1371.6724 | 1372.7939 | -1.1215 | 1 | 7 | 29 | 8 | R.VRVHDLHVGITK.R |
|  | 10994 | **692.8251** | **1383.6356** | **1384.5929** | **-0.9573** | **0** | **10** | **13** | **3** | **K.DTGEYACVTGGQK.T** |
|  | 11247 | 467.2449 | 1398.7130 | 1398.6119 | 0.1011 | 0 | 4 | 54 | 3 | R.CLAENSMGVSSTK.A |
|  | 11257 | **467.3706** | **1399.0900** | **1399.8221** | **-0.7321** | **1** | **3** | **43** | **8** | **R.TSAMLTVRALPIK.F** |
|  | 11340 | 469.0967 | 1404.2681 | 1404.6966 | -0.4284 | 1 | 2 | 49 | 9 | R.AGKTMAIAAQGACR.S 11361 |
|  | 11451 | 471.2372 | 1410.6899 | 1411.7055 | -1.0156 | 0 | 5 | 44 | 3 | R.HQESIEGATATLR.C |
|  | 11531 | **472.7565** | **1415.2477** | **1415.8170** | **-0.5693** | **1** | **(2)** | **52** | **4** | **R.TSAMLTVRALPIK.F** |
|  | 12290 | **487.2949** | **1458.8628** | **1458.7314** | **0.1314** | **0** | **2** | **85** | **10** | **R.LQSLEQETGDIAR.L** |
|  | 12291 | 487.2949 | 1458.8628 | 1458.7574 | 0.1054 | 1 | 7 | 24 | 4 | R.SLMSYKSILVMR.S |
|  | 12787 | 497.2384 | 1488.6933 | 1489.8075 | -1.1142 | 1 | 6 | 30 | 3 | R.ILRLMPADAGVYR.C |
|  | 13177 | **756.3513** | **1510.6880** | **1510.8831** | **-0.1951** | **1** | **6** | **32** | **3** | **R.TVAELAVQGNLLRK.L** |
|  | 13705 | 514.9953 | 1541.9641 | 1541.8963 | 0.0678 | 1 | 1 | 75 | 8 | K.SILVMRSIPELLR.G |
|  | 13742 | **515.7653** | **1544.2741** | **1544.8576** | **-0.5835** | **0** | **7** | **17** | **1** | **K.VEGRPVPTVHWLR.E** |
|  | 13910 | **519.1341** | **1554.3804** | **1554.7534** | **-0.3730** | **1** | **3** | **43** | **5** | **K.FKMSLEGQMAELR.I** |
|  | 13926 | **519.2864** | **1554.8373** | **1553.6893** | **1.1481** | **1** | **3** | **65** | **3** | **R.DDAGLYECVSRGGR.I** |
|  | 15012 | **541.9654** | **1622.8743** | **1622.7433** | **0.1311** | **1** | **1** | **95** | **10** | **R.TDTVSYVFRNMMK.L** |
|  | 15186 | **818.5800** | **1635.1455** | **1634.9620** | **0.1835** | **1** | **2** | **50** | **6** | **R.HLLKGGYIAGALPGLR.E** |
|  | 15341 | **549.7213** | **1646.1419** | **1645.8643** | **0.2776** | **1** | **(0)** | **77** | **9** | **K.MSLEGQMAELRILR.L** |
|  | 15623 | **554.8499** | **1661.5279** | **1661.8593** | **-0.3314** | **1** | **4** | **29** | **2** | **K.MSLEGQMAELRILR.L** |
|  | 15906 | **840.8801** | **1679.7456** | **1680.8907** | **-1.1451** | **1** | **1** | **93** | **1** | **R.LRHQESIEGATATLR.C** |
|  | 16215 | 567.4080 | 1699.2021 | 1699.7512 | -0.5492 | 1 | 1 | 57 | 10 | R.GFFGCETPDDKTQAK.L |
|  | 16857 | **581.2741** | **1740.8005** | **1740.9734** | **-0.1729** | **1** | **2** | **58** | **4** | **K.TAASLRVTEPEVTIVR.G** 16866 |
|  | 17243 | 886.4230 | 1770.8314 | 1771.8307 | -0.9993 | 0 | (1) | 88 | 6 | K.YDVVCEGTMAMLVIR.G |
|  | 17322 | 592.9548 | 1775.8427 | 1774.9618 | 0.8809 | 1 | 14 | 4.2 | 1 | R.VTGTPKPVISWYKDGK.A 17295 |
|  | 17544 | 895.4117 | 1788.8089 | 1787.8256 | 0.9833 | 0 | 6 | 29 | 2 | K.YDVVCEGTMAMLVIR.G 17543 |
|  | 17644 | 598.7994 | 1793.3765 | 1792.9795 | 0.3970 | 1 | 5 | 22 | 2 | R.SQGATRELLIHQLEAK.D |
|  | 17651 | 598.9493 | 1793.8260 | 1793.8553 | -0.0293 | 1 | 4 | 44 | 3 | R.RTCSTMTGPVHFTVGK.S |
|  | 17752 | **901.4049** | **1800.7951** | **1800.8424** | **-0.0473** | **1** | **1** | **82** | **5** | **R.EAMRAEGAPASPPSTGTR.T** |
|  | 18144 | 914.4797 | 1826.9448 | 1826.9601 | -0.0153 | 0 | 1 | 87 | 8 | R.VTFLAGDMVTSAFLTVR.G |
|  | 18770 | **625.1436** | **1872.4090** | **1871.9265** | **0.4825** | **0** | **2** | **41** | **6** | **R.VGAEGQDFPVQVEEVAAK.F** |
|  | 18999 | **945.5447** | **1889.0748** | **1890.0098** | **-0.9350** | **0** | **3** | **51** | **1** | **R.LTVLGLPDPPEDAEVVAR.S** |
|  | 20059 | 987.9419 | 1973.8692 | 1975.0098 | -1.1406 | 1 | 1 | 75 | 8 | -.MDQPQFSGAPRFLTRPK.A |
|  | 20241 | **498.7573** | **1991.0001** | **1991.0734** | **-0.0734** | **1** | **4** | **35** | **3** | **R.MEVRAAPGLTANKPPAAAAR.E** |
|  | 20824 | 682.3677 | 2044.0812 | 2043.0419 | 1.0393 | 1 | 3 | 47 | 1 | K.EKVTLECELSRPNVDVR.W |
|  | 21101 | **1034.9668** | **2067.9190** | **2066.9691** | **0.9499** | **1** | **2** | **53** | **1** | **R.LVVQQAGKTDAGDYSCEAR.G** |
|  | 22457 | **738.0698** | **2211.1875** | **2210.0505** | **1.1370** | **0** | **5** | **27** | **1** | **R.SLFYHQAGESPEHGALAPGSR.R** |
|  | 22682 | 1116.0297 | 2230.0448 | 2229.0736 | 0.9712 | 0 | 11 | 6.9 | 2 | K.DATLSCQIVGNPTPQVSWEK.D 22681 |
|  | 24200 | 610.0450 | 2436.1508 | 2435.1639 | 0.9869 | 0 | 1 | 52 | 8 | K.VTLEDAGTVSFHVGTCSSEAQLK.V |
|  | 24399 | **822.3887** | **2464.1442** | **2465.0904** | **-0.9462** | **0** | **2** | **46** | **6** | **K.LIISDVHAEDEDTYTCDAGDVK.T** |
|  | 24423 | **823.4073** | **2467.2002** | **2468.2345** | **-1.0343** | **0** | **2** | **47** | **2** | **R.VGDTAMFCVELAVPVGPVHWLR.N** |
|  | 24488 | 620.0757 | 2476.2739 | 2477.2988 | -1.0250 | 1 | 3 | 36 | 8 | R.FQEMFATLGIGVEIKLVEQGPR.R |
|  | 26710 | **941.7573** | **2822.2501** | **2823.3749** | **-1.1248** | **0** | **0** | **51** | **6** | **K.VQAEAGAIATLSCEVAQAQTEVTWYK.D** |
|  | 27201 | **975.8002** | **2924.3787** | **2924.3069** | **0.0718** | **1** | **6** | **16** | **1** | **K.EMKQQEGPMFSHTFGDTEAQVGDALR.L** |
|  | 29215 | **848.9121** | **3391.6191** | **3390.4955** | **1.1236** | **1** | **2** | **26** | **9** | **R.DIGRFHSSFLQELQQCDTDDDVAMCFIK.N** |
|  | 29256 | 1137.2014 | 3408.5824 | 3408.7525 | -0.1701 | 1 | 4 | 16 | 5 | K.EALISTFLQGTTQAISAQGLETASFADLGGQRK.E |
|  | 29632 | **891.4553** | **3561.7919** | **3561.5044** | **0.2875** | **0** | **5** | **13** | **2** | **K.TMEVLEGESCSFECVLSHESASDPAMWTVGGK.T** |
|  | 31419 | **1063.7494** | **5313.7106** | **5314.4724** | **-0.7619** | **0** | **4** | **5.5** | **3** | **R.AGAQACFTCTLSEAVPVGEASWYINGAAVQPDDSDWTVTADGSHHALLLR.S** |

| **785.** | A0FGR8\|ESYT2_HUMAN    **Mass:** 102807   **Score:** 38     **Queries matched:** 17   **emPAI:** 0.11 |
| --- | --- |
|  | Extended synaptotagmin-2 OS=Homo sapiens GN=ESYT2 PE=1 SV=1 |

|  | **Query** | **Observed** | **Mr(expt)** | **Mr(calc)** | **Delta** | **Miss** | **Score** | **Expect** | **Rank** | **Peptide** |
| --- | --- | --- | --- | --- | --- | --- | --- | --- | --- | --- |
|  | 1158 | **639.3044** | **638.2971** | **638.3275** | **-0.0304** | **0** | **2** | **83** | **8** | **K.DTYLK.G** |
|  | 2023 | **415.7192** | **829.4237** | **830.4610** | **-1.0372** | **1** | **8** | **37** | **10** | **K.AQESKIR.Y** |
|  | 4763 | **514.3074** | **1026.6003** | **1026.6073** | **-0.0070** | **1** | **25** | **0.51** | **1** | **R.VIKENLSPK.W** |
|  | 4883 | **518.2003** | **1034.3860** | **1034.5583** | **-0.1723** | **1** | **3** | **89** | **9** | **R.MYLLPDKR.R** |
|  | 5541 | 541.3065 | 1080.5985 | 1080.5200 | 0.0785 | 0 | 3 | 77 | 10 | R.TPTFASSSQR.R |
|  | 5884 | **553.2993** | **1104.5841** | **1104.5927** | **-0.0087** | **1** | **19** | **2.2** | **1** | **K.GKSDPYGIIR.V** |
|  | 11507 | **472.2843** | **1413.8312** | **1412.7558** | **1.0753** | **1** | **9** | **17** | **2** | **R.AGVKSIQIHGTMR.V** |
|  | 12430 | **734.8372** | **1467.6598** | **1468.7885** | **-1.1288** | **0** | **0** | **1.1e+002** | **5** | **R.SSSSLLASPGHISVK.E** |
|  | 13372 | **508.2894** | **1521.8464** | **1521.8627** | **-0.0164** | **1** | **4** | **49** | **5** | **K.VDVGQQPLRINGVK.V** |
|  | 13465 | **510.2550** | **1527.7433** | **1527.7542** | **-0.0109** | **0** | **30** | **0.14** | **1** | **R.ERPPDHQHSAQVK.R** 13464 |
|  | 15286 | **548.2783** | **1641.8131** | **1641.8223** | **-0.0092** | **0** | **4** | **46** | **7** | **K.AQPPEAGPQGLHDLGR.S** |
|  | 16935 | **872.9454** | **1743.8762** | **1743.8865** | **-0.0103** | **0** | **13** | **5.1** | **1** | **R.LEWLTLMPNASNLDK.V** |
|  | 19329 | **640.6422** | **1918.9048** | **1918.9670** | **-0.0622** | **0** | **4** | **44** | **3** | **K.VPLSQLLTSEDMTVSQR.F** |
|  | 20546 | 673.7203 | 2018.1392 | 2018.0473 | 0.0919 | 1 | 4 | 37 | 9 | R.IHFIEAQDLQGKDTYLK.G |
|  | 24150 | 810.1216 | 2427.3431 | 2428.3804 | -1.0373 | 0 | 1 | 52 | 4 | R.VILEPLIGDMPLVGALSIFFLR.K |
|  | 27975 | **1029.4927** | **3085.4562** | **3084.3765** | **1.0797** | **0** | **2** | **31** | **1** | **K.SHMSGSPGPGGSNTAPSTPVIGGSDKPGMEEK.A** |

| **786.** | Q9UQV4\|LAMP3_HUMAN    **Mass:** 44661    **Score:** 38     **Queries matched:** 6   **emPAI:** 0.17 |
| --- | --- |
|  | Lysosome-associated membrane glycoprotein 3 OS=Homo sapiens GN=LAMP3 PE=1 SV=3 |

|  | **Query** | **Observed** | **Mr(expt)** | **Mr(calc)** | **Delta** | **Miss** | **Score** | **Expect** | **Rank** | **Peptide** |
| --- | --- | --- | --- | --- | --- | --- | --- | --- | --- | --- |
|  | 10232 | 448.9043 | 1343.6910 | 1343.7119 | -0.0209 | 0 | 4 | 49 | 8 | K.AEMGIQLIVQDK.E |
|  | 15548 | **553.6166** | **1657.8281** | **1657.8399** | **-0.0118** | **0** | **21** | **0.89** | **1** | **K.HAVVMFQTAVGHSFK.C** |
|  | 15806 | 558.7833 | 1673.3282 | 1673.8348 | -0.5067 | 0 | (4) | 27 | 7 | K.HAVVMFQTAVGHSFK.C |
|  | 18776 | 625.3051 | 1872.8935 | 1874.0005 | -1.1070 | 1 | 2 | 69 | 1 | R.LCIKAEMGIQLIVQDK.E |
|  | 21856 | **716.3727** | **2146.0962** | **2146.1092** | **-0.0130** | **1** | **35** | **0.027** | **1** | **K.AEMGIQLIVQDKESVFSPR.R** |
|  | 29394 | 1155.8984 | 3464.6735 | 3465.7650 | -1.0915 | 1 | 5 | 12 | 5 | K.HAVVMFQTAVGHSFKCVSEQSLQLSAHLQVK.T |

| **787.** | P17931\|LEG3_HUMAN    **Mass:** 26193    **Score:** 38     **Queries matched:** 12   **emPAI:** 0.31 |
| --- | --- |
|  | Galectin-3 OS=Homo sapiens GN=LGALS3 PE=1 SV=5 |

|  | **Query** | **Observed** | **Mr(expt)** | **Mr(calc)** | **Delta** | **Miss** | **Score** | **Expect** | **Rank** | **Peptide** |
| --- | --- | --- | --- | --- | --- | --- | --- | --- | --- | --- |
|  | 1313 | 703.3932 | 702.3860 | 702.3912 | -0.0052 | 0 | 33 | 0.13 | 1 | K.LNEISK.L |
|  | 2489 | 431.7398 | 861.4649 | 861.4708 | -0.0059 | 0 | 40 | 0.02 | 1 | R.IALDFQR.G 2475 |
|  | 2632 | **437.2400** | **872.4654** | **873.4093** | **-0.9439** | **0** | **26** | **0.6** | **1** | **K.LDNNWGR.E** |
|  | 4229 | **495.2773** | **988.5400** | **988.5488** | **-0.0088** | **1** | **29** | **0.23** | **1** | **R.RVIVCNTK.L** |
|  | 8850 | **425.2048** | **1272.5926** | **1272.6000** | **-0.0074** | **0** | **10** | **13** | **1** | **R.GNDVAFHFNPR.F** 8823 |
|  | 9878 | 442.2443 | 1323.7111 | 1323.7187 | -0.0076 | 0 | 29 | 0.16 | 1 | K.IQVLVEPDHFK.V |
|  | 12940 | **749.3855** | **1496.7564** | **1496.7664** | **-0.0099** | **0** | **22** | **0.77** | **1** | **R.QSVFPFESGKPFK.I** |
|  | 15496 | **552.6005** | **1654.7797** | **1655.9392** | **-1.1595** | **0** | **5** | **34** | **8** | **R.MLITILGTVKPNANR.I** |
|  | 24222 | **814.1306** | **2439.3700** | **2440.2407** | **-0.8706** | **1** | **4** | **25** | **4** | **K.LNEISKLGISGDIDLTSASYTMI.-** |
|  | 24668 | **834.0879** | **2499.2420** | **2499.3995** | **-0.1575** | **1** | **1** | **54** | **2** | **R.MLITILGTVKPNANRIALDFQR.G** |

| **788.** | P11169\|GTR3_HUMAN    **Mass:** 54345    **Score:** 38     **Queries matched:** 10   **emPAI:** 0.07 |
| --- | --- |
|  | Solute carrier family 2, facilitated glucose transporter member 3 OS=Homo sapiens GN=SLC2A3 PE=1 SV=1 |

|  | **Query** | **Observed** | **Mr(expt)** | **Mr(calc)** | **Delta** | **Miss** | **Score** | **Expect** | **Rank** | **Peptide** |
| --- | --- | --- | --- | --- | --- | --- | --- | --- | --- | --- |
|  | 1814 | 407.2260 | 812.4374 | 813.4457 | -1.0083 | 1 | 3 | 80 | 4 | K.VPETRGR.T |
|  | 4501 | 503.2898 | 1004.5651 | 1003.6066 | 0.9585 | 1 | 7 | 33 | 7 | K.IIKEFINK.T |
|  | 5863 | 552.7719 | 1103.5293 | 1103.6339 | -0.1046 | 0 | 14 | 7.2 | 3 | K.QVTVLELFR.V |
|  | 6616 | **579.3201** | **1156.6256** | **1157.5214** | **-0.8958** | **0** | **38** | **0.025** | **1** | **R.AFEGQAHGADR.S** |
|  | 11626 | **474.5738** | **1420.6997** | **1419.6374** | **1.0623** | **0** | **4** | **57** | **10** | **K.DGVMEMNSIEPAK.E** |
|  | 12172 | 727.3378 | 1452.6610 | 1451.6272 | 1.0338 | 0 | (1) | 91 | 4 | K.DGVMEMNSIEPAK.E |
|  | 23331 | 1157.5846 | 2313.1546 | 2313.1353 | 0.0193 | 0 | 0 | 69 | 3 | R.NSMLIVNLLAVTGGCFMGLCK.V |
|  | 24547 | 829.7572 | 2486.2498 | 2485.2314 | 1.0184 | 1 | 2 | 46 | 4 | R.RNSMLIVNLLAVTGGCFMGLCK.V |
|  | 25531 | 1313.7139 | 2625.4132 | 2625.3403 | 0.0729 | 0 | 2 | 41 | 5 | R.TLHMIGLGGMAFCSTLMTVSLLLK.D |
|  | 26463 | **928.4783** | **2782.4132** | **2781.4414** | **0.9718** | **1** | **4** | **22** | **1** | **R.RTLHMIGLGGMAFCSTLMTVSLLLK.D** |

| **789.** | Q8TDB8\|GTR14_HUMAN    **Mass:** 56854    **Score:** 38     **Queries matched:** 9   **emPAI:** 0.07 |
| --- | --- |
|  | Solute carrier family 2, facilitated glucose transporter member 14 OS=Homo sapiens GN=SLC2A14 PE=2 SV=1 |

|  | **Query** | **Observed** | **Mr(expt)** | **Mr(calc)** | **Delta** | **Miss** | **Score** | **Expect** | **Rank** | **Peptide** |
| --- | --- | --- | --- | --- | --- | --- | --- | --- | --- | --- |
|  | 1814 | 407.2260 | 812.4374 | 813.4457 | -1.0083 | 1 | 3 | 80 | 4 | K.VPETRGR.T |
|  | 5863 | 552.7719 | 1103.5293 | 1103.6339 | -0.1046 | 0 | 14 | 7.2 | 3 | K.QVTVLELFR.V |
|  | 6616 | 579.3201 | 1156.6256 | 1157.5214 | -0.8958 | 0 | 38 | 0.025 | 1 | R.AFEGQAHGADR.S |
|  | 7975 | **410.7394** | **1229.1964** | **1228.6524** | **0.5440** | **1** | **0** | **95** | **4** | **K.EENATRILQR.L** |
|  | 10920 | 690.8480 | 1379.6814 | 1379.6061 | 0.0753 | 0 | 6 | 37 | 6 | K.DGVMGMNSIEPAK.E |
|  | 20628 | **676.3720** | **2026.0942** | **2024.9031** | **1.1911** | **1** | **2** | **59** | **6** | **K.DGVMGMNSIEPAKETTTNV.-** |
|  | 25531 | 1313.7139 | 2625.4132 | 2625.3403 | 0.0729 | 0 | 2 | 41 | 5 | R.TLHMIGLGGMAFCSTLMTVSLLLK.N |
|  | 26463 | 928.4783 | 2782.4132 | 2781.4414 | 0.9718 | 1 | 4 | 22 | 1 | R.RTLHMIGLGGMAFCSTLMTVSLLLK.N |
|  | 29632 | 891.4553 | 3561.7919 | 3562.8417 | -1.0498 | 1 | 1 | 29 | 3 | R.NSMLIVNLLAATGGCLMGLCKIAESVEMLILGR.L |

| **790.** | P51571\|SSRD_HUMAN    **Mass:** 19158    **Score:** 38     **Queries matched:** 9   **emPAI:** 0.20 |
| --- | --- |
|  | Translocon-associated protein subunit delta OS=Homo sapiens GN=SSR4 PE=1 SV=1 |

|  | **Query** | **Observed** | **Mr(expt)** | **Mr(calc)** | **Delta** | **Miss** | **Score** | **Expect** | **Rank** | **Peptide** |
| --- | --- | --- | --- | --- | --- | --- | --- | --- | --- | --- |
|  | 8660 | **632.3098** | **1262.6049** | **1261.6091** | **0.9958** | **0** | **25** | **0.45** | **1** | **R.YQVSWSLDHK.S** 8637 |
|  | 10540 | **680.8563** | **1359.6980** | **1358.7055** | **0.9924** | **1** | **22** | **0.91** | **2** | **K.QFPVTRGQDVGR.Y** |
|  | 10956 | 461.2333 | 1380.6781 | 1380.6707 | 0.0074 | 0 | 4 | 58 | 3 | R.VQNMALYADVGGK.Q 10936 10944 |
|  | 15183 | **818.4377** | **1634.8609** | **1634.8199** | **0.0411** | **1** | **11** | **8.8** | **1** | **K.NRVQNMALYADVGGK.Q** |
|  | 22050 | 722.7070 | 2165.0993 | 2165.1117 | -0.0124 | 0 | 38 | 0.013 | 1 | R.NNEDISIIPPLFTVSVDHR.G |
|  | 23490 | 778.8956 | 2333.6649 | 2333.1189 | 0.5460 | 1 | 0 | 46 | 3 | R.YQVSWSLDHKSAHAGTYEVR.F |

| **791.** | Q5JWF2\|GNAS1_HUMAN    **Mass:** 111697   **Score:** 38     **Queries matched:** 20   **emPAI:** 0.07 |
| --- | --- |
|  | Guanine nucleotide-binding protein G(s) subunit alpha isoforms XLas OS=Homo sapiens GN=GNAS PE=1 SV=2 |

|  | **Query** | **Observed** | **Mr(expt)** | **Mr(calc)** | **Delta** | **Miss** | **Score** | **Expect** | **Rank** | **Peptide** |
| --- | --- | --- | --- | --- | --- | --- | --- | --- | --- | --- |
|  | 750 | 486.8631 | 485.8558 | 486.3166 | -0.4607 | 0 | 5 | 52 | 5 | K.VLAGK.S |
|  | 1997 | **414.7479** | **827.4813** | **828.4454** | **-0.9641** | **1** | **2** | **1.1e+002** | **10** | **K.VSGAPDKR.E** |
|  | 3392 | 465.2698 | 928.5251 | 928.4726 | 0.0525 | 0 | 23 | 0.83 | 1 | R.AASAAPASGAR.R 3372 3391 3393 3394 3398 |
|  | 5181 | 529.2853 | 1056.5560 | 1056.6179 | -0.0619 | 0 | 28 | 0.31 | 1 | R.LLLLGAGESGK.S |
|  | 6427 | 573.7903 | 1145.5660 | 1146.7012 | -1.1352 | 0 | 10 | 15 | 4 | R.TISVILFLNK.Q |
|  | 8281 | **415.8938** | **1244.6595** | **1243.6574** | **1.0021** | **1** | **8** | **23** | **5** | **K.SIWNNRWLR.T** |
|  | 12473 | 491.2423 | 1470.7051 | 1471.7031 | -0.9980 | 0 | 4 | 52 | 9 | R.NFLVQAFGGCFGR.S |
|  | 14390 | 529.3709 | 1585.0909 | 1584.9450 | 0.1459 | 1 | 1 | 67 | 4 | R.LLLLGAGESGKSTIVK.Q |
|  | 19986 | **656.8590** | **1967.5552** | **1968.0152** | **-0.4600** | **1** | **0** | **56** | **8** | **R.NLLRNFLVQAFGGCFGR.S** |
|  | 21676 | **532.2604** | **2125.0124** | **2125.0118** | **0.0006** | **1** | **7** | **19** | **2** | **K.MGYMCTHRLLLLGAGESGK.S** |
|  | 24913 | **847.4097** | **2539.2074** | **2540.1826** | **-0.9753** | **1** | **2** | **50** | **10** | **K.QMRILHVNGFNGEGGEEDPQAAR.S** |
|  | 25250 | **859.0936** | **2574.2589** | **2574.2676** | **-0.0087** | **1** | **33** | **0.035** | **1** | **R.SNEYQLIDCAQYFLDKIDVIK.Q** 25249 |
|  | 26141 | **679.3805** | **2713.4929** | **2714.2089** | **-0.7160** | **1** | **2** | **33** | **3** | **R.ERAERPPVEEEAAEMEGAADAAEGGK.V** |
|  | 29287 | **1141.2595** | **3420.7567** | **3420.7711** | **-0.0143** | **1** | **17** | **0.73** | **1** | **K.NNLKEAIETIVAAMSNLVPPVELANPENQFR.V** |

|  | **Proteins matching a subset of these peptides:** |
| --- | --- |

|  | P63092\|GNAS2_HUMAN    **Mass:** 46092    **Score:** 38     **Queries matched:** 9 |
| --- | --- |
|  | Guanine nucleotide-binding protein G(s) subunit alpha isoforms short OS=Homo sapiens GN=GNAS PE=1 SV=1 |

| **792.** | P01906\|HA26_HUMAN    **Mass:** 28243    **Score:** 38     **Queries matched:** 8   **emPAI:** 0.13 |
| --- | --- |
|  | HLA class II histocompatibility antigen, DQ(6) alpha chain OS=Homo sapiens GN=HLA-DQA2 PE=2 SV=2 |

|  | **Query** | **Observed** | **Mr(expt)** | **Mr(calc)** | **Delta** | **Miss** | **Score** | **Expect** | **Rank** | **Peptide** |
| --- | --- | --- | --- | --- | --- | --- | --- | --- | --- | --- |
|  | 8968 | **427.1859** | **1278.5360** | **1279.6561** | **-1.1201** | **0** | **8** | **24** | **9** | **K.FISFDPQSALR.N** |
|  | 10616 | 455.7241 | 1364.1504 | 1364.6799 | -0.5294 | 0 | (2) | 59 | 9 | K.ETVWQLPMFSK.F 10617 |
|  | 10920 | 690.8480 | 1379.6814 | 1380.6748 | -0.9934 | 0 | 3 | 67 | 10 | K.ETVWQLPMFSK.F |
|  | 11882 | 479.2542 | 1434.7408 | 1434.7507 | -0.0099 | 0 | 38 | 0.021 | 1 | K.VEHWGLDEPLLK.H 11868 11869 |
|  | 18877 | 627.9896 | 1880.9469 | 1879.9614 | 0.9854 | 1 | 1 | 85 | 6 | K.FISFDPQSALRNMAVGK.H |

| **793.** | P01908\|HA21_HUMAN    **Mass:** 28316    **Score:** 38     **Queries matched:** 4   **emPAI:** 0.13 |
| --- | --- |
|  | HLA class II histocompatibility antigen, DQ(1) alpha chain OS=Homo sapiens PE=2 SV=2 |

|  | **Query** | **Observed** | **Mr(expt)** | **Mr(calc)** | **Delta** | **Miss** | **Score** | **Expect** | **Rank** | **Peptide** |
| --- | --- | --- | --- | --- | --- | --- | --- | --- | --- | --- |
|  | 11882 | 479.2542 | 1434.7408 | 1434.7507 | -0.0099 | 0 | 38 | 0.021 | 1 | K.VEHWGLDEPLLK.H 11868 11869 |
|  | 14254 | 789.4358 | 1576.8571 | 1575.8661 | 0.9911 | 0 | 1 | 87 | 9 | R.FDPQFALTNIAVLK.H |

|  | **Proteins matching the same set of peptides:** |
| --- | --- |

|  | P04225\|HA24_HUMAN    **Mass:** 26020    **Score:** 38     **Queries matched:** 4 |
| --- | --- |
|  | HLA class II histocompatibility antigen, DQ(4) alpha chain (Fragment) OS=Homo sapiens GN=HLA-DQA1 PE=1 SV=1 |

|  | P04226\|HA22_HUMAN    **Mass:** 28147    **Score:** 38     **Queries matched:** 4 |
| --- | --- |
|  | HLA class II histocompatibility antigen, DQ(2) alpha chain OS=Homo sapiens GN=HLA-DQA1 PE=2 SV=1 |

| **794.** | Q02543\|RL18A_HUMAN    **Mass:** 21034    **Score:** 38     **Queries matched:** 7   **emPAI:** 0.18 |
| --- | --- |
|  | 60S ribosomal protein L18a OS=Homo sapiens GN=RPL18A PE=1 SV=2 |

|  | **Query** | **Observed** | **Mr(expt)** | **Mr(calc)** | **Delta** | **Miss** | **Score** | **Expect** | **Rank** | **Peptide** |
| --- | --- | --- | --- | --- | --- | --- | --- | --- | --- | --- |
|  | 942 | 550.2479 | 549.2406 | 548.2377 | 1.0030 | 0 | 14 | 9.3 | 1 | R.DMGAR.H |
|  | 981 | **564.9656** | **563.9584** | **564.2326** | **-0.2742** | **0** | **(9)** | **18** | **2** | **R.DMGAR.H** |
|  | 2509 | 432.2154 | 862.4162 | 862.4695 | -0.0532 | 1 | 13 | 9 | 7 | -.MKASGTLR.E |
|  | 3369 | 464.2543 | 926.4941 | 926.5007 | -0.0066 | 0 | 16 | 3.7 | 1 | R.AHSIQIMK.V |
|  | 4522 | **504.3049** | **1006.5953** | **1006.6076** | **-0.0123** | **1** | **17** | **3.3** | **1** | **K.IKFPLPHR.V** |
|  | 12203 | **728.3425** | **1454.6705** | **1454.6824** | **-0.0119** | **0** | **38** | **0.021** | **1** | **R.DLTTAGAVTQCYR.D** |
|  | 20970 | **686.3809** | **2056.1208** | **2055.0095** | **1.1112** | **1** | **4** | **37** | **4** | **K.SSGEIVYCGQVFEKSPLR.V** |

| **795.** | Q9NV96\|CC50A_HUMAN    **Mass:** 41057    **Score:** 38     **Queries matched:** 8   **emPAI:** 0.09 |
| --- | --- |
|  | Cell cycle control protein 50A OS=Homo sapiens GN=TMEM30A PE=1 SV=1 |

|  | **Query** | **Observed** | **Mr(expt)** | **Mr(calc)** | **Delta** | **Miss** | **Score** | **Expect** | **Rank** | **Peptide** |
| --- | --- | --- | --- | --- | --- | --- | --- | --- | --- | --- |
|  | 4898 | **518.2897** | **1034.5649** | **1034.4880** | **0.0769** | **0** | **9** | **21** | **1** | **R.NSSNTADITI.-** |
|  | 9162 | **645.8210** | **1289.6275** | **1290.7044** | **-1.0769** | **1** | **4** | **55** | **3** | **R.KSDLHPTLPAGR.Y** 9163 |
|  | 10162 | **670.9193** | **1339.8241** | **1338.6676** | **1.1565** | **0** | **3** | **69** | **2** | **R.MILSTISWMGGK.N** |
|  | 12999 | 750.8580 | 1499.7014 | 1499.7117 | -0.0103 | 1 | 38 | 0.022 | 1 | K.FRNPPGGDNLEER.F |
|  | 20391 | **1002.9829** | **2003.9513** | **2002.9556** | **0.9957** | **1** | **5** | **32** | **4** | **K.SRDDSQLNGDSSALLNPSK.E** |
|  | 23507 | **779.4241** | **2335.2506** | **2335.0209** | **0.2297** | **1** | **1** | **61** | **1** | **M.AMNYNAKDEVDGGPPCAPGGTAK.T** |
|  | 24514 | **828.4036** | **2482.1889** | **2482.0563** | **0.1326** | **1** | **2** | **47** | **2** | **-.MAMNYNAKDEVDGGPPCAPGGTAK.T** |

| **796.** | P30519\|HMOX2_HUMAN    **Mass:** 36181    **Score:** 38     **Queries matched:** 19   **emPAI:** 0.22 |
| --- | --- |
|  | Heme oxygenase 2 OS=Homo sapiens GN=HMOX2 PE=1 SV=2 |

|  | **Query** | **Observed** | **Mr(expt)** | **Mr(calc)** | **Delta** | **Miss** | **Score** | **Expect** | **Rank** | **Peptide** |
| --- | --- | --- | --- | --- | --- | --- | --- | --- | --- | --- |
|  | 4970 | **521.2786** | **1040.5427** | **1041.4839** | **-0.9412** | **1** | **12** | **10** | **2** | **K.EGTKEAHDR.A** |
|  | 5096 | 525.2559 | 1048.4972 | 1048.5045 | -0.0073 | 0 | 23 | 0.88 | 1 | R.MNALDLNMK.T |
|  | 9038 | **642.2797** | **1282.5448** | **1282.6227** | **-0.0780** | **0** | **2** | **81** | **10** | **R.YMGDLSGGQVLK.K** |
|  | 9227 | 647.2993 | 1292.5840 | 1293.6421 | -1.0581 | 1 | 10 | 15 | 3 | R.MNALDLNMKTK.E 9232 9270 |
|  | 9516 | 655.2991 | 1308.5837 | 1309.6370 | -1.0533 | 1 | (6) | 39 | 9 | R.MNALDLNMKTK.E |
|  | 14544 | **532.2880** | **1593.8422** | **1594.6846** | **-0.8424** | **0** | **3** | **58** | **2** | **M.SAEVETSEGVDESEK.K** |
|  | 16091 | 564.9684 | 1691.8835 | 1692.8174 | -0.9340 | 1 | 5 | 35 | 7 | K.ENQMRMADLSELLK.E 16092 |
|  | 18724 | 935.4875 | 1868.9604 | 1869.8149 | -0.8545 | 1 | 4 | 37 | 4 | -.MSAEVETSEGVDESEKK.N 18725 |
|  | 19364 | 961.7358 | 1921.4571 | 1921.9502 | -0.4931 | 1 | 3 | 30 | 2 | K.GALEGSSCPFRTAMAVLR.K 19378 |
|  | 24085 | 806.4224 | 2416.2453 | 2417.1321 | -0.8869 | 0 | 3 | 41 | 3 | K.AFEYNMQIFNELDQAGSTLAR.E |
|  | 27298 | **982.4880** | **2944.4421** | **2944.4607** | **-0.0186** | **1** | **27** | **0.12** | **1** | **R.ALKLPSTGEGTQFYLFENVDNAQQFK.Q** 27299 |
|  | 28502 | **1067.8488** | **3200.5244** | **3200.5448** | **-0.0203** | **1** | **26** | **0.12** | **1** | **R.IVEEANKAFEYNMQIFNELDQAGSTLAR.E** 28503 |

| **797.** | Q08722\|CD47_HUMAN    **Mass:** 35590    **Score:** 38     **Queries matched:** 3   **emPAI:** 0.11 |
| --- | --- |
|  | Leukocyte surface antigen CD47 OS=Homo sapiens GN=CD47 PE=1 SV=1 |

|  | **Query** | **Observed** | **Mr(expt)** | **Mr(calc)** | **Delta** | **Miss** | **Score** | **Expect** | **Rank** | **Peptide** |
| --- | --- | --- | --- | --- | --- | --- | --- | --- | --- | --- |
|  | 3401 | **465.2831** | **928.5515** | **928.5593** | **-0.0078** | **0** | **38** | **0.032** | **1** | **K.IEVSQLLK.G** |
|  | 10363 | **451.2618** | **1350.7637** | **1349.7190** | **1.0447** | **1** | **6** | **32** | **3** | **R.EGETIIELKYR.V** |
|  | 12994 | 750.8250 | 1499.6355 | 1499.8559 | -0.2204 | 1 | 8 | 18 | 4 | K.IEVSQLLKGDASLK.M |

| **798.** | Q969X5\|ERGI1_HUMAN    **Mass:** 32971    **Score:** 37     **Queries matched:** 3   **emPAI:** 0.11 |
| --- | --- |
|  | Endoplasmic reticulum-Golgi intermediate compartment protein 1 OS=Homo sapiens GN=ERGIC1 PE=1 SV=1 |

|  | **Query** | **Observed** | **Mr(expt)** | **Mr(calc)** | **Delta** | **Miss** | **Score** | **Expect** | **Rank** | **Peptide** |
| --- | --- | --- | --- | --- | --- | --- | --- | --- | --- | --- |
|  | 2132 | **419.2196** | **836.4247** | **836.4293** | **-0.0046** | **1** | **21** | **1.4** | **1** | **M.PFDFRR.F** |
|  | 13343 | 760.8775 | 1519.7404 | 1519.7419 | -0.0015 | 1 | 3 | 63 | 4 | K.QRYSYQYTVANK.E |
|  | 24687 | **834.7539** | **2501.2397** | **2500.2459** | **0.9938** | **0** | **37** | **0.013** | **1** | **K.LSFGDTLQVQNIHGAFNALGGADR.L** |

| **799.** | O15254\|ACOX3_HUMAN    **Mass:** 78321    **Score:** 37     **Queries matched:** 8   **emPAI:** 0.05 |
| --- | --- |
|  | Peroxisomal acyl-coenzyme A oxidase 3 OS=Homo sapiens GN=ACOX3 PE=2 SV=2 |

|  | **Query** | **Observed** | **Mr(expt)** | **Mr(calc)** | **Delta** | **Miss** | **Score** | **Expect** | **Rank** | **Peptide** |
| --- | --- | --- | --- | --- | --- | --- | --- | --- | --- | --- |
|  | 2983 | 451.2271 | 900.4397 | 901.5021 | -1.0624 | 0 | 15 | 6.4 | 8 | R.HLTYIQK.I |
|  | 6327 | **570.3040** | **1138.5935** | **1137.5778** | **1.0157** | **0** | **5** | **48** | **5** | **R.FGASLGSLSSGR.V** |
|  | 8279 | **623.3297** | **1244.6449** | **1243.6561** | **0.9888** | **0** | **14** | **6.6** | **1** | **K.NLWGAVLQESK.V** |
|  | 15377 | **824.9093** | **1647.8040** | **1647.7828** | **0.0213** | **0** | **2** | **75** | **4** | **K.LGQNGLDNGFAMFHK.V** |
|  | 19558 | **646.3195** | **1935.9366** | **1935.9798** | **-0.0432** | **0** | **1** | **69** | **2** | **K.VPALIQCLGMYDSSLAAK.Y** |
|  | 20097 | **660.4121** | **1978.2143** | **1979.0088** | **-0.7944** | **1** | **1** | **59** | **8** | **K.FWVGNMGKTATHAVVFAK.L** |
|  | 21204 | **693.3895** | **2077.1468** | **2076.3398** | **0.8070** | **1** | **11** | **7.5** | **1** | **R.VSIVSLAILNLKLAVAIALR.F** |
|  | 30096 | **1259.9542** | **3776.8408** | **3776.8495** | **-0.0087** | **1** | **37** | **0.0062** | **1** | **K.SVDFLDAYPGILDQKFEVSSVADCLDSAVALAAYK.W** |

| **800.** | Q92820\|GGH_HUMAN    **Mass:** 36340    **Score:** 37     **Queries matched:** 5   **emPAI:** 0.10 |
| --- | --- |
|  | Gamma-glutamyl hydrolase OS=Homo sapiens GN=GGH PE=1 SV=2 |

|  | **Query** | **Observed** | **Mr(expt)** | **Mr(calc)** | **Delta** | **Miss** | **Score** | **Expect** | **Rank** | **Peptide** |
| --- | --- | --- | --- | --- | --- | --- | --- | --- | --- | --- |
|  | 12703 | 742.8453 | 1483.6761 | 1483.7671 | -0.0910 | 1 | 13 | 6.4 | 3 | K.KFFNVLTTNTDGK.I |
|  | 13718 | 515.2967 | 1542.8682 | 1543.8358 | -0.9676 | 0 | 2 | 78 | 5 | K.SINGILFPGGSVDLR.R |
|  | 14596 | **533.7386** | **1598.1939** | **1598.7763** | **-0.5823** | **1** | **3** | **38** | **8** | **K.WSLSVKNFTMNEK.L** |
|  | 25678 | **532.0847** | **2655.3869** | **2654.2938** | **1.0931** | **1** | **2** | **42** | **5** | **K.FFNVLTTNTDGKIEFISTMEGYK.Y** |
|  | 26344 | **920.8148** | **2759.4226** | **2759.4356** | **-0.0130** | **0** | **37** | **0.011** | **1** | **R.MFQNFPTELLLSLAVEPLTANFHK.W** |

| **801.** | P24158\|PRTN3_HUMAN    **Mass:** 28245    **Score:** 37     **Queries matched:** 4   **emPAI:** 0.13 |
| --- | --- |
|  | Myeloblastin OS=Homo sapiens GN=PRTN3 PE=1 SV=3 |

|  | **Query** | **Observed** | **Mr(expt)** | **Mr(calc)** | **Delta** | **Miss** | **Score** | **Expect** | **Rank** | **Peptide** |
| --- | --- | --- | --- | --- | --- | --- | --- | --- | --- | --- |
|  | 9162 | 645.8210 | 1289.6275 | 1289.7568 | -0.1293 | 0 | 31 | 0.11 | 1 | R.LVNVVLGAHNVR.T 9163 9182 |
|  | 24039 | 804.4561 | 2410.3465 | 2411.3835 | -1.0369 | 0 | 3 | 33 | 6 | -.MAHRPPSPALASVLLALLLSGAAR.A |

| **802.** | Q9UH99\|UN84B_HUMAN    **Mass:** 80490    **Score:** 37     **Queries matched:** 19   **emPAI:** 0.05 |
| --- | --- |
|  | Protein unc-84 homolog B OS=Homo sapiens GN=UNC84B PE=1 SV=3 |

|  | **Query** | **Observed** | **Mr(expt)** | **Mr(calc)** | **Delta** | **Miss** | **Score** | **Expect** | **Rank** | **Peptide** |
| --- | --- | --- | --- | --- | --- | --- | --- | --- | --- | --- |
|  | 72 | **402.1769** | **401.1696** | **402.1975** | **-1.0279** | **0** | **8** | **65** | **1** | **R.GGGGR.V** 74 77 78 79 84 85 86 87 88 |
|  | 2132 | 419.2196 | 836.4247 | 836.3624 | 0.0623 | 0 | 11 | 13 | 6 | R.GTGGSESSR.A |
|  | 5110 | 525.7916 | 1049.5687 | 1048.5203 | 1.0484 | 1 | 5 | 45 | 5 | R.FRVHGEPAH.- |
|  | 5349 | 535.3189 | 1068.6231 | 1068.6152 | 0.0079 | 1 | 12 | 10 | 2 | R.GGGGRVGLLQR.E |
|  | 8021 | 616.8495 | 1231.6844 | 1230.6819 | 1.0025 | 0 | 2 | 96 | 7 | R.EAAASLSLTLQK.E |
|  | 12606 | **739.8956** | **1477.7767** | **1476.7281** | **1.0486** | **1** | **2** | **91** | **1** | **R.GTGGSESSRASGLVGR.K** |
|  | 16990 | **584.2921** | **1749.8545** | **1748.8845** | **0.9700** | **1** | **2** | **72** | **3** | **R.RLEALAAEFSSNWQK.E** |
|  | 17303 | 888.4401 | 1774.8657 | 1773.8217 | 1.0440 | 1 | 4 | 41 | 5 | R.DSSPHFQAEQRVMSR.V |
|  | 21221 | **1040.9822** | **2079.9498** | **2079.1687** | **0.7811** | **1** | **3** | **45** | **3** | **R.RLEDQLAGLQQELAALALK.Q** |
|  | 29321 | **1144.8733** | **3431.5980** | **3431.6133** | **-0.0152** | **0** | **37** | **0.0075** | **1** | **K.FTYDQDGEPIQTFHFQAPTMATYQVVELR.I** |

| **803.** | P59190\|RAB15_HUMAN    **Mass:** 24660    **Score:** 37     **Queries matched:** 9   **emPAI:** 0.33 |
| --- | --- |
|  | Ras-related protein Rab-15 OS=Homo sapiens GN=RAB15 PE=1 SV=1 |

|  | **Query** | **Observed** | **Mr(expt)** | **Mr(calc)** | **Delta** | **Miss** | **Score** | **Expect** | **Rank** | **Peptide** |
| --- | --- | --- | --- | --- | --- | --- | --- | --- | --- | --- |
|  | 3541 | 470.7629 | 939.5113 | 939.4814 | 0.0299 | 0 | 15 | 4.3 | 5 | K.QYDVLFR.L |
|  | 5394 | 536.3203 | 1070.6261 | 1070.6336 | -0.0075 | 0 | 45 | 0.006 | 1 | R.LLLIGDSGVGK.T 5392 |
|  | 6493 | **575.8832** | **1149.7518** | **1148.6111** | **1.1407** | **1** | **6** | **31** | **1** | **K.MKTIEVDGIK.V** |
|  | 9695 | 658.8284 | 1315.6422 | 1315.6521 | -0.0099 | 0 | 18 | 2.6 | 1 | R.IQIWDTAGQER.Y 9685 |
|  | 17743 | **601.0430** | **1800.1073** | **1798.9690** | **1.1383** | **1** | **9** | **11** | **1** | **K.ESFTRLTELVLQAHR.K** |
|  | 22094 | 1086.0475 | 2170.0804 | 2170.9187 | -0.8383 | 0 | 1 | 63 | 4 | K.EYGMDFYETSACTNLNIK.E |
|  | 27094 | **968.0948** | **2901.2627** | **2901.3661** | **-0.1034** | **1** | **7** | **11** | **1** | **R.MRASNELALAELEEEEGKPEGPANSSK.T** |

| **804.** | Q58FF3\|ENPLL_HUMAN    **Mass:** 46343    **Score:** 37     **Queries matched:** 10   **emPAI:** 0.17 |
| --- | --- |
|  | Putative endoplasmin-like protein OS=Homo sapiens GN=HSP90B2P PE=5 SV=1 |

|  | **Query** | **Observed** | **Mr(expt)** | **Mr(calc)** | **Delta** | **Miss** | **Score** | **Expect** | **Rank** | **Peptide** |
| --- | --- | --- | --- | --- | --- | --- | --- | --- | --- | --- |
|  | 1333 | 707.3767 | 706.3694 | 705.3810 | 0.9885 | 0 | 19 | 2.3 | 2 | R.FQNVAK.E |
|  | 1752 | 405.1950 | 808.3754 | 807.4127 | 0.9627 | 0 | 6 | 41 | 7 | K.EFGTNIK.L |
|  | 2671 | 438.7295 | 875.4445 | 875.4501 | -0.0056 | 0 | 22 | 1.4 | 1 | K.TFEINPR.H |
|  | 6324 | 570.2897 | 1138.5649 | 1138.5731 | -0.0082 | 0 | 35 | 0.041 | 1 | K.LGVIEDHSNR.T |
|  | 6399 | 572.8155 | 1143.6164 | 1142.5608 | 1.0557 | 1 | 26 | 0.38 | 1 | R.GLFDEYGSKK.S |
|  | 9820 | **441.2962** | **1320.8667** | **1321.6700** | **-0.8033** | **1** | **9** | **16** | **2** | **R.DTMPKNLNFVK.G** |
|  | 12847 | 498.0203 | 1491.0390 | 1491.8310 | -0.7921 | 1 | 14 | 3.2 | 3 | K.TFEINPRHPLIR.D |
|  | 22995 | **758.2184** | **2271.6335** | **2271.1940** | **0.4395** | **1** | **3** | **26** | **2** | **K.SILFVPTFVPRGLFDEYGSK.K** 22996 |
|  | 29357 | **1151.5674** | **3451.6803** | **3451.5703** | **0.1101** | **1** | **4** | **18** | **3** | **K.AMVSQCLTESLCALVASQYGWSGNMERIMK.A** |

| **805.** | Q14571\|ITPR2_HUMAN    **Mass:** 311060   **Score:** 37     **Queries matched:** 51   **emPAI:** 0.02 |
| --- | --- |
|  | Inositol 1,4,5-trisphosphate receptor type 2 OS=Homo sapiens GN=ITPR2 PE=1 SV=2 |

|  | **Query** | **Observed** | **Mr(expt)** | **Mr(calc)** | **Delta** | **Miss** | **Score** | **Expect** | **Rank** | **Peptide** |
| --- | --- | --- | --- | --- | --- | --- | --- | --- | --- | --- |
|  | 1059 | 603.3407 | 602.3334 | 602.3275 | 0.0059 | 0 | 18 | 5.3 | 3 | R.EDVLK.G |
|  | 1387 | 731.4252 | 730.4179 | 730.4225 | -0.0046 | 0 | 9 | 31 | 8 | R.TLAEVAK.N |
|  | 1549 | **397.2195** | **792.4245** | **792.4051** | **0.0194** | **1** | **5** | **54** | **8** | **R.EMLEKK.D** |
|  | 1624 | 400.2350 | 798.4555 | 799.4076 | -0.9521 | 0 | 9 | 19 | 1 | R.DGVPPTSK.K |
|  | 1823 | 407.7058 | 813.3971 | 812.4392 | 0.9579 | 0 | 5 | 54 | 9 | R.DSTLHLK.E |
|  | 2116 | **418.7558** | **835.4971** | **835.3858** | **0.1113** | **0** | **12** | **11** | **8** | **K.AGEGSMLR.L** |
|  | 2325 | 425.7166 | 849.4187 | 848.4314 | 0.9874 | 0 | 9 | 25 | 10 | R.ELVDVMK.N |
|  | 3076 | **453.7824** | **905.5501** | **904.5018** | **1.0483** | **0** | **6** | **50** | **9** | **R.AEVLQAFK.Q** |
|  | 3249 | 459.7278 | 917.4411 | 918.4923 | -1.0512 | 0 | 19 | 2.7 | 2 | R.ELQNFLR.N |
|  | 4435 | **501.2813** | **1000.5479** | **1000.5665** | **-0.0186** | **1** | **3** | **1e+002** | **1** | **R.TLAEVAKNR.G** |
|  | 5060 | 523.8058 | 1045.5971 | 1044.6001 | 0.9970 | 0 | 42 | 0.012 | 1 | K.LLLAIMESR.H |
|  | 5614 | **543.8300** | **1085.6454** | **1084.5158** | **1.1296** | **1** | **4** | **61** | **10** | **R.IRCGAFMSK.L** |
|  | 6070 | 560.2959 | 1118.5772 | 1117.5815 | 0.9958 | 1 | 9 | 19 | 10 | R.AAMGWRLSAR.S 6073 |
|  | 6896 | **589.7725** | **1177.5304** | **1178.5779** | **-1.0475** | **0** | **9** | **19** | **1** | **K.GQLTEASSATSK.A** |
|  | 7515 | **604.2468** | **1206.4790** | **1205.6656** | **0.8134** | **0** | **3** | **65** | **9** | **K.EIETFVSLLR.R** |
|  | 7779 | **407.2234** | **1218.6483** | **1218.6972** | **-0.0490** | **1** | **14** | **5.7** | **2** | **R.FLQTIVKADGK.Y** |
|  | 8405 | **1251.1110** | **1250.1037** | **1250.6196** | **-0.5159** | **0** | **1** | **80** | **2** | **R.NNPALFWFSR.H** |
|  | 10690 | **456.8817** | **1367.6234** | **1367.6391** | **-0.0157** | **1** | **3** | **66** | **10** | **K.DDFTMEVDRLK.N** |
|  | 11282 | 701.3320 | 1400.6495 | 1400.7260 | -0.0764 | 1 | 4 | 49 | 2 | K.LRSEGDNIVVGDK.V |
|  | 11287 | 701.3676 | 1400.7205 | 1399.7671 | 0.9535 | 1 | 4 | 53 | 6 | K.ADLDQLRLTVEK.S |
|  | 11298 | **468.1966** | **1401.5679** | **1401.7212** | **-0.1532** | **1** | **8** | **19** | **1** | **K.KLENGTITQNER.R** |
|  | 12490 | **736.9246** | **1471.8347** | **1471.8398** | **-0.0052** | **0** | **16** | **3.5** | **1** | **R.EQNILAQVFGILK.A** |
|  | 12788 | **497.2411** | **1488.7014** | **1488.7970** | **-0.0955** | **1** | **2** | **79** | **8** | **R.MDLVLQLKNNASK.L** |
|  | 13089 | **502.7945** | **1505.3616** | **1504.6576** | **0.7040** | **1** | **4** | **29** | **5** | **K.RDDDNELMTSGPR.M** |
|  | 13356 | **507.7538** | **1520.2395** | **1520.6525** | **-0.4130** | **1** | **(0)** | **82** | **7** | **K.RDDDNELMTSGPR.M** |
|  | 14258 | **526.8052** | **1577.3937** | **1576.7998** | **0.5940** | **0** | **4** | **35** | **6** | **K.YYANHTAQIEIVR.H** |
|  | 14521 | 531.9179 | 1592.7317 | 1591.8491 | 0.8826 | 1 | 1 | 1e+002 | 7 | R.SLQEKLESTMSLVK.Q |
|  | 15442 | 826.4599 | 1650.9052 | 1651.6930 | -0.7878 | 1 | 6 | 29 | 2 | R.DDDNELMTSGPRMR.V |
|  | 15644 | **555.2472** | **1662.7197** | **1661.7515** | **0.9683** | **1** | **6** | **26** | **2** | **K.SHSNMVQRAAMGWR.L** |
|  | 15744 | 557.2984 | 1668.8734 | 1667.6879 | 1.1854 | 1 | (1) | 90 | 8 | R.DDDNELMTSGPRMR.V |
|  | 16075 | 846.4020 | 1690.7894 | 1690.7993 | -0.0099 | 0 | 1 | 82 | 10 | R.ASFPILLHMMCSER.D |
|  | 16541 | **574.3310** | **1719.9711** | **1718.9216** | **1.0495** | **0** | **3** | **55** | **3** | **K.DVGHNIYILAHQLAR.H** |
|  | 17169 | **882.8190** | **1763.6235** | **1762.8851** | **0.7384** | **1** | **4** | **35** | **2** | **R.KFALTMEFVEEYLK.E** |
|  | 18071 | **608.2903** | **1821.8492** | **1820.8583** | **0.9909** | **0** | **2** | **75** | **6** | **R.TIHGVGEMMTQMVLSR.G** |
|  | 20152 | **662.3276** | **1983.9609** | **1984.9867** | **-1.0258** | **1** | **3** | **42** | **1** | **K.YYANHTAQIEIVRHDR.T** |
|  | 20240 | 498.7552 | 1990.9919 | 1990.1615 | 0.8304 | 1 | 2 | 52 | 8 | K.IIEILQFILSVRLDYR.I |
|  | 20326 | 667.1732 | 1998.4978 | 1998.9792 | -0.4814 | 1 | 2 | 38 | 3 | K.LLLAIMESRHDSENAER.I |
|  | 20406 | 669.3674 | 2005.0805 | 2003.9946 | 1.0859 | 1 | 5 | 27 | 2 | K.QLSGQLAELKEQMTEQR.K |
|  | 21142 | **691.3356** | **2070.9849** | **2072.0646** | **-1.0797** | **0** | **(3)** | **45** | **4** | **K.SAEEVTMSPAIAIMQPILR.F** |
|  | 21320 | **523.0295** | **2088.0888** | **2088.0595** | **0.0293** | **0** | **4** | **36** | **7** | **K.SAEEVTMSPAIAIMQPILR.F** 21319 |
|  | 22053 | **1083.9980** | **2165.9815** | **2166.1395** | **-0.1579** | **0** | **1** | **61** | **4** | **R.TLLAILDIVQAPMSSYFER.L** |
|  | 22105 | 724.6330 | 2170.8772 | 2171.9933 | -1.1161 | 0 | 3 | 39 | 3 | K.DPTEYTGPESYVAQMIVEK.N |
|  | 22196 | **728.3585** | **2182.0536** | **2180.9790** | **1.0746** | **1** | **4** | **36** | **1** | **R.MRAMSLVSNEGDSEQNEIR.S** |
|  | 22314 | 734.3204 | 2199.9395 | 2199.0729 | 0.8666 | 0 | 2 | 49 | 5 | K.LLQQMLKPGSDPDEGDEALK.Y |
|  | 23411 | **582.0121** | **2324.0192** | **2323.1062** | **0.9130** | **0** | **3** | **39** | **8** | **R.VSLDAAGNEGSWFYIHPFWK.L** |
|  | 23855 | 795.0295 | 2382.0666 | 2383.1617 | -1.0951 | 1 | 6 | 18 | 2 | K.VKDPTEYTGPESYVAQMIVEK.N |
|  | 24822 | 842.0481 | 2523.1225 | 2523.1478 | -0.0253 | 0 | 1 | 56 | 2 | R.TPVTGSHQVPTMTLTTMMEACAK.E |
|  | 26366 | 922.1540 | 2763.4401 | 2763.2190 | 0.2211 | 0 | 1 | 50 | 7 | K.CQDMVMTELINGGEDVLIFYNDR.A |
|  | 27884 | **765.8574** | **3059.4006** | **3058.4740** | **0.9266** | **0** | **0** | **48** | **5** | **R.HLCTNTWVTSTSIPIDTDEERPVMLK.I** |

| **806.** | Q9NZR1\|TMOD2_HUMAN    **Mass:** 39571    **Score:** 37     **Queries matched:** 11   **emPAI:** 0.09 |
| --- | --- |
|  | Tropomodulin-2 OS=Homo sapiens GN=TMOD2 PE=1 SV=1 |

|  | **Query** | **Observed** | **Mr(expt)** | **Mr(calc)** | **Delta** | **Miss** | **Score** | **Expect** | **Rank** | **Peptide** |
| --- | --- | --- | --- | --- | --- | --- | --- | --- | --- | --- |
|  | 2414 | 429.1604 | 856.3063 | 857.4719 | -1.1656 | 1 | 7 | 39 | 10 | K.NNDLVRK.K 2449 |
|  | 3321 | 462.2424 | 922.4703 | 922.5600 | -0.0897 | 0 | 9 | 18 | 3 | K.NIPIPTLR.E |
|  | 9984 | 665.3200 | 1328.6255 | 1327.6673 | 0.9581 | 1 | 37 | 0.027 | 1 | K.FGYQFTKQGPR.T |
|  | 12783 | 744.8889 | 1487.7633 | 1486.7780 | 0.9853 | 1 | 3 | 66 | 9 | R.EFAKALETNTHVK.K |
|  | 16002 | 844.4142 | 1686.8138 | 1687.8377 | -1.0239 | 1 | 4 | 50 | 5 | K.ENDTLTEIKIDNQR.Q |
|  | 19010 | **631.0328** | **1890.0765** | **1888.9604** | **1.1161** | **1** | **3** | **47** | **1** | **R.EHLLMYLEKEALEQK.D** |
|  | 20415 | **669.8590** | **2006.5552** | **2005.9931** | **0.5621** | **1** | **1** | **55** | **1** | **K.AATGPFDREHLLMYLEK.E** |
|  | 23170 | **765.0531** | **2292.1375** | **2292.0726** | **0.0649** | **0** | **0** | **75** | **3** | **R.QQLGTAVEMEIAQMLEENSR.I** |
|  | 30211 | **959.4834** | **3833.9045** | **3833.9574** | **-0.0529** | **0** | **1** | **29** | **4** | **K.VTLDPELEEALASASDTELYDLAAVLGVHNLLNNPK.F** |
|  | 30695 | 1044.2001 | 4172.7712 | 4173.1416 | -0.3704 | 1 | 0 | 21 | 5 | K.VKPVFEEPPNPTNVEISLQQMKANDPSLQEVNLNNIK.N |

| **807.** | P16422\|EPCAM_HUMAN    **Mass:** 35594    **Score:** 37     **Queries matched:** 7   **emPAI:** 0.11 |
| --- | --- |
|  | Epithelial cell adhesion molecule OS=Homo sapiens GN=EPCAM PE=1 SV=2 |

|  | **Query** | **Observed** | **Mr(expt)** | **Mr(calc)** | **Delta** | **Miss** | **Score** | **Expect** | **Rank** | **Peptide** |
| --- | --- | --- | --- | --- | --- | --- | --- | --- | --- | --- |
|  | 3074 | **453.7814** | **905.5482** | **904.3531** | **1.1951** | **0** | **8** | **32** | **8** | **K.EMGEMHR.E** 3072 3076 |
|  | 10320 | 450.5858 | 1348.7354 | 1347.5547 | 1.1807 | 1 | 2 | 1.1e+002 | 3 | K.EMGEMHRELNA.- |
|  | 10628 | **455.8879** | **1364.6420** | **1364.6354** | **0.0066** | **1** | **6** | **32** | **9** | **K.DTEITCSERVR.T** |
|  | 26311 | **918.4096** | **2752.2070** | **2752.2286** | **-0.0216** | **0** | **37** | **0.011** | **1** | **R.AKPEGALQNNDGLYDPDCDESGLFK.A** |
|  | 29181 | **1128.4834** | **3382.4284** | **3382.7660** | **-0.3376** | **1** | **1** | **31** | **9** | **R.YQLDPKFITSILYENNVITIDLVQNSSQK.T** |

| **808.** | Q14103\|HNRPD_HUMAN    **Mass:** 38581    **Score:** 37     **Queries matched:** 12   **emPAI:** 0.10 |
| --- | --- |
|  | Heterogeneous nuclear ribonucleoprotein D0 OS=Homo sapiens GN=HNRNPD PE=1 SV=1 |

|  | **Query** | **Observed** | **Mr(expt)** | **Mr(calc)** | **Delta** | **Miss** | **Score** | **Expect** | **Rank** | **Peptide** |
| --- | --- | --- | --- | --- | --- | --- | --- | --- | --- | --- |
|  | 981 | 564.9656 | 563.9584 | 563.2816 | 0.6768 | 0 | 8 | 24 | 3 | R.GGFAGR.A |
|  | 1960 | **413.2636** | **824.5127** | **824.4504** | **0.0622** | **1** | **4** | **60** | **5** | **K.EHKLNGK.V** |
|  | 4603 | 507.7521 | 1013.4897 | 1013.5618 | -0.0721 | 1 | 8 | 25 | 5 | K.LDPITGRSR.G |
|  | 5045 | **523.2886** | **1044.5626** | **1044.5716** | **-0.0090** | **1** | **10** | **19** | **3** | **K.KYHNVGLSK.C** |
|  | 9462 | 653.2922 | 1304.5699 | 1305.6214 | -1.0515 | 1 | 9 | 18 | 1 | R.RGGHQNSYKPY.- 9496 |
|  | 12781 | **744.8786** | **1487.7426** | **1487.7508** | **-0.0081** | **0** | **37** | **0.025** | **1** | **K.IFVGGLSPDTPEEK.I** |
|  | 17092 | 879.4533 | 1756.8921 | 1756.9359 | -0.0439 | 1 | 23 | 0.54 | 2 | K.IFVGGLSPDTPEEKIR.E 17093 |
|  | 20027 | **658.2654** | **1971.7745** | **1970.8566** | **0.9179** | **1** | **4** | **34** | **2** | **K.IDASKNEEDEGHSNSSPR.H** |
|  | 21997 | **720.9083** | **2159.7032** | **2159.9721** | **-0.2690** | **0** | **3** | **32** | **7** | **R.EYFGGFGEVESIELPMDNK.T** |
|  | 31645 | **1115.3793** | **5571.8600** | **5572.4277** | **-0.5677** | **0** | **1** | **6.6** | **1** | **M.SEEQFGGDGAAAAATAAVGGSAGEQEGAMVAATQGAAAAAGSGAGTGGGTASGGTEGGSAESEGAK.I** |

| **809.** | O43402\|CX4NB_HUMAN    **Mass:** 24214    **Score:** 37     **Queries matched:** 4   **emPAI:** 0.16 |
| --- | --- |
|  | Neighbor of COX4 OS=Homo sapiens GN=COX4NB PE=1 SV=1 |

|  | **Query** | **Observed** | **Mr(expt)** | **Mr(calc)** | **Delta** | **Miss** | **Score** | **Expect** | **Rank** | **Peptide** |
| --- | --- | --- | --- | --- | --- | --- | --- | --- | --- | --- |
|  | 9079 | 643.3355 | 1284.6563 | 1284.6674 | -0.0110 | 1 | 37 | 0.028 | 1 | R.VKDASPNQVAEK.V |
|  | 14176 | **524.6196** | **1570.8371** | **1569.7973** | **1.0397** | **0** | **2** | **91** | **4** | **K.YPHCAVNGLLVAEK.Q** |
|  | 18315 | **920.9581** | **1839.9016** | **1839.8924** | **0.0092** | **0** | **3** | **52** | **9** | **R.IAEGFSDTALIMVDNTK.F** |
|  | 30696 | **1045.1798** | **4176.6901** | **4176.9343** | **-0.2442** | **1** | **1** | **13** | **7** | **R.IAEGFSDTALIMVDNTKFTMDCVAPTIHVYEHHENR.W** |

| **810.** | P18124\|RL7_HUMAN    **Mass:** 29264    **Score:** 37     **Queries matched:** 22   **emPAI:** 0.28 |
| --- | --- |
|  | 60S ribosomal protein L7 OS=Homo sapiens GN=RPL7 PE=1 SV=1 |

|  | **Query** | **Observed** | **Mr(expt)** | **Mr(calc)** | **Delta** | **Miss** | **Score** | **Expect** | **Rank** | **Peptide** |
| --- | --- | --- | --- | --- | --- | --- | --- | --- | --- | --- |
|  | 1458 | 767.3107 | 766.3035 | 766.3578 | -0.0543 | 1 | 8 | 28 | 4 | R.MARMAR.K |
|  | 1463 | 771.4301 | 770.4228 | 770.4286 | -0.0058 | 0 | 19 | 2.4 | 3 | R.GINGVSPK.V |
|  | 1687 | **402.7049** | **803.3952** | **803.4323** | **-0.0372** | **0** | **14** | **8.8** | **7** | **K.ASINMLR.I** 1692 1718 1720 |
|  | 2598 | **435.7468** | **869.4790** | **868.5858** | **0.8932** | **1** | **9** | **18** | **2** | **R.KVLQLLR.L** |
|  | 2897 | **446.7583** | **891.5020** | **892.4953** | **-0.9932** | **1** | **7** | **35** | **7** | **K.FAQKMLR.K** |
|  | 5139 | 527.2890 | 1052.5634 | 1052.5655 | -0.0020 | 0 | 7 | 33 | 1 | R.QIFNGTFVK.L |
|  | 6108 | **561.3213** | **1120.6280** | **1120.6240** | **0.0040** | **1** | **29** | **0.21** | **1** | **K.SVNELIYKR.G** |
|  | 7569 | **605.3289** | **1208.6433** | **1209.6969** | **-1.0536** | **1** | **3** | **78** | **4** | **K.EVPAVPETLKK.K** |
|  | 7571 | **605.3319** | **1208.6493** | **1209.6969** | **-1.0476** | **1** | **2** | **90** | **4** | **K.KEVPAVPETLK.K** |
|  | 9918 | **443.0377** | **1326.0912** | **1325.7779** | **0.3133** | **1** | **5** | **26** | **3** | **K.RIALTDNALIAR.S** 9887 9949 |
|  | 12786 | **745.3488** | **1488.6831** | **1487.7117** | **0.9714** | **1** | **4** | **48** | **2** | **K.KTTHFVEGGDAGNR.E** |
|  | 13669 | **514.2665** | **1539.7778** | **1539.7874** | **-0.0096** | **1** | **9** | **16** | **7** | **R.FKEANNFLWPFK.L** 13651 |
|  | 15632 | 831.9434 | 1661.8722 | 1661.8817 | -0.0095 | 0 | 37 | 0.023 | 1 | R.IVEPYIAWGYPNLK.S |
|  | 21611 | **705.9940** | **2114.9601** | **2114.9730** | **-0.0129** | **1** | **9** | **11** | **1** | **K.TTHFVEGGDAGNREDQINR.L** 21610 |
|  | 23410 | **582.0117** | **2324.0178** | **2325.1497** | **-1.1319** | **1** | **3** | **35** | **9** | **K.YGIICMEDLIHEIYTVGKR.F** |

| **811.** | P17813\|EGLN_HUMAN    **Mass:** 71559    **Score:** 37     **Queries matched:** 7   **emPAI:** 0.05 |
| --- | --- |
|  | Endoglin OS=Homo sapiens GN=ENG PE=1 SV=2 |

|  | **Query** | **Observed** | **Mr(expt)** | **Mr(calc)** | **Delta** | **Miss** | **Score** | **Expect** | **Rank** | **Peptide** |
| --- | --- | --- | --- | --- | --- | --- | --- | --- | --- | --- |
|  | 3792 | **479.2625** | **956.5103** | **956.5192** | **-0.0088** | **0** | **4** | **75** | **4** | **R.TLEWRPR.T** |
|  | 6601 | **579.0114** | **1156.0083** | **1155.5269** | **0.4814** | **0** | **7** | **22** | **6** | **K.TGSQDQEVHR.T** |
|  | 11311 | 702.3367 | 1402.6588 | 1402.7602 | -0.1015 | 0 | 16 | 3.6 | 2 | K.TGTLSCTVALRPK.T |
|  | 12602 | 739.8514 | 1477.6883 | 1477.8140 | -0.1257 | 0 | 10 | 12 | 2 | R.LQTSPAPIQTTPPK.D 12603 |
|  | 15957 | **842.8956** | **1683.7767** | **1683.7886** | **-0.0119** | **0** | **37** | **0.022** | **1** | **K.GNCVSLLSPSPEGDPR.F** |
|  | 19050 | 474.5119 | 1894.0186 | 1894.0160 | 0.0027 | 0 | 8 | 14 | 6 | R.GPITSAAELNDPQSILLR.L |

| **812.** | Q8IVE3\|PKHH2_HUMAN    **Mass:** 169720   **Score:** 37     **Queries matched:** 33   **emPAI:** 0.02 |
| --- | --- |
|  | Pleckstrin homology domain-containing family H member 2 OS=Homo sapiens GN=PLEKHH2 PE=2 SV=2 |

|  | **Query** | **Observed** | **Mr(expt)** | **Mr(calc)** | **Delta** | **Miss** | **Score** | **Expect** | **Rank** | **Peptide** |
| --- | --- | --- | --- | --- | --- | --- | --- | --- | --- | --- |
|  | 972 | 563.0458 | 562.0386 | 561.2507 | 0.7879 | 0 | 1 | 1.4e+002 | 7 | R.NADSR.T |
|  | 1129 | 630.3877 | 629.3804 | 630.3588 | -0.9784 | 0 | 10 | 24 | 6 | K.IIEEK.A |
|  | 1540 | **396.8585** | **791.7025** | **792.4242** | **-0.7217** | **1** | **7** | **24** | **6** | **K.KFQSQR.L** |
|  | 2095 | 417.7297 | 833.4448 | 834.4963 | -1.0515 | 0 | 10 | 22 | 9 | R.IYLTVAR.K |
|  | 2991 | 451.2496 | 900.4846 | 899.4573 | 1.0273 | 1 | 9 | 26 | 5 | R.SRSGPGSPR.A 2998 |
|  | 3720 | **476.2819** | **950.5492** | **949.5444** | **1.0048** | **1** | **3** | **76** | **10** | **K.KSSTVSTLK.L** |
|  | 3875 | **482.1488** | **962.2830** | **962.5913** | **-0.3083** | **1** | **13** | **7.5** | **1** | **R.IYLTVARK.W** 3869 |
|  | 5077 | 1047.5593 | 1046.5521 | 1046.5608 | -0.0087 | 0 | 4 | 67 | 9 | K.QTVQLTTEK.H |
|  | 5461 | 538.8049 | 1075.5953 | 1076.6342 | -1.0389 | 1 | 5 | 53 | 7 | R.IYAVAKSGIR.M |
|  | 8410 | 417.7423 | 1250.2050 | 1249.5206 | 0.6844 | 0 | (10) | 9.9 | 8 | K.DMEEMEIPEK.S 8408 |
|  | 8692 | 633.3418 | 1264.6690 | 1265.5155 | -0.8465 | 0 | 37 | 0.029 | 1 | K.DMEEMEIPEK.S 8719 |
|  | 8709 | **633.8196** | **1265.6247** | **1266.6317** | **-1.0069** | **0** | **1** | **1e+002** | **2** | **R.HLSQPQLSSDR.M** |
|  | 10887 | 689.8490 | 1377.6834 | 1378.6212 | -0.9377 | 1 | 5 | 49 | 6 | K.KEQDSSSDELNK.K |
|  | 11463 | 471.2898 | 1410.8475 | 1411.6150 | -0.7676 | 1 | 4 | 47 | 7 | R.YRDGCSEEQLR.Q |
|  | 11662 | **474.9747** | **1421.9023** | **1422.7328** | **-0.8305** | **1** | **9** | **15** | **1** | **K.RHLSQPQLSSDR.M** 11660 11666 |
|  | 13227 | **757.9340** | **1513.8534** | **1513.8100** | **0.0434** | **1** | **2** | **70** | **2** | **K.ALNSPLGKGNSELSK.K** |
|  | 13788 | **516.7900** | **1547.3483** | **1546.7951** | **0.5532** | **1** | **1** | **61** | **6** | **K.LKAANIQTSESETR.L** |
|  | 14488 | **531.2832** | **1590.8278** | **1589.9042** | **0.9236** | **1** | **5** | **37** | **7** | **K.KVAYSKPPTPPLHR.F** 14463 14495 |
|  | 14836 | **807.9013** | **1613.7879** | **1613.8348** | **-0.0468** | **1** | **2** | **82** | **3** | **R.LSSLTFGCFLSRAR.S** |
|  | 14939 | 810.3740 | 1618.7335 | 1618.8171 | -0.0836 | 0 | 11 | 9.4 | 4 | R.MMGSQPLLSSSRPTK.G |
|  | 15447 | **551.5005** | **1651.4798** | **1650.8069** | **0.6729** | **0** | **(4)** | **34** | **5** | **R.MMGSQPLLSSSRPTK.G** |
|  | 19387 | 642.0388 | 1923.0946 | 1924.0088 | -0.9141 | 0 | 1 | 64 | 6 | R.VQAANPLSLQPEGKPTMK.G |
|  | 28231 | 786.8781 | 3143.4831 | 3143.3552 | 0.1279 | 1 | 5 | 17 | 3 | R.TSFATDGGISQNSGAPVSDWSSDEEDGSKGR.S |
|  | 29055 | **836.6488** | **3342.5661** | **3341.6449** | **0.9212** | **1** | **1** | **38** | **3** | **R.LDYSSSSSEANTPSPILTPALMPKHPNSLSGK.G** |
|  | 30336 | 981.5017 | 3921.9777 | 3921.0386 | 0.9391 | 0 | 2 | 15 | 3 | K.LFLAKPITPSSLGSTFLWLAVHEDGLSLLEYNSMR.L |

| **813.** | Q9BZW7\|TSG10_HUMAN    **Mass:** 82111    **Score:** 37     **Queries matched:** 25   **emPAI:** 0.05 |
| --- | --- |
|  | Testis-specific gene 10 protein OS=Homo sapiens GN=TSGA10 PE=1 SV=1 |

|  | **Query** | **Observed** | **Mr(expt)** | **Mr(calc)** | **Delta** | **Miss** | **Score** | **Expect** | **Rank** | **Peptide** |
| --- | --- | --- | --- | --- | --- | --- | --- | --- | --- | --- |
|  | 3056 | 453.2256 | 904.4366 | 905.4706 | -1.0340 | 0 | 21 | 1.7 | 7 | K.ETISTVEK.E |
|  | 3116 | **455.2449** | **908.4752** | **908.4637** | **0.0115** | **1** | **6** | **43** | **6** | **K.EKTISGMK.N** |
|  | 3502 | **469.2552** | **936.4959** | **935.4018** | **1.0941** | **0** | **4** | **56** | **2** | **K.DQEIEMR.E** |
|  | 5905 | **554.2938** | **1106.5731** | **1107.6036** | **-1.0306** | **0** | **4** | **70** | **9** | **R.HLAEIQGNVK.V** |
|  | 6133 | **562.3232** | **1122.6318** | **1123.5292** | **-0.8974** | **1** | **3** | **73** | **5** | **R.MTTERDSLR.E** |
|  | 6978 | **395.2063** | **1182.5970** | **1182.7084** | **-0.1114** | **1** | **2** | **95** | **9** | **K.ELLNRQLVAK.D** |
|  | 7241 | 598.3053 | 1194.5960 | 1194.5624 | 0.0336 | 1 | 37 | 0.03 | 2 | R.EELKCMLEK.Y 7240 |
|  | 8997 | **641.3098** | **1280.6051** | **1280.7201** | **-0.1150** | **1** | **2** | **92** | **2** | **K.SPKSTTAHAILR.R** |
|  | 9776 | **660.8079** | **1319.6012** | **1318.6802** | **0.9209** | **1** | **3** | **68** | **9** | **K.SVVKMEEELQK.V** |
|  | 10261 | 673.3651 | 1344.7157 | 1345.6586 | -0.9429 | 1 | 2 | 84 | 5 | K.ARQSEADNNTLK.L |
|  | 11393 | **704.4074** | **1406.8003** | **1406.5550** | **0.2453** | **0** | **1** | **95** | **9** | **R.MEQMSNMTLMK.E** |
|  | 12518 | **492.2563** | **1473.7471** | **1474.7641** | **-1.0169** | **1** | **3** | **64** | **8** | **K.IDNFTRQNIAQR.E** |
|  | 13609 | 768.9038 | 1535.7931 | 1536.7494 | -0.9563 | 1 | 7 | 27 | 4 | K.MEEELQKVQFEK.V |
|  | 13866 | 776.7971 | 1551.5797 | 1552.7443 | -1.1646 | 1 | (1) | 83 | 5 | K.MEEELQKVQFEK.V |
|  | 14305 | 527.6158 | 1579.8257 | 1578.7889 | 1.0368 | 1 | 5 | 37 | 10 | R.DKEYQSQIALQEK.E |
|  | 14843 | 807.9287 | 1613.8429 | 1614.7849 | -0.9420 | 0 | 7 | 25 | 5 | R.QLDETNDELAQIAR.E |
|  | 15923 | **841.4226** | **1680.8305** | **1680.8431** | **-0.0125** | **1** | **6** | **26** | **4** | **K.LNDTHNELNDIKQK.V** |
|  | 17032 | **877.4459** | **1752.8773** | **1753.8482** | **-0.9709** | **0** | **2** | **76** | **10** | **R.ENELDSAHSEIELLR.S** |
|  | 17450 | **595.3012** | **1782.8816** | **1781.7672** | **1.1144** | **1** | **8** | **18** | **1** | **K.SEESENRQMMEQLR.K** |
|  | 17639 | **897.4269** | **1792.8393** | **1792.8955** | **-0.0561** | **1** | **0** | **1e+002** | **6** | **K.AENNSLRLLYENTEK.D** |
|  | 18854 | 470.7428 | 1878.9422 | 1877.9339 | 1.0083 | 1 | 11 | 7.5 | 1 | K.TISGMKNIIAEMEQASR.Q |
|  | 22388 | 1103.5852 | 2205.1559 | 2203.9626 | 1.1933 | 0 | 2 | 54 | 2 | R.QNYSSNAYHMSSTMKPNTK.C |
|  | 23989 | 602.0718 | 2404.2580 | 2403.1998 | 1.0582 | 1 | 3 | 40 | 9 | R.SQMANERISMQNLEALLVANR.D |
|  | 28628 | **1076.7802** | **3227.3186** | **3227.3913** | **-0.0726** | **1** | **2** | **23** | **3** | **R.IEELECTVHNLDDERMEQMSNMTLMK.E** |

| **814.** | O60488\|ACSL4_HUMAN    **Mass:** 80220    **Score:** 37     **Queries matched:** 18   **emPAI:** 0.09 |
| --- | --- |
|  | Long-chain-fatty-acid--CoA ligase 4 OS=Homo sapiens GN=ACSL4 PE=1 SV=2 |

|  | **Query** | **Observed** | **Mr(expt)** | **Mr(calc)** | **Delta** | **Miss** | **Score** | **Expect** | **Rank** | **Peptide** |
| --- | --- | --- | --- | --- | --- | --- | --- | --- | --- | --- |
|  | 1418 | **750.3829** | **749.3756** | **749.3378** | **0.0378** | **0** | **5** | **65** | **7** | **R.EAANAMK.L** |
|  | 1617 | **399.9902** | **797.9659** | **798.4712** | **-0.5053** | **0** | **3** | **54** | **3** | **K.ALLGGNVR.M** |
|  | 3670 | **474.9032** | **947.7918** | **947.5804** | **0.2115** | **1** | **1** | **97** | **5** | **K.KLILGNYK.W** |
|  | 6082 | **560.7696** | **1119.5246** | **1118.6005** | **0.9241** | **0** | **8** | **24** | **2** | **K.TALLDISCVK.H** |
|  | 6457 | 575.1019 | 1148.1892 | 1147.5655 | 0.6237 | 1 | 3 | 47 | 6 | K.EIREAANAMK.L |
|  | 8177 | **414.2559** | **1239.7458** | **1239.5706** | **0.1751** | **0** | **6** | **33** | **3** | **K.WMNYLEVNR.R** |
|  | 11327 | **468.9110** | **1403.7110** | **1402.7205** | **0.9906** | **0** | **13** | **6.6** | **1** | **K.AKPTSDKPGSPYR.S** |
|  | 11736 | 714.3626 | 1426.7105 | 1426.7932 | -0.0827 | 1 | 8 | 24 | 9 | K.HIIYVDNKAINK.A 11735 |
|  | 14885 | 808.9016 | 1615.7887 | 1616.7352 | -0.9465 | 0 | 3 | 67 | 9 | R.EILSEENEMQPNGK.V |
|  | 15526 | **553.2515** | **1656.7328** | **1656.8545** | **-0.1217** | **1** | **0** | **1.1e+002** | **6** | **K.VQEMNYIQKTLFK.I** |
|  | 17252 | 886.9025 | 1771.7904 | 1771.0104 | 0.7800 | 1 | 7 | 20 | 3 | R.RVNNFGSGLTALGLKPK.N |
|  | 21737 | **533.5159** | **2130.0344** | **2130.0494** | **-0.0150** | **1** | **41** | **0.0071** | **1** | **K.LKDWQEGGYTINDKPNPR.G** |
|  | 22000 | **1081.4878** | **2160.9610** | **2159.9333** | **1.0277** | **0** | **0** | **75** | **3** | **K.GVMMHHSNLIAGMTGQCER.I** |
|  | 24890 | **846.0459** | **2535.1159** | **2535.1311** | **-0.0153** | **0** | **18** | **1** | **1** | **R.WFCTGDIGEFHPDGCLQIIDR.K** |
|  | 25581 | 1319.0894 | 2636.1642 | 2636.3376 | -0.1734 | 1 | 0 | 56 | 3 | K.GDCTVLKPTLMAAVPEIMDRIYK.N |
|  | 28069 | 778.6527 | 3110.5817 | 3110.5925 | -0.0107 | 1 | 13 | 2.6 | 1 | R.SVTHFDSLAVIDIPGADTLDKLFDHAVSK.F 28070 |

| **815.** | P10768\|ESTD_HUMAN    **Mass:** 31956    **Score:** 37     **Queries matched:** 6   **emPAI:** 0.12 |
| --- | --- |
|  | S-formylglutathione hydrolase OS=Homo sapiens GN=ESD PE=1 SV=2 |

|  | **Query** | **Observed** | **Mr(expt)** | **Mr(calc)** | **Delta** | **Miss** | **Score** | **Expect** | **Rank** | **Peptide** |
| --- | --- | --- | --- | --- | --- | --- | --- | --- | --- | --- |
|  | 1328 | 707.2164 | 706.2091 | 705.3810 | 0.8281 | 1 | 9 | 24 | 1 | K.NPGKYK.S |
|  | 7219 | **597.8240** | **1193.6335** | **1192.6678** | **0.9657** | **1** | **(7)** | **28** | **1** | **K.MKFAVYLPPK.A** |
|  | 7581 | 605.7921 | 1209.5697 | 1208.6627 | 0.9069 | 1 | 14 | 5.5 | 2 | K.MKFAVYLPPK.A |
|  | 23279 | **1154.9908** | **2307.9671** | **2307.1244** | **0.8428** | **0** | **4** | **32** | **2** | **K.SGYHQSASEHGLVVIAPDTSPR.G** 23278 |
|  | 30659 | **1380.3401** | **4137.9984** | **4137.0252** | **0.9732** | **1** | **37** | **0.0058** | **1** | **K.SYPGSQLDILIDQGKDDQFLLDGQLLPDNFIAACTEK.K** |

| **816.** | O95436\|NPT2B_HUMAN    **Mass:** 77305    **Score:** 37     **Queries matched:** 19   **emPAI:** 0.05 |
| --- | --- |
|  | Sodium-dependent phosphate transport protein 2B OS=Homo sapiens GN=SLC34A2 PE=1 SV=2 |

|  | **Query** | **Observed** | **Mr(expt)** | **Mr(calc)** | **Delta** | **Miss** | **Score** | **Expect** | **Rank** | **Peptide** |
| --- | --- | --- | --- | --- | --- | --- | --- | --- | --- | --- |
|  | 1373 | **729.3946** | **728.3873** | **728.4796** | **-0.0923** | **0** | **18** | **3.3** | **1** | **K.ILGSVLK.G** 1374 1376 |
|  | 1851 | **408.7422** | **815.4698** | **814.4913** | **0.9785** | **0** | **10** | **23** | **1** | **K.GQVATVIK.K** |
|  | 3465 | 467.2887 | 932.5628 | 932.5695 | -0.0067 | 0 | 10 | 21 | 9 | K.VITKPFTK.L |
|  | 3785 | **478.8073** | **955.5999** | **955.6066** | **-0.0066** | **1** | **37** | **0.035** | **1** | **K.LIVQLDKK.V** |
|  | 5050 | **523.7274** | **1045.4402** | **1045.4474** | **-0.0072** | **0** | **25** | **0.54** | **1** | **K.FTGCFQMR.C** |
|  | 5662 | **545.7731** | **1089.5317** | **1088.6012** | **0.9305** | **1** | **9** | **21** | **3** | **R.MAKGLGNISAK.Y** 5652 |
|  | 5863 | 552.7719 | 1103.5293 | 1104.5961 | -1.0668 | 1 | (8) | 26 | 10 | R.MAKGLGNISAK.Y |
|  | 8334 | **624.3142** | **1246.6137** | **1246.6227** | **-0.0090** | **0** | **26** | **0.39** | **1** | **K.VISQIAMNDEK.A** |
|  | 12069 | **482.9223** | **1445.7451** | **1445.7548** | **-0.0097** | **1** | **9** | **17** | **2** | **K.VISQIAMNDEKAK.N** 12086 |
|  | 13411 | **763.3179** | **1524.6212** | **1524.9603** | **-0.3391** | **1** | **8** | **19** | **1** | **K.ILGSVLKGQVATVIK.K** |
|  | 13747 | 773.4134 | 1544.8122 | 1544.7471 | 0.0652 | 0 | 8 | 19 | 6 | K.YLEGAAGQQPTAPDK.S 13741 |
|  | 20412 | **669.6799** | **2006.0180** | **2004.8405** | **1.1775** | **0** | **2** | **50** | **3** | **K.CCEDLEEAQEGQDVPVK.A** |
|  | 26886 | 714.8480 | 2855.3627 | 2855.5249 | -0.1621 | 0 | 1 | 49 | 5 | R.AAIPIIMGANIGTSITNTIVALMQVGDR.S |
|  | 30084 | **1257.6274** | **3769.8605** | **3769.8826** | **-0.0221** | **0** | **2** | **24** | **1** | **K.IELLPSYSTATLIDEPTEVDDPWNLPTLQDSGIK.W** |

| **817.** | P18031\|PTN1_HUMAN    **Mass:** 50505    **Score:** 37     **Queries matched:** 13   **emPAI:** 0.07 |
| --- | --- |
|  | Tyrosine-protein phosphatase non-receptor type 1 OS=Homo sapiens GN=PTPN1 PE=1 SV=1 |

|  | **Query** | **Observed** | **Mr(expt)** | **Mr(calc)** | **Delta** | **Miss** | **Score** | **Expect** | **Rank** | **Peptide** |
| --- | --- | --- | --- | --- | --- | --- | --- | --- | --- | --- |
|  | 1712 | **403.2272** | **804.4399** | **803.4324** | **1.0075** | **0** | **6** | **53** | **5** | **R.GVVMLNR.V** |
|  | 3095 | 454.7447 | 907.4748 | 906.4844 | 0.9903 | 1 | 11 | 12 | 8 | R.VMEKGSLK.C |
|  | 4817 | **516.2766** | **1030.5387** | **1030.5706** | **-0.0319** | **1** | **5** | **53** | **5** | **K.SRGVVMLNR.V** |
|  | 6060 | 559.9462 | 1117.8778 | 1117.4611 | 0.4167 | 0 | 1 | 92 | 4 | R.HEASDFPCR.V |
|  | 7845 | **612.3304** | **1222.6462** | **1222.6241** | **0.0222** | **1** | **1** | **1.2e+002** | **4** | **R.ILEPHNGKCR.E** |
|  | 8279 | 623.3297 | 1244.6449 | 1244.6547 | -0.0098 | 0 | 7 | 29 | 3 | R.MGLIQTADQLR.F 8288 |
|  | 9168 | **430.8867** | **1289.6383** | **1290.6788** | **-1.0405** | **1** | **6** | **40** | **7** | **R.GVVMLNRVMEK.G** |
|  | 10628 | 455.8879 | 1364.6420 | 1365.6677 | -1.0257 | 0 | 9 | 19 | 3 | K.SGSWAAIYQDIR.H |
|  | 13660 | **770.7010** | **1539.3874** | **1539.7028** | **-0.3154** | **0** | **5** | **25** | **2** | **K.FIMGDSSVQDQWK.E** 13680 |
|  | 14217 | 788.3901 | 1574.7656 | 1573.7947 | 0.9709 | 0 | 37 | 0.027 | 2 | R.QLELENLTTQETR.E |
|  | 22684 | **558.5302** | **2230.0915** | **2231.0753** | **-0.9838** | **0** | **4** | **36** | **8** | **R.ESGSLSPEHGPVVVHCSAGIGR.S** |

| **818.** | P18428\|LBP_HUMAN    **Mass:** 53521    **Score:** 36     **Queries matched:** 8   **emPAI:** 0.07 |
| --- | --- |
|  | Lipopolysaccharide-binding protein OS=Homo sapiens GN=LBP PE=1 SV=3 |

|  | **Query** | **Observed** | **Mr(expt)** | **Mr(calc)** | **Delta** | **Miss** | **Score** | **Expect** | **Rank** | **Peptide** |
| --- | --- | --- | --- | --- | --- | --- | --- | --- | --- | --- |
|  | 3299 | **461.2273** | **920.4400** | **920.4459** | **-0.0060** | **0** | **12** | **11** | **2** | **R.ICEMIQK.S** |
|  | 4498 | 503.2768 | 1004.5390 | 1005.5607 | -1.0217 | 1 | 4 | 64 | 9 | K.FQKVLESR.I 4525 |
|  | 8722 | **634.3182** | **1266.6218** | **1267.6304** | **-1.0086** | **0** | **2** | **82** | **3** | **R.ATAQMLEVMFK.G** |
|  | 20585 | 675.3259 | 2022.9560 | 2023.0019 | -0.0460 | 1 | 2 | 66 | 5 | R.ATAQMLEVMFKGEIFHR.N |
|  | 28957 | **831.6761** | **3322.6753** | **3321.7325** | **0.9428** | **1** | **4** | **16** | **1** | **K.VGLFNAELLEALLNYYILNTFYPKFNDK.L** 28943 |
|  | 30082 | 1257.2888 | 3768.8446 | 3768.8622 | -0.0176 | 0 | 36 | 0.008 | 1 | K.SVSSDLQPYLQTLPVTTEIDSFADIDYSLVEAPR.A |

| **819.** | P16520\|GBB3_HUMAN    **Mass:** 38166    **Score:** 36     **Queries matched:** 12   **emPAI:** 0.10 |
| --- | --- |
|  | Guanine nucleotide-binding protein G(I)/G(S)/G(T) subunit beta-3 OS=Homo sapiens GN=GNB3 PE=1 SV=1 |

|  | **Query** | **Observed** | **Mr(expt)** | **Mr(calc)** | **Delta** | **Miss** | **Score** | **Expect** | **Rank** | **Peptide** |
| --- | --- | --- | --- | --- | --- | --- | --- | --- | --- | --- |
|  | 1725 | 403.7397 | 805.4649 | 804.4970 | 0.9679 | 0 | 7 | 36 | 5 | K.VHAIPLR.S 1701 |
|  | 4012 | 487.2831 | 972.5515 | 972.5240 | 0.0276 | 1 | 12 | 12 | 3 | R.QEAEQLKK.Q 4013 4016 4023 |
|  | 4546 | 505.7235 | 1009.4323 | 1008.4368 | 0.9955 | 0 | 8 | 22 | 4 | -.MGEMEQLR.Q |
|  | 4651 | 509.2787 | 1016.5428 | 1016.5502 | -0.0074 | 0 | 36 | 0.051 | 1 | K.LLVSASQDGK.L 4637 4650 |
|  | 13875 | 776.8790 | 1551.7435 | 1552.6762 | -0.9327 | 0 | 2 | 73 | 3 | R.ELSAHTGYLSCCR.F |
|  | 18161 | **610.3339** | **1827.9799** | **1827.9090** | **0.0709** | **1** | **2** | **69** | **2** | **R.GHLAKIYAMHWATDSK.L** |

| **820.** | P60602\|ROMO1_HUMAN    **Mass:** 8405     **Score:** 36     **Queries matched:** 16   **emPAI:** 0.49 |
| --- | --- |
|  | Reactive oxygen species modulator 1 OS=Homo sapiens GN=ROMO1 PE=1 SV=1 |

|  | **Query** | **Observed** | **Mr(expt)** | **Mr(calc)** | **Delta** | **Miss** | **Score** | **Expect** | **Rank** | **Peptide** |
| --- | --- | --- | --- | --- | --- | --- | --- | --- | --- | --- |
|  | 4857 | 517.2712 | 1032.5279 | 1032.5386 | -0.0107 | 1 | 5 | 62 | 5 | R.GRELMGGIGK.T |
|  | 18671 | **932.9300** | **1863.8454** | **1863.8574** | **-0.0120** | **0** | **36** | **0.025** | **1** | **M.PVAVGPYGQSQPSCFDR.V** |
|  | 21515 | 703.6509 | 2107.9308 | 2108.9516 | -1.0208 | 0 | 5 | 25 | 2 | K.TMMQSGGTFGTFMAIGMGIR.C |
|  | 21547 | 704.3883 | 2110.1431 | 2108.9516 | 1.1915 | 0 | (1) | 66 | 2 | K.TMMQSGGTFGTFMAIGMGIR.C |
|  | 21669 | 708.9681 | 2123.8824 | 2124.9465 | -1.0641 | 0 | (2) | 45 | 4 | K.TMMQSGGTFGTFMAIGMGIR.C 21674 |
|  | 21824 | **715.0397** | **2142.0974** | **2140.9414** | **1.1559** | **0** | **(2)** | **56** | **1** | **K.TMMQSGGTFGTFMAIGMGIR.C** |
|  | 23110 | 762.7441 | 2285.2106 | 2284.9772 | 0.2334 | 1 | 6 | 20 | 5 | K.TMMQSGGTFGTFMAIGMGIRC.- |
|  | 24842 | **632.7745** | **2527.0691** | **2526.1351** | **0.9340** | **0** | **(4)** | **22** | **2** | **K.MGFVMGCAVGMAAGALFGTFSCLR.I** |
|  | 24936 | 848.5294 | 2542.5664 | 2542.1300 | 0.4365 | 0 | 5 | 17 | 1 | K.MGFVMGCAVGMAAGALFGTFSCLR.I |
|  | 27206 | **732.3537** | **2925.3857** | **2926.3520** | **-0.9663** | **1** | **(0)** | **50** | **8** | **R.ELMGGIGKTMMQSGGTFGTFMAIGMGIR.C** |
|  | 27211 | **976.4581** | **2926.3526** | **2926.3520** | **0.0006** | **1** | **5** | **16** | **1** | **R.ELMGGIGKTMMQSGGTFGTFMAIGMGIR.C** |
|  | 27281 | 981.8173 | 2942.4300 | 2942.3469 | 0.0831 | 1 | (1) | 40 | 4 | R.ELMGGIGKTMMQSGGTFGTFMAIGMGIR.C |
|  | 27664 | 754.6235 | 3014.4648 | 3015.3720 | -0.9072 | 1 | 2 | 30 | 10 | K.MGFVMGCAVGMAAGALFGTFSCLRIGMR.G 27665 |
|  | 27745 | 758.6227 | 3030.4616 | 3031.3669 | -0.9053 | 1 | (2) | 34 | 4 | K.MGFVMGCAVGMAAGALFGTFSCLRIGMR.G |

| **821.** | Q9NVJ2\|ARL8B_HUMAN    **Mass:** 21753    **Score:** 36     **Queries matched:** 8   **emPAI:** 0.18 |
| --- | --- |
|  | ADP-ribosylation factor-like protein 8B OS=Homo sapiens GN=ARL8B PE=1 SV=1 |

|  | **Query** | **Observed** | **Mr(expt)** | **Mr(calc)** | **Delta** | **Miss** | **Score** | **Expect** | **Rank** | **Peptide** |
| --- | --- | --- | --- | --- | --- | --- | --- | --- | --- | --- |
|  | 2085 | 417.2428 | 832.4711 | 831.4450 | 1.0261 | 1 | 12 | 14 | 6 | R.EKIEASR.N 2087 |
|  | 4745 | 513.7283 | 1025.4420 | 1026.4705 | -1.0285 | 1 | 9 | 18 | 7 | R.FRSMWER.Y |
|  | 4969 | **521.2734** | **1040.5323** | **1040.5403** | **-0.0080** | **0** | **36** | **0.039** | **1** | **K.IWDIGGQPR.F** |
|  | 6806 | **586.3284** | **1170.6422** | **1169.6040** | **1.0382** | **1** | **1** | **1.2e+002** | **7** | **K.RDLPNALDEK.Q** |
|  | 15457 | 826.9282 | 1651.8419 | 1652.8998 | -1.0580 | 1 | 2 | 71 | 5 | K.GNVTIKIWDIGGQPR.F |
|  | 21232 | **521.0284** | **2080.0844** | **2080.0953** | **-0.0109** | **1** | **6** | **24** | **6** | **K.EKDNIDITLQWLIQHSK.S** |
|  | 28060 | **777.9392** | **3107.7277** | **3107.7455** | **-0.0177** | **1** | **17** | **0.76** | **1** | **K.IEASRNELHNLLDKPQLQGIPVLVLGNK.R** |

| **822.** | Q96BM9\|ARL8A_HUMAN    **Score:** 36     **Queries matched:** 6 |
| --- | --- |
|  | ADP-ribosylation factor-like protein 8A OS=Homo sapiens GN=ARL8A PE=1 SV=1 |

|  | **Query** | **Observed** | **Mr(expt)** | **Mr(calc)** | **Delta** | **Miss** | **Score** | **Expect** | **Rank** | **Peptide** |
| --- | --- | --- | --- | --- | --- | --- | --- | --- | --- | --- |
|  | 2341 | **426.6852** | **851.3559** | **851.4575** | **-0.1016** | **0** | **3** | **79** | **10** | **-.MIALFNK.L** |
|  | 4745 | 513.7283 | 1025.4420 | 1026.4705 | -1.0285 | 1 | 9 | 18 | 7 | R.FRSMWER.Y |
|  | 4969 | 521.2734 | 1040.5323 | 1040.5403 | -0.0080 | 0 | 36 | 0.039 | 1 | K.LWDIGGQPR.F |
|  | 15457 | 826.9282 | 1651.8419 | 1652.8998 | -1.0580 | 1 | 2 | 71 | 5 | K.GNVTIKLWDIGGQPR.F |
|  | 15820 | **558.9615** | **1673.8628** | **1674.8610** | **-0.9982** | **1** | **4** | **46** | **2** | **K.ELIEKMNLSAIQDR.E** |
|  | 21232 | 521.0284 | 2080.0844 | 2080.0953 | -0.0109 | 1 | 6 | 24 | 6 | K.EKDNIDITLQWLIQHSK.S |

| **823.** | O95299\|NDUAA_HUMAN    **Mass:** 41067    **Score:** 36     **Queries matched:** 12   **emPAI:** 0.09 |
| --- | --- |
|  | NADH dehydrogenase [ubiquinone] 1 alpha subcomplex subunit 10, mitochondrial OS=Homo sapiens GN=NDUFA10 PE=1 SV=1 |

|  | **Query** | **Observed** | **Mr(expt)** | **Mr(calc)** | **Delta** | **Miss** | **Score** | **Expect** | **Rank** | **Peptide** |
| --- | --- | --- | --- | --- | --- | --- | --- | --- | --- | --- |
|  | 5895 | **553.8698** | **1105.7251** | **1106.5859** | **-0.8609** | **0** | **2** | **1.1e+002** | **5** | **K.VVEDIEYLK.F** |
|  | 7385 | **601.2767** | **1200.5388** | **1200.7190** | **-0.1802** | **1** | **5** | **54** | **5** | **R.LLKLAATSASAR.V** 7384 |
|  | 7554 | 604.9549 | 1207.8952 | 1208.6111 | -0.7158 | 1 | 5 | 31 | 2 | K.KTFLPEMSEK.C 7551 7571 |
|  | 10357 | **451.2494** | **1350.7263** | **1350.7520** | **-0.0258** | **1** | **6** | **33** | **8** | **R.VLHQFRELPGR.K** 10349 10358 |
|  | 15178 | **545.8127** | **1634.4162** | **1634.8391** | **-0.4229** | **1** | **2** | **49** | **2** | **K.LRYGMWHFLLGDK.A** |
|  | 22227 | **730.1515** | **2187.4326** | **2187.0340** | **0.3986** | **1** | **1** | **48** | **2** | **K.TFLPEMSEKCEVLQYSAR.E** |
|  | 24348 | 819.1068 | 2454.2984 | 2454.3118 | -0.0134 | 0 | 36 | 0.017 | 1 | R.LLQYSDALEHLLTTGQGVVLER.S |

| **824.** | O76024\|WFS1_HUMAN    **Mass:** 101381   **Score:** 36     **Queries matched:** 13   **emPAI:** 0.07 |
| --- | --- |
|  | Wolframin OS=Homo sapiens GN=WFS1 PE=1 SV=1 |

|  | **Query** | **Observed** | **Mr(expt)** | **Mr(calc)** | **Delta** | **Miss** | **Score** | **Expect** | **Rank** | **Peptide** |
| --- | --- | --- | --- | --- | --- | --- | --- | --- | --- | --- |
|  | 1985 | 414.2559 | 826.4972 | 827.5229 | -1.0257 | 1 | 2 | 1.1e+002 | 10 | R.EAVKLLR.R |
|  | 3611 | 472.7524 | 943.4903 | 943.5702 | -0.0800 | 0 | 3 | 90 | 3 | R.TLTDLLLR.F |
|  | 6133 | 562.3232 | 1122.6318 | 1121.5287 | 1.1031 | 1 | 4 | 64 | 4 | R.AWKETNMAR.T |
|  | 9144 | **645.2935** | **1288.5724** | **1288.6371** | **-0.0647** | **1** | **7** | **31** | **5** | **R.GITSENEREVR.Q** |
|  | 11898 | 718.8497 | 1435.6848 | 1435.8035 | -0.1187 | 1 | 6 | 36 | 2 | R.ASSEFKSVLLSLR.Q |
|  | 12367 | **732.4199** | **1462.8252** | **1461.6960** | **1.1291** | **0** | **29** | **0.17** | **1** | **R.DAAAPAEPQAQHTR.S** 12360 |
|  | 16285 | **853.3966** | **1704.7787** | **1704.8292** | **-0.0505** | **1** | **2** | **66** | **4** | **R.DAAAPAEPQAQHTRSR.E** |
|  | 22933 | **755.1196** | **2262.3369** | **2262.0540** | **0.2829** | **0** | **4** | **26** | **3** | **K.GVIPSSLFLQDDEDDDELAGK.S** |
|  | 23154 | **573.5438** | **2290.1462** | **2290.0787** | **0.0675** | **1** | **4** | **33** | **5** | **R.ADGTGPTKGDMEIPFEEVLER.A** |
|  | 26340 | 690.5981 | 2758.3635 | 2757.2778 | 1.0856 | 0 | 1 | 48 | 10 | R.VTDIDNSAESAINMLPFFIGDWMR.C |
|  | 26402 | **555.4753** | **2772.3403** | **2772.3072** | **0.0331** | **1** | **0** | **54** | **6** | **-.MDSNTAPLGPSCPQPPPAPQPQARSR.L** |
|  | 28332 | 1057.5121 | 3169.5144 | 3169.5303 | -0.0159 | 1 | 27 | 0.11 | 1 | K.GVIPSSLFLQDDEDDDELAGKSPEDLPLR.L |

| **825.** | Q01814\|AT2B2_HUMAN    **Mass:** 137987   **Score:** 36     **Queries matched:** 33   **emPAI:** 0.05 |
| --- | --- |
|  | Plasma membrane calcium-transporting ATPase 2 OS=Homo sapiens GN=ATP2B2 PE=1 SV=2 |

|  | **Query** | **Observed** | **Mr(expt)** | **Mr(calc)** | **Delta** | **Miss** | **Score** | **Expect** | **Rank** | **Peptide** |
| --- | --- | --- | --- | --- | --- | --- | --- | --- | --- | --- |
|  | 2983 | 451.2271 | 900.4397 | 901.4981 | -1.0584 | 1 | 15 | 5.6 | 7 | K.EAGRLTQK.E |
|  | 3351 | **463.5364** | **925.0582** | **925.4981** | **-0.4399** | **0** | **0** | **88** | **9** | **K.ILNGAGEPR.V** |
|  | 3571 | 472.2629 | 942.5112 | 943.5702 | -1.0590 | 1 | 2 | 1.1e+002 | 3 | R.GTEAVVKIK.E |
|  | 3944 | 484.7474 | 967.4802 | 966.4923 | 0.9879 | 0 | 15 | 5 | 1 | R.NVFDGIFR.N 3945 |
|  | 4254 | 496.2115 | 990.4085 | 991.3843 | -0.9757 | 0 | 14 | 7.4 | 6 | K.SAEGGDADDR.K |
|  | 7157 | 596.2887 | 1190.5628 | 1190.5714 | -0.0085 | 0 | 35 | 0.051 | 1 | R.MVTGDNINTAR.A 7177 |
|  | 7343 | 400.2387 | 1197.6944 | 1197.6942 | 0.0002 | 1 | 15 | 4.5 | 1 | R.GLNRIQTQIR.V 7304 7332 7336 |
|  | 7631 | 607.2716 | 1212.5287 | 1211.6510 | 0.8777 | 1 | 6 | 40 | 2 | R.SSPTDKHTLVK.G 7635 7648 |
|  | 7991 | 411.2052 | 1230.5939 | 1230.5840 | 0.0099 | 1 | 7 | 30 | 6 | R.NEKGEIEQER.I 8000 |
|  | 8589 | 420.8481 | 1259.5223 | 1259.6002 | -0.0779 | 0 | 5 | 49 | 9 | K.VIEPMACDGLR.T |
|  | 8752 | **634.8785** | **1267.7424** | **1267.6118** | **0.1306** | **1** | **16** | **3** | **1** | **R.SQMPEEKLYK.V** 8738 |
|  | 10493 | 679.8007 | 1357.5868 | 1358.7571 | -1.1704 | 1 | 2 | 91 | 2 | R.GQILWFRGLNR.I |
|  | 11342 | 469.2191 | 1404.6354 | 1403.6901 | 0.9453 | 1 | 29 | 0.15 | 1 | K.KVIEPMACDGLR.T 11331 11349 |
|  | 14657 | 535.3050 | 1602.8931 | 1601.7409 | 1.1523 | 0 | 1 | 87 | 8 | R.TSIHNFMAHPEFR.I |
|  | 15278 | 821.4200 | 1640.8255 | 1640.8370 | -0.0115 | 0 | 26 | 0.28 | 1 | R.QVVAVTGDGTNDGPALK.K 15272 |
|  | 19834 | **652.8645** | **1955.5717** | **1954.7785** | **0.7931** | **0** | **4** | **24** | **1** | **R.NESSHGGEFGCTMEELR.S** |
|  | 21753 | **711.8863** | **2132.6370** | **2132.2205** | **0.4165** | **0** | **3** | **35** | **2** | **R.AGQVVQIPVAEIVVGDIAQVK.Y** |
|  | 24818 | **841.4610** | **2521.3612** | **2522.0649** | **-0.7038** | **1** | **4** | **26** | **1** | **K.QQDGAAAMEMQPLKSAEGGDADDR.K** |
|  | 30527 | **1353.9298** | **4058.7676** | **4058.1737** | **0.5939** | **1** | **3** | **11** | **1** | **K.AVQMLWVNLIMDTFASLALATEPPTETLLLRKPYGR.N** 30528 |
|  | 31013 | **910.0375** | **4545.1513** | **4546.2394** | **-1.0880** | **1** | **0** | **19** | **3** | **K.DPMLLSGTHVMEGSGRMLVTAVGVNSQTGIIFTLLGAGGEEEEK.K** |

| **826.** | P11488\|GNAT1_HUMAN    **Mass:** 40471    **Score:** 35     **Queries matched:** 9   **emPAI:** 0.19 |
| --- | --- |
|  | Guanine nucleotide-binding protein G(t) subunit alpha-1 OS=Homo sapiens GN=GNAT1 PE=1 SV=5 |

|  | **Query** | **Observed** | **Mr(expt)** | **Mr(calc)** | **Delta** | **Miss** | **Score** | **Expect** | **Rank** | **Peptide** |
| --- | --- | --- | --- | --- | --- | --- | --- | --- | --- | --- |
|  | 1899 | 410.7395 | 819.4645 | 818.3770 | 1.0875 | 0 | 9 | 26 | 2 | M.GAGASAEEK.H |
|  | 3113 | 455.2127 | 908.4109 | 908.4175 | -0.0066 | 0 | 33 | 0.081 | 1 | R.MFDVGGQR.S |
|  | 5181 | 529.2853 | 1056.5560 | 1056.6179 | -0.0619 | 0 | 28 | 0.31 | 1 | K.LLLLGAGESGK.S |
|  | 10000 | 665.8217 | 1329.6289 | 1329.6095 | 0.0194 | 1 | (2) | 89 | 3 | -.MGAGASAEEKHSR.E |
|  | 10258 | 673.3621 | 1344.7097 | 1345.6044 | -0.8947 | 1 | 5 | 45 | 3 | -.MGAGASAEEKHSR.E |
|  | 10961 | **691.3682** | **1380.7218** | **1379.7701** | **0.9517** | **0** | **5** | **41** | **1** | **K.FVFDAVTDIIIK.E** |
|  | 14390 | 529.3709 | 1585.0909 | 1584.9450 | 0.1459 | 1 | 1 | 67 | 4 | K.LLLLGAGESGKSTIVK.Q |
|  | 18853 | **627.3184** | **1878.9333** | **1878.8525** | **0.0807** | **1** | **2** | **62** | **5** | **R.KLMHMADTIEEGTMPK.E** |
|  | 25865 | **893.0758** | **2676.2056** | **2675.2427** | **0.9629** | **1** | **0** | **57** | **3** | **K.LMHMADTIEEGTMPKEMSDIIQR.L** |

| **827.** | P19087\|GNAT2_HUMAN    **Mass:** 40720    **Score:** 35     **Queries matched:** 7   **emPAI:** 0.19 |
| --- | --- |
|  | Guanine nucleotide-binding protein G(t) subunit alpha-2 OS=Homo sapiens GN=GNAT2 PE=2 SV=4 |

|  | **Query** | **Observed** | **Mr(expt)** | **Mr(calc)** | **Delta** | **Miss** | **Score** | **Expect** | **Rank** | **Peptide** |
| --- | --- | --- | --- | --- | --- | --- | --- | --- | --- | --- |
|  | 3113 | 455.2127 | 908.4109 | 908.4175 | -0.0066 | 0 | 33 | 0.081 | 1 | R.MFDVGGQR.S |
|  | 5181 | 529.2853 | 1056.5560 | 1056.6179 | -0.0619 | 0 | 28 | 0.31 | 1 | K.LLLLGAGESGK.S |
|  | 6166 | **563.7557** | **1125.4969** | **1124.5978** | **0.8991** | **1** | **10** | **16** | **6** | **K.FSVKDLNFR.M** |
|  | 10961 | 691.3682 | 1380.7218 | 1379.7701 | 0.9517 | 0 | 5 | 41 | 1 | K.FVFDAVTDIIIK.E |
|  | 11405 | 470.2500 | 1407.7283 | 1408.6504 | -0.9221 | 1 | 6 | 34 | 3 | -.MGSGASAEDKELAK.R |
|  | 14390 | 529.3709 | 1585.0909 | 1584.9450 | 0.1459 | 1 | 1 | 67 | 4 | K.LLLLGAGESGKSTIVK.Q |
|  | 17078 | 586.3246 | 1755.9519 | 1756.0611 | -0.1091 | 0 | 1 | 86 | 8 | K.AIIYGNVLQSILAIIR.A |

| **828.** | A8MTJ3\|GNAT3_HUMAN    **Mass:** 40844    **Score:** 35     **Queries matched:** 7   **emPAI:** 0.19 |
| --- | --- |
|  | Guanine nucleotide-binding protein G(t) subunit alpha-3 OS=Homo sapiens GN=GNAT3 PE=2 SV=2 |

|  | **Query** | **Observed** | **Mr(expt)** | **Mr(calc)** | **Delta** | **Miss** | **Score** | **Expect** | **Rank** | **Peptide** |
| --- | --- | --- | --- | --- | --- | --- | --- | --- | --- | --- |
|  | 3113 | 455.2127 | 908.4109 | 908.4175 | -0.0066 | 0 | 33 | 0.081 | 1 | R.MFDVGGQR.S |
|  | 5181 | 529.2853 | 1056.5560 | 1056.6179 | -0.0619 | 0 | 28 | 0.31 | 1 | K.LLLLGAGESGK.S |
|  | 10961 | 691.3682 | 1380.7218 | 1379.7701 | 0.9517 | 0 | 5 | 41 | 1 | K.FVFDAVTDIIIK.E |
|  | 14390 | 529.3709 | 1585.0909 | 1584.9450 | 0.1459 | 1 | 1 | 67 | 4 | K.LLLLGAGESGKSTIVK.Q |
|  | 20458 | 671.9625 | 2012.8657 | 2011.9131 | 0.9526 | 1 | 1 | 65 | 6 | K.IIHKNGYSEQECMEFK.A |
|  | 25748 | 534.0580 | 2665.2535 | 2664.3251 | 0.9284 | 1 | 3 | 32 | 2 | R.QLYAMANTLEDGGMTPQLAEVIKR.L |
|  | 25889 | **537.0884** | **2680.4055** | **2680.3200** | **0.0855** | **1** | **(1)** | **43** | **8** | **R.QLYAMANTLEDGGMTPQLAEVIKR.L** |

| **829.** | Q6NUM9\|RETST_HUMAN    **Mass:** 67461    **Score:** 35     **Queries matched:** 11   **emPAI:** 0.06 |
| --- | --- |
|  | All-trans-retinol 13,14-reductase OS=Homo sapiens GN=RETSAT PE=2 SV=2 |

|  | **Query** | **Observed** | **Mr(expt)** | **Mr(calc)** | **Delta** | **Miss** | **Score** | **Expect** | **Rank** | **Peptide** |
| --- | --- | --- | --- | --- | --- | --- | --- | --- | --- | --- |
|  | 1065 | **604.9551** | **603.9479** | **603.2976** | **0.6502** | **0** | **14** | **6.8** | **1** | **K.NLDSR.I** 1064 1066 1067 |
|  | 2925 | **448.2559** | **894.4973** | **893.3913** | **1.1061** | **0** | **0** | **1.5e+002** | **6** | **R.MEEGSIGR.F** |
|  | 3422 | 466.2599 | 930.5052 | 930.5175 | -0.0123 | 0 | 13 | 8.7 | 9 | K.LFPQLEGK.V |
|  | 12087 | 483.2362 | 1446.6867 | 1445.7402 | 0.9465 | 1 | 1 | 1.1e+002 | 8 | K.EKFPQEEAIIDK.Y |
|  | 20212 | **664.2819** | **1989.8239** | **1989.0565** | **0.7674** | **1** | **1** | **65** | **8** | **K.ATVQSVLLDSAGKACGVSVK.K** 20214 |
|  | 30134 | 1264.3228 | 3789.9464 | 3788.9845 | 0.9619 | 0 | 35 | 0.0092 | 1 | R.AQSPIPNLYLTGQDIFTCGLVGALQGALLCSSAILK.R |
|  | 30846 | 1445.3864 | 4333.1372 | 4333.1035 | 0.0337 | 1 | 1 | 18 | 6 | R.MEEGSIGRFILDQITEGQLDWAPLSSPFDIMVLEGPNGR.K |

| **830.** | Q6NUQ4\|TM214_HUMAN    **Mass:** 77957    **Score:** 35     **Queries matched:** 17   **emPAI:** 0.05 |
| --- | --- |
|  | Transmembrane protein 214 OS=Homo sapiens GN=TMEM214 PE=1 SV=2 |

|  | **Query** | **Observed** | **Mr(expt)** | **Mr(calc)** | **Delta** | **Miss** | **Score** | **Expect** | **Rank** | **Peptide** |
| --- | --- | --- | --- | --- | --- | --- | --- | --- | --- | --- |
|  | 3746 | 477.5526 | 953.0906 | 953.5155 | -0.4249 | 0 | 4 | 37 | 7 | R.RPGVGAGAGGR.G |
|  | 4190 | 493.7821 | 985.5496 | 985.5556 | -0.0060 | 0 | 27 | 0.36 | 2 | K.LTNQELLR.K 4175 |
|  | 6921 | 590.8102 | 1179.6059 | 1178.6845 | 0.9214 | 0 | 15 | 4.7 | 1 | R.LLLMHPNLTK.G 6897 6913 |
|  | 7357 | **400.5730** | **1198.6973** | **1199.6411** | **-0.9438** | **1** | **11** | **12** | **4** | **R.RALGEANGVWK.Y** 7361 |
|  | 8954 | 426.7168 | 1277.1287 | 1276.6160 | 0.5126 | 0 | 3 | 49 | 8 | R.SHSSFQASLTGR.L |
|  | 8985 | 427.5354 | 1279.5844 | 1280.6810 | -1.0967 | 1 | 7 | 24 | 3 | R.RPGVGAGAGGRGGGR.N 9035 |
|  | 19449 | **643.6453** | **1927.9140** | **1928.8027** | **-0.8887** | **1** | **5** | **32** | **2** | **R.KGSSNNQDVVTCDMACK.G** |
|  | 20335 | **500.7492** | **1998.9679** | **1998.0795** | **0.8884** | **1** | **6** | **23** | **5** | **R.LLLMHPNLTKGFGMIGPK.D** |
|  | 23179 | 765.3810 | 2293.1213 | 2293.1599 | -0.0386 | 0 | 10 | 7.3 | 1 | K.CLTIMWALGQAGFANLTEGLK.V |
|  | 24267 | **815.7713** | **2444.2921** | **2444.3063** | **-0.0142** | **0** | **35** | **0.021** | **1** | **K.HLSQSSLLLEHLLSSWEQIPK.K** |
|  | 24925 | 848.0975 | 2541.2708 | 2542.3981 | -1.1274 | 1 | 2 | 45 | 4 | K.SLSPFAITYLDRLLLMHPNLTK.G |
|  | 26372 | **922.8110** | **2765.4111** | **2764.5979** | **0.8132** | **1** | **0** | **58** | **8** | **R.LPWTRLLLLLLVFAVGFLCHDLR.S** |

| **831.** | P84103\|SFRS3_HUMAN    **Mass:** 19546    **Score:** 35     **Queries matched:** 5   **emPAI:** 0.20 |
| --- | --- |
|  | Splicing factor, arginine/serine-rich 3 OS=Homo sapiens GN=SFRS3 PE=1 SV=1 |

|  | **Query** | **Observed** | **Mr(expt)** | **Mr(calc)** | **Delta** | **Miss** | **Score** | **Expect** | **Rank** | **Peptide** |
| --- | --- | --- | --- | --- | --- | --- | --- | --- | --- | --- |
|  | 2357 | **427.1813** | **852.3480** | **852.4242** | **-0.0763** | **0** | **4** | **53** | **1** | **R.GPPPSWGR.R** |
|  | 4293 | 497.2628 | 992.5110 | 993.3896 | -0.8786 | 0 | 5 | 49 | 7 | R.DSCPLDCK.V |
|  | 5003 | **522.2661** | **1042.5177** | **1042.5236** | **-0.0059** | **0** | **35** | **0.051** | **1** | **R.AFGYYGPLR.S** |
|  | 18802 | 625.9760 | 1874.9062 | 1875.9439 | -1.0377 | 1 | 15 | 3.1 | 2 | K.VYVGNLGNNGNKTELER.A 18801 |

| **832.** | Q9BY67\|CADM1_HUMAN    **Mass:** 48935    **Score:** 35     **Queries matched:** 5   **emPAI:** 0.08 |
| --- | --- |
|  | Cell adhesion molecule 1 OS=Homo sapiens GN=CADM1 PE=1 SV=2 |

|  | **Query** | **Observed** | **Mr(expt)** | **Mr(calc)** | **Delta** | **Miss** | **Score** | **Expect** | **Rank** | **Peptide** |
| --- | --- | --- | --- | --- | --- | --- | --- | --- | --- | --- |
|  | 4058 | **488.3044** | **974.5943** | **973.5266** | **1.0677** | **0** | **10** | **20** | **6** | **R.NLMIDIQK.D** |
|  | 11454 | 471.2385 | 1410.6936 | 1411.7347 | -1.0412 | 0 | 8 | 19 | 5 | R.FQLLNFSSSELK.V |
|  | 17466 | **892.8861** | **1783.7577** | **1783.8159** | **-0.0582** | **1** | **2** | **62** | **4** | **K.TDNGTYRCEASNIVGK.A** |
|  | 25981 | **900.4526** | **2698.3359** | **2698.3459** | **-0.0099** | **0** | **31** | **0.053** | **1** | **R.EGDALELTCEAIGKPQPVMVTWVR.V** 25980 |

| **833.** | P51608\|MECP2_HUMAN    **Mass:** 52580    **Score:** 35     **Queries matched:** 17   **emPAI:** 0.07 |
| --- | --- |
|  | Methyl-CpG-binding protein 2 OS=Homo sapiens GN=MECP2 PE=1 SV=1 |

|  | **Query** | **Observed** | **Mr(expt)** | **Mr(calc)** | **Delta** | **Miss** | **Score** | **Expect** | **Rank** | **Peptide** |
| --- | --- | --- | --- | --- | --- | --- | --- | --- | --- | --- |
|  | 2052 | **416.2591** | **830.5036** | **831.4637** | **-0.9600** | **0** | **16** | **5.1** | **1** | **M.VAGMLGLR.E** |
|  | 4069 | **488.7604** | **975.5063** | **974.5032** | **1.0031** | **1** | **10** | **18** | **3** | **R.SSSASSPPKK.E** |
|  | 4232 | **495.2886** | **988.5627** | **988.5189** | **0.0438** | **0** | **5** | **63** | **10** | **K.AATSEGVQVK.R** |
|  | 5950 | **555.7787** | **1109.5428** | **1110.5961** | **-1.0533** | **0** | **2** | **82** | **2** | **K.VELIAYFEK.V** |
|  | 8765 | 635.3023 | 1268.5901 | 1268.7088 | -0.1187 | 0 | 7 | 26 | 2 | R.KPGSVVAAAAAEAK.K |
|  | 10834 | 459.1706 | 1374.4899 | 1373.6721 | 0.8178 | 0 | 2 | 65 | 7 | K.DIVSSSMPRPNR.E |
|  | 13308 | 759.9119 | 1517.8092 | 1517.7209 | 0.0883 | 1 | 35 | 0.039 | 2 | R.EEKSEDQDLQGLK.D 13309 |
|  | 13518 | 511.0238 | 1530.0496 | 1530.6984 | -0.6488 | 1 | 14 | 3.6 | 1 | R.GGSLESDGCPKEPAK.T 13519 |
|  | 13793 | **516.9225** | **1547.7456** | **1548.7818** | **-1.0361** | **0** | **6** | **30** | **4** | **K.AEGGGATTSTQVMVIK.R** 13816 |
|  | 19466 | 965.4677 | 1928.9209 | 1928.8963 | 0.0246 | 0 | 1 | 84 | 9 | K.AETSEGSGSAPAVPEASASPK.Q 19467 |
|  | 22215 | **729.4205** | **2185.2398** | **2186.0386** | **-0.7988** | **1** | **2** | **44** | **5** | **K.DIVSSSMPRPNREEPVDSR.T** |
|  | 25466 | **653.8165** | **2611.2370** | **2611.2622** | **-0.0252** | **1** | **3** | **35** | **4** | **K.MPFQTSPGGKAEGGGATTSTQVMVIK.R** |
|  | 30788 | **1424.3435** | **4270.0087** | **4270.0879** | **-0.0792** | **1** | **1** | **17** | **3** | **K.APVPLLPPLPPPPPEPESSEDPTSPPEPQDLSSSVCKEEK.M** |

| **834.** | Q9BUL8\|PDC10_HUMAN    **Mass:** 24686    **Score:** 35     **Queries matched:** 10   **emPAI:** 0.15 |
| --- | --- |
|  | Programmed cell death protein 10 OS=Homo sapiens GN=PDCD10 PE=1 SV=1 |

|  | **Query** | **Observed** | **Mr(expt)** | **Mr(calc)** | **Delta** | **Miss** | **Score** | **Expect** | **Rank** | **Peptide** |
| --- | --- | --- | --- | --- | --- | --- | --- | --- | --- | --- |
|  | 2747 | **440.7507** | **879.4868** | **880.4879** | **-1.0011** | **1** | **9** | **21** | **7** | **R.RALEHQK.K** 2745 |
|  | 3426 | 466.2826 | 930.5507 | 930.3497 | 0.2010 | 0 | 1 | 1.5e+002 | 5 | R.MTMEEMK.N |
|  | 5384 | 536.2788 | 1070.5431 | 1071.6036 | -1.0606 | 0 | 3 | 1e+002 | 3 | R.VNLSAAQTLR.A |
|  | 7398 | 401.2366 | 1200.6881 | 1201.4963 | -0.8083 | 1 | 7 | 28 | 7 | -.MRMTMEEMK.N |
|  | 7756 | **610.1820** | **1218.3495** | **1217.4912** | **0.8582** | **1** | **(1)** | **96** | **1** | **-.MRMTMEEMK.N** |
|  | 12061 | 723.8434 | 1445.6722 | 1446.8446 | -1.1724 | 1 | 35 | 0.042 | 2 | R.FLQTIKDIASAIK.E 12101 |
|  | 13688 | 771.4152 | 1540.8159 | 1539.8256 | 0.9902 | 1 | 1 | 90 | 7 | K.QILSKIPDEINDR.V |
|  | 16239 | 851.4428 | 1700.8709 | 1701.8607 | -0.9898 | 1 | 2 | 68 | 5 | K.AEKENPGLTQDIIMK.I |

| **835.** | P50995\|ANX11_HUMAN    **Mass:** 54697    **Score:** 35     **Queries matched:** 10   **emPAI:** 0.07 |
| --- | --- |
|  | Annexin A11 OS=Homo sapiens GN=ANXA11 PE=1 SV=1 |

|  | **Query** | **Observed** | **Mr(expt)** | **Mr(calc)** | **Delta** | **Miss** | **Score** | **Expect** | **Rank** | **Peptide** |
| --- | --- | --- | --- | --- | --- | --- | --- | --- | --- | --- |
|  | 2050 | 416.2589 | 830.5033 | 830.4498 | 0.0536 | 0 | 10 | 21 | 7 | K.TLEEAIR.S |
|  | 4553 | **506.1010** | **1010.1873** | **1009.4716** | **0.7157** | **0** | **5** | **34** | **3** | **K.SELSGNFEK.T** |
|  | 4870 | **517.3302** | **1032.6458** | **1033.4577** | **-0.8119** | **0** | **35** | **0.054** | **1** | **R.SDTSGHFQR.L** 4885 |
|  | 10112 | **669.3574** | **1336.7002** | **1335.6055** | **1.0947** | **0** | **5** | **48** | **7** | **R.DAQELYAAGENR.L** |
|  | 14752 | 804.8847 | 1607.7548 | 1606.7582 | 0.9966 | 0 | 20 | 1.1 | 2 | R.EMSGDLEEGMLAVVK.C |
|  | 17931 | **605.2790** | **1812.8153** | **1812.8278** | **-0.0125** | **1** | **6** | **28** | **1** | **K.SLYHDISGDTSGDYRK.I** |
|  | 21081 | **689.3008** | **2064.8807** | **2065.9552** | **-1.0745** | **1** | **4** | **36** | **2** | **R.DAQELYAAGENRLGTDESK.F** |
|  | 21503 | **703.1091** | **2106.3054** | **2107.2003** | **-0.8949** | **1** | **4** | **24** | **7** | **K.TILALMKTPVLFDIYEIK.E** |
|  | 22179 | 1090.5466 | 2179.0787 | 2179.9480 | -0.8693 | 1 | 1 | 62 | 4 | R.MYGKSLYHDISGDTSGDYR.K |

| **836.** | Q02878\|RL6_HUMAN    **Mass:** 32765    **Score:** 35     **Queries matched:** 16   **emPAI:** 0.12 |
| --- | --- |
|  | 60S ribosomal protein L6 OS=Homo sapiens GN=RPL6 PE=1 SV=3 |

|  | **Query** | **Observed** | **Mr(expt)** | **Mr(calc)** | **Delta** | **Miss** | **Score** | **Expect** | **Rank** | **Peptide** |
| --- | --- | --- | --- | --- | --- | --- | --- | --- | --- | --- |
|  | 776 | **503.1034** | **502.0961** | **503.2452** | **-1.1491** | **0** | **9** | **48** | **3** | **K.NGGTR.V** 777 |
|  | 1235 | 675.1210 | 674.1138 | 673.3759 | 0.7379 | 1 | 7 | 40 | 1 | K.KVDAGGK.V |
|  | 3963 | **485.7785** | **969.5424** | **969.5495** | **-0.0070** | **0** | **35** | **0.047** | **1** | **K.AVDSQILPK.I** |
|  | 4309 | 497.7498 | 993.4851 | 993.4920 | -0.0069 | 0 | 10 | 15 | 2 | K.HLTDAYFK.K |
|  | 5519 | 540.7661 | 1079.5177 | 1079.6564 | -0.1387 | 1 | 9 | 21 | 5 | R.NPVLVRGIGR.Y |
|  | 6319 | **570.2681** | **1138.5216** | **1138.5295** | **-0.0079** | **0** | **19** | **2** | **1** | **R.YYPTEDVPR.K** |
|  | 7267 | 399.5266 | 1195.5579 | 1194.5880 | 0.9699 | 1 | 9 | 17 | 5 | K.EKYEITEQR.K 7245 7248 |
|  | 8720 | 634.3061 | 1266.5976 | 1266.6244 | -0.0268 | 1 | 10 | 13 | 3 | R.YYPTEDVPRK.L |
|  | 9063 | **428.9195** | **1283.7366** | **1283.7449** | **-0.0084** | **0** | **10** | **14** | **2** | **K.VLATVTKPVGGDK.N** |
|  | 10037 | **667.0429** | **1332.0713** | **1331.5994** | **0.4719** | **0** | **3** | **47** | **1** | **R.HQEGEIFDTEK.E** |
|  | 13429 | **763.4663** | **1524.9181** | **1524.9239** | **-0.0059** | **0** | **8** | **17** | **1** | **R.ASITPGTILIILTGR.H** |
|  | 17155 | **588.4039** | **1762.1900** | **1763.0669** | **-0.8769** | **0** | **1** | **61** | **5** | **K.QLASGLLLVTGPLVLNR.V** |
|  | 23606 | **588.5466** | **2350.1574** | **2349.4512** | **0.7062** | **1** | **2** | **42** | **3** | **R.VVFLKQLASGLLLVTGPLVLNR.V** |

| **837.** | Q9H4M9\|EHD1_HUMAN    **Mass:** 60646    **Score:** 35     **Queries matched:** 13   **emPAI:** 0.06 |
| --- | --- |
|  | EH domain-containing protein 1 OS=Homo sapiens GN=EHD1 PE=1 SV=2 |

|  | **Query** | **Observed** | **Mr(expt)** | **Mr(calc)** | **Delta** | **Miss** | **Score** | **Expect** | **Rank** | **Peptide** |
| --- | --- | --- | --- | --- | --- | --- | --- | --- | --- | --- |
|  | 1234 | **675.1207** | **674.1135** | **673.3759** | **0.7376** | **0** | **4** | **70** | **2** | **K.ITGANAK.K** |
|  | 10302 | **450.2432** | **1347.7077** | **1347.6452** | **0.0625** | **0** | **15** | **5** | **1** | **K.ADQIETQQLMR.V** 10290 |
|  | 10608 | 682.8520 | 1363.6894 | 1363.7360 | -0.0466 | 1 | 9 | 17 | 4 | R.KLNAFGNAFLNR.F |
|  | 11100 | 695.8426 | 1389.6706 | 1389.6810 | -0.0103 | 0 | 1 | 99 | 7 | R.QEESLMPSQVVK.G |
|  | 11946 | 720.4497 | 1438.8847 | 1438.8296 | 0.0551 | 1 | 9 | 15 | 2 | R.VDRIILLFDAHK.L |
|  | 12421 | 489.9142 | 1466.7206 | 1466.7075 | 0.0131 | 0 | 1 | 93 | 9 | K.MQELLQTQDFSK.F |
|  | 12940 | 749.3855 | 1496.7564 | 1495.7558 | 1.0006 | 1 | 13 | 6.6 | 2 | R.KLFEAEEQDLFK.D |
|  | 24322 | **614.0399** | **2452.1306** | **2451.2171** | **0.9134** | **1** | **2** | **41** | **4** | **K.LLDTVDDMLANDIARLMVMVR.Q** |
|  | 25138 | **639.3201** | **2553.2514** | **2553.2719** | **-0.0205** | **1** | **5** | **21** | **6** | **K.ADQIETQQLMRVYGALMWSLGK.I** |
|  | 28788 | **1091.5513** | **3271.6320** | **3271.6548** | **-0.0228** | **0** | **35** | **0.015** | **1** | **R.IGPEPTTDSFIAVMHGPTEGVVPGNALVVDPR.R** |
|  | 30228 | **769.9308** | **3844.6179** | **3843.9731** | **0.6448** | **1** | **1** | **20** | **6** | **R.IGPEPTTDSFIAVMHGPTEGVVPGNALVVDPRRPFR.K** |
|  | 31452 | **1789.1405** | **5364.3997** | **5365.4463** | **-1.0466** | **1** | **2** | **6.9** | **5** | **K.GGAFDGTMNGPFGHGYGEGAGEGIDDVEWVVGKDKPTYDEIFYTLSPVNGK.I** |

| **838.** | O43760\|SNG2_HUMAN    **Mass:** 25079    **Score:** 35     **Queries matched:** 7   **emPAI:** 0.15 |
| --- | --- |
|  | Synaptogyrin-2 OS=Homo sapiens GN=SYNGR2 PE=1 SV=1 |

|  | **Query** | **Observed** | **Mr(expt)** | **Mr(calc)** | **Delta** | **Miss** | **Score** | **Expect** | **Rank** | **Peptide** |
| --- | --- | --- | --- | --- | --- | --- | --- | --- | --- | --- |
|  | 1927 | **412.2165** | **822.4185** | **821.4032** | **1.0154** | **0** | **35** | **0.049** | **1** | **K.AGGSFDLR.R** 1903 |
|  | 6642 | 580.3155 | 1158.6164 | 1157.6557 | 0.9607 | 0 | 18 | 2.8 | 1 | R.FLTQPQVVAR.A 6645 6646 |
|  | 17775 | **601.6562** | **1801.9467** | **1802.8257** | **-0.8790** | **1** | **1** | **81** | **3** | **-.MESGAYGAAKAGGSFDLR.R** |
|  | 29597 | 888.4337 | 3549.7058 | 3550.8500 | -1.1443 | 1 | 3 | 18 | 4 | R.YGSAIGVLAFLASAFFLVVDAYFPQISNATDRK.Y |

| **839.** | Q9UBC5\|MYO1A_HUMAN    **Mass:** 119238   **Score:** 35     **Queries matched:** 23   **emPAI:** 0.03 |
| --- | --- |
|  | Myosin-Ia OS=Homo sapiens GN=MYO1A PE=1 SV=1 |

|  | **Query** | **Observed** | **Mr(expt)** | **Mr(calc)** | **Delta** | **Miss** | **Score** | **Expect** | **Rank** | **Peptide** |
| --- | --- | --- | --- | --- | --- | --- | --- | --- | --- | --- |
|  | 1313 | 703.3932 | 702.3860 | 702.3912 | -0.0052 | 0 | 28 | 0.42 | 4 | R.INESIK.V |
|  | 2098 | 417.7552 | 833.4958 | 833.4872 | 0.0086 | 0 | 11 | 14 | 8 | K.AQHPLLR.S |
|  | 5461 | 538.8049 | 1075.5953 | 1075.6026 | -0.0073 | 0 | 18 | 2.6 | 1 | R.YLGLLENVR.V |
|  | 8636 | **421.5331** | **1261.5774** | **1262.7421** | **-1.1646** | **1** | **11** | **11** | **1** | **K.SMVQKFLLGLK.N** 8648 8650 8652 8667 |
|  | 9030 | **428.2832** | **1281.8277** | **1282.6009** | **-0.7733** | **1** | **12** | **8** | **1** | **R.ALCSRTMETAK.E** 9028 |
|  | 10231 | 448.9001 | 1343.6783 | 1342.6227 | 1.0556 | 0 | 6 | 33 | 3 | K.VDYFDNGIICK.L |
|  | 10907 | 460.5877 | 1378.7411 | 1378.6841 | 0.0570 | 0 | 15 | 4.2 | 1 | R.GQFSSDLVATQAR.Y |
|  | 12450 | **490.7382** | **1469.1927** | **1468.7959** | **0.3968** | **1** | **3** | **41** | **9** | **K.SSVAILMKNLYSK.S** |
|  | 13165 | **504.3049** | **1509.8930** | **1509.7827** | **0.1102** | **1** | **2** | **67** | **3** | **R.SPKTLFYLEEQR.R** |
|  | 13509 | 510.9062 | 1529.6968 | 1529.6391 | 0.0577 | 0 | 12 | 7 | 2 | R.QYDHTMGLSCFR.I |
|  | 14612 | 800.8879 | 1599.7613 | 1598.8893 | 0.8721 | 1 | 0 | 1.1e+002 | 8 | K.QASLKRPPTAGAQFK.S |
|  | 15277 | **547.9436** | **1640.8090** | **1639.8127** | **0.9963** | **0** | **2** | **76** | **9** | **K.VLGELSMSSGELAFGK.T** |
|  | 18282 | **613.3140** | **1836.9203** | **1835.9563** | **0.9639** | **1** | **2** | **70** | **5** | **K.EKVVTALNVMQAQYAR.D** 18290 |
|  | 18586 | 929.4856 | 1856.9566 | 1856.9632 | -0.0065 | 0 | 35 | 0.033 | 1 | K.EQLLQSNPVLEAFGNAK.T 18593 |
|  | 25563 | 879.0839 | 2634.2298 | 2635.3105 | -1.0807 | 1 | 5 | 22 | 1 | K.KASYPQSVPIPFCGDYIGLQGNPK.L |
|  | 25582 | **660.0658** | **2636.2341** | **2636.3190** | **-0.0849** | **0** | **7** | **12** | **2** | **R.GILAMLDEECLRPGVVSDSTFLAK.L** |

| **840.** | P53634\|CATC_HUMAN    **Mass:** 52607    **Score:** 35     **Queries matched:** 4   **emPAI:** 0.07 |
| --- | --- |
|  | Dipeptidyl-peptidase 1 OS=Homo sapiens GN=CTSC PE=1 SV=1 |

|  | **Query** | **Observed** | **Mr(expt)** | **Mr(calc)** | **Delta** | **Miss** | **Score** | **Expect** | **Rank** | **Peptide** |
| --- | --- | --- | --- | --- | --- | --- | --- | --- | --- | --- |
|  | 2203 | **421.0574** | **840.1002** | **839.4025** | **0.6977** | **1** | **6** | **26** | **10** | **K.YKEEGSK.V** |
|  | 15130 | **816.4042** | **1630.7939** | **1629.6961** | **1.0979** | **0** | **35** | **0.039** | **1** | **K.NSWGTGWGENGYFR.I** |
|  | 28874 | 1101.2040 | 3300.5901 | 3301.3579 | -0.7678 | 1 | 1 | 34 | 6 | K.EDCFRYYSSEYHYVGGFYGGCNEALMK.L 28873 |

| **841.** | Q12846\|STX4_HUMAN    **Mass:** 34273    **Score:** 35     **Queries matched:** 15   **emPAI:** 0.11 |
| --- | --- |
|  | Syntaxin-4 OS=Homo sapiens GN=STX4 PE=1 SV=2 |

|  | **Query** | **Observed** | **Mr(expt)** | **Mr(calc)** | **Delta** | **Miss** | **Score** | **Expect** | **Rank** | **Peptide** |
| --- | --- | --- | --- | --- | --- | --- | --- | --- | --- | --- |
|  | 1486 | **786.9435** | **785.9363** | **785.4508** | **0.4855** | **1** | **0** | **1.3e+002** | **1** | **K.NVERIR.R** |
|  | 2609 | 436.2505 | 870.4864 | 870.5035 | -0.0171 | 1 | 16 | 4.6 | 5 | K.QLGREIR.L |
|  | 3820 | **480.2662** | **958.5179** | **957.5243** | **0.9936** | **1** | **7** | **41** | **8** | **R.DEIKQLGR.E** |
|  | 4444 | **501.5766** | **1001.1386** | **1000.5301** | **0.6085** | **0** | **8** | **19** | **2** | **R.QALNEISAR.H** |
|  | 6329 | 570.3208 | 1138.6270 | 1138.5731 | 0.0540 | 0 | 6 | 36 | 5 | R.HSEIQQLER.S |
|  | 11334 | **703.1091** | **1404.2036** | **1403.7997** | **0.4039** | **1** | **2** | **57** | **8** | **K.ERVALVVHPGTAR.L** 11356 |
|  | 13669 | 514.2665 | 1539.7778 | 1539.6437 | 0.1341 | 0 | 16 | 3.1 | 2 | K.EEADENYNSVNTR.M |
|  | 17726 | **600.6766** | **1799.0081** | **1799.9339** | **-0.9258** | **0** | **2** | **58** | **3** | **K.QQVTILATPLPEESMK.Q** 17761 |
|  | 22394 | **736.3395** | **2205.9966** | **2206.0138** | **-0.0172** | **1** | **35** | **0.026** | **1** | **K.AIEPQKEEADENYNSVNTR.M** |
|  | 24985 | 850.3463 | 2548.0169 | 2548.6006 | -0.5836 | 1 | 5 | 19 | 5 | K.KVLIAICVSITVVLLAVIIGVTVVG.- |
|  | 25596 | **660.8674** | **2639.4406** | **2638.2407** | **1.1999** | **0** | **1** | **49** | **4** | **R.ELHDIFTFLATEVEMQGEMINR.I** |
|  | 27555 | 749.8735 | 2995.4648 | 2994.4579 | 1.0069 | 1 | (4) | 23 | 3 | R.SIRELHDIFTFLATEVEMQGEMINR.I |
|  | 27559 | 749.8743 | 2995.4680 | 2994.4579 | 1.0101 | 1 | 5 | 19 | 2 | R.SIRELHDIFTFLATEVEMQGEMINR.I |

| **842.** | Q5VT99\|LRC38_HUMAN    **Mass:** 32802    **Score:** 35     **Queries matched:** 4   **emPAI:** 0.11 |
| --- | --- |
|  | Leucine-rich repeat-containing protein 38 OS=Homo sapiens GN=LRRC38 PE=2 SV=1 |

|  | **Query** | **Observed** | **Mr(expt)** | **Mr(calc)** | **Delta** | **Miss** | **Score** | **Expect** | **Rank** | **Peptide** |
| --- | --- | --- | --- | --- | --- | --- | --- | --- | --- | --- |
|  | 2601 | 435.7712 | 869.5278 | 869.5447 | -0.0169 | 1 | 35 | 0.05 | 1 | R.KLLVAGNR.I 2604 |
|  | 13103 | **503.2240** | **1506.6503** | **1507.8035** | **-1.1532** | **0** | **3** | **54** | **9** | **R.GLPSVPDPFPLDVR.K** |
|  | 16339 | **854.9237** | **1707.8329** | **1708.0359** | **-0.2030** | **1** | **8** | **20** | **1** | **R.SLSVAALAALPALRSLR.L** |

| **843.** | O60613\|SEP15_HUMAN    **Mass:** 18236    **Score:** 35     **Queries matched:** 3   **emPAI:** 0.21 |
| --- | --- |
|  | 15 kDa selenoprotein OS=Homo sapiens GN=SEP15 PE=1 SV=3 |

|  | **Query** | **Observed** | **Mr(expt)** | **Mr(calc)** | **Delta** | **Miss** | **Score** | **Expect** | **Rank** | **Peptide** |
| --- | --- | --- | --- | --- | --- | --- | --- | --- | --- | --- |
|  | 11554 | **709.3298** | **1416.6450** | **1416.7990** | **-0.1540** | **1** | **2** | **79** | **6** | **K.LGRFPQVQAFVR.S** |
|  | 12488 | 491.6088 | 1471.8047 | 1471.7606 | 0.0441 | 0 | 2 | 89 | 3 | M.AAGPSGCLVPAFGLR.L |
|  | 29145 | **1124.5236** | **3370.5489** | **3370.5632** | **-0.0144** | **0** | **35** | **0.013** | **1** | **R.ELGFSSNLLCSSCDLLGQFNLLQLDPDCR.G** |

| **844.** | Q3B8N2\|LEG9B_HUMAN    **Score:** 35     **Queries matched:** 5 |
| --- | --- |
|  | Galectin-9B OS=Homo sapiens GN=LGALS9B PE=2 SV=3 |

|  | **Query** | **Observed** | **Mr(expt)** | **Mr(calc)** | **Delta** | **Miss** | **Score** | **Expect** | **Rank** | **Peptide** |
| --- | --- | --- | --- | --- | --- | --- | --- | --- | --- | --- |
|  | 1189 | 649.2006 | 648.1933 | 648.3418 | -0.1485 | 0 | 0 | 2.3e+002 | 9 | K.MPFVR.G |
|  | 13586 | 768.3338 | 1534.6530 | 1533.6808 | 0.9723 | 0 | 1 | 1e+002 | 9 | R.NTQINNSWGSEER.S |
|  | 23877 | 796.0455 | 2385.1148 | 2386.1131 | -0.9984 | 0 | 35 | 0.026 | 2 | R.FAVDFQTGFSGNDIAFHFNPR.F |
|  | 25304 | 1291.6307 | 2581.2469 | 2580.2440 | 1.0029 | 1 | 1 | 48 | 3 | K.MPFVRGQSFSVWILCEAHCLK.V |
|  | 26707 | **565.2875** | **2821.4013** | **2821.2737** | **0.1277** | **1** | **0** | **55** | **3** | **K.MHMPFQKGMPFDLCFLVQSSDFK.V** |

|  | **Proteins matching the same set of peptides:** |
| --- | --- |

|  | Q6DKI2\|LEG9C_HUMAN    **Score:** 35     **Queries matched:** 5 |
| --- | --- |

| **845.** | Q99442\|SEC62_HUMAN    **Mass:** 46175    **Score:** 34     **Queries matched:** 7   **emPAI:** 0.08 |
| --- | --- |
|  | Translocation protein SEC62 OS=Homo sapiens GN=SEC62 PE=1 SV=1 |

|  | **Query** | **Observed** | **Mr(expt)** | **Mr(calc)** | **Delta** | **Miss** | **Score** | **Expect** | **Rank** | **Peptide** |
| --- | --- | --- | --- | --- | --- | --- | --- | --- | --- | --- |
|  | 1453 | **766.3519** | **765.3447** | **765.3480** | **-0.0033** | **0** | **22** | **1.1** | **1** | **R.FNCPTK.S** |
|  | 3455 | 467.2007 | 932.3869 | 932.3956 | -0.0087 | 0 | 28 | 0.27 | 1 | K.STNMMGHR.V |
|  | 4678 | **510.3247** | **1018.6347** | **1019.4957** | **-0.8610** | **0** | **2** | **1.3e+002** | **1** | **K.AVDCLLDSK.W** |
|  | 10775 | 686.8470 | 1371.6794 | 1371.6881 | -0.0087 | 1 | 34 | 0.052 | 1 | R.IQEVGEPSKEEK.A |
|  | 11443 | **471.0326** | **1410.0760** | **1409.6284** | **0.4476** | **0** | **4** | **35** | **2** | **K.VGPGNHGTEGSGGER.H** |
|  | 15808 | **558.7985** | **1673.3737** | **1673.9691** | **-0.5954** | **0** | **1** | **59** | **8** | **R.CILFLIIWLITGGR.H** |
|  | 19618 | **648.2953** | **1941.8642** | **1942.9710** | **-1.1068** | **1** | **1** | **69** | **3** | **R.VDYFIASKAVDCLLDSK.W** |

| **846.** | Q9BX97\|PLVAP_HUMAN    **Mass:** 51132    **Score:** 34     **Queries matched:** 14   **emPAI:** 0.07 |
| --- | --- |
|  | Plasmalemma vesicle-associated protein OS=Homo sapiens GN=PLVAP PE=2 SV=1 |

|  | **Query** | **Observed** | **Mr(expt)** | **Mr(calc)** | **Delta** | **Miss** | **Score** | **Expect** | **Rank** | **Peptide** |
| --- | --- | --- | --- | --- | --- | --- | --- | --- | --- | --- |
|  | 1409 | 746.1623 | 745.1551 | 744.3766 | 0.7785 | 0 | 12 | 10 | 6 | R.EAEQLR.M |
|  | 1969 | **413.7272** | **825.4398** | **825.3691** | **0.0708** | **0** | **3** | **67** | **6** | **K.FEMDLR.N** |
|  | 2453 | 430.7156 | 859.4167 | 860.3988 | -0.9821 | 0 | 15 | 7.1 | 8 | R.ENSDLQR.Q |
|  | 2983 | 451.2271 | 900.4397 | 901.4617 | -1.0220 | 1 | 24 | 0.83 | 2 | K.EAQAREAK.L |
|  | 6558 | 577.7543 | 1153.4940 | 1153.6053 | -0.1113 | 0 | 16 | 3.9 | 2 | R.YMAAIILSEK.Q |
|  | 7113 | **397.2108** | **1188.6107** | **1187.6033** | **1.0074** | **1** | **5** | **48** | **6** | **R.DNLAKELEEK.K** |
|  | 7158 | 596.2889 | 1190.5632 | 1190.5713 | -0.0081 | 1 | 4 | 63 | 9 | R.EAKLQAECSR.Q |
|  | 8346 | 624.3542 | 1246.6939 | 1247.5717 | -0.8777 | 0 | 1 | 1.3e+002 | 9 | M.GLAMEHGGSYAR.A |
|  | 10883 | **460.2336** | **1377.6789** | **1378.5679** | **-0.8890** | **0** | **5** | **44** | **9** | **R.ACDHMPSLMSSK.V** |
|  | 10917 | **690.7950** | **1379.5754** | **1378.5679** | **1.0075** | **0** | **(1)** | **1.1e+002** | **2** | **R.ACDHMPSLMSSK.V** |
|  | 13595 | **512.7835** | **1535.3285** | **1534.6690** | **0.6595** | **1** | **1** | **65** | **5** | **R.RACDHMPSLMSSK.V** |
|  | 19641 | **648.8939** | **1943.6598** | **1942.8951** | **0.7647** | **1** | **2** | **40** | **1** | **K.DMNKSCDALLFMLNQK.V** |
|  | 23475 | **778.0652** | **2331.1737** | **2331.1859** | **-0.0121** | **0** | **34** | **0.028** | **1** | **R.SLDNLGYNLYHPLGSELASIR.R** |
|  | 28994 | 1111.2225 | 3330.6458 | 3331.6515 | -1.0058 | 1 | 6 | 11 | 3 | K.SQPMMPVSRPMGPVPNPQPIDPASLEEFKR.K |

| **847.** | P22894\|MMP8_HUMAN    **Mass:** 53550    **Score:** 34     **Queries matched:** 10   **emPAI:** 0.07 |
| --- | --- |
|  | Neutrophil collagenase OS=Homo sapiens GN=MMP8 PE=1 SV=1 |

|  | **Query** | **Observed** | **Mr(expt)** | **Mr(calc)** | **Delta** | **Miss** | **Score** | **Expect** | **Rank** | **Peptide** |
| --- | --- | --- | --- | --- | --- | --- | --- | --- | --- | --- |
|  | 1698 | **403.0457** | **804.0769** | **803.4323** | **0.6446** | **1** | **8** | **23** | **3** | **K.LKEMQR.F** |
|  | 2375 | 427.7398 | 853.4649 | 852.4745 | 0.9904 | 0 | 5 | 43 | 6 | R.GEILFFK.D |
|  | 13383 | 762.3561 | 1522.6976 | 1521.7041 | 0.9935 | 0 | 11 | 10 | 1 | K.TYFFVNDQFWR.Y 13365 |
|  | 14866 | 539.2805 | 1614.8196 | 1614.8518 | -0.0323 | 1 | 11 | 10 | 1 | R.YYAFDLIAQRVTR.V |
|  | 18503 | **926.4817** | **1850.9488** | **1850.9567** | **-0.0079** | **0** | **34** | **0.039** | **1** | **K.DAFELWSVASPLIFTR.I** |
|  | 22974 | **757.3886** | **2269.1438** | **2269.1089** | **0.0349** | **1** | **0** | **72** | **2** | **R.QFMEPGYPKSISGAFPGIESK.V** |
|  | 23370 | **1160.5608** | **2319.1070** | **2319.1172** | **-0.0101** | **0** | **17** | **1.6** | **1** | **K.DISNYGFPSSVQAIDAAVFYR.S** |
|  | 26275 | 687.3196 | 2745.2495 | 2746.2764 | -1.0270 | 1 | 7 | 13 | 1 | K.EMQRFFGLNVTGKPNEETLDMMK.K |
|  | 28939 | **1107.5660** | **3319.6763** | **3319.6415** | **0.0348** | **1** | **1** | **29** | **1** | **K.SISGAFPGIESKVDAVFQQEHFFHVFSGPR.Y** |

| **848.** | P39023\|RL3_HUMAN    **Mass:** 46365    **Score:** 34     **Queries matched:** 22   **emPAI:** 0.08 |
| --- | --- |
|  | 60S ribosomal protein L3 OS=Homo sapiens GN=RPL3 PE=1 SV=2 |

|  | **Query** | **Observed** | **Mr(expt)** | **Mr(calc)** | **Delta** | **Miss** | **Score** | **Expect** | **Rank** | **Peptide** |
| --- | --- | --- | --- | --- | --- | --- | --- | --- | --- | --- |
|  | 2419 | **429.2393** | **856.4640** | **857.3953** | **-0.9313** | **1** | **2** | **1.3e+002** | **9** | **K.DFSSMKK.Y** |
|  | 2709 | **439.3738** | **876.7331** | **876.3614** | **0.3718** | **0** | **4** | **48** | **3** | **K.WQDEDGK.K** |
|  | 3238 | **459.1706** | **916.3267** | **915.5753** | **0.7514** | **1** | **0** | **1.8e+002** | **9** | **R.KSLLVQTK.R** |
|  | 3763 | 478.2568 | 954.4991 | 954.5069 | -0.0078 | 0 | 28 | 0.26 | 1 | R.VIAHTQMR.L |
|  | 3994 | 486.7593 | 971.5040 | 970.5018 | 1.0022 | 0 | (15) | 5.5 | 1 | R.VIAHTQMR.L |
|  | 7948 | **615.2443** | **1228.4740** | **1227.6571** | **0.8168** | **1** | **4** | **55** | **7** | **R.EVDRPGSKVNK.K** |
|  | 8482 | 628.3334 | 1254.6522 | 1253.6265 | 1.0257 | 1 | 1 | 1.2e+002 | 8 | K.GYHHRTEINK.K |
|  | 10186 | **671.7867** | **1341.5588** | **1341.5684** | **-0.0096** | **0** | **34** | **0.051** | **1** | **K.NNASTDYDLSDK.S** |
|  | 17038 | 585.3205 | 1752.9397 | 1751.9101 | 1.0296 | 1 | 6 | 30 | 9 | K.AGMTHIVREVDRPGSK.V |
|  | 18310 | **614.0782** | **1839.2127** | **1839.9513** | **-0.7385** | **1** | **3** | **39** | **3** | **K.KAHLMEIQVNGGTVAEK.L** |
|  | 26019 | **1351.5826** | **2701.1507** | **2701.3633** | **-0.2126** | **0** | **2** | **39** | **4** | **R.LEQQVPVNQVFGQDEMIDVIGVTK.G** 25993 25997 26001 26008 26009 26012 26016 26028 26036 26038 |
|  | 27636 | 1004.4554 | 3010.3443 | 3009.6461 | 0.6982 | 1 | 2 | 29 | 4 | K.KEVVEAVTIVETPPMVVVGIVGYVETPR.G |

| **849.** | P12821\|ACE_HUMAN    **Mass:** 150418   **Score:** 34     **Queries matched:** 24   **emPAI:** 0.05 |
| --- | --- |
|  | Angiotensin-converting enzyme OS=Homo sapiens GN=ACE PE=1 SV=1 |

|  | **Query** | **Observed** | **Mr(expt)** | **Mr(calc)** | **Delta** | **Miss** | **Score** | **Expect** | **Rank** | **Peptide** |
| --- | --- | --- | --- | --- | --- | --- | --- | --- | --- | --- |
|  | 926 | 546.2509 | 545.2436 | 545.2921 | -0.0485 | 1 | 12 | 22 | 6 | R.DKAGR.A |
|  | 1740 | **404.6955** | **807.3764** | **807.4028** | **-0.0263** | **0** | **11** | **14** | **2** | **R.WGVFSGR.T** |
|  | 1893 | **410.7325** | **819.4505** | **820.3974** | **-0.9468** | **1** | **5** | **57** | **10** | **-.MGAASGRR.G** |
|  | 2850 | **444.2518** | **886.4890** | **886.4872** | **0.0018** | **1** | **7** | **42** | **6** | **K.KVQDLER.A** 2847 |
|  | 5584 | 542.8183 | 1083.6220 | 1083.6288 | -0.0067 | 0 | 22 | 0.9 | 1 | R.TLGSANLPLAK.R |
|  | 6261 | **567.2808** | **1132.5470** | **1132.5546** | **-0.0077** | **0** | **8** | **25** | **1** | **K.SMLEKPTDGR.E** |
|  | 10248 | **449.2250** | **1344.6532** | **1345.6891** | **-1.0359** | **1** | **10** | **15** | **1** | **R.WGVFSGRTPPSR.Y** |
|  | 10564 | **681.8131** | **1361.6117** | **1361.6193** | **-0.0076** | **0** | **27** | **0.25** | **1** | **R.YNFDWWYLR.T** |
|  | 11132 | 464.9422 | 1391.8048 | 1392.7435 | -0.9387 | 1 | 1 | 1e+002 | 6 | R.IYSTAKVCLPNK.T |
|  | 11595 | **710.3753** | **1418.7361** | **1418.7340** | **0.0020** | **1** | **0** | **1.3e+002** | **5** | **R.TKYQGICPPVTR.N** |
|  | 12516 | 492.2527 | 1473.7362 | 1474.7303 | -0.9942 | 0 | 4 | 47 | 6 | R.AALPAQELEEYNK.I |
|  | 13393 | **762.8420** | **1523.6694** | **1523.6793** | **-0.0099** | **0** | **33** | **0.065** | **1** | **K.ENYNQEWWSLR.L** |
|  | 14598 | **800.1575** | **1598.3005** | **1597.8385** | **0.4620** | **0** | **1** | **67** | **1** | **K.DMVGLDALDAQPLLK.Y** |
|  | 16160 | **849.3827** | **1696.7509** | **1696.7614** | **-0.0104** | **0** | **22** | **0.65** | **1** | **R.SMYETPSLEQDLER.L** 16159 |
|  | 17414 | 890.9425 | 1779.8704 | 1780.0029 | -0.1324 | 1 | 2 | 70 | 4 | K.ILLQKNMQIANHTLK.Y |
|  | 22242 | 731.0263 | 2190.0571 | 2190.0705 | -0.0134 | 1 | 14 | 3.8 | 1 | R.RQEEAALLSQEFAEAWGQK.A |
|  | 25967 | **674.8460** | **2695.3547** | **2695.3718** | **-0.0171** | **0** | **2** | **35** | **4** | **R.GANPGFHEAIGDVLALSVSTPEHLHK.I** |
|  | 28811 | **820.1918** | **3276.7380** | **3276.7540** | **-0.0160** | **1** | **24** | **0.17** | **1** | **K.VLQAGSSRPWQEVLKDMVGLDALDAQPLLK.Y** |
|  | 29538 | **1176.2148** | **3525.6227** | **3525.6472** | **-0.0245** | **0** | **2** | **21** | **2** | **R.VAEEFFTSLELSPMPPEFWEGSMLEKPADGR.E** |
|  | 30801 | **1433.7782** | **4298.3128** | **4299.1319** | **-0.8191** | **0** | **1** | **16** | **4** | **K.LGFSRPWPEAMQLITGQPNMSASAMLSYFKPLLDWLR.T** |
|  | 31243 | **983.6888** | **4913.4078** | **4914.4733** | **-1.0655** | **1** | **1** | **12** | **1** | **R.LATAMKLGFSRPWPEAMQLITGQPNMSASAMLSYFKPLLDWLR.T** |
|  | 31742 | **1161.1314** | **5800.6204** | **5801.7204** | **-1.1000** | **0** | **3** | **3.7** | **1** | **R.GPIPAHLLGDMWAQSWENIYDMVVPFPDKPNLDVTSTMLQQGWNATHMFR.V** |

| **850.** | P36543\|VATE1_HUMAN    **Mass:** 26186    **Score:** 34     **Queries matched:** 10   **emPAI:** 0.15 |
| --- | --- |
|  | V-type proton ATPase subunit E 1 OS=Homo sapiens GN=ATP6V1E1 PE=1 SV=1 |

|  | **Query** | **Observed** | **Mr(expt)** | **Mr(calc)** | **Delta** | **Miss** | **Score** | **Expect** | **Rank** | **Peptide** |
| --- | --- | --- | --- | --- | --- | --- | --- | --- | --- | --- |
|  | 4067 | **488.7566** | **975.4987** | **974.4419** | **1.0569** | **0** | **9** | **22** | **8** | **K.IMEYYEK.K** |
|  | 6049 | **559.7701** | **1117.5257** | **1117.5992** | **-0.0735** | **1** | **7** | **33** | **4** | **R.GALFGANANRK.F** |
|  | 6408 | **573.3205** | **1144.6264** | **1145.6404** | **-1.0140** | **1** | **7** | **30** | **2** | **K.IKVSNTLESR.L** |
|  | 9483 | 435.9355 | 1304.7846 | 1304.6329 | 0.1518 | 0 | 6 | 30 | 9 | K.IQMSNLMNQAR.L 9482 |
|  | 9830 | **661.4572** | **1320.8999** | **1320.6278** | **0.2721** | **0** | **(3)** | **53** | **4** | **K.IQMSNLMNQAR.L** |
|  | 13713 | **772.3973** | **1542.7800** | **1542.7898** | **-0.0098** | **0** | **34** | **0.049** | **1** | **R.LDLIAQQMMPEVR.G** |
|  | 14269 | 527.1537 | 1578.4394 | 1577.8017 | 0.6377 | 1 | 2 | 53 | 7 | K.IQMSNLMNQARLK.V |
|  | 14392 | 793.8256 | 1585.6366 | 1585.8311 | -0.1945 | 1 | 7 | 23 | 4 | R.ARDDLITDLLNEAK.Q 14393 |

| **851.** | P03973\|SLPI_HUMAN    **Mass:** 15228    **Score:** 34     **Queries matched:** 5   **emPAI:** 0.26 |
| --- | --- |
|  | Antileukoproteinase OS=Homo sapiens GN=SLPI PE=1 SV=2 |

|  | **Query** | **Observed** | **Mr(expt)** | **Mr(calc)** | **Delta** | **Miss** | **Score** | **Expect** | **Rank** | **Peptide** |
| --- | --- | --- | --- | --- | --- | --- | --- | --- | --- | --- |
|  | 2406 | 429.0863 | 856.1580 | 855.4637 | 0.6943 | 1 | 11 | 8.6 | 4 | K.AGVCPPKK.S |
|  | 5959 | **556.2821** | **1110.5496** | **1109.4304** | **1.1192** | **0** | **17** | **3.1** | **1** | **R.CCPDTCGIK.C** |
|  | 10994 | 692.8251 | 1383.6356 | 1383.6453 | -0.0097 | 0 | 34 | 0.056 | 1 | K.CLDPVDTPNPTR.R |
|  | 11117 | **464.7205** | **1391.1397** | **1390.5172** | **0.6226** | **1** | **4** | **35** | **1** | **R.DLKCCMGMCGK.S** |
|  | 19247 | **637.9522** | **1910.8348** | **1909.8451** | **0.9897** | **1** | **8** | **17** | **1** | **R.YKKPECQSDWQCPGK.K** |

| **852.** | P50416\|CPT1A_HUMAN    **Mass:** 88995    **Score:** 34     **Queries matched:** 24   **emPAI:** 0.13 |
| --- | --- |
|  | Carnitine O-palmitoyltransferase 1, liver isoform OS=Homo sapiens GN=CPT1A PE=1 SV=2 |

|  | **Query** | **Observed** | **Mr(expt)** | **Mr(calc)** | **Delta** | **Miss** | **Score** | **Expect** | **Rank** | **Peptide** |
| --- | --- | --- | --- | --- | --- | --- | --- | --- | --- | --- |
|  | 1366 | 723.4456 | 722.4383 | 722.4691 | -0.0308 | 0 | 13 | 7.6 | 7 | R.LPVPAVK.D |
|  | 3475 | **467.7596** | **933.5047** | **934.5487** | **-1.0440** | **1** | **2** | **1.1e+002** | **5** | **K.LFKLASEK.H** |
|  | 5504 | **540.2302** | **1078.4458** | **1078.4535** | **-0.0078** | **0** | **39** | **0.018** | **1** | **R.EMEQQMQR.I** 5503 |
|  | 6232 | 566.2994 | 1130.5843 | 1130.5931 | -0.0088 | 1 | 16 | 4.4 | 2 | K.NKQSLDAVEK.A |
|  | 6935 | **591.2896** | **1180.5647** | **1179.6144** | **0.9503** | **1** | **3** | **79** | **6** | **R.ATKIWMGMVK.I** |
|  | 8409 | 417.7297 | 1250.1672 | 1249.6853 | 0.4820 | 0 | 5 | 33 | 10 | R.YLQSVRPLMK.E |
|  | 10545 | **681.2720** | **1360.5294** | **1360.5388** | **-0.0094** | **0** | **15** | **4.2** | **1** | **R.SCTTESCDFVR.A** |
|  | 11171 | 465.6140 | 1393.8203 | 1393.6006 | 0.2197 | 0 | 3 | 65 | 8 | K.FCLTYEASMTR.L |
|  | 11691 | **475.4319** | **1423.2738** | **1423.7468** | **-0.4730** | **1** | **5** | **30** | **9** | **K.IWMGMVKIFSGR.K** |
|  | 12436 | **490.2625** | **1467.7656** | **1466.8470** | **0.9186** | **1** | **5** | **40** | **3** | **R.AGNAIHAILLYRR.K** |
|  | 13436 | **763.8708** | **1525.7270** | **1525.7372** | **-0.0102** | **0** | **14** | **5.2** | **1** | **R.ILDNTSEPQPGEAR.L** 13421 |
|  | 13718 | 515.2967 | 1542.8682 | 1541.7144 | 1.1538 | 0 | 1 | 1e+002 | 9 | R.IPGEETDTIQHMR.D |
|  | 14038 | 521.6078 | 1561.8017 | 1561.7923 | 0.0095 | 0 | 3 | 60 | 6 | R.MTALAQDFAVGLGPR.L |
|  | 18114 | **609.3201** | **1824.9386** | **1824.7845** | **0.1541** | **1** | **1** | **88** | **9** | **K.DMGKFCLTYEASMTR.L** |
|  | 20007 | **657.6774** | **1970.0105** | **1970.0030** | **0.0074** | **1** | **1** | **69** | **1** | **K.EAMTDIITLFGLSSNSKK.-** |
|  | 23179 | 765.3810 | 2293.1213 | 2294.0420 | -0.9207 | 1 | 3 | 40 | 4 | R.MFNTSRIPGEETDTIQHMR.D |
|  | 23946 | 599.8078 | 2395.2021 | 2394.2139 | 0.9882 | 1 | 8 | 11 | 1 | R.ILDNTSEPQPGEARLAALTAGDR.V |
|  | 24514 | 828.4036 | 2482.1889 | 2482.2097 | -0.0208 | 1 | 1 | 59 | 3 | R.LLGSTIPLCSAQWERMFNTSR.I |
|  | 28353 | **1058.7902** | **3173.3487** | **3173.3659** | **-0.0173** | **1** | **15** | **1.4** | **1** | **K.AAFFVTLDETEEGYRSEDPDTSMDSYAK.S** |
|  | 28535 | **1070.2013** | **3207.5820** | **3208.6161** | **-1.0341** | **1** | **2** | **28** | **1** | **R.GRGPLMVNSNYYAMDLLYILPTHIQAAR.A** |
|  | 30594 | 1365.0176 | 4092.0309 | 4093.1884 | -1.1574 | 1 | 3 | 14 | 4 | K.NGIITGVYPASPSSWLIVVVGVMTTMYAKIDPSLGIIAK.I |
|  | 30937 | **1106.2850** | **4421.1110** | **4421.1315** | **-0.0204** | **0** | **22** | **0.12** | **1** | **R.LQWDIPGECQEVIETSLNTANLLANDVDFHSFPFVAFGK.G** |

| **853.** | P09669\|COX6C_HUMAN    **Mass:** 8776     **Score:** 34     **Queries matched:** 5   **emPAI:** 0.46 |
| --- | --- |
|  | Cytochrome c oxidase subunit 6C OS=Homo sapiens GN=COX6C PE=1 SV=2 |

|  | **Query** | **Observed** | **Mr(expt)** | **Mr(calc)** | **Delta** | **Miss** | **Score** | **Expect** | **Rank** | **Peptide** |
| --- | --- | --- | --- | --- | --- | --- | --- | --- | --- | --- |
|  | 3050 | **453.2080** | **904.4015** | **904.4079** | **-0.0064** | **0** | **34** | **0.08** | **1** | **K.AYADFYR.N** 3049 |
|  | 12225 | **486.2642** | **1455.7707** | **1455.7690** | **0.0017** | **1** | **5** | **37** | **2** | **-.MAPEVLPKPRMR.G** 12228 |
|  | 22401 | **552.5377** | **2206.1215** | **2206.2085** | **-0.0869** | **1** | **3** | **39** | **5** | **R.NHMAVAFVLSLGVAALYKFR.V** |

| **854.** | Q01518\|CAP1_HUMAN    **Mass:** 52222    **Score:** 34     **Queries matched:** 13   **emPAI:** 0.07 |
| --- | --- |
|  | Adenylyl cyclase-associated protein 1 OS=Homo sapiens GN=CAP1 PE=1 SV=4 |

|  | **Query** | **Observed** | **Mr(expt)** | **Mr(calc)** | **Delta** | **Miss** | **Score** | **Expect** | **Rank** | **Peptide** |
| --- | --- | --- | --- | --- | --- | --- | --- | --- | --- | --- |
|  | 1212 | 661.3826 | 660.3754 | 660.3629 | 0.0125 | 0 | 21 | 2.2 | 2 | K.VQVMGK.V |
|  | 2807 | 443.0380 | 884.0615 | 883.4803 | 0.5812 | 0 | 6 | 27 | 4 | K.QVAYIYK.C 2799 2817 2825 2829 2830 |
|  | 6501 | **576.2842** | **1150.5538** | **1150.5077** | **0.0461** | **0** | **5** | **43** | **7** | **K.TDGCHAYLSK.N** |
|  | 8104 | **619.2819** | **1236.5493** | **1237.5431** | **-0.9938** | **0** | **3** | **73** | **5** | **-.MADMQNLVER.L** |
|  | 12828 | 745.8991 | 1489.7837 | 1488.7354 | 1.0482 | 1 | 7 | 26 | 9 | M.ADMQNLVERLER.A |
|  | 12869 | **747.3108** | **1492.6071** | **1493.5915** | **-0.9843** | **0** | **5** | **37** | **2** | **K.EMNDAAMFYTNR.V** |
|  | 13479 | **510.5747** | **1528.7023** | **1528.8647** | **-0.1624** | **1** | **3** | **69** | **9** | **K.VQVMGKVPTISINK.T** |
|  | 23607 | **1176.1124** | **2350.2103** | **2350.2209** | **-0.0106** | **0** | **34** | **0.032** | **1** | **K.AGAAPYVQAFDSLLAGPVAEYLK.I** |

| **855.** | P13763\|HB2Q_HUMAN    **Mass:** 29557    **Score:** 34     **Queries matched:** 13   **emPAI:** 0.13 |
| --- | --- |
|  | HLA class II histocompatibility antigen, DP(W2) beta chain OS=Homo sapiens GN=HLA-DPB1 PE=2 SV=1 |

|  | **Query** | **Observed** | **Mr(expt)** | **Mr(calc)** | **Delta** | **Miss** | **Score** | **Expect** | **Rank** | **Peptide** |
| --- | --- | --- | --- | --- | --- | --- | --- | --- | --- | --- |
|  | 3034 | 452.3924 | 902.7702 | 902.4345 | 0.3358 | 0 | 0 | 1.3e+002 | 6 | K.DILEEER.A |
|  | 5369 | **536.2454** | **1070.4763** | **1070.5906** | **-0.1143** | **0** | **(3)** | **85** | **8** | **M.MVLQVSAAPR.T** 5378 |
|  | 5636 | 544.7886 | 1087.5626 | 1086.5856 | 0.9770 | 0 | 6 | 46 | 6 | M.MVLQVSAAPR.T |
|  | 7439 | **602.3001** | **1202.5855** | **1201.6311** | **0.9544** | **0** | **(2)** | **95** | **4** | **-.MMVLQVSAAPR.T** |
|  | 7762 | 407.1793 | 1218.5160 | 1217.6260 | 0.8899 | 0 | 12 | 10 | 1 | -.MMVLQVSAAPR.T |
|  | 7777 | **610.3258** | **1218.6371** | **1217.6260** | **1.0110** | **0** | **(8)** | **22** | **2** | **-.MMVLQVSAAPR.T** 7728 |
|  | 9271 | 648.3187 | 1294.6228 | 1294.6306 | -0.0078 | 0 | 1 | 1.1e+002 | 6 | R.ATPENYLFQGR.Q |
|  | 13241 | **758.3660** | **1514.7175** | **1514.7300** | **-0.0125** | **0** | **34** | **0.055** | **1** | **R.HNYELGGPMTLQR.R** 13242 |
|  | 16005 | **563.2818** | **1686.8236** | **1686.8260** | **-0.0024** | **1** | **3** | **59** | **10** | **R.HNYELGGPMTLQRR.V** |
|  | 26479 | **929.7919** | **2786.3538** | **2786.4075** | **-0.0537** | **0** | **1** | **43** | **2** | **K.GPLQHHNLLVCHVTDFYPGSIQVR.W** |

|  | **Proteins matching a subset of these peptides:** |
| --- | --- |

|  | P04440\|HB2P_HUMAN    **Mass:** 29426    **Score:** 34     **Queries matched:** 12 |
| --- | --- |
|  | HLA class II histocompatibility antigen, DP(W4) beta chain OS=Homo sapiens GN=HLA-DPB1 PE=2 SV=1 |

|  | P04232\|HB2T_HUMAN    **Mass:** 20638    **Score:** 34     **Queries matched:** 5 |
| --- | --- |
|  | HLA class II histocompatibility antigen, SB beta chain (Fragment) OS=Homo sapiens GN=HLA-DPB1 PE=2 SV=1 |

| **856.** | P18669\|PGAM1_HUMAN    **Mass:** 28900    **Score:** 34     **Queries matched:** 10   **emPAI:** 0.28 |
| --- | --- |
|  | Phosphoglycerate mutase 1 OS=Homo sapiens GN=PGAM1 PE=1 SV=2 |

|  | **Query** | **Observed** | **Mr(expt)** | **Mr(calc)** | **Delta** | **Miss** | **Score** | **Expect** | **Rank** | **Peptide** |
| --- | --- | --- | --- | --- | --- | --- | --- | --- | --- | --- |
|  | 4047 | 488.2459 | 974.4773 | 974.4855 | -0.0082 | 0 | 7 | 36 | 10 | K.AMEAVAAQGK.A 4054 |
|  | 7856 | **612.7960** | **1223.5775** | **1222.6571** | **0.9204** | **1** | **5** | **48** | **3** | **K.HGEAQVKIWR.R** |
|  | 14162 | 524.2842 | 1569.8307 | 1570.8216 | -0.9909 | 1 | 6 | 29 | 5 | R.LNERHYGGLTGLNK.A |
|  | 15945 | **842.4543** | **1682.8940** | **1682.9032** | **-0.0092** | **0** | **22** | **0.61** | **1** | **R.ALPFWNEEIVPQIK.E** |
|  | 19554 | **646.3025** | **1935.8856** | **1934.8833** | **1.0024** | **1** | **1** | **72** | **5** | **R.DAGYEFDICFTSVQKR.A** |
|  | 21585 | **1057.4925** | **2112.9705** | **2114.1194** | **-1.1488** | **0** | **1** | **65** | **7** | **K.NLKPIKPMQFLGDEETVR.K** |
|  | 21951 | **719.3866** | **2155.1380** | **2155.1500** | **-0.0120** | **0** | **18** | **1.5** | **1** | **R.TLWTVLDAIDQMWLPVVR.T** 21943 |
|  | 24127 | **809.0508** | **2424.1307** | **2424.1478** | **-0.0171** | **1** | **31** | **0.059** | **1** | **R.YADLTEDQLPSCESLKDTIAR.A** |

| **857.** | Q08379\|GOGA2_HUMAN    **Mass:** 112217   **Score:** 34     **Queries matched:** 22   **emPAI:** 0.03 |
| --- | --- |
|  | Golgin subfamily A member 2 OS=Homo sapiens GN=GOLGA2 PE=1 SV=2 |

|  | **Query** | **Observed** | **Mr(expt)** | **Mr(calc)** | **Delta** | **Miss** | **Score** | **Expect** | **Rank** | **Peptide** |
| --- | --- | --- | --- | --- | --- | --- | --- | --- | --- | --- |
|  | 1303 | 701.3879 | 700.3806 | 700.2962 | 0.0844 | 0 | 8 | 40 | 6 | K.ECHQK.Q |
|  | 1529 | **395.8878** | **789.7611** | **789.3439** | **0.4172** | **0** | **7** | **31** | **7** | **R.EMQNPR.E** 1514 1515 |
|  | 1834 | **408.2214** | **814.4283** | **815.4865** | **-1.0582** | **1** | **6** | **54** | **4** | **R.QSKLAAAK.K** |
|  | 3374 | **464.7279** | **927.4413** | **926.4821** | **0.9592** | **0** | **7** | **34** | **6** | **R.NSPGVPTGAK.K** |
|  | 3617 | 945.1171 | 944.1099 | 943.5338 | 0.5760 | 1 | 11 | 11 | 3 | K.KLGELQEK.L |
|  | 4132 | 491.2792 | 980.5438 | 979.4974 | 1.0464 | 1 | 0 | 1.4e+002 | 7 | R.DKYAENLK.G |
|  | 5896 | **554.1727** | **1106.3308** | **1106.5277** | **-0.1970** | **1** | **1** | **1.1e+002** | **4** | **R.LAQDKEEMK.V** |
|  | 7889 | **613.3762** | **1224.7379** | **1224.7441** | **-0.0063** | **0** | **34** | **0.06** | **1** | **K.LLELQELVLR.L** |
|  | 10274 | **449.7417** | **1346.2032** | **1346.6976** | **-0.4944** | **1** | **5** | **29** | **9** | **R.ALQGAMEKLQSR.F** 10301 |
|  | 10310 | 675.2675 | 1348.5204 | 1347.7034 | 0.8170 | 0 | 1 | 1.1e+002 | 10 | K.EQLAELQSGFVK.L |
|  | 10799 | 687.3409 | 1372.6672 | 1372.7020 | -0.0348 | 0 | 8 | 22 | 9 | K.AGMQLNLEELQK.K |
|  | 11243 | **467.2339** | **1398.6799** | **1398.7215** | **-0.0416** | **0** | **6** | **37** | **2** | **R.LEAATQQNQQLR.A** |
|  | 13250 | 505.9213 | 1514.7420 | 1513.7156 | 1.0263 | 1 | 8 | 19 | 9 | R.FMELMQEKADLK.E |
|  | 13291 | 759.3514 | 1516.6882 | 1516.7919 | -0.1037 | 1 | 14 | 5.5 | 2 | K.AGMQLNLEELQKK.L |
|  | 13324 | **507.2738** | **1518.7997** | **1518.6919** | **0.1078** | **0** | **8** | **19** | **2** | **R.MQQMSEQVHTLR.E** |
|  | 13711 | 772.3812 | 1542.7479 | 1542.7824 | -0.0345 | 0 | 4 | 48 | 3 | R.DNPTAQQIMQLLR.E |
|  | 16437 | **429.4717** | **1713.8579** | **1714.9213** | **-1.0635** | **1** | **8** | **16** | **2** | **R.VGELERALSAVSTQQK.K** 16459 |
|  | 24530 | **828.9202** | **2483.7387** | **2484.2213** | **-0.4826** | **1** | **0** | **44** | **6** | **R.AQLEAHLGQVMESVRQLQMER.D** |

| **858.** | Q9BVK6\|TMED9_HUMAN    **Mass:** 27374    **Score:** 33     **Queries matched:** 10   **emPAI:** 0.30 |
| --- | --- |
|  | Transmembrane emp24 domain-containing protein 9 OS=Homo sapiens GN=TMED9 PE=1 SV=2 |

|  | **Query** | **Observed** | **Mr(expt)** | **Mr(calc)** | **Delta** | **Miss** | **Score** | **Expect** | **Rank** | **Peptide** |
| --- | --- | --- | --- | --- | --- | --- | --- | --- | --- | --- |
|  | 1453 | 766.3519 | 765.3447 | 766.3861 | -1.0415 | 0 | 8 | 30 | 8 | R.TQLYDK.Q |
|  | 5823 | 551.3133 | 1100.6120 | 1100.6189 | -0.0069 | 1 | 15 | 4.8 | 1 | K.DKLSELQLR.V 5832 |
|  | 6000 | **558.2841** | **1114.5536** | **1113.5641** | **0.9895** | **0** | **4** | **67** | **4** | **K.FSLFAGGMLR.V** 5989 |
|  | 19239 | **637.6993** | **1910.0760** | **1909.9132** | **0.1628** | **0** | **5** | **32** | **1** | **R.EEYQPATPGLGMFVEVK.D** 19226 |
|  | 21263 | **1043.4777** | **2084.9408** | **2084.9547** | **-0.0139** | **0** | **29** | **0.12** | **1** | **K.CFIEEIPDETMVIGNYR.T** |
|  | 21992 | **720.3987** | **2158.1744** | **2159.0971** | **-0.9227** | **1** | **24** | **0.35** | **1** | **R.QLVEQVEQIQKEQNYQR.W** |
|  | 23720 | **788.4465** | **2362.3176** | **2361.3791** | **0.9385** | **1** | **0** | **62** | **3** | **M.AVELGVLLVRPRPGTGLGRVMR.T** |

| **859.** | Q12840\|KIF5A_HUMAN    **Mass:** 118161   **Score:** 33     **Queries matched:** 29   **emPAI:** 0.03 |
| --- | --- |
|  | Kinesin heavy chain isoform 5A OS=Homo sapiens GN=KIF5A PE=1 SV=2 |

|  | **Query** | **Observed** | **Mr(expt)** | **Mr(calc)** | **Delta** | **Miss** | **Score** | **Expect** | **Rank** | **Peptide** |
| --- | --- | --- | --- | --- | --- | --- | --- | --- | --- | --- |
|  | 1549 | 397.2195 | 792.4245 | 793.3640 | -0.9395 | 1 | 9 | 24 | 2 | K.EGAMKDK.R 1568 |
|  | 2519 | 864.4074 | 863.4001 | 862.4218 | 0.9783 | 1 | 8 | 27 | 6 | K.EAKEGAMK.D |
|  | 3960 | 485.7575 | 969.5005 | 969.5243 | -0.0238 | 1 | 1 | 1.1e+002 | 6 | R.IAPEERQK.Y |
|  | 4630 | 508.7725 | 1015.5305 | 1015.5372 | -0.0067 | 1 | 10 | 20 | 3 | R.CELPKLEK.R |
|  | 5492 | **539.7819** | **1077.5493** | **1077.5819** | **-0.0326** | **0** | **3** | **77** | **4** | **K.LFVQDVTTR.V** |
|  | 5970 | 556.8124 | 1111.6102 | 1111.5622 | 0.0480 | 1 | 5 | 47 | 9 | R.HEQSKQDLK.G |
|  | 6021 | 558.7940 | 1115.5733 | 1116.5900 | -1.0167 | 1 | 11 | 12 | 4 | R.EAHHRQLAR.L |
|  | 7522 | **604.2825** | **1206.5505** | **1205.6768** | **0.8737** | **1** | **4** | **54** | **3** | **R.KLFVQDVTTR.V** |
|  | 7544 | **403.4529** | **1207.3369** | **1206.6496** | **0.6873** | **0** | **5** | **31** | **4** | **K.LYLVDLAGSEK.V** 7528 |
|  | 9708 | 658.8701 | 1315.7257 | 1315.6983 | 0.0274 | 1 | 11 | 12 | 9 | K.TIDELKDLNQK.L |
|  | 10476 | **453.2234** | **1356.6483** | **1355.7673** | **0.8809** | **0** | **5** | **43** | **4** | **R.FRPLNQAEILR.G** |
|  | 12858 | 746.8978 | 1491.7810 | 1491.7603 | 0.0207 | 0 | 9 | 16 | 3 | K.VATMLSLESELQR.L 12867 12868 |
|  | 13643 | **513.5936** | **1537.7591** | **1536.7090** | **1.0501** | **1** | **4** | **52** | **1** | **K.QENMETEQKLSGK.L** |
|  | 13814 | **775.4022** | **1548.7898** | **1547.8195** | **0.9703** | **0** | **33** | **0.061** | **1** | **K.ISFLENNLEQLTK.V** |
|  | 16034 | 844.9414 | 1687.8683 | 1688.8403 | -0.9720 | 0 | 2 | 69 | 2 | K.QQMLDQEELLVSTR.G |
|  | 16243 | 851.4529 | 1700.8913 | 1700.7312 | 0.1602 | 0 | 4 | 41 | 3 | K.SAEMEPEDSGGIHSQK.Q 16242 |
|  | 16699 | **865.7896** | **1729.5647** | **1728.9821** | **0.5826** | **1** | **2** | **54** | **1** | **K.LHDPQLMGIIPRIAR.D** |
|  | 18099 | 912.9898 | 1823.9651 | 1823.8328 | 0.1323 | 1 | 5 | 31 | 4 | -.MAETNNECSIKVLCR.F 18098 18100 18101 |
|  | 22707 | 1117.5560 | 2233.0975 | 2232.0692 | 1.0283 | 1 | 0 | 74 | 5 | K.QQMLDQEELLVSTRGDNEK.V |
|  | 25444 | 869.4706 | 2605.3901 | 2604.2794 | 1.1106 | 1 | 1 | 47 | 5 | R.IARDIFNHIYSMDENLEFHIK.V |
|  | 27744 | 758.6226 | 3030.4614 | 3031.3184 | -0.8570 | 1 | 0 | 49 | 6 | R.TTMFICCSPSSYNDAETKSTLMFGQR.A |

| **860.** | O60282\|KIF5C_HUMAN    **Mass:** 109997   **Score:** 33     **Queries matched:** 24   **emPAI:** 0.03 |
| --- | --- |
|  | Kinesin heavy chain isoform 5C OS=Homo sapiens GN=KIF5C PE=1 SV=1 |

|  | **Query** | **Observed** | **Mr(expt)** | **Mr(calc)** | **Delta** | **Miss** | **Score** | **Expect** | **Rank** | **Peptide** |
| --- | --- | --- | --- | --- | --- | --- | --- | --- | --- | --- |
|  | 1526 | 395.7163 | 789.4181 | 788.4280 | 0.9901 | 0 | 3 | 1.2e+002 | 4 | R.DLLDVSK.T |
|  | 2890 | 446.2687 | 890.5229 | 889.4505 | 1.0724 | 0 | 6 | 44 | 6 | K.GLEETVSR.E |
|  | 3811 | **479.8337** | **957.6528** | **957.5858** | **0.0670** | **1** | **1** | **1.2e+002** | **10** | **R.VKALESALK.E** |
|  | 4630 | 508.7725 | 1015.5305 | 1015.5372 | -0.0067 | 1 | 10 | 20 | 3 | R.CELPKLEK.R |
|  | 5325 | 534.3037 | 1066.5929 | 1066.5658 | 0.0270 | 1 | 18 | 2.4 | 2 | K.LSSDYNKLK.I |
|  | 5681 | **546.3176** | **1090.6206** | **1091.5975** | **-0.9769** | **0** | **4** | **68** | **7** | **K.LFVQDLTTR.V** |
|  | 7197 | **597.2769** | **1192.5392** | **1192.5394** | **-0.0002** | **1** | **1** | **1.2e+002** | **4** | **K.IEDQEREMK.L** |
|  | 7544 | 403.4529 | 1207.3369 | 1206.6496 | 0.6873 | 0 | 5 | 31 | 4 | K.LYLVDLAGSEK.V 7528 |
|  | 8962 | **427.0736** | **1278.1989** | **1277.5670** | **0.6319** | **0** | **(0)** | **90** | **8** | **K.QLESAQMDSNR.K** |
|  | 9038 | 642.2797 | 1282.5448 | 1282.7972 | -0.2525 | 1 | 3 | 67 | 8 | K.RATEILNLLLK.D |
|  | 9250 | **432.0754** | **1293.2043** | **1293.5619** | **-0.3576** | **0** | **1** | **76** | **10** | **K.QLESAQMDSNR.K** |
|  | 12901 | **748.3554** | **1494.6963** | **1494.7613** | **-0.0650** | **0** | **4** | **53** | **7** | **K.NVIQHLEMELNR.W** |
|  | 13157 | **504.2655** | **1509.7748** | **1508.6889** | **1.0859** | **1** | **7** | **27** | **2** | **R.SKQLESAQMDSNR.K** |
|  | 13281 | **506.2803** | **1515.8190** | **1516.7005** | **-0.8815** | **0** | **4** | **51** | **3** | **K.DDEINQQSQLAEK.L** |
|  | 13814 | 775.4022 | 1548.7898 | 1547.8195 | 0.9703 | 0 | 33 | 0.061 | 1 | K.ISFLENNLEQLTK.V |
|  | 14885 | 808.9016 | 1615.7887 | 1615.8165 | -0.0279 | 1 | 17 | 2.5 | 2 | K.TRANEQLTDELAQK.T 14884 |
|  | 15952 | **562.2394** | **1683.6965** | **1682.7248** | **0.9717** | **1** | **8** | **16** | **3** | **-.MADPAECSIKVMCR.F** |
|  | 16302 | **569.6161** | **1705.8264** | **1704.8053** | **1.0211** | **0** | **4** | **43** | **1** | **R.QLEESQDSLSEELAK.L** |
|  | 16724 | 866.4468 | 1730.8790 | 1731.8679 | -0.9889 | 0 | 1 | 86 | 6 | K.NTVSVNLELTAEEWK.K |
|  | 19142 | **635.3467** | **1903.0184** | **1903.9673** | **-0.9489** | **1** | **1** | **69** | **3** | **K.LKQQMLDQDELLASTR.R** |
|  | 21625 | **706.3781** | **2116.1123** | **2115.9920** | **0.1203** | **1** | **0** | **83** | **3** | **R.QLDDKDDEINQQSQLAEK.L** |
|  | 24759 | 838.7866 | 2513.3379 | 2514.2676 | -0.9297 | 1 | 2 | 47 | 1 | K.TLADVNGVIEEEFTMARLYISK.M |

| **861.** | P33176\|KINH_HUMAN    **Mass:** 110358   **Score:** 33     **Queries matched:** 23   **emPAI:** 0.03 |
| --- | --- |
|  | Kinesin-1 heavy chain OS=Homo sapiens GN=KIF5B PE=1 SV=1 |

|  | **Query** | **Observed** | **Mr(expt)** | **Mr(calc)** | **Delta** | **Miss** | **Score** | **Expect** | **Rank** | **Peptide** |
| --- | --- | --- | --- | --- | --- | --- | --- | --- | --- | --- |
|  | 1526 | 395.7163 | 789.4181 | 788.4280 | 0.9901 | 0 | 3 | 1.2e+002 | 4 | R.DLLDVSK.T |
|  | 3006 | 451.7310 | 901.4475 | 902.3916 | -0.9441 | 0 | 15 | 6.3 | 1 | K.EMTNHQK.K 3007 |
|  | 3811 | 479.8337 | 957.6528 | 957.5858 | 0.0670 | 1 | 1 | 1.2e+002 | 10 | R.VKALESALK.E |
|  | 4627 | **508.3089** | **1014.6032** | **1014.4916** | **0.1116** | **1** | **5** | **56** | **9** | **K.EMTNHQKK.R** |
|  | 4630 | 508.7725 | 1015.5305 | 1015.5372 | -0.0067 | 1 | 10 | 20 | 3 | R.CELPKLEK.R |
|  | 5515 | 540.3129 | 1078.6112 | 1079.5355 | -0.9243 | 0 | 6 | 42 | 8 | R.AAEMMASLLK.D |
|  | 7544 | 403.4529 | 1207.3369 | 1206.6496 | 0.6873 | 0 | 5 | 31 | 4 | K.LYLVDLAGSEK.V 7528 |
|  | 7957 | 615.2987 | 1228.5829 | 1227.5917 | 0.9911 | 1 | 7 | 35 | 6 | R.AQEKVHEMEK.E |
|  | 8128 | **619.5244** | **1237.0341** | **1236.5954** | **0.4387** | **1** | **1** | **81** | **9** | **K.MMLEQERLR.V** |
|  | 8444 | **627.3235** | **1252.6325** | **1251.6315** | **1.0011** | **1** | **1** | **1.2e+002** | **5** | **K.RAAEMMASLLK.D** |
|  | 12793 | 497.2544 | 1488.7413 | 1488.6739 | 0.0673 | 1 | 1 | 1.1e+002 | 9 | R.RDQDNMQAELNR.L |
|  | 13814 | 775.4022 | 1548.7898 | 1547.8195 | 0.9703 | 0 | 33 | 0.061 | 1 | K.ISFLENNLEQLTK.V |
|  | 16357 | **855.4599** | **1708.9052** | **1708.8519** | **0.0534** | **1** | **2** | **67** | **3** | **K.TKEYELLSDELNQK.S** |
|  | 16785 | **434.7440** | **1734.9467** | **1735.7869** | **-0.8401** | **1** | **3** | **49** | **9** | **K.MEENEKELAACQLR.I** 16786 |
|  | 17593 | 895.9472 | 1789.8798 | 1789.8669 | 0.0130 | 0 | 4 | 44 | 3 | K.NTVCVNVELTAEQWK.K |
|  | 17615 | 896.4455 | 1790.8764 | 1789.8992 | 0.9772 | 1 | 4 | 44 | 4 | K.TQMLDQEELLASTRR.D |
|  | 17675 | 599.4346 | 1795.2821 | 1794.8094 | 0.4726 | 0 | 5 | 21 | 1 | K.QPEGTGMIDEEFTVAR.L |
|  | 21639 | **707.1030** | **2118.2873** | **2118.0350** | **0.2522** | **1** | **2** | **41** | **1** | **K.THTMEGKLHDPEGMGIIPR.I** |
|  | 22476 | **738.9556** | **2213.8449** | **2213.9569** | **-0.1120** | **1** | **2** | **37** | **1** | **R.FVCSPDEVMDTIDEGKSNR.H** |
|  | 25441 | **652.0994** | **2604.3684** | **2604.3138** | **0.0545** | **1** | **2** | **36** | **6** | **R.AAEMMASLLKDLAEIGIAVGNNDVK.Q** |

| **862.** | Q8NEW0\|ZNT7_HUMAN    **Mass:** 41941    **Score:** 33     **Queries matched:** 6   **emPAI:** 0.09 |
| --- | --- |
|  | Zinc transporter 7 OS=Homo sapiens GN=SLC30A7 PE=1 SV=1 |

|  | **Query** | **Observed** | **Mr(expt)** | **Mr(calc)** | **Delta** | **Miss** | **Score** | **Expect** | **Rank** | **Peptide** |
| --- | --- | --- | --- | --- | --- | --- | --- | --- | --- | --- |
|  | 1370 | **726.3718** | **725.3645** | **724.3908** | **0.9737** | **0** | **3** | **58** | **4** | **K.FNLFGK.I** |
|  | 9675 | 439.2352 | 1314.6837 | 1313.6326 | 1.0511 | 0 | 6 | 40 | 9 | R.QLYVQIDFAAM.- |
|  | 17552 | **597.3558** | **1789.0457** | **1788.9331** | **0.1125** | **1** | **0** | **94** | **8** | **-.MLPLSIKDDEYKPPK.F** |
|  | 17962 | **908.4506** | **1814.8866** | **1814.8985** | **-0.0119** | **0** | **33** | **0.051** | **1** | **R.TPPLLENSLPQCYQR.V** |
|  | 18750 | 936.4969 | 1870.9792 | 1869.9850 | 0.9943 | 0 | 3 | 53 | 5 | R.WILSQTHNIFTQAGVR.Q |
|  | 25819 | **891.1261** | **2670.3565** | **2669.1882** | **1.1683** | **0** | **1** | **44** | **6** | **K.HGAAHSHDHAHGHGHFHSHDGPSLK.E** |

Bottom of Form

**Mascot:**  http://www.matrixscience.com/

Top scoring peptide matches to query 72
File7030 Spectrum1621 scans: 1325
Score greater than 21 indicates homology
Score greater than 39 indicates identity

**Score	Expect	Delta Hit Protein	Peptide**
 8.4	65	-1.0279 802	Q9UH99|UN84B_HUMAN GGGGR

Top scoring peptide matches to query 74
File7030 Spectrum1786 scans: 1480
Score greater than 19 indicates homology
Score greater than 39 indicates identity

**Score	Expect	Delta Hit Protein	Peptide**
 6.9	92	-1.0278 802	Q9UH99|UN84B_HUMAN GGGGR

Top scoring peptide matches to query 77
File7030 Spectrum794 scans: 704
Score greater than 15 indicates homology
Score greater than 39 indicates identity

**Score	Expect	Delta Hit Protein	Peptide**
 2.1 2.8e+002	-0.9618 802	Q9UH99|UN84B_HUMAN GGGGR

Top scoring peptide matches to query 78
File7030 Spectrum1199 scans: 1000
Score greater than 19 indicates homology
Score greater than 39 indicates identity

**Score	Expect	Delta Hit Protein	Peptide**
 6.5	1e+002	-0.9618 802	Q9UH99|UN84B_HUMAN GGGGR

Top scoring peptide matches to query 79
File7030 Spectrum1016 scans: 868
Score greater than 14 indicates homology
Score greater than 39 indicates identity

**Score	Expect	Delta Hit Protein	Peptide**
 1.5 3.2e+002	-0.9616 802	Q9UH99|UN84B_HUMAN GGGGR

Top scoring peptide matches to query 84
File7030 Spectrum1014 scans: 866
Score greater than 14 indicates homology
Score greater than 40 indicates identity

**Score	Expect	Delta Hit Protein	Peptide**
 5.0 1.8e+002	0.0218 802	Q9UH99|UN84B_HUMAN GGGGR
 1.6	4e+002	-0.9510	K.DGGVG.-

Top scoring peptide matches to query 85
File7030 Spectrum3956 scans: 3350
Score greater than 16 indicates homology
Score greater than 39 indicates identity

**Score	Expect	Delta Hit Protein	Peptide**
 5.4 1.4e+002	0.8248 802	Q9UH99|UN84B_HUMAN GGGGR
 3.2 2.3e+002	-0.1480	K.DGGVG.-

Top scoring peptide matches to query 86
File7030 Spectrum1930 scans: 1616
Score greater than 14 indicates homology
Score greater than 39 indicates identity

**Score	Expect	Delta Hit Protein	Peptide**
 4.5 1.7e+002	0.8249 802	Q9UH99|UN84B_HUMAN GGGGR
 1.8 3.2e+002	-0.1479	K.DGGVG.-

Top scoring peptide matches to query 87
File7030 Spectrum2068 scans: 1749
[truncated: 5,831,112 more chars]
