# Supplementary material for: Dataset from proteomic analysis of human liver, lung, kidney and intestine microsomes
Source: Data Brief. 2018 Mar 30;18:831–4. doi: 10.1016/j.dib.2018.03.124 (PMC5996730; doi:10.1016/j.dib.2018.03.124)
Supplement: Supplementary file 3 — Supplementary material [file mmc3.doc]

Mascot Search Results

**User	: zhihong**

**Email	: peng_zhihong@hotmail.com**

**Search title	: intestine**

**MS data file	: intestine 2.mgf**

**Database	: SwissProt 101509 (510076 sequences; 179409349 residues)**

**Taxonomy	: Homo sapiens (human) (20335 sequences)**

**Timestamp	: 8 May 2013 at 18:36:52 GMT**

**Warning	: Invalid value (10+) specified for the "CHARGE" parameter**

**Warning	: Invalid value () specified for the "CHARGE" parameter**

**Warning	: Invalid value (12+) specified for the "CHARGE" parameter**

**Warning	: Invalid value () specified for the "CHARGE" parameter**

**Warning	: Invalid value (12+) specified for the "CHARGE" parameter**

**Warning	: Invalid value () specified for the "CHARGE" parameter**

**Warning	: Invalid value (13+) specified for the "CHARGE" parameter**

**Warning	: Invalid value () specified for the "CHARGE" parameter**

**Warning	: Invalid value (11+) specified for the "CHARGE" parameter**

**Warning	: Invalid value () specified for the "CHARGE" parameter**

**Warning	: Error 61 has been detected 86 times and only the first 10 messages have been output**

**Enzyme	: Trypsin**

**Fixed modifications	: Carbamidomethyl (C)**

**Variable modifications : Oxidation (M)**

**Mass values	: Monoisotopic**

**Protein Mass	: Unrestricted**

**Peptide Mass Tolerance : ± 1.2 Da**

**Fragment Mass Tolerance: ± 0.6 Da**

**Max Missed Cleavages	: 1**

**Instrument type	: Default**

**Number of queries	: 32142**

| **Protein hits           :** | **P63261\|ACTG_HUMAN** | Actin, cytoplasmic 2 OS=Homo sapiens GN=ACTG1 PE=1 SV=1 |
| --- | --- | --- |
|  | **P60709\|ACTB_HUMAN** | Actin, cytoplasmic 1 OS=Homo sapiens GN=ACTB PE=1 SV=1 |
|  | **Q9Y6R7\|FCGBP_HUMAN** | IgGFc-binding protein OS=Homo sapiens GN=FCGBP PE=1 SV=3 |
|  | **Q6S8J3\|POTEE_HUMAN** | POTE ankyrin domain family member E OS=Homo sapiens GN=POTEE PE=1 SV=3 |
|  | **A8K7I4\|CLCA1_HUMAN** | Calcium-activated chloride channel regulator 1 OS=Homo sapiens GN=CLCA1 PE=1 SV=2 |
|  | **P14410\|SUIS_HUMAN** | Sucrase-isomaltase, intestinal OS=Homo sapiens GN=SI PE=1 SV=5 |
|  | **P55157\|MTP_HUMAN** | Microsomal triglyceride transfer protein large subunit OS=Homo sapiens GN=MTTP PE=1 SV=1 |
|  | **P15144\|AMPN_HUMAN** | Aminopeptidase N OS=Homo sapiens GN=ANPEP PE=1 SV=4 |
|  | **P09848\|LPH_HUMAN** | Lactase-phlorizin hydrolase OS=Homo sapiens GN=LCT PE=1 SV=2 |
|  | **P07237\|PDIA1_HUMAN** | Protein disulfide-isomerase OS=Homo sapiens GN=P4HB PE=1 SV=3 |
|  | **P05023\|AT1A1_HUMAN** | Sodium/potassium-transporting ATPase subunit alpha-1 OS=Homo sapiens GN=ATP1A1 PE=1 SV=1 |
|  | **P11021\|GRP78_HUMAN** | 78 kDa glucose-regulated protein OS=Homo sapiens GN=HSPA5 PE=1 SV=2 |
|  | **Q00610\|CLH1_HUMAN** | Clathrin heavy chain 1 OS=Homo sapiens GN=CLTC PE=1 SV=5 |
|  | **P06576\|ATPB_HUMAN** | ATP synthase subunit beta, mitochondrial OS=Homo sapiens GN=ATP5B PE=1 SV=3 |
|  | **P35579\|MYH9_HUMAN** | Myosin-9 OS=Homo sapiens GN=MYH9 PE=1 SV=4 |
|  | **Q9ULC5\|ACSL5_HUMAN** | Long-chain-fatty-acid--CoA ligase 5 OS=Homo sapiens GN=ACSL5 PE=1 SV=1 |
|  | **Q9UBC5\|MYO1A_HUMAN** | Myosin-Ia OS=Homo sapiens GN=MYO1A PE=1 SV=1 |
|  | **P21796\|VDAC1_HUMAN** | Voltage-dependent anion-selective channel protein 1 OS=Homo sapiens GN=VDAC1 PE=1 SV=2 |
|  | **P09327\|VILI_HUMAN** | Villin-1 OS=Homo sapiens GN=VIL1 PE=1 SV=4 |
|  | **P25705\|ATPA_HUMAN** | ATP synthase subunit alpha, mitochondrial OS=Homo sapiens GN=ATP5A1 PE=1 SV=1 |
|  | **P40939\|ECHA_HUMAN** | Trifunctional enzyme subunit alpha, mitochondrial OS=Homo sapiens GN=HADHA PE=1 SV=2 |
|  | **P05062\|ALDOB_HUMAN** | Fructose-bisphosphate aldolase B OS=Homo sapiens GN=ALDOB PE=1 SV=2 |
|  | **Q7Z406\|MYH14_HUMAN** | Myosin-14 OS=Homo sapiens GN=MYH14 PE=1 SV=1 |
|  | **P13637\|AT1A3_HUMAN** | Sodium/potassium-transporting ATPase subunit alpha-3 OS=Homo sapiens GN=ATP1A3 PE=1 SV=3 |
|  | **P68371\|TBB2C_HUMAN** | Tubulin beta-2C chain OS=Homo sapiens GN=TUBB2C PE=1 SV=1 |
|  | **P35221\|CTNA1_HUMAN** | Catenin alpha-1 OS=Homo sapiens GN=CTNNA1 PE=1 SV=1 |
|  | **P14625\|ENPL_HUMAN** | Endoplasmin OS=Homo sapiens GN=HSP90B1 PE=1 SV=1 |
|  | **P31327\|CPSM_HUMAN** | Carbamoyl-phosphate synthase [ammonia], mitochondrial OS=Homo sapiens GN=CPS1 PE=1 SV=2 |
|  | **P30101\|PDIA3_HUMAN** | Protein disulfide-isomerase A3 OS=Homo sapiens GN=PDIA3 PE=1 SV=4 |
|  | **P07437\|TBB5_HUMAN** | Tubulin beta chain OS=Homo sapiens GN=TUBB PE=1 SV=2 |
|  | **P31930\|QCR1_HUMAN** | Cytochrome b-c1 complex subunit 1, mitochondrial OS=Homo sapiens GN=UQCRC1 PE=1 SV=3 |
|  | **P04350\|TBB4_HUMAN** | Tubulin beta-4 chain OS=Homo sapiens GN=TUBB4 PE=1 SV=2 |
|  | **P50993\|AT1A2_HUMAN** | Sodium/potassium-transporting ATPase subunit alpha-2 OS=Homo sapiens GN=ATP1A2 PE=1 SV=1 |
|  | **O43451\|MGA_HUMAN** | Maltase-glucoamylase, intestinal OS=Homo sapiens GN=MGAM PE=1 SV=5 |
|  | **P08473\|NEP_HUMAN** | Neprilysin OS=Homo sapiens GN=MME PE=1 SV=2 |
|  | **P27797\|CALR_HUMAN** | Calreticulin OS=Homo sapiens GN=CALR PE=1 SV=1 |
|  | **O60716\|CTND1_HUMAN** | Catenin delta-1 OS=Homo sapiens GN=CTNND1 PE=1 SV=1 |
|  | **P10809\|CH60_HUMAN** | 60 kDa heat shock protein, mitochondrial OS=Homo sapiens GN=HSPD1 PE=1 SV=2 |
|  | **P07355\|ANXA2_HUMAN** | Annexin A2 OS=Homo sapiens GN=ANXA2 PE=1 SV=2 |
|  | **O43707\|ACTN4_HUMAN** | Alpha-actinin-4 OS=Homo sapiens GN=ACTN4 PE=1 SV=2 |
|  | **P31040\|DHSA_HUMAN** | Succinate dehydrogenase [ubiquinone] flavoprotein subunit, mitochondrial OS=Homo sapiens GN=SDHA PE=1 SV=2 |
|  | **P68133\|ACTS_HUMAN** | Actin, alpha skeletal muscle OS=Homo sapiens GN=ACTA1 PE=1 SV=1 |
|  | **P68032\|ACTC_HUMAN** | Actin, alpha cardiac muscle 1 OS=Homo sapiens GN=ACTC1 PE=1 SV=1 |
|  | **P04406\|G3P_HUMAN** | Glyceraldehyde-3-phosphate dehydrogenase OS=Homo sapiens GN=GAPDH PE=1 SV=3 |
|  | **Q9Y4L1\|HYOU1_HUMAN** | Hypoxia up-regulated protein 1 OS=Homo sapiens GN=HYOU1 PE=1 SV=1 |
|  | **Q14764\|MVP_HUMAN** | Major vault protein OS=Homo sapiens GN=MVP PE=1 SV=4 |
|  | **P08684\|CP3A4_HUMAN** | Cytochrome P450 3A4 OS=Homo sapiens GN=CYP3A4 PE=1 SV=4 |
|  | **P68363\|TBA1B_HUMAN** | Tubulin alpha-1B chain OS=Homo sapiens GN=TUBA1B PE=1 SV=1 |
|  | **P21397\|AOFA_HUMAN** | Amine oxidase [flavin-containing] A OS=Homo sapiens GN=MAOA PE=1 SV=1 |
|  | **Q13885\|TBB2A_HUMAN** | Tubulin beta-2A chain OS=Homo sapiens GN=TUBB2A PE=1 SV=1 |
|  | **Q9BQE3\|TBA1C_HUMAN** | Tubulin alpha-1C chain OS=Homo sapiens GN=TUBA1C PE=1 SV=1 |
|  | **Q71U36\|TBA1A_HUMAN** | Tubulin alpha-1A chain OS=Homo sapiens GN=TUBA1A PE=1 SV=1 |
|  | **Q14651\|PLSI_HUMAN** | Plastin-1 OS=Homo sapiens GN=PLS1 PE=1 SV=2 |
|  | **P62736\|ACTA_HUMAN** | Actin, aortic smooth muscle OS=Homo sapiens GN=ACTA2 PE=1 SV=1 |
|  | **P63267\|ACTH_HUMAN** | Actin, gamma-enteric smooth muscle OS=Homo sapiens GN=ACTG2 PE=1 SV=1 |
|  | **Q14697\|GANAB_HUMAN** | Neutral alpha-glucosidase AB OS=Homo sapiens GN=GANAB PE=1 SV=3 |
|  | **P13667\|PDIA4_HUMAN** | Protein disulfide-isomerase A4 OS=Homo sapiens GN=PDIA4 PE=1 SV=2 |
|  | **P09110\|THIK_HUMAN** | 3-ketoacyl-CoA thiolase, peroxisomal OS=Homo sapiens GN=ACAA1 PE=1 SV=2 |
|  | **Q3LXA3\|DHAK_HUMAN** | Bifunctional ATP-dependent dihydroxyacetone kinase/FAD-AMP lyase (cyclizing) OS=Homo sapiens GN=DAK PE=1 SV=1 |
|  | **Q9UGM3\|DMBT1_HUMAN** | Deleted in malignant brain tumors 1 protein OS=Homo sapiens GN=DMBT1 PE=1 SV=2 |
|  | **P16422\|EPCAM_HUMAN** | Epithelial cell adhesion molecule OS=Homo sapiens GN=EPCAM PE=1 SV=2 |
|  | **P09923\|PPBI_HUMAN** | Intestinal alkaline phosphatase OS=Homo sapiens GN=ALPI PE=1 SV=2 |
|  | **P56470\|LEG4_HUMAN** | Galectin-4 OS=Homo sapiens GN=LGALS4 PE=1 SV=1 |
|  | **P68366\|TBA4A_HUMAN** | Tubulin alpha-4A chain OS=Homo sapiens GN=TUBA4A PE=1 SV=1 |
|  | **P45880\|VDAC2_HUMAN** | Voltage-dependent anion-selective channel protein 2 OS=Homo sapiens GN=VDAC2 PE=1 SV=2 |
|  | **O94832\|MYO1D_HUMAN** | Myosin-Id OS=Homo sapiens GN=MYO1D PE=1 SV=2 |
|  | **Q13748\|TBA3C_HUMAN** | Tubulin alpha-3C/D chain OS=Homo sapiens GN=TUBA3C PE=1 SV=3 |
|  | **P62873\|GBB1_HUMAN** | Guanine nucleotide-binding protein G(I)/G(S)/G(T) subunit beta-1 OS=Homo sapiens GN=GNB1 PE=1 SV=3 |
|  | **P16152\|CBR1_HUMAN** | Carbonyl reductase [NADPH] 1 OS=Homo sapiens GN=CBR1 PE=1 SV=3 |
|  | **P22695\|QCR2_HUMAN** | Cytochrome b-c1 complex subunit 2, mitochondrial OS=Homo sapiens GN=UQCRC2 PE=1 SV=3 |
|  | **O00748\|EST2_HUMAN** | Carboxylesterase 2 OS=Homo sapiens GN=CES2 PE=1 SV=1 |
|  | **P37059\|DHB2_HUMAN** | Estradiol 17-beta-dehydrogenase 2 OS=Homo sapiens GN=HSD17B2 PE=1 SV=1 |
|  | **P51659\|DHB4_HUMAN** | Peroxisomal multifunctional enzyme type 2 OS=Homo sapiens GN=HSD17B4 PE=1 SV=3 |
|  | **P54868\|HMCS2_HUMAN** | Hydroxymethylglutaryl-CoA synthase, mitochondrial OS=Homo sapiens GN=HMGCS2 PE=1 SV=1 |
|  | **P05026\|AT1B1_HUMAN** | Sodium/potassium-transporting ATPase subunit beta-1 OS=Homo sapiens GN=ATP1B1 PE=1 SV=1 |
|  | **P04181\|OAT_HUMAN** | Ornithine aminotransferase, mitochondrial OS=Homo sapiens GN=OAT PE=1 SV=1 |
|  | **O95497\|VNN1_HUMAN** | Pantetheinase OS=Homo sapiens GN=VNN1 PE=1 SV=2 |
|  | **Q12864\|CAD17_HUMAN** | Cadherin-17 OS=Homo sapiens GN=CDH17 PE=1 SV=2 |
|  | **P0C0S8\|H2A1_HUMAN** | Histone H2A type 1 OS=Homo sapiens GN=HIST1H2AG PE=1 SV=2 |
|  | **P50120\|RET2_HUMAN** | Retinol-binding protein 2 OS=Homo sapiens GN=RBP2 PE=1 SV=3 |
|  | **P12821\|ACE_HUMAN** | Angiotensin-converting enzyme OS=Homo sapiens GN=ACE PE=1 SV=1 |
|  | **Q6PEY2\|TBA3E_HUMAN** | Tubulin alpha-3E chain OS=Homo sapiens GN=TUBA3E PE=1 SV=2 |
|  | **O60218\|AK1BA_HUMAN** | Aldo-keto reductase family 1 member B10 OS=Homo sapiens GN=AKR1B10 PE=1 SV=1 |
|  | **A5A3E0\|POTEF_HUMAN** | POTE ankyrin domain family member F OS=Homo sapiens GN=POTEF PE=1 SV=2 |
|  | **O95994\|AGR2_HUMAN** | Anterior gradient protein 2 homolog OS=Homo sapiens GN=AGR2 PE=1 SV=1 |
|  | **P35222\|CTNB1_HUMAN** | Catenin beta-1 OS=Homo sapiens GN=CTNNB1 PE=1 SV=1 |
|  | **O75795\|UDB17_HUMAN** | UDP-glucuronosyltransferase 2B17 OS=Homo sapiens GN=UGT2B17 PE=2 SV=1 |
|  | **Q562R1\|ACTBL_HUMAN** | Beta-actin-like protein 2 OS=Homo sapiens GN=ACTBL2 PE=1 SV=2 |
|  | **P00813\|ADA_HUMAN** | Adenosine deaminase OS=Homo sapiens GN=ADA PE=1 SV=3 |
|  | **P06733\|ENOA_HUMAN** | Alpha-enolase OS=Homo sapiens GN=ENO1 PE=1 SV=2 |
|  | **Q16822\|PCKGM_HUMAN** | Phosphoenolpyruvate carboxykinase [GTP], mitochondrial OS=Homo sapiens GN=PCK2 PE=1 SV=2 |
|  | **Q07075\|AMPE_HUMAN** | Glutamyl aminopeptidase OS=Homo sapiens GN=ENPEP PE=1 SV=2 |
|  | **P23528\|COF1_HUMAN** | Cofilin-1 OS=Homo sapiens GN=CFL1 PE=1 SV=3 |
|  | **Q9NY65\|TBA8_HUMAN** | Tubulin alpha-8 chain OS=Homo sapiens GN=TUBA8 PE=1 SV=1 |
|  | **P55084\|ECHB_HUMAN** | Trifunctional enzyme subunit beta, mitochondrial OS=Homo sapiens GN=HADHB PE=1 SV=3 |
|  | **P11142\|HSP7C_HUMAN** | Heat shock cognate 71 kDa protein OS=Homo sapiens GN=HSPA8 PE=1 SV=1 |
|  | **P04844\|RPN2_HUMAN** | Dolichyl-diphosphooligosaccharide--protein glycosyltransferase subunit 2 OS=Homo sapiens GN=RPN2 PE=1 SV=3 |
|  | **P22760\|AAAD_HUMAN** | Arylacetamide deacetylase OS=Homo sapiens GN=AADAC PE=1 SV=4 |
|  | **P62879\|GBB2_HUMAN** | Guanine nucleotide-binding protein G(I)/G(S)/G(T) subunit beta-2 OS=Homo sapiens GN=GNB2 PE=1 SV=3 |
|  | **Q92597\|NDRG1_HUMAN** | Protein NDRG1 OS=Homo sapiens GN=NDRG1 PE=1 SV=1 |
|  | **A6NMY6\|AXA2L_HUMAN** | Putative annexin A2-like protein OS=Homo sapiens GN=ANXA2P2 PE=5 SV=2 |
|  | **P13866\|SC5A1_HUMAN** | Sodium/glucose cotransporter 1 OS=Homo sapiens GN=SLC5A1 PE=1 SV=1 |
|  | **Q13733\|AT1A4_HUMAN** | Sodium/potassium-transporting ATPase subunit alpha-4 OS=Homo sapiens GN=ATP1A4 PE=1 SV=3 |
|  | **P15311\|EZRI_HUMAN** | Ezrin OS=Homo sapiens GN=EZR PE=1 SV=4 |
|  | **P62805\|H4_HUMAN** | Histone H4 OS=Homo sapiens GN=HIST1H4A PE=1 SV=2 |
|  | **O75369\|FLNB_HUMAN** | Filamin-B OS=Homo sapiens GN=FLNB PE=1 SV=1 |
|  | **P35749\|MYH11_HUMAN** | Myosin-11 OS=Homo sapiens GN=MYH11 PE=1 SV=3 |
|  | **P27216\|ANX13_HUMAN** | Annexin A13 OS=Homo sapiens GN=ANXA13 PE=1 SV=2 |
|  | **O75907\|DGAT1_HUMAN** | Diacylglycerol O-acyltransferase 1 OS=Homo sapiens GN=DGAT1 PE=2 SV=2 |
|  | **P63104\|1433Z_HUMAN** | 14-3-3 protein zeta/delta OS=Homo sapiens GN=YWHAZ PE=1 SV=1 |
|  | **P49748\|ACADV_HUMAN** | Very long-chain specific acyl-CoA dehydrogenase, mitochondrial OS=Homo sapiens GN=ACADVL PE=1 SV=1 |
|  | **P12532\|KCRU_HUMAN** | Creatine kinase U-type, mitochondrial OS=Homo sapiens GN=CKMT1A PE=1 SV=1 |
|  | **P16435\|NCPR_HUMAN** | NADPH--cytochrome P450 reductase OS=Homo sapiens GN=POR PE=1 SV=2 |
|  | **Q15084\|PDIA6_HUMAN** | Protein disulfide-isomerase A6 OS=Homo sapiens GN=PDIA6 PE=1 SV=1 |
|  | **P58876\|H2B1D_HUMAN** | Histone H2B type 1-D OS=Homo sapiens GN=HIST1H2BD PE=1 SV=2 |
|  | **Q99880\|H2B1L_HUMAN** | Histone H2B type 1-L OS=Homo sapiens GN=HIST1H2BL PE=1 SV=3 |
|  | **Q99877\|H2B1N_HUMAN** | Histone H2B type 1-N OS=Homo sapiens GN=HIST1H2BN PE=1 SV=3 |
|  | **P05091\|ALDH2_HUMAN** | Aldehyde dehydrogenase, mitochondrial OS=Homo sapiens GN=ALDH2 PE=1 SV=2 |
|  | **P42765\|THIM_HUMAN** | 3-ketoacyl-CoA thiolase, mitochondrial OS=Homo sapiens GN=ACAA2 PE=1 SV=2 |
|  | **P14618\|KPYM_HUMAN** | Pyruvate kinase isozymes M1/M2 OS=Homo sapiens GN=PKM2 PE=1 SV=4 |
|  | **Q9BYX7\|ACTBM_HUMAN** | Beta-actin-like protein 3 OS=Homo sapiens GN=ACTBL3 PE=1 SV=1 |
|  | **Q99805\|TM9S2_HUMAN** | Transmembrane 9 superfamily member 2 OS=Homo sapiens GN=TM9SF2 PE=1 SV=1 |
|  | **P09467\|F16P1_HUMAN** | Fructose-1,6-bisphosphatase 1 OS=Homo sapiens GN=FBP1 PE=1 SV=4 |
|  | **Q8TD06\|AGR3_HUMAN** | Anterior gradient protein 3 homolog OS=Homo sapiens GN=AGR3 PE=1 SV=1 |
|  | **Q5VTE0\|EF1A3_HUMAN** | Putative elongation factor 1-alpha-like 3 OS=Homo sapiens GN=EEF1AL3 PE=5 SV=1 |
|  | **P12104\|FABPI_HUMAN** | Fatty acid-binding protein, intestinal OS=Homo sapiens GN=FABP2 PE=1 SV=2 |
|  | **P16104\|H2AX_HUMAN** | Histone H2A.x OS=Homo sapiens GN=H2AFX PE=1 SV=2 |
|  | **P06727\|APOA4_HUMAN** | Apolipoprotein A-IV OS=Homo sapiens GN=APOA4 PE=1 SV=3 |
|  | **Q8IUE6\|H2A2B_HUMAN** | Histone H2A type 2-B OS=Homo sapiens GN=HIST2H2AB PE=1 SV=3 |
|  | **P13639\|EF2_HUMAN** | Elongation factor 2 OS=Homo sapiens GN=EEF2 PE=1 SV=4 |
|  | **P33778\|H2B1B_HUMAN** | Histone H2B type 1-B OS=Homo sapiens GN=HIST1H2BB PE=1 SV=2 |
|  | **Q8N257\|H2B3B_HUMAN** | Histone H2B type 3-B OS=Homo sapiens GN=HIST3H2BB PE=1 SV=3 |
|  | **P05141\|ADT2_HUMAN** | ADP/ATP translocase 2 OS=Homo sapiens GN=SLC25A5 PE=1 SV=6 |
|  | **P30086\|PEBP1_HUMAN** | Phosphatidylethanolamine-binding protein 1 OS=Homo sapiens GN=PEBP1 PE=1 SV=3 |
|  | **P09211\|GSTP1_HUMAN** | Glutathione S-transferase P OS=Homo sapiens GN=GSTP1 PE=1 SV=2 |
|  | **P07148\|FABPL_HUMAN** | Fatty acid-binding protein, liver OS=Homo sapiens GN=FABP1 PE=1 SV=1 |
|  | **Q6FI13\|H2A2A_HUMAN** | Histone H2A type 2-A OS=Homo sapiens GN=HIST2H2AA3 PE=1 SV=3 |
|  | **P09525\|ANXA4_HUMAN** | Annexin A4 OS=Homo sapiens GN=ANXA4 PE=1 SV=4 |
|  | **P48047\|ATPO_HUMAN** | ATP synthase subunit O, mitochondrial OS=Homo sapiens GN=ATP5O PE=1 SV=1 |
|  | **Q13509\|TBB3_HUMAN** | Tubulin beta-3 chain OS=Homo sapiens GN=TUBB3 PE=1 SV=2 |
|  | **P35232\|PHB_HUMAN** | Prohibitin OS=Homo sapiens GN=PHB PE=1 SV=1 |
|  | **O43895\|XPP2_HUMAN** | Xaa-Pro aminopeptidase 2 OS=Homo sapiens GN=XPNPEP2 PE=1 SV=3 |
|  | **P24462\|CP3A7_HUMAN** | Cytochrome P450 3A7 OS=Homo sapiens GN=CYP3A7 PE=1 SV=2 |
|  | **Q96A33\|CCD47_HUMAN** | Coiled-coil domain-containing protein 47 OS=Homo sapiens GN=CCDC47 PE=1 SV=1 |
|  | **Q16820\|MEP1B_HUMAN** | Meprin A subunit beta OS=Homo sapiens GN=MEP1B PE=1 SV=2 |
|  | **P06744\|G6PI_HUMAN** | Glucose-6-phosphate isomerase OS=Homo sapiens GN=GPI PE=1 SV=4 |
|  | **Q9NYU2\|UGGG1_HUMAN** | UDP-glucose:glycoprotein glucosyltransferase 1 OS=Homo sapiens GN=UGCGL1 PE=1 SV=3 |
|  | **P29401\|TKT_HUMAN** | Transketolase OS=Homo sapiens GN=TKT PE=1 SV=3 |
|  | **P23229\|ITA6_HUMAN** | Integrin alpha-6 OS=Homo sapiens GN=ITGA6 PE=1 SV=4 |
|  | **P22309\|UD11_HUMAN** | UDP-glucuronosyltransferase 1-1 OS=Homo sapiens GN=UGT1A1 PE=1 SV=1 |
|  | **P12236\|ADT3_HUMAN** | ADP/ATP translocase 3 OS=Homo sapiens GN=SLC25A6 PE=1 SV=4 |
|  | **P52272\|HNRPM_HUMAN** | Heterogeneous nuclear ribonucleoprotein M OS=Homo sapiens GN=HNRNPM PE=1 SV=3 |
|  | **P12235\|ADT1_HUMAN** | ADP/ATP translocase 1 OS=Homo sapiens GN=SLC25A4 PE=1 SV=4 |
|  | **P52209\|6PGD_HUMAN** | 6-phosphogluconate dehydrogenase, decarboxylating OS=Homo sapiens GN=PGD PE=1 SV=3 |
|  | **P29992\|GNA11_HUMAN** | Guanine nucleotide-binding protein subunit alpha-11 OS=Homo sapiens GN=GNA11 PE=1 SV=2 |
|  | **Q6UX06\|OLFM4_HUMAN** | Olfactomedin-4 OS=Homo sapiens GN=OLFM4 PE=1 SV=1 |
|  | **P16615\|AT2A2_HUMAN** | Sarcoplasmic/endoplasmic reticulum calcium ATPase 2 OS=Homo sapiens GN=ATP2A2 PE=1 SV=1 |
|  | **P00167\|CYB5_HUMAN** | Cytochrome b5 OS=Homo sapiens GN=CYB5A PE=1 SV=2 |
|  | **P01834\|IGKC_HUMAN** | Ig kappa chain C region OS=Homo sapiens GN=IGKC PE=1 SV=1 |
|  | **Q16134\|ETFD_HUMAN** | Electron transfer flavoprotein-ubiquinone oxidoreductase, mitochondrial OS=Homo sapiens GN=ETFDH PE=1 SV=1 |
|  | **P14854\|CX6B1_HUMAN** | Cytochrome c oxidase subunit 6B1 OS=Homo sapiens GN=COX6B1 PE=1 SV=2 |
|  | **P61586\|RHOA_HUMAN** | Transforming protein RhoA OS=Homo sapiens GN=RHOA PE=1 SV=1 |
|  | **Q9H3R2\|MUC13_HUMAN** | Mucin-13 OS=Homo sapiens GN=MUC13 PE=1 SV=1 |
|  | **Q9UM54\|MYO6_HUMAN** | Myosin-VI OS=Homo sapiens GN=MYO6 PE=1 SV=4 |
|  | **A1L0T0\|ILVBL_HUMAN** | Acetolactate synthase-like protein OS=Homo sapiens GN=ILVBL PE=1 SV=2 |
|  | **Q6NUK1\|SCMC1_HUMAN** | Calcium-binding mitochondrial carrier protein SCaMC-1 OS=Homo sapiens GN=SLC25A24 PE=1 SV=2 |
|  | **Q13724\|MOGS_HUMAN** | Mannosyl-oligosaccharide glucosidase OS=Homo sapiens GN=MOGS PE=1 SV=5 |
|  | **Q2TB90\|HKDC1_HUMAN** | Putative hexokinase HKDC1 OS=Homo sapiens GN=HKDC1 PE=2 SV=2 |
|  | **O75396\|SC22B_HUMAN** | Vesicle-trafficking protein SEC22b OS=Homo sapiens GN=SEC22B PE=1 SV=3 |
|  | **P54855\|UDB15_HUMAN** | UDP-glucuronosyltransferase 2B15 OS=Homo sapiens GN=UGT2B15 PE=1 SV=2 |
|  | **Q99798\|ACON_HUMAN** | Aconitate hydratase, mitochondrial OS=Homo sapiens GN=ACO2 PE=1 SV=2 |
|  | **Q99795\|GPA33_HUMAN** | Cell surface A33 antigen OS=Homo sapiens GN=GPA33 PE=1 SV=1 |
|  | **Q6P1M0\|S27A4_HUMAN** | Long-chain fatty acid transport protein 4 OS=Homo sapiens GN=SLC27A4 PE=1 SV=1 |
|  | **P01877\|IGHA2_HUMAN** | Ig alpha-2 chain C region OS=Homo sapiens GN=IGHA2 PE=1 SV=3 |
|  | **P01876\|IGHA1_HUMAN** | Ig alpha-1 chain C region OS=Homo sapiens GN=IGHA1 PE=1 SV=2 |
|  | **P08134\|RHOC_HUMAN** | Rho-related GTP-binding protein RhoC OS=Homo sapiens GN=RHOC PE=1 SV=1 |
|  | **Q9H0U4\|RAB1B_HUMAN** | Ras-related protein Rab-1B OS=Homo sapiens GN=RAB1B PE=1 SV=1 |
|  | **P39023\|RL3_HUMAN** | 60S ribosomal protein L3 OS=Homo sapiens GN=RPL3 PE=1 SV=2 |
|  | **P05387\|RLA2_HUMAN** | 60S acidic ribosomal protein P2 OS=Homo sapiens GN=RPLP2 PE=1 SV=1 |
|  | **P35580\|MYH10_HUMAN** | Myosin-10 OS=Homo sapiens GN=MYH10 PE=1 SV=3 |
|  | **Q01082\|SPTB2_HUMAN** | Spectrin beta chain, brain 1 OS=Homo sapiens GN=SPTBN1 PE=1 SV=2 |
|  | **P04040\|CATA_HUMAN** | Catalase OS=Homo sapiens GN=CAT PE=1 SV=3 |
|  | **P07099\|HYEP_HUMAN** | Epoxide hydrolase 1 OS=Homo sapiens GN=EPHX1 PE=1 SV=1 |
|  | **Q9UL46\|PSME2_HUMAN** | Proteasome activator complex subunit 2 OS=Homo sapiens GN=PSME2 PE=1 SV=3 |
|  | **P01833\|PIGR_HUMAN** | Polymeric immunoglobulin receptor OS=Homo sapiens GN=PIGR PE=1 SV=4 |
|  | **Q16836\|HCDH_HUMAN** | Hydroxyacyl-coenzyme A dehydrogenase, mitochondrial OS=Homo sapiens GN=HADH PE=1 SV=2 |
|  | **P62820\|RAB1A_HUMAN** | Ras-related protein Rab-1A OS=Homo sapiens GN=RAB1A PE=1 SV=3 |
|  | **O14745\|NHRF1_HUMAN** | Na(+)/H(+) exchange regulatory cofactor NHE-RF1 OS=Homo sapiens GN=SLC9A3R1 PE=1 SV=4 |
|  | **P08238\|HS90B_HUMAN** | Heat shock protein HSP 90-beta OS=Homo sapiens GN=HSP90AB1 PE=1 SV=4 |
|  | **P62937\|PPIA_HUMAN** | Peptidyl-prolyl cis-trans isomerase A OS=Homo sapiens GN=PPIA PE=1 SV=2 |
|  | **P20648\|ATP4A_HUMAN** | Potassium-transporting ATPase alpha chain 1 OS=Homo sapiens GN=ATP4A PE=2 SV=5 |
|  | **P55072\|TERA_HUMAN** | Transitional endoplasmic reticulum ATPase OS=Homo sapiens GN=VCP PE=1 SV=4 |
|  | **P62158\|CALM_HUMAN** | Calmodulin OS=Homo sapiens GN=CALM1 PE=1 SV=2 |
|  | **O60844\|ZG16_HUMAN** | Zymogen granule membrane protein 16 OS=Homo sapiens GN=ZG16 PE=1 SV=1 |
|  | **P0C0S5\|H2AZ_HUMAN** | Histone H2A.Z OS=Homo sapiens GN=H2AFZ PE=1 SV=2 |
|  | **P23284\|PPIB_HUMAN** | Peptidyl-prolyl cis-trans isomerase B OS=Homo sapiens GN=PPIB PE=1 SV=2 |
|  | **P07900\|HS90A_HUMAN** | Heat shock protein HSP 90-alpha OS=Homo sapiens GN=HSP90AA1 PE=1 SV=5 |
|  | **P27824\|CALX_HUMAN** | Calnexin OS=Homo sapiens GN=CANX PE=1 SV=2 |
|  | **Q01970\|PLCB3_HUMAN** | 1-phosphatidylinositol-4,5-bisphosphate phosphodiesterase beta-3 OS=Homo sapiens GN=PLCB3 PE=1 SV=2 |
|  | **O75828\|CBR3_HUMAN** | Carbonyl reductase [NADPH] 3 OS=Homo sapiens GN=CBR3 PE=1 SV=3 |
|  | **Q95604\|1C17_HUMAN** | HLA class I histocompatibility antigen, Cw-17 alpha chain OS=Homo sapiens GN=HLA-C PE=1 SV=1 |
|  | **Q16819\|MEP1A_HUMAN** | Meprin A subunit alpha OS=Homo sapiens GN=MEP1A PE=2 SV=2 |
|  | **P04439\|1A03_HUMAN** | HLA class I histocompatibility antigen, A-3 alpha chain OS=Homo sapiens GN=HLA-A PE=1 SV=2 |
|  | **P30447\|1A23_HUMAN** | HLA class I histocompatibility antigen, A-23 alpha chain OS=Homo sapiens GN=HLA-A PE=1 SV=1 |
|  | **P05534\|1A24_HUMAN** | HLA class I histocompatibility antigen, A-24 alpha chain OS=Homo sapiens GN=HLA-A PE=1 SV=2 |
|  | **P16188\|1A30_HUMAN** | HLA class I histocompatibility antigen, A-30 alpha chain OS=Homo sapiens GN=HLA-A PE=1 SV=2 |
|  | **P30443\|1A01_HUMAN** | HLA class I histocompatibility antigen, A-1 alpha chain OS=Homo sapiens GN=HLA-A PE=1 SV=1 |
|  | **O75310\|UDB11_HUMAN** | UDP-glucuronosyltransferase 2B11 OS=Homo sapiens GN=UGT2B11 PE=2 SV=1 |
|  | **P42330\|AK1C3_HUMAN** | Aldo-keto reductase family 1 member C3 OS=Homo sapiens GN=AKR1C3 PE=1 SV=3 |
|  | **P50416\|CPT1A_HUMAN** | Carnitine O-palmitoyltransferase 1, liver isoform OS=Homo sapiens GN=CPT1A PE=1 SV=2 |
|  | **O75874\|IDHC_HUMAN** | Isocitrate dehydrogenase [NADP] cytoplasmic OS=Homo sapiens GN=IDH1 PE=1 SV=2 |
|  | **O95154\|ARK73_HUMAN** | Aflatoxin B1 aldehyde reductase member 3 OS=Homo sapiens GN=AKR7A3 PE=1 SV=2 |
|  | **O75306\|NDUS2_HUMAN** | NADH dehydrogenase [ubiquinone] iron-sulfur protein 2, mitochondrial OS=Homo sapiens GN=NDUFS2 PE=1 SV=2 |
|  | **P28288\|ABCD3_HUMAN** | ATP-binding cassette sub-family D member 3 OS=Homo sapiens GN=ABCD3 PE=1 SV=1 |
|  | **P40926\|MDHM_HUMAN** | Malate dehydrogenase, mitochondrial OS=Homo sapiens GN=MDH2 PE=1 SV=3 |
|  | **P04843\|RPN1_HUMAN** | Dolichyl-diphosphooligosaccharide--protein glycosyltransferase subunit 1 OS=Homo sapiens GN=RPN1 PE=1 SV=1 |
|  | **Q96I99\|SUCB2_HUMAN** | Succinyl-CoA ligase [GDP-forming] subunit beta, mitochondrial OS=Homo sapiens GN=SUCLG2 PE=1 SV=2 |
|  | **P22626\|ROA2_HUMAN** | Heterogeneous nuclear ribonucleoproteins A2/B1 OS=Homo sapiens GN=HNRNPA2B1 PE=1 SV=2 |
|  | **P08865\|RSSA_HUMAN** | 40S ribosomal protein SA OS=Homo sapiens GN=RPSA PE=1 SV=4 |
|  | **P08758\|ANXA5_HUMAN** | Annexin A5 OS=Homo sapiens GN=ANXA5 PE=1 SV=2 |
|  | **P20674\|COX5A_HUMAN** | Cytochrome c oxidase subunit 5A, mitochondrial OS=Homo sapiens GN=COX5A PE=1 SV=2 |
|  | **Q16891\|IMMT_HUMAN** | Mitochondrial inner membrane protein OS=Homo sapiens GN=IMMT PE=1 SV=1 |
|  | **P40925\|MDHC_HUMAN** | Malate dehydrogenase, cytoplasmic OS=Homo sapiens GN=MDH1 PE=1 SV=4 |
|  | **P08183\|MDR1_HUMAN** | Multidrug resistance protein 1 OS=Homo sapiens GN=ABCB1 PE=1 SV=3 |
|  | **P60174\|TPIS_HUMAN** | Triosephosphate isomerase OS=Homo sapiens GN=TPI1 PE=1 SV=2 |
|  | **Q15436\|SC23A_HUMAN** | Protein transport protein Sec23A OS=Homo sapiens GN=SEC23A PE=1 SV=2 |
|  | **Q9Y2T3\|GUAD_HUMAN** | Guanine deaminase OS=Homo sapiens GN=GDA PE=1 SV=1 |
|  | **P30084\|ECHM_HUMAN** | Enoyl-CoA hydratase, mitochondrial OS=Homo sapiens GN=ECHS1 PE=1 SV=4 |
|  | **Q01518\|CAP1_HUMAN** | Adenylyl cyclase-associated protein 1 OS=Homo sapiens GN=CAP1 PE=1 SV=4 |
|  | **P18859\|ATP5J_HUMAN** | ATP synthase-coupling factor 6, mitochondrial OS=Homo sapiens GN=ATP5J PE=1 SV=1 |
|  | **P00352\|AL1A1_HUMAN** | Retinal dehydrogenase 1 OS=Homo sapiens GN=ALDH1A1 PE=1 SV=2 |
|  | **P21695\|GPDA_HUMAN** | Glycerol-3-phosphate dehydrogenase [NAD+], cytoplasmic OS=Homo sapiens GN=GPD1 PE=1 SV=4 |
|  | **P27338\|AOFB_HUMAN** | Amine oxidase [flavin-containing] B OS=Homo sapiens GN=MAOB PE=1 SV=3 |
|  | **P08107\|HSP71_HUMAN** | Heat shock 70 kDa protein 1 OS=Homo sapiens GN=HSPA1A PE=1 SV=5 |
|  | **P30508\|1C12_HUMAN** | HLA class I histocompatibility antigen, Cw-12 alpha chain OS=Homo sapiens GN=HLA-C PE=1 SV=2 |
|  | **Q29960\|1C16_HUMAN** | HLA class I histocompatibility antigen, Cw-16 alpha chain OS=Homo sapiens GN=HLA-C PE=2 SV=1 |
|  | **P38646\|GRP75_HUMAN** | Stress-70 protein, mitochondrial OS=Homo sapiens GN=HSPA9 PE=1 SV=2 |
|  | **P05451\|REG1A_HUMAN** | Lithostathine-1-alpha OS=Homo sapiens GN=REG1A PE=1 SV=3 |
|  | **P00338\|LDHA_HUMAN** | L-lactate dehydrogenase A chain OS=Homo sapiens GN=LDHA PE=1 SV=2 |
|  | **Q9NZ08\|ERAP1_HUMAN** | Endoplasmic reticulum aminopeptidase 1 OS=Homo sapiens GN=ERAP1 PE=1 SV=3 |
|  | **Q96KP4\|CNDP2_HUMAN** | Cytosolic non-specific dipeptidase OS=Homo sapiens GN=CNDP2 PE=1 SV=2 |
|  | **P50224\|ST1A3_HUMAN** | Sulfotransferase 1A3/1A4 OS=Homo sapiens GN=SULT1A3 PE=1 SV=1 |
|  | **Q9BY64\|UDB28_HUMAN** | UDP-glucuronosyltransferase 2B28 OS=Homo sapiens GN=UGT2B28 PE=1 SV=1 |
|  | **Q3SYC2\|MOGT2_HUMAN** | 2-acylglycerol O-acyltransferase 2 OS=Homo sapiens GN=MOGAT2 PE=1 SV=2 |
|  | **P84077\|ARF1_HUMAN** | ADP-ribosylation factor 1 OS=Homo sapiens GN=ARF1 PE=1 SV=2 |
|  | **P19075\|TSN8_HUMAN** | Tetraspanin-8 OS=Homo sapiens GN=TSPAN8 PE=1 SV=1 |
|  | **P01892\|1A02_HUMAN** | HLA class I histocompatibility antigen, A-2 alpha chain OS=Homo sapiens GN=HLA-A PE=1 SV=1 |
|  | **P10316\|1A69_HUMAN** | HLA class I histocompatibility antigen, A-69 alpha chain OS=Homo sapiens GN=HLA-A PE=1 SV=2 |
|  | **Q6NUM9\|RETST_HUMAN** | All-trans-retinol 13,14-reductase OS=Homo sapiens GN=RETSAT PE=2 SV=2 |
|  | **O00264\|PGRC1_HUMAN** | Membrane-associated progesterone receptor component 1 OS=Homo sapiens GN=PGRMC1 PE=1 SV=3 |
|  | **P14923\|PLAK_HUMAN** | Junction plakoglobin OS=Homo sapiens GN=JUP PE=1 SV=3 |
|  | **P18085\|ARF4_HUMAN** | ADP-ribosylation factor 4 OS=Homo sapiens GN=ARF4 PE=1 SV=3 |
|  | **P27635\|RL10_HUMAN** | 60S ribosomal protein L10 OS=Homo sapiens GN=RPL10 PE=1 SV=4 |
|  | **P12830\|CADH1_HUMAN** | Cadherin-1 OS=Homo sapiens GN=CDH1 PE=1 SV=3 |
|  | **P12814\|ACTN1_HUMAN** | Alpha-actinin-1 OS=Homo sapiens GN=ACTN1 PE=1 SV=2 |
|  | **O43704\|ST1B1_HUMAN** | Sulfotransferase family cytosolic 1B member 1 OS=Homo sapiens GN=SULT1B1 PE=1 SV=2 |
|  | **P31946\|1433B_HUMAN** | 14-3-3 protein beta/alpha OS=Homo sapiens GN=YWHAB PE=1 SV=3 |
|  | **P26232\|CTNA2_HUMAN** | Catenin alpha-2 OS=Homo sapiens GN=CTNNA2 PE=1 SV=5 |
|  | **P62424\|RL7A_HUMAN** | 60S ribosomal protein L7a OS=Homo sapiens GN=RPL7A PE=1 SV=2 |
|  | **Q29940\|1B59_HUMAN** | HLA class I histocompatibility antigen, B-59 alpha chain OS=Homo sapiens GN=HLA-B PE=2 SV=1 |
|  | **P18465\|1B57_HUMAN** | HLA class I histocompatibility antigen, B-57 alpha chain OS=Homo sapiens GN=HLA-B PE=1 SV=1 |
|  | **P30464\|1B15_HUMAN** | HLA class I histocompatibility antigen, B-15 alpha chain OS=Homo sapiens GN=HLA-B PE=1 SV=2 |
|  | **P49411\|EFTU_HUMAN** | Elongation factor Tu, mitochondrial OS=Homo sapiens GN=TUFM PE=1 SV=2 |
|  | **Q02218\|ODO1_HUMAN** | 2-oxoglutarate dehydrogenase E1 component, mitochondrial OS=Homo sapiens GN=OGDH PE=1 SV=3 |
|  | **P30049\|ATPD_HUMAN** | ATP synthase subunit delta, mitochondrial OS=Homo sapiens GN=ATP5D PE=1 SV=2 |
|  | **P60660\|MYL6_HUMAN** | Myosin light polypeptide 6 OS=Homo sapiens GN=MYL6 PE=1 SV=2 |
|  | **P10314\|1A32_HUMAN** | HLA class I histocompatibility antigen, A-32 alpha chain OS=Homo sapiens GN=HLA-A PE=2 SV=2 |
|  | **P16189\|1A31_HUMAN** | HLA class I histocompatibility antigen, A-31 alpha chain OS=Homo sapiens GN=HLA-A PE=2 SV=2 |
|  | **P30512\|1A29_HUMAN** | HLA class I histocompatibility antigen, A-29 alpha chain OS=Homo sapiens GN=HLA-A PE=2 SV=2 |
|  | **P18462\|1A25_HUMAN** | HLA class I histocompatibility antigen, A-25 alpha chain OS=Homo sapiens GN=HLA-A PE=2 SV=1 |
|  | **P62917\|RL8_HUMAN** | 60S ribosomal protein L8 OS=Homo sapiens GN=RPL8 PE=1 SV=2 |
|  | **P42704\|LPPRC_HUMAN** | Leucine-rich PPR motif-containing protein, mitochondrial OS=Homo sapiens GN=LRPPRC PE=1 SV=3 |
|  | **Q9Y277\|VDAC3_HUMAN** | Voltage-dependent anion-selective channel protein 3 OS=Homo sapiens GN=VDAC3 PE=1 SV=1 |
|  | **Q6P5W5\|S39A4_HUMAN** | Zinc transporter ZIP4 OS=Homo sapiens GN=SLC39A4 PE=1 SV=2 |
|  | **P34931\|HS71L_HUMAN** | Heat shock 70 kDa protein 1L OS=Homo sapiens GN=HSPA1L PE=1 SV=2 |
|  | **P36542\|ATPG_HUMAN** | ATP synthase subunit gamma, mitochondrial OS=Homo sapiens GN=ATP5C1 PE=1 SV=1 |
|  | **Q12797\|ASPH_HUMAN** | Aspartyl/asparaginyl beta-hydroxylase OS=Homo sapiens GN=ASPH PE=1 SV=3 |
|  | **P30048\|PRDX3_HUMAN** | Thioredoxin-dependent peroxide reductase, mitochondrial OS=Homo sapiens GN=PRDX3 PE=1 SV=3 |
|  | **P10620\|MGST1_HUMAN** | Microsomal glutathione S-transferase 1 OS=Homo sapiens GN=MGST1 PE=1 SV=1 |
|  | **Q8WWU7\|ITLN2_HUMAN** | Intelectin-2 OS=Homo sapiens GN=ITLN2 PE=2 SV=1 |
|  | **Q96JP2\|MY15B_HUMAN** | Putative myosin-XVB OS=Homo sapiens GN=MYO15B PE=5 SV=2 |
|  | **Q99623\|PHB2_HUMAN** | Prohibitin-2 OS=Homo sapiens GN=PHB2 PE=1 SV=2 |
|  | **Q9BYF1\|ACE2_HUMAN** | Angiotensin-converting enzyme 2 OS=Homo sapiens GN=ACE2 PE=1 SV=2 |
|  | **P54652\|HSP72_HUMAN** | Heat shock-related 70 kDa protein 2 OS=Homo sapiens GN=HSPA2 PE=1 SV=1 |
|  | **P08574\|CY1_HUMAN** | Cytochrome c1, heme protein, mitochondrial OS=Homo sapiens GN=CYC1 PE=1 SV=2 |
|  | **O75947\|ATP5H_HUMAN** | ATP synthase subunit d, mitochondrial OS=Homo sapiens GN=ATP5H PE=1 SV=3 |
|  | **P30685\|1B35_HUMAN** | HLA class I histocompatibility antigen, B-35 alpha chain OS=Homo sapiens GN=HLA-B PE=1 SV=1 |
|  | **P18464\|1B51_HUMAN** | HLA class I histocompatibility antigen, B-51 alpha chain OS=Homo sapiens GN=HLA-B PE=1 SV=1 |
|  | **Q6UWM9\|UD2A3_HUMAN** | UDP-glucuronosyltransferase 2A3 OS=Homo sapiens GN=UGT2A3 PE=2 SV=1 |
|  | **P16444\|DPEP1_HUMAN** | Dipeptidase 1 OS=Homo sapiens GN=DPEP1 PE=1 SV=3 |
|  | **P00403\|COX2_HUMAN** | Cytochrome c oxidase subunit 2 OS=Homo sapiens GN=MT-CO2 PE=1 SV=1 |
|  | **O14983\|AT2A1_HUMAN** | Sarcoplasmic/endoplasmic reticulum calcium ATPase 1 OS=Homo sapiens GN=ATP2A1 PE=1 SV=1 |
|  | **Q15907\|RB11B_HUMAN** | Ras-related protein Rab-11B OS=Homo sapiens GN=RAB11B PE=1 SV=4 |
|  | **P62753\|RS6_HUMAN** | 40S ribosomal protein S6 OS=Homo sapiens GN=RPS6 PE=1 SV=1 |
|  | **P19105\|ML12A_HUMAN** | Myosin regulatory light chain 12A OS=Homo sapiens GN=MYL12A PE=1 SV=2 |
|  | **O14950\|ML12B_HUMAN** | Myosin regulatory light chain 12B OS=Homo sapiens GN=MYL12B PE=1 SV=2 |
|  | **Q9BS26\|ERP44_HUMAN** | Endoplasmic reticulum resident protein ERp44 OS=Homo sapiens GN=ERP44 PE=1 SV=1 |
|  | **P08263\|GSTA1_HUMAN** | Glutathione S-transferase A1 OS=Homo sapiens GN=GSTA1 PE=1 SV=3 |
|  | **P61978\|HNRPK_HUMAN** | Heterogeneous nuclear ribonucleoprotein K OS=Homo sapiens GN=HNRNPK PE=1 SV=1 |
|  | **P08195\|4F2_HUMAN** | 4F2 cell-surface antigen heavy chain OS=Homo sapiens GN=SLC3A2 PE=1 SV=3 |
|  | **Q6PIF6\|MYO7B_HUMAN** | Myosin-VIIb OS=Homo sapiens GN=MYO7B PE=2 SV=2 |
|  | **Q00325\|MPCP_HUMAN** | Phosphate carrier protein, mitochondrial OS=Homo sapiens GN=SLC25A3 PE=1 SV=2 |
|  | **P14314\|GLU2B_HUMAN** | Glucosidase 2 subunit beta OS=Homo sapiens GN=PRKCSH PE=1 SV=2 |
|  | **O95471\|CLD7_HUMAN** | Claudin-7 OS=Homo sapiens GN=CLDN7 PE=1 SV=3 |
|  | **P24844\|MYL9_HUMAN** | Myosin regulatory light polypeptide 9 OS=Homo sapiens GN=MYL9 PE=1 SV=4 |
|  | **Q15067\|ACOX1_HUMAN** | Peroxisomal acyl-coenzyme A oxidase 1 OS=Homo sapiens GN=ACOX1 PE=1 SV=3 |
|  | **Q12907\|LMAN2_HUMAN** | Vesicular integral-membrane protein VIP36 OS=Homo sapiens GN=LMAN2 PE=1 SV=1 |
|  | **P13804\|ETFA_HUMAN** | Electron transfer flavoprotein subunit alpha, mitochondrial OS=Homo sapiens GN=ETFA PE=1 SV=1 |
|  | **P28331\|NDUS1_HUMAN** | NADH-ubiquinone oxidoreductase 75 kDa subunit, mitochondrial OS=Homo sapiens GN=NDUFS1 PE=1 SV=3 |
|  | **Q92928\|RAB1C_HUMAN** | Putative Ras-related protein Rab-1C OS=Homo sapiens GN=RAB1C PE=5 SV=2 |
|  | **P39656\|OST48_HUMAN** | Dolichyl-diphosphooligosaccharide--protein glycosyltransferase 48 kDa subunit OS=Homo sapiens GN=DDOST PE=1 SV=4 |
|  | **O43826\|G6PT1_HUMAN** | Glucose-6-phosphate translocase OS=Homo sapiens GN=SLC37A4 PE=1 SV=1 |
|  | **O00217\|NDUS8_HUMAN** | NADH dehydrogenase [ubiquinone] iron-sulfur protein 8, mitochondrial OS=Homo sapiens GN=NDUFS8 PE=1 SV=1 |
|  | **P50395\|GDIB_HUMAN** | Rab GDP dissociation inhibitor beta OS=Homo sapiens GN=GDI2 PE=1 SV=2 |
|  | **P17931\|LEG3_HUMAN** | Galectin-3 OS=Homo sapiens GN=LGALS3 PE=1 SV=5 |
|  | **P61204\|ARF3_HUMAN** | ADP-ribosylation factor 3 OS=Homo sapiens GN=ARF3 PE=1 SV=2 |
|  | **O00299\|CLIC1_HUMAN** | Chloride intracellular channel protein 1 OS=Homo sapiens GN=CLIC1 PE=1 SV=4 |
|  | **P62841\|RS15_HUMAN** | 40S ribosomal protein S15 OS=Homo sapiens GN=RPS15 PE=1 SV=2 |
|  | **P07954\|FUMH_HUMAN** | Fumarate hydratase, mitochondrial OS=Homo sapiens GN=FH PE=1 SV=3 |
|  | **P54709\|AT1B3_HUMAN** | Sodium/potassium-transporting ATPase subunit beta-3 OS=Homo sapiens GN=ATP1B3 PE=1 SV=1 |
|  | **P62701\|RS4X_HUMAN** | 40S ribosomal protein S4, X isoform OS=Homo sapiens GN=RPS4X PE=1 SV=2 |
|  | **P62834\|RAP1A_HUMAN** | Ras-related protein Rap-1A OS=Homo sapiens GN=RAP1A PE=1 SV=1 |
|  | **O60613\|SEP15_HUMAN** | 15 kDa selenoprotein OS=Homo sapiens GN=SEP15 PE=1 SV=3 |
|  | **P80365\|DHI2_HUMAN** | Corticosteroid 11-beta-dehydrogenase isozyme 2 OS=Homo sapiens GN=HSD11B2 PE=1 SV=2 |
|  | **P16662\|UD2B7_HUMAN** | UDP-glucuronosyltransferase 2B7 OS=Homo sapiens GN=UGT2B7 PE=1 SV=1 |
|  | **Q8TE67\|ES8L3_HUMAN** | Epidermal growth factor receptor kinase substrate 8-like protein 3 OS=Homo sapiens GN=EPS8L3 PE=1 SV=2 |
|  | **P24752\|THIL_HUMAN** | Acetyl-CoA acetyltransferase, mitochondrial OS=Homo sapiens GN=ACAT1 PE=1 SV=1 |
|  | **P00441\|SODC_HUMAN** | Superoxide dismutase [Cu-Zn] OS=Homo sapiens GN=SOD1 PE=1 SV=2 |
|  | **P27487\|DPP4_HUMAN** | Dipeptidyl peptidase 4 OS=Homo sapiens GN=DPP4 PE=1 SV=2 |
|  | **P54920\|SNAA_HUMAN** | Alpha-soluble NSF attachment protein OS=Homo sapiens GN=NAPA PE=1 SV=3 |
|  | **P06748\|NPM_HUMAN** | Nucleophosmin OS=Homo sapiens GN=NPM1 PE=1 SV=2 |
|  | **Q8NBQ5\|DHB11_HUMAN** | Estradiol 17-beta-dehydrogenase 11 OS=Homo sapiens GN=HSD17B11 PE=1 SV=2 |
|  | **Q07021\|C1QBP_HUMAN** | Complement component 1 Q subcomponent-binding protein, mitochondrial OS=Homo sapiens GN=C1QBP PE=1 SV=1 |
|  | **P53597\|SUCA_HUMAN** | Succinyl-CoA ligase [GDP-forming] subunit alpha, mitochondrial OS=Homo sapiens GN=SUCLG1 PE=1 SV=4 |
|  | **P31150\|GDIA_HUMAN** | Rab GDP dissociation inhibitor alpha OS=Homo sapiens GN=GDI1 PE=1 SV=2 |
|  | **Q14914\|PTGR1_HUMAN** | Prostaglandin reductase 1 OS=Homo sapiens GN=PTGR1 PE=1 SV=2 |
|  | **P24539\|AT5F1_HUMAN** | ATP synthase subunit b, mitochondrial OS=Homo sapiens GN=ATP5F1 PE=1 SV=2 |
|  | **P12956\|KU70_HUMAN** | ATP-dependent DNA helicase 2 subunit 1 OS=Homo sapiens GN=XRCC6 PE=1 SV=2 |
|  | **Q93084\|AT2A3_HUMAN** | Sarcoplasmic/endoplasmic reticulum calcium ATPase 3 OS=Homo sapiens GN=ATP2A3 PE=1 SV=2 |
|  | **P04632\|CPNS1_HUMAN** | Calpain small subunit 1 OS=Homo sapiens GN=CAPNS1 PE=1 SV=1 |
|  | **P62241\|RS8_HUMAN** | 40S ribosomal protein S8 OS=Homo sapiens GN=RPS8 PE=1 SV=2 |
|  | **P01893\|HLAH_HUMAN** | Putative HLA class I histocompatibility antigen, alpha chain H OS=Homo sapiens GN=HLA-H PE=5 SV=2 |
|  | **P61224\|RAP1B_HUMAN** | Ras-related protein Rap-1b OS=Homo sapiens GN=RAP1B PE=1 SV=1 |
|  | **Q9UJS0\|CMC2_HUMAN** | Calcium-binding mitochondrial carrier protein Aralar2 OS=Homo sapiens GN=SLC25A13 PE=1 SV=2 |
|  | **P50225\|ST1A1_HUMAN** | Sulfotransferase 1A1 OS=Homo sapiens GN=SULT1A1 PE=1 SV=2 |
|  | **P50226\|ST1A2_HUMAN** | Sulfotransferase 1A2 OS=Homo sapiens GN=SULT1A2 PE=1 SV=1 |
|  | **P22314\|UBA1_HUMAN** | Ubiquitin-like modifier-activating enzyme 1 OS=Homo sapiens GN=UBA1 PE=1 SV=3 |
|  | **P46782\|RS5_HUMAN** | 40S ribosomal protein S5 OS=Homo sapiens GN=RPS5 PE=1 SV=4 |
|  | **P61313\|RL15_HUMAN** | 60S ribosomal protein L15 OS=Homo sapiens GN=RPL15 PE=1 SV=2 |
|  | **P09972\|ALDOC_HUMAN** | Fructose-bisphosphate aldolase C OS=Homo sapiens GN=ALDOC PE=1 SV=2 |
|  | **P07737\|PROF1_HUMAN** | Profilin-1 OS=Homo sapiens GN=PFN1 PE=1 SV=2 |
|  | **P05187\|PPB1_HUMAN** | Alkaline phosphatase, placental type OS=Homo sapiens GN=ALPP PE=1 SV=2 |
|  | **Q8NBX0\|SCPDH_HUMAN** | Probable saccharopine dehydrogenase OS=Homo sapiens GN=SCCPDH PE=1 SV=1 |
|  | **P17066\|HSP76_HUMAN** | Heat shock 70 kDa protein 6 OS=Homo sapiens GN=HSPA6 PE=1 SV=2 |
|  | **Q9BQS7\|HEPH_HUMAN** | Hephaestin OS=Homo sapiens GN=HEPH PE=2 SV=3 |
|  | **P18124\|RL7_HUMAN** | 60S ribosomal protein L7 OS=Homo sapiens GN=RPL7 PE=1 SV=1 |
|  | **Q07065\|CKAP4_HUMAN** | Cytoskeleton-associated protein 4 OS=Homo sapiens GN=CKAP4 PE=1 SV=2 |
|  | **P10321\|1C07_HUMAN** | HLA class I histocompatibility antigen, Cw-7 alpha chain OS=Homo sapiens GN=HLA-C PE=1 SV=3 |
|  | **Q31612\|1B73_HUMAN** | HLA class I histocompatibility antigen, B-73 alpha chain OS=Homo sapiens GN=HLA-B PE=1 SV=1 |
|  | **Q3ZCM7\|TBB8_HUMAN** | Tubulin beta-8 chain OS=Homo sapiens GN=TUBB8 PE=1 SV=2 |
|  | **P32969\|RL9_HUMAN** | 60S ribosomal protein L9 OS=Homo sapiens GN=RPL9 PE=1 SV=1 |
|  | **Q9UQQ1\|NALDL_HUMAN** | N-acetylated-alpha-linked acidic dipeptidase-like protein OS=Homo sapiens GN=NAALADL1 PE=2 SV=1 |
|  | **O15144\|ARPC2_HUMAN** | Actin-related protein 2/3 complex subunit 2 OS=Homo sapiens GN=ARPC2 PE=1 SV=1 |
|  | **P30486\|1B48_HUMAN** | HLA class I histocompatibility antigen, B-48 alpha chain OS=Homo sapiens GN=HLA-B PE=2 SV=1 |
|  | **P23396\|RS3_HUMAN** | 40S ribosomal protein S3 OS=Homo sapiens GN=RPS3 PE=1 SV=2 |
|  | **P37108\|SRP14_HUMAN** | Signal recognition particle 14 kDa protein OS=Homo sapiens GN=SRP14 PE=1 SV=2 |
|  | **P19338\|NUCL_HUMAN** | Nucleolin OS=Homo sapiens GN=NCL PE=1 SV=3 |
|  | **Q6ZN80\|MGAL1_HUMAN** | Putative maltase-glucoamylase-like protein FLJ16351 OS=Homo sapiens PE=2 SV=1 |
|  | **P20020\|AT2B1_HUMAN** | Plasma membrane calcium-transporting ATPase 1 OS=Homo sapiens GN=ATP2B1 PE=1 SV=3 |
|  | **P34913\|HYES_HUMAN** | Epoxide hydrolase 2 OS=Homo sapiens GN=EPHX2 PE=1 SV=2 |
|  | **Q9UJZ1\|STML2_HUMAN** | Stomatin-like protein 2 OS=Homo sapiens GN=STOML2 PE=1 SV=1 |
|  | **O75264\|YS019_HUMAN** | Transmembrane protein HSPC323 OS=Homo sapiens GN=HSPC323 PE=2 SV=2 |
|  | **Q99867\|TBB4Q_HUMAN** | Putative tubulin beta-4q chain OS=Homo sapiens GN=TUBB4Q PE=5 SV=1 |
|  | **Q8NHP1\|ARK74_HUMAN** | Aflatoxin B1 aldehyde reductase member 4 OS=Homo sapiens GN=AKR7L PE=2 SV=5 |
|  | **P84085\|ARF5_HUMAN** | ADP-ribosylation factor 5 OS=Homo sapiens GN=ARF5 PE=1 SV=2 |
|  | **P55795\|HNRH2_HUMAN** | Heterogeneous nuclear ribonucleoprotein H2 OS=Homo sapiens GN=HNRNPH2 PE=1 SV=1 |
|  | **P31943\|HNRH1_HUMAN** | Heterogeneous nuclear ribonucleoprotein H OS=Homo sapiens GN=HNRNPH1 PE=1 SV=4 |
|  | **P00558\|PGK1_HUMAN** | Phosphoglycerate kinase 1 OS=Homo sapiens GN=PGK1 PE=1 SV=3 |
|  | **P10599\|THIO_HUMAN** | Thioredoxin OS=Homo sapiens GN=TXN PE=1 SV=3 |
|  | **O95299\|NDUAA_HUMAN** | NADH dehydrogenase [ubiquinone] 1 alpha subcomplex subunit 10, mitochondrial OS=Homo sapiens GN=NDUFA10 PE=1 SV=1 |
|  | **P61019\|RAB2A_HUMAN** | Ras-related protein Rab-2A OS=Homo sapiens GN=RAB2A PE=1 SV=1 |
|  | **O15173\|PGRC2_HUMAN** | Membrane-associated progesterone receptor component 2 OS=Homo sapiens GN=PGRMC2 PE=1 SV=1 |
|  | **Q8WWA0\|ITLN1_HUMAN** | Intelectin-1 OS=Homo sapiens GN=ITLN1 PE=1 SV=1 |
|  | **Q9P0L0\|VAPA_HUMAN** | Vesicle-associated membrane protein-associated protein A OS=Homo sapiens GN=VAPA PE=1 SV=3 |
|  | **P63244\|GBLP_HUMAN** | Guanine nucleotide-binding protein subunit beta-2-like 1 OS=Homo sapiens GN=GNB2L1 PE=1 SV=3 |
|  | **Q13423\|NNTM_HUMAN** | NAD(P) transhydrogenase, mitochondrial OS=Homo sapiens GN=NNT PE=1 SV=3 |
|  | **P30040\|ERP29_HUMAN** | Endoplasmic reticulum protein ERp29 OS=Homo sapiens GN=ERP29 PE=1 SV=4 |
|  | **P33897\|ABCD1_HUMAN** | ATP-binding cassette sub-family D member 1 OS=Homo sapiens GN=ABCD1 PE=1 SV=2 |
|  | **Q92520\|FAM3C_HUMAN** | Protein FAM3C OS=Homo sapiens GN=FAM3C PE=1 SV=1 |
|  | **A6NIZ1\|RP1BL_HUMAN** | Ras-related protein Rap-1b-like protein OS=Homo sapiens PE=2 SV=1 |
|  | **P36537\|UDB10_HUMAN** | UDP-glucuronosyltransferase 2B10 OS=Homo sapiens GN=UGT2B10 PE=1 SV=1 |
|  | **P50148\|GNAQ_HUMAN** | Guanine nucleotide-binding protein G(q) subunit alpha OS=Homo sapiens GN=GNAQ PE=1 SV=4 |
|  | **Q04826\|1B40_HUMAN** | HLA class I histocompatibility antigen, B-40 alpha chain OS=Homo sapiens GN=HLA-B PE=1 SV=1 |
|  | **P30485\|1B47_HUMAN** | HLA class I histocompatibility antigen, B-47 alpha chain OS=Homo sapiens GN=HLA-B PE=2 SV=1 |
|  | **P58499\|FAM3B_HUMAN** | Protein FAM3B OS=Homo sapiens GN=FAM3B PE=1 SV=2 |
|  | **Q9TNN7\|1C05_HUMAN** | HLA class I histocompatibility antigen, Cw-5 alpha chain OS=Homo sapiens GN=HLA-C PE=2 SV=1 |
|  | **Q29963\|1C06_HUMAN** | HLA class I histocompatibility antigen, Cw-6 alpha chain OS=Homo sapiens GN=HLA-C PE=1 SV=2 |
|  | **Q07654\|TFF3_HUMAN** | Trefoil factor 3 OS=Homo sapiens GN=TFF3 PE=1 SV=1 |
|  | **Q13011\|ECH1_HUMAN** | Delta(3,5)-Delta(2,4)-dienoyl-CoA isomerase, mitochondrial OS=Homo sapiens GN=ECH1 PE=1 SV=2 |
|  | **O95169\|NDUB8_HUMAN** | NADH dehydrogenase [ubiquinone] 1 beta subcomplex subunit 8, mitochondrial OS=Homo sapiens GN=NDUFB8 PE=1 SV=1 |
|  | **Q9NQC3\|RTN4_HUMAN** | Reticulon-4 OS=Homo sapiens GN=RTN4 PE=1 SV=2 |
|  | **Q14103\|HNRPD_HUMAN** | Heterogeneous nuclear ribonucleoprotein D0 OS=Homo sapiens GN=HNRNPD PE=1 SV=1 |
|  | **Q9BUF5\|TBB6_HUMAN** | Tubulin beta-6 chain OS=Homo sapiens GN=TUBB6 PE=1 SV=1 |
|  | **P61513\|RL37A_HUMAN** | 60S ribosomal protein L37a OS=Homo sapiens GN=RPL37A PE=1 SV=2 |
|  | **Q13813\|SPTA2_HUMAN** | Spectrin alpha chain, brain OS=Homo sapiens GN=SPTAN1 PE=1 SV=3 |
|  | **Q8NHW5\|RLA0L_HUMAN** | 60S acidic ribosomal protein P0-like OS=Homo sapiens PE=2 SV=1 |
|  | **P05388\|RLA0_HUMAN** | 60S acidic ribosomal protein P0 OS=Homo sapiens GN=RPLP0 PE=1 SV=1 |
|  | **P54707\|AT12A_HUMAN** | Potassium-transporting ATPase alpha chain 2 OS=Homo sapiens GN=ATP12A PE=2 SV=3 |
|  | **P51149\|RAB7A_HUMAN** | Ras-related protein Rab-7a OS=Homo sapiens GN=RAB7A PE=1 SV=1 |
|  | **O75489\|NDUS3_HUMAN** | NADH dehydrogenase [ubiquinone] iron-sulfur protein 3, mitochondrial OS=Homo sapiens GN=NDUFS3 PE=1 SV=1 |
|  | **Q53GQ0\|DHB12_HUMAN** | Estradiol 17-beta-dehydrogenase 12 OS=Homo sapiens GN=HSD17B12 PE=1 SV=2 |
|  | **P30085\|KCY_HUMAN** | UMP-CMP kinase OS=Homo sapiens GN=CMPK1 PE=1 SV=3 |
|  | **O43488\|ARK72_HUMAN** | Aflatoxin B1 aldehyde reductase member 2 OS=Homo sapiens GN=AKR7A2 PE=1 SV=3 |
|  | **P30041\|PRDX6_HUMAN** | Peroxiredoxin-6 OS=Homo sapiens GN=PRDX6 PE=1 SV=3 |
|  | **Q02817\|MUC2_HUMAN** | Mucin-2 OS=Homo sapiens GN=MUC2 PE=1 SV=2 |
|  | **P30504\|1C04_HUMAN** | HLA class I histocompatibility antigen, Cw-4 alpha chain OS=Homo sapiens GN=HLA-C PE=1 SV=1 |
|  | **Q9HBB8\|MUCDL_HUMAN** | Mucin and cadherin-like protein OS=Homo sapiens GN=MUPCDH PE=1 SV=2 |
|  | **Q13576\|IQGA2_HUMAN** | Ras GTPase-activating-like protein IQGAP2 OS=Homo sapiens GN=IQGAP2 PE=1 SV=3 |
|  | **Q9NY59\|NSMA2_HUMAN** | Sphingomyelin phosphodiesterase 3 OS=Homo sapiens GN=SMPD3 PE=1 SV=1 |
|  | **Q5JPE7\|NOMO2_HUMAN** | Nodal modulator 2 OS=Homo sapiens GN=NOMO2 PE=1 SV=1 |
|  | **Q9HCY8\|S10AE_HUMAN** | Protein S100-A14 OS=Homo sapiens GN=S100A14 PE=1 SV=1 |
|  | **Q9Y6C9\|MTCH2_HUMAN** | Mitochondrial carrier homolog 2 OS=Homo sapiens GN=MTCH2 PE=1 SV=1 |
|  | **O75356\|ENTP5_HUMAN** | Ectonucleoside triphosphate diphosphohydrolase 5 OS=Homo sapiens GN=ENTPD5 PE=1 SV=1 |
|  | **A6NNZ2\|TBB8B_HUMAN** | Tubulin beta-8 chain B OS=Homo sapiens PE=1 SV=1 |
|  | **Q9HAV0\|GBB4_HUMAN** | Guanine nucleotide-binding protein subunit beta-4 OS=Homo sapiens GN=GNB4 PE=1 SV=3 |
|  | **Q9UBI6\|GBG12_HUMAN** | Guanine nucleotide-binding protein G(I)/G(S)/G(O) subunit gamma-12 OS=Homo sapiens GN=GNG12 PE=1 SV=3 |
|  | **P19404\|NDUV2_HUMAN** | NADH dehydrogenase [ubiquinone] flavoprotein 2, mitochondrial OS=Homo sapiens GN=NDUFV2 PE=1 SV=2 |
|  | **P23634\|AT2B4_HUMAN** | Plasma membrane calcium-transporting ATPase 4 OS=Homo sapiens GN=ATP2B4 PE=1 SV=2 |
|  | **P60842\|IF4A1_HUMAN** | Eukaryotic initiation factor 4A-I OS=Homo sapiens GN=EIF4A1 PE=1 SV=1 |
|  | **Q9Y2Q3\|GSTK1_HUMAN** | Glutathione S-transferase kappa 1 OS=Homo sapiens GN=GSTK1 PE=1 SV=3 |
|  | **Q01524\|DEF6_HUMAN** | Defensin-6 OS=Homo sapiens GN=DEFA6 PE=1 SV=1 |
|  | **O43772\|MCAT_HUMAN** | Mitochondrial carnitine/acylcarnitine carrier protein OS=Homo sapiens GN=SLC25A20 PE=1 SV=1 |
|  | **Q08426\|ECHP_HUMAN** | Peroxisomal bifunctional enzyme OS=Homo sapiens GN=EHHADH PE=1 SV=3 |
|  | **P00395\|COX1_HUMAN** | Cytochrome c oxidase subunit 1 OS=Homo sapiens GN=MT-CO1 PE=1 SV=1 |
|  | **Q96GC9\|TMM49_HUMAN** | Transmembrane protein 49 OS=Homo sapiens GN=TMEM49 PE=2 SV=1 |
|  | **Q9UBS4\|DJB11_HUMAN** | DnaJ homolog subfamily B member 11 OS=Homo sapiens GN=DNAJB11 PE=1 SV=1 |
|  | **Q09160\|1A80_HUMAN** | HLA class I histocompatibility antigen, A-80 alpha chain OS=Homo sapiens GN=HLA-A PE=2 SV=1 |
|  | **P37802\|TAGL2_HUMAN** | Transgelin-2 OS=Homo sapiens GN=TAGLN2 PE=1 SV=3 |
|  | **P05787\|K2C8_HUMAN** | Keratin, type II cytoskeletal 8 OS=Homo sapiens GN=KRT8 PE=1 SV=7 |
|  | **P01903\|2DRA_HUMAN** | HLA class II histocompatibility antigen, DR alpha chain OS=Homo sapiens GN=HLA-DRA PE=1 SV=1 |
|  | **Q06830\|PRDX1_HUMAN** | Peroxiredoxin-1 OS=Homo sapiens GN=PRDX1 PE=1 SV=1 |
|  | **Q8N5K1\|CISD2_HUMAN** | CDGSH iron sulfur domain-containing protein 2 OS=Homo sapiens GN=CISD2 PE=1 SV=1 |
|  | **P53675\|CLH2_HUMAN** | Clathrin heavy chain 2 OS=Homo sapiens GN=CLTCL1 PE=1 SV=2 |
|  | **Q9NS69\|TOM22_HUMAN** | Mitochondrial import receptor subunit TOM22 homolog OS=Homo sapiens GN=TOMM22 PE=1 SV=3 |
|  | **Q9H8H3\|MET7A_HUMAN** | Methyltransferase-like protein 7A OS=Homo sapiens GN=METTL7A PE=1 SV=1 |
|  | **P30461\|1B13_HUMAN** | HLA class I histocompatibility antigen, B-13 alpha chain OS=Homo sapiens GN=HLA-B PE=2 SV=1 |
|  | **P30466\|1B18_HUMAN** | HLA class I histocompatibility antigen, B-18 alpha chain OS=Homo sapiens GN=HLA-B PE=1 SV=1 |
|  | **P18463\|1B37_HUMAN** | HLA class I histocompatibility antigen, B-37 alpha chain OS=Homo sapiens GN=HLA-B PE=2 SV=1 |
|  | **P30475\|1B39_HUMAN** | HLA class I histocompatibility antigen, B-39 alpha chain OS=Homo sapiens GN=HLA-B PE=2 SV=1 |
|  | **P62277\|RS13_HUMAN** | 40S ribosomal protein S13 OS=Homo sapiens GN=RPS13 PE=1 SV=2 |
|  | **Q07020\|RL18_HUMAN** | 60S ribosomal protein L18 OS=Homo sapiens GN=RPL18 PE=1 SV=2 |
|  | **Q9Y6U3\|ADSV_HUMAN** | Adseverin OS=Homo sapiens GN=SCIN PE=1 SV=4 |
|  | **P61604\|CH10_HUMAN** | 10 kDa heat shock protein, mitochondrial OS=Homo sapiens GN=HSPE1 PE=1 SV=2 |
|  | **P15880\|RS2_HUMAN** | 40S ribosomal protein S2 OS=Homo sapiens GN=RPS2 PE=1 SV=2 |
|  | **O14880\|MGST3_HUMAN** | Microsomal glutathione S-transferase 3 OS=Homo sapiens GN=MGST3 PE=1 SV=1 |
|  | **P50990\|TCPQ_HUMAN** | T-complex protein 1 subunit theta OS=Homo sapiens GN=CCT8 PE=1 SV=4 |
|  | **P28070\|PSB4_HUMAN** | Proteasome subunit beta type-4 OS=Homo sapiens GN=PSMB4 PE=1 SV=4 |
|  | **P16144\|ITB4_HUMAN** | Integrin beta-4 OS=Homo sapiens GN=ITGB4 PE=1 SV=4 |
|  | **P17174\|AATC_HUMAN** | Aspartate aminotransferase, cytoplasmic OS=Homo sapiens GN=GOT1 PE=1 SV=3 |
|  | **P62745\|RHOB_HUMAN** | Rho-related GTP-binding protein RhoB OS=Homo sapiens GN=RHOB PE=1 SV=1 |
|  | **P61981\|1433G_HUMAN** | 14-3-3 protein gamma OS=Homo sapiens GN=YWHAG PE=1 SV=2 |
|  | **O14653\|GOSR2_HUMAN** | Golgi SNAP receptor complex member 2 OS=Homo sapiens GN=GOSR2 PE=1 SV=2 |
|  | **O95831\|AIFM1_HUMAN** | Apoptosis-inducing factor 1, mitochondrial OS=Homo sapiens GN=AIFM1 PE=1 SV=1 |
|  | **Q16706\|MA2A1_HUMAN** | Alpha-mannosidase 2 OS=Homo sapiens GN=MAN2A1 PE=1 SV=2 |
|  | **P61163\|ACTZ_HUMAN** | Alpha-centractin OS=Homo sapiens GN=ACTR1A PE=1 SV=1 |
|  | **Q9HAW7\|UD17_HUMAN** | UDP-glucuronosyltransferase 1-7 OS=Homo sapiens GN=UGT1A7 PE=1 SV=2 |
|  | **Q9HAW9\|UD18_HUMAN** | UDP-glucuronosyltransferase 1-8 OS=Homo sapiens GN=UGT1A8 PE=1 SV=1 |
|  | **Q9HAW8\|UD110_HUMAN** | UDP-glucuronosyltransferase 1-10 OS=Homo sapiens GN=UGT1A10 PE=1 SV=1 |
|  | **Q14240\|IF4A2_HUMAN** | Eukaryotic initiation factor 4A-II OS=Homo sapiens GN=EIF4A2 PE=1 SV=2 |
|  | **Q02338\|BDH_HUMAN** | D-beta-hydroxybutyrate dehydrogenase, mitochondrial OS=Homo sapiens GN=BDH1 PE=1 SV=3 |
|  | **O43837\|IDH3B_HUMAN** | Isocitrate dehydrogenase [NAD] subunit beta, mitochondrial OS=Homo sapiens GN=IDH3B PE=1 SV=2 |
|  | **P46776\|RL27A_HUMAN** | 60S ribosomal protein L27a OS=Homo sapiens GN=RPL27A PE=1 SV=2 |
|  | **Q15165\|PON2_HUMAN** | Serum paraoxonase/arylesterase 2 OS=Homo sapiens GN=PON2 PE=1 SV=2 |
|  | **O95716\|RAB3D_HUMAN** | Ras-related protein Rab-3D OS=Homo sapiens GN=RAB3D PE=1 SV=1 |
|  | **Q96E17\|RAB3C_HUMAN** | Ras-related protein Rab-3C OS=Homo sapiens GN=RAB3C PE=2 SV=1 |
|  | **P20336\|RAB3A_HUMAN** | Ras-related protein Rab-3A OS=Homo sapiens GN=RAB3A PE=1 SV=1 |
|  | **P20337\|RAB3B_HUMAN** | Ras-related protein Rab-3B OS=Homo sapiens GN=RAB3B PE=1 SV=2 |
|  | **P61106\|RAB14_HUMAN** | Ras-related protein Rab-14 OS=Homo sapiens GN=RAB14 PE=1 SV=4 |
|  | **Q16698\|DECR_HUMAN** | 2,4-dienoyl-CoA reductase, mitochondrial OS=Homo sapiens GN=DECR1 PE=1 SV=1 |
|  | **Q96AG4\|LRC59_HUMAN** | Leucine-rich repeat-containing protein 59 OS=Homo sapiens GN=LRRC59 PE=1 SV=1 |
|  | **P48735\|IDHP_HUMAN** | Isocitrate dehydrogenase [NADP], mitochondrial OS=Homo sapiens GN=IDH2 PE=1 SV=2 |
|  | **P48643\|TCPE_HUMAN** | T-complex protein 1 subunit epsilon OS=Homo sapiens GN=CCT5 PE=1 SV=1 |
|  | **P35606\|COPB2_HUMAN** | Coatomer subunit beta' OS=Homo sapiens GN=COPB2 PE=1 SV=2 |
|  | **Q9H2U2\|IPYR2_HUMAN** | Inorganic pyrophosphatase 2, mitochondrial OS=Homo sapiens GN=PPA2 PE=1 SV=2 |
|  | **Q16795\|NDUA9_HUMAN** | NADH dehydrogenase [ubiquinone] 1 alpha subcomplex subunit 9, mitochondrial OS=Homo sapiens GN=NDUFA9 PE=1 SV=2 |
|  | **Q9NW15\|ANO10_HUMAN** | Anoctamin-10 OS=Homo sapiens GN=ANO10 PE=2 SV=2 |
|  | **Q15043\|S39AE_HUMAN** | Zinc transporter ZIP14 OS=Homo sapiens GN=SLC39A14 PE=1 SV=2 |
|  | **O00151\|PDLI1_HUMAN** | PDZ and LIM domain protein 1 OS=Homo sapiens GN=PDLIM1 PE=1 SV=4 |
|  | **Q9BWM7\|SFXN3_HUMAN** | Sideroflexin-3 OS=Homo sapiens GN=SFXN3 PE=2 SV=1 |
|  | **Q02318\|CP27A_HUMAN** | Sterol 26-hydroxylase, mitochondrial OS=Homo sapiens GN=CYP27A1 PE=1 SV=1 |
|  | **P52815\|RM12_HUMAN** | 39S ribosomal protein L12, mitochondrial OS=Homo sapiens GN=MRPL12 PE=1 SV=2 |
|  | **Q9Y4W6\|AFG32_HUMAN** | AFG3-like protein 2 OS=Homo sapiens GN=AFG3L2 PE=1 SV=2 |
|  | **Q9C002\|NMES1_HUMAN** | Normal mucosa of esophagus-specific gene 1 protein OS=Homo sapiens GN=NMES1 PE=2 SV=1 |
|  | **Q9BX93\|PG12B_HUMAN** | Group XIIB secretory phospholipase A2-like protein OS=Homo sapiens GN=PLA2G12B PE=1 SV=2 |
|  | **O75477\|ERLN1_HUMAN** | Erlin-1 OS=Homo sapiens GN=ERLIN1 PE=1 SV=1 |
|  | **P18669\|PGAM1_HUMAN** | Phosphoglycerate mutase 1 OS=Homo sapiens GN=PGAM1 PE=1 SV=2 |
|  | **P52565\|GDIR1_HUMAN** | Rho GDP-dissociation inhibitor 1 OS=Homo sapiens GN=ARHGDIA PE=1 SV=3 |
|  | **O95292\|VAPB_HUMAN** | Vesicle-associated membrane protein-associated protein B/C OS=Homo sapiens GN=VAPB PE=1 SV=3 |
|  | **O00161\|SNP23_HUMAN** | Synaptosomal-associated protein 23 OS=Homo sapiens GN=SNAP23 PE=1 SV=1 |
|  | **P35613\|BASI_HUMAN** | Basigin OS=Homo sapiens GN=BSG PE=1 SV=2 |
|  | **Q96K78\|GP128_HUMAN** | Probable G-protein coupled receptor 128 OS=Homo sapiens GN=GPR128 PE=1 SV=2 |
|  | **Q14156\|EFR3A_HUMAN** | Protein EFR3 homolog A OS=Homo sapiens GN=EFR3A PE=1 SV=2 |
|  | **P61006\|RAB8A_HUMAN** | Ras-related protein Rab-8A OS=Homo sapiens GN=RAB8A PE=1 SV=1 |
|  | **Q9Y536\|PAL4B_HUMAN** | Peptidylprolyl cis-trans isomerase A-like 4B OS=Homo sapiens GN=PPIAL4B PE=1 SV=1 |
|  | **O43795\|MYO1B_HUMAN** | Myosin-Ib OS=Homo sapiens GN=MYO1B PE=1 SV=3 |
|  | **P20340\|RAB6A_HUMAN** | Ras-related protein Rab-6A OS=Homo sapiens GN=RAB6A PE=1 SV=3 |
|  | **P12955\|PEPD_HUMAN** | Xaa-Pro dipeptidase OS=Homo sapiens GN=PEPD PE=1 SV=3 |
|  | **Q6ZMR3\|LDH6A_HUMAN** | L-lactate dehydrogenase A-like 6A OS=Homo sapiens GN=LDHAL6A PE=2 SV=1 |
|  | **P07195\|LDHB_HUMAN** | L-lactate dehydrogenase B chain OS=Homo sapiens GN=LDHB PE=1 SV=2 |
|  | **P07864\|LDHC_HUMAN** | L-lactate dehydrogenase C chain OS=Homo sapiens GN=LDHC PE=2 SV=4 |
|  | **Q9UBM7\|DHCR7_HUMAN** | 7-dehydrocholesterol reductase OS=Homo sapiens GN=DHCR7 PE=1 SV=1 |
|  | **P51148\|RAB5C_HUMAN** | Ras-related protein Rab-5C OS=Homo sapiens GN=RAB5C PE=1 SV=2 |
|  | **P61769\|B2MG_HUMAN** | Beta-2-microglobulin OS=Homo sapiens GN=B2M PE=1 SV=1 |
|  | **Q99943\|PLCA_HUMAN** | 1-acyl-sn-glycerol-3-phosphate acyltransferase alpha OS=Homo sapiens GN=AGPAT1 PE=2 SV=2 |
|  | **Q9UNL2\|SSRG_HUMAN** | Translocon-associated protein subunit gamma OS=Homo sapiens GN=SSR3 PE=1 SV=1 |
|  | **Q9Y281\|COF2_HUMAN** | Cofilin-2 OS=Homo sapiens GN=CFL2 PE=1 SV=1 |
|  | **P00505\|AATM_HUMAN** | Aspartate aminotransferase, mitochondrial OS=Homo sapiens GN=GOT2 PE=1 SV=2 |
|  | **P13797\|PLST_HUMAN** | Plastin-3 OS=Homo sapiens GN=PLS3 PE=1 SV=4 |
|  | **Q9NY84\|VNN3_HUMAN** | Vascular non-inflammatory molecule 3 OS=Homo sapiens GN=VNN3 PE=2 SV=2 |
|  | **Q9UHB6\|LIMA1_HUMAN** | LIM domain and actin-binding protein 1 OS=Homo sapiens GN=LIMA1 PE=1 SV=1 |
|  | **Q96FQ6\|S10AG_HUMAN** | Protein S100-A16 OS=Homo sapiens GN=S100A16 PE=1 SV=1 |
|  | **Q58FG0\|HS905_HUMAN** | Putative heat shock protein HSP 90-alpha A5 OS=Homo sapiens GN=HSP90AA5P PE=1 SV=1 |
|  | **Q3ZCQ8\|TIM50_HUMAN** | Mitochondrial import inner membrane translocase subunit TIM50 OS=Homo sapiens GN=TIMM50 PE=1 SV=2 |
|  | **P62829\|RL23_HUMAN** | 60S ribosomal protein L23 OS=Homo sapiens GN=RPL23 PE=1 SV=1 |
|  | **P54819\|KAD2_HUMAN** | Adenylate kinase 2, mitochondrial OS=Homo sapiens GN=AK2 PE=1 SV=2 |
|  | **Q9Y394\|DHRS7_HUMAN** | Dehydrogenase/reductase SDR family member 7 OS=Homo sapiens GN=DHRS7 PE=1 SV=1 |
|  | **P30501\|1C02_HUMAN** | HLA class I histocompatibility antigen, Cw-2 alpha chain OS=Homo sapiens GN=HLA-C PE=1 SV=1 |
|  | **Q06323\|PSME1_HUMAN** | Proteasome activator complex subunit 1 OS=Homo sapiens GN=PSME1 PE=1 SV=1 |
|  | **Q8WY22\|BRI3B_HUMAN** | BRI3-binding protein OS=Homo sapiens GN=BRI3BP PE=1 SV=1 |
|  | **Q92841\|DDX17_HUMAN** | Probable ATP-dependent RNA helicase DDX17 OS=Homo sapiens GN=DDX17 PE=1 SV=1 |
|  | **P21399\|ACOC_HUMAN** | Cytoplasmic aconitate hydratase OS=Homo sapiens GN=ACO1 PE=1 SV=3 |
|  | **Q9BWS9\|CHID1_HUMAN** | Chitinase domain-containing protein 1 OS=Homo sapiens GN=CHID1 PE=1 SV=1 |
|  | **Q9HCS2\|CP4FC_HUMAN** | Cytochrome P450 4F12 OS=Homo sapiens GN=CYP4F12 PE=1 SV=1 |
|  | **Q9HBI6\|CP4FB_HUMAN** | Cytochrome P450 4F11 OS=Homo sapiens GN=CYP4F11 PE=2 SV=2 |
|  | **Q96AB3\|ISOC2_HUMAN** | Isochorismatase domain-containing protein 2, mitochondrial OS=Homo sapiens GN=ISOC2 PE=1 SV=1 |
|  | **P04899\|GNAI2_HUMAN** | Guanine nucleotide-binding protein G(i), alpha-2 subunit OS=Homo sapiens GN=GNAI2 PE=1 SV=3 |
|  | **O95342\|ABCBB_HUMAN** | Bile salt export pump OS=Homo sapiens GN=ABCB11 PE=1 SV=1 |
|  | **P84098\|RL19_HUMAN** | 60S ribosomal protein L19 OS=Homo sapiens GN=RPL19 PE=1 SV=1 |
|  | **Q9UDW1\|QCR9_HUMAN** | Cytochrome b-c1 complex subunit 9 OS=Homo sapiens GN=UQCR10 PE=1 SV=3 |
|  | **P46781\|RS9_HUMAN** | 40S ribosomal protein S9 OS=Homo sapiens GN=RPS9 PE=1 SV=3 |
|  | **P55327\|TPD52_HUMAN** | Tumor protein D52 OS=Homo sapiens GN=TPD52 PE=1 SV=2 |
|  | **O60361\|NDK8_HUMAN** | Putative nucleoside diphosphate kinase OS=Homo sapiens GN=NME2P1 PE=5 SV=1 |
|  | **P62330\|ARF6_HUMAN** | ADP-ribosylation factor 6 OS=Homo sapiens GN=ARF6 PE=1 SV=2 |
|  | **P07910\|HNRPC_HUMAN** | Heterogeneous nuclear ribonucleoproteins C1/C2 OS=Homo sapiens GN=HNRNPC PE=1 SV=4 |
|  | **O60812\|HNRCL_HUMAN** | Heterogeneous nuclear ribonucleoprotein C-like 1 OS=Homo sapiens GN=HNRNPCL1 PE=1 SV=1 |
|  | **P50213\|IDH3A_HUMAN** | Isocitrate dehydrogenase [NAD] subunit alpha, mitochondrial OS=Homo sapiens GN=IDH3A PE=1 SV=1 |
|  | **O95168\|NDUB4_HUMAN** | NADH dehydrogenase [ubiquinone] 1 beta subcomplex subunit 4 OS=Homo sapiens GN=NDUFB4 PE=1 SV=3 |
|  | **P00480\|OTC_HUMAN** | Ornithine carbamoyltransferase, mitochondrial OS=Homo sapiens GN=OTC PE=1 SV=3 |
|  | **P09651\|ROA1_HUMAN** | Heterogeneous nuclear ribonucleoprotein A1 OS=Homo sapiens GN=HNRNPA1 PE=1 SV=4 |
|  | **Q13162\|PRDX4_HUMAN** | Peroxiredoxin-4 OS=Homo sapiens GN=PRDX4 PE=1 SV=1 |
|  | **P62847\|RS24_HUMAN** | 40S ribosomal protein S24 OS=Homo sapiens GN=RPS24 PE=1 SV=1 |
|  | **P10768\|ESTD_HUMAN** | S-formylglutathione hydrolase OS=Homo sapiens GN=ESD PE=1 SV=2 |
|  | **P48230\|T4S4_HUMAN** | Transmembrane 4 L6 family member 4 OS=Homo sapiens GN=TM4SF4 PE=1 SV=1 |
|  | **P00367\|DHE3_HUMAN** | Glutamate dehydrogenase 1, mitochondrial OS=Homo sapiens GN=GLUD1 PE=1 SV=2 |
|  | **P35754\|GLRX1_HUMAN** | Glutaredoxin-1 OS=Homo sapiens GN=GLRX PE=1 SV=2 |
|  | **Q08477\|CP4F3_HUMAN** | Leukotriene-B(4) omega-hydroxylase 2 OS=Homo sapiens GN=CYP4F3 PE=2 SV=2 |
|  | **Q10567\|AP1B1_HUMAN** | AP-1 complex subunit beta-1 OS=Homo sapiens GN=AP1B1 PE=1 SV=1 |
|  | **Q07837\|SLC31_HUMAN** | Neutral and basic amino acid transport protein rBAT OS=Homo sapiens GN=SLC3A1 PE=1 SV=2 |
|  | **Q9H8Y8\|GORS2_HUMAN** | Golgi reassembly-stacking protein 2 OS=Homo sapiens GN=GORASP2 PE=1 SV=3 |
|  | **Q9NZ45\|CISD1_HUMAN** | CDGSH iron sulfur domain-containing protein 1 OS=Homo sapiens GN=CISD1 PE=1 SV=1 |
|  | **P20711\|DDC_HUMAN** | Aromatic-L-amino-acid decarboxylase OS=Homo sapiens GN=DDC PE=1 SV=1 |
|  | **Q9HDC9\|APMAP_HUMAN** | Adipocyte plasma membrane-associated protein OS=Homo sapiens GN=APMAP PE=1 SV=2 |
|  | **P35609\|ACTN2_HUMAN** | Alpha-actinin-2 OS=Homo sapiens GN=ACTN2 PE=1 SV=1 |
|  | **Q9H0C2\|ADT4_HUMAN** | ADP/ATP translocase 4 OS=Homo sapiens GN=SLC25A31 PE=1 SV=1 |
|  | **Q7Z7H5\|TMED4_HUMAN** | Transmembrane emp24 domain-containing protein 4 OS=Homo sapiens GN=TMED4 PE=1 SV=1 |
|  | **P78329\|CP4F2_HUMAN** | Leukotriene-B(4) omega-hydroxylase 1 OS=Homo sapiens GN=CYP4F2 PE=1 SV=1 |
|  | **P09601\|HMOX1_HUMAN** | Heme oxygenase 1 OS=Homo sapiens GN=HMOX1 PE=1 SV=1 |
|  | **A6NHG4\|DDTL_HUMAN** | D-dopachrome decarboxylase-like protein OS=Homo sapiens GN=DDTL PE=2 SV=1 |
|  | **Q9NZ01\|TECR_HUMAN** | Trans-2,3-enoyl-CoA reductase OS=Homo sapiens GN=TECR PE=1 SV=1 |
|  | **P10606\|COX5B_HUMAN** | Cytochrome c oxidase subunit 5B, mitochondrial OS=Homo sapiens GN=COX5B PE=1 SV=2 |
|  | **Q13183\|S13A2_HUMAN** | Solute carrier family 13 member 2 OS=Homo sapiens GN=SLC13A2 PE=2 SV=1 |
|  | **O94905\|ERLN2_HUMAN** | Erlin-2 OS=Homo sapiens GN=ERLIN2 PE=1 SV=1 |
|  | **P35241\|RADI_HUMAN** | Radixin OS=Homo sapiens GN=RDX PE=1 SV=1 |
|  | **Q08043\|ACTN3_HUMAN** | Alpha-actinin-3 OS=Homo sapiens GN=ACTN3 PE=1 SV=2 |
|  | **O15258\|RER1_HUMAN** | Protein RER1 OS=Homo sapiens GN=RER1 PE=1 SV=1 |
|  | **P49257\|LMAN1_HUMAN** | Protein ERGIC-53 OS=Homo sapiens GN=LMAN1 PE=1 SV=2 |
|  | **Q96DG6\|CMBL_HUMAN** | Carboxymethylenebutenolidase homolog OS=Homo sapiens GN=CMBL PE=1 SV=1 |
|  | **P23786\|CPT2_HUMAN** | Carnitine O-palmitoyltransferase 2, mitochondrial OS=Homo sapiens GN=CPT2 PE=1 SV=2 |
|  | **Q8NBS9\|TXND5_HUMAN** | Thioredoxin domain-containing protein 5 OS=Homo sapiens GN=TXNDC5 PE=1 SV=2 |
|  | **Q15437\|SC23B_HUMAN** | Protein transport protein Sec23B OS=Homo sapiens GN=SEC23B PE=1 SV=2 |
|  | **Q8IVT2\|CS021_HUMAN** | Uncharacterized protein C19orf21 OS=Homo sapiens GN=C19orf21 PE=1 SV=1 |
|  | **P26885\|FKBP2_HUMAN** | FK506-binding protein 2 OS=Homo sapiens GN=FKBP2 PE=1 SV=2 |
|  | **O15127\|SCAM2_HUMAN** | Secretory carrier-associated membrane protein 2 OS=Homo sapiens GN=SCAMP2 PE=1 SV=2 |
|  | **O75390\|CISY_HUMAN** | Citrate synthase, mitochondrial OS=Homo sapiens GN=CS PE=1 SV=2 |
|  | **P47914\|RL29_HUMAN** | 60S ribosomal protein L29 OS=Homo sapiens GN=RPL29 PE=1 SV=2 |
|  | **P35268\|RL22_HUMAN** | 60S ribosomal protein L22 OS=Homo sapiens GN=RPL22 PE=1 SV=2 |
|  | **P43304\|GPDM_HUMAN** | Glycerol-3-phosphate dehydrogenase, mitochondrial OS=Homo sapiens GN=GPD2 PE=1 SV=3 |
|  | **P20815\|CP3A5_HUMAN** | Cytochrome P450 3A5 OS=Homo sapiens GN=CYP3A5 PE=1 SV=1 |
|  | **P35503\|UD13_HUMAN** | UDP-glucuronosyltransferase 1-3 OS=Homo sapiens GN=UGT1A3 PE=1 SV=1 |
|  | **P35504\|UD15_HUMAN** | UDP-glucuronosyltransferase 1-5 OS=Homo sapiens GN=UGT1A5 PE=1 SV=1 |
|  | **P19224\|UD16_HUMAN** | UDP-glucuronosyltransferase 1-6 OS=Homo sapiens GN=UGT1A6 PE=1 SV=2 |
|  | **P22310\|UD14_HUMAN** | UDP-glucuronosyltransferase 1-4 OS=Homo sapiens GN=UGT1A4 PE=1 SV=1 |
|  | **O60656\|UD19_HUMAN** | UDP-glucuronosyltransferase 1-9 OS=Homo sapiens GN=UGT1A9 PE=1 SV=1 |
|  | **P28066\|PSA5_HUMAN** | Proteasome subunit alpha type-5 OS=Homo sapiens GN=PSMA5 PE=1 SV=3 |
|  | **O14672\|ADA10_HUMAN** | Disintegrin and metalloproteinase domain-containing protein 10 OS=Homo sapiens GN=ADAM10 PE=1 SV=1 |
|  | **P10696\|PPBN_HUMAN** | Alkaline phosphatase, placental-like OS=Homo sapiens GN=ALPPL2 PE=1 SV=4 |
|  | **P62899\|RL31_HUMAN** | 60S ribosomal protein L31 OS=Homo sapiens GN=RPL31 PE=1 SV=1 |
|  | **Q99714\|HCD2_HUMAN** | 3-hydroxyacyl-CoA dehydrogenase type-2 OS=Homo sapiens GN=HSD17B10 PE=1 SV=3 |
|  | **P15531\|NDKA_HUMAN** | Nucleoside diphosphate kinase A OS=Homo sapiens GN=NME1 PE=1 SV=1 |
|  | **P80404\|GABT_HUMAN** | 4-aminobutyrate aminotransferase, mitochondrial OS=Homo sapiens GN=ABAT PE=1 SV=3 |
|  | **Q969Z3\|MOSC2_HUMAN** | MOSC domain-containing protein 2, mitochondrial OS=Homo sapiens GN=MOSC2 PE=1 SV=1 |
|  | **Q1KMD3\|HNRL2_HUMAN** | Heterogeneous nuclear ribonucleoprotein U-like protein 2 OS=Homo sapiens GN=HNRNPUL2 PE=1 SV=1 |
|  | **P26038\|MOES_HUMAN** | Moesin OS=Homo sapiens GN=MSN PE=1 SV=3 |
|  | **P62280\|RS11_HUMAN** | 40S ribosomal protein S11 OS=Homo sapiens GN=RPS11 PE=1 SV=3 |
|  | **P55011\|S12A2_HUMAN** | Solute carrier family 12 member 2 OS=Homo sapiens GN=SLC12A2 PE=1 SV=1 |
|  | **P16401\|H15_HUMAN** | Histone H1.5 OS=Homo sapiens GN=HIST1H1B PE=1 SV=3 |
|  | **Q9BV23\|ABHD6_HUMAN** | Monoacylglycerol lipase ABHD6 OS=Homo sapiens GN=ABHD6 PE=2 SV=1 |
|  | **O60701\|UGDH_HUMAN** | UDP-glucose 6-dehydrogenase OS=Homo sapiens GN=UGDH PE=1 SV=1 |
|  | **P13073\|COX41_HUMAN** | Cytochrome c oxidase subunit 4 isoform 1, mitochondrial OS=Homo sapiens GN=COX4I1 PE=1 SV=1 |
|  | **P51571\|SSRD_HUMAN** | Translocon-associated protein subunit delta OS=Homo sapiens GN=SSR4 PE=1 SV=1 |
|  | **Q9Y5M8\|SRPRB_HUMAN** | Signal recognition particle receptor subunit beta OS=Homo sapiens GN=SRPRB PE=1 SV=3 |
|  | **Q07954\|LRP1_HUMAN** | Prolow-density lipoprotein receptor-related protein 1 OS=Homo sapiens GN=LRP1 PE=1 SV=1 |
|  | **Q86Y39\|NDUAB_HUMAN** | NADH dehydrogenase [ubiquinone] 1 alpha subcomplex subunit 11 OS=Homo sapiens GN=NDUFA11 PE=1 SV=3 |
|  | **Q12931\|TRAP1_HUMAN** | Heat shock protein 75 kDa, mitochondrial OS=Homo sapiens GN=TRAP1 PE=1 SV=3 |
|  | **P01842\|LAC_HUMAN** | Ig lambda chain C regions OS=Homo sapiens GN=IGLC1 PE=1 SV=1 |
|  | **P61158\|ARP3_HUMAN** | Actin-related protein 3 OS=Homo sapiens GN=ACTR3 PE=1 SV=3 |
|  | **Q9UKU0\|ACSL6_HUMAN** | Long-chain-fatty-acid--CoA ligase 6 OS=Homo sapiens GN=ACSL6 PE=2 SV=4 |
|  | **O95182\|NDUA7_HUMAN** | NADH dehydrogenase [ubiquinone] 1 alpha subcomplex subunit 7 OS=Homo sapiens GN=NDUFA7 PE=1 SV=3 |
|  | **P05556\|ITB1_HUMAN** | Integrin beta-1 OS=Homo sapiens GN=ITGB1 PE=1 SV=2 |
|  | **O95881\|TXD12_HUMAN** | Thioredoxin domain-containing protein 12 OS=Homo sapiens GN=TXNDC12 PE=1 SV=1 |
|  | **O95470\|SGPL1_HUMAN** | Sphingosine-1-phosphate lyase 1 OS=Homo sapiens GN=SGPL1 PE=1 SV=3 |
|  | **Q02878\|RL6_HUMAN** | 60S ribosomal protein L6 OS=Homo sapiens GN=RPL6 PE=1 SV=3 |
|  | **O94855\|SC24D_HUMAN** | Protein transport protein Sec24D OS=Homo sapiens GN=SEC24D PE=1 SV=2 |
|  | **Q9H853\|TBA4B_HUMAN** | Putative tubulin-like protein alpha-4B OS=Homo sapiens GN=TUBA4B PE=5 SV=2 |
|  | **Q15181\|IPYR_HUMAN** | Inorganic pyrophosphatase OS=Homo sapiens GN=PPA1 PE=1 SV=2 |
|  | **P56556\|NDUA6_HUMAN** | NADH dehydrogenase [ubiquinone] 1 alpha subcomplex subunit 6 OS=Homo sapiens GN=NDUFA6 PE=1 SV=2 |
|  | **P61026\|RAB10_HUMAN** | Ras-related protein Rab-10 OS=Homo sapiens GN=RAB10 PE=1 SV=1 |
|  | **Q92930\|RAB8B_HUMAN** | Ras-related protein Rab-8B OS=Homo sapiens GN=RAB8B PE=1 SV=2 |
|  | **Q15286\|RAB35_HUMAN** | Ras-related protein Rab-35 OS=Homo sapiens GN=RAB35 PE=1 SV=1 |
|  | **Q9H082\|RB33B_HUMAN** | Ras-related protein Rab-33B OS=Homo sapiens GN=RAB33B PE=1 SV=1 |
|  | **Q6IQ22\|RAB12_HUMAN** | Putative Ras-related protein Rab-12 OS=Homo sapiens GN=RAB12 PE=5 SV=3 |
|  | **Q14964\|RB39A_HUMAN** | Ras-related protein Rab-39A OS=Homo sapiens GN=RAB39 PE=2 SV=2 |
|  | **Q96AX2\|RAB37_HUMAN** | Ras-related protein Rab-37 OS=Homo sapiens GN=RAB37 PE=1 SV=3 |
|  | **P61018\|RAB4B_HUMAN** | Ras-related protein Rab-4B OS=Homo sapiens GN=RAB4B PE=1 SV=1 |
|  | **Q9NRW1\|RAB6B_HUMAN** | Ras-related protein Rab-6B OS=Homo sapiens GN=RAB6B PE=1 SV=1 |
|  | **P59190\|RAB15_HUMAN** | Ras-related protein Rab-15 OS=Homo sapiens GN=RAB15 PE=1 SV=1 |
|  | **Q86YS6\|RAB43_HUMAN** | Ras-related protein Rab-43 OS=Homo sapiens GN=RAB43 PE=1 SV=1 |
|  | **Q96DA2\|RB39B_HUMAN** | Ras-related protein Rab-39B OS=Homo sapiens GN=RAB39B PE=1 SV=1 |
|  | **P20338\|RAB4A_HUMAN** | Ras-related protein Rab-4A OS=Homo sapiens GN=RAB4A PE=1 SV=2 |
|  | **Q15771\|RAB30_HUMAN** | Ras-related protein Rab-30 OS=Homo sapiens GN=RAB30 PE=1 SV=2 |
|  | **P61421\|VA0D1_HUMAN** | V-type proton ATPase subunit d 1 OS=Homo sapiens GN=ATP6V0D1 PE=1 SV=1 |
|  | **O75380\|NDUS6_HUMAN** | NADH dehydrogenase [ubiquinone] iron-sulfur protein 6, mitochondrial OS=Homo sapiens GN=NDUFS6 PE=1 SV=1 |
|  | **Q12965\|MYO1E_HUMAN** | Myosin-Ie OS=Homo sapiens GN=MYO1E PE=1 SV=2 |
|  | **O00160\|MYO1F_HUMAN** | Myosin-If OS=Homo sapiens GN=MYO1F PE=1 SV=2 |
|  | **P20645\|MPRD_HUMAN** | Cation-dependent mannose-6-phosphate receptor OS=Homo sapiens GN=M6PR PE=1 SV=1 |
|  | **P21912\|DHSB_HUMAN** | Succinate dehydrogenase [ubiquinone] iron-sulfur subunit, mitochondrial OS=Homo sapiens GN=SDHB PE=1 SV=3 |
|  | **O00159\|MYO1C_HUMAN** | Myosin-Ic OS=Homo sapiens GN=MYO1C PE=1 SV=3 |
|  | **P67809\|YBOX1_HUMAN** | Nuclease-sensitive element-binding protein 1 OS=Homo sapiens GN=YBX1 PE=1 SV=3 |
|  | **Q09666\|AHNK_HUMAN** | Neuroblast differentiation-associated protein AHNAK OS=Homo sapiens GN=AHNAK PE=1 SV=2 |
|  | **P14550\|AK1A1_HUMAN** | Alcohol dehydrogenase [NADP+] OS=Homo sapiens GN=AKR1A1 PE=1 SV=3 |
|  | **P17858\|K6PL_HUMAN** | 6-phosphofructokinase, liver type OS=Homo sapiens GN=PFKL PE=1 SV=6 |
|  | **Q6UX53\|MET7B_HUMAN** | Methyltransferase-like protein 7B OS=Homo sapiens GN=METTL7B PE=2 SV=2 |
|  | **P11177\|ODPB_HUMAN** | Pyruvate dehydrogenase E1 component subunit beta, mitochondrial OS=Homo sapiens GN=PDHB PE=1 SV=3 |
|  | **O43520\|AT8B1_HUMAN** | Probable phospholipid-transporting ATPase IC OS=Homo sapiens GN=ATP8B1 PE=1 SV=3 |
|  | **P23141\|EST1_HUMAN** | Liver carboxylesterase 1 OS=Homo sapiens GN=CES1 PE=1 SV=2 |
|  | **Q14165\|MLEC_HUMAN** | Malectin OS=Homo sapiens GN=MLEC PE=1 SV=1 |
|  | **P32119\|PRDX2_HUMAN** | Peroxiredoxin-2 OS=Homo sapiens GN=PRDX2 PE=1 SV=5 |
|  | **Q06210\|GFPT1_HUMAN** | Glucosamine--fructose-6-phosphate aminotransferase [isomerizing] 1 OS=Homo sapiens GN=GFPT1 PE=1 SV=3 |
|  | **Q9Y4P3\|TBL2_HUMAN** | Transducin beta-like protein 2 OS=Homo sapiens GN=TBL2 PE=1 SV=1 |
|  | **P62910\|RL32_HUMAN** | 60S ribosomal protein L32 OS=Homo sapiens GN=RPL32 PE=1 SV=2 |
|  | **Q96GE6\|CALL4_HUMAN** | Calmodulin-like protein 4 OS=Homo sapiens GN=CALML4 PE=2 SV=3 |
|  | **P14174\|MIF_HUMAN** | Macrophage migration inhibitory factor OS=Homo sapiens GN=MIF PE=1 SV=4 |
|  | **P00918\|CAH2_HUMAN** | Carbonic anhydrase 2 OS=Homo sapiens GN=CA2 PE=1 SV=2 |
|  | **P35558\|PCKGC_HUMAN** | Phosphoenolpyruvate carboxykinase, cytosolic [GTP] OS=Homo sapiens GN=PCK1 PE=1 SV=3 |
|  | **O95837\|GNA14_HUMAN** | Guanine nucleotide-binding protein subunit alpha-14 OS=Homo sapiens GN=GNA14 PE=2 SV=1 |
|  | **P03891\|NU2M_HUMAN** | NADH-ubiquinone oxidoreductase chain 2 OS=Homo sapiens GN=MT-ND2 PE=1 SV=2 |
|  | **P30050\|RL12_HUMAN** | 60S ribosomal protein L12 OS=Homo sapiens GN=RPL12 PE=1 SV=1 |
|  | **P14927\|QCR7_HUMAN** | Cytochrome b-c1 complex subunit 7 OS=Homo sapiens GN=UQCRB PE=1 SV=2 |
|  | **Q9H115\|SNAB_HUMAN** | Beta-soluble NSF attachment protein OS=Homo sapiens GN=NAPB PE=1 SV=2 |
|  | **Q6DD88\|ATLA3_HUMAN** | Atlastin-3 OS=Homo sapiens GN=ATL3 PE=1 SV=1 |
|  | **Q96QV6\|H2A1A_HUMAN** | Histone H2A type 1-A OS=Homo sapiens GN=HIST1H2AA PE=1 SV=3 |
|  | **P02647\|APOA1_HUMAN** | Apolipoprotein A-I OS=Homo sapiens GN=APOA1 PE=1 SV=1 |
|  | **P51648\|AL3A2_HUMAN** | Fatty aldehyde dehydrogenase OS=Homo sapiens GN=ALDH3A2 PE=1 SV=1 |
|  | **Q9H6S3\|ES8L2_HUMAN** | Epidermal growth factor receptor kinase substrate 8-like protein 2 OS=Homo sapiens GN=EPS8L2 PE=1 SV=2 |
|  | **O15551\|CLD3_HUMAN** | Claudin-3 OS=Homo sapiens GN=CLDN3 PE=1 SV=1 |
|  | **P51572\|BAP31_HUMAN** | B-cell receptor-associated protein 31 OS=Homo sapiens GN=BCAP31 PE=1 SV=3 |
|  | **P0C7M2\|RA1L3_HUMAN** | Putative heterogeneous nuclear ribonucleoprotein A1-like protein 3 OS=Homo sapiens GN=HNRPA1L3 PE=5 SV=1 |
|  | **Q32P51\|RA1L2_HUMAN** | Heterogeneous nuclear ribonucleoprotein A1-like protein 2 OS=Homo sapiens GN=HNRNPA1L2 PE=2 SV=2 |
|  | **Q01523\|DEF5_HUMAN** | Defensin-5 OS=Homo sapiens GN=DEFA5 PE=1 SV=1 |
|  | **P06133\|UD2B4_HUMAN** | UDP-glucuronosyltransferase 2B4 OS=Homo sapiens GN=UGT2B4 PE=1 SV=2 |
|  | **P56134\|ATPK_HUMAN** | ATP synthase subunit f, mitochondrial OS=Homo sapiens GN=ATP5J2 PE=1 SV=3 |
|  | **Q99653\|CHP1_HUMAN** | Calcium-binding protein p22 OS=Homo sapiens GN=CHP PE=1 SV=3 |
|  | **P01871\|IGHM_HUMAN** | Ig mu chain C region OS=Homo sapiens GN=IGHM PE=1 SV=3 |
|  | **P55145\|MANF_HUMAN** | Mesencephalic astrocyte-derived neurotrophic factor OS=Homo sapiens GN=MANF PE=1 SV=2 |
|  | **P02768\|ALBU_HUMAN** | Serum albumin OS=Homo sapiens GN=ALB PE=1 SV=2 |
|  | **P62269\|RS18_HUMAN** | 40S ribosomal protein S18 OS=Homo sapiens GN=RPS18 PE=1 SV=3 |
|  | **P49585\|PCY1A_HUMAN** | Choline-phosphate cytidylyltransferase A OS=Homo sapiens GN=PCYT1A PE=1 SV=2 |
|  | **Q96C19\|EFHD2_HUMAN** | EF-hand domain-containing protein D2 OS=Homo sapiens GN=EFHD2 PE=1 SV=1 |
|  | **P36871\|PGM1_HUMAN** | Phosphoglucomutase-1 OS=Homo sapiens GN=PGM1 PE=1 SV=3 |
|  | **P07602\|SAP_HUMAN** | Proactivator polypeptide OS=Homo sapiens GN=PSAP PE=1 SV=2 |
|  | **P25325\|THTM_HUMAN** | 3-mercaptopyruvate sulfurtransferase OS=Homo sapiens GN=MPST PE=1 SV=3 |
|  | **Q08257\|QOR_HUMAN** | Quinone oxidoreductase OS=Homo sapiens GN=CRYZ PE=1 SV=1 |
|  | **P42766\|RL35_HUMAN** | 60S ribosomal protein L35 OS=Homo sapiens GN=RPL35 PE=1 SV=2 |
|  | **Q8WUD1\|RAB2B_HUMAN** | Ras-related protein Rab-2B OS=Homo sapiens GN=RAB2B PE=1 SV=1 |
|  | **Q14344\|GNA13_HUMAN** | Guanine nucleotide-binding protein subunit alpha-13 OS=Homo sapiens GN=GNA13 PE=1 SV=2 |
|  | **P38159\|HNRPG_HUMAN** | Heterogeneous nuclear ribonucleoprotein G OS=Homo sapiens GN=RBMX PE=1 SV=3 |
|  | **Q03518\|TAP1_HUMAN** | Antigen peptide transporter 1 OS=Homo sapiens GN=TAP1 PE=1 SV=2 |
|  | **Q9Y2J2\|E41L3_HUMAN** | Band 4.1-like protein 3 OS=Homo sapiens GN=EPB41L3 PE=1 SV=2 |
|  | **P15259\|PGAM2_HUMAN** | Phosphoglycerate mutase 2 OS=Homo sapiens GN=PGAM2 PE=1 SV=3 |
|  | **P52597\|HNRPF_HUMAN** | Heterogeneous nuclear ribonucleoprotein F OS=Homo sapiens GN=HNRNPF PE=1 SV=3 |
|  | **Q14CN2\|CLCA4_HUMAN** | Calcium-activated chloride channel regulator 4 OS=Homo sapiens GN=CLCA4 PE=1 SV=2 |
|  | **Q9NZA1\|CLIC5_HUMAN** | Chloride intracellular channel protein 5 OS=Homo sapiens GN=CLIC5 PE=1 SV=3 |
|  | **Q86Y82\|STX12_HUMAN** | Syntaxin-12 OS=Homo sapiens GN=STX12 PE=1 SV=1 |
|  | **O15533\|TPSN_HUMAN** | Tapasin OS=Homo sapiens GN=TAPBP PE=1 SV=1 |
|  | **P62854\|RS26_HUMAN** | 40S ribosomal protein S26 OS=Homo sapiens GN=RPS26 PE=1 SV=3 |
|  | **Q9H9B4\|SFXN1_HUMAN** | Sideroflexin-1 OS=Homo sapiens GN=SFXN1 PE=1 SV=4 |
|  | **Q9Y4X1\|UD2A1_HUMAN** | UDP-glucuronosyltransferase 2A1 OS=Homo sapiens GN=UGT2A1 PE=1 SV=1 |
|  | **O43169\|CYB5B_HUMAN** | Cytochrome b5 type B OS=Homo sapiens GN=CYB5B PE=1 SV=2 |
|  | **P21217\|FUT3_HUMAN** | Galactoside 3(4)-L-fucosyltransferase OS=Homo sapiens GN=FUT3 PE=2 SV=1 |
|  | **P51993\|FUT6_HUMAN** | Alpha-(1,3)-fucosyltransferase OS=Homo sapiens GN=FUT6 PE=2 SV=1 |
|  | **P50995\|ANX11_HUMAN** | Annexin A11 OS=Homo sapiens GN=ANXA11 PE=1 SV=1 |
|  | **Q9Y3Y2\|CA077_HUMAN** | Uncharacterized protein C1orf77 OS=Homo sapiens GN=C1orf77 PE=1 SV=2 |
|  | **Q01105\|SET_HUMAN** | Protein SET OS=Homo sapiens GN=SET PE=1 SV=3 |
|  | **Q01813\|K6PP_HUMAN** | 6-phosphofructokinase type C OS=Homo sapiens GN=PFKP PE=1 SV=2 |
|  | **P38919\|IF4A3_HUMAN** | Eukaryotic initiation factor 4A-III OS=Homo sapiens GN=EIF4A3 PE=1 SV=4 |
|  | **P17693\|HLAG_HUMAN** | HLA class I histocompatibility antigen, alpha chain G OS=Homo sapiens GN=HLA-G PE=1 SV=1 |
|  | **P46778\|RL21_HUMAN** | 60S ribosomal protein L21 OS=Homo sapiens GN=RPL21 PE=1 SV=2 |
|  | **Q92485\|ASM3B_HUMAN** | Acid sphingomyelinase-like phosphodiesterase 3b OS=Homo sapiens GN=SMPDL3B PE=2 SV=2 |
|  | **Q9H299\|SH3L3_HUMAN** | SH3 domain-binding glutamic acid-rich-like protein 3 OS=Homo sapiens GN=SH3BGRL3 PE=1 SV=1 |
|  | **Q9NX40\|OCAD1_HUMAN** | OCIA domain-containing protein 1 OS=Homo sapiens GN=OCIAD1 PE=1 SV=1 |
|  | **P17301\|ITA2_HUMAN** | Integrin alpha-2 OS=Homo sapiens GN=ITGA2 PE=1 SV=1 |
|  | **P30511\|HLAF_HUMAN** | HLA class I histocompatibility antigen, alpha chain F OS=Homo sapiens GN=HLA-F PE=2 SV=2 |
|  | **P06753\|TPM3_HUMAN** | Tropomyosin alpha-3 chain OS=Homo sapiens GN=TPM3 PE=1 SV=1 |
|  | **Q03154\|ACY1_HUMAN** | Aminoacylase-1 OS=Homo sapiens GN=ACY1 PE=1 SV=1 |
|  | **Q9BYZ8\|REG4_HUMAN** | Regenerating islet-derived protein 4 OS=Homo sapiens GN=REG4 PE=1 SV=1 |
|  | **P62750\|RL23A_HUMAN** | 60S ribosomal protein L23a OS=Homo sapiens GN=RPL23A PE=1 SV=1 |
|  | **Q16720\|AT2B3_HUMAN** | Plasma membrane calcium-transporting ATPase 3 OS=Homo sapiens GN=ATP2B3 PE=1 SV=3 |
|  | **Q9Y639\|NPTN_HUMAN** | Neuroplastin OS=Homo sapiens GN=NPTN PE=1 SV=1 |
|  | **Q96L21\|RL10L_HUMAN** | 60S ribosomal protein L10-like OS=Homo sapiens GN=RPL10L PE=1 SV=3 |
|  | **P49720\|PSB3_HUMAN** | Proteasome subunit beta type-3 OS=Homo sapiens GN=PSMB3 PE=1 SV=2 |
|  | **O75083\|WDR1_HUMAN** | WD repeat-containing protein 1 OS=Homo sapiens GN=WDR1 PE=1 SV=4 |
|  | **O94826\|TOM70_HUMAN** | Mitochondrial import receptor subunit TOM70 OS=Homo sapiens GN=TOMM70A PE=1 SV=1 |
|  | **Q03519\|TAP2_HUMAN** | Antigen peptide transporter 2 OS=Homo sapiens GN=TAP2 PE=1 SV=1 |
|  | **P21439\|MDR3_HUMAN** | Multidrug resistance protein 3 OS=Homo sapiens GN=ABCB4 PE=1 SV=2 |
|  | **P13929\|ENOB_HUMAN** | Beta-enolase OS=Homo sapiens GN=ENO3 PE=1 SV=4 |
|  | **P09104\|ENOG_HUMAN** | Gamma-enolase OS=Homo sapiens GN=ENO2 PE=1 SV=3 |
|  | **Q8NFQ8\|TOIP2_HUMAN** | Torsin-1A-interacting protein 2 OS=Homo sapiens GN=TOR1AIP2 PE=1 SV=1 |
|  | **P62263\|RS14_HUMAN** | 40S ribosomal protein S14 OS=Homo sapiens GN=RPS14 PE=1 SV=3 |
|  | **P98073\|ENTK_HUMAN** | Enteropeptidase OS=Homo sapiens GN=PRSS7 PE=2 SV=2 |
|  | **P63010\|AP2B1_HUMAN** | AP-2 complex subunit beta OS=Homo sapiens GN=AP2B1 PE=1 SV=1 |
|  | **Q9Y6N5\|SQRD_HUMAN** | Sulfide:quinone oxidoreductase, mitochondrial OS=Homo sapiens GN=SQRDL PE=1 SV=1 |
|  | **P09681\|GIP_HUMAN** | Gastric inhibitory polypeptide OS=Homo sapiens GN=GIP PE=1 SV=1 |
|  | **Q8WUM4\|PDC6I_HUMAN** | Programmed cell death 6-interacting protein OS=Homo sapiens GN=PDCD6IP PE=1 SV=1 |
|  | **P18621\|RL17_HUMAN** | 60S ribosomal protein L17 OS=Homo sapiens GN=RPL17 PE=1 SV=3 |
|  | **P78371\|TCPB_HUMAN** | T-complex protein 1 subunit beta OS=Homo sapiens GN=CCT2 PE=1 SV=4 |
|  | **P22732\|GTR5_HUMAN** | Solute carrier family 2, facilitated glucose transporter member 5 OS=Homo sapiens GN=SLC2A5 PE=1 SV=1 |
|  | **P14649\|MYL6B_HUMAN** | Myosin light chain 6B OS=Homo sapiens GN=MYL6B PE=1 SV=1 |
|  | **Q99729\|ROAA_HUMAN** | Heterogeneous nuclear ribonucleoprotein A/B OS=Homo sapiens GN=HNRNPAB PE=1 SV=2 |
|  | **P04075\|ALDOA_HUMAN** | Fructose-bisphosphate aldolase A OS=Homo sapiens GN=ALDOA PE=1 SV=2 |
|  | **P06396\|GELS_HUMAN** | Gelsolin OS=Homo sapiens GN=GSN PE=1 SV=1 |
|  | **P61247\|RS3A_HUMAN** | 40S ribosomal protein S3a OS=Homo sapiens GN=RPS3A PE=1 SV=2 |
|  | **P62244\|RS15A_HUMAN** | 40S ribosomal protein S15a OS=Homo sapiens GN=RPS15A PE=1 SV=2 |
|  | **O96000\|NDUBA_HUMAN** | NADH dehydrogenase [ubiquinone] 1 beta subcomplex subunit 10 OS=Homo sapiens GN=NDUFB10 PE=1 SV=3 |
|  | **Q58FF3\|ENPLL_HUMAN** | Putative endoplasmin-like protein OS=Homo sapiens GN=HSP90B2P PE=5 SV=1 |
|  | **O75746\|CMC1_HUMAN** | Calcium-binding mitochondrial carrier protein Aralar1 OS=Homo sapiens GN=SLC25A12 PE=1 SV=2 |
|  | **Q9UI47\|CTNA3_HUMAN** | Catenin alpha-3 OS=Homo sapiens GN=CTNNA3 PE=1 SV=2 |
|  | **P14406\|CX7A2_HUMAN** | Cytochrome c oxidase polypeptide 7A2, mitochondrial OS=Homo sapiens GN=COX7A2 PE=1 SV=1 |
|  | **P12074\|CX6A1_HUMAN** | Cytochrome c oxidase subunit 6A1, mitochondrial OS=Homo sapiens GN=COX6A1 PE=1 SV=4 |
|  | **Q8WWI5\|CTL1_HUMAN** | Choline transporter-like protein 1 OS=Homo sapiens GN=SLC44A1 PE=1 SV=1 |
|  | **Q9Y624\|JAM1_HUMAN** | Junctional adhesion molecule A OS=Homo sapiens GN=F11R PE=1 SV=1 |
|  | **P01908\|HA21_HUMAN** | HLA class II histocompatibility antigen, DQ(1) alpha chain OS=Homo sapiens PE=2 SV=2 |
|  | **P26373\|RL13_HUMAN** | 60S ribosomal protein L13 OS=Homo sapiens GN=RPL13 PE=1 SV=4 |
|  | **P53618\|COPB_HUMAN** | Coatomer subunit beta OS=Homo sapiens GN=COPB1 PE=1 SV=3 |
|  | **P09960\|LKHA4_HUMAN** | Leukotriene A-4 hydrolase OS=Homo sapiens GN=LTA4H PE=1 SV=2 |
|  | **P05783\|K1C18_HUMAN** | Keratin, type I cytoskeletal 18 OS=Homo sapiens GN=KRT18 PE=1 SV=2 |
|  | **P62081\|RS7_HUMAN** | 40S ribosomal protein S7 OS=Homo sapiens GN=RPS7 PE=1 SV=1 |
|  | **P60468\|SC61B_HUMAN** | Protein transport protein Sec61 subunit beta OS=Homo sapiens GN=SEC61B PE=1 SV=2 |
|  | **P46059\|S15A1_HUMAN** | Solute carrier family 15 member 1 OS=Homo sapiens GN=SLC15A1 PE=2 SV=1 |
|  | **Q99828\|CIB1_HUMAN** | Calcium and integrin-binding protein 1 OS=Homo sapiens GN=CIB1 PE=1 SV=4 |
|  | **P16219\|ACADS_HUMAN** | Short-chain specific acyl-CoA dehydrogenase, mitochondrial OS=Homo sapiens GN=ACADS PE=1 SV=1 |
|  | **P13010\|KU86_HUMAN** | ATP-dependent DNA helicase 2 subunit 2 OS=Homo sapiens GN=XRCC5 PE=1 SV=3 |
|  | **Q13838\|UAP56_HUMAN** | Spliceosome RNA helicase BAT1 OS=Homo sapiens GN=BAT1 PE=1 SV=1 |
|  | **O75964\|ATP5L_HUMAN** | ATP synthase subunit g, mitochondrial OS=Homo sapiens GN=ATP5L PE=1 SV=3 |
|  | **O15484\|CAN5_HUMAN** | Calpain-5 OS=Homo sapiens GN=CAPN5 PE=2 SV=2 |
|  | **Q6P1A2\|MBOA5_HUMAN** | Lysophospholipid acyltransferase 5 OS=Homo sapiens GN=LPCAT3 PE=1 SV=1 |
|  | **P08754\|GNAI3_HUMAN** | Guanine nucleotide-binding protein G(k) subunit alpha OS=Homo sapiens GN=GNAI3 PE=1 SV=3 |
|  | **P78310\|CXAR_HUMAN** | Coxsackievirus and adenovirus receptor OS=Homo sapiens GN=CXADR PE=1 SV=1 |
|  | **P57105\|SYJ2B_HUMAN** | Synaptojanin-2-binding protein OS=Homo sapiens GN=SYNJ2BP PE=1 SV=2 |
|  | **Q9ULC6\|PADI1_HUMAN** | Protein-arginine deiminase type-1 OS=Homo sapiens GN=PADI1 PE=1 SV=2 |
|  | **Q9BUP3\|HTAI2_HUMAN** | Oxidoreductase HTATIP2 OS=Homo sapiens GN=HTATIP2 PE=1 SV=1 |
|  | **P53007\|TXTP_HUMAN** | Tricarboxylate transport protein, mitochondrial OS=Homo sapiens GN=SLC25A1 PE=1 SV=2 |
|  | **Q15005\|SPCS2_HUMAN** | Signal peptidase complex subunit 2 OS=Homo sapiens GN=SPCS2 PE=1 SV=3 |
|  | **Q30154\|2B51_HUMAN** | HLA class II histocompatibility antigen, DRB5 beta chain OS=Homo sapiens GN=HLA-DRB5 PE=1 SV=1 |
|  | **P04229\|2B11_HUMAN** | HLA class II histocompatibility antigen, DRB1-1 beta chain OS=Homo sapiens GN=HLA-DRB1 PE=1 SV=2 |
|  | **P20039\|2B1B_HUMAN** | HLA class II histocompatibility antigen, DRB1-11 beta chain OS=Homo sapiens GN=HLA-DRB1 PE=2 SV=1 |
|  | **Q15717\|ELAV1_HUMAN** | ELAV-like protein 1 OS=Homo sapiens GN=ELAVL1 PE=1 SV=2 |
|  | **Q9UDY2\|ZO2_HUMAN** | Tight junction protein ZO-2 OS=Homo sapiens GN=TJP2 PE=1 SV=1 |
|  | **P49821\|NDUV1_HUMAN** | NADH dehydrogenase [ubiquinone] flavoprotein 1, mitochondrial OS=Homo sapiens GN=NDUFV1 PE=1 SV=4 |
|  | **O15143\|ARC1B_HUMAN** | Actin-related protein 2/3 complex subunit 1B OS=Homo sapiens GN=ARPC1B PE=1 SV=3 |
|  | **Q9H0X4\|ITFG3_HUMAN** | Protein ITFG3 OS=Homo sapiens GN=ITFG3 PE=1 SV=1 |
|  | **P62249\|RS16_HUMAN** | 40S ribosomal protein S16 OS=Homo sapiens GN=RPS16 PE=1 SV=2 |
|  | **Q9Y2R5\|RT17_HUMAN** | 28S ribosomal protein S17, mitochondrial OS=Homo sapiens GN=MRPS17 PE=1 SV=1 |
|  | **O43280\|TREA_HUMAN** | Trehalase OS=Homo sapiens GN=TREH PE=1 SV=2 |
|  | **P26641\|EF1G_HUMAN** | Elongation factor 1-gamma OS=Homo sapiens GN=EEF1G PE=1 SV=3 |
|  | **Q9P035\|PTAD1_HUMAN** | Protein tyrosine phosphatase-like protein PTPLAD1 OS=Homo sapiens GN=PTPLAD1 PE=1 SV=2 |
|  | **O15372\|EIF3H_HUMAN** | Eukaryotic translation initiation factor 3 subunit H OS=Homo sapiens GN=EIF3H PE=1 SV=1 |
|  | **Q01814\|AT2B2_HUMAN** | Plasma membrane calcium-transporting ATPase 2 OS=Homo sapiens GN=ATP2B2 PE=1 SV=2 |
|  | **Q58FG1\|HS904_HUMAN** | Putative heat shock protein HSP 90-alpha A4 OS=Homo sapiens GN=HSP90AA4P PE=5 SV=1 |
|  | **Q96G23\|LASS2_HUMAN** | LAG1 longevity assurance homolog 2 OS=Homo sapiens GN=LASS2 PE=1 SV=1 |
|  | **Q53TN4\|CYBR1_HUMAN** | Cytochrome b reductase 1 OS=Homo sapiens GN=CYBRD1 PE=1 SV=1 |
|  | **P50440\|GATM_HUMAN** | Glycine amidinotransferase, mitochondrial OS=Homo sapiens GN=GATM PE=1 SV=1 |
|  | **P22307\|NLTP_HUMAN** | Non-specific lipid-transfer protein OS=Homo sapiens GN=SCP2 PE=1 SV=2 |
|  | **Q9Y3B3\|TMED7_HUMAN** | Transmembrane emp24 domain-containing protein 7 OS=Homo sapiens GN=TMED7 PE=1 SV=2 |
|  | **Q9NRV9\|HEBP1_HUMAN** | Heme-binding protein 1 OS=Homo sapiens GN=HEBP1 PE=1 SV=1 |
|  | **P49326\|FMO5_HUMAN** | Dimethylaniline monooxygenase [N-oxide-forming] 5 OS=Homo sapiens GN=FMO5 PE=2 SV=2 |
|  | **P47985\|UCRI_HUMAN** | Cytochrome b-c1 complex subunit Rieske, mitochondrial OS=Homo sapiens GN=UQCRFS1 PE=1 SV=2 |
|  | **Q92604\|LGAT1_HUMAN** | Acyl-CoA:lysophosphatidylglycerol acyltransferase 1 OS=Homo sapiens GN=LPGAT1 PE=2 SV=1 |
|  | **O15145\|ARPC3_HUMAN** | Actin-related protein 2/3 complex subunit 3 OS=Homo sapiens GN=ARPC3 PE=1 SV=3 |
|  | **Q96SQ9\|CP2S1_HUMAN** | Cytochrome P450 2S1 OS=Homo sapiens GN=CYP2S1 PE=2 SV=2 |
|  | **Q9UBX3\|DIC_HUMAN** | Mitochondrial dicarboxylate carrier OS=Homo sapiens GN=SLC25A10 PE=1 SV=2 |
|  | **Q2M2H8\|MGAL2_HUMAN** | Putative maltase-glucoamylase-like protein LOC93432 OS=Homo sapiens PE=2 SV=2 |
|  | **Q969H8\|CS010_HUMAN** | UPF0556 protein C19orf10 OS=Homo sapiens GN=C19orf10 PE=1 SV=1 |
|  | **Q8N0Y7\|PGAM4_HUMAN** | Probable phosphoglycerate mutase 4 OS=Homo sapiens GN=PGAM4 PE=1 SV=1 |
|  | **P60903\|S10AA_HUMAN** | Protein S100-A10 OS=Homo sapiens GN=S100A10 PE=1 SV=2 |
|  | **Q9P2E9\|RRBP1_HUMAN** | Ribosome-binding protein 1 OS=Homo sapiens GN=RRBP1 PE=1 SV=4 |
|  | **O14907\|TX1B3_HUMAN** | Tax1-binding protein 3 OS=Homo sapiens GN=TAX1BP3 PE=1 SV=2 |
|  | **Q96HR9\|REEP6_HUMAN** | Receptor expression-enhancing protein 6 OS=Homo sapiens GN=REEP6 PE=1 SV=1 |
|  | **P11234\|RALB_HUMAN** | Ras-related protein Ral-B OS=Homo sapiens GN=RALB PE=1 SV=1 |
|  | **P11233\|RALA_HUMAN** | Ras-related protein Ral-A OS=Homo sapiens GN=RALA PE=1 SV=1 |
|  | **Q9Y376\|CAB39_HUMAN** | Calcium-binding protein 39 OS=Homo sapiens GN=CAB39 PE=1 SV=1 |
|  | **Q8WU67\|ABHD3_HUMAN** | Abhydrolase domain-containing protein 3 OS=Homo sapiens GN=ABHD3 PE=2 SV=2 |
|  | **Q9HCU5\|PREB_HUMAN** | Prolactin regulatory element-binding protein OS=Homo sapiens GN=PREB PE=1 SV=2 |
|  | **P46977\|STT3A_HUMAN** | Dolichyl-diphosphooligosaccharide--protein glycosyltransferase subunit STT3A OS=Homo sapiens GN=STT3A PE=1 SV=2 |
|  | **P04233\|HG2A_HUMAN** | HLA class II histocompatibility antigen gamma chain OS=Homo sapiens GN=CD74 PE=1 SV=3 |
|  | **B1AK53\|ESPN_HUMAN** | Espin OS=Homo sapiens GN=ESPN PE=1 SV=1 |
|  | **P40227\|TCPZ_HUMAN** | T-complex protein 1 subunit zeta OS=Homo sapiens GN=CCT6A PE=1 SV=3 |
|  | **Q6UWP2\|DHR11_HUMAN** | Dehydrogenase/reductase SDR family member 11 OS=Homo sapiens GN=DHRS11 PE=1 SV=1 |
|  | **P13640\|MT1G_HUMAN** | Metallothionein-1G OS=Homo sapiens GN=MT1G PE=1 SV=2 |
|  | **P02795\|MT2_HUMAN** | Metallothionein-2 OS=Homo sapiens GN=MT2A PE=1 SV=1 |
|  | **Q9H0U3\|MAGT1_HUMAN** | Magnesium transporter protein 1 OS=Homo sapiens GN=MAGT1 PE=1 SV=1 |
|  | **P13796\|PLSL_HUMAN** | Plastin-2 OS=Homo sapiens GN=LCP1 PE=1 SV=5 |
|  | **Q9HBL7\|CI046_HUMAN** | Transmembrane protein C9orf46 OS=Homo sapiens GN=C9orf46 PE=2 SV=1 |
|  | **P54886\|P5CS_HUMAN** | Delta-1-pyrroline-5-carboxylate synthetase OS=Homo sapiens GN=ALDH18A1 PE=1 SV=2 |
|  | **Q15800\|ERG25_HUMAN** | C-4 methylsterol oxidase OS=Homo sapiens GN=SC4MOL PE=1 SV=1 |
|  | **Q58FF8\|H90B2_HUMAN** | Putative heat shock protein HSP 90-beta 2 OS=Homo sapiens GN=HSP90AB2P PE=1 SV=2 |
|  | **Q14568\|HS902_HUMAN** | Putative heat shock protein HSP 90-alpha A2 OS=Homo sapiens GN=HSP90AA2 PE=1 SV=2 |
|  | **Q9BVK6\|TMED9_HUMAN** | Transmembrane emp24 domain-containing protein 9 OS=Homo sapiens GN=TMED9 PE=1 SV=2 |
|  | **Q15366\|PCBP2_HUMAN** | Poly(rC)-binding protein 2 OS=Homo sapiens GN=PCBP2 PE=1 SV=1 |
|  | **P57721\|PCBP3_HUMAN** | Poly(rC)-binding protein 3 OS=Homo sapiens GN=PCBP3 PE=1 SV=1 |
|  | **Q15365\|PCBP1_HUMAN** | Poly(rC)-binding protein 1 OS=Homo sapiens GN=PCBP1 PE=1 SV=2 |
|  | **O14979\|HNRDL_HUMAN** | Heterogeneous nuclear ribonucleoprotein D-like OS=Homo sapiens GN=HNRPDL PE=1 SV=3 |
|  | **P36873\|PP1G_HUMAN** | Serine/threonine-protein phosphatase PP1-gamma catalytic subunit OS=Homo sapiens GN=PPP1CC PE=1 SV=1 |
|  | **P62140\|PP1B_HUMAN** | Serine/threonine-protein phosphatase PP1-beta catalytic subunit OS=Homo sapiens GN=PPP1CB PE=1 SV=3 |
|  | **P62136\|PP1A_HUMAN** | Serine/threonine-protein phosphatase PP1-alpha catalytic subunit OS=Homo sapiens GN=PPP1CA PE=1 SV=1 |
|  | **P08237\|K6PF_HUMAN** | 6-phosphofructokinase, muscle type OS=Homo sapiens GN=PFKM PE=1 SV=2 |
|  | **P63000\|RAC1_HUMAN** | Ras-related C3 botulinum toxin substrate 1 OS=Homo sapiens GN=RAC1 PE=1 SV=1 |
|  | **P60763\|RAC3_HUMAN** | Ras-related C3 botulinum toxin substrate 3 OS=Homo sapiens GN=RAC3 PE=1 SV=1 |
|  | **P15153\|RAC2_HUMAN** | Ras-related C3 botulinum toxin substrate 2 OS=Homo sapiens GN=RAC2 PE=1 SV=1 |
|  | **P16520\|GBB3_HUMAN** | Guanine nucleotide-binding protein G(I)/G(S)/G(T) subunit beta-3 OS=Homo sapiens GN=GNB3 PE=1 SV=1 |
|  | **O60664\|M6PBP_HUMAN** | Mannose-6-phosphate receptor-binding protein 1 OS=Homo sapiens GN=M6PRBP1 PE=1 SV=2 |
|  | **Q93050\|VPP1_HUMAN** | V-type proton ATPase 116 kDa subunit a isoform 1 OS=Homo sapiens GN=ATP6V0A1 PE=1 SV=3 |
|  | **Q10588\|BST1_HUMAN** | ADP-ribosyl cyclase 2 OS=Homo sapiens GN=BST1 PE=1 SV=2 |
|  | **P31146\|COR1A_HUMAN** | Coronin-1A OS=Homo sapiens GN=CORO1A PE=1 SV=4 |
|  | **P10412\|H14_HUMAN** | Histone H1.4 OS=Homo sapiens GN=HIST1H1E PE=1 SV=2 |
|  | **P16403\|H12_HUMAN** | Histone H1.2 OS=Homo sapiens GN=HIST1H1C PE=1 SV=2 |
|  | **P16402\|H13_HUMAN** | Histone H1.3 OS=Homo sapiens GN=HIST1H1D PE=1 SV=2 |
|  | **Q969Q0\|RL36L_HUMAN** | 60S ribosomal protein L36a-like OS=Homo sapiens GN=RPL36AL PE=1 SV=3 |
|  | **Q9Y3I0\|CV028_HUMAN** | UPF0027 protein C22orf28 OS=Homo sapiens GN=C22orf28 PE=1 SV=1 |
|  | **Q9NUU6\|F105A_HUMAN** | Protein FAM105A OS=Homo sapiens GN=FAM105A PE=2 SV=1 |
|  | **P61626\|LYSC_HUMAN** | Lysozyme C OS=Homo sapiens GN=LYZ PE=1 SV=1 |
|  | **P20339\|RAB5A_HUMAN** | Ras-related protein Rab-5A OS=Homo sapiens GN=RAB5A PE=1 SV=2 |
|  | **P61020\|RAB5B_HUMAN** | Ras-related protein Rab-5B OS=Homo sapiens GN=RAB5B PE=1 SV=1 |
|  | **P22223\|CADH3_HUMAN** | Cadherin-3 OS=Homo sapiens GN=CDH3 PE=1 SV=2 |
|  | **P62988\|UBIQ_HUMAN** | Ubiquitin OS=Homo sapiens GN=RPS27A PE=1 SV=1 |
|  | **P51970\|NDUA8_HUMAN** | NADH dehydrogenase [ubiquinone] 1 alpha subcomplex subunit 8 OS=Homo sapiens GN=NDUFA8 PE=1 SV=3 |
|  | **P62258\|1433E_HUMAN** | 14-3-3 protein epsilon OS=Homo sapiens GN=YWHAE PE=1 SV=1 |
|  | **Q9Y6M7\|S4A7_HUMAN** | Sodium bicarbonate cotransporter 3 OS=Homo sapiens GN=SLC4A7 PE=1 SV=2 |
|  | **Q8NCR9\|CLRN3_HUMAN** | Clarin-3 OS=Homo sapiens GN=CLRN3 PE=2 SV=1 |
|  | **Q9NX14\|NDUBB_HUMAN** | NADH dehydrogenase [ubiquinone] 1 beta subcomplex subunit 11, mitochondrial OS=Homo sapiens GN=NDUFB11 PE=1 SV=1 |
|  | **Q06520\|ST2A1_HUMAN** | Bile salt sulfotransferase OS=Homo sapiens GN=SULT2A1 PE=1 SV=3 |
|  | **P28838\|AMPL_HUMAN** | Cytosol aminopeptidase OS=Homo sapiens GN=LAP3 PE=1 SV=3 |
|  | **O94760\|DDAH1_HUMAN** | N(G),N(G)-dimethylarginine dimethylaminohydrolase 1 OS=Homo sapiens GN=DDAH1 PE=1 SV=3 |
|  | **Q96C12\|ARMC5_HUMAN** | Armadillo repeat-containing protein 5 OS=Homo sapiens GN=ARMC5 PE=2 SV=2 |
|  | **Q9NVH1\|DJC11_HUMAN** | DnaJ homolog subfamily C member 11 OS=Homo sapiens GN=DNAJC11 PE=1 SV=2 |
|  | **P51857\|AK1D1_HUMAN** | 3-oxo-5-beta-steroid 4-dehydrogenase OS=Homo sapiens GN=AKR1D1 PE=1 SV=1 |
|  | **P40616\|ARL1_HUMAN** | ADP-ribosylation factor-like protein 1 OS=Homo sapiens GN=ARL1 PE=1 SV=1 |
|  | **Q96A08\|H2B1A_HUMAN** | Histone H2B type 1-A OS=Homo sapiens GN=HIST1H2BA PE=1 SV=3 |
|  | **Q9P2P6\|STAR9_HUMAN** | StAR-related lipid transfer protein 9 OS=Homo sapiens GN=STARD9 PE=2 SV=2 |
|  | **P53985\|MOT1_HUMAN** | Monocarboxylate transporter 1 OS=Homo sapiens GN=SLC16A1 PE=1 SV=2 |
|  | **P16591\|FER_HUMAN** | Proto-oncogene tyrosine-protein kinase FER OS=Homo sapiens GN=FER PE=1 SV=2 |
|  | **P51991\|ROA3_HUMAN** | Heterogeneous nuclear ribonucleoprotein A3 OS=Homo sapiens GN=HNRNPA3 PE=1 SV=2 |
|  | **P50454\|SERPH_HUMAN** | Serpin H1 OS=Homo sapiens GN=SERPINH1 PE=1 SV=2 |
|  | **P38405\|GNAL_HUMAN** | Guanine nucleotide-binding protein G(olf) subunit alpha OS=Homo sapiens GN=GNAL PE=1 SV=1 |
|  | **Q5JWF2\|GNAS1_HUMAN** | Guanine nucleotide-binding protein G(s) subunit alpha isoforms XLas OS=Homo sapiens GN=GNAS PE=1 SV=2 |
|  | **P63092\|GNAS2_HUMAN** | Guanine nucleotide-binding protein G(s) subunit alpha isoforms short OS=Homo sapiens GN=GNAS PE=1 SV=1 |
|  | **P99999\|CYC_HUMAN** | Cytochrome c OS=Homo sapiens GN=CYCS PE=1 SV=2 |
|  | **Q99735\|MGST2_HUMAN** | Microsomal glutathione S-transferase 2 OS=Homo sapiens GN=MGST2 PE=1 SV=1 |
|  | **O43181\|NDUS4_HUMAN** | NADH dehydrogenase [ubiquinone] iron-sulfur protein 4, mitochondrial OS=Homo sapiens GN=NDUFS4 PE=1 SV=1 |
|  | **Q9BVC6\|TM109_HUMAN** | Transmembrane protein 109 OS=Homo sapiens GN=TMEM109 PE=1 SV=1 |
|  | **O75251\|NDUS7_HUMAN** | NADH dehydrogenase [ubiquinone] iron-sulfur protein 7, mitochondrial OS=Homo sapiens GN=NDUFS7 PE=1 SV=3 |
|  | **Q9NTJ5\|SAC1_HUMAN** | Phosphatidylinositide phosphatase SAC1 OS=Homo sapiens GN=SACM1L PE=1 SV=2 |
|  | **Q15125\|EBP_HUMAN** | 3-beta-hydroxysteroid-Delta(8),Delta(7)-isomerase OS=Homo sapiens GN=EBP PE=1 SV=3 |
|  | **Q8N766\|K0090_HUMAN** | Uncharacterized protein KIAA0090 OS=Homo sapiens GN=KIAA0090 PE=1 SV=1 |
|  | **Q96N66\|MBOA7_HUMAN** | Lysophospholipid acyltransferase 7 OS=Homo sapiens GN=MBOAT7 PE=1 SV=2 |
|  | **P38117\|ETFB_HUMAN** | Electron transfer flavoprotein subunit beta OS=Homo sapiens GN=ETFB PE=1 SV=3 |
|  | **A2A3L6\|TTC24_HUMAN** | Tetratricopeptide repeat protein 24 OS=Homo sapiens GN=TTC24 PE=2 SV=1 |
|  | **P20618\|PSB1_HUMAN** | Proteasome subunit beta type-1 OS=Homo sapiens GN=PSMB1 PE=1 SV=2 |
|  | **Q04760\|LGUL_HUMAN** | Lactoylglutathione lyase OS=Homo sapiens GN=GLO1 PE=1 SV=4 |
|  | **P08708\|RS17_HUMAN** | 40S ribosomal protein S17 OS=Homo sapiens GN=RPS17 PE=1 SV=2 |
|  | **Q13557\|KCC2D_HUMAN** | Calcium/calmodulin-dependent protein kinase type II delta chain OS=Homo sapiens GN=CAMK2D PE=1 SV=3 |
|  | **A0FGR8\|ESYT2_HUMAN** | Extended synaptotagmin-2 OS=Homo sapiens GN=ESYT2 PE=1 SV=1 |
|  | **P63096\|GNAI1_HUMAN** | Guanine nucleotide-binding protein G(i), alpha-1 subunit OS=Homo sapiens GN=GNAI1 PE=1 SV=2 |
|  | **Q9P0J0\|NDUAD_HUMAN** | NADH dehydrogenase [ubiquinone] 1 alpha subcomplex subunit 13 OS=Homo sapiens GN=NDUFA13 PE=1 SV=3 |
|  | **Q8WTW3\|COG1_HUMAN** | Conserved oligomeric Golgi complex subunit 1 OS=Homo sapiens GN=COG1 PE=1 SV=1 |
|  | **A6NL28\|TPM3L_HUMAN** | Putative tropomyosin alpha-3 chain-like protein OS=Homo sapiens PE=5 SV=2 |
|  | **P24390\|ERD21_HUMAN** | ER lumen protein retaining receptor 1 OS=Homo sapiens GN=KDELR1 PE=1 SV=1 |
|  | **Q9C0B2\|K1751_HUMAN** | Uncharacterized protein KIAA1751 OS=Homo sapiens GN=KIAA1751 PE=2 SV=2 |
|  | **P49368\|TCPG_HUMAN** | T-complex protein 1 subunit gamma OS=Homo sapiens GN=CCT3 PE=1 SV=4 |
|  | **Q7KZF4\|SND1_HUMAN** | Staphylococcal nuclease domain-containing protein 1 OS=Homo sapiens GN=SND1 PE=1 SV=1 |
|  | **Q99525\|H4G_HUMAN** | Histone H4-like protein type G OS=Homo sapiens GN=HIST1H4G PE=2 SV=1 |
|  | **Q69YW2\|CA095_HUMAN** | Uncharacterized membrane protein C1orf95 OS=Homo sapiens GN=C1orf95 PE=2 SV=1 |
|  | **P01911\|2B1E_HUMAN** | HLA class II histocompatibility antigen, DRB1-15 beta chain OS=Homo sapiens GN=HLA-DRB1 PE=1 SV=2 |
|  | **P01912\|HB2B_HUMAN** | HLA class II histocompatibility antigen, DR-1 beta chain OS=Homo sapiens GN=HLA-DRB1 PE=1 SV=2 |
|  | **P22061\|PIMT_HUMAN** | Protein-L-isoaspartate(D-aspartate) O-methyltransferase OS=Homo sapiens GN=PCMT1 PE=1 SV=3 |
|  | **P61160\|ARP2_HUMAN** | Actin-related protein 2 OS=Homo sapiens GN=ACTR2 PE=1 SV=1 |
|  | **P49448\|DHE4_HUMAN** | Glutamate dehydrogenase 2, mitochondrial OS=Homo sapiens GN=GLUD2 PE=1 SV=2 |
|  | **P43243\|MATR3_HUMAN** | Matrin-3 OS=Homo sapiens GN=MATR3 PE=1 SV=2 |
|  | **Q16851\|UGPA_HUMAN** | UTP--glucose-1-phosphate uridylyltransferase OS=Homo sapiens GN=UGP2 PE=1 SV=5 |
|  | **Q14802\|FXYD3_HUMAN** | FXYD domain-containing ion transport regulator 3 OS=Homo sapiens GN=FXYD3 PE=2 SV=1 |
|  | **Q9BSE5\|SPEB_HUMAN** | Agmatinase, mitochondrial OS=Homo sapiens GN=AGMAT PE=1 SV=2 |
|  | **Q9UGV2\|NDRG3_HUMAN** | Protein NDRG3 OS=Homo sapiens GN=NDRG3 PE=1 SV=2 |
|  | **Q8NI51\|CTCFL_HUMAN** | Transcriptional repressor CTCFL OS=Homo sapiens GN=CTCFL PE=1 SV=1 |
|  | **Q8IYR2\|SMYD4_HUMAN** | SET and MYND domain-containing protein 4 OS=Homo sapiens GN=SMYD4 PE=2 SV=2 |
|  | **Q6PXP3\|GTR7_HUMAN** | Solute carrier family 2, facilitated glucose transporter member 7 OS=Homo sapiens GN=SLC2A7 PE=2 SV=2 |
|  | **P40429\|RL13A_HUMAN** | 60S ribosomal protein L13a OS=Homo sapiens GN=RPL13A PE=1 SV=2 |
|  | **Q6NVV1\|R13AX_HUMAN** | Putative 60S ribosomal protein L13a-like MGC87657 OS=Homo sapiens PE=5 SV=1 |
|  | **Q99102\|MUC4_HUMAN** | Mucin-4 OS=Homo sapiens GN=MUC4 PE=1 SV=2 |
|  | **Q8NBJ7\|SUMF2_HUMAN** | Sulfatase-modifying factor 2 OS=Homo sapiens GN=SUMF2 PE=1 SV=1 |
|  | **Q6XQN6\|PNCB_HUMAN** | Nicotinate phosphoribosyltransferase OS=Homo sapiens GN=NAPRT1 PE=1 SV=2 |
|  | **P68036\|UB2L3_HUMAN** | Ubiquitin-conjugating enzyme E2 L3 OS=Homo sapiens GN=UBE2L3 PE=1 SV=1 |
|  | **Q969X5\|ERGI1_HUMAN** | Endoplasmic reticulum-Golgi intermediate compartment protein 1 OS=Homo sapiens GN=ERGIC1 PE=1 SV=1 |
|  | **P11310\|ACADM_HUMAN** | Medium-chain specific acyl-CoA dehydrogenase, mitochondrial OS=Homo sapiens GN=ACADM PE=1 SV=1 |
|  | **Q8NI27\|THOC2_HUMAN** | THO complex subunit 2 OS=Homo sapiens GN=THOC2 PE=1 SV=2 |
|  | **P62888\|RL30_HUMAN** | 60S ribosomal protein L30 OS=Homo sapiens GN=RPL30 PE=1 SV=2 |
|  | **P31949\|S10AB_HUMAN** | Protein S100-A11 OS=Homo sapiens GN=S100A11 PE=1 SV=2 |
|  | **Q04941\|PLP2_HUMAN** | Proteolipid protein 2 OS=Homo sapiens GN=PLP2 PE=1 SV=1 |
|  | **P21281\|VATB2_HUMAN** | V-type proton ATPase subunit B, brain isoform OS=Homo sapiens GN=ATP6V1B2 PE=1 SV=3 |
|  | **P15924\|DESP_HUMAN** | Desmoplakin OS=Homo sapiens GN=DSP PE=1 SV=3 |
|  | **Q9Y619\|ORNT1_HUMAN** | Mitochondrial ornithine transporter 1 OS=Homo sapiens GN=SLC25A15 PE=1 SV=1 |
|  | **Q9BSJ8\|ESYT1_HUMAN** | Extended synaptotagmin-1 OS=Homo sapiens GN=ESYT1 PE=1 SV=1 |
|  | **Q9BPW8\|NIPS1_HUMAN** | Protein NipSnap homolog 1 OS=Homo sapiens GN=NIPSNAP1 PE=1 SV=1 |
|  | **O94874\|K0776_HUMAN** | UPF0555 protein KIAA0776 OS=Homo sapiens GN=KIAA0776 PE=1 SV=2 |
|  | **Q9Y2B0\|CNPY2_HUMAN** | Protein canopy homolog 2 OS=Homo sapiens GN=CNPY2 PE=1 SV=1 |
|  | **P0C7P4\|UCRIL_HUMAN** | Cytochrome b-c1 complex subunit Rieske-like protein 1 OS=Homo sapiens GN=UQCRFSL1 PE=2 SV=1 |
|  | **P46779\|RL28_HUMAN** | 60S ribosomal protein L28 OS=Homo sapiens GN=RPL28 PE=1 SV=3 |
|  | **P04220\|MUCB_HUMAN** | Ig mu heavy chain disease protein OS=Homo sapiens PE=1 SV=1 |
|  | **P22059\|OSBP1_HUMAN** | Oxysterol-binding protein 1 OS=Homo sapiens GN=OSBP PE=1 SV=1 |
|  | **Q13492\|PICAL_HUMAN** | Phosphatidylinositol-binding clathrin assembly protein OS=Homo sapiens GN=PICALM PE=1 SV=2 |
|  | **P11766\|ADHX_HUMAN** | Alcohol dehydrogenase class-3 OS=Homo sapiens GN=ADH5 PE=1 SV=4 |
|  | **P25786\|PSA1_HUMAN** | Proteasome subunit alpha type-1 OS=Homo sapiens GN=PSMA1 PE=1 SV=1 |
|  | **O15031\|PLXB2_HUMAN** | Plexin-B2 OS=Homo sapiens GN=PLXNB2 PE=1 SV=3 |
|  | **Q13636\|RAB31_HUMAN** | Ras-related protein Rab-31 OS=Homo sapiens GN=RAB31 PE=1 SV=1 |
|  | **Q9P225\|DYH2_HUMAN** | Dynein heavy chain 2, axonemal OS=Homo sapiens GN=DNAH2 PE=1 SV=3 |
|  | **P13747\|HLAE_HUMAN** | HLA class I histocompatibility antigen, alpha chain E OS=Homo sapiens GN=HLA-E PE=1 SV=3 |
|  | **Q96HE7\|ERO1A_HUMAN** | ERO1-like protein alpha OS=Homo sapiens GN=ERO1L PE=1 SV=2 |
|  | **P14210\|HGF_HUMAN** | Hepatocyte growth factor OS=Homo sapiens GN=HGF PE=1 SV=2 |
|  | **Q7L0R7\|RNF44_HUMAN** | RING finger protein 44 OS=Homo sapiens GN=RNF44 PE=2 SV=1 |
|  | **P09493\|TPM1_HUMAN** | Tropomyosin alpha-1 chain OS=Homo sapiens GN=TPM1 PE=1 SV=2 |
|  | **Q71UM5\|RS27L_HUMAN** | 40S ribosomal protein S27-like protein OS=Homo sapiens GN=RPS27L PE=1 SV=3 |
|  | **P83731\|RL24_HUMAN** | 60S ribosomal protein L24 OS=Homo sapiens GN=RPL24 PE=1 SV=1 |
|  | **Q9ULV0\|MYO5B_HUMAN** | Myosin-Vb OS=Homo sapiens GN=MYO5B PE=1 SV=2 |

Top of Form

Select Summary Report

|  | Peptide Summary Select Summary (protein hits) Select Summary (unassigned) Export Search Results |  | Help |
| --- | --- | --- | --- |
|  | Significance threshold p< | Max. number of hits |  |
|  | Standard scoring  MudPIT scoring | Ions score or expect cut-off | Show sub-sets |
|  | Show pop-ups  Suppress pop-ups | Sort unassigned Decreasing Score Increasing query / Mr Decreasing Intensity | Require bold red |

Bottom of Form

Top of Form

| **1.** | P63261\|ACTG_HUMAN    **Mass:** 42108    **Score:** 5817   **Queries matched:** 234   **emPAI:** 18.82 |
| --- | --- |
|  | Actin, cytoplasmic 2 OS=Homo sapiens GN=ACTG1 PE=1 SV=1 |

|  | **Query** | **Observed** | **Mr(expt)** | **Mr(calc)** | **Delta** | **Miss** | **Score** | **Expect** | **Rank** | **Peptide** |
| --- | --- | --- | --- | --- | --- | --- | --- | --- | --- | --- |
|  | 1426 | **631.3726** | **630.3653** | **630.3701** | **-0.0048** | **0** | **10** | **33** | **3** | **K.ILTER.G** 1421 |
|  | 1491 | **644.8003** | **643.7931** | **643.3653** | **0.4278** | **0** | **18** | **3** | **2** | **R.LDLAGR.D** |
|  | 2106 | **398.2369** | **794.4593** | **794.4650** | **-0.0057** | **0** | **16** | **4** | **1** | **K.IIAPPER.K** 2107 2108 |
|  | 2167 | **400.7687** | **799.5229** | **799.5280** | **-0.0050** | **1** | **24** | **0.83** | **2** | **K.RGILTLK.Y** |
|  | 3777 | **453.2261** | **904.4377** | **904.4436** | **-0.0059** | **1** | **16** | **4.8** | **5** | **K.CDVDIRK.D** |
|  | 4032 | **462.2841** | **922.5536** | **922.5600** | **-0.0063** | **1** | **25** | **0.52** | **1** | **K.IIAPPERK.Y** |
|  | 4771 | **488.7246** | **975.4346** | **975.4410** | **-0.0064** | **0** | **65** | **6.7e-005** | **1** | **K.AGFAGDDAPR.A** 4770 4780 |
|  | 5079 | **499.7437** | **997.4728** | **997.4790** | **-0.0062** | **0** | **29** | **0.18** | **1** | **R.DLTDYLMK.I** 5080 |
|  | 6866 | **566.7635** | **1131.5124** | **1131.5197** | **-0.0072** | **0** | **46** | **0.0037** | **1** | **R.GYSFTTTAER.E** 6863 6864 6867 6868 6872 |
|  | 7246 | **581.3093** | **1160.6041** | **1160.6111** | **-0.0070** | **0** | **39** | **0.022** | **1** | **K.EITALAPSTMK.I** 7227 7244 |
|  | 7368 | **586.2852** | **1170.5558** | **1170.5638** | **-0.0080** | **0** | **59** | **0.0002** | **1** | **R.HQGVMVGMGQK.D** 7365 7366 7367 7369 7370 7371 7379 7382 |
|  | 7612 | **594.2825** | **1186.5505** | **1186.5587** | **-0.0082** | **0** | **(52)** | **0.00089** | **1** | **R.HQGVMVGMGQK.D** 7611 |
|  | 7613 | **594.2828** | **1186.5511** | **1186.5587** | **-0.0076** | **0** | **(48)** | **0.0023** | **1** | **R.HQGVMVGMGQK.D** 7614 7631 |
|  | 7824 | **599.7609** | **1197.5072** | **1197.5150** | **-0.0078** | **0** | **27** | **0.29** | **1** | **K.DSYVGDEAQSK.R** 7825 |
|  | 7850 | **599.8526** | **1197.6906** | **1197.6982** | **-0.0076** | **0** | **65** | **4.6e-005** | **1** | **R.AVFPSIVGRPR.H** 7847 7848 7849 |
|  | 7942 | **602.2798** | **1202.5450** | **1202.5536** | **-0.0086** | **0** | **(26)** | **0.37** | **1** | **R.HQGVMVGMGQK.D** 7940 7941 |
|  | 10805 | **677.8108** | **1353.6070** | **1353.6161** | **-0.0090** | **1** | **44** | **0.0052** | **1** | **K.DSYVGDEAQSKR.G** 10804 10819 10829 |
|  | 13487 | **505.9182** | **1514.7328** | **1514.7419** | **-0.0091** | **0** | **46** | **0.0032** | **1** | **K.IWHHTFYNELR.V** 13481 13482 13483 13484 13485 13486 13490 13491 13492 13493 |
|  | 13503 | **758.8499** | **1515.6853** | **1515.6954** | **-0.0101** | **0** | **40** | **0.014** | **1** | **K.QEYDESGPSIVHR.K** 13474 13502 13504 13509 13528 |
|  | 14439 | **790.8826** | **1579.7506** | **1579.7949** | **-0.0443** | **1** | **2** | **77** | **4** | **R.MQKEITALAPSTMK.I** 14449 |
|  | 15103 | **541.9487** | **1622.8244** | **1622.8338** | **-0.0094** | **1** | **14** | **5.1** | **2** | **R.LDLAGRDLTDYLMK.I** |
|  | 16837 | **872.9008** | **1743.7870** | **1743.8792** | **-0.0922** | **1** | **3** | **52** | **4** | **K.ILTERGYSFTTTAER.E** |
|  | 17483 | **895.9439** | **1789.8733** | **1789.8846** | **-0.0114** | **0** | **73** | **6e-006** | **1** | **K.SYELPDGQVITIGNER.F** 17481 17482 17485 17486 17487 17488 |
|  | 19628 | **977.5291** | **1953.0437** | **1953.0571** | **-0.0134** | **0** | **62** | **6e-005** | **1** | **R.VAPEEHPVLLTEAPLNPK.A** 19625 19626 19627 19630 19631 |
|  | 22339 | **1108.0324** | **2214.0501** | **2214.0627** | **-0.0125** | **0** | **97** | **1.5e-008** | **1** | **K.DLYANTVLSGGTTMYPGIADR.M** 22318 22325 22326 22327 22328 22329 22330 22331 22332 22333 22334 22337 22340 22341 22342 22343 22346 22348 22349 22354 22360 22362 22363 22365 22366 22368 22372 |
|  | 22484 | **1116.0291** | **2230.0435** | **2230.0576** | **-0.0141** | **0** | **(68)** | **1.2e-005** | **1** | **K.DLYANTVLSGGTTMYPGIADR.M** 22483 |
|  | 23356 | **1172.0791** | **2342.1436** | **2342.1576** | **-0.0140** | **1** | **87** | **1.4e-007** | **1** | **R.KDLYANTVLSGGTTMYPGIADR.M** 23354 23355 23357 23358 23359 |
|  | 24885 | **1275.5845** | **2549.1544** | **2549.1665** | **-0.0121** | **0** | **133** | **2.8e-012** | **1** | **K.LCYVALDFEQEMATAASSSSLEK.S** 24847 24848 24849 24850 24851 24852 24853 24854 24855 24856 24857 24858 24859 24860 24861 24862 24864 24865 24866 24867 24868 24870 24872 24873 24874 24875 24878 24879 24880 24881 24882 24883 24884 24886 24888 24889 24890 24892 24893 24894 24896 24897 24898 24899 24903 24904 24906 24907 24911 24912 24916 24919 24920 24923 24924 24925 24928 24929 24930 24931 24932 24936 24937 24940 24948 24949 24950 24951 24953 24954 |
|  | 26379 | **936.4367** | **2806.2883** | **2806.3041** | **-0.0158** | **1** | **40** | **0.0065** | **1** | **K.EKLCYVALDFEQEMATAASSSSLEK.S** |
|  | 26391 | **937.1342** | **2808.3808** | **2808.2694** | **0.1114** | **1** | **1** | **49** | **1** | **M.EEEIAALVIDNGSGMCKAGFAGDDAPR.A** |
|  | 28157 | **1062.2048** | **3183.5927** | **3182.6071** | **0.9856** | **0** | **83** | **2.2e-007** | **1** | **R.TTGIVMDSGDGVTHTVPIYEGYALPHAILR.L** 28137 28138 28139 28140 28141 28142 28145 28147 28148 28152 |
|  | 28202 | **1067.2007** | **3198.5802** | **3198.6020** | **-0.0218** | **0** | **(45)** | **0.0017** | **1** | **R.TTGIVMDSGDGVTHTVPIYEGYALPHAILR.L** 28203 |
|  | 28327 | **1077.8208** | **3230.4406** | **3230.4545** | **-0.0139** | **0** | **62** | **2.7e-005** | **1** | **R.CPEALFQPSFLGMESCGIHETTFNSIMK.C** 28312 28314 28316 28317 28318 28319 28320 28321 28322 28323 28324 28325 28326 28332 28333 |
|  | 28418 | **1083.1512** | **3246.4319** | **3246.4494** | **-0.0175** | **0** | **(37)** | **0.0086** | **1** | **R.CPEALFQPSFLGMESCGIHETTFNSIMK.C** |
|  | 28420 | **1083.1519** | **3246.4338** | **3246.4494** | **-0.0157** | **0** | **(26)** | **0.11** | **1** | **R.CPEALFQPSFLGMESCGIHETTFNSIMK.C** 28419 |
|  | 28460 | **1085.2080** | **3252.6022** | **3252.6021** | **0.0001** | **0** | **74** | **1.9e-006** | **1** | **K.MTQIMFETFNTPAMYVAIQAVLSLYASGR.T** 28441 28442 28444 28450 28451 28453 28457 28461 |

| **2.** | P60709\|ACTB_HUMAN    **Mass:** 42052    **Score:** 5817   **Queries matched:** 234   **emPAI:** 18.82 |
| --- | --- |
|  | Actin, cytoplasmic 1 OS=Homo sapiens GN=ACTB PE=1 SV=1 |

|  | **Query** | **Observed** | **Mr(expt)** | **Mr(calc)** | **Delta** | **Miss** | **Score** | **Expect** | **Rank** | **Peptide** |
| --- | --- | --- | --- | --- | --- | --- | --- | --- | --- | --- |
|  | 1426 | 631.3726 | 630.3653 | 630.3701 | -0.0048 | 0 | 10 | 33 | 3 | K.ILTER.G 1421 |
|  | 1491 | 644.8003 | 643.7931 | 643.3653 | 0.4278 | 0 | 18 | 3 | 2 | R.LDLAGR.D |
|  | 2106 | 398.2369 | 794.4593 | 794.4650 | -0.0057 | 0 | 16 | 4 | 1 | K.IIAPPER.K 2107 2108 |
|  | 2167 | 400.7687 | 799.5229 | 799.5280 | -0.0050 | 1 | 24 | 0.83 | 2 | K.RGILTLK.Y |
|  | 3777 | 453.2261 | 904.4377 | 904.4436 | -0.0059 | 1 | 16 | 4.8 | 5 | K.CDVDIRK.D |
|  | 4032 | 462.2841 | 922.5536 | 922.5600 | -0.0063 | 1 | 25 | 0.52 | 1 | K.IIAPPERK.Y |
|  | 4771 | 488.7246 | 975.4346 | 975.4410 | -0.0064 | 0 | 65 | 6.7e-005 | 1 | K.AGFAGDDAPR.A 4770 4780 |
|  | 5079 | 499.7437 | 997.4728 | 997.4790 | -0.0062 | 0 | 29 | 0.18 | 1 | R.DLTDYLMK.I 5080 |
|  | 6866 | 566.7635 | 1131.5124 | 1131.5197 | -0.0072 | 0 | 46 | 0.0037 | 1 | R.GYSFTTTAER.E 6863 6864 6867 6868 6872 |
|  | 7246 | 581.3093 | 1160.6041 | 1160.6111 | -0.0070 | 0 | 39 | 0.022 | 1 | K.EITALAPSTMK.I 7227 7244 |
|  | 7368 | 586.2852 | 1170.5558 | 1170.5638 | -0.0080 | 0 | 59 | 0.0002 | 1 | R.HQGVMVGMGQK.D 7365 7366 7367 7369 7370 7371 7379 7382 |
|  | 7612 | 594.2825 | 1186.5505 | 1186.5587 | -0.0082 | 0 | (52) | 0.00089 | 1 | R.HQGVMVGMGQK.D 7611 |
|  | 7613 | 594.2828 | 1186.5511 | 1186.5587 | -0.0076 | 0 | (48) | 0.0023 | 1 | R.HQGVMVGMGQK.D 7614 7631 |
|  | 7824 | 599.7609 | 1197.5072 | 1197.5150 | -0.0078 | 0 | 27 | 0.29 | 1 | K.DSYVGDEAQSK.R 7825 |
|  | 7850 | 599.8526 | 1197.6906 | 1197.6982 | -0.0076 | 0 | 65 | 4.6e-005 | 1 | R.AVFPSIVGRPR.H 7847 7848 7849 |
|  | 7942 | 602.2798 | 1202.5450 | 1202.5536 | -0.0086 | 0 | (26) | 0.37 | 1 | R.HQGVMVGMGQK.D 7940 7941 |
|  | 10805 | 677.8108 | 1353.6070 | 1353.6161 | -0.0090 | 1 | 44 | 0.0052 | 1 | K.DSYVGDEAQSKR.G 10804 10819 10829 |
|  | 13487 | 505.9182 | 1514.7328 | 1514.7419 | -0.0091 | 0 | 46 | 0.0032 | 1 | K.IWHHTFYNELR.V 13481 13482 13483 13484 13485 13486 13490 13491 13492 13493 |
|  | 13503 | 758.8499 | 1515.6853 | 1515.6954 | -0.0101 | 0 | 40 | 0.014 | 1 | K.QEYDESGPSIVHR.K 13474 13502 13504 13509 13528 |
|  | 14439 | 790.8826 | 1579.7506 | 1579.7949 | -0.0443 | 1 | 2 | 77 | 4 | R.MQKEITALAPSTMK.I 14449 |
|  | 15103 | 541.9487 | 1622.8244 | 1622.8338 | -0.0094 | 1 | 14 | 5.1 | 2 | R.LDLAGRDLTDYLMK.I |
|  | 16837 | 872.9008 | 1743.7870 | 1743.8792 | -0.0922 | 1 | 3 | 52 | 4 | K.ILTERGYSFTTTAER.E |
|  | 17483 | 895.9439 | 1789.8733 | 1789.8846 | -0.0114 | 0 | 73 | 6e-006 | 1 | K.SYELPDGQVITIGNER.F 17481 17482 17485 17486 17487 17488 |
|  | 19628 | 977.5291 | 1953.0437 | 1953.0571 | -0.0134 | 0 | 62 | 6e-005 | 1 | R.VAPEEHPVLLTEAPLNPK.A 19625 19626 19627 19630 19631 |
|  | 22339 | 1108.0324 | 2214.0501 | 2214.0627 | -0.0125 | 0 | 97 | 1.5e-008 | 1 | K.DLYANTVLSGGTTMYPGIADR.M 22318 22325 22326 22327 22328 22329 22330 22331 22332 22333 22334 22337 22340 22341 22342 22343 22346 22348 22349 22354 22360 22362 22363 22365 22366 22368 22372 |
|  | 22484 | 1116.0291 | 2230.0435 | 2230.0576 | -0.0141 | 0 | (68) | 1.2e-005 | 1 | K.DLYANTVLSGGTTMYPGIADR.M 22483 |
|  | 23356 | 1172.0791 | 2342.1436 | 2342.1576 | -0.0140 | 1 | 87 | 1.4e-007 | 1 | R.KDLYANTVLSGGTTMYPGIADR.M 23354 23355 23357 23358 23359 |
|  | 24885 | 1275.5845 | 2549.1544 | 2549.1665 | -0.0121 | 0 | 133 | 2.8e-012 | 1 | K.LCYVALDFEQEMATAASSSSLEK.S 24847 24848 24849 24850 24851 24852 24853 24854 24855 24856 24857 24858 24859 24860 24861 24862 24864 24865 24866 24867 24868 24870 24872 24873 24874 24875 24878 24879 24880 24881 24882 24883 24884 24886 24888 24889 24890 24892 24893 24894 24896 24897 24898 24899 24903 24904 24906 24907 24911 24912 24916 24919 24920 24923 24924 24925 24928 24929 24930 24931 24932 24936 24937 24940 24948 24949 24950 24951 24953 24954 |
|  | 26379 | 936.4367 | 2806.2883 | 2806.3041 | -0.0158 | 1 | 40 | 0.0065 | 1 | K.EKLCYVALDFEQEMATAASSSSLEK.S |
|  | 26830 | **725.5831** | **2898.3032** | **2899.2423** | **-0.9391** | **1** | **0** | **50** | **8** | **-.MDDDIAALVVDNGSGMCKAGFAGDDAPR.A** |
|  | 28157 | 1062.2048 | 3183.5927 | 3182.6071 | 0.9856 | 0 | 83 | 2.2e-007 | 1 | R.TTGIVMDSGDGVTHTVPIYEGYALPHAILR.L 28137 28138 28139 28140 28141 28142 28145 28147 28148 28152 |
|  | 28202 | 1067.2007 | 3198.5802 | 3198.6020 | -0.0218 | 0 | (45) | 0.0017 | 1 | R.TTGIVMDSGDGVTHTVPIYEGYALPHAILR.L 28203 |
|  | 28327 | 1077.8208 | 3230.4406 | 3230.4545 | -0.0139 | 0 | 62 | 2.7e-005 | 1 | R.CPEALFQPSFLGMESCGIHETTFNSIMK.C 28312 28314 28316 28317 28318 28319 28320 28321 28322 28323 28324 28325 28326 28332 28333 |
|  | 28418 | 1083.1512 | 3246.4319 | 3246.4494 | -0.0175 | 0 | (37) | 0.0086 | 1 | R.CPEALFQPSFLGMESCGIHETTFNSIMK.C |
|  | 28420 | 1083.1519 | 3246.4338 | 3246.4494 | -0.0157 | 0 | (26) | 0.11 | 1 | R.CPEALFQPSFLGMESCGIHETTFNSIMK.C 28419 |
|  | 28460 | 1085.2080 | 3252.6022 | 3252.6021 | 0.0001 | 0 | 74 | 1.9e-006 | 1 | K.MTQIMFETFNTPAMYVAIQAVLSLYASGR.T 28441 28442 28444 28450 28451 28453 28457 28461 |

| **3.** | Q9Y6R7\|FCGBP_HUMAN    **Mass:** 596443   **Score:** 5317   **Queries matched:** 333   **emPAI:** 0.83 |
| --- | --- |
|  | IgGFc-binding protein OS=Homo sapiens GN=FCGBP PE=1 SV=3 |

|  | **Query** | **Observed** | **Mr(expt)** | **Mr(calc)** | **Delta** | **Miss** | **Score** | **Expect** | **Rank** | **Peptide** |
| --- | --- | --- | --- | --- | --- | --- | --- | --- | --- | --- |
|  | 1506 | **649.2015** | **648.1943** | **647.3312** | **0.8630** | **0** | **9** | **31** | **3** | **R.DILCK.A** |
|  | 1833 | **735.3652** | **734.3580** | **734.3633** | **-0.0053** | **0** | **22** | **1.3** | **1** | **K.AEMIGSK.I** 1835 |
|  | 2259 | **403.7093** | **805.4040** | **805.4082** | **-0.0042** | **0** | **32** | **0.12** | **1** | **R.VYQSGPR.A** 2255 2260 2261 |
|  | 2273 | **807.3511** | **806.3439** | **806.3493** | **-0.0055** | **0** | **15** | **5.3** | **1** | **R.NCWATR.G** |
|  | 2456 | **822.4159** | **821.4087** | **821.4139** | **-0.0053** | **0** | **22** | **1.1** | **1** | **R.LCGMLTK.L** 2457 |
|  | 2792 | **844.4209** | **843.4136** | **843.4199** | **-0.0062** | **0** | **12** | **15** | **2** | **K.NAAGDLQR.L** |
|  | 3036 | **430.7424** | **859.4703** | **859.4763** | **-0.0060** | **0** | **50** | **0.0021** | **1** | **R.ISVAQGASK.A** 3037 3051 3058 |
|  | 3573 | **446.2580** | **890.5015** | **889.4869** | **1.0146** | **0** | **15** | **6.6** | **1** | **R.ISVTQGASK.A** 3553 3563 3565 3569 3576 |
|  | 3718 | **451.2629** | **900.5113** | **899.4937** | **1.0176** | **1** | **5** | **58** | **3** | **R.QGRDAVVR.T** |
|  | 4174 | **467.7516** | **933.4886** | **933.4953** | **-0.0067** | **0** | **27** | **0.35** | **1** | **R.NMVLQTTK.G** |
|  | 4574 | **481.7492** | **961.4839** | **961.4903** | **-0.0064** | **0** | **32** | **0.13** | **1** | **K.VTVNGVDMK.L** 4586 4587 |
|  | 4576 | **481.7533** | **961.4920** | **961.4981** | **-0.0061** | **0** | **25** | **0.63** | **1** | **R.GNPAVSYVR.V** 4575 |
|  | 4629 | **483.7815** | **965.5484** | **965.5546** | **-0.0062** | **0** | **19** | **2** | **1** | **K.LVDPQGPLK.D** 4627 |
|  | 4655 | **484.7407** | **967.4668** | **967.4723** | **-0.0055** | **0** | **48** | **0.0021** | **1** | **R.GSQAVSYTR.S** 4647 4648 4649 4650 4652 4653 4654 4665 |
|  | 5040 | **498.7649** | **995.5153** | **994.5196** | **0.9957** | **0** | **26** | **0.37** | **1** | **R.VLVENEHR.G** 5023 5032 |
|  | 5071 | **499.3397** | **996.6649** | **997.4829** | **-0.8180** | **0** | **49** | **0.0018** | **1** | **R.GSQTVSYTR.A** 5049 5066 5081 5082 5097 5098 |
|  | 5130 | **501.3355** | **1000.6565** | **999.5059** | **1.1506** | **1** | **11** | **14** | **5** | **K.CPPELEKK.Y** |
|  | 5603 | **519.2712** | **1036.5278** | **1036.5342** | **-0.0064** | **0** | **21** | **1.2** | **1** | **K.FYPAGDVLR.V** 5592 5594 5602 |
|  | 6647 | **558.2973** | **1114.5800** | **1115.5281** | **-0.9480** | **0** | **1** | **1.4e+002** | **1** | **K.NECGILADPK.G** |
|  | 6720 | **560.7871** | **1119.5595** | **1119.5673** | **-0.0077** | **0** | **42** | **0.011** | **1** | **R.AYSHSVSLTR.G** 6719 |
|  | 7468 | **589.7274** | **1177.4402** | **1177.4492** | **-0.0091** | **0** | **23** | **0.7** | **1** | **R.GSCPTCPEDR.L** 7469 7471 |
|  | 7858 | **600.2494** | **1198.4842** | **1199.6622** | **-1.1780** | **1** | **14** | **6.3** | **2** | **R.SRLPVSLSEGR.L** 7857 |
|  | 7866 | **600.3265** | **1198.6385** | **1198.6459** | **-0.0073** | **0** | **24** | **0.69** | **2** | **K.AGVQVWLGANGK.V** |
|  | 8078 | **605.3289** | **1208.6433** | **1208.6513** | **-0.0080** | **0** | **54** | **0.00064** | **1** | **K.VAVIVSNDHAGK.L** 8076 8077 |
|  | 8398 | **613.2808** | **1224.5471** | **1224.5557** | **-0.0086** | **0** | **64** | **5.9e-005** | **1** | **K.AIGYATAADCGR.T** 8397 8399 |
|  | 8676 | **620.3250** | **1238.6355** | **1238.6442** | **-0.0087** | **0** | **50** | **0.0014** | **1** | **R.VVAEVQICHGK.T** 8677 |
|  | 8840 | **416.9168** | **1247.7285** | **1247.7350** | **-0.0065** | **1** | **17** | **3.3** | **1** | **R.RVSYVGLVTVR.A** |
|  | 9050 | **630.8309** | **1259.6472** | **1259.6550** | **-0.0078** | **0** | **30** | **0.16** | **1** | **R.VAYDLVYYVR.V** 9060 |
|  | 9354 | **638.3212** | **1274.6279** | **1274.6367** | **-0.0088** | **0** | **56** | **0.00035** | **1** | **K.LTYNHGGITGSR.G** 9353 |
|  | 9441 | **640.7853** | **1279.5561** | **1279.5649** | **-0.0088** | **0** | **37** | **0.026** | **1** | **K.AGCVAESTAVCR.A** 9427 9439 9440 |
|  | 9513 | **642.7845** | **1283.5545** | **1283.5638** | **-0.0093** | **0** | **28** | **0.22** | **1** | **R.QCVYDLCAQK.G** |
|  | 10371 | **665.3210** | **1328.6274** | **1328.7300** | **-0.1026** | **0** | **41** | **0.012** | **1** | **K.LDSLVAQQLQSK.N** |
|  | 10418 | **666.8444** | **1331.6743** | **1331.6834** | **-0.0091** | **0** | **72** | **1.1e-005** | **1** | **R.GEVGFVLVDNQR.S** 10412 10417 10445 |
|  | 10687 | **674.3134** | **1346.6121** | **1346.4326** | **0.1796** | **0** | **11** | **12** | **2** | **R.CFEGCECDDR.F** |
|  | 11520 | **698.2896** | **1394.5647** | **1394.5741** | **-0.0095** | **0** | **36** | **0.033** | **1** | **K.GCVLDVCMGGGDR.D** |
|  | 11776 | **705.3867** | **1408.7589** | **1408.7674** | **-0.0085** | **0** | **70** | **1.3e-005** | **1** | **K.AISGLTIDGHAVGAK.L** 11777 11779 |
|  | 11823 | **471.2894** | **1410.8464** | **1410.5690** | **0.2773** | **0** | **(21)** | **1.1** | **1** | **K.GCVLDVCMGGGDR.D** |
|  | 11831 | **706.8705** | **1411.7265** | **1411.7347** | **-0.0082** | **0** | **62** | **8.7e-005** | **1** | **R.YDLAFVVASQATK.L** |
|  | 11962 | **710.8340** | **1419.6534** | **1419.7180** | **-0.0646** | **1** | **5** | **38** | **10** | **R.QWKVTVNGVDMK.L** |
|  | 12042 | **712.8498** | **1423.6850** | **1423.6943** | **-0.0093** | **0** | **70** | **1.3e-005** | **1** | **R.GATTSPGVYELSSR.C** 12041 |
|  | 12537 | **486.2602** | **1455.7589** | **1454.7994** | **0.9595** | **1** | **3** | **67** | **8** | **R.QKAGVQVWLGANGK.V** |
|  | 13691 | **510.7738** | **1529.2997** | **1529.6715** | **-0.3718** | **0** | **1** | **62** | **9** | **R.SPANCPLSCPANSR.Y** |
|  | 14149 | **780.8945** | **1559.7744** | **1559.7845** | **-0.0101** | **0** | **63** | **5.6e-005** | **1** | **R.FAVLQENVAWGNGR.V** 14151 14162 14163 |
|  | 14220 | **783.3098** | **1564.6049** | **1563.6341** | **0.9709** | **0** | **39** | **0.013** | **1** | **R.CTCNGATHQVTCR.D** 14203 14221 14222 14223 14224 14225 14229 14230 |
|  | 14266 | **784.4387** | **1566.8629** | **1566.8730** | **-0.0101** | **0** | **72** | **8e-006** | **1** | **R.VNGVLTALPVSVADGR.I** 14265 14279 14280 14281 14282 14283 |
|  | 14925 | **807.3782** | **1612.7419** | **1613.8447** | **-1.1028** | **0** | **50** | **0.0011** | **1** | **K.SVPGCEGVALVVAQTK.A** 14946 |
|  | 15214 | **815.8511** | **1629.6877** | **1629.6988** | **-0.0111** | **0** | **86** | **2.7e-007** | **1** | **R.LEDGVQACHATGCGR.C** 15212 15213 |
|  | 15366 | **820.3582** | **1638.7019** | **1637.7290** | **0.9729** | **0** | **27** | **0.21** | **1** | **R.CSVQNGLLGCYPDR.F** 15365 15367 |
|  | 15676 | **830.9036** | **1659.7927** | **1658.8199** | **0.9728** | **0** | **11** | **9.7** | **1** | **R.CLANGGIHYITLDGR.V** |
|  | 15710 | **832.3782** | **1662.7418** | **1662.7784** | **-0.0366** | **1** | **3** | **51** | **7** | **K.LDPQGAVRDCVYDR.C** |
|  | 15913 | **838.9609** | **1675.9073** | **1675.9145** | **-0.0072** | **0** | **41** | **0.0094** | **1** | **K.EFAVVAGAAGASVSVTLK.G** 15894 15905 15917 15919 15940 |
|  | 16528 | **574.3517** | **1720.0332** | **1719.7352** | **0.2980** | **1** | **16** | **2.4** | **1** | **R.RCTCNGATHQVTCR.D** 16533 |
|  | 17577 | **899.9534** | **1797.8922** | **1797.9043** | **-0.0122** | **0** | **76** | **2.3e-006** | **1** | **K.VTASSPVAVLSGHSCAQK.H** 17576 17578 17579 |
|  | 18019 | **915.9470** | **1829.8795** | **1829.8908** | **-0.0113** | **0** | **89** | **1.4e-007** | **1** | **R.ASQHGSDVVIETDFGLR.V** 18017 18018 |
|  | 18162 | **920.4923** | **1838.9699** | **1839.8673** | **-0.8973** | **0** | **7** | **22** | **2** | **R.YLPVNSSLLTSDCSER.C** |
|  | 18235 | **922.8898** | **1843.7651** | **1843.7764** | **-0.0113** | **0** | **38** | **0.015** | **1** | **K.DPCHGVTCRPQETCK.E** 18234 |
|  | 18993 | **950.9123** | **1899.8101** | **1899.8237** | **-0.0136** | **0** | **109** | **1.1e-009** | **1** | **R.TCQGSCAALSGLTGCTTR.C** 18994 18995 |
|  | 19867 | **988.4421** | **1974.8696** | **1974.8822** | **-0.0126** | **0** | **83** | **4.8e-007** | **1** | **R.YYPLGEVFYPGPECER.R** 19865 19866 19868 |
|  | 20360 | **1012.9474** | **2023.8802** | **2023.8948** | **-0.0146** | **1** | **82** | **5.3e-007** | **1** | **K.GCVLDVCMGGGDRDILCK.A** 20346 20359 |
|  | 20407 | **1015.0273** | **2028.0400** | **2028.0503** | **-0.0103** | **0** | **83** | **4.6e-007** | **1** | **R.LLFDGDAHLLMSIPSPFR.G** 20406 20409 |
|  | 21065 | **1046.0366** | **2090.0587** | **2090.0731** | **-0.0144** | **0** | **112** | **6.1e-010** | **1** | **R.SLAAYTAACQAAGVAVKPWR.T** 21048 21066 21067 |
|  | 21176 | **1050.4147** | **2098.8148** | **2098.8289** | **-0.0141** | **0** | **103** | **3.6e-009** | **1** | **R.CSCSSSSGLTCQAAGCPPGR.V** 21175 21177 |
|  | 21251 | **1053.4785** | **2104.9425** | **2104.9558** | **-0.0133** | **0** | **59** | **0.00011** | **1** | **K.EEFCGLLSSPTGPLSSCHK.L** 21252 |
|  | 21696 | **1075.5325** | **2149.0504** | **2149.0626** | **-0.0122** | **0** | **13** | **4.5** | **1** | **K.ALASYVAACQAAGVVIEDWR.A** 21697 21711 21715 21722 21730 21735 |
|  | 21792 | **1077.5444** | **2153.0743** | **2152.0742** | **1.0001** | **0** | **90** | **9e-008** | **1** | **K.ALLVADFGLQVSYDWNWR.V** 21740 21752 21754 21755 21756 21758 21759 21760 21761 21766 21774 21775 21777 21790 |
|  | 22007 | **1088.9850** | **2175.9554** | **2175.9711** | **-0.0157** | **0** | **108** | **1.2e-009** | **1** | **R.TVLSPVEPSCEGMQCAAGQR.C** 22005 22006 |
|  | 22512 | **1117.5797** | **2233.1449** | **2233.1590** | **-0.0142** | **0** | **110** | **8.2e-010** | **1** | **R.VTLQPYNVAQLQSSVDLSGSK.V** 22511 22513 22514 22515 |
|  | 22621 | **1125.9936** | **2249.9727** | **2249.9874** | **-0.0147** | **0** | **70** | **6.4e-006** | **1** | **R.YYPLGQTFYPGPGCDSLCR.C** 22619 22620 22622 |
|  | 23798 | **1204.5356** | **2407.0567** | **2407.0719** | **-0.0152** | **0** | **70** | **6.6e-006** | **1** | **R.VDVTLPSSYHGAVCGLCGNMDR.N** 23796 23797 23799 23800 |
|  | 23862 | **806.7045** | **2417.0918** | **2417.1039** | **-0.0121** | **0** | **16** | **1.7** | **1** | **R.FLLSQGVCIPVQDCGCTHNGR.Y** 23884 |
|  | 24008 | **1216.6095** | **2431.2044** | **2431.2206** | **-0.0162** | **0** | **97** | **1.3e-008** | **1** | **K.TEAVGQVHIFFQDGMVTLTPNK.G** 24005 24006 24010 24011 24012 |
|  | 24042 | **812.3868** | **2434.1387** | **2434.2560** | **-0.1173** | **1** | **2** | **43** | **1** | **K.VTVRPGESVMVNISAKAEMIGSK.I** |
|  | 24102 | **612.2637** | **2445.0256** | **2445.0413** | **-0.0157** | **0** | **19** | **0.75** | **1** | **R.TDSFCPLHCPAHSHYSICTR.T** 24101 |
|  | 24126 | **1224.1400** | **2446.2655** | **2446.2802** | **-0.0148** | **0** | **98** | **1.1e-008** | **1** | **R.LLISSLSESPASVSILSQADNTSK.K** 24127 24128 |
|  | 24734 | **1263.0851** | **2524.1556** | **2524.1726** | **-0.0170** | **1** | **113** | **3.4e-010** | **1** | **K.YQKEEFCGLLSSPTGPLSSCHK.L** 24735 24736 24737 |
|  | 25314 | **1310.5757** | **2619.1368** | **2619.1556** | **-0.0188** | **0** | **60** | **5.8e-005** | **1** | **R.VTVPGNYYQLMCGLCGNYNGDPK.D** 25308 25313 25315 25316 |
|  | 25395 | **1318.0631** | **2634.1117** | **2634.1301** | **-0.0185** | **0** | **29** | **0.067** | **1** | **R.VTVPGNYYQQMCGLCGNYNGDPK.D** |
|  | 25586 | **890.3984** | **2668.1735** | **2668.1897** | **-0.0163** | **0** | **56** | **0.00017** | **1** | **R.VPAAYAGSLCGLCGNYNQDPADDLK.A** 25585 |
|  | 25689 | **1342.1010** | **2682.1873** | **2682.2054** | **-0.0180** | **0** | **61** | **5.3e-005** | **1** | **R.VPAAYAASLCGLCGNYNQDPADDLK.A** 25688 25690 |
|  | 25705 | **672.3187** | **2685.2455** | **2685.2615** | **-0.0159** | **0** | **55** | **0.00023** | **1** | **R.FDFMGTCVYVLAQTCGTRPGLHR.F** |
|  | 25946 | **905.6738** | **2713.9995** | **2714.0149** | **-0.0154** | **0** | **78** | **7.5e-007** | **1** | **R.CECGPGGHVTCQEGAACGPHEECR.L** |
|  | 26349 | **1399.1766** | **2796.3387** | **2795.2870** | **1.0517** | **0** | **4** | **24** | **2** | **R.FDFMGTCTYLLVGSCGQNAALPAFR.V** |
|  | 26655 | **957.7059** | **2870.0958** | **2870.1160** | **-0.0202** | **1** | **82** | **2.9e-007** | **1** | **R.RCECGPGGHVTCQEGAACGPHEECR.L** 26653 26654 26656 |
|  | 26664 | **1436.7192** | **2871.4239** | **2871.4405** | **-0.0165** | **0** | **61** | **4.4e-005** | **1** | **R.NEVTYDPYLVLIPDVAAYCPAYVVK.S** 26665 26666 |
|  | 27034 | **980.1252** | **2937.3537** | **2937.3749** | **-0.0212** | **1** | **72** | **3.3e-006** | **1** | **R.LRVPAAYAGSLCGLCGNYNQDPADDLK.A** 27037 27038 27041 |
|  | 27139 | **984.7978** | **2951.3715** | **2951.3905** | **-0.0190** | **1** | **86** | **1.4e-007** | **1** | **R.LRVPAAYAASLCGLCGNYNQDPADDLK.A** |
|  | 27332 | **1002.1175** | **3003.3306** | **3003.3494** | **-0.0187** | **0** | **37** | **0.0088** | **1** | **K.GMVCQEHSCKPGQVCQPSGGILSCVTK.D** |
|  | 27354 | **1005.4750** | **3013.4031** | **3013.4243** | **-0.0211** | **0** | **55** | **0.00016** | **1** | **K.VVVCQEHSCKPGQVCQPSGGILSCVTK.D** 27355 27356 |
|  | 27424 | **1009.8074** | **3026.4005** | **3026.4195** | **-0.0190** | **0** | **51** | **0.00036** | **1** | **K.VVVCQEHSCKPGQVCQPSGGILSCVNK.D** 27422 27423 |
|  | 27808 | **1037.4210** | **3109.2412** | **3109.2600** | **-0.0188** | **0** | **65** | **1.1e-005** | **1** | **K.LCGACGNFDGDQTNDWHDSQEKPAMEK.W** 27807 |
|  | 28128 | **1061.4380** | **3181.2921** | **3181.3145** | **-0.0223** | **0** | **78** | **5.8e-007** | **1** | **R.EGCVCDAGFVLSGDTCVPVGQCGCLHDGR.Y** 28129 |
|  | 28186 | **1597.8086** | **3193.6026** | **3193.6084** | **-0.0058** | **0** | **58** | **8.4e-005** | **1** | **R.EYPGQVLVDDVLQYLPFQAADGQVQVFR.Q** 28185 |
|  | 28371 | **1080.7736** | **3239.2989** | **3239.3200** | **-0.0211** | **0** | **70** | **3.6e-006** | **1** | **R.EGCVCDAGFVLSGDTCVPVGQCGCLHDDR.Y** 28370 |
|  | 28864 | **1128.5359** | **3382.5858** | **3382.6009** | **-0.0151** | **0** | **36** | **0.0098** | **1** | **R.CLLPGQSGPLCDALATYAAACQAAGATVHPWR.S** 28863 28865 |
|  | 28902 | **1130.8257** | **3389.4552** | **3389.4790** | **-0.0237** | **0** | **61** | **3.2e-005** | **1** | **R.GGGQAANALAFGNSWQEETRPGCGATEPGDCPK.L** 28903 28908 |
|  | 29164 | **1163.9075** | **3488.7006** | **3488.7260** | **-0.0254** | **0** | **56** | **0.0001** | **1** | **K.HTTCNHVVEQLLPTSAWGTHYVVPTLASQSR.Y** 29165 29166 29168 |
|  | 29225 | **1171.8061** | **3512.3966** | **3512.4160** | **-0.0193** | **0** | **64** | **1.3e-005** | **1** | **K.GCGEGCGPQGCPVCLAEETAPYESNEACGQLR.N** 29224 |
|  | 29366 | **891.4246** | **3561.6691** | **3561.6909** | **-0.0217** | **0** | **36** | **0.0094** | **1** | **R.VYDLHGSCSYVLAQVCHPKPGDEDFSIVLEK.N** 29364 29365 |
|  | 29511 | **1205.7940** | **3614.3600** | **3614.3828** | **-0.0227** | **0** | **82** | **1.5e-007** | **1** | **K.LDDGDYLCEDGCQNNCPACTPGQAQHYEGDR.L** 29510 29512 |
|  | 29881 | **934.4349** | **3733.7107** | **3733.7328** | **-0.0221** | **0** | **20** | **0.32** | **1** | **K.LDGPFAVCHDTLDPRPFLEQCVYDLCVVGGER.L** 29882 |
|  | 30028 | **1263.9105** | **3788.7097** | **3788.7392** | **-0.0295** | **0** | **33** | **0.017** | **1** | **R.LEQYEGPGFCGPLAPGTGGPFTTCHAHVPPESFFK.G** 30030 30031 30032 30033 30034 |
|  | 30360 | **1324.9440** | **3971.8101** | **3971.1309** | **0.6792** | **0** | **1** | **20** | **1** | **R.GLQAGDVVEFEVRPSWPLYLSANVGIQVLLFGTGAIR.N** |
|  | 30708 | **1416.6195** | **4246.8367** | **4246.8525** | **-0.0158** | **0** | **1** | **17** | **1** | **R.FQDQVCGLCGNYNGDPADDFLTPDGALAPDAVEFASSWK.L** 30709 |
|  | 30781 | **866.1420** | **4325.6735** | **4325.7031** | **-0.0297** | **1** | **3** | **8.2** | **1** | **R.CECGPGGHVTCQEGAACGPHEECRLEDGVQACHATGCGR.C** |
|  | 30909 | **1485.7450** | **4454.2132** | **4453.2039** | **1.0093** | **0** | **35** | **0.0074** | **1** | **K.LTWEAVPGSEFSYAEVELGTADMIHTAEATTNLGLLTFGLAK.A** 30880 30882 30884 30886 30887 30888 30890 30894 30897 30898 30903 30908 30910 |
|  | 31085 | **1548.2769** | **4641.8088** | **4641.8382** | **-0.0294** | **0** | **41** | **0.0011** | **1** | **R.RPDFCPFQCPAHSHYELCGDSCPGSCPSLSAPEGCESACR.E** 31084 31088 |
|  | 31101 | **1163.4757** | **4649.8737** | **4649.9008** | **-0.0271** | **0** | **32** | **0.008** | **1** | **R.RPDFCPLQCPAHSHYELCGDSCPVSCPSLSAPEGCESACR.E** 31099 31100 31102 |
|  | 31235 | **1211.8815** | **4843.4968** | **4843.5073** | **-0.0106** | **0** | **21** | **0.12** | **1** | **K.LQVDGVFVTLPFQLDSLLHAHLSGADVVVTTTSGLSLAFDGDSFVR.L** 31236 31237 |
|  | 31247 | **971.4374** | **4852.1508** | **4852.1853** | **-0.0345** | **1** | **10** | **1.6** | **1** | **K.VVVCQEHSCKPGQVCQPSGGILSCVNKDPCHGVTCRPQETCK.E** 31248 |
|  | 31384 | **990.6367** | **4948.1469** | **4948.1779** | **-0.0310** | **1** | **35** | **0.0043** | **1** | **R.GSCPTCPEDRLEQYEGPGFCGPLAPGTGGPFTTCHAHVPPESFFK.G** 31385 31386 31388 31389 |
|  | 31673 | **1368.8240** | **5471.2668** | **5471.2968** | **-0.0301** | **1** | **13** | **0.42** | **1** | **R.EGCVCDAGFVLSGDTCVPVGQCGCLHDDRYYPLGQTFYPGPGCDSLCR.C** 31674 31675 |
|  | 31889 | **1409.0021** | **5631.9792** | **5632.0093** | **-0.0301** | **0** | **34** | **0.0035** | **1** | **K.IFQHAVVIHSDYAISVQALNAKPDTAELTLLRPIQALGTEYFVLTPPGTSAR.N** |
|  | 31997 | **1595.6661** | **6378.6355** | **6377.6755** | **0.9600** | **0** | **23** | **0.026** | **1** | **R.AQVGCEITCPENSHYEVCGPPCPASCPSPAPLTTPAVCEGPCVEGCQCDAGFVLSADR.C** 31996 |

| **4.** | Q6S8J3\|POTEE_HUMAN    **Mass:** 122882   **Score:** 3878   **Queries matched:** 139   **emPAI:** 0.34 |
| --- | --- |
|  | POTE ankyrin domain family member E OS=Homo sapiens GN=POTEE PE=1 SV=3 |

|  | **Query** | **Observed** | **Mr(expt)** | **Mr(calc)** | **Delta** | **Miss** | **Score** | **Expect** | **Rank** | **Peptide** |
| --- | --- | --- | --- | --- | --- | --- | --- | --- | --- | --- |
|  | 1426 | 631.3726 | 630.3653 | 630.3701 | -0.0048 | 0 | 10 | 33 | 3 | K.ILTER.G 1421 |
|  | 1491 | 644.8003 | 643.7931 | 643.3653 | 0.4278 | 0 | 18 | 3 | 2 | R.LDLAGR.E |
|  | 2167 | 400.7687 | 799.5229 | 799.5280 | -0.0050 | 1 | 24 | 0.83 | 2 | K.RGILTLK.Y |
|  | 4771 | 488.7246 | 975.4346 | 975.4410 | -0.0064 | 0 | 65 | 6.7e-005 | 1 | K.AGFAGDDAPR.A 4770 4780 |
|  | 7769 | **398.8939** | **1193.6599** | **1192.5831** | **1.0768** | **0** | **16** | **3.5** | **3** | **K.EIAALAPSMMK.I** |
|  | 7850 | 599.8526 | 1197.6906 | 1197.6982 | -0.0076 | 0 | 65 | 4.6e-005 | 1 | R.AVFPSIVGRPR.Q 7847 7848 7849 |
|  | 8825 | **624.6529** | **1247.2912** | **1247.5209** | **-0.2297** | **0** | **8** | **18** | **1** | **R.QQGMMGGMHQK.E** |
|  | 12142 | **715.4202** | **1428.8259** | **1429.7785** | **-0.9526** | **1** | **(1)** | **1e+002** | **7** | **K.EIAALAPSMMKIR.I** |
|  | 12676 | **488.5925** | **1462.7556** | **1461.7683** | **0.9873** | **1** | **4** | **54** | **8** | **K.EIAALAPSMMKIR.I** |
|  | 12988 | **495.2776** | **1482.8109** | **1482.6734** | **0.1375** | **0** | **6** | **33** | **4** | **R.ELENFMAIEEMK.K** |
|  | 13098 | **497.2987** | **1488.8742** | **1489.6103** | **-0.7361** | **0** | **11** | **9.6** | **2** | **K.SNVGASGDHDDSAMK.T** 13078 |
|  | 13261 | **750.3462** | **1498.6778** | **1498.6683** | **0.0095** | **0** | **(3)** | **59** | **5** | **R.ELENFMAIEEMK.K** |
|  | 13487 | 505.9182 | 1514.7328 | 1514.7419 | -0.0091 | 0 | 46 | 0.0032 | 1 | K.IWHHTFYNELR.V 13481 13482 13483 13484 13485 13486 13490 13491 13492 13493 |
|  | 13503 | 758.8499 | 1515.6853 | 1515.6954 | -0.0101 | 0 | 40 | 0.014 | 1 | K.QEYDESGPSIVHR.K 13474 13502 13504 13509 13528 |
|  | 13778 | **512.9346** | **1535.7821** | **1535.6158** | **0.1662** | **0** | **7** | **25** | **2** | **K.SNVGTSGDHDDSAMK.T** |
|  | 13944 | **774.3884** | **1546.7622** | **1546.8025** | **-0.0403** | **1** | **10** | **14** | **3** | **K.DLIVMLRDTDVNK.K** |
|  | 13957 | **774.8814** | **1547.7483** | **1547.7873** | **-0.0391** | **1** | **15** | **4** | **4** | **R.MQKEIAALAPSMMK.I** 13967 |
|  | 14204 | **522.2353** | **1563.6840** | **1564.7477** | **-1.0636** | **0** | **4** | **49** | **3** | **-.MVVEVDSMPAASSVK.K** |
|  | 14218 | **522.3247** | **1563.9523** | **1563.7822** | **0.1701** | **1** | **(4)** | **46** | **3** | **R.MQKEIAALAPSMMK.I** |
|  | 14219 | **522.4940** | **1564.4602** | **1563.7822** | **0.6780** | **1** | **(4)** | **36** | **3** | **R.MQKEIAALAPSMMK.I** 14214 |
|  | 14877 | **537.5832** | **1609.7277** | **1609.8424** | **-0.1146** | **0** | **0** | **1.1e+002** | **10** | **R.TALHLASANGNSEVVK.L** |
|  | 15186 | **543.3829** | **1627.1268** | **1626.7633** | **0.3635** | **1** | **6** | **22** | **4** | **R.ELENFMAIEEMKK.H** |
|  | 16563 | **862.4316** | **1722.8487** | **1722.8532** | **-0.0044** | **1** | **11** | **9.4** | **3** | **R.EEIAMLRLELDTMK.H** 16566 |
|  | 16944 | **876.7690** | **1751.5234** | **1750.9539** | **0.5696** | **1** | **12** | **4.4** | **2** | **K.LMAKALLLYGADIESK.N** 16941 |
|  | 17143 | **589.3338** | **1764.9796** | **1765.9435** | **-0.9639** | **1** | **0** | **1e+002** | **5** | **K.RTALHLASANGNSEVVK.L** |
|  | 17483 | 895.9439 | 1789.8733 | 1789.8846 | -0.0114 | 0 | 73 | 6e-006 | 1 | K.SYELPDGQVITIGNER.F 17481 17482 17485 17486 17487 17488 |
|  | 18107 | **918.4613** | **1834.9080** | **1834.7752** | **0.1329** | **1** | **2** | **65** | **5** | **R.GSGKSNVGASGDHDDSAMK.T** |
|  | 19084 | **636.3518** | **1906.0336** | **1905.8487** | **0.1849** | **1** | **4** | **35** | **5** | **K.SNVGTSGDHDDSAMKTLR.S** |
|  | 19508 | **648.3791** | **1942.1156** | **1941.8448** | **0.2708** | **1** | **0** | **79** | **3** | **K.DGDRELENFMAIEEMK.K** |
|  | 20046 | **665.3938** | **1993.1596** | **1993.8550** | **-0.6954** | **0** | **1** | **69** | **8** | **K.YPMEHGIITNWDDMEK.I** |
|  | 20815 | **688.3658** | **2062.0755** | **2062.9477** | **-0.8721** | **1** | **3** | **45** | **8** | **K.GSENSQPEKMSQELEINK.D** 20825 |
|  | 22405 | **741.3660** | **2221.0763** | **2220.2226** | **0.8537** | **1** | **4** | **29** | **3** | **R.TALHLASANGNSEVVKLLLDR.R** |
|  | 24885 | 1275.5845 | 2549.1544 | 2549.1665 | -0.0121 | 0 | 133 | 2.8e-012 | 1 | K.LCYVALDFEQEMATAASSSSLEK.S 24847 24848 24849 24850 24851 24852 24853 24854 24855 24856 24857 24858 24859 24860 24861 24862 24864 24865 24866 24867 24868 24870 24872 24873 24874 24875 24878 24879 24880 24881 24882 24883 24884 24886 24888 24889 24890 24892 24893 24894 24896 24897 24898 24899 24903 24904 24906 24907 24911 24912 24916 24919 24920 24923 24924 24925 24928 24929 24930 24931 24932 24936 24937 24940 24948 24949 24950 24951 24953 24954 |
|  | 25888 | **677.0647** | **2704.2297** | **2703.2455** | **0.9842** | **1** | **0** | **58** | **5** | **K.DLYTNTVLSGGTTMYPGMAHRMQK.E** |
|  | 26379 | 936.4367 | 2806.2883 | 2806.3041 | -0.0158 | 1 | 40 | 0.0065 | 1 | K.EKLCYVALDFEQEMATAASSSSLEK.S |
|  | 26593 | **714.8475** | **2855.3610** | **2854.3035** | **1.0575** | **0** | **1** | **43** | **7** | **K.ALQLNELTMDDDTAVLVIDNGSGMCK.A** |

| **5.** | A8K7I4\|CLCA1_HUMAN    **Mass:** 100847   **Score:** 3464   **Queries matched:** 219   **emPAI:** 4.62 |
| --- | --- |
|  | Calcium-activated chloride channel regulator 1 OS=Homo sapiens GN=CLCA1 PE=1 SV=2 |

|  | **Query** | **Observed** | **Mr(expt)** | **Mr(calc)** | **Delta** | **Miss** | **Score** | **Expect** | **Rank** | **Peptide** |
| --- | --- | --- | --- | --- | --- | --- | --- | --- | --- | --- |
|  | 1232 | **587.3104** | **586.3031** | **585.3598** | **0.9433** | **0** | **17** | **4.9** | **1** | **R.IQAVR.C** 1220 |
|  | 1535 | **653.3455** | **652.3382** | **652.3432** | **-0.0050** | **0** | **20** | **1.4** | **1** | **K.LETYK.N** 1540 1541 |
|  | 1594 | **669.2983** | **668.2910** | **668.2952** | **-0.0042** | **0** | **19** | **1.6** | **1** | **R.CTFNK.V** |
|  | 1918 | **760.3453** | **759.3380** | **759.4068** | **-0.0688** | **1** | **10** | **26** | **4** | **K.RFYFK.N** |
|  | 2079 | **397.2295** | **792.4444** | **792.4494** | **-0.0050** | **0** | **29** | **0.25** | **1** | **R.SAFTVIR.K** 2078 |
|  | 2225 | **402.7239** | **803.4331** | **803.4389** | **-0.0057** | **0** | **36** | **0.051** | **1** | **R.SIQLESK.G** |
|  | 2304 | **809.4350** | **808.4277** | **808.4331** | **-0.0053** | **0** | **28** | **0.28** | **1** | **K.VTGLYEK.G** 2287 2300 2301 |
|  | 2546 | **414.7353** | **827.4560** | **827.4613** | **-0.0053** | **0** | **72** | **1.1e-005** | **1** | **R.ALGGVNAAR.R** 2547 2548 2574 2579 |
|  | 2765 | **421.2454** | **840.4763** | **840.4817** | **-0.0054** | **0** | **41** | **0.013** | **1** | **R.QGASPILR.A** 2770 |
|  | 2848 | **424.7317** | **847.4488** | **847.4552** | **-0.0064** | **0** | **26** | **0.47** | **1** | **K.ADYVRPK.L** 2852 2853 2854 2863 2877 2882 |
|  | 2876 | **425.2242** | **848.4339** | **848.4392** | **-0.0054** | **0** | **35** | **0.069** | **1** | **K.QGGFVVDK.N** |
|  | 2972 | **428.7165** | **855.4185** | **855.4239** | **-0.0054** | **0** | **23** | **0.77** | **1** | **K.FYLSNGR.I** |
|  | 3400 | **440.6995** | **879.3844** | **879.3869** | **-0.0024** | **0** | **20** | **1.7** | **1** | **K.SGSMATGNR.L** 3401 3402 |
|  | 3542 | **445.2376** | **888.4607** | **888.4665** | **-0.0057** | **0** | **30** | **0.22** | **1** | **K.SEISNIAR.V** 3527 |
|  | 3551 | **445.7377** | **889.4608** | **889.4658** | **-0.0049** | **0** | **28** | **0.31** | **1** | **R.STWEVIR.D** 3550 3557 |
|  | 4545 | **480.2808** | **958.5471** | **958.5521** | **-0.0051** | **0** | **47** | **0.0037** | **1** | **R.IVCLVLDK.S** |
|  | 4980 | **496.3388** | **990.6631** | **989.4930** | **1.1701** | **0** | **4** | **63** | **5** | **K.LAEYGPQGR.A** |
|  | 5332 | **509.2972** | **1016.5799** | **1016.5866** | **-0.0067** | **0** | **57** | **0.00037** | **1** | **R.ISTSILDLR.D** 5334 |
|  | 5888 | **530.7101** | **1059.4056** | **1059.4114** | **-0.0058** | **0** | **32** | **0.11** | **1** | **K.CQGGSCYTK.R** 5889 5904 |
|  | 6158 | **539.3026** | **1076.5907** | **1076.5978** | **-0.0072** | **1** | **28** | **0.24** | **1** | **K.TKADYVRPK.L** 6157 |
|  | 6231 | **542.1302** | **1082.2458** | **1082.6309** | **-0.3850** | **1** | **8** | **16** | **1** | **K.VRALGGVNAAR.R** |
|  | 6397 | **548.2631** | **1094.5117** | **1094.5179** | **-0.0062** | **0** | **45** | **0.0042** | **1** | **K.GCEFVLQSR.Q** 6396 6410 |
|  | 6692 | **559.7974** | **1117.5802** | **1117.5880** | **-0.0078** | **1** | **43** | **0.0089** | **1** | **K.KLAEYGPQGR.A** 6693 6713 |
|  | 7709 | **596.3003** | **1190.5861** | **1191.6248** | **-1.0387** | **1** | **13** | **8.7** | **2** | **K.QGGFVVDKNTK.M** |
|  | 7968 | **602.8383** | **1203.6621** | **1203.6685** | **-0.0064** | **0** | **36** | **0.04** | **1** | **K.MAYLQIPGIAK.V** |
|  | 8015 | **603.8070** | **1205.5995** | **1205.6074** | **-0.0080** | **0** | **32** | **0.09** | **1** | **R.CSAGITGTNVVK.K** |
|  | 8127 | **606.3388** | **1210.6631** | **1210.6710** | **-0.0079** | **0** | **32** | **0.09** | **1** | **R.IHLTPDFIAGK.K** 8128 8129 |
|  | 8620 | **619.2733** | **1236.5321** | **1236.5411** | **-0.0090** | **0** | **40** | **0.013** | **1** | **R.YFTTYDTNGR.Y** 8619 8621 8647 8649 |
|  | 9167 | **633.3263** | **1264.6380** | **1264.6465** | **-0.0085** | **0** | **46** | **0.0034** | **1** | **R.AFVHEWAHLR.W** 9168 9169 |
|  | 9499 | **642.3679** | **1282.7212** | **1282.7285** | **-0.0073** | **0** | **44** | **0.0057** | **1** | **K.NVAILIPETWK.T** 9471 9501 9506 |
|  | 9589 | **644.8552** | **1287.6958** | **1287.7034** | **-0.0076** | **0** | **59** | **0.00017** | **1** | **R.ASVTALIESVNGK.T** 9578 9582 9590 9617 9618 |
|  | 10463 | **667.8541** | **1333.6936** | **1333.7024** | **-0.0088** | **1** | **16** | **3.4** | **1** | **R.CSAGITGTNVVKK.C** |
|  | 10553 | **447.2602** | **1338.7587** | **1338.7660** | **-0.0073** | **1** | **28** | **0.2** | **1** | **R.IHLTPDFIAGKK.L** |
|  | 13472 | **758.3180** | **1514.6214** | **1514.6314** | **-0.0100** | **0** | **73** | **6.6e-006** | **1** | **R.WGVFDEYNNDEK.F** 13470 13471 13473 13501 |
|  | 14184 | **521.7861** | **1562.3364** | **1562.8490** | **-0.5126** | **1** | **5** | **27** | **6** | **K.NTKMAYLQIPGIAK.V** |
|  | 15857 | **837.4353** | **1672.8560** | **1672.8679** | **-0.0118** | **1** | **103** | **5.5e-009** | **1** | **K.RLPAAASGGTSICSGLR.S** 15856 15858 15859 15860 15861 |
|  | 16083 | **845.4252** | **1688.8358** | **1687.8628** | **0.9729** | **0** | **24** | **0.4** | **1** | **K.TVTLELLDNGAGADATK.D** 16082 |
|  | 16768 | **870.4392** | **1738.8637** | **1738.8811** | **-0.0174** | **0** | **4** | **44** | **1** | **K.DMVTQASLYLLEATGK.R** |
|  | 17672 | **602.9933** | **1805.9582** | **1805.9676** | **-0.0094** | **1** | **39** | **0.013** | **1** | **K.DTSKFPSPLVVYANIR.Q** 17671 17673 |
|  | 18416 | **928.4874** | **1854.9602** | **1854.9687** | **-0.0085** | **0** | **98** | **1.5e-008** | **1** | **K.YSLQASSQTLTLTVTSR.A** 18391 18393 18394 18403 18405 18406 18407 18408 18409 18411 18412 18413 18414 18415 18417 18420 18423 18424 18426 18427 18430 18431 18432 18433 18434 |
|  | 20401 | **676.9267** | **2027.7583** | **2028.8814** | **-1.1231** | **1** | **1** | **60** | **4** | **K.DDGVYSRYFTTYDTNGR.Y** |
|  | 23237 | **1164.5972** | **2327.1798** | **2327.1944** | **-0.0146** | **0** | **65** | **2.5e-005** | **1** | **K.TTPMTTQPPNPTFSLLQIGQR.I** 23232 23233 23234 23235 23236 23238 23239 |
|  | 23366 | **1172.5951** | **2343.1756** | **2343.1893** | **-0.0137** | **0** | **(53)** | **0.00036** | **1** | **K.TTPMTTQPPNPTFSLLQIGQR.I** 23365 |
|  | 23429 | **785.0170** | **2352.0291** | **2352.0447** | **-0.0156** | **1** | **32** | **0.04** | **1** | **R.WGVFDEYNNDEKFYLSNGR.I** |
|  | 24151 | **817.0983** | **2448.2730** | **2448.2860** | **-0.0130** | **0** | **79** | **9e-007** | **1** | **K.QSGAIIHTVALGPSAAQELEELSK.M** 24149 24150 24152 24153 |
|  | 24210 | **819.4325** | **2455.2757** | **2455.2893** | **-0.0137** | **1** | **6** | **17** | **1** | **K.KTTPMTTQPPNPTFSLLQIGQR.I** |
|  | 24376 | **1241.1010** | **2480.1873** | **2480.2031** | **-0.0157** | **1** | **98** | **1.2e-008** | **1** | **K.TVTLELLDNGAGADATKDDGVYSR.Y** 24373 24374 24375 24377 24378 24379 24380 24381 24382 |
|  | 25889 | **902.4172** | **2704.2297** | **2704.2374** | **-0.0077** | **0** | **51** | **0.00053** | **1** | **K.ASIMFAQHVDSIVEFCTEQNHNK.E** 25862 25864 25865 25866 25870 25873 25876 25901 |
|  | 26356 | **1399.7365** | **2797.4583** | **2797.4691** | **-0.0107** | **0** | **62** | **3.6e-005** | **1** | **K.DTLFLITWTTQPPQILLWDPSGQK.Q** 26347 26357 26358 |
|  | 27204 | **1487.1786** | **2972.3426** | **2972.3597** | **-0.0171** | **0** | **72** | **2.9e-006** | **1** | **K.YPTDGSEIVLLTDGEDNTISGCFNEVK.Q** 27205 27210 |
|  | 27395 | **1511.6470** | **3021.2794** | **3021.2968** | **-0.0174** | **0** | **69** | **5.1e-006** | **1** | **K.NADVLVAESTPPGNDEPYTEQMGNCGEK.G** 27390 27391 27393 27394 27398 27399 |
|  | 27449 | **1514.2615** | **3026.5084** | **3026.5237** | **-0.0153** | **0** | **64** | **2.1e-005** | **1** | **R.TSSGGSFVASDVPNAPIPDLFPPGQITDLK.A** 27446 27447 27448 27450 27453 27455 27456 27457 27465 |
|  | 27786 | **1034.4866** | **3100.4379** | **3100.4547** | **-0.0168** | **1** | **104** | **1.9e-009** | **1** | **K.KYPTDGSEIVLLTDGEDNTISGCFNEVK.Q** |
|  | 28783 | **1122.1541** | **3363.4403** | **3363.4619** | **-0.0216** | **1** | **38** | **0.005** | **1** | **K.NADVLVAESTPPGNDEPYTEQMGNCGEKGER.I** 28788 |
|  | 29267 | **1176.5591** | **3526.6554** | **3526.6747** | **-0.0193** | **0** | **82** | **2.2e-007** | **1** | **K.MTGGLQTYASDQVQNNGLIDAFGALSSGNGAVSQR.S** 29265 29270 29273 29274 29275 29276 29277 29280 |
|  | 29671 | **1219.5147** | **3655.5221** | **3655.6294** | **-0.1073** | **1** | **5** | **9.2** | **1** | **K.LETYKNADVLVAESTPPGNDEPYTEQMGNCGEK.G** |
|  | 31113 | **1554.1161** | **4659.3264** | **4658.3439** | **0.9825** | **0** | **66** | **4.8e-006** | **1** | **R.LNQAGQLFLLQTVELGSWVGMVTFDSAAHVQSELIQINSGSDR.D** 31108 |
|  | 31504 | **1297.6599** | **5186.6105** | **5186.6347** | **-0.0241** | **1** | **75** | **4.8e-007** | **1** | **R.LNQAGQLFLLQTVELGSWVGMVTFDSAAHVQSELIQINSGSDRDTLAK.R** 31502 31503 31505 31506 31507 31508 31509 31510 31511 31512 31514 31515 31517 31519 31520 31521 31522 |
|  | 31535 | **1301.6615** | **5202.6169** | **5202.6296** | **-0.0127** | **1** | **(24)** | **0.055** | **1** | **R.LNQAGQLFLLQTVELGSWVGMVTFDSAAHVQSELIQINSGSDRDTLAK.R** |

| **6.** | P14410\|SUIS_HUMAN    **Mass:** 210698   **Score:** 2803   **Queries matched:** 224   **emPAI:** 1.97 |
| --- | --- |
|  | Sucrase-isomaltase, intestinal OS=Homo sapiens GN=SI PE=1 SV=5 |

|  | **Query** | **Observed** | **Mr(expt)** | **Mr(calc)** | **Delta** | **Miss** | **Score** | **Expect** | **Rank** | **Peptide** |
| --- | --- | --- | --- | --- | --- | --- | --- | --- | --- | --- |
|  | 1095 | **560.3351** | **559.3278** | **558.3126** | **1.0152** | **0** | **10** | **35** | **5** | **K.DIGVR.G** |
|  | 1160 | **573.3511** | **572.3438** | **571.3442** | **0.9996** | **0** | **19** | **3.7** | **1** | **K.LNLGR.N** |
|  | 1261 | **594.3201** | **593.3128** | **593.3173** | **-0.0045** | **0** | **23** | **0.65** | **1** | **R.GYINK.S** |
|  | 1470 | **641.3189** | **640.3116** | **640.3254** | **-0.0138** | **0** | **22** | **0.71** | **1** | **K.LMYSK.T** |
|  | 1568 | **660.3666** | **659.3594** | **659.3854** | **-0.0260** | **0** | **8** | **46** | **5** | **K.SLDVVK.E** |
|  | 1981 | **785.3885** | **784.3812** | **784.3868** | **-0.0056** | **0** | **22** | **1** | **1** | **R.HDLSWK.T** |
|  | 2157 | **400.2654** | **798.5162** | **799.5028** | **-0.9866** | **1** | **10** | **15** | **6** | **K.RGIVISR.S** |
|  | 2217 | **804.3828** | **803.3755** | **803.3814** | **-0.0058** | **0** | **24** | **0.9** | **1** | **R.DQPPGYK.L** |
|  | 2286 | **404.6660** | **807.3174** | **807.3221** | **-0.0047** | **0** | **16** | **4.4** | **1** | **R.WDNMDK.S** 2285 2293 |
|  | 2559 | **829.4116** | **828.4043** | **828.4090** | **-0.0046** | **0** | **10** | **22** | **6** | **K.ITDPNNR.R** 2564 |
|  | 2825 | **423.7258** | **845.4370** | **844.4192** | **1.0178** | **0** | **13** | **9.9** | **2** | **R.TDGLHFR.T** |
|  | 3204 | **434.7081** | **867.4017** | **867.4086** | **-0.0069** | **0** | **8** | **24** | **8** | **R.STYPTSGR.W** 3211 3230 |
|  | 3390 | **440.2421** | **878.4696** | **878.4749** | **-0.0053** | **0** | **41** | **0.013** | **1** | **R.LYDVEIK.E** 3389 |
|  | 3449 | **442.2160** | **882.4175** | **882.4236** | **-0.0060** | **0** | **22** | **0.99** | **1** | **K.TYPAFER.G** 3448 |
|  | 3571 | **446.2468** | **890.4790** | **890.4861** | **-0.0071** | **1** | **7** | **36** | **1** | **K.IYDPQKK.R** |
|  | 3614 | **448.2182** | **894.4219** | **894.4269** | **-0.0050** | **0** | **38** | **0.024** | **1** | **K.NDMLQFK.I** 3599 3627 |
|  | 4233 | **470.2386** | **938.4626** | **938.4682** | **-0.0056** | **0** | **28** | **0.24** | **1** | **R.NHNIANTR.R** 4228 4230 4231 4232 4234 4235 4236 4238 4240 4241 4243 4260 |
|  | 5042 | **498.7816** | **995.5486** | **995.5553** | **-0.0066** | **0** | **55** | **0.00041** | **1** | **R.LGSLHVWGK.G** |
|  | 5555 | **517.7635** | **1033.5124** | **1033.5193** | **-0.0068** | **0** | **45** | **0.0057** | **1** | **R.GQQNDVFVK.W** 5554 5556 |
|  | 5772 | **526.2568** | **1050.4991** | **1050.5056** | **-0.0064** | **0** | **45** | **0.0047** | **1** | **R.VDMYLPADK.I** 5773 |
|  | 5790 | **527.2395** | **1052.4644** | **1052.4716** | **-0.0071** | **0** | **24** | **0.55** | **1** | **R.WFDYHTGK.D** |
|  | 6093 | **537.2873** | **1072.5600** | **1072.5665** | **-0.0065** | **0** | **24** | **0.79** | **1** | **K.ENPFGIQIR.R** |
|  | 6305 | **544.7820** | **1087.5495** | **1087.5563** | **-0.0068** | **1** | **26** | **0.48** | **1** | **R.FRHDLSWK.T** |
|  | 6566 | **554.7626** | **1107.5107** | **1106.5145** | **0.9962** | **0** | **33** | **0.085** | **1** | **R.TSTAEWWAR.E** |
|  | 7174 | **578.7762** | **1155.5378** | **1155.5448** | **-0.0070** | **0** | **21** | **1.1** | **1** | **R.EIVDFYNEK.M** 7187 |
|  | 7256 | **581.7946** | **1161.5747** | **1161.5818** | **-0.0072** | **0** | **29** | **0.22** | **1** | **R.YEVPHQYVK.E** 7255 |
|  | 8010 | **603.8025** | **1205.5904** | **1205.5982** | **-0.0078** | **0** | **33** | **0.083** | **1** | **R.AHVAFPDFFR.T** 8012 |
|  | 8690 | **620.7770** | **1239.5395** | **1239.5480** | **-0.0084** | **0** | **68** | **2.2e-005** | **1** | **R.QDNSYSVNSAR.Y** 8689 8708 8711 |
|  | 9865 | **651.8623** | **1301.7100** | **1301.7191** | **-0.0090** | **0** | **79** | **1.7e-006** | **1** | **K.ILGLTDSVTEVR.V** 9864 9866 9899 |
|  | 10153 | **440.2320** | **1317.6741** | **1317.6830** | **-0.0088** | **1** | **47** | **0.0031** | **1** | **R.RYEVPHQYVK.E** 10154 10178 10182 |
|  | 10554 | **447.2668** | **1338.7786** | **1338.7871** | **-0.0084** | **1** | **29** | **0.17** | **1** | **R.IKLPSDPISTLR.V** 10555 |
|  | 12004 | **711.8233** | **1421.6320** | **1421.6432** | **-0.0111** | **0** | **46** | **0.0031** | **1** | **K.TICMDAVQNWGK.Q** |
|  | 12117 | **714.8848** | **1427.7550** | **1428.8340** | **-1.0790** | **0** | **56** | **0.00033** | **1** | **K.YVIILDPAISIGR.R** 12139 12140 12143 12144 12145 |
|  | 12165 | **716.3264** | **1430.6383** | **1430.6480** | **-0.0097** | **0** | **42** | **0.0087** | **1** | **R.WGGHWLGDNYAR.W** 12166 12167 |
|  | 12299 | **720.3991** | **1438.7837** | **1437.6381** | **1.1456** | **0** | **(24)** | **0.52** | **1** | **K.TICMDAVQNWGK.Q** |
|  | 13299 | **751.8618** | **1501.7091** | **1501.7176** | **-0.0085** | **0** | **40** | **0.014** | **1** | **R.WMQLGAFYPFSR.N** |
|  | 13549 | **760.3610** | **1518.7074** | **1517.7125** | **0.9948** | **0** | **51** | **0.001** | **1** | **R.WMQLGAFYPYSR.N** 13536 |
|  | 13727 | **766.8986** | **1531.7826** | **1531.7922** | **-0.0097** | **0** | **63** | **6.2e-005** | **1** | **K.LNYPPFTPDILDK.L** 13726 13746 |
|  | 13823 | **770.8496** | **1539.6845** | **1539.6929** | **-0.0083** | **0** | **60** | **0.00013** | **1** | **R.DLNWNTWGMFTR.D** 13822 13824 13849 |
|  | 15394 | **820.8944** | **1639.7741** | **1638.8076** | **0.9666** | **1** | **(4)** | **41** | **3** | **K.NDMLQFKIYDPQK.K** |
|  | 15527 | **825.4229** | **1648.8311** | **1648.8421** | **-0.0109** | **0** | **68** | **1.6e-005** | **1** | **K.GTTPVNAVTLTYNGNK.N** 15522 15528 15533 |
|  | 15606 | **828.3968** | **1654.7791** | **1654.8025** | **-0.0233** | **1** | **6** | **28** | **1** | **K.NDMLQFKIYDPQK.K** |
|  | 15662 | **830.4462** | **1658.8779** | **1657.8187** | **1.0592** | **1** | **7** | **24** | **2** | **R.RWMQLGAFYPFSR.N** |
|  | 15812 | **836.3647** | **1670.7149** | **1670.7246** | **-0.0097** | **0** | **65** | **3.2e-005** | **1** | **R.FNCYPDADLATEQK.C** 15811 15827 |
|  | 15817 | **836.4038** | **1670.7931** | **1670.8040** | **-0.0109** | **0** | **61** | **8.6e-005** | **1** | **K.EFTGPTVSDTLYDVK.V** 15815 15816 |
|  | 15992 | **561.3737** | **1681.0993** | **1680.9167** | **0.1826** | **0** | **2** | **55** | **8** | **R.YTLLPFLYTLFYK.A** |
|  | 16415 | **571.6030** | **1711.7873** | **1711.7889** | **-0.0016** | **1** | **8** | **17** | **5** | **K.RDLNWNTWGMFTR.D** |
|  | 17019 | **878.9492** | **1755.8839** | **1755.9342** | **-0.0503** | **1** | **1** | **86** | **8** | **R.VDMYLPADKIGLHLR.G** |
|  | 17124 | **882.9267** | **1763.8388** | **1763.8512** | **-0.0124** | **0** | **74** | **4.5e-006** | **1** | **K.NSLPFNEDTTNMILR.I** 17134 |
|  | 18154 | **920.4380** | **1838.8615** | **1838.8727** | **-0.0111** | **0** | **45** | **0.003** | **1** | **R.NDELNYPPYFPELTK.R** 18152 18153 18155 18156 |
|  | 19218 | **959.4933** | **1916.9720** | **1916.9844** | **-0.0123** | **0** | **86** | **2.8e-007** | **1** | **R.GGYIIPIQEPDVTTTASR.K** 19217 19219 19220 |
|  | 19411 | **645.3384** | **1932.9933** | **1933.0309** | **-0.0376** | **1** | **31** | **0.079** | **1** | **R.LYDVEIKENPFGIQIR.R** |
|  | 20456 | **1016.9758** | **2031.9370** | **2031.9506** | **-0.0136** | **0** | **67** | **1.8e-005** | **1** | **R.INCIPEQFPTEGICAQR.G** 20452 20453 20454 20455 20470 |
|  | 20776 | **1030.5107** | **2059.0069** | **2059.9529** | **-0.9460** | **1** | **2** | **56** | **4** | **K.LMYSKTICMDAVQNWGK.Q** |
|  | 20915 | **1037.9858** | **2073.9571** | **2073.9691** | **-0.0119** | **0** | **65** | **3.2e-005** | **1** | **R.GGHILPCQEPAQNTFYSR.Q** 20914 20916 |
|  | 21927 | **723.1811** | **2166.5214** | **2166.1103** | **0.4112** | **1** | **1** | **45** | **4** | **R.YSSMGITADLQLNTANARIK.L** |
|  | 22076 | **1093.9872** | **2185.9598** | **2185.9725** | **-0.0127** | **0** | **86** | **2.1e-007** | **1** | **R.EAGIPFDTQVTDIDYMEDK.K** 22077 |
|  | 22286 | **1106.0459** | **2210.0772** | **2210.0896** | **-0.0123** | **0** | **69** | **1.1e-005** | **1** | **R.QLDFTIGEAFQDLPQFVDK.I** 22290 |
|  | 22653 | **1128.5787** | **2255.1429** | **2255.1548** | **-0.0119** | **0** | **68** | **1.4e-005** | **1** | **R.TVGGILDFYMFLGPTPEVATK.Q** 22649 22654 22655 22659 22660 22661 22663 22664 22666 22668 22669 22670 22671 22684 22686 22688 |
|  | 23082 | **1152.5859** | **2303.1573** | **2303.1685** | **-0.0112** | **0** | **63** | **3.9e-005** | **1** | **R.YEVPVPLNIPTTPISTYEDR.L** 23080 23081 |
|  | 23150 | **1158.0339** | **2314.0533** | **2314.0675** | **-0.0142** | **1** | **60** | **7.1e-005** | **1** | **R.EAGIPFDTQVTDIDYMEDKK.D** 23149 |
|  | 24236 | **1230.6345** | **2459.2545** | **2459.2696** | **-0.0151** | **1** | **43** | **0.0035** | **1** | **K.RYEVPVPLNIPTTPISTYEDR.L** 24235 |
|  | 24419 | **1244.1351** | **2486.2557** | **2486.2693** | **-0.0136** | **0** | **86** | **1.6e-007** | **1** | **K.TLFDTSIGPLVYSDQYLQISAR.L** 24420 24421 24423 24424 24425 24427 24428 24429 24454 |
|  | 24570 | **1251.5941** | **2501.1737** | **2501.1897** | **-0.0160** | **0** | **75** | **2.1e-006** | **1** | **K.QYDVHSLYGYSMAIATEQAVQK.V** 24569 |
|  | 24604 | **836.4154** | **2506.2244** | **2507.1863** | **-0.9619** | **1** | **1** | **53** | **5** | **R.QDPASWNETFAEMSRNILNIR.Y** |
|  | 24712 | **1260.5748** | **2519.1351** | **2519.1492** | **-0.0141** | **0** | **60** | **7.1e-005** | **1** | **K.QGADTVSAYIPDAIWYDYESGAK.R** 24711 24713 24722 |
|  | 25262 | **870.0721** | **2607.1944** | **2606.4180** | **0.7764** | **1** | **1** | **59** | **2** | **K.IGLHLRGGYIIPIQEPDVTTTASR.K** |
|  | 25292 | **1309.0986** | **2616.1827** | **2616.1993** | **-0.0166** | **0** | **60** | **6.2e-005** | **1** | **R.NHNSDGYEHQDPAFFGQNSLLVK.S** 25289 25290 25291 25294 25302 |
|  | 25432 | **1320.6606** | **2639.3067** | **2639.3173** | **-0.0105** | **0** | **98** | **8.9e-009** | **1** | **R.VIWDSWLPGFAFNDQFIQISTR.L** 25431 25433 25434 |
|  | 26223 | **924.7852** | **2771.3337** | **2771.3465** | **-0.0128** | **0** | **45** | **0.002** | **1** | **K.QYHEVIGHPVMPAYWALGFQLCR.Y** |
|  | 26240 | **925.4699** | **2773.3879** | **2773.4035** | **-0.0156** | **0** | **45** | **0.0019** | **1** | **R.IPSPTLFGNDINSVLFTTQNQTPNR.F** |
|  | 26688 | **1438.2246** | **2874.4347** | **2874.4448** | **-0.0102** | **0** | **64** | **2e-005** | **1** | **K.SFGVFLMNSNAMEIFIQPTPIVTYR.V** 26680 26685 26693 26704 26705 |
|  | 26928 | **973.7902** | **2918.3487** | **2918.3624** | **-0.0138** | **0** | **65** | **1.8e-005** | **1** | **K.DFTYDQVAFNGLPQFVQDLHDHGQK.Y** 26927 26929 26935 |
|  | 27084 | **737.8873** | **2947.5200** | **2946.3645** | **1.1555** | **1** | **4** | **21** | **1** | **R.NHNSDGYEHQDPAFFGQNSLLVKSSR.Q** |
|  | 27309 | **1499.6708** | **2997.3270** | **2997.3412** | **-0.0142** | **0** | **67** | **9.6e-006** | **1** | **R.ELYDAMVAANIPYDVQYTDIDYMER.Q** 27305 27306 27308 |
|  | 27589 | **762.6172** | **3046.4397** | **3046.4574** | **-0.0177** | **1** | **27** | **0.11** | **1** | **K.KDFTYDQVAFNGLPQFVQDLHDHGQK.Y** 27594 |
|  | 27824 | **1039.1156** | **3114.3250** | **3114.3448** | **-0.0198** | **0** | **1** | **38** | **3** | **R.DQLPGDNNNNLYGHQTFFMCIEDTSGK.S** 27823 |
|  | 28600 | **1654.3464** | **3306.6783** | **3306.6900** | **-0.0117** | **0** | **55** | **0.00014** | **1** | **K.QFLWGPAFMVTPVLEPYVQTVNAYVPNAR.W** 28599 |
|  | 30014 | **1262.5795** | **3784.7166** | **3785.8795** | **-1.1629** | **1** | **2** | **20** | **7** | **R.VAENNQPMNAHSNFTYDASNQVLLIADLKLNLGR.N** 30011 30012 |
|  | 30285 | **1313.2438** | **3936.7095** | **3936.8121** | **-0.1026** | **1** | **1** | **19** | **4** | **K.TICMDAVQNWGKQYDVHSLYGYSMAIATEQAVQK.V** |
|  | 30966 | **646.0220** | **4515.1029** | **4516.2544** | **-1.1515** | **1** | **1** | **17** | **1** | **K.ILGLTDSVTEVRVAENNQPMNAHSNFTYDASNQVLLIADLK.L** |
|  | 31301 | **1220.3224** | **4877.2605** | **4877.2894** | **-0.0289** | **0** | **42** | **0.00088** | **1** | **K.LNSYGFHPYYMALEEEGNAHGVFLLNSNAMDVTFQPTPALTYR.T** 31300 31303 31305 31308 31309 31311 31313 31316 |
|  | 31375 | **1235.3894** | **4937.5285** | **4937.5466** | **-0.0181** | **0** | **32** | **0.0078** | **1** | **R.VTGGILDFYILLGDTPEQVVQQYQQLVGLPAMPAYWNLGFQLSR.W** 31374 |
|  | 31701 | **1844.1349** | **5529.3828** | **5528.8271** | **0.5557** | **1** | **0** | **9.2** | **3** | **R.VTGGILDFYILLGDTPEQVVQQYQQLVGLPAMPAYWNLGFQLSRWNYK.S** |

| **7.** | P55157\|MTP_HUMAN    **Mass:** 99916    **Score:** 2419   **Queries matched:** 137   **emPAI:** 3.77 |
| --- | --- |
|  | Microsomal triglyceride transfer protein large subunit OS=Homo sapiens GN=MTTP PE=1 SV=1 |

|  | **Query** | **Observed** | **Mr(expt)** | **Mr(calc)** | **Delta** | **Miss** | **Score** | **Expect** | **Rank** | **Peptide** |
| --- | --- | --- | --- | --- | --- | --- | --- | --- | --- | --- |
|  | 968 | **531.3465** | **530.3392** | **530.3428** | **-0.0035** | **0** | **12** | **20** | **7** | **R.ALISK.F** 967 |
|  | 1511 | **649.2020** | **648.1948** | **647.3238** | **0.8709** | **1** | **11** | **21** | **10** | **K.KEDTR.M** |
|  | 2294 | **405.1943** | **808.3741** | **808.3789** | **-0.0048** | **0** | **14** | **7** | **6** | **R.FEMPASK.I** |
|  | 2584 | **415.7156** | **829.4167** | **829.4195** | **-0.0028** | **0** | **13** | **11** | **5** | **R.IYHQNR.K** 2583 |
|  | 2776 | **421.7682** | **841.5218** | **841.5273** | **-0.0055** | **0** | **21** | **1.4** | **1** | **K.LILGGLEK.A** 2774 |
|  | 2914 | **426.2540** | **850.4935** | **850.4986** | **-0.0051** | **0** | **38** | **0.028** | **1** | **R.MYLLALK.N** 2915 |
|  | 2972 | 428.7165 | 855.4185 | 856.5382 | -1.1197 | 1 | 11 | 13 | 6 | K.LKAVVEAK.K |
|  | 3191 | **434.2176** | **866.4207** | **866.4935** | **-0.0728** | **0** | **(14)** | **5.7** | **6** | **R.MYLLALK.N** |
|  | 3258 | **435.7291** | **869.4436** | **869.4494** | **-0.0058** | **0** | **31** | **0.14** | **1** | **K.ATSVTTYK.I** |
|  | 3570 | **446.2272** | **890.4398** | **890.4458** | **-0.0059** | **0** | **14** | **8.4** | **1** | **K.GSIGSSDIR.E** 3569 |
|  | 4204 | **469.2373** | **936.4600** | **936.4665** | **-0.0064** | **0** | **(39)** | **0.021** | **1** | **K.LQDSVGYR.I** 4205 |
|  | 4681 | **970.6232** | **969.6159** | **969.6222** | **-0.0063** | **1** | **55** | **0.00046** | **1** | **K.KLILGGLEK.A** 4683 4698 |
|  | 6154 | **539.2782** | **1076.5418** | **1076.5502** | **-0.0084** | **0** | **28** | **0.27** | **1** | **K.YLQPDNLSK.A** |
|  | 6631 | **557.3441** | **1112.6736** | **1112.6805** | **-0.0069** | **1** | **47** | **0.0033** | **1** | **K.KEEILQILK.M** 6612 6630 |
|  | 6892 | **567.7495** | **1133.4845** | **1133.4924** | **-0.0079** | **0** | **28** | **0.26** | **1** | **K.EMVAHNYDR.F** 6893 6894 6895 |
|  | 6923 | **568.7604** | **1135.5062** | **1135.5114** | **-0.0052** | **1** | **57** | **0.00035** | **1** | **R.KLCQNEGCK.L** 6920 6922 6928 |
|  | 7044 | **573.7914** | **1145.5682** | **1146.5880** | **-1.0198** | **0** | **57** | **0.00036** | **1** | **K.SDSSIILQER.F** |
|  | 7305 | **583.8079** | **1165.6013** | **1165.6091** | **-0.0078** | **1** | **21** | **1.3** | **2** | **K.FKGSIGSSDIR.E** 7317 |
|  | 7957 | **602.7887** | **1203.5628** | **1203.5706** | **-0.0078** | **0** | **34** | **0.06** | **1** | **K.GCPSLSELWR.S** |
|  | 7992 | **603.3257** | **1204.6368** | **1204.6452** | **-0.0083** | **1** | **47** | **0.0034** | **1** | **R.KYLQPDNLSK.A** 7991 |
|  | 8506 | **411.2213** | **1230.6421** | **1229.7165** | **0.9256** | **1** | **5** | **54** | **7** | **R.LMSGKQAAAIIK.A** |
|  | 9023 | **629.8533** | **1257.6920** | **1257.6982** | **-0.0062** | **0** | **43** | **0.0078** | **1** | **R.NFLAFIQHLR.T** |
|  | 9516 | **642.8136** | **1283.6126** | **1283.6218** | **-0.0092** | **0** | **17** | **2.5** | **1** | **K.GHTTGLSLNNDR.L** |
|  | 9764 | **648.8470** | **1295.6795** | **1295.6874** | **-0.0078** | **0** | **53** | **0.00072** | **1** | **R.SNLNIFQYIGK.A** 9765 |
|  | 9886 | **652.3460** | **1302.6773** | **1302.6853** | **-0.0080** | **0** | **13** | **6.9** | **1** | **K.MLSASGDPISVVK.G** 9887 9902 |
|  | 9996 | **655.8508** | **1309.6870** | **1308.6925** | **0.9945** | **0** | **54** | **0.00062** | **1** | **K.LTYSTEVLLDR.G** 9977 |
|  | 10063 | **657.8189** | **1313.6233** | **1313.6324** | **-0.0091** | **0** | **58** | **0.00022** | **1** | **K.DVNVENVNQQR.G** 10064 |
|  | 10185 | **660.3881** | **1318.7616** | **1318.6803** | **0.0813** | **0** | **(12)** | **8.4** | **1** | **K.MLSASGDPISVVK.G** |
|  | 10422 | **666.8772** | **1331.7398** | **1331.7483** | **-0.0084** | **0** | **79** | **2e-006** | **1** | **R.ETVMIITGTLVR.K** 10421 |
|  | 10468 | **667.8759** | **1333.7371** | **1333.7428** | **-0.0056** | **0** | **49** | **0.002** | **1** | **K.YMLAIVQDILR.F** 10467 |
|  | 10549 | **670.3388** | **1338.6629** | **1338.6707** | **-0.0078** | **0** | **27** | **0.25** | **1** | **R.YDLPFITDEVK.K** |
|  | 10971 | **682.9078** | **1363.8010** | **1363.8075** | **-0.0065** | **0** | **28** | **0.22** | **1** | **K.NALLPEGIPSLLK.Y** 10968 |
|  | 11058 | **685.4560** | **1368.8974** | **1367.7119** | **1.1856** | **1** | **2** | **63** | **1** | **K.EDTRMYLLALK.N** |
|  | 11196 | **689.3126** | **1376.6107** | **1376.6208** | **-0.0101** | **0** | **58** | **0.00024** | **1** | **R.SGSSSAYTGYIER.S** 11195 |
|  | 12493 | **726.8972** | **1451.7799** | **1451.7885** | **-0.0086** | **1** | **37** | **0.023** | **1** | **R.RSNLNIFQYIGK.A** 12494 |
|  | 12729 | **489.9265** | **1466.7577** | **1466.7657** | **-0.0080** | **1** | **35** | **0.039** | **1** | **R.YDLPFITDEVKK.T** 12728 |
|  | 13578 | **761.3937** | **1520.7728** | **1520.7835** | **-0.0107** | **0** | **65** | **4.1e-005** | **1** | **R.SGFTTPNQVLGVSSK.A** 13579 |
|  | 15122 | **812.8810** | **1623.7474** | **1623.7570** | **-0.0095** | **0** | **61** | **8.7e-005** | **1** | **K.VVFAPQPDSTSSGWF.-** |
|  | 16213 | **849.9531** | **1697.8917** | **1697.9022** | **-0.0105** | **0** | **62** | **7e-005** | **1** | **K.NILLSIGELPQEMNK.Y** 16214 |
|  | 16274 | **851.8962** | **1701.7778** | **1701.7880** | **-0.0102** | **0** | **58** | **0.00019** | **1** | **R.NPDGDDDQLIQITMK.D** 16273 |
|  | 17928 | **913.0004** | **1823.9862** | **1822.9928** | **0.9933** | **0** | **75** | **3.3e-006** | **1** | **R.VTVVITTDITVDSSFVK.A** 17903 17907 17929 17930 17931 17932 |
|  | 18285 | **924.4719** | **1846.9293** | **1846.9400** | **-0.0107** | **0** | **82** | **6.7e-007** | **1** | **K.YTAIPIVGQVFQSHCK.G** 18284 18286 18287 |
|  | 19032 | **951.9892** | **1901.9638** | **1901.9734** | **-0.0096** | **0** | **37** | **0.022** | **1** | **R.SASTYSLDILYSGSGILR.R** |
|  | 19068 | **953.4843** | **1904.9541** | **1904.9666** | **-0.0125** | **0** | **93** | **4.5e-008** | **1** | **R.TAAAAIILNNNPSYMDVK.N** 19067 19070 19071 19079 19083 |
|  | 19234 | **960.0453** | **1918.0760** | **1916.9156** | **1.1605** | **0** | **19** | **1.2** | **2** | **K.EFYSYQNEAVAIENIK.R** 19233 |
|  | 20349 | **1012.4824** | **2022.9502** | **2022.9622** | **-0.0120** | **0** | **71** | **7.4e-006** | **1** | **R.FLYACGFASHPNEELLR.A** 20347 20348 |
|  | 20978 | **694.7274** | **2081.1603** | **2081.1745** | **-0.0143** | **0** | **43** | **0.0045** | **1** | **K.ENLEALQRPTLLHLIHGK.V** |
|  | 21243 | **1053.0958** | **2104.1771** | **2104.1892** | **-0.0121** | **0** | **99** | **1.1e-008** | **1** | **K.GLILLIDHSQELQLQSGLK.A** 21241 21254 |
|  | 21663 | **1073.0400** | **2144.0655** | **2144.0789** | **-0.0134** | **1** | **105** | **3e-009** | **1** | **K.VKEFYSYQNEAVAIENIK.R** 21648 21664 21665 |
|  | 23536 | **1183.0081** | **2364.0016** | **2364.0184** | **-0.0169** | **0** | **85** | **2e-007** | **1** | **K.ESVLAGCEFPLHQENSEMCK.V** 23535 23537 |
|  | 25357 | **1314.1271** | **2626.2396** | **2626.2545** | **-0.0149** | **0** | **90** | **6.4e-008** | **1** | **R.GLASLFQTQLSSGTTNEVDISGNCK.V** 25356 |
|  | 25437 | **1320.6622** | **2639.3099** | **2639.3053** | **0.0046** | **0** | **28** | **0.1** | **1** | **K.ANIEVQGGLAIDISGAMEFSLWYR.E** |
|  | 27088 | **1474.7676** | **2947.5206** | **2947.5331** | **-0.0125** | **0** | **82** | **3.2e-007** | **1** | **K.IEDSFVIAVLAEETHNFGLNFLQTIK.G** 27082 27087 27089 27091 27097 27101 27102 27105 27112 27114 27121 27123 |
|  | 28039 | **1582.8095** | **3163.6043** | **3163.6176** | **-0.0133** | **0** | **107** | **1e-009** | **1** | **K.EVLPQLVDAVTSAQTSDSLEAILDFLDFK.S** 28033 28034 28035 28047 28049 28050 28054 28056 28060 28061 28067 |
|  | 31028 | **1144.2898** | **4573.1301** | **4572.1175** | **1.0125** | **1** | **8** | **3** | **1** | **K.AGLETSTETEAGLEFISTVQFSQYPFLVCMQMDKDEAPFR.Q** 31022 |
|  | 31917 | **1893.5325** | **5677.5756** | **5677.7658** | **-0.1902** | **1** | **4** | **3.2** | **2** | **R.VTVVITTDITVDSSFVKAGLETSTETEAGLEFISTVQFSQYPFLVCMQMDK.D** |

| **8.** | P15144\|AMPN_HUMAN    **Mass:** 109870   **Score:** 2300   **Queries matched:** 127   **emPAI:** 2.75 |
| --- | --- |
|  | Aminopeptidase N OS=Homo sapiens GN=ANPEP PE=1 SV=4 |

|  | **Query** | **Observed** | **Mr(expt)** | **Mr(calc)** | **Delta** | **Miss** | **Score** | **Expect** | **Rank** | **Peptide** |
| --- | --- | --- | --- | --- | --- | --- | --- | --- | --- | --- |
|  | 812 | **503.3154** | **502.3081** | **502.3115** | **-0.0034** | **0** | **12** | **23** | **3** | **K.GSLVK.D** |
|  | 1307 | **603.3610** | **602.3537** | **601.3548** | **0.9990** | **0** | **13** | **14** | **3** | **K.GASVLR.M** 1298 |
|  | 3714 | **451.2500** | **900.4854** | **900.4916** | **-0.0062** | **0** | **39** | **0.024** | **1** | **R.ALEQALEK.T** 3693 3710 3713 3731 |
|  | 3848 | **455.7177** | **909.4209** | **909.4266** | **-0.0057** | **0** | **43** | **0.0076** | **1** | **R.SEVYGPMK.N** 3865 |
|  | 5282 | **507.7519** | **1013.4893** | **1013.5982** | **-0.1089** | **1** | **13** | **8.8** | **5** | **R.KIQTQLQR.D** 5281 |
|  | 5288 | **507.7976** | **1013.5807** | **1013.5869** | **-0.0063** | **0** | **45** | **0.0047** | **1** | **R.SIQLPTTVR.D** 5289 |
|  | 6242 | **542.7363** | **1083.4581** | **1083.4655** | **-0.0074** | **0** | **38** | **0.026** | **1** | **R.SEYMEGNVR.K** |
|  | 6735 | **561.3030** | **1120.5914** | **1120.5989** | **-0.0075** | **0** | **28** | **0.25** | **1** | **R.DHSAIPVINR.A** |
|  | 7641 | **594.8234** | **1187.6323** | **1187.6047** | **0.0276** | **0** | **7** | **32** | **10** | **K.LNYTLSQGHR.V** |
|  | 7935 | **601.8226** | **1201.6306** | **1201.6376** | **-0.0070** | **0** | **58** | **0.00028** | **1** | **K.DLTALSNMLPK.G** 7934 |
|  | 8301 | **610.3516** | **1218.6886** | **1217.6326** | **1.0560** | **0** | **(1)** | **1.1e+002** | **3** | **K.DLTALSNMLPK.G** |
|  | 8382 | **612.7639** | **1223.5131** | **1223.5207** | **-0.0075** | **0** | **67** | **2.8e-005** | **1** | **R.VNYDEENWR.K** 8381 |
|  | 8406 | **613.3386** | **1224.6626** | **1224.6714** | **-0.0088** | **0** | **50** | **0.0015** | **1** | **K.EATDVIIIHSK.K** 8396 |
|  | 8624 | **619.3123** | **1236.6101** | **1236.6172** | **-0.0072** | **0** | **40** | **0.013** | **1** | **K.DLMVLNDVYR.V** 8613 |
|  | 9004 | **629.3545** | **1256.6944** | **1256.7030** | **-0.0086** | **0** | **20** | **1.7** | **1** | **K.QVTPLFIHFR.N** |
|  | 9232 | **635.2663** | **1268.5180** | **1268.5269** | **-0.0089** | **0** | **55** | **0.0004** | **1** | **K.DNEETGFGSGTR.A** 9246 |
|  | 9855 | **434.8813** | **1301.6220** | **1302.6932** | **-1.0712** | **0** | **13** | **6.9** | **1** | **R.LPNTLKPDSYR.V** 9853 9857 9858 9876 9899 |
|  | 10351 | **664.7820** | **1327.5494** | **1327.5577** | **-0.0083** | **0** | **35** | **0.043** | **1** | **K.SFPCFDEPAMK.A** |
|  | 10764 | **676.8104** | **1351.6062** | **1351.6156** | **-0.0095** | **1** | **40** | **0.014** | **1** | **R.VNYDEENWRK.I** |
|  | 10923 | **681.3416** | **1360.6687** | **1360.6769** | **-0.0082** | **0** | **68** | **2.6e-005** | **1** | **K.VVATTQMQAADAR.K** 10916 10922 |
|  | 11200 | **689.3386** | **1376.6626** | **1376.6718** | **-0.0092** | **0** | **(53)** | **0.00076** | **1** | **K.VVATTQMQAADAR.K** |
|  | 11635 | **701.8458** | **1401.6770** | **1401.6850** | **-0.0080** | **0** | **37** | **0.029** | **1** | **R.MLSSFLSEDVFK.Q** 11632 11647 |
|  | 12365 | **482.2654** | **1443.7745** | **1443.7834** | **-0.0089** | **0** | **24** | **0.48** | **1** | **R.VTLRPYLTPNDR.G** 12366 12367 |
|  | 12375 | **723.1858** | **1444.3571** | **1443.7068** | **0.6504** | **1** | **1** | **70** | **2** | **R.SEVYGPMKNYLK.K** |
|  | 12672 | **732.3638** | **1462.7130** | **1462.7205** | **-0.0075** | **0** | **39** | **0.015** | **1** | **R.QQQDYWLIDVR.A** |
|  | 12731 | **734.3912** | **1466.7678** | **1466.7769** | **-0.0091** | **0** | **49** | **0.0017** | **1** | **R.YLSYTLNPDLIR.K** 12730 12732 12738 |
|  | 12937 | **740.8885** | **1479.7624** | **1478.7405** | **1.0219** | **0** | **10** | **12** | **2** | **K.EVVLQWFTENSK.-** |
|  | 13087 | **745.3881** | **1488.7617** | **1488.7719** | **-0.0102** | **1** | **90** | **1.4e-007** | **1** | **R.KVVATTQMQAADAR.K** 13073 13085 13086 13089 |
|  | 13343 | **753.3859** | **1504.7573** | **1504.7668** | **-0.0095** | **1** | **(59)** | **0.00016** | **1** | **R.KVVATTQMQAADAR.K** 13341 |
|  | 14912 | **806.9204** | **1611.8263** | **1611.8369** | **-0.0106** | **0** | **62** | **7.9e-005** | **1** | **R.AQIINDAFNLASAHK.V** 14910 14911 14913 |
|  | 15425 | **821.8990** | **1641.7835** | **1642.8825** | **-1.0989** | **1** | **4** | **42** | **1** | **R.SIQLPTTVRDIMNR.W** |
|  | 16573 | **862.9144** | **1723.8142** | **1723.8264** | **-0.0122** | **0** | **59** | **0.00012** | **1** | **R.ENSLLFDPLSSSSSNK.E** 16574 16575 |
|  | 17464 | **895.4130** | **1788.8115** | **1788.8570** | **-0.0455** | **0** | **7** | **19** | **3** | **R.FSTEYELQQLEQFK.K** |
|  | 18340 | **926.4467** | **1850.8788** | **1849.9210** | **0.9578** | **1** | **19** | **1.2** | **2** | **K.ENKEVVLQWFTENSK.-** |
|  | 19255 | **960.9593** | **1919.9041** | **1919.9127** | **-0.0086** | **0** | **23** | **0.45** | **1** | **R.QYMPWEAALSSLSYFK.L** |
|  | 19565 | **973.9928** | **1945.9710** | **1944.9581** | **1.0129** | **1** | **4** | **42** | **9** | **R.RFSTEYELQQLEQFK.K** |
|  | 19850 | **987.4674** | **1972.9201** | **1972.9326** | **-0.0124** | **0** | **37** | **0.019** | **1** | **K.QWMENPNNNPIHPNLR.S** 19849 |
|  | 22984 | **1147.0603** | **2292.1060** | **2292.1175** | **-0.0115** | **0** | **132** | **5.5e-012** | **1** | **K.LFNDYGGGSFSFSNLIQAVTR.R** 22979 22983 22985 22987 22989 22992 22994 22996 23006 23007 23008 |
|  | 23910 | **807.7415** | **2420.2026** | **2420.2125** | **-0.0099** | **1** | **21** | **0.56** | **1** | **K.KLFNDYGGGSFSFSNLIQAVTR.R** |
|  | 24975 | **1277.5981** | **2553.1817** | **2553.1958** | **-0.0141** | **0** | **89** | **7.9e-008** | **1** | **K.SDQIGLPDFNAGAMENWGLVTYR.E** 24973 24974 24976 24978 |
|  | 25711 | **1344.0505** | **2686.0865** | **2686.1017** | **-0.0152** | **0** | **125** | **1.8e-011** | **1** | **K.DSQYEMDSEFEGELADDLAGFYR.S** 25710 25712 25714 25725 25738 25746 |
|  | 26801 | **1447.1234** | **2892.2323** | **2892.2449** | **-0.0127** | **0** | **98** | **8.7e-009** | **1** | **R.STVYCNAIAQGGEEEWDFAWEQFR.N** 26797 26798 26800 26802 |
|  | 26944 | **974.1758** | **2919.5057** | **2919.5230** | **-0.0173** | **1** | **36** | **0.013** | **1** | **R.GVGGSQPPDIDKTELVEPTEYLVVHLK.G** 26942 26943 26945 26946 26947 |
|  | 28342 | **1078.8816** | **3233.6229** | **3233.6357** | **-0.0128** | **0** | **62** | **2.7e-005** | **1** | **K.QDATSTIISITNNVIGQGLVWDFVQSNWK.K** 28343 |
|  | 28775 | **1121.5797** | **3361.7173** | **3361.7307** | **-0.0134** | **1** | **74** | **1.6e-006** | **1** | **R.KQDATSTIISITNNVIGQGLVWDFVQSNWK.K** 28773 28774 28776 |
|  | 30190 | **1287.6412** | **3859.9019** | **3859.9189** | **-0.0171** | **0** | **56** | **7e-005** | **1** | **R.VMAVDALASSHPLSTPASEINTPAQISELFDAISYSK.G** 30184 30185 30187 30188 30189 30193 |
|  | 30210 | **1292.9714** | **3875.8925** | **3875.9139** | **-0.0214** | **0** | **(55)** | **8.9e-005** | **1** | **R.VMAVDALASSHPLSTPASEINTPAQISELFDAISYSK.G** |
|  | 30420 | **1349.9307** | **4046.7702** | **4046.7893** | **-0.0192** | **0** | **10** | **2.4** | **1** | **R.EIPENLMDQYSEVNAISTACSNGVPECEEMVSGLFK.Q** |
|  | 31244 | **1616.5060** | **4846.4961** | **4847.1670** | **-0.6708** | **1** | **1** | **12** | **7** | **K.QWMENPNNNPIHPNLRSTVYCNAIAQGGEEEWDFAWEQFR.N** |

| **9.** | P09848\|LPH_HUMAN    **Mass:** 219519   **Score:** 2137   **Queries matched:** 136   **emPAI:** 0.73 |
| --- | --- |
|  | Lactase-phlorizin hydrolase OS=Homo sapiens GN=LCT PE=1 SV=2 |

|  | **Query** | **Observed** | **Mr(expt)** | **Mr(calc)** | **Delta** | **Miss** | **Score** | **Expect** | **Rank** | **Peptide** |
| --- | --- | --- | --- | --- | --- | --- | --- | --- | --- | --- |
|  | 1564 | **659.4040** | **658.3968** | **658.4126** | **-0.0158** | **1** | **13** | **14** | **10** | **K.ALKTAR.L** |
|  | 1568 | 660.3666 | 659.3594 | 659.3643 | -0.0049 | 0 | 12 | 15 | 2 | K.VVWEK.F |
|  | 1796 | **727.4088** | **726.4015** | **726.4064** | **-0.0049** | **0** | **9** | **19** | **2** | **R.IYYLR.T** |
|  | 1958 | **773.4097** | **772.4025** | **772.4443** | **-0.0418** | **0** | **28** | **0.36** | **2** | **R.SLAAGLNK.S** |
|  | 1986 | **786.4797** | **785.4724** | **785.4799** | **-0.0075** | **0** | **22** | **1.3** | **5** | **R.ILNWLK.E** |
|  | 2022 | **395.2342** | **788.4539** | **787.4188** | **1.0351** | **0** | **13** | **12** | **2** | **R.GVASIADR.S** |
|  | 2762 | **421.2294** | **840.4442** | **840.4494** | **-0.0051** | **0** | **19** | **1.9** | **1** | **K.FYASVVR.C** 2768 |
|  | 3285 | **436.7424** | **871.4702** | **871.4763** | **-0.0061** | **0** | **15** | **6.6** | **1** | **R.ILPDGTTR.Y** 3282 |
|  | 3416 | **441.2467** | **880.4788** | **879.5218** | **0.9570** | **0** | **6** | **38** | **5** | **K.VFIFNLK.L** |
|  | 4102 | **465.2271** | **928.4396** | **928.4767** | **-0.0371** | **0** | **13** | **9** | **1** | **R.GVSIWDPR.R** |
|  | 4424 | **476.2581** | **950.5016** | **950.5073** | **-0.0056** | **0** | **27** | **0.35** | **1** | **K.TYINEALK.A** 4422 4449 |
|  | 4805 | **490.2604** | **978.5062** | **977.5770** | **0.9292** | **1** | **15** | **4.7** | **1** | **R.IVQHKTPR.L** |
|  | 5331 | **509.2854** | **1016.5562** | **1015.5774** | **0.9788** | **1** | **1** | **1.4e+002** | **4** | **R.SLAAGLNKSR.L** |
|  | 5462 | **1028.4622** | **1027.4549** | **1027.4611** | **-0.0062** | **0** | **30** | **0.16** | **1** | **R.EDEFLYGR.F** |
|  | 5632 | **520.7178** | **1039.4211** | **1039.4281** | **-0.0069** | **0** | **14** | **5.1** | **1** | **R.NGDYPDTMK.W** 5635 5642 |
|  | 5740 | **525.1033** | **1048.1921** | **1047.4729** | **0.7192** | **0** | **1** | **1e+002** | **9** | **K.LPDCPSTMK.N** |
|  | 5805 | **1054.4760** | **1053.4687** | **1053.4767** | **-0.0080** | **0** | **32** | **0.087** | **1** | **R.VYHTYDEK.Y** 5804 5806 5823 |
|  | 5822 | **528.3102** | **1054.6059** | **1055.4230** | **-0.8170** | **0** | **(2)** | **89** | **9** | **R.NGDYPDTMK.W** |
|  | 6030 | **535.7793** | **1069.5440** | **1068.4546** | **1.0895** | **0** | **8** | **26** | **8** | **K.NGDYNEVMK.T** |
|  | 6184 | **540.2639** | **1078.5131** | **1078.5182** | **-0.0051** | **0** | **5** | **55** | **7** | **R.LPSFTEEEK.R** |
|  | 6571 | **554.8020** | **1107.5894** | **1106.6852** | **0.9043** | **1** | **4** | **60** | **4** | **K.VKVFIFNLK.L** |
|  | 6987 | **571.2978** | **1140.5810** | **1140.5887** | **-0.0077** | **0** | **49** | **0.0017** | **1** | **R.SELQHLATSR.L** 6986 6998 |
|  | 7160 | **578.2790** | **1154.5434** | **1154.5509** | **-0.0075** | **0** | **38** | **0.024** | **1** | **R.DLFYHGTFR.D** |
|  | 7615 | **594.2969** | **1186.5792** | **1185.7094** | **0.8698** | **1** | **0** | **1.5e+002** | **8** | **R.IAHAVIKAHAR.V** |
|  | 8074 | **605.3071** | **1208.5997** | **1208.6077** | **-0.0080** | **0** | **30** | **0.13** | **1** | **R.AFTFPSEVPSK.A** 8073 |
|  | 9995 | **655.8507** | **1309.6869** | **1308.6139** | **1.0730** | **0** | **10** | **14** | **2** | **R.SWPDSGSFWLK.M** 9971 |
|  | 10233 | **661.8279** | **1321.6412** | **1321.6514** | **-0.0102** | **1** | **56** | **0.00032** | **1** | **K.SRLPEFTESEK.R** 10234 10236 |
|  | 10405 | **666.3525** | **1330.6904** | **1329.5797** | **1.1107** | **0** | **20** | **1.4** | **1** | **R.DPSNQEDVEAAR.R** |
|  | 10608 | **448.2308** | **1341.6706** | **1340.7088** | **0.9618** | **1** | **8** | **21** | **2** | **K.TYINEALKAYR.L** |
|  | 11894 | **708.3649** | **1414.7153** | **1414.7245** | **-0.0092** | **1** | **40** | **0.015** | **1** | **R.FKEYADVLFQR.L** 11900 11911 |
|  | 12212 | **717.3353** | **1432.6561** | **1432.7602** | **-0.1041** | **1** | **3** | **65** | **8** | **R.KSAYFFTSIIEK.N** |
|  | 12429 | **724.8892** | **1447.7639** | **1447.8511** | **-0.0872** | **1** | **0** | **1.3e+002** | **8** | **K.RLLPPNTVNLPSK.V** |
|  | 12516 | **485.5920** | **1453.7542** | **1453.7637** | **-0.0095** | **1** | **0** | **1.2e+002** | **10** | **R.ASQLQTLSDAHRK.A** |
|  | 12677 | **732.3851** | **1462.7556** | **1463.7773** | **-1.0217** | **1** | **5** | **39** | **4** | **K.VRAFTFPSEVPSK.A** |
|  | 12839 | **737.8654** | **1473.7162** | **1473.7252** | **-0.0090** | **0** | **60** | **0.00014** | **1** | **R.YINEAGLNYYVR.L** 12838 |
|  | 13077 | **745.3664** | **1488.7182** | **1488.7110** | **0.0073** | **1** | **0** | **1.2e+002** | **7** | **K.AHARVYHTYDEK.Y** |
|  | 13410 | **755.8622** | **1509.7098** | **1509.7212** | **-0.0114** | **0** | **17** | **2.3** | **1** | **K.GSADFLGLSHYTSR.L** |
|  | 13934 | **516.3124** | **1545.9155** | **1546.9195** | **-1.0040** | **1** | **6** | **30** | **4** | **R.LLPPNTVNLPSKVR.A** 13951 |
|  | 14185 | **782.2904** | **1562.5663** | **1562.7590** | **-0.1927** | **1** | **2** | **66** | **2** | **R.VWEAFANQSRAER.D** |
|  | 14905 | **806.8791** | **1611.7436** | **1611.7529** | **-0.0093** | **0** | **78** | **1.9e-006** | **1** | **R.SLIDGFEGPSGYSQR.F** |
|  | 15133 | **812.9333** | **1623.8520** | **1623.8620** | **-0.0100** | **0** | **74** | **4.3e-006** | **1** | **K.GVISLSLSTHWAEPK.S** 15129 15131 15132 |
|  | 15208 | **815.4173** | **1628.8200** | **1628.8311** | **-0.0111** | **0** | **83** | **5.4e-007** | **1** | **K.GLSIWDTFSHTPLR.V** 15207 |
|  | 15665 | **830.4678** | **1658.9210** | **1658.9031** | **0.0179** | **1** | **8** | **16** | **4** | **R.IYYLRTYINEALK.A** |
|  | 16777 | **870.9157** | **1739.8167** | **1740.8505** | **-1.0337** | **0** | **74** | **4.2e-006** | **1** | **R.YYTEVITNNGMPLAR.E** 16791 16793 16812 |
|  | 16847 | **872.9079** | **1743.8012** | **1743.8117** | **-0.0105** | **0** | **85** | **3.6e-007** | **1** | **K.AHAEAWHLYNDVYR.A** 16845 16846 16858 |
|  | 16907 | **583.9490** | **1748.8253** | **1747.8682** | **0.9571** | **0** | **6** | **29** | **5** | **R.FGLHFVNYSDPSLPR.I** |
|  | 17508 | **896.8882** | **1791.7618** | **1791.7734** | **-0.0116** | **0** | **77** | **2.2e-006** | **1** | **R.VENDAIGDVACDSYHK.I** 17507 |
|  | 17976 | **914.4514** | **1826.8881** | **1826.8967** | **-0.0085** | **0** | **92** | **6.1e-008** | **1** | **R.YVQFMGGWFAHPIFK.N** 17975 17977 |
|  | 19336 | **964.5082** | **1927.0019** | **1927.0163** | **-0.0144** | **0** | **92** | **5.8e-008** | **1** | **K.IAEDLVTLQNLGVSHYR.F** 19335 19337 19339 |
|  | 19863 | **987.9943** | **1973.9741** | **1972.9854** | **0.9887** | **0** | **76** | **2.4e-006** | **1** | **R.ASQGGVISITISSDWAEPR.D** 19843 19862 |
|  | 20128 | **667.8748** | **2000.6025** | **2000.0116** | **0.5909** | **1** | **5** | **21** | **1** | **R.ADGKGLSIWDTFSHTPLR.V** 20117 |
|  | 21323 | **1055.6388** | **2109.2630** | **2108.9651** | **0.2980** | **0** | **28** | **0.1** | **1** | **K.EEYNDPPIYVTENGVSQR.E** 21328 21329 21330 |
|  | 21591 | **712.9973** | **2135.9699** | **2137.0303** | **-1.0603** | **0** | **1** | **62** | **6** | **R.IFPMGHGSSPSLPGVAYYNK.L** |
|  | 22193 | **1101.0254** | **2200.0362** | **2200.1164** | **-0.0802** | **0** | **3** | **40** | **2** | **K.VFLSWAQLLPAGSTQNPDEK.T** 22192 |
|  | 23443 | **1177.5059** | **2352.9972** | **2353.0110** | **-0.0138** | **0** | **110** | **6.3e-010** | **1** | **R.GYTVWSAMDNFEWATGFSER.F** 23441 23442 23444 23446 23450 23462 23464 23465 23466 |
|  | 23719 | **1197.5537** | **2393.0929** | **2392.1198** | **0.9731** | **0** | **3** | **37** | **1** | **R.GYVAWSLMDNFEWLNGYTVK.F** |
|  | 24675 | **1259.1426** | **2516.2706** | **2516.1132** | **0.1574** | **0** | **22** | **0.45** | **1** | **R.DDFLWGVSSSAYQIEGAWDADGK.G** 24681 |
|  | 26323 | **931.4646** | **2791.3720** | **2791.3851** | **-0.0131** | **1** | **74** | **2.3e-006** | **1** | **R.GKVPIYLAGNGMPIGESENLFDDSLR.V** |
|  | 26710 | **959.8128** | **2876.4166** | **2876.4344** | **-0.0178** | **1** | **56** | **0.00013** | **1** | **R.ILNWLKEEYNDPPIYVTENGVSQR.E** 26713 26714 |
|  | 27403 | **1512.7046** | **3023.3946** | **3023.4026** | **-0.0080** | **0** | **85** | **1.8e-007** | **1** | **K.FWMTFNEPMYLAWLGYGSGEFPPGVK.D** |
|  | 27412 | **1513.1888** | **3024.3631** | **3024.3836** | **-0.0205** | **0** | **102** | **2.8e-009** | **1** | **K.EEYGDIPIYITENGVGLTNPNTEDTDR.I** 27411 27416 27417 |
|  | 27906 | **1569.6395** | **3137.2645** | **3137.2866** | **-0.0221** | **0** | **53** | **0.00021** | **1** | **R.LNPPSYEDDQEMAEEEDPSWPSTAMNR.A** 27907 27908 27909 |
|  | 27957 | **1051.7643** | **3152.2710** | **3153.2815** | **-1.0105** | **0** | **(1)** | **31** | **8** | **R.LNPPSYEDDQEMAEEEDPSWPSTAMNR.A** |
|  | 29493 | **1205.1674** | **3612.4803** | **3611.5740** | **0.9062** | **0** | **2** | **20** | **3** | **K.SMSCSLTGSLALQPDQQQDHETTDSSPASAYQR.V** 29492 |
|  | 29832 | **930.4456** | **3717.7534** | **3717.7715** | **-0.0181** | **0** | **39** | **0.0043** | **1** | **K.LWVTFHEPWVMSYAGYGTGQHPPGISDPGVASFK.V** 29831 |
|  | 30044 | **1264.9523** | **3791.8350** | **3791.8530** | **-0.0180** | **1** | **51** | **0.00027** | **1** | **R.LLNWIKEEYGDIPIYITENGVGLTNPNTEDTDR.I** 30043 30049 30050 30052 30054 |
|  | 30998 | **1518.1175** | **4551.3308** | **4551.3379** | **-0.0071** | **0** | **38** | **0.0031** | **1** | **K.FWITLNEPFVIAYQGYGYGTAAPGVSNRPGTAPYIVGHNLIK.A** 30996 30997 |

| **10.** | P07237\|PDIA1_HUMAN    **Mass:** 57480    **Score:** 2025   **Queries matched:** 114   **emPAI:** 6.00 |
| --- | --- |
|  | Protein disulfide-isomerase OS=Homo sapiens GN=P4HB PE=1 SV=3 |

|  | **Query** | **Observed** | **Mr(expt)** | **Mr(calc)** | **Delta** | **Miss** | **Score** | **Expect** | **Rank** | **Peptide** |
| --- | --- | --- | --- | --- | --- | --- | --- | --- | --- | --- |
|  | 869 | **515.3262** | **514.3189** | **514.3479** | **-0.0290** | **0** | **19** | **3.1** | **1** | **K.VLVGK.N** |
|  | 1332 | **608.3361** | **607.3288** | **607.3329** | **-0.0041** | **0** | **19** | **1.9** | **1** | **K.LSNFK.T** 1331 1334 |
|  | 1643 | **678.3772** | **677.3699** | **677.3748** | **-0.0049** | **0** | **7** | **39** | **2** | **R.GYPTIK.F** |
|  | 1646 | **680.3572** | **679.3499** | **679.3541** | **-0.0042** | **0** | **11** | **13** | **1** | **R.TLDGFK.K** |
|  | 1750 | **711.0934** | **710.0862** | **709.3646** | **0.7215** | **0** | **5** | **32** | **1** | **K.LGETYK.D** |
|  | 1890 | **753.3723** | **752.3650** | **752.3705** | **-0.0055** | **0** | **32** | **0.099** | **1** | **K.TAAESFK.G** |
|  | 2046 | **395.7423** | **789.4700** | **788.3664** | **1.1036** | **0** | **7** | **41** | **1** | **R.NGDTASPK.E** |
|  | 2302 | **809.4103** | **808.4030** | **807.4491** | **0.9540** | **1** | **20** | **1.5** | **2** | **R.TLDGFKK.F** |
|  | 2909 | **851.0955** | **850.0882** | **849.3716** | **0.7166** | **0** | **6** | **29** | **8** | **K.DVESDSAK.Q** |
|  | 3059 | **431.2344** | **860.4542** | **861.4596** | **-1.0054** | **0** | **34** | **0.092** | **1** | **K.ALAPEYAK.A** 3060 3078 3079 |
|  | 3850 | **455.7212** | **909.4279** | **909.4345** | **-0.0066** | **0** | **15** | **5** | **1** | **K.FFPASADR.T** 3849 3851 |
|  | 4569 | **481.7261** | **961.4377** | **961.4440** | **-0.0063** | **0** | **27** | **0.36** | **1** | **R.ITEFCHR.F** |
|  | 4630 | **483.7837** | **965.5528** | **965.5586** | **-0.0058** | **0** | **43** | **0.0075** | **1** | **R.ILEFFGLK.K** 4631 4632 |
|  | 4975 | **496.2738** | **990.5331** | **990.5386** | **-0.0055** | **0** | **26** | **0.42** | **1** | **K.ENLLDFIK.H** 4955 |
|  | 5141 | **501.7790** | **1001.5435** | **1001.5505** | **-0.0070** | **1** | **31** | **0.15** | **1** | **K.LKAEGSEIR.L** 5140 5142 5143 5152 5158 |
|  | 5615 | **519.7793** | **1037.5440** | **1037.5505** | **-0.0065** | **0** | **25** | **0.5** | **1** | **K.DHENIVIAK.M** 5597 |
|  | 5975 | **533.7581** | **1065.5017** | **1065.5091** | **-0.0074** | **0** | **15** | **4.4** | **1** | **R.TVIDYNGER.T** |
|  | 6207 | **541.3388** | **1080.6629** | **1080.6695** | **-0.0066** | **0** | **42** | **0.0089** | **1** | **K.THILLFLPK.S** |
|  | 6385 | **547.4689** | **1092.9233** | **1093.6535** | **-0.7302** | **1** | **5** | **32** | **2** | **R.ILEFFGLKK.E** |
|  | 7209 | **579.7944** | **1157.5742** | **1157.5829** | **-0.0087** | **0** | **55** | **0.00049** | **1** | **K.SNFAEALAAHK.Y** |
|  | 7671 | **595.3377** | **1188.6607** | **1188.6502** | **0.0105** | **1** | **7** | **31** | **1** | **K.ALAPEYAKAAGK.L** |
|  | 8018 | **603.8141** | **1205.6136** | **1205.6213** | **-0.0077** | **0** | **58** | **0.00026** | **1** | **R.LITLEEEMTK.Y** |
|  | 8152 | **607.2684** | **1212.5223** | **1212.5299** | **-0.0076** | **0** | **33** | **0.069** | **1** | **K.NFEDVAFDEK.K** |
|  | 8695 | **414.2010** | **1239.5812** | **1238.6044** | **0.9768** | **1** | **11** | **11** | **2** | **K.FFRNGDTASPK.E** |
|  | 9240 | **635.3419** | **1268.6693** | **1268.7638** | **-0.0946** | **0** | **6** | **39** | **7** | **R.ALLCLAVAALVR.A** |
|  | 9560 | **429.5636** | **1285.6690** | **1285.6778** | **-0.0089** | **1** | **49** | **0.0017** | **1** | **R.KSNFAEALAAHK.Y** 9558 9559 |
|  | 9691 | **647.2989** | **1292.5833** | **1292.5918** | **-0.0085** | **0** | **57** | **0.00029** | **1** | **K.MDSTANEVEAVK.V** 9690 9692 |
|  | 9993 | **655.8394** | **1309.6642** | **1308.5867** | **1.0774** | **0** | **(17)** | **2.8** | **1** | **K.MDSTANEVEAVK.V** 9968 |
|  | 12044 | **712.8876** | **1423.7607** | **1423.7711** | **-0.0104** | **1** | **50** | **0.0014** | **1** | **K.YQLDKDGVVLFK.K** |
|  | 12089 | **476.2799** | **1425.8179** | **1424.8649** | **0.9530** | **1** | **8** | **21** | **6** | **R.RALLCLAVAALVR.A** |
|  | 12475 | **726.3489** | **1450.6833** | **1450.6939** | **-0.0106** | **0** | **54** | **0.00052** | **1** | **K.YKPESEELTAER.I** 12474 12476 |
|  | 12624 | **730.8677** | **1459.7209** | **1458.6991** | **1.0219** | **1** | **5** | **46** | **1** | **K.SVSDYDGKLSNFK.T** |
|  | 16358 | **570.2967** | **1707.8682** | **1708.8672** | **-0.9990** | **1** | **0** | **1.1e+002** | **5** | **K.VLVGKNFEDVAFDEK.K** |
|  | 16632 | **577.3046** | **1728.8921** | **1728.9046** | **-0.0126** | **1** | **48** | **0.0018** | **1** | **K.LGETYKDHENIVIAK.M** 16633 |
|  | 17353 | **890.9144** | **1779.8142** | **1779.8275** | **-0.0133** | **0** | **69** | **1.3e-005** | **1** | **K.VDATEESDLAQQYGVR.G** 17354 |
|  | 18071 | **917.4539** | **1832.8933** | **1832.9057** | **-0.0124** | **0** | **75** | **3.1e-006** | **1** | **K.ILFIFIDSDHTDNQR.I** 18072 18073 18075 18079 |
|  | 19155 | **957.4204** | **1912.8263** | **1912.8389** | **-0.0126** | **0** | **80** | **9e-007** | **1** | **K.NVFVEFYAPWCGHCK.Q** 19156 19157 |
|  | 19751 | **983.0200** | **1964.0254** | **1964.0367** | **-0.0113** | **0** | **92** | **6e-008** | **1** | **K.HNQLPLVIEFTEQTAPK.I** 19749 19750 19752 19754 |
|  | 20208 | **1005.5063** | **2008.9980** | **2009.0105** | **-0.0125** | **1** | **94** | **3.8e-008** | **1** | **R.NNFEGEVTKENLLDFIK.H** 20209 20211 20212 |
|  | 20580 | **511.2376** | **2040.9215** | **2040.9339** | **-0.0124** | **1** | **11** | **7.2** | **1** | **K.KNVFVEFYAPWCGHCK.Q** |
|  | 23254 | **777.4092** | **2329.2059** | **2328.1267** | **1.0792** | **1** | **1** | **59** | **3** | **K.DHENIVIAKMDSTANEVEAVK.V** |
|  | 23878 | **1209.6198** | **2417.2249** | **2417.2413** | **-0.0163** | **1** | **45** | **0.0022** | **1** | **K.IKPHLMSQELPEDWDKQPVK.V** 23876 23879 23891 |
|  | 25938 | **1357.1639** | **2712.3133** | **2712.3283** | **-0.0150** | **0** | **86** | **1.6e-007** | **1** | **K.QFLQAAEAIDDIPFGITSNSDVFSK.Y** 25930 25937 25939 25940 25944 |
|  | 26997 | **1468.2440** | **2934.4735** | **2934.4862** | **-0.0127** | **0** | **97** | **1.1e-008** | **1** | **R.TGPAATTLPDGAAAESLVESSEVAVIGFFK.D** 26991 26992 26993 26994 26995 26996 26999 27000 27001 27005 27006 27007 27008 27011 27012 27017 27020 27023 27024 27029 |
|  | 27750 | **1546.2942** | **3090.5738** | **3090.5873** | **-0.0135** | **1** | **53** | **0.00028** | **1** | **K.RTGPAATTLPDGAAAESLVESSEVAVIGFFK.D** |
|  | 29941 | **942.4651** | **3765.8315** | **3765.8473** | **-0.0158** | **1** | **35** | **0.012** | **1** | **R.TGPAATTLPDGAAAESLVESSEVAVIGFFKDVESDSAK.Q** 29940 29942 |

| **11.** | P05023\|AT1A1_HUMAN    **Mass:** 114135   **Score:** 1943   **Queries matched:** 119   **emPAI:** 2.35 |
| --- | --- |
|  | Sodium/potassium-transporting ATPase subunit alpha-1 OS=Homo sapiens GN=ATP1A1 PE=1 SV=1 |

|  | **Query** | **Observed** | **Mr(expt)** | **Mr(calc)** | **Delta** | **Miss** | **Score** | **Expect** | **Rank** | **Peptide** |
| --- | --- | --- | --- | --- | --- | --- | --- | --- | --- | --- |
|  | 1312 | **604.3374** | **603.3301** | **603.3340** | **-0.0039** | **0** | **8** | **39** | **3** | **R.GLTSAR.A** |
|  | 1443 | **633.3343** | **632.3270** | **632.3316** | **-0.0046** | **0** | **25** | **0.93** | **1** | **K.NCLVK.N** |
|  | 2250 | **805.4991** | **804.4918** | **804.3402** | **0.1516** | **0** | **7** | **38** | **7** | **R.VDWDDR.W** |
|  | 2324 | **406.1986** | **810.3825** | **810.3872** | **-0.0046** | **0** | **36** | **0.036** | **1** | **K.YGTDLSR.G** 2340 |
|  | 3076 | **862.4392** | **861.4319** | **861.4378** | **-0.0059** | **0** | **34** | **0.083** | **1** | **R.IISANGCK.V** 3075 3091 |
|  | 3370 | **439.7147** | **877.4149** | **877.4215** | **-0.0065** | **1** | **37** | **0.037** | **1** | **R.DMDELKK.E** |
|  | 3541 | **445.2373** | **888.4600** | **887.4865** | **0.9735** | **0** | **17** | **4.4** | **2** | **K.YQLSIHK.N** 3533 |
|  | 3564 | **446.0373** | **890.0600** | **889.4617** | **0.5982** | **0** | **26** | **0.32** | **1** | **K.TGTLTQNR.M** |
|  | 4246 | **470.2452** | **938.4758** | **938.4821** | **-0.0063** | **1** | **17** | **2.6** | **1** | **R.KYGTDLSR.G** 4231 |
|  | 4807 | **490.2610** | **978.5074** | **978.5135** | **-0.0061** | **0** | **27** | **0.31** | **1** | **R.GIVVYTGDR.T** |
|  | 4851 | **491.7661** | **981.5175** | **981.5243** | **-0.0068** | **0** | **48** | **0.0026** | **1** | **K.LSLDELHR.K** 4852 |
|  | 5286 | **507.7699** | **1013.5252** | **1013.5328** | **-0.0075** | **0** | **39** | **0.021** | **1** | **R.CSSILLHGK.E** |
|  | 5611 | **519.7522** | **1037.4898** | **1037.4964** | **-0.0066** | **0** | **32** | **0.09** | **1** | **R.NSVFQQGMK.N** |
|  | 6002 | **534.8079** | **1067.6013** | **1068.5676** | **-0.9663** | **1** | **15** | **4.4** | **4** | **K.GGDRIPADLR.I** 6022 |
|  | 6250 | **542.8004** | **1083.5863** | **1083.5938** | **-0.0075** | **1** | **16** | **3.7** | **1** | **R.RRPGGWVEK.E** |
|  | 6258 | **543.2701** | **1084.5257** | **1084.5335** | **-0.0078** | **0** | **13** | **8.2** | **1** | **K.ACVVHGSDLK.D** 6261 |
|  | 6292 | **544.2970** | **1086.5794** | **1086.5856** | **-0.0061** | **0** | **34** | **0.079** | **1** | **K.LIIVEGCQR.Q** 6293 |
|  | 6594 | **555.8129** | **1109.6112** | **1109.6193** | **-0.0081** | **1** | **32** | **0.091** | **1** | **K.LSLDELHRK.Y** |
|  | 6914 | **568.2944** | **1134.5743** | **1134.5822** | **-0.0079** | **0** | **39** | **0.018** | **1** | **K.YHTEIVFAR.T** 6916 |
|  | 7763 | **597.8008** | **1193.5870** | **1193.5975** | **-0.0105** | **1** | **10** | **16** | **1** | **R.RNSVFQQGMK.N** |
|  | 8396 | 613.2679 | 1224.5212 | 1224.5301 | -0.0089 | 0 | 37 | 0.029 | 1 | K.CIELCCGSVK.E |
|  | 8505 | **616.3259** | **1230.6372** | **1230.6456** | **-0.0084** | **0** | **38** | **0.022** | **1** | **R.AVAGDASESALLK.C** 8493 8499 |
|  | 8577 | **618.3002** | **1234.5859** | **1235.6986** | **-1.1127** | **0** | **11** | **13** | **2** | **R.LNIPVSQVNPR.D** |
|  | 9083 | **631.3383** | **1260.6621** | **1260.6714** | **-0.0093** | **0** | **40** | **0.015** | **1** | **K.IVEIPFNSTNK.Y** 9079 9084 9089 |
|  | 9221 | **634.8564** | **1267.6982** | **1267.7071** | **-0.0089** | **0** | **27** | **0.29** | **1** | **K.NMVPQQALVIR.N** 9219 9220 |
|  | 9455 | **1281.5680** | **1280.5607** | **1279.6343** | **0.9264** | **1** | **2** | **80** | **10** | **R.NSVFQQGMKNK.I** |
|  | 9520 | **642.8542** | **1283.6939** | **1283.7020** | **-0.0080** | **0** | **(19)** | **1.6** | **2** | **K.NMVPQQALVIR.N** |
|  | 11285 | **691.3743** | **1380.7341** | **1380.7435** | **-0.0094** | **0** | **66** | **3.2e-005** | **1** | **K.VIMVTGDHPITAK.A** 11283 11284 11286 |
|  | 11571 | **699.4022** | **1396.7898** | **1396.7384** | **0.0513** | **0** | **(13)** | **6.3** | **1** | **K.VIMVTGDHPITAK.A** |
|  | 11726 | **704.3330** | **1406.6515** | **1406.6599** | **-0.0084** | **0** | **61** | **0.00011** | **1** | **K.DMTSEQLDDILK.Y** |
|  | 11995 | **711.3441** | **1420.6737** | **1420.6834** | **-0.0097** | **1** | **60** | **0.00013** | **1** | **K.YEPAAVSEQGDKK.G** 11996 |
|  | 13112 | **745.8742** | **1489.7339** | **1489.7446** | **-0.0108** | **0** | **79** | **1.6e-006** | **1** | **K.ADIGVAMGIAGSDVSK.Q** 13113 13114 13131 |
|  | 13845 | **514.3140** | **1539.9203** | **1538.7875** | **1.1327** | **1** | **3** | **61** | **10** | **R.GIVVYTGDRTVMGR.I** 13840 |
|  | 13984 | **517.3012** | **1548.8818** | **1549.8576** | **-0.9758** | **1** | **5** | **39** | **10** | **R.LNIPVSQVNPRDAK.A** |
|  | 14480 | **792.3911** | **1582.7677** | **1583.8671** | **-1.0994** | **0** | **77** | **2.4e-006** | **1** | **R.AVFQANQENLPILK.R** 14497 14498 14512 |
|  | 14836 | **804.4086** | **1606.8026** | **1606.8137** | **-0.0111** | **0** | **19** | **1.7** | **1** | **K.NPNTSEPQHLLVMK.G** 14837 |
|  | 15023 | **540.2840** | **1617.8302** | **1617.8396** | **-0.0094** | **1** | **18** | **1.9** | **1** | **K.KADIGVAMGIAGSDVSK.Q** |
|  | 15042 | **810.3734** | **1618.7323** | **1618.7435** | **-0.0112** | **0** | **74** | **4.7e-006** | **1** | **K.VDNSSLTGESEPQTR.S** 15017 15040 15043 15056 |
|  | 15414 | **547.9315** | **1640.7726** | **1640.7143** | **0.0583** | **1** | **4** | **47** | **6** | **K.CIELCCGSVKEMR.E** |
|  | 16042 | **843.8933** | **1685.7721** | **1685.7832** | **-0.0111** | **0** | **75** | **3.8e-006** | **1** | **R.NIAFFSTNCVEGTAR.G** 16043 16050 |
|  | 16197 | **849.4251** | **1696.8357** | **1695.9090** | **0.9267** | **1** | **0** | **1.1e+002** | **1** | **K.NMVPQQALVIRNGEK.M** |
|  | 16947 | **876.9259** | **1751.8372** | **1751.8478** | **-0.0106** | **0** | **60** | **0.00012** | **1** | **K.DAFQNAYLELGGLGER.V** 16948 |
|  | 17757 | **906.4728** | **1810.9311** | **1810.9425** | **-0.0114** | **0** | **107** | **2.1e-009** | **1** | **R.QGAIVAVTGDGVNDSPALK.K** 17756 17761 |
|  | 18011 | **915.4595** | **1828.9045** | **1828.9167** | **-0.0121** | **0** | **103** | **4.8e-009** | **1** | **K.GVGIISEGNETVEDIAAR.L** 18009 18010 18012 |
|  | 18029 | **915.9751** | **1829.9356** | **1829.9445** | **-0.0088** | **0** | **84** | **3.7e-007** | **1** | **K.MSINAEEVVVGDLVEVK.G** 18027 18028 18032 18033 18052 |
|  | 18081 | **917.4658** | **1832.9171** | **1832.9309** | **-0.0138** | **0** | **28** | **0.17** | **1** | **R.DGPNALTPPPTTPEWIK.F** |
|  | 18129 | **919.4411** | **1836.8676** | **1837.0132** | **-0.1455** | **1** | **3** | **55** | **8** | **R.SAGIKVIMVTGDHPITAK.A** |
|  | 18266 | **616.3117** | **1845.9133** | **1845.9394** | **-0.0261** | **0** | **(1)** | **93** | **7** | **K.MSINAEEVVVGDLVEVK.G** |
|  | 19289 | **962.9550** | **1923.8954** | **1923.9095** | **-0.0142** | **0** | **59** | **0.00011** | **1** | **K.NLEAVETLGSTSTICSDK.T** 19290 |
|  | 19468 | **970.5204** | **1939.0262** | **1939.0375** | **-0.0112** | **1** | **50** | **0.001** | **1** | **R.QGAIVAVTGDGVNDSPALKK.A** 19467 |
|  | 20493 | **679.3719** | **2035.0940** | **2035.0594** | **0.0346** | **1** | **3** | **43** | **5** | **K.IMESFKNMVPQQALVIR.N** |
|  | 23164 | **1159.0046** | **2315.9947** | **2316.0083** | **-0.0136** | **0** | **111** | **5.5e-010** | **1** | **R.WINDVEDSYGQQWTYEQR.K** 23163 23165 |
|  | 24246 | **821.3798** | **2461.1175** | **2462.1707** | **-1.0533** | **1** | **1** | **61** | **2** | **R.IISANGCKVDNSSLTGESEPQTR.S** |
|  | 24267 | **1232.6003** | **2463.1861** | **2463.1951** | **-0.0090** | **0** | **129** | **9.1e-012** | **1** | **K.QAADMILLDDNFASIVTGVEEGR.L** 24262 24264 24266 24279 24283 |
|  | 24343 | **619.2994** | **2473.1684** | **2473.1829** | **-0.0145** | **1** | **10** | **7.9** | **1** | **K.ACVVHGSDLKDMTSEQLDDILK.Y** |
|  | 24359 | **496.2619** | **2476.2733** | **2476.2896** | **-0.0164** | **1** | **7** | **14** | **5** | **K.YQLSIHKNPNTSEPQHLLVMK.G** |
|  | 26342 | **932.7148** | **2795.1225** | **2795.3607** | **-0.2382** | **1** | **3** | **23** | **2** | **K.NLEAVETLGSTSTICSDKTGTLTQNR.M** |
|  | 26487 | **1417.6901** | **2833.3656** | **2833.3770** | **-0.0114** | **1** | **57** | **0.0001** | **1** | **K.EQPLDEELKDAFQNAYLELGGLGER.V** 26486 26488 26497 |
|  | 27795 | **1035.4496** | **3103.3269** | **3102.3380** | **0.9889** | **1** | **72** | **2.5e-006** | **1** | **R.VDWDDRWINDVEDSYGQQWTYEQR.K** |
|  | 28362 | **1080.1381** | **3237.3924** | **3237.4132** | **-0.0208** | **0** | **74** | **1.7e-006** | **1** | **R.MTVAHMWFDNQIHEADTTENQSGVSFDK.T** 28360 |

| **12.** | P11021\|GRP78_HUMAN    **Mass:** 72402    **Score:** 1587   **Queries matched:** 95   **emPAI:** 4.21 |
| --- | --- |
|  | 78 kDa glucose-regulated protein OS=Homo sapiens GN=HSPA5 PE=1 SV=2 |

|  | **Query** | **Observed** | **Mr(expt)** | **Mr(calc)** | **Delta** | **Miss** | **Score** | **Expect** | **Rank** | **Peptide** |
| --- | --- | --- | --- | --- | --- | --- | --- | --- | --- | --- |
|  | 1804 | **728.4614** | **727.4542** | **727.4592** | **-0.0051** | **0** | **40** | **0.019** | **1** | **K.IQQLVK.E** 1803 1805 |
|  | 3753 | **452.2386** | **902.4626** | **902.4684** | **-0.0058** | **0** | **37** | **0.038** | **1** | **R.VMEHFIK.L** 3754 |
|  | 3965 | **459.7545** | **917.4944** | **917.5004** | **-0.0060** | **0** | **31** | **0.16** | **1** | **R.STMKPVQK.V** 3966 3978 |
|  | 3984 | **460.3096** | **918.6047** | **917.4706** | **1.1341** | **0** | **5** | **57** | **5** | **K.VLEDSDLK.K** |
|  | 4833 | **981.4824** | **980.4751** | **980.4814** | **-0.0063** | **0** | **32** | **0.089** | **1** | **K.ETAEAYLGK.K** |
|  | 4897 | **493.7583** | **985.5020** | **985.5080** | **-0.0060** | **0** | **22** | **1.1** | **2** | **R.LTPEEIER.M** |
|  | 5057 | **499.2598** | **996.5051** | **996.5101** | **-0.0050** | **0** | **56** | **0.00039** | **1** | **R.ALSSQHQAR.I** 5054 5055 5056 5058 5059 5066 5067 5068 5073 |
|  | 5706 | **523.7863** | **1045.5579** | **1045.5655** | **-0.0076** | **1** | **17** | **3.4** | **2** | **K.VLEDSDLKK.S** |
|  | 6108 | **537.7772** | **1073.5398** | **1073.5465** | **-0.0068** | **0** | **29** | **0.22** | **1** | **K.ITITNDQNR.L** 6101 |
|  | 7713 | **596.3181** | **1190.6217** | **1190.6295** | **-0.0078** | **0** | **45** | **0.005** | **1** | **K.VYEGERPLTK.D** 7712 |
|  | 8099 | **605.7921** | **1209.5697** | **1209.5778** | **-0.0082** | **1** | **37** | **0.029** | **1** | **K.EFFNGKEPSR.G** 8100 |
|  | 8248 | **609.3152** | **1216.6158** | **1216.6234** | **-0.0076** | **0** | **53** | **0.00085** | **1** | **K.DAGTIAGLNVMR.I** 8247 |
|  | 8452 | **614.8128** | **1227.6111** | **1227.6207** | **-0.0097** | **0** | **47** | **0.003** | **1** | **R.VEIIANDQGNR.I** 8436 8453 |
|  | 8567 | **412.2323** | **1233.6752** | **1232.6183** | **1.0568** | **0** | **(7)** | **27** | **1** | **K.DAGTIAGLNVMR.I** |
|  | 8697 | **620.8119** | **1239.6092** | **1239.6169** | **-0.0077** | **1** | **50** | **0.0014** | **1** | **K.MKETAEAYLGK.K** 8699 |
|  | 10045 | **657.3094** | **1312.6043** | **1312.6122** | **-0.0078** | **0** | **52** | **0.00079** | **1** | **K.FEELNMDLFR.S** |
|  | 11569 | **699.3939** | **1396.7732** | **1396.7813** | **-0.0081** | **0** | **48** | **0.002** | **1** | **K.ELEEIVQPIISK.L** 11568 |
|  | 12158 | **715.8451** | **1429.6757** | **1429.6838** | **-0.0080** | **0** | **67** | **3e-005** | **1** | **R.TWNDPSVQQDIK.F** |
|  | 13440 | **504.9192** | **1511.7357** | **1511.7442** | **-0.0086** | **1** | **38** | **0.022** | **1** | **R.AKFEELNMDLFR.S** 13435 13439 |
|  | 13779 | **768.8983** | **1535.7821** | **1535.7905** | **-0.0084** | **0** | **51** | **0.001** | **1** | **K.TFAPEEISAMVLTK.M** |
|  | 14085 | **778.3954** | **1554.7763** | **1554.7862** | **-0.0099** | **1** | **43** | **0.0066** | **1** | **K.NGRVEIIANDQGNR.I** 14099 14100 |
|  | 14130 | **520.2886** | **1557.8439** | **1556.9323** | **0.9115** | **0** | **2** | **82** | **8** | **K.LSLVAAMLLLLSAAR.A** |
|  | 14247 | **783.8884** | **1565.7622** | **1565.7726** | **-0.0104** | **0** | **53** | **0.00054** | **1** | **R.ITPSYVAFTPEGER.L** |
|  | 14564 | **794.9260** | **1587.8375** | **1587.8468** | **-0.0093** | **1** | **58** | **0.00019** | **1** | **K.KSDIDEIVLVGGSTR.I** 14563 |
|  | 14794 | **803.4327** | **1604.8509** | **1603.8570** | **0.9939** | **0** | **54** | **0.00043** | **1** | **K.TKPYIQVDIGGGQTK.T** 14785 |
|  | 15661 | **830.4460** | **1658.8775** | **1658.8879** | **-0.0104** | **0** | **46** | **0.0026** | **1** | **R.IINEPTAAAIAYGLDK.R** 15683 15687 |
|  | 15777 | **556.9916** | **1667.9529** | **1669.0324** | **-1.0795** | **1** | **2** | **72** | **10** | **M.KLSLVAAMLLLLSAAR.A** |
|  | 15927 | **839.4020** | **1676.7894** | **1676.8006** | **-0.0112** | **0** | **68** | **1.7e-005** | **1** | **K.NQLTSNPENTVFDAK.R** 15925 15926 15950 15952 |
|  | 17800 | **908.4957** | **1814.9769** | **1814.9890** | **-0.0121** | **1** | **98** | **1.5e-008** | **1** | **R.IINEPTAAAIAYGLDKR.E** 17801 |
|  | 18051 | **611.3402** | **1830.9988** | **1832.0627** | **-1.0639** | **1** | **4** | **38** | **1** | **-.MKLSLVAAMLLLLSAAR.A** |
|  | 18123 | **918.9643** | **1835.9140** | **1835.9265** | **-0.0125** | **0** | **82** | **7.6e-007** | **1** | **K.SQIFSTASDNQPTVTIK.V** 18122 18124 |
|  | 18802 | **629.6359** | **1885.8860** | **1886.9639** | **-1.0780** | **0** | **18** | **1.6** | **1** | **K.VTHAVVTVPAYFNDAQR.Q** 18817 |
|  | 19414 | **645.3390** | **1932.9951** | **1933.0058** | **-0.0106** | **0** | **14** | **4.5** | **1** | **K.DNHLLGTFDLTGIPPAPR.G** 19413 |
|  | 19747 | **983.0117** | **1964.0088** | **1964.0215** | **-0.0127** | **1** | **104** | **3.6e-009** | **1** | **K.KSQIFSTASDNQPTVTIK.V** 19748 19765 |
|  | 19859 | **987.9523** | **1973.8901** | **1973.9007** | **-0.0106** | **0** | **97** | **1.7e-008** | **1** | **K.IEWLESHQDADIEDFK.A** 19858 19869 |
|  | 20254 | **504.7688** | **2015.0461** | **2015.0589** | **-0.0128** | **1** | **9** | **13** | **1** | **K.KVTHAVVTVPAYFNDAQR.Q** |
|  | 21895 | **1082.9930** | **2163.9715** | **2163.9848** | **-0.0133** | **0** | **113** | **3.8e-010** | **1** | **R.IEIESFYEGEDFSETLTR.A** 21894 21896 21897 |
|  | 21999 | **1088.4930** | **2174.9715** | **2174.9855** | **-0.0140** | **1** | **94** | **3.6e-008** | **1** | **K.LYGSAGPPPTGEEDTAEKDEL.-** 21994 21995 21996 21998 |
|  | 31354 | **1225.8656** | **4899.4333** | **4898.4570** | **0.9763** | **0** | **17** | **0.37** | **1** | **R.GINPDEAVAYGAAVQAGVLSGDQDTGDLVLLDVCPLTLGIETVGGVMTK.L** 31340 31347 31348 31349 31350 31351 |

| **13.** | Q00610\|CLH1_HUMAN    **Mass:** 193260   **Score:** 1535   **Queries matched:** 91   **emPAI:** 0.93 |
| --- | --- |
|  | Clathrin heavy chain 1 OS=Homo sapiens GN=CLTC PE=1 SV=5 |

|  | **Query** | **Observed** | **Mr(expt)** | **Mr(calc)** | **Delta** | **Miss** | **Score** | **Expect** | **Rank** | **Peptide** |
| --- | --- | --- | --- | --- | --- | --- | --- | --- | --- | --- |
|  | 1699 | **699.1810** | **698.1737** | **697.3105** | **0.8632** | **0** | **9** | **16** | **2** | **K.MYDAAK.L** |
|  | 1728 | **706.4451** | **705.4379** | **705.4425** | **-0.0046** | **0** | **27** | **0.39** | **1** | **K.IVLYAK.K** |
|  | 3137 | **864.4258** | **863.4186** | **862.4218** | **0.9967** | **0** | **5** | **53** | **6** | **K.QSVELCK.K** 3081 |
|  | 3333 | **438.2392** | **874.4638** | **874.4694** | **-0.0056** | **0** | **31** | **0.16** | **1** | **R.AMLSANIR.Q** |
|  | 3551 | 445.7377 | 889.4608 | 889.4505 | 0.0104 | 0 | 8 | 33 | 8 | K.LDASESLR.K |
|  | 4217 | **469.7422** | **937.4699** | **937.4756** | **-0.0057** | **0** | **18** | **2.2** | **1** | **K.EAIDSYIK.A** |
|  | 4425 | **476.2682** | **950.5218** | **949.5167** | **1.0051** | **1** | **7** | **31** | **7** | **K.FKPQKMR.E** |
|  | 5026 | **498.2762** | **994.5379** | **994.5335** | **0.0044** | **1** | **13** | **8.4** | **4** | **K.EYLTKVDK.L** |
|  | 5662 | **521.9990** | **1041.9834** | **1042.4356** | **-0.4522** | **0** | **5** | **33** | **5** | **R.ENPYYDSR.V** 5655 |
|  | 6015 | **535.2728** | **1068.5310** | **1068.5386** | **-0.0076** | **0** | **27** | **0.31** | **1** | **R.AHIAQLCEK.A** 6016 |
|  | 6062 | **536.3101** | **1070.6057** | **1070.6124** | **-0.0068** | **0** | **40** | **0.02** | **1** | **K.HDVVFLITK.Y** 6063 |
|  | 8220 | **608.8055** | **1215.5965** | **1215.6036** | **-0.0071** | **0** | **10** | **15** | **1** | **R.EHLELFWSR.V** |
|  | 8430 | **614.0776** | **1226.1407** | **1225.7547** | **0.3861** | **0** | **10** | **9.1** | **1** | **K.QLPLVKPYLR.S** |
|  | 8526 | **616.8233** | **1231.6320** | **1231.6408** | **-0.0088** | **1** | **34** | **0.057** | **1** | **K.VDKLDASESLR.K** |
|  | 8605 | **618.8328** | **1235.6511** | **1235.6584** | **-0.0073** | **0** | **3** | **73** | **6** | **K.TLQIFNIEMK.S** |
|  | 9176 | **422.6543** | **1264.9410** | **1265.6880** | **-0.7470** | **1** | **4** | **40** | **5** | **R.IAAYLFKGNNR.W** |
|  | 9761 | **648.8336** | **1295.6527** | **1295.6622** | **-0.0095** | **0** | **33** | **0.059** | **1** | **K.LLYNNVSNFGR.L** |
|  | 9909 | **652.8513** | **1303.6881** | **1303.6520** | **0.0361** | **0** | **3** | **70** | **1** | **R.NNLAGAEELFAR.K** |
|  | 10453 | **667.8158** | **1333.6170** | **1333.6262** | **-0.0092** | **0** | **32** | **0.087** | **1** | **K.IYIDSNNNPER.F** 10468 |
|  | 10517 | **669.3434** | **1336.6722** | **1336.6809** | **-0.0087** | **0** | **15** | **4.8** | **1** | **R.VVGAMQLYSVDR.K** |
|  | 10800 | **677.4238** | **1352.8330** | **1352.8391** | **-0.0061** | **0** | **42** | **0.0075** | **1** | **R.NLQNLLILTAIK.A** 10779 10781 |
|  | 12688 | **732.8668** | **1463.7191** | **1463.7296** | **-0.0105** | **0** | **18** | **2** | **1** | **R.ALEHFTDLYDIK.R** |
|  | 12755 | **490.3149** | **1467.9228** | **1466.7803** | **1.1425** | **1** | **7** | **22** | **2** | **K.TLQIFNIEMKSK.M** 12727 |
|  | 13267 | **750.4158** | **1498.8170** | **1498.8256** | **-0.0086** | **0** | **10** | **11** | **1** | **K.WLLLTGISAQQNR.V** |
|  | 13368 | **754.3768** | **1506.7390** | **1506.7501** | **-0.0111** | **0** | **65** | **4.2e-005** | **1** | **K.VIQCFAETGQVQK.I** |
|  | 14006 | **776.3754** | **1550.7363** | **1550.7464** | **-0.0101** | **0** | **44** | **0.0046** | **1** | **R.GQFSTDELVAEVEK.R** |
|  | 16184 | **566.3079** | **1695.9019** | **1695.0043** | **0.8977** | **1** | **4** | **44** | **3** | **R.NLQNLLILTAIKADR.T** |
|  | 16234 | **850.4434** | **1698.8723** | **1698.8770** | **-0.0047** | **0** | **58** | **0.00016** | **1** | **R.FDFVHDLVLYLYR.N** |
|  | 16341 | **854.4252** | **1706.8359** | **1706.8475** | **-0.0116** | **1** | **40** | **0.011** | **1** | **R.GQFSTDELVAEVEKR.N** 16343 |
|  | 16418 | **856.9051** | **1711.7956** | **1710.8473** | **0.9483** | **0** | **31** | **0.079** | **1** | **R.AHMGMFTELAILYSK.F** 16417 |
|  | 16465 | **572.9584** | **1715.8533** | **1715.8631** | **-0.0098** | **0** | **31** | **0.083** | **1** | **K.VSQPIEGHAASFAQFK.M** |
|  | 16608 | **576.7942** | **1727.3607** | **1726.8422** | **0.5185** | **0** | **(2)** | **42** | **6** | **R.AHMGMFTELAILYSK.F** |
|  | 17057 | **879.9379** | **1757.8613** | **1757.8736** | **-0.0123** | **1** | **86** | **3e-007** | **1** | **R.KFNALFAQGNYSEAAK.V** 17056 |
|  | 17109 | **882.4408** | **1762.8670** | **1762.8295** | **0.0376** | **1** | **11** | **9.8** | **3** | **K.EDKLECSEELGDLVK.S** |
|  | 17287 | **888.9141** | **1775.8136** | **1775.8260** | **-0.0125** | **0** | **60** | **0.00011** | **1** | **R.IHEGCEEPATHNALAK.I** 17293 17302 |
|  | 18283 | **616.6486** | **1846.9239** | **1846.9359** | **-0.0121** | **0** | **1** | **90** | **3** | **K.YESLELCRPVLQQGR.K** |
|  | 19494 | **971.9533** | **1941.8921** | **1941.9068** | **-0.0147** | **0** | **54** | **0.00041** | **1** | **R.TSIDAYDNFDNISLAQR.L** 19495 |
|  | 19532 | **973.0245** | **1944.0344** | **1944.0469** | **-0.0125** | **0** | **58** | **0.00014** | **1** | **K.LHIIEVGTPPTGNQPFPK.K** 19531 |
|  | 19567 | **974.0062** | **1945.9979** | **1946.0070** | **-0.0091** | **0** | **48** | **0.0015** | **1** | **K.AFMTADLPNELIELLEK.I** 19568 |
|  | 19953 | **662.3180** | **1983.9321** | **1983.9409** | **-0.0088** | **0** | **0** | **86** | **2** | **R.HNIMDFAMPYFIQVMK.E** |
|  | 20764 | **1029.4855** | **2056.9564** | **2056.1317** | **0.8247** | **1** | **2** | **61** | **8** | **K.SVDPTLALSVYLRANVPNK.V** |
|  | 20857 | **690.3416** | **2068.0030** | **2066.9778** | **1.0252** | **1** | **3** | **44** | **5** | **K.MFDRHSSLAGCQIINYR.T** |
|  | 21461 | **1061.0186** | **2120.0225** | **2120.0313** | **-0.0088** | **0** | **72** | **6.1e-006** | **1** | **K.DTELAEELLQWFLQEEK.R** |
|  | 21748 | **1076.9971** | **2151.9796** | **2151.9929** | **-0.0133** | **0** | **85** | **2.9e-007** | **1** | **R.GQCDLELINVCNENSLFK.S** 21749 |
|  | 22157 | **1098.0702** | **2194.1258** | **2194.1052** | **0.0206** | **0** | **51** | **0.00066** | **1** | **K.VGEQAQVVIIDMNDPSNPIR.R** 22149 |
|  | 22185 | **1100.0470** | **2198.0794** | **2198.0895** | **-0.0101** | **0** | **37** | **0.016** | **1** | **K.YHEQLSTQSLIELFESFK.S** |
|  | 23436 | **1177.1215** | **2352.2284** | **2352.2438** | **-0.0154** | **0** | **104** | **2.7e-009** | **1** | **R.ISGETIFVTAPHEATAGIIGVNR.K** 23435 |
|  | 23468 | **1178.0698** | **2354.1251** | **2354.1390** | **-0.0139** | **0** | **95** | **2.2e-008** | **1** | **K.SVNESLNNLFITEEDYQALR.T** 23469 |
|  | 23489 | **1179.0857** | **2356.1568** | **2355.2119** | **0.9449** | **1** | **0** | **73** | **6** | **R.AHMGMFTELAILYSKFKPQK.M** |
|  | 23570 | **592.8181** | **2367.2434** | **2367.2547** | **-0.0113** | **1** | **11** | **5.4** | **1** | **R.KFDVNTSAVQVLIEHIGNLDR.A** |
|  | 24274 | **822.0737** | **2463.1992** | **2463.2142** | **-0.0150** | **0** | **52** | **0.00051** | **1** | **R.LAELEEFINGPNNAHIQQVGDR.C** |
|  | 25907 | **903.1141** | **2706.3204** | **2706.3363** | **-0.0160** | **1** | **31** | **0.053** | **1** | **K.KAVDVFFPPEAQNDFPVAMQISEK.H** |
|  | 26038 | **911.8206** | **2732.4400** | **2732.5339** | **-0.0938** | **0** | **2** | **35** | **2** | **R.AIQFYLEFKPLLLNDLLMVLSPR.L** |
|  | 26082 | **1370.6156** | **2739.2166** | **2739.2334** | **-0.0167** | **0** | **77** | **1.2e-006** | **1** | **K.ADDPSSYMEVVQAANTSGNWEELVK.Y** |
|  | 26326 | **931.7873** | **2792.3400** | **2792.3486** | **-0.0086** | **0** | **22** | **0.37** | **1** | **K.SFEGLFYFLGSIVNFSQDPDVHFK.Y** |
|  | 27646 | **1021.4736** | **3061.3991** | **3062.3426** | **-0.9435** | **1** | **2** | **31** | **3** | **K.YEEYDNAIITMMNHPTDAWKEGQFK.D** |
|  | 27687 | **1536.2463** | **3070.4781** | **3070.4923** | **-0.0142** | **0** | **83** | **2.7e-007** | **1** | **R.LDNYDAPDIANIAISNELFEEAFAIFR.K** |
|  | 27873 | **1043.5026** | **3127.4859** | **3127.5041** | **-0.0183** | **0** | **75** | **1.5e-006** | **1** | **R.LLEMNLMHAPQVADAILGNQMFTHYDR.A** 27872 27874 27875 |
|  | 27931 | **1049.1864** | **3144.5374** | **3144.5550** | **-0.0176** | **0** | **93** | **2.4e-008** | **1** | **R.FQEHLQLQNLGINPANIGFSTLTMESDK.F** 27930 |
|  | 28109 | **1058.8530** | **3173.5373** | **3173.5492** | **-0.0119** | **0** | **48** | **0.0008** | **1** | **K.WISLNTVALVTDNAVYHWSMEGESQPVK.M** |
|  | 28172 | **1594.3362** | **3186.6578** | **3186.6714** | **-0.0135** | **0** | **68** | **7e-006** | **1** | **R.FQSVPAQPGQTSPLLQYFGILLDQGQLNK.Y** 28170 28171 |
|  | 28998 | **1142.6065** | **3424.7975** | **3423.8072** | **0.9904** | **1** | **14** | **1.7** | **1** | **R.KGQVLSVCVEEENIIPYITNVLQNPDLALR.M** |
|  | 29183 | **1166.5870** | **3496.7393** | **3496.7449** | **-0.0056** | **0** | **93** | **2.1e-008** | **1** | **R.AVVHTHLLNPEWLVNYFGSLSVEDSLECLR.A** |
|  | 31442 | **1254.8987** | **5015.5656** | **5015.5967** | **-0.0311** | **1** | **7** | **2.7** | **1** | **R.FQSVPAQPGQTSPLLQYFGILLDQGQLNKYESLELCRPVLQQGR.K** 31444 |

| **14.** | P06576\|ATPB_HUMAN    **Mass:** 56525    **Score:** 1521   **Queries matched:** 84   **emPAI:** 3.93 |
| --- | --- |
|  | ATP synthase subunit beta, mitochondrial OS=Homo sapiens GN=ATP5B PE=1 SV=3 |

|  | **Query** | **Observed** | **Mr(expt)** | **Mr(calc)** | **Delta** | **Miss** | **Score** | **Expect** | **Rank** | **Peptide** |
| --- | --- | --- | --- | --- | --- | --- | --- | --- | --- | --- |
|  | 1482 | **643.3732** | **642.3659** | **642.4064** | **-0.0406** | **1** | **23** | **1** | **1** | **R.GPIKTK.Q** |
|  | 1971 | **778.8836** | **777.8763** | **778.4225** | **-0.5462** | **0** | **30** | **0.14** | **1** | **K.ILQDYK.S** 1972 |
|  | 3682 | **450.2604** | **898.5063** | **898.5124** | **-0.0060** | **0** | **31** | **0.13** | **1** | **K.VLDSGAPIK.I** |
|  | 4758 | **488.2818** | **974.5490** | **974.5549** | **-0.0059** | **0** | **56** | **0.00043** | **1** | **K.IGLFGGAGVGK.T** |
|  | 4866 | **492.2465** | **982.4784** | **982.5560** | **-0.0775** | **0** | **4** | **56** | **6** | **R.VAAAPASGALR.R** |
|  | 6303 | **544.3415** | **1086.6684** | **1085.4989** | **1.1695** | **1** | **2** | **1e+002** | **10** | **K.ADKLAEEHSS.-** |
|  | 6311 | **544.8179** | **1087.6212** | **1087.6277** | **-0.0065** | **0** | **8** | **29** | **1** | **K.VVDLLAPYAK.G** |
|  | 9080 | **631.3012** | **1260.5877** | **1261.6336** | **-1.0459** | **0** | **3** | **69** | **3** | **R.TIAMDGTEGLVR.G** |
|  | 9399 | **639.3110** | **1276.6074** | **1277.6286** | **-1.0212** | **0** | **(2)** | **94** | **6** | **R.TIAMDGTEGLVR.G** |
|  | 11343 | **693.3536** | **1384.6926** | **1384.7020** | **-0.0094** | **0** | **62** | **8.3e-005** | **1** | **R.IMNVIGEPIDER.G** |
|  | 11714 | **703.8393** | **1405.6640** | **1405.6739** | **-0.0098** | **0** | **70** | **1.4e-005** | **1** | **K.AHGGYSVFAGVGER.T** |
|  | 12243 | **718.3759** | **1434.7373** | **1434.7467** | **-0.0094** | **0** | **69** | **1.7e-005** | **1** | **R.FTQAGSEVSALLGR.I** 12244 12260 |
|  | 12562 | **729.4199** | **1456.8253** | **1456.8323** | **-0.0070** | **0** | **66** | **3e-005** | **1** | **K.TVLIMELINNVAK.A** 12546 12560 12563 12567 12585 |
|  | 12818 | **737.4159** | **1472.8173** | **1472.8272** | **-0.0099** | **0** | **(2)** | **90** | **4** | **K.TVLIMELINNVAK.A** |
|  | 14744 | **801.4066** | **1600.7986** | **1600.8031** | **-0.0046** | **0** | **25** | **0.35** | **2** | **K.VALVYGQMNEPPGAR.A** 14745 |
|  | 15541 | **825.9569** | **1649.8991** | **1649.9101** | **-0.0109** | **0** | **84** | **4.4e-007** | **1** | **R.LVLEVAQHLGESTVR.T** 15542 |
|  | 17797 | **908.4326** | **1814.8507** | **1814.8621** | **-0.0114** | **0** | **65** | **2.9e-005** | **1** | **R.IMDPNIVGSEHYDVAR.G** 17798 |
|  | 18062 | **611.6372** | **1831.8898** | **1830.8570** | **1.0328** | **0** | **(0)** | **1e+002** | **8** | **R.IMDPNIVGSEHYDVAR.G** |
|  | 19235 | **640.3663** | **1918.0770** | **1918.0888** | **-0.0118** | **1** | **57** | **0.00018** | **1** | **K.VLDSGAPIKIPVGPETLGR.I** 19233 19234 |
|  | 19270 | **961.4833** | **1920.9520** | **1920.9581** | **-0.0061** | **0** | **114** | **4.2e-010** | **1** | **R.DQEGQDVLLFIDNIFR.F** 19269 |
|  | 19982 | **994.5148** | **1987.0151** | **1987.0262** | **-0.0111** | **0** | **28** | **0.14** | **1** | **R.AIAELGIYPAVDPLDSTSR.I** 19981 |
|  | 20335 | **1012.0038** | **2021.9930** | **2022.0033** | **-0.0103** | **0** | **51** | **0.0007** | **1** | **R.FLSQPFQVAEVFTGHMGK.L** 20336 |
|  | 20794 | **1030.9955** | **2059.9764** | **2059.9884** | **-0.0120** | **0** | **68** | **1.4e-005** | **1** | **R.EGNDLYHEMIESGVINLK.D** 20793 20795 |
|  | 21207 | **1052.0740** | **2102.1334** | **2103.0419** | **-0.9085** | **1** | **3** | **40** | **5** | **K.DATSKVALVYGQMNEPPGAR.A** |
|  | 23031 | **766.4044** | **2296.1913** | **2297.0668** | **-0.8755** | **0** | **0** | **77** | **9** | **R.IPSAVGYQPTLATDMGTMQER.I** |
|  | 23180 | **1159.5685** | **2317.1224** | **2317.1372** | **-0.0148** | **1** | **62** | **4.7e-005** | **1** | **R.TREGNDLYHEMIESGVINLK.D** 23179 |
|  | 23904 | **807.4495** | **2419.3266** | **2420.2733** | **-0.9468** | **1** | **3** | **35** | **3** | **K.IPVGPETLGRIMNVIGEPIDER.G** |
|  | 25624 | **1338.1874** | **2674.3602** | **2674.3735** | **-0.0133** | **1** | **101** | **4.7e-009** | **1** | **K.SLQDIIAILGMDELSEEDKLTVSR.A** 25620 25621 25622 25623 25625 25626 25627 25628 25630 25631 25632 25639 25642 25645 25647 25649 25650 25651 25653 25657 |
|  | 27574 | **1015.5029** | **3043.4868** | **3043.5034** | **-0.0167** | **0** | **61** | **4.2e-005** | **1** | **K.QFAPIHAEAPEFMEMSVEQEILVTGIK.V** 27575 27576 27577 |
|  | 28745 | **1117.5474** | **3349.6203** | **3349.6329** | **-0.0126** | **0** | **62** | **2.5e-005** | **1** | **K.GFQQILAGEYDHLPEQAFYMVGPIEEAVAK.A** 28741 28742 28743 28744 28746 28747 28748 28749 |
|  | 29821 | **1238.9605** | **3713.8595** | **3713.8789** | **-0.0193** | **0** | **48** | **0.00059** | **1** | **K.GSITSVQAIYVPADDLTDPAPATTFAHLDATTVLSR.A** 29820 |
|  | 30165 | **1281.6576** | **3841.9509** | **3841.9738** | **-0.0229** | **1** | **84** | **1.3e-007** | **1** | **K.KGSITSVQAIYVPADDLTDPAPATTFAHLDATTVLSR.A** 30167 |

| **15.** | P35579\|MYH9_HUMAN    **Mass:** 227646   **Score:** 1472   **Queries matched:** 111   **emPAI:** 0.64 |
| --- | --- |
|  | Myosin-9 OS=Homo sapiens GN=MYH9 PE=1 SV=4 |

|  | **Query** | **Observed** | **Mr(expt)** | **Mr(calc)** | **Delta** | **Miss** | **Score** | **Expect** | **Rank** | **Peptide** |
| --- | --- | --- | --- | --- | --- | --- | --- | --- | --- | --- |
|  | 970 | **533.1628** | **532.1555** | **531.3017** | **0.8539** | **0** | **10** | **28** | **1** | **R.IGQSK.V** |
|  | 1179 | **577.1241** | **576.1168** | **575.2551** | **0.8617** | **0** | **2** | **1.2e+002** | **4** | **K.DQADK.A** |
|  | 1181 | **577.2529** | **576.2456** | **575.3101** | **0.9355** | **0** | **(18)** | **3.1** | **9** | **K.NMALK.K** 1182 |
|  | 1259 | **593.3448** | **592.3375** | **591.3050** | **1.0325** | **0** | **25** | **0.54** | **1** | **K.NMALK.K** |
|  | 1333 | **609.3198** | **608.3125** | **608.3170** | **-0.0045** | **0** | **2** | **79** | **9** | **K.SFVEK.V** |
|  | 1503 | **648.3270** | **647.3197** | **646.3802** | **0.9395** | **0** | **8** | **41** | **8** | **K.FQKPK.Q** |
|  | 1533 | **653.3270** | **652.3197** | **651.3228** | **0.9969** | **0** | **15** | **4.7** | **6** | **R.DYVQK.A** |
|  | 1780 | **719.3887** | **718.3815** | **717.4207** | **0.9608** | **1** | **11** | **18** | **3** | **K.MQLAKK.E** |
|  | 2309 | **809.4545** | **808.4472** | **807.4491** | **0.9981** | **0** | **15** | **4.9** | **1** | **R.TVGQLYK.E** 2303 |
|  | 3007 | **429.7295** | **857.4445** | **858.4447** | **-1.0002** | **0** | **15** | **6.4** | **2** | **R.GELANEVK.V** |
|  | 3090 | **863.0862** | **862.0789** | **861.4742** | **0.6047** | **1** | **0** | **1.3e+002** | **2** | **R.KLQAQMK.D** |
|  | 3118 | **432.2414** | **862.4682** | **861.4014** | **1.0668** | **0** | **3** | **1.1e+002** | **6** | **-.MAQQAADK.Y** |
|  | 3950 | **459.2352** | **916.4558** | **916.4614** | **-0.0055** | **0** | **16** | **4.4** | **2** | **K.DLEGLSQR.H** |
|  | 4043 | **462.7480** | **923.4815** | **923.4865** | **-0.0050** | **0** | **16** | **3.6** | **1** | **R.VVFQEFR.Q** |
|  | 4538 | **480.2587** | **958.5029** | **958.5083** | **-0.0054** | **1** | **16** | **4.7** | **3** | **K.VNKDDIQK.M** |
|  | 4705 | **486.7683** | **971.5220** | **971.5287** | **-0.0067** | **0** | **6** | **45** | **7** | **R.EQEVNILK.K** |
|  | 4894 | **493.3027** | **984.5908** | **985.5556** | **-0.9648** | **1** | **12** | **9.3** | **4** | **K.GALALEEKR.R** |
|  | 5696 | **523.2863** | **1044.5579** | **1044.5637** | **-0.0058** | **0** | **28** | **0.27** | **1** | **R.LEVNLQAMK.A** |
|  | 6504 | **552.2996** | **1102.5846** | **1102.5917** | **-0.0071** | **1** | **29** | **0.2** | **1** | **K.RQQQLTAMK.V** 6503 |
|  | 7152 | **577.7815** | **1153.5484** | **1153.5550** | **-0.0066** | **0** | **38** | **0.024** | **1** | **K.VMQEQGTHPK.F** 7164 |
|  | 7169 | **578.3310** | **1154.6474** | **1154.6560** | **-0.0086** | **1** | **11** | **11** | **3** | **R.RGDLPFVVPR.R** |
|  | 7636 | **396.8672** | **1187.5797** | **1188.5775** | **-0.9978** | **1** | **8** | **26** | **5** | **R.HEEKVAAYDK.L** |
|  | 7729 | **596.8237** | **1191.6329** | **1192.6088** | **-0.9759** | **0** | **5** | **54** | **8** | **K.ALELDSNLYR.I** |
|  | 7959 | **602.7933** | **1203.5720** | **1203.5805** | **-0.0085** | **0** | **22** | **0.95** | **1** | **R.ALEQQVEEMK.T** |
|  | 8163 | **607.3099** | **1212.6052** | **1211.5782** | **1.0270** | **0** | **3** | **81** | **8** | **K.DLEAHIDSANK.N** |
|  | 8351 | **611.3470** | **1220.6794** | **1219.6448** | **1.0346** | **1** | **10** | **17** | **2** | **K.KFDQLLAEEK.T** |
|  | 8371 | **612.3187** | **1222.6229** | **1222.6306** | **-0.0077** | **0** | **44** | **0.0055** | **1** | **R.AGVLAHLEEER.D** |
|  | 8904 | **418.2190** | **1251.6352** | **1250.5891** | **1.0461** | **1** | **7** | **25** | **7** | **K.QVEDEKNSFR.E** |
|  | 9001 | **629.3394** | **1256.6643** | **1256.6724** | **-0.0081** | **1** | **62** | **9e-005** | **1** | **K.KEEELQAALAR.V** 9000 |
|  | 9025 | **420.3174** | **1257.9305** | **1257.5772** | **0.3533** | **0** | **8** | **16** | **3** | **K.THEAQIQEMR.Q** |
|  | 9044 | **630.8172** | **1259.6198** | **1258.7132** | **0.9066** | **1** | **0** | **1.4e+002** | **9** | **K.LQLEKVTTEAK.L** |
|  | 9276 | **636.3421** | **1270.6696** | **1271.6721** | **-1.0024** | **1** | **6** | **33** | **7** | **K.RDLGEELEALK.T** |
|  | 9372 | **638.3942** | **1274.7738** | **1273.6918** | **1.0820** | **0** | **6** | **33** | **7** | **R.YEILTPNSIPK.G** |
|  | 9922 | **653.3341** | **1304.6535** | **1304.6612** | **-0.0076** | **0** | **2** | **89** | **3** | **K.EQADFAIEALAK.A** |
|  | 9981 | **655.3733** | **1308.7320** | **1308.5946** | **0.1375** | **1** | **4** | **52** | **4** | **R.NAEQYKDQADK.A** |
|  | 10940 | **682.3330** | **1362.6515** | **1362.5899** | **0.0615** | **1** | **5** | **45** | **10** | **R.KGAGDGSDEEVDGK.A** |
|  | 11633 | **468.2270** | **1401.6591** | **1400.7147** | **0.9445** | **1** | **2** | **88** | **5** | **R.EEILAQAKENEK.K** 11628 |
|  | 11807 | **706.3163** | **1410.6180** | **1411.8035** | **-1.1854** | **1** | **21** | **0.95** | **2** | **K.KVEAQLQELQVK.F** |
|  | 12887 | **739.3628** | **1476.7111** | **1475.7289** | **0.9822** | **1** | **5** | **45** | **2** | **R.VEEEAAQKNMALK.K** |
|  | 13045 | **743.9493** | **1485.8841** | **1485.8919** | **-0.0078** | **0** | **39** | **0.016** | **1** | **R.VISGVLQLGNIVFK.K** |
|  | 13697 | **765.8815** | **1529.7484** | **1529.7573** | **-0.0089** | **0** | **50** | **0.0013** | **1** | **K.IAQLEEQLDNETK.E** |
|  | 14002 | **776.3459** | **1550.6772** | **1550.6882** | **-0.0110** | **0** | **45** | **0.0039** | **1** | **R.EDQSILCTGESGAGK.T** |
|  | 14046 | **777.4078** | **1552.8011** | **1552.8395** | **-0.0384** | **1** | **10** | **11** | **3** | **K.QIATLHAQVADMKK.K** 14026 |
|  | 14114 | **779.4303** | **1556.8460** | **1557.8515** | **-1.0054** | **1** | **1** | **1e+002** | **9** | **R.QRYEILTPNSIPK.G** |
|  | 14243 | **783.8367** | **1565.6589** | **1564.6675** | **0.9914** | **0** | **68** | **1.6e-005** | **1** | **R.ELEDATETADAMNR.E** |
|  | 14327 | **786.9083** | **1571.8021** | **1570.8468** | **0.9554** | **0** | **12** | **8.9** | **1** | **K.VSHLLGINVTDFTR.G** |
|  | 14505 | **529.2563** | **1584.7470** | **1584.7566** | **-0.0095** | **1** | **68** | **2e-005** | **1** | **K.NKHEAMITDLEER.L** 14504 |
|  | 14602 | **796.3493** | **1590.6840** | **1590.6944** | **-0.0103** | **0** | **75** | **3.9e-006** | **1** | **R.NTDQASMPDNTAAQK.V** 14603 |
|  | 14810 | **536.2308** | **1605.6705** | **1606.6893** | **-1.0188** | **0** | **(8)** | **18** | **2** | **R.NTDQASMPDNTAAQK.V** |
|  | 14972 | **808.4075** | **1614.8005** | **1614.8109** | **-0.0104** | **0** | **13** | **6.6** | **1** | **R.IMGIPEEEQMGLLR.V** |
|  | 15220 | **544.2600** | **1629.7580** | **1630.8058** | **-1.0478** | **0** | **(6)** | **29** | **7** | **R.IMGIPEEEQMGLLR.V** 15231 |
|  | 15490 | **549.7710** | **1646.2912** | **1646.8008** | **-0.5096** | **0** | **(2)** | **53** | **4** | **R.IMGIPEEEQMGLLR.V** 15500 |
|  | 15510 | **412.9692** | **1647.8479** | **1648.7362** | **-0.8884** | **1** | **4** | **45** | **10** | **R.QVREMEAELEDER.K** |
|  | 15587 | **827.4815** | **1652.9485** | **1652.7781** | **0.1704** | **0** | **42** | **0.0065** | **1** | **R.IAEFTTNLTEEEEK.S** |
|  | 15832 | **836.9203** | **1671.8261** | **1671.8369** | **-0.0107** | **0** | **67** | **2.3e-005** | **1** | **K.NFINNPLAQADWAAK.K** 15831 |
|  | 15891 | **559.2911** | **1674.8516** | **1675.8715** | **-1.0199** | **1** | **5** | **39** | **7** | **R.LEVNLQAMKAQFER.D** |
|  | 16600 | **863.9730** | **1725.9314** | **1725.9413** | **-0.0100** | **0** | **61** | **7.6e-005** | **1** | **R.QLLQANPILEAFGNAK.T** |
|  | 16934 | **876.4268** | **1750.8390** | **1750.8481** | **-0.0091** | **0** | **22** | **0.67** | **1** | **R.LTEMETLQSQLMAEK.L** |
|  | 16976 | **585.3173** | **1752.9301** | **1753.7750** | **-0.8448** | **1** | **3** | **52** | **7** | **R.QEEEMMAKEEELVK.V** 16989 |
|  | 17173 | **590.3090** | **1767.9051** | **1766.8430** | **1.0620** | **0** | **(2)** | **71** | **5** | **R.LTEMETLQSQLMAEK.L** |
|  | 17188 | **590.6168** | **1768.8286** | **1768.8414** | **-0.0127** | **1** | **5** | **38** | **3** | **K.KQELEEICHDLEAR.V** |
|  | 18162 | 920.4923 | 1838.9699 | 1838.9010 | 0.0690 | 0 | 26 | 0.28 | 1 | K.HSQAVEELAEQLEQTK.R |
|  | 18596 | **935.4807** | **1868.9469** | **1868.9592** | **-0.0123** | **0** | **98** | **1.7e-008** | **1** | **K.ANLQIDQINTDLNLER.S** |
|  | 18829 | **944.9634** | **1887.9122** | **1887.9247** | **-0.0125** | **1** | **78** | **1.7e-006** | **1** | **K.KLEEEQIILEDQNCK.L** 18828 18848 |
|  | 19380 | **966.0837** | **1930.1529** | **1929.9043** | **0.2486** | **0** | **2** | **59** | **8** | **R.HEMPPHIYAITDTAYR.S** 19379 |
|  | 19548 | **973.5017** | **1944.9887** | **1945.0004** | **-0.0116** | **0** | **51** | **0.00076** | **1** | **K.LQVELDNVTGLLSQSDSK.S** 19551 |
|  | 19593 | **650.6653** | **1948.9740** | **1948.9854** | **-0.0114** | **0** | **37** | **0.019** | **1** | **R.LQQELDDLLVDLDHQR.Q** |
|  | 19612 | **651.4102** | **1951.2088** | **1951.9686** | **-0.7598** | **1** | **1** | **55** | **2** | **R.NTNPNFVRCIIPNHEK.K** |
|  | 19715 | **981.4571** | **1960.8997** | **1960.9113** | **-0.0115** | **0** | **123** | **4.4e-011** | **1** | **K.TQLEELEDELQATEDAK.L** 19716 |
|  | 20062 | **666.0032** | **1994.9877** | **1995.0021** | **-0.0144** | **1** | **64** | **4.2e-005** | **1** | **K.HSQAVEELAEQLEQTKR.V** |
|  | 20063 | **499.7608** | **1995.0141** | **1995.0254** | **-0.0113** | **0** | **8** | **17** | **2** | **R.TFHIFYYLLSGAGEHLK.T** |
|  | 20276 | **1009.5307** | **2017.0468** | **2017.0554** | **-0.0086** | **0** | **68** | **1.4e-005** | **1** | **R.IIGLDQVAGMSETALPGAFK.T** 20275 |
|  | 21499 | **709.3402** | **2124.9988** | **2125.0110** | **-0.0122** | **0** | **3** | **41** | **5** | **K.NMDPLNDNIATLLHQSSDK.F** |
|  | 21625 | **714.3518** | **2140.0334** | **2141.0059** | **-0.9725** | **0** | **(2)** | **54** | **4** | **K.NMDPLNDNIATLLHQSSDK.F** |
|  | 22278 | **737.3673** | **2209.0799** | **2208.2379** | **0.8420** | **1** | **4** | **35** | **8** | **K.VSHLLGINVTDFTRGILTPR.I** |
|  | 22830 | **759.0772** | **2274.2098** | **2274.2042** | **0.0056** | **1** | **4** | **34** | **8** | **R.IIGLDQVAGMSETALPGAFKTR.K** |
|  | 22938 | **762.7043** | **2285.0910** | **2285.1031** | **-0.0121** | **0** | **41** | **0.006** | **1** | **K.VEDMAELTCLNEASVLHNLK.E** |
|  | 23044 | **767.3312** | **2298.9717** | **2298.9879** | **-0.0162** | **1** | **2** | **41** | **5** | **R.SMMQDREDQSILCTGESGAGK.T** |
|  | 23064 | **768.3344** | **2301.9814** | **2301.0981** | **0.8833** | **0** | **(4)** | **29** | **10** | **K.VEDMAELTCLNEASVLHNLK.E** |
|  | 23267 | **1167.0248** | **2332.0350** | **2332.0488** | **-0.0138** | **0** | **129** | **8.2e-012** | **1** | **K.MQQNIQELEEQLEEEESAR.Q** 23268 |
|  | 23669 | **1193.1129** | **2384.2113** | **2384.2263** | **-0.0151** | **0** | **8** | **11** | **1** | **R.INFDVNGYIVGANIETYLLEK.S** |
|  | 24323 | **1236.5825** | **2471.1505** | **2471.1663** | **-0.0158** | **0** | **92** | **3.8e-008** | **1** | **R.IAQLEEELEEEQGNTELINDR.L** 24325 |
|  | 24506 | **1247.0839** | **2492.1532** | **2492.1667** | **-0.0135** | **0** | **90** | **8e-008** | **1** | **K.DFSALESQLQDTQELLQEENR.Q** 24507 24508 |
|  | 24847 | 1275.5797 | 2549.1449 | 2549.2465 | -0.1016 | 1 | 2 | 43 | 3 | K.QLAAENRLTEMETLQSQLMAEK.L 24880 |
|  | 24878 | 850.7252 | 2549.1538 | 2549.2465 | -0.0926 | 1 | (1) | 48 | 6 | K.QLAAENRLTEMETLQSQLMAEK.L |
|  | 27368 | **1006.4940** | **3016.4601** | **3016.4724** | **-0.0123** | **1** | **44** | **0.0023** | **1** | **R.DLGEELEALKTELEDTLDSTAAQQELR.S** |
|  | 27474 | **1516.2117** | **3030.4088** | **3030.4757** | **-0.0669** | **1** | **8** | **8** | **2** | **K.NMDPLNDNIATLLHQSSDKFVSELWK.D** |

| **16.** | Q9ULC5\|ACSL5_HUMAN    **Mass:** 76855    **Score:** 1463   **Queries matched:** 99   **emPAI:** 2.92 |
| --- | --- |
|  | Long-chain-fatty-acid--CoA ligase 5 OS=Homo sapiens GN=ACSL5 PE=1 SV=1 |

|  | **Query** | **Observed** | **Mr(expt)** | **Mr(calc)** | **Delta** | **Miss** | **Score** | **Expect** | **Rank** | **Peptide** |
| --- | --- | --- | --- | --- | --- | --- | --- | --- | --- | --- |
|  | 1317 | **605.0900** | **604.0828** | **603.2976** | **0.7851** | **0** | **1** | **1.5e+002** | **6** | **K.QVSDR.A** |
|  | 1325 | **605.3659** | **604.3586** | **603.3592** | **0.9995** | **0** | **20** | **2.5** | **1** | **K.LAVSSK.F** |
|  | 1582 | **665.3728** | **664.3655** | **664.3544** | **0.0111** | **0** | **17** | **4.4** | **7** | **K.GTNVFK.G** 1580 |
|  | 1687 | **695.3133** | **694.3060** | **695.3642** | **-1.0582** | **0** | **15** | **4.9** | **2** | **R.WLSYK.Q** |
|  | 1885 | **751.3932** | **750.3859** | **750.3912** | **-0.0053** | **0** | **23** | **0.86** | **1** | **K.TFEQVK.A** |
|  | 2060 | **396.7319** | **791.4493** | **791.4541** | **-0.0048** | **1** | **31** | **0.16** | **1** | **K.FKELQK.G** 2061 |
|  | 2240 | **805.4073** | **804.4001** | **804.4051** | **-0.0050** | **0** | **34** | **0.083** | **1** | **R.LLADDMK.T** |
|  | 2808 | **423.2250** | **844.4354** | **844.4403** | **-0.0049** | **0** | **15** | **6.9** | **2** | **K.IQDSLGGR.V** 2807 |
|  | 3396 | **440.6986** | **879.3827** | **878.5225** | **0.8602** | **1** | **4** | **64** | **6** | **K.LAVSSKFK.E** |
|  | 4008 | **461.2399** | **920.4653** | **920.4715** | **-0.0062** | **0** | **32** | **0.12** | **1** | **K.IENIYNR.S** 4007 4009 4018 |
|  | 4167 | **467.7031** | **933.3916** | **933.3981** | **-0.0065** | **0** | **27** | **0.42** | **1** | **R.HDSFWDK.L** 4168 4169 4184 |
|  | 4392 | **474.9769** | **947.9393** | **948.4916** | **-0.5523** | **1** | **3** | **65** | **5** | **K.GYLKDPEK.T** |
|  | 5614 | **519.7687** | **1037.5228** | **1037.5294** | **-0.0066** | **0** | **57** | **0.00032** | **1** | **R.VGFFQGDIR.L** 5613 |
|  | 5824 | **528.3234** | **1054.6322** | **1054.6386** | **-0.0065** | **0** | **62** | **0.0001** | **1** | **K.ALVLIGNVEK.G** 5825 |
|  | 6088 | **537.2546** | **1072.4947** | **1072.5012** | **-0.0064** | **0** | **50** | **0.0021** | **1** | **K.TMYEVFQR.G** 6089 |
|  | 6533 | **553.7532** | **1105.4918** | **1104.6907** | **0.8011** | **1** | **9** | **24** | **4** | **K.FLLKLAVSSK.F** |
|  | 9631 | **645.8253** | **1289.6360** | **1289.6438** | **-0.0078** | **0** | **51** | **0.0011** | **1** | **R.AEYLGSCLLHK.G** 9632 9639 |
|  | 10243 | **661.8437** | **1321.6728** | **1321.6813** | **-0.0084** | **0** | **82** | **8.2e-007** | **1** | **R.IVQAVVYSCGAR.V** 10240 10241 |
|  | 10558 | **447.2717** | **1338.7933** | **1338.8024** | **-0.0091** | **0** | **16** | **3.2** | **1** | **K.TLKPTLFPAVPR.L** 10556 |
|  | 10839 | **678.8460** | **1355.6773** | **1355.7660** | **-0.0886** | **1** | **14** | **5.5** | **1** | **R.EAILEDLQKIGK.E** |
|  | 12635 | **731.3421** | **1460.6696** | **1460.6783** | **-0.0087** | **0** | **64** | **5.2e-005** | **1** | **R.TQIDSLYEHIQD.-** 12633 12634 |
|  | 12636 | **731.3525** | **1460.6905** | **1460.7003** | **-0.0098** | **0** | **49** | **0.0017** | **1** | **K.ADIAMVICDTPQK.A** 12637 |
|  | 12907 | **740.3430** | **1478.6714** | **1477.6984** | **0.9730** | **0** | **19** | **1.5** | **1** | **R.GLAVSDNGPCLGYR.K** 12908 12909 |
|  | 13698 | **766.3051** | **1530.5955** | **1530.6079** | **-0.0123** | **0** | **59** | **0.00015** | **1** | **K.NNDLTSCCFSDAK.T** 13699 13717 13718 |
|  | 13743 | **767.3934** | **1532.7722** | **1532.7796** | **-0.0074** | **0** | **29** | **0.15** | **1** | **K.VIILMDPFDDDLK.Q** 13742 |
|  | 14231 | **783.3669** | **1564.7192** | **1564.7304** | **-0.0112** | **0** | **39** | **0.016** | **1** | **K.GSFEELCQNQVVR.E** 14232 |
|  | 16381 | **855.4647** | **1708.9148** | **1708.9261** | **-0.0113** | **0** | **75** | **3.3e-006** | **1** | **R.SQPVLQIFVHGESLR.S** 16380 16382 16384 16385 16390 |
|  | 16540 | **861.4462** | **1720.8779** | **1720.8883** | **-0.0104** | **0** | **84** | **4.3e-007** | **1** | **K.SGIEILSLYDAENLGK.E** 16539 16541 |
|  | 17820 | **909.4707** | **1816.9268** | **1816.9393** | **-0.0125** | **1** | **52** | **0.00064** | **1** | **K.VIILMDPFDDDLKQR.G** 17821 17834 |
|  | 18795 | **943.4951** | **1884.9757** | **1884.9880** | **-0.0123** | **0** | **73** | **5.1e-006** | **1** | **K.GAMITHQNIVSNAAAFLK.C** 18796 |
|  | 19184 | **957.9885** | **1913.9624** | **1913.9744** | **-0.0120** | **0** | **76** | **2.5e-006** | **1** | **R.VIVTGAAPMSTSVMTFFR.A** 19183 19185 19186 19187 19188 |
|  | 19380 | 966.0837 | 1930.1529 | 1929.9693 | 0.1837 | 0 | (4) | 37 | 2 | R.VIVTGAAPMSTSVMTFFR.A |
|  | 19821 | **985.9575** | **1969.9004** | **1969.9130** | **-0.0126** | **0** | **117** | **1.8e-010** | **1** | **K.TQEALDSDGWLHTGDIGR.W** 19819 19820 |
|  | 20121 | **1001.0436** | **2000.0726** | **2000.0830** | **-0.0104** | **0** | **48** | **0.0015** | **1** | **R.SSLVGVVVPDTDVLPSFAAK.L** 20118 20119 20120 |
|  | 22965 | **573.5438** | **2290.1462** | **2290.1593** | **-0.0131** | **1** | **6** | **21** | **4** | **K.SGIEILSLYDAENLGKEHFR.K** |
|  | 23316 | **1169.6320** | **2337.2494** | **2336.2780** | **0.9714** | **0** | **23** | **0.39** | **1** | **K.AIFLHPEPFSIENGLLTPTLK.A** 23296 23297 23317 23318 |
|  | 24234 | **1230.5494** | **2459.0843** | **2459.0985** | **-0.0141** | **0** | **128** | **1.2e-011** | **1** | **K.LEDVADMNYFTVNNEGEVCIK.G** 24232 24233 |
|  | 24759 | **843.7509** | **2528.2307** | **2528.2469** | **-0.0162** | **0** | **47** | **0.0013** | **1** | **R.KPVPPSPEDLSVICFTSGTTGDPK.G** 24756 24757 24758 24766 |
|  | 27634 | **765.8572** | **3059.3996** | **3059.4157** | **-0.0161** | **0** | **12** | **3.2** | **1** | **K.CVEHAYEPTPDDVAISYLPLAHMFER.I** |
|  | 31173 | **1571.6841** | **4712.0304** | **4712.0539** | **-0.0235** | **0** | **49** | **0.00019** | **1** | **R.AAMGCQVYEAYGQTECTGGCTFTLPGDWTSGHVGVPLACNYVK.L** |
|  | 31706 | **1385.4419** | **5537.7385** | **5537.7680** | **-0.0295** | **0** | **22** | **0.073** | **1** | **K.SSPDQFVGIFAQNRPEWIISELACYTYSMVAVPLYDTLGPEAIVHIVNK.A** |
|  | 31947 | **1472.6389** | **5886.5266** | **5885.9477** | **0.5789** | **1** | **0** | **7.5** | **6** | **K.GYKSSPDQFVGIFAQNRPEWIISELACYTYSMVAVPLYDTLGPEAIVHIVNK.A** |

| **17.** | Q9UBC5\|MYO1A_HUMAN    **Mass:** 119238   **Score:** 1366   **Queries matched:** 116   **emPAI:** 1.91 |
| --- | --- |
|  | Myosin-Ia OS=Homo sapiens GN=MYO1A PE=1 SV=1 |

|  | **Query** | **Observed** | **Mr(expt)** | **Mr(calc)** | **Delta** | **Miss** | **Score** | **Expect** | **Rank** | **Peptide** |
| --- | --- | --- | --- | --- | --- | --- | --- | --- | --- | --- |
|  | 1398 | **623.3942** | **622.3870** | **623.3279** | **-0.9409** | **0** | **21** | **1.4** | **1** | **K.NLYSK.S** 1402 |
|  | 1665 | **688.3735** | **687.3663** | **686.3599** | **1.0064** | **0** | **16** | **6.7** | **1** | **R.DQLSPK.Q** |
|  | 1689 | **696.3513** | **695.3440** | **695.3160** | **0.0280** | **0** | **8** | **21** | **4** | **R.TMETAK.E** |
|  | 2239 | **403.2070** | **804.3994** | **805.4042** | **-1.0048** | **1** | **4** | **85** | **10** | **R.GNGKTSSR.I** |
|  | 2620 | **833.4247** | **832.4174** | **833.4872** | **-1.0697** | **0** | **19** | **2.7** | **1** | **K.AQHPLLR.S** 2621 |
|  | 3306 | **873.4731** | **872.4658** | **872.4716** | **-0.0058** | **0** | **12** | **15** | **1** | **R.AVLDATQR.Q** |
|  | 3585 | **446.7451** | **891.4756** | **890.4610** | **1.0146** | **0** | **30** | **0.18** | **1** | **K.NNDLLFR.D** |
|  | 3817 | **454.7176** | **907.4206** | **908.4828** | **-1.0622** | **0** | **12** | **11** | **2** | **K.LIEHNQR.G** 3838 |
|  | 4153 | **466.7711** | **931.5276** | **932.4756** | **-0.9480** | **0** | **9** | **24** | **2** | **K.TWPAAPYK.C** |
|  | 4383 | **474.7362** | **947.4577** | **947.4647** | **-0.0070** | **0** | **21** | **1.3** | **1** | **R.THYQLMR.K** |
|  | 4636 | **484.2461** | **966.4777** | **966.4844** | **-0.0067** | **0** | **16** | **3.9** | **1** | **K.LCASELFK.G** |
|  | 4955 | 495.7687 | 989.5229 | 989.5294 | -0.0065 | 1 | 25 | 0.58 | 2 | R.FRDQLSPK.Q |
|  | 5931 | **531.7871** | **1061.5597** | **1061.5658** | **-0.0061** | **0** | **57** | **0.00034** | **1** | **R.LFDWIVNR.I** 5932 |
|  | 6080 | **536.7949** | **1071.5752** | **1071.5825** | **-0.0074** | **0** | **38** | **0.031** | **1** | **K.RPPTAGAQFK.S** 6063 6079 6104 |
|  | 6588 | **555.7620** | **1109.5094** | **1110.6397** | **-1.1303** | **1** | **24** | **0.51** | **2** | **K.GHVILTDTKK.S** 6589 |
|  | 6811 | **564.7817** | **1127.5488** | **1127.5571** | **-0.0083** | **0** | **56** | **0.00042** | **1** | **K.VVQGPAGGDNSK.L** 6798 6812 6813 6815 6818 6840 |
|  | 6828 | **565.2581** | **1128.5017** | **1128.5234** | **-0.0217** | **0** | **4** | **69** | **5** | **K.GSHCLEVTVQ.-** |
|  | 7171 | **578.3547** | **1154.6948** | **1154.7023** | **-0.0075** | **0** | **56** | **0.00037** | **1** | **R.LQQLATLIQK.I** 7170 |
|  | 7834 | **599.8046** | **1197.5947** | **1197.6030** | **-0.0083** | **0** | **37** | **0.032** | **1** | **K.TLFYLEEQR.R** |
|  | 7858 | 600.2494 | 1198.4842 | 1198.4925 | -0.0082 | 0 | 46 | 0.0036 | 1 | R.VDGMDDASSFR.A 7857 |
|  | 8186 | **607.8184** | **1213.6222** | **1213.6302** | **-0.0081** | **0** | **25** | **0.53** | **1** | **K.NNLPSTNVLDK.T** |
|  | 8197 | **405.8627** | **1214.5664** | **1214.4874** | **0.0790** | **0** | **(8)** | **26** | **6** | **R.VDGMDDASSFR.A** |
|  | 8235 | **608.8705** | **1215.7265** | **1215.7339** | **-0.0074** | **0** | **14** | **6** | **1** | **K.ASVLLIQAFVR.G** |
|  | 8376 | **408.5619** | **1222.6638** | **1222.5216** | **0.1422** | **0** | **5** | **42** | **7** | **K.YMDIEFDFK.G** |
|  | 8606 | **618.8365** | **1235.6585** | **1235.6662** | **-0.0077** | **0** | **7** | **29** | **5** | **K.SQILISSWFR.G** |
|  | 8805 | **623.8539** | **1245.6933** | **1245.7003** | **-0.0069** | **0** | **30** | **0.14** | **1** | **R.QVLEVTSMVLK.L** |
|  | 8997 | **629.3116** | **1256.6086** | **1256.6183** | **-0.0097** | **1** | **45** | **0.0051** | **1** | **K.KGSHCLEVTVQ.-** |
|  | 9148 | **422.2443** | **1263.7110** | **1262.7421** | **0.9689** | **1** | **12** | **10** | **2** | **K.SMVQKFLLGLK.N** 9134 9137 9142 |
|  | 9432 | **427.2471** | **1278.7196** | **1278.7370** | **-0.0174** | **1** | **(6)** | **37** | **5** | **K.SMVQKFLLGLK.N** |
|  | 9973 | **655.3217** | **1308.6289** | **1308.6357** | **-0.0068** | **0** | **52** | **0.00081** | **1** | **R.CIKPNEHQQR.G** 9974 |
|  | 10026 | **656.7900** | **1311.5654** | **1311.5745** | **-0.0091** | **0** | **4** | **52** | **10** | **R.STWPHWNGGDR.E** |
|  | 10413 | **444.7279** | **1331.1620** | **1330.7721** | **0.3899** | **1** | **0** | **99** | **10** | **R.YLGLLENVRVR.R** |
|  | 10619 | **672.3144** | **1342.6143** | **1342.6227** | **-0.0084** | **0** | **55** | **0.00044** | **1** | **K.VDYFDNGIICK.L** |
|  | 10979 | **455.8973** | **1364.6700** | **1363.7612** | **0.9088** | **1** | **6** | **34** | **4** | **R.KSQILISSWFR.G** |
|  | 11006 | **683.8877** | **1365.7608** | **1364.7374** | **1.0235** | **0** | **0** | **1.2e+002** | **7** | **K.LQQVFIEMTLK.E** |
|  | 11237 | **690.3442** | **1378.6739** | **1378.6841** | **-0.0102** | **0** | **66** | **3.3e-005** | **1** | **R.GQFSSDLVATQAR.Y** 11238 |
|  | 11363 | **693.8454** | **1385.6762** | **1385.6861** | **-0.0098** | **0** | **61** | **0.00011** | **1** | **K.GGEEGPVLMAEAVK.K** |
|  | 12327 | **721.3787** | **1440.7428** | **1440.7500** | **-0.0072** | **0** | **71** | **1.1e-005** | **1** | **R.SEAALTLADFIYK.S** |
|  | 12685 | **732.8483** | **1463.6821** | **1463.6926** | **-0.0105** | **0** | **55** | **0.00039** | **1** | **R.DQCILITGESGSGK.T** |
|  | 12775 | **735.5534** | **1469.0923** | **1468.7959** | **0.2964** | **1** | **4** | **35** | **5** | **K.SSVAILMKNLYSK.S** 12741 |
|  | 13464 | **505.5523** | **1513.6350** | **1513.6442** | **-0.0092** | **0** | **8** | **21** | **2** | **R.QYDHTMGLSCFR.I** |
|  | 14103 | **778.9229** | **1555.8311** | **1554.7065** | **1.1247** | **1** | **8** | **21** | **2** | **R.FGKYMDIEFDFK.G** |
|  | 14154 | **780.9307** | **1559.8469** | **1559.8559** | **-0.0090** | **0** | **58** | **0.00017** | **1** | **K.GSPLGGVITNYLLEK.S** 14147 14152 14153 |
|  | 14192 | **782.4137** | **1562.8128** | **1562.8239** | **-0.0110** | **0** | **92** | **7.1e-008** | **1** | **K.VVTALNVMQAQYAR.D** 14193 |
|  | 14415 | **526.9423** | **1577.8050** | **1578.8188** | **-1.0138** | **0** | **(2)** | **82** | **2** | **K.VVTALNVMQAQYAR.D** |
|  | 14538 | **529.7733** | **1586.2981** | **1586.9297** | **-0.6315** | **1** | **2** | **55** | **5** | **K.ASVLLIQAFVRGWK.A** |
|  | 15127 | **812.9107** | **1623.8069** | **1623.8178** | **-0.0109** | **0** | **64** | **4.9e-005** | **1** | **K.VLGELSMSSGELAFGK.T** 15128 15153 |
|  | 15152 | **813.4773** | **1624.9400** | **1624.7481** | **0.1919** | **0** | **47** | **0.0021** | **1** | **R.DTTGYAYLNHEVSR.V** |
|  | 15179 | **814.4347** | **1626.8548** | **1626.8651** | **-0.0102** | **1** | **65** | **3.7e-005** | **1** | **K.LKGGEEGPVLMAEAVK.K** 15180 15194 |
|  | 15427 | **821.9036** | **1641.7926** | **1642.8600** | **-1.0674** | **1** | **(0)** | **1.1e+002** | **10** | **K.LKGGEEGPVLMAEAVK.K** |
|  | 16027 | **842.9900** | **1683.9654** | **1683.9771** | **-0.0117** | **0** | **25** | **0.33** | **2** | **K.IVIGLDNVAGVSVTSLK.D** 16028 |
|  | 16091 | **845.8950** | **1689.7755** | **1689.7879** | **-0.0124** | **0** | **55** | **0.00038** | **1** | **R.EIGEMVGLNSEEVER.A** 16092 16107 16112 |
|  | 16703 | **868.4116** | **1734.8086** | **1734.8207** | **-0.0121** | **1** | **82** | **6.5e-007** | **1** | **R.DRDQCILITGESGSGK.T** |
|  | 17657 | **602.3222** | **1803.9448** | **1802.9890** | **0.9557** | **1** | **5** | **37** | **3** | **K.GSPLGGVITNYLLEKSR.L** |
|  | 17768 | **907.0051** | **1811.9957** | **1812.0033** | **-0.0076** | **0** | **81** | **7.9e-007** | **1** | **K.GDFLLVSEHVIELLTK.M** 17767 17769 |
|  | 18120 | **918.9564** | **1835.8982** | **1835.9087** | **-0.0106** | **0** | **69** | **1.6e-005** | **1** | **R.AVQSAMAVIGFSEEEIR.Q** |
|  | 18348 | **618.0417** | **1851.1034** | **1851.9036** | **-0.8002** | **0** | **(9)** | **12** | **2** | **R.AVQSAMAVIGFSEEEIR.Q** |
|  | 18398 | **619.2019** | **1854.5839** | **1853.8445** | **0.7394** | **1** | **2** | **45** | **6** | **R.STWPHWNGGDREGVEK.V** |
|  | 18462 | **929.4836** | **1856.9527** | **1856.9632** | **-0.0105** | **0** | **63** | **5.5e-005** | **1** | **K.EQLLQSNPVLEAFGNAK.T** 18477 |
|  | 21398 | **1057.5593** | **2113.1041** | **2113.1168** | **-0.0127** | **0** | **53** | **0.00044** | **1** | **K.LGNVLVADEFQASGIPASGIR.D** |
|  | 21452 | **1060.5575** | **2119.1004** | **2119.1102** | **-0.0098** | **0** | **63** | **4.8e-005** | **1** | **R.NFHIFYQLLAGADEQLLK.A** 21441 21453 |
|  | 21850 | **1080.0535** | **2158.0924** | **2158.1059** | **-0.0135** | **1** | **106** | **2e-009** | **1** | **K.VTYNVTSFIDKNNDLLFR.D** |
|  | 24008 | 1216.6095 | 2431.2044 | 2431.3263 | -0.1219 | 1 | 5 | 26 | 2 | R.NFHIFYQLLAGADEQLLKALK.L 24010 24011 24012 |
|  | 24100 | **490.0122** | **2445.0244** | **2444.1617** | **0.8627** | **1** | **4** | **24** | **9** | **K.CLSTANQELQQLFYQWKCK.R** |
|  | 24614 | **1254.6069** | **2507.1993** | **2507.2155** | **-0.0162** | **0** | **35** | **0.019** | **1** | **K.ASYPQSVPIPFCGDYIGLQGNPK.L** |
|  | 25328 | **1311.1620** | **2620.3094** | **2620.3241** | **-0.0146** | **0** | **52** | **0.0004** | **1** | **R.GILAMLDEECLRPGVVSDSTFLAK.L** 25329 25330 |
|  | 25757 | **1345.7534** | **2689.4923** | **2689.5041** | **-0.0118** | **0** | **76** | **1.3e-006** | **1** | **M.PLLEGSVGVEDLVLLEPLVEESLLK.N** 25759 25760 |
|  | 27665 | **1022.8455** | **3065.5146** | **3065.5287** | **-0.0141** | **0** | **45** | **0.0017** | **1** | **K.YQDYTFYELKPHIYALANVAYQSLR.D** 27664 |
|  | 28679 | **1664.8735** | **3327.7325** | **3327.7431** | **-0.0106** | **0** | **44** | **0.0017** | **1** | **K.EIYTYIGNVVISVNPYQQLPIYGPEFIAK.Y** |

| **18.** | P21796\|VDAC1_HUMAN    **Mass:** 30868    **Score:** 1310   **Queries matched:** 43   **emPAI:** 4.66 |
| --- | --- |
|  | Voltage-dependent anion-selective channel protein 1 OS=Homo sapiens GN=VDAC1 PE=1 SV=2 |

|  | **Query** | **Observed** | **Mr(expt)** | **Mr(calc)** | **Delta** | **Miss** | **Score** | **Expect** | **Rank** | **Peptide** |
| --- | --- | --- | --- | --- | --- | --- | --- | --- | --- | --- |
|  | 2648 | **417.7295** | **833.4445** | **833.4494** | **-0.0050** | **0** | **32** | **0.11** | **1** | **K.VTGSLETK.Y** |
|  | 2949 | **427.7393** | **853.4640** | **853.4698** | **-0.0058** | **0** | **16** | **3.2** | **2** | **K.GYGFGLIK.L** |
|  | 7117 | **576.7808** | **1151.5471** | **1152.6139** | **-1.0668** | **1** | **6** | **37** | **2** | **K.VTGSLETKYR.W** |
|  | 8164 | **607.3099** | **1212.6053** | **1212.6139** | **-0.0086** | **0** | **65** | **4.7e-005** | **1** | **R.VTQSNFAVGYK.T** 8162 |
|  | 11147 | **687.8283** | **1373.6419** | **1373.6503** | **-0.0084** | **0** | **27** | **0.31** | **1** | **R.WTEYGLTFTEK.W** 11170 |
|  | 11603 | **700.8339** | **1399.6533** | **1399.6620** | **-0.0087** | **0** | **86** | **3e-007** | **1** | **K.LTFDSSFSPNTGK.K** 11589 11602 |
|  | 11861 | **707.8143** | **1413.6141** | **1413.6235** | **-0.0094** | **0** | **54** | **0.0005** | **1** | **K.YQIDPDACFSAK.V** 11843 |
|  | 13664 | **764.8803** | **1527.7459** | **1527.7569** | **-0.0110** | **1** | **47** | **0.0026** | **1** | **K.LTFDSSFSPNTGKK.N** 13665 13667 |
|  | 19556 | **649.4468** | **1945.3185** | **1945.0017** | **0.3168** | **1** | **4** | **28** | **1** | **K.KLETAVNLAWTAGNSNTR.F** |
|  | 19697 | **980.4348** | **1958.8549** | **1958.8705** | **-0.0156** | **0** | **95** | **2.8e-008** | **1** | **K.SENGLEFTSSGSANTETTK.V** 19698 |
|  | 21209 | **1052.0876** | **2102.1607** | **2102.1735** | **-0.0128** | **0** | **76** | **2e-006** | **1** | **K.VNNSSLIGLGYTQTLKPGIK.L** 21205 21206 21207 21208 21210 21211 21222 |
|  | 21856 | **720.6616** | **2158.9630** | **2158.9776** | **-0.0145** | **0** | **43** | **0.0046** | **1** | **R.EHINLGCDMDFDIAGPSIR.G** |
|  | 22001 | **1088.5228** | **2175.0311** | **2175.0444** | **-0.0133** | **0** | **135** | **2.7e-012** | **1** | **K.WNTDNTLGTEITVEDQLAR.G** 22000 22002 |
|  | 22091 | **1095.0065** | **2187.9984** | **2188.0131** | **-0.0147** | **1** | **97** | **1.5e-008** | **1** | **K.TKSENGLEFTSSGSANTETTK.V** 22090 22100 22101 22102 22103 |
|  | 23886 | **1210.0890** | **2418.1634** | **2417.1725** | **0.9909** | **0** | **95** | **2.3e-008** | **1** | **R.GALVLGYEGWLAGYQMNFETAK.S** 23866 23868 |
|  | 25218 | **1300.5900** | **2599.1654** | **2599.1827** | **-0.0173** | **0** | **116** | **1.4e-010** | **1** | **K.TDEFQLHTNVNDGTEFGGSIYQK.V** 25219 25220 25229 |

| **19.** | P09327\|VILI_HUMAN    **Mass:** 93093    **Score:** 1275   **Queries matched:** 105   **emPAI:** 2.62 |
| --- | --- |
|  | Villin-1 OS=Homo sapiens GN=VIL1 PE=1 SV=4 |

|  | **Query** | **Observed** | **Mr(expt)** | **Mr(calc)** | **Delta** | **Miss** | **Score** | **Expect** | **Rank** | **Peptide** |
| --- | --- | --- | --- | --- | --- | --- | --- | --- | --- | --- |
|  | 1492 | **645.3882** | **644.3810** | **644.3857** | **-0.0047** | **0** | **14** | **10** | **2** | **K.LSAQVK.G** |
|  | 1660 | **686.4343** | **685.4270** | **684.4283** | **0.9987** | **0** | **20** | **2.2** | **1** | **K.QGLVIR.K** |
|  | 1725 | **706.3500** | **705.3428** | **705.3480** | **-0.0052** | **0** | **32** | **0.13** | **1** | **K.GGVASGMK.H** |
|  | 1892 | **754.3566** | **753.3494** | **753.3545** | **-0.0051** | **0** | **25** | **0.46** | **1** | **K.SYEDLK.A** 1891 |
|  | 2013 | **789.4204** | **788.4131** | **788.4181** | **-0.0049** | **0** | **26** | **0.53** | **1** | **K.AFEVPAR.A** 2014 |
|  | 2365 | **407.7358** | **813.4570** | **812.5232** | **0.9338** | **1** | **8** | **28** | **8** | **K.QGLVIRK.G** |
|  | 2647 | **417.7276** | **833.4406** | **833.4429** | **-0.0023** | **1** | **19** | **2.5** | **1** | **R.KGGVASGMK.H** |
|  | 2874 | **425.2186** | **848.4227** | **849.4378** | **-1.0151** | **1** | **(7)** | **39** | **3** | **R.KGGVASGMK.H** 2907 |
|  | 3582 | **446.7287** | **891.4428** | **891.4484** | **-0.0055** | **0** | **23** | **0.97** | **1** | **K.MVADTISR.T** |
|  | 3641 | **449.2073** | **896.4001** | **896.4062** | **-0.0061** | **0** | **21** | **1.1** | **1** | **R.LFECSNK.T** |
|  | 3674 | **899.4885** | **898.4813** | **898.4872** | **-0.0060** | **0** | **23** | **0.78** | **1** | **K.THTVGSVAK.V** 3673 3680 |
|  | 5177 | **503.1056** | **1004.1967** | **1004.5688** | **-0.3721** | **1** | **0** | **1.2e+002** | **7** | **-.MTKLSAQVK.G** |
|  | 6109 | **537.7889** | **1073.5633** | **1074.5458** | **-0.9825** | **0** | **32** | **0.12** | **1** | **K.YNGEPVQIR.V** 6138 6139 |
|  | 6156 | **539.2983** | **1076.5821** | **1075.5914** | **0.9908** | **0** | **25** | **0.55** | **1** | **R.GDVFLLDLGK.L** |
|  | 6424 | **549.2708** | **1096.5269** | **1096.5335** | **-0.0066** | **0** | **50** | **0.0014** | **1** | **R.MVVYQGGTSR.T** 6429 6431 |
|  | 6595 | **555.8205** | **1109.6264** | **1109.6332** | **-0.0068** | **0** | **32** | **0.098** | **1** | **R.DPETPIIVVK.Q** |
|  | 7652 | **595.2902** | **1188.5659** | **1188.5735** | **-0.0076** | **0** | **56** | **0.00045** | **1** | **R.TNNLETGPSTR.L** 7647 7651 7680 |
|  | 7836 | **400.2121** | **1197.6144** | **1197.6216** | **-0.0072** | **0** | **9** | **20** | **4** | **K.EPPHLMSIFK.G** |
|  | 9158 | **633.2874** | **1264.5603** | **1264.5684** | **-0.0081** | **0** | **32** | **0.081** | **1** | **R.EVQGNESEAFR.G** 9175 |
|  | 9171 | **633.3562** | **1264.6978** | **1265.6285** | **-0.9307** | **1** | **3** | **67** | **8** | **K.MVADTISRTEK.Q** |
|  | 9261 | **635.8311** | **1269.6477** | **1269.6573** | **-0.0096** | **0** | **44** | **0.0062** | **1** | **K.LMEVMNHVLGK.R** 9262 |
|  | 9724 | **432.2579** | **1293.7518** | **1293.6928** | **0.0590** | **1** | **7** | **27** | **6** | **K.KAAATTAQEYLK.T** |
|  | 9894 | **435.2683** | **1302.7829** | **1301.6472** | **1.1358** | **0** | **(9)** | **17** | **7** | **K.LMEVMNHVLGK.R** 9850 |
|  | 9986 | **437.5166** | **1309.5279** | **1308.7289** | **0.7990** | **0** | **8** | **22** | **1** | **K.AAVPDTVVEPALK.A** |
|  | 10007 | **437.7541** | **1310.2406** | **1309.6561** | **0.5845** | **1** | **3** | **43** | **4** | **K.GRMVVYQGGTSR.T** |
|  | 10119 | **658.8568** | **1315.6989** | **1315.7070** | **-0.0081** | **1** | **34** | **0.063** | **1** | **K.KGAMSHALNFIK.A** |
|  | 10698 | **674.4033** | **1346.7921** | **1346.6215** | **0.1706** | **0** | **22** | **0.97** | **1** | **K.HVETNSYDVQR.L** 10687 |
|  | 10699 | **449.9388** | **1346.7945** | **1347.6493** | **-0.8548** | **0** | **6** | **35** | **5** | **R.NVVAGEVEMSWK.S** |
|  | 10808 | **677.8328** | **1353.6511** | **1354.7344** | **-1.0833** | **0** | **77** | **2.9e-006** | **1** | **R.IENLELVPVDSK.W** |
|  | 11046 | **685.3414** | **1368.6683** | **1369.6514** | **-0.9831** | **1** | **7** | **27** | **1** | **K.WSNTKSYEDLK.A** |
|  | 11204 | **689.3545** | **1376.6944** | **1376.7048** | **-0.0104** | **0** | **70** | **1.3e-005** | **1** | **R.LFQVQGTGANNTK.A** 11193 11205 |
|  | 11361 | **462.8829** | **1385.6268** | **1386.7442** | **-1.1174** | **1** | **9** | **16** | **3** | **K.GAMSHALNFIKAK.Q** |
|  | 11851 | **707.3737** | **1412.7328** | **1412.7412** | **-0.0084** | **0** | **55** | **0.00039** | **1** | **K.LIIQWNGPESTR.M** 11849 11867 |
|  | 12345 | **481.7677** | **1442.2812** | **1441.7534** | **0.5278** | **1** | **2** | **51** | **5** | **K.LMEVMNHVLGKR.R** |
|  | 12639 | **731.3605** | **1460.7064** | **1460.7147** | **-0.0083** | **0** | **6** | **38** | **1** | **R.DWSQITAEVTSPK.V** |
|  | 12736 | **734.4127** | **1466.8108** | **1466.8205** | **-0.0097** | **1** | **54** | **0.00049** | **1** | **K.RLQEENLVITPR.L** |
|  | 12874 | **739.3358** | **1476.6571** | **1476.6667** | **-0.0097** | **0** | **76** | **3.5e-006** | **1** | **K.MVDDGSGEVQVWR.I** 12875 |
|  | 12937 | 740.8885 | 1479.7624 | 1479.7722 | -0.0097 | 0 | 15 | 3.8 | 1 | R.ANFLNSNDVFVLK.T |
|  | 12947 | **741.3606** | **1480.7066** | **1480.7157** | **-0.0091** | **1** | **46** | **0.003** | **1** | **K.SYEDLKAELGNSR.D** 12929 12948 |
|  | 14091 | **519.2885** | **1554.8437** | **1554.8518** | **-0.0081** | **0** | **38** | **0.02** | **1** | **K.GSLNITTPGLQIWR.I** |
|  | 14444 | **527.6164** | **1579.8273** | **1579.8358** | **-0.0085** | **1** | **18** | **2** | **1** | **K.SFNRGDVFLLDLGK.L** |
|  | 14543 | **794.4043** | **1586.7940** | **1586.8053** | **-0.0112** | **0** | **76** | **3e-006** | **1** | **K.LYHVSDSEGNLVVR.E** 14544 14545 14546 |
|  | 15931 | **839.4144** | **1676.8142** | **1676.8257** | **-0.0115** | **0** | **64** | **4.4e-005** | **1** | **R.TYVGVVDGENELASPK.L** 15934 |
|  | 16795 | **581.2948** | **1740.8626** | **1741.8895** | **-1.0270** | **1** | **0** | **97** | **5** | **R.VPMGKEPPHLMSIFK.G** |
|  | 16862 | **873.4743** | **1744.9340** | **1744.9472** | **-0.0131** | **1** | **27** | **0.19** | **1** | **K.THPSGRDPETPIIVVK.Q** 16864 |
|  | 18792 | **943.4761** | **1884.9377** | **1884.9516** | **-0.0139** | **0** | **54** | **0.00037** | **1** | **K.FDATSMHVKPQVAAQQK.M** 18790 18791 18793 |
|  | 20776 | 1030.5107 | 2059.0069 | 2059.0197 | -0.0128 | 0 | 94 | 3.5e-008 | 1 | K.QVVVEGQEPANFWMALGGK.A 20777 |
|  | 22040 | **546.0304** | **2180.0925** | **2181.2270** | **-1.1345** | **1** | **18** | **1.3** | **1** | **K.LSAQVKGSLNITTPGLQIWR.I** |
|  | 22110 | **1095.5664** | **2189.1183** | **2189.1150** | **0.0032** | **1** | **6** | **23** | **3** | **R.LQEENLVITPRLFECSNK.T** |
|  | 22418 | **741.9486** | **2222.8240** | **2222.8602** | **-0.0362** | **1** | **4** | **25** | **1** | **K.TQSCCYLWCGKGCSGDER.E** |
|  | 22723 | **754.3623** | **2260.0651** | **2260.0742** | **-0.0091** | **0** | **42** | **0.0049** | **1** | **K.QGHEPPTFTGWFLAWDPFK.W** |
|  | 23884 | 807.0318 | 2418.0736 | 2417.2049 | 0.8686 | 1 | 2 | 44 | 5 | R.TEKQVVVEGQEPANFWMALGGK.A |
|  | 26805 | **724.1069** | **2892.3986** | **2892.4116** | **-0.0130** | **1** | **37** | **0.011** | **1** | **R.KEEHLSIEDFTQAFGMTPAAFSALPR.W** 26803 |
|  | 27044 | **1469.7028** | **2937.3910** | **2937.4145** | **-0.0235** | **0** | **84** | **2.1e-007** | **1** | **K.QYPPSTQVEVQNDGAESAVFQQLFQK.W** |
|  | 27195 | **991.1599** | **2970.4579** | **2970.4757** | **-0.0178** | **0** | **78** | **8.4e-007** | **1** | **R.EVATRPLTQDLLSHEDCYILDQGGLK.I** 27193 27194 |
|  | 28834 | **1125.2095** | **3372.6066** | **3372.6233** | **-0.0167** | **0** | **13** | **2.1** | **1** | **R.IEAMQMVPVPSSTFGSFFDGDCYIILAIHK.T** |
|  | 30542 | **1373.6512** | **4117.9319** | **4117.9514** | **-0.0194** | **0** | **43** | **0.0012** | **1** | **R.FLATEIPDFNQDDLEEDDVFLLDVWDQVFFWIGK.H** 30538 30539 30541 |
|  | 30576 | **1380.9631** | **4139.8676** | **4139.8946** | **-0.0270** | **0** | **47** | **0.00043** | **1** | **K.TASSLSYDIHYWIGQDSSLDEQGAAAIYTTQMDDFLK.G** 30577 30578 30579 30580 30581 |
|  | 30682 | **1406.3887** | **4216.1442** | **4216.1692** | **-0.0250** | **0** | **86** | **5.8e-008** | **1** | **K.VDVFNANSNLSSGPLPIFPLEQLVNKPVEELPEGVDPSR.K** 30683 30685 30686 |

| **20.** | P25705\|ATPA_HUMAN    **Mass:** 59828    **Score:** 1256   **Queries matched:** 74   **emPAI:** 2.77 |
| --- | --- |
|  | ATP synthase subunit alpha, mitochondrial OS=Homo sapiens GN=ATP5A1 PE=1 SV=1 |

|  | **Query** | **Observed** | **Mr(expt)** | **Mr(calc)** | **Delta** | **Miss** | **Score** | **Expect** | **Rank** | **Peptide** |
| --- | --- | --- | --- | --- | --- | --- | --- | --- | --- | --- |
|  | 1194 | **581.2750** | **580.2677** | **580.3445** | **-0.0768** | **0** | **22** | **0.73** | **1** | **R.VHGLR.N** 1196 |
|  | 1261 | 594.3201 | 593.3128 | 594.3013 | -0.9885 | 0 | 8 | 21 | 3 | R.GYLDK.L |
|  | 1655 | **685.4302** | **684.4229** | **684.4283** | **-0.0053** | **0** | **7** | **36** | **8** | **R.VAAAVVR.A** |
|  | 2011 | **395.2129** | **788.4112** | **788.4141** | **-0.0029** | **0** | **18** | **3.7** | **1** | **R.VGSAAQTR.A** |
|  | 2816 | **423.2558** | **844.4970** | **844.5018** | **-0.0048** | **0** | **52** | **0.0014** | **1** | **R.STVAQLVK.R** |
|  | 3134 | **864.4073** | **863.4001** | **863.4059** | **-0.0058** | **0** | **33** | **0.096** | **1** | **R.LTDADAMK.Y** |
|  | 3584 | **446.7448** | **891.4751** | **891.4814** | **-0.0063** | **0** | **33** | **0.11** | **1** | **K.LELAQYR.E** |
|  | 3875 | **456.7400** | **911.4654** | **910.3668** | **1.0986** | **0** | **8** | **25** | **2** | **R.FNDGSDEK.K** |
|  | 5116 | **500.7900** | **999.5655** | **999.5713** | **-0.0058** | **0** | **46** | **0.0049** | **1** | **R.VLSIGDGIAR.V** 5115 5117 |
|  | 5129 | **501.3054** | **1000.5962** | **1000.6029** | **-0.0068** | **1** | **35** | **0.061** | **1** | **K.RSTVAQLVK.R** |
|  | 5367 | **510.7572** | **1019.4999** | **1019.5070** | **-0.0070** | **1** | **37** | **0.037** | **1** | **K.RLTDADAMK.Y** 5368 |
|  | 5443 | **513.7974** | **1025.5802** | **1025.5869** | **-0.0068** | **0** | **33** | **0.076** | **1** | **K.AVDSLVPIGR.G** |
|  | 5958 | **533.1542** | **1064.2938** | **1063.5518** | **0.7420** | **1** | **(2)** | **73** | **7** | **R.AMKQVAGTMK.L** |
|  | 6183 | **540.2624** | **1078.5103** | **1079.5467** | **-1.0364** | **1** | **7** | **34** | **2** | **R.AMKQVAGTMK.L** |
|  | 7084 | **575.3068** | **1148.5991** | **1148.6077** | **-0.0086** | **1** | **25** | **0.48** | **2** | **R.GYLDKLEPSK.I** |
|  | 7372 | **586.3156** | **1170.6165** | **1170.6245** | **-0.0079** | **0** | **48** | **0.0022** | **1** | **R.VVDALGNAIDGK.G** |
|  | 10124 | **658.8699** | **1315.7253** | **1315.7347** | **-0.0094** | **0** | **57** | **0.00031** | **1** | **K.TSIAIDTIINQK.R** 10123 10125 |
|  | 12040 | **712.8374** | **1423.6602** | **1422.6660** | **0.9942** | **0** | **50** | **0.0013** | **1** | **K.TGTAEMSSILEER.I** 12026 12027 |
|  | 12286 | **719.9222** | **1437.8299** | **1437.8416** | **-0.0117** | **0** | **51** | **0.001** | **1** | **K.GIRPAINVGLSVSR.V** 12277 12287 |
|  | 12292 | **720.3248** | **1438.6350** | **1438.6610** | **-0.0260** | **0** | **(18)** | **1.8** | **2** | **K.TGTAEMSSILEER.I** |
|  | 14020 | **776.4210** | **1550.8275** | **1550.8344** | **-0.0069** | **1** | **81** | **1e-006** | **1** | **K.LKEIVTNFLAGFEA.-** 14016 14018 14019 |
|  | 14372 | **788.3915** | **1574.7684** | **1574.7788** | **-0.0104** | **0** | **96** | **3.3e-008** | **1** | **R.ILGADTSVDLEETGR.V** 14361 14370 14371 14382 |
|  | 15137 | **812.9441** | **1623.8736** | **1623.8832** | **-0.0096** | **0** | **67** | **2.4e-005** | **1** | **R.TGAIVDVPVGEELLGR.V** 15121 15136 |
|  | 15768 | **834.3956** | **1666.7767** | **1666.7872** | **-0.0105** | **0** | **30** | **0.1** | **1** | **R.NVQAEEMVEFSSGLK.G** 15774 |
|  | 16074 | **563.6085** | **1687.8036** | **1688.9322** | **-1.1286** | **1** | **4** | **41** | **1** | **R.AGLVSRNALGSSFIAAR.N** |
|  | 17360 | **594.3202** | **1779.9387** | **1779.9843** | **-0.0456** | **1** | **0** | **96** | **2** | **K.RTGAIVDVPVGEELLGR.V** |
|  | 20831 | **689.3343** | **2064.9810** | **2064.0428** | **0.9382** | **1** | **0** | **83** | **4** | **R.EAYPGDVFYLHSRLLER.A** |
|  | 21219 | **1052.5176** | **2103.0206** | **2103.0307** | **-0.0101** | **0** | **70** | **8.2e-006** | **1** | **K.GMSLNLEPDNVGVVVFGNDK.L** 21218 21220 21221 21230 |
|  | 21472 | **1061.0944** | **2120.1742** | **2119.0256** | **1.1486** | **0** | **(4)** | **34** | **3** | **K.GMSLNLEPDNVGVVVFGNDK.L** |
|  | 22591 | **749.4137** | **2245.2193** | **2245.1161** | **0.1032** | **1** | **4** | **29** | **2** | **R.VHGLRNVQAEEMVEFSSGLK.G** |
|  | 23109 | **1155.0794** | **2308.1441** | **2308.1521** | **-0.0080** | **0** | **124** | **3.4e-011** | **1** | **K.QGQYSPMAIEEQVAVIYAGVR.G** 23106 23108 23110 |
|  | 23306 | **1169.5826** | **2337.1507** | **2337.1601** | **-0.0094** | **0** | **133** | **3.9e-012** | **1** | **R.EVAAFAQFGSDLDAATQQLLSR.G** 23290 23301 23303 23305 23310 |
|  | 23557 | **1184.1251** | **2366.2357** | **2366.2495** | **-0.0138** | **0** | **73** | **3.6e-006** | **1** | **K.FENAFLSHVVSQHQALLGTIR.A** 23556 23558 23569 |
|  | 28893 | **1694.7867** | **3387.5589** | **3387.5791** | **-0.0202** | **0** | **50** | **0.00045** | **1** | **K.YTIVVSATASDAAPLQYLAPYSGCSMGEYFR.D** 28892 28895 28897 |
|  | 31466 | **1277.3873** | **5105.5202** | **5105.5471** | **-0.0269** | **0** | **7** | **2.7** | **1** | **K.MNDAFGGGSLTALPVIETQAGDVSAYIPTNVISITDGQIFLETELFYK.G** 31468 |

| **21.** | P40939\|ECHA_HUMAN    **Mass:** 83688    **Score:** 1162   **Queries matched:** 81   **emPAI:** 2.36 |
| --- | --- |
|  | Trifunctional enzyme subunit alpha, mitochondrial OS=Homo sapiens GN=HADHA PE=1 SV=2 |

|  | **Query** | **Observed** | **Mr(expt)** | **Mr(calc)** | **Delta** | **Miss** | **Score** | **Expect** | **Rank** | **Peptide** |
| --- | --- | --- | --- | --- | --- | --- | --- | --- | --- | --- |
|  | 1157 | **572.9952** | **571.9880** | **571.3693** | **0.6186** | **1** | **11** | **14** | **1** | **K.KISPK.R** |
|  | 1603 | **670.4819** | **669.4746** | **669.4789** | **-0.0043** | **0** | **14** | **5.2** | **8** | **K.VIIVVK.D** |
|  | 1770 | **716.4085** | **715.4012** | **714.4025** | **0.9988** | **0** | **17** | **4.7** | **9** | **K.GDVAVVR.I** |
|  | 2016 | **395.2154** | **788.4163** | **787.4440** | **0.9723** | **1** | **4** | **86** | **9** | **R.DKGLVEK.L** |
|  | 2172 | **801.3722** | **800.3649** | **800.3704** | **-0.0055** | **0** | **40** | **0.022** | **1** | **K.YEAAYGK.Q** |
|  | 3238 | **869.4490** | **868.4417** | **867.4450** | **0.9967** | **0** | **24** | **0.58** | **1** | **K.HVAEDLGK.V** 3212 3245 3247 |
|  | 3856 | **455.7323** | **909.4500** | **910.4443** | **-0.9943** | **1** | **4** | **55** | **9** | **R.SRGYICR.N** |
|  | 4041 | **462.7436** | **923.4726** | **923.4786** | **-0.0060** | **0** | **33** | **0.078** | **1** | **K.FGELVMTK.E** |
|  | 4104 | **465.2366** | **928.4586** | **928.4654** | **-0.0068** | **1** | **35** | **0.059** | **1** | **K.KYEAAYGK.Q** |
|  | 4140 | **466.2503** | **930.4860** | **930.4923** | **-0.0063** | **0** | **12** | **12** | **1** | **R.THINYGVK.G** 4123 4142 |
|  | 4766 | **488.3161** | **974.6176** | **974.5033** | **0.1143** | **0** | **25** | **0.54** | **1** | **K.DATLTALDR.G** |
|  | 5086 | **998.5455** | **997.5382** | **997.5444** | **-0.0062** | **0** | **26** | **0.35** | **1** | **R.ILQEGVDPK.K** 5080 |
|  | 5270 | **507.2339** | **1012.4533** | **1012.4614** | **-0.0081** | **0** | **22** | **1** | **1** | **K.DGPGFYTTR.C** |
|  | 5639 | **520.7709** | **1039.5272** | **1039.5338** | **-0.0067** | **0** | **34** | **0.062** | **1** | **R.FVDLYGAQK.I** 5638 |
|  | 6697 | **559.8028** | **1117.5910** | **1117.5979** | **-0.0069** | **0** | **66** | **4.4e-005** | **1** | **K.DTSASAVAVGLK.Q** 6696 |
|  | 7312 | **584.2790** | **1166.5434** | **1165.6091** | **0.9343** | **0** | **1** | **1.3e+002** | **8** | **R.NFTGSSALLTR.T** |
|  | 9514 | **642.7996** | **1283.5846** | **1283.6431** | **-0.0586** | **1** | **2** | **77** | **5** | **K.FGELVMTKESK.A** |
|  | 10158 | **659.8642** | **1317.7138** | **1317.7214** | **-0.0075** | **0** | **17** | **2.9** | **1** | **K.MQLLEIITTEK.T** |
|  | 10246 | **441.7294** | **1322.1663** | **1321.6192** | **0.5471** | **0** | **1** | **66** | **9** | **R.CLAPMMSEVIR.I** |
|  | 10459 | **667.8267** | **1333.6389** | **1333.7163** | **-0.0774** | **0** | **(6)** | **35** | **3** | **K.MQLLEIITTEK.T** |
|  | 11473 | **464.9011** | **1391.6814** | **1391.6908** | **-0.0094** | **0** | **30** | **0.14** | **1** | **K.VIGMHYFSPVDK.M** |
|  | 11807 | 706.3163 | 1410.6180 | 1410.6263 | -0.0083 | 0 | 63 | 6.9e-005 | 1 | K.SEVSSDEDIQFR.L |
|  | 12534 | **728.8525** | **1455.6904** | **1455.7470** | **-0.0566** | **1** | **1** | **1e+002** | **10** | **K.HVAEDLGKVFGER.F** |
|  | 13071 | **745.3293** | **1488.6440** | **1488.7581** | **-0.1141** | **0** | **5** | **45** | **5** | **K.ALMGLYHGQVLCK.K** |
|  | 13572 | **1521.7385** | **1520.7313** | **1520.7392** | **-0.0079** | **0** | **45** | **0.004** | **1** | **K.DLNSDMDSILASLK.L** 13563 |
|  | 13791 | **769.3817** | **1536.7489** | **1536.7341** | **0.0148** | **0** | **(2)** | **75** | **8** | **K.DLNSDMDSILASLK.L** |
|  | 14165 | **781.3948** | **1560.7750** | **1560.7826** | **-0.0076** | **0** | **70** | **1.2e-005** | **1** | **K.MVGVPAALDMMLTGR.S** 14164 |
|  | 14398 | **789.3982** | **1576.7818** | **1576.7919** | **-0.0101** | **0** | **70** | **1.2e-005** | **1** | **R.FGGGNPELLTQMVSK.G** 14397 14399 |
|  | 14646 | **797.7803** | **1593.5460** | **1592.7725** | **0.7735** | **0** | **(4)** | **40** | **4** | **K.MVGVPAALDMMLTGR.S** |
|  | 14852 | **402.9471** | **1607.7592** | **1608.7674** | **-1.0082** | **0** | **(6)** | **27** | **4** | **K.MVGVPAALDMMLTGR.S** |
|  | 15162 | **813.9308** | **1625.8470** | **1625.8552** | **-0.0082** | **0** | **27** | **0.25** | **1** | **R.TIEYLEEVAITFAK.G** |
|  | 15228 | **815.9183** | **1629.8221** | **1629.8322** | **-0.0101** | **0** | **60** | **0.00011** | **1** | **K.TLQEVTQLSQEAQR.I** 15223 15227 15245 |
|  | 15501 | **550.2551** | **1647.7436** | **1648.8342** | **-1.0906** | **1** | **1** | **98** | **7** | **R.KDLNSDMDSILASLK.L** |
|  | 15965 | **840.4279** | **1678.8412** | **1678.8488** | **-0.0076** | **0** | **74** | **4.8e-006** | **1** | **K.ADMVIEAVFEDLSLK.H** 15962 15964 |
|  | 18192 | **921.9061** | **1841.7976** | **1841.8102** | **-0.0126** | **0** | **67** | **2.2e-005** | **1** | **K.TGIEQGSDAGYLCESQK.F** 18193 18199 |
|  | 19408 | **645.3266** | **1932.9580** | **1932.9947** | **-0.0368** | **1** | **(1)** | **81** | **6** | **K.MVGVPAALDMMLTGRSIR.A** |
|  | 19602 | **650.9698** | **1949.8875** | **1948.9897** | **0.8979** | **1** | **4** | **39** | **2** | **K.MVGVPAALDMMLTGRSIR.A** |
|  | 20179 | **1004.0599** | **2006.1052** | **2006.1161** | **-0.0109** | **0** | **82** | **5.3e-007** | **1** | **K.TVLGTPEVLLGALPGAGGTQR.L** |
|  | 20304 | **1011.0574** | **2020.1002** | **2020.1139** | **-0.0137** | **0** | **119** | **1.1e-010** | **1** | **K.HLAILGAGLMGAGIAQVSVDK.G** 20305 20306 |
|  | 20802 | **1031.4873** | **2060.9600** | **2060.9691** | **-0.0091** | **0** | **91** | **7.4e-008** | **1** | **R.DSIFSNLTGQLDYQGFEK.A** |
|  | 20855 | **518.0076** | **2068.0012** | **2068.0160** | **-0.0148** | **1** | **6** | **20** | **2** | **K.QFTPCQLLADHANSPNKK.F** 20857 |
|  | 21577 | **1068.1065** | **2134.1983** | **2134.2110** | **-0.0127** | **1** | **55** | **0.00027** | **1** | **R.KTVLGTPEVLLGALPGAGGTQR.L** |
|  | 22821 | **758.7399** | **2273.1978** | **2273.2089** | **-0.0112** | **0** | **41** | **0.0059** | **1** | **K.MGLVDQLVEPLGPGLKPPEER.T** 22822 |
|  | 23895 | **1210.5570** | **2419.0995** | **2419.1114** | **-0.0119** | **0** | **85** | **2.2e-007** | **1** | **K.ELHSEFSEVMNEIWASDQIR.S** 23896 23897 23898 |
|  | 25182 | **1297.6519** | **2593.2892** | **2593.3066** | **-0.0174** | **0** | **60** | **6.9e-005** | **1** | **R.SAVLISSKPGCFIAGADINMLAACK.T** 25183 25184 |
|  | 26507 | **1418.7470** | **2835.4793** | **2835.4906** | **-0.0113** | **1** | **117** | **1.2e-010** | **1** | **K.KLDSLTTSFGFPVGAATLVDEVGVDVAK.H** 26503 26506 |
|  | 27681 | **1023.5205** | **3067.5397** | **3067.5536** | **-0.0139** | **0** | **54** | **0.00021** | **1** | **K.EVEAVIPDHCIFASNTSALPISEIAAVSK.R** 27676 27677 27679 27680 |

| **22.** | P05062\|ALDOB_HUMAN    **Mass:** 39961    **Score:** 1052   **Queries matched:** 73   **emPAI:** 3.20 |
| --- | --- |
|  | Fructose-bisphosphate aldolase B OS=Homo sapiens GN=ALDOB PE=1 SV=2 |

|  | **Query** | **Observed** | **Mr(expt)** | **Mr(calc)** | **Delta** | **Miss** | **Score** | **Expect** | **Rank** | **Peptide** |
| --- | --- | --- | --- | --- | --- | --- | --- | --- | --- | --- |
|  | 1657 | **685.4552** | **684.4479** | **684.4534** | **-0.0055** | **0** | **21** | **1.3** | **1** | **K.GIVVGIK.L** |
|  | 1921 | **762.3217** | **761.3144** | **762.4640** | **-1.1495** | **0** | **7** | **41** | **1** | **K.VLAAVYK.A** |
|  | 2566 | **415.2112** | **828.4078** | **828.4130** | **-0.0052** | **0** | **22** | **1.4** | **1** | **K.LSFSYGR.A** 2565 |
|  | 3185 | **433.7552** | **865.4958** | **864.4341** | **1.0617** | **1** | **6** | **43** | **7** | **K.KDGVDFGK.W** |
|  | 4607 | **482.7178** | **963.4211** | **963.4266** | **-0.0055** | **0** | **61** | **0.00015** | **1** | **R.AMANCQAAK.G** 4602 4603 4604 4606 4608 4609 4610 4617 |
|  | 4959 | **495.7993** | **989.5840** | **989.4414** | **0.1426** | **0** | **8** | **28** | **10** | **K.VENTEENR.R** |
|  | 6911 | **568.2843** | **1134.5540** | **1135.5226** | **-0.9686** | **1** | **6** | **37** | **1** | **K.RAMANCQAAK.G** |
|  | 8408 | **409.2289** | **1224.6649** | **1225.5761** | **-0.9112** | **1** | **6** | **33** | **8** | **K.EATQEAFMKR.A** 8426 |
|  | 8720 | **621.3231** | **1240.6317** | **1240.6412** | **-0.0095** | **0** | **57** | **0.0003** | **1** | **K.LDQGGAPLAGTNK.E** 8721 |
|  | 8771 | **622.7986** | **1243.5826** | **1242.6720** | **0.9106** | **0** | **19** | **2** | **2** | **R.ALQASALAAWGGK.A** 8760 |
|  | 11932 | **709.8547** | **1417.6948** | **1417.7049** | **-0.0101** | **0** | **43** | **0.0075** | **1** | **K.ETTIQGLDGLSER.C** 11931 |
|  | 12089 | 476.2799 | 1425.8179 | 1424.7524 | 1.0655 | 1 | 9 | 18 | 4 | M.AHRFPALTQEQK.K |
|  | 12556 | **729.3619** | **1456.7092** | **1457.7725** | **-1.0633** | **0** | **38** | **0.019** | **2** | **K.ELSEIAQSIVANGK.G** 12571 12602 |
|  | 13109 | **745.8619** | **1489.7093** | **1489.7195** | **-0.0102** | **0** | **77** | **2.7e-006** | **1** | **K.GILAADESVGTMGNR.L** 13110 |
|  | 13351 | **753.8581** | **1505.7016** | **1505.7144** | **-0.0128** | **0** | **(35)** | **0.039** | **2** | **K.GILAADESVGTMGNR.L** 13347 13365 |
|  | 17443 | **894.4525** | **1786.8903** | **1786.9036** | **-0.0132** | **0** | **54** | **0.00044** | **1** | **K.YTPEQVAMATVTALHR.T** 17429 |
|  | 19202 | **639.3360** | **1914.9862** | **1914.9986** | **-0.0124** | **1** | **45** | **0.003** | **1** | **K.KYTPEQVAMATVTALHR.T** |
|  | 21636 | **1071.5264** | **2141.0382** | **2141.0535** | **-0.0153** | **0** | **109** | **1.2e-009** | **1** | **R.IADQCPSSLAIQENANALAR.Y** 21634 21635 |
|  | 24990 | **1279.0609** | **2556.1073** | **2556.1227** | **-0.0155** | **0** | **58** | **0.0001** | **1** | **K.GQYVHTGSSGAASTQSLFTACYTY.-** 24991 |
|  | 27271 | **748.3744** | **2989.4685** | **2989.4902** | **-0.0218** | **0** | **70** | **4.9e-006** | **1** | **K.ALNDHHVYLEGTLLKPNMVTAGHACTK.K** 27273 27274 |
|  | 27529 | **1013.1975** | **3036.5707** | **3036.5808** | **-0.0101** | **0** | **62** | **3e-005** | **1** | **R.EILFSVDSSINQSIGGVILFHETLYQK.D** 27514 27517 27521 27524 27525 27526 27527 27528 27530 27531 27532 27536 27538 27540 27544 27549 |
|  | 30428 | **1351.6288** | **4051.8645** | **4051.8932** | **-0.0287** | **0** | **61** | **2.3e-005** | **1** | **R.YASICQQNGLVPIVEPEVIPDGDHDLEHCQYVTEK.V** 30429 30430 30432 30433 30434 |
|  | 30472 | **1361.0188** | **4080.0346** | **4080.0522** | **-0.0177** | **0** | **74** | **1.1e-006** | **1** | **R.TVPAAVPGICFLSGGMSEEDATLNLNAINLCPLPKPWK.L** 30465 30467 30470 30475 30479 |

| **23.** | Q7Z406\|MYH14_HUMAN    **Mass:** 228889   **Score:** 985    **Queries matched:** 111   **emPAI:** 0.56 |
| --- | --- |
|  | Myosin-14 OS=Homo sapiens GN=MYH14 PE=1 SV=1 |

|  | **Query** | **Observed** | **Mr(expt)** | **Mr(calc)** | **Delta** | **Miss** | **Score** | **Expect** | **Rank** | **Peptide** |
| --- | --- | --- | --- | --- | --- | --- | --- | --- | --- | --- |
|  | 895 | **518.2900** | **517.2828** | **516.3271** | **0.9556** | **0** | **15** | **12** | **1** | **R.ILSGK.L** |
|  | 956 | **529.3418** | **528.3345** | **528.3384** | **-0.0038** | **0** | **1** | **1.8e+002** | **2** | **K.AAVLR.A** |
|  | 1333 | 609.3198 | 608.3125 | 608.3170 | -0.0045 | 0 | 2 | 79 | 9 | K.SFVEK.V |
|  | 1533 | 653.3270 | 652.3197 | 651.3228 | 0.9969 | 0 | 15 | 4.7 | 6 | R.DYVQK.A |
|  | 1945 | **768.3584** | **767.3511** | **767.3306** | **0.0205** | **0** | **7** | **35** | **6** | **K.MQAQMK.E** |
|  | 2309 | 809.4545 | 808.4472 | 807.4491 | 0.9981 | 0 | 15 | 4.9 | 1 | R.TVGQLYK.E 2303 |
|  | 2391 | **408.7420** | **815.4694** | **814.5025** | **0.9669** | **1** | **12** | **14** | **10** | **R.AKLALGSR.V** |
|  | 2582 | **415.7114** | **829.4082** | **830.3995** | **-0.9913** | **1** | **6** | **54** | **3** | **R.AGDGERAR.A** |
|  | 2983 | **428.7632** | **855.5117** | **855.5178** | **-0.0060** | **0** | **13** | **8.6** | **1** | **R.LVLDQLR.C** |
|  | 3010 | **429.7532** | **857.4918** | **856.4879** | **1.0040** | **1** | **3** | **1e+002** | **5** | **R.RVADQLR.D** |
|  | 3020 | **430.2502** | **858.4859** | **858.4559** | **0.0300** | **1** | **4** | **83** | **3** | **K.GRQELEK.L** |
|  | 3245 | 435.2680 | 868.5215 | 868.4403 | 0.0812 | 0 | 17 | 2.7 | 1 | K.SVHELER.A 3212 |
|  | 3415 | **441.2466** | **880.4786** | **879.4306** | **1.0480** | **1** | **6** | **35** | **10** | **R.KMQAQMK.E** |
|  | 3416 | 441.2467 | 880.4788 | 879.4306 | 1.0482 | 1 | (4) | 58 | 6 | R.KMQAQMK.E |
|  | 3627 | 896.4321 | 895.4248 | 895.4255 | -0.0007 | 1 | (6) | 40 | 6 | R.KMQAQMK.E 3644 |
|  | 3956 | **459.2655** | **916.5164** | **916.5229** | **-0.0065** | **0** | **20** | **2** | **1** | **R.QLVSTLEK.K** |
|  | 3962 | **459.7188** | **917.4229** | **916.4250** | **0.9979** | **0** | **13** | **9** | **2** | **R.LGEEDAGAR.A** 3960 |
|  | 4529 | **479.7539** | **957.4931** | **957.4992** | **-0.0060** | **0** | **16** | **4.2** | **6** | **R.QQQQSALR.V** |
|  | 4594 | **482.2655** | **962.5164** | **962.5106** | **0.0058** | **0** | **17** | **3.2** | **1** | **K.MTIAALESK.L** |
|  | 5120 | **500.8023** | **999.5900** | **999.5964** | **-0.0064** | **0** | **8** | **30** | **5** | **R.LEVTVQALK.T** |
|  | 5168 | **502.7631** | **1003.5116** | **1004.5138** | **-1.0022** | **1** | **7** | **32** | **9** | **K.KTLEEETR.I** |
|  | 5171 | **502.7869** | **1003.5593** | **1003.5121** | **0.0472** | **0** | **10** | **17** | **3** | **M.AAVTMSVPGR.K** |
|  | 5344 | **509.7494** | **1017.4843** | **1017.4913** | **-0.0070** | **0** | **30** | **0.19** | **1** | **R.AMEAEAAGLR.E** |
|  | 5897 | **530.7947** | **1059.5749** | **1059.5811** | **-0.0062** | **0** | **3** | **95** | **3** | **R.AELEALLSSK.D** |
|  | 6130 | **538.2873** | **1074.5601** | **1074.5669** | **-0.0068** | **0** | **37** | **0.037** | **1** | **R.AELSSLQTAR.Q** |
|  | 6871 | **566.7903** | **1131.5661** | **1130.5567** | **1.0094** | **0** | **52** | **0.00096** | **1** | **R.LQEELAASDR.A** 6854 |
|  | 6872 | 566.8233 | 1131.6320 | 1131.6070 | 0.0250 | 1 | 11 | 14 | 6 | M.AAVTMSVPGRK.A |
|  | 7030 | **573.3156** | **1144.6167** | **1144.5360** | **0.0807** | **0** | **8** | **27** | **6** | **R.EQLEEEAAAR.E** |
|  | 7112 | **576.7526** | **1151.4906** | **1151.5869** | **-0.0964** | **1** | **3** | **75** | **9** | **R.CIVPNHEKR.A** |
|  | 7465 | **589.3629** | **1176.7113** | **1176.6503** | **0.0610** | **0** | **9** | **19** | **3** | **R.LLGLGVTDFSR.A** 7459 7480 |
|  | 7592 | **593.8083** | **1185.6020** | **1185.6102** | **-0.0082** | **0** | **35** | **0.048** | **1** | **K.VQELQQQSAR.E** 7579 |
|  | 8351 | 611.3470 | 1220.6794 | 1219.6448 | 1.0346 | 1 | 10 | 17 | 2 | R.KFDQLLAEEK.A |
|  | 8423 | **613.8241** | **1225.6336** | **1225.6237** | **0.0099** | **1** | **2** | **1e+002** | **9** | **R.DQIQRMNPPK.F** |
|  | 9001 | 629.3394 | 1256.6643 | 1256.6724 | -0.0081 | 1 | 62 | 9e-005 | 1 | R.KEEELQAALAR.A 9000 |
|  | 9033 | **630.2999** | **1258.5853** | **1257.6969** | **0.8885** | **0** | **6** | **37** | **2** | **R.YEILTPNAIPK.G** |
|  | 9044 | 630.8172 | 1259.6198 | 1258.7132 | 0.9066 | 1 | 0 | 1.4e+002 | 9 | K.LQLEKVTTEAK.M |
|  | 9583 | **430.2266** | **1287.6579** | **1288.6557** | **-0.9979** | **1** | **4** | **62** | **9** | **R.VRAMEAEAAGLR.E** |
|  | 9922 | 653.3341 | 1304.6535 | 1304.6612 | -0.0076 | 0 | 2 | 89 | 3 | K.EQADFALEALAK.A |
|  | 10998 | **683.8347** | **1365.6549** | **1365.6637** | **-0.0088** | **1** | **13** | **7.3** | **1** | **R.TSREEIFSQNR.E** |
|  | 11039 | **457.2050** | **1368.5931** | **1368.6642** | **-0.0711** | **1** | **5** | **39** | **10** | **R.VMQRNCAAYLK.L** 11074 |
|  | 11437 | **464.2476** | **1389.7209** | **1389.7616** | **-0.0407** | **0** | **4** | **50** | **9** | **K.VTDIIVSFQAAAR.G** 11461 |
|  | 11833 | **706.8941** | **1411.7736** | **1411.7823** | **-0.0087** | **0** | **3** | **71** | **3** | **K.VIQYLAHVASSPK.G** |
|  | 12432 | **483.7500** | **1448.2282** | **1448.7848** | **-0.5566** | **1** | **3** | **44** | **10** | **R.IHEAAVQELRQR.H** |
|  | 12452 | **725.8465** | **1449.6784** | **1449.6888** | **-0.0104** | **0** | **61** | **0.0001** | **1** | **R.DQADFSVLHYAGK.V** |
|  | 12737 | **734.4150** | **1466.8154** | **1467.8158** | **-1.0003** | **1** | **37** | **0.023** | **1** | **R.RLELQLQEVQGR.A** |
|  | 12866 | **738.8527** | **1475.6909** | **1475.7005** | **-0.0096** | **0** | **39** | **0.017** | **1** | **R.FLTNGPSSSPGQER.E** 12877 12878 |
|  | 13308 | **752.3522** | **1502.6898** | **1503.7351** | **-1.0453** | **1** | **31** | **0.093** | **1** | **K.AQMASAGQGKEEAVK.Q** 13320 13321 |
|  | 13376 | **503.3153** | **1506.9242** | **1505.8276** | **1.0966** | **0** | **4** | **49** | **3** | **R.MVSAVLQFGNIALK.R** 13360 |
|  | 13832 | **770.8975** | **1539.7805** | **1539.8773** | **-0.0968** | **1** | **9** | **17** | **10** | **K.KVIQYLAHVASSPK.G** |
|  | 13862 | **771.8914** | **1541.7682** | **1541.8565** | **-0.0884** | **1** | **7** | **23** | **4** | **R.QRYEILTPNAIPK.G** |
|  | 13911 | **773.3808** | **1544.7470** | **1543.7478** | **0.9993** | **0** | **7** | **23** | **9** | **K.LAQAEEQLEQETR.E** 13893 |
|  | 13974 | **517.2592** | **1548.7558** | **1549.7923** | **-1.0364** | **0** | **5** | **36** | **6** | **R.LMATLSNTNPSFVR.C** 14008 |
|  | 14002 | 776.3459 | 1550.6772 | 1550.6882 | -0.0110 | 0 | 45 | 0.0039 | 1 | R.EDQSILCTGESGAGK.T |
|  | 14136 | **780.3887** | **1558.7628** | **1558.7739** | **-0.0112** | **0** | **74** | **5.2e-006** | **1** | **R.ELQTAQAQLSEWR.R** |
|  | 14371 | 788.3910 | 1574.7674 | 1573.8199 | 0.9475 | 1 | 3 | 64 | 3 | R.AELEALLSSKDDVGK.S |
|  | 14373 | **788.4086** | **1574.8027** | **1574.8126** | **-0.0099** | **0** | **57** | **0.00024** | **1** | **K.MIQALELDPNLYR.V** |
|  | 14787 | **803.3566** | **1604.6986** | **1604.7100** | **-0.0114** | **0** | **16** | **2.9** | **1** | **R.NTDQATMPDNTAAQK.L** |
|  | 14860 | **805.3483** | **1608.6820** | **1608.6937** | **-0.0117** | **0** | **19** | **1.5** | **1** | **R.ELEDVTESAESMNR.E** 14873 |
|  | 15147 | **542.5968** | **1624.7686** | **1624.6886** | **0.0800** | **0** | **(7)** | **22** | **5** | **R.ELEDVTESAESMNR.E** 15146 |
|  | 15231 | 815.9291 | 1629.8437 | 1629.8548 | -0.0111 | 0 | 40 | 0.011 | 1 | R.VLGFSHEEIISMLR.M |
|  | 15490 | 549.7710 | 1646.2912 | 1645.8498 | 0.4414 | 0 | (1) | 59 | 7 | R.VLGFSHEEIISMLR.M |
|  | 16126 | **846.9362** | **1691.8578** | **1691.8254** | **0.0324** | **0** | **0** | **1.1e+002** | **8** | **K.FEEDLLLLEDQNSK.L** |
|  | 16600 | 863.9730 | 1725.9314 | 1725.9413 | -0.0100 | 0 | 61 | 7.6e-005 | 1 | R.QLLQANPILEAFGNAK.T |
|  | 16724 | **579.3433** | **1735.0080** | **1733.9074** | **1.1006** | **1** | **3** | **46** | **2** | **K.VAQEQGGHPKFQRPR.H** |
|  | 16911 | **875.4421** | **1748.8697** | **1748.8805** | **-0.0108** | **0** | **46** | **0.0029** | **1** | **R.HGQALGELAEQLEQAR.R** |
|  | 17618 | **901.4947** | **1800.9749** | **1800.9833** | **-0.0083** | **0** | **87** | **1.9e-007** | **1** | **K.LLLQVESLTTELSAER.S** 17617 |
|  | 17920 | **608.9686** | **1823.8839** | **1823.8955** | **-0.0116** | **0** | **27** | **0.22** | **1** | **R.HEVPPHVYAVTEGAYR.S** |
|  | 18013 | **610.6427** | **1828.9063** | **1828.0571** | **0.8492** | **1** | **1** | **83** | **4** | **R.LLGLGVTDFSRALLTPR.I** |
|  | 18058 | **916.9360** | **1831.8574** | **1831.8687** | **-0.0113** | **0** | **86** | **2.8e-007** | **1** | **R.AQVTELEDELTAAEDAK.L** |
|  | 18487 | **930.4286** | **1858.8426** | **1858.8544** | **-0.0118** | **0** | **84** | **4.3e-007** | **1** | **R.EAQAALAEAQEDLESER.V** 18486 |
|  | 18488 | **930.4465** | **1858.8784** | **1858.8908** | **-0.0124** | **0** | **82** | **6.4e-007** | **1** | **R.AQAELENVSGALNEAESK.T** 18489 |
|  | 18842 | **945.3987** | **1888.7828** | **1889.8537** | **-1.0709** | **1** | **0** | **85** | **3** | **R.ERNTDQATMPDNTAAQK.L** |
|  | 19398 | **966.9448** | **1931.8751** | **1931.8894** | **-0.0144** | **0** | **44** | **0.004** | **1** | **R.LQQELDDATMDLEQQR.Q** 19399 |
|  | 19593 | 650.6653 | 1948.9740 | 1947.8844 | 1.0896 | 0 | (2) | 59 | 8 | R.LQQELDDATMDLEQQR.Q |
|  | 22195 | **734.3644** | **2200.0713** | **2200.0872** | **-0.0159** | **0** | **68** | **1.4e-005** | **1** | **R.LQQHIQELEAHLEAEEGAR.Q** |
|  | 22529 | **1118.5823** | **2235.1500** | **2233.9869** | **1.1631** | **1** | **7** | **15** | **3** | **R.QAQQDRDEMADEVANGNLSK.A** 22518 |
|  | 22797 | **568.7753** | **2271.0722** | **2272.2480** | **-1.1758** | **1** | **4** | **35** | **5** | **R.KAPPRPGPVPEAAQPFLFTPR.G** |
|  | 22908 | **761.3457** | **2281.0153** | **2281.0314** | **-0.0162** | **1** | **15** | **2.3** | **1** | **R.SMLQDREDQSILCTGESGAGK.T** |
|  | 22937 | **762.6948** | **2285.0625** | **2285.0780** | **-0.0155** | **0** | **8** | **13** | **1** | **K.AEDMAELTCLNEASVLHNLR.E** |
|  | 22955 | **764.0239** | **2289.0500** | **2289.0655** | **-0.0155** | **1** | **6** | **22** | **2** | **R.RLDGESSELQEQMVEQQQR.A** |
|  | 23702 | **797.7421** | **2390.2046** | **2390.2190** | **-0.0145** | **0** | **40** | **0.0075** | **1** | **K.DVEGIVGLEQVSSLGDGPPGGRPR.R** |
|  | 24177 | **818.3978** | **2452.1717** | **2452.1845** | **-0.0128** | **0** | **32** | **0.049** | **1** | **K.LQQLFNHTMFVLEQEEYQR.E** |
|  | 24339 | **1237.5723** | **2473.1300** | **2473.1456** | **-0.0156** | **0** | **98** | **1.2e-008** | **1** | **R.LGQLEEELEEEQSNSELLNDR.Y** 24340 24341 |
|  | 27570 | **761.8685** | **3043.4450** | **3043.4581** | **-0.0131** | **1** | **4** | **20** | **1** | **R.DLGEELEALRGELEDTLDSTNAQQELR.S** |

| **24.** | P13637\|AT1A3_HUMAN    **Mass:** 113102   **Score:** 981    **Queries matched:** 56   **emPAI:** 0.57 |
| --- | --- |
|  | Sodium/potassium-transporting ATPase subunit alpha-3 OS=Homo sapiens GN=ATP1A3 PE=1 SV=3 |

|  | **Query** | **Observed** | **Mr(expt)** | **Mr(calc)** | **Delta** | **Miss** | **Score** | **Expect** | **Rank** | **Peptide** |
| --- | --- | --- | --- | --- | --- | --- | --- | --- | --- | --- |
|  | 1443 | 633.3343 | 632.3270 | 632.3316 | -0.0046 | 0 | 25 | 0.93 | 1 | K.NCLVK.N |
|  | 3564 | 446.0373 | 890.0600 | 889.4617 | 0.5982 | 0 | 26 | 0.32 | 1 | K.TGTLTQNR.M |
|  | 5611 | 519.7522 | 1037.4898 | 1037.4964 | -0.0066 | 0 | 32 | 0.09 | 1 | R.NSVFQQGMK.N |
|  | 6292 | 544.2970 | 1086.5794 | 1086.5856 | -0.0061 | 0 | 34 | 0.079 | 1 | K.LIIVEGCQR.Q 6293 |
|  | 7143 | **577.3082** | **1152.6018** | **1152.5267** | **0.0751** | **1** | **1** | **1.2e+002** | **3** | **K.MSVEEVCRK.Y** |
|  | 7763 | 597.8008 | 1193.5870 | 1193.5975 | -0.0105 | 1 | 10 | 16 | 1 | R.RNSVFQQGMK.N |
|  | 8577 | 618.3002 | 1234.5859 | 1235.6986 | -1.1127 | 0 | 11 | 13 | 2 | R.LNIPVSQVNPR.D |
|  | 9221 | 634.8564 | 1267.6982 | 1267.7071 | -0.0089 | 0 | 27 | 0.29 | 1 | K.NMVPQQALVIR.E 9219 9220 |
|  | 9355 | **425.8835** | **1274.6287** | **1274.6354** | **-0.0067** | **0** | **11** | **11** | **2** | **R.DVAGDASESALLK.C** |
|  | 9398 | **639.3052** | **1276.5959** | **1275.7009** | **0.8950** | **1** | **(3)** | **69** | **7** | **R.YLLVMKGAPER.I** |
|  | 9455 | 1281.5680 | 1280.5607 | 1279.6343 | 0.9264 | 1 | 2 | 80 | 10 | R.NSVFQQGMKNK.I |
|  | 9520 | 642.8542 | 1283.6939 | 1283.7020 | -0.0080 | 0 | (19) | 1.6 | 2 | K.NMVPQQALVIR.E |
|  | 9666 | **431.2499** | **1290.7280** | **1291.6958** | **-0.9678** | **1** | **7** | **28** | **8** | **R.YLLVMKGAPER.I** |
|  | 11285 | 691.3743 | 1380.7341 | 1380.7435 | -0.0094 | 0 | 66 | 3.2e-005 | 1 | K.VIMVTGDHPITAK.A 11283 11284 11286 |
|  | 11571 | 699.4022 | 1396.7898 | 1396.7384 | 0.0513 | 0 | (13) | 6.3 | 1 | K.VIMVTGDHPITAK.A |
|  | 12172 | **716.3534** | **1430.6923** | **1430.7365** | **-0.0441** | **1** | **1** | **99** | **5** | **K.RDVAGDASESALLK.C** |
|  | 13112 | 745.8742 | 1489.7339 | 1489.7446 | -0.0108 | 0 | 79 | 1.6e-006 | 1 | K.ADIGVAMGIAGSDVSK.Q 13113 13114 13131 |
|  | 13984 | 517.3012 | 1548.8818 | 1549.8576 | -0.9758 | 1 | 5 | 39 | 10 | R.LNIPVSQVNPRDAK.A |
|  | 15023 | 540.2840 | 1617.8302 | 1617.8396 | -0.0094 | 1 | 18 | 1.9 | 1 | K.KADIGVAMGIAGSDVSK.Q |
|  | 15042 | 810.3734 | 1618.7323 | 1618.7435 | -0.0112 | 0 | 74 | 4.7e-006 | 1 | K.VDNSSLTGESEPQTR.S 15017 15040 15043 15056 |
|  | 17150 | **589.3901** | **1765.1486** | **1765.8635** | **-0.7149** | **0** | **1** | **62** | **10** | **K.EAFQNAYLELGGLGER.V** |
|  | 17757 | 906.4728 | 1810.9311 | 1810.9425 | -0.0114 | 0 | 107 | 2.1e-009 | 1 | R.QGAIVAVTGDGVNDSPALK.K 17756 17761 |
|  | 18011 | 915.4595 | 1828.9045 | 1828.9167 | -0.0121 | 0 | 103 | 4.8e-009 | 1 | K.GVGIISEGNETVEDIAAR.L 18009 18010 18012 |
|  | 18129 | 919.4411 | 1836.8676 | 1837.0132 | -0.1455 | 1 | 3 | 55 | 8 | R.SAGIKVIMVTGDHPITAK.A |
|  | 19289 | 962.9550 | 1923.8954 | 1923.9095 | -0.0142 | 0 | 59 | 0.00011 | 1 | K.NLEAVETLGSTSTICSDK.T 19290 |
|  | 19468 | 970.5204 | 1939.0262 | 1939.0375 | -0.0112 | 1 | 50 | 0.001 | 1 | R.QGAIVAVTGDGVNDSPALKK.A 19467 |
|  | 20493 | 679.3719 | 2035.0940 | 2035.0594 | 0.0346 | 1 | 3 | 43 | 5 | K.IMESFKNMVPQQALVIR.E |
|  | 24267 | 1232.6003 | 2463.1861 | 2463.1951 | -0.0090 | 0 | 129 | 9.1e-012 | 1 | K.QAADMILLDDNFASIVTGVEEGR.L 24262 24264 24266 24279 24283 |
|  | 26342 | 932.7148 | 2795.1225 | 2795.3607 | -0.2382 | 1 | 3 | 23 | 2 | K.NLEAVETLGSTSTICSDKTGTLTQNR.M |
|  | 30522 | **1371.3523** | **4111.0350** | **4111.8277** | **-0.7926** | **1** | **3** | **13** | **8** | **K.TGTLTQNRMTVAHMWFDNQIHEADTTEDQSGTSFDK.S** |
|  | 30643 | **1388.6764** | **4163.0073** | **4161.8945** | **1.1128** | **1** | **6** | **6.5** | **2** | **K.GFAFDCDDVNFTTDNLCFVGLMSMIDPPRAAVPDAVGK.C** |

| **25.** | P68371\|TBB2C_HUMAN    **Mass:** 50255    **Score:** 958    **Queries matched:** 51   **emPAI:** 2.90 |
| --- | --- |
|  | Tubulin beta-2C chain OS=Homo sapiens GN=TUBB2C PE=1 SV=1 |

|  | **Query** | **Observed** | **Mr(expt)** | **Mr(calc)** | **Delta** | **Miss** | **Score** | **Expect** | **Rank** | **Peptide** |
| --- | --- | --- | --- | --- | --- | --- | --- | --- | --- | --- |
|  | 1837 | **737.3976** | **736.3904** | **737.3456** | **-0.9553** | **0** | **9** | **21** | **2** | **R.GSQQYR.A** |
|  | 2296 | **405.2068** | **808.3991** | **807.3399** | **1.0593** | **0** | **8** | **28** | **7** | **R.EEYPDR.I** |
|  | 5466 | **514.7600** | **1027.5053** | **1027.5121** | **-0.0067** | **0** | **25** | **0.47** | **1** | **K.TAVCDIPPR.G** 5464 5477 5478 |
|  | 5627 | **520.2973** | **1038.5800** | **1038.5862** | **-0.0062** | **0** | **60** | **0.00016** | **1** | **R.YLTVAAVFR.G** 5628 |
|  | 5960 | **533.2140** | **1064.4134** | **1064.4201** | **-0.0067** | **0** | **11** | **12** | **1** | **K.NMMAACDPR.H** 5970 |
|  | 6152 | **539.2660** | **1076.5174** | **1076.5250** | **-0.0076** | **1** | **25** | **0.51** | **1** | **K.IREEYPDR.I** |
|  | 8471 | **615.2986** | **1228.5827** | **1228.5910** | **-0.0083** | **0** | **66** | **3.7e-005** | **1** | **R.ISEQFTAMFR.R** |
|  | 8778 | **415.7271** | **1244.1593** | **1244.5860** | **-0.4266** | **0** | **(10)** | **11** | **1** | **R.ISEQFTAMFR.R** |
|  | 10180 | **660.3506** | **1318.6867** | **1318.6955** | **-0.0088** | **0** | **67** | **3e-005** | **1** | **R.IMNTFSVVPSPK.V** 10151 |
|  | 10304 | **663.8410** | **1325.6674** | **1325.7126** | **-0.0451** | **1** | **5** | **41** | **5** | **K.TAVCDIPPRGLK.M** |
|  | 10356 | **664.8233** | **1327.6320** | **1327.6408** | **-0.0088** | **0** | **53** | **0.00068** | **1** | **R.INVYYNEATGGK.Y** 10355 |
|  | 11355 | **462.7160** | **1385.1261** | **1384.6921** | **0.4339** | **1** | **4** | **34** | **7** | **K.RISEQFTAMFR.R** |
|  | 12166 | 716.3267 | 1430.6389 | 1430.5965 | 0.0423 | 1 | 8 | 21 | 2 | K.NMMAACDPRHGR.Y 12164 12165 |
|  | 12402 | **723.8434** | **1445.6722** | **1445.6820** | **-0.0098** | **0** | **54** | **0.00051** | **1** | **K.EVDEQMLNVQNK.N** 12404 |
|  | 12637 | 731.3526 | 1460.6906 | 1461.6769 | -0.9863 | 0 | (10) | 13 | 2 | K.EVDEQMLNVQNK.N 12636 |
|  | 14745 | 801.4085 | 1600.8023 | 1600.8131 | -0.0107 | 0 | 51 | 0.001 | 1 | R.AVLVDLEPGTMDSVR.S |
|  | 15059 | **540.9470** | **1619.8192** | **1619.8283** | **-0.0090** | **0** | **28** | **0.19** | **1** | **R.LHFFMPGFAPLTSR.G** |
|  | 16113 | **846.4318** | **1690.8491** | **1690.8600** | **-0.0109** | **0** | **45** | **0.0035** | **1** | **R.ALTVPELTQQMFDAK.N** 16114 |
|  | 16178 | **848.9148** | **1695.8150** | **1695.8257** | **-0.0106** | **0** | **54** | **0.00046** | **1** | **K.NSSYFVEWIPNNVK.T** 16177 16189 |
|  | 17883 | **911.9587** | **1821.9028** | **1821.9156** | **-0.0127** | **0** | **54** | **0.00051** | **1** | **R.EIVHLQAGQCGNQIGAK.F** 17882 17884 |
|  | 18460 | **929.4695** | **1856.9244** | **1856.9342** | **-0.0098** | **0** | **77** | **2.2e-006** | **2** | **K.MSATFIGNSTAIQELFK.R** 18473 |
|  | 18642 | **937.1213** | **1872.2280** | **1872.9291** | **-0.7011** | **0** | **(2)** | **46** | **1** | **K.MSATFIGNSTAIQELFK.R** |
|  | 19481 | **647.6556** | **1939.9451** | **1938.8849** | **1.0602** | **1** | **5** | **29** | **6** | **R.MSMKEVDEQMLNVQNK.N** |
|  | 19688 | **979.9890** | **1957.9635** | **1957.9745** | **-0.0111** | **0** | **82** | **5.4e-007** | **1** | **K.GHYTEGAELVDSVLDVVR.K** |
|  | 25910 | **1354.6663** | **2707.3180** | **2707.3310** | **-0.0130** | **0** | **136** | **1.6e-012** | **1** | **K.LTTPTYGDLNHLVSATMSGVTTCLR.F** 25908 25911 |
|  | 26354 | **933.4486** | **2797.3240** | **2797.3361** | **-0.0121** | **0** | **85** | **1.8e-007** | **1** | **R.SGPFGQIFRPDNFVFGQSGAGNNWAK.G** 26352 26353 26359 |
|  | 27829 | **1039.4714** | **3115.3925** | **3115.4159** | **-0.0234** | **0** | **48** | **0.00075** | **1** | **K.FWEVISDEHGIDPTGTYHGDSDLQLER.I** |
|  | 28611 | **1104.5131** | **3310.5174** | **3310.5268** | **-0.0095** | **0** | **79** | **5.2e-007** | **1** | **K.EAESCDCLQGFQLTHSLGGGTGSGMGTLLISK.I** 28610 |

| **26.** | P35221\|CTNA1_HUMAN    **Mass:** 100693   **Score:** 956    **Queries matched:** 61   **emPAI:** 1.06 |
| --- | --- |
|  | Catenin alpha-1 OS=Homo sapiens GN=CTNNA1 PE=1 SV=1 |

|  | **Query** | **Observed** | **Mr(expt)** | **Mr(calc)** | **Delta** | **Miss** | **Score** | **Expect** | **Rank** | **Peptide** |
| --- | --- | --- | --- | --- | --- | --- | --- | --- | --- | --- |
|  | 1118 | **562.0571** | **561.0499** | **560.2554** | **0.7944** | **0** | **10** | **19** | **1** | **K.NGNEK.E** 1101 |
|  | 2524 | **827.4280** | **826.4208** | **825.4232** | **0.9975** | **0** | **27** | **0.31** | **1** | **K.ASYVASTK.Y** 2508 |
|  | 2584 | 415.7156 | 829.4167 | 830.4610 | -1.0443 | 1 | 13 | 11 | 2 | K.KIAEAGSR.M |
|  | 3290 | **872.5037** | **871.4964** | **871.5015** | **-0.0051** | **0** | **22** | **1.2** | **2** | **K.VVEDGILK.L** 3293 |
|  | 5183 | **503.2608** | **1004.5070** | **1004.5138** | **-0.0068** | **0** | **17** | **3.8** | **1** | **K.NTSDVISAAK.K** |
|  | 5503 | **515.7851** | **1029.5556** | **1029.5641** | **-0.0084** | **0** | **44** | **0.0061** | **1** | **K.LLSNTVMPR.F** |
|  | 5508 | **516.2649** | **1030.5152** | **1030.5229** | **-0.0077** | **0** | **10** | **18** | **1** | **R.IVAECNAVR.Q** |
|  | 6712 | **560.3209** | **1118.6272** | **1118.6084** | **0.0188** | **0** | **14** | **6.5** | **4** | **K.QDLLAYLQR.I** |
|  | 6889 | **567.5985** | **1133.1825** | **1132.6088** | **0.5737** | **1** | **5** | **36** | **5** | **K.NTSDVISAAKK.I** |
|  | 8233 | **608.8356** | **1215.6567** | **1215.6645** | **-0.0078** | **0** | **52** | **0.00099** | **1** | **K.NLMNAVVQTVK.A** |
|  | 8552 | **617.3415** | **1232.6684** | **1231.6595** | **1.0090** | **0** | **(3)** | **77** | **4** | **K.NLMNAVVQTVK.A** |
|  | 9330 | **637.3266** | **1272.6386** | **1271.6543** | **0.9843** | **0** | **15** | **5.2** | **3** | **R.AIMAQLPQEQK.A** |
|  | 10127 | **439.7130** | **1316.1173** | **1316.6870** | **-0.5697** | **1** | **7** | **19** | **7** | **R.DQMAAARGILQK.N** |
|  | 10128 | **439.7300** | **1316.1683** | **1315.6666** | **0.5017** | **1** | **4** | **40** | **9** | **R.ERIVAECNAVR.Q** |
|  | 10968 | 682.8745 | 1363.7343 | 1363.7421 | -0.0077 | 0 | 44 | 0.005 | 1 | R.LLILADMADVYK.L |
|  | 11029 | **684.8622** | **1367.7098** | **1367.7197** | **-0.0099** | **0** | **11** | **9.6** | **1** | **K.HVNPVQALSEFK.A** |
|  | 11143 | **458.8609** | **1373.5610** | **1373.5704** | **-0.0094** | **0** | **20** | **1.4** | **1** | **R.TIADHCPDSACK.Q** |
|  | 11213 | **689.8412** | **1377.6678** | **1377.6776** | **-0.0098** | **0** | **70** | **1.3e-005** | **1** | **K.IAEQVASFQEEK.S** |
|  | 11262 | **461.0073** | **1380.0000** | **1379.7370** | **0.2630** | **0** | **(2)** | **59** | **7** | **R.LLILADMADVYK.L** |
|  | 12383 | **723.3628** | **1444.7111** | **1444.7198** | **-0.0087** | **0** | **33** | **0.063** | **1** | **K.WDDSGNDIIVLAK.Q** 12384 |
|  | 12437 | **725.3472** | **1448.6798** | **1448.6895** | **-0.0098** | **0** | **50** | **0.0013** | **1** | **R.NAGNEQDLGIQYK.A** |
|  | 12517 | **485.5969** | **1453.7690** | **1454.7915** | **-1.0225** | **1** | **10** | **12** | **2** | **R.AIMAQLPQEQKAK.I** 12547 |
|  | 13608 | **762.8303** | **1523.6461** | **1523.6562** | **-0.0101** | **0** | **29** | **0.15** | **1** | **K.AAAGEFADDPCSSVK.R** 13609 |
|  | 13725 | **766.8967** | **1531.7788** | **1531.7882** | **-0.0094** | **0** | **46** | **0.003** | **1** | **K.QIIVDPLSFSEER.F** |
|  | 17829 | **909.9387** | **1817.8628** | **1817.8755** | **-0.0128** | **0** | **69** | **1.4e-005** | **1** | **R.TSVQTEDDQLIAGQSAR.A** 17830 |
|  | 18214 | **615.2807** | **1842.8203** | **1843.9039** | **-1.0837** | **1** | **2** | **64** | **5** | **-.MTAVHAGNINFKWDPK.S** |
|  | 18739 | **941.4503** | **1880.8861** | **1880.8972** | **-0.0111** | **0** | **58** | **0.00015** | **1** | **R.LESIISGAALMADSSCTR.D** 18738 18740 |
|  | 18991 | **950.9012** | **1899.7878** | **1900.9353** | **-1.1475** | **1** | **0** | **84** | **5** | **K.HVNPVQALSEFKAMDSI.-** |
|  | 19112 | **637.2760** | **1908.8062** | **1908.9363** | **-0.1302** | **1** | **1** | **71** | **3** | **R.QALQDLLSEYMGNAGRK.E** |
|  | 19663 | **653.0015** | **1955.9826** | **1955.9952** | **-0.0127** | **0** | **15** | **2.8** | **1** | **K.AHVLAASVEQATENFLEK.G** 19664 |
|  | 19813 | **985.5557** | **1969.0969** | **1969.1096** | **-0.0127** | **0** | **50** | **0.00093** | **1** | **R.LLEPLVTQVTTLVNTNSK.G** 19815 |
|  | 20188 | **669.9926** | **2006.9560** | **2007.0248** | **-0.0687** | **1** | **5** | **31** | **1** | **K.YQKSQGMASLNLPAVSWK.M** |
|  | 22309 | **738.3699** | **2212.0880** | **2211.9776** | **0.1103** | **1** | **1** | **59** | **2** | **K.QGDLMKAAAGEFADDPCSSVK.R** |
|  | 23116 | **1155.5427** | **2309.0709** | **2309.0886** | **-0.0177** | **0** | **74** | **3e-006** | **1** | **R.VIHVVTSEMDNYEPGVYTEK.V** 23115 23117 |
|  | 24227 | **1230.1146** | **2458.2147** | **2458.2315** | **-0.0168** | **0** | **96** | **1.7e-008** | **1** | **K.NVPILYTASQACLQHPDVAAYK.A** |
|  | 25141 | **1293.6710** | **2585.3275** | **2585.3371** | **-0.0096** | **0** | **101** | **5e-009** | **1** | **K.AEVQNLGGELVVSGVDSAMSLIQAAK.N** 25140 25142 |
|  | 25443 | **660.8791** | **2639.4873** | **2638.4905** | **0.9967** | **1** | **0** | **52** | **4** | **R.TLAVERLLEPLVTQVTTLVNTNSK.G** |
|  | 25927 | **1356.2147** | **2710.4149** | **2710.4251** | **-0.0103** | **0** | **87** | **1.1e-007** | **1** | **K.AVMDHVSDSFLETNVPLLVLIEAAK.N** 25925 |
|  | 26399 | **937.8317** | **2810.4732** | **2809.4830** | **0.9902** | **0** | **46** | **0.0014** | **1** | **R.MSASQLEALCPQVINAALALAAKPQSK.L** 26390 |
|  | 26408 | **938.5042** | **2812.4908** | **2812.5004** | **-0.0096** | **1** | **9** | **6.7** | **1** | **K.VKAEVQNLGGELVVSGVDSAMSLIQAAK.N** |
|  | 28204 | **1067.5284** | **3199.5635** | **3199.5772** | **-0.0137** | **0** | **61** | **4e-005** | **1** | **R.VLTDAVDDITSIDDFLAVSENHILEDVNK.C** |
|  | 28434 | **813.8969** | **3251.5585** | **3252.6700** | **-1.1115** | **1** | **2** | **32** | **6** | **K.AVMDHVSDSFLETNVPLLVLIEAAKNGNEK.E** |
|  | 30507 | **1368.6385** | **4102.8938** | **4102.9216** | **-0.0278** | **0** | **108** | **4.4e-010** | **1** | **K.QLQQAVTGISNAAQATASDDASQHQGGGGGELAYALNNFDK.Q** 30508 |

| **27.** | P14625\|ENPL_HUMAN    **Mass:** 92696    **Score:** 953    **Queries matched:** 65   **emPAI:** 1.46 |
| --- | --- |
|  | Endoplasmin OS=Homo sapiens GN=HSP90B1 PE=1 SV=1 |

|  | **Query** | **Observed** | **Mr(expt)** | **Mr(calc)** | **Delta** | **Miss** | **Score** | **Expect** | **Rank** | **Peptide** |
| --- | --- | --- | --- | --- | --- | --- | --- | --- | --- | --- |
|  | 1499 | **646.4086** | **645.4013** | **645.4061** | **-0.0048** | **0** | **20** | **2.8** | **2** | **K.VIVTSK.H** |
|  | 1727 | **706.3829** | **705.3757** | **705.3810** | **-0.0053** | **0** | **34** | **0.087** | **1** | **R.FQNVAK.E** 1733 |
|  | 1767 | **715.4301** | **714.4228** | **715.4228** | **-1.0000** | **0** | **21** | **1.7** | **1** | **K.NLGTIAK.S** 1774 |
|  | 2645 | **417.7261** | **833.4377** | **832.4589** | **0.9789** | **1** | **7** | **39** | **8** | **R.IERMLR.L** |
|  | 3142 | **432.7469** | **863.4793** | **863.4786** | **0.0007** | **1** | **6** | **49** | **8** | **R.KTLDMIK.K** |
|  | 3333 | 438.2392 | 874.4638 | 875.4501 | -0.9863 | 0 | 22 | 1.5 | 5 | K.TFEINPR.H |
|  | 3981 | **460.2690** | **918.5233** | **918.5287** | **-0.0053** | **1** | **23** | **0.98** | **1** | **K.NKEIFLR.E** |
|  | 4342 | **473.2995** | **944.5845** | **944.5906** | **-0.0061** | **0** | **24** | **0.83** | **1** | **R.GTTITLVLK.E** |
|  | 4597 | **482.2940** | **962.5735** | **962.5800** | **-0.0066** | **0** | **32** | **0.11** | **2** | **K.LIINSLYK.N** |
|  | 4848 | **491.7425** | **981.4704** | **981.4767** | **-0.0063** | **0** | **26** | **0.42** | **2** | **K.SGTSEFLNK.M** 4847 4858 |
|  | 5003 | **497.2632** | **992.5119** | **992.5179** | **-0.0059** | **0** | **18** | **2.7** | **1** | **R.SGYLLPDTK.A** |
|  | 5169 | **502.7756** | **1003.5367** | **1003.5451** | **-0.0084** | **1** | **8** | **26** | **1** | **K.KTFEINPR.H** |
|  | 5519 | **516.3103** | **1030.6060** | **1030.4906** | **0.1155** | **0** | **17** | **3.9** | **1** | **K.IYFMAGSSR.K** |
|  | 6263 | **543.2921** | **1084.5695** | **1084.5764** | **-0.0069** | **0** | **47** | **0.0031** | **1** | **R.LSLNIDPDAK.V** |
|  | 6960 | **570.2896** | **1138.5647** | **1138.5731** | **-0.0084** | **0** | **51** | **0.0012** | **1** | **K.LGVIEDHSNR.T** |
|  | 9352 | **638.3206** | **1274.6266** | **1274.6354** | **-0.0088** | **0** | **61** | **0.00011** | **1** | **R.ELISNASDALDK.I** 9383 |
|  | 9601 | **645.3001** | **1288.5855** | **1288.5935** | **-0.0080** | **0** | **52** | **0.00096** | **1** | **K.DISTNYYASQK.K** 9600 |
|  | 9946 | **654.3275** | **1306.6405** | **1305.6427** | **0.9978** | **0** | **5** | **39** | **1** | **K.EFEPLLNWMK.D** |
|  | 11044 | **457.2226** | **1368.6459** | **1368.7509** | **-0.1050** | **1** | **7** | **25** | **7** | **R.MMKLIINSLYK.N** |
|  | 13018 | **743.3755** | **1484.7365** | **1484.7471** | **-0.0106** | **0** | **77** | **2.7e-006** | **1** | **K.GVVDSDDLPLNVSR.E** |
|  | 13047 | **496.3375** | **1485.9906** | **1486.7061** | **-0.7155** | **1** | **3** | **57** | **2** | **K.FAFQAEVNRMMK.L** 13049 |
|  | 13305 | **751.9356** | **1501.8567** | **1500.7970** | **1.0597** | **1** | **5** | **42** | **6** | **R.MLRLSLNIDPDAK.V** |
|  | 13452 | **757.3880** | **1512.7614** | **1512.7719** | **-0.0104** | **0** | **70** | **1.1e-005** | **1** | **K.NLLHVTDTGVGMTR.E** 13444 13453 13454 |
|  | 13478 | **758.3544** | **1514.6943** | **1514.7041** | **-0.0098** | **1** | **44** | **0.0046** | **1** | **K.IADDKYNDTFWK.E** 13479 13491 |
|  | 13624 | **763.3626** | **1524.7107** | **1524.7195** | **-0.0088** | **0** | **33** | **0.064** | **1** | **K.EEASDYLELDTIK.N** |
|  | 13897 | **772.8417** | **1543.6689** | **1543.8205** | **-0.1516** | **1** | **41** | **0.0092** | **1** | **R.ELISNASDALDKIR.L** 13903 |
|  | 14233 | **522.5806** | **1564.7199** | **1564.7595** | **-0.0397** | **1** | **10** | **11** | **1** | **K.EFEPLLNWMKDK.A** 14223 |
|  | 14658 | **798.3606** | **1594.7066** | **1594.7160** | **-0.0094** | **0** | **42** | **0.0074** | **1** | **R.VFITDDFHDMMPK.Y** 14657 14662 |
|  | 16930 | **584.6096** | **1750.8070** | **1750.8171** | **-0.0101** | **1** | **16** | **2.8** | **1** | **R.RVFITDDFHDMMPK.Y** 16927 |
|  | 17415 | **893.4462** | **1784.8779** | **1784.8904** | **-0.0125** | **0** | **47** | **0.0022** | **1** | **R.EEEAIQLDGLNASQIR.E** |
|  | 17653 | **602.3161** | **1803.9265** | **1804.7851** | **-0.8586** | **0** | **4** | **38** | **3** | **R.ADDEVDVDGTVEEDLGK.S** |
|  | 18529 | **932.4655** | **1862.9163** | **1861.9284** | **0.9880** | **1** | **23** | **0.57** | **1** | **R.EAVEKEFEPLLNWMK.D** |
|  | 18713 | **939.9708** | **1877.9271** | **1877.9352** | **-0.0081** | **0** | **80** | **9.8e-007** | **1** | **K.YSQFINFPIYVWSSK.T** 18724 |
|  | 20633 | **1023.5273** | **2045.0401** | **2045.0528** | **-0.0127** | **0** | **121** | **7.7e-011** | **1** | **R.LISLTDENALSGNEELTVK.I** 20632 |
|  | 21298 | **703.5721** | **2107.6944** | **2106.9150** | **0.7794** | **1** | **1** | **46** | **10** | **K.TETVEEPMEEEEAAKEEK.E** |
|  | 22715 | **754.0208** | **2259.0406** | **2259.0556** | **-0.0150** | **0** | **79** | **9.7e-007** | **1** | **R.FQSSHHPTDITSLDQYVER.M** 22714 22716 |
|  | 23476 | **1178.5308** | **2355.0470** | **2355.0623** | **-0.0154** | **0** | **77** | **1.5e-006** | **1** | **R.LTESPCALVASQYGWSGNMER.I** 23475 23477 |
|  | 24811 | **848.0980** | **2541.2721** | **2541.2838** | **-0.0118** | **0** | **40** | **0.007** | **1** | **K.TVWDWELMNDIKPIWQRPSK.E** |
|  | 26013 | **1364.6648** | **2727.3150** | **2727.3311** | **-0.0161** | **1** | **25** | **0.2** | **1** | **R.TDDEVVQREEEAIQLDGLNASQIR.E** |
|  | 27078 | **1474.6920** | **2947.3695** | **2947.3837** | **-0.0142** | **0** | **43** | **0.0025** | **1** | **K.GYEVIYLTEPVDEYCIQALPEFDGK.R** |
|  | 27797 | **1035.4963** | **3103.4672** | **3103.4848** | **-0.0176** | **1** | **53** | **0.00022** | **1** | **K.GYEVIYLTEPVDEYCIQALPEFDGKR.F** |
|  | 28658 | **1109.1864** | **3324.5374** | **3324.5497** | **-0.0123** | **0** | **4** | **19** | **1** | **K.MTEAQEDGQSTSELIGQFGVGFYSAFLVADK.V** |

| **28.** | P31327\|CPSM_HUMAN    **Mass:** 165975   **Score:** 944    **Queries matched:** 92   **emPAI:** 0.66 |
| --- | --- |
|  | Carbamoyl-phosphate synthase [ammonia], mitochondrial OS=Homo sapiens GN=CPS1 PE=1 SV=2 |

|  | **Query** | **Observed** | **Mr(expt)** | **Mr(calc)** | **Delta** | **Miss** | **Score** | **Expect** | **Rank** | **Peptide** |
| --- | --- | --- | --- | --- | --- | --- | --- | --- | --- | --- |
|  | 1313 | **604.3381** | **603.3308** | **602.3388** | **0.9920** | **0** | **18** | **4.3** | **1** | **K.NVVSGK.T** |
|  | 1371 | **616.3649** | **615.3576** | **614.3500** | **1.0076** | **0** | **20** | **2.7** | **2** | **K.NNVIR.L** |
|  | 1684 | **693.3514** | **692.3442** | **691.4268** | **0.9173** | **0** | **7** | **39** | **9** | **R.ILTAFK.V** |
|  | 1993 | **788.4578** | **787.4505** | **787.4552** | **-0.0047** | **0** | **14** | **9.1** | **1** | **R.TSINVVR.H** 2009 |
|  | 2458 | **411.7136** | **821.4127** | **821.4184** | **-0.0057** | **0** | **17** | **3.2** | **1** | **K.SLFHYR.Q** |
|  | 2946 | **854.4385** | **853.4313** | **853.4368** | **-0.0055** | **0** | **25** | **0.48** | **1** | **K.AMLSTGFK.I** |
|  | 3015 | **430.2269** | **858.4393** | **858.4447** | **-0.0053** | **0** | **14** | **8.9** | **7** | **K.LNEINEK.I** |
|  | 3071 | **431.7178** | **861.4210** | **861.4266** | **-0.0055** | **0** | **7** | **40** | **10** | **R.DILNMEK.T** |
|  | 3167 | **433.2214** | **864.4283** | **864.4341** | **-0.0058** | **0** | **15** | **5.1** | **7** | **K.FLEEATR.V** |
|  | 3783 | **453.2609** | **904.5072** | **904.5018** | **0.0054** | **0** | **16** | **4.3** | **1** | **K.LFAEAVQK.S** |
|  | 4040 | **462.7342** | **923.4538** | **922.4760** | **0.9779** | **0** | **10** | **15** | **5** | **K.YLESNGIK.V** |
|  | 4781 | **489.2304** | **976.4463** | **976.4535** | **-0.0072** | **0** | **(4)** | **74** | **3** | **R.EVEMDAVGK.D** |
|  | 4985 | **496.7733** | **991.5320** | **992.4485** | **-0.9164** | **0** | **9** | **20** | **7** | **R.EVEMDAVGK.D** |
|  | 4988 | **497.0209** | **992.0273** | **992.5291** | **-0.5017** | **1** | **4** | **42** | **10** | **K.KFLEEATR.V** |
|  | 5005 | **497.2768** | **992.5390** | **991.5702** | **0.9688** | **1** | **8** | **27** | **4** | **R.GVLKEYGVK.V** |
|  | 5346 | **509.7679** | **1017.5212** | **1017.5277** | **-0.0065** | **0** | **42** | **0.012** | **1** | **K.SVGEVMAIGR.T** |
|  | 5553 | **517.7380** | **1033.4615** | **1033.5226** | **-0.0611** | **0** | **(12)** | **10** | **2** | **K.SVGEVMAIGR.T** 5537 5552 |
|  | 5583 | **518.7372** | **1035.4599** | **1035.5237** | **-0.0637** | **0** | **11** | **14** | **2** | **K.EIEYEVVR.D** 5595 |
|  | 5605 | **519.2766** | **1036.5387** | **1036.5110** | **0.0276** | **0** | **19** | **2.1** | **1** | **R.ETLMDLSTK.A** 5598 |
|  | 6002 | 534.8079 | 1067.6013 | 1067.6087 | -0.0074 | 1 | 20 | 1.4 | 1 | R.LRDADPILR.C |
|  | 7084 | 575.3068 | 1148.5991 | 1148.5682 | 0.0309 | 1 | 25 | 0.43 | 1 | K.MRDILNMEK.T |
|  | 7237 | **581.2871** | **1160.5597** | **1161.5931** | **-1.0334** | **0** | **24** | **0.64** | **2** | **K.FVHDNYVIR.R** 7238 7260 |
|  | 8250 | **609.3179** | **1216.6212** | **1216.6088** | **0.0124** | **0** | **15** | **4.6** | **2** | **K.SLGQWLQEEK.V** |
|  | 8343 | **611.3256** | **1220.6367** | **1219.6482** | **0.9885** | **1** | **3** | **76** | **8** | **R.DILNMEKTLK.G** |
|  | 8349 | **611.3372** | **1220.6599** | **1220.6652** | **-0.0053** | **0** | **41** | **0.014** | **1** | **R.SIFSAVLDELK.V** |
|  | 8497 | **615.8253** | **1229.6361** | **1229.6438** | **-0.0077** | **0** | **51** | **0.0013** | **1** | **K.IMGTSPLQIDR.A** |
|  | 9064 | **421.0563** | **1260.1472** | **1259.6597** | **0.4875** | **1** | **3** | **57** | **7** | **K.APMFSWPRLR.D** |
|  | 9788 | **649.8012** | **1297.5877** | **1297.6554** | **-0.0676** | **0** | **10** | **15** | **5** | **K.LYFEELSLER.I** |
|  | 9923 | **435.8975** | **1304.6707** | **1304.6031** | **0.0677** | **1** | **5** | **43** | **7** | **R.EVEMDAVGKDGR.V** |
|  | 9932 | **653.8401** | **1305.6656** | **1305.6751** | **-0.0095** | **0** | **22** | **0.86** | **1** | **K.AADTIGYPVMIR.S** |
|  | 10170 | **660.3309** | **1318.6473** | **1319.7271** | **-1.0798** | **1** | **5** | **48** | **9** | **K.AMLSTGFKIPQK.G** |
|  | 10293 | **663.3759** | **1324.7371** | **1324.7099** | **0.0272** | **0** | **18** | **2** | **3** | **R.GQNQPVLNITNK.Q** 10275 10292 |
|  | 10963 | **682.8611** | **1363.7077** | **1363.7169** | **-0.0092** | **0** | **40** | **0.015** | **1** | **K.AFAMTNQILVEK.S** 10943 10982 |
|  | 11301 | **691.8629** | **1381.7113** | **1381.7201** | **-0.0089** | **0** | **63** | **7e-005** | **1** | **K.AQTAHIVLEDGTK.M** 11302 |
|  | 11956 | **710.3733** | **1418.7320** | **1418.7405** | **-0.0085** | **0** | **59** | **0.00018** | **1** | **K.AVNTLNEALEFAK.S** |
|  | 13004 | **742.9334** | **1483.8521** | **1483.8623** | **-0.0102** | **0** | **20** | **1.3** | **1** | **K.GILIGIQQSFRPR.F** |
|  | 13135 | **746.8405** | **1491.6665** | **1491.6763** | **-0.0098** | **0** | **75** | **4.3e-006** | **1** | **K.AIDDNMSLDEIEK.L** |
|  | 14384 | **788.9045** | **1575.7944** | **1576.8096** | **-1.0152** | **1** | **4** | **45** | **8** | **R.QLFSDKLNEINEK.I** |
|  | 14559 | **794.9044** | **1587.7942** | **1587.8045** | **-0.0104** | **0** | **78** | **2e-006** | **1** | **R.FLGVAEQLHNEGFK.L** 14558 14560 |
|  | 14584 | **795.4202** | **1588.8259** | **1588.8348** | **-0.0089** | **0** | **77** | **2.5e-006** | **1** | **K.IAPSFAVESIEDALK.A** 14585 14587 |
|  | 14633 | **796.8903** | **1591.7660** | **1591.7777** | **-0.0117** | **0** | **45** | **0.0037** | **1** | **R.SAYALGGLGSGICPNR.E** 14634 |
|  | 14649 | **797.8850** | **1593.7553** | **1593.7668** | **-0.0115** | **1** | **72** | **6.7e-006** | **1** | **K.GLNSESMTEETLKR.A** 14650 |
|  | 14875 | **805.8699** | **1609.7252** | **1609.7617** | **-0.0365** | **1** | **(7)** | **23** | **7** | **K.GLNSESMTEETLKR.A** |
|  | 15342 | **410.2189** | **1636.8466** | **1636.8277** | **0.0190** | **1** | **(6)** | **29** | **5** | **R.IGSSMKSVGEVMAIGR.T** |
|  | 15578 | **551.9312** | **1652.7716** | **1651.7876** | **0.9841** | **1** | **1** | **89** | **9** | **K.FVEGAREVEMDAVGK.D** |
|  | 15588 | **551.9940** | **1652.9602** | **1652.8226** | **0.1377** | **1** | **6** | **25** | **6** | **R.IGSSMKSVGEVMAIGR.T** |
|  | 16263 | **567.7808** | **1700.3206** | **1700.8515** | **-0.5309** | **1** | **1** | **62** | **7** | **K.IMGTSPLQIDRAEDR.S** |
|  | 16677 | **867.4167** | **1732.8189** | **1732.8308** | **-0.0119** | **0** | **40** | **0.012** | **1** | **K.IEFEGQPVDFVDPNK.Q** 16676 |
|  | 16896 | **874.8916** | **1747.7686** | **1747.7797** | **-0.0111** | **0** | **37** | **0.02** | **1** | **K.TSACFEPSLDYMVTK.I** |
|  | 16910 | **583.9581** | **1748.8524** | **1748.8635** | **-0.0111** | **0** | **35** | **0.032** | **1** | **R.GAEVHLVPWNHDFTK.M** |
|  | 19066 | **477.2452** | **1904.9515** | **1904.9646** | **-0.0131** | **1** | **11** | **7.2** | **1** | **K.RGAEVHLVPWNHDFTK.M** |
|  | 20235 | **504.0317** | **2012.0979** | **2012.1095** | **-0.0116** | **0** | **3** | **41** | **3** | **K.HLPTLDHPIIPADYVAIK.A** |
|  | 20393 | **1014.5364** | **2027.0583** | **2027.0721** | **-0.0138** | **0** | **84** | **3.6e-007** | **1** | **K.TVLMNPNIASVQTNEVGLK.Q** 20395 |
|  | 20643 | **683.0557** | **2046.1452** | **2046.9939** | **-0.8487** | **1** | **4** | **33** | **4** | **K.MSMANRGQNQPVLNITNK.Q** |
|  | 23708 | **1197.0886** | **2392.1627** | **2392.2196** | **-0.0569** | **1** | **6** | **21** | **2** | **K.TLGVDFIDVATKVMIGENVDEK.H** 23709 |
|  | 23748 | **800.3898** | **2398.1477** | **2398.2124** | **-0.0647** | **1** | **2** | **44** | **2** | **R.ETLMDLSTKAFAMTNQILVEK.S** |
|  | 23837 | **805.6927** | **2414.0562** | **2414.0705** | **-0.0143** | **0** | **30** | **0.071** | **1** | **R.CEMASTGEVACFGEGIHTAFLK.A** |
|  | 25914 | **1354.7001** | **2707.3856** | **2707.4003** | **-0.0147** | **0** | **110** | **6.8e-010** | **1** | **K.MEYDGILIAGGPGNPALAEPLIQNVR.K** 25913 25915 |
|  | 26426 | **939.8102** | **2816.4087** | **2816.4232** | **-0.0145** | **1** | **38** | **0.0089** | **1** | **K.IEFEGQPVDFVDPNKQNLIAEVSTK.D** |
|  | 26636 | **1434.2376** | **2866.4605** | **2866.4746** | **-0.0141** | **0** | **84** | **2.3e-007** | **1** | **K.GQILTMANPIIGNGGAPDTTALDELGLSK.Y** 26641 |
|  | 28890 | **1129.8709** | **3386.5907** | **3386.6096** | **-0.0188** | **0** | **80** | **4.4e-007** | **1** | **K.GYSFGHPSSVAGEVVFNTGLGGYPEAITDPAYK.G** |
|  | 28995 | **856.1631** | **3420.6235** | **3420.6404** | **-0.0169** | **1** | **4** | **17** | **2** | **K.SVDYPCLLRPSYVLSGSAMNVVFSEDEMKK.F** |
|  | 29485 | **1203.5034** | **3607.4884** | **3607.6335** | **-0.1450** | **1** | **27** | **0.059** | **1** | **K.TVVVNCNPETVSTDFDECDKLYFEELSLER.I** |
|  | 29686 | **1221.8677** | **3662.5812** | **3661.7399** | **0.8413** | **1** | **2** | **20** | **3** | **K.MKGYSFGHPSSVAGEVVFNTGLGGYPEAITDPAYK.G** |
|  | 29894 | **1247.9552** | **3740.8438** | **3740.8679** | **-0.0242** | **0** | **50** | **0.00039** | **1** | **R.VISHAISEHVEDAGVHSGDATLMLPTQTISQGAIEK.V** 29895 |
|  | 30355 | **1323.9785** | **3968.9137** | **3967.9340** | **0.9797** | **0** | **12** | **1.7** | **1** | **K.LFATEATSDWLNANNVPATPVAWPSQEGQNPSLSSIR.K** |
|  | 30910 | 1485.7457 | 4454.2154 | 4454.2481 | -0.0328 | 1 | 2 | 16 | 8 | R.GAEVHLVPWNHDFTKMEYDGILIAGGPGNPALAEPLIQNVR.K |

| **29.** | P30101\|PDIA3_HUMAN    **Mass:** 57146    **Score:** 935    **Queries matched:** 72   **emPAI:** 3.27 |
| --- | --- |
|  | Protein disulfide-isomerase A3 OS=Homo sapiens GN=PDIA3 PE=1 SV=4 |

|  | **Query** | **Observed** | **Mr(expt)** | **Mr(calc)** | **Delta** | **Miss** | **Score** | **Expect** | **Rank** | **Peptide** |
| --- | --- | --- | --- | --- | --- | --- | --- | --- | --- | --- |
|  | 1285 | **601.0270** | **600.0198** | **599.3279** | **0.6919** | **0** | **12** | **9.2** | **1** | **K.NLEPK.Y** |
|  | 2019 | **395.2191** | **788.4236** | **787.4188** | **1.0048** | **1** | **6** | **54** | **4** | **R.DGKALER.F** |
|  | 3179 | **866.4554** | **865.4481** | **865.4545** | **-0.0064** | **1** | **21** | **1.2** | **1** | **K.YKELGEK.L** 3187 3197 |
|  | 3264 | **435.7472** | **869.4799** | **868.5018** | **0.9781** | **0** | **29** | **0.19** | **1** | **K.DPNIVIAK.M** |
|  | 3361 | **439.2453** | **876.4760** | **876.4817** | **-0.0057** | **0** | **43** | **0.0091** | **1** | **K.LNFAVASR.K** 3350 3351 3362 3364 3371 3372 3373 |
|  | 4807 | 490.2610 | 978.5074 | 979.4943 | -0.9869 | 1 | 8 | 27 | 4 | R.NRVMMVAK.K |
|  | 5643 | **521.2361** | **1040.4577** | **1039.5662** | **0.8915** | **0** | **0** | **1.5e+002** | **4** | **R.TADGIVSHLK.K** |
|  | 6246 | **542.7839** | **1083.5532** | **1083.5601** | **-0.0069** | **0** | **37** | **0.031** | **1** | **K.YGVSGYPTLK.I** 6245 |
|  | 7381 | **586.7702** | **1171.5258** | **1171.5332** | **-0.0073** | **0** | **42** | **0.011** | **1** | **K.FVMQEEFSR.D** 7380 |
|  | 7709 | 596.3003 | 1190.5861 | 1190.5931 | -0.0070 | 0 | 59 | 0.00018 | 1 | R.LAPEYEAAATR.L 7708 |
|  | 7821 | **399.9089** | **1196.7050** | **1196.7128** | **-0.0079** | **1** | **46** | **0.0037** | **1** | **K.LSKDPNIVIAK.M** |
|  | 8596 | **618.7556** | **1235.4967** | **1235.5054** | **-0.0088** | **0** | **31** | **0.12** | **1** | **R.DGEEAGAYDGPR.T** |
|  | 10587 | **671.3415** | **1340.6684** | **1340.6765** | **-0.0081** | **0** | **46** | **0.0037** | **1** | **R.GFPTIYFSPANK.K** 10585 10586 |
|  | 10693 | **674.3501** | **1346.6856** | **1346.6942** | **-0.0086** | **1** | **66** | **3.8e-005** | **1** | **K.RLAPEYEAAATR.L** |
|  | 10885 | **680.3287** | **1358.6428** | **1358.6507** | **-0.0079** | **0** | **42** | **0.0094** | **1** | **R.FLQDYFDGNLK.R** |
|  | 11026 | **684.8307** | **1367.6469** | **1367.6569** | **-0.0099** | **0** | **31** | **0.11** | **1** | **K.SEPIPESNDGPVK.V** 11027 |
|  | 11069 | **685.8475** | **1369.6804** | **1369.6878** | **-0.0074** | **0** | **13** | **7.2** | **1** | **R.ELSDFISYLQR.E** 11062 |
|  | 11551 | **699.2886** | **1396.5627** | **1396.5711** | **-0.0084** | **0** | **73** | **6.1e-006** | **1** | **K.VDCTANTNTCNK.Y** 11549 11552 11553 11554 11581 |
|  | 12770 | **735.3881** | **1468.7617** | **1468.7714** | **-0.0098** | **1** | **16** | **2.9** | **1** | **R.GFPTIYFSPANKK.L** |
|  | 13294 | **501.3357** | **1500.9852** | **1501.6871** | **-0.7019** | **1** | **5** | **34** | **9** | **K.GEKFVMQEEFSR.D** |
|  | 13494 | **505.9220** | **1514.7441** | **1514.7518** | **-0.0077** | **1** | **12** | **8** | **1** | **R.FLQDYFDGNLKR.Y** 13471 13472 |
|  | 14430 | **790.4143** | **1578.8139** | **1578.8253** | **-0.0114** | **0** | **41** | **0.01** | **1** | **R.EATNPPVIQEEKPK.K** |
|  | 15046 | **810.3906** | **1618.7667** | **1618.7766** | **-0.0099** | **0** | **1** | **93** | **5** | **K.DLLIAYYDVDYEK.N** |
|  | 15470 | **823.4105** | **1644.8064** | **1644.8736** | **-0.0672** | **1** | **3** | **54** | **4** | **K.FLDAGHKLNFAVASR.K** |
|  | 15559 | **551.2903** | **1650.8490** | **1651.0297** | **-0.1807** | **1** | **7** | **21** | **1** | **R.RLALFPGVALLLAAAR.L** |
|  | 15565 | **826.8818** | **1651.7490** | **1651.7590** | **-0.0100** | **1** | **62** | **6.8e-005** | **1** | **K.IFRDGEEAGAYDGPR.T** 15564 |
|  | 15730 | **832.8772** | **1663.7398** | **1663.7512** | **-0.0113** | **0** | **18** | **1.9** | **1** | **K.MDATANDVPSPYEVR.G** |
|  | 23386 | **1174.5317** | **2347.0489** | **2347.0645** | **-0.0155** | **0** | **95** | **2.3e-008** | **1** | **K.DASIVGFFDDSFSEAHSEFLK.A** 23387 23388 23389 |
|  | 25095 | **1288.1484** | **2574.2823** | **2574.2966** | **-0.0143** | **0** | **107** | **1.3e-009** | **1** | **K.TFSHELSDFGLESTAGEIPVVAIR.T** 25092 25093 25094 |
|  | 25126 | **861.7291** | **2582.1656** | **2582.1756** | **-0.0101** | **0** | **22** | **0.42** | **1** | **R.ISDTGSAGLMLVEFFAPWCGHCK.R** |
|  | 25217 | **867.3948** | **2599.1627** | **2598.1706** | **0.9921** | **0** | **(3)** | **32** | **2** | **R.ISDTGSAGLMLVEFFAPWCGHCK.R** |
|  | 25856 | **676.6020** | **2702.3789** | **2702.3915** | **-0.0127** | **1** | **12** | **3.8** | **1** | **R.KTFSHELSDFGLESTAGEIPVVAIR.T** 25855 |
|  | 26554 | **949.7928** | **2846.3565** | **2846.3731** | **-0.0166** | **1** | **45** | **0.002** | **1** | **K.FIQENIFGICPHMTEDNKDLIQGK.D** |
|  | 27039 | **980.1267** | **2937.3581** | **2937.3709** | **-0.0128** | **1** | **123** | **2.5e-011** | **1** | **K.FISDKDASIVGFFDDSFSEAHSEFLK.A** 27035 27036 27040 |
|  | 29296 | **1177.2425** | **3528.7058** | **3528.7273** | **-0.0215** | **0** | **88** | **6.5e-008** | **1** | **R.FAHTNVESLVNEYDDNGEGIILFRPSHLTNK.F** 29284 29295 29297 |

| **30.** | P07437\|TBB5_HUMAN    **Mass:** 50095    **Score:** 909    **Queries matched:** 50   **emPAI:** 2.65 |
| --- | --- |
|  | Tubulin beta chain OS=Homo sapiens GN=TUBB PE=1 SV=2 |

|  | **Query** | **Observed** | **Mr(expt)** | **Mr(calc)** | **Delta** | **Miss** | **Score** | **Expect** | **Rank** | **Peptide** |
| --- | --- | --- | --- | --- | --- | --- | --- | --- | --- | --- |
|  | 1837 | 737.3976 | 736.3904 | 737.3456 | -0.9553 | 0 | 9 | 21 | 2 | R.GSQQYR.A |
|  | 2296 | 405.2068 | 808.3991 | 807.3399 | 1.0593 | 0 | 8 | 28 | 7 | R.EEYPDR.I |
|  | 5466 | 514.7600 | 1027.5053 | 1027.5121 | -0.0067 | 0 | 25 | 0.47 | 1 | K.TAVCDIPPR.G 5464 5477 5478 |
|  | 5627 | 520.2973 | 1038.5800 | 1038.5862 | -0.0062 | 0 | 60 | 0.00016 | 1 | R.YLTVAAVFR.G 5628 |
|  | 5960 | 533.2140 | 1064.4134 | 1064.4201 | -0.0067 | 0 | 11 | 12 | 1 | K.NMMAACDPR.H 5970 |
|  | 6152 | 539.2660 | 1076.5174 | 1076.5250 | -0.0076 | 1 | 25 | 0.51 | 1 | K.IREEYPDR.I |
|  | 8471 | 615.2986 | 1228.5827 | 1228.5910 | -0.0083 | 0 | 66 | 3.7e-005 | 1 | R.ISEQFTAMFR.R |
|  | 8778 | 415.7271 | 1244.1593 | 1244.5860 | -0.4266 | 0 | (10) | 11 | 1 | R.ISEQFTAMFR.R |
|  | 9839 | **651.3180** | **1300.6215** | **1300.6299** | **-0.0084** | **0** | **61** | **0.00011** | **1** | **R.ISVYYNEATGGK.Y** |
|  | 10180 | 660.3506 | 1318.6867 | 1318.6955 | -0.0088 | 0 | 67 | 3e-005 | 1 | R.IMNTFSVVPSPK.V 10151 |
|  | 10304 | 663.8410 | 1325.6674 | 1325.7126 | -0.0451 | 1 | 5 | 41 | 5 | K.TAVCDIPPRGLK.M |
|  | 11355 | 462.7160 | 1385.1261 | 1384.6921 | 0.4339 | 1 | 4 | 34 | 7 | K.RISEQFTAMFR.R |
|  | 12166 | 716.3267 | 1430.6389 | 1430.5965 | 0.0423 | 1 | 8 | 21 | 2 | K.NMMAACDPRHGR.Y 12164 12165 |
|  | 12402 | 723.8434 | 1445.6722 | 1445.6820 | -0.0098 | 0 | 54 | 0.00051 | 1 | K.EVDEQMLNVQNK.N 12404 |
|  | 12637 | 731.3526 | 1460.6906 | 1461.6769 | -0.9863 | 0 | (10) | 13 | 2 | K.EVDEQMLNVQNK.N 12636 |
|  | 14975 | **808.4165** | **1614.8184** | **1614.8287** | **-0.0103** | **0** | **33** | **0.062** | **1** | **R.AILVDLEPGTMDSVR.S** 14974 |
|  | 15059 | 540.9470 | 1619.8192 | 1619.8283 | -0.0090 | 0 | 28 | 0.19 | 1 | R.LHFFMPGFAPLTSR.G |
|  | 15266 | **544.9023** | **1631.6850** | **1630.8236** | **0.8614** | **0** | **(6)** | **28** | **2** | **R.AILVDLEPGTMDSVR.S** 15251 |
|  | 15660 | **830.4460** | **1658.8774** | **1658.8879** | **-0.0105** | **0** | **11** | **9.7** | **1** | **R.ALTVPELTQQVFDAK.N** |
|  | 16178 | 848.9148 | 1695.8150 | 1695.8257 | -0.0106 | 0 | 54 | 0.00046 | 1 | K.NSSYFVEWIPNNVK.T 16177 16189 |
|  | 17883 | 911.9587 | 1821.9028 | 1821.9156 | -0.0127 | 0 | 54 | 0.00051 | 1 | R.EIVHIQAGQCGNQIGAK.F 17882 17884 |
|  | 18599 | **935.4893** | **1868.9640** | **1868.9706** | **-0.0066** | **0** | **91** | **7e-008** | **1** | **K.MAVTFIGNSTAIQELFK.R** |
|  | 19481 | 647.6556 | 1939.9451 | 1938.8849 | 1.0602 | 1 | 5 | 29 | 6 | R.MSMKEVDEQMLNVQNK.N |
|  | 19688 | 979.9890 | 1957.9635 | 1957.9745 | -0.0111 | 0 | 82 | 5.4e-007 | 1 | K.GHYTEGAELVDSVLDVVR.K |
|  | 25910 | 1354.6663 | 2707.3180 | 2707.3310 | -0.0130 | 0 | 136 | 1.6e-012 | 1 | K.LTTPTYGDLNHLVSATMSGVTTCLR.F 25908 25911 |
|  | 26354 | 933.4486 | 2797.3240 | 2797.3361 | -0.0121 | 0 | 85 | 1.8e-007 | 1 | R.SGPFGQIFRPDNFVFGQSGAGNNWAK.G 26352 26353 26359 |
|  | 27789 | **1034.8009** | **3101.3809** | **3101.4003** | **-0.0194** | **0** | **52** | **0.00029** | **1** | **K.FWEVISDEHGIDPTGTYHGDSDLQLDR.I** |
|  | 28611 | 1104.5131 | 3310.5174 | 3310.5268 | -0.0095 | 0 | 79 | 5.2e-007 | 1 | K.EAESCDCLQGFQLTHSLGGGTGSGMGTLLISK.I 28610 |

| **31.** | P31930\|QCR1_HUMAN    **Mass:** 53297    **Score:** 840    **Queries matched:** 49   **emPAI:** 1.10 |
| --- | --- |
|  | Cytochrome b-c1 complex subunit 1, mitochondrial OS=Homo sapiens GN=UQCRC1 PE=1 SV=3 |

|  | **Query** | **Observed** | **Mr(expt)** | **Mr(calc)** | **Delta** | **Miss** | **Score** | **Expect** | **Rank** | **Peptide** |
| --- | --- | --- | --- | --- | --- | --- | --- | --- | --- | --- |
|  | 1536 | **653.3458** | **652.3386** | **652.3068** | **0.0318** | **0** | **18** | **2.2** | **2** | **R.FETEK.N** |
|  | 1925 | **762.4305** | **761.4232** | **761.3854** | **0.0378** | **0** | **1** | **1.9e+002** | **3** | **M.AASVVCR.A** 1920 |
|  | 2299 | **405.2086** | **808.4026** | **808.4079** | **-0.0053** | **0** | **25** | **0.55** | **1** | **R.FTGSEIR.H** 2300 2301 |
|  | 2308 | **405.2270** | **808.4394** | **808.4443** | **-0.0049** | **0** | **15** | **5.6** | **2** | **R.SLLTYGR.R** |
|  | 3628 | **448.7232** | **895.4319** | **895.4374** | **-0.0055** | **0** | **30** | **0.16** | **1** | **R.SGMFWLR.F** |
|  | 4687 | **486.2637** | **970.5128** | **970.5196** | **-0.0068** | **0** | **26** | **0.44** | **1** | **K.NRPGSALEK.E** 4689 4692 |
|  | 5411 | **512.7548** | **1023.4951** | **1023.5025** | **-0.0074** | **0** | **16** | **3.9** | **1** | **R.EHTAYYIK.A** |
|  | 8084 | **605.4215** | **1208.8283** | **1209.4754** | **-0.6470** | **0** | **(4)** | **47** | **4** | **R.EMQENDASMR.D** 8096 |
|  | 8433 | **409.7305** | **1226.1696** | **1225.4703** | **0.6993** | **0** | **(8)** | **15** | **2** | **R.EMQENDASMR.D** |
|  | 8434 | **614.2727** | **1226.5309** | **1225.4703** | **1.0606** | **0** | **(6)** | **42** | **2** | **R.EMQENDASMR.D** |
|  | 8745 | **415.1906** | **1242.5501** | **1241.4652** | **1.0849** | **0** | **12** | **10** | **3** | **R.EMQENDASMR.D** |
|  | 10250 | **662.3093** | **1322.6041** | **1322.6136** | **-0.0095** | **0** | **75** | **4.6e-006** | **1** | **R.LCTSATESEVAR.G** 10251 |
|  | 14457 | **527.9248** | **1580.7526** | **1579.7783** | **0.9743** | **0** | **2** | **70** | **8** | **K.NNGAGYFLEHLAFK.G** |
|  | 15963 | **560.6207** | **1678.8404** | **1679.7573** | **-0.9169** | **0** | **2** | **68** | **3** | **K.EVESMGAHLNAYSTR.E** |
|  | 17185 | **885.4083** | **1768.8020** | **1768.8099** | **-0.0079** | **0** | **66** | **2.6e-005** | **1** | **K.IDDMMFVLQGQWMR.L** 17184 |
|  | 17598 | **600.9474** | **1799.8203** | **1800.7997** | **-0.9794** | **0** | **(4)** | **41** | **2** | **K.IDDMMFVLQGQWMR.L** |
|  | 17609 | **601.3022** | **1800.8849** | **1800.7997** | **0.0852** | **0** | **(3)** | **59** | **5** | **K.IDDMMFVLQGQWMR.L** |
|  | 19708 | **654.3296** | **1959.9669** | **1960.0346** | **-0.0677** | **1** | **0** | **93** | **10** | **-.MAASVVCRAATAGAQVLLR.A** |
|  | 20403 | **676.9860** | **2027.9362** | **2027.9453** | **-0.0091** | **1** | **22** | **0.55** | **1** | **R.MKIDDMMFVLQGQWMR.L** 20409 |
|  | 20712 | **1027.4952** | **2052.9759** | **2052.9899** | **-0.0139** | **0** | **91** | **7.4e-008** | **1** | **R.NALVSHLDGTTPVCEDIGR.S** 20711 |
|  | 20788 | **687.5076** | **2059.5011** | **2059.9352** | **-0.4341** | **1** | **(1)** | **51** | **7** | **R.MKIDDMMFVLQGQWMR.L** |
|  | 21079 | **1046.5574** | **2091.1002** | **2091.1146** | **-0.0145** | **0** | **91** | **7.2e-008** | **1** | **R.MVLAAAGGVEHQQLLDLAQK.H** 21080 21081 |
|  | 21294 | **703.3973** | **2107.1702** | **2107.1096** | **0.0606** | **0** | **(5)** | **24** | **1** | **R.MVLAAAGGVEHQQLLDLAQK.H** 21293 |
|  | 23455 | **1177.5757** | **2353.1368** | **2353.1525** | **-0.0157** | **0** | **59** | **8.6e-005** | **1** | **K.HLGGIPWTYAEDAVPTLTPCR.F** 23456 23457 |
|  | 23506 | **1180.5791** | **2359.1436** | **2359.1577** | **-0.0140** | **0** | **101** | **6.4e-009** | **1** | **K.AVELLGDIVQNCSLEDSQIEK.E** 23507 23516 |
|  | 24024 | **811.4276** | **2431.2608** | **2431.3005** | **-0.0397** | **1** | **5** | **24** | **3** | **K.APRMVLAAAGGVEHQQLLDLAQK.H** |
|  | 25845 | **1351.6227** | **2701.2308** | **2701.2482** | **-0.0174** | **0** | **101** | **4.9e-009** | **1** | **K.YIYDQCPAVAGYGPIEQLPDYNR.I** 25846 |
|  | 27885 | **1565.7759** | **3129.5372** | **3129.5520** | **-0.0148** | **0** | **56** | **0.00012** | **1** | **R.DVVFNYLHATAFQGTPLAQAVEGPSENVR.K** |
|  | 31976 | **1227.6073** | **6133.0001** | **6133.0293** | **-0.0292** | **1** | **69** | **8.5e-007** | **1** | **R.HRDDALPFAHVAIAVEGPGWASPDNVALQVANAIIGHYDCTYGGGVHLSSPLASGAVANK.L** 31975 31977 |

| **32.** | P04350\|TBB4_HUMAN    **Mass:** 50010    **Score:** 838    **Queries matched:** 46   **emPAI:** 2.16 |
| --- | --- |
|  | Tubulin beta-4 chain OS=Homo sapiens GN=TUBB4 PE=1 SV=2 |

|  | **Query** | **Observed** | **Mr(expt)** | **Mr(calc)** | **Delta** | **Miss** | **Score** | **Expect** | **Rank** | **Peptide** |
| --- | --- | --- | --- | --- | --- | --- | --- | --- | --- | --- |
|  | 1837 | 737.3976 | 736.3904 | 737.3456 | -0.9553 | 0 | 9 | 21 | 2 | R.GSQQYR.A |
|  | 2083 | **397.2539** | **792.4931** | **791.3450** | **1.1482** | **0** | **8** | **30** | **1** | **R.EEFPDR.I** 2074 |
|  | 5466 | 514.7600 | 1027.5053 | 1027.5121 | -0.0067 | 0 | 25 | 0.47 | 1 | K.TAVCDIPPR.G 5464 5477 5478 |
|  | 5627 | 520.2973 | 1038.5800 | 1038.5862 | -0.0062 | 0 | 60 | 0.00016 | 1 | R.YLTVAAVFR.G 5628 |
|  | 5960 | 533.2140 | 1064.4134 | 1064.4201 | -0.0067 | 0 | 11 | 12 | 1 | K.NMMAACDPR.H 5970 |
|  | 8471 | 615.2986 | 1228.5827 | 1228.5910 | -0.0083 | 0 | 66 | 3.7e-005 | 1 | R.ISEQFTAMFR.R |
|  | 8778 | 415.7271 | 1244.1593 | 1244.5860 | -0.4266 | 0 | (10) | 11 | 1 | R.ISEQFTAMFR.R |
|  | 10180 | 660.3506 | 1318.6867 | 1318.6955 | -0.0088 | 0 | 67 | 3e-005 | 1 | R.IMNTFSVVPSPK.V 10151 |
|  | 10304 | 663.8410 | 1325.6674 | 1325.7126 | -0.0451 | 1 | 5 | 41 | 5 | K.TAVCDIPPRGLK.M |
|  | 11355 | 462.7160 | 1385.1261 | 1384.6921 | 0.4339 | 1 | 4 | 34 | 7 | K.RISEQFTAMFR.R |
|  | 12166 | 716.3267 | 1430.6389 | 1430.5965 | 0.0423 | 1 | 8 | 21 | 2 | K.NMMAACDPRHGR.Y 12164 12165 |
|  | 14745 | 801.4085 | 1600.8023 | 1600.8131 | -0.0107 | 0 | 51 | 0.001 | 1 | R.AVLVDLEPGTMDSVR.S |
|  | 15059 | 540.9470 | 1619.8192 | 1619.8283 | -0.0090 | 0 | 28 | 0.19 | 1 | R.LHFFMPGFAPLTSR.G |
|  | 16113 | 846.4318 | 1690.8491 | 1690.8600 | -0.0109 | 0 | 45 | 0.0035 | 1 | R.ALTVPELTQQMFDAK.N 16114 |
|  | 16178 | 848.9148 | 1695.8150 | 1695.8257 | -0.0106 | 0 | 54 | 0.00046 | 1 | K.NSSYFVEWIPNNVK.T 16177 16189 |
|  | 17883 | 911.9587 | 1821.9028 | 1821.9156 | -0.0127 | 0 | 54 | 0.00051 | 1 | R.EIVHLQAGQCGNQIGAK.F 17882 17884 |
|  | 18004 | **610.6196** | **1828.8371** | **1828.8744** | **-0.0373** | **0** | **11** | **8.3** | **1** | **R.INVYYNEATGGNYVPR.A** |
|  | 18460 | 929.4695 | 1856.9244 | 1856.9342 | -0.0098 | 0 | 79 | 1.3e-006 | 1 | K.MAATFIGNSTAIQELFK.R 18473 |
|  | 18994 | 950.9130 | 1899.8115 | 1900.8580 | -1.0465 | 1 | (0) | 87 | 8 | R.MSMKEVDEQMLSVQSK.N |
|  | 19023 | **951.8894** | **1901.7642** | **1900.8580** | **0.9062** | **1** | **1** | **78** | **4** | **R.MSMKEVDEQMLSVQSK.N** |
|  | 20236 | **671.8511** | **2012.5316** | **2013.0353** | **-0.5037** | **1** | **6** | **15** | **1** | **K.MAATFIGNSTAIQELFKR.I** |
|  | 25910 | 1354.6663 | 2707.3180 | 2707.3310 | -0.0130 | 0 | 136 | 1.6e-012 | 1 | K.LTTPTYGDLNHLVSATMSGVTTCLR.F 25908 25911 |
|  | 26354 | 933.4486 | 2797.3240 | 2797.3361 | -0.0121 | 0 | 85 | 1.8e-007 | 1 | R.SGPFGQIFRPDNFVFGQSGAGNNWAK.G 26352 26353 26359 |
|  | 27829 | 1039.4714 | 3115.3925 | 3115.4159 | -0.0234 | 0 | 48 | 0.00075 | 1 | K.FWEVISDEHGIDPTGTYHGDSDLQLER.I |
|  | 28611 | 1104.5131 | 3310.5174 | 3310.5268 | -0.0095 | 0 | 79 | 5.2e-007 | 1 | K.EAESCDCLQGFQLTHSLGGGTGSGMGTLLISK.I 28610 |

| **33.** | P50993\|AT1A2_HUMAN    **Mass:** 113505   **Score:** 837    **Queries matched:** 53   **emPAI:** 0.52 |
| --- | --- |
|  | Sodium/potassium-transporting ATPase subunit alpha-2 OS=Homo sapiens GN=ATP1A2 PE=1 SV=1 |

|  | **Query** | **Observed** | **Mr(expt)** | **Mr(calc)** | **Delta** | **Miss** | **Score** | **Expect** | **Rank** | **Peptide** |
| --- | --- | --- | --- | --- | --- | --- | --- | --- | --- | --- |
|  | 1443 | 633.3343 | 632.3270 | 632.3316 | -0.0046 | 0 | 25 | 0.93 | 1 | K.NCLVK.N |
|  | 3564 | 446.0373 | 890.0600 | 889.4617 | 0.5982 | 0 | 26 | 0.32 | 1 | K.TGTLTQNR.M |
|  | 5611 | 519.7522 | 1037.4898 | 1037.4964 | -0.0066 | 0 | 32 | 0.09 | 1 | R.NSVFQQGMK.N |
|  | 5840 | **529.2236** | **1056.4326** | **1057.5114** | **-1.0788** | **1** | **10** | **16** | **7** | **K.SSKIMDSFK.N** |
|  | 6258 | 543.2701 | 1084.5257 | 1084.5335 | -0.0078 | 0 | 13 | 8.2 | 1 | K.ACVVHGSDLK.D 6261 |
|  | 6292 | 544.2970 | 1086.5794 | 1086.5856 | -0.0061 | 0 | 34 | 0.079 | 1 | K.LIIVEGCQR.Q 6293 |
|  | 7763 | 597.8008 | 1193.5870 | 1193.5975 | -0.0105 | 1 | 10 | 16 | 1 | R.RNSVFQQGMK.N |
|  | 9221 | 634.8564 | 1267.6982 | 1267.7071 | -0.0089 | 0 | 27 | 0.29 | 1 | K.NMVPQQALVIR.E 9219 9220 |
|  | 9407 | **426.5761** | **1276.7063** | **1276.6147** | **0.0917** | **0** | **10** | **16** | **2** | **R.DTAGDASESALLK.C** |
|  | 9455 | 1281.5680 | 1280.5607 | 1279.6343 | 0.9264 | 1 | 2 | 80 | 10 | R.NSVFQQGMKNK.I |
|  | 9520 | 642.8542 | 1283.6939 | 1283.7020 | -0.0080 | 0 | (19) | 1.6 | 2 | K.NMVPQQALVIR.E |
|  | 11285 | 691.3743 | 1380.7341 | 1380.7435 | -0.0094 | 0 | 66 | 3.2e-005 | 1 | K.VIMVTGDHPITAK.A 11283 11284 11286 |
|  | 11571 | 699.4022 | 1396.7898 | 1396.7384 | 0.0513 | 0 | (13) | 6.3 | 1 | K.VIMVTGDHPITAK.A |
|  | 11840 | **471.7472** | **1412.2198** | **1412.7082** | **-0.4884** | **1** | **4** | **37** | **6** | **R.EAKACVVHGSDLK.D** |
|  | 14911 | 538.2826 | 1611.8261 | 1611.8402 | -0.0141 | 1 | 4 | 49 | 8 | R.LNIPMSQVNPREAK.A |
|  | 15042 | 810.3734 | 1618.7323 | 1618.7435 | -0.0112 | 0 | 74 | 4.7e-006 | 1 | K.VDNSSLTGESEPQTR.S 15017 15040 15043 15056 |
|  | 15775 | **834.9836** | **1667.9526** | **1666.7881** | **1.1645** | **0** | **5** | **34** | **2** | **K.LCFVGLMSMIDPPR.A** |
|  | 16882 | **583.1085** | **1746.3037** | **1745.9458** | **0.3579** | **1** | **8** | **13** | **1** | **R.TVMGRIATLASGLEVGR.T** 16886 16887 |
|  | 17757 | 906.4728 | 1810.9311 | 1810.9425 | -0.0114 | 0 | 107 | 2.1e-009 | 1 | R.QGAIVAVTGDGVNDSPALK.K 17756 17761 |
|  | 18011 | 915.4595 | 1828.9045 | 1828.9167 | -0.0121 | 0 | 103 | 4.8e-009 | 1 | K.GVGIISEGNETVEDIAAR.L 18009 18010 18012 |
|  | 18129 | 919.4411 | 1836.8676 | 1837.0132 | -0.1455 | 1 | 3 | 55 | 8 | R.SAGIKVIMVTGDHPITAK.A |
|  | 18830 | **630.3144** | **1887.9213** | **1886.9659** | **0.9554** | **0** | **3** | **49** | **2** | **K.MQINAEEVVVGDLVEVK.G** |
|  | 19289 | 962.9550 | 1923.8954 | 1923.9095 | -0.0142 | 0 | 59 | 0.00011 | 1 | K.NLEAVETLGSTSTICSDK.T 19290 |
|  | 19468 | 970.5204 | 1939.0262 | 1939.0375 | -0.0112 | 1 | 50 | 0.001 | 1 | R.QGAIVAVTGDGVNDSPALKK.A 19467 |
|  | 22821 | 758.7399 | 2273.1978 | 2272.1369 | 1.0609 | 1 | 1 | 65 | 9 | K.MQINAEEVVVGDLVEVKGGDR.V |
|  | 24267 | 1232.6003 | 2463.1861 | 2463.1951 | -0.0090 | 0 | 129 | 9.1e-012 | 1 | K.QAADMILLDDNFASIVTGVEEGR.L 24262 24264 24266 24279 24283 |
|  | 26342 | 932.7148 | 2795.1225 | 2795.3607 | -0.2382 | 1 | 3 | 23 | 2 | K.NLEAVETLGSTSTICSDKTGTLTQNR.M |
|  | 29452 | **1197.2338** | **3588.6795** | **3587.6847** | **0.9948** | **1** | **1** | **30** | **5** | **K.EMQDAFQNAYMELGGLGERVLGFCQLNLPSGK.F** |

| **34.** | O43451\|MGA_HUMAN    **Mass:** 211031   **Score:** 823    **Queries matched:** 67   **emPAI:** 0.34 |
| --- | --- |
|  | Maltase-glucoamylase, intestinal OS=Homo sapiens GN=MGAM PE=1 SV=5 |

|  | **Query** | **Observed** | **Mr(expt)** | **Mr(calc)** | **Delta** | **Miss** | **Score** | **Expect** | **Rank** | **Peptide** |
| --- | --- | --- | --- | --- | --- | --- | --- | --- | --- | --- |
|  | 1489 | **644.4048** | **643.3975** | **643.4017** | **-0.0042** | **0** | **12** | **15** | **3** | **R.GVVITR.S** |
|  | 2217 | 804.3828 | 803.3755 | 803.3814 | -0.0058 | 0 | 24 | 0.9 | 1 | R.DQPPGYK.K |
|  | 2305 | **405.2257** | **808.4369** | **807.3731** | **1.0638** | **1** | **8** | **25** | **6** | **R.MKADGMR.V** |
|  | 2396 | **408.7604** | **815.5063** | **814.4449** | **1.0614** | **0** | **10** | **22** | **3** | **R.HYLNIR.Y** |
|  | 3331 | **438.2315** | **874.4484** | **874.4549** | **-0.0065** | **0** | **13** | **10** | **4** | **R.LDVTYHK.N** |
|  | 3727 | **451.2683** | **900.5219** | **900.5392** | **-0.0173** | **1** | **8** | **29** | **2** | **R.ISTRLPSK.Y** |
|  | 3850 | 455.7212 | 909.4279 | 908.4426 | 0.9853 | 0 | 3 | 68 | 9 | K.NEMLQFK.I |
|  | 3875 | 456.7400 | 911.4654 | 911.4573 | 0.0081 | 0 | 8 | 24 | 1 | R.NHNTIGTR.R |
|  | 5341 | **509.7431** | **1017.4716** | **1016.4848** | **0.9868** | **0** | **15** | **5.7** | **4** | **K.VEMELPGDK.I** |
|  | 5379 | **511.2605** | **1020.5065** | **1020.5128** | **-0.0063** | **0** | **17** | **3.8** | **1** | **R.GVEDDVFIK.Y** |
|  | 5732 | **1048.5601** | **1047.5528** | **1047.5601** | **-0.0073** | **0** | **38** | **0.027** | **1** | **R.QLDFTLSPK.F** 5733 |
|  | 6137 | **538.7524** | **1075.4902** | **1074.6186** | **0.8716** | **1** | **6** | **42** | **7** | **R.QPFSIKVTR.R** |
|  | 8523 | **616.8061** | **1231.5976** | **1231.6026** | **-0.0050** | **0** | **25** | **0.49** | **1** | **R.AYVAFPDFFR.N** 8538 |
|  | 9064 | 421.0563 | 1260.1472 | 1260.5445 | -0.3973 | 0 | 3 | 47 | 3 | R.YEYGTLDNMR.E 9059 9072 |
|  | 9475 | **641.8224** | **1281.6302** | **1281.6387** | **-0.0085** | **0** | **11** | **11** | **2** | **R.INCIPDQPPTK.A** |
|  | 9943 | **436.5424** | **1306.6053** | **1307.6734** | **-1.0681** | **1** | **9** | **16** | **3** | **R.NARNVTAYFPR.A** |
|  | 11083 | **686.3155** | **1370.6164** | **1370.6255** | **-0.0091** | **0** | **40** | **0.015** | **1** | **K.YLYGFGETEHR.S** |
|  | 11252 | **460.7375** | **1379.1907** | **1379.7384** | **-0.5476** | **1** | **2** | **60** | **8** | **K.FAGFPALINRMK.A** |
|  | 11469 | **696.8208** | **1391.6270** | **1391.6358** | **-0.0087** | **1** | **8** | **24** | **1** | **R.RDFTYDSVDFK.G** |
|  | 12652 | **731.8789** | **1461.7431** | **1462.7892** | **-1.0461** | **1** | **1** | **1.1e+002** | **6** | **R.GVVITRSTFPSSGR.W** |
|  | 12796 | **736.4299** | **1470.8453** | **1470.7831** | **0.0622** | **1** | **3** | **69** | **2** | **R.SFILTRSTFAGSGK.F** 12782 |
|  | 13299 | 751.8618 | 1501.7091 | 1501.7176 | -0.0085 | 0 | 40 | 0.014 | 1 | R.WMQLGAFYPFSR.N |
|  | 14205 | **522.2354** | **1563.6844** | **1563.6929** | **-0.0085** | **0** | **14** | **5** | **2** | **R.DLEWHTWGMFSR.D** |
|  | 14662 | 532.6356 | 1594.8849 | 1594.8943 | -0.0095 | 0 | 4 | 42 | 2 | K.TLPAPLDHINLHVR.G |
|  | 15303 | **818.3704** | **1634.7262** | **1634.7365** | **-0.0103** | **0** | **22** | **0.78** | **1** | **R.WYDYYTGVDINAR.G** |
|  | 15662 | 830.4462 | 1658.8779 | 1657.8187 | 1.0592 | 1 | 7 | 24 | 2 | R.RWMQLGAFYPFSR.N |
|  | 16508 | **860.4434** | **1718.8723** | **1719.7940** | **-0.9217** | **1** | **5** | **40** | **3** | **R.RDLEWHTWGMFSR.D** |
|  | 16844 | **872.9049** | **1743.7953** | **1743.8063** | **-0.0111** | **0** | **60** | **0.00012** | **1** | **R.EIEELYNNPQNPER.S** 16842 |
|  | 17845 | **910.4647** | **1818.9149** | **1818.9264** | **-0.0115** | **0** | **77** | **2.5e-006** | **1** | **K.SSVYANAFPSTPVNPLR.L** 17846 |
|  | 18534 | **622.2957** | **1863.8653** | **1862.8733** | **0.9920** | **0** | **23** | **0.56** | **1** | **R.DASLNHPPYMPHLESR.D** 18530 18531 |
|  | 20612 | **1022.9940** | **2043.9734** | **2043.9862** | **-0.0128** | **0** | **80** | **9.7e-007** | **1** | **K.HNGVPSQTSPTVTYDSNLK.V** 20611 |
|  | 20686 | **1026.5299** | **2051.0453** | **2051.0588** | **-0.0136** | **0** | **63** | **4.5e-005** | **1** | **R.GGYILPWQEPALNTHLSR.Q** 20684 20685 20687 20693 |
|  | 22612 | **750.6712** | **2248.9918** | **2249.0059** | **-0.0141** | **0** | **28** | **0.13** | **1** | **R.AAQLPYDVQHADIDYMDER.R** |
|  | 22756 | **1133.5478** | **2265.0811** | **2265.0954** | **-0.0142** | **1** | **49** | **0.0009** | **1** | **R.GVEDDVFIKYPNDGDIVWGK.V** 22757 22758 |
|  | 23396 | **1175.0391** | **2348.0636** | **2348.0783** | **-0.0148** | **0** | **95** | **2.2e-008** | **1** | **K.VMAYVPDAVWYDYETGSQVR.W** 23395 |
|  | 24295 | **1234.1610** | **2466.3075** | **2466.3159** | **-0.0084** | **0** | **93** | **3.1e-008** | **1** | **R.VLFDSSIGPLLFADQFLQLSTR.L** |
|  | 24795 | **1268.6466** | **2535.2787** | **2534.2516** | **1.0271** | **0** | **4** | **26** | **2** | **K.STGTIIWDSQLLGFTFSDMFIR.I** |
|  | 25604 | **1337.0717** | **2672.1288** | **2672.1458** | **-0.0170** | **0** | **95** | **1.7e-008** | **1** | **K.FDGMWIDMNEPSSFVNGAVSPGCR.D** 25605 25612 25613 25614 |
|  | 26448 | **1411.6935** | **2821.3724** | **2821.3882** | **-0.0158** | **0** | **76** | **1.6e-006** | **1** | **K.NLPSSPVFGSNVDNVLLTAEYQTSNR.F** |
|  | 27252 | **1493.7694** | **2985.5243** | **2985.5409** | **-0.0166** | **0** | **42** | **0.0039** | **1** | **R.YEVPVPLNIPSMPSSTPEGQLYDVLIK.K** 27253 |
|  | 28471 | **1086.2301** | **3255.6685** | **3255.6849** | **-0.0164** | **1** | **33** | **0.022** | **1** | **K.NRYEVPVPLNIPSMPSSTPEGQLYDVLIK.K** |
|  | 31213 | **1205.1329** | **4816.5026** | **4816.5229** | **-0.0203** | **0** | **18** | **0.23** | **1** | **K.AHTEGVTVVRPLLHEFVSDQVTWDIDSQFLLGPAFLVSPVLER.N** 31214 |
|  | 31486 | **1284.1146** | **5132.4294** | **5132.4509** | **-0.0215** | **0** | **3** | **6.3** | **1** | **K.TLCMESQQILPDGSLVQHYNVHNLYGWSQTRPTYEAVQEVTGQR.G** |
|  | 31491 | **1287.8497** | **5147.3698** | **5148.4458** | **-1.0760** | **0** | **(3)** | **6.1** | **1** | **K.TLCMESQQILPDGSLVQHYNVHNLYGWSQTRPTYEAVQEVTGQR.G** |

| **35.** | P08473\|NEP_HUMAN    **Mass:** 86144    **Score:** 797    **Queries matched:** 59   **emPAI:** 1.52 |
| --- | --- |
|  | Neprilysin OS=Homo sapiens GN=MME PE=1 SV=2 |

|  | **Query** | **Observed** | **Mr(expt)** | **Mr(calc)** | **Delta** | **Miss** | **Score** | **Expect** | **Rank** | **Peptide** |
| --- | --- | --- | --- | --- | --- | --- | --- | --- | --- | --- |
|  | 863 | **514.8105** | **513.8032** | **514.3479** | **-0.5446** | **0** | **14** | **4.7** | **4** | **K.ALAIK.E** |
|  | 1759 | **713.4145** | **712.4072** | **712.4119** | **-0.0047** | **0** | **12** | **10** | **5** | **R.GGEPLLK.L** |
|  | 2806 | **845.4424** | **844.4352** | **843.4814** | **0.9538** | **0** | **29** | **0.26** | **1** | **K.AIAQLNSK.Y** 2808 |
|  | 3666 | **899.4563** | **898.4490** | **898.4548** | **-0.0058** | **0** | **24** | **0.64** | **1** | **R.AYQNYIK.K** |
|  | 5047 | **499.2264** | **996.4383** | **996.5029** | **-0.0645** | **0** | **3** | **69** | **9** | **R.YGNFDILR.D** |
|  | 5160 | **502.2961** | **1002.5777** | **1001.5142** | **1.0635** | **0** | **6** | **42** | **10** | **R.NVIPETSSR.Y** 5136 |
|  | 5263 | **506.7369** | **1011.4592** | **1011.4661** | **-0.0069** | **0** | **43** | **0.0082** | **1** | **K.YGASWTAEK.A** |
|  | 6554 | **554.2758** | **1106.5370** | **1106.5430** | **-0.0060** | **0** | **47** | **0.0033** | **1** | **R.NDPMLLYNK.M** |
|  | 6588 | 555.7620 | 1109.5094 | 1109.5175 | -0.0081 | 1 | 29 | 0.17 | 1 | K.NSYMNPEKK.C |
|  | 6589 | 555.7622 | 1109.5099 | 1109.5175 | -0.0076 | 1 | 37 | 0.028 | 1 | R.KNSYMNPEK.K |
|  | 6831 | **565.2689** | **1128.5232** | **1128.5312** | **-0.0081** | **0** | **22** | **1.2** | **1** | **K.TDVHSPGNFR.I** |
|  | 7252 | **581.7840** | **1161.5534** | **1161.5601** | **-0.0066** | **0** | **38** | **0.024** | **1** | **R.DLQNLMSWR.F** 7253 |
|  | 9198 | **634.3364** | **1266.6582** | **1266.6642** | **-0.0060** | **0** | **62** | **8.6e-005** | **1** | **R.FIMDLVSSLSR.T** 9193 9197 9199 |
|  | 9528 | **429.0878** | **1284.2416** | **1283.6397** | **0.6018** | **0** | **6** | **21** | **8** | **R.LYVEAAFAGESK.H** |
|  | 9679 | **646.8657** | **1291.7169** | **1291.7248** | **-0.0079** | **0** | **66** | **3.9e-005** | **1** | **K.HVVEDLIAQIR.E** 9678 |
|  | 10012 | **656.2776** | **1310.5407** | **1310.5489** | **-0.0081** | **0** | **13** | **6.4** | **1** | **R.DYYECTGIYK.E** |
|  | 12435 | **725.3413** | **1448.6681** | **1448.6783** | **-0.0103** | **0** | **68** | **1.9e-005** | **1** | **R.IGYPDDIVSNDNK.L** 12428 12458 |
|  | 12534 | 728.8525 | 1455.6904 | 1455.6994 | -0.0090 | 0 | 61 | 9.5e-005 | 1 | K.ALYGTTSETATWR.R 12535 |
|  | 12665 | **488.3164** | **1461.9273** | **1460.8239** | **1.1034** | **1** | **5** | **36** | **5** | **K.KVLINLFVGTDDK.N** |
|  | 15608 | **828.4060** | **1654.7975** | **1654.7885** | **0.0089** | **1** | **1** | **1e+002** | **5** | **K.YSARDLQNLMSWR.F** |
|  | 16968 | **877.4359** | **1752.8573** | **1752.9509** | **-0.0936** | **1** | **6** | **25** | **2** | **R.DELEVVLKDVLQEPK.T** 16971 |
|  | 17639 | **902.4372** | **1802.8598** | **1802.8720** | **-0.0122** | **0** | **28** | **0.19** | **1** | **K.SESQMDITDINTPKPK.K** |
|  | 19277 | **962.0109** | **1922.0072** | **1922.0149** | **-0.0077** | **1** | **66** | **2.6e-005** | **1** | **R.YGNFDILRDELEVVLK.D** |
|  | 19992 | **498.0008** | **1987.9741** | **1987.9884** | **-0.0144** | **1** | **28** | **0.13** | **1** | **M.GKSESQMDITDINTPKPK.K** 19977 19993 |
|  | 20075 | **998.9504** | **1995.8863** | **1995.8963** | **-0.0100** | **0** | **75** | **3.1e-006** | **1** | **K.DGDLVDWWTQQSASNFK.E** |
|  | 20438 | **1015.9572** | **2029.8999** | **2029.9125** | **-0.0127** | **0** | **73** | **4.4e-006** | **1** | **R.LIQNMDATTEPCTDFFK.Y** 20437 20439 |
|  | 20951 | **1040.4929** | **2078.9713** | **2078.9844** | **-0.0131** | **0** | **98** | **1.3e-008** | **1** | **R.IIGTLQNSAEFSEAFHCR.K** 20950 20952 |
|  | 21798 | **1078.0115** | **2154.0084** | **2154.0191** | **-0.0107** | **0** | **16** | **2.1** | **1** | **R.EVFIQTLDDLTWMDAETK.K** |
|  | 21847 | **1080.0377** | **2158.0609** | **2158.0735** | **-0.0126** | **0** | **51** | **0.00064** | **1** | **K.LLPDIYGWPVATENWEQK.Y** 21846 21848 |
|  | 22915 | **761.7084** | **2282.1035** | **2282.1140** | **-0.0106** | **1** | **13** | **3.6** | **1** | **R.EVFIQTLDDLTWMDAETKK.R** 22916 |
|  | 23775 | **601.5719** | **2402.2585** | **2402.1899** | **0.0686** | **1** | **7** | **15** | **2** | **K.EIANATAKPEDRNDPMLLYNK.M** |
|  | 24557 | **834.0512** | **2499.1318** | **2499.1455** | **-0.0137** | **1** | **46** | **0.0016** | **1** | **R.NFNKDGDLVDWWTQQSASNFK.E** |
|  | 27596 | **1016.8267** | **3047.4582** | **3047.4763** | **-0.0182** | **1** | **45** | **0.0015** | **1** | **K.LNNEYLELNYKEDEYFENIIQNLK.F** |
|  | 27607 | **1017.8411** | **3050.5016** | **3050.5113** | **-0.0097** | **0** | **48** | **0.00082** | **1** | **K.QLFFLNFAQVWCGTYRPEYAVNSIK.T** |
|  | 31227 | **1211.5927** | **4842.3415** | **4842.3613** | **-0.0198** | **0** | **30** | **0.02** | **1** | **R.NQIVFPAGILQPPFFSAQQSNSLNYGGIGMVIGHEITHGFDDNGR.N** 31231 31232 31233 |
|  | 31956 | **1496.3658** | **5981.4343** | **5980.9431** | **0.4912** | **0** | **4** | **2.2** | **1** | **K.MTLAQIQNNFSLEINGKPFSWLNFTNEIMSTVNISITNEEDVVVYAPEYLTK.L** |

| **36.** | P27797\|CALR_HUMAN    **Mass:** 48283    **Score:** 793    **Queries matched:** 58   **emPAI:** 2.30 |
| --- | --- |
|  | Calreticulin OS=Homo sapiens GN=CALR PE=1 SV=1 |

|  | **Query** | **Observed** | **Mr(expt)** | **Mr(calc)** | **Delta** | **Miss** | **Score** | **Expect** | **Rank** | **Peptide** |
| --- | --- | --- | --- | --- | --- | --- | --- | --- | --- | --- |
|  | 1072 | **554.2762** | **553.2689** | **552.2544** | **1.0145** | **0** | **11** | **11** | **1** | **K.SDFGK.F** 1068 |
|  | 4747 | **488.2429** | **974.4713** | **974.4781** | **-0.0068** | **0** | **26** | **0.52** | **1** | **K.GLQTSQDAR.F** 4744 4748 4749 4750 4751 4764 |
|  | 5361 | **510.2838** | **1018.5529** | **1018.5600** | **-0.0070** | **0** | **14** | **7.7** | **2** | **K.VHVIFNYK.G** |
|  | 6845 | **565.7495** | **1129.4845** | **1129.4928** | **-0.0083** | **1** | **36** | **0.045** | **1** | **K.FYGDEEKDK.G** 6853 |
|  | 11793 | **705.8126** | **1409.6107** | **1409.6212** | **-0.0105** | **0** | **5** | **41** | **1** | **K.EQFLDGDGWTSR.W** |
|  | 12862 | **492.8860** | **1475.6362** | **1475.6463** | **-0.0101** | **0** | **21** | **1.1** | **1** | **K.HEQNIDCGGGYVK.L** 12863 |
|  | 14833 | **804.3860** | **1606.7574** | **1606.7667** | **-0.0093** | **0** | **63** | **5.4e-005** | **1** | **R.FYALSASFEPFSNK.G** 14834 |
|  | 17600 | **900.9181** | **1799.8217** | **1799.8326** | **-0.0108** | **1** | **46** | **0.0024** | **1** | **K.IKDPDASKPEDWDER.A** 17598 17599 |
|  | 24691 | **1260.0927** | **2518.1707** | **2518.1864** | **-0.0156** | **1** | **73** | **3.6e-006** | **1** | **K.IDNSQVESGSLEDDWDFLPPKK.I** 24690 24692 24693 24694 24695 |
|  | 26179 | **920.7529** | **2759.2368** | **2759.2562** | **-0.0194** | **0** | **24** | **0.2** | **1** | **K.IDDPTDSKPEDWDKPEHIPDPDAK.K** 26180 |
|  | 26593 | 714.8475 | 2855.3610 | 2855.3800 | -0.0190 | 1 | 34 | 0.02 | 1 | R.CKDDEFTHLYTLIVRPDNTYEVK.I 26595 |
|  | 27160 | **987.1310** | **2958.3713** | **2958.3883** | **-0.0170** | **1** | **39** | **0.0078** | **1** | **R.AKIDDPTDSKPEDWDKPEHIPDPDAK.K** 27158 27159 |
|  | 27163 | **1480.6405** | **2959.2664** | **2959.2858** | **-0.0193** | **0** | **58** | **7.2e-005** | **1** | **K.KPEDWDEEMDGEWEPPVIQNPEYK.G** 27162 27164 27165 |
|  | 28501 | **1634.7433** | **3267.4720** | **3267.4884** | **-0.0164** | **0** | **46** | **0.001** | **1** | **K.SGTIFDNFLITNDEAYAEEFGNETWGVTK.A** 28503 28504 28505 |
|  | 29072 | **1152.8373** | **3455.4900** | **3455.5108** | **-0.0208** | **0** | **50** | **0.00034** | **1** | **K.LFPNSLDQTDMHGDSEYNIMFGPDICGPGTK.K** 29069 29070 29071 29073 |
|  | 30616 | **1383.6648** | **4147.9725** | **4147.9844** | **-0.0118** | **0** | **68** | **4.3e-006** | **1** | **K.GTWIHPEIDNPEYSPDPSIYAYDNFGVLGLDLWQVK.S** 30601 30603 30605 30606 30607 30608 30611 30612 30614 30617 30618 |

| **37.** | O60716\|CTND1_HUMAN    **Mass:** 108674   **Score:** 787    **Queries matched:** 46   **emPAI:** 0.89 |
| --- | --- |
|  | Catenin delta-1 OS=Homo sapiens GN=CTNND1 PE=1 SV=1 |

|  | **Query** | **Observed** | **Mr(expt)** | **Mr(calc)** | **Delta** | **Miss** | **Score** | **Expect** | **Rank** | **Peptide** |
| --- | --- | --- | --- | --- | --- | --- | --- | --- | --- | --- |
|  | 2736 | **420.7604** | **839.5063** | **838.3610** | **1.1453** | **0** | **10** | **14** | **2** | **K.DEWFSR.G** 2717 |
|  | 2883 | **849.5381** | **848.5308** | **848.5371** | **-0.0063** | **0** | **11** | **16** | **2** | **R.IYISLLK.E** 2884 |
|  | 4787 | **489.2712** | **976.5279** | **976.4879** | **0.0400** | **0** | **13** | **9** | **2** | **K.GGPPPPNWR.Q** |
|  | 5339 | **509.7249** | **1017.4352** | **1017.5455** | **-0.1102** | **0** | **1** | **1.5e+002** | **4** | **R.GSLASLDSLR.K** |
|  | 5377 | **511.2519** | **1020.4893** | **1020.4876** | **0.0017** | **0** | **5** | **53** | **1** | **R.FVGDADLER.Q** |
|  | 6096 | **537.2975** | **1072.5804** | **1072.5876** | **-0.0072** | **1** | **16** | **4.8** | **1** | **K.LRETQGIEK.L** |
|  | 9479 | **428.2336** | **1281.6791** | **1281.6863** | **-0.0072** | **1** | **13** | **6.2** | **1** | **K.KEVHLGACGALK.N** |
|  | 9542 | **429.2127** | **1284.6163** | **1285.6415** | **-1.0252** | **0** | **10** | **15** | **3** | **R.QDVYGPQPQVR.V** |
|  | 11764 | **705.3128** | **1408.6111** | **1408.6219** | **-0.0108** | **0** | **35** | **0.036** | **1** | **K.SLDNNYSTPNER.G** 11765 |
|  | 12444 | **725.3863** | **1448.7580** | **1448.7664** | **-0.0083** | **0** | **49** | **0.0015** | **1** | **R.GYELLFQPEVVR.I** |
|  | 14244 | **783.8718** | **1565.7290** | **1565.7409** | **-0.0119** | **0** | **50** | **0.0013** | **1** | **K.SNAAAYLQHLCYR.N** 14245 |
|  | 14703 | **533.6304** | **1597.8693** | **1597.9919** | **-0.1227** | **1** | **1** | **89** | **10** | **K.LKGIPVLVGLLDHPK.K** |
|  | 14754 | **534.9075** | **1601.7006** | **1601.7110** | **-0.0104** | **0** | **34** | **0.049** | **1** | **R.FHPEPYGLEDDQR.S** 14753 |
|  | 15571 | **826.9282** | **1651.8419** | **1651.8529** | **-0.0110** | **0** | **63** | **6.1e-005** | **1** | **K.ALSAIADLLTNEHER.V** 15548 15572 |
|  | 16173 | **848.8961** | **1695.7776** | **1696.8645** | **-1.0869** | **1** | **2** | **65** | **8** | **R.APSRQDVYGPQPQVR.V** |
|  | 18617 | **624.6529** | **1870.9368** | **1869.9472** | **0.9896** | **1** | **38** | **0.014** | **1** | **R.GKKPIEDPANDTVDFPK.R** |
|  | 18786 | **943.4592** | **1884.9039** | **1884.9152** | **-0.0113** | **0** | **92** | **6.4e-008** | **1** | **R.HIEWESVLTNTAGCLR.N** 18787 |
|  | 19247 | **960.9437** | **1919.8728** | **1919.8861** | **-0.0133** | **0** | **89** | **1.2e-007** | **1** | **R.SQSSHSYDDSTLPLIDR.N** |
|  | 19653 | **652.8635** | **1955.5686** | **1955.9847** | **-0.4161** | **1** | **4** | **27** | **3** | **R.VSPQDANPLMANGTLTRR.H** |
|  | 19844 | **987.4104** | **1972.8062** | **1972.8187** | **-0.0125** | **0** | **64** | **3.7e-005** | **1** | **R.HYEDGYPGGSDNYGSLSR.V** 19857 |
|  | 20540 | **1020.4489** | **2038.8833** | **2039.0582** | **-0.1749** | **1** | **1** | **74** | **7** | **R.VRVSPQDANPLMANGTLTR.R** |
|  | 21683 | **1074.4142** | **2146.8138** | **2146.8282** | **-0.0144** | **0** | **99** | **8.8e-009** | **1** | **R.SMGYDDLDYGMMSDYGTAR.R** 21682 |
|  | 22688 | 753.0577 | 2256.1514 | 2256.1532 | -0.0018 | 1 | 2 | 52 | 7 | K.ESKTPAILEASAGAIQNLCAGR.W |
|  | 23596 | **1188.0370** | **2374.0594** | **2374.0760** | **-0.0166** | **0** | **96** | **1.6e-008** | **1** | **R.YQEAAPNVANNTGPHAASCFGAK.K** 23597 23603 |
|  | 24349 | **1238.1013** | **2474.1881** | **2474.1999** | **-0.0118** | **0** | **87** | **1.3e-007** | **1** | **R.DMDLTEVITGTLWNLSSHDSIK.M** 24350 24352 |
|  | 26208 | **692.8477** | **2767.3618** | **2767.3752** | **-0.0134** | **0** | **9** | **8.6** | **1** | **K.MEIVDHALHALTDEVIIPHSGWER.E** |
|  | 26216 | **924.1555** | **2769.4446** | **2770.2854** | **-0.8409** | **1** | **2** | **41** | **5** | **-.MDDSEVESTASILASVKEQEAQFEK.L** |
|  | 30391 | **1339.3292** | **4014.9658** | **4014.9810** | **-0.0152** | **0** | **26** | **0.069** | **1** | **K.NLPGGQQNSSWNFSEDTVISILNTINEVIAENLEAAK.K** |
|  | 30482 | **1361.8782** | **4082.6127** | **4081.8508** | **0.7619** | **0** | **1** | **16** | **4** | **K.GTTPLMQDEGQESLEEELDVLVLDDEGGQVSYPSMQK.I** |
|  | 30596 | **1382.0269** | **4143.0588** | **4143.0760** | **-0.0172** | **1** | **86** | **6e-008** | **1** | **K.NLPGGQQNSSWNFSEDTVISILNTINEVIAENLEAAKK.L** |
|  | 30669 | **1399.0392** | **4194.0957** | **4194.9348** | **-0.8391** | **1** | **6** | **5.5** | **2** | **K.GTTPLMQDEGQESLEEELDVLVLDDEGGQVSYPSMQKI.-** |
|  | 31333 | **1222.6190** | **4886.4470** | **4887.5042** | **-1.0573** | **1** | **2** | **10** | **2** | **K.HAIPNLVKNLPGGQQNSSWNFSEDTVISILNTINEVIAENLEAAK.K** |

| **38.** | P10809\|CH60_HUMAN    **Mass:** 61187    **Score:** 769    **Queries matched:** 58   **emPAI:** 1.57 |
| --- | --- |
|  | 60 kDa heat shock protein, mitochondrial OS=Homo sapiens GN=HSPD1 PE=1 SV=2 |

|  | **Query** | **Observed** | **Mr(expt)** | **Mr(calc)** | **Delta** | **Miss** | **Score** | **Expect** | **Rank** | **Peptide** |
| --- | --- | --- | --- | --- | --- | --- | --- | --- | --- | --- |
|  | 793 | **502.2947** | **501.2875** | **501.2911** | **-0.0036** | **0** | **15** | **13** | **8** | **K.NIGAK.L** 788 |
|  | 1375 | **616.3982** | **615.3909** | **615.3956** | **-0.0047** | **0** | **12** | **19** | **1** | **K.GVITVK.D** |
|  | 1671 | **689.3779** | **688.3706** | **688.3756** | **-0.0050** | **0** | **12** | **16** | **8** | **K.DGVTVAK.S** |
|  | 1983 | **785.5083** | **784.5010** | **784.5058** | **-0.0048** | **0** | **14** | **7.4** | **8** | **K.IGIEIIK.R** |
|  | 2803 | **844.7811** | **843.7739** | **843.4888** | **0.2851** | **0** | **5** | **46** | **2** | **K.IPAMTIAK.N** 2800 |
|  | 2951 | **427.7428** | **853.4711** | **854.4610** | **-0.9899** | **0** | **15** | **4.5** | **4** | **K.GANPVEIR.R** |
|  | 3727 | 451.2683 | 900.5219 | 900.5280 | -0.0061 | 0 | 23 | 1 | 1 | K.LSDGVAVLK.V 3704 |
|  | 3883 | **457.2162** | **912.4178** | **911.5804** | **0.8374** | **0** | **14** | **6.4** | **1** | **K.VGLQVVAVK.A** |
|  | 4551 | **480.7557** | **959.4969** | **959.5036** | **-0.0067** | **0** | **56** | **0.00051** | **1** | **R.VTDALNATR.A** 4536 |
|  | 4822 | **490.3407** | **978.6669** | **977.4930** | **1.1739** | **1** | **8** | **23** | **9** | **K.DVKFGADAR.A** |
|  | 5316 | **508.8258** | **1015.6371** | **1016.5866** | **-0.9495** | **1** | **2** | **1.2e+002** | **4** | **K.VTKDGVTVAK.S** |
|  | 7682 | **595.8043** | **1189.5941** | **1189.6012** | **-0.0072** | **0** | **11** | **12** | **1** | **K.EIGNIISDAMK.K** |
|  | 8545 | **617.2971** | **1232.5796** | **1232.5885** | **-0.0089** | **0** | **58** | **0.00025** | **1** | **K.VGGTSDVEVNEK.K** |
|  | 10171 | **440.5568** | **1318.6486** | **1317.6962** | **0.9524** | **1** | **7** | **31** | **2** | **K.EIGNIISDAMKK.V** |
|  | 10548 | **670.3294** | **1338.6443** | **1338.7772** | **-0.1329** | **1** | **4** | **56** | **6** | **R.VLAPHLTRAYAK.D** |
|  | 10647 | **672.8568** | **1343.6991** | **1343.7085** | **-0.0094** | **0** | **62** | **9.4e-005** | **1** | **R.TVIIEQSWGSPK.V** |
|  | 13324 | **752.8779** | **1503.7412** | **1503.7490** | **-0.0078** | **0** | **64** | **4.6e-005** | **1** | **K.TLNDELEIIEGMK.F** 13323 |
|  | 14105 | **778.9535** | **1555.8925** | **1555.9007** | **-0.0082** | **1** | **48** | **0.0018** | **1** | **R.GVMLAVDAVIAELKK.Q** |
|  | 14725 | **534.2584** | **1599.7534** | **1599.9018** | **-0.1484** | **1** | **1** | **98** | **6** | **R.RGVMLAVDAVIAELK.K** |
|  | 15218 | **815.8736** | **1629.7326** | **1629.9011** | **-0.1685** | **1** | **33** | **0.059** | **2** | **K.VGEVIVTKDDAMLLK.G** 15233 |
|  | 16023 | **842.9512** | **1683.8879** | **1683.8978** | **-0.0099** | **0** | **55** | **0.00037** | **1** | **R.AAVEEGIVLGGGCALLR.C** |
|  | 16106 | **564.3503** | **1690.0292** | **1689.6078** | **0.4214** | **0** | **(7)** | **20** | **2** | **K.DPGMGAMGGMGGGMGGGMF.-** |
|  | 16313 | **569.2550** | **1704.7432** | **1705.6027** | **-0.8596** | **0** | **36** | **0.029** | **1** | **K.DPGMGAMGGMGGGMGGGMF.-** |
|  | 16315 | **569.2681** | **1704.7826** | **1705.6027** | **-0.8202** | **0** | **(2)** | **68** | **4** | **K.DPGMGAMGGMGGGMGGGMF.-** |
|  | 16333 | **569.7653** | **1706.2740** | **1705.6027** | **0.6712** | **0** | **(3)** | **40** | **7** | **K.DPGMGAMGGMGGGMGGGMF.-** 16332 |
|  | 16535 | **574.5780** | **1720.7122** | **1721.5977** | **-0.8855** | **0** | **(4)** | **47** | **1** | **K.DPGMGAMGGMGGGMGGGMF.-** |
|  | 16542 | **574.6338** | **1720.8795** | **1721.5977** | **-0.7181** | **0** | **(2)** | **79** | **8** | **K.DPGMGAMGGMGGGMGGGMF.-** |
|  | 16552 | **861.9291** | **1721.8437** | **1721.5977** | **0.2461** | **0** | **(2)** | **64** | **3** | **K.DPGMGAMGGMGGGMGGGMF.-** |
|  | 20643 | 683.0557 | 2046.1452 | 2046.1585 | -0.0134 | 1 | 65 | 2.7e-005 | 1 | K.KISSIQSIVPALEIANAHR.K |
|  | 20927 | **692.7316** | **2075.1729** | **2075.7879** | **-0.6151** | **1** | **(1)** | **67** | **8** | **K.EEKDPGMGAMGGMGGGMGGGMF.-** |
|  | 21075 | **697.9916** | **2090.9531** | **2091.7829** | **-0.8298** | **1** | **(5)** | **27** | **1** | **K.EEKDPGMGAMGGMGGGMGGGMF.-** |
|  | 21083 | **698.3362** | **2091.9869** | **2091.7829** | **0.2040** | **1** | **10** | **9.7** | **3** | **K.EEKDPGMGAMGGMGGGMGGGMF.-** 21072 21086 21123 |
|  | 21101 | **698.3513** | **2092.0320** | **2091.7829** | **0.2491** | **1** | **(3)** | **47** | **2** | **K.EEKDPGMGAMGGMGGGMGGGMF.-** |
|  | 21127 | **1047.4625** | **2092.9105** | **2091.7829** | **1.1276** | **1** | **(2)** | **53** | **6** | **K.EEKDPGMGAMGGMGGGMGGGMF.-** 21128 |
|  | 21285 | **703.3289** | **2106.9649** | **2107.7778** | **-0.8129** | **1** | **(5)** | **26** | **5** | **K.EEKDPGMGAMGGMGGGMGGGMF.-** |
|  | 21299 | **703.5721** | **2107.6946** | **2107.7778** | **-0.0832** | **1** | **(3)** | **32** | **2** | **K.EEKDPGMGAMGGMGGGMGGGMF.-** |
|  | 21316 | **703.9848** | **2108.9326** | **2107.7778** | **1.1548** | **1** | **(4)** | **35** | **4** | **K.EEKDPGMGAMGGMGGGMGGGMF.-** |
|  | 21375 | **705.0485** | **2112.1237** | **2112.1323** | **-0.0085** | **0** | **55** | **0.00025** | **1** | **R.ALMLQGVDLLADAVAVTMGPK.G** 21374 21387 |
|  | 23018 | **765.7206** | **2294.1399** | **2294.1529** | **-0.0130** | **1** | **5** | **23** | **2** | **R.IQEIIEQLDVTTSEYEKEK.L** |
|  | 24608 | **1254.5509** | **2507.0872** | **2507.1018** | **-0.0146** | **0** | **109** | **8.7e-010** | **1** | **K.IMQSSSEVGYDAMAGDFVNMVEK.G** 24609 24610 24620 |
|  | 25016 | **1280.6195** | **2559.2245** | **2559.2413** | **-0.0168** | **0** | **152** | **4.6e-014** | **1** | **K.LVQDVANNTNEEAGDGTTTATVLAR.S** 25014 |

| **39.** | P07355\|ANXA2_HUMAN    **Mass:** 38808    **Score:** 768    **Queries matched:** 60   **emPAI:** 4.77 |
| --- | --- |
|  | Annexin A2 OS=Homo sapiens GN=ANXA2 PE=1 SV=2 |

|  | **Query** | **Observed** | **Mr(expt)** | **Mr(calc)** | **Delta** | **Miss** | **Score** | **Expect** | **Rank** | **Peptide** |
| --- | --- | --- | --- | --- | --- | --- | --- | --- | --- | --- |
|  | 1323 | **605.3397** | **604.3324** | **604.3367** | **-0.0042** | **0** | **20** | **2.3** | **1** | **R.IMVSR.S** |
|  | 1861 | **745.4591** | **744.4518** | **744.4568** | **-0.0049** | **0** | **30** | **0.27** | **1** | **K.LMVALAK.G** |
|  | 1893 | **756.3911** | **755.3838** | **755.3524** | **0.0315** | **0** | **33** | **0.087** | **1** | **R.LYDSMK.G** |
|  | 2274 | **807.3513** | **806.3440** | **807.4603** | **-1.1163** | **0** | **18** | **3** | **2** | **R.SVPHLQK.V** |
|  | 2438 | **410.6824** | **819.3502** | **820.4000** | **-1.0499** | **0** | **2** | **1.1e+002** | **9** | **R.SEVDMLK.I** |
|  | 3027 | **430.2997** | **858.5849** | **858.5174** | **0.0674** | **1** | **16** | **5.7** | **5** | **K.KELASALK.S** 3022 3029 |
|  | 3404 | **880.4352** | **879.4280** | **879.4338** | **-0.0058** | **0** | **42** | **0.0095** | **1** | **R.DLYDAGVK.R** 3417 |
|  | 4277 | **471.7094** | **941.4043** | **940.4688** | **0.9355** | **1** | **(7)** | **33** | **6** | **R.LYDSMKGK.G** |
|  | 4506 | **479.2400** | **956.4654** | **956.4637** | **0.0017** | **1** | **12** | **11** | **5** | **R.LYDSMKGK.G** 4518 |
|  | 4767 | **488.3164** | **974.6182** | **973.5742** | **1.0440** | **1** | **12** | **12** | **1** | **K.LMVALAKGR.R** |
|  | 5565 | **518.2646** | **1034.5146** | **1034.5219** | **-0.0073** | **0** | **22** | **0.94** | **1** | **K.WISIMTER.S** 5566 |
|  | 6273 | **543.7426** | **1085.4707** | **1085.4778** | **-0.0071** | **0** | **46** | **0.0045** | **1** | **K.AYTNFDAER.D** |
|  | 6330 | **545.3241** | **1088.6336** | **1089.5852** | **-0.9516** | **1** | **11** | **14** | **1** | **R.SEVDMLKIR.S** |
|  | 6409 | **1096.4915** | **1095.4842** | **1095.4907** | **-0.0065** | **0** | **41** | **0.012** | **1** | **K.ALLYLCGGDD.-** |
|  | 6486 | **551.7813** | **1101.5479** | **1101.6692** | **-0.1213** | **1** | **11** | **15** | **3** | **K.VLIRIMVSR.S** |
|  | 6598 | **556.2769** | **1110.5392** | **1110.5458** | **-0.0066** | **0** | **56** | **0.00032** | **1** | **R.QDIAFAYQR.R** 6589 |
|  | 7401 | **587.3105** | **1172.6064** | **1171.5431** | **1.0634** | **1** | **6** | **39** | **6** | **K.EMYKTDLEK.D** |
|  | 8226 | **406.2101** | **1215.6085** | **1216.5944** | **-0.9859** | **0** | **6** | **44** | **9** | **-.MSTVHEILCK.L** |
|  | 8746 | **622.2825** | **1242.5504** | **1243.6156** | **-1.0653** | **0** | **66** | **3.9e-005** | **1** | **R.TNQELQEINR.V** 8773 8797 |
|  | 10788 | **677.3307** | **1352.6469** | **1352.6572** | **-0.0103** | **1** | **27** | **0.29** | **1** | **K.DIISDTSGDFRK.L** |
|  | 11997 | **711.3453** | **1420.6761** | **1420.6874** | **-0.0113** | **0** | **37** | **0.029** | **1** | **K.SLYYYIQQDTK.G** |
|  | 12025 | **712.3339** | **1422.6532** | **1422.7211** | **-0.0679** | **1** | **11** | **9.8** | **2** | **R.IMVSRSEVDMLK.I** 12017 12020 |
|  | 12620 | **730.8359** | **1459.6572** | **1459.6653** | **-0.0081** | **0** | **49** | **0.0019** | **1** | **K.SYSPYDMLESIR.K** 12621 |
|  | 13867 | **771.9235** | **1541.8324** | **1541.8413** | **-0.0090** | **0** | **88** | **2.1e-007** | **1** | **K.GVDEVTIVNILTNR.S** 13865 |
|  | 14556 | **794.8823** | **1587.7501** | **1587.7603** | **-0.0102** | **1** | **37** | **0.023** | **1** | **K.SYSPYDMLESIRK.E** |
|  | 15281 | **545.1676** | **1632.4810** | **1631.8341** | **0.6469** | **1** | **3** | **42** | **5** | **K.GTDVPKWISIMTER.S** |
|  | 15544 | **825.9893** | **1649.9641** | **1649.9716** | **-0.0075** | **0** | **67** | **1.9e-005** | **1** | **K.SALSGHLETVILGLLK.T** 15545 15546 |
|  | 17321 | **889.9331** | **1777.8517** | **1776.8564** | **0.9953** | **0** | **58** | **0.00015** | **1** | **K.GLGTDEDSLIEIICSR.T** 17304 17305 |
|  | 17746 | **906.4306** | **1810.8466** | **1810.8585** | **-0.0118** | **1** | **57** | **0.00018** | **1** | **K.TDLEKDIISDTSGDFR.K** 17747 |
|  | 18244 | **922.9484** | **1843.8823** | **1843.8952** | **-0.0129** | **0** | **73** | **5.4e-006** | **1** | **K.LSLEGDHSTPPSAYGSVK.A** 18219 18243 18245 |
|  | 19101 | **954.9387** | **1907.8628** | **1907.8748** | **-0.0121** | **0** | **56** | **0.00023** | **1** | **R.AEDGSVIDYELIDQDAR.D** 19102 |
|  | 20821 | **1032.9879** | **2063.9613** | **2063.9760** | **-0.0147** | **1** | **77** | **1.9e-006** | **1** | **R.RAEDGSVIDYELIDQDAR.D** 20820 |
|  | 21804 | **1078.0304** | **2154.0462** | **2154.0593** | **-0.0130** | **1** | **46** | **0.0022** | **1** | **K.AYTNFDAERDALNIETAIK.T** |
|  | 26521 | **1419.7102** | **2837.4059** | **2837.4170** | **-0.0112** | **0** | **76** | **1.6e-006** | **1** | **K.GDLENAFLNLVQCIQNKPLYFADR.L** 26520 26522 26524 |
|  | 28187 | **1065.5437** | **3193.6093** | **3193.6230** | **-0.0137** | **1** | **46** | **0.0011** | **1** | **K.EVKGDLENAFLNLVQCIQNKPLYFADR.L** |

| **40.** | O43707\|ACTN4_HUMAN    **Mass:** 105245   **Score:** 768    **Queries matched:** 49   **emPAI:** 1.29 |
| --- | --- |
|  | Alpha-actinin-4 OS=Homo sapiens GN=ACTN4 PE=1 SV=2 |

|  | **Query** | **Observed** | **Mr(expt)** | **Mr(calc)** | **Delta** | **Miss** | **Score** | **Expect** | **Rank** | **Peptide** |
| --- | --- | --- | --- | --- | --- | --- | --- | --- | --- | --- |
|  | 2332 | **406.2531** | **810.4917** | **810.4963** | **-0.0047** | **0** | **27** | **0.27** | **1** | **K.VQQLVPK.R** 2331 |
|  | 2497 | **825.4404** | **824.4332** | **824.4392** | **-0.0060** | **0** | **23** | **0.64** | **1** | **R.LDHLAEK.F** |
|  | 3077 | **431.7295** | **861.4445** | **860.5344** | **0.9100** | **1** | **11** | **18** | **2** | **R.RVHKPPK.V** |
|  | 3141 | **432.7422** | **863.4698** | **863.4752** | **-0.0055** | **0** | **10** | **18** | **1** | **K.ALDFIASK.G** |
|  | 3720 | **451.2631** | **900.5116** | **899.4647** | **1.0469** | **1** | **5** | **57** | **7** | **K.EAMLKHR.D** |
|  | 7866 | 600.3265 | 1198.6385 | 1198.6234 | 0.0152 | 0 | 3 | 73 | 10 | R.DLLLDPAWEK.Q |
|  | 8963 | **419.3133** | **1254.9180** | **1255.5833** | **-0.6654** | **0** | **7** | **20** | **4** | **K.DHGGALGPEEFK.A** 8935 |
|  | 9821 | **434.2306** | **1299.6700** | **1298.6619** | **1.0082** | **0** | **7** | **25** | **5** | **R.HRPELIEYDK.L** 9823 |
|  | 9837 | **434.5367** | **1300.5884** | **1300.5982** | **-0.0098** | **0** | **33** | **0.065** | **1** | **K.HTNYTMEHIR.V** 9848 |
|  | 10289 | **663.3396** | **1324.6646** | **1324.6735** | **-0.0088** | **1** | **45** | **0.0041** | **1** | **K.RDHALLEEQSK.Q** |
|  | 10766 | **676.8121** | **1351.6096** | **1351.6190** | **-0.0094** | **0** | **45** | **0.0041** | **1** | **K.GISQEQMQEFR.A** |
|  | 11111 | **686.8945** | **1371.7745** | **1371.7795** | **-0.0050** | **0** | **10** | **13** | **1** | **K.LMLLLEVISGER.L** |
|  | 11376 | **693.8876** | **1385.7606** | **1385.7667** | **-0.0061** | **0** | **73** | **6.9e-006** | **1** | **R.VGWEQLLTTIAR.T** 11375 |
|  | 12001 | **474.7404** | **1421.1994** | **1420.6986** | **0.5008** | **0** | **2** | **56** | **9** | **K.GYEEWLLNEIR.R** |
|  | 12134 | **715.3807** | **1428.7469** | **1428.7572** | **-0.0103** | **0** | **50** | **0.0014** | **1** | **R.TINEVENQILTR.D** 12133 |
|  | 13019 | **743.3785** | **1484.7425** | **1484.7524** | **-0.0099** | **0** | **64** | **5.1e-005** | **1** | **K.NVNVQNFHISWK.D** |
|  | 13468 | **757.9012** | **1513.7878** | **1513.7988** | **-0.0110** | **0** | **49** | **0.0017** | **1** | **K.LVSIGAEEIVDGNAK.M** 13470 |
|  | 13795 | **769.3860** | **1536.7575** | **1536.7671** | **-0.0096** | **0** | **76** | **3.3e-006** | **1** | **R.FAIQDISVEETSAK.E** |
|  | 13957 | 774.8814 | 1547.7483 | 1547.7580 | -0.0097 | 1 | 43 | 0.0068 | 1 | K.HRDYETATLSDIK.A |
|  | 14276 | **784.8839** | **1567.7531** | **1567.8470** | **-0.0939** | **1** | **4** | **52** | **2** | **R.HRPELIEYDKLR.K** |
|  | 15892 | **838.4485** | **1674.8824** | **1674.8941** | **-0.0116** | **1** | **59** | **0.00014** | **1** | **K.VLAGDKNFITAEELR.R** 15893 |
|  | 17254 | **887.4627** | **1772.9107** | **1772.9210** | **-0.0102** | **0** | **72** | **7.3e-006** | **1** | **R.QFASQANVVGPWIQTK.M** 17253 |
|  | 17511 | **896.9272** | **1791.8398** | **1791.8502** | **-0.0103** | **0** | **69** | **1.5e-005** | **1** | **R.MAPYQGPDAVPGALDYK.S** |
|  | 17807 | **908.9348** | **1815.8551** | **1815.8672** | **-0.0122** | **0** | **56** | **0.00028** | **1** | **K.MLDAEDIVNTARPDEK.A** 17810 |
|  | 18076 | **611.9735** | **1832.8987** | **1831.8622** | **1.0365** | **0** | **(2)** | **71** | **3** | **K.MLDAEDIVNTARPDEK.A** |
|  | 19038 | **952.4672** | **1902.9198** | **1902.9323** | **-0.0125** | **1** | **125** | **3.3e-011** | **1** | **R.KDDPVTNLNNAFEVAEK.Y** 19039 |
|  | 19259 | **961.0023** | **1919.9900** | **1919.0000** | **0.9900** | **0** | **65** | **2.9e-005** | **1** | **K.LSGSNPYTTVTPQIINSK.W** 19243 |
|  | 19266 | **641.3120** | **1920.9140** | **1919.9225** | **0.9916** | **1** | **12** | **6.8** | **2** | **K.AGTQIENIDEDFRDGLK.L** |
|  | 19532 | 973.0245 | 1944.0344 | 1942.9670 | 1.0674 | 0 | 0 | 87 | 5 | R.ISIEMNGTLEDQLSHLK.Q |
|  | 20187 | **669.9895** | **2006.9467** | **2006.9560** | **-0.0094** | **0** | **18** | **1.3** | **1** | **K.AIMTYVSSFYHAFSGAQK.A** 20179 |
|  | 20790 | **1030.9764** | **2059.9383** | **2059.9520** | **-0.0137** | **0** | **89** | **1.1e-007** | **1** | **K.VLAVNQENEHLMEDYEK.L** 20791 |
|  | 22204 | **735.0067** | **2201.9983** | **2202.0130** | **-0.0147** | **1** | **23** | **0.39** | **1** | **R.ASFNHFDKDHGGALGPEEFK.A** 22203 |
|  | 28653 | **1108.9152** | **3323.7237** | **3323.7401** | **-0.0165** | **0** | **60** | **4e-005** | **1** | **R.SIVDYKPNLDLLEQQHQLIQEALIFDNK.H** 28654 |

| **41.** | P31040\|DHSA_HUMAN    **Mass:** 73672    **Score:** 768    **Queries matched:** 48   **emPAI:** 1.09 |
| --- | --- |
|  | Succinate dehydrogenase [ubiquinone] flavoprotein subunit, mitochondrial OS=Homo sapiens GN=SDHA PE=1 SV=2 |

|  | **Query** | **Observed** | **Mr(expt)** | **Mr(calc)** | **Delta** | **Miss** | **Score** | **Expect** | **Rank** | **Peptide** |
| --- | --- | --- | --- | --- | --- | --- | --- | --- | --- | --- |
|  | 1110 | **561.3312** | **560.3239** | **560.2918** | **0.0321** | **0** | **16** | **7** | **1** | **K.DLASR.D** |
|  | 1406 | **626.3939** | **625.3866** | **624.3092** | **1.0774** | **0** | **10** | **12** | **3** | **K.GGQAHR.C** |
|  | 1735 | **708.3878** | **707.3805** | **707.3854** | **-0.0049** | **0** | **24** | **0.73** | **1** | **K.LYGDLK.H** 1732 |
|  | 2265 | **403.8886** | **805.7626** | **806.4286** | **-0.6660** | **0** | **12** | **8.2** | **3** | **R.AFGGQSLK.F** |
|  | 4230 | 470.2382 | 938.4619 | 939.3361 | -0.8742 | 0 | 11 | 13 | 6 | R.CCCVADR.T 4256 |
|  | 4816 | **490.2967** | **978.5789** | **977.5076** | **1.0713** | **1** | **4** | **58** | **9** | **-.MSGVRGLSR.L** |
|  | 4934 | **495.2366** | **988.4586** | **988.4396** | **0.0190** | **1** | **6** | **48** | **5** | **R.EGRGCGPEK.D** |
|  | 6391 | **547.7795** | **1093.5445** | **1093.5516** | **-0.0071** | **0** | **60** | **0.00016** | **1** | **K.NTVVATGGYGR.T** |
|  | 7228 | **580.7845** | **1159.5544** | **1159.5556** | **-0.0012** | **0** | **4** | **73** | **7** | **K.SMQNHAAVFR.V** |
|  | 7755 | **597.3677** | **1192.7209** | **1191.6281** | **1.0928** | **1** | **9** | **19** | **2** | **R.TSELRLSMQK.S** 7702 |
|  | 9384 | **638.8260** | **1275.6374** | **1275.6459** | **-0.0085** | **0** | **30** | **0.13** | **1** | **K.HTLSYVDVGTGK.V** |
|  | 10420 | **666.8752** | **1331.7358** | **1331.7449** | **-0.0091** | **0** | **55** | **0.00047** | **1** | **K.VTLEYRPVIDK.T** |
|  | 10811 | **452.2439** | **1353.7100** | **1353.7153** | **-0.0054** | **0** | **6** | **34** | **1** | **R.TGHSLLHTLYGR.S** |
|  | 10995 | **456.2196** | **1365.6369** | **1366.7357** | **-1.0988** | **1** | **5** | **45** | **8** | **K.IYQRAFGGQSLK.F** |
|  | 11671 | **468.9177** | **1403.7312** | **1403.7409** | **-0.0097** | **1** | **36** | **0.035** | **1** | **R.KHTLSYVDVGTGK.V** 11673 11688 |
|  | 12655 | **731.9160** | **1461.8175** | **1460.7657** | **1.0518** | **1** | **4** | **55** | **9** | **R.VGSVLQEGCGKISK.L** |
|  | 12821 | **737.4216** | **1472.8287** | **1472.8351** | **-0.0064** | **0** | **94** | **4.6e-008** | **1** | **R.LGANSLLDLVVFGR.A** 12820 12822 |
|  | 15183 | **814.4728** | **1626.9310** | **1626.8035** | **0.1275** | **0** | **5** | **38** | **2** | **K.TLNEADCATVPPAIR.S** |
|  | 17748 | **906.4376** | **1810.8606** | **1810.8737** | **-0.0132** | **0** | **55** | **0.00033** | **1** | **R.IDEYDYSKPIQGQQK.K** 17749 17750 17751 |
|  | 19457 | **647.0018** | **1937.9835** | **1936.9928** | **0.9907** | **0** | **24** | **0.37** | **1** | **K.VPPIKPNAGEESVMNLDK.L** |
|  | 20840 | **689.6841** | **2066.0306** | **2066.0432** | **-0.0127** | **1** | **20** | **0.78** | **1** | **K.VRIDEYDYSKPIQGQQK.K** |
|  | 22415 | **741.4571** | **2221.3494** | **2222.1729** | **-0.8234** | **1** | **2** | **41** | **6** | **K.VPPIKPNAGEESVMNLDKLR.F** |
|  | 22745 | **1132.9908** | **2263.9671** | **2263.9838** | **-0.0167** | **0** | **105** | **2.3e-009** | **1** | **R.TYFSCTSAHTSTGDGTAMITR.A** 22744 22746 |
|  | 26340 | **699.6024** | **2794.3806** | **2794.3973** | **-0.0167** | **1** | **28** | **0.096** | **1** | **R.GCGPEKDHVYLQLHHLPPEQLATR.L** |
|  | 26902 | **972.4838** | **2914.4297** | **2914.4461** | **-0.0165** | **0** | **25** | **0.19** | **1** | **K.VSDSISAQYPVVDHEFDAVVVGAGGAGLR.A** 26901 |
|  | 27132 | **984.4521** | **2950.3346** | **2950.3563** | **-0.0216** | **0** | **61** | **3.9e-005** | **1** | **R.HVNGQDQIVPGLYACGEAACASVHGANR.L** 27128 27129 27135 27136 27137 |
|  | 28799 | **1122.8552** | **3365.5438** | **3365.5632** | **-0.0193** | **0** | **57** | **7.4e-005** | **1** | **R.AGLPCQDLEFVQFHPTGIYGAGCLITEGCR.G** 28798 |
|  | 30065 | **1266.5801** | **3796.7184** | **3796.7137** | **0.0047** | **0** | **41** | **0.0026** | **1** | **K.GSDWLGDQDAIHYMTEQAPAAVVELENYGMPFSR.T** 30066 |

| **42.** | P68133\|ACTS_HUMAN    **Mass:** 42366    **Score:** 755    **Queries matched:** 80   **emPAI:** 2.27 |
| --- | --- |
|  | Actin, alpha skeletal muscle OS=Homo sapiens GN=ACTA1 PE=1 SV=1 |

|  | **Query** | **Observed** | **Mr(expt)** | **Mr(calc)** | **Delta** | **Miss** | **Score** | **Expect** | **Rank** | **Peptide** |
| --- | --- | --- | --- | --- | --- | --- | --- | --- | --- | --- |
|  | 1426 | 631.3726 | 630.3653 | 630.3701 | -0.0048 | 0 | 10 | 33 | 3 | K.ILTER.G 1421 |
|  | 1491 | 644.8003 | 643.7931 | 643.3653 | 0.4278 | 0 | 18 | 3 | 2 | R.LDLAGR.D |
|  | 2106 | 398.2369 | 794.4593 | 794.4650 | -0.0057 | 0 | 16 | 4 | 1 | K.IIAPPER.K 2107 2108 |
|  | 2167 | 400.7687 | 799.5229 | 799.5280 | -0.0050 | 1 | 24 | 0.83 | 2 | K.RGILTLK.Y |
|  | 4032 | 462.2841 | 922.5536 | 922.5600 | -0.0063 | 1 | 25 | 0.52 | 1 | K.IIAPPERK.Y |
|  | 4771 | 488.7246 | 975.4346 | 975.4410 | -0.0064 | 0 | 65 | 6.7e-005 | 1 | K.AGFAGDDAPR.A 4770 4780 |
|  | 5079 | 499.7437 | 997.4728 | 997.4790 | -0.0062 | 0 | 29 | 0.18 | 1 | R.DLTDYLMK.I 5080 |
|  | 7246 | 581.3093 | 1160.6041 | 1160.6111 | -0.0070 | 0 | 39 | 0.022 | 1 | K.EITALAPSTMK.I 7227 7244 |
|  | 7368 | 586.2852 | 1170.5558 | 1170.5638 | -0.0080 | 0 | 59 | 0.0002 | 1 | R.HQGVMVGMGQK.D 7365 7366 7367 7369 7370 7371 7379 7382 |
|  | 7612 | 594.2825 | 1186.5505 | 1186.5587 | -0.0082 | 0 | (52) | 0.00089 | 1 | R.HQGVMVGMGQK.D 7611 |
|  | 7613 | 594.2828 | 1186.5511 | 1186.5587 | -0.0076 | 0 | (48) | 0.0023 | 1 | R.HQGVMVGMGQK.D 7614 7631 |
|  | 7824 | 599.7609 | 1197.5072 | 1197.5150 | -0.0078 | 0 | 27 | 0.29 | 1 | K.DSYVGDEAQSK.R 7825 |
|  | 7850 | 599.8526 | 1197.6906 | 1197.6982 | -0.0076 | 0 | 65 | 4.6e-005 | 1 | R.AVFPSIVGRPR.H 7847 7848 7849 |
|  | 7942 | 602.2798 | 1202.5450 | 1202.5536 | -0.0086 | 0 | (26) | 0.37 | 1 | R.HQGVMVGMGQK.D 7940 7941 |
|  | 10805 | 677.8108 | 1353.6070 | 1353.6161 | -0.0090 | 1 | 44 | 0.0052 | 1 | K.DSYVGDEAQSKR.G 10804 10819 10829 |
|  | 13259 | **750.3368** | **1498.6590** | **1499.7005** | **-1.0414** | **0** | **12** | **6.9** | **1** | **K.QEYDEAGPSIVHR.K** 13258 |
|  | 13487 | 505.9182 | 1514.7328 | 1514.7419 | -0.0091 | 0 | 46 | 0.0032 | 1 | K.IWHHTFYNELR.V 13481 13482 13483 13484 13485 13486 13490 13491 13492 13493 |
|  | 14439 | 790.8826 | 1579.7506 | 1579.7949 | -0.0443 | 1 | 2 | 77 | 4 | R.MQKEITALAPSTMK.I 14449 |
|  | 15103 | 541.9487 | 1622.8244 | 1622.8338 | -0.0094 | 1 | 14 | 5.1 | 2 | R.LDLAGRDLTDYLMK.I |
|  | 17483 | 895.9439 | 1789.8733 | 1789.8846 | -0.0114 | 0 | 73 | 6e-006 | 1 | K.SYELPDGQVITIGNER.F 17481 17482 17485 17486 17487 17488 |
|  | 19701 | **980.4697** | **1958.9248** | **1959.9036** | **-0.9789** | **0** | **21** | **0.73** | **1** | **K.YPIEHGIITNWDDMEK.I** |
|  | 22723 | 754.3623 | 2260.0651 | 2261.0093 | -0.9442 | 0 | 3 | 43 | 9 | K.DLYANNVMSGGTTMYPGIADR.M |
|  | 24953 | 1276.0880 | 2550.1615 | 2551.1458 | -0.9843 | 0 | 44 | 0.0024 | 2 | K.LCYVALDFENEMATAASSSSLEK.S 24948 24949 24950 24951 24954 24960 24961 24962 24966 |
|  | 25567 | **889.0652** | **2664.1737** | **2664.1982** | **-0.0245** | **1** | **0** | **57** | **2** | **K.DLYANNVMSGGTTMYPGIADRMQK.E** |
|  | 29079 | **865.1610** | **3456.6149** | **3456.6349** | **-0.0200** | **1** | **6** | **9.5** | **1** | **K.YPIEHGIITNWDDMEKIWHHTFYNELR.V** |

| **43.** | P68032\|ACTC_HUMAN    **Mass:** 42334    **Score:** 755    **Queries matched:** 79   **emPAI:** 2.28 |
| --- | --- |
|  | Actin, alpha cardiac muscle 1 OS=Homo sapiens GN=ACTC1 PE=1 SV=1 |

|  | **Query** | **Observed** | **Mr(expt)** | **Mr(calc)** | **Delta** | **Miss** | **Score** | **Expect** | **Rank** | **Peptide** |
| --- | --- | --- | --- | --- | --- | --- | --- | --- | --- | --- |
|  | 1426 | 631.3726 | 630.3653 | 630.3701 | -0.0048 | 0 | 10 | 33 | 3 | K.ILTER.G 1421 |
|  | 1491 | 644.8003 | 643.7931 | 643.3653 | 0.4278 | 0 | 18 | 3 | 2 | R.LDLAGR.D |
|  | 2106 | 398.2369 | 794.4593 | 794.4650 | -0.0057 | 0 | 16 | 4 | 1 | K.IIAPPER.K 2107 2108 |
|  | 2167 | 400.7687 | 799.5229 | 799.5280 | -0.0050 | 1 | 24 | 0.83 | 2 | K.RGILTLK.Y |
|  | 4032 | 462.2841 | 922.5536 | 922.5600 | -0.0063 | 1 | 25 | 0.52 | 1 | K.IIAPPERK.Y |
|  | 4771 | 488.7246 | 975.4346 | 975.4410 | -0.0064 | 0 | 65 | 6.7e-005 | 1 | K.AGFAGDDAPR.A 4770 4780 |
|  | 5079 | 499.7437 | 997.4728 | 997.4790 | -0.0062 | 0 | 29 | 0.18 | 1 | R.DLTDYLMK.I 5080 |
|  | 7246 | 581.3093 | 1160.6041 | 1160.6111 | -0.0070 | 0 | 39 | 0.022 | 1 | K.EITALAPSTMK.I 7227 7244 |
|  | 7368 | 586.2852 | 1170.5558 | 1170.5638 | -0.0080 | 0 | 59 | 0.0002 | 1 | R.HQGVMVGMGQK.D 7365 7366 7367 7369 7370 7371 7379 7382 |
|  | 7612 | 594.2825 | 1186.5505 | 1186.5587 | -0.0082 | 0 | (52) | 0.00089 | 1 | R.HQGVMVGMGQK.D 7611 |
|  | 7613 | 594.2828 | 1186.5511 | 1186.5587 | -0.0076 | 0 | (48) | 0.0023 | 1 | R.HQGVMVGMGQK.D 7614 7631 |
|  | 7824 | 599.7609 | 1197.5072 | 1197.5150 | -0.0078 | 0 | 27 | 0.29 | 1 | K.DSYVGDEAQSK.R 7825 |
|  | 7850 | 599.8526 | 1197.6906 | 1197.6982 | -0.0076 | 0 | 65 | 4.6e-005 | 1 | R.AVFPSIVGRPR.H 7847 7848 7849 |
|  | 7942 | 602.2798 | 1202.5450 | 1202.5536 | -0.0086 | 0 | (26) | 0.37 | 1 | R.HQGVMVGMGQK.D 7940 7941 |
|  | 10805 | 677.8108 | 1353.6070 | 1353.6161 | -0.0090 | 1 | 44 | 0.0052 | 1 | K.DSYVGDEAQSKR.G 10804 10819 10829 |
|  | 13259 | 750.3368 | 1498.6590 | 1499.7005 | -1.0414 | 0 | 12 | 6.9 | 1 | K.QEYDEAGPSIVHR.K 13258 |
|  | 13487 | 505.9182 | 1514.7328 | 1514.7419 | -0.0091 | 0 | 46 | 0.0032 | 1 | K.IWHHTFYNELR.V 13481 13482 13483 13484 13485 13486 13490 13491 13492 13493 |
|  | 14439 | 790.8826 | 1579.7506 | 1579.7949 | -0.0443 | 1 | 2 | 77 | 4 | R.MQKEITALAPSTMK.I 14449 |
|  | 15103 | 541.9487 | 1622.8244 | 1622.8338 | -0.0094 | 1 | 14 | 5.1 | 2 | R.LDLAGRDLTDYLMK.I |
|  | 17483 | 895.9439 | 1789.8733 | 1789.8846 | -0.0114 | 0 | 73 | 6e-006 | 1 | K.SYELPDGQVITIGNER.F 17481 17482 17485 17486 17487 17488 |
|  | 19701 | 980.4697 | 1958.9248 | 1959.9036 | -0.9789 | 0 | 21 | 0.73 | 1 | K.YPIEHGIITNWDDMEK.I |
|  | 24953 | 1276.0880 | 2550.1615 | 2551.1458 | -0.9843 | 0 | 44 | 0.0024 | 2 | K.LCYVALDFENEMATAASSSSLEK.S 24948 24949 24950 24951 24954 24960 24961 24962 24966 |
|  | 25371 | **877.4372** | **2629.2897** | **2630.2469** | **-0.9571** | **1** | **1** | **47** | **2** | **K.DLYANNVLSGGTTMYPGIADRMQK.E** |
|  | 29079 | 865.1610 | 3456.6149 | 3456.6349 | -0.0200 | 1 | 6 | 9.5 | 1 | K.YPIEHGIITNWDDMEKIWHHTFYNELR.V |

| **44.** | P04406\|G3P_HUMAN    **Mass:** 36201    **Score:** 728    **Queries matched:** 48   **emPAI:** 2.62 |
| --- | --- |
|  | Glyceraldehyde-3-phosphate dehydrogenase OS=Homo sapiens GN=GAPDH PE=1 SV=3 |

|  | **Query** | **Observed** | **Mr(expt)** | **Mr(calc)** | **Delta** | **Miss** | **Score** | **Expect** | **Rank** | **Peptide** |
| --- | --- | --- | --- | --- | --- | --- | --- | --- | --- | --- |
|  | 1842 | **739.3565** | **738.3492** | **738.3548** | **-0.0056** | **0** | **24** | **0.5** | **1** | **K.YDNSLK.I** |
|  | 2104 | **398.2102** | **794.4059** | **794.4109** | **-0.0050** | **0** | **27** | **0.34** | **1** | **K.LTGMAFR.V** |
|  | 3264 | 435.7472 | 869.4799 | 868.5018 | 0.9781 | 0 | 5 | 45 | 8 | K.VIPELNGK.L |
|  | 3817 | 454.7176 | 907.4206 | 908.4828 | -1.0622 | 0 | 28 | 0.25 | 1 | K.AGAHLQGGAK.R 3833 3836 3837 3838 |
|  | 7896 | **601.3029** | **1200.5913** | **1200.5995** | **-0.0082** | **0** | **69** | **1.8e-005** | **1** | **R.VVDLMAHMASK.E** 7894 7899 |
|  | 11819 | **706.3941** | **1410.7736** | **1410.7831** | **-0.0094** | **0** | **49** | **0.0017** | **1** | **R.GALQNIIPASTGAAK.A** 11818 11831 |
|  | 14930 | **807.4495** | **1612.8844** | **1612.8937** | **-0.0093** | **0** | **96** | **3.1e-008** | **1** | **K.LVINGNPITIFQER.D** 14950 14951 14952 |
|  | 17108 | **882.3995** | **1762.7845** | **1762.7951** | **-0.0105** | **0** | **71** | **9.1e-006** | **1** | **K.LISWYDNEFGYSNR.V** 17106 17107 |
|  | 18091 | **917.9468** | **1833.8790** | **1832.9124** | **0.9666** | **0** | **51** | **0.00083** | **1** | **K.IISNASCTTNCLAPLAK.V** 18077 18080 |
|  | 22307 | **1107.0512** | **2212.0877** | **2212.1020** | **-0.0143** | **0** | **67** | **1.6e-005** | **1** | **R.VIISAPSADAPMFVMGVNHEK.Y** 22308 22309 22310 |
|  | 22457 | **743.3497** | **2227.0272** | **2228.0970** | **-1.0698** | **0** | **(0)** | **81** | **8** | **R.VIISAPSADAPMFVMGVNHEK.Y** |
|  | 22848 | **1139.0172** | **2276.0199** | **2276.0307** | **-0.0109** | **0** | **76** | **2.1e-006** | **1** | **K.WGDAGAEYVVESTGVFTTMEK.A** 22845 22849 22859 22860 22862 22863 22873 22879 |
|  | 23211 | **1162.1189** | **2322.2232** | **2322.1824** | **0.0408** | **1** | **1** | **63** | **8** | **K.LTGMAFRVPTANVSVVDLTCR.L** |
|  | 25201 | **865.7878** | **2594.3417** | **2594.3527** | **-0.0110** | **0** | **44** | **0.0029** | **1** | **K.VIHDNFGIVEGLMTTVHAITATQK.T** 25196 25198 25199 |
|  | 28601 | **1103.5216** | **3307.5430** | **3307.5570** | **-0.0140** | **0** | **37** | **0.0091** | **1** | **K.VDIVAINDPFIDLNYMVYMFQYDSTHGK.F** 28602 |
|  | 30412 | **1009.9724** | **4035.8603** | **4035.8875** | **-0.0272** | **0** | **42** | **0.0019** | **1** | **K.GILGYTEHQVVSSDFNSDTHSSTFDAGAGIALNDHFVK.L** 30410 30411 |

| **45.** | Q9Y4L1\|HYOU1_HUMAN    **Mass:** 111494   **Score:** 720    **Queries matched:** 46   **emPAI:** 0.48 |
| --- | --- |
|  | Hypoxia up-regulated protein 1 OS=Homo sapiens GN=HYOU1 PE=1 SV=1 |

|  | **Query** | **Observed** | **Mr(expt)** | **Mr(calc)** | **Delta** | **Miss** | **Score** | **Expect** | **Rank** | **Peptide** |
| --- | --- | --- | --- | --- | --- | --- | --- | --- | --- | --- |
|  | 1296 | **602.4030** | **601.3957** | **600.3595** | **1.0362** | **0** | **8** | **49** | **2** | **K.LAELR.K** |
|  | 1888 | **752.5126** | **751.5053** | **751.3357** | **0.1696** | **0** | **4** | **58** | **8** | **K.MMALDR.E** |
|  | 2409 | **409.2398** | **816.4651** | **817.4844** | **-1.0192** | **1** | **10** | **21** | **2** | **R.AMAKLLR.E** 2428 |
|  | 2551 | **414.7604** | **827.5063** | **827.5116** | **-0.0053** | **0** | **11** | **14** | **3** | **R.VQEVLLK.A** |
|  | 3114 | **432.2326** | **862.4506** | **863.3960** | **-0.9454** | **0** | **6** | **45** | **7** | **R.MGPYPQR.K** |
|  | 4125 | **465.7629** | **929.5112** | **929.5182** | **-0.0070** | **1** | **39** | **0.025** | **1** | **K.AVGKEELGK.N** |
|  | 5184 | **503.2758** | **1004.5371** | **1004.5443** | **-0.0072** | **0** | **24** | **0.77** | **1** | **R.YFQHLLGK.Q** |
|  | 5195 | **503.7788** | **1005.5430** | **1005.5495** | **-0.0065** | **0** | **8** | **27** | **1** | **R.EVQYLLNK.A** |
|  | 8749 | **415.2027** | **1242.5862** | **1243.7070** | **-1.1209** | **1** | **4** | **58** | **10** | **R.AVLQAARMAGLK.V** |
|  | 8771 | 622.7986 | 1243.5826 | 1243.5907 | -0.0081 | 0 | 37 | 0.028 | 1 | R.FFGDSAASMAIK.N |
|  | 9027 | **630.2561** | **1258.4976** | **1259.5856** | **-1.0880** | **0** | **(2)** | **98** | **6** | **R.FFGDSAASMAIK.N** |
|  | 11039 | 457.2050 | 1368.5931 | 1368.7990 | -0.2058 | 1 | 7 | 27 | 4 | K.FTKPRPRPKDK.N 11049 |
|  | 13511 | **506.2689** | **1515.7849** | **1514.7875** | **0.9974** | **1** | **3** | **59** | **4** | **K.TKEAGMQPQLQIR.G** 13510 |
|  | 14485 | **792.4303** | **1582.8460** | **1582.7813** | **0.0647** | **1** | **(4)** | **45** | **2** | **R.FFGDSAASMAIKNPK.A** |
|  | 14722 | **800.7733** | **1599.5321** | **1598.7763** | **0.7558** | **1** | **5** | **29** | **2** | **R.FFGDSAASMAIKNPK.A** 14707 |
|  | 15619 | **828.8783** | **1655.7420** | **1655.7501** | **-0.0081** | **0** | **28** | **0.18** | **1** | **R.VEFEELCADLFER.V** |
|  | 16027 | 842.9900 | 1683.9654 | 1682.8151 | 1.1503 | 0 | 68 | 1.7e-005 | 1 | K.AANSLEAFIFETQDK.L |
|  | 16348 | **570.0104** | **1707.0093** | **1706.0164** | **0.9929** | **0** | **11** | **8.9** | **1** | **K.VAIVKPGVPMEIVLNK.E** |
|  | 16711 | **868.4412** | **1734.8679** | **1734.8788** | **-0.0110** | **0** | **44** | **0.0045** | **1** | **K.LGNTISSLFGGGTTPDAK.E** |
|  | 16772 | **580.6494** | **1738.9262** | **1738.8746** | **0.0516** | **1** | **6** | **26** | **1** | **K.MMALDREVQYLLNK.A** |
|  | 19479 | **970.9573** | **1939.9001** | **1940.9188** | **-1.0186** | **1** | **1** | **73** | **6** | **K.NGTRAEPPLNASASDQGEK.V** |
|  | 21140 | **1048.0573** | **2094.0999** | **2094.1109** | **-0.0110** | **0** | **115** | **2.6e-010** | **1** | **K.VLQLINDNTATALSYGVFR.R** 21141 |
|  | 22025 | **1090.0447** | **2178.0748** | **2178.0878** | **-0.0130** | **0** | **73** | **4.7e-006** | **1** | **K.LSAASTWLEDEGVGATTVMLK.E** 22026 |
|  | 22401 | **556.2736** | **2221.0654** | **2221.2681** | **-0.2028** | **1** | **3** | **40** | **7** | **K.NATLAEQAKLPATEKPVLLSK.D** |
|  | 22769 | **1134.0581** | **2266.1017** | **2266.1113** | **-0.0096** | **0** | **85** | **2.5e-007** | **1** | **R.LIPEMDQIFTEVEMTTLEK.V** 22764 22768 |
|  | 23349 | **1172.0550** | **2342.0955** | **2341.1060** | **0.9895** | **0** | **92** | **4.3e-008** | **1** | **R.VESVFETLVEDSAEEESTLTK.L** |
|  | 24180 | **1227.1065** | **2452.1983** | **2451.2203** | **0.9781** | **1** | **0** | **69** | **2** | **K.LSAASTWLEDEGVGATTVMLKEK.L** |
|  | 24488 | **831.4635** | **2491.3687** | **2492.2030** | **-0.8343** | **1** | **2** | **42** | **6** | **K.SEAQKPSEKAEAGPEGVAPAPEGEK.K** |
|  | 24714 | **840.7247** | **2519.1522** | **2519.1672** | **-0.0150** | **0** | **16** | **1.8** | **1** | **K.TVLSANADHMAQIEGLMDDVDFK.A** |
|  | 24898 | 1275.5848 | 2549.1551 | 2550.2709 | -1.1158 | 1 | 2 | 38 | 2 | K.GARLIPEMDQIFTEVEMTTLEK.V |
|  | 25998 | **909.4217** | **2725.2434** | **2725.2561** | **-0.0127** | **0** | **46** | **0.0014** | **1** | **R.YSHDFNFHINYGDLGFLGPEDLR.V** |
|  | 27899 | **1568.3153** | **3134.6161** | **3134.6282** | **-0.0121** | **0** | **105** | **1.3e-009** | **1** | **R.VPGPVQQALQSAEMSLDEIEQVILVGGATR.V** 27897 27898 27901 |
|  | 28540 | **1094.8361** | **3281.4864** | **3280.5219** | **0.9645** | **1** | **1** | **39** | **5** | **K.VETGSEPGDTEPLELGGPGAEPEQKEQSTGQK.R** |
|  | 29028 | **1146.5760** | **3436.7063** | **3435.5343** | **1.1720** | **0** | **2** | **28** | **3** | **K.DINTTAQNIMFYDMGSGSTVCTIVTYQMVK.T** |
|  | 29690 | **1832.4180** | **3662.8214** | **3661.9133** | **0.9081** | **1** | **3** | **16** | **8** | **R.RVCWALVAVLLADLLALSDTLAVMSVDLGSESMK.V** |

| **46.** | Q14764\|MVP_HUMAN    **Mass:** 99551    **Score:** 719    **Queries matched:** 52   **emPAI:** 1.00 |
| --- | --- |
|  | Major vault protein OS=Homo sapiens GN=MVP PE=1 SV=4 |

|  | **Query** | **Observed** | **Mr(expt)** | **Mr(calc)** | **Delta** | **Miss** | **Score** | **Expect** | **Rank** | **Peptide** |
| --- | --- | --- | --- | --- | --- | --- | --- | --- | --- | --- |
|  | 897 | **518.3204** | **517.3132** | **516.3020** | **1.0112** | **0** | **11** | **30** | **5** | **K.AIASR.V** |
|  | 2624 | **417.2285** | **832.4423** | **832.4629** | **-0.0206** | **0** | **8** | **31** | **6** | **R.VLFAPMR.M** |
|  | 2932 | **427.2268** | **852.4391** | **852.4453** | **-0.0062** | **0** | **38** | **0.026** | **1** | **R.HADLEIR.L** |
|  | 3404 | 880.4352 | 879.4280 | 880.5130 | -1.0851 | 0 | (10) | 17 | 4 | K.SLQPLAPR.N 3417 |
|  | 4534 | **480.2403** | **958.4660** | **957.4992** | **0.9669** | **1** | **6** | **44** | **2** | **R.LEQEARGR.L** 4523 |
|  | 4984 | **496.7711** | **991.5276** | **991.5338** | **-0.0062** | **0** | **17** | **3.3** | **1** | **R.ELELVYAR.A** |
|  | 5108 | **500.3521** | **998.6897** | **997.5781** | **1.1116** | **1** | **7** | **27** | **5** | **R.QNQALRLR.A** |
|  | 5312 | **508.7811** | **1015.5477** | **1015.5549** | **-0.0072** | **0** | **23** | **0.94** | **1** | **R.AQLELEVSK.A** 5314 |
|  | 5529 | **516.7606** | **1031.5066** | **1031.5135** | **-0.0069** | **0** | **30** | **0.16** | **1** | **K.ILDQSEAEK.A** |
|  | 6850 | **565.8030** | **1129.5914** | **1129.5979** | **-0.0065** | **0** | **64** | **6.3e-005** | **1** | **R.IEGEGSVLQAK.L** |
|  | 7240 | **581.2970** | **1160.5794** | **1160.6554** | **-0.0759** | **1** | **0** | **1.6e+002** | **6** | **R.VEVGPKTYIR.Q** |
|  | 8720 | 621.3231 | 1240.6317 | 1241.6979 | -1.0662 | 1 | 14 | 6.2 | 2 | K.AQQLAEVEVKK.F |
|  | 9556 | **643.8130** | **1285.6114** | **1285.6190** | **-0.0076** | **0** | **15** | **5** | **1** | **R.TAVFGFETSEAK.G** |
|  | 9597 | **430.2997** | **1287.8774** | **1287.6670** | **0.2104** | **1** | **8** | **21** | **5** | **R.QKILDQSEAEK.A** |
|  | 9874 | **652.3099** | **1302.6053** | **1302.6139** | **-0.0086** | **0** | **58** | **0.00022** | **1** | **R.HYCTVANPVSR.D** 9873 |
|  | 10971 | 682.9078 | 1363.8010 | 1364.6460 | -0.8450 | 1 | 9 | 19 | 6 | K.ALLDFEDKDGDK.V |
|  | 11490 | **697.3363** | **1392.6580** | **1392.6674** | **-0.0094** | **0** | **70** | **1.2e-005** | **1** | **R.GAVASVTFDDFHK.N** 11491 |
|  | 12514 | **727.8488** | **1453.6831** | **1453.6912** | **-0.0081** | **0** | **36** | **0.034** | **1** | **K.LFSVPDFVGDACK.A** |
|  | 12868 | **492.9052** | **1475.6938** | **1474.7351** | **0.9587** | **1** | **4** | **51** | **4** | **R.QDNERVLFAPMR.M** |
|  | 13025 | **743.4356** | **1484.8567** | **1485.6834** | **-0.8268** | **0** | **18** | **1.8** | **1** | **R.ALQPLEEGEDEEK.V** |
|  | 13885 | **772.4508** | **1542.8871** | **1541.8049** | **1.0822** | **0** | **24** | **0.45** | **1** | **K.AQALAIETEAELQR.V** |
|  | 16774 | **870.5123** | **1739.0101** | **1739.0193** | **-0.0091** | **1** | **121** | **7.2e-011** | **1** | **R.KEVEVVEIIQATIIR.Q** 16753 |
|  | 17799 | **908.4749** | **1814.9353** | **1814.9454** | **-0.0102** | **0** | **16** | **2.8** | **1** | **R.LAQDPFPLYPGEVLEK.D** |
|  | 18621 | **936.5472** | **1871.0798** | **1871.0880** | **-0.0082** | **0** | **49** | **0.0011** | **1** | **K.DITPLQVVLPNTALHLK.A** 18641 |
|  | 18660 | **937.8451** | **1873.6757** | **1872.9792** | **0.6965** | **0** | **12** | **4.6** | **3** | **R.SVQLAIEITTNSQEAAAK.H** |
|  | 19369 | **965.9852** | **1929.9559** | **1929.9684** | **-0.0124** | **0** | **79** | **1.3e-006** | **1** | **R.QAIPLDENEGIYVQDVK.T** 19370 |
|  | 20167 | **1003.4991** | **2004.9836** | **2004.9945** | **-0.0109** | **0** | **5** | **29** | **1** | **K.VVAGDEWLFEGPGTYIPR.K** |
|  | 20990 | **1042.0184** | **2082.0223** | **2082.0343** | **-0.0120** | **0** | **77** | **1.9e-006** | **1** | **R.AVIGSTYMLTQDEVLWEK.E** 20989 |
|  | 21742 | **718.0313** | **2151.0719** | **2151.0861** | **-0.0142** | **0** | **41** | **0.0069** | **1** | **R.IPPYHYIHVLDQNSNVSR.V** 21743 |
|  | 25747 | **896.7908** | **2687.3507** | **2687.3629** | **-0.0122** | **0** | **14** | **2.6** | **1** | **R.ALCLLLGPDFFTDVITIETADHAR.L** |
|  | 26402 | **938.1370** | **2811.3891** | **2811.4039** | **-0.0149** | **0** | **36** | **0.017** | **1** | **R.DQAVFPQNGLVVSSVDVQSVEPVDQR.T** |
|  | 27839 | **1040.5267** | **3118.5584** | **3118.5796** | **-0.0212** | **0** | **85** | **1.5e-007** | **1** | **R.VASGPSPGEGISPQSAQAPQAPGDNHVVPVLR.-** |
|  | 28520 | **1637.8366** | **3273.6585** | **3273.6704** | **-0.0118** | **0** | **77** | **9.7e-007** | **1** | **K.STLITDGSTPINLFNTAFGLLGMGPEGQPLGR.R** 28519 |
|  | 28702 | **1667.8639** | **3333.7132** | **3333.7245** | **-0.0113** | **0** | **66** | **1.2e-005** | **1** | **K.SFFLQPGEQLEQGIQDVYVLSEQQGLLLR.A** 28701 |
|  | 29715 | **918.0094** | **3668.0085** | **3668.0229** | **-0.0144** | **1** | **18** | **0.4** | **1** | **R.LAQDPFPLYPGEVLEKDITPLQVVLPNTALHLK.A** 29712 |
|  | 30336 | **990.7574** | **3959.0007** | **3958.9960** | **0.0047** | **1** | **2** | **20** | **5** | **K.GPDGMALPRPRDQAVFPQNGLVVSSVDVQSVEPVDQR.T** |
|  | 31632 | **1333.9158** | **5331.6340** | **5330.6446** | **0.9894** | **0** | **31** | **0.011** | **1** | **R.TGEEWLVTVQDTEAHVPDVHEEVLGVVPITTLGPHNYCVILDPVGPDGK.N** 31633 |
|  | 31681 | **1098.3507** | **5486.7172** | **5486.7457** | **-0.0286** | **1** | **7** | **2.3** | **1** | **R.RTGEEWLVTVQDTEAHVPDVHEEVLGVVPITTLGPHNYCVILDPVGPDGK.N** |

| **47.** | P08684\|CP3A4_HUMAN    **Mass:** 57705    **Score:** 697    **Queries matched:** 53   **emPAI:** 1.12 |
| --- | --- |
|  | Cytochrome P450 3A4 OS=Homo sapiens GN=CYP3A4 PE=1 SV=4 |

|  | **Query** | **Observed** | **Mr(expt)** | **Mr(calc)** | **Delta** | **Miss** | **Score** | **Expect** | **Rank** | **Peptide** |
| --- | --- | --- | --- | --- | --- | --- | --- | --- | --- | --- |
|  | 1096 | **560.3808** | **559.3735** | **558.3741** | **0.9994** | **0** | **23** | **1.6** | **1** | **K.TVLVK.E** |
|  | 2578 | **415.2532** | **828.4919** | **827.4752** | **1.0167** | **0** | **5** | **60** | **9** | **K.ETQIPLK.L** |
|  | 2930 | **853.4177** | **852.4104** | **853.4190** | **-1.0086** | **0** | **13** | **7.7** | **2** | **R.FALMNMK.L** |
|  | 7386 | **586.8259** | **1171.6373** | **1171.6448** | **-0.0075** | **0** | **41** | **0.012** | **1** | **R.EAETGKPVTLK.D** |
|  | 7417 | **588.2571** | **1174.4997** | **1174.5077** | **-0.0080** | **0** | **20** | **1.8** | **1** | **K.ECYSVFTNR.R** |
|  | 9104 | **631.8223** | **1261.6301** | **1261.6376** | **-0.0075** | **0** | **17** | **3.3** | **1** | **K.DVEINGMFIPK.G** 9105 9121 |
|  | 11017 | **684.3561** | **1366.6976** | **1366.7067** | **-0.0091** | **0** | **22** | **0.84** | **1** | **R.VLQNFSFKPCK.E** |
|  | 11225 | **689.8887** | **1377.7629** | **1376.6459** | **1.1170** | **0** | **1** | **1e+002** | **9** | **K.SAISIAEDEEWK.R** |
|  | 11691 | **703.3827** | **1404.7508** | **1405.7929** | **-1.0421** | **1** | **11** | **10** | **5** | **R.LRSLLSPTFTSGK.L** |
|  | 12262 | **479.7539** | **1436.2397** | **1435.8043** | **0.4354** | **1** | **13** | **4.1** | **4** | **R.FALMNMKLALIR.V** |
|  | 12330 | **721.3984** | **1440.7823** | **1440.7911** | **-0.0088** | **0** | **19** | **1.5** | **1** | **K.GVVVMIPSYALHR.D** 12329 12341 |
|  | 12543 | **486.2649** | **1455.7728** | **1456.7861** | **-1.0132** | **0** | **(2)** | **86** | **7** | **K.GVVVMIPSYALHR.D** |
|  | 13737 | **767.3759** | **1532.7373** | **1532.7470** | **-0.0098** | **1** | **54** | **0.00054** | **1** | **K.SAISIAEDEEWKR.L** 13720 13721 13736 |
|  | 14995 | **808.9016** | **1615.7885** | **1616.7295** | **-0.9410** | **1** | **6** | **31** | **4** | **R.NCIGMRFALMNMK.L** |
|  | 15025 | **540.2929** | **1617.8567** | **1616.7295** | **1.1272** | **1** | **(1)** | **92** | **6** | **R.NCIGMRFALMNMK.L** |
|  | 15336 | **819.4098** | **1636.8050** | **1636.8130** | **-0.0080** | **0** | **82** | **7.2e-007** | **1** | **R.VDFLQLMIDSQNSK.E** 15335 15355 |
|  | 16108 | **564.3505** | **1690.0296** | **1690.0393** | **-0.0097** | **0** | **31** | **0.072** | **1** | **K.LSLGGLLQPEKPVVLK.V** 16105 16106 16107 |
|  | 16284 | **851.9592** | **1701.9038** | **1701.9124** | **-0.0086** | **0** | **77** | **2.5e-006** | **1** | **K.EMVPIIAQYGDVLVR.N** 16283 |
|  | 17745 | **906.4283** | **1810.8421** | **1810.8526** | **-0.0105** | **0** | **31** | **0.085** | **1** | **K.DNIDPYIYTPFGSGPR.N** 17743 17744 |
|  | 19372 | **483.4975** | **1929.9609** | **1929.9731** | **-0.0122** | **1** | **6** | **27** | **3** | **K.HRVDFLQLMIDSQNSK.E** |
|  | 19516 | **648.7004** | **1943.0795** | **1943.0914** | **-0.0119** | **1** | **42** | **0.0065** | **1** | **K.LKEMVPIIAQYGDVLVR.N** |
|  | 20715 | **1027.4968** | **2052.9791** | **2052.9905** | **-0.0114** | **1** | **74** | **3.3e-006** | **1** | **K.NKDNIDPYIYTPFGSGPR.N** 20714 |
|  | 22729 | **754.7515** | **2261.2328** | **2261.2936** | **-0.0608** | **1** | **1** | **62** | **2** | **K.KLGIPGPTPLPFLGNILSYHK.G** |
|  | 23533 | **788.7626** | **2363.2659** | **2364.1267** | **-0.8608** | **1** | **1** | **57** | **3** | **R.VDFLQLMIDSQNSKETESHK.A** |
|  | 23709 | 1197.0895 | 2392.1644 | 2392.1773 | -0.0129 | 0 | 88 | 1.1e-007 | 1 | K.VWGFYDGQQPVLAITDPDMIK.T 23708 23710 |
|  | 24557 | 834.0512 | 2499.1318 | 2499.5040 | -0.3722 | 1 | 0 | 67 | 7 | K.ETQIPLKLSLGGLLQPEKPVVLK.V |
|  | 25813 | **1349.6547** | **2697.2948** | **2697.3030** | **-0.0082** | **0** | **70** | **6.6e-006** | **1** | **K.APPTYDTVLQMEYLDMVVNETLR.L** 25807 25808 25809 25810 25811 25812 25814 |
|  | 30129 | **1274.9291** | **3821.7654** | **3820.7778** | **0.9876** | **0** | **4** | **13** | **2** | **K.DVFGAYSMDVITSTSFGVNIDSLNNPQDPFVENTK.K** |

| **48.** | P68363\|TBA1B_HUMAN    **Mass:** 50804    **Score:** 688    **Queries matched:** 37   **emPAI:** 2.58 |
| --- | --- |
|  | Tubulin alpha-1B chain OS=Homo sapiens GN=TUBA1B PE=1 SV=1 |

|  | **Query** | **Observed** | **Mr(expt)** | **Mr(calc)** | **Delta** | **Miss** | **Score** | **Expect** | **Rank** | **Peptide** |
| --- | --- | --- | --- | --- | --- | --- | --- | --- | --- | --- |
|  | 1369 | **615.3498** | **614.3426** | **613.3435** | **0.9990** | **0** | **22** | **1.5** | **1** | **R.GDVVPK.D** |
|  | 5307 | **508.2902** | **1014.5658** | **1014.5709** | **-0.0051** | **0** | **43** | **0.0084** | **1** | **K.DVNAAIATIK.T** |
|  | 8847 | **625.2756** | **1248.5366** | **1248.5453** | **-0.0087** | **0** | **29** | **0.17** | **1** | **K.YMACCLLYR.G** |
|  | 11801 | **470.9266** | **1409.7580** | **1409.7667** | **-0.0087** | **0** | **13** | **6.6** | **1** | **R.QLFHPEQLITGK.E** |
|  | 13059 | **744.4392** | **1486.8639** | **1486.8719** | **-0.0080** | **0** | **64** | **5.2e-005** | **1** | **R.LISQIVSSITASLR.F** 13061 |
|  | 14494 | **792.8747** | **1583.7348** | **1583.7443** | **-0.0095** | **0** | **2** | **71** | **1** | **R.SIQFVDWCPTGFK.V** |
|  | 16269 | **851.4518** | **1700.8890** | **1700.8985** | **-0.0095** | **0** | **61** | **9.5e-005** | **1** | **R.AVFVDLEPTVIDEVR.T** |
|  | 16490 | **859.9390** | **1717.8634** | **1717.8747** | **-0.0113** | **0** | **37** | **0.021** | **1** | **R.NLDIERPTYTNLNR.L** 16491 |
|  | 16792 | **581.2870** | **1740.8391** | **1739.8454** | **0.9938** | **1** | **3** | **53** | **2** | **K.RSIQFVDWCPTGFK.V** |
|  | 17026 | **878.9797** | **1755.9449** | **1755.9559** | **-0.0110** | **0** | **36** | **0.025** | **1** | **R.IHFPLATYAPVISAEK.A** 17025 17028 17029 |
|  | 17925 | **912.9903** | **1823.9660** | **1823.9782** | **-0.0121** | **0** | **56** | **0.00029** | **1** | **K.VGINYQPPTVVPGGDLAK.V** 17924 17926 |
|  | 18536 | **932.9506** | **1863.8866** | **1863.8971** | **-0.0105** | **0** | **91** | **8.5e-008** | **1** | **R.AVCMLSNTTAIAEAWAR.L** |
|  | 20182 | **1004.4432** | **2006.8719** | **2006.8858** | **-0.0139** | **0** | **83** | **4.7e-007** | **1** | **K.TIGGGDDSFNTFFSETGAGK.H** 20183 20184 20191 |
|  | 23245 | **1165.5043** | **2328.9940** | **2329.0110** | **-0.0170** | **0** | **77** | **1.2e-006** | **1** | **R.AFVHWYVGEGMEEGEFSEAR.E** 23246 |
|  | 23808 | **1205.1003** | **2408.1861** | **2408.2012** | **-0.0151** | **0** | **123** | **3.3e-011** | **1** | **R.FDGALNVDLTEFQTNLVPYPR.I** 23809 23811 23812 |
|  | 26118 | **917.4299** | **2749.2680** | **2749.2840** | **-0.0160** | **0** | **32** | **0.04** | **1** | **K.AYHEQLSVAEITNACFEPANQMVK.C** |
|  | 28280 | **805.1068** | **3216.3981** | **3216.4168** | **-0.0187** | **1** | **22** | **0.27** | **1** | **R.AFVHWYVGEGMEEGEFSEAREDMAALEK.D** |
|  | 28905 | **1130.8749** | **3389.6028** | **3389.6173** | **-0.0145** | **0** | **77** | **9.2e-007** | **1** | **K.LADQCTGLQGFLVFHSFGGGTGSGFTSLLMER.L** 28906 |
|  | 29243 | **880.4315** | **3517.6967** | **3517.7123** | **-0.0156** | **1** | **6** | **9.9** | **2** | **R.KLADQCTGLQGFLVFHSFGGGTGSGFTSLLMER.L** 29242 |
|  | 30755 | **1074.9904** | **4295.9323** | **4295.9497** | **-0.0173** | **0** | **10** | **2.3** | **1** | **R.ECISIHVGQAGVQIGNACWELYCLEHGIQPDGQMPSDK.T** |
|  | 31692 | **1380.3986** | **5517.5651** | **5516.5891** | **0.9761** | **0** | **0** | **9.1** | **7** | **K.LEFSIYPAPQVSTAVVEPYNSILTTHTTLEHSDCAFMVDNEAIYDICR.R** |

| **49.** | P21397\|AOFA_HUMAN    **Mass:** 60157    **Score:** 679    **Queries matched:** 45   **emPAI:** 1.32 |
| --- | --- |
|  | Amine oxidase [flavin-containing] A OS=Homo sapiens GN=MAOA PE=1 SV=1 |

|  | **Query** | **Observed** | **Mr(expt)** | **Mr(calc)** | **Delta** | **Miss** | **Score** | **Expect** | **Rank** | **Peptide** |
| --- | --- | --- | --- | --- | --- | --- | --- | --- | --- | --- |
|  | 2010 | **395.2128** | **788.4110** | **788.4908** | **-0.0798** | **1** | **12** | **15** | **3** | **K.YKLLPR.S** |
|  | 2110 | **398.2411** | **794.4677** | **794.4762** | **-0.0086** | **1** | **7** | **28** | **4** | **K.LHKEIR.K** |
|  | 2274 | 807.3513 | 806.3440 | 807.4279 | -1.0839 | 1 | 15 | 6 | 6 | K.EAFWKK.K |
|  | 2523 | **414.2015** | **826.3885** | **827.4939** | **-1.1054** | **0** | **(10)** | **14** | **4** | **R.LPMGAVIK.C** |
|  | 2796 | **422.7310** | **843.4474** | **843.4888** | **-0.0414** | **0** | **12** | **14** | **5** | **R.LPMGAVIK.C** 2801 |
|  | 3630 | **448.7280** | **895.4414** | **895.4473** | **-0.0059** | **0** | **27** | **0.33** | **1** | **K.ICELYAK.V** |
|  | 4093 | **464.7735** | **927.5325** | **926.3336** | **1.1989** | **0** | **4** | **61** | **5** | **K.CMMYYK.E** |
|  | 4444 | **476.7497** | **951.4848** | **951.4913** | **-0.0065** | **0** | **17** | **3.4** | **1** | **K.ELGIETYK.V** |
|  | 5419 | **512.7748** | **1023.5351** | **1023.5423** | **-0.0071** | **1** | **20** | **1.5** | **1** | **K.KICELYAK.V** |
|  | 6752 | **562.2784** | **1122.5422** | **1121.5466** | **0.9957** | **0** | **57** | **0.00032** | **1** | **K.FVGGSGQVSER.I** 6742 |
|  | 6858 | **566.3040** | **1130.5935** | **1130.6005** | **-0.0071** | **0** | **54** | **0.00065** | **1** | **R.IMDLLGDQVK.L** |
|  | 7576 | **593.3078** | **1184.6010** | **1184.6077** | **-0.0067** | **0** | **34** | **0.06** | **1** | **R.IFFAGTETATK.W** 7571 7593 |
|  | 8502 | **616.2899** | **1230.5652** | **1229.5928** | **0.9724** | **0** | **5** | **55** | **5** | **K.DIWVQEPESK.D** |
|  | 8848 | **625.3031** | **1248.5916** | **1249.6415** | **-1.0499** | **1** | **51** | **0.0012** | **1** | **R.KFVGGSGQVSER.I** 8869 8892 |
|  | 9432 | 427.2471 | 1278.7196 | 1279.7023 | -0.9828 | 1 | 7 | 27 | 3 | R.LSKELGIETYK.V |
|  | 9561 | **643.8679** | **1285.7212** | **1285.7242** | **-0.0030** | **1** | **1** | **1.1e+002** | **7** | **R.EVLNGLGKVTEK.D** |
|  | 10965 | **682.8704** | **1363.7262** | **1363.7360** | **-0.0099** | **0** | **20** | **1.3** | **1** | **K.IHFRPELPAER.N** 10966 10989 |
|  | 11609 | **700.9066** | **1399.7987** | **1399.8075** | **-0.0088** | **0** | **6** | **31** | **2** | **K.YVINAIPPTLTAK.I** |
|  | 13848 | **771.3325** | **1540.6504** | **1540.6616** | **-0.0113** | **0** | **81** | **9.2e-007** | **1** | **K.WSGYMEGAVEAGER.A** 13869 |
|  | 15695 | **831.4733** | **1660.9320** | **1660.9400** | **-0.0080** | **0** | **77** | **2.4e-006** | **1** | **K.LLTEYGVSVLVLEAR.D** 15682 15694 |
|  | 16057 | **844.4251** | **1686.8357** | **1686.8465** | **-0.0108** | **1** | **73** | **5.5e-006** | **1** | **K.VTEKDIWVQEPESK.D** |
|  | 16227 | **850.4207** | **1698.8269** | **1698.8366** | **-0.0097** | **0** | **78** | **2e-006** | **1** | **K.DVPAVEITHTFWER.N** 16225 |
|  | 16734 | **579.6298** | **1735.8676** | **1735.8169** | **0.0507** | **1** | **5** | **32** | **5** | **R.LPMGAVIKCMMYYK.E** |
|  | 18108 | **918.4618** | **1834.9090** | **1834.9213** | **-0.0123** | **0** | **50** | **0.0011** | **1** | **K.VLGSQEALHPVHYEEK.N** 18107 |
|  | 21593 | **1068.9952** | **2135.9759** | **2135.9912** | **-0.0153** | **1** | **33** | **0.044** | **1** | **K.EIPTDAPWEAQHADKWDK.M** |
|  | 22104 | **1095.5071** | **2188.9996** | **2189.0138** | **-0.0142** | **0** | **88** | **1.2e-007** | **1** | **R.NEHVDYVDVGGAYVGPTQNR.I** 22105 22106 22107 |
|  | 22691 | **1129.0983** | **2256.1820** | **2256.1936** | **-0.0117** | **0** | **82** | **5e-007** | **1** | **K.ASIAGHMFDVVVIGGGISGLSAAK.L** 22705 |
|  | 27589 | 762.6172 | 3046.4397 | 3047.5056 | -1.0659 | 1 | 5 | 15 | 2 | -.MENQEKASIAGHMFDVVVIGGGISGLSAAK.L |

| **50.** | Q13885\|TBB2A_HUMAN    **Mass:** 50274    **Score:** 660    **Queries matched:** 48   **emPAI:** 1.73 |
| --- | --- |
|  | Tubulin beta-2A chain OS=Homo sapiens GN=TUBB2A PE=1 SV=1 |

|  | **Query** | **Observed** | **Mr(expt)** | **Mr(calc)** | **Delta** | **Miss** | **Score** | **Expect** | **Rank** | **Peptide** |
| --- | --- | --- | --- | --- | --- | --- | --- | --- | --- | --- |
|  | 1837 | 737.3976 | 736.3904 | 737.3456 | -0.9553 | 0 | 9 | 21 | 2 | R.GSQQYR.A |
|  | 2296 | 405.2068 | 808.3991 | 807.3399 | 1.0593 | 0 | 8 | 28 | 7 | R.EEYPDR.I |
|  | 5466 | 514.7600 | 1027.5053 | 1027.5121 | -0.0067 | 0 | 25 | 0.47 | 1 | K.TAVCDIPPR.G 5464 5477 5478 |
|  | 5960 | 533.2140 | 1064.4134 | 1064.4201 | -0.0067 | 0 | 11 | 12 | 1 | K.NMMAACDPR.H 5970 |
|  | 6152 | 539.2660 | 1076.5174 | 1076.5250 | -0.0076 | 1 | 25 | 0.51 | 1 | K.IREEYPDR.I |
|  | 8471 | 615.2986 | 1228.5827 | 1228.5910 | -0.0083 | 0 | 66 | 3.7e-005 | 1 | R.ISEQFTAMFR.R |
|  | 8778 | 415.7271 | 1244.1593 | 1244.5860 | -0.4266 | 0 | (10) | 11 | 1 | R.ISEQFTAMFR.R |
|  | 10304 | 663.8410 | 1325.6674 | 1325.7126 | -0.0451 | 1 | 5 | 41 | 5 | K.TAVCDIPPRGLK.M |
|  | 10823 | **678.3283** | **1354.6421** | **1354.6517** | **-0.0096** | **0** | **44** | **0.0055** | **1** | **R.INVYYNEAAGNK.Y** |
|  | 11010 | **684.1195** | **1366.2243** | **1366.6625** | **-0.4381** | **0** | **2** | **53** | **6** | **R.IMNTFSVMPSPK.V** |
|  | 11307 | **692.0279** | **1382.0412** | **1382.6574** | **-0.6162** | **0** | **(2)** | **60** | **1** | **R.IMNTFSVMPSPK.V** 11297 |
|  | 11355 | 462.7160 | 1385.1261 | 1384.6921 | 0.4339 | 1 | 4 | 34 | 7 | K.RISEQFTAMFR.R |
|  | 12166 | 716.3267 | 1430.6389 | 1430.5965 | 0.0423 | 1 | 8 | 21 | 2 | K.NMMAACDPRHGR.Y 12164 12165 |
|  | 12402 | 723.8434 | 1445.6722 | 1445.6820 | -0.0098 | 0 | 54 | 0.00051 | 1 | K.EVDEQMLNVQNK.N 12404 |
|  | 12637 | 731.3526 | 1460.6906 | 1461.6769 | -0.9863 | 0 | (10) | 13 | 2 | K.EVDEQMLNVQNK.N 12636 |
|  | 14975 | 808.4165 | 1614.8184 | 1614.8287 | -0.0103 | 0 | 33 | 0.062 | 1 | R.AILVDLEPGTMDSVR.S 14974 |
|  | 15059 | 540.9470 | 1619.8192 | 1619.8283 | -0.0090 | 0 | 28 | 0.19 | 1 | R.LHFFMPGFAPLTSR.G |
|  | 15266 | 544.9023 | 1631.6850 | 1630.8236 | 0.8614 | 0 | (6) | 28 | 2 | R.AILVDLEPGTMDSVR.S 15251 |
|  | 16178 | 848.9148 | 1695.8150 | 1695.8257 | -0.0106 | 0 | 54 | 0.00046 | 1 | K.NSSYFVEWIPNNVK.T 16177 16189 |
|  | 17883 | 911.9587 | 1821.9028 | 1821.9156 | -0.0127 | 0 | 54 | 0.00051 | 1 | R.EIVHIQAGQCGNQIGAK.F 17882 17884 |
|  | 18460 | 929.4695 | 1856.9244 | 1856.9342 | -0.0098 | 0 | 77 | 2.2e-006 | 2 | K.MSATFIGNSTAIQELFK.R 18473 |
|  | 18642 | 937.1213 | 1872.2280 | 1872.9291 | -0.7011 | 0 | (2) | 46 | 1 | K.MSATFIGNSTAIQELFK.R |
|  | 19481 | 647.6556 | 1939.9451 | 1938.8849 | 1.0602 | 1 | 5 | 29 | 6 | R.MSMKEVDEQMLNVQNK.N |
|  | 19688 | 979.9890 | 1957.9635 | 1957.9745 | -0.0111 | 0 | 82 | 5.4e-007 | 1 | K.GHYTEGAELVDSVLDVVR.K |
|  | 25910 | 1354.6663 | 2707.3180 | 2707.3310 | -0.0130 | 0 | 136 | 1.6e-012 | 1 | K.LTTPTYGDLNHLVSATMSGVTTCLR.F 25908 25911 |
|  | 26354 | 933.4486 | 2797.3240 | 2797.3361 | -0.0121 | 0 | 85 | 1.8e-007 | 1 | R.SGPFGQIFRPDNFVFGQSGAGNNWAK.G 26352 26353 26359 |
|  | 27789 | 1034.8009 | 3101.3809 | 3101.4003 | -0.0194 | 0 | 27 | 0.095 | 2 | K.FWEVISDEHGIDPTGSYHGDSDLQLER.I |

|  | **Proteins matching a subset of these peptides:** |
| --- | --- |

|  | Q9BVA1\|TBB2B_HUMAN    **Mass:** 50377    **Score:** 638    **Queries matched:** 47 |
| --- | --- |
|  | Tubulin beta-2B chain OS=Homo sapiens GN=TUBB2B PE=1 SV=1 |

| **51.** | Q9BQE3\|TBA1C_HUMAN    **Mass:** 50548    **Score:** 659    **Queries matched:** 37   **emPAI:** 2.61 |
| --- | --- |
|  | Tubulin alpha-1C chain OS=Homo sapiens GN=TUBA1C PE=1 SV=1 |

|  | **Query** | **Observed** | **Mr(expt)** | **Mr(calc)** | **Delta** | **Miss** | **Score** | **Expect** | **Rank** | **Peptide** |
| --- | --- | --- | --- | --- | --- | --- | --- | --- | --- | --- |
|  | 1369 | 615.3498 | 614.3426 | 613.3435 | 0.9990 | 0 | 22 | 1.5 | 1 | R.GDVVPK.D |
|  | 5307 | 508.2902 | 1014.5658 | 1014.5709 | -0.0051 | 0 | 43 | 0.0084 | 1 | K.DVNAAIATIK.T |
|  | 8847 | 625.2756 | 1248.5366 | 1248.5453 | -0.0087 | 0 | 29 | 0.17 | 1 | K.YMACCLLYR.G |
|  | 11801 | 470.9266 | 1409.7580 | 1409.7667 | -0.0087 | 0 | 13 | 6.6 | 1 | R.QLFHPEQLITGK.E |
|  | 13059 | 744.4392 | 1486.8639 | 1486.8719 | -0.0080 | 0 | 64 | 5.2e-005 | 1 | R.LISQIVSSITASLR.F 13061 |
|  | 14696 | **799.8823** | **1597.7500** | **1597.7599** | **-0.0100** | **0** | **38** | **0.02** | **1** | **R.TIQFVDWCPTGFK.V** |
|  | 16269 | 851.4518 | 1700.8890 | 1700.8985 | -0.0095 | 0 | 61 | 9.5e-005 | 1 | R.AVFVDLEPTVIDEVR.T |
|  | 16490 | 859.9390 | 1717.8634 | 1717.8747 | -0.0113 | 0 | 37 | 0.021 | 1 | R.NLDIERPTYTNLNR.L 16491 |
|  | 17026 | 878.9797 | 1755.9449 | 1755.9559 | -0.0110 | 0 | 36 | 0.025 | 1 | R.IHFPLATYAPVISAEK.A 17025 17028 17029 |
|  | 17925 | 912.9903 | 1823.9660 | 1823.9782 | -0.0121 | 0 | 56 | 0.00029 | 1 | K.VGINYQPPTVVPGGDLAK.V 17924 17926 |
|  | 20182 | 1004.4432 | 2006.8719 | 2006.8858 | -0.0139 | 0 | 83 | 4.7e-007 | 1 | K.TIGGGDDSFNTFFSETGAGK.H 20183 20184 20191 |
|  | 23245 | 1165.5043 | 2328.9940 | 2329.0110 | -0.0170 | 0 | 77 | 1.2e-006 | 1 | R.AFVHWYVGEGMEEGEFSEAR.E 23246 |
|  | 23509 | **787.4150** | **2359.2231** | **2359.1413** | **0.0818** | **1** | **1** | **65** | **3** | **R.AVCMLSNTTAVAEAWARLDHK.F** |
|  | 23808 | 1205.1003 | 2408.1861 | 2408.2012 | -0.0151 | 0 | 123 | 3.3e-011 | 1 | R.FDGALNVDLTEFQTNLVPYPR.I 23809 23811 23812 |
|  | 26194 | **922.1013** | **2763.2821** | **2763.2996** | **-0.0175** | **0** | **52** | **0.00038** | **1** | **K.AYHEQLTVAEITNACFEPANQMVK.C** |
|  | 28280 | 805.1068 | 3216.3981 | 3216.4168 | -0.0187 | 1 | 22 | 0.27 | 1 | R.AFVHWYVGEGMEEGEFSEAREDMAALEK.D |
|  | 28608 | **1103.8486** | **3308.5241** | **3307.5060** | **1.0181** | **1** | **1** | **32** | **6** | **K.AYHEQLTVAEITNACFEPANQMVKCDPR.H** |
|  | 28905 | 1130.8749 | 3389.6028 | 3389.6173 | -0.0145 | 0 | 77 | 9.2e-007 | 1 | K.LADQCTGLQGFLVFHSFGGGTGSGFTSLLMER.L 28906 |
|  | 29243 | 880.4315 | 3517.6967 | 3517.7123 | -0.0156 | 1 | 6 | 9.9 | 2 | R.KLADQCTGLQGFLVFHSFGGGTGSGFTSLLMER.L 29242 |
|  | 30755 | 1074.9904 | 4295.9323 | 4295.9497 | -0.0173 | 0 | 10 | 2.3 | 1 | R.ECISIHVGQAGVQIGNACWELYCLEHGIQPDGQMPSDK.T |
|  | 31692 | 1380.3986 | 5517.5651 | 5516.5891 | 0.9761 | 0 | 0 | 9.1 | 7 | K.LEFSIYPAPQVSTAVVEPYNSILTTHTTLEHSDCAFMVDNEAIYDICR.R |

| **52.** | Q71U36\|TBA1A_HUMAN    **Mass:** 50788    **Score:** 659    **Queries matched:** 34   **emPAI:** 2.33 |
| --- | --- |
|  | Tubulin alpha-1A chain OS=Homo sapiens GN=TUBA1A PE=1 SV=1 |

|  | **Query** | **Observed** | **Mr(expt)** | **Mr(calc)** | **Delta** | **Miss** | **Score** | **Expect** | **Rank** | **Peptide** |
| --- | --- | --- | --- | --- | --- | --- | --- | --- | --- | --- |
|  | 1369 | 615.3498 | 614.3426 | 613.3435 | 0.9990 | 0 | 22 | 1.5 | 1 | R.GDVVPK.D |
|  | 5307 | 508.2902 | 1014.5658 | 1014.5709 | -0.0051 | 0 | 43 | 0.0084 | 1 | K.DVNAAIATIK.T |
|  | 8847 | 625.2756 | 1248.5366 | 1248.5453 | -0.0087 | 0 | 29 | 0.17 | 1 | K.YMACCLLYR.G |
|  | 11801 | 470.9266 | 1409.7580 | 1409.7667 | -0.0087 | 0 | 13 | 6.6 | 1 | R.QLFHPEQLITGK.E |
|  | 14696 | 799.8823 | 1597.7500 | 1597.7599 | -0.0100 | 0 | 38 | 0.02 | 1 | R.TIQFVDWCPTGFK.V |
|  | 16269 | 851.4518 | 1700.8890 | 1700.8985 | -0.0095 | 0 | 61 | 9.5e-005 | 1 | R.AVFVDLEPTVIDEVR.T |
|  | 16490 | 859.9390 | 1717.8634 | 1717.8747 | -0.0113 | 0 | 37 | 0.021 | 1 | R.NLDIERPTYTNLNR.L 16491 |
|  | 17026 | 878.9797 | 1755.9449 | 1755.9559 | -0.0110 | 0 | 36 | 0.025 | 1 | R.IHFPLATYAPVISAEK.A 17025 17028 17029 |
|  | 17925 | 912.9903 | 1823.9660 | 1823.9782 | -0.0121 | 0 | 56 | 0.00029 | 1 | K.VGINYQPPTVVPGGDLAK.V 17924 17926 |
|  | 18536 | 932.9506 | 1863.8866 | 1863.8971 | -0.0105 | 0 | 91 | 8.5e-008 | 1 | R.AVCMLSNTTAIAEAWAR.L |
|  | 20182 | 1004.4432 | 2006.8719 | 2006.8858 | -0.0139 | 0 | 83 | 4.7e-007 | 1 | K.TIGGGDDSFNTFFSETGAGK.H 20183 20184 20191 |
|  | 23245 | 1165.5043 | 2328.9940 | 2329.0110 | -0.0170 | 0 | 77 | 1.2e-006 | 1 | R.AFVHWYVGEGMEEGEFSEAR.E 23246 |
|  | 23808 | 1205.1003 | 2408.1861 | 2408.2012 | -0.0151 | 0 | 123 | 3.3e-011 | 1 | R.FDGALNVDLTEFQTNLVPYPR.I 23809 23811 23812 |
|  | 26118 | 917.4299 | 2749.2680 | 2749.2840 | -0.0160 | 0 | 32 | 0.04 | 1 | K.AYHEQLSVAEITNACFEPANQMVK.C |
|  | 28280 | 805.1068 | 3216.3981 | 3216.4168 | -0.0187 | 1 | 22 | 0.27 | 1 | R.AFVHWYVGEGMEEGEFSEAREDMAALEK.D |
|  | 28905 | 1130.8749 | 3389.6028 | 3389.6173 | -0.0145 | 0 | 77 | 9.2e-007 | 1 | K.LADQCTGLQGFLVFHSFGGGTGSGFTSLLMER.L 28906 |
|  | 29243 | 880.4315 | 3517.6967 | 3517.7123 | -0.0156 | 1 | 6 | 9.9 | 2 | R.KLADQCTGLQGFLVFHSFGGGTGSGFTSLLMER.L 29242 |
|  | 30755 | 1074.9904 | 4295.9323 | 4295.9497 | -0.0173 | 0 | 10 | 2.3 | 1 | R.ECISIHVGQAGVQIGNACWELYCLEHGIQPDGQMPSDK.T |
|  | 31692 | 1380.3986 | 5517.5651 | 5516.5891 | 0.9761 | 0 | 0 | 9.1 | 7 | K.LEFSIYPAPQVSTAVVEPYNSILTTHTTLEHSDCAFMVDNEAIYDICR.R |

| **53.** | Q14651\|PLSI_HUMAN    **Mass:** 70608    **Score:** 656    **Queries matched:** 52   **emPAI:** 2.09 |
| --- | --- |
|  | Plastin-1 OS=Homo sapiens GN=PLS1 PE=1 SV=2 |

|  | **Query** | **Observed** | **Mr(expt)** | **Mr(calc)** | **Delta** | **Miss** | **Score** | **Expect** | **Rank** | **Peptide** |
| --- | --- | --- | --- | --- | --- | --- | --- | --- | --- | --- |
|  | 1020 | **545.3256** | **544.3183** | **543.3493** | **0.9690** | **1** | **17** | **7** | **1** | **R.KIGAR.I** |
|  | 1946 | **769.4039** | **768.3967** | **768.4018** | **-0.0051** | **0** | **24** | **0.52** | **1** | **K.TSISSFK.D** 1945 1948 |
|  | 1973 | **779.4353** | **778.4280** | **778.4337** | **-0.0057** | **0** | **5** | **52** | **4** | **K.YAISVAR.K** |
|  | 3168 | **433.2217** | **864.4288** | **865.4658** | **-1.0370** | **1** | **10** | **18** | **5** | **K.DISKTFR.K** |
|  | 3532 | **888.4882** | **887.4809** | **887.4865** | **-0.0056** | **0** | **21** | **1.5** | **1** | **K.WVNQTLK.S** 3533 |
|  | 4325 | **472.7755** | **943.5365** | **942.5386** | **0.9979** | **0** | **36** | **0.051** | **1** | **K.VNDEIIIK.W** |
|  | 4351 | **473.7607** | **945.5069** | **945.5131** | **-0.0062** | **0** | **18** | **3.2** | **1** | **K.ILSVADSNK.D** |
|  | 5234 | **505.2946** | **1008.5746** | **1007.4883** | **1.0863** | **0** | **12** | **9.3** | **1** | **M.ENSTTTISR.E** |
|  | 6020 | **535.3128** | **1068.6111** | **1068.6179** | **-0.0068** | **0** | **9** | **21** | **6** | **K.LSPEELLLR.W** |
|  | 6111 | **537.7914** | **1073.5683** | **1073.5757** | **-0.0074** | **0** | **5** | **57** | **3** | **K.EASLPLPGYK.V** |
|  | 6127 | **538.2730** | **1074.5313** | **1074.5379** | **-0.0066** | **0** | **58** | **0.00031** | **1** | **R.AGLMLQEADK.L** |
|  | 6340 | **545.8008** | **1089.5870** | **1088.5900** | **0.9970** | **0** | **44** | **0.0069** | **1** | **K.SLADGILLCK.M** |
|  | 8801 | **623.8309** | **1245.6472** | **1245.6565** | **-0.0093** | **1** | **31** | **0.11** | **1** | **K.ILSVADSNKDGK.I** 8786 |
|  | 11769 | **705.3333** | **1408.6521** | **1408.6656** | **-0.0136** | **0** | **7** | **22** | **1** | **K.IENCNYAVELGK.N** |
|  | 11870 | **707.8910** | **1413.7675** | **1413.7768** | **-0.0093** | **0** | **54** | **0.00058** | **1** | **R.AYFHLLNQIAPK.G** 11871 |
|  | 11964 | **474.2282** | **1419.6628** | **1418.6430** | **1.0198** | **0** | **7** | **27** | **10** | **K.MVMTVFACLMGK.G** |
|  | 13073 | 497.2392 | 1488.6957 | 1488.7056 | -0.0099 | 1 | 2 | 83 | 9 | R.ENLSDEDKLNNAK.Y |
|  | 13720 | 766.8614 | 1531.7082 | 1531.7188 | -0.0106 | 0 | 36 | 0.034 | 1 | K.MINLSEPDTIDER.A 13721 |
|  | 13738 | **767.3867** | **1532.7588** | **1532.7691** | **-0.0103** | **1** | **76** | **2.9e-006** | **1** | **R.AGLMLQEADKLGCK.Q** |
|  | 13791 | 769.3817 | 1536.7489 | 1536.7606 | -0.0117 | 1 | 43 | 0.0054 | 1 | K.KIENCNYAVELGK.N 13793 |
|  | 14721 | **533.9678** | **1598.8817** | **1598.8919** | **-0.0103** | **0** | **33** | **0.053** | **1** | **R.IYALPDDLVEVKPK.M** 14699 14720 |
|  | 15398 | **820.9038** | **1639.7931** | **1639.8028** | **-0.0097** | **0** | **32** | **0.067** | **1** | **K.HLIPMNPNDDSLFK.S** 15396 15397 |
|  | 16231 | **850.4308** | **1698.8470** | **1698.8538** | **-0.0068** | **0** | **60** | **0.00012** | **1** | **K.ISFEEFVSLMQELK.S** 16230 |
|  | 18226 | **461.7824** | **1843.1003** | **1843.1084** | **-0.0081** | **0** | **26** | **0.22** | **2** | **K.EGKPHLVLGLLWQIIK.V** |
|  | 21834 | **1079.5472** | **2157.0799** | **2157.0874** | **-0.0075** | **0** | **80** | **8.4e-007** | **1** | **R.NEALIALLNEGEELEELMK.L** 21835 |
|  | 21906 | **1083.1078** | **2164.2010** | **2164.2103** | **-0.0093** | **0** | **76** | **2.2e-006** | **1** | **K.SISTSLPVLDLIDAIAPNAVR.Q** 21905 21907 |
|  | 22500 | **1117.5214** | **2233.0282** | **2233.0426** | **-0.0145** | **0** | **104** | **3.4e-009** | **1** | **K.IDIDNSGYVSDYELQDLFK.E** 22502 |
|  | 23472 | **1178.0856** | **2354.1566** | **2354.1714** | **-0.0148** | **1** | **46** | **0.002** | **1** | **K.GGEDGPAIAIDLSGINETNDLKR.A** |
|  | 24251 | **1231.6482** | **2461.2818** | **2461.2952** | **-0.0133** | **1** | **43** | **0.0037** | **1** | **R.YTLNVLSDLGEGEKVNDEIIIK.W** 24252 |
|  | 25375 | **877.7576** | **2630.2511** | **2630.2666** | **-0.0156** | **0** | **27** | **0.12** | **1** | **R.WVNYHLTNAGWHTISNFSQDIK.D** 25373 |
|  | 25805 | **1349.6119** | **2697.2093** | **2697.2253** | **-0.0160** | **0** | **67** | **1.1e-005** | **1** | **R.EGITAIGGTSTISSEGTQHSYSEEEK.V** 25803 25804 |
|  | 26446 | **941.4116** | **2821.2130** | **2821.5371** | **-0.3241** | **1** | **1** | **47** | **1** | **K.SISTSLPVLDLIDAIAPNAVRQEMIR.R** |

| **54.** | P62736\|ACTA_HUMAN    **Mass:** 42381    **Score:** 656    **Queries matched:** 68   **emPAI:** 1.76 |
| --- | --- |
|  | Actin, aortic smooth muscle OS=Homo sapiens GN=ACTA2 PE=1 SV=1 |

|  | **Query** | **Observed** | **Mr(expt)** | **Mr(calc)** | **Delta** | **Miss** | **Score** | **Expect** | **Rank** | **Peptide** |
| --- | --- | --- | --- | --- | --- | --- | --- | --- | --- | --- |
|  | 1426 | 631.3726 | 630.3653 | 630.3701 | -0.0048 | 0 | 10 | 33 | 3 | K.ILTER.G 1421 |
|  | 1491 | 644.8003 | 643.7931 | 643.3653 | 0.4278 | 0 | 18 | 3 | 2 | R.LDLAGR.D |
|  | 2106 | 398.2369 | 794.4593 | 794.4650 | -0.0057 | 0 | 16 | 4 | 1 | K.IIAPPER.K 2107 2108 |
|  | 2167 | 400.7687 | 799.5229 | 799.5280 | -0.0050 | 1 | 24 | 0.83 | 2 | K.RGILTLK.Y |
|  | 4032 | 462.2841 | 922.5536 | 922.5600 | -0.0063 | 1 | 25 | 0.52 | 1 | K.IIAPPERK.Y |
|  | 4771 | 488.7246 | 975.4346 | 975.4410 | -0.0064 | 0 | 65 | 6.7e-005 | 1 | K.AGFAGDDAPR.A 4770 4780 |
|  | 5079 | 499.7437 | 997.4728 | 997.4790 | -0.0062 | 0 | 29 | 0.18 | 1 | R.DLTDYLMK.I 5080 |
|  | 7246 | 581.3093 | 1160.6041 | 1160.6111 | -0.0070 | 0 | 39 | 0.022 | 1 | K.EITALAPSTMK.I 7227 7244 |
|  | 7368 | 586.2852 | 1170.5558 | 1170.5638 | -0.0080 | 0 | 59 | 0.0002 | 1 | R.HQGVMVGMGQK.D 7365 7366 7367 7369 7370 7371 7379 7382 |
|  | 7612 | 594.2825 | 1186.5505 | 1186.5587 | -0.0082 | 0 | (52) | 0.00089 | 1 | R.HQGVMVGMGQK.D 7611 |
|  | 7613 | 594.2828 | 1186.5511 | 1186.5587 | -0.0076 | 0 | (48) | 0.0023 | 1 | R.HQGVMVGMGQK.D 7614 7631 |
|  | 7824 | 599.7609 | 1197.5072 | 1197.5150 | -0.0078 | 0 | 27 | 0.29 | 1 | K.DSYVGDEAQSK.R 7825 |
|  | 7850 | 599.8526 | 1197.6906 | 1197.6982 | -0.0076 | 0 | 65 | 4.6e-005 | 1 | R.AVFPSIVGRPR.H 7847 7848 7849 |
|  | 7942 | 602.2798 | 1202.5450 | 1202.5536 | -0.0086 | 0 | (26) | 0.37 | 1 | R.HQGVMVGMGQK.D 7940 7941 |
|  | 10805 | 677.8108 | 1353.6070 | 1353.6161 | -0.0090 | 1 | 44 | 0.0052 | 1 | K.DSYVGDEAQSKR.G 10804 10819 10829 |
|  | 13259 | 750.3368 | 1498.6590 | 1499.7005 | -1.0414 | 0 | 12 | 6.9 | 1 | K.QEYDEAGPSIVHR.K 13258 |
|  | 14439 | 790.8826 | 1579.7506 | 1579.7949 | -0.0443 | 1 | 2 | 77 | 4 | R.MQKEITALAPSTMK.I 14449 |
|  | 15103 | 541.9487 | 1622.8244 | 1622.8338 | -0.0094 | 1 | 14 | 5.1 | 2 | R.LDLAGRDLTDYLMK.I |
|  | 17483 | 895.9439 | 1789.8733 | 1789.8846 | -0.0114 | 0 | 73 | 6e-006 | 1 | K.SYELPDGQVITIGNER.F 17481 17482 17485 17486 17487 17488 |
|  | 19701 | 980.4697 | 1958.9248 | 1959.9036 | -0.9789 | 0 | 21 | 0.73 | 1 | K.YPIEHGIITNWDDMEK.I |
|  | 22967 | **764.5195** | **2290.5368** | **2289.8858** | **0.6510** | **0** | **3** | **30** | **1** | **-.MCEEEDSTALVCDNGSGLCK.A** |
|  | 24953 | 1276.0880 | 2550.1615 | 2551.1458 | -0.9843 | 0 | 44 | 0.0024 | 2 | K.LCYVALDFENEMATAASSSSLEK.S 24948 24949 24950 24951 24954 24960 24961 24962 24966 |
|  | 25371 | 877.4372 | 2629.2897 | 2630.2469 | -0.9571 | 1 | 1 | 47 | 2 | K.DLYANNVLSGGTTMYPGIADRMQK.E |

| **55.** | P63267\|ACTH_HUMAN    **Mass:** 42249    **Score:** 656    **Queries matched:** 66   **emPAI:** 1.77 |
| --- | --- |
|  | Actin, gamma-enteric smooth muscle OS=Homo sapiens GN=ACTG2 PE=1 SV=1 |

|  | **Query** | **Observed** | **Mr(expt)** | **Mr(calc)** | **Delta** | **Miss** | **Score** | **Expect** | **Rank** | **Peptide** |
| --- | --- | --- | --- | --- | --- | --- | --- | --- | --- | --- |
|  | 1426 | 631.3726 | 630.3653 | 630.3701 | -0.0048 | 0 | 10 | 33 | 3 | K.ILTER.G 1421 |
|  | 1491 | 644.8003 | 643.7931 | 643.3653 | 0.4278 | 0 | 18 | 3 | 2 | R.LDLAGR.D |
|  | 2106 | 398.2369 | 794.4593 | 794.4650 | -0.0057 | 0 | 16 | 4 | 1 | K.IIAPPER.K 2107 2108 |
|  | 2167 | 400.7687 | 799.5229 | 799.5280 | -0.0050 | 1 | 24 | 0.83 | 2 | K.RGILTLK.Y |
|  | 4032 | 462.2841 | 922.5536 | 922.5600 | -0.0063 | 1 | 25 | 0.52 | 1 | K.IIAPPERK.Y |
|  | 4771 | 488.7246 | 975.4346 | 975.4410 | -0.0064 | 0 | 65 | 6.7e-005 | 1 | K.AGFAGDDAPR.A 4770 4780 |
|  | 5079 | 499.7437 | 997.4728 | 997.4790 | -0.0062 | 0 | 29 | 0.18 | 1 | R.DLTDYLMK.I 5080 |
|  | 7246 | 581.3093 | 1160.6041 | 1160.6111 | -0.0070 | 0 | 39 | 0.022 | 1 | K.EITALAPSTMK.I 7227 7244 |
|  | 7368 | 586.2852 | 1170.5558 | 1170.5638 | -0.0080 | 0 | 59 | 0.0002 | 1 | R.HQGVMVGMGQK.D 7365 7366 7367 7369 7370 7371 7379 7382 |
|  | 7612 | 594.2825 | 1186.5505 | 1186.5587 | -0.0082 | 0 | (52) | 0.00089 | 1 | R.HQGVMVGMGQK.D 7611 |
|  | 7613 | 594.2828 | 1186.5511 | 1186.5587 | -0.0076 | 0 | (48) | 0.0023 | 1 | R.HQGVMVGMGQK.D 7614 7631 |
|  | 7824 | 599.7609 | 1197.5072 | 1197.5150 | -0.0078 | 0 | 27 | 0.29 | 1 | K.DSYVGDEAQSK.R 7825 |
|  | 7850 | 599.8526 | 1197.6906 | 1197.6982 | -0.0076 | 0 | 65 | 4.6e-005 | 1 | R.AVFPSIVGRPR.H 7847 7848 7849 |
|  | 7942 | 602.2798 | 1202.5450 | 1202.5536 | -0.0086 | 0 | (26) | 0.37 | 1 | R.HQGVMVGMGQK.D 7940 7941 |
|  | 10805 | 677.8108 | 1353.6070 | 1353.6161 | -0.0090 | 1 | 44 | 0.0052 | 1 | K.DSYVGDEAQSKR.G 10804 10819 10829 |
|  | 14439 | 790.8826 | 1579.7506 | 1579.7949 | -0.0443 | 1 | 2 | 77 | 4 | R.MQKEITALAPSTMK.I 14449 |
|  | 15103 | 541.9487 | 1622.8244 | 1622.8338 | -0.0094 | 1 | 14 | 5.1 | 2 | R.LDLAGRDLTDYLMK.I |
|  | 17483 | 895.9439 | 1789.8733 | 1789.8846 | -0.0114 | 0 | 73 | 6e-006 | 1 | K.SYELPDGQVITIGNER.F 17481 17482 17485 17486 17487 17488 |
|  | 19701 | 980.4697 | 1958.9248 | 1959.9036 | -0.9789 | 0 | 21 | 0.73 | 1 | K.YPIEHGIITNWDDMEK.I |
|  | 24953 | 1276.0880 | 2550.1615 | 2551.1458 | -0.9843 | 0 | 44 | 0.0024 | 2 | K.LCYVALDFENEMATAASSSSLEK.S 24948 24949 24950 24951 24954 24960 24961 24962 24966 |
|  | 25371 | 877.4372 | 2629.2897 | 2630.2469 | -0.9571 | 1 | 1 | 47 | 2 | K.DLYANNVLSGGTTMYPGIADRMQK.E |
|  | 30432 | 1351.9561 | 4052.8463 | 4052.0142 | 0.8321 | 0 | 0 | 25 | 8 | K.YSVWIGGSILASLSTFQQMWISKPEYDEAGPSIVHR.K |

| **56.** | Q14697\|GANAB_HUMAN    **Mass:** 107263   **Score:** 635    **Queries matched:** 61   **emPAI:** 1.11 |
| --- | --- |
|  | Neutral alpha-glucosidase AB OS=Homo sapiens GN=GANAB PE=1 SV=3 |

|  | **Query** | **Observed** | **Mr(expt)** | **Mr(calc)** | **Delta** | **Miss** | **Score** | **Expect** | **Rank** | **Peptide** |
| --- | --- | --- | --- | --- | --- | --- | --- | --- | --- | --- |
|  | 1485 | **644.3149** | **643.3076** | **643.3442** | **-0.0366** | **0** | **6** | **60** | **5** | **R.FPQPR.T** |
|  | 2753 | **421.0563** | **840.0981** | **839.4072** | **0.6909** | **1** | **4** | **36** | **7** | **K.NMTRFR.I** |
|  | 3307 | **437.2403** | **872.4660** | **872.4603** | **0.0056** | **0** | **24** | **0.76** | **1** | **R.LDLLEDR.S** 3317 3327 |
|  | 4117 | **465.2733** | **928.5321** | **929.5116** | **-0.9795** | **0** | **0** | **1.6e+002** | **6** | **-.MAAVAAVAAR.R** |
|  | 4694 | **486.2706** | **970.5267** | **971.5764** | **-1.0496** | **0** | **38** | **0.029** | **1** | **R.SLLLSVNAR.G** 4711 |
|  | 4791 | **489.2752** | **976.5359** | **976.4839** | **0.0521** | **0** | **16** | **4.2** | **1** | **R.AHAHLDTGR.R** |
|  | 5208 | **504.2756** | **1006.5367** | **1006.4720** | **0.0647** | **0** | **20** | **1.9** | **1** | **K.VTEGGEPYR.L** |
|  | 6266 | **543.3106** | **1084.6067** | **1085.6127** | **-1.0061** | **1** | **5** | **56** | **6** | **-.MAAVAAVAARR.R** |
|  | 6298 | **544.3151** | **1086.6156** | **1085.6709** | **0.9446** | **0** | **16** | **4.5** | **2** | **K.IILTARPFR.L** |
|  | 6518 | **552.8389** | **1103.6633** | **1103.6703** | **-0.0070** | **0** | **43** | **0.0091** | **1** | **K.LVAIVDPHIK.V** |
|  | 6821 | **564.7900** | **1127.5654** | **1127.5723** | **-0.0069** | **0** | **41** | **0.012** | **1** | **R.GLLEFEHQR.A** 6839 |
|  | 7031 | **573.3206** | **1144.6267** | **1144.6353** | **-0.0086** | **0** | **15** | **5.3** | **1** | **R.SIRPGLSPYR.A** |
|  | 8555 | **411.9275** | **1232.7606** | **1231.7652** | **0.9953** | **1** | **11** | **12** | **1** | **R.KLVAIVDPHIK.V** |
|  | 8854 | **625.3312** | **1248.6479** | **1247.6510** | **0.9969** | **1** | **11** | **12** | **1** | **R.LKVTEGGEPYR.L** |
|  | 9605 | **645.3268** | **1288.6391** | **1289.6550** | **-1.0159** | **1** | **4** | **61** | **6** | **R.FPQPRTMLER.L** |
|  | 9868 | **652.2695** | **1302.5245** | **1302.5333** | **-0.0088** | **1** | **8** | **24** | **1** | **K.TCEESSFCKR.Q** |
|  | 10177 | **440.5652** | **1318.6739** | **1318.6816** | **-0.0077** | **0** | **34** | **0.054** | **1** | **R.SGGMERPFVLAR.A** |
|  | 10785 | **677.3220** | **1352.6295** | **1351.8439** | **0.7856** | **0** | **1** | **1.1e+002** | **10** | **K.VLLVLELQGLQK.N** |
|  | 10821 | **678.2925** | **1354.5704** | **1354.5803** | **-0.0099** | **0** | **23** | **0.68** | **1** | **K.DAQHYGGWEHR.D** |
|  | 15118 | **542.2550** | **1623.7432** | **1622.6705** | **1.0727** | **1** | **2** | **82** | **5** | **R.SNFKTCEESSFCK.R** |
|  | 15725 | **832.5212** | **1663.0279** | **1663.0396** | **-0.0117** | **0** | **66** | **2.4e-005** | **1** | **R.VVIIGAGKPAAVVLQTK.G** 15719 15724 15726 |
|  | 15970 | **560.7994** | **1679.3763** | **1679.9359** | **-0.5596** | **1** | **1** | **55** | **8** | **R.YRVPDVLVADPPIAR.L** 15978 |
|  | 16782 | **870.9617** | **1739.9089** | **1739.9206** | **-0.0117** | **0** | **25** | **0.36** | **1** | **R.LSFQHDPETSVLVLR.K** 16783 |
|  | 18382 | **927.7368** | **1853.4591** | **1854.0761** | **-0.6170** | **1** | **1** | **62** | **3** | **K.VLLVLELQGLQKNMTR.F** |
|  | 18653 | **625.3354** | **1872.9843** | **1872.9958** | **-0.0115** | **1** | **30** | **0.095** | **1** | **R.REPWLLPSQHNDIIR.D** 18652 |
|  | 19323 | **964.4225** | **1926.8304** | **1926.8452** | **-0.0147** | **0** | **82** | **6e-007** | **1** | **K.MMDYLQGSGETPQTDVR.W** 19326 |
|  | 19533 | **649.0190** | **1944.0353** | **1942.8401** | **1.1952** | **0** | **(3)** | **43** | **2** | **K.MMDYLQGSGETPQTDVR.W** |
|  | 19559 | **973.9485** | **1945.8824** | **1945.8959** | **-0.0135** | **0** | **87** | **2e-007** | **1** | **R.FGAVWTGDNTAEWDHLK.I** 19563 |
|  | 21662 | **537.0157** | **2144.0337** | **2144.0473** | **-0.0136** | **0** | **31** | **0.072** | **1** | **R.DVHNIYGLYVHMATADGLR.Q** |
|  | 21797 | **718.9966** | **2153.9681** | **2153.9825** | **-0.0144** | **1** | **36** | **0.019** | **1** | **R.VSQGSKDPAEGDGAQPEETPR.D** |
|  | 23694 | **797.4336** | **2389.2791** | **2389.2907** | **-0.0115** | **0** | **22** | **0.41** | **1** | **K.HHGPQTLYLPVTLSSIPVFQR.G** |
|  | 24474 | **830.7474** | **2489.2203** | **2489.1567** | **0.0636** | **1** | **2** | **39** | **4** | **K.TLFGKMMDYLQGSGETPQTDVR.W** |
|  | 24554 | **1250.1888** | **2498.3631** | **2498.3744** | **-0.0113** | **0** | **40** | **0.0063** | **1** | **R.ALLDSLQLGPDSLTVHLIHEVTK.V** 24553 24555 |
|  | 24786 | **845.7662** | **2534.2769** | **2534.2918** | **-0.0149** | **0** | **26** | **0.16** | **1** | **R.QYASLTGTQALPPLFSLGYHQSR.W** |
|  | 25195 | **865.7864** | **2594.3375** | **2594.3455** | **-0.0080** | **0** | **4** | **26** | **1** | **R.WMSETGIIDVFLLLGPSISDVFR.Q** |
|  | 26627 | **1433.5704** | **2865.1263** | **2865.1436** | **-0.0172** | **0** | **66** | **1.2e-005** | **1** | **R.DGSDYEGWCWPGSAGYPDFTNPTMR.A** 26626 26628 |
|  | 27344 | **1004.1398** | **3009.3975** | **3009.4145** | **-0.0170** | **0** | **62** | **3.5e-005** | **1** | **R.FSFSGNTLVSSSADPEGHFETPIWIER.V** |
|  | 29598 | **912.4647** | **3645.8295** | **3645.8442** | **-0.0147** | **0** | **46** | **0.00083** | **1** | **R.LYNLDVFQYELYNPMALYGSVPVLLAHNPHR.D** 29599 29600 |
|  | 30319 | **791.5734** | **3952.8304** | **3952.8513** | **-0.0208** | **0** | **36** | **0.0065** | **1** | **K.THSDSKPYGPMSVGLDFSLPGMEHVYGIPEHADNLR.L** 30318 |
|  | 30758 | **860.7905** | **4298.9160** | **4297.9287** | **0.9872** | **1** | **7** | **4.1** | **1** | **R.WNYRDEADVLEVDQGFDDHNLPCDVIWLDIEHADGK.R** |
|  | 31483 | **1709.6815** | **5126.0227** | **5126.6216** | **-0.5988** | **1** | **4** | **3.9** | **1** | **R.WMSETGIIDVFLLLGPSISDVFRQYASLTGTQALPPLFSLGYHQSR.W** |
|  | 32038 | **1449.7174** | **7243.5507** | **7243.5706** | **-0.0200** | **0** | **18** | **0.069** | **1** | **R.EGIPVMRPLWVQYPQDVTTFNIDDQYLLGDALLVHPVSDSGAHGVQVYLPGQGEVWYDIQSYQK.H** |

| **57.** | P13667\|PDIA4_HUMAN    **Mass:** 73229    **Score:** 627    **Queries matched:** 37   **emPAI:** 1.00 |
| --- | --- |
|  | Protein disulfide-isomerase A4 OS=Homo sapiens GN=PDIA4 PE=1 SV=2 |

|  | **Query** | **Observed** | **Mr(expt)** | **Mr(calc)** | **Delta** | **Miss** | **Score** | **Expect** | **Rank** | **Peptide** |
| --- | --- | --- | --- | --- | --- | --- | --- | --- | --- | --- |
|  | 790 | **501.3365** | **500.3292** | **500.3322** | **-0.0030** | **0** | **17** | **4.9** | **1** | **K.VVVGK.T** |
|  | 4737 | **487.7473** | **973.4801** | **973.4869** | **-0.0068** | **0** | **30** | **0.18** | **1** | **K.FIEEHATK.L** |
|  | 6194 | **540.7405** | **1079.4664** | **1080.4836** | **-1.0172** | **0** | **19** | **1.9** | **1** | **K.GQAVDYEGSR.T** 6209 |
|  | 6789 | **563.7894** | **1125.5642** | **1125.5706** | **-0.0064** | **0** | **40** | **0.016** | **1** | **R.FDVSGYPTIK.I** |
|  | 6883 | **567.2840** | **1132.5534** | **1132.5612** | **-0.0077** | **0** | **43** | **0.0077** | **1** | **K.VDATAETDLAK.R** 6902 |
|  | 7147 | **577.3129** | **1152.6113** | **1152.6767** | **-0.0654** | **1** | **6** | **36** | **3** | **K.YALPLVGHRK.V** |
|  | 7684 | **595.8183** | **1189.6220** | **1189.6302** | **-0.0082** | **0** | **103** | **8.5e-009** | **1** | **K.IDATSASVLASR.F** 7685 7686 |
|  | 7739 | **597.2756** | **1192.5367** | **1193.5578** | **-1.0210** | **0** | **20** | **1.7** | **1** | **K.GRPYDYNGPR.E** |
|  | 9085 | **631.3394** | **1260.6642** | **1260.6714** | **-0.0072** | **0** | **32** | **0.085** | **1** | **K.QLEPVYNSLAK.K** 9091 |
|  | 9608 | **645.3342** | **1288.6539** | **1288.6623** | **-0.0084** | **1** | **53** | **0.00072** | **1** | **K.VDATAETDLAKR.F** |
|  | 9650 | **646.2783** | **1290.5420** | **1290.5510** | **-0.0091** | **0** | **41** | **0.01** | **1** | **K.MDATANDVPSDR.Y** |
|  | 10134 | **439.8886** | **1316.6438** | **1316.6513** | **-0.0075** | **0** | **18** | **2.6** | **1** | **K.FHHTFSTEIAK.F** 10136 10143 |
|  | 12339 | **721.8825** | **1441.7505** | **1441.7599** | **-0.0094** | **0** | **75** | **4.3e-006** | **1** | **K.VSQGQLVVMQPEK.F** 12340 |
|  | 12611 | **730.4407** | **1458.8668** | **1457.7548** | **1.1120** | **0** | **(5)** | **40** | **2** | **K.VSQGQLVVMQPEK.F** |
|  | 13298 | **751.8605** | **1501.7064** | **1501.7161** | **-0.0097** | **1** | **12** | **9.1** | **1** | **K.FEGGDRDLEHLSK.F** |
|  | 15171 | **814.3985** | **1626.7824** | **1626.7930** | **-0.0105** | **0** | **12** | **7.2** | **1** | **K.VEGFPTIYFAPSGDK.K** |
|  | 15543 | **550.9949** | **1649.9628** | **1649.8699** | **0.0929** | **1** | **8** | **18** | **1** | **K.VVVGKTFDSIVMDPK.K** |
|  | 16700 | **868.3887** | **1734.7628** | **1733.7713** | **0.9915** | **0** | **30** | **0.096** | **1** | **R.SHMMDVQGSTQDSAIK.D** |
|  | 17389 | **892.4267** | **1782.8388** | **1782.8498** | **-0.0110** | **0** | **75** | **3.2e-006** | **1** | **K.YGIVDYMIEQSGPPSK.E** |
|  | 20080 | **666.3378** | **1995.9917** | **1994.9019** | **1.0898** | **0** | **1** | **68** | **3** | **K.DTVLLEFYAPWCGHCK.Q** |
|  | 20540 | 1020.4489 | 2038.8833 | 2038.8980 | -0.0148 | 0 | 86 | 2.1e-007 | 1 | K.GESDPAYQQYQDAANNLR.E 20541 |
|  | 21586 | **1068.5595** | **2135.1043** | **2135.1151** | **-0.0107** | **0** | **30** | **0.093** | **1** | **R.EVSQPDWTPPPEVTLVLTK.E** 21585 |
|  | 22602 | **1124.0453** | **2246.0760** | **2246.0914** | **-0.0153** | **1** | **118** | **1.4e-010** | **1** | **K.DLGLSESGEDVNAAILDESGKK.F** 22600 22601 |
|  | 23020 | **1148.5598** | **2295.1051** | **2295.0260** | **0.0791** | **1** | **1** | **55** | **4** | **K.YEPRSHMMDVQGSTQDSAIK.D** |
|  | 23291 | **779.7070** | **2336.0991** | **2336.1141** | **-0.0150** | **1** | **45** | **0.0027** | **1** | **R.SHMMDVQGSTQDSAIKDFVLK.Y** |

| **58.** | P09110\|THIK_HUMAN    **Mass:** 44834    **Score:** 619    **Queries matched:** 31   **emPAI:** 1.06 |
| --- | --- |
|  | 3-ketoacyl-CoA thiolase, peroxisomal OS=Homo sapiens GN=ACAA1 PE=1 SV=2 |

|  | **Query** | **Observed** | **Mr(expt)** | **Mr(calc)** | **Delta** | **Miss** | **Score** | **Expect** | **Rank** | **Peptide** |
| --- | --- | --- | --- | --- | --- | --- | --- | --- | --- | --- |
|  | 2085 | **397.4028** | **792.7910** | **792.4051** | **0.3859** | **1** | **4** | **48** | **1** | **R.LMEKEK.A** 2073 |
|  | 2936 | **853.5834** | **852.5761** | **851.5229** | **1.0532** | **1** | **2** | **90** | **3** | **K.LRLPPEK.V** |
|  | 10349 | **443.2708** | **1326.7905** | **1325.8031** | **0.9875** | **1** | **20** | **1.3** | **1** | **R.QVITLLNELKR.R** 10318 |
|  | 11245 | **690.4128** | **1378.8110** | **1378.8184** | **-0.0074** | **0** | **18** | **2.3** | **1** | **K.AEELGLPILGVLR.S** |
|  | 11908 | **708.8597** | **1415.7049** | **1415.7191** | **-0.0141** | **1** | **3** | **70** | **4** | **R.GNPGNITSRLMEK.E** |
|  | 13170 | **747.3784** | **1492.7423** | **1492.7522** | **-0.0099** | **1** | **61** | **9.6e-005** | **1** | **R.EKQDTFALASQQK.A** 13169 13171 |
|  | 17640 | **902.4714** | **1802.9282** | **1802.9336** | **-0.0054** | **0** | **60** | **0.00011** | **1** | **K.DTTPDELLSAVMTAVLK.D** 17641 17656 |
|  | 17650 | **902.9262** | **1803.8379** | **1803.8495** | **-0.0116** | **0** | **12** | **7.2** | **1** | **R.DCLIPMGITSENVAER.F** |
|  | 19210 | **959.0041** | **1915.9936** | **1916.0051** | **-0.0114** | **0** | **73** | **5.2e-006** | **1** | **K.VNPLGGAVALGHPLGCTGAR.Q** 19209 |
|  | 20257 | **672.9901** | **2015.9485** | **2015.9623** | **-0.0138** | **0** | **23** | **0.5** | **1** | **K.GCFQAEIVPVTTTVHDDK.G** |
|  | 20464 | **678.3775** | **2032.1107** | **2030.9877** | **1.1230** | **1** | **1** | **75** | **5** | **K.ARDCLIPMGITSENVAER.F** |
|  | 21181 | **1050.5647** | **2099.1148** | **2099.1263** | **-0.0114** | **0** | **116** | **2.4e-010** | **1** | **R.IAQFLSDIPETVPLSTVNR.Q** 21189 |
|  | 21687 | **716.8909** | **2147.6508** | **2147.8922** | **-0.2414** | **0** | **2** | **36** | **4** | **R.NGSYDIGMACGVESMSLADR.G** |
|  | 21877 | **1081.5315** | **2161.0484** | **2161.0611** | **-0.0127** | **0** | **101** | **6e-009** | **1** | **K.DGSTTAGNSSQVSDGAAAILLAR.R** 21876 |
|  | 22507 | **1117.5620** | **2233.1095** | **2233.1260** | **-0.0165** | **0** | **76** | **2e-006** | **1** | **R.SITVTQDEGIRPSTTMEGLAK.L** 22506 |
|  | 23165 | 773.0061 | 2315.9965 | 2317.1622 | -1.1657 | 1 | 4 | 29 | 2 | K.DGSTTAGNSSQVSDGAAAILLARR.S |
|  | 24052 | **1219.1085** | **2436.2025** | **2437.0753** | **-0.8728** | **0** | **0** | **62** | **2** | **R.AYGVVSMCIGTGMGAAAVFEYPGN.-** |
|  | 25354 | **1313.7133** | **2625.4120** | **2625.4240** | **-0.0121** | **0** | **43** | **0.003** | **1** | **R.SYAVVGVPPDIMGIGPAYAIPVALQK.A** 25353 |
|  | 27172 | **988.5065** | **2962.4978** | **2962.5117** | **-0.0139** | **0** | **44** | **0.0023** | **1** | **K.DVNLRPEQLGDICVGNVLQPGAGAIMAR.I** 27171 |

| **59.** | Q3LXA3\|DHAK_HUMAN    **Mass:** 59282    **Score:** 619    **Queries matched:** 44   **emPAI:** 0.95 |
| --- | --- |
|  | Bifunctional ATP-dependent dihydroxyacetone kinase/FAD-AMP lyase (cyclizing) OS=Homo sapiens GN=DAK PE=1 SV=1 |

|  | **Query** | **Observed** | **Mr(expt)** | **Mr(calc)** | **Delta** | **Miss** | **Score** | **Expect** | **Rank** | **Peptide** |
| --- | --- | --- | --- | --- | --- | --- | --- | --- | --- | --- |
|  | 1989 | **787.3101** | **786.3028** | **786.4600** | **-0.1572** | **0** | **32** | **0.15** | **1** | **K.QVNVVTK.A** |
|  | 2947 | **427.7371** | **853.4596** | **853.4294** | **0.0303** | **0** | **2** | **87** | **4** | **R.ASYISSAR.L** |
|  | 3519 | **444.2505** | **886.4864** | **886.4912** | **-0.0048** | **0** | **8** | **35** | **3** | **R.AIQEWLK.E** |
|  | 3905 | **457.7970** | **913.5794** | **913.5848** | **-0.0054** | **0** | **38** | **0.026** | **1** | **K.LSVLLLEK.M** 3882 |
|  | 4396 | **475.2377** | **948.4608** | **947.4559** | **1.0048** | **0** | **7** | **39** | **1** | **K.SAEAAAEATK.N** 4380 |
|  | 5009 | **497.3003** | **992.5860** | **992.4848** | **0.1011** | **0** | **3** | **91** | **9** | **K.MATADEIVK.L** |
|  | 8257 | **406.5659** | **1216.6760** | **1215.6823** | **0.9937** | **1** | **10** | **16** | **4** | **R.VALRSDLDSLK.G** |
|  | 8306 | **610.3638** | **1218.7131** | **1217.6689** | **1.0442** | **1** | **9** | **18** | **2** | **R.IKMATADEIVK.L** 8298 |
|  | 11460 | **696.3763** | **1390.7381** | **1390.7456** | **-0.0075** | **0** | **22** | **0.95** | **1** | **K.EGPPPASPAQLLSK.L** |
|  | 12315 | **481.1834** | **1440.5283** | **1439.8712** | **0.6571** | **0** | **11** | **8.4** | **5** | **R.AVAQAGTVGTLLIVK.N** 12302 |
|  | 14620 | **531.2952** | **1590.8639** | **1590.8729** | **-0.0091** | **0** | **30** | **0.13** | **1** | **R.LEQPDPGAVAAAAILR.A** 14617 |
|  | 16687 | **578.6545** | **1732.9418** | **1733.8002** | **-0.8584** | **1** | **1** | **85** | **9** | **K.SAEAAAEATKNMEAGAGR.A** |
|  | 19479 | 970.9573 | 1939.9001 | 1939.9123 | -0.0122 | 0 | 121 | 7.8e-011 | 1 | R.VAPAEPQEAPDSTAAGGSASK.R 19478 19489 |
|  | 19576 | **649.9899** | **1946.9478** | **1946.9560** | **-0.0082** | **0** | **39** | **0.013** | **1** | **R.TMLDSLWAAGQELQAWK.S** 19575 |
|  | 19890 | **989.4686** | **1976.9226** | **1976.9335** | **-0.0110** | **0** | **32** | **0.063** | **1** | **K.TSLPAWSAAMDAGLEAMQK.Y** |
|  | 21678 | **716.5201** | **2146.5384** | **2146.1746** | **0.3638** | **1** | **12** | **3.6** | **1** | **R.AVAQAGTVGTLLIVKNYTGDR.L** 21677 21679 21680 21685 |
|  | 22111 | **1095.5664** | **2189.1183** | **2189.1290** | **-0.0107** | **0** | **86** | **2e-007** | **1** | **R.AEGIPVEMVVIGDDSAFTVLK.K** 22102 22110 |
|  | 22449 | **1114.0828** | **2226.1510** | **2226.1644** | **-0.0135** | **0** | **99** | **1.1e-008** | **1** | **K.LIDAETTAAAWPNVAAVSITGR.K** 22450 |
|  | 23181 | **773.4115** | **2317.2127** | **2317.2239** | **-0.0113** | **1** | **(4)** | **29** | **2** | **R.AEGIPVEMVVIGDDSAFTVLKK.A** |
|  | 23276 | **584.3123** | **2333.2199** | **2333.2189** | **0.0011** | **1** | **5** | **21** | **2** | **R.AEGIPVEMVVIGDDSAFTVLKK.A** |
|  | 23590 | **791.4476** | **2371.3211** | **2371.3297** | **-0.0087** | **0** | **67** | **1.3e-005** | **1** | **K.GMLTGVIAGAVFTSPAVGSILAAIR.A** 23589 |
|  | 25630 | 892.4612 | 2674.3619 | 2673.3684 | 0.9935 | 1 | 3 | 27 | 3 | R.EQARAEGIPVEMVVIGDDSAFTVLK.K 25625 |
|  | 27932 | **1049.5262** | **3145.5569** | **3145.5761** | **-0.0192** | **0** | **107** | **9.3e-010** | **1** | **K.LVNSVAGCADDALAGLVACNPNLQLLQGHR.V** 27933 |
|  | 28518 | **1092.2249** | **3273.6527** | **3273.6710** | **-0.0183** | **1** | **68** | **6.5e-006** | **1** | **K.KLVNSVAGCADDALAGLVACNPNLQLLQGHR.V** |
|  | 29442 | **897.1771** | **3584.6791** | **3584.8668** | **-0.1876** | **1** | **3** | **19** | **1** | **K.LVNSVAGCADDALAGLVACNPNLQLLQGHRVALR.S** |
|  | 31709 | **1847.8667** | **5540.5783** | **5539.7572** | **0.8211** | **1** | **1** | **7.9** | **2** | **K.LMLDHMTNTTNASHVPVQPGSSVVMMVNNLGGLSFLELGIIADATVRSLEGR.G** |

| **60.** | Q9UGM3\|DMBT1_HUMAN    **Mass:** 268039   **Score:** 600    **Queries matched:** 17   **emPAI:** 0.09 |
| --- | --- |
|  | Deleted in malignant brain tumors 1 protein OS=Homo sapiens GN=DMBT1 PE=1 SV=2 |

|  | **Query** | **Observed** | **Mr(expt)** | **Mr(calc)** | **Delta** | **Miss** | **Score** | **Expect** | **Rank** | **Peptide** |
| --- | --- | --- | --- | --- | --- | --- | --- | --- | --- | --- |
|  | 1814 | **731.1769** | **730.1697** | **729.3770** | **0.7927** | **0** | **6** | **43** | **3** | **R.LVNGDGR.C** |
|  | 5700 | **523.7732** | **1045.5319** | **1045.5087** | **0.0232** | **1** | **4** | **63** | **3** | **R.LVNGGDRCR.G** |
|  | 9328 | **425.2042** | **1272.5908** | **1273.5509** | **-0.9601** | **1** | **3** | **66** | **6** | **R.AYDPSSRCYR.G** |
|  | 11622 | **701.5563** | **1401.0981** | **1400.7194** | **0.3787** | **1** | **5** | **31** | **2** | **R.SSPLIARVCDGAR.G** |
|  | 12601 | **730.3759** | **1458.7371** | **1458.7467** | **-0.0096** | **0** | **69** | **1.8e-005** | **1** | **R.FGQGSGPIVLDDVR.C** 12600 12616 |
|  | 13910 | **773.3602** | **1544.7059** | **1544.7154** | **-0.0095** | **0** | **69** | **1.4e-005** | **1** | **R.QLGCGWATSAPGNAR.F** |
|  | 14368 | **788.3563** | **1574.6980** | **1574.7082** | **-0.0102** | **0** | **38** | **0.017** | **1** | **R.QLGCGWAMSAPGNAR.F** |
|  | 14597 | **531.1882** | **1590.5427** | **1590.7031** | **-0.1604** | **0** | **(2)** | **61** | **10** | **R.QLGCGWAMSAPGNAR.F** |
|  | 21147 | **699.3126** | **2094.9160** | **2093.9259** | **0.9901** | **1** | **5** | **28** | **2** | **R.VCDGARGSFTSSSNFMSIR.F** |
|  | 23677 | **1193.9794** | **2385.9442** | **2385.9591** | **-0.0149** | **0** | **123** | **3.3e-011** | **1** | **R.GSWGTVCDDSWDTSDANVVCR.Q** 23678 |
|  | 23831 | **1207.4845** | **2412.9544** | **2412.9700** | **-0.0155** | **0** | **91** | **4.8e-008** | **1** | **R.GSWGTVCDDSWDTNDANVVCR.Q** |
|  | 24468 | **1245.5002** | **2488.9859** | **2489.0013** | **-0.0153** | **0** | **121** | **4.5e-011** | **1** | **R.GSWGTVCDDYWDTNDANVVCR.Q** 24469 |
|  | 27261 | **997.1375** | **2988.3907** | **2987.4018** | **0.9889** | **0** | **10** | **4.8** | **1** | **R.QLGCGWAMSAPGNAQFGQGSGPIVLDDVR.C** |

| **61.** | P16422\|EPCAM_HUMAN    **Mass:** 35594    **Score:** 596    **Queries matched:** 29   **emPAI:** 1.73 |
| --- | --- |
|  | Epithelial cell adhesion molecule OS=Homo sapiens GN=EPCAM PE=1 SV=2 |

|  | **Query** | **Observed** | **Mr(expt)** | **Mr(calc)** | **Delta** | **Miss** | **Score** | **Expect** | **Rank** | **Peptide** |
| --- | --- | --- | --- | --- | --- | --- | --- | --- | --- | --- |
|  | 1109 | **561.3217** | **560.3144** | **559.3329** | **0.9814** | **0** | **13** | **17** | **4** | **R.TALQK.E** |
|  | 1521 | **650.3325** | **649.3252** | **649.3291** | **-0.0039** | **0** | **14** | **8.1** | **5** | **K.CLVMK.A** |
|  | 1580 | 665.3572 | 664.3500 | 665.3241 | -0.9741 | 0 | (8) | 30 | 9 | K.CLVMK.A |
|  | 3764 | **452.7266** | **903.4387** | **903.4450** | **-0.0063** | **0** | **10** | **17** | **2** | **K.GESLFHSK.K** |
|  | 10194 | **660.8223** | **1319.6300** | **1319.6404** | **-0.0105** | **0** | **50** | **0.0015** | **1** | **K.LAVNCFVNNNR.Q** |
|  | 10411 | **444.6325** | **1330.8758** | **1331.5598** | **-0.6840** | **1** | **7** | **24** | **8** | **K.EMGEMHRELNA.-** 10413 |
|  | 10694 | **449.9033** | **1346.6880** | **1347.5547** | **-0.8668** | **1** | **(7)** | **31** | **3** | **K.EMGEMHRELNA.-** |
|  | 12513 | **727.8199** | **1453.6253** | **1453.6355** | **-0.0101** | **1** | **41** | **0.01** | **1** | **R.TDKDTEITCSER.V** |
|  | 18853 | **945.9365** | **1889.8584** | **1889.8683** | **-0.0099** | **0** | **105** | **3.1e-009** | **1** | **K.TQNDVDIADVAYYFEK.D** 18868 |
|  | 19475 | **970.9278** | **1939.8410** | **1939.8550** | **-0.0140** | **0** | **82** | **5.5e-007** | **1** | **R.QCQCTSVGAQNTVICSK.L** 19474 19476 19477 |
|  | 26140 | **1377.1113** | **2752.2081** | **2752.2286** | **-0.0205** | **0** | **68** | **8.5e-006** | **1** | **R.AKPEGALQNNDGLYDPDCDESGLFK.A** 26141 26152 26153 |
|  | 26643 | **1435.1871** | **2868.3597** | **2868.3739** | **-0.0142** | **0** | **51** | **0.00047** | **1** | **K.MDLTVNGEQLDLDPGQTLIYYVDEK.A** 26644 26650 |
|  | 26877 | **970.4434** | **2908.3084** | **2908.3297** | **-0.0213** | **1** | **32** | **0.033** | **1** | **R.RAKPEGALQNNDGLYDPDCDESGLFK.A** |
|  | 27312 | **1000.1533** | **2997.4381** | **2996.4689** | **0.9693** | **1** | **34** | **0.021** | **1** | **K.KMDLTVNGEQLDLDPGQTLIYYVDEK.A** 27302 27303 27304 27305 27311 |

| **62.** | P09923\|PPBI_HUMAN    **Mass:** 57119    **Score:** 595    **Queries matched:** 30   **emPAI:** 1.27 |
| --- | --- |
|  | Intestinal alkaline phosphatase OS=Homo sapiens GN=ALPI PE=1 SV=2 |

|  | **Query** | **Observed** | **Mr(expt)** | **Mr(calc)** | **Delta** | **Miss** | **Score** | **Expect** | **Rank** | **Peptide** |
| --- | --- | --- | --- | --- | --- | --- | --- | --- | --- | --- |
|  | 1659 | **686.4152** | **685.4079** | **685.4486** | **-0.0407** | **1** | **24** | **0.81** | **1** | **R.ILKGQK.N** |
|  | 1944 | **767.3627** | **766.3554** | **766.3610** | **-0.0056** | **0** | **28** | **0.29** | **1** | **K.TYNVDR.Q** |
|  | 2951 | 427.7428 | 853.4711 | 853.5385 | -0.0674 | 1 | 13 | 6.4 | 8 | K.KLQPIQK.V |
|  | 2984 | **428.7633** | **855.5121** | **854.5086** | **1.0035** | **1** | **8** | **27** | **2** | **R.LLSRNPR.G** |
|  | 3979 | **460.2606** | **918.5066** | **918.5135** | **-0.0069** | **0** | **58** | **0.00027** | **1** | **K.SVGVVTTTR.V** |
|  | 5945 | **532.2893** | **1062.5639** | **1062.5710** | **-0.0070** | **0** | **56** | **0.00041** | **1** | **R.GSSIFGLAPSK.A** |
|  | 6659 | **558.3562** | **1114.6978** | **1114.5982** | **0.0997** | **1** | **1** | **1.1e+002** | **10** | **R.QAAEALDAAKK.L** |
|  | 6688 | **559.7805** | **1117.5464** | **1117.5550** | **-0.0086** | **0** | **44** | **0.0073** | **1** | **R.GNEVISVMNR.A** |
|  | 7024 | **573.2909** | **1144.5673** | **1143.5713** | **0.9960** | **0** | **71** | **1.4e-005** | **1** | **R.GFYLFVEGGR.I** |
|  | 7862 | **600.3046** | **1198.5947** | **1198.6016** | **-0.0069** | **0** | **26** | **0.35** | **1** | **K.LGPETPLAMDR.F** |
|  | 10168 | **660.2826** | **1318.5507** | **1318.6993** | **-0.1486** | **0** | **25** | **0.48** | **1** | **K.ANFQTIGLSAAAR.F** |
|  | 12978 | **742.3121** | **1482.6097** | **1482.6198** | **-0.0100** | **0** | **57** | **0.00025** | **1** | **R.NWYSDADMPASAR.Q** 12977 |
|  | 13206 | **499.3396** | **1494.9970** | **1494.8745** | **0.1225** | **0** | **7** | **18** | **9** | **-.MQGPWVLLLLGLR.L** |
|  | 13247 | **500.2798** | **1497.8175** | **1497.7609** | **0.0566** | **1** | **7** | **24** | **3** | **K.NGKLGPETPLAMDR.F** |
|  | 13259 | 750.3368 | 1498.6590 | 1498.6147 | 0.0444 | 0 | (3) | 60 | 8 | R.NWYSDADMPASAR.Q |
|  | 13416 | **504.2708** | **1509.7905** | **1510.8694** | **-1.0789** | **0** | **(3)** | **56** | **8** | **-.MQGPWVLLLLGLR.L** |
|  | 14629 | **796.8835** | **1591.7524** | **1591.8206** | **-0.0682** | **1** | **9** | **17** | **5** | **R.GSSIFGLAPSKAQDSK.A** |
|  | 16356 | **854.9233** | **1707.8321** | **1707.8441** | **-0.0120** | **0** | **61** | **9.5e-005** | **1** | **R.VQHASPAGTYAHTVNR.N** 16355 |
|  | 16936 | **876.4292** | **1750.8438** | **1750.8560** | **-0.0121** | **0** | **72** | **6.7e-006** | **1** | **R.QVPDSAATATAYLCGVK.A** 16935 |
|  | 19671 | **979.0543** | **1956.0940** | **1956.1044** | **-0.0105** | **0** | **87** | **1.6e-007** | **1** | **K.NLILFLGDGLGVPTVTATR.I** |
|  | 24213 | **1229.5300** | **2457.0455** | **2456.0777** | **0.9678** | **0** | **33** | **0.031** | **1** | **K.YMFPMGTPDPEYPADASQNGIR.L** 24214 |
|  | 25153 | **863.4157** | **2587.2253** | **2587.2410** | **-0.0157** | **1** | **72** | **4e-006** | **1** | **K.YEIHRDPTLDPSLMEMTEAALR.L** |
|  | 26110 | **1374.6574** | **2747.3001** | **2747.3146** | **-0.0145** | **0** | **80** | **6.3e-007** | **1** | **R.TELMQASLDQSVTHLMGLFEPGDTK.Y** 26112 26113 |
|  | 28434 | 813.8969 | 3251.5585 | 3251.5735 | -0.0150 | 0 | 9 | 5.3 | 1 | R.AGQLTSEEDTLTLVTADHSHVFSFGGYTLR.G |

| **63.** | P56470\|LEG4_HUMAN    **Mass:** 36032    **Score:** 580    **Queries matched:** 44   **emPAI:** 2.29 |
| --- | --- |
|  | Galectin-4 OS=Homo sapiens GN=LGALS4 PE=1 SV=1 |

|  | **Query** | **Observed** | **Mr(expt)** | **Mr(calc)** | **Delta** | **Miss** | **Score** | **Expect** | **Rank** | **Peptide** |
| --- | --- | --- | --- | --- | --- | --- | --- | --- | --- | --- |
|  | 1790 | **721.3941** | **720.3868** | **720.3919** | **-0.0050** | **0** | **9** | **22** | **2** | **R.LSAFQR.V** |
|  | 1855 | **743.4363** | **742.4290** | **742.5065** | **-0.0775** | **1** | **22** | **1.2** | **3** | **R.RTIIIK.G** |
|  | 2376 | **408.2376** | **814.4607** | **814.4661** | **-0.0054** | **0** | **14** | **8.5** | **1** | **R.LQGGLTAR.R** |
|  | 2418 | **818.4349** | **817.4276** | **817.4334** | **-0.0058** | **0** | **23** | **1.1** | **1** | **K.GYVPPTGK.S** 2421 |
|  | 2513 | **413.7238** | **825.4331** | **825.4385** | **-0.0054** | **0** | **15** | **4.1** | **1** | **K.SFAINFK.V** |
|  | 2621 | 417.2164 | 832.4182 | 832.4225 | -0.0043 | 0 | 18 | 3 | 1 | R.MGNGTVVR.N 2639 |
|  | 5906 | **531.2165** | **1060.4184** | **1061.5870** | **-1.1685** | **0** | **53** | **0.00081** | **1** | **K.VVFNTLQGGK.W** 5935 5936 5944 |
|  | 11374 | **693.8810** | **1385.7475** | **1385.8282** | **-0.0807** | **1** | **6** | **33** | **3** | **R.TIIIKGYVPPTGK.S** |
|  | 11962 | 710.8340 | 1419.6534 | 1419.6630 | -0.0096 | 0 | 73 | 6.2e-006 | 1 | R.NSLLNGSWGSEEK.K |
|  | 12247 | **718.3812** | **1434.7479** | **1434.7579** | **-0.0100** | **0** | **34** | **0.054** | **1** | **K.VGSSGDIALHINPR.M** 12248 12249 12250 12252 |
|  | 15532 | **825.9137** | **1649.8128** | **1649.7950** | **0.0178** | **0** | **34** | **0.04** | **1** | **K.VVVNGNPFYEYGHR.L** 15533 15551 |
|  | 15880 | **838.3970** | **1674.7795** | **1673.8063** | **0.9732** | **0** | **15** | **4** | **1** | **K.VYANGQHLFDFAHR.L** 15866 |
|  | 18891 | **946.9932** | **1891.9719** | **1891.9779** | **-0.0060** | **0** | **2** | **59** | **1** | **R.VDTLEIQGDVTLSYVQI.-** |
|  | 19542 | **649.3320** | **1944.9743** | **1944.9847** | **-0.0104** | **0** | **55** | **0.00033** | **1** | **K.ITHNPFGPGQFFDLSIR.C** 19540 19541 19543 |
|  | 20905 | **1037.5400** | **2073.0655** | **2073.0796** | **-0.0141** | **1** | **81** | **6.4e-007** | **1** | **K.KITHNPFGPGQFFDLSIR.C** 20906 20909 |
|  | 24533 | **1248.6000** | **2495.1854** | **2495.2023** | **-0.0169** | **0** | **69** | **9e-006** | **1** | **R.FFVNFVVGQDPGSDVAFHFNPR.F** 24526 24528 24534 24535 24537 24538 24539 24540 |
|  | 25497 | **884.7700** | **2651.2882** | **2651.3034** | **-0.0152** | **1** | **53** | **0.00031** | **1** | **K.RFFVNFVVGQDPGSDVAFHFNPR.F** 25496 |
|  | 28381 | **811.8845** | **3243.5087** | **3243.5203** | **-0.0116** | **1** | **1** | **32** | **2** | **R.FFVNFVVGQDPGSDVAFHFNPRFDGWDK.V** |

| **64.** | P68366\|TBA4A_HUMAN    **Mass:** 50634    **Score:** 562    **Queries matched:** 30   **emPAI:** 1.91 |
| --- | --- |
|  | Tubulin alpha-4A chain OS=Homo sapiens GN=TUBA4A PE=1 SV=1 |

|  | **Query** | **Observed** | **Mr(expt)** | **Mr(calc)** | **Delta** | **Miss** | **Score** | **Expect** | **Rank** | **Peptide** |
| --- | --- | --- | --- | --- | --- | --- | --- | --- | --- | --- |
|  | 1369 | 615.3498 | 614.3426 | 613.3435 | 0.9990 | 0 | 22 | 1.5 | 1 | R.GDVVPK.D |
|  | 4891 | **493.2842** | **984.5537** | **984.5604** | **-0.0066** | **0** | **23** | **0.8** | **1** | **K.DVNAAIAAIK.T** |
|  | 8847 | 625.2756 | 1248.5366 | 1248.5453 | -0.0087 | 0 | 29 | 0.17 | 1 | K.YMACCLLYR.G |
|  | 11801 | 470.9266 | 1409.7580 | 1409.7667 | -0.0087 | 0 | 13 | 6.6 | 1 | R.QLFHPEQLITGK.E |
|  | 13059 | 744.4392 | 1486.8639 | 1486.8719 | -0.0080 | 0 | 64 | 5.2e-005 | 1 | R.LISQIVSSITASLR.F 13061 |
|  | 14494 | 792.8747 | 1583.7348 | 1583.7443 | -0.0095 | 0 | 2 | 71 | 1 | R.SIQFVDWCPTGFK.V |
|  | 16458 | **858.4598** | **1714.9050** | **1714.9142** | **-0.0091** | **0** | **74** | **4.8e-006** | **1** | **R.AVFVDLEPTVIDEIR.N** 16457 |
|  | 16490 | 859.9390 | 1717.8634 | 1717.8747 | -0.0113 | 0 | 37 | 0.021 | 1 | R.NLDIERPTYTNLNR.L 16491 |
|  | 16792 | 581.2870 | 1740.8391 | 1739.8454 | 0.9938 | 1 | 3 | 53 | 2 | K.RSIQFVDWCPTGFK.V |
|  | 17026 | 878.9797 | 1755.9449 | 1755.9559 | -0.0110 | 0 | 36 | 0.025 | 1 | R.IHFPLATYAPVISAEK.A 17025 17028 17029 |
|  | 17925 | 912.9903 | 1823.9660 | 1823.9782 | -0.0121 | 0 | 56 | 0.00029 | 1 | K.VGINYQPPTVVPGGDLAK.V 17924 17926 |
|  | 17946 | **609.3189** | **1824.9347** | **1824.9734** | **-0.0387** | **1** | **8** | **15** | **4** | **R.GHYTIGKEIIDPVLDR.I** |
|  | 18536 | 932.9506 | 1863.8866 | 1863.8971 | -0.0105 | 0 | 91 | 8.5e-008 | 1 | R.AVCMLSNTTAIAEAWAR.L |
|  | 23245 | 1165.5043 | 2328.9940 | 2329.0110 | -0.0170 | 0 | 77 | 1.2e-006 | 1 | R.AFVHWYVGEGMEEGEFSEAR.E 23246 |
|  | 23808 | 1205.1003 | 2408.1861 | 2408.2012 | -0.0151 | 0 | 123 | 3.3e-011 | 1 | R.FDGALNVDLTEFQTNLVPYPR.I 23809 23811 23812 |
|  | 26118 | 917.4299 | 2749.2680 | 2749.2840 | -0.0160 | 0 | 32 | 0.04 | 1 | K.AYHEQLSVAEITNACFEPANQMVK.C |
|  | 28280 | 805.1068 | 3216.3981 | 3216.4168 | -0.0187 | 1 | 22 | 0.27 | 1 | R.AFVHWYVGEGMEEGEFSEAREDMAALEK.D |
|  | 31692 | 1380.3986 | 5517.5651 | 5516.5891 | 0.9761 | 0 | 0 | 9.1 | 7 | K.LEFSIYPAPQVSTAVVEPYNSILTTHTTLEHSDCAFMVDNEAIYDICR.R |

| **65.** | P45880\|VDAC2_HUMAN    **Mass:** 32060    **Score:** 556    **Queries matched:** 23   **emPAI:** 1.18 |
| --- | --- |
|  | Voltage-dependent anion-selective channel protein 2 OS=Homo sapiens GN=VDAC2 PE=1 SV=2 |

|  | **Query** | **Observed** | **Mr(expt)** | **Mr(calc)** | **Delta** | **Miss** | **Score** | **Expect** | **Rank** | **Peptide** |
| --- | --- | --- | --- | --- | --- | --- | --- | --- | --- | --- |
|  | 2858 | **424.7370** | **847.4595** | **847.4651** | **-0.0056** | **0** | **25** | **0.59** | **1** | **K.VTGTLETK.Y** 2835 |
|  | 4255 | **470.7322** | **939.4498** | **939.4563** | **-0.0064** | **0** | **23** | **0.69** | **1** | **R.NNFAVGYR.T** 4254 |
|  | 9700 | **647.3337** | **1292.6528** | **1292.6612** | **-0.0084** | **0** | **17** | **3.2** | **1** | **K.YQLDPTASISAK.V** |
|  | 12113 | **714.8486** | **1427.6826** | **1427.6933** | **-0.0107** | **0** | **72** | **7.7e-006** | **1** | **K.LTFDTTFSPNTGK.K** |
|  | 19086 | **954.3936** | **1906.7725** | **1906.7851** | **-0.0125** | **0** | **65** | **2.7e-005** | **1** | **K.SCSGVEFSTSGSSNTDTGK.V** 19087 |
|  | 21207 | 1052.0740 | 2102.1334 | 2102.1484 | -0.0150 | 0 | 105 | 2.5e-009 | 1 | K.VNNSSLIGVGYTQTLRPGVK.L 21201 21205 21206 21208 21209 21210 21211 21222 |
|  | 24696 | **1260.1010** | **2518.1873** | **2518.2010** | **-0.0136** | **0** | **109** | **9.5e-010** | **1** | **K.WNTDNTLGTEIAIEDQICQGLK.L** 24697 24705 |
|  | 24750 | **1264.5798** | **2527.1451** | **2527.1616** | **-0.0165** | **0** | **87** | **1.2e-007** | **1** | **R.TGDFQLHTNVNDGTEFGGSIYQK.V** 24749 24751 |

| **66.** | O94832\|MYO1D_HUMAN    **Mass:** 116927   **Score:** 553    **Queries matched:** 51   **emPAI:** 0.45 |
| --- | --- |
|  | Myosin-Id OS=Homo sapiens GN=MYO1D PE=1 SV=2 |

|  | **Query** | **Observed** | **Mr(expt)** | **Mr(calc)** | **Delta** | **Miss** | **Score** | **Expect** | **Rank** | **Peptide** |
| --- | --- | --- | --- | --- | --- | --- | --- | --- | --- | --- |
|  | 2356 | **407.2213** | **812.4280** | **811.4262** | **1.0018** | **1** | **7** | **31** | **9** | **K.QYKVMK.T** |
|  | 3070 | **431.7163** | **861.4180** | **860.5153** | **0.9027** | **1** | **6** | **53** | **4** | **R.VKNMLLK.S** |
|  | 3074 | **431.7219** | **861.4292** | **860.3851** | **1.0442** | **0** | **13** | **9.4** | **5** | **K.NMWPEGK.L** 3066 |
|  | 3569 | 446.2269 | 890.4392 | 889.5120 | 0.9272 | 0 | 15 | 5.9 | 2 | K.LSITEVTK.R |
|  | 3712 | **451.2454** | **900.4762** | **901.5498** | **-1.0736** | **1** | **8** | **28** | **4** | **K.IFIRTPR.T** |
|  | 4284 | **472.2054** | **942.3962** | **942.5386** | **-0.1424** | **0** | **4** | **69** | **9** | **R.INDIIEVK.N** |
|  | 5429 | **513.2869** | **1024.5593** | **1024.5665** | **-0.0072** | **0** | **25** | **0.48** | **1** | **R.VIVQQPGER.S** |
|  | 5710 | **524.2418** | **1046.4691** | **1047.4985** | **-1.0294** | **0** | **21** | **1.3** | **1** | **K.NYDTTIHGK.N** |
|  | 6287 | **544.2784** | **1086.5423** | **1085.5870** | **0.9554** | **0** | **14** | **7.8** | **1** | **K.DQLVVFHTK.D** 6294 |
|  | 6575 | **555.2609** | **1108.5072** | **1108.5149** | **-0.0077** | **0** | **70** | **1.4e-005** | **1** | **K.SSINDAAEFR.V** |
|  | 6580 | **555.3093** | **1108.6040** | **1108.5513** | **0.0527** | **1** | **5** | **46** | **4** | **R.DTIEQYKGR.E** |
|  | 7904 | **601.3343** | **1200.6540** | **1200.6649** | **-0.0108** | **1** | **15** | **4.9** | **1** | **K.VAAVEMLKGQR.A** 7926 |
|  | 8313 | **610.7929** | **1219.5713** | **1219.7037** | **-0.1324** | **1** | **8** | **25** | **7** | **K.ALLYRTVATGR.D** |
|  | 9969 | **655.3096** | **1308.6047** | **1308.6132** | **-0.0085** | **0** | **60** | **0.00013** | **1** | **K.SNCVLEAFGNAK.T** 9988 |
|  | 11984 | **474.3256** | **1419.9549** | **1420.7496** | **-0.7947** | **1** | **13** | **5.1** | **1** | **K.RLMYNSSNPVLK.N** 11979 |
|  | 12047 | **712.9125** | **1423.8105** | **1423.8187** | **-0.0082** | **0** | **72** | **8.6e-006** | **1** | **R.IGELVGVLVNHFK.S** 12048 |
|  | 13312 | **752.3827** | **1502.7508** | **1502.7617** | **-0.0109** | **0** | **75** | **4.3e-006** | **1** | **K.FVVDGDTPLIENGK.V** 13299 13322 |
|  | 13601 | **508.6005** | **1522.7796** | **1522.7892** | **-0.0096** | **1** | **36** | **0.034** | **1** | **R.RFEEALQTIFNR.W** 13612 |
|  | 14293 | **785.4179** | **1568.8211** | **1568.8311** | **-0.0099** | **0** | **55** | **0.00036** | **1** | **R.HQVEYLGLLENVR.V** |
|  | 14579 | **795.3942** | **1588.7738** | **1587.9534** | **0.8204** | **1** | **7** | **26** | **2** | **R.AQMLIRIVLFLQK.V** |
|  | 15380 | **820.4205** | **1638.8264** | **1638.8365** | **-0.0102** | **0** | **31** | **0.09** | **2** | **K.GDPIGGHINNYLLEK.S** 15356 |
|  | 15538 | **825.9533** | **1649.8919** | **1649.9028** | **-0.0109** | **0** | **53** | **0.00051** | **1** | **K.VIGFKPEEIQTVYK.I** 15537 15539 |
|  | 15923 | **839.3769** | **1676.7392** | **1675.9145** | **0.8248** | **0** | **17** | **2.5** | **2** | **K.TIPLYNLTGLSVSNGK.D** |
|  | 17383 | **891.9526** | **1781.8906** | **1781.8988** | **-0.0082** | **0** | **77** | **2.2e-006** | **1** | **R.HYAGDVVYSVIGFIDK.N** |
|  | 20360 | 1012.9474 | 2023.8802 | 2024.0367 | -0.1565 | 1 | 3 | 49 | 5 | R.HYAGDVVYSVIGFIDKNK.D |
|  | 20677 | **684.3154** | **2049.9243** | **2050.1133** | **-0.1890** | **1** | **1** | **63** | **1** | **K.VMKTIPLYNLTGLSVSNGK.D** |
|  | 21519 | **1064.5112** | **2127.0079** | **2127.0208** | **-0.0128** | **0** | **102** | **5.7e-009** | **1** | **R.SFHSFYQLLQGGSEQMLR.S** 21520 21521 |
|  | 21609 | **1070.0037** | **2137.9928** | **2139.0129** | **-1.0201** | **1** | **0** | **81** | **3** | **R.LMYNSSNPVLKNMWPEGK.L** |
|  | 22216 | **735.3715** | **2203.0926** | **2202.0159** | **1.0766** | **0** | **3** | **41** | **2** | **K.ADFVLMDTVSMPEFMANLR.L** |
|  | 23432 | **785.0662** | **2352.1767** | **2352.1862** | **-0.0096** | **0** | **27** | **0.16** | **1** | **K.HIDYFNNQIIVDLVEQQHK.G** |
|  | 23517 | **591.0800** | **2360.2910** | **2359.1154** | **1.1755** | **1** | **6** | **19** | **2** | **K.MISEFTWPNHDLPSDKEAVK.K** |
|  | 23551 | **592.3202** | **2365.2519** | **2364.2763** | **0.9756** | **1** | **0** | **71** | **4** | **R.VVADAMKVIGFKPEEIQTVYK.I** |
|  | 23569 | 592.8094 | 2367.2087 | 2368.1845 | -0.9758 | 1 | 5 | 25 | 5 | K.YIMQYIAAITNPSQRAEVER.V |
|  | 24428 | 829.7601 | 2486.2584 | 2487.1960 | -0.9376 | 1 | 1 | 54 | 3 | K.ADFVLMDTVSMPEFMANLRLR.F |
|  | 26403 | **704.0928** | **2812.3420** | **2812.3530** | **-0.0111** | **1** | **0** | **55** | **5** | **K.YMDINFDFKGDPIGGHINNYLLEK.S** |
|  | 27382 | **1007.4988** | **3019.4747** | **3019.4927** | **-0.0180** | **0** | **58** | **7.4e-005** | **1** | **R.AWEGNYLASKPDTPQTSGTFVPVANELK.R** 27381 |

| **67.** | Q13748\|TBA3C_HUMAN    **Mass:** 50612    **Score:** 553    **Queries matched:** 27   **emPAI:** 1.52 |
| --- | --- |
|  | Tubulin alpha-3C/D chain OS=Homo sapiens GN=TUBA3C PE=1 SV=3 |

|  | **Query** | **Observed** | **Mr(expt)** | **Mr(calc)** | **Delta** | **Miss** | **Score** | **Expect** | **Rank** | **Peptide** |
| --- | --- | --- | --- | --- | --- | --- | --- | --- | --- | --- |
|  | 1369 | 615.3498 | 614.3426 | 613.3435 | 0.9990 | 0 | 22 | 1.5 | 1 | R.GDVVPK.D |
|  | 5307 | 508.2902 | 1014.5658 | 1014.5709 | -0.0051 | 0 | 43 | 0.0084 | 1 | K.DVNAAIATIK.T |
|  | 11801 | 470.9266 | 1409.7580 | 1409.7667 | -0.0087 | 0 | 13 | 6.6 | 1 | R.QLFHPEQLITGK.E |
|  | 14696 | 799.8823 | 1597.7500 | 1597.7599 | -0.0100 | 0 | 38 | 0.02 | 1 | R.TIQFVDWCPTGFK.V |
|  | 16490 | 859.9390 | 1717.8634 | 1717.8747 | -0.0113 | 0 | 37 | 0.021 | 1 | R.NLDIERPTYTNLNR.L 16491 |
|  | 17026 | 878.9797 | 1755.9449 | 1755.9559 | -0.0110 | 0 | 36 | 0.025 | 1 | R.IHFPLATYAPVISAEK.A 17025 17028 17029 |
|  | 17925 | 912.9903 | 1823.9660 | 1823.9782 | -0.0121 | 0 | 56 | 0.00029 | 1 | K.VGINYQPPTVVPGGDLAK.V 17924 17926 |
|  | 18530 | 621.9794 | 1862.9165 | 1861.8347 | 1.0817 | 1 | 5 | 32 | 4 | K.YMACCMLYRGDVVPK.D |
|  | 18536 | 932.9506 | 1863.8866 | 1863.8971 | -0.0105 | 0 | 91 | 8.5e-008 | 1 | R.AVCMLSNTTAIAEAWAR.L |
|  | 20182 | 1004.4432 | 2006.8719 | 2006.8858 | -0.0139 | 0 | 83 | 4.7e-007 | 1 | K.TIGGGDDSFNTFFSETGAGK.H 20183 20184 20191 |
|  | 23245 | 1165.5043 | 2328.9940 | 2329.0110 | -0.0170 | 0 | 77 | 1.2e-006 | 1 | R.AFVHWYVGEGMEEGEFSEAR.E 23246 |
|  | 23808 | 1205.1003 | 2408.1861 | 2408.2012 | -0.0151 | 0 | 123 | 3.3e-011 | 1 | R.FDGALNVDLTEFQTNLVPYPR.I 23809 23811 23812 |
|  | 26118 | 917.4299 | 2749.2680 | 2749.2840 | -0.0160 | 0 | 32 | 0.04 | 1 | K.AYHEQLSVAEITNACFEPANQMVK.C |
|  | 30755 | 1074.9904 | 4295.9323 | 4295.9497 | -0.0173 | 0 | 10 | 2.3 | 1 | R.ECISIHVGQAGVQIGNACWELYCLEHGIQPDGQMPSDK.T |

| **68.** | P62873\|GBB1_HUMAN    **Mass:** 38151    **Score:** 550    **Queries matched:** 33   **emPAI:** 1.81 |
| --- | --- |
|  | Guanine nucleotide-binding protein G(I)/G(S)/G(T) subunit beta-1 OS=Homo sapiens GN=GNB1 PE=1 SV=3 |

|  | **Query** | **Observed** | **Mr(expt)** | **Mr(calc)** | **Delta** | **Miss** | **Score** | **Expect** | **Rank** | **Peptide** |
| --- | --- | --- | --- | --- | --- | --- | --- | --- | --- | --- |
|  | 2250 | 805.4991 | 804.4918 | 804.4970 | -0.0052 | 0 | 42 | 0.012 | 1 | K.VHAIPLR.S 2218 2233 2237 2244 2251 2258 |
|  | 5229 | **505.2587** | **1008.5028** | **1008.5101** | **-0.0072** | **0** | **54** | **0.00068** | **1** | **R.AGVLAGHDNR.V** 5226 5228 5230 5231 5232 5235 |
|  | 5329 | **509.2790** | **1016.5434** | **1016.5502** | **-0.0068** | **0** | **30** | **0.17** | **1** | **R.LLVSASQDGK.L** 5328 |
|  | 8401 | **613.2935** | **1224.5724** | **1224.5809** | **-0.0085** | **0** | **70** | **1.6e-005** | **1** | **R.LFVSGACDASAK.L** |
|  | 10213 | **661.3418** | **1320.6690** | **1320.6067** | **0.0623** | **1** | **2** | **91** | **7** | **K.LWDVREGMCR.Q** |
|  | 10496 | **668.8042** | **1335.5938** | **1335.6030** | **-0.0091** | **0** | **72** | **8.2e-006** | **1** | **K.IYAMHWGTDSR.L** |
|  | 10757 | **451.2634** | **1350.7685** | **1351.5979** | **-0.8295** | **0** | **(8)** | **19** | **2** | **K.IYAMHWGTDSR.L** |
|  | 10792 | **677.3515** | **1352.6884** | **1352.6976** | **-0.0092** | **0** | **53** | **0.00065** | **1** | **K.LIIWDSYTTNK.V** 10809 |
|  | 13597 | **762.3354** | **1522.6562** | **1522.6657** | **-0.0095** | **0** | **66** | **2.8e-005** | **1** | **R.ELAGHTGYLSCCR.F** 13596 |
|  | 20245 | **672.3198** | **2013.9375** | **2014.9742** | **-1.0367** | **0** | **3** | **43** | **1** | **K.ACADATLSQITNNIDPVGR.I** |
|  | 21507 | **1064.0085** | **2126.0025** | **2126.0143** | **-0.0117** | **0** | **93** | **3.8e-008** | **1** | **R.LLLAGYDDFNCNVWDALK.A** 21508 21510 |
|  | 23848 | **1208.5548** | **2415.0951** | **2415.1087** | **-0.0136** | **0** | **95** | **2.2e-008** | **1** | **R.VSCLGVTDDGMAVATGSWDSFLK.I** 23847 23849 |
|  | 29121 | **1157.8501** | **3470.5285** | **3470.5403** | **-0.0119** | **0** | **34** | **0.012** | **1** | **R.SSWVMTCAYAPSGNYVACGGLDNICSIYNLK.T** |
|  | 29253 | **1174.8314** | **3521.4724** | **3520.5049** | **0.9676** | **0** | **27** | **0.059** | **1** | **R.QTFTGHESDINAICFFPNGNAFATGSDDATCR.L** |

| **69.** | P16152\|CBR1_HUMAN    **Mass:** 30641    **Score:** 547    **Queries matched:** 23   **emPAI:** 2.20 |
| --- | --- |
|  | Carbonyl reductase [NADPH] 1 OS=Homo sapiens GN=CBR1 PE=1 SV=3 |

|  | **Query** | **Observed** | **Mr(expt)** | **Mr(calc)** | **Delta** | **Miss** | **Score** | **Expect** | **Rank** | **Peptide** |
| --- | --- | --- | --- | --- | --- | --- | --- | --- | --- | --- |
|  | 2802 | **422.7634** | **843.5123** | **843.5178** | **-0.0055** | **0** | **35** | **0.067** | **1** | **K.IGVTVLSR.I** |
|  | 3183 | **433.7496** | **865.4847** | **865.4545** | **0.0302** | **1** | **8** | **25** | **5** | **K.FVEDTKK.G** |
|  | 6136 | **538.7521** | **1075.4897** | **1075.4968** | **-0.0071** | **0** | **39** | **0.021** | **1** | **K.SCSPELQQK.F** 6137 |
|  | 8005 | **402.6952** | **1205.0637** | **1205.6438** | **-0.5801** | **0** | **9** | **14** | **4** | **R.VVNVSSIMSVR.A** 8022 |
|  | 9460 | **641.3046** | **1280.5947** | **1280.6037** | **-0.0090** | **0** | **29** | **0.19** | **1** | **K.EGWPSSAYGVTK.I** |
|  | 12578 | **729.8659** | **1457.7173** | **1457.7272** | **-0.0099** | **0** | **57** | **0.00024** | **1** | **K.ILLNACCPGWVR.T** 12579 |
|  | 13283 | **501.2430** | **1500.7073** | **1501.8650** | **-1.1577** | **1** | **4** | **47** | **2** | **R.VVNVSSIMSVRALK.S** |
|  | 14717 | **800.4053** | **1598.7960** | **1598.8053** | **-0.0093** | **0** | **60** | **0.00013** | **1** | **R.FHQLDIDDLQSIR.A** 14715 14716 |
|  | 15574 | **826.9339** | **1651.8532** | **1651.8642** | **-0.0109** | **0** | **95** | **3.6e-008** | **1** | **R.GQAAVQQLQAEGLSPR.F** 15575 |
|  | 16367 | **570.3373** | **1707.9902** | **1707.8237** | **0.1665** | **0** | **5** | **35** | **4** | **R.SETITEEELVGLMNK.F** |
|  | 16551 | **574.9532** | **1721.8377** | **1720.8931** | **0.9447** | **1** | **1** | **85** | **8** | **R.DLCRLFSGDVVLTAR.D** |
|  | 17341 | **890.4630** | **1778.9115** | **1778.9203** | **-0.0088** | **0** | **59** | **0.00013** | **1** | **K.EYGGLDVLVNNAGIAFK.V** |
|  | 19092 | **954.5096** | **1907.0047** | **1907.0152** | **-0.0105** | **1** | **159** | **1.4e-014** | **1** | **R.KEYGGLDVLVNNAGIAFK.V** |
|  | 20060 | **998.5003** | **1994.9861** | **1994.9983** | **-0.0122** | **1** | **79** | **1.1e-006** | **1** | **K.FRSETITEEELVGLMNK.F** 20061 |
|  | 28023 | **1055.5126** | **3163.5159** | **3163.5350** | **-0.0191** | **0** | **42** | **0.003** | **1** | **K.SPEEGAETPVYLALLPPDAEGPHGQFVSEK.R** 28024 |

| **70.** | P22695\|QCR2_HUMAN    **Mass:** 48584    **Score:** 540    **Queries matched:** 50   **emPAI:** 2.04 |
| --- | --- |
|  | Cytochrome b-c1 complex subunit 2, mitochondrial OS=Homo sapiens GN=UQCRC2 PE=1 SV=3 |

|  | **Query** | **Observed** | **Mr(expt)** | **Mr(calc)** | **Delta** | **Miss** | **Score** | **Expect** | **Rank** | **Peptide** |
| --- | --- | --- | --- | --- | --- | --- | --- | --- | --- | --- |
|  | 1810 | **730.4200** | **729.4127** | **729.4021** | **0.0106** | **0** | **18** | **4** | **1** | **R.GIEAVGGK.L** |
|  | 1867 | **747.4313** | **746.4241** | **746.4286** | **-0.0046** | **0** | **9** | **28** | **4** | **K.LSVTATR.E** |
|  | 1896 | **758.3887** | **757.3814** | **758.4286** | **-1.0473** | **0** | **4** | **95** | **7** | **R.GGLGLSGAK.A** 1912 |
|  | 2071 | **397.2045** | **792.3944** | **792.4494** | **-0.0550** | **1** | **6** | **49** | **10** | **K.KFVSGQK.S** 2069 |
|  | 2896 | **425.7446** | **849.4746** | **849.4807** | **-0.0061** | **0** | **22** | **1.1** | **1** | **R.LTSSLTTK.G** |
|  | 6572 | **554.8211** | **1107.6277** | **1106.6196** | **1.0080** | **1** | **3** | **75** | **8** | **K.LLTRAGSFSR.F** |
|  | 8578 | **618.3091** | **1234.6037** | **1234.6128** | **-0.0091** | **0** | **29** | **0.19** | **1** | **K.QVAEQFLNMR.G** |
|  | 9098 | **631.8071** | **1261.5996** | **1262.6731** | **-1.0735** | **1** | **2** | **91** | **5** | **R.GGLGLSGAKANYR.G** |
|  | 10307 | **663.8524** | **1325.6903** | **1325.6979** | **-0.0076** | **0** | **35** | **0.044** | **1** | **R.WEVADLQPQLK.I** |
|  | 11337 | **693.3088** | **1384.6030** | **1384.6115** | **-0.0085** | **0** | **43** | **0.0065** | **1** | **R.ENMAYTVECLR.G** 11360 |
|  | 12434 | **725.3350** | **1448.6554** | **1449.7324** | **-1.0771** | **0** | **60** | **0.00012** | **1** | **R.GSNTTSHLHQAVAK.A** 12460 12461 12462 12465 12477 |
|  | 12437 | 725.3472 | 1448.6798 | 1449.8378 | -1.1580 | 0 | 0 | 1.3e+002 | 5 | R.MALIGLGVSHPVLK.Q |
|  | 12586 | **730.3005** | **1458.5865** | **1457.8202** | **0.7663** | **1** | **4** | **51** | **2** | **R.GIEAVGGKLSVTATR.E** |
|  | 12969 | **741.9016** | **1481.7887** | **1481.7990** | **-0.0104** | **1** | **47** | **0.0022** | **1** | **R.RWEVADLQPQLK.I** 12970 |
|  | 14823 | **803.9177** | **1605.8208** | **1605.8335** | **-0.0128** | **1** | **57** | **0.00026** | **1** | **K.RGSNTTSHLHQAVAK.A** 14797 14798 14799 14800 14822 |
|  | 15224 | **815.9179** | **1629.8213** | **1629.8322** | **-0.0109** | **0** | **63** | **5.8e-005** | **1** | **K.TIAQGNLSNTDVQAAK.N** 15226 15256 |
|  | 15745 | **833.4101** | **1664.8056** | **1664.8158** | **-0.0102** | **0** | **62** | **6.5e-005** | **1** | **R.YEDFSNLGTTHLLR.L** 15744 |
|  | 16859 | **873.4060** | **1744.7975** | **1744.8090** | **-0.0116** | **0** | **71** | **8.3e-006** | **1** | **K.SMAASGNLGHTPFVDEL.-** 16860 |
|  | 19121 | **955.4799** | **1908.9453** | **1908.9581** | **-0.0128** | **0** | **60** | **0.0001** | **1** | **K.ATAAPAGAPPQPQDLEFTK.L** 19122 |
|  | 22904 | **1140.5780** | **2279.1414** | **2279.1508** | **-0.0093** | **0** | **11** | **6.4** | **1** | **R.GDVDILMEFLLNVTTAPEFR.R** |
|  | 23408 | **784.0676** | **2349.1809** | **2349.1978** | **-0.0169** | **0** | **59** | **9.3e-005** | **1** | **K.AVAFQNPQTHVIENLHAAAYR.N** |
|  | 30126 | **1274.3256** | **3819.9549** | **3818.9843** | **0.9706** | **1** | **2** | **19** | **1** | **K.ATAAPAGAPPQPQDLEFTKLPNGLVIASLENYSPVSR.I** |
|  | 30157 | **1280.9539** | **3839.8398** | **3839.8530** | **-0.0133** | **0** | **51** | **0.00027** | **1** | **K.ATQQPFDVSAFNASYSDSGLFGIYTISQATAAGDVIK.A** 30153 30154 30156 30159 |
|  | 30486 | **1022.2587** | **4085.0058** | **4084.0403** | **0.9656** | **0** | **22** | **0.18** | **1** | **R.EQNGDSLVHAAFVAESAVAGSAEANAFSVLQHVLGAGPHVK.R** 30488 30489 |
|  | 31693 | **1840.2024** | **5517.5853** | **5516.7041** | **0.8813** | **1** | **1** | **8.2** | **1** | **K.LKAGYLMSVESSECFLEEVGSQALVAGSYMPPSTVLQQIDSVANADIINAAK.K** |

| **71.** | O00748\|EST2_HUMAN    **Mass:** 62109    **Score:** 533    **Queries matched:** 47   **emPAI:** 1.26 |
| --- | --- |
|  | Carboxylesterase 2 OS=Homo sapiens GN=CES2 PE=1 SV=1 |

|  | **Query** | **Observed** | **Mr(expt)** | **Mr(calc)** | **Delta** | **Miss** | **Score** | **Expect** | **Rank** | **Peptide** |
| --- | --- | --- | --- | --- | --- | --- | --- | --- | --- | --- |
|  | 4075 | **464.2243** | **926.4340** | **926.4399** | **-0.0058** | **0** | **20** | **1.5** | **1** | **K.YWANFAR.N** |
|  | 4985 | 496.7733 | 991.5320 | 991.5385 | -0.0065 | 0 | 20 | 1.6 | 1 | K.NIRPPHMK.A 4969 |
|  | 5528 | **516.7580** | **1031.5014** | **1031.5076** | **-0.0062** | **0** | **25** | **0.58** | **1** | **R.SFFGGNYIK.F** 5527 |
|  | 9193 | 634.2900 | 1266.5654 | 1266.5728 | -0.0074 | 0 | 21 | 1 | 2 | K.FTEEEEQLSR.K 9192 |
|  | 9268 | **636.3049** | **1270.5952** | **1270.6041** | **-0.0089** | **0** | **46** | **0.0039** | **1** | **K.IQELEEPEER.H** 9269 |
|  | 9788 | 649.8012 | 1297.5877 | 1297.5972 | -0.0095 | 1 | 23 | 0.63 | 1 | R.IYDTQKEMDR.E |
|  | 11522 | **698.3362** | **1394.6579** | **1394.6677** | **-0.0098** | **1** | **35** | **0.041** | **1** | **K.FTEEEEQLSRK.M** |
|  | 11596 | **700.3879** | **1398.7613** | **1398.7694** | **-0.0080** | **0** | **71** | **1.1e-005** | **1** | **K.MIPGVVDGVFLPR.H** 11594 |
|  | 11632 | 468.2269 | 1401.6589 | 1401.6677 | -0.0089 | 0 | 24 | 0.52 | 1 | K.ADHGDELPFVFR.S 11631 |
|  | 12862 | 492.8860 | 1475.6362 | 1475.7038 | -0.0676 | 1 | 9 | 17 | 6 | K.EMDREASQAALQK.M |
|  | 13521 | **758.9349** | **1515.8552** | **1515.8660** | **-0.0108** | **1** | **68** | **1.9e-005** | **1** | **K.SKEEILAINKPFK.M** 13522 13523 |
|  | 14078 | **778.3754** | **1554.7363** | **1554.7467** | **-0.0104** | **0** | **31** | **0.099** | **1** | **R.FAPPEPPESWSGVR.D** 14076 14079 |
|  | 14392 | **526.2953** | **1575.8640** | **1575.8733** | **-0.0093** | **0** | **46** | **0.0028** | **1** | **R.TTHTGQVLGSLVHVK.G** 14390 |
|  | 16749 | **869.9180** | **1737.8215** | **1737.8336** | **-0.0120** | **0** | **73** | **4.9e-006** | **1** | **R.WVQQNIAHFGGNPDR.V** 16750 16751 |
|  | 17211 | **886.4420** | **1770.8695** | **1770.8802** | **-0.0107** | **0** | **96** | **2.7e-008** | **1** | **K.HATGNWGYLDQVAALR.W** 17212 |
|  | 18038 | **916.0113** | **1830.0080** | **1830.0147** | **-0.0067** | **0** | **31** | **0.082** | **1** | **K.MLTLLMLPPTFGDLLR.E** 18037 |
|  | 26312 | **930.4825** | **2788.4256** | **2788.4867** | **-0.0611** | **1** | **4** | **25** | **1** | **R.EASQAALQKMLTLLMLPPTFGDLLR.E** |
|  | 28984 | **1708.8766** | **3415.7386** | **3415.7565** | **-0.0178** | **0** | **59** | **4.8e-005** | **1** | **R.HPQELLASADFQPVPSIVGVNNNEFGWLIPK.V** 28981 28982 28983 28985 28987 28989 |
|  | 29554 | **1210.9232** | **3629.7478** | **3629.7651** | **-0.0173** | **0** | **26** | **0.087** | **1** | **R.NGNPNGEGLPHWPLFDQEEQYLQLNLQPAVGR.A** 29553 29558 29559 29560 29561 29562 |
|  | 31053 | **1534.6950** | **4601.0630** | **4601.0760** | **-0.0130** | **0** | **69** | **2.2e-006** | **1** | **R.EEYIGDNGDPQTLQAQFQEMMADSMFVIPALQVAHFQCSR.A** |

| **72.** | P37059\|DHB2_HUMAN    **Mass:** 43441    **Score:** 522    **Queries matched:** 34   **emPAI:** 1.93 |
| --- | --- |
|  | Estradiol 17-beta-dehydrogenase 2 OS=Homo sapiens GN=HSD17B2 PE=1 SV=1 |

|  | **Query** | **Observed** | **Mr(expt)** | **Mr(calc)** | **Delta** | **Miss** | **Score** | **Expect** | **Rank** | **Peptide** |
| --- | --- | --- | --- | --- | --- | --- | --- | --- | --- | --- |
|  | 5221 | **504.7920** | **1007.5695** | **1007.5764** | **-0.0069** | **0** | **40** | **0.015** | **1** | **R.DIQHAILAK.S** 5199 5208 5220 |
|  | 7861 | **600.2860** | **1198.5575** | **1198.5838** | **-0.0264** | **0** | **3** | **83** | **6** | **K.AAVTMFSSVMR.L** |
|  | 8139 | **404.8672** | **1211.5797** | **1211.5870** | **-0.0073** | **0** | **28** | **0.24** | **1** | **R.HFGQDKPMPR.A** 8138 |
|  | 8477 | **410.5681** | **1228.6825** | **1227.5819** | **1.1007** | **0** | **(11)** | **13** | **4** | **R.HFGQDKPMPR.A** |
|  | 10426 | **666.8951** | **1331.7756** | **1331.7813** | **-0.0056** | **0** | **63** | **6.5e-005** | **1** | **R.NFLLLINSLASK.D** 10425 |
|  | 13320 | 752.8613 | 1503.7081 | 1503.7174 | -0.0093 | 0 | 84 | 4.6e-007 | 1 | R.LVNVSSMGGGAPMER.L 13335 |
|  | 13545 | **760.3239** | **1518.6333** | **1519.7123** | **-1.0790** | **0** | **(38)** | **0.021** | **1** | **R.LVNVSSMGGGAPMER.L** 13572 |
|  | 13775 | **768.8760** | **1535.7374** | **1535.7072** | **0.0302** | **0** | **(3)** | **64** | **2** | **R.LVNVSSMGGGAPMER.L** 13773 13803 |
|  | 16427 | **856.9977** | **1711.9808** | **1711.9906** | **-0.0098** | **0** | **49** | **0.0013** | **1** | **R.LSVLQMDITKPVQIK.D** 16397 16425 16426 16428 |
|  | 16484 | **573.3146** | **1716.9221** | **1716.8400** | **0.0821** | **1** | **2** | **73** | **10** | **K.GRLVNVSSMGGGAPMER.L** |
|  | 16601 | **864.4265** | **1726.8383** | **1726.8495** | **-0.0111** | **0** | **78** | **1.7e-006** | **1** | **K.AVLVTGGDCGLGHALCK.Y** |
|  | 16625 | **577.1310** | **1728.3711** | **1727.9855** | **0.3856** | **0** | **(6)** | **17** | **2** | **R.LSVLQMDITKPVQIK.D** |
|  | 18672 | **938.4876** | **1874.9607** | **1874.9738** | **-0.0131** | **0** | **44** | **0.0041** | **1** | **K.VASIQPGGFLTNIAGTSDK.W** |
|  | 21454 | **707.3782** | **2119.1129** | **2119.1102** | **0.0027** | **1** | **2** | **54** | **10** | **R.DIQHAILAKSPFAYYTPGK.G** |
|  | 25824 | **1351.1504** | **2700.2862** | **2700.3031** | **-0.0169** | **0** | **60** | **5.5e-005** | **1** | **K.DILDHLPAEVQEDYGQDYILAQR.N** 25823 25825 25847 |
|  | 25920 | **1355.6650** | **2709.3155** | **2709.3286** | **-0.0131** | **0** | **96** | **1.7e-008** | **1** | **K.YLDELGFTVFAGVLNENGPGAEELR.R** |
|  | 26866 | **1454.2466** | **2906.4786** | **2906.4888** | **-0.0102** | **0** | **89** | **6.7e-008** | **1** | **R.GLWAVINNAGVLGFPTDGELLLMTDYK.Q** 26865 |

| **73.** | P51659\|DHB4_HUMAN    **Mass:** 80092    **Score:** 521    **Queries matched:** 43   **emPAI:** 0.72 |
| --- | --- |
|  | Peroxisomal multifunctional enzyme type 2 OS=Homo sapiens GN=HSD17B4 PE=1 SV=3 |

|  | **Query** | **Observed** | **Mr(expt)** | **Mr(calc)** | **Delta** | **Miss** | **Score** | **Expect** | **Rank** | **Peptide** |
| --- | --- | --- | --- | --- | --- | --- | --- | --- | --- | --- |
|  | 1376 | **616.4244** | **615.4171** | **615.3340** | **0.0831** | **0** | **8** | **52** | **10** | **K.GGNIGAK.W** |
|  | 1807 | **729.4569** | **728.4496** | **728.4545** | **-0.0048** | **0** | **12** | **12** | **8** | **R.TLGAIVR.Q** |
|  | 1931 | **763.1146** | **762.1073** | **761.4072** | **0.7002** | **0** | **2** | **1e+002** | **1** | **K.VYQGPAK.G** |
|  | 3665 | **450.2256** | **898.4366** | **899.5188** | **-1.0823** | **1** | **10** | **15** | **3** | **K.VVEEIRR.R** |
|  | 4045 | **462.9033** | **923.7920** | **924.5181** | **-0.7261** | **1** | **1** | **72** | **3** | **K.AFFSGRLK.A** |
|  | 5313 | **508.7812** | **1015.5478** | **1015.4909** | **0.0569** | **1** | **12** | **12** | **3** | **R.AAWEHMKK.Q** |
|  | 6753 | **562.2784** | **1122.5422** | **1122.5492** | **-0.0070** | **0** | **36** | **0.042** | **1** | **K.NHPMTPEAVK.A** 6751 |
|  | 7926 | 401.2849 | 1200.8327 | 1199.6775 | 1.1552 | 1 | 5 | 42 | 8 | R.WERTLGAIVR.Q |
|  | 9787 | **433.5364** | **1297.5874** | **1298.7208** | **-1.1334** | **1** | **2** | **86** | **7** | **R.VHLRGSFQVTR.A** |
|  | 10619 | 672.3144 | 1342.6143 | 1342.7034 | -0.0890 | 0 | 18 | 2.1 | 2 | K.VNAVFEWHITK.G |
|  | 11794 | **705.8180** | **1409.6215** | **1409.6310** | **-0.0095** | **0** | **64** | **5.1e-005** | **1** | **K.AVANYDSVEEGEK.V** 11795 |
|  | 12126 | **715.3324** | **1428.6502** | **1428.6593** | **-0.0091** | **0** | **78** | **2e-006** | **1** | **K.IDSEGGVSANHTSR.A** 12128 |
|  | 12724 | **734.3701** | **1466.7257** | **1466.7365** | **-0.0108** | **0** | **20** | **1.1** | **1** | **R.ATSTATSGFAGAIGQK.L** 12745 |
|  | 13214 | **748.9394** | **1495.8643** | **1495.8722** | **-0.0078** | **0** | **71** | **9.6e-006** | **1** | **K.LGLLGLANSLAIEGR.K** 13194 13215 13232 |
|  | 13935 | **516.3547** | **1546.0424** | **1546.7852** | **-0.7428** | **1** | **3** | **45** | **4** | **R.RVLQQFADNDVSR.F** |
|  | 15262 | **544.9019** | **1631.6839** | **1631.9102** | **-0.2263** | **1** | **1** | **1e+002** | **9** | **R.GNIMLSQKLQMILK.D** |
|  | 15468 | **549.2726** | **1644.7961** | **1643.9108** | **0.8853** | **1** | **6** | **29** | **5** | **R.FDGRVVLVTGAGAGLGR.A** |
|  | 16767 | **580.6213** | **1738.8420** | **1738.8533** | **-0.0113** | **1** | **50** | **0.001** | **1** | **R.KSNIHCNTIAPNAGSR.M** |
|  | 16831 | **581.9688** | **1742.8846** | **1743.9520** | **-1.0674** | **1** | **6** | **24** | **3** | **R.GALVVVNDLGGDFKGVGK.G** |
|  | 19643 | **489.5212** | **1954.0559** | **1954.0676** | **-0.0117** | **0** | **17** | **1.9** | **1** | **K.VLHGEQYLELYKPLPR.A** |
|  | 21409 | **529.5340** | **2114.1069** | **2113.1644** | **0.9425** | **1** | **2** | **61** | **10** | **K.TALDAFGRIDVVVNNAGILR.D** |
|  | 23415 | **1176.0637** | **2350.1129** | **2350.1263** | **-0.0134** | **0** | **99** | **1e-008** | **1** | **R.IIMTSSASGIYGNFGQANYSAAK.L** 23414 |
|  | 24282 | **822.4031** | **2464.1876** | **2464.1985** | **-0.0109** | **0** | **32** | **0.04** | **1** | **K.FIYEGSSDFSCLPTFGVIIGQK.S** |
|  | 24538 | 832.7372 | 2495.1897 | 2495.2267 | -0.0370 | 1 | 2 | 45 | 9 | R.FAKPVYPGQTLQTEMWKEGNR.I 24533 24539 |
|  | 24792 | **1268.6339** | **2535.2533** | **2535.2704** | **-0.0172** | **0** | **97** | **1.4e-008** | **1** | **K.VQETGDIVISNAYVDLAPTSGTSAK.T** 24793 24796 |
|  | 25863 | **902.1330** | **2703.3772** | **2703.3869** | **-0.0098** | **0** | **37** | **0.012** | **1** | **K.LPPFSYAYTELEAIMYALGVGASIK.D** |
|  | 26445 | **565.0712** | **2820.3198** | **2820.4103** | **-0.0905** | **1** | **3** | **29** | **5** | **K.GAADTTIILSDEDFMEVVLGKLDPQK.A** |
|  | 26634 | **956.1796** | **2865.5171** | **2865.5348** | **-0.0178** | **0** | **4** | **19** | **2** | **K.VAVAIPNRPPDAVLTDTTSLNQAALYR.L** |
|  | 26723 | **960.8084** | **2879.4032** | **2879.4222** | **-0.0190** | **0** | **39** | **0.0076** | **1** | **K.ICDFENASKPQSIQESTGSIIEVLSK.I** |
|  | 27583 | **1015.5240** | **3043.5501** | **3043.5616** | **-0.0115** | **1** | **58** | **7.4e-005** | **1** | **K.LPPFSYAYTELEAIMYALGVGASIKDPK.D** |
|  | 30357 | **993.4933** | **3969.9441** | **3969.9625** | **-0.0184** | **0** | **9** | **3.8** | **1** | **R.LSGDWNPLHIDPNFASLAGFDKPILHGLCTFGFSAR.R** |

| **74.** | P54868\|HMCS2_HUMAN    **Mass:** 57113    **Score:** 506    **Queries matched:** 31   **emPAI:** 0.76 |
| --- | --- |
|  | Hydroxymethylglutaryl-CoA synthase, mitochondrial OS=Homo sapiens GN=HMGCS2 PE=1 SV=1 |

|  | **Query** | **Observed** | **Mr(expt)** | **Mr(calc)** | **Delta** | **Miss** | **Score** | **Expect** | **Rank** | **Peptide** |
| --- | --- | --- | --- | --- | --- | --- | --- | --- | --- | --- |
|  | 2526 | **414.2434** | **826.4723** | **825.5436** | **0.9287** | **1** | **3** | **79** | **8** | **R.LLTPVKR.I** |
|  | 2880 | **425.2590** | **848.5035** | **848.3487** | **0.1548** | **0** | **1** | **1.4e+002** | **9** | **R.CYTSYR.K** |
|  | 3561 | **445.9170** | **889.8195** | **890.4862** | **-0.6667** | **0** | **4** | **47** | **5** | **K.GLEAFGGLK.L** 3552 |
|  | 3610 | **447.9905** | **893.9664** | **893.4243** | **0.5421** | **0** | **5** | **32** | **4** | **K.YNNVEAGK.Y** |
|  | 4276 | **471.3338** | **940.6530** | **940.3960** | **0.2570** | **0** | **16** | **3.5** | **2** | **K.ASQDMFDK.K** |
|  | 4330 | **473.2478** | **944.4811** | **943.5239** | **0.9572** | **1** | **12** | **13** | **8** | **K.KIQNQWK.Q** |
|  | 4388 | **474.8930** | **947.7715** | **947.5222** | **0.2493** | **1** | **0** | **1.1e+002** | **7** | **K.MVQKSLAR.L** |
|  | 5016 | **497.7661** | **993.5176** | **993.5244** | **-0.0067** | **0** | **31** | **0.14** | **1** | **K.YTVGLGQTR.M** |
|  | 5705 | **523.7859** | **1045.5572** | **1045.5655** | **-0.0083** | **0** | **40** | **0.018** | **1** | **K.LVSSTSDLPK.R** 5692 |
|  | 6008 | **535.2492** | **1068.4839** | **1068.4910** | **-0.0071** | **1** | **24** | **0.55** | **1** | **K.ASQDMFDKK.T** |
|  | 9547 | **643.3368** | **1284.6590** | **1283.6357** | **1.0233** | **0** | **8** | **20** | **3** | **R.VSQDAAPGSPLDK.L** |
|  | 9871 | **435.1951** | **1302.5635** | **1303.5979** | **-1.0344** | **1** | **10** | **16** | **2** | **R.ALDRCYTSYR.K** |
|  | 16760 | **870.3836** | **1738.7527** | **1738.7654** | **-0.0128** | **0** | **62** | **6.2e-005** | **1** | **K.CVSPEEFTEIMNQR.E** |
|  | 18771 | **942.9627** | **1883.9109** | **1883.9206** | **-0.0098** | **0** | **87** | **2.1e-007** | **1** | **R.IGAFSYGSGLAASFFSFR.V** 18772 |
|  | 21021 | **696.3261** | **2085.9565** | **2086.1647** | **-0.2082** | **1** | **3** | **41** | **1** | **R.AVQETSLTPARLLPVAHQR.F** |
|  | 21934 | **1084.5090** | **2167.0035** | **2167.0143** | **-0.0108** | **0** | **97** | **1.6e-008** | **1** | **R.LMFNDFLSASSDTQTSLYK.G** 21933 21935 |
|  | 23738 | **1199.0726** | **2396.1307** | **2396.1437** | **-0.0130** | **0** | **81** | **5.9e-007** | **1** | **K.VNFSPPGDTNSLFPGTWYLER.V** 23736 23737 |
|  | 25348 | **1313.6617** | **2625.3089** | **2625.3214** | **-0.0125** | **0** | **50** | **0.0006** | **1** | **K.DVGILALEVYFPAQYVDQTDLEK.Y** 25349 25350 25351 |
|  | 26760 | **963.1188** | **2886.3347** | **2886.3534** | **-0.0187** | **0** | **45** | **0.0017** | **1** | **R.GTHMENVYDFYKPNLASEYPIVDGK.L** 26761 |
|  | 28902 | 1130.8257 | 3389.4552 | 3388.6043 | 0.8509 | 1 | 3 | 18 | 2 | K.QAGSDRPFTLDDLQYMIFHTPFCKMVQK.S |

| **75.** | P05026\|AT1B1_HUMAN    **Mass:** 35438    **Score:** 501    **Queries matched:** 30   **emPAI:** 1.48 |
| --- | --- |
|  | Sodium/potassium-transporting ATPase subunit beta-1 OS=Homo sapiens GN=ATP1B1 PE=1 SV=1 |

|  | **Query** | **Observed** | **Mr(expt)** | **Mr(calc)** | **Delta** | **Miss** | **Score** | **Expect** | **Rank** | **Peptide** |
| --- | --- | --- | --- | --- | --- | --- | --- | --- | --- | --- |
|  | 1879 | **750.3400** | **749.3327** | **748.3239** | **1.0088** | **1** | **6** | **45** | **6** | **R.DEDKDK.V** |
|  | 3175 | **433.7151** | **865.4156** | **866.4246** | **-1.0090** | **1** | **6** | **44** | **6** | **K.YKDSAQR.D** |
|  | 4172 | **467.7317** | **933.4488** | **933.4556** | **-0.0067** | **1** | **39** | **0.023** | **1** | **K.AKEEGSWK.K** |
|  | 5778 | **526.2785** | **1050.5424** | **1050.5498** | **-0.0074** | **1** | **12** | **9.8** | **3** | **K.FIWNSEKK.E** |
|  | 5785 | **526.7541** | **1051.4937** | **1050.5498** | **0.9439** | **1** | **7** | **27** | **2** | **K.KFIWNSEK.K** |
|  | 8488 | **615.7814** | **1229.5482** | **1229.5564** | **-0.0082** | **0** | **67** | **3.5e-005** | **1** | **K.AYGENIGYSEK.D** 8489 |
|  | 9882 | **652.3314** | **1302.6483** | **1302.6568** | **-0.0085** | **0** | **24** | **0.62** | **1** | **K.TEISFRPNDPK.S** 9881 |
|  | 10013 | **656.2856** | **1310.5566** | **1309.6224** | **0.9342** | **0** | **3** | **61** | **1** | **K.NESLETYPVMK.Y** |
|  | 10308 | **663.8526** | **1325.6906** | **1325.6979** | **-0.0073** | **0** | **59** | **0.00019** | **1** | **K.SYEAYVLNIVR.F** 10309 |
|  | 12319 | **481.2417** | **1440.7034** | **1439.8282** | **0.8751** | **1** | **4** | **52** | **7** | **K.EGKPCIIIKLNR.V** |
|  | 13082 | **745.3723** | **1488.7299** | **1488.7395** | **-0.0096** | **0** | **42** | **0.0092** | **1** | **K.YNPNVLPVQCTGK.R** 13081 |
|  | 13097 | **745.4361** | **1488.8576** | **1488.8664** | **-0.0088** | **0** | **64** | **4.9e-005** | **1** | **R.VAPPGLTQIPQIQK.T** 13095 |
|  | 13160 | **747.2959** | **1492.5772** | **1492.9242** | **-0.3469** | **1** | **6** | **30** | **9** | **K.LNRVLGFKPKPPK.N** 13159 |
|  | 13278 | **751.3441** | **1500.6737** | **1500.6844** | **-0.0108** | **1** | **58** | **0.00019** | **1** | **K.AYGENIGYSEKDR.F** 13279 |
|  | 18195 | **461.4690** | **1841.8467** | **1842.9879** | **-1.1412** | **1** | **0** | **1e+002** | **9** | **K.SYEAYVLNIVRFLEK.Y** |
|  | 18374 | **927.3755** | **1852.7364** | **1852.7495** | **-0.0131** | **0** | **78** | **1.5e-006** | **1** | **R.DDMIFEDCGDVPSEPK.E** 18375 18376 |
|  | 26228 | **925.1411** | **2772.4015** | **2773.5127** | **-1.1111** | **1** | **1** | **45** | **2** | **R.VAPPGLTQIPQIQKTEISFRPNDPK.S** |
|  | 26475 | **1415.1753** | **2828.3360** | **2828.3486** | **-0.0126** | **0** | **69** | **7.5e-006** | **1** | **K.VGNVEYFGLGNSPGFPLQYYPYYGK.L** 26471 26473 26474 |

| **76.** | P04181\|OAT_HUMAN    **Mass:** 48846    **Score:** 499    **Queries matched:** 33   **emPAI:** 0.94 |
| --- | --- |
|  | Ornithine aminotransferase, mitochondrial OS=Homo sapiens GN=OAT PE=1 SV=1 |

|  | **Query** | **Observed** | **Mr(expt)** | **Mr(calc)** | **Delta** | **Miss** | **Score** | **Expect** | **Rank** | **Peptide** |
| --- | --- | --- | --- | --- | --- | --- | --- | --- | --- | --- |
|  | 1526 | **651.3502** | **650.3429** | **649.3105** | **1.0325** | **0** | **12** | **11** | **3** | **R.NELMK.L** |
|  | 3425 | **441.2963** | **880.5781** | **880.4807** | **0.0974** | **1** | **9** | **19** | **7** | **R.KWGYTVK.G** |
|  | 4261 | **470.8103** | **939.6060** | **939.6117** | **-0.0056** | **0** | **23** | **0.8** | **1** | **K.GLLNAIVIK.E** |
|  | 6448 | **550.2896** | **1098.5647** | **1098.6298** | **-0.0651** | **1** | **0** | **1.4e+002** | **8** | **M.FSKLAHLQR.F** |
|  | 8036 | **604.3054** | **1206.5962** | **1206.6033** | **-0.0071** | **0** | **39** | **0.018** | **1** | **K.GIYLWDVEGR.K** |
|  | 8623 | **413.1968** | **1236.5686** | **1236.6404** | **-0.0718** | **0** | **10** | **13** | **2** | **K.IVFAAGNFWGR.T** 8627 8633 8655 |
|  | 9792 | **433.5707** | **1297.6904** | **1297.7969** | **-0.1065** | **1** | **9** | **18** | **7** | **K.GLLNAIVIKETK.D** |
|  | 13677 | **510.5616** | **1528.6628** | **1528.8209** | **-0.1581** | **1** | **4** | **55** | **3** | **R.GVHSSVASATSVATKK.T** 13669 |
|  | 14159 | **781.3724** | **1560.7302** | **1560.7420** | **-0.0118** | **0** | **53** | **0.00062** | **1** | **K.TVQGPPTSDDIFER.E** |
|  | 15906 | **838.8971** | **1675.7796** | **1675.7909** | **-0.0113** | **0** | **32** | **0.078** | **1** | **K.VLPMNTGVEAGETACK.L** |
|  | 16054 | **844.4079** | **1686.8012** | **1686.8974** | **-0.0962** | **1** | **5** | **35** | **10** | **R.NELMKLPSDVVTAVR.G** 16061 |
|  | 16140 | **847.4318** | **1692.8491** | **1691.7859** | **1.0632** | **0** | **(14)** | **4.5** | **2** | **K.VLPMNTGVEAGETACK.L** |
|  | 16734 | 579.6298 | 1735.8676 | 1735.8794 | -0.0118 | 0 | 33 | 0.06 | 1 | K.YGAHNYHPLPVALER.G 16735 16740 |
|  | 18849 | **473.2657** | **1889.0338** | **1888.0643** | **0.9695** | **1** | **2** | **64** | **9** | **R.LRDNGLLAKPTHGDIIR.F** |
|  | 19614 | **976.9627** | **1951.9109** | **1951.9203** | **-0.0095** | **0** | **71** | **6.8e-006** | **1** | **R.AFYNNVLGEYEEYITK.L** 19615 |
|  | 20271 | **673.3333** | **2016.9779** | **2016.0132** | **0.9647** | **1** | **4** | **38** | **2** | **K.VLPMNTGVEAGETACKLAR.K** |
|  | 21182 | **1050.5723** | **2099.1300** | **2099.1415** | **-0.0115** | **0** | **95** | **2.5e-008** | **1** | **R.WLAVDYENVRPDIVLLGK.A** 21175 21183 21191 |
|  | 21628 | **1071.0466** | **2140.0787** | **2140.0899** | **-0.0112** | **0** | **103** | **4.8e-009** | **1** | **R.VAIAALEVLEEENLAENADK.L** 21629 |
|  | 23667 | **795.7018** | **2384.0835** | **2384.1008** | **-0.0173** | **1** | **38** | **0.013** | **1** | **R.KYFDFLSSYSAVNQGHCHPK.I** 23668 |
|  | 26383 | **936.5211** | **2806.5414** | **2805.5487** | **0.9927** | **1** | **80** | **5.9e-007** | **1** | **R.VAIAALEVLEEENLAENADKLGIILR.N** |

| **77.** | O95497\|VNN1_HUMAN    **Mass:** 57716    **Score:** 499    **Queries matched:** 21   **emPAI:** 0.65 |
| --- | --- |
|  | Pantetheinase OS=Homo sapiens GN=VNN1 PE=1 SV=2 |

|  | **Query** | **Observed** | **Mr(expt)** | **Mr(calc)** | **Delta** | **Miss** | **Score** | **Expect** | **Rank** | **Peptide** |
| --- | --- | --- | --- | --- | --- | --- | --- | --- | --- | --- |
|  | 5699 | **523.7653** | **1045.5160** | **1045.5226** | **-0.0066** | **0** | **21** | **1.5** | **1** | **R.EEALALMNR.N** |
|  | 5913 | **531.2689** | **1060.5233** | **1060.5302** | **-0.0069** | **0** | **37** | **0.039** | **1** | **R.FGQTPVQER.L** |
|  | 11776 | 705.3867 | 1408.7589 | 1409.6973 | -0.9384 | 0 | 4 | 53 | 4 | K.LTGVAGNYTVCQK.D |
|  | 13001 | **495.5893** | **1483.7460** | **1483.7089** | **0.0371** | **1** | **4** | **53** | **2** | **K.KMTGSGIYAPNSSR.A** 13006 |
|  | 13090 | **497.2617** | **1488.7632** | **1488.7725** | **-0.0092** | **0** | **34** | **0.048** | **1** | **R.VNFLASNIHYPSK.K** |
|  | 15710 | 832.3782 | 1662.7418 | 1662.7526 | -0.0108 | 0 | 75 | 3.7e-006 | 1 | R.YQYNTDVVFDSQGK.L 15709 |
|  | 15743 | **833.4009** | **1664.7873** | **1664.7981** | **-0.0107** | **0** | **59** | **0.00015** | **1** | **K.QNLFMGENQFNVPK.E** |
|  | 17573 | **899.8917** | **1797.7688** | **1797.7799** | **-0.0112** | **0** | **98** | **1.6e-008** | **1** | **K.TTNLNTCGDSAETASTR.F** 17572 |
|  | 20466 | **678.3949** | **2032.1629** | **2032.1721** | **-0.0092** | **0** | **23** | **0.45** | **1** | **R.LFSLKPTSGPVLTVTLFGR.L** 20465 |
|  | 25321 | **1310.6779** | **2619.3412** | **2618.2435** | **1.0977** | **0** | **4** | **26** | **3** | **K.MSENIPNEVYALGAFDGLHTVEGR.Y** 25319 |
|  | 26435 | **1410.1461** | **2818.2777** | **2818.2908** | **-0.0131** | **0** | **67** | **1e-005** | **1** | **R.DSLYPYLEDIPDPEVNWIPCNNR.N** 26434 |
|  | 29761 | **1228.9431** | **3683.8075** | **3683.8220** | **-0.0144** | **0** | **102** | **2.1e-009** | **1** | **R.NLDILEGAITSAADQGAHIIVTPEDAIYGWNFNR.D** 29759 29762 |
|  | 30254 | **783.3791** | **3911.8591** | **3911.8739** | **-0.0148** | **0** | **4** | **12** | **1** | **K.DFHVDTIVFPTAWMNVLPHLSAVEFHSAWAMGMR.V** |

| **78.** | Q12864\|CAD17_HUMAN    **Mass:** 92547    **Score:** 492    **Queries matched:** 31   **emPAI:** 0.73 |
| --- | --- |
|  | Cadherin-17 OS=Homo sapiens GN=CDH17 PE=1 SV=2 |

|  | **Query** | **Observed** | **Mr(expt)** | **Mr(calc)** | **Delta** | **Miss** | **Score** | **Expect** | **Rank** | **Peptide** |
| --- | --- | --- | --- | --- | --- | --- | --- | --- | --- | --- |
|  | 3699 | **900.5686** | **899.5613** | **898.5640** | **0.9973** | **0** | **18** | **2.8** | **6** | **K.ILYHIIK.G** |
|  | 5354 | **510.2527** | **1018.4908** | **1017.5342** | **0.9565** | **0** | **14** | **7** | **2** | **K.VSEDVAIGTK.V** |
|  | 6070 | **536.7631** | **1071.5117** | **1071.5196** | **-0.0079** | **0** | **10** | **21** | **2** | **R.EGSQELNPAK.N** |
|  | 7037 | **573.7308** | **1145.4470** | **1145.5757** | **-0.1287** | **0** | **0** | **1.5e+002** | **7** | **K.DAYVFYAVAK.D** |
|  | 9826 | **650.8485** | **1299.6825** | **1298.6870** | **0.9954** | **0** | **23** | **0.72** | **1** | **K.AENPEPLVFGVK.Y** |
|  | 10962 | **682.8518** | **1363.6891** | **1363.6619** | **0.0271** | **0** | **13** | **6.9** | **1** | **K.DPEGLDISYSLR.G** 10961 |
|  | 13138 | **746.8518** | **1491.6889** | **1491.6994** | **-0.0105** | **0** | **55** | **0.00038** | **1** | **R.WNDPGAQYSLVDK.E** |
|  | 13944 | 774.3884 | 1546.7622 | 1546.7740 | -0.0118 | 0 | 37 | 0.024 | 1 | K.DINDNRPTFLQSK.Y |
|  | 16908 | **875.4200** | **1748.8254** | **1748.8369** | **-0.0115** | **1** | **56** | **0.00031** | **1** | **R.WNDPGAQYSLVDKEK.L** 16907 |
|  | 17173 | 590.3090 | 1767.9051 | 1767.9156 | -0.0105 | 0 | 20 | 1 | 1 | K.IDHVTGEIFSVAPLDR.E |
|  | 18821 | **944.4901** | **1886.9657** | **1886.9778** | **-0.0121** | **0** | **60** | **9.4e-005** | **1** | **K.DEYGKPLSYPLEIHVK.V** 18820 18822 |
|  | 22554 | **1120.5207** | **2239.0269** | **2239.0797** | **-0.0528** | **0** | **51** | **0.00058** | **1** | **R.FPFSIDQEGDIYVTQPLDR.E** 22555 |
|  | 24705 | 1260.1431 | 2518.2716 | 2518.2856 | -0.0141 | 0 | 85 | 2.3e-007 | 1 | K.FTLIVTDVNEAPQFSQHVFQAK.V 24704 |
|  | 25345 | **876.0883** | **2625.2430** | **2625.2599** | **-0.0169** | **1** | **48** | **0.00088** | **1** | **R.FPFSIDQEGDIYVTQPLDREEK.D** 25346 25357 |
|  | 26736 | **962.1252** | **2883.3539** | **2883.3709** | **-0.0170** | **1** | **57** | **0.00011** | **1** | **K.VKDINDNPPTCPSPVTVFEVQENER.L** |
|  | 26872 | **970.1257** | **2907.3554** | **2907.3747** | **-0.0193** | **1** | **39** | **0.0061** | **1** | **R.LGNSIGTLTAHDRDEENTANSFLNYR.I** |
|  | 28930 | **1132.9031** | **3395.6874** | **3395.7031** | **-0.0157** | **0** | **43** | **0.0023** | **1** | **R.VQVVATEVGGSSLSSVSEFHLILMDVNDNPPR.L** 28918 28919 28927 28928 28933 |
|  | 29101 | **1157.2037** | **3468.5894** | **3468.6085** | **-0.0191** | **0** | **88** | **6.5e-008** | **1** | **K.DYTGLFFCHPLSAPGSLIFEATDDDQHLFR.G** |

| **79.** | P0C0S8\|H2A1_HUMAN    **Mass:** 14083    **Score:** 483    **Queries matched:** 24   **emPAI:** 3.36 |
| --- | --- |
|  | Histone H2A type 1 OS=Homo sapiens GN=HIST1H2AG PE=1 SV=2 |

|  | **Query** | **Observed** | **Mr(expt)** | **Mr(calc)** | **Delta** | **Miss** | **Score** | **Expect** | **Rank** | **Peptide** |
| --- | --- | --- | --- | --- | --- | --- | --- | --- | --- | --- |
|  | 820 | **505.2373** | **504.2301** | **503.2816** | **0.9485** | **1** | **3** | **1.5e+002** | **7** | **M.SGRGK.Q** |
|  | 2684 | **419.2129** | **836.4112** | **836.4140** | **-0.0028** | **1** | **16** | **4.2** | **1** | **R.KGNYAER.V** 2697 2698 2699 2716 |
|  | 2897 | **425.7636** | **849.5127** | **849.5184** | **-0.0058** | **0** | **32** | **0.13** | **1** | **R.HLQLAIR.N** 2888 2898 2899 2901 |
|  | 4323 | **472.7661** | **943.5175** | **943.5240** | **-0.0064** | **0** | **53** | **0.00095** | **1** | **R.AGLQFPVGR.V** 4330 |
|  | 16139 | **424.2194** | **1692.8486** | **1691.8954** | **0.9531** | **1** | **12** | **7.5** | **2** | **R.HLQLAIRNDEELNK.L** |
|  | 19379 | 966.0837 | 1930.1529 | 1930.1615 | -0.0086 | 0 | 62 | 5.7e-005 | 1 | K.VTIAQGGVLPNIQAVLLPK.K 19375 19376 19378 19380 19381 |
|  | 26905 | **1458.2919** | **2914.5692** | **2914.5804** | **-0.0112** | **0** | **125** | **1.4e-011** | **1** | **R.VGAGAPVYLAAVLEYLTAEILELAGNAAR.D** 26904 26906 26907 |

|  | **Proteins matching the same set of peptides:** |
| --- | --- |

|  | Q9BTM1\|H2AJ_HUMAN    **Mass:** 14011    **Score:** 483    **Queries matched:** 24 |
| --- | --- |
|  | Histone H2A.J OS=Homo sapiens GN=H2AFJ PE=1 SV=1 |

|  | Q96KK5\|H2A1H_HUMAN    **Mass:** 13898    **Score:** 483    **Queries matched:** 24 |
| --- | --- |
|  | Histone H2A type 1-H OS=Homo sapiens GN=HIST1H2AH PE=1 SV=3 |

|  | Q93077\|H2A1C_HUMAN    **Mass:** 14097    **Score:** 483    **Queries matched:** 24 |
| --- | --- |
|  | Histone H2A type 1-C OS=Homo sapiens GN=HIST1H2AC PE=1 SV=3 |

|  | Q99878\|H2A1J_HUMAN    **Mass:** 13928    **Score:** 483    **Queries matched:** 24 |
| --- | --- |
|  | Histone H2A type 1-J OS=Homo sapiens GN=HIST1H2AJ PE=1 SV=3 |

|  | **Proteins matching a subset of these peptides:** |
| --- | --- |

|  | Q7L7L0\|H2A3_HUMAN    **Mass:** 14113    **Score:** 483    **Queries matched:** 19 |
| --- | --- |
|  | Histone H2A type 3 OS=Homo sapiens GN=HIST3H2A PE=1 SV=3 |

|  | P04908\|H2A1B_HUMAN    **Mass:** 14127    **Score:** 483    **Queries matched:** 19 |
| --- | --- |
|  | Histone H2A type 1-B/E OS=Homo sapiens GN=HIST1H2AB PE=1 SV=2 |

|  | P20671\|H2A1D_HUMAN    **Mass:** 14099    **Score:** 483    **Queries matched:** 19 |
| --- | --- |
|  | Histone H2A type 1-D OS=Homo sapiens GN=HIST1H2AD PE=1 SV=2 |

| **80.** | P50120\|RET2_HUMAN    **Mass:** 15868    **Score:** 483    **Queries matched:** 28   **emPAI:** 1.99 |
| --- | --- |
|  | Retinol-binding protein 2 OS=Homo sapiens GN=RBP2 PE=1 SV=3 |

|  | **Query** | **Observed** | **Mr(expt)** | **Mr(calc)** | **Delta** | **Miss** | **Score** | **Expect** | **Rank** | **Peptide** |
| --- | --- | --- | --- | --- | --- | --- | --- | --- | --- | --- |
|  | 1232 | 587.3104 | 586.3031 | 585.3962 | 0.9069 | 1 | 17 | 4.9 | 1 | R.KIAVR.L 1220 |
|  | 1758 | **713.3613** | **712.3541** | **711.3552** | **0.9989** | **0** | **2** | **90** | **2** | **K.TTSTFR.N** |
|  | 5381 | **511.2661** | **1020.5177** | **1020.5240** | **-0.0063** | **0** | **50** | **0.0017** | **1** | **K.ALDIDFATR.K** |
|  | 7094 | **575.7684** | **1149.5222** | **1149.5302** | **-0.0080** | **0** | **51** | **0.0012** | **1** | **K.VIDQDGDNFK.T** 7092 7093 |
|  | 16457 | 858.4598 | 1714.9050 | 1715.8917 | -0.9867 | 0 | 8 | 17 | 4 | K.ALVTWEGDVLVCVQK.G |
|  | 16545 | **574.7784** | **1721.3135** | **1720.8632** | **0.4503** | **1** | **24** | **0.32** | **1** | **R.LTQTKVIDQDGDNFK.T** |
|  | 20553 | **1020.9527** | **2039.8908** | **2039.9000** | **-0.0092** | **0** | **102** | **5.7e-009** | **1** | **R.NYDVDFTVGVEFDEYTK.S** 20548 20549 20551 20554 20555 20556 20557 20558 20559 20561 20562 20573 20579 |
|  | 23106 | 1155.0533 | 2308.0921 | 2307.9048 | 0.1873 | 0 | 4 | 30 | 3 | R.DQNGTWEMESNENFEGYMK.A |
|  | 24392 | **1242.0815** | **2482.1485** | **2482.1621** | **-0.0136** | **1** | **44** | **0.0026** | **1** | **K.QWIEGDKLYLELTCGDQVCR.Q** 24390 24391 24393 |

| **81.** | P12821\|ACE_HUMAN    **Mass:** 150418   **Score:** 483    **Queries matched:** 58   **emPAI:** 0.44 |
| --- | --- |
|  | Angiotensin-converting enzyme OS=Homo sapiens GN=ACE PE=1 SV=1 |

|  | **Query** | **Observed** | **Mr(expt)** | **Mr(calc)** | **Delta** | **Miss** | **Score** | **Expect** | **Rank** | **Peptide** |
| --- | --- | --- | --- | --- | --- | --- | --- | --- | --- | --- |
|  | 1449 | **634.3548** | **633.3475** | **633.3520** | **-0.0044** | **0** | **12** | **14** | **8** | **R.LATAMK.L** 1448 |
|  | 2246 | **403.2276** | **804.4406** | **804.4024** | **0.0382** | **1** | **10** | **20** | **4** | **-.MGAASGRR.G** 2257 |
|  | 2298 | **405.2079** | **808.4013** | **807.4028** | **0.9985** | **0** | **29** | **0.22** | **1** | **R.WGVFSGR.T** |
|  | 2459 | **411.7139** | **821.4133** | **820.3974** | **1.0160** | **1** | **(4)** | **63** | **3** | **-.MGAASGRR.G** |
|  | 3513 | **444.0417** | **886.0688** | **886.4872** | **-0.4184** | **1** | **3** | **71** | **6** | **K.KVQDLER.A** |
|  | 3882 | 457.2050 | 912.3954 | 912.4011 | -0.0057 | 0 | 13 | 7 | 1 | K.CDIYQSK.E |
|  | 4501 | **479.2159** | **956.4173** | **956.4239** | **-0.0067** | **0** | **16** | **4.8** | **2** | **K.FVEEYDR.T** |
|  | 4813 | **490.2810** | **978.5474** | **978.5538** | **-0.0064** | **0** | **34** | **0.063** | **1** | **R.AILQFYPK.Y** 4812 |
|  | 5279 | **507.7429** | **1013.4712** | **1013.4778** | **-0.0066** | **0** | **23** | **0.78** | **1** | **R.SEGPLPDSGR.V** 5299 |
|  | 5562 | **518.2372** | **1034.4599** | **1034.4669** | **-0.0070** | **0** | **0** | **1.5e+002** | **4** | **R.TQGDFDPGAK.F** |
|  | 6254 | **542.8183** | **1083.6220** | **1083.6288** | **-0.0067** | **0** | **21** | **1.2** | **1** | **R.TLGSANLPLAK.R** 6234 |
|  | 6882 | **567.2809** | **1132.5473** | **1132.5546** | **-0.0073** | **0** | **26** | **0.39** | **1** | **K.SMLEKPTDGR.E** |
|  | 7436 | **588.8180** | **1175.6215** | **1175.6298** | **-0.0083** | **0** | **58** | **0.00025** | **1** | **K.YVELINQAAR.L** 7437 |
|  | 8694 | **414.2010** | **1239.5811** | **1239.7299** | **-0.1487** | **1** | **6** | **35** | **1** | **R.TLGSANLPLAKR.Q** |
|  | 10123 | 658.8698 | 1315.7251 | 1314.7085 | 1.0166 | 0 | 9 | 18 | 2 | K.FHIPSSVPYIR.Y |
|  | 10765 | **676.8106** | **1351.6067** | **1351.6157** | **-0.0090** | **0** | **14** | **5** | **1** | **R.LNGYVDAGDSWR.S** 10768 |
|  | 10934 | **681.8134** | **1361.6121** | **1361.6193** | **-0.0071** | **0** | **7** | **31** | **1** | **R.YNFDWWYLR.T** |
|  | 11904 | **708.8072** | **1415.5998** | **1415.6106** | **-0.0108** | **0** | **22** | **0.77** | **2** | **K.QDGFTDTGAYWR.S** |
|  | 11940 | **709.8988** | **1417.7830** | **1418.7340** | **-0.9510** | **1** | **9** | **16** | **2** | **R.TKYQGICPPVTR.N** |
|  | 11978 | **474.2891** | **1419.8455** | **1419.7358** | **0.1097** | **0** | **13** | **5.6** | **1** | **K.FDVNQLQNTTIK.R** 11965 11968 11984 |
|  | 12842 | **492.2529** | **1473.7370** | **1474.7303** | **-0.9934** | **0** | **2** | **88** | **10** | **R.AALPAQELEEYNK.I** |
|  | 13610 | **762.8423** | **1523.6700** | **1523.6793** | **-0.0093** | **0** | **30** | **0.11** | **1** | **K.ENYNQEWWSLR.L** |
|  | 13746 | 767.4000 | 1532.7855 | 1531.7995 | 0.9860 | 0 | 6 | 31 | 8 | R.VSFLGLDLDAQQAR.V |
|  | 13957 | 774.8814 | 1547.7483 | 1547.8308 | -0.0825 | 1 | 11 | 11 | 8 | R.KFDVNQLQNTTIK.R |
|  | 14401 | **526.6199** | **1576.8380** | **1575.8369** | **1.0011** | **1** | **6** | **28** | **4** | **K.FDVNQLQNTTIKR.I** |
|  | 14425 | **527.2603** | **1578.7591** | **1578.7539** | **0.0052** | **0** | **3** | **58** | **3** | **R.HSHGPQFGSEVELR.H** |
|  | 16170 | **848.8728** | **1695.7310** | **1696.7614** | **-1.0303** | **0** | **36** | **0.03** | **1** | **R.SMYETPSLEQDLER.L** 16187 |
|  | 16200 | **849.4645** | **1696.9145** | **1696.9260** | **-0.0115** | **0** | **54** | **0.00049** | **1** | **K.VLQAGSSRPWQEVLK.D** |
|  | 16933 | **876.4261** | **1750.8376** | **1750.8467** | **-0.0091** | **1** | **84** | **4.8e-007** | **1** | **R.KYEDLLWAWEGWR.D** |
|  | 16954 | **877.3851** | **1752.7556** | **1752.7678** | **-0.0123** | **0** | **24** | **0.39** | **1** | **R.EVVCHASAWDFYNR.K** |
|  | 16985 | **585.7771** | **1754.3095** | **1753.9192** | **0.3903** | **0** | **1** | **60** | **5** | **K.IAFIPFSYLVDQWR.W** |
|  | 17118 | **588.8215** | **1763.4426** | **1764.0080** | **-0.5654** | **1** | **1** | **65** | **4** | **K.ILLQKNMQIANHTLK.Y** |
|  | 17687 | **904.4009** | **1806.7872** | **1806.7995** | **-0.0123** | **0** | **41** | **0.0083** | **1** | **K.EAGYEGPLHQCDIYR.S** |
|  | 21424 | **1059.0746** | **2116.1346** | **2116.1469** | **-0.0123** | **0** | **55** | **0.00027** | **1** | **R.LFQELQPLYLNLHAYVR.R** 21425 |
|  | 21460 | **707.6777** | **2120.0112** | **2120.0208** | **-0.0096** | **0** | **29** | **0.11** | **1** | **K.TATCWSLDPDLTNILASSR.S** |
|  | 22122 | **1096.0366** | **2190.0587** | **2190.0705** | **-0.0118** | **1** | **102** | **5.9e-009** | **1** | **R.RQEEAALLSQEFAEAWGQK.A** 22120 22121 |
|  | 23132 | **771.3987** | **2311.1742** | **2312.2027** | **-1.0285** | **1** | **32** | **0.048** | **1** | **K.MALEKIAFLPFGYLVDQWR.W** |
|  | 25773 | **898.4321** | **2692.2746** | **2692.2915** | **-0.0169** | **0** | **51** | **0.00046** | **1** | **K.HLHSLNLLSSEGGSDEHDINFLMK.M** |
|  | 25790 | **540.0784** | **2695.3555** | **2695.3718** | **-0.0163** | **0** | **24** | **0.27** | **1** | **R.GANPGFHEAIGDVLALSVSTPEHLHK.I** |
|  | 26443 | **941.1133** | **2820.3180** | **2820.3363** | **-0.0183** | **0** | **46** | **0.0015** | **1** | **R.VTMDQLSTVHHEMGHIQYYLQYK.D** 26444 |
|  | 29264 | **1176.2168** | **3525.6286** | **3525.6472** | **-0.0186** | **0** | **41** | **0.003** | **1** | **R.VAEEFFTSLELSPMPPEFWEGSMLEKPADGR.E** 29265 |
|  | 31944 | **1471.6847** | **5882.7097** | **5882.7441** | **-0.0345** | **0** | **16** | **0.18** | **1** | **K.YFQPVTQWLQEQNQQNGEVLGWPEYQWHPPLPDNYPEGIDLVTDEAEASK.F** |

| **82.** | Q6PEY2\|TBA3E_HUMAN    **Mass:** 50568    **Score:** 482    **Queries matched:** 27   **emPAI:** 1.19 |
| --- | --- |
|  | Tubulin alpha-3E chain OS=Homo sapiens GN=TUBA3E PE=1 SV=2 |

|  | **Query** | **Observed** | **Mr(expt)** | **Mr(calc)** | **Delta** | **Miss** | **Score** | **Expect** | **Rank** | **Peptide** |
| --- | --- | --- | --- | --- | --- | --- | --- | --- | --- | --- |
|  | 1369 | 615.3498 | 614.3426 | 613.3435 | 0.9990 | 0 | 22 | 1.5 | 1 | R.GDVVPK.D |
|  | 5307 | 508.2902 | 1014.5658 | 1014.5709 | -0.0051 | 0 | 43 | 0.0084 | 1 | K.DVNAAIATIK.T |
|  | 10962 | 682.8518 | 1363.6891 | 1363.7322 | -0.0431 | 1 | 6 | 36 | 9 | R.LVHKFDLMYAK.W |
|  | 11243 | **460.5877** | **1378.7412** | **1379.7271** | **-0.9859** | **1** | **(6)** | **38** | **8** | **R.LVHKFDLMYAK.W** |
|  | 11801 | 470.9266 | 1409.7580 | 1409.7667 | -0.0087 | 0 | 13 | 6.6 | 1 | R.QLFHPEQLITGK.E |
|  | 14696 | 799.8823 | 1597.7500 | 1597.7599 | -0.0100 | 0 | 38 | 0.02 | 1 | R.TIQFVDWCPTGFK.V |
|  | 16490 | 859.9390 | 1717.8634 | 1717.8747 | -0.0113 | 0 | 37 | 0.021 | 1 | R.NLDIERPTYTNLNR.L 16491 |
|  | 17026 | 878.9797 | 1755.9449 | 1755.9559 | -0.0110 | 0 | 36 | 0.025 | 1 | R.IHFPLATYAPVISAEK.A 17025 17028 17029 |
|  | 17925 | 912.9903 | 1823.9660 | 1823.9782 | -0.0121 | 0 | 56 | 0.00029 | 1 | K.VGINYQPPTVVPGGDLAK.V 17924 17926 |
|  | 18530 | 621.9794 | 1862.9165 | 1861.8347 | 1.0817 | 1 | 5 | 32 | 4 | K.YMACCMLYRGDVVPK.D |
|  | 18536 | 932.9506 | 1863.8866 | 1863.8971 | -0.0105 | 0 | 91 | 8.5e-008 | 1 | R.AVCMLSNTTAIAEAWAR.L |
|  | 20182 | 1004.4432 | 2006.8719 | 2006.8858 | -0.0139 | 0 | 83 | 4.7e-007 | 1 | K.TIGGGDDSFNTFFSETGAGK.H 20183 20184 20191 |
|  | 23808 | 1205.1003 | 2408.1861 | 2408.2012 | -0.0151 | 0 | 123 | 3.3e-011 | 1 | R.FDGALNVDLTEFQTNLVPYPR.I 23809 23811 23812 |
|  | 26118 | 917.4299 | 2749.2680 | 2749.2840 | -0.0160 | 0 | 32 | 0.04 | 1 | K.AYHEQLSVAEITNACFEPANQMVK.C |
|  | 30755 | 1074.9904 | 4295.9323 | 4295.9497 | -0.0173 | 0 | 10 | 2.3 | 1 | R.ECISIHVGQAGVQIGNACWELYCLEHGIQPDGQMPSDK.T |

| **83.** | O60218\|AK1BA_HUMAN    **Mass:** 36226    **Score:** 478    **Queries matched:** 39   **emPAI:** 2.62 |
| --- | --- |
|  | Aldo-keto reductase family 1 member B10 OS=Homo sapiens GN=AKR1B10 PE=1 SV=1 |

|  | **Query** | **Observed** | **Mr(expt)** | **Mr(calc)** | **Delta** | **Miss** | **Score** | **Expect** | **Rank** | **Peptide** |
| --- | --- | --- | --- | --- | --- | --- | --- | --- | --- | --- |
|  | 1423 | **630.3890** | **629.3818** | **629.3497** | **0.0321** | **0** | **11** | **20** | **4** | **K.SVTPAR.I** |
|  | 1608 | **673.3830** | **672.3757** | **672.4170** | **-0.0413** | **1** | **26** | **0.59** | **2** | **K.VKEAVK.V** |
|  | 1706 | **700.3841** | **699.3768** | **699.3816** | **-0.0048** | **0** | **16** | **5.2** | **1** | **R.FHIQR.N** |
|  | 2375 | **408.2373** | **814.4600** | **814.4912** | **-0.0312** | **1** | **7** | **45** | **9** | **K.IQEKAVK.R** |
|  | 3280 | **436.2690** | **870.5235** | **870.5287** | **-0.0052** | **0** | **33** | **0.092** | **1** | **K.TAAQVLIR.F** 3278 |
|  | 3369 | **439.7130** | **877.4115** | **877.4182** | **-0.0066** | **0** | **37** | **0.034** | **1** | **K.SGDDLFPK.D** |
|  | 4763 | **488.3158** | **974.6170** | **973.4829** | **1.1342** | **1** | **7** | **36** | **5** | **K.DDKGNAIGGK.A** |
|  | 6542 | **553.8106** | **1105.6065** | **1105.6131** | **-0.0066** | **1** | **42** | **0.011** | **1** | **K.REDLFIVSK.L** 6540 |
|  | 9681 | **431.6001** | **1291.7786** | **1290.6932** | **1.0854** | **1** | **6** | **32** | **5** | **K.EAVKVAIDAGYR.H** |
|  | 10752 | **676.3621** | **1350.7097** | **1350.7184** | **-0.0087** | **0** | **11** | **11** | **2** | **R.IVENIQVFDFK.L** |
|  | 14034 | **518.2900** | **1551.8481** | **1551.8998** | **-0.0517** | **1** | **3** | **66** | **3** | **K.TAAQVLIRFHIQR.N** |
|  | 14384 | 788.9045 | 1575.7944 | 1575.8045 | -0.0101 | 0 | 70 | 1.1e-005 | 1 | K.ALGVSNFSHFQIEK.L 14386 14393 |
|  | 14700 | **799.9262** | **1597.8377** | **1598.8854** | **-1.0477** | **1** | **11** | **8.9** | **2** | **K.MPIVGLGTWKSPLGK.V** |
|  | 15146 | 813.3910 | 1624.7675 | 1624.7766 | -0.0091 | 0 | 44 | 0.0046 | 1 | K.LSDEEMATILSFNR.N 15145 15147 |
|  | 19908 | **990.0197** | **1978.0249** | **1978.0353** | **-0.0104** | **0** | **39** | **0.013** | **1** | **K.LSYLDVYLIHWPQGFK.S** 19907 |
|  | 21167 | **1050.0112** | **2098.0079** | **2096.9949** | **1.0130** | **1** | **1** | **69** | **3** | **K.LSDEEMATILSFNRNWR.A** |
|  | 22503 | **745.3627** | **2233.0664** | **2233.0837** | **-0.0174** | **0** | **25** | **0.26** | **1** | **K.YKPVTNQVECHPYLTQEK.L** 22504 22505 |
|  | 22695 | **1129.4817** | **2256.9488** | **2256.9633** | **-0.0145** | **0** | **54** | **0.00026** | **1** | **R.ACNVLQSSHLEDYPFDAEY.-** 22694 22696 |
|  | 22752 | **1133.5465** | **2265.0785** | **2265.0875** | **-0.0090** | **0** | **94** | **3e-008** | **1** | **K.ATFLDAWEAMEELVDEGLVK.A** 22753 22754 22755 |
|  | 24778 | **844.7144** | **2531.1213** | **2531.1387** | **-0.0174** | **0** | **59** | **7.1e-005** | **1** | **R.HIDCAYVYQNEHEVGEAIQEK.I** 24777 |
|  | 26717 | **960.5004** | **2878.4793** | **2878.4059** | **0.0734** | **1** | **1** | **44** | **7** | **K.GNAIGGKATFLDAWEAMEELVDEGLVK.A** |
|  | 28356 | **1079.5470** | **3235.6192** | **3235.6401** | **-0.0209** | **0** | **40** | **0.0049** | **1** | **K.GITVTAYSPLGSPDRPWAKPEDPSLLEDPK.I** 28355 28357 |

| **84.** | A5A3E0\|POTEF_HUMAN    **Mass:** 123020   **Score:** 477    **Queries matched:** 67   **emPAI:** 0.23 |
| --- | --- |
|  | POTE ankyrin domain family member F OS=Homo sapiens GN=POTEF PE=1 SV=2 |

|  | **Query** | **Observed** | **Mr(expt)** | **Mr(calc)** | **Delta** | **Miss** | **Score** | **Expect** | **Rank** | **Peptide** |
| --- | --- | --- | --- | --- | --- | --- | --- | --- | --- | --- |
|  | 1491 | 644.8003 | 643.7931 | 643.3653 | 0.4278 | 0 | 18 | 3 | 2 | R.LDLAGR.E |
|  | 1501 | **647.3463** | **646.3390** | **645.3446** | **0.9944** | **1** | **23** | **1.5** | **2** | **K.QDKQK.R** |
|  | 2167 | 400.7687 | 799.5229 | 799.5280 | -0.0050 | 1 | 24 | 0.83 | 2 | K.RGILTLK.Y |
|  | 4771 | 488.7246 | 975.4346 | 975.4410 | -0.0064 | 0 | 65 | 6.7e-005 | 1 | K.AGFAGDDAPR.A 4770 4780 |
|  | 7769 | 398.8939 | 1193.6599 | 1192.5831 | 1.0768 | 0 | 16 | 3.5 | 3 | K.EIAALAPSMMK.I |
|  | 7816 | **599.3235** | **1196.6325** | **1196.6149** | **0.0176** | **0** | **13** | **7.1** | **5** | **K.DILHENSTLR.E** |
|  | 7850 | 599.8526 | 1197.6906 | 1197.6982 | -0.0076 | 0 | 65 | 4.6e-005 | 1 | R.AVFPSIVGRPR.Q 7847 7848 7849 |
|  | 8825 | 624.6529 | 1247.2912 | 1247.5209 | -0.2297 | 0 | 8 | 18 | 1 | R.QQGMMGGMHQK.E |
|  | 12142 | 715.4202 | 1428.8259 | 1429.7785 | -0.9526 | 1 | (1) | 1e+002 | 7 | K.EIAALAPSMMKIR.I |
|  | 12629 | **487.7855** | **1460.3346** | **1459.6790** | **0.6555** | **0** | **3** | **42** | **9** | **K.ISSENSNPEQDLK.L** |
|  | 12676 | 488.5925 | 1462.7556 | 1461.7683 | 0.9873 | 1 | 4 | 54 | 8 | K.EIAALAPSMMKIR.I |
|  | 12988 | 495.2776 | 1482.8109 | 1482.6734 | 0.1375 | 0 | 6 | 33 | 4 | R.ELENFMAIEEMK.K |
|  | 13098 | 497.2987 | 1488.8742 | 1489.6103 | -0.7361 | 0 | 11 | 9.6 | 2 | K.SNVGASGDHDDSAMK.T 13078 |
|  | 13261 | 750.3462 | 1498.6778 | 1498.6683 | 0.0095 | 0 | (3) | 59 | 5 | R.ELENFMAIEEMK.K |
|  | 13487 | 505.9182 | 1514.7328 | 1514.7419 | -0.0091 | 0 | 46 | 0.0032 | 1 | K.IWHHTFYNELR.V 13481 13482 13483 13484 13485 13486 13490 13491 13492 13493 |
|  | 13503 | 758.8499 | 1515.6853 | 1515.6954 | -0.0101 | 0 | 40 | 0.014 | 1 | K.QEYDESGPSIVHR.K 13474 13502 13504 13509 13528 |
|  | 13778 | 512.9346 | 1535.7821 | 1535.6158 | 0.1662 | 0 | 7 | 25 | 2 | K.SNVGTSGDHDDSAMK.T |
|  | 13944 | 774.3884 | 1546.7622 | 1546.8025 | -0.0403 | 1 | 10 | 14 | 3 | K.DLIVMLRDTDVNK.Q |
|  | 13957 | 774.8814 | 1547.7483 | 1547.7873 | -0.0391 | 1 | 15 | 4 | 4 | R.MQKEIAALAPSMMK.I 13967 |
|  | 14204 | 522.2353 | 1563.6840 | 1564.7477 | -1.0636 | 0 | 4 | 49 | 3 | -.MVVEVDSMPAASSVK.K |
|  | 14218 | 522.3247 | 1563.9523 | 1563.7822 | 0.1701 | 1 | (4) | 46 | 3 | R.MQKEIAALAPSMMK.I |
|  | 14219 | 522.4940 | 1564.4602 | 1563.7822 | 0.6780 | 1 | (4) | 36 | 3 | R.MQKEIAALAPSMMK.I 14214 |
|  | 14877 | 537.5832 | 1609.7277 | 1609.8424 | -0.1146 | 0 | 0 | 1.1e+002 | 10 | R.TALHLASANGNSEVVK.L |
|  | 15186 | 543.3829 | 1627.1268 | 1626.7633 | 0.3635 | 1 | 6 | 22 | 4 | R.ELENFMAIEEMKK.H |
|  | 16563 | 862.4316 | 1722.8487 | 1722.8532 | -0.0044 | 1 | 11 | 9.4 | 3 | R.EEIAMLRLELDTMK.H 16566 |
|  | 16944 | 876.7690 | 1751.5234 | 1750.9539 | 0.5696 | 1 | 12 | 4.4 | 2 | K.LMAKALLLYGADIESK.N 16941 |
|  | 17143 | 589.3338 | 1764.9796 | 1765.9435 | -0.9639 | 1 | 0 | 1e+002 | 5 | K.RTALHLASANGNSEVVK.L |
|  | 17483 | 895.9439 | 1789.8733 | 1789.8846 | -0.0114 | 0 | 73 | 6e-006 | 1 | K.SYELPDGQVITIGNER.F 17481 17482 17485 17486 17487 17488 |
|  | 18107 | 918.4613 | 1834.9080 | 1834.7752 | 0.1329 | 1 | 2 | 65 | 5 | R.GSGKSNVGASGDHDDSAMK.T |
|  | 19084 | 636.3518 | 1906.0336 | 1905.8487 | 0.1849 | 1 | 4 | 35 | 5 | K.SNVGTSGDHDDSAMKTLR.S |
|  | 19508 | 648.3791 | 1942.1156 | 1941.8448 | 0.2708 | 1 | 0 | 79 | 3 | K.DGDRELENFMAIEEMK.K |
|  | 20046 | 665.3938 | 1993.1596 | 1993.8550 | -0.6954 | 0 | 1 | 69 | 8 | K.YPMEHGIITNWDDMEK.I |
|  | 22405 | 741.3660 | 2221.0763 | 2220.2226 | 0.8537 | 1 | 4 | 29 | 3 | R.TALHLASANGNSEVVKLLLDR.R |
|  | 25888 | 677.0647 | 2704.2297 | 2703.2455 | 0.9842 | 1 | 0 | 58 | 5 | K.DLYTNTVLSGGTTMYPGMAHRMQK.E |
|  | 26593 | 714.8475 | 2855.3610 | 2854.3035 | 1.0575 | 0 | 1 | 43 | 7 | K.ALQLNELTMDDDTAVLVIDNGSGMCK.A |
|  | 27316 | **1500.1398** | **2998.2650** | **2999.4598** | **-1.1948** | **1** | **1** | **39** | **2** | **K.HRSTHVGFPENLTNGATAGNGDDGLIPPR.K** |

| **85.** | O95994\|AGR2_HUMAN    **Mass:** 20024    **Score:** 469    **Queries matched:** 19   **emPAI:** 2.43 |
| --- | --- |
|  | Anterior gradient protein 2 homolog OS=Homo sapiens GN=AGR2 PE=1 SV=1 |

|  | **Query** | **Observed** | **Mr(expt)** | **Mr(calc)** | **Delta** | **Miss** | **Score** | **Expect** | **Rank** | **Peptide** |
| --- | --- | --- | --- | --- | --- | --- | --- | --- | --- | --- |
|  | 9217 | **634.8185** | **1267.6224** | **1267.6309** | **-0.0085** | **0** | **30** | **0.13** | **1** | **K.HLSPDGQYVPR.I** 9193 9245 |
|  | 9404 | **639.3457** | **1276.6768** | **1276.6850** | **-0.0081** | **0** | **56** | **0.00033** | **1** | **R.IMFVDPSLTVR.A** 9403 |
|  | 9703 | **647.3424** | **1292.6701** | **1292.6799** | **-0.0097** | **0** | **(18)** | **2.5** | **1** | **R.IMFVDPSLTVR.A** |
|  | 19483 | **970.9811** | **1939.9477** | **1939.9601** | **-0.0123** | **0** | **92** | **5.9e-008** | **1** | **R.LYAYEPADTALLLDNMK.K** 19482 |
|  | 20069 | **998.5307** | **1995.0468** | **1995.0564** | **-0.0096** | **0** | **81** | **7.7e-007** | **1** | **K.LAEQFVLLNLVYETTDK.H** 20064 |
|  | 20859 | **690.3549** | **2068.0428** | **2068.0550** | **-0.0123** | **1** | **14** | **3.8** | **1** | **R.LYAYEPADTALLLDNMKK.A** |
|  | 22190 | **1101.0248** | **2200.0350** | **2200.0477** | **-0.0127** | **0** | **70** | **7.7e-006** | **1** | **R.GWGDQLIWTQTYEEALYK.S** 22189 22192 22193 |
|  | 25063 | **857.1000** | **2568.2781** | **2568.2941** | **-0.0160** | **0** | **44** | **0.0028** | **1** | **K.TSNKPLMIIHHLDECPHSQALK.K** 25062 |
|  | 25799 | **540.2819** | **2696.3729** | **2696.3890** | **-0.0161** | **1** | **4** | **22** | **1** | **K.TSNKPLMIIHHLDECPHSQALKK.V** |
|  | 28414 | **812.1728** | **3244.6621** | **3244.6768** | **-0.0148** | **1** | **14** | **1.8** | **1** | **K.LAEQFVLLNLVYETTDKHLSPDGQYVPR.I** |

| **86.** | P35222\|CTNB1_HUMAN    **Mass:** 86069    **Score:** 466    **Queries matched:** 40   **emPAI:** 0.80 |
| --- | --- |
|  | Catenin beta-1 OS=Homo sapiens GN=CTNNB1 PE=1 SV=1 |

|  | **Query** | **Observed** | **Mr(expt)** | **Mr(calc)** | **Delta** | **Miss** | **Score** | **Expect** | **Rank** | **Peptide** |
| --- | --- | --- | --- | --- | --- | --- | --- | --- | --- | --- |
|  | 2427 | **819.4135** | **818.4062** | **818.4134** | **-0.0072** | **0** | **40** | **0.019** | **1** | **R.NLSDAATK.Q** |
|  | 2767 | **421.2862** | **840.5578** | **839.5593** | **0.9986** | **0** | **10** | **15** | **4** | **R.LVQLLVR.A** 2737 2760 |
|  | 5313 | 508.7812 | 1015.5478 | 1015.5372 | 0.0106 | 0 | 5 | 53 | 8 | R.LAEPSQMLK.H |
|  | 6251 | **542.8006** | **1083.5866** | **1082.5906** | **0.9960** | **0** | **26** | **0.43** | **1** | **K.AAVMVHQLSK.K** |
|  | 6496 | **1103.1073** | **1102.1000** | **1102.5805** | **-0.4805** | **0** | **3** | **60** | **2** | **R.SPQMVSAIVR.T** |
|  | 9864 | 651.8491 | 1301.6836 | 1301.6914 | -0.0079 | 0 | 54 | 0.00053 | 1 | R.LVQNCLWTLR.N |
|  | 11233 | **690.3097** | **1378.6048** | **1378.6147** | **-0.0099** | **0** | **44** | **0.0055** | **1** | **R.TMQNTNDVETAR.C** 11237 |
|  | 11310 | **692.3215** | **1382.6284** | **1382.6361** | **-0.0077** | **0** | **51** | **0.0012** | **1** | **R.HQEAEMAQNAVR.L** 11312 |
|  | 11346 | **693.3625** | **1384.7104** | **1384.7198** | **-0.0094** | **0** | **67** | **2.8e-005** | **1** | **K.LLNDEDQVVVNK.A** |
|  | 11535 | **466.2376** | **1395.6911** | **1394.6096** | **1.0815** | **0** | **(4)** | **55** | **7** | **R.TMQNTNDVETAR.C** |
|  | 12548 | **486.3002** | **1455.8788** | **1454.8643** | **1.0145** | **1** | **6** | **31** | **5** | **R.LAGGLQKMVALLNK.T** |
|  | 12936 | **494.2532** | **1479.7378** | **1480.7674** | **-1.0296** | **0** | **10** | **13** | **5** | **R.NEGVATYAAAVLFR.M** |
|  | 14191 | **782.4056** | **1562.7967** | **1561.7779** | **1.0189** | **0** | **45** | **0.0041** | **1** | **K.MMVCQVGGIEALVR.T** |
|  | 15125 | **812.8854** | **1623.7562** | **1623.7675** | **-0.0113** | **0** | **72** | **7.7e-006** | **1** | **R.TSMGGTQQQFVEGVR.M** |
|  | 15397 | 547.6047 | 1639.7924 | 1639.7625 | 0.0299 | 0 | (7) | 23 | 4 | R.TSMGGTQQQFVEGVR.M 15385 |
|  | 17145 | **883.5176** | **1765.0206** | **1765.0284** | **-0.0078** | **0** | **60** | **0.0001** | **1** | **K.LIILASGGPQALVNIMR.T** |
|  | 17376 | **594.8421** | **1781.5045** | **1781.0233** | **0.4812** | **0** | **(5)** | **24** | **2** | **K.LIILASGGPQALVNIMR.T** 17359 17363 17381 |
|  | 17439 | **894.4040** | **1786.7934** | **1785.8679** | **0.9255** | **1** | **14** | **3.8** | **2** | **R.AGDREDITEPAICALR.H** |
|  | 20592 | **1021.5117** | **2041.0089** | **2041.0228** | **-0.0140** | **0** | **93** | **4.6e-008** | **1** | **K.HAVVNLINYQDDAELATR.A** 20593 |
|  | 21171 | **700.4199** | **2098.2379** | **2098.8679** | **-0.6300** | **0** | **10** | **8.2** | **1** | **-.MATQADLMELDMAMEPDR.K** 21179 |
|  | 22282 | **737.6044** | **2209.7915** | **2210.9680** | **-1.1765** | **1** | **0** | **57** | **6** | **-.MATQADLMELDMAMEPDRK.A** |
|  | 22902 | **570.7807** | **2279.0937** | **2278.1440** | **0.9497** | **0** | **4** | **34** | **3** | **K.EAAEAIEAEGATAPLTELLHSR.N** |
|  | 23983 | **1214.6786** | **2427.3426** | **2427.3526** | **-0.0099** | **0** | **68** | **1.1e-005** | **1** | **R.GLNTIPLFVQLLYSPIENIQR.V** 23984 |
|  | 24274 | 822.0737 | 2463.1992 | 2463.1846 | 0.0146 | 1 | 1 | 55 | 7 | R.SPQMVSAIVRTMQNTNDVETAR.C |
|  | 26876 | **970.4415** | **2908.3026** | **2908.6902** | **-0.3876** | **1** | **1** | **38** | **7** | **R.IVIRGLNTIPLFVQLLYSPIENIQR.V** |
|  | 27729 | **1029.1638** | **3084.4696** | **3084.4901** | **-0.0205** | **0** | **55** | **0.00014** | **1** | **K.AAVSHWQQQSYLDSGIHSGATTTAPSLSGK.G** |
|  | 28209 | **1068.1622** | **3201.4649** | **3201.4859** | **-0.0211** | **0** | **46** | **0.0011** | **1** | **R.AAMFPETLDEGMQIPSTQFDAAHPTNVQR.L** |
|  | 29326 | **886.1651** | **3540.6313** | **3539.8633** | **0.7680** | **1** | **7** | **8.3** | **1** | **K.MLGSPVDSVLFYAITTLHNLLLHQEGAKMAVR.L** |
|  | 30660 | **1394.3490** | **4180.0252** | **4180.0490** | **-0.0239** | **0** | **81** | **1.9e-007** | **1** | **K.QEGMEGLLGTLVQLLGSDDINVVTCAAGILSNLTCNNYK.N** |

| **87.** | O75795\|UDB17_HUMAN    **Mass:** 61625    **Score:** 464    **Queries matched:** 48   **emPAI:** 1.71 |
| --- | --- |
|  | UDP-glucuronosyltransferase 2B17 OS=Homo sapiens GN=UGT2B17 PE=2 SV=1 |

|  | **Query** | **Observed** | **Mr(expt)** | **Mr(calc)** | **Delta** | **Miss** | **Score** | **Expect** | **Rank** | **Peptide** |
| --- | --- | --- | --- | --- | --- | --- | --- | --- | --- | --- |
|  | 825 | **506.0474** | **505.0401** | **504.2907** | **0.7494** | **0** | **4** | **79** | **1** | **K.SSAIK.L** |
|  | 1526 | 651.3502 | 650.3429 | 649.3105 | 1.0325 | 0 | 12 | 11 | 3 | K.ENIMK.L |
|  | 3463 | **442.7263** | **883.4380** | **883.4440** | **-0.0059** | **0** | **8** | **24** | **1** | **R.WTYSISK.N** |
|  | 3716 | **451.2560** | **900.4975** | **900.5029** | **-0.0053** | **0** | **46** | **0.0045** | **1** | **K.GAALSVDIR.T** |
|  | 3964 | **459.7441** | **917.4737** | **917.4793** | **-0.0056** | **0** | **40** | **0.02** | **1** | **K.AEMWLIR.T** |
|  | 4961 | **496.0763** | **990.1381** | **989.5328** | **0.6053** | **1** | **7** | **25** | **9** | **K.ENIMKLSR.I** |
|  | 5200 | **504.2530** | **1006.4914** | **1005.5277** | **0.9637** | **1** | **(6)** | **46** | **7** | **K.ENIMKLSR.I** |
|  | 5499 | **515.7647** | **1029.5149** | **1028.5179** | **0.9970** | **0** | **35** | **0.06** | **1** | **R.FSVGYTVEK.N** 5479 5480 |
|  | 5731 | **524.7836** | **1047.5527** | **1047.5600** | **-0.0073** | **0** | **37** | **0.036** | **1** | **K.SVINDPIYK.E** 5734 |
|  | 5908 | **531.2651** | **1060.5156** | **1060.5223** | **-0.0067** | **0** | **26** | **0.43** | **1** | **K.LCEDAVLNK.K** 5909 |
|  | 6288 | **544.2803** | **1086.5460** | **1086.5782** | **-0.0322** | **0** | **7** | **33** | **8** | **K.KPNTLGSNTR.L** 6280 |
|  | 6469 | **550.8162** | **1099.6178** | **1099.6237** | **-0.0059** | **0** | **30** | **0.18** | **1** | **K.TILEELVQR.G** |
|  | 7206 | **579.7574** | **1157.5002** | **1157.5063** | **-0.0061** | **0** | **44** | **0.0072** | **1** | **K.NDLEDFFMK.M** |
|  | 7662 | **595.3117** | **1188.6089** | **1188.6172** | **-0.0084** | **1** | **34** | **0.062** | **1** | **K.LCEDAVLNKK.L** |
|  | 10984 | **455.9054** | **1364.6945** | **1363.7129** | **0.9815** | **1** | **4** | **51** | **7** | **R.TMSSRDLLNALK.S** |
|  | 11919 | **709.3783** | **1416.7420** | **1416.7514** | **-0.0093** | **0** | **53** | **0.00069** | **1** | **K.WLPQNDLLGHPK.T** 11920 |
|  | 12517 | 485.5969 | 1453.7690 | 1453.7790 | -0.0100 | 0 | 30 | 0.13 | 1 | R.IHHDQPVKPLDR.A 12518 12519 12520 |
|  | 12683 | **488.7570** | **1463.2491** | **1462.7562** | **0.4929** | **1** | **7** | **15** | **2** | **K.GAALSVDIRTMSSR.D** |
|  | 13759 | **512.2678** | **1533.7815** | **1533.7899** | **-0.0085** | **1** | **6** | **29** | **1** | **R.FDGKKPNTLGSNTR.L** |
|  | 15719 | 832.4338 | 1662.8530 | 1662.8651 | -0.0120 | 1 | 38 | 0.019 | 1 | K.SVINDPIYKENIMK.L 15721 |
|  | 16348 | 570.0104 | 1707.0093 | 1706.7433 | 0.2661 | 1 | 4 | 42 | 4 | K.NDLEDFFMKMFDR.W |
|  | 19027 | **951.9767** | **1901.9388** | **1901.9498** | **-0.0110** | **0** | **24** | **0.42** | **1** | **K.VLVWPTEYSHWINMK.T** 19040 |
|  | 24592 | **1253.1022** | **2504.1898** | **2504.2046** | **-0.0148** | **0** | **77** | **1.3e-006** | **1** | **K.WDQFYSEVLGRPTTLFETMGK.A** 24590 24593 24594 24595 |
|  | 27936 | **1574.2463** | **3146.4781** | **3146.4915** | **-0.0134** | **0** | **41** | **0.0042** | **1** | **K.NGGGFLFPPSYVPVVMSELSDQMIFMER.I** |
|  | 28526 | **1638.3666** | **3274.7186** | **3274.7311** | **-0.0125** | **0** | **79** | **4.9e-007** | **1** | **K.FDVLLADAVNPCGELLAELLNIPFLYSLR.F** 28524 28525 |
|  | 29735 | **612.9839** | **3671.8597** | **3671.8752** | **-0.0155** | **0** | **11** | **2.9** | **1** | **R.TYWDFEFPRPFLPNVDFVGGLHCKPAKPLPK.E** 29734 |
|  | 30620 | **1038.2572** | **4148.9997** | **4148.0401** | **0.9596** | **0** | **15** | **0.75** | **1** | **K.AFITHGGTNGIYEAIYHGIPMVGIPLFADQHDNIAHMK.A** 30621 30622 30623 |

| **88.** | Q562R1\|ACTBL_HUMAN    **Mass:** 42318    **Score:** 463    **Queries matched:** 51   **emPAI:** 0.81 |
| --- | --- |
|  | Beta-actin-like protein 2 OS=Homo sapiens GN=ACTBL2 PE=1 SV=2 |

|  | **Query** | **Observed** | **Mr(expt)** | **Mr(calc)** | **Delta** | **Miss** | **Score** | **Expect** | **Rank** | **Peptide** |
| --- | --- | --- | --- | --- | --- | --- | --- | --- | --- | --- |
|  | 1426 | 631.3726 | 630.3653 | 630.3701 | -0.0048 | 0 | 10 | 33 | 3 | K.ILTER.G 1421 |
|  | 1491 | 644.8003 | 643.7931 | 643.3653 | 0.4278 | 0 | 18 | 3 | 2 | R.LDLAGR.D |
|  | 2106 | 398.2369 | 794.4593 | 794.4650 | -0.0057 | 0 | 16 | 4 | 1 | K.IIAPPER.K 2107 2108 |
|  | 3777 | 453.2261 | 904.4377 | 904.4436 | -0.0059 | 1 | 16 | 4.8 | 5 | K.CDVDIRK.D |
|  | 4032 | 462.2841 | 922.5536 | 922.5600 | -0.0063 | 1 | 25 | 0.52 | 1 | K.IIAPPERK.Y |
|  | 5079 | 499.7437 | 997.4728 | 997.4790 | -0.0062 | 0 | 29 | 0.18 | 1 | R.DLTDYLMK.I 5080 |
|  | 7368 | 586.2852 | 1170.5558 | 1170.5638 | -0.0080 | 0 | 59 | 0.0002 | 1 | R.HQGVMVGMGQK.D 7365 7366 7367 7369 7370 7371 7379 7382 |
|  | 7612 | 594.2825 | 1186.5505 | 1186.5587 | -0.0082 | 0 | (52) | 0.00089 | 1 | R.HQGVMVGMGQK.D 7611 |
|  | 7613 | 594.2828 | 1186.5511 | 1186.5587 | -0.0076 | 0 | (48) | 0.0023 | 1 | R.HQGVMVGMGQK.D 7614 7631 |
|  | 7942 | 602.2798 | 1202.5450 | 1202.5536 | -0.0086 | 0 | (26) | 0.37 | 1 | R.HQGVMVGMGQK.D 7940 7941 |
|  | 8469 | **615.2747** | **1228.5349** | **1229.6703** | **-1.1354** | **0** | **7** | **34** | **2** | **R.AVFPSMIGRPR.H** 8468 |
|  | 15103 | 541.9487 | 1622.8244 | 1622.8338 | -0.0094 | 1 | 14 | 5.1 | 2 | R.LDLAGRDLTDYLMK.I |
|  | 17483 | 895.9439 | 1789.8733 | 1789.8846 | -0.0114 | 0 | 73 | 6e-006 | 1 | R.SYELPDGQVITIGNER.F 17481 17482 17485 17486 17487 17488 |
|  | 19625 | 652.0216 | 1953.0428 | 1953.0571 | -0.0143 | 0 | 26 | 0.25 | 2 | R.VAPDEHPILLTEAPLNPK.I 19626 19627 19628 19629 19630 19631 |
|  | 20756 | **686.4156** | **2056.2249** | **2055.8911** | **0.3338** | **0** | **1** | **52** | **1** | **-.MTDNELSALVVDNGSGMCK.A** |
|  | 22363 | 739.3583 | 2215.0530 | 2216.0419 | -0.9889 | 0 | 6 | 18 | 3 | K.DLYANTVLSGGSTMYPGIADR.M 22365 |
|  | 23362 | **586.7835** | **2343.1049** | **2344.1369** | **-1.0320** | **1** | **1** | **59** | **9** | **R.KDLYANTVLSGGSTMYPGIADR.M** |
|  | 28232 | **803.8564** | **3211.3964** | **3210.6384** | **0.7581** | **0** | **0** | **37** | **3** | **R.TTGIVMDSGDGVTHIVPIYEGYALPHAILR.L** |
|  | 28360 | 1080.1368 | 3237.3887 | 3238.5865 | -1.1978 | 0 | 2 | 29 | 4 | K.MTQIMFEAFNTPAMYVAIQAVLSLYASGR.T |
|  | 29114 | **868.4133** | **3469.6242** | **3470.6030** | **-0.9788** | **1** | **3** | **20** | **3** | **K.YPIEHGVVTNWDDMEKIWYHTFYNELR.V** |

| **89.** | P00813\|ADA_HUMAN    **Mass:** 41024    **Score:** 459    **Queries matched:** 29   **emPAI:** 1.85 |
| --- | --- |
|  | Adenosine deaminase OS=Homo sapiens GN=ADA PE=1 SV=3 |

|  | **Query** | **Observed** | **Mr(expt)** | **Mr(calc)** | **Delta** | **Miss** | **Score** | **Expect** | **Rank** | **Peptide** |
| --- | --- | --- | --- | --- | --- | --- | --- | --- | --- | --- |
|  | 1867 | 747.4313 | 746.4241 | 746.3997 | 0.0244 | 0 | 8 | 38 | 8 | K.VVELCK.K |
|  | 3674 | 899.4885 | 898.4813 | 898.5348 | -0.0536 | 1 | 4 | 64 | 5 | K.RLNINAAK.S |
|  | 4226 | **470.2111** | **938.4077** | **938.4136** | **-0.0059** | **0** | **6** | **33** | **7** | **R.SILCCMR.H** |
|  | 6848 | **565.7897** | **1129.5649** | **1128.5927** | **0.9722** | **0** | **17** | **3.6** | **1** | **R.YSPHLLANSK.V** |
|  | 8523 | 616.8061 | 1231.5976 | 1231.5067 | 0.0909 | 0 | 2 | 1e+002 | 9 | R.DMGFTEEEFK.R |
|  | 8555 | 411.9275 | 1232.7606 | 1232.6223 | 0.1382 | 0 | 3 | 67 | 5 | -.MAQTPAFDKPK.V |
|  | 9847 | **651.7933** | **1301.5721** | **1301.5809** | **-0.0088** | **0** | **33** | **0.071** | **1** | **K.STLDTDYQMTK.R** |
|  | 11303 | **691.8633** | **1381.7120** | **1381.7202** | **-0.0081** | **0** | **36** | **0.033** | **1** | **R.TVHAGEVGSAEVVK.E** |
|  | 12668 | **732.3213** | **1462.6280** | **1462.6373** | **-0.0093** | **0** | **16** | **3** | **1** | **K.FDYYMPAIAGCR.E** |
|  | 18804 | **943.9547** | **1885.8947** | **1885.9071** | **-0.0123** | **0** | **79** | **1.4e-006** | **1** | **R.LGHGYHTLEDQALYNR.L** 18803 18805 |
|  | 19786 | **984.4648** | **1966.9150** | **1966.9272** | **-0.0122** | **0** | **77** | **1.9e-006** | **1** | **K.NDQANYSLNTDDPLIFK.S** 19787 |
|  | 21697 | 717.3574 | 2149.0504 | 2150.1583 | -1.1079 | 1 | 2 | 56 | 3 | R.TVHAGEVGSAEVVKEAVDILK.T |
|  | 24059 | **610.5783** | **2438.2841** | **2438.2958** | **-0.0116** | **0** | **7** | **15** | **4** | **K.VELHVHLDGSIKPETILYYGR.R** |
|  | 27762 | **1031.5698** | **3091.6876** | **3091.6991** | **-0.0115** | **0** | **80** | **4.3e-007** | **1** | **R.GIALPANTAEGLLNVIGMDKPLTLPDFLAK.F** 27759 27761 |
|  | 28428 | **1084.1705** | **3249.4898** | **3250.4976** | **-1.0078** | **1** | **0** | **42** | **5** | **K.NDQANYSLNTDDPLIFKSTLDTDYQMTK.R** |
|  | 28726 | **1673.3268** | **3344.6390** | **3344.6525** | **-0.0135** | **0** | **78** | **6.9e-007** | **1** | **K.VEPIPWNQAEGDLTPDEVVALVGQGLQEGER.D** 28725 28728 |
|  | 29866 | **1242.6325** | **3724.8755** | **3724.8948** | **-0.0193** | **0** | **78** | **5.2e-007** | **1** | **K.YQQQTVVAIDLAGDETIPGSSLLPGHVQAYQEAVK.S** |
|  | 29953 | **755.1644** | **3770.7858** | **3770.8086** | **-0.0228** | **1** | **42** | **0.0022** | **1** | **R.LRQENMHFEICPWSSYLTGAWKPDTEHAVIR.L** |
|  | 30232 | **1297.9799** | **3890.9178** | **3890.9327** | **-0.0149** | **1** | **24** | **0.13** | **1** | **K.VEPIPWNQAEGDLTPDEVVALVGQGLQEGERDFGVK.A** 30231 |
|  | 30745 | **1426.4193** | **4276.2361** | **4275.1924** | **1.0437** | **1** | **2** | **14** | **1** | **K.YQQQTVVAIDLAGDETIPGSSLLPGHVQAYQEAVKSGIHR.T** |
|  | 30998 | 1518.1175 | 4551.3308 | 4552.3208 | -0.9900 | 1 | 5 | 5.7 | 2 | R.GIALPANTAEGLLNVIGMDKPLTLPDFLAKFDYYMPAIAGCR.E |

| **90.** | P06733\|ENOA_HUMAN    **Mass:** 47481    **Score:** 459    **Queries matched:** 38   **emPAI:** 1.13 |
| --- | --- |
|  | Alpha-enolase OS=Homo sapiens GN=ENO1 PE=1 SV=2 |

|  | **Query** | **Observed** | **Mr(expt)** | **Mr(calc)** | **Delta** | **Miss** | **Score** | **Expect** | **Rank** | **Peptide** |
| --- | --- | --- | --- | --- | --- | --- | --- | --- | --- | --- |
|  | 1809 | **730.4155** | **729.4082** | **729.4385** | **-0.0303** | **1** | **9** | **29** | **1** | **K.NVIKEK.Y** |
|  | 1943 | **766.3687** | **765.3614** | **765.3657** | **-0.0043** | **0** | **22** | **1.1** | **1** | **R.EIFDSR.G** |
|  | 2243 | **403.2129** | **804.4112** | **805.4446** | **-1.0334** | **0** | **9** | **24** | **8** | **K.YNQLLR.I** |
|  | 3483 | **443.0397** | **884.0648** | **884.4426** | **-0.3777** | **1** | **6** | **28** | **2** | **R.YMGKGVSK.A** |
|  | 3766 | **452.7319** | **903.4492** | **903.4549** | **-0.0057** | **0** | **15** | **6** | **1** | **R.IEEELGSK.A** |
|  | 4549 | **480.7503** | **959.4861** | **959.4924** | **-0.0063** | **0** | **22** | **1.3** | **1** | **K.LNVTEQEK.I** |
|  | 5199 | 504.2509 | 1006.4873 | 1006.4940 | -0.0066 | 0 | 17 | 3.6 | 2 | K.SCNCLLLK.V 5204 |
|  | 6071 | **536.7653** | **1071.5160** | **1071.5237** | **-0.0077** | **1** | **16** | **5.2** | **1** | **R.SGKYDLDFK.S** |
|  | 6332 | **545.3615** | **1088.7085** | **1087.5873** | **1.1212** | **1** | **8** | **24** | **1** | **K.KLNVTEQEK.I** |
|  | 7013 | **572.3076** | **1142.6007** | **1142.6084** | **-0.0077** | **0** | **54** | **0.00063** | **1** | **R.IGAEVYHNLK.N** |
|  | 9444 | **640.7927** | **1279.5709** | **1279.5788** | **-0.0079** | **0** | **33** | **0.064** | **1** | **K.LMIEMDGTENK.S** |
|  | 11717 | **703.8570** | **1405.6994** | **1405.7089** | **-0.0095** | **0** | **28** | **0.23** | **1** | **R.GNPTVEVDLFTSK.G** |
|  | 12063 | **713.3619** | **1424.7092** | **1424.7187** | **-0.0095** | **0** | **48** | **0.0022** | **1** | **R.YISPDQLADLYK.S** |
|  | 13414 | **504.2530** | **1509.7371** | **1510.7007** | **-0.9636** | **1** | **(4)** | **47** | **9** | **K.LMIEMDGTENKSK.F** |
|  | 13638 | **509.6262** | **1525.8567** | **1526.6956** | **-0.8390** | **1** | **13** | **6.4** | **3** | **K.LMIEMDGTENKSK.F** |
|  | 13829 | **770.8912** | **1539.7678** | **1539.7756** | **-0.0078** | **0** | **90** | **1.4e-007** | **1** | **K.VVIGMDVAASEFFR.S** 13823 13824 13828 |
|  | 13845 | 514.3140 | 1539.9203 | 1540.7569 | -0.8366 | 0 | 3 | 60 | 9 | K.LAQANGWGVMVSHR.S |
|  | 16087 | **564.2622** | **1689.7646** | **1690.8889** | **-1.1243** | **1** | **7** | **24** | **1** | **K.YNQLLRIEEELGSK.A** |
|  | 17652 | **902.9704** | **1803.9262** | **1803.9366** | **-0.0104** | **0** | **54** | **0.0004** | **1** | **R.AAVPSGASTGIYEALELR.D** |
|  | 17989 | **610.1827** | **1827.5262** | **1827.9189** | **-0.3927** | **1** | **2** | **42** | **2** | **K.VVIGMDVAASEFFRSGK.Y** 18000 |
|  | 19462 | **647.3201** | **1938.9384** | **1938.9696** | **-0.0312** | **0** | **1** | **77** | **2** | **K.LAMQEFMILPVGAANFR.E** |
|  | 19704 | **980.9599** | **1959.9052** | **1959.9174** | **-0.0121** | **0** | **72** | **5.8e-006** | **1** | **K.DATNVGDEGGFAPNILENK.E** 19705 |
|  | 20462 | **1017.0253** | **2032.0361** | **2032.0477** | **-0.0116** | **0** | **89** | **1e-007** | **1** | **K.FTASAGIQVVGDDLTVTNPK.R** 20474 |
|  | 24629 | **1255.5547** | **2509.0948** | **2509.1074** | **-0.0126** | **0** | **70** | **6.6e-006** | **1** | **K.DYPVVSIEDPFDQDDWGAWQK.F** 24630 24631 24632 |
|  | 27244 | **995.4358** | **2983.2857** | **2984.3869** | **-1.1011** | **1** | **38** | **0.008** | **1** | **K.SFIKDYPVVSIEDPFDQDDWGAWQK.F** |
|  | 27349 | **753.8890** | **3011.5268** | **3010.5625** | **0.9644** | **0** | **14** | **2** | **1** | **R.HIADLAGNSEVILPVPAFNVINGGSHAGNK.L** 27350 27351 |

| **91.** | Q16822\|PCKGM_HUMAN    **Mass:** 71447    **Score:** 453    **Queries matched:** 37   **emPAI:** 1.03 |
| --- | --- |
|  | Phosphoenolpyruvate carboxykinase [GTP], mitochondrial OS=Homo sapiens GN=PCK2 PE=1 SV=2 |

|  | **Query** | **Observed** | **Mr(expt)** | **Mr(calc)** | **Delta** | **Miss** | **Score** | **Expect** | **Rank** | **Peptide** |
| --- | --- | --- | --- | --- | --- | --- | --- | --- | --- | --- |
|  | 1475 | **642.3348** | **641.3276** | **640.3656** | **0.9619** | **0** | **7** | **32** | **7** | **K.GAQLPR.I** |
|  | 4463 | **477.2839** | **952.5532** | **952.5593** | **-0.0061** | **0** | **6** | **38** | **3** | **R.ETPIGLVPK.E** |
|  | 4548 | **480.7273** | **959.4400** | **959.4461** | **-0.0061** | **0** | **44** | **0.0075** | **1** | **R.DFVEHSAR.L** 4556 |
|  | 4565 | **481.2468** | **960.4791** | **960.4851** | **-0.0060** | **0** | **22** | **1.2** | **1** | **R.VLDWICR.R** |
|  | 5483 | **515.2699** | **1028.5252** | **1029.5455** | **-1.0202** | **0** | **54** | **0.00062** | **1** | **K.EGALDLSGLR.A** 5501 |
|  | 6410 | 548.7501 | 1095.4856 | 1095.4920 | -0.0064 | 0 | 31 | 0.12 | 1 | K.YNNCWLAR.T |
|  | 8227 | **608.8159** | **1215.6172** | **1214.5891** | **1.0281** | **1** | **4** | **68** | **3** | **R.SESTAAAEHKGK.I** |
|  | 9278 | **636.3427** | **1270.6709** | **1270.6768** | **-0.0060** | **0** | **42** | **0.01** | **1** | **K.EVLAELEALER.R** |
|  | 10451 | **445.4711** | **1333.3916** | **1332.7289** | **0.6626** | **0** | **1** | **71** | **7** | **R.AIDTTQLFSLPK.D** |
|  | 12069 | **713.4025** | **1424.7905** | **1424.7987** | **-0.0082** | **0** | **84** | **4.9e-007** | **1** | **R.VLSGDLGQLPTGIR.D** 12068 12078 |
|  | 12450 | **725.8217** | **1449.6289** | **1449.6381** | **-0.0092** | **0** | **39** | **0.015** | **1** | **K.VECVGDDIAWMR.F** |
|  | 12472 | **484.3207** | **1449.9402** | **1449.7405** | **0.1997** | **0** | **10** | **11** | **3** | **K.GVPLVYEAFNWR.H** |
|  | 13758 | **767.8862** | **1533.7578** | **1533.7675** | **-0.0097** | **0** | **37** | **0.025** | **1** | **R.SYLTEQVNQDLPK.E** |
|  | 14499 | **792.9362** | **1583.8578** | **1584.8268** | **-0.9691** | **0** | **19** | **1.4** | **1** | **K.TNLAMMRPALPGWK.V** |
|  | 14813 | **803.8703** | **1605.7260** | **1605.7358** | **-0.0098** | **0** | **45** | **0.0034** | **1** | **R.GQLGNWMSPADFQR.A** 14812 |
|  | 15209 | **543.9479** | **1628.8218** | **1628.8774** | **-0.0556** | **0** | **3** | **67** | **6** | **R.LGTPVLQALGDGDFVK.C** |
|  | 16210 | **849.9329** | **1697.8513** | **1697.8624** | **-0.0112** | **0** | **63** | **5.5e-005** | **1** | **R.EIISFGSGYGGNSLLGK.K** |
|  | 16590 | **576.1375** | **1725.3907** | **1724.8767** | **0.5140** | **0** | **1** | **60** | **3** | **R.IGVQLTDSAYVVASMR.I** |
|  | 17629 | **601.6096** | **1801.8070** | **1801.8172** | **-0.0102** | **0** | **30** | **0.1** | **1** | **R.DEAGHFLWPGFGENAR.V** |
|  | 17951 | **609.3290** | **1824.9653** | **1825.9574** | **-0.9921** | **1** | **1** | **79** | **2** | **R.EIISFGSGYGGNSLLGKK.C** 17940 |
|  | 19699 | **653.9764** | **1958.9073** | **1957.9183** | **0.9890** | **1** | **32** | **0.064** | **1** | **R.RDEAGHFLWPGFGENAR.V** 19687 19701 |
|  | 21605 | **1069.5466** | **2137.0787** | **2137.0878** | **-0.0090** | **0** | **1** | **69** | **1** | **R.DEGWLAEHMLILGITSPAGK.K** |
|  | 26077 | **1370.1619** | **2738.3092** | **2738.3196** | **-0.0105** | **0** | **79** | **6.9e-007** | **1** | **R.QCPIMDPAWEAPEGVPIDAIIFGGR.R** 26076 |
|  | 26164 | **689.8377** | **2755.3217** | **2754.3146** | **1.0072** | **0** | **(0)** | **56** | **9** | **R.QCPIMDPAWEAPEGVPIDAIIFGGR.R** |
|  | 26373 | **935.7684** | **2804.2833** | **2804.3011** | **-0.0178** | **0** | **52** | **0.00041** | **1** | **K.CLHSVGQPLTGQGEPVSQWPCNPEK.T** |
|  | 26945 | 730.8837 | 2919.5058 | 2919.3540 | 0.1518 | 1 | 3 | 28 | 5 | R.FPGCMQGRTMYVLPFSMGPVGSPLSR.I |
|  | 27656 | **1022.5277** | **3064.5613** | **3064.4391** | **0.1222** | **1** | **2** | **31** | **3** | **K.TNLAMMRPALPGWKVECVGDDIAWMR.F** |
|  | 29147 | **1160.9019** | **3479.6838** | **3479.7025** | **-0.0187** | **0** | **35** | **0.014** | **1** | **R.LCQPEGIHICDGTEAENTATLTLLEQQGLIR.K** |

| **92.** | Q07075\|AMPE_HUMAN    **Mass:** 109717   **Score:** 445    **Queries matched:** 47   **emPAI:** 0.54 |
| --- | --- |
|  | Glutamyl aminopeptidase OS=Homo sapiens GN=ENPEP PE=1 SV=2 |

|  | **Query** | **Observed** | **Mr(expt)** | **Mr(calc)** | **Delta** | **Miss** | **Score** | **Expect** | **Rank** | **Peptide** |
| --- | --- | --- | --- | --- | --- | --- | --- | --- | --- | --- |
|  | 1939 | **764.4496** | **763.4423** | **763.4480** | **-0.0057** | **0** | **28** | **0.32** | **2** | **R.YLDLLK.D** |
|  | 4597 | 482.2940 | 962.5735 | 962.5800 | -0.0066 | 0 | 32 | 0.1 | 1 | K.LLYGLASVK.N |
|  | 4661 | **485.2473** | **968.4800** | **967.4797** | **1.0003** | **0** | **3** | **69** | **4** | **R.SSVLGFACK.M** |
|  | 6170 | **539.7947** | **1077.5749** | **1077.5819** | **-0.0070** | **0** | **41** | **0.014** | **1** | **K.TQDVFTVIR.Y** 6169 |
|  | 6363 | **547.2587** | **1092.5028** | **1093.5080** | **-1.0052** | **0** | **3** | **85** | **1** | **R.YISYNSYGK.N** |
|  | 8261 | **609.7952** | **1217.5759** | **1217.5829** | **-0.0070** | **0** | **19** | **1.8** | **2** | **R.EENFLPWQR.V** |
|  | 8351 | 611.3470 | 1220.6794 | 1219.6421 | 1.0373 | 1 | 6 | 37 | 6 | R.YTLNNRNLGR.I |
|  | 9751 | **432.8838** | **1295.6295** | **1294.6227** | **1.0067** | **0** | **5** | **41** | **6** | **K.EYGALSNMPVAK.E** 9708 9752 |
|  | 11151 | **687.8447** | **1373.6749** | **1373.6840** | **-0.0091** | **1** | **30** | **0.15** | **1** | **K.REENFLPWQR.V** |
|  | 11356 | **462.7312** | **1385.1718** | **1385.7568** | **-0.5850** | **1** | **5** | **28** | **3** | **R.YLWLHLRETR.I** |
|  | 11812 | **706.3396** | **1410.6646** | **1410.6739** | **-0.0093** | **0** | **42** | **0.008** | **1** | **K.SIVATDHEPTDAR.K** 11824 |
|  | 11926 | **473.2799** | **1416.8179** | **1417.7388** | **-0.9209** | **0** | **5** | **40** | **2** | **R.QMGYPVLNVNGVK.N** |
|  | 12967 | **741.8429** | **1481.6712** | **1481.6787** | **-0.0074** | **0** | **79** | **1.5e-006** | **1** | **K.TSDFWAALEEASR.L** 12966 |
|  | 14586 | **530.6160** | **1588.8263** | **1588.9188** | **-0.0925** | **1** | **2** | **86** | **7** | **R.AQLLDYKVALNLTK.Y** |
|  | 15381 | **410.7141** | **1638.8273** | **1637.8373** | **0.9900** | **1** | **6** | **28** | **7** | **R.VKSIVATDHEPTDAR.K** |
|  | 15930 | **839.4138** | **1676.8131** | **1676.8232** | **-0.0101** | **0** | **20** | **1.2** | **1** | **R.MLEDWIKPENFQK.G** 15929 |
|  | 17544 | **599.3419** | **1795.0039** | **1794.8536** | **0.1503** | **1** | **1** | **73** | **10** | **K.NAKTSDFWAALEEASR.L** |
|  | 18226 | 461.7824 | 1843.1003 | 1841.9887 | 1.1116 | 1 | 30 | 0.093 | 1 | R.EQVLETVKNNIEWLK.Q |
|  | 18266 | 616.3117 | 1845.9133 | 1845.9070 | 0.0063 | 0 | 5 | 36 | 1 | R.VISAVTYIISMFEDDK.E |
|  | 19019 | **951.5138** | **1901.0130** | **1901.0258** | **-0.0128** | **0** | **34** | **0.04** | **1** | **R.ISNSGKPLTIYVQPEQK.H** 19018 |
|  | 20273 | **673.3406** | **2017.0001** | **2018.0619** | **-1.0618** | **1** | **0** | **88** | **8** | **R.QMGYPVLNVNGVKNITQK.R** |
|  | 20769 | **686.9937** | **2057.9593** | **2057.1269** | **0.8324** | **1** | **4** | **40** | **2** | **K.RISNSGKPLTIYVQPEQK.H** |
|  | 20998 | **1042.5198** | **2083.0250** | **2083.0374** | **-0.0124** | **0** | **53** | **0.00044** | **1** | **R.ANPSQPPSDLGYTWNIPVK.W** 20999 |
|  | 22493 | **744.7115** | **2231.1126** | **2231.1222** | **-0.0096** | **0** | **23** | **0.41** | **1** | **R.VNYEVATWDSIATALSLNHK.T** |
|  | 22524 | **1118.5380** | **2235.0614** | **2235.0683** | **-0.0070** | **0** | **48** | **0.0011** | **1** | **K.NMAWNWIQLNWDYLVNR.Y** 22526 |
|  | 24308 | **823.9890** | **2468.9450** | **2468.1828** | **0.7622** | **1** | **0** | **56** | **7** | **K.EVMDTWTRQMGYPVLNVNGVK.N** |
|  | 25747 | 896.7908 | 2687.3507 | 2686.2593 | 1.0914 | 1 | 2 | 44 | 10 | R.MLEDWIKPENFQKGCQMYLEK.Y |
|  | 26093 | **1371.6895** | **2741.3643** | **2741.3775** | **-0.0131** | **0** | **84** | **2.4e-007** | **1** | **R.IVTIAEPFNTELQLWQMESFFAK.Y** 26092 26094 26095 |
|  | 29636 | **1217.9581** | **3650.8526** | **3649.7399** | **1.1127** | **0** | **3** | **20** | **5** | **K.ELYPMIEEYFQGQVKPIADSLGWNDAGDHVTK.L** 29634 |
|  | 31270 | **1622.1216** | **4863.3429** | **4863.3610** | **-0.0181** | **0** | **58** | **3e-005** | **1** | **R.DQMLLEDVLPVQEDDSLMSSHPIIVTVTTPDEITSVFDGISYSK.G** 31267 31269 31271 |
|  | 31667 | **1366.4119** | **5461.6184** | **5461.6414** | **-0.0230** | **1** | **21** | **0.09** | **1** | **R.VISAVTYIISMFEDDKELYPMIEEYFQGQVKPIADSLGWNDAGDHVTK.L** |

| **93.** | P23528\|COF1_HUMAN    **Mass:** 18719    **Score:** 444    **Queries matched:** 33   **emPAI:** 6.82 |
| --- | --- |
|  | Cofilin-1 OS=Homo sapiens GN=CFL1 PE=1 SV=3 |

|  | **Query** | **Observed** | **Mr(expt)** | **Mr(calc)** | **Delta** | **Miss** | **Score** | **Expect** | **Rank** | **Peptide** |
| --- | --- | --- | --- | --- | --- | --- | --- | --- | --- | --- |
|  | 1566 | **659.4397** | **658.4324** | **658.4377** | **-0.0053** | **1** | **17** | **5.8** | **1** | **K.KLTGIK.H** 1563 |
|  | 2148 | **400.2021** | **798.3896** | **798.3945** | **-0.0050** | **0** | **17** | **3.2** | **1** | **K.MIYASSK.D** |
|  | 3918 | **915.5083** | **914.5010** | **914.5073** | **-0.0062** | **0** | **48** | **0.0031** | **1** | **K.NIILEEGK.E** |
|  | 5166 | **502.6408** | **1003.2669** | **1003.5186** | **-0.2516** | **1** | **8** | **20** | **1** | **K.SSTPEEVKK.R** 5182 |
|  | 5190 | **503.2945** | **1004.5744** | **1003.5186** | **1.0559** | **1** | **8** | **29** | **8** | **R.KSSTPEEVK.K** |
|  | 5552 | 517.7380 | 1033.4614 | 1033.4685 | -0.0071 | 1 | 28 | 0.26 | 1 | K.MLPDKDCR.Y 5539 5553 5564 5572 5573 5578 |
|  | 8539 | **617.1330** | **1232.2514** | **1232.6435** | **-0.3920** | **0** | **(5)** | **36** | **2** | **-.MASGVAVSDGVIK.V** |
|  | 8871 | **625.8362** | **1249.6579** | **1248.6384** | **1.0195** | **0** | **6** | **35** | **10** | **-.MASGVAVSDGVIK.V** 8836 |
|  | 9976 | **655.3403** | **1308.6660** | **1308.6748** | **-0.0088** | **1** | **27** | **0.26** | **1** | **K.AVLFCLSEDKK.N** 9953 |
|  | 10566 | **670.8887** | **1339.7628** | **1339.7711** | **-0.0083** | **0** | **87** | **2.6e-007** | **1** | **K.LGGSAVISLEGKPL.-** 10565 |
|  | 13547 | **760.3395** | **1518.6645** | **1518.6773** | **-0.0127** | **0** | **26** | **0.32** | **1** | **K.HELQANCYEEVK.D** |
|  | 17479 | **895.9035** | **1789.7924** | **1789.8053** | **-0.0129** | **1** | **83** | **5.4e-007** | **1** | **K.HELQANCYEEVKDR.C** 17478 17480 |
|  | 18109 | **612.6462** | **1834.9167** | **1835.9451** | **-1.0284** | **1** | **4** | **51** | **8** | **M.ASGVAVSDGVIKVFNDMK.V** |
|  | 19960 | **662.3694** | **1984.0863** | **1982.9805** | **1.1058** | **1** | **1** | **71** | **5** | **-.MASGVAVSDGVIKVFNDMK.V** |
|  | 20006 | **995.5340** | **1989.0534** | **1989.0611** | **-0.0077** | **1** | **69** | **1.1e-005** | **1** | **K.KEDLVFIFWAPESAPLK.S** 19997 20016 |
|  | 20457 | **678.3258** | **2031.9556** | **2031.0095** | **0.9461** | **1** | **1** | **71** | **4** | **K.LTGIKHELQANCYEEVK.D** |
|  | 21916 | **1083.5464** | **2165.0782** | **2165.0892** | **-0.0110** | **0** | **103** | **4.4e-009** | **1** | **K.EILVGDVGQTVDDPYATFVK.M** |
|  | 27650 | **1531.7908** | **3061.5670** | **3061.5860** | **-0.0190** | **1** | **56** | **0.00013** | **1** | **K.NIILEEGKEILVGDVGQTVDDPYATFVK.M** |

| **94.** | Q9NY65\|TBA8_HUMAN    **Mass:** 50746    **Score:** 439    **Queries matched:** 20   **emPAI:** 1.18 |
| --- | --- |
|  | Tubulin alpha-8 chain OS=Homo sapiens GN=TUBA8 PE=1 SV=1 |

|  | **Query** | **Observed** | **Mr(expt)** | **Mr(calc)** | **Delta** | **Miss** | **Score** | **Expect** | **Rank** | **Peptide** |
| --- | --- | --- | --- | --- | --- | --- | --- | --- | --- | --- |
|  | 1369 | 615.3498 | 614.3426 | 613.3435 | 0.9990 | 0 | 22 | 1.5 | 1 | R.GDVVPK.D |
|  | 11801 | 470.9266 | 1409.7580 | 1409.7667 | -0.0087 | 0 | 13 | 6.6 | 1 | R.QLFHPEQLITGK.E |
|  | 13059 | 744.4392 | 1486.8639 | 1486.8719 | -0.0080 | 0 | 64 | 5.2e-005 | 1 | R.LISQIVSSITASLR.F 13061 |
|  | 14696 | 799.8823 | 1597.7500 | 1597.7599 | -0.0100 | 0 | 38 | 0.02 | 1 | R.TIQFVDWCPTGFK.V |
|  | 16269 | 851.4518 | 1700.8890 | 1700.8655 | 0.0235 | 0 | 10 | 11 | 2 | R.AVMIDLEPTVVDEVR.A |
|  | 16490 | 859.9390 | 1717.8634 | 1717.8747 | -0.0113 | 0 | 37 | 0.021 | 1 | R.NLDIERPTYTNLNR.L 16491 |
|  | 17925 | 912.9903 | 1823.9660 | 1823.9782 | -0.0121 | 0 | 56 | 0.00029 | 1 | K.VGINYQPPTVVPGGDLAK.V 17924 17926 |
|  | 18530 | 621.9794 | 1862.9165 | 1861.8347 | 1.0817 | 1 | 5 | 32 | 4 | K.YMACCMLYRGDVVPK.D |
|  | 18536 | 932.9506 | 1863.8866 | 1863.8971 | -0.0105 | 0 | 91 | 8.5e-008 | 1 | R.AVCMLSNTTAIAEAWAR.L |
|  | 23245 | 1165.5043 | 2328.9940 | 2329.0110 | -0.0170 | 0 | 77 | 1.2e-006 | 1 | R.AFVHWYVGEGMEEGEFSEAR.E 23246 |
|  | 23808 | 1205.1003 | 2408.1861 | 2408.2012 | -0.0151 | 0 | 123 | 3.3e-011 | 1 | R.FDGALNVDLTEFQTNLVPYPR.I 23809 23811 23812 |
|  | 26216 | 924.1555 | 2769.4446 | 2770.2578 | -0.8133 | 0 | 0 | 59 | 10 | K.AYHEQLSVAEITSSCFEPNSQMVK.C |

| **95.** | P55084\|ECHB_HUMAN    **Mass:** 51547    **Score:** 439    **Queries matched:** 45   **emPAI:** 0.88 |
| --- | --- |
|  | Trifunctional enzyme subunit beta, mitochondrial OS=Homo sapiens GN=HADHB PE=1 SV=3 |

|  | **Query** | **Observed** | **Mr(expt)** | **Mr(calc)** | **Delta** | **Miss** | **Score** | **Expect** | **Rank** | **Peptide** |
| --- | --- | --- | --- | --- | --- | --- | --- | --- | --- | --- |
|  | 1127 | **564.3106** | **563.3033** | **562.2962** | **1.0070** | **0** | **13** | **12** | **2** | **K.DTVTK.D** 1125 |
|  | 2810 | **423.2340** | **844.4535** | **844.4589** | **-0.0054** | **0** | **42** | **0.015** | **1** | **R.LVMAAANR.L** 2811 |
|  | 2925 | **426.7604** | **851.5061** | **851.5116** | **-0.0055** | **0** | **14** | **5.6** | **1** | **K.VGLPPLEK.F** 2926 |
|  | 3067 | **431.2607** | **860.5069** | **860.4538** | **0.0531** | **0** | **(7)** | **45** | **5** | **R.LVMAAANR.L** 3068 |
|  | 3867 | **456.2522** | **910.4898** | **911.5552** | **-1.0654** | **0** | **19** | **1.6** | **1** | **K.TLAKPNIR.N** |
|  | 4497 | **478.7767** | **955.5389** | **955.5451** | **-0.0062** | **0** | **16** | **3.8** | **1** | **R.NVVVVDGVR.T** 4479 4500 |
|  | 4742 | **487.7855** | **973.5564** | **973.5630** | **-0.0066** | **1** | **24** | **0.8** | **1** | **R.KLMLDLNK.A** 4721 4744 4757 4767 |
|  | 4800 | **489.7726** | **977.5306** | **977.5368** | **-0.0062** | **0** | **29** | **0.2** | **1** | **K.ALAMGYKPK.A** 4799 |
|  | 4964 | **496.2393** | **990.4640** | **989.5579** | **0.9061** | **1** | **(9)** | **20** | **3** | **R.KLMLDLNK.A** 4956 |
|  | 5003 | 497.2632 | 992.5119 | 993.5317 | -1.0198 | 0 | (4) | 64 | 3 | K.ALAMGYKPK.A |
|  | 5989 | **534.2650** | **1066.5153** | **1066.5229** | **-0.0076** | **0** | **15** | **4.7** | **1** | **K.DLMPHDLAR.A** 5973 |
|  | 11912 | **708.8858** | **1415.7570** | **1415.7660** | **-0.0090** | **0** | **62** | **8.8e-005** | **1** | **K.DQLLLGPTYATPK.V** 11911 11913 |
|  | 13529 | **759.3920** | **1516.7694** | **1516.7773** | **-0.0079** | **0** | **15** | **4.4** | **1** | **K.AQDEGLLSDVVPFK.V** |
|  | 13913 | **773.3827** | **1544.7509** | **1544.7617** | **-0.0107** | **0** | **73** | **6.8e-006** | **1** | **K.DNGIRPSSLEQMAK.L** |
|  | 13968 | **517.2309** | **1548.6709** | **1549.8035** | **-1.1326** | **0** | **1** | **91** | **4** | **R.FSIRPLSCSSQLR.A** |
|  | 14172 | **521.3044** | **1560.8913** | **1560.7566** | **0.1347** | **0** | **(1)** | **85** | **6** | **K.DNGIRPSSLEQMAK.L** |
|  | 16123 | **846.8377** | **1691.6609** | **1691.6708** | **-0.0099** | **0** | **71** | **7.2e-006** | **1** | **K.AMDSDWFAENYMGR.K** 16124 |
|  | 16126 | 846.9362 | 1691.8578 | 1692.8431 | -0.9853 | 1 | 3 | 58 | 3 | K.TSNVAREAALGAGFSDK.T |
|  | 16912 | **583.9640** | **1748.8701** | **1747.8239** | **1.0462** | **1** | **7** | **22** | **1** | **K.AYLRDFMYVSQDPK.D** |
|  | 18346 | **926.5051** | **1850.9957** | **1851.0030** | **-0.0073** | **0** | **45** | **0.003** | **1** | **K.EVVDYIIFGTVIQEVK.T** 18344 |
|  | 21572 | **1067.9907** | **2133.9669** | **2133.9803** | **-0.0134** | **0** | **90** | **9.4e-008** | **1** | **K.FNNWGGSLSLGHPFGATGCR.L** 21582 |
|  | 25458 | **882.1067** | **2643.2982** | **2642.2938** | **1.0044** | **1** | **2** | **43** | **6** | **R.DFMYVSQDPKDQLLLGPTYATPK.V** |
|  | 25894 | **902.4262** | **2704.2568** | **2704.2737** | **-0.0169** | **0** | **35** | **0.02** | **1** | **K.EGGQYGLVAACAAGGQGHAMIVEAYPK.-** 25893 |
|  | 29391 | **1191.6103** | **3571.8092** | **3571.8306** | **-0.0214** | **0** | **93** | **2e-008** | **1** | **K.LKPAFIKPYGTVTAANSSFLTDGASAMLIMAEEK.A** 29393 29397 |

| **96.** | P11142\|HSP7C_HUMAN    **Mass:** 71082    **Score:** 417    **Queries matched:** 47   **emPAI:** 1.38 |
| --- | --- |
|  | Heat shock cognate 71 kDa protein OS=Homo sapiens GN=HSPA8 PE=1 SV=1 |

|  | **Query** | **Observed** | **Mr(expt)** | **Mr(calc)** | **Delta** | **Miss** | **Score** | **Expect** | **Rank** | **Peptide** |
| --- | --- | --- | --- | --- | --- | --- | --- | --- | --- | --- |
|  | 1958 | 773.4097 | 772.4025 | 772.4079 | -0.0054 | 0 | 35 | 0.066 | 1 | K.DNNLLGK.F |
|  | 1961 | **774.4308** | **773.4235** | **773.4283** | **-0.0048** | **0** | **27** | **0.47** | **1** | **R.NTTIPTK.Q** |
|  | 2227 | **804.4412** | **803.4339** | **803.4389** | **-0.0050** | **0** | **23** | **1.2** | **1** | **K.ITITNDK.G** |
|  | 2619 | **833.4131** | **832.4058** | **832.4290** | **-0.0232** | **1** | **3** | **94** | **8** | **K.KDISENK.R** |
|  | 7812 | **399.7613** | **1196.2620** | **1196.6554** | **-0.3934** | **0** | **6** | **24** | **1** | **K.FELTGIPPAPR.G** |
|  | 8452 | 614.8128 | 1227.6111 | 1227.6207 | -0.0097 | 0 | 47 | 0.003 | 1 | K.VEIIANDQGNR.T 8436 8453 |
|  | 8579 | **618.3121** | **1234.6096** | **1234.6169** | **-0.0073** | **0** | **32** | **0.1** | **1** | **R.MVNHFIAEFK.R** |
|  | 8905 | **626.8296** | **1251.6446** | **1251.6533** | **-0.0086** | **1** | **15** | **4.1** | **1** | **K.MKEIAEAYLGK.T** 8888 |
|  | 8915 | **627.3079** | **1252.6013** | **1252.6088** | **-0.0075** | **0** | **32** | **0.092** | **1** | **R.FEELNADLFR.G** |
|  | 9220 | 634.8561 | 1267.6977 | 1267.6482 | 0.0495 | 1 | (9) | 18 | 8 | K.MKEIAEAYLGK.T |
|  | 9251 | **424.0562** | **1269.1468** | **1269.5547** | **-0.4079** | **0** | **7** | **18** | **3** | **R.FDDAVVQSDMK.H** 9282 |
|  | 11433 | **464.2330** | **1389.6772** | **1390.7180** | **-1.0408** | **1** | **5** | **44** | **3** | **R.MVNHFIAEFKR.K** 11445 |
|  | 11740 | **704.8468** | **1407.6790** | **1406.7129** | **0.9662** | **1** | **(2)** | **87** | **7** | **R.MVNHFIAEFKR.K** |
|  | 11797 | **705.8332** | **1409.6518** | **1409.6609** | **-0.0091** | **1** | **35** | **0.044** | **1** | **R.RFDDAVVQSDMK.H** |
|  | 12078 | 713.8774 | 1425.7402 | 1425.6558 | 0.0844 | 1 | (3) | 65 | 5 | R.RFDDAVVQSDMK.H |
|  | 12344 | **481.7492** | **1442.2259** | **1442.7803** | **-0.5544** | **1** | **5** | **28** | **10** | **K.ELEKVCNPIITK.L** |
|  | 12936 | 494.2532 | 1479.7378 | 1479.7470 | -0.0092 | 1 | 31 | 0.11 | 1 | R.ARFEELNADLFR.G 12935 |
|  | 12957 | **741.4027** | **1480.7909** | **1480.7998** | **-0.0089** | **0** | **53** | **0.00067** | **1** | **K.SQIHDIVLVGGSTR.I** 12956 |
|  | 13054 | **744.3494** | **1486.6842** | **1486.6940** | **-0.0098** | **0** | **50** | **0.0013** | **1** | **R.TTPSYVAFTDTER.L** |
|  | 14343 | **525.1035** | **1572.2887** | **1572.7420** | **-0.4532** | **1** | **8** | **13** | **2** | **K.EEFEHQQKELEK.V** 14331 14356 |
|  | 14993 | **808.8926** | **1615.7707** | **1615.7804** | **-0.0096** | **0** | **47** | **0.0023** | **1** | **K.SFYPEEVSSMVLTK.M** |
|  | 15176 | **543.2846** | **1626.8320** | **1626.9053** | **-0.0733** | **1** | **2** | **66** | **6** | **R.QATKDAGTIAGLNVLR.I** |
|  | 15523 | **825.3964** | **1648.7783** | **1648.7879** | **-0.0096** | **0** | **47** | **0.0021** | **1** | **K.NQVAMNPTNTVFDAK.R** |
|  | 15661 | 830.4460 | 1658.8775 | 1658.8879 | -0.0104 | 0 | 46 | 0.0026 | 1 | R.IINEPTAAAIAYGLDK.K 15683 15687 |
|  | 16111 | **846.3604** | **1690.7063** | **1690.7183** | **-0.0121** | **0** | **28** | **0.16** | **1** | **K.STAGDTHLGGEDFDNR.M** 16110 |
|  | 17447 | **894.4931** | **1786.9716** | **1786.9828** | **-0.0112** | **1** | **38** | **0.015** | **1** | **R.IINEPTAAAIAYGLDKK.V** 17430 |
|  | 19931 | **661.3336** | **1980.9790** | **1980.9905** | **-0.0115** | **0** | **38** | **0.017** | **1** | **K.TVTNAVVTVPAYFNDSQR.Q** 19936 |
|  | 22720 | **1130.5707** | **2259.1268** | **2259.1383** | **-0.0115** | **0** | **53** | **0.00038** | **1** | **K.SINPDEAVAYGAAVQAAILSGDK.S** 22719 |
|  | 23841 | **805.7280** | **2414.1623** | **2415.2158** | **-1.0535** | **1** | **0** | **75** | **4** | **K.HWPFMVVNDAGRPKVQVEYK.G** |
|  | 26236 | **1387.6584** | **2773.3023** | **2773.3195** | **-0.0172** | **0** | **100** | **5.7e-009** | **1** | **K.QTQTFTTYSDNQPGVLIQVYEGER.A** 26235 |
|  | 28463 | **1085.2245** | **3252.6516** | **3253.7731** | **-1.1215** | **0** | **0** | **44** | **8** | **K.SENVQDLLLLDVTPLSLGIETAGGVMTVLIK.R** |

| **97.** | P04844\|RPN2_HUMAN    **Mass:** 69355    **Score:** 416    **Queries matched:** 24   **emPAI:** 0.97 |
| --- | --- |
|  | Dolichyl-diphosphooligosaccharide--protein glycosyltransferase subunit 2 OS=Homo sapiens GN=RPN2 PE=1 SV=3 |

|  | **Query** | **Observed** | **Mr(expt)** | **Mr(calc)** | **Delta** | **Miss** | **Score** | **Expect** | **Rank** | **Peptide** |
| --- | --- | --- | --- | --- | --- | --- | --- | --- | --- | --- |
|  | 1642 | **678.3771** | **677.3698** | **677.3748** | **-0.0050** | **0** | **20** | **1.8** | **1** | **R.VTYPAK.A** |
|  | 6035 | **535.7868** | **1069.5590** | **1069.5669** | **-0.0078** | **0** | **22** | **0.98** | **1** | **K.QEIQHLFR.E** |
|  | 6192 | **540.3284** | **1078.6423** | **1077.5818** | **1.0605** | **0** | **3** | **83** | **10** | **R.YIANTVELR.V** |
|  | 11106 | **686.8669** | **1371.7192** | **1371.7245** | **-0.0053** | **0** | **43** | **0.0065** | **1** | **R.SIVEEIEDLVAR.L** 11105 |
|  | 12941 | **740.9258** | **1479.8371** | **1479.8449** | **-0.0078** | **0** | **4** | **50** | **3** | **K.NPILWNVADVVIK.F** |
|  | 15318 | **818.9219** | **1635.8292** | **1634.8338** | **0.9954** | **0** | **36** | **0.031** | **1** | **K.EDQVIQLMNAIFSK.K** 15309 |
|  | 18167 | **920.5245** | **1839.0345** | **1839.0466** | **-0.0120** | **0** | **92** | **6.7e-008** | **1** | **R.LQVTNVLSQPLTQATVK.L** |
|  | 20614 | **1023.0003** | **2043.9861** | **2043.9976** | **-0.0115** | **0** | **91** | **8.3e-008** | **1** | **K.TSFTPVGDVFELNFMNVK.F** 20616 |
|  | 23098 | **769.7134** | **2306.1183** | **2306.1291** | **-0.0108** | **0** | **25** | **0.28** | **1** | **K.NFESLSEAFSVASAAAVLSHNR.Y** |
|  | 24588 | **835.4318** | **2503.2736** | **2502.2867** | **0.9869** | **0** | **34** | **0.027** | **1** | **R.YHVPVVVVPEGSASDTHEQAILR.L** |
|  | 24767 | **1265.6578** | **2529.3011** | **2529.3174** | **-0.0163** | **1** | **76** | **1.8e-006** | **1** | **K.ISTEVGITNVDLSTVDKDQSIAPK.T** 24768 |
|  | 25267 | **870.4515** | **2608.3326** | **2608.3456** | **-0.0130** | **0** | **21** | **0.53** | **1** | **K.EETVLATVQALQTASHLSQQADLR.S** |
|  | 27063 | **981.8314** | **2942.4723** | **2942.4881** | **-0.0159** | **1** | **71** | **4.6e-006** | **1** | **K.LMDHVGTEPSIKEDQVIQLMNAIFSK.K** |
|  | 28462 | **1085.2245** | **3252.6516** | **3252.6666** | **-0.0150** | **0** | **35** | **0.015** | **1** | **K.ASLDRPFTNLESAFYSIVGLSSLGAQVPDAK.K** 28463 28464 |
|  | 29462 | **1199.3015** | **3594.8827** | **3594.8974** | **-0.0147** | **1** | **68** | **6.3e-006** | **1** | **K.NPILWNVADVVIKFPEEEAPSTVLSQNLFTPK.Q** |
|  | 30123 | **1274.3020** | **3819.8842** | **3819.9010** | **-0.0168** | **0** | **62** | **2.3e-005** | **1** | **K.GTFIADSHQNFALFFQLVDVNTGAELTPHQTFVR.L** |
|  | 30398 | **1342.0142** | **4023.0207** | **4021.8721** | **1.1486** | **1** | **3** | **14** | **5** | **K.TSFTPVGDVFELNFMNVKFSSGYYDFLVEVEGDNR.Y** |
|  | 30721 | **1419.4149** | **4255.2229** | **4255.2376** | **-0.0146** | **0** | **9** | **2.3** | **1** | **K.DLLLAAVSEDSSVTQIYHAVAALSGFGLPLASQEALSALTAR.L** |

| **98.** | P22760\|AAAD_HUMAN    **Mass:** 45833    **Score:** 415    **Queries matched:** 24   **emPAI:** 0.60 |
| --- | --- |
|  | Arylacetamide deacetylase OS=Homo sapiens GN=AADAC PE=1 SV=4 |

|  | **Query** | **Observed** | **Mr(expt)** | **Mr(calc)** | **Delta** | **Miss** | **Score** | **Expect** | **Rank** | **Peptide** |
| --- | --- | --- | --- | --- | --- | --- | --- | --- | --- | --- |
|  | 2635 | **417.5520** | **833.0894** | **833.4032** | **-0.3137** | **0** | **22** | **0.85** | **1** | **K.YGVNPER.I** 2639 |
|  | 3346 | **438.7478** | **875.4810** | **874.5025** | **0.9785** | **0** | **34** | **0.072** | **1** | **K.FNNILVR.V** |
|  | 6009 | **535.2501** | **1068.4856** | **1068.4910** | **-0.0054** | **0** | **6** | **37** | **3** | **R.DDGLMYVTR.L** |
|  | 8604 | **618.8290** | **1235.6435** | **1235.6510** | **-0.0075** | **0** | **84** | **6.3e-007** | **1** | **R.LDAVVVSTNYR.L** 8603 |
|  | 11730 | **469.9142** | **1406.7207** | **1406.7307** | **-0.0099** | **0** | **16** | **3** | **1** | **R.QHVPVESSHLFK.F** |
|  | 16904 | **875.4079** | **1748.8012** | **1748.8118** | **-0.0105** | **0** | **61** | **7.8e-005** | **1** | **K.GHVYNNPNYGSSELAK.K** 16903 |
|  | 16914 | **875.8887** | **1749.7628** | **1748.9613** | **0.8014** | **1** | **0** | **1e+002** | **10** | **K.FINWSSLLPERFIK.G** |
|  | 17772 | **605.0089** | **1812.0049** | **1812.0046** | **0.0003** | **1** | **9** | **13** | **1** | **K.ISHRLINQYIEWLK.E** |
|  | 18562 | **933.9887** | **1865.9629** | **1864.9432** | **1.0197** | **1** | **9** | **12** | **2** | **R.WTADRLDAVVVSTNYR.L** 18561 |
|  | 19136 | **956.4680** | **1910.9215** | **1910.9315** | **-0.0100** | **0** | **8** | **15** | **1** | **K.YHFPIQFEDVYNALR.W** 19137 |
|  | 22148 | **1097.4823** | **2192.9500** | **2193.1980** | **-0.2479** | **1** | **1** | **70** | **4** | **K.LRGLPLTYVITCQYDLLR.D** |
|  | 25028 | **641.0842** | **2560.3078** | **2560.3148** | **-0.0070** | **0** | **37** | **0.013** | **1** | **K.TIQNLATFVELLGLHHFMDSFK.V** |
|  | 25341 | **1313.1649** | **2624.3153** | **2624.3293** | **-0.0141** | **0** | **129** | **9e-012** | **1** | **R.IGISGDSAGGNLAAAVTQQLLDDPDVK.I** 25340 25342 |
|  | 27273 | 598.9015 | 2989.4711 | 2990.4882 | -1.0171 | 1 | 1 | 38 | 3 | R.GLPLTYVITCQYDLLRDDGLMYVTR.L |
|  | 29059 | **1726.8961** | **3451.7777** | **3451.7915** | **-0.0138** | **0** | **42** | **0.0022** | **1** | **K.IQSLIYPALQPLDVDLPSYQENSNFLFLSK.S** 29060 29061 |

| **99.** | P62879\|GBB2_HUMAN    **Mass:** 38048    **Score:** 408    **Queries matched:** 34   **emPAI:** 1.33 |
| --- | --- |
|  | Guanine nucleotide-binding protein G(I)/G(S)/G(T) subunit beta-2 OS=Homo sapiens GN=GNB2 PE=1 SV=3 |

|  | **Query** | **Observed** | **Mr(expt)** | **Mr(calc)** | **Delta** | **Miss** | **Score** | **Expect** | **Rank** | **Peptide** |
| --- | --- | --- | --- | --- | --- | --- | --- | --- | --- | --- |
|  | 1592 | **668.3137** | **667.3064** | **667.2418** | **0.0646** | **0** | **5** | **34** | **2** | **R.DSMCR.Q** |
|  | 2250 | 805.4991 | 804.4918 | 804.4970 | -0.0052 | 0 | 42 | 0.012 | 1 | K.VHAIPLR.S 2218 2233 2237 2244 2251 2258 |
|  | 5229 | 505.2587 | 1008.5028 | 1008.5101 | -0.0072 | 0 | 54 | 0.00068 | 1 | R.AGVLAGHDNR.V 5226 5228 5230 5231 5232 5235 |
|  | 5329 | 509.2790 | 1016.5434 | 1016.5502 | -0.0068 | 0 | 30 | 0.17 | 1 | R.LLVSASQDGK.L 5328 |
|  | 8937 | **628.2986** | **1254.5827** | **1254.5914** | **-0.0087** | **0** | **38** | **0.021** | **1** | **R.TFVSGACDASIK.L** 8938 |
|  | 10496 | 668.8042 | 1335.5938 | 1335.6030 | -0.0091 | 0 | 72 | 8.2e-006 | 1 | K.IYAMHWGTDSR.L |
|  | 10757 | 451.2634 | 1350.7685 | 1351.5979 | -0.8295 | 0 | (8) | 19 | 2 | K.IYAMHWGTDSR.L |
|  | 10792 | 677.3515 | 1352.6884 | 1352.6976 | -0.0092 | 0 | 53 | 0.00065 | 1 | K.LIIWDSYTTNK.V 10809 |
|  | 13969 | **775.3431** | **1548.6717** | **1548.6813** | **-0.0096** | **0** | **14** | **4.8** | **1** | **R.ELPGHTGYLSCCR.F** 13968 13977 |
|  | 19391 | **966.4725** | **1930.9305** | **1930.9419** | **-0.0114** | **0** | **59** | **0.00014** | **1** | **K.ACGDSTLTQITAGLDPVGR.I** |
|  | 21841 | **1079.9938** | **2157.9730** | **2157.9863** | **-0.0133** | **0** | **67** | **1.6e-005** | **1** | **R.LLLAGYDDFNCNIWDAMK.G** 21842 21857 |
|  | 23848 | 1208.5548 | 2415.0951 | 2415.1087 | -0.0136 | 0 | 95 | 2.2e-008 | 1 | R.VSCLGVTDDGMAVATGSWDSFLK.I 23847 23849 |
|  | 24353 | **825.7701** | **2474.2884** | **2474.1358** | **0.1526** | **1** | **12** | **4.1** | **2** | **R.SGRLLLAGYDDFNCNIWDAMK.G** |

| **100.** | Q92597\|NDRG1_HUMAN    **Mass:** 43264    **Score:** 406    **Queries matched:** 24   **emPAI:** 2.19 |
| --- | --- |
|  | Protein NDRG1 OS=Homo sapiens GN=NDRG1 PE=1 SV=1 |

|  | **Query** | **Observed** | **Mr(expt)** | **Mr(calc)** | **Delta** | **Miss** | **Score** | **Expect** | **Rank** | **Peptide** |
| --- | --- | --- | --- | --- | --- | --- | --- | --- | --- | --- |
|  | 3305 | **437.2350** | **872.4555** | **873.3941** | **-0.9386** | **0** | **6** | **59** | **3** | **R.SHTSEGTR.S** |
|  | 10537 | **669.8242** | **1337.6338** | **1337.6423** | **-0.0085** | **0** | **56** | **0.00031** | **1** | **R.TASGSSVTSLDGTR.S** |
|  | 14326 | **786.8743** | **1571.7340** | **1571.7436** | **-0.0096** | **0** | **40** | **0.013** | **1** | **K.MADCGGLPQISQPAK.L** |
|  | 14445 | **790.9219** | **1579.8293** | **1579.8392** | **-0.0099** | **0** | **48** | **0.0019** | **1** | **K.SIIGMGTGAGAYILTR.F** 14448 |
|  | 14686 | **533.3085** | **1596.9038** | **1595.8341** | **1.0696** | **0** | **(3)** | **51** | **10** | **K.SIIGMGTGAGAYILTR.F** 14666 |
|  | 16518 | **860.8958** | **1719.7769** | **1719.7886** | **-0.0117** | **0** | **50** | **0.0012** | **1** | **K.EEMQSNVEVVHTYR.Q** 16517 |
|  | 17954 | **913.9033** | **1825.7920** | **1824.7997** | **0.9922** | **0** | **95** | **3.4e-008** | **1** | **K.YFVQGMGYMPSASMTR.L** 17937 |
|  | 18185 | **921.4963** | **1840.9781** | **1840.7946** | **0.1835** | **0** | **(2)** | **72** | **1** | **K.YFVQGMGYMPSASMTR.L** |
|  | 18201 | **921.9730** | **1841.9314** | **1841.9444** | **-0.0131** | **0** | **49** | **0.0012** | **1** | **R.EMQDVDLAEVKPLVEK.G** 18202 |
|  | 19746 | **983.0041** | **1963.9936** | **1964.0050** | **-0.0114** | **0** | **66** | **2.6e-005** | **1** | **K.GNRPVILTYHDIGMNHK.T** 19745 |
|  | 21012 | **1043.5389** | **2085.0633** | **2085.0717** | **-0.0084** | **0** | **15** | **2.8** | **1** | **K.ISGWTQALPDMVVSHLFGK.E** 21013 |
|  | 21190 | **701.0470** | **2100.1192** | **2101.0667** | **-0.9475** | **0** | **(3)** | **49** | **5** | **K.ISGWTQALPDMVVSHLFGK.E** |
|  | 23604 | **792.7057** | **2375.0954** | **2375.1102** | **-0.0147** | **0** | **52** | **0.00042** | **1** | **R.SHTSEGAHLDITPNSGAAGNSAGPK.S** |
|  | 24568 | **834.7315** | **2501.1725** | **2500.1589** | **1.0136** | **1** | **8** | **10** | **1** | **K.LAEAFKYFVQGMGYMPSASMTR.L** |
|  | 25053 | **856.4183** | **2566.2332** | **2566.2499** | **-0.0167** | **0** | **21** | **0.53** | **1** | **R.QHIVNDMNPGNLHLFINAYNSR.R** |
|  | 29909 | **938.7129** | **3750.8225** | **3750.8411** | **-0.0187** | **0** | **40** | **0.0032** | **1** | **K.GETITGLLQEFDVQEQDIETLHGSVHVTLCGTPK.G** |
|  | 30766 | **1077.0299** | **4304.0905** | **4304.1127** | **-0.0222** | **0** | **40** | **0.0024** | **1** | **R.DLEIERPMPGTHTVTLQCPALLVVGDSSPAVDAVVECNSK.L** |

| **101.** | A6NMY6\|AXA2L_HUMAN    **Mass:** 38806    **Score:** 406    **Queries matched:** 42   **emPAI:** 2.03 |
| --- | --- |
|  | Putative annexin A2-like protein OS=Homo sapiens GN=ANXA2P2 PE=5 SV=2 |

|  | **Query** | **Observed** | **Mr(expt)** | **Mr(calc)** | **Delta** | **Miss** | **Score** | **Expect** | **Rank** | **Peptide** |
| --- | --- | --- | --- | --- | --- | --- | --- | --- | --- | --- |
|  | 1323 | 605.3397 | 604.3324 | 604.3367 | -0.0042 | 0 | 20 | 2.3 | 1 | R.IMVSR.S |
|  | 1861 | 745.4591 | 744.4518 | 744.4568 | -0.0049 | 0 | 30 | 0.27 | 1 | K.LMVALAK.G |
|  | 2274 | 807.3513 | 806.3440 | 807.4603 | -1.1163 | 0 | 18 | 3 | 2 | R.SVPHLQK.V |
|  | 2438 | 410.6824 | 819.3502 | 820.4000 | -1.0499 | 0 | 2 | 1.1e+002 | 9 | R.SEVDMLK.I |
|  | 3027 | 430.2997 | 858.5849 | 858.5174 | 0.0674 | 1 | 16 | 5.7 | 5 | K.KELASALK.S 3022 3029 |
|  | 4767 | 488.3164 | 974.6182 | 973.5742 | 1.0440 | 1 | 12 | 12 | 1 | K.LMVALAKGR.R |
|  | 5565 | 518.2646 | 1034.5146 | 1034.5219 | -0.0073 | 0 | 22 | 0.94 | 1 | K.WISIMTER.S 5566 |
|  | 6273 | 543.7426 | 1085.4707 | 1085.4778 | -0.0071 | 0 | 46 | 0.0045 | 1 | K.AYTNFDAER.D |
|  | 6330 | 545.3241 | 1088.6336 | 1089.5852 | -0.9516 | 1 | 11 | 14 | 1 | R.SEVDMLKIR.S |
|  | 6409 | 1096.4915 | 1095.4842 | 1095.4907 | -0.0065 | 0 | 41 | 0.012 | 1 | K.ALLYLCGGDD.- |
|  | 6486 | 551.7813 | 1101.5479 | 1101.6692 | -0.1213 | 1 | 11 | 15 | 3 | K.VLIRIMVSR.S |
|  | 7401 | 587.3105 | 1172.6064 | 1171.5431 | 1.0634 | 1 | 6 | 39 | 6 | K.EMYKTDLEK.D |
|  | 8226 | 406.2101 | 1215.6085 | 1216.5944 | -0.9859 | 0 | 6 | 44 | 9 | -.MSTVHEILCK.L |
|  | 8746 | 622.2825 | 1242.5504 | 1243.6156 | -1.0653 | 0 | 66 | 3.9e-005 | 1 | R.TNQELQEINR.V 8773 8797 |
|  | 10788 | 677.3307 | 1352.6469 | 1352.6572 | -0.0103 | 1 | 27 | 0.29 | 1 | K.DIISDTSGDFRK.L |
|  | 11997 | 711.3453 | 1420.6761 | 1420.6874 | -0.0113 | 0 | 37 | 0.029 | 1 | K.SLYYYIQQDTK.G |
|  | 12025 | 712.3339 | 1422.6532 | 1422.7211 | -0.0679 | 1 | 11 | 9.8 | 2 | R.IMVSRSEVDMLK.I 12017 12020 |
|  | 12620 | 730.8359 | 1459.6572 | 1459.6653 | -0.0081 | 0 | 49 | 0.0019 | 1 | K.SYSPYDMLESIR.K 12621 |
|  | 13099 | **497.2989** | **1488.8748** | **1487.7732** | **1.1015** | **0** | **5** | **43** | **7** | **K.GDLENAFLNLVQR.I** |
|  | 14556 | 794.8823 | 1587.7501 | 1587.7603 | -0.0102 | 1 | 37 | 0.023 | 1 | K.SYSPYDMLESIRK.E |
|  | 15281 | 545.1676 | 1632.4810 | 1631.8341 | 0.6469 | 1 | 3 | 42 | 5 | K.GTDVPKWISIMTER.S |
|  | 15544 | 825.9893 | 1649.9641 | 1649.9716 | -0.0075 | 0 | 67 | 1.9e-005 | 1 | K.SALSGHLETVILGLLK.T 15545 15546 |
|  | 17321 | 889.9331 | 1777.8517 | 1776.8564 | 0.9953 | 0 | 58 | 0.00015 | 1 | K.GLGTDEDSLIEIICSR.T 17304 17305 |
|  | 17746 | 906.4306 | 1810.8466 | 1810.8585 | -0.0118 | 1 | 57 | 0.00018 | 1 | K.TDLEKDIISDTSGDFR.K 17747 |
|  | 18244 | 922.9484 | 1843.8823 | 1843.8952 | -0.0129 | 0 | 73 | 5.4e-006 | 1 | K.LSLEGDHSTPPSAYGSVK.A 18219 18243 18245 |
|  | 21804 | 1078.0304 | 2154.0462 | 2154.0593 | -0.0130 | 1 | 46 | 0.0022 | 1 | K.AYTNFDAERDALNIETAIK.T |

| **102.** | P13866\|SC5A1_HUMAN    **Mass:** 74305    **Score:** 406    **Queries matched:** 30   **emPAI:** 0.55 |
| --- | --- |
|  | Sodium/glucose cotransporter 1 OS=Homo sapiens GN=SLC5A1 PE=1 SV=1 |

|  | **Query** | **Observed** | **Mr(expt)** | **Mr(calc)** | **Delta** | **Miss** | **Score** | **Expect** | **Rank** | **Peptide** |
| --- | --- | --- | --- | --- | --- | --- | --- | --- | --- | --- |
|  | 2641 | **417.7158** | **833.4170** | **833.4218** | **-0.0048** | **0** | **11** | **17** | **4** | **R.LCWSLR.N** |
|  | 4983 | **496.7709** | **991.5272** | **991.4876** | **0.0397** | **0** | **30** | **0.19** | **1** | **R.ADSFHIFR.D** |
|  | 5802 | **527.6195** | **1053.2245** | **1053.4437** | **-0.2192** | **0** | **8** | **16** | **2** | **-.MDSSTWSPK.T** |
|  | 6204 | **541.2889** | **1080.5633** | **1080.5717** | **-0.0083** | **0** | **26** | **0.36** | **1** | **R.GTVGGFFLAGR.S** |
|  | 6973 | **570.7773** | **1139.5400** | **1139.5467** | **-0.0067** | **0** | **16** | **3.8** | **1** | **K.GGCILCGYLK.L** |
|  | 7943 | **401.8584** | **1202.5535** | **1203.6281** | **-1.0747** | **1** | **(8)** | **27** | **2** | **R.ASEKELMIAGR.L** |
|  | 8291 | **610.3152** | **1218.6158** | **1219.6230** | **-1.0072** | **1** | **9** | **22** | **2** | **R.ASEKELMIAGR.L** |
|  | 8578 | 618.3091 | 1234.6037 | 1234.6380 | -0.0343 | 0 | 9 | 21 | 6 | K.AGVVTMPEYLR.K |
|  | 8715 | **621.3008** | **1240.5871** | **1240.5315** | **0.0556** | **1** | **1** | **1e+002** | **10** | **K.MTEEEEKAMK.M** 8724 |
|  | 9652 | **646.3016** | **1290.5887** | **1290.5948** | **-0.0061** | **0** | **3** | **65** | **4** | **K.IACVVPSECEK.Y** |
|  | 10940 | 682.3330 | 1362.6515 | 1362.6602 | -0.0087 | 0 | 78 | 2.3e-006 | 1 | K.MTDTSEKPLWR.T 10941 10955 |
|  | 11234 | **690.3207** | **1378.6268** | **1378.7279** | **-0.1011** | **1** | **7** | **26** | **5** | **K.AGVVTMPEYLRK.R** |
|  | 11891 | **708.3622** | **1414.7099** | **1414.7191** | **-0.0092** | **0** | **40** | **0.012** | **1** | **K.ETIEIETQVPEK.K** 11890 |
|  | 13882 | **772.4095** | **1542.8045** | **1542.8141** | **-0.0095** | **1** | **68** | **1.9e-005** | **1** | **K.ETIEIETQVPEKK.K** 13881 |
|  | 16222 | **850.3987** | **1698.7829** | **1698.7948** | **-0.0119** | **0** | **71** | **9.3e-006** | **1** | **R.IDLDAEEENIQEGPK.E** 16250 |
|  | 16314 | **853.3981** | **1704.7816** | **1704.7930** | **-0.0114** | **0** | **56** | **0.00025** | **1** | **R.AYDLFCGLEQHGAPK.M** |
|  | 16964 | **877.4344** | **1752.8543** | **1752.8621** | **-0.0078** | **0** | **76** | **3e-006** | **1** | **K.LMPMFIMVMPGMISR.I** 16965 16967 |
|  | 17624 | **601.3992** | **1801.1759** | **1800.8469** | **0.3290** | **0** | **(5)** | **23** | **3** | **K.LMPMFIMVMPGMISR.I** |
|  | 20537 | **680.5820** | **2038.7241** | **2038.0115** | **0.7126** | **1** | **2** | **45** | **7** | **R.ILYTEKIACVVPSECEK.Y** |
|  | 23169 | **1159.1001** | **2316.1856** | **2316.1970** | **-0.0114** | **0** | **39** | **0.0087** | **1** | **K.VGCTNIAYPTLVVELMPNGLR.G** |
|  | 24688 | **840.1412** | **2517.4019** | **2516.2737** | **1.1282** | **1** | **0** | **54** | **6** | **K.LMPMFIMVMPGMISRILYTEK.I** |
|  | 27767 | **1032.8350** | **3095.4831** | **3095.5033** | **-0.0203** | **1** | **59** | **6.8e-005** | **1** | **R.IDLDAEEENIQEGPKETIEIETQVPEK.K** |

| **103.** | Q13733\|AT1A4_HUMAN    **Mass:** 115119   **Score:** 398    **Queries matched:** 31   **emPAI:** 0.25 |
| --- | --- |
|  | Sodium/potassium-transporting ATPase subunit alpha-4 OS=Homo sapiens GN=ATP1A4 PE=1 SV=3 |

|  | **Query** | **Observed** | **Mr(expt)** | **Mr(calc)** | **Delta** | **Miss** | **Score** | **Expect** | **Rank** | **Peptide** |
| --- | --- | --- | --- | --- | --- | --- | --- | --- | --- | --- |
|  | 1254 | **591.3050** | **590.2977** | **589.3071** | **0.9906** | **0** | **12** | **15** | **6** | **K.DIQSK.Q** |
|  | 1443 | 633.3343 | 632.3270 | 632.3316 | -0.0046 | 0 | 25 | 0.93 | 1 | K.NCLVK.N |
|  | 3564 | 446.0373 | 890.0600 | 889.4617 | 0.5982 | 0 | 26 | 0.32 | 1 | K.TGTLTQNR.M |
|  | 4911 | **494.2866** | **986.5587** | **987.4332** | **-0.8745** | **0** | **3** | **98** | **7** | **K.EVVMDDHK.L** |
|  | 6292 | 544.2970 | 1086.5794 | 1086.5856 | -0.0061 | 0 | 34 | 0.079 | 1 | K.LIIVEGCQR.L 6293 |
|  | 9139 | **632.6740** | **1263.3335** | **1262.6354** | **0.6981** | **0** | **1** | **70** | **4** | **R.ATTGDASESALLK.F** |
|  | 9221 | 634.8564 | 1267.6982 | 1267.7071 | -0.0089 | 0 | 27 | 0.29 | 1 | K.NMVPQQALVIR.G 9219 9220 |
|  | 9520 | 642.8542 | 1283.6939 | 1283.7020 | -0.0080 | 0 | (19) | 1.6 | 2 | K.NMVPQQALVIR.G |
|  | 11285 | 691.3743 | 1380.7341 | 1380.7435 | -0.0094 | 0 | 66 | 3.2e-005 | 1 | K.VIMVTGDHPITAK.A 11283 11284 11286 |
|  | 11571 | 699.4022 | 1396.7898 | 1396.7384 | 0.0513 | 0 | (13) | 6.3 | 1 | K.VIMVTGDHPITAK.A |
|  | 11950 | **710.3458** | **1418.6771** | **1418.7365** | **-0.0594** | **1** | **4** | **55** | **8** | **K.RATTGDASESALLK.F** |
|  | 14157 | **521.2361** | **1560.6866** | **1561.7082** | **-1.0216** | **0** | **4** | **50** | **3** | **K.FIEQSYSSVAEMR.E** |
|  | 17150 | 589.3901 | 1765.1486 | 1765.8635 | -0.7149 | 0 | 1 | 62 | 10 | K.EAFQNAYLELGGLGER.V |
|  | 18129 | 919.4411 | 1836.8676 | 1837.0132 | -0.1455 | 1 | 3 | 55 | 8 | R.SAGIKVIMVTGDHPITAK.A |
|  | 19289 | 962.9550 | 1923.8954 | 1923.9095 | -0.0142 | 0 | 59 | 0.00011 | 1 | K.NLEAVETLGSTSTICSDK.T 19290 |
|  | 19512 | **648.6410** | **1942.9011** | **1942.0445** | **0.8566** | **0** | **2** | **66** | **5** | **K.MQINVQEVVLGDLVEIK.G** |
|  | 20493 | 679.3719 | 2035.0940 | 2035.0594 | 0.0346 | 1 | 3 | 43 | 5 | K.IMESFKNMVPQQALVIR.G |
|  | 24267 | 1232.6003 | 2463.1861 | 2463.1951 | -0.0090 | 0 | 129 | 9.1e-012 | 1 | K.QAADMILLDDNFASIVTGVEEGR.L 24262 24264 24266 24279 24283 |
|  | 26342 | 932.7148 | 2795.1225 | 2795.3607 | -0.2382 | 1 | 3 | 23 | 2 | K.NLEAVETLGSTSTICSDKTGTLTQNR.M |

| **104.** | P15311\|EZRI_HUMAN    **Mass:** 69484    **Score:** 396    **Queries matched:** 43   **emPAI:** 0.77 |
| --- | --- |
|  | Ezrin OS=Homo sapiens GN=EZR PE=1 SV=4 |

|  | **Query** | **Observed** | **Mr(expt)** | **Mr(calc)** | **Delta** | **Miss** | **Score** | **Expect** | **Rank** | **Peptide** |
| --- | --- | --- | --- | --- | --- | --- | --- | --- | --- | --- |
|  | 1917 | **759.4394** | **758.4322** | **759.4425** | **-1.0103** | **1** | **(16)** | **6.3** | **4** | **R.MAALRAK.E** |
|  | 1966 | **776.8287** | **775.8215** | **775.4374** | **0.3841** | **1** | **23** | **0.8** | **1** | **R.MAALRAK.E** |
|  | 2012 | **395.2133** | **788.4120** | **787.4188** | **0.9932** | **0** | **10** | **24** | **6** | **K.VSAQEVR.K** |
|  | 2271 | **404.0305** | **806.0465** | **805.4004** | **0.6461** | **0** | **10** | **14** | **7** | **K.EELMLR.L** 2269 2272 |
|  | 2473 | **412.2588** | **822.5030** | **822.5076** | **-0.0046** | **0** | **8** | **27** | **6** | **M.PKPINVR.V** |
|  | 3655 | **449.7140** | **897.4135** | **897.4192** | **-0.0056** | **0** | **21** | **1.2** | **1** | **K.SGYLSSER.L** |
|  | 3884 | **457.2214** | **912.4283** | **913.5232** | **-1.0949** | **0** | **6** | **41** | **7** | **K.IALLEEAR.R** |
|  | 3953 | **459.2532** | **916.4918** | **916.4502** | **0.0417** | **0** | **12** | **11** | **8** | **K.EAQDDLVK.T** |
|  | 4558 | **480.7983** | **959.5821** | **958.5851** | **0.9970** | **0** | **15** | **6.1** | **1** | **K.FVIKPIDK.K** |
|  | 4862 | **492.2437** | **982.4729** | **982.4212** | **0.0518** | **1** | **12** | **11** | **3** | **K.EQMMREK.E** |
|  | 4923 | **494.7715** | **987.5285** | **987.4733** | **0.0552** | **1** | **6** | **45** | **7** | **K.RAQEEAER.L** |
|  | 5156 | **502.2788** | **1002.5431** | **1002.5498** | **-0.0067** | **1** | **20** | **2** | **1** | **R.KENPLQFK.F** |
|  | 5717 | **524.2749** | **1046.5352** | **1046.5430** | **-0.0077** | **1** | **9** | **22** | **9** | **R.EKEELMLR.L** |
|  | 6514 | **552.7924** | **1103.5703** | **1103.5764** | **-0.0061** | **0** | **50** | **0.0017** | **1** | **K.IGFPWSEIR.N** |
|  | 6646 | **558.2513** | **1114.4880** | **1115.5822** | **-1.0942** | **1** | **14** | **5.8** | **1** | **R.AKEAQDDLVK.T** 6683 |
|  | 6718 | **560.7811** | **1119.5477** | **1119.5560** | **-0.0083** | **1** | **1** | **1.4e+002** | **3** | **K.QRIDEFEAL.-** |
|  | 10968 | 682.8745 | 1363.7343 | 1363.7394 | -0.0051 | 1 | 8 | 21 | 4 | R.LIPQRVMDQHK.L |
|  | 11275 | **461.2349** | **1380.6828** | **1379.7343** | **0.9485** | **1** | **(4)** | **58** | **5** | **R.LIPQRVMDQHK.L** |
|  | 11906 | **472.8921** | **1415.6544** | **1415.6640** | **-0.0097** | **1** | **7** | **25** | **3** | **R.AQEEAERLEADR.M** |
|  | 12803 | **736.8931** | **1471.7716** | **1471.7817** | **-0.0101** | **1** | **55** | **0.00039** | **1** | **R.RKPDTIEVQQMK.A** 12804 |
|  | 13166 | **747.3445** | **1492.6744** | **1492.6841** | **-0.0097** | **0** | **67** | **2.4e-005** | **1** | **R.THNDIIHNENMR.Q** 13165 |
|  | 13357 | **753.8881** | **1505.7616** | **1504.7998** | **0.9618** | **1** | **4** | **48** | **8** | **K.SGYLSSERLIPQR.V** |
|  | 13905 | **515.6282** | **1543.8627** | **1542.8558** | **1.0068** | **1** | **7** | **25** | **3** | **K.LTPKIGFPWSEIR.N** |
|  | 15554 | **826.4067** | **1650.7988** | **1650.8100** | **-0.0112** | **0** | **91** | **9.4e-008** | **1** | **K.SQEQLAAELAEYTAK.I** |
|  | 15788 | **835.3991** | **1668.7837** | **1668.7936** | **-0.0100** | **0** | **53** | **0.00058** | **1** | **R.EVWYFGLHYVDNK.G** 15789 |
|  | 17303 | **593.2854** | **1776.8344** | **1776.8473** | **-0.0129** | **0** | **20** | **1.1** | **1** | **R.ILQLCMGNHELYMR.R** 17288 |
|  | 19392 | **966.5399** | **1931.0653** | **1930.9959** | **0.0693** | **1** | **1** | **82** | **1** | **R.QLLTLSSELSQARDENK.R** |
|  | 19881 | **989.0151** | **1976.0156** | **1976.0255** | **-0.0099** | **0** | **10** | **8.6** | **1** | **K.GTDLWLGVDALGLNIYEK.D** |
|  | 20514 | **1019.5008** | **2036.9870** | **2036.9942** | **-0.0072** | **0** | **49** | **0.0011** | **1** | **K.FYPEDVAEELIQDITQK.L** 20513 |
|  | 22534 | **746.3795** | **2236.1166** | **2236.1263** | **-0.0097** | **1** | **9** | **11** | **1** | **R.AKFYPEDVAEELIQDITQK.L** |
|  | 23280 | **1168.0878** | **2334.1610** | **2334.1743** | **-0.0133** | **1** | **78** | **1.1e-006** | **1** | **K.GTDLWLGVDALGLNIYEKDDK.L** 23281 |
|  | 24836 | **850.1041** | **2547.2904** | **2546.3227** | **0.9676** | **1** | **0** | **62** | **3** | **K.SQEQLAAELAEYTAKIALLEEAR.R** |
|  | 26453 | **1412.2031** | **2822.3917** | **2822.4048** | **-0.0131** | **0** | **64** | **2.6e-005** | **1** | **K.EGILSDEIYCPPETAVLLGSYAVQAK.F** |
|  | 27372 | **755.3551** | **3017.3913** | **3016.5362** | **0.8551** | **1** | **0** | **51** | **3** | **-.MPKPINVRVTTMDAELEFAIQPNTTGK.Q** |

| **105.** | P62805\|H4_HUMAN    **Mass:** 11360    **Score:** 395    **Queries matched:** 30   **emPAI:** 10.15 |
| --- | --- |
|  | Histone H4 OS=Homo sapiens GN=HIST1H4A PE=1 SV=2 |

|  | **Query** | **Observed** | **Mr(expt)** | **Mr(calc)** | **Delta** | **Miss** | **Score** | **Expect** | **Rank** | **Peptide** |
| --- | --- | --- | --- | --- | --- | --- | --- | --- | --- | --- |
|  | 742 | **488.2478** | **487.2405** | **487.2867** | **-0.0462** | **1** | **6** | **94** | **8** | **K.GGAKR.H** 748 |
|  | 820 | 505.2373 | 504.2301 | 503.2816 | 0.9485 | 1 | 3 | 1.5e+002 | 7 | M.SGRGK.G |
|  | 884 | **516.3115** | **515.3042** | **515.3180** | **-0.0137** | **1** | **3** | **1.7e+002** | **9** | **R.GGVKR.I** |
|  | 4942 | **495.2898** | **988.5651** | **988.5706** | **-0.0054** | **0** | **52** | **0.0011** | **1** | **K.VFLENVIR.D** 4940 4941 |
|  | 6900 | **567.7711** | **1133.5276** | **1133.5353** | **-0.0077** | **0** | **45** | **0.0057** | **1** | **R.DAVTYTEHAK.R** 6879 6883 6901 6902 |
|  | 7503 | **590.8105** | **1179.6064** | **1179.6135** | **-0.0071** | **0** | **58** | **0.00022** | **1** | **R.ISGLIYEETR.G** 7502 |
|  | 9971 | 655.3107 | 1308.6068 | 1309.6952 | -1.0884 | 0 | 63 | 7.5e-005 | 1 | K.TVTAMDVVYALK.R 9995 |
|  | 10292 | 663.3752 | 1324.7359 | 1324.7463 | -0.0103 | 0 | 38 | 0.024 | 1 | R.DNIQGITKPAIR.R 10275 10293 10294 10295 10297 |
|  | 10504 | **446.2428** | **1335.7065** | **1335.7146** | **-0.0081** | **1** | **19** | **1.6** | **1** | **K.RISGLIYEETR.G** 10515 |
|  | 12283 | **719.8980** | **1437.7813** | **1437.7901** | **-0.0088** | **1** | **121** | **9.2e-011** | **1** | **R.KTVTAMDVVYALK.R** 12282 |
|  | 12715 | **733.9005** | **1465.7865** | **1465.7963** | **-0.0098** | **1** | **72** | **7.3e-006** | **1** | **K.TVTAMDVVYALKR.Q** 12713 12716 |
|  | 12979 | **495.2238** | **1482.6495** | **1481.7912** | **0.8583** | **1** | **(5)** | **35** | **6** | **K.TVTAMDVVYALKR.Q** |

| **106.** | O75369\|FLNB_HUMAN    **Mass:** 280188   **Score:** 394    **Queries matched:** 68   **emPAI:** 0.17 |
| --- | --- |
|  | Filamin-B OS=Homo sapiens GN=FLNB PE=1 SV=1 |

|  | **Query** | **Observed** | **Mr(expt)** | **Mr(calc)** | **Delta** | **Miss** | **Score** | **Expect** | **Rank** | **Peptide** |
| --- | --- | --- | --- | --- | --- | --- | --- | --- | --- | --- |
|  | 793 | 502.2947 | 501.2875 | 501.2911 | -0.0036 | 0 | 14 | 14 | 9 | R.LNGAK.G |
|  | 1287 | **601.0272** | **600.0199** | **600.3595** | **-0.3396** | **1** | **2** | **77** | **4** | **K.KQTPK.Q** |
|  | 2805 | **423.1375** | **844.2604** | **844.4767** | **-0.2162** | **1** | **6** | **47** | **5** | **K.ISGEGRVK.E** |
|  | 3383 | **440.0295** | **878.0445** | **877.4141** | **0.6304** | **0** | **6** | **29** | **10** | **R.ATQTGDASK.C** |
|  | 3895 | **457.5773** | **913.1401** | **912.5141** | **0.6260** | **1** | **4** | **43** | **3** | **R.ASGRGLQPK.G** |
|  | 4869 | **492.2765** | **982.5385** | **982.5560** | **-0.0174** | **0** | **15** | **4.4** | **2** | **K.IAGPGLGSGVR.A** 4853 4871 |
|  | 5153 | **502.2491** | **1002.4837** | **1001.5141** | **0.9695** | **0** | **4** | **68** | **8** | **K.AEVSIQNNK.D** |
|  | 5201 | **504.2631** | **1006.5116** | **1006.5196** | **-0.0080** | **0** | **16** | **4.2** | **1** | **K.IQQNTFTR.W** |
|  | 5529 | 516.7606 | 1031.5066 | 1032.5386 | -1.0320 | 1 | 9 | 20 | 9 | R.IDIQMKNR.M |
|  | 5692 | 523.2668 | 1044.5190 | 1044.5274 | -0.0084 | 0 | 9 | 24 | 1 | R.GIEPTGNMVK.Q |
|  | 6127 | 538.2730 | 1074.5313 | 1074.5669 | -0.0356 | 1 | 8 | 25 | 8 | K.AQGDASKVTAK.G |
|  | 6130 | 538.2873 | 1074.5601 | 1074.4982 | 0.0620 | 0 | 7 | 38 | 6 | K.AEITFDDHK.N |
|  | 6274 | **543.7767** | **1085.5388** | **1086.6186** | **-1.0798** | **1** | **7** | **36** | **6** | **K.VKVFGPGVER.S** |
|  | 6493 | **551.7999** | **1101.5853** | **1101.5931** | **-0.0078** | **0** | **14** | **7.4** | **1** | **K.VNQPASFAIR.L** |
|  | 6573 | **555.2546** | **1108.4947** | **1107.5965** | **0.8983** | **0** | **5** | **41** | **2** | **K.IFFAGDTIPK.S** |
|  | 6715 | **560.7687** | **1119.5228** | **1119.5309** | **-0.0081** | **0** | **33** | **0.089** | **1** | **K.IFAQDGEGQR.I** |
|  | 7090 | **575.3300** | **1148.6454** | **1149.6142** | **-0.9689** | **0** | **3** | **84** | **8** | **K.VVASGPGLEHGK.V** |
|  | 7284 | **583.1077** | **1164.2009** | **1163.6047** | **0.5962** | **0** | **30** | **0.099** | **1** | **R.VNIGQGSHPQK.V** |
|  | 7400 | **587.3079** | **1172.6013** | **1172.6111** | **-0.0098** | **0** | **9** | **20** | **1** | **R.VTEAEIVPMGK.N** |
|  | 7900 | **601.3187** | **1200.6229** | **1199.6510** | **0.9719** | **1** | **5** | **49** | **7** | **R.VKVEPAVDTSR.I** |
|  | 8114 | **605.8520** | **1209.6894** | **1209.6942** | **-0.0048** | **1** | **7** | **27** | **8** | **K.IAGPGLGSGVRAR.V** |
|  | 8115 | **605.8677** | **1209.7209** | **1209.7194** | **0.0016** | **1** | **6** | **37** | **1** | **K.VKIAGPGLGSGVR.A** |
|  | 8316 | **610.8127** | **1219.6108** | **1219.6812** | **-0.0704** | **1** | **2** | **86** | **2** | **K.VKVLPTYDASK.V** 8345 |
|  | 8511 | **411.2441** | **1230.7104** | **1231.6574** | **-0.9470** | **1** | **8** | **26** | **3** | **R.KYHQRPTFR.Q** |
|  | 9391 | **638.8347** | **1275.6548** | **1275.6837** | **-0.0289** | **0** | **15** | **5.1** | **1** | **R.AWGPGLHGGIVGR.S** |
|  | 9810 | **433.9162** | **1298.7269** | **1298.7347** | **-0.0078** | **0** | **17** | **2.5** | **1** | **K.VTVLFAGQHISK.S** 9798 |
|  | 10315 | **443.0379** | **1326.0919** | **1326.7255** | **-0.6337** | **1** | **8** | **15** | **10** | **K.LNGLENRVEVGK.D** |
|  | 10625 | **448.5664** | **1342.6773** | **1341.6830** | **0.9943** | **0** | **28** | **0.19** | **1** | **K.YGGELVPHFPAR.V** |
|  | 11612 | **467.7217** | **1400.1434** | **1400.7260** | **-0.5826** | **0** | **5** | **25** | **3** | **R.IGNLQTDLSDGLR.L** |
|  | 11976 | **474.2846** | **1419.8318** | **1419.7511** | **0.0808** | **1** | **3** | **66** | **8** | **K.VFGPGIEGKDVFR.E** |
|  | 12619 | **730.8355** | **1459.6565** | **1459.6653** | **-0.0089** | **0** | **22** | **0.83** | **1** | **R.TFEMSDFIVDTR.D** |
|  | 13022 | **743.3962** | **1484.7779** | **1484.7657** | **0.0122** | **1** | **11** | **11** | **2** | **R.GIEPTGNMVKQPAK.F** 13021 |
|  | 13153 | **747.0171** | **1492.0196** | **1491.6374** | **0.3822** | **0** | **7** | **19** | **2** | **R.MDGTYACSYTPVK.A** |
|  | 13551 | **760.3757** | **1518.7368** | **1518.7467** | **-0.0099** | **0** | **22** | **0.91** | **1** | **R.GEAGVPAEFSIWTR.E** |
|  | 14170 | **781.4177** | **1560.8209** | **1560.8300** | **-0.0091** | **0** | **36** | **0.032** | **1** | **K.VLFASQEIPASPFR.V** |
|  | 15083 | **541.6018** | **1621.7836** | **1621.7948** | **-0.0112** | **1** | **33** | **0.052** | **1** | **R.VEVGKDQEFTVDTR.G** |
|  | 15508 | **824.9198** | **1647.8250** | **1647.8369** | **-0.0119** | **0** | **40** | **0.011** | **1** | **K.HVGNQQYNVTYVVK.E** 15526 |
|  | 15743 | 833.4009 | 1664.7873 | 1664.7755 | 0.0119 | 1 | 7 | 23 | 4 | K.DQEFTVDTRGAGGQGK.L |
|  | 15832 | 836.9203 | 1671.8261 | 1671.8291 | -0.0029 | 1 | 5 | 40 | 4 | K.AFVGQKSSFLVDCSK.A |
|  | 17024 | **878.9744** | **1755.9342** | **1755.9447** | **-0.0106** | **0** | **12** | **7.4** | **1** | **K.VSYFPTVPGVYIVSTK.F** |
|  | 17799 | 908.4749 | 1814.9353 | 1814.8947 | 0.0406 | 0 | 2 | 69 | 3 | K.VMYTPMAPGNYLISVK.Y |
|  | 18536 | 932.9506 | 1863.8866 | 1863.9149 | -0.0283 | 1 | 5 | 31 | 6 | K.IFAQDGEGQRIDIQMK.N |
|  | 19998 | **663.8350** | **1988.4831** | **1988.8997** | **-0.4166** | **1** | **0** | **54** | **10** | **R.SSTETCYSAIPKASSDASK.V** |
|  | 20120 | 1001.0435 | 2000.0724 | 1999.9269 | 0.1455 | 0 | 4 | 37 | 4 | K.IPEINSSDMSAHVTSPSGR.V 20129 |
|  | 20141 | **501.5176** | **2002.0412** | **2002.0537** | **-0.0125** | **0** | **12** | **6.4** | **1** | **K.HTIAVVWGGVNIPHSPYR.V** |
|  | 20258 | **1008.9846** | **2015.9547** | **2015.9663** | **-0.0116** | **0** | **68** | **1.5e-005** | **1** | **R.YMIGVTYGGDDIPLSPYR.I** 20268 |
|  | 20635 | **1023.5330** | **2045.0514** | **2045.0641** | **-0.0127** | **0** | **112** | **5.6e-010** | **1** | **R.LVSPGSANETSSILVESVTR.S** 20634 |
|  | 20913 | **692.3232** | **2073.9479** | **2074.9630** | **-1.0151** | **1** | **1** | **64** | **3** | **R.GLSEGRTFEMSDFIVDTR.D** |
|  | 21498 | **532.2457** | **2124.9538** | **2124.1175** | **0.8363** | **1** | **5** | **26** | **4** | **R.DAGEGLLAVQITDQEGKPKR.A** |
|  | 23020 | 1148.5598 | 2295.1051 | 2295.1167 | -0.0116 | 0 | 63 | 3.6e-005 | 1 | K.DGTYAVTYVPLTAGMYTLTMK.Y |
|  | 23636 | **596.0477** | **2380.1616** | **2380.1733** | **-0.0117** | **1** | **3** | **39** | **6** | **K.ADIEMPFDPSKVVASGPGLEHGK.V** |
|  | 23976 | **810.0552** | **2427.1439** | **2427.1596** | **-0.0157** | **0** | **41** | **0.0053** | **1** | **K.AGSNMLLIGVHGPTTPCEEVSMK.H** |
|  | 24461 | **830.4434** | **2488.3083** | **2487.2758** | **1.0325** | **1** | **3** | **32** | **4** | **K.AEGPGLSKAGVENGKPTHFTVYTK.G** |
|  | 24833 | **1274.1512** | **2546.2879** | **2546.3017** | **-0.0137** | **0** | **56** | **0.00018** | **1** | **K.VTASGPGLSSYGVPASLPVDFAIDAR.D** |
|  | 27845 | **1041.1469** | **3120.4187** | **3121.4269** | **-1.0081** | **1** | **0** | **42** | **5** | **K.MDCQETPEGYKVMYTPMAPGNYLISVK.Y** |
|  | 27957 | 1051.7643 | 3152.2710 | 3153.4167 | -1.1457 | 1 | (0) | 35 | 10 | K.MDCQETPEGYKVMYTPMAPGNYLISVK.Y |
|  | 28642 | **1108.5327** | **3322.5763** | **3323.5340** | **-0.9576** | **0** | **1** | **34** | **1** | **K.ALGALVDSCAPGLCPDWESWDPQKPVDNAR.E** |
|  | 30262 | **980.4788** | **3917.8862** | **3917.9323** | **-0.0462** | **0** | **1** | **23** | **4** | **K.GPGLEAVGNIANKPTYFDIYTAGAGVGDIGVEVEDPQGK.N** |
|  | 30949 | **1500.1398** | **4497.3975** | **4496.3075** | **1.0900** | **1** | **1** | **14** | **6** | **K.VTASGPGLSSYGVPASLPVDFAIDARDAGEGLLAVQITDQEGKPK.R** |

| **107.** | P35749\|MYH11_HUMAN    **Mass:** 228054   **Score:** 389    **Queries matched:** 67   **emPAI:** 0.10 |
| --- | --- |
|  | Myosin-11 OS=Homo sapiens GN=MYH11 PE=1 SV=3 |

|  | **Query** | **Observed** | **Mr(expt)** | **Mr(calc)** | **Delta** | **Miss** | **Score** | **Expect** | **Rank** | **Peptide** |
| --- | --- | --- | --- | --- | --- | --- | --- | --- | --- | --- |
|  | 970 | 533.1628 | 532.1555 | 531.3017 | 0.8539 | 0 | 10 | 28 | 1 | R.IGQSK.I |
|  | 1092 | **559.4482** | **558.4410** | **558.3125** | **0.1284** | **0** | **6** | **74** | **1** | **K.NNALK.K** |
|  | 1182 | 577.2718 | 576.2646 | 575.2663 | 0.9983 | 0 | 8 | 37 | 4 | K.NESAR.Q |
|  | 1234 | **587.3912** | **586.3839** | **587.3279** | **-0.9440** | **0** | **23** | **1.2** | **4** | **R.DVVQK.A** |
|  | 1333 | 609.3198 | 608.3125 | 608.3170 | -0.0045 | 0 | 2 | 79 | 9 | K.SFVEK.L |
|  | 1503 | 648.3270 | 647.3197 | 646.3802 | 0.9395 | 0 | 8 | 41 | 8 | K.FQKPK.Q |
|  | 1780 | 719.3887 | 718.3815 | 717.4207 | 0.9608 | 1 | 11 | 18 | 3 | K.MQLAKK.E |
|  | 1945 | 768.3584 | 767.3511 | 767.4218 | -0.0707 | 0 | 11 | 13 | 2 | K.FLFVDK.N |
|  | 2309 | 809.4545 | 808.4472 | 807.4491 | 0.9981 | 0 | 15 | 4.9 | 1 | R.TVGQLYK.E 2303 |
|  | 2413 | **409.4393** | **816.8641** | **816.3977** | **0.4664** | **0** | **8** | **22** | **4** | **R.NALQDEK.R** |
|  | 3090 | 863.0862 | 862.0789 | 861.4742 | 0.6047 | 1 | 0 | 1.3e+002 | 2 | R.KLQAQMK.D |
|  | 3607 | **447.7480** | **893.4815** | **893.4494** | **0.0321** | **0** | **12** | **11** | **6** | **R.DEIFATAK.E** |
|  | 3684 | **450.2680** | **898.5215** | **898.5348** | **-0.0133** | **1** | **17** | **2.8** | **2** | **K.QRALAAAAK.K** 3670 |
|  | 3981 | 460.2690 | 918.5233 | 918.3930 | 0.1303 | 0 | 12 | 11 | 4 | R.LEEEEDR.G |
|  | 4138 | **466.2389** | **930.4633** | **929.5182** | **0.9452** | **0** | **5** | **55** | **9** | **R.QLVSNLEK.K** |
|  | 4222 | **469.7896** | **937.5645** | **937.5022** | **0.0624** | **0** | **2** | **95** | **6** | **R.IVFQEFR.Q** |
|  | 4396 | 475.2377 | 948.4608 | 949.4036 | -0.9428 | 1 | 2 | 1e+002 | 8 | K.CSDGERAR.A |
|  | 5696 | 523.2863 | 1044.5579 | 1044.5637 | -0.0058 | 0 | 14 | 7.2 | 2 | R.LEVNMQALK.G |
|  | 6096 | 537.2975 | 1072.5804 | 1072.6128 | -0.0324 | 1 | 8 | 28 | 8 | R.EQEVTVLKK.A |
|  | 6504 | 552.2996 | 1102.5846 | 1102.5917 | -0.0071 | 1 | 29 | 0.2 | 1 | K.RQQQLTAMK.V 6503 |
|  | 8182 | **405.2994** | **1212.8762** | **1213.5509** | **-0.6747** | **0** | **6** | **25** | **3** | **R.SHEAQVQEMR.Q** |
|  | 8351 | 611.3470 | 1220.6794 | 1219.6448 | 1.0346 | 1 | 10 | 17 | 2 | R.KFDQLLAEEK.N |
|  | 8492 | **615.7869** | **1229.5592** | **1228.7027** | **0.8565** | **1** | **1** | **1.1e+002** | **9** | **K.LQLEKVTAEAK.I** |
|  | 8869 | 417.5520 | 1249.6342 | 1248.6211 | 1.0131 | 1 | 4 | 53 | 9 | R.GNETSFVPSRR.S |
|  | 9001 | 629.3394 | 1256.6643 | 1256.6724 | -0.0081 | 1 | 62 | 9e-005 | 1 | K.KEEELQAALAR.L 9000 |
|  | 9276 | 636.3421 | 1270.6696 | 1271.6721 | -1.0024 | 1 | 6 | 33 | 7 | K.RDLGEELEALK.T |
|  | 9309 | **636.8474** | **1271.6803** | **1271.6721** | **0.0082** | **0** | **4** | **53** | **6** | **K.LEAQVQELQSK.C** |
|  | 11069 | 685.8475 | 1369.6804 | 1370.7405 | -1.0601 | 1 | 4 | 49 | 3 | K.EILLQVEDERK.M |
|  | 11271 | **691.3381** | **1380.6616** | **1381.6759** | **-1.0143** | **1** | **9** | **17** | **2** | **K.MTESSLPSASKTK.K** 11273 |
|  | 11548 | **699.2822** | **1396.5498** | **1396.6326** | **-0.0828** | **1** | **2** | **83** | **6** | **K.RALETQMEEMK.T** |
|  | 13032 | **496.2269** | **1485.6588** | **1485.6994** | **-0.0406** | **1** | **2** | **74** | **9** | **R.SHEAQVQEMRQK.H** |
|  | 13438 | **756.8572** | **1511.6999** | **1511.7211** | **-0.0212** | **1** | **5** | **35** | **3** | **K.MLKAEMEDLVSSK.D** |
|  | 13860 | **514.7596** | **1541.2569** | **1541.7685** | **-0.5116** | **0** | **6** | **20** | **7** | **K.IAQLEEQVEQEAR.E** |
|  | 14002 | 776.3459 | 1550.6772 | 1550.6882 | -0.0110 | 0 | 45 | 0.0039 | 1 | R.EDQSILCTGESGAGK.T |
|  | 14027 | **776.8448** | **1551.6750** | **1550.6518** | **1.0232** | **0** | **5** | **41** | **7** | **R.ELDEATESNEAMGR.E** |
|  | 14298 | **524.2418** | **1569.7036** | **1570.7773** | **-1.0737** | **1** | **5** | **36** | **5** | **K.NKHESMISELEVR.L** |
|  | 14329 | **524.9512** | **1571.8319** | **1570.7306** | **1.1013** | **0** | **4** | **57** | **5** | **K.ITDVIMAFQAMCR.G** |
|  | 14517 | **397.2228** | **1584.8621** | **1585.8134** | **-0.9512** | **1** | **3** | **56** | **5** | **K.HESMISELEVRLK.K** |
|  | 14602 | 796.3493 | 1590.6840 | 1590.6944 | -0.0103 | 0 | 75 | 3.9e-006 | 1 | R.NTDQASMPDNTAAQK.V 14603 |
|  | 14810 | 536.2308 | 1605.6705 | 1606.6893 | -1.0188 | 0 | (8) | 18 | 2 | R.NTDQASMPDNTAAQK.V |
|  | 15230 | **544.2867** | **1629.8384** | **1630.8103** | **-0.9719** | **0** | **8** | **19** | **1** | **K.NFINSPVAQADWAAK.R** |
|  | 15704 | **831.9428** | **1661.8709** | **1661.8559** | **0.0151** | **1** | **13** | **5.3** | **3** | **R.LEVNMQALKGQFER.D** |
|  | 16600 | 863.9730 | 1725.9314 | 1725.9413 | -0.0100 | 0 | 61 | 7.6e-005 | 1 | K.QLLQANPILEAFGNAK.T |
|  | 16887 | 874.4332 | 1746.8519 | 1745.8407 | 1.0112 | 1 | 1 | 85 | 5 | K.SFVEKLCTEQGSHPK.F |
|  | 16917 | **584.2922** | **1749.8549** | **1750.8964** | **-1.0415** | **1** | **1** | **81** | **4** | **K.HLPIYSEKIVDMYK.G** |
|  | 17449 | **596.8117** | **1787.4133** | **1786.9114** | **0.5019** | **1** | **2** | **42** | **9** | **K.NFINSPVAQADWAAKR.L** |
|  | 17491 | **597.6547** | **1789.9422** | **1789.8945** | **0.0477** | **1** | **1** | **94** | **7** | **R.ISDLTTNLAEEEEKAK.N** |
|  | 18136 | **613.4894** | **1837.4463** | **1836.9444** | **0.5019** | **1** | **5** | **21** | **1** | **R.YEILAANAIPKGFMDGK.Q** |
|  | 18844 | **630.6019** | **1888.7838** | **1888.0094** | **0.7744** | **1** | **1** | **72** | **4** | **R.LVWVPSEKQGFEAASIK.E** |
|  | 19715 | 981.4571 | 1960.8997 | 1960.9113 | -0.0115 | 0 | 123 | 4.4e-011 | 1 | K.TQLEELEDELQATEDAK.L 19716 |
|  | 20153 | **668.7888** | **2003.3446** | **2002.9881** | **0.3565** | **1** | **6** | **15** | **3** | **K.LEDEILVMDDQNNKLSK.E** |
|  | 20273 | 673.3406 | 2017.0001 | 2017.9626 | -0.9625 | 0 | 1 | 76 | 3 | K.SLEADLMQLQEDLAAAER.A |
|  | 20296 | **674.0273** | **2019.0602** | **2018.9830** | **0.0772** | **1** | **(3)** | **44** | **5** | **K.LEDEILVMDDQNNKLSK.E** |
|  | 20573 | 1021.0319 | 2040.0493 | 2041.0327 | -0.9834 | 1 | 4 | 37 | 1 | K.EEELQAALARLDDEIAQK.N |
|  | 20583 | **1021.4736** | **2040.9327** | **2039.9999** | **0.9328** | **1** | **8** | **15** | **1** | **K.RHEMPPHIYAIADTAYR.S** |
|  | 20605 | **681.9905** | **2042.9496** | **2042.0830** | **0.8666** | **1** | **0** | **84** | **4** | **K.VKPLLQVTRQEEEMQAK.E** |
|  | 22908 | 761.3457 | 2281.0153 | 2281.0314 | -0.0162 | 1 | 15 | 2.3 | 1 | R.SMLQDREDQSILCTGESGAGK.T |
|  | 26066 | **913.4018** | **2737.1835** | **2737.2170** | **-0.0335** | **1** | **2** | **34** | **1** | **R.IAQLEEELEEEQGNMEAMSDRVR.K** |
|  | 28615 | **1104.8646** | **3311.5720** | **3311.5616** | **0.0105** | **1** | **11** | **3.6** | **2** | **K.VDYNASAWLTKNMDPLNDNVTSLLNASSDK.F** 28616 |

| **108.** | P27216\|ANX13_HUMAN    **Mass:** 35669    **Score:** 389    **Queries matched:** 39   **emPAI:** 2.02 |
| --- | --- |
|  | Annexin A13 OS=Homo sapiens GN=ANXA13 PE=1 SV=2 |

|  | **Query** | **Observed** | **Mr(expt)** | **Mr(calc)** | **Delta** | **Miss** | **Score** | **Expect** | **Rank** | **Peptide** |
| --- | --- | --- | --- | --- | --- | --- | --- | --- | --- | --- |
|  | 1237 | **588.3675** | **587.3602** | **586.3802** | **0.9800** | **0** | **8** | **53** | **3** | **R.IVVTR.A** |
|  | 2276 | **807.3970** | **806.3898** | **806.3956** | **-0.0059** | **0** | **18** | **2.7** | **1** | **K.SLSDMVR.S** |
|  | 2462 | **411.7553** | **821.4961** | **822.3905** | **-0.8945** | **0** | **(7)** | **31** | **2** | **K.SLSDMVR.S** |
|  | 2667 | **418.2530** | **834.4915** | **834.4963** | **-0.0048** | **0** | **37** | **0.038** | **1** | **K.AYLTLVR.C** |
|  | 3018 | **859.4711** | **858.4638** | **858.4698** | **-0.0060** | **0** | **29** | **0.29** | **1** | **K.ELEEVLK.S** |
|  | 3467 | **885.0679** | **884.0607** | **883.3672** | **0.6935** | **0** | **1** | **80** | **6** | **R.SDTSGDFR.K** |
|  | 3784 | **453.3408** | **904.6671** | **905.6062** | **-0.9391** | **1** | **5** | **53** | **1** | **R.KLLVALLH.-** |
|  | 4706 | **486.7683** | **971.5220** | **971.5287** | **-0.0067** | **0** | **16** | **4.6** | **3** | **R.AEVDLQGIK.A** |
|  | 5024 | **498.2634** | **994.5122** | **994.4356** | **0.0767** | **0** | **29** | **0.18** | **1** | **K.DLYDAGEGR.W** 5012 5019 |
|  | 5237 | **505.7397** | **1009.4649** | **1009.4716** | **-0.0067** | **0** | **12** | **9** | **6** | **K.SELSGNFEK.T** |
|  | 5262 | **506.7346** | **1011.4547** | **1011.4621** | **-0.0074** | **1** | **24** | **0.57** | **1** | **R.SDTSGDFRK.L** |
|  | 6793 | **563.8472** | **1125.6799** | **1125.6870** | **-0.0071** | **0** | **69** | **1.9e-005** | **1** | **K.ILVSLLQANR.N** 6791 |
|  | 7237 | 581.2871 | 1160.5597 | 1160.5673 | -0.0077 | 0 | 45 | 0.0048 | 1 | K.GAGTDEETLIR.I |
|  | 8723 | **414.5531** | **1240.6375** | **1241.6074** | **-0.9699** | **1** | **4** | **61** | **7** | **K.YQKSLSDMVR.S** 8744 |
|  | 8956 | **628.3733** | **1254.7321** | **1253.7819** | **0.9502** | **1** | **9** | **17** | **3** | **K.KILVSLLQANR.N** |
|  | 10470 | **667.9755** | **1333.9365** | **1332.7401** | **1.1964** | **1** | **1** | **81** | **4** | **K.EIIAIKEAYQR.L** |
|  | 10773 | **676.8783** | **1351.7420** | **1351.7500** | **-0.0079** | **0** | **23** | **0.61** | **1** | **R.ATFQAYQILIGK.D** 10752 |
|  | 11224 | **460.2559** | **1377.7458** | **1378.7344** | **-0.9885** | **1** | **5** | **41** | **6** | **K.ATYGKELEEVLK.S** |
|  | 13136 | **746.8497** | **1491.6848** | **1491.6954** | **-0.0106** | **1** | **40** | **0.015** | **1** | **K.ASSPQGFDVDRDAK.K** |
|  | 13973 | **775.3847** | **1548.7548** | **1548.7631** | **-0.0083** | **1** | **23** | **0.63** | **1** | **R.SLESDVKGDTSGNLK.K** |
|  | 14185 | 782.2904 | 1562.5663 | 1562.5766 | -0.0103 | 0 | 38 | 0.015 | 1 | R.CAQDCEDYFAER.L |
|  | 14636 | **796.8968** | **1591.7791** | **1591.7882** | **-0.0091** | **0** | **81** | **8.9e-007** | **1** | **R.WGTDELAFNEVLAK.R** 14635 |
|  | 15252 | **816.4208** | **1630.8270** | **1630.8348** | **-0.0078** | **0** | **93** | **5.9e-008** | **1** | **K.GMGTNEAAIIEILSGR.T** 15250 |
|  | 15484 | **549.6299** | **1645.8680** | **1645.8787** | **-0.0107** | **0** | **25** | **0.34** | **1** | **K.TALALLDRPSEYAAR.Q** 15498 |
|  | 15496 | **824.4167** | **1646.8188** | **1646.8297** | **-0.0109** | **0** | **(43)** | **0.0062** | **1** | **K.GMGTNEAAIIEILSGR.T** 15481 |
|  | 16097 | **564.2844** | **1689.8314** | **1688.7489** | **1.0825** | **1** | **7** | **23** | **1** | **R.NEGDDVDKDLAGQDAK.D** |
|  | 18341 | **926.4688** | **1850.9231** | **1849.9309** | **0.9922** | **1** | **49** | **0.0013** | **1** | **K.ELEEVLKSELSGNFEK.T** 18319 18335 |
|  | 20164 | **669.2872** | **2004.8399** | **2005.9925** | **-1.1526** | **1** | **3** | **42** | **9** | **K.ACKGMGTNEAAIIEILSGR.T** |

| **109.** | O75907\|DGAT1_HUMAN    **Mass:** 55699    **Score:** 383    **Queries matched:** 16   **emPAI:** 0.68 |
| --- | --- |
|  | Diacylglycerol O-acyltransferase 1 OS=Homo sapiens GN=DGAT1 PE=2 SV=2 |

|  | **Query** | **Observed** | **Mr(expt)** | **Mr(calc)** | **Delta** | **Miss** | **Score** | **Expect** | **Rank** | **Peptide** |
| --- | --- | --- | --- | --- | --- | --- | --- | --- | --- | --- |
|  | 1985 | **786.3727** | **785.3655** | **785.3014** | **0.0641** | **0** | **(9)** | **23** | **3** | **K.DMDYSR.I** |
|  | 2212 | **402.2438** | **802.4731** | **801.2963** | **1.1768** | **0** | **12** | **14** | **2** | **K.DMDYSR.I** |
|  | 6347 | **546.2908** | **1090.5670** | **1090.5746** | **-0.0076** | **0** | **9** | **22** | **5** | **R.HFYKPMLR.R** |
|  | 12524 | **728.3299** | **1454.6452** | **1454.6539** | **-0.0086** | **0** | **49** | **0.0017** | **1** | **K.DGDAGVGSGHWELR.C** 12525 |
|  | 17894 | **912.4115** | **1822.8084** | **1821.8170** | **0.9915** | **0** | **96** | **3e-008** | **1** | **R.LQDSLFSSDSGFSNYR.G** 17880 |
|  | 18059 | **916.9360** | **1831.8575** | **1831.8700** | **-0.0125** | **0** | **81** | **7.5e-007** | **1** | **R.DAAAGPDVGAAGDAPAPAPNK.D** |
|  | 20198 | **670.3146** | **2007.9221** | **2007.9358** | **-0.0137** | **0** | **50** | **0.0009** | **1** | **R.TGSRPSSHGGGGPAAAEEEVR.D** 20202 |
|  | 20301 | **1010.9827** | **2019.9509** | **2019.9650** | **-0.0141** | **0** | **78** | **1.6e-006** | **1** | **K.ASSAAAPHTVSYPDNLTYR.D** 20299 20300 |
|  | 21902 | **722.3630** | **2164.0671** | **2164.0369** | **0.0302** | **1** | **9** | **11** | **3** | **R.RTGSRPSSHGGGGPAAAEEEVR.D** |
|  | 26057 | **912.7562** | **2735.2467** | **2735.2557** | **-0.0091** | **0** | **7** | **13** | **1** | **R.DWWNSESVTYFWQNWNIPVHK.W** |
|  | 28511 | **1090.5044** | **3268.4913** | **3268.5134** | **-0.0220** | **1** | **54** | **0.00018** | **1** | **R.DAAAGPDVGAAGDAPAPAPNKDGDAGVGSGHWELR.C** |

| **110.** | P63104\|1433Z_HUMAN    **Mass:** 27899    **Score:** 380    **Queries matched:** 17   **emPAI:** 1.15 |
| --- | --- |
|  | 14-3-3 protein zeta/delta OS=Homo sapiens GN=YWHAZ PE=1 SV=1 |

|  | **Query** | **Observed** | **Mr(expt)** | **Mr(calc)** | **Delta** | **Miss** | **Score** | **Expect** | **Rank** | **Peptide** |
| --- | --- | --- | --- | --- | --- | --- | --- | --- | --- | --- |
|  | 3811 | **454.2629** | **906.5113** | **906.5174** | **-0.0062** | **0** | **20** | **1.8** | **2** | **R.NLLSVAYK.N** |
|  | 4144 | **466.7147** | **931.4149** | **931.4222** | **-0.0072** | **1** | **16** | **4.3** | **1** | **K.MKGDYYR.Y** |
|  | 6509 | **552.7081** | **1103.4016** | **1103.4086** | **-0.0070** | **0** | **59** | **0.0002** | **1** | **R.YDDMAACMK.S** |
|  | 6583 | **555.3574** | **1108.7002** | **1107.5495** | **1.1507** | **0** | **21** | **1.1** | **1** | **K.EMQPTHPIR.L** |
|  | 6931 | **568.7974** | **1135.5803** | **1135.3984** | **0.1819** | **0** | **(6)** | **37** | **9** | **R.YDDMAACMK.S** |
|  | 7104 | **576.2781** | **1150.5416** | **1150.5506** | **-0.0090** | **0** | **10** | **14** | **2** | **R.YLAEVAAGDDK.K** |
|  | 9423 | **640.3257** | **1278.6368** | **1278.6456** | **-0.0087** | **1** | **30** | **0.15** | **1** | **R.YLAEVAAGDDKK.G** 9425 |
|  | 13956 | **774.8554** | **1547.6961** | **1547.7063** | **-0.0102** | **0** | **55** | **0.00042** | **1** | **K.SVTEQGAELSNEER.N** 13955 13957 |
|  | 20586 | **1021.4914** | **2040.9682** | **2039.9800** | **0.9882** | **0** | **105** | **2.6e-009** | **1** | **K.GIVDQSQQAYQEAFEISK.K** 20569 20570 |
|  | 21551 | **1066.4939** | **2130.9732** | **2130.9844** | **-0.0112** | **0** | **74** | **3.3e-006** | **1** | **K.TAFDEAIAELDTLSEESYK.D** 21552 |
|  | 28586 | **1101.5450** | **3301.6133** | **3301.6275** | **-0.0142** | **1** | **65** | **1.4e-005** | **1** | **K.TAFDEAIAELDTLSEESYKDSTLIMQLLR.D** |

| **111.** | P49748\|ACADV_HUMAN    **Mass:** 70745    **Score:** 378    **Queries matched:** 30   **emPAI:** 0.58 |
| --- | --- |
|  | Very long-chain specific acyl-CoA dehydrogenase, mitochondrial OS=Homo sapiens GN=ACADVL PE=1 SV=1 |

|  | **Query** | **Observed** | **Mr(expt)** | **Mr(calc)** | **Delta** | **Miss** | **Score** | **Expect** | **Rank** | **Peptide** |
| --- | --- | --- | --- | --- | --- | --- | --- | --- | --- | --- |
|  | 2837 | **424.2400** | **846.4655** | **847.5167** | **-1.0513** | **0** | **7** | **39** | **1** | **K.GILLFGTK.A** |
|  | 3503 | **443.7256** | **885.4367** | **885.4419** | **-0.0051** | **0** | **26** | **0.52** | **1** | **K.SFAVGMFK.G** |
|  | 4089 | **464.7554** | **927.4962** | **927.5025** | **-0.0063** | **0** | **20** | **1.5** | **1** | **K.ELVEPVSR.F** |
|  | 5151 | **502.2446** | **1002.4746** | **1002.4804** | **-0.0058** | **0** | **11** | **16** | **5** | **R.TSAVPSPCGK.Y** |
|  | 7004 | **571.8131** | **1141.6117** | **1142.6520** | **-1.0403** | **1** | **2** | **90** | **3** | **R.QLLRLGGGSSR.L** |
|  | 7568 | **593.1536** | **1184.2926** | **1184.5422** | **-0.2496** | **0** | **8** | **17** | **1** | **K.SDSHPSDALTR.K** |
|  | 7841 | **599.8285** | **1197.6425** | **1197.6506** | **-0.0080** | **0** | **29** | **0.19** | **1** | **K.IHNFGLIQEK.L** |
|  | 8128 | 404.5618 | 1210.6635 | 1211.5791 | -0.9156 | 0 | 15 | 4.4 | 3 | R.FGMAAALAGTMR.G |
|  | 9707 | **647.7856** | **1293.5567** | **1293.6281** | **-0.0714** | **1** | **8** | **22** | **6** | **-.MQAARMAASLGR.Q** |
|  | 10121 | **658.8655** | **1315.7165** | **1315.7248** | **-0.0083** | **0** | **58** | **0.00023** | **1** | **K.GIVNEQFLLQR.L** |
|  | 11849 | 707.3728 | 1412.7310 | 1411.6732 | 1.0578 | 0 | 22 | 0.83 | 2 | K.ASNTAEVFFDGVR.V |
|  | 11986 | **711.0920** | **1420.1694** | **1419.6742** | **0.4952** | **0** | **24** | **0.33** | **1** | **R.SLSEGHPTAQHEK.M** 11970 |
|  | 12393 | **723.4352** | **1444.8559** | **1443.8198** | **1.0361** | **1** | **31** | **0.11** | **1** | **K.KGIVNEQFLLQR.L** |
|  | 13812 | **513.7413** | **1538.2020** | **1537.8729** | **0.3291** | **1** | **4** | **29** | **7** | **K.IHNFGLIQEKLAR.M** |
|  | 14640 | **797.3864** | **1592.7583** | **1592.8675** | **-0.1092** | **1** | **10** | **12** | **2** | **R.IFRIFEGTNDILR.L** 14650 |
|  | 18057 | **916.9322** | **1831.8499** | **1831.7977** | **0.0522** | **0** | **5** | **33** | **1** | **K.VTDECIQIMGGMGFMK.E** |
|  | 18343 | **926.5002** | **1850.9859** | **1850.9931** | **-0.0071** | **0** | **63** | **5.1e-005** | **1** | **K.LWISNGGLADIFTVFAK.T** 18364 |
|  | 20315 | **1011.4670** | **2020.9194** | **2020.9312** | **-0.0118** | **0** | **55** | **0.00028** | **1** | **R.EGMAALQSDPWQQELYR.N** 20314 |
|  | 23188 | **773.7314** | **2318.1723** | **2317.1606** | **1.0118** | **1** | **1** | **55** | **8** | **K.VAMHILNNGRFGMAAALAGTMR.G** |
|  | 23827 | **804.7783** | **2411.3131** | **2412.3012** | **-0.9881** | **1** | **1** | **58** | **4** | **K.ELSGLGSALKNPFGNAGLLLGEAGK.Q** |
|  | 25404 | **1318.6534** | **2635.2923** | **2635.3064** | **-0.0141** | **0** | **92** | **3.9e-008** | **1** | **K.ELGAFGLQVPSELGGVGLCNTQYAR.L** 25411 |
|  | 26250 | **926.5002** | **2776.4789** | **2777.4428** | **-0.9639** | **1** | **33** | **0.026** | **1** | **K.YYTLNGSKLWISNGGLADIFTVFAK.T** |
|  | 26279 | **1391.6926** | **2781.3707** | **2781.3861** | **-0.0154** | **0** | **62** | **4e-005** | **1** | **K.GQLTTDQVFPYPSVLNEEQTQFLK.E** 26278 26280 |

| **112.** | P12532\|KCRU_HUMAN    **Mass:** 47406    **Score:** 378    **Queries matched:** 23   **emPAI:** 0.84 |
| --- | --- |
|  | Creatine kinase U-type, mitochondrial OS=Homo sapiens GN=CKMT1A PE=1 SV=1 |

|  | **Query** | **Observed** | **Mr(expt)** | **Mr(calc)** | **Delta** | **Miss** | **Score** | **Expect** | **Rank** | **Peptide** |
| --- | --- | --- | --- | --- | --- | --- | --- | --- | --- | --- |
|  | 1404 | **625.1445** | **624.1372** | **623.3755** | **0.7617** | **0** | **15** | **3.3** | **1** | **R.AGVHIK.L** 1398 |
|  | 3305 | 437.2350 | 872.4555 | 872.3664 | 0.0890 | 0 | 9 | 28 | 1 | R.SGYFDER.Y |
|  | 8111 | **605.8419** | **1209.6692** | **1208.5893** | **1.0799** | **1** | **3** | **65** | **3** | **R.VISMEKGGNMK.R** |
|  | 13988 | **775.4764** | **1548.9382** | **1547.7984** | **1.1399** | **1** | **4** | **41** | **5** | **R.LYPPSAEYPDLRK.H** |
|  | 14385 | **526.2722** | **1575.7947** | **1575.8045** | **-0.0099** | **1** | **6** | **32** | **6** | **R.RLYPPSAEYPDLR.K** 14399 |
|  | 15466 | **823.3972** | **1644.7799** | **1644.7896** | **-0.0097** | **0** | **37** | **0.024** | **1** | **K.SFLIWVNEEDHTR.V** 15446 |
|  | 16819 | **872.4080** | **1742.8015** | **1743.9566** | **-1.1551** | **1** | **2** | **73** | **8** | **-.MAGPFSRLLSARPGLR.L** |
|  | 19818 | **657.4251** | **1969.2535** | **1968.9410** | **0.3125** | **1** | **1** | **51** | **5** | **R.KHNNCMASHLTPAVYAR.L** |
|  | 20333 | **1011.9835** | **2021.9525** | **2021.9655** | **-0.0130** | **0** | **94** | **3.7e-008** | **1** | **R.GTGGVDTAATGGVFDISNLDR.L** 20334 |
|  | 24258 | **821.7497** | **2462.2272** | **2462.2363** | **-0.0090** | **0** | **83** | **3.9e-007** | **1** | **K.SEVELVQLVIDGVNYLIDCER.R** 24256 24257 |
|  | 26283 | **928.4478** | **2782.3216** | **2782.3385** | **-0.0169** | **0** | **13** | **3.1** | **1** | **K.TTPTGWTLDQCIQTGVDNPGHPFIK.T** |
|  | 26977 | **1465.6879** | **2929.3612** | **2929.3692** | **-0.0080** | **0** | **66** | **1.4e-005** | **1** | **K.TVGMVAGDEETYEVFADLFDPVIQER.H** 26974 26975 |
|  | 30096 | **1267.9587** | **3800.8544** | **3800.8753** | **-0.0209** | **0** | **78** | **5.2e-007** | **1** | **R.LSEMTEAEQQQLIDDHFLFDKPVSPLLTAAGMAR.D** 30095 30098 |

| **113.** | P16435\|NCPR_HUMAN    **Mass:** 77097    **Score:** 370    **Queries matched:** 38   **emPAI:** 0.60 |
| --- | --- |
|  | NADPH--cytochrome P450 reductase OS=Homo sapiens GN=POR PE=1 SV=2 |

|  | **Query** | **Observed** | **Mr(expt)** | **Mr(calc)** | **Delta** | **Miss** | **Score** | **Expect** | **Rank** | **Peptide** |
| --- | --- | --- | --- | --- | --- | --- | --- | --- | --- | --- |
|  | 1560 | **659.3422** | **658.3349** | **657.3922** | **0.9428** | **1** | **11** | **20** | **2** | **K.AGRINK.G** |
|  | 2497 | 825.4404 | 824.4332 | 824.3916 | 0.0416 | 0 | 20 | 1.4 | 2 | R.ESSFVEK.M |
|  | 2511 | **413.6945** | **825.3745** | **825.4457** | **-0.0712** | **1** | **8** | **24** | **6** | **R.LSKDAHR.Y** |
|  | 3937 | **458.7511** | **915.4876** | **915.4926** | **-0.0050** | **0** | **35** | **0.062** | **1** | **K.GVATNWLR.A** |
|  | 4152 | **466.7701** | **931.5257** | **931.5313** | **-0.0057** | **0** | **18** | **3.1** | **1** | **R.ALVPMFVR.K** |
|  | 4266 | **941.4668** | **940.4595** | **941.4099** | **-0.9504** | **0** | **16** | **4.1** | **1** | **K.VYMGEMGR.L** 4277 |
|  | 4333 | **473.2566** | **944.4986** | **944.5039** | **-0.0054** | **1** | **25** | **0.62** | **1** | **R.KLNQGTER.H** |
|  | 4441 | **476.3318** | **950.6491** | **951.5178** | **-0.8687** | **0** | **10** | **16** | **1** | **K.FAVFGLGNK.T** |
|  | 4835 | **491.2576** | **980.5006** | **980.4597** | **0.0409** | **1** | **10** | **17** | **5** | **R.KMASSSGEGK.E** 4830 |
|  | 5102 | **500.2994** | **998.5842** | **998.5913** | **-0.0071** | **0** | **3** | **87** | **10** | **K.VYVQHLLK.Q** |
|  | 5171 | 502.7869 | 1003.5593 | 1003.5662 | -0.0069 | 0 | 36 | 0.045 | 1 | K.IQTLTSSVR.E |
|  | 5873 | **530.2760** | **1058.5374** | **1059.6263** | **-1.0888** | **1** | **7** | **34** | **3** | **R.ALVPMFVRK.S** |
|  | 6036 | **535.8032** | **1069.5918** | **1069.5992** | **-0.0074** | **1** | **21** | **1.2** | **1** | **K.RLEQLGAQR.I** |
|  | 6234 | 542.2681 | 1082.5217 | 1081.5193 | 1.0025 | 1 | 4 | 56 | 6 | K.GRYSLDVWS.- |
|  | 6602 | **556.2821** | **1110.5496** | **1110.5570** | **-0.0074** | **1** | **20** | **1.5** | **1** | **K.QDREHLWK.L** |
|  | 7813 | **599.2673** | **1196.5200** | **1196.5284** | **-0.0084** | **0** | **7** | **27** | **1** | **K.TYEHFNAMGK.Y** |
|  | 11456 | **696.3642** | **1390.7138** | **1390.7205** | **-0.0066** | **0** | **15** | **4.6** | **3** | **R.DGALTQLNVAFSR.E** |
|  | 12028 | **712.3415** | **1422.6684** | **1422.6779** | **-0.0095** | **0** | **47** | **0.0027** | **1** | **K.SYENQKPPFDAK.N** |
|  | 12592 | **730.3431** | **1458.6716** | **1458.6813** | **-0.0097** | **0** | **43** | **0.0073** | **1** | **K.EVGETLLYYGCR.R** 12591 |
|  | 13792 | **769.3818** | **1536.7490** | **1536.7606** | **-0.0116** | **0** | **58** | **0.00021** | **1** | **R.HLMHLELDISDSK.I** |
|  | 15340 | **819.4251** | **1636.8357** | **1636.8461** | **-0.0104** | **0** | **61** | **9.2e-005** | **1** | **R.TALTYYLDITNPPR.T** 15358 |
|  | 16291 | **568.6522** | **1702.9348** | **1701.7821** | **1.1528** | **1** | **3** | **57** | **1** | **K.TYEHFNAMGKYVDK.R** |
|  | 24110 | **1224.0866** | **2446.1585** | **2446.1765** | **-0.0179** | **0** | **36** | **0.02** | **1** | **R.YESGDHVAVYPANDSALVNQLGK.I** |
|  | 24181 | **1227.1079** | **2452.2013** | **2452.2121** | **-0.0109** | **0** | **19** | **0.79** | **1** | **R.TNVLYELAQYASEPSEQELLR.K** 24180 |
|  | 24190 | **1227.5706** | **2453.1266** | **2453.1387** | **-0.0121** | **0** | **116** | **1.6e-010** | **1** | **R.IFELGLGDDDGNLEEDFITWR.E** 24189 24192 |
|  | 24217 | **1229.6553** | **2457.2960** | **2457.3090** | **-0.0130** | **0** | **72** | **5.1e-006** | **1** | **K.ATTPVIMVGPGTGVAPFIGFIQER.A** 24218 24219 |
|  | 25572 | **534.0670** | **2665.2984** | **2664.1915** | **1.1070** | **0** | **4** | **28** | **5** | **R.EQFWPAVCEHFGVEATGEESSIR.Q** |
|  | 25951 | **906.1213** | **2715.3420** | **2715.3616** | **-0.0196** | **1** | **68** | **9.5e-006** | **1** | **K.IRYESGDHVAVYPANDSALVNQLGK.I** |
|  | 27323 | **1001.1657** | **3000.4753** | **2999.6055** | **0.8698** | **1** | **1** | **40** | **1** | **K.ATTPVIMVGPGTGVAPFIGFIQERAWLR.Q** |

| **114.** | Q15084\|PDIA6_HUMAN    **Mass:** 48490    **Score:** 366    **Queries matched:** 24   **emPAI:** 0.56 |
| --- | --- |
|  | Protein disulfide-isomerase A6 OS=Homo sapiens GN=PDIA6 PE=1 SV=1 |

|  | **Query** | **Observed** | **Mr(expt)** | **Mr(calc)** | **Delta** | **Miss** | **Score** | **Expect** | **Rank** | **Peptide** |
| --- | --- | --- | --- | --- | --- | --- | --- | --- | --- | --- |
|  | 1581 | **665.3574** | **664.3501** | **664.3544** | **-0.0043** | **0** | **27** | **0.4** | **1** | **K.IFGSNK.N** |
|  | 1626 | **676.3572** | **675.3500** | **675.3551** | **-0.0052** | **0** | **16** | **5.8** | **1** | **R.SDIVSR.A** |
|  | 7696 | **596.2740** | **1190.5335** | **1190.5428** | **-0.0093** | **0** | **6** | **41** | **1** | **K.NRPEDYQGGR.T** 7697 |
|  | 11351 | **693.3729** | **1384.7312** | **1385.7514** | **-1.0203** | **0** | **91** | **1.2e-007** | **1** | **R.TGEAIVDAALSALR.Q** 11368 11372 11373 |
|  | 13000 | **742.8513** | **1483.6880** | **1482.7103** | **0.9777** | **0** | **6** | **34** | **3** | **K.GSFSEQGINEFLR.E** |
|  | 13466 | **757.8727** | **1513.7309** | **1513.7412** | **-0.0103** | **0** | **26** | **0.34** | **1** | **K.NLEPEWAAAASEVK.E** |
|  | 14977 | **808.4207** | **1614.8267** | **1614.8366** | **-0.0098** | **0** | **53** | **0.0006** | **1** | **R.GSTAPVGGGAFPTIVER.E** 14976 |
|  | 18388 | **618.9615** | **1853.8626** | **1854.0574** | **-0.1948** | **1** | **1** | **77** | **5** | **R.TGEAIVDAALSALRQLVK.D** |
|  | 25006 | **853.7269** | **2558.1590** | **2558.2837** | **-0.1247** | **1** | **3** | **31** | **1** | **K.NRPEDYQGGRTGEAIVDAALSALR.Q** |
|  | 25118 | **861.1043** | **2580.2911** | **2580.3085** | **-0.0174** | **1** | **10** | **6.5** | **1** | **K.VGAVDADKHHSLGGQYGVQGFPTIK.I** |
|  | 25412 | **1319.1798** | **2636.3451** | **2636.3585** | **-0.0134** | **0** | **58** | **0.00011** | **1** | **R.ALDLFSDNAPPPELLEIINEDIAK.R** 25403 25404 25411 25413 25414 |
|  | 26168 | **1379.6309** | **2757.2472** | **2757.2604** | **-0.0132** | **1** | **72** | **3.7e-006** | **1** | **R.DGELPVEDDIDLSDVELDDLGKDEL.-** |
|  | 26332 | **931.8239** | **2792.4499** | **2792.4596** | **-0.0097** | **1** | **46** | **0.0016** | **1** | **R.ALDLFSDNAPPPELLEIINEDIAKR.T** 26331 |

| **115.** | P58876\|H2B1D_HUMAN    **Mass:** 13928    **Score:** 365    **Queries matched:** 35   **emPAI:** 1.71 |
| --- | --- |
|  | Histone H2B type 1-D OS=Homo sapiens GN=HIST1H2BD PE=1 SV=2 |

|  | **Query** | **Observed** | **Mr(expt)** | **Mr(calc)** | **Delta** | **Miss** | **Score** | **Expect** | **Rank** | **Peptide** |
| --- | --- | --- | --- | --- | --- | --- | --- | --- | --- | --- |
|  | 1143 | **571.3412** | **570.3339** | **569.3173** | **1.0166** | **0** | **9** | **19** | **7** | **K.SAPAPK.K** |
|  | 2387 | **408.7295** | **815.4444** | **815.4501** | **-0.0057** | **0** | **22** | **1.3** | **2** | **R.EIQTAVR.L** |
|  | 2539 | **414.7127** | **827.4107** | **827.4137** | **-0.0030** | **0** | **32** | **0.11** | **1** | **K.HAVSEGTK.A** 2534 2557 2561 2562 2563 2564 |
|  | 4465 | **477.3016** | **952.5887** | **952.5957** | **-0.0070** | **0** | **39** | **0.017** | **1** | **R.LLLPGELAK.H** 4466 4467 |
|  | 6940 | **1137.5386** | **1136.5313** | **1136.5390** | **-0.0077** | **0** | **9** | **17** | **1** | **K.ESYSVYVYK.V** |
|  | 7343 | **585.2951** | **1168.5756** | **1167.5884** | **0.9872** | **0** | **11** | **11** | **1** | **K.QVHPDTGISSK.A** 7326 7352 |
|  | 9163 | **633.3196** | **1264.6247** | **1264.6339** | **-0.0092** | **1** | **54** | **0.00052** | **1** | **R.KESYSVYVYK.V** 9140 9164 |
|  | 9218 | **634.8256** | **1267.6367** | **1268.6434** | **-1.0068** | **1** | **12** | **7.8** | **6** | **-.MPEPTKSAPAPK.K** 9255 9261 |
|  | 12125 | **715.3318** | **1428.6490** | **1428.7473** | **-0.0983** | **1** | **6** | **32** | **3** | **R.IAGEASRLAHYNK.R** |
|  | 16824 | **872.4091** | **1742.8036** | **1742.8120** | **-0.0084** | **0** | **113** | **5.2e-010** | **1** | **K.AMGIMNSFVNDIFER.I** 16819 16820 16822 16823 16835 16836 16837 16839 16840 16841 |
|  | 17071 | **880.4634** | **1758.9122** | **1758.8069** | **0.1053** | **0** | **(4)** | **46** | **1** | **K.AMGIMNSFVNDIFER.I** |

|  | **Proteins matching a subset of these peptides:** |
| --- | --- |

|  | Q5QNW6\|H2B2F_HUMAN    **Mass:** 13912    **Score:** 365    **Queries matched:** 32 |
| --- | --- |
|  | Histone H2B type 2-F OS=Homo sapiens GN=HIST2H2BF PE=1 SV=3 |

|  | O60814\|H2B1K_HUMAN    **Mass:** 13882    **Score:** 365    **Queries matched:** 32 |
| --- | --- |
|  | Histone H2B type 1-K OS=Homo sapiens GN=HIST1H2BK PE=1 SV=3 |

|  | P62807\|H2B1C_HUMAN    **Mass:** 13811    **Score:** 365    **Queries matched:** 32 |
| --- | --- |
|  | Histone H2B type 1-C/E/F/G/I OS=Homo sapiens GN=HIST1H2BC PE=1 SV=3 |

|  | Q93079\|H2B1H_HUMAN    **Mass:** 13884    **Score:** 365    **Queries matched:** 32 |
| --- | --- |
|  | Histone H2B type 1-H OS=Homo sapiens GN=HIST1H2BH PE=1 SV=3 |

|  | P57053\|H2BFS_HUMAN    **Mass:** 13936    **Score:** 365    **Queries matched:** 31 |
| --- | --- |
|  | Histone H2B type F-S OS=Homo sapiens GN=H2BFS PE=1 SV=2 |

|  | Q99879\|H2B1M_HUMAN    **Mass:** 13981    **Score:** 365    **Queries matched:** 31 |
| --- | --- |
|  | Histone H2B type 1-M OS=Homo sapiens GN=HIST1H2BM PE=1 SV=3 |

| **116.** | Q99880\|H2B1L_HUMAN    **Mass:** 13944    **Score:** 365    **Queries matched:** 33   **emPAI:** 1.69 |
| --- | --- |
|  | Histone H2B type 1-L OS=Homo sapiens GN=HIST1H2BL PE=1 SV=3 |

|  | **Query** | **Observed** | **Mr(expt)** | **Mr(calc)** | **Delta** | **Miss** | **Score** | **Expect** | **Rank** | **Peptide** |
| --- | --- | --- | --- | --- | --- | --- | --- | --- | --- | --- |
|  | 1084 | **558.3552** | **557.3479** | **556.3220** | **1.0259** | **0** | **11** | **17** | **2** | **M.PELAK.S** |
|  | 1143 | 571.3412 | 570.3339 | 569.3173 | 1.0166 | 0 | 9 | 19 | 7 | K.SAPAPK.K |
|  | 2387 | 408.7295 | 815.4444 | 815.4501 | -0.0057 | 0 | 22 | 1.3 | 2 | R.EIQTAVR.L |
|  | 2539 | 414.7127 | 827.4107 | 827.4137 | -0.0030 | 0 | 32 | 0.11 | 1 | K.HAVSEGTK.A 2534 2557 2561 2562 2563 2564 |
|  | 4465 | 477.3016 | 952.5887 | 952.5957 | -0.0070 | 0 | 39 | 0.017 | 1 | R.LLLPGELAK.H 4466 4467 |
|  | 6940 | 1137.5386 | 1136.5313 | 1136.5390 | -0.0077 | 0 | 9 | 17 | 1 | K.ESYSVYVYK.V |
|  | 7343 | 585.2951 | 1168.5756 | 1167.5884 | 0.9872 | 0 | 11 | 11 | 1 | K.QVHPDTGISSK.A 7326 7352 |
|  | 8934 | **628.2241** | **1254.4337** | **1254.6642** | **-0.2305** | **1** | **3** | **61** | **7** | **-.MPELAKSAPAPK.K** |
|  | 9163 | 633.3196 | 1264.6247 | 1264.6339 | -0.0092 | 1 | 54 | 0.00052 | 1 | R.KESYSVYVYK.V 9140 9164 |
|  | 16824 | 872.4091 | 1742.8036 | 1742.8120 | -0.0084 | 0 | 113 | 5.2e-010 | 1 | K.AMGIMNSFVNDIFER.I 16819 16820 16822 16823 16835 16836 16837 16839 16840 16841 |
|  | 17071 | 880.4634 | 1758.9122 | 1758.8069 | 0.1053 | 0 | (4) | 46 | 1 | K.AMGIMNSFVNDIFER.I |

| **117.** | Q99877\|H2B1N_HUMAN    **Mass:** 13914    **Score:** 365    **Queries matched:** 34   **emPAI:** 1.71 |
| --- | --- |
|  | Histone H2B type 1-N OS=Homo sapiens GN=HIST1H2BN PE=1 SV=3 |

|  | **Query** | **Observed** | **Mr(expt)** | **Mr(calc)** | **Delta** | **Miss** | **Score** | **Expect** | **Rank** | **Peptide** |
| --- | --- | --- | --- | --- | --- | --- | --- | --- | --- | --- |
|  | 1143 | 571.3412 | 570.3339 | 569.3173 | 1.0166 | 0 | 9 | 19 | 7 | K.SAPAPK.K |
|  | 2387 | 408.7295 | 815.4444 | 815.4501 | -0.0057 | 0 | 22 | 1.3 | 2 | R.EIQTAVR.L |
|  | 2539 | 414.7127 | 827.4107 | 827.4137 | -0.0030 | 0 | 32 | 0.11 | 1 | K.HAVSEGTK.A 2534 2557 2561 2562 2563 2564 |
|  | 4465 | 477.3016 | 952.5887 | 952.5957 | -0.0070 | 0 | 39 | 0.017 | 1 | R.LLLPGELAK.H 4466 4467 |
|  | 6940 | 1137.5386 | 1136.5313 | 1136.5390 | -0.0077 | 0 | 9 | 17 | 1 | K.ESYSVYVYK.V |
|  | 7343 | 585.2951 | 1168.5756 | 1167.5884 | 0.9872 | 0 | 11 | 11 | 1 | K.QVHPDTGISSK.A 7326 7352 |
|  | 8691 | **620.7792** | **1239.5439** | **1238.6329** | **0.9111** | **1** | **6** | **33** | **5** | **-.MPEPSKSAPAPK.K** |
|  | 8934 | 628.2241 | 1254.4337 | 1254.6278 | -0.1941 | 1 | (4) | 50 | 5 | -.MPEPSKSAPAPK.K |
|  | 9163 | 633.3196 | 1264.6247 | 1264.6339 | -0.0092 | 1 | 54 | 0.00052 | 1 | R.KESYSVYVYK.V 9140 9164 |
|  | 12125 | 715.3318 | 1428.6490 | 1428.7473 | -0.0983 | 1 | 6 | 32 | 3 | R.IAGEASRLAHYNK.R |
|  | 16824 | 872.4091 | 1742.8036 | 1742.8120 | -0.0084 | 0 | 113 | 5.2e-010 | 1 | K.AMGIMNSFVNDIFER.I 16819 16820 16822 16823 16835 16836 16837 16839 16840 16841 |
|  | 17071 | 880.4634 | 1758.9122 | 1758.8069 | 0.1053 | 0 | (4) | 46 | 1 | K.AMGIMNSFVNDIFER.I |

| **118.** | P05091\|ALDH2_HUMAN    **Mass:** 56859    **Score:** 363    **Queries matched:** 31   **emPAI:** 0.77 |
| --- | --- |
|  | Aldehyde dehydrogenase, mitochondrial OS=Homo sapiens GN=ALDH2 PE=1 SV=2 |

|  | **Query** | **Observed** | **Mr(expt)** | **Mr(calc)** | **Delta** | **Miss** | **Score** | **Expect** | **Rank** | **Peptide** |
| --- | --- | --- | --- | --- | --- | --- | --- | --- | --- | --- |
|  | 1044 | **547.3414** | **546.3342** | **546.3377** | **-0.0036** | **0** | **20** | **3.3** | **2** | **K.TVTVK.V** |
|  | 1323 | 605.3397 | 604.3324 | 604.2704 | 0.0620 | 0 | 13 | 13 | 8 | K.EDVDK.A |
|  | 2391 | 408.7420 | 815.4694 | 815.4753 | -0.0059 | 0 | 18 | 3.7 | 2 | R.VTLELGGK.S 2382 2398 |
|  | 2572 | **415.2399** | **828.4652** | **828.4705** | **-0.0052** | **0** | **16** | **5** | **1** | **R.LADLIER.D** |
|  | 4589 | **482.2449** | **962.4752** | **961.4869** | **0.9883** | **0** | **9** | **23** | **8** | **R.VVGNPFDSK.T** |
|  | 4704 | **486.7635** | **971.5125** | **971.5764** | **-0.0639** | **1** | **17** | **3.9** | **1** | **K.RVTLELGGK.S** |
|  | 6488 | **551.7836** | **1101.5526** | **1101.5601** | **-0.0075** | **0** | **51** | **0.0014** | **1** | **K.LLCGGGIAADR.G** |
|  | 7604 | **593.8392** | **1185.6638** | **1185.6717** | **-0.0079** | **0** | **59** | **0.00023** | **1** | **R.VIQVAAGSSNLK.R** |
|  | 7995 | **603.3295** | **1204.6445** | **1204.6200** | **0.0245** | **1** | **9** | **18** | **1** | **K.SRVVGNPFDSK.T** |
|  | 10268 | **662.8469** | **1323.6793** | **1324.7826** | **-1.1034** | **1** | **7** | **25** | **3** | **R.LLNRLADLIER.D** 10299 |
|  | 10614 | **671.8890** | **1341.7635** | **1341.7728** | **-0.0093** | **1** | **2** | **86** | **1** | **R.VIQVAAGSSNLKR.V** |
|  | 11052 | **685.3927** | **1368.7708** | **1368.7799** | **-0.0091** | **0** | **37** | **0.026** | **1** | **K.LGPALATGNVVVMK.V** 11020 |
|  | 11366 | **693.8470** | **1385.6794** | **1384.7748** | **0.9046** | **0** | **(1)** | **1.1e+002** | **3** | **K.LGPALATGNVVVMK.V** |
|  | 11936 | **709.8667** | **1417.7188** | **1418.7479** | **-1.0291** | **0** | **15** | **3.9** | **1** | **K.EEIFGPVMQILK.F** |
|  | 13349 | **753.8521** | **1505.6897** | **1505.6998** | **-0.0101** | **0** | **57** | **0.00024** | **1** | **K.TEQGPQVDETQFK.K** 13370 |
|  | 13703 | **766.3704** | **1530.7263** | **1530.7355** | **-0.0092** | **0** | **17** | **2.5** | **1** | **K.TIPIDGDFFSYTR.H** |
|  | 14713 | **800.3934** | **1598.7722** | **1598.7828** | **-0.0106** | **0** | **77** | **2.4e-006** | **1** | **R.ELGEYGLQAYTEVK.T** |
|  | 17464 | 895.4130 | 1788.8115 | 1788.8206 | -0.0092 | 0 | 75 | 3.1e-006 | 1 | R.TFVQEDIYDEFVER.S |
|  | 22079 | **1094.0366** | **2186.0587** | **2186.0718** | **-0.0131** | **0** | **53** | **0.00047** | **1** | **R.GYFIQPTVFGDVQDGMTIAK.E** |
|  | 22226 | **735.3922** | **2203.1548** | **2202.0667** | **1.0881** | **0** | **(2)** | **59** | **6** | **R.GYFIQPTVFGDVQDGMTIAK.E** |
|  | 25966 | **907.4621** | **2719.3645** | **2719.3767** | **-0.0122** | **0** | **13** | **3** | **1** | **R.HEPVGVCGQIIPWNFPLLMQAWK.L** |
|  | 26493 | **945.7773** | **2834.3100** | **2834.3280** | **-0.0180** | **1** | **3** | **30** | **6** | **K.TFPTVNPSTGEVICQVAEGDKEDVDK.A** |
|  | 27977 | **1053.2250** | **3156.6531** | **3156.6668** | **-0.0137** | **0** | **35** | **0.014** | **1** | **R.TYLAALETLDNGKPYVISYLVDLDMVLK.C** |
|  | 29007 | **857.9565** | **3427.7971** | **3427.7949** | **0.0022** | **1** | **3** | **19** | **1** | **R.DRTYLAALETLDNGKPYVISYLVDLDMVLK.C** |
|  | 30219 | **1294.9484** | **3881.8233** | **3881.8359** | **-0.0127** | **1** | **69** | **3.8e-006** | **1** | **K.DLDKANYLSQALQAGTVWVNCYDVFGAQSPFGGYK.M** |
|  | 31230 | **1211.7513** | **4842.9763** | **4843.5436** | **-0.5674** | **1** | **1** | **12** | **6** | **K.VAEQTPLTALYVANLIKEAGFPPGVVNIVPGFGPTAGAAIASHEDVDK.V** |

| **119.** | P42765\|THIM_HUMAN    **Mass:** 42354    **Score:** 362    **Queries matched:** 21   **emPAI:** 1.14 |
| --- | --- |
|  | 3-ketoacyl-CoA thiolase, mitochondrial OS=Homo sapiens GN=ACAA2 PE=1 SV=2 |

|  | **Query** | **Observed** | **Mr(expt)** | **Mr(calc)** | **Delta** | **Miss** | **Score** | **Expect** | **Rank** | **Peptide** |
| --- | --- | --- | --- | --- | --- | --- | --- | --- | --- | --- |
|  | 1194 | 581.2750 | 580.2677 | 580.3445 | -0.0768 | 0 | 22 | 0.73 | 1 | R.HVGLR.V 1196 |
|  | 3563 | 446.0371 | 890.0597 | 889.4756 | 0.5841 | 0 | 8 | 23 | 2 | R.SLDLDISK.T |
|  | 4379 | **474.3466** | **946.6786** | **945.5760** | **1.1026** | **1** | **7** | **31** | **5** | **R.GVFVVAAKR.T** |
|  | 4486 | **478.2558** | **954.4971** | **954.5035** | **-0.0064** | **0** | **25** | **0.52** | **1** | **K.HNFTPLAR.I** |
|  | 6748 | **561.9860** | **1121.9574** | **1122.6073** | **-0.6500** | **0** | **2** | **61** | **1** | **R.TPFGAYGGLLK.D** |
|  | 7645 | **594.8421** | **1187.6696** | **1187.6775** | **-0.0078** | **0** | **48** | **0.0025** | **1** | **R.ITAHLVHELR.R** 7674 |
|  | 15591 | **827.8723** | **1653.7299** | **1653.7417** | **-0.0117** | **1** | **30** | **0.11** | **1** | **R.EECDKYALQSQQR.W** |
|  | 18111 | **918.4665** | **1834.9184** | **1833.9446** | **0.9739** | **0** | **49** | **0.0014** | **1** | **K.TNVNGGAIALGHPLGGSGSR.I** 18099 18109 18110 |
|  | 19634 | **977.9399** | **1953.8653** | **1953.8778** | **-0.0125** | **0** | **54** | **0.00037** | **1** | **K.AANDAGYFNDEMAPIEVK.T** |
|  | 23106 | 1155.0533 | 2308.0921 | 2308.1045 | -0.0124 | 0 | 87 | 1.7e-007 | 1 | K.DMDLVEVNEAFAPQYLAVER.S 23107 |
|  | 24659 | **839.4152** | **2515.2238** | **2515.2402** | **-0.0164** | **0** | **6** | **17** | **3** | **K.DGTVTAGNASGVADGAGAVIIASEDAVK.K** |
|  | 25895 | **1353.1987** | **2704.3829** | **2704.3969** | **-0.0139** | **0** | **52** | **0.0004** | **1** | **R.IVGYFVSGCDPSIMGIGPVPAISGALK.K** |
|  | 26126 | **1376.1907** | **2750.3668** | **2750.3797** | **-0.0129** | **0** | **73** | **2.8e-006** | **1** | **K.VSPETVDSVIMGNVLQSSSDAIYLAR.H** |
|  | 27916 | **1047.1923** | **3138.5550** | **3138.5729** | **-0.0180** | **0** | **60** | **5.3e-005** | **1** | **K.LEDSLWVSLTDQHVQLPMAMTAENLAVK.H** |
|  | 28197 | **800.1333** | **3196.5041** | **3195.5645** | **0.9396** | **1** | **4** | **18** | **1** | **K.DMDLVEVNEAFAPQYLAVERSLDLDISK.T** |

| **120.** | P14618\|KPYM_HUMAN    **Mass:** 58470    **Score:** 354    **Queries matched:** 26   **emPAI:** 0.54 |
| --- | --- |
|  | Pyruvate kinase isozymes M1/M2 OS=Homo sapiens GN=PKM2 PE=1 SV=4 |

|  | **Query** | **Observed** | **Mr(expt)** | **Mr(calc)** | **Delta** | **Miss** | **Score** | **Expect** | **Rank** | **Peptide** |
| --- | --- | --- | --- | --- | --- | --- | --- | --- | --- | --- |
|  | 752 | **490.2640** | **489.2567** | **488.2958** | **0.9609** | **0** | **15** | **12** | **4** | **K.GATLK.I** |
|  | 1627 | **676.3828** | **675.3755** | **675.3803** | **-0.0048** | **0** | **14** | **9.6** | **1** | **R.SVETLK.E** |
|  | 1776 | **717.4086** | **716.4013** | **716.4069** | **-0.0056** | **0** | **21** | **2** | **1** | **K.VVEVGSK.I** |
|  | 1827 | **734.3561** | **733.3488** | **733.3541** | **-0.0053** | **0** | **31** | **0.18** | **1** | **K.SGMNVAR.L** |
|  | 2734 | **840.4036** | **839.3963** | **839.5229** | **-0.1266** | **0** | **10** | **15** | **3** | **R.APIIAVTR.N** |
|  | 3213 | **434.7416** | **867.4687** | **867.4749** | **-0.0062** | **0** | **11** | **10** | **2** | **R.MQHLIAR.E** |
|  | 5355 | **510.2582** | **1018.5017** | **1018.5083** | **-0.0066** | **0** | **11** | **13** | **3** | **K.GDYPLEAVR.M** |
|  | 6004 | **534.9075** | **1067.8004** | **1068.4627** | **-0.6623** | **1** | **4** | **39** | **3** | **K.MMIGRCNR.A** |
|  | 6251 | 542.8006 | 1083.5866 | 1082.6448 | 0.9419 | 1 | 2 | 97 | 7 | K.GPEIRTGLIK.G |
|  | 7816 | 599.3235 | 1196.6325 | 1196.6401 | -0.0075 | 0 | 33 | 0.08 | 1 | R.LDIDSPPITAR.N |
|  | 8542 | **617.1337** | **1232.2528** | **1231.6595** | **0.5933** | **1** | **5** | **36** | **9** | **K.NICKVVEVGSK.I** |
|  | 12654 | **488.2789** | **1461.8150** | **1461.8079** | **0.0071** | **0** | **4** | **48** | **6** | **K.IYVDDGLISLQVK.Q** |
|  | 15323 | **818.9456** | **1635.8767** | **1635.8832** | **-0.0065** | **0** | **20** | **1.1** | **1** | **K.GVNLPGAAVDLPAVSEK.D** |
|  | 17338 | **890.4356** | **1778.8565** | **1778.8687** | **-0.0121** | **0** | **69** | **1.4e-005** | **1** | **K.GADFLVTEVENGGSLGSK.K** 17355 |
|  | 18491 | **930.4498** | **1858.8850** | **1858.8924** | **-0.0074** | **0** | **107** | **2.4e-009** | **1** | **K.FGVEQDVDMVFASFIR.K** |
|  | 18682 | **938.9835** | **1875.9525** | **1875.9620** | **-0.0095** | **0** | **41** | **0.0074** | **1** | **R.AGKPVICATQMLESMIK.K** |
|  | 21986 | **1088.0565** | **2174.0985** | **2174.1107** | **-0.0122** | **0** | **124** | **3.7e-011** | **1** | **R.LAPITSDPTEATAVGAVEASFK.C** 21987 |
|  | 22901 | **760.5380** | **2278.5921** | **2278.1529** | **0.4392** | **0** | **2** | **33** | **2** | **K.GDVVIVLTGWRPGSGFTNTMR.V** |
|  | 24284 | **822.4299** | **2464.2680** | **2464.2849** | **-0.0170** | **0** | **21** | **0.56** | **1** | **R.TATESFASDPILYRPVAVALDTK.G** 24285 |
|  | 24512 | **1247.5691** | **2493.1236** | **2493.1363** | **-0.0127** | **0** | **25** | **0.23** | **1** | **R.AEGSDVANAVLDGADCIMLSGETAK.G** |
|  | 24994 | **853.0930** | **2556.2571** | **2556.2683** | **-0.0112** | **1** | **11** | **5.6** | **1** | **R.GIFPVLCKDPVQEAWAEDVDLR.V** |
|  | 29678 | **1221.0745** | **3660.2016** | **3659.6741** | **0.5274** | **0** | **2** | **18** | **1** | **-.MSKPHSEAGTAFIQTQQLHAAMADTFLEHMCR.L** |
|  | 31258 | **1214.7975** | **4855.1609** | **4854.2986** | **0.8623** | **1** | **1** | **12** | **1** | **-.MSKPHSEAGTAFIQTQQLHAAMADTFLEHMCRLDIDSPPITAR.N** |

| **121.** | Q9BYX7\|ACTBM_HUMAN    **Mass:** 42331    **Score:** 353    **Queries matched:** 37   **emPAI:** 0.53 |
| --- | --- |
|  | Beta-actin-like protein 3 OS=Homo sapiens GN=ACTBL3 PE=1 SV=1 |

|  | **Query** | **Observed** | **Mr(expt)** | **Mr(calc)** | **Delta** | **Miss** | **Score** | **Expect** | **Rank** | **Peptide** |
| --- | --- | --- | --- | --- | --- | --- | --- | --- | --- | --- |
|  | 1426 | 631.3726 | 630.3653 | 630.3701 | -0.0048 | 0 | 10 | 33 | 3 | K.ILTER.G 1421 |
|  | 1491 | 644.8003 | 643.7931 | 643.3653 | 0.4278 | 0 | 18 | 3 | 2 | R.LDLAGR.E |
|  | 3832 | **455.2456** | **908.4767** | **908.4967** | **-0.0200** | **0** | **5** | **54** | **3** | **K.TTFNSIVK.S** |
|  | 5451 | **514.2915** | **1026.5684** | **1027.4896** | **-0.9211** | **0** | **12** | **9.3** | **2** | **R.ELTDYLMK.I** |
|  | 10362 | **443.5863** | **1327.7370** | **1328.5424** | **-0.8054** | **0** | **12** | **9.5** | **2** | **R.HQGMMEGMHQK.E** |
|  | 12164 | 477.8854 | 1430.6345 | 1429.8214 | 0.8131 | 1 | 6 | 37 | 2 | K.EITALAPSIMKIK.I 12174 12182 |
|  | 13487 | 505.9182 | 1514.7328 | 1514.7419 | -0.0091 | 0 | 46 | 0.0032 | 1 | K.IWHHTFYNELR.V 13481 13482 13483 13484 13485 13486 13490 13491 13492 13493 |
|  | 13503 | 758.8499 | 1515.6853 | 1515.6954 | -0.0101 | 0 | 40 | 0.014 | 1 | K.QEYDESGPSIVHR.K 13474 13502 13504 13509 13528 |
|  | 17483 | 895.9439 | 1789.8733 | 1789.8846 | -0.0114 | 0 | 73 | 6e-006 | 1 | K.SYELPDGQVITIGNER.F 17481 17482 17485 17486 17487 17488 |
|  | 20046 | 665.3938 | 1993.1596 | 1993.8550 | -0.6954 | 0 | 1 | 69 | 8 | K.YPMEHGIITNWDDMEK.I |
|  | 29124 | **1157.8890** | **3470.6453** | **3469.6078** | **1.0375** | **1** | **3** | **18** | **1** | **K.AGFAGDDAPQAVFPSIVGRPRHQGMMEGMHQK.E** 29118 29123 |

| **122.** | Q99805\|TM9S2_HUMAN    **Mass:** 76809    **Score:** 353    **Queries matched:** 21   **emPAI:** 0.33 |
| --- | --- |
|  | Transmembrane 9 superfamily member 2 OS=Homo sapiens GN=TM9SF2 PE=1 SV=1 |

|  | **Query** | **Observed** | **Mr(expt)** | **Mr(calc)** | **Delta** | **Miss** | **Score** | **Expect** | **Rank** | **Peptide** |
| --- | --- | --- | --- | --- | --- | --- | --- | --- | --- | --- |
|  | 1735 | 708.3878 | 707.3805 | 707.4218 | -0.0413 | 0 | 15 | 5.4 | 8 | K.IYSVVK.V |
|  | 2838 | **424.3023** | **846.5901** | **847.4803** | **-0.8903** | **1** | **2** | **1.2e+002** | **8** | **K.LEPKSFK.H** |
|  | 4188 | **468.2530** | **934.4915** | **934.4984** | **-0.0070** | **0** | **10** | **20** | **5** | **K.NAIEHPVR.T** |
|  | 9936 | **436.2479** | **1305.7219** | **1305.7306** | **-0.0087** | **0** | **5** | **39** | **3** | **K.LVHGDIFRPPR.K** |
|  | 10146 | **440.0301** | **1317.0684** | **1316.7717** | **0.2966** | **1** | **4** | **42** | **7** | **R.LPVLSPPRWPR.L** |
|  | 10244 | **441.5652** | **1321.6738** | **1322.7493** | **-1.0754** | **1** | **9** | **18** | **3** | **-.MSARLPVLSPPR.W** |
|  | 12234 | **479.2105** | **1434.6098** | **1434.6198** | **-0.0100** | **0** | **20** | **1.3** | **1** | **K.DACVISSDFHER.D** |
|  | 13441 | **504.9202** | **1511.7388** | **1510.7456** | **0.9931** | **0** | **7** | **27** | **6** | **R.DTFYIFNHVDIK.I** |
|  | 14468 | **528.2571** | **1581.7496** | **1581.7610** | **-0.0114** | **0** | **29** | **0.16** | **1** | **K.IYYHVVETGSMGAR.L** |
|  | 15456 | **549.0074** | **1644.0005** | **1644.0702** | **-0.0697** | **0** | **2** | **62** | **9** | **R.LLLLSLLLLGAVPGPR.R** |
|  | 17455 | **894.9028** | **1787.7910** | **1787.8011** | **-0.0101** | **0** | **56** | **0.00026** | **1** | **R.FCNPGFPIGCYITDK.G** 17454 |
|  | 17621 | **601.3872** | **1801.1398** | **1800.1713** | **0.9685** | **1** | **0** | **76** | **2** | **R.LLLLSLLLLGAVPGPRR.S** |
|  | 20934 | **1039.4186** | **2076.8226** | **2076.8371** | **-0.0145** | **0** | **127** | **1.6e-011** | **1** | **R.YNQMDSTEDAQEEFGWK.L** 20933 |
|  | 21690 | **716.9931** | **2147.9575** | **2147.9728** | **-0.0154** | **0** | **42** | **0.0051** | **1** | **K.HTHIDKPDCSGPPMDISNK.A** |
|  | 21914 | **722.6909** | **2165.0509** | **2163.9677** | **1.0832** | **0** | **(3)** | **40** | **2** | **K.HTHIDKPDCSGPPMDISNK.A** |
|  | 24590 | 835.5329 | 2503.5769 | 2504.3284 | -0.7515 | 0 | 6 | 13 | 1 | R.GALMTCAVVLWVLLGTPAGYVAAR.F |
|  | 26158 | **1378.1158** | **2754.2171** | **2754.2371** | **-0.0199** | **0** | **42** | **0.0035** | **1** | **R.LDSVESVLPYEYTAFDFCQASEGK.R** |
|  | 29194 | **1169.5231** | **3505.5474** | **3505.5642** | **-0.0168** | **0** | **72** | **2.1e-006** | **1** | **K.SMLLNYQHHWIVDNMPVTWCYDVEDGQR.F** |
|  | 29607 | **1217.6106** | **3649.8100** | **3649.6541** | **0.1559** | **1** | **2** | **26** | **4** | **K.KSMLLNYQHHWIVDNMPVTWCYDVEDGQR.F** |

| **123.** | P09467\|F16P1_HUMAN    **Mass:** 37190    **Score:** 346    **Queries matched:** 19   **emPAI:** 0.78 |
| --- | --- |
|  | Fructose-1,6-bisphosphatase 1 OS=Homo sapiens GN=FBP1 PE=1 SV=4 |

|  | **Query** | **Observed** | **Mr(expt)** | **Mr(calc)** | **Delta** | **Miss** | **Score** | **Expect** | **Rank** | **Peptide** |
| --- | --- | --- | --- | --- | --- | --- | --- | --- | --- | --- |
|  | 1720 | **703.4045** | **702.3972** | **702.4024** | **-0.0052** | **0** | **13** | **13** | **1** | **K.AISSAVR.K** |
|  | 4559 | **480.9364** | **959.8582** | **960.4665** | **-0.6082** | **1** | **0** | **1.3e+002** | **2** | **K.VYEKHSAQ.-** |
|  | 5041 | **498.7691** | **995.5236** | **994.4906** | **1.0330** | **1** | **11** | **11** | **10** | **R.FVMEEGRK.A** |
|  | 8536 | **617.0577** | **1232.1008** | **1232.5972** | **-0.4964** | **0** | **(2)** | **59** | **4** | **R.YVGSMVADVHR.T** |
|  | 8862 | **417.2441** | **1248.7105** | **1248.5921** | **0.1184** | **0** | **5** | **51** | **3** | **R.YVGSMVADVHR.T** |
|  | 10613 | **448.2562** | **1341.7468** | **1341.6928** | **0.0539** | **1** | **10** | **14** | **2** | **K.GKIYSLNEGYAK.D** |
|  | 11935 | **709.8616** | **1417.7086** | **1418.6942** | **-0.9857** | **1** | **4** | **50** | **4** | **K.KFPPDNSAPYGAR.Y** |
|  | 12500 | **727.3467** | **1452.6788** | **1452.6885** | **-0.0097** | **0** | **45** | **0.0044** | **1** | **K.DFDPAVTEYIQR.K** |
|  | 14793 | **803.4283** | **1604.8421** | **1604.8522** | **-0.0101** | **0** | **47** | **0.0023** | **1** | **K.EAVLDVIPTDIHQR.A** 14792 |
|  | 16669 | **866.9659** | **1731.9172** | **1731.9263** | **-0.0091** | **1** | **90** | **1.2e-007** | **1** | **K.KLDVLSNDLVMNMLK.S** |
|  | 17077 | **880.9013** | **1759.7881** | **1759.7983** | **-0.0102** | **0** | **82** | **6.9e-007** | **1** | **R.LLYECNPMAYVMEK.A** 17076 |
|  | 17655 | **902.9732** | **1803.9319** | **1803.9400** | **-0.0082** | **0** | **91** | **8.9e-008** | **1** | **R.GTGELTQLLNSLCTAVK.A** 17667 |
|  | 20884 | **1036.5354** | **2071.0562** | **2071.0698** | **-0.0136** | **0** | **80** | **8e-007** | **1** | **K.AGIAHLYGIAGSTNVTGDQVK.K** |
|  | 22199 | **734.3921** | **2200.1544** | **2199.1648** | **0.9897** | **1** | **29** | **0.11** | **1** | **R.KAGIAHLYGIAGSTNVTGDQVK.K** 22188 |
|  | 25616 | **892.1056** | **2673.2949** | **2674.2003** | **-0.9054** | **1** | **0** | **55** | **2** | **-.MADQAPFDTDVNTLTRFVMEEGR.K** |

| **124.** | Q8TD06\|AGR3_HUMAN    **Mass:** 19273    **Score:** 343    **Queries matched:** 20   **emPAI:** 1.98 |
| --- | --- |
|  | Anterior gradient protein 3 homolog OS=Homo sapiens GN=AGR3 PE=1 SV=1 |

|  | **Query** | **Observed** | **Mr(expt)** | **Mr(calc)** | **Delta** | **Miss** | **Score** | **Expect** | **Rank** | **Peptide** |
| --- | --- | --- | --- | --- | --- | --- | --- | --- | --- | --- |
|  | 4277 | 471.7094 | 941.4043 | 940.4654 | 0.9389 | 0 | 10 | 16 | 2 | R.LYTYEPR.D 4266 |
|  | 4473 | **477.7740** | **953.5334** | **953.5406** | **-0.0073** | **0** | **11** | **11** | **3** | **K.RPPQTLSR.G** 4457 |
|  | 7603 | **593.8294** | **1185.6443** | **1184.6475** | **0.9968** | **0** | **48** | **0.0026** | **1** | **R.DLPLLIENMK.K** |
|  | 8788 | **623.3107** | **1244.6068** | **1244.6149** | **-0.0082** | **0** | **13** | **7.3** | **1** | **K.NLSPDGQYVPR.I** |
|  | 9404 | 639.3457 | 1276.6768 | 1276.6850 | -0.0081 | 0 | 56 | 0.00033 | 1 | R.IMFVDPSLTVR.A 9403 |
|  | 9703 | 647.3424 | 1292.6701 | 1292.6799 | -0.0097 | 0 | (18) | 2.5 | 1 | R.IMFVDPSLTVR.A |
|  | 14631 | **796.8890** | **1591.7635** | **1591.7738** | **-0.0103** | **0** | **62** | **7.4e-005** | **1** | **K.FIMLNLMHETTDK.N** |
|  | 14833 | 804.3860 | 1606.7574 | 1607.7687 | -1.0113 | 0 | (4) | 48 | 6 | K.FIMLNLMHETTDK.N 14834 |
|  | 17319 | **889.9167** | **1777.8189** | **1777.8305** | **-0.0115** | **0** | **96** | **2.6e-008** | **1** | **K.VFAQNEEIQEMAQNK.F** 17318 17320 |
|  | 21490 | **709.0092** | **2124.0058** | **2123.0972** | **0.9086** | **1** | **2** | **53** | **3** | **R.LYTYEPRDLPLLIENMK.K** |
|  | 24601 | **1253.5757** | **2505.1368** | **2505.1489** | **-0.0121** | **0** | **69** | **8.3e-006** | **1** | **R.GWGDDITWVQTYEEGLFYAQK.S** 24600 |
|  | 24812 | **636.3255** | **2541.2729** | **2540.2879** | **0.9850** | **1** | **3** | **33** | **4** | **K.SKKPLMVIHHLEDCQYSQALK.K** |
|  | 26439 | **940.7955** | **2819.3648** | **2818.3782** | **0.9866** | **1** | **42** | **0.0038** | **1** | **K.FIMLNLMHETTDKNLSPDGQYVPR.I** |

| **125.** | Q5VTE0\|EF1A3_HUMAN    **Mass:** 50495    **Score:** 343    **Queries matched:** 38   **emPAI:** 1.04 |
| --- | --- |
|  | Putative elongation factor 1-alpha-like 3 OS=Homo sapiens GN=EEF1AL3 PE=5 SV=1 |

|  | **Query** | **Observed** | **Mr(expt)** | **Mr(calc)** | **Delta** | **Miss** | **Score** | **Expect** | **Rank** | **Peptide** |
| --- | --- | --- | --- | --- | --- | --- | --- | --- | --- | --- |
|  | 3277 | **436.2635** | **870.5124** | **869.5334** | **0.9789** | **0** | **13** | **8.2** | **4** | **K.QLIVGVNK.M** 3267 |
|  | 3903 | **457.7841** | **913.5537** | **913.5597** | **-0.0060** | **0** | **15** | **5.4** | **2** | **R.QTVAVGVIK.A** |
|  | 4197 | **468.7578** | **935.5011** | **935.5076** | **-0.0065** | **1** | **18** | **2.5** | **4** | **K.RYEEIVK.E** |
|  | 5432 | **1025.6041** | **1024.5969** | **1024.6030** | **-0.0061** | **0** | **21** | **1.1** | **1** | **K.IGGIGTVPVGR.V** 5409 5431 |
|  | 6721 | **560.7994** | **1119.5842** | **1119.5924** | **-0.0082** | **0** | **26** | **0.46** | **2** | **K.STTTGHLIYK.C** |
|  | 7355 | **585.7771** | **1169.5396** | **1169.5387** | **0.0010** | **1** | **13** | **8.3** | **5** | **K.EAAEMGKGSFK.Y** |
|  | 9471 | 641.7799 | 1281.5453 | 1281.5547 | -0.0094 | 0 | 12 | 9 | 1 | K.MDSTEPPYSQK.R |
|  | 9794 | **433.5846** | **1297.7319** | **1297.5496** | **0.1822** | **0** | **(1)** | **1e+002** | **4** | **K.MDSTEPPYSQK.R** |
|  | 10073 | **438.9157** | **1313.7254** | **1313.7343** | **-0.0089** | **0** | **29** | **0.19** | **1** | **R.EHALLAYTLGVK.Q** |
|  | 10126 | **658.8964** | **1315.7783** | **1315.7282** | **0.0501** | **1** | **8** | **25** | **6** | **R.DMRQTVAVGVIK.A** |
|  | 10429 | **445.0339** | **1332.0799** | **1331.7231** | **0.3568** | **1** | **(1)** | **87** | **9** | **R.DMRQTVAVGVIK.A** |
|  | 11668 | **702.8624** | **1403.7102** | **1403.7197** | **-0.0096** | **0** | **55** | **0.00038** | **1** | **K.YYVTIIDAPGHR.D** 11669 |
|  | 21920 | **722.8296** | **2165.4669** | **2165.2572** | **0.2097** | **1** | **0** | **55** | **3** | **R.EHALLAYTLGVKQLIVGVNK.M** |
|  | 24653 | **1258.1870** | **2514.3595** | **2514.3768** | **-0.0173** | **0** | **61** | **4.9e-005** | **1** | **R.VETGVLKPGMVVTFAPVNVTTEVK.S** 24654 24655 |
|  | 27289 | **999.1312** | **2994.3719** | **2994.3926** | **-0.0207** | **0** | **15** | **1.6** | **1** | **K.SGDAAIVDMVPGKPMCVESFSDYPPLGR.F** |
|  | 27389 | **1007.8565** | **3020.5475** | **3020.5601** | **-0.0126** | **0** | **51** | **0.00037** | **1** | **K.DGNASGTTLLEALDCILPPTRPTDKPLR.L** 27386 27388 |
|  | 27947 | **788.1672** | **3148.6396** | **3148.6550** | **-0.0154** | **1** | **45** | **0.0014** | **1** | **R.KDGNASGTTLLEALDCILPPTRPTDKPLR.L** |
|  | 29004 | **1714.9052** | **3427.7957** | **3427.7003** | **0.0954** | **1** | **0** | **33** | **4** | **R.DFIKNMITGTSQADCAVLIVAAGVGEFEAGISK.N** |
|  | 29377 | **1784.3303** | **3566.6461** | **3566.6639** | **-0.0178** | **0** | **50** | **0.00035** | **1** | **K.IGYNPDTVAFVPISGWNGDNMLEPSANMPWFK.G** 29376 29379 29380 29381 29382 29383 |
|  | 29422 | **896.9106** | **3583.6135** | **3582.6588** | **0.9546** | **0** | **(1)** | **31** | **3** | **K.IGYNPDTVAFVPISGWNGDNMLEPSANMPWFK.G** |
|  | 29780 | **1232.5837** | **3694.7294** | **3694.7589** | **-0.0295** | **1** | **41** | **0.0025** | **1** | **K.KIGYNPDTVAFVPISGWNGDNMLEPSANMPWFK.G** 29781 29783 |
|  | 30287 | **1313.3353** | **3936.9842** | **3937.8596** | **-0.8755** | **1** | **3** | **15** | **2** | **K.IGYNPDTVAFVPISGWNGDNMLEPSANMPWFKGWK.V** |

|  | **Proteins matching the same set of peptides:** |
| --- | --- |

|  | P68104\|EF1A1_HUMAN    **Mass:** 50451    **Score:** 343    **Queries matched:** 38 |
| --- | --- |
|  | Elongation factor 1-alpha 1 OS=Homo sapiens GN=EEF1A1 PE=1 SV=1 |

| **126.** | P12104\|FABPI_HUMAN    **Mass:** 15198    **Score:** 340    **Queries matched:** 16   **emPAI:** 2.13 |
| --- | --- |
|  | Fatty acid-binding protein, intestinal OS=Homo sapiens GN=FABP2 PE=1 SV=2 |

|  | **Query** | **Observed** | **Mr(expt)** | **Mr(calc)** | **Delta** | **Miss** | **Score** | **Expect** | **Rank** | **Peptide** |
| --- | --- | --- | --- | --- | --- | --- | --- | --- | --- | --- |
|  | 1917 | 759.4394 | 758.4322 | 759.4313 | -0.9991 | 0 | 25 | 0.72 | 1 | K.MGVNIVK.R |
|  | 3438 | **441.7203** | **881.4260** | **880.4766** | **0.9494** | **0** | **3** | **66** | **4** | **K.LAAHDNLK.L** |
|  | 4965 | **496.2427** | **990.4709** | **990.4771** | **-0.0062** | **0** | **24** | **0.78** | **1** | **R.GTWSLEGNK.L** |
|  | 5155 | **502.2711** | **1002.5276** | **1002.5346** | **-0.0069** | **0** | **41** | **0.016** | **1** | **K.LTITQEGNK.F** 5154 |
|  | 5233 | **505.2897** | **1008.5649** | **1008.5716** | **-0.0067** | **1** | **39** | **0.02** | **1** | **R.KLAAHDNLK.L** 5226 |
|  | 8520 | **616.7924** | **1231.5703** | **1231.5793** | **-0.0090** | **0** | **76** | **4.1e-006** | **1** | **R.TDNGNELNTVR.E** 8521 |
|  | 9601 | 645.3001 | 1288.5855 | 1289.5598 | -0.9742 | 1 | 5 | 46 | 8 | R.SENYDKFMEK.M 9600 |
|  | 11406 | **694.8433** | **1387.6721** | **1387.6804** | **-0.0083** | **1** | **27** | **0.31** | **1** | **K.RTDNGNELNTVR.E** |
|  | 21806 | **1078.0369** | **2154.0592** | **2154.0732** | **-0.0140** | **0** | **81** | **7.5e-007** | **1** | **R.EIIGDELVQTYVYEGVEAK.R** 21807 21808 21809 |

| **127.** | P16104\|H2AX_HUMAN    **Mass:** 15135    **Score:** 340    **Queries matched:** 15   **emPAI:** 2.16 |
| --- | --- |
|  | Histone H2A.x OS=Homo sapiens GN=H2AFX PE=1 SV=2 |

|  | **Query** | **Observed** | **Mr(expt)** | **Mr(calc)** | **Delta** | **Miss** | **Score** | **Expect** | **Rank** | **Peptide** |
| --- | --- | --- | --- | --- | --- | --- | --- | --- | --- | --- |
|  | 820 | 505.2373 | 504.2301 | 503.2816 | 0.9485 | 1 | 3 | 1.5e+002 | 7 | M.SGRGK.T |
|  | 2897 | 425.7636 | 849.5127 | 849.5184 | -0.0058 | 0 | 32 | 0.13 | 1 | R.HLQLAIR.N 2888 2898 2899 2901 |
|  | 3644 | 449.2338 | 896.4530 | 896.3876 | 0.0654 | 0 | 3 | 80 | 7 | K.ATQASQEY.- |
|  | 4323 | 472.7661 | 943.5175 | 943.5240 | -0.0064 | 0 | 53 | 0.00095 | 1 | R.AGLQFPVGR.V 4330 |
|  | 16139 | 424.2194 | 1692.8486 | 1691.8954 | 0.9531 | 1 | 12 | 7.5 | 2 | R.HLQLAIRNDEELNK.L |
|  | 22792 | **757.7943** | **2270.3611** | **2270.3726** | **-0.0115** | **0** | **31** | **0.048** | **1** | **K.LLGGVTIAQGGVLPNIQAVLLPK.K** |
|  | 26905 | 1458.2919 | 2914.5692 | 2914.5804 | -0.0112 | 0 | 125 | 1.4e-011 | 1 | R.VGAGAPVYLAAVLEYLTAEILELAGNAAR.D 26904 26906 26907 |

| **128.** | P06727\|APOA4_HUMAN    **Mass:** 45371    **Score:** 337    **Queries matched:** 39   **emPAI:** 1.80 |
| --- | --- |
|  | Apolipoprotein A-IV OS=Homo sapiens GN=APOA4 PE=1 SV=3 |

|  | **Query** | **Observed** | **Mr(expt)** | **Mr(calc)** | **Delta** | **Miss** | **Score** | **Expect** | **Rank** | **Peptide** |
| --- | --- | --- | --- | --- | --- | --- | --- | --- | --- | --- |
|  | 4754 | **488.2559** | **974.4973** | **974.5032** | **-0.0059** | **0** | **44** | **0.0073** | **1** | **R.ISASAEELR.Q** |
|  | 4775 | **488.7610** | **975.5074** | **975.5138** | **-0.0064** | **0** | **20** | **1.8** | **1** | **R.QLTPYAQR.M** 4781 |
|  | 4869 | 492.2765 | 982.5385 | 982.5447 | -0.0062 | 0 | 46 | 0.004 | 1 | R.LAPLAEDVR.G 4868 |
|  | 5282 | 507.7519 | 1013.4893 | 1014.5458 | -1.0565 | 1 | 10 | 18 | 8 | K.ELEELRAR.L 5296 5309 |
|  | 6234 | 542.2681 | 1082.5217 | 1082.5284 | -0.0067 | 0 | 8 | 22 | 3 | R.LTPYADEFK.V |
|  | 7801 | **598.7785** | **1195.5424** | **1195.5509** | **-0.0085** | **0** | **14** | **5.7** | **1** | **R.VEPYGENFNK.A** 7802 |
|  | 7925 | **401.2848** | **1200.8325** | **1201.6414** | **-0.8089** | **1** | **11** | **9.7** | **4** | **K.ARISASAEELR.Q** 7913 |
|  | 8584 | **618.3368** | **1234.6590** | **1234.6670** | **-0.0079** | **0** | **43** | **0.0067** | **1** | **R.LLPHANEVSQK.I** |
|  | 9014 | **629.8368** | **1257.6590** | **1257.6677** | **-0.0087** | **1** | **27** | **0.3** | **1** | **K.IDQTVEELRR.S** 9015 |
|  | 9553 | **643.6765** | **1285.3383** | **1285.6738** | **-0.3355** | **1** | **6** | **22** | **6** | **R.GNLRGNTEGLQK.S** |
|  | 9567 | **644.3318** | **1286.6490** | **1286.6579** | **-0.0088** | **0** | **56** | **0.00034** | **1** | **R.TQVNTQAEQLR.R** 9566 |
|  | 9822 | **434.2307** | **1299.6702** | **1299.6783** | **-0.0080** | **1** | **39** | **0.016** | **1** | **K.IDQNVEELKGR.L** 9821 |
|  | 10384 | **444.2187** | **1329.6342** | **1328.7300** | **0.9043** | **1** | **3** | **73** | **8** | **K.VKIDQTVEELR.R** |
|  | 10737 | **675.8256** | **1349.6367** | **1349.6463** | **-0.0096** | **0** | **53** | **0.00076** | **1** | **R.SLAPYAQDTQEK.L** 10736 |
|  | 10767 | **676.8283** | **1351.6421** | **1351.6520** | **-0.0100** | **1** | **38** | **0.021** | **1** | **R.RVEPYGENFNK.A** 10745 10768 |
|  | 12311 | **480.9364** | **1439.7874** | **1438.7932** | **0.9941** | **1** | **34** | **0.052** | **1** | **K.KLVPFATELHER.L** 12299 |
|  | 12352 | **722.3818** | **1442.7490** | **1442.7590** | **-0.0100** | **1** | **14** | **5** | **1** | **R.TQVNTQAEQLRR.Q** 12351 |
|  | 12650 | **488.2514** | **1461.7323** | **1461.8052** | **-0.0729** | **1** | **15** | **4** | **1** | **R.ARLLPHANEVSQK.I** |
|  | 14126 | **779.9013** | **1557.7879** | **1557.7973** | **-0.0094** | **0** | **58** | **0.00019** | **1** | **K.LNHQLEGLTFQMK.K** |
|  | 15131 | 542.2912 | 1623.8518 | 1622.9144 | 0.9374 | 1 | 0 | 1.1e+002 | 8 | K.LVPFATELHERLAK.D |
|  | 15296 | **817.9174** | **1633.8202** | **1633.8311** | **-0.0110** | **0** | **77** | **2.3e-006** | **1** | **K.SELTQQLNALFQDK.L** |
|  | 17666 | **602.6401** | **1804.8984** | **1804.9108** | **-0.0124** | **0** | **54** | **0.00047** | **1** | **K.LGPHAGDVEGHLSFLEK.D** |
|  | 17767 | 605.0054 | 1811.9943 | 1811.1397 | 0.8546 | 1 | 4 | 43 | 6 | M.FLKAVVLTLALVAVAGAR.A |
|  | 19330 | **964.4720** | **1926.9295** | **1926.9435** | **-0.0140** | **0** | **91** | **8.4e-008** | **1** | **K.SLAELGGHLDQQVEEFR.R** |
|  | 19699 | 653.9764 | 1958.9073 | 1958.1751 | 0.7322 | 1 | 3 | 47 | 3 | -.MFLKAVVLTLALVAVAGAR.A |

| **129.** | Q8IUE6\|H2A2B_HUMAN    **Mass:** 13987    **Score:** 335    **Queries matched:** 16   **emPAI:** 1.69 |
| --- | --- |
|  | Histone H2A type 2-B OS=Homo sapiens GN=HIST2H2AB PE=1 SV=3 |

|  | **Query** | **Observed** | **Mr(expt)** | **Mr(calc)** | **Delta** | **Miss** | **Score** | **Expect** | **Rank** | **Peptide** |
| --- | --- | --- | --- | --- | --- | --- | --- | --- | --- | --- |
|  | 820 | 505.2373 | 504.2301 | 503.2816 | 0.9485 | 1 | 3 | 1.5e+002 | 7 | M.SGRGK.Q |
|  | 2674 | **418.7558** | **835.4970** | **835.5028** | **-0.0058** | **0** | **29** | **0.22** | **1** | **R.HLQLAVR.N** 2675 |
|  | 2684 | 419.2129 | 836.4112 | 836.4140 | -0.0028 | 1 | 16 | 4.2 | 1 | R.KGNYAER.V 2697 2698 2699 2716 |
|  | 4323 | 472.7661 | 943.5175 | 943.5240 | -0.0064 | 0 | 53 | 0.00095 | 1 | R.AGLQFPVGR.V 4330 |
|  | 10122 | **439.5809** | **1315.7208** | **1314.8248** | **0.8960** | **1** | **9** | **20** | **4** | **R.IIPRHLQLAVR.N** |
|  | 22792 | 757.7943 | 2270.3611 | 2270.3726 | -0.0115 | 0 | 31 | 0.048 | 1 | K.LLGGVTIAQGGVLPNIQAVLLPK.K |
|  | 26905 | 1458.2919 | 2914.5692 | 2914.5804 | -0.0112 | 0 | 125 | 1.4e-011 | 1 | R.VGAGAPVYLAAVLEYLTAEILELAGNAAR.D 26904 26906 26907 |

| **130.** | P13639\|EF2_HUMAN    **Mass:** 96246    **Score:** 334    **Queries matched:** 27   **emPAI:** 0.35 |
| --- | --- |
|  | Elongation factor 2 OS=Homo sapiens GN=EEF2 PE=1 SV=4 |

|  | **Query** | **Observed** | **Mr(expt)** | **Mr(calc)** | **Delta** | **Miss** | **Score** | **Expect** | **Rank** | **Peptide** |
| --- | --- | --- | --- | --- | --- | --- | --- | --- | --- | --- |
|  | 1279 | **600.3089** | **599.3016** | **600.3707** | **-1.0691** | **1** | **4** | **74** | **4** | **K.KANIR.N** |
|  | 2693 | **419.2420** | **836.4695** | **836.4248** | **0.0447** | **0** | **10** | **16** | **6** | **R.TILMMGR.Y** |
|  | 2931 | **427.2127** | **852.4109** | **852.4197** | **-0.0088** | **0** | **(10)** | **15** | **1** | **R.TILMMGR.Y** |
|  | 3633 | **448.7319** | **895.4493** | **895.4143** | **0.0350** | **1** | **17** | **3** | **1** | **K.KVEDMMK.K** |
|  | 3649 | **449.2360** | **896.4574** | **895.4143** | **1.0431** | **1** | **(1)** | **1.2e+002** | **6** | **K.KVEDMMK.K** |
|  | 3888 | **457.2486** | **912.4826** | **911.4092** | **1.0734** | **1** | **(4)** | **68** | **10** | **K.KVEDMMK.K** |
|  | 5768 | **526.2364** | **1050.4582** | **1049.5506** | **0.9077** | **1** | **6** | **36** | **7** | **K.KSDPVVSYR.E** |
|  | 6555 | **554.2771** | **1106.5396** | **1106.6336** | **-0.0939** | **0** | **20** | **1.8** | **1** | **R.VFSGLVSTGLK.V** |
|  | 9012 | **420.2047** | **1257.5924** | **1256.7274** | **0.8649** | **1** | **4** | **58** | **10** | **K.EGKPLLKAVMR.R** 8999 |
|  | 11625 | **468.1686** | **1401.4839** | **1401.7980** | **-0.3140** | **1** | **4** | **43** | **8** | **K.KEDLYLKPIQR.T** |
|  | 14255 | **523.2606** | **1566.7599** | **1566.7712** | **-0.0113** | **1** | **13** | **5.9** | **1** | **K.DLEEDHACIPIKK.S** |
|  | 14648 | **797.8794** | **1593.7442** | **1593.7556** | **-0.0114** | **0** | **56** | **0.00027** | **1** | **R.ETVSEESNVLCLSK.S** |
|  | 17591 | **900.4465** | **1798.8785** | **1798.8890** | **-0.0105** | **0** | **85** | **3.3e-007** | **1** | **K.AYLPVNESFGFTADLR.S** 17601 |
|  | 17768 | 907.0051 | 1811.9957 | 1812.8824 | -0.8867 | 1 | (0) | 90 | 7 | K.GPLMMYISKMVPTSDK.G |
|  | 18016 | **610.6605** | **1828.9596** | **1828.8773** | **0.0822** | **1** | **2** | **61** | **3** | **K.GPLMMYISKMVPTSDK.G** 17997 |
|  | 19717 | **981.4859** | **1960.9572** | **1960.9717** | **-0.0145** | **0** | **79** | **1.2e-006** | **1** | **R.GHVFEESQVAGTPMFVVK.A** |
|  | 21563 | **711.6852** | **2132.0337** | **2133.0677** | **-1.0340** | **1** | **5** | **29** | **1** | **K.RGHVFEESQVAGTPMFVVK.A** |
|  | 21653 | **715.4202** | **2143.2389** | **2142.0705** | **1.1683** | **1** | **4** | **34** | **2** | **K.ARPFPDGLAEDIDKGEVSAR.Q** |
|  | 22219 | **1102.5550** | **2203.0955** | **2203.1048** | **-0.0093** | **0** | **56** | **0.00022** | **1** | **K.STAISLFYELSENDLNFIK.Q** |
|  | 22393 | **1110.5751** | **2219.1356** | **2219.1474** | **-0.0118** | **0** | **48** | **0.0014** | **1** | **R.ALLELQLEPEELYQTFQR.I** 22392 22394 |
|  | 26364 | **934.4720** | **2800.3943** | **2800.4032** | **-0.0089** | **0** | **31** | **0.043** | **1** | **K.DGAGFLINLIDSPGHVDFSSEVTAALR.V** |
|  | 27262 | **1495.2725** | **2988.5304** | **2988.5453** | **-0.0149** | **0** | **60** | **5.1e-005** | **1** | **R.LMEPIYLVEIQCPEQVVGGIYGVLNR.K** |

| **131.** | P33778\|H2B1B_HUMAN    **Mass:** 13942    **Score:** 334    **Queries matched:** 31   **emPAI:** 1.10 |
| --- | --- |
|  | Histone H2B type 1-B OS=Homo sapiens GN=HIST1H2BB PE=1 SV=2 |

|  | **Query** | **Observed** | **Mr(expt)** | **Mr(calc)** | **Delta** | **Miss** | **Score** | **Expect** | **Rank** | **Peptide** |
| --- | --- | --- | --- | --- | --- | --- | --- | --- | --- | --- |
|  | 1143 | 571.3412 | 570.3339 | 569.3173 | 1.0166 | 0 | 9 | 19 | 7 | K.SAPAPK.K |
|  | 2387 | 408.7295 | 815.4444 | 815.4501 | -0.0057 | 0 | 22 | 1.3 | 2 | R.EIQTAVR.L |
|  | 2539 | 414.7127 | 827.4107 | 827.4137 | -0.0030 | 0 | 32 | 0.11 | 1 | K.HAVSEGTK.A 2534 2557 2561 2562 2563 2564 |
|  | 4465 | 477.3016 | 952.5887 | 952.5957 | -0.0070 | 0 | 39 | 0.017 | 1 | R.LLLPGELAK.H 4466 4467 |
|  | 7106 | **576.2811** | **1150.5476** | **1150.5546** | **-0.0070** | **0** | **6** | **35** | **1** | **K.ESYSIYVYK.V** |
|  | 7343 | 585.2951 | 1168.5756 | 1167.5884 | 0.9872 | 0 | 11 | 11 | 1 | K.QVHPDTGISSK.A 7326 7352 |
|  | 8691 | 620.7792 | 1239.5439 | 1238.6329 | 0.9111 | 1 | 6 | 33 | 5 | -.MPEPSKSAPAPK.K |
|  | 8934 | 628.2241 | 1254.4337 | 1254.6278 | -0.1941 | 1 | (4) | 50 | 5 | -.MPEPSKSAPAPK.K |
|  | 12125 | 715.3318 | 1428.6490 | 1428.7473 | -0.0983 | 1 | 6 | 32 | 3 | R.IAGEASRLAHYNK.R |
|  | 16824 | 872.4091 | 1742.8036 | 1742.8120 | -0.0084 | 0 | 113 | 5.2e-010 | 1 | K.AMGIMNSFVNDIFER.I 16819 16820 16822 16823 16835 16836 16837 16839 16840 16841 |
|  | 17071 | 880.4634 | 1758.9122 | 1758.8069 | 0.1053 | 0 | (4) | 46 | 1 | K.AMGIMNSFVNDIFER.I |

|  | **Proteins matching a subset of these peptides:** |
| --- | --- |

|  | P06899\|H2B1J_HUMAN    **Mass:** 13896    **Score:** 334    **Queries matched:** 29 |
| --- | --- |
|  | Histone H2B type 1-J OS=Homo sapiens GN=HIST1H2BJ PE=1 SV=3 |

|  | Q16778\|H2B2E_HUMAN    **Mass:** 13912    **Score:** 334    **Queries matched:** 29 |
| --- | --- |
|  | Histone H2B type 2-E OS=Homo sapiens GN=HIST2H2BE PE=1 SV=3 |

|  | P23527\|H2B1O_HUMAN    **Mass:** 13898    **Score:** 334    **Queries matched:** 29 |
| --- | --- |
|  | Histone H2B type 1-O OS=Homo sapiens GN=HIST1H2BO PE=1 SV=3 |

| **132.** | Q8N257\|H2B3B_HUMAN    **Mass:** 13900    **Score:** 334    **Queries matched:** 31   **emPAI:** 1.11 |
| --- | --- |
|  | Histone H2B type 3-B OS=Homo sapiens GN=HIST3H2BB PE=1 SV=3 |

|  | **Query** | **Observed** | **Mr(expt)** | **Mr(calc)** | **Delta** | **Miss** | **Score** | **Expect** | **Rank** | **Peptide** |
| --- | --- | --- | --- | --- | --- | --- | --- | --- | --- | --- |
|  | 1143 | 571.3412 | 570.3339 | 569.3173 | 1.0166 | 0 | 9 | 19 | 7 | K.SAPAPK.K |
|  | 2539 | 414.7127 | 827.4107 | 827.4137 | -0.0030 | 0 | 32 | 0.11 | 1 | K.HAVSEGTK.A 2534 2557 2561 2562 2563 2564 |
|  | 4465 | 477.3016 | 952.5887 | 952.5957 | -0.0070 | 0 | 39 | 0.017 | 1 | R.LLLPGELAK.H 4466 4467 |
|  | 7106 | 576.2811 | 1150.5476 | 1150.5546 | -0.0070 | 0 | 6 | 35 | 1 | K.ESYSIYVYK.V |
|  | 7343 | 585.2951 | 1168.5756 | 1167.5884 | 0.9872 | 0 | 11 | 11 | 1 | K.QVHPDTGISSK.A 7326 7352 |
|  | 8424 | **613.8243** | **1225.6340** | **1224.6172** | **1.0168** | **1** | **(1)** | **1.1e+002** | **7** | **-.MPDPSKSAPAPK.K** |
|  | 8693 | **620.7975** | **1239.5804** | **1240.6122** | **-1.0317** | **1** | **6** | **33** | **10** | **-.MPDPSKSAPAPK.K** 8711 |
|  | 16745 | **869.4826** | **1736.9506** | **1736.0196** | **0.9311** | **1** | **3** | **59** | **3** | **R.EVQTAVRLLLPGELAK.H** |
|  | 16824 | 872.4091 | 1742.8036 | 1742.8120 | -0.0084 | 0 | 113 | 5.2e-010 | 1 | K.AMGIMNSFVNDIFER.I 16819 16820 16822 16823 16835 16836 16837 16839 16840 16841 |
|  | 17071 | 880.4634 | 1758.9122 | 1758.8069 | 0.1053 | 0 | (4) | 46 | 1 | K.AMGIMNSFVNDIFER.I |

| **133.** | P05141\|ADT2_HUMAN    **Mass:** 33102    **Score:** 332    **Queries matched:** 38   **emPAI:** 1.94 |
| --- | --- |
|  | ADP/ATP translocase 2 OS=Homo sapiens GN=SLC25A5 PE=1 SV=6 |

|  | **Query** | **Observed** | **Mr(expt)** | **Mr(calc)** | **Delta** | **Miss** | **Score** | **Expect** | **Rank** | **Peptide** |
| --- | --- | --- | --- | --- | --- | --- | --- | --- | --- | --- |
|  | 817 | **504.2427** | **503.2355** | **502.2499** | **0.9855** | **0** | **12** | **25** | **8** | **K.AGAER.E** 802 807 |
|  | 2733 | **840.3472** | **839.3399** | **839.3452** | **-0.0053** | **0** | **29** | **0.17** | **1** | **R.MMMQSGR.K** 2752 |
|  | 2979 | **856.4830** | **855.4757** | **855.4814** | **-0.0057** | **0** | **20** | **1.5** | **1** | **K.TAVAPIER.V** 2981 |
|  | 2982 | **428.7509** | **855.4871** | **855.4926** | **-0.0055** | **0** | **9** | **21** | **7** | **R.GNLANVIR.Y** |
|  | 2990 | **429.0875** | **856.1604** | **855.3401** | **0.8203** | **0** | **(9)** | **14** | **6** | **R.MMMQSGR.K** |
|  | 2991 | **429.0876** | **856.1607** | **855.3401** | **0.8206** | **0** | **(2)** | **70** | **3** | **R.MMMQSGR.K** |
|  | 3056 | **431.2256** | **860.4367** | **860.4426** | **-0.0058** | **0** | **31** | **0.15** | **1** | **R.GLGDCLVK.I** 3057 |
|  | 3739 | **451.7433** | **901.4720** | **901.4770** | **-0.0050** | **0** | **38** | **0.032** | **1** | **K.GAWSNVLR.G** 3737 3738 |
|  | 6730 | **561.2871** | **1120.5595** | **1120.5665** | **-0.0070** | **0** | **27** | **0.35** | **1** | **K.EQGVLSFWR.G** |
|  | 6872 | 566.8233 | 1131.6320 | 1131.6400 | -0.0080 | 1 | 26 | 0.46 | 1 | K.QIFLGGVDKR.T 6874 |
|  | 6935 | **568.8394** | **1135.6643** | **1135.6713** | **-0.0070** | **0** | **38** | **0.025** | **1** | **K.LLLQVQHASK.Q** 6909 6934 |
|  | 8297 | **610.3333** | **1218.6521** | **1218.6608** | **-0.0088** | **0** | **18** | **2.8** | **1** | **K.DFLAGGVAAAISK.T** |
|  | 9140 | 632.8073 | 1263.6001 | 1264.6849 | -1.0849 | 1 | 4 | 60 | 7 | R.GLGDCLVKIYK.S |
|  | 9212 | **634.7562** | **1267.4979** | **1266.6972** | **0.8007** | **1** | **3** | **65** | **10** | **K.YKQIFLGGVDK.R** 9213 |
|  | 12409 | **723.8707** | **1445.7269** | **1445.7343** | **-0.0074** | **0** | **59** | **0.00017** | **1** | **R.YFPTQALNFAFK.D** 12418 |
|  | 15157 | **542.9452** | **1625.8137** | **1626.8327** | **-1.0190** | **0** | **2** | **78** | **9** | **R.GMGGAFVLVLYDEIK.K** |
|  | 16072 | **844.8683** | **1687.7221** | **1687.7334** | **-0.0113** | **0** | **45** | **0.0034** | **1** | **K.GTDIMYTGTLDCWR.K** |
|  | 17803 | **908.9157** | **1815.8167** | **1815.8284** | **-0.0117** | **1** | **80** | **9.6e-007** | **1** | **R.KGTDIMYTGTLDCWR.K** 17802 |
|  | 19322 | **643.2836** | **1926.8291** | **1926.0363** | **0.7927** | **0** | **2** | **53** | **2** | **K.GLYQGFNVSVQGIIIYR.A** |
|  | 26343 | **1398.6686** | **2795.3226** | **2795.3377** | **-0.0151** | **0** | **87** | **1.3e-007** | **1** | **R.YFAGNLASGGAAGATSLCFVYPLDFAR.T** 26345 26346 26348 26349 |
|  | 27608 | **1018.1352** | **3051.3837** | **3052.4865** | **-1.1028** | **1** | **1** | **38** | **7** | **R.YFAGNLASGGAAGATSLCFVYPLDFARTR.L** |

| **134.** | P30086\|PEBP1_HUMAN    **Mass:** 21158    **Score:** 331    **Queries matched:** 17   **emPAI:** 2.78 |
| --- | --- |
|  | Phosphatidylethanolamine-binding protein 1 OS=Homo sapiens GN=PEBP1 PE=1 SV=3 |

|  | **Query** | **Observed** | **Mr(expt)** | **Mr(calc)** | **Delta** | **Miss** | **Score** | **Expect** | **Rank** | **Peptide** |
| --- | --- | --- | --- | --- | --- | --- | --- | --- | --- | --- |
|  | 1556 | **658.3726** | **657.3653** | **657.3697** | **-0.0044** | **0** | **24** | **0.96** | **1** | **M.PVDLSK.W** |
|  | 3498 | **443.2708** | **884.5270** | **884.5331** | **-0.0061** | **0** | **26** | **0.38** | **1** | **K.VLTPTQVK.N** 3495 |
|  | 4207 | **469.2499** | **936.4852** | **936.4916** | **-0.0064** | **0** | **21** | **1.1** | **1** | **K.LYEQLSGK.-** |
|  | 6510 | **552.7081** | **1103.4016** | **1102.5077** | **0.8939** | **0** | **16** | **3.7** | **2** | **K.CDEPILSNR.S** |
|  | 14151 | 780.9125 | 1559.8104 | 1559.8195 | -0.0091 | 0 | 71 | 1e-005 | 1 | K.LYTLVLTDPDAPSR.K |
|  | 15278 | **816.8974** | **1631.7802** | **1631.7903** | **-0.0101** | **0** | **63** | **6.1e-005** | **1** | **K.NRPTSISWDGLDSGK.L** 15277 15279 |
|  | 16079 | **563.6420** | **1687.9041** | **1687.9145** | **-0.0104** | **1** | **0** | **1.1e+002** | **8** | **K.LYTLVLTDPDAPSRK.D** |
|  | 16361 | **854.9511** | **1707.8875** | **1707.8984** | **-0.0109** | **0** | **51** | **0.00097** | **1** | **R.YVWLVYEQDRPLK.C** 16362 |
|  | 19591 | **975.4694** | **1948.9243** | **1948.9378** | **-0.0135** | **0** | **86** | **2.4e-007** | **1** | **K.GNDISSGTVLSDYVGSGPPK.G** 19592 |
|  | 26327 | **931.7997** | **2792.3772** | **2792.3956** | **-0.0183** | **1** | **18** | **1** | **1** | **R.YVWLVYEQDRPLKCDEPILSNR.S** |
|  | 29125 | **1157.9033** | **3470.6881** | **3470.7106** | **-0.0225** | **0** | **59** | **4.8e-005** | **1** | **K.WSGPLSLQEVDEQPQHPLHVTYAGAAVDELGK.V** 29126 |

| **135.** | P09211\|GSTP1_HUMAN    **Mass:** 23569    **Score:** 329    **Queries matched:** 8   **emPAI:** 0.57 |
| --- | --- |
|  | Glutathione S-transferase P OS=Homo sapiens GN=GSTP1 PE=1 SV=2 |

|  | **Query** | **Observed** | **Mr(expt)** | **Mr(calc)** | **Delta** | **Miss** | **Score** | **Expect** | **Rank** | **Peptide** |
| --- | --- | --- | --- | --- | --- | --- | --- | --- | --- | --- |
|  | 2221 | **402.7048** | **803.3950** | **802.4232** | **0.9719** | **1** | **9** | **26** | **6** | **R.GRCAALR.M** |
|  | 13980 | **517.2893** | **1548.8461** | **1549.8405** | **-0.9944** | **1** | **7** | **23** | **1** | **M.PPYTVVYFPVRGR.C** |
|  | 18766 | **942.4724** | **1882.9303** | **1882.9425** | **-0.0122** | **0** | **128** | **1.5e-011** | **1** | **K.FQDGDLTLYQSNTILR.H** 18765 |
|  | 21414 | **529.7533** | **2114.9841** | **2115.9743** | **-0.9902** | **0** | **9** | **11** | **7** | **K.DQQEAALVDMVNDGVEDLR.C** |
|  | 21503 | **1063.5776** | **2125.1407** | **2125.1531** | **-0.0124** | **0** | **63** | **4.3e-005** | **1** | **K.ALPGQLKPFETLLSQNQGGK.T** 21504 |
|  | 21802 | **1078.0265** | **2154.0384** | **2154.0521** | **-0.0136** | **1** | **70** | **8.8e-006** | **1** | **K.YISLIYTNYEAGKDDYVK.A** |

| **136.** | P07148\|FABPL_HUMAN    **Mass:** 14256    **Score:** 328    **Queries matched:** 23   **emPAI:** 4.50 |
| --- | --- |
|  | Fatty acid-binding protein, liver OS=Homo sapiens GN=FABP1 PE=1 SV=1 |

|  | **Query** | **Observed** | **Mr(expt)** | **Mr(calc)** | **Delta** | **Miss** | **Score** | **Expect** | **Rank** | **Peptide** |
| --- | --- | --- | --- | --- | --- | --- | --- | --- | --- | --- |
|  | 2477 | **824.4458** | **823.4385** | **823.4440** | **-0.0054** | **0** | **32** | **0.095** | **1** | **K.FTITAGSK.V** 2478 2479 |
|  | 5484 | **515.2797** | **1028.5449** | **1029.5455** | **-1.0006** | **0** | **47** | **0.0032** | **1** | **K.GVSEIVQNGK.H** 5510 |
|  | 6489 | **1102.5658** | **1101.5585** | **1101.5666** | **-0.0081** | **0** | **42** | **0.012** | **1** | **K.TVVQLEGDNK.L** |
|  | 8114 | 605.8520 | 1209.6894 | 1209.6969 | -0.0074 | 0 | 48 | 0.0023 | 1 | K.AIGLPEELIQK.G |
|  | 10355 | 664.8231 | 1327.6317 | 1328.7300 | -1.0983 | 1 | 6 | 36 | 4 | K.VKTVVQLEGDNK.L 10356 |
|  | 17097 | **881.8866** | **1761.7586** | **1761.8032** | **-0.0445** | **0** | **56** | **0.00028** | **1** | **K.YQLQSQENFEAFMK.A** 17096 17098 |
|  | 17303 | 593.2854 | 1776.8344 | 1777.7981 | -0.9637 | 0 | (10) | 11 | 2 | K.YQLQSQENFEAFMK.A |
|  | 23652 | **1191.6036** | **2381.1927** | **2380.2196** | **0.9731** | **0** | **87** | **1.6e-007** | **1** | **K.SVTELNGDIITNTMTLGDIVFK.R** 23649 23651 23653 23654 23655 23656 |
|  | 24085 | **1222.0454** | **2442.0763** | **2442.0930** | **-0.0168** | **0** | **79** | **8.6e-007** | **1** | **K.VIQNEFTVGEECELETMTGEK.V** |
|  | 24802 | **846.4435** | **2536.3086** | **2536.3207** | **-0.0121** | **1** | **22** | **0.43** | **1** | **K.SVTELNGDIITNTMTLGDIVFKR.I** 24801 |

| **137.** | Q6FI13\|H2A2A_HUMAN    **Mass:** 14087    **Score:** 328    **Queries matched:** 22   **emPAI:** 3.36 |
| --- | --- |
|  | Histone H2A type 2-A OS=Homo sapiens GN=HIST2H2AA3 PE=1 SV=3 |

|  | **Query** | **Observed** | **Mr(expt)** | **Mr(calc)** | **Delta** | **Miss** | **Score** | **Expect** | **Rank** | **Peptide** |
| --- | --- | --- | --- | --- | --- | --- | --- | --- | --- | --- |
|  | 820 | 505.2373 | 504.2301 | 503.2816 | 0.9485 | 1 | 3 | 1.5e+002 | 7 | M.SGRGK.Q |
|  | 2684 | 419.2129 | 836.4112 | 836.4140 | -0.0028 | 1 | 16 | 4.2 | 1 | R.KGNYAER.V 2697 2698 2699 2716 |
|  | 2897 | 425.7636 | 849.5127 | 849.5184 | -0.0058 | 0 | 32 | 0.13 | 1 | R.HLQLAIR.N 2888 2898 2899 2901 |
|  | 4323 | 472.7661 | 943.5175 | 943.5240 | -0.0064 | 0 | 53 | 0.00095 | 1 | R.AGLQFPVGR.V 4330 |
|  | 16139 | 424.2194 | 1692.8486 | 1691.8954 | 0.9531 | 1 | 12 | 7.5 | 2 | R.HLQLAIRNDEELNK.L |
|  | 19379 | 966.0837 | 1930.1529 | 1930.1615 | -0.0086 | 0 | 62 | 5.7e-005 | 1 | K.VTIAQGGVLPNIQAVLLPK.K 19375 19376 19378 19380 19381 |
|  | 26984 | **1467.2700** | **2932.5255** | **2932.5368** | **-0.0113** | **0** | **127** | **1.1e-011** | **1** | **R.VGAGAPVYMAAVLEYLTAEILELAGNAAR.D** 26985 |

|  | **Proteins matching the same set of peptides:** |
| --- | --- |

|  | Q16777\|H2A2C_HUMAN    **Mass:** 13980    **Score:** 328    **Queries matched:** 22 |
| --- | --- |
|  | Histone H2A type 2-C OS=Homo sapiens GN=HIST2H2AC PE=1 SV=4 |

| **138.** | P09525\|ANXA4_HUMAN    **Mass:** 36088    **Score:** 327    **Queries matched:** 22   **emPAI:** 1.44 |
| --- | --- |
|  | Annexin A4 OS=Homo sapiens GN=ANXA4 PE=1 SV=4 |

|  | **Query** | **Observed** | **Mr(expt)** | **Mr(calc)** | **Delta** | **Miss** | **Score** | **Expect** | **Rank** | **Peptide** |
| --- | --- | --- | --- | --- | --- | --- | --- | --- | --- | --- |
|  | 644 | **462.0821** | **461.0748** | **460.2646** | **0.8103** | **0** | **3** | **1.2e+002** | **7** | **K.GGTVK.A** |
|  | 5041 | 498.7691 | 995.5236 | 994.5270 | 0.9966 | 0 | 39 | 0.018 | 1 | K.FLTVLCSR.N |
|  | 6635 | **557.7238** | **1113.4331** | **1114.5843** | **-1.1512** | **1** | **8** | **25** | **2** | **R.NTAQRQEIR.T** |
|  | 6686 | **1118.4879** | **1117.4806** | **1117.4863** | **-0.0056** | **0** | **14** | **6.5** | **1** | **R.SDTSFMFQR.V** |
|  | 7413 | **587.8028** | **1173.5910** | **1173.5990** | **-0.0079** | **0** | **71** | **1.5e-005** | **1** | **K.GLGTDDNTLIR.V** 7415 |
|  | 11084 | **686.3314** | **1370.6482** | **1370.6579** | **-0.0097** | **0** | **48** | **0.002** | **1** | **R.ISQTYQQQYGR.S** |
|  | 11234 | 690.3207 | 1378.6268 | 1378.6365 | -0.0097 | 0 | 61 | 0.00011 | 1 | R.DEGNYLDDALVR.Q |
|  | 13770 | **512.7494** | **1535.2263** | **1535.7614** | **-0.5350** | **1** | **1** | **70** | **7** | **K.SMKGLGTDDNTLIR.V** |
|  | 14304 | **524.2722** | **1569.7948** | **1569.8052** | **-0.0104** | **1** | **37** | **0.028** | **1** | **R.NHLLHVFDEYKR.I** |
|  | 15690 | **831.4060** | **1660.7975** | **1660.8090** | **-0.0116** | **0** | **110** | **1.2e-009** | **1** | **K.GAGTDEGCLIEILASR.T** 15692 15704 |
|  | 15752 | **833.9263** | **1665.8381** | **1665.8461** | **-0.0080** | **0** | **43** | **0.0055** | **1** | **K.SETSGSFEDALLAIVK.C** |
|  | 16129 | **846.9393** | **1691.8640** | **1691.8730** | **-0.0090** | **0** | **84** | **4.5e-007** | **1** | **K.GLGTDEDAIISVLAYR.N** 16130 |
|  | 16893 | **583.3519** | **1747.0340** | **1745.9094** | **1.1245** | **1** | **(2)** | **69** | **5** | **K.GLGTDDNTLIRVMVSR.A** |
|  | 17104 | **588.3669** | **1762.0790** | **1761.9043** | **0.1746** | **1** | **2** | **64** | **9** | **K.GLGTDDNTLIRVMVSR.A** |
|  | 20346 | 675.3218 | 2022.9435 | 2022.9793 | -0.0358 | 1 | 3 | 46 | 10 | K.GGTVKAASGFNAMEDAQTLR.K |
|  | 20531 | **680.3568** | **2038.0486** | **2038.0405** | **0.0081** | **1** | **4** | **37** | **3** | **K.AMKGLGTDEDAIISVLAYR.N** |
|  | 23188 | 773.7314 | 2318.1723 | 2318.1866 | -0.0143 | 1 | 36 | 0.019 | 1 | R.VLVSLSAGGRDEGNYLDDALVR.Q |
|  | 30198 | **1289.9797** | **3866.9174** | **3866.9322** | **-0.0148** | **1** | **18** | **0.48** | **1** | **R.DLIDDLKSELSGNFEQVIVGMMTPTVLYDVQELR.R** |

| **139.** | P48047\|ATPO_HUMAN    **Mass:** 23377    **Score:** 326    **Queries matched:** 24   **emPAI:** 2.35 |
| --- | --- |
|  | ATP synthase subunit O, mitochondrial OS=Homo sapiens GN=ATP5O PE=1 SV=1 |

|  | **Query** | **Observed** | **Mr(expt)** | **Mr(calc)** | **Delta** | **Miss** | **Score** | **Expect** | **Rank** | **Peptide** |
| --- | --- | --- | --- | --- | --- | --- | --- | --- | --- | --- |
|  | 3060 | 861.4617 | 860.4544 | 860.4603 | -0.0059 | 0 | 9 | 30 | 2 | K.SLNDITAK.E |
|  | 5427 | **513.2814** | **1024.5483** | **1024.6281** | **-0.0797** | **1** | **4** | **54** | **10** | **R.VAQILKEPK.V** |
|  | 5867 | **529.8942** | **1057.7739** | **1058.5542** | **-0.7803** | **0** | **(8)** | **25** | **6** | **-.MAAPAVSGLSR.Q** 5873 5894 |
|  | 6114 | **537.8119** | **1073.6092** | **1074.5492** | **-0.9399** | **0** | **8** | **30** | **2** | **-.MAAPAVSGLSR.Q** |
|  | 6541 | **553.8106** | **1105.6065** | **1105.6132** | **-0.0066** | **0** | **61** | **0.00014** | **1** | **K.SFLSQGQVLK.L** 6536 |
|  | 7037 | 573.7308 | 1145.4470 | 1144.5764 | 0.8706 | 0 | 22 | 1.1 | 1 | R.YATALYSAASK.Q |
|  | 7229 | **580.7872** | **1159.5598** | **1159.6601** | **-0.1003** | **0** | **6** | **40** | **9** | **K.VAASVLNPYVK.R** |
|  | 9017 | **629.8412** | **1257.6678** | **1257.6751** | **-0.0073** | **0** | **41** | **0.013** | **1** | **K.TDPSILGGMIVR.I** 9016 |
|  | 12590 | **730.3252** | **1458.6358** | **1457.7773** | **0.8586** | **1** | **3** | **61** | **3** | **-.MAAPAVSGLSRQVR.C** |
|  | 13499 | **758.5594** | **1515.1042** | **1514.7729** | **0.3314** | **1** | **3** | **46** | **2** | **R.YATALYSAASKQNK.L** |
|  | 18633 | **937.0076** | **1872.0006** | **1872.0105** | **-0.0099** | **0** | **60** | **8.9e-005** | **1** | **R.FSPLTTNLINLLAENGR.L** 18613 18634 18654 18655 |
|  | 20680 | **1026.4970** | **2050.9793** | **2050.9928** | **-0.0135** | **0** | **110** | **1e-009** | **1** | **R.LSNTQGVVSAFSTMMSVHR.G** 20682 |
|  | 20851 | **690.3195** | **2067.9366** | **2066.9878** | **0.9488** | **0** | **(11)** | **6.6** | **1** | **R.LSNTQGVVSAFSTMMSVHR.G** |
|  | 23178 | **1159.5681** | **2317.1217** | **2317.1359** | **-0.0142** | **0** | **56** | **0.0002** | **1** | **R.GEVPCTVTSASPLEEATLSELK.T** 23177 |

| **140.** | Q13509\|TBB3_HUMAN    **Mass:** 50856    **Score:** 326    **Queries matched:** 26   **emPAI:** 0.76 |
| --- | --- |
|  | Tubulin beta-3 chain OS=Homo sapiens GN=TUBB3 PE=1 SV=2 |

|  | **Query** | **Observed** | **Mr(expt)** | **Mr(calc)** | **Delta** | **Miss** | **Score** | **Expect** | **Rank** | **Peptide** |
| --- | --- | --- | --- | --- | --- | --- | --- | --- | --- | --- |
|  | 1837 | 737.3976 | 736.3904 | 737.3456 | -0.9553 | 0 | 9 | 21 | 2 | R.GSQQYR.A |
|  | 2296 | 405.2068 | 808.3991 | 807.3399 | 1.0593 | 0 | 8 | 28 | 7 | R.EEYPDR.I |
|  | 5960 | 533.2140 | 1064.4134 | 1064.4201 | -0.0067 | 0 | 11 | 12 | 1 | K.NMMAACDPR.H 5970 |
|  | 8471 | 615.2986 | 1228.5827 | 1228.5910 | -0.0083 | 0 | 66 | 3.7e-005 | 1 | R.ISEQFTAMFR.R |
|  | 8778 | 415.7271 | 1244.1593 | 1244.5860 | -0.4266 | 0 | (10) | 11 | 1 | R.ISEQFTAMFR.R |
|  | 10180 | 660.3506 | 1318.6867 | 1318.6955 | -0.0088 | 0 | 67 | 3e-005 | 1 | R.IMNTFSVVPSPK.V 10151 |
|  | 11355 | 462.7160 | 1385.1261 | 1384.6921 | 0.4339 | 1 | 4 | 34 | 7 | K.RISEQFTAMFR.R |
|  | 12166 | 716.3267 | 1430.6389 | 1430.5965 | 0.0423 | 1 | 8 | 21 | 2 | K.NMMAACDPRHGR.Y 12164 12165 |
|  | 14975 | 808.4165 | 1614.8184 | 1614.8287 | -0.0103 | 0 | 33 | 0.062 | 1 | R.AILVDLEPGTMDSVR.S 14974 |
|  | 15266 | 544.9023 | 1631.6850 | 1630.8236 | 0.8614 | 0 | (6) | 28 | 2 | R.AILVDLEPGTMDSVR.S 15251 |
|  | 16113 | 846.4318 | 1690.8491 | 1690.8600 | -0.0109 | 0 | 45 | 0.0035 | 1 | R.ALTVPELTQQMFDAK.N 16114 |
|  | 16178 | 848.9148 | 1695.8150 | 1695.8257 | -0.0106 | 0 | 54 | 0.00046 | 1 | K.NSSYFVEWIPNNVK.V 16177 16189 |
|  | 17883 | 911.9587 | 1821.9028 | 1821.9156 | -0.0127 | 0 | 54 | 0.00051 | 1 | R.EIVHIQAGQCGNQIGAK.F 17882 17884 |
|  | 18572 | **934.4942** | **1866.9738** | **1866.8889** | **0.0849** | **1** | **1** | **72** | **10** | **R.MSMKEVDEQMLAIQSK.N** |
|  | 19688 | 979.9890 | 1957.9635 | 1957.9745 | -0.0111 | 0 | 82 | 5.4e-007 | 1 | K.GHYTEGAELVDSVLDVVR.K |

| **141.** | P35232\|PHB_HUMAN    **Mass:** 29843    **Score:** 325    **Queries matched:** 19   **emPAI:** 2.71 |
| --- | --- |
|  | Prohibitin OS=Homo sapiens GN=PHB PE=1 SV=1 |

|  | **Query** | **Observed** | **Mr(expt)** | **Mr(calc)** | **Delta** | **Miss** | **Score** | **Expect** | **Rank** | **Peptide** |
| --- | --- | --- | --- | --- | --- | --- | --- | --- | --- | --- |
|  | 5417 | **1024.5244** | **1023.5171** | **1022.4920** | **1.0251** | **0** | **4** | **62** | **3** | **K.EFTEAVEAK.Q** |
|  | 7081 | **575.2950** | **1148.5755** | **1148.5826** | **-0.0071** | **0** | **73** | **7.2e-006** | **1** | **R.FDAGELITQR.E** |
|  | 7581 | **593.3292** | **1184.6438** | **1184.6513** | **-0.0076** | **0** | **43** | **0.0073** | **1** | **K.DLQNVNITLR.I** |
|  | 7667 | **397.2194** | **1188.6364** | **1188.6350** | **0.0015** | **1** | **7** | **34** | **10** | **K.KAAIISAEGDSK.A** |
|  | 7811 | **399.7609** | **1196.2610** | **1195.6271** | **0.6339** | **1** | **5** | **33** | **3** | **-.MAAKVFESIGK.F** |
|  | 11545 | **466.2825** | **1395.8256** | **1395.8350** | **-0.0095** | **0** | **45** | **0.0039** | **1** | **R.ILFRPVASQLPR.I** 11544 |
|  | 11825 | **471.3338** | **1410.9796** | **1409.8103** | **1.1693** | **1** | **4** | **38** | **10** | **R.SRPRNVPVITGSK.D** |
|  | 12357 | **722.8281** | **1443.6416** | **1443.6518** | **-0.0102** | **0** | **52** | **0.00077** | **1** | **R.IFTSIGEDYDER.V** 12358 |
|  | 14824 | **803.9197** | **1605.8248** | **1605.8362** | **-0.0114** | **1** | **89** | **1.5e-007** | **1** | **R.KLEAAEDIAYQLSR.S** 14825 |
|  | 18377 | **927.4199** | **1852.8252** | **1854.0251** | **-1.1999** | **0** | **33** | **0.051** | **1** | **R.NITYLPAGQSVLLQLPQ.-** 18394 |
|  | 20094 | **999.5419** | **1997.0693** | **1997.0793** | **-0.0100** | **0** | **98** | **1.4e-008** | **1** | **K.AAELIANSLATAGDGLIELR.K** |
|  | 27856 | **1041.8814** | **3122.6222** | **3122.6376** | **-0.0154** | **0** | **59** | **6.3e-005** | **1** | **R.GVQDIVVGEGTHFLIPWVQKPIIFDCR.S** 27857 27858 |
|  | 27891 | **627.1171** | **3130.5490** | **3130.6564** | **-0.1074** | **1** | **5** | **16** | **2** | **K.VFESIGKFGLALAVAGGVVNSALYNVDAGHR.A** |

| **142.** | O43895\|XPP2_HUMAN    **Mass:** 76090    **Score:** 322    **Queries matched:** 30   **emPAI:** 0.40 |
| --- | --- |
|  | Xaa-Pro aminopeptidase 2 OS=Homo sapiens GN=XPNPEP2 PE=1 SV=3 |

|  | **Query** | **Observed** | **Mr(expt)** | **Mr(calc)** | **Delta** | **Miss** | **Score** | **Expect** | **Rank** | **Peptide** |
| --- | --- | --- | --- | --- | --- | --- | --- | --- | --- | --- |
|  | 897 | 518.3204 | 517.3132 | 516.3020 | 1.0112 | 0 | 11 | 30 | 4 | K.VSGVR.S |
|  | 1257 | **593.2918** | **592.2845** | **591.3380** | **0.9465** | **0** | **16** | **4.4** | **3** | **R.LFANK.S** |
|  | 1708 | **701.4152** | **700.4079** | **700.4119** | **-0.0040** | **0** | **12** | **15** | **2** | **K.EQALLK.A** |
|  | 4487 | **478.2620** | **954.5095** | **953.5406** | **0.9688** | **1** | **14** | **6.4** | **3** | **K.VGPELQRR.Q** 4457 4458 4473 4482 4489 |
|  | 5475 | **515.2455** | **1028.4764** | **1029.5818** | **-1.1054** | **1** | **3** | **84** | **9** | **K.NSKEQALLK.A** |
|  | 6452 | **550.3235** | **1098.6324** | **1098.6397** | **-0.0073** | **0** | **47** | **0.0031** | **1** | **R.VLIGNIDLSR.L** |
|  | 6931 | 568.7974 | 1135.5803 | 1134.5815 | 0.9987 | 1 | 6 | 37 | 8 | K.VSGVRSQMQK.H |
|  | 12729 | 489.9265 | 1466.7577 | 1465.6937 | 1.0640 | 0 | 4 | 45 | 10 | K.GTVDEFSGAEIVDK.F 12727 |
|  | 13015 | **743.3650** | **1484.7154** | **1484.7255** | **-0.0101** | **0** | **43** | **0.0072** | **1** | **K.LVTDTYSPVMMTK.A** |
|  | 13273 | **750.8472** | **1499.6799** | **1500.7204** | **-1.0405** | **0** | **(1)** | **1.1e+002** | **5** | **K.LVTDTYSPVMMTK.A** |
|  | 19479 | 970.9573 | 1939.9001 | 1941.0030 | -1.1029 | 1 | 1 | 79 | 10 | R.AWITGFTGSAGTAVVTMKK.A |
|  | 19824 | **657.6774** | **1970.0103** | **1969.0092** | **1.0011** | **1** | **2** | **63** | **10** | **R.RAWITGFTGSAGTAVVTMK.K** |
|  | 21336 | **1056.0775** | **2110.1405** | **2109.1470** | **0.9935** | **0** | **82** | **5e-007** | **1** | **K.EVGTTPIVTWLLTEIPAGGR.V** 21321 21322 |
|  | 21486 | **1062.5700** | **2123.1253** | **2123.1375** | **-0.0121** | **0** | **76** | **2e-006** | **1** | **R.NLIDVSLLSPEHLQYLNR.Y** 21487 |
|  | 23130 | **1156.5756** | **2311.1366** | **2309.9946** | **1.1420** | **1** | **1** | **58** | **3** | **R.YWTQAERQMDCNWELHK.E** |
|  | 23568 | **790.0632** | **2367.1679** | **2367.1787** | **-0.0108** | **1** | **13** | **3.9** | **1** | **K.TKYPGSYLTFEVVSFVPYDR.N** |
|  | 25071 | **1285.6178** | **2569.2210** | **2569.2377** | **-0.0166** | **0** | **108** | **1.1e-009** | **1** | **R.ASDIPYNPFFYSYTLLTDSSIR.L** 25073 |
|  | 27720 | **1028.8207** | **3083.4402** | **3082.4520** | **0.9883** | **0** | **21** | **0.39** | **1** | **R.GEEQFSSGPSFETISASGLNAALAHYSPTK.E** |
|  | 28883 | **847.4097** | **3385.6096** | **3385.6215** | **-0.0119** | **1** | **27** | **0.085** | **1** | **K.FRGEEQFSSGPSFETISASGLNAALAHYSPTK.E** |
|  | 31207 | **1598.6325** | **4792.8755** | **4793.4632** | **-0.5877** | **1** | **0** | **10** | **3** | **K.VPTAVLLSALEETAWLFNLRASDIPYNPFFYSYTLLTDSSIR.L** |

| **143.** | P24462\|CP3A7_HUMAN    **Mass:** 57831    **Score:** 320    **Queries matched:** 26   **emPAI:** 0.55 |
| --- | --- |
|  | Cytochrome P450 3A7 OS=Homo sapiens GN=CYP3A7 PE=1 SV=2 |

|  | **Query** | **Observed** | **Mr(expt)** | **Mr(calc)** | **Delta** | **Miss** | **Score** | **Expect** | **Rank** | **Peptide** |
| --- | --- | --- | --- | --- | --- | --- | --- | --- | --- | --- |
|  | 1096 | 560.3808 | 559.3735 | 558.3741 | 0.9994 | 0 | 23 | 1.6 | 1 | K.TVLVK.E |
|  | 2578 | 415.2532 | 828.4919 | 827.4752 | 1.0167 | 0 | 5 | 60 | 9 | K.ETQIPLK.L |
|  | 6416 | **548.8056** | **1095.5966** | **1096.6604** | **-1.0638** | **1** | **4** | **56** | **5** | **K.ETQIPLKLR.F** |
|  | 6739 | **561.3737** | **1120.7329** | **1120.6856** | **0.0473** | **1** | **5** | **46** | **3** | **K.VISFLTKSVK.Q** |
|  | 7386 | 586.8259 | 1171.6373 | 1171.6448 | -0.0075 | 0 | 41 | 0.012 | 1 | R.EAETGKPVTLK.H |
|  | 7417 | 588.2571 | 1174.4997 | 1174.5077 | -0.0080 | 0 | 20 | 1.8 | 1 | K.ECYSVFTNR.R |
|  | 9104 | 631.8223 | 1261.6301 | 1261.6376 | -0.0075 | 0 | 17 | 3.3 | 1 | K.DVEINGMFIPK.G 9105 9121 |
|  | 11017 | 684.3561 | 1366.6976 | 1366.7067 | -0.0091 | 0 | 22 | 0.84 | 1 | R.VLQNFSFKPCK.E |
|  | 11435 | **464.2452** | **1389.7138** | **1389.8166** | **-0.1028** | **1** | **0** | **1.2e+002** | **8** | **R.FALVNMKLALVR.V** |
|  | 11691 | 703.3827 | 1404.7508 | 1405.7929 | -1.0421 | 1 | 11 | 10 | 5 | R.IRSLLSPTFTSGK.L |
|  | 15336 | 819.4098 | 1636.8050 | 1636.8130 | -0.0080 | 0 | 82 | 7.2e-007 | 1 | R.VDFLQLMIDSQNSK.D 15335 15355 |
|  | 16284 | 851.9592 | 1701.9038 | 1701.9124 | -0.0086 | 0 | 77 | 2.5e-006 | 1 | K.EMVPIIAQYGDVLVR.N 16283 |
|  | 17700 | **603.3419** | **1807.0038** | **1805.9498** | **1.0539** | **0** | **7** | **23** | **1** | **K.GVVVMIPSYVLHHDPK.Y** |
|  | 17745 | 906.4283 | 1810.8421 | 1810.8526 | -0.0105 | 0 | 31 | 0.085 | 1 | K.DNIDPYIYTPFGSGPR.N 17743 17744 |
|  | 19372 | 483.4975 | 1929.9609 | 1929.9731 | -0.0122 | 1 | 6 | 27 | 3 | K.HRVDFLQLMIDSQNSK.D |
|  | 19516 | 648.7004 | 1943.0795 | 1943.0914 | -0.0119 | 1 | 42 | 0.0065 | 1 | K.LKEMVPIIAQYGDVLVR.N |
|  | 20715 | 1027.4968 | 2052.9791 | 2052.9905 | -0.0114 | 1 | 74 | 3.3e-006 | 1 | K.NKDNIDPYIYTPFGSGPR.N 20714 |
|  | 31142 | **1173.2203** | **4688.8523** | **4688.3975** | **0.4547** | **0** | **2** | **9.6** | **1** | **K.ALSDLELMAQSIIFIFAGYETTSSVLSFIIYELATHPDVQQK.V** |

| **144.** | Q96A33\|CCD47_HUMAN    **Mass:** 56123    **Score:** 314    **Queries matched:** 23   **emPAI:** 0.47 |
| --- | --- |
|  | Coiled-coil domain-containing protein 47 OS=Homo sapiens GN=CCDC47 PE=1 SV=1 |

|  | **Query** | **Observed** | **Mr(expt)** | **Mr(calc)** | **Delta** | **Miss** | **Score** | **Expect** | **Rank** | **Peptide** |
| --- | --- | --- | --- | --- | --- | --- | --- | --- | --- | --- |
|  | 4516 | **479.2737** | **956.5329** | **956.5403** | **-0.0074** | **1** | **45** | **0.0057** | **1** | **R.RLEEAALR.R** |
|  | 6727 | **561.2684** | **1120.5223** | **1119.4754** | **1.0469** | **0** | **5** | **58** | **6** | **R.IMNEEDPEK.Q** |
|  | 7616 | **396.5412** | **1186.6019** | **1187.5856** | **-0.9837** | **0** | **12** | **11** | **5** | **K.IMQEEGQPLK.L** |
|  | 11404 | **694.7881** | **1387.5616** | **1388.6241** | **-1.0625** | **1** | **8** | **20** | **4** | **K.ERIMNEEDPEK.Q** |
|  | 12175 | **716.3718** | **1430.7291** | **1430.7374** | **-0.0083** | **0** | **34** | **0.053** | **1** | **R.MMRPVSDQVQIK.V** |
|  | 14716 | 800.4053 | 1598.7960 | 1599.8018 | -1.0058 | 1 | 1 | 1e+002 | 4 | K.NSRLAQAWFNTHR.E |
|  | 16434 | **571.9170** | **1712.7291** | **1712.7386** | **-0.0094** | **0** | **18** | **1.6** | **1** | **K.EMQDLSEFCSDKPK.S** |
|  | 16604 | **864.4883** | **1726.9621** | **1725.8971** | **1.0650** | **1** | **2** | **68** | **2** | **K.IMQEEGQPLKLPDTK.R** |
|  | 18340 | 926.4467 | 1850.8788 | 1850.8898 | -0.0110 | 0 | 75 | 3e-006 | 1 | R.ELLESNFTLVGDDGTNK.E 18356 |
|  | 18403 | 928.4772 | 1854.9399 | 1854.9516 | -0.0117 | 0 | 36 | 0.029 | 1 | R.TLLFTFNVPGSGNTYPK.D |
|  | 21149 | **1048.5234** | **2095.0323** | **2095.0403** | **-0.0080** | **0** | **38** | **0.015** | **1** | **K.DMEALLPLMNMVIYSIDK.A** 21150 |
|  | 21347 | **1057.0045** | **2111.9945** | **2111.0352** | **0.9593** | **0** | **(2)** | **56** | **4** | **K.DMEALLPLMNMVIYSIDK.A** |
|  | 22255 | **552.5512** | **2206.1755** | **2206.9875** | **-0.8120** | **1** | **1** | **58** | **7** | **K.VTMNDEDMDTYVFAVGTRK.A** |
|  | 23132 | 771.3987 | 2311.1742 | 2310.1673 | 1.0069 | 1 | 10 | 8.7 | 2 | K.DMEALLPLMNMVIYSIDKAK.K |
|  | 25083 | **1287.0876** | **2572.1607** | **2572.1747** | **-0.0139** | **0** | **93** | **3.1e-008** | **1** | **K.YGLPDSLAILSEMGEVTDGMMDTK.M** 25084 25085 |
|  | 26939 | **730.8527** | **2919.3818** | **2919.4014** | **-0.0196** | **1** | **5** | **17** | **1** | **K.MVHFLTHYADKIESVHFSDQFSGPK.I** |
|  | 26985 | 734.1393 | 2932.5283 | 2931.3551 | 1.1731 | 1 | 3 | 25 | 6 | K.SGAKYGLPDSLAILSEMGEVTDGMMDTK.M |
|  | 27172 | 988.5065 | 2962.4978 | 2963.3450 | -0.8472 | 1 | (2) | 36 | 7 | K.SGAKYGLPDSLAILSEMGEVTDGMMDTK.M |
|  | 30365 | **796.5802** | **3977.8646** | **3978.8036** | **-0.9390** | **1** | **1** | **21** | **2** | **K.YGLPDSLAILSEMGEVTDGMMDTKMVHFLTHYADK.I** |

| **145.** | Q16820\|MEP1B_HUMAN    **Mass:** 80500    **Score:** 312    **Queries matched:** 20   **emPAI:** 0.50 |
| --- | --- |
|  | Meprin A subunit beta OS=Homo sapiens GN=MEP1B PE=1 SV=2 |

|  | **Query** | **Observed** | **Mr(expt)** | **Mr(calc)** | **Delta** | **Miss** | **Score** | **Expect** | **Rank** | **Peptide** |
| --- | --- | --- | --- | --- | --- | --- | --- | --- | --- | --- |
|  | 1028 | **546.2509** | **545.2436** | **544.3333** | **0.9104** | **0** | **12** | **24** | **6** | **R.ILSGR.E** 1022 1025 |
|  | 5349 | **509.7842** | **1017.5539** | **1017.5607** | **-0.0068** | **0** | **4** | **78** | **3** | **K.GVILNAFER.Y** |
|  | 7300 | **583.7502** | **1165.4859** | **1165.4935** | **-0.0075** | **0** | **44** | **0.0055** | **1** | **K.GSGCWSSVGNR.R** 7298 7299 |
|  | 7354 | **585.7674** | **1169.5202** | **1169.5274** | **-0.0072** | **0** | **52** | **0.00088** | **1** | **R.MDFSDSDLLK.L** |
|  | 7618 | **594.3127** | **1186.6108** | **1185.5224** | **1.0885** | **0** | **(1)** | **1.2e+002** | **5** | **R.MDFSDSDLLK.L** |
|  | 9096 | **631.7887** | **1261.5628** | **1261.5721** | **-0.0092** | **0** | **34** | **0.057** | **1** | **K.QELSIGANCDR.I** |
|  | 12489 | **726.8422** | **1451.6698** | **1451.6794** | **-0.0096** | **0** | **48** | **0.002** | **1** | **R.GGGYGTSAFITHER.L** |
|  | 14856 | **536.9304** | **1607.7693** | **1607.7805** | **-0.0112** | **1** | **33** | **0.058** | **1** | **R.RGGGYGTSAFITHER.L** |
|  | 16288 | **852.3265** | **1702.6385** | **1702.6504** | **-0.0119** | **0** | **59** | **0.00011** | **1** | **R.CQSGEDWWYMGER.C** 16287 |
|  | 20185 | **669.9780** | **2006.9123** | **2007.9637** | **-1.0514** | **0** | **8** | **13** | **2** | **R.EYSADNVDGNLTLVEEIK.E** |
|  | 21049 | **1045.5324** | **2089.0501** | **2089.0651** | **-0.0150** | **1** | **90** | **8.6e-008** | **1** | **R.KGSGASLGGLSIDDINLSETR.C** |
|  | 21862 | **1080.5573** | **2159.0999** | **2158.0405** | **1.0595** | **0** | **1** | **74** | **10** | **R.WPHTIPYVLEDSLEMNAK.G** |
|  | 22836 | **759.3683** | **2275.0832** | **2275.0983** | **-0.0151** | **0** | **50** | **0.00071** | **1** | **K.TCIDFKPWAGETNYISVFK.G** 22865 |
|  | 29039 | **1147.8268** | **3440.4585** | **3440.4779** | **-0.0194** | **0** | **73** | **1.6e-006** | **1** | **R.EHNFNTYSDDISDSLNVPYDYTSVMHYSK.T** |

| **146.** | P06744\|G6PI_HUMAN    **Mass:** 63335    **Score:** 312    **Queries matched:** 27   **emPAI:** 0.41 |
| --- | --- |
|  | Glucose-6-phosphate isomerase OS=Homo sapiens GN=GPI PE=1 SV=4 |

|  | **Query** | **Observed** | **Mr(expt)** | **Mr(calc)** | **Delta** | **Miss** | **Score** | **Expect** | **Rank** | **Peptide** |
| --- | --- | --- | --- | --- | --- | --- | --- | --- | --- | --- |
|  | 1225 | **586.9156** | **585.9083** | **586.3802** | **-0.4719** | **1** | **1** | **1.1e+002** | **1** | **K.QLAKK.I** |
|  | 1639 | **677.3937** | **676.3864** | **677.3530** | **-0.9666** | **0** | **20** | **2.5** | **6** | **-.MAALTR.D** |
|  | 1794 | **724.7210** | **723.7137** | **724.3214** | **-0.6077** | **0** | **5** | **27** | **7** | **R.MFNGEK.I** |
|  | 2919 | **426.7112** | **851.4079** | **851.4137** | **-0.0058** | **0** | **22** | **0.91** | **1** | **K.INYTEGR.A** |
|  | 3378 | **439.7791** | **877.5436** | **877.5498** | **-0.0062** | **0** | **17** | **4** | **1** | **R.AVLHVALR.N** |
|  | 4793 | **489.2997** | **976.5849** | **977.4930** | **-0.9081** | **1** | **10** | **19** | **3** | **R.LFDANKDR.F** |
|  | 4982 | **496.7602** | **991.5058** | **991.5127** | **-0.0069** | **0** | **32** | **0.1** | **1** | **K.EWFLQAAK.D** |
|  | 5711 | **524.2426** | **1046.4705** | **1047.5746** | **-1.1041** | **1** | **6** | **43** | **8** | **R.MLVDLAKSR.G** |
|  | 6129 | **538.2823** | **1074.5500** | **1075.5332** | **-0.9832** | **0** | **(6)** | **49** | **8** | **K.NLVTEDVMR.M** |
|  | 6377 | **547.3035** | **1092.5924** | **1091.5281** | **1.0643** | **0** | **11** | **13** | **1** | **K.NLVTEDVMR.M** 6351 |
|  | 8269 | **609.8234** | **1217.6322** | **1217.6404** | **-0.0083** | **0** | **27** | **0.29** | **1** | **K.HFVALSTNTTK.V** |
|  | 14756 | **801.9119** | **1601.8092** | **1601.8202** | **-0.0110** | **0** | **52** | **0.00071** | **1** | **R.VWYVSNIDGTHIAK.T** 14757 |
|  | 15607 | **828.4037** | **1654.7928** | **1654.8050** | **-0.0122** | **0** | **75** | **3.8e-006** | **1** | **K.TFTTQETITNAETAK.E** |
|  | 16294 | **852.4761** | **1702.9376** | **1702.9440** | **-0.0064** | **0** | **66** | **2.6e-005** | **1** | **K.ILLANFLAQTEALMR.G** 16293 |
|  | 16372 | **855.3425** | **1708.6705** | **1707.8944** | **0.7761** | **0** | **13** | **4.4** | **2** | **K.VFEGNRPTNSIVFTK.L** |
|  | 18052 | 916.5068 | 1830.9990 | 1831.0091 | -0.0101 | 0 | 40 | 0.0095 | 1 | K.TLAQLNPESSLFIIASK.T |
|  | 18135 | **613.3307** | **1836.9704** | **1835.9314** | **1.0390** | **0** | **1** | **88** | **7** | **K.LTPFMLGALVAMYEHK.I** |
|  | 19043 | **952.4944** | **1902.9742** | **1904.0553** | **-1.0811** | **1** | **0** | **94** | **1** | **K.ILLANFLAQTEALMRGK.S** |
|  | 19830 | **657.8565** | **1970.5477** | **1970.9983** | **-0.4506** | **1** | **1** | **53** | **8** | **R.SNTPILVDGKDVMPEVNK.V** |
|  | 23248 | **1165.5156** | **2329.0167** | **2329.0263** | **-0.0096** | **0** | **96** | **1.9e-008** | **1** | **K.EFGIDPQNMFEFWDWVGGR.Y** 23247 |
|  | 23378 | **782.5679** | **2344.6820** | **2345.0212** | **-0.3392** | **0** | **(1)** | **36** | **3** | **K.EFGIDPQNMFEFWDWVGGR.Y** 23375 |
|  | 24992 | **853.0668** | **2556.1787** | **2556.1896** | **-0.0110** | **1** | **27** | **0.12** | **1** | **K.VKEFGIDPQNMFEFWDWVGGR.Y** |

| **147.** | Q9NYU2\|UGGG1_HUMAN    **Mass:** 177819   **Score:** 312    **Queries matched:** 42   **emPAI:** 0.20 |
| --- | --- |
|  | UDP-glucose:glycoprotein glucosyltransferase 1 OS=Homo sapiens GN=UGCGL1 PE=1 SV=3 |

|  | **Query** | **Observed** | **Mr(expt)** | **Mr(calc)** | **Delta** | **Miss** | **Score** | **Expect** | **Rank** | **Peptide** |
| --- | --- | --- | --- | --- | --- | --- | --- | --- | --- | --- |
|  | 729 | **485.3050** | **484.2977** | **484.3009** | **-0.0032** | **0** | **20** | **0.98** | **1** | **K.GPIAK.F** |
|  | 1397 | **623.3581** | **622.3508** | **621.3486** | **1.0022** | **0** | **26** | **0.41** | **1** | **K.NFITK.M** |
|  | 1971 | 778.8836 | 777.8763 | 778.4377 | -0.5614 | 0 | 18 | 2 | 4 | R.IIWGYK.I 1972 |
|  | 2511 | 413.6945 | 825.3745 | 825.4232 | -0.0487 | 1 | 7 | 31 | 8 | K.STEYKAK.D |
|  | 3444 | **441.7500** | **881.4854** | **880.4879** | **0.9975** | **0** | **3** | **73** | **7** | **K.SHIQQLR.V** |
|  | 4365 | **474.2790** | **946.5434** | **945.4821** | **1.0614** | **0** | **11** | **15** | **1** | **R.HYIFNPR.K** |
|  | 5218 | **504.5847** | **1007.1548** | **1007.5288** | **-0.3739** | **0** | **2** | **71** | **5** | **R.FTILDSQGK.T** |
|  | 5648 | **521.2970** | **1040.5794** | **1040.5866** | **-0.0071** | **0** | **56** | **0.00042** | **1** | **K.VDALLSAQPK.G** |
|  | 6014 | **535.2708** | **1068.5269** | **1068.5352** | **-0.0083** | **0** | **10** | **15** | **3** | **R.WLHQQTEK.Q** |
|  | 8832 | **624.7997** | **1247.5849** | **1246.5441** | **1.0408** | **1** | **7** | **27** | **7** | **R.EMDGYRFWK.S** |
|  | 9212 | 634.7562 | 1267.4979 | 1267.6673 | -0.1694 | 1 | 10 | 14 | 1 | K.SNAGKINYVFR.H |
|  | 9337 | **425.5479** | **1273.6219** | **1274.7194** | **-1.0975** | **1** | **7** | **32** | **10** | **R.AITKTAVSSELR.T** |
|  | 9403 | 639.3457 | 1276.6768 | 1276.7247 | -0.0478 | 1 | 10 | 16 | 5 | R.IMMLSVLKNTK.T |
|  | 10507 | **446.2690** | **1335.7851** | **1335.7245** | **0.0606** | **1** | **3** | **61** | **5** | **K.ADSKAITTSLTTK.W** 10494 |
|  | 10545 | **670.3237** | **1338.6329** | **1338.6415** | **-0.0086** | **0** | **19** | **1.6** | **1** | **K.EISYENTQISR.A** 10544 |
|  | 11009 | **456.3151** | **1365.9235** | **1365.7876** | **0.1359** | **1** | **5** | **29** | **5** | **R.FLRIMMLSVLK.N** |
|  | 13350 | **753.8580** | **1505.7014** | **1504.7378** | **0.9636** | **1** | **8** | **21** | **4** | **R.AKTIDLCNNPMTK.E** |
|  | 14361 | 787.9163 | 1573.8181 | 1572.8260 | 0.9921 | 0 | 54 | 0.00045 | 1 | R.AIWAALQTQTSNAAK.N 14351 |
|  | 15237 | **815.9915** | **1629.9684** | **1629.9746** | **-0.0062** | **0** | **7** | **22** | **2** | **K.ILFLDVLFPLVVDK.F** |
|  | 15703 | **831.9284** | **1661.8423** | **1661.8526** | **-0.0103** | **0** | **58** | **0.00017** | **1** | **K.VWQLQDLSFQTAAR.I** |
|  | 17790 | **605.5485** | **1813.6236** | **1812.8572** | **0.7663** | **1** | **4** | **27** | **3** | **R.VFMNCQSKLSDMPLK.S** |
|  | 19927 | **661.2828** | **1980.8265** | **1981.9754** | **-1.1489** | **1** | **1** | **72** | **8** | **K.MDFILSHAVYCRDVLK.L** |
|  | 23219 | **775.4764** | **2323.4073** | **2323.1478** | **0.2596** | **1** | **2** | **39** | **6** | **R.ISMINNPAKEISYENTQISR.A** |
|  | 23874 | **806.7464** | **2417.2174** | **2416.0644** | **1.1530** | **1** | **1** | **53** | **3** | **-.MGCKGDASGACAAGALPVTGVCYK.M** 23859 |
|  | 24237 | **820.7706** | **2459.2901** | **2459.2961** | **-0.0060** | **0** | **20** | **0.67** | **1** | **R.YNSWPSSLQELLRPTFPGVIR.Q** 24238 |
|  | 25076 | **1285.6511** | **2569.2877** | **2569.2675** | **0.0201** | **0** | **47** | **0.0013** | **1** | **R.GYYEQTGVGPLPVVLFNGMPFER.E** 25060 25062 25064 |
|  | 25461 | **882.4232** | **2644.2478** | **2644.2657** | **-0.0179** | **0** | **23** | **0.33** | **1** | **R.IYSHDGTDSPPDADEVVIVLNNFK.S** |
|  | 26845 | **726.5922** | **2902.3395** | **2902.3562** | **-0.0167** | **0** | **3** | **31** | **1** | **R.AYNYVAQEVDDYHAFQTLTHIYNK.V** |
|  | 26963 | **975.8427** | **2924.5061** | **2924.5171** | **-0.0110** | **0** | **60** | **5.4e-005** | **1** | **R.IIGPLEDSELFNQDDFHLLENIILK.T** |
|  | 27604 | **1526.2436** | **3050.4727** | **3050.4882** | **-0.0154** | **0** | **85** | **1.5e-007** | **1** | **K.FLDMPQSPLFTLNLNTPESWMVESVR.T** 27605 |
|  | 29091 | **1155.2271** | **3462.6593** | **3462.6838** | **-0.0244** | **0** | **82** | **2.6e-007** | **1** | **R.GQYQGLSQDPNSLSNLDQDLPNNMIHQVPIK.S** |
|  | 30406 | **807.6054** | **4032.9904** | **4033.0012** | **-0.0108** | **1** | **20** | **0.26** | **1** | **R.KNLHNMVFIVDPAHETTAELMNTAEMFLSNHIPLR.I** |

| **148.** | P29401\|TKT_HUMAN    **Mass:** 68519    **Score:** 310    **Queries matched:** 24   **emPAI:** 0.37 |
| --- | --- |
|  | Transketolase OS=Homo sapiens GN=TKT PE=1 SV=3 |

|  | **Query** | **Observed** | **Mr(expt)** | **Mr(calc)** | **Delta** | **Miss** | **Score** | **Expect** | **Rank** | **Peptide** |
| --- | --- | --- | --- | --- | --- | --- | --- | --- | --- | --- |
|  | 1150 | **572.3729** | **571.3656** | **571.3693** | **-0.0037** | **0** | **17** | **4.2** | **1** | **K.LQALK.D** |
|  | 1183 | **577.2754** | **576.2681** | **575.2663** | **1.0018** | **0** | **8** | **35** | **1** | **K.DTANR.L** 1182 |
|  | 2403 | **409.2221** | **816.4296** | **817.4182** | **-0.9885** | **0** | **9** | **25** | **9** | **R.GITGVEDK.E** |
|  | 3920 | **458.2819** | **914.5493** | **914.5549** | **-0.0056** | **1** | **26** | **0.49** | **1** | **R.KLILDSAR.A** 3909 3910 3912 3927 3928 |
|  | 7488 | **590.3254** | **1178.6362** | **1177.6244** | **1.0118** | **0** | **3** | **73** | **10** | **K.ESWHGKPLPK.N** 7490 |
|  | 10491 | **446.0371** | **1335.0896** | **1334.6076** | **0.4820** | **1** | **2** | **58** | **5** | **K.SQDPRNPHNDR.F** |
|  | 15978 | 840.9700 | 1679.9255 | 1680.8795 | -0.9540 | 1 | 1 | 84 | 7 | K.LGHASDRIIALDGDTK.N |
|  | 18770 | **942.9582** | **1883.9018** | **1883.9153** | **-0.0135** | **0** | **28** | **0.17** | **1** | **R.SVPTSTVFYPSDGVATEK.A** |
|  | 20294 | **1010.5322** | **2019.0498** | **2019.0636** | **-0.0139** | **0** | **79** | **1.3e-006** | **1** | **K.ILATPPQEDAPSVDIANIR.M** 20295 |
|  | 20338 | **1012.0079** | **2022.0012** | **2022.0091** | **-0.0080** | **0** | **64** | **3.9e-005** | **1** | **K.NMAEQIIQEIYSQIQSK.K** 20339 |
|  | 20854 | **690.3379** | **2067.9918** | **2068.0048** | **-0.0129** | **0** | **10** | **8.6** | **1** | **R.LGQSDPAPLQHQMDIYQK.R** |
|  | 24613 | **836.7369** | **2507.1888** | **2507.2040** | **-0.0152** | **0** | **38** | **0.0096** | **1** | **R.TSRPENAIIYNNNEDFQVGQAK.V** |
|  | 27048 | **980.4653** | **2938.3742** | **2938.5110** | **-0.1368** | **1** | **2** | **33** | **3** | **K.ILATPPQEDAPSVDIANIRMPSLPSYK.V** |
|  | 29917 | **938.9783** | **3751.8842** | **3751.9057** | **-0.0215** | **0** | **2** | **23** | **1** | **R.ILTVEDHYYEGGIGEAVSSAVVGEPGITVTHLAVNR.V** |
|  | 30346 | **1323.2564** | **3966.7472** | **3966.7678** | **-0.0206** | **0** | **98** | **3.7e-009** | **1** | **R.MAAISESNINLCGSHCGVSIGEDGPSQMALEDLAMFR.S** |

| **149.** | P23229\|ITA6_HUMAN    **Mass:** 127751   **Score:** 308    **Queries matched:** 30   **emPAI:** 0.26 |
| --- | --- |
|  | Integrin alpha-6 OS=Homo sapiens GN=ITGA6 PE=1 SV=4 |

|  | **Query** | **Observed** | **Mr(expt)** | **Mr(calc)** | **Delta** | **Miss** | **Score** | **Expect** | **Rank** | **Peptide** |
| --- | --- | --- | --- | --- | --- | --- | --- | --- | --- | --- |
|  | 2720 | **419.7765** | **837.5384** | **837.5436** | **-0.0052** | **0** | **27** | **0.31** | **1** | **R.LLLVGAPR.A** |
|  | 3532 | 888.4882 | 887.4809 | 887.4461 | 0.0349 | 1 | 5 | 67 | 10 | K.QIDDNRK.F |
|  | 5442 | **513.7925** | **1025.5704** | **1025.5770** | **-0.0066** | **0** | **12** | **9.2** | **3** | **R.WNNVKPIR.L** |
|  | 5811 | **527.8185** | **1053.6224** | **1053.6295** | **-0.0070** | **0** | **17** | **2.7** | **1** | **R.SRPVINIQK.T** |
|  | 6938 | **569.2553** | **1136.4959** | **1135.6098** | **0.8861** | **1** | **5** | **52** | **6** | **K.SGLSSRVQFR.N** |
|  | 7135 | **577.2773** | **1152.5400** | **1151.5757** | **0.9643** | **0** | **13** | **7.4** | **1** | **R.CYVLSQNLR.I** |
|  | 7686 | 595.8192 | 1189.6237 | 1190.5932 | -0.9694 | 0 | 3 | 78 | 9 | K.DEITFVSGAPR.A |
|  | 9145 | **422.2404** | **1263.6994** | **1263.7411** | **-0.0417** | **1** | **8** | **25** | **2** | **R.ANHSGAVVLLKR.D** |
|  | 11456 | 696.3642 | 1390.7138 | 1389.7252 | 0.9886 | 0 | 39 | 0.018 | 1 | R.AFIDVTAAAENIR.L |
|  | 11569 | 699.3939 | 1396.7732 | 1396.6219 | 0.1513 | 1 | 8 | 20 | 7 | R.NPTKDGDDAHEAK.L 11563 |
|  | 13280 | **751.3542** | **1500.6938** | **1500.7031** | **-0.0093** | **0** | **72** | **7.4e-006** | **1** | **K.FGSCQQGVAATFTK.D** |
|  | 14191 | 782.4056 | 1562.7967 | 1561.8868 | 0.9099 | 1 | 2 | 80 | 9 | K.EISNGKWLLYLVK.V |
|  | 16453 | **858.4387** | **1714.8629** | **1714.8737** | **-0.0108** | **0** | **48** | **0.0017** | **1** | **K.LETTSNQDNLAPITAK.A** 16452 16458 |
|  | 16472 | **859.3652** | **1716.7158** | **1716.8651** | **-0.1493** | **1** | **5** | **34** | **3** | **R.QKTACGAPSGICLQVK.S** |
|  | 16658 | **866.4455** | **1730.8764** | **1730.8879** | **-0.0115** | **0** | **37** | **0.02** | **1** | **K.LIATFPDTLTYSAYR.E** 16659 |
|  | 17635 | **601.8024** | **1802.3855** | **1802.9275** | **-0.5420** | **1** | **1** | **60** | **5** | **R.LGAAFNLDTREDNVIR.K** |
|  | 18476 | **620.3245** | **1857.9517** | **1857.8316** | **0.1202** | **0** | **1** | **81** | **9** | **R.DGEVGGAVYVYMNQQGR.W** 18471 |
|  | 18540 | **622.3350** | **1863.9832** | **1862.9989** | **0.9843** | **0** | **4** | **39** | **8** | **R.VNSLPEVLPILNSDEPK.T** 18544 |
|  | 18660 | 937.8451 | 1873.6757 | 1873.6884 | -0.0126 | 0 | 74 | 2.8e-006 | 1 | R.IEDDMDGGDWSFCDGR.L |
|  | 19627 | 977.5289 | 1953.0432 | 1951.9211 | 1.1221 | 1 | 2 | 56 | 3 | R.GHEKFGSCQQGVAATFTK.D |
|  | 20379 | **1014.0017** | **2025.9889** | **2026.0007** | **-0.0119** | **0** | **98** | **1.4e-008** | **1** | **R.NSYPDVAVGSLSDSVTIFR.S** |
|  | 24715 | **1260.5900** | **2519.1654** | **2519.1816** | **-0.0162** | **0** | **59** | **8.1e-005** | **1** | **K.NIGDINQDGYPDIAVGAPYDDLGK.V** |
|  | 26204 | **923.0637** | **2766.1692** | **2766.3112** | **-0.1420** | **1** | **4** | **20** | **2** | **R.KYGDPGSLFGFSLAMHWQLQPEDK.R** |
|  | 28206 | **800.9004** | **3199.5725** | **3199.5826** | **-0.0101** | **0** | **9** | **5.6** | **2** | **K.SAHLLPEHIFDGEGLASSFGYDVAVVDLNK.D** |

| **150.** | P22309\|UD11_HUMAN    **Mass:** 60180    **Score:** 307    **Queries matched:** 15   **emPAI:** 0.62 |
| --- | --- |
|  | UDP-glucuronosyltransferase 1-1 OS=Homo sapiens GN=UGT1A1 PE=1 SV=1 |

|  | **Query** | **Observed** | **Mr(expt)** | **Mr(calc)** | **Delta** | **Miss** | **Score** | **Expect** | **Rank** | **Peptide** |
| --- | --- | --- | --- | --- | --- | --- | --- | --- | --- | --- |
|  | 4553 | **480.7598** | **959.5051** | **959.5110** | **-0.0059** | **0** | **46** | **0.0043** | **1** | **K.AMAIADALGK.I** |
|  | 5204 | 504.2660 | 1006.5175 | 1006.5236 | -0.0061 | 0 | 6 | 39 | 3 | K.TYPVPFQR.E |
|  | 5607 | **519.2922** | **1036.5699** | **1036.5553** | **0.0146** | **1** | **11** | **11** | **3** | **K.AVINDKSYK.E** 5603 |
|  | 5635 | 520.7592 | 1039.5039 | 1039.5120 | -0.0081 | 1 | 26 | 0.35 | 1 | K.SYKENIMR.L |
|  | 5836 | **528.7754** | **1055.5363** | **1055.5069** | **0.0294** | **1** | **(2)** | **1.1e+002** | **8** | **K.SYKENIMR.L** |
|  | 16950 | **876.9799** | **1751.9453** | **1751.9570** | **-0.0117** | **0** | **37** | **0.02** | **1** | **R.GHEIVVLAPDASLYIR.D** |
|  | 19596 | **975.5161** | **1949.0177** | **1949.0258** | **-0.0082** | **0** | **83** | **5.1e-007** | **1** | **R.EVTVQDLLSSASVWLFR.S** 19597 19598 |
|  | 22217 | **1102.5538** | **2203.0931** | **2203.1042** | **-0.0111** | **0** | **52** | **0.00056** | **1** | **K.GAGVTLNVLEMTSEDLENALK.A** |
|  | 22422 | **1113.0279** | **2224.0413** | **2224.0549** | **-0.0136** | **0** | **89** | **9.6e-008** | **1** | **K.ESFVSLGHNVFENDSFLQR.V** 22421 |
|  | 23185 | **773.4149** | **2317.2228** | **2317.1929** | **0.0298** | **0** | **10** | **8.2** | **1** | **K.DRPVEPLDLAVFWVEFVMR.H** |
|  | 25955 | **906.1672** | **2715.4799** | **2715.4894** | **-0.0095** | **0** | **41** | **0.0049** | **1** | **K.ILLIPVDGSHWLSMLGAIQQLQQR.G** |

| **151.** | P12236\|ADT3_HUMAN    **Mass:** 33073    **Score:** 305    **Queries matched:** 35   **emPAI:** 1.94 |
| --- | --- |
|  | ADP/ATP translocase 3 OS=Homo sapiens GN=SLC25A6 PE=1 SV=4 |

|  | **Query** | **Observed** | **Mr(expt)** | **Mr(calc)** | **Delta** | **Miss** | **Score** | **Expect** | **Rank** | **Peptide** |
| --- | --- | --- | --- | --- | --- | --- | --- | --- | --- | --- |
|  | 2733 | 840.3472 | 839.3399 | 839.3452 | -0.0053 | 0 | 29 | 0.17 | 1 | R.MMMQSGR.K 2752 |
|  | 2979 | 856.4830 | 855.4757 | 855.4814 | -0.0057 | 0 | 20 | 1.5 | 1 | K.TAVAPIER.V 2981 |
|  | 2982 | 428.7509 | 855.4871 | 855.4926 | -0.0055 | 0 | 9 | 21 | 7 | R.GNLANVIR.Y |
|  | 2990 | 429.0875 | 856.1604 | 855.3401 | 0.8203 | 0 | (9) | 14 | 6 | R.MMMQSGR.K |
|  | 2991 | 429.0876 | 856.1607 | 855.3401 | 0.8206 | 0 | (2) | 70 | 3 | R.MMMQSGR.K |
|  | 3056 | 431.2256 | 860.4367 | 860.4426 | -0.0058 | 0 | 31 | 0.15 | 1 | R.GLGDCLVK.I 3057 |
|  | 3304 | **437.2298** | **872.4450** | **873.4246** | **-0.9796** | **0** | **17** | **4.1** | **1** | **K.HTQFWR.Y** 3322 |
|  | 3739 | 451.7433 | 901.4720 | 901.4770 | -0.0050 | 0 | 38 | 0.032 | 1 | K.GAWSNVLR.G 3737 3738 |
|  | 4001 | **461.0073** | **920.0000** | **920.4716** | **-0.4715** | **1** | **11** | **9.6** | **5** | **K.IFRDEGGK.A** |
|  | 6730 | 561.2871 | 1120.5595 | 1120.5665 | -0.0070 | 0 | 27 | 0.35 | 1 | K.EQGVLSFWR.G |
|  | 6772 | **562.8160** | **1123.6174** | **1124.5536** | **-0.9362** | **0** | **1** | **1.1e+002** | **8** | **-.MTEQAISFAK.D** |
|  | 6935 | 568.8394 | 1135.6643 | 1135.6713 | -0.0070 | 0 | 38 | 0.025 | 1 | K.LLLQVQHASK.Q 6909 6934 |
|  | 8559 | **412.1891** | **1233.5454** | **1232.6765** | **0.8690** | **0** | **18** | **2.4** | **1** | **K.DFLAGGIAAAISK.T** |
|  | 9212 | 634.7562 | 1267.4979 | 1266.6972 | 0.8007 | 1 | 3 | 65 | 10 | K.YKQIFLGGVDK.H 9213 |
|  | 12409 | 723.8707 | 1445.7269 | 1445.7343 | -0.0074 | 0 | 59 | 0.00017 | 1 | R.YFPTQALNFAFK.D 12418 |
|  | 15157 | 542.9452 | 1625.8137 | 1626.8327 | -1.0190 | 0 | 2 | 78 | 9 | R.GMGGAFVLVLYDELK.K |
|  | 15444 | **822.8553** | **1643.6960** | **1643.7072** | **-0.0112** | **0** | **28** | **0.18** | **1** | **K.GADIMYTGTVDCWR.K** |
|  | 17222 | **886.9022** | **1771.7899** | **1771.8022** | **-0.0123** | **1** | **81** | **8.1e-007** | **1** | **R.KGADIMYTGTVDCWR.K** 17223 |
|  | 26343 | 1398.6686 | 2795.3226 | 2795.3377 | -0.0151 | 0 | 87 | 1.3e-007 | 1 | R.YFAGNLASGGAAGATSLCFVYPLDFAR.T 26345 26346 26348 26349 |
|  | 27608 | 1018.1352 | 3051.3837 | 3052.4865 | -1.1028 | 1 | 1 | 38 | 7 | R.YFAGNLASGGAAGATSLCFVYPLDFARTR.L |

| **152.** | P52272\|HNRPM_HUMAN    **Mass:** 77749    **Score:** 304    **Queries matched:** 93   **emPAI:** 0.45 |
| --- | --- |
|  | Heterogeneous nuclear ribonucleoprotein M OS=Homo sapiens GN=HNRNPM PE=1 SV=3 |

|  | **Query** | **Observed** | **Mr(expt)** | **Mr(calc)** | **Delta** | **Miss** | **Score** | **Expect** | **Rank** | **Peptide** |
| --- | --- | --- | --- | --- | --- | --- | --- | --- | --- | --- |
|  | 1951 | **771.4093** | **770.4020** | **769.2986** | **1.1034** | **0** | **2** | **1.1e+002** | **4** | **K.MEESMK.K** |
|  | 2014 | 395.2139 | 788.4132 | 788.3851 | 0.0282 | 0 | 2 | 1.6e+002 | 9 | R.MAAPIDR.V |
|  | 2250 | 805.4991 | 804.4918 | 804.3622 | 0.1296 | 0 | 8 | 35 | 4 | R.MGPVMDR.M 2229 |
|  | 2294 | 405.1943 | 808.3741 | 808.3749 | -0.0008 | 1 | 14 | 7 | 6 | K.MENGKSK.G 2296 |
|  | 2694 | **419.2662** | **836.5179** | **836.3884** | **0.1295** | **0** | **7** | **30** | **2** | **R.MGLVMDR.M** |
|  | 2714 | **419.7217** | **837.4289** | **836.3521** | **1.0769** | **0** | **(6)** | **42** | **4** | **R.MGPVMDR.M** |
|  | 2730 | **420.2430** | **838.4715** | **838.3677** | **0.1038** | **0** | **8** | **22** | **1** | **R.MGLSMER.M** |
|  | 3211 | 434.7104 | 867.4063 | 867.3579 | 0.0484 | 0 | 12 | 9.3 | 1 | R.MGQTMER.I 3204 3212 |
|  | 3482 | **443.0397** | **884.0648** | **883.3528** | **0.7120** | **0** | **(7)** | **20** | **5** | **R.MGQTMER.I** |
|  | 3600 | **447.7164** | **893.4183** | **892.4589** | **0.9594** | **0** | **7** | **32** | **9** | **K.ACQIFVR.N** |
|  | 3849 | 910.4343 | 909.4271 | 908.4426 | 0.9845 | 0 | 10 | 16 | 5 | R.GCAVVEFK.M |
|  | 4507 | **479.2471** | **956.4796** | **955.4004** | **1.0792** | **0** | **(2)** | **1e+002** | **4** | **R.FGSGMNMGR.I** |
|  | 4693 | **486.2659** | **970.5173** | **971.3953** | **-0.8780** | **0** | **(12)** | **11** | **8** | **R.FGSGMNMGR.I** 4686 |
|  | 4718 | **487.2669** | **972.5193** | **971.3953** | **1.1240** | **0** | **12** | **13** | **4** | **R.FGSGMNMGR.I** 4688 4719 |
|  | 6649 | **558.3035** | **1114.5925** | **1113.6393** | **0.9532** | **0** | **1** | **1.2e+002** | **6** | **R.INEILSNALK.R** |
|  | 6673 | **559.2816** | **1116.5487** | **1116.4805** | **0.0682** | **0** | **(1)** | **1.3e+002** | **3** | **R.MGAGLGHGMDR.V** |
|  | 6683 | 559.3779 | 1116.7412 | 1115.6339 | 1.1073 | 1 | 9 | 18 | 5 | K.WQSLKDLVK.E 6646 |
|  | 6903 | **567.7718** | **1133.5291** | **1132.4754** | **1.0538** | **0** | **2** | **1.1e+002** | **9** | **R.MGAGLGHGMDR.V** |
|  | 6964 | **570.3088** | **1138.6031** | **1139.5249** | **-0.9218** | **1** | **(2)** | **1e+002** | **4** | **R.MMNGMKLSGR.E** |
|  | 6989 | **571.3047** | **1140.5948** | **1139.5249** | **1.0699** | **1** | **(6)** | **39** | **2** | **R.MMNGMKLSGR.E** |
|  | 7054 | **574.2786** | **1146.5427** | **1145.4450** | **1.0977** | **1** | **0** | **1.5e+002** | **7** | **R.ACRMMNGMK.L** |
|  | 7188 | **579.2844** | **1156.5542** | **1155.5199** | **1.0343** | **1** | **(2)** | **1e+002** | **2** | **R.MMNGMKLSGR.E** |
|  | 7196 | **579.3209** | **1156.6273** | **1155.5199** | **1.1074** | **1** | **19** | **2.2** | **1** | **R.MMNGMKLSGR.E** 7159 7168 7184 |
|  | 7199 | **579.3212** | **1156.6279** | **1155.5199** | **1.1081** | **1** | **(13)** | **9.1** | **2** | **R.MMNGMKLSGR.E** 7195 |
|  | 7658 | **595.3027** | **1188.5909** | **1187.5791** | **1.0118** | **0** | **12** | **9.8** | **4** | **R.MVPAGMGAGLER.M** |
|  | 7979 | **402.2883** | **1203.8430** | **1203.5740** | **0.2690** | **0** | **(12)** | **7.9** | **3** | **R.MVPAGMGAGLER.M** |
|  | 7980 | **603.0793** | **1204.1441** | **1203.5740** | **0.5701** | **0** | **(2)** | **66** | **5** | **R.MVPAGMGAGLER.M** |
|  | 8071 | **605.2914** | **1208.5683** | **1208.6224** | **-0.0540** | **0** | **6** | **32** | **5** | **K.EVFSMAGVVVR.A** |
|  | 8323 | **407.7351** | **1220.1835** | **1219.5689** | **0.6146** | **0** | **(5)** | **32** | **3** | **R.MVPAGMGAGLER.M** |
|  | 12035 | **475.2605** | **1422.7596** | **1423.6548** | **-0.8952** | **1** | **5** | **39** | **9** | **R.MGSVERMGSGIER.M** |
|  | 12078 | 713.8774 | 1425.7402 | 1425.7504 | -0.0102 | 0 | 32 | 0.094 | 1 | R.LGSTVFVANLDYK.V |
|  | 12098 | **714.3554** | **1426.6961** | **1426.7061** | **-0.0099** | **0** | **40** | **0.014** | **1** | **R.MGPAMGPALGAGIER.M** |
|  | 12613 | **487.3202** | **1458.9389** | **1458.6959** | **0.2430** | **0** | **(8)** | **19** | **5** | **R.MGPAMGPALGAGIER.M** |
|  | 13205 | **499.3337** | **1494.9792** | **1495.6945** | **-0.7153** | **1** | **7** | **21** | **2** | **R.MGLVMDRMGSVER.M** |
|  | 13225 | **749.3417** | **1496.6688** | **1495.6945** | **0.9743** | **1** | **(3)** | **64** | **2** | **R.MGLVMDRMGSVER.M** |
|  | 13460 | **505.2956** | **1512.8649** | **1511.6895** | **1.1755** | **1** | **(5)** | **38** | **2** | **R.MGLVMDRMGSVER.M** |
|  | 14232 | 783.3669 | 1564.7193 | 1565.7290 | -1.0097 | 1 | 1 | 1e+002 | 8 | R.MGQTMERIGSGVER.M |
|  | 14348 | **525.2634** | **1572.7683** | **1571.8090** | **0.9593** | **1** | **1** | **95** | **6** | **R.MAAPIDRVGQTIER.M** |
|  | 14939 | **538.9284** | **1613.7634** | **1612.7701** | **0.9933** | **0** | **25** | **0.38** | **1** | **R.MGPLGLDHMASSIER.M** |
|  | 15679 | **830.9252** | **1659.8358** | **1659.7306** | **0.1052** | **1** | **0** | **1e+002** | **3** | **R.GCAVVEFKMEESMK.K** |
|  | 15939 | **420.2194** | **1676.8487** | **1677.7926** | **-0.9439** | **1** | **7** | **22** | **2** | **R.MATGLERMGANNLER.M** |
|  | 16438 | **857.8381** | **1713.6616** | **1713.6732** | **-0.0116** | **0** | **26** | **0.25** | **1** | **K.MGGMEGPFGGGMENMGR.F** |
|  | 16648 | **865.9326** | **1729.8507** | **1729.6681** | **0.1826** | **0** | **(1)** | **86** | **5** | **K.MGGMEGPFGGGMENMGR.F** |
|  | 16970 | **877.4372** | **1752.8598** | **1751.8651** | **0.9947** | **0** | **52** | **0.00073** | **1** | **K.VGEVTYVELLMDAEGK.S** 16937 16944 |
|  | 17310 | **593.3256** | **1776.9549** | **1777.6528** | **-0.6980** | **0** | **(3)** | **59** | **6** | **K.MGGMEGPFGGGMENMGR.F** |
|  | 18442 | **619.5248** | **1855.5525** | **1854.8717** | **0.6809** | **1** | **6** | **19** | **1** | **R.IGSGVERMGAGMGFGLER.M** |
|  | 18578 | **623.3472** | **1867.0199** | **1866.8418** | **0.1781** | **0** | **1** | **73** | **5** | **K.MEEESGAPGVPSGNGAPGPK.G** |
|  | 18947 | **632.9952** | **1895.9639** | **1894.9861** | **0.9778** | **1** | **6** | **22** | **1** | **R.GEIIAKQGGGGGGGSVPGIER.M** |
|  | 19056 | **952.9558** | **1903.8971** | **1903.8451** | **0.0519** | **1** | **2** | **61** | **4** | **R.LGGAGMERMGAGLGHGMDR.V** 19064 |
|  | 19264 | **641.2913** | **1920.8520** | **1919.8400** | **1.0119** | **1** | **(0)** | **94** | **4** | **R.LGGAGMERMGAGLGHGMDR.V** |
|  | 20163 | **1003.4263** | **2004.8380** | **2005.9206** | **-1.0826** | **1** | **(5)** | **27** | **1** | **R.MVPAGMGAGLERMGPVMDR.M** |
|  | 20170 | **669.3922** | **2005.1546** | **2005.9206** | **-0.7660** | **1** | **6** | **22** | **1** | **R.MVPAGMGAGLERMGPVMDR.M** |
|  | 20177 | **669.6805** | **2006.0196** | **2005.9206** | **0.0990** | **1** | **(0)** | **82** | **4** | **R.MVPAGMGAGLERMGPVMDR.M** |
|  | 20194 | **670.0420** | **2007.1041** | **2007.9362** | **-0.8321** | **1** | **(2)** | **58** | **2** | **R.MGLSMERMVPAGMGAGLER.M** |
|  | 20312 | **506.2285** | **2020.8847** | **2021.9155** | **-1.0308** | **1** | **(3)** | **48** | **8** | **R.MVPAGMGAGLERMGPVMDR.M** |
|  | 20360 | 1012.9474 | 2023.8802 | 2023.9311 | -0.0509 | 1 | 4 | 38 | 3 | R.MGLSMERMVPAGMGAGLER.M |
|  | 20482 | **678.9847** | **2033.9322** | **2033.9457** | **-0.0135** | **0** | **46** | **0.0021** | **1** | **R.GNFGGSFAGSFGGAGGHAPGVAR.K** |
|  | 20510 | **1019.4429** | **2036.8712** | **2037.9104** | **-1.0392** | **1** | **(3)** | **38** | **3** | **R.MVPAGMGAGLERMGPVMDR.M** |
|  | 21621 | **714.0138** | **2139.0195** | **2139.9717** | **-0.9522** | **1** | **2** | **53** | **3** | **K.FNECGHVLYADIKMENGK.S** |
|  | 21653 | 715.4202 | 2143.2389 | 2143.9449 | -0.7060 | 1 | 2 | 44 | 5 | R.MGLAMGGGGGASFDRAIEMER.G |
|  | 22018 | **1089.5226** | **2177.0306** | **2177.0432** | **-0.0125** | **0** | **102** | **5.6e-009** | **1** | **K.GIGMGNIGPAGMGMEGIGFGINK.M** 22019 |
|  | 22269 | **737.0593** | **2208.1560** | **2209.0330** | **-0.8770** | **0** | **(2)** | **54** | **1** | **K.GIGMGNIGPAGMGMEGIGFGINK.M** |
|  | 22443 | **742.9334** | **2225.7782** | **2225.0279** | **0.7503** | **0** | **(7)** | **12** | **1** | **K.GIGMGNIGPAGMGMEGIGFGINK.M** |
|  | 23173 | **773.3668** | **2317.0785** | **2317.0977** | **-0.0192** | **1** | **4** | **30** | **3** | **R.MGANSLERMGPAMGPALGAGIER.M** |
|  | 23603 | 792.6869 | 2375.0388 | 2375.1032 | -0.0643 | 1 | 1 | 59 | 8 | R.MGSGIERMGPLGLDHMASSIER.M |
|  | 25488 | **884.4400** | **2650.2982** | **2651.0630** | **-0.7648** | **1** | **1** | **51** | **2** | **K.MGGMEGPFGGGMENMGRFGSGMNMGR.I** |
|  | 26032 | **683.8660** | **2731.4348** | **2731.0376** | **0.3972** | **1** | **(0)** | **55** | **7** | **K.MGGMEGPFGGGMENMGRFGSGMNMGR.I** |
|  | 26271 | **927.7864** | **2780.3373** | **2779.2885** | **1.0488** | **1** | **0** | **59** | **7** | **R.AIEMERGNFGGSFAGSFGGAGGHAPGVAR.K** |
|  | 26603 | **953.0916** | **2856.2530** | **2855.2823** | **0.9707** | **1** | **2** | **30** | **1** | **R.MGPAMGPALGAGIERMGLAMGGGGGASFDR.A** |
|  | 29692 | **916.7184** | **3662.8444** | **3662.8589** | **-0.0145** | **0** | **13** | **1.6** | **1** | **R.GIGTVTFEQSIEAVQAISMFNGQLLFDRPMHVK.M** 29694 |
|  | 30285 | 1313.2438 | 3936.7095 | 3936.6854 | 0.0241 | 1 | (4) | 9.3 | 1 | K.GIGMGNIGPAGMGMEGIGFGINKMGGMEGPFGGGMENMGR.F |
|  | 30318 | 791.5727 | 3952.8271 | 3952.6803 | 0.1467 | 1 | 8 | 4.2 | 3 | K.GIGMGNIGPAGMGMEGIGFGINKMGGMEGPFGGGMENMGR.F 30319 |
|  | 30356 | **993.2996** | **3969.1694** | **3968.6753** | **0.4941** | **1** | **(6)** | **5.7** | **1** | **K.GIGMGNIGPAGMGMEGIGFGINKMGGMEGPFGGGMENMGR.F** |
|  | 30669 | 1399.0392 | 4194.0957 | 4194.1210 | -0.0253 | 0 | 82 | 1.4e-007 | 1 | K.VMATTGGMGMGPGGPGMITIPPSILNNPNIPNEIIHALQAGR.L |
|  | 30689 | **1057.5065** | **4225.9968** | **4226.0599** | **-0.0631** | **1** | **0** | **24** | **4** | **R.GIGTVTFEQSIEAVQAISMFNGQLLFDRPMHVKMDER.A** |

| **153.** | P12235\|ADT1_HUMAN    **Mass:** 33271    **Score:** 304    **Queries matched:** 32   **emPAI:** 1.36 |
| --- | --- |
|  | ADP/ATP translocase 1 OS=Homo sapiens GN=SLC25A4 PE=1 SV=4 |

|  | **Query** | **Observed** | **Mr(expt)** | **Mr(calc)** | **Delta** | **Miss** | **Score** | **Expect** | **Rank** | **Peptide** |
| --- | --- | --- | --- | --- | --- | --- | --- | --- | --- | --- |
|  | 810 | **503.2911** | **502.2838** | **501.2659** | **1.0179** | **0** | **4** | **1.4e+002** | **6** | **K.GAAQR.E** |
|  | 2733 | 840.3472 | 839.3399 | 839.3452 | -0.0053 | 0 | 29 | 0.17 | 1 | R.MMMQSGR.K 2752 |
|  | 2979 | 856.4830 | 855.4757 | 855.4814 | -0.0057 | 0 | 20 | 1.5 | 1 | K.TAVAPIER.V 2981 |
|  | 2982 | 428.7509 | 855.4871 | 855.4926 | -0.0055 | 0 | 9 | 21 | 7 | R.GNLANVIR.Y |
|  | 2990 | 429.0875 | 856.1604 | 855.3401 | 0.8203 | 0 | (9) | 14 | 6 | R.MMMQSGR.K |
|  | 2991 | 429.0876 | 856.1607 | 855.3401 | 0.8206 | 0 | (2) | 70 | 3 | R.MMMQSGR.K |
|  | 3739 | 451.7433 | 901.4720 | 901.4770 | -0.0050 | 0 | 38 | 0.032 | 1 | K.GAWSNVLR.G 3737 3738 |
|  | 5192 | **503.3030** | **1004.5915** | **1003.5451** | **1.0464** | **0** | **8** | **28** | **1** | **K.QLFLGGVDR.H** 5175 |
|  | 6386 | **547.7248** | **1093.4351** | **1093.5767** | **-0.1416** | **1** | **13** | **7.9** | **2** | **K.QISAEKQYK.G** |
|  | 6935 | 568.8394 | 1135.6643 | 1135.6713 | -0.0070 | 0 | 38 | 0.025 | 1 | K.LLLQVQHASK.Q 6909 6934 |
|  | 8047 | **604.3333** | **1206.6519** | **1206.5492** | **0.1028** | **0** | **6** | **42** | **10** | **-.MGDHAWSFLK.D** |
|  | 12409 | 723.8707 | 1445.7269 | 1445.7343 | -0.0074 | 0 | 59 | 0.00017 | 1 | R.YFPTQALNFAFK.D 12418 |
|  | 15157 | 542.9452 | 1625.8137 | 1626.8327 | -1.0190 | 0 | 2 | 78 | 9 | R.GMGGAFVLVLYDEIK.K |
|  | 15444 | 822.8553 | 1643.6960 | 1643.7072 | -0.0112 | 0 | 28 | 0.18 | 1 | K.GADIMYTGTVDCWR.K |
|  | 17222 | 886.9022 | 1771.7899 | 1771.8022 | -0.0123 | 1 | 81 | 8.1e-007 | 1 | R.KGADIMYTGTVDCWR.K 17223 |
|  | 17497 | **597.9719** | **1790.8939** | **1792.0206** | **-1.1267** | **1** | **1** | **89** | **7** | **K.LLLQVQHASKQISAEK.Q** |
|  | 19322 | 643.2836 | 1926.8291 | 1926.0363 | 0.7927 | 0 | 2 | 53 | 2 | R.GLYQGFNVSVQGIIIYR.A |
|  | 26343 | 1398.6686 | 2795.3226 | 2795.3377 | -0.0151 | 0 | 87 | 1.3e-007 | 1 | R.YFAGNLASGGAAGATSLCFVYPLDFAR.T 26345 26346 26348 26349 |
|  | 27608 | 1018.1352 | 3051.3837 | 3052.4865 | -1.1028 | 1 | 1 | 38 | 7 | R.YFAGNLASGGAAGATSLCFVYPLDFARTR.L |

| **154.** | P52209\|6PGD_HUMAN    **Mass:** 53619    **Score:** 302    **Queries matched:** 22   **emPAI:** 0.31 |
| --- | --- |
|  | 6-phosphogluconate dehydrogenase, decarboxylating OS=Homo sapiens GN=PGD PE=1 SV=3 |

|  | **Query** | **Observed** | **Mr(expt)** | **Mr(calc)** | **Delta** | **Miss** | **Score** | **Expect** | **Rank** | **Peptide** |
| --- | --- | --- | --- | --- | --- | --- | --- | --- | --- | --- |
|  | 1260 | **594.2950** | **593.2878** | **592.2890** | **0.9987** | **0** | **(2)** | **92** | **1** | **K.EMVSK.L** |
|  | 1341 | **610.1820** | **609.1747** | **608.2840** | **0.8907** | **0** | **6** | **30** | **8** | **K.EMVSK.L** |
|  | 2178 | **401.2428** | **800.4710** | **800.4756** | **-0.0046** | **0** | **15** | **6.4** | **1** | **K.VVGAQSLK.E** |
|  | 4378 | **474.3258** | **946.6370** | **947.5440** | **-0.9070** | **0** | **7** | **39** | **4** | **K.TIFQGIAAK.V** |
|  | 4879 | **492.7398** | **983.4650** | **983.4713** | **-0.0062** | **1** | **4** | **63** | **9** | **K.FQFDGDKK.S** |
|  | 6729 | **561.2737** | **1120.5328** | **1120.5400** | **-0.0072** | **0** | **52** | **0.00096** | **1** | **K.VDDFLANEAK.G** 6725 |
|  | 7720 | **596.7916** | **1191.5687** | **1191.5772** | **-0.0085** | **0** | **26** | **0.39** | **1** | **K.AGQAVDDFIEK.L** |
|  | 10745 | 676.2835 | 1350.5523 | 1350.5623 | -0.0099 | 0 | 20 | 1.4 | 2 | K.SAVENCQDSWR.R 10768 |
|  | 11445 | 464.5511 | 1390.6314 | 1390.7490 | -0.1176 | 1 | 5 | 47 | 5 | K.VVGAQSLKEMVSK.L |
|  | 12121 | **714.9099** | **1427.8053** | **1426.7642** | **1.0410** | **0** | **2** | **81** | **5** | **K.IISYAQGFMLLR.Q** |
|  | 13768 | **512.5867** | **1534.7382** | **1535.7831** | **-1.0449** | **1** | **7** | **27** | **10** | **R.TVSKVDDFLANEAK.G** |
|  | 14611 | **796.4020** | **1590.7894** | **1590.8002** | **-0.0108** | **0** | **67** | **2.4e-005** | **1** | **K.GILFVGSGVSGGEEGAR.Y** 14612 |
|  | 17729 | **604.3091** | **1809.9054** | **1808.9937** | **0.9117** | **1** | **2** | **68** | **6** | **K.EAWPHIKTIFQGIAAK.V** |
|  | 18641 | 937.0479 | 1872.0811 | 1871.0768 | 1.0044 | 1 | 2 | 62 | 4 | R.IILLVKAGQAVDDFIEK.L |
|  | 20079 | **666.3351** | **1995.9834** | **1996.9829** | **-0.9995** | **1** | **1** | **70** | **5** | **R.YGPSLMPGGNKEAWPHIK.T** |
|  | 21862 | 1080.5573 | 2159.0999 | 2159.1110 | -0.0111 | 0 | 115 | 2.8e-010 | 1 | K.LVPLLDTGDIIIDGGNSEYR.D 21863 |
|  | 22846 | **759.6563** | **2275.9471** | **2275.9627** | **-0.0156** | **0** | **36** | **0.019** | **1** | **K.VGTGEPCCDWVGDEGAGHFVK.M** |
|  | 26389 | **937.1098** | **2808.3076** | **2808.3252** | **-0.0176** | **0** | **5** | **19** | **2** | **R.AVSTGVQAGIPMPCFTTALSFYDGYR.H** |

| **155.** | P29992\|GNA11_HUMAN    **Mass:** 42382    **Score:** 302    **Queries matched:** 23   **emPAI:** 1.14 |
| --- | --- |
|  | Guanine nucleotide-binding protein subunit alpha-11 OS=Homo sapiens GN=GNA11 PE=1 SV=2 |

|  | **Query** | **Observed** | **Mr(expt)** | **Mr(calc)** | **Delta** | **Miss** | **Score** | **Expect** | **Rank** | **Peptide** |
| --- | --- | --- | --- | --- | --- | --- | --- | --- | --- | --- |
|  | 3054 | **431.2132** | **860.4118** | **860.4175** | **-0.0057** | **0** | **22** | **1.5** | **1** | **R.MVDVGGQR.S** |
|  | 5851 | **529.3129** | **1056.6112** | **1056.6179** | **-0.0067** | **0** | **42** | **0.011** | **1** | **K.DTILQLNLK.E** |
|  | 8156 | **607.3018** | **1212.5890** | **1212.6826** | **-0.0936** | **1** | **1** | **1e+002** | **6** | **R.INAEIEKQLR.R** |
|  | 9110 | **631.8373** | **1261.6601** | **1260.6826** | **0.9775** | **1** | **1** | **1.1e+002** | **9** | **R.DAQAAREFILK.M** |
|  | 9442 | **640.7906** | **1279.5666** | **1279.5755** | **-0.0088** | **0** | **50** | **0.0014** | **1** | **K.MFVDLNPDSDK.I** |
|  | 12551 | **486.3614** | **1456.0624** | **1456.8501** | **-0.7876** | **1** | **8** | **13** | **3** | **R.ELKLLLLGTGESGK.S** |
|  | 12835 | **492.2443** | **1473.7111** | **1473.7212** | **-0.0100** | **1** | **23** | **0.6** | **1** | **R.IIHGAGYSEEDKR.G** 12836 12840 12841 |
|  | 13586 | **761.8936** | **1521.7725** | **1521.7827** | **-0.0102** | **0** | **52** | **0.00084** | **1** | **K.VTTFEHQYVSAIK.T** |
|  | 16064 | **844.4674** | **1686.9203** | **1686.9305** | **-0.0102** | **0** | **60** | **0.00011** | **1** | **R.IATLGYLPTQQDVLR.V** 16065 |
|  | 17367 | **891.3882** | **1780.7618** | **1780.7726** | **-0.0108** | **0** | **70** | **1e-005** | **1** | **K.TLWEDPGIQECYDR.R** |
|  | 17583 | **899.9674** | **1797.9203** | **1797.9270** | **-0.0067** | **0** | **63** | **5.2e-005** | **1** | **K.LVYQNIFTAMQAMIR.A** |
|  | 21426 | **706.4020** | **2116.1841** | **2116.9149** | **-0.7308** | **1** | **3** | **41** | **2** | **M.TLESMMACCLSDEVKESK.R** |
|  | 22166 | **1098.5339** | **2195.0533** | **2195.0688** | **-0.0154** | **0** | **58** | **0.00012** | **1** | **K.ILYSHLVDYFPEFDGPQR.D** 22167 22173 |
|  | 22928 | **1142.6166** | **2283.2186** | **2283.2303** | **-0.0117** | **0** | **39** | **0.0097** | **1** | **R.TIITYPWFQNSSVILFLNK.K** 22929 |
|  | 23827 | 804.7783 | 2411.3131 | 2411.3253 | -0.0122 | 1 | 5 | 24 | 2 | R.TIITYPWFQNSSVILFLNKK.D |
|  | 24460 | **830.3573** | **2488.0501** | **2487.2688** | **0.7813** | **1** | **2** | **34** | **2** | **K.LVYQNIFTAMQAMIRAMETLK.I** |

| **156.** | Q6UX06\|OLFM4_HUMAN    **Mass:** 57529    **Score:** 293    **Queries matched:** 18   **emPAI:** 0.37 |
| --- | --- |
|  | Olfactomedin-4 OS=Homo sapiens GN=OLFM4 PE=1 SV=1 |

|  | **Query** | **Observed** | **Mr(expt)** | **Mr(calc)** | **Delta** | **Miss** | **Score** | **Expect** | **Rank** | **Peptide** |
| --- | --- | --- | --- | --- | --- | --- | --- | --- | --- | --- |
|  | 2961 | **428.2345** | **854.4545** | **854.4684** | **-0.0139** | **0** | **7** | **27** | **6** | **K.LDIVMHK.M** 2981 |
|  | 2982 | 428.7509 | 855.4871 | 855.4490 | 0.0381 | 0 | 8 | 25 | 8 | R.LLEYYR.L |
|  | 6257 | **543.2507** | **1084.4868** | **1084.4938** | **-0.0070** | **0** | **18** | **2.8** | **1** | **R.DYSPQHPNK.G** |
|  | 8811 | **416.2846** | **1245.8318** | **1245.7002** | **0.1316** | **1** | **7** | **24** | **8** | **K.EMEKLVIQLK.E** |
|  | 9309 | 636.8474 | 1271.6803 | 1271.6874 | -0.0071 | 0 | 10 | 14 | 1 | R.LEFTAHVLSQK.F |
|  | 9378 | **638.8055** | **1275.5964** | **1275.6037** | **-0.0072** | **0** | **50** | **0.0015** | **1** | **R.GFSYLYGAWGR.D** 9379 |
|  | 12631 | **731.3353** | **1460.6561** | **1460.7412** | **-0.0851** | **0** | **2** | **76** | **4** | **K.GLYWVAPLNTDGR.L** |
|  | 13244 | **500.2561** | **1497.7465** | **1497.8515** | **-0.1050** | **1** | **1** | **91** | **8** | **K.LETLDKNNVLAIR.R** |
|  | 16817 | **872.3856** | **1742.7567** | **1742.7675** | **-0.0108** | **0** | **48** | **0.0018** | **1** | **R.TEEIFYYYDTNTGK.E** |
|  | 17718 | **905.4871** | **1808.9596** | **1808.9672** | **-0.0076** | **0** | **26** | **0.25** | **1** | **R.LYNTLDDLLLYINAR.E** 17721 |
|  | 20542 | **1020.4553** | **2038.8961** | **2038.9089** | **-0.0128** | **0** | **57** | **0.00019** | **1** | **R.GTCQCSVSLPDTTFPVDR.V** 20543 |
|  | 24685 | **1259.6398** | **2517.2650** | **2517.2791** | **-0.0141** | **0** | **134** | **2.7e-012** | **1** | **K.LYVYNDGYLLNYDLSVLQKPQ.-** 24676 24686 |

| **157.** | P16615\|AT2A2_HUMAN    **Mass:** 116336   **Score:** 293    **Queries matched:** 46   **emPAI:** 0.45 |
| --- | --- |
|  | Sarcoplasmic/endoplasmic reticulum calcium ATPase 2 OS=Homo sapiens GN=ATP2A2 PE=1 SV=1 |

|  | **Query** | **Observed** | **Mr(expt)** | **Mr(calc)** | **Delta** | **Miss** | **Score** | **Expect** | **Rank** | **Peptide** |
| --- | --- | --- | --- | --- | --- | --- | --- | --- | --- | --- |
|  | 2607 | **416.2588** | **830.5031** | **831.4702** | **-0.9671** | **0** | **10** | **22** | **4** | **R.IEVASSVK.L** |
|  | 2703 | **419.3144** | **836.6142** | **835.4300** | **1.1842** | **1** | **4** | **56** | **8** | **K.VYRQDR.K** |
|  | 2840 | **424.6559** | **847.2973** | **846.4382** | **0.8591** | **0** | **6** | **50** | **8** | **K.GTAVAICR.R** |
|  | 2930 | 853.4177 | 852.4104 | 852.4163 | -0.0060 | 0 | 31 | 0.12 | 1 | R.AIYNNMK.Q |
|  | 3237 | **435.2233** | **868.4320** | **868.4113** | **0.0207** | **0** | **(8)** | **20** | **1** | **R.AIYNNMK.Q** |
|  | 4971 | **496.2660** | **990.5175** | **989.5216** | **0.9959** | **0** | **31** | **0.15** | **1** | **R.VIMITGDNK.G** |
|  | 5546 | **517.3055** | **1032.5965** | **1032.4989** | **0.0977** | **0** | **10** | **20** | **4** | **K.HTDPVPDPR.A** |
|  | 6090 | **537.2575** | **1072.5005** | **1071.5560** | **0.9445** | **0** | **9** | **26** | **4** | **R.NAENAIEALK.E** 6053 |
|  | 6549 | **554.2529** | **1106.4912** | **1106.4993** | **-0.0081** | **0** | **43** | **0.0084** | **1** | **R.EWGSGSDTLR.C** 6548 |
|  | 8371 | 612.3187 | 1222.6229 | 1222.5136 | 0.1093 | 0 | 4 | 58 | 7 | R.DEMVATEQER.T |
|  | 8482 | **615.5700** | **1229.1255** | **1229.5975** | **-0.4721** | **1** | **4** | **44** | **3** | **R.CFARVEPSHK.S** 8498 |
|  | 9689 | **647.2882** | **1292.5619** | **1292.6877** | **-0.1258** | **1** | **3** | **69** | **2** | **K.FVARNYLEPGK.E** 9712 |
|  | 9918 | **653.3021** | **1304.5897** | **1304.5997** | **-0.0100** | **0** | **5** | **43** | **3** | **R.EFDELNPSAQR.D** 9911 |
|  | 10544 | 670.3234 | 1338.6323 | 1338.6602 | -0.0279 | 0 | 3 | 71 | 6 | K.NMLFSGTNIAAGK.A |
|  | 10968 | 682.8745 | 1363.7343 | 1363.7289 | 0.0055 | 0 | 13 | 7.4 | 3 | K.EPLISGWLFFR.Y |
|  | 11237 | 690.3442 | 1378.6739 | 1379.6980 | -1.0240 | 0 | 13 | 7.7 | 4 | R.CLALATHDNPLR.R |
|  | 12453 | **725.8491** | **1449.6836** | **1450.7602** | **-1.0766** | **1** | **4** | **47** | **6** | **K.KNMLFSGTNIAAGK.A** |
|  | 12728 | 734.3859 | 1466.7572 | 1466.7551 | 0.0020 | 1 | (4) | 51 | 3 | K.KNMLFSGTNIAAGK.A |
|  | 12867 | **738.8541** | **1475.6936** | **1475.7038** | **-0.0102** | **1** | **8** | **22** | **1** | **K.IRDEMVATEQER.T** 12868 |
|  | 13158 | **747.2254** | **1492.4362** | **1491.7537** | **0.6825** | **1** | **1** | **75** | **3** | **R.ANACNSVIKQLMK.K** |
|  | 13322 | 752.8751 | 1503.7356 | 1502.8126 | 0.9229 | 1 | 3 | 64 | 5 | K.KAEIGIAMGSGTAVAK.T |
|  | 13542 | **507.0747** | **1518.2024** | **1518.8076** | **-0.6052** | **1** | **(0)** | **81** | **10** | **K.KAEIGIAMGSGTAVAK.T** |
|  | 13632 | **763.8530** | **1525.6915** | **1525.7017** | **-0.0102** | **0** | **57** | **0.00025** | **1** | **K.SMSVYCTPNKPSR.T** |
|  | 13917 | **773.8729** | **1545.7313** | **1544.8232** | **0.9080** | **0** | **18** | **1.8** | **2** | **K.AMGVVVATGVNTEIGK.I** |
|  | 14161 | **781.3769** | **1560.7392** | **1561.7082** | **-0.9690** | **0** | **32** | **0.07** | **1** | **R.EEMHLEDSANFIK.Y** |
|  | 14210 | **782.8974** | **1563.7802** | **1563.7893** | **-0.0090** | **1** | **67** | **2.5e-005** | **1** | **R.RIGIFGQDEDVTSK.A** |
|  | 14362 | **787.9305** | **1573.8464** | **1573.8563** | **-0.0099** | **0** | **61** | **0.0001** | **1** | **R.VDQSILTGESVSVIK.H** |
|  | 14400 | **526.6100** | **1576.8081** | **1577.7031** | **-0.8950** | **0** | **(5)** | **39** | **3** | **R.EEMHLEDSANFIK.Y** |
|  | 15058 | **810.9055** | **1619.7964** | **1619.8076** | **-0.0113** | **0** | **51** | **0.00091** | **1** | **K.VGEATETALTCLVEK.M** |
|  | 19522 | **972.5779** | **1943.1413** | **1944.1118** | **-0.9705** | **1** | **3** | **42** | **1** | **K.ISLPVILMDETLKFVAR.N** |
|  | 19616 | **976.9629** | **1951.9112** | **1951.9231** | **-0.0119** | **0** | **71** | **6.8e-006** | **1** | **R.SLPSVETLGCTSVICSDK.T** 19617 |
|  | 22127 | **1096.0968** | **2190.1790** | **2190.1896** | **-0.0106** | **1** | **6** | **20** | **1** | **K.DIVPGDIVEIAVGDKVPADIR.L** |
|  | 23951 | **1213.0746** | **2424.1346** | **2424.1479** | **-0.0133** | **0** | **37** | **0.014** | **1** | **K.TASEMVLADDNFSTIVAAVEEGR.A** 23949 23952 |
|  | 24327 | **824.7548** | **2471.2425** | **2471.2544** | **-0.0119** | **0** | **16** | **1.6** | **1** | **K.TVEEVLGHFGVNESTGLSLEQVK.K** |
|  | 25941 | **1357.1660** | **2712.3175** | **2713.3190** | **-1.0015** | **1** | **1** | **52** | **4** | **K.VGEATETALTCLVEKMNVFDTELK.G** |
|  | 26886 | **970.8201** | **2909.4384** | **2908.4528** | **0.9856** | **1** | **23** | **0.27** | **1** | **K.IVEFLQSFDEITAMTGDGVNDAPALKK.A** 26887 |

| **158.** | P00167\|CYB5_HUMAN    **Mass:** 15321    **Score:** 293    **Queries matched:** 18   **emPAI:** 1.48 |
| --- | --- |
|  | Cytochrome b5 OS=Homo sapiens GN=CYB5A PE=1 SV=2 |

|  | **Query** | **Observed** | **Mr(expt)** | **Mr(calc)** | **Delta** | **Miss** | **Score** | **Expect** | **Rank** | **Peptide** |
| --- | --- | --- | --- | --- | --- | --- | --- | --- | --- | --- |
|  | 1840 | **738.3981** | **737.3908** | **737.3959** | **-0.0052** | **0** | **32** | **0.11** | **1** | **K.VYDLTK.F** |
|  | 1848 | **740.4252** | **739.4180** | **740.3051** | **-0.8871** | **0** | **13** | **6.2** | **1** | **R.LYMAED.-** |
|  | 6753 | 562.2784 | 1122.5422 | 1122.4863 | 0.0559 | 0 | 6 | 41 | 9 | -.MAEQSDEAVK.Y 6751 |
|  | 7590 | **593.7996** | **1185.5847** | **1185.5917** | **-0.0070** | **0** | **27** | **0.29** | **1** | **K.YYTLEEIQK.H** |
|  | 13427 | **756.3726** | **1510.7307** | **1510.7416** | **-0.0109** | **0** | **46** | **0.003** | **1** | **K.FLEEHPGGEEVLR.E** 13428 13429 13431 |
|  | 15344 | **819.4306** | **1636.8466** | **1636.8573** | **-0.0107** | **0** | **49** | **0.0015** | **1** | **K.TFIIGELHPDDRPK.L** 15341 15343 15345 15346 |
|  | 22242 | **1103.4606** | **2204.9066** | **2204.9207** | **-0.0141** | **0** | **113** | **3.3e-010** | **1** | **R.EQAGGDATENFEDVGHSTDAR.E** 22243 22252 |
|  | 30562 | **1377.7209** | **4130.1410** | **4130.1550** | **-0.0140** | **0** | **74** | **1e-006** | **1** | **K.LNKPPETLITTIDSSSSWWTNWVIPAISAVAVALMYR.L** |

| **159.** | P01834\|IGKC_HUMAN    **Mass:** 11773    **Score:** 289    **Queries matched:** 8   **emPAI:** 3.28 |
| --- | --- |
|  | Ig kappa chain C region OS=Homo sapiens GN=IGKC PE=1 SV=1 |

|  | **Query** | **Observed** | **Mr(expt)** | **Mr(calc)** | **Delta** | **Miss** | **Score** | **Expect** | **Rank** | **Peptide** |
| --- | --- | --- | --- | --- | --- | --- | --- | --- | --- | --- |
|  | 3536 | **445.0338** | **888.0529** | **887.4865** | **0.5665** | **1** | **0** | **1.2e+002** | **8** | **R.EAKVQWK.V** |
|  | 13301 | **751.8769** | **1501.7393** | **1501.7512** | **-0.0118** | **0** | **27** | **0.26** | **1** | **K.DSTYSLSSTLTLSK.A** |
|  | 17566 | **899.4462** | **1796.8779** | **1796.8880** | **-0.0100** | **0** | **56** | **0.00025** | **1** | **K.SGTASVVCLLNNFYPR.E** |
|  | 18669 | **938.4607** | **1874.9068** | **1874.9197** | **-0.0128** | **0** | **62** | **6.1e-005** | **1** | **K.VYACEVTHQGLSSPVTK.S** |
|  | 19549 | **973.5110** | **1945.0074** | **1945.0197** | **-0.0123** | **0** | **58** | **0.00015** | **1** | **-.TVAAPSVFIFPPSDEQLK.S** 19551 |
|  | 21580 | **1068.4807** | **2134.9469** | **2134.9614** | **-0.0146** | **0** | **102** | **5.1e-009** | **1** | **K.VDNALQSGNSQESVTEQDSK.D** 21579 |

| **160.** | Q16134\|ETFD_HUMAN    **Mass:** 69262    **Score:** 288    **Queries matched:** 19   **emPAI:** 0.37 |
| --- | --- |
|  | Electron transfer flavoprotein-ubiquinone oxidoreductase, mitochondrial OS=Homo sapiens GN=ETFDH PE=1 SV=1 |

|  | **Query** | **Observed** | **Mr(expt)** | **Mr(calc)** | **Delta** | **Miss** | **Score** | **Expect** | **Rank** | **Peptide** |
| --- | --- | --- | --- | --- | --- | --- | --- | --- | --- | --- |
|  | 5365 | **510.7287** | **1019.4429** | **1019.4494** | **-0.0065** | **0** | **18** | **3.1** | **1** | **R.WEGVNMER.F** |
|  | 5599 | **519.2634** | **1036.5122** | **1035.4444** | **1.0678** | **0** | **(2)** | **90** | **6** | **R.WEGVNMER.F** |
|  | 6648 | **558.3027** | **1114.5909** | **1114.5982** | **-0.0073** | **0** | **35** | **0.049** | **1** | **K.GIATNDVGIQK.D** |
|  | 8808 | **416.2588** | **1245.7546** | **1246.6856** | **-0.9309** | **1** | **9** | **20** | **1** | **K.KNYLPLCAIR.W** |
|  | 9085 | 631.3394 | 1260.6642 | 1259.6510 | 1.0132 | 0 | 9 | 19 | 5 | R.ALNEGGFQSIPK.L |
|  | 9234 | **635.3212** | **1268.6278** | **1268.6361** | **-0.0083** | **0** | **36** | **0.032** | **1** | **K.GAPLNTPVTEDR.F** 9233 |
|  | 9655 | **646.3136** | **1290.6126** | **1290.6204** | **-0.0078** | **0** | **35** | **0.045** | **1** | **R.NLSIYDGPEQR.F** |
|  | 9717 | **432.2201** | **1293.6384** | **1293.6466** | **-0.0083** | **0** | **7** | **25** | **5** | **R.VDHTVGWPLDR.H** |
|  | 11101 | **458.2329** | **1371.6770** | **1370.7306** | **0.9464** | **1** | **2** | **80** | **7** | **K.ATFERGLELHAK.V** |
|  | 13816 | **770.3867** | **1538.7589** | **1538.7664** | **-0.0075** | **0** | **3** | **63** | **1** | **K.VTIFAEGCHGHLAK.Q** |
|  | 19955 | **662.3300** | **1983.9682** | **1983.9836** | **-0.0154** | **0** | **2** | **59** | **2** | **K.AAQIGAHTLSGACLDPGAFK.E** |
|  | 21977 | **1087.4982** | **2172.9818** | **2172.9939** | **-0.0121** | **0** | **67** | **1.8e-005** | **1** | **R.FCPAGVYEFVPVEQGDGFR.L** |
|  | 22616 | **1125.5789** | **2249.1432** | **2249.1539** | **-0.0107** | **0** | **117** | **1.5e-010** | **1** | **K.SGILAAESIFNQLTSENLQSK.T** 22614 22615 |
|  | 24781 | **845.1312** | **2532.3719** | **2532.3787** | **-0.0069** | **1** | **16** | **1.6** | **1** | **K.YRIPVPILPGLPMNNHGNYIVR.L** |
|  | 26792 | **964.8183** | **2891.4331** | **2891.2855** | **0.1476** | **1** | **3** | **29** | **4** | **K.TCDIKDPSQNINWVVPEGGGGPAYNGM.-** |
|  | 30302 | **1315.7118** | **3944.1135** | **3943.9091** | **0.2045** | **0** | **7** | **5.8** | **1** | **R.LGHLVSWMGEQAEALGVEVYPGYAAAEVLFHDDGSVK.G** |

| **161.** | P14854\|CX6B1_HUMAN    **Mass:** 10414    **Score:** 284    **Queries matched:** 12   **emPAI:** 4.08 |
| --- | --- |
|  | Cytochrome c oxidase subunit 6B1 OS=Homo sapiens GN=COX6B1 PE=1 SV=2 |

|  | **Query** | **Observed** | **Mr(expt)** | **Mr(calc)** | **Delta** | **Miss** | **Score** | **Expect** | **Rank** | **Peptide** |
| --- | --- | --- | --- | --- | --- | --- | --- | --- | --- | --- |
|  | 2254 | **806.3994** | **805.3921** | **805.3970** | **-0.0049** | **0** | **19** | **2.6** | **2** | **R.AEGTFPGK.I** |
|  | 3963 | **459.7315** | **917.4484** | **918.4811** | **-1.0327** | **1** | **21** | **1.7** | **1** | **R.AEGTFPGKI.-** 3973 |
|  | 5188 | **503.2915** | **1004.5685** | **1003.4835** | **1.0850** | **0** | **12** | **12** | **3** | **R.FPNQNQTR.N** |
|  | 12761 | **735.3221** | **1468.6297** | **1468.6405** | **-0.0108** | **0** | **33** | **0.068** | **1** | **K.GGDISVCEWYQR.V** 12762 |
|  | 14026 | 776.8362 | 1551.6579 | 1551.6677 | -0.0098 | 0 | 52 | 0.00079 | 1 | R.NCWQNYLDFHR.C 14002 14025 |
|  | 22784 | **1135.5066** | **2268.9986** | **2269.0110** | **-0.0124** | **0** | **112** | **5.3e-010** | **1** | **R.VYQSLCPTSWVTDWDEQR.A** 22785 22789 |

| **162.** | P61586\|RHOA_HUMAN    **Mass:** 22096    **Score:** 284    **Queries matched:** 18   **emPAI:** 2.59 |
| --- | --- |
|  | Transforming protein RhoA OS=Homo sapiens GN=RHOA PE=1 SV=1 |

|  | **Query** | **Observed** | **Mr(expt)** | **Mr(calc)** | **Delta** | **Miss** | **Score** | **Expect** | **Rank** | **Peptide** |
| --- | --- | --- | --- | --- | --- | --- | --- | --- | --- | --- |
|  | 1329 | **606.7065** | **605.6992** | **605.2591** | **0.4401** | **0** | **4** | **56** | **9** | **R.DMANR.I** |
|  | 1916 | **759.4308** | **758.4235** | **758.3963** | **0.0272** | **0** | **9** | **33** | **5** | **K.WTPEVK.H** |
|  | 4846 | **491.7334** | **981.4522** | **981.4590** | **-0.0068** | **0** | **16** | **3.5** | **1** | **R.EVFEMATR.A** 4857 |
|  | 6308 | **544.7883** | **1087.5621** | **1087.5696** | **-0.0075** | **0** | **51** | **0.0015** | **1** | **K.LVIVGDGACGK.T** 6280 6307 |
|  | 8243 | **406.5327** | **1216.5762** | **1215.6646** | **0.9117** | **1** | **5** | **56** | **4** | **K.KLVIVGDGACGK.T** |
|  | 10433 | **667.2951** | **1332.5756** | **1332.5842** | **-0.0086** | **0** | **62** | **9.2e-005** | **1** | **R.IGAFGYMECSAK.T** |
|  | 12100 | **714.3648** | **1426.7151** | **1426.7238** | **-0.0087** | **1** | **23** | **0.65** | **1** | **K.MKQEPVKPEEGR.D** |
|  | 14420 | **527.1569** | **1578.4489** | **1577.7218** | **0.7271** | **1** | **6** | **20** | **5** | **R.IGAFGYMECSAKTK.D** 14419 |
|  | 14840 | **804.4351** | **1606.8557** | **1606.8654** | **-0.0097** | **0** | **76** | **3e-006** | **1** | **K.HFCPNVPIILVGNK.K** |
|  | 16721 | **579.3232** | **1734.9479** | **1734.9603** | **-0.0124** | **1** | **46** | **0.0029** | **1** | **K.HFCPNVPIILVGNKK.D** |
|  | 20195 | **1004.9594** | **2007.9041** | **2007.9174** | **-0.0132** | **0** | **53** | **0.00042** | **1** | **K.QVELALWDTAGQEDYDR.L** |
|  | 26237 | **1387.6605** | **2773.3065** | **2772.3171** | **0.9894** | **0** | **65** | **2e-005** | **1** | **K.DQFPEVYVPTVFENYVADIEVDGK.Q** 26226 |
|  | 29092 | **1155.5641** | **3463.6704** | **3463.6891** | **-0.0186** | **0** | **62** | **2.6e-005** | **1** | **R.LRPLSYPDTDVILMCFSIDSPDSLENIPEK.W** |

| **163.** | Q9H3R2\|MUC13_HUMAN    **Mass:** 55710    **Score:** 281    **Queries matched:** 22   **emPAI:** 0.57 |
| --- | --- |
|  | Mucin-13 OS=Homo sapiens GN=MUC13 PE=1 SV=1 |

|  | **Query** | **Observed** | **Mr(expt)** | **Mr(calc)** | **Delta** | **Miss** | **Score** | **Expect** | **Rank** | **Peptide** |
| --- | --- | --- | --- | --- | --- | --- | --- | --- | --- | --- |
|  | 1047 | **548.2829** | **547.2756** | **546.3166** | **0.9590** | **0** | **11** | **24** | **1** | **K.VFPGK.I** |
|  | 2216 | **402.2890** | **802.5635** | **801.4821** | **1.0814** | **1** | **6** | **50** | **9** | **K.VRITASR.D** |
|  | 6319 | **545.2383** | **1088.4621** | **1088.4709** | **-0.0088** | **0** | **15** | **5.3** | **1** | **R.HSSMPRPDY.-** 6315 |
|  | 8394 | **613.2622** | **1224.5097** | **1224.5193** | **-0.0096** | **0** | **47** | **0.003** | **1** | **R.DSQMQNPYSR.H** |
|  | 14439 | 790.8826 | 1579.7506 | 1579.7617 | -0.0111 | 0 | 54 | 0.0005 | 1 | K.ISVTVSETFDPEEK.H |
|  | 14889 | **806.3995** | **1610.7844** | **1610.7941** | **-0.0096** | **0** | **53** | **0.00065** | **1** | **R.STGFTNLGAEGSVFPK.V** |
|  | 14994 | **539.5975** | **1615.7708** | **1615.7842** | **-0.0134** | **0** | **20** | **1.1** | **1** | **R.SSSSNFLNYDLTLR.C** 14991 |
|  | 17183 | **590.4301** | **1768.2685** | **1768.8162** | **-0.5477** | **1** | **3** | **35** | **4** | **R.ITASRDSQMQNPYSR.H** |
|  | 19963 | **993.4697** | **1984.9249** | **1984.9378** | **-0.0129** | **0** | **89** | **1.1e-007** | **1** | **K.HIEEENLIDEDFQNLK.L** 19969 |
|  | 20176 | **669.6552** | **2005.9438** | **2005.9567** | **-0.0129** | **0** | **16** | **2.2** | **1** | **K.HSMAYQDLHSEITSLFK.D** |
|  | 21542 | **1066.0262** | **2130.0379** | **2130.0528** | **-0.0148** | **0** | **92** | **5.3e-008** | **1** | **K.SDLQRPNPQSPFCVASSLK.C** 21543 |
|  | 22642 | **752.0734** | **2253.1983** | **2254.1229** | **-0.9247** | **1** | **3** | **45** | **5** | **K.HIEEENLIDEDFQNLKLR.S** |
|  | 24306 | **1235.4796** | **2468.9447** | **2467.9791** | **0.9656** | **0** | **20** | **0.59** | **1** | **K.SGGAPECACVPGYQEDANGNCQK.C** 24307 24308 |
|  | 28926 | **1132.9017** | **3395.6834** | **3395.0353** | **0.6481** | **1** | **3** | **21** | **1** | **K.FQLILTIVGTIAGIVILSMIIALIVTARSNNK.T** |
|  | 28963 | **1137.6251** | **3409.8535** | **3411.0302** | **-1.1767** | **1** | **(2)** | **25** | **5** | **K.FQLILTIVGTIAGIVILSMIIALIVTARSNNK.T** 28964 |

| **164.** | Q9UM54\|MYO6_HUMAN    **Mass:** 150965   **Score:** 280    **Queries matched:** 57   **emPAI:** 0.13 |
| --- | --- |
|  | Myosin-VI OS=Homo sapiens GN=MYO6 PE=1 SV=4 |

|  | **Query** | **Observed** | **Mr(expt)** | **Mr(calc)** | **Delta** | **Miss** | **Score** | **Expect** | **Rank** | **Peptide** |
| --- | --- | --- | --- | --- | --- | --- | --- | --- | --- | --- |
|  | 1477 | **642.7714** | **641.7641** | **641.3173** | **0.4469** | **0** | **24** | **0.48** | **1** | **R.YFANK.E** |
|  | 1644 | **679.5075** | **678.5002** | **677.3860** | **1.1142** | **1** | **2** | **1.2e+002** | **8** | **R.DKFIR.E** |
|  | 1964 | **776.4246** | **775.4173** | **775.4374** | **-0.0201** | **1** | **10** | **21** | **6** | **K.MQKTIR.M** |
|  | 2326 | **406.2100** | **810.4054** | **809.4872** | **0.9182** | **1** | **11** | **11** | **1** | **K.SKLAVHR.N** |
|  | 2712 | **419.7039** | **837.3932** | **837.4345** | **-0.0413** | **0** | **11** | **13** | **2** | **R.STGASFIR.C** |
|  | 5021 | **498.2529** | **994.4912** | **994.4971** | **-0.0059** | **0** | **9** | **19** | **3** | **R.SLDSYPVSK.N** |
|  | 5235 | 505.2948 | 1008.5750 | 1008.5062 | 0.0688 | 1 | 7 | 30 | 9 | K.YMPDKLAR.L |
|  | 5638 | 520.7707 | 1039.5268 | 1038.5029 | 1.0239 | 1 | 5 | 49 | 9 | R.YLNRGCTR.Y |
|  | 5852 | **529.3129** | **1056.6113** | **1056.6179** | **-0.0066** | **0** | **6** | **42** | **2** | **K.TQLNLLLDK.L** |
|  | 5983 | **534.2598** | **1066.5050** | **1065.4978** | **1.0072** | **0** | **6** | **35** | **6** | **K.EEQELYQK.E** |
|  | 6200 | **541.2712** | **1080.5278** | **1080.5352** | **-0.0074** | **0** | **2** | **1e+002** | **3** | **K.LQQFFNER.I** |
|  | 7393 | **587.2673** | **1172.5200** | **1171.5557** | **0.9643** | **0** | **3** | **84** | **2** | **R.VNHWLTCSR.W** |
|  | 8039 | **604.3068** | **1206.5991** | **1206.5550** | **0.0441** | **1** | **6** | **41** | **4** | **R.IQEEMEKER.K** |
|  | 8293 | **610.3176** | **1218.6207** | **1219.6925** | **-1.0718** | **1** | **3** | **84** | **5** | **K.AGKLSFISVGNK.F** |
|  | 8445 | **614.7765** | **1227.5384** | **1226.6619** | **0.8766** | **0** | **49** | **0.0019** | **1** | **R.IQAEVEAQLAR.Q** 8437 |
|  | 8964 | **419.3135** | **1254.9186** | **1254.7296** | **0.1890** | **1** | **4** | **47** | **8** | **R.GPAVLATKAAAGTK.K** |
|  | 10262 | **662.8107** | **1323.6069** | **1323.6163** | **-0.0093** | **0** | **49** | **0.0015** | **1** | **K.STMMTQEQIQK.E** |
|  | 10292 | 663.3752 | 1324.7359 | 1325.8031 | -1.0671 | 1 | 10 | 14 | 7 | K.TQLNLLLDKLR.S 10304 10330 10343 |
|  | 10426 | 666.8951 | 1331.7756 | 1331.7813 | -0.0057 | 1 | 18 | 2.4 | 3 | K.FKTQLNLLLDK.L 10425 |
|  | 10558 | 447.2717 | 1338.7933 | 1339.6112 | -0.8179 | 0 | (7) | 25 | 7 | K.STMMTQEQIQK.E |
|  | 10690 | **449.8912** | **1346.6518** | **1346.7115** | **-0.0597** | **0** | **8** | **23** | **7** | **K.NLEISIDTLMAK.I** |
|  | 11220 | **460.2455** | **1377.7146** | **1378.7205** | **-1.0058** | **1** | **6** | **32** | **5** | **K.SDPDHLAELVKR.V** |
|  | 11984 | 474.3256 | 1419.9549 | 1419.7609 | 0.1940 | 1 | 8 | 14 | 5 | R.ILKEEQELYQK.E |
|  | 12457 | **725.8589** | **1449.7032** | **1449.7133** | **-0.0101** | **0** | **78** | **2.2e-006** | **1** | **K.MSQSIIVSGESGAGK.T** |
|  | 12692 | **733.3355** | **1464.6565** | **1465.7083** | **-1.0518** | **0** | **(5)** | **39** | **3** | **K.MSQSIIVSGESGAGK.T** |
|  | 12874 | 739.3358 | 1476.6571 | 1475.8382 | 0.8189 | 1 | 6 | 36 | 5 | R.VMLTTAGGTKGTVIK.V 12892 |
|  | 14468 | 528.2571 | 1581.7496 | 1580.7902 | 0.9594 | 1 | 5 | 41 | 6 | K.IKSTMMTQEQIQK.E |
|  | 14542 | **529.9384** | **1586.7934** | **1585.8001** | **0.9933** | **0** | **6** | **34** | **6** | **K.TVYSHLFDHVVNR.V** |
|  | 14679 | **533.2687** | **1596.7842** | **1596.7851** | **-0.0009** | **1** | **(2)** | **77** | **9** | **K.IKSTMMTQEQIQK.E** |
|  | 14785 | 535.6225 | 1603.8457 | 1603.8855 | -0.0398 | 1 | 5 | 42 | 3 | K.NLEISIDTLMAKIK.S |
|  | 15086 | **811.9289** | **1621.8432** | **1620.8512** | **0.9921** | **0** | **85** | **3.8e-007** | **1** | **K.SSVVGGFVSHYLLEK.S** 15071 15072 |
|  | 15991 | **561.3570** | **1681.0491** | **1681.8675** | **-0.8184** | **1** | **1** | **73** | **4** | **K.ALGLNENDYKFGLTK.V** |
|  | 16110 | 564.5758 | 1690.7056 | 1690.9188 | -0.2132 | 1 | 1 | 83 | 5 | R.STGASFIRCIKPNLK.M |
|  | 16214 | 849.9534 | 1697.8923 | 1697.8373 | 0.0550 | 1 | 5 | 33 | 3 | K.FIRELFESSTNNNK.D |
|  | 16473 | **859.3872** | **1716.7599** | **1715.9491** | **0.8108** | **1** | **(5)** | **32** | **1** | **K.QIKNLEISIDTLMAK.I** |
|  | 16663 | **866.8809** | **1731.7473** | **1731.7588** | **-0.0115** | **0** | **76** | **2.8e-006** | **1** | **R.YLTESYGTGQDIDDR.I** |
|  | 16665 | **578.2675** | **1731.7806** | **1731.9440** | **-0.1635** | **1** | **6** | **28** | **3** | **K.QIKNLEISIDTLMAK.I** 16672 |
|  | 17702 | **603.3674** | **1807.0803** | **1806.9662** | **0.1141** | **0** | **2** | **67** | **6** | **R.QARPTYATAMLQSLLK.-** |
|  | 18998 | **634.3135** | **1899.9186** | **1901.0444** | **-1.1258** | **0** | **7** | **18** | **1** | **R.QMELHPDKPPILLVAGK.D** |
|  | 19543 | 649.3321 | 1944.9746 | 1945.8583 | -0.8837 | 1 | 8 | 14 | 2 | K.MTPEQMAKEMSEFLSR.G 19542 19575 |
|  | 20325 | **1011.8969** | **2021.7791** | **2022.0520** | **-0.2729** | **1** | **1** | **58** | **2** | **K.EYDALVKSSEELLSALQK.K** |
|  | 20998 | 1042.5198 | 2083.0250 | 2084.0803 | -1.0553 | 1 | 4 | 39 | 3 | R.DALAKTVYSHLFDHVVNR.V 20999 |
|  | 30463 | **1020.5077** | **4078.0017** | **4077.9452** | **0.0564** | **0** | **4** | **12** | **2** | **-.MEDGKPVWAPHPTDGFQMGNIVDIGPDSLTIEPLNQK.G** |
|  | 30708 | 1416.6195 | 4246.8367 | 4247.0667 | -0.2300 | 1 | 0 | 21 | 3 | -.MEDGKPVWAPHPTDGFQMGNIVDIGPDSLTIEPLNQKGK.T |
|  | 31497 | **1291.8182** | **5163.2439** | **5163.3810** | **-0.1371** | **1** | **1** | **8.4** | **1** | **K.MTSHHFEGAQILSQLQCSGMVSVLDLMQGGYPSRASFHELYNMYK.K** |

| **165.** | A1L0T0\|ILVBL_HUMAN    **Mass:** 68452    **Score:** 279    **Queries matched:** 23   **emPAI:** 0.45 |
| --- | --- |
|  | Acetolactate synthase-like protein OS=Homo sapiens GN=ILVBL PE=1 SV=2 |

|  | **Query** | **Observed** | **Mr(expt)** | **Mr(calc)** | **Delta** | **Miss** | **Score** | **Expect** | **Rank** | **Peptide** |
| --- | --- | --- | --- | --- | --- | --- | --- | --- | --- | --- |
|  | 6746 | **561.8008** | **1121.5871** | **1121.5941** | **-0.0070** | **0** | **69** | **1.9e-005** | **1** | **R.HGGENVAAVLR.A** |
|  | 8927 | **627.7999** | **1253.5852** | **1253.5935** | **-0.0083** | **0** | **45** | **0.0049** | **1** | **K.VLHDAQQQCR.D** |
|  | 11348 | **693.3632** | **1384.7119** | **1384.7973** | **-0.0854** | **1** | **4** | **58** | **4** | **K.AAMGLGARGLLLSR.E** |
|  | 16897 | **874.9392** | **1747.8637** | **1747.8750** | **-0.0112** | **0** | **5** | **38** | **1** | **R.AAVETLGVPCFLGGMAR.G** |
|  | 21199 | **701.3868** | **2101.1385** | **2100.1442** | **0.9943** | **0** | **20** | **0.95** | **1** | **R.FIFTLVGGHISPLLVACEK.L** |
|  | 22056 | **1092.6079** | **2183.2013** | **2183.2162** | **-0.0149** | **0** | **58** | **0.00013** | **1** | **R.LSGTVGVAAVTAGPGLTNTVTAVK.N** 22057 |
|  | 22143 | **731.7197** | **2192.1372** | **2192.1524** | **-0.0153** | **1** | **50** | **0.00085** | **1** | **R.HKIPVMALVGNDAGWTQISR.E** |
|  | 22311 | **554.0349** | **2212.1105** | **2212.2249** | **-0.1144** | **1** | **6** | **19** | **2** | **R.AKRPLMVLGSQALLTPTSADK.L** 22307 22310 |
|  | 22438 | **1113.5570** | **2225.0995** | **2225.1117** | **-0.0122** | **0** | **37** | **0.017** | **2** | **K.LVEGLQGQTWAPDWVEELR.E** 22437 22439 22447 |
|  | 23105 | **770.3621** | **2308.0644** | **2308.0794** | **-0.0150** | **0** | **5** | **25** | **1** | **R.EQVPSLGSNVACGLAYTDYHK.A** |
|  | 23561 | **1184.1377** | **2366.2608** | **2366.2740** | **-0.0131** | **0** | **88** | **1.2e-007** | **1** | **K.NAQMAQSPILLLGGAASTLLQNR.G** |
|  | 30515 | **1027.5660** | **4106.2351** | **4106.1261** | **0.1090** | **1** | **2** | **13** | **2** | **R.DIVPTLRAAMAAAQSGTPGPVFVELPVDVLYPYFMVQK.E** |
|  | 30984 | **1133.7333** | **4530.9040** | **4531.4796** | **-0.5756** | **1** | **0** | **16** | **1** | **R.LSGTVGVAAVTAGPGLTNTVTAVKNAQMAQSPILLLGGAASTLLQNR.G** |
|  | 31264 | **1215.3903** | **4857.5319** | **4857.5488** | **-0.0168** | **0** | **38** | **0.0024** | **1** | **K.AAMPVAQHLNPVQVLQLVEETLPDNSILVVDGGDFVGTAAHLVQPR.G** 31262 31263 31265 |

| **166.** | Q6NUK1\|SCMC1_HUMAN    **Mass:** 53548    **Score:** 279    **Queries matched:** 18   **emPAI:** 0.60 |
| --- | --- |
|  | Calcium-binding mitochondrial carrier protein SCaMC-1 OS=Homo sapiens GN=SLC25A24 PE=1 SV=2 |

|  | **Query** | **Observed** | **Mr(expt)** | **Mr(calc)** | **Delta** | **Miss** | **Score** | **Expect** | **Rank** | **Peptide** |
| --- | --- | --- | --- | --- | --- | --- | --- | --- | --- | --- |
|  | 3958 | **459.2657** | **916.5168** | **916.4865** | **0.0303** | **0** | **6** | **50** | **9** | **K.LLTEEGQK.I** |
|  | 4959 | 495.7993 | 989.5840 | 989.4778 | 0.1062 | 1 | 13 | 8.9 | 4 | K.SLDKNNDGK.I |
|  | 5507 | **516.2606** | **1030.5067** | **1029.5099** | **0.9967** | **0** | **25** | **0.59** | **1** | **K.IMMQVHGSK.S** |
|  | 6897 | **567.7627** | **1133.5108** | **1133.5182** | **-0.0073** | **0** | **11** | **14** | **3** | **K.FWAYEQYK.K** |
|  | 10672 | **673.3650** | **1344.7154** | **1344.7765** | **-0.0611** | **1** | **1** | **1.2e+002** | **4** | **R.IISKEGIPGLYR.G** |
|  | 10912 | **681.2997** | **1360.5849** | **1359.6639** | **0.9210** | **1** | **0** | **1.4e+002** | **4** | **K.IMMQVHGSKSDK.M** |
|  | 10921 | **681.3343** | **1360.6540** | **1361.5922** | **-0.9381** | **0** | **67** | **3e-005** | **1** | **K.TGQYSGIYDCAK.K** |
|  | 11316 | **692.3542** | **1382.6939** | **1382.7041** | **-0.0102** | **0** | **16** | **3.3** | **1** | **R.NLGIPLGQDAEEK.I** |
|  | 12895 | **493.2935** | **1476.8586** | **1477.7163** | **-0.8576** | **1** | **4** | **59** | **3** | **K.LDFEEFMKYLK.D** |
|  | 17370 | **891.4750** | **1780.9355** | **1780.9433** | **-0.0078** | **0** | **95** | **3.2e-008** | **1** | **K.VLPAVGISYVVYENMK.Q** 17371 |
|  | 22399 | **1111.0446** | **2220.0746** | **2220.0853** | **-0.0108** | **0** | **61** | **6.8e-005** | **1** | **R.MQAQAMLEGSPQLNMVGLFR.R** 22400 22406 |
|  | 24493 | **624.0315** | **2492.0969** | **2493.2290** | **-1.1322** | **1** | **2** | **41** | **3** | **R.TRMQAQAMLEGSPQLNMVGLFR.R** |
|  | 24832 | **849.7361** | **2546.1866** | **2546.2024** | **-0.0158** | **1** | **24** | **0.27** | **1** | **K.HSTGIDIGDSLTIPDEFTEDEKK.S** |
|  | 25496 | 663.8289 | 2651.2866 | 2652.2750 | -0.9884 | 1 | 2 | 38 | 7 | R.FISGSMAGATAQTFIYPMEVMKTR.L |
|  | 28591 | **1102.2379** | **3303.6919** | **3304.6618** | **-0.9699** | **0** | **15** | **1.2** | **1** | **K.DSVNPGVMVLLGCGALSSTCGQLASYPLALVR.T** |

| **167.** | Q13724\|MOGS_HUMAN    **Mass:** 92032    **Score:** 279    **Queries matched:** 16   **emPAI:** 0.13 |
| --- | --- |
|  | Mannosyl-oligosaccharide glucosidase OS=Homo sapiens GN=MOGS PE=1 SV=5 |

|  | **Query** | **Observed** | **Mr(expt)** | **Mr(calc)** | **Delta** | **Miss** | **Score** | **Expect** | **Rank** | **Peptide** |
| --- | --- | --- | --- | --- | --- | --- | --- | --- | --- | --- |
|  | 442 | **443.0379** | **442.0307** | **442.2288** | **-0.1982** | **0** | **4** | **17** | **1** | **R.GGPGR.R** |
|  | 4489 | 478.2987 | 954.5829 | 953.5406 | 1.0423 | 1 | 13 | 7.3 | 1 | R.RAVPAEGVR.T 4473 4474 |
|  | 4998 | **497.2472** | **992.4798** | **992.5232** | **-0.0434** | **0** | **10** | **16** | **1** | **R.WVLAWYR.A** |
|  | 9323 | **637.2495** | **1272.4845** | **1272.6098** | **-0.1254** | **0** | **31** | **0.12** | **1** | **R.SLAASSSFYGQR.N** |
|  | 9480 | **641.8684** | **1281.7223** | **1281.7292** | **-0.0070** | **0** | **4** | **56** | **1** | **R.LGPLLDILADSR.H** |
|  | 14133 | **520.3191** | **1557.9354** | **1557.9467** | **-0.0113** | **0** | **9** | **15** | **1** | **K.AVQLKPRPPQGLVR.V** |
|  | 18391 | 927.9824 | 1853.9502 | 1854.9952 | -1.0450 | 1 | 0 | 99 | 2 | R.QHIQDGALRLTTEFVK.R |
|  | 19277 | 962.0109 | 1922.0072 | 1922.0666 | -0.0594 | 0 | 7 | 19 | 7 | K.VDPALFPPVPLFTAVPSR.S |
|  | 24661 | **1258.6265** | **2515.2384** | **2515.2457** | **-0.0074** | **0** | **97** | **1.4e-008** | **1** | **K.YGSYNVFWTSNPGLPLLTEMVK.S** 24660 |
|  | 26645 | **1435.2402** | **2868.4659** | **2868.4797** | **-0.0138** | **0** | **97** | **1.2e-008** | **1** | **R.VTVEPQDSGTSALPLVSLFFYVVTDGK.E** 26646 |
|  | 26981 | **978.0745** | **2931.2016** | **2930.5456** | **0.6559** | **1** | **4** | **18** | **6** | **K.ALPRLHAWFSWLHQSQAGPLPLSYR.W** |
|  | 27055 | **981.2228** | **2940.6465** | **2940.6589** | **-0.0124** | **0** | **19** | **0.51** | **1** | **R.VVGRPQPQLQYVDALGYVSLFPLLLR.L** |

| **168.** | Q2TB90\|HKDC1_HUMAN    **Mass:** 103760   **Score:** 276    **Queries matched:** 40   **emPAI:** 0.32 |
| --- | --- |
|  | Putative hexokinase HKDC1 OS=Homo sapiens GN=HKDC1 PE=2 SV=2 |

|  | **Query** | **Observed** | **Mr(expt)** | **Mr(calc)** | **Delta** | **Miss** | **Score** | **Expect** | **Rank** | **Peptide** |
| --- | --- | --- | --- | --- | --- | --- | --- | --- | --- | --- |
|  | 1221 | **586.3630** | **585.3557** | **584.3282** | **1.0275** | **0** | **8** | **27** | **1** | **K.ELAPR.C** |
|  | 1438 | **632.3682** | **631.3609** | **631.3177** | **0.0432** | **0** | **8** | **50** | **1** | **K.EDQIK.K** |
|  | 2605 | **831.4967** | **830.4894** | **829.4909** | **0.9985** | **0** | **19** | **3.1** | **1** | **R.ILQETVK.E** |
|  | 2734 | 840.4036 | 839.3963 | 839.4613 | -0.0651 | 0 | 17 | 3.1 | 1 | K.SHGLATVR.M |
|  | 2749 | **421.0561** | **840.0976** | **839.5956** | **0.5020** | **1** | **3** | **48** | **9** | **R.VLLVKIR.S** |
|  | 3357 | **439.2103** | **876.4060** | **876.4124** | **-0.0064** | **0** | **22** | **1.2** | **1** | **K.EVCGAVSR.R** 3355 |
|  | 3383 | 440.0295 | 878.0445 | 878.4684 | -0.4239 | 0 | 9 | 17 | 2 | K.MLPTFVR.A |
|  | 3572 | **446.2478** | **890.4811** | **890.4862** | **-0.0051** | **0** | **31** | **0.15** | **1** | **K.AGLLFGGEK.S** |
|  | 3902 | **457.7840** | **913.5535** | **913.5596** | **-0.0061** | **0** | **21** | **1.3** | **1** | **K.GAALITAVAK.R** |
|  | 5777 | **526.2768** | **1050.5390** | **1050.5458** | **-0.0067** | **1** | **28** | **0.23** | **1** | **K.YKEGLANTR.E** |
|  | 5900 | **530.8262** | **1059.6378** | **1059.6441** | **-0.0063** | **0** | **12** | **10** | **1** | **R.VLALFQLTR.E** |
|  | 5999 | **534.7787** | **1067.5429** | **1067.5499** | **-0.0069** | **0** | **22** | **1** | **1** | **R.FLLSESGSTK.G** |
|  | 6036 | 535.8032 | 1069.5918 | 1069.6608 | -0.0690 | 1 | 11 | 13 | 2 | K.GAALITAVAKR.L |
|  | 6141 | **538.7706** | **1075.5266** | **1074.5306** | **0.9960** | **0** | **37** | **0.034** | **1** | **R.GVQDTDVVSR.L** |
|  | 7574 | **593.2903** | **1184.5661** | **1184.5747** | **-0.0086** | **0** | **33** | **0.082** | **1** | **R.TTVGMDGTLYK.I** |
|  | 8361 | **408.2347** | **1221.6824** | **1220.6587** | **1.0237** | **1** | **12** | **9.6** | **1** | **K.MAKAGLLFGGEK.S** |
|  | 9355 | 425.8835 | 1274.6287 | 1273.6918 | 0.9369 | 0 | 9 | 16 | 6 | K.LEEGVLLSWTK.K |
|  | 11101 | 458.2329 | 1371.6770 | 1371.6526 | 0.0243 | 0 | 8 | 21 | 2 | K.MTSGMYLGEIVR.Q 11100 |
|  | 11528 | **465.9332** | **1394.7777** | **1395.8085** | **-1.0309** | **1** | **14** | **5.3** | **1** | **R.ILQETVKELAPR.C** 11535 |
|  | 14840 | 804.4351 | 1606.8557 | 1606.7676 | 0.0881 | 0 | 1 | 86 | 7 | -.MFAVHLMAFYFSK.L |
|  | 14862 | **805.3986** | **1608.7826** | **1608.6760** | **0.1066** | **0** | **3** | **57** | **8** | **K.ATDCEGEDVVDMLR.E** |
|  | 15146 | 813.3910 | 1624.7675 | 1624.6709 | 0.0967 | 0 | (1) | 86 | 7 | K.ATDCEGEDVVDMLR.E |
|  | 15161 | **813.9274** | **1625.8402** | **1626.8875** | **-1.0473** | **1** | **6** | **29** | **2** | **R.RAAQLCGAGLAAIVEK.R** |
|  | 15239 | **544.3415** | **1630.0026** | **1630.8573** | **-0.8547** | **1** | **3** | **56** | **9** | **K.GAAMVTAVASRVQAQR.K** |
|  | 15627 | **828.9100** | **1655.8054** | **1655.8130** | **-0.0076** | **0** | **30** | **0.11** | **1** | **K.GASLPLGFTFSFPCR.Q** |
|  | 15971 | **840.8963** | **1679.7780** | **1679.7899** | **-0.0118** | **0** | **17** | **2.4** | **1** | **R.MLPTYVCGLPDGTEK.G** 15977 |
|  | 16179 | **566.2792** | **1695.8157** | **1695.7848** | **0.0309** | **0** | **(2)** | **67** | **9** | **R.MLPTYVCGLPDGTEK.G** |
|  | 16480 | **573.2980** | **1716.8721** | **1716.9062** | **-0.0341** | **1** | **0** | **1e+002** | **7** | **M.FAVHLMAFYFSKLK.E** |
|  | 17285 | **444.9605** | **1775.8131** | **1775.9240** | **-0.1110** | **1** | **5** | **33** | **7** | **K.DTNPTAAVKMLPTFVR.A** |
|  | 17712 | **904.9731** | **1807.9316** | **1807.8484** | **0.0832** | **1** | **1** | **87** | **8** | **R.YEKMTSGMYLGEIVR.Q** |
|  | 20845 | **689.9059** | **2066.6958** | **2065.9296** | **0.7662** | **1** | **2** | **40** | **8** | **K.ATDCEGEDVVDMLREAIK.R** |
|  | 21041 | **1045.0057** | **2087.9969** | **2088.0098** | **-0.0129** | **0** | **70** | **9.9e-006** | **1** | **R.HVQMESQFYPTPNEIIR.G** 21039 |
|  | 22782 | **1135.4910** | **2268.9674** | **2268.9780** | **-0.0106** | **0** | **96** | **1.8e-008** | **1** | **R.MCINTEWGAFGDDGALEDIR.T** |
|  | 23129 | **771.3810** | **2311.1213** | **2312.2120** | **-1.0907** | **1** | **5** | **26** | **1** | **K.MTSGMYLGEIVRQILIDLTK.Q** |
|  | 26926 | **973.7581** | **2918.2525** | **2917.2647** | **0.9878** | **1** | **84** | **1.9e-007** | **1** | **R.MCINTEWGAFGDDGALEDIRTEFDR.E** |

| **169.** | O75396\|SC22B_HUMAN    **Mass:** 24896    **Score:** 274    **Queries matched:** 20   **emPAI:** 1.35 |
| --- | --- |
|  | Vesicle-trafficking protein SEC22b OS=Homo sapiens GN=SEC22B PE=1 SV=3 |

|  | **Query** | **Observed** | **Mr(expt)** | **Mr(calc)** | **Delta** | **Miss** | **Score** | **Expect** | **Rank** | **Peptide** |
| --- | --- | --- | --- | --- | --- | --- | --- | --- | --- | --- |
|  | 3939 | **458.7829** | **915.5512** | **915.5575** | **-0.0064** | **0** | **14** | **8.7** | **1** | **M.VLLTMIAR.V** |
|  | 4312 | **472.3204** | **942.6263** | **943.4723** | **-0.8460** | **0** | **12** | **11** | **9** | **K.LNEQSPTR.C** |
|  | 4932 | **495.2238** | **988.4330** | **989.5029** | **-1.0699** | **0** | **6** | **45** | **10** | **R.GEALSALDSK.A** 4971 |
|  | 5911 | **531.2668** | **1060.5190** | **1060.5876** | **-0.0686** | **1** | **18** | **2.7** | **2** | **K.ANNLSSLSKK.Y** |
|  | 6078 | **536.7873** | **1071.5601** | **1071.5672** | **-0.0071** | **1** | **48** | **0.0032** | **1** | **R.KLNEQSPTR.C** 6055 6076 6084 6085 |
|  | 6194 | 540.7405 | 1079.4664 | 1078.5879 | 0.8785 | 0 | 2 | 90 | 6 | -.MVLLTMIAR.V |
|  | 8059 | **604.7949** | **1207.5752** | **1207.5833** | **-0.0081** | **0** | **48** | **0.0024** | **1** | **R.DLQQYQSQAK.Q** 8057 |
|  | 11869 | **707.8858** | **1413.7570** | **1413.7650** | **-0.0079** | **0** | **65** | **3.9e-005** | **1** | **R.IMVANIEEVLQR.G** |
|  | 14534 | **793.9047** | **1585.7949** | **1585.8060** | **-0.0111** | **0** | **82** | **7.1e-007** | **1** | **R.NLGSINTELQDVQR.I** |
|  | 19847 | **987.4588** | **1972.9030** | **1972.9160** | **-0.0130** | **0** | **65** | **3e-005** | **1** | **R.VADGLPLAASMQEDEQSGR.D** 19848 |
|  | 23612 | **595.0380** | **2376.1230** | **2376.1386** | **-0.0156** | **1** | **61** | **5.8e-005** | **1** | **K.KLAFAYLEDLHSEFDEQHGK.K** |
|  | 23775 | 601.5719 | 2402.2585 | 2401.2682 | 0.9903 | 1 | 1 | 55 | 7 | K.KVPTVSRPYSFIEFDTFIQK.T |
|  | 29194 | 1169.5231 | 3505.5474 | 3506.6383 | -1.0909 | 0 | 2 | 21 | 2 | R.CTLEAGAMTFHYIIEQGVCYLVLCEAAFPK.K |

| **170.** | P54855\|UDB15_HUMAN    **Mass:** 61461    **Score:** 273    **Queries matched:** 28   **emPAI:** 0.70 |
| --- | --- |
|  | UDP-glucuronosyltransferase 2B15 OS=Homo sapiens GN=UGT2B15 PE=1 SV=2 |

|  | **Query** | **Observed** | **Mr(expt)** | **Mr(calc)** | **Delta** | **Miss** | **Score** | **Expect** | **Rank** | **Peptide** |
| --- | --- | --- | --- | --- | --- | --- | --- | --- | --- | --- |
|  | 825 | 506.0474 | 505.0401 | 504.2907 | 0.7494 | 0 | 4 | 79 | 1 | K.SSAIK.L |
|  | 3716 | 451.2560 | 900.4975 | 900.5029 | -0.0053 | 0 | 46 | 0.0045 | 1 | K.GAALSVDIR.T |
|  | 3964 | 459.7441 | 917.4737 | 917.4793 | -0.0056 | 0 | 40 | 0.02 | 1 | K.AEMWLIR.T |
|  | 4784 | **489.2638** | **976.5130** | **975.5171** | **0.9959** | **1** | **9** | **26** | **9** | **K.ENVMKLSR.I** |
|  | 4984 | 496.7711 | 991.5276 | 991.5120 | 0.0155 | 1 | (7) | 37 | 4 | K.ENVMKLSR.I |
|  | 6288 | 544.2803 | 1086.5460 | 1086.5782 | -0.0322 | 0 | 7 | 33 | 8 | K.KPNTLGSNTR.L 6280 |
|  | 6469 | 550.8162 | 1099.6178 | 1099.6237 | -0.0059 | 0 | 30 | 0.18 | 1 | K.TILEELVQR.G |
|  | 10984 | 455.9054 | 1364.6945 | 1363.7129 | 0.9815 | 1 | 4 | 51 | 7 | R.TMSSRDLLNALK.S |
|  | 11919 | 709.3783 | 1416.7420 | 1416.7514 | -0.0093 | 0 | 53 | 0.00069 | 1 | K.WLPQNDLLGHPK.T 11920 |
|  | 12683 | 488.7570 | 1463.2491 | 1462.7562 | 0.4929 | 1 | 7 | 15 | 2 | K.GAALSVDIRTMSSR.D |
|  | 13759 | 512.2678 | 1533.7815 | 1533.7899 | -0.0085 | 1 | 6 | 29 | 1 | R.FDGKKPNTLGSNTR.L |
|  | 19027 | 951.9767 | 1901.9388 | 1901.9498 | -0.0110 | 0 | 24 | 0.42 | 1 | K.VLVWPTEYSHWINMK.T 19040 |
|  | 24592 | 1253.1022 | 2504.1898 | 2504.2046 | -0.0148 | 0 | 77 | 1.3e-006 | 1 | K.WDQFYSEVLGRPTTLFETMGK.A 24590 24593 24594 24595 |
|  | 26354 | 933.4486 | 2797.3240 | 2796.3992 | 0.9248 | 1 | 2 | 34 | 3 | R.IHHDQPMKPLDRAVFWIEFVMR.H |
|  | 27936 | 1574.2463 | 3146.4781 | 3146.4915 | -0.0134 | 0 | 41 | 0.0042 | 1 | K.NGGGFLFPPSYVPVVMSELSDQMIFMER.I |
|  | 29735 | 612.9839 | 3671.8597 | 3671.8752 | -0.0155 | 0 | 11 | 2.9 | 1 | R.TYWDFEFPRPFLPNVDFVGGLHCKPAKPLPK.E 29734 |
|  | 30620 | 1038.2572 | 4148.9997 | 4148.0401 | 0.9596 | 0 | 15 | 0.75 | 1 | K.AFITHGGTNGIYEAIYHGIPMVGIPLFADQHDNIAHMK.A 30621 30622 30623 |

| **171.** | Q99798\|ACON_HUMAN    **Mass:** 86113    **Score:** 272    **Queries matched:** 42   **emPAI:** 0.59 |
| --- | --- |
|  | Aconitate hydratase, mitochondrial OS=Homo sapiens GN=ACO2 PE=1 SV=2 |

|  | **Query** | **Observed** | **Mr(expt)** | **Mr(calc)** | **Delta** | **Miss** | **Score** | **Expect** | **Rank** | **Peptide** |
| --- | --- | --- | --- | --- | --- | --- | --- | --- | --- | --- |
|  | 861 | **514.3058** | **513.2985** | **514.3227** | **-1.0242** | **0** | **12** | **10** | **3** | **K.ALGVR.Q** 870 |
|  | 869 | 515.3262 | 514.3189 | 514.3479 | -0.0290 | 0 | 15 | 7.3 | 5 | K.VIGVK.L |
|  | 991 | **539.3455** | **538.3382** | **538.2976** | **0.0406** | **0** | **7** | **24** | **2** | **R.HLGGR.A** |
|  | 2060 | 396.7319 | 791.4493 | 791.3847 | 0.0646 | 1 | 31 | 0.16 | 1 | R.MKELQQ.- 2061 |
|  | 2698 | 419.3138 | 836.6131 | 836.4868 | 0.1263 | 0 | 12 | 9.2 | 4 | K.QALAHGLK.C |
|  | 2941 | **853.7800** | **852.7727** | **853.4657** | **-0.6931** | **0** | **23** | **0.49** | **1** | **R.IHETNLK.K** |
|  | 3000 | **429.2127** | **856.4109** | **855.5290** | **0.8819** | **1** | **7** | **40** | **8** | **K.NINIVRK.R** |
|  | 4195 | **468.7523** | **935.4901** | **934.4872** | **1.0029** | **0** | **35** | **0.049** | **1** | **R.DGYAQILR.D** |
|  | 4854 | **491.7846** | **981.5545** | **981.5607** | **-0.0062** | **1** | **10** | **15** | **3** | **R.IHETNLKK.Q** |
|  | 5992 | **534.2739** | **1066.5333** | **1066.5407** | **-0.0074** | **0** | **30** | **0.15** | **1** | **K.NTIVTSYNR.N** |
|  | 7082 | **575.2954** | **1148.5763** | **1149.6216** | **-1.0453** | **0** | **6** | **39** | **2** | **-.MAPYSLLVTR.L** 7109 |
|  | 7360 | **585.8415** | **1169.6685** | **1169.6768** | **-0.0082** | **0** | **29** | **0.17** | **1** | **R.LNRPLTLSEK.I** 7359 |
|  | 7843 | **599.8336** | **1197.6526** | **1196.5925** | **1.0601** | **0** | **0** | **1.4e+002** | **7** | **R.LEAPDADELPK.G** |
|  | 7860 | **600.2819** | **1198.5492** | **1198.6809** | **-0.1317** | **0** | **8** | **27** | **6** | **K.DLEDLQILIK.V** |
|  | 8292 | **610.3156** | **1218.6165** | **1218.6245** | **-0.0079** | **0** | **14** | **5.6** | **1** | **K.LTGSLSGWSSPK.D** |
|  | 11044 | 457.2226 | 1368.6459 | 1367.8752 | 0.7707 | 1 | 7 | 24 | 5 | K.DVILKVAGILTVK.G |
|  | 11854 | **707.3833** | **1412.7520** | **1411.7459** | **1.0061** | **1** | **11** | **10** | **1** | **K.VAEKEGWPLDIR.V** |
|  | 12673 | **732.3733** | **1462.7321** | **1462.7416** | **-0.0095** | **0** | **39** | **0.016** | **1** | **K.SQFTITPGSEQIR.A** |
|  | 14095 | **519.5857** | **1555.7352** | **1555.7453** | **-0.0101** | **0** | **19** | **1.6** | **1** | **K.CTTDHISAAGPWLK.F** |
|  | 14108 | **779.3514** | **1556.6882** | **1556.6995** | **-0.0113** | **0** | **50** | **0.0014** | **1** | **K.FNPETDYLTGTDGK.K** 14127 |
|  | 14744 | 801.4066 | 1600.7986 | 1600.7845 | 0.0140 | 0 | 41 | 0.01 | 1 | R.NAVTQEFGPVPDTAR.Y |
|  | 15767 | **834.3807** | **1666.7468** | **1666.7587** | **-0.0119** | **0** | **9** | **15** | **1** | **R.WVVIGDENYGEGSSR.E** |
|  | 16025 | **842.9556** | **1683.8967** | **1684.9036** | **-1.0069** | **1** | **2** | **71** | **9** | **K.WDGKDLEDLQILIK.V** |
|  | 16032 | **843.3986** | **1684.7827** | **1684.7944** | **-0.0117** | **1** | **26** | **0.3** | **1** | **K.FNPETDYLTGTDGKK.F** |
|  | 16916 | **584.2790** | **1749.8153** | **1750.8672** | **-1.0519** | **1** | **1** | **89** | **5** | **K.CKSQFTITPGSEQIR.A** |
|  | 16968 | 877.4359 | 1752.8573 | 1752.8682 | -0.0110 | 0 | 40 | 0.011 | 1 | K.DINQEVYNFLATAGAK.Y 16969 16971 |
|  | 18183 | **921.4497** | **1840.8849** | **1840.8955** | **-0.0107** | **0** | **59** | **0.00014** | **1** | **K.IVYGHLDDPASQEIER.G** |
|  | 18253 | **923.4026** | **1844.7906** | **1844.8033** | **-0.0127** | **0** | **87** | **2e-007** | **1** | **R.VGLIGSCTNSSYEDMGR.S** 18254 |
|  | 19623 | **651.8033** | **1952.3880** | **1952.0003** | **0.3877** | **1** | **2** | **44** | **9** | **R.AKDINQEVYNFLATAGAK.Y** |
|  | 21963 | **1086.4957** | **2170.9769** | **2171.0252** | **-0.0483** | **0** | **9** | **10** | **2** | **R.DLGGIVLANACGPCIGQWDR.K** |
|  | 22717 | **754.0461** | **2259.1164** | **2259.1318** | **-0.0154** | **0** | **18** | **1.2** | **1** | **K.VAVPSTIHCDHLIEAQVGGEK.D** |
|  | 23408 | 784.0676 | 2349.1809 | 2350.0939 | -0.9131 | 0 | 6 | 20 | 2 | K.VAMSHFEPNEYIHYDLLEK.N |
|  | 31002 | **1139.7710** | **4555.0549** | **4555.0665** | **-0.0116** | **0** | **11** | **1.1** | **1** | **K.GGTGAIVEYHGPGVDSISCTGMATICNMGAEIGATTSVFPYNHR.M** 31001 |

| **172.** | Q99795\|GPA33_HUMAN    **Mass:** 36237    **Score:** 271    **Queries matched:** 16   **emPAI:** 0.64 |
| --- | --- |
|  | Cell surface A33 antigen OS=Homo sapiens GN=GPA33 PE=1 SV=1 |

|  | **Query** | **Observed** | **Mr(expt)** | **Mr(calc)** | **Delta** | **Miss** | **Score** | **Expect** | **Rank** | **Peptide** |
| --- | --- | --- | --- | --- | --- | --- | --- | --- | --- | --- |
|  | 4855 | **491.7847** | **981.5548** | **981.5607** | **-0.0059** | **0** | **41** | **0.011** | **1** | **K.LLLTHTER.V** |
|  | 10900 | **680.8178** | **1359.6209** | **1359.6306** | **-0.0097** | **0** | **19** | **2.1** | **1** | **R.EAYEEPPEQLR.E** 10898 |
|  | 11698 | **703.4303** | **1404.8460** | **1405.7102** | **-0.8642** | **1** | **14** | **5.4** | **3** | **K.NYIHGELYKNR.V** |
|  | 15126 | **542.2694** | **1623.7862** | **1624.6601** | **-0.8739** | **1** | **13** | **5.3** | **2** | **R.EEEDDYRQEEQR.S** |
|  | 16173 | 848.8961 | 1695.7776 | 1695.7887 | -0.0111 | 0 | 46 | 0.0031 | 1 | K.SVTLPCTYHTSTSSR.E 16174 |
|  | 21939 | **723.4185** | **2167.2337** | **2167.0328** | **0.2009** | **1** | **2** | **43** | **1** | **R.ASQGKSVTLPCTYHTSTSSR.E** |
|  | 23730 | **1198.1263** | **2394.2381** | **2394.2543** | **-0.0162** | **0** | **109** | **8.1e-010** | **1** | **R.YNILNQEQPLAQPASGQPVSLK.N** 23728 23729 23731 |
|  | 24959 | **851.1200** | **2550.3382** | **2550.3554** | **-0.0172** | **1** | **51** | **0.00052** | **1** | **K.RYNILNQEQPLAQPASGQPVSLK.N** |
|  | 28956 | **852.6984** | **3406.7646** | **3406.7840** | **-0.0194** | **0** | **5** | **11** | **1** | **R.LLVLVPPSKPECGIEGETIIGNNIQLTCQSK.E** 28958 |
|  | 31208 | **959.7679** | **4793.8030** | **4794.1542** | **-0.3512** | **1** | **3** | **5.5** | **1** | **K.NRVSISNNAEQSDASITIDQLTMADNGTYECSVSLMSDLEGNTK.S** |

| **173.** | Q6P1M0\|S27A4_HUMAN    **Mass:** 72930    **Score:** 267    **Queries matched:** 19   **emPAI:** 0.28 |
| --- | --- |
|  | Long-chain fatty acid transport protein 4 OS=Homo sapiens GN=SLC27A4 PE=1 SV=1 |

|  | **Query** | **Observed** | **Mr(expt)** | **Mr(calc)** | **Delta** | **Miss** | **Score** | **Expect** | **Rank** | **Peptide** |
| --- | --- | --- | --- | --- | --- | --- | --- | --- | --- | --- |
|  | 2590 | **415.7396** | **829.4647** | **830.4432** | **-0.9785** | **0** | **13** | **12** | **7** | **R.MALGNGLR.Q** |
|  | 4653 | 484.7404 | 967.4662 | 966.4519 | 1.0143 | 1 | 3 | 70 | 5 | R.DRTGDTFR.W |
|  | 5112 | **500.7788** | **999.5431** | **999.5501** | **-0.0070** | **0** | **8** | **28** | **2** | **R.YLLNQPPR.E** |
|  | 6571 | 554.8020 | 1107.5894 | 1106.6488 | 0.9406 | 0 | 4 | 64 | 5 | R.ILSFVYPIR.L |
|  | 6605 | **556.3157** | **1110.6169** | **1109.5214** | **1.0955** | **0** | **5** | **40** | **5** | **R.EAENQHQVR.M** |
|  | 8010 | 603.8025 | 1205.5904 | 1205.5903 | 0.0001 | 0 | 1 | 1.1e+002 | 6 | R.MAALVYYGFR.M 8012 |
|  | 10902 | **454.2236** | **1359.6489** | **1358.8034** | **0.8455** | **1** | **9** | **21** | **2** | **R.RTVPILFASTVR.R** |
|  | 12840 | 492.2463 | 1473.7170 | 1474.9024 | -1.1854 | 1 | 4 | 52 | 7 | R.ILSFVYPIRLVR.V |
|  | 13193 | **748.3702** | **1494.7259** | **1495.7168** | **-0.9908** | **1** | **11** | **11** | **5** | **R.RFDGYLNQGANNK.K** |
|  | 13994 | **775.8802** | **1549.7458** | **1549.7559** | **-0.0100** | **0** | **108** | **2.2e-009** | **1** | **R.GLASGDVAAIFMENR.N** |
|  | 16506 | **860.3880** | **1718.7614** | **1718.7716** | **-0.0101** | **0** | **39** | **0.013** | **1** | **R.AGMAAVASPTGNCDLER.F** |
|  | 17974 | **914.4412** | **1826.8678** | **1826.8799** | **-0.0121** | **0** | **88** | **1.6e-007** | **1** | **R.QLDEYSSSVANFLQAR.G** 17973 |
|  | 18878 | **946.9656** | **1891.9166** | **1891.9276** | **-0.0110** | **1** | **66** | **2.8e-005** | **1** | **R.WKGENVSTTEVEGTLSR.L** |
|  | 21062 | **1046.0193** | **2090.0240** | **2090.0354** | **-0.0114** | **0** | **39** | **0.012** | **1** | **R.LLDMADVAVYGVEVPGTEGR.A** |
|  | 21917 | **722.7047** | **2165.0921** | **2165.1045** | **-0.0123** | **1** | **13** | **4** | **1** | **K.EGFDPAIVKDPLFYLDAQK.G** |
|  | 24880 | 850.7253 | 2549.1540 | 2550.2206 | -1.0666 | 1 | 1 | 55 | 5 | R.AGMAAVASPTGNCDLERFAQVLEK.E |
|  | 26817 | **965.8463** | **2894.5169** | **2895.4411** | **-0.9242** | **1** | **2** | **33** | **3** | **R.GLASGDVAAIFMENRNEFVGLWLGMAK.L** |

| **174.** | P01877\|IGHA2_HUMAN    **Mass:** 37301    **Score:** 267    **Queries matched:** 20   **emPAI:** 1.16 |
| --- | --- |
|  | Ig alpha-2 chain C region OS=Homo sapiens GN=IGHA2 PE=1 SV=3 |

|  | **Query** | **Observed** | **Mr(expt)** | **Mr(calc)** | **Delta** | **Miss** | **Score** | **Expect** | **Rank** | **Peptide** |
| --- | --- | --- | --- | --- | --- | --- | --- | --- | --- | --- |
|  | 2581 | **415.7114** | **829.4082** | **829.4116** | **-0.0035** | **0** | **16** | **5.5** | **1** | **K.SVTCHVK.H** |
|  | 3635 | **448.7321** | **895.4497** | **895.4552** | **-0.0054** | **0** | **34** | **0.058** | **1** | **K.YLTWASR.Q** |
|  | 4258 | **470.7428** | **939.4710** | **939.4774** | **-0.0064** | **0** | **35** | **0.045** | **1** | **K.SAVQGPPER.D** 4251 |
|  | 8167 | **607.3154** | **1212.6162** | **1212.6251** | **-0.0089** | **0** | **52** | **0.00091** | **1** | **R.WLQGSQELPR.E** 8169 8180 |
|  | 13436 | **756.8463** | **1511.6781** | **1511.6893** | **-0.0112** | **0** | **68** | **2e-005** | **1** | **R.DASGATFTWTPSSGK.S** |
|  | 18114 | **918.4733** | **1834.9321** | **1834.9425** | **-0.0104** | **0** | **110** | **1.1e-009** | **1** | **R.QEPSQGTTTFAVTSILR.V** 18113 |
|  | 22266 | **737.0255** | **2208.0547** | **2208.0344** | **0.0203** | **0** | **3** | **39** | **9** | **K.GDTFSCMVGHEALPLAFTQK.T** |
|  | 23293 | **779.7125** | **2336.1156** | **2336.1293** | **-0.0138** | **1** | **29** | **0.097** | **1** | **K.KGDTFSCMVGHEALPLAFTQK.T** 23292 23294 |
|  | 23553 | **789.4273** | **2365.2601** | **2364.0912** | **1.1689** | **0** | **5** | **21** | **1** | **R.MAGKPTHVNVSVVMAEVDGTCY.-** |
|  | 26876 | 970.4415 | 2908.3026 | 2908.3208 | -0.0182 | 0 | 26 | 0.14 | 1 | K.HYTNPSQDVTVPCPVPPPPPCCHPR.L |
|  | 29402 | **1191.9579** | **3572.8518** | **3572.8661** | **-0.0142** | **0** | **36** | **0.0096** | **1** | **K.SGNTFRPEVHLLPPPSEELALNELVTLTCLAR.G** 29400 29401 29403 |

| **175.** | P01876\|IGHA1_HUMAN    **Mass:** 38486    **Score:** 266    **Queries matched:** 20   **emPAI:** 1.10 |
| --- | --- |
|  | Ig alpha-1 chain C region OS=Homo sapiens GN=IGHA1 PE=1 SV=2 |

|  | **Query** | **Observed** | **Mr(expt)** | **Mr(calc)** | **Delta** | **Miss** | **Score** | **Expect** | **Rank** | **Peptide** |
| --- | --- | --- | --- | --- | --- | --- | --- | --- | --- | --- |
|  | 2581 | 415.7114 | 829.4082 | 829.4116 | -0.0035 | 0 | 16 | 5.5 | 1 | K.SVTCHVK.H |
|  | 3635 | 448.7321 | 895.4497 | 895.4552 | -0.0054 | 0 | 34 | 0.058 | 1 | K.YLTWASR.Q |
|  | 4258 | 470.7428 | 939.4710 | 939.4774 | -0.0064 | 0 | 35 | 0.045 | 1 | K.SAVQGPPER.D 4251 |
|  | 8167 | 607.3154 | 1212.6162 | 1212.6251 | -0.0089 | 0 | 52 | 0.00091 | 1 | R.WLQGSQELPR.E 8169 8180 |
|  | 11160 | **688.3088** | **1374.6031** | **1374.6126** | **-0.0094** | **0** | **56** | **0.00035** | **1** | **K.TFTCTAAYPESK.T** 11161 |
|  | 18114 | 918.4733 | 1834.9321 | 1834.9425 | -0.0104 | 0 | 110 | 1.1e-009 | 1 | R.QEPSQGTTTFAVTSILR.V 18113 |
|  | 22266 | 737.0255 | 2208.0547 | 2208.0344 | 0.0203 | 0 | 3 | 39 | 9 | K.GDTFSCMVGHEALPLAFTQK.T |
|  | 23293 | 779.7125 | 2336.1156 | 2336.1293 | -0.0138 | 1 | 29 | 0.097 | 1 | K.KGDTFSCMVGHEALPLAFTQK.T 23292 23294 |
|  | 23525 | **788.0579** | **2361.1518** | **2362.1297** | **-0.9780** | **0** | **5** | **23** | **4** | **R.LAGKPTHVNVSVVMAEVDGTCY.-** |
|  | 29402 | 1191.9579 | 3572.8518 | 3572.8661 | -0.0142 | 0 | 36 | 0.0096 | 1 | K.SGNTFRPEVHLLPPPSEELALNELVTLTCLAR.G 29400 29401 29403 |

| **176.** | P08134\|RHOC_HUMAN    **Mass:** 22334    **Score:** 265    **Queries matched:** 15   **emPAI:** 1.58 |
| --- | --- |
|  | Rho-related GTP-binding protein RhoC OS=Homo sapiens GN=RHOC PE=1 SV=1 |

|  | **Query** | **Observed** | **Mr(expt)** | **Mr(calc)** | **Delta** | **Miss** | **Score** | **Expect** | **Rank** | **Peptide** |
| --- | --- | --- | --- | --- | --- | --- | --- | --- | --- | --- |
|  | 1329 | 606.7065 | 605.6992 | 605.2591 | 0.4401 | 0 | 4 | 56 | 9 | R.DMANR.I |
|  | 1916 | 759.4308 | 758.4235 | 758.3963 | 0.0272 | 0 | 9 | 33 | 5 | K.WTPEVK.H |
|  | 4846 | 491.7334 | 981.4522 | 981.4590 | -0.0068 | 0 | 16 | 3.5 | 1 | R.EVFEMATR.A 4857 |
|  | 6308 | 544.7883 | 1087.5621 | 1087.5696 | -0.0075 | 0 | 51 | 0.0015 | 1 | K.LVIVGDGACGK.T 6280 6307 |
|  | 8243 | 406.5327 | 1216.5762 | 1215.6646 | 0.9117 | 1 | 5 | 56 | 4 | K.KLVIVGDGACGK.T |
|  | 14840 | 804.4351 | 1606.8557 | 1606.8654 | -0.0097 | 0 | 76 | 3e-006 | 1 | K.HFCPNVPIILVGNK.K |
|  | 16721 | 579.3232 | 1734.9479 | 1734.9603 | -0.0124 | 1 | 46 | 0.0029 | 1 | K.HFCPNVPIILVGNKK.D |
|  | 20195 | 1004.9594 | 2007.9041 | 2007.9174 | -0.0132 | 0 | 53 | 0.00042 | 1 | K.QVELALWDTAGQEDYDR.L |
|  | 26296 | **1394.1671** | **2786.3197** | **2786.3327** | **-0.0130** | **0** | **62** | **3.5e-005** | **1** | **K.DQFPEVYVPTVFENYIADIEVDGK.Q** 26297 26298 |
|  | 29092 | 1155.5641 | 3463.6704 | 3463.6891 | -0.0186 | 0 | 62 | 2.6e-005 | 1 | R.LRPLSYPDTDVILMCFSIDSPDSLENIPEK.W |

| **177.** | Q9H0U4\|RAB1B_HUMAN    **Mass:** 22328    **Score:** 264    **Queries matched:** 18   **emPAI:** 1.58 |
| --- | --- |
|  | Ras-related protein Rab-1B OS=Homo sapiens GN=RAB1B PE=1 SV=1 |

|  | **Query** | **Observed** | **Mr(expt)** | **Mr(calc)** | **Delta** | **Miss** | **Score** | **Expect** | **Rank** | **Peptide** |
| --- | --- | --- | --- | --- | --- | --- | --- | --- | --- | --- |
|  | 1480 | **643.2714** | **642.2641** | **642.4064** | **-0.1424** | **0** | **19** | **2.4** | **1** | **K.LLVGNK.S** |
|  | 6064 | **536.3201** | **1070.6257** | **1070.6336** | **-0.0079** | **0** | **15** | **5.6** | **1** | **K.LLLIGDSGVGK.S** |
|  | 6285 | **544.2767** | **1086.5389** | **1086.5458** | **-0.0069** | **0** | **16** | **4.8** | **5** | **K.QWLQEIDR.Y** |
|  | 10115 | **658.8291** | **1315.6436** | **1315.6521** | **-0.0084** | **0** | **53** | **0.00078** | **1** | **K.LQIWDTAGQER.F** 10116 |
|  | 12318 | **721.3586** | **1440.7027** | **1440.7143** | **-0.0116** | **0** | **50** | **0.0014** | **1** | **R.MGPGAASGGERPNLK.I** 12313 12319 12320 |
|  | 12579 | 729.8665 | 1457.7185 | 1456.7092 | 1.0092 | 0 | (1) | 1e+002 | 4 | R.MGPGAASGGERPNLK.I |
|  | 16578 | **862.9362** | **1723.8579** | **1723.8669** | **-0.0090** | **0** | **25** | **0.37** | **1** | **K.EFADSLGIPFLETSAK.N** 16557 |
|  | 18580 | **934.9427** | **1867.8708** | **1867.8808** | **-0.0100** | **0** | **71** | **7.9e-006** | **1** | **K.NATNVEQAFMTMAAEIK.K** |
|  | 18767 | **942.9406** | **1883.8667** | **1883.8757** | **-0.0090** | **0** | **(62)** | **7.2e-005** | **2** | **K.NATNVEQAFMTMAAEIK.K** 18768 |
|  | 20413 | **1015.1899** | **2028.3653** | **2027.9656** | **0.3998** | **1** | **1** | **51** | **1** | **K.NATNVEQAFMTMAAEIKK.R** |
|  | 21964 | **1086.4994** | **2170.9842** | **2170.9947** | **-0.0104** | **0** | **81** | **6.7e-007** | **1** | **R.FADDTYTESYISTIGVDFK.I** 21963 |

| **178.** | P39023\|RL3_HUMAN    **Mass:** 46365    **Score:** 263    **Queries matched:** 26   **emPAI:** 0.72 |
| --- | --- |
|  | 60S ribosomal protein L3 OS=Homo sapiens GN=RPL3 PE=1 SV=2 |

|  | **Query** | **Observed** | **Mr(expt)** | **Mr(calc)** | **Delta** | **Miss** | **Score** | **Expect** | **Rank** | **Peptide** |
| --- | --- | --- | --- | --- | --- | --- | --- | --- | --- | --- |
|  | 1787 | **720.3658** | **719.3585** | **719.3636** | **-0.0051** | **0** | **10** | **19** | **3** | **K.GCVVGTK.K** |
|  | 3586 | **446.7551** | **891.4956** | **890.5048** | **0.9908** | **1** | **9** | **26** | **5** | **K.KAFMGPLK.K** |
|  | 3868 | **456.2557** | **910.4968** | **911.4059** | **-0.9090** | **0** | **1** | **1.1e+002** | **5** | **R.FQTMEEK.K** |
|  | 3915 | **458.2520** | **914.4895** | **915.5753** | **-1.0858** | **1** | **22** | **1.2** | **1** | **R.KSLLVQTK.R** 3953 |
|  | 4868 | 492.2715 | 982.5283 | 982.5349 | -0.0065 | 0 | 47 | 0.0028 | 1 | R.HGSLGFLPR.K |
|  | 8470 | **615.2807** | **1228.5468** | **1227.6571** | **0.8897** | **1** | **4** | **63** | **2** | **R.EVDRPGSKVNK.K** |
|  | 10975 | **683.2969** | **1364.5793** | **1364.7135** | **-0.1342** | **1** | **2** | **77** | **2** | **R.KVACIGAWHPAR.V** |
|  | 12235 | **718.3245** | **1434.6345** | **1434.6449** | **-0.0104** | **0** | **56** | **0.00033** | **1** | **K.TVFAEHISDECK.R** |
|  | 13933 | **516.3109** | **1545.9109** | **1546.9130** | **-1.0021** | **1** | **6** | **27** | **7** | **R.VIAHTQMRLLPLR.Q** |
|  | 14606 | **796.3755** | **1590.7364** | **1590.7460** | **-0.0096** | **1** | **32** | **0.075** | **1** | **K.TVFAEHISDECKR.R** |
|  | 16199 | **849.4488** | **1696.8831** | **1695.8614** | **1.0218** | **0** | **18** | **1.9** | **1** | **K.AHLMEIQVNGGTVAEK.L** 16195 |
|  | 17179 | **590.3373** | **1767.9900** | **1767.9050** | **0.0850** | **1** | **2** | **66** | **5** | **K.AGMTHIVREVDRPGSK.V** 17180 17181 |
|  | 17469 | **597.3012** | **1788.8816** | **1789.9080** | **-1.0264** | **1** | **9** | **14** | **1** | **K.YCQVIRVIAHTQMR.L** |
|  | 18169 | **614.0776** | **1839.2111** | **1839.9513** | **-0.7402** | **1** | **7** | **13** | **2** | **K.KAHLMEIQVNGGTVAEK.L** |
|  | 24052 | 1219.1085 | 2436.2025 | 2436.2148 | -0.0123 | 0 | 69 | 9.2e-006 | 1 | K.SINPLGGFVHYGEVTNDFVMLK.G 24053 |
|  | 25708 | **1343.6836** | **2685.3526** | **2685.3684** | **-0.0158** | **0** | **84** | **2.7e-007** | **1** | **R.LEQQVPVNQVFGQDEMIDVIGVTK.G** 25707 |
|  | 25832 | **1351.5819** | **2701.1493** | **2701.3633** | **-0.2140** | **0** | **(1)** | **43** | **3** | **R.LEQQVPVNQVFGQDEMIDVIGVTK.G** 25828 25831 |
|  | 26828 | **966.9827** | **2897.9262** | **2897.5460** | **0.3801** | **0** | **0** | **36** | **3** | **K.EVVEAVTIVETPPMVVVGIVGYVETPR.G** |

| **179.** | P05387\|RLA2_HUMAN    **Mass:** 11658    **Score:** 262    **Queries matched:** 9   **emPAI:** 3.34 |
| --- | --- |
|  | 60S acidic ribosomal protein P2 OS=Homo sapiens GN=RPLP2 PE=1 SV=1 |

|  | **Query** | **Observed** | **Mr(expt)** | **Mr(calc)** | **Delta** | **Miss** | **Score** | **Expect** | **Rank** | **Peptide** |
| --- | --- | --- | --- | --- | --- | --- | --- | --- | --- | --- |
|  | 8982 | **628.8416** | **1255.6687** | **1255.6772** | **-0.0085** | **0** | **35** | **0.044** | **1** | **K.NIEDVIAQGIGK.L** |
|  | 17228 | **886.9496** | **1771.8846** | **1771.8952** | **-0.0106** | **1** | **66** | **3e-005** | **1** | **K.ILDSVGIEADDDRLNK.V** 17226 17227 |
|  | 18564 | **934.4321** | **1866.8497** | **1867.9679** | **-1.1182** | **0** | **49** | **0.0011** | **1** | **R.YVASYLLAALGGNSSPSAK.D** 18597 |
|  | 26244 | **1387.7106** | **2773.4066** | **2773.4246** | **-0.0180** | **0** | **89** | **6.9e-008** | **1** | **K.LASVPAGGAVAVSAAPGSAAPAAGSAPAAAEEK.K** 26242 26243 |

| **180.** | P35580\|MYH10_HUMAN    **Mass:** 229827   **Score:** 261    **Queries matched:** 52   **emPAI:** 0.07 |
| --- | --- |
|  | Myosin-10 OS=Homo sapiens GN=MYH10 PE=1 SV=3 |

|  | **Query** | **Observed** | **Mr(expt)** | **Mr(calc)** | **Delta** | **Miss** | **Score** | **Expect** | **Rank** | **Peptide** |
| --- | --- | --- | --- | --- | --- | --- | --- | --- | --- | --- |
|  | 970 | 533.1628 | 532.1555 | 531.3017 | 0.8539 | 0 | 10 | 28 | 1 | R.IGQSK.I |
|  | 1092 | 559.4482 | 558.4410 | 558.3125 | 0.1284 | 0 | 6 | 74 | 1 | K.NNALK.V |
|  | 1415 | **628.3733** | **627.3661** | **628.4020** | **-1.0359** | **1** | **2** | **87** | **8** | **R.ARLAAK.K** |
|  | 1533 | 653.3270 | 652.3197 | 651.3228 | 0.9969 | 0 | 15 | 4.7 | 6 | R.DYVQK.A |
|  | 1627 | 676.3828 | 675.3755 | 675.3803 | -0.0048 | 0 | 9 | 27 | 4 | R.EVSTLK.N |
|  | 2309 | 809.4545 | 808.4472 | 807.4491 | 0.9981 | 0 | 15 | 4.9 | 1 | R.TVGQLYK.E 2303 |
|  | 2993 | **429.0878** | **856.1610** | **855.4198** | **0.7412** | **1** | **9** | **16** | **9** | **K.AESEHKR.K** |
|  | 3090 | 863.0862 | 862.0789 | 861.4742 | 0.6047 | 1 | 0 | 1.3e+002 | 2 | R.KLQAQMK.D |
|  | 3752 | **452.2244** | **902.4343** | **902.5073** | **-0.0730** | **0** | **13** | **9.9** | **5** | **K.ATISALEAK.I** |
|  | 4151 | **466.7671** | **931.5197** | **930.4883** | **1.0314** | **1** | **10** | **18** | **5** | **K.VSEGDRLR.V** |
|  | 4222 | 469.7896 | 937.5645 | 937.5022 | 0.0624 | 0 | 2 | 95 | 6 | R.IVFQEFR.Q |
|  | 4912 | **494.2935** | **986.5724** | **985.5444** | **1.0280** | **0** | **3** | **1e+002** | **7** | **K.LQELEGAVK.S** |
|  | 5023 | 498.2632 | 994.5117 | 993.4880 | 1.0238 | 0 | 4 | 63 | 9 | R.GGPISFSSSR.S |
|  | 6096 | 537.2975 | 1072.5804 | 1072.5764 | 0.0040 | 1 | 11 | 16 | 3 | R.EQEVAELKK.A |
|  | 6367 | **547.2668** | **1092.5190** | **1091.5029** | **1.0161** | **1** | **3** | **74** | **10** | **K.EQMEKANAR.M** |
|  | 7203 | **579.5328** | **1157.0510** | **1157.4580** | **-0.4070** | **0** | **1** | **96** | **1** | **R.ADMEDLMSSK.D** |
|  | 8255 | **609.3309** | **1216.6472** | **1215.5956** | **1.0516** | **1** | **8** | **25** | **1** | **K.SDNARQQLER.Q** |
|  | 8351 | 611.3470 | 1220.6794 | 1219.6448 | 1.0346 | 1 | 10 | 17 | 2 | K.KFDQLLAEEK.S |
|  | 8371 | 612.3187 | 1222.6229 | 1222.6306 | -0.0077 | 0 | 44 | 0.0055 | 1 | R.AGVLAHLEEER.D |
|  | 8492 | 615.7869 | 1229.5592 | 1228.7027 | 0.8565 | 1 | 1 | 1.1e+002 | 9 | K.LQLEKVTAEAK.I |
|  | 8838 | **416.9039** | **1247.6899** | **1247.5816** | **0.1083** | **0** | **10** | **13** | **4** | **R.ALEQQVEEMR.T** |
|  | 9033 | 630.2999 | 1258.5853 | 1257.6969 | 0.8885 | 0 | 6 | 37 | 2 | R.YEILTPNAIPK.G |
|  | 9704 | **647.4996** | **1292.9847** | **1293.6426** | **-0.6579** | **1** | **0** | **96** | **4** | **R.GGPISFSSSRSGR.R** |
|  | 10005 | **437.7089** | **1310.1048** | **1310.5813** | **-0.4764** | **0** | **3** | **43** | **9** | **K.EIFMQVEDER.R** 10012 10019 |
|  | 10725 | **675.3666** | **1348.7186** | **1347.6340** | **1.0846** | **1** | **4** | **57** | **2** | **K.VEGELEEMERK.H** 10716 |
|  | 11849 | 707.3728 | 1412.7310 | 1413.7827 | -1.0517 | 0 | 6 | 35 | 7 | K.QVLALQSQLADTK.K |
|  | 12114 | **714.8531** | **1427.6916** | **1427.7368** | **-0.0452** | **1** | **9** | **16** | **2** | **K.DLEAQIEAANKAR.D** |
|  | 12300 | **480.6037** | **1438.7892** | **1439.7045** | **-0.9153** | **0** | **7** | **27** | **8** | **K.SDLLLEGFNNYR.F** |
|  | 12503 | **485.2639** | **1452.7698** | **1451.7885** | **0.9814** | **0** | **10** | **12** | **2** | **K.VIQYLAHVASSHK.G** 12483 12484 |
|  | 13862 | 771.8914 | 1541.7682 | 1541.8565 | -0.0884 | 1 | 7 | 23 | 4 | R.QRYEILTPNAIPK.G |
|  | 14002 | 776.3459 | 1550.6772 | 1550.6882 | -0.0110 | 0 | 45 | 0.0039 | 1 | R.EDQSILCTGESGAGK.T |
|  | 14670 | **532.9060** | **1595.6962** | **1594.8541** | **0.8421** | **0** | **15** | **3.6** | **3** | **K.ITDIIIFFQAVCR.G** |
|  | 14811 | **803.8584** | **1605.7022** | **1605.8362** | **-0.1339** | **1** | **1** | **99** | **7** | **K.FDQLLAEEKSISAR.Y** |
|  | 14977 | 808.4207 | 1614.8267 | 1614.7849 | 0.0419 | 1 | 10 | 13 | 4 | K.QVRELEAELEDER.K 14976 |
|  | 15551 | 826.3910 | 1650.7675 | 1651.9297 | -1.1622 | 1 | 1 | 95 | 5 | K.VVSSVLQFGNISFKK.E |
|  | 16468 | **572.9968** | **1715.9685** | **1715.8842** | **0.0842** | **1** | **10** | **11** | **2** | **K.TFVEKLVQEQGSHSK.F** |
|  | 17310 | 593.3256 | 1776.9549 | 1775.9206 | 1.0342 | 1 | 6 | 27 | 2 | R.AVIYNPATQADWTAKK.L |
|  | 19612 | 651.4102 | 1951.2088 | 1951.9686 | -0.7598 | 1 | 1 | 55 | 2 | R.NTNPNFVRCIIPNHEK.R |
|  | 19715 | 981.4571 | 1960.8997 | 1960.9113 | -0.0115 | 0 | 123 | 4.4e-011 | 1 | R.TQLEELEDELQATEDAK.L 19716 |
|  | 21020 | **696.3229** | **2085.9468** | **2085.9702** | **-0.0234** | **0** | **2** | **61** | **4** | **R.QLHLEGASLELSDDDTESK.T** |
|  | 21212 | **701.7299** | **2102.1678** | **2103.0194** | **-0.8517** | **0** | **1** | **67** | **7** | **R.IVGLDQVTGMTETAFGSAYK.T** |
|  | 22938 | 762.7043 | 2285.0910 | 2285.1031 | -0.0121 | 0 | 41 | 0.006 | 1 | K.VEDMAELTCLNEASVLHNLK.D |
|  | 23064 | 768.3344 | 2301.9814 | 2301.0981 | 0.8833 | 0 | (4) | 29 | 10 | K.VEDMAELTCLNEASVLHNLK.D |
|  | 23516 | 787.7372 | 2360.1897 | 2360.1913 | -0.0016 | 1 | 4 | 28 | 4 | K.DERTFHIFYQLLSGAGEHLK.S |

| **181.** | Q01082\|SPTB2_HUMAN    **Mass:** 275237   **Score:** 260    **Queries matched:** 66   **emPAI:** 0.05 |
| --- | --- |
|  | Spectrin beta chain, brain 1 OS=Homo sapiens GN=SPTBN1 PE=1 SV=2 |

|  | **Query** | **Observed** | **Mr(expt)** | **Mr(calc)** | **Delta** | **Miss** | **Score** | **Expect** | **Rank** | **Peptide** |
| --- | --- | --- | --- | --- | --- | --- | --- | --- | --- | --- |
|  | 1053 | **549.3380** | **548.3307** | **547.3078** | **1.0229** | **1** | **7** | **41** | **6** | **K.KASSR.S** |
|  | 1085 | **558.3567** | **557.3494** | **558.3489** | **-0.9995** | **0** | **13** | **9.8** | **4** | **K.SLLAR.K** |
|  | 1375 | 616.3982 | 615.3909 | 615.3816 | 0.0093 | 1 | 11 | 22 | 3 | K.RITAR.K |
|  | 2210 | **402.2437** | **802.4727** | **801.4596** | **1.0132** | **0** | **10** | **22** | **8** | **K.LISDINK.A** |
|  | 3583 | **446.7448** | **891.4750** | **890.4345** | **1.0405** | **0** | **16** | **4.8** | **1** | **R.EASLGEASK.L** |
|  | 4035 | **462.7184** | **923.4222** | **924.5392** | **-1.1170** | **1** | **8** | **26** | **2** | **K.EKLTGLHK.M** |
|  | 4134 | **466.2376** | **930.4607** | **929.4566** | **1.0041** | **0** | **8** | **28** | **2** | **R.QLQEDAAR.L** |
|  | 4512 | **479.2573** | **956.5000** | **957.5243** | **-1.0244** | **0** | **5** | **56** | **5** | **K.INAVVETGR.R** |
|  | 4678 | **485.7846** | **969.5546** | **968.5767** | **0.9779** | **0** | **8** | **23** | **9** | **R.VAVVNQIAR.Q** |
|  | 4860 | **492.2355** | **982.4564** | **981.5144** | **0.9420** | **0** | **9** | **17** | **2** | **K.WVNSHLAR.V** |
|  | 4936 | **495.2530** | **988.4915** | **988.4574** | **0.0342** | **0** | **3** | **1e+002** | **6** | **R.DTGNIGQER.V** |
|  | 5064 | **499.2972** | **996.5798** | **996.4764** | **0.1034** | **0** | **7** | **26** | **1** | **K.ILSSDDYGK.D** |
|  | 5181 | **503.2516** | **1004.4886** | **1005.5859** | **-1.0972** | **1** | **8** | **26** | **4** | **K.VDKLYAGLK.D** |
|  | 5305 | **508.2893** | **1014.5641** | **1014.5709** | **-0.0069** | **0** | **7** | **32** | **3** | **K.LLEVLSGER.L** |
|  | 5760 | **525.7789** | **1049.5432** | **1049.4811** | **0.0620** | **0** | **5** | **46** | **6** | **K.SLLDACESR.R** |
|  | 6525 | **553.2703** | **1104.5261** | **1104.5539** | **-0.0278** | **1** | **3** | **84** | **5** | **K.FRFFSMVR.D** |
|  | 7641 | 594.8234 | 1187.6323 | 1188.5735 | -0.9412 | 0 | 11 | 13 | 3 | R.LVSDGNINSDR.I |
|  | 8313 | 610.7929 | 1219.5713 | 1219.6131 | -0.0419 | 1 | 10 | 16 | 5 | R.ANNQKVYMPR.E |
|  | 8407 | **409.2288** | **1224.6645** | **1225.7142** | **-1.0497** | **1** | **9** | **18** | **8** | **R.ELALRNELIR.Q** |
|  | 8527 | **616.8271** | **1231.6397** | **1231.6019** | **0.0378** | **0** | **2** | **1.1e+002** | **4** | **R.DGMAFNALIHK.H** |
|  | 8756 | **622.3350** | **1242.6555** | **1242.7547** | **-0.0992** | **1** | **2** | **92** | **3** | **K.DLTSVNILLKK.Q** |
|  | 8934 | 628.2241 | 1254.4337 | 1254.6357 | -0.2020 | 0 | 3 | 64 | 9 | K.HRPDLIDFDK.L |
|  | 9100 | **631.8076** | **1261.6007** | **1261.7064** | **-0.1057** | **1** | **3** | **77** | **10** | **K.DLTSVMRLLSK.H** |
|  | 9147 | **632.8627** | **1263.7109** | **1263.7187** | **-0.0078** | **0** | **2** | **92** | **8** | **K.HLLGVEDLLQK.H** |
|  | 10697 | **674.3850** | **1346.7553** | **1346.7598** | **-0.0044** | **0** | **3** | **70** | **6** | **R.LWEYLLELLR.A** |
|  | 11019 | **456.7813** | **1367.3222** | **1366.7568** | **0.5654** | **0** | **1** | **65** | **5** | **K.TALPAQSAATLPAR.T** |
|  | 11186 | **459.7188** | **1376.1344** | **1376.7299** | **-0.5955** | **1** | **6** | **26** | **4** | **K.LYAGLKDLAEER.R** |
|  | 11974 | **474.2844** | **1419.8313** | **1420.6439** | **-0.8126** | **0** | **5** | **45** | **6** | **K.QQMLENQMEVR.K** |
|  | 12565 | **486.7311** | **1457.1716** | **1457.7766** | **-0.6050** | **0** | **7** | **16** | **2** | **K.QLWGLLIEETEK.R** |
|  | 13141 | **498.2584** | **1491.7534** | **1491.7066** | **0.0468** | **1** | **9** | **18** | **6** | **R.EFARDTGNIGQER.V** |
|  | 13242 | **500.2324** | **1497.6753** | **1496.6817** | **0.9936** | **0** | **1** | **1e+002** | **10** | **K.AELFTQSCADLDK.W** |
|  | 13690 | **765.4286** | **1528.8427** | **1528.7344** | **0.1084** | **0** | **1** | **1e+002** | **7** | **K.EQWLNNMQIPEK.L** |
|  | 14231 | 783.3669 | 1564.7192 | 1564.7337 | -0.0145 | 1 | 11 | 8.7 | 3 | K.KQQMLENQMEVR.K 14210 |
|  | 14435 | **790.4898** | **1578.9651** | **1578.7898** | **0.1753** | **1** | **13** | **4.6** | **2** | **K.SAKDALLLWCQMK.T** |
|  | 14516 | **397.2227** | **1584.8617** | **1585.8563** | **-0.9946** | **1** | **8** | **18** | **4** | **R.EIGQSVDEVEKLIK.R** |
|  | 14980 | **539.2983** | **1614.8732** | **1613.8777** | **0.9955** | **1** | **11** | **10** | **1** | **K.QLWGLLIEETEKR.H** |
|  | 15935 | **839.4199** | **1676.8252** | **1675.7590** | **1.0661** | **1** | **2** | **79** | **10** | **R.HEAFEKSAATWDER.F** |
|  | 16336 | **569.9037** | **1706.6894** | **1706.7094** | **-0.0199** | **0** | **1** | **76** | **5** | **K.EGEDMIAEEHFGSEK.I** |
|  | 16352 | **854.8954** | **1707.7763** | **1707.7886** | **-0.0123** | **0** | **82** | **6.9e-007** | **1** | **R.TQETPSAQMEGFLNR.K** |
|  | 16549 | **574.9426** | **1721.8061** | **1720.8156** | **0.9905** | **0** | **11** | **9.7** | **3** | **K.LLDPEDISVDHPDEK.S** |
|  | 16565 | **575.2904** | **1722.8494** | **1723.7835** | **-0.9341** | **0** | **(4)** | **48** | **1** | **R.TQETPSAQMEGFLNR.K** |
|  | 16945 | **584.9459** | **1751.8158** | **1752.8391** | **-1.0233** | **0** | **5** | **37** | **2** | **K.GEQVSQNGLPAEQGSPR.M** 16978 |
|  | 17307 | **889.4441** | **1776.8736** | **1776.8853** | **-0.0117** | **0** | **17** | **2** | **1** | **R.SQNIVTDSSSLSAEAIR.Q** |
|  | 18375 | 927.3755 | 1852.7364 | 1851.8785 | 0.8579 | 1 | 1 | 72 | 8 | R.TQETPSAQMEGFLNRK.H |
|  | 18818 | **629.9918** | **1886.9536** | **1886.9659** | **-0.0123** | **0** | **3** | **45** | **7** | **K.EGMQLISEKPETEAVVK.E** |
|  | 20668 | **1025.4796** | **2048.9447** | **2048.9572** | **-0.0125** | **0** | **71** | **6.7e-006** | **1** | **R.TQTAIASEDMPNTLTEAEK.L** 20667 |
|  | 20679 | **684.6659** | **2050.9760** | **2049.9855** | **0.9905** | **0** | **13** | **4.5** | **1** | **K.IVSSSDVGHDEYSTQSLVK.K** |
|  | 21862 | 1080.5573 | 2159.0999 | 2158.0809 | 1.0191 | 1 | 1 | 70 | 9 | K.SIITYVVTYYHYFSKMK.A |
|  | 22373 | **739.6952** | **2216.0639** | **2216.1484** | **-0.0845** | **1** | **0** | **73** | **7** | **R.VAVVNQIARQLMHSGHPSEK.E** |
|  | 22403 | **741.3636** | **2221.0689** | **2219.9650** | **1.1040** | **0** | **(2)** | **56** | **4** | **R.DMGEMVTQGQTDAQYMFLR.Q** |
|  | 22524 | 1118.5380 | 2235.0614 | 2235.0527 | 0.0087 | 1 | 4 | 30 | 3 | R.FFSMVRDLMLWMEDVIR.Q |
|  | 22531 | **1118.5930** | **2235.1715** | **2235.9599** | **-0.7884** | **0** | **9** | **8.9** | **1** | **R.DMGEMVTQGQTDAQYMFLR.Q** 22530 |
|  | 22830 | 759.0772 | 2274.2098 | 2273.1440 | 1.0658 | 1 | 4 | 33 | 6 | R.LFQLNREVDDLEQWIAER.E |
|  | 23185 | 773.4149 | 2317.2228 | 2318.1212 | -0.8985 | 0 | 2 | 49 | 4 | K.DALLSALSIQNYHLECNETK.S |
|  | 23300 | **780.0562** | **2337.1468** | **2337.1601** | **-0.0133** | **0** | **74** | **3.2e-006** | **1** | **K.HQILEQAVEDYAETVHQLSK.T** |
|  | 24987 | **852.7391** | **2555.1956** | **2555.0913** | **0.1043** | **1** | **4** | **30** | **1** | **K.MRDMGEMVTQGQTDAQYMFLR.Q** |
|  | 25289 | 655.0527 | 2616.1818 | 2617.2918 | -1.1100 | 1 | 3 | 32 | 6 | R.FESLEPEMNNQASRVAVVNQIAR.Q 25294 |
|  | 26376 | **936.1199** | **2805.3380** | **2805.3569** | **-0.0190** | **0** | **54** | **0.00027** | **1** | **R.VDTVNHLADELINSGHSDAATIAEWK.D** |
|  | 27319 | **1000.8237** | **2999.4494** | **2999.4586** | **-0.0092** | **0** | **16** | **1.4** | **1** | **R.LEEASLLHQFQADADDIDAWMLDILK.I** |
|  | 27367 | **755.1088** | **3016.4062** | **3015.4535** | **0.9527** | **0** | **(2)** | **31** | **3** | **R.LEEASLLHQFQADADDIDAWMLDILK.I** |
|  | 30391 | 1339.3292 | 4014.9658 | 4013.9467 | 1.0191 | 1 | 0 | 27 | 6 | K.DVAEEIANYRPTLDTLHEQASALPQEHAESPDVRGR.L |

| **182.** | P04040\|CATA_HUMAN    **Mass:** 59947    **Score:** 259    **Queries matched:** 28   **emPAI:** 0.83 |
| --- | --- |
|  | Catalase OS=Homo sapiens GN=CAT PE=1 SV=3 |

|  | **Query** | **Observed** | **Mr(expt)** | **Mr(calc)** | **Delta** | **Miss** | **Score** | **Expect** | **Rank** | **Peptide** |
| --- | --- | --- | --- | --- | --- | --- | --- | --- | --- | --- |
|  | 3544 | **445.2594** | **888.5042** | **888.4818** | **0.0224** | **1** | **6** | **49** | **3** | **R.DPRGFAVK.F** |
|  | 4501 | 479.2159 | 956.4173 | 956.5443 | -0.1271 | 1 | 12 | 11 | 4 | K.VFEHIGKK.T |
|  | 4577 | **481.7661** | **961.5176** | **961.5233** | **-0.0057** | **0** | **33** | **0.09** | **1** | **K.DAQIFIQK.K** |
|  | 4736 | **974.4840** | **973.4767** | **973.4828** | **-0.0062** | **0** | **51** | **0.0015** | **1** | **K.NLSVEDAAR.L** 4739 4760 |
|  | 5751 | **525.2813** | **1048.5481** | **1048.5553** | **-0.0072** | **0** | **21** | **1.3** | **1** | **R.DLFNAIATGK.Y** |
|  | 9672 | **646.8049** | **1291.5952** | **1291.6044** | **-0.0092** | **0** | **11** | **10** | **1** | **R.LSQEDPDYGIR.D** |
|  | 9990 | **655.8319** | **1309.6493** | **1309.6925** | **-0.0432** | **1** | **1** | **1e+002** | **3** | **K.RLCENIAGHLK.D** |
|  | 11822 | **471.2860** | **1410.8361** | **1411.7109** | **-0.8748** | **1** | **5** | **36** | **3** | **R.LFAYPDTHRHR.L** |
|  | 11864 | **707.8578** | **1413.7010** | **1413.7100** | **-0.0090** | **0** | **72** | **7.7e-006** | **1** | **K.ADVLTTGAGNPVGDK.L** |
|  | 12949 | **741.3680** | **1480.7214** | **1480.7310** | **-0.0096** | **0** | **1** | **93** | **2** | **R.AFYVNVLNEEQR.K** |
|  | 13000 | 742.8513 | 1483.6880 | 1482.6951 | 0.9929 | 0 | 36 | 0.033 | 1 | R.FSTVAGESGSADTVR.D |
|  | 13167 | **747.3477** | **1492.6809** | **1492.6906** | **-0.0098** | **0** | **67** | **2.5e-005** | **1** | **R.FNTANDDNVTQVR.A** |
|  | 14999 | **539.6193** | **1615.8360** | **1615.8165** | **0.0194** | **1** | **3** | **56** | **4** | **K.TDQGIKNLSVEDAAR.L** |
|  | 16358 | 570.2967 | 1707.8682 | 1707.8805 | -0.0123 | 0 | 14 | 4.6 | 1 | K.NAIHTFVQSGSHLAAR.E |
|  | 16419 | **856.9118** | **1711.8090** | **1711.8206** | **-0.0116** | **0** | **46** | **0.0031** | **1** | **K.GAGAFGYFEVTHDITK.Y** |
|  | 17208 | **591.2887** | **1770.8443** | **1770.7744** | **0.0699** | **1** | **1** | **92** | **5** | **M.ADSRDPASDQMQHWK.E** |
|  | 18349 | **618.0419** | **1851.1040** | **1850.8759** | **0.2281** | **1** | **8** | **13** | **1** | **R.FSTVAGESGSADTVRDPR.G** 18339 18354 |
|  | 23533 | 788.7626 | 2363.2659 | 2363.2809 | -0.0150 | 1 | 31 | 0.049 | 1 | K.ADVLTTGAGNPVGDKLNVITVGPR.G |
|  | 24681 | 1259.6030 | 2517.1915 | 2517.1965 | -0.0050 | 0 | 85 | 2.1e-007 | 1 | K.FYTEDGNWDLVGNNTPIFFIR.D |
|  | 27357 | **754.3752** | **3013.4716** | **3013.4933** | **-0.0217** | **1** | **25** | **0.17** | **1** | **K.NFTEVHPDYGSHIQALLDKYNAEKPK.N** |
|  | 30401 | **1007.7512** | **4026.9755** | **4026.9945** | **-0.0190** | **1** | **2** | **20** | **3** | **K.FYTEDGNWDLVGNNTPIFFIRDPILFPSFIHSQK.R** |
|  | 31068 | **1542.9925** | **4625.9558** | **4625.9911** | **-0.0352** | **0** | **37** | **0.0028** | **1** | **R.DGPMCMQDNQGGAPNYYPNSFGAPEQQPSALEHSIQYSGEVR.R** 31069 |
|  | 31089 | **929.5714** | **4642.8204** | **4641.9860** | **0.8344** | **0** | **(2)** | **8** | **5** | **R.DGPMCMQDNQGGAPNYYPNSFGAPEQQPSALEHSIQYSGEVR.R** |

| **183.** | P07099\|HYEP_HUMAN    **Mass:** 53143    **Score:** 257    **Queries matched:** 17   **emPAI:** 0.61 |
| --- | --- |
|  | Epoxide hydrolase 1 OS=Homo sapiens GN=EPHX1 PE=1 SV=1 |

|  | **Query** | **Observed** | **Mr(expt)** | **Mr(calc)** | **Delta** | **Miss** | **Score** | **Expect** | **Rank** | **Peptide** |
| --- | --- | --- | --- | --- | --- | --- | --- | --- | --- | --- |
|  | 2469 | **412.2242** | **822.4339** | **822.4388** | **-0.0049** | **0** | **9** | **20** | **1** | **K.VISYWR.N** |
|  | 2662 | **418.2348** | **834.4551** | **834.4599** | **-0.0048** | **0** | **27** | **0.42** | **1** | **R.FLGLTER.D** 2666 |
|  | 3912 | 458.2385 | 914.4624 | 914.4684 | -0.0060 | 0 | 44 | 0.0073 | 1 | K.VFYSLMR.E |
|  | 4031 | **462.2653** | **922.5161** | **921.4668** | **1.0493** | **0** | **6** | **36** | **5** | **K.GFNSVATAR.I** |
|  | 5771 | **526.2529** | **1050.4913** | **1050.4982** | **-0.0069** | **0** | **25** | **0.51** | **1** | **R.YLEDGGLER.K** |
|  | 6855 | **566.2929** | **1130.5711** | **1130.5794** | **-0.0082** | **0** | **7** | **34** | **1** | **K.LISYSYMVR.G** |
|  | 16339 | **854.3942** | **1706.7738** | **1706.7860** | **-0.0122** | **0** | **74** | **4.4e-006** | **1** | **K.VETSDEEIHDLHQR.I** |
|  | 20111 | **1000.4917** | **1998.9688** | **1998.9799** | **-0.0111** | **0** | **93** | **4.5e-008** | **1** | **R.GGHFAAFEEPELLAQDIR.K** 20110 20112 |
|  | 22893 | **760.3925** | **2278.1556** | **2278.1674** | **-0.0119** | **0** | **3** | **39** | **4** | **K.VYVPTGFSAFPFELLHTPEK.W** |
|  | 26241 | **925.4706** | **2773.3899** | **2773.3997** | **-0.0098** | **0** | **5** | **20** | **1** | **K.FSLDDLLTNVMLYWTTGTIISSQR.F** |
|  | 26843 | **968.1680** | **2901.4823** | **2901.4946** | **-0.0124** | **1** | **27** | **0.11** | **1** | **R.KFSLDDLLTNVMLYWTTGTIISSQR.F** |
|  | 27655 | **1021.8040** | **3062.3902** | **3062.4080** | **-0.0178** | **0** | **21** | **0.37** | **1** | **K.NHGLSDEHVFEVICPSIPGYGFSEASSK.K** 27654 |
|  | 28917 | **1132.5509** | **3394.6309** | **3394.6479** | **-0.0170** | **0** | **43** | **0.0021** | **1** | **R.LGFQEFYIQGGDWGSLICTNMAQLVPSHVK.G** |

| **184.** | Q9UL46\|PSME2_HUMAN    **Mass:** 27515    **Score:** 256    **Queries matched:** 24   **emPAI:** 0.47 |
| --- | --- |
|  | Proteasome activator complex subunit 2 OS=Homo sapiens GN=PSME2 PE=1 SV=3 |

|  | **Query** | **Observed** | **Mr(expt)** | **Mr(calc)** | **Delta** | **Miss** | **Score** | **Expect** | **Rank** | **Peptide** |
| --- | --- | --- | --- | --- | --- | --- | --- | --- | --- | --- |
|  | 1142 | **571.3162** | **570.3089** | **569.3537** | **0.9552** | **0** | **27** | **0.31** | **1** | **K.IVNPK.G** 1143 1144 |
|  | 2653 | **417.7374** | **833.4602** | **832.4477** | **1.0126** | **0** | **11** | **17** | **4** | **R.AMVLDLR.A** |
|  | 3971 | **460.0153** | **918.0161** | **917.4575** | **0.5586** | **0** | **6** | **31** | **7** | **-.MAKPCGVR.L** |
|  | 5389 | **511.7631** | **1021.5117** | **1020.4699** | **1.0418** | **0** | **4** | **72** | **10** | **K.CGFLPGNEK.V** |
|  | 5768 | 526.2364 | 1050.4582 | 1049.4600 | 0.9982 | 0 | 7 | 29 | 5 | K.ETHVMDYR.A |
|  | 8741 | **621.8521** | **1241.6897** | **1241.6040** | **0.0856** | **1** | **8** | **21** | **5** | **K.YFSERGDAVAK.A** 8758 |
|  | 10770 | **676.8697** | **1351.7249** | **1351.7347** | **-0.0098** | **1** | **28** | **0.23** | **1** | **K.TKVEAFQTTISK.Y** 10771 |
|  | 11068 | **457.5671** | **1369.6794** | **1368.7653** | **0.9141** | **0** | **11** | **12** | **2** | **R.APLDIPIPDPPPK.D** 11061 |
|  | 11596 | 700.3879 | 1398.7613 | 1399.7354 | -0.9741 | 1 | 8 | 20 | 9 | M.AKPCGVRLSGEAR.K 11594 11611 |
|  | 13761 | **512.2835** | **1533.8287** | **1533.7311** | **0.0976** | **0** | **6** | **32** | **8** | **K.IEDGNDFGVAIQEK.V** |
|  | 16054 | 844.4079 | 1686.8012 | 1685.8049 | 0.9963 | 0 | 76 | 3e-006 | 1 | R.QNLFQEAEEFLYR.F |
|  | 16609 | **576.9547** | **1727.8421** | **1727.8591** | **-0.0169** | **1** | **2** | **61** | **4** | **R.ALVHERDEAAYGELR.A** |
|  | 18961 | **633.3248** | **1896.9525** | **1896.9621** | **-0.0096** | **0** | **22** | **0.66** | **1** | **R.AFYAELYHIISSNLEK.I** |
|  | 23051 | **767.6211** | **2299.8416** | **2299.1637** | **0.6779** | **1** | **3** | **30** | **1** | **R.QNLFQEAEEFLYRFLPQK.I** |
|  | 24773 | **1266.1823** | **2530.3499** | **2530.3642** | **-0.0143** | **0** | **114** | **2.6e-010** | **1** | **K.IIYLNQLLQEDSLNVADLTSLR.A** 24774 24775 |

| **185.** | P01833\|PIGR_HUMAN    **Mass:** 84429    **Score:** 254    **Queries matched:** 24   **emPAI:** 0.35 |
| --- | --- |
|  | Polymeric immunoglobulin receptor OS=Homo sapiens GN=PIGR PE=1 SV=4 |

|  | **Query** | **Observed** | **Mr(expt)** | **Mr(calc)** | **Delta** | **Miss** | **Score** | **Expect** | **Rank** | **Peptide** |
| --- | --- | --- | --- | --- | --- | --- | --- | --- | --- | --- |
|  | 1253 | **590.4249** | **589.4177** | **588.3343** | **1.0833** | **1** | **9** | **38** | **1** | **R.KAAGSR.D** |
|  | 1680 | **691.3363** | **690.3290** | **689.3959** | **0.9331** | **0** | **14** | **10** | **1** | **R.TTVEIK.I** |
|  | 1918 | 760.3453 | 759.3380 | 759.3439 | -0.0059 | 0 | 15 | 8.1 | 1 | K.FSSYEK.Y |
|  | 3757 | **452.2405** | **902.4665** | **902.4722** | **-0.0057** | **1** | **19** | **2.5** | **1** | **K.RAPAFEGR.I** 3755 3756 3759 |
|  | 4901 | **494.1052** | **986.1958** | **985.4062** | **0.7896** | **0** | **9** | **16** | **2** | **K.EEAEMAYK.D** |
|  | 8116 | **606.2549** | **1210.4952** | **1210.5037** | **-0.0085** | **0** | **34** | **0.05** | **1** | **K.AFVNCDENSR.L** |
|  | 8448 | **614.8048** | **1227.5949** | **1227.7551** | **-0.1601** | **0** | **17** | **3** | **1** | **R.LVSLTLNLVTR.A** |
|  | 8860 | **417.2432** | **1248.7077** | **1249.6667** | **-0.9590** | **0** | **9** | **17** | **2** | **K.DGSFSVVITGLR.K** |
|  | 11627 | **701.8312** | **1401.6478** | **1401.6962** | **-0.0484** | **0** | **9** | **19** | **2** | **R.CPLLVDSEGWVK.A** |
|  | 14590 | **398.2217** | **1588.8577** | **1589.7685** | **-0.9108** | **1** | **7** | **24** | **4** | **K.ADAAPDEKVLDSGFR.E** |
|  | 15636 | **829.4394** | **1656.8643** | **1656.8029** | **0.0615** | **0** | **43** | **0.0057** | **2** | **R.GGCITLISSEGYVSSK.Y** |
|  | 16016 | **562.2808** | **1683.8205** | **1682.9454** | **0.8750** | **1** | **6** | **31** | **4** | **R.TTVEIKIIEGEPNLK.V** |
|  | 16330 | **853.8995** | **1705.7844** | **1705.7941** | **-0.0097** | **0** | **89** | **1.4e-007** | **1** | **R.QSSGENCDVVVNTLGK.R** |
|  | 21952 | **1085.9791** | **2169.9437** | **2169.9597** | **-0.0159** | **1** | **50** | **0.00088** | **1** | **R.LSDAGQYLCQAGDDSNSNKK.N** 21951 |
|  | 22566 | **1121.0845** | **2240.1544** | **2240.1688** | **-0.0144** | **0** | **65** | **2.6e-005** | **1** | **K.NADLQVLKPEPELVYEDLR.G** 22567 |
|  | 22585 | **749.1355** | **2244.3847** | **2244.3933** | **-0.0087** | **0** | **18** | **0.96** | **1** | **R.ALVSTLVPLGLVLAVGAVAVGVAR.A** |
|  | 22919 | **571.5698** | **2282.2499** | **2282.0784** | **0.1716** | **1** | **2** | **47** | **8** | **K.FLCRQSSGENCDVVVNTLGK.R** |
|  | 29551 | **1210.9082** | **3629.7028** | **3629.7209** | **-0.0181** | **0** | **51** | **0.0003** | **1** | **R.YLCGAHSDGQLQEGSPIQAWQLFVNEESTIPR.S** 29550 |

| **186.** | Q16836\|HCDH_HUMAN    **Mass:** 34313    **Score:** 254    **Queries matched:** 17   **emPAI:** 0.87 |
| --- | --- |
|  | Hydroxyacyl-coenzyme A dehydrogenase, mitochondrial OS=Homo sapiens GN=HADH PE=1 SV=2 |

|  | **Query** | **Observed** | **Mr(expt)** | **Mr(calc)** | **Delta** | **Miss** | **Score** | **Expect** | **Rank** | **Peptide** |
| --- | --- | --- | --- | --- | --- | --- | --- | --- | --- | --- |
|  | 1526 | 651.3502 | 650.3429 | 649.3435 | 0.9994 | 0 | 12 | 11 | 3 | K.NELFK.R |
|  | 7323 | **584.7800** | **1167.5454** | **1168.5571** | **-1.0118** | **0** | **9** | **18** | **3** | **R.SVSSSSTASASAK.K** |
|  | 9850 | 434.8809 | 1301.6209 | 1301.6373 | -0.0164 | 1 | 1 | 1e+002 | 6 | -.MAFVTRQFMR.S |
|  | 11255 | **690.8139** | **1379.6132** | **1379.6238** | **-0.0106** | **1** | **34** | **0.053** | **1** | **R.GDASKEDIDTAMK.L** 11250 |
|  | 15039 | **810.3691** | **1618.7236** | **1618.8330** | **-0.1094** | **0** | **2** | **71** | **7** | **R.FAGLHFFNPVPVMK.L** |
|  | 23490 | **1179.0857** | **2356.1568** | **2356.1661** | **-0.0093** | **0** | **76** | **2e-006** | **1** | **K.LGAGYPMGPFELLDYVGLDTTK.F** 23488 23489 23491 |
|  | 26137 | **918.1287** | **2751.3643** | **2751.3828** | **-0.0184** | **0** | **18** | **0.96** | **1** | **K.FAAEHTIFASNTSSLQITSIANATTR.Q** |
|  | 26181 | **921.0864** | **2760.2373** | **2760.2966** | **-0.0593** | **0** | **23** | **0.25** | **1** | **K.FIVDGWHEMDAENPLHQPSPSLNK.L** |
|  | 27724 | **1028.8782** | **3083.6127** | **3083.6238** | **-0.0111** | **0** | **37** | **0.0086** | **1** | **K.TLSTIATSTDAASVVHSTDLVVEAIVENLK.V** 27725 |
|  | 28526 | 1638.3666 | 3274.7186 | 3275.5618 | -0.8432 | 1 | 2 | 25 | 4 | K.EDIDTAMKLGAGYPMGPFELLDYVGLDTTK.F |
|  | 29491 | **903.9885** | **3611.9250** | **3611.9346** | **-0.0095** | **0** | **52** | **0.00022** | **1** | **K.HVTVIGGGLMGAGIAQVAAATGHTVVLVDQTEDILAK.S** 29488 |

| **187.** | P62820\|RAB1A_HUMAN    **Mass:** 22891    **Score:** 252    **Queries matched:** 19   **emPAI:** 1.17 |
| --- | --- |
|  | Ras-related protein Rab-1A OS=Homo sapiens GN=RAB1A PE=1 SV=3 |

|  | **Query** | **Observed** | **Mr(expt)** | **Mr(calc)** | **Delta** | **Miss** | **Score** | **Expect** | **Rank** | **Peptide** |
| --- | --- | --- | --- | --- | --- | --- | --- | --- | --- | --- |
|  | 1480 | 643.2714 | 642.2641 | 642.4064 | -0.1424 | 0 | 19 | 2.4 | 1 | K.LLVGNK.C |
|  | 3636 | **448.7370** | **895.4594** | **895.4651** | **-0.0057** | **0** | **22** | **1** | **1** | **K.VVDYTTAK.E** |
|  | 5420 | **512.7836** | **1023.5527** | **1023.5601** | **-0.0074** | **1** | **45** | **0.0054** | **1** | **K.KVVDYTTAK.E** 5433 |
|  | 5713 | **524.2430** | **1046.4714** | **1045.4862** | **0.9852** | **0** | **7** | **33** | **1** | **R.MGPGATAGGAEK.S** |
|  | 6064 | 536.3201 | 1070.6257 | 1070.6336 | -0.0079 | 0 | 15 | 5.6 | 1 | K.LLLIGDSGVGK.S |
|  | 6285 | 544.2767 | 1086.5389 | 1086.5458 | -0.0069 | 0 | 16 | 4.8 | 5 | K.QWLQEIDR.Y |
|  | 7937 | **401.5603** | **1201.6591** | **1201.5873** | **0.0717** | **1** | **12** | **9** | **1** | **K.RMGPGATAGGAEK.S** |
|  | 8243 | 406.5327 | 1216.5762 | 1217.5823 | -1.0060 | 1 | (5) | 53 | 2 | K.RMGPGATAGGAEK.S |
|  | 10115 | 658.8291 | 1315.6436 | 1315.6521 | -0.0084 | 0 | 53 | 0.00078 | 1 | K.LQIWDTAGQER.F 10116 |
|  | 13143 | **498.2632** | **1491.7676** | **1492.6544** | **-0.8868** | **0** | **7** | **28** | **3** | **M.SSMNPEYDYLFK.L** |
|  | 15376 | **547.2779** | **1638.8118** | **1639.6898** | **-0.8780** | **0** | **6** | **30** | **3** | **-.MSSMNPEYDYLFK.L** |
|  | 16578 | 862.9362 | 1723.8579 | 1723.8669 | -0.0090 | 0 | 25 | 0.37 | 1 | K.EFADSLGIPFLETSAK.N 16557 |
|  | 18767 | 942.9406 | 1883.8667 | 1883.8757 | -0.0090 | 0 | 87 | 2.3e-007 | 1 | K.NATNVEQSFMTMAAEIK.K 18768 |
|  | 21964 | 1086.4994 | 2170.9842 | 2170.9947 | -0.0104 | 0 | 81 | 6.7e-007 | 1 | R.FADDTYTESYISTIGVDFK.I 21963 |

| **188.** | O14745\|NHRF1_HUMAN    **Mass:** 39130    **Score:** 247    **Queries matched:** 21   **emPAI:** 0.58 |
| --- | --- |
|  | Na(+)/H(+) exchange regulatory cofactor NHE-RF1 OS=Homo sapiens GN=SLC9A3R1 PE=1 SV=4 |

|  | **Query** | **Observed** | **Mr(expt)** | **Mr(calc)** | **Delta** | **Miss** | **Score** | **Expect** | **Rank** | **Peptide** |
| --- | --- | --- | --- | --- | --- | --- | --- | --- | --- | --- |
|  | 1534 | **653.3298** | **652.3225** | **651.3084** | **1.0141** | **0** | **21** | **1.1** | **1** | **R.LCTMK.K** |
|  | 1955 | **772.4266** | **771.4193** | **771.4239** | **-0.0046** | **0** | **25** | **0.73** | **1** | **K.AGLLAGDR.L** |
|  | 2102 | **398.2048** | **794.3951** | **795.3983** | **-1.0032** | **1** | **10** | **15** | **5** | **R.LCTMKK.G** |
|  | 2456 | 822.4159 | 821.4087 | 821.3775 | 0.0311 | 0 | 10 | 18 | 5 | R.LCCLEK.G |
|  | 3932 | **458.7179** | **915.4212** | **914.4022** | **1.0190** | **0** | **7** | **41** | **3** | **R.ETDEFFK.K** |
|  | 5429 | 513.2869 | 1024.5593 | 1025.5393 | -0.9800 | 0 | 8 | 25 | 10 | R.LVEPGSPAEK.A |
|  | 6706 | **560.2722** | **1118.5298** | **1117.5338** | **0.9959** | **1** | **7** | **36** | **3** | **K.RAPQMDWSK.K** |
|  | 8473 | **615.3178** | **1228.6209** | **1228.6299** | **-0.0090** | **0** | **14** | **6.1** | **1** | **R.LVEVNGENVEK.E** 8448 |
|  | 10456 | **667.8214** | **1333.6281** | **1333.6370** | **-0.0089** | **0** | **34** | **0.053** | **1** | **R.IVEVNGVCMEGK.Q** 10455 |
|  | 11601 | **700.8314** | **1399.6483** | **1399.6579** | **-0.0096** | **0** | **53** | **0.00062** | **1** | **R.SVDPDSPAEASGLR.A** |
|  | 13353 | **753.8658** | **1505.7170** | **1505.7263** | **-0.0093** | **1** | **57** | **0.00023** | **1** | **K.KGPSGYGFNLHSDK.S** |
|  | 14882 | **537.6158** | **1609.8257** | **1609.8352** | **-0.0095** | **1** | **5** | **37** | **4** | **K.LLVVDRETDEFFK.K** |
|  | 15161 | 813.9274 | 1625.8402 | 1625.8512 | -0.0110 | 0 | 63 | 6.3e-005 | 1 | R.LLVVDPETDEQLQK.L 15160 |
|  | 23012 | **765.3903** | **2293.1490** | **2293.1662** | **-0.0173** | **1** | **19** | **1** | **1** | **R.LVEVNGENVEKETHQQVVSR.I** |
|  | 25149 | **1294.5922** | **2587.1698** | **2587.1898** | **-0.0201** | **0** | **84** | **2.4e-007** | **1** | **R.AQEAPGQAEPPAAAEVQGAGNENEPR.E** 25148 25150 |
|  | 29659 | **1826.8138** | **3651.6131** | **3651.7863** | **-0.1732** | **1** | **5** | **9.8** | **3** | **R.EALAEAALESPRPALVRSASSDTSEELNSQDSPPK.Q** |

| **189.** | P08238\|HS90B_HUMAN    **Mass:** 83554    **Score:** 245    **Queries matched:** 32   **emPAI:** 0.35 |
| --- | --- |
|  | Heat shock protein HSP 90-beta OS=Homo sapiens GN=HSP90AB1 PE=1 SV=4 |

|  | **Query** | **Observed** | **Mr(expt)** | **Mr(calc)** | **Delta** | **Miss** | **Score** | **Expect** | **Rank** | **Peptide** |
| --- | --- | --- | --- | --- | --- | --- | --- | --- | --- | --- |
|  | 3092 | **863.0865** | **862.0792** | **862.4218** | **-0.3426** | **0** | **21** | **1.1** | **1** | **R.EMLQQSK.I** |
|  | 3518 | **444.2483** | **886.4820** | **885.5396** | **0.9425** | **1** | **13** | **11** | **9** | **R.RLSELLR.Y** |
|  | 3560 | **445.8990** | **889.7834** | **889.4141** | **0.3693** | **1** | **4** | **56** | **6** | **K.AEADKNDK.A** |
|  | 3567 | **446.2129** | **890.4113** | **890.4174** | **-0.0061** | **0** | **11** | **16** | **3** | **K.FYEAFSK.N** |
|  | 4347 | **473.7424** | **945.4703** | **945.5429** | **-0.0726** | **1** | **10** | **18** | **9** | **R.IMKAQALR.D** |
|  | 5703 | **523.7759** | **1045.5373** | **1044.5927** | **0.9446** | **1** | **10** | **16** | **3** | **K.VEKVTISNR.L** |
|  | 6985 | **571.2835** | **1140.5523** | **1140.5523** | **0.0000** | **0** | **2** | **96** | **6** | **K.LGIHEDSTNR.R** |
|  | 7104 | 576.2781 | 1150.5416 | 1150.5506 | -0.0090 | 0 | 12 | 9.1 | 1 | K.YIDQEELNK.T |
|  | 7239 | **581.2903** | **1160.5661** | **1159.5761** | **0.9900** | **0** | **20** | **1.8** | **1** | **K.SIYYITGESK.E** |
|  | 7545 | **592.3403** | **1182.6660** | **1181.5937** | **1.0723** | **1** | **0** | **1.3e+002** | **7** | **K.FENLCKLMK.E** |
|  | 8261 | 609.7952 | 1217.5759 | 1216.6849 | 0.8910 | 1 | 20 | 1.6 | 1 | R.EMLQQSKILK.V |
|  | 8613 | 618.9980 | 1235.9814 | 1235.6299 | 0.3516 | 1 | 4 | 35 | 2 | R.RAPFDLFENK.K |
|  | 8741 | 621.8521 | 1241.6897 | 1241.6979 | -0.0083 | 0 | 43 | 0.0066 | 1 | K.ADLINNLGTIAK.S 8742 8756 |
|  | 8826 | **624.7519** | **1247.4892** | **1247.4984** | **-0.0092** | **0** | **9** | **17** | **1** | **R.DNSTMGYMMAK.K** |
|  | 9134 | 421.9743 | 1262.9011 | 1263.4934 | -0.5923 | 0 | (6) | 28 | 6 | R.DNSTMGYMMAK.K |
|  | 9136 | **422.0405** | **1263.0996** | **1263.4934** | **-0.3938** | **0** | **(9)** | **12** | **2** | **R.DNSTMGYMMAK.K** 9161 9168 |
|  | 9352 | 638.3206 | 1274.6266 | 1274.6354 | -0.0088 | 0 | 61 | 0.00011 | 1 | R.ELISNASDALDK.I 9383 |
|  | 9446 | **427.5716** | **1279.6930** | **1279.4883** | **0.2048** | **0** | **(6)** | **32** | **8** | **R.DNSTMGYMMAK.K** |
|  | 10716 | 675.3210 | 1348.6275 | 1347.6572 | 0.9704 | 0 | 1 | 1.2e+002 | 10 | K.HFSVEGQLEFR.A |
|  | 10726 | **675.3670** | **1348.7194** | **1348.7272** | **-0.0078** | **0** | **47** | **0.0031** | **1** | **R.TLTLVDTGIGMTK.A** |
|  | 13456 | **757.3917** | **1512.7688** | **1512.7784** | **-0.0096** | **0** | **74** | **4.5e-006** | **1** | **R.GVVDSEDLPLNISR.E** |
|  | 13661 | **764.4358** | **1526.8571** | **1526.7365** | **0.1206** | **0** | **23** | **0.59** | **1** | **K.SLTNDWEDHLAVK.H** |
|  | 13897 | 772.8417 | 1543.6689 | 1543.8205 | -0.1516 | 1 | 41 | 0.0092 | 1 | R.ELISNASDALDKIR.Y 13903 |
|  | 14835 | **804.3886** | **1606.7627** | **1606.6846** | **0.0781** | **1** | **7** | **23** | **4** | **K.EISDDEAEEEKGEK.E** |
|  | 17384 | **594.9837** | **1781.9293** | **1781.9424** | **-0.0131** | **0** | **44** | **0.0046** | **1** | **K.HLEINPDHPIVETLR.Q** |
|  | 18278 | **924.3959** | **1846.7773** | **1846.7897** | **-0.0124** | **0** | **71** | **7.4e-006** | **1** | **R.NPDDITQEEYGEFYK.S** |

| **190.** | P62937\|PPIA_HUMAN    **Mass:** 18229    **Score:** 243    **Queries matched:** 26   **emPAI:** 2.17 |
| --- | --- |
|  | Peptidyl-prolyl cis-trans isomerase A OS=Homo sapiens GN=PPIA PE=1 SV=2 |

|  | **Query** | **Observed** | **Mr(expt)** | **Mr(calc)** | **Delta** | **Miss** | **Score** | **Expect** | **Rank** | **Peptide** |
| --- | --- | --- | --- | --- | --- | --- | --- | --- | --- | --- |
|  | 1662 | **687.3984** | **686.3911** | **685.3912** | **0.9999** | **0** | **25** | **0.75** | **1** | **K.HVVFGK.V** 1661 |
|  | 1689 | 696.3513 | 695.3440 | 695.3490 | -0.0050 | 0 | 7 | 25 | 7 | K.SIYGEK.F |
|  | 2843 | **424.7085** | **847.4024** | **847.4076** | **-0.0051** | **0** | **4** | **87** | **9** | **K.TEWLDGK.H** |
|  | 5816 | **528.2708** | **1054.5271** | **1054.5335** | **-0.0064** | **0** | **50** | **0.0014** | **1** | **R.VSFELFADK.V** |
|  | 6704 | **560.2459** | **1118.4773** | **1118.5278** | **-0.0505** | **0** | **5** | **54** | **8** | **K.ITIADCGQLE.-** |
|  | 7154 | **577.7865** | **1153.5584** | **1153.5655** | **-0.0071** | **0** | **15** | **4.5** | **2** | **K.FEDENFILK.H** 7153 |
|  | 8834 | **624.8157** | **1247.6168** | **1246.6227** | **0.9941** | **1** | **63** | **7.5e-005** | **1** | **K.KITIADCGQLE.-** 8818 |
|  | 9707 | 647.7856 | 1293.5567 | 1293.5693 | -0.0126 | 0 | (5) | 39 | 9 | K.EGMNIVEAMER.F |
|  | 9980 | **437.2477** | **1308.7213** | **1309.5642** | **-0.8429** | **0** | **11** | **10** | **1** | **K.EGMNIVEAMER.F** |
|  | 11243 | 460.5877 | 1378.7412 | 1378.7496 | -0.0084 | 1 | 6 | 35 | 7 | R.VSFELFADKVPK.T 11244 |
|  | 12026 | 712.3354 | 1422.6562 | 1422.7103 | -0.0541 | 1 | 2 | 76 | 7 | K.TAENFRALSTGEK.G |
|  | 13342 | **502.5834** | **1504.7283** | **1504.7377** | **-0.0095** | **1** | **28** | **0.2** | **1** | **K.VKEGMNIVEAMER.F** |
|  | 13798 | **769.3933** | **1536.7721** | **1536.7276** | **0.0445** | **1** | **(1)** | **99** | **2** | **K.VKEGMNIVEAMER.F** |
|  | 14695 | **799.8712** | **1597.7279** | **1597.7381** | **-0.0103** | **0** | **58** | **0.00018** | **1** | **R.IIPGFMCQGGDFTR.H** 14694 |
|  | 14948 | **807.9384** | **1613.8621** | **1613.7331** | **0.1291** | **0** | **(6)** | **32** | **4** | **R.IIPGFMCQGGDFTR.H** |
|  | 17053 | **879.8738** | **1757.7331** | **1756.7872** | **0.9459** | **1** | **2** | **62** | **2** | **K.EGMNIVEAMERFGSR.N** |
|  | 18045 | **916.4542** | **1830.8939** | **1830.9039** | **-0.0101** | **1** | **55** | **0.00036** | **1** | **K.SIYGEKFEDENFILK.H** 18044 |
|  | 26318 | **931.1086** | **2790.3041** | **2790.3218** | **-0.0177** | **0** | **33** | **0.031** | **1** | **K.HTGPGILSMANAGPNTNGSQFFICTAK.T** 26321 |
|  | 27873 | 1043.5026 | 3127.4859 | 3128.5529 | -1.0671 | 1 | 3 | 22 | 6 | -.MVNPTVFFDIAVDGEPLGRVSFELFADK.V |

| **191.** | P20648\|ATP4A_HUMAN    **Mass:** 115756   **Score:** 241    **Queries matched:** 29   **emPAI:** 0.10 |
| --- | --- |
|  | Potassium-transporting ATPase alpha chain 1 OS=Homo sapiens GN=ATP4A PE=2 SV=5 |

|  | **Query** | **Observed** | **Mr(expt)** | **Mr(calc)** | **Delta** | **Miss** | **Score** | **Expect** | **Rank** | **Peptide** |
| --- | --- | --- | --- | --- | --- | --- | --- | --- | --- | --- |
|  | 1522 | **650.3469** | **649.3396** | **649.3105** | **0.0292** | **0** | **22** | **1.3** | **1** | **K.LENMK.K** 1514 |
|  | 3564 | 446.0373 | 890.0600 | 889.4617 | 0.5982 | 0 | 26 | 0.32 | 1 | K.TGTLTQNR.M |
|  | 4060 | **463.5787** | **925.1429** | **925.4981** | **-0.3552** | **0** | **2** | **56** | **10** | **R.VPVDQVNR.K** |
|  | 8262 | **609.7962** | **1217.5778** | **1216.6299** | **0.9479** | **0** | **4** | **56** | **5** | **R.IASLASGVENEK.T** |
|  | 10287 | **663.3088** | **1324.6031** | **1323.6717** | **0.9314** | **0** | **(2)** | **89** | **8** | **K.AESDIMHLRPR.N** 10291 |
|  | 10587 | 671.3415 | 1340.6684 | 1339.6666 | 1.0018 | 0 | 10 | 15 | 3 | K.AESDIMHLRPR.N 10585 |
|  | 11285 | 691.3743 | 1380.7341 | 1380.7435 | -0.0094 | 0 | 66 | 3.2e-005 | 1 | R.VIMVTGDHPITAK.A 11283 11284 11286 |
|  | 11571 | 699.4022 | 1396.7898 | 1396.7384 | 0.0513 | 0 | (13) | 6.3 | 1 | R.VIMVTGDHPITAK.A |
|  | 12822 | 737.4216 | 1472.8287 | 1473.6922 | -0.8635 | 0 | 0 | 1.1e+002 | 9 | K.FSELTLGNAMGYR.D |
|  | 13006 | 495.7051 | 1484.0935 | 1484.8562 | -0.7627 | 1 | 2 | 52 | 5 | K.RIVIGDASETALLK.F |
|  | 13408 | **755.8467** | **1509.6788** | **1510.8290** | **-1.1502** | **1** | **0** | **1.1e+002** | **2** | **R.VPADIRILAAQGCK.V** |
|  | 14350 | **787.4092** | **1572.8038** | **1573.8134** | **-1.0096** | **1** | **4** | **47** | **8** | **K.KADIGVAMGIAGSDAAK.N** |
|  | 15042 | 810.3734 | 1618.7323 | 1618.7435 | -0.0112 | 0 | 74 | 4.7e-006 | 1 | K.VDNSSLTGESEPQTR.S 15017 15040 15043 15056 |
|  | 24275 | **822.3976** | **2464.1709** | **2464.1904** | **-0.0195** | **0** | **0** | **65** | **4** | **K.NAADMILLDDNFASIVTGVEQGR.L** |
|  | 24352 | 825.7382 | 2474.1928 | 2474.1570 | 0.0359 | 1 | 3 | 39 | 10 | K.DMDPSELVEALRTHPEMVFAR.T |
|  | 24587 | **835.3508** | **2503.0305** | **2504.2073** | **-1.1768** | **1** | **(0)** | **53** | **3** | **R.ACVINGMQLKDMDPSELVEALR.T** |
|  | 24589 | **835.4454** | **2503.3145** | **2504.2073** | **-0.8928** | **1** | **1** | **51** | **5** | **R.ACVINGMQLKDMDPSELVEALR.T** |
|  | 28608 | 1103.8486 | 3308.5241 | 3307.6758 | 0.8482 | 1 | 1 | 32 | 7 | K.NAADMILLDDNFASIVTGVEQGRLIFDNLK.K |
|  | 29584 | **910.1400** | **3636.5309** | **3635.6411** | **0.8898** | **0** | **0** | **26** | **3** | **K.DYPPGYAFDVEAMNFPSSGLCFAGLVSMIDPPR.A** |

| **192.** | P55072\|TERA_HUMAN    **Mass:** 89950    **Score:** 241    **Queries matched:** 33   **emPAI:** 0.50 |
| --- | --- |
|  | Transitional endoplasmic reticulum ATPase OS=Homo sapiens GN=VCP PE=1 SV=4 |

|  | **Query** | **Observed** | **Mr(expt)** | **Mr(calc)** | **Delta** | **Miss** | **Score** | **Expect** | **Rank** | **Peptide** |
| --- | --- | --- | --- | --- | --- | --- | --- | --- | --- | --- |
|  | 2670 | **418.7011** | **835.3876** | **836.5232** | **-1.1356** | **0** | **6** | **42** | **8** | **K.AIGVKPPR.G** 2694 |
|  | 5533 | **516.7922** | **1031.5699** | **1031.5499** | **0.0200** | **0** | **11** | **15** | **4** | **K.GDDLSTAILK.Q** |
|  | 5569 | **518.2785** | **1034.5424** | **1033.4287** | **1.1137** | **0** | **11** | **13** | **4** | **R.DHFEEAMR.F** |
|  | 5749 | **525.2757** | **1048.5368** | **1048.5441** | **-0.0072** | **0** | **33** | **0.083** | **1** | **K.DVDLEFLAK.M** |
|  | 5774 | **526.2625** | **1050.5105** | **1050.5168** | **-0.0063** | **0** | **52** | **0.00087** | **1** | **K.MDELQLFR.G** |
|  | 5902 | **531.1867** | **1060.3589** | **1060.5261** | **-0.1673** | **1** | **7** | **32** | **10** | **R.RSVSDNDIR.K** |
|  | 7387 | **586.8337** | **1171.6528** | **1171.6601** | **-0.0073** | **0** | **24** | **0.61** | **1** | **R.GILLYGPPGTGK.T** |
|  | 8717 | **621.3074** | **1240.6002** | **1241.6252** | **-1.0250** | **0** | **34** | **0.05** | **1** | **R.EVDIGIPDATGR.L** |
|  | 13833 | **770.9003** | **1539.7861** | **1539.7967** | **-0.0106** | **0** | **48** | **0.002** | **1** | **R.LGDVISIQPCPDVK.Y** |
|  | 14410 | **789.8944** | **1577.7741** | **1577.7871** | **-0.0130** | **0** | **4** | **49** | **1** | **K.AIANECQANFISIK.G** |
|  | 15204 | **815.4019** | **1628.7892** | **1628.7981** | **-0.0089** | **1** | **71** | **9.3e-006** | **1** | **R.KYEMFAQTLQQSR.G** |
|  | 17523 | **897.0440** | **1792.0735** | **1792.9393** | **-0.8658** | **1** | **7** | **19** | **2** | **K.MDELQLFRGDTVLLK.G** |
|  | 17601 | 900.9478 | 1799.8809 | 1798.7978 | 1.0831 | 0 | 8 | 19 | 4 | K.MTNGFSGADLTEICQR.A 17588 |
|  | 17602 | **601.0211** | **1800.0415** | **1798.9829** | **1.0587** | **1** | **7** | **18** | **3** | **R.LDQLIYIPLPDEKSR.V** |
|  | 17732 | **905.9968** | **1809.9791** | **1809.9876** | **-0.0085** | **0** | **15** | **3.4** | **1** | **K.NAPAIIFIDELDAIAPK.R** |
|  | 17899 | **912.4568** | **1822.8990** | **1822.9101** | **-0.0111** | **0** | **41** | **0.0093** | **1** | **R.ELQELVQYPVEHPDK.F** |
|  | 18156 | 920.4385 | 1838.8625 | 1838.9118 | -0.0492 | 1 | 2 | 63 | 10 | R.VINQILTEMDGMSTKK.N |
|  | 18179 | **614.3465** | **1840.0177** | **1839.7867** | **0.2310** | **0** | **5** | **31** | **6** | **R.EAVCIVLSDDTCSDEK.I** |
|  | 18821 | 944.4901 | 1886.9657 | 1887.9764 | -1.0108 | 1 | 3 | 54 | 5 | R.LGDVISIQPCPDVKYGK.R |
|  | 20254 | 504.7688 | 2015.0461 | 2014.9645 | 0.0816 | 1 | 7 | 19 | 2 | K.FGMTPSKGVLFYGPPGCGK.T |
|  | 21947 | **1085.5588** | **2169.1031** | **2169.1165** | **-0.0133** | **0** | **90** | **8.7e-008** | **1** | **R.LIVDEAINEDNSVVSLSQPK.M** 21948 21957 |
|  | 23236 | 1164.5972 | 2327.1798 | 2327.0699 | 0.1099 | 1 | 3 | 42 | 6 | R.QTNPSAMEVEEDDPVPEIRR.D 23235 |
|  | 24549 | **833.4263** | **2497.2570** | **2497.2701** | **-0.0131** | **1** | **0** | **64** | **3** | **R.ETVVEVPQVTWEDIGGLEDVKR.E** |
|  | 24688 | 840.1412 | 2517.4019 | 2517.4180 | -0.0161 | 0 | 22 | 0.4 | 1 | K.NVFIIGATNRPDIIDPAILRPGR.L |
|  | 28618 | **1104.8871** | **3311.6394** | **3311.6496** | **-0.0102** | **1** | **2** | **27** | **1** | **R.AVANETGAFFFLINGPEIMSKLAGESESNLR.K** |
|  | 30088 | **760.7678** | **3798.8024** | **3799.8799** | **-1.0775** | **1** | **0** | **30** | **2** | **K.LADDVDLEQVANETHGHVGADLAALCSEAALQAIRK.K** 30095 |
|  | 30877 | **741.3488** | **4442.0493** | **4443.0556** | **-1.0063** | **1** | **0** | **19** | **2** | **K.MDLIDLEDETIDAEVMNSLAVTMDDFRWALSQSNPSALR.E** |

| **193.** | P62158\|CALM_HUMAN    **Mass:** 16827    **Score:** 240    **Queries matched:** 19   **emPAI:** 2.46 |
| --- | --- |
|  | Calmodulin OS=Homo sapiens GN=CALM1 PE=1 SV=2 |

|  | **Query** | **Observed** | **Mr(expt)** | **Mr(calc)** | **Delta** | **Miss** | **Score** | **Expect** | **Rank** | **Peptide** |
| --- | --- | --- | --- | --- | --- | --- | --- | --- | --- | --- |
|  | 2243 | 403.2129 | 804.4112 | 804.4164 | -0.0052 | 0 | 18 | 3.6 | 1 | K.ELGTVMR.S |
|  | 9166 | **422.5495** | **1264.6267** | **1264.6048** | **0.0219** | **0** | **4** | **51** | **2** | **K.DGNGYISAAELR.H** |
|  | 14669 | **798.8549** | **1595.6952** | **1595.7063** | **-0.0111** | **1** | **38** | **0.02** | **1** | **K.DTDSEEEIREAFR.V** 14670 14681 |
|  | 15578 | 551.9312 | 1652.7716 | 1651.7763 | 0.9953 | 0 | 1 | 88 | 8 | -.MADQLTEEQIAEFK.E |
|  | 16980 | **877.9328** | **1753.8510** | **1753.8635** | **-0.0124** | **1** | **76** | **2.8e-006** | **1** | **R.VFDKDGNGYISAAELR.H** 16976 16988 16989 16990 16991 |
|  | 18242 | **922.9444** | **1843.8742** | **1843.8840** | **-0.0097** | **1** | **60** | **0.0001** | **1** | **K.EAFSLFDKDGDGTITTK.E** 18239 18241 |
|  | 24471 | **1245.5371** | **2489.0597** | **2489.0726** | **-0.0130** | **0** | **96** | **1.6e-008** | **1** | **R.EADIDGDGQVNYEEFVQMMTAK.-** 24472 |
|  | 30447 | **1357.2822** | **4068.8249** | **4068.8391** | **-0.0142** | **0** | **16** | **0.67** | **1** | **R.SLGQNPTEAELQDMINEVDADGNGTIDFPEFLTMMAR.K** 30453 |

| **194.** | O60844\|ZG16_HUMAN    **Mass:** 18336    **Score:** 240    **Queries matched:** 15   **emPAI:** 4.59 |
| --- | --- |
|  | Zymogen granule membrane protein 16 OS=Homo sapiens GN=ZG16 PE=1 SV=1 |

|  | **Query** | **Observed** | **Mr(expt)** | **Mr(calc)** | **Delta** | **Miss** | **Score** | **Expect** | **Rank** | **Peptide** |
| --- | --- | --- | --- | --- | --- | --- | --- | --- | --- | --- |
|  | 4405 | **475.2864** | **948.5583** | **948.5644** | **-0.0062** | **1** | **38** | **0.031** | **1** | **K.KLVFVTDK.G** |
|  | 5609 | **519.7502** | **1037.4858** | **1037.4931** | **-0.0072** | **0** | **47** | **0.0031** | **1** | **K.VWSDYVGGR.N** |
|  | 10230 | **661.7739** | **1321.5333** | **1321.5422** | **-0.0089** | **0** | **61** | **0.0001** | **1** | **R.SSSYSGEYGSGGGK.R** 10258 |
|  | 11397 | **463.2528** | **1386.7367** | **1385.6728** | **1.0639** | **1** | **1** | **1.1e+002** | **7** | **R.YGKVWSDYVGGR.N** |
|  | 12897 | **739.8237** | **1477.6328** | **1477.6433** | **-0.0106** | **1** | **51** | **0.00096** | **1** | **R.SSSYSGEYGSGGGKR.F** |
|  | 16423 | **856.9330** | **1711.8514** | **1711.8642** | **-0.0128** | **0** | **49** | **0.0013** | **1** | **R.FSHSGNQLDGPITALR.V** 16400 16408 16424 |
|  | 19294 | **962.9906** | **1923.9666** | **1923.9803** | **-0.0136** | **0** | **16** | **2.4** | **1** | **K.DSGTSFNAVPLHPNTVLR.F** |
|  | 22774 | **1134.5718** | **2267.1290** | **2267.1434** | **-0.0144** | **0** | **66** | **2.2e-005** | **1** | **R.NGDLEEIFLHPGESVIQVSGK.Y** 22773 |
|  | 23274 | **1167.5561** | **2333.0977** | **2333.1110** | **-0.0133** | **0** | **86** | **2e-007** | **1** | **R.SGSLIDAIGLHWDVYPTSCSR.C** 23275 |

| **195.** | P0C0S5\|H2AZ_HUMAN    **Mass:** 13545    **Score:** 240    **Queries matched:** 10   **emPAI:** 1.77 |
| --- | --- |
|  | Histone H2A.Z OS=Homo sapiens GN=H2AFZ PE=1 SV=2 |

|  | **Query** | **Observed** | **Mr(expt)** | **Mr(calc)** | **Delta** | **Miss** | **Score** | **Expect** | **Rank** | **Peptide** |
| --- | --- | --- | --- | --- | --- | --- | --- | --- | --- | --- |
|  | 2897 | 425.7636 | 849.5127 | 849.5184 | -0.0058 | 0 | 32 | 0.13 | 1 | R.HLQLAIR.G 2888 2898 2899 2901 |
|  | 4323 | 472.7661 | 943.5175 | 943.5240 | -0.0064 | 0 | 53 | 0.00095 | 1 | R.AGLQFPVGR.I 4330 |
|  | 26815 | **1448.2643** | **2894.5140** | **2894.5276** | **-0.0136** | **0** | **107** | **1.2e-009** | **1** | **R.VGATAAVYSAAILEYLTAEVLELAGNASK.D** 26816 26817 |

|  | **Proteins matching the same set of peptides:** |
| --- | --- |

|  | Q71UI9\|H2AV_HUMAN    **Mass:** 13501    **Score:** 240    **Queries matched:** 10 |
| --- | --- |
|  | Histone H2A.V OS=Homo sapiens GN=H2AFV PE=1 SV=3 |

| **196.** | P23284\|PPIB_HUMAN    **Mass:** 23785    **Score:** 238    **Queries matched:** 23   **emPAI:** 1.84 |
| --- | --- |
|  | Peptidyl-prolyl cis-trans isomerase B OS=Homo sapiens GN=PPIB PE=1 SV=2 |

|  | **Query** | **Observed** | **Mr(expt)** | **Mr(calc)** | **Delta** | **Miss** | **Score** | **Expect** | **Rank** | **Peptide** |
| --- | --- | --- | --- | --- | --- | --- | --- | --- | --- | --- |
|  | 1662 | 687.3984 | 686.3911 | 685.3912 | 0.9999 | 0 | 25 | 0.75 | 1 | K.HVVFGK.V 1661 |
|  | 2036 | **790.4045** | **789.3972** | **789.4021** | **-0.0049** | **0** | **26** | **0.62** | **1** | **K.TAWLDGK.H** 2013 2014 |
|  | 5531 | **516.7797** | **1031.5449** | **1030.5481** | **0.9968** | **0** | **58** | **0.00026** | **1** | **K.VLEGMEVVR.K** 5514 |
|  | 6948 | **569.7653** | **1137.5160** | **1136.5866** | **0.9294** | **1** | **4** | **55** | **2** | **R.FPDENFKLK.H** |
|  | 9544 | **643.3279** | **1284.6412** | **1285.5761** | **-0.9349** | **0** | **9** | **18** | **1** | **K.DFMIQGGDFTR.G** |
|  | 10961 | 682.8518 | 1363.6891 | 1363.6983 | -0.0093 | 0 | 62 | 9e-005 | 1 | K.TVDNFVALATGEK.G 10962 |
|  | 11134 | **687.3828** | **1372.7511** | **1372.7602** | **-0.0091** | **1** | **28** | **0.21** | **1** | **K.IEVEKPFAIAKE.-** 11149 |
|  | 12556 | 729.3619 | 1456.7092 | 1456.7198 | -0.0106 | 0 | 62 | 7.7e-005 | 1 | K.DTNGSQFFITTVK.T 12571 12572 12573 |
|  | 12831 | **737.8433** | **1473.6720** | **1473.6823** | **-0.0103** | **0** | **59** | **0.00015** | **1** | **K.HYGPGWVSMANAGK.D** 12846 |
|  | 14322 | **786.4217** | **1570.8289** | **1570.8389** | **-0.0099** | **1** | **74** | **4.7e-006** | **1** | **R.DKPLKDVIIADCGK.I** 14329 |
|  | 15158 | **813.9145** | **1625.8145** | **1625.8236** | **-0.0090** | **1** | **36** | **0.029** | **1** | **R.VIKDFMIQGGDFTR.G** 15157 |

| **197.** | P07900\|HS90A_HUMAN    **Mass:** 85006    **Score:** 237    **Queries matched:** 31   **emPAI:** 0.47 |
| --- | --- |
|  | Heat shock protein HSP 90-alpha OS=Homo sapiens GN=HSP90AA1 PE=1 SV=5 |

|  | **Query** | **Observed** | **Mr(expt)** | **Mr(calc)** | **Delta** | **Miss** | **Score** | **Expect** | **Rank** | **Peptide** |
| --- | --- | --- | --- | --- | --- | --- | --- | --- | --- | --- |
|  | 1572 | **661.3661** | **660.3588** | **659.4218** | **0.9370** | **0** | **10** | **26** | **1** | **K.VTVITK.H** |
|  | 1868 | **747.4567** | **746.4494** | **746.4538** | **-0.0044** | **0** | **23** | **1** | **1** | **K.TLVSVTK.E** |
|  | 3092 | 863.0865 | 862.0792 | 862.4218 | -0.3426 | 0 | 21 | 1.1 | 1 | R.EMLQQSK.I |
|  | 3560 | 445.8990 | 889.7834 | 889.4141 | 0.3693 | 1 | 4 | 56 | 6 | K.AEADKNDK.S |
|  | 4347 | 473.7424 | 945.4703 | 945.5429 | -0.0726 | 1 | 10 | 18 | 9 | R.IMKAQALR.D |
|  | 4382 | **474.7241** | **947.4336** | **947.4389** | **-0.0052** | **0** | **4** | **74** | **4** | **K.FYEQFSK.N** |
|  | 7104 | 576.2781 | 1150.5416 | 1150.5506 | -0.0090 | 0 | 12 | 9.1 | 1 | K.YIDQEELNK.T |
|  | 7326 | 584.7976 | 1167.5807 | 1167.5632 | 0.0174 | 0 | 17 | 3.2 | 1 | K.LGIHEDSQNR.K 7327 |
|  | 7545 | 592.3403 | 1182.6660 | 1181.5937 | 1.0723 | 1 | 0 | 1.3e+002 | 7 | K.FENLCKIMK.D |
|  | 7629 | **594.7504** | **1187.4863** | **1187.4951** | **-0.0088** | **0** | **48** | **0.0025** | **1** | **R.DNSTMGYMAAK.K** |
|  | 7969 | **602.8389** | **1203.6632** | **1203.4900** | **0.1732** | **0** | **(1)** | **1.2e+002** | **8** | **R.DNSTMGYMAAK.K** |
|  | 8261 | 609.7952 | 1217.5759 | 1216.6849 | 0.8910 | 1 | 20 | 1.6 | 1 | R.EMLQQSKILK.V |
|  | 8341 | **611.3104** | **1220.6062** | **1219.4849** | **1.1213** | **0** | **(3)** | **74** | **8** | **R.DNSTMGYMAAK.K** |
|  | 8385 | **612.8124** | **1223.6103** | **1223.6186** | **-0.0083** | **0** | **35** | **0.047** | **1** | **K.HIYYITGETK.D** |
|  | 8741 | 621.8521 | 1241.6897 | 1241.6979 | -0.0083 | 0 | 43 | 0.0066 | 1 | K.ADLINNLGTIAK.S 8742 8756 |
|  | 9659 | **646.3174** | **1290.6202** | **1290.6303** | **-0.0101** | **0** | **18** | **2.3** | **1** | **R.ELISNSSDALDK.I** |
|  | 10442 | **667.3448** | **1332.6751** | **1331.5849** | **1.0902** | **1** | **8** | **24** | **8** | **R.DNSTMGYMAAKK.H** |
|  | 10715 | **675.3105** | **1348.6064** | **1347.5799** | **1.0266** | **1** | **(1)** | **1.2e+002** | **7** | **R.DNSTMGYMAAKK.H** |
|  | 10716 | 675.3210 | 1348.6275 | 1347.6572 | 0.9704 | 0 | 1 | 1.2e+002 | 10 | K.HFSVEGQLEFR.A |
|  | 10726 | 675.3670 | 1348.7194 | 1348.7272 | -0.0078 | 0 | 47 | 0.0031 | 1 | R.TLTIVDTGIGMTK.A |
|  | 11481 | **465.0193** | **1392.0362** | **1392.5892** | **-0.5530** | **1** | **4** | **34** | **2** | **R.DKEVSDDEAEEK.E** |
|  | 13456 | 757.3917 | 1512.7688 | 1512.7784 | -0.0096 | 0 | 74 | 4.5e-006 | 1 | R.GVVDSEDLPLNISR.E |
|  | 13661 | 764.4358 | 1526.8571 | 1526.7365 | 0.1206 | 0 | 23 | 0.59 | 1 | K.SLTNDWEDHLAVK.H |
|  | 16846 | 582.2744 | 1743.8012 | 1742.8080 | 0.9933 | 1 | 2 | 72 | 4 | K.AQALRDNSTMGYMAAK.K |
|  | 17324 | **889.9722** | **1777.9299** | **1777.9403** | **-0.0104** | **0** | **71** | **7.6e-006** | **1** | **K.HSQFIGYPITLFVEK.E** |
|  | 17433 | **596.3157** | **1785.9254** | **1785.9373** | **-0.0120** | **0** | **28** | **0.17** | **1** | **K.HLEINPDHSIIETLR.Q** |
|  | 18066 | **917.3879** | **1832.7613** | **1832.7741** | **-0.0127** | **0** | **66** | **2.3e-005** | **1** | **R.NPDDITNEEYGEFYK.S** |
|  | 25101 | **859.7572** | **2576.2498** | **2576.2655** | **-0.0157** | **0** | **26** | **0.18** | **1** | **K.HGLEVIYMIEPIDEYCVQQLK.E** |

| **198.** | P27824\|CALX_HUMAN    **Mass:** 67982    **Score:** 235    **Queries matched:** 30   **emPAI:** 0.53 |
| --- | --- |
|  | Calnexin OS=Homo sapiens GN=CANX PE=1 SV=2 |

|  | **Query** | **Observed** | **Mr(expt)** | **Mr(calc)** | **Delta** | **Miss** | **Score** | **Expect** | **Rank** | **Peptide** |
| --- | --- | --- | --- | --- | --- | --- | --- | --- | --- | --- |
|  | 2631 | **417.2446** | **832.4746** | **831.4755** | **0.9990** | **0** | **16** | **4.8** | **1** | **K.LHFIFR.H** |
|  | 2729 | **839.4678** | **838.4606** | **838.4072** | **0.0533** | **0** | **8** | **21** | **1** | **K.TGIYEEK.H** 2711 2712 |
|  | 3755 | 452.2399 | 902.4652 | 901.4545 | 1.0107 | 1 | 12 | 14 | 4 | K.TYFTDKK.T |
|  | 4582 | **482.2245** | **962.4344** | **961.5457** | **0.8887** | **1** | **9** | **23** | **4** | **R.AKHHAISAK.L** |
|  | 5919 | **531.2996** | **1060.5846** | **1060.5917** | **-0.0071** | **0** | **22** | **1.1** | **1** | **R.GTLSGWILSK.A** 5920 |
|  | 7084 | 575.3068 | 1148.5991 | 1148.5197 | 0.0794 | 1 | 8 | 25 | 7 | K.KDDTDDEIAK.Y |
|  | 9817 | **434.2152** | **1299.6239** | **1300.7173** | **-1.0934** | **1** | **7** | **25** | **3** | **K.LPGDKGLVLMSR.A** 9821 |
|  | 11844 | **707.3171** | **1412.6196** | **1412.6282** | **-0.0086** | **1** | **24** | **0.54** | **1** | **K.YDGKWEVEEMK.E** |
|  | 13067 | **744.8550** | **1487.6954** | **1487.7045** | **-0.0091** | **0** | **74** | **4.8e-006** | **1** | **R.IVDDWANDGWGLK.K** |
|  | 14995 | 808.9016 | 1615.7885 | 1615.7995 | -0.0109 | 1 | 45 | 0.0039 | 1 | R.IVDDWANDGWGLKK.A 14996 14997 |
|  | 16734 | 579.6298 | 1735.8676 | 1734.8253 | 1.0423 | 0 | 4 | 41 | 7 | K.IPNPDFFEDLEPFR.M |
|  | 18527 | **621.9776** | **1862.9110** | **1862.9203** | **-0.0093** | **1** | **44** | **0.0042** | **1** | **R.KIPNPDFFEDLEPFR.M** 18526 |
|  | 21285 | 703.3289 | 2106.9649 | 2106.9746 | -0.0096 | 0 | 34 | 0.036 | 1 | K.IPDPEAVKPDDWDEDAPAK.I |
|  | 22457 | 743.3497 | 2227.0272 | 2228.1201 | -1.0929 | 0 | 0 | 78 | 5 | K.WKPPMIDNPSYQGIWKPR.K |
|  | 23833 | **805.3994** | **2413.1764** | **2413.2365** | **-0.0601** | **1** | **3** | **39** | **3** | **K.GKWKPPMIDNPSYQGIWKPR.K** |
|  | 29422 | 896.9106 | 3583.6135 | 3584.6778 | -1.0644 | 1 | 2 | 22 | 2 | R.MTPFSAIGLELWSMTSDIFFDNFIICADRR.I |
|  | 29791 | **926.2267** | **3700.8776** | **3700.8963** | **-0.0187** | **0** | **37** | **0.0065** | **1** | **K.LNKPFLFDTKPLIVQYEVNFQNGIECGGAYVK.L** 29793 |
|  | 30872 | **1479.7465** | **4436.2176** | **4436.2322** | **-0.0147** | **0** | **69** | **2.7e-006** | **1** | **K.THLYTLILNPDNSFEILVDQSVVNSGNLLNDMTPPVNPSR.E** 30871 30873 |
|  | 30887 | 1485.4034 | 4453.1885 | 4452.2271 | 0.9614 | 0 | (3) | 12 | 9 | K.THLYTLILNPDNSFEILVDQSVVNSGNLLNDMTPPVNPSR.E |
|  | 31833 | **1392.0971** | **5564.3591** | **5564.3950** | **-0.0359** | **0** | **31** | **0.0072** | **1** | **K.IPDEEATKPEGWLDDEPEYVPDPDAEKPEDWDEDMDGEWEAPQIANPR.C** |

| **199.** | Q01970\|PLCB3_HUMAN    **Mass:** 139511   **Score:** 234    **Queries matched:** 36   **emPAI:** 0.08 |
| --- | --- |
|  | 1-phosphatidylinositol-4,5-bisphosphate phosphodiesterase beta-3 OS=Homo sapiens GN=PLCB3 PE=1 SV=2 |

|  | **Query** | **Observed** | **Mr(expt)** | **Mr(calc)** | **Delta** | **Miss** | **Score** | **Expect** | **Rank** | **Peptide** |
| --- | --- | --- | --- | --- | --- | --- | --- | --- | --- | --- |
|  | 1702 | **699.4103** | **698.4030** | **697.3105** | **1.0925** | **0** | **3** | **84** | **9** | **K.MFSADK.K** |
|  | 2401 | **409.2184** | **816.4222** | **815.4977** | **0.9245** | **1** | **17** | **3.9** | **6** | **K.AVTLTRR.L** |
|  | 2471 | **412.2466** | **822.4785** | **822.4824** | **-0.0038** | **1** | **14** | **6.3** | **5** | **R.RLEHLR.Q** |
|  | 3482 | 443.0397 | 884.0648 | 883.5352 | 0.5296 | 1 | 6 | 28 | 7 | R.QALQRLR.E 3495 |
|  | 4001 | 461.0073 | 920.0000 | 920.4603 | -0.4603 | 0 | 12 | 7.8 | 3 | R.IAAFEEGGK.F |
|  | 4446 | **476.7632** | **951.5119** | **950.4127** | **1.0992** | **1** | **6** | **41** | **7** | **K.EMNEREK.K** |
|  | 5649 | **521.3040** | **1040.5935** | **1040.5363** | **0.0572** | **1** | **12** | **11** | **2** | **K.RHNSISEAK.M** |
|  | 6980 | **570.8237** | **1139.6329** | **1139.5472** | **0.0857** | **1** | **9** | **19** | **7** | **K.RYQEFQNR.Q** |
|  | 7163 | **578.2893** | **1154.5641** | **1153.5339** | **1.0302** | **0** | **4** | **57** | **2** | **R.SGYHYVCLR.N** |
|  | 7165 | **578.3059** | **1154.5973** | **1154.6044** | **-0.0071** | **0** | **20** | **1.5** | **1** | **R.HITESVNSIR.R** |
|  | 7899 | 401.2130 | 1200.6173 | 1201.6302 | -1.0129 | 1 | 3 | 73 | 9 | K.KEAELTEINR.R |
|  | 8832 | 624.7997 | 1247.5849 | 1248.6826 | -1.0977 | 1 | 8 | 23 | 4 | K.VISGQFLSDRK.V |
|  | 9826 | 650.8485 | 1299.6825 | 1300.6921 | -1.0096 | 0 | 5 | 41 | 5 | K.LAMNILAQNASR.N |
|  | 10582 | **671.3139** | **1340.6132** | **1340.7048** | **-0.0916** | **0** | **8** | **23** | **3** | **R.LLDGLAQAQAEGR.C** 10581 |
|  | 10978 | **683.3411** | **1364.6676** | **1363.6983** | **0.9692** | **0** | **12** | **8.8** | **4** | **R.EVVLDANTTQFK.R** 10988 |
|  | 11016 | **456.5712** | **1366.6918** | **1365.7728** | **0.9189** | **1** | **4** | **51** | **5** | **K.LQVNQDGRIPVK.N** |
|  | 11469 | 696.8208 | 1391.6270 | 1391.7018 | -0.0747 | 1 | 3 | 67 | 5 | R.HRPSAGGPDSAGRK.R |
|  | 14532 | **793.8959** | **1585.7773** | **1585.7882** | **-0.0109** | **0** | **65** | **3.7e-005** | **1** | **K.LLAQLAQECQEQR.A** 14533 |
|  | 16955 | **585.2593** | **1752.7562** | **1751.9682** | **0.7880** | **0** | **4** | **39** | **2** | **R.LNEVLYPPLRPSQAR.L** 16975 |
|  | 19921 | **661.0037** | **1979.9893** | **1980.0025** | **-0.0131** | **0** | **13** | **4.6** | **1** | **R.RPPGPTTSPASTSLSSPGQR.D** |
|  | 19956 | **662.3416** | **1984.0030** | **1984.9966** | **-0.9936** | **1** | **4** | **40** | **5** | **K.SLGDEGLNRGPYVLGPADR.E** |
|  | 20192 | **1004.5319** | **2007.0493** | **2007.0636** | **-0.0144** | **0** | **91** | **7.8e-008** | **1** | **R.LVAGQQQVLQQLAEEEPK.L** 20193 20198 |
|  | 20479 | **678.7075** | **2033.1007** | **2032.0023** | **1.0985** | **1** | **1** | **73** | **2** | **R.QALLWGCRCVELDVWK.G** |
|  | 23296 | 779.7624 | 2336.2653 | 2337.2151 | -0.9497 | 1 | 2 | 52 | 9 | K.GKPYLTLEQLMDFINQKQR.D |
|  | 23843 | **604.5526** | **2414.1813** | **2415.2506** | **-1.0693** | **1** | **2** | **51** | **8** | **R.HDRLVAGQQQVLQQLAEEEPK.L** |
|  | 28559 | **1097.5575** | **3289.6507** | **3290.6711** | **-1.0204** | **1** | **1** | **40** | **2** | **R.DVLEAIAETAFKTSPYPVILSFENHVDSAK.Q** |
|  | 28993 | **1140.9442** | **3419.8108** | **3418.8210** | **0.9898** | **1** | **5** | **12** | **1** | **R.SIFGDALLIEPLDKYPLAPGVPLPSPQDLMGR.I** |
|  | 28998 | 1142.6065 | 3424.7975 | 3423.6942 | 1.1033 | 1 | 4 | 16 | 2 | R.LEEKLMTVVSGPDPVNTVFLNFMAVQDDTAK.V |
|  | 30783 | **1443.3691** | **4327.0856** | **4327.0994** | **-0.0138** | **0** | **63** | **1.2e-005** | **1** | **R.NEANQPLCLPALLIYTEASDYIPDDHQDYAEALINPIK.H** |

| **200.** | O75828\|CBR3_HUMAN    **Mass:** 31230    **Score:** 234    **Queries matched:** 14   **emPAI:** 0.77 |
| --- | --- |
|  | Carbonyl reductase [NADPH] 3 OS=Homo sapiens GN=CBR3 PE=1 SV=3 |

|  | **Query** | **Observed** | **Mr(expt)** | **Mr(calc)** | **Delta** | **Miss** | **Score** | **Expect** | **Rank** | **Peptide** |
| --- | --- | --- | --- | --- | --- | --- | --- | --- | --- | --- |
|  | 2802 | 422.7634 | 843.5123 | 843.5178 | -0.0055 | 0 | 35 | 0.067 | 1 | K.LGVTVLSR.I |
|  | 9610 | **430.5631** | **1288.6674** | **1287.6969** | **0.9705** | **0** | **2** | **96** | **9** | **R.VVNISSLQCLR.A** |
|  | 10360 | **664.8440** | **1327.6734** | **1326.6788** | **0.9946** | **0** | **2** | **89** | **4** | **R.ILVNACCPGPVK.T** |
|  | 14717 | 800.4053 | 1598.7960 | 1598.8053 | -0.0093 | 0 | 60 | 0.00013 | 1 | R.FHQLDIDDLQSIR.A 14715 14716 |
|  | 15561 | **551.3153** | **1650.9241** | **1650.9893** | **-0.0652** | **1** | **9** | **15** | **2** | **R.VALVTGANRGIGLAIAR.E** 15555 |
|  | 15574 | 826.9339 | 1651.8532 | 1651.8642 | -0.0109 | 0 | 95 | 3.6e-008 | 1 | R.GQAAVQQLQAEGLSPR.F 15575 |
|  | 16921 | **875.9517** | **1749.8889** | **1749.8832** | **0.0057** | **1** | **1** | **83** | **5** | **R.ELCRQFSGDVVLTAR.D** |
|  | 17341 | 890.4630 | 1778.9115 | 1777.9363 | 0.9752 | 0 | 48 | 0.0017 | 2 | K.EYGGLNVLVNNAAVAFK.S |
|  | 18390 | **618.9897** | **1853.9474** | **1852.8587** | **1.0887** | **1** | **1** | **92** | **9** | **K.SDDPMPFDIKAEMTLK.T** |
|  | 19092 | 954.5096 | 1907.0047 | 1906.0312 | 0.9735 | 1 | 40 | 0.01 | 2 | R.KEYGGLNVLVNNAAVAFK.S |

| **201.** | Q95604\|1C17_HUMAN    **Mass:** 41612    **Score:** 233    **Queries matched:** 11   **emPAI:** 0.54 |
| --- | --- |
|  | HLA class I histocompatibility antigen, Cw-17 alpha chain OS=Homo sapiens GN=HLA-C PE=1 SV=1 |

|  | **Query** | **Observed** | **Mr(expt)** | **Mr(calc)** | **Delta** | **Miss** | **Score** | **Expect** | **Rank** | **Peptide** |
| --- | --- | --- | --- | --- | --- | --- | --- | --- | --- | --- |
|  | 1094 | **560.3320** | **559.3248** | **558.3125** | **1.0122** | **0** | **20** | **3.5** | **10** | **K.LEAAR.E** |
|  | 12099 | **714.3628** | **1426.7111** | **1425.7365** | **0.9747** | **0** | **3** | **68** | **4** | **K.WAAVVVPSGQEQR.Y** |
|  | 13341 | 753.3655 | 1504.7165 | 1504.7270 | -0.0105 | 0 | 57 | 0.00025 | 1 | R.SWTAADTAAQISQR.K |
|  | 15206 | **815.4121** | **1628.8097** | **1628.8199** | **-0.0102** | **0** | **72** | **7.9e-006** | **1** | **R.FIAVGYVDDTQFVR.F** 15203 15205 |
|  | 18119 | **459.9797** | **1835.8896** | **1835.9027** | **-0.0131** | **0** | **13** | **5.4** | **2** | **K.THVTHHPVSDHEATLR.C** |
|  | 21343 | **1056.5170** | **2111.0194** | **2111.0299** | **-0.0105** | **0** | **45** | **0.003** | **1** | **R.CWALGFYPAEITLTWQR.D** 21342 |
|  | 25408 | **1319.0913** | **2636.1681** | **2636.1838** | **-0.0158** | **0** | **68** | **1.1e-005** | **1** | **R.DGEDQTQDTELVETRPAGDGTFQK.W** 25407 |

| **202.** | Q16819\|MEP1A_HUMAN    **Mass:** 85449    **Score:** 233    **Queries matched:** 20   **emPAI:** 0.35 |
| --- | --- |
|  | Meprin A subunit alpha OS=Homo sapiens GN=MEP1A PE=2 SV=2 |

|  | **Query** | **Observed** | **Mr(expt)** | **Mr(calc)** | **Delta** | **Miss** | **Score** | **Expect** | **Rank** | **Peptide** |
| --- | --- | --- | --- | --- | --- | --- | --- | --- | --- | --- |
|  | 1281 | **600.3425** | **599.3353** | **599.3391** | **-0.0038** | **0** | **4** | **66** | **1** | **K.LQSPR.F** |
|  | 1447 | **634.2997** | **633.2925** | **632.3897** | **0.9027** | **0** | **11** | **18** | **3** | **R.ILYPK.R** |
|  | 2339 | **406.6998** | **811.3850** | **810.3872** | **0.9979** | **0** | **10** | **15** | **3** | **R.ESSGYLR.L** |
|  | 2974 | **856.4504** | **855.4432** | **855.4491** | **-0.0059** | **0** | **29** | **0.21** | **1** | **R.YLFQGTK.G** 2976 |
|  | 3119 | **432.2423** | **862.4699** | **862.3781** | **0.0919** | **0** | **6** | **51** | **10** | **R.DDSTGNVR.K** |
|  | 3668 | **450.2428** | **898.4711** | **899.4825** | **-1.0114** | **1** | **8** | **24** | **5** | **K.GDKLQSPR.F** |
|  | 4090 | **464.7629** | **927.5113** | **926.5814** | **0.9299** | **1** | **2** | **1e+002** | **9** | **R.LVVWVRR.D** |
|  | 8313 | 610.7929 | 1219.5713 | 1219.5802 | -0.0089 | 0 | 43 | 0.0069 | 1 | K.ANICGMIQGTR.D |
|  | 13676 | **510.5434** | **1528.6083** | **1528.6188** | **-0.0105** | **0** | **19** | **1.4** | **1** | **R.VGTYHTDCNCFR.S** |
|  | 13898 | **772.8799** | **1543.7452** | **1543.6878** | **0.0574** | **0** | **8** | **20** | **5** | **R.CISGHAFFYTGER.C** |
|  | 14576 | **795.3470** | **1588.6794** | **1588.6907** | **-0.0113** | **0** | **55** | **0.0004** | **1** | **K.VQTFQGDDDHNWK.I** 14575 |
|  | 17831 | **909.9578** | **1817.9011** | **1817.9134** | **-0.0123** | **0** | **64** | **4.6e-005** | **1** | **R.SIDLGWSGFISHQMLK.R** 17832 |
|  | 20874 | **1035.9750** | **2069.9354** | **2069.9483** | **-0.0129** | **0** | **64** | **3.4e-005** | **1** | **R.FYNSEGYGFGVTLYPNSR.E** 20873 |
|  | 22732 | **755.0541** | **2262.1406** | **2262.1545** | **-0.0139** | **0** | **58** | **0.00011** | **1** | **K.AIIEHEILHALGFYHEQSR.T** |
|  | 24206 | **819.3873** | **2455.1402** | **2455.1590** | **-0.0189** | **0** | **44** | **0.0026** | **1** | **R.LAFHVCSGENDAILEWPVENR.Q** |
|  | 32007 | **1616.7960** | **6463.1550** | **6463.8387** | **-0.6837** | **1** | **2** | **4.3** | **1** | **K.ANICGMIQGTRDDTDWAHQDSAQAGEVDHTLLGQCTGAGYFMQFSTSSGSAEEAALLESR.I** |

| **203.** | P04439\|1A03_HUMAN    **Mass:** 41100    **Score:** 231    **Queries matched:** 20   **emPAI:** 0.55 |
| --- | --- |
|  | HLA class I histocompatibility antigen, A-3 alpha chain OS=Homo sapiens GN=HLA-A PE=1 SV=2 |

|  | **Query** | **Observed** | **Mr(expt)** | **Mr(calc)** | **Delta** | **Miss** | **Score** | **Expect** | **Rank** | **Peptide** |
| --- | --- | --- | --- | --- | --- | --- | --- | --- | --- | --- |
|  | 5040 | 498.7649 | 995.5153 | 995.4308 | 0.0845 | 0 | 16 | 3.9 | 3 | R.FDSDAASQR.M 5054 |
|  | 11481 | 465.0193 | 1392.0362 | 1392.6707 | -0.6345 | 0 | (3) | 43 | 4 | R.SWTAADMAAQITK.R |
|  | 11799 | **705.8859** | **1409.7572** | **1408.6656** | **1.0915** | **0** | **5** | **43** | **1** | **R.SWTAADMAAQITK.R** 11768 11769 |
|  | 12099 | 714.3628 | 1426.7111 | 1426.7205 | -0.0093 | 0 | 11 | 9.6 | 1 | K.WAAVVVPSGEEQR.Y |
|  | 12742 | **490.2472** | **1467.7197** | **1466.7266** | **0.9931** | **1** | **21** | **1.1** | **1** | **R.KWEAAHEAEQLR.A** |
|  | 13614 | **508.9797** | **1523.9174** | **1524.6627** | **-0.7453** | **1** | **7** | **23** | **1** | **R.FDSDAASQRMEPR.A** 13613 |
|  | 15206 | 815.4121 | 1628.8097 | 1628.8199 | -0.0102 | 0 | 72 | 7.9e-006 | 1 | R.FIAVGYVDDTQFVR.F 15203 15205 |
|  | 18745 | **628.2999** | **1881.8780** | **1881.8904** | **-0.0124** | **0** | **41** | **0.0072** | **1** | **K.THMTHHPISDHEATLR.C** |
|  | 20661 | **513.0184** | **2048.0446** | **2048.0626** | **-0.0179** | **0** | **15** | **3.2** | **1** | **R.YTCHVQHEGLPKPLTLR.W** |
|  | 21343 | 1056.5170 | 2111.0194 | 2111.0299 | -0.0105 | 0 | 45 | 0.003 | 1 | R.CWALGFYPAEITLTWQR.D 21342 |
|  | 23392 | **587.7993** | **2347.1682** | **2347.0234** | **0.1448** | **0** | **1** | **60** | **8** | **K.GGSYTQAASSDSAQGSDVSLTACK.V** |
|  | 25408 | 1319.0913 | 2636.1681 | 2636.1838 | -0.0158 | 0 | 68 | 1.1e-005 | 1 | R.DGEDQTQDTELVETRPAGDGTFQK.W 25407 |

|  | **Proteins matching a subset of these peptides:** |
| --- | --- |

|  | P13746\|1A11_HUMAN    **Mass:** 41196    **Score:** 231    **Queries matched:** 19 |
| --- | --- |
|  | HLA class I histocompatibility antigen, A-11 alpha chain OS=Homo sapiens GN=HLA-A PE=1 SV=1 |

| **204.** | P30447\|1A23_HUMAN    **Mass:** 40992    **Score:** 231    **Queries matched:** 17   **emPAI:** 0.55 |
| --- | --- |
|  | HLA class I histocompatibility antigen, A-23 alpha chain OS=Homo sapiens GN=HLA-A PE=1 SV=1 |

|  | **Query** | **Observed** | **Mr(expt)** | **Mr(calc)** | **Delta** | **Miss** | **Score** | **Expect** | **Rank** | **Peptide** |
| --- | --- | --- | --- | --- | --- | --- | --- | --- | --- | --- |
|  | 1188 | **579.3209** | **578.3136** | **577.2456** | **1.0680** | **0** | **10** | **19** | **6** | **R.NSSDR.K** |
|  | 2373 | **408.2321** | **814.4497** | **813.3729** | **1.0768** | **0** | **4** | **89** | **4** | **K.AHSQTDR.E** |
|  | 5040 | 498.7649 | 995.5153 | 995.4308 | 0.0845 | 0 | 16 | 3.9 | 3 | R.FDSDAASQR.M 5054 |
|  | 10365 | **443.7359** | **1328.1860** | **1327.6996** | **0.4863** | **1** | **13** | **4.5** | **1** | **K.WEAARVAEQLR.A** |
|  | 12099 | 714.3628 | 1426.7111 | 1426.7205 | -0.0093 | 0 | 11 | 9.6 | 1 | K.WAAVVVPSGEEQR.Y |
|  | 13614 | 508.9797 | 1523.9174 | 1524.6627 | -0.7453 | 1 | 7 | 23 | 1 | R.FDSDAASQRMEPR.A 13613 |
|  | 15206 | 815.4121 | 1628.8097 | 1628.8199 | -0.0102 | 0 | 72 | 7.9e-006 | 1 | R.FIAVGYVDDTQFVR.F 15203 15205 |
|  | 18745 | 628.2999 | 1881.8780 | 1881.8904 | -0.0124 | 0 | 41 | 0.0072 | 1 | K.THMTHHPISDHEATLR.C |
|  | 20661 | 513.0184 | 2048.0446 | 2048.0626 | -0.0179 | 0 | 15 | 3.2 | 1 | R.YTCHVQHEGLPKPLTLR.W |
|  | 21343 | 1056.5170 | 2111.0194 | 2111.0299 | -0.0105 | 0 | 45 | 0.003 | 1 | R.CWALGFYPAEITLTWQR.D 21342 |
|  | 25408 | 1319.0913 | 2636.1681 | 2636.1838 | -0.0158 | 0 | 68 | 1.1e-005 | 1 | R.DGEDQTQDTELVETRPAGDGTFQK.W 25407 |

| **205.** | P05534\|1A24_HUMAN    **Mass:** 40948    **Score:** 231    **Queries matched:** 21   **emPAI:** 0.55 |
| --- | --- |
|  | HLA class I histocompatibility antigen, A-24 alpha chain OS=Homo sapiens GN=HLA-A PE=1 SV=2 |

|  | **Query** | **Observed** | **Mr(expt)** | **Mr(calc)** | **Delta** | **Miss** | **Score** | **Expect** | **Rank** | **Peptide** |
| --- | --- | --- | --- | --- | --- | --- | --- | --- | --- | --- |
|  | 1188 | 579.3209 | 578.3136 | 577.2456 | 1.0680 | 0 | 10 | 19 | 6 | R.NSSDR.K |
|  | 2373 | 408.2321 | 814.4497 | 813.3729 | 1.0768 | 0 | 4 | 89 | 4 | K.AHSQTDR.E |
|  | 5040 | 498.7649 | 995.5153 | 995.4308 | 0.0845 | 0 | 16 | 3.9 | 3 | R.FDSDAASQR.M 5054 |
|  | 11481 | 465.0193 | 1392.0362 | 1392.6707 | -0.6345 | 0 | (3) | 43 | 4 | R.SWTAADMAAQITK.R |
|  | 11799 | 705.8859 | 1409.7572 | 1408.6656 | 1.0915 | 0 | 5 | 43 | 1 | R.SWTAADMAAQITK.R 11768 11769 |
|  | 12099 | 714.3628 | 1426.7111 | 1426.7205 | -0.0093 | 0 | 11 | 9.6 | 1 | K.WAAVVVPSGEEQR.Y |
|  | 13614 | 508.9797 | 1523.9174 | 1524.6627 | -0.7453 | 1 | 7 | 23 | 1 | R.FDSDAASQRMEPR.A 13613 |
|  | 15206 | 815.4121 | 1628.8097 | 1628.8199 | -0.0102 | 0 | 72 | 7.9e-006 | 1 | R.FIAVGYVDDTQFVR.F 15203 15205 |
|  | 18745 | 628.2999 | 1881.8780 | 1881.8904 | -0.0124 | 0 | 41 | 0.0072 | 1 | K.THMTHHPISDHEATLR.C |
|  | 19083 | 636.3424 | 1906.0054 | 1905.9254 | 0.0800 | 1 | 5 | 32 | 4 | K.EDLRSWTAADMAAQITK.R |
|  | 20661 | 513.0184 | 2048.0446 | 2048.0626 | -0.0179 | 0 | 15 | 3.2 | 1 | R.YTCHVQHEGLPKPLTLR.W |
|  | 21343 | 1056.5170 | 2111.0194 | 2111.0299 | -0.0105 | 0 | 45 | 0.003 | 1 | R.CWALGFYPAEITLTWQR.D 21342 |
|  | 25408 | 1319.0913 | 2636.1681 | 2636.1838 | -0.0158 | 0 | 68 | 1.1e-005 | 1 | R.DGEDQTQDTELVETRPAGDGTFQK.W 25407 |

| **206.** | P16188\|1A30_HUMAN    **Mass:** 41164    **Score:** 231    **Queries matched:** 16   **emPAI:** 0.55 |
| --- | --- |
|  | HLA class I histocompatibility antigen, A-30 alpha chain OS=Homo sapiens GN=HLA-A PE=1 SV=2 |

|  | **Query** | **Observed** | **Mr(expt)** | **Mr(calc)** | **Delta** | **Miss** | **Score** | **Expect** | **Rank** | **Peptide** |
| --- | --- | --- | --- | --- | --- | --- | --- | --- | --- | --- |
|  | 5040 | 498.7649 | 995.5153 | 995.4308 | 0.0845 | 0 | 16 | 3.9 | 3 | R.FDSDAASQR.M 5054 |
|  | 12099 | 714.3628 | 1426.7111 | 1426.7205 | -0.0093 | 0 | 11 | 9.6 | 1 | K.WAAVVVPSGEEQR.Y |
|  | 13614 | 508.9797 | 1523.9174 | 1524.6627 | -0.7453 | 1 | 7 | 23 | 1 | R.FDSDAASQRMEPR.A 13613 |
|  | 15206 | 815.4121 | 1628.8097 | 1628.8199 | -0.0102 | 0 | 72 | 7.9e-006 | 1 | R.FIAVGYVDDTQFVR.F 15203 15205 |
|  | 18745 | 628.2999 | 1881.8780 | 1881.8904 | -0.0124 | 0 | 41 | 0.0072 | 1 | K.THMTHHPISDHEATLR.C |
|  | 20661 | 513.0184 | 2048.0446 | 2048.0626 | -0.0179 | 0 | 15 | 3.2 | 1 | R.YTCHVQHEGLPKPLTLR.W |
|  | 21343 | 1056.5170 | 2111.0194 | 2111.0299 | -0.0105 | 0 | 45 | 0.003 | 1 | R.CWALGFYPAEITLTWQR.D 21342 |
|  | 23392 | 587.7993 | 2347.1682 | 2347.0234 | 0.1448 | 0 | 1 | 60 | 8 | K.GGSYTQAASSDSAQGSDVSLTACK.V |
|  | 24274 | 822.0737 | 2463.1992 | 2464.3260 | -1.1269 | 0 | 1 | 63 | 9 | R.TLLLLLSGALALTHTWAGSHSMR.Y |
|  | 25408 | 1319.0913 | 2636.1681 | 2636.1838 | -0.0158 | 0 | 68 | 1.1e-005 | 1 | R.DGEDQTQDTELVETRPAGDGTFQK.W 25407 |

| **207.** | P30443\|1A01_HUMAN    **Mass:** 41105    **Score:** 231    **Queries matched:** 16   **emPAI:** 0.55 |
| --- | --- |
|  | HLA class I histocompatibility antigen, A-1 alpha chain OS=Homo sapiens GN=HLA-A PE=1 SV=1 |

|  | **Query** | **Observed** | **Mr(expt)** | **Mr(calc)** | **Delta** | **Miss** | **Score** | **Expect** | **Rank** | **Peptide** |
| --- | --- | --- | --- | --- | --- | --- | --- | --- | --- | --- |
|  | 2373 | 408.2321 | 814.4497 | 813.3729 | 1.0768 | 0 | 4 | 89 | 4 | K.AHSQTDR.A |
|  | 11481 | 465.0193 | 1392.0362 | 1392.6707 | -0.6345 | 0 | (3) | 43 | 4 | R.SWTAADMAAQITK.R |
|  | 11799 | 705.8859 | 1409.7572 | 1408.6656 | 1.0915 | 0 | 5 | 43 | 1 | R.SWTAADMAAQITK.R 11768 11769 |
|  | 12099 | 714.3628 | 1426.7111 | 1426.7205 | -0.0093 | 0 | 11 | 9.6 | 1 | K.WAAVVVPSGEEQR.Y |
|  | 15206 | 815.4121 | 1628.8097 | 1628.8199 | -0.0102 | 0 | 72 | 7.9e-006 | 1 | R.FIAVGYVDDTQFVR.F 15203 15205 |
|  | 18745 | 628.2999 | 1881.8780 | 1881.8904 | -0.0124 | 0 | 41 | 0.0072 | 1 | K.THMTHHPISDHEATLR.C |
|  | 20661 | 513.0184 | 2048.0446 | 2048.0626 | -0.0179 | 0 | 15 | 3.2 | 1 | R.YTCHVQHEGLPKPLTLR.W |
|  | 21343 | 1056.5170 | 2111.0194 | 2111.0299 | -0.0105 | 0 | 45 | 0.003 | 1 | R.CWALGFYPAEITLTWQR.D 21342 |
|  | 23392 | 587.7993 | 2347.1682 | 2347.0234 | 0.1448 | 0 | 1 | 60 | 8 | K.GGSYTQAASSDSAQGSDVSLTACK.V |
|  | 25408 | 1319.0913 | 2636.1681 | 2636.1838 | -0.0158 | 0 | 68 | 1.1e-005 | 1 | R.DGEDQTQDTELVETRPAGDGTFQK.W 25407 |

|  | **Proteins matching the same set of peptides:** |
| --- | --- |

|  | P30455\|1A36_HUMAN    **Mass:** 41193    **Score:** 231    **Queries matched:** 16 |
| --- | --- |
|  | HLA class I histocompatibility antigen, A-36 alpha chain OS=Homo sapiens GN=HLA-A PE=1 SV=1 |

| **208.** | O75310\|UDB11_HUMAN    **Mass:** 61455    **Score:** 231    **Queries matched:** 24   **emPAI:** 0.34 |
| --- | --- |
|  | UDP-glucuronosyltransferase 2B11 OS=Homo sapiens GN=UGT2B11 PE=2 SV=1 |

|  | **Query** | **Observed** | **Mr(expt)** | **Mr(calc)** | **Delta** | **Miss** | **Score** | **Expect** | **Rank** | **Peptide** |
| --- | --- | --- | --- | --- | --- | --- | --- | --- | --- | --- |
|  | 683 | **473.2791** | **472.2719** | **472.2758** | **-0.0039** | **0** | **13** | **13** | **5** | **K.GAAVR.L** 688 |
|  | 1526 | 651.3502 | 650.3429 | 649.3105 | 1.0325 | 0 | 12 | 11 | 3 | K.ENIMK.L |
|  | 4010 | **461.2456** | **920.4767** | **919.4586** | **1.0181** | **0** | **6** | **40** | **7** | **K.ADIWLMR.N** |
|  | 4961 | 496.0763 | 990.1381 | 989.5328 | 0.6053 | 1 | 7 | 25 | 9 | K.ENIMKLSR.I |
|  | 5200 | 504.2530 | 1006.4914 | 1005.5277 | 0.9637 | 1 | (6) | 46 | 7 | K.ENIMKLSR.I |
|  | 6469 | 550.8162 | 1099.6178 | 1098.6761 | 0.9417 | 1 | 15 | 4.8 | 2 | K.TILKELVQR.G |
|  | 7580 | **593.3279** | **1184.6412** | **1183.6125** | **1.0287** | **0** | **2** | **91** | **5** | **K.FEVYPTSLTK.T** |
|  | 9315 | **425.0561** | **1272.1464** | **1272.5479** | **-0.4015** | **0** | **10** | **11** | **6** | **K.LSDQMTFMER.V** 9343 |
|  | 11919 | 709.3783 | 1416.7420 | 1416.7514 | -0.0093 | 0 | 53 | 0.00069 | 1 | K.WIPQNDLLGHPK.T 11920 |
|  | 12271 | **719.4207** | **1436.8269** | **1436.8715** | **-0.0446** | **1** | **32** | **0.089** | **3** | **R.ANVIATALAKIPQK.V** 12272 |
|  | 12388 | **723.3916** | **1444.7686** | **1444.7787** | **-0.0100** | **0** | **35** | **0.039** | **1** | **R.IQHDQPVKPLDR.A** |
|  | 13483 | 505.9178 | 1514.7315 | 1515.7061 | -0.9747 | 1 | 9 | 15 | 3 | K.LSDQMTFMERVK.N |
|  | 16139 | 424.2194 | 1692.8486 | 1692.8756 | -0.0271 | 1 | 6 | 30 | 7 | K.TVINDPLYKENIMK.L |
|  | 17571 | **600.2493** | **1797.7260** | **1797.8819** | **-0.1559** | **0** | **7** | **19** | **3** | **R.LDFNTMSSTDLLNALK.T** |
|  | 24592 | 1253.1022 | 2504.1898 | 2504.2046 | -0.0148 | 0 | 77 | 1.3e-006 | 1 | K.WDQFYSEVLGRPTTLFETMGK.A 24590 24593 24594 24595 |
|  | 26969 | **976.5400** | **2926.5981** | **2926.3854** | **0.2127** | **0** | **2** | **28** | **5** | **K.DSFWLYFSQEQEILWELYDIFR.N** |

| **209.** | P42330\|AK1C3_HUMAN    **Mass:** 37220    **Score:** 230    **Queries matched:** 11   **emPAI:** 0.47 |
| --- | --- |
|  | Aldo-keto reductase family 1 member C3 OS=Homo sapiens GN=AKR1C3 PE=1 SV=3 |

|  | **Query** | **Observed** | **Mr(expt)** | **Mr(calc)** | **Delta** | **Miss** | **Score** | **Expect** | **Rank** | **Peptide** |
| --- | --- | --- | --- | --- | --- | --- | --- | --- | --- | --- |
|  | 1658 | **685.4557** | **684.4485** | **684.4534** | **-0.0049** | **0** | **30** | **0.16** | **1** | **R.GVVVLAK.S** |
|  | 4990 | **993.0438** | **992.0365** | **991.4757** | **0.5608** | **1** | **6** | **28** | **1** | **M.DSKQQCVK.L** |
|  | 5000 | **497.2560** | **992.4975** | **992.5039** | **-0.0065** | **0** | **40** | **0.017** | **1** | **K.SIGVSNFNR.R** |
|  | 7208 | **579.7769** | **1157.5393** | **1157.5717** | **-0.0324** | **1** | **15** | **5.5** | **1** | **K.REDIFYTSK.L** |
|  | 16320 | **569.3004** | **1704.8794** | **1705.9363** | **-1.0568** | **1** | **1** | **90** | **8** | **K.SKDIVLVAYSALGSQR.D** |
|  | 21331 | **704.3194** | **2109.9364** | **2109.9513** | **-0.0149** | **0** | **10** | **7.7** | **1** | **K.YKPVCNQVECHPYFNR.S** |
|  | 22024 | **727.0292** | **2178.0659** | **2178.0817** | **-0.0158** | **0** | **76** | **2.4e-006** | **1** | **R.HIDSAHLYNNEEQVGLAIR.S** |
|  | 22529 | 1118.5823 | 2235.1500 | 2235.1609 | -0.0109 | 0 | 90 | 7.7e-008 | 1 | R.WVDPNSPVLLEDPVLCALAK.K 22528 |
|  | 23833 | 805.3994 | 2413.1764 | 2413.1889 | -0.0125 | 0 | 7 | 14 | 2 | K.LNDGHFMPVLGFGTYAPPEVPR.S |
|  | 25578 | **1334.0501** | **2666.0855** | **2666.0986** | **-0.0130** | **0** | **53** | **0.00028** | **1** | **R.NLHYFNSDSFASHPNYPYSDEY.-** |

| **210.** | P50416\|CPT1A_HUMAN    **Mass:** 88995    **Score:** 229    **Queries matched:** 34   **emPAI:** 0.33 |
| --- | --- |
|  | Carnitine O-palmitoyltransferase 1, liver isoform OS=Homo sapiens GN=CPT1A PE=1 SV=2 |

|  | **Query** | **Observed** | **Mr(expt)** | **Mr(calc)** | **Delta** | **Miss** | **Score** | **Expect** | **Rank** | **Peptide** |
| --- | --- | --- | --- | --- | --- | --- | --- | --- | --- | --- |
|  | 1316 | **605.0897** | **604.0824** | **603.2976** | **0.7847** | **0** | **2** | **1.3e+002** | **5** | **K.DTVNR.Y** |
|  | 1793 | **724.4501** | **723.4428** | **722.4691** | **0.9737** | **0** | **12** | **9** | **7** | **R.LPVPAVK.D** |
|  | 2499 | **825.4518** | **824.4445** | **824.4504** | **-0.0059** | **0** | **5** | **48** | **6** | **R.LSHEALR.Q** |
|  | 2895 | **425.7420** | **849.4695** | **849.4749** | **-0.0053** | **0** | **21** | **1.3** | **3** | **R.LQWYLK.L** |
|  | 3523 | **444.2684** | **886.5222** | **886.3868** | **0.1355** | **0** | **3** | **1.1e+002** | **10** | **K.HQHMYR.L** |
|  | 4309 | **472.2871** | **942.5595** | **943.4810** | **-0.9215** | **1** | **5** | **55** | **7** | **R.VPWARCR.Q** |
|  | 4740 | **487.7682** | **973.5219** | **973.5273** | **-0.0054** | **0** | **36** | **0.044** | **1** | **K.SFTFVVFK.N** |
|  | 5445 | **513.8099** | **1025.6052** | **1025.6121** | **-0.0069** | **0** | **17** | **2.8** | **1** | **K.IDPSLGIIAK.I** 5424 5444 |
|  | 5586 | **518.7662** | **1035.5178** | **1035.5019** | **0.0159** | **0** | **2** | **1.1e+002** | **8** | **R.LAMTGSGIDR.H** |
|  | 5690 | **523.2607** | **1044.5069** | **1044.5141** | **-0.0072** | **0** | **17** | **3.4** | **1** | **K.VWLYHDGR.L** |
|  | 7307 | **583.8230** | **1165.6314** | **1165.6383** | **-0.0068** | **0** | **4** | **60** | **6** | **K.YLAVESPFLK.E** |
|  | 9994 | **437.5650** | **1309.6732** | **1310.7459** | **-1.0727** | **0** | **28** | **0.23** | **1** | **R.AGNAIHAILLYR.R** |
|  | 10642 | **672.8278** | **1343.6410** | **1343.6503** | **-0.0094** | **0** | **65** | **4.2e-005** | **1** | **R.AMVDPAQTVEQR.L** 10643 |
|  | 10837 | **678.8446** | **1355.6746** | **1355.6834** | **-0.0087** | **0** | **10** | **14** | **1** | **K.GDINPNIPYPTR.L** |
|  | 11209 | **689.4226** | **1376.8305** | **1377.6057** | **-0.7752** | **0** | **7** | **29** | **6** | **K.FCLTYEASMTR.L** |
|  | 11752 | **470.3055** | **1407.8947** | **1408.8402** | **-0.9455** | **1** | **9** | **14** | **2** | **K.IDPSLGIIAKINR.T** |
|  | 12067 | **475.9369** | **1424.7889** | **1423.7468** | **1.0421** | **1** | **7** | **28** | **8** | **K.IWMGMVKIFSGR.K** 12039 |
|  | 12369 | **482.2760** | **1443.8062** | **1444.5664** | **-0.7602** | **0** | **8** | **23** | **2** | **R.SEDPDTSMDSYAK.S** |
|  | 12778 | **735.8274** | **1469.6402** | **1469.6490** | **-0.0088** | **0** | **72** | **8.1e-006** | **1** | **R.TLETANCMSSQTK.N** |
|  | 13635 | **763.8700** | **1525.7254** | **1525.7372** | **-0.0118** | **0** | **77** | **2.6e-006** | **1** | **R.ILDNTSEPQPGEAR.L** 13636 |
|  | 13859 | **514.7595** | **1541.2567** | **1541.7144** | **-0.4577** | **0** | **6** | **22** | **2** | **R.IPGEETDTIQHMR.D** |
|  | 16649 | **865.9433** | **1729.8720** | **1729.8821** | **-0.0101** | **0** | **21** | **0.84** | **1** | **R.LLGSTIPLCSAQWER.M** 16650 |
|  | 17832 | 909.9588 | 1817.9030 | 1818.9774 | -1.0744 | 1 | 8 | 17 | 2 | K.DTVNRYLQSVRPLMK.E 17831 |
|  | 17957 | **913.9592** | **1825.9038** | **1825.9131** | **-0.0094** | **0** | **12** | **5.9** | **1** | **K.EAMTDIITLFGLSSNSK.K** |
|  | 22610 | **750.4158** | **2248.2255** | **2247.2450** | **0.9805** | **0** | **0** | **72** | **6** | **K.NVVSGVLFGTGLWVALIVTMR.Y** |
|  | 28106 | **1058.7898** | **3173.3475** | **3173.3659** | **-0.0184** | **1** | **53** | **0.00018** | **1** | **K.AAFFVTLDETEEGYRSEDPDTSMDSYAK.S** |
|  | 30460 | **816.4123** | **4077.0254** | **4077.1934** | **-0.1681** | **1** | **3** | **14** | **3** | **K.NGIITGVYPASPSSWLIVVVGVMTTMYAKIDPSLGIIAK.I** |

| **211.** | O75874\|IDHC_HUMAN    **Mass:** 46915    **Score:** 229    **Queries matched:** 31   **emPAI:** 1.15 |
| --- | --- |
|  | Isocitrate dehydrogenase [NADP] cytoplasmic OS=Homo sapiens GN=IDH1 PE=1 SV=2 |

|  | **Query** | **Observed** | **Mr(expt)** | **Mr(calc)** | **Delta** | **Miss** | **Score** | **Expect** | **Rank** | **Peptide** |
| --- | --- | --- | --- | --- | --- | --- | --- | --- | --- | --- |
|  | 1979 | **783.4418** | **782.4345** | **782.4399** | **-0.0054** | **0** | **13** | **7.9** | **1** | **K.GLPNVQR.S** |
|  | 3763 | **452.7106** | **903.4066** | **902.4399** | **0.9668** | **0** | **23** | **0.89** | **1** | **K.IWYEHR.L** |
|  | 3913 | **458.2449** | **914.4752** | **913.5637** | **0.9116** | **0** | **11** | **16** | **1** | **R.IIWELIK.E** |
|  | 4779 | **488.7791** | **975.5436** | **975.5502** | **-0.0066** | **0** | **44** | **0.0082** | **1** | **R.NILGGTVFR.E** 4777 4778 |
|  | 10437 | **667.3217** | **1332.6289** | **1332.6608** | **-0.0320** | **1** | **11** | **10** | **2** | **K.QMWKSPNGTIR.N** 10447 |
|  | 10584 | **671.3367** | **1340.6589** | **1340.6684** | **-0.0095** | **0** | **31** | **0.1** | **1** | **K.TVEAEAAHGTVTR.H** |
|  | 12274 | **479.9667** | **1436.8782** | **1436.8867** | **-0.0086** | **0** | **46** | **0.0032** | **1** | **R.LVSGWVKPIIIGR.H** |
|  | 12292 | 720.3248 | 1438.6350 | 1437.6987 | 0.9362 | 0 | 23 | 0.57 | 1 | K.VEITYTPSDGTQK.V |
|  | 12385 | **723.3643** | **1444.7140** | **1444.7238** | **-0.0098** | **1** | **31** | **0.099** | **1** | **R.FKDIFQEIYDK.Q** |
|  | 13396 | **755.3278** | **1508.6411** | **1508.6493** | **-0.0083** | **0** | **54** | **0.00044** | **1** | **R.SDYLNTFEFMDK.L** 13383 13384 13386 13413 |
|  | 13619 | **509.2353** | **1524.6840** | **1524.6443** | **0.0397** | **0** | **(1)** | **1e+002** | **8** | **R.SDYLNTFEFMDK.L** |
|  | 16158 | **848.3822** | **1694.7498** | **1694.7604** | **-0.0105** | **0** | **20** | **1.1** | **1** | **K.ISGGSVVEMQGDEMTR.I** 16150 |
|  | 17563 | **899.4219** | **1796.8293** | **1796.8403** | **-0.0110** | **0** | **46** | **0.0026** | **1** | **K.SIEDFAHSSFQMALSK.G** 17565 17579 |
|  | 17919 | **608.9559** | **1823.8458** | **1822.8553** | **0.9905** | **1** | **(1)** | **83** | **7** | **K.KISGGSVVEMQGDEMTR.I** |
|  | 18167 | 920.5245 | 1839.0345 | 1838.8503 | 0.1843 | 1 | 1 | 76 | 5 | K.KISGGSVVEMQGDEMTR.I |
|  | 18707 | **939.9654** | **1877.9162** | **1877.9272** | **-0.0109** | **0** | **73** | **4.8e-006** | **1** | **K.GQETSTNPIASIFAWTR.G** 18708 |
|  | 23790 | **1203.6150** | **2405.2154** | **2405.2267** | **-0.0113** | **0** | **23** | **0.39** | **1** | **K.LIFPYVELDLHSYDLGIENR.D** 23789 |
|  | 25004 | **853.7203** | **2558.1390** | **2558.1570** | **-0.0180** | **0** | **26** | **0.15** | **1** | **K.VTYLVHNFEEGGGVAMGMYNQDK.S** 25005 |

| **212.** | O95154\|ARK73_HUMAN    **Mass:** 37582    **Score:** 229    **Queries matched:** 22   **emPAI:** 0.61 |
| --- | --- |
|  | Aflatoxin B1 aldehyde reductase member 3 OS=Homo sapiens GN=AKR7A3 PE=1 SV=2 |

|  | **Query** | **Observed** | **Mr(expt)** | **Mr(calc)** | **Delta** | **Miss** | **Score** | **Expect** | **Rank** | **Peptide** |
| --- | --- | --- | --- | --- | --- | --- | --- | --- | --- | --- |
|  | 1460 | **636.3673** | **635.3600** | **634.3438** | **1.0161** | **0** | **19** | **2.2** | **1** | **R.AFLER.G** |
|  | 3361 | 439.2453 | 876.4760 | 876.4599 | 0.0161 | 1 | 13 | 8.8 | 8 | -.MSRQLSR.A 3371 3373 |
|  | 9382 | **638.8245** | **1275.6344** | **1274.6401** | **0.9943** | **1** | **43** | **0.007** | **1** | **R.RMDAPTSAAVTR.A** 9345 |
|  | 9656 | **646.3151** | **1290.6156** | **1290.6350** | **-0.0195** | **1** | **(3)** | **71** | **7** | **R.RMDAPTSAAVTR.A** |
|  | 11992 | **711.3149** | **1420.6152** | **1420.6234** | **-0.0082** | **0** | **36** | **0.03** | **2** | **R.FFGNTWAEMYR.N** 12006 12007 12008 |
|  | 12608 | **487.2953** | **1458.8641** | **1458.7435** | **0.1206** | **0** | **2** | **86** | **10** | **R.ARPATVLGAMEMGR.R** |
|  | 14277 | **784.9228** | **1567.8310** | **1567.8399** | **-0.0088** | **0** | **53** | **0.00064** | **1** | **R.FYAFNPLAGGLLTGK.Y** 14278 |
|  | 16697 | **867.9437** | **1733.8728** | **1734.8723** | **-0.9995** | **1** | **(3)** | **53** | **6** | **R.MDAPTSAAVTRAFLER.G** 16718 |
|  | 16919 | **875.9356** | **1749.8565** | **1750.8672** | **-1.0107** | **1** | **7** | **21** | **3** | **R.MDAPTSAAVTRAFLER.G** |
|  | 18560 | **933.9662** | **1865.9179** | **1865.9305** | **-0.0126** | **0** | **84** | **3.9e-007** | **1** | **K.ALQAAYGASAPSMTSATLR.W** 18559 |
|  | 18761 | **628.6353** | **1882.8841** | **1881.9254** | **0.9587** | **0** | **(3)** | **53** | **6** | **K.ALQAAYGASAPSMTSATLR.W** |
|  | 22187 | **1100.4429** | **2198.8712** | **2199.0783** | **-0.2071** | **0** | **0** | **70** | **1** | **K.SNGWILPTVYQGMYNAITR.Q** |
|  | 23748 | 800.3898 | 2398.1477 | 2398.1627 | -0.0151 | 0 | 33 | 0.036 | 1 | R.VDLFYLHMPDHSTPVEETLR.A |

| **213.** | O75306\|NDUS2_HUMAN    **Mass:** 52911    **Score:** 228    **Queries matched:** 20   **emPAI:** 0.31 |
| --- | --- |
|  | NADH dehydrogenase [ubiquinone] iron-sulfur protein 2, mitochondrial OS=Homo sapiens GN=NDUFS2 PE=1 SV=2 |

|  | **Query** | **Observed** | **Mr(expt)** | **Mr(calc)** | **Delta** | **Miss** | **Score** | **Expect** | **Rank** | **Peptide** |
| --- | --- | --- | --- | --- | --- | --- | --- | --- | --- | --- |
|  | 4540 | **480.2672** | **958.5199** | **958.5270** | **-0.0070** | **0** | **31** | **0.15** | **1** | **R.IIAQCLNK.M** |
|  | 6117 | **537.9966** | **1073.9786** | **1074.6662** | **-0.6876** | **0** | **1** | **90** | **6** | **K.LLNIRPPPR.A** |
|  | 8225 | **406.2100** | **1215.6081** | **1216.6135** | **-1.0055** | **0** | **6** | **41** | **9** | **K.CDPHIGLLHR.G** 8259 |
|  | 11205 | 689.3583 | 1376.7020 | 1376.5602 | 0.1419 | 1 | 9 | 17 | 3 | R.GDCYDRYLCR.V |
|  | 11746 | **470.2425** | **1407.7056** | **1406.7261** | **0.9794** | **1** | **8** | **18** | **2** | **R.LVMELSGEMVRK.C** 11719 |
|  | 12280 | **719.8808** | **1437.7470** | **1436.8140** | **0.9331** | **1** | **1** | **99** | **2** | **K.IKAPGFAHLAGLDK.M** |
|  | 12580 | **729.8709** | **1457.7273** | **1457.7361** | **-0.0089** | **0** | **59** | **0.00016** | **1** | **R.LDELEELLTNNR.I** 12581 12599 |
|  | 19491 | **648.0144** | **1941.0214** | **1941.0333** | **-0.0119** | **0** | **17** | **2** | **1** | **K.NITLNFGPQHPAAHGVLR.L** |
|  | 22317 | **1107.5133** | **2213.0121** | **2213.0277** | **-0.0156** | **0** | **69** | **1.1e-005** | **1** | **K.TQPYDVYDQVEFDVPVGSR.G** 22316 22318 |
|  | 24166 | **1226.5671** | **2451.1197** | **2451.1318** | **-0.0120** | **0** | **83** | **3.2e-007** | **1** | **R.QWQPDVEWAQQFGGAVMYPSK.E** |
|  | 24298 | **823.3236** | **2466.9490** | **2467.1267** | **-0.1777** | **0** | **(3)** | **30** | **6** | **R.QWQPDVEWAQQFGGAVMYPSK.E** |
|  | 24549 | 833.4263 | 2497.2570 | 2497.3554 | -0.0984 | 1 | 7 | 15 | 1 | K.DTIVKNITLNFGPQHPAAHGVLR.L |
|  | 28099 | **793.8944** | **3171.5483** | **3171.5634** | **-0.0151** | **0** | **12** | **3.2** | **1** | **R.MHAAYIRPGGVHQDLPLGLMDDIYQFSK.N** 28098 |

| **214.** | P28288\|ABCD3_HUMAN    **Mass:** 75941    **Score:** 226    **Queries matched:** 20   **emPAI:** 0.21 |
| --- | --- |
|  | ATP-binding cassette sub-family D member 3 OS=Homo sapiens GN=ABCD3 PE=1 SV=1 |

|  | **Query** | **Observed** | **Mr(expt)** | **Mr(calc)** | **Delta** | **Miss** | **Score** | **Expect** | **Rank** | **Peptide** |
| --- | --- | --- | --- | --- | --- | --- | --- | --- | --- | --- |
|  | 1376 | 616.4244 | 615.4171 | 614.3574 | 1.0597 | 0 | 8 | 50 | 7 | K.IMVPR.T |
|  | 2437 | **410.5695** | **819.1245** | **818.3705** | **0.7540** | **0** | **8** | **20** | **1** | **K.MGNLDNR.I** |
|  | 2666 | 835.4984 | 834.4911 | 834.3654 | 0.1257 | 0 | (5) | 54 | 4 | K.MGNLDNR.I |
|  | 3580 | **446.7269** | **891.4392** | **891.4450** | **-0.0058** | **0** | **21** | **1.5** | **1** | **R.DLNFEVR.S** |
|  | 6114 | 537.8119 | 1073.6092 | 1073.6154 | -0.0062 | 0 | 24 | 0.75 | 1 | R.ITELMQVLK.D |
|  | 10631 | **672.3735** | **1342.7325** | **1342.7398** | **-0.0072** | **0** | **4** | **48** | **1** | **R.VLGELWPLFGGR.L** |
|  | 11857 | **707.6946** | **1413.3746** | **1413.6558** | **-0.2812** | **1** | **6** | **23** | **2** | **K.YERTMVSQQEK.G** |
|  | 14161 | 781.3769 | 1560.7392 | 1561.7372 | -0.9980 | 1 | 2 | 71 | 10 | R.DQVIYPDGREDQK.R |
|  | 16212 | **849.9387** | **1697.8628** | **1697.8737** | **-0.0109** | **0** | **71** | **9.2e-006** | **1** | **K.EYLDNVQLGHILER.E** |
|  | 16998 | **585.9802** | **1754.9188** | **1753.9396** | **0.9792** | **1** | **8** | **19** | **2** | **R.ITELMQVLKDLNHGK.Y** |
|  | 17107 | 882.3995 | 1762.7845 | 1762.9366 | -0.1521 | 0 | 2 | 72 | 6 | K.FDHVPLATPNGDVLIR.D 17108 |
|  | 17989 | 610.1827 | 1827.5262 | 1827.0076 | 0.5186 | 0 | 3 | 35 | 1 | R.NSSLAGAAFLLLCLLHK.R |
|  | 21136 | **1047.9520** | **2093.8895** | **2093.9001** | **-0.0105** | **0** | **95** | **2.4e-008** | **1** | **R.EGGWDSVQDWMDVLSGGEK.Q** |
|  | 23147 | **1157.8182** | **2313.6219** | **2313.1059** | **0.5160** | **1** | **2** | **34** | **4** | **K.STHSELLEDYYQSGRMLLR.M** |
|  | 23960 | **809.4467** | **2425.3182** | **2425.1770** | **0.1412** | **0** | **1** | **52** | **1** | **R.TYCDVWMIQNGTLIESGIIGR.S** |
|  | 24160 | **817.4722** | **2449.3947** | **2449.3919** | **0.0028** | **1** | **0** | **58** | **3** | **K.RYLLNFIAAMPLISLVNNFLK.Y** |
|  | 24816 | **1271.7421** | **2541.4696** | **2541.4781** | **-0.0086** | **0** | **53** | **0.00032** | **1** | **K.GIEGVQVIPLIPGAGEIIIADNIIK.F** 24814 24815 |

| **215.** | P40926\|MDHM_HUMAN    **Mass:** 35937    **Score:** 225    **Queries matched:** 26   **emPAI:** 0.64 |
| --- | --- |
|  | Malate dehydrogenase, mitochondrial OS=Homo sapiens GN=MDH2 PE=1 SV=3 |

|  | **Query** | **Observed** | **Mr(expt)** | **Mr(calc)** | **Delta** | **Miss** | **Score** | **Expect** | **Rank** | **Peptide** |
| --- | --- | --- | --- | --- | --- | --- | --- | --- | --- | --- |
|  | 2473 | 412.2588 | 822.5030 | 821.4283 | 1.0747 | 1 | 6 | 35 | 10 | K.KGEDFVK.T |
|  | 4084 | **464.7321** | **927.4497** | **927.4563** | **-0.0066** | **0** | **29** | **0.21** | **1** | **K.HGVYNPNK.I** 4085 4087 4088 4098 4099 4115 |
|  | 4983 | 496.7709 | 991.5272 | 991.5338 | -0.0066 | 0 | 20 | 1.9 | 2 | R.ANTFVAELK.G |
|  | 6095 | **537.2917** | **1072.5689** | **1072.5764** | **-0.0075** | **0** | **17** | **3.8** | **1** | **R.IQEAGTEVVK.A** |
|  | 9760 | **648.8334** | **1295.6522** | **1295.7673** | **-0.1151** | **0** | **6** | **36** | **10** | **M.LSALARPASAALR.R** |
|  | 10341 | **664.3357** | **1326.6568** | **1326.6642** | **-0.0074** | **0** | **36** | **0.031** | **1** | **R.FVFSLVDAMNGK.E** |
|  | 10524 | **669.5179** | **1337.0213** | **1337.7126** | **-0.6913** | **0** | **15** | **3.1** | **1** | **K.GCDVVVIPAGVPR.K** |
|  | 12100 | 714.3648 | 1426.7151 | 1426.8078 | -0.0927 | 0 | 4 | 53 | 4 | -.MLSALARPASAALR.R |
|  | 12778 | 735.8274 | 1469.6402 | 1469.6933 | -0.0530 | 0 | 6 | 36 | 4 | K.AGAGSATLSMAYAGAR.F |
|  | 13077 | 745.3664 | 1488.7182 | 1488.7283 | -0.0100 | 0 | 41 | 0.01 | 1 | K.GYLGPEQLPDCLK.G |
|  | 14150 | **780.8995** | **1559.7844** | **1559.7944** | **-0.0100** | **0** | **3** | **55** | **6** | **K.VDFPQDQLTALTGR.I** |
|  | 17521 | **897.0436** | **1792.0726** | **1792.0822** | **-0.0096** | **0** | **71** | **8.5e-006** | **1** | **K.VAVLGASGGIGQPLSLLLK.N** 17523 17528 |
|  | 19851 | **987.4746** | **1972.9347** | **1972.9452** | **-0.0105** | **0** | **81** | **7.8e-007** | **1** | **K.SQETECTYFSTPLLLGK.K** 19862 |
|  | 23549 | **1183.6277** | **2365.2408** | **2364.2325** | **1.0083** | **0** | **42** | **0.0045** | **1** | **R.LTLYDIAHTPGVAADLSHIETK.A** 23540 |
|  | 24827 | **849.4072** | **2545.1997** | **2545.4955** | **-0.2959** | **1** | **0** | **66** | **4** | **K.VAVLGASGGIGQPLSLLLKNSPLVSR.L** |
|  | 31485 | **1283.3839** | **5129.5065** | **5129.5334** | **-0.0269** | **1** | **9** | **1.7** | **1** | **R.DDLFNTNATIVATLTAACAQHCPEAMICVIANPVNSTIPITAEVFKK.H** |

| **216.** | P04843\|RPN1_HUMAN    **Mass:** 68641    **Score:** 224    **Queries matched:** 37   **emPAI:** 0.45 |
| --- | --- |
|  | Dolichyl-diphosphooligosaccharide--protein glycosyltransferase subunit 1 OS=Homo sapiens GN=RPN1 PE=1 SV=1 |

|  | **Query** | **Observed** | **Mr(expt)** | **Mr(calc)** | **Delta** | **Miss** | **Score** | **Expect** | **Rank** | **Peptide** |
| --- | --- | --- | --- | --- | --- | --- | --- | --- | --- | --- |
|  | 895 | 518.2900 | 517.2828 | 516.3271 | 0.9556 | 0 | 15 | 12 | 1 | K.LISGK.R |
|  | 1608 | 673.3830 | 672.3757 | 672.3806 | -0.0049 | 0 | 23 | 1.3 | 9 | K.LDAQVK.E |
|  | 1770 | 716.4085 | 715.4012 | 716.4068 | -1.0056 | 0 | 19 | 3.2 | 3 | R.QELVTK.I |
|  | 1808 | **730.4049** | **729.3976** | **728.3453** | **1.0523** | **0** | **13** | **12** | **4** | **K.DPAAEAR.M** |
|  | 2478 | 412.7267 | 823.4389 | 822.4487 | 0.9902 | 0 | 13 | 7 | 2 | R.LDFSITK.D 2477 |
|  | 2734 | 840.4036 | 839.3963 | 839.4025 | -0.0062 | 0 | 16 | 4.1 | 2 | R.NVESYTK.L 2758 |
|  | 2739 | **421.0011** | **839.9876** | **839.5116** | **0.4760** | **0** | **10** | **9.3** | **7** | **K.IILPEGAK.N** |
|  | 3841 | **455.2531** | **908.4916** | **908.4967** | **-0.0051** | **0** | **18** | **2.5** | **1** | **K.IDHILDAL.-** |
|  | 5165 | **502.3393** | **1002.6640** | **1003.4756** | **-0.8117** | **1** | **(7)** | **33** | **3** | **K.DPAAEARMK.V** 5166 |
|  | 5322 | **509.2376** | **1016.4607** | **1016.4676** | **-0.0068** | **0** | **24** | **0.75** | **1** | **R.HFDETVNR.Y** 5317 5321 5323 |
|  | 5933 | **531.7892** | **1061.5638** | **1061.5717** | **-0.0079** | **1** | **21** | **1.4** | **1** | **R.DISTLNSGKK.S** 5934 |
|  | 9236 | **635.3233** | **1268.6320** | **1268.6990** | **-0.0669** | **1** | **1** | **1.2e+002** | **10** | **K.HTGAVLKGPFSR.Y** |
|  | 9258 | **635.7968** | **1269.5791** | **1269.5877** | **-0.0087** | **0** | **7** | **28** | **4** | **R.DVPAYSQDTFK.V** |
|  | 9695 | **647.3043** | **1292.5941** | **1292.6031** | **-0.0090** | **1** | **34** | **0.061** | **1** | **R.LKTEGSDLCDR.V** 9694 9696 |
|  | 10017 | **656.3105** | **1310.6064** | **1310.6143** | **-0.0078** | **0** | **23** | **0.65** | **1** | **R.SEDLLDYGPFR.D** 10031 |
|  | 10618 | **672.3062** | **1342.5979** | **1342.7456** | **-0.1477** | **1** | **5** | **44** | **1** | **K.ELVLKSAVEAER.L** |
|  | 11437 | 464.2476 | 1389.7209 | 1390.7126 | -0.9917 | 1 | 7 | 27 | 3 | R.VSEMQKLDAQVK.E |
|  | 12258 | **718.8458** | **1435.6771** | **1434.6990** | **0.9781** | **0** | **14** | **5.3** | **1** | **K.NIEIDSPYEISR.A** |
|  | 15585 | **827.4440** | **1652.8735** | **1652.8846** | **-0.0111** | **0** | **54** | **0.00048** | **1** | **K.VTAEVVLAHLGGGSTSR.A** |
|  | 20013 | **996.0032** | **1989.9919** | **1990.0048** | **-0.0129** | **0** | **81** | **6.6e-007** | **1** | **R.FVDHVFDEQVIDSLTVK.I** 20015 |
|  | 22416 | **741.6855** | **2222.0346** | **2222.0473** | **-0.0127** | **0** | **18** | **1.3** | **1** | **K.QFVVFEGNHYFYSPYPTK.T** |
|  | 26732 | **721.3836** | **2881.5053** | **2881.5226** | **-0.0172** | **0** | **36** | **0.014** | **1** | **K.ISVIVETVYTHVLHPYPTQITQSEK.Q** 26733 |
|  | 27302 | 999.8187 | 2996.4344 | 2996.4596 | -0.0252 | 0 | 43 | 0.0028 | 1 | K.THYIVGYNLPSYEYLYNLGDQYALK.M 27303 27304 |

| **217.** | Q96I99\|SUCB2_HUMAN    **Mass:** 46824    **Score:** 224    **Queries matched:** 37   **emPAI:** 0.47 |
| --- | --- |
|  | Succinyl-CoA ligase [GDP-forming] subunit beta, mitochondrial OS=Homo sapiens GN=SUCLG2 PE=1 SV=2 |

|  | **Query** | **Observed** | **Mr(expt)** | **Mr(calc)** | **Delta** | **Miss** | **Score** | **Expect** | **Rank** | **Peptide** |
| --- | --- | --- | --- | --- | --- | --- | --- | --- | --- | --- |
|  | 1493 | **645.3884** | **644.3811** | **644.3857** | **-0.0046** | **0** | **14** | **12** | **4** | **K.AVASVAK.K** |
|  | 1900 | **758.4464** | **757.4391** | **756.4381** | **1.0010** | **0** | **16** | **5.7** | **8** | **K.LLTADPK.V** |
|  | 1982 | **785.4576** | **784.4504** | **784.4555** | **-0.0052** | **0** | **23** | **0.93** | **1** | **K.AQILAGGR.G** |
|  | 3576 | 446.2691 | 890.5236 | 890.4280 | 0.0957 | 0 | 7 | 40 | 10 | K.LMSDNGVR.V |
|  | 4566 | **481.2473** | **960.4799** | **960.4876** | **-0.0077** | **0** | **35** | **0.06** | **1** | **K.SQAADQITK.L** |
|  | 4700 | **486.3614** | **970.7083** | **969.5719** | **1.1363** | **1** | **7** | **25** | **4** | **K.AQILAGGRGK.G** |
|  | 5346 | 509.7679 | 1017.5212 | 1018.5229 | -1.0017 | 1 | 6 | 47 | 8 | K.KLMSDNGVR.V |
|  | 5725 | **524.3271** | **1046.6396** | **1045.5226** | **1.1170** | **0** | **5** | **57** | **7** | **-.MASPVAAQAGK.L** 5710 |
|  | 7480 | 589.8628 | 1177.7110 | 1177.7771 | -0.0661 | 1 | 11 | 12 | 3 | K.LLRALALRPR.F |
|  | 7725 | **596.8142** | **1191.6139** | **1190.6183** | **0.9956** | **0** | **12** | **9** | **1** | **K.EQIDIFEGIK.D** |
|  | 8222 | **608.8078** | **1215.6010** | **1215.6095** | **-0.0085** | **0** | **51** | **0.0011** | **1** | **R.LEGTNVQEAQK.I** 8190 |
|  | 8387 | **612.8151** | **1223.6156** | **1223.6220** | **-0.0064** | **0** | **2** | **90** | **4** | **R.ETYLAILMDR.S** |
|  | 8435 | **614.2901** | **1226.5656** | **1225.5649** | **1.0008** | **1** | **5** | **44** | **1** | **R.QKDIFAMDDK.S** 8397 |
|  | 8691 | 620.7792 | 1239.5439 | 1239.5520 | -0.0081 | 0 | 11 | 9.8 | 1 | K.INFDDNAEFR.Q |
|  | 9627 | **645.8129** | **1289.6112** | **1289.6510** | **-0.0398** | **1** | **5** | **50** | **6** | **K.LMSDNGVRVQR.F** |
|  | 9669 | **431.2580** | **1290.7521** | **1290.6642** | **0.0879** | **0** | **8** | **20** | **3** | **R.MAENLGFVGPLK.S** 9662 9668 |
|  | 9737 | **648.3306** | **1294.6467** | **1293.8020** | **0.8447** | **1** | **5** | **39** | **2** | **R.ELELKVPLVVR.L** |
|  | 10392 | **444.4888** | **1330.4446** | **1329.6048** | **0.8398** | **0** | **2** | **73** | **5** | **K.SENEPIENEAAK.Y** |
|  | 12701 | **489.2863** | **1464.8370** | **1463.7521** | **1.0850** | **1** | **6** | **32** | **1** | **R.RWLNLQEYQSK.K** |
|  | 13212 | **748.8831** | **1495.7517** | **1495.7055** | **0.0462** | **1** | **2** | **83** | **10** | **K.INFDDNAEFRQK.D** |
|  | 14636 | 796.8968 | 1591.7791 | 1591.8392 | -0.0600 | 1 | 16 | 2.7 | 2 | K.QMIGYNLATKQTPK.E 14635 |
|  | 14671 | **798.8932** | **1595.7719** | **1595.7831** | **-0.0112** | **0** | **19** | **1.4** | **1** | **R.FFVADTANEALEAAK.R** |
|  | 17872 | **607.9765** | **1820.9077** | **1820.9672** | **-0.0596** | **1** | **1** | **98** | **6** | **K.EAQVYQAFKLLTADPK.V** |
|  | 20737 | **1028.0458** | **2054.0770** | **2054.0895** | **-0.0125** | **0** | **58** | **0.00014** | **1** | **K.ILNNSGLPITSAIDLEDAAK.K** 20738 |
|  | 22440 | **742.7082** | **2225.1027** | **2225.2756** | **-0.1729** | **1** | **2** | **47** | **10** | **R.ALALRPRFLAAGSQAVQLTSR.R** |
|  | 22763 | **756.3398** | **2265.9975** | **2265.0107** | **0.9869** | **1** | **12** | **4.6** | **1** | **K.DIFAMDDKSENEPIENEAAK.Y** |
|  | 24553 | 833.7944 | 2498.3613 | 2498.3931 | -0.0318 | 0 | 2 | 41 | 8 | K.VEAILVNIFGGIVNCAIIANGITK.A |
|  | 26120 | **1375.6437** | **2749.2728** | **2749.2905** | **-0.0177** | **0** | **83** | **3.3e-007** | **1** | **K.IDATQVEVNPFGETPEGQVVCFDAK.I** 26119 26121 |

| **218.** | P22626\|ROA2_HUMAN    **Mass:** 37464    **Score:** 223    **Queries matched:** 18   **emPAI:** 0.46 |
| --- | --- |
|  | Heterogeneous nuclear ribonucleoproteins A2/B1 OS=Homo sapiens GN=HNRNPA2B1 PE=1 SV=2 |

|  | **Query** | **Observed** | **Mr(expt)** | **Mr(calc)** | **Delta** | **Miss** | **Score** | **Expect** | **Rank** | **Peptide** |
| --- | --- | --- | --- | --- | --- | --- | --- | --- | --- | --- |
|  | 3080 | **431.7356** | **861.4567** | **860.5484** | **0.9083** | **1** | **12** | **14** | **4** | **K.KLFVGGIK.E** |
|  | 4995 | **497.2434** | **992.4723** | **992.4783** | **-0.0060** | **0** | **13** | **8.9** | **1** | **K.LTDCVVMR.D** |
|  | 5847 | **529.2944** | **1056.5743** | **1056.5815** | **-0.0072** | **0** | **25** | **0.57** | **1** | **K.TLETVPLER.K** |
|  | 6280 | 544.2419 | 1086.4692 | 1086.4770 | -0.0078 | 0 | 7 | 32 | 4 | R.NYYEQWGK.L |
|  | 7640 | **594.8233** | **1187.6320** | **1187.6398** | **-0.0077** | **0** | **54** | **0.00067** | **1** | **K.IDTIEIITDR.Q** 7641 |
|  | 8646 | **619.6621** | **1237.3097** | **1236.5405** | **0.7692** | **0** | **4** | **35** | **3** | **R.QEMQEVQSSR.S** |
|  | 11766 | **705.3150** | **1408.6154** | **1409.6800** | **-1.0645** | **0** | **8** | **19** | **5** | **K.YHTINGHNAEVR.K** 11811 |
|  | 12656 | **488.2821** | **1461.8245** | **1460.7545** | **1.0700** | **1** | **5** | **37** | **2** | **-.MEKTLETVPLER.K** |
|  | 16156 | **848.3808** | **1694.7470** | **1694.7577** | **-0.0107** | **0** | **66** | **2.6e-005** | **1** | **R.GFGFVTFDDHDPVDK.I** |
|  | 17593 | **900.4625** | **1798.9105** | **1797.9149** | **0.9956** | **0** | **85** | **3.2e-007** | **1** | **K.LFIGGLSFETTEESLR.N** 17580 |
|  | 18732 | **627.6572** | **1879.9497** | **1878.9588** | **0.9909** | **1** | **7** | **20** | **5** | **K.LFVGGIKEDTEEHHLR.D** 18725 |
|  | 19318 | **964.0068** | **1925.9991** | **1926.0098** | **-0.0107** | **1** | **4** | **37** | **1** | **R.KLFIGGLSFETTEESLR.N** |
|  | 22108 | **1095.5176** | **2189.0206** | **2188.8981** | **0.1225** | **0** | **54** | **0.00031** | **1** | **R.NMGGPYGGGNYGPGGSGGSGGYGGR.S** |
|  | 26539 | **711.3430** | **2841.3427** | **2842.3167** | **-0.9740** | **0** | **0** | **57** | **2** | **R.GFGFVTFSSMAEVDAAMAARPHSIDGR.V** |

| **219.** | P08865\|RSSA_HUMAN    **Mass:** 32947    **Score:** 222    **Queries matched:** 13   **emPAI:** 0.24 |
| --- | --- |
|  | 40S ribosomal protein SA OS=Homo sapiens GN=RPSA PE=1 SV=4 |

|  | **Query** | **Observed** | **Mr(expt)** | **Mr(calc)** | **Delta** | **Miss** | **Score** | **Expect** | **Rank** | **Peptide** |
| --- | --- | --- | --- | --- | --- | --- | --- | --- | --- | --- |
|  | 6933 | **568.8369** | **1135.6591** | **1134.6284** | **1.0307** | **0** | **7** | **36** | **4** | **K.SDGIYIINLK.R** |
|  | 8080 | **403.8886** | **1208.6439** | **1207.5941** | **1.0498** | **0** | **4** | **55** | **8** | **-.MSGALDVLQMK.E** 8070 |
|  | 14939 | 538.9284 | 1613.7634 | 1613.9253 | -0.1619 | 1 | 3 | 59 | 7 | R.AVLKFAAATGATPIAGR.F 14934 |
|  | 16208 | **849.9283** | **1697.8420** | **1697.8526** | **-0.0106** | **0** | **75** | **3.9e-006** | **1** | **R.FTPGTFTNQIQAAFR.E** 16209 |
|  | 16456 | **858.4575** | **1714.9005** | **1714.7897** | **0.1108** | **1** | **0** | **1.1e+002** | **9** | **R.DPEEIEKEEQAAAEK.A** |
|  | 16785 | **870.9727** | **1739.9308** | **1739.9417** | **-0.0110** | **0** | **82** | **6.8e-007** | **1** | **R.AIVAIENPADVSVISSR.N** 16786 |
|  | 25296 | **873.0917** | **2616.2532** | **2616.2683** | **-0.0151** | **0** | **24** | **0.26** | **1** | **K.FLAAGTHLGGTNLDFQMEQYIYK.R** |
|  | 26732 | 721.3836 | 2881.5053 | 2882.4828 | -0.9774 | 1 | 9 | 7.4 | 2 | K.FAAATGATPIAGRFTPGTFTNQIQAAFR.E |
|  | 27294 | **999.4911** | **2995.4514** | **2995.4709** | **-0.0195** | **0** | **9** | **6.9** | **1** | **R.ADHQPLTEASYVNLPTIALCNTDSPLR.Y** |

| **220.** | P08758\|ANXA5_HUMAN    **Mass:** 35971    **Score:** 220    **Queries matched:** 9   **emPAI:** 0.35 |
| --- | --- |
|  | Annexin A5 OS=Homo sapiens GN=ANXA5 PE=1 SV=2 |

|  | **Query** | **Observed** | **Mr(expt)** | **Mr(calc)** | **Delta** | **Miss** | **Score** | **Expect** | **Rank** | **Peptide** |
| --- | --- | --- | --- | --- | --- | --- | --- | --- | --- | --- |
|  | 5119 | **500.7922** | **999.5699** | **1000.5917** | **-1.0218** | **0** | **46** | **0.0041** | **1** | **K.VLTEIIASR.T** |
|  | 5285 | **507.7573** | **1013.5001** | **1013.5069** | **-0.0068** | **0** | **6** | **45** | **3** | **R.LYDAYELK.H** |
|  | 6538 | **553.7922** | **1105.5699** | **1105.5768** | **-0.0068** | **0** | **0** | **1.7e+002** | **10** | **R.SEIDLFNIR.K** |
|  | 7018 | **572.8034** | **1143.5923** | **1142.6845** | **0.9077** | **0** | **5** | **48** | **10** | **K.LIVALMKPSR.L** |
|  | 9606 | **645.3274** | **1288.6402** | **1289.6326** | **-0.9923** | **0** | **4** | **52** | **8** | **K.NFATSLYSMIK.G** |
|  | 16309 | **852.9501** | **1703.8856** | **1703.8941** | **-0.0085** | **0** | **88** | **1.7e-007** | **1** | **K.GLGTDEESILTLLTSR.S** 16308 |
|  | 30062 | **1265.9716** | **3794.8929** | **3794.9149** | **-0.0220** | **1** | **65** | **1.1e-005** | **1** | **R.MLVVLLQANRDPDAGIDEAQVEQDAQALFQAGELK.W** 30064 |

| **221.** | P20674\|COX5A_HUMAN    **Mass:** 16923    **Score:** 220    **Queries matched:** 17   **emPAI:** 1.81 |
| --- | --- |
|  | Cytochrome c oxidase subunit 5A, mitochondrial OS=Homo sapiens GN=COX5A PE=1 SV=2 |

|  | **Query** | **Observed** | **Mr(expt)** | **Mr(calc)** | **Delta** | **Miss** | **Score** | **Expect** | **Rank** | **Peptide** |
| --- | --- | --- | --- | --- | --- | --- | --- | --- | --- | --- |
|  | 1953 | **771.4676** | **770.4603** | **770.4650** | **-0.0047** | **0** | **32** | **0.1** | **1** | **K.IIDAALR.A** 1950 |
|  | 4303 | **472.2844** | **942.5543** | **942.5750** | **-0.0206** | **1** | **15** | **5.7** | **3** | **R.ILEVVKDK.A** |
|  | 5389 | 511.7631 | 1021.5117 | 1021.5193 | -0.0076 | 0 | 46 | 0.0041 | 1 | R.LNDFASTVR.I 5376 5395 |
|  | 7189 | **579.2899** | **1156.5652** | **1157.6339** | **-1.0687** | **1** | **4** | **67** | **10** | **K.IIDAALRACR.R** |
|  | 15877 | **838.3895** | **1674.7644** | **1675.8491** | **-1.0847** | **0** | **84** | **5e-007** | **1** | **K.GINTLVTYDMVPEPK.I** 15911 |
|  | 17843 | **607.3104** | **1818.9093** | **1819.9390** | **-1.0297** | **1** | **2** | **77** | **3** | **R.KGINTLVTYDMVPEPK.I** |
|  | 20697 | **1027.0068** | **2051.9991** | **2052.0105** | **-0.0114** | **0** | **48** | **0.0014** | **1** | **R.WVTYFNKPDIDAWELR.K** 20696 |
|  | 22039 | **727.7042** | **2180.0907** | **2180.1055** | **-0.0148** | **1** | **1** | **68** | **5** | **R.WVTYFNKPDIDAWELRK.G** |
|  | 29008 | **1143.6075** | **3427.8008** | **3427.8126** | **-0.0118** | **1** | **37** | **0.0068** | **1** | **K.EIYPYVIQELRPTLNELGISTPEELGLDKV.-** 29004 29005 29006 |

| **222.** | Q16891\|IMMT_HUMAN    **Mass:** 84026    **Score:** 219    **Queries matched:** 33   **emPAI:** 0.41 |
| --- | --- |
|  | Mitochondrial inner membrane protein OS=Homo sapiens GN=IMMT PE=1 SV=1 |

|  | **Query** | **Observed** | **Mr(expt)** | **Mr(calc)** | **Delta** | **Miss** | **Score** | **Expect** | **Rank** | **Peptide** |
| --- | --- | --- | --- | --- | --- | --- | --- | --- | --- | --- |
|  | 1775 | **717.3264** | **716.3191** | **716.4068** | **-0.0877** | **0** | **9** | **30** | **1** | **R.TVEGALK.E** |
|  | 2571 | **829.4725** | **828.4652** | **828.4705** | **-0.0053** | **0** | **21** | **1.5** | **1** | **K.SIQSGPLK.I** 2578 |
|  | 3900 | **457.7592** | **913.5039** | **913.5093** | **-0.0055** | **1** | **18** | **2.9** | **1** | **R.RIDQLNR.E** 3882 |
|  | 5041 | 498.7691 | 995.5236 | 994.5005 | 1.0231 | 0 | 13 | 7.6 | 6 | K.ISSVSEVMK.E |
|  | 5552 | 517.7380 | 1033.4614 | 1034.5430 | -1.0816 | 1 | 10 | 16 | 3 | K.MKSVIENAK.K |
|  | 5793 | **527.2603** | **1052.5061** | **1052.5138** | **-0.0078** | **0** | **7** | **27** | **1** | **R.GVYSEETLR.A** |
|  | 5877 | **530.2909** | **1058.5673** | **1058.5720** | **-0.0046** | **1** | **6** | **52** | **8** | **K.KVQAAQSEAK.V** |
|  | 6410 | 548.7501 | 1095.4856 | 1094.6236 | 0.8619 | 1 | 10 | 15 | 4 | R.FYAVQKLAR.R |
|  | 7141 | **577.3047** | **1152.5948** | **1152.5887** | **0.0061** | **0** | **16** | **4.1** | **1** | **R.ERPPEEVAAR.L** |
|  | 7457 | **589.3287** | **1176.6429** | **1176.6251** | **0.0178** | **1** | **4** | **69** | **10** | **K.FVNQLKGESR.R** |
|  | 8098 | **605.7792** | **1209.5439** | **1209.5513** | **-0.0074** | **0** | **37** | **0.029** | **1** | **K.SEFEQNLSEK.L** 8097 |
|  | 8786 | 623.2988 | 1244.5831 | 1244.5885 | -0.0054 | 0 | 28 | 0.24 | 1 | R.YSTSGSSGLTTGK.I |
|  | 11972 | **474.2625** | **1419.7656** | **1418.6579** | **1.1077** | **1** | **1** | **99** | **5** | **K.WDSHFRESVEK.T** |
|  | 12903 | **739.8964** | **1477.7783** | **1477.7889** | **-0.0106** | **0** | **40** | **0.014** | **1** | **K.EVAGAKPHITAAEGK.L** |
|  | 13400 | **503.9387** | **1508.7942** | **1508.8021** | **-0.0079** | **0** | **25** | **0.4** | **1** | **K.LHNMIVDLDNVVK.K** 13397 |
|  | 13586 | 761.8936 | 1521.7725 | 1521.8667 | -0.0942 | 1 | 5 | 39 | 7 | R.KAHQLWLSVEALK.Y |
|  | 13615 | **509.0098** | **1524.0077** | **1524.7970** | **-0.7894** | **0** | **(4)** | **40** | **2** | **K.LHNMIVDLDNVVK.K** |
|  | 13660 | **764.4122** | **1526.8098** | **1526.8205** | **-0.0107** | **0** | **63** | **6.7e-005** | **1** | **K.VVSQYHELVVQAR.D** 13652 |
|  | 14029 | **518.2646** | **1551.7719** | **1550.7953** | **0.9766** | **1** | **4** | **47** | **3** | **R.AFDSAVAKALEHHR.S** |
|  | 14484 | **792.4153** | **1582.8161** | **1582.8243** | **-0.0081** | **0** | **34** | **0.051** | **1** | **R.ELDSITPEVLPGWK.G** 14469 |
|  | 16755 | **580.3047** | **1737.8922** | **1738.9254** | **-1.0331** | **1** | **3** | **56** | **2** | **K.RELDSITPEVLPGWK.G** 16748 |
|  | 17897 | **912.4415** | **1822.8684** | **1822.8809** | **-0.0125** | **0** | **88** | **1.9e-007** | **1** | **R.GIEQAVQSHAVAEEEAR.K** |
|  | 18479 | **930.0055** | **1857.9965** | **1858.0063** | **-0.0097** | **0** | **58** | **0.00015** | **1** | **K.LFEMVLGPAAYNVPLPK.K** |
|  | 18618 | **936.4957** | **1870.9769** | **1870.9888** | **-0.0118** | **0** | **45** | **0.0032** | **1** | **K.TSSAETPTIPLGSAVEAIK.A** 18607 |
|  | 25572 | 534.0670 | 2665.2984 | 2665.3130 | -0.0145 | 1 | 21 | 0.51 | 1 | K.GMSVSDLADKLSTDDLNSLIAHAHR.R |

| **223.** | P40925\|MDHC_HUMAN    **Mass:** 36631    **Score:** 219    **Queries matched:** 18   **emPAI:** 0.63 |
| --- | --- |
|  | Malate dehydrogenase, cytoplasmic OS=Homo sapiens GN=MDH1 PE=1 SV=4 |

|  | **Query** | **Observed** | **Mr(expt)** | **Mr(calc)** | **Delta** | **Miss** | **Score** | **Expect** | **Rank** | **Peptide** |
| --- | --- | --- | --- | --- | --- | --- | --- | --- | --- | --- |
|  | 1694 | **698.3531** | **697.3458** | **698.3963** | **-1.0505** | **0** | **12** | **7.7** | **2** | **K.SAPSIPK.E** |
|  | 3169 | **865.4377** | **864.4305** | **864.4375** | **-0.0070** | **0** | **10** | **19** | **3** | **K.LSSAMSAAK.A** |
|  | 3958 | 459.2657 | 916.5168 | 915.5025 | 1.0143 | 0 | 15 | 6.8 | 2 | K.LGVTANDVK.N 3957 |
|  | 5004 | **497.2704** | **992.5262** | **992.5324** | **-0.0063** | **1** | **(14)** | **7** | **1** | **R.KLSSAMSAAK.A** 4989 |
|  | 5228 | 505.2586 | 1008.5027 | 1008.5273 | -0.0247 | 1 | 15 | 4.5 | 3 | R.KLSSAMSAAK.A 5226 5231 5232 5235 |
|  | 5437 | **513.7341** | **1025.4536** | **1025.4600** | **-0.0064** | **0** | **42** | **0.01** | **1** | **K.ENFSCLTR.L** |
|  | 7275 | **582.8000** | **1163.5854** | **1163.5935** | **-0.0081** | **0** | **54** | **0.00068** | **1** | **K.GEFVTTVQQR.G** |
|  | 12463 | **725.8695** | **1449.7245** | **1449.7351** | **-0.0107** | **1** | **40** | **0.014** | **1** | **K.DVIATDKEDVAFK.D** |
|  | 16280 | **568.2919** | **1701.8538** | **1702.9366** | **-1.0828** | **1** | **1** | **90** | **6** | **K.GEFVTTVQQRGAAVIK.A** |
|  | 16937 | 876.4416 | 1750.8687 | 1750.8777 | -0.0090 | 1 | 46 | 0.0031 | 1 | K.EVGVYEALKDDSWLK.G |
|  | 17044 | **879.4592** | **1756.9039** | **1756.9142** | **-0.0103** | **0** | **90** | **1.1e-007** | **1** | **K.VIVVGNPANTNCLTASK.S** 17043 |

| **224.** | P08183\|MDR1_HUMAN    **Mass:** 141788   **Score:** 217    **Queries matched:** 39   **emPAI:** 0.20 |
| --- | --- |
|  | Multidrug resistance protein 1 OS=Homo sapiens GN=ABCB1 PE=1 SV=3 |

|  | **Query** | **Observed** | **Mr(expt)** | **Mr(calc)** | **Delta** | **Miss** | **Score** | **Expect** | **Rank** | **Peptide** |
| --- | --- | --- | --- | --- | --- | --- | --- | --- | --- | --- |
|  | 2540 | **414.7127** | **827.4107** | **826.5137** | **0.8971** | **1** | **4** | **70** | **9** | **K.QRIAIAR.A** |
|  | 2804 | **423.0523** | **844.0901** | **844.4290** | **-0.3389** | **0** | **10** | **13** | **8** | **K.INEGIGDK.I** |
|  | 3140 | **432.7295** | **863.4445** | **862.4582** | **0.9863** | **0** | **14** | **7.7** | **3** | **K.MLSGQALK.D** |
|  | 4672 | **485.7269** | **969.4392** | **968.5226** | **0.9166** | **0** | **15** | **4.8** | **2** | **R.TCIVIAHR.L** |
|  | 5872 | **530.2739** | **1058.5333** | **1057.5768** | **0.9565** | **0** | **15** | **6** | **1** | **R.VVSQEEIVR.A** |
|  | 6813 | 564.7820 | 1127.5495 | 1127.6299 | -0.0803 | 1 | 16 | 4.3 | 3 | K.VVQEALDKAR.E 6811 6815 6818 |
|  | 6848 | 565.7897 | 1129.5649 | 1128.6000 | 0.9649 | 1 | 2 | 94 | 8 | R.GAQLSGGQKQR.I |
|  | 6883 | 567.2840 | 1132.5534 | 1131.6136 | 0.9399 | 0 | 22 | 1.1 | 3 | R.TVVSLTQEQK.F |
|  | 8555 | 411.9275 | 1232.7606 | 1233.5998 | -0.8393 | 1 | 4 | 66 | 4 | R.YMVFRSMLR.Q |
|  | 9372 | 638.3942 | 1274.7738 | 1273.6626 | 1.1111 | 1 | 37 | 0.026 | 1 | K.VGDKGTQLSGGQK.Q 9373 |
|  | 10353 | **664.8086** | **1327.6026** | **1327.7459** | **-0.1433** | **1** | **9** | **16** | **2** | **R.VVSQEEIVRAAK.E** 10358 |
|  | 10477 | **668.2856** | **1334.5566** | **1333.6660** | **0.8906** | **0** | **37** | **0.029** | **1** | **K.GQTLALVGSSGCGK.S** |
|  | 11822 | 471.2860 | 1410.8361 | 1410.7619 | 0.0742 | 1 | 4 | 52 | 8 | K.LPHKFDTLVGER.G |
|  | 12669 | **732.3298** | **1462.6450** | **1461.7610** | **0.8840** | **1** | **29** | **0.16** | **1** | **K.KGQTLALVGSSGCGK.S** 12661 |
|  | 12943 | **494.2937** | **1479.8592** | **1478.7616** | **1.0975** | **0** | **10** | **12** | **2** | **K.IIDNKPSIDSYSK.S** 12897 12904 12921 12944 |
|  | 13324 | 752.8779 | 1503.7412 | 1504.7443 | -1.0031 | 1 | 7 | 23 | 5 | R.ENVTMDEIEKAVK.E |
|  | 13755 | **512.2479** | **1533.7219** | **1534.8395** | **-1.1176** | **1** | **5** | **37** | **7** | **R.FYDPLAGKVLLDGK.E** |
|  | 15002 | **539.6297** | **1615.8673** | **1615.8794** | **-0.0122** | **1** | **20** | **1.3** | **1** | **R.VKEHGTHQQLLAQK.G** |
|  | 15682 | 830.9335 | 1659.8524 | 1660.8308 | -0.9784 | 0 | 7 | 23 | 3 | R.NADVIAGFDDGVIVEK.G |
|  | 15693 | **831.4116** | **1660.8086** | **1660.8203** | **-0.0117** | **0** | **63** | **6.3e-005** | **1** | **K.VQSGQTVALVGNSGCGK.S** |
|  | 16763 | **870.3930** | **1738.7715** | **1738.7985** | **-0.0270** | **1** | **2** | **72** | **2** | **R.SMLRQDVSWFDDPK.N** 16784 |
|  | 17112 | **882.4616** | **1762.9087** | **1761.9010** | **1.0077** | **1** | **7** | **24** | **2** | **K.FDTLVGERGAQLSGGQK.Q** |
|  | 18925 | **947.9401** | **1893.8656** | **1893.8779** | **-0.0123** | **0** | **96** | **2.5e-008** | **1** | **R.LYDPTEGMVSVDGQDIR.T** |
|  | 20757 | **686.4401** | **2056.2984** | **2057.1520** | **-0.8537** | **1** | **7** | **11** | **1** | **K.ELLAYAKAGAVAEEVLAAIR.T** |
|  | 23752 | **1200.1574** | **2398.3001** | **2398.3108** | **-0.0106** | **0** | **40** | **0.0068** | **1** | **R.EIIGVVSQEPVLFATTIAENIR.Y** 23753 |
|  | 27741 | **1030.4982** | **3088.4727** | **3088.4924** | **-0.0197** | **0** | **55** | **0.00015** | **1** | **R.AHLGIVSQEPILFDCSIAENIAYGDNSR.V** |
|  | 31479 | **1024.2853** | **5116.3900** | **5116.5092** | **-0.1192** | **0** | **1** | **9.6** | **1** | **K.LYMVVGTLAAIIHGAGLPLMMLVFGEMTDIFANAGNLEDLMSNITNR.S** |

| **225.** | P60174\|TPIS_HUMAN    **Mass:** 26938    **Score:** 216    **Queries matched:** 19   **emPAI:** 1.87 |
| --- | --- |
|  | Triosephosphate isomerase OS=Homo sapiens GN=TPI1 PE=1 SV=2 |

|  | **Query** | **Observed** | **Mr(expt)** | **Mr(calc)** | **Delta** | **Miss** | **Score** | **Expect** | **Rank** | **Peptide** |
| --- | --- | --- | --- | --- | --- | --- | --- | --- | --- | --- |
|  | 6942 | **569.2857** | **1136.5569** | **1136.5648** | **-0.0079** | **0** | **28** | **0.27** | **1** | **K.IAVAAQNCYK.V** |
|  | 8591 | **618.3599** | **1234.7052** | **1233.5949** | **1.1102** | **0** | **13** | **6.7** | **2** | **K.SNVSDAVAQSTR.I** |
|  | 10303 | **663.8350** | **1325.6554** | **1325.6649** | **-0.0096** | **0** | **71** | **9.9e-006** | **1** | **R.IIYGGSVTGATCK.E** 10311 |
|  | 12575 | **729.8599** | **1457.7052** | **1457.7151** | **-0.0099** | **0** | **37** | **0.029** | **1** | **R.HVFGESDELIGQK.V** 12576 12577 |
|  | 12712 | **489.5779** | **1465.7120** | **1465.7161** | **-0.0041** | **0** | **19** | **1.5** | **1** | **K.TATPQQAQEVHEK.L** 12710 |
|  | 13870 | **514.9633** | **1541.8681** | **1541.8777** | **-0.0095** | **1** | **11** | **9.1** | **2** | **R.KQSLGELIGTLNAAK.V** |
|  | 14528 | **793.8673** | **1585.7199** | **1585.7307** | **-0.0108** | **0** | **34** | **0.045** | **1** | **K.DCGATWVVLGHSER.R** |
|  | 14772 | **802.4454** | **1602.8763** | **1601.8817** | **0.9946** | **0** | **64** | **4.7e-005** | **1** | **K.VVLAYEPVWAIGTGK.T** 14761 |
|  | 14943 | **538.9426** | **1613.8059** | **1613.8162** | **-0.0103** | **1** | **37** | **0.025** | **1** | **R.RHVFGESDELIGQK.V** 14942 |
|  | 15085 | **811.9131** | **1621.8117** | **1620.8181** | **0.9936** | **0** | **55** | **0.00039** | **1** | **K.VTNGAFTGEISPGMIK.D** |
|  | 27472 | **758.1481** | **3028.5632** | **3028.5757** | **-0.0125** | **0** | **29** | **0.065** | **1** | **K.ELASQPDVDGFLVGGASLKPEFVDIINAK.Q** 27465 27473 |

| **226.** | Q15436\|SC23A_HUMAN    **Mass:** 87018    **Score:** 214    **Queries matched:** 22   **emPAI:** 0.23 |
| --- | --- |
|  | Protein transport protein Sec23A OS=Homo sapiens GN=SEC23A PE=1 SV=2 |

|  | **Query** | **Observed** | **Mr(expt)** | **Mr(calc)** | **Delta** | **Miss** | **Score** | **Expect** | **Rank** | **Peptide** |
| --- | --- | --- | --- | --- | --- | --- | --- | --- | --- | --- |
|  | 3066 | 431.2580 | 860.5014 | 861.3803 | -0.8789 | 0 | 17 | 3.9 | 3 | K.DMHGQFK.M 3076 |
|  | 4530 | **479.7605** | **957.5064** | **956.4828** | **1.0237** | **0** | **10** | **20** | **2** | **K.HFEALANR.A** |
|  | 5414 | **512.7620** | **1023.5095** | **1022.5185** | **0.9909** | **0** | **4** | **64** | **2** | **R.DPWPVPQGK.R** |
|  | 5792 | **527.2564** | **1052.4983** | **1051.5372** | **0.9611** | **0** | **3** | **76** | **9** | **K.MGFGGTLEIK.T** |
|  | 7152 | 577.7815 | 1153.5484 | 1153.6244 | -0.0760 | 1 | 7 | 29 | 7 | K.QTFQRVFTK.D |
|  | 7266 | **582.2861** | **1162.5576** | **1161.6063** | **0.9512** | **0** | **5** | **50** | **2** | **K.QLQEMLGLSK.V** |
|  | 10351 | 664.7820 | 1327.5494 | 1327.6157 | -0.0663 | 1 | 12 | 9.7 | 5 | R.SWHDIDKDNAK.Y |
|  | 10435 | **667.3052** | **1332.5959** | **1332.6058** | **-0.0099** | **0** | **63** | **7.3e-005** | **1** | **R.YIDTEHGGSQAR.F** 10430 |
|  | 11829 | **706.8699** | **1411.7252** | **1411.7130** | **0.0122** | **1** | **3** | **64** | **6** | **K.MGFGGTLEIKTSR.E** |
|  | 13919 | **516.2649** | **1545.7728** | **1544.8576** | **0.9153** | **1** | **0** | **1.1e+002** | **7** | **R.DPWPVPQGKRPLR.S** |
|  | 15253 | **816.4236** | **1630.8326** | **1629.8396** | **0.9930** | **0** | **74** | **4.4e-006** | **1** | **K.IDMNLTDLLGELQR.D** |
|  | 15302 | **818.3351** | **1634.6557** | **1634.6671** | **-0.0114** | **0** | **37** | **0.02** | **1** | **K.SGYQDMPEYENFR.H** |
|  | 15928 | **559.9376** | **1676.7910** | **1675.8814** | **0.9096** | **1** | **4** | **44** | **2** | **K.DLSAKQLQEMLGLSK.V** 15945 |
|  | 21616 | **1070.4769** | **2138.9393** | **2138.9545** | **-0.0152** | **0** | **99** | **9.5e-009** | **1** | **R.SSFLQVFNNSPDESSYYR.H** |
|  | 22902 | 570.7807 | 2279.0937 | 2279.1000 | -0.0063 | 0 | (2) | 45 | 6 | R.IMMFIGGPATQGPGMVVGDELK.T |
|  | 23018 | 765.7206 | 2294.1399 | 2295.0949 | -0.9550 | 0 | 3 | 37 | 3 | R.IMMFIGGPATQGPGMVVGDELK.T |
|  | 27102 | 737.8881 | 2947.5234 | 2946.4294 | 1.0940 | 0 | 0 | 53 | 9 | R.NWADAQTQIQNIAASFDQEAAAILMAR.L |
|  | 27964 | **1051.8715** | **3152.5926** | **3152.6077** | **-0.0152** | **0** | **46** | **0.0012** | **1** | **K.ICGLSPTTTLAIYFEVVNQHNAPIPQGGR.G** |
|  | 28776 | 841.4371 | 3361.7194 | 3362.5694 | -0.8499 | 1 | 2 | 29 | 5 | K.ISGAIGPCVSLNSKGPCVSENEIGTGGTCQWK.I |

| **227.** | Q9Y2T3\|GUAD_HUMAN    **Mass:** 51484    **Score:** 214    **Queries matched:** 17   **emPAI:** 0.32 |
| --- | --- |
|  | Guanine deaminase OS=Homo sapiens GN=GDA PE=1 SV=1 |

|  | **Query** | **Observed** | **Mr(expt)** | **Mr(calc)** | **Delta** | **Miss** | **Score** | **Expect** | **Rank** | **Peptide** |
| --- | --- | --- | --- | --- | --- | --- | --- | --- | --- | --- |
|  | 6548 | 554.2529 | 1106.4912 | 1106.5608 | -0.0696 | 0 | 7 | 33 | 6 | R.NIEEVYVGGK.Q |
|  | 6801 | **564.2844** | **1126.5543** | **1126.5619** | **-0.0076** | **0** | **12** | **9.2** | **1** | **R.DHLLGVSDSGK.I** |
|  | 11971 | **710.8653** | **1419.7160** | **1419.7245** | **-0.0085** | **0** | **32** | **0.075** | **1** | **K.IVFLEEASQQEK.L** 11970 |
|  | 12033 | **475.2416** | **1422.7028** | **1423.6377** | **-0.9348** | **0** | **6** | **32** | **7** | **K.EWCFKPCEIR.E** |
|  | 12447 | **725.4258** | **1448.8370** | **1447.7168** | **1.1202** | **0** | **0** | **1.3e+002** | **6** | **R.DLHIQSHISENR.D** |
|  | 13670 | **510.2637** | **1527.7693** | **1526.7486** | **1.0207** | **0** | **9** | **17** | **2** | **M.CAAQMPPLAHIFR.G** |
|  | 15242 | **816.3525** | **1630.6904** | **1630.7007** | **-0.0103** | **0** | **65** | **4e-005** | **1** | **K.VCMDLNDTFPEYK.E** |
|  | 15244 | **816.3839** | **1630.7531** | **1630.7627** | **-0.0096** | **0** | **70** | **1.3e-005** | **1** | **R.FQNIDFAEEVYTR.V** |
|  | 15650 | **553.7793** | **1658.3161** | **1657.7891** | **0.5270** | **0** | **4** | **27** | **6** | **-.MCAAQMPPLAHIFR.G** 15642 |
|  | 19665 | **653.0085** | **1956.0036** | **1955.9332** | **0.0704** | **0** | **1** | **79** | **5** | **R.FSLSCSETLMGELGNIAK.T** |
|  | 21693 | **717.3353** | **2148.9842** | **2148.9860** | **-0.0019** | **1** | **1** | **66** | **6** | **R.AFVGKVCMDLNDTFPEYK.E** |
|  | 22381 | **740.3675** | **2218.0806** | **2218.0978** | **-0.0172** | **1** | **6** | **19** | **1** | **R.DLHIQSHISENRDEVEAVK.N** |
|  | 28884 | **1129.5928** | **3385.7565** | **3385.7769** | **-0.0204** | **1** | **78** | **6.3e-007** | **1** | **R.LATLGGSQALGLDGEIGNFEVGKEFDAILINPK.A** 28888 |
|  | 29046 | **861.9662** | **3443.8359** | **3444.6352** | **-0.7993** | **1** | **0** | **34** | **4** | **M.CAAQMPPLAHIFRGTFVHSTWTCPMEVLR.D** |

| **228.** | P30084\|ECHM_HUMAN    **Mass:** 31823    **Score:** 212    **Queries matched:** 12   **emPAI:** 0.40 |
| --- | --- |
|  | Enoyl-CoA hydratase, mitochondrial OS=Homo sapiens GN=ECHS1 PE=1 SV=4 |

|  | **Query** | **Observed** | **Mr(expt)** | **Mr(calc)** | **Delta** | **Miss** | **Score** | **Expect** | **Rank** | **Peptide** |
| --- | --- | --- | --- | --- | --- | --- | --- | --- | --- | --- |
|  | 5245 | **506.2414** | **1010.4682** | **1010.4743** | **-0.0061** | **0** | **11** | **11** | **1** | **K.EGMTAFVEK.R** |
|  | 10819 | 678.2153 | 1354.4160 | 1353.6268 | 0.7892 | 0 | 1 | 78 | 3 | K.SLAMEMVLTGDR.I |
|  | 11383 | **463.2300** | **1386.6681** | **1387.7792** | **-1.1111** | **1** | **2** | **89** | **3** | **-.MAALRVLLSCVR.G** |
|  | 12718 | **733.9206** | **1465.8266** | **1465.8365** | **-0.0099** | **0** | **69** | **1.6e-005** | **1** | **K.NNTVGLIQLNRPK.A** |
|  | 16145 | **565.3362** | **1692.9869** | **1692.8539** | **0.1330** | **1** | **(2)** | **67** | **7** | **R.AVGKSLAMEMVLTGDR.I** |
|  | 16388 | **570.7812** | **1709.3217** | **1708.8488** | **0.4730** | **1** | **4** | **29** | **4** | **R.AVGKSLAMEMVLTGDR.I** |
|  | 16730 | **868.8605** | **1735.7065** | **1735.7182** | **-0.0116** | **0** | **36** | **0.026** | **1** | **K.EMQNLSFQDCYSSK.F** |
|  | 19990 | **663.6573** | **1987.9502** | **1987.9594** | **-0.0092** | **0** | **14** | **3.6** | **1** | **K.ICPVETLVEEAIQCAEK.I** |
|  | 20688 | **513.7737** | **2051.0659** | **2051.0027** | **0.0632** | **1** | **4** | **36** | **4** | **K.SLAMEMVLTGDRISAQDAK.Q** |
|  | 21494 | **1063.0674** | **2124.1202** | **2124.1328** | **-0.0125** | **0** | **99** | **9.8e-009** | **1** | **K.AQFAQPEILIGTIPGAGGTQR.L** 21493 |
|  | 21632 | **714.4457** | **2140.3152** | **2139.9605** | **0.3547** | **1** | **6** | **15** | **1** | **K.EMQNLSFQDCYSSKFLK.H** |

| **229.** | Q01518\|CAP1_HUMAN    **Mass:** 52222    **Score:** 212    **Queries matched:** 27   **emPAI:** 0.41 |
| --- | --- |
|  | Adenylyl cyclase-associated protein 1 OS=Homo sapiens GN=CAP1 PE=1 SV=4 |

|  | **Query** | **Observed** | **Mr(expt)** | **Mr(calc)** | **Delta** | **Miss** | **Score** | **Expect** | **Rank** | **Peptide** |
| --- | --- | --- | --- | --- | --- | --- | --- | --- | --- | --- |
|  | 1116 | **561.4855** | **560.4782** | **560.3030** | **0.1752** | **1** | **7** | **57** | **5** | **K.NRGSK.L** 1108 |
|  | 3470 | **443.0379** | **884.0612** | **883.4803** | **0.5809** | **0** | **14** | **3.9** | **1** | **K.QVAYIYK.C** 3465 3474 3477 3479 3480 3482 |
|  | 8145 | **606.8414** | **1211.6683** | **1211.6761** | **-0.0078** | **1** | **15** | **4.4** | **1** | **K.KEPAVLELEGK.K** |
|  | 8341 | 611.3104 | 1220.6062 | 1221.5482 | -0.9420 | 0 | 3 | 79 | 9 | -.MADMQNLVER.L 8365 |
|  | 8589 | **618.3500** | **1234.6854** | **1234.5863** | **0.0991** | **0** | **9** | **19** | **1** | **K.NSLDCEIVSAK.S** |
|  | 9068 | **421.0566** | **1260.1478** | **1260.6860** | **-0.5382** | **1** | **5** | **29** | **6** | **K.CVNTTLQIKGK.I** |
|  | 9655 | 646.3136 | 1290.6126 | 1290.6602 | -0.0475 | 1 | 15 | 5 | 2 | K.INSITVDNCKK.L |
|  | 10716 | 675.3210 | 1348.6275 | 1347.6816 | 0.9459 | 1 | 2 | 98 | 6 | K.GKINSITVDNCK.K |
|  | 11298 | **691.8232** | **1381.6319** | **1381.6408** | **-0.0089** | **0** | **54** | **0.00062** | **1** | **R.LEAVSHTSDMHR.G** |
|  | 12082 | **713.9069** | **1425.7993** | **1425.8079** | **-0.0086** | **0** | **27** | **0.28** | **1** | **K.LSDLLAPISEQIK.E** |
|  | 13195 | **499.2596** | **1494.7570** | **1493.5915** | **1.1655** | **0** | **8** | **17** | **1** | **K.EMNDAAMFYTNR.V** |
|  | 15062 | **811.0630** | **1620.1114** | **1619.7759** | **0.3355** | **1** | **1** | **68** | **1** | **-.MADMQNLVERLER.A** |
|  | 17041 | **879.4400** | **1756.8654** | **1756.8778** | **-0.0123** | **0** | **47** | **0.0019** | **1** | **R.ALLVTASQCQQPAENK.L** 17042 |
|  | 17149 | **589.3629** | **1765.0669** | **1764.8689** | **0.1980** | **1** | **4** | **35** | **1** | **R.AVGRLEAVSHTSDMHR.G** |
|  | 17836 | **607.2620** | **1818.7641** | **1817.8440** | **0.9200** | **1** | **2** | **64** | **6** | **K.EMNDAAMFYTNRVLK.E** |
|  | 20891 | **1037.0139** | **2072.0133** | **2072.0273** | **-0.0140** | **0** | **67** | **1.8e-005** | **1** | **R.VENQENVSNLVIEDTELK.Q** 20892 |
|  | 26395 | **1406.1565** | **2810.2984** | **2810.3109** | **-0.0125** | **0** | **49** | **0.0007** | **1** | **K.SSEMNVLIPTEGGDFNEFPVPEQFK.T** |

| **230.** | P18859\|ATP5J_HUMAN    **Mass:** 12580    **Score:** 211    **Queries matched:** 11   **emPAI:** 1.99 |
| --- | --- |
|  | ATP synthase-coupling factor 6, mitochondrial OS=Homo sapiens GN=ATP5J PE=1 SV=1 |

|  | **Query** | **Observed** | **Mr(expt)** | **Mr(calc)** | **Delta** | **Miss** | **Score** | **Expect** | **Rank** | **Peptide** |
| --- | --- | --- | --- | --- | --- | --- | --- | --- | --- | --- |
|  | 3200 | **434.2571** | **866.4996** | **867.4926** | **-0.9930** | **0** | **13** | **7.1** | **8** | **R.SAVSVHLR.R** 3193 3228 |
|  | 5895 | **530.7838** | **1059.5531** | **1059.5600** | **-0.0070** | **0** | **16** | **4.8** | **1** | **K.FEVIEKPQA.-** 5917 |
|  | 15909 | **838.9238** | **1675.8331** | **1675.8457** | **-0.0126** | **1** | **26** | **0.29** | **1** | **K.FEDPKFEVIEKPQA.-** |
|  | 20960 | **1040.9686** | **2079.9227** | **2079.9345** | **-0.0118** | **0** | **97** | **1.6e-008** | **1** | **R.QTSGGPVDASSEYQQELER.E** 20959 |
|  | 22533 | **746.3472** | **2236.0197** | **2236.0356** | **-0.0159** | **1** | **55** | **0.00025** | **1** | **K.RQTSGGPVDASSEYQQELER.E** 22527 |
|  | 23745 | **600.2865** | **2397.1169** | **2396.0453** | **1.0716** | **1** | **3** | **42** | **6** | **K.QMFGNADMNTFPTFKFEDPK.F** |

| **231.** | P00352\|AL1A1_HUMAN    **Mass:** 55454    **Score:** 210    **Queries matched:** 35   **emPAI:** 0.79 |
| --- | --- |
|  | Retinal dehydrogenase 1 OS=Homo sapiens GN=ALDH1A1 PE=1 SV=2 |

|  | **Query** | **Observed** | **Mr(expt)** | **Mr(calc)** | **Delta** | **Miss** | **Score** | **Expect** | **Rank** | **Peptide** |
| --- | --- | --- | --- | --- | --- | --- | --- | --- | --- | --- |
|  | 1044 | 547.3414 | 546.3342 | 546.3377 | -0.0036 | 0 | 20 | 3.3 | 2 | K.TVTVK.I |
|  | 1323 | 605.3397 | 604.3324 | 604.2704 | 0.0620 | 0 | 13 | 13 | 8 | K.EDVDK.A |
|  | 1532 | **653.3209** | **652.3136** | **651.3592** | **0.9545** | **0** | **17** | **2.5** | **1** | **K.IQYTK.I** |
|  | 2391 | 408.7420 | 815.4694 | 815.4753 | -0.0059 | 0 | 18 | 3.7 | 2 | R.VTLELGGK.S 2382 2398 |
|  | 2517 | **413.7583** | **825.5020** | **824.3334** | **1.1686** | **0** | **13** | **7.3** | **1** | **R.TMDASER.G** |
|  | 2572 | 415.2399 | 828.4652 | 828.4705 | -0.0052 | 0 | 16 | 5 | 1 | K.LADLIER.D |
|  | 4704 | 486.7635 | 971.5125 | 971.5764 | -0.0639 | 1 | 17 | 3.9 | 1 | K.RVTLELGGK.S |
|  | 5602 | 519.2711 | 1036.5277 | 1037.4560 | -0.9283 | 1 | 6 | 36 | 3 | R.TMDASERGR.L |
|  | 6658 | **558.3335** | **1114.6524** | **1114.6597** | **-0.0073** | **1** | **36** | **0.037** | **1** | **K.ILDLIESGKK.E** 6648 6653 |
|  | 10963 | 682.8611 | 1363.7077 | 1363.6839 | 0.0238 | 0 | 19 | 1.8 | 2 | R.LLLATMESMNGGK.L |
|  | 11545 | 466.2825 | 1395.8256 | 1395.6738 | 0.1518 | 0 | (10) | 12 | 5 | R.LLLATMESMNGGK.L 11546 11551 |
|  | 11936 | 709.8667 | 1417.7188 | 1417.7275 | -0.0087 | 0 | 8 | 24 | 2 | K.EEIFGPVQQIMK.F |
|  | 13900 | **772.8858** | **1543.7570** | **1543.7671** | **-0.0101** | **0** | **28** | **0.22** | **1** | **R.TIPIDGNFFTYTR.H** |
|  | 14581 | **795.3967** | **1588.7789** | **1588.7886** | **-0.0097** | **0** | **64** | **4.7e-005** | **1** | **R.ANNTFYGLSAGVFTK.D** 14580 |
|  | 15464 | **823.3968** | **1644.7790** | **1644.7896** | **-0.0106** | **0** | **65** | **3.9e-005** | **1** | **K.IFINNEWHDSVSGK.K** 15465 15467 |
|  | 15545 | 550.9955 | 1649.9648 | 1650.8069 | -0.8421 | 1 | 5 | 33 | 6 | R.DRLLLATMESMNGGK.L |
|  | 16240 | **850.8873** | **1699.7601** | **1699.7729** | **-0.0128** | **0** | **24** | **0.4** | **1** | **R.ELGEYGFHEYTEVK.T** |
|  | 16439 | **857.9216** | **1713.8286** | **1713.8396** | **-0.0110** | **0** | **53** | **0.00058** | **1** | **K.LYSNAYLNDLAGCIK.T** 16440 |
|  | 20003 | **995.4593** | **1988.9041** | **1988.9302** | **-0.0261** | **0** | **16** | **2.2** | **1** | **K.GYFVQPTVFSNVTDEMR.I** |
|  | 20109 | **667.3154** | **1998.9243** | **1998.0323** | **0.8920** | **1** | **20** | **0.87** | **1** | **K.IQGRTIPIDGNFFTYTR.H** |
|  | 23919 | **808.0693** | **2421.1860** | **2422.2665** | **-1.0805** | **1** | **2** | **42** | **2** | **-.MSSSGTPDLPVLLTDLKIQYTK.I** |
|  | 27672 | **1023.1738** | **3066.4995** | **3066.5121** | **-0.0126** | **0** | **23** | **0.26** | **1** | **K.EAGFPPGVVNIVPGYGPTAGAAISSHMDIDK.V** 27665 27671 |
|  | 28160 | **1062.2564** | **3183.7472** | **3183.7690** | **-0.0217** | **0** | **65** | **1.4e-005** | **1** | **K.IGPALSCGNTVVVKPAEQTPLTALHVASLIK.E** |

| **232.** | P21695\|GPDA_HUMAN    **Mass:** 38171    **Score:** 210    **Queries matched:** 15   **emPAI:** 0.76 |
| --- | --- |
|  | Glycerol-3-phosphate dehydrogenase [NAD+], cytoplasmic OS=Homo sapiens GN=GPD1 PE=1 SV=4 |

|  | **Query** | **Observed** | **Mr(expt)** | **Mr(calc)** | **Delta** | **Miss** | **Score** | **Expect** | **Rank** | **Peptide** |
| --- | --- | --- | --- | --- | --- | --- | --- | --- | --- | --- |
|  | 956 | 529.3418 | 528.3345 | 528.3384 | -0.0038 | 0 | 1 | 1.8e+002 | 2 | K.AAVIR.L |
|  | 3586 | 446.7551 | 891.4956 | 890.4974 | 0.9982 | 1 | 13 | 10 | 1 | R.KVAEAFAR.T |
|  | 5305 | 508.2893 | 1014.5641 | 1014.5709 | -0.0069 | 0 | 30 | 0.17 | 1 | K.LISEVIGER.L |
|  | 8924 | **627.7832** | **1253.5518** | **1254.6352** | **-1.0833** | **0** | **5** | **44** | **10** | **R.LGLMEMIAFAK.L** 8925 |
|  | 14101 | **778.9068** | **1555.7990** | **1555.8107** | **-0.0116** | **0** | **61** | **0.0001** | **1** | **K.IVGGNAAQLAQFDPR.V** |
|  | 15756 | **833.9542** | **1665.8938** | **1665.9050** | **-0.0112** | **1** | **56** | **0.0003** | **1** | **K.KLTEIINTQHENVK.Y** 15754 |
|  | 20838 | **689.6697** | **2065.9874** | **2064.9973** | **0.9901** | **1** | **35** | **0.027** | **1** | **K.FCETTIGCKDPAQGQLLK.E** |
|  | 21666 | **1073.0450** | **2144.0755** | **2144.0857** | **-0.0102** | **0** | **41** | **0.0072** | **1** | **R.LGIPMSVLMGANIASEVADEK.F** 21669 |
|  | 22015 | **726.4460** | **2176.3163** | **2176.0755** | **0.2408** | **0** | **(3)** | **37** | **4** | **R.LGIPMSVLMGANIASEVADEK.F** |
|  | 25681 | **671.0435** | **2680.1450** | **2680.1621** | **-0.0171** | **0** | **13** | **2.5** | **1** | **K.VCYEGQPVGEFIHCLQNHPEHM.-** |
|  | 28963 | 1137.6251 | 3409.8535 | 3409.8650 | -0.0115 | 0 | 66 | 8.9e-006 | 1 | K.LPPNVVAVPDVVQAAEDADILIFVVPHQFIGK.I 28964 |

| **233.** | P27338\|AOFB_HUMAN    **Mass:** 59238    **Score:** 210    **Queries matched:** 28   **emPAI:** 0.63 |
| --- | --- |
|  | Amine oxidase [flavin-containing] B OS=Homo sapiens GN=MAOB PE=1 SV=3 |

|  | **Query** | **Observed** | **Mr(expt)** | **Mr(calc)** | **Delta** | **Miss** | **Score** | **Expect** | **Rank** | **Peptide** |
| --- | --- | --- | --- | --- | --- | --- | --- | --- | --- | --- |
|  | 1376 | 616.4244 | 615.4171 | 615.3704 | 0.0467 | 1 | 8 | 47 | 4 | R.NQKVK.Y |
|  | 3630 | 448.7280 | 895.4414 | 895.4473 | -0.0059 | 0 | 27 | 0.33 | 1 | K.LCELYAK.V |
|  | 4148 | **466.7440** | **931.4735** | **931.4797** | **-0.0062** | **0** | **47** | **0.0041** | **1** | **R.IMDLLGDR.V** |
|  | 4444 | 476.7497 | 951.4848 | 951.4913 | -0.0065 | 0 | 17 | 3.4 | 1 | K.ELGLETYK.V |
|  | 5419 | 512.7748 | 1023.5351 | 1023.5423 | -0.0071 | 1 | 20 | 1.5 | 1 | K.KLCELYAK.V |
|  | 6752 | 562.2784 | 1122.5422 | 1121.5466 | 0.9957 | 0 | 57 | 0.00032 | 1 | K.FVGGSGQVSER.I 6742 |
|  | 8848 | 625.3031 | 1248.5916 | 1249.6415 | -1.0499 | 1 | 51 | 0.0012 | 1 | R.KFVGGSGQVSER.I 8869 8892 |
|  | 14596 | **531.1867** | **1590.5383** | **1589.7906** | **0.7477** | **0** | **4** | **34** | **3** | **K.CDVVVVGGGISGMAAAK.L** 14622 |
|  | 15148 | **813.3944** | **1624.7743** | **1624.7845** | **-0.0103** | **0** | **51** | **0.001** | **1** | **K.YVDLGGSYVGPTQNR.I** 15149 |
|  | 15954 | **560.3002** | **1677.8787** | **1677.8573** | **0.0214** | **1** | **8** | **18** | **2** | **K.ELGLETYKVNEVER.L** 15948 15951 |
|  | 17763 | **906.9667** | **1811.9188** | **1811.9305** | **-0.0117** | **0** | **42** | **0.0064** | **1** | **K.VLGSLEALEPVHYEEK.N** 17762 |
|  | 19226 | **959.9592** | **1917.9039** | **1917.9142** | **-0.0103** | **0** | **61** | **8.4e-005** | **1** | **R.ENVLVETLNHEMYEAK.Y** |
|  | 20843 | **689.8487** | **2066.5242** | **2065.9959** | **0.5284** | **1** | **(3)** | **27** | **1** | **-.MSNKCDVVVVGGGISGMAAAK.L** |
|  | 20984 | **694.9366** | **2081.7881** | **2081.9908** | **-0.2027** | **1** | **6** | **18** | **1** | **-.MSNKCDVVVVGGGISGMAAAK.L** 21002 |
|  | 25100 | **859.4424** | **2575.3053** | **2574.1770** | **1.1283** | **1** | **0** | **69** | **3** | **R.EIPSDAPWKAPLAEEWDNMTMK.E** |
|  | 28299 | **1613.7844** | **3225.5543** | **3225.5717** | **-0.0175** | **0** | **38** | **0.0072** | **1** | **K.IPEDEIWQSEPESVDVPAQPITTTFLER.H** |
|  | 28725 | 1115.8854 | 3344.6343 | 3343.5204 | 1.1139 | 1 | 1 | 33 | 5 | R.QPVDRIYFAGTETATHWSGYMEGAVEAGER.A |
|  | 30509 | **1368.9576** | **4103.8511** | **4105.0354** | **-1.1843** | **1** | **1** | **23** | **7** | **R.EILHAMGKIPEDEIWQSEPESVDVPAQPITTTFLER.H** |
|  | 30971 | **1506.7759** | **4517.3058** | **4516.1389** | **1.1669** | **1** | **1** | **14** | **3** | **K.DYCGTMIIDGEEAPVAYTLDDTKPEGNYAAIMGFILAHKAR.K** |

| **234.** | P08107\|HSP71_HUMAN    **Mass:** 70294    **Score:** 209    **Queries matched:** 29   **emPAI:** 0.51 |
| --- | --- |
|  | Heat shock 70 kDa protein 1 OS=Homo sapiens GN=HSPA1A PE=1 SV=5 |

|  | **Query** | **Observed** | **Mr(expt)** | **Mr(calc)** | **Delta** | **Miss** | **Score** | **Expect** | **Rank** | **Peptide** |
| --- | --- | --- | --- | --- | --- | --- | --- | --- | --- | --- |
|  | 1724 | **705.3886** | **704.3813** | **703.3501** | **1.0312** | **0** | **10** | **20** | **5** | **K.DISQNK.R** |
|  | 1755 | **713.0680** | **712.0607** | **711.4643** | **0.5964** | **1** | **5** | **29** | **6** | **R.IPKVQK.L** |
|  | 2227 | 804.4412 | 803.4339 | 803.4389 | -0.0050 | 0 | 23 | 1.2 | 1 | K.ITITNDK.G |
|  | 6578 | **555.2967** | **1108.5789** | **1108.5665** | **0.0124** | **0** | **18** | **2.3** | **1** | **K.LLQDFFNGR.D** |
|  | 7819 | **599.3470** | **1196.6794** | **1196.6877** | **-0.0083** | **0** | **9** | **20** | **2** | **K.DAGVIAGLNVLR.I** |
|  | 8329 | **611.2651** | **1220.5156** | **1221.5700** | **-1.0544** | **0** | **10** | **16** | **4** | **K.FGDPVVQSDMK.H** |
|  | 8452 | 614.8128 | 1227.6111 | 1227.6207 | -0.0097 | 0 | 47 | 0.003 | 1 | K.VEIIANDQGNR.T 8436 8453 |
|  | 10738 | **675.8354** | **1349.6562** | **1349.6649** | **-0.0087** | **1** | **22** | **1** | **1** | **R.KFGDPVVQSDMK.H** |
|  | 12697 | **733.4055** | **1464.7964** | **1464.8049** | **-0.0085** | **0** | **24** | **0.45** | **1** | **K.AQIHDLVLVGGSTR.I** |
|  | 13054 | 744.3494 | 1486.6842 | 1486.6940 | -0.0098 | 0 | 50 | 0.0013 | 1 | R.TTPSYVAFTDTER.L |
|  | 13878 | **772.3791** | **1542.7437** | **1541.7296** | **1.0141** | **1** | **16** | **2.8** | **1** | **R.ARFEELCSDLFR.S** |
|  | 14435 | 790.4898 | 1578.9651 | 1578.8154 | 0.1496 | 1 | 0 | 93 | 10 | K.LLQDFFNGRDLNK.S |
|  | 15148 | 813.3944 | 1624.7743 | 1624.9260 | -0.1518 | 1 | 11 | 8.7 | 2 | R.QATKDAGVIAGLNVLR.I |
|  | 15645 | **829.9228** | **1657.8310** | **1657.8424** | **-0.0113** | **0** | **21** | **0.92** | **1** | **K.NQVALNPQNTVFDAK.R** 15646 |
|  | 15876 | **838.3638** | **1674.7130** | **1674.7234** | **-0.0104** | **0** | **55** | **0.00037** | **1** | **K.ATAGDTHLGGEDFDNR.L** |
|  | 15972 | **560.9504** | **1679.8295** | **1679.8420** | **-0.0125** | **0** | **48** | **0.0018** | **1** | **K.HWPFQVINDGDKPK.V** |
|  | 16055 | **563.2787** | **1686.8142** | **1687.8239** | **-1.0097** | **1** | **1** | **96** | **5** | **R.VSAKNALESYAFNMK.S** 16077 |
|  | 16073 | **844.8687** | **1687.7229** | **1686.8940** | **0.8288** | **0** | **21** | **0.79** | **1** | **R.IINEPTAAAIAYGLDR.T** 16072 |
|  | 23079 | **768.7191** | **2303.1354** | **2303.1467** | **-0.0114** | **0** | **5** | **24** | **1** | **K.SINPDEAVAYGAAVQAAILMGDK.S** |
|  | 26230 | **925.1461** | **2772.4165** | **2773.3956** | **-0.9791** | **1** | **(0)** | **56** | **5** | **R.DLNKSINPDEAVAYGAAVQAAILMGDK.S** |
|  | 26312 | 930.4825 | 2788.4256 | 2789.3905 | -0.9649 | 1 | 0 | 56 | 8 | R.DLNKSINPDEAVAYGAAVQAAILMGDK.S |
|  | 28124 | **1590.8688** | **3179.7230** | **3179.7363** | **-0.0133** | **0** | **57** | **8.1e-005** | **1** | **K.SENVQDLLLLDVAPLSLGLETAGGVMTALIK.R** 28123 |
|  | 28486 | **1087.5414** | **3259.6023** | **3259.6223** | **-0.0200** | **1** | **6** | **12** | **1** | **K.MKEIAEAYLGYPVTNAVITVPAYFNDSQR.Q** |

| **235.** | P30508\|1C12_HUMAN    **Mass:** 41316    **Score:** 207    **Queries matched:** 11   **emPAI:** 0.42 |
| --- | --- |
|  | HLA class I histocompatibility antigen, Cw-12 alpha chain OS=Homo sapiens GN=HLA-C PE=1 SV=2 |

|  | **Query** | **Observed** | **Mr(expt)** | **Mr(calc)** | **Delta** | **Miss** | **Score** | **Expect** | **Rank** | **Peptide** |
| --- | --- | --- | --- | --- | --- | --- | --- | --- | --- | --- |
|  | 1195 | **581.3248** | **580.3175** | **580.2969** | **0.0206** | **0** | **1** | **1e+002** | **5** | **R.AEHPK.T** |
|  | 12099 | 714.3628 | 1426.7111 | 1426.7205 | -0.0093 | 0 | 11 | 9.6 | 1 | K.WAAVVVPSGEEQR.Y |
|  | 15206 | 815.4121 | 1628.8097 | 1628.8199 | -0.0102 | 0 | 72 | 7.9e-006 | 1 | R.FIAVGYVDDTQFVR.F 15203 15205 |
|  | 18119 | 459.9797 | 1835.8896 | 1835.9027 | -0.0131 | 0 | 13 | 5.4 | 2 | K.THVTHHPVSDHEATLR.C |
|  | 20661 | 513.0184 | 2048.0446 | 2049.0102 | -0.9656 | 0 | 14 | 4 | 2 | R.YTCHVQHEGLPEPLTLR.W |
|  | 21343 | 1056.5170 | 2111.0194 | 2111.0299 | -0.0105 | 0 | 45 | 0.003 | 1 | R.CWALGFYPAEITLTWQR.D 21342 |
|  | 25408 | 1319.0913 | 2636.1681 | 2636.1838 | -0.0158 | 0 | 68 | 1.1e-005 | 1 | R.DGEDQTQDTELVETRPAGDGTFQK.W 25407 |

| **236.** | Q29960\|1C16_HUMAN    **Mass:** 41240    **Score:** 207    **Queries matched:** 13   **emPAI:** 0.42 |
| --- | --- |
|  | HLA class I histocompatibility antigen, Cw-16 alpha chain OS=Homo sapiens GN=HLA-C PE=2 SV=1 |

|  | **Query** | **Observed** | **Mr(expt)** | **Mr(calc)** | **Delta** | **Miss** | **Score** | **Expect** | **Rank** | **Peptide** |
| --- | --- | --- | --- | --- | --- | --- | --- | --- | --- | --- |
|  | 1195 | 581.3248 | 580.3175 | 580.2969 | 0.0206 | 0 | 1 | 1e+002 | 5 | R.AEHPK.T |
|  | 10064 | 657.8190 | 1313.6234 | 1314.6429 | -1.0195 | 1 | 4 | 61 | 8 | K.WEAARAAEQQR.A 10079 |
|  | 12099 | 714.3628 | 1426.7111 | 1426.7205 | -0.0093 | 0 | 11 | 9.6 | 1 | K.WAAVVVPSGEEQR.Y |
|  | 13150 | **498.3366** | **1491.9880** | **1492.6940** | **-0.7060** | **0** | **2** | **58** | **9** | **R.SCTAADTAAQITQR.K** |
|  | 15206 | 815.4121 | 1628.8097 | 1628.8199 | -0.0102 | 0 | 72 | 7.9e-006 | 1 | R.FIAVGYVDDTQFVR.F 15203 15205 |
|  | 20661 | 513.0184 | 2048.0446 | 2049.0102 | -0.9656 | 0 | 14 | 4 | 2 | R.YTCHVQHEGLPEPLTLR.W |
|  | 21343 | 1056.5170 | 2111.0194 | 2111.0299 | -0.0105 | 0 | 45 | 0.003 | 1 | R.CWALGFYPAEITLTWQR.D 21342 |
|  | 25408 | 1319.0913 | 2636.1681 | 2636.1838 | -0.0158 | 0 | 68 | 1.1e-005 | 1 | R.DGEDQTQDTELVETRPAGDGTFQK.W 25407 |

| **237.** | P38646\|GRP75_HUMAN    **Mass:** 73920    **Score:** 207    **Queries matched:** 54   **emPAI:** 0.55 |
| --- | --- |
|  | Stress-70 protein, mitochondrial OS=Homo sapiens GN=HSPA9 PE=1 SV=2 |

|  | **Query** | **Observed** | **Mr(expt)** | **Mr(calc)** | **Delta** | **Miss** | **Score** | **Expect** | **Rank** | **Peptide** |
| --- | --- | --- | --- | --- | --- | --- | --- | --- | --- | --- |
|  | 615 | **459.0116** | **458.0043** | **458.2601** | **-0.2558** | **0** | **6** | **52** | **1** | **R.AAAAR.L** 608 612 613 617 618 620 |
|  | 1290 | **601.3630** | **600.3557** | **600.3595** | **-0.0038** | **0** | **12** | **16** | **5** | **R.ELLAR.K** |
|  | 1308 | **604.3007** | **603.2934** | **603.3380** | **-0.0446** | **0** | **19** | **3.1** | **1** | **K.NVPFK.I** 1309 |
|  | 1646 | 680.3572 | 679.3499 | 679.3323 | 0.0176 | 0 | 8 | 22 | 2 | -.MISASR.A |
|  | 1961 | 774.4308 | 773.4235 | 773.4283 | -0.0048 | 0 | 27 | 0.47 | 1 | R.NTTIPTK.K |
|  | 2840 | 424.6559 | 847.2973 | 846.4018 | 0.8955 | 0 | (6) | 54 | 9 | K.DNMALQR.V |
|  | 3109 | **432.2173** | **862.4200** | **862.3967** | **0.0233** | **0** | **6** | **50** | **9** | **K.DNMALQR.V** |
|  | 3712 | 451.2454 | 900.4762 | 900.4415 | 0.0347 | 0 | 10 | 21 | 1 | K.LFEMAYK.K |
|  | 3724 | **451.2634** | **900.5123** | **899.5229** | **0.9894** | **1** | **6** | **50** | **5** | **R.HIVKEFK.R** 3718 |
|  | 4527 | **479.7480** | **957.4814** | **957.4879** | **-0.0066** | **0** | **70** | **1.7e-005** | **1** | **K.VLENAEGAR.T** |
|  | 4796 | **489.7283** | **977.4420** | **977.4488** | **-0.0067** | **0** | **16** | **4.7** | **4** | **K.AMQDAEVSK.S** |
|  | 4829 | **490.7748** | **979.5351** | **978.4328** | **1.1023** | **0** | **9** | **18** | **2** | **K.DDIENMVK.N** |
|  | 7084 | 575.3068 | 1148.5991 | 1147.5469 | 1.0522 | 1 | 12 | 9.2 | 4 | R.KDSETGENIR.Q |
|  | 9247 | **635.3832** | **1268.7519** | **1269.7405** | **-0.9885** | **1** | **4** | **56** | **7** | **K.LINRNTTIPTK.K** |
|  | 9529 | **429.0879** | **1284.2418** | **1284.6786** | **-0.4368** | **1** | **1** | **79** | **9** | **K.QAKVLENAEGAR.T** |
|  | 9642 | **645.8392** | **1289.6638** | **1289.6728** | **-0.0090** | **0** | **40** | **0.015** | **1** | **K.VQQTVQDLFGR.A** 9609 |
|  | 10454 | **667.8201** | **1333.6257** | **1332.6310** | **0.9947** | **0** | **51** | **0.0012** | **1** | **K.ETAENYLGHTAK.N** |
|  | 10931 | **681.3705** | **1360.7265** | **1360.7351** | **-0.0085** | **0** | **23** | **0.66** | **1** | **R.AQFEGIVTDLIR.R** |
|  | 11675 | **702.8824** | **1403.7503** | **1404.6555** | **-0.9051** | **1** | **2** | **93** | **7** | **K.DDIENMVKNAEK.Y** |
|  | 12414 | **483.0226** | **1446.0458** | **1445.7548** | **0.2910** | **0** | **(7)** | **19** | **6** | **K.SDIGEVILVGGMTR.M** |
|  | 12456 | **725.8570** | **1449.6994** | **1449.7100** | **-0.0106** | **0** | **39** | **0.016** | **1** | **R.TTPSVVAFTADGER.L** |
|  | 12649 | **488.2468** | **1461.7185** | **1461.7497** | **-0.0313** | **0** | **8** | **20** | **5** | **K.SDIGEVILVGGMTR.M** 12637 |
|  | 14051 | **777.4207** | **1552.8269** | **1552.8323** | **-0.0054** | **0** | **48** | **0.0021** | **1** | **K.LYSPSQIGAFVLMK.M** |
|  | 14276 | 784.8839 | 1567.7531 | 1567.7631 | -0.0099 | 0 | 2 | 88 | 3 | R.QAVTNPNNTFYATK.R |
|  | 14630 | **796.8850** | **1591.7555** | **1591.7664** | **-0.0109** | **1** | **30** | **0.11** | **1** | **K.MKETAENYLGHTAK.N** |
|  | 14638 | **796.9760** | **1591.9375** | **1591.9450** | **-0.0075** | **0** | **6** | **28** | **1** | **K.LLGQFTLIGIPPAPR.G** |
|  | 15473 | **823.4385** | **1644.8625** | **1644.8723** | **-0.0097** | **0** | **17** | **2.3** | **1** | **R.VINEPTAAALAYGLDK.S** |
|  | 15717 | **832.4144** | **1662.8143** | **1661.8559** | **0.9584** | **1** | **2** | **79** | **5** | **R.MPKVQQTVQDLFGR.A** |
|  | 16149 | **847.9232** | **1693.8318** | **1693.8424** | **-0.0106** | **0** | **10** | **12** | **1** | **K.NAVITVPAYFNDSQR.Q** |
|  | 16426 | 571.6674 | 1711.9804 | 1712.7271 | -0.7467 | 1 | 10 | 11 | 1 | K.MASEREGSGSSGTGEQK.E 16442 |
|  | 17831 | 909.9578 | 1817.9011 | 1817.9379 | -0.0369 | 1 | 2 | 65 | 4 | K.SDIGEVILVGGMTRMPK.V |
|  | 18108 | 918.4618 | 1834.9090 | 1833.9329 | 0.9762 | 1 | (1) | 86 | 8 | K.SDIGEVILVGGMTRMPK.V |
|  | 18447 | **619.6363** | **1855.8872** | **1855.8986** | **-0.0113** | **0** | **30** | **0.098** | **1** | **R.VEAVNMAEGIIHDTETK.M** 18415 |
|  | 18628 | **624.9907** | **1871.9503** | **1871.8935** | **0.0569** | **0** | **(3)** | **47** | **3** | **R.VEAVNMAEGIIHDTETK.M** 18632 18648 |
|  | 20737 | 1028.0458 | 2054.0770 | 2054.9545 | -0.8775 | 0 | 17 | 1.5 | 2 | K.STNGDTFLGGEDFDQALLR.H |
|  | 20936 | **1039.5095** | **2077.0045** | **2077.0184** | **-0.0139** | **0** | **57** | **0.00018** | **1** | **K.GAVVGIDLGTTNSCVAVMEGK.Q** |
|  | 21098 | **698.3511** | **2092.0316** | **2093.0133** | **-0.9817** | **0** | **(2)** | **51** | **1** | **K.GAVVGIDLGTTNSCVAVMEGK.Q** 21093 |
|  | 24964 | **851.4515** | **2551.3328** | **2551.3282** | **0.0046** | **1** | **2** | **37** | **1** | **R.GVPQIEVTFDIDANGIVHVSAKDK.G** |
|  | 31048 | **1530.2929** | **4587.8567** | **4588.4892** | **-0.6325** | **0** | **1** | **12** | **8** | **K.AVNPDEAVAIGAAIQGGVLAGDVTDVLLLDVTPLSLGIETLGGVFTK.L** |

| **238.** | P05451\|REG1A_HUMAN    **Mass:** 19118    **Score:** 206    **Queries matched:** 10   **emPAI:** 0.74 |
| --- | --- |
|  | Lithostathine-1-alpha OS=Homo sapiens GN=REG1A PE=1 SV=3 |

|  | **Query** | **Observed** | **Mr(expt)** | **Mr(calc)** | **Delta** | **Miss** | **Score** | **Expect** | **Rank** | **Peptide** |
| --- | --- | --- | --- | --- | --- | --- | --- | --- | --- | --- |
|  | 3076 | 862.4392 | 861.4319 | 861.3538 | 0.0780 | 0 | 0 | 1.9e+002 | 10 | K.DVPCEDK.F |
|  | 9191 | **634.2858** | **1266.5570** | **1266.5663** | **-0.0093** | **0** | **13** | **6.7** | **2** | **R.ISCPEGTNAYR.S** |
|  | 12259 | **479.5672** | **1435.6799** | **1435.6884** | **-0.0086** | **0** | **16** | **3** | **1** | **R.WHWSSGSLVSYK.S** 12258 |
|  | 15243 | **816.3629** | **1630.7113** | **1629.7167** | **0.9946** | **1** | **5** | **37** | **2** | **K.DVPCEDKFSFVCK.F** |
|  | 19359 | **965.4464** | **1928.8783** | **1928.8905** | **-0.0122** | **0** | **42** | **0.0062** | **1** | **K.ESGTDDFNVWIGLHDPK.K** |
|  | 25733 | **1344.1342** | **2686.2538** | **2686.2697** | **-0.0160** | **0** | **91** | **4.9e-008** | **1** | **K.SWGIGAPSSVNPGYCVSLTSSTGFQK.W** 25734 25735 |
|  | 30170 | **1281.9530** | **3842.8372** | **3842.8495** | **-0.0123** | **0** | **53** | **0.00015** | **1** | **R.ETWVDADLYCQNMNSGNLVSVLTQAEGAFVASLIK.E** |

| **239.** | P00338\|LDHA_HUMAN    **Mass:** 36950    **Score:** 206    **Queries matched:** 27   **emPAI:** 1.17 |
| --- | --- |
|  | L-lactate dehydrogenase A chain OS=Homo sapiens GN=LDHA PE=1 SV=2 |

|  | **Query** | **Observed** | **Mr(expt)** | **Mr(calc)** | **Delta** | **Miss** | **Score** | **Expect** | **Rank** | **Peptide** |
| --- | --- | --- | --- | --- | --- | --- | --- | --- | --- | --- |
|  | 1829 | **734.4145** | **733.4073** | **733.4123** | **-0.0050** | **0** | **32** | **0.15** | **1** | **R.NVNIFK.F** |
|  | 1852 | **742.4518** | **741.4445** | **741.4497** | **-0.0052** | **0** | **7** | **30** | **2** | **R.LNLVQR.N** |
|  | 3890 | **457.2919** | **912.5692** | **912.5756** | **-0.0064** | **0** | **29** | **0.21** | **1** | **K.LVIITAGAR.Q** |
|  | 5243 | **506.2371** | **1010.4596** | **1010.4669** | **-0.0072** | **0** | **30** | **0.14** | **1** | **K.DYNVTANSK.L** 5244 |
|  | 5255 | **506.2830** | **1010.5514** | **1010.5583** | **-0.0069** | **0** | **11** | **11** | **1** | **R.VHPVSTMIK.G** |
|  | 6712 | 560.3209 | 1118.6272 | 1118.6335 | -0.0064 | 0 | 15 | 5.5 | 2 | K.DQLIYNLLK.E |
|  | 7549 | **592.5642** | **1183.1137** | **1182.6543** | **0.4594** | **1** | **8** | **14** | **1** | **R.RVHPVSTMIK.G** |
|  | 8784 | **623.2872** | **1244.5599** | **1245.6717** | **-1.1118** | **1** | **5** | **50** | **1** | **K.KSADTLWGIQK.E** |
|  | 8831 | **624.7997** | **1247.5848** | **1247.5928** | **-0.0080** | **0** | **60** | **0.00013** | **1** | **R.VIGSGCNLDSAR.F** 8830 8832 |
|  | 9124 | **632.3207** | **1262.6269** | **1263.6711** | **-1.0441** | **0** | **5** | **50** | **9** | **K.QVVESAYEVIK.L** 9152 |
|  | 11179 | **688.8456** | **1375.6766** | **1374.7354** | **0.9412** | **1** | **1** | **1.1e+002** | **3** | **K.VTLTSEEEARLK.K** |
|  | 13185 | **498.9391** | **1493.7954** | **1494.7678** | **-0.9725** | **1** | **5** | **39** | **9** | **K.IVSGKDYNVTANSK.L** |
|  | 15283 | **817.3899** | **1632.7652** | **1632.7752** | **-0.0100** | **0** | **37** | **0.023** | **1** | **K.GEMMDLQHGSLFLR.T** |
|  | 17042 | 879.4400 | 1756.8654 | 1756.9359 | -0.0705 | 1 | 0 | 1.1e+002 | 4 | K.EVHKQVVESAYEVIK.L |
|  | 18662 | **625.6549** | **1873.9429** | **1873.9542** | **-0.0113** | **1** | **21** | **0.8** | **1** | **K.LKGEMMDLQHGSLFLR.T** 18661 |
|  | 19517 | **972.5599** | **1943.1052** | **1943.1132** | **-0.0080** | **0** | **21** | **0.73** | **1** | **K.LLIVSNPVDILTYVAWK.I** |
|  | 20903 | **1037.5372** | **2073.0599** | **2073.0742** | **-0.0143** | **1** | **77** | **1.6e-006** | **1** | **K.DQLIYNLLKEEQTPQNK.I** |
|  | 21374 | 1057.0691 | 2112.1236 | 2111.0609 | 1.0628 | 0 | 57 | 0.00018 | 1 | K.GYTSWAIGLSVADLAESIMK.N |
|  | 23433 | **785.0749** | **2352.2030** | **2352.2399** | **-0.0369** | **1** | **1** | **65** | **3** | **K.LKGYTSWAIGLSVADLAESIMK.N** |
|  | 26949 | **974.1815** | **2919.5227** | **2919.5416** | **-0.0189** | **1** | **12** | **3.8** | **1** | **K.GLYGIKDDVFLSVPCILGQNGISDLVK.V** |
|  | 29714 | **917.9529** | **3667.7824** | **3667.8028** | **-0.0204** | **0** | **22** | **0.2** | **1** | **R.LGVHPLSCHGWVLGEHGDSSVPVWSGMNVAGVSLK.T** 29712 |

| **240.** | Q9NZ08\|ERAP1_HUMAN    **Mass:** 107736   **Score:** 204    **Queries matched:** 31   **emPAI:** 0.35 |
| --- | --- |
|  | Endoplasmic reticulum aminopeptidase 1 OS=Homo sapiens GN=ERAP1 PE=1 SV=3 |

|  | **Query** | **Observed** | **Mr(expt)** | **Mr(calc)** | **Delta** | **Miss** | **Score** | **Expect** | **Rank** | **Peptide** |
| --- | --- | --- | --- | --- | --- | --- | --- | --- | --- | --- |
|  | 752 | 490.2640 | 489.2567 | 488.2343 | 1.0224 | 0 | 11 | 27 | 8 | K.GAGER.L |
|  | 1131 | **567.3575** | **566.3502** | **565.2707** | **1.0795** | **0** | **6** | **26** | **1** | **K.SSASSK.L** |
|  | 1411 | **627.4653** | **626.4581** | **627.3163** | **-0.8582** | **0** | **4** | **47** | **2** | **R.NVHMK.Q** |
|  | 2618 | **417.2068** | **832.3991** | **831.4450** | **0.9541** | **1** | **5** | **67** | **6** | **R.TKEGELR.I** |
|  | 4093 | 464.7735 | 927.5325 | 928.3531 | -0.8206 | 0 | 5 | 55 | 4 | K.GMDGFCSR.S |
|  | 5576 | **518.3050** | **1034.5954** | **1034.6012** | **-0.0057** | **0** | **52** | **0.00097** | **1** | **K.ALDLSLYLK.H** 5565 |
|  | 5585 | **1036.5244** | **1035.5171** | **1034.5760** | **0.9411** | **0** | **16** | **4.4** | **1** | **K.SGIVQYLQK.H** |
|  | 5709 | **523.8001** | **1045.5855** | **1046.5365** | **-0.9509** | **0** | **16** | **4.2** | **7** | **K.GACILNMLR.E** |
|  | 7663 | **595.3144** | **1188.6143** | **1188.5663** | **0.0481** | **0** | **4** | **59** | **8** | **K.YQFSLSSTEK.S** |
|  | 8675 | **620.3245** | **1238.6345** | **1237.6853** | **0.9492** | **0** | **2** | **86** | **7** | **R.HLAISNMPLVK.S** |
|  | 8685 | **620.7748** | **1239.5351** | **1239.5441** | **-0.0090** | **0** | **23** | **0.65** | **1** | **R.DMNEVETQFK.A** |
|  | 8914 | **627.2996** | **1252.5847** | **1252.5944** | **-0.0097** | **0** | **39** | **0.019** | **1** | **K.TMMNTWTLQK.G** |
|  | 11514 | **697.8077** | **1393.6009** | **1393.6110** | **-0.0101** | **0** | **29** | **0.17** | **1** | **K.QTWTDEGSVSER.M** |
|  | 11672 | **702.8730** | **1403.7314** | **1403.7409** | **-0.0094** | **0** | **57** | **0.00027** | **1** | **R.ILASTQFEPTAAR.M** |
|  | 14295 | **785.5073** | **1569.0000** | **1568.7947** | **0.2053** | **1** | **7** | **19** | **2** | **K.GFFSSLKENGSQLR.C** |
|  | 14363 | **787.9445** | **1573.8744** | **1573.8828** | **-0.0084** | **0** | **46** | **0.0033** | **1** | **R.ASLINNAFQLVSIGK.L** |
|  | 14661 | **399.7284** | **1594.8845** | **1593.7933** | **1.0912** | **1** | **1** | **89** | **9** | **K.SQIEFALCRTQNK.E** |
|  | 16920 | **584.2983** | **1749.8730** | **1748.8808** | **0.9923** | **0** | **27** | **0.22** | **1** | **K.FMEFVSVSVTHPELK.V** |
|  | 20312 | 506.2285 | 2020.8847 | 2020.8987 | -0.0140 | 0 | 11 | 6.6 | 1 | R.SQHSSSSSHWHQEGVDVK.T 20314 20315 |
|  | 20510 | 1019.4429 | 2036.8712 | 2036.8819 | -0.0108 | 0 | 82 | 5.7e-007 | 1 | K.NEDLWDSMASICPTDGVK.G 20511 |
|  | 22145 | **549.0417** | **2192.1377** | **2193.0082** | **-0.8705** | **0** | **8** | **14** | **1** | **R.CVQQTIETIEENIGWMDK.N** |
|  | 23525 | 788.0579 | 2361.1518 | 2361.1641 | -0.0124 | 0 | 17 | 1.6 | 1 | K.GSDGAPDTGYLWHVPLTFITSK.S |
|  | 23688 | **1195.0933** | **2388.1720** | **2388.1857** | **-0.0138** | **0** | **9** | **9.7** | **1** | **K.HETEIMPVFQGLNELIPMYK.L** 23689 |
|  | 23764 | **801.0433** | **2400.1082** | **2400.1202** | **-0.0121** | **0** | **41** | **0.006** | **1** | **K.FELGSSSIAHMVMGTTNQFSTR.T** |
|  | 23878 | 1209.6198 | 2417.2249 | 2416.1151 | 1.1098 | 0 | (2) | 49 | 4 | K.FELGSSSIAHMVMGTTNQFSTR.T |
|  | 30560 | **1033.0295** | **4128.0891** | **4127.1772** | **0.9119** | **0** | **1** | **23** | **5** | **R.QEQIALLAPEPLLVGLPYTVVIHYAGNLSETFHGFYK.S** |

| **241.** | Q96KP4\|CNDP2_HUMAN    **Mass:** 53187    **Score:** 203    **Queries matched:** 23   **emPAI:** 0.50 |
| --- | --- |
|  | Cytosolic non-specific dipeptidase OS=Homo sapiens GN=CNDP2 PE=1 SV=2 |

|  | **Query** | **Observed** | **Mr(expt)** | **Mr(calc)** | **Delta** | **Miss** | **Score** | **Expect** | **Rank** | **Peptide** |
| --- | --- | --- | --- | --- | --- | --- | --- | --- | --- | --- |
|  | 1460 | 636.3673 | 635.3600 | 634.3438 | 1.0161 | 0 | 11 | 14 | 8 | K.FAELR.S |
|  | 5784 | **526.7320** | **1051.4494** | **1051.4570** | **-0.0076** | **0** | **11** | **11** | **2** | **K.YIDENQDR.Y** |
|  | 6611 | **556.8026** | **1111.5907** | **1111.5986** | **-0.0079** | **0** | **11** | **11** | **1** | **K.TGQEIPVNVR.F** |
|  | 10101 | **658.3729** | **1314.7313** | **1313.7191** | **1.0122** | **0** | **3** | **74** | **4** | **K.QLGGSVELVDIGK.Q** |
|  | 13274 | **750.8910** | **1499.7674** | **1499.7773** | **-0.0098** | **0** | **40** | **0.014** | **1** | **K.WVAIQSVSAWPEK.R** 13275 |
|  | 14204 | 522.2353 | 1563.6840 | 1562.8127 | 0.8714 | 1 | 6 | 30 | 1 | R.AMKTVFGVEPDLTR.E |
|  | 14299 | **524.2426** | **1569.7058** | **1569.8726** | **-0.1668** | **1** | **11** | **9.7** | **1** | **K.QLGGSVELVDIGKQK.L** 14313 |
|  | 14444 | 527.6164 | 1579.8273 | 1578.8076 | 1.0198 | 1 | (4) | 52 | 8 | R.AMKTVFGVEPDLTR.E |
|  | 16657 | **866.3746** | **1730.7346** | **1731.7879** | **-1.0533** | **0** | **16** | **2.7** | **1** | **K.LYDDIDFDIEEFAK.D** |
|  | 16697 | 867.9437 | 1733.8728 | 1733.8836 | -0.0108 | 0 | 47 | 0.0024 | 1 | R.EGGSIPVTLTFQEATGK.N 16696 |
|  | 17762 | 906.9667 | 1811.9188 | 1811.9934 | -0.0746 | 1 | 4 | 41 | 2 | K.LAKWVAIQSVSAWPEK.R 17763 |
|  | 18281 | **924.4481** | **1846.8817** | **1845.8244** | **1.0573** | **0** | **2** | **65** | **10** | **K.DVDYVCISDNYWLGK.K** |
|  | 22237 | **1103.0714** | **2204.1283** | **2204.1399** | **-0.0116** | **0** | **70** | **9.1e-006** | **1** | **R.LVPNMTPEVVGEQVTSYLTK.K** 22236 |
|  | 22393 | 1110.5751 | 2219.1356 | 2219.1123 | 0.0233 | 1 | 0 | 76 | 5 | R.WRYPSLSLHGIEGAFSGSGAK.T |
|  | 27817 | **623.5183** | **3112.5552** | **3112.5685** | **-0.0134** | **1** | **35** | **0.015** | **1** | **K.DLHSGVYGGSVHEAMTDLILLMGSLVDKR.G** 27818 27819 |
|  | 28632 | **1106.5267** | **3316.5584** | **3316.5711** | **-0.0127** | **0** | **38** | **0.0071** | **1** | **K.TVCIYGHLDVQPAALEDGWDSEPFTLVER.D** |

| **242.** | P50224\|ST1A3_HUMAN    **Mass:** 34288    **Score:** 203    **Queries matched:** 14   **emPAI:** 0.68 |
| --- | --- |
|  | Sulfotransferase 1A3/1A4 OS=Homo sapiens GN=SULT1A3 PE=1 SV=1 |

|  | **Query** | **Observed** | **Mr(expt)** | **Mr(calc)** | **Delta** | **Miss** | **Score** | **Expect** | **Rank** | **Peptide** |
| --- | --- | --- | --- | --- | --- | --- | --- | --- | --- | --- |
|  | 4501 | 479.2159 | 956.4173 | 957.4080 | -0.9907 | 0 | 18 | 2.7 | 1 | R.FDADYAEK.M |
|  | 5486 | **515.2902** | **1028.5659** | **1027.4579** | **1.1080** | **0** | **7** | **35** | **4** | **K.MAGCSLSFR.S** |
|  | 5695 | **523.2839** | **1044.5533** | **1044.6080** | **-0.0547** | **1** | **1** | **1.5e+002** | **10** | **K.VVYVARNPK.D** |
|  | 14947 | **538.9521** | **1613.8344** | **1612.7522** | **1.0823** | **0** | **6** | **32** | **2** | **K.AHPEPGTWDSFLEK.F** |
|  | 15608 | 828.4060 | 1654.7975 | 1654.8065 | -0.0090 | 0 | 46 | 0.0031 | 1 | R.THPVLYLFYEDMK.E 15625 |
|  | 17436 | **894.0206** | **1786.0266** | **1786.0352** | **-0.0087** | **0** | **51** | **0.00077** | **1** | **K.SHLPLALLPQTLLDQK.V** |
|  | 19238 | **960.4421** | **1918.8697** | **1917.9870** | **0.8828** | **0** | **5** | **30** | **1** | **-.MELIQDTSRPPLEYVK.G** |
|  | 21485 | **708.6824** | **2123.0253** | **2123.0397** | **-0.0145** | **1** | **16** | **2.3** | **1** | **R.THPVLYLFYEDMKENPK.R** |
|  | 23334 | **1171.0669** | **2340.1192** | **2340.1308** | **-0.0115** | **0** | **104** | **3.2e-009** | **1** | **K.SGTTWVSQILDMIYQGGDLEK.C** 23335 |
|  | 25862 | 902.1273 | 2703.3600 | 2703.3756 | -0.0156 | 1 | 26 | 0.18 | 1 | R.VPFLEVNDPGEPSGLETLKDTPPPR.L |
|  | 27185 | **989.8419** | **2966.5038** | **2966.5178** | **-0.0140** | **0** | **59** | **7e-005** | **1** | **K.YFAEALGPLQSFQARPDDLLINTYPK.S** |
|  | 30989 | **1137.0055** | **4543.9929** | **4543.0718** | **0.9210** | **1** | **1** | **15** | **5** | **K.FMAGEVSYGSWYQHVQEWWELSRTHPVLYLFYEDMK.E** |

| **243.** | Q9BY64\|UDB28_HUMAN    **Mass:** 61493    **Score:** 201    **Queries matched:** 17   **emPAI:** 0.12 |
| --- | --- |
|  | UDP-glucuronosyltransferase 2B28 OS=Homo sapiens GN=UGT2B28 PE=1 SV=1 |

|  | **Query** | **Observed** | **Mr(expt)** | **Mr(calc)** | **Delta** | **Miss** | **Score** | **Expect** | **Rank** | **Peptide** |
| --- | --- | --- | --- | --- | --- | --- | --- | --- | --- | --- |
|  | 683 | 473.2791 | 472.2719 | 472.2758 | -0.0039 | 0 | 13 | 13 | 5 | K.GAAVR.L 688 |
|  | 4010 | 461.2456 | 920.4767 | 919.4586 | 1.0181 | 0 | 6 | 40 | 7 | K.ADIWLMR.N |
|  | 6469 | 550.8162 | 1099.6178 | 1098.6761 | 0.9417 | 1 | 15 | 4.8 | 2 | K.TILKELVQR.G |
|  | 9315 | 425.0561 | 1272.1464 | 1272.5479 | -0.4015 | 0 | 10 | 11 | 6 | K.LSDQMTFMER.V 9343 |
|  | 11516 | **697.8632** | **1393.7119** | **1392.7765** | **0.9354** | **0** | **4** | **58** | **8** | **K.WIPQNDLLGLPK.T** |
|  | 12271 | 719.4207 | 1436.8269 | 1436.8715 | -0.0446 | 1 | 32 | 0.089 | 3 | R.ANVIATALAKIPQK.V 12272 |
|  | 13483 | 505.9178 | 1514.7315 | 1515.7061 | -0.9747 | 1 | 9 | 15 | 3 | K.LSDQMTFMERVK.N |
|  | 17900 | **912.4662** | **1822.9178** | **1821.9059** | **1.0120** | **1** | **10** | **12** | **1** | **R.AVFWIEFVMCHKGAK.H** |
|  | 24592 | 1253.1022 | 2504.1898 | 2504.2046 | -0.0148 | 0 | 77 | 1.3e-006 | 1 | K.WDQFYSEVLGRPTTLFETMGK.A 24590 24593 24594 24595 |
|  | 25353 | 1313.7124 | 2625.4102 | 2626.3564 | -0.9461 | 1 | 1 | 48 | 5 | K.LEVYPTSLTKTEFENIIMQQVK.R |

| **244.** | Q3SYC2\|MOGT2_HUMAN    **Mass:** 38456    **Score:** 201    **Queries matched:** 15   **emPAI:** 0.75 |
| --- | --- |
|  | 2-acylglycerol O-acyltransferase 2 OS=Homo sapiens GN=MOGAT2 PE=1 SV=2 |

|  | **Query** | **Observed** | **Mr(expt)** | **Mr(calc)** | **Delta** | **Miss** | **Score** | **Expect** | **Rank** | **Peptide** |
| --- | --- | --- | --- | --- | --- | --- | --- | --- | --- | --- |
|  | 1838 | **737.4362** | **736.4289** | **736.4344** | **-0.0055** | **0** | **22** | **0.98** | **1** | **R.HIQAIR.C** |
|  | 3535 | **445.0337** | **888.0528** | **887.4348** | **0.6180** | **0** | **10** | **13** | **1** | **K.TAELDPSR.N** |
|  | 5251 | **506.2688** | **1010.5231** | **1009.5305** | **0.9927** | **0** | **5** | **43** | **3** | **K.ESAAHILNR.K** 5256 |
|  | 13105 | **745.8361** | **1489.6577** | **1489.6660** | **-0.0083** | **0** | **30** | **0.12** | **1** | **K.FNIPADQHLEFC.-** 13106 |
|  | 13576 | **761.3779** | **1520.7413** | **1520.7486** | **-0.0073** | **0** | **62** | **9.1e-005** | **1** | **M.VEFAPLFMPWER.R** 13575 13577 |
|  | 16388 | 570.7812 | 1709.3217 | 1708.9810 | 0.3407 | 1 | 2 | 49 | 8 | R.LQKIMGISLPLFHGR.G |
|  | 17809 | **908.9451** | **1815.8756** | **1815.8864** | **-0.0108** | **0** | **45** | **0.0034** | **1** | **K.TLHPSEEEVNQLHQR.Y** 17808 |
|  | 23902 | **605.8238** | **2419.2663** | **2420.2005** | **-0.9342** | **1** | **4** | **29** | **2** | **R.DYIMSAGLVTSEKESAAHILNR.K** |
|  | 26625 | **955.8660** | **2864.5763** | **2864.5872** | **-0.0110** | **0** | **57** | **0.00011** | **1** | **K.GGGNLLGIIVGGAQEALDARPGSFTLLLR.N** |
|  | 29468 | **1200.2773** | **3597.8102** | **3597.8256** | **-0.0154** | **0** | **50** | **0.00032** | **1** | **R.LALTHGAPLVPIFSFGENDLFDQIPNSSGSWLR.Y** |

| **245.** | P84077\|ARF1_HUMAN    **Mass:** 20741    **Score:** 200    **Queries matched:** 20   **emPAI:** 1.34 |
| --- | --- |
|  | ADP-ribosylation factor 1 OS=Homo sapiens GN=ARF1 PE=1 SV=2 |

|  | **Query** | **Observed** | **Mr(expt)** | **Mr(calc)** | **Delta** | **Miss** | **Score** | **Expect** | **Rank** | **Peptide** |
| --- | --- | --- | --- | --- | --- | --- | --- | --- | --- | --- |
|  | 2763 | **421.2294** | **840.4443** | **839.5130** | **0.9313** | **0** | **8** | **24** | **8** | **K.IRPLWR.H** |
|  | 4760 | 488.2868 | 974.5590 | 975.4695 | -0.9104 | 0 | 5 | 67 | 5 | R.MLAEDELR.D |
|  | 6295 | **544.3089** | **1086.6032** | **1086.6107** | **-0.0075** | **0** | **38** | **0.027** | **1** | **R.ILMVGLDAAGK.T** |
|  | 6491 | **551.7946** | **1101.5746** | **1102.6056** | **-1.0311** | **0** | **(1)** | **1.5e+002** | **5** | **R.ILMVGLDAAGK.T** |
|  | 8806 | **416.2394** | **1245.6965** | **1245.6135** | **0.0830** | **1** | **14** | **6.5** | **1** | **R.VNEAREELMR.M** |
|  | 14235 | **783.3787** | **1564.7428** | **1564.7522** | **-0.0094** | **0** | **50** | **0.001** | **1** | **K.NISFTVWDVGGQDK.I** |
|  | 15219 | **815.8859** | **1629.7573** | **1629.7668** | **-0.0095** | **0** | **8** | **19** | **1** | **K.QDLPNAMNAAEITDK.L** 15225 |
|  | 15491 | **549.8032** | **1646.3877** | **1645.7617** | **0.6259** | **0** | **(1)** | **62** | **5** | **K.QDLPNAMNAAEITDK.L** |
|  | 15605 | **414.7019** | **1654.7786** | **1655.8858** | **-1.1071** | **1** | **4** | **45** | **9** | **-.MGNIFANLFKGLFGK.K** |
|  | 15750 | **556.2821** | **1665.8245** | **1665.7702** | **0.0543** | **1** | **8** | **16** | **1** | **R.EELMRMLAEDELR.D** |
|  | 17881 | **608.3079** | **1821.9019** | **1822.0274** | **-0.1255** | **1** | **4** | **51** | **1** | **R.ILMVGLDAAGKTTILYK.L** 17891 |
|  | 21757 | **718.3621** | **2152.0644** | **2152.0338** | **0.0306** | **0** | **5** | **30** | **2** | **R.HYFQNTQGLIFVVDSNDR.E** |
|  | 23212 | **1162.1194** | **2322.2242** | **2322.2359** | **-0.0117** | **0** | **46** | **0.0018** | **1** | **K.LGEIVTTIPTIGFNVETVEYK.N** 23210 23211 23213 |
|  | 24055 | **813.3949** | **2437.1629** | **2437.1775** | **-0.0146** | **1** | **42** | **0.0041** | **1** | **R.HYFQNTQGLIFVVDSNDRER.V** |
|  | 27890 | **1566.2245** | **3130.4344** | **3130.4454** | **-0.0110** | **0** | **61** | **4e-005** | **1** | **R.NWYIQATCATSGDGLYEGLDWLSNQLR.N** |

| **246.** | P19075\|TSN8_HUMAN    **Mass:** 26711    **Score:** 200    **Queries matched:** 11   **emPAI:** 0.30 |
| --- | --- |
|  | Tetraspanin-8 OS=Homo sapiens GN=TSPAN8 PE=1 SV=1 |

|  | **Query** | **Observed** | **Mr(expt)** | **Mr(calc)** | **Delta** | **Miss** | **Score** | **Expect** | **Rank** | **Peptide** |
| --- | --- | --- | --- | --- | --- | --- | --- | --- | --- | --- |
|  | 2246 | 403.2276 | 804.4406 | 804.4164 | 0.0242 | 0 | 12 | 15 | 2 | M.AGVSACIK.Y 2235 |
|  | 5557 | **517.7682** | **1033.5219** | **1033.5291** | **-0.0072** | **0** | **39** | **0.022** | **1** | **K.LLSATGESEK.Q** 5558 |
|  | 8614 | **619.2012** | **1236.3878** | **1236.5669** | **-0.1792** | **0** | **4** | **47** | **1** | **K.QRPCQSYNGK.Q** |
|  | 16995 | **878.4470** | **1754.8794** | **1754.8879** | **-0.0086** | **0** | **90** | **1.1e-007** | **1** | **K.QFQEAIIVFQEEFK.C** 16994 16996 16997 17004 17009 |

| **247.** | P01892\|1A02_HUMAN    **Mass:** 41181    **Score:** 200    **Queries matched:** 17   **emPAI:** 0.84 |
| --- | --- |
|  | HLA class I histocompatibility antigen, A-2 alpha chain OS=Homo sapiens GN=HLA-A PE=1 SV=1 |

|  | **Query** | **Observed** | **Mr(expt)** | **Mr(calc)** | **Delta** | **Miss** | **Score** | **Expect** | **Rank** | **Peptide** |
| --- | --- | --- | --- | --- | --- | --- | --- | --- | --- | --- |
|  | 5040 | 498.7649 | 995.5153 | 995.4308 | 0.0845 | 0 | 16 | 3.9 | 3 | R.FDSDAASQR.M 5054 |
|  | 5928 | **531.7654** | **1061.5162** | **1062.5683** | **-1.0521** | **1** | **7** | **34** | **10** | **K.VKAHSQTHR.V** |
|  | 10652 | **673.2642** | **1344.5139** | **1344.5227** | **-0.0088** | **0** | **23** | **0.64** | **1** | **R.MYGCDVGSDWR.F** |
|  | 12099 | 714.3628 | 1426.7111 | 1425.7365 | 0.9747 | 0 | 3 | 68 | 4 | K.WAAVVVPSGQEQR.Y |
|  | 13614 | 508.9797 | 1523.9174 | 1524.6627 | -0.7453 | 1 | 7 | 23 | 1 | R.FDSDAASQRMEPR.A 13613 |
|  | 14374 | **525.9420** | **1574.8041** | **1573.8113** | **0.9927** | **1** | **23** | **0.64** | **1** | **K.HKWEAAHVAEQLR.A** |
|  | 15206 | 815.4121 | 1628.8097 | 1628.8199 | -0.0102 | 0 | 72 | 7.9e-006 | 1 | R.FIAVGYVDDTQFVR.F 15203 15205 |
|  | 18194 | **614.9559** | **1841.8460** | **1841.8591** | **-0.0132** | **0** | **31** | **0.087** | **1** | **K.THMTHHAVSDHEATLR.C** 18195 18196 |
|  | 20661 | 513.0184 | 2048.0446 | 2048.0626 | -0.0179 | 0 | 15 | 3.2 | 1 | R.YTCHVQHEGLPKPLTLR.W |
|  | 25408 | 1319.0913 | 2636.1681 | 2636.1838 | -0.0158 | 0 | 68 | 1.1e-005 | 1 | R.DGEDQTQDTELVETRPAGDGTFQK.W 25407 |

| **248.** | P10316\|1A69_HUMAN    **Mass:** 41236    **Score:** 200    **Queries matched:** 17   **emPAI:** 0.84 |
| --- | --- |
|  | HLA class I histocompatibility antigen, A-69 alpha chain OS=Homo sapiens GN=HLA-A PE=1 SV=2 |

|  | **Query** | **Observed** | **Mr(expt)** | **Mr(calc)** | **Delta** | **Miss** | **Score** | **Expect** | **Rank** | **Peptide** |
| --- | --- | --- | --- | --- | --- | --- | --- | --- | --- | --- |
|  | 5040 | 498.7649 | 995.5153 | 995.4308 | 0.0845 | 0 | 16 | 3.9 | 3 | R.FDSDAASQR.M 5054 |
|  | 10652 | 673.2642 | 1344.5139 | 1344.5227 | -0.0088 | 0 | 23 | 0.64 | 1 | R.MYGCDVGSDWR.F |
|  | 12099 | 714.3628 | 1426.7111 | 1425.7365 | 0.9747 | 0 | 3 | 68 | 4 | K.WAAVVVPSGQEQR.Y |
|  | 13614 | 508.9797 | 1523.9174 | 1524.6627 | -0.7453 | 1 | 7 | 23 | 1 | R.FDSDAASQRMEPR.A 13613 |
|  | 14374 | 525.9420 | 1574.8041 | 1573.8113 | 0.9927 | 1 | 23 | 0.64 | 1 | K.HKWEAAHVAEQLR.A |
|  | 15206 | 815.4121 | 1628.8097 | 1628.8199 | -0.0102 | 0 | 72 | 7.9e-006 | 1 | R.FIAVGYVDDTQFVR.F 15203 15205 |
|  | 18194 | 614.9559 | 1841.8460 | 1841.8591 | -0.0132 | 0 | 31 | 0.087 | 1 | K.THMTHHAVSDHEATLR.C 18195 18196 |
|  | 20661 | 513.0184 | 2048.0446 | 2048.0626 | -0.0179 | 0 | 15 | 3.2 | 1 | R.YTCHVQHEGLPKPLTLR.W |
|  | 25408 | 1319.0913 | 2636.1681 | 2636.1838 | -0.0158 | 0 | 68 | 1.1e-005 | 1 | R.DGEDQTQDTELVETRPAGDGTFQK.W 25407 |
|  | 29926 | **1252.9806** | **3755.9199** | **3754.9617** | **0.9582** | **1** | **1** | **28** | **3** | **R.TLVLLLSGALALTQTWAGSHSMRYFYTSVSRPGR.G** |

|  | **Proteins matching a subset of these peptides:** |
| --- | --- |

|  | P01891\|1A68_HUMAN    **Mass:** 41168    **Score:** 192    **Queries matched:** 14 |
| --- | --- |
|  | HLA class I histocompatibility antigen, A-68 alpha chain OS=Homo sapiens GN=HLA-A PE=1 SV=4 |

| **249.** | Q6NUM9\|RETST_HUMAN    **Mass:** 67461    **Score:** 200    **Queries matched:** 27   **emPAI:** 0.24 |
| --- | --- |
|  | All-trans-retinol 13,14-reductase OS=Homo sapiens GN=RETSAT PE=2 SV=2 |

|  | **Query** | **Observed** | **Mr(expt)** | **Mr(calc)** | **Delta** | **Miss** | **Score** | **Expect** | **Rank** | **Peptide** |
| --- | --- | --- | --- | --- | --- | --- | --- | --- | --- | --- |
|  | 1771 | **716.4247** | **715.4174** | **715.4228** | **-0.0054** | **0** | **22** | **1.4** | **2** | **R.AGGAVLTK.A** |
|  | 3317 | 437.2606 | 872.5066 | 872.4828 | 0.0238 | 1 | 10 | 21 | 9 | K.NLDSRIR.A |
|  | 3351 | 439.1413 | 876.2680 | 877.3963 | -1.1283 | 0 | (6) | 45 | 6 | R.MEEGSIGR.F |
|  | 3610 | 447.9905 | 893.9664 | 893.3913 | 0.5751 | 0 | 8 | 15 | 2 | R.MEEGSIGR.F |
|  | 5755 | **525.3107** | **1048.6068** | **1048.5917** | **0.0151** | **1** | **9** | **21** | **3** | **K.AYIQGLKEK.F** |
|  | 6386 | 547.7248 | 1093.4351 | 1093.4434 | -0.0082 | 0 | 22 | 0.89 | 1 | K.AGGCCHTFGK.N |
|  | 6487 | **551.7819** | **1101.5493** | **1101.5091** | **0.0402** | **1** | **24** | **0.64** | **1** | **K.RGSDYETFK.N** |
|  | 8242 | **609.2949** | **1216.5753** | **1217.6040** | **-1.0287** | **0** | **2** | **91** | **1** | **K.QAFSANQVPEK.L** |
|  | 8492 | 615.7869 | 1229.5592 | 1230.5590 | -0.9998 | 1 | 10 | 16 | 2 | R.KEYPMYSGEK.A 8488 |
|  | 9670 | **646.3856** | **1290.7567** | **1290.7660** | **-0.0093** | **0** | **35** | **0.048** | **1** | **K.VVSSGAPHAILLK.F** |
|  | 11783 | **705.4258** | **1408.8371** | **1408.8442** | **-0.0071** | **0** | **40** | **0.013** | **1** | **K.FLPLPVVQLLDR.C** 11784 |
|  | 11800 | **470.9264** | **1409.7573** | **1409.7224** | **0.0348** | **0** | **4** | **54** | **6** | **K.NSFVEASMSVVLK.L** |
|  | 12445 | **483.9405** | **1448.7996** | **1448.8099** | **-0.0104** | **1** | **7** | **30** | **1** | **K.RPPAPLVTDKEAR.K** |
|  | 19992 | 498.0008 | 1987.9741 | 1989.0565 | -1.0824 | 1 | 6 | 22 | 7 | K.ATVQSVLLDSAGKACGVSVK.K |
|  | 20651 | **683.3678** | **2047.0816** | **2046.3043** | **0.7773** | **0** | **2** | **64** | **8** | **M.WLPLVLLLAVLLLAVLCK.V** |
|  | 21467 | **1061.0453** | **2120.0760** | **2120.0902** | **-0.0142** | **0** | **86** | **2.4e-007** | **1** | **K.VESVTAGSPLTNQFYLAAPR.G** 21468 |
|  | 23322 | **780.4253** | **2338.2542** | **2337.1199** | **1.1344** | **1** | **1** | **64** | **8** | **R.GSDYETFKNSFVEASMSVVLK.L** 23297 23318 |
|  | 24164 | **817.9459** | **2450.8158** | **2450.1648** | **0.6509** | **1** | **0** | **47** | **2** | **K.NGLEFDTGIHYIGRMEEGSIGR.F** |
|  | 25949 | **1358.6497** | **2715.2848** | **2715.2964** | **-0.0116** | **0** | **27** | **0.14** | **1** | **R.STMIMLIPTAYEWFEEWQAELK.G** |
|  | 29099 | **867.9406** | **3467.7331** | **3466.7456** | **0.9875** | **0** | **4** | **15** | **1** | **K.GHELVNIYCPIVVSNAGLFNTYEHLLPGNAR.C** |
|  | 30039 | **1263.9971** | **3788.9694** | **3788.9845** | **-0.0151** | **0** | **51** | **0.00024** | **1** | **R.AQSPIPNLYLTGQDIFTCGLVGALQGALLCSSAILK.R** |
|  | 30794 | **1445.3851** | **4333.1336** | **4333.1035** | **0.0301** | **1** | **2** | **12** | **5** | **R.MEEGSIGRFILDQITEGQLDWAPLSSPFDIMVLEGPNGR.K** |

| **250.** | O00264\|PGRC1_HUMAN    **Mass:** 21772    **Score:** 199    **Queries matched:** 8   **emPAI:** 0.91 |
| --- | --- |
|  | Membrane-associated progesterone receptor component 1 OS=Homo sapiens GN=PGRMC1 PE=1 SV=3 |

|  | **Query** | **Observed** | **Mr(expt)** | **Mr(calc)** | **Delta** | **Miss** | **Score** | **Expect** | **Rank** | **Peptide** |
| --- | --- | --- | --- | --- | --- | --- | --- | --- | --- | --- |
|  | 3042 | **430.7466** | **859.4785** | **858.4997** | **0.9789** | **0** | **11** | **17** | **4** | **R.ILMAINGK.V** |
|  | 5413 | **1024.5066** | **1023.4993** | **1023.5059** | **-0.0066** | **0** | **31** | **0.13** | **1** | **R.GLATFCLDK.E** |
|  | 13506 | **758.8605** | **1515.7064** | **1515.7147** | **-0.0083** | **0** | **46** | **0.0029** | **1** | **K.FYGPEGPYGVFAGR.D** |
|  | 15448 | **822.9066** | **1643.7986** | **1643.8096** | **-0.0111** | **1** | **47** | **0.0023** | **1** | **R.KFYGPEGPYGVFAGR.D** 15462 |
|  | 17473 | **597.3122** | **1788.9147** | **1788.9192** | **-0.0045** | **1** | **2** | **74** | **6** | **R.FDGVQDPRILMAINGK.V** |
|  | 29529 | **1208.5515** | **3622.6327** | **3622.6475** | **-0.0148** | **1** | **93** | **1.8e-008** | **1** | **K.EALKDEYDDLSDLTAAQQETLSDWESQFTFK.Y** 29528 |

| **251.** | P14923\|PLAK_HUMAN    **Mass:** 82434    **Score:** 199    **Queries matched:** 19   **emPAI:** 0.30 |
| --- | --- |
|  | Junction plakoglobin OS=Homo sapiens GN=JUP PE=1 SV=3 |

|  | **Query** | **Observed** | **Mr(expt)** | **Mr(calc)** | **Delta** | **Miss** | **Score** | **Expect** | **Rank** | **Peptide** |
| --- | --- | --- | --- | --- | --- | --- | --- | --- | --- | --- |
|  | 2501 | **413.2401** | **824.4657** | **824.3889** | **0.0768** | **0** | **3** | **64** | **4** | **K.AHQDAQR.H** |
|  | 6286 | **1087.5466** | **1086.5394** | **1086.6437** | **-0.1044** | **0** | **5** | **59** | **7** | **R.LNYGIPAIVK.L** |
|  | 8597 | **618.7919** | **1235.5693** | **1235.5782** | **-0.0089** | **1** | **22** | **0.83** | **1** | **R.ISEDKNPDYR.K** |
|  | 9864 | 651.8491 | 1301.6836 | 1301.6914 | -0.0079 | 0 | 54 | 0.00053 | 1 | R.LVQNCLWTLR.N |
|  | 10580 | **671.3132** | **1340.6118** | **1340.7188** | **-0.1070** | **0** | **6** | **33** | **6** | **K.LLNDEDPVVVTK.A** |
|  | 10763 | **676.8049** | **1351.5953** | **1351.6038** | **-0.0085** | **0** | **24** | **0.55** | **1** | **R.TMQNTSDLDTAR.C** 10746 |
|  | 12446 | **725.4094** | **1448.8042** | **1448.8140** | **-0.0098** | **0** | **1** | **1.2e+002** | **3** | **K.LLNQPNQWPLVK.A** |
|  | 13403 | **504.1991** | **1509.5755** | **1509.6877** | **-0.1122** | **0** | **8** | **18** | **5** | **-.MEVMNLMEQPIK.V** 13417 |
|  | 14711 | **800.3924** | **1598.7702** | **1598.7801** | **-0.0099** | **0** | **53** | **0.00055** | **1** | **R.HVAAGTQQPYTDGVR.M** 14710 14712 |
|  | 16800 | **581.3094** | **1740.9063** | **1740.0298** | **0.8766** | **1** | **5** | **32** | **5** | **R.EGLLAIFKSGGIPALVR.M** |
|  | 21812 | **1078.0685** | **2154.1224** | **2154.1367** | **-0.0143** | **0** | **65** | **2.9e-005** | **1** | **R.NLALCPANHAPLQEAAVIPR.L** 21813 |
|  | 23384 | **1174.1495** | **2346.2845** | **2346.2947** | **-0.0102** | **0** | **8** | **11** | **1** | **R.LNTIPLFVQLLYSSVENIQR.V** 23385 |
|  | 28489 | **1088.5414** | **3262.6023** | **3262.6174** | **-0.0150** | **0** | **63** | **2.1e-005** | **1** | **K.ILVNQLSVDDVNVLTCATGTLSNLTCNNSK.N** |

| **252.** | P18085\|ARF4_HUMAN    **Mass:** 20612    **Score:** 198    **Queries matched:** 21   **emPAI:** 1.35 |
| --- | --- |
|  | ADP-ribosylation factor 4 OS=Homo sapiens GN=ARF4 PE=1 SV=3 |

|  | **Query** | **Observed** | **Mr(expt)** | **Mr(calc)** | **Delta** | **Miss** | **Score** | **Expect** | **Rank** | **Peptide** |
| --- | --- | --- | --- | --- | --- | --- | --- | --- | --- | --- |
|  | 4926 | **494.7755** | **987.5364** | **987.5423** | **-0.0059** | **0** | **36** | **0.044** | **1** | **K.MLLVDELR.D** 4940 |
|  | 5187 | **503.2907** | **1004.5668** | **1003.5372** | **1.0296** | **0** | **(7)** | **36** | **9** | **K.MLLVDELR.D** |
|  | 6206 | **541.3305** | **1080.6465** | **1079.5975** | **1.0490** | **0** | **1** | **1.2e+002** | **3** | **M.GLTISSLFSR.L** 6200 |
|  | 6295 | 544.3089 | 1086.6032 | 1086.6107 | -0.0075 | 0 | 38 | 0.027 | 1 | R.ILMVGLDAAGK.T |
|  | 6491 | 551.7946 | 1101.5746 | 1102.6056 | -1.0311 | 0 | (1) | 1.5e+002 | 5 | R.ILMVGLDAAGK.T |
|  | 13303 | **751.8984** | **1501.7823** | **1501.8109** | **-0.0285** | **1** | **7** | **28** | **4** | **K.QMRILMVGLDAAGK.T** |
|  | 13538 | **759.8886** | **1517.7625** | **1517.8058** | **-0.0432** | **1** | **(3)** | **61** | **7** | **K.QMRILMVGLDAAGK.T** |
|  | 13639 | **763.9682** | **1525.9218** | **1524.8664** | **1.0554** | **1** | **10** | **11** | **2** | **M.GLTISSLFSRLFGK.K** |
|  | 15712 | **832.3820** | **1662.7495** | **1662.7593** | **-0.0098** | **0** | **87** | **2.1e-007** | **1** | **K.QDLPNAMAISEMTDK.L** |
|  | 15973 | **560.9511** | **1679.8315** | **1678.7542** | **1.0773** | **0** | **(3)** | **61** | **3** | **K.QDLPNAMAISEMTDK.L** |
|  | 17881 | 608.3079 | 1821.9019 | 1822.0274 | -0.1255 | 1 | 4 | 51 | 1 | R.ILMVGLDAAGKTTILYK.L 17891 |
|  | 21757 | 718.3621 | 2152.0644 | 2152.0338 | 0.0306 | 0 | 5 | 30 | 2 | K.HYFQNTQGLIFVVDSNDR.E |
|  | 23212 | 1162.1194 | 2322.2242 | 2322.2359 | -0.0117 | 0 | 46 | 0.0018 | 1 | K.LGEIVTTIPTIGFNVETVEYK.N 23210 23211 23213 |
|  | 24055 | 813.3949 | 2437.1629 | 2437.1775 | -0.0146 | 1 | 42 | 0.0041 | 1 | K.HYFQNTQGLIFVVDSNDRER.I |
|  | 24133 | **816.4775** | **2446.4108** | **2446.2196** | **0.1912** | **1** | **4** | **23** | **1** | **K.QDLPNAMAISEMTDKLGLQSLR.N** |

| **253.** | P27635\|RL10_HUMAN    **Mass:** 25044    **Score:** 198    **Queries matched:** 22   **emPAI:** 1.69 |
| --- | --- |
|  | 60S ribosomal protein L10 OS=Homo sapiens GN=RPL10 PE=1 SV=4 |

|  | **Query** | **Observed** | **Mr(expt)** | **Mr(calc)** | **Delta** | **Miss** | **Score** | **Expect** | **Rank** | **Peptide** |
| --- | --- | --- | --- | --- | --- | --- | --- | --- | --- | --- |
|  | 1271 | **597.3676** | **596.3603** | **596.3646** | **-0.0042** | **0** | **7** | **21** | **2** | **K.IHISK.K** |
|  | 1821 | **732.4307** | **731.4234** | **730.4337** | **0.9897** | **1** | **15** | **7.4** | **8** | **R.TKLQNK.E** |
|  | 4824 | **490.7149** | **979.4152** | **979.4215** | **-0.0063** | **0** | **42** | **0.0087** | **1** | **K.MLSCAGADR.L** 4823 4839 |
|  | 5026 | 498.2762 | 994.5379 | 995.4164 | -0.8786 | 0 | (10) | 14 | 7 | K.MLSCAGADR.L 5039 |
|  | 6639 | **557.8018** | **1113.5891** | **1113.5965** | **-0.0074** | **1** | **40** | **0.017** | **1** | **K.RLIPDGCGVK.Y** |
|  | 6728 | **561.2712** | **1120.5279** | **1121.6305** | **-1.1026** | **1** | **24** | **0.67** | **1** | **K.EHVIEALRR.A** 6759 |
|  | 7642 | **594.8236** | **1187.6327** | **1187.6411** | **-0.0084** | **0** | **43** | **0.0075** | **1** | **R.GAFGKPQGTVAR.V** |
|  | 11115 | **458.2946** | **1371.8621** | **1372.8092** | **-0.9470** | **1** | **4** | **49** | **3** | **R.LHPFHVIRINK.M** |
|  | 13247 | 500.2798 | 1497.8175 | 1496.8497 | 0.9678 | 1 | 9 | 15 | 2 | R.VHIGQVIMSIRTK.L 13217 |
|  | 13896 | **772.8280** | **1543.6414** | **1543.6501** | **-0.0086** | **0** | **64** | **4.5e-005** | **1** | **K.FNADEFEDMVAEK.R** |
|  | 14135 | **780.3754** | **1558.7363** | **1559.6450** | **-0.9087** | **0** | **(0)** | **1.2e+002** | **8** | **K.FNADEFEDMVAEK.R** |
|  | 16239 | **567.5875** | **1699.7406** | **1699.7512** | **-0.0106** | **1** | **40** | **0.0099** | **1** | **K.FNADEFEDMVAEKR.L** 16238 |
|  | 24053 | 813.0750 | 2436.2032 | 2437.3376 | -1.1344 | 1 | 2 | 40 | 3 | R.GAFGKPQGTVARVHIGQVIMSIR.T |
|  | 27715 | **1028.1244** | **3081.3513** | **3081.3695** | **-0.0182** | **0** | **49** | **0.00061** | **1** | **K.VDEFPLCGHMVSDEYEQLSSEALEAAR.I** 27716 |
|  | 28540 | 1094.8361 | 3281.4864 | 3280.5016 | 0.9848 | 1 | 89 | 6e-008 | 1 | K.AKVDEFPLCGHMVSDEYEQLSSEALEAAR.I |

| **254.** | P12830\|CADH1_HUMAN    **Mass:** 97852    **Score:** 197    **Queries matched:** 17   **emPAI:** 0.30 |
| --- | --- |
|  | Cadherin-1 OS=Homo sapiens GN=CDH1 PE=1 SV=3 |

|  | **Query** | **Observed** | **Mr(expt)** | **Mr(calc)** | **Delta** | **Miss** | **Score** | **Expect** | **Rank** | **Peptide** |
| --- | --- | --- | --- | --- | --- | --- | --- | --- | --- | --- |
|  | 2566 | 415.2112 | 828.4078 | 828.4341 | -0.0263 | 0 | 5 | 60 | 10 | K.VTEPLDR.E 2564 |
|  | 6628 | **557.3256** | **1112.6367** | **1112.5938** | **0.0429** | **0** | **8** | **25** | **4** | **R.GLDARPEVTR.N** |
|  | 9257 | **635.7624** | **1269.5102** | **1269.5183** | **-0.0081** | **1** | **41** | **0.011** | **1** | **K.KLADMYGGGEDD.-** |
|  | 9805 | **650.3351** | **1298.6557** | **1298.6653** | **-0.0095** | **0** | **21** | **1.2** | **2** | **R.NDVAPTLMSVPR.Y** |
|  | 12203 | **716.8909** | **1431.7672** | **1430.7729** | **0.9943** | **0** | **66** | **3.3e-005** | **1** | **R.NTGVISVVTTGLDR.E** |
|  | 13137 | **498.2359** | **1491.6858** | **1492.7959** | **-1.1101** | **1** | **4** | **53** | **6** | **K.MALEVGDYKINLK.L** |
|  | 13394 | **503.7788** | **1508.3145** | **1508.7908** | **-0.4763** | **1** | **(2)** | **55** | **1** | **K.MALEVGDYKINLK.L** |
|  | 13591 | **508.2843** | **1521.8312** | **1521.7358** | **0.0953** | **1** | **5** | **38** | **5** | **R.VLGRVNFEDCTGR.Q** 13596 |
|  | 13865 | 771.9226 | 1541.8307 | 1542.8042 | -0.9735 | 1 | 5 | 34 | 3 | R.ETGWLKVTEPLDR.E |
|  | 16403 | **856.4598** | **1710.9051** | **1710.9152** | **-0.0101** | **0** | **47** | **0.0023** | **1** | **K.GQVPENEANVVITTLK.V** |
|  | 23548 | **1183.6174** | **2365.2203** | **2365.2318** | **-0.0115** | **0** | **74** | **3.2e-006** | **1** | **K.VFYSITGQGADTPPVGVFIIER.E** 23547 |
|  | 27281 | **998.4106** | **2992.2099** | **2992.2271** | **-0.0172** | **0** | **35** | **0.012** | **1** | **R.DNVYYYDEEGGGEEDQDFDLSQLHR.G** |
|  | 30706 | **1415.6880** | **4244.0421** | **4244.0550** | **-0.0129** | **0** | **41** | **0.0018** | **1** | **K.VTDADAPNTPAWEAVYTILNDDGGQFVVTTNPVNNDGILK.T** |
|  | 31545 | **870.0740** | **5214.4002** | **5213.5139** | **0.8862** | **1** | **2** | **6.9** | **1** | **R.ERIATYTLFSHAVSSNGNAVEDPMEILITVTDQNDNKPEFTQEVFK.G** |

| **255.** | P12814\|ACTN1_HUMAN    **Mass:** 103563   **Score:** 197    **Queries matched:** 24   **emPAI:** 0.23 |
| --- | --- |
|  | Alpha-actinin-1 OS=Homo sapiens GN=ACTN1 PE=1 SV=2 |

|  | **Query** | **Observed** | **Mr(expt)** | **Mr(calc)** | **Delta** | **Miss** | **Score** | **Expect** | **Rank** | **Peptide** |
| --- | --- | --- | --- | --- | --- | --- | --- | --- | --- | --- |
|  | 2497 | 825.4404 | 824.4332 | 824.4392 | -0.0060 | 0 | 23 | 0.64 | 1 | R.LDHLAEK.F |
|  | 2568 | **415.2274** | **828.4402** | **829.4181** | **-0.9779** | **0** | **9** | **23** | **7** | **K.ATLPDADK.E** |
|  | 3141 | 432.7422 | 863.4698 | 863.4752 | -0.0055 | 0 | 10 | 18 | 1 | K.ALDFIASK.G |
|  | 5883 | **530.5510** | **1059.0874** | **1059.5383** | **-0.4509** | **0** | **1** | **87** | **4** | **R.IMSIVDPNR.L** |
|  | 7866 | 600.3265 | 1198.6385 | 1198.6234 | 0.0152 | 0 | 3 | 73 | 10 | R.DLLLDPAWEK.Q |
|  | 9837 | 434.5367 | 1300.5884 | 1300.5982 | -0.0098 | 0 | 33 | 0.065 | 1 | K.HTNYTMEHIR.V 9848 |
|  | 11111 | 686.8945 | 1371.7745 | 1371.7795 | -0.0050 | 0 | 10 | 13 | 1 | K.LMLLLEVISGER.L |
|  | 11376 | 693.8876 | 1385.7606 | 1385.7667 | -0.0061 | 0 | 73 | 6.9e-006 | 1 | R.VGWEQLLTTIAR.T 11375 |
|  | 12001 | 474.7404 | 1421.1994 | 1420.6986 | 0.5008 | 0 | 2 | 56 | 9 | K.GYEEWLLNEIR.R |
|  | 12134 | 715.3807 | 1428.7469 | 1428.7572 | -0.0103 | 0 | 50 | 0.0014 | 1 | R.TINEVENQILTR.D 12133 |
|  | 13199 | **499.2840** | **1494.8302** | **1495.8034** | **-0.9733** | **1** | **6** | **27** | **7** | **R.QYEKSIVNYKPK.I** 13231 |
|  | 13795 | 769.3860 | 1536.7575 | 1536.7671 | -0.0096 | 0 | 76 | 3.3e-006 | 1 | R.FAIQDISVEETSAK.E |
|  | 15561 | 551.3153 | 1650.9241 | 1651.7624 | -0.8383 | 1 | 11 | 9.5 | 1 | R.DAKGISQEQMNEFR.A |
|  | 16851 | **582.2964** | **1743.8673** | **1744.7462** | **-0.8788** | **0** | **3** | **51** | **9** | **R.ETADTDTADQVMASFK.I** |
|  | 17070 | **587.3107** | **1758.9104** | **1758.8458** | **0.0646** | **0** | **2** | **71** | **6** | **K.MLDAEDIVGTARPDEK.A** |
|  | 18849 | 473.2657 | 1889.0338 | 1889.9371 | -0.9033 | 1 | 3 | 51 | 3 | R.KDDPLTNLNTAFDVAEK.Y |
|  | 20187 | 669.9895 | 2006.9467 | 2006.9560 | -0.0094 | 0 | 18 | 1.3 | 1 | K.AIMTYVSSFYHAFSGAQK.A 20179 |
|  | 24466 | **830.4720** | **2488.3941** | **2488.2519** | **0.1422** | **1** | **0** | **59** | **8** | **K.YLDIPKMLDAEDIVGTARPDEK.A** |
|  | 30257 | **783.9930** | **3914.9288** | **3914.6312** | **0.2976** | **1** | **4** | **11** | **3** | **-.MDHYDSQQTNDYMQPEEDWDRDLLLDPAWEK.Q** |

| **256.** | O43704\|ST1B1_HUMAN    **Mass:** 35048    **Score:** 197    **Queries matched:** 10   **emPAI:** 0.11 |
| --- | --- |
|  | Sulfotransferase family cytosolic 1B member 1 OS=Homo sapiens GN=SULT1B1 PE=1 SV=2 |

|  | **Query** | **Observed** | **Mr(expt)** | **Mr(calc)** | **Delta** | **Miss** | **Score** | **Expect** | **Rank** | **Peptide** |
| --- | --- | --- | --- | --- | --- | --- | --- | --- | --- | --- |
|  | 3123 | **432.2579** | **862.5012** | **861.4345** | **1.0667** | **1** | **16** | **5.1** | **6** | **R.KGTAGDWK.N** 3074 |
|  | 3873 | **456.2901** | **910.5656** | **909.5396** | **1.0260** | **1** | **4** | **57** | **8** | **K.NPSPRIVK.T** |
|  | 6906 | **568.2511** | **1134.4876** | **1133.6445** | **0.8432** | **0** | **4** | **61** | **4** | **K.THLPTDLLPK.S** |
|  | 10588 | **447.9016** | **1340.6831** | **1340.6911** | **-0.0080** | **0** | **5** | **45** | **2** | **R.IIHHTSFEVMK.D** |
|  | 10833 | **678.8254** | **1355.6362** | **1355.7305** | **-0.0943** | **0** | **6** | **36** | **3** | **K.VPMLEMTLPGLR.T** |
|  | 11104 | **458.2464** | **1371.7174** | **1371.7254** | **-0.0080** | **0** | **(4)** | **57** | **10** | **K.VPMLEMTLPGLR.T** |
|  | 23285 | **1168.5649** | **2335.1153** | **2335.1253** | **-0.0100** | **0** | **114** | **3.2e-010** | **1** | **K.SGTTWVSEIIDMILNDGDIEK.C** 23284 |
|  | 25337 | **1312.6089** | **2623.2032** | **2623.2509** | **-0.0477** | **1** | **2** | **38** | **4** | **K.SGTTWVSEIIDMILNDGDIEKCK.R** |

| **257.** | P31946\|1433B_HUMAN    **Mass:** 28179    **Score:** 197    **Queries matched:** 11   **emPAI:** 0.46 |
| --- | --- |
|  | 14-3-3 protein beta/alpha OS=Homo sapiens GN=YWHAB PE=1 SV=3 |

|  | **Query** | **Observed** | **Mr(expt)** | **Mr(calc)** | **Delta** | **Miss** | **Score** | **Expect** | **Rank** | **Peptide** |
| --- | --- | --- | --- | --- | --- | --- | --- | --- | --- | --- |
|  | 3760 | **452.2576** | **902.5006** | **902.5073** | **-0.0067** | **0** | **18** | **3.4** | **1** | **R.VISSIEQK.T** 3753 3754 |
|  | 3811 | 454.2629 | 906.5113 | 906.5174 | -0.0062 | 0 | 20 | 1.8 | 2 | R.NLLSVAYK.N |
|  | 5292 | **508.2113** | **1014.4080** | **1014.4150** | **-0.0070** | **0** | **12** | **12** | **1** | **R.YDDMAAAMK.A** |
|  | 6583 | 555.3574 | 1108.7002 | 1107.5495 | 1.1507 | 0 | 21 | 1.1 | 1 | K.EMQPTHPIR.L |
|  | 11939 | **709.8932** | **1417.7718** | **1418.7518** | **-0.9799** | **1** | **0** | **1.3e+002** | **10** | **R.SSWRVISSIEQK.T** |
|  | 14693 | **799.8688** | **1597.7230** | **1597.7332** | **-0.0102** | **0** | **66** | **3e-005** | **1** | **K.AVTEQGHELSNEER.N** 14692 |
|  | 21845 | **1080.0089** | **2158.0033** | **2158.0178** | **-0.0146** | **0** | **105** | **2.7e-009** | **1** | **K.QTTVSNSQQAYQEAFEISK.K** 21844 |

| **258.** | P26232\|CTNA2_HUMAN    **Mass:** 106045   **Score:** 196    **Queries matched:** 39   **emPAI:** 0.19 |
| --- | --- |
|  | Catenin alpha-2 OS=Homo sapiens GN=CTNNA2 PE=1 SV=5 |

|  | **Query** | **Observed** | **Mr(expt)** | **Mr(calc)** | **Delta** | **Miss** | **Score** | **Expect** | **Rank** | **Peptide** |
| --- | --- | --- | --- | --- | --- | --- | --- | --- | --- | --- |
|  | 2524 | 827.4280 | 826.4208 | 825.4232 | 0.9975 | 0 | 27 | 0.31 | 1 | K.ASYVASTK.Y 2508 |
|  | 2584 | 415.7156 | 829.4167 | 830.4610 | -1.0443 | 1 | 13 | 11 | 2 | K.KIAEAGSR.M |
|  | 2869 | **425.0573** | **848.1001** | **848.4174** | **-0.3174** | **1** | **8** | **23** | **7** | **R.KQGETMR.I** |
|  | 3148 | **432.8838** | **863.7530** | **864.4123** | **-0.6594** | **1** | **(3)** | **54** | **8** | **R.KQGETMR.I** |
|  | 5508 | 516.2649 | 1030.5152 | 1030.5229 | -0.0077 | 0 | 10 | 18 | 1 | R.IVAECNAVR.Q |
|  | 5834 | **528.7682** | **1055.5219** | **1054.5659** | **0.9561** | **0** | **12** | **11** | **3** | **K.GDPLNIAIDK.M** |
|  | 5934 | 531.7894 | 1061.5643 | 1060.5587 | 1.0056 | 0 | 19 | 2.3 | 1 | K.LLSETVMPR.F 5918 5933 |
|  | 6712 | 560.3209 | 1118.6272 | 1118.6084 | 0.0188 | 0 | 14 | 6.5 | 4 | K.QDLLAYLQR.I |
|  | 7078 | **575.2841** | **1148.5537** | **1147.5655** | **0.9882** | **1** | **7** | **30** | **7** | **R.DEMAAARGALK.K** |
|  | 8260 | **406.6999** | **1217.0778** | **1216.6849** | **0.3929** | **0** | **12** | **6.4** | **1** | **K.NLMNAVVLTVK.A** 8264 |
|  | 9678 | 431.5793 | 1291.7160 | 1292.6911 | -0.9750 | 1 | 19 | 1.8 | 3 | K.EMVKLNYVAAR.R |
|  | 10128 | 439.7300 | 1316.1683 | 1315.6666 | 0.5017 | 1 | 4 | 40 | 9 | R.ERIVAECNAVR.Q |
|  | 12383 | 723.3628 | 1444.7111 | 1444.7198 | -0.0087 | 0 | 33 | 0.063 | 1 | K.WDDSGNDIIVLAK.Q 12384 |
|  | 12525 | 485.8892 | 1454.6457 | 1455.7755 | -1.1298 | 1 | 6 | 34 | 10 | R.AIMAQLPQEEKAK.I |
|  | 14760 | **534.9646** | **1601.8720** | **1601.7872** | **0.0848** | **0** | **3** | **62** | **2** | **K.NATMLYTASQAFLR.H** |
|  | 16054 | 844.4079 | 1686.8012 | 1685.9386 | 0.8627 | 1 | 5 | 34 | 8 | K.VLEATKLLSETVMPR.F |
|  | 16281 | **568.2944** | **1701.8615** | **1701.9335** | **-0.0720** | **1** | **(2)** | **70** | **6** | **K.VLEATKLLSETVMPR.F** |
|  | 16286 | **568.3280** | **1701.9622** | **1702.8196** | **-0.8574** | **0** | **4** | **42** | **6** | **K.CVIALQEGDVDTLDR.T** |
|  | 16302 | **568.8522** | **1703.5347** | **1702.8964** | **0.6383** | **1** | **1** | **56** | **10** | **-.MTSATSPIILKWDPK.S** |
|  | 16654 | **866.0067** | **1729.9987** | **1729.8821** | **0.1166** | **1** | **3** | **47** | **3** | **K.KNATMLYTASQAFLR.H** 16651 |
|  | 17829 | 909.9387 | 1817.8628 | 1817.8755 | -0.0128 | 0 | 69 | 1.4e-005 | 1 | R.TSVQTEDDQLIAGQSAR.A 17830 |
|  | 18739 | 941.4503 | 1880.8861 | 1880.8972 | -0.0111 | 0 | 58 | 0.00015 | 1 | R.LESIISGAALMADSSCTR.D 18738 18740 |
|  | 19663 | 653.0015 | 1955.9826 | 1955.0112 | 0.9713 | 0 | 10 | 9.7 | 2 | K.AHVLAASVEQATQNFLEK.G |
|  | 19813 | 985.5557 | 1969.0969 | 1969.1096 | -0.0127 | 0 | 50 | 0.00093 | 1 | R.LLEPLVTQVTTLVNTSNK.G 19815 |
|  | 20204 | **670.3729** | **2008.0969** | **2008.1027** | **-0.0057** | **1** | **4** | **39** | **3** | **K.NLMNAVVLTVKASYVASTK.Y** |
|  | 21965 | **724.7221** | **2171.1445** | **2171.2170** | **-0.0725** | **1** | **(2)** | **60** | **4** | **R.ALLSAVTRLLILADMADVMR.L** |
|  | 22086 | **1094.8630** | **2187.7115** | **2187.2119** | **0.4996** | **1** | **5** | **18** | **1** | **R.ALLSAVTRLLILADMADVMR.L** |
|  | 28308 | **808.1395** | **3228.5290** | **3229.6077** | **-1.0787** | **1** | **0** | **44** | **6** | **K.GHTGIGELAAALNEFDNKIILDPMTFSEAR.F** |
|  | 28454 | **1085.2046** | **3252.5919** | **3251.7482** | **0.8437** | **1** | **1** | **36** | **1** | **K.LVRMAATQIDSLCPQVINAALTLAARPQSK.V** |

| **259.** | P62424\|RL7A_HUMAN    **Mass:** 30148    **Score:** 196    **Queries matched:** 21   **emPAI:** 1.03 |
| --- | --- |
|  | 60S ribosomal protein L7a OS=Homo sapiens GN=RPL7A PE=1 SV=2 |

|  | **Query** | **Observed** | **Mr(expt)** | **Mr(calc)** | **Delta** | **Miss** | **Score** | **Expect** | **Rank** | **Peptide** |
| --- | --- | --- | --- | --- | --- | --- | --- | --- | --- | --- |
|  | 1160 | 573.3511 | 572.3438 | 571.3554 | 0.9884 | 1 | 17 | 6.2 | 4 | K.ARLGR.L |
|  | 2878 | **425.2422** | **848.4698** | **849.5297** | **-1.0599** | **1** | **6** | **47** | **1** | **R.LGRLVHR.K** |
|  | 3758 | **452.2439** | **902.4733** | **901.5233** | **0.9500** | **0** | **1** | **1.6e+002** | **7** | **R.QTATQLLK.L** |
|  | 4820 | **490.3149** | **978.6152** | **978.6226** | **-0.0074** | **1** | **9** | **21** | **1** | **K.KVAPAPAVVK.K** |
|  | 5953 | **532.7820** | **1063.5494** | **1063.5563** | **-0.0069** | **0** | **34** | **0.061** | **1** | **R.HWGGNVLGPK.S** |
|  | 6103 | **537.3178** | **1072.6211** | **1072.6281** | **-0.0070** | **1** | **29** | **0.24** | **1** | **K.KVVNPLFEK.R** |
|  | 6978 | **570.8182** | **1139.6218** | **1139.7026** | **-0.0808** | **1** | **9** | **18** | **1** | **K.GALAKLVEAIR.T** 6990 |
|  | 8311 | **610.7732** | **1219.5319** | **1219.6574** | **-0.1255** | **1** | **6** | **36** | **8** | **R.RHWGGNVLGPK.S** 8315 |
|  | 10673 | **673.3654** | **1344.7163** | **1344.7249** | **-0.0086** | **0** | **73** | **6.8e-006** | **1** | **R.AGVNTVTTLVENK.K** 10674 |
|  | 12570 | **729.8296** | **1457.6446** | **1457.6535** | **-0.0089** | **1** | **23** | **0.66** | **1** | **R.TNYNDRYDEIR.R** 12569 |
|  | 14283 | 784.9308 | 1567.8471 | 1568.8311 | -0.9839 | 0 | 41 | 0.011 | 1 | K.VPPAINQFTQALDR.Q 14281 |
|  | 16242 | **850.8876** | **1699.7607** | **1699.7723** | **-0.0116** | **0** | **6** | **30** | **1** | **K.TCTTVAFTQVNSEDK.G** |
|  | 17735 | **604.3403** | **1809.9990** | **1810.0101** | **-0.0111** | **1** | **42** | **0.0068** | **1** | **R.LKVPPAINQFTQALDR.Q** 17736 |
|  | 21625 | 714.3518 | 2140.0334 | 2140.0470 | -0.0136 | 1 | 16 | 2 | 1 | K.TCTTVAFTQVNSEDKGALAK.L |
|  | 25738 | 1344.2448 | 2686.4749 | 2686.4880 | -0.0131 | 0 | 80 | 6.6e-007 | 1 | K.AQLVVIAHDVDPIELVVFLPALCR.K |

| **260.** | Q29940\|1B59_HUMAN    **Mass:** 40844    **Score:** 194    **Queries matched:** 17   **emPAI:** 0.55 |
| --- | --- |
|  | HLA class I histocompatibility antigen, B-59 alpha chain OS=Homo sapiens GN=HLA-B PE=2 SV=1 |

|  | **Query** | **Observed** | **Mr(expt)** | **Mr(calc)** | **Delta** | **Miss** | **Score** | **Expect** | **Rank** | **Peptide** |
| --- | --- | --- | --- | --- | --- | --- | --- | --- | --- | --- |
|  | 995 | **542.1287** | **541.1214** | **542.3176** | **-1.1963** | **0** | **13** | **4.3** | **1** | **R.VTAPR.T** 1006 |
|  | 10365 | 443.7359 | 1328.1860 | 1327.6996 | 0.4863 | 1 | 13 | 4.5 | 1 | K.WEAARVAEQLR.A |
|  | 12099 | 714.3628 | 1426.7111 | 1426.7205 | -0.0093 | 0 | 11 | 9.6 | 1 | K.WAAVVVPSGEEQR.Y |
|  | 14933 | **538.7706** | **1613.2899** | **1613.8162** | **-0.5263** | **1** | **2** | **52** | **2** | **R.NTQIFKTNTQTYR.E** |
|  | 15206 | 815.4121 | 1628.8097 | 1628.8199 | -0.0102 | 0 | 72 | 7.9e-006 | 1 | R.FIAVGYVDDTQFVR.F 15203 15205 |
|  | 18332 | **617.6424** | **1849.9054** | **1849.9183** | **-0.0130** | **0** | **32** | **0.074** | **1** | **K.THVTHHPISDHEATLR.C** 18320 |
|  | 20661 | 513.0184 | 2048.0446 | 2048.0626 | -0.0179 | 0 | 15 | 3.2 | 1 | R.YTCHVQHEGLPKPLTLR.W |
|  | 21343 | 1056.5170 | 2111.0194 | 2111.0299 | -0.0105 | 0 | 45 | 0.003 | 1 | R.CWALGFYPAEITLTWQR.D 21342 |
|  | 22490 | **744.6667** | **2230.9784** | **2230.9938** | **-0.0154** | **0** | **35** | **0.024** | **1** | **R.DGEDQTQDTELVETRPAGDR.T** 22489 22491 |
|  | 26805 | 724.1069 | 2892.3986 | 2892.1868 | 0.2118 | 0 | 2 | 36 | 6 | R.YYNQSEAGSHTWQTMYGCDLGPDGR.L |

|  | **Proteins matching a subset of these peptides:** |
| --- | --- |

|  | P30492\|1B54_HUMAN    **Mass:** 40640    **Score:** 194    **Queries matched:** 15 |
| --- | --- |
|  | HLA class I histocompatibility antigen, B-54 alpha chain OS=Homo sapiens GN=HLA-B PE=2 SV=1 |

|  | P30495\|1B56_HUMAN    **Mass:** 40738    **Score:** 194    **Queries matched:** 15 |
| --- | --- |
|  | HLA class I histocompatibility antigen, B-56 alpha chain OS=Homo sapiens GN=HLA-B PE=2 SV=1 |

|  | P30493\|1B55_HUMAN    **Mass:** 40756    **Score:** 194    **Queries matched:** 14 |
| --- | --- |
|  | HLA class I histocompatibility antigen, B-55 alpha chain OS=Homo sapiens GN=HLA-B PE=1 SV=1 |

| **261.** | P18465\|1B57_HUMAN    **Mass:** 40541    **Score:** 194    **Queries matched:** 16   **emPAI:** 0.56 |
| --- | --- |
|  | HLA class I histocompatibility antigen, B-57 alpha chain OS=Homo sapiens GN=HLA-B PE=1 SV=1 |

|  | **Query** | **Observed** | **Mr(expt)** | **Mr(calc)** | **Delta** | **Miss** | **Score** | **Expect** | **Rank** | **Peptide** |
| --- | --- | --- | --- | --- | --- | --- | --- | --- | --- | --- |
|  | 995 | 542.1287 | 541.1214 | 542.3176 | -1.1963 | 0 | 13 | 4.3 | 1 | R.VTAPR.T 1006 |
|  | 10365 | 443.7359 | 1328.1860 | 1327.6996 | 0.4863 | 1 | 13 | 4.5 | 1 | K.WEAARVAEQLR.A |
|  | 11973 | **474.2790** | **1419.8151** | **1419.6565** | **0.1587** | **1** | **4** | **55** | **6** | **R.FDSDAASPRMAPR.A** |
|  | 12099 | 714.3628 | 1426.7111 | 1426.7205 | -0.0093 | 0 | 11 | 9.6 | 1 | K.WAAVVVPSGEEQR.Y |
|  | 15206 | 815.4121 | 1628.8097 | 1628.8199 | -0.0102 | 0 | 72 | 7.9e-006 | 1 | R.FIAVGYVDDTQFVR.F 15203 15205 |
|  | 18332 | 617.6424 | 1849.9054 | 1849.9183 | -0.0130 | 0 | 32 | 0.074 | 1 | K.THVTHHPISDHEATLR.C 18320 |
|  | 20661 | 513.0184 | 2048.0446 | 2048.0626 | -0.0179 | 0 | 15 | 3.2 | 1 | R.YTCHVQHEGLPKPLTLR.W |
|  | 21343 | 1056.5170 | 2111.0194 | 2111.0299 | -0.0105 | 0 | 45 | 0.003 | 1 | R.CWALGFYPAEITLTWQR.D 21342 |
|  | 22490 | 744.6667 | 2230.9784 | 2230.9938 | -0.0154 | 0 | 35 | 0.024 | 1 | R.DGEDQTQDTELVETRPAGDR.T 22489 22491 |

|  | **Proteins matching a subset of these peptides:** |
| --- | --- |

|  | P30484\|1B46_HUMAN    **Mass:** 40700    **Score:** 194    **Queries matched:** 15 |
| --- | --- |
|  | HLA class I histocompatibility antigen, B-46 alpha chain OS=Homo sapiens GN=HLA-B PE=2 SV=1 |

| **262.** | P30464\|1B15_HUMAN    **Mass:** 40648    **Score:** 194    **Queries matched:** 16   **emPAI:** 0.56 |
| --- | --- |
|  | HLA class I histocompatibility antigen, B-15 alpha chain OS=Homo sapiens GN=HLA-B PE=1 SV=2 |

|  | **Query** | **Observed** | **Mr(expt)** | **Mr(calc)** | **Delta** | **Miss** | **Score** | **Expect** | **Rank** | **Peptide** |
| --- | --- | --- | --- | --- | --- | --- | --- | --- | --- | --- |
|  | 995 | 542.1287 | 541.1214 | 542.3176 | -1.1963 | 0 | 13 | 4.3 | 1 | R.VTAPR.T 1006 |
|  | 11047 | **457.2375** | **1368.6906** | **1367.6793** | **1.0112** | **1** | **10** | **13** | **4** | **K.TNTQTYRESLR.N** |
|  | 11973 | 474.2790 | 1419.8151 | 1419.6565 | 0.1587 | 1 | 4 | 55 | 6 | R.FDSDAASPRMAPR.A |
|  | 12099 | 714.3628 | 1426.7111 | 1426.7205 | -0.0093 | 0 | 11 | 9.6 | 1 | K.WAAVVVPSGEEQR.Y |
|  | 15206 | 815.4121 | 1628.8097 | 1628.8199 | -0.0102 | 0 | 72 | 7.9e-006 | 1 | R.FIAVGYVDDTQFVR.F 15203 15205 |
|  | 18332 | 617.6424 | 1849.9054 | 1849.9183 | -0.0130 | 0 | 32 | 0.074 | 1 | K.THVTHHPISDHEATLR.C 18320 |
|  | 20661 | 513.0184 | 2048.0446 | 2048.0626 | -0.0179 | 0 | 15 | 3.2 | 1 | R.YTCHVQHEGLPKPLTLR.W |
|  | 21343 | 1056.5170 | 2111.0194 | 2111.0299 | -0.0105 | 0 | 45 | 0.003 | 1 | R.CWALGFYPAEITLTWQR.D 21342 |
|  | 22490 | 744.6667 | 2230.9784 | 2230.9938 | -0.0154 | 0 | 35 | 0.024 | 1 | R.DGEDQTQDTELVETRPAGDR.T 22489 22491 |

| **263.** | P49411\|EFTU_HUMAN    **Mass:** 49852    **Score:** 194    **Queries matched:** 20   **emPAI:** 0.44 |
| --- | --- |
|  | Elongation factor Tu, mitochondrial OS=Homo sapiens GN=TUFM PE=1 SV=2 |

|  | **Query** | **Observed** | **Mr(expt)** | **Mr(calc)** | **Delta** | **Miss** | **Score** | **Expect** | **Rank** | **Peptide** |
| --- | --- | --- | --- | --- | --- | --- | --- | --- | --- | --- |
|  | 3334 | **438.2392** | **874.4638** | **873.4630** | **1.0009** | **0** | **2** | **1.3e+002** | **7** | **R.QPMILEK.G** |
|  | 5532 | **516.7842** | **1031.5539** | **1031.5611** | **-0.0072** | **0** | **30** | **0.18** | **1** | **R.GTVVTGTLER.G** |
|  | 6484 | **551.7728** | **1101.5310** | **1101.5376** | **-0.0066** | **0** | **(3)** | **79** | **4** | **K.ELAMPGEDLK.F** |
|  | 6696 | 559.8022 | 1117.5898 | 1117.5325 | 0.0573 | 0 | 10 | 16 | 7 | K.ELAMPGEDLK.F |
|  | 11097 | **686.8253** | **1371.6360** | **1371.6452** | **-0.0093** | **1** | **34** | **0.054** | **1** | **K.KGDECELLGHSK.N** |
|  | 12988 | 495.2776 | 1482.8109 | 1482.8195 | -0.0085 | 0 | 7 | 25 | 2 | R.QIGVEHVVVYVNK.A |
|  | 12998 | **742.7082** | **1483.4018** | **1482.8154** | **0.5864** | **1** | **1** | **67** | **8** | **R.AEAGDNLGALVRGLK.R** |
|  | 15031 | **540.3164** | **1617.9274** | **1617.9389** | **-0.0115** | **0** | **2** | **73** | **5** | **R.GLVMVKPGSIKPHQK.V** |
|  | 15854 | **837.4291** | **1672.8436** | **1672.8533** | **-0.0096** | **0** | **64** | **4.2e-005** | **1** | **R.GITINAAHVEYSTAAR.H** |
|  | 17197 | **590.9227** | **1769.7464** | **1769.7580** | **-0.0116** | **0** | **24** | **0.42** | **1** | **R.HYAHTDCPGHADYVK.N** |
|  | 19088 | **954.4792** | **1906.9439** | **1906.9558** | **-0.0118** | **0** | **76** | **2.3e-006** | **1** | **R.TIGTGLVTNTLAMTEEEK.N** 19076 19089 |
|  | 20404 | **676.9902** | **2027.9487** | **2027.0734** | **0.8752** | **1** | **2** | **53** | **2** | **-.MAAATLLRATPHFSGLAAGR.T** |
|  | 20815 | 688.3658 | 2062.0755 | 2060.9936 | 1.0819 | 0 | 3 | 42 | 5 | K.ADAVQDSEMVELVELEIR.E |
|  | 21531 | **1065.0804** | **2128.1463** | **2128.1568** | **-0.0105** | **0** | **16** | **2** | **1** | **R.DLEKPFLLPVEAVYSVPGR.G** |
|  | 22442 | **742.8513** | **2225.5320** | **2225.2433** | **0.2887** | **1** | **2** | **38** | **1** | **R.ATPHFSGLAAGRTFLLQGLLR.L** |
|  | 26400 | **938.1257** | **2811.3552** | **2811.3718** | **-0.0166** | **0** | **51** | **0.00047** | **1** | **K.NMITGTAPLDGCILVVAANDGPMPQTR.E** 26401 |
|  | 29285 | **1177.2167** | **3528.6282** | **3529.7535** | **-1.1253** | **1** | **2** | **21** | **1** | **R.HKPFVSHFMPVMFSLTWDMACRIILPPEK.E** |

| **264.** | Q02218\|ODO1_HUMAN    **Mass:** 117059   **Score:** 194    **Queries matched:** 36   **emPAI:** 0.17 |
| --- | --- |
|  | 2-oxoglutarate dehydrogenase E1 component, mitochondrial OS=Homo sapiens GN=OGDH PE=1 SV=3 |

|  | **Query** | **Observed** | **Mr(expt)** | **Mr(calc)** | **Delta** | **Miss** | **Score** | **Expect** | **Rank** | **Peptide** |
| --- | --- | --- | --- | --- | --- | --- | --- | --- | --- | --- |
|  | 2627 | **417.2432** | **832.4718** | **832.4225** | **0.0493** | **1** | **7** | **43** | **4** | **R.GEMVKNR.T** |
|  | 4103 | **465.2363** | **928.4581** | **928.4654** | **-0.0073** | **0** | **17** | **3.6** | **1** | **K.VYYDLTR.E** |
|  | 5265 | **506.8077** | **1011.6008** | **1011.4985** | **0.1023** | **0** | **7** | **28** | **4** | **R.DPAAAPATGNK.K** |
|  | 6325 | **545.2872** | **1088.5598** | **1088.5648** | **-0.0050** | **0** | **23** | **0.93** | **1** | **R.DMVGQVAITR.I** |
|  | 6494 | **551.8930** | **1101.7715** | **1102.5706** | **-0.7991** | **1** | **3** | **74** | **9** | **M.FHLRTCAAK.L** |
|  | 6781 | **563.3187** | **1124.6229** | **1124.6302** | **-0.0073** | **1** | **23** | **0.74** | **1** | **K.KTHLTELQR.L** 6773 |
|  | 6967 | **570.3373** | **1138.6601** | **1138.7186** | **-0.0585** | **1** | **9** | **17** | **2** | **R.LNVLANVIRK.E** 6968 6982 |
|  | 8867 | **625.8214** | **1249.6281** | **1249.6060** | **0.0222** | **1** | **9** | **17** | **4** | **-.MFHLRTCAAK.L** |
|  | 10418 | 666.8444 | 1331.6743 | 1331.6980 | -0.0237 | 1 | 14 | 5.7 | 6 | K.ARDMVGQVAITR.I 10439 |
|  | 11545 | 466.2825 | 1395.8256 | 1395.7286 | 0.0970 | 0 | 9 | 15 | 7 | R.LLDTAFDLDVFK.N |
|  | 11688 | 703.3740 | 1404.7335 | 1403.5663 | 1.1671 | 0 | 5 | 45 | 5 | K.AEQFYCGDTEGK.K |
|  | 13246 | **500.2793** | **1497.8161** | **1496.7597** | **1.0564** | **1** | **6** | **32** | **3** | **R.HHVLHDQNVDKR.T** |
|  | 13747 | **511.9437** | **1532.8092** | **1531.6613** | **1.1479** | **1** | **4** | **54** | **6** | **K.AEQFYCGDTEGKK.V** |
|  | 14786 | **535.7793** | **1604.3161** | **1604.8787** | **-0.5626** | **1** | **2** | **48** | **7** | **R.TTISRAKPVWYAGR.D** |
|  | 15665 | 830.4678 | 1658.9210 | 1658.9283 | -0.0073 | 0 | 24 | 0.42 | 1 | R.IEQLSPFPFDLLLK.E 15663 |
|  | 16284 | 851.9592 | 1701.9038 | 1700.9283 | 0.9754 | 1 | 4 | 44 | 5 | K.RFGLEGCEVLIPALK.T 16283 |
|  | 17481 | 895.9439 | 1789.8731 | 1790.8080 | -0.9348 | 0 | 9 | 15 | 3 | K.SSENGVDYVIMGMPHR.G 17482 17483 |
|  | 17710 | **452.9871** | **1807.9194** | **1806.8029** | **1.1165** | **0** | **(3)** | **59** | **3** | **K.SSENGVDYVIMGMPHR.G** |
|  | 19492 | **648.0645** | **1941.1717** | **1941.9142** | **-0.7425** | **1** | **2** | **50** | **3** | **R.QKFETPGIMQFTNEEK.R** |
|  | 20516 | **510.2564** | **2036.9963** | **2035.9204** | **1.0759** | **1** | **6** | **21** | **1** | **K.SSENGVDYVIMGMPHRGR.L** |
|  | 21218 | 1052.5169 | 2103.0191 | 2104.0913 | -1.0721 | 0 | 3 | 45 | 3 | R.GSLAAVAHAQSLVEAQPNVDK.L |
|  | 22048 | **546.5238** | **2182.0661** | **2180.9329** | **1.1332** | **0** | **4** | **31** | **5** | **R.NGHNEMDEPMFTQPLMYK.Q** |
|  | 23317 | 1169.6320 | 2337.2494 | 2337.0340 | 0.2154 | 1 | 1 | 59 | 3 | R.RNGHNEMDEPMFTQPLMYK.Q |
|  | 23607 | **1188.6262** | **2375.2379** | **2375.2519** | **-0.0140** | **0** | **91** | **5.4e-008** | **1** | **R.NITLSLVANPSHLEAADPVVMGK.T** 23606 |
|  | 26978 | **977.4762** | **2929.4068** | **2929.4232** | **-0.0165** | **1** | **43** | **0.0026** | **1** | **K.YAELLVSQGVVNQPEYEEEISKYDK.I** |
|  | 29543 | **1210.2225** | **3627.6458** | **3627.6664** | **-0.0206** | **0** | **17** | **0.67** | **1** | **K.EANFDINQLYDCNWVVVNCSTPGNFFHVLR.R** |
|  | 30225 | **1295.6399** | **3883.8978** | **3882.8881** | **1.0098** | **0** | **70** | **3.3e-006** | **1** | **R.SMSCPSTGLTEDILTHIGNVASSVPVENFTIHGGLSR.I** |

| **265.** | P30049\|ATPD_HUMAN    **Mass:** 17479    **Score:** 194    **Queries matched:** 11   **emPAI:** 0.49 |
| --- | --- |
|  | ATP synthase subunit delta, mitochondrial OS=Homo sapiens GN=ATP5D PE=1 SV=2 |

|  | **Query** | **Observed** | **Mr(expt)** | **Mr(calc)** | **Delta** | **Miss** | **Score** | **Expect** | **Rank** | **Peptide** |
| --- | --- | --- | --- | --- | --- | --- | --- | --- | --- | --- |
|  | 4897 | 493.7583 | 985.5020 | 985.5444 | -0.0424 | 0 | 34 | 0.075 | 1 | R.IEANEALVK.A |
|  | 12197 | **716.8535** | **1431.6925** | **1430.7001** | **0.9924** | **0** | **87** | **2.4e-007** | **1** | **K.AQAELVGTADEATR.A** |
|  | 13771 | **512.7568** | **1535.2485** | **1535.9082** | **-0.6597** | **1** | **5** | **26** | **3** | **-.MLPAALLRRPGLGR.L** |
|  | 29437 | **1195.6039** | **3583.7898** | **3583.7967** | **-0.0069** | **0** | **54** | **0.00015** | **1** | **K.YFVSSGSIAVNADSSVQLLAEEAVTLDMLDLGAAK.A** 29423 29425 29426 29427 29430 29434 29444 |

| **266.** | P60660\|MYL6_HUMAN    **Mass:** 17090    **Score:** 193    **Queries matched:** 13   **emPAI:** 3.18 |
| --- | --- |
|  | Myosin light polypeptide 6 OS=Homo sapiens GN=MYL6 PE=1 SV=2 |

|  | **Query** | **Observed** | **Mr(expt)** | **Mr(calc)** | **Delta** | **Miss** | **Score** | **Expect** | **Rank** | **Peptide** |
| --- | --- | --- | --- | --- | --- | --- | --- | --- | --- | --- |
|  | 1414 | **628.3623** | **627.3550** | **626.3752** | **0.9799** | **0** | **6** | **38** | **7** | **K.VLGNPK.S** |
|  | 5027 | **498.2945** | **994.5745** | **994.5811** | **-0.0066** | **0** | **49** | **0.0017** | **1** | **R.HVLVTLGEK.M** |
|  | 8860 | 417.2432 | 1248.7077 | 1248.5768 | 0.1308 | 0 | 5 | 42 | 7 | K.EGNGTVMGAEIR.H |
|  | 10581 | 671.3138 | 1340.6130 | 1340.6217 | -0.0087 | 0 | 33 | 0.062 | 1 | K.ILYSQCGDVMR.A 10582 |
|  | 10812 | **677.8649** | **1353.7153** | **1353.7252** | **-0.0099** | **0** | **40** | **0.014** | **1** | **R.ALGQNPTNAEVLK.V** |
|  | 16559 | **862.4143** | **1722.8139** | **1721.8407** | **0.9733** | **1** | **27** | **0.23** | **1** | **R.VFDKEGNGTVMGAEIR.H** |
|  | 17426 | **893.9097** | **1785.8048** | **1785.8169** | **-0.0122** | **1** | **78** | **1.6e-006** | **1** | **K.NKDQGTYEDYVEGLR.V** 17427 |
|  | 18823 | **944.5001** | **1886.9857** | **1886.9964** | **-0.0108** | **0** | **57** | **0.00022** | **1** | **K.VLDFEHFLPMLQTVAK.N** 18824 18825 18836 |

| **267.** | P10314\|1A32_HUMAN    **Mass:** 41307    **Score:** 192    **Queries matched:** 16   **emPAI:** 0.54 |
| --- | --- |
|  | HLA class I histocompatibility antigen, A-32 alpha chain OS=Homo sapiens GN=HLA-A PE=2 SV=2 |

|  | **Query** | **Observed** | **Mr(expt)** | **Mr(calc)** | **Delta** | **Miss** | **Score** | **Expect** | **Rank** | **Peptide** |
| --- | --- | --- | --- | --- | --- | --- | --- | --- | --- | --- |
|  | 2373 | 408.2321 | 814.4497 | 813.3729 | 1.0768 | 0 | 4 | 89 | 4 | K.AHSQTDR.E |
|  | 5040 | 498.7649 | 995.5153 | 995.4308 | 0.0845 | 0 | 16 | 3.9 | 3 | R.FDSDAASQR.M 5054 |
|  | 9796 | **433.7320** | **1298.1743** | **1298.6327** | **-0.4584** | **1** | **5** | **32** | **5** | **K.AHSQTDRESLR.I** |
|  | 10365 | 443.7359 | 1328.1860 | 1327.6996 | 0.4863 | 1 | 13 | 4.5 | 1 | K.WEAARVAEQLR.A |
|  | 13614 | 508.9797 | 1523.9174 | 1524.6627 | -0.7453 | 1 | 7 | 23 | 1 | R.FDSDAASQRMEPR.A 13613 |
|  | 15206 | 815.4121 | 1628.8097 | 1628.8199 | -0.0102 | 0 | 72 | 7.9e-006 | 1 | R.FIAVGYVDDTQFVR.F 15203 15205 |
|  | 18194 | 614.9559 | 1841.8460 | 1841.8591 | -0.0132 | 0 | 31 | 0.087 | 1 | K.THMTHHAVSDHEATLR.C 18195 18196 |
|  | 20661 | 513.0184 | 2048.0446 | 2048.0626 | -0.0179 | 0 | 15 | 3.2 | 1 | R.YTCHVQHEGLPKPLTLR.W |
|  | 25408 | 1319.0913 | 2636.1681 | 2636.1838 | -0.0158 | 0 | 68 | 1.1e-005 | 1 | R.DGEDQTQDTELVETRPAGDGTFQK.W 25407 |

|  | **Proteins matching a subset of these peptides:** |
| --- | --- |

|  | P30459\|1A74_HUMAN    **Mass:** 41150    **Score:** 192    **Queries matched:** 15 |
| --- | --- |
|  | HLA class I histocompatibility antigen, A-74 alpha chain OS=Homo sapiens GN=HLA-A PE=1 SV=1 |

| **268.** | P16189\|1A31_HUMAN    **Mass:** 41263    **Score:** 192    **Queries matched:** 15   **emPAI:** 0.54 |
| --- | --- |
|  | HLA class I histocompatibility antigen, A-31 alpha chain OS=Homo sapiens GN=HLA-A PE=2 SV=2 |

|  | **Query** | **Observed** | **Mr(expt)** | **Mr(calc)** | **Delta** | **Miss** | **Score** | **Expect** | **Rank** | **Peptide** |
| --- | --- | --- | --- | --- | --- | --- | --- | --- | --- | --- |
|  | 2509 | **413.5326** | **825.0507** | **825.4093** | **-0.3586** | **0** | **6** | **25** | **6** | **K.AHSQIDR.V** |
|  | 5040 | 498.7649 | 995.5153 | 995.4308 | 0.0845 | 0 | 16 | 3.9 | 3 | R.FDSDAASQR.M 5054 |
|  | 10365 | 443.7359 | 1328.1860 | 1327.6996 | 0.4863 | 1 | 13 | 4.5 | 1 | K.WEAARVAEQLR.A |
|  | 13614 | 508.9797 | 1523.9174 | 1524.6627 | -0.7453 | 1 | 7 | 23 | 1 | R.FDSDAASQRMEPR.A 13613 |
|  | 15206 | 815.4121 | 1628.8097 | 1628.8199 | -0.0102 | 0 | 72 | 7.9e-006 | 1 | R.FIAVGYVDDTQFVR.F 15203 15205 |
|  | 18194 | 614.9559 | 1841.8460 | 1841.8591 | -0.0132 | 0 | 31 | 0.087 | 1 | K.THMTHHAVSDHEATLR.C 18195 18196 |
|  | 20661 | 513.0184 | 2048.0446 | 2048.0626 | -0.0179 | 0 | 15 | 3.2 | 1 | R.YTCHVQHEGLPKPLTLR.W |
|  | 25408 | 1319.0913 | 2636.1681 | 2636.1838 | -0.0158 | 0 | 68 | 1.1e-005 | 1 | R.DGEDQTQDTELVETRPAGDGTFQK.W 25407 |

|  | **Proteins matching the same set of peptides:** |
| --- | --- |

|  | P16190\|1A33_HUMAN    **Mass:** 41151    **Score:** 192    **Queries matched:** 15 |
| --- | --- |
|  | HLA class I histocompatibility antigen, A-33 alpha chain OS=Homo sapiens GN=HLA-A PE=1 SV=3 |

| **269.** | P30512\|1A29_HUMAN    **Mass:** 41122    **Score:** 192    **Queries matched:** 16   **emPAI:** 0.55 |
| --- | --- |
|  | HLA class I histocompatibility antigen, A-29 alpha chain OS=Homo sapiens GN=HLA-A PE=2 SV=2 |

|  | **Query** | **Observed** | **Mr(expt)** | **Mr(calc)** | **Delta** | **Miss** | **Score** | **Expect** | **Rank** | **Peptide** |
| --- | --- | --- | --- | --- | --- | --- | --- | --- | --- | --- |
|  | 5040 | 498.7649 | 995.5153 | 995.4308 | 0.0845 | 0 | 16 | 3.9 | 3 | R.FDSDAASQR.M 5054 |
|  | 10365 | 443.7359 | 1328.1860 | 1327.6996 | 0.4863 | 1 | 13 | 4.5 | 1 | K.WEAARVAEQLR.A |
|  | 13614 | 508.9797 | 1523.9174 | 1524.6627 | -0.7453 | 1 | 7 | 23 | 1 | R.FDSDAASQRMEPR.A 13613 |
|  | 15206 | 815.4121 | 1628.8097 | 1628.8199 | -0.0102 | 0 | 72 | 7.9e-006 | 1 | R.FIAVGYVDDTQFVR.F 15203 15205 |
|  | 18194 | 614.9559 | 1841.8460 | 1841.8591 | -0.0132 | 0 | 31 | 0.087 | 1 | K.THMTHHAVSDHEATLR.C 18195 18196 |
|  | 20661 | 513.0184 | 2048.0446 | 2048.0626 | -0.0179 | 0 | 15 | 3.2 | 1 | R.YTCHVQHEGLPKPLTLR.W |
|  | 25408 | 1319.0913 | 2636.1681 | 2636.1838 | -0.0158 | 0 | 68 | 1.1e-005 | 1 | R.DGEDQTQDTELVETRPAGDGTFQK.W 25407 |
|  | 26950 | **974.4291** | **2920.2654** | **2920.1963** | **0.0691** | **0** | **4** | **19** | **1** | **R.GYYNQSEAGSHTIQMMYGCHVGSDGR.F** |
|  | 26953 | **974.5107** | **2920.5104** | **2920.1963** | **0.3141** | **0** | **(1)** | **47** | **1** | **R.GYYNQSEAGSHTIQMMYGCHVGSDGR.F** |

| **270.** | P18462\|1A25_HUMAN    **Mass:** 41477    **Score:** 192    **Queries matched:** 17   **emPAI:** 0.54 |
| --- | --- |
|  | HLA class I histocompatibility antigen, A-25 alpha chain OS=Homo sapiens GN=HLA-A PE=2 SV=1 |

|  | **Query** | **Observed** | **Mr(expt)** | **Mr(calc)** | **Delta** | **Miss** | **Score** | **Expect** | **Rank** | **Peptide** |
| --- | --- | --- | --- | --- | --- | --- | --- | --- | --- | --- |
|  | 2373 | 408.2321 | 814.4497 | 813.3729 | 1.0768 | 0 | 4 | 89 | 4 | K.AHSQTDR.E |
|  | 5040 | 498.7649 | 995.5153 | 995.4308 | 0.0845 | 0 | 16 | 3.9 | 3 | R.FDSDAASQR.M 5054 |
|  | 9796 | 433.7320 | 1298.1743 | 1298.6327 | -0.4584 | 1 | 5 | 32 | 5 | K.AHSQTDRESLR.I |
|  | 13614 | 508.9797 | 1523.9174 | 1524.6627 | -0.7453 | 1 | 7 | 23 | 1 | R.FDSDAASQRMEPR.A 13613 |
|  | 14307 | **524.2791** | **1569.8155** | **1569.7324** | **0.0831** | **1** | **6** | **28** | **2** | **R.KWETAHEAEQWR.A** |
|  | 15206 | 815.4121 | 1628.8097 | 1628.8199 | -0.0102 | 0 | 72 | 7.9e-006 | 1 | R.FIAVGYVDDTQFVR.F 15203 15205 |
|  | 18194 | 614.9559 | 1841.8460 | 1841.8591 | -0.0132 | 0 | 31 | 0.087 | 1 | K.THMTHHAVSDHEATLR.C 18195 18196 |
|  | 20661 | 513.0184 | 2048.0446 | 2048.0626 | -0.0179 | 0 | 15 | 3.2 | 1 | R.YTCHVQHEGLPKPLTLR.W |
|  | 25408 | 1319.0913 | 2636.1681 | 2636.1838 | -0.0158 | 0 | 68 | 1.1e-005 | 1 | R.DGEDQTQDTELVETRPAGDGTFQK.W 25407 |
|  | 29926 | 1252.9806 | 3755.9199 | 3754.9617 | 0.9582 | 1 | 1 | 28 | 3 | R.TLVLLLSGALALTQTWAGSHSMRYFYTSVSRPGR.G |

|  | **Proteins matching a subset of these peptides:** |
| --- | --- |

|  | P30450\|1A26_HUMAN    **Mass:** 41321    **Score:** 192    **Queries matched:** 16 |
| --- | --- |
|  | HLA class I histocompatibility antigen, A-26 alpha chain OS=Homo sapiens GN=HLA-A PE=1 SV=2 |

|  | P30456\|1A43_HUMAN    **Mass:** 41292    **Score:** 192    **Queries matched:** 16 |
| --- | --- |
|  | HLA class I histocompatibility antigen, A-43 alpha chain OS=Homo sapiens GN=HLA-A PE=2 SV=1 |

|  | P30453\|1A34_HUMAN    **Mass:** 41314    **Score:** 192    **Queries matched:** 15 |
| --- | --- |
|  | HLA class I histocompatibility antigen, A-34 alpha chain OS=Homo sapiens GN=HLA-A PE=1 SV=1 |

|  | P30457\|1A66_HUMAN    **Mass:** 41341    **Score:** 192    **Queries matched:** 15 |
| --- | --- |
|  | HLA class I histocompatibility antigen, A-66 alpha chain OS=Homo sapiens GN=HLA-A PE=1 SV=1 |

| **271.** | P62917\|RL8_HUMAN    **Mass:** 28235    **Score:** 191    **Queries matched:** 19   **emPAI:** 0.46 |
| --- | --- |
|  | 60S ribosomal protein L8 OS=Homo sapiens GN=RPL8 PE=1 SV=2 |

|  | **Query** | **Observed** | **Mr(expt)** | **Mr(calc)** | **Delta** | **Miss** | **Score** | **Expect** | **Rank** | **Peptide** |
| --- | --- | --- | --- | --- | --- | --- | --- | --- | --- | --- |
|  | 810 | 503.2911 | 502.2838 | 501.3023 | 0.9815 | 1 | 4 | 1.4e+002 | 6 | R.KGAAR.L |
|  | 1020 | 545.3256 | 544.3183 | 543.3493 | 0.9690 | 1 | 9 | 39 | 6 | R.GKLAR.A |
|  | 1379 | **618.3428** | **617.3355** | **616.3333** | **1.0022** | **0** | **24** | **1.3** | **1** | **R.HGYIK.G** |
|  | 2521 | **413.9450** | **825.8754** | **825.5324** | **0.3430** | **0** | **12** | **6.4** | **2** | **R.IDKPILK.A** |
|  | 2543 | **414.7289** | **827.4433** | **826.5388** | **0.9045** | **1** | **11** | **15** | **2** | **R.KVGLIAAR.R** 2518 2519 2533 |
|  | 3303 | **437.2297** | **872.4448** | **873.5032** | **-1.0584** | **1** | **7** | **39** | **10** | **K.KVISSANR.A** |
|  | 4264 | **471.2205** | **940.4264** | **940.5454** | **-0.1191** | **0** | **39** | **0.021** | **1** | **R.AVVGVVAGGGR.I** 4279 |
|  | 5839 | **529.1312** | **1056.2479** | **1056.6444** | **-0.3965** | **1** | **5** | **40** | **8** | **R.GAPLAKVVFR.D** |
|  | 16077 | 844.9098 | 1687.8050 | 1687.8165 | -0.0115 | 0 | 76 | 2.7e-006 | 1 | R.ASGNYATVISHNPETK.K 16074 16075 16076 |
|  | 22565 | **747.7008** | **2240.0806** | **2240.0936** | **-0.0130** | **0** | **7** | **16** | **1** | **R.TELFIAAEGIHTGQFVYCGK.K** |
|  | 26887 | 970.8213 | 2909.4420 | 2908.4627 | 0.9793 | 1 | 0 | 54 | 6 | R.GVAMNPVEHPFGGGNHQHIGKPSTIRR.D |
|  | 28920 | **1132.5695** | **3394.6866** | **3394.7047** | **-0.0182** | **1** | **76** | **1e-006** | **1** | **K.KAQLNIGNVLPVGTMPEGTIVCCLEEKPGDR.G** |

| **272.** | P42704\|LPPRC_HUMAN    **Mass:** 159003   **Score:** 191    **Queries matched:** 46   **emPAI:** 0.17 |
| --- | --- |
|  | Leucine-rich PPR motif-containing protein, mitochondrial OS=Homo sapiens GN=LRPPRC PE=1 SV=3 |

|  | **Query** | **Observed** | **Mr(expt)** | **Mr(calc)** | **Delta** | **Miss** | **Score** | **Expect** | **Rank** | **Peptide** |
| --- | --- | --- | --- | --- | --- | --- | --- | --- | --- | --- |
|  | 827 | **506.2578** | **505.2506** | **504.2908** | **0.9598** | **0** | **7** | **53** | **2** | **K.ASTVK.S** |
|  | 1011 | **544.3177** | **543.3104** | **542.3540** | **0.9564** | **0** | **10** | **31** | **5** | **M.AALLR.S** |
|  | 1103 | **561.2664** | **560.2591** | **559.3190** | **0.9401** | **1** | **10** | **32** | **6** | **R.ARSAR.L** |
|  | 2762 | 421.2294 | 840.4442 | 841.5385 | -1.0943 | 0 | 8 | 25 | 8 | R.LIITQVR.R |
|  | 3000 | 429.2127 | 856.4109 | 857.5083 | -1.0974 | 1 | 6 | 43 | 9 | R.LDLSVRR.T |
|  | 3017 | **430.2380** | **858.4615** | **859.4586** | **-0.9970** | **0** | **10** | **23** | **10** | **K.DILIACR.L** |
|  | 3863 | **456.2024** | **910.3903** | **910.3967** | **-0.0064** | **0** | **3** | **62** | **8** | **K.VFNDTCR.S** |
|  | 4466 | 477.3020 | 952.5895 | 952.5416 | 0.0479 | 1 | 17 | 2.7 | 9 | K.ILGFMKTK.D 4460 |
|  | 5080 | 499.7438 | 997.4730 | 997.6396 | -0.1666 | 1 | 9 | 20 | 10 | R.LIITQVRR.D |
|  | 5203 | **504.2659** | **1006.5173** | **1005.5858** | **0.9315** | **1** | **15** | **5.9** | **10** | **R.LYAIAAKEK.D** |
|  | 5834 | 528.7682 | 1055.5219 | 1055.5287 | -0.0068 | 0 | 12 | 9.3 | 2 | K.VYLQNEYK.F |
|  | 7808 | **399.7249** | **1196.1530** | **1196.7605** | **-0.6074** | **1** | **8** | **16** | **6** | **K.TLRLLAEILR.E** |
|  | 9324 | **637.2900** | **1272.5654** | **1271.6470** | **0.9184** | **0** | **27** | **0.33** | **1** | **K.TVLDQQQTPSR.L** 9276 9279 |
|  | 9442 | 640.7906 | 1279.5666 | 1279.7023 | -0.1357 | 1 | 10 | 13 | 4 | R.DYLKEAVTTLK.T |
|  | 11207 | **459.9229** | **1376.7469** | **1375.7282** | **1.0187** | **1** | **7** | **26** | **6** | **K.MNVKIPENIYR.G** |
|  | 11665 | **702.8374** | **1403.6602** | **1403.6715** | **-0.0112** | **0** | **29** | **0.17** | **1** | **R.CVANNQVETLEK.L** 11698 |
|  | 14047 | **518.6078** | **1552.8017** | **1553.8453** | **-1.0436** | **0** | **2** | **72** | **9** | **R.NLLESYHVPELIK.D** |
|  | 14428 | **790.4035** | **1578.7924** | **1579.7817** | **-0.9892** | **1** | **3** | **61** | **1** | **R.QYFHQLEKMNVK.I** |
|  | 14853 | **536.9271** | **1607.7595** | **1606.8065** | **0.9531** | **1** | **5** | **36** | **1** | **R.DQMYYNLLKLYK.I** |
|  | 15498 | 824.4252 | 1646.8358 | 1645.8675 | 0.9683 | 1 | 4 | 53 | 5 | K.DVTSAKALYEHLTAK.N |
|  | 15542 | 825.9569 | 1649.8993 | 1649.8447 | 0.0546 | 1 | 2 | 70 | 3 | K.LVELTQKLFECDR.D |
|  | 15715 | **832.3876** | **1662.7607** | **1662.7705** | **-0.0098** | **0** | **80** | **1.1e-006** | **1** | **R.AGDMENAENILTVMR.D** |
|  | 15722 | **555.2967** | **1662.8684** | **1661.8777** | **0.9907** | **0** | **6** | **32** | **3** | **K.LGAVYDVSHYNALLK.V** |
|  | 15951 | 839.9032 | 1677.7918 | 1678.7654 | -0.9736 | 0 | (12) | 7.9 | 2 | R.AGDMENAENILTVMR.D |
|  | 16180 | **566.2871** | **1695.8395** | **1695.8138** | **0.0257** | **0** | **2** | **70** | **4** | **R.LIASYCNVGDIEGASK.I** |
|  | 16456 | 858.4575 | 1714.9005 | 1714.9083 | -0.0078 | 0 | 59 | 0.00015 | 1 | K.VIEPQYFGLAYLFR.K 16455 |
|  | 17649 | **902.9230** | **1803.8314** | **1803.8423** | **-0.0109** | **0** | **51** | **0.00085** | **1** | **K.LLMSEDYFTQAMEVK.A** |
|  | 18604 | **624.3143** | **1869.9212** | **1869.0459** | **0.8753** | **1** | **4** | **38** | **4** | **K.ASTVKSVLELIPELNEK.E** |
|  | 18747 | **941.9747** | **1881.9349** | **1881.9472** | **-0.0123** | **0** | **58** | **0.00018** | **1** | **K.EQNIVFNAETYSNLIK.L** 18748 |
|  | 18751 | **628.3372** | **1881.9898** | **1880.9744** | **1.0154** | **1** | **2** | **63** | **5** | **R.ADAVWNKIQEENVIPR.E** |
|  | 19333 | **643.3311** | **1926.9715** | **1927.0965** | **-0.1249** | **0** | **1** | **71** | **4** | **R.CGAIAEQTPILLLFLLR.N** |
|  | 20185 | 669.9780 | 2006.9123 | 2006.9230 | -0.0107 | 1 | 23 | 0.48 | 1 | K.LFECDRDQMYYNLLK.L |
|  | 22596 | **1124.0248** | **2246.0350** | **2246.0484** | **-0.0134** | **0** | **7** | **15** | **1** | **K.NNNIDAAIENIENMLTSENK.V** |
|  | 23366 | 1172.5951 | 2343.1756 | 2343.1529 | 0.0227 | 1 | 1 | 65 | 5 | R.VTYQRLIASYCNVGDIEGASK.I 23365 |
|  | 24147 | **817.0843** | **2448.2312** | **2448.2457** | **-0.0145** | **1** | **7** | **14** | **1** | **K.SVLELIPELNEKEEAYNSLMK.S** |
|  | 25987 | **908.7854** | **2723.3344** | **2724.4244** | **-1.0900** | **1** | **3** | **29** | **2** | **K.LKEVQMHSFPLQFTLHCALLANK.T** |
|  | 26128 | **917.8304** | **2750.4695** | **2749.2793** | **1.1902** | **0** | **1** | **43** | **8** | **R.GPTEAVGYFLYNLIDSMSDSEVQAK.E** |
|  | 27219 | **992.1269** | **2973.3588** | **2974.5076** | **-1.1487** | **1** | **1** | **39** | **2** | **K.GAYDIFLNAKEQNIVFNAETYSNLIK.L** |
|  | 27969 | **789.9349** | **3155.7107** | **3155.7230** | **-0.0124** | **0** | **12** | **2.6** | **1** | **K.QLHEAIVTLGLAEPSTNISFPLVTVHLEK.G** |

| **273.** | Q9Y277\|VDAC3_HUMAN    **Mass:** 30981    **Score:** 190    **Queries matched:** 15   **emPAI:** 0.58 |
| --- | --- |
|  | Voltage-dependent anion-selective channel protein 3 OS=Homo sapiens GN=VDAC3 PE=1 SV=1 |

|  | **Query** | **Observed** | **Mr(expt)** | **Mr(calc)** | **Delta** | **Miss** | **Score** | **Expect** | **Rank** | **Peptide** |
| --- | --- | --- | --- | --- | --- | --- | --- | --- | --- | --- |
|  | 2416 | **409.5527** | **817.0908** | **816.3878** | **0.7029** | **0** | **6** | **40** | **10** | **K.NFSAGGHK.V** |
|  | 3337 | **438.2487** | **874.4829** | **873.4055** | **1.0775** | **0** | **3** | **1.1e+002** | **8** | **K.GYGFGMVK.I** |
|  | 8930 | **627.8233** | **1253.6320** | **1253.6404** | **-0.0084** | **0** | **9** | **16** | **1** | **K.LSQNNFALGYK.A** |
|  | 10383 | **665.8223** | **1329.6301** | **1329.6387** | **-0.0086** | **0** | **40** | **0.014** | **1** | **K.VCNYGLTFTQK.W** |
|  | 11939 | 709.8932 | 1417.7718 | 1417.7817 | -0.0099 | 0 | 31 | 0.12 | 1 | K.LTLDTIFVPNTGK.K |
|  | 16622 | **577.1246** | **1728.3519** | **1728.7633** | **-0.4115** | **1** | **0** | **66** | **6** | **-.MCNTPTYCDLGKAAK.D** |
|  | 19102 | 954.9387 | 1907.8628 | 1906.8697 | 0.9931 | 0 | 15 | 3.3 | 2 | K.WNTDNTLGTEISWENK.L 19101 |
|  | 20008 | **664.2819** | **1989.8239** | **1989.8375** | **-0.0135** | **0** | **6** | **21** | **2** | **K.SCSGVEFSTSGHAYTDTGK.A** |
|  | 21193 | **1051.0853** | **2100.1561** | **2100.1691** | **-0.0130** | **0** | **93** | **4.3e-008** | **1** | **K.VNNASLIGLGYTQTLRPGVK.L** 21192 21201 |
|  | 24696 | 1260.1010 | 2518.1873 | 2518.2339 | -0.0466 | 1 | 47 | 0.0015 | 2 | K.WNTDNTLGTEISWENKLAEGLK.L 24697 |
|  | 24785 | **845.7284** | **2534.1635** | **2534.1826** | **-0.0191** | **0** | **6** | **16** | **1** | **K.AADFQLHTHVNDGTEFGGSIYQK.V** |

| **274.** | Q6P5W5\|S39A4_HUMAN    **Mass:** 69233    **Score:** 187    **Queries matched:** 9   **emPAI:** 0.11 |
| --- | --- |
|  | Zinc transporter ZIP4 OS=Homo sapiens GN=SLC39A4 PE=1 SV=2 |

|  | **Query** | **Observed** | **Mr(expt)** | **Mr(calc)** | **Delta** | **Miss** | **Score** | **Expect** | **Rank** | **Peptide** |
| --- | --- | --- | --- | --- | --- | --- | --- | --- | --- | --- |
|  | 712 | **478.2912** | **477.2840** | **476.2343** | **1.0497** | **0** | **1** | **1.4e+002** | **1** | **R.GASSR.D** |
|  | 3265 | **435.7585** | **869.5025** | **869.5083** | **-0.0058** | **1** | **24** | **0.67** | **1** | **R.RLSPELR.L** 3232 |
|  | 7425 | **588.3669** | **1174.7193** | **1173.5924** | **1.1269** | **1** | **13** | **8** | **2** | **R.MQARAAGQTPK.T** 7412 |
|  | 18710 | **939.9700** | **1877.9255** | **1877.9370** | **-0.0115** | **0** | **91** | **8.7e-008** | **1** | **R.ADLVAEESPELLNPEPR.R** 18709 |
|  | 28671 | **1109.5818** | **3325.7235** | **3326.7293** | **-1.0057** | **0** | **43** | **0.002** | **1** | **K.TACVDIPQLLEEAVGAGAPGSAGGVLAALLDHVR.S** 28672 |

| **275.** | P34931\|HS71L_HUMAN    **Mass:** 70730    **Score:** 186    **Queries matched:** 23   **emPAI:** 0.29 |
| --- | --- |
|  | Heat shock 70 kDa protein 1L OS=Homo sapiens GN=HSPA1L PE=1 SV=2 |

|  | **Query** | **Observed** | **Mr(expt)** | **Mr(calc)** | **Delta** | **Miss** | **Score** | **Expect** | **Rank** | **Peptide** |
| --- | --- | --- | --- | --- | --- | --- | --- | --- | --- | --- |
|  | 1724 | 705.3886 | 704.3813 | 703.3501 | 1.0312 | 0 | 10 | 20 | 5 | K.DISQNK.R |
|  | 2227 | 804.4412 | 803.4339 | 803.4389 | -0.0050 | 0 | 23 | 1.2 | 1 | K.ITITNDK.G |
|  | 7819 | 599.3470 | 1196.6794 | 1196.6877 | -0.0083 | 0 | 9 | 20 | 2 | K.DAGVIAGLNVLR.I |
|  | 8452 | 614.8128 | 1227.6111 | 1227.6207 | -0.0097 | 0 | 47 | 0.003 | 1 | K.VEIIANDQGNR.T 8436 8453 |
|  | 12697 | 733.4055 | 1464.7964 | 1464.8413 | -0.0449 | 1 | 24 | 0.45 | 1 | K.AKIHDIVLVGGSTR.I |
|  | 13054 | 744.3494 | 1486.6842 | 1486.6940 | -0.0098 | 0 | 50 | 0.0013 | 1 | R.TTPSYVAFTDTER.L |
|  | 15148 | 813.3944 | 1624.7743 | 1624.9260 | -0.1518 | 1 | 11 | 8.7 | 2 | R.QATKDAGVIAGLNVLR.I |
|  | 15661 | 830.4460 | 1658.8775 | 1658.8879 | -0.0104 | 0 | 46 | 0.0026 | 1 | R.IINEPTAAAIAYGLDK.G 15683 15687 |
|  | 15876 | 838.3638 | 1674.7130 | 1674.7234 | -0.0104 | 0 | 55 | 0.00037 | 1 | K.ATAGDTHLGGEDFDNR.L |
|  | 15926 | 839.4018 | 1676.7890 | 1675.7988 | 0.9902 | 0 | 3 | 60 | 3 | K.NQVAMNPQNTVFDAK.R |
|  | 21740 | 1076.5413 | 2151.0680 | 2152.0510 | -0.9831 | 1 | 1 | 64 | 6 | R.FEELCADLFRGTLEPVEK.A 21754 21792 |
|  | 23079 | 768.7191 | 2303.1354 | 2303.1467 | -0.0114 | 0 | 5 | 24 | 1 | K.SINPDEAVAYGAAVQAAILMGDK.S |
|  | 23516 | 787.7372 | 2360.1897 | 2360.1794 | 0.0103 | 1 | 6 | 21 | 3 | R.LIGDAAKNQVAMNPQNTVFDAK.R |
|  | 26230 | 925.1461 | 2772.4165 | 2773.3956 | -0.9791 | 1 | (0) | 56 | 5 | R.DLNKSINPDEAVAYGAAVQAAILMGDK.S |
|  | 26312 | 930.4825 | 2788.4256 | 2789.3905 | -0.9649 | 1 | 0 | 56 | 8 | R.DLNKSINPDEAVAYGAAVQAAILMGDK.S |
|  | 26625 | 955.8660 | 2864.5763 | 2865.6137 | -1.0374 | 0 | 6 | 14 | 3 | K.VQDLLLLDVAPLSLGLETAGGVMTALIK.R |
|  | 26957 | **974.5287** | **2920.5642** | **2919.5270** | **1.0372** | **0** | **1** | **45** | **4** | **R.HVLIFDLGGGTFDVSILTIDDGIFEVK.A** |

| **276.** | P36542\|ATPG_HUMAN    **Mass:** 33032    **Score:** 185    **Queries matched:** 25   **emPAI:** 0.72 |
| --- | --- |
|  | ATP synthase subunit gamma, mitochondrial OS=Homo sapiens GN=ATP5C1 PE=1 SV=1 |

|  | **Query** | **Observed** | **Mr(expt)** | **Mr(calc)** | **Delta** | **Miss** | **Score** | **Expect** | **Rank** | **Peptide** |
| --- | --- | --- | --- | --- | --- | --- | --- | --- | --- | --- |
|  | 1250 | **590.3295** | **589.3222** | **589.3257** | **-0.0036** | **0** | **30** | **0.3** | **1** | **K.MVAAAK.Y** |
|  | 1687 | 695.3133 | 694.3060 | 695.3854 | -1.0794 | 0 | 15 | 4.9 | 2 | R.SVISYK.T |
|  | 3149 | **432.8838** | **863.7531** | **863.4865** | **0.2666** | **0** | **0** | **1.1e+002** | **10** | **K.LTLTFNR.T** |
|  | 4042 | **462.7437** | **923.4728** | **922.4066** | **1.0662** | **0** | **1** | **1.2e+002** | **7** | **K.NASEMIDK.L** |
|  | 4463 | 477.2839 | 952.5532 | 951.4881 | 1.0651 | 1 | 5 | 44 | 7 | K.SMKMVAAAK.Y |
|  | 4729 | **487.3202** | **972.6259** | **971.4924** | **1.1336** | **1** | **9** | **22** | **7** | **K.ADIKGPEDK.K** |
|  | 4819 | **490.3102** | **978.6058** | **979.5273** | **-0.9215** | **1** | **(3)** | **76** | **7** | **K.MVAAAKYAR.A** 4830 |
|  | 5062 | **499.2840** | **996.5534** | **995.5222** | **1.0312** | **1** | **9** | **19** | **9** | **K.MVAAAKYAR.A** |
|  | 5723 | **524.2842** | **1046.5538** | **1046.5608** | **-0.0070** | **0** | **66** | **4.5e-005** | **1** | **K.SEVATLTAAGK.E** |
|  | 5896 | **530.7854** | **1059.5562** | **1059.5634** | **-0.0072** | **0** | **24** | **0.71** | **1** | **K.EVMLVGIGDK.I** |
|  | 8173 | **607.3174** | **1212.6203** | **1212.6285** | **-0.0082** | **0** | **59** | **0.00019** | **1** | **R.GLCGAIHSSIAK.Q** 8172 |
|  | 8381 | 612.7637 | 1223.5128 | 1223.6986 | -0.1858 | 1 | 8 | 24 | 3 | K.KHLLIGVSSDR.G 8382 |
|  | 9661 | **646.3302** | **1290.6458** | **1291.6561** | **-1.0102** | **0** | **37** | **0.029** | **1** | **R.THSDQFLVAFK.E** 9684 |
|  | 10311 | 663.8645 | 1325.7144 | 1325.7231 | -0.0086 | 0 | 76 | 3.1e-006 | 1 | R.IYGLGSLALYEK.A 10310 |
|  | 12452 | 725.8465 | 1449.6784 | 1449.7497 | -0.0713 | 1 | 9 | 18 | 2 | K.QMKSEVATLTAAGK.E 12467 |
|  | 14748 | **534.6198** | **1600.8376** | **1599.8225** | **1.0151** | **1** | **5** | **42** | **8** | **R.GLCGAIHSSIAKQMK.S** |
|  | 20791 | 687.6535 | 2059.9387 | 2059.8786 | 0.0601 | 1 | 4 | 39 | 4 | K.ESTTSEQSARMTAMDNASK.N |
|  | 21223 | **702.2110** | **2103.6112** | **2104.1086** | **-0.4974** | **1** | **5** | **19** | **1** | **K.SEVATLTAAGKEVMLVGIGDK.I** |
|  | 28821 | **1124.5665** | **3370.6778** | **3370.6973** | **-0.0195** | **0** | **16** | **0.97** | **1** | **R.KPPTFGDASVIALELLNSGYEFDEGSIIFNK.F** |

| **277.** | Q12797\|ASPH_HUMAN    **Mass:** 86266    **Score:** 185    **Queries matched:** 13   **emPAI:** 0.04 |
| --- | --- |
|  | Aspartyl/asparaginyl beta-hydroxylase OS=Homo sapiens GN=ASPH PE=1 SV=3 |

|  | **Query** | **Observed** | **Mr(expt)** | **Mr(calc)** | **Delta** | **Miss** | **Score** | **Expect** | **Rank** | **Peptide** |
| --- | --- | --- | --- | --- | --- | --- | --- | --- | --- | --- |
|  | 3125 | **432.2788** | **862.5430** | **861.4318** | **1.1112** | **1** | **11** | **17** | **3** | **K.HGGHKNGR.K** |
|  | 5205 | **504.2708** | **1006.5270** | **1006.5997** | **-0.0727** | **0** | **6** | **38** | **4** | **R.MHLGLVIPK.E** |
|  | 7738 | **398.4728** | **1192.3965** | **1191.5805** | **0.8160** | **1** | **2** | **76** | **10** | **R.DEGLAVMDKAK.G** |
|  | 8003 | **603.3823** | **1204.7500** | **1204.6452** | **0.1048** | **1** | **4** | **67** | **10** | **R.GKIEEAVNAFK.E** |
|  | 11168 | **688.3658** | **1374.7170** | **1373.7555** | **0.9615** | **0** | **7** | **27** | **2** | **R.LVQLFPNDTSLK.N** |
|  | 12850 | **738.3556** | **1474.6967** | **1473.8191** | **0.8777** | **1** | **6** | **33** | **2** | **K.AQNKIAESIPYLK.E** |
|  | 13282 | **501.2423** | **1500.7049** | **1500.7936** | **-0.0887** | **1** | **4** | **56** | **3** | **K.AKGLFLPEDENLR.E** |
|  | 14149 | 780.8945 | 1559.7744 | 1558.7991 | 0.9753 | 1 | 9 | 15 | 6 | K.GLFLPEDENLREK.G |
|  | 18992 | **950.9012** | **1899.7878** | **1899.8011** | **-0.0132** | **0** | **105** | **3.1e-009** | **1** | **K.LGIYDADGDGDFDVDDAK.V** 18991 |
|  | 23962 | **809.7095** | **2426.1068** | **2426.1179** | **-0.0111** | **0** | **5** | **21** | **1** | **K.VLIFDDSFEHEVWQDASSFR.L** |
|  | 25390 | **878.6979** | **2633.0718** | **2632.2540** | **0.8178** | **1** | **3** | **30** | **4** | **K.YSIMHPGTHVWPHTGPTNCRLR.M** |
|  | 31657 | **1807.6240** | **5419.8502** | **5420.3481** | **-0.4979** | **0** | **3** | **4.4** | **3** | **K.EQIQSLLHEMVHAEHVEGEDLQQEDGPTGEPQQEDDEFLMATDVDDR.F** |

| **278.** | P30048\|PRDX3_HUMAN    **Mass:** 28017    **Score:** 184    **Queries matched:** 10   **emPAI:** 0.66 |
| --- | --- |
|  | Thioredoxin-dependent peroxide reductase, mitochondrial OS=Homo sapiens GN=PRDX3 PE=1 SV=3 |

|  | **Query** | **Observed** | **Mr(expt)** | **Mr(calc)** | **Delta** | **Miss** | **Score** | **Expect** | **Rank** | **Peptide** |
| --- | --- | --- | --- | --- | --- | --- | --- | --- | --- | --- |
|  | 802 | 503.1060 | 502.0987 | 502.2863 | -0.1876 | 0 | 12 | 25 | 1 | R.ASVAR.H |
|  | 4456 | **477.2336** | **952.4527** | **951.4549** | **0.9978** | **0** | **19** | **1.9** | **1** | **K.DLSLDDFK.G** |
|  | 6931 | 568.7974 | 1135.5803 | 1136.5713 | -0.9911 | 1 | 8 | 23 | 4 | K.DLSLDDFKGK.Y |
|  | 8020 | **603.8289** | **1205.6433** | **1205.6517** | **-0.0084** | **0** | **35** | **0.048** | **1** | **K.HLSVNDLPVGR.S** |
|  | 12653 | **731.8949** | **1461.7752** | **1461.7827** | **-0.0075** | **0** | **75** | **4.1e-006** | **1** | **R.DYGVLLEGSGLALR.G** |
|  | 14232 | 783.3669 | 1564.7193 | 1565.7719 | -1.0526 | 0 | 1 | 84 | 7 | R.TSLTNLLCSGSSQAK.L |
|  | 19539 | **649.3312** | **1944.9719** | **1946.0585** | **-1.0866** | **1** | **2** | **56** | **4** | **K.QISRDYGVLLEGSGLALR.G** |
|  | 28879 | **1129.2028** | **3384.5865** | **3384.6085** | **-0.0220** | **0** | **72** | **2.4e-006** | **1** | **K.AFQYVETHGEVCPANWTPDSPTIKPSPAASK.E** 28877 28880 |

| **279.** | P10620\|MGST1_HUMAN    **Mass:** 17644    **Score:** 183    **Queries matched:** 11   **emPAI:** 2.28 |
| --- | --- |
|  | Microsomal glutathione S-transferase 1 OS=Homo sapiens GN=MGST1 PE=1 SV=1 |

|  | **Query** | **Observed** | **Mr(expt)** | **Mr(calc)** | **Delta** | **Miss** | **Score** | **Expect** | **Rank** | **Peptide** |
| --- | --- | --- | --- | --- | --- | --- | --- | --- | --- | --- |
|  | 12008 | 711.8282 | 1421.6418 | 1421.6505 | -0.0087 | 0 | 55 | 0.0004 | 1 | K.MMLMSTATAFYR.L 11992 12006 12007 |
|  | 14459 | **791.3847** | **1580.7548** | **1580.7657** | **-0.0109** | **1** | **47** | **0.0021** | **1** | **R.KVFANPEDCVAFGK.G** |
|  | 18949 | **949.0136** | **1896.0125** | **1896.0257** | **-0.0132** | **0** | **60** | **0.00011** | **1** | **R.IYHTIAYLTPLPQPNR.A** 18950 |
|  | 26384 | **936.6785** | **2807.0136** | **2806.3809** | **0.6327** | **0** | **28** | **0.065** | **1** | **M.VDLTQVMDDEVFMAFASYATIILSK.M** 26380 |
|  | 29796 | **926.7492** | **3702.9677** | **3702.9773** | **-0.0096** | **0** | **54** | **0.00014** | **1** | **R.AHLNDLENIIPFLGIGLLYSLSGPDPSTAILHFR.L** |
|  | 30831 | **1098.1711** | **4388.6555** | **4389.0461** | **-0.3907** | **1** | **0** | **15** | **1** | **-.MVDLTQVMDDEVFMAFASYATIILSKMMLMSTATAFYR.L** |

| **280.** | Q8WWU7\|ITLN2_HUMAN    **Mass:** 36929    **Score:** 182    **Queries matched:** 8   **emPAI:** 0.34 |
| --- | --- |
|  | Intelectin-2 OS=Homo sapiens GN=ITLN2 PE=2 SV=1 |

|  | **Query** | **Observed** | **Mr(expt)** | **Mr(calc)** | **Delta** | **Miss** | **Score** | **Expect** | **Rank** | **Peptide** |
| --- | --- | --- | --- | --- | --- | --- | --- | --- | --- | --- |
|  | 8662 | **619.9519** | **1237.8892** | **1238.6297** | **-0.7405** | **1** | **6** | **26** | **3** | **-.MLSMLRTMTR.L** |
|  | 8939 | **628.3029** | **1254.5912** | **1254.6246** | **-0.0335** | **1** | **(3)** | **67** | **3** | **-.MLSMLRTMTR.L** |
|  | 9271 | **636.3179** | **1270.6213** | **1270.6196** | **0.0017** | **1** | **(2)** | **82** | **6** | **-.MLSMLRTMTR.L** |
|  | 9646 | **430.9033** | **1289.6882** | **1288.6928** | **0.9954** | **0** | **31** | **0.12** | **1** | **R.LGHNLFGIYQK.Y** |
|  | 15923 | 839.3769 | 1676.7392 | 1676.7505 | -0.0112 | 0 | 34 | 0.04 | 1 | R.EFETCAFSFSSLPR.S |
|  | 16883 | **583.1091** | **1746.3054** | **1746.8835** | **-0.5781** | **1** | **1** | **64** | **9** | **R.VFNNERAANALCAGIK.V** |
|  | 22747 | **755.6848** | **2264.0324** | **2265.0525** | **-1.0200** | **1** | **3** | **41** | **6** | **K.CWNDNGPAIPVVYDFGDAKK.T** |
|  | 30657 | **1393.5895** | **4177.7466** | **4177.7726** | **-0.0259** | **1** | **171** | **1.5e-016** | **1** | **K.ADYPEGDGNWANYNTFGSAEAATSDDYKNPGYYDIQAK.D** |

| **281.** | Q96JP2\|MY15B_HUMAN    **Mass:** 168096   **Score:** 182    **Queries matched:** 27   **emPAI:** 0.07 |
| --- | --- |
|  | Putative myosin-XVB OS=Homo sapiens GN=MYO15B PE=5 SV=2 |

|  | **Query** | **Observed** | **Mr(expt)** | **Mr(calc)** | **Delta** | **Miss** | **Score** | **Expect** | **Rank** | **Peptide** |
| --- | --- | --- | --- | --- | --- | --- | --- | --- | --- | --- |
|  | 631 | **460.2628** | **459.2555** | **459.2441** | **0.0114** | **0** | **6** | **97** | **1** | **K.QGGAK.A** |
|  | 3417 | 881.5146 | 880.5074 | 880.4337 | 0.0736 | 1 | 1 | 1.2e+002 | 10 | R.MRGFQAR.K |
|  | 3761 | **452.2576** | **902.5007** | **901.4729** | **1.0278** | **1** | **11** | **15** | **6** | **R.ISQNERR.K** |
|  | 5281 | 507.7519 | 1013.4892 | 1014.4739 | -0.9847 | 0 | 3 | 91 | 9 | R.GMAALCQHK.L |
|  | 5620 | **520.2512** | **1038.4878** | **1037.5618** | **0.9260** | **0** | **1** | **1.4e+002** | **10** | **R.SQALVDLHR.S** |
|  | 6441 | **549.9246** | **1097.8346** | **1098.5921** | **-0.7575** | **1** | **1** | **87** | **3** | **K.IDPKDEALAK.L** |
|  | 7120 | **576.7866** | **1151.5586** | **1152.6251** | **-1.0665** | **0** | **6** | **42** | **2** | **K.SEQLVLHTAR.A** |
|  | 7389 | **586.9143** | **1171.8139** | **1171.5047** | **0.3093** | **0** | **6** | **30** | **6** | **R.DNWANYFSR.F** |
|  | 7806 | **598.8185** | **1195.6224** | **1196.6765** | **-1.0541** | **0** | **3** | **76** | **1** | **K.APLQLGPSSSIK.E** |
|  | 8798 | **623.3466** | **1244.6786** | **1243.5979** | **1.0807** | **1** | **5** | **54** | **6** | **R.KMGEGQAEARPA.-** |
|  | 9343 | 425.5762 | 1273.7067 | 1273.7142 | -0.0076 | 0 | 8 | 24 | 6 | R.KPPTPPEKPQR.D |
|  | 11131 | **687.3630** | **1372.7114** | **1372.7133** | **-0.0018** | **1** | **4** | **57** | **3** | **R.ALERVPSMELGR.L** |
|  | 11256 | **690.8505** | **1379.6865** | **1379.7633** | **-0.0768** | **1** | **1** | **1e+002** | **9** | **K.SEQLVLHTARAR.A** |
|  | 11785 | **470.6422** | **1408.9046** | **1407.7834** | **1.1212** | **0** | **4** | **41** | **6** | **K.VTQGPGLRPDQLK.I** |
|  | 12505 | **727.4053** | **1452.7960** | **1453.8140** | **-1.0180** | **1** | **1** | **90** | **8** | **K.APLQLGPSSSIKEK.Q** |
|  | 12677 | 732.3851 | 1462.7556 | 1463.7229 | -0.9673 | 1 | 6 | 31 | 2 | K.ETAAKGHGQGPAQGR.G 12676 |
|  | 14948 | 807.9384 | 1613.8621 | 1613.8737 | -0.0115 | 0 | 86 | 3.2e-007 | 1 | K.LLGALEQSQLASGATR.A |
|  | 16654 | 866.0067 | 1729.9987 | 1729.0025 | 0.9962 | 1 | 76 | 2.7e-006 | 1 | R.AIEALVELFLNELKK.D 16642 16643 |
|  | 16850 | **872.9386** | **1743.8626** | **1743.8751** | **-0.0125** | **0** | **60** | **0.0001** | **1** | **R.ASEAASQASPSAVTSKPR.K** |
|  | 23034 | **766.5350** | **2296.5833** | **2296.0607** | **0.5225** | **1** | **2** | **34** | **5** | **R.ETSEEAEDRPYQPKSFQQK.R** |
|  | 23470 | **1178.0740** | **2354.1334** | **2355.1891** | **-1.0557** | **1** | **6** | **17** | **3** | **R.QQQARASEAASQASPSAVTSKPR.K** 23477 |
|  | 26599 | **714.8903** | **2855.5319** | **2854.3643** | **1.1676** | **1** | **1** | **42** | **1** | **R.SAPQPPEYPGRPDMAVPGWSAMVRSR.L** |
|  | 29873 | **746.7670** | **3728.7985** | **3729.6446** | **-0.8462** | **0** | **0** | **31** | **1** | **K.EASGVGGGHSPMCPPHCHMPPGPAGEWPGATVQQPR.Q** |

| **282.** | Q99623\|PHB2_HUMAN    **Mass:** 33276    **Score:** 182    **Queries matched:** 27   **emPAI:** 1.12 |
| --- | --- |
|  | Prohibitin-2 OS=Homo sapiens GN=PHB2 PE=1 SV=2 |

|  | **Query** | **Observed** | **Mr(expt)** | **Mr(calc)** | **Delta** | **Miss** | **Score** | **Expect** | **Rank** | **Peptide** |
| --- | --- | --- | --- | --- | --- | --- | --- | --- | --- | --- |
|  | 1164 | **574.2851** | **573.2778** | **572.3282** | **0.9496** | **0** | **19** | **3.5** | **1** | **M.AQNLK.D** |
|  | 1636 | **677.3595** | **676.3522** | **676.3578** | **-0.0056** | **0** | **19** | **3.1** | **1** | **R.GMGTALK.L** |
|  | 1786 | **720.3654** | **719.3581** | **719.3636** | **-0.0054** | **0** | **6** | **56** | **4** | **-.MAQNLK.D** |
|  | 1819 | **731.6047** | **730.5974** | **730.3973** | **0.2001** | **0** | **1** | **1.7e+002** | **1** | **R.AAQNISK.T** |
|  | 2845 | **848.4490** | **847.4418** | **847.4473** | **-0.0055** | **0** | **22** | **1.1** | **1** | **K.MLGEALSK.N** 2846 |
|  | 3172 | **433.2636** | **864.5126** | **863.4422** | **1.0704** | **0** | **(6)** | **43** | **7** | **K.MLGEALSK.N** |
|  | 3564 | 446.0373 | 890.0600 | 889.4617 | 0.5982 | 0 | 9 | 16 | 6 | K.TIATSQNR.I |
|  | 3756 | 452.2401 | 902.4656 | 903.5025 | -1.0369 | 1 | 17 | 4 | 2 | R.GSDSLIKGK.K 3783 |
|  | 3769 | **452.7557** | **903.4969** | **903.5025** | **-0.0057** | **1** | **22** | **1.1** | **3** | **R.KISSPTGSK.D** 3772 3773 |
|  | 5030 | **498.3364** | **994.6582** | **993.4767** | **1.1815** | **0** | **6** | **32** | **6** | **R.LGLDYEER.V** |
|  | 5843 | **529.2700** | **1056.5255** | **1056.5312** | **-0.0057** | **0** | **34** | **0.072** | **1** | **K.QVAQQEAQR.A** |
|  | 7454 | **589.3160** | **1176.6175** | **1176.6251** | **-0.0076** | **0** | **63** | **8.7e-005** | **1** | **K.FNASQLITQR.A** |
|  | 8200 | **608.3106** | **1214.6065** | **1214.6142** | **-0.0077** | **0** | **53** | **0.00082** | **1** | **K.IVQAEGEAEAAK.M** 8193 |
|  | 8240 | **609.2947** | **1216.5749** | **1216.5837** | **-0.0087** | **0** | **28** | **0.25** | **1** | **R.ESVFTVEGGHR.A** |
|  | 9532 | **643.2843** | **1284.5540** | **1283.7020** | **0.8521** | **1** | **3** | **76** | **8** | **R.LPAGPRGMGTALK.L** |
|  | 13571 | **507.9173** | **1520.7301** | **1519.8068** | **0.9233** | **1** | **2** | **80** | **4** | **K.MLGEALSKNPGYIK.L** |
|  | 18379 | **927.4918** | **1852.9691** | **1852.9796** | **-0.0105** | **0** | **59** | **0.00014** | **1** | **R.IGGVQQDTILAEGLHFR.I** 18392 |
|  | 18835 | **944.9827** | **1887.9508** | **1887.9625** | **-0.0117** | **0** | **56** | **0.00025** | **1** | **R.VLSRPNAQELPSMYQR.L** 18833 18834 |
|  | 22211 | **1102.5391** | **2203.0636** | **2203.2054** | **-0.1419** | **1** | **2** | **58** | **1** | **R.IPWFQYPIIYDIRARPR.K** |

| **283.** | Q9BYF1\|ACE2_HUMAN    **Mass:** 92860    **Score:** 181    **Queries matched:** 19   **emPAI:** 0.22 |
| --- | --- |
|  | Angiotensin-converting enzyme 2 OS=Homo sapiens GN=ACE2 PE=1 SV=2 |

|  | **Query** | **Observed** | **Mr(expt)** | **Mr(calc)** | **Delta** | **Miss** | **Score** | **Expect** | **Rank** | **Peptide** |
| --- | --- | --- | --- | --- | --- | --- | --- | --- | --- | --- |
|  | 1695 | **698.3829** | **697.3757** | **697.3799** | **-0.0042** | **0** | **21** | **1.1** | **2** | **R.QYFLK.V** |
|  | 3474 | 443.0381 | 884.0616 | 883.4222 | 0.6394 | 0 | 11 | 7.6 | 3 | R.SSVAYAMR.Q 3462 |
|  | 4344 | **473.3052** | **944.5958** | **944.5291** | **0.0667** | **1** | **5** | **57** | **9** | **R.TEVEKAIR.M** |
|  | 9263 | **635.8424** | **1269.6701** | **1269.6758** | **-0.0056** | **0** | **73** | **7.8e-006** | **1** | **R.ISFNFFVTAPK.N** 9235 |
|  | 11241 | **690.3578** | **1378.7011** | **1379.6568** | **-0.9557** | **0** | **1** | **1e+002** | **5** | **R.SGENPYASIDISK.G** |
|  | 12453 | 725.8491 | 1449.6836 | 1449.6922 | -0.0086 | 0 | 27 | 0.25 | 1 | K.NQMILFGEEDVR.V |
|  | 14574 | **530.5510** | **1588.6311** | **1587.6378** | **0.9932** | **0** | **7** | **21** | **1** | **R.ANHYEDYGDYWR.G** |
|  | 14910 | 806.9200 | 1611.8254 | 1612.8461 | -1.0206 | 0 | 8 | 20 | 2 | K.SEPWTLALENVVGAK.N |
|  | 15941 | **839.4509** | **1676.8873** | **1676.8556** | **0.0317** | **1** | **4** | **47** | **6** | **K.VKNQMILFGEEDVR.V** |
|  | 16372 | 855.3425 | 1708.6705 | 1707.7013 | 0.9692 | 0 | 26 | 0.22 | 1 | R.GDYEVNGVDGYDYSR.G |
|  | 16913 | **875.8752** | **1749.7358** | **1749.7457** | **-0.0099** | **0** | **65** | **3.1e-005** | **1** | **K.AYEWNDNEMYLFR.S** |
|  | 21965 | 724.7221 | 2171.1445 | 2172.1386 | -0.9941 | 1 | 0 | 82 | 8 | K.LQLQALQQNGSSVLSEDKSK.R |
|  | 26982 | **978.1430** | **2931.4072** | **2931.4234** | **-0.0162** | **0** | **61** | **4.1e-005** | **1** | **K.LMNAYPSYISPIGCLPAHLLGDMWGR.F** |
|  | 27112 | 983.5154 | 2947.5245 | 2947.4183 | 0.1062 | 0 | (1) | 44 | 4 | K.LMNAYPSYISPIGCLPAHLLGDMWGR.F |
|  | 27670 | **1023.1710** | **3066.4912** | **3065.5896** | **0.9017** | **1** | **1** | **45** | **1** | **K.WSAFLKEQSTLAQMYPLQEIQNLTVK.L** |
|  | 29237 | **1173.1999** | **3516.5780** | **3516.5959** | **-0.0179** | **0** | **6** | **10** | **1** | **K.VCNPDNPQECLLLEPGLNEIMANSLDYNER.L** |
|  | 30142 | **1276.2826** | **3825.8259** | **3825.8461** | **-0.0202** | **0** | **34** | **0.011** | **1** | **R.FWTNLYSLTVPFGQKPNIDVTDAMVDQAWDAQR.I** |

| **284.** | P54652\|HSP72_HUMAN    **Mass:** 70263    **Score:** 181    **Queries matched:** 24   **emPAI:** 0.43 |
| --- | --- |
|  | Heat shock-related 70 kDa protein 2 OS=Homo sapiens GN=HSPA2 PE=1 SV=1 |

|  | **Query** | **Observed** | **Mr(expt)** | **Mr(calc)** | **Delta** | **Miss** | **Score** | **Expect** | **Rank** | **Peptide** |
| --- | --- | --- | --- | --- | --- | --- | --- | --- | --- | --- |
|  | 1958 | 773.4097 | 772.4025 | 772.4079 | -0.0054 | 0 | 35 | 0.066 | 1 | K.DNNLLGK.F |
|  | 1961 | 774.4308 | 773.4235 | 773.4283 | -0.0048 | 0 | 27 | 0.47 | 1 | R.NTTIPTK.Q |
|  | 2227 | 804.4412 | 803.4339 | 803.4389 | -0.0050 | 0 | 23 | 1.2 | 1 | K.ITITNDK.G |
|  | 8452 | 614.8128 | 1227.6111 | 1227.6207 | -0.0097 | 0 | 47 | 0.003 | 1 | K.VEIIANDQGNR.T 8436 8453 |
|  | 8472 | **615.2988** | **1228.5831** | **1228.6776** | **-0.0944** | **0** | **3** | **75** | **3** | **K.DAGTITGLNVLR.I** |
|  | 8915 | 627.3079 | 1252.6013 | 1252.6088 | -0.0075 | 0 | 32 | 0.092 | 1 | R.FEELNADLFR.G |
|  | 9550 | **429.2669** | **1284.7790** | **1285.5496** | **-0.7706** | **0** | **9** | **19** | **2** | **K.FEDATVQSDMK.H** |
|  | 10312 | **663.9360** | **1325.8575** | **1324.6696** | **1.1879** | **1** | **1** | **98** | **2** | **K.MKEIAEAYLGGK.V** |
|  | 11861 | 707.8143 | 1413.6141 | 1413.6446 | -0.0305 | 1 | 10 | 13 | 3 | R.KFEDATVQSDMK.H |
|  | 12936 | 494.2532 | 1479.7378 | 1479.7470 | -0.0092 | 1 | 31 | 0.11 | 1 | R.ARFEELNADLFR.G 12935 |
|  | 13054 | 744.3494 | 1486.6842 | 1486.6940 | -0.0098 | 0 | 50 | 0.0013 | 1 | R.TTPSYVAFTDTER.L |
|  | 15661 | 830.4460 | 1658.8775 | 1658.8879 | -0.0104 | 0 | 46 | 0.0026 | 1 | R.IINEPTAAAIAYGLDK.K 15683 15687 |
|  | 16111 | 846.3604 | 1690.7063 | 1690.7183 | -0.0121 | 0 | 28 | 0.16 | 1 | K.STAGDTHLGGEDFDNR.M 16110 |
|  | 17447 | 894.4931 | 1786.9716 | 1786.9828 | -0.0112 | 1 | 38 | 0.015 | 1 | R.IINEPTAAAIAYGLDKK.G 17430 |
|  | 17751 | 604.6279 | 1810.8618 | 1812.0105 | -1.1487 | 1 | 3 | 53 | 6 | K.LDKGQIQEIVLVGGSTR.I |
|  | 18120 | 918.9564 | 1835.8982 | 1834.8996 | 0.9986 | 1 | 5 | 34 | 5 | K.NQVAMNPTNTIFDAKR.L |
|  | 21669 | 716.0335 | 2145.0785 | 2144.0273 | 1.0512 | 1 | 5 | 27 | 3 | K.NALESYTYNIKQTVEDEK.L |

| **285.** | P08574\|CY1_HUMAN    **Mass:** 35709    **Score:** 180    **Queries matched:** 19   **emPAI:** 1.02 |
| --- | --- |
|  | Cytochrome c1, heme protein, mitochondrial OS=Homo sapiens GN=CYC1 PE=1 SV=2 |

|  | **Query** | **Observed** | **Mr(expt)** | **Mr(calc)** | **Delta** | **Miss** | **Score** | **Expect** | **Rank** | **Peptide** |
| --- | --- | --- | --- | --- | --- | --- | --- | --- | --- | --- |
|  | 3373 | 439.7373 | 877.4600 | 876.4487 | 1.0113 | 0 | 18 | 3.2 | 3 | -.MAAAAASLR.G |
|  | 3852 | **455.7235** | **909.4323** | **909.4379** | **-0.0055** | **0** | **21** | **1.2** | **1** | **K.DVCTFLR.W** |
|  | 4682 | **486.2182** | **970.4219** | **971.5916** | **-1.1697** | **1** | **13** | **8.3** | **2** | **R.KLAYRPPK.-** |
|  | 9805 | 650.3351 | 1298.6557 | 1297.6990 | 0.9567 | 0 | 47 | 0.0027 | 1 | R.GLLSSLDHTSIR.R 9792 |
|  | 11776 | 705.3867 | 1408.7589 | 1407.8310 | 0.9279 | 1 | 1 | 92 | 7 | M.AAAAASLRGVVLGPR.G |
|  | 13283 | 501.2430 | 1500.7073 | 1500.8260 | -0.1187 | 1 | 3 | 71 | 6 | R.TPQAVALSSKSGLSR.G 13289 |
|  | 13582 | **761.4379** | **1520.8613** | **1519.6977** | **1.1636** | **0** | **20** | **1.4** | **1** | **R.HLVGVCYTEDEAK.E** |
|  | 13806 | **513.3103** | **1536.9091** | **1536.8558** | **0.0532** | **0** | **4** | **48** | **6** | **R.GLLCSARPGQLPLR.T** |
|  | 13845 | 514.3140 | 1539.9203 | 1538.8715 | 1.0488 | 1 | 6 | 29 | 4 | -.MAAAAASLRGVVLGPR.G |
|  | 19178 | **957.9668** | **1913.9190** | **1913.9312** | **-0.0121** | **0** | **46** | **0.0023** | **1** | **K.LFDYFPKPYPNSEAAR.A** 19177 |
|  | 23241 | **776.8925** | **2327.6556** | **2327.2563** | **0.3993** | **1** | **1** | **41** | **2** | **R.MGLKMLMMMALLVPLVYTIK.R** |
|  | 25474 | **883.3982** | **2647.1727** | **2647.1894** | **-0.0167** | **0** | **47** | **0.0014** | **1** | **K.ELAAEVEVQDGPNEDGEMFMRPGK.L** |
|  | 25504 | **1327.6250** | **2653.2354** | **2653.2483** | **-0.0128** | **0** | **69** | **8.4e-006** | **1** | **R.HGGEDYVFSLLTGYCEPPTGVSLR.E** |
|  | 30599 | **1037.9637** | **4147.8259** | **4148.8765** | **-1.0506** | **1** | **25** | **0.072** | **1** | **R.HLVGVCYTEDEAKELAAEVEVQDGPNEDGEMFMRPGK.L** |
|  | 30821 | **1095.5248** | **4378.0700** | **4378.0854** | **-0.0154** | **0** | **37** | **0.0052** | **1** | **R.EGLYFNPYFPGQAIAMAPPIYTDVLEFDDGTPATMSQIAK.D** 30823 |

| **286.** | O75947\|ATP5H_HUMAN    **Mass:** 18537    **Score:** 179    **Queries matched:** 15   **emPAI:** 1.57 |
| --- | --- |
|  | ATP synthase subunit d, mitochondrial OS=Homo sapiens GN=ATP5H PE=1 SV=3 |

|  | **Query** | **Observed** | **Mr(expt)** | **Mr(calc)** | **Delta** | **Miss** | **Score** | **Expect** | **Rank** | **Peptide** |
| --- | --- | --- | --- | --- | --- | --- | --- | --- | --- | --- |
|  | 1259 | 593.3448 | 592.3375 | 591.3380 | 0.9995 | 0 | 25 | 0.54 | 1 | K.FNALK.V |
|  | 2111 | **398.4462** | **794.8778** | **794.3666** | **0.5112** | **1** | **15** | **3** | **1** | **K.EMEKMK.N** |
|  | 2537 | **414.7124** | **827.4102** | **826.3564** | **1.0538** | **1** | **(9)** | **22** | **3** | **K.EMEKMK.N** |
|  | 4997 | **497.2453** | **992.4760** | **992.4815** | **-0.0055** | **0** | **23** | **0.87** | **2** | **K.AGLVDDFEK.K** |
|  | 6732 | **561.2887** | **1120.5628** | **1120.5764** | **-0.0136** | **1** | **27** | **0.31** | **1** | **K.AGLVDDFEKK.F** |
|  | 7301 | **583.7757** | **1165.5368** | **1165.5437** | **-0.0069** | **0** | **52** | **0.00097** | **1** | **K.SCAEWVSLSK.A** 7302 |
|  | 15123 | **812.8825** | **1623.7505** | **1623.7628** | **-0.0123** | **1** | **65** | **3.6e-005** | **1** | **K.YTAQVDAEEKEDVK.S** 15124 |
|  | 16017 | **842.9219** | **1683.8293** | **1683.8409** | **-0.0116** | **1** | **34** | **0.042** | **1** | **K.KYPYWPHQPIENL.-** 16018 |
|  | 18631 | **936.9922** | **1871.9698** | **1871.9781** | **-0.0083** | **0** | **16** | **2.5** | **1** | **K.TIDWVAFAEIIPQNQK.A** 18650 |
|  | 24277 | **1233.0930** | **2464.1715** | **2464.1832** | **-0.0117** | **0** | **84** | **2.6e-007** | **1** | **K.NLIPFDQMTIEDLNEAFPETK.L** 24276 |

| **287.** | P30685\|1B35_HUMAN    **Mass:** 40715    **Score:** 179    **Queries matched:** 17   **emPAI:** 0.42 |
| --- | --- |
|  | HLA class I histocompatibility antigen, B-35 alpha chain OS=Homo sapiens GN=HLA-B PE=1 SV=1 |

|  | **Query** | **Observed** | **Mr(expt)** | **Mr(calc)** | **Delta** | **Miss** | **Score** | **Expect** | **Rank** | **Peptide** |
| --- | --- | --- | --- | --- | --- | --- | --- | --- | --- | --- |
|  | 995 | 542.1287 | 541.1214 | 542.3176 | -1.1963 | 0 | 13 | 4.3 | 1 | R.VTAPR.T 1006 |
|  | 10365 | 443.7359 | 1328.1860 | 1327.6996 | 0.4863 | 1 | 13 | 4.5 | 1 | K.WEAARVAEQLR.A |
|  | 11047 | 457.2375 | 1368.6906 | 1367.6793 | 1.0112 | 1 | 10 | 13 | 4 | K.TNTQTYRESLR.N |
|  | 12099 | 714.3628 | 1426.7111 | 1426.7205 | -0.0093 | 0 | 11 | 9.6 | 1 | K.WAAVVVPSGEEQR.Y |
|  | 12433 | **483.7814** | **1448.3225** | **1447.6692** | **0.6533** | **1** | **1** | **70** | **2** | **R.FDSDAASPRTEPR.A** |
|  | 14933 | 538.7706 | 1613.2899 | 1613.8162 | -0.5263 | 1 | 2 | 52 | 2 | R.NTQIFKTNTQTYR.E |
|  | 15206 | 815.4121 | 1628.8097 | 1628.8199 | -0.0102 | 0 | 72 | 7.9e-006 | 1 | R.FIAVGYVDDTQFVR.F 15203 15205 |
|  | 18119 | 459.9797 | 1835.8896 | 1835.9027 | -0.0131 | 0 | 13 | 5.4 | 2 | K.THVTHHPVSDHEATLR.C |
|  | 20661 | 513.0184 | 2048.0446 | 2048.0626 | -0.0179 | 0 | 15 | 3.2 | 1 | R.YTCHVQHEGLPKPLTLR.W |
|  | 21343 | 1056.5170 | 2111.0194 | 2111.0299 | -0.0105 | 0 | 45 | 0.003 | 1 | R.CWALGFYPAEITLTWQR.D 21342 |
|  | 22490 | 744.6667 | 2230.9784 | 2230.9938 | -0.0154 | 0 | 35 | 0.024 | 1 | R.DGEDQTQDTELVETRPAGDR.T 22489 22491 |

|  | **Proteins matching a subset of these peptides:** |
| --- | --- |

|  | P30491\|1B53_HUMAN    **Mass:** 40755    **Score:** 179    **Queries matched:** 16 |
| --- | --- |
|  | HLA class I histocompatibility antigen, B-53 alpha chain OS=Homo sapiens GN=HLA-B PE=1 SV=1 |

|  | P10319\|1B58_HUMAN    **Mass:** 40597    **Score:** 179    **Queries matched:** 15 |
| --- | --- |
|  | HLA class I histocompatibility antigen, B-58 alpha chain OS=Homo sapiens GN=HLA-B PE=2 SV=1 |

|  | P30498\|1B78_HUMAN    **Mass:** 40738    **Score:** 179    **Queries matched:** 14 |
| --- | --- |
|  | HLA class I histocompatibility antigen, B-78 alpha chain OS=Homo sapiens GN=HLA-B PE=2 SV=1 |

| **288.** | P18464\|1B51_HUMAN    **Mass:** 40826    **Score:** 179    **Queries matched:** 16   **emPAI:** 0.42 |
| --- | --- |
|  | HLA class I histocompatibility antigen, B-51 alpha chain OS=Homo sapiens GN=HLA-B PE=1 SV=1 |

|  | **Query** | **Observed** | **Mr(expt)** | **Mr(calc)** | **Delta** | **Miss** | **Score** | **Expect** | **Rank** | **Peptide** |
| --- | --- | --- | --- | --- | --- | --- | --- | --- | --- | --- |
|  | 995 | 542.1287 | 541.1214 | 542.3176 | -1.1963 | 0 | 13 | 4.3 | 1 | R.VTAPR.T 1006 |
|  | 12099 | 714.3628 | 1426.7111 | 1426.7205 | -0.0093 | 0 | 11 | 9.6 | 1 | K.WAAVVVPSGEEQR.Y |
|  | 12433 | 483.7814 | 1448.3225 | 1447.6692 | 0.6533 | 1 | 1 | 70 | 2 | R.FDSDAASPRTEPR.A |
|  | 14933 | 538.7706 | 1613.2899 | 1613.8162 | -0.5263 | 1 | 2 | 52 | 2 | R.NTQIFKTNTQTYR.E |
|  | 15206 | 815.4121 | 1628.8097 | 1628.8199 | -0.0102 | 0 | 72 | 7.9e-006 | 1 | R.FIAVGYVDDTQFVR.F 15203 15205 |
|  | 18119 | 459.9797 | 1835.8896 | 1835.9027 | -0.0131 | 0 | 13 | 5.4 | 2 | K.THVTHHPVSDHEATLR.C |
|  | 20661 | 513.0184 | 2048.0446 | 2048.0626 | -0.0179 | 0 | 15 | 3.2 | 1 | R.YTCHVQHEGLPKPLTLR.W |
|  | 21343 | 1056.5170 | 2111.0194 | 2111.0299 | -0.0105 | 0 | 45 | 0.003 | 1 | R.CWALGFYPAEITLTWQR.D 21342 |
|  | 22490 | 744.6667 | 2230.9784 | 2230.9938 | -0.0154 | 0 | 35 | 0.024 | 1 | R.DGEDQTQDTELVETRPAGDR.T 22489 22491 |
|  | 26817 | 965.8463 | 2894.5169 | 2894.1661 | 0.3508 | 0 | 1 | 44 | 6 | R.YYNQSEAGSHTWQTMYGCDVGPDGR.L |

|  | **Proteins matching a subset of these peptides:** |
| --- | --- |

|  | P30490\|1B52_HUMAN    **Mass:** 40781    **Score:** 179    **Queries matched:** 15 |
| --- | --- |
|  | HLA class I histocompatibility antigen, B-52 alpha chain OS=Homo sapiens GN=HLA-B PE=2 SV=1 |

| **289.** | Q6UWM9\|UD2A3_HUMAN    **Mass:** 60929    **Score:** 179    **Queries matched:** 29   **emPAI:** 0.81 |
| --- | --- |
|  | UDP-glucuronosyltransferase 2A3 OS=Homo sapiens GN=UGT2A3 PE=2 SV=1 |

|  | **Query** | **Observed** | **Mr(expt)** | **Mr(calc)** | **Delta** | **Miss** | **Score** | **Expect** | **Rank** | **Peptide** |
| --- | --- | --- | --- | --- | --- | --- | --- | --- | --- | --- |
|  | 2121 | **398.8745** | **795.7344** | **795.3949** | **0.3395** | **0** | **14** | **3.4** | **1** | **R.MTFLER.V** 2114 |
|  | 4572 | **481.7369** | **961.4591** | **961.4651** | **-0.0060** | **0** | **30** | **0.2** | **1** | **R.ISVGGNMER.S** 4570 4571 4574 |
|  | 4784 | 489.2638 | 976.5130 | 975.4920 | 1.0211 | 1 | 9 | 26 | 9 | K.ENAMRLSR.I 4790 |
|  | 4785 | **489.2707** | **976.5268** | **977.4600** | **-0.9333** | **0** | **(7)** | **38** | **10** | **R.ISVGGNMER.S** |
|  | 5962 | **533.2623** | **1064.5100** | **1064.5172** | **-0.0072** | **0** | **(11)** | **14** | **1** | **K.TMTSEDLLR.A** |
|  | 6207 | 541.3388 | 1080.6629 | 1080.5121 | 0.1508 | 0 | 11 | 11 | 2 | K.TMTSEDLLR.A |
|  | 6238 | **542.3391** | **1082.6637** | **1082.6699** | **-0.0063** | **0** | **68** | **2.5e-005** | **1** | **K.VILEELIVR.G** 6231 |
|  | 9334 | **425.2608** | **1272.7605** | **1272.5921** | **0.1684** | **0** | **12** | **9.7** | **2** | **K.FEVVHMPQDR.T** 9331 |
|  | 12271 | 719.4207 | 1436.8269 | 1436.8351 | -0.0082 | 0 | 47 | 0.0027 | 1 | K.ANIIASALAQIPQK.V 12272 |
|  | 12517 | 485.5969 | 1453.7690 | 1453.7790 | -0.0100 | 0 | 30 | 0.13 | 1 | R.IHHDQPVKPLDR.A 12518 12519 12520 |
|  | 13303 | 751.8984 | 1501.7823 | 1501.7923 | -0.0099 | 0 | 29 | 0.16 | 1 | K.ALGRPTTLCETVGK.A |
|  | 14937 | **807.8872** | **1613.7599** | **1613.7719** | **-0.0120** | **1** | **65** | **3.8e-005** | **1** | **R.TVITDSSYKENAMR.L** |
|  | 17709 | **904.9639** | **1807.9132** | **1807.9257** | **-0.0125** | **0** | **25** | **0.4** | **1** | **R.LYDWIPQNDLLGHPK.T** |
|  | 20533 | **680.3571** | **2038.0495** | **2037.0683** | **0.9812** | **1** | **0** | **85** | **9** | **R.LYDWIPQNDLLGHPKTK.A** |
|  | 20678 | **684.3561** | **2050.0464** | **2051.0800** | **-1.0336** | **0** | **1** | **70** | **1** | **R.GHEVTVLTHSKPSLIDYR.K** |
|  | 26903 | **1458.2681** | **2914.5216** | **2914.5328** | **-0.0112** | **0** | **58** | **8.4e-005** | **1** | **R.TEENEIFVDLALNVLPGLSTWQSVIK.L** |
|  | 30524 | **823.4062** | **4111.9946** | **4112.0111** | **-0.0165** | **0** | **20** | **0.3** | **1** | **K.AFITHGGMNGIYEAIYHGVPMVGVPIFGDQLDNIAHMK.A** 30526 |

| **290.** | P16444\|DPEP1_HUMAN    **Mass:** 46101    **Score:** 178    **Queries matched:** 11   **emPAI:** 0.26 |
| --- | --- |
|  | Dipeptidase 1 OS=Homo sapiens GN=DPEP1 PE=1 SV=3 |

|  | **Query** | **Observed** | **Mr(expt)** | **Mr(calc)** | **Delta** | **Miss** | **Score** | **Expect** | **Rank** | **Peptide** |
| --- | --- | --- | --- | --- | --- | --- | --- | --- | --- | --- |
|  | 8439 | **409.8714** | **1226.5924** | **1226.6078** | **-0.0154** | **0** | **5** | **49** | **9** | **R.TLEQMDVVHR.M** |
|  | 14022 | **518.0098** | **1551.0075** | **1550.8416** | **0.1658** | **0** | **5** | **33** | **4** | **R.ANLTTLAGTHTNIPK.L** 14033 |
|  | 15989 | **561.3242** | **1680.9507** | **1681.9437** | **-0.9930** | **0** | **1** | **92** | **8** | **R.LGVLIDLAHVSVATMK.A** |
|  | 18106 | **918.4519** | **1834.8892** | **1833.8971** | **0.9921** | **0** | **71** | **8.4e-006** | **1** | **R.MYPETFLYVTSSAGIR.Q** 18067 |
|  | 22727 | **1131.5111** | **2261.0077** | **2261.0212** | **-0.0135** | **0** | **69** | **9.6e-006** | **1** | **R.AGFVGGQFWSVYTPCDTQNK.D** 22728 |
|  | 25206 | **866.0768** | **2595.2087** | **2595.2176** | **-0.0089** | **0** | **9** | **7.7** | **1** | **R.DSPVIDGHNDLPWQLLDMFNNR.L** |
|  | 26126 | 1376.1907 | 2750.3668 | 2750.3408 | 0.0260 | 1 | 3 | 32 | 3 | R.LVKQTDSLVMVNFYNNYISCTNK.A |
|  | 30459 | **1359.9529** | **4076.8368** | **4076.8599** | **-0.0231** | **0** | **47** | **0.00054** | **1** | **R.YLTLTHSCNTPWADNWLVDTGDSEPQSQGLSPFGQR.V** |

| **291.** | P00403\|COX2_HUMAN    **Mass:** 25719    **Score:** 177    **Queries matched:** 16   **emPAI:** 0.51 |
| --- | --- |
|  | Cytochrome c oxidase subunit 2 OS=Homo sapiens GN=MT-CO2 PE=1 SV=1 |

|  | **Query** | **Observed** | **Mr(expt)** | **Mr(calc)** | **Delta** | **Miss** | **Score** | **Expect** | **Rank** | **Peptide** |
| --- | --- | --- | --- | --- | --- | --- | --- | --- | --- | --- |
|  | 2794 | **422.7272** | **843.4398** | **843.4450** | **-0.0052** | **0** | **11** | **15** | **1** | **R.LLDVDNR.V** 2809 |
|  | 6557 | **554.2840** | **1106.5534** | **1105.6859** | **0.8675** | **0** | **6** | **46** | **8** | **R.VVLPIEAPIR.M** |
|  | 7328 | **584.7976** | **1167.5807** | **1168.5838** | **-1.0032** | **0** | **3** | **66** | **6** | **K.IFEMGPVFTL.-** 7331 |
|  | 18341 | 926.4688 | 1850.9231 | 1850.9335 | -0.0105 | 0 | 37 | 0.021 | 2 | R.ILYMTDEVNDPSLTIK.S |
|  | 22446 | **1114.0780** | **2226.1414** | **2226.1541** | **-0.0126** | **0** | **66** | **1.9e-005** | **1** | **R.MMITSQDVLHSWAVPTLGLK.T** 22437 22438 22439 22440 22447 |
|  | 28684 | **833.4213** | **3329.6562** | **3329.8244** | **-0.1681** | **1** | **1** | **33** | **2** | **R.VVLPIEAPIRMMITSQDVLHSWAVPTLGLK.T** |
|  | 30864 | **1476.3980** | **4426.1720** | **4425.1960** | **0.9761** | **0** | **14** | **0.73** | **1** | **R.LNQTTFTATRPGVYYGQCSEICGANHSFMPIVLELIPLK.I** 30856 30858 |

| **292.** | O14983\|AT2A1_HUMAN    **Mass:** 111550   **Score:** 176    **Queries matched:** 35   **emPAI:** 0.22 |
| --- | --- |
|  | Sarcoplasmic/endoplasmic reticulum calcium ATPase 1 OS=Homo sapiens GN=ATP2A1 PE=1 SV=1 |

|  | **Query** | **Observed** | **Mr(expt)** | **Mr(calc)** | **Delta** | **Miss** | **Score** | **Expect** | **Rank** | **Peptide** |
| --- | --- | --- | --- | --- | --- | --- | --- | --- | --- | --- |
|  | 2930 | 853.4177 | 852.4104 | 852.4163 | -0.0060 | 0 | 31 | 0.12 | 1 | R.AIYNNMK.Q |
|  | 3237 | 435.2233 | 868.4320 | 868.4113 | 0.0207 | 0 | (8) | 20 | 1 | R.AIYNNMK.Q |
|  | 3555 | **445.7683** | **889.5221** | **888.4777** | **1.0444** | **1** | **5** | **71** | **6** | **K.SSRAAVGNK.M** |
|  | 4377 | **474.3256** | **946.6366** | **946.5521** | **0.0845** | **1** | **11** | **15** | **5** | **K.EKIMAVIK.E** |
|  | 4971 | 496.2660 | 990.5175 | 989.5216 | 0.9959 | 0 | 31 | 0.15 | 1 | R.VIMITGDNK.G |
|  | 5164 | **502.3313** | **1002.6480** | **1003.4869** | **-0.8388** | **0** | **9** | **23** | **1** | **R.ANACNSVIR.Q** |
|  | 5318 | **508.9797** | **1015.9449** | **1016.5549** | **-0.6100** | **1** | **9** | **16** | **1** | **K.GTAIAICRR.I** 5319 |
|  | 6090 | 537.2575 | 1072.5005 | 1071.5560 | 0.9445 | 0 | 9 | 26 | 4 | R.NAENAIEALK.E 6053 |
|  | 6653 | 558.3152 | 1114.6159 | 1114.7074 | -0.0914 | 1 | 5 | 47 | 9 | R.ILAIKSTTLR.V 6661 |
|  | 7037 | 573.7308 | 1145.4470 | 1144.4893 | 0.9577 | 0 | 1 | 1.1e+002 | 4 | K.SMSVYCSPAK.S |
|  | 7252 | 581.7840 | 1161.5534 | 1161.5812 | -0.0278 | 0 | 9 | 20 | 7 | K.EVTGSIQLCR.D |
|  | 10544 | 670.3234 | 1338.6323 | 1338.6602 | -0.0279 | 0 | 3 | 71 | 6 | K.NMLFSGTNIAAGK.A |
|  | 10968 | 682.8745 | 1363.7343 | 1363.7289 | 0.0055 | 0 | 13 | 7.4 | 3 | K.EPLISGWLFFR.Y |
|  | 12453 | 725.8491 | 1449.6836 | 1450.7602 | -1.0766 | 1 | 4 | 47 | 6 | K.KNMLFSGTNIAAGK.A |
|  | 12554 | **486.5652** | **1456.6739** | **1455.6698** | **1.0041** | **0** | **9** | **16** | **1** | **K.TGTLTTNQMSVCK.M** |
|  | 12615 | **487.3207** | **1458.9402** | **1458.6595** | **0.2807** | **1** | **3** | **61** | **9** | **K.SMSVYCSPAKSSR.A** |
|  | 12728 | 734.3859 | 1466.7572 | 1466.7551 | 0.0020 | 1 | (4) | 51 | 3 | K.KNMLFSGTNIAAGK.A |
|  | 13322 | 752.8751 | 1503.7356 | 1502.8126 | 0.9229 | 1 | 3 | 64 | 5 | K.KAEIGIAMGSGTAVAK.T |
|  | 13339 | **753.3115** | **1504.6084** | **1503.7650** | **0.8434** | **1** | **4** | **46** | **6** | **R.ANACNSVIRQLMK.K** |
|  | 13404 | **755.8193** | **1509.6241** | **1509.7610** | **-0.1368** | **1** | **2** | **71** | **8** | **K.MNVFNTDVRSLSK.V** |
|  | 13542 | 507.0747 | 1518.2024 | 1518.8076 | -0.6052 | 1 | (0) | 81 | 10 | K.KAEIGIAMGSGTAVAK.T |
|  | 14177 | **781.7061** | **1561.3977** | **1560.8247** | **0.5730** | **0** | **3** | **44** | **2** | **K.VGEATETALTTLVEK.M** |
|  | 14362 | 787.9305 | 1573.8464 | 1573.8563 | -0.0099 | 0 | 61 | 0.0001 | 1 | R.VDQSILTGESVSVIK.H |
|  | 18032 | 915.9753 | 1829.9360 | 1830.8782 | -0.9422 | 1 | 0 | 93 | 6 | R.DQMAATEQDKTPLQQK.L |
|  | 18047 | **611.3104** | **1830.9093** | **1831.9648** | **-1.0556** | **1** | **7** | **21** | **3** | **R.VIMITGDNKGTAIAICR.R** |
|  | 18304 | **617.1330** | **1848.3772** | **1847.9597** | **0.4174** | **1** | **(1)** | **54** | **4** | **R.VIMITGDNKGTAIAICR.R** |
|  | 19616 | 976.9629 | 1951.9112 | 1951.9231 | -0.0119 | 0 | 71 | 6.8e-006 | 1 | R.SLPSVETLGCTSVICSDK.T 19617 |
|  | 23951 | 1213.0746 | 2424.1346 | 2424.1479 | -0.0133 | 0 | 37 | 0.014 | 1 | K.TASEMVLADDNFSTIVAAVEEGR.A 23949 23952 |
|  | 27958 | **1577.1436** | **3152.2725** | **3152.4972** | **-0.2247** | **1** | **1** | **32** | **5** | **M.EAAHAKTTEECLAYFGVSETTGLTPDQVK.R** |

| **293.** | Q15907\|RB11B_HUMAN    **Mass:** 24588    **Score:** 176    **Queries matched:** 11   **emPAI:** 0.33 |
| --- | --- |
|  | Ras-related protein Rab-11B OS=Homo sapiens GN=RAB11B PE=1 SV=4 |

|  | **Query** | **Observed** | **Mr(expt)** | **Mr(calc)** | **Delta** | **Miss** | **Score** | **Expect** | **Rank** | **Peptide** |
| --- | --- | --- | --- | --- | --- | --- | --- | --- | --- | --- |
|  | 1672 | **689.3892** | **688.3819** | **688.3868** | **-0.0049** | **0** | **22** | **1.7** | **2** | **K.SNLLSR.F** |
|  | 3020 | 430.2502 | 858.4859 | 857.4243 | 1.0616 | 0 | 2 | 1.3e+002 | 7 | R.AVPTDEAR.A |
|  | 5675 | **522.3049** | **1042.5952** | **1042.6023** | **-0.0071** | **0** | **18** | **2.9** | **1** | **K.VVLIGDSGVGK.S** |
|  | 7228 | 580.7845 | 1159.5544 | 1159.5622 | -0.0078 | 0 | 18 | 2.4 | 1 | K.HLTYENVER.W |
|  | 9621 | **645.3731** | **1288.7317** | **1288.7391** | **-0.0074** | **0** | **63** | **6.4e-005** | **1** | **R.GAVGALLVYDIAK.H** |
|  | 15151 | **542.6121** | **1624.8144** | **1624.8243** | **-0.0099** | **0** | **18** | **1.7** | **1** | **R.DHADSNIVIMLVGNK.S** 15143 15150 |
|  | 15421 | **547.9799** | **1640.9179** | **1640.8192** | **0.0987** | **0** | **(3)** | **62** | **10** | **R.DHADSNIVIMLVGNK.S** |
|  | 23244 | **1165.0569** | **2328.0992** | **2328.1121** | **-0.0129** | **0** | **137** | **1.4e-012** | **1** | **K.NNLSFIETSALDSTNVEEAFK.N** |
|  | 26700 | **959.1533** | **2874.4380** | **2874.3923** | **0.0457** | **1** | **2** | **32** | **2** | **R.AFAEKNNLSFIETSALDSTNVEEAFK.N** |

| **294.** | P62753\|RS6_HUMAN    **Mass:** 28834    **Score:** 174    **Queries matched:** 14   **emPAI:** 0.45 |
| --- | --- |
|  | 40S ribosomal protein S6 OS=Homo sapiens GN=RPS6 PE=1 SV=1 |

|  | **Query** | **Observed** | **Mr(expt)** | **Mr(calc)** | **Delta** | **Miss** | **Score** | **Expect** | **Rank** | **Peptide** |
| --- | --- | --- | --- | --- | --- | --- | --- | --- | --- | --- |
|  | 1177 | **576.4089** | **575.4016** | **574.3438** | **1.0578** | **0** | **5** | **75** | **5** | **R.LSSLR.A** |
|  | 4921 | **494.7554** | **987.4962** | **987.4873** | **0.0089** | **0** | **18** | **2.7** | **4** | **K.LIEVDDER.K** |
|  | 5526 | **516.7404** | **1031.4663** | **1031.4719** | **-0.0056** | **0** | **14** | **7.5** | **1** | **K.GHSCYRPR.R** 5525 |
|  | 6671 | **559.1301** | **1116.2456** | **1115.5822** | **0.6634** | **1** | **5** | **33** | **7** | **K.LIEVDDERK.L** |
|  | 9518 | **642.8388** | **1283.6631** | **1283.6722** | **-0.0091** | **0** | **17** | **2.6** | **1** | **K.DIPGLTDTTVPR.R** |
|  | 10481 | **668.3354** | **1334.6562** | **1334.6653** | **-0.0090** | **0** | **33** | **0.064** | **1** | **K.LNISFPATGCQK.L** 10492 10509 |
|  | 12677 | 732.3851 | 1462.7556 | 1462.7602 | -0.0046 | 1 | 5 | 42 | 5 | M.KLNISFPATGCQK.L |
|  | 14689 | **799.7518** | **1597.4891** | **1597.8312** | **-0.3421** | **1** | **3** | **39** | **3** | **K.GEKDIPGLTDTTVPR.R** 14704 |
|  | 15057 | **810.8768** | **1619.7390** | **1619.7501** | **-0.0111** | **0** | **83** | **5.9e-007** | **1** | **R.MATEVAADALGEEWK.G** |
|  | 17961 | **914.0195** | **1826.0245** | **1826.0336** | **-0.0090** | **0** | **114** | **4.3e-010** | **1** | **R.GCIVDANLSVLNLVIVK.K** |

| **295.** | P19105\|ML12A_HUMAN    **Mass:** 19839    **Score:** 172    **Queries matched:** 9   **emPAI:** 1.03 |
| --- | --- |
|  | Myosin regulatory light chain 12A OS=Homo sapiens GN=MYL12A PE=1 SV=2 |

|  | **Query** | **Observed** | **Mr(expt)** | **Mr(calc)** | **Delta** | **Miss** | **Score** | **Expect** | **Rank** | **Peptide** |
| --- | --- | --- | --- | --- | --- | --- | --- | --- | --- | --- |
|  | 5563 | **518.2575** | **1034.5005** | **1034.5066** | **-0.0062** | **0** | **29** | **0.22** | **1** | **R.ELLTTMGDR.F** |
|  | 11904 | 708.8072 | 1415.5998 | 1414.6252 | 0.9746 | 0 | 38 | 0.019 | 1 | R.FTDEEVDELYR.E |
|  | 14938 | **538.9281** | **1613.7625** | **1613.8566** | **-0.0941** | **1** | **5** | **38** | **3** | **K.GNFNYIEFTRILK.H** |
|  | 20151 | **1002.5089** | **2003.0031** | **2002.9670** | **0.0362** | **1** | **51** | **0.00077** | **1** | **R.DGFIDKEDLHDMLASLGK.N** 20146 |
|  | 21059 | **1045.9907** | **2089.9669** | **2089.9779** | **-0.0110** | **0** | **96** | **2.3e-008** | **1** | **R.ATSNVFAMFDQSQIQEFK.E** 21060 |
|  | 28814 | **1123.1412** | **3366.4019** | **3365.5180** | **0.8839** | **1** | **3** | **16** | **1** | **R.NAFACFDEEATGTIQEDYLRELLTTMGDR.F** |
|  | 29065 | **1152.6144** | **3454.8213** | **3455.5070** | **-0.6857** | **0** | **1** | **32** | **1** | **K.NPTDEYLDAMMNEAPGPINFTMFLTMFGEK.L** |

| **296.** | O14950\|ML12B_HUMAN    **Mass:** 19824    **Score:** 172    **Queries matched:** 9   **emPAI:** 1.03 |
| --- | --- |
|  | Myosin regulatory light chain 12B OS=Homo sapiens GN=MYL12B PE=1 SV=2 |

|  | **Query** | **Observed** | **Mr(expt)** | **Mr(calc)** | **Delta** | **Miss** | **Score** | **Expect** | **Rank** | **Peptide** |
| --- | --- | --- | --- | --- | --- | --- | --- | --- | --- | --- |
|  | 5563 | 518.2575 | 1034.5005 | 1034.5066 | -0.0062 | 0 | 29 | 0.22 | 1 | R.ELLTTMGDR.F |
|  | 11904 | 708.8072 | 1415.5998 | 1414.6252 | 0.9746 | 0 | 38 | 0.019 | 1 | R.FTDEEVDELYR.E |
|  | 14938 | 538.9281 | 1613.7625 | 1613.8566 | -0.0941 | 1 | 5 | 38 | 3 | K.GNFNYIEFTRILK.H |
|  | 20151 | 1002.5089 | 2003.0031 | 2002.9670 | 0.0362 | 1 | 51 | 0.00077 | 1 | R.DGFIDKEDLHDMLASLGK.N 20146 |
|  | 21059 | 1045.9907 | 2089.9669 | 2089.9779 | -0.0110 | 0 | 96 | 2.3e-008 | 1 | R.ATSNVFAMFDQSQIQEFK.E 21060 |
|  | 28814 | 1123.1412 | 3366.4019 | 3365.5180 | 0.8839 | 1 | 3 | 16 | 1 | R.NAFACFDEEATGTIQEDYLRELLTTMGDR.F |
|  | 31083 | **1547.4708** | **4639.3907** | **4639.0903** | **0.3004** | **1** | **2** | **11** | **1** | **K.NPTDAYLDAMMNEAPGPINFTMFLTMFGEKLNGTDPEDVIR.N** |

| **297.** | Q9BS26\|ERP44_HUMAN    **Mass:** 47341    **Score:** 172    **Queries matched:** 12   **emPAI:** 0.26 |
| --- | --- |
|  | Endoplasmic reticulum resident protein ERp44 OS=Homo sapiens GN=ERP44 PE=1 SV=1 |

|  | **Query** | **Observed** | **Mr(expt)** | **Mr(calc)** | **Delta** | **Miss** | **Score** | **Expect** | **Rank** | **Peptide** |
| --- | --- | --- | --- | --- | --- | --- | --- | --- | --- | --- |
|  | 1750 | 711.0934 | 710.0862 | 710.2914 | -0.2052 | 0 | 4 | 36 | 2 | R.NGMMMK.R |
|  | 3667 | **450.2427** | **898.4708** | **898.3823** | **0.0885** | **1** | **2** | **88** | **2** | **R.NGMMMKR.E** |
|  | 4411 | **475.8327** | **949.6508** | **948.5644** | **1.0865** | **1** | **9** | **21** | **5** | **R.ISKYPTLK.L** |
|  | 7016 | **572.3478** | **1142.6810** | **1142.5399** | **0.1412** | **1** | **3** | **77** | **2** | **K.LFRNGMMMK.R** |
|  | 12346 | **722.3130** | **1442.6114** | **1442.6209** | **-0.0094** | **0** | **63** | **6.4e-005** | **1** | **R.VDCDQHSDIAQR.Y** 12347 |
|  | 13181 | **498.9340** | **1493.7801** | **1494.8017** | **-1.0216** | **0** | **5** | **37** | **2** | **-.MHPAVFLSLPDLR.C** |
|  | 17336 | **890.4174** | **1778.8202** | **1778.8322** | **-0.0121** | **0** | **70** | **1.2e-005** | **1** | **K.EDTESLEIFQNEVAR.Q** 17337 |
|  | 24752 | **632.8123** | **2527.2202** | **2527.3369** | **-0.1168** | **1** | **1** | **51** | **8** | **R.HPLLHIQKTPADCPVIAIDSFR.H** |
|  | 29343 | **1184.2424** | **3549.7055** | **3549.7238** | **-0.0184** | **1** | **54** | **0.00016** | **1** | **R.FSQMLHPIFEEASDVIKEEFPNENQVVFAR.V** 29345 |

| **298.** | P08263\|GSTA1_HUMAN    **Mass:** 25672    **Score:** 171    **Queries matched:** 22   **emPAI:** 0.51 |
| --- | --- |
|  | Glutathione S-transferase A1 OS=Homo sapiens GN=GSTA1 PE=1 SV=3 |

|  | **Query** | **Observed** | **Mr(expt)** | **Mr(calc)** | **Delta** | **Miss** | **Score** | **Expect** | **Rank** | **Peptide** |
| --- | --- | --- | --- | --- | --- | --- | --- | --- | --- | --- |
|  | 3277 | 436.2635 | 870.5124 | 870.5175 | -0.0051 | 0 | 16 | 4 | 1 | R.ISNLPTVK.K |
|  | 3290 | 872.5037 | 871.4964 | 870.5538 | 0.9426 | 1 | 7 | 38 | 8 | K.DAKLALIK.E |
|  | 3704 | 901.4398 | 900.4325 | 900.4381 | -0.0057 | 0 | 14 | 8.3 | 1 | R.YFPAFEK.V |
|  | 3711 | **451.2453** | **900.4761** | **900.4818** | **-0.0056** | **0** | **28** | **0.33** | **1** | **K.FLQPGSPR.K** 3710 3713 3736 3742 |
|  | 3991 | **460.7375** | **919.4605** | **919.4664** | **-0.0059** | **0** | **5** | **54** | **2** | **K.LHYFNAR.G** |
|  | 5488 | **515.3264** | **1028.6381** | **1028.5767** | **0.0614** | **1** | **8** | **27** | **2** | **K.KFLQPGSPR.K** |
|  | 5702 | **523.7737** | **1045.5329** | **1045.5404** | **-0.0074** | **1** | **29** | **0.2** | **1** | **K.SAEDLDKLR.N** 5691 |
|  | 8238 | **609.2946** | **1216.5746** | **1216.5836** | **-0.0091** | **0** | **57** | **0.00033** | **1** | **K.SHGQDYLVGNK.L** 8263 |
|  | 14127 | 779.9018 | 1557.7890 | 1556.8311 | 0.9579 | 1 | 3 | 64 | 5 | K.VLKSHGQDYLVGNK.L |
|  | 22323 | **1108.0090** | **2214.0035** | **2214.0159** | **-0.0124** | **0** | **101** | **6.3e-009** | **1** | **R.NDGYLMFQQVPMVEIDGMK.L** 22324 22354 |
|  | 24559 | **834.2344** | **2499.6813** | **2499.1960** | **0.4853** | **1** | **3** | **21** | **1** | **K.LRNDGYLMFQQVPMVEIDGMK.L** |
|  | 27800 | **1036.2212** | **3105.6417** | **3105.6525** | **-0.0108** | **0** | **12** | **3.2** | **1** | **R.ADIHLVELLYYVEELDSSLISSFPLLK.A** 27799 |
|  | 27923 | **1571.8060** | **3141.5975** | **3141.6051** | **-0.0076** | **0** | **23** | **0.23** | **1** | **R.ALIDMYIEGIADLGEMILLLPVCPPEEK.D** |

|  | **Proteins matching a subset of these peptides:** |
| --- | --- |

|  | P09210\|GSTA2_HUMAN    **Mass:** 25648    **Score:** 166    **Queries matched:** 19 |
| --- | --- |
|  | Glutathione S-transferase A2 OS=Homo sapiens GN=GSTA2 PE=1 SV=4 |

| **299.** | P61978\|HNRPK_HUMAN    **Mass:** 51230    **Score:** 169    **Queries matched:** 22   **emPAI:** 0.52 |
| --- | --- |
|  | Heterogeneous nuclear ribonucleoprotein K OS=Homo sapiens GN=HNRNPK PE=1 SV=1 |

|  | **Query** | **Observed** | **Mr(expt)** | **Mr(calc)** | **Delta** | **Miss** | **Score** | **Expect** | **Rank** | **Peptide** |
| --- | --- | --- | --- | --- | --- | --- | --- | --- | --- | --- |
|  | 1806 | **729.4102** | **728.4029** | **728.4181** | **-0.0151** | **0** | **21** | **1.5** | **1** | **K.NAGAVIGK.G** 1802 |
|  | 3274 | **436.2265** | **870.4385** | **870.4712** | **-0.0327** | **0** | **9** | **24** | **4** | **R.GPPPPPPGR.G** 3269 |
|  | 5076 | **998.2856** | **997.2783** | **996.4335** | **0.8448** | **0** | **5** | **37** | **1** | **R.GGDLMAYDR.R** |
|  | 5094 | **500.2533** | **998.4920** | **997.3811** | **1.1109** | **0** | **8** | **26** | **3** | **R.DYDDMSPR.R** |
|  | 5266 | **507.0747** | **1012.1349** | **1012.4284** | **-0.2935** | **0** | **(5)** | **35** | **2** | **R.GGDLMAYDR.R** |
|  | 5303 | **508.2843** | **1014.5541** | **1013.3760** | **1.1781** | **0** | **(3)** | **84** | **5** | **R.DYDDMSPR.R** |
|  | 5458 | **514.3084** | **1026.6021** | **1026.5723** | **0.0299** | **1** | **5** | **49** | **9** | **R.RGPPPPPPGR.G** |
|  | 5799 | **527.3206** | **1052.6267** | **1052.6342** | **-0.0075** | **0** | **32** | **0.09** | **1** | **R.VVLIGGKPDR.V** |
|  | 6596 | **555.8343** | **1109.6540** | **1109.5876** | **0.0664** | **1** | **7** | **29** | **5** | **R.GGRPMPPSRR.D** |
|  | 7634 | **396.8544** | **1187.5413** | **1187.6346** | **-0.0933** | **1** | **0** | **1.5e+002** | **4** | **R.RGRPVGFPMR.G** |
|  | 9024 | **420.2430** | **1257.7073** | **1258.5677** | **-0.8604** | **0** | **8** | **21** | **3** | **K.IDEPLEGSEDR.I** 9022 |
|  | 10560 | **670.8232** | **1339.6318** | **1339.7962** | **-0.1644** | **0** | **3** | **73** | **9** | **K.IILDLISESPIK.G** |
|  | 11974 | 474.2844 | 1419.8313 | 1420.8303 | -0.9990 | 1 | 5 | 43 | 4 | R.ARNLPLPPPPPPR.G 12017 |
|  | 13967 | 775.3246 | 1548.6346 | 1548.6450 | -0.0104 | 0 | 52 | 0.00068 | 1 | K.LFQECCPHSTDR.V |
|  | 17351 | **890.8973** | **1779.7801** | **1779.7911** | **-0.0110** | **0** | **51** | **0.00091** | **1** | **R.TDYNASVSVPDSSGPER.I** |
|  | 19211 | **959.0148** | **1916.0150** | **1916.0255** | **-0.0105** | **0** | **57** | **0.00021** | **1** | **R.GSYGDLGGPIITTQVTIPK.D** |
|  | 25162 | **1295.1912** | **2588.3678** | **2588.3810** | **-0.0132** | **0** | **73** | **3e-006** | **1** | **R.IITITGTQDQIQNAQYLLQNSVK.Q** 25161 |

| **300.** | P08195\|4F2_HUMAN    **Mass:** 68180    **Score:** 169    **Queries matched:** 20   **emPAI:** 0.30 |
| --- | --- |
|  | 4F2 cell-surface antigen heavy chain OS=Homo sapiens GN=SLC3A2 PE=1 SV=3 |

|  | **Query** | **Observed** | **Mr(expt)** | **Mr(calc)** | **Delta** | **Miss** | **Score** | **Expect** | **Rank** | **Peptide** |
| --- | --- | --- | --- | --- | --- | --- | --- | --- | --- | --- |
|  | 4877 | **492.7146** | **983.4146** | **983.4209** | **-0.0063** | **0** | **10** | **16** | **1** | **R.HWDQNER.F** 4880 |
|  | 7451 | **589.3055** | **1176.5965** | **1175.6662** | **0.9303** | **0** | **6** | **41** | **5** | **K.LEPHEGLLLR.F** |
|  | 11092 | **686.4180** | **1370.8215** | **1370.8286** | **-0.0070** | **0** | **52** | **0.00085** | **1** | **R.LLTSFLPAQLLR.L** 11093 |
|  | 11274 | **691.3474** | **1380.6803** | **1379.6932** | **0.9870** | **1** | **4** | **58** | **2** | **K.EDFDSLLQSAKK.K** |
|  | 11927 | **473.2862** | **1416.8367** | **1416.8452** | **-0.0086** | **1** | **23** | **0.68** | **1** | **R.LKLEPHEGLLLR.F** 11914 |
|  | 13342 | 502.5834 | 1504.7283 | 1504.7522 | -0.0239 | 0 | 8 | 19 | 3 | K.GQSEDPGSLLSLFR.R 13319 |
|  | 16542 | 574.6338 | 1720.8795 | 1719.7920 | 1.0876 | 0 | 2 | 73 | 6 | K.QPMNAASGAAMSLAGAEK.N |
|  | 17187 | **885.4152** | **1768.8159** | **1768.8268** | **-0.0109** | **0** | **27** | **0.2** | **1** | **R.GENSWFSTQVDTVATK.V** |
|  | 18364 | 926.9925 | 1851.9704 | 1851.9764 | -0.0060 | 0 | 10 | 11 | 3 | -.MELQPPEASIAVVSIPR.Q |
|  | 18561 | 933.9887 | 1865.9629 | 1865.9748 | -0.0119 | 0 | 58 | 0.00016 | 1 | R.IGDLQAFQGHGAGNLAGLK.G 18562 |
|  | 18813 | **944.4116** | **1886.8086** | **1886.7874** | **0.0212** | **0** | **0** | **88** | **7** | **K.NAEVTGTMSQDTEVDMK.E** |
|  | 22021 | **726.7125** | **2177.1156** | **2177.1270** | **-0.0114** | **1** | **6** | **23** | **1** | **K.VKDALEFWLQAGVDGFQVR.D** |
|  | 22364 | **739.3586** | **2215.0539** | **2215.1089** | **-0.0550** | **1** | **1** | **62** | **4** | **K.QPMNAASGAAMSLAGAEKNGLVK.I** |
|  | 22974 | **764.7194** | **2291.1364** | **2291.1487** | **-0.0123** | **0** | **40** | **0.0083** | **1** | **R.SLLHGDFHAFSAGPGLFSYIR.H** |
|  | 23119 | **1155.5941** | **2309.1737** | **2309.1863** | **-0.0126** | **1** | **29** | **0.11** | **1** | **K.ADLLLSTQPGREEGSPLELER.L** |

| **301.** | Q6PIF6\|MYO7B_HUMAN    **Mass:** 243612   **Score:** 168    **Queries matched:** 59   **emPAI:** 0.09 |
| --- | --- |
|  | Myosin-VIIb OS=Homo sapiens GN=MYO7B PE=2 SV=2 |

|  | **Query** | **Observed** | **Mr(expt)** | **Mr(calc)** | **Delta** | **Miss** | **Score** | **Expect** | **Rank** | **Peptide** |
| --- | --- | --- | --- | --- | --- | --- | --- | --- | --- | --- |
|  | 1836 | **736.3937** | **735.3865** | **734.4075** | **0.9789** | **1** | **10** | **17** | **3** | **R.RDAFVK.G** |
|  | 2245 | **403.2275** | **804.4405** | **803.4654** | **0.9751** | **1** | **26** | **0.55** | **1** | **R.KSGFPIR.Y** 2220 2246 2247 |
|  | 3106 | **432.2163** | **862.4181** | **861.4232** | **0.9949** | **0** | **6** | **51** | **5** | **R.EVSDWVK.K** |
|  | 3976 | **460.2470** | **918.4795** | **918.4658** | **0.0137** | **1** | **3** | **99** | **8** | **K.DKTVEEAK.V** |
|  | 4365 | 474.2790 | 946.5434 | 945.5647 | 0.9787 | 0 | 7 | 38 | 10 | K.LILVGFER.L |
|  | 4895 | **493.7572** | **985.4998** | **985.6172** | **-0.1173** | **1** | **13** | **8.8** | **2** | **K.KGDLLVLTK.K** |
|  | 5335 | **509.3027** | **1016.5908** | **1016.5866** | **0.0042** | **1** | **8** | **30** | **9** | **K.ISDKVISQK.E** |
|  | 5457 | **514.3077** | **1026.6009** | **1026.5862** | **0.0147** | **0** | **2** | **87** | **9** | **K.LWLNISPGK.D** |
|  | 5991 | **534.2669** | **1066.5193** | **1065.4873** | **1.0320** | **0** | **(7)** | **33** | **8** | **R.SSQQGSSVMR.Q** |
|  | 6066 | **536.3405** | **1070.6663** | **1069.5768** | **1.0896** | **0** | **6** | **44** | **10** | **R.ELVPENLTR.L** |
|  | 6212 | **541.7662** | **1081.5178** | **1081.4822** | **0.0356** | **0** | **11** | **12** | **2** | **R.SSQQGSSVMR.Q** 6215 |
|  | 6618 | **557.0399** | **1112.0653** | **1112.6091** | **-0.5438** | **1** | **6** | **24** | **2** | **R.QAKAGNHLFK.S** |
|  | 6660 | **558.3568** | **1114.6991** | **1115.5757** | **-0.8766** | **1** | **5** | **46** | **8** | **R.GKLLAPDCSR.R** 6679 |
|  | 6677 | **559.3094** | **1116.6042** | **1116.6114** | **-0.0071** | **0** | **32** | **0.093** | **1** | **R.FGVLLPNAMR.M** |
|  | 6943 | **569.3004** | **1136.5863** | **1136.5536** | **0.0327** | **1** | **10** | **14** | **3** | **R.LMSSEEWKK.S** |
|  | 7141 | 577.3047 | 1152.5948 | 1152.5485 | 0.0464 | 1 | (6) | 38 | 7 | R.LMSSEEWKK.S |
|  | 7171 | 578.3547 | 1154.6948 | 1154.6812 | 0.0137 | 1 | 18 | 2.4 | 4 | R.KLWLNISPGK.D |
|  | 7481 | **589.8986** | **1177.7826** | **1176.5961** | **1.1865** | **0** | **27** | **0.25** | **1** | **R.WASLVTAACAK.A** |
|  | 9131 | **632.3469** | **1262.6792** | **1263.6935** | **-1.0143** | **0** | **8** | **22** | **2** | **K.LLEVQHQELR.D** |
|  | 9934 | **653.8437** | **1305.6728** | **1305.6816** | **-0.0088** | **0** | **48** | **0.0023** | **1** | **R.EIFNLELAETK.L** 9945 |
|  | 10154 | 659.8444 | 1317.6742 | 1317.6785 | -0.0043 | 0 | 7 | 31 | 10 | K.DMVSMAVLPLAR.A |
|  | 10259 | **662.3679** | **1322.7213** | **1322.7782** | **-0.0569** | **1** | **6** | **31** | **8** | **R.LQAIARSQPLAR.Q** 10275 |
|  | 10272 | **442.2439** | **1323.7098** | **1322.6111** | **1.0987** | **0** | **11** | **12** | **3** | **R.YSGMMETVHIR.K** 10276 |
|  | 10629 | **672.3630** | **1342.7114** | **1341.7292** | **0.9822** | **0** | **5** | **45** | **9** | **R.EDAIHLAGLIYK.A** |
|  | 11241 | 690.3578 | 1378.7011 | 1377.6962 | 1.0049 | 0 | 1 | 1.1e+002 | 6 | R.FPQGTDLTMLQK.L |
|  | 11743 | **470.2405** | **1407.6995** | **1408.7575** | **-1.0580** | **1** | **8** | **19** | **2** | **K.AFLQPKNIHDAR.F** 11751 |
|  | 12981 | **742.3466** | **1482.6787** | **1482.6959** | **-0.0172** | **1** | **7** | **23** | **2** | **R.YSGMMETVHIRK.S** |
|  | 13369 | **754.3798** | **1506.7450** | **1506.7541** | **-0.0091** | **0** | **27** | **0.26** | **1** | **R.FMGDLPEPVLYAR.S** 13363 |
|  | 13819 | **770.4109** | **1538.8073** | **1537.9192** | **0.8882** | **1** | **2** | **84** | **5** | **K.TGVAIGGIIKEAKPGK.V** |
|  | 13968 | 517.2309 | 1548.6709 | 1548.8671 | -0.1962 | 1 | 6 | 32 | 1 | R.AVVVIQAHARGMAAR.R |
|  | 14852 | 402.9471 | 1607.7592 | 1607.6780 | 0.0812 | 1 | 9 | 15 | 1 | R.CEQMAQERGESQR.Q |
|  | 15218 | 815.8736 | 1629.7326 | 1629.7444 | -0.0117 | 0 | 45 | 0.0034 | 1 | R.SIYDTVTDTEMVEK.V |
|  | 15751 | **556.2861** | **1665.8366** | **1665.8250** | **0.0116** | **0** | **4** | **41** | **5** | **K.ILQFSDSESWDVIK.L** |
|  | 17278 | **592.6347** | **1774.8823** | **1775.9240** | **-1.0417** | **1** | **1** | **95** | **10** | **R.QMTLGITDVWLRTDK.D** |
|  | 17778 | **907.4539** | **1812.8933** | **1812.9046** | **-0.0113** | **0** | **47** | **0.002** | **1** | **K.YIDIYFNPSGVIEGAR.I** |
|  | 19441 | **646.3728** | **1936.0966** | **1935.9798** | **0.1168** | **0** | **3** | **49** | **2** | **K.MLLELSFPEVMGLATNR.E** |
|  | 19766 | **656.2712** | **1965.7919** | **1964.8985** | **0.8934** | **1** | **2** | **50** | **10** | **K.FWSLGSGRDHMMDAIAR.C** |
|  | 19970 | **663.0170** | **1986.0291** | **1985.9701** | **0.0590** | **1** | **1** | **71** | **7** | **R.SSQQGSSVMRQIHDTLGR.E** |
|  | 20631 | **682.6793** | **2045.0161** | **2045.0290** | **-0.0129** | **1** | **61** | **8.1e-005** | **1** | **R.KQPPHQVEVEAAEQNVSR.I** |
|  | 21025 | **696.3560** | **2086.0462** | **2087.0396** | **-0.9933** | **1** | **9** | **11** | **1** | **K.QGLLASENWTLGQNDRTGK.T** |
|  | 23579 | **790.7429** | **2369.2068** | **2369.1685** | **0.0382** | **1** | **0** | **69** | **6** | **K.IYFPNDTSEMLEVVANTRVR.D** |
|  | 24313 | **824.0734** | **2469.1983** | **2469.2063** | **-0.0081** | **1** | **1** | **56** | **4** | **R.GVWSGEYSFEKEEELVELLAR.H** |
|  | 25078 | **857.4709** | **2569.3908** | **2570.2257** | **-0.8349** | **1** | **0** | **61** | **4** | **R.FMGDLPEPVLYARSSQQGSSVMR.Q** |
|  | 25337 | 1312.6089 | 2623.2032 | 2623.2343 | -0.0311 | 1 | 2 | 35 | 2 | K.EFFTPWHDSREDPVSTELIYR.Q 25336 |
|  | 26319 | **931.1129** | **2790.3169** | **2790.3323** | **-0.0154** | **0** | **64** | **2.3e-005** | **1** | **K.VFGFLPAMIGGQEGQASPHFEDLESK.T** |
|  | 27882 | **1044.1860** | **3129.5363** | **3129.4834** | **0.0529** | **1** | **2** | **33** | **1** | **R.EMCMHIAHKQGLSDHLGFSLQVAVYDK.F** |
|  | 28352 | **809.6649** | **3234.6303** | **3233.6022** | **1.0282** | **1** | **0** | **42** | **6** | **R.LLCETSLGYKMDDLLTSYVQQLLSAMNK.Q** |
|  | 28495 | **1089.5178** | **3265.5316** | **3266.5237** | **-0.9921** | **1** | **4** | **20** | **3** | **K.EHWIRAEDFGVLSPMHPNSVQGVDDMIR.L** |

| **302.** | Q00325\|MPCP_HUMAN    **Mass:** 40525    **Score:** 168    **Queries matched:** 27   **emPAI:** 0.42 |
| --- | --- |
|  | Phosphate carrier protein, mitochondrial OS=Homo sapiens GN=SLC25A3 PE=1 SV=2 |

|  | **Query** | **Observed** | **Mr(expt)** | **Mr(calc)** | **Delta** | **Miss** | **Score** | **Expect** | **Rank** | **Peptide** |
| --- | --- | --- | --- | --- | --- | --- | --- | --- | --- | --- |
|  | 1167 | **575.3107** | **574.3034** | **574.2962** | **0.0072** | **0** | **14** | **12** | **4** | **K.EEGLK.A** |
|  | 1563 | 659.4039 | 658.3966 | 658.4014 | -0.0048 | 1 | 13 | 13 | 2 | K.KLGLTQ.- |
|  | 2480 | **412.7308** | **823.4471** | **822.4487** | **0.9984** | **0** | **16** | **3.6** | **1** | **R.TVEALYK.F** |
|  | 2775 | **421.7632** | **841.5119** | **841.5174** | **-0.0055** | **0** | **19** | **2.2** | **1** | **K.FVVPKPR.S** |
|  | 2823 | **423.7119** | **845.4092** | **844.4113** | **0.9979** | **0** | **21** | **1.8** | **1** | **R.MQVDPQK.Y** 2804 |
|  | 4108 | **465.2518** | **928.4891** | **928.4953** | **-0.0062** | **0** | **10** | **19** | **5** | **K.GVAPLWMR.Q** |
|  | 4252 | **470.6973** | **939.3801** | **938.4531** | **0.9270** | **1** | **11** | **12** | **1** | **R.DAAPKMYK.E** |
|  | 4316 | **472.5935** | **943.1725** | **943.5087** | **-0.3362** | **1** | **6** | **31** | **2** | **K.EDGVRGLAK.G** |
|  | 5045 | **499.2066** | **996.3986** | **996.4950** | **-0.0963** | **1** | **5** | **42** | **3** | **K.MYKEEGLK.A** |
|  | 5335 | 509.3027 | 1016.5908 | 1016.5978 | -0.0070 | 1 | 37 | 0.041 | 1 | K.GSSASLVLKR.L |
|  | 6698 | **559.8206** | **1117.6266** | **1117.6343** | **-0.0077** | **1** | **18** | **2.7** | **1** | **K.EKGSSASLVLK.R** |
|  | 7117 | 576.7808 | 1151.5471 | 1151.5645 | -0.0174 | 1 | 2 | 99 | 10 | R.MQVDPQKYK.G |
|  | 10928 | **681.3578** | **1360.7010** | **1360.7099** | **-0.0089** | **0** | **51** | **0.0011** | **1** | **R.IQTQPGYANTLR.D** 10927 |
|  | 12921 | 493.9455 | 1478.8146 | 1478.7702 | 0.0444 | 1 | 11 | 10 | 1 | R.SSSPGPTGQPRRPR.N 12914 12943 |
|  | 15478 | **823.7396** | **1645.4647** | **1645.9556** | **-0.4909** | **1** | **1** | **57** | **7** | **R.TVEALYKFVVPKPR.S** |
|  | 17796 | **908.4267** | **1814.8388** | **1814.8484** | **-0.0096** | **0** | **52** | **0.00064** | **1** | **K.GWAPTFLGYSMQGLCK.F** 17795 |
|  | 18192 | 921.9061 | 1841.7976 | 1842.9588 | -1.1612 | 1 | 2 | 58 | 7 | R.IQTQPGYANTLRDAAPK.M 18199 |
|  | 19976 | **994.4763** | **1986.9381** | **1986.9509** | **-0.0128** | **0** | **90** | **1e-007** | **1** | **K.VLYSNMLGEENTYLWR.T** 19982 |
|  | 25522 | **1330.1594** | **2658.3043** | **2658.3250** | **-0.0208** | **0** | **22** | **0.39** | **1** | **R.TSLYLAASASAEFFADIALAPMEAAK.V** |
|  | 28136 | **1061.7805** | **3182.3197** | **3181.6534** | **0.6664** | **1** | **0** | **39** | **1** | **M.FSSVAHLARANPFNTPHLQLVHDGLGDLR.S** |

| **303.** | P14314\|GLU2B_HUMAN    **Mass:** 60357    **Score:** 168    **Queries matched:** 23   **emPAI:** 0.61 |
| --- | --- |
|  | Glucosidase 2 subunit beta OS=Homo sapiens GN=PRKCSH PE=1 SV=2 |

|  | **Query** | **Observed** | **Mr(expt)** | **Mr(calc)** | **Delta** | **Miss** | **Score** | **Expect** | **Rank** | **Peptide** |
| --- | --- | --- | --- | --- | --- | --- | --- | --- | --- | --- |
|  | 1205 | **583.2867** | **582.2794** | **582.2836** | **-0.0042** | **0** | **11** | **6.8** | **1** | **K.FSAMK.Y** |
|  | 2154 | **400.2538** | **798.4930** | **798.4963** | **-0.0033** | **0** | **14** | **7** | **1** | **K.LVSQKPK.L** |
|  | 3938 | **458.7577** | **915.5009** | **915.5065** | **-0.0057** | **0** | **45** | **0.0059** | **1** | **K.ILIEDWK.K** |
|  | 5104 | **500.3097** | **998.6049** | **998.6124** | **-0.0075** | **1** | **23** | **0.82** | **1** | **K.LIELQAGKK.S** |
|  | 5105 | **500.3099** | **998.6053** | **998.6124** | **-0.0071** | **1** | **10** | **15** | **2** | **K.KLIELQAGK.K** |
|  | 7211 | **579.8077** | **1157.6008** | **1157.6080** | **-0.0072** | **0** | **17** | **3.3** | **2** | **K.LWEEQLAAAK.A** 7187 |
|  | 8034 | **604.2990** | **1206.5835** | **1206.5914** | **-0.0079** | **1** | **45** | **0.0045** | **1** | **R.SLKDMEESIR.N** 8056 |
|  | 9656 | 646.3151 | 1290.6156 | 1290.6238 | -0.0082 | 0 | 38 | 0.022 | 1 | R.ESLQQMAEVTR.E 9657 |
|  | 10096 | **439.2315** | **1314.6725** | **1314.6779** | **-0.0054** | **1** | **35** | **0.044** | **1** | **R.TVKEEAEKPER.E** 10079 10081 |
|  | 10532 | **669.8094** | **1337.6043** | **1337.6133** | **-0.0089** | **0** | **57** | **0.00028** | **1** | **K.ETMVTSTTEPSR.C** 10533 |
|  | 12428 | 724.8498 | 1447.6851 | 1447.6943 | -0.0091 | 0 | 14 | 5.8 | 2 | K.AQQEQELAADAFK.E |
|  | 14024 | **776.8286** | **1551.6427** | **1551.6525** | **-0.0098** | **0** | **60** | **0.00013** | **1** | **K.YEQGTGCWQGPNR.S** |
|  | 17557 | **599.7609** | **1796.2610** | **1795.8523** | **0.4087** | **1** | **1** | **52** | **6** | **R.ESLQQMAEVTREGFR.L** |
|  | 21936 | **723.3474** | **2167.0202** | **2167.0335** | **-0.0132** | **0** | **11** | **6** | **1** | **K.LGGSPTSLGTWGSWIGPDHDK.F** |
|  | 26029 | **683.8301** | **2731.2912** | **2731.3065** | **-0.0152** | **1** | **35** | **0.019** | **1** | **K.LGGSPTSLGTWGSWIGPDHDKFSAMK.Y** |
|  | 27558 | **1014.7103** | **3041.1090** | **3040.1250** | **0.9840** | **0** | **28** | **0.055** | **1** | **R.VNDGVCDCCDGTDEYNSGVICENTCK.E** |
|  | 28838 | **1125.2429** | **3372.7069** | **3372.3818** | **0.3251** | **0** | **1** | **33** | **5** | **R.CEYLMELMTPAACPEPPPEAPTEDDHDEL.-** |

| **304.** | O95471\|CLD7_HUMAN    **Mass:** 22831    **Score:** 167    **Queries matched:** 3   **emPAI:** 0.17 |
| --- | --- |
|  | Claudin-7 OS=Homo sapiens GN=CLDN7 PE=1 SV=3 |

|  | **Query** | **Observed** | **Mr(expt)** | **Mr(calc)** | **Delta** | **Miss** | **Score** | **Expect** | **Rank** | **Peptide** |
| --- | --- | --- | --- | --- | --- | --- | --- | --- | --- | --- |
|  | 20021 | **996.4047** | **1990.7949** | **1990.8080** | **-0.0131** | **0** | **113** | **4e-010** | **1** | **K.GLWMDCVTQSTGMMSCK.M** 20022 |
|  | 26337 | **699.5942** | **2794.3479** | **2793.4124** | **0.9355** | **1** | **4** | **26** | **3** | **R.ALMVVSLVLGFLAMFVATMGMKCTR.C** |

| **305.** | P24844\|MYL9_HUMAN    **Mass:** 19871    **Score:** 167    **Queries matched:** 6   **emPAI:** 0.70 |
| --- | --- |
|  | Myosin regulatory light polypeptide 9 OS=Homo sapiens GN=MYL9 PE=1 SV=4 |

|  | **Query** | **Observed** | **Mr(expt)** | **Mr(calc)** | **Delta** | **Miss** | **Score** | **Expect** | **Rank** | **Peptide** |
| --- | --- | --- | --- | --- | --- | --- | --- | --- | --- | --- |
|  | 1042 | **547.2689** | **546.2616** | **547.3329** | **-1.0714** | **1** | **7** | **63** | **8** | **K.AKTTK.K** |
|  | 5563 | 518.2575 | 1034.5005 | 1034.5066 | -0.0062 | 0 | 29 | 0.22 | 1 | R.ELLTTMGDR.F |
|  | 20151 | 1002.5089 | 2003.0031 | 2002.9670 | 0.0362 | 1 | 51 | 0.00077 | 1 | R.DGFIDKEDLHDMLASLGK.N 20146 |
|  | 21059 | 1045.9907 | 2089.9669 | 2089.9779 | -0.0110 | 0 | 96 | 2.3e-008 | 1 | R.ATSNVFAMFDQSQIQEFK.E 21060 |

| **306.** | Q15067\|ACOX1_HUMAN    **Mass:** 74889    **Score:** 166    **Queries matched:** 20   **emPAI:** 0.21 |
| --- | --- |
|  | Peroxisomal acyl-coenzyme A oxidase 1 OS=Homo sapiens GN=ACOX1 PE=1 SV=3 |

|  | **Query** | **Observed** | **Mr(expt)** | **Mr(calc)** | **Delta** | **Miss** | **Score** | **Expect** | **Rank** | **Peptide** |
| --- | --- | --- | --- | --- | --- | --- | --- | --- | --- | --- |
|  | 2127 | **399.2327** | **796.4508** | **796.3459** | **0.1049** | **0** | **6** | **39** | **8** | **R.ENMLMK.Y** |
|  | 4792 | **489.2997** | **976.5849** | **976.5957** | **-0.0108** | **1** | **16** | **5.1** | **1** | **K.LFSEKLLK.I** |
|  | 5352 | **510.2385** | **1018.4624** | **1019.4672** | **-1.0048** | **0** | **8** | **27** | **1** | **K.SYDQVHSGK.L** |
|  | 6240 | **542.5089** | **1083.0033** | **1083.7016** | **-0.6983** | **1** | **24** | **0.4** | **1** | **R.VKELLTLIR.S** |
|  | 7925 | 401.2848 | 1200.8325 | 1201.6165 | -0.7840 | 0 | 9 | 15 | 7 | K.LTYGTMVFVR.S |
|  | 8415 | **613.6446** | **1225.2747** | **1225.7394** | **-0.4646** | **1** | **1** | **80** | **8** | **R.LVEIAAKNLQK.E** |
|  | 8956 | 628.3733 | 1254.7321 | 1253.7455 | 0.9866 | 1 | 5 | 42 | 9 | K.IQDKAIQAVLR.S |
|  | 12224 | **478.7763** | **1433.3072** | **1432.6510** | **0.6561** | **0** | **11** | **7.9** | **2** | **K.FGYDEIDNGYLK.M** |
|  | 13296 | **501.3375** | **1500.9906** | **1500.7936** | **0.1970** | **0** | **3** | **47** | **3** | **K.EVAWNLTSVDLVR.A** |
|  | 13390 | **754.9380** | **1507.8614** | **1507.8722** | **-0.0108** | **0** | **26** | **0.32** | **1** | **K.TSNHAIVLAQLITK.G** |
|  | 16188 | **849.3925** | **1696.7704** | **1696.7807** | **-0.0103** | **0** | **38** | **0.016** | **1** | **R.EFGIADPDEIMWFK.K** |
|  | 19347 | **643.6765** | **1928.0075** | **1928.0731** | **-0.0656** | **0** | **6** | **24** | **7** | **R.EIGTHKPLPGITVGDIGPK.F** 19363 |
|  | 20115 | **667.3448** | **1999.0127** | **1999.9172** | **-0.9045** | **1** | **0** | **94** | **8** | **K.MREFGIADPDEIMWFK.K** |
|  | 21210 | 701.7276 | 2102.1610 | 2101.9163 | 0.2446 | 1 | 2 | 53 | 7 | K.FGYDEIDNGYLKMDNHR.I |
|  | 23727 | **1198.1050** | **2394.1954** | **2394.2067** | **-0.0113** | **0** | **35** | **0.025** | **1** | **R.SDAVALVDAFDFQDVTLGSVLGR.Y** |
|  | 24353 | 825.7701 | 2474.2884 | 2474.3016 | -0.0132 | 0 | 62 | 4.5e-005 | 1 | R.INEGIGQGDLSELPELHALTAGLK.A |
|  | 25853 | **901.7574** | **2702.2505** | **2702.2646** | **-0.0141** | **0** | **20** | **0.64** | **1** | **R.EIENMILNDPDFQHEDLNFLTR.S** 25854 |
|  | 26586 | **1428.2057** | **2854.3968** | **2854.4124** | **-0.0156** | **0** | **94** | **2.2e-008** | **1** | **R.GLETTATYDPETQEFILNSPTVTSIK.W** |

| **307.** | Q12907\|LMAN2_HUMAN    **Mass:** 40545    **Score:** 166    **Queries matched:** 9   **emPAI:** 0.30 |
| --- | --- |
|  | Vesicular integral-membrane protein VIP36 OS=Homo sapiens GN=LMAN2 PE=1 SV=1 |

|  | **Query** | **Observed** | **Mr(expt)** | **Mr(calc)** | **Delta** | **Miss** | **Score** | **Expect** | **Rank** | **Peptide** |
| --- | --- | --- | --- | --- | --- | --- | --- | --- | --- | --- |
|  | 1814 | 731.1769 | 730.1697 | 729.3657 | 0.8040 | 0 | 7 | 39 | 1 | R.LTPDER.S |
|  | 3295 | **437.0524** | **872.0902** | **872.4505** | **-0.3603** | **0** | **8** | **25** | **4** | **R.SGPLTGWR.V** |
|  | 5714 | **524.2626** | **1046.5106** | **1046.5179** | **-0.0073** | **0** | **22** | **1.2** | **1** | **K.NCIDITGVR.L** |
|  | 11216 | **689.8487** | **1377.6828** | **1376.6970** | **0.9859** | **1** | **3** | **71** | **4** | **R.GRLTVMTDLEDK.N** |
|  | 12073 | **713.8198** | **1425.6250** | **1425.6347** | **-0.0097** | **0** | **63** | **6e-005** | **1** | **R.WTELAGCTADFR.N** 12074 |
|  | 23174 | **1159.5476** | **2317.0807** | **2317.0936** | **-0.0130** | **0** | **99** | **9e-009** | **1** | **K.LFQLMVEHTPDEESIDWTK.I** 23173 |
|  | 29872 | **933.2015** | **3728.7768** | **3728.7967** | **-0.0199** | **0** | **10** | **3.1** | **1** | **R.EHSLIKPYQGVGSSSMPLWDFQGSTMLTSQYVR.L** |

| **308.** | P13804\|ETFA_HUMAN    **Mass:** 35400    **Score:** 166    **Queries matched:** 15   **emPAI:** 0.66 |
| --- | --- |
|  | Electron transfer flavoprotein subunit alpha, mitochondrial OS=Homo sapiens GN=ETFA PE=1 SV=1 |

|  | **Query** | **Observed** | **Mr(expt)** | **Mr(calc)** | **Delta** | **Miss** | **Score** | **Expect** | **Rank** | **Peptide** |
| --- | --- | --- | --- | --- | --- | --- | --- | --- | --- | --- |
|  | 1422 | **630.3887** | **629.3815** | **629.3860** | **-0.0046** | **0** | **8** | **39** | **7** | **R.AASLLR.F** |
|  | 4713 | **486.8050** | **971.5954** | **970.5924** | **1.0030** | **1** | **6** | **44** | **1** | **K.VVVSGGRGLK.S** 4705 |
|  | 7374 | **586.3234** | **1170.6322** | **1170.6397** | **-0.0075** | **0** | **19** | **1.8** | **1** | **K.VLVAQHDVYK.G** |
|  | 8259 | 406.6998 | 1217.0776 | 1216.6499 | 0.4277 | 1 | 7 | 22 | 1 | -.MFRAAAPGQLR.R |
|  | 9637 | **645.8356** | **1289.6566** | **1289.6649** | **-0.0084** | **0** | **72** | **8.8e-006** | **1** | **R.LGGEVSCLVAGTK.C** |
|  | 11289 | **691.4149** | **1380.8152** | **1380.8228** | **-0.0076** | **0** | **21** | **1.2** | **1** | **K.LEVAPISDIIAIK.S** |
|  | 15215 | **815.8573** | **1629.7000** | **1629.7118** | **-0.0118** | **0** | **56** | **0.0003** | **1** | **R.GTSFDAAATSGGSASSEK.A** 15211 |
|  | 17290 | **888.9305** | **1775.8465** | **1775.8577** | **-0.0112** | **0** | **37** | **0.023** | **1** | **K.ASSTSPVEISEWLDQK.L** 17289 17304 |
|  | 19055 | **952.9555** | **1903.8965** | **1903.9098** | **-0.0134** | **0** | **53** | **0.00053** | **1** | **R.AAVDAGFVPNDMQVGQTGK.I** 19056 |
|  | 27076 | **983.1949** | **2946.5630** | **2946.5742** | **-0.0112** | **1** | **16** | **1.1** | **1** | **K.TIVAINKDPEAPIFQVADYGIVADLFK.V** |

| **309.** | P28331\|NDUS1_HUMAN    **Mass:** 80443    **Score:** 165    **Queries matched:** 42   **emPAI:** 0.50 |
| --- | --- |
|  | NADH-ubiquinone oxidoreductase 75 kDa subunit, mitochondrial OS=Homo sapiens GN=NDUFS1 PE=1 SV=3 |

|  | **Query** | **Observed** | **Mr(expt)** | **Mr(calc)** | **Delta** | **Miss** | **Score** | **Expect** | **Rank** | **Peptide** |
| --- | --- | --- | --- | --- | --- | --- | --- | --- | --- | --- |
|  | 1847 | **740.4251** | **739.4178** | **739.4592** | **-0.0414** | **0** | **10** | **15** | **4** | **K.NIGPLVK.T** |
|  | 2162 | **400.7236** | **799.4327** | **799.4374** | **-0.0047** | **0** | **32** | **0.12** | **1** | **K.VGMQIPR.F** 2156 2180 |
|  | 2809 | 423.2286 | 844.4427 | 844.4477 | -0.0049 | 0 | (6) | 61 | 1 | R.LTEPMVR.N |
|  | 3044 | **430.7505** | **859.4864** | **860.4426** | **-0.9562** | **0** | **12** | **14** | **7** | **R.LTEPMVR.N** 3064 |
|  | 4831 | **490.7947** | **979.5749** | **979.5815** | **-0.0066** | **0** | **9** | **18** | **3** | **K.VAVTPPGLAR.E** |
|  | 5120 | 500.8023 | 999.5900 | 998.6124 | 0.9776 | 1 | 5 | 51 | 7 | K.ALVGLSKSPK.G |
|  | 5378 | **511.2533** | **1020.4920** | **1020.4984** | **-0.0063** | **0** | **11** | **14** | **1** | **R.MCLVEIEK.A** |
|  | 5585 | 1036.5244 | 1035.5171 | 1036.4933 | -0.9762 | 0 | (9) | 19 | 6 | R.MCLVEIEK.A |
|  | 5951 | **532.7765** | **1063.5384** | **1063.5451** | **-0.0066** | **0** | **36** | **0.037** | **1** | **R.FEAPLFNAR.I** |
|  | 7432 | **588.7955** | **1175.5765** | **1175.5865** | **-0.0100** | **0** | **5** | **47** | **4** | **K.VVAACAMPVMK.G** 7443 |
|  | 8558 | **617.7694** | **1233.5243** | **1233.5336** | **-0.0093** | **0** | **19** | **2** | **2** | **K.DFYMTDSISR.A** |
|  | 9200 | **423.2286** | **1266.6641** | **1265.5791** | **1.0850** | **1** | **10** | **15** | **2** | **R.CIQCTRCIR.F** |
|  | 10420 | 666.8752 | 1331.7358 | 1332.6781 | -0.9423 | 1 | 9 | 18 | 7 | R.MCLVEIEKAPK.V 10460 |
|  | 10607 | **448.2307** | **1341.6703** | **1341.7802** | **-0.1099** | **1** | **12** | **9.2** | **3** | **K.NIGPLVKTIMTR.C** |
|  | 10807 | **677.8148** | **1353.6151** | **1353.6235** | **-0.0084** | **0** | **20** | **1.4** | **1** | **R.GNDMQVGTYIEK.M** |
|  | 11079 | **457.7840** | **1370.3303** | **1369.6184** | **0.7119** | **0** | **(1)** | **73** | **3** | **R.GNDMQVGTYIEK.M** |
|  | 12057 | **713.3311** | **1424.6475** | **1424.7735** | **-0.1260** | **1** | **24** | **0.55** | **1** | **R.NRLEEVSPNLVR.Y** |
|  | 14855 | **804.8909** | **1607.7672** | **1607.7791** | **-0.0119** | **0** | **95** | **3.7e-008** | **1** | **R.FASEIAGVDDLGTTGR.G** 14835 |
|  | 15461 | **823.3567** | **1644.6988** | **1644.7090** | **-0.0101** | **0** | **34** | **0.044** | **1** | **R.MHEDINEEWISDK.T** |
|  | 16339 | 854.3942 | 1706.7738 | 1707.7974 | -1.0236 | 1 | 2 | 75 | 5 | K.VGMQIPRFCYHER.L |
|  | 16449 | **572.3478** | **1714.0215** | **1712.9607** | **1.0608** | **1** | **1** | **87** | **8** | **K.EAKKPMVVLGSSALQR.N** |
|  | 17548 | **898.6074** | **1795.2003** | **1795.0091** | **0.1912** | **0** | **3** | **38** | **2** | **K.DVAAIAGGLVDAEALVALK.D** |
|  | 17573 | 899.8917 | 1797.7688 | 1797.0108 | 0.7580 | 1 | 5 | 28 | 3 | R.NDGAAILAAVSSIAQKIR.M 17572 |
|  | 18164 | **920.4960** | **1838.9775** | **1838.9890** | **-0.0115** | **0** | **45** | **0.0034** | **1** | **K.ILQDIASGSHPFSQVLK.E** 18154 18165 |
|  | 21120 | **1047.0275** | **2092.0404** | **2092.0510** | **-0.0107** | **0** | **35** | **0.027** | **1** | **R.ALSEIAGMTLPYDTLDQVR.N** 21119 |
|  | 23516 | 787.7372 | 2360.1897 | 2360.1900 | -0.0003 | 0 | 18 | 1.2 | 1 | K.VALIGSPVDLTYTYDHLGDSPK.I 23510 |
|  | 24339 | 1237.5723 | 2473.1300 | 2474.3203 | -1.1903 | 1 | 3 | 34 | 2 | K.IIRALSEIAGMTLPYDTLDQVR.N |
|  | 28305 | **1076.8607** | **3227.5603** | **3226.5757** | **0.9846** | **0** | **32** | **0.025** | **1** | **K.DCFIIYQGHHGDVGAPIADVILPGAAYTEK.S** |
|  | 30207 | **969.6821** | **3874.6992** | **3873.8892** | **0.8100** | **0** | **0** | **25** | **4** | **K.MFMSELSGNIIDICPVGALTSKPYAFTARPWETR.K** |
|  | 30756 | **1433.5959** | **4297.7660** | **4297.7942** | **-0.0282** | **0** | **27** | **0.038** | **1** | **R.EGVMEFLLANHPLDCPICDQGGECDLQDQSMMFGNDR.S** 30757 |

| **310.** | Q92928\|RAB1C_HUMAN    **Mass:** 22231    **Score:** 165    **Queries matched:** 16   **emPAI:** 0.89 |
| --- | --- |
|  | Putative Ras-related protein Rab-1C OS=Homo sapiens GN=RAB1C PE=5 SV=2 |

|  | **Query** | **Observed** | **Mr(expt)** | **Mr(calc)** | **Delta** | **Miss** | **Score** | **Expect** | **Rank** | **Peptide** |
| --- | --- | --- | --- | --- | --- | --- | --- | --- | --- | --- |
|  | 1480 | 643.2714 | 642.2641 | 642.4064 | -0.1424 | 0 | 19 | 2.4 | 1 | K.LLVGNK.S |
|  | 6064 | 536.3201 | 1070.6257 | 1070.6336 | -0.0079 | 0 | 15 | 5.6 | 1 | K.LLLIGDSGVGK.S |
|  | 6285 | 544.2767 | 1086.5389 | 1086.5458 | -0.0069 | 0 | 16 | 4.8 | 5 | K.QWLQEIDR.H |
|  | 10115 | 658.8291 | 1315.6436 | 1315.6521 | -0.0084 | 0 | 53 | 0.00078 | 1 | K.LQIWDTAGQER.F 10116 |
|  | 14517 | 397.2228 | 1584.8621 | 1584.7678 | 0.0943 | 0 | 11 | 9.6 | 1 | K.QMGPGAASGGERPNLK.I 14497 14498 14512 |
|  | 16578 | 862.9362 | 1723.8579 | 1723.8669 | -0.0090 | 0 | 25 | 0.37 | 1 | K.EFADSLGIPFLETSAK.N 16557 |
|  | 18580 | 934.9427 | 1867.8708 | 1867.8808 | -0.0100 | 0 | 71 | 7.9e-006 | 1 | K.NATNVEQAFMTMAAEIK.K |
|  | 18767 | 942.9406 | 1883.8667 | 1883.8757 | -0.0090 | 0 | (62) | 7.2e-005 | 2 | K.NATNVEQAFMTMAAEIK.K 18768 |
|  | 20413 | 1015.1899 | 2028.3653 | 2027.9656 | 0.3998 | 1 | 1 | 51 | 1 | K.NATNVEQAFMTMAAEIKK.Q |
|  | 27247 | **746.8489** | **2983.3667** | **2984.3903** | **-1.0236** | **1** | **8** | **8.7** | **2** | **K.QMGPGAASGGERPNLKIDSTPVKPAGGGCC.-** |

| **311.** | P39656\|OST48_HUMAN    **Mass:** 50940    **Score:** 164    **Queries matched:** 14   **emPAI:** 0.42 |
| --- | --- |
|  | Dolichyl-diphosphooligosaccharide--protein glycosyltransferase 48 kDa subunit OS=Homo sapiens GN=DDOST PE=1 SV=4 |

|  | **Query** | **Observed** | **Mr(expt)** | **Mr(calc)** | **Delta** | **Miss** | **Score** | **Expect** | **Rank** | **Peptide** |
| --- | --- | --- | --- | --- | --- | --- | --- | --- | --- | --- |
|  | 1655 | 685.4302 | 684.4229 | 684.4170 | 0.0059 | 0 | 13 | 9.4 | 1 | K.APTIVGK.S |
|  | 4967 | **496.2480** | **990.4814** | **989.4964** | **0.9850** | **1** | **9** | **23** | **3** | **R.KMEPSTAAR.A** 4961 |
|  | 5709 | 523.8001 | 1045.5855 | 1045.5920 | -0.0065 | 0 | 33 | 0.083 | 1 | K.SSLNPILFR.G |
|  | 7351 | **585.3505** | **1168.6865** | **1168.6928** | **-0.0063** | **0** | **9** | **17** | **1** | **K.NTLLIAGLQAR.N** 7350 |
|  | 10010 | **656.2712** | **1310.5279** | **1311.6863** | **-1.1584** | **0** | **4** | **47** | **6** | **K.LPDVYGVFQFK.V** |
|  | 15819 | **836.4154** | **1670.8163** | **1670.8264** | **-0.0101** | **0** | **53** | **0.00058** | **1** | **R.YSQTGNYELAVALSR.W** |
|  | 18105 | **918.4468** | **1834.8790** | **1834.8890** | **-0.0100** | **0** | **46** | **0.0032** | **1** | **K.WVPFDGDDIQLEFVR.I** 18104 |
|  | 27798 | **1036.1990** | **3105.5751** | **3105.5870** | **-0.0119** | **0** | **15** | **1.7** | **1** | **R.VGETAPPNAYTVTDLVEYSIVIQQLSNGK.W** |
|  | 28409 | **1082.5460** | **3244.6162** | **3244.6364** | **-0.0202** | **0** | **56** | **0.00012** | **1** | **K.TAVIDHHNYDISDLGQHTLIVADTENLLK.A** |
|  | 30794 | 1445.3851 | 4333.1336 | 4333.1617 | -0.0282 | 0 | 66 | 5.3e-006 | 1 | R.GVGMVADPDNPLVLDILTGSSTSYSFFPDKPITQYPHAVGK.N 30796 |

| **312.** | O43826\|G6PT1_HUMAN    **Mass:** 46729    **Score:** 163    **Queries matched:** 11   **emPAI:** 0.26 |
| --- | --- |
|  | Glucose-6-phosphate translocase OS=Homo sapiens GN=SLC37A4 PE=1 SV=1 |

|  | **Query** | **Observed** | **Mr(expt)** | **Mr(calc)** | **Delta** | **Miss** | **Score** | **Expect** | **Rank** | **Peptide** |
| --- | --- | --- | --- | --- | --- | --- | --- | --- | --- | --- |
|  | 4177 | **467.7741** | **933.5336** | **932.4815** | **1.0521** | **0** | **4** | **82** | **1** | **R.VTVTSDSPK.L** |
|  | 5729 | **524.7580** | **1047.5014** | **1047.5097** | **-0.0083** | **0** | **61** | **0.00014** | **1** | **K.AGLSNYGNPR.H** 5728 |
|  | 8218 | **608.7927** | **1215.5709** | **1214.5965** | **0.9744** | **1** | **9** | **21** | **3** | **R.NLDPMPSEGKK.G** |
|  | 11533 | **698.8433** | **1395.6720** | **1395.6817** | **-0.0097** | **0** | **75** | **4e-006** | **1** | **K.FVSGVLSDQMSAR.W** |
|  | 18628 | 624.9907 | 1871.9503 | 1872.9105 | -0.9602 | 0 | 3 | 46 | 2 | K.DDLGFITSSQSAAYAISK.F |
|  | 27365 | **1006.1732** | **3015.4978** | **3014.5019** | **0.9959** | **0** | **0** | **50** | **1** | **K.GQSALVGSSYMSALEVGGLVGSIAAGYLSDR.A** |
|  | 28054 | 1055.5429 | 3163.6067 | 3163.5579 | 0.0489 | 1 | 4 | 18 | 1 | R.HGLLLFMMAGMTVSMYLFRVTVTSDSPK.L |
|  | 30103 | **1269.6345** | **3805.8817** | **3805.9012** | **-0.0195** | **1** | **52** | **0.00018** | **1** | **K.TFSFVMPSLVEEIPLDKDDLGFITSSQSAAYAISK.F** 30104 |
|  | 30128 | **1274.5988** | **3820.7744** | **3821.8961** | **-1.1217** | **1** | **(1)** | **25** | **6** | **K.TFSFVMPSLVEEIPLDKDDLGFITSSQSAAYAISK.F** |

| **313.** | O00217\|NDUS8_HUMAN    **Mass:** 24203    **Score:** 162    **Queries matched:** 12   **emPAI:** 0.34 |
| --- | --- |
|  | NADH dehydrogenase [ubiquinone] iron-sulfur protein 8, mitochondrial OS=Homo sapiens GN=NDUFS8 PE=1 SV=1 |

|  | **Query** | **Observed** | **Mr(expt)** | **Mr(calc)** | **Delta** | **Miss** | **Score** | **Expect** | **Rank** | **Peptide** |
| --- | --- | --- | --- | --- | --- | --- | --- | --- | --- | --- |
|  | 3492 | **443.2017** | **884.3889** | **884.3950** | **-0.0061** | **0** | **5** | **53** | **1** | **R.YDIDMTK.C** |
|  | 5009 | 497.3003 | 992.5860 | 992.4675 | 0.1184 | 1 | 4 | 63 | 5 | R.RYPSGEER.C |
|  | 8563 | **617.8169** | **1233.6192** | **1233.6353** | **-0.0161** | **0** | **3** | **84** | **3** | **R.SLHSSAVAATYK.Y** |
|  | 8999 | 629.3336 | 1256.6527 | 1256.6587 | -0.0060 | 0 | 39 | 0.021 | 1 | R.GLGMTLSYLFR.E |
|  | 9016 | 629.8410 | 1257.6674 | 1258.5863 | -0.9189 | 1 | 7 | 30 | 8 | R.TTRYDIDMTK.C |
|  | 13272 | **750.8072** | **1499.5998** | **1499.6095** | **-0.0096** | **0** | **12** | **6.7** | **1** | **K.YVNMQDPEMDMK.S** |
|  | 13511 | 506.2689 | 1515.7849 | 1515.6044 | 0.1806 | 0 | (7) | 25 | 2 | K.YVNMQDPEMDMK.S 13474 13503 |
|  | 20589 | **1021.5002** | **2040.9859** | **2040.9972** | **-0.0113** | **0** | **91** | **6.4e-008** | **1** | **K.LCEAICPAQAITIEAEPR.A** 20588 |
|  | 24016 | **1216.6292** | **2431.2437** | **2432.2926** | **-1.0489** | **1** | **4** | **31** | **1** | **R.TLLWTELFRGLGMTLSYLFR.E** |

| **314.** | P50395\|GDIB_HUMAN    **Mass:** 51087    **Score:** 161    **Queries matched:** 23   **emPAI:** 0.33 |
| --- | --- |
|  | Rab GDP dissociation inhibitor beta OS=Homo sapiens GN=GDI2 PE=1 SV=2 |

|  | **Query** | **Observed** | **Mr(expt)** | **Mr(calc)** | **Delta** | **Miss** | **Score** | **Expect** | **Rank** | **Peptide** |
| --- | --- | --- | --- | --- | --- | --- | --- | --- | --- | --- |
|  | 869 | 515.3262 | 514.3189 | 514.3479 | -0.0290 | 0 | 15 | 7.3 | 5 | K.VIGVK.S |
|  | 3804 | **454.2111** | **906.4076** | **905.4494** | **0.9582** | **0** | **16** | **4.3** | **1** | **R.TFEGIDPK.K** |
|  | 4219 | **469.7473** | **937.4801** | **937.4869** | **-0.0068** | **0** | **10** | **16** | **4** | **K.LYSESLAR.Y** |
|  | 6789 | 563.7894 | 1125.5642 | 1124.5900 | 0.9742 | 0 | 16 | 3.9 | 5 | K.MLLYTEVTR.Y |
|  | 6824 | **564.8027** | **1127.5908** | **1126.5441** | **1.0467** | **0** | **2** | **1.1e+002** | **1** | **K.IPGSPPESMGR.G** |
|  | 7479 | **589.8421** | **1177.6696** | **1178.6845** | **-1.0149** | **0** | **2** | **1.1e+002** | **9** | **R.VICILSHPIK.N** |
|  | 8268 | **609.8198** | **1217.6250** | **1216.7067** | **0.9182** | **0** | **4** | **64** | **4** | **K.FVSISDLLVPK.D** |
|  | 9279 | 636.3518 | 1270.6891 | 1270.6558 | 0.0333 | 1 | 3 | 77 | 6 | K.VTEGSFVYKGGK.I |
|  | 10267 | **442.2313** | **1323.6720** | **1323.7286** | **-0.0566** | **0** | **7** | **26** | **4** | **K.YIAIVSTTVETK.E** |
|  | 10841 | **452.9104** | **1355.7095** | **1355.6616** | **0.0479** | **1** | **7** | **31** | **2** | **K.IPGSPPESMGRGR.D** 10846 |
|  | 10982 | 683.3495 | 1364.6844 | 1364.6936 | -0.0092 | 0 | 42 | 0.0091 | 1 | K.DLGTESQIFISR.T |
|  | 12799 | **736.8571** | **1471.6996** | **1471.7711** | **-0.0715** | **1** | **4** | **49** | **6** | **R.KFLVYVANFDEK.D** |
|  | 13420 | **504.2787** | **1509.8143** | **1509.7320** | **0.0824** | **1** | **8** | **20** | **7** | **R.CKQLICDPSYVK.D** |
|  | 15101 | **812.4033** | **1622.7921** | **1621.6752** | **1.1169** | **1** | **8** | **20** | **4** | **K.RMTGSEFDFEEMK.R** |
|  | 17513 | **896.9383** | **1791.8620** | **1790.9277** | **0.9344** | **1** | **0** | **1.1e+002** | **4** | **K.MLLYTEVTRYLDFK.V** |
|  | 19023 | 951.8894 | 1901.7642 | 1901.8102 | -0.0459 | 0 | 12 | 5.2 | 1 | R.TDDYLDQPCYETINR.I |
|  | 19213 | **639.9392** | **1916.7956** | **1916.8098** | **-0.0142** | **0** | **2** | **59** | **3** | **R.TYDATTHFETTCDDIK.N** 19221 |
|  | 20086 | **666.3525** | **1996.0356** | **1994.9870** | **1.0486** | **0** | **1** | **76** | **9** | **K.VPSTEAEALASSLMGLFEK.R** |
|  | 21735 | 1076.0404 | 2150.0663 | 2150.0797 | -0.0134 | 0 | 61 | 6.1e-005 | 1 | K.FDLGQDVIDFTGHALALYR.T 21736 |
|  | 22896 | **760.3950** | **2278.1631** | **2278.1746** | **-0.0116** | **1** | **76** | **2e-006** | **1** | **K.KFDLGQDVIDFTGHALALYR.T** |

| **315.** | P17931\|LEG3_HUMAN    **Mass:** 26193    **Score:** 161    **Queries matched:** 14   **emPAI:** 1.25 |
| --- | --- |
|  | Galectin-3 OS=Homo sapiens GN=LGALS3 PE=1 SV=5 |

|  | **Query** | **Observed** | **Mr(expt)** | **Mr(calc)** | **Delta** | **Miss** | **Score** | **Expect** | **Rank** | **Peptide** |
| --- | --- | --- | --- | --- | --- | --- | --- | --- | --- | --- |
|  | 2604 | **416.2479** | **830.4812** | **830.4861** | **-0.0050** | **1** | **40** | **0.021** | **1** | **K.KLNEISK.L** 2601 |
|  | 3321 | **437.7090** | **873.4034** | **873.4093** | **-0.0059** | **0** | **30** | **0.22** | **1** | **K.LDNNWGR.E** 3312 3319 3320 |
|  | 4939 | **495.2779** | **988.5413** | **988.5488** | **-0.0075** | **1** | **36** | **0.049** | **1** | **R.RVIVCNTK.L** |
|  | 9327 | **637.3026** | **1272.5907** | **1272.6000** | **-0.0093** | **0** | **69** | **1.9e-005** | **1** | **R.GNDVAFHFNPR.F** |
|  | 10272 | 442.2439 | 1323.7098 | 1323.7187 | -0.0089 | 0 | 27 | 0.26 | 1 | K.IQVLVEPDHFK.V |
|  | 13229 | **749.3860** | **1496.7574** | **1496.7664** | **-0.0089** | **0** | **57** | **0.00027** | **1** | **R.QSVFPFESGKPFK.I** 13230 |
|  | 15405 | **820.9748** | **1639.9351** | **1639.9443** | **-0.0092** | **0** | **61** | **8.2e-005** | **1** | **R.MLITILGTVKPNANR.I** 15372 15422 |

| **316.** | P61204\|ARF3_HUMAN    **Mass:** 20645    **Score:** 159    **Queries matched:** 23   **emPAI:** 0.98 |
| --- | --- |
|  | ADP-ribosylation factor 3 OS=Homo sapiens GN=ARF3 PE=1 SV=2 |

|  | **Query** | **Observed** | **Mr(expt)** | **Mr(calc)** | **Delta** | **Miss** | **Score** | **Expect** | **Rank** | **Peptide** |
| --- | --- | --- | --- | --- | --- | --- | --- | --- | --- | --- |
|  | 2763 | 421.2294 | 840.4443 | 839.5130 | 0.9313 | 0 | 8 | 24 | 8 | K.IRPLWR.H |
|  | 4760 | 488.2868 | 974.5590 | 975.4695 | -0.9104 | 0 | 5 | 67 | 5 | R.MLAEDELR.D |
|  | 6295 | 544.3089 | 1086.6032 | 1086.6107 | -0.0075 | 0 | 38 | 0.027 | 1 | R.ILMVGLDAAGK.T |
|  | 6491 | 551.7946 | 1101.5746 | 1102.6056 | -1.0311 | 0 | (1) | 1.5e+002 | 5 | R.ILMVGLDAAGK.T |
|  | 6541 | 553.8106 | 1105.6065 | 1105.5954 | 0.0112 | 0 | 20 | 1.8 | 2 | -.MGNIFGNLLK.S 6536 |
|  | 6750 | **562.2648** | **1122.5150** | **1121.5903** | **0.9247** | **0** | **(9)** | **21** | **3** | **-.MGNIFGNLLK.S** 6755 |
|  | 8806 | 416.2394 | 1245.6965 | 1245.6135 | 0.0830 | 1 | 14 | 6.5 | 1 | R.VNEAREELMR.M |
|  | 12821 | 737.4216 | 1472.8287 | 1472.8715 | -0.0428 | 1 | 1 | 1e+002 | 9 | M.GNIFGNLLKSLIGK.K |
|  | 14235 | 783.3787 | 1564.7428 | 1564.7522 | -0.0094 | 0 | 50 | 0.001 | 1 | K.NISFTVWDVGGQDK.I |
|  | 15219 | 815.8859 | 1629.7573 | 1629.7668 | -0.0095 | 0 | 8 | 19 | 1 | K.QDLPNAMNAAEITDK.L 15225 |
|  | 15491 | 549.8032 | 1646.3877 | 1645.7617 | 0.6259 | 0 | (1) | 62 | 5 | K.QDLPNAMNAAEITDK.L |
|  | 15750 | 556.2821 | 1665.8245 | 1665.7702 | 0.0543 | 1 | 8 | 16 | 1 | R.EELMRMLAEDELR.D |
|  | 17881 | 608.3079 | 1821.9019 | 1822.0274 | -0.1255 | 1 | 4 | 51 | 1 | R.ILMVGLDAAGKTTILYK.L 17891 |
|  | 21757 | 718.3621 | 2152.0644 | 2152.0338 | 0.0306 | 0 | 5 | 30 | 2 | R.HYFQNTQGLIFVVDSNDR.E |
|  | 23212 | 1162.1194 | 2322.2242 | 2322.2359 | -0.0117 | 0 | 46 | 0.0018 | 1 | K.LGEIVTTIPTIGFNVETVEYK.N 23210 23211 23213 |
|  | 24055 | 813.3949 | 2437.1629 | 2437.1775 | -0.0146 | 1 | 42 | 0.0041 | 1 | R.HYFQNTQGLIFVVDSNDRER.V |

| **317.** | O00299\|CLIC1_HUMAN    **Mass:** 27248    **Score:** 158    **Queries matched:** 13   **emPAI:** 0.69 |
| --- | --- |
|  | Chloride intracellular channel protein 1 OS=Homo sapiens GN=CLIC1 PE=1 SV=4 |

|  | **Query** | **Observed** | **Mr(expt)** | **Mr(calc)** | **Delta** | **Miss** | **Score** | **Expect** | **Rank** | **Peptide** |
| --- | --- | --- | --- | --- | --- | --- | --- | --- | --- | --- |
|  | 4506 | 479.2400 | 956.4654 | 956.4715 | -0.0062 | 0 | 31 | 0.14 | 1 | R.YLSNAYAR.E 4499 |
|  | 6181 | **540.2562** | **1078.4979** | **1077.5026** | **0.9953** | **0** | **8** | **25** | **5** | **K.IGNCPFSQR.L** |
|  | 9467 | **641.3336** | **1280.6527** | **1280.6613** | **-0.0086** | **0** | **58** | **0.0002** | **1** | **K.GVTFNVTTVDTK.R** |
|  | 10354 | **664.8211** | **1327.6277** | **1327.6368** | **-0.0091** | **0** | **48** | **0.0021** | **1** | **K.NSNPALNDNLEK.G** |
|  | 13918 | **516.2606** | **1545.7600** | **1546.7701** | **-1.0101** | **0** | **0** | **1.1e+002** | **5** | **-.MAEEQPQVELFVK.A** |
|  | 15741 | **555.7060** | **1664.0961** | **1663.7737** | **0.3225** | **1** | **4** | **35** | **1** | **K.AGSDGAKIGNCPFSQR.L** |
|  | 19406 | **967.4772** | **1932.9398** | **1931.9662** | **0.9735** | **0** | **5** | **31** | **1** | **K.FLDGNELTLADCNLLPK.L** 19412 |
|  | 20793 | 1030.9955 | 2059.9764 | 2060.0612 | -0.0848 | 1 | 5 | 33 | 8 | R.KFLDGNELTLADCNLLPK.L |
|  | 23132 | 771.3987 | 2311.1742 | 2311.2650 | -0.0908 | 1 | 3 | 35 | 10 | R.LFMVLWLKGVTFNVTTVDTK.R |
|  | 25081 | **1287.0573** | **2572.0999** | **2572.1163** | **-0.0163** | **0** | **65** | **2e-005** | **1** | **R.EEFASTCPDDEEIELAYEQVAK.A** 25082 |

| **318.** | P62841\|RS15_HUMAN    **Mass:** 17029    **Score:** 157    **Queries matched:** 8   **emPAI:** 0.51 |
| --- | --- |
|  | 40S ribosomal protein S15 OS=Homo sapiens GN=RPS15 PE=1 SV=2 |

|  | **Query** | **Observed** | **Mr(expt)** | **Mr(calc)** | **Delta** | **Miss** | **Score** | **Expect** | **Rank** | **Peptide** |
| --- | --- | --- | --- | --- | --- | --- | --- | --- | --- | --- |
|  | 2626 | **417.2431** | **832.4716** | **833.3953** | **-0.9236** | **0** | **13** | **11** | **4** | **-.MAEVEQK.K** |
|  | 10416 | **666.8438** | **1331.6731** | **1331.6807** | **-0.0076** | **0** | **25** | **0.46** | **1** | **K.HGRPGIGATHSSR.F** |
|  | 10793 | **677.3536** | **1352.6927** | **1352.7010** | **-0.0082** | **0** | **19** | **1.7** | **1** | **K.EAPPMEKPEVVK.T** 10798 |
|  | 25151 | **1294.6168** | **2587.2191** | **2587.2298** | **-0.0107** | **0** | **90** | **7.2e-008** | **1** | **R.GVDLDQLLDMSYEQLMQLYSAR.Q** 25152 25159 |
|  | 28085 | **792.9123** | **3167.6201** | **3167.6365** | **-0.0165** | **0** | **14** | **1.7** | **1** | **K.TFNQVEIKPEMIGHYLGEFSITYKPVK.H** |

| **319.** | P07954\|FUMH_HUMAN    **Mass:** 54773    **Score:** 157    **Queries matched:** 17   **emPAI:** 0.39 |
| --- | --- |
|  | Fumarate hydratase, mitochondrial OS=Homo sapiens GN=FH PE=1 SV=3 |

|  | **Query** | **Observed** | **Mr(expt)** | **Mr(calc)** | **Delta** | **Miss** | **Score** | **Expect** | **Rank** | **Peptide** |
| --- | --- | --- | --- | --- | --- | --- | --- | --- | --- | --- |
|  | 1381 | **619.2018** | **618.1946** | **618.3337** | **-0.1391** | **0** | **6** | **65** | **4** | **K.NGSTLK.E** |
|  | 2119 | **398.7226** | **795.4306** | **795.4351** | **-0.0046** | **0** | **7** | **30** | **6** | **K.NVLHSAR.L** |
|  | 2203 | **402.2052** | **802.3958** | **801.4531** | **0.9428** | **1** | **13** | **10** | **1** | **R.IKAAMPR.I** |
|  | 2884 | 425.2730 | 848.5314 | 847.4803 | 1.0511 | 0 | 9 | 22 | 1 | K.EFAQIIK.I |
|  | 4530 | 479.7605 | 957.5064 | 956.4716 | 1.0349 | 0 | 27 | 0.37 | 1 | K.YYGAQTVR.S |
|  | 7961 | **602.8115** | **1203.6084** | **1203.6169** | **-0.0085** | **0** | **3** | **74** | **4** | **R.AIEMLGGELGSK.I** |
|  | 13108 | **745.8546** | **1489.6945** | **1489.7049** | **-0.0103** | **0** | **5** | **43** | **2** | **R.AAAEVNQDYGLDPK.I** |
|  | 13421 | **504.2879** | **1509.8418** | **1509.7576** | **0.0842** | **1** | **1** | **88** | **3** | **K.VPNDKYYGAQTVR.S** |
|  | 15497 | **824.4169** | **1646.8193** | **1645.8345** | **0.9848** | **1** | **4** | **49** | **2** | **K.IANAIMKAADEVAEGK.L** |
|  | 17112 | 882.4616 | 1762.9087 | 1762.9214 | -0.0127 | 0 | 42 | 0.0068 | 1 | R.IYELAAGGTAVGTGLNTR.I |
|  | 21492 | **1063.0400** | **2124.0655** | **2124.0772** | **-0.0117** | **0** | **44** | **0.0036** | **1** | **R.SGLGELILPENEPGSSIMPGK.V** 21491 |
|  | 22708 | **753.7182** | **2258.1328** | **2258.1439** | **-0.0111** | **0** | **47** | **0.0017** | **1** | **K.LMNESLMLVTALNPHIGYDK.A** 22707 |
|  | 24302 | **823.4155** | **2467.2246** | **2466.2318** | **0.9928** | **0** | **16** | **1.7** | **1** | **K.ETAIELGYLTAEQFDEWVKPK.D** |
|  | 26128 | 917.8304 | 2750.4695 | 2750.3077 | 0.1618 | 0 | 2 | 39 | 3 | K.FEALAAHDALVELSGAMNTTACSLMK.I |
|  | 28512 | **1090.8627** | **3269.5662** | **3269.5888** | **-0.0226** | **0** | **66** | **1.1e-005** | **1** | **K.LNDHFPLVVWQTGSGTQTNMNVNEVISNR.A** |

| **320.** | P54709\|AT1B3_HUMAN    **Mass:** 31834    **Score:** 156    **Queries matched:** 12   **emPAI:** 0.57 |
| --- | --- |
|  | Sodium/potassium-transporting ATPase subunit beta-3 OS=Homo sapiens GN=ATP1B3 PE=1 SV=1 |

|  | **Query** | **Observed** | **Mr(expt)** | **Mr(calc)** | **Delta** | **Miss** | **Score** | **Expect** | **Rank** | **Peptide** |
| --- | --- | --- | --- | --- | --- | --- | --- | --- | --- | --- |
|  | 2422 | **818.4360** | **817.4288** | **816.4341** | **0.9947** | **0** | **10** | **21** | **10** | **K.IDGSANLK.S** |
|  | 4846 | 491.7334 | 981.4522 | 980.5477 | 0.9045 | 1 | 11 | 12 | 2 | R.VMFKITAR.A |
|  | 7433 | **588.8041** | **1175.5936** | **1174.5982** | **0.9954** | **0** | **33** | **0.077** | **1** | **K.SLNQSLAEWK.L** |
|  | 7480 | 589.8628 | 1177.7110 | 1177.7183 | -0.0072 | 0 | 44 | 0.0058 | 1 | R.IIGLKPEGVPR.I |
|  | 15193 | **814.9243** | **1627.8340** | **1626.8406** | **0.9934** | **0** | **68** | **2.1e-005** | **1** | **K.LFIYNPTTGEFLGR.T** 15174 15175 |
|  | 15690 | 831.4060 | 1660.7975 | 1661.8294 | -1.0320 | 1 | 8 | 19 | 4 | K.EVTVECKIDGSANLK.S |
|  | 16045 | **843.9086** | **1685.8026** | **1685.8148** | **-0.0122** | **1** | **61** | **8.9e-005** | **1** | **R.SDPTSYAGYIEDLKK.F** |
|  | 24175 | **1226.6650** | **2451.3155** | **2451.3274** | **-0.0119** | **0** | **15** | **1.9** | **1** | **K.LHVGYLQPLVAVQVSFAPNNTGK.E** |
|  | 25776 | **898.4685** | **2692.3837** | **2692.3935** | **-0.0098** | **0** | **3** | **30** | **5** | **R.DQIPSPGLMVFPKPVTALEYTFSR.S** |
|  | 27696 | **1025.5376** | **3073.5910** | **3072.4691** | **1.1219** | **1** | **3** | **23** | **2** | **K.NEDIPNVAVYPHNGMIDLKYFPYYGK.K** |

| **321.** | P62701\|RS4X_HUMAN    **Mass:** 29807    **Score:** 154    **Queries matched:** 16   **emPAI:** 0.43 |
| --- | --- |
|  | 40S ribosomal protein S4, X isoform OS=Homo sapiens GN=RPS4X PE=1 SV=2 |

|  | **Query** | **Observed** | **Mr(expt)** | **Mr(calc)** | **Delta** | **Miss** | **Score** | **Expect** | **Rank** | **Peptide** |
| --- | --- | --- | --- | --- | --- | --- | --- | --- | --- | --- |
|  | 2063 | **396.7504** | **791.4862** | **791.4905** | **-0.0043** | **1** | **18** | **3** | **1** | **R.KIFVGTK.G** |
|  | 2556 | **415.2027** | **828.3908** | **828.3952** | **-0.0044** | **0** | **22** | **1.2** | **1** | **K.HWMLDK.L** |
|  | 4959 | 495.7993 | 989.5840 | 989.5910 | -0.0070 | 0 | 59 | 0.00023 | 1 | R.LSNIFVIGK.G 4958 |
|  | 6108 | 537.7772 | 1073.5398 | 1073.5717 | -0.0319 | 1 | 11 | 13 | 4 | R.LTIAEERDK.R 6114 |
|  | 8203 | **608.3360** | **1214.6574** | **1214.6520** | **0.0054** | **0** | **2** | **1.1e+002** | **10** | **K.GIPHLVTHDAR.T** |
|  | 11552 | 699.2886 | 1396.5627 | 1396.7463 | -0.1835 | 1 | 5 | 43 | 9 | R.FAVHRITPEEAK.Y |
|  | 12387 | **723.3729** | **1444.7313** | **1444.7409** | **-0.0096** | **0** | **66** | **3.6e-005** | **1** | **K.VNDTIQIDLETGK.I** 12375 |
|  | 14529 | **793.8781** | **1585.7417** | **1584.8849** | **0.8568** | **1** | **7** | **27** | **3** | **K.GIPHLVTHDARTIR.Y** |
|  | 17378 | **891.9117** | **1781.8089** | **1781.8189** | **-0.0100** | **0** | **76** | **2.9e-006** | **1** | **K.FDTGNLCMVTGGANLGR.I** 17379 17396 |
|  | 17568 | **450.2528** | **1796.9823** | **1797.8138** | **-0.8316** | **0** | **(7)** | **18** | **10** | **K.FDTGNLCMVTGGANLGR.I** |
|  | 21624 | **714.3478** | **2140.0215** | **2140.0874** | **-0.0659** | **1** | **3** | **46** | **6** | **K.VRTDITYPAGFMDVISIDK.T** |

| **322.** | P62834\|RAP1A_HUMAN    **Mass:** 21316    **Score:** 154    **Queries matched:** 12   **emPAI:** 1.70 |
| --- | --- |
|  | Ras-related protein Rap-1A OS=Homo sapiens GN=RAP1A PE=1 SV=1 |

|  | **Query** | **Observed** | **Mr(expt)** | **Mr(calc)** | **Delta** | **Miss** | **Score** | **Expect** | **Rank** | **Peptide** |
| --- | --- | --- | --- | --- | --- | --- | --- | --- | --- | --- |
|  | 4894 | 493.3027 | 984.5908 | 984.5968 | -0.0060 | 0 | 50 | 0.0015 | 1 | K.LVVLGSGGVGK.S |
|  | 9013 | **420.2085** | **1257.6036** | **1257.5513** | **0.0523** | **0** | **7** | **31** | **8** | **K.YDPTIEDSYR.K** |
|  | 11362 | **693.8259** | **1385.6372** | **1385.6463** | **-0.0091** | **1** | **27** | **0.28** | **1** | **K.YDPTIEDSYRK.Q** 11356 11359 |
|  | 13182 | **747.8976** | **1493.7807** | **1493.7878** | **-0.0071** | **0** | **71** | **9.7e-006** | **1** | **K.INVNEIFYDLVR.Q** |
|  | 14723 | **800.8427** | **1599.6709** | **1599.6810** | **-0.0101** | **0** | **64** | **4.5e-005** | **1** | **R.QWCNCAFLESSAK.S** |
|  | 15636 | 829.4394 | 1656.8643 | 1656.8757 | -0.0113 | 1 | 49 | 0.0014 | 1 | R.VKDTEDVPMILVGNK.C |
|  | 15746 | **833.4597** | **1664.9048** | **1664.9138** | **-0.0090** | **0** | **34** | **0.049** | **1** | **K.SALTVQFVQGIFVEK.Y** |
|  | 15834 | **558.3147** | **1671.9223** | **1672.8706** | **-0.9483** | **1** | **(5)** | **33** | **3** | **R.VKDTEDVPMILVGNK.C** |
|  | 23528 | **788.4244** | **2362.2513** | **2363.0621** | **-0.8108** | **1** | **1** | **52** | **5** | **K.DTEDVPMILVGNKCDLEDER.V** |
|  | 29852 | **931.6119** | **3722.4184** | **3722.6759** | **-0.2574** | **1** | **1** | **19** | **3** | **K.QVEVDCQQCMLEILDTAGTEQFTAMRDLYMK.N** |

| **323.** | O60613\|SEP15_HUMAN    **Mass:** 18236    **Score:** 153    **Queries matched:** 3   **emPAI:** 0.47 |
| --- | --- |
|  | 15 kDa selenoprotein OS=Homo sapiens GN=SEP15 PE=1 SV=3 |

|  | **Query** | **Observed** | **Mr(expt)** | **Mr(calc)** | **Delta** | **Miss** | **Score** | **Expect** | **Rank** | **Peptide** |
| --- | --- | --- | --- | --- | --- | --- | --- | --- | --- | --- |
|  | 14478 | **792.3606** | **1582.7066** | **1582.7151** | **-0.0085** | **0** | **49** | **0.0015** | **1** | **K.WNTDSVEEFLSEK.L** |
|  | 15020 | **809.8862** | **1617.7578** | **1618.7960** | **-1.0382** | **0** | **1** | **1e+002** | **6** | **-.MAAGPSGCLVPAFGLR.L** |
|  | 17022 | **878.9655** | **1755.9163** | **1755.9254** | **-0.0091** | **0** | **130** | **1e-011** | **1** | **K.LLDDNGNIAEELSILK.W** |

| **324.** | P80365\|DHI2_HUMAN    **Mass:** 44612    **Score:** 153    **Queries matched:** 17   **emPAI:** 0.50 |
| --- | --- |
|  | Corticosteroid 11-beta-dehydrogenase isozyme 2 OS=Homo sapiens GN=HSD11B2 PE=1 SV=2 |

|  | **Query** | **Observed** | **Mr(expt)** | **Mr(calc)** | **Delta** | **Miss** | **Score** | **Expect** | **Rank** | **Peptide** |
| --- | --- | --- | --- | --- | --- | --- | --- | --- | --- | --- |
|  | 1123 | **563.3259** | **562.3187** | **561.2983** | **1.0204** | **1** | **6** | **60** | **8** | **R.SSRGR.I** |
|  | 10264 | **662.8274** | **1323.6402** | **1323.6493** | **-0.0091** | **0** | **28** | **0.2** | **1** | **R.AVLITGCDSGFGK.E** |
|  | 11225 | 689.8887 | 1377.7629 | 1376.8364 | 0.9265 | 1 | 3 | 75 | 6 | R.LARPQRLPVATR.A |
|  | 14537 | **529.6205** | **1585.8396** | **1585.8498** | **-0.0101** | **0** | **34** | **0.046** | **1** | **R.LLQMDLTKPGDISR.V** |
|  | 14775 | **535.3202** | **1602.9389** | **1601.8447** | **1.0942** | **0** | **(2)** | **75** | **9** | **R.LLQMDLTKPGDISR.V** |
|  | 16975 | 585.3132 | 1752.9179 | 1752.8716 | 0.0462 | 1 | 2 | 61 | 9 | R.AVLITGCDSGFGKETAK.K |
|  | 18548 | **622.6497** | **1864.9273** | **1863.9380** | **0.9893** | **0** | **34** | **0.044** | **1** | **K.DYIEHLHGQFLHSLR.L** |
|  | 18613 | 624.6384 | 1870.8935 | 1871.0298 | -0.1364 | 1 | 8 | 17 | 1 | R.LRLLQMDLTKPGDISR.V |
|  | 20537 | 680.5820 | 2038.7241 | 2039.1779 | -0.4538 | 1 | 1 | 46 | 8 | R.KQLLLANLPQELLQAYGK.D |
|  | 23331 | **1171.0559** | **2340.0973** | **2340.1130** | **-0.0158** | **0** | **72** | **4.7e-006** | **1** | **R.IVTVGSPAGDMPYPCLGAYGTSK.A** 23332 23333 |
|  | 23947 | **808.8926** | **2423.6561** | **2424.3046** | **-0.6485** | **0** | **1** | **35** | **3** | **R.LAMSDLTPVVDAITDALLAARPR.R** 23959 |
|  | 25654 | **892.4836** | **2674.4289** | **2673.4411** | **0.9878** | **1** | **9** | **6.7** | **1** | **K.KLDSMGFTVLATVLELNSPGAIELR.T** |
|  | 28792 | **1122.8107** | **3365.4102** | **3365.7226** | **-0.3124** | **1** | **1** | **26** | **3** | **K.AAVALLMDTFSCELLPWGVKVSIIQPGCFK.T** |
|  | 29025 | **859.6786** | **3434.6853** | **3433.7015** | **0.9838** | **0** | **14** | **1.6** | **1** | **K.AHTTSTGLWGLVNNAGHNEVVADAELSPVATFR.S** |

| **325.** | P16662\|UD2B7_HUMAN    **Mass:** 61168    **Score:** 151    **Queries matched:** 21   **emPAI:** 0.51 |
| --- | --- |
|  | UDP-glucuronosyltransferase 2B7 OS=Homo sapiens GN=UGT2B7 PE=1 SV=1 |

|  | **Query** | **Observed** | **Mr(expt)** | **Mr(calc)** | **Delta** | **Miss** | **Score** | **Expect** | **Rank** | **Peptide** |
| --- | --- | --- | --- | --- | --- | --- | --- | --- | --- | --- |
|  | 683 | 473.2791 | 472.2719 | 472.2758 | -0.0039 | 0 | 13 | 13 | 5 | R.GAAVR.V 688 |
|  | 4784 | 489.2638 | 976.5130 | 975.5171 | 0.9959 | 1 | 9 | 26 | 9 | K.ENVMKLSR.I |
|  | 4984 | 496.7711 | 991.5276 | 991.5120 | 0.0155 | 1 | (7) | 37 | 4 | K.ENVMKLSR.I |
|  | 6469 | 550.8162 | 1099.6178 | 1099.6237 | -0.0059 | 0 | 11 | 15 | 3 | K.TILDELIQR.G |
|  | 11919 | 709.3783 | 1416.7420 | 1416.7514 | -0.0093 | 0 | 53 | 0.00069 | 1 | K.WIPQNDLLGHPK.T 11920 |
|  | 12388 | 723.3916 | 1444.7686 | 1444.7787 | -0.0100 | 0 | 35 | 0.039 | 1 | R.IQHDQPVKPLDR.A |
|  | 13776 | **768.8846** | **1535.7547** | **1535.7653** | **-0.0106** | **1** | **28** | **0.18** | **1** | **R.VINDPSYKENVMK.L** |
|  | 13946 | **516.5950** | **1546.7631** | **1546.7740** | **-0.0109** | **0** | **33** | **0.056** | **1** | **R.FDGNKPDTLGLNTR.L** 13945 |
|  | 17169 | **884.9385** | **1767.8625** | **1767.8713** | **-0.0088** | **0** | **92** | **6.2e-008** | **1** | **R.VDFNTMSSTDLLNALK.R** 17170 |
|  | 17387 | **595.2432** | **1782.7077** | **1783.8662** | **-1.1586** | **0** | **(2)** | **57** | **4** | **R.VDFNTMSSTDLLNALK.R** 17403 17408 |
|  | 21201 | 701.3934 | 2101.1585 | 2101.1069 | 0.0516 | 1 | 1 | 65 | 8 | K.VLWRFDGNKPDTLGLNTR.L |
|  | 25990 | **908.9279** | **2723.7618** | **2723.4370** | **0.3248** | **1** | **0** | **39** | **4** | **R.IQHDQPVKPLDRAVFWIEFVMR.H** |
|  | 28608 | 1103.8486 | 3308.5241 | 3309.4476 | -0.9235 | 0 | 1 | 31 | 4 | K.EMEDFVQSSGENGVVVFSLGSMVSNMTEER.A |
|  | 29088 | **865.9232** | **3459.6635** | **3460.6506** | **-0.9871** | **1** | **4** | **16** | **2** | **K.HSGGFIFPPSYVPVVMSELTDQMTFMERVK.N** |
|  | 30463 | 1020.5077 | 4078.0017 | 4078.0233 | -0.0217 | 0 | 45 | 0.00078 | 1 | R.AFITHGGANGIYEAIYHGIPMVGIPLFADQPDNIAHMK.A |

| **326.** | Q8TE67\|ES8L3_HUMAN    **Mass:** 67219    **Score:** 151    **Queries matched:** 24   **emPAI:** 0.38 |
| --- | --- |
|  | Epidermal growth factor receptor kinase substrate 8-like protein 3 OS=Homo sapiens GN=EPS8L3 PE=1 SV=2 |

|  | **Query** | **Observed** | **Mr(expt)** | **Mr(calc)** | **Delta** | **Miss** | **Score** | **Expect** | **Rank** | **Peptide** |
| --- | --- | --- | --- | --- | --- | --- | --- | --- | --- | --- |
|  | 1028 | 546.2509 | 545.2436 | 545.2558 | -0.0121 | 0 | 8 | 59 | 10 | K.NEAGR.S |
|  | 1996 | **395.0747** | **788.1349** | **788.4214** | **-0.2866** | **1** | **8** | **27** | **6** | **R.RMLGISP.-** 2035 |
|  | 2667 | 418.2530 | 834.4915 | 835.3970 | -0.9055 | 0 | 18 | 2.9 | 2 | -.MSRPSSR.A |
|  | 5138 | **501.7734** | **1001.5322** | **1001.5393** | **-0.0071** | **0** | **22** | **1.1** | **1** | **R.ELTVVQGEK.L** |
|  | 5974 | **533.7494** | **1065.4842** | **1065.4913** | **-0.0071** | **0** | **45** | **0.0047** | **1** | **K.LFEMDAQGR.V** 5973 |
|  | 6243 | **542.7817** | **1083.5488** | **1083.5560** | **-0.0072** | **0** | **10** | **15** | **1** | **R.VQGPEDALQK.L** |
|  | 7218 | **580.3047** | **1158.5948** | **1158.5265** | **0.0683** | **1** | **5** | **59** | **6** | **R.RSSSPEDPER.D** |
|  | 8494 | **615.8042** | **1229.5938** | **1229.6040** | **-0.0102** | **0** | **5** | **45** | **3** | **R.LGSTSHFPQEK.T** 8495 |
|  | 8771 | 622.7986 | 1243.5826 | 1244.7088 | -1.1262 | 0 | 17 | 2.9 | 3 | R.TLGSLTGSQLLR.I |
|  | 11048 | **685.3531** | **1368.6916** | **1368.6997** | **-0.0081** | **0** | **5** | **45** | **2** | **K.ALEEELEQRPR.L** |
|  | 12160 | **715.8763** | **1429.7380** | **1429.7453** | **-0.0073** | **0** | **67** | **2.6e-005** | **1** | **R.DGWLQLLDIETK.E** |
|  | 12993 | **495.2975** | **1482.8705** | **1482.8082** | **0.0623** | **0** | **5** | **37** | **7** | **K.ETSAPELVHILFK.S** 12987 |
|  | 15575 | 826.9341 | 1651.8536 | 1651.8140 | 0.0396 | 1 | 2 | 76 | 8 | K.MQVLYEFEARNPR.E |
|  | 18928 | **632.3205** | **1893.9397** | **1893.9553** | **-0.0156** | **0** | **28** | **0.18** | **1** | **R.IRPGELQMLCPQEAPR.I** |
|  | 22158 | **732.4025** | **2194.1858** | **2195.2354** | **-1.0496** | **1** | **1** | **67** | **3** | **R.LATWLKETSAPELVHILFK.S** |
|  | 23696 | **598.3343** | **2389.3081** | **2390.2302** | **-0.9221** | **1** | **2** | **39** | **9** | **K.ALEEELEQRPRLGGLQPGQDR.W** |
|  | 25398 | **879.4319** | **2635.2738** | **2635.2878** | **-0.0140** | **0** | **20** | **0.66** | **1** | **R.LSSRPEEVTDWLQAENFSTATVR.T** |
|  | 25682 | **1341.1714** | **2680.3282** | **2680.3457** | **-0.0174** | **0** | **60** | **5.6e-005** | **1** | **R.SGYIPSNILEPLQPGTPGTQGQSPSR.V** |
|  | 26227 | **925.1092** | **2772.3057** | **2772.3692** | **-0.0634** | **0** | **0** | **50** | **4** | **K.THNHDPQPGDPNSRPSSPKPAQPALK.M** |
|  | 30843 | **1468.6771** | **4403.0095** | **4402.0342** | **0.9753** | **0** | **44** | **0.00085** | **1** | **R.ADWTGDEPLPYQPTFSDDWQLPEPSSQAPLGYQDPVSLR.R** |

| **327.** | P24752\|THIL_HUMAN    **Mass:** 45456    **Score:** 150    **Queries matched:** 30   **emPAI:** 0.37 |
| --- | --- |
|  | Acetyl-CoA acetyltransferase, mitochondrial OS=Homo sapiens GN=ACAT1 PE=1 SV=1 |

|  | **Query** | **Observed** | **Mr(expt)** | **Mr(calc)** | **Delta** | **Miss** | **Score** | **Expect** | **Rank** | **Peptide** |
| --- | --- | --- | --- | --- | --- | --- | --- | --- | --- | --- |
|  | 727 | **484.3827** | **483.3755** | **484.3009** | **-0.9254** | **0** | **4** | **29** | **1** | **K.AGIPK.E** |
|  | 2577 | **415.2525** | **828.4904** | **828.4957** | **-0.0053** | **0** | **20** | **2** | **1** | **K.LEDLIVK.D** |
|  | 2775 | 421.7632 | 841.5119 | 840.4705 | 1.0414 | 0 | 11 | 12 | 3 | K.GQPDVVVK.E |
|  | 3169 | 865.4377 | 864.4305 | 864.4341 | -0.0037 | 0 | 13 | 9.8 | 2 | R.GSTPYGGVK.L |
|  | 4683 | 486.2483 | 970.4821 | 969.5495 | 0.9326 | 1 | 7 | 37 | 7 | K.AGIPKEEVK.E |
|  | 4743 | **974.5645** | **973.5572** | **972.5604** | **0.9968** | **0** | **21** | **1.7** | **1** | **K.EVVIVSATR.T** |
|  | 4922 | **494.7713** | **987.5281** | **988.4899** | **-0.9618** | **0** | **17** | **3.9** | **6** | **K.MLEIDPQK.V** |
|  | 6302 | **544.3413** | **1086.6681** | **1087.6502** | **-0.9821** | **0** | **6** | **49** | **1** | **R.IVGHLTHALK.Q** |
|  | 7846 | **400.2351** | **1197.6835** | **1196.7353** | **0.9482** | **1** | **13** | **7** | **1** | **M.AVLAALLRSGAR.S** 7808 |
|  | 7866 | 600.3265 | 1198.6385 | 1198.6921 | -0.0536 | 0 | 32 | 0.088 | 1 | K.LGSIAIQGAIEK.A 7849 |
|  | 10149 | **440.1978** | **1317.5716** | **1317.5805** | **-0.0089** | **0** | **(7)** | **27** | **4** | **K.IHMGSCAENTAK.K** |
|  | 10349 | 443.2708 | 1326.7905 | 1327.7758 | -0.9853 | 1 | 10 | 15 | 9 | -.MAVLAALLRSGAR.S 10357 |
|  | 10487 | **445.9170** | **1334.7292** | **1333.5755** | **1.1538** | **0** | **12** | **9.5** | **1** | **K.IHMGSCAENTAK.K** 10455 |
|  | 10630 | **672.3634** | **1342.7122** | **1343.7707** | **-1.0585** | **1** | **(7)** | **28** | **2** | **-.MAVLAALLRSGAR.S** |
|  | 11620 | **701.3934** | **1400.7723** | **1401.7868** | **-1.0145** | **0** | **23** | **0.64** | **1** | **K.FGNEVIPVTVTVK.G** 11644 |
|  | 12660 | **488.3048** | **1461.8926** | **1461.6704** | **0.2222** | **1** | **8** | **20** | **4** | **K.IHMGSCAENTAKK.L** |
|  | 18689 | **939.4377** | **1876.8608** | **1876.8737** | **-0.0129** | **0** | **70** | **9.3e-006** | **1** | **K.EAYMGNVLQGGEGQAPTR.Q** 18690 |
|  | 19480 | **970.9649** | **1939.9152** | **1940.0401** | **-0.1249** | **0** | **6** | **25** | **1** | **R.QAVLGAGLPISTPCTTINK.V** |
|  | 24704 | 840.4307 | 2518.2702 | 2518.3141 | -0.0440 | 1 | 4 | 28 | 3 | K.KEDIAMWEVNEAFSLVVLANIK.M |
|  | 26253 | **695.3395** | **2777.3291** | **2776.3549** | **0.9742** | **0** | **2** | **37** | **6** | **K.ENGTVTAANASTLNDGAAALVLMTADAAK.R** |
|  | 26430 | **940.1715** | **2817.4927** | **2817.5027** | **-0.0100** | **0** | **56** | **0.00013** | **1** | **R.IVAFADAAVEPIDFPIAPVYAASMVLK.D** 26429 |
|  | 26710 | 959.8128 | 2876.4166 | 2876.4637 | -0.0471 | 1 | 1 | 47 | 6 | K.MLEIDPQKVNINGGAVSLGHPIGMSGAR.I |
|  | 30989 | 1137.0055 | 4543.9929 | 4544.9186 | -0.9257 | 1 | 0 | 16 | 9 | K.VCASGMKAIMMASQSLMCGHQDVMVAGGMESMSNVPYVMNR.G |

| **328.** | P00441\|SODC_HUMAN    **Mass:** 16154    **Score:** 149    **Queries matched:** 7   **emPAI:** 1.36 |
| --- | --- |
|  | Superoxide dismutase [Cu-Zn] OS=Homo sapiens GN=SOD1 PE=1 SV=2 |

|  | **Query** | **Observed** | **Mr(expt)** | **Mr(calc)** | **Delta** | **Miss** | **Score** | **Expect** | **Rank** | **Peptide** |
| --- | --- | --- | --- | --- | --- | --- | --- | --- | --- | --- |
|  | 1378 | **618.3163** | **617.3090** | **617.3020** | **0.0070** | **0** | **9** | **36** | **3** | **K.ADDLGK.G** |
|  | 1673 | **689.3968** | **688.3896** | **688.3942** | **-0.0046** | **0** | **21** | **2.3** | **10** | **K.AVCVLK.G** |
|  | 13285 | **751.3809** | **1500.7472** | **1500.7573** | **-0.0101** | **0** | **54** | **0.00051** | **1** | **K.GDGPVQGIINFEQK.E** 13273 |
|  | 29244 | **880.6547** | **3518.5898** | **3518.6175** | **-0.0277** | **0** | **48** | **0.00059** | **1** | **K.GLTEGLHGFHVHEFGDNTAGCTSAGPHFNPLSR.K** |
|  | 29839 | **930.9540** | **3719.7868** | **3719.8061** | **-0.0193** | **1** | **81** | **3e-007** | **1** | **R.HVGDLGNVTADKDGVADVSIEDSVISLSGDHCIIGR.T** 29838 |

| **329.** | P27487\|DPP4_HUMAN    **Mass:** 88907    **Score:** 149    **Queries matched:** 24   **emPAI:** 0.33 |
| --- | --- |
|  | Dipeptidyl peptidase 4 OS=Homo sapiens GN=DPP4 PE=1 SV=2 |

|  | **Query** | **Observed** | **Mr(expt)** | **Mr(calc)** | **Delta** | **Miss** | **Score** | **Expect** | **Rank** | **Peptide** |
| --- | --- | --- | --- | --- | --- | --- | --- | --- | --- | --- |
|  | 2499 | 825.4518 | 824.4445 | 825.3691 | -0.9246 | 0 | 3 | 69 | 7 | K.MGFVDNK.R |
|  | 2980 | **428.7452** | **855.4757** | **855.4814** | **-0.0057** | **0** | **10** | **15** | **1** | **K.AGAVNPTVK.F** |
|  | 3570 | 446.2272 | 890.4398 | 891.5542 | -1.1143 | 1 | 4 | 81 | 8 | R.LKLYSLR.W |
|  | 4639 | **484.2553** | **966.4960** | **966.5022** | **-0.0062** | **0** | **19** | **2** | **2** | **K.IQLSDYTK.V** |
|  | 4870 | **492.2818** | **982.5490** | **981.4702** | **1.0788** | **1** | **10** | **15** | **3** | **K.MGFVDNKR.I** |
|  | 6201 | **541.2776** | **1080.5407** | **1079.5247** | **1.0160** | **0** | **1** | **1.1e+002** | **8** | **K.IISNEEGYR.H** |
|  | 6500 | **552.2785** | **1102.5424** | **1102.5506** | **-0.0081** | **0** | **47** | **0.0034** | **1** | **R.VLEDNSALDK.M** |
|  | 6918 | **568.3108** | **1134.6071** | **1135.5105** | **-0.9034** | **1** | **5** | **55** | **2** | **K.GTDDATADSRK.T** |
|  | 7419 | **588.2948** | **1174.5750** | **1174.5838** | **-0.0088** | **0** | **49** | **0.002** | **1** | **K.MLQNVQMPSK.K** |
|  | 8790 | **623.3354** | **1244.6562** | **1244.6652** | **-0.0090** | **1** | **41** | **0.011** | **1** | **R.KTYTLTDYLK.N** 8793 |
|  | 10786 | **451.8847** | **1352.6324** | **1352.6401** | **-0.0077** | **0** | **25** | **0.41** | **1** | **R.WISDHEYLYK.Q** |
|  | 13282 | 501.2423 | 1500.7049 | 1500.7144 | -0.0094 | 0 | 4 | 54 | 2 | R.QHIEMSTTGWVGR.F |
|  | 13406 | **755.8226** | **1509.6306** | **1509.6412** | **-0.0106** | **0** | **45** | **0.0034** | **1** | **R.WEYYDSVYTER.Y** 13405 |
|  | 20585 | **681.3255** | **2040.9547** | **2040.9694** | **-0.0147** | **0** | **31** | **0.074** | **1** | **R.FRPSEPHFTLDGNSFYK.I** |
|  | 23335 | 1171.0901 | 2340.1656 | 2340.1750 | -0.0094 | 0 | 49 | 0.00086 | 1 | R.LNWATYLASTENIIVASFDGR.G 23334 23336 |
|  | 25583 | **667.8334** | **2667.3047** | **2667.3115** | **-0.0069** | **1** | **1** | **50** | **3** | **K.YYQLRCSGPGLPLYTLHSSVNDK.G** |
|  | 26255 | **926.9187** | **2777.7343** | **2777.4000** | **0.3343** | **1** | **2** | **25** | **8** | **K.RIAIWGWSYGGYVTSMVLGSGSGVFK.C** |
|  | 27475 | **1011.1450** | **3030.4132** | **3029.5247** | **0.8886** | **1** | **3** | **24** | **1** | **K.ADTVFRLNWATYLASTENIIVASFDGR.G** |
|  | 27608 | 1018.1352 | 3051.3837 | 3050.3321 | 1.0517 | 1 | 3 | 23 | 3 | R.IQNYSVMDICDYDESSGRWNCLVAR.Q 27609 |

| **330.** | P54920\|SNAA_HUMAN    **Mass:** 33667    **Score:** 149    **Queries matched:** 7   **emPAI:** 0.24 |
| --- | --- |
|  | Alpha-soluble NSF attachment protein OS=Homo sapiens GN=NAPA PE=1 SV=3 |

|  | **Query** | **Observed** | **Mr(expt)** | **Mr(calc)** | **Delta** | **Miss** | **Score** | **Expect** | **Rank** | **Peptide** |
| --- | --- | --- | --- | --- | --- | --- | --- | --- | --- | --- |
|  | 8912 | **627.2909** | **1252.5672** | **1252.5757** | **-0.0085** | **0** | **9** | **19** | **1** | **K.IEEACEIYAR.A** |
|  | 12631 | 731.3353 | 1460.6561 | 1459.6619 | 0.9942 | 0 | 22 | 0.82 | 1 | K.YEELFPAFSDSR.E |
|  | 17574 | **600.2753** | **1797.8042** | **1798.9287** | **-1.1246** | **1** | **6** | **23** | **1** | **R.AIEIYTDMGRFTIAAK.H** |
|  | 20938 | **1039.5216** | **2077.0287** | **2077.0401** | **-0.0115** | **0** | **90** | **9.9e-008** | **1** | **K.AIDIYEQVGTNAMDSPLLK.Y** 20937 |
|  | 24093 | **1222.5377** | **2443.0609** | **2442.1862** | **0.8747** | **0** | **1** | **54** | **8** | **K.NWSAAGNAFCQAAQLHLQLQSK.H** |
|  | 25031 | **854.7107** | **2561.1102** | **2561.1306** | **-0.0203** | **1** | **62** | **3.6e-005** | **1** | **K.AIAHYEQSADYYKGEESNSSANK.C** |

| **331.** | P06748\|NPM_HUMAN    **Mass:** 32726    **Score:** 149    **Queries matched:** 12   **emPAI:** 0.39 |
| --- | --- |
|  | Nucleophosmin OS=Homo sapiens GN=NPM1 PE=1 SV=2 |

|  | **Query** | **Observed** | **Mr(expt)** | **Mr(calc)** | **Delta** | **Miss** | **Score** | **Expect** | **Rank** | **Peptide** |
| --- | --- | --- | --- | --- | --- | --- | --- | --- | --- | --- |
|  | 2475 | **412.6961** | **823.3776** | **822.4236** | **0.9541** | **1** | **2** | **94** | **7** | **K.GQESFKK.Q** |
|  | 3528 | **444.7127** | **887.4109** | **886.4872** | **0.9237** | **1** | **10** | **22** | **6** | **K.SIRDTPAK.N** |
|  | 5393 | **512.2444** | **1022.4742** | **1022.4821** | **-0.0079** | **1** | **30** | **0.18** | **1** | **K.ADKDYHFK.V** |
|  | 6850 | 565.8030 | 1129.5914 | 1129.5979 | -0.0065 | 1 | 25 | 0.48 | 2 | K.GPSSVEDIKAK.M |
|  | 10893 | **680.5820** | **1359.1494** | **1359.6758** | **-0.5264** | **1** | **1** | **86** | **3** | **K.FINYVKNCFR.M** |
|  | 10935 | **681.8151** | **1361.6157** | **1360.7020** | **0.9137** | **1** | **2** | **94** | **4** | **K.MQASIEKGGSLPK.V** |
|  | 14274 | **784.8637** | **1567.7127** | **1567.7226** | **-0.0099** | **0** | **54** | **0.00045** | **1** | **K.VDNDENEHQLSLR.T** |
|  | 22453 | **1114.1067** | **2226.1988** | **2226.2083** | **-0.0094** | **0** | **69** | **9.5e-006** | **1** | **K.MSVQPTVSLGGFEITPPVVLR.L** 22452 |
|  | 27036 | 980.1265 | 2937.3578 | 2938.2315 | -0.8738 | 0 | 1 | 38 | 3 | -.MEDSMDMDMSPLRPQNYLFGCELK.A |
|  | 27849 | **1041.5078** | **3121.5016** | **3121.3501** | **0.1515** | **1** | **3** | **23** | **1** | **M.EDSMDMDMSPLRPQNYLFGCELKADK.D** |
|  | 27853 | **781.4185** | **3121.6450** | **3121.3501** | **0.2949** | **1** | **(2)** | **33** | **3** | **M.EDSMDMDMSPLRPQNYLFGCELKADK.D** |

| **332.** | Q8NBQ5\|DHB11_HUMAN    **Mass:** 33285    **Score:** 149    **Queries matched:** 16   **emPAI:** 0.90 |
| --- | --- |
|  | Estradiol 17-beta-dehydrogenase 11 OS=Homo sapiens GN=HSD17B11 PE=1 SV=2 |

|  | **Query** | **Observed** | **Mr(expt)** | **Mr(calc)** | **Delta** | **Miss** | **Score** | **Expect** | **Rank** | **Peptide** |
| --- | --- | --- | --- | --- | --- | --- | --- | --- | --- | --- |
|  | 3388 | **440.2385** | **878.4624** | **877.4731** | **0.9892** | **0** | **11** | **15** | **3** | **K.AFLPAMTK.N** |
|  | 4281 | **942.4873** | **941.4800** | **941.4858** | **-0.0058** | **0** | **18** | **2.4** | **1** | **R.LTAYEFAK.L** 4280 |
|  | 5118 | **500.7920** | **999.5694** | **999.5753** | **-0.0059** | **0** | **34** | **0.066** | **1** | **K.LVLWDINK.H** |
|  | 7322 | **584.7702** | **1167.5258** | **1168.6274** | **-1.1016** | **0** | **33** | **0.07** | **1** | **R.LMHGILTEQK.M** 7336 |
|  | 7516 | **395.0747** | **1182.2023** | **1182.6648** | **-0.4625** | **1** | **10** | **9** | **3** | **R.LTAYEFAKLK.S** |
|  | 7628 | **594.6350** | **1187.2553** | **1186.6056** | **0.6497** | **1** | **0** | **98** | **4** | **K.FDAVIGYKMK.A** |
|  | 14331 | 786.9415 | 1571.8685 | 1571.8770 | -0.0085 | 0 | 36 | 0.034 | 1 | K.TLTDELAALQITGVK.T 14330 |
|  | 16126 | 846.9362 | 1691.8578 | 1691.8672 | -0.0094 | 0 | 76 | 3.1e-006 | 1 | K.TFEVNVLAHFWTTK.A 16127 |
|  | 18146 | **920.0045** | **1837.9945** | **1838.0012** | **-0.0067** | **0** | **49** | **0.0014** | **1** | **K.MIFIPSSIAFLTTLER.I** |
|  | 19453 | **969.9856** | **1937.9566** | **1937.9694** | **-0.0128** | **0** | **39** | **0.011** | **1** | **K.NPSTSLGPTLEPEEVVNR.L** 19454 |
|  | 22457 | 743.3497 | 2227.0272 | 2226.0375 | 0.9896 | 1 | 3 | 39 | 1 | K.VHTFVVDCSNREDIYSSAK.K |

| **333.** | Q07021\|C1QBP_HUMAN    **Mass:** 31742    **Score:** 148    **Queries matched:** 9   **emPAI:** 0.25 |
| --- | --- |
|  | Complement component 1 Q subcomponent-binding protein, mitochondrial OS=Homo sapiens GN=C1QBP PE=1 SV=1 |

|  | **Query** | **Observed** | **Mr(expt)** | **Mr(calc)** | **Delta** | **Miss** | **Score** | **Expect** | **Rank** | **Peptide** |
| --- | --- | --- | --- | --- | --- | --- | --- | --- | --- | --- |
|  | 4504 | **479.2334** | **956.4522** | **957.5607** | **-1.1085** | **0** | **7** | **37** | **8** | **R.VLGSSVAGLR.A** |
|  | 16192 | **849.4166** | **1696.8187** | **1696.8308** | **-0.0121** | **1** | **71** | **9.3e-006** | **1** | **K.AFVDFLSDEIKEER.K** 16193 |
|  | 19111 | **637.2454** | **1908.7143** | **1907.7019** | **1.0123** | **0** | **1** | **60** | **6** | **R.GPCACGCGCGSLHTDGDK.A** |
|  | 22944 | **1144.0815** | **2286.1485** | **2286.1631** | **-0.0146** | **0** | **50** | **0.00082** | **1** | **K.VEEQEPELTSTPNFVVEVIK.N** 22943 22945 |
|  | 27835 | **1040.1370** | **3117.3891** | **3117.4026** | **-0.0135** | **0** | **18** | **0.76** | **1** | **K.DTNYTLNTDSLDWALYDHLMDFLADR.G** |
|  | 27904 | **1046.1339** | **3135.3799** | **3135.3979** | **-0.0180** | **0** | **14** | **1.7** | **1** | **K.ALVLDCHYPEDEVGQEDEAESDIFSIR.E** |

| **334.** | P53597\|SUCA_HUMAN    **Mass:** 36626    **Score:** 146    **Queries matched:** 21   **emPAI:** 0.48 |
| --- | --- |
|  | Succinyl-CoA ligase [GDP-forming] subunit alpha, mitochondrial OS=Homo sapiens GN=SUCLG1 PE=1 SV=4 |

|  | **Query** | **Observed** | **Mr(expt)** | **Mr(calc)** | **Delta** | **Miss** | **Score** | **Expect** | **Rank** | **Peptide** |
| --- | --- | --- | --- | --- | --- | --- | --- | --- | --- | --- |
|  | 2563 | 415.2103 | 828.4061 | 828.4705 | -0.0644 | 0 | 4 | 78 | 5 | K.LVGGTTPGK.G |
|  | 2973 | **428.7234** | **855.4322** | **856.5243** | **-1.0920** | **1** | **5** | **56** | **10** | **K.GRIGIVSR.S** |
|  | 5397 | **512.2654** | **1022.5162** | **1022.5219** | **-0.0057** | **0** | **8** | **28** | **4** | **K.IICQGFTGK.Q** |
|  | 6446 | **550.2769** | **1098.5393** | **1097.5652** | **0.9741** | **0** | **5** | **52** | **3** | **R.MGHAGAIIAGGK.G** 6429 |
|  | 6689 | **559.7864** | **1117.5582** | **1117.6066** | **-0.0484** | **0** | **7** | **32** | **5** | **K.IGIMPGHIHK.K** |
|  | 7020 | **572.9955** | **1143.9765** | **1143.6400** | **0.3365** | **0** | **11** | **9.3** | **1** | **R.SFLLPQNGIR.H** 7021 |
|  | 8668 | **620.2931** | **1238.5717** | **1237.6713** | **0.9004** | **1** | **17** | **2.5** | **1** | **R.RMGHAGAIIAGGK.G** 8673 |
|  | 14261 | **784.4278** | **1566.8410** | **1566.8519** | **-0.0108** | **0** | **63** | **6.4e-005** | **1** | **K.GGQTHLGLPVFNTVK.E** 14260 14262 |
|  | 16148 | **847.9047** | **1693.7948** | **1693.8060** | **-0.0112** | **0** | **26** | **0.28** | **1** | **K.QGTFHSQQALEYGTK.L** |
|  | 16576 | **862.9216** | **1723.8287** | **1723.8386** | **-0.0099** | **0** | **32** | **0.063** | **1** | **R.LIGPNCPGVINPGECK.I** |
|  | 23913 | **1211.1503** | **2420.2860** | **2420.2985** | **-0.0125** | **0** | **69** | **8.7e-006** | **1** | **K.ISALQSAGVVVSMSPAQLGTTIYK.E** 23914 |
|  | 25555 | **888.1030** | **2661.2871** | **2661.4119** | **-0.1248** | **1** | **1** | **54** | **2** | **M.TATLAAAADIATMVSGSSGLAAARLLSR.S** |
|  | 26323 | 931.4646 | 2791.3720 | 2792.4524 | -1.0804 | 1 | 5 | 21 | 2 | -.MTATLAAAADIATMVSGSSGLAAARLLSR.S 26329 26330 |

| **335.** | P31150\|GDIA_HUMAN    **Mass:** 51177    **Score:** 146    **Queries matched:** 12   **emPAI:** 0.24 |
| --- | --- |
|  | Rab GDP dissociation inhibitor alpha OS=Homo sapiens GN=GDI1 PE=1 SV=2 |

|  | **Query** | **Observed** | **Mr(expt)** | **Mr(calc)** | **Delta** | **Miss** | **Score** | **Expect** | **Rank** | **Peptide** |
| --- | --- | --- | --- | --- | --- | --- | --- | --- | --- | --- |
|  | 4219 | 469.7473 | 937.4801 | 937.4869 | -0.0068 | 0 | 10 | 16 | 4 | K.LYSESLAR.Y |
|  | 6789 | 563.7894 | 1125.5642 | 1124.5900 | 0.9742 | 0 | 16 | 3.9 | 5 | K.MLLYTEVTR.Y |
|  | 10923 | 681.3416 | 1360.6687 | 1360.5791 | 0.0895 | 0 | 7 | 31 | 6 | R.MAGTAFDFENMK.R |
|  | 11469 | 696.8208 | 1391.6270 | 1392.5690 | -0.9419 | 0 | (6) | 38 | 3 | R.MAGTAFDFENMK.R |
|  | 12997 | **495.3874** | **1483.1404** | **1483.6613** | **-0.5209** | **0** | **1** | **67** | **4** | **K.TFEGVDPQTTSMR.D** |
|  | 15271 | **408.9293** | **1631.6879** | **1631.8090** | **-0.1210** | **1** | **13** | **6.3** | **3** | **K.RFQLLEGPPESMGR.G** 15250 |
|  | 17106 | 882.3989 | 1762.7833 | 1763.8335 | -1.0502 | 1 | 2 | 71 | 3 | R.CKQLICDPSYIPDR.V |
|  | 17513 | 896.9383 | 1791.8620 | 1790.9277 | 0.9344 | 1 | 0 | 1.1e+002 | 4 | K.MLLYTEVTRYLDFK.V |
|  | 21735 | 1076.0404 | 2150.0663 | 2150.0797 | -0.0134 | 0 | 61 | 6.1e-005 | 1 | K.FDLGQDVIDFTGHALALYR.T 21736 |
|  | 22896 | 760.3950 | 2278.1631 | 2278.1746 | -0.0116 | 1 | 76 | 2e-006 | 1 | R.KFDLGQDVIDFTGHALALYR.T |

| **336.** | Q14914\|PTGR1_HUMAN    **Mass:** 36075    **Score:** 146    **Queries matched:** 16   **emPAI:** 0.35 |
| --- | --- |
|  | Prostaglandin reductase 1 OS=Homo sapiens GN=PTGR1 PE=1 SV=2 |

|  | **Query** | **Observed** | **Mr(expt)** | **Mr(calc)** | **Delta** | **Miss** | **Score** | **Expect** | **Rank** | **Peptide** |
| --- | --- | --- | --- | --- | --- | --- | --- | --- | --- | --- |
|  | 1528 | **651.5529** | **650.5456** | **649.3581** | **1.1875** | **1** | **8** | **22** | **5** | **-.MVRTK.T** |
|  | 4550 | **480.7503** | **959.4861** | **959.4924** | **-0.0063** | **0** | **6** | **47** | **7** | **K.VVGAVGSDEK.V** |
|  | 4934 | 495.2366 | 988.4586 | 987.4886 | 0.9700 | 1 | 8 | 29 | 4 | R.WQGDARQK.A |
|  | 7538 | **395.2157** | **1182.6252** | **1182.6972** | **-0.0720** | **1** | **8** | **23** | **2** | **K.VVESKNVALPK.G** 7524 |
|  | 9406 | **426.5696** | **1276.6871** | **1275.6922** | **0.9949** | **1** | **20** | **1.6** | **1** | **K.TVESLEETLKK.A** |
|  | 9709 | **647.7872** | **1293.5599** | **1293.5693** | **-0.0094** | **0** | **44** | **0.0058** | **1** | **K.EGDTMMGQQVAK.V** 9708 9721 |
|  | 10528 | **446.7761** | **1337.3064** | **1337.6761** | **-0.3697** | **0** | **3** | **47** | **9** | **R.IAICGAISTYNR.T** |
|  | 13768 | 512.5867 | 1534.7382 | 1534.7483 | -0.0102 | 1 | 25 | 0.38 | 1 | R.LKEGDTMMGQQVAK.V 13767 |
|  | 15579 | **827.3932** | **1652.7718** | **1652.7835** | **-0.0117** | **0** | **41** | **0.0093** | **1** | **K.HFVGYPTNSDFELK.T** |
|  | 20895 | **1037.0667** | **2072.1187** | **2072.1306** | **-0.0119** | **0** | **18** | **1.3** | **1** | **R.TGPLPPGPPPEIVIYQELR.M** |
|  | 22116 | **1096.0128** | **2190.0111** | **2190.0199** | **-0.0088** | **0** | **78** | **1.5e-006** | **1** | **K.EYIIEGFENMPAAFMGMLK.G** 22115 |

| **337.** | P24539\|AT5F1_HUMAN    **Mass:** 28947    **Score:** 146    **Queries matched:** 10   **emPAI:** 0.63 |
| --- | --- |
|  | ATP synthase subunit b, mitochondrial OS=Homo sapiens GN=ATP5F1 PE=1 SV=2 |

|  | **Query** | **Observed** | **Mr(expt)** | **Mr(calc)** | **Delta** | **Miss** | **Score** | **Expect** | **Rank** | **Peptide** |
| --- | --- | --- | --- | --- | --- | --- | --- | --- | --- | --- |
|  | 6153 | **539.2743** | **1076.5340** | **1076.5403** | **-0.0063** | **0** | **22** | **1** | **1** | **R.HYLFDVQR.N** |
|  | 11317 | **692.3597** | **1382.7049** | **1382.7154** | **-0.0105** | **0** | **65** | **4.5e-005** | **1** | **K.HVVQSISTQQEK.E** 11318 |
|  | 13350 | 753.8580 | 1505.7014 | 1505.7119 | -0.0105 | 0 | 25 | 0.37 | 1 | R.LDYHISVQNMMR.R 13352 |
|  | 16165 | **848.4312** | **1694.8479** | **1694.8587** | **-0.0109** | **0** | **53** | **0.00058** | **1** | **K.QASIQHIQNAIDTEK.S** 16161 16164 |
|  | 19311 | **642.6766** | **1925.0079** | **1925.0218** | **-0.0139** | **1** | **38** | **0.015** | **1** | **K.HVVQSISTQQEKETIAK.C** |
|  | 25778 | **898.5914** | **2692.7525** | **2693.5228** | **-0.7703** | **1** | **1** | **33** | **1** | **R.VVLSAAATAAPSLKNAAFLGPGVLQATR.T** |

| **338.** | P12956\|KU70_HUMAN    **Mass:** 70084    **Score:** 145    **Queries matched:** 15   **emPAI:** 0.17 |
| --- | --- |
|  | ATP-dependent DNA helicase 2 subunit 1 OS=Homo sapiens GN=XRCC6 PE=1 SV=2 |

|  | **Query** | **Observed** | **Mr(expt)** | **Mr(calc)** | **Delta** | **Miss** | **Score** | **Expect** | **Rank** | **Peptide** |
| --- | --- | --- | --- | --- | --- | --- | --- | --- | --- | --- |
|  | 1064 | **551.3150** | **550.3077** | **550.3115** | **-0.0038** | **0** | **10** | **17** | **1** | **R.AYGLK.S** |
|  | 2326 | 406.2100 | 810.4054 | 809.4032 | 1.0022 | 0 | 11 | 11 | 1 | R.SQIYGSR.Q |
|  | 3628 | 448.7232 | 895.4319 | 895.4473 | -0.0154 | 1 | 11 | 12 | 9 | R.KMPFTEK.I |
|  | 5196 | **503.7832** | **1005.5518** | **1004.5542** | **0.9976** | **0** | **15** | **5.7** | **2** | **R.ILELDQFK.G** |
|  | 5205 | 504.2708 | 1006.5270 | 1006.5335 | -0.0065 | 0 | 17 | 3.4 | 1 | R.LGSLVDEFK.E |
|  | 7406 | **587.4091** | **1172.8036** | **1171.6812** | **1.1224** | **1** | **63** | **6.6e-005** | **1** | **K.KQELLEALTK.H** |
|  | 8043 | **604.3185** | **1206.6224** | **1206.6496** | **-0.0272** | **0** | **2** | **88** | **3** | **R.DSLIFLVDASK.A** |
|  | 13561 | **760.8620** | **1519.7094** | **1519.7195** | **-0.0100** | **0** | **12** | **8.7** | **1** | **K.ELVYPPDYNPEGK.V** |
|  | 14350 | 787.4092 | 1572.8038 | 1572.8147 | -0.0109 | 0 | 67 | 2.8e-005 | 1 | K.NIYVLQELDNPGAK.R 14364 |
|  | 19025 | **951.9297** | **1901.8448** | **1901.8578** | **-0.0129** | **0** | **73** | **5.2e-006** | **1** | **R.IMLFTNEDNPHGNDSAK.A** |
|  | 19379 | 966.0837 | 1930.1529 | 1929.9982 | 0.1547 | 1 | 2 | 50 | 2 | K.AGDLRDTGIFLDLMHLK.K |
|  | 20376 | **1013.9580** | **2025.9013** | **2025.0782** | **0.8231** | **1** | **5** | **30** | **2** | **K.IISSDRDLLAVVFYGTEK.D** |
|  | 21555 | **711.3586** | **2131.0539** | **2131.1354** | **-0.0815** | **0** | **3** | **48** | **3** | **K.IQVTPPGFQLVFLPFADDK.R** |
|  | 29329 | **1181.5977** | **3541.7712** | **3541.5629** | **0.2083** | **0** | **4** | **16** | **2** | **R.FQDMMGHGSDYSLSEVLWVCANLFSDVQFK.M** |

| **339.** | Q93084\|AT2A3_HUMAN    **Mass:** 115444   **Score:** 144    **Queries matched:** 19   **emPAI:** 0.06 |
| --- | --- |
|  | Sarcoplasmic/endoplasmic reticulum calcium ATPase 3 OS=Homo sapiens GN=ATP2A3 PE=1 SV=2 |

|  | **Query** | **Observed** | **Mr(expt)** | **Mr(calc)** | **Delta** | **Miss** | **Score** | **Expect** | **Rank** | **Peptide** |
| --- | --- | --- | --- | --- | --- | --- | --- | --- | --- | --- |
|  | 2840 | 424.6559 | 847.2973 | 846.4382 | 0.8591 | 0 | 6 | 50 | 8 | K.GTAVAICR.R |
|  | 9160 | **633.3097** | **1264.6048** | **1265.7707** | **-1.1659** | **1** | **3** | **65** | **9** | **K.VPADLRLIEIK.S** 9179 |
|  | 10542 | **447.1784** | **1338.5134** | **1339.7030** | **-1.1896** | **1** | **5** | **38** | **4** | **R.CLALATRDAPPR.K** |
|  | 10622 | **672.3282** | **1342.6418** | **1341.7365** | **0.9054** | **1** | **5** | **39** | **1** | **R.VGSRTAPLTPTSR.E** |
|  | 11377 | **463.0054** | **1385.9945** | **1385.7125** | **0.2819** | **1** | **6** | **24** | **1** | **R.AIYSNMKQFIR.Y** 11371 |
|  | 12671 | **488.5741** | **1462.7006** | **1461.7650** | **0.9356** | **1** | **2** | **79** | **10** | **K.MFVKGAPESVIER.C** |
|  | 13322 | 752.8751 | 1503.7356 | 1502.8126 | 0.9229 | 1 | 3 | 64 | 5 | K.KAEIGIAMGSGTAVAK.S |
|  | 13396 | 755.3278 | 1508.6411 | 1508.7293 | -0.0882 | 0 | 14 | 4.7 | 2 | K.MNVFDTDLQALSR.V 13400 |
|  | 13542 | 507.0747 | 1518.2024 | 1518.8076 | -0.6052 | 1 | (0) | 81 | 10 | K.KAEIGIAMGSGTAVAK.S |
|  | 13573 | **761.3759** | **1520.7371** | **1521.7973** | **-1.0602** | **0** | **10** | **13** | **4** | **-.MEAAHLLPAADVLR.H** |
|  | 13626 | **509.2756** | **1524.8050** | **1524.7242** | **0.0807** | **0** | **(3)** | **56** | **8** | **K.MNVFDTDLQALSR.V** |
|  | 15058 | 810.9055 | 1619.7964 | 1619.8076 | -0.0113 | 0 | 51 | 0.00091 | 1 | K.VGEATETALTCLVEK.M |
|  | 19616 | 976.9629 | 1951.9112 | 1951.9231 | -0.0119 | 0 | 71 | 6.8e-006 | 1 | R.SLPSVETLGCTSVICSDK.T 19617 |
|  | 21460 | 707.6777 | 2120.0112 | 2120.1325 | -0.1213 | 1 | 4 | 33 | 3 | K.STTLRVDQSILTGESVSVTK.H |
|  | 31245 | **1618.2664** | **4851.7773** | **4852.2453** | **-0.4680** | **1** | **1** | **9.5** | **6** | **K.TGTLTTNQMSVCRMFVVAEADAGSCLLHEFTISGTTYTPEGEVR.Q** |

| **340.** | P04632\|CPNS1_HUMAN    **Mass:** 28469    **Score:** 144    **Queries matched:** 11   **emPAI:** 0.28 |
| --- | --- |
|  | Calpain small subunit 1 OS=Homo sapiens GN=CAPNS1 PE=1 SV=1 |

|  | **Query** | **Observed** | **Mr(expt)** | **Mr(calc)** | **Delta** | **Miss** | **Score** | **Expect** | **Rank** | **Peptide** |
| --- | --- | --- | --- | --- | --- | --- | --- | --- | --- | --- |
|  | 2309 | 809.4545 | 808.4472 | 807.4279 | 1.0193 | 0 | 6 | 39 | 6 | R.WQAIYK.Q |
|  | 24075 | **611.0605** | **2440.2129** | **2440.0424** | **0.1705** | **1** | **3** | **37** | **2** | **R.RYSDESGNMDFDNFISCLVR.L** |
|  | 24129 | **816.4334** | **2446.2782** | **2446.2856** | **-0.0074** | **0** | **4** | **32** | **1** | **R.ILGGVISAISEAAAQYNPEPPPPR.T** |
|  | 24727 | **1262.1226** | **2522.2306** | **2522.2396** | **-0.0091** | **0** | **140** | **7.3e-013** | **1** | **R.LFAQLAGDDMEVSATELMNILNK.V** 24728 |
|  | 29415 | **1194.2560** | **3579.7461** | **3580.6535** | **-0.9074** | **0** | **(3)** | **20** | **2** | **K.GGGGGGGGGGGLGGGLGNVLGGLISGAGGGGGGGGGGGGGGGGGGGGTAMR.I** |
|  | 29466 | **1200.1368** | **3597.3887** | **3596.6485** | **0.7402** | **0** | **7** | **5.1** | **1** | **K.GGGGGGGGGGGLGGGLGNVLGGLISGAGGGGGGGGGGGGGGGGGGGGTAMR.I** 29469 |
|  | 30990 | **1515.6722** | **4543.9949** | **4545.1918** | **-1.1969** | **1** | **5** | **5.7** | **2** | **M.FLVNSFLKGGGGGGGGGGGLGGGLGNVLGGLISGAGGGGGGGGGGGGGGGGGGGGTAMR.I** |
|  | 31116 | **1554.4530** | **4660.3372** | **4660.2373** | **0.0999** | **1** | **5** | **5.9** | **2** | **-.MFLVNSFLKGGGGGGGGGGGLGGGLGNVLGGLISGAGGGGGGGGGGGGGGGGGGGGTAMR.I** |
|  | 31131 | **780.3889** | **4676.2894** | **4676.2322** | **0.0572** | **1** | **(4)** | **7.3** | **2** | **-.MFLVNSFLKGGGGGGGGGGGLGGGLGNVLGGLISGAGGGGGGGGGGGGGGGGGGGGTAMR.I** |

| **341.** | P62241\|RS8_HUMAN    **Mass:** 24475    **Score:** 142    **Queries matched:** 15   **emPAI:** 0.79 |
| --- | --- |
|  | 40S ribosomal protein S8 OS=Homo sapiens GN=RPS8 PE=1 SV=2 |

|  | **Query** | **Observed** | **Mr(expt)** | **Mr(calc)** | **Delta** | **Miss** | **Score** | **Expect** | **Rank** | **Peptide** |
| --- | --- | --- | --- | --- | --- | --- | --- | --- | --- | --- |
|  | 887 | **517.3309** | **516.3237** | **517.2972** | **-0.9736** | **1** | **13** | **19** | **5** | **K.TGGKR.K** 891 907 |
|  | 2544 | **414.7300** | **827.4454** | **827.4766** | **-0.0312** | **1** | **7** | **34** | **10** | **K.RKPYHK.K** |
|  | 3400 | 440.6995 | 879.3844 | 879.5403 | -0.1558 | 1 | 7 | 35 | 8 | R.IHTVRVR.G 3395 |
|  | 4663 | **485.2529** | **968.4912** | **968.4967** | **-0.0055** | **0** | **26** | **0.38** | **1** | **K.ELEFYLR.K** |
|  | 8309 | **610.7673** | **1219.5201** | **1218.6357** | **0.8845** | **0** | **8** | **25** | **2** | **K.YELGRPAANTK.I** |
|  | 13357 | 753.8881 | 1505.7616 | 1505.7726 | -0.0110 | 0 | 59 | 0.00016 | 1 | K.ISSLLEEQFQQGK.L 13358 |
|  | 15051 | **540.6008** | **1618.7805** | **1618.7892** | **-0.0087** | **0** | **20** | **1.2** | **1** | **R.QWYESHYALPLGR.K** 15050 |
|  | 16494 | **859.9518** | **1717.8891** | **1717.8999** | **-0.0107** | **0** | **29** | **0.14** | **1** | **R.IIDVVYNASNNELVR.T** 16496 |
|  | 17438 | **894.3718** | **1786.7291** | **1786.7403** | **-0.0112** | **0** | **65** | **2.9e-005** | **1** | **R.LDVGNFSWGSECCTR.K** |

| **342.** | P01893\|HLAH_HUMAN    **Mass:** 41052    **Score:** 142    **Queries matched:** 14   **emPAI:** 0.42 |
| --- | --- |
|  | Putative HLA class I histocompatibility antigen, alpha chain H OS=Homo sapiens GN=HLA-H PE=5 SV=2 |

|  | **Query** | **Observed** | **Mr(expt)** | **Mr(calc)** | **Delta** | **Miss** | **Score** | **Expect** | **Rank** | **Peptide** |
| --- | --- | --- | --- | --- | --- | --- | --- | --- | --- | --- |
|  | 1936 | **763.1170** | **762.1097** | **762.3694** | **-0.2597** | **0** | **0** | **1.4e+002** | **7** | **R.NTQICK.A** |
|  | 11481 | 465.0193 | 1392.0362 | 1392.6707 | -0.6345 | 0 | (3) | 43 | 4 | R.SWTAADMAAQITK.R |
|  | 11799 | 705.8859 | 1409.7572 | 1408.6656 | 1.0915 | 0 | 5 | 43 | 1 | R.SWTAADMAAQITK.R 11768 11769 |
|  | 12099 | 714.3628 | 1426.7111 | 1426.7205 | -0.0093 | 0 | 11 | 9.6 | 1 | K.WAAVVVPSGEEQR.Y |
|  | 15470 | 823.4105 | 1644.8064 | 1644.8148 | -0.0084 | 0 | 4 | 42 | 2 | R.FISVGYVDDTQFVR.F |
|  | 18745 | 628.2999 | 1881.8780 | 1881.8904 | -0.0124 | 0 | 41 | 0.0072 | 1 | K.THMTHHPISDHEATLR.C |
|  | 20661 | 513.0184 | 2048.0446 | 2049.0102 | -0.9656 | 0 | 14 | 4 | 2 | R.YTCHVQHEGLPEPLTLR.W |
|  | 21343 | 1056.5170 | 2111.0194 | 2111.0299 | -0.0105 | 0 | 45 | 0.003 | 1 | R.CWALGFYPAEITLTWQR.D 21342 |
|  | 23700 | **598.5371** | **2390.1191** | **2390.1550** | **-0.0359** | **1** | **4** | **27** | **7** | **R.ADPPKTHMTHHPISDHEATLR.C** |
|  | 25408 | 1319.0913 | 2636.1681 | 2636.1838 | -0.0158 | 0 | 68 | 1.1e-005 | 1 | R.DGEDQTQDTELVETRPAGDGTFQK.W 25407 |

| **343.** | P61224\|RAP1B_HUMAN    **Mass:** 21040    **Score:** 141    **Queries matched:** 11   **emPAI:** 1.31 |
| --- | --- |
|  | Ras-related protein Rap-1b OS=Homo sapiens GN=RAP1B PE=1 SV=1 |

|  | **Query** | **Observed** | **Mr(expt)** | **Mr(calc)** | **Delta** | **Miss** | **Score** | **Expect** | **Rank** | **Peptide** |
| --- | --- | --- | --- | --- | --- | --- | --- | --- | --- | --- |
|  | 2481 | **412.7346** | **823.4546** | **824.4868** | **-1.0323** | **1** | **5** | **45** | **8** | **K.TPVPGKAR.K** |
|  | 4894 | 493.3027 | 984.5908 | 984.5968 | -0.0060 | 0 | 50 | 0.0015 | 1 | K.LVVLGSGGVGK.S |
|  | 9013 | 420.2085 | 1257.6036 | 1257.5513 | 0.0523 | 0 | 7 | 31 | 8 | K.YDPTIEDSYR.K |
|  | 11362 | 693.8259 | 1385.6372 | 1385.6463 | -0.0091 | 1 | 27 | 0.28 | 1 | K.YDPTIEDSYRK.Q 11356 11359 |
|  | 13182 | 747.8976 | 1493.7807 | 1493.7878 | -0.0071 | 0 | 71 | 9.7e-006 | 1 | K.INVNEIFYDLVR.Q |
|  | 14057 | **777.8486** | **1553.6827** | **1553.6933** | **-0.0105** | **0** | **64** | **4.6e-005** | **1** | **R.QWNNCAFLESSAK.S** |
|  | 15746 | 833.4597 | 1664.9048 | 1664.9138 | -0.0090 | 0 | 34 | 0.049 | 1 | K.SALTVQFVQGIFVEK.Y |
|  | 29696 | **1222.5665** | **3664.6778** | **3665.6721** | **-0.9944** | **1** | **6** | **9** | **1** | **K.QVEVDAQQCMLEILDTAGTEQFTAMRDLYMK.N** 29697 |

| **344.** | Q9UJS0\|CMC2_HUMAN    **Mass:** 74528    **Score:** 141    **Queries matched:** 22   **emPAI:** 0.28 |
| --- | --- |
|  | Calcium-binding mitochondrial carrier protein Aralar2 OS=Homo sapiens GN=SLC25A13 PE=1 SV=2 |

|  | **Query** | **Observed** | **Mr(expt)** | **Mr(calc)** | **Delta** | **Miss** | **Score** | **Expect** | **Rank** | **Peptide** |
| --- | --- | --- | --- | --- | --- | --- | --- | --- | --- | --- |
|  | 329 | **430.7264** | **429.7191** | **430.2288** | **-0.5097** | **0** | **2** | **1.4e+002** | **3** | **K.GAGAR.V** |
|  | 676 | **472.3208** | **471.3135** | **470.2489** | **1.0646** | **0** | **1** | **1.3e+002** | **5** | **K.AIGGGP.-** |
|  | 1549 | **656.4037** | **655.3965** | **656.3969** | **-1.0005** | **0** | **1** | **1e+002** | **8** | **R.LQVAAR.A** |
|  | 4033 | **462.2850** | **922.5554** | **921.4854** | **1.0699** | **1** | **21** | **1.3** | **1** | **K.GAKACFLR.D** |
|  | 4390 | **474.9753** | **947.9359** | **947.4746** | **0.4614** | **0** | **8** | **21** | **2** | **R.MTLADIER.I** |
|  | 4593 | **482.2637** | **962.5128** | **962.5185** | **-0.0057** | **0** | **46** | **0.0048** | **1** | **K.LTVNDFVR.D** |
|  | 7103 | **576.2759** | **1150.5372** | **1150.5447** | **-0.0075** | **0** | **31** | **0.12** | **1** | **R.YEGFFGLYR.G** |
|  | 7433 | 588.8041 | 1175.5936 | 1176.5921 | -0.9985 | 1 | 7 | 32 | 4 | R.GRMTLADIER.I |
|  | 9346 | **425.7343** | **1274.1811** | **1274.7347** | **-0.5536** | **1** | **3** | **44** | **10** | **K.AIKLTVNDFVR.D** |
|  | 9427 | 640.3346 | 1278.6546 | 1278.6456 | 0.0090 | 1 | 11 | 11 | 3 | K.AGKGEVTFEDVK.Q |
|  | 10469 | **667.9018** | **1333.7890** | **1333.7969** | **-0.0079** | **0** | **2** | **85** | **3** | **R.GLLPQLLGVAPEK.A** 10462 |
|  | 15475 | **549.3360** | **1644.9862** | **1646.0130** | **-1.0269** | **1** | **4** | **38** | **3** | **R.GLLPQLLGVAPEKAIK.L** |
|  | 19799 | **985.0173** | **1968.0201** | **1968.0316** | **-0.0115** | **0** | **77** | **1.9e-006** | **1** | **R.IAPLEEGTLPFNLAEAQR.Q** 19810 |
|  | 21255 | **702.7347** | **2105.1822** | **2104.1569** | **1.0254** | **0** | **2** | **56** | **4** | **R.FGLGSVAGAVGATAVYPIDLVK.T** |
|  | 21808 | 1078.0371 | 2154.0597 | 2153.0656 | 0.9941 | 0 | 8 | 14 | 2 | R.WFYIDFGGVKPMGSEPVPK.S 21807 |
|  | 23512 | **591.0472** | **2360.1596** | **2361.3056** | **-1.1460** | **1** | **0** | **71** | **5** | **R.FGLGSVAGAVGATAVYPIDLVKTR.M** |
|  | 23565 | **789.7613** | **2366.2620** | **2367.0841** | **-0.8221** | **1** | **2** | **47** | **4** | **K.YASIEKNGEFFMSPNDFVTR.Y** |
|  | 24015 | **811.4199** | **2431.2378** | **2430.2583** | **0.9794** | **0** | **24** | **0.28** | **1** | **R.HLTYAEFTQFLLEIQLEHAK.Q** |
|  | 25121 | **1291.5984** | **2581.1822** | **2580.2934** | **0.8888** | **0** | **35** | **0.019** | **1** | **K.FGQVTPMEVDILFQLADLYEPR.G** |

| **345.** | P50225\|ST1A1_HUMAN    **Mass:** 34289    **Score:** 140    **Queries matched:** 13   **emPAI:** 0.23 |
| --- | --- |
|  | Sulfotransferase 1A1 OS=Homo sapiens GN=SULT1A1 PE=1 SV=2 |

|  | **Query** | **Observed** | **Mr(expt)** | **Mr(calc)** | **Delta** | **Miss** | **Score** | **Expect** | **Rank** | **Peptide** |
| --- | --- | --- | --- | --- | --- | --- | --- | --- | --- | --- |
|  | 4501 | 479.2159 | 956.4173 | 957.4080 | -0.9907 | 0 | 18 | 2.7 | 1 | R.FDADYAEK.M |
|  | 5486 | 515.2902 | 1028.5659 | 1027.4579 | 1.1080 | 0 | 7 | 35 | 4 | K.MAGCSLSFR.S |
|  | 15415 | **821.3945** | **1640.7744** | **1640.7835** | **-0.0091** | **0** | **10** | **12** | **1** | **K.VHPEPGTWDSFLEK.F** |
|  | 15608 | 828.4060 | 1654.7975 | 1654.8065 | -0.0090 | 0 | 46 | 0.0031 | 1 | R.THPVLYLFYEDMK.E 15625 |
|  | 17602 | 601.0211 | 1800.0415 | 1800.0509 | -0.0094 | 0 | 18 | 1.5 | 1 | K.THLPLALLPQTLLDQK.V |
|  | 18358 | **926.9672** | **1851.9198** | **1852.9353** | **-1.0155** | **1** | **1** | **79** | **7** | **K.APGIPSGMETLKDTPAPR.L** |
|  | 19238 | 960.4421 | 1918.8697 | 1917.9870 | 0.8828 | 0 | 5 | 30 | 1 | -.MELIQDTSRPPLEYVK.G |
|  | 21485 | 708.6824 | 2123.0253 | 2123.0397 | -0.0145 | 1 | 16 | 2.3 | 1 | R.THPVLYLFYEDMKENPK.R |
|  | 23334 | 1171.0669 | 2340.1192 | 2340.1308 | -0.0115 | 0 | 104 | 3.2e-009 | 1 | K.SGTTWVSQILDMIYQGGDLEK.C 23335 |
|  | 26945 | 730.8837 | 2919.5058 | 2918.3123 | 1.1936 | 0 | 4 | 23 | 2 | K.FMVGEVSYGSWYQHVQEWWELSR.T |
|  | 27050 | **980.8386** | **2939.4940** | **2939.5069** | **-0.0129** | **0** | **2** | **35** | **1** | **K.YFAEALGPLQSFQARPDDLLISTYPK.S** |

| **346.** | P50226\|ST1A2_HUMAN    **Mass:** 34377    **Score:** 140    **Queries matched:** 13   **emPAI:** 0.23 |
| --- | --- |
|  | Sulfotransferase 1A2 OS=Homo sapiens GN=SULT1A2 PE=1 SV=1 |

|  | **Query** | **Observed** | **Mr(expt)** | **Mr(calc)** | **Delta** | **Miss** | **Score** | **Expect** | **Rank** | **Peptide** |
| --- | --- | --- | --- | --- | --- | --- | --- | --- | --- | --- |
|  | 4501 | 479.2159 | 956.4173 | 957.4080 | -0.9907 | 0 | 18 | 2.7 | 1 | R.FDADYAEK.M |
|  | 5233 | 505.2897 | 1008.5649 | 1008.6080 | -0.0431 | 1 | 13 | 7.6 | 8 | K.NTPAPRLLK.T |
|  | 5486 | 515.2902 | 1028.5659 | 1027.4579 | 1.1080 | 0 | 7 | 35 | 4 | K.MAGCSLSFR.S |
|  | 13439 | 756.8748 | 1511.7350 | 1511.6537 | 0.0813 | 0 | 2 | 75 | 2 | R.EFMDHSISPFMR.K |
|  | 15608 | 828.4060 | 1654.7975 | 1654.8065 | -0.0090 | 0 | 46 | 0.0031 | 1 | R.THPVLYLFYEDMK.E 15625 |
|  | 17602 | 601.0211 | 1800.0415 | 1800.0509 | -0.0094 | 0 | 18 | 1.5 | 1 | K.THLPLALLPQTLLDQK.V |
|  | 19238 | 960.4421 | 1918.8697 | 1917.9870 | 0.8828 | 0 | 5 | 30 | 1 | -.MELIQDTSRPPLEYVK.G |
|  | 21485 | 708.6824 | 2123.0253 | 2123.0397 | -0.0145 | 1 | 16 | 2.3 | 1 | R.THPVLYLFYEDMKENPK.R |
|  | 23334 | 1171.0669 | 2340.1192 | 2340.1308 | -0.0115 | 0 | 104 | 3.2e-009 | 1 | K.SGTTWVSQILDMIYQGGDLEK.C 23335 |
|  | 27050 | 980.8386 | 2939.4940 | 2939.5069 | -0.0129 | 0 | 2 | 35 | 1 | K.YFAEALGPLQSFQARPDDLLISTYPK.S |
|  | 30989 | 1137.0055 | 4543.9929 | 4543.0718 | 0.9210 | 1 | 1 | 15 | 5 | K.FMAGEVSYGSWYQHVQEWWELSRTHPVLYLFYEDMK.E |

| **347.** | P22314\|UBA1_HUMAN    **Mass:** 118858   **Score:** 140    **Queries matched:** 22   **emPAI:** 0.17 |
| --- | --- |
|  | Ubiquitin-like modifier-activating enzyme 1 OS=Homo sapiens GN=UBA1 PE=1 SV=3 |

|  | **Query** | **Observed** | **Mr(expt)** | **Mr(calc)** | **Delta** | **Miss** | **Score** | **Expect** | **Rank** | **Peptide** |
| --- | --- | --- | --- | --- | --- | --- | --- | --- | --- | --- |
|  | 793 | 502.2947 | 501.2875 | 500.3322 | 0.9553 | 0 | 19 | 5.4 | 5 | K.LIAGK.I 786 790 |
|  | 1395 | **623.3359** | **622.3287** | **621.3850** | **0.9437** | **1** | **14** | **5.8** | **7** | **K.KISFK.S** |
|  | 3937 | 458.7511 | 915.4876 | 915.4385 | 0.0492 | 1 | 10 | 19 | 10 | R.RCVYYR.K |
|  | 4875 | **492.3000** | **982.5854** | **983.4600** | **-0.8746** | **0** | **3** | **82** | **7** | **R.DEFEGLFK.Q** |
|  | 5356 | **510.2633** | **1018.5121** | **1017.4451** | **1.0671** | **0** | **47** | **0.0039** | **1** | **R.VGEFCHNR.G** 5340 |
|  | 8757 | **415.2274** | **1242.6604** | **1242.7183** | **-0.0579** | **0** | **3** | **73** | **3** | **R.KPLLESGTLGTK.G** |
|  | 8877 | **417.7260** | **1250.1560** | **1249.5575** | **0.5986** | **0** | **11** | **7.1** | **1** | **R.AENYDIPSADR.H** |
|  | 9896 | **435.2686** | **1302.7838** | **1303.6442** | **-0.8604** | **0** | **16** | **3.5** | **1** | **R.LDQPMTEIVSR.V** 9912 |
|  | 12382 | **723.3334** | **1444.6522** | **1444.6617** | **-0.0095** | **0** | **27** | **0.26** | **1** | **K.DNPGVVTCLDEAR.H** 12398 |
|  | 17712 | 904.9731 | 1807.9316 | 1807.9428 | -0.0112 | 0 | 72 | 7.9e-006 | 1 | R.ALPAVQQNNLDEDLIR.K |
|  | 18122 | 612.9785 | 1835.9137 | 1834.8771 | 1.0366 | 0 | 3 | 53 | 2 | R.FEVQGLQPNGEEMTLK.Q 18124 |
|  | 18763 | **942.4710** | **1882.9275** | **1882.9384** | **-0.0109** | **0** | **35** | **0.031** | **1** | **R.NEEDAAELVALAQAVNAR.A** |
|  | 19105 | **954.9963** | **1907.9780** | **1907.9881** | **-0.0100** | **0** | **36** | **0.025** | **1** | **K.SLVASLAEPDFVVTDFAK.F** |
|  | 24536 | **624.8042** | **2495.1877** | **2495.2150** | **-0.0273** | **0** | **3** | **36** | **3** | **K.LEITMLSQGVSMLYSFFMPAAK.L** |
|  | 28311 | **646.7335** | **3228.6309** | **3227.4540** | **1.1769** | **0** | **2** | **32** | **5** | **R.HGFESGDFVSFSEVQGMVELNGNQPMEIK.V** |
|  | 28411 | **1082.5470** | **3244.6192** | **3243.4489** | **1.1703** | **0** | **(1)** | **39** | **5** | **R.HGFESGDFVSFSEVQGMVELNGNQPMEIK.V** |

| **348.** | P46782\|RS5_HUMAN    **Mass:** 23033    **Score:** 139    **Queries matched:** 12   **emPAI:** 0.58 |
| --- | --- |
|  | 40S ribosomal protein S5 OS=Homo sapiens GN=RPS5 PE=1 SV=4 |

|  | **Query** | **Observed** | **Mr(expt)** | **Mr(calc)** | **Delta** | **Miss** | **Score** | **Expect** | **Rank** | **Peptide** |
| --- | --- | --- | --- | --- | --- | --- | --- | --- | --- | --- |
|  | 807 | 503.1244 | 502.1171 | 502.2863 | -0.1692 | 0 | 11 | 34 | 2 | R.AGTVR.R |
|  | 1100 | **561.0923** | **560.0850** | **559.3078** | **0.7772** | **1** | **4** | **92** | **2** | **R.NNGKK.L** |
|  | 4684 | **486.2602** | **970.5059** | **971.4858** | **-0.9799** | **0** | **4** | **62** | **4** | **K.AQCPIVER.L** |
|  | 6969 | **570.3934** | **1138.7723** | **1139.6411** | **-0.8688** | **1** | **6** | **32** | **8** | **R.QAVDVSPLRR.V** |
|  | 7448 | **589.2637** | **1176.5129** | **1176.5202** | **-0.0073** | **0** | **29** | **0.2** | **1** | **R.LTNSMMMHGR.N** |
|  | 7751 | **398.5581** | **1192.6525** | **1192.5151** | **0.1374** | **0** | **(4)** | **60** | **1** | **R.LTNSMMMHGR.N** |
|  | 9126 | **632.3370** | **1262.6595** | **1261.6567** | **1.0028** | **1** | **6** | **36** | **1** | **K.YAKYLPHSAGR.Y** |
|  | 15224 | 815.9179 | 1629.8213 | 1630.8236 | -1.0023 | 0 | 10 | 14 | 2 | K.TIAECLADELINAAK.G |
|  | 15353 | **819.8074** | **1637.6003** | **1637.7072** | **-0.1069** | **1** | **1** | **65** | **1** | **R.LTNSMMMHGRNNGK.K** |
|  | 20927 | 692.7316 | 2075.1729 | 2075.0734 | 0.0994 | 1 | 3 | 42 | 5 | R.VNQAIWLLCTGAREAAFR.N |
|  | 23207 | **1162.0708** | **2322.1270** | **2322.1380** | **-0.0109** | **0** | **97** | **1.5e-008** | **1** | **K.WSTDDVQINDISLQDYIAVK.E** |
|  | 27657 | **767.1752** | **3064.6716** | **3064.6822** | **-0.0106** | **0** | **62** | **2.7e-005** | **1** | **K.HAFEIIHLLTGENPLQVLVNAIINSGPR.E** |

| **349.** | P61313\|RL15_HUMAN    **Mass:** 24245    **Score:** 139    **Queries matched:** 16   **emPAI:** 1.08 |
| --- | --- |
|  | 60S ribosomal protein L15 OS=Homo sapiens GN=RPL15 PE=1 SV=2 |

|  | **Query** | **Observed** | **Mr(expt)** | **Mr(calc)** | **Delta** | **Miss** | **Score** | **Expect** | **Rank** | **Peptide** |
| --- | --- | --- | --- | --- | --- | --- | --- | --- | --- | --- |
|  | 3380 | **440.0291** | **878.0436** | **878.4280** | **-0.3844** | **1** | **9** | **14** | **4** | **K.KQSDVMR.F** |
|  | 5203 | 504.2659 | 1006.5173 | 1006.5236 | -0.0063 | 0 | 38 | 0.027 | 1 | K.YIQELWR.K 5202 |
|  | 5247 | **506.2561** | **1010.4976** | **1010.5046** | **-0.0070** | **0** | **14** | **5.3** | **1** | **K.FHHTIGGSR.R** 5248 5250 |
|  | 6361 | **547.2529** | **1092.4913** | **1092.5346** | **-0.0433** | **1** | **5** | **50** | **5** | **R.EMRGLTSAGR.K** |
|  | 11476 | **696.8827** | **1391.7508** | **1391.7157** | **0.0351** | **1** | **8** | **20** | **5** | **K.FARSLQSVAEER.A** |
|  | 13328 | **752.9097** | **1503.8048** | **1503.8126** | **-0.0078** | **0** | **28** | **0.18** | **1** | **K.FFEVILIDPFHK.A** 13327 |
|  | 15673 | **830.8908** | **1659.7669** | **1659.7780** | **-0.0111** | **0** | **57** | **0.00021** | **1** | **R.VLNSYWVGEDSTYK.F** 15674 |
|  | 16324 | **853.4551** | **1704.8956** | **1704.9060** | **-0.0104** | **0** | **51** | **0.00089** | **1** | **K.GATYGKPVHHGVNQLK.F** 16323 |
|  | 16521 | **574.2790** | **1719.8151** | **1718.9216** | **0.8935** | **1** | **6** | **29** | **8** | **R.RNPDTQWITKPVHK.H** |
|  | 18210 | **615.0621** | **1842.1644** | **1842.0336** | **0.1308** | **1** | **1** | **60** | **8** | **R.QLSALHRAPRPTRPDK.A** |

| **350.** | P09972\|ALDOC_HUMAN    **Mass:** 39830    **Score:** 139    **Queries matched:** 14   **emPAI:** 0.09 |
| --- | --- |
|  | Fructose-bisphosphate aldolase C OS=Homo sapiens GN=ALDOC PE=1 SV=2 |

|  | **Query** | **Observed** | **Mr(expt)** | **Mr(calc)** | **Delta** | **Miss** | **Score** | **Expect** | **Rank** | **Peptide** |
| --- | --- | --- | --- | --- | --- | --- | --- | --- | --- | --- |
|  | 1218 | **585.3762** | **584.3689** | **583.3693** | **0.9995** | **0** | **26** | **0.37** | **1** | **R.IVAPGK.G** |
|  | 1657 | 685.4552 | 684.4479 | 684.4534 | -0.0055 | 0 | 21 | 1.3 | 1 | K.GIVVGIK.V |
|  | 1921 | 762.3217 | 761.3144 | 762.4640 | -1.1495 | 0 | 7 | 41 | 1 | K.VLAAVYK.A |
|  | 3057 | 861.4448 | 860.4375 | 860.4691 | -0.0315 | 1 | 17 | 4.5 | 6 | K.WRCVLK.I |
|  | 5345 | **509.7579** | **1017.5013** | **1017.4880** | **0.0133** | **0** | **18** | **2.9** | **4** | **K.DDNGVPFVR.T** |
|  | 7925 | 401.2848 | 1200.8325 | 1199.6411 | 1.1915 | 0 | 14 | 5.1 | 3 | R.ALQASALNAWR.G 7871 7906 |
|  | 9239 | **635.3419** | **1268.6693** | **1269.7656** | **-1.0964** | **1** | **8** | **25** | **6** | **R.TIQDKGIVVGIK.V** |
|  | 12517 | 485.5969 | 1453.7690 | 1454.7517 | -0.9827 | 1 | 6 | 34 | 6 | M.PHSYPALSAEQKK.E |
|  | 12851 | **738.3574** | **1474.7003** | **1473.6922** | **1.0081** | **0** | **4** | **59** | **1** | **-.MPHSYPALSAEQK.K** |
|  | 14530 | **793.8781** | **1585.7417** | **1585.7922** | **-0.0506** | **1** | **2** | **83** | **5** | **-.MPHSYPALSAEQKK.E** |
|  | 22806 | **1137.0597** | **2272.1048** | **2272.1183** | **-0.0135** | **0** | **91** | **6.7e-008** | **1** | **K.GVVPLAGTDGETTTQGLDGLSER.C** 22807 |

| **351.** | P07737\|PROF1_HUMAN    **Mass:** 15216    **Score:** 138    **Queries matched:** 14   **emPAI:** 1.49 |
| --- | --- |
|  | Profilin-1 OS=Homo sapiens GN=PFN1 PE=1 SV=2 |

|  | **Query** | **Observed** | **Mr(expt)** | **Mr(calc)** | **Delta** | **Miss** | **Score** | **Expect** | **Rank** | **Peptide** |
| --- | --- | --- | --- | --- | --- | --- | --- | --- | --- | --- |
|  | 3559 | **445.8835** | **889.7525** | **889.5307** | **0.2218** | **0** | **7** | **27** | **6** | **K.TLVLLMGK.E** 3560 3567 |
|  | 7101 | **576.2555** | **1150.4964** | **1150.6458** | **-0.1494** | **1** | **13** | **7.2** | **5** | **K.EGVHGGLINKK.C** |
|  | 8166 | **607.3104** | **1212.6062** | **1212.6139** | **-0.0077** | **0** | **21** | **1.1** | **1** | **K.DSPSVWAAVPGK.T** 8165 8169 |
|  | 8544 | **617.2843** | **1232.5540** | **1233.7003** | **-1.1462** | **1** | **6** | **35** | **3** | **K.TDKTLVLLMGK.E** |
|  | 11241 | 690.3578 | 1378.7011 | 1378.7093 | -0.0081 | 0 | 42 | 0.0081 | 1 | K.STGGAPTFNVTVTK.T 11237 |
|  | 12780 | **735.8785** | **1469.7425** | **1469.7515** | **-0.0089** | **0** | **51** | **0.00097** | **1** | **R.SSFYVNGLTLGGQK.C** |
|  | 15141 | **542.5089** | **1624.5049** | **1624.7403** | **-0.2354** | **0** | **5** | **25** | **4** | **R.DSLLQDGEFSMDLR.T** |
|  | 15439 | **822.4666** | **1642.9187** | **1642.9294** | **-0.0108** | **0** | **69** | **1.5e-005** | **1** | **K.TFVNITPAEVGVLVGK.D** 15438 |

| **352.** | P05187\|PPB1_HUMAN    **Mass:** 58259    **Score:** 137    **Queries matched:** 25   **emPAI:** 0.28 |
| --- | --- |
|  | Alkaline phosphatase, placental type OS=Homo sapiens GN=ALPP PE=1 SV=2 |

|  | **Query** | **Observed** | **Mr(expt)** | **Mr(calc)** | **Delta** | **Miss** | **Score** | **Expect** | **Rank** | **Peptide** |
| --- | --- | --- | --- | --- | --- | --- | --- | --- | --- | --- |
|  | 1659 | 686.4152 | 685.4079 | 685.4486 | -0.0407 | 1 | 24 | 0.81 | 1 | R.ILKGQK.K |
|  | 2984 | 428.7633 | 855.5121 | 854.5086 | 1.0035 | 1 | 8 | 27 | 2 | R.LLSRNPR.G |
|  | 3979 | 460.2606 | 918.5066 | 918.5135 | -0.0069 | 0 | 58 | 0.00027 | 1 | K.SVGVVTTTR.V |
|  | 4048 | **463.2282** | **924.4419** | **923.5116** | **0.9303** | **0** | **7** | **28** | **7** | **R.FPYVALSK.T** |
|  | 4131 | **466.2321** | **930.4497** | **929.4818** | **0.9680** | **0** | **12** | **13** | **1** | **R.EAAEALGAAK.K** |
|  | 6688 | 559.7805 | 1117.5464 | 1117.5550 | -0.0086 | 0 | 44 | 0.0073 | 1 | R.GNEVISVMNR.A |
|  | 7423 | **588.3113** | **1174.6080** | **1174.6670** | **-0.0590** | **1** | **5** | **52** | **5** | **K.AGKSVGVVTTTR.V** |
|  | 8991 | **419.7763** | **1256.3069** | **1255.7037** | **0.6032** | **1** | **5** | **32** | **8** | **K.NLVQEWLAKR.Q** |
|  | 9919 | **653.3212** | **1304.6279** | **1304.6837** | **-0.0558** | **0** | **3** | **67** | **2** | **K.GNFQTIGLSAAAR.F** |
|  | 12482 | **484.7315** | **1451.1725** | **1450.6477** | **0.5248** | **0** | **7** | **15** | **1** | **R.NWYSDADVPASAR.Q** 12485 |
|  | 12789 | **736.4019** | **1470.7892** | **1469.7548** | **1.0344** | **1** | **1** | **1.1e+002** | **6** | **K.DKLGPEIPLAMDR.F** |
|  | 16356 | 854.9233 | 1707.8321 | 1707.8441 | -0.0120 | 0 | 61 | 9.5e-005 | 1 | R.VQHASPAGTYAHTVNR.N 16355 |
|  | 18412 | 928.4858 | 1854.9570 | 1855.0244 | -0.0673 | 1 | 2 | 58 | 5 | R.KAYTVLLYGNGPGYVLK.D 18406 18407 18409 18411 18413 18415 18416 18426 18430 18434 |

| **353.** | Q8NBX0\|SCPDH_HUMAN    **Mass:** 47464    **Score:** 137    **Queries matched:** 13   **emPAI:** 0.26 |
| --- | --- |
|  | Probable saccharopine dehydrogenase OS=Homo sapiens GN=SCCPDH PE=1 SV=1 |

|  | **Query** | **Observed** | **Mr(expt)** | **Mr(calc)** | **Delta** | **Miss** | **Score** | **Expect** | **Rank** | **Peptide** |
| --- | --- | --- | --- | --- | --- | --- | --- | --- | --- | --- |
|  | 3275 | **871.5188** | **870.5115** | **870.5538** | **-0.0423** | **1** | **12** | **12** | **2** | **R.VLEKAALK.L** |
|  | 6610 | **556.8006** | **1111.5866** | **1111.6250** | **-0.0384** | **1** | **3** | **68** | **4** | **R.LPWAVAGRSR.E** |
|  | 7884 | **400.8920** | **1199.6542** | **1198.6571** | **0.9971** | **1** | **6** | **37** | **6** | **R.SSRLPWAVAGR.S** |
|  | 12096 | **714.3445** | **1426.6745** | **1426.6841** | **-0.0096** | **0** | **57** | **0.00025** | **1** | **K.SAIYGFGDQSNLR.K** |
|  | 12844 | **492.2818** | **1473.8236** | **1474.8871** | **-1.0636** | **0** | **8** | **19** | **2** | **R.NVSNLKPVPLIGPK.L** |
|  | 14931 | **807.4669** | **1612.9192** | **1612.7919** | **0.1272** | **0** | **8** | **17** | **2** | **K.GYSIPFMGSDVSVVR.R** |
|  | 15234 | **544.3104** | **1629.9094** | **1628.7869** | **1.1226** | **0** | **(5)** | **42** | **6** | **K.GYSIPFMGSDVSVVR.R** |
|  | 23759 | **1200.6209** | **2399.2271** | **2399.2373** | **-0.0101** | **0** | **96** | **2e-008** | **1** | **K.GVYIIGSSGFDSIPADLGVIYTR.N** 23749 |
|  | 27047 | **980.1684** | **2937.4834** | **2938.4932** | **-1.0099** | **0** | **4** | **23** | **1** | **K.GPEAGYVATPIAMVQAAMTLLSDASHLPK.A** |
|  | 27780 | **775.6291** | **3098.4875** | **3097.5179** | **0.9696** | **0** | **27** | **0.093** | **1** | **K.MNGTLTAVESFLTIHSGPEGLSIHDGTWK.S** |
|  | 29712 | 917.9523 | 3667.7800 | 3667.8776 | -0.0976 | 1 | 5 | 11 | 3 | K.ICTQVKGPEAGYVATPIAMVQAAMTLLSDASHLPK.A 29714 |

| **354.** | P17066\|HSP76_HUMAN    **Mass:** 71440    **Score:** 137    **Queries matched:** 20   **emPAI:** 0.22 |
| --- | --- |
|  | Heat shock 70 kDa protein 6 OS=Homo sapiens GN=HSPA6 PE=1 SV=2 |

|  | **Query** | **Observed** | **Mr(expt)** | **Mr(calc)** | **Delta** | **Miss** | **Score** | **Expect** | **Rank** | **Peptide** |
| --- | --- | --- | --- | --- | --- | --- | --- | --- | --- | --- |
|  | 752 | 490.2640 | 489.2567 | 488.2343 | 1.0224 | 0 | 11 | 27 | 8 | R.GAGER.N |
|  | 1613 | **674.4667** | **673.4594** | **672.3806** | **1.0788** | **0** | **5** | **81** | **3** | **R.LVGDAAK.S** |
|  | 1755 | 713.0680 | 712.0607 | 711.4643 | 0.5964 | 1 | 5 | 29 | 6 | R.IPKVQK.L |
|  | 2227 | 804.4412 | 803.4339 | 803.4389 | -0.0050 | 0 | 23 | 1.2 | 1 | K.ITITNDK.G |
|  | 2705 | **419.3145** | **836.6145** | **836.3633** | **0.2512** | **1** | **8** | **23** | **8** | **K.MQDKCR.E** |
|  | 5339 | 509.7249 | 1017.4352 | 1017.5091 | -0.0739 | 0 | 1 | 1.5e+002 | 4 | K.GSLQEESLR.D |
|  | 6312 | **544.8260** | **1087.6374** | **1088.4846** | **-0.8472** | **1** | **5** | **55** | **7** | **K.AEDEAQRDR.V** |
|  | 8452 | 614.8128 | 1227.6111 | 1227.6207 | -0.0097 | 0 | 47 | 0.003 | 1 | R.VEILANDQGNR.T 8436 8453 |
|  | 9028 | **420.5290** | **1258.5651** | **1257.5547** | **1.0104** | **0** | **2** | **1e+002** | **8** | **K.FADTTVQSDMK.H** |
|  | 9091 | 631.3729 | 1260.7312 | 1260.6310 | 0.1002 | 1 | 14 | 5.4 | 1 | K.GSLQEESLRDK.I |
|  | 13054 | 744.3494 | 1486.6842 | 1486.6940 | -0.0098 | 0 | 50 | 0.0013 | 1 | R.TTPSYVAFTDTER.L |
|  | 13878 | 772.3791 | 1542.7437 | 1541.7296 | 1.0141 | 1 | 16 | 2.8 | 1 | R.ARFEELCSDLFR.S |
|  | 15545 | 550.9955 | 1649.9648 | 1648.9049 | 1.0599 | 1 | 5 | 36 | 8 | R.VAAKNSLEAHVFHVK.G |
|  | 15876 | 838.3638 | 1674.7130 | 1674.7234 | -0.0104 | 0 | 55 | 0.00037 | 1 | K.ATAGDTHLGGEDFDNR.L |
|  | 16073 | 844.8687 | 1687.7229 | 1686.8940 | 0.8288 | 0 | 21 | 0.79 | 1 | R.IINEPTAAAIAYGLDR.R 16072 |
|  | 17181 | 590.3544 | 1768.0413 | 1767.9016 | 0.1397 | 1 | 2 | 60 | 3 | K.SQAALNPHNTVFDAKR.L |
|  | 21625 | 714.3518 | 2140.0334 | 2141.0641 | -1.0307 | 1 | 3 | 40 | 2 | R.TTPSYVAFTDTERLVGDAAK.S |

|  | **Proteins matching a subset of these peptides:** |
| --- | --- |

|  | P48741\|HSP77_HUMAN    **Mass:** 40448    **Score:** 133    **Queries matched:** 10 |
| --- | --- |
|  | Putative heat shock 70 kDa protein 7 OS=Homo sapiens GN=HSPA7 PE=5 SV=2 |

| **355.** | Q9BQS7\|HEPH_HUMAN    **Mass:** 131221   **Score:** 136    **Queries matched:** 26   **emPAI:** 0.12 |
| --- | --- |
|  | Hephaestin OS=Homo sapiens GN=HEPH PE=2 SV=3 |

|  | **Query** | **Observed** | **Mr(expt)** | **Mr(calc)** | **Delta** | **Miss** | **Score** | **Expect** | **Rank** | **Peptide** |
| --- | --- | --- | --- | --- | --- | --- | --- | --- | --- | --- |
|  | 2503 | **413.2641** | **824.5136** | **823.4228** | **1.0908** | **0** | **8** | **22** | **4** | **R.IGGTYWK.V** |
|  | 5822 | 528.3102 | 1054.6059 | 1053.4913 | 1.1146 | 0 | 2 | 91 | 10 | R.GLTMYQGER.V |
|  | 6116 | **537.8392** | **1073.6638** | **1073.6709** | **-0.0072** | **0** | **21** | **1.4** | **1** | **R.HLGILGPVIR.A** |
|  | 6598 | 556.2769 | 1110.5392 | 1110.5418 | -0.0026 | 0 | 12 | 9.5 | 8 | R.GALDGNSPPQR.Q |
|  | 8201 | **608.3162** | **1214.6179** | **1214.6255** | **-0.0076** | **1** | **31** | **0.12** | **1** | **K.NLREPGSISDK.F** |
|  | 13122 | **497.8007** | **1490.3804** | **1490.7109** | **-0.3305** | **0** | **5** | **25** | **3** | **K.SCSMAPPVDLLTGK.V** |
|  | 14088 | **519.2855** | **1554.8346** | **1554.7209** | **0.1137** | **1** | **4** | **46** | **9** | **K.GILEPHGGRSDMDR.E** |
|  | 14848 | **804.8679** | **1607.7213** | **1607.7315** | **-0.0102** | **0** | **8** | **17** | **1** | **K.DSEGSLYPDGSSGPLK.A** |
|  | 14942 | 807.9097 | 1613.8048 | 1614.7175 | -0.9127 | 1 | 2 | 83 | 10 | R.SWEREWHNQSEK.D |
|  | 15080 | **811.8809** | **1621.7472** | **1621.7584** | **-0.0112** | **0** | **34** | **0.041** | **1** | **R.LLSEDIEGFQDSNR.M** 15083 |
|  | 16479 | **573.2979** | **1716.8719** | **1717.8743** | **-1.0024** | **1** | **3** | **55** | **5** | **K.VKSCSMAPPVDLLTGK.V** 16498 |
|  | 17574 | 600.2753 | 1797.8042 | 1797.8580 | -0.0539 | 0 | 0 | 91 | 10 | R.AIYNVSQCPGHQATPR.Q |
|  | 17601 | 900.9478 | 1799.8809 | 1799.8988 | -0.0179 | 1 | 10 | 11 | 2 | K.LYANLRGLTMYQGER.V |
|  | 18237 | **922.9174** | **1843.8203** | **1843.8311** | **-0.0108** | **0** | **53** | **0.00054** | **1** | **K.SWYSNANQAAAMLDFR.L** |
|  | 19380 | 966.0837 | 1930.1529 | 1929.9903 | 0.1626 | 1 | (3) | 47 | 4 | R.DIEEGNVKMLGMQIPIK.N 19384 |
|  | 19558 | **973.9286** | **1945.8426** | **1945.9853** | **-0.1426** | **1** | **3** | **47** | **4** | **R.DIEEGNVKMLGMQIPIK.N** |
|  | 20796 | **1031.0408** | **2060.0670** | **2060.0790** | **-0.0120** | **0** | **89** | **1.3e-007** | **1** | **R.NVITNQPLDSDIVASSFLK.S** |
|  | 23378 | 782.5679 | 2344.6820 | 2344.0760 | 0.6060 | 1 | 1 | 41 | 5 | R.EWHNQSEKDSYGYIFLSNK.D |
|  | 23695 | **598.3271** | **2389.2793** | **2390.2329** | **-0.9537** | **1** | **2** | **47** | **4** | **R.NVITNQPLDSDIVASSFLKSDK.N** |
|  | 29385 | **892.9385** | **3567.7251** | **3566.7399** | **0.9851** | **0** | **5** | **12** | **1** | **K.GDTVAWHLLGLGTETDVHGVMFQGNTVQLQGMR.K** |
|  | 29866 | 1242.6325 | 3724.8755 | 3725.7251 | -0.8496 | 0 | 0 | 32 | 7 | K.GAAMLFPHTFVMAIMQPDNLGTFEIYCQAGSHR.E |
|  | 30560 | 1033.0295 | 4128.0891 | 4128.1096 | -0.0205 | 1 | 17 | 0.57 | 1 | K.EYKDDSYTDEVAQPAWLGFLGPVLQAEVGDVILIHLK.N |
|  | 30748 | **1429.2904** | **4284.8494** | **4285.9628** | **-1.1134** | **1** | **3** | **12** | **2** | **K.GAAMLFPHTFVMAIMQPDNLGTFEIYCQAGSHREAGMR.A** |

| **356.** | P18124\|RL7_HUMAN    **Mass:** 29264    **Score:** 136    **Queries matched:** 20   **emPAI:** 0.84 |
| --- | --- |
|  | 60S ribosomal protein L7 OS=Homo sapiens GN=RPL7 PE=1 SV=1 |

|  | **Query** | **Observed** | **Mr(expt)** | **Mr(calc)** | **Delta** | **Miss** | **Score** | **Expect** | **Rank** | **Peptide** |
| --- | --- | --- | --- | --- | --- | --- | --- | --- | --- | --- |
|  | 1789 | **721.3834** | **720.3761** | **720.3806** | **-0.0045** | **0** | **19** | **2.1** | **1** | **R.NFAELK.I** |
|  | 2222 | **402.7117** | **803.4089** | **803.4323** | **-0.0234** | **0** | **16** | **5.7** | **1** | **K.ASINMLR.I** 2247 |
|  | 3582 | 446.7287 | 891.4428 | 891.4596 | -0.0168 | 1 | 4 | 68 | 2 | R.TEIRMAR.M |
|  | 3596 | **447.2428** | **892.4711** | **892.4953** | **-0.0242** | **1** | **8** | **26** | **6** | **K.FAQKMLR.K** |
|  | 4622 | **483.2656** | **964.5166** | **964.5229** | **-0.0063** | **0** | **28** | **0.29** | **1** | **K.SVNELIYK.R** |
|  | 5784 | 526.7320 | 1051.4494 | 1052.5655 | -1.1161 | 0 | 9 | 18 | 3 | R.QIFNGTFVK.L 5798 |
|  | 7363 | **586.2816** | **1170.5487** | **1169.6768** | **0.8719** | **0** | **9** | **18** | **2** | **R.IALTDNALIAR.S** |
|  | 7717 | **596.7304** | **1191.4463** | **1191.5924** | **-0.1461** | **0** | **6** | **36** | **6** | **K.AGNFYVPAEPK.L** |
|  | 10211 | **661.3310** | **1320.6474** | **1319.6874** | **0.9601** | **1** | **3** | **76** | **8** | **R.KAGNFYVPAEPK.L** |
|  | 10883 | **453.8845** | **1358.6317** | **1359.6168** | **-0.9850** | **0** | **1** | **1.1e+002** | **8** | **K.TTHFVEGGDAGNR.E** |
|  | 15704 | 831.9428 | 1661.8709 | 1661.8817 | -0.0108 | 0 | 66 | 2.6e-005 | 1 | R.IVEPYIAWGYPNLK.S |
|  | 21413 | **1058.4874** | **2114.9603** | **2114.9730** | **-0.0127** | **1** | **49** | **0.001** | **1** | **K.TTHFVEGGDAGNREDQINR.L** 21412 21414 |
|  | 21784 | **718.3806** | **2152.1200** | **2153.0537** | **-0.9336** | **0** | **58** | **0.00012** | **1** | **K.YGIICMEDLIHEIYTVGK.R** 21786 21787 21788 |

| **357.** | Q07065\|CKAP4_HUMAN    **Mass:** 66097    **Score:** 134    **Queries matched:** 23   **emPAI:** 0.12 |
| --- | --- |
|  | Cytoskeleton-associated protein 4 OS=Homo sapiens GN=CKAP4 PE=1 SV=2 |

|  | **Query** | **Observed** | **Mr(expt)** | **Mr(calc)** | **Delta** | **Miss** | **Score** | **Expect** | **Rank** | **Peptide** |
| --- | --- | --- | --- | --- | --- | --- | --- | --- | --- | --- |
|  | 2045 | **395.7421** | **789.4696** | **790.3457** | **-0.8761** | **0** | **11** | **19** | **3** | **K.EAADTER.L** |
|  | 2971 | **428.7165** | **855.4185** | **856.5018** | **-1.0833** | **0** | **19** | **2.2** | **1** | **K.LQNEILK.D** |
|  | 4335 | **473.2683** | **944.5220** | **943.5087** | **1.0134** | **1** | **17** | **4.2** | **7** | **R.RLEEELR.Q** |
|  | 7968 | 602.8383 | 1203.6621 | 1202.6506 | 1.0115 | 1 | 7 | 33 | 5 | K.EAVKEIQTSAK.S |
|  | 9230 | **423.7612** | **1268.2619** | **1268.6109** | **-0.3491** | **1** | **6** | **23** | **6** | **R.GSKGGHGAASPSEK.G** 9256 |
|  | 11869 | 707.8858 | 1413.7570 | 1413.7100 | 0.0471 | 0 | 8 | 22 | 8 | R.EELGQGLQGVEQK.V |
|  | 11897 | **708.3875** | **1414.7605** | **1413.7212** | **1.0393** | **1** | **2** | **82** | **5** | **K.GLLDDLRNDLDR.L** |
|  | 15231 | 815.9291 | 1629.8437 | 1628.8733 | 0.9704 | 1 | 3 | 66 | 4 | K.RSVGELPSTVESLQK.V |
|  | 15470 | 823.4105 | 1644.8064 | 1645.8159 | -1.0095 | 1 | 3 | 64 | 5 | K.VASLEESEGNKQDLK.A 15478 |
|  | 16221 | **850.3863** | **1698.7580** | **1697.8696** | **0.8884** | **1** | **8** | **17** | **5** | **R.QREELGQGLQGVEQK.V** |
|  | 17212 | 886.4422 | 1770.8698 | 1771.8298 | -0.9600 | 0 | 1 | 99 | 5 | R.STLQTMESDIYTEVR.E |
|  | 17554 | **898.9755** | **1795.9365** | **1795.9469** | **-0.0104** | **0** | **67** | **2.1e-005** | **1** | **K.VQSLQATFGTFESILR.S** |
|  | 18176 | **614.3029** | **1839.8868** | **1838.9309** | **0.9559** | **0** | **12** | **6** | **1** | **R.LQHVEDGVLSMQVASAR.Q** 18180 |
|  | 19057 | **952.9573** | **1903.9000** | **1903.9123** | **-0.0123** | **0** | **86** | **2.3e-007** | **1** | **R.LEGLGSSEADQDGLASTVR.S** |
|  | 21845 | 1080.0089 | 2158.0033 | 2158.9992 | -0.9959 | 1 | 9 | 11 | 2 | K.GGHGAASPSEKGAHPSGGADDVAK.K |
|  | 22856 | **759.6868** | **2276.0385** | **2276.2263** | **-0.1879** | **1** | **0** | **79** | **4** | **R.LTELTKSINDNIAIFTEVQK.R** |
|  | 26487 | 1417.6901 | 2833.3656 | 2834.2738 | -0.9083 | 1 | 3 | 27 | 3 | R.EWDMEALRSTLQTMESDIYTEVR.E 26492 26493 26497 |

| **358.** | P10321\|1C07_HUMAN    **Mass:** 41136    **Score:** 133    **Queries matched:** 10   **emPAI:** 0.42 |
| --- | --- |
|  | HLA class I histocompatibility antigen, Cw-7 alpha chain OS=Homo sapiens GN=HLA-C PE=1 SV=3 |

|  | **Query** | **Observed** | **Mr(expt)** | **Mr(calc)** | **Delta** | **Miss** | **Score** | **Expect** | **Rank** | **Peptide** |
| --- | --- | --- | --- | --- | --- | --- | --- | --- | --- | --- |
|  | 1094 | 560.3320 | 559.3248 | 558.3125 | 1.0122 | 0 | 20 | 3.5 | 10 | K.LEAAR.A |
|  | 7345 | **585.2987** | **1168.5829** | **1167.6248** | **0.9581** | **1** | **9** | **19** | **1** | **K.ETLQRAEPPK.T** |
|  | 12099 | 714.3628 | 1426.7111 | 1425.7365 | 0.9747 | 0 | 3 | 68 | 4 | K.WAAVVVPSGQEQR.Y |
|  | 15470 | 823.4105 | 1644.8064 | 1644.8148 | -0.0084 | 0 | 4 | 42 | 2 | R.FISVGYVDDTQFVR.F |
|  | 18332 | 617.6424 | 1849.9054 | 1849.9183 | -0.0130 | 0 | 32 | 0.074 | 1 | K.THVTHHPLSDHEATLR.C 18320 |
|  | 21343 | 1056.5170 | 2111.0194 | 2111.0299 | -0.0105 | 0 | 45 | 0.003 | 1 | R.CWALGFYPAEITLTWQR.D 21342 |
|  | 25408 | 1319.0913 | 2636.1681 | 2636.1838 | -0.0158 | 0 | 68 | 1.1e-005 | 1 | R.DGEDQTQDTELVETRPAGDGTFQK.W 25407 |

| **359.** | Q31612\|1B73_HUMAN    **Mass:** 40809    **Score:** 133    **Queries matched:** 12   **emPAI:** 0.42 |
| --- | --- |
|  | HLA class I histocompatibility antigen, B-73 alpha chain OS=Homo sapiens GN=HLA-B PE=1 SV=1 |

|  | **Query** | **Observed** | **Mr(expt)** | **Mr(calc)** | **Delta** | **Miss** | **Score** | **Expect** | **Rank** | **Peptide** |
| --- | --- | --- | --- | --- | --- | --- | --- | --- | --- | --- |
|  | 1936 | 763.1170 | 762.1097 | 762.3694 | -0.2597 | 0 | 0 | 1.4e+002 | 7 | R.NTQICK.A |
|  | 2630 | **417.2441** | **832.4737** | **832.4299** | **0.0438** | **0** | **4** | **87** | **3** | **-.MLVMAPR.T** |
|  | 10365 | 443.7359 | 1328.1860 | 1327.6996 | 0.4863 | 1 | 13 | 4.5 | 1 | K.WEAARVAEQLR.A |
|  | 12099 | 714.3628 | 1426.7111 | 1425.7365 | 0.9747 | 0 | 3 | 68 | 4 | K.WAAVVVPSGQEQR.Y |
|  | 15669 | **554.2529** | **1659.7368** | **1658.8305** | **0.9063** | **0** | **1** | **91** | **8** | **R.FITVGYVDDTQFVR.F** |
|  | 18332 | 617.6424 | 1849.9054 | 1849.9183 | -0.0130 | 0 | 32 | 0.074 | 1 | K.THVTHHPISDHEATLR.C 18320 |
|  | 21185 | **700.7486** | **2099.2240** | **2098.0484** | **1.1756** | **1** | **4** | **28** | **2** | **R.GEPRFITVGYVDDTQFVR.F** |
|  | 21343 | 1056.5170 | 2111.0194 | 2111.0299 | -0.0105 | 0 | 45 | 0.003 | 1 | R.CWALGFYPAEITLTWQR.D 21342 |
|  | 25408 | 1319.0913 | 2636.1681 | 2636.1838 | -0.0158 | 0 | 68 | 1.1e-005 | 1 | R.DGEDQTQDTELVETRPAGDGTFQK.W 25407 |

| **360.** | Q3ZCM7\|TBB8_HUMAN    **Mass:** 50257    **Score:** 133    **Queries matched:** 26   **emPAI:** 0.33 |
| --- | --- |
|  | Tubulin beta-8 chain OS=Homo sapiens GN=TUBB8 PE=1 SV=2 |

|  | **Query** | **Observed** | **Mr(expt)** | **Mr(calc)** | **Delta** | **Miss** | **Score** | **Expect** | **Rank** | **Peptide** |
| --- | --- | --- | --- | --- | --- | --- | --- | --- | --- | --- |
|  | 1837 | 737.3976 | 736.3904 | 737.3456 | -0.9553 | 0 | 9 | 21 | 2 | R.GSQQYR.A |
|  | 2296 | 405.2068 | 808.3991 | 807.3399 | 1.0593 | 0 | 8 | 28 | 7 | R.EEYPDR.I |
|  | 5466 | 514.7600 | 1027.5053 | 1027.5121 | -0.0067 | 0 | 25 | 0.47 | 1 | K.TAVCDIPPR.G 5464 5477 5478 |
|  | 5960 | 533.2140 | 1064.4134 | 1064.4201 | -0.0067 | 0 | 11 | 12 | 1 | K.NMMAACDPR.H 5970 |
|  | 6152 | 539.2660 | 1076.5174 | 1076.5250 | -0.0076 | 1 | 25 | 0.51 | 1 | K.IREEYPDR.I |
|  | 8618 | **619.2731** | **1236.5316** | **1237.6931** | **-1.1615** | **1** | **6** | **38** | **5** | **R.YLTAAAIFRGR.M** |
|  | 10304 | 663.8410 | 1325.6674 | 1325.7126 | -0.0451 | 1 | 5 | 41 | 5 | K.TAVCDIPPRGLK.M |
|  | 10596 | **671.4405** | **1340.8664** | **1341.6313** | **-0.7649** | **0** | **4** | **44** | **5** | **R.INVYYNEASGGR.Y** |
|  | 12166 | 716.3267 | 1430.6389 | 1430.5965 | 0.0423 | 1 | 8 | 21 | 2 | K.NMMAACDPRHGR.Y 12164 12165 |
|  | 14745 | 801.4085 | 1600.8023 | 1600.8131 | -0.0107 | 0 | 51 | 0.001 | 1 | R.AVLVDLEPGTMDSVR.S |
|  | 15059 | 540.9470 | 1619.8192 | 1619.8283 | -0.0090 | 0 | 28 | 0.19 | 1 | R.LHFFMPGFAPLTSR.G |
|  | 17979 | **609.9809** | **1826.9209** | **1827.9513** | **-1.0304** | **0** | **2** | **60** | **6** | **R.EIVLTQIGQCGNQIGAK.F** |
|  | 18466 | **620.0762** | **1857.2067** | **1856.9169** | **0.2898** | **1** | **6** | **20** | **1** | **R.INVYYNEASGGRYVPR.A** 18474 |
|  | 21923 | **723.0158** | **2166.0254** | **2166.0085** | **0.0169** | **1** | **0** | **77** | **2** | **K.GHYTEGAELMESVMDVVRK.E** |
|  | 25855 | 901.7996 | 2702.3769 | 2703.3360 | -0.9592 | 0 | 3 | 30 | 4 | K.LPTPTYGDLNHLVSATMSGVTTCLR.F 25853 25890 |
|  | 28611 | 1104.5131 | 3310.5174 | 3310.5268 | -0.0095 | 0 | 79 | 5.2e-007 | 1 | K.EAESCDCLQGFQLTHSLGGGTGSGMGTLLLSK.I 28610 |

| **361.** | P32969\|RL9_HUMAN    **Mass:** 21964    **Score:** 132    **Queries matched:** 11   **emPAI:** 0.38 |
| --- | --- |
|  | 60S ribosomal protein L9 OS=Homo sapiens GN=RPL9 PE=1 SV=1 |

|  | **Query** | **Observed** | **Mr(expt)** | **Mr(calc)** | **Delta** | **Miss** | **Score** | **Expect** | **Rank** | **Peptide** |
| --- | --- | --- | --- | --- | --- | --- | --- | --- | --- | --- |
|  | 1096 | 560.3808 | 559.3735 | 558.3741 | 0.9994 | 0 | 23 | 1.6 | 1 | R.TVIVK.G |
|  | 2387 | 408.7295 | 815.4444 | 815.4865 | -0.0421 | 1 | 15 | 7.4 | 7 | R.KELATVR.T |
|  | 7359 | 585.8415 | 1169.6685 | 1169.5968 | 0.0717 | 0 | 9 | 17 | 5 | K.FLDGIYVSEK.G |
|  | 9791 | **649.8489** | **1297.6833** | **1297.6918** | **-0.0085** | **1** | **5** | **48** | **8** | **R.KFLDGIYVSEK.G** |
|  | 10386 | **665.8291** | **1329.6436** | **1329.6533** | **-0.0097** | **0** | **13** | **7.8** | **1** | **R.TICSHVQNMIK.G** |
|  | 14700 | 799.9262 | 1597.8377 | 1597.8464 | -0.0086 | 0 | 70 | 1.2e-005 | 1 | R.DFNHINVELSLLGK.K |
|  | 20927 | 692.7316 | 2075.1729 | 2076.0609 | -0.8880 | 1 | 3 | 41 | 3 | R.TICSHVQNMIKGVTLGFR.Y |
|  | 21372 | **1057.0669** | **2112.1192** | **2112.1314** | **-0.0122** | **0** | **57** | **0.00017** | **1** | **K.TILSNQTVDIPENVDITLK.G** 21373 |
|  | 23224 | **776.3681** | **2326.0825** | **2325.2540** | **0.8285** | **1** | **2** | **51** | **2** | **K.TILSNQTVDIPENVDITLKGR.T** |
|  | 24406 | **829.1033** | **2484.2882** | **2483.3172** | **0.9709** | **0** | **12** | **4.6** | **1** | **R.SVYAHFPINVVIQENGSLVEIR.N** |

| **362.** | Q9UQQ1\|NALDL_HUMAN    **Mass:** 80800    **Score:** 132    **Queries matched:** 20   **emPAI:** 0.14 |
| --- | --- |
|  | N-acetylated-alpha-linked acidic dipeptidase-like protein OS=Homo sapiens GN=NAALADL1 PE=2 SV=1 |

|  | **Query** | **Observed** | **Mr(expt)** | **Mr(calc)** | **Delta** | **Miss** | **Score** | **Expect** | **Rank** | **Peptide** |
| --- | --- | --- | --- | --- | --- | --- | --- | --- | --- | --- |
|  | 1746 | **710.3898** | **709.3826** | **708.3265** | **1.0561** | **0** | **5** | **44** | **7** | **-.MQWTK.V** |
|  | 2404 | **409.2222** | **816.4298** | **815.4865** | **0.9434** | **0** | **14** | **9.1** | **3** | **R.TAGSVILR.L** |
|  | 5012 | 497.6351 | 993.2556 | 992.5039 | 0.7517 | 0 | 12 | 7.9 | 1 | R.EPHLASSPR.D |
|  | 5295 | **508.2479** | **1014.4813** | **1015.5410** | **-1.0597** | **1** | **16** | **4.8** | **3** | **R.ENLRELSR.E** 5320 |
|  | 6006 | **535.2364** | **1068.4582** | **1068.5087** | **-0.0505** | **0** | **12** | **9.8** | **8** | **K.VSDYSETLR.S** |
|  | 6100 | **537.2994** | **1072.5842** | **1071.6036** | **0.9806** | **0** | **13** | **8.9** | **1** | **R.NSSNVLGIIR.G** |
|  | 7264 | **582.2787** | **1162.5429** | **1161.5778** | **0.9651** | **0** | **9** | **19** | **3** | **K.FEAEAAALGQR.I** |
|  | 7308 | **583.8231** | **1165.6316** | **1165.6383** | **-0.0067** | **0** | **11** | **11** | **1** | **R.LSDSFFLPLK.V** |
|  | 11164 | **688.3532** | **1374.6917** | **1374.6999** | **-0.0082** | **0** | **57** | **0.00026** | **1** | **R.MLNDQLMLLER.T** |
|  | 11432 | **464.1945** | **1389.5616** | **1390.6948** | **-1.1333** | **0** | **(10)** | **12** | **3** | **R.MLNDQLMLLER.T** 11433 |
|  | 13348 | **753.8404** | **1505.6662** | **1505.6746** | **-0.0084** | **0** | **56** | **0.00028** | **1** | **R.DTASGSEAWAEVQR.Q** |
|  | 14165 | 781.3948 | 1560.7750 | 1561.8576 | -1.0826 | 1 | 4 | 53 | 6 | K.LEGTIALTRYGGVGR.G |
|  | 14492 | **396.9298** | **1583.6903** | **1582.9155** | **0.7748** | **1** | **11** | **8.8** | **3** | **R.LELRNSSNVLGIIR.G** |
|  | 15343 | 819.4306 | 1636.8466 | 1637.9101 | -1.0634 | 1 | 1 | 1e+002 | 5 | R.ISTLQKGSPDPLQVR.M |
|  | 24488 | 831.4635 | 2491.3687 | 2492.3387 | -0.9700 | 1 | 2 | 37 | 3 | K.LQERTVAYINVDISVFANATLR.V |
|  | 28022 | **1055.2344** | **3162.6813** | **3162.6925** | **-0.0111** | **0** | **70** | **4.2e-006** | **1** | **R.SFLQAAQQDLGALLEQHSISLGPLVTAVEK.F** |
|  | 31131 | 780.3889 | 4676.2894 | 4677.2293 | -0.9399 | 0 | 1 | 14 | 4 | K.DPESGLDSAEAGTYEVLLSFPSQEQPNVVDIVGPTGGIIHSCHR.T |
|  | 32104 | **1208.3713** | **8451.5484** | **8451.0466** | **0.5018** | **1** | **1** | **1.2** | **1** | **K.DPESGLDSAEATTYEVLLSFPSQEQPNVVDIVGPTGGIIHSCHRTEENVTGEQGGPDVVQPYAAYAPSGTPQGLLVYANR.G** |

| **363.** | O15144\|ARPC2_HUMAN    **Mass:** 34426    **Score:** 132    **Queries matched:** 14   **emPAI:** 0.11 |
| --- | --- |
|  | Actin-related protein 2/3 complex subunit 2 OS=Homo sapiens GN=ARPC2 PE=1 SV=1 |

|  | **Query** | **Observed** | **Mr(expt)** | **Mr(calc)** | **Delta** | **Miss** | **Score** | **Expect** | **Rank** | **Peptide** |
| --- | --- | --- | --- | --- | --- | --- | --- | --- | --- | --- |
|  | 3831 | **455.2456** | **908.4766** | **908.4967** | **-0.0201** | **1** | **1** | **1.3e+002** | **10** | **R.AKTSDFLK.V** |
|  | 4084 | 464.7321 | 927.4497 | 927.4524 | -0.0027 | 0 | 9 | 22 | 9 | K.VFMQEFK.E |
|  | 6331 | **545.3257** | **1088.6368** | **1087.5451** | **1.0917** | **0** | **5** | **54** | **4** | **R.DYLHYHIK.C** |
|  | 6735 | 561.3030 | 1120.5914 | 1120.6526 | -0.0612 | 1 | 2 | 1.1e+002 | 9 | K.TKVMVSISLK.F |
|  | 7865 | **400.5501** | **1198.6283** | **1197.6176** | **1.0107** | **0** | **7** | **32** | **1** | **K.DSIVHQAGMLK.R** |
|  | 8182 | 405.2994 | 1212.8762 | 1213.6125 | -0.7363 | 0 | (4) | 45 | 8 | K.DSIVHQAGMLK.R |
|  | 9246 | 635.3828 | 1268.7511 | 1267.6996 | 1.0514 | 1 | 7 | 26 | 1 | K.VLNRARPDAEK.K |
|  | 10256 | **662.3529** | **1322.6913** | **1322.6830** | **0.0083** | **0** | **5** | **40** | **2** | **K.ELQAHGADELLK.R** |
|  | 12938 | **494.2631** | **1479.7674** | **1478.7841** | **0.9832** | **1** | **2** | **81** | **3** | **K.ELQAHGADELLKR.V** |
|  | 19929 | **661.3093** | **1980.9062** | **1980.1619** | **0.7443** | **1** | **3** | **48** | **4** | **M.ILLEVNNRIIEETLALK.F** |
|  | 21519 | 1064.5112 | 2127.0079 | 2127.1973 | -0.1894 | 1 | 13 | 4.7 | 3 | -.MILLEVNNRIIEETLALK.F 21520 |
|  | 27737 | **1544.7697** | **3087.5247** | **3087.5441** | **-0.0193** | **0** | **83** | **2.3e-007** | **1** | **R.VYGSFLVNPESGYNVSLLYDLENLPASK.D** 27738 |

| **364.** | P30486\|1B48_HUMAN    **Mass:** 40679    **Score:** 132    **Queries matched:** 13   **emPAI:** 0.56 |
| --- | --- |
|  | HLA class I histocompatibility antigen, B-48 alpha chain OS=Homo sapiens GN=HLA-B PE=2 SV=1 |

|  | **Query** | **Observed** | **Mr(expt)** | **Mr(calc)** | **Delta** | **Miss** | **Score** | **Expect** | **Rank** | **Peptide** |
| --- | --- | --- | --- | --- | --- | --- | --- | --- | --- | --- |
|  | 1094 | 560.3320 | 559.3248 | 558.3125 | 1.0122 | 0 | 20 | 3.5 | 10 | K.LEAAR.V |
|  | 2630 | 417.2441 | 832.4737 | 832.4299 | 0.0438 | 0 | 4 | 87 | 3 | -.MLVMAPR.T |
|  | 11047 | 457.2375 | 1368.6906 | 1367.6793 | 1.0112 | 1 | 10 | 13 | 4 | K.TNTQTYRESLR.N |
|  | 13341 | 753.3655 | 1504.7165 | 1504.7270 | -0.0105 | 0 | 57 | 0.00025 | 1 | R.SWTAADTAAQISQR.K |
|  | 15470 | 823.4105 | 1644.8064 | 1644.8148 | -0.0084 | 0 | 4 | 42 | 2 | R.FISVGYVDDTQFVR.F |
|  | 18332 | 617.6424 | 1849.9054 | 1849.9183 | -0.0130 | 0 | 32 | 0.074 | 1 | K.THVTHHPISDHEATLR.C 18320 |
|  | 20661 | 513.0184 | 2048.0446 | 2048.0626 | -0.0179 | 0 | 15 | 3.2 | 1 | R.YTCHVQHEGLPKPLTLR.W |
|  | 21343 | 1056.5170 | 2111.0194 | 2111.0299 | -0.0105 | 0 | 45 | 0.003 | 1 | R.CWALGFYPAEITLTWQR.D 21342 |
|  | 22490 | 744.6667 | 2230.9784 | 2230.9938 | -0.0154 | 0 | 35 | 0.024 | 1 | R.DGEDQTQDTELVETRPAGDR.T 22489 22491 |

|  | **Proteins matching a subset of these peptides:** |
| --- | --- |

|  | Q31610\|1B81_HUMAN    **Mass:** 40717    **Score:** 132    **Queries matched:** 12 |
| --- | --- |
|  | HLA class I histocompatibility antigen, B-81 alpha chain OS=Homo sapiens GN=HLA-B PE=2 SV=1 |

| **365.** | P23396\|RS3_HUMAN    **Mass:** 26842    **Score:** 132    **Queries matched:** 16   **emPAI:** 0.30 |
| --- | --- |
|  | 40S ribosomal protein S3 OS=Homo sapiens GN=RPS3 PE=1 SV=2 |

|  | **Query** | **Observed** | **Mr(expt)** | **Mr(calc)** | **Delta** | **Miss** | **Score** | **Expect** | **Rank** | **Peptide** |
| --- | --- | --- | --- | --- | --- | --- | --- | --- | --- | --- |
|  | 1761 | **714.3268** | **713.3196** | **713.4436** | **-0.1240** | **0** | **20** | **1.6** | **1** | **R.QGVLGIK.V** |
|  | 2155 | **400.2643** | **798.5139** | **797.5123** | **1.0016** | **0** | **7** | **35** | **7** | **K.LLGGLAVR.R** 2144 |
|  | 3539 | **445.2172** | **888.4199** | **887.4712** | **0.9487** | **0** | **6** | **53** | **9** | **R.TQNVLGEK.G** |
|  | 3625 | **448.7084** | **895.4022** | **895.4804** | **-0.0782** | **0** | **18** | **2.3** | **4** | **K.FVADGIFK.A** |
|  | 5421 | **512.7916** | **1023.5686** | **1023.5753** | **-0.0067** | **1** | **10** | **17** | **1** | **R.KFVADGIFK.A** |
|  | 9609 | 645.3348 | 1288.6550 | 1287.6605 | 0.9945 | 0 | 11 | 11 | 2 | R.GLCAIAQAESLR.Y |
|  | 12025 | 712.3339 | 1422.6532 | 1422.6627 | -0.0095 | 0 | 65 | 4e-005 | 1 | R.ELAEDGYSGVEVR.V |
|  | 12768 | **735.3732** | **1468.7319** | **1469.7613** | **-1.0294** | **0** | **15** | **4.2** | **1** | **K.DEILPTTPISEQK.G** |
|  | 14275 | **784.8818** | **1567.7490** | **1567.8432** | **-0.0942** | **1** | **3** | **68** | **4** | **K.IMLPWDPTGKIGPK.K** |
|  | 14349 | **787.3977** | **1572.7809** | **1572.7970** | **-0.0161** | **0** | **16** | **3.5** | **1** | **K.GGKPEPPAMPQPVPTA.-** |
|  | 16245 | **850.8950** | **1699.7754** | **1700.8378** | **-1.0624** | **1** | **1** | **85** | **8** | **R.ACYGVLRFIMESGAK.G** |
|  | 24299 | **1234.5920** | **2467.1695** | **2467.1842** | **-0.0147** | **0** | **90** | **8.3e-008** | **1** | **K.FVDGLMIHSGDPVNYYVDTAVR.H** |
|  | 24395 | **828.4060** | **2482.1962** | **2483.1791** | **-0.9829** | **0** | **(1)** | **63** | **6** | **K.FVDGLMIHSGDPVNYYVDTAVR.H** |
|  | 27791 | **776.3527** | **3101.3815** | **3101.5757** | **-0.1942** | **1** | **5** | **16** | **2** | **K.FVDGLMIHSGDPVNYYVDTAVRHVLLR.Q** 27790 |

| **366.** | P37108\|SRP14_HUMAN    **Mass:** 14675    **Score:** 131    **Queries matched:** 8   **emPAI:** 0.27 |
| --- | --- |
|  | Signal recognition particle 14 kDa protein OS=Homo sapiens GN=SRP14 PE=1 SV=2 |

|  | **Query** | **Observed** | **Mr(expt)** | **Mr(calc)** | **Delta** | **Miss** | **Score** | **Expect** | **Rank** | **Peptide** |
| --- | --- | --- | --- | --- | --- | --- | --- | --- | --- | --- |
|  | 1650 | **684.3988** | **683.3915** | **682.4377** | **0.9538** | **0** | **1** | **1e+002** | **3** | **R.TKPIPK.K** |
|  | 9025 | 420.3174 | 1257.9305 | 1257.6176 | 0.3129 | 0 | 5 | 31 | 9 | K.FQMAYSNLLR.A 9033 |
|  | 15942 | **839.4512** | **1676.8878** | **1676.8985** | **-0.0107** | **0** | **82** | **6.6e-007** | **1** | **M.VLLESEQFLTELTR.L** 15941 |
|  | 19836 | **658.3151** | **1971.9235** | **1970.9706** | **0.9529** | **1** | **0** | **85** | **6** | **K.FQMAYSNLLRANMDGLK.K** |
|  | 23566 | **789.7615** | **2366.2628** | **2367.2030** | **-0.9402** | **0** | **0** | **74** | **9** | **K.AAAAAAAAAPAAAATAPTTAATTAATAAQ.-** |
|  | 25212 | **520.4521** | **2597.2241** | **2596.3456** | **0.8785** | **1** | **4** | **27** | **6** | **K.TKAAAAAAAAAPAAAATAPTTAATTAATAAQ.-** |

| **367.** | P19338\|NUCL_HUMAN    **Mass:** 76625    **Score:** 131    **Queries matched:** 22   **emPAI:** 0.10 |
| --- | --- |
|  | Nucleolin OS=Homo sapiens GN=NCL PE=1 SV=3 |

|  | **Query** | **Observed** | **Mr(expt)** | **Mr(calc)** | **Delta** | **Miss** | **Score** | **Expect** | **Rank** | **Peptide** |
| --- | --- | --- | --- | --- | --- | --- | --- | --- | --- | --- |
|  | 996 | **542.1290** | **541.1218** | **540.3635** | **0.7582** | **0** | **6** | **22** | **6** | **K.VVPVK.A** 992 |
|  | 1058 | **551.0203** | **550.0130** | **549.2660** | **0.7470** | **0** | **10** | **14** | **9** | **R.GGFGGR.G** |
|  | 1184 | **577.3271** | **576.3198** | **576.3054** | **0.0145** | **0** | **14** | **8.7** | **1** | **R.IGMTR.K** |
|  | 1467 | **639.3777** | **638.3704** | **639.3414** | **-0.9710** | **0** | **25** | **0.4** | **1** | **K.MAPPPK.E** |
|  | 1847 | 740.4251 | 739.4178 | 739.4956 | -0.0778 | 1 | 8 | 21 | 5 | K.VVPVKAK.N |
|  | 1878 | **750.3342** | **749.3270** | **749.3741** | **-0.0472** | **1** | **9** | **25** | **2** | **K.EMAKQK.A** |
|  | 2801 | 422.7581 | 843.5017 | 843.5065 | -0.0049 | 0 | 9 | 25 | 2 | K.ALELTGLK.V |
|  | 3489 | **443.0399** | **884.0652** | **883.5491** | **0.5161** | **1** | **3** | **49** | **2** | **K.KVAVATPAK.K** 3484 |
|  | 5026 | 498.2762 | 994.5379 | 994.4356 | 0.1023 | 0 | 10 | 14 | 8 | K.NSTWSGESK.T |
|  | 5841 | **529.2582** | **1056.5019** | **1056.5968** | **-0.0948** | **0** | **13** | **8.8** | **1** | **K.VTLDWAKPK.G** |
|  | 6826 | **564.9457** | **1127.8768** | **1127.5683** | **0.3085** | **1** | **13** | **6** | **1** | **R.GSPNARSQPSK.T** |
|  | 7235 | **581.2817** | **1160.5488** | **1159.5761** | **0.9727** | **0** | **7** | **35** | **1** | **R.SISLYYTGEK.G** |
|  | 7482 | **589.9789** | **1177.9433** | **1177.5615** | **0.3818** | **0** | **0** | **99** | **4** | **K.EVFEDAAEIR.L** |
|  | 11519 | **465.5856** | **1393.7351** | **1393.7426** | **-0.0075** | **1** | **6** | **31** | **2** | **R.LELQGPRGSPNAR.S** |
|  | 12404 | 723.8439 | 1445.6733 | 1446.7718 | -1.0985 | 1 | 13 | 6.8 | 4 | K.NLPYKVTQDELK.E |
|  | 14388 | **526.2871** | **1575.8393** | **1576.7039** | **-0.8645** | **1** | **6** | **30** | **4** | **K.AAKEAMEDGEIDGNK.V** |
|  | 15242 | 816.3525 | 1630.6904 | 1630.9294 | -0.2390 | 1 | 4 | 43 | 8 | K.ALELTGLKVFGNEIK.L |
|  | 17721 | 604.2780 | 1809.8120 | 1808.8621 | 0.9499 | 1 | 2 | 64 | 10 | K.SKGYAFIEFASFEDAK.E |
|  | 23130 | 1156.5756 | 2311.1366 | 2311.1485 | -0.0119 | 0 | 27 | 0.15 | 1 | K.VEGTEPTTAFNLFVGNLNFNK.S |
|  | 24565 | **1251.1315** | **2500.2484** | **2500.2584** | **-0.0101** | **0** | **125** | **2e-011** | **1** | **K.TLVLSNLSYSATEETLQEVFEK.A** |

| **368.** | Q6ZN80\|MGAL1_HUMAN    **Mass:** 74110    **Score:** 131    **Queries matched:** 12   **emPAI:** 0.16 |
| --- | --- |
|  | Putative maltase-glucoamylase-like protein FLJ16351 OS=Homo sapiens PE=2 SV=1 |

|  | **Query** | **Observed** | **Mr(expt)** | **Mr(calc)** | **Delta** | **Miss** | **Score** | **Expect** | **Rank** | **Peptide** |
| --- | --- | --- | --- | --- | --- | --- | --- | --- | --- | --- |
|  | 4468 | **477.3563** | **952.6981** | **953.5004** | **-0.8023** | **0** | **(7)** | **21** | **3** | **-.MFLSISTR.L** |
|  | 4674 | **485.7476** | **969.4806** | **969.4953** | **-0.0148** | **0** | **11** | **12** | **8** | **-.MFLSISTR.L** 4671 |
|  | 8523 | 616.8061 | 1231.5976 | 1231.6026 | -0.0050 | 0 | 25 | 0.49 | 1 | R.AYVAFPDFFR.N 8538 |
|  | 12159 | **477.5728** | **1429.6965** | **1430.6804** | **-0.9838** | **1** | **7** | **30** | **8** | **R.STFPSSGRWGGHR.L** |
|  | 13299 | 751.8618 | 1501.7091 | 1501.7176 | -0.0085 | 0 | 40 | 0.014 | 1 | R.WMQLGAFYPFSR.N |
|  | 17550 | **898.8834** | **1795.7522** | **1795.7649** | **-0.0128** | **0** | **61** | **7.2e-005** | **1** | **R.WYDYSTGTSSTSTGQR.K** |
|  | 23038 | **1149.5221** | **2297.0296** | **2297.0423** | **-0.0127** | **0** | **81** | **6.6e-007** | **1** | **K.FDGLWIDMNEPSNFVDGSVR.G** |
|  | 23078 | **576.7868** | **2303.1181** | **2302.9657** | **0.1524** | **0** | **10** | **7** | **1** | **R.GCSNEMLNNPPYMPYLESR.D** |
|  | 30691 | **1410.0394** | **4227.0965** | **4226.9201** | **0.1764** | **1** | **1** | **22** | **8** | **R.YGYQNDAEISSLYDAMVAAQIPYDVQHVDIDYMNRK.L** |
|  | 31491 | 1287.8497 | 5147.3698 | 5147.4142 | -0.0444 | 0 | 0 | 11 | 3 | K.TLCMESQQILPDSSPVEHYNVHNLYGWSQTRPTYEAVQEVTGQR.G |

| **369.** | P20020\|AT2B1_HUMAN    **Mass:** 139637   **Score:** 130    **Queries matched:** 31   **emPAI:** 0.14 |
| --- | --- |
|  | Plasma membrane calcium-transporting ATPase 1 OS=Homo sapiens GN=ATP2B1 PE=1 SV=3 |

|  | **Query** | **Observed** | **Mr(expt)** | **Mr(calc)** | **Delta** | **Miss** | **Score** | **Expect** | **Rank** | **Peptide** |
| --- | --- | --- | --- | --- | --- | --- | --- | --- | --- | --- |
|  | 996 | 542.1290 | 541.1218 | 541.3224 | -0.2006 | 0 | 5 | 28 | 7 | K.ANLPK.K |
|  | 2109 | **398.2373** | **794.4600** | **794.3559** | **0.1042** | **0** | **5** | **51** | **1** | **R.DYQDVR.N** |
|  | 2529 | **414.2688** | **826.5230** | **826.3933** | **0.1297** | **0** | **9** | **19** | **6** | **K.EAGHGTQK.E** 2520 |
|  | 3828 | **455.2452** | **908.4759** | **908.4386** | **0.0374** | **0** | **4** | **56** | **1** | **K.TGTLTMNR.M** |
|  | 3974 | **460.2462** | **918.4779** | **918.5076** | **-0.0296** | **0** | **8** | **31** | **3** | **R.GQILWFR.G** |
|  | 4518 | 479.2832 | 956.5518 | 955.4400 | 1.1119 | 0 | 9 | 22 | 2 | K.FFDIDSGR.N |
|  | 4886 | **493.2556** | **984.4965** | **984.5029** | **-0.0063** | **0** | **8** | **27** | **3** | **K.VYTFNSVR.K** |
|  | 7682 | 595.8043 | 1189.5941 | 1189.5939 | 0.0002 | 0 | 8 | 25 | 6 | K.GIIDSTVSDQR.Q |
|  | 7709 | 596.3003 | 1190.5861 | 1190.5714 | 0.0148 | 0 | 11 | 13 | 8 | R.MVTGDNINTAR.A 7708 |
|  | 9861 | **651.8354** | **1301.6562** | **1301.6649** | **-0.0087** | **0** | **29** | **0.18** | **1** | **K.TVIEPMASEGLR.T** |
|  | 9937 | **653.8759** | **1305.7373** | **1304.7704** | **0.9669** | **1** | **2** | **87** | **7** | **R.IFSKGASEIILK.K** |
|  | 10184 | **660.3678** | **1318.7210** | **1317.6598** | **1.0612** | **0** | **(4)** | **60** | **1** | **K.TVIEPMASEGLR.T** |
|  | 11164 | 688.3532 | 1374.6917 | 1374.7289 | -0.0371 | 1 | 6 | 32 | 3 | R.ALMELRSTDALR.K |
|  | 12117 | 714.8848 | 1427.7550 | 1427.8361 | -0.0811 | 1 | 13 | 7.6 | 4 | R.KPYGRNKPLISR.T 12144 |
|  | 13866 | **514.9511** | **1541.8315** | **1542.6806** | **-0.8491** | **0** | **1** | **89** | **4** | **-.MGDMANNSVAYSGVK.N** |
|  | 14752 | **534.8122** | **1601.4147** | **1601.7409** | **-0.3261** | **0** | **6** | **22** | **1** | **R.SSIHNFMTHPEFR.I** |
|  | 15416 | **821.4199** | **1640.8253** | **1640.8370** | **-0.0117** | **0** | **47** | **0.0022** | **1** | **R.QVVAVTGDGTNDGPALK.K** 15417 15418 |
|  | 15622 | **828.8894** | **1655.7642** | **1655.7751** | **-0.0108** | **0** | **45** | **0.0035** | **1** | **K.TSPNEGLSGNPADLER.R** 15605 |
|  | 16013 | **842.8819** | **1683.7492** | **1683.8250** | **-0.0758** | **0** | **2** | **67** | **4** | **K.DPLLLSGTHVMEGSGR.M** 16005 |
|  | 18742 | **941.4688** | **1880.9231** | **1879.9415** | **0.9816** | **0** | **27** | **0.2** | **1** | **K.EASDIILTDDNFTSIVK.A** |
|  | 19191 | **958.0068** | **1913.9990** | **1914.0098** | **-0.0108** | **0** | **74** | **3.6e-006** | **1** | **K.YGDLLPADGILIQGNDLK.I** 19190 |
|  | 21472 | 1061.0944 | 2120.1742 | 2120.1841 | -0.0100 | 0 | 2 | 57 | 6 | R.GGQVIQIPVADITVGDIAQVK.Y |
|  | 30487 | **681.8417** | **4085.0068** | **4085.1548** | **-0.1480** | **1** | **2** | **17** | **1** | **R.NVYDSISKFLQFQLTVNVVAVIVAFTGACITQDSPLK.A** |

| **370.** | P34913\|HYES_HUMAN    **Mass:** 63316    **Score:** 130    **Queries matched:** 22   **emPAI:** 0.33 |
| --- | --- |
|  | Epoxide hydrolase 2 OS=Homo sapiens GN=EPHX2 PE=1 SV=2 |

|  | **Query** | **Observed** | **Mr(expt)** | **Mr(calc)** | **Delta** | **Miss** | **Score** | **Expect** | **Rank** | **Peptide** |
| --- | --- | --- | --- | --- | --- | --- | --- | --- | --- | --- |
|  | 897 | 518.3204 | 517.3132 | 516.3020 | 1.0112 | 0 | 11 | 30 | 5 | K.AISAR.K |
|  | 1334 | 609.3199 | 608.3127 | 607.3329 | 0.9797 | 0 | 12 | 8.5 | 1 | K.NFSIK.E 1332 |
|  | 1843 | **739.3568** | **738.3495** | **739.4228** | **-1.0734** | **0** | **37** | **0.028** | **1** | **R.NPPVVSK.M** 1844 |
|  | 2290 | **404.7336** | **807.4526** | **806.4030** | **1.0496** | **0** | **13** | **9** | **5** | **R.VLAMDMK.G** |
|  | 2469 | 412.2242 | 822.4339 | 822.3979 | 0.0359 | 0 | (3) | 74 | 10 | R.VLAMDMK.G |
|  | 2821 | **423.4551** | **844.8955** | **844.4039** | **0.4916** | **0** | **11** | **12** | **1** | **K.GGPEGATTR.L** |
|  | 4872 | **492.2895** | **982.5644** | **981.4841** | **1.0803** | **0** | **7** | **32** | **3** | **K.EMVTFLDK.L** |
|  | 9399 | 639.3110 | 1276.6074 | 1276.6155 | -0.0081 | 0 | 13 | 7.6 | 1 | R.DGLAQLMCELK.M |
|  | 12354 | **722.4034** | **1442.7922** | **1441.8578** | **0.9344** | **1** | **3** | **73** | **9** | **R.KILIPALMVTAEK.D** |
|  | 13870 | 514.9633 | 1541.8681 | 1541.8534 | 0.0148 | 0 | 5 | 41 | 6 | K.INRPMLQAALMLR.K |
|  | 13878 | 772.3791 | 1542.7437 | 1541.8534 | 0.8904 | 0 | (2) | 86 | 10 | K.INRPMLQAALMLR.K |
|  | 16564 | **862.4317** | **1722.8488** | **1722.8577** | **-0.0089** | **0** | **43** | **0.0063** | **1** | **K.GFTTAILTNTWLDDR.A** |
|  | 19227 | **959.9606** | **1917.9066** | **1917.9182** | **-0.0117** | **0** | **0** | **1e+002** | **2** | **R.MVTEEEIQFYVQQFK.K** |
|  | 23820 | **1206.1279** | **2410.2413** | **2410.2566** | **-0.0153** | **0** | **55** | **0.00019** | **1** | **R.AVASLNTPFIPANPNMSPLESIK.A** 23819 23821 |
|  | 24155 | **817.4003** | **2449.1790** | **2449.1923** | **-0.0133** | **0** | **11** | **5.6** | **1** | **K.DFVLVPQMSQHMEDWIPHLK.R** |
|  | 27869 | **1043.1660** | **3126.4762** | **3126.4934** | **-0.0172** | **0** | **27** | **0.085** | **1** | **K.ANPVFDYQLYFQEPGVAEAELEQNLSR.T** |
|  | 29056 | **863.4537** | **3449.7856** | **3449.7436** | **0.0420** | **0** | **6** | **10** | **1** | **K.VTGIQLLNTPAPLPTSCNPSDMSHGYVTVKPR.V** |
|  | 29874 | **746.7945** | **3728.9361** | **3728.9496** | **-0.0135** | **1** | **23** | **0.17** | **1** | **K.ILIPALMVTAEKDFVLVPQMSQHMEDWIPHLK.R** |

| **371.** | Q9UJZ1\|STML2_HUMAN    **Mass:** 38624    **Score:** 130    **Queries matched:** 13   **emPAI:** 0.20 |
| --- | --- |
|  | Stomatin-like protein 2 OS=Homo sapiens GN=STOML2 PE=1 SV=1 |

|  | **Query** | **Observed** | **Mr(expt)** | **Mr(calc)** | **Delta** | **Miss** | **Score** | **Expect** | **Rank** | **Peptide** |
| --- | --- | --- | --- | --- | --- | --- | --- | --- | --- | --- |
|  | 1549 | 656.4037 | 655.3965 | 656.4082 | -1.0117 | 1 | 1 | 1e+002 | 8 | M.LARAAR.G |
|  | 2237 | 403.1946 | 804.3746 | 803.4436 | 0.9311 | 1 | 4 | 94 | 9 | -.MLARAAR.G |
|  | 5930 | **531.7711** | **1061.5276** | **1061.5353** | **-0.0077** | **0** | **5** | **56** | **10** | **R.ATVLESEGTR.E** |
|  | 14205 | 522.2354 | 1563.6844 | 1563.7385 | -0.0541 | 1 | 6 | 28 | 4 | R.VKESMQMQVEAER.R |
|  | 15896 | **838.4520** | **1674.8895** | **1674.0080** | **0.8815** | **0** | **7** | **22** | **1** | **R.ILEPGLNILIPVLDR.I** |
|  | 18846 | **945.4380** | **1888.8614** | **1888.9014** | **-0.0400** | **1** | **1** | **81** | **5** | **R.DVQGTDASLDEELDRVK.M** |
|  | 20240 | **1007.5356** | **2013.0567** | **2013.0684** | **-0.0116** | **0** | **22** | **0.54** | **1** | **R.NTVVLFVPQQEAWVVER.M** 20239 |
|  | 20797 | **1031.0408** | **2060.0670** | **2060.0385** | **0.0284** | **1** | **1** | **81** | **4** | **R.ATVLESEGTRESAINVAEGK.K** |
|  | 23250 | **1165.5455** | **2329.0765** | **2329.0896** | **-0.0131** | **0** | **85** | **2.3e-007** | **1** | **K.ASYGVEDPEYAVTQLAQTTMR.S** 23251 |
|  | 23497 | **786.9083** | **2357.7032** | **2357.2314** | **0.4717** | **1** | **1** | **46** | **10** | **R.NTVVLFVPQQEAWVVERMGR.F** |
|  | 27657 | 767.1752 | 3064.6716 | 3065.5049 | -0.8334 | 0 | 17 | 0.93 | 2 | K.DSNTILLPSNPGDVTSMVAQAMGVYGALTK.A |

| **372.** | O75264\|YS019_HUMAN    **Mass:** 15012    **Score:** 130    **Queries matched:** 8   **emPAI:** 1.00 |
| --- | --- |
|  | Transmembrane protein HSPC323 OS=Homo sapiens GN=HSPC323 PE=2 SV=2 |

|  | **Query** | **Observed** | **Mr(expt)** | **Mr(calc)** | **Delta** | **Miss** | **Score** | **Expect** | **Rank** | **Peptide** |
| --- | --- | --- | --- | --- | --- | --- | --- | --- | --- | --- |
|  | 2735 | **420.7431** | **839.4716** | **838.3569** | **1.1147** | **0** | **6** | **39** | **4** | **K.EPGDHER.A** |
|  | 16663 | 866.8809 | 1731.7473 | 1731.8526 | -0.1053 | 1 | 4 | 39 | 3 | K.TAKEGESNLGLDLEEK.E |
|  | 16808 | **871.8804** | **1741.7462** | **1741.7577** | **-0.0115** | **1** | **67** | **1.9e-005** | **1** | **R.MESNLYQDQSEDKR.E** 16807 16809 16810 |
|  | 22630 | **751.6750** | **2252.0031** | **2252.0193** | **-0.0161** | **1** | **57** | **0.00015** | **1** | **K.EGESNLGLDLEEKEPGDHER.A** |
|  | 27407 | **1008.9846** | **3023.9320** | **3023.6001** | **0.3319** | **0** | **1** | **30** | **1** | **-.METLGALLVLEFLLLSPVEAQQATEHR.L** |

| **373.** | Q99867\|TBB4Q_HUMAN    **Mass:** 48917    **Score:** 129    **Queries matched:** 10   **emPAI:** 0.16 |
| --- | --- |
|  | Putative tubulin beta-4q chain OS=Homo sapiens GN=TUBB4Q PE=5 SV=1 |

|  | **Query** | **Observed** | **Mr(expt)** | **Mr(calc)** | **Delta** | **Miss** | **Score** | **Expect** | **Rank** | **Peptide** |
| --- | --- | --- | --- | --- | --- | --- | --- | --- | --- | --- |
|  | 2296 | 405.2068 | 808.3991 | 807.3399 | 1.0593 | 0 | 8 | 28 | 7 | R.EEYPDR.I |
|  | 6152 | 539.2660 | 1076.5174 | 1076.5250 | -0.0076 | 1 | 25 | 0.51 | 1 | K.IREEYPDR.I |
|  | 10148 | **659.7482** | **1317.4819** | **1317.5996** | **-0.1177** | **1** | **1** | **1.1e+002** | **8** | **R.QCGAGNNWAKGR.Y** |
|  | 14745 | 801.4085 | 1600.8023 | 1600.8131 | -0.0107 | 0 | 51 | 0.001 | 1 | R.AVLVDLEPGTMDSVR.S |
|  | 18227 | **615.3779** | **1843.1120** | **1843.8332** | **-0.7212** | **0** | **(1)** | **69** | **2** | **R.YTEGAELMESVMDVVR.K** 18214 |
|  | 18251 | **615.7810** | **1844.3212** | **1843.8332** | **0.4880** | **0** | **5** | **23** | **1** | **R.YTEGAELMESVMDVVR.K** |
|  | 28611 | 1104.5131 | 3310.5174 | 3310.5268 | -0.0095 | 0 | 79 | 5.2e-007 | 1 | K.EAESCDCLQGFQLTHSLGGGTGSGMGTLLLSK.I 28610 |
|  | 31452 | **1689.0008** | **5063.9807** | **5065.1164** | **-1.1357** | **1** | **1** | **6.8** | **2** | **R.KAFLHWYTGEGMDEMEFTEAESNMNDLVSEYQQYQDATAEGEGV.-** |

| **374.** | Q8NHP1\|ARK74_HUMAN    **Mass:** 37290    **Score:** 129    **Queries matched:** 15   **emPAI:** 0.21 |
| --- | --- |
|  | Aflatoxin B1 aldehyde reductase member 4 OS=Homo sapiens GN=AKR7L PE=2 SV=5 |

|  | **Query** | **Observed** | **Mr(expt)** | **Mr(calc)** | **Delta** | **Miss** | **Score** | **Expect** | **Rank** | **Peptide** |
| --- | --- | --- | --- | --- | --- | --- | --- | --- | --- | --- |
|  | 1460 | 636.3673 | 635.3600 | 634.3438 | 1.0161 | 0 | 19 | 2.2 | 1 | R.AFLER.G |
|  | 3361 | 439.2453 | 876.4760 | 876.4599 | 0.0161 | 1 | 13 | 8.8 | 8 | -.MSRQLSR.A 3371 3373 |
|  | 5638 | 520.7707 | 1039.5268 | 1038.4586 | 1.0682 | 1 | 7 | 33 | 4 | R.MGSSDCRVK.I |
|  | 9382 | 638.8245 | 1275.6344 | 1274.6401 | 0.9943 | 1 | 43 | 0.007 | 1 | R.RMDAPTSAAVTR.A 9345 |
|  | 9656 | 646.3151 | 1290.6156 | 1290.6350 | -0.0195 | 1 | (3) | 71 | 7 | R.RMDAPTSAAVTR.A |
|  | 12608 | 487.2953 | 1458.8641 | 1458.7435 | 0.1206 | 0 | 2 | 86 | 10 | R.ARPATVLGAMEMGR.R |
|  | 14496 | **792.9191** | **1583.8236** | **1583.8348** | **-0.0112** | **0** | **61** | **9.8e-005** | **1** | **R.FYAYNPLAGGLLTGK.Y** 14482 14511 |
|  | 16697 | 867.9437 | 1733.8728 | 1734.8723 | -0.9995 | 1 | (3) | 53 | 6 | R.MDAPTSAAVTRAFLER.G 16718 |
|  | 16919 | 875.9356 | 1749.8565 | 1750.8672 | -1.0107 | 1 | 7 | 21 | 3 | R.MDAPTSAAVTRAFLER.G |

| **375.** | P84085\|ARF5_HUMAN    **Mass:** 20631    **Score:** 128    **Queries matched:** 19   **emPAI:** 0.67 |
| --- | --- |
|  | ADP-ribosylation factor 5 OS=Homo sapiens GN=ARF5 PE=1 SV=2 |

|  | **Query** | **Observed** | **Mr(expt)** | **Mr(calc)** | **Delta** | **Miss** | **Score** | **Expect** | **Rank** | **Peptide** |
| --- | --- | --- | --- | --- | --- | --- | --- | --- | --- | --- |
|  | 2701 | **419.3143** | **836.6140** | **835.5028** | **1.1112** | **0** | **9** | **18** | **7** | **K.LGLQHLR.S** |
|  | 2763 | 421.2294 | 840.4443 | 839.5130 | 0.9313 | 0 | 8 | 24 | 8 | K.IRPLWR.H |
|  | 6195 | **540.7406** | **1079.4666** | **1078.6359** | **0.8307** | **1** | **4** | **65** | **6** | **K.LGLQHLRSR.T** 6168 6193 |
|  | 6295 | 544.3089 | 1086.6032 | 1086.6107 | -0.0075 | 0 | 38 | 0.027 | 1 | R.ILMVGLDAAGK.T |
|  | 6491 | 551.7946 | 1101.5746 | 1102.6056 | -1.0311 | 0 | (1) | 1.5e+002 | 5 | R.ILMVGLDAAGK.T |
|  | 7839 | **400.2123** | **1197.6150** | **1196.6223** | **0.9927** | **0** | **7** | **33** | **9** | **-.MGLTVSALFSR.I** |
|  | 13303 | 751.8984 | 1501.7823 | 1501.8109 | -0.0285 | 1 | 7 | 28 | 4 | K.QMRILMVGLDAAGK.T |
|  | 13538 | 759.8886 | 1517.7625 | 1517.8058 | -0.0432 | 1 | (3) | 61 | 7 | K.QMRILMVGLDAAGK.T |
|  | 17881 | 608.3079 | 1821.9019 | 1822.0274 | -0.1255 | 1 | 4 | 51 | 1 | R.ILMVGLDAAGKTTILYK.L 17891 |
|  | 21757 | 718.3621 | 2152.0644 | 2152.0338 | 0.0306 | 0 | 5 | 30 | 2 | R.HYFQNTQGLIFVVDSNDR.E |
|  | 23212 | 1162.1194 | 2322.2242 | 2322.2359 | -0.0117 | 0 | 46 | 0.0018 | 1 | K.LGEIVTTIPTIGFNVETVEYK.N 23210 23211 23213 |
|  | 24055 | 813.3949 | 2437.1629 | 2437.1775 | -0.0146 | 1 | 42 | 0.0041 | 1 | R.HYFQNTQGLIFVVDSNDRER.V |
|  | 24625 | **837.1755** | **2508.5048** | **2508.2465** | **0.2583** | **1** | **0** | **49** | **4** | **K.QDMPNAMPVSELTDKLGLQHLR.S** |

| **376.** | P55795\|HNRH2_HUMAN    **Mass:** 49517    **Score:** 128    **Queries matched:** 13   **emPAI:** 0.16 |
| --- | --- |
|  | Heterogeneous nuclear ribonucleoprotein H2 OS=Homo sapiens GN=HNRNPH2 PE=1 SV=1 |

|  | **Query** | **Observed** | **Mr(expt)** | **Mr(calc)** | **Delta** | **Miss** | **Score** | **Expect** | **Rank** | **Peptide** |
| --- | --- | --- | --- | --- | --- | --- | --- | --- | --- | --- |
|  | 1980 | **784.5820** | **783.5748** | **783.4167** | **0.1581** | **0** | **3** | **68** | **1** | **R.YVEVFK.S** |
|  | 2062 | **396.7324** | **791.4502** | **792.3800** | **-0.9298** | **0** | **7** | **37** | **9** | **M.MLSTEGR.E** |
|  | 4050 | **463.2324** | **924.4503** | **923.4205** | **1.0299** | **0** | **8** | **22** | **6** | **-.MMLSTEGR.E** |
|  | 4240 | 470.2401 | 938.4657 | 939.4154 | -0.9496 | 0 | (6) | 35 | 10 | -.MMLSTEGR.E |
|  | 4271 | **471.2623** | **940.5100** | **939.4154** | **1.0946** | **0** | **(2)** | **98** | **9** | **-.MMLSTEGR.E** |
|  | 6375 | **547.2921** | **1092.5697** | **1091.5724** | **0.9973** | **0** | **11** | **12** | **1** | **R.VHIEIGPDGR.V** 6372 |
|  | 10542 | 447.1784 | 1338.5134 | 1339.6633 | -1.1499 | 1 | 4 | 48 | 5 | R.AEVRTHYDPPR.K |
|  | 15140 | **542.3879** | **1624.1418** | **1624.8685** | **-0.7267** | **1** | **4** | **33** | **2** | **K.IQNGTSGIRFIYTR.E** |
|  | 16030 | **843.3839** | **1684.7533** | **1683.7601** | **0.9931** | **0** | **58** | **0.00017** | **1** | **K.HTGPNSPDTANDGFVR.L** 16013 |
|  | 18182 | **921.4423** | **1840.8700** | **1840.8843** | **-0.0143** | **0** | **83** | **5.1e-007** | **1** | **R.STGEAFVQFASQEIAEK.A** |
|  | 21467 | 1061.0453 | 2120.0760 | 2119.9909 | 0.0851 | 0 | 2 | 56 | 3 | R.EGRPSGEAFVELESEEEVK.L |

| **377.** | P31943\|HNRH1_HUMAN    **Mass:** 49484    **Score:** 128    **Queries matched:** 12   **emPAI:** 0.16 |
| --- | --- |
|  | Heterogeneous nuclear ribonucleoprotein H OS=Homo sapiens GN=HNRNPH1 PE=1 SV=4 |

|  | **Query** | **Observed** | **Mr(expt)** | **Mr(calc)** | **Delta** | **Miss** | **Score** | **Expect** | **Rank** | **Peptide** |
| --- | --- | --- | --- | --- | --- | --- | --- | --- | --- | --- |
|  | 1980 | 784.5820 | 783.5748 | 783.4167 | 0.1581 | 0 | 3 | 68 | 1 | R.YVEVFK.S |
|  | 6375 | 547.2921 | 1092.5697 | 1091.5724 | 0.9973 | 0 | 11 | 12 | 1 | R.VHIEIGPDGR.V 6372 |
|  | 10251 | 662.3094 | 1322.6043 | 1322.6541 | -0.0497 | 0 | 20 | 1.3 | 2 | M.MLGTEGGEGFVVK.V 10245 10250 |
|  | 10535 | **669.8175** | **1337.6204** | **1338.6490** | **-1.0285** | **0** | **(5)** | **47** | **9** | **M.MLGTEGGEGFVVK.V** |
|  | 10542 | 447.1784 | 1338.5134 | 1339.6633 | -1.1499 | 1 | 4 | 48 | 5 | R.AEVRTHYDPPR.K |
|  | 16030 | 843.3839 | 1684.7533 | 1683.7601 | 0.9931 | 0 | 58 | 0.00017 | 1 | K.HTGPNSPDTANDGFVR.L 16013 |
|  | 18182 | 921.4423 | 1840.8700 | 1840.8843 | -0.0143 | 0 | 83 | 5.1e-007 | 1 | R.STGEAFVQFASQEIAEK.A |
|  | 20078 | **998.9870** | **1995.9594** | **1995.9690** | **-0.0096** | **0** | **1** | **69** | **2** | **R.ATENDIYNFFSPLNPVR.V** |

| **378.** | P00558\|PGK1_HUMAN    **Mass:** 44985    **Score:** 127    **Queries matched:** 20   **emPAI:** 0.62 |
| --- | --- |
|  | Phosphoglycerate kinase 1 OS=Homo sapiens GN=PGK1 PE=1 SV=3 |

|  | **Query** | **Observed** | **Mr(expt)** | **Mr(calc)** | **Delta** | **Miss** | **Score** | **Expect** | **Rank** | **Peptide** |
| --- | --- | --- | --- | --- | --- | --- | --- | --- | --- | --- |
|  | 3767 | **452.7406** | **903.4667** | **903.4735** | **-0.0068** | **0** | **16** | **5.4** | **1** | **K.ALMDEVVK.A** |
|  | 4404 | **475.2840** | **948.5534** | **948.5314** | **0.0220** | **1** | **4** | **65** | **9** | **K.IVKDLMSK.A** |
|  | 7903 | **601.3303** | **1200.6461** | **1200.6536** | **-0.0075** | **0** | **24** | **0.58** | **1** | **K.IQLINNMLDK.V** 7936 |
|  | 10508 | **446.2690** | **1335.7852** | **1334.6864** | **1.0988** | **1** | **6** | **31** | **5** | **K.ALMDEVVKATSR.G** |
|  | 11016 | 456.5712 | 1366.6918 | 1366.7027 | -0.0109 | 0 | 13 | 6.7 | 1 | R.AHSSMVGVNLPQK.A |
|  | 12481 | **726.4460** | **1450.8775** | **1449.7472** | **1.1303** | **1** | **4** | **50** | **1** | **R.VVMRVDFNVPMK.N** |
|  | 15304 | **545.9330** | **1634.7771** | **1633.7849** | **0.9922** | **0** | **27** | **0.24** | **1** | **K.LGDVYVNDAFGTAHR.A** |
|  | 16979 | **877.8918** | **1753.7691** | **1753.7798** | **-0.0106** | **0** | **25** | **0.35** | **1** | **R.GCITIIGGGDTATCCAK.W** |
|  | 17177 | **884.9966** | **1767.9786** | **1767.9883** | **-0.0097** | **0** | **43** | **0.005** | **1** | **K.ALESPERPFLAILGGAK.V** 17178 |
|  | 19769 | **983.9689** | **1965.9232** | **1965.9353** | **-0.0121** | **0** | **59** | **0.00011** | **1** | **K.VLNNMEIGTSLFDEEGAK.I** |
|  | 20644 | **512.5437** | **2046.1457** | **2046.9905** | **-0.8448** | **1** | **7** | **17** | **2** | **R.VDFNVPMKNNQITNNQR.I** |
|  | 21232 | **1053.0278** | **2104.0411** | **2104.0531** | **-0.0119** | **0** | **58** | **0.00015** | **1** | **K.QIVWNGPVGVFEWEAFAR.G** |
|  | 25249 | **869.0792** | **2604.2158** | **2604.2312** | **-0.0154** | **1** | **18** | **1** | **1** | **K.TGQATVASGIPAGWMGLDCGPESSKK.Y** |
|  | 26273 | **696.1047** | **2780.3896** | **2781.3789** | **-0.9893** | **1** | **5** | **21** | **3** | **K.DCVGPEVEKACANPAAGSVILLENLR.F** |
|  | 27247 | 746.8489 | 2983.3667 | 2982.4770 | 0.8896 | 1 | 1 | 37 | 7 | K.LGDVYVNDAFGTAHRAHSSMVGVNLPQK.A |
|  | 28490 | **816.6793** | **3262.6882** | **3262.7094** | **-0.0212** | **1** | **13** | **2.2** | **1** | **K.SVVLMSHLGRPDGVPMPDKYSLEPVAVELK.S** 28491 |
|  | 29848 | **931.4332** | **3721.7036** | **3722.7882** | **-1.0846** | **1** | **2** | **24** | **8** | **K.VNEMIIGGGMAFTFLKVLNNMEIGTSLFDEEGAK.I** |

| **379.** | P10599\|THIO_HUMAN    **Mass:** 12015    **Score:** 126    **Queries matched:** 8   **emPAI:** 1.36 |
| --- | --- |
|  | Thioredoxin OS=Homo sapiens GN=TXN PE=1 SV=3 |

|  | **Query** | **Observed** | **Mr(expt)** | **Mr(calc)** | **Delta** | **Miss** | **Score** | **Expect** | **Rank** | **Peptide** |
| --- | --- | --- | --- | --- | --- | --- | --- | --- | --- | --- |
|  | 3819 | **454.7241** | **907.4336** | **907.4399** | **-0.0063** | **0** | **60** | **0.00015** | **1** | **K.VGEFSGANK.E** 3818 3820 3843 |
|  | 9001 | 629.3394 | 1256.6643 | 1257.6816 | -1.0173 | 1 | 11 | 13 | 10 | K.EKLEATINELV.- 9000 |
|  | 10499 | **668.8180** | **1335.6215** | **1335.6306** | **-0.0091** | **0** | **66** | **3.3e-005** | **1** | **K.TAFQEALDAAGDK.L** |
|  | 12675 | **732.3848** | **1462.7551** | **1462.7642** | **-0.0091** | **0** | **25** | **0.37** | **1** | **K.MIKPFFHSLSEK.Y** |

| **380.** | O95299\|NDUAA_HUMAN    **Mass:** 41067    **Score:** 126    **Queries matched:** 19   **emPAI:** 0.69 |
| --- | --- |
|  | NADH dehydrogenase [ubiquinone] 1 alpha subcomplex subunit 10, mitochondrial OS=Homo sapiens GN=NDUFA10 PE=1 SV=1 |

|  | **Query** | **Observed** | **Mr(expt)** | **Mr(calc)** | **Delta** | **Miss** | **Score** | **Expect** | **Rank** | **Peptide** |
| --- | --- | --- | --- | --- | --- | --- | --- | --- | --- | --- |
|  | 3693 | 450.7338 | 899.4531 | 900.5280 | -1.0749 | 1 | 21 | 1.3 | 3 | K.LAKEIAEK.L 3714 3731 |
|  | 6784 | **563.6085** | **1125.2024** | **1124.5284** | **0.6739** | **0** | **12** | **6.9** | **1** | **K.CEVLQYSAR.E** |
|  | 7932 | **601.8079** | **1201.6012** | **1200.7190** | **0.8822** | **1** | **6** | **39** | **1** | **R.LLKLAATSASAR.V** 7892 |
|  | 8587 | **618.3439** | **1234.6732** | **1234.6809** | **-0.0077** | **1** | **20** | **1.4** | **1** | **K.KVVEDIEYLK.F** 8565 |
|  | 9387 | **638.8275** | **1275.6403** | **1275.6493** | **-0.0090** | **0** | **35** | **0.042** | **1** | **R.VITVDGNICTGK.G** 9386 |
|  | 9524 | **428.9195** | **1283.7366** | **1283.6768** | **0.0597** | **1** | **3** | **60** | **1** | **R.GIHSSVQCKLR.Y** |
|  | 10066 | **657.8320** | **1313.6494** | **1314.6793** | **-1.0299** | **1** | **4** | **53** | **7** | **K.QDNRTLYHLR.L** |
|  | 10382 | **665.7886** | **1329.5627** | **1328.5885** | **0.9743** | **0** | **17** | **2.6** | **1** | **K.YSPGYNTEVGDK.W** |
|  | 10759 | **451.2638** | **1350.7697** | **1350.7520** | **0.0177** | **1** | **5** | **39** | **9** | **R.VLHQFRELPGR.K** |
|  | 11313 | **692.3232** | **1382.6319** | **1381.6489** | **0.9830** | **0** | **0** | **1.4e+002** | **7** | **R.YGMWHFLLGDK.A** |
|  | 11945 | **710.3324** | **1418.6502** | **1418.6612** | **-0.0110** | **1** | **26** | **0.32** | **1** | **R.KQCVDHYNEVK.S** |
|  | 15192 | **814.9058** | **1627.7970** | **1627.8093** | **-0.0123** | **0** | **48** | **0.0018** | **1** | **K.ITSAYLQDIENAYK.K** |
|  | 24199 | **1228.1571** | **2454.2996** | **2454.3118** | **-0.0122** | **0** | **89** | **9e-008** | **1** | **R.LLQYSDALEHLLTTGQGVVLER.S** |
|  | 29353 | **889.9770** | **3555.8791** | **3555.8977** | **-0.0186** | **1** | **16** | **0.88** | **1** | **R.LLVQDKFEVLNYTSIPIFLPEVTIGAHQTDR.V** |

| **381.** | P61019\|RAB2A_HUMAN    **Mass:** 23702    **Score:** 125    **Queries matched:** 8   **emPAI:** 0.81 |
| --- | --- |
|  | Ras-related protein Rab-2A OS=Homo sapiens GN=RAB2A PE=1 SV=1 |

|  | **Query** | **Observed** | **Mr(expt)** | **Mr(calc)** | **Delta** | **Miss** | **Score** | **Expect** | **Rank** | **Peptide** |
| --- | --- | --- | --- | --- | --- | --- | --- | --- | --- | --- |
|  | 5403 | **512.2835** | **1022.5525** | **1021.4943** | **1.0582** | **0** | **6** | **40** | **8** | **-.MAYAYLFK.Y** |
|  | 10936 | **681.8353** | **1361.6561** | **1361.6649** | **-0.0088** | **0** | **19** | **1.6** | **1** | **R.EHGLIFMETSAK.T** |
|  | 13179 | **747.8700** | **1493.7254** | **1493.7362** | **-0.0107** | **0** | **48** | **0.0018** | **1** | **K.TASNVEEAFINTAK.E** |
|  | 13993 | **775.8786** | **1549.7426** | **1549.7525** | **-0.0099** | **0** | **62** | **8.4e-005** | **1** | **K.LQIWDTAGQESFR.S** 13975 |
|  | 16485 | **573.3469** | **1717.0188** | **1716.8287** | **0.1900** | **0** | **4** | **39** | **3** | **R.QHSNSNMVIMLIGNK.S** |
|  | 16489 | **573.6064** | **1717.7973** | **1717.8060** | **-0.0087** | **0** | **5** | **34** | **1** | **R.DTFNHLTTWLEDAR.Q** |
|  | 17079 | **880.9385** | **1759.8625** | **1759.8741** | **-0.0115** | **0** | **65** | **3.2e-005** | **1** | **K.IQEGVFDINNEANGIK.I** |

| **382.** | O15173\|PGRC2_HUMAN    **Mass:** 23861    **Score:** 124    **Queries matched:** 10   **emPAI:** 0.81 |
| --- | --- |
|  | Membrane-associated progesterone receptor component 2 OS=Homo sapiens GN=PGRMC2 PE=1 SV=1 |

|  | **Query** | **Observed** | **Mr(expt)** | **Mr(calc)** | **Delta** | **Miss** | **Score** | **Expect** | **Rank** | **Peptide** |
| --- | --- | --- | --- | --- | --- | --- | --- | --- | --- | --- |
|  | 1959 | **773.4210** | **772.4137** | **771.3551** | **1.0585** | **0** | **23** | **1.1** | **1** | **K.YDYVGR.L** |
|  | 5046 | **499.2230** | **996.4314** | **996.4375** | **-0.0061** | **0** | **7** | **31** | **8** | **R.EWEMQFK.E** |
|  | 5196 | 503.7832 | 1005.5518 | 1006.5083 | -0.9565 | 0 | 29 | 0.24 | 1 | R.DFSLEQLR.Q |
|  | 5413 | 1024.5066 | 1023.4993 | 1023.5059 | -0.0066 | 0 | 31 | 0.13 | 1 | R.GLATFCLDK.D |
|  | 12801 | **736.8651** | **1471.7157** | **1471.7248** | **-0.0092** | **0** | **64** | **5.5e-005** | **1** | **K.FYGPAGPYGIFAGR.D** |
|  | 16817 | 872.3856 | 1742.7567 | 1743.8733 | -1.1166 | 1 | 2 | 65 | 8 | K.GSKFYGPAGPYGIFAGR.D 16852 |
|  | 18392 | 618.9980 | 1853.9721 | 1852.9391 | 1.0330 | 1 | 5 | 37 | 7 | R.RGLGAGAGAGEESPATSLPR.M |
|  | 24971 | **1277.5830** | **2553.1515** | **2553.1653** | **-0.0138** | **1** | **69** | **9e-006** | **1** | **K.DALRDEYDDLSDLNAVQMESVR.E** 24970 |

| **383.** | Q8WWA0\|ITLN1_HUMAN    **Mass:** 35510    **Score:** 123    **Queries matched:** 11   **emPAI:** 0.50 |
| --- | --- |
|  | Intelectin-1 OS=Homo sapiens GN=ITLN1 PE=1 SV=1 |

|  | **Query** | **Observed** | **Mr(expt)** | **Mr(calc)** | **Delta** | **Miss** | **Score** | **Expect** | **Rank** | **Peptide** |
| --- | --- | --- | --- | --- | --- | --- | --- | --- | --- | --- |
|  | 7918 | **401.2846** | **1200.8319** | **1200.5928** | **0.2391** | **0** | **11** | **10** | **5** | **R.EFTAGFVQFR.V** |
|  | 11712 | **703.8176** | **1405.6206** | **1405.6296** | **-0.0090** | **0** | **15** | **3.8** | **1** | **K.EWTCSSSPSLPR.S** |
|  | 20773 | **687.3278** | **2058.9615** | **2058.9721** | **-0.0106** | **1** | **38** | **0.016** | **1** | **K.EIKDECPSAFDGLYFLR.T** 20772 |
|  | 21780 | **1077.0491** | **2152.0836** | **2152.0953** | **-0.0117** | **0** | **57** | **0.00018** | **1** | **R.TDTGFLQTLGHNLFGIYQK.Y** 21779 21781 |
|  | 29832 | 930.4456 | 3717.7534 | 3716.6658 | 1.0876 | 1 | 1 | 28 | 8 | R.TENGVIYQTFCDMTSGGGGWTLVASVHENDMRGK.C |
|  | 29879 | **1867.1158** | **3732.2171** | **3732.6607** | **-0.4436** | **1** | **(0)** | **24** | **8** | **R.TENGVIYQTFCDMTSGGGGWTLVASVHENDMRGK.C** |
|  | 30642 | **1388.2701** | **4161.7886** | **4161.8140** | **-0.0254** | **1** | **69** | **2.7e-006** | **1** | **K.AVYPEGDGNWANYNTFGSAEAATSDDYKNPGYYDIQAK.D** |
|  | 31441 | **1003.4123** | **5012.0254** | **5012.0675** | **-0.0422** | **0** | **1** | **7.3** | **1** | **R.VTGCNTEHHCIGGGGYFPEASPQQCGDFSGFDWSGYGTHVGYSSSR.E** |

| **384.** | Q9P0L0\|VAPA_HUMAN    **Mass:** 28103    **Score:** 123    **Queries matched:** 21   **emPAI:** 0.66 |
| --- | --- |
|  | Vesicle-associated membrane protein-associated protein A OS=Homo sapiens GN=VAPA PE=1 SV=3 |

|  | **Query** | **Observed** | **Mr(expt)** | **Mr(calc)** | **Delta** | **Miss** | **Score** | **Expect** | **Rank** | **Peptide** |
| --- | --- | --- | --- | --- | --- | --- | --- | --- | --- | --- |
|  | 1031 | **546.3209** | **545.3136** | **544.2969** | **1.0167** | **0** | **12** | **24** | **4** | **K.TTAPR.R** |
|  | 1249 | **590.3257** | **589.3185** | **588.2867** | **1.0317** | **0** | **9** | **36** | **2** | **R.DEGLR.L** |
|  | 2670 | 418.7011 | 835.3876 | 835.3932 | -0.0056 | 0 | 11 | 13 | 2 | R.LQGEMMK.L 2669 |
|  | 3234 | **435.2161** | **868.4177** | **867.3830** | **1.0347** | **0** | **(10)** | **16** | **3** | **R.LQGEMMK.L** |
|  | 4985 | 496.7733 | 991.5320 | 991.4943 | 0.0377 | 1 | 9 | 20 | 8 | K.RLQGEMMK.L 4969 |
|  | 5025 | **498.2691** | **994.5236** | **994.5308** | **-0.0072** | **1** | **18** | **2.5** | **2** | **R.HLRDEGLR.L** |
|  | 13711 | **766.4059** | **1530.7972** | **1530.7711** | **0.0261** | **1** | **3** | **56** | **5** | **K.EAKPDELMDSKLR.C** |
|  | 14249 | **783.9246** | **1565.8346** | **1565.8454** | **-0.0108** | **1** | **49** | **0.0017** | **1** | **K.FKGPFTDVVTTNLK.L** |
|  | 14424 | **527.2564** | **1578.7474** | **1579.7334** | **-0.9860** | **1** | **7** | **26** | **1** | **R.LQGEMMKLSEENR.H** |
|  | 15030 | **809.9425** | **1617.8704** | **1616.8774** | **0.9931** | **0** | **59** | **0.00014** | **1** | **K.HEQILVLDPPTDLK.F** 15012 15013 |
|  | 17278 | 592.6347 | 1774.8823 | 1774.8962 | -0.0139 | 1 | 24 | 0.42 | 1 | R.KVAHSDKPGSTSTASFR.D |
|  | 23126 | **1156.5520** | **2311.0894** | **2311.1017** | **-0.0123** | **0** | **13** | **3.8** | **1** | **K.FMVQTIFAPPNTSDMEAVWK.E** 23127 |
|  | 24979 | **852.0762** | **2553.2069** | **2554.2771** | **-1.0702** | **1** | **2** | **45** | **5** | **-.MASASGAMAKHEQILVLDPPTDLK.F** 24980 24987 |
|  | 29467 | **1200.2380** | **3597.6923** | **3597.7120** | **-0.0197** | **0** | **46** | **0.0008** | **1** | **R.YCVRPNSGIIDPGSTVTVSVMLQPFDYDPNEK.S** |

| **385.** | P63244\|GBLP_HUMAN    **Mass:** 35511    **Score:** 123    **Queries matched:** 12   **emPAI:** 0.35 |
| --- | --- |
|  | Guanine nucleotide-binding protein subunit beta-2-like 1 OS=Homo sapiens GN=GNB2L1 PE=1 SV=3 |

|  | **Query** | **Observed** | **Mr(expt)** | **Mr(calc)** | **Delta** | **Miss** | **Score** | **Expect** | **Rank** | **Peptide** |
| --- | --- | --- | --- | --- | --- | --- | --- | --- | --- | --- |
|  | 1324 | **605.3616** | **604.3544** | **603.3592** | **0.9952** | **1** | **11** | **22** | **8** | **R.DKTIK.L** |
|  | 5772 | 526.2568 | 1050.4991 | 1049.5579 | 0.9412 | 1 | 8 | 24 | 5 | R.DKTIIMWK.L |
|  | 7738 | 398.4728 | 1192.3965 | 1191.5520 | 0.8445 | 0 | 8 | 19 | 3 | R.DETNYGIPQR.A |
|  | 10624 | **672.3343** | **1342.6541** | **1343.7384** | **-1.0842** | **1** | **9** | **16** | **3** | **K.LVKVWNLANCK.L** |
|  | 11775 | **705.3727** | **1408.7309** | **1407.7214** | **1.0095** | **1** | **1** | **1.1e+002** | **3** | **-.MTEQMTLRGTLK.G** |
|  | 12864 | **738.8382** | **1475.6618** | **1475.6715** | **-0.0096** | **0** | **53** | **0.00065** | **1** | **K.DGQAMLWDLNEGK.H** 12865 |
|  | 17458 | **894.9960** | **1787.9775** | **1787.9880** | **-0.0105** | **1** | **58** | **0.00017** | **1** | **K.IIVDELKQEVISTSSK.A** 17459 |
|  | 22837 | **759.3726** | **2275.0959** | **2275.1055** | **-0.0097** | **0** | **7** | **16** | **1** | **K.HLYTLDGGDIINALCFSPNR.Y** |
|  | 25366 | **876.7690** | **2627.2851** | **2626.2962** | **0.9889** | **0** | **19** | **0.8** | **1** | **K.GHNGWVTQIATTPQFPDMILSASR.D** |
|  | 27452 | **1009.8438** | **3026.5094** | **3025.5444** | **0.9650** | **1** | **7** | **11** | **1** | **R.GTLKGHNGWVTQIATTPQFPDMILSASR.D** |

| **386.** | Q13423\|NNTM_HUMAN    **Mass:** 114564   **Score:** 123    **Queries matched:** 24   **emPAI:** 0.14 |
| --- | --- |
|  | NAD(P) transhydrogenase, mitochondrial OS=Homo sapiens GN=NNT PE=1 SV=3 |

|  | **Query** | **Observed** | **Mr(expt)** | **Mr(calc)** | **Delta** | **Miss** | **Score** | **Expect** | **Rank** | **Peptide** |
| --- | --- | --- | --- | --- | --- | --- | --- | --- | --- | --- |
|  | 1449 | 634.3548 | 633.3475 | 633.3520 | -0.0045 | 0 | 15 | 8.3 | 1 | R.GTVVMK.D |
|  | 2148 | 400.2021 | 798.3896 | 799.3608 | -0.9713 | 0 | 9 | 22 | 8 | R.MLDMFK.R |
|  | 4423 | **476.2577** | **950.5008** | **949.4902** | **1.0106** | **1** | **9** | **19** | **4** | **K.MLTEQGKK.V** 4432 |
|  | 4885 | **493.2448** | **984.4750** | **983.6015** | **0.8735** | **1** | **2** | **1.1e+002** | **1** | **K.LLKAISPDK.D** |
|  | 4920 | **494.7552** | **987.4959** | **988.5077** | **-1.0118** | **0** | **5** | **61** | **8** | **K.TVAELEAEK.A** |
|  | 6050 | **536.2308** | **1070.4470** | **1071.6176** | **-1.1706** | **0** | **6** | **43** | **1** | **K.EVLASDLVVK.V** |
|  | 8482 | 615.5700 | 1229.1255 | 1229.6438 | -0.5183 | 0 | 17 | 2.3 | 1 | K.TTVLAMDQVPR.V |
|  | 9743 | **432.7295** | **1295.1667** | **1294.7609** | **0.4059** | **0** | **13** | **4.7** | **4** | **R.VALSPAGVQNLVK.Q** |
|  | 10309 | 663.8528 | 1325.6911 | 1326.7871 | -1.0960 | 1 | 3 | 67 | 5 | K.EVLASDLVVKVR.A |
|  | 13612 | 762.9544 | 1523.8943 | 1523.9035 | -0.0093 | 0 | 54 | 0.00046 | 1 | K.ILIVGGGVAGLASAGAAK.S |
|  | 14129 | **520.2770** | **1557.8091** | **1556.6777** | **1.1315** | **1** | **3** | **64** | **5** | **K.ESGEGQGGYAKEMSK.E** |
|  | 14387 | **526.2806** | **1575.8201** | **1574.9872** | **0.8329** | **1** | **3** | **56** | **4** | **K.LQGLLKSAPLLLPGR.H** |
|  | 14676 | **533.1525** | **1596.4358** | **1595.9498** | **0.4860** | **1** | **1** | **59** | **10** | **K.EVDILISTALIPGKK.A** |
|  | 15427 | 821.9036 | 1641.7926 | 1641.8032 | -0.0106 | 0 | 41 | 0.0094 | 1 | R.MATQASTLYSNNITK.L |
|  | 20255 | **672.7134** | **2015.1185** | **2016.2095** | **-1.0910** | **1** | **7** | **17** | **1** | **K.VPPAKILIVGGGVAGLASAGAAK.S** 20257 |
|  | 21048 | 697.3547 | 2089.0422 | 2088.0197 | 1.0225 | 0 | 2 | 59 | 3 | R.VTIAQGYDALSSMANIAGYK.A |
|  | 22247 | **1103.5864** | **2205.1583** | **2205.1681** | **-0.0098** | **0** | **38** | **0.012** | **1** | **K.TSGTLISFIYPAQNPELLNK.L** |
|  | 22646 | **752.3522** | **2254.0347** | **2254.2831** | **-0.2484** | **1** | **6** | **21** | **1** | **K.ILIVGGGVAGLASAGAAKSMGAIVR.G** |
|  | 26459 | **942.4840** | **2824.4300** | **2824.4469** | **-0.0169** | **0** | **50** | **0.00055** | **1** | **R.SLGVGYAAVDNPIFYKPNTAMLLGDAK.K** |
|  | 27309 | 1499.6708 | 2997.3270 | 2996.5430 | 0.7841 | 1 | 3 | 25 | 2 | K.RSLGVGYAAVDNPIFYKPNTAMLLGDAK.K |
|  | 28290 | **1075.1764** | **3222.5073** | **3223.6448** | **-1.1374** | **1** | **0** | **43** | **2** | **R.VTIAQGYDALSSMANIAGYKAVVLAANHFGR.F** |
|  | 30534 | **1030.0114** | **4116.0163** | **4117.0460** | **-1.0297** | **0** | **0** | **23** | **2** | **R.SLANVILGGYGTTSTAGGKPMEISGTHTEINLDNAIDMIR.E** |

| **387.** | P30040\|ERP29_HUMAN    **Mass:** 29032    **Score:** 122    **Queries matched:** 14   **emPAI:** 0.44 |
| --- | --- |
|  | Endoplasmic reticulum protein ERp29 OS=Homo sapiens GN=ERP29 PE=1 SV=4 |

|  | **Query** | **Observed** | **Mr(expt)** | **Mr(calc)** | **Delta** | **Miss** | **Score** | **Expect** | **Rank** | **Peptide** |
| --- | --- | --- | --- | --- | --- | --- | --- | --- | --- | --- |
|  | 1483 | **643.3835** | **642.3762** | **642.3813** | **-0.0051** | **0** | **9** | **23** | **2** | **K.VGAIQR.W** |
|  | 4605 | **482.7176** | **963.4207** | **962.4742** | **0.9464** | **0** | **6** | **40** | **2** | **K.LNMELSEK.Y** |
|  | 6030 | 535.7793 | 1069.5440 | 1069.6396 | -0.0956 | 1 | 14 | 5.7 | 2 | K.VGAIQRWLK.G |
|  | 6130 | 538.2873 | 1074.5601 | 1074.5305 | 0.0296 | 0 | 9 | 24 | 2 | K.QGQDNLSSVK.E |
|  | 6919 | **568.3280** | **1134.6414** | **1133.6444** | **0.9970** | **0** | **17** | **3.3** | **1** | **K.SLNILTAFQK.K** |
|  | 10196 | **660.8316** | **1319.6486** | **1319.6550** | **-0.0064** | **0** | **4** | **64** | **1** | **K.ESYPVFYLFR.D** |
|  | 14849 | **804.8757** | **1607.7369** | **1607.7468** | **-0.0099** | **0** | **6** | **33** | **2** | **R.DGDFENPVPYTGAVK.V** |
|  | 16386 | **855.4744** | **1708.9343** | **1707.7774** | **1.1569** | **0** | **24** | **0.45** | **1** | **K.ILDQGEDFPASEMTR.I** 16351 |
|  | 17083 | **587.7952** | **1760.3639** | **1760.9672** | **-0.6033** | **1** | **2** | **51** | **5** | **K.EELQKSLNILTAFQK.K** |
|  | 25128 | **1292.1345** | **2582.2545** | **2582.2662** | **-0.0117** | **0** | **75** | **1.9e-006** | **1** | **K.GQGVYLGMPGCLPVYDALAGEFIR.A** 25129 |
|  | 28970 | **1138.2090** | **3411.6051** | **3411.6239** | **-0.0188** | **1** | **65** | **1.2e-005** | **1** | **R.LAENSASSDDLLVAEVGISDYGDKLNMELSEK.Y** |
|  | 29666 | **1218.6260** | **3652.8561** | **3652.0691** | **0.7870** | **1** | **1** | **28** | **4** | **-.MAAAVPRAAFLSPLLPLLLGFLLLSAPHGGSGLHTK.G** |

| **388.** | P33897\|ABCD1_HUMAN    **Mass:** 83398    **Score:** 121    **Queries matched:** 12   **emPAI:** 0.09 |
| --- | --- |
|  | ATP-binding cassette sub-family D member 1 OS=Homo sapiens GN=ABCD1 PE=1 SV=2 |

|  | **Query** | **Observed** | **Mr(expt)** | **Mr(calc)** | **Delta** | **Miss** | **Score** | **Expect** | **Rank** | **Peptide** |
| --- | --- | --- | --- | --- | --- | --- | --- | --- | --- | --- |
|  | 1639 | 677.3937 | 676.3864 | 676.3908 | -0.0044 | 0 | 22 | 1.5 | 2 | K.IFQAAK.D |
|  | 5604 | **519.2740** | **1036.5335** | **1035.5349** | **0.9987** | **1** | **1** | **1.2e+002** | **9** | **K.FEKLDSAAR.L** |
|  | 9004 | 629.3545 | 1256.6944 | 1256.6724 | 0.0220 | 0 | 9 | 18 | 10 | R.NLLTAAADAIER.I |
|  | 11654 | **702.3373** | **1402.6601** | **1402.6688** | **-0.0087** | **0** | **70** | **1.4e-005** | **1** | **R.ELEDAQAGSGTIGR.S** 11660 |
|  | 13793 | 513.2574 | 1536.7505 | 1536.8624 | -0.1118 | 0 | 0 | 1.1e+002 | 10 | R.ETGLLALHSAALVSR.T |
|  | 15263 | **816.8493** | **1631.6840** | **1631.8091** | **-0.1251** | **0** | **4** | **44** | **1** | **R.LWYVMLEQFLMK.Y** |
|  | 17441 | **894.4518** | **1786.8891** | **1786.8427** | **0.0464** | **0** | **5** | **33** | **3** | **K.YHTHLLQFDGEGGWK.F** 17442 |
|  | 18146 | 920.0045 | 1837.9945 | 1837.8516 | 0.1429 | 1 | 4 | 47 | 3 | R.DQVIYPDSVEDMQRK.G |
|  | 18358 | 926.9672 | 1851.9198 | 1851.9326 | -0.0129 | 0 | 74 | 4e-006 | 1 | R.GLQAPAGEPTQEASGVAAAK.A |
|  | 30309 | **1317.3718** | **3949.0937** | **3949.8516** | **-0.7579** | **1** | **1** | **19** | **7** | **K.GYSEQDLEAILDVVHLHHILQREGGWEAMCDWK.D** |

| **389.** | Q92520\|FAM3C_HUMAN    **Mass:** 24950    **Score:** 120    **Queries matched:** 7   **emPAI:** 0.33 |
| --- | --- |
|  | Protein FAM3C OS=Homo sapiens GN=FAM3C PE=1 SV=1 |

|  | **Query** | **Observed** | **Mr(expt)** | **Mr(calc)** | **Delta** | **Miss** | **Score** | **Expect** | **Rank** | **Peptide** |
| --- | --- | --- | --- | --- | --- | --- | --- | --- | --- | --- |
|  | 2222 | 402.7117 | 803.4089 | 803.4137 | -0.0048 | 0 | 10 | 20 | 5 | R.SALDTAAR.S |
|  | 4496 | **956.5459** | **955.5386** | **955.5451** | **-0.0064** | **0** | **46** | **0.0043** | **1** | **R.GINVALANGK.T** |
|  | 8176 | **607.3389** | **1212.6632** | **1213.6455** | **-0.9823** | **1** | **1** | **1.1e+002** | **5** | **K.TKSPFEQHIK.N** |
|  | 11273 | 461.2326 | 1380.6761 | 1379.6656 | 1.0105 | 1 | 2 | 93 | 10 | R.DNWVFCGGKGIK.T |
|  | 19797 | **984.9758** | **1967.9371** | **1967.9510** | **-0.0139** | **0** | **65** | **2.8e-005** | **1** | **K.AIQDGTIVLMGTYDDGATK.L** 19798 |
|  | 20483 | **678.9916** | **2033.9529** | **2033.0034** | **0.9495** | **1** | **4** | **40** | **1** | **K.ICLEDNVLMSGVKNNVGR.G** |

| **390.** | A6NIZ1\|RP1BL_HUMAN    **Mass:** 21140    **Score:** 120    **Queries matched:** 6   **emPAI:** 0.65 |
| --- | --- |
|  | Ras-related protein Rap-1b-like protein OS=Homo sapiens PE=2 SV=1 |

|  | **Query** | **Observed** | **Mr(expt)** | **Mr(calc)** | **Delta** | **Miss** | **Score** | **Expect** | **Rank** | **Peptide** |
| --- | --- | --- | --- | --- | --- | --- | --- | --- | --- | --- |
|  | 2481 | 412.7346 | 823.4546 | 824.4868 | -1.0323 | 1 | 5 | 45 | 8 | K.TPVPGKAR.K |
|  | 9013 | 420.2085 | 1257.6036 | 1257.5513 | 0.0523 | 0 | 7 | 31 | 8 | K.YDPTIEDSYR.E |
|  | 13182 | 747.8976 | 1493.7807 | 1493.7878 | -0.0071 | 0 | 71 | 9.7e-006 | 1 | K.INVNEIFYDLVR.Q |
|  | 14057 | 777.8486 | 1553.6827 | 1553.6933 | -0.0105 | 0 | 64 | 4.6e-005 | 1 | R.QWNNCAFLESSAK.S |
|  | 15746 | 833.4597 | 1664.9048 | 1664.9138 | -0.0090 | 0 | 34 | 0.049 | 1 | K.SALTVQFVQGIFVEK.Y |
|  | 27883 | **1044.1863** | **3129.5370** | **3128.4100** | **1.1270** | **0** | **2** | **29** | **4** | **R.EQVEVDAQQCMLEILDTAGTEQFTAMR.D** |

| **391.** | P36537\|UDB10_HUMAN    **Mass:** 61190    **Score:** 119    **Queries matched:** 22   **emPAI:** 0.27 |
| --- | --- |
|  | UDP-glucuronosyltransferase 2B10 OS=Homo sapiens GN=UGT2B10 PE=1 SV=1 |

|  | **Query** | **Observed** | **Mr(expt)** | **Mr(calc)** | **Delta** | **Miss** | **Score** | **Expect** | **Rank** | **Peptide** |
| --- | --- | --- | --- | --- | --- | --- | --- | --- | --- | --- |
|  | 683 | 473.2791 | 472.2719 | 472.2758 | -0.0039 | 0 | 13 | 13 | 5 | K.GAAVR.V 688 |
|  | 1526 | 651.3502 | 650.3429 | 649.3105 | 1.0325 | 0 | 12 | 11 | 3 | K.ENIMK.L |
|  | 4010 | 461.2456 | 920.4767 | 919.4586 | 1.0181 | 0 | 6 | 40 | 7 | K.ADIWLMR.N |
|  | 4961 | 496.0763 | 990.1381 | 989.5328 | 0.6053 | 1 | 7 | 25 | 9 | K.ENIMKLSR.I |
|  | 5200 | 504.2530 | 1006.4914 | 1005.5277 | 0.9637 | 1 | (6) | 46 | 7 | K.ENIMKLSR.I |
|  | 5727 | **524.6195** | **1047.2243** | **1047.5535** | **-0.3292** | **1** | **1** | **94** | **3** | **R.KADIWLMR.N** |
|  | 6469 | 550.8162 | 1099.6178 | 1098.6761 | 0.9417 | 1 | 15 | 4.8 | 2 | K.TILKELVQR.G |
|  | 9315 | 425.0561 | 1272.1464 | 1272.5479 | -0.4015 | 0 | 10 | 11 | 6 | K.LSDQMTFMER.V 9343 |
|  | 11919 | 709.3783 | 1416.7420 | 1416.7514 | -0.0093 | 0 | 53 | 0.00069 | 1 | K.WIPQNDLLGHPK.T 11920 |
|  | 12271 | 719.4207 | 1436.8269 | 1436.8715 | -0.0446 | 1 | 32 | 0.089 | 3 | R.ANVIATALAKIPQK.V 12272 |
|  | 12388 | 723.3916 | 1444.7686 | 1444.7787 | -0.0100 | 0 | 35 | 0.039 | 1 | R.IQHDQPVKPLDR.A |
|  | 13483 | 505.9178 | 1514.7315 | 1515.7061 | -0.9747 | 1 | 9 | 15 | 3 | K.LSDQMTFMERVK.N |
|  | 17169 | 884.9385 | 1767.8625 | 1767.8713 | -0.0088 | 0 | 92 | 6.2e-008 | 1 | R.VDFNTMSSTDLLNALK.T 17170 |
|  | 17387 | 595.2432 | 1782.7077 | 1783.8662 | -1.1586 | 0 | (2) | 57 | 4 | R.VDFNTMSSTDLLNALK.T 17403 17408 |
|  | 25990 | 908.9279 | 2723.7618 | 2723.4370 | 0.3248 | 1 | 0 | 39 | 4 | R.IQHDQPVKPLDRAVFWIEFVMR.H |

| **392.** | P50148\|GNAQ_HUMAN    **Mass:** 42400    **Score:** 119    **Queries matched:** 18   **emPAI:** 0.40 |
| --- | --- |
|  | Guanine nucleotide-binding protein G(q) subunit alpha OS=Homo sapiens GN=GNAQ PE=1 SV=4 |

|  | **Query** | **Observed** | **Mr(expt)** | **Mr(calc)** | **Delta** | **Miss** | **Score** | **Expect** | **Rank** | **Peptide** |
| --- | --- | --- | --- | --- | --- | --- | --- | --- | --- | --- |
|  | 3054 | 431.2132 | 860.4118 | 860.4175 | -0.0057 | 0 | 22 | 1.5 | 1 | R.MVDVGGQR.S |
|  | 5851 | 529.3129 | 1056.6112 | 1056.6179 | -0.0067 | 0 | 42 | 0.011 | 1 | K.DTILQLNLK.E |
|  | 6050 | 536.2308 | 1070.4470 | 1070.5033 | -0.0563 | 0 | 2 | 1.2e+002 | 3 | K.YYLNDLDR.V |
|  | 8429 | **613.9706** | **1225.9267** | **1225.5939** | **0.3329** | **1** | **3** | **46** | **6** | **R.REYQLSDSTK.Y** |
|  | 9110 | 631.8373 | 1261.6601 | 1260.6826 | 0.9775 | 1 | 1 | 1.1e+002 | 9 | R.DAQAAREFILK.M |
|  | 9442 | 640.7906 | 1279.5666 | 1279.5755 | -0.0088 | 0 | 50 | 0.0014 | 1 | K.MFVDLNPDSDK.I |
|  | 12551 | 486.3614 | 1456.0624 | 1456.8501 | -0.7876 | 1 | 8 | 13 | 3 | R.ELKLLLLGTGESGK.S |
|  | 12884 | **493.2369** | **1476.6887** | **1475.7005** | **0.9883** | **1** | **0** | **1.3e+002** | **10** | **R.IIHGSGYSDEDKR.G** |
|  | 16926 | **875.9741** | **1749.9337** | **1750.7944** | **-0.8607** | **1** | **5** | **37** | **2** | **K.QMRIIHGSGYSDEDK.R** |
|  | 17583 | 899.9674 | 1797.9203 | 1797.9270 | -0.0067 | 0 | 63 | 5.2e-005 | 1 | K.LVYQNIFTAMQAMIR.A |
|  | 18654 | 937.5000 | 1872.9854 | 1871.8137 | 1.1718 | 0 | 4 | 39 | 10 | -.MTLESIMACCLSEEAK.E 18655 |
|  | 19069 | **953.4843** | **1904.9541** | **1903.8035** | **1.1505** | **0** | **(3)** | **51** | **1** | **-.MTLESIMACCLSEEAK.E** |
|  | 21776 | **718.3646** | **2152.0719** | **2151.0735** | **0.9983** | **1** | **4** | **31** | **1** | **R.EVDVEKVSAFENPYVDAIK.S** |
|  | 22928 | 1142.6166 | 2283.2186 | 2283.2303 | -0.0117 | 0 | 39 | 0.0097 | 1 | R.TIITYPWFQNSSVILFLNK.K 22929 |
|  | 23827 | 804.7783 | 2411.3131 | 2411.3253 | -0.0122 | 1 | 5 | 24 | 2 | R.TIITYPWFQNSSVILFLNKK.D |
|  | 27218 | **992.1266** | **2973.3579** | **2972.3902** | **0.9677** | **1** | **3** | **23** | **4** | **K.DLLEEKIMYSHLVDYFPEYDGPQR.D** |

| **393.** | Q04826\|1B40_HUMAN    **Mass:** 40822    **Score:** 118    **Queries matched:** 16   **emPAI:** 0.55 |
| --- | --- |
|  | HLA class I histocompatibility antigen, B-40 alpha chain OS=Homo sapiens GN=HLA-B PE=1 SV=1 |

|  | **Query** | **Observed** | **Mr(expt)** | **Mr(calc)** | **Delta** | **Miss** | **Score** | **Expect** | **Rank** | **Peptide** |
| --- | --- | --- | --- | --- | --- | --- | --- | --- | --- | --- |
|  | 995 | 542.1287 | 541.1214 | 542.3176 | -1.1963 | 0 | 13 | 4.3 | 1 | R.VTAPR.T 1006 |
|  | 5014 | **497.7372** | **993.4599** | **994.4356** | **-0.9757** | **0** | **4** | **64** | **10** | **R.FDSDATSPR.K** |
|  | 10365 | 443.7359 | 1328.1860 | 1327.6996 | 0.4863 | 1 | 13 | 4.5 | 1 | K.WEAARVAEQLR.A |
|  | 11047 | 457.2375 | 1368.6906 | 1367.6793 | 1.0112 | 1 | 10 | 13 | 4 | K.TNTQTYRESLR.N |
|  | 12099 | 714.3628 | 1426.7111 | 1426.7205 | -0.0093 | 0 | 11 | 9.6 | 1 | K.WAAVVVPSGEEQR.Y |
|  | 15451 | **822.9308** | **1643.8470** | **1643.8559** | **-0.0089** | **0** | **34** | **0.047** | **1** | **R.FITVGYVDDTLFVR.F** 15452 |
|  | 18332 | 617.6424 | 1849.9054 | 1849.9183 | -0.0130 | 0 | 32 | 0.074 | 1 | K.THVTHHPISDHEATLR.C 18320 |
|  | 20661 | 513.0184 | 2048.0446 | 2048.0626 | -0.0179 | 0 | 15 | 3.2 | 1 | R.YTCHVQHEGLPKPLTLR.W |
|  | 21343 | 1056.5170 | 2111.0194 | 2111.0299 | -0.0105 | 0 | 45 | 0.003 | 1 | R.CWALGFYPAEITLTWQR.D 21342 |
|  | 22490 | 744.6667 | 2230.9784 | 2230.9938 | -0.0154 | 0 | 35 | 0.024 | 1 | R.DGEDQTQDTELVETRPAGDR.T 22489 22491 |

|  | **Proteins matching a subset of these peptides:** |
| --- | --- |

|  | P30479\|1B41_HUMAN    **Mass:** 40856    **Score:** 118    **Queries matched:** 15 |
| --- | --- |
|  | HLA class I histocompatibility antigen, B-41 alpha chain OS=Homo sapiens GN=HLA-B PE=1 SV=1 |

|  | P30483\|1B45_HUMAN    **Mass:** 40674    **Score:** 118    **Queries matched:** 15 |
| --- | --- |
|  | HLA class I histocompatibility antigen, B-45 alpha chain OS=Homo sapiens GN=HLA-B PE=2 SV=1 |

|  | P30488\|1B50_HUMAN    **Mass:** 40801    **Score:** 118    **Queries matched:** 15 |
| --- | --- |
|  | HLA class I histocompatibility antigen, B-50 alpha chain OS=Homo sapiens GN=HLA-B PE=2 SV=1 |

|  | P30487\|1B49_HUMAN    **Mass:** 40841    **Score:** 118    **Queries matched:** 14 |
| --- | --- |
|  | HLA class I histocompatibility antigen, B-49 alpha chain OS=Homo sapiens GN=HLA-B PE=2 SV=2 |

|  | P30481\|1B44_HUMAN    **Mass:** 40798    **Score:** 103    **Queries matched:** 12 |
| --- | --- |
|  | HLA class I histocompatibility antigen, B-44 alpha chain OS=Homo sapiens GN=HLA-B PE=1 SV=1 |

| **394.** | P30485\|1B47_HUMAN    **Mass:** 40888    **Score:** 118    **Queries matched:** 17   **emPAI:** 0.55 |
| --- | --- |
|  | HLA class I histocompatibility antigen, B-47 alpha chain OS=Homo sapiens GN=HLA-B PE=2 SV=1 |

|  | **Query** | **Observed** | **Mr(expt)** | **Mr(calc)** | **Delta** | **Miss** | **Score** | **Expect** | **Rank** | **Peptide** |
| --- | --- | --- | --- | --- | --- | --- | --- | --- | --- | --- |
|  | 995 | 542.1287 | 541.1214 | 542.3176 | -1.1963 | 0 | 13 | 4.3 | 1 | R.VTAPR.T 1006 |
|  | 5014 | 497.7372 | 993.4599 | 994.4356 | -0.9757 | 0 | 4 | 64 | 10 | R.FDSDATSPR.K |
|  | 7124 | **576.8024** | **1151.5903** | **1152.4836** | **-0.8933** | **0** | **1** | **1.1e+002** | **3** | **R.GYHQDAYDGK.D** |
|  | 10365 | 443.7359 | 1328.1860 | 1327.6996 | 0.4863 | 1 | 13 | 4.5 | 1 | K.WEAARVAEQLR.A |
|  | 12099 | 714.3628 | 1426.7111 | 1426.7205 | -0.0093 | 0 | 11 | 9.6 | 1 | K.WAAVVVPSGEEQR.Y |
|  | 14620 | 531.2952 | 1590.8639 | 1591.7599 | -0.8961 | 1 | 7 | 22 | 5 | R.MFGCDVGPDGRLLR.G |
|  | 15451 | 822.9308 | 1643.8470 | 1643.8559 | -0.0089 | 0 | 34 | 0.047 | 1 | R.FITVGYVDDTLFVR.F 15452 |
|  | 18332 | 617.6424 | 1849.9054 | 1849.9183 | -0.0130 | 0 | 32 | 0.074 | 1 | K.THVTHHPISDHEATLR.C 18320 |
|  | 20661 | 513.0184 | 2048.0446 | 2048.0626 | -0.0179 | 0 | 15 | 3.2 | 1 | R.YTCHVQHEGLPKPLTLR.W |
|  | 21343 | 1056.5170 | 2111.0194 | 2111.0299 | -0.0105 | 0 | 45 | 0.003 | 1 | R.CWALGFYPAEITLTWQR.D 21342 |
|  | 22490 | 744.6667 | 2230.9784 | 2230.9938 | -0.0154 | 0 | 35 | 0.024 | 1 | R.DGEDQTQDTELVETRPAGDR.T 22489 22491 |

|  | **Proteins matching a subset of these peptides:** |
| --- | --- |

|  | P03989\|1B27_HUMAN    **Mass:** 40802    **Score:** 118    **Queries matched:** 15 |
| --- | --- |
|  | HLA class I histocompatibility antigen, B-27 alpha chain OS=Homo sapiens GN=HLA-B PE=1 SV=2 |

| **395.** | P58499\|FAM3B_HUMAN    **Mass:** 26307    **Score:** 118    **Queries matched:** 6   **emPAI:** 0.31 |
| --- | --- |
|  | Protein FAM3B OS=Homo sapiens GN=FAM3B PE=1 SV=2 |

|  | **Query** | **Observed** | **Mr(expt)** | **Mr(calc)** | **Delta** | **Miss** | **Score** | **Expect** | **Rank** | **Peptide** |
| --- | --- | --- | --- | --- | --- | --- | --- | --- | --- | --- |
|  | 3061 | **861.4772** | **860.4699** | **860.4756** | **-0.0057** | **0** | **16** | **5.6** | **1** | **K.FIQSAAPK.S** |
|  | 5088 | **499.8003** | **997.5861** | **997.5920** | **-0.0059** | **0** | **10** | **14** | **2** | **R.SIGERPVLK.A** |
|  | 13108 | 745.8546 | 1489.6945 | 1489.8980 | -0.2035 | 1 | 5 | 44 | 3 | R.SIGERPVLKAPVPK.R |
|  | 18280 | **924.4481** | **1846.8817** | **1846.8923** | **-0.0107** | **0** | **51** | **0.00088** | **1** | **R.YSGWPAEIQIEGCIPK.E** 18281 |
|  | 20953 | **1040.4966** | **2078.9786** | **2077.9925** | **0.9861** | **0** | **73** | **4.2e-006** | **1** | **K.ICFEDNLLMGEQLGNVAR.G** |

| **396.** | Q9TNN7\|1C05_HUMAN    **Mass:** 41342    **Score:** 118    **Queries matched:** 10   **emPAI:** 0.30 |
| --- | --- |
|  | HLA class I histocompatibility antigen, Cw-5 alpha chain OS=Homo sapiens GN=HLA-C PE=2 SV=1 |

|  | **Query** | **Observed** | **Mr(expt)** | **Mr(calc)** | **Delta** | **Miss** | **Score** | **Expect** | **Rank** | **Peptide** |
| --- | --- | --- | --- | --- | --- | --- | --- | --- | --- | --- |
|  | 1195 | 581.3248 | 580.3175 | 580.2969 | 0.0206 | 0 | 1 | 1e+002 | 5 | R.AEHPK.T |
|  | 12099 | 714.3628 | 1426.7111 | 1426.7205 | -0.0093 | 0 | 11 | 9.6 | 1 | K.WAAVVVPSGEEQR.Y |
|  | 13936 | **774.2357** | **1546.4568** | **1545.7899** | **0.6668** | **1** | **5** | **28** | **3** | **R.SWTAADKAAQITQR.K** |
|  | 18119 | 459.9797 | 1835.8896 | 1835.9027 | -0.0131 | 0 | 13 | 5.4 | 2 | K.THVTHHPVSDHEATLR.C |
|  | 19922 | **991.0371** | **1980.0597** | **1979.9588** | **0.1008** | **1** | **1** | **77** | **5** | **K.DYIALNEDLRSWTAADK.A** |
|  | 20661 | 513.0184 | 2048.0446 | 2049.0102 | -0.9656 | 0 | 14 | 4 | 2 | R.YTCHVQHEGLPEPLTLR.W |
|  | 21343 | 1056.5170 | 2111.0194 | 2111.0299 | -0.0105 | 0 | 45 | 0.003 | 1 | R.CWALGFYPAEITLTWQR.D 21342 |
|  | 25408 | 1319.0913 | 2636.1681 | 2636.1838 | -0.0158 | 0 | 68 | 1.1e-005 | 1 | R.DGEDQTQDTELVETRPAGDGTFQK.W 25407 |

| **397.** | Q29963\|1C06_HUMAN    **Mass:** 41399    **Score:** 118    **Queries matched:** 9   **emPAI:** 0.30 |
| --- | --- |
|  | HLA class I histocompatibility antigen, Cw-6 alpha chain OS=Homo sapiens GN=HLA-C PE=1 SV=2 |

|  | **Query** | **Observed** | **Mr(expt)** | **Mr(calc)** | **Delta** | **Miss** | **Score** | **Expect** | **Rank** | **Peptide** |
| --- | --- | --- | --- | --- | --- | --- | --- | --- | --- | --- |
|  | 1195 | 581.3248 | 580.3175 | 580.2969 | 0.0206 | 0 | 1 | 1e+002 | 5 | R.AEHPK.T |
|  | 12099 | 714.3628 | 1426.7111 | 1426.7205 | -0.0093 | 0 | 11 | 9.6 | 1 | K.WAAVVVPSGEEQR.Y |
|  | 15470 | 823.4105 | 1644.8064 | 1644.8148 | -0.0084 | 0 | 4 | 42 | 2 | R.FISVGYVDDTQFVR.F |
|  | 18119 | 459.9797 | 1835.8896 | 1835.9027 | -0.0131 | 0 | 13 | 5.4 | 2 | K.THVTHHPVSDHEATLR.C |
|  | 20661 | 513.0184 | 2048.0446 | 2049.0102 | -0.9656 | 0 | 14 | 4 | 2 | R.YTCHVQHEGLPEPLTLR.W |
|  | 21343 | 1056.5170 | 2111.0194 | 2111.0299 | -0.0105 | 0 | 45 | 0.003 | 1 | R.CWALGFYPAEITLTWQR.D 21342 |
|  | 25408 | 1319.0913 | 2636.1681 | 2636.1838 | -0.0158 | 0 | 68 | 1.1e-005 | 1 | R.DGEDQTQDTELVETRPAGDGTFQK.W 25407 |

| **398.** | Q07654\|TFF3_HUMAN    **Mass:** 9091     **Score:** 118    **Queries matched:** 12   **emPAI:** 3.37 |
| --- | --- |
|  | Trefoil factor 3 OS=Homo sapiens GN=TFF3 PE=1 SV=1 |

|  | **Query** | **Observed** | **Mr(expt)** | **Mr(calc)** | **Delta** | **Miss** | **Score** | **Expect** | **Rank** | **Peptide** |
| --- | --- | --- | --- | --- | --- | --- | --- | --- | --- | --- |
|  | 3702 | **451.1653** | **900.3159** | **900.3218** | **-0.0059** | **0** | **37** | **0.036** | **1** | **R.GCCFDSR.I** 3701 |
|  | 9300 | **636.8013** | **1271.5880** | **1271.5969** | **-0.0089** | **0** | **49** | **0.0019** | **1** | **R.VDCGYPHVTPK.E** 9299 9301 9302 9303 9304 9305 9306 |
|  | 13875 | **772.3644** | **1542.7142** | **1542.7249** | **-0.0107** | **1** | **32** | **0.081** | **1** | **K.DRVDCGYPHVTPK.E** 13876 |

| **399.** | Q13011\|ECH1_HUMAN    **Mass:** 36136    **Score:** 118    **Queries matched:** 18   **emPAI:** 0.22 |
| --- | --- |
|  | Delta(3,5)-Delta(2,4)-dienoyl-CoA isomerase, mitochondrial OS=Homo sapiens GN=ECH1 PE=1 SV=2 |

|  | **Query** | **Observed** | **Mr(expt)** | **Mr(calc)** | **Delta** | **Miss** | **Score** | **Expect** | **Rank** | **Peptide** |
| --- | --- | --- | --- | --- | --- | --- | --- | --- | --- | --- |
|  | 1872 | **749.1600** | **748.1527** | **748.3650** | **-0.2123** | **1** | **3** | **83** | **1** | **K.RNAMNK.V** |
|  | 3115 | **432.2327** | **862.4509** | **863.4865** | **-1.0356** | **0** | **6** | **52** | **2** | **K.VNLLYSR.D** |
|  | 5517 | **516.2894** | **1030.5643** | **1030.5706** | **-0.0062** | **1** | **11** | **15** | **1** | **-.MAAGIVASRR.L** |
|  | 5733 | 524.7840 | 1047.5534 | 1046.5655 | 0.9880 | 1 | (9) | 22 | 4 | -.MAAGIVASRR.L 5720 |
|  | 11949 | **710.3434** | **1418.6722** | **1417.6984** | **0.9738** | **1** | **6** | **31** | **3** | **R.DADCRAVVISGAGK.M** |
|  | 12140 | 715.4152 | 1428.8159 | 1427.6537 | 1.1622 | 1 | 6 | 30 | 5 | R.EMVECFNKISR.D |
|  | 12749 | **490.2785** | **1467.8137** | **1467.6698** | **0.1439** | **0** | **1** | **91** | **9** | **K.MMADEALGSGLVSR.V** 12755 12760 |
|  | 14204 | 522.2353 | 1563.6840 | 1563.7749 | -0.0908 | 1 | (3) | 65 | 7 | R.KMMADEALGSGLVSR.V |
|  | 14424 | 527.2564 | 1578.7474 | 1579.7698 | -1.0224 | 1 | (1) | 85 | 6 | R.KMMADEALGSGLVSR.V |
|  | 14664 | **532.6850** | **1595.0332** | **1595.7647** | **-0.7315** | **1** | **7** | **18** | **5** | **R.KMMADEALGSGLVSR.V** 14657 |
|  | 16661 | **866.4684** | **1730.9223** | **1730.9315** | **-0.0092** | **0** | **42** | **0.0062** | **1** | **K.VIGNQSLVNELAFTAR.K** |
|  | 21155 | **1049.0031** | **2095.9915** | **2095.9992** | **-0.0077** | **0** | **102** | **5.9e-009** | **1** | **K.MFTAGIDLMDMASDILQPK.G** |
|  | 23184 | **773.4130** | **2317.2171** | **2317.2239** | **-0.0068** | **1** | **10** | **7.7** | **1** | **R.VFPDKEVMLDAALALAAEISSK.S** |
|  | 30140 | **1276.2737** | **3825.7992** | **3826.8658** | **-1.0666** | **1** | **3** | **17** | **4** | **K.VNLLYSRDHSVAESLNYVASWNMSMLQTQDLVK.S** |

| **400.** | O95169\|NDUB8_HUMAN    **Mass:** 21865    **Score:** 118    **Queries matched:** 17   **emPAI:** 0.18 |
| --- | --- |
|  | NADH dehydrogenase [ubiquinone] 1 beta subcomplex subunit 8, mitochondrial OS=Homo sapiens GN=NDUFB8 PE=1 SV=1 |

|  | **Query** | **Observed** | **Mr(expt)** | **Mr(calc)** | **Delta** | **Miss** | **Score** | **Expect** | **Rank** | **Peptide** |
| --- | --- | --- | --- | --- | --- | --- | --- | --- | --- | --- |
|  | 333 | **430.8864** | **429.8792** | **430.2540** | **-0.3748** | **0** | **7** | **46** | **1** | **R.AAAAK.K** 336 355 356 359 |
|  | 3011 | **429.7551** | **857.4957** | **856.4589** | **1.0368** | **0** | **6** | **52** | **2** | **R.NVMPLGAR.T** 2973 |
|  | 8398 | 613.2808 | 1224.5471 | 1225.6931 | -1.1461 | 0 | 0 | 1.3e+002 | 9 | R.AGVLGVQWLQR.A |
|  | 12799 | 736.8571 | 1471.6996 | 1471.7095 | -0.0100 | 0 | 17 | 2.5 | 1 | K.QYPYNNLYLER.G |
|  | 16963 | **585.2895** | **1752.8468** | **1753.9774** | **-1.1305** | **1** | **(0)** | **1.1e+002** | **10** | **-.MAVARAGVLGVQWLQR.A** |
|  | 17203 | **885.9476** | **1769.8807** | **1769.9723** | **-0.0916** | **1** | **15** | **3.4** | **2** | **-.MAVARAGVLGVQWLQR.A** 17197 |
|  | 18354 | 618.2954 | 1851.8644 | 1850.8444 | 1.0200 | 1 | 1 | 75 | 5 | R.TASHMTKDMFPGPYPR.T 18339 |
|  | 21542 | 1066.0262 | 2130.0379 | 2129.9200 | 0.1180 | 0 | 1 | 67 | 5 | R.LNWGEPMHWHLDMYNR.N |
|  | 22274 | **1105.4402** | **2208.8658** | **2208.8834** | **-0.0175** | **0** | **75** | **2e-006** | **1** | **R.VEDYEPYPDDGMGYGDYPK.L** 22273 |

| **401.** | Q9NQC3\|RTN4_HUMAN    **Mass:** 130250   **Score:** 117    **Queries matched:** 23   **emPAI:** 0.09 |
| --- | --- |
|  | Reticulon-4 OS=Homo sapiens GN=RTN4 PE=1 SV=2 |

|  | **Query** | **Observed** | **Mr(expt)** | **Mr(calc)** | **Delta** | **Miss** | **Score** | **Expect** | **Rank** | **Peptide** |
| --- | --- | --- | --- | --- | --- | --- | --- | --- | --- | --- |
|  | 946 | **528.2426** | **527.2353** | **526.3479** | **0.8874** | **0** | **5** | **45** | **5** | **K.IPGLK.R** 945 |
|  | 2020 | **395.2265** | **788.4385** | **787.4440** | **0.9946** | **0** | **6** | **60** | **2** | **K.AQIVTEK.N** |
|  | 3593 | **447.2188** | **892.4231** | **891.4484** | **0.9747** | **1** | **6** | **44** | **7** | **K.NVKDAMAK.I** 3571 3594 |
|  | 3624 | **448.7049** | **895.3952** | **894.4195** | **0.9757** | **1** | **5** | **50** | **7** | **K.DSFNEKR.V** |
|  | 4313 | **472.3205** | **942.6264** | **943.4148** | **-0.7883** | **0** | **23** | **0.8** | **1** | **K.SDEGHPFR.A** |
|  | 5022 | **498.2584** | **994.5023** | **993.4437** | **1.0586** | **0** | **(11)** | **12** | **1** | **R.SSAENMDLK.E** |
|  | 5233 | 505.2897 | 1008.5649 | 1009.4386 | -0.8737 | 0 | 16 | 3.7 | 5 | R.SSAENMDLK.E 5254 5258 |
|  | 6567 | **554.7712** | **1107.5278** | **1108.4706** | **-0.9428** | **0** | **3** | **78** | **2** | **K.EDSDMLAAGGK.I** 6585 |
|  | 6791 | 563.8468 | 1125.6790 | 1125.6142 | 0.0649 | 0 | 8 | 25 | 5 | K.AESAVIVANPR.E |
|  | 11171 | **459.2489** | **1374.7249** | **1375.7599** | **-1.0350** | **0** | **4** | **49** | **3** | **R.LFLVDDLVDSLK.F** |
|  | 12129 | **477.2336** | **1428.6791** | **1429.6514** | **-0.9723** | **0** | **3** | **67** | **7** | **R.EEYADFKPFER.V** |
|  | 14190 | **782.3782** | **1562.7418** | **1562.7511** | **-0.0093** | **0** | **49** | **0.0015** | **1** | **K.YSNSALGHVNCTIK.E** 14189 14206 |
|  | 14838 | **804.4122** | **1606.8098** | **1606.8216** | **-0.0118** | **0** | **83** | **5.6e-007** | **1** | **R.HQAQIDHYLGLANK.N** 14839 |
|  | 19703 | **654.2784** | **1959.8133** | **1960.9789** | **-1.1656** | **1** | **4** | **31** | **3** | **K.YSNSALGHVNCTIKELR.R** |

| **402.** | Q14103\|HNRPD_HUMAN    **Mass:** 38581    **Score:** 117    **Queries matched:** 17   **emPAI:** 0.20 |
| --- | --- |
|  | Heterogeneous nuclear ribonucleoprotein D0 OS=Homo sapiens GN=HNRNPD PE=1 SV=1 |

|  | **Query** | **Observed** | **Mr(expt)** | **Mr(calc)** | **Delta** | **Miss** | **Score** | **Expect** | **Rank** | **Peptide** |
| --- | --- | --- | --- | --- | --- | --- | --- | --- | --- | --- |
|  | 1045 | **548.2822** | **547.2750** | **547.3152** | **-0.0402** | **1** | **17** | **5.3** | **2** | **R.AKAMK.T** |
|  | 4673 | **485.7363** | **969.4580** | **969.4628** | **-0.0048** | **0** | **13** | **7.4** | **1** | **R.HSEAATAQR.E** |
|  | 5363 | **510.3000** | **1018.5855** | **1018.4946** | **0.0908** | **0** | **12** | **11** | **6** | **R.GFCFITFK.E** |
|  | 5707 | **523.7906** | **1045.5666** | **1044.5716** | **0.9950** | **1** | **10** | **16** | **3** | **K.KYHNVGLSK.C** |
|  | 7302 | 583.7759 | 1165.5372 | 1166.5642 | -1.0270 | 0 | 20 | 1.3 | 2 | K.FGEVVDCTLK.L 7323 |
|  | 10824 | **678.3327** | **1354.6508** | **1354.6591** | **-0.0083** | **0** | **16** | **3.5** | **1** | **K.MFIGGLSWDTTK.K** |
|  | 12981 | 742.3466 | 1482.6787 | 1482.7541 | -0.0754 | 1 | 3 | 67 | 9 | K.MFIGGLSWDTTKK.D |
|  | 13068 | **744.8780** | **1487.7414** | **1487.7508** | **-0.0094** | **0** | **42** | **0.0088** | **1** | **K.IFVGGLSPDTPEEK.I** |
|  | 16647 | **865.9271** | **1729.8396** | **1729.8385** | **0.0010** | **1** | **2** | **77** | **5** | **R.GFCFITFKEEEPVK.K** 16646 |
|  | 17672 | 602.9933 | 1805.9582 | 1806.8499 | -0.8916 | 1 | 7 | 22 | 4 | K.DYFSKFGEVVDCTLK.L |
|  | 19826 | **657.6856** | **1970.0350** | **1970.8566** | **-0.8216** | **1** | **1** | **68** | **2** | **K.IDASKNEEDEGHSNSSPR.H** |
|  | 21866 | **1080.9872** | **2159.9598** | **2159.9721** | **-0.0123** | **0** | **79** | **9.3e-007** | **1** | **R.EYFGGFGEVESIELPMDNK.T** 21867 21870 |
|  | 31841 | **1858.5604** | **5572.6594** | **5572.4277** | **0.2318** | **0** | **4** | **3.7** | **1** | **M.SEEQFGGDGAAAAATAAVGGSAGEQEGAMVAATQGAAAAAGSGAGTGGGTASGGTEGGSAESEGAK.I** |

| **403.** | Q9BUF5\|TBB6_HUMAN    **Mass:** 50281    **Score:** 117    **Queries matched:** 22   **emPAI:** 0.33 |
| --- | --- |
|  | Tubulin beta-6 chain OS=Homo sapiens GN=TUBB6 PE=1 SV=1 |

|  | **Query** | **Observed** | **Mr(expt)** | **Mr(calc)** | **Delta** | **Miss** | **Score** | **Expect** | **Rank** | **Peptide** |
| --- | --- | --- | --- | --- | --- | --- | --- | --- | --- | --- |
|  | 1837 | 737.3976 | 736.3904 | 737.3456 | -0.9553 | 0 | 9 | 21 | 2 | R.GSQQYR.A |
|  | 2083 | 397.2539 | 792.4931 | 791.3450 | 1.1482 | 0 | 8 | 30 | 1 | R.EEFPDR.I 2074 |
|  | 5960 | 533.2140 | 1064.4134 | 1064.4201 | -0.0067 | 0 | 11 | 12 | 1 | R.NMMAACDPR.H 5970 |
|  | 11010 | 684.1195 | 1366.2243 | 1366.6625 | -0.4381 | 0 | 2 | 53 | 6 | R.IMNTFSVMPSPK.V |
|  | 11307 | 692.0279 | 1382.0412 | 1382.6574 | -0.6162 | 0 | (2) | 60 | 1 | R.IMNTFSVMPSPK.V 11297 |
|  | 12166 | 716.3267 | 1430.6389 | 1430.5965 | 0.0423 | 1 | 8 | 21 | 2 | R.NMMAACDPRHGR.Y 12164 12165 |
|  | 15059 | 540.9470 | 1619.8192 | 1619.8283 | -0.0090 | 0 | 28 | 0.19 | 1 | R.LHFFMPGFAPLTSR.G |
|  | 16178 | 848.9148 | 1695.8150 | 1695.8257 | -0.0106 | 0 | 54 | 0.00046 | 1 | K.NSSYFVEWIPNNVK.V 16177 16189 |
|  | 18460 | 929.4695 | 1856.9244 | 1856.9342 | -0.0098 | 0 | 75 | 3.5e-006 | 3 | K.MASTFIGNSTAIQELFK.R 18473 |
|  | 18642 | 937.1213 | 1872.2280 | 1872.9291 | -0.7011 | 0 | (2) | 46 | 1 | K.MASTFIGNSTAIQELFK.R |
|  | 20335 | 1012.0038 | 2021.9930 | 2020.9631 | 1.0299 | 1 | 1 | 66 | 3 | R.GPMSMKEVDEQMLAIQSK.N |
|  | 21238 | **702.3736** | **2104.0990** | **2104.0623** | **0.0367** | **1** | **3** | **45** | **4** | **K.YVPRAALVDLEPGTMDSVR.S** |
|  | 25486 | **1326.1547** | **2650.2948** | **2650.3273** | **-0.0325** | **0** | **43** | **0.0031** | **1** | **K.LTTPTYGDLNHLVSATMSGVTTSLR.F** 25488 |

| **404.** | P61513\|RL37A_HUMAN    **Mass:** 10497    **Score:** 117    **Queries matched:** 7   **emPAI:** 1.63 |
| --- | --- |
|  | 60S ribosomal protein L37a OS=Homo sapiens GN=RPL37A PE=1 SV=2 |

|  | **Query** | **Observed** | **Mr(expt)** | **Mr(calc)** | **Delta** | **Miss** | **Score** | **Expect** | **Rank** | **Peptide** |
| --- | --- | --- | --- | --- | --- | --- | --- | --- | --- | --- |
|  | 5388 | **511.7034** | **1021.3922** | **1021.3998** | **-0.0076** | **0** | **8** | **26** | **1** | **K.YTCSFCGK.T** 5386 |
|  | 5798 | 527.3024 | 1052.5902 | 1052.5978 | -0.0076 | 1 | 31 | 0.13 | 1 | K.KIEISQHAK.Y 5784 |
|  | 11680 | **703.3074** | **1404.6003** | **1404.6101** | **-0.0098** | **0** | **46** | **0.003** | **1** | **R.AVGIWHCGSCMK.T** |
|  | 17960 | **913.9611** | **1825.9077** | **1825.9211** | **-0.0134** | **0** | **58** | **0.00015** | **1** | **K.TVAGGAWTYNTTSAVTVK.S** 17959 |

| **405.** | Q13813\|SPTA2_HUMAN    **Mass:** 285163   **Score:** 116    **Queries matched:** 61   **emPAI:** 0.08 |
| --- | --- |
|  | Spectrin alpha chain, brain OS=Homo sapiens GN=SPTAN1 PE=1 SV=3 |

|  | **Query** | **Observed** | **Mr(expt)** | **Mr(calc)** | **Delta** | **Miss** | **Score** | **Expect** | **Rank** | **Peptide** |
| --- | --- | --- | --- | --- | --- | --- | --- | --- | --- | --- |
|  | 890 | **517.3691** | **516.3618** | **515.2816** | **1.0802** | **0** | **3** | **1.7e+002** | **2** | **K.AAAQR.K** |
|  | 1611 | **673.4028** | **672.3956** | **673.3759** | **-0.9803** | **0** | **7** | **56** | **9** | **R.DALLSR.R** |
|  | 1906 | **758.5673** | **757.5600** | **757.4082** | **0.1518** | **0** | **4** | **76** | **1** | **R.QQVLDR.Y** |
|  | 3713 | 451.2454 | 900.4762 | 901.4505 | -0.9743 | 0 | 15 | 6.5 | 7 | K.DPTNIQSK.H 3711 |
|  | 3929 | **458.3303** | **914.6461** | **913.5345** | **1.1116** | **1** | **13** | **9.1** | **1** | **K.KQEALVAR.Y** |
|  | 4126 | **465.7894** | **929.5642** | **929.4818** | **0.0825** | **0** | **0** | **1.9e+002** | **6** | **K.AINVQEEK.I** |
|  | 4521 | **479.5535** | **957.0924** | **957.4879** | **-0.3955** | **0** | **10** | **12** | **8** | **K.EPIAASTNR.G** 4515 |
|  | 4894 | 493.3027 | 984.5908 | 985.5556 | -0.9648 | 0 | 9 | 20 | 7 | R.IDGITIQAR.Q 4910 |
|  | 5300 | **508.2729** | **1014.5312** | **1014.5458** | **-0.0146** | **0** | **8** | **30** | **2** | **R.DLTGVQNLR.K** |
|  | 6075 | **536.7871** | **1071.5595** | **1071.5672** | **-0.0077** | **1** | **17** | **3.8** | **1** | **K.KLDPAQSASR.E** 6055 6076 6077 6085 |
|  | 6173 | **539.8076** | **1077.6006** | **1076.5648** | **1.0358** | **1** | **2** | **1e+002** | **9** | **K.EAGSVSLRMK.Q** |
|  | 6562 | **554.3022** | **1106.5898** | **1107.5812** | **-0.9913** | **0** | **4** | **65** | **4** | **K.ITALDEFATK.L** |
|  | 6714 | **560.7526** | **1119.4906** | **1118.5720** | **0.9186** | **0** | **30** | **0.18** | **1** | **R.VNEVNQFAAK.L** |
|  | 7996 | **603.3304** | **1204.6462** | **1203.6711** | **0.9751** | **0** | **5** | **50** | **6** | **R.DLSSVQTLLTK.Q** 7980 |
|  | 8189 | **405.7065** | **1214.0976** | **1214.6255** | **-0.5279** | **1** | **9** | **12** | **3** | **R.EKEPIAASTNR.G** |
|  | 8244 | **609.3006** | **1216.5866** | **1215.6360** | **0.9507** | **0** | **7** | **36** | **7** | **R.WSQLLANSAAR.K** |
|  | 8475 | **410.5680** | **1228.6821** | **1227.6724** | **1.0097** | **1** | **8** | **29** | **9** | **K.KLLEAQSHFR.K** |
|  | 8619 | 619.2732 | 1236.5318 | 1235.6510 | 0.8808 | 1 | 21 | 0.99 | 1 | K.KLLVGSEDYGR.D |
|  | 8676 | 620.3250 | 1238.6355 | 1239.7551 | -1.1196 | 1 | 7 | 24 | 4 | K.DLIGVQNLLKK.H |
|  | 9431 | **640.3520** | **1278.6894** | **1278.6819** | **0.0075** | **0** | **20** | **1.4** | **2** | **R.ALSSEGKPYVTK.E** |
|  | 9578 | 644.7769 | 1287.5393 | 1287.5943 | -0.0550 | 0 | 6 | 35 | 2 | R.QQVAPTDDETGK.E |
|  | 9650 | 646.2783 | 1290.5420 | 1290.5728 | -0.0308 | 0 | 9 | 19 | 6 | R.DVEDEETWIR.E |
|  | 9764 | 648.8470 | 1295.6795 | 1294.6629 | 1.0166 | 1 | 8 | 19 | 5 | K.DPTNIQSKHQK.H 9732 |
|  | 9860 | **651.8251** | **1301.6357** | **1301.6463** | **-0.0106** | **0** | **59** | **0.00017** | **1** | **K.VLETAEDIQER.R** |
|  | 10252 | **662.3158** | **1322.6170** | **1323.6895** | **-1.0725** | **0** | **3** | **72** | **7** | **R.SQLLGSAHEVQR.F** |
|  | 10655 | **449.2143** | **1344.6211** | **1345.6415** | **-1.0204** | **1** | **3** | **67** | **9** | **K.DWWKVEVNDR.Q** |
|  | 12196 | **478.2376** | **1431.6911** | **1432.6722** | **-0.9811** | **0** | **10** | **13** | **2** | **R.DVDEIEAWISEK.L** |
|  | 13135 | 746.8405 | 1491.6665 | 1491.6729 | -0.0064 | 1 | 4 | 53 | 5 | R.DADETKEWIEEK.N |
|  | 13193 | 748.3702 | 1494.7259 | 1494.7361 | -0.0102 | 0 | 45 | 0.0041 | 1 | R.MQHNLEQQIQAR.N 13209 |
|  | 13301 | 751.8769 | 1501.7393 | 1500.7208 | 1.0185 | 0 | 11 | 9.3 | 2 | R.EANELQQWINEK.E |
|  | 13530 | **759.3923** | **1516.7700** | **1516.7409** | **0.0291** | **1** | **3** | **67** | **1** | **R.DAEELEKWIQEK.L** |
|  | 13627 | **509.2791** | **1524.8155** | **1524.6878** | **0.1277** | **1** | **4** | **50** | **9** | **K.EKEQLMASDDFGR.D** |
|  | 14353 | **525.2928** | **1572.8565** | **1571.8453** | **1.0112** | **1** | **(3)** | **65** | **10** | **R.IRGVIDMGNSLIER.G** |
|  | 14557 | **530.2577** | **1587.7512** | **1587.8403** | **-0.0890** | **1** | **4** | **48** | **6** | **R.IRGVIDMGNSLIER.G** |
|  | 14589 | **398.2212** | **1588.8557** | **1587.7682** | **1.0875** | **1** | **6** | **28** | **7** | **K.KHEAFETDFTVHK.D** |
|  | 15043 | 810.3736 | 1618.7326 | 1618.7376 | -0.0049 | 0 | 3 | 54 | 9 | R.QFQDAGHFDAENIK.K 15024 |
|  | 15513 | **412.9830** | **1647.9029** | **1648.7991** | **-0.8962** | **1** | **4** | **46** | **4** | **K.CTELNQAWSSLGKR.A** |
|  | 16037 | **562.6670** | **1684.9791** | **1683.8805** | **1.0987** | **1** | **6** | **24** | **3** | **K.KHQALQAEIAGHEPR.I** 16023 |
|  | 17586 | **900.0057** | **1797.9969** | **1797.9009** | **0.0960** | **0** | **0** | **94** | **9** | **K.HQAFEAEVQANSGAIVK.L** |
|  | 18810 | **629.6785** | **1886.0138** | **1884.9429** | **1.0709** | **1** | **3** | **44** | **5** | **M.DPSGVKVLETAEDIQER.R** |
|  | 18981 | **633.7414** | **1898.2023** | **1898.8655** | **-0.6632** | **0** | **3** | **37** | **3** | **R.DGHVSLQEYMAFMISR.E** |
|  | 20815 | 688.3658 | 2062.0755 | 2061.0742 | 1.0013 | 0 | 6 | 22 | 3 | R.LEAELAAHEPAIQGVLDTGK.K |
|  | 21190 | 701.0470 | 2100.1192 | 2101.0990 | -0.9798 | 1 | 2 | 53 | 6 | R.LMVHTVATFNSIKELNER.W |
|  | 23016 | **765.7147** | **2294.1222** | **2293.1325** | **0.9896** | **1** | **33** | **0.039** | **1** | **R.EAFLNTEDKGDSLDSVEALIK.K** 23008 |
|  | 23192 | **774.3403** | **2319.9990** | **2320.1659** | **-0.1669** | **1** | **4** | **29** | **1** | **R.SSLSSAQADFNQLAELDRQIK.S** |
|  | 23231 | **776.7094** | **2327.1064** | **2327.1254** | **-0.0190** | **0** | **18** | **1.2** | **1** | **K.NQALNTDNYGHDLASVQALQR.K** |
|  | 23278 | **778.8827** | **2333.6264** | **2334.1638** | **-0.5374** | **1** | **3** | **23** | **2** | **K.EQLMASDDFGRDLASVQALLR.K** |
|  | 25098 | **859.4036** | **2575.1889** | **2575.2125** | **-0.0237** | **0** | **38** | **0.011** | **1** | **K.YTEHSTVGLAQQWDQLDQLGMR.M** |

| **406.** | Q8NHW5\|RLA0L_HUMAN    **Mass:** 34514    **Score:** 116    **Queries matched:** 10   **emPAI:** 0.23 |
| --- | --- |
|  | 60S acidic ribosomal protein P0-like OS=Homo sapiens PE=2 SV=1 |

|  | **Query** | **Observed** | **Mr(expt)** | **Mr(calc)** | **Delta** | **Miss** | **Score** | **Expect** | **Rank** | **Peptide** |
| --- | --- | --- | --- | --- | --- | --- | --- | --- | --- | --- |
|  | 2221 | 402.7048 | 803.3950 | 802.4119 | 0.9831 | 0 | 9 | 27 | 8 | K.QMQQIR.M |
|  | 4353 | **473.8952** | **945.7758** | **945.5681** | **0.2077** | **1** | **8** | **21** | **10** | **R.GKVVVLMGK.N** |
|  | 8326 | **407.7439** | **1220.2099** | **1220.6149** | **-0.4050** | **0** | **8** | **15** | **9** | **R.GHLENNPALEK.L** |
|  | 9182 | **633.8072** | **1265.5998** | **1265.6074** | **-0.0076** | **0** | **13** | **7.7** | **2** | **K.CFIVGADNVGSK.Q** |
|  | 10050 | **657.3548** | **1312.6950** | **1312.7027** | **-0.0077** | **0** | **43** | **0.0069** | **1** | **K.TSFFQALGITTK.I** |
|  | 11528 | 465.9332 | 1394.7777 | 1393.7244 | 1.0533 | 1 | 7 | 25 | 9 | K.VVVLMGKNTMMR.K |
|  | 11788 | **470.7424** | **1409.2053** | **1409.7193** | **-0.5140** | **1** | **(2)** | **51** | **8** | **K.VVVLMGKNTMMR.K** |
|  | 22032 | **1090.5465** | **2179.0785** | **2179.0943** | **-0.0158** | **0** | **61** | **7.3e-005** | **1** | **R.AGAIAPCEVTVPAQNTGLGPEK.T** 22030 22031 |

| **407.** | P05388\|RLA0_HUMAN    **Mass:** 34423    **Score:** 116    **Queries matched:** 12   **emPAI:** 0.23 |
| --- | --- |
|  | 60S acidic ribosomal protein P0 OS=Homo sapiens GN=RPLP0 PE=1 SV=1 |

|  | **Query** | **Observed** | **Mr(expt)** | **Mr(calc)** | **Delta** | **Miss** | **Score** | **Expect** | **Rank** | **Peptide** |
| --- | --- | --- | --- | --- | --- | --- | --- | --- | --- | --- |
|  | 2221 | 402.7048 | 803.3950 | 802.4119 | 0.9831 | 0 | 9 | 27 | 8 | K.QMQQIR.M |
|  | 8326 | 407.7439 | 1220.2099 | 1220.6149 | -0.4050 | 0 | 8 | 15 | 9 | R.GHLENNPALEK.L |
|  | 9182 | 633.8072 | 1265.5998 | 1265.6074 | -0.0076 | 0 | 13 | 7.7 | 2 | K.CFIVGADNVGSK.Q |
|  | 10050 | 657.3548 | 1312.6950 | 1312.7027 | -0.0077 | 0 | 43 | 0.0069 | 1 | K.TSFFQALGITTK.I |
|  | 11003 | **456.2522** | **1365.7348** | **1365.6931** | **0.0417** | **1** | **10** | **15** | **2** | **K.AVVLMGKNTMMR.K** |
|  | 11298 | 691.8232 | 1381.6319 | 1381.6880 | -0.0561 | 1 | (5) | 40 | 8 | K.AVVLMGKNTMMR.K |
|  | 18938 | **948.4987** | **1894.9828** | **1894.9928** | **-0.0100** | **0** | **1** | **72** | **1** | **R.VLALSVETDYTFPLAEK.V** |
|  | 22032 | 1090.5465 | 2179.0785 | 2179.0943 | -0.0158 | 0 | 61 | 7.3e-005 | 1 | R.AGAIAPCEVTVPAQNTGLGPEK.T 22030 22031 |
|  | 26135 | **1376.6567** | **2751.2989** | **2751.4595** | **-0.1606** | **0** | **0** | **57** | **2** | **K.AFLADPSAFVAAAPVAAATTAAPAAAAAPAK.V** 26136 |

| **408.** | P54707\|AT12A_HUMAN    **Mass:** 116292   **Score:** 116    **Queries matched:** 26   **emPAI:** 0.10 |
| --- | --- |
|  | Potassium-transporting ATPase alpha chain 2 OS=Homo sapiens GN=ATP12A PE=2 SV=3 |

|  | **Query** | **Observed** | **Mr(expt)** | **Mr(calc)** | **Delta** | **Miss** | **Score** | **Expect** | **Rank** | **Peptide** |
| --- | --- | --- | --- | --- | --- | --- | --- | --- | --- | --- |
|  | 1443 | 633.3343 | 632.3270 | 632.3316 | -0.0046 | 0 | 25 | 0.93 | 1 | K.NCLVK.N |
|  | 3564 | 446.0373 | 890.0600 | 889.4617 | 0.5982 | 0 | 26 | 0.32 | 1 | K.TGTLTQNR.M |
|  | 4436 | **476.2801** | **950.5456** | **950.4127** | **0.1328** | **0** | **2** | **1e+002** | **5** | **K.AESDIMNR.K** |
|  | 5632 | 520.7178 | 1039.4211 | 1040.5251 | -1.1040 | 0 | 3 | 73 | 9 | K.GGDQIPADIR.V |
|  | 6292 | 544.2970 | 1086.5794 | 1086.5856 | -0.0061 | 0 | 34 | 0.079 | 1 | K.LIIVEGCQR.Q 6293 |
|  | 6793 | 563.8472 | 1125.6799 | 1126.6346 | -0.9547 | 0 | 8 | 25 | 9 | R.LNIAVEQVNK.R |
|  | 9574 | **644.4641** | **1286.9135** | **1286.7082** | **0.2054** | **0** | **11** | **8.1** | **1** | **K.AVIGDASETALLK.F** 9594 |
|  | 9703 | 647.3424 | 1292.6701 | 1293.6573 | -0.9872 | 1 | 3 | 79 | 3 | R.FLMVMKGAPER.I |
|  | 10429 | 445.0339 | 1332.0799 | 1331.7119 | 0.3680 | 1 | 7 | 22 | 1 | R.DAKAAVVTGMELK.D |
|  | 11285 | 691.3743 | 1380.7341 | 1380.7435 | -0.0094 | 0 | 66 | 3.2e-005 | 1 | K.VIMVTGDHPITAK.A 11283 11284 11286 |
|  | 11571 | 699.4022 | 1396.7898 | 1396.7384 | 0.0513 | 0 | (13) | 6.3 | 1 | K.VIMVTGDHPITAK.A |
|  | 12157 | **477.5517** | **1429.6332** | **1428.6919** | **0.9413** | **0** | **2** | **91** | **9** | **K.YGTDIIMGLSSTR.A** |
|  | 13591 | 508.2843 | 1521.8312 | 1522.7701 | -0.9390 | 0 | 7 | 23 | 1 | K.FSEVILGDVMEIR.K |
|  | 13802 | **513.2746** | **1536.8020** | **1536.7460** | **0.0560** | **1** | **1** | **91** | **10** | **R.VEWEKDYVNDLK.D** |
|  | 16810 | 871.8810 | 1741.7475 | 1742.7967 | -1.0492 | 0 | 3 | 50 | 9 | K.CSTIMINGEEHPLDK.S |
|  | 18129 | 919.4411 | 1836.8676 | 1837.0132 | -0.1455 | 1 | 3 | 55 | 8 | R.SAGIKVIMVTGDHPITAK.A |
|  | 22902 | 570.7807 | 2279.0937 | 2280.2073 | -1.1136 | 1 | 3 | 44 | 5 | K.AIAKSVGIISANSETVEDIAHR.L |
|  | 24956 | **851.0778** | **2550.2115** | **2550.2669** | **-0.0555** | **1** | **3** | **35** | **2** | **K.NCLVKNLEAVETLGSTSIICSDK.T** |
|  | 26319 | 931.1129 | 2790.3169 | 2791.4677 | -1.1508 | 1 | 2 | 36 | 9 | K.AVIGDASETALLKFSEVILGDVMEIR.K |
|  | 28170 | 1594.3350 | 3186.6554 | 3185.7165 | 0.9389 | 1 | 1 | 38 | 8 | R.EYLEWTGYTAFFVGILVQQIADLIIRK.T |
|  | 30955 | **1504.3110** | **4509.9113** | **4511.0837** | **-1.1724** | **1** | **1** | **15** | **1** | **K.TGTLTQNRMTVAHLWFDNQIFVADTSEDHSNQVFDQSSR.T** |

| **409.** | P51149\|RAB7A_HUMAN    **Mass:** 23760    **Score:** 116    **Queries matched:** 9   **emPAI:** 0.56 |
| --- | --- |
|  | Ras-related protein Rab-7a OS=Homo sapiens GN=RAB7A PE=1 SV=1 |

|  | **Query** | **Observed** | **Mr(expt)** | **Mr(calc)** | **Delta** | **Miss** | **Score** | **Expect** | **Rank** | **Peptide** |
| --- | --- | --- | --- | --- | --- | --- | --- | --- | --- | --- |
|  | 1291 | **601.4076** | **600.4003** | **599.4370** | **0.9633** | **1** | **20** | **2.7** | **1** | **K.KVLLK.V** |
|  | 1985 | 786.3727 | 785.3655 | 785.3708 | -0.0053 | 0 | 10 | 18 | 1 | K.FSNQYK.A |
|  | 3130 | **432.3091** | **862.6037** | **862.3855** | **0.2183** | **0** | **7** | **40** | **1** | **K.EVMVDDR.L** |
|  | 7830 | **599.7975** | **1197.5804** | **1196.5859** | **0.9945** | **0** | **28** | **0.24** | **1** | **K.TSLMNQYVNK.K** |
|  | 9487 | **642.3129** | **1282.6113** | **1282.6193** | **-0.0080** | **0** | **6** | **36** | **2** | **K.NNIPYFETSAK.E** |
|  | 14582 | **795.4130** | **1588.8115** | **1588.8209** | **-0.0094** | **0** | **44** | **0.0046** | **1** | **K.EAINVEQAFQTIAR.N** |
|  | 15493 | **824.4068** | **1646.7990** | **1646.8086** | **-0.0096** | **0** | **99** | **1.7e-008** | **1** | **R.LVTMQIWDTAGQER.F** 15516 |
|  | 26420 | **939.1427** | **2814.4063** | **2815.4116** | **-1.0053** | **1** | **2** | **33** | **4** | **R.LVTMQIWDTAGQERFQSLGVAFYR.G** |

| **410.** | O75489\|NDUS3_HUMAN    **Mass:** 30337    **Score:** 116    **Queries matched:** 12   **emPAI:** 0.42 |
| --- | --- |
|  | NADH dehydrogenase [ubiquinone] iron-sulfur protein 3, mitochondrial OS=Homo sapiens GN=NDUFS3 PE=1 SV=1 |

|  | **Query** | **Observed** | **Mr(expt)** | **Mr(calc)** | **Delta** | **Miss** | **Score** | **Expect** | **Rank** | **Peptide** |
| --- | --- | --- | --- | --- | --- | --- | --- | --- | --- | --- |
|  | 2127 | 399.2327 | 796.4508 | 797.4283 | -0.9775 | 0 | 6 | 37 | 6 | R.QPPESLK.L 2151 |
|  | 4533 | **480.2336** | **958.4527** | **959.4461** | **-0.9934** | **0** | **5** | **57** | **5** | **R.DHTNAQFK.S** |
|  | 9760 | 648.8334 | 1295.6522 | 1294.6557 | 0.9964 | 0 | 28 | 0.22 | 1 | K.DFPLSGYVELR.Y |
|  | 13038 | **743.8937** | **1485.7728** | **1485.7827** | **-0.0099** | **0** | **42** | **0.0094** | **1** | **R.VVAEPVELAQEFR.K** |
|  | 13443 | **504.9307** | **1511.7704** | **1511.7804** | **-0.0101** | **0** | **11** | **10** | **3** | **R.ESAGADTRPTVRPR.N** |
|  | 14008 | 776.3780 | 1550.7414 | 1550.7518 | -0.0104 | 0 | 72 | 8e-006 | 1 | R.ILTDYGFEGHPFR.K 14007 |
|  | 14721 | 533.9678 | 1598.8817 | 1598.9045 | -0.0228 | 1 | 8 | 16 | 3 | R.LWWRGILGASALTR.G |
|  | 16779 | **870.9198** | **1739.8250** | **1739.8308** | **-0.0057** | **0** | **40** | **0.01** | **1** | **K.FDLNSPWEAFPVYR.Q** 16778 |
|  | 21125 | **1047.1491** | **2092.2835** | **2092.9829** | **-0.6994** | **0** | **15** | **2.4** | **1** | **R.EIWDMFGVFFANHPDLR.R** |

| **411.** | Q53GQ0\|DHB12_HUMAN    **Mass:** 34416    **Score:** 115    **Queries matched:** 18   **emPAI:** 0.68 |
| --- | --- |
|  | Estradiol 17-beta-dehydrogenase 12 OS=Homo sapiens GN=HSD17B12 PE=1 SV=2 |

|  | **Query** | **Observed** | **Mr(expt)** | **Mr(calc)** | **Delta** | **Miss** | **Score** | **Expect** | **Rank** | **Peptide** |
| --- | --- | --- | --- | --- | --- | --- | --- | --- | --- | --- |
|  | 1919 | **760.4263** | **759.4190** | **759.4239** | **-0.0049** | **0** | **14** | **8.8** | **1** | **K.TVGLQSR.T** |
|  | 3169 | 865.4377 | 864.4305 | 864.4197 | 0.0107 | 0 | (3) | 83 | 6 | K.IVMNMNK.S |
|  | 3419 | **441.2668** | **880.5189** | **880.4146** | **0.1043** | **0** | **10** | **14** | **3** | **K.IVMNMNK.S** |
|  | 5354 | 510.2527 | 1018.4908 | 1017.5342 | 0.9565 | 0 | 14 | 6.6 | 1 | K.LDQVSSEIK.E |
|  | 7161 | **578.2839** | **1154.5533** | **1154.6594** | **-0.1061** | **1** | **8** | **23** | **6** | **K.HGMKVVLISR.S** |
|  | 9910 | **652.8533** | **1303.6920** | **1303.6992** | **-0.0072** | **0** | **16** | **3.6** | **1** | **K.MININILSVCK.M** |
|  | 11104 | 458.2464 | 1371.7174 | 1372.7207 | -1.0033 | 0 | 6 | 36 | 4 | K.MTQLVLPGMVER.S |
|  | 11684 | **703.3310** | **1404.6474** | **1404.7105** | **-0.0631** | **0** | **(0)** | **1.3e+002** | **4** | **K.MTQLVLPGMVER.S** |
|  | 14530 | 793.8781 | 1585.7417 | 1585.7512 | -0.0095 | 0 | 40 | 0.011 | 1 | R.TIAVDFASEDIYDK.I 14529 |
|  | 14782 | **802.9174** | **1603.8202** | **1603.8426** | **-0.0224** | **1** | **5** | **42** | **5** | **K.MTQLVLPGMVERSK.G** |
|  | 15614 | **828.4611** | **1654.9076** | **1653.9131** | **0.9945** | **0** | **38** | **0.016** | **1** | **K.GVFVQSVLPYFVATK.L** |
|  | 17989 | 610.1827 | 1827.5262 | 1826.9302 | 0.5960 | 1 | 1 | 56 | 3 | R.TIAVDFASEDIYDKIK.T |
|  | 24541 | **624.8333** | **2495.3039** | **2495.3213** | **-0.0174** | **0** | **3** | **32** | **1** | **M.ESALPAAGFLYWVGAGTVAYLALR.I** |
|  | 24584 | **1252.1898** | **2502.3651** | **2502.3767** | **-0.0116** | **0** | **54** | **0.00026** | **1** | **K.GAILNISSGSGMLPVPLLTIYSATK.T** |
|  | 25871 | **677.0625** | **2704.2209** | **2703.4458** | **0.7751** | **0** | **2** | **45** | **3** | **R.TNGYLIHALMGSIISNLPSWIYLK.I** |
|  | 26212 | **1385.1883** | **2768.3621** | **2768.3770** | **-0.0149** | **0** | **65** | **2.1e-005** | **1** | **R.VWGVGNEAGVGPGLGEWAVVTGSTDGIGK.S** 26211 |

| **412.** | P30085\|KCY_HUMAN    **Mass:** 22436    **Score:** 115    **Queries matched:** 12   **emPAI:** 0.88 |
| --- | --- |
|  | UMP-CMP kinase OS=Homo sapiens GN=CMPK1 PE=1 SV=3 |

|  | **Query** | **Observed** | **Mr(expt)** | **Mr(calc)** | **Delta** | **Miss** | **Score** | **Expect** | **Rank** | **Peptide** |
| --- | --- | --- | --- | --- | --- | --- | --- | --- | --- | --- |
|  | 7720 | 596.7916 | 1191.5687 | 1191.5367 | 0.0320 | 1 | 5 | 44 | 7 | R.SDDNRESLEK.R |
|  | 8054 | **604.3666** | **1206.7187** | **1205.6557** | **1.0630** | **1** | **1** | **1.1e+002** | **9** | **K.NKFLIDGFPR.N** |
|  | 10793 | 677.3536 | 1352.6927 | 1352.5700 | 0.1227 | 0 | 8 | 21 | 4 | R.EMDQTMAANAQK.N |
|  | 12913 | **493.9214** | **1478.7423** | **1478.7518** | **-0.0094** | **0** | **47** | **0.0025** | **1** | **K.YGYTHLSAGELLR.D** 12905 12912 12919 |
|  | 13564 | **760.8778** | **1519.7411** | **1519.7518** | **-0.0107** | **1** | **84** | **5.8e-007** | **1** | **R.KNPDSQYGELIEK.Y** |
|  | 15381 | 410.7141 | 1638.8273 | 1637.9967 | 0.8306 | 1 | 6 | 28 | 8 | K.EGKIVPVEITISLLK.R |
|  | 22717 | 754.0461 | 2259.1164 | 2258.2028 | 0.9136 | 1 | 1 | 58 | 6 | -.MKPLVVFVLGGPGAGKGTQCAR.I |
|  | 23578 | **1185.6097** | **2369.2049** | **2369.2188** | **-0.0139** | **0** | **32** | **0.046** | **1** | **R.IQTYLQSTKPIIDLYEEMGK.V** 23579 |

| **413.** | O43488\|ARK72_HUMAN    **Mass:** 40020    **Score:** 115    **Queries matched:** 12   **emPAI:** 0.20 |
| --- | --- |
|  | Aflatoxin B1 aldehyde reductase member 2 OS=Homo sapiens GN=AKR7A2 PE=1 SV=3 |

|  | **Query** | **Observed** | **Mr(expt)** | **Mr(calc)** | **Delta** | **Miss** | **Score** | **Expect** | **Rank** | **Peptide** |
| --- | --- | --- | --- | --- | --- | --- | --- | --- | --- | --- |
|  | 1460 | 636.3673 | 635.3600 | 634.3438 | 1.0161 | 0 | 19 | 2.2 | 1 | R.AFLER.G |
|  | 8213 | **406.0264** | **1215.0573** | **1214.6190** | **0.4383** | **1** | **7** | **22** | **2** | **R.RMDAPASAAAVR.A** |
|  | 9178 | **422.7634** | **1265.2685** | **1265.6108** | **-0.3423** | **0** | **(2)** | **61** | **5** | **R.VASVLGTMEMGR.R** |
|  | 9502 | **642.3690** | **1282.7235** | **1281.6057** | **1.1178** | **0** | **2** | **79** | **3** | **R.VASVLGTMEMGR.R** |
|  | 14496 | 792.9191 | 1583.8236 | 1583.8348 | -0.0112 | 0 | 61 | 9.8e-005 | 1 | R.FYAYNPLAGGLLTGK.Y 14482 14511 |
|  | 17656 | 902.9747 | 1803.9348 | 1803.9479 | -0.0131 | 0 | 19 | 1.4 | 1 | K.ALQAAYGASAPSVTSAALR.W 17663 17665 |
|  | 28225 | **802.9188** | **3207.6462** | **3207.6676** | **-0.0214** | **1** | **1** | **31** | **2** | **K.EHHFEAIALVEKALQAAYGASAPSVTSAALR.W** |
|  | 29541 | **1210.0890** | **3627.2451** | **3626.6982** | **0.5470** | **1** | **2** | **18** | **1** | **R.GHTELDTAFMYSDGQSETILGGLGLGLGGGDCRVK.I** |

| **414.** | P30041\|PRDX6_HUMAN    **Mass:** 25133    **Score:** 114    **Queries matched:** 6   **emPAI:** 0.33 |
| --- | --- |
|  | Peroxiredoxin-6 OS=Homo sapiens GN=PRDX6 PE=1 SV=3 |

|  | **Query** | **Observed** | **Mr(expt)** | **Mr(calc)** | **Delta** | **Miss** | **Score** | **Expect** | **Rank** | **Peptide** |
| --- | --- | --- | --- | --- | --- | --- | --- | --- | --- | --- |
|  | 1322 | **605.0920** | **604.0848** | **604.2857** | **-0.2009** | **0** | **4** | **77** | **1** | **R.YTPQP.-** |
|  | 10850 | **453.2252** | **1356.6538** | **1355.8136** | **0.8402** | **1** | **3** | **74** | **7** | **R.VVISLQLTAEKR.V** |
|  | 11521 | **698.3284** | **1394.6422** | **1394.6500** | **-0.0079** | **0** | **4** | **51** | **6** | **R.DFTPVCTTELGR.A** |
|  | 18776 | **942.9987** | **1883.9829** | **1883.9914** | **-0.0085** | **1** | **41** | **0.008** | **1** | **R.ELAILLGMLDPAEKDEK.G** |
|  | 21161 | **1049.5443** | **2097.0741** | **2097.0855** | **-0.0114** | **0** | **93** | **4.7e-008** | **1** | **M.PGGLLLGDVAPNFEANTTVGR.I** |
|  | 22538 | **746.4777** | **2236.4112** | **2237.2056** | **-0.7944** | **1** | **1** | **45** | **3** | **R.NVKLIALSIDSVEDHLAWSK.D** |

| **415.** | Q02817\|MUC2_HUMAN    **Mass:** 552218   **Score:** 114    **Queries matched:** 22   **emPAI:** 0.03 |
| --- | --- |
|  | Mucin-2 OS=Homo sapiens GN=MUC2 PE=1 SV=2 |

|  | **Query** | **Observed** | **Mr(expt)** | **Mr(calc)** | **Delta** | **Miss** | **Score** | **Expect** | **Rank** | **Peptide** |
| --- | --- | --- | --- | --- | --- | --- | --- | --- | --- | --- |
|  | 2049 | **396.1920** | **790.3694** | **789.4055** | **0.9640** | **0** | **11** | **16** | **6** | **R.TATLCPK.T** |
|  | 2140 | **399.5376** | **797.0607** | **796.4191** | **0.6416** | **1** | **2** | **61** | **7** | **K.LEDKHR.V** |
|  | 2253 | **806.3729** | **805.3656** | **804.4858** | **0.8798** | **1** | **8** | **31** | **6** | **K.KNAVVFK.S** |
|  | 4460 | 477.2601 | 952.5057 | 953.4203 | -0.9146 | 0 | 11 | 10 | 1 | R.SNNDFTTR.D |
|  | 5351 | **509.9053** | **1017.7960** | **1016.6018** | **1.1942** | **1** | **2** | **80** | **9** | **R.QAVALPYKK.Y** |
|  | 5604 | 519.2740 | 1036.5335 | 1037.5982 | -1.0646 | 0 | 6 | 36 | 1 | K.RPAVTVPGGGK.T |
|  | 6129 | 538.2823 | 1074.5500 | 1073.5539 | 0.9961 | 0 | 31 | 0.14 | 1 | R.LIGQSCTAPK.I |
|  | 10393 | **666.2908** | **1330.5670** | **1330.5758** | **-0.0088** | **0** | **33** | **0.079** | **1** | **K.TCGCVGPDNVPR.E** |
|  | 11810 | **706.3342** | **1410.6539** | **1410.6636** | **-0.0097** | **0** | **14** | **5** | **1** | **K.VQCDVSVGFICK.N** |
|  | 12946 | **494.5642** | **1480.6709** | **1481.6695** | **-0.9986** | **0** | **7** | **27** | **2** | **R.QCSHAGGRPGNWR.T** 12955 |
|  | 13077 | 745.3664 | 1488.7182 | 1487.8382 | 0.8801 | 1 | 5 | 43 | 4 | K.QTCLKTVVLLADK.K |
|  | 14753 | 801.8575 | 1601.7004 | 1601.9002 | -0.1998 | 1 | 2 | 73 | 3 | K.RPAVTVPGGGKTTPHK.D |
|  | 15362 | **820.3347** | **1638.6549** | **1638.6654** | **-0.0105** | **0** | **36** | **0.023** | **1** | **R.EYQACGPAEEPTCK.S** |
|  | 15692 | 831.4072 | 1660.7999 | 1661.8083 | -1.0084 | 1 | 7 | 21 | 5 | R.CPKDRPIYEEDLK.K |
|  | 17445 | **894.4615** | **1786.9084** | **1786.8396** | **0.0689** | **1** | **2** | **64** | **2** | **K.GVMLWGWREHVCNK.D** |
|  | 17495 | **896.4109** | **1790.8073** | **1790.8114** | **-0.0040** | **0** | **48** | **0.0016** | **1** | **K.NCVCLEGGSGIICQPK.R** |
|  | 17565 | 599.9511 | 1796.8313 | 1796.8848 | -0.0535 | 0 | 6 | 23 | 4 | K.TVHMMPMQVQVQVNR.Q |
|  | 17874 | **608.2512** | **1821.7318** | **1821.7444** | **-0.0126** | **1** | **0** | **93** | **2** | **K.AQALDHSCSCCKEEK.T** |
|  | 22135 | **731.3637** | **2191.0691** | **2190.1545** | **0.9146** | **1** | **2** | **52** | **2** | **K.RPDNQHVILKPGDFKSDPK.N** |
|  | 26204 | 923.0637 | 2766.1692 | 2766.1861 | -0.0170 | 0 | 53 | 0.00027 | 1 | K.INQPDVVCEDPEEEVAPASCSEHR.A |
|  | 29856 | **931.8939** | **3723.5463** | **3723.5732** | **-0.0269** | **0** | **5** | **8.3** | **1** | **K.TCPGNLVYLESGSPCMDTCSHLEVSSLCEEHR.M** |

| **416.** | P30504\|1C04_HUMAN    **Mass:** 41368    **Score:** 114    **Queries matched:** 8   **emPAI:** 0.09 |
| --- | --- |
|  | HLA class I histocompatibility antigen, Cw-4 alpha chain OS=Homo sapiens GN=HLA-C PE=1 SV=1 |

|  | **Query** | **Observed** | **Mr(expt)** | **Mr(calc)** | **Delta** | **Miss** | **Score** | **Expect** | **Rank** | **Peptide** |
| --- | --- | --- | --- | --- | --- | --- | --- | --- | --- | --- |
|  | 1195 | 581.3248 | 580.3175 | 580.2969 | 0.0206 | 0 | 1 | 1e+002 | 5 | R.AEHPK.T |
|  | 12099 | 714.3628 | 1426.7111 | 1426.7205 | -0.0093 | 0 | 11 | 9.6 | 1 | K.WAAVVVPSGEEQR.Y |
|  | 15206 | 815.4121 | 1628.8097 | 1628.8199 | -0.0102 | 0 | 72 | 7.9e-006 | 1 | R.FIAVGYVDDTQFVR.F 15203 15205 |
|  | 18119 | 459.9797 | 1835.8896 | 1835.9027 | -0.0131 | 0 | 13 | 5.4 | 2 | K.THVTHHPVSDHEATLR.C |
|  | 20661 | 513.0184 | 2048.0446 | 2049.0102 | -0.9656 | 0 | 14 | 4 | 2 | R.YTCHVQHEGLPEPLTLR.W |
|  | 30334 | **1320.3988** | **3958.1746** | **3958.2450** | **-0.0705** | **1** | **1** | **21** | **2** | **R.WKPSSQPTIPIVGIVAGLAVLAVLAVLGAMVAVVMCRR.K** |

| **417.** | Q9HBB8\|MUCDL_HUMAN    **Mass:** 88444    **Score:** 114    **Queries matched:** 10   **emPAI:** 0.04 |
| --- | --- |
|  | Mucin and cadherin-like protein OS=Homo sapiens GN=MUPCDH PE=1 SV=2 |

|  | **Query** | **Observed** | **Mr(expt)** | **Mr(calc)** | **Delta** | **Miss** | **Score** | **Expect** | **Rank** | **Peptide** |
| --- | --- | --- | --- | --- | --- | --- | --- | --- | --- | --- |
|  | 802 | 503.1060 | 502.0987 | 502.2863 | -0.1877 | 0 | 9 | 45 | 3 | R.GTVAR.G |
|  | 3873 | 456.2901 | 910.5656 | 909.4708 | 1.0947 | 0 | 3 | 64 | 10 | R.FPQSLYR.G |
|  | 8686 | **620.7755** | **1239.5365** | **1240.7252** | **-1.1887** | **1** | **16** | **3.1** | **2** | **R.GTVARGAGAGVVVK.D** 8688 |
|  | 12421 | **483.2733** | **1446.7981** | **1446.8156** | **-0.0175** | **0** | **10** | **13** | **4** | **R.SVPSPMTFLLLVK.G** |
|  | 17583 | 899.9674 | 1797.9203 | 1798.9061 | -0.9858 | 0 | 2 | 58 | 7 | K.VNSTVIPETQLQAEDR.D |
|  | 20846 | **1034.4965** | **2066.9784** | **2066.9909** | **-0.0125** | **0** | **78** | **1.3e-006** | **1** | **R.IQAQDPEFSDLNSAITYR.I** 20847 20852 |
|  | 26337 | 699.5942 | 2794.3479 | 2793.4450 | 0.9029 | 1 | 5 | 18 | 2 | R.YSVTQVTVEAVAAAGSPPRFPQSLYR.G |

| **418.** | Q13576\|IQGA2_HUMAN    **Mass:** 181071   **Score:** 114    **Queries matched:** 35   **emPAI:** 0.11 |
| --- | --- |
|  | Ras GTPase-activating-like protein IQGAP2 OS=Homo sapiens GN=IQGAP2 PE=1 SV=3 |

|  | **Query** | **Observed** | **Mr(expt)** | **Mr(calc)** | **Delta** | **Miss** | **Score** | **Expect** | **Rank** | **Peptide** |
| --- | --- | --- | --- | --- | --- | --- | --- | --- | --- | --- |
|  | 1597 | **670.3835** | **669.3763** | **669.2792** | **0.0971** | **0** | **10** | **13** | **3** | **K.FNMDK.Y** |
|  | 1671 | 689.3779 | 688.3706 | 687.3915 | 0.9791 | 0 | 17 | 4.9 | 4 | R.NLGSVAK.V |
|  | 1815 | **731.4153** | **730.4081** | **731.3701** | **-0.9621** | **0** | **9** | **31** | **1** | **K.EIIDDK.S** |
|  | 2923 | **426.7209** | **851.4273** | **851.4324** | **-0.0051** | **0** | **23** | **0.83** | **1** | **K.MVVSFNR.G** |
|  | 3142 | 432.7469 | 863.4793 | 862.4119 | 1.0674 | 1 | 7 | 35 | 3 | R.KEYMHR.R |
|  | 6979 | **570.8185** | **1139.6225** | **1139.6127** | **0.0098** | **1** | **3** | **77** | **8** | **K.IQAFWKGYK.Q** |
|  | 7572 | **593.2755** | **1184.5365** | **1185.6717** | **-1.1353** | **0** | **2** | **97** | **4** | **K.GIAEQTVVTLR.N** |
|  | 7883 | **600.8289** | **1199.6433** | **1199.6622** | **-0.0189** | **1** | **7** | **32** | **9** | **K.LEASIENLRR.V** 7908 |
|  | 9137 | 422.0406 | 1263.0999 | 1263.6306 | -0.5307 | 1 | 5 | 31 | 1 | K.SQKLGDSESVSK.V |
|  | 10990 | **683.4045** | **1364.7945** | **1364.8027** | **-0.0082** | **0** | **5** | **44** | **2** | **K.LGIAPQIQDLLGK.V** |
|  | 12113 | 714.8486 | 1427.6826 | 1428.6845 | -1.0019 | 0 | 0 | 1.3e+002 | 8 | R.TLEQTGHVSSENK.Y |
|  | 12458 | 725.8640 | 1449.7133 | 1449.7650 | -0.0516 | 1 | 7 | 24 | 2 | K.LIFQMPQNKSTK.F 12451 |
|  | 12760 | 490.4935 | 1468.4587 | 1468.6504 | -0.1917 | 0 | 1 | 71 | 4 | K.VDFTEEEISNMR.K |
|  | 13071 | 745.3293 | 1488.6440 | 1488.8188 | -0.1748 | 0 | 2 | 77 | 9 | K.SLIINTNPVEVYK.A |
|  | 14931 | 807.4669 | 1612.9192 | 1612.7403 | 0.1789 | 1 | 0 | 1.1e+002 | 6 | K.VDFTEEEISNMRK.E |
|  | 15634 | **553.2844** | **1656.8314** | **1655.8229** | **1.0086** | **1** | **2** | **66** | **6** | **K.ELEKYGIQMPSFSK.I** |
|  | 15689 | **831.4036** | **1660.7926** | **1660.8056** | **-0.0131** | **0** | **76** | **3e-006** | **1** | **K.AWVNQLETQTGEASK.L** |
|  | 16509 | **860.4437** | **1718.8729** | **1717.8887** | **0.9842** | **0** | **24** | **0.51** | **1** | **K.GVLLDIDDLQTNQFK.N** |
|  | 18640 | **937.0394** | **1872.0643** | **1872.0720** | **-0.0077** | **0** | **9** | **13** | **1** | **K.VLNSIISSLDLLPYGLR.Y** |
|  | 18813 | 944.4116 | 1886.8086 | 1886.9561 | -0.1475 | 0 | 11 | 7.6 | 2 | K.QWVTLVVDVNQCLEGK.K |
|  | 19432 | **646.3151** | **1935.9234** | **1936.9643** | **-1.0409** | **1** | **2** | **57** | **7** | **R.FQATSSGPILREEFEAR.K** |
|  | 19462 | 647.3201 | 1938.9384 | 1940.0579 | -1.1195 | 1 | 1 | 73 | 1 | K.SKVDQVQDIVTGNPTVIK.M |
|  | 20085 | **666.3524** | **1996.0353** | **1996.9888** | **-0.9535** | **1** | **1** | **71** | **3** | **K.HSQSMIEDAQLPLEQKK.R** |
|  | 21399 | **705.3755** | **2113.1046** | **2112.9269** | **0.1777** | **1** | **7** | **18** | **1** | **R.YGSIVDDERLSAEEMDER.R** |
|  | 22551 | **747.2972** | **2238.8699** | **2238.1532** | **0.7167** | **1** | **5** | **21** | **3** | **K.NVTFDIIATEDVGIFDVRSK.F** |
|  | 23392 | 587.7993 | 2347.1682 | 2347.3515 | -0.1833 | 1 | 3 | 36 | 5 | K.VLNSIISSLDLLPYGLRYIAK.V 23391 |
|  | 23485 | **1179.0603** | **2356.1060** | **2356.1183** | **-0.0122** | **0** | **39** | **0.01** | **1** | **K.VLWLDEIQQAVDEANVDEDR.A** 23486 |
|  | 25728 | **1344.0824** | **2686.1502** | **2685.3142** | **0.8361** | **1** | **1** | **51** | **2** | **K.RWMEVCLVEELPPTTELEEGLR.N** |
|  | 28495 | 1089.5178 | 3265.5316 | 3265.5521 | -0.0205 | 0 | 47 | 0.0008 | 1 | R.NQPGNTLTEILETPATAQQEVDHATDMVSR.A |
|  | 29137 | **869.1936** | **3472.7453** | **3472.7627** | **-0.0174** | **0** | **5** | **13** | **1** | **R.TLETLLLPTANISDVDPAHAQHYQDVLYHAK.S** |
|  | 30324 | **791.8038** | **3953.9828** | **3954.8687** | **-0.8860** | **1** | **0** | **26** | **10** | **R.NQPGNTLTEILETPATAQQEVDHATDMVSRAMIDSR.T** |

| **419.** | Q9NY59\|NSMA2_HUMAN    **Mass:** 72347    **Score:** 113    **Queries matched:** 15   **emPAI:** 0.16 |
| --- | --- |
|  | Sphingomyelin phosphodiesterase 3 OS=Homo sapiens GN=SMPD3 PE=1 SV=1 |

|  | **Query** | **Observed** | **Mr(expt)** | **Mr(calc)** | **Delta** | **Miss** | **Score** | **Expect** | **Rank** | **Peptide** |
| --- | --- | --- | --- | --- | --- | --- | --- | --- | --- | --- |
|  | 812 | 503.3154 | 502.3081 | 502.3115 | -0.0034 | 0 | 12 | 23 | 3 | K.ASVVK.K |
|  | 1425 | **631.3508** | **630.3435** | **630.4064** | **-0.0629** | **1** | **23** | **1.6** | **1** | **R.AATKLK.E** |
|  | 3984 | 460.3096 | 918.6047 | 917.4454 | 1.1593 | 0 | 4 | 79 | 7 | K.VLESEEGR.R |
|  | 4473 | 477.7740 | 953.5334 | 953.5083 | 0.0251 | 0 | 12 | 8.2 | 1 | R.RPYIYSR.L 4487 4489 |
|  | 6107 | **537.7766** | **1073.5387** | **1073.5465** | **-0.0078** | **1** | **19** | **2.1** | **1** | **K.VLESEEGRR.E** |
|  | 8185 | **607.8058** | **1213.5971** | **1213.6051** | **-0.0080** | **0** | **18** | **2.6** | **1** | **K.VQVGSTPQDQR.I** |
|  | 9295 | **424.7369** | **1271.1887** | **1271.6874** | **-0.4986** | **0** | **8** | **16** | **3** | **K.GLAGGAALLSEWK.G** |
|  | 13196 | **499.2692** | **1494.7858** | **1495.8035** | **-1.0177** | **1** | **2** | **74** | **10** | **R.LAASFIPTTYEKR.Q** |
|  | 15096 | **812.3783** | **1622.7420** | **1621.8134** | **0.9287** | **1** | **3** | **57** | **6** | **K.CNDDALASKGALFLK.V** |
|  | 18311 | **925.4461** | **1848.8777** | **1848.8829** | **-0.0052** | **0** | **33** | **0.054** | **1** | **R.CGQLDLLQDWLADFR.K** |
|  | 25755 | **897.4496** | **2689.3269** | **2689.3421** | **-0.0152** | **0** | **31** | **0.053** | **1** | **K.AEVEEFSFITQLSGLTDHLPVAMR.L** |
|  | 29676 | **1220.5796** | **3658.7169** | **3657.7509** | **0.9661** | **0** | **57** | **6.4e-005** | **1** | **R.LGPGEEKPWAIGTLLDTNGLYDEDVCTPDNLQK.V** 29675 |

| **420.** | Q5JPE7\|NOMO2_HUMAN    **Mass:** 140435   **Score:** 113    **Queries matched:** 21   **emPAI:** 0.08 |
| --- | --- |
|  | Nodal modulator 2 OS=Homo sapiens GN=NOMO2 PE=1 SV=1 |

|  | **Query** | **Observed** | **Mr(expt)** | **Mr(calc)** | **Delta** | **Miss** | **Score** | **Expect** | **Rank** | **Peptide** |
| --- | --- | --- | --- | --- | --- | --- | --- | --- | --- | --- |
|  | 3472 | **443.0380** | **884.0614** | **883.4222** | **0.6392** | **0** | **8** | **18** | **3** | **R.QTGYMLR.C** |
|  | 6661 | 558.3570 | 1114.6994 | 1114.5983 | 0.1012 | 0 | 8 | 26 | 1 | K.IQSTVTQPGGK.F 6648 |
|  | 7838 | **400.2121** | **1197.6146** | **1197.6216** | **-0.0071** | **0** | **17** | **3** | **1** | **R.FCLSKPGVYK.V** |
|  | 8127 | 606.3388 | 1210.6631 | 1210.6822 | -0.0192 | 0 | 9 | 19 | 6 | K.HITLIFNPTR.K 8129 |
|  | 10541 | **447.1157** | **1338.3253** | **1338.7772** | **-0.4519** | **1** | **1** | **77** | **10** | **K.HITLIFNPTRK.L** |
|  | 10681 | **673.7961** | **1345.5777** | **1346.6943** | **-1.1165** | **1** | **8** | **25** | **3** | **R.GERITFDVAPSR.L** 10682 10704 |
|  | 11225 | 689.8887 | 1377.7629 | 1377.7729 | -0.0100 | 0 | 13 | 7.8 | 1 | K.GQPLGPAGVQVSLR.N |
|  | 16115 | **846.4329** | **1690.8512** | **1689.8574** | **0.9938** | **0** | **21** | **0.93** | **1** | **R.DGENYVVLLDSTLPR.S** |
|  | 17678 | **603.0793** | **1806.2162** | **1805.0523** | **1.1639** | **1** | **7** | **15** | **1** | **K.VLSKGQPLGPAGVQVSLR.N** |
|  | 19708 | 654.3296 | 1959.9669 | 1959.9764 | -0.0095 | 1 | 1 | 84 | 5 | K.VNAMTFTFDNVLPGKYK.I |
|  | 19732 | **982.4677** | **1962.9207** | **1962.9357** | **-0.0149** | **0** | **95** | **3.1e-008** | **1** | **R.FEPSSQMIEVQEGQNLK.I** |
|  | 21004 | **695.7031** | **2084.0876** | **2083.0950** | **0.9926** | **0** | **11** | **6.7** | **1** | **K.AEDDQPLPGVLLSLSGGLFR.S** |
|  | 22079 | 1094.0366 | 2186.0587 | 2186.1154 | -0.0567 | 0 | 0 | 77 | 3 | K.IAPNTPQLADIVATGFSVCGR.I 22081 |
|  | 25092 | 859.1008 | 2574.2805 | 2575.3489 | -1.0684 | 1 | 7 | 14 | 5 | K.MMDVTVTIKSSIDSEPALVLGPLK.S |
|  | 26126 | 1376.1907 | 2750.3668 | 2751.4119 | -1.0452 | 0 | 3 | 31 | 2 | R.QINQFDLSGNVITSSEYLPTLWVK.L |
|  | 27845 | 1041.1469 | 3120.4187 | 3120.4361 | -0.0174 | 0 | 46 | 0.0011 | 1 | R.MTKPPVQEMVDELQGPFSYDFSYWAR.S |

|  | **Proteins matching a subset of these peptides:** |
| --- | --- |

|  | Q15155\|NOMO1_HUMAN    **Mass:** 135237   **Score:** 113    **Queries matched:** 19 |
| --- | --- |
|  | Nodal modulator 1 OS=Homo sapiens GN=NOMO1 PE=1 SV=4 |

|  | P69849\|NOMO3_HUMAN    **Mass:** 135067   **Score:** 113    **Queries matched:** 16 |
| --- | --- |
|  | Nodal modulator 3 OS=Homo sapiens GN=NOMO3 PE=2 SV=1 |

| **421.** | Q9HCY8\|S10AE_HUMAN    **Mass:** 11826    **Score:** 113    **Queries matched:** 5   **emPAI:** 0.78 |
| --- | --- |
|  | Protein S100-A14 OS=Homo sapiens GN=S100A14 PE=1 SV=1 |

|  | **Query** | **Observed** | **Mr(expt)** | **Mr(calc)** | **Delta** | **Miss** | **Score** | **Expect** | **Rank** | **Peptide** |
| --- | --- | --- | --- | --- | --- | --- | --- | --- | --- | --- |
|  | 5704 | **523.7778** | **1045.5411** | **1044.5451** | **0.9960** | **0** | **9** | **22** | **2** | **K.ETLTPSELR.D** |
|  | 7473 | **589.7730** | **1177.5313** | **1177.5397** | **-0.0084** | **0** | **28** | **0.28** | **1** | **K.IANLGSCNDSK.L** |
|  | 8867 | 625.8214 | 1249.6281 | 1249.6343 | -0.0061 | 0 | 76 | 3.6e-006 | 1 | R.SFWELIGEAAK.S |
|  | 9142 | 422.2117 | 1263.6132 | 1264.5836 | -0.9705 | 0 | 9 | 19 | 3 | K.NFHQYSVEGGK.E |
|  | 15766 | **834.3547** | **1666.6949** | **1666.7070** | **-0.0121** | **0** | **61** | **7.8e-005** | **1** | **R.SANAEDAQEFSDVER.A** |

| **422.** | Q9Y6C9\|MTCH2_HUMAN    **Mass:** 33936    **Score:** 113    **Queries matched:** 8   **emPAI:** 0.69 |
| --- | --- |
|  | Mitochondrial carrier homolog 2 OS=Homo sapiens GN=MTCH2 PE=1 SV=1 |

|  | **Query** | **Observed** | **Mr(expt)** | **Mr(calc)** | **Delta** | **Miss** | **Score** | **Expect** | **Rank** | **Peptide** |
| --- | --- | --- | --- | --- | --- | --- | --- | --- | --- | --- |
|  | 4567 | **481.2736** | **960.5326** | **960.5393** | **-0.0067** | **0** | **34** | **0.07** | **1** | **R.GLFTGLTPR.L** |
|  | 9455 | 1281.5680 | 1280.5607 | 1280.6547 | -0.0940 | 1 | 4 | 58 | 4 | R.SMVQFIGRESK.Y |
|  | 13334 | **502.3313** | **1503.9721** | **1503.8086** | **0.1635** | **0** | **2** | **60** | **8** | **R.EEGILGFFAGLVPR.L** |
|  | 16940 | **876.5008** | **1750.9870** | **1750.9982** | **-0.0111** | **0** | **39** | **0.014** | **1** | **K.VLIQVGYEPLPPTIGR.N** |
|  | 17295 | **593.0054** | **1775.9943** | **1776.0046** | **-0.0104** | **0** | **33** | **0.054** | **1** | **R.SAATLITHPFHVITLR.S** |
|  | 23470 | 1178.0740 | 2354.1334 | 2354.1502 | -0.0169 | 1 | 53 | 0.0004 | 1 | K.VLQHYQESDKGEELGPGNVQK.E |
|  | 23508 | **787.3948** | **2359.1625** | **2359.1743** | **-0.0118** | **0** | **43** | **0.0044** | **1** | **R.QVCQLPGLFSYAQHIASIDGR.R** |
|  | 25415 | **879.7906** | **2636.3499** | **2637.3757** | **-1.0258** | **0** | **0** | **65** | **8** | **-.MADAASQVLLGSGLTILSQPLMYVK.V** |

| **423.** | O75356\|ENTP5_HUMAN    **Mass:** 47886    **Score:** 112    **Queries matched:** 12   **emPAI:** 0.35 |
| --- | --- |
|  | Ectonucleoside triphosphate diphosphohydrolase 5 OS=Homo sapiens GN=ENTPD5 PE=1 SV=1 |

|  | **Query** | **Observed** | **Mr(expt)** | **Mr(calc)** | **Delta** | **Miss** | **Score** | **Expect** | **Rank** | **Peptide** |
| --- | --- | --- | --- | --- | --- | --- | --- | --- | --- | --- |
|  | 735 | **487.3203** | **486.3131** | **486.3166** | **-0.0035** | **0** | **17** | **6** | **1** | **K.GGILK.V** |
|  | 1241 | **589.1233** | **588.1160** | **587.3391** | **0.7769** | **0** | **2** | **1.6e+002** | **9** | **K.ATAGLR.L** |
|  | 6910 | **568.2799** | **1134.5453** | **1133.6233** | **0.9219** | **0** | **6** | **41** | **7** | **R.IHVYTFVQK.M** |
|  | 9798 | 650.2628 | 1298.5110 | 1298.5700 | -0.0591 | 0 | 17 | 2.5 | 1 | R.AVDTDMIDYEK.G |
|  | 12230 | **717.8704** | **1433.7263** | **1433.7343** | **-0.0080** | **0** | **32** | **0.092** | **1** | **R.WLEAEWIFGGVK.Y** |
|  | 12341 | 721.8909 | 1441.7673 | 1441.7776 | -0.0103 | 0 | 82 | 7.8e-007 | 1 | K.QGAETVQGLLEVAK.D |
|  | 16179 | 566.2792 | 1695.8157 | 1695.9097 | -0.0940 | 1 | 3 | 50 | 3 | K.LYTHSYLGFGLKAAR.L |
|  | 25120 | **861.1075** | **2580.3006** | **2579.3119** | **0.9887** | **0** | **25** | **0.23** | **1** | **R.QETVGTLDLGGASTQITFLPQFEK.T** |
|  | 27314 | **1000.1845** | **2997.5317** | **2997.5522** | **-0.0205** | **0** | **16** | **1.4** | **1** | **K.MPGQLPILEGEVFDSVKPGLSAFVDQPK.Q** |
|  | 28194 | **799.8989** | **3195.5666** | **3195.5772** | **-0.0105** | **0** | **38** | **0.0068** | **1** | **K.GSVSIMDGSDEGILAWVTVNFLTGQLHGHR.Q** 28193 |
|  | 30367 | **1327.5494** | **3979.6265** | **3980.0255** | **-0.3990** | **1** | **0** | **20** | **3** | **K.SPFLVPKGSVSIMDGSDEGILAWVTVNFLTGQLHGHR.Q** |

| **424.** | A6NNZ2\|TBB8B_HUMAN    **Mass:** 50168    **Score:** 112    **Queries matched:** 18   **emPAI:** 0.24 |
| --- | --- |
|  | Tubulin beta-8 chain B OS=Homo sapiens PE=1 SV=1 |

|  | **Query** | **Observed** | **Mr(expt)** | **Mr(calc)** | **Delta** | **Miss** | **Score** | **Expect** | **Rank** | **Peptide** |
| --- | --- | --- | --- | --- | --- | --- | --- | --- | --- | --- |
|  | 1837 | 737.3976 | 736.3904 | 737.3456 | -0.9553 | 0 | 9 | 21 | 2 | R.GSQQYR.A |
|  | 2296 | 405.2068 | 808.3991 | 807.3399 | 1.0593 | 0 | 8 | 28 | 7 | R.EEYPDR.I |
|  | 5466 | 514.7600 | 1027.5053 | 1027.5121 | -0.0067 | 0 | 25 | 0.47 | 1 | K.TAVCDIPPR.G 5464 5477 5478 |
|  | 5960 | 533.2140 | 1064.4134 | 1064.4201 | -0.0067 | 0 | 11 | 12 | 1 | K.NMMAACDPR.H 5970 |
|  | 6152 | 539.2660 | 1076.5174 | 1076.5250 | -0.0076 | 1 | 25 | 0.51 | 1 | K.IREEYPDR.I |
|  | 10304 | 663.8410 | 1325.6674 | 1325.7126 | -0.0451 | 1 | 5 | 41 | 5 | K.TAVCDIPPRGLK.M |
|  | 15059 | 540.9470 | 1619.8192 | 1619.8283 | -0.0090 | 0 | 28 | 0.19 | 1 | R.LHFFMPGFAPLTSR.G |
|  | 25855 | 901.7996 | 2702.3769 | 2703.3360 | -0.9592 | 0 | 3 | 30 | 4 | K.LPTPTYGDLNHLVSATMSGVTTCLR.F 25853 25890 |
|  | 28139 | 796.6538 | 3182.5861 | 3183.4828 | -0.8966 | 0 | 4 | 18 | 2 | K.MSATFIGNNAAIQELFTCVSEQFTAMFR.R |
|  | 28611 | 1104.5131 | 3310.5174 | 3310.5268 | -0.0095 | 0 | 79 | 5.2e-007 | 1 | K.EAESCDCLQGFQLTHSLGGGTGSGMGTLLISK.I 28610 |
|  | 31045 | **1147.7341** | **4586.9074** | **4587.1812** | **-0.2738** | **1** | **1** | **14** | **1** | **R.AVLVDLEPGTMDSVHSGPFGQVFRPDNFISGQCGAGNNWAKGR.Y** |

| **425.** | Q9HAV0\|GBB4_HUMAN    **Mass:** 38284    **Score:** 112    **Queries matched:** 19   **emPAI:** 0.45 |
| --- | --- |
|  | Guanine nucleotide-binding protein subunit beta-4 OS=Homo sapiens GN=GNB4 PE=1 SV=3 |

|  | **Query** | **Observed** | **Mr(expt)** | **Mr(calc)** | **Delta** | **Miss** | **Score** | **Expect** | **Rank** | **Peptide** |
| --- | --- | --- | --- | --- | --- | --- | --- | --- | --- | --- |
|  | 5229 | 505.2587 | 1008.5028 | 1008.5101 | -0.0072 | 0 | 54 | 0.00068 | 1 | R.AGVLAGHDNR.V 5226 5228 5230 5231 5232 5235 |
|  | 5329 | 509.2790 | 1016.5434 | 1016.5502 | -0.0068 | 0 | 30 | 0.17 | 1 | R.LLVSASQDGK.L 5328 |
|  | 10792 | 677.3515 | 1352.6884 | 1352.6976 | -0.0092 | 0 | 53 | 0.00065 | 1 | K.LIIWDSYTTNK.M 10809 |
|  | 13969 | 775.3431 | 1548.6717 | 1548.6813 | -0.0096 | 0 | 14 | 4.8 | 1 | R.ELPGHTGYLSCCR.F 13968 13977 |
|  | 16222 | 850.3987 | 1698.7829 | 1697.8445 | 0.9385 | 1 | 4 | 43 | 8 | R.QEAEQLRNQIQDAR.K |
|  | 21821 | **719.3654** | **2155.0744** | **2156.0248** | **-0.9504** | **0** | **4** | **37** | **1** | **R.LLLAGYDDFNCNVWDTLK.G** 21818 |
|  | 22094 | **1095.0549** | **2188.0953** | **2187.1510** | **0.9443** | **1** | **2** | **54** | **5** | **K.LIIWDSYTTNKMHAIPLR.S** |
|  | 29121 | 1157.8501 | 3470.5285 | 3470.5403 | -0.0119 | 0 | 34 | 0.012 | 1 | R.SSWVMTCAYAPSGNYVACGGLDNICSIYNLK.T |

| **426.** | Q9UBI6\|GBG12_HUMAN    **Mass:** 8115     **Score:** 111    **Queries matched:** 11   **emPAI:** 2.42 |
| --- | --- |
|  | Guanine nucleotide-binding protein G(I)/G(S)/G(O) subunit gamma-12 OS=Homo sapiens GN=GNG12 PE=1 SV=3 |

|  | **Query** | **Observed** | **Mr(expt)** | **Mr(calc)** | **Delta** | **Miss** | **Score** | **Expect** | **Rank** | **Peptide** |
| --- | --- | --- | --- | --- | --- | --- | --- | --- | --- | --- |
|  | 1857 | **744.4308** | **743.4236** | **743.4290** | **-0.0054** | **0** | **3** | **1.1e+002** | **6** | **R.TVQQLR.L** |
|  | 3726 | **451.2638** | **900.5131** | **899.5301** | **0.9831** | **1** | **12** | **13** | **3** | **R.RTVQQLR.L** 3679 3718 3723 |
|  | 7045 | **573.7929** | **1145.5711** | **1145.5789** | **-0.0077** | **0** | **58** | **0.00027** | **1** | **K.TASTNNIAQAR.R** 7062 |
|  | 14652 | **532.2631** | **1593.7675** | **1594.7733** | **-1.0058** | **1** | **3** | **56** | **7** | **-.MSSKTASTNNIAQAR.R** |
|  | 15363 | **820.3396** | **1638.6646** | **1638.6766** | **-0.0120** | **0** | **56** | **0.00024** | **1** | **K.ASADLMSYCEEHAR.S** 15364 |
|  | 16603 | **864.4597** | **1726.9048** | **1726.9141** | **-0.0094** | **0** | **27** | **0.23** | **1** | **R.SDPLLIGIPTSENPFK.D** |

| **427.** | P19404\|NDUV2_HUMAN    **Mass:** 27659    **Score:** 111    **Queries matched:** 9   **emPAI:** 0.47 |
| --- | --- |
|  | NADH dehydrogenase [ubiquinone] flavoprotein 2, mitochondrial OS=Homo sapiens GN=NDUFV2 PE=1 SV=2 |

|  | **Query** | **Observed** | **Mr(expt)** | **Mr(calc)** | **Delta** | **Miss** | **Score** | **Expect** | **Rank** | **Peptide** |
| --- | --- | --- | --- | --- | --- | --- | --- | --- | --- | --- |
|  | 947 | **528.2779** | **527.2706** | **527.3431** | **-0.0725** | **0** | **0** | **1.4e+002** | **2** | **R.KPVGK.Y** |
|  | 8250 | 609.3179 | 1216.6212 | 1216.6299 | -0.0087 | 0 | 48 | 0.0026 | 1 | R.NSDSILEAIQK.K |
|  | 9003 | **629.3524** | **1256.6902** | **1256.6976** | **-0.0074** | **1** | **12** | **9.8** | **1** | **K.LGIKVGETTPDK.L** |
|  | 9165 | **422.5492** | **1264.6257** | **1265.6741** | **-1.0485** | **1** | **4** | **53** | **6** | **R.ARAAGLTAHWGR.H** |
|  | 15620 | **828.8853** | **1655.7561** | **1655.7654** | **-0.0093** | **0** | **51** | **0.00083** | **1** | **R.VYEVATFYTMYNR.K** 15621 |
|  | 21044 | **697.3099** | **2088.9080** | **2090.0103** | **-1.1023** | **1** | **1** | **66** | **4** | **R.SGRFSCEPAGGLTSLTEPPK.G** |
|  | 23733 | **799.6880** | **2396.0421** | **2396.0557** | **-0.0135** | **1** | **8** | **11** | **1** | **R.DTPENNPDTPFDFTPENYKR.I** |
|  | 25617 | **1337.6550** | **2673.2955** | **2673.3109** | **-0.0154** | **1** | **50** | **0.00062** | **1** | **R.FSCEPAGGLTSLTEPPKGPGFGVQAGL.-** |

| **428.** | P23634\|AT2B4_HUMAN    **Mass:** 139030   **Score:** 111    **Queries matched:** 30   **emPAI:** 0.08 |
| --- | --- |
|  | Plasma membrane calcium-transporting ATPase 4 OS=Homo sapiens GN=ATP2B4 PE=1 SV=2 |

|  | **Query** | **Observed** | **Mr(expt)** | **Mr(calc)** | **Delta** | **Miss** | **Score** | **Expect** | **Rank** | **Peptide** |
| --- | --- | --- | --- | --- | --- | --- | --- | --- | --- | --- |
|  | 1206 | **583.3227** | **582.3154** | **582.3125** | **0.0029** | **0** | **8** | **15** | **1** | **K.IHGEK.N** |
|  | 2079 | 397.2295 | 792.4444 | 792.4164 | 0.0280 | 0 | 15 | 6.4 | 4 | K.SMSTVIR.N |
|  | 2288 | **404.6981** | **807.3817** | **808.4113** | **-1.0296** | **0** | **(4)** | **71** | **10** | **K.SMSTVIR.N** |
|  | 3785 | **453.3409** | **904.6673** | **903.4960** | **1.1713** | **1** | **1** | **1.1e+002** | **9** | **K.LMELRSR.D** |
|  | 3828 | 455.2452 | 908.4759 | 908.4386 | 0.0374 | 0 | 4 | 56 | 1 | K.TGTLTMNR.M |
|  | 3974 | 460.2462 | 918.4779 | 918.5076 | -0.0296 | 0 | 8 | 31 | 3 | R.GQILWFR.G |
|  | 4518 | 479.2832 | 956.5518 | 955.4400 | 1.1119 | 0 | 9 | 22 | 2 | K.FFDIDSGR.K |
|  | 4886 | 493.2556 | 984.4965 | 984.5029 | -0.0063 | 0 | 8 | 27 | 3 | K.VYTFNSVR.K |
|  | 5132 | **501.3357** | **1000.6568** | **1000.6029** | **0.0539** | **1** | **8** | **28** | **6** | **K.SVLQGKLTR.L** |
|  | 7166 | **578.3185** | **1154.6224** | **1153.6204** | **1.0020** | **1** | **5** | **45** | **1** | **K.EGGLPRQVGNK.T** |
|  | 7582 | **593.3292** | **1184.6439** | **1184.5608** | **0.0831** | **0** | **8** | **28** | **8** | **R.QNMGQHLDVK.L** |
|  | 7709 | 596.3003 | 1190.5861 | 1190.5714 | 0.0148 | 0 | 11 | 13 | 8 | R.MVTGDNINTAR.A 7708 |
|  | 9548 | **643.3533** | **1284.6920** | **1285.5873** | **-0.8954** | **1** | **4** | **62** | **7** | **R.NPNGGFRMYSK.G** |
|  | 10277 | **442.2745** | **1323.8016** | **1324.6670** | **-0.8654** | **1** | **12** | **8** | **1** | **R.RQNMGQHLDVK.L** |
|  | 11316 | 692.3542 | 1382.6939 | 1382.7227 | -0.0288 | 1 | 3 | 65 | 5 | R.MYSKGASEIILR.K |
|  | 11615 | **701.3204** | **1400.6263** | **1399.6943** | **0.9320** | **1** | **0** | **1.3e+002** | **5** | **K.EAGHGTTKEEITK.D** |
|  | 13754 | **512.2318** | **1533.6734** | **1534.7674** | **-1.0940** | **1** | **4** | **44** | **3** | **K.SMSTVIRNPNGGFR.M** |
|  | 15081 | **541.5988** | **1621.7744** | **1622.8239** | **-1.0495** | **0** | **6** | **28** | **4** | **R.MTVVQAYIGGIHYR.Q** |
|  | 15416 | 821.4199 | 1640.8253 | 1640.8370 | -0.0117 | 0 | 47 | 0.0022 | 1 | R.QVVAVTGDGTNDGPALK.K 15417 15418 |
|  | 17103 | **588.3427** | **1762.0063** | **1760.9533** | **1.0530** | **1** | **2** | **66** | **6** | **K.HTLVKGIIDSTVGEHR.Q** |
|  | 18742 | 941.4688 | 1880.9231 | 1879.9415 | 0.9816 | 0 | 27 | 0.2 | 1 | K.EASDIILTDDNFTSIVK.A |
|  | 19191 | 958.0068 | 1913.9990 | 1914.0098 | -0.0108 | 0 | 74 | 3.6e-006 | 1 | K.YGDLLPADGILIQGNDLK.I 19190 |
|  | 19413 | 645.3388 | 1932.9946 | 1931.9847 | 1.0099 | 1 | 3 | 49 | 6 | K.QAGITVRMVTGDNINTAR.A |
|  | 20017 | **664.3619** | **1990.0638** | **1990.9200** | **-0.8562** | **1** | **2** | **55** | **4** | **-.MTNPSDRVLPANSMAESR.E** |
|  | 28702 | 1667.8639 | 3333.7132 | 3333.7240 | -0.0108 | 0 | 1 | 34 | 2 | K.AVQMLWVNLIMDTFASLALATEPPTESLLK.R |
|  | 30487 | 681.8417 | 4085.0068 | 4085.1548 | -0.1480 | 1 | 2 | 17 | 1 | R.NVYDSISKFLQFQLTVNVVAVIVAFTGACITQDSPLK.A |

| **429.** | P60842\|IF4A1_HUMAN    **Mass:** 46353    **Score:** 111    **Queries matched:** 16   **emPAI:** 0.26 |
| --- | --- |
|  | Eukaryotic initiation factor 4A-I OS=Homo sapiens GN=EIF4A1 PE=1 SV=1 |

|  | **Query** | **Observed** | **Mr(expt)** | **Mr(calc)** | **Delta** | **Miss** | **Score** | **Expect** | **Rank** | **Peptide** |
| --- | --- | --- | --- | --- | --- | --- | --- | --- | --- | --- |
|  | 3443 | **441.7424** | **881.4702** | **880.3709** | **1.0993** | **0** | **3** | **64** | **4** | **-.MSASQDSR.S** |
|  | 3829 | **455.2455** | **908.4765** | **909.4378** | **-0.9614** | **0** | **6** | **40** | **3** | **R.VFDMLNR.R** |
|  | 5997 | **534.3673** | **1066.7201** | **1065.5389** | **1.1811** | **1** | **10** | **13** | **4** | **R.VFDMLNRR.Y** |
|  | 6025 | **535.3394** | **1068.6643** | **1067.5400** | **1.1243** | **0** | **10** | **16** | **2** | **R.QFYINVER.E** 5994 |
|  | 6980 | 570.8237 | 1139.6329 | 1139.6662 | -0.0333 | 0 | 9 | 17 | 5 | K.ATQALVLAPTR.E |
|  | 7343 | 585.2951 | 1168.5756 | 1168.5764 | -0.0008 | 0 | 3 | 72 | 9 | K.DQIYDIFQK.L |
|  | 14072 | **778.3561** | **1554.6977** | **1554.7058** | **-0.0081** | **0** | **71** | **1e-005** | **1** | **K.MFVLDEADEMLSR.G** |
|  | 14588 | **530.6256** | **1588.8550** | **1587.8944** | **0.9606** | **1** | **1** | **89** | **7** | **R.SGSSRVLITTDLLAR.G** |
|  | 15024 | 540.2921 | 1617.8543 | 1617.8661 | -0.0118 | 0 | 32 | 0.067 | 1 | K.LQMEAPHIIVGTPGR.V |
|  | 17978 | **914.4666** | **1826.9187** | **1826.9315** | **-0.0128** | **0** | **57** | **0.00021** | **1** | **R.GIYAYGFEKPSAIQQR.A** |
|  | 19859 | 987.9523 | 1973.8901 | 1974.9431 | -1.0529 | 1 | 0 | 83 | 7 | K.YIKMFVLDEADEMLSR.G 19858 |
|  | 21662 | 537.0157 | 2144.0337 | 2143.1273 | 0.9063 | 0 | 2 | 60 | 9 | R.GIDVQQVSLVINYDLPTNR.E |
|  | 25147 | **863.1337** | **2586.3794** | **2586.3938** | **-0.0145** | **1** | **18** | **1** | **1** | **K.LNSNTQVVLLSATMPSDVLEVTKK.F** |
|  | 30661 | **1394.9724** | **4181.8954** | **4181.8430** | **0.0524** | **1** | **1** | **17** | **6** | **R.SRDNGPDGMEPEGVIESNWNEIVDSFDDMNLSESLLR.G** |

| **430.** | Q9Y2Q3\|GSTK1_HUMAN    **Mass:** 25594    **Score:** 111    **Queries matched:** 17   **emPAI:** 0.74 |
| --- | --- |
|  | Glutathione S-transferase kappa 1 OS=Homo sapiens GN=GSTK1 PE=1 SV=3 |

|  | **Query** | **Observed** | **Mr(expt)** | **Mr(calc)** | **Delta** | **Miss** | **Score** | **Expect** | **Rank** | **Peptide** |
| --- | --- | --- | --- | --- | --- | --- | --- | --- | --- | --- |
|  | 5431 | 513.3054 | 1024.5963 | 1023.5059 | 1.0904 | 0 | 5 | 46 | 5 | K.GLYMANDLK.L |
|  | 9235 | 635.3217 | 1268.6289 | 1268.6798 | -0.0509 | 0 | 1 | 1.2e+002 | 6 | R.MELLAHLLGEK.W |
|  | 9977 | 655.3494 | 1308.6842 | 1307.6808 | 1.0033 | 0 | 4 | 55 | 3 | K.WMGPIPPAVNAR.L |
|  | 10290 | **663.3524** | **1324.6902** | **1323.6758** | **1.0144** | **0** | **(0)** | **1.3e+002** | **8** | **K.WMGPIPPAVNAR.L** |
|  | 11010 | 684.1195 | 1366.2243 | 1365.7670 | 0.4574 | 0 | 3 | 48 | 5 | R.HHLQIPIHFPK.D |
|  | 12198 | **716.8537** | **1431.6928** | **1431.7027** | **-0.0099** | **0** | **63** | **6.8e-005** | **1** | **K.AGMSAEQAQGLLEK.I** |
|  | 12436 | **725.3451** | **1448.6756** | **1447.6977** | **0.9780** | **0** | **(1)** | **1e+002** | **5** | **K.AGMSAEQAQGLLEK.I** |
|  | 16612 | **864.9308** | **1727.8470** | **1727.8577** | **-0.0107** | **0** | **13** | **5** | **2** | **R.NEDITEPQSILAAAEK.A** 16602 16613 16614 |
|  | 17204 | **885.9528** | **1769.8910** | **1769.9022** | **-0.0112** | **0** | **40** | **0.01** | **1** | **R.FLTAVNLEHPEMLEK.A** 17214 |
|  | 24658 | **839.1296** | **2514.3671** | **2514.3780** | **-0.0109** | **0** | **45** | **0.0018** | **1** | **R.YQNIWNINLQLRPSLITGIMK.D** |
|  | 24812 | 636.3255 | 2541.2729 | 2542.3552 | -1.0823 | 1 | 9 | 8.1 | 1 | R.MELLAHLLGEKWMGPIPPAVNAR.L |
|  | 25752 | **897.1066** | **2688.2981** | **2688.3119** | **-0.0138** | **0** | **31** | **0.053** | **1** | **R.YGAFGLPITVAHVDGQTHMLFGSDR.M** 25749 |

| **431.** | Q01524\|DEF6_HUMAN    **Mass:** 11310    **Score:** 110    **Queries matched:** 7   **emPAI:** 0.35 |
| --- | --- |
|  | Defensin-6 OS=Homo sapiens GN=DEFA6 PE=1 SV=1 |

|  | **Query** | **Observed** | **Mr(expt)** | **Mr(calc)** | **Delta** | **Miss** | **Score** | **Expect** | **Rank** | **Peptide** |
| --- | --- | --- | --- | --- | --- | --- | --- | --- | --- | --- |
|  | 4414 | **476.1965** | **950.3784** | **950.3851** | **-0.0067** | **0** | **26** | **0.44** | **1** | **R.AFTCHCR.R** |
|  | 7504 | **591.2249** | **1180.4353** | **1179.5156** | **0.9197** | **0** | **30** | **0.13** | **1** | **K.AYEADAQEQR.G** |
|  | 13625 | **763.3936** | **1524.7727** | **1523.7467** | **1.0260** | **0** | **3** | **61** | **2** | **K.AEPLQAEDDPLQAK.A** |
|  | 19811 | **657.3520** | **1969.0341** | **1970.1962** | **-1.1620** | **1** | **4** | **38** | **3** | **-.MRTLTILTAVLLVALQAK.A** |
|  | 21023 | **1043.9994** | **2085.9842** | **2085.9239** | **0.0603** | **0** | **4** | **36** | **2** | **R.GANDQDFAVSFAEDASSSLR.A** |
|  | 23673 | **1193.4904** | **2384.9662** | **2384.9824** | **-0.0162** | **0** | **110** | **6e-010** | **1** | **R.SCYSTEYSYGTCTVMGINHR.F** 23672 |

| **432.** | O43772\|MCAT_HUMAN    **Mass:** 33264    **Score:** 109    **Queries matched:** 15   **emPAI:** 0.38 |
| --- | --- |
|  | Mitochondrial carnitine/acylcarnitine carrier protein OS=Homo sapiens GN=SLC25A20 PE=1 SV=1 |

|  | **Query** | **Observed** | **Mr(expt)** | **Mr(calc)** | **Delta** | **Miss** | **Score** | **Expect** | **Rank** | **Peptide** |
| --- | --- | --- | --- | --- | --- | --- | --- | --- | --- | --- |
|  | 3561 | 445.9170 | 889.8195 | 889.5055 | 0.3140 | 0 | (5) | 43 | 4 | K.GTVLTLMR.D |
|  | 3795 | **453.7661** | **905.5177** | **905.5004** | **0.0173** | **0** | **5** | **55** | **2** | **K.GTVLTLMR.D** |
|  | 3799 | **453.7844** | **905.5542** | **906.4745** | **-0.9203** | **0** | **2** | **1.3e+002** | **9** | **K.GFNAVMIR.A** |
|  | 3816 | **454.5790** | **907.1435** | **907.4763** | **-0.3328** | **0** | **11** | **8.5** | **3** | **R.EGITGLYR.G** |
|  | 5486 | 515.2902 | 1028.5659 | 1027.4644 | 1.1014 | 0 | 11 | 12 | 1 | K.YTGTLDCAK.K |
|  | 7136 | **577.2781** | **1152.5416** | **1152.6291** | **-0.0875** | **1** | **5** | **52** | **8** | **K.KLYQEFGIR.G** |
|  | 10773 | 676.8783 | 1351.7420 | 1350.7693 | 0.9727 | 1 | 4 | 60 | 9 | R.GIYKGTVLTLMR.D |
|  | 13588 | **761.9045** | **1521.7944** | **1521.8038** | **-0.0094** | **1** | **26** | **0.28** | **1** | **R.ELIRDEGVTSLYK.G** |
|  | 18211 | **922.3892** | **1842.7638** | **1842.8797** | **-0.1159** | **0** | **14** | **3.6** | **1** | **R.AFPANAACFLGFEVAMK.F** |
|  | 19443 | **969.4387** | **1936.8629** | **1936.8739** | **-0.0110** | **0** | **74** | **3.5e-006** | **1** | **R.DVPASGMYFMTYEWLK.N** 19444 |
|  | 19826 | 657.6856 | 1970.0350 | 1968.8638 | 1.1712 | 0 | (1) | 82 | 3 | R.DVPASGMYFMTYEWLK.N |
|  | 25337 | 1312.6089 | 2623.2032 | 2623.2200 | -0.0167 | 0 | 45 | 0.0022 | 1 | R.LQTQPPSLPGQPPMYSGTFDCFR.K 25336 |
|  | 26594 | **572.0800** | **2855.3634** | **2856.3537** | **-0.9902** | **1** | **6** | **16** | **1** | **K.GTVLTLMRDVPASGMYFMTYEWLK.N** |

| **433.** | Q08426\|ECHP_HUMAN    **Mass:** 80072    **Score:** 109    **Queries matched:** 23   **emPAI:** 0.15 |
| --- | --- |
|  | Peroxisomal bifunctional enzyme OS=Homo sapiens GN=EHHADH PE=1 SV=3 |

|  | **Query** | **Observed** | **Mr(expt)** | **Mr(calc)** | **Delta** | **Miss** | **Score** | **Expect** | **Rank** | **Peptide** |
| --- | --- | --- | --- | --- | --- | --- | --- | --- | --- | --- |
|  | 1454 | **635.1334** | **634.1261** | **633.3697** | **0.7563** | **0** | **9** | **24** | **2** | **R.LTSSVK.E** |
|  | 3282 | 436.2816 | 870.5486 | 871.5014 | -0.9528 | 0 | 6 | 46 | 6 | R.ILADEALK.L 3276 |
|  | 9491 | **642.3236** | **1282.6327** | **1282.7245** | **-0.0918** | **1** | **7** | **30** | **1** | **R.ARIPVIAVDSDK.N** |
|  | 10359 | **664.8438** | **1327.6731** | **1326.6779** | **0.9951** | **0** | **20** | **1.3** | **1** | **K.VVNSDPVEEAIR.F** 10360 |
|  | 13446 | **504.9548** | **1511.8426** | **1510.8970** | **0.9456** | **1** | **6** | **33** | **7** | **R.ILADEALKLGILDK.V** |
|  | 13751 | **767.4529** | **1532.8912** | **1531.7743** | **1.1169** | **1** | **8** | **17** | **1** | **R.FAQRVSDQPLESR.R** |
|  | 15310 | **818.4354** | **1634.8562** | **1634.8668** | **-0.0106** | **0** | **64** | **4.1e-005** | **1** | **R.AVQAAVQYPYEVGIK.K** |
|  | 15373 | **547.2639** | **1638.7697** | **1639.9158** | **-1.1460** | **1** | **4** | **45** | **2** | **M.AEYTRLHNALALIR.L** |
|  | 17018 | **878.9414** | **1755.8683** | **1755.8679** | **0.0004** | **0** | **3** | **55** | **4** | **R.QNPDIPQLEPSDYLK.K** |
|  | 18104 | 918.4464 | 1834.8781 | 1833.9407 | 0.9374 | 1 | 2 | 67 | 5 | K.AIVICGAEGKFSAGADIR.G |
|  | 19213 | 639.9392 | 1916.7956 | 1917.0102 | -0.2146 | 0 | 1 | 76 | 7 | K.TASARPVSSVGVVGLGTMGR.G |
|  | 19292 | **642.3182** | **1923.9329** | **1923.0101** | **0.9228** | **1** | **4** | **35** | **6** | **K.KEEELFLYLLQSGQAR.A** |
|  | 21127 | 1047.4625 | 2092.9105 | 2093.0602 | -0.1497 | 0 | 7 | 17 | 1 | K.ELGGVDLVIEAVFEEMSLK.K 21126 21128 |
|  | 22236 | 1103.0713 | 2204.1280 | 2205.1602 | -1.0322 | 1 | 8 | 13 | 5 | K.ELGGVDLVIEAVFEEMSLKK.Q 22237 |
|  | 24486 | **1246.6621** | **2491.3097** | **2491.3244** | **-0.0147** | **0** | **12** | **4.1** | **1** | **K.LLEVIPSQYSSPTTIATVMNLSK.K** 24485 |
|  | 25063 | 857.1000 | 2568.2781 | 2569.2846 | -1.0065 | 1 | 1 | 49 | 6 | R.TISQDEILERCLYSLINEAFR.I |
|  | 30140 | 1276.2737 | 3825.7992 | 3825.8123 | -0.0131 | 0 | 71 | 2.3e-006 | 1 | R.MLNPYYNQAYFLLEEGSKPEEVDQVLEEFGFK.M |

| **434.** | P00395\|COX1_HUMAN    **Mass:** 57060    **Score:** 108    **Queries matched:** 8   **emPAI:** 0.13 |
| --- | --- |
|  | Cytochrome c oxidase subunit 1 OS=Homo sapiens GN=MT-CO1 PE=1 SV=1 |

|  | **Query** | **Observed** | **Mr(expt)** | **Mr(calc)** | **Delta** | **Miss** | **Score** | **Expect** | **Rank** | **Peptide** |
| --- | --- | --- | --- | --- | --- | --- | --- | --- | --- | --- |
|  | 5530 | **516.7626** | **1031.5107** | **1031.5189** | **-0.0082** | **0** | **8** | **31** | **4** | **R.WLFSTNHK.D** |
|  | 14600 | **796.3101** | **1590.6056** | **1589.8024** | **0.8031** | **0** | **1** | **80** | **2** | **K.VFSWLATLHGSNMK.W** |
|  | 30013 | **1262.5767** | **3784.7082** | **3784.7325** | **-0.0244** | **0** | **56** | **9.1e-005** | **1** | **K.VLMVEEPSMNLEWLYGCPPPYHTFEEPVYMK.S** 30011 30012 30014 |
|  | 30093 | **1267.6054** | **3799.7942** | **3800.7275** | **-0.9332** | **0** | **(16)** | **0.75** | **1** | **K.VLMVEEPSMNLEWLYGCPPPYHTFEEPVYMK.S** |
|  | 30857 | **1107.3007** | **4425.1735** | **4426.0369** | **-0.8633** | **1** | **3** | **12** | **1** | **K.KEPFGYMGMVWAMMSIGFLGFIVWAHHMFTVGMDVDTR.A** |

| **435.** | Q96GC9\|TMM49_HUMAN    **Mass:** 46607    **Score:** 108    **Queries matched:** 8   **emPAI:** 0.08 |
| --- | --- |
|  | Transmembrane protein 49 OS=Homo sapiens GN=TMEM49 PE=2 SV=1 |

|  | **Query** | **Observed** | **Mr(expt)** | **Mr(calc)** | **Delta** | **Miss** | **Score** | **Expect** | **Rank** | **Peptide** |
| --- | --- | --- | --- | --- | --- | --- | --- | --- | --- | --- |
|  | 2862 | **424.8176** | **847.6207** | **847.3719** | **0.2488** | **1** | **3** | **84** | **8** | **K.NCDQRR.V** |
|  | 5974 | 533.7494 | 1065.4842 | 1066.6427 | -1.1584 | 0 | 9 | 18 | 10 | K.IFVIITFSK.H 5973 |
|  | 10544 | 670.3234 | 1338.6323 | 1337.5452 | 1.0871 | 1 | 3 | 72 | 7 | -.MAENGKNCDQR.R |
|  | 26187 | **921.7971** | **2762.3695** | **2761.5670** | **0.8026** | **1** | **0** | **57** | **1** | **R.QPLITLQYFSLEILVILKEWTSK.L** |
|  | 26350 | **700.1080** | **2796.4030** | **2796.3549** | **0.0481** | **1** | **3** | **33** | **9** | **R.IEACMWGIGTAIGELPPYFMARAAR.L** |
|  | 29085 | **1153.8120** | **3458.4142** | **3458.4368** | **-0.0226** | **0** | **74** | **1.4e-006** | **1** | **R.LSGAEPDDEEYQEFEEMLEHAESAQDFASR.A** 29086 |

| **436.** | Q9UBS4\|DJB11_HUMAN    **Mass:** 40774    **Score:** 107    **Queries matched:** 8   **emPAI:** 0.09 |
| --- | --- |
|  | DnaJ homolog subfamily B member 11 OS=Homo sapiens GN=DNAJB11 PE=1 SV=1 |

|  | **Query** | **Observed** | **Mr(expt)** | **Mr(calc)** | **Delta** | **Miss** | **Score** | **Expect** | **Rank** | **Peptide** |
| --- | --- | --- | --- | --- | --- | --- | --- | --- | --- | --- |
|  | 2578 | 415.2532 | 828.4919 | 828.4454 | 0.0465 | 0 | 11 | 17 | 2 | R.TTQLGPGR.F 2552 |
|  | 3057 | 861.4448 | 860.4375 | 860.4967 | -0.0592 | 1 | 15 | 7.3 | 8 | R.SASIKDIK.K 3064 |
|  | 8717 | 621.3074 | 1240.6002 | 1240.6663 | -0.0661 | 0 | 19 | 1.6 | 2 | R.TLEVEIEPGVR.D |
|  | 19942 | **992.4276** | **1982.8405** | **1982.8536** | **-0.0131** | **0** | **75** | **2.9e-006** | **1** | **R.FQMTQEVVCDECPNVK.L** 19943 |
|  | 24669 | **839.7012** | **2516.0819** | **2515.0962** | **0.9857** | **0** | **13** | **3.1** | **1** | **R.DGMEYPFIGEGEPHVDGEPGDLR.F** |

| **437.** | Q09160\|1A80_HUMAN    **Mass:** 41051    **Score:** 107    **Queries matched:** 10   **emPAI:** 0.30 |
| --- | --- |
|  | HLA class I histocompatibility antigen, A-80 alpha chain OS=Homo sapiens GN=HLA-A PE=2 SV=1 |

|  | **Query** | **Observed** | **Mr(expt)** | **Mr(calc)** | **Delta** | **Miss** | **Score** | **Expect** | **Rank** | **Peptide** |
| --- | --- | --- | --- | --- | --- | --- | --- | --- | --- | --- |
|  | 9253 | **424.0566** | **1269.1480** | **1269.7081** | **-0.5601** | **1** | **4** | **35** | **8** | **K.WAAVVVPSGKEK.R** |
|  | 11481 | 465.0193 | 1392.0362 | 1392.6707 | -0.6345 | 0 | (3) | 43 | 4 | R.SWTAADMAAQITK.R |
|  | 11799 | 705.8859 | 1409.7572 | 1408.6656 | 1.0915 | 0 | 5 | 43 | 1 | R.SWTAADMAAQITK.R 11768 11769 |
|  | 18745 | 628.2999 | 1881.8780 | 1881.8904 | -0.0124 | 0 | 41 | 0.0072 | 1 | K.THMTHHPISDHEATLR.C |
|  | 20661 | 513.0184 | 2048.0446 | 2049.0102 | -0.9656 | 0 | 14 | 4 | 2 | R.YTCHVQHEGLPEPLTLR.W |
|  | 25408 | 1319.0913 | 2636.1681 | 2636.1838 | -0.0158 | 0 | 68 | 1.1e-005 | 1 | R.DGEDQTQDTELVETRPAGDGTFQK.W 25407 |
|  | 26962 | **975.8258** | **2924.4556** | **2924.1978** | **0.2578** | **0** | **0** | **56** | **4** | **R.GYYNQSEDGSHTIQIMYGCDVGSDGR.F** |

| **438.** | P37802\|TAGL2_HUMAN    **Mass:** 22548    **Score:** 107    **Queries matched:** 13   **emPAI:** 0.60 |
| --- | --- |
|  | Transgelin-2 OS=Homo sapiens GN=TAGLN2 PE=1 SV=3 |

|  | **Query** | **Observed** | **Mr(expt)** | **Mr(calc)** | **Delta** | **Miss** | **Score** | **Expect** | **Rank** | **Peptide** |
| --- | --- | --- | --- | --- | --- | --- | --- | --- | --- | --- |
|  | 4841 | **491.2790** | **980.5434** | **980.5152** | **0.0282** | **0** | **9** | **19** | **2** | **K.DVGRPQPGR.E** |
|  | 6584 | **555.4071** | **1108.7996** | **1108.6101** | **0.1895** | **1** | **3** | **57** | **2** | **R.KDVGRPQPGR.E** |
|  | 6995 | **571.3283** | **1140.6421** | **1139.6009** | **1.0412** | **1** | **2** | **1.1e+002** | **6** | **K.KIQASTMAFK.Q** |
|  | 8205 | **608.3442** | **1214.6738** | **1214.6805** | **-0.0067** | **0** | **22** | **1** | **1** | **R.TLMNLGGLAVAR.D** |
|  | 8275 | **610.1809** | **1218.3473** | **1217.6187** | **0.7286** | **0** | **5** | **38** | **1** | **K.NVIGLQMGTNR.G** |
|  | 8506 | 411.2213 | 1230.6421 | 1230.6754 | -0.0333 | 0 | (5) | 48 | 5 | R.TLMNLGGLAVAR.D |
|  | 12198 | 716.8537 | 1431.6928 | 1431.7470 | -0.0542 | 1 | 10 | 13 | 4 | R.GPAYGLSREVQQK.I |
|  | 14647 | **797.8578** | **1593.7010** | **1593.7100** | **-0.0090** | **0** | **23** | **0.55** | **1** | **R.DDGLFSGDPNWFPK.K** |
|  | 16159 | **848.4196** | **1694.8247** | **1693.8093** | **1.0153** | **0** | **8** | **16** | **1** | **K.QMEQISQFLQAAER.Y** |
|  | 21179 | 1050.5123 | 2099.0101 | 2099.0211 | -0.0110 | 0 | 112 | 6.2e-010 | 1 | R.YGINTTDIFQTVDLWEGK.N |
|  | 23844 | **805.7536** | **2414.2390** | **2414.2515** | **-0.0126** | **1** | **6** | **18** | **1** | **K.DGTVLCELINALYPEGQAPVKK.I** |
|  | 25287 | **872.4581** | **2614.3524** | **2614.2050** | **0.1474** | **1** | **3** | **34** | **4** | **K.NVIGLQMGTNRGASQAGMTGYGMPR.Q** |
|  | 27224 | **744.5983** | **2974.3642** | **2974.3953** | **-0.0311** | **1** | **0** | **47** | **5** | **R.YGINTTDIFQTVDLWEGKNMACVQR.T** |

| **439.** | P05787\|K2C8_HUMAN    **Mass:** 53671    **Score:** 107    **Queries matched:** 24   **emPAI:** 0.31 |
| --- | --- |
|  | Keratin, type II cytoskeletal 8 OS=Homo sapiens GN=KRT8 PE=1 SV=7 |

|  | **Query** | **Observed** | **Mr(expt)** | **Mr(calc)** | **Delta** | **Miss** | **Score** | **Expect** | **Rank** | **Peptide** |
| --- | --- | --- | --- | --- | --- | --- | --- | --- | --- | --- |
|  | 1463 | **637.3876** | **636.3803** | **636.3152** | **0.0650** | **0** | **11** | **12** | **9** | **K.MLETK.W** |
|  | 2418 | 818.4349 | 817.4276 | 817.4294 | -0.0018 | 1 | 9 | 27 | 5 | K.IETRDGK.L |
|  | 3253 | **435.7165** | **869.4184** | **869.4243** | **-0.0059** | **0** | **26** | **0.44** | **1** | **R.ISSSSFSR.V** |
|  | 5693 | **523.2756** | **1044.5367** | **1044.5451** | **-0.0084** | **0** | **8** | **25** | **1** | **R.QLETLGQEK.L** |
|  | 6180 | **540.2558** | **1078.4970** | **1078.5043** | **-0.0073** | **0** | **25** | **0.53** | **1** | **K.AQYEDIANR.S** |
|  | 7136 | 577.2781 | 1152.5416 | 1152.5485 | -0.0069 | 0 | 6 | 35 | 3 | R.EYQELMNVK.L |
|  | 7350 | 585.3502 | 1168.6858 | 1168.5434 | 0.1424 | 0 | (1) | 1.1e+002 | 5 | R.EYQELMNVK.L |
|  | 7355 | 585.7771 | 1169.5396 | 1168.5434 | 0.9963 | 0 | 40 | 0.014 | 1 | R.AEAESMYQIK.Y |
|  | 7403 | **587.3183** | **1172.6220** | **1172.6289** | **-0.0068** | **0** | **13** | **8.5** | **1** | **K.LVSESSDVLPK.-** |
|  | 7566 | **395.7132** | **1184.1177** | **1184.5383** | **-0.4206** | **0** | **(7)** | **23** | **4** | **R.AEAESMYQIK.Y** |
|  | 8441 | **614.3465** | **1226.6784** | **1227.5441** | **-0.8657** | **1** | **2** | **92** | **8** | **K.KDVDEAYMNK.V** |
|  | 8876 | **417.7125** | **1250.1156** | **1250.6367** | **-0.5211** | **1** | **6** | **23** | **8** | **K.VSTSGPRAFSSR.S** |
|  | 10645 | **672.8372** | **1343.6599** | **1343.6681** | **-0.0082** | **0** | **19** | **1.7** | **1** | **R.ASLEAAIADAEQR.G** 10628 10644 |
|  | 11054 | **685.4309** | **1368.8473** | **1367.6642** | **1.1830** | **0** | **5** | **40** | **3** | **R.TEMENEFVLIK.K** |
|  | 11551 | 699.2886 | 1396.5627 | 1396.7350 | -0.1723 | 1 | 10 | 12 | 4 | K.TLNNKFASFIDK.V 11549 11552 |
|  | 12849 | **738.3365** | **1474.6584** | **1474.6908** | **-0.0324** | **0** | **36** | **0.031** | **1** | **R.LESGMQNMSIHTK.T** |
|  | 13107 | **745.8488** | **1489.6829** | **1490.6857** | **-1.0028** | **0** | **(6)** | **32** | **8** | **R.LESGMQNMSIHTK.T** |
|  | 13984 | 517.3012 | 1548.8818 | 1549.7922 | -0.9104 | 1 | 5 | 36 | 9 | R.QLREYQELMNVK.L |
|  | 17513 | 896.9383 | 1791.8620 | 1791.8713 | -0.0092 | 0 | 53 | 0.00053 | 1 | K.LEAELGNMQGLVEDFK.N |
|  | 30267 | **1309.0153** | **3924.0240** | **3924.0415** | **-0.0176** | **0** | **54** | **0.00012** | **1** | **R.GGLGGGYGGASGMGGITAVTVNQSLLSPLVLEVDPNIQAVR.T** |

| **440.** | P01903\|2DRA_HUMAN    **Mass:** 28760    **Score:** 107    **Queries matched:** 9   **emPAI:** 0.64 |
| --- | --- |
|  | HLA class II histocompatibility antigen, DR alpha chain OS=Homo sapiens GN=HLA-DRA PE=1 SV=1 |

|  | **Query** | **Observed** | **Mr(expt)** | **Mr(calc)** | **Delta** | **Miss** | **Score** | **Expect** | **Rank** | **Peptide** |
| --- | --- | --- | --- | --- | --- | --- | --- | --- | --- | --- |
|  | 3974 | 460.2462 | 918.4779 | 918.4844 | -0.0065 | 0 | 35 | 0.062 | 1 | K.ANLEIMTK.R |
|  | 4301 | **472.2814** | **942.5482** | **943.4875** | **-0.9394** | **1** | **7** | **33** | **10** | **R.EDHLFRK.F** |
|  | 10693 | 674.3501 | 1346.6856 | 1346.6904 | -0.0048 | 0 | 7 | 31 | 10 | R.EPNVLICFIDK.F |
|  | 12107 | **476.7632** | **1427.2679** | **1427.7925** | **-0.5246** | **0** | **2** | **62** | **10** | **K.FTPPVVNVTWLR.N** |
|  | 12245 | **479.2544** | **1434.7413** | **1434.7507** | **-0.0095** | **0** | **52** | **0.00091** | **1** | **R.VEHWGLDEPLLK.H** |
|  | 17631 | **901.9803** | **1801.9460** | **1800.9734** | **0.9726** | **0** | **63** | **5.8e-005** | **1** | **R.NGKPVTTGVSETVFLPR.E** |
|  | 21840 | **720.3309** | **2157.9710** | **2157.9830** | **-0.0120** | **0** | **23** | **0.39** | **1** | **K.FHYLPFLPSTEDVYDCR.V** |
|  | 24394 | **828.3968** | **2482.1687** | **2482.2492** | **-0.0805** | **1** | **4** | **29** | **4** | **R.LEEFGRFASFEAQGALANIAVDK.A** |
|  | 26804 | **579.4866** | **2892.3968** | **2893.5486** | **-1.1518** | **0** | **3** | **27** | **1** | **-.MAISGVPVLGFFIIAVLMSAQESWAIK.E** |

| **441.** | Q06830\|PRDX1_HUMAN    **Mass:** 22324    **Score:** 107    **Queries matched:** 24   **emPAI:** 0.88 |
| --- | --- |
|  | Peroxiredoxin-1 OS=Homo sapiens GN=PRDX1 PE=1 SV=1 |

|  | **Query** | **Observed** | **Mr(expt)** | **Mr(calc)** | **Delta** | **Miss** | **Score** | **Expect** | **Rank** | **Peptide** |
| --- | --- | --- | --- | --- | --- | --- | --- | --- | --- | --- |
|  | 1126 | **564.3095** | **563.3022** | **562.2711** | **1.0312** | **0** | **13** | **13** | **1** | **M.SSGNAK.I** |
|  | 2428 | 410.2115 | 818.4084 | 818.4134 | -0.0050 | 0 | 13 | 9.8 | 3 | R.SVDETLR.L |
|  | 3601 | **894.4257** | **893.4184** | **893.4243** | **-0.0059** | **0** | **28** | **0.26** | **1** | **K.ADEGISFR.G** 3600 3602 |
|  | 3994 | **460.7509** | **919.4872** | **919.5015** | **-0.0143** | **0** | **29** | **0.24** | **1** | **R.GLFIIDDK.G** 3995 |
|  | 4253 | **940.4562** | **939.4490** | **939.4549** | **-0.0059** | **0** | **50** | **0.0016** | **1** | **K.DISLSDYK.G** |
|  | 4807 | 490.2610 | 978.5074 | 979.5239 | -1.0166 | 0 | 8 | 25 | 3 | K.IGHPAPNFK.A |
|  | 6562 | 554.3022 | 1106.5898 | 1106.5972 | -0.0073 | 0 | 6 | 45 | 2 | R.TIAQDYGVLK.A |
|  | 7273 | **582.7871** | **1163.5597** | **1163.5645** | **-0.0048** | **0** | **25** | **0.53** | **1** | **K.ATAVMPDGQFK.D** 7264 |
|  | 7488 | 590.3254 | 1178.6362 | 1179.5594 | -0.9232 | 0 | (4) | 68 | 9 | K.ATAVMPDGQFK.D |
|  | 7814 | **399.8479** | **1196.5219** | **1195.6237** | **0.8981** | **0** | **8** | **24** | **6** | **R.LVQAFQFTDK.H** |
|  | 13613 | 508.9797 | 1523.9172 | 1523.7845 | 0.1328 | 1 | 4 | 51 | 4 | M.SSGNAKIGHPAPNFK.A |
|  | 15561 | 551.3153 | 1650.9241 | 1649.9577 | 0.9664 | 1 | 8 | 17 | 4 | K.GILRQITVNDLPVGR.S |
|  | 16926 | 875.9741 | 1749.9337 | 1749.9447 | -0.0110 | 1 | 58 | 0.00017 | 1 | K.KQGGLGPMNIPLVSDPK.R 16921 16927 |
|  | 17142 | **589.3284** | **1764.9633** | **1765.9397** | **-0.9764** | **1** | **(1)** | **81** | **6** | **K.KQGGLGPMNIPLVSDPK.R** |
|  | 20064 | 998.5294 | 1995.0442 | 1996.0266 | -0.9824 | 1 | 3 | 43 | 3 | R.SVDETLRLVQAFQFTDK.H |
|  | 20220 | **671.1440** | **2010.4101** | **2011.0698** | **-0.6597** | **1** | **1** | **49** | **2** | **R.QITVNDLPVGRSVDETLR.L** |
|  | 23785 | **802.7282** | **2405.1628** | **2405.1798** | **-0.0170** | **0** | **39** | **0.01** | **1** | **K.HGEVCPAGWKPGSDTIKPDVQK.S** 23784 |

| **442.** | Q8N5K1\|CISD2_HUMAN    **Mass:** 15497    **Score:** 107    **Queries matched:** 6   **emPAI:** 0.96 |
| --- | --- |
|  | CDGSH iron sulfur domain-containing protein 2 OS=Homo sapiens GN=CISD2 PE=1 SV=1 |

|  | **Query** | **Observed** | **Mr(expt)** | **Mr(calc)** | **Delta** | **Miss** | **Score** | **Expect** | **Rank** | **Peptide** |
| --- | --- | --- | --- | --- | --- | --- | --- | --- | --- | --- |
|  | 2210 | 402.2437 | 802.4727 | 801.4596 | 1.0132 | 0 | 10 | 21 | 6 | K.DSLINLK.I |
|  | 5164 | 502.3313 | 1002.6480 | 1002.5498 | 0.0982 | 0 | 8 | 27 | 3 | R.LTVSEWLR.L |
|  | 8543 | **617.2654** | **1232.5162** | **1232.5244** | **-0.0082** | **0** | **20** | **1.5** | **1** | **K.TFPACDGSHNK.H** |
|  | 9550 | 429.2669 | 1284.7790 | 1285.7030 | -0.9240 | 0 | 11 | 12 | 1 | R.LPVPESITGFAR.L |
|  | 15053 | **810.4350** | **1618.8554** | **1618.8679** | **-0.0124** | **0** | **84** | **4.5e-007** | **1** | **K.HNELTGDNVGPLILK.K** |
|  | 15658 | **830.4292** | **1658.8438** | **1658.8549** | **-0.0111** | **0** | **42** | **0.0072** | **1** | **K.VVNEINIEDLCLTK.A** |

| **443.** | P53675\|CLH2_HUMAN    **Mass:** 189020   **Score:** 106    **Queries matched:** 40   **emPAI:** 0.10 |
| --- | --- |
|  | Clathrin heavy chain 2 OS=Homo sapiens GN=CLTCL1 PE=1 SV=2 |

|  | **Query** | **Observed** | **Mr(expt)** | **Mr(calc)** | **Delta** | **Miss** | **Score** | **Expect** | **Rank** | **Peptide** |
| --- | --- | --- | --- | --- | --- | --- | --- | --- | --- | --- |
|  | 1728 | 706.4451 | 705.4379 | 705.4425 | -0.0046 | 0 | 27 | 0.39 | 1 | K.IVLYAK.K |
|  | 2105 | **398.2102** | **794.4059** | **795.4967** | **-1.0908** | **0** | **8** | **22** | **3** | **M.AQILPVR.F** |
|  | 6015 | 535.2728 | 1068.5310 | 1068.5386 | -0.0076 | 0 | 27 | 0.31 | 1 | R.AHIAQLCEK.A 6016 |
|  | 6986 | 571.2977 | 1140.5809 | 1140.4506 | 0.1303 | 0 | 7 | 32 | 5 | R.ESSCYNPER.V 6993 7002 |
|  | 8605 | 618.8328 | 1235.6511 | 1235.6584 | -0.0073 | 0 | 3 | 73 | 6 | K.TLQIFNIEMK.S |
|  | 9031 | **630.2972** | **1258.5799** | **1257.6928** | **0.8871** | **1** | **2** | **91** | **8** | **K.VDKLDALESLR.K** |
|  | 10517 | 669.3434 | 1336.6722 | 1336.6809 | -0.0087 | 0 | 15 | 4.8 | 1 | R.VVGAMQLYSVDR.K |
|  | 10800 | 677.4238 | 1352.8330 | 1352.8391 | -0.0061 | 0 | 42 | 0.0075 | 1 | R.NLQNLLILTAIK.A 10779 10781 |
|  | 10812 | 677.8649 | 1353.7153 | 1353.8132 | -0.0979 | 0 | 7 | 27 | 9 | K.AGQLPLVKPYLR.S |
|  | 12755 | 490.3149 | 1467.9228 | 1466.7803 | 1.1425 | 1 | 7 | 22 | 2 | K.TLQIFNIEMKSK.M 12727 |
|  | 14006 | 776.3754 | 1550.7363 | 1550.7464 | -0.0101 | 0 | 44 | 0.0046 | 1 | R.GQFSTDELVAEVEK.R |
|  | 15151 | 542.6121 | 1624.8144 | 1625.8552 | -1.0408 | 0 | 3 | 61 | 5 | R.ESYIETELIFALAK.T |
|  | 16184 | 566.3079 | 1695.9019 | 1695.0043 | 0.8977 | 1 | 4 | 44 | 3 | R.NLQNLLILTAIKADR.T |
|  | 16254 | **850.9416** | **1699.8686** | **1699.9105** | **-0.0418** | **0** | **1** | **93** | **3** | **R.QLIDQVVQTALSETR.D** |
|  | 16341 | 854.4252 | 1706.8359 | 1706.8475 | -0.0116 | 1 | 40 | 0.011 | 1 | R.GQFSTDELVAEVEKR.N 16343 |
|  | 16418 | 856.9051 | 1711.7956 | 1710.8473 | 0.9483 | 0 | 31 | 0.079 | 1 | R.AHMGMFTELAILYSK.F 16417 |
|  | 16501 | **573.7771** | **1718.3095** | **1718.8199** | **-0.5104** | **1** | **3** | **40** | **6** | **K.GNNWWAQSVELCKK.D** |
|  | 16608 | 576.7942 | 1727.3607 | 1726.8422 | 0.5185 | 0 | (2) | 42 | 6 | R.AHMGMFTELAILYSK.F |
|  | 17109 | 882.4408 | 1762.8670 | 1762.8295 | 0.0376 | 1 | 11 | 9.8 | 3 | K.EDKLECSEELGDLVK.T |
|  | 17211 | 886.4420 | 1770.8695 | 1770.8107 | 0.0588 | 1 | 5 | 32 | 2 | K.DHLYKDAMQHAAESR.D |
|  | 18412 | 928.4858 | 1854.9570 | 1856.0116 | -1.0546 | 1 | 2 | 70 | 6 | R.RQLIDQVVQTALSETR.D |
|  | 18559 | 933.9662 | 1865.9179 | 1865.9094 | 0.0086 | 1 | 3 | 56 | 6 | R.SNNWEDLVKFLQMAR.K |
|  | 19230 | **640.3184** | **1917.9333** | **1917.0472** | **0.8860** | **0** | **1** | **80** | **4** | **K.LHIIEVGQPAAGNQPFVK.K** |
|  | 19567 | 974.0062 | 1945.9979 | 1946.0070 | -0.0091 | 0 | 48 | 0.0015 | 1 | K.AFMTADLPNELIELLEK.I 19568 |
|  | 20670 | **1025.5376** | **2049.0606** | **2048.1193** | **0.9413** | **0** | **1** | **65** | **3** | **R.GYFEELILLLEAALGLER.A** |
|  | 22225 | **735.3877** | **2203.1413** | **2202.0661** | **1.0752** | **0** | **9** | **10** | **1** | **K.VGEQAQVTIIDMSDPMAPIR.R** |
|  | 22671 | 1128.5840 | 2255.1534 | 2254.0753 | 1.0781 | 0 | 2 | 51 | 2 | R.ASIDAYDNFDNISLAQQLEK.H |
|  | 23489 | 1179.0857 | 2356.1568 | 2355.2119 | 0.9449 | 1 | 0 | 73 | 6 | R.AHMGMFTELAILYSKFKPQK.M |
|  | 29848 | 931.4332 | 3721.7036 | 3722.8793 | -1.1758 | 1 | 2 | 23 | 7 | K.VGEQAQVTIIDMSDPMAPIRRPISAESAIMNPASK.V |
|  | 30073 | **1267.2010** | **3798.5813** | **3798.6890** | **-0.1076** | **1** | **2** | **18** | **8** | **R.VSELEDFINGPNNAHIQQVGDRCYEEGMYEAAK.L** |
|  | 30380 | **800.3784** | **3996.8554** | **3995.9502** | **0.9052** | **1** | **0** | **27** | **6** | **R.VMEYISRLDNYDALDIASIAVSSALYEEAFTVFHK.F** |

| **444.** | Q9NS69\|TOM22_HUMAN    **Mass:** 15512    **Score:** 106    **Queries matched:** 8   **emPAI:** 0.56 |
| --- | --- |
|  | Mitochondrial import receptor subunit TOM22 homolog OS=Homo sapiens GN=TOMM22 PE=1 SV=3 |

|  | **Query** | **Observed** | **Mr(expt)** | **Mr(calc)** | **Delta** | **Miss** | **Score** | **Expect** | **Rank** | **Peptide** |
| --- | --- | --- | --- | --- | --- | --- | --- | --- | --- | --- |
|  | 11214 | **689.8416** | **1377.6687** | **1377.6751** | **-0.0064** | **0** | **55** | **0.0004** | **1** | **R.LWGLTEMFPER.V** |
|  | 14109 | **779.3879** | **1556.7612** | **1556.7729** | **-0.0117** | **0** | **61** | **9.7e-005** | **1** | **K.LQMEQQQQLQQR.Q** 14125 |
|  | 21128 | 1047.4628 | 2092.9110 | 2093.0463 | -0.1353 | 0 | 3 | 44 | 2 | -.MAAAVAAAGAGEPQSPDELLPK.G 21126 21127 |
|  | 21335 | **704.3564** | **2110.0473** | **2109.0412** | **1.0061** | **0** | **(2)** | **51** | **9** | **-.MAAAVAAAGAGEPQSPDELLPK.G** |
|  | 22835 | **1138.5239** | **2275.0333** | **2274.2406** | **0.7927** | **0** | **2** | **47** | **2** | **R.QILLGPNTGLSGGMPGALPSLPGK.I** |

| **445.** | Q9H8H3\|MET7A_HUMAN    **Mass:** 28814    **Score:** 106    **Queries matched:** 5   **emPAI:** 0.45 |
| --- | --- |
|  | Methyltransferase-like protein 7A OS=Homo sapiens GN=METTL7A PE=1 SV=1 |

|  | **Query** | **Observed** | **Mr(expt)** | **Mr(calc)** | **Delta** | **Miss** | **Score** | **Expect** | **Rank** | **Peptide** |
| --- | --- | --- | --- | --- | --- | --- | --- | --- | --- | --- |
|  | 4119 | **465.2792** | **928.5438** | **927.5250** | **1.0188** | **1** | **2** | **1.1e+002** | **9** | **K.NQERILR.E** |
|  | 12212 | 717.3353 | 1432.6561 | 1432.6657 | -0.0096 | 0 | 32 | 0.081 | 1 | R.VTCIDPNPNFEK.F |
|  | 14237 | **783.3980** | **1564.7813** | **1564.7919** | **-0.0106** | **0** | **90** | **1.2e-007** | **1** | **K.LSLLEVGCGTGANFK.F** |
|  | 15099 | **812.3986** | **1622.7826** | **1622.7940** | **-0.0115** | **0** | **32** | **0.078** | **1** | **R.ELFSNLQEFAGPSGK.L** 15127 |

| **446.** | P30461\|1B13_HUMAN    **Mass:** 40791    **Score:** 105    **Queries matched:** 17   **emPAI:** 0.42 |
| --- | --- |
|  | HLA class I histocompatibility antigen, B-13 alpha chain OS=Homo sapiens GN=HLA-B PE=2 SV=1 |

|  | **Query** | **Observed** | **Mr(expt)** | **Mr(calc)** | **Delta** | **Miss** | **Score** | **Expect** | **Rank** | **Peptide** |
| --- | --- | --- | --- | --- | --- | --- | --- | --- | --- | --- |
|  | 995 | 542.1287 | 541.1214 | 542.3176 | -1.1963 | 0 | 13 | 4.3 | 1 | R.VTAPR.T 1006 |
|  | 5014 | 497.7372 | 993.4599 | 994.4356 | -0.9757 | 0 | 4 | 64 | 10 | R.FDSDATSPR.M |
|  | 10365 | 443.7359 | 1328.1860 | 1327.6996 | 0.4863 | 1 | 13 | 4.5 | 1 | K.WEAARVAEQLR.A |
|  | 12099 | 714.3628 | 1426.7111 | 1426.7205 | -0.0093 | 0 | 11 | 9.6 | 1 | K.WAAVVVPSGEEQR.Y |
|  | 12464 | **484.2489** | **1449.7249** | **1449.6671** | **0.0578** | **1** | **7** | **27** | **1** | **R.FDSDATSPRMAPR.A** |
|  | 15669 | 554.2529 | 1659.7368 | 1658.8305 | 0.9063 | 0 | 1 | 91 | 8 | R.FITVGYVDDTQFVR.F |
|  | 18332 | 617.6424 | 1849.9054 | 1849.9183 | -0.0130 | 0 | 32 | 0.074 | 1 | K.THVTHHPISDHEATLR.C 18320 |
|  | 20661 | 513.0184 | 2048.0446 | 2048.0626 | -0.0179 | 0 | 15 | 3.2 | 1 | R.YTCHVQHEGLPKPLTLR.W |
|  | 21185 | 700.7486 | 2099.2240 | 2098.0484 | 1.1756 | 1 | 4 | 28 | 2 | R.GEPRFITVGYVDDTQFVR.F |
|  | 21343 | 1056.5170 | 2111.0194 | 2111.0299 | -0.0105 | 0 | 45 | 0.003 | 1 | R.CWALGFYPAEITLTWQR.D 21342 |
|  | 22490 | 744.6667 | 2230.9784 | 2230.9938 | -0.0154 | 0 | 35 | 0.024 | 1 | R.DGEDQTQDTELVETRPAGDR.T 22489 22491 |
|  | 26805 | 724.1069 | 2892.3986 | 2892.1868 | 0.2118 | 0 | 2 | 36 | 6 | R.YYNQSEAGSHTWQTMYGCDLGPDGR.L |

| **447.** | P30466\|1B18_HUMAN    **Mass:** 40535    **Score:** 105    **Queries matched:** 15   **emPAI:** 0.42 |
| --- | --- |
|  | HLA class I histocompatibility antigen, B-18 alpha chain OS=Homo sapiens GN=HLA-B PE=1 SV=1 |

|  | **Query** | **Observed** | **Mr(expt)** | **Mr(calc)** | **Delta** | **Miss** | **Score** | **Expect** | **Rank** | **Peptide** |
| --- | --- | --- | --- | --- | --- | --- | --- | --- | --- | --- |
|  | 995 | 542.1287 | 541.1214 | 542.3176 | -1.1963 | 0 | 13 | 4.3 | 1 | R.VTAPR.T 1006 |
|  | 10365 | 443.7359 | 1328.1860 | 1327.6996 | 0.4863 | 1 | 13 | 4.5 | 1 | K.WEAARVAEQLR.A |
|  | 11047 | 457.2375 | 1368.6906 | 1367.6793 | 1.0112 | 1 | 10 | 13 | 4 | K.TNTQTYRESLR.N |
|  | 12099 | 714.3628 | 1426.7111 | 1426.7205 | -0.0093 | 0 | 11 | 9.6 | 1 | K.WAAVVVPSGEEQR.Y |
|  | 12433 | 483.7814 | 1448.3225 | 1447.6692 | 0.6533 | 1 | 1 | 70 | 2 | R.FDSDAASPRTEPR.A |
|  | 14553 | **530.2165** | **1587.6278** | **1586.8093** | **0.8185** | **0** | **3** | **55** | **5** | **R.FISVGYVDGTQFVR.F** |
|  | 18332 | 617.6424 | 1849.9054 | 1849.9183 | -0.0130 | 0 | 32 | 0.074 | 1 | K.THVTHHPISDHEATLR.C 18320 |
|  | 20661 | 513.0184 | 2048.0446 | 2048.0626 | -0.0179 | 0 | 15 | 3.2 | 1 | R.YTCHVQHEGLPKPLTLR.W |
|  | 21343 | 1056.5170 | 2111.0194 | 2111.0299 | -0.0105 | 0 | 45 | 0.003 | 1 | R.CWALGFYPAEITLTWQR.D 21342 |
|  | 22490 | 744.6667 | 2230.9784 | 2230.9938 | -0.0154 | 0 | 35 | 0.024 | 1 | R.DGEDQTQDTELVETRPAGDR.T 22489 22491 |

| **448.** | P18463\|1B37_HUMAN    **Mass:** 40716    **Score:** 105    **Queries matched:** 16   **emPAI:** 0.42 |
| --- | --- |
|  | HLA class I histocompatibility antigen, B-37 alpha chain OS=Homo sapiens GN=HLA-B PE=2 SV=1 |

|  | **Query** | **Observed** | **Mr(expt)** | **Mr(calc)** | **Delta** | **Miss** | **Score** | **Expect** | **Rank** | **Peptide** |
| --- | --- | --- | --- | --- | --- | --- | --- | --- | --- | --- |
|  | 995 | 542.1287 | 541.1214 | 542.3176 | -1.1963 | 0 | 13 | 4.3 | 1 | R.VTAPR.T 1006 |
|  | 7107 | **576.2840** | **1150.5534** | **1149.4543** | **1.0991** | **0** | **4** | **68** | **10** | **R.MSGCDVGPDGR.L** 7105 |
|  | 12099 | 714.3628 | 1426.7111 | 1426.7205 | -0.0093 | 0 | 11 | 9.6 | 1 | K.WAAVVVPSGEEQR.Y |
|  | 12433 | 483.7814 | 1448.3225 | 1447.6692 | 0.6533 | 1 | 1 | 70 | 2 | R.FDSDAASPRTEPR.A |
|  | 13940 | **774.3403** | **1546.6660** | **1547.7185** | **-1.0525** | **1** | **6** | **31** | **1** | **R.MSGCDVGPDGRLLR.G** |
|  | 15470 | 823.4105 | 1644.8064 | 1644.8148 | -0.0084 | 0 | 4 | 42 | 2 | R.FISVGYVDDTQFVR.F |
|  | 18332 | 617.6424 | 1849.9054 | 1849.9183 | -0.0130 | 0 | 32 | 0.074 | 1 | K.THVTHHPISDHEATLR.C 18320 |
|  | 20661 | 513.0184 | 2048.0446 | 2048.0626 | -0.0179 | 0 | 15 | 3.2 | 1 | R.YTCHVQHEGLPKPLTLR.W |
|  | 21343 | 1056.5170 | 2111.0194 | 2111.0299 | -0.0105 | 0 | 45 | 0.003 | 1 | R.CWALGFYPAEITLTWQR.D 21342 |
|  | 22490 | 744.6667 | 2230.9784 | 2230.9938 | -0.0154 | 0 | 35 | 0.024 | 1 | R.DGEDQTQDTELVETRPAGDR.T 22489 22491 |

|  | **Proteins matching a subset of these peptides:** |
| --- | --- |

|  | Q29718\|1B82_HUMAN    **Mass:** 40681    **Score:** 105    **Queries matched:** 12 |
| --- | --- |
|  | HLA class I histocompatibility antigen, B-82 alpha chain OS=Homo sapiens GN=HLA-B PE=2 SV=1 |

| **449.** | P30475\|1B39_HUMAN    **Mass:** 40645    **Score:** 105    **Queries matched:** 13   **emPAI:** 0.42 |
| --- | --- |
|  | HLA class I histocompatibility antigen, B-39 alpha chain OS=Homo sapiens GN=HLA-B PE=2 SV=1 |

|  | **Query** | **Observed** | **Mr(expt)** | **Mr(calc)** | **Delta** | **Miss** | **Score** | **Expect** | **Rank** | **Peptide** |
| --- | --- | --- | --- | --- | --- | --- | --- | --- | --- | --- |
|  | 1936 | 763.1170 | 762.1097 | 762.3694 | -0.2597 | 0 | 0 | 1.4e+002 | 7 | R.NTQICK.T |
|  | 2630 | 417.2441 | 832.4737 | 832.4299 | 0.0438 | 0 | 4 | 87 | 3 | -.MLVMAPR.T |
|  | 10365 | 443.7359 | 1328.1860 | 1327.6996 | 0.4863 | 1 | 13 | 4.5 | 1 | K.WEAARVAEQLR.T |
|  | 12099 | 714.3628 | 1426.7111 | 1426.7205 | -0.0093 | 0 | 11 | 9.6 | 1 | K.WAAVVVPSGEEQR.Y |
|  | 15470 | 823.4105 | 1644.8064 | 1644.8148 | -0.0084 | 0 | 4 | 42 | 2 | R.FISVGYVDDTQFVR.F |
|  | 18332 | 617.6424 | 1849.9054 | 1849.9183 | -0.0130 | 0 | 32 | 0.074 | 1 | K.THVTHHPISDHEATLR.C 18320 |
|  | 20661 | 513.0184 | 2048.0446 | 2048.0626 | -0.0179 | 0 | 15 | 3.2 | 1 | R.YTCHVQHEGLPKPLTLR.W |
|  | 21343 | 1056.5170 | 2111.0194 | 2111.0299 | -0.0105 | 0 | 45 | 0.003 | 1 | R.CWALGFYPAEITLTWQR.D 21342 |
|  | 22490 | 744.6667 | 2230.9784 | 2230.9938 | -0.0154 | 0 | 35 | 0.024 | 1 | R.DGEDQTQDTELVETRPAGDR.T 22489 22491 |

|  | **Proteins matching the same set of peptides:** |
| --- | --- |

|  | Q95365\|1B38_HUMAN    **Mass:** 40733    **Score:** 105    **Queries matched:** 13 |
| --- | --- |
|  | HLA class I histocompatibility antigen, B-38 alpha chain OS=Homo sapiens GN=HLA-B PE=2 SV=1 |

|  | **Proteins matching a subset of these peptides:** |
| --- | --- |

|  | Q29836\|1B67_HUMAN    **Mass:** 40602    **Score:** 105    **Queries matched:** 12 |
| --- | --- |
|  | HLA class I histocompatibility antigen, B-67 alpha chain OS=Homo sapiens GN=HLA-B PE=1 SV=1 |

|  | P30462\|1B14_HUMAN    **Mass:** 40675    **Score:** 105    **Queries matched:** 12 |
| --- | --- |
|  | HLA class I histocompatibility antigen, B-14 alpha chain OS=Homo sapiens GN=HLA-B PE=1 SV=1 |

|  | P01889\|1B07_HUMAN    **Mass:** 40777    **Score:** 105    **Queries matched:** 11 |
| --- | --- |
|  | HLA class I histocompatibility antigen, B-7 alpha chain OS=Homo sapiens GN=HLA-B PE=1 SV=3 |

|  | P30460\|1B08_HUMAN    **Mass:** 40648    **Score:** 105    **Queries matched:** 11 |
| --- | --- |
|  | HLA class I histocompatibility antigen, B-8 alpha chain OS=Homo sapiens GN=HLA-B PE=1 SV=1 |

|  | P30480\|1B42_HUMAN    **Mass:** 40650    **Score:** 105    **Queries matched:** 11 |
| --- | --- |
|  | HLA class I histocompatibility antigen, B-42 alpha chain OS=Homo sapiens GN=HLA-B PE=1 SV=1 |

| **450.** | P62277\|RS13_HUMAN    **Mass:** 17212    **Score:** 105    **Queries matched:** 15   **emPAI:** 1.25 |
| --- | --- |
|  | 40S ribosomal protein S13 OS=Homo sapiens GN=RPS13 PE=1 SV=2 |

|  | **Query** | **Observed** | **Mr(expt)** | **Mr(calc)** | **Delta** | **Miss** | **Score** | **Expect** | **Rank** | **Peptide** |
| --- | --- | --- | --- | --- | --- | --- | --- | --- | --- | --- |
|  | 873 | **515.3265** | **514.3193** | **514.3227** | **-0.0035** | **0** | **20** | **2.4** | **1** | **K.AVAVR.K** 860 861 |
|  | 1581 | 665.3574 | 664.3501 | 664.3544 | -0.0043 | 0 | 11 | 15 | 7 | R.FVTGNK.I |
|  | 2788 | **422.2659** | **842.5172** | **842.5225** | **-0.0053** | **0** | **27** | **0.38** | **1** | **R.LILIESR.I** |
|  | 6441 | 549.9246 | 1097.8346 | 1097.5240 | 0.3105 | 0 | 1 | 94 | 4 | K.YESSTASALVA.- |
|  | 8917 | **418.5664** | **1252.6774** | **1252.7503** | **-0.0729** | **0** | **14** | **5.1** | **7** | **K.GLTPSQIGVILR.D** 8903 8908 |
|  | 11291 | **461.2862** | **1380.8367** | **1380.8453** | **-0.0085** | **1** | **42** | **0.0078** | **1** | **K.KGLTPSQIGVILR.D** 11290 |
|  | 12279 | **719.8695** | **1437.7245** | **1437.7351** | **-0.0106** | **1** | **79** | **1.4e-006** | **1** | **K.LTSDDVKEQIYK.L** |
|  | 16130 | 846.9393 | 1691.8640 | 1692.9086 | -1.0447 | 0 | 35 | 0.037 | 1 | K.GLAPDLPEDLYHLIK.K 16141 |
|  | 19099 | **636.9490** | **1907.8253** | **1908.0356** | **-0.2103** | **1** | **1** | **76** | **8** | **K.SKGLAPDLPEDLYHLIK.K** |

| **451.** | Q07020\|RL18_HUMAN    **Mass:** 21735    **Score:** 105    **Queries matched:** 15   **emPAI:** 0.63 |
| --- | --- |
|  | 60S ribosomal protein L18 OS=Homo sapiens GN=RPL18 PE=1 SV=2 |

|  | **Query** | **Observed** | **Mr(expt)** | **Mr(calc)** | **Delta** | **Miss** | **Score** | **Expect** | **Rank** | **Peptide** |
| --- | --- | --- | --- | --- | --- | --- | --- | --- | --- | --- |
|  | 1700 | **699.3982** | **698.3910** | **698.3963** | **-0.0053** | **0** | **19** | **2.2** | **1** | **R.VQEVPK.L** |
|  | 2362 | **407.7346** | **813.4547** | **812.4504** | **1.0043** | **1** | **8** | **25** | **4** | **K.LPGRENK.T** |
|  | 3605 | **447.7349** | **893.4552** | **893.4607** | **-0.0055** | **0** | **3** | **76** | **4** | **K.SQDIYLR.L** |
|  | 4219 | 469.7473 | 937.4801 | 937.5497 | -0.0697 | 1 | 5 | 49 | 9 | K.LYRFLAR.R |
|  | 6980 | 570.8237 | 1139.6329 | 1139.6411 | -0.0082 | 0 | 22 | 0.83 | 1 | R.TNRPPLSLSR.M 7001 |
|  | 8871 | 625.8362 | 1249.6579 | 1249.6667 | -0.0087 | 0 | 49 | 0.002 | 1 | R.TNSTFNQVVLK.R |
|  | 10672 | 673.3650 | 1344.7154 | 1344.7249 | -0.0095 | 0 | 26 | 0.33 | 1 | K.TAVVVGTITDDVR.V 10642 10643 |
|  | 10688 | **449.8787** | **1346.6142** | **1346.7922** | **-0.1780** | **1** | **10** | **15** | **5** | **K.SQDIYLRLLVK.L** |
|  | 11730 | 469.9142 | 1406.7207 | 1405.7678 | 0.9530 | 1 | 6 | 34 | 8 | R.RTNSTFNQVVLK.R 11721 |
|  | 12626 | **730.8987** | **1459.7829** | **1459.7922** | **-0.0093** | **0** | **82** | **8.7e-007** | **1** | **K.ILTFDQLALDSPK.G** 12639 |

| **452.** | Q9Y6U3\|ADSV_HUMAN    **Mass:** 80895    **Score:** 104    **Queries matched:** 24   **emPAI:** 0.25 |
| --- | --- |
|  | Adseverin OS=Homo sapiens GN=SCIN PE=1 SV=4 |

|  | **Query** | **Observed** | **Mr(expt)** | **Mr(calc)** | **Delta** | **Miss** | **Score** | **Expect** | **Rank** | **Peptide** |
| --- | --- | --- | --- | --- | --- | --- | --- | --- | --- | --- |
|  | 1045 | 548.2822 | 547.2750 | 547.3152 | -0.0402 | 1 | 17 | 5.3 | 2 | R.KAAMK.T |
|  | 1324 | 605.3616 | 604.3544 | 603.3591 | 0.9952 | 1 | 12 | 14 | 1 | K.KESLK.S |
|  | 1454 | 635.1334 | 634.1261 | 633.3446 | 0.7815 | 1 | 5 | 55 | 6 | K.NGTSKK.G |
|  | 1840 | 738.3981 | 737.3908 | 737.3959 | -0.0052 | 0 | 13 | 8.7 | 4 | K.VYVTEK.V |
|  | 4114 | **465.2588** | **928.5031** | **928.5090** | **-0.0059** | **0** | **34** | **0.081** | **1** | **K.ANQVATGIR.Y** |
|  | 4498 | **478.7949** | **955.5753** | **956.5192** | **-0.9439** | **0** | **14** | **6** | **1** | **K.QAGLQVWR.I** |
|  | 5585 | 1036.5244 | 1035.5171 | 1035.5600 | -0.0429 | 0 | 11 | 12 | 3 | K.GAEYVASVLK.C |
|  | 6189 | **540.2974** | **1078.5803** | **1078.5883** | **-0.0080** | **1** | **31** | **0.12** | **1** | **K.KGGQAPAPPTR.L** |
|  | 7175 | **578.7841** | **1155.5536** | **1154.5502** | **1.0033** | **1** | **3** | **76** | **4** | **R.LYGCSNKTGR.F** |
|  | 8151 | **607.2634** | **1212.5123** | **1212.6727** | **-0.1604** | **1** | **10** | **14** | **5** | **R.AGKQAGLQVWR.I** |
|  | 11104 | 458.2464 | 1371.7174 | 1370.7055 | 1.0119 | 1 | 7 | 30 | 2 | K.VEIWRVENNGR.I |
|  | 14415 | 526.9423 | 1577.8050 | 1577.7950 | 0.0099 | 0 | 0 | 1.1e+002 | 7 | R.GQIIYTWQGANATR.D |
|  | 14558 | 794.9042 | 1587.7939 | 1587.7239 | 0.0701 | 0 | 11 | 11 | 4 | K.TAEEFLQQMNYSK.N |
|  | 15461 | 823.3567 | 1644.6988 | 1645.7626 | -1.0638 | 1 | (2) | 67 | 7 | K.MAKLYMVSDASGSMR.V |
|  | 15939 | 420.2194 | 1676.8487 | 1677.7524 | -0.9037 | 1 | 4 | 46 | 9 | K.MAKLYMVSDASGSMR.V |
|  | 18133 | **613.3267** | **1836.9583** | **1836.9694** | **-0.0110** | **0** | **6** | **26** | **2** | **K.AGGVASGLNHVLTNDLTAK.R** |
|  | 18607 | 935.9932 | 1869.9718 | 1869.9836 | -0.0119 | 0 | 40 | 0.0097 | 1 | K.NTQIQVLPEGGETPIFK.Q 18618 |
|  | 21159 | **699.8322** | **2096.4748** | **2095.9810** | **0.4938** | **0** | **4** | **23** | **2** | **K.DYQTSPLLETQAEDHPPR.L** |
|  | 21315 | **703.9813** | **2108.9221** | **2108.9368** | **-0.0146** | **0** | **34** | **0.032** | **1** | **K.LHSSPQMAAQHNMVDDGSGK.V** 21314 |
|  | 24967 | **851.7435** | **2552.2088** | **2552.2242** | **-0.0154** | **0** | **48** | **0.001** | **1** | **K.VLGEKPELPDGGDDDDIIADISNR.K** |
|  | 26328 | **699.1018** | **2792.3779** | **2792.3123** | **0.0656** | **1** | **0** | **62** | **8** | **K.LHSSPQMAAQHNMVDDGSGKVEIWR.V** |
|  | 28224 | **1070.1804** | **3207.5194** | **3207.5290** | **-0.0096** | **0** | **7** | **9.2** | **1** | **R.VTVVAEENPFSMAMLLSEECFILDHGAAK.Q** |

| **453.** | P61604\|CH10_HUMAN    **Mass:** 10925    **Score:** 104    **Queries matched:** 19   **emPAI:** 2.50 |
| --- | --- |
|  | 10 kDa heat shock protein, mitochondrial OS=Homo sapiens GN=HSPE1 PE=1 SV=2 |

|  | **Query** | **Observed** | **Mr(expt)** | **Mr(calc)** | **Delta** | **Miss** | **Score** | **Expect** | **Rank** | **Peptide** |
| --- | --- | --- | --- | --- | --- | --- | --- | --- | --- | --- |
|  | 2817 | **423.2574** | **844.5003** | **843.4524** | **1.0479** | **0** | **20** | **2.1** | **1** | **K.GGIMLPEK.S** 2796 |
|  | 3050 | **430.9033** | **859.7921** | **859.4473** | **0.3448** | **0** | **(6)** | **35** | **1** | **K.GGIMLPEK.S** 3019 3043 |
|  | 3809 | **454.2528** | **906.4911** | **906.4963** | **-0.0053** | **0** | **18** | **2.6** | **2** | **K.FLPLFDR.V** 3810 |
|  | 5275 | **507.2815** | **1012.5484** | **1012.5553** | **-0.0069** | **0** | **46** | **0.0041** | **1** | **K.GGEIQPVSVK.V** 5276 |
|  | 5575 | **518.2999** | **1034.5852** | **1034.5913** | **-0.0061** | **1** | **14** | **6.9** | **2** | **R.KFLPLFDR.V** |
|  | 9044 | 630.8172 | 1259.6198 | 1259.6544 | -0.0345 | 1 | 0 | 1.4e+002 | 8 | K.GGIMLPEKSQGK.V |
|  | 10102 | **658.3779** | **1314.7413** | **1314.7507** | **-0.0094** | **0** | **59** | **0.00019** | **1** | **K.VLQATVVAVGSGSK.G** |
|  | 13262 | **750.3582** | **1498.7019** | **1499.8672** | **-1.1653** | **1** | **6** | **34** | **1** | **K.VLQATVVAVGSGSKGK.G** 13271 |
|  | 13297 | **501.5753** | **1501.7041** | **1502.8609** | **-1.1568** | **1** | **7** | **25** | **9** | **K.FLPLFDRVLVER.S** |
|  | 13687 | **765.3990** | **1528.7835** | **1528.7926** | **-0.0090** | **1** | **59** | **0.00016** | **1** | **K.VVLDDKDYFLFR.D** 13686 |
|  | 14112 | **779.4138** | **1556.8130** | **1557.7827** | **-0.9698** | **1** | **3** | **60** | **4** | **K.DYFLFRDGDILGK.Y** |
|  | 15222 | **544.2784** | **1629.8135** | **1630.8600** | **-1.0465** | **1** | **7** | **25** | **1** | **R.SAAETVTKGGIMLPEK.S** |

| **454.** | P15880\|RS2_HUMAN    **Mass:** 31590    **Score:** 104    **Queries matched:** 20   **emPAI:** 0.40 |
| --- | --- |
|  | 40S ribosomal protein S2 OS=Homo sapiens GN=RPS2 PE=1 SV=2 |

|  | **Query** | **Observed** | **Mr(expt)** | **Mr(calc)** | **Delta** | **Miss** | **Score** | **Expect** | **Rank** | **Peptide** |
| --- | --- | --- | --- | --- | --- | --- | --- | --- | --- | --- |
|  | 875 | **515.3266** | **514.3193** | **515.2928** | **-0.9735** | **1** | **7** | **40** | **5** | **R.GRGAR.G** |
|  | 1658 | 685.4557 | 684.4485 | 684.4534 | -0.0049 | 0 | 17 | 3.3 | 8 | R.GAIILAK.L |
|  | 1916 | 759.4308 | 758.4235 | 758.4286 | -0.0051 | 0 | 18 | 4 | 1 | K.EVATAIR.G |
|  | 2012 | 395.2133 | 788.4120 | 789.4167 | -1.0047 | 0 | 10 | 21 | 5 | R.CGSVLVR.L |
|  | 3777 | 453.2261 | 904.4377 | 905.4317 | -0.9940 | 0 | 16 | 5 | 6 | K.EWMPVTK.L 3804 |
|  | 4226 | 470.2111 | 938.4077 | 938.6025 | -0.1948 | 1 | 6 | 33 | 7 | K.LSIVPVRR.G |
|  | 5430 | **513.2997** | **1024.5849** | **1024.5917** | **-0.0068** | **0** | **27** | **0.27** | **1** | **R.GTGIVSAPVPK.K** |
|  | 6235 | **542.2694** | **1082.5241** | **1082.5945** | **-0.0703** | **1** | **11** | **13** | **3** | **K.THTRVSVQR.T** |
|  | 6466 | **550.7813** | **1099.5481** | **1099.6172** | **-0.0691** | **1** | **7** | **35** | **6** | **K.IMPVQKQTR.A** |
|  | 6958 | **570.2751** | **1138.5356** | **1138.5441** | **-0.0085** | **0** | **36** | **0.036** | **1** | **R.GCTATLGNFAK.A** |
|  | 10437 | 667.3217 | 1332.6289 | 1332.6384 | -0.0095 | 1 | 34 | 0.054 | 1 | K.AEDKEWMPVTK.L 10438 |
|  | 12069 | 713.4025 | 1424.7905 | 1424.8715 | -0.0810 | 1 | 12 | 7.4 | 3 | K.EVATAIRGAIILAK.L 12068 |
|  | 12123 | **715.2830** | **1428.5515** | **1427.7733** | **0.7782** | **1** | **3** | **55** | **10** | **R.VSVQRTQAPAVATT.-** |
|  | 16461 | **858.8862** | **1715.7579** | **1715.7681** | **-0.0102** | **0** | **92** | **6.9e-008** | **1** | **K.LLMMAGIDDCYTSAR.G** |
|  | 16480 | 573.2980 | 1716.8721 | 1715.8995 | 0.9726 | 0 | 2 | 70 | 5 | K.AFVAIGDYNGHVGLGVK.C |
|  | 16663 | 866.8809 | 1731.7473 | 1731.7630 | -0.0157 | 0 | (0) | 1e+002 | 7 | K.LLMMAGIDDCYTSAR.G |
|  | 18782 | **629.2937** | **1884.8593** | **1884.7843** | **0.0750** | **0** | **4** | **43** | **4** | **-.MADDAGAAGGPGGPGGPGMGNR.G** |

| **455.** | O14880\|MGST3_HUMAN    **Mass:** 16734    **Score:** 103    **Queries matched:** 18   **emPAI:** 2.49 |
| --- | --- |
|  | Microsomal glutathione S-transferase 3 OS=Homo sapiens GN=MGST3 PE=1 SV=1 |

|  | **Query** | **Observed** | **Mr(expt)** | **Mr(calc)** | **Delta** | **Miss** | **Score** | **Expect** | **Rank** | **Peptide** |
| --- | --- | --- | --- | --- | --- | --- | --- | --- | --- | --- |
|  | 891 | 517.7327 | 516.7254 | 516.3271 | 0.3983 | 0 | 12 | 16 | 3 | M.AVLSK.E 887 |
|  | 1713 | **702.3727** | **701.3654** | **701.3708** | **-0.0054** | **0** | **30** | **0.27** | **1** | **K.SGLGSGPK.C** 1719 |
|  | 14878 | **805.8851** | **1609.7557** | **1609.7664** | **-0.0107** | **0** | **45** | **0.0041** | **1** | **R.VLYAYGYYTGEPSK.R** 14879 |
|  | 17153 | **883.9342** | **1765.8538** | **1765.8675** | **-0.0137** | **1** | **37** | **0.02** | **1** | **R.VLYAYGYYTGEPSKR.S** 17154 |
|  | 25575 | **889.7014** | **2666.0824** | **2666.4142** | **-0.3318** | **0** | **0** | **48** | **4** | **K.EYGFVLLTGAASFIMVAHLAINVSK.A** |
|  | 25692 | **895.0742** | **2682.2007** | **2682.2206** | **-0.0200** | **0** | **20** | **0.58** | **1** | **K.VEYPIMYSTDPENGHIFNCIQR.A** 25691 |
|  | 26669 | **718.8760** | **2871.4751** | **2871.4861** | **-0.0110** | **0** | **10** | **5.5** | **1** | **R.AHQNTLEVYPPFLFFLAVGGVYHPR.I** 26670 |
|  | 27219 | 992.1269 | 2973.3588 | 2973.3789 | -0.0201 | 1 | 47 | 0.001 | 1 | K.YKVEYPIMYSTDPENGHIFNCIQR.A 27221 27222 27223 27224 |

| **456.** | P50990\|TCPQ_HUMAN    **Mass:** 60153    **Score:** 102    **Queries matched:** 23   **emPAI:** 0.20 |
| --- | --- |
|  | T-complex protein 1 subunit theta OS=Homo sapiens GN=CCT8 PE=1 SV=4 |

|  | **Query** | **Observed** | **Mr(expt)** | **Mr(calc)** | **Delta** | **Miss** | **Score** | **Expect** | **Rank** | **Peptide** |
| --- | --- | --- | --- | --- | --- | --- | --- | --- | --- | --- |
|  | 2331 | 811.4981 | 810.4908 | 810.4422 | 0.0486 | 0 | 6 | 32 | 9 | -.MALHVPK.A |
|  | 5503 | 515.7851 | 1029.5556 | 1030.5560 | -1.0003 | 1 | 10 | 16 | 5 | R.LNSKWDLR.R |
|  | 5953 | 532.7820 | 1063.5494 | 1064.5139 | -0.9644 | 0 | 12 | 11 | 2 | R.AVDDGVNTFK.V |
|  | 7108 | **576.2843** | **1150.5540** | **1149.5818** | **0.9722** | **0** | **14** | **5.6** | **1** | **K.FAEAFEAIPR.A** |
|  | 7605 | **593.8395** | **1185.6644** | **1184.5383** | **1.1261** | **0** | **6** | **40** | **6** | **K.TAEELMNFSK.G** |
|  | 8562 | **412.2079** | **1233.6018** | **1232.5707** | **1.0311** | **0** | **7** | **29** | **3** | **K.GEENLMDAQVK.A** |
|  | 10465 | **667.8748** | **1333.7350** | **1332.7401** | **0.9948** | **0** | **47** | **0.0026** | **1** | **K.LFVTNDAATILR.E** 10444 10447 |
|  | 10689 | **674.3145** | **1346.6145** | **1346.7016** | **-0.0872** | **1** | **9** | **18** | **5** | **K.APGFAQMLKEGAK.H** |
|  | 13416 | 504.2708 | 1509.7905 | 1509.8515 | -0.0610 | 1 | 12 | 7.7 | 1 | K.RLVPGGGATEIELAK.Q 13400 13412 |
|  | 13752 | **767.5396** | **1533.0647** | **1533.8151** | **-0.7505** | **1** | **1** | **67** | **6** | **R.AVDDGVNTFKVLTR.D** |
|  | 13810 | **769.8877** | **1537.7608** | **1537.7698** | **-0.0089** | **0** | **67** | **2.3e-005** | **1** | **K.DMLEAGILDTYLGK.Y** |
|  | 14049 | **777.4096** | **1552.8047** | **1553.7647** | **-0.9600** | **0** | **(1)** | **1e+002** | **2** | **K.DMLEAGILDTYLGK.Y** |
|  | 14258 | **523.2820** | **1566.8243** | **1566.8365** | **-0.0123** | **1** | **27** | **0.23** | **1** | **K.HEKEDGAISTIVLR.G** |
|  | 16961 | **585.2891** | **1752.8454** | **1753.9371** | **-1.0918** | **1** | **5** | **34** | **1** | **-.MALHVPKAPGFAQMLK.E** |
|  | 18020 | **610.9682** | **1829.8828** | **1830.9186** | **-1.0358** | **1** | **4** | **38** | **3** | **R.TSIMSKQYGNEVFLAK.L** |
|  | 18077 | 917.4576 | 1832.9006 | 1833.9659 | -1.0653 | 0 | 1 | 80 | 2 | K.ILGSGISSSSVLHGMVFK.K 18091 |
|  | 18923 | **947.9096** | **1893.8047** | **1893.9506** | **-0.1459** | **0** | **1** | **81** | **2** | **R.IGLSVSEVIEGYEIACR.K** |
|  | 21607 | **713.4144** | **2137.2215** | **2137.0812** | **0.1403** | **1** | **0** | **69** | **9** | **K.VADMALHYANKYNIMLVR.L** |

| **457.** | P28070\|PSB4_HUMAN    **Mass:** 29242    **Score:** 102    **Queries matched:** 12   **emPAI:** 0.44 |
| --- | --- |
|  | Proteasome subunit beta type-4 OS=Homo sapiens GN=PSMB4 PE=1 SV=4 |

|  | **Query** | **Observed** | **Mr(expt)** | **Mr(calc)** | **Delta** | **Miss** | **Score** | **Expect** | **Rank** | **Peptide** |
| --- | --- | --- | --- | --- | --- | --- | --- | --- | --- | --- |
|  | 4078 | **464.2452** | **926.4759** | **925.4327** | **1.0431** | **0** | **6** | **42** | **2** | **-.MEAFLGSR.S** |
|  | 13711 | 766.4059 | 1530.7972 | 1530.8076 | -0.0104 | 0 | 27 | 0.25 | 1 | R.TQNPMVTGTSVLGVK.F |
|  | 13948 | **774.4047** | **1546.7948** | **1546.8025** | **-0.0078** | **0** | **(7)** | **26** | **1** | **R.TQNPMVTGTSVLGVK.F** |
|  | 18745 | 628.2999 | 1881.8780 | 1882.8771 | -0.9991 | 0 | 0 | 94 | 6 | R.IPSTPDSFMDPASALYR.G |
|  | 20242 | **504.2891** | **2013.1271** | **2012.0037** | **1.1234** | **0** | **3** | **47** | **4** | **K.FEGGVVIAADMLGSYGSLAR.F** |
|  | 20894 | **519.0246** | **2072.0693** | **2071.1096** | **0.9597** | **1** | **1** | **66** | **10** | **R.GPITRTQNPMVTGTSVLGVK.F** |
|  | 22157 | 1098.0702 | 2194.1258 | 2193.0048 | 1.1210 | 0 | 39 | 0.0096 | 2 | R.VNNSTMLGASGDYADFQYLK.Q 22149 |
|  | 22293 | **1106.0487** | **2210.0829** | **2208.9997** | **1.0831** | **0** | **(4)** | **34** | **2** | **R.VNNSTMLGASGDYADFQYLK.Q** |
|  | 24315 | **1235.6213** | **2469.2281** | **2470.1852** | **-0.9571** | **1** | **2** | **50** | **1** | **-.MEAFLGSRSGLWAGGPAPGQFYR.I** |
|  | 25383 | **1316.6100** | **2631.2054** | **2631.2163** | **-0.0109** | **0** | **72** | **3.7e-006** | **1** | **K.GVEIEGPLSTETNWDIAHMISGFE.-** 25382 |

| **458.** | P16144\|ITB4_HUMAN    **Mass:** 205729   **Score:** 102    **Queries matched:** 40   **emPAI:** 0.09 |
| --- | --- |
|  | Integrin beta-4 OS=Homo sapiens GN=ITGB4 PE=1 SV=4 |

|  | **Query** | **Observed** | **Mr(expt)** | **Mr(calc)** | **Delta** | **Miss** | **Score** | **Expect** | **Rank** | **Peptide** |
| --- | --- | --- | --- | --- | --- | --- | --- | --- | --- | --- |
|  | 1640 | **677.4423** | **676.4350** | **676.3504** | **0.0847** | **0** | **7** | **46** | **1** | **R.LSASSGR.S** |
|  | 2417 | **409.7210** | **817.4274** | **816.4242** | **1.0032** | **0** | **13** | **11** | **9** | **R.TGSFHIR.R** |
|  | 3876 | **456.7420** | **911.4695** | **912.6008** | **-1.1313** | **0** | **3** | **67** | **9** | **R.LVNITIIK.E** |
|  | 4796 | 489.7283 | 977.4420 | 976.5263 | 0.9157 | 1 | 11 | 13 | 9 | K.VKMVDELK.R |
|  | 5324 | **509.2515** | **1016.4884** | **1016.5688** | **-0.0804** | **0** | **3** | **91** | **9** | **R.MLLIENLR.E** |
|  | 6486 | 551.7813 | 1101.5479 | 1101.5567 | -0.0088 | 0 | 45 | 0.0055 | 1 | R.HVTQEFVSR.T |
|  | 7218 | 580.3047 | 1158.5948 | 1158.5928 | 0.0021 | 1 | (6) | 46 | 2 | R.RSQMSPQGLR.V |
|  | 7425 | 588.3669 | 1174.7193 | 1174.5877 | 0.1317 | 1 | 13 | 7.4 | 1 | R.RSQMSPQGLR.V 7409 7422 |
|  | 8732 | **621.4160** | **1240.8175** | **1240.6135** | **0.2040** | **0** | **6** | **32** | **2** | **-.MAGPRPSPWAR.L** |
|  | 8885 | **626.2717** | **1250.5289** | **1250.6004** | **-0.0714** | **0** | **8** | **20** | **1** | **R.SEHSHSTTLPR.D** |
|  | 9074 | **631.1107** | **1260.2068** | **1259.7238** | **0.4830** | **0** | **3** | **48** | **1** | **R.LVFSALGPTSLR.V** |
|  | 12098 | 714.3554 | 1426.6961 | 1426.8031 | -0.1070 | 0 | 9 | 19 | 3 | K.LLELQEVDSLLR.G |
|  | 13404 | 755.8193 | 1509.6241 | 1509.6341 | -0.0099 | 0 | 39 | 0.016 | 1 | R.SCVQCQAWGTGEK.K |
|  | 13775 | 768.8760 | 1535.7374 | 1535.7468 | -0.0093 | 0 | 38 | 0.018 | 1 | K.NVISLTEDVDEFR.N |
|  | 15144 | **813.3884** | **1624.7623** | **1624.7733** | **-0.0110** | **0** | **36** | **0.031** | **1** | **R.DVVSFEQPEFSVSR.G** |
|  | 15394 | 820.8944 | 1639.7741 | 1639.7842 | -0.0100 | 0 | 5 | 40 | 2 | K.VAPGYYTLTADQDAR.G |
|  | 15406 | **547.6525** | **1639.9356** | **1639.9257** | **0.0099** | **1** | **4** | **48** | **9** | **K.LLELQEVDSLLRGR.Q** |
|  | 16103 | **564.3465** | **1690.0177** | **1690.9294** | **-0.9117** | **1** | **2** | **61** | **8** | **R.LTVPGLSENVPYKFK.V** 16112 |
|  | 16555 | **574.9841** | **1721.9306** | **1722.9087** | **-0.9781** | **1** | **1** | **83** | **2** | **K.KQDHTIVDTVLMAPR.S** |
|  | 16817 | 872.3856 | 1742.7567 | 1741.8127 | 0.9440 | 0 | 9 | 13 | 2 | R.ENLMASDHLDTPMLR.S |
|  | 17058 | **440.4750** | **1757.8709** | **1757.8076** | **0.0633** | **0** | **(3)** | **55** | **8** | **R.ENLMASDHLDTPMLR.S** |
|  | 17330 | **593.8018** | **1778.3835** | **1777.8846** | **0.4988** | **1** | **3** | **34** | **2** | **K.NVISLTEDVDEFRNK.L** |
|  | 19917 | **660.8378** | **1979.4917** | **1979.1164** | **0.3753** | **1** | **2** | **45** | **1** | **K.QLLVEAIDVPAGTATLGRR.L** |
|  | 20355 | **675.3740** | **2023.1001** | **2024.1517** | **-1.0517** | **1** | **6** | **24** | **5** | **K.ELQVKLLELQEVDSLLR.G** |
|  | 20921 | **692.6609** | **2074.9608** | **2074.9742** | **-0.0134** | **0** | **33** | **0.04** | **1** | **R.ALEHVDGTHVCQLPEDQK.G** |
|  | 21849 | **720.3655** | **2158.0748** | **2158.9841** | **-0.9094** | **0** | **0** | **88** | **7** | **R.TLTTSGTLSTHMDQQFFQT.-** |
|  | 22224 | **735.3863** | **2203.1370** | **2204.0749** | **-0.9379** | **1** | **2** | **53** | **1** | **R.VKYWIQGDSESEAHLLDSK.V** |
|  | 22706 | **753.6702** | **2257.9887** | **2257.0831** | **0.9056** | **1** | **3** | **38** | **2** | **R.ENLMASDHLDTPMLRSGNLK.G** |
|  | 25875 | **677.0638** | **2704.2263** | **2705.2868** | **-1.0605** | **0** | **0** | **62** | **1** | **R.SFTSQMLSSQPPPHGDLGAPQNPNAK.A** |
|  | 26395 | 1406.1565 | 2810.2984 | 2811.3960 | -1.0976 | 1 | 3 | 28 | 2 | R.ESIVVMESSFQITEETQIDTTLRR.S 26407 |
|  | 28031 | **791.9080** | **3163.6030** | **3163.5105** | **0.0925** | **1** | **1** | **39** | **1** | **R.SFTSQMLSSQPPPHGDLGAPQNPNAKAAGSR.K** |
|  | 28954 | **852.4316** | **3405.6975** | **3405.7092** | **-0.0118** | **0** | **8** | **6.5** | **1** | **K.LHTYFPVSSLGVLQEDSSNIVELLEEAFNR.I** |
|  | 29782 | **1232.5989** | **3694.7748** | **3694.7104** | **0.0644** | **1** | **1** | **29** | **3** | **R.RPNGDIVGYLVTCEMAQGGGPATAFRVDGDSPESR.L** |
|  | 29813 | **1238.2313** | **3711.6721** | **3710.7471** | **0.9250** | **1** | **5** | **11** | **7** | **R.MDFAFPGSTNSLHRMTTTSAAAYGTHLSPHVPHR.V** |
|  | 30239 | **1300.3593** | **3898.0559** | **3898.8645** | **-0.8086** | **0** | **3** | **12** | **3** | **K.RPMSIPIIPDIPIVDAQSGEDYDSFLMYSDDVLR.S** |
|  | 30366 | **1326.9800** | **3977.9181** | **3977.9007** | **0.0175** | **0** | **3** | **14** | **2** | **R.DIGWRPDSTHLLVFSTESAFHYEADGANVLAGIMSR.N** |

| **459.** | P17174\|AATC_HUMAN    **Mass:** 46447    **Score:** 102    **Queries matched:** 14   **emPAI:** 0.08 |
| --- | --- |
|  | Aspartate aminotransferase, cytoplasmic OS=Homo sapiens GN=GOT1 PE=1 SV=3 |

|  | **Query** | **Observed** | **Mr(expt)** | **Mr(calc)** | **Delta** | **Miss** | **Score** | **Expect** | **Rank** | **Peptide** |
| --- | --- | --- | --- | --- | --- | --- | --- | --- | --- | --- |
|  | 3063 | **431.2499** | **860.4853** | **861.4708** | **-0.9855** | **0** | **24** | **0.9** | **1** | **R.IGADFLAR.W** 3111 |
|  | 4380 | 474.5632 | 947.1118 | 947.5189 | -0.4070 | 0 | 6 | 30 | 1 | K.VNLGVGAYR.T |
|  | 5807 | **527.7465** | **1053.4784** | **1054.5923** | **-1.1140** | **0** | **2** | **88** | **5** | **K.HIYLLPSGR.I** |
|  | 6747 | **561.8204** | **1121.6263** | **1120.5764** | **1.0499** | **0** | **10** | **17** | **5** | **K.QVEYLVNEK.H** |
|  | 8386 | **612.8141** | **1223.6136** | **1222.6162** | **0.9974** | **1** | **1** | **1.1e+002** | **9** | **K.TMADRILTMR.S** |
|  | 9291 | **424.7325** | **1271.1757** | **1270.6994** | **0.4763** | **0** | **8** | **15** | **1** | **R.VGGVQSLGGTGALR.I** 9290 |
|  | 15215 | 815.8573 | 1629.7000 | 1629.8283 | -0.1283 | 0 | 7 | 25 | 3 | K.EPESILQVLSQMEK.I |
|  | 15655 | **830.3573** | **1658.7000** | **1659.8468** | **-1.1467** | **0** | **9** | **14** | **1** | **K.NLDYVATSIHEAVTK.I** |
|  | 20335 | 1012.0038 | 2021.9930 | 2023.1143 | -1.1213 | 0 | 25 | 0.29 | 2 | M.APPSVFAEVPQAQPVLVFK.L |
|  | 22508 | **1117.5665** | **2233.1185** | **2233.1266** | **-0.0081** | **0** | **68** | **1.2e-005** | **1** | **R.IVASTLSNPELFEEWTGNVK.T** 22509 |
|  | 23750 | **800.4127** | **2398.2163** | **2398.2281** | **-0.0117** | **0** | **14** | **3.1** | **1** | **K.IANDNSLNHEYLPILGLAEFR.S** |

| **460.** | P62745\|RHOB_HUMAN    **Mass:** 22565    **Score:** 101    **Queries matched:** 5   **emPAI:** 0.37 |
| --- | --- |
|  | Rho-related GTP-binding protein RhoB OS=Homo sapiens GN=RHOB PE=1 SV=1 |

|  | **Query** | **Observed** | **Mr(expt)** | **Mr(calc)** | **Delta** | **Miss** | **Score** | **Expect** | **Rank** | **Peptide** |
| --- | --- | --- | --- | --- | --- | --- | --- | --- | --- | --- |
|  | 1127 | 564.3106 | 563.3033 | 562.2347 | 1.0686 | 0 | 12 | 14 | 7 | R.TDDGR.A |
|  | 3154 | **432.8841** | **863.7536** | **863.4535** | **0.3001** | **1** | **1** | **1.1e+002** | **7** | **R.TELARMK.Q** |
|  | 20195 | 1004.9594 | 2007.9041 | 2007.9174 | -0.0132 | 0 | 53 | 0.00042 | 1 | K.QVELALWDTAGQEDYDR.L |
|  | 26237 | 1387.6605 | 2773.3065 | 2773.3011 | 0.0054 | 0 | 57 | 0.00011 | 2 | K.DEFPEVYVPTVFENYVADIEVDGK.Q 26226 |

| **461.** | P61981\|1433G_HUMAN    **Mass:** 28456    **Score:** 101    **Queries matched:** 10   **emPAI:** 0.28 |
| --- | --- |
|  | 14-3-3 protein gamma OS=Homo sapiens GN=YWHAG PE=1 SV=2 |

|  | **Query** | **Observed** | **Mr(expt)** | **Mr(calc)** | **Delta** | **Miss** | **Score** | **Expect** | **Rank** | **Peptide** |
| --- | --- | --- | --- | --- | --- | --- | --- | --- | --- | --- |
|  | 3760 | 452.2576 | 902.5006 | 902.5073 | -0.0067 | 0 | 18 | 3.4 | 1 | R.VISSIEQK.T 3753 3754 |
|  | 3811 | 454.2629 | 906.5113 | 906.5174 | -0.0062 | 0 | 20 | 1.8 | 2 | R.NLLSVAYK.N |
|  | 4144 | 466.7147 | 931.4149 | 931.4222 | -0.0072 | 1 | 16 | 4.3 | 1 | K.MKGDYYR.Y |
|  | 5292 | 508.2113 | 1014.4080 | 1014.4150 | -0.0070 | 0 | 12 | 12 | 1 | R.YDDMAAAMK.N |
|  | 11939 | 709.8932 | 1417.7718 | 1418.7518 | -0.9799 | 1 | 0 | 1.3e+002 | 10 | R.SSWRVISSIEQK.T |
|  | 15438 | 822.4666 | 1642.9187 | 1642.7798 | 0.1389 | 0 | 1 | 93 | 10 | K.NVTELNEPLSNEER.N |
|  | 27348 | **1004.8297** | **3011.4673** | **3011.4851** | **-0.0178** | **0** | **59** | **7.3e-005** | **1** | **R.LGLALNYSVFYYEIQNAPEQACHLAK.T** |
|  | 28585 | **1101.5393** | **3301.5961** | **3300.6071** | **0.9890** | **1** | **64** | **1.9e-005** | **1** | **K.TAFDDAIAELDTLNEDSYKDSTLIMQLLR.D** |

| **462.** | O14653\|GOSR2_HUMAN    **Mass:** 24874    **Score:** 101    **Queries matched:** 5   **emPAI:** 0.15 |
| --- | --- |
|  | Golgi SNAP receptor complex member 2 OS=Homo sapiens GN=GOSR2 PE=1 SV=2 |

|  | **Query** | **Observed** | **Mr(expt)** | **Mr(calc)** | **Delta** | **Miss** | **Score** | **Expect** | **Rank** | **Peptide** |
| --- | --- | --- | --- | --- | --- | --- | --- | --- | --- | --- |
|  | 5507 | 516.2606 | 1030.5067 | 1029.5567 | 0.9500 | 1 | 6 | 49 | 2 | R.QREELLSR.T |
|  | 8768 | **622.3972** | **1242.7799** | **1243.6019** | **-0.8221** | **0** | **1** | **1.1e+002** | **1** | **-.MDPLFQQTHK.Q** |
|  | 17082 | **880.9696** | **1759.9246** | **1759.9324** | **-0.0078** | **0** | **78** | **1.8e-006** | **1** | **K.ILDIANMLGLSNTVMR.L** 17081 |
|  | 17522 | **598.3651** | **1792.0733** | **1791.9223** | **0.1510** | **0** | **(1)** | **86** | **3** | **K.ILDIANMLGLSNTVMR.L** |

| **463.** | O95831\|AIFM1_HUMAN    **Mass:** 67144    **Score:** 101    **Queries matched:** 20   **emPAI:** 0.18 |
| --- | --- |
|  | Apoptosis-inducing factor 1, mitochondrial OS=Homo sapiens GN=AIFM1 PE=1 SV=1 |

|  | **Query** | **Observed** | **Mr(expt)** | **Mr(calc)** | **Delta** | **Miss** | **Score** | **Expect** | **Rank** | **Peptide** |
| --- | --- | --- | --- | --- | --- | --- | --- | --- | --- | --- |
|  | 862 | **514.3060** | **513.2988** | **514.2500** | **-0.9512** | **0** | **15** | **4.6** | **1** | **R.DPGAR.V** 864 871 872 874 876 877 |
|  | 1243 | **589.1240** | **588.1167** | **587.3391** | **0.7776** | **1** | **5** | **80** | **1** | **R.REGVK.V** |
|  | 6534 | **553.7712** | **1105.5279** | **1105.5364** | **-0.0084** | **0** | **54** | **0.00073** | **1** | **K.SATEQSGTGIR.S** 6535 |
|  | 10133 | **659.3112** | **1316.6079** | **1315.7095** | **0.8983** | **1** | **7** | **31** | **1** | **R.SLSAIDRAGAEVK.S** |
|  | 10604 | **448.2226** | **1341.6460** | **1341.6538** | **-0.0078** | **0** | **11** | **11** | **1** | **R.VEHHDHAVVSGR.L** |
|  | 12375 | 723.1858 | 1444.3571 | 1443.7973 | 0.5598 | 0 | 1 | 76 | 5 | R.ALGTEVIQLFPEK.G |
|  | 15101 | 812.4033 | 1622.7921 | 1622.8014 | -0.0093 | 0 | 39 | 0.014 | 1 | K.ILPEYLSNWTMEK.V 15100 |
|  | 15639 | **829.4893** | **1656.9640** | **1657.8159** | **-0.8519** | **1** | **1** | **90** | **5** | **K.KAALSASEGEEVPQDK.A** |
|  | 20530 | **680.3542** | **2038.0407** | **2039.1813** | **-1.1405** | **1** | **0** | **79** | **10** | **K.VMPNAIVQSVGVSSGKLLIK.L** |
|  | 21637 | **714.8848** | **2141.6325** | **2142.0125** | **-0.3800** | **1** | **5** | **18** | **3** | **K.GNMGKILPEYLSNWTMEK.V** |
|  | 22494 | **744.7408** | **2231.2007** | **2231.2161** | **-0.0154** | **1** | **44** | **0.003** | **1** | **R.KVETDHIVAAVGLEPNVELAK.T** 22495 |

| **464.** | Q16706\|MA2A1_HUMAN    **Mass:** 131742   **Score:** 101    **Queries matched:** 20   **emPAI:** 0.06 |
| --- | --- |
|  | Alpha-mannosidase 2 OS=Homo sapiens GN=MAN2A1 PE=1 SV=2 |

|  | **Query** | **Observed** | **Mr(expt)** | **Mr(calc)** | **Delta** | **Miss** | **Score** | **Expect** | **Rank** | **Peptide** |
| --- | --- | --- | --- | --- | --- | --- | --- | --- | --- | --- |
|  | 778 | **498.3360** | **497.3288** | **498.2914** | **-0.9627** | **0** | **8** | **13** | **5** | **R.LPGGR.F** |
|  | 1581 | 665.3574 | 664.3501 | 664.3908 | -0.0407 | 1 | 15 | 5.9 | 2 | K.YKINK.F |
|  | 1776 | 717.4086 | 716.4013 | 715.4017 | 0.9996 | 0 | 10 | 25 | 2 | R.VHYAVK.K |
|  | 3712 | 451.2454 | 900.4762 | 900.4487 | 0.0275 | 0 | 10 | 21 | 2 | R.IMESHLR.A |
|  | 4248 | **470.2652** | **938.5159** | **937.4691** | **1.0468** | **0** | **4** | **62** | **8** | **R.MLLDQYR.K** |
|  | 6485 | **551.7763** | **1101.5379** | **1100.5462** | **0.9918** | **0** | **6** | **43** | **3** | **R.GLEQGIQDNK.I** |
|  | 8856 | **417.2253** | **1248.6542** | **1249.6588** | **-1.0046** | **1** | **9** | **19** | **6** | **R.EIAMKISSDIK.S** |
|  | 9858 | 434.8815 | 1301.6227 | 1302.6172 | -0.9946 | 1 | 3 | 73 | 4 | R.MDRIMESHLR.A |
|  | 12133 | 715.3806 | 1428.7467 | 1429.8656 | -1.1190 | 1 | 5 | 44 | 7 | K.ITANLFRILLEK.R |
|  | 12351 | 481.9235 | 1442.7487 | 1443.8086 | -1.0598 | 1 | 6 | 33 | 2 | R.TKVLLAPLGDDFR.Y |
|  | 13226 | **499.9141** | **1496.7204** | **1497.8150** | **-1.0947** | **0** | **10** | **13** | **4** | **R.LLAENNEIISNIR.D** |
|  | 14128 | **779.9049** | **1557.7953** | **1557.8038** | **-0.0086** | **0** | **53** | **0.00061** | **1** | **K.FLSSSLYTALTEAR.R** |
|  | 14976 | 808.4205 | 1614.8265 | 1613.7838 | 1.0427 | 0 | 11 | 9.8 | 2 | R.FYTDLNGYQIQPR.M |
|  | 15930 | 839.4138 | 1676.8131 | 1677.8032 | -0.9901 | 0 | 1 | 85 | 6 | R.EGSFPQGQLSMLQEK.I |
|  | 18121 | **612.9738** | **1835.8994** | **1836.9768** | **-1.0774** | **1** | **5** | **36** | **6** | **K.GTGLFCSTTQGKILVQK.L** 18122 |
|  | 22502 | 745.3505 | 2233.0296 | 2232.0786 | 0.9510 | 0 | 6 | 21 | 1 | R.SGWAIDPFGHSPTMAYLLNR.A |
|  | 22507 | 1117.5620 | 2233.1095 | 2234.0691 | -0.9597 | 0 | 1 | 61 | 3 | R.FGCPWGVPPETIHPGNVQSR.A |
|  | 26535 | **947.4567** | **2839.3484** | **2838.4408** | **0.9076** | **0** | **2** | **38** | **6** | **K.SVSYPSLLSHITSSLMNHPVIPMANK.F** |
|  | 28544 | **1095.5531** | **3283.6375** | **3283.6547** | **-0.0172** | **0** | **72** | **2.8e-006** | **1** | **K.FSSPTLELQGEFSPLQSSLPCDIHLVNLR.T** |

| **465.** | P61163\|ACTZ_HUMAN    **Mass:** 42701    **Score:** 101    **Queries matched:** 9   **emPAI:** 0.18 |
| --- | --- |
|  | Alpha-centractin OS=Homo sapiens GN=ACTR1A PE=1 SV=1 |

|  | **Query** | **Observed** | **Mr(expt)** | **Mr(calc)** | **Delta** | **Miss** | **Score** | **Expect** | **Rank** | **Peptide** |
| --- | --- | --- | --- | --- | --- | --- | --- | --- | --- | --- |
|  | 1491 | 644.8003 | 643.7931 | 643.3653 | 0.4278 | 0 | 18 | 3 | 2 | R.IDIAGR.D |
|  | 3815 | **454.5635** | **907.1125** | **907.4182** | **-0.3057** | **1** | **9** | **13** | **8** | **K.SDMDLRR.T** |
|  | 6427 | **549.3069** | **1096.5992** | **1095.4832** | **1.1160** | **1** | **9** | **17** | **2** | **K.KEYEEDGAR.S** |
|  | 16024 | **842.9520** | **1683.8895** | **1682.9243** | **0.9652** | **0** | **94** | **4.9e-008** | **1** | **R.TLFSNIVLSGGSTLFK.G** 16005 16019 |
|  | 17261 | **592.2831** | **1773.8276** | **1772.7893** | **1.0383** | **0** | **5** | **39** | **2** | **K.EGYDFHSSSEFEIVK.A** |
|  | 19104 | **636.9773** | **1907.9100** | **1909.0244** | **-1.1143** | **1** | **5** | **32** | **1** | **K.HVRVMAGALEGDIFIGPK.A** |
|  | 24783 | **845.4289** | **2533.2648** | **2533.2813** | **-0.0164** | **0** | **27** | **0.14** | **1** | **K.DQLQTFSEEHPVLLTEAPLNPR.K** |

| **466.** | Q9HAW7\|UD17_HUMAN    **Mass:** 60578    **Score:** 100    **Queries matched:** 13   **emPAI:** 0.20 |
| --- | --- |
|  | UDP-glucuronosyltransferase 1-7 OS=Homo sapiens GN=UGT1A7 PE=1 SV=2 |

|  | **Query** | **Observed** | **Mr(expt)** | **Mr(calc)** | **Delta** | **Miss** | **Score** | **Expect** | **Rank** | **Peptide** |
| --- | --- | --- | --- | --- | --- | --- | --- | --- | --- | --- |
|  | 4553 | 480.7598 | 959.5051 | 959.5110 | -0.0059 | 0 | 46 | 0.0043 | 1 | K.AMAIADALGK.I |
|  | 5607 | 519.2922 | 1036.5699 | 1036.5553 | 0.0146 | 1 | 11 | 11 | 3 | K.AVINDKSYK.E 5603 |
|  | 5635 | 520.7592 | 1039.5039 | 1039.5120 | -0.0081 | 1 | 26 | 0.35 | 1 | K.SYKENIMR.L |
|  | 5836 | 528.7754 | 1055.5363 | 1055.5069 | 0.0294 | 1 | (2) | 1.1e+002 | 8 | K.SYKENIMR.L |
|  | 6250 | 542.8004 | 1083.5863 | 1084.5012 | -0.9149 | 0 | 12 | 11 | 3 | R.EFMVFADAR.W |
|  | 14394 | **789.3527** | **1576.6908** | **1577.6845** | **-0.9938** | **0** | **2** | **68** | **3** | **K.TYSTSYTLEDQDR.E** |
|  | 22217 | 1102.5538 | 2203.0931 | 2203.1042 | -0.0111 | 0 | 52 | 0.00056 | 1 | K.GAGVTLNVLEMTSEDLENALK.A |
|  | 23066 | **1152.0767** | **2302.1388** | **2302.1490** | **-0.0102** | **0** | **57** | **0.00014** | **1** | **K.LLVVPMDGSHWFTMQSVVEK.L** 23065 |
|  | 23185 | 773.4149 | 2317.2228 | 2317.1929 | 0.0298 | 0 | 10 | 8.2 | 1 | K.DRPVEPLDLAVFWVEFVMR.H |
|  | 26350 | 700.1080 | 2796.4030 | 2797.5023 | -1.0993 | 1 | 4 | 26 | 5 | K.LLVVPMDGSHWFTMQSVVEKLILR.G 26351 |

| **467.** | Q9HAW9\|UD18_HUMAN    **Mass:** 60444    **Score:** 100    **Queries matched:** 13   **emPAI:** 0.20 |
| --- | --- |
|  | UDP-glucuronosyltransferase 1-8 OS=Homo sapiens GN=UGT1A8 PE=1 SV=1 |

|  | **Query** | **Observed** | **Mr(expt)** | **Mr(calc)** | **Delta** | **Miss** | **Score** | **Expect** | **Rank** | **Peptide** |
| --- | --- | --- | --- | --- | --- | --- | --- | --- | --- | --- |
|  | 4553 | 480.7598 | 959.5051 | 959.5110 | -0.0059 | 0 | 46 | 0.0043 | 1 | K.AMAIADALGK.I |
|  | 5607 | 519.2922 | 1036.5699 | 1036.5553 | 0.0146 | 1 | 11 | 11 | 3 | K.AVINDKSYK.E 5603 |
|  | 5635 | 520.7592 | 1039.5039 | 1039.5120 | -0.0081 | 1 | 26 | 0.35 | 1 | K.SYKENIMR.L |
|  | 5836 | 528.7754 | 1055.5363 | 1055.5069 | 0.0294 | 1 | (2) | 1.1e+002 | 8 | K.SYKENIMR.L |
|  | 22217 | 1102.5538 | 2203.0931 | 2203.1042 | -0.0111 | 0 | 52 | 0.00056 | 1 | K.GAGVTLNVLEMTSEDLENALK.A |
|  | 23044 | 767.3312 | 2298.9717 | 2300.1035 | -1.1318 | 0 | 6 | 19 | 3 | K.ESSFDAVFLDPFDACGLIVAK.Y |
|  | 23066 | 1152.0767 | 2302.1388 | 2302.1490 | -0.0102 | 0 | 57 | 0.00014 | 1 | K.LLVVPMDGSHWFTMQSVVEK.L 23065 |
|  | 23185 | 773.4149 | 2317.2228 | 2317.1929 | 0.0298 | 0 | 10 | 8.2 | 1 | K.DRPVEPLDLAVFWVEFVMR.H |
|  | 26350 | 700.1080 | 2796.4030 | 2797.5023 | -1.0993 | 1 | 4 | 26 | 5 | K.LLVVPMDGSHWFTMQSVVEKLILR.G 26351 |
|  | 28633 | **1106.5814** | **3316.7224** | **3316.7343** | **-0.0119** | **0** | **0** | **41** | **3** | **K.NALEIASEILQTPVTAYDLYSHTSIWLLR.T** |

| **468.** | Q9HAW8\|UD110_HUMAN    **Mass:** 60568    **Score:** 100    **Queries matched:** 14   **emPAI:** 0.20 |
| --- | --- |
|  | UDP-glucuronosyltransferase 1-10 OS=Homo sapiens GN=UGT1A10 PE=1 SV=1 |

|  | **Query** | **Observed** | **Mr(expt)** | **Mr(calc)** | **Delta** | **Miss** | **Score** | **Expect** | **Rank** | **Peptide** |
| --- | --- | --- | --- | --- | --- | --- | --- | --- | --- | --- |
|  | 4553 | 480.7598 | 959.5051 | 959.5110 | -0.0059 | 0 | 46 | 0.0043 | 1 | K.AMAIADALGK.I |
|  | 5607 | 519.2922 | 1036.5699 | 1036.5553 | 0.0146 | 1 | 11 | 11 | 3 | K.AVINDKSYK.E 5603 |
|  | 5635 | 520.7592 | 1039.5039 | 1039.5120 | -0.0081 | 1 | 26 | 0.35 | 1 | K.SYKENIMR.L |
|  | 5836 | 528.7754 | 1055.5363 | 1055.5069 | 0.0294 | 1 | (2) | 1.1e+002 | 8 | K.SYKENIMR.L |
|  | 14394 | 789.3527 | 1576.6908 | 1576.7005 | -0.0097 | 0 | 3 | 56 | 2 | K.TYSTSYTLEDQNR.E |
|  | 22217 | 1102.5538 | 2203.0931 | 2203.1042 | -0.0111 | 0 | 52 | 0.00056 | 1 | K.GAGVTLNVLEMTSEDLENALK.A |
|  | 23066 | 1152.0767 | 2302.1388 | 2302.1490 | -0.0102 | 0 | 57 | 0.00014 | 1 | K.LLVVPMDGSHWFTMQSVVEK.L 23065 |
|  | 23185 | 773.4149 | 2317.2228 | 2317.1929 | 0.0298 | 0 | 10 | 8.2 | 1 | K.DRPVEPLDLAVFWVEFVMR.H |
|  | 26350 | 700.1080 | 2796.4030 | 2797.5023 | -1.0993 | 1 | 4 | 26 | 5 | K.LLVVPMDGSHWFTMQSVVEKLILR.G 26351 |
|  | 28633 | 1106.5814 | 3316.7224 | 3316.7343 | -0.0119 | 0 | 0 | 41 | 3 | R.NALEIASEILQTPVTAYDLYSHTSIWLLR.T |
|  | 30515 | 1027.5660 | 4106.2351 | 4105.9125 | 0.3226 | 1 | 1 | 16 | 7 | R.GIFCHHLEEGAQCPAPLSYVPNDLLGFSDAMTFKER.V |

| **469.** | Q14240\|IF4A2_HUMAN    **Mass:** 46601    **Score:** 100    **Queries matched:** 12   **emPAI:** 0.17 |
| --- | --- |
|  | Eukaryotic initiation factor 4A-II OS=Homo sapiens GN=EIF4A2 PE=1 SV=2 |

|  | **Query** | **Observed** | **Mr(expt)** | **Mr(calc)** | **Delta** | **Miss** | **Score** | **Expect** | **Rank** | **Peptide** |
| --- | --- | --- | --- | --- | --- | --- | --- | --- | --- | --- |
|  | 3829 | 455.2455 | 908.4765 | 909.4378 | -0.9614 | 0 | 6 | 40 | 3 | R.VFDMLNR.R |
|  | 5997 | 534.3673 | 1066.7201 | 1065.5389 | 1.1811 | 1 | 10 | 13 | 4 | R.VFDMLNRR.Y |
|  | 6025 | 535.3394 | 1068.6643 | 1067.5400 | 1.1243 | 0 | 10 | 16 | 2 | K.QFYINVER.E 5994 |
|  | 13910 | 773.3602 | 1544.7059 | 1543.8471 | 0.8588 | 0 | 7 | 23 | 6 | K.LQAEAPHIVVGTPGR.V 13914 |
|  | 14072 | 778.3561 | 1554.6977 | 1554.7058 | -0.0081 | 0 | 71 | 1e-005 | 1 | K.MFVLDEADEMLSR.G |
|  | 14190 | 782.3782 | 1562.7418 | 1562.7035 | 0.0383 | 0 | 7 | 23 | 3 | R.DFTVSALHGDMDQK.E |
|  | 14588 | 530.6256 | 1588.8550 | 1587.8944 | 0.9606 | 1 | 1 | 89 | 7 | R.SGSSRVLITTDLLAR.G |
|  | 17978 | 914.4666 | 1826.9187 | 1826.9315 | -0.0128 | 0 | 57 | 0.00021 | 1 | R.GIYAYGFEKPSAIQQR.A |
|  | 21662 | 537.0157 | 2144.0337 | 2143.1273 | 0.9063 | 0 | 2 | 60 | 9 | R.GIDVQQVSLVINYDLPTNR.E |
|  | 30956 | **1504.3118** | **4509.9135** | **4510.9012** | **-0.9878** | **1** | **1** | **16** | **2** | **-.MSGGSADYNREHGGPEGMDPDGVIESNWNEIVDNFDDMNLK.E** |

| **470.** | Q02338\|BDH_HUMAN    **Mass:** 38532    **Score:** 100    **Queries matched:** 14   **emPAI:** 0.20 |
| --- | --- |
|  | D-beta-hydroxybutyrate dehydrogenase, mitochondrial OS=Homo sapiens GN=BDH1 PE=1 SV=3 |

|  | **Query** | **Observed** | **Mr(expt)** | **Mr(calc)** | **Delta** | **Miss** | **Score** | **Expect** | **Rank** | **Peptide** |
| --- | --- | --- | --- | --- | --- | --- | --- | --- | --- | --- |
|  | 2963 | **428.2532** | **854.4918** | **854.4246** | **0.0672** | **1** | **13** | **7.7** | **2** | **K.DKGHDGVK.E** 2966 |
|  | 3237 | 435.2233 | 868.4320 | 867.4160 | 1.0160 | 0 | 4 | 52 | 6 | R.SPYCITK.F |
|  | 6637 | **557.7800** | **1113.5454** | **1114.5369** | **-0.9915** | **0** | **1** | **1.1e+002** | **3** | **R.YEMYPLGVK.V** |
|  | 8239 | **406.5322** | **1216.5748** | **1216.6234** | **-0.0486** | **1** | **8** | **27** | **3** | **R.LPGKTLSACDR.E** |
|  | 8361 | 408.2347 | 1221.6824 | 1220.6951 | 0.9873 | 1 | 10 | 14 | 3 | R.MTKSFLPLIR.R 8337 |
|  | 9815 | **650.7989** | **1299.5833** | **1299.5918** | **-0.0084** | **0** | **40** | **0.015** | **1** | **K.FGVEAFSDCLR.Y** |
|  | 10495 | **668.7888** | **1335.5631** | **1335.6783** | **-0.1152** | **1** | **1** | **1.1e+002** | **3** | **R.RTYASAAEPVGSK.A** |
|  | 16609 | 576.9547 | 1727.8421 | 1727.8553 | -0.0132 | 0 | 1 | 79 | 5 | K.AVLVTGCDSGFGFSLAK.H |
|  | 16662 | **578.0390** | **1731.0952** | **1730.9138** | **0.1814** | **1** | **4** | **33** | **5** | **K.QVAEVNLWGTVRMTK.S** |
|  | 21047 | **697.3541** | **2089.0406** | **2089.0522** | **-0.0117** | **0** | **13** | **4.4** | **1** | **R.MQIMTHLPGAISDMIYIR.-** |
|  | 25766 | **1346.2102** | **2690.4059** | **2690.4167** | **-0.0108** | **0** | **63** | **3e-005** | **1** | **K.VSVVEPGNFIAATSLYSPESIQAIAK.K** 25764 |

| **471.** | O43837\|IDH3B_HUMAN    **Mass:** 42442    **Score:** 100    **Queries matched:** 11   **emPAI:** 0.09 |
| --- | --- |
|  | Isocitrate dehydrogenase [NAD] subunit beta, mitochondrial OS=Homo sapiens GN=IDH3B PE=1 SV=2 |

|  | **Query** | **Observed** | **Mr(expt)** | **Mr(calc)** | **Delta** | **Miss** | **Score** | **Expect** | **Rank** | **Peptide** |
| --- | --- | --- | --- | --- | --- | --- | --- | --- | --- | --- |
|  | 7872 | **600.4665** | **1198.9185** | **1198.6054** | **0.3131** | **0** | **0** | **1e+002** | **5** | **R.GLSTSAAAHAASR.S** |
|  | 8017 | **603.8138** | **1205.6130** | **1205.6880** | **-0.0750** | **0** | **3** | **80** | **6** | **R.HNNLDLVIIR.E** |
|  | 10010 | 656.2712 | 1310.5279 | 1309.6448 | 0.8831 | 1 | 5 | 44 | 4 | K.GELASYDMRLR.R |
|  | 10895 | **454.2090** | **1359.6050** | **1359.7445** | **-0.1395** | **1** | **9** | **17** | **4** | **-.MAALSGVRWLTR.A** |
|  | 14991 | 808.6408 | 1615.2669 | 1614.8803 | 0.3866 | 1 | 1 | 64 | 3 | K.VAIIGKIHTPMEYK.G |
|  | 16985 | 585.7771 | 1754.3095 | 1753.9376 | 0.3719 | 1 | 1 | 62 | 6 | R.WLTRALVSAGNPGAWR.G |
|  | 17810 | 908.9691 | 1815.9237 | 1815.9335 | -0.0098 | 0 | 100 | 1.2e-008 | 1 | R.NIANPTAMLLSASNMLR.H |
|  | 18292 | **616.7787** | **1847.3144** | **1847.9233** | **-0.6089** | **0** | **(0)** | **69** | **3** | **R.NIANPTAMLLSASNMLR.H** |
|  | 23617 | **793.3901** | **2377.1484** | **2378.2203** | **-1.0719** | **1** | **3** | **40** | **1** | **R.ALVSAGNPGAWRGLSTSAAAHAASR.S** |
|  | 26407 | 938.4862 | 2812.4368 | 2811.4272 | 1.0096 | 1 | 4 | 25 | 1 | R.HPFAQAVGRNIANPTAMLLSASNMLR.H |
|  | 29672 | **915.1676** | **3656.6413** | **3656.8112** | **-0.1699** | **1** | **4** | **13** | **1** | **R.NIANPTAMLLSASNMLRHLNLEYHSSMIADAVK.K** |

| **472.** | P46776\|RL27A_HUMAN    **Mass:** 16665    **Score:** 99     **Queries matched:** 11   **emPAI:** 0.52 |
| --- | --- |
|  | 60S ribosomal protein L27a OS=Homo sapiens GN=RPL27A PE=1 SV=2 |

|  | **Query** | **Observed** | **Mr(expt)** | **Mr(calc)** | **Delta** | **Miss** | **Score** | **Expect** | **Rank** | **Peptide** |
| --- | --- | --- | --- | --- | --- | --- | --- | --- | --- | --- |
|  | 996 | 542.1290 | 541.1218 | 541.3588 | -0.2370 | 1 | 5 | 28 | 7 | K.GKLPK.Q |
|  | 1648 | **683.4768** | **682.4695** | **682.4378** | **0.0318** | **0** | **11** | **10** | **7** | **K.QPVIVK.A** |
|  | 1775 | 717.3264 | 716.3191 | 716.4068 | -0.0877 | 1 | 9 | 30 | 1 | R.AEEKIK.S |
|  | 6606 | **556.3234** | **1110.6323** | **1110.6397** | **-0.0074** | **0** | **57** | **0.00026** | **1** | **K.TGAAPIIDVVR.S** 6604 6613 |
|  | 10769 | **676.8430** | **1351.6715** | **1352.7776** | **-1.1061** | **1** | **0** | **1.2e+002** | **5** | **K.NKTGAAPIIDVVR.S** |
|  | 11316 | 692.3542 | 1382.6939 | 1382.6805 | 0.0134 | 1 | 4 | 62 | 4 | K.YHPGYFGKVGMK.H |
|  | 12010 | **711.8331** | **1421.6517** | **1421.6609** | **-0.0092** | **0** | **47** | **0.0027** | **1** | **R.NQSFCPTVNLDK.L** 12009 |
|  | 14409 | **789.8830** | **1577.7514** | **1577.7620** | **-0.0106** | **1** | **21** | **0.93** | **1** | **K.RNQSFCPTVNLDK.L** |

| **473.** | Q15165\|PON2_HUMAN    **Mass:** 39658    **Score:** 99     **Queries matched:** 14   **emPAI:** 0.20 |
| --- | --- |
|  | Serum paraoxonase/arylesterase 2 OS=Homo sapiens GN=PON2 PE=1 SV=2 |

|  | **Query** | **Observed** | **Mr(expt)** | **Mr(calc)** | **Delta** | **Miss** | **Score** | **Expect** | **Rank** | **Peptide** |
| --- | --- | --- | --- | --- | --- | --- | --- | --- | --- | --- |
|  | 2891 | **425.7344** | **849.4542** | **849.4596** | **-0.0054** | **0** | **11** | **15** | **3** | **K.NTVEIFK.F** 2905 |
|  | 18267 | **923.9644** | **1845.9142** | **1845.9261** | **-0.0119** | **0** | **58** | **0.00017** | **1** | **K.LFVYDPNNPPSSEVLR.I** |
|  | 19589 | **650.3851** | **1948.1334** | **1947.9374** | **0.1960** | **1** | **2** | **58** | **1** | **M.GAWVGCGLAGDRAGFLGER.L** |
|  | 28536 | **1093.8617** | **3278.5632** | **3277.5925** | **0.9707** | **0** | **2** | **29** | **4** | **K.VLELDTLVDNLSIDPSSGDIWVGCHPNGQK.L** |
|  | 29513 | **904.6884** | **3614.7243** | **3614.7470** | **-0.0227** | **0** | **12** | **1.9** | **1** | **K.HELLPSVNDITAVGPAHFYATNDHYFSDPFLK.Y** 29500 |
|  | 29925 | **1252.6014** | **3754.7825** | **3754.7944** | **-0.0119** | **0** | **62** | **2e-005** | **1** | **R.GFDLASFNPHGISTFIDNDDTVYLFVVNHPEFK.N** 29923 29924 |
|  | 30329 | **792.0026** | **3954.9767** | **3954.0051** | **0.9717** | **1** | **0** | **28** | **3** | **K.VVAEGFDSANGINISPDDKYIYVADILAHEIHVLEK.H** |
|  | 30538 | 1373.6493 | 4117.9260 | 4118.1034 | -0.1774 | 1 | 4 | 11 | 2 | R.EVESVDLPHCHLIKGIEAGSEDIDILPNGLAFFSVGLK.F 30539 30542 |

| **474.** | O95716\|RAB3D_HUMAN    **Mass:** 24480    **Score:** 99     **Queries matched:** 14   **emPAI:** 0.33 |
| --- | --- |
|  | Ras-related protein Rab-3D OS=Homo sapiens GN=RAB3D PE=1 SV=1 |

|  | **Query** | **Observed** | **Mr(expt)** | **Mr(calc)** | **Delta** | **Miss** | **Score** | **Expect** | **Rank** | **Peptide** |
| --- | --- | --- | --- | --- | --- | --- | --- | --- | --- | --- |
|  | 4921 | 494.7554 | 987.4962 | 987.5025 | -0.0063 | 0 | 21 | 1.6 | 1 | R.TITTAYYR.G |
|  | 5511 | **516.2711** | **1030.5277** | **1029.4839** | **1.0438** | **0** | **5** | **52** | **7** | **M.ASAGDTQAGPR.D** |
|  | 6462 | **550.7324** | **1099.4502** | **1099.6601** | **-0.2099** | **0** | **10** | **16** | **7** | **K.LLLIGNSSVGK.T** 6477 |
|  | 7439 | **588.8261** | **1175.6375** | **1176.5193** | **-0.8818** | **0** | **11** | **13** | **3** | **-.MASAGDTQAGPR.D** 7453 |
|  | 10115 | 658.8291 | 1315.6436 | 1315.6521 | -0.0084 | 0 | 53 | 0.00078 | 1 | K.LQIWDTAGQER.Y 10116 |
|  | 11168 | 688.3658 | 1374.7170 | 1374.7255 | -0.0085 | 1 | 5 | 41 | 6 | K.ENINVKQVFER.L 11164 |
|  | 12676 | 488.5925 | 1462.7556 | 1463.6027 | -0.8471 | 0 | 4 | 56 | 9 | R.DAADQNFDYMFK.L |
|  | 16345 | **854.4330** | **1706.8515** | **1706.8628** | **-0.0113** | **0** | **60** | **0.00012** | **1** | **K.TYSWDNAQVILVGNK.C** |
|  | 25336 | 1312.6089 | 2623.2032 | 2622.1115 | 1.0917 | 1 | 0 | 59 | 3 | -.MASAGDTQAGPRDAADQNFDYMFK.L |
|  | 30509 | 1368.9576 | 4103.8511 | 4102.9808 | 0.8702 | 1 | 3 | 14 | 3 | R.TITTAYYRGAMGFLLMYDIANQESFAAVQDWATQIK.T |

| **475.** | Q96E17\|RAB3C_HUMAN    **Mass:** 26164    **Score:** 99     **Queries matched:** 13   **emPAI:** 0.31 |
| --- | --- |
|  | Ras-related protein Rab-3C OS=Homo sapiens GN=RAB3C PE=2 SV=1 |

|  | **Query** | **Observed** | **Mr(expt)** | **Mr(calc)** | **Delta** | **Miss** | **Score** | **Expect** | **Rank** | **Peptide** |
| --- | --- | --- | --- | --- | --- | --- | --- | --- | --- | --- |
|  | 4669 | **485.3528** | **968.6911** | **969.3168** | **-0.6257** | **0** | **4** | **44** | **4** | **K.CDMEDER.V** |
|  | 4921 | 494.7554 | 987.4962 | 987.5025 | -0.0063 | 0 | 21 | 1.6 | 1 | R.TITTAYYR.G |
|  | 6462 | 550.7324 | 1099.4502 | 1099.6601 | -0.2099 | 0 | 10 | 16 | 7 | K.LLIIGNSSVGK.T 6477 |
|  | 10115 | 658.8291 | 1315.6436 | 1315.6521 | -0.0084 | 0 | 53 | 0.00078 | 1 | K.LQIWDTAGQER.Y 10116 |
|  | 14130 | 520.2886 | 1557.8439 | 1557.6664 | 0.1775 | 0 | (2) | 88 | 10 | R.HEAPMQMASAQDAR.Y |
|  | 14355 | **525.3103** | **1572.9091** | **1573.6613** | **-0.7522** | **0** | **11** | **9.5** | **1** | **R.HEAPMQMASAQDAR.Y** |
|  | 14418 | **527.1549** | **1578.4427** | **1578.7447** | **-0.3020** | **0** | **3** | **39** | **5** | **K.MSESLETDPAITAAK.Q** |
|  | 15362 | 820.3347 | 1638.6549 | 1638.6978 | -0.0429 | 1 | 3 | 48 | 2 | K.CDMEDERVISTER.G |
|  | 16345 | 854.4330 | 1706.8515 | 1706.8628 | -0.0113 | 0 | 60 | 0.00012 | 1 | K.TYSWDNAQVILVGNK.C |
|  | 18246 | **922.9612** | **1843.9078** | **1844.8080** | **-0.9002** | **1** | **3** | **57** | **5** | **-.MRHEAPMQMASAQDAR.Y** |
|  | 18514 | **621.3673** | **1861.0799** | **1860.8029** | **0.2770** | **1** | **(1)** | **76** | **9** | **-.MRHEAPMQMASAQDAR.Y** |

| **476.** | P20336\|RAB3A_HUMAN    **Mass:** 25196    **Score:** 99     **Queries matched:** 7   **emPAI:** 0.32 |
| --- | --- |
|  | Ras-related protein Rab-3A OS=Homo sapiens GN=RAB3A PE=1 SV=1 |

|  | **Query** | **Observed** | **Mr(expt)** | **Mr(calc)** | **Delta** | **Miss** | **Score** | **Expect** | **Rank** | **Peptide** |
| --- | --- | --- | --- | --- | --- | --- | --- | --- | --- | --- |
|  | 4669 | 485.3528 | 968.6911 | 969.3168 | -0.6257 | 0 | 4 | 44 | 4 | K.CDMEDER.V |
|  | 4921 | 494.7554 | 987.4962 | 987.5025 | -0.0063 | 0 | 21 | 1.6 | 1 | R.TITTAYYR.G |
|  | 6462 | 550.7324 | 1099.4502 | 1099.6601 | -0.2099 | 0 | 10 | 16 | 7 | K.ILIIGNSSVGK.T 6477 |
|  | 10115 | 658.8291 | 1315.6436 | 1315.6521 | -0.0084 | 0 | 53 | 0.00078 | 1 | K.LQIWDTAGQER.Y 10116 |
|  | 16345 | 854.4330 | 1706.8515 | 1706.8628 | -0.0113 | 0 | 60 | 0.00012 | 1 | K.TYSWDNAQVLLVGNK.C |

| **477.** | P20337\|RAB3B_HUMAN    **Mass:** 24970    **Score:** 99     **Queries matched:** 12   **emPAI:** 0.33 |
| --- | --- |
|  | Ras-related protein Rab-3B OS=Homo sapiens GN=RAB3B PE=1 SV=2 |

|  | **Query** | **Observed** | **Mr(expt)** | **Mr(calc)** | **Delta** | **Miss** | **Score** | **Expect** | **Rank** | **Peptide** |
| --- | --- | --- | --- | --- | --- | --- | --- | --- | --- | --- |
|  | 4921 | 494.7554 | 987.4962 | 987.5025 | -0.0063 | 0 | 21 | 1.6 | 1 | R.TITTAYYR.G |
|  | 6462 | 550.7324 | 1099.4502 | 1099.6601 | -0.2099 | 0 | 10 | 16 | 7 | K.LLIIGNSSVGK.T 6477 |
|  | 10115 | 658.8291 | 1315.6436 | 1315.6521 | -0.0084 | 0 | 53 | 0.00078 | 1 | K.LQIWDTAGQER.Y 10116 |
|  | 15266 | 544.9023 | 1631.6850 | 1631.7283 | -0.0433 | 0 | (2) | 80 | 9 | R.LSDTPPLLQQNCSC.- 15262 15265 15269 15273 |
|  | 16345 | 854.4330 | 1706.8515 | 1706.8628 | -0.0113 | 0 | 60 | 0.00012 | 1 | K.TYSWDNAQVILVGNK.C |
|  | 19815 | 657.3741 | 1969.1004 | 1969.9785 | -0.8781 | 0 | 3 | 48 | 8 | K.GQLLAEQLGFDFFEASAK.E |

| **478.** | P61106\|RAB14_HUMAN    **Mass:** 24110    **Score:** 99     **Queries matched:** 7   **emPAI:** 0.55 |
| --- | --- |
|  | Ras-related protein Rab-14 OS=Homo sapiens GN=RAB14 PE=1 SV=4 |

|  | **Query** | **Observed** | **Mr(expt)** | **Mr(calc)** | **Delta** | **Miss** | **Score** | **Expect** | **Rank** | **Peptide** |
| --- | --- | --- | --- | --- | --- | --- | --- | --- | --- | --- |
|  | 10115 | 658.8291 | 1315.6436 | 1315.6521 | -0.0084 | 0 | 53 | 0.00078 | 1 | K.LQIWDTAGQER.F 10116 |
|  | 14481 | **792.3939** | **1582.7733** | **1583.7330** | **-0.9597** | **0** | **5** | **35** | **5** | **-.MATAPYNYSYIFK.Y** |
|  | 15056 | 810.8657 | 1619.7169 | 1620.7995 | -1.0826 | 1 | 3 | 54 | 5 | K.TGENVEDAFLEAAKK.I |
|  | 15111 | **812.4702** | **1622.9259** | **1622.9355** | **-0.0097** | **0** | **59** | **0.00013** | **1** | **R.NLTNPNTVIILIGNK.A** |
|  | 18137 | **613.6140** | **1837.8200** | **1836.8288** | **0.9913** | **0** | **33** | **0.053** | **1** | **K.FMADCPHTIGVEFGTR.I** 18130 |

| **479.** | Q16698\|DECR_HUMAN    **Mass:** 36330    **Score:** 99     **Queries matched:** 11   **emPAI:** 0.22 |
| --- | --- |
|  | 2,4-dienoyl-CoA reductase, mitochondrial OS=Homo sapiens GN=DECR1 PE=1 SV=1 |

|  | **Query** | **Observed** | **Mr(expt)** | **Mr(calc)** | **Delta** | **Miss** | **Score** | **Expect** | **Rank** | **Peptide** |
| --- | --- | --- | --- | --- | --- | --- | --- | --- | --- | --- |
|  | 6612 | 556.8228 | 1111.6311 | 1111.6390 | -0.0079 | 0 | 16 | 3.6 | 3 | R.FNVIQPGPIK.T 6623 6630 |
|  | 7658 | 595.3027 | 1188.5909 | 1188.5961 | -0.0052 | 0 | 16 | 3.6 | 1 | K.AMLPPNSFQGK.V |
|  | 7983 | **603.2904** | **1204.5663** | **1203.5852** | **0.9810** | **1** | **7** | **29** | **1** | **K.EMIGRIPCGR.L** |
|  | 10458 | **667.8267** | **1333.6389** | **1333.6474** | **-0.0085** | **0** | **60** | **0.00015** | **1** | **K.ATAEQISSQTGNK.V** |
|  | 14794 | 803.4327 | 1604.8509 | 1603.7763 | 1.0746 | 0 | 7 | 22 | 7 | R.DPDMVQNTVSELIK.V |
|  | 18550 | **933.4812** | **1864.9478** | **1863.9547** | **0.9932** | **0** | **67** | **2.3e-005** | **1** | **K.GMTTLLSSLGAQCVIASR.K** |
|  | 20369 | **675.9991** | **2024.9756** | **2023.9851** | **0.9905** | **1** | **28** | **0.13** | **1** | **K.FDGGEEVLISGEFNDLRK.V** |
|  | 23828 | **603.8376** | **2411.3213** | **2412.1816** | **-0.8603** | **1** | **3** | **33** | **7** | **K.ATAEQISSQTGNKVHAIQCDVR.D** |
|  | 27552 | **1014.1223** | **3039.3449** | **3038.5893** | **0.7556** | **1** | **0** | **49** | **2** | **K.VAFITGGGTGLGKGMTTLLSSLGAQCVIASR.K** |

| **480.** | Q96AG4\|LRC59_HUMAN    **Mass:** 35308    **Score:** 99     **Queries matched:** 11   **emPAI:** 0.22 |
| --- | --- |
|  | Leucine-rich repeat-containing protein 59 OS=Homo sapiens GN=LRRC59 PE=1 SV=1 |

|  | **Query** | **Observed** | **Mr(expt)** | **Mr(calc)** | **Delta** | **Miss** | **Score** | **Expect** | **Rank** | **Peptide** |
| --- | --- | --- | --- | --- | --- | --- | --- | --- | --- | --- |
|  | 1720 | 703.4045 | 702.3972 | 702.4064 | -0.0092 | 0 | 7 | 49 | 9 | R.SWAVLK.L |
|  | 1898 | **758.4106** | **757.4034** | **756.3766** | **1.0268** | **0** | **11** | **18** | **4** | **K.EANQAPK.S** |
|  | 1912 | 759.1600 | 758.1528 | 758.4035 | -0.2507 | 1 | 1 | 1.5e+002 | 8 | K.GGNLRDK.L |
|  | 6902 | 567.7713 | 1133.5280 | 1133.5750 | -0.0470 | 0 | 9 | 20 | 4 | K.ATILDLSCNK.L |
|  | 7027 | **573.3068** | **1144.5990** | **1143.6037** | **0.9953** | **0** | **40** | **0.016** | **1** | **K.LQQLPADFGR.L** |
|  | 11359 | 693.8202 | 1385.6259 | 1385.7415 | -0.1156 | 1 | 6 | 35 | 8 | K.NKLQQLPADFGR.L 11360 |
|  | 13749 | **767.4361** | **1532.8576** | **1532.8674** | **-0.0098** | **0** | **91** | **9.8e-008** | **1** | **R.LVNLQHLDLLNNK.L** |
|  | 18437 | **619.5241** | **1855.5505** | **1856.0077** | **-0.4572** | **1** | **6** | **19** | **1** | **K.ELAALPKATILDLSCNK.L** |
|  | 23140 | **771.7274** | **2312.1604** | **2312.1747** | **-0.0143** | **1** | **16** | **1.9** | **1** | **R.DKLDGNELDLSLSDLNEVPVK.E** 23141 |

| **481.** | P48735\|IDHP_HUMAN    **Mass:** 51333    **Score:** 98     **Queries matched:** 22   **emPAI:** 0.32 |
| --- | --- |
|  | Isocitrate dehydrogenase [NADP], mitochondrial OS=Homo sapiens GN=IDH2 PE=1 SV=2 |

|  | **Query** | **Observed** | **Mr(expt)** | **Mr(calc)** | **Delta** | **Miss** | **Score** | **Expect** | **Rank** | **Peptide** |
| --- | --- | --- | --- | --- | --- | --- | --- | --- | --- | --- |
|  | 1580 | 665.3572 | 664.3500 | 665.3748 | -1.0249 | 0 | 13 | 11 | 2 | K.YSVAVK.C |
|  | 3615 | **448.2307** | **894.4469** | **893.4243** | **1.0226** | **0** | **59** | **0.0002** | **1** | **K.ATDFVADR.A** 3603 |
|  | 3763 | 452.7106 | 903.4066 | 902.4399 | 0.9668 | 0 | 23 | 0.89 | 1 | K.IWYEHR.L |
|  | 4369 | **474.2862** | **946.5578** | **946.5640** | **-0.0062** | **0** | **12** | **10** | **4** | **R.IIWQFIK.E** |
|  | 4779 | 488.7791 | 975.5436 | 975.5502 | -0.0066 | 0 | 44 | 0.0082 | 1 | R.NILGGTVFR.E 4777 4778 |
|  | 7155 | **577.7871** | **1153.5597** | **1153.5655** | **-0.0059** | **0** | **2** | **82** | **9** | **K.DIFQEIFDK.H** |
|  | 8454 | **614.8316** | **1227.6486** | **1227.6571** | **-0.0085** | **1** | **8** | **24** | **1** | **R.GKLDGNQDLIR.F** |
|  | 8656 | **413.5338** | **1237.5794** | **1238.6805** | **-1.1011** | **1** | **5** | **43** | **9** | **R.EPIICKNIPR.L** 8669 |
|  | 9463 | **427.8789** | **1280.6150** | **1280.6435** | **-0.0285** | **1** | **10** | **13** | **5** | **K.MVFTPKDGSGVK.E** 9455 9473 |
|  | 11315 | **692.3518** | **1382.6891** | **1382.6976** | **-0.0086** | **0** | **46** | **0.0037** | **1** | **K.DLAGCIHGLSNVK.L** 11313 |
|  | 11761 | **705.2353** | **1408.4560** | **1409.6531** | **-1.1970** | **0** | **4** | **38** | **5** | **K.VCVETVESGAMTK.D** |
|  | 15878 | **559.2664** | **1674.7773** | **1675.7909** | **-1.0137** | **0** | **5** | **33** | **2** | **K.VAKPVVEMDGDEMTR.I** |
|  | 16370 | **570.4553** | **1708.3441** | **1707.7808** | **0.5634** | **0** | **(3)** | **37** | **1** | **K.VAKPVVEMDGDEMTR.I** |
|  | 25414 | 1319.1815 | 2636.3485 | 2636.3342 | 0.0143 | 1 | 0 | 66 | 4 | K.VAKPVVEMDGDEMTRIIWQFIK.E |
|  | 26960 | **975.4758** | **2923.4057** | **2923.4200** | **-0.0143** | **1** | **14** | **2.5** | **1** | **K.YFDLGLPNRDQTDDQVTIDSALATQK.Y** |

| **482.** | P48643\|TCPE_HUMAN    **Mass:** 60089    **Score:** 98     **Queries matched:** 29   **emPAI:** 0.20 |
| --- | --- |
|  | T-complex protein 1 subunit epsilon OS=Homo sapiens GN=CCT5 PE=1 SV=1 |

|  | **Query** | **Observed** | **Mr(expt)** | **Mr(calc)** | **Delta** | **Miss** | **Score** | **Expect** | **Rank** | **Peptide** |
| --- | --- | --- | --- | --- | --- | --- | --- | --- | --- | --- |
|  | 1894 | **757.3978** | **756.3905** | **756.3952** | **-0.0047** | **0** | **39** | **0.025** | **1** | **K.SHIMAAK.A** |
|  | 2175 | **801.4409** | **800.4336** | **800.4505** | **-0.0168** | **1** | **11** | **17** | **1** | **K.VEGKVGGR.L** |
|  | 4048 | 463.2282 | 924.4419 | 923.4600 | 0.9819 | 0 | 7 | 31 | 9 | R.FSELTAEK.L |
|  | 4891 | 493.2842 | 984.5537 | 983.6379 | 0.9158 | 1 | 9 | 20 | 10 | K.LIKGVIVDK.D 4880 |
|  | 6663 | **558.5414** | **1115.0683** | **1115.6372** | **-0.5689** | **1** | **4** | **41** | **4** | **R.MILKIDDIR.K** |
|  | 6867 | 566.7635 | 1131.5124 | 1131.6322 | -0.1197 | 1 | (2) | 1e+002 | 7 | R.MILKIDDIR.K |
|  | 7344 | **585.2968** | **1168.5791** | **1167.5659** | **1.0131** | **0** | **10** | **13** | **1** | **K.LDVTSVEDYK.A** |
|  | 7531 | **592.3131** | **1182.6117** | **1182.6179** | **-0.0063** | **0** | **12** | **8.8** | **1** | **R.SLHDALCVIR.N** 7555 7556 |
|  | 8510 | **616.3621** | **1230.7096** | **1229.5863** | **1.1233** | **1** | **2** | **89** | **9** | **K.DFSHPQMPKK.V** 8486 |
|  | 9607 | **430.5576** | **1288.6510** | **1289.6762** | **-1.0251** | **0** | **7** | **31** | **6** | **K.QQISLATQMVR.M** 9616 |
|  | 14793 | 803.4283 | 1604.8421 | 1604.7902 | 0.0519 | 1 | 4 | 48 | 8 | R.TSLGPNGLDKMMVDK.D |
|  | 15177 | **543.2847** | **1626.8324** | **1627.8789** | **-1.0466** | **1** | **2** | **66** | **3** | **R.LMGLEALKSHIMAAK.A** 15205 |
|  | 15770 | **834.4499** | **1666.8852** | **1666.8930** | **-0.0078** | **0** | **68** | **1.7e-005** | **1** | **K.LGFAGLVQEISFGTTK.D** 15772 |
|  | 17092 | **881.4748** | **1760.9350** | **1759.8887** | **1.0464** | **1** | **2** | **72** | **2** | **K.AVANTMRTSLGPNGLDK.M** |
|  | 19119 | **637.3173** | **1908.9301** | **1910.0149** | **-1.0848** | **1** | **5** | **32** | **3** | **K.LGFAGLVQEISFGTTKDK.M** |
|  | 20101 | **667.0316** | **1998.0729** | **1998.0170** | **0.0558** | **1** | **2** | **63** | **1** | **R.IADGYEQAARVAIEHLDK.I** |
|  | 25664 | **1339.1108** | **2676.2071** | **2677.2211** | **-1.0140** | **0** | **1** | **46** | **4** | **K.DGDVTVTNDGATILSMMDVDHQIAK.L** |
|  | 26232 | **463.0949** | **2772.5260** | **2773.3567** | **-0.8308** | **1** | **0** | **50** | **4** | **-.MASMGTLAFDEYGRPFLIIKDQDR.K** |
|  | 27816 | **1038.5155** | **3112.5247** | **3112.5412** | **-0.0165** | **0** | **54** | **0.00022** | **1** | **K.SQDDEIGDGTTGVVVLAGALLEEAEQLLDR.G** 27817 27819 |
|  | 28427 | **813.3781** | **3249.4831** | **3249.5026** | **-0.0195** | **1** | **1** | **36** | **3** | **K.MMVDKDGDVTVTNDGATILSMMDVDHQIAK.L** |

| **483.** | P35606\|COPB2_HUMAN    **Mass:** 103278   **Score:** 98     **Queries matched:** 19   **emPAI:** 0.11 |
| --- | --- |
|  | Coatomer subunit beta' OS=Homo sapiens GN=COPB2 PE=1 SV=2 |

|  | **Query** | **Observed** | **Mr(expt)** | **Mr(calc)** | **Delta** | **Miss** | **Score** | **Expect** | **Rank** | **Peptide** |
| --- | --- | --- | --- | --- | --- | --- | --- | --- | --- | --- |
|  | 1491 | 644.8003 | 643.7931 | 643.4017 | 0.3914 | 1 | 15 | 5.1 | 6 | R.LDIKR.K |
|  | 2567 | **415.2188** | **828.4231** | **828.3324** | **0.0908** | **0** | **6** | **52** | **9** | **R.DFSMADK.V** |
|  | 4624 | **483.6155** | **965.2163** | **965.4607** | **-0.2443** | **0** | **3** | **52** | **6** | **K.IWDYQNK.T** |
|  | 4676 | **485.7787** | **969.5428** | **968.4386** | **1.1042** | **1** | **4** | **66** | **1** | **R.RDFSMADK.V** |
|  | 5761 | **525.7791** | **1049.5437** | **1049.5506** | **-0.0069** | **0** | **13** | **7.5** | **1** | **R.TYLPSQVSR.V** |
|  | 6427 | 549.3069 | 1096.5992 | 1096.6240 | -0.0248 | 1 | 5 | 41 | 7 | K.DGERLPLAVK.D |
|  | 7137 | **577.2978** | **1152.5810** | **1152.5887** | **-0.0077** | **0** | **48** | **0.0025** | **1** | **K.HSEVQQANLK.A** |
|  | 9031 | 630.2972 | 1258.5799 | 1259.7125 | -1.1326 | 0 | 2 | 98 | 9 | R.FELALQLGELK.I |
|  | 9560 | 429.5636 | 1285.6690 | 1285.7394 | -0.0704 | 1 | 5 | 45 | 5 | K.WKQLAELAISK.C |
|  | 9936 | 436.2479 | 1305.7219 | 1305.7081 | 0.0138 | 1 | 5 | 47 | 6 | R.LVKIWDYQNK.T |
|  | 10520 | **669.3922** | **1336.7697** | **1336.6007** | **0.1690** | **0** | **45** | **0.0038** | **1** | **K.DNNQFASASLDR.T** |
|  | 13560 | **507.5159** | **1519.5258** | **1519.7089** | **-0.1832** | **0** | **2** | **57** | **4** | **K.NWVVTGADDMQIR.V** |
|  | 13567 | **507.7429** | **1520.2068** | **1519.6878** | **0.5191** | **0** | **3** | **47** | **4** | **R.VHMFEAHSDYIR.C** |
|  | 13767 | 768.3344 | 1534.6543 | 1535.7038 | -1.0496 | 1 | 7 | 23 | 3 | R.NVMEEGKDFQPSR.S |
|  | 14171 | **521.3040** | **1560.8902** | **1560.8222** | **0.0681** | **1** | **4** | **48** | **10** | **R.DFSMADKVLPTIPK.E** |
|  | 15279 | 544.9341 | 1631.7804 | 1631.8090 | -0.0286 | 1 | 1 | 96 | 10 | R.KNWVVTGADDMQIR.V |
|  | 20251 | **672.6617** | **2014.9632** | **2014.9749** | **-0.0116** | **0** | **0** | **85** | **5** | **K.VWQLGSSSPNFTLEGHEK.G** |
|  | 20823 | **1033.0042** | **2063.9937** | **2064.0051** | **-0.0114** | **0** | **51** | **0.00079** | **1** | **K.AAESLADPTEYENLFPGLK.E** |
|  | 24784 | **845.7144** | **2534.1214** | **2534.1384** | **-0.0169** | **0** | **7** | **12** | **1** | **K.GVNCIDYYSGGDKPYLISGADDR.L** |

| **484.** | Q9H2U2\|IPYR2_HUMAN    **Mass:** 38409    **Score:** 97     **Queries matched:** 10   **emPAI:** 0.21 |
| --- | --- |
|  | Inorganic pyrophosphatase 2, mitochondrial OS=Homo sapiens GN=PPA2 PE=1 SV=2 |

|  | **Query** | **Observed** | **Mr(expt)** | **Mr(calc)** | **Delta** | **Miss** | **Score** | **Expect** | **Rank** | **Peptide** |
| --- | --- | --- | --- | --- | --- | --- | --- | --- | --- | --- |
|  | 1176 | **576.3757** | **575.3684** | **574.3512** | **1.0172** | **0** | **13** | **12** | **1** | **K.ALLMK.K** |
|  | 3554 | **445.7682** | **889.5218** | **889.5273** | **-0.0055** | **0** | **31** | **0.14** | **1** | **K.AFALEVIK.S** 3557 |
|  | 4898 | **493.7604** | **985.5061** | **986.4967** | **-0.9906** | **0** | **8** | **29** | **10** | **R.TGAPAAACLR.L** |
|  | 9683 | **646.9993** | **1291.9840** | **1291.6594** | **0.3246** | **1** | **5** | **31** | **3** | **R.WTNAKMEIATK.E** |
|  | 10826 | **678.3566** | **1354.6987** | **1354.7092** | **-0.0105** | **0** | **48** | **0.0022** | **1** | **K.LIAINANDPEASK.F** |
|  | 10914 | **681.3040** | **1360.5935** | **1361.7013** | **-1.1078** | **1** | **3** | **72** | **2** | **K.EPMNPIKQYVK.D** |
|  | 11047 | 457.2375 | 1368.6906 | 1368.7660 | -0.0754 | 1 | 10 | 15 | 8 | R.LLRTGAPAAACLR.L 11072 |
|  | 17418 | **893.4811** | **1784.9476** | **1784.9560** | **-0.0084** | **0** | **69** | **1.4e-005** | **1** | **K.ILGILALIDEGETDWK.L** |

| **485.** | Q16795\|NDUA9_HUMAN    **Mass:** 42654    **Score:** 97     **Queries matched:** 15   **emPAI:** 0.18 |
| --- | --- |
|  | NADH dehydrogenase [ubiquinone] 1 alpha subcomplex subunit 9, mitochondrial OS=Homo sapiens GN=NDUFA9 PE=1 SV=2 |

|  | **Query** | **Observed** | **Mr(expt)** | **Mr(calc)** | **Delta** | **Miss** | **Score** | **Expect** | **Rank** | **Peptide** |
| --- | --- | --- | --- | --- | --- | --- | --- | --- | --- | --- |
|  | 4514 | **479.2636** | **956.5127** | **956.5192** | **-0.0065** | **0** | **8** | **27** | **4** | **R.YVVNHLGR.M** |
|  | 5906 | 531.2165 | 1060.4184 | 1061.6015 | -1.1831 | 1 | 8 | 26 | 7 | R.VVRVLSMSR.S |
|  | 7269 | **582.3113** | **1162.6080** | **1162.6169** | **-0.0088** | **0** | **32** | **0.1** | **1** | **R.MGSQVIIPYR.C** |
|  | 8095 | **605.5485** | **1209.0824** | **1208.5760** | **0.5063** | **0** | **1** | **72** | **2** | **R.FLNSFASMHR.F** |
|  | 9032 | **630.2975** | **1258.5804** | **1258.5870** | **-0.0066** | **0** | **29** | **0.17** | **1** | **K.NFDFEDVFVK.I** |
|  | 9309 | 636.8474 | 1271.6803 | 1270.7074 | 0.9729 | 0 | 7 | 31 | 3 | R.FGPIPLGSLGWK.T |
|  | 9523 | **642.8752** | **1283.7358** | **1283.6557** | **0.0801** | **0** | **10** | **14** | **3** | **R.QLHHALMPHGK.G** 9526 |
|  | 11509 | **697.4195** | **1392.8244** | **1391.7673** | **1.0571** | **0** | **6** | **34** | **1** | **K.FIHVSHLNANIK.S** |
|  | 11578 | **699.8605** | **1397.7064** | **1396.7310** | **0.9754** | **1** | **8** | **19** | **2** | **K.GIVNAVKDPDANGK.S** 11577 |
|  | 13021 | 743.3962 | 1484.7779 | 1484.7875 | -0.0095 | 0 | 59 | 0.00015 | 1 | R.WLSAEIEDVKPAK.T 13022 |
|  | 19231 | **640.3372** | **1917.9897** | **1917.8785** | **0.1111** | **1** | **1** | **87** | **9** | **R.DWETKNFDFEDVFVK.I** |
|  | 27646 | 1021.4736 | 3061.3991 | 3062.5113 | -1.1123 | 1 | 1 | 43 | 6 | R.SAITAIATSVCHGPPCRQLHHALMPHGK.G |

| **486.** | Q9NW15\|ANO10_HUMAN    **Mass:** 76793    **Score:** 96     **Queries matched:** 15   **emPAI:** 0.05 |
| --- | --- |
|  | Anoctamin-10 OS=Homo sapiens GN=ANO10 PE=2 SV=2 |

|  | **Query** | **Observed** | **Mr(expt)** | **Mr(calc)** | **Delta** | **Miss** | **Score** | **Expect** | **Rank** | **Peptide** |
| --- | --- | --- | --- | --- | --- | --- | --- | --- | --- | --- |
|  | 1184 | 577.3271 | 576.3198 | 576.3271 | -0.0073 | 0 | 7 | 41 | 7 | K.LYPGK.S |
|  | 2398 | 408.9307 | 815.8469 | 815.4752 | 0.3717 | 0 | 5 | 48 | 6 | K.LVTENLK.E |
|  | 2848 | 424.7317 | 847.4488 | 847.4626 | -0.0138 | 0 | 6 | 54 | 10 | R.WGTLLMK.R |
|  | 6232 | **542.2550** | **1082.4954** | **1082.5033** | **-0.0078** | **0** | **9** | **19** | **2** | **K.LEDTWYTR.F** |
|  | 7269 | 582.3113 | 1162.6080 | 1162.5176 | 0.0904 | 1 | 7 | 34 | 6 | K.EEPMESGKEK.A |
|  | 11492 | **465.2325** | **1392.6757** | **1391.6755** | **1.0002** | **1** | **7** | **27** | **5** | **K.DEKMIPGYPQAK.L** 11505 |
|  | 12148 | **477.2839** | **1428.8298** | **1427.7772** | **1.0526** | **0** | **4** | **55** | **4** | **R.LESAYQNHLILK.V** |
|  | 13010 | **495.7583** | **1484.2531** | **1484.8748** | **-0.6218** | **1** | **7** | **17** | **2** | **K.IRMLLGAEAVGLVK.E** 13035 |
|  | 14430 | 790.4143 | 1578.8139 | 1577.8276 | 0.9864 | 1 | 6 | 28 | 4 | K.MIPGYPQAKLYPGK.S |
|  | 21167 | 1050.0112 | 2098.0079 | 2098.0218 | -0.0139 | 0 | 96 | 2.2e-008 | 1 | K.YEQETLENQNLYLVGASK.I |
|  | 22036 | **727.4122** | **2179.2149** | **2179.2253** | **-0.0104** | **0** | **7** | **16** | **1** | **R.LLTSGIVIQVFPLHDSEALK.K** |
|  | 26186 | **691.5831** | **2762.3034** | **2761.4637** | **0.8397** | **0** | **0** | **54** | **3** | **K.VTLSALDTSESSFTPLVVIELAQDVK.E** |
|  | 26877 | 970.4434 | 2908.3084 | 2909.3000 | -0.9916 | 1 | 4 | 20 | 2 | R.QNFKGFDDNNDDFLTMAECQFIIK.H |

| **487.** | Q15043\|S39AE_HUMAN    **Mass:** 54902    **Score:** 96     **Queries matched:** 5   **emPAI:** 0.14 |
| --- | --- |
|  | Zinc transporter ZIP14 OS=Homo sapiens GN=SLC39A14 PE=1 SV=2 |

|  | **Query** | **Observed** | **Mr(expt)** | **Mr(calc)** | **Delta** | **Miss** | **Score** | **Expect** | **Rank** | **Peptide** |
| --- | --- | --- | --- | --- | --- | --- | --- | --- | --- | --- |
|  | 9124 | 632.3207 | 1262.6269 | 1262.7095 | -0.0826 | 0 | 4 | 52 | 10 | K.ALLNHLDVGVGR.G |
|  | 12477 | 726.3676 | 1450.7207 | 1450.7304 | -0.0097 | 0 | 51 | 0.0009 | 1 | R.YGEGDSLTLQQLK.A |
|  | 20715 | 1027.4968 | 2052.9791 | 2052.9150 | 0.0641 | 0 | 1 | 75 | 5 | K.NEHHHGHSHYASESLPSK.K |
|  | 23204 | **1161.5706** | **2321.1266** | **2320.1369** | **0.9897** | **0** | **72** | **4.6e-006** | **1** | **R.IGSSELQEFCPTILQQLDSR.A** |
|  | 29705 | **1834.2929** | **3666.5711** | **3666.0181** | **0.5531** | **0** | **0** | **26** | **4** | **K.GSILIPFIIQNLGLLTGFTIMVVLTMYSGQIQIG.-** |

| **488.** | O00151\|PDLI1_HUMAN    **Mass:** 36505    **Score:** 96     **Queries matched:** 7   **emPAI:** 0.10 |
| --- | --- |
|  | PDZ and LIM domain protein 1 OS=Homo sapiens GN=PDLIM1 PE=1 SV=4 |

|  | **Query** | **Observed** | **Mr(expt)** | **Mr(calc)** | **Delta** | **Miss** | **Score** | **Expect** | **Rank** | **Peptide** |
| --- | --- | --- | --- | --- | --- | --- | --- | --- | --- | --- |
|  | 687 | **474.2792** | **473.2719** | **472.3009** | **0.9710** | **0** | **7** | **68** | **9** | **R.LVGGK.D** |
|  | 7421 | **588.3026** | **1174.5905** | **1174.5982** | **-0.0077** | **0** | **14** | **7** | **2** | **K.DFEQPLAISR.V** |
|  | 14470 | **528.2761** | **1581.8064** | **1582.8024** | **-0.9961** | **1** | **4** | **46** | **10** | **K.MLQEKQELNEPPK.Q** |
|  | 14682 | **799.4285** | **1596.8425** | **1597.7406** | **-0.8981** | **0** | **0** | **1.1e+002** | **2** | **R.SAMPFTASPASSTTAR.V** |
|  | 25359 | **876.4268** | **2626.2585** | **2627.3026** | **-1.0442** | **1** | **3** | **28** | **2** | **R.HPYKMNLASEPQEVLHIGSAHNR.S** |
|  | 28615 | 1104.8646 | 3311.5720 | 3311.5874 | -0.0154 | 0 | 63 | 2.4e-005 | 1 | K.AALANLCIGDVITAIDGENTSNMTHLEAQNR.I 28616 |

| **489.** | Q9BWM7\|SFXN3_HUMAN    **Mass:** 35823    **Score:** 95     **Queries matched:** 14   **emPAI:** 0.22 |
| --- | --- |
|  | Sideroflexin-3 OS=Homo sapiens GN=SFXN3 PE=2 SV=1 |

|  | **Query** | **Observed** | **Mr(expt)** | **Mr(calc)** | **Delta** | **Miss** | **Score** | **Expect** | **Rank** | **Peptide** |
| --- | --- | --- | --- | --- | --- | --- | --- | --- | --- | --- |
|  | 3517 | **444.2373** | **886.4600** | **887.5341** | **-1.0741** | **0** | **12** | **13** | **4** | **K.HLPPLVGR.F** 3546 |
|  | 11089 | **686.4146** | **1370.8147** | **1370.7517** | **0.0629** | **0** | **0** | **1.2e+002** | **4** | **R.NLLLSGAQLEASR.N** |
|  | 14461 | **791.4089** | **1580.8032** | **1580.8158** | **-0.0126** | **0** | **31** | **0.1** | **1** | **R.ELQVGIPVADEAGQR.L** |
|  | 15393 | **410.9469** | **1639.7584** | **1639.8239** | **-0.0656** | **0** | **4** | **51** | **8** | **-.MGELPLDINIQEPR.W** |
|  | 15417 | 821.4283 | 1640.8421 | 1640.8522 | -0.0101 | 0 | 62 | 8.1e-005 | 1 | R.AGVVTPGITEDQLWR.A 15402 15418 |
|  | 17978 | 914.4666 | 1826.9187 | 1826.8475 | 0.0711 | 1 | 6 | 23 | 2 | R.AKYVYDSAFHPDTGEK.V 17972 |
|  | 18540 | 622.3350 | 1863.9832 | 1864.9755 | -0.9923 | 1 | 8 | 17 | 4 | R.QRELQVGIPVADEAGQR.L |
|  | 20033 | **665.0162** | **1992.0267** | **1992.0892** | **-0.0625** | **0** | **1** | **74** | **9** | **R.QLGTAYVSATTGAVATALGLK.S** |
|  | 23755 | **800.4476** | **2398.3209** | **2399.0452** | **-0.7243** | **0** | **2** | **46** | **7** | **R.MSAQVPMNMTITGCMLTFYR.K** |
|  | 23765 | **801.0574** | **2400.1503** | **2399.0452** | **1.1051** | **0** | **(1)** | **63** | **5** | **R.MSAQVPMNMTITGCMLTFYR.K** |

| **490.** | Q02318\|CP27A_HUMAN    **Mass:** 60595    **Score:** 95     **Queries matched:** 16   **emPAI:** 0.13 |
| --- | --- |
|  | Sterol 26-hydroxylase, mitochondrial OS=Homo sapiens GN=CYP27A1 PE=1 SV=1 |

|  | **Query** | **Observed** | **Mr(expt)** | **Mr(calc)** | **Delta** | **Miss** | **Score** | **Expect** | **Rank** | **Peptide** |
| --- | --- | --- | --- | --- | --- | --- | --- | --- | --- | --- |
|  | 2868 | **425.0563** | **848.0980** | **848.3997** | **-0.3016** | **0** | **5** | **39** | **6** | **-.MAALGCAR.L** |
|  | 6322 | **545.2667** | **1088.5188** | **1087.6502** | **0.8686** | **1** | **8** | **26** | **5** | **K.KVGLQFLQR.Q** |
|  | 6680 | **559.3162** | **1116.6178** | **1117.5848** | **-0.9671** | **1** | **11** | **12** | **8** | **-.MAALGCARLR.W** |
|  | 11815 | **471.2493** | **1410.7262** | **1409.7990** | **0.9271** | **1** | **5** | **43** | **8** | **R.SLEEIPRLGQLR.F** |
|  | 11902 | **472.7704** | **1415.2894** | **1414.7853** | **0.5041** | **0** | **10** | **7.7** | **2** | **R.IAELEMQLLLAR.L** 11885 11901 |
|  | 13532 | **759.4475** | **1516.8805** | **1516.7351** | **0.1454** | **0** | **20** | **1.2** | **2** | **R.YLDGWNAIFSFGK.K** |
|  | 26099 | **915.1233** | **2742.3480** | **2742.3574** | **-0.0094** | **0** | **48** | **0.001** | **1** | **R.LLKPAEAALYTDAFNEVIDDFMTR.L** |
|  | 28422 | **1083.8729** | **3248.5969** | **3248.6097** | **-0.0128** | **0** | **53** | **0.00025** | **1** | **R.EAMGSLPELLMAGVDTTSNTLTWALYHLSK.D** 28423 28424 |
|  | 28429 | **650.9257** | **3249.5922** | **3250.5436** | **-0.9513** | **0** | **1** | **32** | **2** | **K.YGPMWMSYLGPQMHVNLASAPLLEQVMR.Q** |
|  | 29099 | 867.9406 | 3467.7331 | 3468.7347 | -1.0016 | 1 | 1 | 32 | 2 | R.QALNQRLLKPAEAALYTDAFNEVIDDFMTR.L |
|  | 29991 | **1258.6491** | **3772.9253** | **3772.9247** | **0.0006** | **1** | **6** | **8.1** | **1** | **K.DPEIQEALHEEVVGVVPAGQVPQHKDFAHMPLLK.A** 29969 |

| **491.** | P52815\|RM12_HUMAN    **Mass:** 21563    **Score:** 94     **Queries matched:** 5   **emPAI:** 0.39 |
| --- | --- |
|  | 39S ribosomal protein L12, mitochondrial OS=Homo sapiens GN=MRPL12 PE=1 SV=2 |

|  | **Query** | **Observed** | **Mr(expt)** | **Mr(calc)** | **Delta** | **Miss** | **Score** | **Expect** | **Rank** | **Peptide** |
| --- | --- | --- | --- | --- | --- | --- | --- | --- | --- | --- |
|  | 6607 | **556.3414** | **1110.6682** | **1110.5101** | **0.1581** | **1** | **2** | **95** | **5** | **R.HMRSSGHQR.C** |
|  | 10917 | **681.3152** | **1360.6159** | **1359.7510** | **0.8649** | **0** | **2** | **90** | **2** | **K.NYIQGINLVQAK.K** |
|  | 25918 | **1355.2616** | **2708.5086** | **2708.5211** | **-0.0125** | **0** | **90** | **5.2e-008** | **1** | **K.IQQLVQDIASLTLLEISDLNELLK.K** |
|  | 26514 | **946.5422** | **2836.6049** | **2836.6161** | **-0.0112** | **1** | **23** | **0.23** | **1** | **K.IQQLVQDIASLTLLEISDLNELLKK.T** |
|  | 29577 | **727.9932** | **3634.9298** | **3633.8382** | **1.0916** | **1** | **2** | **20** | **2** | **K.IQDVGLVPMGGVMSGAVPAAAAQEAVEEDIPIAKER.T** |

| **492.** | Q9Y4W6\|AFG32_HUMAN    **Mass:** 88984    **Score:** 94     **Queries matched:** 25   **emPAI:** 0.04 |
| --- | --- |
|  | AFG3-like protein 2 OS=Homo sapiens GN=AFG3L2 PE=1 SV=2 |

|  | **Query** | **Observed** | **Mr(expt)** | **Mr(calc)** | **Delta** | **Miss** | **Score** | **Expect** | **Rank** | **Peptide** |
| --- | --- | --- | --- | --- | --- | --- | --- | --- | --- | --- |
|  | 1714 | **702.3847** | **701.3774** | **700.4119** | **0.9655** | **0** | **9** | **31** | **1** | **R.LEVVNK.R** |
|  | 1981 | 785.3885 | 784.3812 | 784.3603 | 0.0209 | 0 | 6 | 39 | 5 | K.EEPPGEK.V |
|  | 2779 | **422.0044** | **841.9942** | **842.5225** | **-0.5283** | **1** | **9** | **15** | **5** | **R.VIGGLEKK.T** 2770 2784 2787 |
|  | 4160 | **467.2229** | **932.4313** | **932.4424** | **-0.0111** | **1** | **4** | **81** | **9** | **R.SSGGGGGGGGKR.G** |
|  | 4253 | 940.4562 | 939.4490 | 940.3929 | -0.9439 | 0 | 8 | 25 | 10 | R.MCMTLGGR.V |
|  | 6001 | **534.8022** | **1067.5898** | **1067.5975** | **-0.0077** | **0** | **3** | **72** | **8** | **K.GAILTGPPGTGK.T** |
|  | 6068 | **536.7107** | **1071.4069** | **1070.5608** | **0.8462** | **0** | **7** | **37** | **9** | **K.TQVLQPEEK.K** |
|  | 6217 | **541.7686** | **1081.5225** | **1082.5397** | **-1.0171** | **0** | **4** | **63** | **8** | **R.VSEEIFFGR.I** |
|  | 6544 | **553.8469** | **1105.6793** | **1104.6291** | **1.0502** | **1** | **2** | **1.1e+002** | **8** | **R.ILINDAYKR.T** |
|  | 7613 | 594.2828 | 1186.5511 | 1185.5818 | 0.9693 | 1 | 1 | 1.3e+002 | 9 | K.GFEKYFPNGK.N |
|  | 8919 | **627.3611** | **1252.7077** | **1252.5758** | **0.1320** | **1** | **12** | **8.5** | **1** | **K.FKDVAGCEEAK.L** |
|  | 9036 | **630.3244** | **1258.6342** | **1259.6874** | **-1.0531** | **1** | **3** | **82** | **3** | **K.QYQDLGAKIPK.G** |
|  | 10554 | 447.2668 | 1338.7786 | 1339.7612 | -0.9826 | 1 | 7 | 25 | 7 | R.QIFIGPPDIKGR.A |
|  | 12899 | **739.8568** | **1477.6989** | **1476.7936** | **0.9053** | **0** | **1** | **1.1e+002** | **3** | **R.NSLLTDIIAAYQR.F** |
|  | 16011 | **562.0358** | **1683.0857** | **1683.8250** | **-0.7394** | **1** | **1** | **67** | **8** | **R.TGRGMGGLFSVGETTAK.V** |
|  | 16154 | **848.2097** | **1694.4049** | **1694.7902** | **-0.3853** | **1** | **5** | **23** | **1** | **K.EQLLDRMCMTLGGR.V** |
|  | 17533 | **598.9573** | **1793.8500** | **1793.8618** | **-0.0118** | **0** | **11** | **8.2** | **1** | **R.QGDMVLEKPYSEATAR.L** |
|  | 18197 | **921.9570** | **1841.8994** | **1841.9119** | **-0.0125** | **0** | **94** | **3.9e-008** | **1** | **R.NLETLQQELGIEGENR.V** |
|  | 19360 | **643.9672** | **1928.8798** | **1929.9982** | **-1.1184** | **1** | **2** | **65** | **1** | **K.NAPCILFIDEIDAVGRK.R** |
|  | 19373 | **644.3365** | **1929.9876** | **1929.9982** | **-0.0106** | **1** | **6** | **29** | **2** | **R.KNAPCILFIDEIDAVGR.K** 19363 |
|  | 20927 | 692.7316 | 2075.1729 | 2074.0994 | 1.0735 | 0 | 8 | 14 | 2 | R.GLQQLLVPGGVGPGEQPCLR.T |

| **493.** | Q9C002\|NMES1_HUMAN    **Mass:** 9611     **Score:** 94     **Queries matched:** 6   **emPAI:** 1.85 |
| --- | --- |
|  | Normal mucosa of esophagus-specific gene 1 protein OS=Homo sapiens GN=NMES1 PE=2 SV=1 |

|  | **Query** | **Observed** | **Mr(expt)** | **Mr(calc)** | **Delta** | **Miss** | **Score** | **Expect** | **Rank** | **Peptide** |
| --- | --- | --- | --- | --- | --- | --- | --- | --- | --- | --- |
|  | 5272 | **507.2751** | **1012.5355** | **1012.5416** | **-0.0060** | **0** | **28** | **0.25** | **1** | **M.SFFQLLMK.R** 5273 |
|  | 5480 | 515.2628 | 1028.5111 | 1028.5365 | -0.0254 | 0 | (4) | 67 | 9 | M.SFFQLLMK.R |
|  | 23409 | **1175.6400** | **2349.2655** | **2349.2804** | **-0.0150** | **0** | **58** | **0.00011** | **1** | **K.LITINQQWKPIEELQNVQR.V** 23410 |
|  | 26232 | 463.0949 | 2772.5260 | 2771.4608 | 1.0652 | 0 | 1 | 37 | 2 | K.ELIPLVVFMTVAAGGASSFAVYSLWK.T |

| **494.** | Q9BX93\|PG12B_HUMAN    **Mass:** 22443    **Score:** 94     **Queries matched:** 2   **emPAI:** 0.17 |
| --- | --- |
|  | Group XIIB secretory phospholipase A2-like protein OS=Homo sapiens GN=PLA2G12B PE=1 SV=2 |

|  | **Query** | **Observed** | **Mr(expt)** | **Mr(calc)** | **Delta** | **Miss** | **Score** | **Expect** | **Rank** | **Peptide** |
| --- | --- | --- | --- | --- | --- | --- | --- | --- | --- | --- |
|  | 22165 | **1098.5249** | **2195.0352** | **2195.0423** | **-0.0070** | **0** | **94** | **3.3e-008** | **1** | **R.GSFESVNSYFDSFLELLGGK.N** |
|  | 30610 | **1037.9989** | **4147.9665** | **4149.0196** | **-1.0531** | **1** | **1** | **21** | **3** | **K.APMPRPGYKPQEPNGCGSYFLGLKVPESMDLGIPAMTK.C** |

| **495.** | O75477\|ERLN1_HUMAN    **Mass:** 39072    **Score:** 94     **Queries matched:** 14   **emPAI:** 0.20 |
| --- | --- |
|  | Erlin-1 OS=Homo sapiens GN=ERLIN1 PE=1 SV=1 |

|  | **Query** | **Observed** | **Mr(expt)** | **Mr(calc)** | **Delta** | **Miss** | **Score** | **Expect** | **Rank** | **Peptide** |
| --- | --- | --- | --- | --- | --- | --- | --- | --- | --- | --- |
|  | 1831 | **734.4390** | **733.4318** | **734.4327** | **-1.0009** | **0** | **28** | **0.35** | **1** | **K.TLIFNK.I** |
|  | 3521 | **444.2605** | **886.5065** | **886.5123** | **-0.0058** | **1** | **16** | **5** | **7** | **K.KAVIEAEK.I** |
|  | 4844 | **491.7083** | **981.4021** | **980.4927** | **0.9094** | **0** | **7** | **31** | **8** | **K.YQAIASNSK.I** |
|  | 5919 | 531.2996 | 1060.5846 | 1060.5513 | 0.0333 | 1 | 6 | 46 | 5 | R.TGRESSLPSK.E |
|  | 9183 | **633.8171** | **1265.6197** | **1265.6074** | **0.0124** | **1** | **5** | **40** | **4** | **R.RNFELMEAEK.T** |
|  | 10535 | 669.8175 | 1337.6204 | 1338.6489 | -1.0285 | 1 | 6 | 37 | 5 | R.NFELMEAEKTK.L |
|  | 10705 | **674.8472** | **1347.6799** | **1347.6882** | **-0.0083** | **0** | **23** | **0.74** | **1** | **R.SVQTTLQTDEVK.N** |
|  | 10802 | **677.7750** | **1353.5354** | **1354.6438** | **-1.1085** | **1** | **(4)** | **46** | **3** | **R.NFELMEAEKTK.L** |
|  | 11027 | 684.8312 | 1367.6479 | 1368.7976 | -1.1497 | 1 | 6 | 34 | 3 | K.AVIEAEKIAQVAK.I 11026 |
|  | 13089 | 497.2614 | 1488.7624 | 1489.7888 | -1.0264 | 1 | 8 | 23 | 9 | K.RISEIEDAAFLAR.E 13073 |
|  | 19763 | **655.9914** | **1964.9523** | **1964.0441** | **0.9082** | **0** | **2** | **55** | **1** | **R.IEVVNMLAPYAVFDIVR.N** |
|  | 21887 | **1082.0100** | **2162.0055** | **2162.0176** | **-0.0122** | **0** | **92** | **5.7e-008** | **1** | **K.IYFGSNIPNMFVDSSCALK.Y** |

| **496.** | P18669\|PGAM1_HUMAN    **Mass:** 28900    **Score:** 93     **Queries matched:** 18   **emPAI:** 1.68 |
| --- | --- |
|  | Phosphoglycerate mutase 1 OS=Homo sapiens GN=PGAM1 PE=1 SV=2 |

|  | **Query** | **Observed** | **Mr(expt)** | **Mr(calc)** | **Delta** | **Miss** | **Score** | **Expect** | **Rank** | **Peptide** |
| --- | --- | --- | --- | --- | --- | --- | --- | --- | --- | --- |
|  | 1250 | 590.3295 | 589.3222 | 589.3071 | 0.0151 | 0 | 16 | 6.9 | 3 | K.AETAAK.H |
|  | 1357 | **614.1743** | **613.1670** | **612.4323** | **0.7348** | **0** | **3** | **62** | **1** | **K.LVLIR.H** |
|  | 4752 | **488.2468** | **974.4790** | **974.4855** | **-0.0065** | **0** | **40** | **0.021** | **1** | **K.AMEAVAAQGK.A** 4758 |
|  | 5875 | **530.2792** | **1058.5438** | **1058.5509** | **-0.0071** | **0** | **37** | **0.041** | **1** | **R.HYGGLTGLNK.A** 5874 |
|  | 7098 | **575.8344** | **1149.6543** | **1149.6618** | **-0.0076** | **0** | **34** | **0.056** | **1** | **R.VLIAAHGNSLR.G** 7078 |
|  | 9948 | **436.5701** | **1306.6885** | **1305.7629** | **0.9255** | **1** | **8** | **21** | **6** | **K.RVLIAAHGNSLR.G** |
|  | 10027 | **656.8007** | **1311.5869** | **1311.5956** | **-0.0087** | **0** | **44** | **0.0053** | **1** | **R.HGESAWNLENR.F** 10028 |
|  | 16025 | 842.9556 | 1683.8967 | 1682.9032 | 0.9935 | 0 | 37 | 0.024 | 1 | R.ALPFWNEEIVPQIK.E |
|  | 18571 | **934.4937** | **1866.9729** | **1867.8509** | **-0.8781** | **0** | **2** | **63** | **5** | **R.YADLTEDQLPSCESLK.D** 18600 |
|  | 19080 | **636.3311** | **1905.9713** | **1906.0173** | **-0.0460** | **1** | **3** | **55** | **4** | **K.LVLIRHGESAWNLENR.F** |
|  | 21822 | **719.3879** | **2155.1418** | **2155.1500** | **-0.0082** | **0** | **32** | **0.059** | **1** | **R.TLWTVLDAIDQMWLPVVR.T** |
|  | 23950 | **809.0516** | **2424.1331** | **2424.1478** | **-0.0147** | **1** | **25** | **0.2** | **1** | **R.YADLTEDQLPSCESLKDTIAR.A** |
|  | 31492 | **1718.0429** | **5151.1067** | **5150.6559** | **0.4508** | **1** | **1** | **7.6** | **1** | **K.HLEGLSEEAIMELNLPTGIPIVYELDKNLKPIKPMQFLGDEETVR.K** |

| **497.** | P52565\|GDIR1_HUMAN    **Mass:** 23250    **Score:** 93     **Queries matched:** 7   **emPAI:** 0.36 |
| --- | --- |
|  | Rho GDP-dissociation inhibitor 1 OS=Homo sapiens GN=ARHGDIA PE=1 SV=3 |

|  | **Query** | **Observed** | **Mr(expt)** | **Mr(calc)** | **Delta** | **Miss** | **Score** | **Expect** | **Rank** | **Peptide** |
| --- | --- | --- | --- | --- | --- | --- | --- | --- | --- | --- |
|  | 1048 | **548.3151** | **547.3078** | **546.2948** | **1.0131** | **0** | **13** | **14** | **5** | **K.GMLAR.G** |
|  | 1848 | 740.4252 | 739.4180 | 739.3025 | 0.1155 | 0 | 4 | 50 | 6 | R.FTDDDK.T |
|  | 3088 | **431.8873** | **861.7600** | **861.4192** | **0.3409** | **1** | **14** | **4.9** | **3** | **K.DDESLRK.Y** |
|  | 9107 | **631.8256** | **1261.6366** | **1260.5445** | **1.0921** | **0** | **4** | **61** | **5** | **K.TDYMVGSYGPR.A** |
|  | 14732 | **534.2613** | **1599.7620** | **1600.7555** | **-0.9935** | **1** | **5** | **35** | **3** | **K.IDKTDYMVGSYGPR.A** |
|  | 16931 | **876.4171** | **1750.8197** | **1750.8301** | **-0.0104** | **0** | **66** | **2.5e-005** | **1** | **R.AEEYEFLTPVEEAPK.G** |
|  | 19222 | **959.5065** | **1916.9985** | **1916.9327** | **0.0658** | **1** | **51** | **0.00073** | **1** | **K.SIQEIQELDKDDESLR.K** |

| **498.** | O95292\|VAPB_HUMAN    **Mass:** 27439    **Score:** 93     **Queries matched:** 13   **emPAI:** 0.48 |
| --- | --- |
|  | Vesicle-associated membrane protein-associated protein B/C OS=Homo sapiens GN=VAPB PE=1 SV=3 |

|  | **Query** | **Observed** | **Mr(expt)** | **Mr(calc)** | **Delta** | **Miss** | **Score** | **Expect** | **Rank** | **Peptide** |
| --- | --- | --- | --- | --- | --- | --- | --- | --- | --- | --- |
|  | 1031 | 546.3209 | 545.3136 | 544.2969 | 1.0167 | 0 | 12 | 24 | 4 | K.TTAPR.R |
|  | 2440 | **820.4719** | **819.4646** | **819.4702** | **-0.0056** | **0** | **29** | **0.23** | **1** | **K.IISTTASK.T** |
|  | 4878 | **492.7390** | **983.4635** | **984.5465** | **-1.0829** | **1** | **10** | **16** | **2** | **K.RLQGEVQR.L** |
|  | 9453 | **641.2715** | **1280.5284** | **1279.6143** | **0.9141** | **0** | **3** | **63** | **6** | **K.SLSSSLDDTEVK.K** |
|  | 11744 | **704.8572** | **1407.6998** | **1407.7093** | **-0.0095** | **1** | **57** | **0.00026** | **1** | **K.SLSSSLDDTEVKK.V** |
|  | 13711 | 766.4059 | 1530.7972 | 1530.7711 | 0.0261 | 1 | 5 | 43 | 3 | K.EAKPEDLMDSKLR.C |
|  | 17782 | **605.3315** | **1812.9726** | **1813.1845** | **-0.2119** | **0** | **0** | **91** | **3** | **R.LLALVVLFFIVGVIIGK.I** |
|  | 20623 | **682.3831** | **2044.1274** | **2044.0589** | **0.0684** | **1** | **3** | **49** | **9** | **R.GPFTDVVTTNLKLGNPTDR.N** |
|  | 23193 | **1161.0123** | **2320.0101** | **2320.0214** | **-0.0113** | **0** | **57** | **0.00016** | **1** | **K.FMVQSMFAPTDTSDMEAVWK.E** |
|  | 25294 | 655.0533 | 2616.1840 | 2617.1651 | -0.9811 | 1 | 0 | 64 | 9 | K.HKFMVQSMFAPTDTSDMEAVWK.E |
|  | 29539 | **907.6619** | **3626.6184** | **3627.5765** | **-0.9581** | **1** | **0** | **32** | **10** | **K.FMVQSMFAPTDTSDMEAVWKEAKPEDLMDSK.L** |
|  | 29889 | **1247.5768** | **3739.7085** | **3740.7927** | **-1.0842** | **1** | **4** | **13** | **1** | **R.RYCVRPNSGIIDAGASINVSVMLQPFDYDPNEK.S** 29890 |

| **499.** | O00161\|SNP23_HUMAN    **Mass:** 23682    **Score:** 93     **Queries matched:** 5   **emPAI:** 0.16 |
| --- | --- |
|  | Synaptosomal-associated protein 23 OS=Homo sapiens GN=SNAP23 PE=1 SV=1 |

|  | **Query** | **Observed** | **Mr(expt)** | **Mr(calc)** | **Delta** | **Miss** | **Score** | **Expect** | **Rank** | **Peptide** |
| --- | --- | --- | --- | --- | --- | --- | --- | --- | --- | --- |
|  | 17685 | **904.3937** | **1806.7729** | **1806.7843** | **-0.0114** | **0** | **61** | **8.6e-005** | **1** | **K.TTWGDGGENSPCNVVSK.Q** 17684 |
|  | 20111 | 1000.4917 | 1998.9688 | 1998.9680 | 0.0008 | 0 | 1 | 69 | 6 | K.DMALNIGNEIDAQNPQIK.R |
|  | 21572 | 1067.9907 | 2133.9669 | 2134.0099 | -0.0431 | 0 | 0 | 87 | 10 | R.EDEMEENLTQVGSILGNLK.D |
|  | 26312 | 930.4825 | 2788.4256 | 2788.3549 | 0.0708 | 1 | 2 | 36 | 2 | R.ITNDAREDEMEENLTQVGSILGNLK.D |

| **500.** | P35613\|BASI_HUMAN    **Mass:** 42573    **Score:** 92     **Queries matched:** 10   **emPAI:** 0.29 |
| --- | --- |
|  | Basigin OS=Homo sapiens GN=BSG PE=1 SV=2 |

|  | **Query** | **Observed** | **Mr(expt)** | **Mr(calc)** | **Delta** | **Miss** | **Score** | **Expect** | **Rank** | **Peptide** |
| --- | --- | --- | --- | --- | --- | --- | --- | --- | --- | --- |
|  | 4601 | **482.7112** | **963.4079** | **963.5362** | **-0.1283** | **1** | **11** | **13** | **4** | **R.NHLTRAPR.V** |
|  | 5280 | **1014.4932** | **1013.4859** | **1013.4931** | **-0.0072** | **0** | **26** | **0.43** | **1** | **R.FFVSSSQGR.S** |
|  | 6098 | **537.2978** | **1072.5810** | **1072.5877** | **-0.0066** | **0** | **33** | **0.11** | **1** | **K.GSDQAIITLR.V** |
|  | 15732 | **832.8961** | **1663.7776** | **1663.7882** | **-0.0107** | **0** | **4** | **42** | **7** | **K.SESVPPVTDWAWYK.I** |
|  | 17191 | **590.6578** | **1768.9515** | **1767.7945** | **1.1570** | **1** | **5** | **34** | **1** | **K.ITDSEDKALMNGSESR.F** |
|  | 17648 | **902.9081** | **1803.8016** | **1803.8131** | **-0.0115** | **0** | **73** | **5.8e-006** | **1** | **K.SSEHINEGETAMLVCK.S** 17647 |
|  | 21411 | **705.9924** | **2114.9553** | **2114.9691** | **-0.0138** | **0** | **25** | **0.29** | **1** | **R.SELHIENLNMEADPGQYR.C** |
|  | 27867 | **1042.7881** | **3125.3424** | **3126.4465** | **-1.1040** | **1** | **1** | **33** | **7** | **R.FFVSSSQGRSELHIENLNMEADPGQYR.C** |
|  | 29230 | **1172.5320** | **3514.5741** | **3514.5922** | **-0.0181** | **0** | **42** | **0.0024** | **1** | **K.VDSDDQWGEYSCVFLPEPMGTANIQLHGPPR.V** |

| **501.** | Q96K78\|GP128_HUMAN    **Mass:** 89992    **Score:** 92     **Queries matched:** 14   **emPAI:** 0.04 |
| --- | --- |
|  | Probable G-protein coupled receptor 128 OS=Homo sapiens GN=GPR128 PE=1 SV=2 |

|  | **Query** | **Observed** | **Mr(expt)** | **Mr(calc)** | **Delta** | **Miss** | **Score** | **Expect** | **Rank** | **Peptide** |
| --- | --- | --- | --- | --- | --- | --- | --- | --- | --- | --- |
|  | 4962 | **496.2179** | **990.4212** | **989.4315** | **0.9897** | **0** | **3** | **89** | **6** | **R.NGGTWENGR.C** |
|  | 6515 | **552.8021** | **1103.5896** | **1103.6373** | **-0.0477** | **0** | **9** | **23** | **2** | **K.VLMLLSSIGR.R** |
|  | 6755 | 562.2991 | 1122.5836 | 1123.5873 | -1.0037 | 1 | 3 | 83 | 4 | R.TKVFQSEASK.V |
|  | 8726 | **414.5570** | **1240.6491** | **1241.5823** | **-0.9331** | **0** | **(10)** | **16** | **2** | **K.DTPNAGNPMAVR.L** |
|  | 9016 | 629.8410 | 1257.6674 | 1257.5772 | 0.0903 | 0 | 11 | 13 | 4 | K.DTPNAGNPMAVR.L 9017 |
|  | 10404 | **666.3524** | **1330.6902** | **1329.6024** | **1.0878** | **0** | **2** | **1e+002** | **5** | **K.TCGFVVYQNDK.L** |
|  | 16678 | **867.4169** | **1732.8192** | **1732.8301** | **-0.0110** | **0** | **69** | **1.4e-005** | **1** | **K.VTIGNCNENLETLEK.Q** 16675 |
|  | 17312 | **593.3363** | **1776.9871** | **1775.9141** | **1.0730** | **1** | **1** | **92** | **6** | **K.MYNFLRSLPTLHER.F** |
|  | 17749 | 906.4376 | 1810.8606 | 1810.8858 | -0.0253 | 1 | 6 | 25 | 4 | R.CNHTTNFAVLMTFKK.D 17748 |
|  | 19413 | 645.3388 | 1932.9946 | 1932.9404 | 0.0542 | 1 | 1 | 92 | 10 | K.TCGFVVYQNDKLFQSK.T |
|  | 25294 | 655.0533 | 2616.1840 | 2615.1719 | 1.0121 | 1 | 5 | 21 | 4 | K.GLRCTIANFCENSTYMGFTFAR.I |

| **502.** | Q14156\|EFR3A_HUMAN    **Mass:** 93777    **Score:** 91     **Queries matched:** 13   **emPAI:** 0.04 |
| --- | --- |
|  | Protein EFR3 homolog A OS=Homo sapiens GN=EFR3A PE=1 SV=2 |

|  | **Query** | **Observed** | **Mr(expt)** | **Mr(calc)** | **Delta** | **Miss** | **Score** | **Expect** | **Rank** | **Peptide** |
| --- | --- | --- | --- | --- | --- | --- | --- | --- | --- | --- |
|  | 1238 | **588.3679** | **587.3606** | **586.3438** | **1.0168** | **0** | **11** | **24** | **4** | **R.ELLGR.A** |
|  | 1504 | **648.3307** | **647.3234** | **647.3135** | **0.0099** | **0** | **4** | **89** | **9** | **K.CMLPK.S** |
|  | 3507 | **443.7559** | **885.4972** | **885.5470** | **-0.0497** | **0** | **9** | **22** | **2** | **R.IQIMLLR.S** |
|  | 4882 | **492.7916** | **983.5686** | **983.4746** | **0.0940** | **1** | **11** | **13** | **8** | **R.QDTSFMKK.N** |
|  | 8416 | **409.4393** | **1225.2962** | **1224.6390** | **0.6571** | **0** | **4** | **39** | **5** | **K.LTFYAVSAPEK.L** |
|  | 12037 | **475.2840** | **1422.8301** | **1423.6943** | **-0.8642** | **0** | **10** | **13** | **1** | **K.IAESLGGSGYSVER.L** |
|  | 14435 | 790.4898 | 1578.9651 | 1578.9708 | -0.0058 | 0 | 91 | 8.2e-008 | 1 | R.AGIIQVLLEAVAIAAK.G |
|  | 15022 | **540.2624** | **1617.7655** | **1616.8046** | **0.9609** | **0** | **5** | **42** | **7** | **R.LSVPYVPQVTDEDR.L** |
|  | 16057 | 844.4251 | 1686.8357 | 1686.9556 | -0.1200 | 0 | 8 | 19 | 4 | K.GSIGPTVLEVFNTLLK.H |
|  | 17992 | **610.2869** | **1827.8390** | **1828.8917** | **-1.0527** | **1** | **11** | **8.1** | **1** | **K.TDMEKLTFYAVSAPEK.L** |
|  | 20080 | 666.3378 | 1995.9917 | 1997.1053 | -1.1137 | 1 | 0 | 92 | 9 | R.IQIMLLRSLLMVTSGYK.A |
|  | 23156 | **772.4508** | **2314.3306** | **2313.1965** | **1.1341** | **0** | **4** | **26** | **2** | **K.VPVFGTSTHTLDISQLGDLGTR.R** |
|  | 26811 | **724.3373** | **2893.3203** | **2892.4830** | **0.8372** | **0** | **3** | **26** | **7** | **K.TIVTALPGSFLDPLLSPSLMEDYELR.Q** |

| **503.** | P61006\|RAB8A_HUMAN    **Mass:** 23824    **Score:** 91     **Queries matched:** 14   **emPAI:** 0.56 |
| --- | --- |
|  | Ras-related protein Rab-8A OS=Homo sapiens GN=RAB8A PE=1 SV=1 |

|  | **Query** | **Observed** | **Mr(expt)** | **Mr(calc)** | **Delta** | **Miss** | **Score** | **Expect** | **Rank** | **Peptide** |
| --- | --- | --- | --- | --- | --- | --- | --- | --- | --- | --- |
|  | 1624 | **675.3809** | **674.3736** | **674.3785** | **-0.0049** | **0** | **22** | **1.5** | **1** | **K.MILGNK.C** |
|  | 2104 | 398.2102 | 794.4059 | 794.4109 | -0.0050 | 0 | 15 | 5.1 | 4 | K.TCVLFR.F |
|  | 2550 | **414.7477** | **827.4808** | **828.3687** | **-0.8879** | **0** | **10** | **20** | **4** | **K.FMETSAK.A** |
|  | 4921 | 494.7554 | 987.4962 | 987.5025 | -0.0063 | 0 | 21 | 1.6 | 1 | R.TITTAYYR.G |
|  | 6064 | 536.3201 | 1070.6257 | 1070.6336 | -0.0079 | 0 | 15 | 5.6 | 1 | K.LLLIGDSGVGK.T |
|  | 10115 | 658.8291 | 1315.6436 | 1315.6521 | -0.0084 | 0 | 53 | 0.00078 | 1 | K.LQIWDTAGQER.F 10116 |
|  | 10580 | 671.3132 | 1340.6118 | 1340.6208 | -0.0090 | 0 | 7 | 30 | 3 | R.NIEEHASADVEK.M |
|  | 11863 | **707.8450** | **1413.6754** | **1413.6848** | **-0.0094** | **0** | **38** | **0.021** | **1** | **K.LEGNSPQGSNQGVK.I** |
|  | 13862 | 771.8914 | 1541.7682 | 1541.7798 | -0.0116 | 1 | 45 | 0.0035 | 1 | K.KLEGNSPQGSNQGVK.I 13875 |
|  | 14415 | 526.9423 | 1577.8050 | 1578.8154 | -1.0105 | 0 | 2 | 84 | 3 | K.ANINVENAFFTLAR.D |
|  | 15799 | **835.5329** | **1669.0512** | **1669.8055** | **-0.7543** | **0** | **3** | **46** | **1** | **R.GAMGIMLVYDITNEK.S** |
|  | 23772 | **801.4252** | **2401.2537** | **2402.1610** | **-0.9073** | **1** | **1** | **65** | **10** | **R.GAMGIMLVYDITNEKSFDNIR.N** |

| **504.** | Q9Y536\|PAL4B_HUMAN    **Mass:** 18398    **Score:** 90     **Queries matched:** 7   **emPAI:** 0.21 |
| --- | --- |
|  | Peptidylprolyl cis-trans isomerase A-like 4B OS=Homo sapiens GN=PPIAL4B PE=1 SV=1 |

|  | **Query** | **Observed** | **Mr(expt)** | **Mr(calc)** | **Delta** | **Miss** | **Score** | **Expect** | **Rank** | **Peptide** |
| --- | --- | --- | --- | --- | --- | --- | --- | --- | --- | --- |
|  | 1689 | 696.3513 | 695.3440 | 695.3490 | -0.0050 | 0 | 7 | 25 | 7 | K.SIYGEK.F |
|  | 2843 | 424.7085 | 847.4024 | 847.4076 | -0.0051 | 0 | 4 | 87 | 9 | K.TEWLDGK.H |
|  | 7068 | **574.7391** | **1147.4637** | **1148.5826** | **-1.1188** | **1** | **5** | **48** | **10** | **K.FDDENLIRK.H** |
|  | 12026 | 712.3354 | 1422.6562 | 1422.7103 | -0.0541 | 1 | 2 | 76 | 7 | K.TAENFRALSTGEK.G |
|  | 14695 | 799.8712 | 1597.7279 | 1597.7381 | -0.0103 | 0 | 58 | 0.00018 | 1 | R.IIPGFMCQGGDFTR.H 14694 |
|  | 14948 | 807.9384 | 1613.8621 | 1613.7331 | 0.1291 | 0 | (6) | 32 | 4 | R.IIPGFMCQGGDFTR.H |

| **505.** | O43795\|MYO1B_HUMAN    **Mass:** 132928   **Score:** 90     **Queries matched:** 23   **emPAI:** 0.03 |
| --- | --- |
|  | Myosin-Ib OS=Homo sapiens GN=MYO1B PE=1 SV=3 |

|  | **Query** | **Observed** | **Mr(expt)** | **Mr(calc)** | **Delta** | **Miss** | **Score** | **Expect** | **Rank** | **Peptide** |
| --- | --- | --- | --- | --- | --- | --- | --- | --- | --- | --- |
|  | 1044 | 547.3414 | 546.3342 | 546.3013 | 0.0328 | 0 | 15 | 9.7 | 9 | R.TVEAK.Q |
|  | 1960 | **774.3759** | **773.3686** | **772.4443** | **0.9244** | **1** | **6** | **56** | **8** | **K.LEDLRK.Q** |
|  | 2689 | **419.2376** | **836.4606** | **835.4473** | **1.0133** | **0** | **6** | **38** | **7** | **K.ASVATLMK.N** |
|  | 2738 | **420.7846** | **839.5545** | **839.4402** | **0.1143** | **1** | **3** | **71** | **8** | **R.RAGYAFR.Q** |
|  | 3849 | 910.4343 | 909.4271 | 909.4821 | -0.0550 | 1 | 11 | 12 | 1 | K.FFRANAGK.K |
|  | 6025 | 535.3394 | 1068.6643 | 1068.5604 | 0.1039 | 0 | 8 | 23 | 3 | K.IYEFTLQR.I 6033 |
|  | 8020 | 603.8289 | 1205.6433 | 1205.6557 | -0.0124 | 0 | 8 | 22 | 2 | K.SQIVIAAWYR.R |
|  | 8376 | 408.5619 | 1222.6638 | 1222.5216 | 0.1422 | 0 | 5 | 42 | 7 | K.YMDIEFDFK.G |
|  | 8497 | 615.8253 | 1229.6361 | 1228.6485 | 0.9876 | 1 | 6 | 36 | 10 | K.NKMPSLSPIDK.N |
|  | 10413 | 444.7279 | 1331.1620 | 1330.7721 | 0.3899 | 1 | 0 | 99 | 10 | R.YLGLLENVRVR.R |
|  | 13661 | 764.4358 | 1526.8571 | 1526.8456 | 0.0115 | 1 | 2 | 78 | 7 | K.SLFPEGNPAKINLK.R |
|  | 14103 | 778.9229 | 1555.8311 | 1554.7065 | 1.1247 | 1 | 8 | 21 | 2 | R.FGKYMDIEFDFK.G |
|  | 16651 | 577.6389 | 1729.8949 | 1728.9774 | 0.9175 | 0 | 1 | 91 | 9 | R.IFLLTNNNLLLADQK.S |
|  | 17374 | **594.7929** | **1781.3569** | **1781.8407** | **-0.4838** | **0** | **1** | **57** | **2** | **K.VSMSSQNDGFFAVHLK.E** |
|  | 18462 | 929.4836 | 1856.9527 | 1856.9632 | -0.0105 | 0 | 63 | 5.5e-005 | 1 | K.EQLLQSNPVLEAFGNAK.T 18477 |
|  | 22268 | **553.0448** | **2208.1501** | **2208.1215** | **0.0286** | **0** | **9** | **9.4** | **1** | **R.NFHVFYQLLSGASEELLNK.L** |
|  | 22586 | **749.1583** | **2244.4530** | **2245.1260** | **-0.6730** | **1** | **0** | **55** | **5** | **K.NELKEICELTGIDQSVLER.A** |
|  | 22706 | 753.6702 | 2257.9887 | 2258.1041 | -0.1155 | 1 | 9 | 10 | 1 | K.GDFLFSSDHLIEMATKLYR.T 22709 |
|  | 26341 | **932.4815** | **2794.4227** | **2793.3973** | **1.0254** | **1** | **1** | **47** | **2** | **K.GPARSGVEVLFNELEIPVEEYSFGR.S** |
|  | 29881 | 934.4349 | 3733.7107 | 3734.8820 | -1.1714 | 1 | 0 | 30 | 4 | K.MEVKTSLLDNMIGVGDMVLLEPLNEETFINNLK.K |

| **506.** | P20340\|RAB6A_HUMAN    **Mass:** 23692    **Score:** 90     **Queries matched:** 10   **emPAI:** 0.35 |
| --- | --- |
|  | Ras-related protein Rab-6A OS=Homo sapiens GN=RAB6A PE=1 SV=3 |

|  | **Query** | **Observed** | **Mr(expt)** | **Mr(calc)** | **Delta** | **Miss** | **Score** | **Expect** | **Rank** | **Peptide** |
| --- | --- | --- | --- | --- | --- | --- | --- | --- | --- | --- |
|  | 3389 | 879.4769 | 878.4696 | 878.4055 | 0.0641 | 0 | 16 | 4.6 | 4 | R.EDMIDIK.L |
|  | 7440 | **588.8312** | **1175.6478** | **1175.6550** | **-0.0072** | **0** | **13** | **7.3** | **1** | **K.LVFLGEQSVGK.T** |
|  | 8878 | **417.7261** | **1250.1566** | **1250.7135** | **-0.5569** | **1** | **10** | **9.6** | **8** | **R.FRSLIPSYIR.D** |
|  | 8899 | **418.2096** | **1251.6069** | **1250.5714** | **1.0356** | **0** | **11** | **12** | **3** | **-.MSTGGDFGNPLR.K** |
|  | 9082 | **631.3337** | **1260.6528** | **1260.6748** | **-0.0220** | **0** | **1** | **1.2e+002** | **6** | **R.GSDVIIMLVGNK.T** |
|  | 10115 | 658.8291 | 1315.6436 | 1315.6521 | -0.0084 | 0 | 53 | 0.00078 | 1 | R.LQLWDTAGQER.F 10116 |
|  | 11276 | **691.3510** | **1380.6875** | **1380.6959** | **-0.0084** | **0** | **47** | **0.0024** | **1** | **K.ELNVMFIETSAK.A** |
|  | 11576 | **699.8549** | **1397.6952** | **1396.6908** | **1.0044** | **0** | **(6)** | **33** | **2** | **K.ELNVMFIETSAK.A** |
|  | 14444 | 527.6164 | 1579.8273 | 1579.8279 | -0.0006 | 1 | 4 | 45 | 5 | K.AKELNVMFIETSAK.A |

| **507.** | P12955\|PEPD_HUMAN    **Mass:** 55311    **Score:** 90     **Queries matched:** 13   **emPAI:** 0.30 |
| --- | --- |
|  | Xaa-Pro dipeptidase OS=Homo sapiens GN=PEPD PE=1 SV=3 |

|  | **Query** | **Observed** | **Mr(expt)** | **Mr(calc)** | **Delta** | **Miss** | **Score** | **Expect** | **Rank** | **Peptide** |
| --- | --- | --- | --- | --- | --- | --- | --- | --- | --- | --- |
|  | 2143 | **399.7429** | **797.4712** | **798.3984** | **-0.9272** | **0** | **6** | **42** | **8** | **K.ISSEAHR.E** |
|  | 6537 | **553.7793** | **1105.5440** | **1104.5485** | **0.9956** | **0** | **1** | **1.2e+002** | **1** | **K.TDMELEVLR.Y** |
|  | 7102 | **576.2556** | **1150.4967** | **1150.5037** | **-0.0070** | **0** | **46** | **0.0041** | **1** | **R.GVNTDSGSVCR.E** 7101 |
|  | 7859 | **600.2753** | **1198.5361** | **1197.5965** | **0.9397** | **0** | **4** | **57** | **2** | **R.LPASHATWMGK.I** |
|  | 9556 | 643.8130 | 1285.6114 | 1285.6448 | -0.0334 | 1 | 2 | 92 | 10 | K.ISSEAHREVMK.A 9568 |
|  | 24067 | **814.3781** | **2440.1125** | **2439.1762** | **0.9363** | **1** | **3** | **37** | **7** | **R.AVMGAMKPGVWWPDMHRLADR.I** 24060 |
|  | 27366 | **755.0748** | **3016.2702** | **3016.2941** | **-0.0238** | **0** | **43** | **0.0023** | **1** | **R.HSSYTCICGSGENSAVLHYGHAGAPNDR.T** |
|  | 27853 | 781.4185 | 3121.6450 | 3121.6547 | -0.0097 | 0 | 45 | 0.0017 | 1 | K.YAVDDVQYVDEIASVLTSQKPSVLLTLR.G 27852 |
|  | 28685 | **833.4322** | **3329.6997** | **3329.7118** | **-0.0122** | **0** | **11** | **3** | **1** | **R.HLQPGMVLTVEPGIYFIDHLLDEALADPAR.A** |

| **508.** | Q6ZMR3\|LDH6A_HUMAN    **Mass:** 36826    **Score:** 90     **Queries matched:** 9   **emPAI:** 0.10 |
| --- | --- |
|  | L-lactate dehydrogenase A-like 6A OS=Homo sapiens GN=LDHAL6A PE=2 SV=1 |

|  | **Query** | **Observed** | **Mr(expt)** | **Mr(calc)** | **Delta** | **Miss** | **Score** | **Expect** | **Rank** | **Peptide** |
| --- | --- | --- | --- | --- | --- | --- | --- | --- | --- | --- |
|  | 3320 | 437.7089 | 873.4032 | 874.4582 | -1.0550 | 0 | 9 | 28 | 4 | K.MPNIVSSK.D |
|  | 7967 | **402.2276** | **1203.6609** | **1203.6135** | **0.0474** | **0** | **11** | **12** | **5** | **K.SAETLWEIQK.E** |
|  | 8690 | 620.7770 | 1239.5395 | 1239.6533 | -0.1138 | 1 | 6 | 33 | 9 | K.KVISSGYEMVK.M |
|  | 8831 | 624.7997 | 1247.5848 | 1247.5928 | -0.0080 | 0 | 60 | 0.00013 | 1 | R.VIGSGCNLDSAR.F 8830 8832 |
|  | 14166 | **521.2656** | **1560.7751** | **1559.8229** | **0.9522** | **1** | **6** | **33** | **2** | **K.VKLTLEEEACLQK.S** |
|  | 19682 | **653.3359** | **1956.9858** | **1957.1288** | **-0.1430** | **0** | **2** | **55** | **5** | **K.LLIVTNPVDILTYVAWK.L** |
|  | 26192 | **691.6305** | **2762.4929** | **2762.4636** | **0.0292** | **1** | **1** | **41** | **5** | **K.MPNIVSSKDYLVTANSNLVIITAGAR.Q** |

| **509.** | P07195\|LDHB_HUMAN    **Mass:** 36900    **Score:** 90     **Queries matched:** 6   **emPAI:** 0.10 |
| --- | --- |
|  | L-lactate dehydrogenase B chain OS=Homo sapiens GN=LDHB PE=1 SV=2 |

|  | **Query** | **Observed** | **Mr(expt)** | **Mr(calc)** | **Delta** | **Miss** | **Score** | **Expect** | **Rank** | **Peptide** |
| --- | --- | --- | --- | --- | --- | --- | --- | --- | --- | --- |
|  | 1027 | **546.2509** | **545.2436** | **544.3221** | **0.9216** | **0** | **7** | **70** | **1** | **K.IVADK.D** |
|  | 1852 | 742.4518 | 741.4445 | 741.4497 | -0.0052 | 0 | 7 | 30 | 2 | R.LNLVQR.N |
|  | 8831 | 624.7997 | 1247.5848 | 1247.5928 | -0.0080 | 0 | 60 | 0.00013 | 1 | R.VIGSGCNLDSAR.F 8830 8832 |
|  | 19584 | **650.3466** | **1948.0179** | **1946.9230** | **1.0948** | **0** | **4** | **40** | **3** | **K.GEMMDLQHGSLFLQTPK.I** |

| **510.** | P07864\|LDHC_HUMAN    **Mass:** 36630    **Score:** 90     **Queries matched:** 7   **emPAI:** 0.10 |
| --- | --- |
|  | L-lactate dehydrogenase C chain OS=Homo sapiens GN=LDHC PE=2 SV=4 |

|  | **Query** | **Observed** | **Mr(expt)** | **Mr(calc)** | **Delta** | **Miss** | **Score** | **Expect** | **Rank** | **Peptide** |
| --- | --- | --- | --- | --- | --- | --- | --- | --- | --- | --- |
|  | 1621 | **675.1239** | **674.1166** | **674.3785** | **-0.2619** | **0** | **0** | **1.8e+002** | **7** | **R.NVAIMK.S** |
|  | 7354 | 585.7674 | 1169.5202 | 1168.6387 | 0.8816 | 1 | 2 | 94 | 10 | R.RVHPVSTMVK.G |
|  | 8831 | 624.7997 | 1247.5848 | 1247.5928 | -0.0080 | 0 | 60 | 0.00013 | 1 | R.VIGSGCNLDSAR.F 8830 8832 |
|  | 16608 | 576.7942 | 1727.3607 | 1726.9326 | 0.4282 | 1 | 5 | 22 | 3 | R.IVIVTAGARQQEGETR.L |
|  | 21957 | 724.3757 | 2170.1054 | 2171.0391 | -0.9337 | 1 | 1 | 70 | 4 | K.LKGEMMDLQHGSLFFSTSK.I |

| **511.** | Q9UBM7\|DHCR7_HUMAN    **Mass:** 55195    **Score:** 89     **Queries matched:** 5   **emPAI:** 0.07 |
| --- | --- |
|  | 7-dehydrocholesterol reductase OS=Homo sapiens GN=DHCR7 PE=1 SV=1 |

|  | **Query** | **Observed** | **Mr(expt)** | **Mr(calc)** | **Delta** | **Miss** | **Score** | **Expect** | **Rank** | **Peptide** |
| --- | --- | --- | --- | --- | --- | --- | --- | --- | --- | --- |
|  | 2752 | 421.0563 | 840.0981 | 840.3912 | -0.2931 | 1 | 6 | 25 | 6 | R.CASKYGR.D 2751 |
|  | 14424 | 527.2564 | 1578.7474 | 1579.8762 | -1.1288 | 1 | 0 | 1.1e+002 | 8 | R.YTAAVPYRLLPGIF.- |
|  | 18165 | 614.0000 | 1838.9782 | 1837.8992 | 1.0789 | 1 | 3 | 48 | 5 | R.KPKVIECSYTSADGQR.H |
|  | 21973 | **1087.0794** | **2172.1441** | **2172.1579** | **-0.0138** | **0** | **89** | **1.1e-007** | **1** | **K.FLPGYVGGIQEGAVTPAGVVNK.Y** |

| **512.** | P51148\|RAB5C_HUMAN    **Mass:** 23696    **Score:** 89     **Queries matched:** 9   **emPAI:** 0.35 |
| --- | --- |
|  | Ras-related protein Rab-5C OS=Homo sapiens GN=RAB5C PE=1 SV=2 |

|  | **Query** | **Observed** | **Mr(expt)** | **Mr(calc)** | **Delta** | **Miss** | **Score** | **Expect** | **Rank** | **Peptide** |
| --- | --- | --- | --- | --- | --- | --- | --- | --- | --- | --- |
|  | 8120 | **606.2874** | **1210.5603** | **1210.5690** | **-0.0088** | **0** | **8** | **23** | **6** | **K.NEPQNATGAPGR.N** |
|  | 8631 | **413.2640** | **1236.7703** | **1236.6323** | **0.1380** | **0** | **6** | **34** | **3** | **R.GGAARPNGPAAGNK.I** |
|  | 10772 | **676.8751** | **1351.7357** | **1350.6204** | **1.1153** | **0** | **41** | **0.01** | **1** | **K.FEIWDTAGQER.Y** |
|  | 14028 | **776.8925** | **1551.7704** | **1551.7789** | **-0.0085** | **0** | **73** | **6.3e-006** | **1** | **K.TAMNVNEIFMAIAK.K** |
|  | 14284 | **523.7732** | **1568.2979** | **1567.7738** | **0.5241** | **0** | **(4)** | **33** | **3** | **K.TAMNVNEIFMAIAK.K** |
|  | 15770 | 834.4499 | 1666.8852 | 1667.8274 | -0.9422 | 1 | 4 | 45 | 7 | -.MAGRGGAARPNGPAAGNK.I 15774 |
|  | 16770 | **580.6484** | **1738.9235** | **1740.0509** | **-1.1274** | **1** | **5** | **34** | **3** | **K.LVLLGESAVGKSSLVLR.F** |
|  | 22433 | **742.5356** | **2224.5849** | **2224.1488** | **0.4361** | **1** | **2** | **38** | **4** | **R.GAQAAIVVYDITNTDTFARAK.N** |

| **513.** | P61769\|B2MG_HUMAN    **Mass:** 13820    **Score:** 89     **Queries matched:** 9   **emPAI:** 0.65 |
| --- | --- |
|  | Beta-2-microglobulin OS=Homo sapiens GN=B2M PE=1 SV=1 |

|  | **Query** | **Observed** | **Mr(expt)** | **Mr(calc)** | **Delta** | **Miss** | **Score** | **Expect** | **Rank** | **Peptide** |
| --- | --- | --- | --- | --- | --- | --- | --- | --- | --- | --- |
|  | 6759 | 562.3048 | 1122.5951 | 1121.6193 | 0.9758 | 0 | 10 | 14 | 2 | R.VNHVTLSQPK.I 6752 |
|  | 7070 | **1148.5508** | **1147.5435** | **1147.5509** | **-0.0074** | **0** | **56** | **0.0004** | **1** | **K.VEHSDLSFSK.D** 7069 |
|  | 24981 | **852.0767** | **2553.2082** | **2553.2210** | **-0.0128** | **0** | **35** | **0.022** | **1** | **K.SNFLNCYVSGFHPSDIEVDLLK.N** 24972 24979 24980 24982 |

| **514.** | Q99943\|PLCA_HUMAN    **Mass:** 32038    **Score:** 88     **Queries matched:** 6   **emPAI:** 0.12 |
| --- | --- |
|  | 1-acyl-sn-glycerol-3-phosphate acyltransferase alpha OS=Homo sapiens GN=AGPAT1 PE=2 SV=2 |

|  | **Query** | **Observed** | **Mr(expt)** | **Mr(calc)** | **Delta** | **Miss** | **Score** | **Expect** | **Rank** | **Peptide** |
| --- | --- | --- | --- | --- | --- | --- | --- | --- | --- | --- |
|  | 7235 | 581.2817 | 1160.5488 | 1161.5778 | -1.0290 | 1 | 1 | 1.3e+002 | 9 | R.GGGDYLKKPGGGG.- |
|  | 9181 | **633.7938** | **1265.5731** | **1264.8053** | **0.7678** | **1** | **4** | **52** | **4** | **K.ILRLMLLHIK.Y** |
|  | 22487 | **744.3992** | **2230.1759** | **2231.0946** | **-0.9187** | **1** | **1** | **76** | **5** | **R.VWVFPEGTRNHNGSMLPFK.R** |
|  | 22510 | **1117.5673** | **2233.1200** | **2233.1260** | **-0.0061** | **0** | **88** | **1.2e-007** | **1** | **R.TGDAISVMSEVAQTLLTQDVR.V** |
|  | 26135 | 1376.6567 | 2751.2989 | 2752.4709 | -1.1720 | 1 | 6 | 17 | 1 | K.MAFYNGWILFLAVLAIPVCAVRGR.N 26136 |

| **515.** | Q9UNL2\|SSRG_HUMAN    **Mass:** 21067    **Score:** 88     **Queries matched:** 4   **emPAI:** 0.18 |
| --- | --- |
|  | Translocon-associated protein subunit gamma OS=Homo sapiens GN=SSR3 PE=1 SV=1 |

|  | **Query** | **Observed** | **Mr(expt)** | **Mr(calc)** | **Delta** | **Miss** | **Score** | **Expect** | **Rank** | **Peptide** |
| --- | --- | --- | --- | --- | --- | --- | --- | --- | --- | --- |
|  | 16340 | **854.4069** | **1706.7992** | **1706.8111** | **-0.0120** | **0** | **88** | **1.7e-007** | **1** | **K.QQSEEDLLLQDFSR.N** |
|  | 20845 | 689.9059 | 2066.6958 | 2065.9916 | 0.7042 | 1 | 1 | 53 | 9 | K.GSSKQQSEEDLLLQDFSR.N |
|  | 27686 | **1024.3488** | **3070.0244** | **3070.6433** | **-0.6188** | **1** | **0** | **34** | **2** | **R.NLSAKSSALFFGNAFIVSAIPIWLYWR.I** |
|  | 28923 | **1132.6038** | **3394.7895** | **3393.7393** | **1.0502** | **0** | **2** | **27** | **1** | **R.IWHMDLIQSAVLYSVMTLVSTYLVAFAYK.N** |

| **516.** | Q9Y281\|COF2_HUMAN    **Mass:** 18839    **Score:** 88     **Queries matched:** 8   **emPAI:** 0.45 |
| --- | --- |
|  | Cofilin-2 OS=Homo sapiens GN=CFL2 PE=1 SV=1 |

|  | **Query** | **Observed** | **Mr(expt)** | **Mr(calc)** | **Delta** | **Miss** | **Score** | **Expect** | **Rank** | **Peptide** |
| --- | --- | --- | --- | --- | --- | --- | --- | --- | --- | --- |
|  | 2148 | 400.2021 | 798.3896 | 798.3945 | -0.0050 | 0 | 17 | 3.2 | 1 | K.MIYASSK.D |
|  | 4098 | 465.0199 | 928.0253 | 928.5229 | -0.4976 | 0 | 7 | 26 | 6 | R.QIIVEEAK.Q 4115 |
|  | 7315 | **584.2983** | **1166.5820** | **1166.5642** | **0.0178** | **0** | **1** | **1.1e+002** | **4** | **K.AVLFCLSDDK.R** |
|  | 10951 | **682.3831** | **1362.7516** | **1361.6861** | **1.0655** | **0** | **1** | **1.1e+002** | **4** | **-.MASGVTVNDEVIK.V** |
|  | 20006 | 995.5340 | 1989.0534 | 1989.0611 | -0.0077 | 1 | 69 | 1.1e-005 | 1 | K.KEDLVFIFWAPESAPLK.S 19997 20016 |

| **517.** | P00505\|AATM_HUMAN    **Mass:** 47844    **Score:** 88     **Queries matched:** 11   **emPAI:** 0.08 |
| --- | --- |
|  | Aspartate aminotransferase, mitochondrial OS=Homo sapiens GN=GOT2 PE=1 SV=2 |

|  | **Query** | **Observed** | **Mr(expt)** | **Mr(calc)** | **Delta** | **Miss** | **Score** | **Expect** | **Rank** | **Peptide** |
| --- | --- | --- | --- | --- | --- | --- | --- | --- | --- | --- |
|  | 7163 | 578.2893 | 1154.5641 | 1153.5186 | 1.0455 | 0 | 0 | 1.4e+002 | 10 | R.DAGMQLQGYR.Y |
|  | 9267 | **636.2803** | **1270.5460** | **1270.6881** | **-0.1421** | **1** | **3** | **69** | **8** | **K.AEAQIAAKNLDK.E** |
|  | 12790 | **491.2732** | **1470.7977** | **1471.8544** | **-1.0567** | **1** | **7** | **26** | **3** | **R.IIGMRTQLVSNLK.K** |
|  | 13236 | **749.4407** | **1496.8668** | **1495.7381** | **1.1287** | **0** | **1** | **98** | **3** | **K.EYLPIGGLAEFCK.A** |
|  | 14542 | 529.9384 | 1586.7934 | 1586.8416 | -0.0482 | 1 | 7 | 24 | 4 | R.DDNGKPYVLPSVRK.A |
|  | 15402 | 820.9409 | 1639.8672 | 1640.7538 | -0.8867 | 1 | 0 | 1.1e+002 | 5 | R.VGAFTMVCKDADEAK.R |
|  | 15480 | **823.8669** | **1645.7193** | **1645.7294** | **-0.0101** | **0** | **88** | **2e-007** | **1** | **K.TCGFDFTGAVEDISK.I** 15466 |
|  | 17996 | **610.3378** | **1827.9917** | **1826.9825** | **1.0092** | **0** | **2** | **70** | **9** | **K.ILIRPMYSNPPLNGAR.I** |
|  | 18506 | **621.2951** | **1860.8635** | **1860.0370** | **0.8265** | **0** | **8** | **16** | **2** | **R.VLPGIAAAFHPGLAAAASAR.A** 18507 |

| **518.** | P13797\|PLST_HUMAN    **Mass:** 71279    **Score:** 87     **Queries matched:** 23   **emPAI:** 0.16 |
| --- | --- |
|  | Plastin-3 OS=Homo sapiens GN=PLS3 PE=1 SV=4 |

|  | **Query** | **Observed** | **Mr(expt)** | **Mr(calc)** | **Delta** | **Miss** | **Score** | **Expect** | **Rank** | **Peptide** |
| --- | --- | --- | --- | --- | --- | --- | --- | --- | --- | --- |
|  | 1426 | 631.3726 | 630.3653 | 629.3748 | 0.9905 | 0 | 9 | 38 | 7 | R.EIIQK.L |
|  | 2350 | **407.2003** | **812.3860** | **812.3851** | **0.0009** | **0** | **1** | **1.2e+002** | **10** | **K.YAVSMAR.R** |
|  | 4299 | **472.2808** | **942.5471** | **941.5334** | **1.0137** | **0** | **5** | **53** | **7** | **K.VNKPPYPK.L** |
|  | 6020 | 535.3128 | 1068.6111 | 1068.6179 | -0.0068 | 0 | 9 | 21 | 6 | K.LSPEELLLR.W |
|  | 6172 | **539.8030** | **1077.5914** | **1076.5284** | **1.0630** | **1** | **21** | **1.2** | **2** | **K.LMLDGDRNK.D** |
|  | 6702 | **560.1185** | **1118.2225** | **1118.5430** | **-0.3205** | **0** | **6** | **28** | **3** | **K.EANMPLPGYK.V** |
|  | 9383 | 638.8257 | 1275.6369 | 1275.6241 | 0.0128 | 1 | 5 | 42 | 3 | K.RAESMLQQADK.L |
|  | 11428 | **463.9301** | **1388.7685** | **1389.7074** | **-0.9389** | **1** | **8** | **25** | **1** | **K.EANMPLPGYKVR.E** |
|  | 11769 | 705.3333 | 1408.6521 | 1408.6656 | -0.0136 | 0 | 7 | 22 | 1 | K.LENCNYAVELGK.H |
|  | 11870 | 707.8910 | 1413.7675 | 1413.7768 | -0.0093 | 0 | 54 | 0.00058 | 1 | K.AYFHLLNQIAPK.G 11871 |
|  | 12178 | **716.3939** | **1430.7733** | **1429.7599** | **1.0134** | **1** | **2** | **83** | **1** | **R.EIIQKLMLDGDR.N** |
|  | 12679 | **732.4025** | **1462.7905** | **1462.6441** | **0.1464** | **0** | **1** | **1e+002** | **10** | **K.MVMTVFACLMGR.G** |
|  | 13791 | 769.3817 | 1536.7489 | 1536.7606 | -0.0117 | 1 | 43 | 0.0054 | 1 | K.KLENCNYAVELGK.H 13793 |
|  | 14096 | **778.8827** | **1555.7509** | **1555.8544** | **-0.1035** | **1** | **6** | **35** | **7** | **K.VNKPPYPKLGANMK.K** |
|  | 14483 | **792.4102** | **1582.8058** | **1582.8202** | **-0.0145** | **1** | **1** | **1e+002** | **1** | **R.TLSEAGKSTSIQSFK.D** 14500 |
|  | 16396 | **571.2740** | **1710.8001** | **1710.7771** | **0.0231** | **0** | **6** | **30** | **2** | **R.IDINMSGFNETDDLK.R** |
|  | 21379 | **705.3190** | **2112.9353** | **2114.0756** | **-1.1403** | **1** | **1** | **72** | **3** | **K.ANDDIIVNWVNRTLSEAGK.S** |
|  | 22474 | **744.0519** | **2229.1340** | **2230.1402** | **-1.0062** | **1** | **1** | **74** | **5** | **R.DGETLEELMKLSPEELLLR.W** 22490 |
|  | 29576 | **1212.6459** | **3634.9158** | **3634.8354** | **0.0803** | **0** | **3** | **18** | **4** | **R.NWMNSLGVNPHVNHLYADLQDALVILQLYER.I** |

| **519.** | Q9NY84\|VNN3_HUMAN    **Mass:** 56766    **Score:** 87     **Queries matched:** 8   **emPAI:** 0.07 |
| --- | --- |
|  | Vascular non-inflammatory molecule 3 OS=Homo sapiens GN=VNN3 PE=2 SV=2 |

|  | **Query** | **Observed** | **Mr(expt)** | **Mr(calc)** | **Delta** | **Miss** | **Score** | **Expect** | **Rank** | **Peptide** |
| --- | --- | --- | --- | --- | --- | --- | --- | --- | --- | --- |
|  | 4012 | **461.2475** | **920.4804** | **920.4716** | **0.0088** | **1** | **5** | **60** | **4** | **R.LGQGSGKFQ.-** |
|  | 4906 | **494.2436** | **986.4726** | **986.5185** | **-0.0459** | **1** | **3** | **91** | **10** | **R.VFEKDPPR.L** |
|  | 11709 | **703.7958** | **1405.5771** | **1406.7041** | **-1.1270** | **0** | **2** | **72** | **6** | **K.DNSIYVVANIGDK.K** |
|  | 12528 | **728.3892** | **1454.7638** | **1454.6929** | **0.0708** | **0** | **2** | **75** | **6** | **K.DSELVTFDTPFGK.F** |
|  | 15710 | 832.3782 | 1662.7418 | 1662.7526 | -0.0108 | 0 | 75 | 3.7e-006 | 1 | R.YQYNTDVVFDSQGK.L 15709 |
|  | 17822 | **606.6503** | **1816.9290** | **1817.9444** | **-1.0154** | **1** | **4** | **44** | **7** | **R.TETPVSKEEALLLMNK.N** |
|  | 31278 | **974.2895** | **4866.4111** | **4865.2887** | **1.1223** | **1** | **3** | **7.3** | **7** | **R.EPTYPAAVDWHAYASSVKPFSSEQSDFLGMIYFDEFTFTKLK.R** |

| **520.** | Q9UHB6\|LIMA1_HUMAN    **Mass:** 85630    **Score:** 87     **Queries matched:** 28   **emPAI:** 0.14 |
| --- | --- |
|  | LIM domain and actin-binding protein 1 OS=Homo sapiens GN=LIMA1 PE=1 SV=1 |

|  | **Query** | **Observed** | **Mr(expt)** | **Mr(calc)** | **Delta** | **Miss** | **Score** | **Expect** | **Rank** | **Peptide** |
| --- | --- | --- | --- | --- | --- | --- | --- | --- | --- | --- |
|  | 1252 | **590.4242** | **589.4169** | **589.3257** | **0.0912** | **0** | **4** | **1e+002** | **1** | **K.MSKPK.W** |
|  | 1636 | 677.3595 | 676.3522 | 676.3868 | -0.0345 | 1 | 9 | 26 | 9 | R.RSSSLK.E |
|  | 1714 | 702.3847 | 701.3774 | 700.2924 | 1.0850 | 0 | 5 | 77 | 6 | K.MMFEK.G |
|  | 1947 | **769.4149** | **768.4076** | **768.4494** | **-0.0418** | **0** | **15** | **4.6** | **1** | **K.TVSPPIR.K** 1948 |
|  | 2693 | 419.2420 | 836.4695 | 835.3824 | 1.0871 | 0 | 10 | 17 | 8 | M.ESSPFNR.R |
|  | 4761 | **488.3047** | **974.5948** | **973.5192** | **1.0756** | **1** | **11** | **15** | **1** | **K.QVENAKASK.K** 4753 |
|  | 5730 | **524.7819** | **1047.5492** | **1047.5560** | **-0.0068** | **1** | **9** | **20** | **1** | **R.LSETSIKDR.M** |
|  | 5796 | **527.2690** | **1052.5234** | **1052.5614** | **-0.0380** | **0** | **5** | **48** | **6** | **R.SPPEALVQGR.Y** |
|  | 6143 | **538.7924** | **1075.5703** | **1074.5743** | **0.9960** | **0** | **44** | **0.007** | **1** | **K.VGVLAASMEAK.A** |
|  | 6222 | **541.7966** | **1081.5786** | **1080.6291** | **0.9495** | **1** | **1** | **1.2e+002** | **7** | **K.SPKTVSPPIR.K** |
|  | 6602 | 556.2821 | 1110.5496 | 1111.5695 | -1.0199 | 1 | 11 | 10 | 4 | R.MAKYQAAVSK.Q |
|  | 8359 | **408.2327** | **1221.6762** | **1222.5434** | **-0.8672** | **1** | **7** | **30** | **8** | **K.KMENCLGESR.H** |
|  | 10092 | **658.3360** | **1314.6574** | **1313.6615** | **0.9959** | **1** | **6** | **33** | **2** | **R.YPHIKDGEDLK.D** 10065 |
|  | 12228 | **478.8891** | **1433.6454** | **1434.7024** | **-1.0570** | **1** | **6** | **37** | **9** | **R.ASSLSESSPPKAMK.K** |
|  | 12333 | **721.4689** | **1440.9233** | **1440.6554** | **0.2679** | **1** | **0** | **1.1e+002** | **9** | **K.YQKAAEETNMEK.K** |
|  | 13532 | 759.4475 | 1516.8805 | 1516.7409 | 0.1395 | 0 | 5 | 37 | 3 | K.SQDVELWEGEVVK.E |
|  | 13943 | **516.5913** | **1546.7521** | **1546.7627** | **-0.0106** | **0** | **11** | **9.5** | **1** | **R.ETPHSPGVEDAPIAK.V** |
|  | 14829 | **536.3325** | **1605.9756** | **1606.7686** | **-0.7930** | **1** | **6** | **23** | **2** | **K.SEISENTDASGKIEK.Y** |
|  | 15481 | 549.5998 | 1645.7775 | 1644.7856 | 0.9920 | 0 | 14 | 4.6 | 1 | R.ADHPPAEVTSHAASGAK.A |
|  | 16394 | **571.2540** | **1710.7403** | **1711.8893** | **-1.1491** | **0** | **2** | **70** | **6** | **R.SRPFTVAASFQSTSVK.S** |
|  | 18945 | **632.9736** | **1895.8991** | **1895.9126** | **-0.0135** | **1** | **52** | **0.00058** | **1** | **K.KWENPGLGAESHTDSLR.N** |
|  | 19451 | **646.9846** | **1937.9320** | **1937.9456** | **-0.0136** | **1** | **34** | **0.037** | **1** | **R.HRADHPPAEVTSHAASGAK.A** 19445 |
|  | 19769 | 983.9689 | 1965.9232 | 1965.9867 | -0.0636 | 0 | 7 | 19 | 2 | K.NENEEILERPAQLANAR.E |
|  | 21603 | **713.3619** | **2137.0638** | **2138.0895** | **-1.0257** | **0** | **1** | **72** | **9** | **R.IAWPPPTELGSSGSALEEGIK.M** |

| **521.** | Q96FQ6\|S10AG_HUMAN    **Mass:** 11851    **Score:** 86     **Queries matched:** 13   **emPAI:** 1.37 |
| --- | --- |
|  | Protein S100-A16 OS=Homo sapiens GN=S100A16 PE=1 SV=1 |

|  | **Query** | **Observed** | **Mr(expt)** | **Mr(calc)** | **Delta** | **Miss** | **Score** | **Expect** | **Rank** | **Peptide** |
| --- | --- | --- | --- | --- | --- | --- | --- | --- | --- | --- |
|  | 9536 | **643.3002** | **1284.5858** | **1284.5946** | **-0.0088** | **0** | **28** | **0.25** | **1** | **K.LIHEQEQQSSS.-** 9535 9537 9538 |
|  | 9721 | 647.8702 | 1293.7259 | 1293.7333 | -0.0073 | 0 | 13 | 6.7 | 1 | K.AVIVLVENFYK.Y |
|  | 10980 | **683.3425** | **1364.6705** | **1364.6797** | **-0.0091** | **0** | **33** | **0.077** | **1** | **K.LIQNLDANHDGR.I** |
|  | 11359 | 693.8202 | 1385.6259 | 1385.6357 | -0.0098 | 0 | 54 | 0.00052 | 1 | K.ELNHMLSDTGNR.K 11360 11361 |
|  | 11652 | **702.2657** | **1402.5168** | **1401.6307** | **0.8862** | **0** | **(2)** | **68** | **5** | **K.ELNHMLSDTGNR.K** |
|  | 13480 | **505.9151** | **1514.7234** | **1513.7307** | **0.9927** | **1** | **26** | **0.3** | **1** | **K.ELNHMLSDTGNRK.A** 13465 |
|  | 20966 | **1041.0455** | **2080.0765** | **2080.0881** | **-0.0116** | **0** | **12** | **5.7** | **1** | **R.ISFDEYWTLIGGITGPIAK.L** |

| **522.** | Q58FG0\|HS905_HUMAN    **Mass:** 38942    **Score:** 85     **Queries matched:** 12   **emPAI:** 0.20 |
| --- | --- |
|  | Putative heat shock protein HSP 90-alpha A5 OS=Homo sapiens GN=HSP90AA5P PE=1 SV=1 |

|  | **Query** | **Observed** | **Mr(expt)** | **Mr(calc)** | **Delta** | **Miss** | **Score** | **Expect** | **Rank** | **Peptide** |
| --- | --- | --- | --- | --- | --- | --- | --- | --- | --- | --- |
|  | 4771 | 488.7246 | 975.4346 | 975.4509 | -0.0163 | 0 | 11 | 16 | 8 | K.QVSDAEAEK.K 4782 |
|  | 7545 | 592.3403 | 1182.6660 | 1181.5937 | 1.0723 | 1 | 0 | 1.3e+002 | 7 | K.FENLCKIMK.D |
|  | 8385 | 612.8124 | 1223.6103 | 1223.6186 | -0.0083 | 0 | 35 | 0.047 | 1 | K.HIYYITGETK.D |
|  | 12162 | **716.0335** | **1430.0523** | **1429.6620** | **0.3904** | **1** | **4** | **40** | **1** | **K.EMPPLRGGDDTSR.M** |
|  | 12381 | **723.2854** | **1444.5562** | **1445.6569** | **-1.1006** | **1** | **(1)** | **98** | **2** | **K.EMPPLRGGDDTSR.M** |
|  | 12488 | **726.8382** | **1451.6618** | **1452.5787** | **-0.9169** | **1** | **2** | **74** | **9** | **R.GGDDTSRMEEVGGSG.-** |
|  | 17246 | **887.4361** | **1772.8576** | **1772.8693** | **-0.0117** | **1** | **1** | **82** | **2** | **K.HFSVEGQLEELKDSR.R** |
|  | 17324 | 889.9722 | 1777.9299 | 1777.9403 | -0.0104 | 0 | 71 | 7.6e-006 | 1 | K.HSQFIGYPITLFVEK.K |
|  | 19066 | 477.2452 | 1904.9515 | 1906.0353 | -1.0838 | 1 | 3 | 54 | 7 | K.HSQFIGYPITLFVEKK.R |
|  | 24343 | 619.2994 | 2473.1684 | 2472.2332 | 0.9352 | 0 | 2 | 43 | 9 | -.MGFHHVGQAGLELLTSGHPALER.R |
|  | 31122 | **1167.7826** | **4667.1013** | **4667.1840** | **-0.0828** | **1** | **2** | **11** | **2** | **R.NPDAITNEEYGEFHQSLTNNWEDHLAVKHFSVEGQLEELK.D** |

| **523.** | Q3ZCQ8\|TIM50_HUMAN    **Mass:** 39850    **Score:** 85     **Queries matched:** 10   **emPAI:** 0.20 |
| --- | --- |
|  | Mitochondrial import inner membrane translocase subunit TIM50 OS=Homo sapiens GN=TIMM50 PE=1 SV=2 |

|  | **Query** | **Observed** | **Mr(expt)** | **Mr(calc)** | **Delta** | **Miss** | **Score** | **Expect** | **Rank** | **Peptide** |
| --- | --- | --- | --- | --- | --- | --- | --- | --- | --- | --- |
|  | 2821 | 423.4551 | 844.8955 | 844.4879 | 0.4077 | 1 | 7 | 32 | 6 | R.SGLRLGSR.G |
|  | 3396 | 440.6986 | 879.3827 | 878.4610 | 0.9217 | 0 | 3 | 87 | 10 | M.AASAAVFSR.L |
|  | 5453 | **514.2952** | **1026.5759** | **1025.4964** | **1.0795** | **0** | **9** | **19** | **3** | **-.MAASAAVFSR.L** |
|  | 7589 | **396.1970** | **1185.5693** | **1185.6353** | **-0.0660** | **0** | **8** | **26** | **2** | **K.TIALNGVEDVR.T** |
|  | 9270 | **636.3164** | **1270.6183** | **1269.6425** | **0.9757** | **1** | **48** | **0.002** | **1** | **R.RAPDQAAEIGSR.G** |
|  | 9728 | **432.3090** | **1293.9052** | **1294.6816** | **-0.7763** | **1** | **6** | **27** | **6** | **-.MAASAAVFSRLR.S** 9733 |
|  | 16626 | **865.4042** | **1728.7939** | **1728.8067** | **-0.0128** | **0** | **62** | **6.8e-005** | **1** | **K.AQGPQQQPGSEGPSYAK.K** |
|  | 18448 | **619.6445** | **1855.9118** | **1856.9017** | **-0.9899** | **1** | **2** | **71** | **3** | **K.AQGPQQQPGSEGPSYAKK.V** |
|  | 31949 | **1474.6211** | **5894.4553** | **5894.9732** | **-0.5179** | **1** | **3** | **2.9** | **2** | **K.DYRQMIIEPTSPCLLPDPLQEPYYQPPYTLVLELTGVLLHPEWSLATGWR.F** |

| **524.** | P62829\|RL23_HUMAN    **Mass:** 14970    **Score:** 85     **Queries matched:** 10   **emPAI:** 0.26 |
| --- | --- |
|  | 60S ribosomal protein L23 OS=Homo sapiens GN=RPL23 PE=1 SV=1 |

|  | **Query** | **Observed** | **Mr(expt)** | **Mr(calc)** | **Delta** | **Miss** | **Score** | **Expect** | **Rank** | **Peptide** |
| --- | --- | --- | --- | --- | --- | --- | --- | --- | --- | --- |
|  | 3557 | 890.5520 | 889.5447 | 889.5498 | -0.0050 | 0 | 17 | 3.7 | 1 | K.VHPAVVIR.Q |
|  | 3696 | **450.7581** | **899.5016** | **899.5076** | **-0.0061** | **0** | **15** | **5.4** | **2** | **K.GSAITGPVAK.E** 3689 |
|  | 4387 | **474.7764** | **947.5382** | **948.5644** | **-1.0261** | **0** | **11** | **14** | **5** | **K.NLYIISVK.G** |
|  | 5699 | 523.7653 | 1045.5160 | 1045.4651 | 0.0509 | 0 | 9 | 22 | 2 | K.ECADLWPR.I |
|  | 12854 | **738.4178** | **1474.8210** | **1474.7524** | **0.0686** | **0** | **3** | **64** | **4** | **R.LPAAGVGDMVMATVK.K** |
|  | 14531 | **793.8812** | **1585.7479** | **1586.8524** | **-1.1045** | **1** | **6** | **29** | **1** | **R.LPAAGVGDMVMATVKK.G** |
|  | 18462 | 929.4836 | 1856.9527 | 1857.9805 | -1.0277 | 1 | 3 | 51 | 3 | R.LNRLPAAGVGDMVMATVK.K |
|  | 19808 | **985.5160** | **1969.0175** | **1969.0303** | **-0.0127** | **0** | **78** | **1.6e-006** | **1** | **R.ISLGLPVGAVINCADNTGAK.N** 19809 |

| **525.** | P54819\|KAD2_HUMAN    **Mass:** 26689    **Score:** 84     **Queries matched:** 8   **emPAI:** 0.31 |
| --- | --- |
|  | Adenylate kinase 2, mitochondrial OS=Homo sapiens GN=AK2 PE=1 SV=2 |

|  | **Query** | **Observed** | **Mr(expt)** | **Mr(calc)** | **Delta** | **Miss** | **Score** | **Expect** | **Rank** | **Peptide** |
| --- | --- | --- | --- | --- | --- | --- | --- | --- | --- | --- |
|  | 4753 | 488.2514 | 974.4882 | 973.4902 | 0.9979 | 0 | 9 | 25 | 3 | K.NLETPLCK.N |
|  | 4823 | 490.4935 | 978.9724 | 978.5862 | 0.3862 | 0 | 2 | 72 | 6 | R.AVLLGPPGAGK.G |
|  | 5569 | 518.2785 | 1034.5424 | 1033.6396 | 0.9028 | 1 | 7 | 36 | 10 | R.ITGRLIHPK.S |
|  | 15220 | 544.2600 | 1629.7580 | 1628.8080 | 0.9501 | 1 | 8 | 19 | 5 | K.EPMKDDITGEPLIR.R 15229 |
|  | 16138 | **565.2900** | **1692.8481** | **1693.8015** | **-0.9534** | **1** | **1** | **79** | **7** | **R.TVRQAEMLDDLMEK.R** |
|  | 20080 | 666.3378 | 1995.9917 | 1996.0054 | -0.0137 | 0 | 37 | 0.018 | 1 | R.LQAYHTQTTPLIEYYR.K |
|  | 24825 | **849.1109** | **2544.3109** | **2544.3224** | **-0.0115** | **0** | **69** | **7.8e-006** | **1** | **R.GIHSAIDASQTPDVVFASILAAFSK.A** |

| **526.** | Q9Y394\|DHRS7_HUMAN    **Mass:** 38673    **Score:** 84     **Queries matched:** 10   **emPAI:** 0.32 |
| --- | --- |
|  | Dehydrogenase/reductase SDR family member 7 OS=Homo sapiens GN=DHRS7 PE=1 SV=1 |

|  | **Query** | **Observed** | **Mr(expt)** | **Mr(calc)** | **Delta** | **Miss** | **Score** | **Expect** | **Rank** | **Peptide** |
| --- | --- | --- | --- | --- | --- | --- | --- | --- | --- | --- |
|  | 1522 | 650.3469 | 649.3396 | 649.3435 | -0.0039 | 0 | 22 | 1.3 | 1 | R.IENFK.S 1514 |
|  | 7357 | **585.8048** | **1169.5949** | **1169.7244** | **-0.1295** | **1** | **7** | **30** | **7** | **K.LGVSLVLSARR.V** |
|  | 7418 | **588.2607** | **1174.5069** | **1174.5142** | **-0.0073** | **0** | **31** | **0.14** | **1** | **K.SGVDADSSYFK.I** |
|  | 11909 | **708.8672** | **1415.7198** | **1415.7191** | **0.0007** | **0** | **4** | **54** | **4** | **R.IDILVNNGGMSQR.S** |
|  | 14186 | **782.3468** | **1562.6790** | **1562.7617** | **-0.0826** | **1** | **9** | **15** | **6** | **K.SGVDADSSYFKIFK.T** |
|  | 14195 | **782.4489** | **1562.8833** | **1562.8919** | **-0.0087** | **0** | **21** | **1** | **1** | **K.LIELNYLGTVSLTK.C** |
|  | 23777 | **802.1191** | **2403.3354** | **2403.3447** | **-0.0093** | **0** | **1** | **50** | **1** | **K.IVTVNSILGIISVPLSIGYCASK.H** |
|  | 28752 | **1117.8811** | **3350.6215** | **3350.6493** | **-0.0278** | **0** | **44** | **0.0016** | **1** | **R.RPEWELTDMVVWVTGASSGIGEELAYQLSK.L** |
|  | 29386 | **1190.9435** | **3569.8086** | **3569.8287** | **-0.0201** | **0** | **50** | **0.00038** | **1** | **R.TELATYPGIIVSNICPGPVQSNIVENSLAGEVTK.T** |

| **527.** | P30501\|1C02_HUMAN    **Mass:** 41468    **Score:** 83     **Queries matched:** 9   **emPAI:** 0.30 |
| --- | --- |
|  | HLA class I histocompatibility antigen, Cw-2 alpha chain OS=Homo sapiens GN=HLA-C PE=1 SV=1 |

|  | **Query** | **Observed** | **Mr(expt)** | **Mr(calc)** | **Delta** | **Miss** | **Score** | **Expect** | **Rank** | **Peptide** |
| --- | --- | --- | --- | --- | --- | --- | --- | --- | --- | --- |
|  | 1195 | 581.3248 | 580.3175 | 580.2969 | 0.0206 | 0 | 1 | 1e+002 | 5 | R.AEHPK.T |
|  | 2203 | 402.2052 | 802.3958 | 802.4119 | -0.0161 | 1 | 6 | 61 | 6 | M.RVMEPR.T |
|  | 12099 | 714.3628 | 1426.7111 | 1426.7205 | -0.0093 | 0 | 11 | 9.6 | 1 | K.WAAVVVPSGEEQR.Y |
|  | 14639 | **797.3464** | **1592.6783** | **1592.6896** | **-0.0112** | **0** | **15** | **3.2** | **1** | **R.WVEQEGPEYWDR.E** |
|  | 17676 | **602.9950** | **1805.9632** | **1805.8121** | **0.1510** | **1** | **4** | **43** | **6** | **R.GRWVEQEGPEYWDR.E** |
|  | 18119 | 459.9797 | 1835.8896 | 1835.9027 | -0.0131 | 0 | 13 | 5.4 | 2 | K.THVTHHPVSDHEATLR.C |
|  | 20661 | 513.0184 | 2048.0446 | 2049.0102 | -0.9656 | 0 | 14 | 4 | 2 | R.YTCHVQHEGLPEPLTLR.W |
|  | 25408 | 1319.0913 | 2636.1681 | 2636.1838 | -0.0158 | 0 | 68 | 1.1e-005 | 1 | R.DGEDQTQDTELVETRPAGDGTFQK.W 25407 |

| **528.** | Q06323\|PSME1_HUMAN    **Mass:** 28876    **Score:** 83     **Queries matched:** 20   **emPAI:** 0.85 |
| --- | --- |
|  | Proteasome activator complex subunit 1 OS=Homo sapiens GN=PSME1 PE=1 SV=1 |

|  | **Query** | **Observed** | **Mr(expt)** | **Mr(calc)** | **Delta** | **Miss** | **Score** | **Expect** | **Rank** | **Peptide** |
| --- | --- | --- | --- | --- | --- | --- | --- | --- | --- | --- |
|  | 2767 | 421.2862 | 840.5578 | 839.5593 | 0.9986 | 0 | 32 | 0.096 | 1 | K.IVVLLQR.L 2737 2754 |
|  | 3270 | **435.8875** | **869.7605** | **869.4607** | **0.2998** | **0** | **0** | **1e+002** | **10** | **R.VQPEAQAK.V** |
|  | 3797 | **453.7820** | **905.5495** | **906.4667** | **-0.9172** | **0** | **9** | **21** | **5** | **R.LMVMEIR.N** |
|  | 7219 | **580.3048** | **1158.5951** | **1158.6033** | **-0.0082** | **0** | **8** | **24** | **5** | **K.LEGFHTQISK.Y** |
|  | 9218 | 634.8256 | 1267.6367 | 1267.6448 | -0.0081 | 0 | 47 | 0.0029 | 1 | K.TENLLGSYFPK.K |
|  | 9394 | **426.2337** | **1275.6794** | **1274.6839** | **0.9955** | **1** | **(9)** | **20** | **8** | **R.DIRLMVMEIR.N** |
|  | 9658 | **431.2132** | **1290.6177** | **1290.6788** | **-0.0611** | **1** | **9** | **18** | **6** | **R.DIRLMVMEIR.N** |
|  | 9923 | 435.8975 | 1304.6707 | 1304.6799 | -0.0091 | 0 | 23 | 0.78 | 1 | K.VFELMTSLHTK.L |
|  | 10865 | **453.3409** | **1357.0009** | **1356.7183** | **0.2826** | **1** | **9** | **14** | **1** | **M.AMLRVQPEAQAK.V** 10864 |
|  | 11271 | 691.3381 | 1380.6616 | 1380.6708 | -0.0092 | 1 | 18 | 2.3 | 1 | K.VDVFREDLCTK.T |
|  | 11527 | **698.3948** | **1394.7750** | **1394.7809** | **-0.0059** | **0** | **21** | **1.1** | **1** | **R.NAYAVLYDIILK.N** |
|  | 11824 | 471.3255 | 1410.9548 | 1411.7307 | -0.7759 | 0 | 8 | 15 | 5 | K.EPALNEANLSNLK.A |
|  | 13328 | 752.9097 | 1503.8048 | 1503.7537 | 0.0510 | 1 | 2 | 73 | 4 | -.MAMLRVQPEAQAK.V |
|  | 13550 | **760.3679** | **1518.7213** | **1518.7314** | **-0.0101** | **0** | **40** | **0.014** | **1** | **R.IEDGNNFGVAVQEK.V** |
|  | 22899 | **760.4271** | **2278.2594** | **2278.2685** | **-0.0092** | **0** | **15** | **2.3** | **1** | **K.DVIEQLNLVTTWLQLQIPR.I** |
|  | 23992 | **810.4322** | **2428.2749** | **2428.2849** | **-0.0100** | **1** | **40** | **0.0072** | **1** | **K.ISELDAFLKEPALNEANLSNLK.A** 23976 |

| **529.** | Q8WY22\|BRI3B_HUMAN    **Mass:** 27932    **Score:** 83     **Queries matched:** 10   **emPAI:** 0.14 |
| --- | --- |
|  | BRI3-binding protein OS=Homo sapiens GN=BRI3BP PE=1 SV=1 |

|  | **Query** | **Observed** | **Mr(expt)** | **Mr(calc)** | **Delta** | **Miss** | **Score** | **Expect** | **Rank** | **Peptide** |
| --- | --- | --- | --- | --- | --- | --- | --- | --- | --- | --- |
|  | 2598 | **416.2294** | **830.4443** | **830.4498** | **-0.0055** | **0** | **16** | **5.3** | **1** | **R.VLESLDR.S** |
|  | 5248 | 506.2561 | 1010.4976 | 1011.5574 | -1.0597 | 1 | 11 | 12 | 2 | M.GARASGGPLAR.A 5247 5260 |
|  | 5706 | 523.7863 | 1045.5579 | 1045.5768 | -0.0188 | 1 | 19 | 2.3 | 1 | R.VLESLDRSK.D |
|  | 7008 | **572.2682** | **1142.5218** | **1142.5978** | **-0.0760** | **1** | **15** | **6** | **5** | **-.MGARASGGPLAR.A** |
|  | 7215 | **580.2706** | **1158.5266** | **1158.5928** | **-0.0662** | **1** | **(6)** | **42** | **4** | **-.MGARASGGPLAR.A** |
|  | 16221 | 850.3863 | 1698.7580 | 1698.7697 | -0.0116 | 0 | 83 | 5.3e-007 | 1 | R.SSPSGPSNPSNPSVEEK.L |
|  | 21023 | 1043.9994 | 2085.9842 | 2085.9967 | -0.0125 | 0 | 14 | 3.3 | 1 | R.TVNTFSQSVSSLFGEDNVR.A |
|  | 30837 | **1467.5803** | **4399.7191** | **4398.5218** | **1.1974** | **1** | **3** | **8.4** | **6** | **R.ALLLVGVVLLAYWFLSLTLGFTFSVLHVVFGRFFWIVR.V** |

| **530.** | Q92841\|DDX17_HUMAN    **Mass:** 72953    **Score:** 83     **Queries matched:** 22   **emPAI:** 0.05 |
| --- | --- |
|  | Probable ATP-dependent RNA helicase DDX17 OS=Homo sapiens GN=DDX17 PE=1 SV=1 |

|  | **Query** | **Observed** | **Mr(expt)** | **Mr(calc)** | **Delta** | **Miss** | **Score** | **Expect** | **Rank** | **Peptide** |
| --- | --- | --- | --- | --- | --- | --- | --- | --- | --- | --- |
|  | 1105 | **561.2952** | **560.2880** | **559.2827** | **1.0053** | **1** | **12** | **19** | **3** | **K.DGGRR.D** |
|  | 2308 | 405.2270 | 808.4394 | 809.4759 | -1.0365 | 1 | 12 | 9.6 | 10 | R.GGGGLPPKK.F |
|  | 3312 | 437.2480 | 872.4814 | 873.4166 | -0.9352 | 1 | 11 | 16 | 5 | R.GGGGGGGGRSR.Y 3309 3314 3321 |
|  | 4460 | 477.2601 | 952.5057 | 951.4345 | 1.0712 | 1 | 1 | 1.1e+002 | 7 | -.MRGGGFGDR.D |
|  | 4648 | 484.7399 | 967.4652 | 968.4312 | -0.9660 | 1 | 6 | 39 | 9 | R.DSASYRDR.S |
|  | 5802 | 527.6195 | 1053.2245 | 1052.4961 | 0.7284 | 0 | 20 | 0.92 | 1 | K.STCIYGGAPK.G |
|  | 7260 | 581.7995 | 1161.5844 | 1162.5652 | -0.9808 | 0 | 16 | 3.8 | 1 | R.DMVGIAQTGSGK.T |
|  | 8436 | 614.2993 | 1226.5840 | 1226.6520 | -0.0681 | 1 | 8 | 26 | 7 | R.GGFGARGGGGLPPK.K 8424 |
|  | 10290 | 663.3524 | 1324.6902 | 1324.6986 | -0.0085 | 0 | 13 | 6.9 | 1 | K.VLEEANQAINPK.L |
|  | 10500 | **668.8190** | **1335.6235** | **1335.6315** | **-0.0080** | **0** | **9** | **19** | **1** | **R.MLDMGFEPQIR.K** |
|  | 11896 | **472.5935** | **1414.7587** | **1415.7085** | **-0.9498** | **0** | **7** | **27** | **5** | **K.GTAYTFFTPGNLK.Q** |
|  | 12407 | **723.8506** | **1445.6866** | **1446.7462** | **-1.0595** | **0** | **(1)** | **1.1e+002** | **2** | **K.LIQLMEEIMAEK.E** |
|  | 12904 | 493.7580 | 1478.2521 | 1478.7360 | -0.4839 | 0 | 7 | 15 | 5 | K.LIQLMEEIMAEK.E |
|  | 13359 | **502.9333** | **1505.7781** | **1504.8249** | **0.9532** | **1** | **2** | **78** | **7** | **R.LIDFLESGKTNLR.R** |
|  | 14786 | 535.7793 | 1604.3161 | 1603.8141 | 0.5020 | 1 | 6 | 22 | 4 | K.STCIYGGAPKGPQIR.D |
|  | 15370 | **547.2587** | **1638.7542** | **1638.8009** | **-0.0467** | **1** | **2** | **78** | **8** | **K.LMQLVDHRGGGGGGGGR.S** |
|  | 16112 | 846.4097 | 1690.8049 | 1690.8162 | -0.0113 | 0 | 83 | 5.5e-007 | 1 | R.ELAQQVQQVADDYGK.C |
|  | 22951 | **763.4660** | **2287.3762** | **2286.9593** | **0.4168** | **1** | **0** | **53** | **2** | **R.TTSSANNPNLMYQDECDRR.L** |

| **531.** | P21399\|ACOC_HUMAN    **Mass:** 98850    **Score:** 83     **Queries matched:** 26   **emPAI:** 0.08 |
| --- | --- |
|  | Cytoplasmic aconitate hydratase OS=Homo sapiens GN=ACO1 PE=1 SV=3 |

|  | **Query** | **Observed** | **Mr(expt)** | **Mr(calc)** | **Delta** | **Miss** | **Score** | **Expect** | **Rank** | **Peptide** |
| --- | --- | --- | --- | --- | --- | --- | --- | --- | --- | --- |
|  | 1109 | 561.3217 | 560.3144 | 559.3329 | 0.9814 | 1 | 13 | 17 | 10 | R.DAVKK.L |
|  | 1250 | 590.3295 | 589.3222 | 589.2860 | 0.0362 | 0 | 16 | 6.9 | 3 | R.DWAAK.G |
|  | 1661 | 687.3727 | 686.3655 | 685.4123 | 0.9532 | 0 | 11 | 19 | 2 | R.QVGVVGK.F |
|  | 2537 | 414.7124 | 827.4102 | 826.4548 | 0.9554 | 0 | 9 | 22 | 4 | K.EPLGVNAK.G 2528 2530 |
|  | 3195 | **434.2340** | **866.4534** | **867.4273** | **-0.9739** | **0** | **1** | **1.4e+002** | **10** | **K.TFQAVMR.F** |
|  | 5177 | 503.1056 | 1004.1967 | 1004.4821 | -0.2855 | 1 | 2 | 77 | 5 | R.RGNDAVMAR.G |
|  | 5329 | 509.2790 | 1016.5434 | 1016.6131 | -0.0696 | 1 | 6 | 45 | 7 | R.LLNRFLNK.Q |
|  | 5776 | **526.2736** | **1050.5327** | **1049.4778** | **1.0549** | **0** | **7** | **29** | **6** | **K.NQDLEFER.N** |
|  | 6384 | **1093.7305** | **1092.7232** | **1091.6564** | **1.0668** | **1** | **4** | **61** | **1** | **K.HLRQVGVVGK.F** |
|  | 6520 | **553.2517** | **1104.4887** | **1103.5104** | **0.9784** | **0** | **12** | **12** | **2** | **K.TVVPCCSGPK.R** |
|  | 8551 | **617.3284** | **1232.6423** | **1232.5509** | **0.0914** | **0** | **4** | **65** | **4** | **K.WGSQAFHNMR.I** |
|  | 10172 | **660.3332** | **1318.6518** | **1319.6218** | **-0.9700** | **1** | **6** | **41** | **6** | **K.NQDLEFERNR.E** |
|  | 12612 | **487.2964** | **1458.8673** | **1458.7718** | **0.0954** | **1** | **7** | **25** | **5** | **R.IDFEKEPLGVNAK.G** 12589 |
|  | 13477 | **505.9041** | **1514.6905** | **1514.7015** | **-0.0110** | **0** | **4** | **47** | **6** | **K.GFQVAPEHHNDHK.T** |
|  | 14607 | **796.3759** | **1590.7371** | **1591.7889** | **-1.0517** | **1** | **2** | **69** | **3** | **R.GNDAVMARGTFANIR.L** |
|  | 16305 | **568.9629** | **1703.8668** | **1702.9327** | **0.9341** | **0** | **2** | **74** | **6** | **R.YTIIIPENLKPQMK.V** |
|  | 16864 | 582.6525 | 1744.9358 | 1744.8858 | 0.0499 | 1 | 5 | 31 | 6 | R.QYVIPGMFKEVYQK.I 16861 |
|  | 18360 | **926.9786** | **1851.9426** | **1851.9519** | **-0.0093** | **0** | **75** | **3.5e-006** | **1** | **K.FVEFFGPGVAQLSIADR.A** |
|  | 18660 | 937.8451 | 1873.6757 | 1873.9057 | -0.2300 | 0 | 38 | 0.012 | 2 | K.IETVNESWNALATPSDK.L |
|  | 21053 | **697.3929** | **2089.1570** | **2089.1684** | **-0.0114** | **0** | **3** | **41** | **3** | **R.IIPPGSGIIHQVNLEYLAR.V** |
|  | 22120 | 731.0266 | 2190.0578 | 2191.1096 | -1.0517 | 1 | 1 | 65 | 2 | -.MSNPFAHLAEPLDPVQPGKK.F |
|  | 29173 | **1164.8917** | **3491.6533** | **3491.6741** | **-0.0208** | **0** | **5** | **11** | **1** | **R.ATIANMCPEYGATAAFFPVDEVSITYLVQTGR.D** |

| **532.** | Q9BWS9\|CHID1_HUMAN    **Mass:** 45083    **Score:** 83     **Queries matched:** 7   **emPAI:** 0.17 |
| --- | --- |
|  | Chitinase domain-containing protein 1 OS=Homo sapiens GN=CHID1 PE=1 SV=1 |

|  | **Query** | **Observed** | **Mr(expt)** | **Mr(calc)** | **Delta** | **Miss** | **Score** | **Expect** | **Rank** | **Peptide** |
| --- | --- | --- | --- | --- | --- | --- | --- | --- | --- | --- |
|  | 1052 | **549.3023** | **548.2950** | **547.2966** | **0.9985** | **1** | **8** | **35** | **6** | **K.SDAKK.A** |
|  | 2802 | 422.7634 | 843.5123 | 843.5066 | 0.0057 | 0 | 12 | 14 | 8 | R.GLVVTDLK.A |
|  | 15039 | 810.3691 | 1618.7236 | 1618.7304 | -0.0068 | 0 | 42 | 0.008 | 1 | R.LLFEDWTYDDFR.N |
|  | 18858 | **631.0355** | **1890.0846** | **1889.9847** | **0.0999** | **1** | **2** | **56** | **1** | **K.TLLEKSQFSDKPVQDR.G** |
|  | 23371 | **1173.0049** | **2343.9952** | **2344.2009** | **-0.2057** | **1** | **3** | **30** | **1** | **R.NVLDSEDEIEELSKTVVQVAK.N** 23378 |
|  | 30282 | **1312.9640** | **3935.8701** | **3935.8829** | **-0.0127** | **0** | **63** | **1.3e-005** | **1** | **K.EFEQLAPVLDGFSLMTYDYSTAHQPGPNAPLSWVR.A** |

| **533.** | Q9HCS2\|CP4FC_HUMAN    **Mass:** 60955    **Score:** 83     **Queries matched:** 4   **emPAI:** 0.06 |
| --- | --- |
|  | Cytochrome P450 4F12 OS=Homo sapiens GN=CYP4F12 PE=1 SV=1 |

|  | **Query** | **Observed** | **Mr(expt)** | **Mr(calc)** | **Delta** | **Miss** | **Score** | **Expect** | **Rank** | **Peptide** |
| --- | --- | --- | --- | --- | --- | --- | --- | --- | --- | --- |
|  | 7014 | **572.3181** | **1142.6217** | **1142.6295** | **-0.0078** | **0** | **15** | **6** | **1** | **R.SITNASAAIAPK.D** |
|  | 10359 | 664.8438 | 1327.6731 | 1326.7846 | 0.8885 | 0 | 4 | 61 | 7 | K.VVLALMLLHFR.F |
|  | 11182 | **688.9026** | **1375.7906** | **1375.7963** | **-0.0056** | **0** | **83** | **7.4e-007** | **1** | **K.TLDFIDVLLLSK.D** |
|  | 14312 | **786.0964** | **1570.1782** | **1569.8263** | **0.3518** | **1** | **2** | **54** | **4** | **R.LVHDFTDAVIRER.R** |

| **534.** | Q9HBI6\|CP4FB_HUMAN    **Mass:** 60843    **Score:** 83     **Queries matched:** 5   **emPAI:** 0.06 |
| --- | --- |
|  | Cytochrome P450 4F11 OS=Homo sapiens GN=CYP4F11 PE=2 SV=2 |

|  | **Query** | **Observed** | **Mr(expt)** | **Mr(calc)** | **Delta** | **Miss** | **Score** | **Expect** | **Rank** | **Peptide** |
| --- | --- | --- | --- | --- | --- | --- | --- | --- | --- | --- |
|  | 11182 | 688.9026 | 1375.7906 | 1375.7963 | -0.0056 | 0 | 83 | 7.4e-007 | 1 | K.TLDFIDVLLLSK.D |
|  | 12159 | 477.5728 | 1429.6965 | 1428.6932 | 1.0033 | 1 | 7 | 25 | 2 | K.SVNIMHDKWQR.L 12124 |
|  | 19765 | 656.0113 | 1965.0120 | 1966.0209 | -1.0088 | 0 | 3 | 44 | 10 | R.MLTPAFHFNILKPYMK.I |
|  | 31536 | **1735.2394** | **5202.6963** | **5203.7548** | **-1.0584** | **1** | **2** | **8.5** | **4** | **-.MPQLSLSWLGLGPVAASPWLLLLLVGGSWLLARVLAWTYTFYDNCR.R** |

| **535.** | Q96AB3\|ISOC2_HUMAN    **Mass:** 22608    **Score:** 82     **Queries matched:** 6   **emPAI:** 0.17 |
| --- | --- |
|  | Isochorismatase domain-containing protein 2, mitochondrial OS=Homo sapiens GN=ISOC2 PE=1 SV=1 |

|  | **Query** | **Observed** | **Mr(expt)** | **Mr(calc)** | **Delta** | **Miss** | **Score** | **Expect** | **Rank** | **Peptide** |
| --- | --- | --- | --- | --- | --- | --- | --- | --- | --- | --- |
|  | 24144 | **816.7649** | **2447.2728** | **2446.3220** | **0.9509** | **1** | **15** | **2.1** | **1** | **K.LIKEPAPDSGLLGLFQGQNSLLH.-** 24115 |
|  | 26101 | **915.1516** | **2742.4330** | **2741.4388** | **0.9942** | **0** | **5** | **20** | **1** | **R.QSGAFLSTSEGLILQLVGDAVHPQFK.E** |
|  | 27583 | 1015.5240 | 3043.5501 | 3044.5753 | -1.0252 | 1 | 1 | 35 | 10 | R.MRQSGAFLSTSEGLILQLVGDAVHPQFK.E |
|  | 29337 | **1182.6409** | **3544.9008** | **3544.9215** | **-0.0207** | **0** | **83** | **1.6e-007** | **1** | **R.LLEVPVMLTEQYPQGLGPTVPELGTEGLRPLAK.T** 29338 |

| **536.** | P04899\|GNAI2_HUMAN    **Mass:** 40995    **Score:** 82     **Queries matched:** 13   **emPAI:** 0.19 |
| --- | --- |
|  | Guanine nucleotide-binding protein G(i), alpha-2 subunit OS=Homo sapiens GN=GNAI2 PE=1 SV=3 |

|  | **Query** | **Observed** | **Mr(expt)** | **Mr(calc)** | **Delta** | **Miss** | **Score** | **Expect** | **Rank** | **Peptide** |
| --- | --- | --- | --- | --- | --- | --- | --- | --- | --- | --- |
|  | 5850 | **529.3127** | **1056.6108** | **1056.6179** | **-0.0071** | **0** | **26** | **0.46** | **1** | **K.LLLLGAGESGK.S** 5844 5849 |
|  | 6591 | **555.7626** | **1109.5106** | **1109.5175** | **-0.0069** | **0** | **37** | **0.028** | **1** | **K.LFDSICNNK.W** |
|  | 9442 | 640.7906 | 1279.5666 | 1280.5932 | -1.0265 | 1 | 2 | 95 | 10 | K.MFDVGGQRSER.K |
|  | 11258 | **460.9072** | **1379.6998** | **1379.7085** | **-0.0088** | **0** | **19** | **1.7** | **2** | **K.TTGIVETHFTFK.D** |
|  | 14205 | 522.2354 | 1563.6844 | 1564.7457 | -1.0613 | 1 | 4 | 44 | 10 | K.DLHFKMFDVGGQR.S |
|  | 14509 | **529.2706** | **1584.7899** | **1584.9450** | **-0.1552** | **1** | **3** | **61** | **4** | **K.LLLLGAGESGKSTIVK.Q** |
|  | 14527 | **793.8569** | **1585.6992** | **1585.7083** | **-0.0091** | **0** | **13** | **6** | **3** | **K.EIYTHFTCATDTK.N** |
|  | 16365 | **570.3245** | **1707.9516** | **1706.9243** | **1.0272** | **0** | **1** | **82** | **10** | **K.NVQFVFDAVTDVIIK.N** |
|  | 16745 | 869.4826 | 1736.9506 | 1735.9542 | 0.9964 | 0 | 72 | 6.2e-006 | 1 | R.AVVYSNTIQSIMAIVK.A |
|  | 18805 | 629.6392 | 1885.8957 | 1884.8168 | 1.0788 | 1 | 5 | 31 | 4 | R.MHESMKLFDSICNNK.W |
|  | 28590 | **826.8949** | **3303.5505** | **3303.5758** | **-0.0253** | **1** | **5** | **12** | **1** | **K.ITHSPLTICFPEYTGANKYDEAASYIQSK.F** |

| **537.** | O95342\|ABCBB_HUMAN    **Mass:** 147440   **Score:** 82     **Queries matched:** 22   **emPAI:** 0.02 |
| --- | --- |
|  | Bile salt export pump OS=Homo sapiens GN=ABCB11 PE=1 SV=1 |

|  | **Query** | **Observed** | **Mr(expt)** | **Mr(calc)** | **Delta** | **Miss** | **Score** | **Expect** | **Rank** | **Peptide** |
| --- | --- | --- | --- | --- | --- | --- | --- | --- | --- | --- |
|  | 893 | **518.2855** | **517.2782** | **516.3020** | **0.9763** | **0** | **8** | **66** | **1** | **K.ISAAR.F** |
|  | 1109 | 561.3217 | 560.3144 | 559.3329 | 0.9814 | 0 | 13 | 17 | 4 | K.TAIQK.A |
|  | 1494 | **645.3884** | **644.3812** | **643.4017** | **0.9795** | **1** | **16** | **7.6** | **7** | **K.LDRIK.G** |
|  | 2540 | 414.7127 | 827.4107 | 826.5137 | 0.8971 | 1 | 4 | 70 | 9 | K.QRIAIAR.A |
|  | 3601 | 894.4257 | 893.4184 | 893.4032 | 0.0152 | 0 | 2 | 1.1e+002 | 9 | K.WDNFQGK.I |
|  | 4134 | 466.2376 | 930.4607 | 929.5294 | 0.9313 | 1 | 4 | 72 | 10 | R.TVAGIGKER.R |
|  | 4672 | 485.7269 | 969.4392 | 968.5226 | 0.9166 | 0 | 15 | 4.8 | 2 | R.TCIVIAHR.L |
|  | 5495 | **515.3518** | **1028.6889** | **1028.5978** | **0.0911** | **0** | **7** | **29** | **6** | **K.STALQLIQR.F** |
|  | 7048 | **573.8127** | **1145.6108** | **1144.5547** | **1.0562** | **1** | **9** | **22** | **4** | **K.VMIDGHDSKK.V** |
|  | 10346 | **664.3685** | **1326.7224** | **1326.7329** | **-0.0105** | **1** | **11** | **11** | **5** | **K.EIPMERVIAAAK.Q** |
|  | 10850 | 453.2252 | 1356.6538 | 1355.8136 | 0.8402 | 0 | 6 | 34 | 1 | R.VISAVVLSATALGR.A |
|  | 12256 | **479.5535** | **1435.6386** | **1435.7936** | **-0.1550** | **1** | **11** | **11** | **8** | **K.ISAARFFQLLDR.Q** |
|  | 15427 | 821.9036 | 1641.7926 | 1641.8185 | -0.0259 | 0 | 4 | 51 | 6 | K.QAQLHDFVMSLPEK.Y |
|  | 15529 | **550.6428** | **1648.9065** | **1649.7872** | **-0.8807** | **0** | **4** | **43** | **5** | **R.AMLGQDIAWFDDLR.N** |
|  | 16691 | **578.8155** | **1733.4246** | **1732.9108** | **0.5139** | **1** | **5** | **24** | **2** | **K.DIPVQEEVEPAPVRR.I** |
|  | 17383 | 891.9526 | 1781.8906 | 1781.8982 | -0.0076 | 1 | 2 | 63 | 4 | K.AYAKAGVVADEVISSMR.T |
|  | 18288 | **924.4728** | **1846.9310** | **1846.9411** | **-0.0101** | **0** | **82** | **6.8e-007** | **1** | **K.ILLLDEATSALDTESEK.T** |
|  | 20614 | 1023.0003 | 2043.9861 | 2042.8826 | 1.1035 | 0 | 3 | 44 | 2 | R.MEIGWFDCNSVGELNTR.F |
|  | 22148 | 1097.4823 | 2192.9500 | 2193.1100 | -0.1599 | 1 | 6 | 19 | 1 | K.AGVVADEVISSMRTVAAFGGEK.R |
|  | 22476 | **1115.6023** | **2229.1900** | **2229.1423** | **0.0477** | **1** | **1** | **63** | **3** | **R.DKQALEMVGQITNEALSNIR.T** |
|  | 23460 | **785.5073** | **2353.5000** | **2353.2868** | **0.2131** | **0** | **3** | **23** | **3** | **K.LSLVILCFFPFLALSGATQTR.M** |
|  | 30502 | **1364.6588** | **4090.9546** | **4092.0337** | **-1.0791** | **0** | **1** | **23** | **3** | **R.LATDASQVQGAAGSQIGMIVNSFTNVTVAMIIAFSFSWK.L** |

| **538.** | P84098\|RL19_HUMAN    **Mass:** 23565    **Score:** 82     **Queries matched:** 10   **emPAI:** 0.16 |
| --- | --- |
|  | 60S ribosomal protein L19 OS=Homo sapiens GN=RPL19 PE=1 SV=1 |

|  | **Query** | **Observed** | **Mr(expt)** | **Mr(calc)** | **Delta** | **Miss** | **Score** | **Expect** | **Rank** | **Peptide** |
| --- | --- | --- | --- | --- | --- | --- | --- | --- | --- | --- |
|  | 1165 | **574.9915** | **573.9842** | **573.3234** | **0.6608** | **0** | **10** | **20** | **6** | **K.NTLAR.R** |
|  | 1482 | 643.3732 | 642.3659 | 641.3319 | 1.0340 | 0 | 13 | 9.5 | 3 | R.HMGIGK.R 1472 |
|  | 2946 | 854.4385 | 853.4313 | 854.4545 | -1.0232 | 1 | 10 | 15 | 2 | K.GRHMGIGK.R 2957 |
|  | 5371 | **510.7828** | **1019.5510** | **1019.5586** | **-0.0076** | **0** | **13** | **9.9** | **1** | **R.ILMEHIHK.L** |
|  | 7704 | **596.2982** | **1190.5819** | **1190.5906** | **-0.0087** | **0** | **29** | **0.21** | **1** | **R.HMYHSLYLK.V** 7705 7706 |
|  | 19498 | **971.9594** | **1941.9043** | **1941.9180** | **-0.0138** | **0** | **82** | **6.3e-007** | **1** | **K.VWLDPNETNEIANANSR.Q** |

| **539.** | Q9UDW1\|QCR9_HUMAN    **Mass:** 7304     **Score:** 82     **Queries matched:** 5   **emPAI:** 0.57 |
| --- | --- |
|  | Cytochrome b-c1 complex subunit 9 OS=Homo sapiens GN=UQCR10 PE=1 SV=3 |

|  | **Query** | **Observed** | **Mr(expt)** | **Mr(calc)** | **Delta** | **Miss** | **Score** | **Expect** | **Rank** | **Peptide** |
| --- | --- | --- | --- | --- | --- | --- | --- | --- | --- | --- |
|  | 1925 | 762.4305 | 761.4232 | 761.4283 | -0.0051 | 0 | 20 | 2.3 | 1 | M.AAATLTSK.L 1920 |
|  | 3597 | **447.5051** | **892.9957** | **892.4688** | **0.5269** | **0** | **4** | **41** | **2** | **-.MAAATLTSK.L** |
|  | 18525 | **932.4228** | **1862.8310** | **1862.8435** | **-0.0125** | **0** | **82** | **6.8e-007** | **1** | **R.AFDQGADAIYDHINEGK.L** 18524 |

| **540.** | P46781\|RS9_HUMAN    **Mass:** 22635    **Score:** 82     **Queries matched:** 20   **emPAI:** 0.87 |
| --- | --- |
|  | 40S ribosomal protein S9 OS=Homo sapiens GN=RPS9 PE=1 SV=3 |

|  | **Query** | **Observed** | **Mr(expt)** | **Mr(calc)** | **Delta** | **Miss** | **Score** | **Expect** | **Rank** | **Peptide** |
| --- | --- | --- | --- | --- | --- | --- | --- | --- | --- | --- |
|  | 2843 | 424.7085 | 847.4024 | 847.5280 | -0.1255 | 1 | 5 | 61 | 4 | K.FTLAKIR.K |
|  | 3107 | **432.2164** | **862.4182** | **863.4865** | **-1.0682** | **1** | **12** | **13** | **2** | **R.KTYVTPR.R** |
|  | 3517 | 444.2373 | 886.4600 | 886.4661 | -0.0061 | 0 | 28 | 0.32 | 1 | K.HIDFSLR.S |
|  | 3998 | **460.7612** | **919.5078** | **919.5127** | **-0.0048** | **0** | **44** | **0.0077** | **1** | **K.LIGEYGLR.N** 3975 |
|  | 4007 | 461.2349 | 920.4552 | 920.4603 | -0.0051 | 0 | 14 | 7.4 | 1 | K.IEDFLER.R |
|  | 4178 | **467.7812** | **933.5479** | **933.5535** | **-0.0056** | **0** | **30** | **0.21** | **1** | **K.LDYILGLK.I** |
|  | 4907 | **494.2612** | **986.5078** | **987.5349** | **-1.0270** | **1** | **19** | **2.4** | **5** | **K.SRLDQELK.L** |
|  | 5363 | 510.3000 | 1018.5855 | 1018.5924 | -0.0069 | 1 | 25 | 0.54 | 1 | R.RLQTQVFK.L 5348 |
|  | 5512 | **516.2758** | **1030.5371** | **1031.5764** | **-1.0393** | **0** | **41** | **0.014** | **1** | **R.LFEGNALLR.R** 5525 5533 |
|  | 7725 | 596.8142 | 1191.6139 | 1192.6889 | -1.0751 | 1 | 11 | 11 | 2 | K.MKLDYILGLK.I |
|  | 7954 | **602.3661** | **1202.7176** | **1201.7043** | **1.0133** | **1** | **4** | **57** | **4** | **K.LGLAKSIHHAR.V** |
|  | 9284 | **636.3732** | **1270.7319** | **1270.7398** | **-0.0079** | **0** | **36** | **0.039** | **1** | **K.QVVNIPSFIVR.L** |
|  | 10346 | 664.3685 | 1326.7224 | 1327.6983 | -0.9759 | 1 | 18 | 2.4 | 2 | R.ELLTLDEKDPR.R 10373 |
|  | 11597 | **700.4199** | **1398.8253** | **1398.8347** | **-0.0094** | **1** | **50** | **0.0013** | **1** | **R.KQVVNIPSFIVR.L** |
|  | 18191 | **614.8809** | **1841.6208** | **1842.0363** | **-0.4156** | **1** | **1** | **60** | **6** | **K.QVVNIPSFIVRLDSQK.H** |

| **541.** | P55327\|TPD52_HUMAN    **Mass:** 24369    **Score:** 81     **Queries matched:** 4   **emPAI:** 0.16 |
| --- | --- |
|  | Tumor protein D52 OS=Homo sapiens GN=TPD52 PE=1 SV=2 |

|  | **Query** | **Observed** | **Mr(expt)** | **Mr(calc)** | **Delta** | **Miss** | **Score** | **Expect** | **Rank** | **Peptide** |
| --- | --- | --- | --- | --- | --- | --- | --- | --- | --- | --- |
|  | 1684 | 693.3514 | 692.3442 | 692.3493 | -0.0052 | 0 | 15 | 6.8 | 1 | K.NSPTFK.S |
|  | 1820 | **732.2934** | **731.2861** | **730.4225** | **0.8636** | **1** | **13** | **11** | **1** | **K.KLEDVK.N** |
|  | 10300 | **663.8151** | **1325.6156** | **1325.6252** | **-0.0096** | **0** | **21** | **1** | **1** | **K.GWQDVTATSAYK.K** |
|  | 15637 | **829.4487** | **1656.8829** | **1656.8934** | **-0.0105** | **0** | **81** | **7.9e-007** | **1** | **K.VEEEIQTLSQVLAAK.E** |

| **542.** | O60361\|NDK8_HUMAN    **Mass:** 15690    **Score:** 81     **Queries matched:** 7   **emPAI:** 0.95 |
| --- | --- |
|  | Putative nucleoside diphosphate kinase OS=Homo sapiens GN=NME2P1 PE=5 SV=1 |

|  | **Query** | **Observed** | **Mr(expt)** | **Mr(calc)** | **Delta** | **Miss** | **Score** | **Expect** | **Rank** | **Peptide** |
| --- | --- | --- | --- | --- | --- | --- | --- | --- | --- | --- |
|  | 5770 | **526.2495** | **1050.4843** | **1050.4917** | **-0.0073** | **0** | **35** | **0.054** | **1** | **R.GDFCIQVGR.N** |
|  | 8785 | **623.2929** | **1244.5711** | **1245.6937** | **-1.1226** | **1** | **0** | **1.4e+002** | **10** | **-.MQCGLVGKIIK.R** |
|  | 17417 | **893.4566** | **1784.8986** | **1784.9091** | **-0.0104** | **0** | **61** | **7.8e-005** | **1** | **R.VMLGETNPADSKPGTIR.G** |
|  | 17623 | **601.3988** | **1801.1746** | **1800.9040** | **0.2706** | **0** | **(0)** | **76** | **7** | **R.VMLGETNPADSKPGTIR.G** |
|  | 21118 | **1047.0264** | **2092.0382** | **2092.0486** | **-0.0104** | **0** | **31** | **0.067** | **1** | **K.YMNSGPVVAMVWEGLNVVK.T** |
|  | 21491 | 1063.0399 | 2124.0653 | 2124.0384 | 0.0269 | 0 | (1) | 64 | 5 | K.YMNSGPVVAMVWEGLNVVK.T |
|  | 23929 | **808.3954** | **2422.1645** | **2422.2137** | **-0.0492** | **1** | **2** | **47** | **1** | **K.YMNSGPVVAMVWEGLNVVKTGR.V** |

|  | **Proteins matching a subset of these peptides:** |
| --- | --- |

|  | P22392\|NDKB_HUMAN    **Mass:** 17401    **Score:** 81     **Queries matched:** 6 |
| --- | --- |
|  | Nucleoside diphosphate kinase B OS=Homo sapiens GN=NME2 PE=1 SV=1 |

| **543.** | P62330\|ARF6_HUMAN    **Mass:** 20183    **Score:** 81     **Queries matched:** 8   **emPAI:** 0.42 |
| --- | --- |
|  | ADP-ribosylation factor 6 OS=Homo sapiens GN=ARF6 PE=1 SV=2 |

|  | **Query** | **Observed** | **Mr(expt)** | **Mr(calc)** | **Delta** | **Miss** | **Score** | **Expect** | **Rank** | **Peptide** |
| --- | --- | --- | --- | --- | --- | --- | --- | --- | --- | --- |
|  | 2763 | 421.2294 | 840.4443 | 839.5130 | 0.9313 | 0 | 8 | 24 | 8 | K.IRPLWR.H |
|  | 9151 | **633.1082** | **1264.2017** | **1263.5884** | **0.6133** | **0** | **0** | **89** | **3** | **K.FNVWDVGGQDK.I** |
|  | 13749 | 767.4361 | 1532.8576 | 1532.8384 | 0.0192 | 1 | 12 | 8.5 | 2 | R.EMRDAIILIFANK.Q |
|  | 17545 | **599.3470** | **1795.0191** | **1793.8618** | **1.1573** | **0** | **2** | **67** | **7** | **K.QDLPDAMKPHEIQEK.L** |
|  | 17856 | **607.5645** | **1819.6717** | **1820.0481** | **-0.3764** | **1** | **7** | **17** | **1** | **R.ILMLGLDAAGKTTILYK.L** |
|  | 18121 | 612.9738 | 1835.8994 | 1836.0430 | -0.1436 | 1 | (5) | 37 | 7 | R.ILMLGLDAAGKTTILYK.L |
|  | 22641 | **1127.5946** | **2253.1746** | **2253.1893** | **-0.0147** | **0** | **35** | **0.026** | **1** | **K.LGQSVTTIPTVGFNVETVTYK.N** |
|  | 27809 | **1555.7069** | **3109.3993** | **3109.4128** | **-0.0135** | **0** | **69** | **6.3e-006** | **1** | **R.NWYVQPSCATSGDGLYEGLTWLTSNYK.S** |

| **544.** | P07910\|HNRPC_HUMAN    **Mass:** 33707    **Score:** 81     **Queries matched:** 19   **emPAI:** 0.37 |
| --- | --- |
|  | Heterogeneous nuclear ribonucleoproteins C1/C2 OS=Homo sapiens GN=HNRNPC PE=1 SV=4 |

|  | **Query** | **Observed** | **Mr(expt)** | **Mr(calc)** | **Delta** | **Miss** | **Score** | **Expect** | **Rank** | **Peptide** |
| --- | --- | --- | --- | --- | --- | --- | --- | --- | --- | --- |
|  | 1816 | **731.4252** | **730.4180** | **730.4225** | **-0.0045** | **0** | **12** | **15** | **4** | **K.ELTQIK.Q** |
|  | 3027 | 430.2997 | 858.5849 | 858.5174 | 0.0674 | 1 | 17 | 4.3 | 3 | K.KELTQIK.Q |
|  | 3514 | **444.2048** | **886.3950** | **886.4007** | **-0.0057** | **0** | **14** | **8.9** | **1** | **R.MYSYPAR.V** |
|  | 3839 | **455.2491** | **908.4837** | **908.2995** | **0.1842** | **0** | **10** | **16** | **2** | **R.DSANGEDDS.-** |
|  | 4309 | 472.2871 | 942.5595 | 942.5651 | -0.0055 | 0 | 15 | 5.2 | 1 | R.VPPPPPIAR.A 4308 4326 |
|  | 5021 | 498.2529 | 994.4912 | 994.4971 | -0.0059 | 0 | 30 | 0.16 | 1 | K.SDVEAIFSK.Y |
|  | 6164 | **539.7400** | **1077.4654** | **1078.4825** | **-1.0171** | **1** | **3** | **81** | **5** | **K.TDPRSMNSR.V** |
|  | 6478 | **551.3149** | **1100.6153** | **1099.6237** | **0.9916** | **1** | **23** | **0.89** | **1** | **K.LKGDDLQAIK.K** |
|  | 6756 | **562.2996** | **1122.5846** | **1122.5921** | **-0.0075** | **1** | **48** | **0.0025** | **1** | **K.KSDVEAIFSK.Y** 6767 |
|  | 7214 | **580.2490** | **1158.4834** | **1158.6132** | **-0.1298** | **0** | **4** | **61** | **6** | **K.VDSLLENLEK.I** |
|  | 7914 | **401.2844** | **1200.8313** | **1201.6051** | **-0.7739** | **1** | **8** | **21** | **9** | **M.ASNVTNKTDPR.S** |
|  | 10126 | 658.8964 | 1315.7783 | 1315.7864 | -0.0081 | 0 | 63 | 6.6e-005 | 1 | R.VFIGNLNTLVVK.K |
|  | 12362 | **482.2584** | **1443.7533** | **1443.8813** | **-0.1281** | **1** | **3** | **60** | **6** | **R.VFIGNLNTLVVKK.S** |
|  | 13600 | **762.3768** | **1522.7391** | **1523.9188** | **-1.1797** | **1** | **3** | **66** | **2** | **R.VPPPPPIARAVVPSK.R** |
|  | 15999 | **561.6399** | **1681.8978** | **1681.9073** | **-0.0094** | **0** | **1** | **84** | **10** | **R.MIAGQVLDINLAAEPK.V** |
|  | 17985 | **610.1809** | **1827.5209** | **1826.9501** | **0.5708** | **1** | **3** | **33** | **2** | **R.MYSYPARVPPPPPIAR.A** |

| **545.** | O60812\|HNRCL_HUMAN    **Mass:** 32180    **Score:** 81     **Queries matched:** 16   **emPAI:** 0.39 |
| --- | --- |
|  | Heterogeneous nuclear ribonucleoprotein C-like 1 OS=Homo sapiens GN=HNRNPCL1 PE=1 SV=1 |

|  | **Query** | **Observed** | **Mr(expt)** | **Mr(calc)** | **Delta** | **Miss** | **Score** | **Expect** | **Rank** | **Peptide** |
| --- | --- | --- | --- | --- | --- | --- | --- | --- | --- | --- |
|  | 1799 | **728.0422** | **727.0350** | **727.3501** | **-0.3151** | **0** | **9** | **14** | **2** | **K.DETHVK.M** 1798 |
|  | 3027 | 430.2997 | 858.5849 | 858.4811 | 0.1038 | 0 | 17 | 4.3 | 3 | K.QELTQIK.Q |
|  | 3912 | 458.2385 | 914.4624 | 913.5094 | 0.9530 | 1 | 6 | 42 | 5 | K.VNRGNAGVK.R |
|  | 5021 | 498.2529 | 994.4912 | 994.4971 | -0.0059 | 0 | 30 | 0.16 | 1 | K.SDVEAIFSK.Y |
|  | 6478 | 551.3149 | 1100.6153 | 1099.6237 | 0.9916 | 1 | 23 | 0.89 | 1 | K.LKGDDLQAIK.Q |
|  | 6547 | **554.2401** | **1106.4655** | **1105.4281** | **1.0375** | **0** | **6** | **42** | **5** | **K.MDPHSMNSR.V** |
|  | 6756 | 562.2996 | 1122.5846 | 1122.5921 | -0.0075 | 1 | 48 | 0.0025 | 1 | K.KSDVEAIFSK.Y 6767 |
|  | 7214 | 580.2490 | 1158.4834 | 1158.6132 | -0.1298 | 0 | 4 | 61 | 6 | K.VDSLLENLEK.I |
|  | 10126 | 658.8964 | 1315.7783 | 1315.7864 | -0.0081 | 0 | 63 | 6.6e-005 | 1 | R.VFIGNLNTLVVK.K |
|  | 12362 | 482.2584 | 1443.7533 | 1443.8813 | -0.1281 | 1 | 3 | 60 | 6 | R.VFIGNLNTLVVKK.S |
|  | 16233 | **567.2975** | **1698.8706** | **1698.9152** | **-0.0445** | **1** | **2** | **63** | **8** | **K.GDDLQAIKQELTQIK.Q** |
|  | 27102 | 737.8881 | 2947.5234 | 2946.4990 | 1.0244 | 1 | (0) | 53 | 8 | R.DYYDGMYSFPARVPPPPPIALAVVPSK.R |
|  | 27170 | **988.5028** | **2962.4866** | **2962.4939** | **-0.0073** | **1** | **3** | **29** | **3** | **R.DYYDGMYSFPARVPPPPPIALAVVPSK.R** |
|  | 30830 | **1463.8921** | **4388.6544** | **4388.8358** | **-0.1813** | **1** | **8** | **2.5** | **1** | **K.DETHVKMESEGGAEDSAEEGDPLDDDVNEDQGDDQLELIK.D** |

| **546.** | P50213\|IDH3A_HUMAN    **Mass:** 40022    **Score:** 81     **Queries matched:** 11   **emPAI:** 0.20 |
| --- | --- |
|  | Isocitrate dehydrogenase [NAD] subunit alpha, mitochondrial OS=Homo sapiens GN=IDH3A PE=1 SV=1 |

|  | **Query** | **Observed** | **Mr(expt)** | **Mr(calc)** | **Delta** | **Miss** | **Score** | **Expect** | **Rank** | **Peptide** |
| --- | --- | --- | --- | --- | --- | --- | --- | --- | --- | --- |
|  | 4563 | **481.2412** | **960.4677** | **960.4739** | **-0.0061** | **0** | **11** | **14** | **1** | **K.WMIPSEAK.E** |
|  | 5612 | **519.7648** | **1037.5150** | **1037.5216** | **-0.0066** | **0** | **20** | **1.6** | **1** | **R.MSDGLFLQK.C** |
|  | 5659 | **521.7861** | **1041.5576** | **1040.5615** | **0.9961** | **0** | **18** | **2.9** | **1** | **R.NVTAIQGPGGK.W** |
|  | 14620 | 531.2952 | 1590.8639 | 1589.8712 | 0.9927 | 0 | 6 | 29 | 8 | K.TPIAAGHPSMNLLLR.K |
|  | 15392 | **547.4689** | **1639.3850** | **1638.8222** | **0.5628** | **1** | **6** | **19** | **1** | **K.ANIMRMSDGLFLQK.C** |
|  | 16697 | 867.9437 | 1733.8728 | 1733.9610 | -0.0883 | 1 | 4 | 43 | 2 | K.TPIAAGHPSMNLLLRK.T |
|  | 18269 | **923.9797** | **1845.9449** | **1845.9514** | **-0.0065** | **0** | **60** | **9.5e-005** | **1** | **K.DMANPTALLLSAVMMLR.H** |
|  | 18706 | **470.4744** | **1877.8686** | **1877.9413** | **-0.0727** | **0** | **(3)** | **53** | **4** | **K.DMANPTALLLSAVMMLR.H** |
|  | 19965 | **993.5057** | **1984.9969** | **1985.1244** | **-0.1275** | **1** | **1** | **69** | **1** | **K.GPLKTPIAAGHPSMNLLLR.K** |
|  | 20682 | 684.6675 | 2050.9806 | 2050.0596 | 0.9210 | 1 | 2 | 63 | 3 | K.APIQWEERNVTAIQGPGGK.W |
|  | 24582 | **835.0804** | **2502.2195** | **2501.2286** | **0.9909** | **0** | **41** | **0.0059** | **1** | **R.ENTEGEYSGIEHVIVDGVVQSIK.L** |

| **547.** | O95168\|NDUB4_HUMAN    **Mass:** 15256    **Score:** 80     **Queries matched:** 6   **emPAI:** 0.26 |
| --- | --- |
|  | NADH dehydrogenase [ubiquinone] 1 beta subcomplex subunit 4 OS=Homo sapiens GN=NDUFB4 PE=1 SV=3 |

|  | **Query** | **Observed** | **Mr(expt)** | **Mr(calc)** | **Delta** | **Miss** | **Score** | **Expect** | **Rank** | **Peptide** |
| --- | --- | --- | --- | --- | --- | --- | --- | --- | --- | --- |
|  | 6084 | 536.8181 | 1071.6217 | 1070.6084 | 1.0133 | 1 | 7 | 41 | 7 | K.LIQEGKLDR.T |
|  | 13925 | **516.2806** | **1545.8201** | **1545.8304** | **-0.0103** | **0** | **11** | **9.1** | **1** | **R.TINVYPNFRPTPK.N** |
|  | 20626 | **1023.4982** | **2044.9818** | **2044.9953** | **-0.0135** | **0** | **72** | **5.9e-006** | **1** | **R.TLPETLDPAEYNISPETR.R** 20627 |
|  | 22197 | **734.3754** | **2200.1043** | **2201.0964** | **-0.9921** | **1** | **3** | **45** | **1** | **R.TLPETLDPAEYNISPETRR.A** 22209 |

| **548.** | P00480\|OTC_HUMAN    **Mass:** 40081    **Score:** 80     **Queries matched:** 18   **emPAI:** 0.56 |
| --- | --- |
|  | Ornithine carbamoyltransferase, mitochondrial OS=Homo sapiens GN=OTC PE=1 SV=3 |

|  | **Query** | **Observed** | **Mr(expt)** | **Mr(calc)** | **Delta** | **Miss** | **Score** | **Expect** | **Rank** | **Peptide** |
| --- | --- | --- | --- | --- | --- | --- | --- | --- | --- | --- |
|  | 1042 | 547.2689 | 546.2616 | 547.2602 | -0.9986 | 0 | 8 | 48 | 4 | K.ENGTK.L |
|  | 4225 | **470.1923** | **938.3701** | **939.4735** | **-1.1034** | **0** | **4** | **63** | **10** | **K.SLGMIFEK.R** |
|  | 4951 | **495.7551** | **989.4957** | **989.5029** | **-0.0072** | **0** | **23** | **0.86** | **2** | **K.QSDLDTLAK.E** |
|  | 5978 | **533.7767** | **1065.5388** | **1065.4978** | **0.0409** | **0** | **13** | **7.7** | **3** | **K.GYEPDASVTK.L** |
|  | 7879 | **400.8756** | **1199.6049** | **1199.6121** | **-0.0072** | **0** | **35** | **0.046** | **1** | **K.FGMHLQAATPK.G** 7878 |
|  | 11721 | 469.7422 | 1406.2048 | 1406.6626 | -0.4577 | 1 | 8 | 13 | 1 | R.NGHNFMVRNFR.C |
|  | 11865 | **707.8606** | **1413.7066** | **1412.7122** | **0.9944** | **0** | **17** | **2.7** | **1** | **R.LQAFQGYQVTMK.T** 11866 |
|  | 14492 | 396.9298 | 1583.6903 | 1584.8082 | -1.1180 | 1 | 10 | 12 | 7 | K.RLQAFQGYQVTMK.T |
|  | 23991 | **810.4121** | **2428.2143** | **2428.2243** | **-0.0099** | **0** | **17** | **1.3** | **1** | **K.GLTLSWIGDGNNILHSIMMSAAK.F** 23989 |
|  | 25116 | **861.0952** | **2580.2638** | **2580.3332** | **-0.0694** | **0** | **1** | **49** | **1** | **K.WTIMAVMVSLLTDYSPQLQKPK.F** |
|  | 26006 | **909.7936** | **2726.3589** | **2727.4016** | **-1.0427** | **1** | **2** | **37** | **6** | **K.WTIMAVMVSLLTDYSPQLQKPKF.-** |
|  | 28913 | **1132.2275** | **3393.6608** | **3393.6762** | **-0.0154** | **0** | **32** | **0.026** | **1** | **K.LLLTNDPLEAAHGGNVLITDTWISMGQEEEK.K** 28918 28919 |
|  | 30176 | **1285.6719** | **3853.9938** | **3854.0141** | **-0.0203** | **0** | **46** | **0.00064** | **1** | **K.EASIPIINGLSDLYHPIQILADYLTLQEHYSSLK.G** |

| **549.** | P09651\|ROA1_HUMAN    **Mass:** 38936    **Score:** 80     **Queries matched:** 16   **emPAI:** 0.32 |
| --- | --- |
|  | Heterogeneous nuclear ribonucleoprotein A1 OS=Homo sapiens GN=HNRNPA1 PE=1 SV=4 |

|  | **Query** | **Observed** | **Mr(expt)** | **Mr(calc)** | **Delta** | **Miss** | **Score** | **Expect** | **Rank** | **Peptide** |
| --- | --- | --- | --- | --- | --- | --- | --- | --- | --- | --- |
|  | 3080 | 431.7356 | 861.4567 | 860.5484 | 0.9083 | 1 | 12 | 14 | 4 | K.KIFVGGIK.E |
|  | 3640 | **448.7788** | **895.5431** | **894.3944** | **1.1487** | **0** | **6** | **40** | **3** | **R.SGSGNFGGGR.G** 3621 |
|  | 7851 | **400.2376** | **1197.6909** | **1196.5091** | **1.1818** | **0** | **12** | **10** | **2** | **K.QEMASASSSQR.G** |
|  | 8297 | 610.3333 | 1218.6521 | 1217.6326 | 1.0195 | 0 | 12 | 10 | 2 | K.IEVIEIMTDR.G |
|  | 8560 | **412.1910** | **1233.5513** | **1233.6275** | **-0.0762** | **0** | **(7)** | **27** | **2** | **K.IEVIEIMTDR.G** |
|  | 14423 | **527.2535** | **1578.7386** | **1579.7624** | **-1.0238** | **1** | **2** | **76** | **8** | **K.ALSKQEMASASSSQR.G** |
|  | 16146 | **847.8488** | **1693.6829** | **1693.6928** | **-0.0099** | **0** | **39** | **0.012** | **1** | **R.NQGGYGGSSSSSSYGSGR.R** |
|  | 16220 | **850.3782** | **1698.7419** | **1698.7526** | **-0.0107** | **0** | **54** | **0.0004** | **1** | **R.GFAFVTFDDHDSVDK.I** |
|  | 18325 | **617.3602** | **1849.0587** | **1849.7939** | **-0.7352** | **1** | **3** | **51** | **2** | **R.NQGGYGGSSSSSSYGSGRR.F** |
|  | 18732 | 627.6572 | 1879.9497 | 1878.9588 | 0.9909 | 1 | 7 | 20 | 5 | K.IFVGGIKEDTEEHHLR.D 18725 |
|  | 19164 | **957.4725** | **1912.9305** | **1911.9942** | **0.9363** | **1** | **29** | **0.12** | **1** | **R.KLFIGGLSFETTDESLR.S** 19141 19149 19163 |

| **550.** | Q13162\|PRDX4_HUMAN    **Mass:** 30749    **Score:** 80     **Queries matched:** 7   **emPAI:** 0.12 |
| --- | --- |
|  | Peroxiredoxin-4 OS=Homo sapiens GN=PRDX4 PE=1 SV=1 |

|  | **Query** | **Observed** | **Mr(expt)** | **Mr(calc)** | **Delta** | **Miss** | **Score** | **Expect** | **Rank** | **Peptide** |
| --- | --- | --- | --- | --- | --- | --- | --- | --- | --- | --- |
|  | 2123 | **399.1852** | **796.3559** | **796.4555** | **-0.0997** | **0** | **7** | **27** | **5** | **R.QGGLGPIR.I** 2116 |
|  | 2428 | 410.2115 | 818.4084 | 818.4134 | -0.0050 | 0 | 13 | 9.8 | 3 | R.SVDETLR.L |
|  | 3994 | 460.7509 | 919.4872 | 919.5015 | -0.0143 | 0 | 29 | 0.24 | 1 | R.GLFIIDDK.G 3995 |
|  | 9687 | **431.7356** | **1292.1851** | **1291.6884** | **0.4966** | **0** | **7** | **21** | **2** | **R.VSVADHSLHLSK.A** |
|  | 24084 | **815.0245** | **2442.0516** | **2442.0659** | **-0.0142** | **1** | **80** | **7e-007** | **1** | **R.TREEECHFYAGGQVYPGEASR.V** |

| **551.** | P62847\|RS24_HUMAN    **Mass:** 15413    **Score:** 80     **Queries matched:** 10   **emPAI:** 0.25 |
| --- | --- |
|  | 40S ribosomal protein S24 OS=Homo sapiens GN=RPS24 PE=1 SV=1 |

|  | **Query** | **Observed** | **Mr(expt)** | **Mr(calc)** | **Delta** | **Miss** | **Score** | **Expect** | **Rank** | **Peptide** |
| --- | --- | --- | --- | --- | --- | --- | --- | --- | --- | --- |
|  | 882 | **516.3111** | **515.3038** | **514.3115** | **0.9923** | **0** | **9** | **44** | **10** | **K.ATVPK.T** |
|  | 2115 | **398.7078** | **795.4011** | **795.4061** | **-0.0050** | **1** | **9** | **21** | **5** | **R.KFMTNR.L** 2103 |
|  | 4411 | 475.8327 | 949.6508 | 948.4699 | 1.1810 | 0 | 12 | 11 | 2 | -.MNDTVTIR.T |
|  | 4734 | **487.3214** | **972.6283** | **972.5352** | **0.0931** | **1** | **6** | **46** | **6** | **R.GTAKANVGAGK.K** |
|  | 8021 | **403.0458** | **1206.1157** | **1205.6187** | **0.4970** | **1** | **9** | **12** | **3** | **-.MNDTVTIRTR.K** |
|  | 8592 | **618.3605** | **1234.7064** | **1235.6696** | **-0.9632** | **0** | **(6)** | **34** | **3** | **K.QMVIDVLHPGK.A** |
|  | 8919 | 627.3611 | 1252.7077 | 1251.6646 | 1.0432 | 0 | 7 | 28 | 2 | K.QMVIDVLHPGK.A |
|  | 15984 | **841.3873** | **1680.7600** | **1680.7705** | **-0.0105** | **0** | **68** | **1.8e-005** | **1** | **K.TTGFGMIYDSLDYAK.K** 15983 |

| **552.** | P10768\|ESTD_HUMAN    **Mass:** 31956    **Score:** 80     **Queries matched:** 7   **emPAI:** 0.25 |
| --- | --- |
|  | S-formylglutathione hydrolase OS=Homo sapiens GN=ESD PE=1 SV=2 |

|  | **Query** | **Observed** | **Mr(expt)** | **Mr(calc)** | **Delta** | **Miss** | **Score** | **Expect** | **Rank** | **Peptide** |
| --- | --- | --- | --- | --- | --- | --- | --- | --- | --- | --- |
|  | 1635 | **677.3346** | **676.3273** | **675.3551** | **0.9722** | **0** | **6** | **53** | **4** | **K.QISSNK.C** |
|  | 6913 | **568.2919** | **1134.5692** | **1134.6067** | **-0.0375** | **1** | **5** | **50** | **7** | **-.MALKQISSNK.C** |
|  | 11942 | **710.3038** | **1418.5931** | **1419.7762** | **-1.1831** | **1** | **1** | **1e+002** | **5** | **K.FAVYLPPKAETGK.C** |
|  | 20136 | **1001.9919** | **2001.9692** | **2001.9805** | **-0.0113** | **0** | **57** | **0.00021** | **1** | **K.SVSAFAPICNPVLCPWGK.K** 20137 |
|  | 20275 | 673.3554 | 2017.0442 | 2017.9536 | -0.9094 | 0 | 5 | 30 | 3 | R.MSIFGHSMGGHGALICALK.N |
|  | 30572 | **1380.0123** | **4137.0152** | **4137.0252** | **-0.0101** | **1** | **41** | **0.0022** | **1** | **K.SYPGSQLDILIDQGKDDQFLLDGQLLPDNFIAACTEK.K** |

| **553.** | P48230\|T4S4_HUMAN    **Mass:** 22351    **Score:** 80     **Queries matched:** 3   **emPAI:** 0.17 |
| --- | --- |
|  | Transmembrane 4 L6 family member 4 OS=Homo sapiens GN=TM4SF4 PE=1 SV=1 |

|  | **Query** | **Observed** | **Mr(expt)** | **Mr(calc)** | **Delta** | **Miss** | **Score** | **Expect** | **Rank** | **Peptide** |
| --- | --- | --- | --- | --- | --- | --- | --- | --- | --- | --- |
|  | 27834 | **1039.7824** | **3116.3252** | **3116.3433** | **-0.0181** | **0** | **57** | **8.2e-005** | **1** | **K.CLMANSTWGYPFHDGDYLNDEALWNK.C** 27833 |
|  | 28171 | 1063.2264 | 3186.6575 | 3187.6668 | -1.0093 | 0 | 0 | 44 | 2 | R.FAMFTSTIFAVVGFLGAGYSFIISAISINK.G |

| **554.** | P00367\|DHE3_HUMAN    **Mass:** 61701    **Score:** 79     **Queries matched:** 22   **emPAI:** 0.26 |
| --- | --- |
|  | Glutamate dehydrogenase 1, mitochondrial OS=Homo sapiens GN=GLUD1 PE=1 SV=2 |

|  | **Query** | **Observed** | **Mr(expt)** | **Mr(calc)** | **Delta** | **Miss** | **Score** | **Expect** | **Rank** | **Peptide** |
| --- | --- | --- | --- | --- | --- | --- | --- | --- | --- | --- |
|  | 1005 | **544.1245** | **543.1172** | **543.2765** | **-0.1593** | **0** | **10** | **22** | **4** | **K.SNAPR.V** |
|  | 2518 | 413.7710 | 825.5275 | 825.5072 | 0.0203 | 1 | 11 | 10 | 4 | K.AGVKINPK.N |
|  | 5060 | **499.2692** | **996.5238** | **996.5314** | **-0.0075** | **0** | **24** | **0.55** | **1** | **K.ALASLMTYK.C** |
|  | 5237 | 505.7397 | 1009.4649 | 1010.5219 | -1.0570 | 1 | 8 | 25 | 10 | R.RFTMELAK.K |
|  | 5260 | 506.6950 | 1011.3755 | 1010.4345 | 0.9409 | 0 | 10 | 14 | 4 | R.EDDPNFFK.M |
|  | 5269 | **1013.4408** | **1012.4335** | **1012.5263** | **-0.0928** | **0** | **(12)** | **10** | **2** | **K.ALASLMTYK.C** |
|  | 5870 | **530.2664** | **1058.5183** | **1058.5257** | **-0.0074** | **0** | **20** | **2.1** | **1** | **K.NLNHVSYGR.L** |
|  | 6186 | **540.2869** | **1078.5593** | **1078.5658** | **-0.0065** | **0** | **22** | **0.99** | **1** | **R.TAAYVNAIEK.V** |
|  | 7782 | **399.2327** | **1194.6762** | **1195.6713** | **-0.9952** | **0** | **9** | **17** | **2** | **K.LQHGSILGFPK.A** |
|  | 8709 | **621.2919** | **1240.5693** | **1240.5823** | **-0.0130** | **0** | **22** | **0.84** | **2** | **R.YSTDVSVDEVK.A** |
|  | 13118 | **745.9636** | **1489.9127** | **1490.7188** | **-0.8061** | **0** | **3** | **53** | **2** | **K.DIVHSGLAYTMER.S** |
|  | 16742 | **869.4442** | **1736.8737** | **1736.8846** | **-0.0109** | **0** | **15** | **3.4** | **1** | **K.HGGTIPIVPTAEFQDR.I** 16738 |
|  | 16896 | 874.8916 | 1747.7686 | 1747.8828 | -0.1142 | 0 | 37 | 0.021 | 2 | K.TFVVQGFGNVGLHSMR.Y |
|  | 18933 | **632.3758** | **1894.1055** | **1893.0658** | **1.0397** | **0** | **1** | **64** | **9** | **R.IIKPCNHVLSLSFPIR.R** |
|  | 19211 | 959.0148 | 1916.0150 | 1914.9146 | 1.1004 | 0 | 6 | 28 | 4 | K.GFIGPGIDVPAPDMSTGER.E |
|  | 19445 | 485.2480 | 1936.9627 | 1935.8996 | 1.0631 | 0 | 3 | 48 | 4 | R.DSNYHLLMSVQESLER.K |
|  | 22573 | **748.0562** | **2241.1468** | **2241.1641** | **-0.0173** | **1** | **13** | **4.5** | **1** | **K.IIAEGANGPTTPEADKIFLER.N** 22579 |
|  | 24474 | 830.7474 | 2489.2203 | 2489.2359 | -0.0156 | 0 | 25 | 0.25 | 1 | K.AKPYEGSILEADCDILIPAASEK.Q |
|  | 26097 | **686.5787** | **2742.2859** | **2741.4138** | **0.8720** | **0** | **3** | **26** | **2** | **R.NIMVIPDLYLNAGGVTVSYFEWLK.N** |
|  | 27474 | 1516.2117 | 3030.4088 | 3030.4256 | -0.0168 | 0 | 65 | 1.5e-005 | 1 | R.GVFHGIENFINEASYMSILGMTPGFGDK.T |

| **555.** | P35754\|GLRX1_HUMAN    **Mass:** 12053    **Score:** 79     **Queries matched:** 4   **emPAI:** 0.33 |
| --- | --- |
|  | Glutaredoxin-1 OS=Homo sapiens GN=GLRX PE=1 SV=2 |

|  | **Query** | **Observed** | **Mr(expt)** | **Mr(calc)** | **Delta** | **Miss** | **Score** | **Expect** | **Rank** | **Peptide** |
| --- | --- | --- | --- | --- | --- | --- | --- | --- | --- | --- |
|  | 5300 | 508.2729 | 1014.5312 | 1015.6179 | -1.0867 | 1 | 8 | 31 | 5 | R.TVPRVFIGK.D |
|  | 8684 | **413.9450** | **1238.8131** | **1238.7234** | **0.0897** | **0** | **5** | **33** | **6** | **R.AQEILSQLPIK.Q** |
|  | 28551 | **1096.8846** | **3287.6321** | **3287.6422** | **-0.0101** | **0** | **63** | **2.2e-005** | **1** | **K.QGLLEFVDITATNHTNEIQDYLQQLTGAR.T** 28552 |

| **556.** | Q08477\|CP4F3_HUMAN    **Mass:** 60663    **Score:** 79     **Queries matched:** 9   **emPAI:** 0.13 |
| --- | --- |
|  | Leukotriene-B(4) omega-hydroxylase 2 OS=Homo sapiens GN=CYP4F3 PE=2 SV=2 |

|  | **Query** | **Observed** | **Mr(expt)** | **Mr(calc)** | **Delta** | **Miss** | **Score** | **Expect** | **Rank** | **Peptide** |
| --- | --- | --- | --- | --- | --- | --- | --- | --- | --- | --- |
|  | 4750 | 488.2446 | 974.4746 | 974.5032 | -0.0287 | 1 | 12 | 13 | 6 | K.KLSDEDIR.A 4748 4751 |
|  | 11182 | 688.9026 | 1375.7906 | 1375.7963 | -0.0056 | 0 | 83 | 7.4e-007 | 1 | K.TLDFIDVLLLSK.D |
|  | 13538 | 759.8886 | 1517.7625 | 1517.7726 | -0.0100 | 0 | 24 | 0.46 | 1 | R.TLPSQGVDDFLQAK.A |
|  | 13864 | **514.9320** | **1541.7742** | **1541.7838** | **-0.0096** | **0** | **27** | **0.26** | **1** | **R.LVHDFTDAVIQER.R** |
|  | 19765 | 656.0113 | 1965.0120 | 1966.0209 | -1.0088 | 0 | 3 | 44 | 10 | R.MLTPAFHFNILKPYMK.I |
|  | 25217 | 867.3948 | 2599.1627 | 2600.3169 | -1.1542 | 1 | 3 | 30 | 1 | K.IFNESVNIMHAKWQLLASEGSAR.L |
|  | 28344 | **1079.0852** | **3234.2338** | **3234.6053** | **-0.3715** | **1** | **1** | **23** | **3** | **K.WQLLASEGSARLDMFEHISLMTLDSLQK.C** |

| **557.** | Q10567\|AP1B1_HUMAN    **Mass:** 105452   **Score:** 79     **Queries matched:** 20   **emPAI:** 0.07 |
| --- | --- |
|  | AP-1 complex subunit beta-1 OS=Homo sapiens GN=AP1B1 PE=1 SV=1 |

|  | **Query** | **Observed** | **Mr(expt)** | **Mr(calc)** | **Delta** | **Miss** | **Score** | **Expect** | **Rank** | **Peptide** |
| --- | --- | --- | --- | --- | --- | --- | --- | --- | --- | --- |
|  | 3793 | **453.7527** | **905.4908** | **904.5052** | **0.9856** | **0** | **5** | **61** | **6** | **K.VIASMTVGK.D** |
|  | 4125 | 465.7629 | 929.5112 | 930.4997 | -0.9885 | 0 | 12 | 11 | 2 | K.AVWLPAMK.A |
|  | 4577 | 481.7661 | 961.5176 | 962.5437 | -1.0260 | 1 | 17 | 3.6 | 7 | K.KGEIFELK.A 4595 |
|  | 5278 | **507.5159** | **1013.0172** | **1013.5757** | **-0.5585** | **0** | **11** | **7.6** | **4** | **R.LLSTDPVAAK.E** |
|  | 5746 | **525.2634** | **1048.5122** | **1048.5951** | **-0.0829** | **1** | **3** | **85** | **4** | **K.KVIASMTVGK.D** |
|  | 7023 | **573.2770** | **1144.5395** | **1145.6478** | **-1.1083** | **1** | **1** | **1.4e+002** | **4** | **K.LEKLDIMIR.L** |
|  | 9430 | **640.3375** | **1278.6604** | **1278.6932** | **-0.0328** | **1** | **1** | **1.1e+002** | **10** | **K.AKGLEISGTFTR.Q** |
|  | 11120 | **458.3056** | **1371.8949** | **1371.6964** | **0.1985** | **1** | **5** | **37** | **8** | **K.VLMKFMEMLSK.D** |
|  | 11323 | **462.1449** | **1383.4129** | **1383.7874** | **-0.3745** | **0** | **1** | **84** | **5** | **K.LTNGIWVLAELR.I** |
|  | 11528 | 465.9332 | 1394.7777 | 1394.7881 | -0.0105 | 0 | 11 | 10 | 3 | R.LSHANSAVVLSAVK.V |
|  | 13631 | **509.3132** | **1524.9179** | **1523.7290** | **1.1889** | **0** | **5** | **35** | **3** | **R.NVEGQDMLYQSLK.L** |
|  | 14282 | 784.9308 | 1567.8471 | 1567.8933 | -0.0462 | 0 | 3 | 64 | 8 | R.LASQANIAQVLAELK.E |
|  | 18745 | 628.2999 | 1881.8780 | 1882.0710 | -0.1930 | 1 | 1 | 84 | 5 | R.LSHANSAVVLSAVKVLMK.F 18763 |
|  | 28611 | 1104.5131 | 3310.5174 | 3309.6295 | 0.8879 | 1 | 1 | 34 | 3 | K.VIASMTVGKDVSALFPDVVNCMQTDNLELK.K |
|  | 29516 | **1205.9482** | **3614.8229** | **3614.8356** | **-0.0127** | **0** | **33** | **0.017** | **1** | **R.IDNADELLESFLEGFHDESTQVQLQLLTAIVK.L** |
|  | 29950 | **1257.6697** | **3769.9872** | **3770.0077** | **-0.0205** | **0** | **45** | **0.00097** | **1** | **R.NSFGLAPAAPLQVHAPLSPNQTVEISLPLSTVGSVMK.M** 29951 |
|  | 30023 | **758.3724** | **3786.8258** | **3786.0026** | **0.8232** | **0** | **(5)** | **12** | **1** | **R.NSFGLAPAAPLQVHAPLSPNQTVEISLPLSTVGSVMK.M** |

| **558.** | Q07837\|SLC31_HUMAN    **Mass:** 79258    **Score:** 79     **Queries matched:** 16   **emPAI:** 0.10 |
| --- | --- |
|  | Neutral and basic amino acid transport protein rBAT OS=Homo sapiens GN=SLC3A1 PE=1 SV=2 |

|  | **Query** | **Observed** | **Mr(expt)** | **Mr(calc)** | **Delta** | **Miss** | **Score** | **Expect** | **Rank** | **Peptide** |
| --- | --- | --- | --- | --- | --- | --- | --- | --- | --- | --- |
|  | 2420 | **409.7211** | **817.4277** | **816.3977** | **1.0300** | **0** | **3** | **1.1e+002** | **8** | **R.ELDGIDR.I** |
|  | 5358 | **510.2637** | **1018.5128** | **1017.4879** | **1.0249** | **0** | **10** | **18** | **6** | **K.EQPDLNFR.N** |
|  | 5715 | **524.2645** | **1046.5145** | **1046.5219** | **-0.0074** | **0** | **22** | **1** | **1** | **K.GVQPYAGMPK.E** |
|  | 6054 | **536.2789** | **1070.5433** | **1070.5244** | **0.0189** | **0** | **6** | **45** | **1** | **R.NPDVQEEIK.E** |
|  | 6531 | **553.3666** | **1104.7186** | **1103.5935** | **1.1251** | **1** | **3** | **91** | **3** | **R.IRLSTNSADK.G** |
|  | 7703 | **596.2915** | **1190.5684** | **1189.5979** | **0.9705** | **0** | **4** | **61** | **6** | **R.GILGSQEPDFK.G** |
|  | 11914 | 709.3155 | 1416.6164 | 1417.5907 | -0.9743 | 0 | 3 | 72 | 4 | R.NQCYFHQFMK.E |
|  | 12104 | **476.6080** | **1426.8023** | **1427.5987** | **-0.7964** | **0** | **4** | **59** | **8** | **R.QTMDQYSTEPGR.Y** |
|  | 13071 | 745.3293 | 1488.6440 | 1488.6555 | -0.0115 | 0 | 67 | 2.3e-005 | 1 | R.FMGTEAYAESIDR.T |
|  | 13444 | 504.9362 | 1511.7869 | 1511.7984 | -0.0115 | 0 | 16 | 2.9 | 1 | K.LIIDFIPNHTSDK.H 13453 |
|  | 14474 | **528.3102** | **1581.9089** | **1581.8362** | **0.0727** | **1** | **4** | **46** | **6** | **R.NPDVQEEIKEILR.F** |
|  | 20364 | **675.7056** | **2024.0951** | **2024.1054** | **-0.0104** | **0** | **33** | **0.048** | **1** | **K.LYQDLSLLHANELLLNR.G** 20363 |
|  | 23108 | 770.3884 | 2308.1435 | 2309.1627 | -1.0192 | 1 | 1 | 67 | 10 | K.GVQPYAGMPKEVLFQFSGQAR.Y |
|  | 24473 | **830.7300** | **2489.1683** | **2489.1784** | **-0.0101** | **0** | **0** | **64** | **3** | **R.EVDPIFGTMEDFENLVAAIHDK.G** |

| **559.** | Q9H8Y8\|GORS2_HUMAN    **Mass:** 47287    **Score:** 78     **Queries matched:** 7   **emPAI:** 0.16 |
| --- | --- |
|  | Golgi reassembly-stacking protein 2 OS=Homo sapiens GN=GORASP2 PE=1 SV=3 |

|  | **Query** | **Observed** | **Mr(expt)** | **Mr(calc)** | **Delta** | **Miss** | **Score** | **Expect** | **Rank** | **Peptide** |
| --- | --- | --- | --- | --- | --- | --- | --- | --- | --- | --- |
|  | 5923 | **531.3462** | **1060.6778** | **1059.5560** | **1.1218** | **1** | **10** | **16** | **8** | **R.LNKDNDTLK.D** |
|  | 10788 | 677.3307 | 1352.6469 | 1352.8027 | -0.1558 | 1 | 10 | 13 | 3 | K.DLLKANVEKPVK.M |
|  | 13732 | **767.3299** | **1532.6452** | **1532.6566** | **-0.0113** | **0** | **13** | **6.5** | **1** | **K.LYVYNTDTDNCR.E** |
|  | 15397 | 547.6047 | 1639.7924 | 1638.9015 | 0.8909 | 0 | 2 | 71 | 6 | K.ISLPGQMAGTPITPLK.D |
|  | 18594 | **935.4692** | **1868.9238** | **1867.9469** | **0.9770** | **0** | **20** | **0.9** | **1** | **R.AGLEPFFDFIVSINGSR.L** |
|  | 20432 | **677.3573** | **2029.0501** | **2028.9865** | **0.0636** | **0** | **2** | **60** | **2** | **M.GSSQSVEIPGGGTEGYHVLR.V** |
|  | 21958 | **1086.0706** | **2170.1266** | **2170.1382** | **-0.0117** | **0** | **78** | **1.4e-006** | **1** | **R.ETSVTPSNLWGGQGLLGVSIR.F** |

| **560.** | Q9NZ45\|CISD1_HUMAN    **Mass:** 12362    **Score:** 78     **Queries matched:** 9   **emPAI:** 0.32 |
| --- | --- |
|  | CDGSH iron sulfur domain-containing protein 1 OS=Homo sapiens GN=CISD1 PE=1 SV=1 |

|  | **Query** | **Observed** | **Mr(expt)** | **Mr(calc)** | **Delta** | **Miss** | **Score** | **Expect** | **Rank** | **Peptide** |
| --- | --- | --- | --- | --- | --- | --- | --- | --- | --- | --- |
|  | 5972 | **533.7322** | **1065.4498** | **1066.5957** | **-1.1459** | **0** | **8** | **26** | **5** | **K.AMINLHIQK.D** |
|  | 9942 | **654.3093** | **1306.6041** | **1306.6129** | **-0.0087** | **1** | **27** | **0.27** | **1** | **K.KFPFCDGAHTK.H** |
|  | 13090 | 497.2617 | 1488.7632 | 1488.6919 | 0.0714 | 0 | 6 | 33 | 6 | K.IVHAFDMEDLGDK.A |
|  | 15309 | 818.4151 | 1634.8156 | 1634.8264 | -0.0107 | 0 | 78 | 1.8e-006 | 1 | K.HNEETGDNVGPLIIK.K 15307 15318 |
|  | 17114 | **882.5365** | **1763.0584** | **1762.9213** | **0.1371** | **1** | **3** | **45** | **4** | **K.HNEETGDNVGPLIIKK.K** |
|  | 19699 | 653.9764 | 1958.9073 | 1958.9044 | 0.0029 | 1 | 5 | 29 | 2 | K.DNPKIVHAFDMEDLGDK.A |
|  | 23797 | 803.3595 | 2407.0567 | 2407.3263 | -0.2697 | 1 | 4 | 30 | 3 | R.VEWIAAVTIAAGTAAIGYLAYKR.F |

| **561.** | P20711\|DDC_HUMAN    **Mass:** 54544    **Score:** 78     **Queries matched:** 17   **emPAI:** 0.14 |
| --- | --- |
|  | Aromatic-L-amino-acid decarboxylase OS=Homo sapiens GN=DDC PE=1 SV=1 |

|  | **Query** | **Observed** | **Mr(expt)** | **Mr(calc)** | **Delta** | **Miss** | **Score** | **Expect** | **Rank** | **Peptide** |
| --- | --- | --- | --- | --- | --- | --- | --- | --- | --- | --- |
|  | 4282 | **471.7689** | **941.5232** | **941.5294** | **-0.0062** | **0** | **34** | **0.058** | **1** | **K.VNEALLQR.I** |
|  | 4495 | **478.7763** | **955.5381** | **954.6226** | **0.9155** | **1** | **9** | **19** | **2** | **R.AGLIGGVKLK.A** |
|  | 5449 | **514.2695** | **1026.5244** | **1025.5254** | **0.9990** | **0** | **5** | **54** | **6** | **R.TVESAHVQR.A** |
|  | 7265 | **582.2820** | **1162.5494** | **1161.6519** | **0.8975** | **1** | **1** | **1.3e+002** | **6** | **R.HWQIPLGRR.F** |
|  | 7764 | **398.8700** | **1193.5881** | **1193.5499** | **0.0382** | **0** | **8** | **26** | **4** | **K.AIPSDGNFAMR.A** |
|  | 8471 | 615.2986 | 1228.5827 | 1228.6427 | -0.0599 | 1 | 6 | 41 | 10 | R.SLKMWFVFR.M |
|  | 9282 | 636.3641 | 1270.7136 | 1271.7285 | -1.0149 | 1 | 1 | 1.2e+002 | 5 | K.KIHLVPCHLR.D |
|  | 12241 | **718.3652** | **1434.7158** | **1434.7289** | **-0.0131** | **1** | **7** | **25** | **4** | **K.LKAIPSDGNFAMR.A** |
|  | 16254 | 850.9416 | 1699.8686 | 1699.8814 | -0.0128 | 0 | 70 | 1e-005 | 1 | R.LQAASPELTQAAIMEK.L 16245 16246 16250 16267 |
|  | 18869 | **946.4735** | **1890.9325** | **1890.8128** | **0.1197** | **0** | **4** | **38** | **1** | **K.EMVDYVANYMEGIEGR.Q** |
|  | 22399 | 1111.0446 | 2220.0746 | 2221.1888 | -1.1143 | 1 | 3 | 43 | 3 | K.VIHRLQAASPELTQAAIMEK.L |
|  | 23365 | 1172.5947 | 2343.1749 | 2343.2183 | -0.0434 | 1 | 1 | 56 | 3 | K.LVAYSSDQAHSSVERAGLIGGVK.L 23366 |

| **562.** | Q9HDC9\|APMAP_HUMAN    **Mass:** 46622    **Score:** 78     **Queries matched:** 11   **emPAI:** 0.17 |
| --- | --- |
|  | Adipocyte plasma membrane-associated protein OS=Homo sapiens GN=APMAP PE=1 SV=2 |

|  | **Query** | **Observed** | **Mr(expt)** | **Mr(calc)** | **Delta** | **Miss** | **Score** | **Expect** | **Rank** | **Peptide** |
| --- | --- | --- | --- | --- | --- | --- | --- | --- | --- | --- |
|  | 2983 | 428.7632 | 855.5117 | 855.5178 | -0.0060 | 0 | 12 | 10 | 5 | K.VLLDQLR.F |
|  | 3615 | 448.2307 | 894.4469 | 893.3913 | 1.0556 | 0 | 12 | 11 | 3 | -.MSEADGLR.Q |
|  | 5996 | **534.3449** | **1066.6753** | **1067.5797** | **-0.9045** | **1** | **1** | **1.1e+002** | **4** | **R.RVYVSGLMK.G** |
|  | 8163 | 607.3099 | 1212.6052 | 1211.7238 | 0.8815 | 1 | 2 | 86 | 9 | R.EVKVLLDQLR.F |
|  | 13539 | **506.9372** | **1517.7897** | **1516.7310** | **1.0586** | **1** | **4** | **53** | **5** | **K.IYFTDSSSKWQR.R** |
|  | 15030 | 809.9425 | 1617.8704 | 1616.8306 | 1.0398 | 1 | 0 | 1.1e+002 | 4 | R.MIFKLFSQETVMK.F |
|  | 15418 | 821.4283 | 1640.8421 | 1641.9189 | -1.0767 | 1 | 2 | 69 | 7 | R.EVKLLLSSETPIEGK.N |
|  | 20749 | **1028.9879** | **2055.9613** | **2055.0789** | **0.8824** | **1** | **1** | **73** | **2** | **K.FVPRYSLVLELSDSGAFR.R** |
|  | 21077 | **698.0268** | **2091.0585** | **2091.0709** | **-0.0123** | **0** | **47** | **0.0016** | **1** | **R.RPLRPQVVTDDDGQAPEAK.D** |
|  | 26674 | **958.8021** | **2873.3844** | **2873.4018** | **-0.0175** | **0** | **50** | **0.00057** | **1** | **R.LFENQLVGPESIAHIGDVMFTGTADGR.V** |
|  | 28764 | **840.4363** | **3357.7160** | **3357.6412** | **0.0748** | **1** | **3** | **22** | **1** | **R.QAERLFENQLVGPESIAHIGDVMFTGTADGR.V** |

| **563.** | P35609\|ACTN2_HUMAN    **Mass:** 104358   **Score:** 78     **Queries matched:** 17   **emPAI:** 0.11 |
| --- | --- |
|  | Alpha-actinin-2 OS=Homo sapiens GN=ACTN2 PE=1 SV=1 |

|  | **Query** | **Observed** | **Mr(expt)** | **Mr(calc)** | **Delta** | **Miss** | **Score** | **Expect** | **Rank** | **Peptide** |
| --- | --- | --- | --- | --- | --- | --- | --- | --- | --- | --- |
|  | 4427 | **476.2684** | **950.5222** | **951.6229** | **-1.1007** | **1** | **7** | **31** | **1** | **K.VKQLVPIR.D** |
|  | 7866 | 600.3265 | 1198.6385 | 1198.6234 | 0.0152 | 0 | 3 | 73 | 10 | R.DLLLDPAWEK.Q |
|  | 8753 | **622.3304** | **1242.6462** | **1242.6357** | **0.0105** | **0** | **7** | **27** | **2** | **R.HRPDLIDYSK.L** |
|  | 9837 | 434.5367 | 1300.5884 | 1300.5982 | -0.0098 | 0 | 33 | 0.065 | 1 | K.HTNYTMEHIR.V 9848 |
|  | 9949 | **436.5801** | **1306.7183** | **1307.6213** | **-0.9030** | **1** | **1** | **1.1e+002** | **8** | **R.TPEKTMQAMQK.K** |
|  | 10766 | 676.8121 | 1351.6096 | 1351.6190 | -0.0094 | 0 | 7 | 25 | 5 | K.GITQEQMNEFR.A |
|  | 11111 | 686.8945 | 1371.7745 | 1371.7795 | -0.0050 | 0 | 10 | 13 | 1 | K.LMLLLEVISGER.L |
|  | 12001 | 474.7404 | 1421.1994 | 1420.6986 | 0.5008 | 0 | 2 | 56 | 9 | K.GYEEWLLNEIR.R |
|  | 13795 | 769.3860 | 1536.7575 | 1536.7671 | -0.0096 | 0 | 76 | 3.3e-006 | 1 | R.FAIQDISVEETSAK.E |
|  | 16790 | **581.2643** | **1740.7710** | **1741.8556** | **-1.0846** | **0** | **5** | **34** | **7** | **K.DDPIGNINLAMEIAEK.H** |
|  | 17045 | **586.6470** | **1756.9193** | **1757.8505** | **-0.9313** | **0** | **(1)** | **91** | **6** | **K.DDPIGNINLAMEIAEK.H** |
|  | 19412 | 967.5042 | 1932.9939 | 1932.9541 | 0.0398 | 1 | 3 | 52 | 4 | K.AGTQIENIEEDFRNGLK.L |
|  | 20762 | **686.6592** | **2056.9559** | **2057.0867** | **-0.1308** | **1** | **3** | **47** | **6** | **R.MEKLLETIDQLHLEFAK.R** |
|  | 21173 | **700.4306** | **2098.2700** | **2097.0775** | **1.1924** | **1** | **1** | **51** | **5** | **K.LNKDDPIGNINLAMEIAEK.H** |
|  | 23977 | **810.0883** | **2427.2431** | **2428.1904** | **-0.9472** | **1** | **1** | **65** | **6** | **K.ATLPEADGERQSIMAIQNEVEK.V** |
|  | 24406 | 829.1033 | 2484.2882 | 2485.2536 | -0.9654 | 1 | 3 | 36 | 10 | R.QFAAQANAIGPWIQNKMEEIAR.S |

| **564.** | Q9H0C2\|ADT4_HUMAN    **Mass:** 35285    **Score:** 77     **Queries matched:** 11   **emPAI:** 0.11 |
| --- | --- |
|  | ADP/ATP translocase 4 OS=Homo sapiens GN=SLC25A31 PE=1 SV=1 |

|  | **Query** | **Observed** | **Mr(expt)** | **Mr(calc)** | **Delta** | **Miss** | **Score** | **Expect** | **Rank** | **Peptide** |
| --- | --- | --- | --- | --- | --- | --- | --- | --- | --- | --- |
|  | 2979 | 856.4830 | 855.4757 | 855.4814 | -0.0057 | 0 | 20 | 1.5 | 1 | K.TAVAPIER.V 2981 |
|  | 2982 | 428.7509 | 855.4871 | 855.4926 | -0.0055 | 0 | 9 | 21 | 7 | R.GNLANVIR.Y |
|  | 3407 | **440.7394** | **879.4643** | **879.5905** | **-0.1263** | **1** | **2** | **1.2e+002** | **9** | **K.GLLPKPKK.T** |
|  | 6294 | 544.3039 | 1086.5932 | 1085.6444 | 0.9488 | 0 | 1 | 1.3e+002 | 5 | K.LLLQVQASSK.Q |
|  | 7545 | 592.3403 | 1182.6660 | 1183.5148 | -0.8488 | 1 | 2 | 87 | 5 | R.MMMQSGEAKR.Q |
|  | 10068 | **657.8419** | **1313.6693** | **1313.6802** | **-0.0109** | **1** | **7** | **31** | **3** | **K.YKQLFMSGVNK.E** |
|  | 12158 | 715.8451 | 1429.6757 | 1430.7670 | -1.0913 | 1 | 14 | 5.3 | 3 | K.IKEFFHIDIGGR.- |
|  | 12409 | 723.8707 | 1445.7269 | 1445.7343 | -0.0074 | 0 | 59 | 0.00017 | 1 | R.YFPTQALNFAFK.D 12418 |
|  | 12997 | 495.3874 | 1483.1404 | 1482.7256 | 0.4149 | 0 | 0 | 74 | 6 | K.IYQHEGISSFFR.G |

| **565.** | Q7Z7H5\|TMED4_HUMAN    **Mass:** 26097    **Score:** 77     **Queries matched:** 11   **emPAI:** 0.50 |
| --- | --- |
|  | Transmembrane emp24 domain-containing protein 4 OS=Homo sapiens GN=TMED4 PE=1 SV=1 |

|  | **Query** | **Observed** | **Mr(expt)** | **Mr(calc)** | **Delta** | **Miss** | **Score** | **Expect** | **Rank** | **Peptide** |
| --- | --- | --- | --- | --- | --- | --- | --- | --- | --- | --- |
|  | 2065 | **793.3272** | **792.3199** | **793.4156** | **-1.0957** | **0** | **5** | **57** | **9** | **R.MALFAGGK.L** |
|  | 2146 | **399.7797** | **797.5448** | **796.4555** | **1.0893** | **0** | **3** | **80** | **3** | **M.AGVGAGPLR.A** |
|  | 5584 | **518.7602** | **1035.5058** | **1034.4992** | **1.0066** | **0** | **10** | **18** | **3** | **R.LTSESTNQR.V** 5564 |
|  | 6183 | 540.2624 | 1078.5103 | 1078.5957 | -0.0854 | 1 | 6 | 39 | 4 | R.MALFAGGKLR.V 6182 |
|  | 6655 | **558.3209** | **1114.6273** | **1114.6346** | **-0.0073** | **1** | **46** | **0.0043** | **1** | **K.DKLTELQLR.A** |
|  | 10591 | **671.3674** | **1340.7202** | **1340.7300** | **-0.0098** | **0** | **38** | **0.021** | **2** | **R.QLLDQVEQIQK.E** |
|  | 10620 | **672.3198** | **1342.6250** | **1342.6962** | **-0.0712** | **1** | **(2)** | **91** | **5** | **-.MAGVGAGPLRAMGR.Q** |
|  | 11181 | **459.5974** | **1375.7704** | **1374.6860** | **1.0844** | **1** | **16** | **3.6** | **1** | **-.MAGVGAGPLRAMGR.Q** |
|  | 22378 | **740.0414** | **2217.1023** | **2217.1178** | **-0.0155** | **0** | **48** | **0.0013** | **1** | **R.VHLDIQVGEHANNYPEIAAK.D** |

| **566.** | P78329\|CP4F2_HUMAN    **Mass:** 60442    **Score:** 77     **Queries matched:** 11   **emPAI:** 0.20 |
| --- | --- |
|  | Leukotriene-B(4) omega-hydroxylase 1 OS=Homo sapiens GN=CYP4F2 PE=1 SV=1 |

|  | **Query** | **Observed** | **Mr(expt)** | **Mr(calc)** | **Delta** | **Miss** | **Score** | **Expect** | **Rank** | **Peptide** |
| --- | --- | --- | --- | --- | --- | --- | --- | --- | --- | --- |
|  | 4750 | 488.2446 | 974.4746 | 974.5032 | -0.0287 | 1 | 12 | 13 | 6 | K.KLSDEDIR.A 4748 4751 |
|  | 7044 | 573.7914 | 1145.5682 | 1146.5669 | -0.9987 | 1 | 21 | 1.4 | 2 | R.FDPENIKER.S |
|  | 7357 | 585.8048 | 1169.5949 | 1169.6128 | -0.0178 | 1 | 6 | 36 | 10 | R.LRCFPQPPR.R |
|  | 11182 | 688.9026 | 1375.7906 | 1375.7963 | -0.0056 | 0 | 83 | 7.4e-007 | 1 | K.TLDFIDVLLLSK.D |
|  | 13538 | 759.8886 | 1517.7625 | 1517.7726 | -0.0100 | 0 | 24 | 0.46 | 1 | R.TLPSQGVDDFLQAK.A |
|  | 13864 | 514.9320 | 1541.7742 | 1541.7838 | -0.0096 | 0 | 27 | 0.26 | 1 | R.LVHDFTDAVIQER.R |
|  | 19765 | 656.0113 | 1965.0120 | 1966.0209 | -1.0088 | 0 | 3 | 44 | 10 | R.MLTPAFHFNILKPYMK.I |
|  | 22133 | **731.3353** | **2190.9842** | **2190.9364** | **0.0478** | **0** | **2** | **58** | **9** | **R.NWFWGHQGMVNPTEEGMR.V** |
|  | 22226 | 735.3922 | 2203.1548 | 2203.1646 | -0.0098 | 0 | 20 | 0.8 | 1 | K.VWMGPISPLLSLCHPDIIR.S |

| **567.** | P09601\|HMOX1_HUMAN    **Mass:** 32798    **Score:** 76     **Queries matched:** 13   **emPAI:** 0.24 |
| --- | --- |
|  | Heme oxygenase 1 OS=Homo sapiens GN=HMOX1 PE=1 SV=1 |

|  | **Query** | **Observed** | **Mr(expt)** | **Mr(calc)** | **Delta** | **Miss** | **Score** | **Expect** | **Rank** | **Peptide** |
| --- | --- | --- | --- | --- | --- | --- | --- | --- | --- | --- |
|  | 1225 | 586.9156 | 585.9083 | 586.3802 | -0.4719 | 1 | 1 | 1.1e+002 | 1 | K.KIAQK.A 1226 |
|  | 7560 | **592.7742** | **1183.5339** | **1182.6105** | **0.9234** | **1** | **6** | **42** | **3** | **K.DQSPSRAPGLR.Q** |
|  | 7850 | 599.8526 | 1197.6906 | 1197.5990 | 0.0917 | 0 | 6 | 37 | 5 | K.VQDSAPVETPR.G 7865 |
|  | 9126 | 632.3370 | 1262.6595 | 1263.5951 | -0.9356 | 0 | 0 | 1.4e+002 | 8 | R.MNSLEMTPAVR.Q |
|  | 13127 | **746.3608** | **1490.7070** | **1490.7333** | **-0.0263** | **1** | **4** | **58** | **7** | **R.SRMNSLEMTPAVR.Q** |
|  | 14157 | 521.2361 | 1560.6866 | 1560.6991 | -0.0124 | 0 | 4 | 54 | 4 | K.EVHTQAENAEFMR.N |
|  | 14693 | 799.8688 | 1597.7230 | 1597.8060 | -0.0830 | 1 | 4 | 43 | 8 | R.ASNKVQDSAPVETPR.G |
|  | 19200 | **639.3304** | **1914.9693** | **1915.9468** | **-0.9775** | **0** | **0** | **94** | **4** | **K.ESPVFAPVYFPEELHR.K** |
|  | 22887 | **760.0613** | **2277.1622** | **2277.1714** | **-0.0093** | **0** | **28** | **0.13** | **1** | **K.LVMASLYHIYVALEEEIER.N** |
|  | 23018 | 765.7206 | 2294.1399 | 2293.1664 | 0.9735 | 0 | (2) | 51 | 7 | K.LVMASLYHIYVALEEEIER.N |
|  | 24192 | 1227.6245 | 2453.2345 | 2453.2478 | -0.0134 | 0 | 70 | 7.7e-006 | 1 | K.ALDLPSSGEGLAFFTFPNIASATK.F |

| **568.** | A6NHG4\|DDTL_HUMAN    **Mass:** 14414    **Score:** 76     **Queries matched:** 4   **emPAI:** 0.27 |
| --- | --- |
|  | D-dopachrome decarboxylase-like protein OS=Homo sapiens GN=DDTL PE=2 SV=1 |

|  | **Query** | **Observed** | **Mr(expt)** | **Mr(calc)** | **Delta** | **Miss** | **Score** | **Expect** | **Rank** | **Peptide** |
| --- | --- | --- | --- | --- | --- | --- | --- | --- | --- | --- |
|  | 12454 | **484.2384** | **1449.6933** | **1449.7041** | **-0.0108** | **0** | **20** | **1.3** | **1** | **R.SHSAHFFEFLTK.E** |
|  | 13265 | **750.3914** | **1498.7682** | **1498.7780** | **-0.0098** | **0** | **54** | **0.00046** | **1** | **M.PFLELDTNLPANR.V** 13264 13275 |

|  | **Proteins matching the same set of peptides:** |
| --- | --- |

|  | P30046\|DOPD_HUMAN    **Mass:** 12818    **Score:** 76     **Queries matched:** 4 |
| --- | --- |
|  | D-dopachrome decarboxylase OS=Homo sapiens GN=DDT PE=1 SV=3 |

| **569.** | Q9NZ01\|TECR_HUMAN    **Mass:** 36410    **Score:** 76     **Queries matched:** 10   **emPAI:** 0.10 |
| --- | --- |
|  | Trans-2,3-enoyl-CoA reductase OS=Homo sapiens GN=TECR PE=1 SV=1 |

|  | **Query** | **Observed** | **Mr(expt)** | **Mr(calc)** | **Delta** | **Miss** | **Score** | **Expect** | **Rank** | **Peptide** |
| --- | --- | --- | --- | --- | --- | --- | --- | --- | --- | --- |
|  | 2832 | **424.0564** | **846.0983** | **845.4131** | **0.6853** | **0** | **4** | **61** | **4** | **K.DEDVLQK.L** |
|  | 5691 | 523.2622 | 1044.5097 | 1044.5175 | -0.0077 | 0 | 12 | 11 | 1 | R.FSHGTMPLR.N |
|  | 7161 | 578.2839 | 1154.5533 | 1154.5621 | -0.0088 | 0 | 9 | 17 | 4 | K.THPQWYPAR.Q |
|  | 8047 | 604.3333 | 1206.6519 | 1206.6608 | -0.0089 | 0 | 13 | 7.2 | 1 | K.VEPHATIAEIK.N 8046 |
|  | 8223 | **608.8098** | **1215.6051** | **1215.6135** | **-0.0085** | **0** | **54** | **0.00069** | **1** | **K.HYEVEILDAK.T** 8224 |
|  | 14186 | 782.3468 | 1562.6790 | 1562.8028 | -0.1237 | 1 | 9 | 15 | 6 | R.FSHGTMPLRNIFK.N |
|  | 19949 | **662.0194** | **1983.0364** | **1983.0499** | **-0.0135** | **1** | **20** | **0.91** | **1** | **K.LCFLDKVEPHATIAEIK.N** 19950 |

| **570.** | P10606\|COX5B_HUMAN    **Mass:** 13915    **Score:** 76     **Queries matched:** 12   **emPAI:** 1.11 |
| --- | --- |
|  | Cytochrome c oxidase subunit 5B, mitochondrial OS=Homo sapiens GN=COX5B PE=1 SV=2 |

|  | **Query** | **Observed** | **Mr(expt)** | **Mr(calc)** | **Delta** | **Miss** | **Score** | **Expect** | **Rank** | **Peptide** |
| --- | --- | --- | --- | --- | --- | --- | --- | --- | --- | --- |
|  | 1963 | **775.4330** | **774.4257** | **774.4309** | **-0.0052** | **0** | **30** | **0.24** | **1** | **R.EIMLAAK.K** |
|  | 5450 | **514.2705** | **1026.5265** | **1027.5774** | **-1.0510** | **0** | **0** | **1.5e+002** | **3** | **R.GAGTLAAQALR.A** |
|  | 7599 | **593.8234** | **1185.6322** | **1185.6394** | **-0.0072** | **0** | **38** | **0.025** | **1** | **K.GLDPYNVLAPK.G** |
|  | 8952 | **419.2410** | **1254.7012** | **1254.7156** | **-0.0145** | **1** | **5** | **43** | **6** | **R.GAGTLAAQALRAR.G** |
|  | 10038 | **438.2556** | **1311.7449** | **1311.6670** | **0.0779** | **0** | **4** | **51** | **8** | **R.EDPNLVPSISNK.R** 10039 |
|  | 10074 | **657.8701** | **1313.7256** | **1313.7343** | **-0.0087** | **1** | **45** | **0.0042** | **1** | **K.KGLDPYNVLAPK.G** 10047 |
|  | 12769 | **735.3877** | **1468.7608** | **1467.7681** | **0.9927** | **1** | **9** | **18** | **1** | **R.EDPNLVPSISNKR.I** 12724 12745 |
|  | 20023 | **996.4457** | **1990.8768** | **1990.8902** | **-0.0134** | **0** | **45** | **0.0031** | **1** | **R.SMASGGGVPTDEEQATGLER.E** |

| **571.** | Q13183\|S13A2_HUMAN    **Mass:** 64995    **Score:** 76     **Queries matched:** 5   **emPAI:** 0.12 |
| --- | --- |
|  | Solute carrier family 13 member 2 OS=Homo sapiens GN=SLC13A2 PE=2 SV=1 |

|  | **Query** | **Observed** | **Mr(expt)** | **Mr(calc)** | **Delta** | **Miss** | **Score** | **Expect** | **Rank** | **Peptide** |
| --- | --- | --- | --- | --- | --- | --- | --- | --- | --- | --- |
|  | 16266 | **851.4179** | **1700.8211** | **1700.8329** | **-0.0118** | **0** | **60** | **0.00011** | **1** | **K.LDNGQALPVTSASSEGR.A** 16276 |
|  | 21445 | **707.3212** | **2118.9419** | **2118.9575** | **-0.0156** | **0** | **38** | **0.013** | **1** | **K.MQEQQQAAYCVIQTEHR.L** |
|  | 22713 | **754.0194** | **2259.0362** | **2260.2038** | **-1.1676** | **1** | **2** | **51** | **3** | **K.MPWNIVLLLGGGYALAKGSER.S** |
|  | 30285 | 1313.2438 | 3936.7095 | 3936.9893 | -0.2799 | 1 | 4 | 10 | 2 | K.GESMVSDGTVAIFIGIIMFIIPSKFPGLTQDPENPGK.L |

| **572.** | O94905\|ERLN2_HUMAN    **Mass:** 38044    **Score:** 76     **Queries matched:** 11   **emPAI:** 0.21 |
| --- | --- |
|  | Erlin-2 OS=Homo sapiens GN=ERLIN2 PE=1 SV=1 |

|  | **Query** | **Observed** | **Mr(expt)** | **Mr(calc)** | **Delta** | **Miss** | **Score** | **Expect** | **Rank** | **Peptide** |
| --- | --- | --- | --- | --- | --- | --- | --- | --- | --- | --- |
|  | 3716 | 451.2560 | 900.4975 | 900.5280 | -0.0305 | 1 | 20 | 2 | 6 | K.KALIEAEK.V |
|  | 4844 | 491.7083 | 981.4021 | 980.5291 | 0.8730 | 1 | 7 | 31 | 6 | K.YKAIASNSK.I |
|  | 10065 | 657.8310 | 1313.6474 | 1313.5921 | 0.0553 | 1 | 10 | 14 | 2 | R.RNYELMESEK.T |
|  | 10705 | 674.8472 | 1347.6799 | 1347.6882 | -0.0083 | 0 | 23 | 0.74 | 1 | K.SVQTTLQTDEVK.N |
|  | 11498 | **697.3704** | **1392.7263** | **1392.8201** | **-0.0938** | **1** | **9** | **18** | **8** | **R.VTKPNIPEAIRR.N** |
|  | 12656 | 488.2821 | 1461.8245 | 1461.7827 | 0.0418 | 1 | 4 | 54 | 6 | K.KISEIEDAAFLAR.E |
|  | 14691 | **799.8582** | **1597.7017** | **1597.7116** | **-0.0099** | **0** | **77** | **2.3e-006** | **1** | **K.DIPNMFMDSAGSVSK.Q** |
|  | 17518 | **598.3544** | **1792.0415** | **1792.9393** | **-0.8978** | **1** | **3** | **49** | **7** | **K.VAQVAEITYGQKVMEK.E** |
|  | 21491 | 1063.0399 | 2124.0653 | 2123.1772 | 0.8880 | 0 | 5 | 31 | 2 | K.LALQQDLTSMAPGLVIQAVR.V |
|  | 24428 | 829.7601 | 2486.2584 | 2486.1458 | 0.1126 | 1 | (0) | 66 | 4 | K.DIPNMFMDSAGSVSKQFEGLADK.L |
|  | 24688 | 840.1412 | 2517.4019 | 2518.1356 | -0.7337 | 1 | 3 | 30 | 3 | K.DIPNMFMDSAGSVSKQFEGLADK.L |

| **573.** | P35241\|RADI_HUMAN    **Mass:** 68635    **Score:** 76     **Queries matched:** 22   **emPAI:** 0.23 |
| --- | --- |
|  | Radixin OS=Homo sapiens GN=RDX PE=1 SV=1 |

|  | **Query** | **Observed** | **Mr(expt)** | **Mr(calc)** | **Delta** | **Miss** | **Score** | **Expect** | **Rank** | **Peptide** |
| --- | --- | --- | --- | --- | --- | --- | --- | --- | --- | --- |
|  | 2034 | **395.6827** | **789.3509** | **790.3643** | **-1.0135** | **0** | **8** | **34** | **6** | **K.QAADQMK.N** |
|  | 2473 | 412.2588 | 822.5030 | 822.5076 | -0.0046 | 0 | 8 | 27 | 6 | M.PKPINVR.V |
|  | 2962 | **428.2530** | **854.4915** | **853.4334** | **1.0581** | **0** | **3** | **80** | **9** | **K.GYSTWLK.L** |
|  | 4558 | 480.7983 | 959.5821 | 958.5851 | 0.9970 | 0 | 15 | 6.1 | 1 | K.FVIKPIDK.K |
|  | 4907 | 494.2612 | 986.5078 | 987.5237 | -1.0158 | 0 | 27 | 0.41 | 3 | K.QIEEQTIK.A 4922 4944 |
|  | 5156 | 502.2788 | 1002.5431 | 1002.5498 | -0.0067 | 1 | 20 | 2 | 1 | K.KENPLQFK.F |
|  | 6514 | 552.7924 | 1103.5703 | 1103.5764 | -0.0061 | 0 | 50 | 0.0017 | 1 | K.IGFPWSEIR.N |
|  | 12803 | 736.8931 | 1471.7716 | 1471.7817 | -0.0101 | 1 | 55 | 0.00039 | 1 | R.RKPDTIEVQQMK.A 12804 |
|  | 13297 | 501.5753 | 1501.7041 | 1501.8617 | -0.1575 | 1 | 32 | 0.086 | 1 | K.QLFDQVVKTVGLR.E |
|  | 13891 | **515.3279** | **1542.9618** | **1541.8525** | **1.1093** | **1** | **7** | **22** | **6** | **K.KQLQALSSELAQAR.D** |
|  | 13905 | 515.6282 | 1543.8627 | 1542.8558 | 1.0068 | 1 | 7 | 25 | 3 | K.LTPKIGFPWSEIR.N |
|  | 15546 | 825.9902 | 1649.9658 | 1649.7099 | 0.2559 | 1 | 4 | 42 | 3 | R.GMLREDSMMEYLK.I 15527 |
|  | 16542 | 574.6338 | 1720.8795 | 1719.8259 | 1.0537 | 0 | (2) | 65 | 5 | R.ILALCMGNHELYMR.R |
|  | 16726 | **579.5326** | **1735.5759** | **1735.8208** | **-0.2448** | **0** | **3** | **44** | **7** | **R.ILALCMGNHELYMR.R** 16738 |
|  | 18838 | **630.3455** | **1888.0146** | **1886.9697** | **1.0448** | **1** | **4** | **38** | **1** | **K.QLQALSSELAQARDETK.K** |
|  | 27372 | 755.3551 | 3017.3913 | 3016.5362 | 0.8551 | 1 | 0 | 51 | 3 | -.MPKPINVRVTTMDAELEFAIQPNTTGK.Q |
|  | 31271 | 1217.0115 | 4864.0168 | 4865.1933 | -1.1765 | 0 | 0 | 12 | 3 | K.TVMSAPPPPPPPPVIPPTENEHDEHDENNAEASAELSNEGVMNHR.S |

| **574.** | Q08043\|ACTN3_HUMAN    **Mass:** 103917   **Score:** 76     **Queries matched:** 23   **emPAI:** 0.04 |
| --- | --- |
|  | Alpha-actinin-3 OS=Homo sapiens GN=ACTN3 PE=1 SV=2 |

|  | **Query** | **Observed** | **Mr(expt)** | **Mr(calc)** | **Delta** | **Miss** | **Score** | **Expect** | **Rank** | **Peptide** |
| --- | --- | --- | --- | --- | --- | --- | --- | --- | --- | --- |
|  | 1084 | 558.3552 | 557.3479 | 556.3220 | 1.0259 | 0 | 9 | 25 | 7 | R.ELPAK.Q |
|  | 2079 | 397.2295 | 792.4444 | 792.4316 | 0.0128 | 1 | 12 | 12 | 7 | R.RMVPYK.G |
|  | 2637 | **417.7009** | **833.3872** | **833.4218** | **-0.0345** | **1** | **16** | **5.1** | **2** | **K.WDMVRK.L** |
|  | 3141 | 432.7422 | 863.4698 | 863.4752 | -0.0055 | 0 | 10 | 18 | 1 | K.ALDFIASK.G |
|  | 6387 | **547.7445** | **1093.4745** | **1092.5233** | **0.9511** | **1** | **7** | **31** | **9** | **R.GKEEMLSQR.D** |
|  | 7866 | 600.3265 | 1198.6385 | 1198.6234 | 0.0152 | 0 | 3 | 73 | 10 | R.DLLLDPAWEK.Q |
|  | 8947 | **628.3372** | **1254.6599** | **1254.6469** | **0.0130** | **1** | **1** | **1.3e+002** | **9** | **K.ASLHEAWTRGK.E** |
|  | 10541 | 447.1157 | 1338.3253 | 1338.5333 | -0.2079 | 0 | 4 | 41 | 3 | K.QNGMMEPDDFR.A |
|  | 10819 | 678.2153 | 1354.4160 | 1354.5282 | -0.1122 | 0 | (1) | 81 | 7 | K.QNGMMEPDDFR.A |
|  | 11111 | 686.8945 | 1371.7745 | 1371.7795 | -0.0050 | 0 | 10 | 13 | 1 | K.LMLLLEVISGER.L |
|  | 13795 | 769.3860 | 1536.7575 | 1536.7671 | -0.0096 | 0 | 76 | 3.3e-006 | 1 | R.FAIQDISVEETSAK.E |
|  | 14418 | 527.1549 | 1578.4427 | 1577.7000 | 0.7427 | 0 | (1) | 66 | 8 | M.MMVMQPEGLGAGEGR.F |
|  | 14868 | **537.2992** | **1608.8757** | **1609.6899** | **-0.8141** | **0** | **2** | **74** | **7** | **M.MMVMQPEGLGAGEGR.F** |
|  | 15283 | 817.3899 | 1632.7652 | 1633.8060 | -1.0407 | 1 | 0 | 1.1e+002 | 6 | R.DAKGLSQEQLNEFR.A |
|  | 16147 | **565.5994** | **1693.7765** | **1692.7456** | **1.0309** | **0** | **4** | **39** | **4** | **-.MMMVMQPEGLGAGEGR.F** |
|  | 16632 | 577.3046 | 1728.8921 | 1728.9006 | -0.0085 | 1 | 1 | 78 | 10 | R.TINEVENQVLTRDAK.G |
|  | 16658 | 866.4455 | 1730.8764 | 1730.9203 | -0.0438 | 1 | 12 | 7 | 2 | K.ILAGDKNYITPEELR.R |
|  | 18677 | **626.2927** | **1875.8563** | **1875.9438** | **-0.0875** | **1** | **5** | **29** | **3** | **R.QQEQNIINYKTNIDR.L** |
|  | 19412 | 967.5042 | 1932.9939 | 1932.9541 | 0.0398 | 1 | 3 | 52 | 4 | K.AGTQIENIEEDFRNGLK.L |
|  | 22394 | 740.7192 | 2219.1359 | 2219.0463 | 0.0896 | 1 | 2 | 48 | 5 | R.TVPWLENRVGEPSMSAMQR.K |
|  | 30114 | **1271.5184** | **3811.5335** | **3811.5317** | **0.0017** | **1** | **3** | **13** | **1** | **-.MMMVMQPEGLGAGEGRFAGGGGGGEYMEQEEDWDR.D** |
|  | 30921 | **744.0299** | **4458.1358** | **4459.0910** | **-0.9552** | **1** | **1** | **16** | **4** | **R.IMTMVDPNAAGVVTFQAFIDFMTRETAETDTTEQVVASFK.I** |
|  | 31032 | **1144.5328** | **4574.1023** | **4574.1192** | **-0.0169** | **1** | **1** | **15** | **1** | **K.MLDAEDIVNTPKPDEKAIMTYVSCFYHAFAGAEQAETAANR.I** |

| **575.** | O15258\|RER1_HUMAN    **Mass:** 23057    **Score:** 75     **Queries matched:** 3   **emPAI:** 0.17 |
| --- | --- |
|  | Protein RER1 OS=Homo sapiens GN=RER1 PE=1 SV=1 |

|  | **Query** | **Observed** | **Mr(expt)** | **Mr(calc)** | **Delta** | **Miss** | **Score** | **Expect** | **Rank** | **Peptide** |
| --- | --- | --- | --- | --- | --- | --- | --- | --- | --- | --- |
|  | 4599 | **482.3234** | **962.6322** | **961.5022** | **1.1300** | **0** | **1** | **1.4e+002** | **8** | **R.YIPFTHGK.R** |
|  | 19035 | **952.4307** | **1902.8469** | **1901.8564** | **0.9905** | **0** | **75** | **2.8e-006** | **1** | **K.VDPSLMEDSDDGPSLPTK.Q** 19024 |

| **576.** | P49257\|LMAN1_HUMAN    **Mass:** 57798    **Score:** 75     **Queries matched:** 16   **emPAI:** 0.13 |
| --- | --- |
|  | Protein ERGIC-53 OS=Homo sapiens GN=LMAN1 PE=1 SV=2 |

|  | **Query** | **Observed** | **Mr(expt)** | **Mr(calc)** | **Delta** | **Miss** | **Score** | **Expect** | **Rank** | **Peptide** |
| --- | --- | --- | --- | --- | --- | --- | --- | --- | --- | --- |
|  | 2302 | 809.4103 | 808.4030 | 807.4126 | 0.9904 | 1 | 10 | 16 | 9 | K.KEEFQK.G |
|  | 3639 | **448.7478** | **895.4810** | **895.4875** | **-0.0066** | **0** | **25** | **0.47** | **1** | **K.TQHEILR.Q** |
|  | 5523 | **516.3124** | **1030.6103** | **1030.6036** | **0.0068** | **1** | **10** | **19** | **4** | **K.EHLHIVKR.D** |
|  | 8973 | **419.3145** | **1254.9218** | **1254.6343** | **0.2875** | **0** | **5** | **34** | **7** | **R.YVSSLTEEISK.R** 8963 |
|  | 9640 | **645.8379** | **1289.6613** | **1290.6833** | **-1.0219** | **1** | **0** | **1.3e+002** | **6** | **R.DFRNKPYPVR.A** |
|  | 11055 | **457.2919** | **1368.8539** | **1368.6772** | **0.1766** | **1** | **6** | **30** | **1** | **K.EPPTPDKEISEK.E** 11053 |
|  | 11170 | 688.3695 | 1374.7243 | 1374.7004 | 0.0240 | 1 | 5 | 43 | 8 | R.ELRQVFEGQNR.I |
|  | 11932 | 709.8547 | 1417.6948 | 1416.7110 | 0.9838 | 0 | 5 | 39 | 5 | R.GDGVGGDPAVALPHR.R |
|  | 12221 | **717.4462** | **1432.8778** | **1431.7140** | **1.1638** | **1** | **4** | **48** | **9** | **R.QLDMILDEQRR.Y** |
|  | 14696 | 799.8823 | 1597.7500 | 1596.7572 | 0.9927 | 0 | 1 | 92 | 5 | K.AAFENWEVEVTFR.V |
|  | 17848 | **607.3161** | **1818.9265** | **1818.9489** | **-0.0225** | **1** | **4** | **44** | **1** | **R.FVRGDGVGGDPAVALPHR.R** |
|  | 22038 | **727.6738** | **2179.9995** | **2180.0134** | **-0.0139** | **0** | **49** | **0.0011** | **1** | **K.GHPDLQGQPAEEIFESVGDR.E** |
|  | 22152 | **732.0309** | **2193.0710** | **2193.0848** | **-0.0138** | **0** | **15** | **2.9** | **1** | **R.GAGMPGQHGQITQQELDTVVK.T** |
|  | 25601 | **891.4367** | **2671.2883** | **2671.3065** | **-0.0182** | **0** | **45** | **0.0021** | **1** | **R.LVSGMQHPGSAGGVYETTQHFIDIK.E** |

| **577.** | Q96DG6\|CMBL_HUMAN    **Mass:** 28372    **Score:** 75     **Queries matched:** 10   **emPAI:** 0.13 |
| --- | --- |
|  | Carboxymethylenebutenolidase homolog OS=Homo sapiens GN=CMBL PE=1 SV=1 |

|  | **Query** | **Observed** | **Mr(expt)** | **Mr(calc)** | **Delta** | **Miss** | **Score** | **Expect** | **Rank** | **Peptide** |
| --- | --- | --- | --- | --- | --- | --- | --- | --- | --- | --- |
|  | 1955 | 772.4266 | 771.4193 | 772.4694 | -1.0501 | 0 | 14 | 8.7 | 9 | R.EISAILK.Y |
|  | 4839 | 491.2682 | 980.5218 | 980.5291 | -0.0073 | 0 | 13 | 8 | 1 | R.EVQVEHIK.A |
|  | 5485 | **515.2867** | **1028.5589** | **1028.5654** | **-0.0065** | **0** | **27** | **0.31** | **1** | **R.NLIEWLNK.Y** 5506 |
|  | 26420 | 939.1427 | 2814.4063 | 2813.5579 | 0.8484 | 1 | 58 | 9.7e-005 | 1 | K.NPTLFIFAENDVVIPLKDVSLLTQK.L 26418 26419 26421 |
|  | 27347 | **753.6436** | **3010.5451** | **3009.4347** | **1.1104** | **1** | **1** | **44** | **1** | **K.QQCHAQKIGIVGFCWGGTAVHHLMMK.Y** |
|  | 27422 | 757.6068 | 3026.3981 | 3025.4296 | 0.9685 | 1 | (0) | 49 | 4 | K.QQCHAQKIGIVGFCWGGTAVHHLMMK.Y |

| **578.** | P23786\|CPT2_HUMAN    **Mass:** 74244    **Score:** 75     **Queries matched:** 22   **emPAI:** 0.10 |
| --- | --- |
|  | Carnitine O-palmitoyltransferase 2, mitochondrial OS=Homo sapiens GN=CPT2 PE=1 SV=2 |

|  | **Query** | **Observed** | **Mr(expt)** | **Mr(calc)** | **Delta** | **Miss** | **Score** | **Expect** | **Rank** | **Peptide** |
| --- | --- | --- | --- | --- | --- | --- | --- | --- | --- | --- |
|  | 3229 | **435.2000** | **868.3854** | **867.3909** | **0.9945** | **0** | **8** | **21** | **3** | **R.CSEAFVR.E** |
|  | 3728 | **451.2687** | **900.5229** | **901.4981** | **-0.9752** | **1** | **24** | **0.8** | **2** | **K.LEDTIRR.Y** |
|  | 5047 | 499.2264 | 996.4383 | 996.6266 | -0.1883 | 1 | 5 | 51 | 7 | -.MVPRLLLR.A |
|  | 5433 | 513.3103 | 1024.6060 | 1023.4920 | 1.1141 | 1 | 10 | 14 | 4 | K.RCSEAFVR.E |
|  | 5785 | 526.7541 | 1051.4937 | 1051.5008 | -0.0071 | 0 | 4 | 65 | 9 | R.EFLQCVEK.A 5792 |
|  | 5942 | **532.2783** | **1062.5421** | **1062.5492** | **-0.0071** | **0** | **16** | **4.4** | **1** | **R.ATNMTVSAIR.F** 5950 |
|  | 10688 | 449.8787 | 1346.6142 | 1346.7935 | -0.1793 | 1 | 10 | 15 | 7 | R.HLFALRHLAAAK.G 10684 |
|  | 10987 | **683.3678** | **1364.7210** | **1364.7300** | **-0.0089** | **0** | **27** | **0.29** | **1** | **R.TETIRPASVYTK.R** |
|  | 11493 | **697.3462** | **1392.6778** | **1392.6820** | **-0.0041** | **1** | **3** | **71** | **3** | **R.NAREFLQCVEK.A** |
|  | 11902 | 472.7704 | 1415.2894 | 1414.8045 | 0.4850 | 1 | 8 | 14 | 6 | R.LFNSTRLPKPSR.D |
|  | 16684 | **867.4617** | **1732.9089** | **1733.7535** | **-0.8446** | **0** | **24** | **0.46** | **1** | **R.HSAGELQQMMVECSK.Y** |
|  | 17649 | 902.9230 | 1803.8314 | 1804.9934 | -1.1621 | 1 | 2 | 65 | 4 | K.LNFELTDALKTGITAAK.E |
|  | 22138 | **731.6038** | **2191.7895** | **2191.1823** | **0.6071** | **1** | **1** | **44** | **8** | **R.SIVPTMHYQDSLPRLPIPK.L** |
|  | 24311 | **1235.6057** | **2469.1969** | **2469.2063** | **-0.0095** | **0** | **27** | **0.16** | **1** | **K.YILSDSSPAPEFPLAYLTSENR.D** 24312 |
|  | 25154 | **1294.6295** | **2587.2445** | **2587.2614** | **-0.0169** | **0** | **68** | **1.1e-005** | **1** | **K.DSTQTPAVTPQSQPATTDSTVTVQK.L** |
|  | 27592 | **762.6382** | **3046.5236** | **3046.5400** | **-0.0164** | **1** | **4** | **19** | **2** | **R.KGNFYIFDVLDQDGNIVSPSEIQAHLK.Y** |
|  | 32025 | **1708.8479** | **6831.3625** | **6831.3708** | **-0.0083** | **0** | **18** | **0.076** | **1** | **K.GIILPELYLDPAYGQINHNVLSTSTLSSPAVNLGGFAPVVSDGFGVGYAVHDNWIGCNVSSYPGR.N** 32024 |

| **579.** | Q8NBS9\|TXND5_HUMAN    **Mass:** 48283    **Score:** 75     **Queries matched:** 10   **emPAI:** 0.08 |
| --- | --- |
|  | Thioredoxin domain-containing protein 5 OS=Homo sapiens GN=TXNDC5 PE=1 SV=2 |

|  | **Query** | **Observed** | **Mr(expt)** | **Mr(calc)** | **Delta** | **Miss** | **Score** | **Expect** | **Rank** | **Peptide** |
| --- | --- | --- | --- | --- | --- | --- | --- | --- | --- | --- |
|  | 1193 | **580.3048** | **579.2975** | **578.3428** | **0.9547** | **0** | **8** | **27** | **3** | **K.VYVAK.V** |
|  | 1643 | 678.3772 | 677.3699 | 677.3748 | -0.0049 | 0 | 7 | 39 | 2 | R.GYPTLK.L |
|  | 1974 | **780.3436** | **779.3364** | **779.4177** | **-0.0814** | **1** | **7** | **38** | **5** | **K.KVDQYK.G** |
|  | 3960 | 459.5974 | 917.1803 | 916.5018 | 0.6785 | 0 | 7 | 25 | 1 | K.EFPGLAGVK.I |
|  | 7127 | **576.8106** | **1151.6067** | **1151.6128** | **-0.0061** | **0** | **4** | **58** | **3** | **R.GYPTLLWFR.D** |
|  | 9554 | **643.7557** | **1285.4968** | **1285.6303** | **-0.1335** | **0** | **1** | **94** | **8** | **R.LQPTWNDLGDK.Y** |
|  | 17085 | **881.3791** | **1760.7437** | **1760.7570** | **-0.0133** | **0** | **75** | **3.5e-006** | **1** | **K.VDCTAHSDVCSAQGVR.G** |
|  | 19767 | **656.2856** | **1965.8349** | **1964.8469** | **0.9880** | **0** | **21** | **0.65** | **1** | **K.VDCTQHYELCSGNQVR.G** |
|  | 22144 | **731.7197** | **2192.1374** | **2192.1477** | **-0.0103** | **0** | **12** | **5.3** | **2** | **K.ALAPTWEQLALGLEHSETVK.I** 22142 |

| **580.** | Q15437\|SC23B_HUMAN    **Mass:** 87393    **Score:** 74     **Queries matched:** 16   **emPAI:** 0.04 |
| --- | --- |
|  | Protein transport protein Sec23B OS=Homo sapiens GN=SEC23B PE=1 SV=2 |

|  | **Query** | **Observed** | **Mr(expt)** | **Mr(calc)** | **Delta** | **Miss** | **Score** | **Expect** | **Rank** | **Peptide** |
| --- | --- | --- | --- | --- | --- | --- | --- | --- | --- | --- |
|  | 1031 | 546.3209 | 545.3136 | 546.3013 | -0.9877 | 0 | 12 | 26 | 5 | K.DLTAK.Q |
|  | 2910 | **851.0955** | **850.0882** | **850.3821** | **-0.2939** | **0** | **0** | **1.1e+002** | **2** | **K.EDPTSFR.L** |
|  | 3657 | **449.7808** | **897.5470** | **896.4174** | **1.1295** | **1** | **7** | **28** | **7** | **K.DNARFMK.K** |
|  | 9534 | **643.2950** | **1284.5754** | **1284.6285** | **-0.0531** | **1** | **1** | **1.1e+002** | **10** | **R.LCQKFGQYNK.E** |
|  | 10435 | 667.3052 | 1332.5959 | 1331.6218 | 0.9741 | 0 | 19 | 1.7 | 2 | R.YINTEHGGSQAR.F 10430 |
|  | 15253 | 816.4236 | 1630.8326 | 1629.8396 | 0.9930 | 0 | 74 | 4.4e-006 | 1 | K.IDMNLTDLLGELQR.D |
|  | 16693 | **578.9360** | **1733.7861** | **1734.7559** | **-0.9698** | **1** | **2** | **68** | **3** | **R.KAGYQDMPEYENFK.H** |
|  | 17166 | **884.9253** | **1767.8361** | **1767.9917** | **-0.1555** | **1** | **5** | **31** | **4** | **R.ELKIAGAIGPCVSLNVK.G** 17176 |
|  | 17201 | **885.9467** | **1769.8789** | **1770.8246** | **-0.9458** | **0** | **2** | **74** | **4** | **-.MATYLEFIQQNEER.D** |
|  | 20143 | **501.5273** | **2002.0800** | **2002.9611** | **-0.8811** | **0** | **1** | **67** | **9** | **R.LSDSFSLYPQFMFHLR.R** |
|  | 20547 | **680.8715** | **2039.5926** | **2040.1475** | **-0.5550** | **1** | **0** | **59** | **2** | **R.LEATRMVVPLACLLTPLK.E** |
|  | 22708 | 753.7182 | 2258.1328 | 2259.1280 | -0.9952 | 0 | 2 | 54 | 9 | R.IMLFTGGPPTQGPGMVVGDELK.I |
|  | 30436 | **1014.4548** | **4053.7902** | **4053.9531** | **-0.1629** | **0** | **3** | **12** | **1** | **K.VNPSQTHNNLYAWGQETGAPILTDDVSLQVFMDHLK.K** |
|  | 30622 | 692.5076 | 4149.0021 | 4149.0558 | -0.0536 | 1 | 4 | 9.6 | 3 | K.DLTAKQIQDMLGLTKPAMPMQQARPAQPQEHPFASSR.F |

| **581.** | Q8IVT2\|CS021_HUMAN    **Mass:** 75482    **Score:** 74     **Queries matched:** 20   **emPAI:** 0.15 |
| --- | --- |
|  | Uncharacterized protein C19orf21 OS=Homo sapiens GN=C19orf21 PE=1 SV=1 |

|  | **Query** | **Observed** | **Mr(expt)** | **Mr(calc)** | **Delta** | **Miss** | **Score** | **Expect** | **Rank** | **Peptide** |
| --- | --- | --- | --- | --- | --- | --- | --- | --- | --- | --- |
|  | 2075 | **397.2194** | **792.4243** | **792.3912** | **0.0331** | **1** | **5** | **52** | **2** | **-.MDRVTR.Y** |
|  | 3410 | **440.7659** | **879.5173** | **878.4359** | **1.0815** | **0** | **7** | **30** | **4** | **K.GAPHSSPAR.G** |
|  | 3880 | **456.8827** | **911.7509** | **911.4825** | **0.2685** | **0** | **3** | **50** | **5** | **K.QEASKPPR.G** |
|  | 4112 | **465.2552** | **928.4959** | **928.4250** | **0.0709** | **0** | **9** | **25** | **8** | **R.LDAGDADPR.R** 4102 4111 4122 |
|  | 8266 | **609.8104** | **1217.6062** | **1216.6234** | **0.9828** | **1** | **10** | **14** | **1** | **K.AATSPKATMSPR.H** 8262 |
|  | 8560 | 412.1910 | 1233.5513 | 1232.6183 | 0.9330 | 1 | (2) | 84 | 10 | K.AATSPKATMSPR.H 8551 |
|  | 10543 | **670.3146** | **1338.6147** | **1339.6732** | **-1.0585** | **0** | **26** | **0.38** | **1** | **R.GTPAGTTPGASQAPK.A** |
|  | 11071 | **685.8625** | **1369.7104** | **1369.7201** | **-0.0097** | **0** | **34** | **0.058** | **1** | **R.HLSESSGKPLSTK.Q** 11049 11072 |
|  | 11845 | **707.3245** | **1412.6345** | **1411.7684** | **0.8661** | **1** | **2** | **74** | **3** | **R.ERWAVIQGQAVR.K** |
|  | 13263 | **750.3834** | **1498.7522** | **1498.7627** | **-0.0105** | **0** | **61** | **9.9e-005** | **1** | **R.ALSSDSILSPAPDAR.A** |
|  | 16179 | 566.2792 | 1695.8157 | 1694.8271 | 0.9886 | 1 | 3 | 53 | 4 | K.QEASKPPRGCPQANR.G |
|  | 18369 | **618.3605** | **1852.0596** | **1851.9228** | **0.1368** | **1** | **6** | **23** | **5** | **R.ASTPDWVSEGPQPGLRR.A** |
|  | 19875 | **659.3403** | **1974.9990** | **1973.8980** | **1.1010** | **1** | **6** | **25** | **1** | **R.GLHSENREDEGWQVYR.L** |

| **582.** | P26885\|FKBP2_HUMAN    **Mass:** 15810    **Score:** 74     **Queries matched:** 6   **emPAI:** 0.25 |
| --- | --- |
|  | FK506-binding protein 2 OS=Homo sapiens GN=FKBP2 PE=1 SV=2 |

|  | **Query** | **Observed** | **Mr(expt)** | **Mr(calc)** | **Delta** | **Miss** | **Score** | **Expect** | **Rank** | **Peptide** |
| --- | --- | --- | --- | --- | --- | --- | --- | --- | --- | --- |
|  | 10419 | **666.8562** | **1331.6978** | **1331.7085** | **-0.0106** | **0** | **25** | **0.5** | **2** | **K.LVIPSELGYGER.G** |
|  | 11340 | **462.5680** | **1384.6821** | **1384.6922** | **-0.0101** | **1** | **6** | **38** | **1** | **R.KGDVLHMHYTGK.L** |
|  | 14421 | **790.3425** | **1578.6705** | **1578.6807** | **-0.0102** | **0** | **74** | **4.3e-006** | **1** | **K.GWDQGLLGMCEGEK.R** |
|  | 16938 | **438.7407** | **1750.9337** | **1750.7767** | **0.1570** | **1** | **5** | **35** | **8** | **K.GWDQGLLGMCEGEKR.K** 16941 |
|  | 20782 | **687.3645** | **2059.0717** | **2060.1187** | **-1.0471** | **0** | **0** | **87** | **4** | **R.VLTVLSICLSAVATATGAEGK.R** |

| **583.** | O15127\|SCAM2_HUMAN    **Mass:** 37082    **Score:** 74     **Queries matched:** 2   **emPAI:** 0.10 |
| --- | --- |
|  | Secretory carrier-associated membrane protein 2 OS=Homo sapiens GN=SCAMP2 PE=1 SV=2 |

|  | **Query** | **Observed** | **Mr(expt)** | **Mr(calc)** | **Delta** | **Miss** | **Score** | **Expect** | **Rank** | **Peptide** |
| --- | --- | --- | --- | --- | --- | --- | --- | --- | --- | --- |
|  | 11503 | **465.2536** | **1392.7390** | **1392.7473** | **-0.0084** | **0** | **9** | **17** | **2** | **R.ELQNTVANLHVR.Q** |
|  | 22230 | **1103.0072** | **2203.9998** | **2204.0134** | **-0.0136** | **0** | **74** | **3.6e-006** | **1** | **R.TGASFQQAQEEFSQGIFSSR.T** |

| **584.** | O75390\|CISY_HUMAN    **Mass:** 51908    **Score:** 73     **Queries matched:** 12   **emPAI:** 0.15 |
| --- | --- |
|  | Citrate synthase, mitochondrial OS=Homo sapiens GN=CS PE=1 SV=2 |

|  | **Query** | **Observed** | **Mr(expt)** | **Mr(calc)** | **Delta** | **Miss** | **Score** | **Expect** | **Rank** | **Peptide** |
| --- | --- | --- | --- | --- | --- | --- | --- | --- | --- | --- |
|  | 1374 | **616.3741** | **615.3669** | **615.3340** | **0.0329** | **1** | **10** | **33** | **5** | **R.KTDPR.Y** |
|  | 6121 | **538.2531** | **1074.4917** | **1073.5651** | **0.9265** | **0** | **5** | **59** | **2** | **K.NASCLVLAAR.H** 6122 |
|  | 6825 | **564.8265** | **1127.6385** | **1126.6499** | **0.9887** | **0** | **4** | **71** | **2** | **R.ALGFPLERPK.S** |
|  | 17019 | 878.9492 | 1755.8839 | 1756.8311 | -0.9472 | 0 | 6 | 27 | 2 | K.TVVGQITVDMMYGGMR.G |
|  | 17096 | 881.8845 | 1761.7545 | 1761.8785 | -0.1240 | 0 | 26 | 0.26 | 2 | K.GLVYETSVLDPDEGIR.F 17097 17101 |
|  | 17467 | **895.4330** | **1788.8515** | **1788.8209** | **0.0306** | **0** | **(4)** | **39** | **2** | **K.TVVGQITVDMMYGGMR.G** |
|  | 25458 | 882.1067 | 2643.2982 | 2643.2964 | 0.0018 | 1 | 1 | 44 | 8 | K.YWELIYEDSMDLIAKLPCVAAK.I |
|  | 28736 | **1116.9060** | **3347.6962** | **3347.7078** | **-0.0116** | **0** | **48** | **0.00075** | **1** | **K.GGEEPLPEGLFWLLVTGHIPTEEQVSWLSK.E** |
|  | 30143 | **1276.5842** | **3826.7309** | **3825.7329** | **0.9979** | **0** | **46** | **0.00078** | **1** | **R.EGSGIGAIDSNLDWSHNFTNMLGYTDHQFTELTR.L** |

| **585.** | P47914\|RL29_HUMAN    **Mass:** 17798    **Score:** 73     **Queries matched:** 5   **emPAI:** 0.22 |
| --- | --- |
|  | 60S ribosomal protein L29 OS=Homo sapiens GN=RPL29 PE=1 SV=2 |

|  | **Query** | **Observed** | **Mr(expt)** | **Mr(calc)** | **Delta** | **Miss** | **Score** | **Expect** | **Rank** | **Peptide** |
| --- | --- | --- | --- | --- | --- | --- | --- | --- | --- | --- |
|  | 1548 | **656.3319** | **655.3246** | **654.4428** | **0.8817** | **0** | **22** | **0.78** | **1** | **K.ALVKPK.E** |
|  | 2351 | **407.2003** | **812.3860** | **811.5028** | **0.8832** | **1** | **5** | **50** | **7** | **R.NGIKKPR.S** |
|  | 4250 | **470.3303** | **938.6461** | **937.5960** | **1.0501** | **1** | **3** | **70** | **3** | **K.EVKPKIPK.G** |
|  | 9969 | 655.3096 | 1308.6047 | 1307.6074 | 0.9973 | 1 | 4 | 54 | 5 | K.MQANNAKAMSAR.A |
|  | 11206 | **689.3732** | **1376.7318** | **1376.7412** | **-0.0094** | **0** | **73** | **6.9e-006** | **1** | **K.AQAAAPASVPAQAPK.R** |

| **586.** | P35268\|RL22_HUMAN    **Mass:** 14835    **Score:** 73     **Queries matched:** 7   **emPAI:** 1.01 |
| --- | --- |
|  | 60S ribosomal protein L22 OS=Homo sapiens GN=RPL22 PE=1 SV=2 |

|  | **Query** | **Observed** | **Mr(expt)** | **Mr(calc)** | **Delta** | **Miss** | **Score** | **Expect** | **Rank** | **Peptide** |
| --- | --- | --- | --- | --- | --- | --- | --- | --- | --- | --- |
|  | 1371 | 616.3649 | 615.3576 | 614.4115 | 0.9461 | 1 | 18 | 4.3 | 5 | K.KQVLK.F |
|  | 8045 | **604.3278** | **1206.6411** | **1206.6496** | **-0.0085** | **0** | **23** | **0.71** | **1** | **K.ITVTSEVPFSK.R** |
|  | 8738 | **621.8392** | **1241.6638** | **1241.6728** | **-0.0090** | **0** | **23** | **0.79** | **1** | **K.AGNLGGGVVTIER.S** 8767 |
|  | 19396 | **644.8343** | **1931.4810** | **1930.7228** | **0.7582** | **0** | **2** | **42** | **3** | **R.YFQINQDEEEEEDED.-** |
|  | 27718 | **1028.4618** | **3082.3635** | **3082.3801** | **-0.0165** | **0** | **49** | **0.00059** | **1** | **K.FTLDCTHPVEDGIMDAANFEQFLQER.I** 27717 |

| **587.** | P43304\|GPDM_HUMAN    **Mass:** 81315    **Score:** 73     **Queries matched:** 21   **emPAI:** 0.05 |
| --- | --- |
|  | Glycerol-3-phosphate dehydrogenase, mitochondrial OS=Homo sapiens GN=GPD2 PE=1 SV=3 |

|  | **Query** | **Observed** | **Mr(expt)** | **Mr(calc)** | **Delta** | **Miss** | **Score** | **Expect** | **Rank** | **Peptide** |
| --- | --- | --- | --- | --- | --- | --- | --- | --- | --- | --- |
|  | 746 | **488.3153** | **487.3080** | **486.2550** | **1.0530** | **0** | **6** | **1e+002** | **6** | **K.AGPSR.T** |
|  | 1539 | **653.4302** | **652.4230** | **651.2898** | **1.1332** | **1** | **9** | **16** | **4** | **R.KMDDK.D** |
|  | 2119 | 398.7226 | 795.4306 | 794.4650 | 0.9655 | 0 | 5 | 48 | 10 | R.VPIPVDR.S |
|  | 2640 | **417.7125** | **833.4104** | **832.4477** | **0.9628** | **0** | **6** | **48** | **9** | **R.IVELMGR.E** |
|  | 3147 | **432.8837** | **863.7528** | **864.4487** | **-0.6959** | **1** | **6** | **27** | **1** | **K.MASVTGKR.W** 3169 |
|  | 6806 | **564.3337** | **1126.6529** | **1127.5757** | **-0.9228** | **1** | **4** | **65** | **8** | **R.MVKEALHER.A** |
|  | 7025 | **573.2979** | **1144.5813** | **1143.6434** | **0.9379** | **0** | **15** | **5.6** | **1** | **R.MNLAIALTAAR.Y** |
|  | 7228 | 580.7845 | 1159.5544 | 1159.6383 | -0.0839 | 0 | (6) | 39 | 3 | R.MNLAIALTAAR.Y |
|  | 7678 | **397.4031** | **1189.1873** | **1189.6853** | **-0.4979** | **1** | **1** | **81** | **8** | **R.VSGSRLAILMK.T** |
|  | 8162 | 607.3098 | 1212.6051 | 1212.5525 | 0.0526 | 0 | 11 | 10 | 3 | K.FLYYEMGYK.S |
|  | 8170 | **607.3161** | **1212.6176** | **1212.6325** | **-0.0148** | **0** | **5** | **44** | **1** | **R.ALEHFPMLQK.D** |
|  | 8847 | 625.2756 | 1248.5366 | 1248.6020 | -0.0654 | 0 | 2 | 85 | 2 | R.SMAEDTINAAVK.T |
|  | 10005 | 437.7089 | 1310.1048 | 1309.6700 | 0.4348 | 1 | 3 | 43 | 8 | K.LDIEQYRMVK.E |
|  | 10537 | 669.8242 | 1337.6338 | 1337.7013 | -0.0675 | 1 | 5 | 42 | 5 | K.AFEVAKMASVTGK.R |
|  | 10797 | **677.3705** | **1352.7265** | **1351.6806** | **1.0460** | **0** | **17** | **2.7** | **1** | **K.LYDLVAGSNCLK.S** |
|  | 15733 | **832.8961** | **1663.7776** | **1663.8352** | **-0.0576** | **1** | **0** | **1.1e+002** | **10** | **K.CVINATGPFTDSVRK.M** |
|  | 16628 | **865.4410** | **1728.8675** | **1728.8756** | **-0.0081** | **0** | **73** | **5.8e-006** | **1** | **R.YGAATANYMEVVSLLK.K** |
|  | 17468 | **597.2911** | **1788.8516** | **1789.8747** | **-1.0231** | **0** | **0** | **1e+002** | **8** | **K.LVGAIVYYDGQHNDAR.M** |
|  | 17567 | **600.0013** | **1796.9822** | **1796.8475** | **0.1347** | **1** | **2** | **61** | **8** | **K.AADCISEPVNREPPSR.E** |
|  | 18946 | **632.9794** | **1895.9165** | **1895.9087** | **0.0077** | **1** | **4** | **35** | **5** | **R.IVELMGRELNWDDYK.K** |

| **588.** | P20815\|CP3A5_HUMAN    **Mass:** 57357    **Score:** 73     **Queries matched:** 16   **emPAI:** 0.13 |
| --- | --- |
|  | Cytochrome P450 3A5 OS=Homo sapiens GN=CYP3A5 PE=1 SV=1 |

|  | **Query** | **Observed** | **Mr(expt)** | **Mr(calc)** | **Delta** | **Miss** | **Score** | **Expect** | **Rank** | **Peptide** |
| --- | --- | --- | --- | --- | --- | --- | --- | --- | --- | --- |
|  | 1096 | 560.3808 | 559.3735 | 558.3741 | 0.9994 | 0 | 23 | 1.6 | 1 | R.TVLVK.E |
|  | 2578 | 415.2532 | 828.4919 | 827.4752 | 1.0167 | 0 | 5 | 60 | 9 | K.ETQIPLK.L |
|  | 2930 | 853.4177 | 852.4104 | 853.4190 | -1.0086 | 0 | 13 | 7.7 | 2 | R.FALMNMK.L |
|  | 6334 | **1090.4318** | **1089.4245** | **1089.4801** | **-0.0556** | **1** | **1** | **1.4e+002** | **6** | **K.FDTECYKK.Y** |
|  | 7417 | 588.2571 | 1174.4997 | 1174.5077 | -0.0080 | 0 | 20 | 1.8 | 1 | K.ECYSVFTNR.R |
|  | 8140 | **404.8731** | **1211.5975** | **1212.7343** | **-1.1368** | **1** | **7** | **29** | **8** | **R.LFPVAIRLER.T** |
|  | 11017 | 684.3561 | 1366.6976 | 1366.7067 | -0.0091 | 0 | 22 | 0.84 | 1 | R.VLQNFSFKPCK.E |
|  | 11225 | 689.8887 | 1377.7629 | 1376.6459 | 1.1170 | 0 | 1 | 1e+002 | 9 | K.SAISLAEDEEWK.R |
|  | 11691 | 703.3827 | 1404.7508 | 1405.7929 | -1.0421 | 1 | 11 | 10 | 5 | R.IRSLLSPTFTSGK.L |
|  | 12262 | 479.7539 | 1436.2397 | 1435.8043 | 0.4354 | 1 | 13 | 4.1 | 4 | R.FALMNMKLALIR.V |
|  | 13737 | 767.3759 | 1532.7373 | 1532.7470 | -0.0098 | 1 | 54 | 0.00054 | 1 | K.SAISLAEDEEWKR.I 13720 13721 13736 |
|  | 14995 | 808.9016 | 1615.7885 | 1616.7295 | -0.9410 | 1 | 6 | 31 | 4 | R.NCIGMRFALMNMK.L |
|  | 15025 | 540.2929 | 1617.8567 | 1616.7295 | 1.1272 | 1 | (1) | 92 | 6 | R.NCIGMRFALMNMK.L |

| **589.** | P35503\|UD13_HUMAN    **Mass:** 60869    **Score:** 72     **Queries matched:** 9   **emPAI:** 0.13 |
| --- | --- |
|  | UDP-glucuronosyltransferase 1-3 OS=Homo sapiens GN=UGT1A3 PE=1 SV=1 |

|  | **Query** | **Observed** | **Mr(expt)** | **Mr(calc)** | **Delta** | **Miss** | **Score** | **Expect** | **Rank** | **Peptide** |
| --- | --- | --- | --- | --- | --- | --- | --- | --- | --- | --- |
|  | 4553 | 480.7598 | 959.5051 | 959.5110 | -0.0059 | 0 | 46 | 0.0043 | 1 | K.AMAIADALGK.I |
|  | 5607 | 519.2922 | 1036.5699 | 1036.5553 | 0.0146 | 1 | 11 | 11 | 3 | K.AVINDKSYK.E 5603 |
|  | 5635 | 520.7592 | 1039.5039 | 1039.5120 | -0.0081 | 1 | 26 | 0.35 | 1 | K.SYKENIMR.L |
|  | 5836 | 528.7754 | 1055.5363 | 1055.5069 | 0.0294 | 1 | (2) | 1.1e+002 | 8 | K.SYKENIMR.L |
|  | 6728 | 561.2712 | 1120.5279 | 1121.6305 | -1.1026 | 1 | 13 | 7.8 | 7 | R.EVLRELHAR.G |
|  | 19648 | **652.6575** | **1954.9508** | **1956.0469** | **-1.0961** | **0** | **10** | **9.7** | **2** | **R.EVSVVDILSHASVWLFR.G** |
|  | 22217 | 1102.5538 | 2203.0931 | 2203.1042 | -0.0111 | 0 | 52 | 0.00056 | 1 | K.GAGVTLNVLEMTSEDLENALK.A |
|  | 23185 | 773.4149 | 2317.2228 | 2317.1929 | 0.0298 | 0 | 10 | 8.2 | 1 | K.DRPVEPLDLAVFWVEFVMR.H |

| **590.** | P35504\|UD15_HUMAN    **Mass:** 60602    **Score:** 72     **Queries matched:** 11   **emPAI:** 0.13 |
| --- | --- |
|  | UDP-glucuronosyltransferase 1-5 OS=Homo sapiens GN=UGT1A5 PE=1 SV=1 |

|  | **Query** | **Observed** | **Mr(expt)** | **Mr(calc)** | **Delta** | **Miss** | **Score** | **Expect** | **Rank** | **Peptide** |
| --- | --- | --- | --- | --- | --- | --- | --- | --- | --- | --- |
|  | 4553 | 480.7598 | 959.5051 | 959.5110 | -0.0059 | 0 | 46 | 0.0043 | 1 | K.AMAIADALGK.I |
|  | 5607 | 519.2922 | 1036.5699 | 1036.5553 | 0.0146 | 1 | 11 | 11 | 3 | K.AVINDKSYK.E 5603 |
|  | 5635 | 520.7592 | 1039.5039 | 1039.5120 | -0.0081 | 1 | 26 | 0.35 | 1 | K.SYKENIMR.L |
|  | 5836 | 528.7754 | 1055.5363 | 1055.5069 | 0.0294 | 1 | (2) | 1.1e+002 | 8 | K.SYKENIMR.L |
|  | 14123 | **520.2504** | **1557.7293** | **1558.7782** | **-1.0489** | **0** | **3** | **60** | **3** | **R.MAIMNNMSLIIHR.S** |
|  | 16872 | **582.9638** | **1745.8696** | **1746.8691** | **-0.9995** | **1** | **6** | **29** | **4** | **R.RMAIMNNMSLIIHR.S** |
|  | 18479 | 930.0055 | 1857.9965 | 1858.0023 | -0.0057 | 0 | 6 | 26 | 2 | R.GHQVVVLTLEVNMYIK.E |
|  | 22217 | 1102.5538 | 2203.0931 | 2203.1042 | -0.0111 | 0 | 52 | 0.00056 | 1 | K.GAGVTLNVLEMTSEDLENALK.A |
|  | 23185 | 773.4149 | 2317.2228 | 2317.1929 | 0.0298 | 0 | 10 | 8.2 | 1 | K.DRPVEPLDLAVFWVEFVMR.H |
|  | 29272 | **1176.8862** | **3527.6369** | **3527.7945** | **-0.1576** | **1** | **3** | **16** | **3** | **R.VKNMLYPLALSYLCHAVSAPYASLASELFQR.E** |

| **591.** | P19224\|UD16_HUMAN    **Mass:** 61338    **Score:** 72     **Queries matched:** 12   **emPAI:** 0.12 |
| --- | --- |
|  | UDP-glucuronosyltransferase 1-6 OS=Homo sapiens GN=UGT1A6 PE=1 SV=2 |

|  | **Query** | **Observed** | **Mr(expt)** | **Mr(calc)** | **Delta** | **Miss** | **Score** | **Expect** | **Rank** | **Peptide** |
| --- | --- | --- | --- | --- | --- | --- | --- | --- | --- | --- |
|  | 4553 | 480.7598 | 959.5051 | 959.5110 | -0.0059 | 0 | 46 | 0.0043 | 1 | K.AMAIADALGK.I |
|  | 5607 | 519.2922 | 1036.5699 | 1036.5553 | 0.0146 | 1 | 11 | 11 | 3 | K.AVINDKSYK.E 5603 |
|  | 5635 | 520.7592 | 1039.5039 | 1039.5120 | -0.0081 | 1 | 26 | 0.35 | 1 | K.SYKENIMR.L |
|  | 5836 | 528.7754 | 1055.5363 | 1055.5069 | 0.0294 | 1 | (2) | 1.1e+002 | 8 | K.SYKENIMR.L |
|  | 12752 | **490.2967** | **1467.8684** | **1468.6484** | **-0.7799** | **0** | **7** | **22** | **2** | **R.YQSFGNNHFAER.S** |
|  | 15058 | 810.9055 | 1619.7964 | 1620.8399 | -1.0435 | 1 | 0 | 1.1e+002 | 7 | R.KIYPVPYDQEELK.N |
|  | 15999 | 561.6399 | 1681.8978 | 1681.8134 | 0.0845 | 1 | 2 | 71 | 7 | R.SPDPVSYIPRCYTK.F |
|  | 21198 | **701.3827** | **2101.1262** | **2101.2147** | **-0.0884** | **1** | **2** | **53** | **5** | **R.GHEIVVVVPEVNLLLKESK.Y** |
|  | 22217 | 1102.5538 | 2203.0931 | 2203.1042 | -0.0111 | 0 | 52 | 0.00056 | 1 | K.GAGVTLNVLEMTSEDLENALK.A |
|  | 23185 | 773.4149 | 2317.2228 | 2317.1929 | 0.0298 | 0 | 10 | 8.2 | 1 | K.DRPVEPLDLAVFWVEFVMR.H |
|  | 29866 | 1242.6325 | 3724.8755 | 3725.8134 | -0.9379 | 1 | 0 | 32 | 9 | R.KDLSQEFEAYINASGEHGIVVFSLGSMVSEIPEK.K |

| **592.** | P22310\|UD14_HUMAN    **Mass:** 60613    **Score:** 72     **Queries matched:** 10   **emPAI:** 0.13 |
| --- | --- |
|  | UDP-glucuronosyltransferase 1-4 OS=Homo sapiens GN=UGT1A4 PE=1 SV=1 |

|  | **Query** | **Observed** | **Mr(expt)** | **Mr(calc)** | **Delta** | **Miss** | **Score** | **Expect** | **Rank** | **Peptide** |
| --- | --- | --- | --- | --- | --- | --- | --- | --- | --- | --- |
|  | 4553 | 480.7598 | 959.5051 | 959.5110 | -0.0059 | 0 | 46 | 0.0043 | 1 | K.AMAIADALGK.I |
|  | 5607 | 519.2922 | 1036.5699 | 1036.5553 | 0.0146 | 1 | 11 | 11 | 3 | K.AVINDKSYK.E 5603 |
|  | 5635 | 520.7592 | 1039.5039 | 1039.5120 | -0.0081 | 1 | 26 | 0.35 | 1 | K.SYKENIMR.L |
|  | 5836 | 528.7754 | 1055.5363 | 1055.5069 | 0.0294 | 1 | (2) | 1.1e+002 | 8 | K.SYKENIMR.L |
|  | 12433 | 483.7814 | 1448.3225 | 1447.6766 | 0.6459 | 0 | 0 | 85 | 4 | K.GTQCPNPSSYIPK.L |
|  | 19893 | **659.9976** | **1976.9710** | **1977.9876** | **-1.0166** | **1** | **(0)** | **89** | **4** | **R.YSRSMAIMNNVSLALHR.C** |
|  | 19911 | **660.4246** | **1978.2519** | **1977.9876** | **0.2642** | **1** | **2** | **45** | **3** | **R.YSRSMAIMNNVSLALHR.C** |
|  | 22217 | 1102.5538 | 2203.0931 | 2203.1042 | -0.0111 | 0 | 52 | 0.00056 | 1 | K.GAGVTLNVLEMTSEDLENALK.A |
|  | 23185 | 773.4149 | 2317.2228 | 2317.1929 | 0.0298 | 0 | 10 | 8.2 | 1 | K.DRPVEPLDLAVFWVEFVMR.H |

| **593.** | O60656\|UD19_HUMAN    **Mass:** 60757    **Score:** 72     **Queries matched:** 8   **emPAI:** 0.13 |
| --- | --- |
|  | UDP-glucuronosyltransferase 1-9 OS=Homo sapiens GN=UGT1A9 PE=1 SV=1 |

|  | **Query** | **Observed** | **Mr(expt)** | **Mr(calc)** | **Delta** | **Miss** | **Score** | **Expect** | **Rank** | **Peptide** |
| --- | --- | --- | --- | --- | --- | --- | --- | --- | --- | --- |
|  | 4553 | 480.7598 | 959.5051 | 959.5110 | -0.0059 | 0 | 46 | 0.0043 | 1 | K.AMAIADALGK.I |
|  | 5607 | 519.2922 | 1036.5699 | 1036.5553 | 0.0146 | 1 | 11 | 11 | 3 | K.AVINDKSYK.E 5603 |
|  | 5635 | 520.7592 | 1039.5039 | 1039.5120 | -0.0081 | 1 | 26 | 0.35 | 1 | K.SYKENIMR.L |
|  | 5836 | 528.7754 | 1055.5363 | 1055.5069 | 0.0294 | 1 | (2) | 1.1e+002 | 8 | K.SYKENIMR.L |
|  | 17864 | **911.3962** | **1820.7779** | **1819.8750** | **0.9030** | **0** | **1** | **86** | **1** | **K.LLVVPMDGSHWFTMR.S** |
|  | 22217 | 1102.5538 | 2203.0931 | 2203.1042 | -0.0111 | 0 | 52 | 0.00056 | 1 | K.GAGVTLNVLEMTSEDLENALK.A |
|  | 23185 | 773.4149 | 2317.2228 | 2317.1929 | 0.0298 | 0 | 10 | 8.2 | 1 | K.DRPVEPLDLAVFWVEFVMR.H |

| **594.** | P28066\|PSA5_HUMAN    **Mass:** 26565    **Score:** 72     **Queries matched:** 4   **emPAI:** 0.14 |
| --- | --- |
|  | Proteasome subunit alpha type-5 OS=Homo sapiens GN=PSMA5 PE=1 SV=3 |

|  | **Query** | **Observed** | **Mr(expt)** | **Mr(calc)** | **Delta** | **Miss** | **Score** | **Expect** | **Rank** | **Peptide** |
| --- | --- | --- | --- | --- | --- | --- | --- | --- | --- | --- |
|  | 8160 | **405.2079** | **1212.6019** | **1213.7645** | **-1.1626** | **1** | **7** | **27** | **3** | **K.EAIKSSLIILK.Q** |
|  | 24345 | **825.4164** | **2473.2273** | **2473.2424** | **-0.0151** | **0** | **72** | **4.6e-006** | **1** | **K.LNATNIELATVQPGQNFHMFTK.E** |
|  | 24461 | 830.4434 | 2488.3083 | 2489.2373 | -0.9290 | 0 | (2) | 39 | 5 | K.LNATNIELATVQPGQNFHMFTK.E |
|  | 30556 | **825.3931** | **4121.9290** | **4120.9007** | **1.0282** | **1** | **3** | **14** | **2** | **K.GPQLFHMDPSGTFVQCDARAIGSASEGAQSSLQEVYHK.S** |

| **595.** | O14672\|ADA10_HUMAN    **Mass:** 86140    **Score:** 72     **Queries matched:** 20   **emPAI:** 0.04 |
| --- | --- |
|  | Disintegrin and metalloproteinase domain-containing protein 10 OS=Homo sapiens GN=ADAM10 PE=1 SV=1 |

|  | **Query** | **Observed** | **Mr(expt)** | **Mr(calc)** | **Delta** | **Miss** | **Score** | **Expect** | **Rank** | **Peptide** |
| --- | --- | --- | --- | --- | --- | --- | --- | --- | --- | --- |
|  | 3571 | 446.2468 | 890.4790 | 890.4345 | 0.0445 | 0 | 5 | 67 | 8 | R.INTTADEK.D |
|  | 4455 | **477.2260** | **952.4373** | **951.4675** | **0.9698** | **0** | **4** | **53** | **7** | **R.LDFHAHGR.H** |
|  | 5428 | **513.2867** | **1024.5588** | **1025.5506** | **-0.9917** | **0** | **9** | **17** | **7** | **R.LVDADGPLAR.L** |
|  | 5946 | **532.2894** | **1062.5643** | **1062.4263** | **0.1380** | **0** | **3** | **93** | **8** | **R.GYCDVFMR.C** |
|  | 7620 | **594.3202** | **1186.6258** | **1187.5346** | **-0.9088** | **0** | **1** | **1.2e+002** | **4** | **R.DTSLFSDEFK.V** |
|  | 10612 | **448.2520** | **1341.7340** | **1341.6823** | **0.0517** | **1** | **3** | **62** | **7** | **R.CRLVDADGPLAR.L** |
|  | 13277 | **500.9516** | **1499.8331** | **1499.8433** | **-0.0102** | **1** | **13** | **6.2** | **1** | **R.RRPPQPIQQPQR.Q** |
|  | 14788 | **803.3829** | **1604.7512** | **1604.7617** | **-0.0105** | **0** | **6** | **29** | **1** | **R.TITLQPGSPCNDFR.G** |
|  | 16253 | **850.9226** | **1699.8305** | **1699.8417** | **-0.0112** | **0** | **72** | **7.4e-006** | **1** | **K.AIDTIYQTTDFSGIR.N** |
|  | 16484 | 573.3146 | 1716.9221 | 1717.8271 | -0.9050 | 1 | 2 | 63 | 7 | R.INTTADEKDPTNPFR.F |
|  | 17456 | **596.9536** | **1787.8390** | **1788.8431** | **-1.0041** | **0** | **1** | **76** | **5** | **R.HYEGLSYNVDSLHQK.H** |
|  | 17518 | 598.3544 | 1792.0415 | 1792.9505 | -0.9091 | 1 | 11 | 7.5 | 1 | K.FSLCSIRNISQVLEK.K |
|  | 21775 | 1077.0420 | 2152.0694 | 2152.1640 | -0.0946 | 1 | 5 | 27 | 2 | K.KSLNTGIITVQNYGSHVPPK.V 21740 21755 21790 21792 |
|  | 26185 | **921.4981** | **2761.4725** | **2762.4941** | **-1.0216** | **1** | **0** | **57** | **5** | **R.VLILLLSWAAGMGGQYGNPLNKYIR.H** |
|  | 26968 | **976.4583** | **2926.3529** | **2926.6289** | **-0.2759** | **1** | **1** | **42** | **2** | **-.MVLLRVLILLLSWAAGMGGQYGNPLNK.Y** |
|  | 30240 | **1300.5900** | **3898.7481** | **3899.5009** | **-0.7528** | **1** | **2** | **19** | **1** | **K.RNNCFVESGQPICGNGMVEQGEECDCGYSDQCK.D** |

| **596.** | P10696\|PPBN_HUMAN    **Mass:** 57626    **Score:** 72     **Queries matched:** 24   **emPAI:** 0.13 |
| --- | --- |
|  | Alkaline phosphatase, placental-like OS=Homo sapiens GN=ALPPL2 PE=1 SV=4 |

|  | **Query** | **Observed** | **Mr(expt)** | **Mr(calc)** | **Delta** | **Miss** | **Score** | **Expect** | **Rank** | **Peptide** |
| --- | --- | --- | --- | --- | --- | --- | --- | --- | --- | --- |
|  | 1659 | 686.4152 | 685.4079 | 685.4486 | -0.0407 | 1 | 24 | 0.81 | 1 | R.ILKGQK.K |
|  | 3979 | 460.2606 | 918.5066 | 918.5135 | -0.0069 | 0 | 58 | 0.00027 | 1 | K.SVGVVTTTR.V |
|  | 4048 | 463.2282 | 924.4419 | 923.5116 | 0.9303 | 0 | 7 | 28 | 7 | R.FPYVALSK.T |
|  | 6688 | 559.7805 | 1117.5464 | 1117.5550 | -0.0086 | 0 | 44 | 0.0073 | 1 | R.GNEVISVMNR.A |
|  | 7423 | 588.3113 | 1174.6080 | 1174.6670 | -0.0590 | 1 | 5 | 52 | 5 | K.AGKSVGVVTTTR.V |
|  | 9919 | 653.3212 | 1304.6279 | 1304.6837 | -0.0558 | 0 | 3 | 67 | 2 | K.GNFQTIGLSAAAR.F |
|  | 12482 | 484.7315 | 1451.1725 | 1450.6477 | 0.5248 | 0 | 7 | 15 | 1 | R.NWYSDADVPASAR.Q 12485 |
|  | 13206 | 499.3396 | 1494.9970 | 1494.8745 | 0.1225 | 0 | 7 | 18 | 9 | -.MQGPWVLLLLGLR.L |
|  | 13416 | 504.2708 | 1509.7905 | 1510.8694 | -1.0789 | 0 | (3) | 56 | 8 | -.MQGPWVLLLLGLR.L |
|  | 18412 | 928.4858 | 1854.9570 | 1855.0244 | -0.0673 | 1 | 2 | 58 | 5 | R.KAYTVLLYGNGPGYVLK.D 18406 18407 18409 18411 18413 18415 18416 18426 18430 18434 |
|  | 24075 | 611.0605 | 2440.2129 | 2439.1740 | 1.0388 | 1 | 1 | 63 | 6 | K.TYSVDKHVPDSGATATAYLCGVK.G |
|  | 25576 | **1334.0494** | **2666.0843** | **2667.1258** | **-1.0414** | **1** | **5** | **17** | **1** | **R.KYMFPMGTPDPEYPDDYSQGGTR.L** |
|  | 29048 | **862.4099** | **3445.6105** | **3446.7003** | **-1.0897** | **1** | **0** | **37** | **1** | **R.YVWNRTELLQASLDPSVTHLMGLFEPGDMK.Y** |

| **597.** | P62899\|RL31_HUMAN    **Mass:** 14454    **Score:** 71     **Queries matched:** 8   **emPAI:** 0.62 |
| --- | --- |
|  | 60S ribosomal protein L31 OS=Homo sapiens GN=RPL31 PE=1 SV=1 |

|  | **Query** | **Observed** | **Mr(expt)** | **Mr(calc)** | **Delta** | **Miss** | **Score** | **Expect** | **Rank** | **Peptide** |
| --- | --- | --- | --- | --- | --- | --- | --- | --- | --- | --- |
|  | 1505 | **648.3416** | **647.3343** | **647.3391** | **-0.0048** | **0** | **8** | **35** | **4** | **R.NVPYR.I** |
|  | 3901 | **457.7659** | **913.5172** | **912.5294** | **0.9879** | **1** | **7** | **34** | **4** | **K.RIHGVGFK.K** |
|  | 4923 | 494.7715 | 987.5285 | 987.5349 | -0.0063 | 0 | 31 | 0.15 | 1 | R.SAINEVVTR.E |
|  | 5326 | **509.2688** | **1016.5230** | **1016.5291** | **-0.0060** | **0** | **9** | **25** | **3** | **R.EYTINIHK.R** |
|  | 7888 | **601.0644** | **1200.1143** | **1200.6575** | **-0.5431** | **1** | **1** | **90** | **4** | **K.GRSAINEVVTR.E** |
|  | 11580 | **699.8727** | **1397.7308** | **1396.6479** | **1.0829** | **1** | **7** | **30** | **1** | **K.FAMKEMGTPDVR.I** |
|  | 15454 | **822.9608** | **1643.9071** | **1643.9175** | **-0.0104** | **0** | **47** | **0.0024** | **1** | **K.LYTLVTYVPVTTFK.N** 15453 |

| **598.** | Q99714\|HCD2_HUMAN    **Mass:** 27134    **Score:** 71     **Queries matched:** 9   **emPAI:** 0.30 |
| --- | --- |
|  | 3-hydroxyacyl-CoA dehydrogenase type-2 OS=Homo sapiens GN=HSD17B10 PE=1 SV=3 |

|  | **Query** | **Observed** | **Mr(expt)** | **Mr(calc)** | **Delta** | **Miss** | **Score** | **Expect** | **Rank** | **Peptide** |
| --- | --- | --- | --- | --- | --- | --- | --- | --- | --- | --- |
|  | 1491 | 644.8003 | 643.7931 | 643.3653 | 0.4278 | 0 | 18 | 2.8 | 1 | R.LDGAIR.M |
|  | 5005 | 497.2768 | 992.5390 | 992.4895 | 0.0495 | 1 | 6 | 47 | 8 | -.MAAACRSVK.G 4981 5009 |
|  | 17413 | **893.4070** | **1784.7994** | **1784.8112** | **-0.0117** | **0** | **52** | **0.00066** | **1** | **R.LVAGEMGQNEPDQGGQR.G** |
|  | 21005 | **1043.0814** | **2084.1483** | **2084.1591** | **-0.0109** | **0** | **37** | **0.018** | **1** | **R.VMTIAPGLFGTPLLTSLPEK.V** 21006 |
|  | 26717 | 960.5004 | 2878.4793 | 2877.5025 | 0.9768 | 0 | 10 | 5.9 | 2 | R.LGDPAEYAHLVQAIIENPFLNGEVIR.L |
|  | 28895 | 1694.7880 | 3387.5614 | 3388.6504 | -1.0890 | 1 | 2 | 26 | 6 | R.VLDVNLMGTFNVIRLVAGEMGQNEPDQGGQR.G |

| **599.** | P15531\|NDKA_HUMAN    **Mass:** 17309    **Score:** 71     **Queries matched:** 6   **emPAI:** 0.50 |
| --- | --- |
|  | Nucleoside diphosphate kinase A OS=Homo sapiens GN=NME1 PE=1 SV=1 |

|  | **Query** | **Observed** | **Mr(expt)** | **Mr(calc)** | **Delta** | **Miss** | **Score** | **Expect** | **Rank** | **Peptide** |
| --- | --- | --- | --- | --- | --- | --- | --- | --- | --- | --- |
|  | 960 | **530.2582** | **529.2510** | **528.3635** | **0.8874** | **0** | **7** | **70** | **4** | **R.LVGLK.F** |
|  | 5770 | 526.2495 | 1050.4843 | 1050.4917 | -0.0073 | 0 | 35 | 0.054 | 1 | R.GDFCIQVGR.N |
|  | 16344 | **854.4315** | **1706.8485** | **1706.9277** | **-0.0792** | **1** | **2** | **63** | **9** | **R.LVGLKFMQASEDLLK.E** |
|  | 17417 | 893.4566 | 1784.8986 | 1784.9091 | -0.0104 | 0 | 61 | 7.8e-005 | 1 | R.VMLGETNPADSKPGTIR.G |
|  | 17623 | 601.3988 | 1801.1746 | 1800.9040 | 0.2706 | 0 | (0) | 76 | 7 | R.VMLGETNPADSKPGTIR.G |
|  | 24110 | 1224.0866 | 2446.1585 | 2445.2297 | 0.9288 | 1 | 2 | 47 | 3 | K.YMHSGPVVAMVWEGLNVVKTGR.V |

| **600.** | P80404\|GABT_HUMAN    **Mass:** 57087    **Score:** 71     **Queries matched:** 15   **emPAI:** 0.07 |
| --- | --- |
|  | 4-aminobutyrate aminotransferase, mitochondrial OS=Homo sapiens GN=ABAT PE=1 SV=3 |

|  | **Query** | **Observed** | **Mr(expt)** | **Mr(calc)** | **Delta** | **Miss** | **Score** | **Expect** | **Rank** | **Peptide** |
| --- | --- | --- | --- | --- | --- | --- | --- | --- | --- | --- |
|  | 4291 | **472.2789** | **942.5433** | **941.5546** | **0.9887** | **0** | **5** | **50** | **3** | **R.QSLLSVAPK.G** 4299 |
|  | 8127 | 606.3388 | 1210.6631 | 1210.7397 | -0.0767 | 1 | 8 | 20 | 7 | K.LRQSLLSVAPK.G 8120 |
|  | 8212 | **406.0262** | **1215.0567** | **1214.5576** | **0.4991** | **1** | **7** | **22** | **4** | **K.KMMTGGFFHK.E** |
|  | 10788 | 677.3307 | 1352.6469 | 1351.8438 | 0.8031 | 0 | 4 | 51 | 8 | K.NLLLAEVINIIK.R |
|  | 13879 | **515.2628** | **1542.7666** | **1542.6912** | **0.0754** | **0** | **5** | **38** | **2** | **K.VDVEFDYDGPLMK.T** |
|  | 13929 | **516.2894** | **1545.8465** | **1546.8943** | **-1.0479** | **1** | **8** | **19** | **3** | **R.LLVPGSRHISQAAAK.V** |
|  | 17205 | **885.9603** | **1769.9060** | **1769.9141** | **-0.0081** | **0** | **48** | **0.0017** | **1** | **K.IDIPSFDWPIAPFPR.L** 17207 |
|  | 22939 | **572.2950** | **2285.1508** | **2285.0867** | **0.0641** | **1** | **2** | **50** | **7** | **-.MASMLLAQRLACSFQHSYR.L** |
|  | 24130 | **816.4454** | **2446.3145** | **2447.2770** | **-0.9625** | **0** | **3** | **35** | **7** | **R.MLDLYSQISSVPIGYSHPALLK.L** |
|  | 28165 | **1062.8125** | **3185.4157** | **3184.4272** | **0.9885** | **1** | **1** | **37** | **4** | **K.GMSQLITMACGSCSNENALKTIFMWYR.S** |
|  | 30513 | **822.2347** | **4106.1370** | **4105.8179** | **0.3191** | **1** | **3** | **11** | **1** | **R.GQRGFSQEELETCMINQAPGCPDYSILSFMGAFHGR.T** |
|  | 30821 | 1095.5248 | 4378.0700 | 4377.9307 | 0.1393 | 1 | 1 | 19 | 9 | K.HGCAFLVDEVQTGGGCTGKFWAHEHWGLDDPADVMTFSK.K |

| **601.** | Q969Z3\|MOSC2_HUMAN    **Mass:** 38569    **Score:** 71     **Queries matched:** 10   **emPAI:** 0.20 |
| --- | --- |
|  | MOSC domain-containing protein 2, mitochondrial OS=Homo sapiens GN=MOSC2 PE=1 SV=1 |

|  | **Query** | **Observed** | **Mr(expt)** | **Mr(calc)** | **Delta** | **Miss** | **Score** | **Expect** | **Rank** | **Peptide** |
| --- | --- | --- | --- | --- | --- | --- | --- | --- | --- | --- |
|  | 3118 | 432.2414 | 862.4682 | 863.3443 | -0.8761 | 0 | 8 | 31 | 1 | R.DCGNEAAK.W |
|  | 3362 | 439.2453 | 876.4761 | 876.4310 | 0.0451 | 1 | 8 | 29 | 4 | K.KVMACPR.C |
|  | 3968 | **459.7732** | **917.5319** | **917.5375** | **-0.0056** | **0** | **11** | **15** | **8** | **K.LWIYPVK.S** |
|  | 4276 | 471.3338 | 940.6530 | 941.5546 | -0.9015 | 1 | 15 | 4.2 | 4 | R.KQPLDTLK.S |
|  | 8321 | **407.7346** | **1220.1821** | **1219.6561** | **0.5261** | **1** | **6** | **25** | **10** | **K.QPLDTLKSYR.L** |
|  | 10533 | 669.8094 | 1337.6043 | 1337.6762 | -0.0718 | 1 | 6 | 31 | 2 | R.LVQFETNMKGR.T |
|  | 15871 | **837.9169** | **1673.8192** | **1673.8295** | **-0.0103** | **0** | **29** | **0.14** | **1** | **R.CILTTVDPDTGVIDR.K** |
|  | 19778 | **656.3932** | **1966.1577** | **1966.8911** | **-0.7333** | **1** | **0** | **72** | **4** | **K.SCKGVPVSEAECTAMGLR.S** |
|  | 30288 | **1313.3358** | **3936.9856** | **3937.0005** | **-0.0149** | **1** | **56** | **8.4e-005** | **1** | **R.KLLPTLDQNFQVAYPDYCPLLIMTDASLVDLNTR.M** 30287 |

| **602.** | Q1KMD3\|HNRL2_HUMAN    **Mass:** 85622    **Score:** 70     **Queries matched:** 14   **emPAI:** 0.04 |
| --- | --- |
|  | Heterogeneous nuclear ribonucleoprotein U-like protein 2 OS=Homo sapiens GN=HNRNPUL2 PE=1 SV=1 |

|  | **Query** | **Observed** | **Mr(expt)** | **Mr(calc)** | **Delta** | **Miss** | **Score** | **Expect** | **Rank** | **Peptide** |
| --- | --- | --- | --- | --- | --- | --- | --- | --- | --- | --- |
|  | 1497 | **646.3746** | **645.3674** | **644.3354** | **1.0320** | **1** | **11** | **20** | **4** | **R.NNRNK.R** |
|  | 2102 | 398.2048 | 794.3951 | 794.3922 | 0.0029 | 1 | 14 | 5.9 | 4 | K.YKEEAR.K |
|  | 4366 | **474.2844** | **946.5542** | **945.4953** | **1.0588** | **0** | **7** | **33** | **4** | **K.VTQNLPMK.E** |
|  | 5087 | **499.7948** | **997.5750** | **998.5873** | **-1.0123** | **1** | **1** | **1.3e+002** | **9** | **R.GLKVDLAQR.L** |
|  | 11661 | **468.7817** | **1403.3234** | **1402.6398** | **0.6836** | **1** | **4** | **37** | **6** | **K.GLEEPEMDPKSR.D** |
|  | 11957 | **710.3737** | **1418.7329** | **1418.6421** | **0.0908** | **1** | **10** | **12** | **4** | **R.MKGLEEPEMDPK.S** 11971 |
|  | 14835 | 804.3886 | 1606.7627 | 1606.8137 | -0.0510 | 0 | 70 | 1.1e-005 | 1 | R.YNVLGAETVLNQMR.M |
|  | 15736 | **832.9047** | **1663.7948** | **1663.8426** | **-0.0478** | **1** | **8** | **19** | **4** | **K.VCFEAKVTQNLPMK.E** 15735 |
|  | 18571 | 934.4937 | 1866.9729 | 1865.9491 | 1.0237 | 1 | 1 | 74 | 7 | R.YNVLGAETVLNQMRMK.G |
|  | 19931 | 661.3336 | 1980.9790 | 1979.9067 | 1.0723 | 1 | 2 | 65 | 6 | R.KEVEGDDVPESIMLEMK.A |
|  | 19949 | 662.0194 | 1983.0364 | 1982.9381 | 0.0983 | 1 | 1 | 67 | 8 | R.NFILDQCNVYNSGQRR.K |
|  | 25649 | 892.4631 | 2674.3674 | 2674.3710 | -0.0036 | 1 | 5 | 20 | 2 | K.TIEECEVILMVGLPGSGKTQWALK.Y |

| **603.** | P26038\|MOES_HUMAN    **Mass:** 67892    **Score:** 70     **Queries matched:** 16   **emPAI:** 0.17 |
| --- | --- |
|  | Moesin OS=Homo sapiens GN=MSN PE=1 SV=3 |

|  | **Query** | **Observed** | **Mr(expt)** | **Mr(calc)** | **Delta** | **Miss** | **Score** | **Expect** | **Rank** | **Peptide** |
| --- | --- | --- | --- | --- | --- | --- | --- | --- | --- | --- |
|  | 1396 | **623.3455** | **622.3382** | **622.2632** | **0.0750** | **0** | **8** | **24** | **1** | **R.EMAEK.E** |
|  | 3937 | 458.7511 | 915.4876 | 915.5138 | -0.0261 | 1 | 10 | 18 | 8 | K.KVTAQDVR.K |
|  | 4558 | 480.7983 | 959.5821 | 958.5851 | 0.9970 | 0 | 15 | 6.1 | 1 | K.FVIKPIDK.K |
|  | 6514 | 552.7924 | 1103.5703 | 1103.5764 | -0.0061 | 0 | 50 | 0.0017 | 1 | K.IGFPWSEIR.N |
|  | 7019 | **572.8102** | **1143.6059** | **1142.6043** | **1.0016** | **1** | **5** | **51** | **7** | **R.RALELEQER.K** |
|  | 7708 | 596.2993 | 1190.5841 | 1189.5649 | 1.0192 | 0 | 8 | 27 | 5 | K.AQMVQEDLEK.T |
|  | 12224 | 478.7763 | 1433.3072 | 1433.6027 | -0.2955 | 0 | 7 | 19 | 9 | K.TANDMIHAENMR.L |
|  | 12803 | 736.8931 | 1471.7716 | 1471.7817 | -0.0101 | 1 | 55 | 0.00039 | 1 | R.RKPDTIEVQQMK.A 12804 |
|  | 13905 | 515.6282 | 1543.8627 | 1542.8558 | 1.0068 | 1 | 7 | 25 | 3 | R.LTPKIGFPWSEIR.N |
|  | 15837 | **558.3320** | **1671.9743** | **1671.8427** | **0.1316** | **1** | **2** | **64** | **9** | **R.QEAEEAKEALLQASR.D** |
|  | 16512 | **573.9997** | **1718.9772** | **1718.8509** | **0.1264** | **0** | **5** | **31** | **4** | **K.TQEQLALEMAELTAR.I** |
|  | 16542 | 574.6338 | 1720.8795 | 1719.8259 | 1.0537 | 0 | (2) | 65 | 5 | R.ILALCMGNHELYMR.R |
|  | 16726 | 579.5326 | 1735.5759 | 1735.8208 | -0.2448 | 0 | 3 | 44 | 7 | R.ILALCMGNHELYMR.R 16738 |
|  | 23919 | 808.0693 | 2421.1860 | 2420.1165 | 1.0695 | 1 | 3 | 37 | 1 | K.ESEAVEWQQKAQMVQEDLEK.T |

| **604.** | P62280\|RS11_HUMAN    **Mass:** 18590    **Score:** 70     **Queries matched:** 8   **emPAI:** 0.46 |
| --- | --- |
|  | 40S ribosomal protein S11 OS=Homo sapiens GN=RPS11 PE=1 SV=3 |

|  | **Query** | **Observed** | **Mr(expt)** | **Mr(calc)** | **Delta** | **Miss** | **Score** | **Expect** | **Rank** | **Peptide** |
| --- | --- | --- | --- | --- | --- | --- | --- | --- | --- | --- |
|  | 2393 | **816.4773** | **815.4700** | **815.4753** | **-0.0053** | **0** | **19** | **2.7** | **1** | **R.VLLGETGK.E** |
|  | 2974 | 856.4504 | 855.4432 | 855.4239 | 0.0193 | 1 | 5 | 51 | 5 | K.YNRFEK.R |
|  | 4403 | **475.2605** | **948.5064** | **947.5804** | **0.9260** | **1** | **2** | **1.2e+002** | **9** | **R.FNVLKVTK.A** |
|  | 5547 | **517.3097** | **1032.6048** | **1031.5910** | **1.0139** | **1** | **5** | **55** | **5** | **K.MQRTIVIR.R** |
|  | 6101 | 537.3100 | 1072.6054 | 1072.6128 | -0.0074 | 1 | 15 | 6.4 | 2 | R.VLLGETGKEK.L |
|  | 10689 | 674.3145 | 1346.6145 | 1346.6224 | -0.0079 | 0 | 31 | 0.13 | 1 | K.NMSVHLSPCFR.D |
|  | 19966 | **993.5127** | **1985.0108** | **1985.0252** | **-0.0143** | **0** | **65** | **3.3e-005** | **1** | **R.DVQIGDIVTVGECRPLSK.T** 19967 |

| **605.** | P55011\|S12A2_HUMAN    **Mass:** 132048   **Score:** 70     **Queries matched:** 34   **emPAI:** 0.06 |
| --- | --- |
|  | Solute carrier family 12 member 2 OS=Homo sapiens GN=SLC12A2 PE=1 SV=1 |

|  | **Query** | **Observed** | **Mr(expt)** | **Mr(calc)** | **Delta** | **Miss** | **Score** | **Expect** | **Rank** | **Peptide** |
| --- | --- | --- | --- | --- | --- | --- | --- | --- | --- | --- |
|  | 1004 | **543.3948** | **542.3876** | **542.3540** | **0.0335** | **0** | **6** | **38** | **3** | **K.GVLVR.C** |
|  | 1105 | 561.2952 | 560.2880 | 559.2714 | 1.0165 | 0 | 12 | 19 | 3 | R.DGGGVR.D 1111 |
|  | 3275 | 871.5188 | 870.5115 | 870.5175 | -0.0059 | 0 | 16 | 4.4 | 1 | K.TATQPLLK.K |
|  | 4011 | **461.2458** | **920.4770** | **920.4273** | **0.0497** | **0** | **8** | **27** | **3** | **K.EMSIDQAK.Y** |
|  | 5580 | **518.3285** | **1034.6425** | **1034.6488** | **-0.0063** | **0** | **17** | **3** | **1** | **K.DLPPILLVR.G** 5571 5584 |
|  | 7140 | **577.3045** | **1152.5944** | **1152.6325** | **-0.0380** | **1** | **6** | **36** | **6** | **R.AMATLLSKFR.I** |
|  | 7489 | **590.3290** | **1178.6435** | **1177.5550** | **1.0886** | **1** | **9** | **22** | **5** | **K.KDWLQADMR.D** 7467 |
|  | 10437 | 667.3217 | 1332.6289 | 1333.6626 | -1.0337 | 0 | 9 | 18 | 4 | R.FQVDLVSENAGR.A 10438 10454 |
|  | 11168 | 688.3658 | 1374.7170 | 1374.8057 | -0.0887 | 1 | 3 | 66 | 9 | R.MKPNTLVLGFKK.D |
|  | 11327 | **692.8230** | **1383.6314** | **1383.6452** | **-0.0138** | **1** | **4** | **56** | **7** | **K.EMSIDQAKYQR.W** |
|  | 11533 | 698.8433 | 1395.6720 | 1394.6534 | 1.0186 | 1 | 15 | 4 | 2 | R.QAMKEMSIDQAK.Y |
|  | 12632 | **487.8946** | **1460.6620** | **1459.6806** | **0.9815** | **0** | **1** | **1e+002** | **10** | **K.DNIYPAFQMFAK.G** |
|  | 12687 | **732.8618** | **1463.7090** | **1463.7191** | **-0.0101** | **0** | **61** | **9.6e-005** | **1** | **R.EGAQYLMQAAGLGR.M** |
|  | 12928 | **494.2364** | **1479.6873** | **1479.7140** | **-0.0267** | **0** | **(1)** | **99** | **4** | **R.EGAQYLMQAAGLGR.M** |
|  | 14073 | **519.2427** | **1554.7064** | **1554.7162** | **-0.0098** | **1** | **3** | **57** | **4** | **R.LHEDDKEQDIADK.M** |
|  | 14267 | **523.2953** | **1566.8642** | **1565.8301** | **1.0341** | **1** | **5** | **40** | **7** | **R.DAVVTYTAESKGVVK.F** |
|  | 14338 | **525.1028** | **1572.2865** | **1572.6903** | **-0.4038** | **0** | **9** | **10** | **2** | **K.QTPADGEASGESEPAK.G** |
|  | 15566 | **551.6003** | **1651.7792** | **1651.8401** | **-0.0609** | **0** | **6** | **29** | **6** | **R.CMLNIWGVMLFIR.L** |
|  | 15936 | **559.9522** | **1676.8348** | **1676.8062** | **0.0286** | **0** | **4** | **50** | **7** | **K.NVGLMICGHVHMGPR.R** |
|  | 16423 | 856.9330 | 1711.8514 | 1712.8403 | -0.9889 | 0 | 14 | 4.3 | 2 | K.EHSILMIDEINDIR.I |
|  | 17941 | **913.4435** | **1824.8725** | **1823.9563** | **0.9162** | **0** | **32** | **0.061** | **1** | **K.EHSSTANIIVMSLPVAR.K** |
|  | 18099 | 612.3178 | 1833.9314 | 1832.9073 | 1.0241 | 1 | 1 | 86 | 10 | K.NVGLMICGHVHMGPRR.Q |
|  | 18305 | **617.1330** | **1848.3772** | **1848.9022** | **-0.5250** | **1** | **(1)** | **56** | **6** | **K.NVGLMICGHVHMGPRR.Q** |
|  | 19794 | **656.9677** | **1967.8811** | **1968.0462** | **-0.1651** | **1** | **4** | **33** | **2** | **K.EHSSTANIIVMSLPVARK.G** |
|  | 27589 | 762.6172 | 3046.4397 | 3046.3680 | 0.0717 | 1 | 2 | 29 | 6 | K.EPFEDGFANGEESTPTRDAVVTYTAESK.G |
|  | 28422 | 1083.8729 | 3248.5969 | 3247.9927 | 0.6042 | 1 | 2 | 26 | 8 | K.AQIVLLVILLLAIGDFVIGTFIPLESKKPK.G |
|  | 30027 | **1263.6661** | **3787.9766** | **3788.7967** | **-0.8201** | **1** | **0** | **29** | **3** | **K.DWLQADMRDVDMYINLFHDAFDIQYGVVVIR.L** |
|  | 30463 | 1020.5077 | 4078.0017 | 4077.1894 | 0.8123 | 1 | 0 | 25 | 7 | K.EHSILMIDEINDIRIIGAITVVILLGISVAGMEWEAK.A |

| **606.** | P16401\|H15_HUMAN    **Mass:** 22566    **Score:** 70     **Queries matched:** 5   **emPAI:** 0.87 |
| --- | --- |
|  | Histone H1.5 OS=Homo sapiens GN=HIST1H1B PE=1 SV=3 |

|  | **Query** | **Observed** | **Mr(expt)** | **Mr(calc)** | **Delta** | **Miss** | **Score** | **Expect** | **Rank** | **Peptide** |
| --- | --- | --- | --- | --- | --- | --- | --- | --- | --- | --- |
|  | 2322 | **811.3892** | **810.3819** | **810.3872** | **-0.0053** | **0** | **27** | **0.28** | **1** | **K.GTGASGSFK.L** |
|  | 6370 | **547.2759** | **1092.5372** | **1092.5451** | **-0.0079** | **0** | **38** | **0.027** | **1** | **K.ALAAGGYDVEK.N** |
|  | 10560 | 670.8232 | 1339.6318 | 1339.7711 | -0.1393 | 1 | 41 | 0.0099 | 1 | R.KATGPPVSELITK.A 10567 |
|  | 13800 | **513.2672** | **1536.7796** | **1537.8100** | **-1.0304** | **1** | **1** | **1e+002** | **8** | **K.GTLVQTKGTGASGSFK.L** |

| **607.** | Q9BV23\|ABHD6_HUMAN    **Mass:** 38762    **Score:** 70     **Queries matched:** 8   **emPAI:** 0.10 |
| --- | --- |
|  | Monoacylglycerol lipase ABHD6 OS=Homo sapiens GN=ABHD6 PE=2 SV=1 |

|  | **Query** | **Observed** | **Mr(expt)** | **Mr(calc)** | **Delta** | **Miss** | **Score** | **Expect** | **Rank** | **Peptide** |
| --- | --- | --- | --- | --- | --- | --- | --- | --- | --- | --- |
|  | 2432 | **410.2239** | **818.4331** | **819.4273** | **-0.9941** | **0** | **4** | **85** | **10** | **R.TLGMQVR.Y** |
|  | 4787 | 489.2712 | 976.5279 | 976.5052 | 0.0227 | 0 | 17 | 3.9 | 1 | K.DMWLSVVK.F |
|  | 6172 | 539.8030 | 1077.5914 | 1076.6117 | 0.9797 | 0 | 24 | 0.69 | 1 | K.LFLEIVSEK.S |
|  | 7617 | **594.3107** | **1186.6069** | **1187.6199** | **-1.0130** | **1** | **4** | **68** | **2** | **R.IPHNNFYRK.L** |
|  | 9493 | **642.3301** | **1282.6456** | **1281.7809** | **0.8647** | **1** | **6** | **37** | **4** | **K.IKVPTQIIWGK.Q** |
|  | 13723 | **766.8855** | **1531.7564** | **1531.7842** | **-0.0277** | **1** | **16** | **3.4** | **5** | **R.SSLDDLSIDGQVKR.I** |
|  | 14578 | **795.3905** | **1588.7664** | **1588.7767** | **-0.0102** | **0** | **70** | **1.2e-005** | **1** | **K.QDQVLDVSGADMLAK.S** |
|  | 27569 | **1015.4800** | **3043.4181** | **3043.6001** | **-0.1819** | **1** | **0** | **46** | **4** | **R.GRPGHKPSILMLHGFSAHKDMWLSVVK.F** |

| **608.** | O60701\|UGDH_HUMAN    **Mass:** 55674    **Score:** 70     **Queries matched:** 9   **emPAI:** 0.14 |
| --- | --- |
|  | UDP-glucose 6-dehydrogenase OS=Homo sapiens GN=UGDH PE=1 SV=1 |

|  | **Query** | **Observed** | **Mr(expt)** | **Mr(calc)** | **Delta** | **Miss** | **Score** | **Expect** | **Rank** | **Peptide** |
| --- | --- | --- | --- | --- | --- | --- | --- | --- | --- | --- |
|  | 1578 | **664.3442** | **663.3370** | **663.2824** | **0.0546** | **0** | **14** | **7.8** | **1** | **K.DTGDTR.E** |
|  | 2100 | **398.1952** | **794.3759** | **794.4360** | **-0.0601** | **1** | **8** | **26** | **8** | **-.MFEIKK.I** |
|  | 6676 | **559.2911** | **1116.5677** | **1116.5775** | **-0.0098** | **0** | **23** | **0.75** | **1** | **R.VTVVDVNESR.I** |
|  | 7945 | **602.3002** | **1202.5858** | **1201.6091** | **0.9767** | **1** | **1** | **1.4e+002** | **10** | **K.ELDYERIHK.K** |
|  | 11094 | **686.4401** | **1370.8656** | **1369.6838** | **1.1818** | **0** | **19** | **1.5** | **1** | **R.VLIGGDETPEGQR.A** 11054 |
|  | 11569 | 699.3939 | 1396.7732 | 1397.7667 | -0.9935 | 1 | 14 | 5.2 | 2 | K.FSLQDPPNKKPK.V |
|  | 12917 | **740.3835** | **1478.7524** | **1478.7617** | **-0.0092** | **0** | **71** | **9.7e-006** | **1** | **K.ILTTNTWSSELSK.L** |
|  | 27862 | **1042.2189** | **3123.6348** | **3123.4700** | **0.1648** | **0** | **2** | **30** | **2** | **R.ISSINSISALCEATGADVEEVATAIGMDQR.I** |

| **609.** | P13073\|COX41_HUMAN    **Mass:** 19621    **Score:** 70     **Queries matched:** 16   **emPAI:** 0.43 |
| --- | --- |
|  | Cytochrome c oxidase subunit 4 isoform 1, mitochondrial OS=Homo sapiens GN=COX4I1 PE=1 SV=1 |

|  | **Query** | **Observed** | **Mr(expt)** | **Mr(calc)** | **Delta** | **Miss** | **Score** | **Expect** | **Rank** | **Peptide** |
| --- | --- | --- | --- | --- | --- | --- | --- | --- | --- | --- |
|  | 1846 | **740.3202** | **739.3130** | **739.3177** | **-0.0047** | **0** | **13** | **6.6** | **2** | **K.WDYEK.N** |
|  | 1946 | 769.4039 | 768.3967 | 769.4082 | -1.0116 | 0 | 3 | 65 | 10 | K.HLSASQK.A |
|  | 2495 | **413.2138** | **824.4131** | **824.3884** | **0.0246** | **1** | **8** | **25** | **5** | **K.RMLDMK.V** |
|  | 4848 | 491.7425 | 981.4704 | 982.4178 | -0.9474 | 0 | 26 | 0.39 | 1 | K.ESFAEMNR.G 4858 |
|  | 4939 | 495.2779 | 988.5413 | 988.5342 | 0.0071 | 1 | 11 | 14 | 7 | K.EWVAKQTK.R |
|  | 8688 | 620.7758 | 1239.5370 | 1239.5441 | -0.0072 | 0 | 58 | 0.00019 | 1 | K.ASWSSLSMDEK.V 8686 |
|  | 9009 | **629.7934** | **1257.5722** | **1257.5812** | **-0.0089** | **1** | **30** | **0.16** | **1** | **K.FKESFAEMNR.G** 9008 |
|  | 9770 | **649.2920** | **1296.5694** | **1296.5775** | **-0.0080** | **1** | **25** | **0.48** | **1** | **K.WDYEKNEWK.K** 9768 |
|  | 11572 | **699.4463** | **1396.8780** | **1396.7575** | **0.1205** | **1** | **4** | **49** | **10** | **R.RDHPLPEVAHVK.H** 11571 |
|  | 12154 | **715.8116** | **1429.6087** | **1429.6184** | **-0.0096** | **0** | **8** | **22** | **1** | **K.SEDFSLPAYMDR.R** |
|  | 21902 | 722.3630 | 2164.0671 | 2163.0789 | 0.9882 | 1 | 42 | 0.0052 | 1 | K.HYVYGPLPQSFDKEWVAK.Q |

| **610.** | P51571\|SSRD_HUMAN    **Mass:** 19158    **Score:** 70     **Queries matched:** 5   **emPAI:** 0.20 |
| --- | --- |
|  | Translocon-associated protein subunit delta OS=Homo sapiens GN=SSR4 PE=1 SV=1 |

|  | **Query** | **Observed** | **Mr(expt)** | **Mr(calc)** | **Delta** | **Miss** | **Score** | **Expect** | **Rank** | **Peptide** |
| --- | --- | --- | --- | --- | --- | --- | --- | --- | --- | --- |
|  | 9099 | **631.8076** | **1261.6006** | **1261.6091** | **-0.0086** | **0** | **19** | **1.7** | **1** | **R.YQVSWSLDHK.S** |
|  | 10874 | **453.7819** | **1358.3238** | **1358.7055** | **-0.3817** | **1** | **6** | **26** | **4** | **K.QFPVTRGQDVGR.Y** |
|  | 10978 | 683.3411 | 1364.6676 | 1364.6758 | -0.0083 | 0 | 70 | 1.4e-005 | 1 | R.VQNMALYADVGGK.Q |
|  | 18632 | 624.9976 | 1871.9710 | 1873.0706 | -1.0996 | 0 | 1 | 72 | 3 | M.AAMASLGALALLLLSSLSR.C |
|  | 21327 | **704.2963** | **2109.8670** | **2109.0677** | **0.7992** | **1** | **2** | **50** | **5** | **R.VQNMALYADVGGKQFPVTR.G** |

| **611.** | Q9Y5M8\|SRPRB_HUMAN    **Mass:** 29912    **Score:** 70     **Queries matched:** 6   **emPAI:** 0.13 |
| --- | --- |
|  | Signal recognition particle receptor subunit beta OS=Homo sapiens GN=SRPRB PE=1 SV=3 |

|  | **Query** | **Observed** | **Mr(expt)** | **Mr(calc)** | **Delta** | **Miss** | **Score** | **Expect** | **Rank** | **Peptide** |
| --- | --- | --- | --- | --- | --- | --- | --- | --- | --- | --- |
|  | 5085 | **499.7758** | **997.5370** | **998.5760** | **-1.0390** | **0** | **11** | **11** | **4** | **K.LIQQQLEK.E** |
|  | 9159 | **633.3060** | **1264.5974** | **1263.6281** | **0.9692** | **0** | **6** | **33** | **3** | **K.NTPSFLIACNK.Q** |
|  | 12024 | **712.3287** | **1422.6429** | **1421.7554** | **0.8875** | **1** | **8** | **23** | **1** | **K.GKEFEFSQLPLK.V** |
|  | 14961 | **539.2273** | **1614.6600** | **1615.7148** | **-1.0548** | **0** | **2** | **72** | **6** | **R.DTQTSITDSCAVYR.V** 14958 |
|  | 17457 | **894.9461** | **1787.8777** | **1787.8901** | **-0.0124** | **0** | **70** | **1.2e-005** | **1** | **R.SAAPSTLDSSSTAPAQLGK.K** |

| **612.** | Q07954\|LRP1_HUMAN    **Mass:** 523119   **Score:** 70     **Queries matched:** 63   **emPAI:** 0.01 |
| --- | --- |
|  | Prolow-density lipoprotein receptor-related protein 1 OS=Homo sapiens GN=LRP1 PE=1 SV=1 |

|  | **Query** | **Observed** | **Mr(expt)** | **Mr(calc)** | **Delta** | **Miss** | **Score** | **Expect** | **Rank** | **Peptide** |
| --- | --- | --- | --- | --- | --- | --- | --- | --- | --- | --- |
|  | 1238 | 588.3679 | 587.3606 | 586.3438 | 1.0168 | 0 | 11 | 24 | 4 | R.ELLGR.G |
|  | 2013 | 789.4204 | 788.4131 | 789.4596 | -1.0465 | 1 | 12 | 16 | 3 | R.TKLVDSK.I |
|  | 2555 | **415.1906** | **828.3667** | **827.4977** | **0.8690** | **1** | **14** | **8.2** | **1** | **K.KQINVAR.L** |
|  | 4144 | 466.7147 | 931.4149 | 931.4732 | -0.0582 | 1 | 10 | 20 | 5 | K.CARMPGLK.G |
|  | 4145 | **466.7268** | **931.4391** | **932.4675** | **-1.0285** | **1** | **2** | **1.2e+002** | **8** | **R.SRLDGTER.V** |
|  | 4261 | 470.8103 | 939.6060 | 938.4756 | 1.1304 | 0 | 8 | 21 | 4 | K.SGHCIPLR.W |
|  | 4658 | **484.7608** | **967.5070** | **966.4341** | **1.0729** | **1** | **6** | **36** | **7** | **R.FKCENNR.C** |
|  | 5658 | **521.7654** | **1041.5162** | **1042.5117** | **-0.9955** | **0** | **1** | **1.3e+002** | **7** | **R.IDAMDVHVK.A** |
|  | 5955 | **532.8175** | **1063.6204** | **1064.4920** | **-0.8716** | **0** | **11** | **13** | **3** | **R.IEAASMSGAGR.R** |
|  | 6870 | **566.7837** | **1131.5528** | **1132.5547** | **-1.0018** | **0** | **11** | **14** | **1** | **R.ILQDDLTCR.A** |
|  | 7973 | **402.2435** | **1203.7087** | **1204.5982** | **-0.8895** | **1** | **6** | **36** | **8** | **R.IEAASMSGAGRR.T** 7960 7998 |
|  | 8607 | **412.8960** | **1235.6661** | **1236.7190** | **-1.0530** | **0** | **4** | **60** | **3** | **R.GVGGAPPTVTLLR.S** 8641 |
|  | 9476 | **428.2242** | **1281.6509** | **1281.6863** | **-0.0354** | **1** | **4** | **47** | **5** | **K.TLIEGKMTHPR.A** 9484 |
|  | 10028 | 438.2029 | 1311.5870 | 1310.6765 | 0.9105 | 1 | 14 | 6.2 | 3 | R.IDAMDVHVKAGR.V 10038 |
|  | 10046 | **657.3256** | **1312.6366** | **1312.5250** | **0.1115** | **0** | **5** | **40** | **5** | **R.CIPMSWTCDK.E** |
|  | 10321 | **443.0390** | **1326.0952** | **1326.6714** | **-0.5762** | **1** | **(4)** | **34** | **9** | **R.IDAMDVHVKAGR.V** 10332 |
|  | 10636 | **448.7232** | **1343.1479** | **1342.6517** | **0.4962** | **0** | **7** | **20** | **8** | **R.FNSTEYQVVTR.V** |
|  | 10965 | 682.8704 | 1363.7262 | 1362.6455 | 1.0806 | 0 | 3 | 77 | 3 | R.EYAGYLLYSER.T 10949 |
|  | 11126 | **687.3196** | **1372.6247** | **1372.6306** | **-0.0059** | **1** | **5** | **48** | **5** | **R.LERAWMDGSHR.D** 11127 11134 |
|  | 12156 | **715.8171** | **1429.6196** | **1428.6126** | **1.0070** | **0** | **1** | **1.1e+002** | **7** | **R.FGTCSQLCNNTK.G** |
|  | 12550 | **486.3361** | **1455.9865** | **1455.7681** | **0.2184** | **1** | **2** | **60** | **4** | **R.LNGTDPIVAADSKR.G** |
|  | 12809 | **491.7793** | **1472.3162** | **1472.6136** | **-0.2975** | **0** | **4** | **34** | **4** | **K.CNASSQFLCSSGR.C** |
|  | 13407 | **755.8460** | **1509.6775** | **1509.6882** | **-0.0107** | **0** | **70** | **1.4e-005** | **1** | **R.MYDAQQQQVGTNK.C** |
|  | 13471 | 758.3180 | 1514.6214 | 1515.7358 | -1.1144 | 1 | 9 | 16 | 3 | R.NLFWTSYDTNKK.Q 13472 |
|  | 14725 | 534.2584 | 1599.7534 | 1600.8434 | -1.0899 | 1 | 5 | 39 | 2 | R.SLPPAAPPTTSNRHR.R |
|  | 14819 | **402.4583** | **1605.8041** | **1604.8998** | **0.9043** | **1** | **7** | **21** | **7** | **K.LNGAYRTVLVSSGLR.E** |
|  | 15324 | **546.2996** | **1635.8769** | **1634.9468** | **0.9301** | **1** | **3** | **56** | **6** | **R.LERGVGGAPPTVTLLR.S** |
|  | 15378 | **547.2797** | **1638.8173** | **1639.7658** | **-0.9485** | **1** | **3** | **61** | **5** | **K.MGTCSKADGSGSVVLR.N** |
|  | 15691 | **554.6070** | **1660.7991** | **1659.8740** | **0.9252** | **1** | **(1)** | **93** | **7** | **R.RMHLNGSNVQVLHR.T** |
|  | 15895 | **559.3038** | **1674.8895** | **1675.8689** | **-0.9794** | **1** | **2** | **72** | **5** | **R.RMHLNGSNVQVLHR.T** |
|  | 16412 | **571.3890** | **1711.1451** | **1711.8642** | **-0.7191** | **1** | **11** | **6.5** | **1** | **R.VNRFNSTEYQVVTR.V** |
|  | 16924 | **875.9523** | **1749.8900** | **1748.7934** | **1.0966** | **0** | **9** | **13** | **2** | **R.VNNGGCSSLCLATPGSR.Q** 16923 16925 |
|  | 17196 | **885.8582** | **1769.7017** | **1770.7917** | **-1.0899** | **0** | **2** | **68** | **6** | **R.MTNGAMNVEIGNPTYK.M** |
|  | 17328 | **593.6599** | **1777.9579** | **1776.9046** | **1.0533** | **0** | **1** | **77** | **8** | **K.ITWPNGLTLDYVTER.I** |
|  | 18236 | **615.5964** | **1843.7673** | **1843.7296** | **0.0377** | **0** | **1** | **82** | **9** | **K.MMPECQCPPHMTGPR.C** |
|  | 21046 | **697.3363** | **2088.9871** | **2089.9449** | **-0.9578** | **0** | **3** | **48** | **1** | **R.EQMIYWTDVTTQGSMIR.R** |
|  | 21505 | **532.2959** | **2125.1545** | **2125.9851** | **-0.8306** | **1** | **6** | **20** | **2** | **R.SDAIYSARYDGSGHMEVLR.G** |
|  | 21989 | **725.8746** | **2174.6021** | **2174.8997** | **-0.2976** | **0** | **6** | **15** | **1** | **K.LYWTDGDNISMANMDGSNR.T** |
|  | 22253 | **736.3397** | **2205.9973** | **2205.8296** | **0.1677** | **0** | **2** | **57** | **2** | **R.LCNGVQDCMDGSDEGPHCR.E** |
|  | 23349 | 1172.0550 | 2342.0955 | 2342.7594 | -0.6639 | 0 | 1 | 59 | 3 | K.CDGEDDCGDNSDENPEECAR.F |
|  | 23517 | 591.0800 | 2360.2910 | 2360.0161 | 0.2748 | 1 | 2 | 44 | 7 | R.GKLYWTDGDNISMANMDGSNR.T |
|  | 23638 | **794.3984** | **2380.1733** | **2379.1430** | **1.0303** | **1** | **1** | **54** | **2** | **K.MPRGIAIDWVAGNVYWTDSGR.D** |
|  | 24479 | **830.9335** | **2489.7786** | **2490.1276** | **-0.3491** | **1** | **3** | **25** | **2** | **K.CEWLCLLSPSGPVCTCPNGKR.L** |
|  | 26786 | **964.4152** | **2890.2238** | **2891.0739** | **-0.8501** | **0** | **6** | **11** | **5** | **R.DNDCVDGSDEPANCTQMTCGVDEFR.C** |
|  | 27136 | 738.8377 | 2951.3217 | 2951.3944 | -0.0727 | 1 | 1 | 38 | 7 | R.HTVDQTRPGAFERETVITMSGDDHPR.A |
|  | 27294 | 999.4911 | 2995.4514 | 2995.7759 | -0.3245 | 1 | 1 | 42 | 2 | M.LTPPLLLLLPLLSALVAAAIDAPKTCSPK.Q |
|  | 27450 | 1514.2616 | 3026.5086 | 3027.4912 | -0.9826 | 1 | 2 | 33 | 2 | R.NLNAPVQPFEDPEHMKNVIALAFDYR.A |
|  | 27517 | 1013.1967 | 3036.5682 | 3035.5029 | 1.0653 | 0 | 2 | 31 | 5 | R.VEGIAVDWIAGNIYWTDQGFDVIEVAR.L 27507 27514 |
|  | 27671 | 1023.1722 | 3066.4949 | 3065.3859 | 1.1090 | 1 | 1 | 40 | 10 | K.LYWTDGDNISMANMDGSNRTLLFSGQK.G |

| **613.** | Q86Y39\|NDUAB_HUMAN    **Mass:** 15071    **Score:** 69     **Queries matched:** 6   **emPAI:** 0.26 |
| --- | --- |
|  | NADH dehydrogenase [ubiquinone] 1 alpha subcomplex subunit 11 OS=Homo sapiens GN=NDUFA11 PE=1 SV=3 |

|  | **Query** | **Observed** | **Mr(expt)** | **Mr(calc)** | **Delta** | **Miss** | **Score** | **Expect** | **Rank** | **Peptide** |
| --- | --- | --- | --- | --- | --- | --- | --- | --- | --- | --- |
|  | 13883 | **772.4264** | **1542.8382** | **1541.8454** | **0.9929** | **0** | **11** | **10** | **1** | **R.VTLNPPGTFLEGVAK.V** |
|  | 18664 | **625.6690** | **1873.9852** | **1872.9581** | **1.0271** | **0** | **4** | **43** | **3** | **K.AYSTTSIASVAGLTAAAYR.V** |
|  | 22916 | 761.7084 | 2282.1035 | 2282.1518 | -0.0483 | 0 | 0 | 73 | 10 | R.THNYGIGAAACVYFGIAASLVK.M |
|  | 23908 | **807.7297** | **2420.1674** | **2420.1794** | **-0.0121** | **0** | **62** | **4.4e-005** | **1** | **R.EKPDDPLNYFLGGCAGGLTLGAR.T** 23909 |
|  | 25359 | 876.4268 | 2626.2585 | 2626.3148 | -0.0564 | 1 | 2 | 36 | 9 | R.THNYGIGAAACVYFGIAASLVKMGR.L |

| **614.** | Q12931\|TRAP1_HUMAN    **Mass:** 80345    **Score:** 69     **Queries matched:** 30   **emPAI:** 0.09 |
| --- | --- |
|  | Heat shock protein 75 kDa, mitochondrial OS=Homo sapiens GN=TRAP1 PE=1 SV=3 |

|  | **Query** | **Observed** | **Mr(expt)** | **Mr(calc)** | **Delta** | **Miss** | **Score** | **Expect** | **Rank** | **Peptide** |
| --- | --- | --- | --- | --- | --- | --- | --- | --- | --- | --- |
|  | 1114 | **561.3568** | **560.3495** | **559.3330** | **1.0166** | **0** | **14** | **13** | **4** | **R.VTNVK.V** |
|  | 1709 | **701.4891** | **700.4818** | **700.4483** | **0.0335** | **0** | **8** | **39** | **5** | **K.VLIQTK.A** |
|  | 1900 | 758.4464 | 757.4391 | 756.4381 | 1.0010 | 0 | 21 | 1.8 | 2 | K.ATDILPK.W |
|  | 2501 | 413.2401 | 824.4657 | 824.3664 | 0.0992 | 0 | 3 | 64 | 4 | K.EFSSEAR.V |
|  | 2542 | **414.7127** | **827.4109** | **828.5433** | **-1.1323** | **1** | **4** | **72** | **7** | **R.KVLIQTK.A** |
|  | 5098 | 500.2798 | 998.5450 | 998.5621 | -0.0171 | 1 | 17 | 3.1 | 2 | R.TTAQLGPRR.N 5081 5082 5097 |
|  | 8407 | 409.2288 | 1224.6645 | 1225.7295 | -1.0650 | 1 | 10 | 13 | 5 | R.ELRALLLWGR.R |
|  | 8944 | **419.2201** | **1254.6384** | **1254.6581** | **-0.0197** | **1** | **6** | **35** | **9** | **R.RNPAWSLQAGR.L** 8966 |
|  | 13147 | **498.3364** | **1491.9873** | **1491.8409** | **0.1464** | **0** | **3** | **51** | **7** | **R.AQLLQPTLEINPR.H** |
|  | 13456 | 757.3917 | 1512.7688 | 1512.7784 | -0.0096 | 0 | 74 | 4.5e-006 | 1 | R.GVVDSEDIPLNLSR.E |
|  | 14429 | **790.4108** | **1578.8070** | **1577.7404** | **1.0666** | **0** | **20** | **1.2** | **1** | **R.MNTLQAIWMMDPK.D** |
|  | 14642 | **797.4013** | **1592.7881** | **1593.7353** | **-0.9473** | **0** | **(8)** | **18** | **2** | **R.MNTLQAIWMMDPK.D** |
|  | 14658 | 798.3606 | 1594.7066 | 1593.7353 | 0.9713 | 0 | (8) | 19 | 3 | R.MNTLQAIWMMDPK.D |
|  | 14889 | 806.3995 | 1610.7844 | 1609.7302 | 1.0542 | 0 | (4) | 49 | 5 | R.MNTLQAIWMMDPK.D |
|  | 15376 | 547.2779 | 1638.8118 | 1637.7355 | 1.0763 | 1 | 4 | 47 | 6 | K.FEDRSPAAECLSEK.E |
|  | 16303 | **568.9247** | **1703.7524** | **1703.8995** | **-0.1471** | **1** | **1** | **80** | **6** | **R.YTLHYKTDAPLNIR.S** |
|  | 16924 | 875.9523 | 1749.8900 | 1749.8364 | 0.0536 | 1 | 3 | 50 | 10 | R.RMNTLQAIWMMDPK.D |
|  | 17974 | 914.4412 | 1826.8678 | 1826.9019 | -0.0341 | 0 | (5) | 32 | 4 | R.LDTHPAMVTVLEMGAAR.H |
|  | 18208 | **615.0619** | **1842.1638** | **1842.8968** | **-0.7330** | **0** | **6** | **19** | **1** | **R.LDTHPAMVTVLEMGAAR.H** 18220 |
|  | 19439 | **646.3520** | **1936.0341** | **1936.9135** | **-0.8793** | **1** | **1** | **85** | **7** | **K.ETEELMAWMRNVLGSR.V** |
|  | 20727 | **685.4449** | **2053.3128** | **2054.2000** | **-0.8872** | **1** | **3** | **31** | **6** | **R.AQLLQPTLEINPRHALIK.K** |
|  | 23324 | **780.5487** | **2338.6243** | **2338.1474** | **0.4769** | **0** | **2** | **32** | **1** | **K.LVSDGQALPEMEIHLQTNAEK.G** |
|  | 23733 | 799.6880 | 2396.0421 | 2396.2093 | -0.1672 | 1 | 1 | 54 | 10 | R.LDTHPAMVTVLEMGAARHFLR.M |
|  | 26053 | **912.5015** | **2734.4826** | **2733.3855** | **1.0971** | **0** | **8** | **8.6** | **1** | **K.GTITIQDTGIGMTQEELVSNLGTIAR.S** |
|  | 26912 | **972.7786** | **2915.3141** | **2914.4357** | **0.8783** | **1** | **3** | **28** | **1** | **K.TDAPLNIRSIFYVPDMKPSMFDVSR.E** |

| **615.** | P01842\|LAC_HUMAN    **Mass:** 11401    **Score:** 69     **Queries matched:** 3   **emPAI:** 0.82 |
| --- | --- |
|  | Ig lambda chain C regions OS=Homo sapiens GN=IGLC1 PE=1 SV=1 |

|  | **Query** | **Observed** | **Mr(expt)** | **Mr(calc)** | **Delta** | **Miss** | **Score** | **Expect** | **Rank** | **Peptide** |
| --- | --- | --- | --- | --- | --- | --- | --- | --- | --- | --- |
|  | 4951 | 495.7551 | 989.4957 | 989.5029 | -0.0072 | 0 | 46 | 0.0043 | 1 | K.AGVETTTPSK.Q |
|  | 16394 | 571.2540 | 1710.7403 | 1710.7519 | -0.0117 | 0 | 4 | 44 | 2 | R.SYSCQVTHEGSTVEK.T |
|  | 22296 | **1106.0743** | **2210.1341** | **2210.1446** | **-0.0104** | **0** | **56** | **0.00019** | **1** | **K.ATLVCLISDFYPGAVTVAWK.A** |

| **616.** | P61158\|ARP3_HUMAN    **Mass:** 47797    **Score:** 69     **Queries matched:** 15   **emPAI:** 0.16 |
| --- | --- |
|  | Actin-related protein 3 OS=Homo sapiens GN=ACTR3 PE=1 SV=3 |

|  | **Query** | **Observed** | **Mr(expt)** | **Mr(calc)** | **Delta** | **Miss** | **Score** | **Expect** | **Rank** | **Peptide** |
| --- | --- | --- | --- | --- | --- | --- | --- | --- | --- | --- |
|  | 4122 | 465.5856 | 929.1567 | 928.4839 | 0.6728 | 1 | 4 | 50 | 8 | K.VGDQAQRR.V |
|  | 4370 | **474.2889** | **946.5631** | **946.4720** | **0.0912** | **0** | **16** | **4.4** | **2** | **K.LSEELSGGR.L** |
|  | 4903 | **494.2364** | **986.4582** | **986.4644** | **-0.0062** | **0** | **2** | **1.2e+002** | **6** | **R.HNPVFGVMS.-** |
|  | 7610 | **396.5233** | **1186.5482** | **1187.5894** | **-1.0412** | **1** | **10** | **14** | **4** | **K.ESAKVGDQAQR.R** |
|  | 10526 | **446.7448** | **1337.2127** | **1337.5921** | **-0.3795** | **0** | **10** | **8.9** | **4** | **K.DYEEIGPSICR.H** |
|  | 11789 | **470.7442** | **1409.2107** | **1408.7714** | **0.4392** | **0** | **1** | **69** | **9** | **R.DITYFIQQLLR.D** |
|  | 13261 | 750.3462 | 1498.6778 | 1498.6875 | -0.0096 | 0 | 51 | 0.0009 | 1 | R.HGIVEDWDLMER.F 13269 13273 |
|  | 17254 | 887.4627 | 1772.9107 | 1771.8676 | 1.0432 | 1 | 1 | 89 | 8 | K.NIVLSGGSTMFRDFGR.R |
|  | 17917 | **608.8189** | **1823.4349** | **1823.8659** | **-0.4310** | **1** | **0** | **71** | **7** | **M.AGRLPACVVDCGTGYTK.L** |
|  | 19670 | **653.0129** | **1956.0168** | **1954.9063** | **1.1105** | **1** | **6** | **24** | **2** | **-.MAGRLPACVVDCGTGYTK.L** |
|  | 24096 | **815.3962** | **2443.1669** | **2443.1795** | **-0.0126** | **0** | **39** | **0.01** | **1** | **K.GVDDLDFFIGDEAIEKPTYATK.W** |
|  | 24394 | 828.3968 | 2482.1687 | 2481.1812 | 0.9875 | 0 | 9 | 8 | 1 | R.AEPEDHYFLLTEPPLNTPENR.E |
|  | 25675 | **893.7455** | **2678.2148** | **2678.2298** | **-0.0150** | **0** | **12** | **3.6** | **1** | **R.YAVWFGGSMLASTPEFYQVCHTK.K** |

| **617.** | Q9UKU0\|ACSL6_HUMAN    **Mass:** 78728    **Score:** 69     **Queries matched:** 27   **emPAI:** 0.10 |
| --- | --- |
|  | Long-chain-fatty-acid--CoA ligase 6 OS=Homo sapiens GN=ACSL6 PE=2 SV=4 |

|  | **Query** | **Observed** | **Mr(expt)** | **Mr(calc)** | **Delta** | **Miss** | **Score** | **Expect** | **Rank** | **Peptide** |
| --- | --- | --- | --- | --- | --- | --- | --- | --- | --- | --- |
|  | 1818 | **731.6043** | **730.5970** | **729.4133** | **1.1837** | **1** | **5** | **71** | **2** | **R.KQAEVR.S** |
|  | 2453 | **411.4187** | **820.8229** | **820.4000** | **0.4229** | **0** | **0** | **1.1e+002** | **2** | **R.LLSDDMK.A** |
|  | 3232 | 435.2104 | 868.4062 | 868.5242 | -0.1181 | 1 | 8 | 20 | 9 | K.AKRPELR.E |
|  | 3518 | 444.2483 | 886.4820 | 886.4872 | -0.0052 | 0 | 22 | 1.4 | 3 | M.QTQEILR.I |
|  | 4007 | 461.2349 | 920.4552 | 919.5127 | 0.9425 | 0 | 4 | 69 | 10 | K.IENIYIR.S |
|  | 4353 | 473.8952 | 945.7758 | 945.4953 | 0.2805 | 0 | 8 | 19 | 9 | K.AILEDMVR.L |
|  | 4579 | **481.8793** | **961.7440** | **961.4902** | **0.2537** | **0** | **(7)** | **27** | **1** | **K.AILEDMVR.L** |
|  | 5614 | 519.7687 | 1037.5228 | 1037.5294 | -0.0066 | 0 | 57 | 0.00032 | 1 | R.VGFFQGDIR.L 5613 |
|  | 5909 | 531.2651 | 1060.5156 | 1059.5495 | 0.9661 | 0 | 6 | 42 | 8 | K.IQASLGGCVR.M |
|  | 6100 | 537.2994 | 1072.5842 | 1073.5903 | -1.0061 | 1 | (9) | 25 | 7 | K.KAILEDMVR.L |
|  | 6319 | 545.2383 | 1088.4621 | 1089.5852 | -1.1231 | 1 | 9 | 21 | 4 | K.KAILEDMVR.L 6327 |
|  | 6661 | 558.3570 | 1114.6994 | 1115.5546 | -0.8552 | 1 | 3 | 71 | 3 | R.TMYQVFRR.G |
|  | 9244 | **635.3768** | **1268.7390** | **1268.7564** | **-0.0175** | **1** | **1** | **98** | **5** | **M.QTQEILRILR.L** |
|  | 10571 | **447.7166** | **1340.1281** | **1339.6693** | **0.4588** | **1** | **4** | **39** | **7** | **K.KQIEELYSISM.-** |
|  | 11316 | 692.3542 | 1382.6939 | 1381.8082 | 0.8858 | 1 | 1 | 1.2e+002 | 10 | K.WLPAGTLKIIDR.K |
|  | 16423 | 856.9330 | 1711.8514 | 1712.8006 | -0.9492 | 0 | 6 | 32 | 4 | K.EALDSDGWLHTGDIGK.W |
|  | 17266 | **887.9697** | **1773.9249** | **1772.9937** | **0.9312** | **1** | **1** | **82** | **6** | **R.ILRLPELGDLGQFFR.S** |
|  | 23204 | 1161.5706 | 2321.1266 | 2320.1886 | 0.9380 | 1 | 4 | 29 | 2 | K.GAMLTHGNVVADFSGFLKVTEK.V |
|  | 23318 | 780.0910 | 2337.2512 | 2337.2151 | 0.0361 | 0 | 31 | 0.066 | 1 | K.AIHIHSDMFSVQNGLLTPTLK.A 23296 23297 23316 23317 23322 |
|  | 30558 | **1376.3689** | **4126.0849** | **4126.8307** | **-0.7458** | **0** | **5** | **8.6** | **1** | **K.SMQAVEDCGQENHQAPVPPQPDDLSIVCFTSGTTGNPK.G** |

| **618.** | O95182\|NDUA7_HUMAN    **Mass:** 12601    **Score:** 69     **Queries matched:** 6   **emPAI:** 0.31 |
| --- | --- |
|  | NADH dehydrogenase [ubiquinone] 1 alpha subcomplex subunit 7 OS=Homo sapiens GN=NDUFA7 PE=1 SV=3 |

|  | **Query** | **Observed** | **Mr(expt)** | **Mr(calc)** | **Delta** | **Miss** | **Score** | **Expect** | **Rank** | **Peptide** |
| --- | --- | --- | --- | --- | --- | --- | --- | --- | --- | --- |
|  | 827 | 506.2578 | 505.2506 | 504.2656 | 0.9850 | 0 | 7 | 53 | 2 | M.ASATR.L |
|  | 4030 | **462.2637** | **922.5128** | **922.4872** | **0.0256** | **1** | **7** | **32** | **5** | **R.YQEISKR.T** |
|  | 16454 | **858.4563** | **1714.8980** | **1714.9101** | **-0.0120** | **0** | **69** | **1.5e-005** | **1** | **K.ALVSGKPAESSAVAATEK.K** 16459 |
|  | 18210 | 615.0621 | 1842.1644 | 1843.0051 | -0.8407 | 1 | 3 | 44 | 6 | K.ALVSGKPAESSAVAATEKK.A 18195 |

| **619.** | P05556\|ITB1_HUMAN    **Mass:** 91664    **Score:** 69     **Queries matched:** 8   **emPAI:** 0.08 |
| --- | --- |
|  | Integrin beta-1 OS=Homo sapiens GN=ITGB1 PE=1 SV=2 |

|  | **Query** | **Observed** | **Mr(expt)** | **Mr(calc)** | **Delta** | **Miss** | **Score** | **Expect** | **Rank** | **Peptide** |
| --- | --- | --- | --- | --- | --- | --- | --- | --- | --- | --- |
|  | 3539 | 445.2172 | 888.4199 | 887.5076 | 0.9123 | 0 | 14 | 7.6 | 1 | K.NVLSLTNK.G |
|  | 5284 | **507.7561** | **1013.4977** | **1014.5710** | **-1.0733** | **0** | **2** | **1e+002** | **1** | **K.SAVTTVVNPK.Y** |
|  | 13774 | **768.7634** | **1535.5123** | **1535.5228** | **-0.0105** | **0** | **60** | **9.1e-005** | **1** | **K.FCECDNFNCDR.S** |
|  | 14651 | **399.4480** | **1593.7629** | **1592.8596** | **0.9033** | **1** | **11** | **9.5** | **1** | **K.TVMPYISTTPAKLR.N** |
|  | 16265 | **851.3862** | **1700.7579** | **1701.8726** | **-1.1147** | **1** | **7** | **20** | **1** | **R.ITSDFRIGFGSFVEK.T** |
|  | 18219 | 615.3095 | 1842.9067 | 1843.9251 | -1.0184 | 1 | 2 | 61 | 4 | R.DKLPQPVQPDPVSHCK.E |
|  | 24316 | **824.0848** | **2469.2327** | **2469.2427** | **-0.0100** | **0** | **28** | **0.12** | **1** | **K.LSENNIQTIFAVTEEFQPVYK.E** 24314 |

| **620.** | O95881\|TXD12_HUMAN    **Mass:** 19365    **Score:** 68     **Queries matched:** 7   **emPAI:** 0.20 |
| --- | --- |
|  | Thioredoxin domain-containing protein 12 OS=Homo sapiens GN=TXNDC12 PE=1 SV=1 |

|  | **Query** | **Observed** | **Mr(expt)** | **Mr(calc)** | **Delta** | **Miss** | **Score** | **Expect** | **Rank** | **Peptide** |
| --- | --- | --- | --- | --- | --- | --- | --- | --- | --- | --- |
|  | 1079 | **557.0183** | **556.0110** | **555.3744** | **0.6366** | **0** | **2** | **62** | **1** | **K.ALKPK.F** |
|  | 11478 | **464.9371** | **1391.7894** | **1391.6793** | **0.1101** | **1** | **5** | **38** | **1** | **K.EAQERLTGDAFR.K** |
|  | 11664 | **702.8160** | **1403.6174** | **1404.7006** | **-1.0832** | **1** | **4** | **52** | **3** | **K.SWCGACKALKPK.F** 11700 |
|  | 15506 | **824.9003** | **1647.7860** | **1647.7967** | **-0.0107** | **0** | **68** | **1.8e-005** | **1** | **K.YFYVSAEQVVQGMK.E** 15505 |
|  | 22892 | **760.3610** | **2278.0611** | **2277.0736** | **0.9875** | **1** | **1** | **64** | **7** | **K.YFYVSAEQVVQGMKEAQER.L** |

| **621.** | O95470\|SGPL1_HUMAN    **Mass:** 64053    **Score:** 68     **Queries matched:** 12   **emPAI:** 0.12 |
| --- | --- |
|  | Sphingosine-1-phosphate lyase 1 OS=Homo sapiens GN=SGPL1 PE=1 SV=3 |

|  | **Query** | **Observed** | **Mr(expt)** | **Mr(calc)** | **Delta** | **Miss** | **Score** | **Expect** | **Rank** | **Peptide** |
| --- | --- | --- | --- | --- | --- | --- | --- | --- | --- | --- |
|  | 7086 | **575.3108** | **1148.6070** | **1147.5981** | **1.0089** | **0** | **(0)** | **1.5e+002** | **4** | **-.MPSTDLLMLK.A** |
|  | 7283 | **583.1074** | **1164.2002** | **1163.5930** | **0.6072** | **0** | **2** | **60** | **7** | **-.MPSTDLLMLK.A** |
|  | 12436 | 725.3451 | 1448.6756 | 1447.6884 | 0.9872 | 0 | 1 | 1.1e+002 | 6 | K.AGYPLEHPFDFR.V |
|  | 12450 | 725.8217 | 1449.6289 | 1448.7367 | 0.8922 | 1 | 2 | 76 | 10 | R.VPLTKMMEVDVR.A |
|  | 16044 | **562.9390** | **1685.7953** | **1685.8447** | **-0.0494** | **1** | **5** | **37** | **2** | **R.DFDIYRLSNLMTAK.G** |
|  | 17225 | **886.9177** | **1771.8208** | **1771.8233** | **-0.0026** | **0** | **21** | **0.95** | **1** | **K.TTGMGAIYGMAQTTVDR.N** |
|  | 17473 | 597.3122 | 1788.9147 | 1787.8182 | 1.0965 | 0 | (2) | 75 | 7 | K.TTGMGAIYGMAQTTVDR.N |
|  | 17613 | **901.4627** | **1800.9107** | **1800.9185** | **-0.0078** | **0** | **65** | **3.2e-005** | **1** | **K.AFEPYLEILEVYSTK.A** |
|  | 17666 | 602.6401 | 1804.8984 | 1803.8132 | 1.0853 | 0 | (3) | 49 | 4 | K.TTGMGAIYGMAQTTVDR.N |
|  | 19974 | **663.2911** | **1986.8514** | **1986.9503** | **-0.0989** | **1** | **1** | **68** | **5** | **K.AKTTGMGAIYGMAQTTVDR.N** |
|  | 22804 | **758.3726** | **2272.0960** | **2272.1065** | **-0.0105** | **0** | **5** | **26** | **1** | **K.AYGDFAWSNPLHPDIFPGLR.K** |
|  | 31595 | **1771.0853** | **5310.2342** | **5309.4625** | **0.7716** | **1** | **1** | **8.3** | **5** | **K.TTGMGAIYGMAQTTVDRNMVAELSSVFLDSLYSTDTVTQGSQMNGSPKPH.-** |

| **622.** | Q02878\|RL6_HUMAN    **Mass:** 32765    **Score:** 68     **Queries matched:** 11   **emPAI:** 0.24 |
| --- | --- |
|  | 60S ribosomal protein L6 OS=Homo sapiens GN=RPL6 PE=1 SV=3 |

|  | **Query** | **Observed** | **Mr(expt)** | **Mr(calc)** | **Delta** | **Miss** | **Score** | **Expect** | **Rank** | **Peptide** |
| --- | --- | --- | --- | --- | --- | --- | --- | --- | --- | --- |
|  | 822 | **505.2694** | **504.2621** | **503.2452** | **1.0169** | **0** | **5** | **1e+002** | **3** | **K.NGGTR.V** |
|  | 1992 | **788.4463** | **787.4390** | **787.4440** | **-0.0049** | **0** | **14** | **9.3** | **4** | **K.IDISNVK.I** |
|  | 3184 | **866.4924** | **865.4852** | **865.4909** | **-0.0058** | **0** | **25** | **0.47** | **1** | **K.FVIATSTK.I** 3183 |
|  | 4215 | **469.7292** | **937.4439** | **937.4505** | **-0.0065** | **0** | **19** | **2.1** | **1** | **K.YEITEQR.K** |
|  | 5016 | 497.7661 | 993.5176 | 993.4920 | 0.0256 | 0 | 6 | 36 | 7 | K.HLTDAYFK.K |
|  | 6956 | **570.2681** | **1138.5217** | **1138.5295** | **-0.0078** | **0** | **3** | **68** | **9** | **R.YYPTEDVPR.K** |
|  | 8114 | 605.8520 | 1209.6894 | 1210.7285 | -1.0391 | 1 | 7 | 28 | 9 | K.AVDSQILPKIK.A |
|  | 13639 | 763.9682 | 1525.9218 | 1524.9239 | 0.9979 | 0 | 39 | 0.012 | 1 | R.ASITPGTILIILTGR.H |
|  | 14577 | **530.5826** | **1588.7261** | **1588.7369** | **-0.0108** | **1** | **9** | **17** | **3** | **R.HQEGEIFDTEKEK.Y** |
|  | 17114 | 882.5365 | 1763.0584 | 1763.0669 | -0.0084 | 0 | 51 | 0.00075 | 1 | K.QLASGLLLVTGPLVLNR.V |

| **623.** | O94855\|SC24D_HUMAN    **Mass:** 114476   **Score:** 68     **Queries matched:** 18   **emPAI:** 0.07 |
| --- | --- |
|  | Protein transport protein Sec24D OS=Homo sapiens GN=SEC24D PE=1 SV=2 |

|  | **Query** | **Observed** | **Mr(expt)** | **Mr(calc)** | **Delta** | **Miss** | **Score** | **Expect** | **Rank** | **Peptide** |
| --- | --- | --- | --- | --- | --- | --- | --- | --- | --- | --- |
|  | 2136 | **399.3459** | **796.6772** | **796.3901** | **0.2870** | **0** | **6** | **26** | **3** | **K.RPYSMK.L** 2127 |
|  | 4268 | **471.2493** | **940.4841** | **940.5018** | **-0.0177** | **0** | **10** | **17** | **2** | **R.VGFITYNK.V** |
|  | 4368 | **474.2846** | **946.5545** | **945.5131** | **1.0415** | **1** | **4** | **78** | **6** | **K.LVNTDKEK.I** |
|  | 5995 | **534.3169** | **1066.6192** | **1065.5641** | **1.0551** | **1** | **0** | **1.5e+002** | **7** | **K.KIGFDAIMR.V** |
|  | 13476 | **758.3524** | **1514.6902** | **1514.7075** | **-0.0174** | **0** | **18** | **1.9** | **1** | **K.SCETDALINFFAK.S** 13475 13506 |
|  | 14424 | 527.2564 | 1578.7474 | 1579.7446 | -0.9972 | 1 | 1 | 94 | 7 | K.STMLPAAVRCSESR.L |
|  | 15255 | **816.4454** | **1630.8763** | **1631.8267** | **-0.9504** | **1** | **4** | **44** | **7** | **R.QQFLNDLRNDIEK.K** |
|  | 16443 | **857.9427** | **1713.8708** | **1713.9090** | **-0.0382** | **0** | **16** | **3** | **1** | **K.LFIFHSSLPTAEAPGK.L** |
|  | 16736 | **868.9589** | **1735.9032** | **1735.9145** | **-0.0113** | **0** | **50** | **0.00099** | **1** | **K.ILFQPQTNVYDSLAK.D** 16737 |
|  | 17886 | **608.3247** | **1821.9521** | **1822.9619** | **-1.0098** | **1** | **2** | **66** | **3** | **R.MIMGIIQQKRPYSMK.L** |
|  | 18846 | 945.4380 | 1888.8614 | 1888.9023 | -0.0408 | 0 | 21 | 0.75 | 1 | K.NCASPSAASQLILPDSMK.V |
|  | 18919 | **632.0328** | **1893.0767** | **1893.9295** | **-0.8528** | **1** | **5** | **32** | **2** | **R.EQPEMVFRQFLVEDK.G** |
|  | 19084 | 636.3518 | 1906.0336 | 1904.8972 | 1.1364 | 0 | (6) | 26 | 4 | K.NCASPSAASQLILPDSMK.V |
|  | 19531 | 973.0245 | 1944.0344 | 1944.9727 | -0.9383 | 0 | 4 | 38 | 3 | R.IHNLGLNCSSQLADLYK.S |

| **624.** | Q9H853\|TBA4B_HUMAN    **Mass:** 27819    **Score:** 67     **Queries matched:** 8   **emPAI:** 0.29 |
| --- | --- |
|  | Putative tubulin-like protein alpha-4B OS=Homo sapiens GN=TUBA4B PE=5 SV=2 |

|  | **Query** | **Observed** | **Mr(expt)** | **Mr(calc)** | **Delta** | **Miss** | **Score** | **Expect** | **Rank** | **Peptide** |
| --- | --- | --- | --- | --- | --- | --- | --- | --- | --- | --- |
|  | 5462 | 1028.4622 | 1027.4549 | 1026.5094 | 0.9455 | 0 | 5 | 56 | 6 | R.QDPSQPLSR.Q |
|  | 6477 | 551.2903 | 1100.5660 | 1100.5145 | 0.0515 | 1 | 18 | 2.4 | 1 | -.MRHQQTER.Q 6457 |
|  | 11801 | 470.9266 | 1409.7580 | 1409.7667 | -0.0087 | 0 | 13 | 6.6 | 1 | R.QIFHPEQLITGK.E |
|  | 13059 | 744.4392 | 1486.8639 | 1486.8719 | -0.0080 | 0 | 64 | 5.2e-005 | 1 | R.LISQIVSSITASLR.F 13061 |
|  | 17206 | **885.9603** | **1769.9060** | **1768.8605** | **1.0455** | **1** | **6** | **29** | **1** | **R.QDPSQPLSRQHGTYR.Q** |
|  | 22115 | 1096.0128 | 2190.0111 | 2190.0303 | -0.0192 | 0 | 0 | 81 | 3 | R.GTGSDVTSFLMEWLSVNYGK.K |

| **625.** | Q15181\|IPYR_HUMAN    **Mass:** 33095    **Score:** 67     **Queries matched:** 7   **emPAI:** 0.24 |
| --- | --- |
|  | Inorganic pyrophosphatase OS=Homo sapiens GN=PPA1 PE=1 SV=2 |

|  | **Query** | **Observed** | **Mr(expt)** | **Mr(calc)** | **Delta** | **Miss** | **Score** | **Expect** | **Rank** | **Peptide** |
| --- | --- | --- | --- | --- | --- | --- | --- | --- | --- | --- |
|  | 1047 | 548.2829 | 547.2756 | 546.3125 | 0.9631 | 1 | 10 | 27 | 5 | K.KTNGK.G |
|  | 9423 | 640.3257 | 1278.6368 | 1277.6438 | 0.9930 | 1 | (7) | 27 | 10 | R.WSNAKMEIATK.D |
|  | 9707 | 647.7856 | 1293.5567 | 1293.6387 | -0.0820 | 1 | 8 | 21 | 5 | R.WSNAKMEIATK.D 9727 9741 |
|  | 15813 | **836.3835** | **1670.7525** | **1670.7644** | **-0.0119** | **0** | **68** | **1.7e-005** | **1** | **K.GISCMNTTLSESPFK.C** |
|  | 24241 | **821.0402** | **2460.0987** | **2460.1135** | **-0.0148** | **0** | **17** | **1.4** | **1** | **K.GYIWNYGAIPQTWEDPGHNDK.H** |

| **626.** | P56556\|NDUA6_HUMAN    **Mass:** 15127    **Score:** 67     **Queries matched:** 2   **emPAI:** 0.26 |
| --- | --- |
|  | NADH dehydrogenase [ubiquinone] 1 alpha subcomplex subunit 6 OS=Homo sapiens GN=NDUFA6 PE=1 SV=2 |

|  | **Query** | **Observed** | **Mr(expt)** | **Mr(calc)** | **Delta** | **Miss** | **Score** | **Expect** | **Rank** | **Peptide** |
| --- | --- | --- | --- | --- | --- | --- | --- | --- | --- | --- |
|  | 18571 | 934.4937 | 1866.9729 | 1866.9840 | -0.0111 | 0 | 49 | 0.0013 | 1 | R.EVPNTVHQFQLDITVK.M 18572 |

| **627.** | P61026\|RAB10_HUMAN    **Mass:** 22755    **Score:** 67     **Queries matched:** 14   **emPAI:** 0.17 |
| --- | --- |
|  | Ras-related protein Rab-10 OS=Homo sapiens GN=RAB10 PE=1 SV=1 |

|  | **Query** | **Observed** | **Mr(expt)** | **Mr(calc)** | **Delta** | **Miss** | **Score** | **Expect** | **Rank** | **Peptide** |
| --- | --- | --- | --- | --- | --- | --- | --- | --- | --- | --- |
|  | 1624 | 675.3809 | 674.3736 | 674.3785 | -0.0049 | 0 | 22 | 1.5 | 1 | R.MLLGNK.C |
|  | 2104 | 398.2102 | 794.4059 | 794.4109 | -0.0050 | 0 | 15 | 5.1 | 4 | K.TCVLFR.F |
|  | 2496 | **413.2194** | **824.4243** | **823.4076** | **1.0168** | **0** | **18** | **2.4** | **1** | **K.SFENISK.W** 2476 |
|  | 2550 | 414.7477 | 827.4808 | 828.4018 | -0.9210 | 0 | 10 | 20 | 4 | R.FFETSAK.A |
|  | 5451 | 514.2915 | 1026.5684 | 1026.5750 | -0.0065 | 1 | 23 | 0.74 | 1 | K.KTYDLLFK.L |
|  | 6064 | 536.3201 | 1070.6257 | 1070.6336 | -0.0079 | 0 | 15 | 5.6 | 1 | K.LLLIGDSGVGK.T |
|  | 9107 | 631.8256 | 1261.6366 | 1260.7078 | 0.9288 | 0 | 6 | 41 | 3 | K.AFLTLAEDILR.K |
|  | 9581 | **644.8146** | **1287.6147** | **1287.6248** | **-0.0101** | **0** | **9** | **17** | **1** | **R.FHTITTSYYR.G** |
|  | 10115 | 658.8291 | 1315.6436 | 1315.6521 | -0.0084 | 0 | 53 | 0.00078 | 1 | K.LQIWDTAGQER.F 10116 |
|  | 14459 | 791.3847 | 1580.7548 | 1581.7895 | -1.0346 | 0 | 9 | 14 | 2 | R.GAMGIMLVYDITNGK.S |
|  | 14700 | 799.9262 | 1597.8377 | 1597.7844 | 0.0534 | 0 | (8) | 20 | 4 | R.GAMGIMLVYDITNGK.S |
|  | 23496 | **590.3143** | **2357.2280** | **2357.1499** | **0.0780** | **1** | **4** | **33** | **8** | **K.TPVKEPNSENVDISSGGGVTGWK.S** |

| **628.** | Q92930\|RAB8B_HUMAN    **Mass:** 23740    **Score:** 67     **Queries matched:** 8   **emPAI:** 0.16 |
| --- | --- |
|  | Ras-related protein Rab-8B OS=Homo sapiens GN=RAB8B PE=1 SV=2 |

|  | **Query** | **Observed** | **Mr(expt)** | **Mr(calc)** | **Delta** | **Miss** | **Score** | **Expect** | **Rank** | **Peptide** |
| --- | --- | --- | --- | --- | --- | --- | --- | --- | --- | --- |
|  | 1624 | 675.3809 | 674.3736 | 674.3785 | -0.0049 | 0 | 22 | 1.5 | 1 | R.MILGNK.C |
|  | 4921 | 494.7554 | 987.4962 | 987.5025 | -0.0063 | 0 | 21 | 1.6 | 1 | R.TITTAYYR.G |
|  | 6064 | 536.3201 | 1070.6257 | 1070.6336 | -0.0079 | 0 | 15 | 5.6 | 1 | K.LLLIGDSGVGK.T |
|  | 10115 | 658.8291 | 1315.6436 | 1315.6521 | -0.0084 | 0 | 53 | 0.00078 | 1 | K.LQIWDTAGQER.F 10116 |
|  | 10214 | **441.2467** | **1320.7183** | **1319.5776** | **1.1407** | **0** | **4** | **59** | **10** | **K.MNDSNSAGAGGPVK.I** |
|  | 12794 | **736.4190** | **1470.8235** | **1469.6313** | **1.1922** | **1** | **3** | **58** | **6** | **R.MILGNKCDMNDK.R** |
|  | 15799 | 835.5329 | 1669.0512 | 1669.8055 | -0.7543 | 0 | 3 | 46 | 1 | R.GAMGIMLVYDITNEK.S |

| **629.** | Q15286\|RAB35_HUMAN    **Mass:** 23296    **Score:** 67     **Queries matched:** 6   **emPAI:** 0.16 |
| --- | --- |
|  | Ras-related protein Rab-35 OS=Homo sapiens GN=RAB35 PE=1 SV=1 |

|  | **Query** | **Observed** | **Mr(expt)** | **Mr(calc)** | **Delta** | **Miss** | **Score** | **Expect** | **Rank** | **Peptide** |
| --- | --- | --- | --- | --- | --- | --- | --- | --- | --- | --- |
|  | 1480 | 643.2714 | 642.2641 | 642.4064 | -0.1424 | 0 | 19 | 2.4 | 1 | R.ILVGNK.N |
|  | 6064 | 536.3201 | 1070.6257 | 1070.6336 | -0.0079 | 0 | 15 | 5.6 | 1 | K.LLIIGDSGVGK.S |
|  | 10115 | 658.8291 | 1315.6436 | 1315.6521 | -0.0084 | 0 | 53 | 0.00078 | 1 | K.LQIWDTAGQER.F 10116 |
|  | 20244 | **672.3135** | **2013.9188** | **2013.8898** | **0.0290** | **1** | **9** | **12** | **2** | **K.RWLHEINQNCDDVCR.I** |
|  | 25557 | **888.4196** | **2662.2369** | **2661.2996** | **0.9372** | **1** | **3** | **32** | **1** | **K.VVETEDAYKFAGQMGIQLFETSAK.E** |

| **630.** | Q9H082\|RB33B_HUMAN    **Mass:** 26043    **Score:** 67     **Queries matched:** 8   **emPAI:** 0.15 |
| --- | --- |
|  | Ras-related protein Rab-33B OS=Homo sapiens GN=RAB33B PE=1 SV=1 |

|  | **Query** | **Observed** | **Mr(expt)** | **Mr(calc)** | **Delta** | **Miss** | **Score** | **Expect** | **Rank** | **Peptide** |
| --- | --- | --- | --- | --- | --- | --- | --- | --- | --- | --- |
|  | 1480 | 643.2714 | 642.2641 | 642.4064 | -0.1424 | 0 | 19 | 2.4 | 1 | R.ILVGNK.C |
|  | 1526 | 651.3502 | 650.3429 | 649.3911 | 0.9518 | 1 | 12 | 11 | 3 | R.SRIFK.I |
|  | 6551 | **554.2609** | **1106.5072** | **1107.5560** | **-1.0489** | **0** | **6** | **45** | **3** | **R.TEATIGVDFR.E** |
|  | 10115 | 658.8291 | 1315.6436 | 1315.6521 | -0.0084 | 0 | 53 | 0.00078 | 1 | K.IQLWDTAGQER.F 10116 |
|  | 17789 | **605.5481** | **1813.6225** | **1813.9356** | **-0.3132** | **1** | **0** | **69** | **5** | **K.CDLRSAIQVPTDLAQK.F** |
|  | 27128 | 738.3665 | 2949.4370 | 2948.4590 | 0.9780 | 1 | 0 | 51 | 8 | R.SAIQVPTDLAQKFADTHSMPLFETSAK.N |
|  | 28423 | 1083.8729 | 3248.5969 | 3247.4761 | 1.1208 | 1 | 2 | 26 | 6 | -.MAEEMESSLEASFSSSGAVSGASGFLPPARSR.I |

| **631.** | Q6IQ22\|RAB12_HUMAN    **Mass:** 27573    **Score:** 67     **Queries matched:** 7   **emPAI:** 0.14 |
| --- | --- |
|  | Putative Ras-related protein Rab-12 OS=Homo sapiens GN=RAB12 PE=5 SV=3 |

|  | **Query** | **Observed** | **Mr(expt)** | **Mr(calc)** | **Delta** | **Miss** | **Score** | **Expect** | **Rank** | **Peptide** |
| --- | --- | --- | --- | --- | --- | --- | --- | --- | --- | --- |
|  | 5053 | **499.2577** | **996.5008** | **997.6284** | **-1.1276** | **0** | **7** | **26** | **1** | **K.LQVIIIGSR.G** |
|  | 5145 | **501.7996** | **1001.5846** | **1000.5739** | **1.0107** | **1** | **10** | **17** | **3** | **K.KMPLDILR.N** |
|  | 6305 | 544.7820 | 1087.5495 | 1087.5622 | -0.0126 | 1 | 13 | 9.8 | 5 | R.EITRQQGEK.F |
|  | 10115 | 658.8291 | 1315.6436 | 1315.6521 | -0.0084 | 0 | 53 | 0.00078 | 1 | R.LQIWDTAGQER.F 10116 |
|  | 16770 | 580.6484 | 1738.9235 | 1738.8823 | 0.0412 | 1 | 4 | 47 | 5 | R.RAGGGGGLGAGSPALSGGQGR.R |
|  | 16775 | **580.6778** | **1739.0116** | **1738.8823** | **0.1293** | **1** | **6** | **23** | **1** | **R.AGGGGGLGAGSPALSGGQGRR.R** |

| **632.** | Q14964\|RB39A_HUMAN    **Score:** 67     **Queries matched:** 7 |
| --- | --- |
|  | Ras-related protein Rab-39A OS=Homo sapiens GN=RAB39 PE=2 SV=2 |

|  | **Query** | **Observed** | **Mr(expt)** | **Mr(calc)** | **Delta** | **Miss** | **Score** | **Expect** | **Rank** | **Peptide** |
| --- | --- | --- | --- | --- | --- | --- | --- | --- | --- | --- |
|  | 1249 | 590.3257 | 589.3185 | 588.3384 | 0.9801 | 0 | 9 | 36 | 2 | R.FPGLR.S |
|  | 5429 | 513.2869 | 1024.5593 | 1024.6433 | -0.0840 | 0 | 14 | 5.8 | 2 | R.IVFLLVGHK.C |
|  | 10115 | 658.8291 | 1315.6436 | 1315.6521 | -0.0084 | 0 | 53 | 0.00078 | 1 | K.LQLWDTAGQER.F 10116 |
|  | 11357 | **462.7480** | **1385.2223** | **1385.6802** | **-0.4579** | **0** | **3** | **46** | **2** | **-.METIWIYQFR.L** |
|  | 14651 | 399.4480 | 1593.7629 | 1594.7839 | -1.0210 | 0 | 9 | 16 | 6 | K.DATNVEESFTILTR.D |
|  | 24306 | 1235.4796 | 2468.9447 | 2468.3138 | 0.6309 | 1 | 1 | 45 | 2 | -.METIWIYQFRLIVIGDSTVGK.S |

| **633.** | Q96AX2\|RAB37_HUMAN    **Mass:** 25028    **Score:** 67     **Queries matched:** 8   **emPAI:** 0.15 |
| --- | --- |
|  | Ras-related protein Rab-37 OS=Homo sapiens GN=RAB37 PE=1 SV=3 |

|  | **Query** | **Observed** | **Mr(expt)** | **Mr(calc)** | **Delta** | **Miss** | **Score** | **Expect** | **Rank** | **Peptide** |
| --- | --- | --- | --- | --- | --- | --- | --- | --- | --- | --- |
|  | 2708 | **419.5458** | **837.0770** | **837.3981** | **-0.3211** | **0** | **7** | **23** | **9** | **K.SSFDNIR.A** |
|  | 6136 | 538.7521 | 1075.4897 | 1076.5285 | -1.0387 | 0 | 12 | 10 | 6 | -.MTGTPGAVATR.D |
|  | 6296 | **544.3090** | **1086.6035** | **1085.6193** | **0.9842** | **1** | **4** | **74** | **9** | **R.NKVVTVDGVR.V** |
|  | 10115 | 658.8291 | 1315.6436 | 1315.6521 | -0.0084 | 0 | 53 | 0.00078 | 1 | K.LQIWDTAGQER.F 10116 |
|  | 10559 | **447.5051** | **1339.4936** | **1339.6660** | **-0.1724** | **0** | **2** | **70** | **7** | **R.EYGVPFLETSAK.T** |
|  | 18062 | 611.6372 | 1831.8898 | 1830.8530 | 1.0368 | 1 | 6 | 29 | 1 | -.MTGTPGAVATRDGEAPER.S |
|  | 21525 | **1064.7815** | **2127.5484** | **2128.0953** | **-0.5469** | **1** | **1** | **50** | **3** | **K.DGAFLSGTFIATVGIDFRNK.V** |

| **634.** | P61018\|RAB4B_HUMAN    **Score:** 67     **Queries matched:** 8 |
| --- | --- |
|  | Ras-related protein Rab-4B OS=Homo sapiens GN=RAB4B PE=1 SV=1 |

|  | **Query** | **Observed** | **Mr(expt)** | **Mr(calc)** | **Delta** | **Miss** | **Score** | **Expect** | **Rank** | **Peptide** |
| --- | --- | --- | --- | --- | --- | --- | --- | --- | --- | --- |
|  | 1609 | **673.3937** | **672.3864** | **671.3966** | **0.9898** | **0** | **11** | **20** | **1** | **R.VVNVGGK.T** |
|  | 5133 | **501.3361** | **1000.6577** | **999.6077** | **1.0500** | **1** | **3** | **80** | **8** | **R.VVNVGGKTVK.L** |
|  | 10115 | 658.8291 | 1315.6436 | 1315.6521 | -0.0084 | 0 | 53 | 0.00078 | 1 | K.LQIWDTAGQER.F 10116 |
|  | 11082 | **686.3134** | **1370.6121** | **1369.6296** | **0.9825** | **0** | **8** | **21** | **3** | **R.MGSGIQYGDASLR.Q** |
|  | 15466 | 823.3972 | 1644.7799 | 1643.8631 | 0.9168 | 1 | 3 | 60 | 9 | K.TVKLQIWDTAGQER.F |
|  | 16920 | 584.2983 | 1749.8730 | 1750.8784 | -1.0054 | 1 | 2 | 64 | 9 | R.MGSGIQYGDASLRQLR.Q |
|  | 17302 | 593.2797 | 1776.8172 | 1775.8690 | 0.9482 | 1 | 2 | 66 | 6 | K.DLDPEREVTFLEASR.F |

| **635.** | Q9NRW1\|RAB6B_HUMAN    **Mass:** 23561    **Score:** 67     **Queries matched:** 7   **emPAI:** 0.16 |
| --- | --- |
|  | Ras-related protein Rab-6B OS=Homo sapiens GN=RAB6B PE=1 SV=1 |

|  | **Query** | **Observed** | **Mr(expt)** | **Mr(calc)** | **Delta** | **Miss** | **Score** | **Expect** | **Rank** | **Peptide** |
| --- | --- | --- | --- | --- | --- | --- | --- | --- | --- | --- |
|  | 7440 | 588.8312 | 1175.6478 | 1175.6550 | -0.0072 | 0 | 13 | 7.3 | 1 | K.LVFLGEQSVGK.T |
|  | 8878 | 417.7261 | 1250.1566 | 1250.7135 | -0.5569 | 1 | 10 | 9.6 | 8 | R.FRSLIPSYIR.D |
|  | 9082 | 631.3337 | 1260.6528 | 1260.6748 | -0.0220 | 0 | 1 | 1.2e+002 | 6 | R.GSDVIIMLVGNK.T |
|  | 10115 | 658.8291 | 1315.6436 | 1315.6521 | -0.0084 | 0 | 53 | 0.00078 | 1 | R.LQLWDTAGQER.F 10116 |
|  | 10735 | **675.6597** | **1349.3049** | **1348.6558** | **0.6491** | **1** | **1** | **78** | **10** | **-.MSAGGDFGNPLRK.F** |
|  | 20475 | **678.6867** | **2033.0383** | **2032.0187** | **1.0196** | **1** | **2** | **61** | **2** | **K.ELSVMFIETSAKTGYNVK.Q** |

| **636.** | P59190\|RAB15_HUMAN    **Mass:** 24660    **Score:** 67     **Queries matched:** 3   **emPAI:** 0.15 |
| --- | --- |
|  | Ras-related protein Rab-15 OS=Homo sapiens GN=RAB15 PE=1 SV=1 |

|  | **Query** | **Observed** | **Mr(expt)** | **Mr(calc)** | **Delta** | **Miss** | **Score** | **Expect** | **Rank** | **Peptide** |
| --- | --- | --- | --- | --- | --- | --- | --- | --- | --- | --- |
|  | 6064 | 536.3201 | 1070.6257 | 1070.6336 | -0.0079 | 0 | 15 | 5.6 | 1 | R.LLLIGDSGVGK.T |
|  | 10115 | 658.8291 | 1315.6436 | 1315.6521 | -0.0084 | 0 | 53 | 0.00078 | 1 | R.IQIWDTAGQER.Y 10116 |

| **637.** | Q86YS6\|RAB43_HUMAN    **Mass:** 23553    **Score:** 67     **Queries matched:** 6   **emPAI:** 0.16 |
| --- | --- |
|  | Ras-related protein Rab-43 OS=Homo sapiens GN=RAB43 PE=1 SV=1 |

|  | **Query** | **Observed** | **Mr(expt)** | **Mr(calc)** | **Delta** | **Miss** | **Score** | **Expect** | **Rank** | **Peptide** |
| --- | --- | --- | --- | --- | --- | --- | --- | --- | --- | --- |
|  | 9200 | 423.2286 | 1266.6641 | 1265.5888 | 1.0753 | 0 | 6 | 39 | 9 | K.DSSNVEEAFLR.V 9178 |
|  | 10115 | 658.8291 | 1315.6436 | 1315.6521 | -0.0084 | 0 | 53 | 0.00078 | 1 | K.LQIWDTAGQER.F 10116 |
|  | 17609 | 601.3022 | 1800.8849 | 1799.9928 | 0.8921 | 1 | 6 | 28 | 2 | K.LVLVGDASVGKTCVVQR.F |
|  | 19009 | **951.3044** | **1900.5943** | **1899.9877** | **0.6067** | **1** | **1** | **50** | **1** | **R.VATELIMRHGGPLFSEK.S** |

|  | **Proteins matching a subset of these peptides:** |
| --- | --- |

|  | A6NDJ8\|RB43L_HUMAN    **Mass:** 20425    **Score:** 67     **Queries matched:** 5 |
| --- | --- |
|  | Putative Rab-43-like protein ENSP00000330714 OS=Homo sapiens PE=5 SV=3 |

| **638.** | Q96DA2\|RB39B_HUMAN    **Score:** 67     **Queries matched:** 4 |
| --- | --- |
|  | Ras-related protein Rab-39B OS=Homo sapiens GN=RAB39B PE=1 SV=1 |

|  | **Query** | **Observed** | **Mr(expt)** | **Mr(calc)** | **Delta** | **Miss** | **Score** | **Expect** | **Rank** | **Peptide** |
| --- | --- | --- | --- | --- | --- | --- | --- | --- | --- | --- |
|  | 10115 | 658.8291 | 1315.6436 | 1315.6521 | -0.0084 | 0 | 53 | 0.00078 | 1 | K.LQIWDTAGQER.F 10116 |
|  | 13550 | 760.3679 | 1518.7213 | 1517.8202 | 0.9011 | 0 | 6 | 37 | 3 | R.NSVGGLLLFDITNR.R |
|  | 14624 | **531.3463** | **1591.0169** | **1591.8206** | **-0.8036** | **1** | **4** | **37** | **7** | **R.DAINVEKAFTDLTR.D** |

| **639.** | P20338\|RAB4A_HUMAN    **Mass:** 24141    **Score:** 67     **Queries matched:** 7   **emPAI:** 0.16 |
| --- | --- |
|  | Ras-related protein Rab-4A OS=Homo sapiens GN=RAB4A PE=1 SV=2 |

|  | **Query** | **Observed** | **Mr(expt)** | **Mr(calc)** | **Delta** | **Miss** | **Score** | **Expect** | **Rank** | **Peptide** |
| --- | --- | --- | --- | --- | --- | --- | --- | --- | --- | --- |
|  | 9322 | **637.2454** | **1272.4762** | **1273.6489** | **-1.1727** | **0** | **0** | **1.2e+002** | **6** | **K.SCLLHQFIEK.K** |
|  | 10115 | 658.8291 | 1315.6436 | 1315.6521 | -0.0084 | 0 | 53 | 0.00078 | 1 | K.LQIWDTAGQER.F 10116 |
|  | 10807 | 677.8148 | 1353.6151 | 1353.6347 | -0.0196 | 0 | 2 | 89 | 5 | R.MGSGIQYGDAALR.Q |
|  | 16699 | **868.3774** | **1734.7403** | **1735.8377** | **-1.0973** | **1** | **2** | **67** | **5** | **K.DLDADREVTFLEASR.F** |
|  | 16916 | 584.2790 | 1749.8153 | 1750.8784 | -1.0631 | 1 | 7 | 25 | 1 | R.MGSGIQYGDAALRQLR.S |
|  | 17351 | 890.8973 | 1779.7801 | 1779.8428 | -0.0627 | 1 | 1 | 92 | 8 | K.FKDDSNHTIGVEFGSK.I |

| **640.** | Q15771\|RAB30_HUMAN    **Mass:** 23386    **Score:** 67     **Queries matched:** 3   **emPAI:** 0.16 |
| --- | --- |
|  | Ras-related protein Rab-30 OS=Homo sapiens GN=RAB30 PE=1 SV=2 |

|  | **Query** | **Observed** | **Mr(expt)** | **Mr(calc)** | **Delta** | **Miss** | **Score** | **Expect** | **Rank** | **Peptide** |
| --- | --- | --- | --- | --- | --- | --- | --- | --- | --- | --- |
|  | 10115 | 658.8291 | 1315.6436 | 1315.6521 | -0.0084 | 0 | 53 | 0.00078 | 1 | K.LQIWDTAGQER.F 10116 |
|  | 24125 | **816.4236** | **2446.2489** | **2446.2276** | **0.0213** | **1** | **8** | **13** | **1** | **-.MSMEDYDFLFKIVLIGNAGVGK.T** |

| **641.** | P61421\|VA0D1_HUMAN    **Mass:** 40759    **Score:** 67     **Queries matched:** 5   **emPAI:** 0.09 |
| --- | --- |
|  | V-type proton ATPase subunit d 1 OS=Homo sapiens GN=ATP6V0D1 PE=1 SV=1 |

|  | **Query** | **Observed** | **Mr(expt)** | **Mr(calc)** | **Delta** | **Miss** | **Score** | **Expect** | **Rank** | **Peptide** |
| --- | --- | --- | --- | --- | --- | --- | --- | --- | --- | --- |
|  | 2981 | 428.7455 | 855.4765 | 855.5065 | -0.0301 | 0 | 5 | 57 | 9 | R.SIAELVPK.C |
|  | 4191 | **468.7002** | **935.3859** | **934.4549** | **0.9310** | **0** | **15** | **5.7** | **2** | **R.FFEHEVK.L** |
|  | 7982 | **603.2513** | **1204.4881** | **1203.6005** | **0.8876** | **1** | **5** | **51** | **9** | **K.MVVEFRHMR.N** |
|  | 9089 | 631.3570 | 1260.6994 | 1260.5662 | 0.1332 | 0 | 3 | 77 | 10 | K.NVADYYPEYK.L |
|  | 27226 | **992.4911** | **2974.4516** | **2974.4672** | **-0.0156** | **0** | **67** | **1.2e-005** | **1** | **K.LHLQSTDYGNFLANEASPLTVSVIDDR.L** |

| **642.** | O75380\|NDUS6_HUMAN    **Mass:** 14045    **Score:** 66     **Queries matched:** 8   **emPAI:** 0.28 |
| --- | --- |
|  | NADH dehydrogenase [ubiquinone] iron-sulfur protein 6, mitochondrial OS=Homo sapiens GN=NDUFS6 PE=1 SV=1 |

|  | **Query** | **Observed** | **Mr(expt)** | **Mr(calc)** | **Delta** | **Miss** | **Score** | **Expect** | **Rank** | **Peptide** |
| --- | --- | --- | --- | --- | --- | --- | --- | --- | --- | --- |
|  | 4287 | **472.2732** | **942.5319** | **942.4052** | **0.1267** | **0** | **5** | **57** | **8** | **M.AAAMTFCR.L** |
|  | 9119 | **421.7632** | **1262.2679** | **1261.5939** | **0.6740** | **0** | **11** | **7.6** | **1** | **K.VTHTGQVYDDK.D** |
|  | 10520 | 669.3922 | 1336.7697 | 1335.6605 | 1.1092 | 1 | 4 | 54 | 4 | R.CFGVRVSPTGEK.V 10509 |
|  | 11741 | **704.8493** | **1407.6840** | **1407.6929** | **-0.0089** | **0** | **66** | **3e-005** | **1** | **R.VIACDGGGGALGHPK.V** 11725 |
|  | 14309 | **524.2839** | **1569.8300** | **1569.7578** | **0.0722** | **1** | **1** | **99** | **9** | **-.MAAAMTFCRLLNR.C** |
|  | 16172 | **566.2646** | **1695.7721** | **1695.7853** | **-0.0132** | **1** | **5** | **36** | **3** | **K.VTHTGQVYDDKDYR.R** |

| **643.** | Q12965\|MYO1E_HUMAN    **Mass:** 127552   **Score:** 66     **Queries matched:** 21   **emPAI:** 0.03 |
| --- | --- |
|  | Myosin-Ie OS=Homo sapiens GN=MYO1E PE=1 SV=2 |

|  | **Query** | **Observed** | **Mr(expt)** | **Mr(calc)** | **Delta** | **Miss** | **Score** | **Expect** | **Rank** | **Peptide** |
| --- | --- | --- | --- | --- | --- | --- | --- | --- | --- | --- |
|  | 1741 | **709.3828** | **708.3755** | **707.4218** | **0.9538** | **0** | **16** | **3.5** | **4** | **R.YAILTK.A** |
|  | 2860 | **424.7464** | **847.4782** | **846.3719** | **1.1063** | **0** | **5** | **66** | **9** | **K.VDDIDDR.R** |
|  | 3948 | **459.0124** | **916.0103** | **915.5290** | **0.4813** | **1** | **2** | **86** | **8** | **R.VIQKSWR.K** |
|  | 4665 | 485.2688 | 968.5230 | 968.5040 | 0.0191 | 0 | 7 | 32 | 6 | K.VPDQGAAGVR.R |
|  | 5221 | 504.7920 | 1007.5695 | 1008.5128 | -0.9433 | 0 | 10 | 16 | 9 | K.IDFADTVTK.Y |
|  | 5778 | 526.2785 | 1050.5424 | 1051.4757 | -0.9332 | 1 | 11 | 13 | 8 | R.QMDSKWGGK.S |
|  | 6094 | **537.2874** | **1072.5603** | **1072.5448** | **0.0155** | **1** | **1** | **1.5e+002** | **5** | **R.VVMRNPGER.S** |
|  | 6654 | **558.3207** | **1114.6268** | **1114.6458** | **-0.0190** | **1** | **8** | **24** | **3** | **K.GRPTTAGSKIK.K** |
|  | 7929 | **401.2857** | **1200.8352** | **1201.5840** | **-0.7487** | **1** | **15** | **3.9** | **2** | **K.KPRDWEESR.V** 7917 |
|  | 8019 | **402.8872** | **1205.6398** | **1205.6768** | **-0.0370** | **1** | **6** | **40** | **7** | **K.ISNFLLEKSR.V** |
|  | 9908 | **652.8426** | **1303.6706** | **1302.5741** | **1.0965** | **0** | **5** | **46** | **3** | **K.ENWGPWSAGGSR.Q** |
|  | 12344 | 481.7492 | 1442.2259 | 1442.7042 | -0.4783 | 1 | 6 | 21 | 6 | K.IDFADTVTKYDR.R 12343 |
|  | 13716 | **766.5364** | **1531.0583** | **1529.8817** | **1.1766** | **1** | **0** | **84** | **1** | **K.QLPLKFSNTLELK.L** |
|  | 13943 | 516.5913 | 1546.7521 | 1547.7250 | -0.9729 | 0 | 2 | 80 | 10 | R.ENQCVIISGESGAGK.T |
|  | 16612 | 864.9308 | 1727.8470 | 1726.7839 | 1.0632 | 0 | 17 | 2.2 | 1 | K.YFEIQFSPGGEPDGGK.I 16613 |
|  | 17947 | **609.3198** | **1824.9375** | **1823.9087** | **1.0287** | **1** | **0** | **92** | **7** | **K.YVQMREEASDLLLNK.K** |
|  | 18223 | **922.5005** | **1842.9865** | **1841.9887** | **0.9979** | **0** | **66** | **3.7e-005** | **1** | **K.DIILQSNPLLEAFGNAK.T** |
|  | 27792 | **1035.1539** | **3102.4400** | **3102.3851** | **0.0549** | **0** | **3** | **26** | **6** | **K.EIEMYQGAAQYENPPHIYALADNMYR.N** |

| **644.** | O00160\|MYO1F_HUMAN    **Mass:** 125466   **Score:** 66     **Queries matched:** 22   **emPAI:** 0.03 |
| --- | --- |
|  | Myosin-If OS=Homo sapiens GN=MYO1F PE=1 SV=2 |

|  | **Query** | **Observed** | **Mr(expt)** | **Mr(calc)** | **Delta** | **Miss** | **Score** | **Expect** | **Rank** | **Peptide** |
| --- | --- | --- | --- | --- | --- | --- | --- | --- | --- | --- |
|  | 1194 | 581.2750 | 580.2677 | 580.3445 | -0.0768 | 0 | 8 | 18 | 6 | R.HVAVR.K |
|  | 2135 | **399.3458** | **796.6771** | **796.4191** | **0.2580** | **0** | **4** | **40** | **1** | **R.NGVPPSAR.G** |
|  | 2752 | 421.0563 | 840.0981 | 839.4290 | 0.6691 | 1 | 6 | 22 | 4 | R.KFDGFAR.T |
|  | 2967 | **428.3031** | **854.5916** | **854.3956** | **0.1960** | **1** | **1** | **1.1e+002** | **9** | **R.KYEEMR.E** |
|  | 3740 | **451.7532** | **901.4919** | **901.4730** | **0.0189** | **1** | **11** | **15** | **9** | **R.RSSQAPTR.A** |
|  | 5504 | **515.8073** | **1029.6001** | **1028.5251** | **1.0750** | **0** | **4** | **63** | **6** | **R.GPPSTSLGASR.R** 5473 |
|  | 6168 | 539.7767 | 1077.5388 | 1076.5978 | 0.9410 | 1 | 4 | 70 | 5 | R.DALAKGLYAR.L 6147 6148 |
|  | 8019 | 402.8872 | 1205.6398 | 1205.6768 | -0.0370 | 1 | 6 | 40 | 7 | K.ISNFLLEKSR.V |
|  | 9611 | **645.3415** | **1288.6685** | **1287.7299** | **0.9386** | **1** | **12** | **10** | **5** | **R.GFGDLAVLKVGGR.T** 9586 |
|  | 11740 | 704.8468 | 1407.6790 | 1406.7379 | 0.9412 | 1 | 4 | 56 | 4 | R.GDERQGVQHLLR.A |
|  | 11900 | 472.7489 | 1415.2248 | 1414.6729 | 0.5519 | 1 | 2 | 56 | 8 | R.VDFADSVTKYDR.R |
|  | 12620 | 730.8359 | 1459.6572 | 1459.7745 | -0.1173 | 1 | 2 | 80 | 9 | K.TVAAKYIMGYISK.V |
|  | 14030 | **518.2664** | **1551.7774** | **1552.7383** | **-0.9608** | **1** | **2** | **90** | **5** | **K.EGWGGGGTRSVTFSR.G** |
|  | 14672 | **798.9154** | **1595.8163** | **1595.8672** | **-0.0509** | **1** | **5** | **37** | **7** | **R.SVTFSRGFGDLAVLK.V** 14666 |
|  | 18192 | 921.9061 | 1841.7976 | 1842.0211 | -0.2235 | 1 | 1 | 86 | 10 | R.TLTVSVGDGLPKSSKPTR.K |
|  | 18223 | 922.5005 | 1842.9865 | 1841.9887 | 0.9979 | 0 | 66 | 3.7e-005 | 1 | K.DIILQSNPLLEAFGNAK.T |
|  | 24407 | **829.1041** | **2484.2906** | **2484.2530** | **0.0376** | **0** | **2** | **43** | **3** | **K.QSGVDDMVLLPQITEDAIAANLR.K** |

| **645.** | P20645\|MPRD_HUMAN    **Mass:** 31487    **Score:** 66     **Queries matched:** 4   **emPAI:** 0.12 |
| --- | --- |
|  | Cation-dependent mannose-6-phosphate receptor OS=Homo sapiens GN=M6PR PE=1 SV=1 |

|  | **Query** | **Observed** | **Mr(expt)** | **Mr(calc)** | **Delta** | **Miss** | **Score** | **Expect** | **Rank** | **Peptide** |
| --- | --- | --- | --- | --- | --- | --- | --- | --- | --- | --- |
|  | 1234 | 587.3912 | 586.3839 | 585.3850 | 0.9989 | 0 | 28 | 0.39 | 1 | R.LVVGAK.G |
|  | 2039 | **790.4157** | **789.4084** | **789.4133** | **-0.0049** | **0** | **19** | **2.8** | **1** | **R.NVPAAYR.G** |
|  | 8361 | 408.2347 | 1221.6824 | 1220.6118 | 1.0706 | 1 | 8 | 27 | 6 | R.RAVVMISCNR.H |
|  | 15183 | 814.4728 | 1626.9310 | 1627.7590 | -0.8280 | 0 | 66 | 2.9e-005 | 1 | R.HTLADNFNPVSEER.G |

| **646.** | P21912\|DHSB_HUMAN    **Mass:** 32407    **Score:** 66     **Queries matched:** 22   **emPAI:** 0.55 |
| --- | --- |
|  | Succinate dehydrogenase [ubiquinone] iron-sulfur subunit, mitochondrial OS=Homo sapiens GN=SDHB PE=1 SV=3 |

|  | **Query** | **Observed** | **Mr(expt)** | **Mr(calc)** | **Delta** | **Miss** | **Score** | **Expect** | **Rank** | **Peptide** |
| --- | --- | --- | --- | --- | --- | --- | --- | --- | --- | --- |
|  | 1222 | **586.3998** | **585.3926** | **584.3282** | **1.0644** | **0** | **15** | **5.7** | **3** | **K.GLNPGK.A** 1218 |
|  | 1856 | **744.3368** | **743.3295** | **743.3346** | **-0.0051** | **0** | **28** | **0.36** | **1** | **K.MMATYK.E** |
|  | 2422 | 818.4360 | 817.4288 | 816.4341 | 0.9947 | 0 | 22 | 1.3 | 1 | R.IDTNLNK.V |
|  | 3764 | 452.7266 | 903.4387 | 903.4194 | 0.0193 | 1 | 6 | 53 | 6 | K.KMMATYK.E |
|  | 4721 | 487.2720 | 972.5295 | 972.5352 | -0.0057 | 1 | 23 | 0.93 | 1 | R.RIDTNLNK.V 4720 |
|  | 6951 | **569.8256** | **1137.6366** | **1137.5818** | **0.0547** | **0** | **12** | **10** | **2** | **K.LQDPFSLYR.C** |
|  | 8224 | 608.8099 | 1215.6052 | 1215.6355 | -0.0303 | 0 | 8 | 27 | 8 | K.CGPMVLDALIK.I |
|  | 8974 | **419.3147** | **1254.9224** | **1254.7044** | **0.2180** | **1** | **6** | **26** | **6** | **R.GAQTAAATAPRIK.K** |
|  | 11258 | 460.9072 | 1379.6998 | 1380.7224 | -1.0226 | 0 | 14 | 6 | 9 | K.YLGPAVLMQAYR.W |
|  | 12562 | 729.4199 | 1456.8253 | 1456.8146 | 0.0107 | 1 | 16 | 2.9 | 4 | K.CGPMVLDALIKIK.N 12560 12563 |
|  | 13992 | **775.7751** | **1549.5356** | **1549.7736** | **-0.2380** | **1** | **5** | **33** | **2** | **K.QQYLQSIEEREK.L** |
|  | 15829 | **836.9089** | **1671.8033** | **1671.8144** | **-0.0111** | **0** | **37** | **0.025** | **1** | **K.DLVPDLSNFYAQYK.S** |
|  | 18255 | **923.4378** | **1844.8611** | **1844.8727** | **-0.0116** | **0** | **43** | **0.0054** | **1** | **K.AGDKPHMQTYEVDLNK.C** 18235 18256 18257 |
|  | 22679 | **1128.9685** | **2255.9225** | **2254.9551** | **0.9673** | **0** | **9** | **9.7** | **1** | **R.EGICGSCAMNINGGNTLACTR.R** |
|  | 24418 | **622.5422** | **2486.1399** | **2486.1536** | **-0.0138** | **1** | **21** | **0.52** | **1** | **R.WDPDKAGDKPHMQTYEVDLNK.C** |

| **647.** | O00159\|MYO1C_HUMAN    **Mass:** 122503   **Score:** 65     **Queries matched:** 19   **emPAI:** 0.06 |
| --- | --- |
|  | Myosin-Ic OS=Homo sapiens GN=MYO1C PE=1 SV=3 |

|  | **Query** | **Observed** | **Mr(expt)** | **Mr(calc)** | **Delta** | **Miss** | **Score** | **Expect** | **Rank** | **Peptide** |
| --- | --- | --- | --- | --- | --- | --- | --- | --- | --- | --- |
|  | 3585 | 446.7451 | 891.4756 | 890.4610 | 1.0146 | 0 | 30 | 0.18 | 1 | K.NNDLLFR.N |
|  | 4020 | **461.7504** | **921.4862** | **920.4828** | **1.0034** | **1** | **9** | **19** | **1** | **K.SLGRGEFR.L** 4003 |
|  | 5343 | **509.7462** | **1017.4779** | **1018.4767** | **-0.9987** | **1** | **1** | **1.5e+002** | **2** | **R.QHMERYR.G** |
|  | 5543 | **517.2969** | **1032.5792** | **1032.5829** | **-0.0036** | **0** | **8** | **29** | **6** | **K.NGHLAVVAPR.L** |
|  | 6338 | **545.7858** | **1089.5571** | **1089.6182** | **-0.0611** | **0** | **8** | **26** | **3** | **K.YLGLLENLR.V** |
|  | 7958 | **602.7891** | **1203.5636** | **1202.6044** | **0.9592** | **1** | **8** | **27** | **3** | **K.KDNYPQSVPR.L** |
|  | 10738 | 675.8354 | 1349.6562 | 1348.6293 | 1.0270 | 0 | 6 | 38 | 3 | R.DQAVMISGESGAGK.T 10741 |
|  | 13305 | 751.9356 | 1501.8567 | 1500.8585 | 0.9981 | 0 | 65 | 4.3e-005 | 1 | K.MSLLQLVEILQSK.E |
|  | 13523 | 758.9352 | 1515.8559 | 1516.8534 | -0.9975 | 0 | (5) | 37 | 8 | K.MSLLQLVEILQSK.E |
|  | 15687 | 830.9471 | 1659.8797 | 1659.8402 | 0.0395 | 1 | 3 | 52 | 3 | K.EPAYVRCIKPNDAK.Q |
|  | 16029 | **562.4653** | **1684.3742** | **1683.9229** | **0.4512** | **0** | **2** | **52** | **9** | **-.MALQVELVPTGEIIR.V** |
|  | 19122 | 955.4799 | 1908.9453 | 1910.0877 | -1.1424 | 0 | 6 | 24 | 2 | R.VLQALGSEPIQYAVPVVK.Y 19121 |
|  | 20218 | **671.0023** | **2009.9850** | **2009.0793** | **0.9056** | **1** | **5** | **31** | **2** | **K.QKGDVVLQSDHVIETLTK.T** |
|  | 21272 | **1054.1434** | **2106.2723** | **2105.8639** | **0.4084** | **1** | **3** | **37** | **1** | **K.ETMCSSKNPIMSQCFDR.S** |
|  | 26266 | **695.8507** | **2779.3737** | **2778.5149** | **0.8588** | **1** | **0** | **57** | **9** | **-.MALQVELVPTGEIIRVVHPHRPCK.L** |
|  | 29453 | **1197.2339** | **3588.6798** | **3588.8424** | **-0.1626** | **1** | **3** | **18** | **5** | **R.VNSININQGSITFAGGPGRDGTIDFTPGSELLITK.A** |

| **648.** | P67809\|YBOX1_HUMAN    **Mass:** 35903    **Score:** 65     **Queries matched:** 12   **emPAI:** 0.35 |
| --- | --- |
|  | Nuclease-sensitive element-binding protein 1 OS=Homo sapiens GN=YBX1 PE=1 SV=3 |

|  | **Query** | **Observed** | **Mr(expt)** | **Mr(calc)** | **Delta** | **Miss** | **Score** | **Expect** | **Rank** | **Peptide** |
| --- | --- | --- | --- | --- | --- | --- | --- | --- | --- | --- |
|  | 1764 | **714.4463** | **713.4390** | **712.3293** | **1.1098** | **0** | **9** | **21** | **5** | **R.NFNYR.R** |
|  | 4412 | **475.8991** | **949.7836** | **950.5199** | **-0.7362** | **1** | **9** | **14** | **3** | **R.GYRPRFR.R** |
|  | 4933 | **495.2352** | **988.4558** | **988.4477** | **0.0082** | **0** | **18** | **2.7** | **2** | **R.FPPYYMR.R** 4926 |
|  | 10580 | 671.3132 | 1340.6118 | 1339.6567 | 0.9550 | 1 | 5 | 48 | 9 | R.QNMYRGYRPR.F 10571 |
|  | 11869 | 707.8858 | 1413.7570 | 1413.7616 | -0.0046 | 1 | 8 | 19 | 7 | K.EDVFVHQTAIKK.N |
|  | 16163 | **848.4311** | **1694.8476** | **1694.8588** | **-0.0112** | **0** | **34** | **0.043** | **1** | **K.GAEAANVTGPGGVPVQGSK.Y** 16165 |
|  | 16851 | 582.2964 | 1743.8673 | 1743.8792 | -0.0118 | 1 | 26 | 0.27 | 1 | R.NDTKEDVFVHQTAIK.K |
|  | 28474 | **1086.4626** | **3256.3661** | **3256.3889** | **-0.0227** | **1** | **50** | **0.00039** | **1** | **R.NYQQNYQNSESGEKNEGSESAPEGQAQQR.R** |
|  | 30932 | **1119.0504** | **4472.1726** | **4471.1375** | **1.0350** | **0** | **0** | **20** | **2** | **M.SSEAETQQPPAAPPAAPALSAADTKPGTTGSGAGSGGPGGLTSAAPAGGDK.K** |

| **649.** | Q09666\|AHNK_HUMAN    **Mass:** 629213   **Score:** 65     **Queries matched:** 192   **emPAI:** 0.02 |
| --- | --- |
|  | Neuroblast differentiation-associated protein AHNAK OS=Homo sapiens GN=AHNAK PE=1 SV=2 |

|  | **Query** | **Observed** | **Mr(expt)** | **Mr(calc)** | **Delta** | **Miss** | **Score** | **Expect** | **Rank** | **Peptide** |
| --- | --- | --- | --- | --- | --- | --- | --- | --- | --- | --- |
|  | 1203 | **583.1080** | **582.1008** | **581.3649** | **0.7358** | **1** | **10** | **6.8** | **2** | **K.HGKLK.F** |
|  | 1503 | 648.3270 | 647.3197 | 646.4166 | 0.9031 | 1 | 8 | 41 | 8 | K.FKKPK.F |
|  | 1507 | **649.2016** | **648.1943** | **649.3291** | **-1.1348** | **1** | **14** | **11** | **1** | **K.MPKMK.M** 1508 |
|  | 1524 | **651.1976** | **650.1903** | **649.3621** | **0.8282** | **1** | **10** | **19** | **6** | **K.FKMPK.L** |
|  | 1619 | **675.1237** | **674.1164** | **673.3646** | **0.7517** | **0** | **5** | **56** | **3** | **K.IEGDLK.G** |
|  | 1666 | **688.3839** | **687.3766** | **686.3711** | **1.0055** | **0** | **14** | **11** | **8** | **K.GLDLGGR.G** |
|  | 1683 | **693.2937** | **692.2864** | **691.3211** | **0.9654** | **0** | **9** | **24** | **2** | **K.VEGEMK.V** |
|  | 1735 | 708.3878 | 707.3805 | 707.3160 | 0.0645 | 0 | 23 | 0.85 | 3 | K.MEGDLK.G 1732 |
|  | 1754 | **712.3689** | **711.3616** | **711.4279** | **-0.0663** | **0** | **8** | **21** | **6** | **K.GPQVALK.G** |
|  | 1959 | 773.4210 | 772.4137 | 771.4491 | 0.9646 | 1 | 10 | 23 | 7 | K.VDVKSPK.T |
|  | 2355 | **407.2083** | **812.4019** | **812.4392** | **-0.0373** | **0** | **8** | **27** | **4** | **K.VPEVDVR.G** |
|  | 2583 | 415.7155 | 829.4165 | 828.4891 | 0.9274 | 1 | (5) | 71 | 9 | K.LNVPKMK.V |
|  | 2796 | 422.7310 | 843.4474 | 842.4498 | 0.9976 | 0 | 10 | 19 | 7 | K.ADLGVSGPK.V |
|  | 2821 | 423.4551 | 844.8955 | 844.4840 | 0.4115 | 1 | 10 | 16 | 4 | K.LNVPKMK.V |
|  | 3066 | 431.2580 | 860.5014 | 859.4473 | 1.0541 | 0 | 21 | 1.8 | 1 | K.MPEINIK.A |
|  | 3160 | **865.0633** | **864.0560** | **863.3881** | **0.6679** | **0** | **0** | **1.1e+002** | **3** | **K.MPDMNIK.A** |
|  | 3447 | **442.0246** | **882.0346** | **882.4633** | **-0.4287** | **0** | **2** | **58** | **7** | **K.IPEMHLK.A** 3439 |
|  | 3577 | **446.2692** | **890.5238** | **889.4328** | **1.0911** | **0** | **(6)** | **44** | **4** | **K.MQVGGDGVK.V** |
|  | 3729 | **451.2735** | **900.5324** | **900.4553** | **0.0771** | **0** | **14** | **7.6** | **2** | **K.ADIDVSGPK.V** |
|  | 3805 | **454.2142** | **906.4139** | **905.4277** | **0.9862** | **0** | **7** | **35** | **7** | **K.MQVGGDGVK.V** |
|  | 3876 | 456.7420 | 911.4695 | 912.5029 | -1.0334 | 0 | 4 | 55 | 6 | K.GGVDVTLPR.V |
|  | 4103 | 465.2363 | 928.4581 | 928.4502 | 0.0079 | 0 | 12 | 12 | 3 | K.GEGPEVDVK.L 4086 4092 |
|  | 4118 | **465.2734** | **928.5322** | **928.4865** | **0.0457** | **0** | **8** | **31** | **6** | **K.ADIEISGPK.V** 4082 |
|  | 4122 | 465.5856 | 929.1567 | 928.4866 | 0.6701 | 0 | 4 | 52 | 10 | K.GDVDVSLPK.L |
|  | 4614 | **482.7480** | **963.4815** | **962.5259** | **0.9556** | **1** | **8** | **26** | **2** | **K.FPTMKVPK.F** |
|  | 4669 | 485.3528 | 968.6911 | 969.4920 | -0.8009 | 0 | 3 | 66 | 10 | K.FGTFGGLGSK.S |
|  | 5142 | 501.7793 | 1001.5440 | 1001.5580 | -0.0140 | 0 | 19 | 2.3 | 2 | K.LQVTMPGIK.V 5141 5152 5162 |
|  | 5627 | 520.2973 | 1038.5800 | 1038.4651 | 0.1149 | 1 | 17 | 3 | 7 | -.MEKEETTR.E 5628 |
|  | 5648 | 521.2970 | 1040.5794 | 1039.6026 | 0.9768 | 1 | 5 | 47 | 9 | K.VKVPEVDVR.G |
|  | 5981 | **534.2584** | **1066.5023** | **1066.6023** | **-0.1000** | **1** | **5** | **52** | **7** | **K.VPDVDIKGPK.V** 5979 5984 |
|  | 6070 | 536.7631 | 1071.5117 | 1071.5634 | -0.0517 | 0 | 7 | 35 | 8 | K.MPSLEISAPK.V |
|  | 6270 | **543.3829** | **1084.7513** | **1085.5539** | **-0.8026** | **0** | **6** | **36** | **1** | **K.LEVPDMNIR.G** |
|  | 6279 | **543.8070** | **1085.5995** | **1084.6128** | **0.9867** | **1** | **4** | **62** | **9** | **K.VPDVELKSAK.M** |
|  | 6569 | **554.7784** | **1107.5422** | **1108.5877** | **-1.0455** | **0** | **4** | **65** | **10** | **R.VSAPEVSVGHK.G** |
|  | 6593 | **555.7950** | **1109.5754** | **1108.5951** | **0.9803** | **1** | **5** | **47** | **8** | **K.MPKFSVSGLK.A** |
|  | 6644 | **558.2506** | **1114.4867** | **1115.5533** | **-1.0666** | **0** | **3** | **78** | **8** | **K.MPDVDISVPK.I** |
|  | 6654 | 558.3207 | 1114.6268 | 1113.6030 | 1.0238 | 1 | 6 | 41 | 10 | K.LKGDVDVSGPK.L |
|  | 6803 | **564.3155** | **1126.6164** | **1125.5562** | **1.0602** | **1** | **4** | **66** | **10** | **K.FKMPEMSIK.A** |
|  | 6927 | **568.7628** | **1135.5110** | **1136.5722** | **-1.0612** | **1** | **(9)** | **19** | **2** | **K.FKMPEMNIK.V** |
|  | 6986 | 571.2977 | 1140.5809 | 1140.5784 | 0.0026 | 1 | 6 | 34 | 9 | K.MPKIHMSGPK.I |
|  | 6994 | **571.3159** | **1140.6173** | **1139.6372** | **0.9801** | **1** | **7** | **30** | **6** | **K.MPEINIKAPK.I** |
|  | 7040 | **573.7773** | **1145.5400** | **1146.5777** | **-1.0377** | **1** | **4** | **61** | **8** | **K.MPEMSIKAPK.I** 7071 7072 |
|  | 7064 | **574.3517** | **1146.6888** | **1146.5777** | **0.1111** | **1** | **(2)** | **1.1e+002** | **9** | **K.MPEMSIKAPK.I** |
|  | 7077 | **575.2764** | **1148.5383** | **1148.6376** | **-0.0993** | **1** | **8** | **25** | **6** | **K.MPDLHLKAPK.I** |
|  | 7154 | 577.7865 | 1153.5584 | 1154.5464 | -0.9879 | 1 | 15 | 4.5 | 3 | K.FKMPDMNIK.A |
|  | 7184 | 579.2406 | 1156.4666 | 1155.6136 | 0.8531 | 1 | 1 | 1.4e+002 | 5 | K.AKGEVDVDVPK.L |
|  | 7212 | **579.8217** | **1157.6289** | **1157.6267** | **0.0022** | **1** | **4** | **68** | **7** | **K.FKIPEMHLK.A** |
|  | 7242 | **581.3022** | **1160.5898** | **1159.5729** | **1.0169** | **1** | **6** | **43** | **3** | **K.MPDMNIKAPK.I** |
|  | 7307 | 583.8230 | 1165.6314 | 1164.6035 | 1.0279 | 1 | 9 | 18 | 1 | K.MPKFSMPSLK.G |
|  | 7330 | **584.7979** | **1167.5811** | **1168.5620** | **-0.9809** | **1** | **11** | **12** | **1** | **K.FKMPEMNIK.V** |
|  | 7418 | 588.2607 | 1174.5069 | 1175.5678 | -1.0609 | 1 | (5) | 57 | 2 | K.MPDMNIKAPK.I |
|  | 7582 | 593.3292 | 1184.6439 | 1185.6250 | -0.9811 | 1 | 12 | 9.7 | 3 | K.MPEMNIKVPK.I 7621 |
|  | 7584 | **593.3604** | **1184.7061** | **1185.6427** | **-0.9366** | **0** | **10** | **17** | **7** | **K.ISMPDVGLNLK.A** |
|  | 7606 | **593.8398** | **1185.6650** | **1184.6190** | **1.0460** | **1** | **1** | **1.2e+002** | **5** | **K.FGTFGGLGSKSK.G** |
|  | 7633 | **594.7619** | **1187.5092** | **1187.6042** | **-0.0950** | **0** | **2** | **1.1e+002** | **9** | **K.MPEMSIKPQK.I** |
|  | 7659 | **595.3043** | **1188.5941** | **1189.6013** | **-1.0072** | **1** | **5** | **53** | **1** | **K.VKGDMDVSVPK.V** |
|  | 7668 | **595.3307** | **1188.6469** | **1187.6584** | **0.9885** | **1** | **1** | **1.2e+002** | **1** | **K.MKGDVVVSLPK.V** |
|  | 7723 | **398.2102** | **1191.6088** | **1190.7023** | **0.9065** | **0** | **13** | **7.5** | **1** | **K.ISIPDVGLHLK.G** |
|  | 7935 | 601.8226 | 1201.6306 | 1201.6376 | -0.0071 | 1 | 15 | 5.5 | 5 | K.VKGDMDISLPK.V 7934 7949 |
|  | 7950 | **602.3370** | **1202.6594** | **1201.6199** | **1.0395** | **1** | **(2)** | **1e+002** | **10** | **K.MPEMNIKVPK.I** |
|  | 8085 | **404.0300** | **1209.0683** | **1209.5675** | **-0.4992** | **1** | **7** | **20** | **2** | **K.FKMPEMHFK.T** 8112 |
|  | 8149 | **405.0418** | **1212.1035** | **1211.6146** | **0.4889** | **0** | **4** | **34** | **3** | **K.VDINAPDVDVR.G** |
|  | 8157 | **405.2067** | **1212.5984** | **1211.5757** | **1.0226** | **1** | **8** | **24** | **2** | **K.FSMPGFKGEGR.E** |
|  | 8185 | 607.8058 | 1213.5971 | 1214.5940 | -0.9968 | 1 | 11 | 12 | 2 | K.MPEMHFKAPK.I 8192 8202 8215 8229 |
|  | 8235 | 608.8705 | 1215.7265 | 1216.5733 | -0.8467 | 1 | 8 | 23 | 2 | K.MPDMHFKAPK.I |
|  | 8302 | **610.3518** | **1218.6889** | **1217.6326** | **1.0564** | **1** | **(10)** | **16** | **2** | **K.VKGDMDISLPK.V** |
|  | 8512 | **616.3733** | **1230.7320** | **1230.5889** | **0.1431** | **1** | **(4)** | **60** | **1** | **K.MPEMHFKAPK.I** |
|  | 8527 | 616.8271 | 1231.6397 | 1230.5195 | 1.1202 | 0 | (3) | 76 | 2 | K.MPDMHVNMPK.I |
|  | 8649 | 619.7659 | 1237.5172 | 1238.6870 | -1.1699 | 1 | 9 | 18 | 2 | K.LPKADIDVSGPK.V |
|  | 8671 | **620.3102** | **1238.6058** | **1238.6143** | **-0.0085** | **0** | **8** | **21** | **1** | **K.GEGPDVDVNLPK.A** |
|  | 8690 | 620.7770 | 1239.5395 | 1239.6347 | -0.0952 | 0 | 11 | 9.8 | 2 | K.GEGPEVDVTLPK.A 8689 8706 |
|  | 8720 | 621.3231 | 1240.6317 | 1240.6663 | -0.0346 | 1 | 9 | 19 | 4 | K.LEGGEVDLKGPK.V |
|  | 8833 | **624.8035** | **1247.5924** | **1246.5144** | **1.0779** | **0** | **8** | **22** | **2** | **K.MPDMHVNMPK.I** |
|  | 8923 | **627.7681** | **1253.5217** | **1252.6299** | **0.8918** | **0** | **1** | **1e+002** | **2** | **K.GEGPEVDVNLPK.A** |
|  | 9067 | **421.0564** | **1260.1473** | **1260.6272** | **-0.4799** | **0** | **1** | **86** | **10** | **K.MSLPDVDLDLK.G** |
|  | 9555 | **643.8104** | **1285.6062** | **1284.6020** | **1.0042** | **0** | **4** | **52** | **4** | **K.GEGPEVDMNLPK.A** |
|  | 9721 | 647.8702 | 1293.7259 | 1293.6275 | 0.0984 | 0 | 3 | 64 | 4 | K.ISMPDVDFNLK.G |
|  | 9824 | **434.2333** | **1299.6782** | **1300.6155** | **-0.9373** | **0** | **8** | **23** | **4** | **K.ISMPDVDLHMK.G** |
|  | 9866 | 651.8628 | 1301.7111 | 1300.6697 | 1.0415 | 1 | 7 | 31 | 8 | K.GPDVKLPDMSVK.T |
|  | 9933 | **653.8420** | **1305.6694** | **1304.7201** | **0.9493** | **0** | **8** | **24** | **1** | **K.VHAPGLNLSGVGGK.M** |
|  | 9957 | **654.8267** | **1307.6388** | **1307.6431** | **-0.0044** | **0** | **0** | **1.3e+002** | **9** | **K.ISMPDIDFNLK.G** |
|  | 9995 | 655.8507 | 1309.6869 | 1310.6363 | -0.9494 | 1 | 8 | 23 | 6 | K.MKMPTFSTPGAK.G |
|  | 10164 | **440.2630** | **1317.7672** | **1316.6105** | **1.1567** | **0** | **(6)** | **35** | **1** | **K.ISMPDVDLHMK.G** |
|  | 10334 | **443.0399** | **1326.0979** | **1325.7303** | **0.3676** | **1** | **5** | **33** | **4** | **K.GGVDVTLPRVEGK.V** |
|  | 10353 | 664.8086 | 1327.6026 | 1326.7217 | 0.8809 | 1 | 6 | 34 | 4 | K.VKMPDVDISVPK.I 10357 |
|  | 10361 | **664.8526** | **1327.6906** | **1328.6460** | **-0.9553** | **0** | **1** | **1e+002** | **5** | **K.GDVDVSVPEVEGK.L** 10366 |
|  | 10389 | **444.2505** | **1329.7296** | **1330.6591** | **-0.9295** | **0** | **5** | **47** | **5** | **K.ISMPDFDLHLK.G** |
|  | 10527 | **446.7551** | **1337.2434** | **1336.7099** | **0.5335** | **1** | **10** | **9.1** | **1** | **R.RVTAYTVDVTGR.E** |
|  | 10945 | **682.3501** | **1362.6856** | **1362.6892** | **-0.0035** | **1** | **1** | **1e+002** | **6** | **K.FGVSTGREGQTPK.A** |
|  | 11056 | **685.4449** | **1368.8752** | **1367.7231** | **1.1521** | **1** | **6** | **32** | **1** | **K.LEVPDMNIRGPK.V** 11017 |
|  | 11564 | **699.3302** | **1396.6458** | **1397.7402** | **-1.0944** | **1** | **2** | **86** | **5** | **K.VEGDLKGPEVDIK.G** |
|  | 12082 | 713.9069 | 1425.7993 | 1425.7715 | 0.0278 | 1 | 7 | 24 | 3 | K.LEGELKGPELDVK.G |
|  | 12514 | 727.8488 | 1453.6831 | 1452.7824 | 0.9007 | 1 | 7 | 25 | 7 | R.GPDVKLEGPDVSLK.G |
|  | 12567 | 486.7926 | 1457.3560 | 1457.7436 | -0.3876 | 1 | (4) | 35 | 7 | K.VEGEMKVPDVDIK.G |
|  | 12603 | **487.2618** | **1458.7635** | **1459.7228** | **-0.9593** | **1** | **2** | **85** | **5** | **K.MEGDLKAPEVDIK.G** |
|  | 12622 | **487.5606** | **1459.6601** | **1459.7229** | **-0.0628** | **1** | **13** | **7.1** | **3** | **K.VEGDMKVPDVDIK.G** 12615 |
|  | 12844 | 492.2818 | 1473.8236 | 1473.7385 | 0.0851 | 1 | 9 | 16 | 1 | K.VEGEMKVPDVDIK.G |
|  | 12915 | **493.9227** | **1478.7463** | **1478.7625** | **-0.0162** | **1** | **7** | **25** | **3** | **K.FKMPEMSIKPQK.I** |
|  | 13069 | **744.9973** | **1487.9799** | **1488.7494** | **-0.7694** | **0** | **28** | **0.15** | **1** | **K.ISMQDVDLSLGSPK.L** |
|  | 13082 | 745.3723 | 1488.7299 | 1487.7541 | 0.9758 | 1 | 2 | 77 | 10 | K.VEGEMKVPDVEIK.G |
|  | 13306 | **501.7790** | **1502.3151** | **1501.7446** | **0.5705** | **1** | **4** | **33** | **3** | **K.VEGEMKVPDVDIR.G** |
|  | 13484 | 758.3731 | 1514.7317 | 1515.7239 | -0.9922 | 0 | 7 | 25 | 4 | K.IEGEMQVPDVDIR.G |
|  | 13535 | **759.8252** | **1517.6358** | **1517.7686** | **-0.1327** | **1** | **1** | **98** | **9** | **K.GKGGVTGSPEASISGSK.G** 13547 |
|  | 13652 | 764.3672 | 1526.7198 | 1526.8014 | -0.0816 | 1 | 7 | 23 | 5 | K.MSLPDVDLDLKGPK.M |
|  | 13852 | **514.5897** | **1540.7472** | **1541.8123** | **-1.0652** | **1** | **3** | **69** | **4** | **K.ISMPDVDLNLKGPK.L** |
|  | 13862 | 771.8914 | 1541.7682 | 1542.7963 | -1.0282 | 1 | (6) | 28 | 8 | K.MSLPDVDLDLKGPK.M |
|  | 14091 | 519.2885 | 1554.8437 | 1555.8093 | -0.9656 | 1 | 8 | 18 | 5 | K.GDVDVSAPKLEGELK.G |
|  | 14114 | 779.4303 | 1556.8460 | 1555.8280 | 1.0181 | 1 | 3 | 68 | 2 | K.ISMPDIDLNLKGPK.V |
|  | 14327 | 786.9083 | 1571.8021 | 1571.8229 | -0.0208 | 1 | 1 | 96 | 4 | K.VSMPDVELNLKSPK.V |
|  | 14479 | **528.5866** | **1582.7380** | **1582.7847** | **-0.0467** | **1** | **2** | **78** | **7** | **K.ISMPDVDLHMKGPK.V** |
|  | 14635 | 796.8967 | 1591.7789 | 1592.8596 | -1.0807 | 1 | 4 | 45 | 8 | K.ISMPDLDLHLKSPK.A |
|  | 14670 | 532.9060 | 1595.6962 | 1596.8334 | -1.1372 | 1 | 1 | 91 | 10 | K.ISMPDFDLHLKGPK.V |
|  | 14718 | **800.4070** | **1598.7995** | **1599.8356** | **-1.0360** | **1** | **4** | **47** | **2** | **K.GDVDVSLPKVESDLK.G** |
|  | 14770 | **535.2880** | **1602.8422** | **1603.8280** | **-0.9857** | **1** | **5** | **37** | **4** | **K.APKISMPDFDLNLK.G** |
|  | 15000 | **808.9253** | **1615.8360** | **1615.8127** | **0.0233** | **1** | **9** | **16** | **1** | **K.GDMDISLPKVEGDLK.G** |
|  | 15197 | **543.6720** | **1627.9942** | **1626.8439** | **1.1502** | **1** | **0** | **1e+002** | **10** | **K.APKISMPDFDLHLK.G** |
|  | 15283 | 817.3899 | 1632.7652 | 1631.8076 | 0.9576 | 1 | 2 | 67 | 4 | K.GDMDISVPKLEGDLK.G |
|  | 15346 | 410.2193 | 1636.8481 | 1636.8647 | -0.0166 | 1 | 3 | 60 | 10 | K.APKIPMPDFDLHLK.G 15334 |
|  | 15985 | **841.4123** | **1680.8100** | **1680.8207** | **-0.0106** | **0** | **5** | **33** | **1** | **K.VDVDVPDVNIEGPDAK.L** |
|  | 16160 | **848.4204** | **1694.8263** | **1694.8363** | **-0.0100** | **0** | **15** | **3.2** | **1** | **K.VDIDVPDVNIEGPDAK.L** |
|  | 16206 | **849.9182** | **1697.8219** | **1697.8360** | **-0.0141** | **0** | **13** | **5.8** | **1** | **K.IDVTAPDVSIEEPEGK.L** |
|  | 16234 | 850.4434 | 1698.8723 | 1699.9178 | -1.0455 | 1 | 1 | 88 | 4 | R.ISMSEVDLNVAAPKVK.G |
|  | 16384 | 570.6458 | 1708.9156 | 1709.8182 | -0.9026 | 1 | 9 | 13 | 4 | K.GEYDMTVPKLEGDLK.G 16385 |
|  | 16399 | **856.4382** | **1710.8619** | **1710.8788** | **-0.0169** | **0** | **32** | **0.072** | **1** | **R.VDIETPNLEGTLTGPR.L** |
|  | 16467 | **572.9955** | **1715.9648** | **1714.8276** | **1.1372** | **1** | **2** | **63** | **7** | **K.MYFPDVEFDIKSPK.F** |
|  | 16563 | 862.4316 | 1722.8487 | 1723.8338 | -0.9851 | 1 | 9 | 16 | 4 | K.GEYDVTMPKVESEIK.V 16566 |
|  | 16613 | 864.9308 | 1727.8470 | 1727.8665 | -0.0195 | 1 | 2 | 74 | 10 | R.ELVGREMGVDVHFPK.A |
|  | 16793 | 871.4272 | 1740.8398 | 1739.8764 | 0.9634 | 1 | 1 | 83 | 7 | K.VEGDMQVPDLDIKGPK.V |
|  | 17462 | **597.2694** | **1788.7862** | **1787.9088** | **0.8774** | **1** | **14** | **4.1** | **1** | **K.MQVGGDGVKVPGIDATTK.L** |
|  | 17652 | 902.9704 | 1803.9262 | 1802.8952 | 1.0311 | 0 | 0 | 1e+002 | 10 | K.VDVNAPDVQAPDWHLK.M |
|  | 17917 | 608.8189 | 1823.4349 | 1823.9451 | -0.5102 | 1 | 0 | 69 | 6 | K.APNISMPDVDLNLKGPK.I |
|  | 18175 | **614.2993** | **1839.8760** | **1838.9812** | **0.8948** | **1** | **5** | **34** | **2** | **K.TPKISMPDIDLNLTGPK.I** |
|  | 18224 | **615.3500** | **1843.0281** | **1841.9159** | **1.1122** | **1** | **6** | **28** | **10** | **K.GDLDIAGPNLEGDFKGPK.V** 18215 |
|  | 18481 | **620.3401** | **1857.9986** | **1857.9506** | **0.0480** | **1** | **4** | **38** | **1** | **K.SPQISMSDIDLNLKGPK.I** |
|  | 18786 | 943.4592 | 1884.9039 | 1884.9065 | -0.0026 | 0 | 1 | 74 | 2 | R.HELTEISNVDVETQSGK.T |
|  | 19045 | **635.3511** | **1903.0316** | **1903.0051** | **0.0265** | **1** | **0** | **86** | **6** | **K.VGIDTPDIDIHGPEGKLK.G** |
|  | 19206 | **639.6523** | **1915.9352** | **1916.9327** | **-0.9975** | **1** | **7** | **19** | **5** | **K.ASLGSLEGEAEAEASSPKGK.F** |
|  | 19329 | **643.3133** | **1926.9180** | **1928.0255** | **-1.1075** | **1** | **5** | **30** | **2** | **K.FSVSGLKAEGPDVAVDLPK.G** |
|  | 19516 | 648.7004 | 1943.0795 | 1943.9510 | -0.8715 | 1 | 5 | 27 | 5 | K.MDVNVGDIDIEGPEGKLK.G |
|  | 20391 | **507.7477** | **2026.9615** | **2026.9749** | **-0.0134** | **0** | **38** | **0.014** | **1** | **K.VDIDAPDVDVHGPDWHLK.M** |
|  | 20493 | 679.3719 | 2035.0940 | 2036.1266 | -1.0326 | 1 | 6 | 23 | 1 | K.VPGIDATTKLNVGAPDVTLR.G 20491 |
|  | 20626 | 1023.4982 | 2044.9818 | 2046.0059 | -1.0241 | 0 | 1 | 74 | 7 | K.VDIDVPDVDVQGPDWHLK.M |
|  | 20653 | **683.4322** | **2047.2749** | **2048.0031** | **-0.7281** | **1** | **5** | **21** | **2** | **K.TGTCRISMSEVDLNVAAPK.V** |
|  | 20825 | 689.0527 | 2064.1364 | 2063.0034 | 1.1330 | 1 | 3 | 45 | 2 | K.FSMPGFKGEGPEVDVNLPK.A |
|  | 20957 | **694.3037** | **2079.8893** | **2081.0139** | **-1.1246** | **1** | **1** | **63** | **6** | **K.FSMPSLKGEGPEFDVNLSK.A** |
|  | 21627 | **714.3648** | **2140.0726** | **2139.1147** | **0.9579** | **1** | **2** | **51** | **4** | **K.MKLPQFGISTPGSDLHVNAK.G** |
|  | 22773 | 756.7169 | 2267.1289 | 2266.2030 | 0.9259 | 1 | 0 | 74 | 2 | K.GLDLGGRGGVQVPAVDISSSLGGR.A |
|  | 23241 | 776.8925 | 2327.6556 | 2327.1910 | 0.4645 | 1 | 0 | 49 | 4 | K.GPKVDIDVPDVNVQGPDWHLK.M |
|  | 23297 | 779.7624 | 2336.2655 | 2337.2176 | -0.9521 | 1 | 1 | 62 | 5 | R.VDIETPNLEGTLTGPRLGSPSGK.T 23322 |
|  | 23576 | **790.5862** | **2368.7367** | **2369.1905** | **-0.4538** | **1** | **2** | **29** | **2** | **K.MPSMNIQTHKISMPDVGLNLK.A** |
|  | 24125 | 816.4236 | 2446.2489 | 2446.1872 | 0.0617 | 1 | 5 | 23 | 2 | K.MPDVHFKSPQISMSDIDLNLK.G |
|  | 24155 | 817.4003 | 2449.1790 | 2448.1818 | 0.9972 | 1 | 3 | 41 | 4 | K.MDIDVPDVEVQGPDWHLKMPK.M |
|  | 24289 | **822.7153** | **2465.1242** | **2464.2883** | **0.8358** | **1** | **5** | **22** | **1** | **K.LEGPDVSLKGPGVDLPSVNLSMPK.V** |
|  | 25113 | **860.4963** | **2578.4672** | **2577.2810** | **1.1862** | **1** | **2** | **39** | **1** | **K.ADIDVSGPKVDVDIPDVNIEGPDAK.L** |
|  | 26390 | 937.1213 | 2808.3420 | 2809.3481 | -1.0060 | 1 | 1 | 47 | 4 | R.GPQVDVKGPFVEAEVPDVDLECPDAK.L |
|  | 30020 | **947.2229** | **3784.8625** | **3784.8731** | **-0.0106** | **0** | **19** | **0.42** | **1** | **K.EGDQIVGATIYFDNLQSGEVTQLLNTMGHHTVGLK.L** |
|  | 30333 | **1320.2041** | **3957.5905** | **3957.9153** | **-0.3249** | **1** | **3** | **11** | **3** | **K.AEGPDVAVDLPKGDINIEGPSMNIEGPDLNVEGPEGGLK.G** |

| **650.** | P14550\|AK1A1_HUMAN    **Mass:** 36892    **Score:** 65     **Queries matched:** 8   **emPAI:** 0.10 |
| --- | --- |
|  | Alcohol dehydrogenase [NADP+] OS=Homo sapiens GN=AKR1A1 PE=1 SV=3 |

|  | **Query** | **Observed** | **Mr(expt)** | **Mr(calc)** | **Delta** | **Miss** | **Score** | **Expect** | **Rank** | **Peptide** |
| --- | --- | --- | --- | --- | --- | --- | --- | --- | --- | --- |
|  | 11094 | 686.4401 | 1370.8656 | 1369.7751 | 1.0904 | 1 | 2 | 70 | 7 | K.VICIPKSITPSR.I |
|  | 12207 | **478.2987** | **1431.8744** | **1430.7010** | **1.1734** | **0** | **2** | **75** | **8** | **-.MAASCVLLHTGQK.M** |
|  | 12853 | **738.3973** | **1474.7800** | **1474.7892** | **-0.0092** | **0** | **65** | **4.3e-005** | **1** | **K.GLVQALGLSNFNSR.Q** |
|  | 12861 | **738.8140** | **1475.6134** | **1475.6643** | **-0.0509** | **0** | **2** | **88** | **5** | **K.VFDFTFSPEEMK.Q** |
|  | 14008 | 776.3780 | 1550.7414 | 1550.7576 | -0.0162 | 0 | 2 | 73 | 7 | R.GLEVTAYSPLGSSDR.A |
|  | 14649 | 797.8850 | 1593.7553 | 1593.8402 | -0.0849 | 1 | 4 | 50 | 6 | R.EELFVTSKLWNTK.H |
|  | 18175 | 614.2993 | 1839.8760 | 1839.9917 | -0.1157 | 1 | 3 | 49 | 9 | K.MPLIGLGTWKSEPGQVK.A |
|  | 24461 | 830.4434 | 2488.3083 | 2488.3213 | -0.0131 | 1 | 16 | 1.7 | 1 | R.AWRDPDEPVLLEEPVVLALAEK.Y |

| **651.** | P17858\|K6PL_HUMAN    **Mass:** 85762    **Score:** 65     **Queries matched:** 19   **emPAI:** 0.24 |
| --- | --- |
|  | 6-phosphofructokinase, liver type OS=Homo sapiens GN=PFKL PE=1 SV=6 |

|  | **Query** | **Observed** | **Mr(expt)** | **Mr(calc)** | **Delta** | **Miss** | **Score** | **Expect** | **Rank** | **Peptide** |
| --- | --- | --- | --- | --- | --- | --- | --- | --- | --- | --- |
|  | 2135 | 399.3458 | 796.6771 | 796.3901 | 0.2870 | 0 | 3 | 53 | 6 | K.MLAQYR.I |
|  | 5779 | **526.2806** | **1050.5467** | **1050.5532** | **-0.0065** | **0** | **43** | **0.0081** | **1** | **R.TFVLEVMGR.H** |
|  | 6913 | 568.2919 | 1134.5692 | 1134.5782 | -0.0090 | 1 | 25 | 0.57 | 1 | K.RFDEATQLR.G |
|  | 7240 | 581.2970 | 1160.5794 | 1160.6223 | -0.0429 | 1 | 0 | 1.6e+002 | 5 | -.MAAVDLEKLR.A |
|  | 9208 | **634.3881** | **1266.7617** | **1265.6988** | **1.0629** | **1** | **4** | **53** | **6** | **R.LMLKMLAQYR.I** |
|  | 9478 | **641.8407** | **1281.6668** | **1280.6911** | **0.9758** | **1** | **5** | **42** | **1** | **R.AVTRMGIYVGAK.V** |
|  | 9502 | 642.3690 | 1282.7235 | 1281.6937 | 1.0298 | 1 | (4) | 58 | 2 | R.LMLKMLAQYR.I |
|  | 10932 | **454.7176** | **1361.1309** | **1360.6479** | **0.4830** | **1** | **0** | **92** | **5** | **K.VNVEHMTEKMK.T** |
|  | 11932 | 709.8547 | 1417.6948 | 1417.7678 | -0.0730 | 1 | 5 | 39 | 6 | K.DLVVQRLGFDTR.V |
|  | 14168 | **781.4006** | **1560.7867** | **1560.7970** | **-0.0103** | **0** | **29** | **0.16** | **1** | **R.VFANAPDSACVIGLK.K** 14149 14163 |
|  | 15497 | 824.4169 | 1646.8193 | 1645.8199 | 0.9994 | 0 | 25 | 0.37 | 1 | R.SEWGSLLEELVAEGK.I |
|  | 16080 | **563.7526** | **1688.2360** | **1688.8920** | **-0.6559** | **1** | **3** | **37** | **6** | **R.VFANAPDSACVIGLKK.K** |
|  | 16393 | **570.9802** | **1709.9187** | **1709.9788** | **-0.0601** | **1** | **5** | **34** | **3** | **R.GSRLNIIIIAEGAIDR.N** |
|  | 17442 | 894.4522 | 1786.8899 | 1786.8996 | -0.0097 | 0 | 28 | 0.17 | 1 | K.AIGVLTSGGDAQGMNAAVR.A 17440 17441 |
|  | 19168 | **638.6595** | **1912.9566** | **1912.9677** | **-0.0110** | **0** | **21** | **0.83** | **1** | **R.IMEVIDAITTTAQSHQR.T** |

| **652.** | Q6UX53\|MET7B_HUMAN    **Mass:** 28269    **Score:** 65     **Queries matched:** 8   **emPAI:** 0.29 |
| --- | --- |
|  | Methyltransferase-like protein 7B OS=Homo sapiens GN=METTL7B PE=2 SV=2 |

|  | **Query** | **Observed** | **Mr(expt)** | **Mr(calc)** | **Delta** | **Miss** | **Score** | **Expect** | **Rank** | **Peptide** |
| --- | --- | --- | --- | --- | --- | --- | --- | --- | --- | --- |
|  | 2819 | **845.7263** | **844.7190** | **844.4191** | **0.2999** | **0** | **13** | **7.9** | **1** | **R.HLQYER.F** |
|  | 3654 | **449.7035** | **897.3924** | **898.5348** | **-1.1425** | **1** | **6** | **38** | **2** | **K.VLQEVRR.V** |
|  | 8130 | **404.5645** | **1210.6718** | **1211.5968** | **-0.9250** | **1** | **10** | **12** | **3** | **K.FLTKSMAENR.H** 8157 |
|  | 12533 | **728.8433** | **1455.6721** | **1455.6817** | **-0.0096** | **0** | **21** | **0.93** | **1** | **R.VTCLDPNPHFEK.F** |
|  | 13897 | 772.8417 | 1543.6689 | 1544.7949 | -1.1259 | 0 | 6 | 33 | 7 | K.SYFPYLMAVLTPK.S |
|  | 16373 | **855.3886** | **1708.7625** | **1708.7726** | **-0.0101** | **0** | **58** | **0.00018** | **1** | **K.DLENAQFSEIQMER.Q** |
|  | 22639 | **752.0149** | **2253.0228** | **2253.0372** | **-0.0143** | **1** | **30** | **0.086** | **1** | **R.ETWKDLENAQFSEIQMER.Q** |

| **653.** | P11177\|ODPB_HUMAN    **Mass:** 39550    **Score:** 64     **Queries matched:** 8   **emPAI:** 0.09 |
| --- | --- |
|  | Pyruvate dehydrogenase E1 component subunit beta, mitochondrial OS=Homo sapiens GN=PDHB PE=1 SV=3 |

|  | **Query** | **Observed** | **Mr(expt)** | **Mr(calc)** | **Delta** | **Miss** | **Score** | **Expect** | **Rank** | **Peptide** |
| --- | --- | --- | --- | --- | --- | --- | --- | --- | --- | --- |
|  | 1863 | **746.3378** | **745.3305** | **744.4381** | **0.8924** | **0** | **20** | **2.5** | **2** | **R.EVSGLLK.R** |
|  | 3735 | **451.7335** | **901.4524** | **900.5392** | **0.9132** | **1** | **13** | **9.5** | **2** | **R.EVSGLLKR.R** 3727 |
|  | 8500 | **616.0731** | **1230.1317** | **1230.5881** | **-0.4564** | **0** | **3** | **55** | **5** | **K.VVSPWNSEDAK.G** |
|  | 16888 | **874.4409** | **1746.8672** | **1746.8763** | **-0.0091** | **0** | **64** | **4e-005** | **1** | **R.IMEGPAFNFLDAPAVR.V** |
|  | 18345 | **618.0057** | **1850.9952** | **1850.8940** | **0.1012** | **0** | **3** | **49** | **7** | **R.TIRPMDMETIEASVMK.T** |
|  | 20757 | 686.4401 | 2056.2984 | 2055.1616 | 1.1368 | 1 | 5 | 18 | 2 | K.ILEDNSIPQVKDIIFAIK.K |
|  | 31487 | **1284.8645** | **5135.4289** | **5135.4939** | **-0.0650** | **0** | **0** | **13** | **4** | **R.IIDTPISEMGFAGIAVGAAMAGLRPICEFMTFNFSMQAIDQVINSAAK.T** |

| **654.** | O43520\|AT8B1_HUMAN    **Mass:** 144802   **Score:** 64     **Queries matched:** 25   **emPAI:** 0.03 |
| --- | --- |
|  | Probable phospholipid-transporting ATPase IC OS=Homo sapiens GN=ATP8B1 PE=1 SV=3 |

|  | **Query** | **Observed** | **Mr(expt)** | **Mr(calc)** | **Delta** | **Miss** | **Score** | **Expect** | **Rank** | **Peptide** |
| --- | --- | --- | --- | --- | --- | --- | --- | --- | --- | --- |
|  | 1828 | **734.3819** | **733.3746** | **733.4156** | **-0.0410** | **0** | **12** | **15** | **1** | **R.MSIIVR.T** |
|  | 2087 | **397.4031** | **792.7915** | **792.4164** | **0.3752** | **1** | **3** | **57** | **6** | **K.IMKNSGK.T** |
|  | 3554 | 445.7682 | 889.5218 | 889.4943 | 0.0275 | 0 | 13 | 11 | 10 | K.AMVVDLVK.R |
|  | 3704 | 901.4398 | 900.4325 | 901.4505 | -1.0180 | 0 | 6 | 50 | 9 | K.DLVDDVAR.H |
|  | 4373 | **474.2999** | **946.5852** | **945.4304** | **1.1548** | **0** | **7** | **39** | **5** | **K.AEEQWQR.R** |
|  | 5344 | 509.7494 | 1017.4843 | 1018.4865 | -1.0023 | 1 | 9 | 24 | 6 | K.MDKEINNR.T |
|  | 6656 | **558.3320** | **1114.6494** | **1115.5319** | **-0.8825** | **1** | **4** | **65** | **8** | **R.VNREAEENR.E** |
|  | 7149 | **577.3309** | **1152.6472** | **1153.5550** | **-0.9078** | **0** | **5** | **43** | **5** | **K.FMAASVASTNR.D** 7162 |
|  | 7595 | **593.8154** | **1185.6162** | **1185.6241** | **-0.0079** | **0** | **13** | **9.1** | **1** | **K.LQDGVPETISK.L** |
|  | 9807 | **650.3466** | **1298.6786** | **1297.6449** | **1.0337** | **1** | **4** | **52** | **2** | **K.KFMAASVASTNR.D** |
|  | 11078 | **457.7592** | **1370.2558** | **1369.6912** | **0.5647** | **0** | **12** | **6.2** | **1** | **K.TGTLTQNIMTFK.K** 11039 11077 |
|  | 11094 | 686.4401 | 1370.8656 | 1370.7657 | 0.0999 | 0 | 2 | 84 | 9 | K.DLILLGATAIEDK.L |
|  | 13453 | 757.3884 | 1512.7623 | 1511.8725 | 0.8898 | 1 | 9 | 15 | 2 | R.LLLVHGRWSYIR.M 13452 |
|  | 14179 | **781.8989** | **1561.7832** | **1561.7948** | **-0.0116** | **0** | **64** | **4.6e-005** | **1** | **R.TQNTITISELGTER.T** |
|  | 15051 | 540.6008 | 1618.7805 | 1617.9355 | 0.8450 | 1 | 3 | 55 | 7 | K.LSLRFPGLYIVGQR.D |
|  | 16662 | 578.0390 | 1731.0952 | 1730.8873 | 0.2079 | 0 | 6 | 23 | 2 | K.AITLAIGDGANDVNMIK.T |
|  | 17919 | 608.9559 | 1823.8458 | 1824.8676 | -1.0218 | 1 | 1 | 79 | 6 | K.FMAASVASTNRDEALDK.V |
|  | 18201 | 921.9730 | 1841.9314 | 1842.9873 | -1.0559 | 1 | 4 | 47 | 4 | K.KAITLAIGDGANDVNMIK.T |
|  | 18487 | 930.4286 | 1858.8426 | 1858.9822 | -0.1396 | 1 | (3) | 56 | 5 | K.KAITLAIGDGANDVNMIK.T 18491 |
|  | 23852 | **605.0326** | **2416.1015** | **2417.1766** | **-1.0751** | **1** | **1** | **56** | **5** | **K.TYKYNAFTFIPMNLFEQFK.R** |

| **655.** | P23141\|EST1_HUMAN    **Mass:** 62766    **Score:** 64     **Queries matched:** 11   **emPAI:** 0.06 |
| --- | --- |
|  | Liver carboxylesterase 1 OS=Homo sapiens GN=CES1 PE=1 SV=2 |

|  | **Query** | **Observed** | **Mr(expt)** | **Mr(calc)** | **Delta** | **Miss** | **Score** | **Expect** | **Rank** | **Peptide** |
| --- | --- | --- | --- | --- | --- | --- | --- | --- | --- | --- |
|  | 1763 | **714.3738** | **713.3665** | **712.4119** | **0.9546** | **0** | **16** | **4.3** | **6** | **K.ENIPLK.L** 1759 |
|  | 10704 | 674.8421 | 1347.6696 | 1346.6677 | 1.0019 | 1 | 4 | 59 | 3 | K.EGASEEEIRLSK.M |
|  | 11611 | 467.7034 | 1400.0883 | 1399.6893 | 0.3990 | 1 | 7 | 19 | 6 | K.MVMKFWANFAR.N |
|  | 15215 | 815.8573 | 1629.7000 | 1629.8191 | -0.1191 | 0 | 4 | 49 | 5 | R.FTPPQPAEPWSFVK.N |
|  | 17198 | **590.9409** | **1769.8009** | **1769.9346** | **-0.1336** | **0** | **3** | **55** | **5** | **K.GDVKPLAEQIAITAGCK.T** |
|  | 17980 | **914.5158** | **1827.0171** | **1826.0223** | **0.9948** | **0** | **(1)** | **71** | **1** | **R.ESQPLLGTVIDGMLLLK.T** |
|  | 18085 | **459.2393** | **1832.9282** | **1831.9792** | **0.9490** | **1** | **10** | **12** | **3** | **K.EGYLQIGANTQAAQKLK.D** |
|  | 18196 | 461.4692 | 1841.8475 | 1842.0172 | -0.1697 | 0 | 5 | 34 | 2 | R.ESQPLLGTVIDGMLLLK.T |
|  | 30037 | **1263.9778** | **3788.9115** | **3788.9261** | **-0.0146** | **0** | **50** | **0.00031** | **1** | **R.WVQDNIASFGGNPGSVTIFGESAGGESVSVLVLSPLAK.N** 30038 |

| **656.** | Q14165\|MLEC_HUMAN    **Mass:** 32385    **Score:** 64     **Queries matched:** 7   **emPAI:** 0.12 |
| --- | --- |
|  | Malectin OS=Homo sapiens GN=MLEC PE=1 SV=1 |

|  | **Query** | **Observed** | **Mr(expt)** | **Mr(calc)** | **Delta** | **Miss** | **Score** | **Expect** | **Rank** | **Peptide** |
| --- | --- | --- | --- | --- | --- | --- | --- | --- | --- | --- |
|  | 3183 | 433.7496 | 865.4847 | 864.5181 | 0.9666 | 0 | 11 | 14 | 2 | R.LNGHVVVK.D |
|  | 3580 | 446.7269 | 891.4392 | 892.4290 | -0.9898 | 0 | 8 | 27 | 7 | K.DLDIFDR.V |
|  | 14628 | **796.8754** | **1591.7362** | **1591.7478** | **-0.0116** | **0** | **64** | **4.6e-005** | **1** | **R.SNPEDQILYQTER.Y** |
|  | 16925 | 584.3040 | 1749.8901 | 1750.8634 | -0.9733 | 0 | 5 | 36 | 10 | K.VCALYIMAGTVDDVPK.L |
|  | 17175 | **590.3254** | **1767.9543** | **1766.8583** | **1.0960** | **0** | **(4)** | **46** | **5** | **K.VCALYIMAGTVDDVPK.L** |
|  | 29687 | **1221.8678** | **3662.5816** | **3662.7976** | **-0.2160** | **0** | **2** | **20** | **8** | **R.TPNPYASDNSSLMFPILVAFGVFIPTLFCLCR.L** 29686 |

| **657.** | P32119\|PRDX2_HUMAN    **Mass:** 22049    **Score:** 64     **Queries matched:** 7   **emPAI:** 0.61 |
| --- | --- |
|  | Peroxiredoxin-2 OS=Homo sapiens GN=PRDX2 PE=1 SV=5 |

|  | **Query** | **Observed** | **Mr(expt)** | **Mr(calc)** | **Delta** | **Miss** | **Score** | **Expect** | **Rank** | **Peptide** |
| --- | --- | --- | --- | --- | --- | --- | --- | --- | --- | --- |
|  | 3083 | **862.4973** | **861.4900** | **861.4960** | **-0.0060** | **0** | **37** | **0.037** | **1** | **R.GLFIIDGK.G** 3084 |
|  | 4036 | **462.7217** | **923.4287** | **923.4348** | **-0.0061** | **0** | **11** | **12** | **2** | **K.TDEGIAYR.G** |
|  | 4717 | **487.2585** | **972.5025** | **971.5440** | **0.9585** | **0** | **16** | **4.4** | **1** | **R.IGKPAPDFK.A** |
|  | 5399 | **512.2680** | **1022.5215** | **1022.5284** | **-0.0069** | **0** | **24** | **0.64** | **1** | **R.LSEDYGVLK.T** |
|  | 7486 | **590.3179** | **1178.6212** | **1178.6295** | **-0.0083** | **1** | **21** | **1.2** | **1** | **R.RLSEDYGVLK.T** |
|  | 16722 | **868.4882** | **1734.9619** | **1733.9676** | **0.9943** | **0** | **53** | **0.0005** | **1** | **K.EGGLGPLNIPLLADVTR.R** |

| **658.** | Q06210\|GFPT1_HUMAN    **Mass:** 79555    **Score:** 64     **Queries matched:** 17   **emPAI:** 0.10 |
| --- | --- |
|  | Glucosamine--fructose-6-phosphate aminotransferase [isomerizing] 1 OS=Homo sapiens GN=GFPT1 PE=1 SV=3 |

|  | **Query** | **Observed** | **Mr(expt)** | **Mr(calc)** | **Delta** | **Miss** | **Score** | **Expect** | **Rank** | **Peptide** |
| --- | --- | --- | --- | --- | --- | --- | --- | --- | --- | --- |
|  | 4144 | 466.7147 | 931.4149 | 930.5572 | 0.8578 | 1 | (10) | 18 | 4 | R.KEIMLGLK.R |
|  | 4377 | 474.3256 | 946.6366 | 946.5521 | 0.0845 | 1 | 11 | 13 | 3 | R.KEIMLGLK.R 4373 |
|  | 7467 | 589.5409 | 1177.0672 | 1177.5826 | -0.5154 | 1 | 1 | 78 | 4 | K.EDTETIKNTK.R |
|  | 8221 | **406.2062** | **1215.5969** | **1215.6905** | **-0.0936** | **0** | **6** | **41** | **7** | **K.LMPVIMIIMR.D** 8226 |
|  | 8259 | 406.6998 | 1217.0776 | 1216.5910 | 0.4866 | 1 | 4 | 42 | 5 | K.LVKYMYDNR.E |
|  | 13438 | 756.8572 | 1511.6999 | 1511.7104 | -0.0104 | 0 | 44 | 0.0053 | 1 | R.ESQDTSFTTLVER.V 13441 |
|  | 15951 | 839.9032 | 1677.7918 | 1677.8032 | -0.0114 | 0 | 44 | 0.0047 | 1 | K.EIFEQPESVVNTMR.G |
|  | 16141 | 565.3140 | 1692.9201 | 1693.7981 | -0.8781 | 0 | (5) | 31 | 2 | K.EIFEQPESVVNTMR.G 16142 |
|  | 16560 | **575.2841** | **1722.8305** | **1722.8763** | **-0.0458** | **1** | **2** | **73** | **4** | **R.GYHYATCLEGALKIK.E** |
|  | 17590 | **600.6201** | **1798.8384** | **1798.8499** | **-0.0116** | **0** | **21** | **0.74** | **1** | **R.WATHGEPSPVNSHPQR.S** |
|  | 19973 | **663.0244** | **1986.0514** | **1985.0391** | **1.0123** | **1** | **0** | **86** | **5** | **R.LPDLIKEVLSMDDEIQK.L** |
|  | 19990 | 663.6573 | 1987.9502 | 1987.0044 | 0.9458 | 1 | 0 | 83 | 4 | R.QGRPVVICDKEDTETIK.N |
|  | 24829 | **849.4366** | **2545.2879** | **2545.2985** | **-0.0106** | **0** | **5** | **23** | **1** | **R.QVLEELTELPVMVELASDFLDR.N** |

| **659.** | Q9Y4P3\|TBL2_HUMAN    **Mass:** 50393    **Score:** 64     **Queries matched:** 21   **emPAI:** 0.07 |
| --- | --- |
|  | Transducin beta-like protein 2 OS=Homo sapiens GN=TBL2 PE=1 SV=1 |

|  | **Query** | **Observed** | **Mr(expt)** | **Mr(calc)** | **Delta** | **Miss** | **Score** | **Expect** | **Rank** | **Peptide** |
| --- | --- | --- | --- | --- | --- | --- | --- | --- | --- | --- |
|  | 663 | **465.2363** | **464.2290** | **464.2231** | **0.0060** | **0** | **10** | **13** | **1** | **K.SSGSK.K** |
|  | 1106 | **561.2989** | **560.2917** | **560.2554** | **0.0363** | **0** | **8** | **47** | **4** | **R.AGEER.S** |
|  | 2594 | **415.8948** | **829.7751** | **830.3817** | **-0.6066** | **1** | **3** | **78** | **3** | **R.EHRSMR.A** |
|  | 8094 | **605.5481** | **1209.0816** | **1208.5860** | **0.4957** | **1** | **0** | **87** | **7** | **R.MASVSKDGTWK.L** |
|  | 10317 | **443.0380** | **1326.0922** | **1326.6279** | **-0.5356** | **0** | **8** | **15** | **5** | **R.FVASCGFTPDVK.V** 10341 |
|  | 11669 | 468.9109 | 1403.7109 | 1403.7534 | -0.0425 | 1 | 3 | 73 | 10 | R.AVRLFHNTPGHR.A |
|  | 13671 | **764.9148** | **1527.8150** | **1527.8257** | **-0.0106** | **0** | **64** | **5.5e-005** | **1** | **R.LQQQLTQAQETLK.S** |
|  | 19357 | **643.9482** | **1928.8227** | **1927.9965** | **0.8262** | **0** | **3** | **50** | **6** | **K.FIMTASSDTTVLIWSLK.G** |
|  | 19373 | 644.3365 | 1929.9876 | 1929.8745 | 0.1132 | 1 | 8 | 17 | 1 | K.REDGGYTFTATPEDFPK.K |
|  | 21168 | **700.3493** | **2098.0261** | **2097.1793** | **0.8467** | **1** | **5** | **31** | **2** | **R.LQQQLTQAQETLKSLGALK.K** |
|  | 21207 | 1052.0740 | 2102.1334 | 2101.1544 | 0.9789 | 1 | 5 | 28 | 4 | K.EKPQQHNFTHRLLAAALK.S 21206 |
|  | 23940 | **808.5825** | **2422.7256** | **2423.1573** | **-0.4318** | **1** | **5** | **13** | **1** | **R.LLAAALKSHSGNISCMDFSSNGK.Y** 23937 23938 |
|  | 24542 | **832.8772** | **2495.6098** | **2495.3496** | **0.2602** | **0** | **9** | **5.9** | **1** | **R.LALSPNAQVLALASGSSIHLYNTR.R** |
|  | 26766 | **963.2483** | **2886.7230** | **2887.5584** | **-0.8354** | **0** | **3** | **24** | **1** | **M.ELSQMSELMGLSVLLGLLALMATAAVAR.G** |
|  | 26932 | **973.8406** | **2918.4999** | **2919.5483** | **-1.0484** | **0** | **(0)** | **52** | **1** | **M.ELSQMSELMGLSVLLGLLALMATAAVAR.G** |
|  | 27608 | 1018.1352 | 3051.3837 | 3050.5888 | 0.7950 | 0 | 7 | 9.9 | 1 | -.MELSQMSELMGLSVLLGLLALMATAAVAR.G |
|  | 27664 | 767.3841 | 3065.5073 | 3066.5837 | -1.0764 | 0 | (0) | 49 | 3 | -.MELSQMSELMGLSVLLGLLALMATAAVAR.G |

| **660.** | P62910\|RL32_HUMAN    **Mass:** 15964    **Score:** 63     **Queries matched:** 5   **emPAI:** 0.24 |
| --- | --- |
|  | 60S ribosomal protein L32 OS=Homo sapiens GN=RPL32 PE=1 SV=2 |

|  | **Query** | **Observed** | **Mr(expt)** | **Mr(calc)** | **Delta** | **Miss** | **Score** | **Expect** | **Rank** | **Peptide** |
| --- | --- | --- | --- | --- | --- | --- | --- | --- | --- | --- |
|  | 2981 | 428.7455 | 855.4765 | 855.4967 | -0.0202 | 0 | 6 | 38 | 4 | K.FLVHNVK.E |
|  | 12692 | 733.3355 | 1464.6565 | 1464.6667 | -0.0102 | 0 | 63 | 5.5e-005 | 1 | K.SYCAEIAHNVSSK.N |
|  | 13129 | **497.9374** | **1490.7903** | **1490.7551** | **0.0352** | **0** | **4** | **48** | **10** | **K.GQILMPNIGYGSNK.K** |
|  | 15052 | **540.6202** | **1618.8387** | **1618.8501** | **-0.0114** | **1** | **10** | **13** | **1** | **K.GQILMPNIGYGSNKK.T** |
|  | 17138 | **589.3066** | **1764.8979** | **1765.9185** | **-1.0206** | **1** | **1** | **80** | **3** | **R.FKGQILMPNIGYGSNK.K** |

| **661.** | Q96GE6\|CALL4_HUMAN    **Mass:** 21983    **Score:** 63     **Queries matched:** 13   **emPAI:** 0.17 |
| --- | --- |
|  | Calmodulin-like protein 4 OS=Homo sapiens GN=CALML4 PE=2 SV=3 |

|  | **Query** | **Observed** | **Mr(expt)** | **Mr(calc)** | **Delta** | **Miss** | **Score** | **Expect** | **Rank** | **Peptide** |
| --- | --- | --- | --- | --- | --- | --- | --- | --- | --- | --- |
|  | 4568 | **481.2868** | **960.5590** | **961.5444** | **-0.9854** | **1** | **5** | **62** | **8** | **R.SKLTSLGEK.L** |
|  | 7177 | **578.8029** | **1155.5912** | **1154.5754** | **1.0158** | **1** | **10** | **17** | **1** | **K.KGYVMASDLR.S** |
|  | 9352 | 638.3206 | 1274.6266 | 1274.6978 | -0.0712 | 0 | 6 | 36 | 5 | K.EILLAMLMVDK.E 9383 |
|  | 11105 | 686.8668 | 1371.7190 | 1370.6613 | 1.0577 | 0 | 0 | 1.4e+002 | 7 | R.CLGASPTPGEVQR.H |
|  | 11944 | **710.3181** | **1418.6217** | **1418.7877** | **-0.1660** | **1** | **(2)** | **81** | **8** | **K.KEILLAMLMVDK.E** |
|  | 11984 | 474.3256 | 1419.9549 | 1418.7877 | 1.1673 | 1 | 6 | 22 | 7 | K.KEILLAMLMVDK.E |
|  | 13991 | **517.3307** | **1548.9702** | **1549.7445** | **-0.7742** | **1** | **10** | **11** | **4** | **K.GTERGSGSSKPTGSSR.G** |
|  | 16450 | **572.3729** | **1714.0968** | **1713.8396** | **0.2572** | **1** | **5** | **26** | **3** | **R.MAKFLSQDQINEYK.E** 16442 |
|  | 27719 | **771.6285** | **3082.4851** | **3082.5005** | **-0.0154** | **0** | **63** | **2.3e-005** | **1** | **R.HLQTHGIDGNGELDFSTFLTIMHMQIK.Q** 27721 |
|  | 27831 | **1039.5201** | **3115.5386** | **3114.4903** | **1.0483** | **0** | **(0)** | **49** | **1** | **R.HLQTHGIDGNGELDFSTFLTIMHMQIK.Q** |

| **662.** | P14174\|MIF_HUMAN    **Mass:** 12639    **Score:** 63     **Queries matched:** 3   **emPAI:** 0.31 |
| --- | --- |
|  | Macrophage migration inhibitory factor OS=Homo sapiens GN=MIF PE=1 SV=4 |

|  | **Query** | **Observed** | **Mr(expt)** | **Mr(calc)** | **Delta** | **Miss** | **Score** | **Expect** | **Rank** | **Peptide** |
| --- | --- | --- | --- | --- | --- | --- | --- | --- | --- | --- |
|  | 9585 | **644.8454** | **1287.6762** | **1286.6805** | **0.9957** | **0** | **46** | **0.0036** | **1** | **M.PMFIVNTNVPR.A** 9568 |
|  | 31939 | **1162.5806** | **5807.8665** | **5806.8797** | **0.9867** | **0** | **8** | **1.2** | **1** | **R.ASVPDGFLSELTQQLAQATGKPPQYIAVHVVPDQLMAFGGSSEPCALCSLHSIGK.I** |

| **663.** | P00918\|CAH2_HUMAN    **Mass:** 29285    **Score:** 63     **Queries matched:** 6   **emPAI:** 0.28 |
| --- | --- |
|  | Carbonic anhydrase 2 OS=Homo sapiens GN=CA2 PE=1 SV=2 |

|  | **Query** | **Observed** | **Mr(expt)** | **Mr(calc)** | **Delta** | **Miss** | **Score** | **Expect** | **Rank** | **Peptide** |
| --- | --- | --- | --- | --- | --- | --- | --- | --- | --- | --- |
|  | 1662 | 687.3984 | 686.3911 | 685.3071 | 1.0840 | 0 | 11 | 17 | 5 | K.YGDFGK.A 1661 |
|  | 2461 | **411.7314** | **821.4483** | **820.4807** | **0.9676** | **1** | **8** | **26** | **10** | **R.QIKASFK.-** |
|  | 4184 | 468.2295 | 934.4444 | 934.4509 | -0.0065 | 0 | 28 | 0.3 | 1 | K.GGPLDGTYR.L |
|  | 4882 | 492.7916 | 983.5686 | 983.5764 | -0.0077 | 0 | 32 | 0.094 | 1 | K.VGSAKPGLQK.V |
|  | 15775 | 834.9836 | 1667.9526 | 1667.9610 | -0.0084 | 0 | 56 | 0.00025 | 1 | K.AVQQPDGLAVLGIFLK.V |

| **664.** | P35558\|PCKGC_HUMAN    **Mass:** 69948    **Score:** 63     **Queries matched:** 15   **emPAI:** 0.05 |
| --- | --- |
|  | Phosphoenolpyruvate carboxykinase, cytosolic [GTP] OS=Homo sapiens GN=PCK1 PE=1 SV=3 |

|  | **Query** | **Observed** | **Mr(expt)** | **Mr(calc)** | **Delta** | **Miss** | **Score** | **Expect** | **Rank** | **Peptide** |
| --- | --- | --- | --- | --- | --- | --- | --- | --- | --- | --- |
|  | 2053 | **396.2133** | **790.4120** | **791.4323** | **-1.0204** | **1** | **9** | **24** | **5** | **R.MASRLAK.E** |
|  | 4283 | **471.7947** | **941.5749** | **942.4672** | **-0.8923** | **0** | **7** | **33** | **4** | **K.FDAQGHLR.A** |
|  | 6068 | 536.7107 | 1071.4069 | 1072.5877 | -1.1807 | 0 | 15 | 5.2 | 1 | K.TVIVTQEQR.D |
|  | 8937 | 628.2986 | 1254.5827 | 1254.6067 | -0.0239 | 0 | 7 | 28 | 4 | K.YLAAAFPSACGK.T |
|  | 11645 | **702.0460** | **1402.0775** | **1402.7126** | **-0.6351** | **0** | **8** | **15** | **1** | **R.LLGQMEEEGILR.R** |
|  | 13620 | **509.2384** | **1524.6935** | **1525.7922** | **-1.0988** | **0** | **6** | **33** | **2** | **-.MPPQLQNGLNLSAK.V** |
|  | 14637 | **796.9055** | **1591.7964** | **1590.7534** | **1.0430** | **0** | **14** | **4.3** | **2** | **K.TNLAMMNPSLPGWK.V** |
|  | 15097 | **541.9232** | **1622.7478** | **1622.7841** | **-0.0363** | **1** | **1** | **98** | **6** | **K.EGKFLWPGFGENSR.V** |
|  | 16210 | 849.9329 | 1697.8513 | 1697.8624 | -0.0112 | 0 | 63 | 5.5e-005 | 1 | R.EIISFGSGYGGNSLLGK.K |
|  | 16355 | 570.2845 | 1707.8316 | 1707.8324 | -0.0008 | 0 | 1 | 90 | 10 | K.GLGHINMMELFSISK.E |
|  | 16913 | 875.8752 | 1749.7358 | 1749.8971 | -0.1613 | 0 | 24 | 0.37 | 2 | K.IGIELTDSPYVVASMR.I |
|  | 17951 | 609.3290 | 1824.9653 | 1825.9574 | -0.9921 | 1 | 1 | 79 | 2 | R.EIISFGSGYGGNSLLGKK.C 17940 |
|  | 19466 | **647.3364** | **1938.9873** | **1939.9787** | **-0.9915** | **0** | **1** | **74** | **5** | **R.TMYVIPFSMGPLGSPLSK.I** |
|  | 23193 | 1161.0123 | 2320.0101 | 2319.0969 | 0.9132 | 0 | 1 | 50 | 4 | K.IIMHDPFAMRPFFGYNFGK.Y |

| **665.** | O95837\|GNA14_HUMAN    **Mass:** 42000    **Score:** 63     **Queries matched:** 5   **emPAI:** 0.09 |
| --- | --- |
|  | Guanine nucleotide-binding protein subunit alpha-14 OS=Homo sapiens GN=GNA14 PE=2 SV=1 |

|  | **Query** | **Observed** | **Mr(expt)** | **Mr(calc)** | **Delta** | **Miss** | **Score** | **Expect** | **Rank** | **Peptide** |
| --- | --- | --- | --- | --- | --- | --- | --- | --- | --- | --- |
|  | 3054 | 431.2132 | 860.4118 | 860.4175 | -0.0057 | 0 | 22 | 1.5 | 1 | R.MVDVGGQR.S |
|  | 11224 | 460.2559 | 1377.7458 | 1376.6572 | 1.0887 | 1 | 6 | 34 | 4 | K.LYQDQNPDKEK.V |
|  | 11857 | 707.6946 | 1413.3746 | 1414.5349 | -1.1603 | 0 | 0 | 85 | 10 | -.MAGCCCLSAEEK.E |
|  | 12551 | 486.3614 | 1456.0624 | 1456.8501 | -0.7876 | 1 | 8 | 13 | 3 | R.ELKLLLLGTGESGK.S |
|  | 17583 | 899.9674 | 1797.9203 | 1797.9270 | -0.0067 | 0 | 63 | 5.2e-005 | 1 | K.LVYQNIFTAMQAMIR.A |

| **666.** | P03891\|NU2M_HUMAN    **Mass:** 38935    **Score:** 63     **Queries matched:** 4   **emPAI:** 0.10 |
| --- | --- |
|  | NADH-ubiquinone oxidoreductase chain 2 OS=Homo sapiens GN=MT-ND2 PE=1 SV=2 |

|  | **Query** | **Observed** | **Mr(expt)** | **Mr(calc)** | **Delta** | **Miss** | **Score** | **Expect** | **Rank** | **Peptide** |
| --- | --- | --- | --- | --- | --- | --- | --- | --- | --- | --- |
|  | 19113 | **955.4114** | **1908.8083** | **1909.0230** | **-0.2147** | **0** | **0** | **83** | **3** | **R.LIYSTSITLLPMSNNVK.M** |
|  | 22061 | **1092.9747** | **2183.9349** | **2184.1534** | **-0.2185** | **1** | **1** | **61** | **2** | **R.LIYSTSITLLPMSNNVKMK.W** |
|  | 26547 | **1423.2368** | **2844.4591** | **2844.6769** | **-0.2178** | **0** | **46** | **0.0014** | **1** | **K.LTWLTPLIPSTLLSLGGLPPLTGFLPK.W** 26549 |

| **667.** | P30050\|RL12_HUMAN    **Mass:** 17979    **Score:** 63     **Queries matched:** 4   **emPAI:** 0.21 |
| --- | --- |
|  | 60S ribosomal protein L12 OS=Homo sapiens GN=RPL12 PE=1 SV=1 |

|  | **Query** | **Observed** | **Mr(expt)** | **Mr(calc)** | **Delta** | **Miss** | **Score** | **Expect** | **Rank** | **Peptide** |
| --- | --- | --- | --- | --- | --- | --- | --- | --- | --- | --- |
|  | 11172 | **688.3753** | **1374.7361** | **1374.6779** | **0.0581** | **1** | **2** | **86** | **8** | **K.VGDDIAKATGDWK.G** |
|  | 15885 | **838.4017** | **1674.7889** | **1674.7995** | **-0.0106** | **0** | **63** | **6.1e-005** | **1** | **K.EILGTAQSVGCNVDGR.H** |
|  | 20638 | **683.0048** | **2045.9925** | **2045.9113** | **0.0812** | **0** | **4** | **39** | **5** | **R.HPHDIIDDINSGAVECPAS.-** |
|  | 23702 | 797.7421 | 2390.2046 | 2391.3849 | -1.1803 | 1 | 2 | 43 | 6 | K.LTIQNRQAQIEVVPSASALIIK.A |

| **668.** | P14927\|QCR7_HUMAN    **Mass:** 13522    **Score:** 63     **Queries matched:** 13   **emPAI:** 0.66 |
| --- | --- |
|  | Cytochrome b-c1 complex subunit 7 OS=Homo sapiens GN=UQCRB PE=1 SV=2 |

|  | **Query** | **Observed** | **Mr(expt)** | **Mr(calc)** | **Delta** | **Miss** | **Score** | **Expect** | **Rank** | **Peptide** |
| --- | --- | --- | --- | --- | --- | --- | --- | --- | --- | --- |
|  | 1832 | **735.3649** | **734.3576** | **734.4439** | **-0.0863** | **0** | **14** | **8.1** | **1** | **K.HQILPK.E** 1836 |
|  | 1916 | 759.4308 | 758.4235 | 758.4075 | 0.0160 | 0 | 7 | 45 | 7 | K.WLDGIR.K |
|  | 4309 | 472.2871 | 942.5595 | 941.5658 | 0.9938 | 1 | 13 | 8.6 | 2 | K.RALDLNLK.H 4308 |
|  | 6876 | **567.2664** | **1132.5183** | **1133.5863** | **-1.0680** | **1** | **10** | **18** | **2** | **-.MAGKQAVSASGK.W** 6878 6893 6895 |
|  | 8544 | 617.2843 | 1232.5540 | 1232.5614 | -0.0074 | 0 | 32 | 0.089 | 1 | K.WYYNAAGFNK.L |
|  | 13031 | **743.8338** | **1485.6530** | **1486.7892** | **-1.1362** | **1** | **3** | **58** | **5** | **K.QAVSASGKWLDGIR.K** |
|  | 17722 | **905.9151** | **1809.8156** | **1809.8268** | **-0.0112** | **1** | **54** | **0.00038** | **1** | **R.DDTIYEDEDVKEAIR.R** 17723 |

| **669.** | Q9H115\|SNAB_HUMAN    **Mass:** 33878    **Score:** 62     **Queries matched:** 7   **emPAI:** 0.11 |
| --- | --- |
|  | Beta-soluble NSF attachment protein OS=Homo sapiens GN=NAPB PE=1 SV=2 |

|  | **Query** | **Observed** | **Mr(expt)** | **Mr(calc)** | **Delta** | **Miss** | **Score** | **Expect** | **Rank** | **Peptide** |
| --- | --- | --- | --- | --- | --- | --- | --- | --- | --- | --- |
|  | 815 | **504.0523** | **503.0450** | **503.2340** | **-0.1889** | **0** | **6** | **82** | **4** | **M.DNAGK.E** |
|  | 3988 | **460.5718** | **919.1291** | **919.4181** | **-0.2891** | **1** | **7** | **26** | **7** | **-.MDNAGKER.E** |
|  | 4890 | **493.2841** | **984.5536** | **983.5222** | **1.0314** | **0** | **11** | **13** | **2** | **K.LHMQLQSK.H** |
|  | 9647 | **645.8524** | **1289.6903** | **1289.5735** | **0.1167** | **0** | **5** | **44** | **2** | **K.SIQGDGEGDGDLK.-** |
|  | 11958 | **710.3740** | **1418.7334** | **1417.6685** | **1.0649** | **1** | **2** | **90** | **5** | **K.KSIQGDGEGDGDLK.-** |
|  | 21465 | **707.6946** | **2120.0619** | **2118.9463** | **1.1157** | **1** | **1** | **72** | **4** | **R.GLFGGNTRIEEACEMYTR.A** |
|  | 25031 | 854.7107 | 2561.1102 | 2561.1306 | -0.0203 | 1 | 62 | 3.6e-005 | 1 | K.AIAHYEQSADYYKGEESNSSANK.C |

| **670.** | Q6DD88\|ATLA3_HUMAN    **Mass:** 60960    **Score:** 62     **Queries matched:** 8   **emPAI:** 0.06 |
| --- | --- |
|  | Atlastin-3 OS=Homo sapiens GN=ATL3 PE=1 SV=1 |

|  | **Query** | **Observed** | **Mr(expt)** | **Mr(calc)** | **Delta** | **Miss** | **Score** | **Expect** | **Rank** | **Peptide** |
| --- | --- | --- | --- | --- | --- | --- | --- | --- | --- | --- |
|  | 989 | **537.2993** | **536.2920** | **535.2788** | **1.0132** | **1** | **6** | **42** | **3** | **K.KMGGK.D** |
|  | 6468 | **550.8159** | **1099.6172** | **1098.6186** | **0.9986** | **1** | **4** | **66** | **9** | **K.QLALDHFKK.T** |
|  | 11149 | 687.8417 | 1373.6688 | 1372.7245 | 0.9443 | 1 | 4 | 51 | 3 | -.MLSPQRVAAAASR.G |
|  | 14346 | **787.3790** | **1572.7434** | **1573.8828** | **-1.1394** | **1** | **2** | **90** | **4** | **R.DLDVVVVSVAGAFRK.G** |
|  | 18063 | **916.9559** | **1831.8973** | **1831.9097** | **-0.0124** | **0** | **62** | **6e-005** | **1** | **K.SMLQATAEANNLAAAASAK.D** |
|  | 18296 | **616.9923** | **1847.9551** | **1847.9047** | **0.0504** | **0** | **(1)** | **89** | **1** | **K.SMLQATAEANNLAAAASAK.D** |
|  | 23362 | 586.7835 | 2343.1049 | 2342.2167 | 0.8883 | 0 | 7 | 15 | 5 | R.LAMDEIFQKPFQTLMFLVR.D |
|  | 25318 | **874.0991** | **2619.2754** | **2618.2864** | **0.9889** | **0** | **10** | **6.1** | **1** | **R.GGSDPETTGIQIWSEVFTVEKPGGK.K** |

| **671.** | Q96QV6\|H2A1A_HUMAN    **Mass:** 14225    **Score:** 62     **Queries matched:** 15   **emPAI:** 1.08 |
| --- | --- |
|  | Histone H2A type 1-A OS=Homo sapiens GN=HIST1H2AA PE=1 SV=3 |

|  | **Query** | **Observed** | **Mr(expt)** | **Mr(calc)** | **Delta** | **Miss** | **Score** | **Expect** | **Rank** | **Peptide** |
| --- | --- | --- | --- | --- | --- | --- | --- | --- | --- | --- |
|  | 820 | 505.2373 | 504.2301 | 503.2816 | 0.9485 | 1 | 3 | 1.5e+002 | 7 | M.SGRGK.Q |
|  | 2684 | 419.2129 | 836.4112 | 836.4140 | -0.0028 | 1 | 16 | 4.2 | 1 | R.KGNYAER.I 2697 2698 2699 2716 |
|  | 2897 | 425.7636 | 849.5127 | 849.5184 | -0.0058 | 0 | 32 | 0.13 | 1 | R.HLQLAIR.N 2888 2898 2899 2901 |
|  | 4323 | 472.7661 | 943.5175 | 943.5240 | -0.0064 | 0 | 53 | 0.00095 | 1 | R.AGLQFPVGR.I 4330 |
|  | 16139 | 424.2194 | 1692.8486 | 1691.8954 | 0.9531 | 1 | 12 | 7.5 | 2 | R.HLQLAIRNDEELNK.L |
|  | 22792 | 757.7943 | 2270.3611 | 2270.3726 | -0.0115 | 0 | 31 | 0.048 | 1 | K.LLGGVTIAQGGVLPNIQAVLLPK.K |

| **672.** | P02647\|APOA1_HUMAN    **Mass:** 30759    **Score:** 62     **Queries matched:** 10   **emPAI:** 0.26 |
| --- | --- |
|  | Apolipoprotein A-I OS=Homo sapiens GN=APOA1 PE=1 SV=1 |

|  | **Query** | **Observed** | **Mr(expt)** | **Mr(calc)** | **Delta** | **Miss** | **Score** | **Expect** | **Rank** | **Peptide** |
| --- | --- | --- | --- | --- | --- | --- | --- | --- | --- | --- |
|  | 1311 | **604.3369** | **603.3296** | **602.2772** | **1.0524** | **0** | **21** | **2.4** | **1** | **K.ENGGAR.L** |
|  | 1761 | 714.3268 | 713.3196 | 713.4296 | -0.1101 | 1 | 15 | 6.3 | 8 | R.QRLAAR.L |
|  | 5261 | **506.7239** | **1011.4333** | **1011.5713** | **-0.1380** | **0** | **7** | **29** | **8** | **K.AKPALEDLR.Q** |
|  | 8897 | **626.8098** | **1251.6049** | **1251.6136** | **-0.0086** | **0** | **9** | **16** | **3** | **K.VQPYLDDFQK.K** |
|  | 9486 | **642.2856** | **1282.5567** | **1282.5652** | **-0.0085** | **0** | **13** | **7.1** | **1** | **K.WQEEMELYR.Q** |
|  | 9847 | 651.7933 | 1301.5721 | 1300.6411 | 0.9310 | 0 | 22 | 0.91 | 2 | R.THLAPYSDELR.Q |
|  | 11368 | 693.8577 | 1385.7008 | 1385.7078 | -0.0070 | 0 | 35 | 0.04 | 1 | K.VSFLSALEEYTK.K |
|  | 11871 | 472.2633 | 1413.7681 | 1414.6953 | -0.9272 | 1 | 8 | 22 | 4 | K.ENGGARLAEYHAK.A |
|  | 14906 | **806.8904** | **1611.7662** | **1611.7781** | **-0.0119** | **0** | **47** | **0.0022** | **1** | **K.LLDNWDSVTSTFSK.L** |
|  | 20008 | 664.2819 | 1989.8239 | 1989.1445 | 0.6794 | 1 | 6 | 19 | 1 | -.MKAAVLTLAVLFLTGSQAR.H |

| **673.** | P51648\|AL3A2_HUMAN    **Mass:** 55269    **Score:** 62     **Queries matched:** 8   **emPAI:** 0.14 |
| --- | --- |
|  | Fatty aldehyde dehydrogenase OS=Homo sapiens GN=ALDH3A2 PE=1 SV=1 |

|  | **Query** | **Observed** | **Mr(expt)** | **Mr(calc)** | **Delta** | **Miss** | **Score** | **Expect** | **Rank** | **Peptide** |
| --- | --- | --- | --- | --- | --- | --- | --- | --- | --- | --- |
|  | 4014 | **461.2706** | **920.5267** | **919.4399** | **1.0868** | **0** | **7** | **33** | **7** | **R.YPPNSQSK.V** 4004 |
|  | 9147 | 632.8627 | 1263.7109 | 1263.7187 | -0.0078 | 0 | 20 | 1.5 | 1 | K.HLTPVTLELGGK.S |
|  | 12214 | **717.3498** | **1432.6851** | **1432.6946** | **-0.0095** | **0** | **49** | **0.0019** | **1** | **K.NVDEAINFINER.E** |
|  | 14291 | **523.9250** | **1568.7531** | **1568.7624** | **-0.0092** | **0** | **34** | **0.046** | **1** | **R.FDHIFYTGNTAVGK.I** |
|  | 21134 | **698.9492** | **2093.8257** | **2093.1554** | **0.6702** | **1** | **0** | **72** | **3** | **K.IVMEAAAKHLTPVTLELGGK.S** |
|  | 21331 | 704.3194 | 2109.9364 | 2109.3541 | 0.5823 | 1 | 2 | 52 | 5 | K.EKLGLLLLTFLGIVAAVLVK.A |
|  | 21516 | **709.8988** | **2126.6746** | **2127.1034** | **-0.4289** | **1** | **4** | **23** | **2** | **K.HLTPVTLELGGKSPCYIDK.D** |

| **674.** | Q9H6S3\|ES8L2_HUMAN    **Mass:** 81197    **Score:** 62     **Queries matched:** 13   **emPAI:** 0.05 |
| --- | --- |
|  | Epidermal growth factor receptor kinase substrate 8-like protein 2 OS=Homo sapiens GN=EPS8L2 PE=1 SV=2 |

|  | **Query** | **Observed** | **Mr(expt)** | **Mr(calc)** | **Delta** | **Miss** | **Score** | **Expect** | **Rank** | **Peptide** |
| --- | --- | --- | --- | --- | --- | --- | --- | --- | --- | --- |
|  | 1420 | **630.2796** | **629.2723** | **628.3908** | **0.8815** | **0** | **5** | **75** | **2** | **R.AVLAQK.I** |
|  | 1524 | 651.1976 | 650.1903 | 649.3911 | 0.7992 | 0 | 30 | 0.17 | 1 | R.GHLVPK.E |
|  | 2661 | **418.2348** | **834.4550** | **834.4236** | **0.0315** | **0** | **6** | **52** | **6** | **R.DAVDFLR.G** |
|  | 3196 | **434.2388** | **866.4630** | **866.4722** | **-0.0092** | **1** | **6** | **39** | **8** | **K.GHQEKIR.Q** |
|  | 3330 | **438.2146** | **874.4146** | **873.3828** | **1.0319** | **0** | **12** | **13** | **9** | **R.ESQEEPR.A** |
|  | 3532 | 888.4882 | 887.4809 | 888.4851 | -1.0042 | 0 | 7 | 39 | 5 | K.MRPQTLK.G |
|  | 7507 | **591.3098** | **1180.6051** | **1181.6629** | **-1.0578** | **1** | **8** | **23** | **1** | **K.ISNIRAQPQR.H** |
|  | 11656 | **702.3707** | **1402.7269** | **1403.7256** | **-0.9987** | **1** | **7** | **26** | **3** | **K.SEAITSVDDAIRK.L** |
|  | 12736 | 734.4127 | 1466.8108 | 1467.7616 | -0.9508 | 1 | 4 | 52 | 9 | K.MRPQTLKGHQEK.I |
|  | 19863 | 987.9943 | 1973.9741 | 1973.0040 | 0.9701 | 0 | 16 | 2.4 | 2 | K.IWTQEMLLQVNDQSLR.L 19854 |
|  | 19989 | **994.9753** | **1987.9360** | **1987.9487** | **-0.0127** | **0** | **62** | **5.8e-005** | **1** | **R.SQPVSQPLTYESGPDEVR.A** |
|  | 20142 | **501.5269** | **2002.0785** | **2001.8772** | **0.2013** | **0** | **5** | **29** | **2** | **K.DELMQHMDEVNDELIR.K** |

| **675.** | O15551\|CLD3_HUMAN    **Mass:** 23816    **Score:** 62     **Queries matched:** 7   **emPAI:** 0.35 |
| --- | --- |
|  | Claudin-3 OS=Homo sapiens GN=CLDN3 PE=1 SV=1 |

|  | **Query** | **Observed** | **Mr(expt)** | **Mr(calc)** | **Delta** | **Miss** | **Score** | **Expect** | **Rank** | **Peptide** |
| --- | --- | --- | --- | --- | --- | --- | --- | --- | --- | --- |
|  | 10519 | **669.3634** | **1336.7122** | **1337.6212** | **-0.9089** | **0** | **20** | **1.4** | **1** | **R.STGPGASLGTGYDR.K** |
|  | 11716 | **703.8466** | **1405.6786** | **1405.6878** | **-0.0092** | **0** | **50** | **0.0014** | **1** | **R.DFYNPVVPEAQK.R** 11715 |
|  | 12711 | **733.8604** | **1465.7061** | **1465.7161** | **-0.0100** | **1** | **5** | **37** | **2** | **R.STGPGASLGTGYDRK.D** |
|  | 14171 | 521.3040 | 1560.8902 | 1561.7889 | -0.8986 | 1 | 5 | 37 | 7 | R.DFYNPVVPEAQKR.E 14172 14182 |

| **676.** | P51572\|BAP31_HUMAN    **Mass:** 28031    **Score:** 62     **Queries matched:** 12   **emPAI:** 0.29 |
| --- | --- |
|  | B-cell receptor-associated protein 31 OS=Homo sapiens GN=BCAP31 PE=1 SV=3 |

|  | **Query** | **Observed** | **Mr(expt)** | **Mr(calc)** | **Delta** | **Miss** | **Score** | **Expect** | **Rank** | **Peptide** |
| --- | --- | --- | --- | --- | --- | --- | --- | --- | --- | --- |
|  | 1998 | **395.2012** | **788.3879** | **789.4609** | **-1.0730** | **1** | **4** | **99** | **7** | **K.LFRAQR.N** |
|  | 2728 | **839.4557** | **838.4484** | **838.4548** | **-0.0064** | **0** | **24** | **0.52** | **1** | **R.LLEEHAK.L** |
|  | 5186 | **503.2901** | **1004.5656** | **1003.5549** | **1.0106** | **1** | **9** | **23** | **2** | **K.LKDELASTK.Q** |
|  | 5509 | **516.2653** | **1030.5161** | **1030.5229** | **-0.0068** | **0** | **45** | **0.006** | **1** | **K.AENQVLAMR.K** |
|  | 5718 | **524.2785** | **1046.5424** | **1046.5179** | **0.0246** | **0** | **(7)** | **33** | **3** | **K.AENQVLAMR.K** |
|  | 7027 | 573.3068 | 1144.5990 | 1143.5594 | 1.0396 | 0 | 9 | 19 | 10 | K.LQAAVDGPMDK.K |
|  | 7433 | 588.8041 | 1175.5936 | 1174.6128 | 0.9808 | 1 | 5 | 48 | 8 | K.AENQVLAMRK.Q |
|  | 9309 | 636.8474 | 1271.6803 | 1271.6544 | 0.0259 | 1 | 4 | 54 | 7 | K.LQAAVDGPMDKK.E |
|  | 9771 | **433.2048** | **1296.5926** | **1296.6020** | **-0.0094** | **1** | **17** | **2.9** | **1** | **K.YMEENDQLKK.G** |
|  | 11627 | 701.8312 | 1401.6478 | 1400.7445 | 0.9033 | 1 | 5 | 42 | 4 | K.LEKAENQVLAMR.K |
|  | 16441 | **857.9288** | **1713.8430** | **1713.8533** | **-0.0103** | **1** | **41** | **0.0081** | **1** | **K.LDVGNAEVKLEEENR.S** |
|  | 18566 | **623.2929** | **1866.8567** | **1865.8665** | **0.9902** | **0** | **15** | **3.3** | **1** | **K.VNLQNNPGAMEHFHMK.L** |

| **677.** | P0C7M2\|RA1L3_HUMAN    **Mass:** 34373    **Score:** 62     **Queries matched:** 17   **emPAI:** 0.23 |
| --- | --- |
|  | Putative heterogeneous nuclear ribonucleoprotein A1-like protein 3 OS=Homo sapiens GN=HNRPA1L3 PE=5 SV=1 |

|  | **Query** | **Observed** | **Mr(expt)** | **Mr(calc)** | **Delta** | **Miss** | **Score** | **Expect** | **Rank** | **Peptide** |
| --- | --- | --- | --- | --- | --- | --- | --- | --- | --- | --- |
|  | 3080 | 431.7356 | 861.4567 | 860.5484 | 0.9083 | 1 | 12 | 14 | 4 | K.KIFVGGIK.E |
|  | 3640 | 448.7788 | 895.5431 | 894.3944 | 1.1487 | 0 | 6 | 40 | 3 | R.SGSGNFGGGR.G 3621 |
|  | 7851 | 400.2376 | 1197.6909 | 1196.5091 | 1.1818 | 0 | 12 | 10 | 2 | K.QEMASASSSQR.G |
|  | 8297 | 610.3333 | 1218.6521 | 1217.6326 | 1.0195 | 0 | 12 | 10 | 2 | K.IEVIEIMTDR.G |
|  | 8560 | 412.1910 | 1233.5513 | 1233.6275 | -0.0762 | 0 | (7) | 27 | 2 | K.IEVIEIMTDR.G |
|  | 14423 | 527.2535 | 1578.7386 | 1579.7624 | -1.0238 | 1 | 2 | 76 | 8 | K.ALSKQEMASASSSQR.G |
|  | 16220 | 850.3782 | 1698.7419 | 1698.7526 | -0.0107 | 0 | 54 | 0.0004 | 1 | R.GFAFVTFDDHDSVDK.I |
|  | 16670 | **578.3131** | **1731.9175** | **1732.7071** | **-0.7896** | **0** | **3** | **57** | **9** | **R.NQGGYGGSSSSSSCVSGR.R** 16693 |
|  | 18732 | 627.6572 | 1879.9497 | 1878.9588 | 0.9909 | 1 | 7 | 20 | 5 | K.IFVGGIKEDTEEHHLR.D 18725 |
|  | 19164 | 957.4725 | 1912.9305 | 1911.9942 | 0.9363 | 1 | 29 | 0.12 | 1 | R.KLFIGGLSFETTDESLR.S 19141 19149 19163 |
|  | 30920 | **892.6341** | **4458.1341** | **4457.7926** | **0.3415** | **1** | **3** | **11** | **3** | **R.AGGRYGGSGDGSNGFGNDGSNFGGGGSYNDFGNYNNQSSNFGPMK.G** |

| **678.** | Q32P51\|RA1L2_HUMAN    **Mass:** 34375    **Score:** 62     **Queries matched:** 15   **emPAI:** 0.23 |
| --- | --- |
|  | Heterogeneous nuclear ribonucleoprotein A1-like protein 2 OS=Homo sapiens GN=HNRNPA1L2 PE=2 SV=2 |

|  | **Query** | **Observed** | **Mr(expt)** | **Mr(calc)** | **Delta** | **Miss** | **Score** | **Expect** | **Rank** | **Peptide** |
| --- | --- | --- | --- | --- | --- | --- | --- | --- | --- | --- |
|  | 3080 | 431.7356 | 861.4567 | 860.5484 | 0.9083 | 1 | 12 | 14 | 4 | K.KIFVGGIK.E |
|  | 7851 | 400.2376 | 1197.6909 | 1196.5091 | 1.1818 | 0 | 12 | 10 | 2 | K.QEMASASSSQR.G |
|  | 8297 | 610.3333 | 1218.6521 | 1217.6326 | 1.0195 | 0 | 12 | 10 | 2 | K.IEVIEIMTDR.G |
|  | 8560 | 412.1910 | 1233.5513 | 1233.6275 | -0.0762 | 0 | (7) | 27 | 2 | K.IEVIEIMTDR.G |
|  | 14590 | 398.2217 | 1588.8577 | 1589.7831 | -0.9254 | 1 | 5 | 38 | 8 | K.ALPKQEMASASSSQR.G |
|  | 14821 | **536.2791** | **1605.8155** | **1605.7780** | **0.0375** | **1** | **(3)** | **54** | **9** | **K.ALPKQEMASASSSQR.G** 14802 |
|  | 16220 | 850.3782 | 1698.7419 | 1698.7526 | -0.0107 | 0 | 54 | 0.0004 | 1 | R.GFAFVTFDDHDSVDK.I |
|  | 18732 | 627.6572 | 1879.9497 | 1878.9588 | 0.9909 | 1 | 7 | 20 | 5 | K.IFVGGIKEDTEEHHLR.D 18725 |
|  | 19164 | 957.4725 | 1912.9305 | 1911.9942 | 0.9363 | 1 | 29 | 0.12 | 1 | R.KLFIGGLSFETTDESLR.S 19141 19149 19163 |
|  | 31927 | **1897.2565** | **5688.7476** | **5688.2439** | **0.5037** | **1** | **1** | **8.3** | **2** | **R.GGFGGSCGGGGYGGSGDGYNGFGNDGSNFGGGGSYNDFGNYNNQSSNFGPMKGGNFGGR.S** |

| **679.** | Q01523\|DEF5_HUMAN    **Mass:** 10407    **Score:** 61     **Queries matched:** 4   **emPAI:** 0.38 |
| --- | --- |
|  | Defensin-5 OS=Homo sapiens GN=DEFA5 PE=1 SV=1 |

|  | **Query** | **Observed** | **Mr(expt)** | **Mr(calc)** | **Delta** | **Miss** | **Score** | **Expect** | **Rank** | **Peptide** |
| --- | --- | --- | --- | --- | --- | --- | --- | --- | --- | --- |
|  | 9707 | 647.7856 | 1293.5567 | 1292.6031 | 0.9537 | 0 | 19 | 1.9 | 2 | R.ESLSGVCEISGR.L |
|  | 22741 | **1132.5448** | **2263.0750** | **2262.0876** | **0.9874** | **0** | **62** | **4.8e-005** | **1** | **K.QSGEDNQDLAISFAGNGLSALR.T** 22739 22740 |

| **680.** | P06133\|UD2B4_HUMAN    **Mass:** 60872    **Score:** 61     **Queries matched:** 13   **emPAI:** 0.27 |
| --- | --- |
|  | UDP-glucuronosyltransferase 2B4 OS=Homo sapiens GN=UGT2B4 PE=1 SV=2 |

|  | **Query** | **Observed** | **Mr(expt)** | **Mr(calc)** | **Delta** | **Miss** | **Score** | **Expect** | **Rank** | **Peptide** |
| --- | --- | --- | --- | --- | --- | --- | --- | --- | --- | --- |
|  | 11919 | 709.3783 | 1416.7420 | 1416.7514 | -0.0093 | 0 | 53 | 0.00069 | 1 | K.WIPQNDLLGHPK.T 11920 |
|  | 12517 | 485.5969 | 1453.7690 | 1453.7790 | -0.0100 | 0 | 30 | 0.13 | 1 | R.IHHDQPVKPLDR.A 12518 12519 12520 |
|  | 13946 | 516.5950 | 1546.7631 | 1546.7740 | -0.0109 | 0 | 33 | 0.056 | 1 | R.FDGNKPDTLGLNTR.L 13945 |
|  | 19040 | 635.3217 | 1902.9433 | 1901.9498 | 0.9935 | 0 | 6 | 23 | 1 | K.VLVWPTEFSHWMNIK.T 19027 |
|  | 21201 | 701.3934 | 2101.1585 | 2101.1069 | 0.0516 | 1 | 1 | 65 | 8 | K.VLWRFDGNKPDTLGLNTR.L |
|  | 22249 | **736.0800** | **2205.2181** | **2206.0940** | **-0.8759** | **0** | **3** | **41** | **9** | **K.GAAVSLDFHTMSSTDLLNALK.T** |
|  | 23697 | **797.4435** | **2389.3086** | **2389.2311** | **0.0775** | **1** | **0** | **63** | **3** | **K.AKGAAVSLDFHTMSSTDLLNALK.T** |

| **681.** | P56134\|ATPK_HUMAN    **Mass:** 11025    **Score:** 61     **Queries matched:** 6   **emPAI:** 0.86 |
| --- | --- |
|  | ATP synthase subunit f, mitochondrial OS=Homo sapiens GN=ATP5J2 PE=1 SV=3 |

|  | **Query** | **Observed** | **Mr(expt)** | **Mr(calc)** | **Delta** | **Miss** | **Score** | **Expect** | **Rank** | **Peptide** |
| --- | --- | --- | --- | --- | --- | --- | --- | --- | --- | --- |
|  | 10071 | **657.8626** | **1313.7107** | **1313.7166** | **-0.0059** | **0** | **48** | **0.0024** | **1** | **K.LGELPSWILMR.D** 10070 |
|  | 12112 | **714.8456** | **1427.6767** | **1427.6834** | **-0.0067** | **0** | **45** | **0.004** | **1** | **R.DFSPSGIFGAFQR.G** |
|  | 12558 | **486.6003** | **1456.7790** | **1456.7054** | **0.0736** | **0** | **2** | **81** | **9** | **-.MASVGECPAPVPVK.D** |
|  | 16239 | 567.5875 | 1699.7406 | 1699.8273 | -0.0868 | 1 | 14 | 4.1 | 3 | -.MASVGECPAPVPVKDK.K |
|  | 26072 | **913.7589** | **2738.2549** | **2739.3843** | **-1.1294** | **1** | **2** | **39** | **1** | **K.LGELPSWILMRDFSPSGIFGAFQR.G** |

| **682.** | Q99653\|CHP1_HUMAN    **Mass:** 22442    **Score:** 61     **Queries matched:** 9   **emPAI:** 0.37 |
| --- | --- |
|  | Calcium-binding protein p22 OS=Homo sapiens GN=CHP PE=1 SV=3 |

|  | **Query** | **Observed** | **Mr(expt)** | **Mr(calc)** | **Delta** | **Miss** | **Score** | **Expect** | **Rank** | **Peptide** |
| --- | --- | --- | --- | --- | --- | --- | --- | --- | --- | --- |
|  | 6560 | **554.2970** | **1106.5794** | **1106.5866** | **-0.0072** | **1** | **2** | **1e+002** | **6** | **-.MGSRASTLLR.D** |
|  | 6943 | 569.3004 | 1136.5863 | 1137.5553 | -0.9691 | 1 | 10 | 14 | 1 | R.LYDLDKDEK.I 6965 |
|  | 9097 | **631.8054** | **1261.5963** | **1261.6051** | **-0.0088** | **0** | **42** | **0.0099** | **1** | **K.ETGFSHSQITR.L** |
|  | 9935 | **653.8464** | **1305.6782** | **1306.7245** | **-1.0463** | **0** | **3** | **66** | **2** | **R.IPELAINPLGDR.I** |
|  | 11852 | **707.3782** | **1412.7419** | **1411.7055** | **1.0364** | **1** | **6** | **36** | **3** | **K.SKDVNGPEPLNSR.S** |
|  | 18934 | **948.4627** | **1894.9109** | **1894.9214** | **-0.0105** | **0** | **48** | **0.0017** | **1** | **R.IINAFFPEGEDQVNFR.G** |
|  | 26921 | **973.4672** | **2917.3798** | **2916.4685** | **0.9114** | **1** | **2** | **39** | **1** | **R.DELLQVLRMMVGVNISDEQLGSIADR.T** |
|  | 30623 | 692.5079 | 4149.0039 | 4147.9202 | 1.0837 | 1 | 2 | 15 | 6 | R.MMVGVNISDEQLGSIADRTIQEADQDGDSAISFTEFVK.V |

| **683.** | P01871\|IGHM_HUMAN    **Mass:** 49960    **Score:** 61     **Queries matched:** 16   **emPAI:** 0.16 |
| --- | --- |
|  | Ig mu chain C region OS=Homo sapiens GN=IGHM PE=1 SV=3 |

|  | **Query** | **Observed** | **Mr(expt)** | **Mr(calc)** | **Delta** | **Miss** | **Score** | **Expect** | **Rank** | **Peptide** |
| --- | --- | --- | --- | --- | --- | --- | --- | --- | --- | --- |
|  | 3112 | **432.2318** | **862.4490** | **861.5171** | **0.9319** | **0** | **11** | **17** | **2** | **K.VTSTLTIK.E** |
|  | 3859 | **455.7447** | **909.4748** | **908.4141** | **1.0607** | **0** | **7** | **27** | **4** | **R.DGFFGNPR.K** |
|  | 4359 | **474.2300** | **946.4455** | **946.5084** | **-0.0629** | **1** | **15** | **6.3** | **1** | **R.VTERTVDK.S** 4380 |
|  | 8851 | **625.3175** | **1248.6204** | **1248.6285** | **-0.0080** | **0** | **34** | **0.054** | **1** | **K.LICQATGFSPR.Q** |
|  | 9412 | **426.7209** | **1277.1409** | **1276.7027** | **0.4382** | **0** | **5** | **32** | **4** | **K.YAATSQVLLPSK.D** |
|  | 13532 | 759.4475 | 1516.8805 | 1516.6650 | 0.2154 | 0 | 3 | 70 | 6 | K.DVMQGTDEHVVCK.V 13522 |
|  | 14708 | **800.3770** | **1598.7395** | **1599.7715** | **-1.0320** | **0** | **14** | **4.5** | **1** | **K.YVTSAPMPEPQAPGR.Y** 14731 14741 |
|  | 14975 | 808.4165 | 1614.8184 | 1615.7664 | -0.9480 | 0 | (6) | 27 | 3 | K.YVTSAPMPEPQAPGR.Y 14957 14974 |
|  | 15010 | **809.4019** | **1616.7892** | **1616.8006** | **-0.0114** | **0** | **55** | **0.00038** | **1** | **K.QVGSGVTTDQVQAEAK.E** |
|  | 15341 | 546.6227 | 1636.8462 | 1636.9229 | -0.0767 | 0 | 8 | 17 | 4 | R.VFAIPPSFASIFLTK.S |

| **684.** | P55145\|MANF_HUMAN    **Mass:** 20700    **Score:** 61     **Queries matched:** 14   **emPAI:** 0.19 |
| --- | --- |
|  | Mesencephalic astrocyte-derived neurotrophic factor OS=Homo sapiens GN=MANF PE=1 SV=2 |

|  | **Query** | **Observed** | **Mr(expt)** | **Mr(calc)** | **Delta** | **Miss** | **Score** | **Expect** | **Rank** | **Peptide** |
| --- | --- | --- | --- | --- | --- | --- | --- | --- | --- | --- |
|  | 709 | **476.2755** | **475.2683** | **474.2550** | **1.0132** | **0** | **3** | **1.4e+002** | **10** | **K.AASAR.T** |
|  | 2233 | 403.0462 | 804.0778 | 803.4137 | 0.6641 | 1 | 12 | 9.6 | 3 | K.AASARTDL.- |
|  | 2354 | **407.2081** | **812.4016** | **812.4068** | **-0.0053** | **0** | **17** | **3.1** | **2** | **R.FYQDLK.D** |
|  | 2799 | **422.7545** | **843.4945** | **843.4524** | **0.0421** | **0** | **17** | **3.7** | **5** | **K.INELMPK.Y** 2798 2813 |
|  | 4682 | 486.2182 | 970.4219 | 971.5473 | -1.1254 | 1 | 4 | 60 | 9 | R.KINELMPK.Y |
|  | 4930 | **495.0607** | **988.1068** | **987.5423** | **0.5646** | **1** | **(3)** | **61** | **8** | **R.KINELMPK.Y** |
|  | 6864 | 566.7632 | 1131.5118 | 1130.6183 | 0.8935 | 0 | 18 | 2.5 | 2 | K.QIDLSTVDLK.K |
|  | 10172 | 660.3332 | 1318.6518 | 1318.6955 | -0.0436 | 1 | 7 | 29 | 3 | K.INELMPKYAPK.A |
|  | 14158 | **781.3583** | **1560.7020** | **1560.7130** | **-0.0110** | **0** | **61** | **0.00011** | **1** | **R.LCYYIGATDDAATK.I** |
|  | 15940 | 839.4383 | 1676.8620 | 1675.8669 | 0.9952 | 0 | 11 | 9.1 | 1 | R.DVTFSPATIENELIK.F |
|  | 19116 | **637.3002** | **1908.8787** | **1909.0785** | **-0.1998** | **0** | **3** | **50** | **5** | **M.WATQGLAVALALSVLPGSR.A** |
|  | 20591 | **681.3416** | **2041.0030** | **2040.1190** | **0.8840** | **0** | **7** | **19** | **1** | **-.MWATQGLAVALALSVLPGSR.A** |

| **685.** | P02768\|ALBU_HUMAN    **Mass:** 71317    **Score:** 60     **Queries matched:** 14   **emPAI:** 0.23 |
| --- | --- |
|  | Serum albumin OS=Homo sapiens GN=ALB PE=1 SV=2 |

|  | **Query** | **Observed** | **Mr(expt)** | **Mr(calc)** | **Delta** | **Miss** | **Score** | **Expect** | **Rank** | **Peptide** |
| --- | --- | --- | --- | --- | --- | --- | --- | --- | --- | --- |
|  | 4562 | **481.1834** | **960.3522** | **959.5552** | **0.7970** | **0** | **14** | **8.1** | **5** | **K.FQNALLVR.Y** |
|  | 6129 | 538.2823 | 1074.5500 | 1074.4917 | 0.0584 | 0 | 6 | 42 | 7 | R.NECFLQHK.D |
|  | 7099 | **575.8845** | **1149.7544** | **1148.6077** | **1.1466** | **0** | **4** | **49** | **6** | **K.LVNEVTEFAK.T** |
|  | 10603 | **671.8171** | **1341.6197** | **1341.6275** | **-0.0078** | **0** | **45** | **0.0046** | **1** | **K.AVMDDFAAFVEK.C** |
|  | 12227 | **717.7646** | **1433.5147** | **1433.5261** | **-0.0114** | **0** | **18** | **1.6** | **1** | **R.ETYGEMADCCAK.Q** |
|  | 12738 | 489.9496 | 1466.8270 | 1466.8358 | -0.0087 | 1 | 34 | 0.048 | 1 | R.RHPDYSVVLLLR.L 12731 12732 |
|  | 13117 | **497.6351** | **1489.8834** | **1489.8980** | **-0.0146** | **1** | **10** | **12** | **2** | **K.QTALVELVKHKPK.A** |
|  | 13922 | **516.2670** | **1545.7793** | **1545.7894** | **-0.0102** | **1** | **15** | **4.3** | **1** | **K.LKECCEKPLLEK.S** 13921 |
|  | 15100 | 812.4030 | 1622.7915 | 1622.9508 | -0.1593 | 1 | 5 | 35 | 4 | R.HPDYSVVLLLRLAK.T |
|  | 15365 | 820.3582 | 1638.7019 | 1638.9305 | -0.2286 | 1 | 9 | 15 | 2 | K.KVPQVSTPTLVEVSR.N |
|  | 24476 | **830.7631** | **2489.2674** | **2489.2777** | **-0.0103** | **0** | **29** | **0.092** | **1** | **K.ALVLIAFAQYLQQCPFEDHVK.L** |

| **686.** | P62269\|RS18_HUMAN    **Mass:** 17708    **Score:** 60     **Queries matched:** 8   **emPAI:** 0.48 |
| --- | --- |
|  | 40S ribosomal protein S18 OS=Homo sapiens GN=RPS18 PE=1 SV=3 |

|  | **Query** | **Observed** | **Mr(expt)** | **Mr(calc)** | **Delta** | **Miss** | **Score** | **Expect** | **Rank** | **Peptide** |
| --- | --- | --- | --- | --- | --- | --- | --- | --- | --- | --- |
|  | 1019 | **545.3015** | **544.2942** | **543.3241** | **0.9700** | **1** | **6** | **76** | **9** | **K.GVGRR.Y** |
|  | 2362 | 407.7346 | 813.4547 | 812.4657 | 0.9890 | 0 | 7 | 34 | 7 | K.FQHILR.V |
|  | 4123 | 465.7506 | 929.4867 | 929.4930 | -0.0063 | 1 | 11 | 16 | 1 | K.LREDLER.L |
|  | 6059 | **536.2991** | **1070.5836** | **1070.5906** | **-0.0070** | **0** | **40** | **0.02** | **1** | **R.VITIMQNPR.Q** 6060 |
|  | 10212 | **661.3363** | **1320.6580** | **1320.6674** | **-0.0093** | **0** | **36** | **0.035** | **1** | **K.YSQVLANGLDNK.L** 10238 |
|  | 14621 | **531.2997** | **1590.8774** | **1589.8525** | **1.0249** | **1** | **3** | **57** | **10** | **K.YSQVLANGLDNKLR.E** |

| **687.** | P49585\|PCY1A_HUMAN    **Mass:** 42047    **Score:** 60     **Queries matched:** 10   **emPAI:** 0.19 |
| --- | --- |
|  | Choline-phosphate cytidylyltransferase A OS=Homo sapiens GN=PCYT1A PE=1 SV=2 |

|  | **Query** | **Observed** | **Mr(expt)** | **Mr(calc)** | **Delta** | **Miss** | **Score** | **Expect** | **Rank** | **Peptide** |
| --- | --- | --- | --- | --- | --- | --- | --- | --- | --- | --- |
|  | 2833 | **424.0566** | **846.0987** | **845.4494** | **0.6492** | **1** | **7** | **28** | **5** | **K.VKDVEEK.S** |
|  | 3510 | **443.7686** | **885.5226** | **886.4946** | **-0.9720** | **0** | **8** | **29** | **8** | **R.MLQAISPK.Q** |
|  | 3863 | 456.2024 | 910.3903 | 909.3684 | 1.0219 | 0 | 9 | 17 | 2 | -.MDAQCSAK.V |
|  | 5736 | **525.1028** | **1048.1910** | **1047.4556** | **0.7354** | **0** | **19** | **1.4** | **1** | **R.YDAVQHCR.Y** |
|  | 6082 | **536.8065** | **1071.5985** | **1070.5291** | **1.0694** | **0** | **6** | **43** | **6** | **R.GTPCERPVR.V** |
|  | 17381 | 594.9591 | 1781.8555 | 1781.8432 | 0.0123 | 1 | 4 | 49 | 2 | R.KEAPGPNGATEEDGVPSK.V |
|  | 17657 | 602.3222 | 1803.9448 | 1803.8693 | 0.0755 | 0 | 3 | 49 | 7 | R.VYADGIFDLFHSGHAR.A |
|  | 18614 | **936.4619** | **1870.9091** | **1870.9175** | **-0.0084** | **0** | **60** | **9.8e-005** | **1** | **R.EFIGSFLEMFGPEGALK.H** |
|  | 19985 | **663.3783** | **1987.1131** | **1987.9574** | **-0.8444** | **1** | **1** | **72** | **8** | **R.WPFSGKTSPPCSPANLSR.H** |
|  | 20020 | **664.5709** | **1990.6909** | **1989.9360** | **0.7549** | **1** | **3** | **32** | **2** | **R.VTMEEASRGTPCERPVR.V** |

| **688.** | Q96C19\|EFHD2_HUMAN    **Mass:** 26794    **Score:** 60     **Queries matched:** 14   **emPAI:** 0.30 |
| --- | --- |
|  | EF-hand domain-containing protein D2 OS=Homo sapiens GN=EFHD2 PE=1 SV=1 |

|  | **Query** | **Observed** | **Mr(expt)** | **Mr(calc)** | **Delta** | **Miss** | **Score** | **Expect** | **Rank** | **Peptide** |
| --- | --- | --- | --- | --- | --- | --- | --- | --- | --- | --- |
|  | 2096 | **397.8539** | **793.6932** | **793.3858** | **0.3075** | **0** | **11** | **8** | **6** | **R.FEEEIK.A** 2097 |
|  | 3410 | 440.7659 | 879.5173 | 878.4167 | 1.1006 | 1 | 5 | 55 | 9 | K.KQAEEMK.Q |
|  | 9106 | **631.8233** | **1261.6320** | **1261.6402** | **-0.0081** | **0** | **36** | **0.037** | **1** | **R.LSEIDVSSEGVK.G** |
|  | 10479 | **668.3209** | **1334.6273** | **1334.6864** | **-0.0591** | **1** | **2** | **94** | **4** | **-.MATDELATKLSR.R** |
|  | 10771 | 451.5826 | 1351.7258 | 1350.6813 | 1.0445 | 1 | (1) | 1e+002 | 4 | -.MATDELATKLSR.R |
|  | 14275 | 784.8818 | 1567.7490 | 1567.7590 | -0.0100 | 0 | 45 | 0.004 | 1 | R.ADLNQGIGEPQSPSR.R |
|  | 17387 | 595.2432 | 1782.7077 | 1781.9532 | 0.7545 | 1 | 0 | 92 | 7 | K.LMMEKLGAPQTHLGLK.N |
|  | 17769 | 605.0062 | 1811.9969 | 1811.8871 | 0.1097 | 1 | 8 | 17 | 3 | R.DGFIDLMELKLMMEK.L 17768 |
|  | 18795 | 943.4951 | 1884.9757 | 1885.8880 | -0.9123 | 1 | 4 | 38 | 6 | K.QYDAGRDGFIDLMELK.L |
|  | 18828 | 630.3109 | 1887.9109 | 1886.9520 | 0.9589 | 1 | 3 | 45 | 4 | R.KAAAGELQEDSGLCVLAR.L |
|  | 31104 | **1163.7500** | **4650.9709** | **4650.1813** | **0.7896** | **1** | **(0)** | **15** | **1** | **R.LQMEGEGGGETPEQPGLNGAAAAAAGAPDEAAEALGSADCELSAKLLR.R** |
|  | 31123 | **778.8584** | **4667.1067** | **4666.1762** | **0.9305** | **1** | **1** | **13** | **6** | **R.LQMEGEGGGETPEQPGLNGAAAAAAGAPDEAAEALGSADCELSAKLLR.R** |

| **689.** | P36871\|PGM1_HUMAN    **Mass:** 61696    **Score:** 60     **Queries matched:** 20   **emPAI:** 0.06 |
| --- | --- |
|  | Phosphoglucomutase-1 OS=Homo sapiens GN=PGM1 PE=1 SV=3 |

|  | **Query** | **Observed** | **Mr(expt)** | **Mr(calc)** | **Delta** | **Miss** | **Score** | **Expect** | **Rank** | **Peptide** |
| --- | --- | --- | --- | --- | --- | --- | --- | --- | --- | --- |
|  | 1840 | 738.3981 | 737.3908 | 737.3959 | -0.0052 | 0 | 13 | 8.7 | 4 | K.VYTVEK.A |
|  | 2787 | 422.2480 | 842.4814 | 841.4770 | 1.0045 | 0 | 8 | 30 | 7 | R.IAAANGIGR.L 2769 |
|  | 3826 | **455.2266** | **908.4387** | **907.4763** | **0.9624** | **0** | **15** | **5.1** | **3** | **R.LIFTDGSR.I** |
|  | 4148 | 466.7440 | 931.4735 | 932.5729 | -1.0994 | 1 | 10 | 19 | 7 | -.MVKIVTVK.T |
|  | 6339 | **545.7923** | **1089.5700** | **1089.5778** | **-0.0078** | **0** | **60** | **0.00018** | **1** | **R.LSGTGSAGATIR.L** |
|  | 6829 | **565.2674** | **1128.5202** | **1128.5274** | **-0.0071** | **0** | **3** | **80** | **10** | **K.FFGNLMDASK.L** |
|  | 7889 | **401.0629** | **1200.1668** | **1200.6099** | **-0.4431** | **0** | **12** | **6.7** | **3** | **R.QEATLVVGGDGR.F** |
|  | 11370 | **462.9212** | **1385.7418** | **1384.7173** | **1.0245** | **0** | **28** | **0.21** | **1** | **R.IDAMHGVVGPYVK.K** 11352 |
|  | 13232 | 749.4061 | 1496.7976 | 1497.8013 | -1.0037 | 1 | 5 | 40 | 4 | R.FYMKEAIQLIAR.I 13231 |
|  | 14826 | **402.4646** | **1605.8294** | **1604.8998** | **0.9296** | **1** | **10** | **11** | **2** | **R.IVFRLSGTGSAGATIR.L** 14822 |
|  | 15531 | **550.9434** | **1649.8084** | **1648.8169** | **0.9915** | **0** | **23** | **0.61** | **1** | **K.TQAYQDQKPGTSGLR.K** 15525 |
|  | 15792 | **557.2837** | **1668.8292** | **1669.8974** | **-1.0682** | **1** | **3** | **55** | **4** | **K.IRIDAMHGVVGPYVK.K** |
|  | 19730 | **655.3107** | **1962.9102** | **1963.1176** | **-0.2074** | **0** | **0** | **92** | **8** | **K.INQDPQVMLAPLISIALK.V** |
|  | 24108 | **816.3629** | **2446.0669** | **2446.3332** | **-0.2663** | **1** | **5** | **24** | **1** | **K.IKAIGGIILTASHNPGGPNGDFGIK.F** |
|  | 31007 | **1142.0354** | **4564.1125** | **4564.1349** | **-0.0224** | **0** | **9** | **2.2** | **1** | **K.ILCEELGAPANSAVNCVPLEDFGGHHPDPNLTYAADLVETMK.S** |

| **690.** | P07602\|SAP_HUMAN    **Mass:** 59899    **Score:** 60     **Queries matched:** 9   **emPAI:** 0.06 |
| --- | --- |
|  | Proactivator polypeptide OS=Homo sapiens GN=PSAP PE=1 SV=2 |

|  | **Query** | **Observed** | **Mr(expt)** | **Mr(calc)** | **Delta** | **Miss** | **Score** | **Expect** | **Rank** | **Peptide** |
| --- | --- | --- | --- | --- | --- | --- | --- | --- | --- | --- |
|  | 6698 | 559.8206 | 1117.6266 | 1118.5642 | -0.9376 | 0 | 8 | 27 | 6 | K.DVVTAAGDMLK.D |
|  | 6900 | 567.7711 | 1133.5276 | 1134.5591 | -1.0315 | 0 | (7) | 36 | 6 | K.DVVTAAGDMLK.D |
|  | 10004 | **656.0113** | **1310.0080** | **1310.5965** | **-0.5885** | **0** | **1** | **76** | **5** | **K.GCSFLPDPYQK.Q** |
|  | 10198 | **660.8388** | **1319.6629** | **1318.7245** | **0.9385** | **1** | **3** | **67** | **2** | **K.LVGYLDRNLEK.N** |
|  | 16640 | **865.5001** | **1728.9857** | **1728.9913** | **-0.0056** | **0** | **60** | **0.00011** | **1** | **K.EIVDSYLPVILDIIK.G** |
|  | 21295 | **703.4303** | **2107.2691** | **2107.9952** | **-0.7261** | **1** | **1** | **60** | **6** | **K.SLPCDICKDVVTAAGDMLK.D** |
|  | 23057 | **768.0237** | **2301.0492** | **2301.3534** | **-0.3042** | **0** | **0** | **69** | **6** | **-.MYALFLLASLLGAALAGPVLGLK.E** |
|  | 23636 | 596.0477 | 2380.1616 | 2380.0821 | 0.0795 | 0 | 7 | 14 | 1 | K.GEMSRPGEVCSALNLCESLQK.H |
|  | 24086 | **815.0438** | **2442.1096** | **2442.1204** | **-0.0107** | **0** | **4** | **33** | **4** | **K.NYISQYSEIAIQMMMHMQPK.E** |

| **691.** | P25325\|THTM_HUMAN    **Mass:** 33443    **Score:** 60     **Queries matched:** 4   **emPAI:** 0.11 |
| --- | --- |
|  | 3-mercaptopyruvate sulfurtransferase OS=Homo sapiens GN=MPST PE=1 SV=3 |

|  | **Query** | **Observed** | **Mr(expt)** | **Mr(calc)** | **Delta** | **Miss** | **Score** | **Expect** | **Rank** | **Peptide** |
| --- | --- | --- | --- | --- | --- | --- | --- | --- | --- | --- |
|  | 4298 | **472.2796** | **942.5446** | **942.5134** | **0.0312** | **0** | **7** | **36** | **8** | **R.QNLPLSSGK.S** 4284 |
|  | 17594 | **900.4833** | **1798.9520** | **1798.9617** | **-0.0097** | **0** | **60** | **0.00011** | **1** | **R.AGQPLQLLDASWYLPK.L** |
|  | 19325 | **643.2843** | **1926.8311** | **1925.9959** | **0.8352** | **1** | **4** | **38** | **7** | **R.QNLPLSSGKSQPAPAEFR.A** |

| **692.** | Q08257\|QOR_HUMAN    **Mass:** 35356    **Score:** 60     **Queries matched:** 17   **emPAI:** 0.22 |
| --- | --- |
|  | Quinone oxidoreductase OS=Homo sapiens GN=CRYZ PE=1 SV=1 |

|  | **Query** | **Observed** | **Mr(expt)** | **Mr(calc)** | **Delta** | **Miss** | **Score** | **Expect** | **Rank** | **Peptide** |
| --- | --- | --- | --- | --- | --- | --- | --- | --- | --- | --- |
|  | 1064 | 551.3150 | 550.3077 | 550.3115 | -0.0038 | 0 | 10 | 17 | 1 | R.AYGLK.I |
|  | 5770 | 526.2495 | 1050.4843 | 1050.5314 | -0.0471 | 1 | (6) | 39 | 9 | -.MATGQKLMR.A |
|  | 5774 | 526.2625 | 1050.5105 | 1050.5314 | -0.0209 | 1 | 21 | 1.1 | 2 | -.MATGQKLMR.A 5761 |
|  | 8363 | **408.2505** | **1221.7297** | **1220.6441** | **1.0856** | **0** | **6** | **35** | **1** | **R.VFEFGGPEVLK.L** |
|  | 15266 | 544.9023 | 1631.6850 | 1632.8485 | -1.1635 | 0 | 7 | 21 | 1 | K.IVLQNGAHEVFNHR.E 15262 15263 15265 15267 15269 15273 |
|  | 22035 | **727.4055** | **2179.1947** | **2179.1168** | **0.0779** | **0** | **0** | **77** | **4** | **K.AGESVLVHGASGGVGLAACQIAR.A** |
|  | 24956 | 851.0778 | 2550.2115 | 2550.2278 | -0.0164 | 0 | 4 | 23 | 1 | R.VFTSSTISGGYAEYALAADHTVYK.L |
|  | 25953 | **906.1398** | **2715.3975** | **2715.4119** | **-0.0145** | **0** | **20** | **0.66** | **1** | **R.KPLLPYTPGSDVAGVIEAVGDNASAFK.K** 25954 |
|  | 29174 | **1165.2272** | **3492.6597** | **3493.7591** | **-1.0994** | **0** | **56** | **9.4e-005** | **1** | **K.EEFQQYAAALQAGMEIGWLKPVIGSQYPLEK.V** |

| **693.** | P42766\|RL35_HUMAN    **Mass:** 14543    **Score:** 59     **Queries matched:** 6   **emPAI:** 0.61 |
| --- | --- |
|  | 60S ribosomal protein L35 OS=Homo sapiens GN=RPL35 PE=1 SV=2 |

|  | **Query** | **Observed** | **Mr(expt)** | **Mr(calc)** | **Delta** | **Miss** | **Score** | **Expect** | **Rank** | **Peptide** |
| --- | --- | --- | --- | --- | --- | --- | --- | --- | --- | --- |
|  | 1477 | 642.7714 | 641.7641 | 641.3537 | 0.4105 | 1 | 13 | 5.8 | 3 | K.FYKGK.K |
|  | 6769 | **562.8029** | **1123.5912** | **1123.5985** | **-0.0074** | **1** | **33** | **0.072** | **1** | **R.LNKHEENLK.T** |
|  | 6842 | **565.3362** | **1128.6579** | **1128.6655** | **-0.0076** | **0** | **9** | **21** | **2** | **K.YKPLDLRPK.K** |
|  | 7014 | 572.3181 | 1142.6217 | 1142.6659 | -0.0442 | 0 | 11 | 13 | 2 | R.VLTVINQTQK.E |
|  | 14104 | **778.9315** | **1555.8484** | **1555.8569** | **-0.0086** | **1** | **58** | **0.00022** | **1** | **K.QLDDLKVELSQLR.V** 14105 |

| **694.** | Q8WUD1\|RAB2B_HUMAN    **Mass:** 24427    **Score:** 59     **Queries matched:** 4   **emPAI:** 0.34 |
| --- | --- |
|  | Ras-related protein Rab-2B OS=Homo sapiens GN=RAB2B PE=1 SV=1 |

|  | **Query** | **Observed** | **Mr(expt)** | **Mr(calc)** | **Delta** | **Miss** | **Score** | **Expect** | **Rank** | **Peptide** |
| --- | --- | --- | --- | --- | --- | --- | --- | --- | --- | --- |
|  | 10936 | 681.8353 | 1361.6561 | 1361.6649 | -0.0088 | 0 | 19 | 1.6 | 1 | R.EHGLIFMETSAK.T |
|  | 13993 | 775.8786 | 1549.7426 | 1549.7525 | -0.0099 | 0 | 62 | 8.4e-005 | 1 | K.LQIWDTAGQESFR.S 13975 |
|  | 16489 | 573.6064 | 1717.7973 | 1717.8060 | -0.0087 | 0 | 3 | 55 | 2 | R.ETFNHLTSWLEDAR.Q |

| **695.** | Q14344\|GNA13_HUMAN    **Mass:** 44364    **Score:** 59     **Queries matched:** 17   **emPAI:** 0.18 |
| --- | --- |
|  | Guanine nucleotide-binding protein subunit alpha-13 OS=Homo sapiens GN=GNA13 PE=1 SV=2 |

|  | **Query** | **Observed** | **Mr(expt)** | **Mr(calc)** | **Delta** | **Miss** | **Score** | **Expect** | **Rank** | **Peptide** |
| --- | --- | --- | --- | --- | --- | --- | --- | --- | --- | --- |
|  | 1308 | 604.3007 | 603.2934 | 603.3380 | -0.0446 | 0 | 19 | 3.1 | 1 | K.NVPFK.M 1309 |
|  | 3054 | 431.2132 | 860.4118 | 860.4175 | -0.0057 | 0 | 22 | 1.5 | 1 | K.MVDVGGQR.S |
|  | 3510 | 443.7686 | 885.5226 | 886.3677 | -0.8451 | 0 | 19 | 2.5 | 1 | K.MMSFDTR.A |
|  | 4451 | **477.1936** | **952.3726** | **951.4484** | **0.9242** | **0** | **9** | **18** | **5** | **-.MADFLPSR.S** |
|  | 5850 | 529.3127 | 1056.6108 | 1056.6179 | -0.0071 | 0 | 26 | 0.46 | 1 | K.ILLLGAGESGK.S 5844 5849 |
|  | 8481 | **615.3787** | **1228.7428** | **1227.5996** | **1.1431** | **0** | **17** | **3.3** | **1** | **R.IIHGQDFDQR.A** |
|  | 9062 | **421.0563** | **1260.1471** | **1260.5955** | **-0.4484** | **0** | **6** | **26** | **4** | **R.APMAAQGMVETR.V** |
|  | 9412 | 426.7209 | 1277.1409 | 1276.5904 | 0.5506 | 0 | (3) | 50 | 6 | R.APMAAQGMVETR.V |
|  | 14672 | 798.9154 | 1595.8163 | 1594.8355 | 0.9808 | 0 | 10 | 13 | 2 | R.EEFRPTIYSNVIK.G |
|  | 16195 | 849.4239 | 1696.8332 | 1696.8818 | -0.0486 | 1 | 2 | 75 | 10 | K.DTILHDNLKQLMLQ.- |
|  | 17089 | **881.4525** | **1760.8903** | **1761.9261** | **-1.0357** | **0** | **28** | **0.19** | **1** | **R.LTESLNIFETIVNNR.V** |
|  | 19334 | **964.5039** | **1926.9933** | **1927.0051** | **-0.0118** | **0** | **53** | **0.00052** | **1** | **K.LGEPDYIPSQQDILLAR.R** |
|  | 22012 | **726.3827** | **2176.1264** | **2176.9374** | **-0.8109** | **1** | **0** | **77** | **2** | **K.MMSFDTRAPMAAQGMVETR.V** |
|  | 24983 | **852.0931** | **2553.2574** | **2553.2724** | **-0.0150** | **0** | **12** | **3.8** | **1** | **R.DQQQKPLYHHFTTAINTENIR.L** |

| **696.** | P38159\|HNRPG_HUMAN    **Mass:** 42306    **Score:** 59     **Queries matched:** 17   **emPAI:** 0.09 |
| --- | --- |
|  | Heterogeneous nuclear ribonucleoprotein G OS=Homo sapiens GN=RBMX PE=1 SV=3 |

|  | **Query** | **Observed** | **Mr(expt)** | **Mr(calc)** | **Delta** | **Miss** | **Score** | **Expect** | **Rank** | **Peptide** |
| --- | --- | --- | --- | --- | --- | --- | --- | --- | --- | --- |
|  | 1743 | **710.3652** | **709.3580** | **710.2984** | **-0.9404** | **0** | **1** | **1e+002** | **8** | **R.DGYGGSR.D** |
|  | 2951 | 427.7428 | 853.4711 | 853.4770 | -0.0059 | 1 | 13 | 7.5 | 9 | R.REPLPSR.R |
|  | 2962 | 428.2530 | 854.4915 | 853.4770 | 1.0145 | 1 | 7 | 32 | 1 | R.EPLPSRR.D 2943 |
|  | 3288 | **436.7502** | **871.4857** | **872.4981** | **-1.0123** | **1** | **2** | **1.2e+002** | **9** | **R.RGPPPPPR.S** |
|  | 3815 | 454.5635 | 907.1125 | 907.3784 | -0.2659 | 0 | 8 | 16 | 10 | R.DYGHSSSR.D 3834 3835 |
|  | 4560 | **480.9364** | **959.8582** | **959.5725** | **0.2857** | **0** | **2** | **83** | **7** | **R.IVEVLLMK.D** |
|  | 4990 | 993.0438 | 992.0365 | 991.3883 | 0.6482 | 0 | 2 | 69 | 6 | R.YDDYSSSR.D |
|  | 8119 | **404.5254** | **1210.5544** | **1209.6506** | **0.9038** | **1** | **3** | **64** | **9** | **K.ALEAVFGKYGR.I** |
|  | 12242 | **718.3695** | **1434.7245** | **1434.7354** | **-0.0110** | **0** | **59** | **0.00017** | **1** | **K.LFIGGLNTETNEK.A** |
|  | 12243 | 718.3759 | 1434.7373 | 1434.7103 | 0.0270 | 0 | 6 | 37 | 8 | K.VEQATKPSFESGR.R |
|  | 13347 | 502.6408 | 1504.9004 | 1504.7052 | 0.1952 | 1 | 6 | 30 | 4 | R.SAPSGPVRSSSGMGGR.A |
|  | 14077 | **519.2525** | **1554.7357** | **1554.7063** | **0.0294** | **1** | **4** | **53** | **5** | **R.GYPPPRDSYSSSSR.G** |
|  | 14830 | **536.3405** | **1605.9995** | **1605.6808** | **0.3187** | **1** | **2** | **55** | **9** | **R.DYTYRDYGHSSSR.D** |
|  | 20986 | **521.4748** | **2081.8703** | **2080.7673** | **1.1030** | **0** | **2** | **51** | **8** | **R.GGHMDDGGYSMNFNMSSSR.G** |

| **697.** | Q03518\|TAP1_HUMAN    **Mass:** 87733    **Score:** 59     **Queries matched:** 17   **emPAI:** 0.09 |
| --- | --- |
|  | Antigen peptide transporter 1 OS=Homo sapiens GN=TAP1 PE=1 SV=2 |

|  | **Query** | **Observed** | **Mr(expt)** | **Mr(calc)** | **Delta** | **Miss** | **Score** | **Expect** | **Rank** | **Peptide** |
| --- | --- | --- | --- | --- | --- | --- | --- | --- | --- | --- |
|  | 1283 | **600.9672** | **599.9599** | **599.2850** | **0.6750** | **0** | **0** | **1.3e+002** | **5** | **R.CPAPR.G** |
|  | 7701 | **596.2885** | **1190.5625** | **1189.5874** | **0.9751** | **1** | **4** | **63** | **7** | **R.DGVRVPMASSR.C** |
|  | 8992 | **629.2937** | **1256.5728** | **1256.5819** | **-0.0091** | **0** | **30** | **0.14** | **1** | **R.EGGTHQQLMEK.K** 9002 9006 |
|  | 9331 | 425.2338 | 1272.6796 | 1272.5768 | 0.1027 | 0 | (2) | 1e+002 | 8 | R.EGGTHQQLMEK.K |
|  | 10662 | **673.3406** | **1344.6667** | **1343.6438** | **1.0229** | **1** | **1** | **1.1e+002** | **7** | **R.VPMASSRCPAPR.G** |
|  | 13020 | **743.3901** | **1484.7656** | **1484.7736** | **-0.0080** | **0** | **53** | **0.00074** | **1** | **R.QVAAVGQEPQVFGR.S** |
|  | 13440 | 504.9192 | 1511.7357 | 1511.7004 | 0.0352 | 1 | 5 | 38 | 4 | R.SFANEEGEAQKFR.E |
|  | 13917 | 773.8729 | 1545.7313 | 1545.7423 | -0.0110 | 0 | 18 | 1.7 | 1 | R.ELISWGAPGSADSTR.L 13911 13919 |
|  | 15737 | **555.6137** | **1663.8193** | **1664.6963** | **-0.8770** | **0** | **3** | **58** | **6** | **K.GCYWAMVQAPADAPE.-** |
|  | 16322 | **427.2309** | **1704.8945** | **1705.8873** | **-0.9928** | **1** | **9** | **14** | **3** | **R.SFRPHRGAESPRPGR.D** |
|  | 17511 | 896.9272 | 1791.8398 | 1791.9380 | -0.0982 | 1 | 2 | 78 | 8 | R.APPSFPPPAASRGGLGGTR.S |
|  | 25899 | **902.8007** | **2705.3803** | **2705.3945** | **-0.0142** | **0** | **18** | **1.1** | **1** | **R.SLQENIAYGLTQKPTMEEITAAAVK.S** |
|  | 28992 | **855.9572** | **3419.7997** | **3419.8201** | **-0.0204** | **0** | **14** | **1.4** | **1** | **K.STVAALLQNLYQPTGGQLLLDGKPLPQYEHR.Y** |

| **698.** | Q9Y2J2\|E41L3_HUMAN    **Mass:** 121458   **Score:** 59     **Queries matched:** 23   **emPAI:** 0.06 |
| --- | --- |
|  | Band 4.1-like protein 3 OS=Homo sapiens GN=EPB41L3 PE=1 SV=2 |

|  | **Query** | **Observed** | **Mr(expt)** | **Mr(calc)** | **Delta** | **Miss** | **Score** | **Expect** | **Rank** | **Peptide** |
| --- | --- | --- | --- | --- | --- | --- | --- | --- | --- | --- |
|  | 1130 | **565.3496** | **564.3423** | **563.3027** | **1.0396** | **1** | **7** | **34** | **1** | **R.SSSKR.Y** 1129 |
|  | 4599 | 482.3234 | 962.6322 | 961.5233 | 1.1089 | 0 | 10 | 18 | 1 | R.AGAPVPEPPK.E 4578 |
|  | 9815 | 650.7989 | 1299.5833 | 1299.7034 | -0.1201 | 0 | 8 | 22 | 8 | K.VVQETVLVEER.R |
|  | 9816 | **434.2067** | **1299.5982** | **1298.7194** | **0.8788** | **1** | **4** | **49** | **8** | **R.KGEEVTPISAIR.H** |
|  | 11149 | 687.8417 | 1373.6688 | 1373.6786 | -0.0098 | 0 | 33 | 0.078 | 1 | K.AVLEQEETAAASR.E |
|  | 12018 | **474.9753** | **1421.9039** | **1421.6787** | **0.2253** | **1** | **7** | **21** | **9** | **R.EVTDKEQEFAAR.A** 12028 |
|  | 13656 | **764.3781** | **1526.7417** | **1527.7317** | **-0.9901** | **1** | **4** | **50** | **1** | **R.DAENQKNWLDPAK.E** 13667 |
|  | 14187 | **782.3755** | **1562.7364** | **1563.7450** | **-1.0086** | **1** | **10** | **12** | **3** | **K.AQELEKTQDDLMK.H** 14203 |
|  | 14749 | **801.4365** | **1600.8584** | **1600.8672** | **-0.0088** | **0** | **53** | **0.00065** | **1** | **K.GISQTNLITTVTPEK.K** |
|  | 16777 | 870.9157 | 1739.8167 | 1739.8288 | -0.0120 | 0 | 5 | 31 | 4 | K.VILLDGSEYTCDVEK.R |
|  | 17689 | **603.2905** | **1806.8496** | **1806.7916** | **0.0579** | **0** | **3** | **61** | **5** | **R.GMTPAEAEMHFLENAK.K** |
|  | 18188 | **614.7956** | **1841.3649** | **1840.8764** | **0.4885** | **0** | **15** | **2.3** | **1** | **R.QEDAPMIEPLVPEETK.Q** |
|  | 18946 | 632.9794 | 1895.9165 | 1895.9299 | -0.0134 | 1 | 7 | 20 | 2 | K.VILLDGSEYTCDVEKR.S |
|  | 21965 | 724.7221 | 2171.1445 | 2170.9888 | 0.1557 | 1 | 2 | 60 | 5 | K.SHRGMTPAEAEMHFLENAK.K 21953 |
|  | 23964 | **809.7318** | **2426.1736** | **2426.0092** | **0.1644** | **0** | **2** | **45** | **2** | **R.TDTAADGETTATESDQEEDAELK.A** |
|  | 24573 | **626.3012** | **2501.1757** | **2501.2815** | **-0.1058** | **1** | **2** | **46** | **4** | **K.IRPGEFEQFESTIGFKLPNHR.A** |
|  | 31594 | **1328.4063** | **5309.5959** | **5309.6812** | **-0.0853** | **0** | **2** | **8.3** | **1** | **K.GTTLFSFSLQLPESFPSLLDDDGYLSFPNLSETNLLPQSLQHYLPIR.S** |

| **699.** | P15259\|PGAM2_HUMAN    **Mass:** 28919    **Score:** 59     **Queries matched:** 15   **emPAI:** 0.63 |
| --- | --- |
|  | Phosphoglycerate mutase 2 OS=Homo sapiens GN=PGAM2 PE=1 SV=3 |

|  | **Query** | **Observed** | **Mr(expt)** | **Mr(calc)** | **Delta** | **Miss** | **Score** | **Expect** | **Rank** | **Peptide** |
| --- | --- | --- | --- | --- | --- | --- | --- | --- | --- | --- |
|  | 1250 | 590.3295 | 589.3222 | 589.3071 | 0.0151 | 0 | 16 | 6.9 | 3 | K.AETAAK.H |
|  | 4752 | 488.2468 | 974.4790 | 974.4855 | -0.0065 | 0 | 40 | 0.021 | 1 | K.AMEAVAAQGK.A 4758 |
|  | 5875 | 530.2792 | 1058.5438 | 1058.5509 | -0.0071 | 0 | 37 | 0.041 | 1 | R.HYGGLTGLNK.A 5874 |
|  | 7098 | 575.8344 | 1149.6543 | 1149.6618 | -0.0076 | 0 | 34 | 0.056 | 1 | R.VLIAAHGNSLR.G 7078 |
|  | 9948 | 436.5701 | 1306.6885 | 1305.7629 | 0.9255 | 1 | 8 | 21 | 6 | K.RVLIAAHGNSLR.G |
|  | 10850 | 453.2252 | 1356.6538 | 1356.5807 | 0.0732 | 0 | 4 | 64 | 6 | R.HGESTWNQENR.F |
|  | 16025 | 842.9556 | 1683.8967 | 1682.9032 | 0.9935 | 0 | 37 | 0.024 | 1 | R.ALPFWNEEIVPQIK.A |
|  | 17098 | 881.9021 | 1761.7896 | 1761.8971 | -0.1074 | 0 | 10 | 11 | 2 | R.YAGLKPGELPTCESLK.D 17097 |
|  | 19217 | 959.4933 | 1916.9720 | 1917.9982 | -1.0262 | 1 | 13 | 5.6 | 2 | R.RYAGLKPGELPTCESLK.D 19219 |
|  | 24308 | 823.9890 | 2468.9450 | 2469.3202 | -0.3752 | 1 | 0 | 56 | 6 | R.AIRTLWAILDGTDQMWLPVVR.T |

| **700.** | P52597\|HNRPF_HUMAN    **Mass:** 45985    **Score:** 58     **Queries matched:** 11   **emPAI:** 0.08 |
| --- | --- |
|  | Heterogeneous nuclear ribonucleoprotein F OS=Homo sapiens GN=HNRNPF PE=1 SV=3 |

|  | **Query** | **Observed** | **Mr(expt)** | **Mr(calc)** | **Delta** | **Miss** | **Score** | **Expect** | **Rank** | **Peptide** |
| --- | --- | --- | --- | --- | --- | --- | --- | --- | --- | --- |
|  | 5377 | 511.2519 | 1020.4893 | 1020.4950 | -0.0057 | 0 | 0 | 1.5e+002 | 5 | R.TEMDWVLK.H |
|  | 6375 | 547.2921 | 1092.5697 | 1091.5724 | 0.9973 | 0 | 11 | 12 | 1 | R.VHIEIGPDGR.V 6372 |
|  | 10202 | **660.8574** | **1319.7002** | **1318.6591** | **1.0410** | **0** | **(1)** | **1e+002** | **9** | **M.MLGPEGGEGFVVK.L** |
|  | 10460 | 667.8465 | 1333.6784 | 1334.6541 | -0.9756 | 0 | 6 | 38 | 1 | M.MLGPEGGEGFVVK.L |
|  | 13441 | 504.9202 | 1511.7388 | 1510.7238 | 1.0149 | 1 | 9 | 17 | 3 | R.ESMGHRYIEVFK.S |
|  | 14588 | 530.6256 | 1588.8550 | 1587.8443 | 1.0107 | 1 | 1 | 90 | 8 | M.MLGPEGGEGFVVKLR.G |
|  | 16385 | 570.6460 | 1708.9162 | 1708.7792 | 0.1370 | 0 | 5 | 37 | 8 | R.QSGEAFVELGSEDDVK.M |
|  | 18569 | **934.4703** | **1866.9260** | **1866.9363** | **-0.0103** | **0** | **58** | **0.00014** | **1** | **K.ITGEAFVQFASQELAEK.A** |
|  | 20078 | 998.9870 | 1995.9594 | 1995.9690 | -0.0096 | 0 | 1 | 69 | 2 | K.ATENDIYNFFSPLNPVR.V |
|  | 21290 | **703.3839** | **2107.1299** | **2106.0541** | **1.0758** | **1** | **1** | **75** | **1** | **K.FMSVQRPGPYDRPGTARR.Y** |

| **701.** | Q14CN2\|CLCA4_HUMAN    **Mass:** 101903   **Score:** 58     **Queries matched:** 15   **emPAI:** 0.11 |
| --- | --- |
|  | Calcium-activated chloride channel regulator 4 OS=Homo sapiens GN=CLCA4 PE=1 SV=2 |

|  | **Query** | **Observed** | **Mr(expt)** | **Mr(calc)** | **Delta** | **Miss** | **Score** | **Expect** | **Rank** | **Peptide** |
| --- | --- | --- | --- | --- | --- | --- | --- | --- | --- | --- |
|  | 2225 | 402.7239 | 803.4331 | 803.4389 | -0.0057 | 0 | 36 | 0.051 | 1 | K.SLQLESK.G |
|  | 2283 | **404.5659** | **807.1173** | **806.3705** | **0.7468** | **0** | **9** | **15** | **2** | **R.CSAGISGR.N** |
|  | 4545 | 480.2808 | 958.5471 | 958.5521 | -0.0051 | 0 | 47 | 0.0037 | 1 | R.IVCLVLDK.S |
|  | 5413 | 1024.5066 | 1023.4993 | 1023.4226 | 0.0767 | 0 | 4 | 65 | 10 | K.CQGGSCLSR.A |
|  | 5684 | **522.7919** | **1043.5692** | **1044.5498** | **-0.9806** | **1** | **3** | **78** | **5** | **R.LNRMNQAAK.H** |
|  | 6781 | 563.3187 | 1124.6229 | 1124.6077 | 0.0152 | 1 | 4 | 54 | 5 | R.IDSTTKLYGK.D |
|  | 8888 | 626.3102 | 1250.6059 | 1249.6455 | 0.9604 | 0 | 0 | 1.3e+002 | 4 | K.VGTWAYNLQAK.A |
|  | 12334 | **481.5512** | **1441.6317** | **1442.8279** | **-1.1962** | **1** | **30** | **0.13** | **1** | **K.ISQRIVCLVLDK.S** |
|  | 13703 | 766.3704 | 1530.7263 | 1531.9086 | -1.1823 | 1 | 7 | 26 | 7 | R.YIIRISASILDLR.D |
|  | 16881 | **583.1084** | **1746.3034** | **1745.8988** | **0.4045** | **0** | **4** | **27** | **3** | **K.IILTWTAPGDNFDVGK.V** |
|  | 17023 | **586.3160** | **1755.9263** | **1756.9141** | **-0.9878** | **1** | **0** | **1e+002** | **9** | **R.AANSSVPPITVNAKMNK.D** |
|  | 22758 | 756.0345 | 2265.0816 | 2265.9956 | -0.9140 | 0 | 1 | 59 | 8 | K.ASIMFMQSIDSVVEFCNEK.T 22757 |
|  | 26499 | **945.9365** | **2834.7876** | **2835.3129** | **-0.5253** | **1** | **3** | **22** | **1** | **K.VQTEKASIMFMQSIDSVVEFCNEK.T** |
|  | 31455 | **1693.8665** | **5078.5776** | **5078.4973** | **0.0802** | **1** | **4** | **5.1** | **1** | **K.DTFFLITWNSLPPSISLWDPSGTIMENFTVDATSKMAYLSIPGTAK.V** |

| **702.** | Q9NZA1\|CLIC5_HUMAN    **Mass:** 46816    **Score:** 58     **Queries matched:** 7   **emPAI:** 0.08 |
| --- | --- |
|  | Chloride intracellular channel protein 5 OS=Homo sapiens GN=CLIC5 PE=1 SV=3 |

|  | **Query** | **Observed** | **Mr(expt)** | **Mr(calc)** | **Delta** | **Miss** | **Score** | **Expect** | **Rank** | **Peptide** |
| --- | --- | --- | --- | --- | --- | --- | --- | --- | --- | --- |
|  | 5105 | 500.3099 | 998.6053 | 997.5345 | 1.0709 | 1 | 7 | 36 | 10 | R.YLKNAYAR.D |
|  | 9468 | **641.3342** | **1280.6539** | **1280.6248** | **0.0291** | **0** | **0** | **1.2e+002** | **8** | **R.ESNTAGIDIFSK.F** |
|  | 11378 | **463.1317** | **1386.3734** | **1385.7011** | **0.6723** | **1** | **0** | **81** | **6** | **K.NTKQQNNAALER.G** |
|  | 12318 | 721.3586 | 1440.7027 | 1441.7637 | -1.0610 | 1 | 8 | 20 | 4 | K.QQNNAALERGLTK.A 12354 |
|  | 13367 | **754.3417** | **1506.6688** | **1506.6798** | **-0.0110** | **0** | **58** | **0.00018** | **1** | **K.GEETSLASVQSEDR.G** |
|  | 19406 | 967.4772 | 1932.9398 | 1932.9503 | -0.0105 | 0 | 5 | 35 | 2 | K.FLDGDELTLADCNLLPK.L |

| **703.** | Q86Y82\|STX12_HUMAN    **Mass:** 31736    **Score:** 58     **Queries matched:** 5   **emPAI:** 0.12 |
| --- | --- |
|  | Syntaxin-12 OS=Homo sapiens GN=STX12 PE=1 SV=1 |

|  | **Query** | **Observed** | **Mr(expt)** | **Mr(calc)** | **Delta** | **Miss** | **Score** | **Expect** | **Rank** | **Peptide** |
| --- | --- | --- | --- | --- | --- | --- | --- | --- | --- | --- |
|  | 5207 | **504.2745** | **1006.5344** | **1006.5117** | **0.0227** | **0** | **10** | **17** | **1** | **K.NLMSQLGTK.Q** |
|  | 20042 | **997.5064** | **1992.9983** | **1992.0388** | **0.9595** | **0** | **58** | **0.00014** | **1** | **K.LQENLQQLQHSTNQLAK.E** 20033 |
|  | 20523 | **680.2873** | **2037.8402** | **2038.0807** | **-0.2405** | **1** | **3** | **37** | **3** | **K.ELGSLPLPLSTSEQRQQR.L** |
|  | 22427 | **742.3749** | **2224.1030** | **2223.0854** | **1.0175** | **1** | **3** | **47** | **2** | **K.ERLMNDFSAALNNFQAVQR.R** |

| **704.** | O15533\|TPSN_HUMAN    **Mass:** 47938    **Score:** 58     **Queries matched:** 3   **emPAI:** 0.16 |
| --- | --- |
|  | Tapasin OS=Homo sapiens GN=TAPBP PE=1 SV=1 |

|  | **Query** | **Observed** | **Mr(expt)** | **Mr(calc)** | **Delta** | **Miss** | **Score** | **Expect** | **Rank** | **Peptide** |
| --- | --- | --- | --- | --- | --- | --- | --- | --- | --- | --- |
|  | 8151 | 607.2634 | 1212.5123 | 1211.5175 | 0.9948 | 0 | 41 | 0.011 | 1 | R.GAPAPHCEMSR.F |
|  | 12555 | **729.3461** | **1456.6777** | **1456.6881** | **-0.0104** | **0** | **47** | **0.0028** | **1** | **K.WASGLTPAQNCPR.A** |
|  | 30099 | **1267.9628** | **3800.8665** | **3800.8760** | **-0.0095** | **0** | **5** | **9.7** | **1** | **R.LGQDALLDLSFAYMPPTSEAASSLAPGPPPFGLEWR.R** |

| **705.** | P62854\|RS26_HUMAN    **Mass:** 13292    **Score:** 58     **Queries matched:** 11   **emPAI:** 0.30 |
| --- | --- |
|  | 40S ribosomal protein S26 OS=Homo sapiens GN=RPS26 PE=1 SV=3 |

|  | **Query** | **Observed** | **Mr(expt)** | **Mr(calc)** | **Delta** | **Miss** | **Score** | **Expect** | **Rank** | **Peptide** |
| --- | --- | --- | --- | --- | --- | --- | --- | --- | --- | --- |
|  | 2245 | 403.2275 | 804.4405 | 805.4559 | -1.0154 | 0 | 16 | 5.6 | 3 | R.GHVQPIR.C 2236 2244 2253 2258 2271 2272 2279 |
|  | 12555 | 729.3461 | 1456.6777 | 1456.8514 | -0.1737 | 1 | 13 | 6.7 | 3 | K.FVIRNIVEAAAVR.D |
|  | 15582 | **827.4170** | **1652.8194** | **1652.8297** | **-0.0103** | **0** | **58** | **0.00019** | **1** | **R.DISEASVFDAYVLPK.L** 15583 |

| **706.** | Q9H9B4\|SFXN1_HUMAN    **Mass:** 35881    **Score:** 58     **Queries matched:** 12   **emPAI:** 0.22 |
| --- | --- |
|  | Sideroflexin-1 OS=Homo sapiens GN=SFXN1 PE=1 SV=4 |

|  | **Query** | **Observed** | **Mr(expt)** | **Mr(calc)** | **Delta** | **Miss** | **Score** | **Expect** | **Rank** | **Peptide** |
| --- | --- | --- | --- | --- | --- | --- | --- | --- | --- | --- |
|  | 2731 | **420.3174** | **838.6203** | **839.4541** | **-0.8338** | **1** | **8** | **21** | **6** | **R.VYFNKGL.-** |
|  | 3377 | **439.7605** | **877.5064** | **877.5134** | **-0.0070** | **0** | **22** | **1.3** | **1** | **K.HVSPLIGR.F** 3392 |
|  | 10045 | 657.3094 | 1312.6043 | 1311.6856 | 0.9187 | 0 | 9 | 16 | 4 | -.MSGELPPNINIK.E |
|  | 13276 | **750.8994** | **1499.7843** | **1499.7943** | **-0.0101** | **0** | **64** | **5.1e-005** | **1** | **R.NILLTNEQLESAR.K** |
|  | 14217 | **522.3246** | **1563.9519** | **1562.8416** | **1.1103** | **1** | **5** | **34** | **2** | **M.SGELPPNINIKEPR.W** |
|  | 16360 | **854.9501** | **1707.8857** | **1707.8944** | **-0.0087** | **0** | **18** | **1.6** | **1** | **R.QGIVPPGLTENELWR.A** |
|  | 21353 | **1057.0057** | **2111.9969** | **2111.0607** | **0.9362** | **1** | **1** | **76** | **1** | **K.VGIPVTDENGNRLGESANAAK.Q** |
|  | 23768 | **601.0454** | **2400.1525** | **2401.0067** | **-0.8542** | **0** | **(3)** | **43** | **7** | **R.MSAQVPMNMTITGCMMTFYR.T** |
|  | 23770 | **801.0735** | **2400.1988** | **2401.0067** | **-0.8079** | **0** | **3** | **42** | **2** | **R.MSAQVPMNMTITGCMMTFYR.T** |
|  | 27728 | **772.1244** | **3084.4685** | **3084.4220** | **0.0465** | **1** | **3** | **23** | **2** | **K.MILIGRMSAQVPMNMTITGCMMTFYR.T** |
|  | 28512 | 1090.8627 | 3269.5662 | 3270.7542 | -1.1880 | 1 | 2 | 26 | 2 | K.QAITQVVVSRILMAAPGMAIPPFIMNTLEK.K |

| **707.** | Q9Y4X1\|UD2A1_HUMAN    **Mass:** 60346    **Score:** 58     **Queries matched:** 15   **emPAI:** 0.20 |
| --- | --- |
|  | UDP-glucuronosyltransferase 2A1 OS=Homo sapiens GN=UGT2A1 PE=1 SV=1 |

|  | **Query** | **Observed** | **Mr(expt)** | **Mr(calc)** | **Delta** | **Miss** | **Score** | **Expect** | **Rank** | **Peptide** |
| --- | --- | --- | --- | --- | --- | --- | --- | --- | --- | --- |
|  | 1560 | 659.3422 | 658.3349 | 657.4061 | 0.9288 | 0 | 16 | 6.5 | 1 | R.IEGVIK.D |
|  | 4784 | 489.2638 | 976.5130 | 975.4920 | 1.0211 | 1 | 9 | 26 | 9 | K.ENAMRLSR.I 4790 |
|  | 12271 | 719.4207 | 1436.8269 | 1436.8351 | -0.0082 | 0 | 47 | 0.0027 | 1 | K.ANLIASALAQIPQK.V 12272 |
|  | 12414 | 483.0226 | 1446.0458 | 1446.6926 | -0.6467 | 1 | 8 | 15 | 4 | R.FSPASTVEKHCGK.V |
|  | 12517 | 485.5969 | 1453.7690 | 1453.7790 | -0.0100 | 0 | 30 | 0.13 | 1 | R.IHHDQPVKPLDR.A 12518 12519 12520 |
|  | 17319 | 889.9167 | 1777.8189 | 1776.8175 | 1.0014 | 0 | 3 | 51 | 8 | K.DFHMVSQEICDGVLK.N |
|  | 22621 | 1125.9936 | 2249.9727 | 2250.0456 | -0.0728 | 0 | 2 | 43 | 2 | R.NFISYHLQDYMFETLWK.S 22620 22622 |
|  | 23590 | 791.4476 | 2371.3211 | 2372.2733 | -0.9523 | 1 | 12 | 4.2 | 2 | K.AKGAAVEVNLNTMTSVDLLSALR.T |

| **708.** | O43169\|CYB5B_HUMAN    **Mass:** 16436    **Score:** 58     **Queries matched:** 6   **emPAI:** 0.24 |
| --- | --- |
|  | Cytochrome b5 type B OS=Homo sapiens GN=CYB5B PE=1 SV=2 |

|  | **Query** | **Observed** | **Mr(expt)** | **Mr(calc)** | **Delta** | **Miss** | **Score** | **Expect** | **Rank** | **Peptide** |
| --- | --- | --- | --- | --- | --- | --- | --- | --- | --- | --- |
|  | 1671 | 689.3779 | 688.3706 | 687.3803 | 0.9903 | 0 | 15 | 8.2 | 6 | R.LEEVAK.R |
|  | 6747 | 561.8204 | 1121.6263 | 1121.6346 | -0.0082 | 0 | 11 | 12 | 1 | K.ELWLVIHGR.V |
|  | 6958 | 570.2751 | 1138.5356 | 1139.4765 | -0.9408 | 0 | 32 | 0.096 | 2 | -.MATAEASGSDGK.G |
|  | 21574 | **712.3488** | **2134.0244** | **2133.0378** | **0.9866** | **0** | **14** | **3.3** | **1** | **K.QYYIGDIHPSDLKPESGSK.D** |
|  | 29539 | 907.6619 | 3626.6184 | 3626.6397 | -0.0213 | 0 | 58 | 5.9e-005 | 1 | R.FLNEHPGGEEVLLEQAGVDASESFEDVGHSSDAR.E |
|  | 30559 | **1376.7459** | **4127.2157** | **4127.9018** | **-0.6861** | **1** | **5** | **8.2** | **10** | **R.FLNEHPGGEEVLLEQAGVDASESFEDVGHSSDAREMLK.Q** |

| **709.** | P21217\|FUT3_HUMAN    **Mass:** 42489    **Score:** 57     **Queries matched:** 8   **emPAI:** 0.09 |
| --- | --- |
|  | Galactoside 3(4)-L-fucosyltransferase OS=Homo sapiens GN=FUT3 PE=2 SV=1 |

|  | **Query** | **Observed** | **Mr(expt)** | **Mr(calc)** | **Delta** | **Miss** | **Score** | **Expect** | **Rank** | **Peptide** |
| --- | --- | --- | --- | --- | --- | --- | --- | --- | --- | --- |
|  | 2879 | **425.2549** | **848.4952** | **847.4228** | **1.0724** | **0** | **11** | **14** | **9** | **R.YLSYFR.W** |
|  | 8856 | 417.2253 | 1248.6542 | 1249.6455 | -0.9913 | 0 | 11 | 12 | 2 | R.YYQSLQAHLK.V |
|  | 11405 | **694.8094** | **1387.6043** | **1386.6891** | **0.9152** | **1** | **10** | **14** | **1** | **R.YLQELDKDHAR.Y** |
|  | 15782 | **557.0399** | **1668.0979** | **1667.8242** | **0.2737** | **0** | **4** | **36** | **5** | **-.MDPLGAAKPQWPWR.R** |
|  | 15955 | **839.9633** | **1677.9120** | **1677.9202** | **-0.0083** | **0** | **57** | **0.00021** | **1** | **R.NALEAWAVPVVLGPSR.S** |
|  | 18252 | **615.8042** | **1844.3908** | **1843.9284** | **0.4624** | **1** | **0** | **68** | **2** | **R.SHKPLPKGTMMETLSR.Y** |
|  | 21571 | **712.3287** | **2133.9644** | **2133.1847** | **0.7797** | **1** | **2** | **58** | **5** | **K.LWRNALEAWAVPVVLGPSR.S** |
|  | 30840 | **1468.0004** | **4400.9793** | **4401.0841** | **-0.1048** | **1** | **3** | **9.7** | **1** | **R.YFNLTMSYRSDSDIFTPYGWLEPWSGQPAHPPLNLSAK.T** |

|  | **Proteins matching a subset of these peptides:** |
| --- | --- |

|  | Q11128\|FUT5_HUMAN    **Mass:** 43437    **Score:** 57     **Queries matched:** 6 |
| --- | --- |
|  | Alpha-(1,3)-fucosyltransferase OS=Homo sapiens GN=FUT5 PE=2 SV=1 |

| **710.** | P51993\|FUT6_HUMAN    **Mass:** 42289    **Score:** 57     **Queries matched:** 8   **emPAI:** 0.09 |
| --- | --- |
|  | Alpha-(1,3)-fucosyltransferase OS=Homo sapiens GN=FUT6 PE=2 SV=1 |

|  | **Query** | **Observed** | **Mr(expt)** | **Mr(calc)** | **Delta** | **Miss** | **Score** | **Expect** | **Rank** | **Peptide** |
| --- | --- | --- | --- | --- | --- | --- | --- | --- | --- | --- |
|  | 2879 | 425.2549 | 848.4952 | 847.4228 | 1.0724 | 0 | 11 | 14 | 9 | R.YLSYFR.W |
|  | 8856 | 417.2253 | 1248.6542 | 1249.6455 | -0.9913 | 0 | 11 | 12 | 2 | R.YYQSLQAHLK.V |
|  | 11405 | 694.8094 | 1387.6043 | 1386.6891 | 0.9152 | 1 | 10 | 14 | 1 | R.YLQELDKDHAR.Y |
|  | 13759 | 512.2678 | 1533.7815 | 1533.7060 | 0.0755 | 0 | 2 | 79 | 7 | R.VSQDDPTVYPNGSR.F |
|  | 15955 | 839.9633 | 1677.9120 | 1677.9202 | -0.0083 | 0 | 57 | 0.00021 | 1 | R.NALEAWAVPVVLGPSR.S |
|  | 18252 | 615.8042 | 1844.3908 | 1843.8920 | 0.4987 | 0 | 0 | 68 | 2 | R.SHKPLPQGTMMETLSR.Y |
|  | 21571 | 712.3287 | 2133.9644 | 2133.1847 | 0.7797 | 1 | 2 | 58 | 5 | K.LWRNALEAWAVPVVLGPSR.S |
|  | 24679 | **840.0532** | **2517.1377** | **2517.2468** | **-0.1092** | **1** | **0** | **63** | **1** | **K.VDVYGRSHKPLPQGTMMETLSR.Y** |

| **711.** | P50995\|ANX11_HUMAN    **Mass:** 54697    **Score:** 57     **Queries matched:** 21   **emPAI:** 0.14 |
| --- | --- |
|  | Annexin A11 OS=Homo sapiens GN=ANXA11 PE=1 SV=1 |

|  | **Query** | **Observed** | **Mr(expt)** | **Mr(calc)** | **Delta** | **Miss** | **Score** | **Expect** | **Rank** | **Peptide** |
| --- | --- | --- | --- | --- | --- | --- | --- | --- | --- | --- |
|  | 1323 | 605.3397 | 604.3324 | 604.3367 | -0.0042 | 0 | 20 | 2.3 | 1 | R.IMVSR.S |
|  | 1367 | **615.2967** | **614.2894** | **613.4527** | **0.8368** | **1** | **13** | **11** | **1** | **R.KILLK.I** |
|  | 2046 | 395.7423 | 789.4700 | 790.4119 | -0.9419 | 1 | (4) | 96 | 4 | K.AMRGAGTK.D |
|  | 2253 | 806.3729 | 805.3656 | 806.4069 | -1.0413 | 1 | 9 | 26 | 5 | K.AMRGAGTK.D |
|  | 5237 | 505.7397 | 1009.4649 | 1009.4716 | -0.0067 | 0 | 12 | 9 | 6 | K.SELSGNFEK.T |
|  | 6447 | **550.2784** | **1098.5422** | **1099.6349** | **-1.0927** | **0** | **7** | **31** | **9** | **R.LLISLSQGNR.D** |
|  | 8104 | **605.8101** | **1209.6057** | **1208.6084** | **0.9973** | **1** | **8** | **24** | **4** | **K.FNAVLCSRSR.A** 8079 |
|  | 10497 | **668.8057** | **1335.5969** | **1335.6055** | **-0.0086** | **0** | **34** | **0.05** | **1** | **R.DAQELYAAGENR.L** 10483 |
|  | 12943 | 494.2937 | 1479.8592 | 1480.6464 | -0.7872 | 0 | 4 | 44 | 9 | R.DESTNVDMSLAQR.D |
|  | 14810 | 536.2308 | 1605.6705 | 1606.7582 | -1.0877 | 0 | (2) | 61 | 7 | R.EMSGDLEEGMLAVVK.C |
|  | 15114 | **542.1291** | **1623.3654** | **1622.7531** | **0.6123** | **0** | **4** | **28** | **3** | **R.EMSGDLEEGMLAVVK.C** 15107 |
|  | 15492 | **549.9246** | **1646.7519** | **1646.8661** | **-0.1143** | **1** | **5** | **41** | **3** | **R.IMVSRSETDLLDIR.S** |
|  | 16289 | **852.4312** | **1702.8477** | **1702.8560** | **-0.0082** | **0** | **54** | **0.00045** | **1** | **K.GVGTDEACLIEILASR.S** 16298 |
|  | 18258 | **923.4553** | **1844.8961** | **1845.8969** | **-1.0008** | **1** | **7** | **22** | **1** | **K.TLEEAIRSDTSGHFQR.L** |
|  | 20596 | **681.3705** | **2041.0898** | **2042.0433** | **-0.9535** | **1** | **1** | **78** | **3** | **R.GTITDAPGFDPLRDAEVLR.K** |
|  | 21612 | **713.6852** | **2138.0337** | **2139.0010** | **-0.9673** | **1** | **4** | **36** | **4** | **K.SICREMSGDLEEGMLAVVK.C** |
|  | 22031 | 1090.5465 | 2179.0785 | 2179.9480 | -0.8696 | 1 | 0 | 80 | 3 | R.MYGKSLYHDISGDTSGDYR.K |

| **712.** | Q9Y3Y2\|CA077_HUMAN    **Mass:** 26380    **Score:** 57     **Queries matched:** 20   **emPAI:** 0.14 |
| --- | --- |
|  | Uncharacterized protein C1orf77 OS=Homo sapiens GN=C1orf77 PE=1 SV=2 |

|  | **Query** | **Observed** | **Mr(expt)** | **Mr(calc)** | **Delta** | **Miss** | **Score** | **Expect** | **Rank** | **Peptide** |
| --- | --- | --- | --- | --- | --- | --- | --- | --- | --- | --- |
|  | 442 | 443.0379 | 442.0307 | 442.2288 | -0.1982 | 0 | 4 | 17 | 1 | R.GGPGR.G |
|  | 619 | **459.0119** | **458.0047** | **458.2601** | **-0.2555** | **0** | **6** | **52** | **1** | **R.GGLGR.G** 616 621 622 625 626 627 |
|  | 878 | **516.3036** | **515.2964** | **515.2816** | **0.0148** | **0** | **12** | **20** | **7** | **R.GGIGGR.G** |
|  | 1058 | 551.0203 | 550.0130 | 549.2660 | 0.7470 | 0 | 10 | 14 | 9 | R.GGFGGR.G |
|  | 1286 | **601.0272** | **600.0199** | **600.3456** | **-0.3257** | **1** | **7** | **29** | **1** | **R.GGVRGR.G** |
|  | 5175 | 503.1043 | 1004.1941 | 1003.4982 | 0.6959 | 1 | 4 | 50 | 8 | R.GAMGRGGIGGR.G |
|  | 6713 | 560.3362 | 1118.6579 | 1118.6230 | 0.0349 | 1 | 6 | 40 | 6 | R.TLLRGGMSLR.G |
|  | 7519 | **592.2632** | **1182.5119** | **1181.5710** | **0.9409** | **1** | **2** | **83** | **5** | **K.STTKMSLNER.F** |
|  | 9035 | **630.3204** | **1258.6262** | **1257.7115** | **0.9147** | **1** | **6** | **37** | **10** | **-.MAAQSAPKVVLK.S** |
|  | 9822 | 434.2307 | 1299.6702 | 1300.7034 | -1.0332 | 1 | 11 | 12 | 9 | R.GGMSLRGQNLLR.G |
|  | 12408 | **723.8549** | **1445.6952** | **1445.7045** | **-0.0093** | **0** | **57** | **0.00026** | **1** | **R.ASMQQQQQLASAR.N** |
|  | 13760 | **512.2678** | **1533.7816** | **1533.9216** | **-0.1399** | **1** | **5** | **39** | **10** | **R.LGRPIGALARGAIGGR.G** 13759 |
|  | 17389 | 892.4267 | 1782.8388 | 1782.9410 | -0.1022 | 0 | 2 | 74 | 8 | R.LAQQMENRPSVQAALK.L |

| **713.** | Q01105\|SET_HUMAN    **Mass:** 33469    **Score:** 57     **Queries matched:** 4   **emPAI:** 0.11 |
| --- | --- |
|  | Protein SET OS=Homo sapiens GN=SET PE=1 SV=3 |

|  | **Query** | **Observed** | **Mr(expt)** | **Mr(calc)** | **Delta** | **Miss** | **Score** | **Expect** | **Rank** | **Peptide** |
| --- | --- | --- | --- | --- | --- | --- | --- | --- | --- | --- |
|  | 2081 | **397.2295** | **792.4445** | **791.3773** | **1.0672** | **0** | **4** | **69** | **7** | **R.SSQTQNK.A** |
|  | 3604 | **447.7345** | **893.4544** | **893.4971** | **-0.0426** | **0** | **7** | **31** | **8** | **R.QSPLPPQK.K** |
|  | 18171 | **920.9011** | **1839.7877** | **1839.7992** | **-0.0115** | **0** | **57** | **0.00019** | **1** | **R.IDFYFDENPYFENK.V** |
|  | 30817 | **1456.4078** | **4366.2017** | **4366.2274** | **-0.0257** | **1** | **0** | **19** | **3** | **R.SELIAKIPNFWVTTFVNHPQVSALLGEEDEEALHYLTR.V** |

| **714.** | Q01813\|K6PP_HUMAN    **Mass:** 86454    **Score:** 57     **Queries matched:** 18   **emPAI:** 0.13 |
| --- | --- |
|  | 6-phosphofructokinase type C OS=Homo sapiens GN=PFKP PE=1 SV=2 |

|  | **Query** | **Observed** | **Mr(expt)** | **Mr(calc)** | **Delta** | **Miss** | **Score** | **Expect** | **Rank** | **Peptide** |
| --- | --- | --- | --- | --- | --- | --- | --- | --- | --- | --- |
|  | 2491 | **413.1818** | **824.3490** | **824.2970** | **0.0519** | **0** | **6** | **38** | **10** | **-.MDADDSR.A** |
|  | 5187 | 503.2907 | 1004.5668 | 1003.5563 | 1.0105 | 1 | 16 | 4.2 | 1 | R.FQDAVRLR.G 5175 5188 |
|  | 5779 | 526.2806 | 1050.5467 | 1050.5532 | -0.0065 | 0 | 43 | 0.0081 | 1 | R.TFVLEVMGR.H |
|  | 7821 | 399.9089 | 1196.7050 | 1197.7631 | -1.0581 | 1 | 13 | 7.4 | 6 | K.LRPLMKILAK.Y |
|  | 7990 | **402.5525** | **1204.6357** | **1205.6478** | **-1.0121** | **1** | **5** | **48** | **5** | **R.AMEWITAKLK.E** |
|  | 10351 | 664.7820 | 1327.5494 | 1326.6680 | 0.8814 | 1 | 7 | 31 | 10 | R.GRSFAGNLNTYK.R |
|  | 16414 | **571.4140** | **1711.2202** | **1710.8611** | **0.3591** | **1** | **1** | **65** | **3** | **K.FTTDDSICVLGISKR.N** |
|  | 17442 | 894.4522 | 1786.8899 | 1786.8996 | -0.0097 | 0 | 28 | 0.17 | 1 | K.AIGVLTSGGDAQGMNAAVR.A 17440 17441 |
|  | 19669 | **979.0154** | **1956.0162** | **1956.9905** | **-0.9743** | **1** | **1** | **68** | **6** | **K.EWSGLLEELARNGQIDK.E** 19658 |
|  | 21436 | **1060.0201** | **2118.0257** | **2118.0382** | **-0.0125** | **0** | **28** | **0.13** | **1** | **K.EIGWTDVGGWTGQGGSILGTK.R** |
|  | 27646 | 1021.4736 | 3061.3991 | 3060.5597 | 0.8394 | 1 | 0 | 50 | 10 | K.AACNLLQRGITNLCVIGGDGSLTGANLFR.K |
|  | 30811 | **1452.4896** | **4354.4470** | **4354.0740** | **0.3730** | **0** | **5** | **5** | **1** | **K.VYFIYEGYQGMVDGGSNIAEADWESVSSILQVGGTIIGSAR.C** |
|  | 31375 | 1235.3894 | 4937.5285 | 4936.4265 | 1.1020 | 1 | 2 | 8.1 | 4 | R.MGVEAVIALLEATPDTPACVVSLNGNHAVRLPLMECVQMTQDVQK.A |

| **715.** | P38919\|IF4A3_HUMAN    **Mass:** 47126    **Score:** 57     **Queries matched:** 21   **emPAI:** 0.08 |
| --- | --- |
|  | Eukaryotic initiation factor 4A-III OS=Homo sapiens GN=EIF4A3 PE=1 SV=4 |

|  | **Query** | **Observed** | **Mr(expt)** | **Mr(calc)** | **Delta** | **Miss** | **Score** | **Expect** | **Rank** | **Peptide** |
| --- | --- | --- | --- | --- | --- | --- | --- | --- | --- | --- |
|  | 2359 | **407.5530** | **813.0913** | **812.5483** | **0.5430** | **1** | **3** | **55** | **5** | **R.AIKQIIK.G** |
|  | 2607 | 416.2588 | 830.5031 | 829.4446 | 1.0585 | 0 | 10 | 22 | 2 | R.ELYIHR.I 2608 |
|  | 5270 | 507.2339 | 1012.4533 | 1013.5618 | -1.1084 | 1 | 2 | 1e+002 | 7 | K.NDDIRILR.D |
|  | 6531 | 553.3666 | 1104.7186 | 1105.5689 | -0.8503 | 1 | 5 | 50 | 2 | R.LLKEEDMTK.V |
|  | 7702 | 596.2888 | 1190.5630 | 1189.5939 | 0.9691 | 0 | 5 | 49 | 2 | R.DVIAQSQSGTGK.T |
|  | 7754 | **597.3518** | **1192.6889** | **1192.5910** | **0.0979** | **1** | **4** | **55** | **2** | **K.VDWLTEKMR.E** |
|  | 10688 | 449.8787 | 1346.6142 | 1347.7584 | -1.1443 | 1 | 10 | 15 | 5 | K.FMTDPIRILVK.R |
|  | 10859 | **679.3719** | **1356.7293** | **1355.6173** | **1.1120** | **0** | **5** | **43** | **3** | **-.MATTATMATSGSAR.K** |
|  | 11115 | 458.2946 | 1371.8621 | 1371.6122 | 0.2499 | 0 | (3) | 71 | 8 | -.MATTATMATSGSAR.K |
|  | 11123 | **458.3307** | **1371.9702** | **1371.6122** | **0.3580** | **0** | **(3)** | **51** | **6** | **-.MATTATMATSGSAR.K** |
|  | 13286 | **501.2569** | **1500.7489** | **1499.7072** | **1.0417** | **1** | **2** | **82** | **8** | **-.MATTATMATSGSARK.R** |
|  | 13767 | 768.3344 | 1534.6543 | 1535.7211 | -1.0668 | 0 | (4) | 48 | 9 | K.MLVLDEADEMLNK.G |
|  | 14027 | 776.8448 | 1551.6750 | 1551.7160 | -0.0410 | 0 | 9 | 14 | 3 | K.MLVLDEADEMLNK.G |
|  | 14263 | **784.4283** | **1566.8420** | **1566.7831** | **0.0589** | **1** | **3** | **64** | **4** | **K.QFFVAVEREEWK.F** |
|  | 14681 | 533.2827 | 1596.8263 | 1596.8373 | -0.0109 | 1 | 9 | 14 | 1 | R.KLDYGQHVVAGTPGR.V |
|  | 15022 | 540.2624 | 1617.7655 | 1616.8634 | 0.9021 | 1 | 8 | 20 | 1 | R.SGASRVLISTDVWAR.G |
|  | 17978 | 914.4666 | 1826.9187 | 1826.9315 | -0.0128 | 0 | 57 | 0.00021 | 1 | R.GIYAYGFEKPSAIQQR.A |
|  | 18340 | 926.4467 | 1850.8788 | 1851.9110 | -1.0323 | 1 | 0 | 92 | 5 | K.MLVLDEADEMLNKGFK.E |
|  | 21146 | **699.3060** | **2094.8961** | **2094.9099** | **-0.0138** | **1** | **1** | **64** | **3** | **R.EANFTVSSMHGDMPQKER.E** |
|  | 30168 | **961.4960** | **3841.9550** | **3841.8913** | **0.0637** | **1** | **1** | **27** | **3** | **R.ELAVQIQKGLLALGDYMNVQCHACIGGTNVGEDIR.K** |

| **716.** | P17693\|HLAG_HUMAN    **Mass:** 38542    **Score:** 57     **Queries matched:** 11   **emPAI:** 0.10 |
| --- | --- |
|  | HLA class I histocompatibility antigen, alpha chain G OS=Homo sapiens GN=HLA-G PE=1 SV=1 |

|  | **Query** | **Observed** | **Mr(expt)** | **Mr(calc)** | **Delta** | **Miss** | **Score** | **Expect** | **Rank** | **Peptide** |
| --- | --- | --- | --- | --- | --- | --- | --- | --- | --- | --- |
|  | 2440 | 820.4719 | 819.4646 | 818.4143 | 1.0503 | 0 | 3 | 87 | 7 | -.MVVMAPR.T |
|  | 7557 | **592.6347** | **1183.2548** | **1183.6019** | **-0.3471** | **1** | **(1)** | **72** | **8** | **K.EMLQRADPPK.T** |
|  | 7717 | 596.7304 | 1191.4463 | 1192.5036 | -1.0574 | 0 | 6 | 33 | 3 | R.GYEQYAYDGK.D |
|  | 7913 | 601.3992 | 1200.7839 | 1199.5968 | 1.1871 | 1 | 6 | 39 | 2 | K.EMLQRADPPK.T |
|  | 11562 | **699.3167** | **1396.6189** | **1395.6816** | **0.9373** | **1** | **3** | **66** | **6** | **R.YLENGKEMLQR.A** |
|  | 12099 | 714.3628 | 1426.7111 | 1426.7205 | -0.0093 | 0 | 11 | 9.6 | 1 | K.WAAVVVPSGEEQR.Y |
|  | 13341 | 753.3655 | 1504.7165 | 1504.7634 | -0.0469 | 1 | 57 | 0.00025 | 1 | R.SWTAADTAAQISKR.K |
|  | 15794 | **557.2984** | **1668.8734** | **1669.8318** | **-0.9584** | **1** | **2** | **67** | **9** | **K.AHAQTDRMNLQTLR.G** |
|  | 24023 | **811.4236** | **2431.2489** | **2430.2081** | **1.0409** | **1** | **2** | **46** | **9** | **R.ADPPKTHVTHHPVFDYEATLR.C** |
|  | 24710 | **840.7107** | **2519.1104** | **2520.3046** | **-1.1942** | **0** | **0** | **66** | **5** | **R.TLFLLLSGALTLTETWAGSHSMR.Y** |
|  | 28104 | **794.1431** | **3172.5432** | **3173.6729** | **-1.1298** | **1** | **1** | **41** | **4** | **M.VVMAPRTLFLLLSGALTLTETWAGSHSMR.Y** |

| **717.** | P46778\|RL21_HUMAN    **Mass:** 18610    **Score:** 56     **Queries matched:** 9   **emPAI:** 0.46 |
| --- | --- |
|  | 60S ribosomal protein L21 OS=Homo sapiens GN=RPL21 PE=1 SV=2 |

|  | **Query** | **Observed** | **Mr(expt)** | **Mr(calc)** | **Delta** | **Miss** | **Score** | **Expect** | **Rank** | **Peptide** |
| --- | --- | --- | --- | --- | --- | --- | --- | --- | --- | --- |
|  | 2089 | **397.7037** | **793.3928** | **794.3956** | **-1.0028** | **1** | **7** | **34** | **7** | **-.MTNTKGK.R** |
|  | 2447 | **411.2269** | **820.4392** | **820.4668** | **-0.0275** | **1** | **9** | **23** | **10** | **K.RQPAPPR.E** |
|  | 8753 | 622.3304 | 1242.6462 | 1242.6543 | -0.0081 | 0 | 26 | 0.37 | 1 | K.HGVVPLATYMR.I |
|  | 9552 | **643.5901** | **1285.1656** | **1285.6271** | **-0.4615** | **1** | **(2)** | **68** | **5** | **K.GMGTVQKGMPHK.C** |
|  | 9558 | 643.8417 | 1285.6688 | 1285.6271 | 0.0417 | 1 | 6 | 38 | 7 | K.GMGTVQKGMPHK.C |
|  | 9839 | 651.3180 | 1300.6215 | 1301.6220 | -1.0005 | 1 | (2) | 93 | 4 | K.GMGTVQKGMPHK.C |
|  | 15404 | **820.9541** | **1639.8936** | **1639.9046** | **-0.0110** | **0** | **47** | **0.0023** | **1** | **R.VYNVTQHAVGIVVNK.Q** 15377 |
|  | 19633 | **652.2716** | **1953.7928** | **1954.0749** | **-0.2820** | **1** | **2** | **55** | **2** | **K.TGRVYNVTQHAVGIVVNK.Q** |

| **718.** | Q92485\|ASM3B_HUMAN    **Mass:** 51237    **Score:** 56     **Queries matched:** 5   **emPAI:** 0.07 |
| --- | --- |
|  | Acid sphingomyelinase-like phosphodiesterase 3b OS=Homo sapiens GN=SMPDL3B PE=2 SV=2 |

|  | **Query** | **Observed** | **Mr(expt)** | **Mr(calc)** | **Delta** | **Miss** | **Score** | **Expect** | **Rank** | **Peptide** |
| --- | --- | --- | --- | --- | --- | --- | --- | --- | --- | --- |
|  | 2325 | **406.2061** | **810.3975** | **810.4348** | **-0.0373** | **0** | **9** | **18** | **5** | **K.LPGPSGAGR.I** |
|  | 21843 | **720.3357** | **2157.9854** | **2158.9776** | **-0.9921** | **0** | **4** | **33** | **4** | **K.DMVTYFMNLSQANAQGTPR.W** |
|  | 24611 | **1254.5747** | **2507.1349** | **2508.2433** | **-1.1084** | **0** | **56** | **0.00015** | **1** | **R.MLYDDAGVPISAMFITPGVTPWK.T** |
|  | 26240 | 925.4699 | 2773.3879 | 2772.3575 | 1.0304 | 1 | 3 | 31 | 4 | R.ATLSLKDMVTYFMNLSQANAQGTPR.W |
|  | 27836 | **780.3657** | **3117.4338** | **3117.4502** | **-0.0164** | **0** | **1** | **43** | **1** | **R.WELEYQLTEAYGVPDASAHSMHTVLDR.I** |

| **719.** | Q9H299\|SH3L3_HUMAN    **Mass:** 10488    **Score:** 56     **Queries matched:** 4   **emPAI:** 0.92 |
| --- | --- |
|  | SH3 domain-binding glutamic acid-rich-like protein 3 OS=Homo sapiens GN=SH3BGRL3 PE=1 SV=1 |

|  | **Query** | **Observed** | **Mr(expt)** | **Mr(calc)** | **Delta** | **Miss** | **Score** | **Expect** | **Rank** | **Peptide** |
| --- | --- | --- | --- | --- | --- | --- | --- | --- | --- | --- |
|  | 5833 | **528.7660** | **1055.5174** | **1055.5248** | **-0.0073** | **0** | **49** | **0.002** | **1** | **R.VYSTSVTGSR.E** 5821 |
|  | 12073 | 713.8198 | 1425.6250 | 1425.7464 | -0.1214 | 1 | 6 | 31 | 8 | R.VYSTSVTGSREIK.S |
|  | 23099 | **769.7143** | **2306.1210** | **2306.1325** | **-0.0114** | **1** | **31** | **0.065** | **1** | **R.IQYQLVDISQDNALRDEMR.A** |

| **720.** | Q9NX40\|OCAD1_HUMAN    **Mass:** 27780    **Score:** 56     **Queries matched:** 2   **emPAI:** 0.14 |
| --- | --- |
|  | OCIA domain-containing protein 1 OS=Homo sapiens GN=OCIAD1 PE=1 SV=1 |

|  | **Query** | **Observed** | **Mr(expt)** | **Mr(calc)** | **Delta** | **Miss** | **Score** | **Expect** | **Rank** | **Peptide** |
| --- | --- | --- | --- | --- | --- | --- | --- | --- | --- | --- |
|  | 10346 | 664.3685 | 1326.7224 | 1325.7303 | 0.9921 | 1 | 56 | 0.00033 | 1 | K.KLENSPLGEALR.S |
|  | 16325 | **427.2312** | **1704.8957** | **1705.7195** | **-0.8238** | **0** | **5** | **35** | **5** | **R.VFAECNDESFWFR.S** |

| **721.** | P17301\|ITA2_HUMAN    **Mass:** 130468   **Score:** 56     **Queries matched:** 14   **emPAI:** 0.03 |
| --- | --- |
|  | Integrin alpha-2 OS=Homo sapiens GN=ITGA2 PE=1 SV=1 |

|  | **Query** | **Observed** | **Mr(expt)** | **Mr(calc)** | **Delta** | **Miss** | **Score** | **Expect** | **Rank** | **Peptide** |
| --- | --- | --- | --- | --- | --- | --- | --- | --- | --- | --- |
|  | 1246 | **589.8028** | **588.7955** | **588.2690** | **0.5266** | **0** | **18** | **3.3** | **1** | **-.MGPER.T** |
|  | 1891 | 753.7855 | 752.7782 | 753.4425 | -0.6643 | 0 | 6 | 26 | 10 | K.FIFSLK.V |
|  | 4189 | **468.3275** | **934.6405** | **934.4872** | **0.1532** | **0** | **7** | **34** | **3** | **K.ILGSDGAFR.S** 4194 |
|  | 5255 | 506.2830 | 1010.5514 | 1010.6164 | -0.0650 | 1 | 4 | 60 | 9 | R.VYLFTIKK.G |
|  | 7414 | **587.8229** | **1173.6313** | **1173.6394** | **-0.0081** | **0** | **7** | **32** | **1** | **K.FVQGLDIGPTK.T** |
|  | 12816 | **737.3900** | **1472.7653** | **1472.7736** | **-0.0082** | **0** | **4** | **52** | **1** | **K.TQVGLIQYANNPR.V** 12831 |
|  | 14059 | **518.9172** | **1553.7297** | **1552.8613** | **0.8684** | **0** | **3** | **63** | **10** | **K.IPLLYDAEIHLTR.S** |
|  | 16729 | **579.5331** | **1735.5776** | **1735.8815** | **-0.3039** | **1** | **3** | **43** | **4** | **K.EKNPLMYLTGVQTDK.A** |
|  | 17755 | **906.4703** | **1810.9260** | **1810.8672** | **0.0588** | **1** | **2** | **65** | **2** | **K.DVHMKGEYFVNVTTR.I** |
|  | 22444 | **743.0483** | **2226.1232** | **2225.1323** | **0.9909** | **1** | **0** | **83** | **6** | **K.DTITDVLLVGAPMYMSDLKK.E** |
|  | 27960 | **1577.1440** | **3152.2735** | **3152.5040** | **-0.2304** | **1** | **1** | **33** | **2** | **K.CPVDLSTATCEKLNLQTSTSIPNVTEMK.T** |
|  | 29097 | **1156.8928** | **3467.6566** | **3467.6813** | **-0.0247** | **0** | **56** | **0.00011** | **1** | **K.NMVVNQAQSCPEHIIYIQEPSDVVNSLDLR.V** |

| **722.** | P30511\|HLAF_HUMAN    **Mass:** 39255    **Score:** 56     **Queries matched:** 7   **emPAI:** 0.10 |
| --- | --- |
|  | HLA class I histocompatibility antigen, alpha chain F OS=Homo sapiens GN=HLA-F PE=2 SV=2 |

|  | **Query** | **Observed** | **Mr(expt)** | **Mr(calc)** | **Delta** | **Miss** | **Score** | **Expect** | **Rank** | **Peptide** |
| --- | --- | --- | --- | --- | --- | --- | --- | --- | --- | --- |
|  | 4984 | 496.7711 | 991.5276 | 990.4771 | 1.0505 | 0 | 6 | 45 | 7 | R.FDSDAAIPR.M |
|  | 8654 | **413.5326** | **1237.5761** | **1236.5986** | **0.9775** | **0** | **9** | **18** | **3** | **K.DYISLNEDLR.S** 8655 |
|  | 12099 | 714.3628 | 1426.7111 | 1426.7205 | -0.0093 | 0 | 11 | 9.6 | 1 | K.WAAVVVPSGEEQR.Y |
|  | 21343 | 1056.5170 | 2111.0194 | 2111.0299 | -0.0105 | 0 | 45 | 0.003 | 1 | R.CWALGFYPAEITLTWQR.D 21342 |
|  | 26214 | **693.1296** | **2768.4892** | **2769.1290** | **-0.6398** | **1** | **6** | **16** | **1** | **R.RYNQSEAGSHTLQGMNGCDMGPDGR.L** |

| **723.** | P06753\|TPM3_HUMAN    **Mass:** 32856    **Score:** 56     **Queries matched:** 15   **emPAI:** 0.39 |
| --- | --- |
|  | Tropomyosin alpha-3 chain OS=Homo sapiens GN=TPM3 PE=1 SV=1 |

|  | **Query** | **Observed** | **Mr(expt)** | **Mr(calc)** | **Delta** | **Miss** | **Score** | **Expect** | **Rank** | **Peptide** |
| --- | --- | --- | --- | --- | --- | --- | --- | --- | --- | --- |
|  | 1244 | **589.3513** | **588.3440** | **588.3483** | **-0.0043** | **0** | **13** | **16** | **1** | **K.ILTDK.L** |
|  | 1648 | 683.4768 | 682.4695 | 681.3190 | 1.1506 | 0 | 8 | 24 | 8 | K.MQMLK.L |
|  | 2090 | **397.7126** | **793.4107** | **793.4190** | **-0.0083** | **1** | **(3)** | **83** | **10** | **K.KMQMLK.L** |
|  | 2092 | **397.7214** | **793.4282** | **793.4190** | **0.0092** | **1** | **9** | **21** | **1** | **K.KMQMLK.L** 2072 |
|  | 3604 | 447.7345 | 893.4544 | 893.4606 | -0.0062 | 1 | 9 | 23 | 3 | R.KYEEVAR.K |
|  | 5630 | **520.3093** | **1038.6040** | **1037.5249** | **1.0791** | **1** | **8** | **27** | **4** | **K.MQMLKLDK.E** |
|  | 6581 | **555.3152** | **1108.6158** | **1108.4819** | **0.1340** | **1** | **4** | **53** | **8** | **K.AADESERGMK.V** |
|  | 6857 | **566.3038** | **1130.5930** | **1130.6005** | **-0.0075** | **0** | **33** | **0.079** | **1** | **K.MELQEIQLK.E** |
|  | 9523 | 642.8752 | 1283.7358 | 1283.7449 | -0.0091 | 1 | 36 | 0.034 | 1 | R.KLVIIEGDLER.T 9529 |
|  | 10114 | **658.8212** | **1315.6279** | **1315.6368** | **-0.0089** | **0** | **30** | **0.14** | **1** | **K.AADAEAEVASLNR.R** 10138 |
|  | 11945 | 710.3324 | 1418.6502 | 1418.7075 | -0.0572 | 1 | 8 | 22 | 5 | K.QLEDELAAMQKK.L |
|  | 15507 | **550.2769** | **1647.8089** | **1647.8025** | **0.0064** | **1** | **5** | **39** | **5** | **K.DEEKMELQEIQLK.E** |

| **724.** | Q03154\|ACY1_HUMAN    **Mass:** 46084    **Score:** 55     **Queries matched:** 12   **emPAI:** 0.37 |
| --- | --- |
|  | Aminoacylase-1 OS=Homo sapiens GN=ACY1 PE=1 SV=1 |

|  | **Query** | **Observed** | **Mr(expt)** | **Mr(calc)** | **Delta** | **Miss** | **Score** | **Expect** | **Rank** | **Peptide** |
| --- | --- | --- | --- | --- | --- | --- | --- | --- | --- | --- |
|  | 1509 | **649.2019** | **648.1946** | **648.2901** | **-0.0955** | **0** | **6** | **55** | **2** | **R.GAQDMK.C** |
|  | 4890 | 493.2841 | 984.5536 | 983.5552 | 0.9983 | 0 | 24 | 0.67 | 1 | R.LHEAVFLR.G |
|  | 6087 | **537.2450** | **1072.4754** | **1072.4825** | **-0.0071** | **0** | **28** | **0.34** | **1** | **K.DSEGYIYAR.G** |
|  | 8414 | **613.6140** | **1225.2133** | **1224.6323** | **0.5810** | **0** | **0** | **91** | **10** | **R.VTSTGRPGHASR.F** |
|  | 11895 | **708.3726** | **1414.7307** | **1414.7391** | **-0.0084** | **0** | **15** | **4.5** | **1** | **R.AVGVPALGFSPMNR.T** |
|  | 20440 | **1015.9739** | **2029.9333** | **2029.9448** | **-0.0115** | **0** | **37** | **0.018** | **1** | **K.DMNLTLEPEIMPAATDNR.Y** |
|  | 22750 | **755.7399** | **2264.1980** | **2263.0651** | **1.1328** | **1** | **0** | **75** | **10** | **R.VTSTGRPGHASRFMEDTAAEK.L** |
|  | 23884 | 807.0318 | 2418.0736 | 2417.1389 | 0.9347 | 1 | 5 | 22 | 3 | R.VCKDMNLTLEPEIMPAATDNR.Y |
|  | 24482 | **831.0604** | **2490.1594** | **2490.1703** | **-0.0109** | **0** | **14** | **2.8** | **1** | **R.AGFALDEGIANPTDAFTVFYSER.S** |
|  | 24755 | **843.7136** | **2528.1189** | **2528.1332** | **-0.0143** | **0** | **28** | **0.095** | **1** | **K.WMHPQVTPTDDSNPWWAAFSR.V** |
|  | 30309 | 1317.3718 | 3949.0937 | 3949.1241 | -0.0305 | 0 | 30 | 0.029 | 1 | K.VEVAPGYVVTVLTWPGTNPTLSSILLNSHTDVVPVFK.E 30310 |

| **725.** | Q9BYZ8\|REG4_HUMAN    **Mass:** 18617    **Score:** 55     **Queries matched:** 12   **emPAI:** 0.46 |
| --- | --- |
|  | Regenerating islet-derived protein 4 OS=Homo sapiens GN=REG4 PE=1 SV=1 |

|  | **Query** | **Observed** | **Mr(expt)** | **Mr(calc)** | **Delta** | **Miss** | **Score** | **Expect** | **Rank** | **Peptide** |
| --- | --- | --- | --- | --- | --- | --- | --- | --- | --- | --- |
|  | 2704 | **419.3145** | **836.6145** | **837.3949** | **-0.7804** | **1** | **(4)** | **51** | **9** | **-.MASRSMR.L** 2702 |
|  | 2965 | **428.2591** | **854.5036** | **853.3898** | **1.1138** | **1** | **(6)** | **39** | **4** | **-.MASRSMR.L** 2934 |
|  | 3251 | **435.2926** | **868.5706** | **869.3847** | **-0.8141** | **1** | **15** | **4.3** | **1** | **-.MASRSMR.L** |
|  | 5981 | 534.2584 | 1066.5023 | 1065.4338 | 1.0685 | 0 | 5 | 44 | 6 | K.SNCYGYFR.K |
|  | 7149 | 577.3309 | 1152.6472 | 1153.5186 | -0.8714 | 1 | 3 | 75 | 9 | R.SWSGKSMGGNK.H |
|  | 13744 | **767.3939** | **1532.7732** | **1532.8782** | **-0.1050** | **1** | **6** | **31** | **6** | **R.SMRLLLLLSCLAK.T** |
|  | 15888 | **559.2841** | **1674.8304** | **1673.9002** | **0.9302** | **1** | **1** | **83** | **4** | **R.SQPIWIGLHDPQKR.Q** |
|  | 18471 | 929.9374 | 1857.8602 | 1856.8668 | 0.9934 | 0 | 16 | 2.8 | 1 | R.QQWQWIDGAMYLYR.S |
|  | 25700 | **896.0389** | **2685.0950** | **2685.1118** | **-0.0168** | **1** | **58** | **8.3e-005** | **1** | **K.HCAEMSSNNNFLTWSSNECNKR.Q** |
|  | 29337 | 1182.6409 | 3544.9008 | 3544.8761 | 0.0247 | 1 | 1 | 22 | 3 | R.LLLLLSCLAKTGVLGDIIMRPSCAPGWFYHK.S |

| **726.** | P62750\|RL23A_HUMAN    **Mass:** 17684    **Score:** 55     **Queries matched:** 6   **emPAI:** 0.22 |
| --- | --- |
|  | 60S ribosomal protein L23a OS=Homo sapiens GN=RPL23A PE=1 SV=1 |

|  | **Query** | **Observed** | **Mr(expt)** | **Mr(calc)** | **Delta** | **Miss** | **Score** | **Expect** | **Rank** | **Peptide** |
| --- | --- | --- | --- | --- | --- | --- | --- | --- | --- | --- |
|  | 6325 | 545.2872 | 1088.5598 | 1088.6091 | -0.0493 | 1 | 5 | 60 | 7 | R.TSPTFRRPK.T |
|  | 8188 | **607.8439** | **1213.6733** | **1213.6819** | **-0.0086** | **1** | **8** | **25** | **5** | **R.NKLDHYAIIK.F** |
|  | 8740 | **414.8981** | **1241.6724** | **1240.6775** | **0.9948** | **0** | **13** | **7.7** | **1** | **K.VNTLIRPDGEK.K** |
|  | 11667 | **702.8494** | **1403.6842** | **1403.6932** | **-0.0090** | **0** | **55** | **0.00039** | **1** | **R.LAPDYDALDVANK.I** |
|  | 17592 | **600.6383** | **1798.8931** | **1799.9669** | **-1.0738** | **1** | **1** | **91** | **5** | **R.LAPDYDALDVANKIGII.-** 17596 |

| **727.** | Q16720\|AT2B3_HUMAN    **Mass:** 135253   **Score:** 55     **Queries matched:** 33   **emPAI:** 0.06 |
| --- | --- |
|  | Plasma membrane calcium-transporting ATPase 3 OS=Homo sapiens GN=ATP2B3 PE=1 SV=3 |

|  | **Query** | **Observed** | **Mr(expt)** | **Mr(calc)** | **Delta** | **Miss** | **Score** | **Expect** | **Rank** | **Peptide** |
| --- | --- | --- | --- | --- | --- | --- | --- | --- | --- | --- |
|  | 2079 | 397.2295 | 792.4444 | 792.4164 | 0.0280 | 0 | 15 | 6.4 | 4 | K.SMSTVIR.M |
|  | 2288 | 404.6981 | 807.3817 | 808.4113 | -1.0296 | 0 | (4) | 71 | 10 | K.SMSTVIR.M |
|  | 2570 | **415.2292** | **828.4439** | **827.4865** | **0.9574** | **0** | **7** | **43** | **4** | **R.DKPLISR.T** 2543 |
|  | 3974 | 460.2462 | 918.4779 | 918.5076 | -0.0296 | 0 | 8 | 31 | 3 | R.GQILWFR.G |
|  | 4886 | 493.2556 | 984.4965 | 984.5029 | -0.0063 | 0 | 8 | 27 | 3 | K.VYTFNSVR.K |
|  | 7709 | 596.3003 | 1190.5861 | 1190.5714 | 0.0148 | 0 | 11 | 13 | 8 | R.MVTGDNINTAR.A 7708 |
|  | 7849 | 400.2375 | 1197.6906 | 1197.6942 | -0.0035 | 1 | 8 | 21 | 3 | R.GLNRIQTQIR.V |
|  | 9096 | 631.7887 | 1261.5628 | 1261.6554 | -0.0925 | 1 | 11 | 13 | 4 | R.EQIPEDKLYK.V |
|  | 9937 | 653.8759 | 1305.7373 | 1304.7704 | 0.9669 | 1 | 2 | 87 | 7 | R.LFSKGASEILLK.K |
|  | 10155 | **440.2385** | **1317.6936** | **1318.7608** | **-1.0673** | **1** | **6** | **35** | **8** | **K.ILPPEKEGALPR.Q** |
|  | 11927 | 473.2862 | 1416.8367 | 1417.7057 | -0.8691 | 1 | 8 | 20 | 9 | R.KIIEPMACDGLR.T 11929 |
|  | 12145 | 715.4211 | 1428.8277 | 1428.8201 | 0.0076 | 1 | 13 | 7.3 | 2 | R.KPYGRDKPLISR.T 12117 12144 |
|  | 12464 | 484.2489 | 1449.7249 | 1448.6639 | 1.0609 | 0 | 3 | 74 | 3 | K.QDGAVAMEMQPLK.S |
|  | 13411 | **504.2442** | **1509.7108** | **1509.6729** | **0.0378** | **1** | **2** | **75** | **3** | **K.GKQQDGAMESSQTK.A** |
|  | 14178 | **781.8798** | **1561.7451** | **1560.7640** | **0.9811** | **1** | **5** | **36** | **1** | **K.KQDGAVAMEMQPLK.S** |
|  | 14414 | **526.9377** | **1577.7914** | **1576.7589** | **1.0325** | **1** | **(3)** | **63** | **6** | **K.KQDGAVAMEMQPLK.S** |
|  | 14525 | **529.3671** | **1585.0794** | **1584.7389** | **0.3405** | **1** | **3** | **41** | **8** | **K.SMSTVIRMPDGGFR.L** |
|  | 15416 | 821.4199 | 1640.8253 | 1640.8370 | -0.0117 | 0 | 47 | 0.0022 | 1 | R.QVVAVTGDGTNDGPALK.K 15417 15418 |
|  | 16104 | **564.3466** | **1690.0179** | **1689.8145** | **0.2034** | **0** | **7** | **18** | **1** | **R.DVPQAGGFGCTLAELR.T** |
|  | 18742 | 941.4688 | 1880.9231 | 1879.9415 | 0.9816 | 0 | 27 | 0.2 | 1 | K.EASDIILTDDNFTSIVK.A |
|  | 19191 | 958.0068 | 1913.9990 | 1914.0098 | -0.0108 | 0 | 27 | 0.21 | 2 | K.YGDLLPADGVLIQANDLK.I 19190 |
|  | 21454 | 707.3782 | 2119.1129 | 2118.9674 | 0.1455 | 1 | 9 | 9.9 | 1 | K.SADKDPMLLSGTHVMEGSGR.M |
|  | 22956 | **764.0409** | **2289.1010** | **2289.1497** | **-0.0487** | **1** | **1** | **69** | **4** | **R.AIAAKCGIIQPGEDFLCLEGK.E** |
|  | 30487 | 681.8417 | 4085.0068 | 4085.1548 | -0.1480 | 1 | 2 | 17 | 1 | R.NVYDSISKFLQFQLTVNVVAVIVAFTGACITQDSPLK.A |
|  | 30991 | **1137.7662** | **4547.0359** | **4548.2186** | **-1.1828** | **1** | **0** | **18** | **6** | **K.DPMLLSGTHVMEGSGRMVVTAVGVNSQTGIIFTLLGAGGEEEEK.K** |

| **728.** | Q9Y639\|NPTN_HUMAN    **Mass:** 31500    **Score:** 55     **Queries matched:** 3   **emPAI:** 0.25 |
| --- | --- |
|  | Neuroplastin OS=Homo sapiens GN=NPTN PE=1 SV=1 |

|  | **Query** | **Observed** | **Mr(expt)** | **Mr(calc)** | **Delta** | **Miss** | **Score** | **Expect** | **Rank** | **Peptide** |
| --- | --- | --- | --- | --- | --- | --- | --- | --- | --- | --- |
|  | 17258 | **592.2465** | **1773.7177** | **1773.7298** | **-0.0120** | **1** | **30** | **0.1** | **1** | **R.SENKNEGQDATMYCK.S** |
|  | 21146 | 699.3060 | 2094.8961 | 2094.9105 | -0.0145 | 0 | 43 | 0.0041 | 1 | R.AEDSGEYHCVYHFVSAPK.A |
|  | 23048 | **767.4000** | **2299.1782** | **2298.1137** | **1.0646** | **1** | **2** | **52** | **7** | **K.ENGMPMDIVNTSGRFFIINK.E** |

| **729.** | Q96L21\|RL10L_HUMAN    **Mass:** 24959    **Score:** 55     **Queries matched:** 14   **emPAI:** 0.33 |
| --- | --- |
|  | 60S ribosomal protein L10-like OS=Homo sapiens GN=RPL10L PE=1 SV=3 |

|  | **Query** | **Observed** | **Mr(expt)** | **Mr(calc)** | **Delta** | **Miss** | **Score** | **Expect** | **Rank** | **Peptide** |
| --- | --- | --- | --- | --- | --- | --- | --- | --- | --- | --- |
|  | 1271 | 597.3676 | 596.3603 | 596.3646 | -0.0042 | 0 | 7 | 21 | 2 | K.IHISK.K |
|  | 4824 | 490.7149 | 979.4152 | 979.4215 | -0.0063 | 0 | 42 | 0.0087 | 1 | K.MLSCAGADR.L 4823 4839 |
|  | 5026 | 498.2762 | 994.5379 | 995.4164 | -0.8786 | 0 | (10) | 14 | 7 | K.MLSCAGADR.L 5039 |
|  | 5111 | **500.7648** | **999.5151** | **999.4630** | **0.0521** | **1** | **9** | **24** | **6** | **K.YMVKSCGR.D** |
|  | 6600 | **556.2781** | **1110.5416** | **1111.5662** | **-1.0246** | **0** | **6** | **32** | **6** | **K.YVPSHGPLDK.W** |
|  | 7642 | 594.8236 | 1187.6327 | 1187.6411 | -0.0084 | 0 | 43 | 0.0075 | 1 | R.GAFGKPQGTVAR.V |
|  | 11115 | 458.2946 | 1371.8621 | 1372.8092 | -0.9470 | 1 | 4 | 49 | 3 | R.LHPFHVIRINK.M |
|  | 13247 | 500.2798 | 1497.8175 | 1496.8497 | 0.9678 | 1 | 9 | 15 | 2 | R.VHIGQVIMSIRTK.L 13217 |
|  | 13896 | 772.8280 | 1543.6414 | 1542.7024 | 0.9390 | 1 | 31 | 0.087 | 2 | K.FNADEFEDMVAKK.C |
|  | 24053 | 813.0750 | 2436.2032 | 2437.3376 | -1.1344 | 1 | 2 | 40 | 3 | R.GAFGKPQGTVARVHIGQVIMSIR.T |

| **730.** | P49720\|PSB3_HUMAN    **Mass:** 23219    **Score:** 55     **Queries matched:** 13   **emPAI:** 0.16 |
| --- | --- |
|  | Proteasome subunit beta type-3 OS=Homo sapiens GN=PSMB3 PE=1 SV=2 |

|  | **Query** | **Observed** | **Mr(expt)** | **Mr(calc)** | **Delta** | **Miss** | **Score** | **Expect** | **Rank** | **Peptide** |
| --- | --- | --- | --- | --- | --- | --- | --- | --- | --- | --- |
|  | 13120 | **497.7475** | **1490.2208** | **1490.6567** | **-0.4360** | **0** | **5** | **29** | **4** | **M.SIMSYNGGAVMAMK.G** |
|  | 14839 | 536.6107 | 1606.8103 | 1605.7023 | 1.1080 | 0 | (4) | 51 | 3 | -.MSIMSYNGGAVMAMK.G |
|  | 15068 | **541.2776** | **1620.8111** | **1621.6972** | **-0.8861** | **0** | **(3)** | **59** | **3** | **-.MSIMSYNGGAVMAMK.G** |
|  | 15091 | **541.7687** | **1622.2844** | **1621.6972** | **0.5872** | **0** | **(1)** | **63** | **7** | **-.MSIMSYNGGAVMAMK.G** |
|  | 15110 | **541.9556** | **1622.8451** | **1621.6972** | **1.1478** | **0** | **(1)** | **97** | **4** | **-.MSIMSYNGGAVMAMK.G** |
|  | 15335 | 819.4098 | 1636.8050 | 1637.6921 | -0.8871 | 0 | (0) | 1.1e+002 | 10 | -.MSIMSYNGGAVMAMK.G |
|  | 15370 | 547.2587 | 1638.7542 | 1637.6921 | 1.0620 | 0 | 4 | 45 | 4 | -.MSIMSYNGGAVMAMK.G |
|  | 15922 | **559.8418** | **1676.5036** | **1675.7732** | **0.7304** | **1** | **1** | **67** | **2** | **M.SIMSYNGGAVMAMKGK.N** |
|  | 17155 | **883.9476** | **1765.8807** | **1765.8927** | **-0.0120** | **0** | **50** | **0.0011** | **1** | **R.FGPYYTEPVIAGLDPK.T** 17156 |
|  | 17696 | **603.3295** | **1806.9668** | **1806.8136** | **0.1531** | **1** | **(3)** | **55** | **5** | **-.MSIMSYNGGAVMAMKGK.N** |
|  | 17884 | 911.9589 | 1821.9032 | 1822.8086 | -0.9054 | 1 | 4 | 48 | 7 | -.MSIMSYNGGAVMAMKGK.N |
|  | 17916 | **608.8162** | **1823.4268** | **1822.8086** | **0.6183** | **1** | **(0)** | **66** | **6** | **-.MSIMSYNGGAVMAMKGK.N** |

| **731.** | O75083\|WDR1_HUMAN    **Mass:** 66836    **Score:** 55     **Queries matched:** 10   **emPAI:** 0.18 |
| --- | --- |
|  | WD repeat-containing protein 1 OS=Homo sapiens GN=WDR1 PE=1 SV=4 |

|  | **Query** | **Observed** | **Mr(expt)** | **Mr(calc)** | **Delta** | **Miss** | **Score** | **Expect** | **Rank** | **Peptide** |
| --- | --- | --- | --- | --- | --- | --- | --- | --- | --- | --- |
|  | 2160 | **400.6922** | **799.3698** | **799.4552** | **-0.0854** | **0** | **6** | **54** | **10** | **R.IAVVGEGR.E** |
|  | 3453 | **442.2313** | **882.4480** | **882.4633** | **-0.0153** | **0** | **14** | **6.1** | **2** | **R.YTSLMLR.D** 3448 |
|  | 7030 | 573.3156 | 1144.6167 | 1144.6241 | -0.0074 | 0 | 16 | 4.5 | 3 | K.VFASLPQVER.G |
|  | 8336 | **611.2822** | **1220.5499** | **1219.5833** | **0.9666** | **1** | **2** | **1e+002** | **7** | **K.DIAWTEDSKR.I** |
|  | 13424 | **504.5250** | **1510.5532** | **1510.8144** | **-0.2611** | **1** | **1** | **83** | **4** | **R.IWDTTQKEHLLK.Y** |
|  | 15020 | 809.8862 | 1617.7578 | 1617.7675 | -0.0097 | 0 | 33 | 0.055 | 1 | K.YAPSGFYIASGDVSGK.L |
|  | 21845 | 1080.0089 | 2158.0033 | 2158.9181 | -0.9148 | 0 | 1 | 70 | 3 | R.MTVDESGQLISCSMDDTVR.Y |
|  | 22266 | 737.0255 | 2208.0547 | 2207.0648 | 0.9899 | 0 | 40 | 0.0087 | 1 | K.FGAVFLWDSGSSVGEITGHNK.V |
|  | 28912 | **1132.2266** | **3393.6579** | **3393.6738** | **-0.0159** | **0** | **29** | **0.06** | **1** | **K.IWDVSVNSVVSTFPMGSTVLDQQLGCLWQK.D** |

| **732.** | O94826\|TOM70_HUMAN    **Mass:** 68096    **Score:** 55     **Queries matched:** 13   **emPAI:** 0.24 |
| --- | --- |
|  | Mitochondrial import receptor subunit TOM70 OS=Homo sapiens GN=TOMM70A PE=1 SV=1 |

|  | **Query** | **Observed** | **Mr(expt)** | **Mr(calc)** | **Delta** | **Miss** | **Score** | **Expect** | **Rank** | **Peptide** |
| --- | --- | --- | --- | --- | --- | --- | --- | --- | --- | --- |
|  | 1114 | 561.3568 | 560.3495 | 559.2966 | 1.0530 | 0 | 12 | 19 | 5 | K.EANVK.L |
|  | 1949 | **770.4356** | **769.4283** | **769.4334** | **-0.0050** | **0** | **33** | **0.069** | **1** | **K.AVELNPK.Y** |
|  | 2206 | **402.2431** | **802.4716** | **801.4232** | **1.0484** | **0** | **7** | **41** | **10** | **K.AIEIDNK.C** |
|  | 3331 | 438.2315 | 874.4484 | 873.4443 | 1.0041 | 0 | 10 | 23 | 10 | K.EGEALEVK.E |
|  | 9145 | 422.2404 | 1263.6994 | 1263.5918 | 0.1077 | 1 | 4 | 57 | 10 | K.WKEVAQDCTK.A |
|  | 10037 | **438.2487** | **1311.7244** | **1310.7306** | **0.9938** | **0** | **25** | **0.46** | **1** | **R.LRPESALAQAQK.C** 10020 |
|  | 10833 | 678.8254 | 1355.6362 | 1355.6470 | -0.0108 | 0 | 41 | 0.013 | 1 | K.NVDLSTFYQNR.A |
|  | 11376 | 693.8876 | 1385.7606 | 1385.7514 | 0.0092 | 1 | 7 | 26 | 8 | K.QDLDRGLELISK.A |
|  | 12120 | **714.9067** | **1427.7988** | **1428.6377** | **-0.8389** | **1** | **3** | **68** | **1** | **R.GNMEKAIDMFNK.A** |
|  | 14099 | 778.8868 | 1555.7590 | 1555.8181 | -0.0590 | 1 | 6 | 35 | 4 | K.NREPLMPSPQFIK.S |
|  | 21573 | **712.3462** | **2134.0167** | **2134.0252** | **-0.0085** | **0** | **31** | **0.073** | **1** | **K.ILLDQVEEAVADFDECIR.L** |
|  | 24769 | **844.1081** | **2529.3024** | **2529.2169** | **0.0855** | **1** | **4** | **27** | **2** | **R.QAYTGNNSSQIQAAMKGFEEVIK.K** |

| **733.** | Q03519\|TAP2_HUMAN    **Mass:** 76186    **Score:** 54     **Queries matched:** 13   **emPAI:** 0.15 |
| --- | --- |
|  | Antigen peptide transporter 2 OS=Homo sapiens GN=TAP2 PE=1 SV=1 |

|  | **Query** | **Observed** | **Mr(expt)** | **Mr(calc)** | **Delta** | **Miss** | **Score** | **Expect** | **Rank** | **Peptide** |
| --- | --- | --- | --- | --- | --- | --- | --- | --- | --- | --- |
|  | 1859 | **744.4313** | **743.4241** | **743.4290** | **-0.0049** | **0** | **17** | **4.4** | **2** | **R.ALVAGASR.A** |
|  | 2540 | 414.7127 | 827.4107 | 826.5137 | 0.8971 | 1 | 4 | 70 | 9 | K.QRLAIAR.A |
|  | 3779 | **453.2303** | **904.4461** | **904.4072** | **0.0389** | **0** | **2** | **1.2e+002** | **9** | **K.EALEQCR.Q** |
|  | 4749 | 488.2433 | 974.4721 | 973.4465 | 1.0256 | 0 | 13 | 8.6 | 3 | K.EQDQVNNK.V 4744 |
|  | 6098 | 537.2978 | 1072.5810 | 1073.6419 | -1.0609 | 1 | 9 | 26 | 6 | K.VLMWRLLK.L 6122 |
|  | 8590 | **618.3542** | **1234.6938** | **1234.7034** | **-0.0095** | **0** | **31** | **0.11** | **1** | **R.AHQILVLQEGK.L** |
|  | 10209 | **661.2828** | **1320.5510** | **1319.5564** | **0.9946** | **0** | **37** | **0.03** | **1** | **R.SFGAEEHEVCR.Y** |
|  | 13432 | **756.4677** | **1510.9209** | **1510.8216** | **0.0993** | **1** | **3** | **51** | **9** | **R.EIQDAVARAGQVVR.E** |
|  | 14884 | **537.7914** | **1610.3525** | **1610.7147** | **-0.3622** | **1** | **2** | **49** | **3** | **R.SFGAEEHEVCRYK.E** |
|  | 19145 | **638.3276** | **1911.9609** | **1913.0696** | **-1.1087** | **0** | **3** | **45** | **2** | **R.LTLLSLLHMPFTIAAEK.V** |
|  | 23499 | **1180.1246** | **2358.2347** | **2357.2413** | **0.9934** | **0** | **34** | **0.03** | **1** | **R.LSSDTTLMSNWLPLNANVLLR.S** |

| **734.** | P21439\|MDR3_HUMAN    **Mass:** 142004   **Score:** 54     **Queries matched:** 26   **emPAI:** 0.08 |
| --- | --- |
|  | Multidrug resistance protein 3 OS=Homo sapiens GN=ABCB4 PE=1 SV=2 |

|  | **Query** | **Observed** | **Mr(expt)** | **Mr(calc)** | **Delta** | **Miss** | **Score** | **Expect** | **Rank** | **Peptide** |
| --- | --- | --- | --- | --- | --- | --- | --- | --- | --- | --- |
|  | 1092 | 559.4482 | 558.4410 | 558.3377 | 0.1033 | 0 | 4 | 1.1e+002 | 5 | K.EIGIK.K |
|  | 1659 | 686.4152 | 685.4079 | 685.4122 | -0.0043 | 0 | 13 | 11 | 8 | K.LLAGNAK.R |
|  | 2540 | 414.7127 | 827.4107 | 826.5137 | 0.8971 | 1 | 4 | 70 | 9 | K.QRIAIAR.A |
|  | 4672 | 485.7269 | 969.4392 | 968.5226 | 0.9166 | 0 | 15 | 4.8 | 2 | R.TCIVIAHR.L |
|  | 5047 | 499.2264 | 996.4383 | 995.6603 | 0.7780 | 1 | 8 | 25 | 1 | R.IAIARALIR.Q |
|  | 6066 | 536.3405 | 1070.6663 | 1069.5412 | 1.1251 | 1 | 6 | 41 | 8 | R.SMAFKAMLR.Q |
|  | 6813 | 564.7820 | 1127.5495 | 1127.6299 | -0.0803 | 1 | 16 | 4.3 | 3 | K.VVQEALDKAR.E 6811 6815 6818 |
|  | 6848 | 565.7897 | 1129.5649 | 1128.6000 | 0.9649 | 1 | 2 | 94 | 8 | R.GAQLSGGQKQR.I |
|  | 7101 | 576.2555 | 1150.4964 | 1149.5700 | 0.9265 | 1 | 22 | 1 | 2 | R.GNVTMDEIKK.A |
|  | 8198 | **405.8704** | **1214.5893** | **1214.5641** | **0.0251** | **0** | **7** | **30** | **7** | **K.EANAYEFIMK.L** |
|  | 8259 | 406.6998 | 1217.0776 | 1217.5975 | -0.5199 | 1 | 4 | 44 | 6 | K.AATRMAPNGWK.S |
|  | 9372 | 638.3942 | 1274.7738 | 1273.6626 | 1.1111 | 1 | 37 | 0.026 | 1 | R.VGDKGTQLSGGQK.Q 9373 |
|  | 10477 | 668.2856 | 1334.5566 | 1333.6660 | 0.8906 | 0 | 37 | 0.029 | 1 | K.GQTLALVGSSGCGK.S |
|  | 11475 | **464.9242** | **1391.7507** | **1392.6972** | **-0.9465** | **0** | **4** | **50** | **10** | **R.FGAYLIVNGHMR.F** |
|  | 12669 | 732.3298 | 1462.6450 | 1461.7610 | 0.8840 | 1 | 29 | 0.16 | 1 | K.KGQTLALVGSSGCGK.S 12661 |
|  | 12726 | **489.9253** | **1466.7540** | **1465.8504** | **0.9036** | **0** | **1** | **93** | **9** | **R.ANVPVLQGLSLEVK.K** |
|  | 14159 | 781.3724 | 1560.7302 | 1560.8260 | -0.0958 | 1 | 5 | 39 | 6 | R.TVIAFGGQNKELER.Y |
|  | 15002 | 539.6297 | 1615.8673 | 1615.8794 | -0.0122 | 1 | 20 | 1.3 | 1 | R.VKEHGTHQQLLAQK.G |
|  | 16658 | 866.4455 | 1730.8764 | 1729.8345 | 1.0419 | 0 | 3 | 53 | 6 | K.GIYFSMVSVQAGTQNL.- |
|  | 17112 | 882.4616 | 1762.9087 | 1761.9010 | 1.0077 | 1 | 7 | 24 | 2 | K.FDTLVGERGAQLSGGQK.Q |
|  | 30664 | **1394.9767** | **4181.9082** | **4180.9692** | **0.9390** | **1** | **15** | **0.69** | **1** | **R.YSDWQDKLFMSLGTIMAIAHGSGLPLMMIVFGEMTDK.F** |

| **735.** | P13929\|ENOB_HUMAN    **Mass:** 47244    **Score:** 54     **Queries matched:** 16   **emPAI:** 0.08 |
| --- | --- |
|  | Beta-enolase OS=Homo sapiens GN=ENO3 PE=1 SV=4 |

|  | **Query** | **Observed** | **Mr(expt)** | **Mr(calc)** | **Delta** | **Miss** | **Score** | **Expect** | **Rank** | **Peptide** |
| --- | --- | --- | --- | --- | --- | --- | --- | --- | --- | --- |
|  | 4604 | 482.7175 | 963.4205 | 963.5324 | -0.1119 | 1 | 17 | 3.6 | 6 | M.AMQKIFAR.E 4602 4603 4606 4607 |
|  | 6688 | 559.7805 | 1117.5464 | 1118.5940 | -1.0476 | 1 | 6 | 41 | 6 | K.KACNCLLLK.V |
|  | 6900 | 567.7711 | 1133.5276 | 1133.5717 | -0.0441 | 0 | 10 | 17 | 4 | K.TAIQAAGYPDK.V 6901 6902 |
|  | 13811 | **770.0015** | **1537.9885** | **1537.7049** | **0.2836** | **1** | **1** | **77** | **10** | **K.YDLDFKSPDDPAR.H** |
|  | 14628 | 796.8754 | 1591.7362 | 1592.8271 | -1.0909 | 1 | 4 | 42 | 8 | R.GNPTVEVDLHTAKGR.F |
|  | 15586 | **551.9651** | **1652.8736** | **1652.8457** | **0.0280** | **1** | **3** | **54** | **8** | **K.EAMRIGAEVYHHLK.G** |
|  | 16183 | **566.3038** | **1695.8895** | **1694.8297** | **1.0598** | **1** | **8** | **17** | **1** | **K.YNQLMRIEEALGDK.A** |
|  | 17652 | 902.9704 | 1803.9262 | 1803.9366 | -0.0104 | 0 | 54 | 0.0004 | 1 | R.AAVPSGASTGIYEALELR.D |
|  | 25734 | 1344.1345 | 2686.2545 | 2687.3265 | -1.0720 | 1 | 13 | 3.3 | 2 | K.TAIQAAGYPDKVVIGMDVAASEFYR.N 25733 |

| **736.** | P09104\|ENOG_HUMAN    **Mass:** 47581    **Score:** 54     **Queries matched:** 9   **emPAI:** 0.08 |
| --- | --- |
|  | Gamma-enolase OS=Homo sapiens GN=ENO2 PE=1 SV=3 |

|  | **Query** | **Observed** | **Mr(expt)** | **Mr(calc)** | **Delta** | **Miss** | **Score** | **Expect** | **Rank** | **Peptide** |
| --- | --- | --- | --- | --- | --- | --- | --- | --- | --- | --- |
|  | 2841 | **424.6959** | **847.3772** | **847.4089** | **-0.0317** | **0** | **9** | **26** | **4** | **R.FAGHNFR.N** 2873 |
|  | 6882 | 567.2809 | 1132.5473 | 1132.6063 | -0.0589 | 1 | 14 | 6.6 | 2 | -.MSIEKIWAR.E |
|  | 11717 | 703.8570 | 1405.6994 | 1405.7089 | -0.0095 | 0 | 18 | 2 | 2 | R.GNPTVEVDLYTAK.G |
|  | 13854 | **771.3868** | **1540.7590** | **1539.7205** | **1.0385** | **1** | **0** | **1.1e+002** | **1** | **K.YDLDFKSPTDPSR.Y** |
|  | 13936 | 774.2357 | 1546.4568 | 1546.7847 | -0.3279 | 1 | 3 | 39 | 10 | R.AVEEKACNCLLLK.V |
|  | 17652 | 902.9704 | 1803.9262 | 1803.9366 | -0.0104 | 0 | 54 | 0.0004 | 1 | R.AAVPSGASTGIYEALELR.D |
|  | 23979 | **810.0948** | **2427.2625** | **2427.1749** | **0.0877** | **1** | **2** | **43** | **1** | **K.LAMQEFMILPVGAESFRDAMR.L** |
|  | 24222 | **820.3585** | **2458.0536** | **2459.1647** | **-1.1112** | **1** | **(0)** | **61** | **6** | **K.LAMQEFMILPVGAESFRDAMR.L** |

| **737.** | Q8NFQ8\|TOIP2_HUMAN    **Mass:** 51460    **Score:** 54     **Queries matched:** 5   **emPAI:** 0.07 |
| --- | --- |
|  | Torsin-1A-interacting protein 2 OS=Homo sapiens GN=TOR1AIP2 PE=1 SV=1 |

|  | **Query** | **Observed** | **Mr(expt)** | **Mr(calc)** | **Delta** | **Miss** | **Score** | **Expect** | **Rank** | **Peptide** |
| --- | --- | --- | --- | --- | --- | --- | --- | --- | --- | --- |
|  | 8935 | 628.2245 | 1254.4344 | 1253.5135 | 0.9209 | 0 | 18 | 1.7 | 1 | K.YCDHENAAFK.D |
|  | 14383 | **526.2562** | **1575.7467** | **1575.7562** | **-0.0096** | **1** | **8** | **19** | **1** | **K.LEEIKENAQDTMR.Q** |
|  | 14782 | 802.9174 | 1603.8202 | 1603.8318 | -0.0116 | 0 | 54 | 0.00045 | 1 | R.LLAPEAGSHPQQTQK.L 14783 |
|  | 24805 | **847.2352** | **2538.6839** | **2539.3547** | **-0.6708** | **1** | **1** | **40** | **7** | **K.FLQKHLNASNPTEPATIIFTAAR.E** |

| **738.** | P62263\|RS14_HUMAN    **Mass:** 16434    **Score:** 54     **Queries matched:** 5   **emPAI:** 0.53 |
| --- | --- |
|  | 40S ribosomal protein S14 OS=Homo sapiens GN=RPS14 PE=1 SV=3 |

|  | **Query** | **Observed** | **Mr(expt)** | **Mr(calc)** | **Delta** | **Miss** | **Score** | **Expect** | **Rank** | **Peptide** |
| --- | --- | --- | --- | --- | --- | --- | --- | --- | --- | --- |
|  | 1258 | **593.3365** | **592.3293** | **591.3050** | **1.0242** | **0** | **14** | **6.6** | **1** | **R.VTGGMK.V** |
|  | 1331 | 608.3361 | 607.3288 | 607.3000 | 0.0289 | 0 | (12) | 11 | 2 | R.VTGGMK.V |
|  | 6383 | **547.3288** | **1092.6430** | **1093.6495** | **-1.0065** | **0** | **27** | **0.3** | **1** | **K.ELGITALHIK.L** |
|  | 12698 | **489.2744** | **1464.8015** | **1464.8161** | **-0.0146** | **1** | **2** | **79** | **9** | **K.TPGPGAQSALRALAR.S** |
|  | 22747 | 755.6848 | 2264.0324 | 2264.0491 | -0.0167 | 1 | 54 | 0.00035 | 1 | K.ADRDESSPYAAMLAAQDVAQR.C |

| **739.** | P98073\|ENTK_HUMAN    **Mass:** 114906   **Score:** 54     **Queries matched:** 11   **emPAI:** 0.07 |
| --- | --- |
|  | Enteropeptidase OS=Homo sapiens GN=PRSS7 PE=2 SV=2 |

|  | **Query** | **Observed** | **Mr(expt)** | **Mr(calc)** | **Delta** | **Miss** | **Score** | **Expect** | **Rank** | **Peptide** |
| --- | --- | --- | --- | --- | --- | --- | --- | --- | --- | --- |
|  | 7885 | **600.8509** | **1199.6872** | **1198.6306** | **1.0566** | **0** | **2** | **89** | **5** | **K.SNLTSPQTVPR.L** |
|  | 12577 | 486.9095 | 1457.7066 | 1457.8276 | -0.1210 | 0 | 15 | 3.9 | 4 | R.MTVLLITNDVLAR.G 12575 |
|  | 13975 | 775.3910 | 1548.7674 | 1548.7784 | -0.0110 | 0 | 56 | 0.00035 | 1 | K.ITSGVTYNPNLQDK.L |
|  | 14670 | 532.9060 | 1595.6962 | 1595.7764 | -0.0802 | 1 | 16 | 2.7 | 2 | K.ESQRGAALGQSHEAR.A |
|  | 16442 | 572.2950 | 1713.8631 | 1714.9049 | -1.0418 | 1 | 7 | 24 | 2 | K.CALPNRPGVYARVSR.F |
|  | 18521 | **621.6629** | **1861.9669** | **1863.0288** | **-1.0619** | **1** | **2** | **62** | **4** | **R.MTVLLITNDVLARGGFK.A** |
|  | 22672 | **752.7253** | **2255.1540** | **2255.8583** | **-0.7043** | **0** | **2** | **57** | **1** | **K.ADLFCDGEVNCPDGSDEDNK.M** |
|  | 23216 | **775.4089** | **2323.2050** | **2323.2133** | **-0.0084** | **0** | **20** | **0.85** | **1** | **K.VLAFDLQQMIDEIFLSSNLK.N** |
|  | 28923 | 1132.6038 | 3394.7895 | 3395.6973 | -0.9078 | 0 | 0 | 37 | 5 | R.FLLTGSSGSFQATHYPKPSETSVVCQWIIR.V |
|  | 30257 | 783.9930 | 3914.9288 | 3914.9124 | 0.0164 | 1 | 2 | 18 | 9 | R.QERVGLLSLPLDPTLEPACLSFWYHMYGENVHK.L |

| **740.** | P63010\|AP2B1_HUMAN    **Mass:** 105398   **Score:** 54     **Queries matched:** 19   **emPAI:** 0.07 |
| --- | --- |
|  | AP-2 complex subunit beta OS=Homo sapiens GN=AP2B1 PE=1 SV=1 |

|  | **Query** | **Observed** | **Mr(expt)** | **Mr(calc)** | **Delta** | **Miss** | **Score** | **Expect** | **Rank** | **Peptide** |
| --- | --- | --- | --- | --- | --- | --- | --- | --- | --- | --- |
|  | 2408 | **409.2354** | **816.4562** | **816.3977** | **0.0585** | **0** | **13** | **12** | **5** | **K.AELNNEK.K** |
|  | 3544 | 445.2594 | 888.5042 | 888.5103 | -0.0061 | 0 | 13 | 9.4 | 1 | K.VIAAMTVGK.D |
|  | 3793 | 453.7527 | 905.4908 | 904.5052 | 0.9856 | 0 | (6) | 50 | 4 | K.VIAAMTVGK.D |
|  | 4577 | 481.7661 | 961.5176 | 962.5437 | -1.0260 | 1 | 17 | 3.6 | 7 | K.KGEIFELK.A 4595 |
|  | 7023 | 573.2770 | 1144.5395 | 1145.6478 | -1.1083 | 1 | 1 | 1.4e+002 | 4 | K.LEKLDIMIR.L |
|  | 11433 | 464.2330 | 1389.6772 | 1388.6646 | 1.0126 | 1 | 3 | 75 | 8 | K.DSDYYNMLLKK.L |
|  | 11528 | 465.9332 | 1394.7777 | 1394.7881 | -0.0105 | 0 | 11 | 10 | 3 | R.LSHANSAVVLSAVK.V |
|  | 12357 | 722.8281 | 1443.6416 | 1443.7722 | -0.1306 | 0 | 7 | 27 | 9 | R.IQPGNPNYTLSLK.C |
|  | 13631 | 509.3132 | 1524.9179 | 1523.7290 | 1.1889 | 0 | 5 | 35 | 3 | R.NVEGQDMLYQSLK.L |
|  | 14282 | 784.9308 | 1567.8471 | 1567.8933 | -0.0462 | 0 | 3 | 64 | 8 | R.LASQANIAQVLAELK.E |
|  | 18745 | 628.2999 | 1881.8780 | 1882.0710 | -0.1930 | 1 | 1 | 84 | 5 | R.LSHANSAVVLSAVKVLMK.F 18763 |
|  | 22744 | 1132.9908 | 2263.9671 | 2264.2780 | -0.3109 | 0 | 3 | 38 | 2 | K.LAPPLVTLLSGEPEVQYVALR.N 22745 |
|  | 24066 | **814.0680** | **2439.1821** | **2439.1298** | **0.0524** | **0** | **3** | **40** | **7** | **K.DVSSLFPDVVNCMQTDNLELK.K** |
|  | 30237 | **1300.3568** | **3898.0486** | **3898.0737** | **-0.0251** | **0** | **40** | **0.0025** | **1** | **K.NSFGVIPSTPLAIHTPLMPNQSIDVSLPLNTLGPVMK.M** 30239 |
|  | 30691 | 1410.0394 | 4227.0965 | 4227.1117 | -0.0152 | 0 | 32 | 0.017 | 1 | R.DLIADSNPMVVANAVAALSEISESHPNSNLLDLNPQNINK.L |

| **741.** | Q9Y6N5\|SQRD_HUMAN    **Mass:** 50214    **Score:** 54     **Queries matched:** 18   **emPAI:** 0.07 |
| --- | --- |
|  | Sulfide:quinone oxidoreductase, mitochondrial OS=Homo sapiens GN=SQRDL PE=1 SV=1 |

|  | **Query** | **Observed** | **Mr(expt)** | **Mr(calc)** | **Delta** | **Miss** | **Score** | **Expect** | **Rank** | **Peptide** |
| --- | --- | --- | --- | --- | --- | --- | --- | --- | --- | --- |
|  | 2922 | **426.7200** | **851.4254** | **850.4548** | **0.9705** | **0** | **6** | **35** | **7** | **R.NLTVNYK.K** |
|  | 3951 | **459.2478** | **916.4810** | **916.4398** | **0.0413** | **0** | **12** | **13** | **6** | **R.LSMYLMK.A** |
|  | 6922 | 568.7600 | 1135.5053 | 1135.6026 | -0.0972 | 1 | 8 | 23 | 4 | K.TWKALQDFK.E |
|  | 8706 | 620.9932 | 1239.9718 | 1239.7009 | 0.2708 | 0 | 7 | 16 | 2 | -.MVPLVAVVSGPR.A 8671 |
|  | 10271 | **662.8550** | **1323.6955** | **1323.7034** | **-0.0079** | **1** | **2** | **87** | **9** | **K.IGSNYSVKTVEK.T** |
|  | 10630 | 672.3634 | 1342.7122 | 1342.7205 | -0.0082 | 0 | 54 | 0.00057 | 1 | K.TAAAVAAQSGILDR.T 10640 |
|  | 18729 | **627.3782** | **1879.1127** | **1879.0567** | **0.0560** | **1** | **5** | **31** | **1** | **R.SKANIIFNTSLGAIFGVK.K** 18733 |
|  | 20280 | **1009.7761** | **2017.5376** | **2017.0051** | **0.5325** | **0** | **1** | **46** | **2** | **R.NHYEVLVLGGGSGGITMAAR.M** 20269 |
|  | 20542 | 1020.4553 | 2038.8961 | 2037.9942 | 0.9019 | 1 | 6 | 21 | 2 | R.RYPNVFGIGDCTNLPTSK.T 20543 |
|  | 23004 | **765.3699** | **2293.0878** | **2292.1355** | **0.9523** | **1** | **8** | **12** | **1** | **R.NHYEVLVLGGGSGGITMAARMK.R** 22975 |
|  | 25153 | 863.4157 | 2587.2253 | 2586.2894 | 0.9359 | 1 | 2 | 42 | 3 | R.VILAEFDYKAEPLETFPFDQSK.E |
|  | 27906 | 1569.6395 | 3137.2645 | 3137.6298 | -0.3653 | 1 | 2 | 23 | 6 | K.VGAENVAIVEPSERHFYQPIWTLVGAGAK.Q |

| **742.** | P09681\|GIP_HUMAN    **Mass:** 17211    **Score:** 54     **Queries matched:** 7   **emPAI:** 0.23 |
| --- | --- |
|  | Gastric inhibitory polypeptide OS=Homo sapiens GN=GIP PE=1 SV=1 |

|  | **Query** | **Observed** | **Mr(expt)** | **Mr(calc)** | **Delta** | **Miss** | **Score** | **Expect** | **Rank** | **Peptide** |
| --- | --- | --- | --- | --- | --- | --- | --- | --- | --- | --- |
|  | 6060 | 536.2991 | 1070.5837 | 1071.5672 | -0.9835 | 0 | 5 | 55 | 9 | R.ALELASQANR.K |
|  | 6189 | 540.2974 | 1078.5803 | 1079.5836 | -1.0033 | 1 | 6 | 38 | 10 | K.VSSPQPRGPR.Y |
|  | 7872 | 600.4665 | 1198.9185 | 1199.6622 | -0.7436 | 1 | 1 | 92 | 4 | R.ALELASQANRK.E |
|  | 12149 | **715.4410** | **1428.8675** | **1427.7480** | **1.1195** | **1** | **2** | **80** | **6** | **R.EARALELASQANR.K** 12142 |
|  | 17975 | 609.9698 | 1826.8875 | 1825.8080 | 1.0795 | 0 | 6 | 23 | 2 | R.YAEGTFISDYSIAMDK.I |
|  | 23055 | **1151.0966** | **2300.1786** | **2300.1868** | **-0.0082** | **0** | **54** | **0.00033** | **1** | **R.DLLIQELLACLLDQTNLCR.L** |

| **743.** | Q8WUM4\|PDC6I_HUMAN    **Mass:** 96590    **Score:** 54     **Queries matched:** 14   **emPAI:** 0.04 |
| --- | --- |
|  | Programmed cell death 6-interacting protein OS=Homo sapiens GN=PDCD6IP PE=1 SV=1 |

|  | **Query** | **Observed** | **Mr(expt)** | **Mr(calc)** | **Delta** | **Miss** | **Score** | **Expect** | **Rank** | **Peptide** |
| --- | --- | --- | --- | --- | --- | --- | --- | --- | --- | --- |
|  | 1013 | **544.9023** | **543.8950** | **543.3380** | **0.5570** | **0** | **5** | **72** | **2** | **R.ALAAAK.K** |
|  | 4774 | **488.7607** | **975.5069** | **974.5396** | **0.9672** | **0** | **5** | **61** | **2** | **K.ETVLSALSR.E** |
|  | 5086 | 998.5455 | 997.5382 | 996.5604 | 0.9778 | 0 | 8 | 26 | 8 | K.ELPELLQR.N |
|  | 7349 | **585.3259** | **1168.6373** | **1168.6452** | **-0.0079** | **0** | **20** | **1.5** | **1** | **K.STPVNVPISQK.F** |
|  | 9144 | **632.8466** | **1263.6787** | **1264.7213** | **-1.0426** | **1** | **5** | **50** | **9** | **-.MATFISVQLKK.T** |
|  | 9257 | 635.7624 | 1269.5102 | 1268.7340 | 0.7762 | 1 | 2 | 88 | 9 | K.DLDPIGKATLVK.S |
|  | 10234 | 661.8280 | 1321.6414 | 1321.6051 | 0.0363 | 1 | 13 | 6.3 | 3 | K.KDNDFIYHDR.V |
|  | 11105 | 686.8668 | 1371.7190 | 1372.7245 | -1.0055 | 1 | 0 | 1.4e+002 | 6 | K.ADLVNRSIAQMR.E |
|  | 12108 | **476.7635** | **1427.2686** | **1426.7093** | **0.5593** | **1** | **13** | **5** | **1** | **K.DAFDKGSLFGGSVK.L** |
|  | 13599 | **762.3507** | **1522.6869** | **1521.8403** | **0.8466** | **1** | **2** | **83** | **5** | **R.VYGGLTTKVQESLK.K** |
|  | 16668 | **578.3000** | **1731.8781** | **1730.8322** | **1.0459** | **1** | **13** | **5.3** | **1** | **R.LLDEEEATDNDLRAK.F** |
|  | 17698 | **603.3304** | **1806.9695** | **1805.9094** | **1.0601** | **0** | **2** | **71** | **4** | **K.MVPVSVQQSLAAYNQR.K** |
|  | 19223 | **959.5543** | **1917.0941** | **1918.0094** | **-0.9154** | **1** | **7** | **18** | **1** | **K.MVPVSVQQSLAAYNQRK.A** |
|  | 21872 | **1081.4763** | **2160.9381** | **2160.9535** | **-0.0154** | **0** | **54** | **0.00034** | **1** | **K.FIQQTYPSGGEEQAQYCR.A** |

| **744.** | P18621\|RL17_HUMAN    **Mass:** 21611    **Score:** 54     **Queries matched:** 7   **emPAI:** 0.18 |
| --- | --- |
|  | 60S ribosomal protein L17 OS=Homo sapiens GN=RPL17 PE=1 SV=3 |

|  | **Query** | **Observed** | **Mr(expt)** | **Mr(calc)** | **Delta** | **Miss** | **Score** | **Expect** | **Rank** | **Peptide** |
| --- | --- | --- | --- | --- | --- | --- | --- | --- | --- | --- |
|  | 5341 | 509.7431 | 1017.4716 | 1017.4781 | -0.0065 | 0 | 20 | 1.8 | 1 | K.QWGWTQGR.W |
|  | 7264 | 582.2787 | 1162.5429 | 1162.5506 | -0.0076 | 0 | 24 | 0.56 | 1 | R.YSLDPENPTK.S 7273 |
|  | 10441 | **667.3439** | **1332.6733** | **1331.7271** | **0.9462** | **1** | **4** | **61** | **4** | **K.KSAEFLLHMLK.N** |
|  | 11080 | **457.7970** | **1370.3691** | **1369.7136** | **0.6555** | **1** | **6** | **22** | **10** | **R.ETAQAIKGMHIR.K** |
|  | 15106 | **812.4279** | **1622.8412** | **1622.8515** | **-0.0103** | **0** | **54** | **0.0005** | **1** | **K.EQIVPKPEEEVAQK.K** 15105 |

| **745.** | P78371\|TCPB_HUMAN    **Mass:** 57794    **Score:** 53     **Queries matched:** 18   **emPAI:** 0.13 |
| --- | --- |
|  | T-complex protein 1 subunit beta OS=Homo sapiens GN=CCT2 PE=1 SV=4 |

|  | **Query** | **Observed** | **Mr(expt)** | **Mr(calc)** | **Delta** | **Miss** | **Score** | **Expect** | **Rank** | **Peptide** |
| --- | --- | --- | --- | --- | --- | --- | --- | --- | --- | --- |
|  | 1187 | **579.3202** | **578.3130** | **578.3176** | **-0.0047** | **0** | **7** | **31** | **3** | **K.IFGSR.V** |
|  | 2430 | **410.2133** | **818.4121** | **818.4320** | **-0.0199** | **0** | **(9)** | **24** | **8** | **K.VLVDMSR.V** |
|  | 2625 | **417.2431** | **832.4716** | **833.4872** | **-1.0155** | **1** | **11** | **15** | **6** | **K.IFGSRVR.V** |
|  | 2655 | **418.2022** | **834.3897** | **834.4269** | **-0.0372** | **0** | **11** | **15** | **3** | **K.VLVDMSR.V** |
|  | 3281 | **436.2816** | **870.5486** | **869.5334** | **1.0152** | **0** | **13** | **9.6** | **2** | **K.LAVEAVLR.L** |
|  | 11721 | 469.7422 | 1406.2048 | 1405.7639 | 0.4409 | 0 | 3 | 40 | 6 | -.MASLSLAPVNIFK.A |
|  | 12874 | 739.3358 | 1476.6571 | 1477.7446 | -1.0875 | 0 | 6 | 33 | 4 | R.QDLMNIAGTTLSSK.L |
|  | 12944 | 494.2940 | 1479.8600 | 1480.7231 | -0.8631 | 1 | 4 | 51 | 9 | R.TPGKEAVAMESYAK.A |
|  | 13532 | 759.4475 | 1516.8805 | 1516.8865 | -0.0060 | 0 | 35 | 0.039 | 1 | R.LTSFIGAIAIGDLVK.S 13507 |
|  | 13965 | **775.2859** | **1548.5573** | **1547.7865** | **0.7708** | **0** | **4** | **42** | **3** | **R.DASLMVTNDGATILK.N** |
|  | 19164 | 957.4725 | 1912.9305 | 1911.9466 | 0.9839 | 0 | 1 | 75 | 3 | K.LGGSLADSYLDEGFLLDK.K |
|  | 19644 | **652.3619** | **1954.0638** | **1953.9756** | **0.0883** | **1** | **8** | **14** | **2** | **K.AAREALLSSAVDHGSDEVK.F** |
|  | 22266 | 737.0255 | 2208.0547 | 2207.1732 | 0.8815 | 1 | 5 | 28 | 4 | R.QDLMNIAGTTLSSKLLTHHK.D |
|  | 23543 | **789.3680** | **2365.0823** | **2365.0977** | **-0.0154** | **0** | **9** | **9.7** | **1** | **R.TVYGGGCSEMLMAHAVTQLANR.T** |
|  | 25740 | **672.6271** | **2686.4795** | **2686.4112** | **0.0683** | **1** | **0** | **59** | **4** | **K.ALRMLPTIIADNAGYDSADLVAQLR.A** |
|  | 28228 | **1070.5273** | **3208.5602** | **3208.5651** | **-0.0049** | **0** | **44** | **0.0015** | **1** | **R.QLIYNYPEQLFGAAGVMAIEHADFAGVER.L** 28227 |

| **746.** | P22732\|GTR5_HUMAN    **Mass:** 55394    **Score:** 53     **Queries matched:** 7   **emPAI:** 0.14 |
| --- | --- |
|  | Solute carrier family 2, facilitated glucose transporter member 5 OS=Homo sapiens GN=SLC2A5 PE=1 SV=1 |

|  | **Query** | **Observed** | **Mr(expt)** | **Mr(calc)** | **Delta** | **Miss** | **Score** | **Expect** | **Rank** | **Peptide** |
| --- | --- | --- | --- | --- | --- | --- | --- | --- | --- | --- |
|  | 1970 | **777.4819** | **776.4747** | **776.4796** | **-0.0049** | **0** | **36** | **0.056** | **1** | **R.YLLIQK.K** |
|  | 2590 | 415.7396 | 829.4647 | 828.5181 | 0.9466 | 1 | 13 | 12 | 5 | K.KALQTLR.G 2589 |
|  | 10798 | 677.3707 | 1352.7268 | 1352.7340 | -0.0072 | 0 | 46 | 0.0032 | 1 | K.TFIEINQIFTK.M |
|  | 12442 | **725.3726** | **1448.7306** | **1448.7398** | **-0.0093** | **1** | **38** | **0.024** | **1** | **K.VSEVYPEKEELK.E** |
|  | 13723 | 766.8855 | 1531.7564 | 1530.7427 | 1.0138 | 1 | 22 | 0.83 | 2 | R.GWDSVDREVAEIR.Q |
|  | 30656 | **1045.2378** | **4176.9221** | **4177.1197** | **-0.1976** | **0** | **0** | **23** | **8** | **R.TGEFMEDFPLTLLWSVTVSMFPFGGFIGSLLVGPLVNK.F** |

| **747.** | P14649\|MYL6B_HUMAN    **Mass:** 22864    **Score:** 53     **Queries matched:** 10   **emPAI:** 0.36 |
| --- | --- |
|  | Myosin light chain 6B OS=Homo sapiens GN=MYL6B PE=1 SV=1 |

|  | **Query** | **Observed** | **Mr(expt)** | **Mr(calc)** | **Delta** | **Miss** | **Score** | **Expect** | **Rank** | **Peptide** |
| --- | --- | --- | --- | --- | --- | --- | --- | --- | --- | --- |
|  | 1414 | 628.3623 | 627.3550 | 626.3752 | 0.9799 | 0 | 6 | 38 | 7 | K.VLGNPK.S |
|  | 5045 | 499.2066 | 996.3986 | 996.5604 | -0.1617 | 0 | 5 | 43 | 4 | R.HVLTTLGEK.M |
|  | 10581 | 671.3138 | 1340.6130 | 1340.6217 | -0.0087 | 0 | 33 | 0.062 | 1 | K.ILYSQCGDVMR.A 10582 |
|  | 10812 | 677.8649 | 1353.7153 | 1353.7252 | -0.0099 | 0 | 40 | 0.014 | 1 | R.ALGQNPTNAEVLK.V |
|  | 10980 | 683.3425 | 1364.6705 | 1363.7459 | 0.9246 | 1 | 2 | 93 | 7 | K.TKAEPAVPQAPQK.T |
|  | 17183 | 590.4301 | 1768.2685 | 1768.9505 | -0.6820 | 1 | 3 | 37 | 5 | K.VMGAELRHVLTTLGEK.M |
|  | 17664 | **602.3832** | **1804.1277** | **1803.8176** | **0.3101** | **1** | **2** | **54** | **6** | **K.NRGQGTYEDYLEGFR.V** |
|  | 18535 | **622.3011** | **1863.8814** | **1863.9917** | **-0.1102** | **1** | **(1)** | **88** | **8** | **R.RVDFETFLPMLQAVAK.N** |
|  | 18742 | 941.4688 | 1880.9231 | 1879.9866 | 0.9365 | 1 | 6 | 28 | 3 | R.RVDFETFLPMLQAVAK.N |

| **748.** | Q99729\|ROAA_HUMAN    **Mass:** 36316    **Score:** 53     **Queries matched:** 8   **emPAI:** 0.10 |
| --- | --- |
|  | Heterogeneous nuclear ribonucleoprotein A/B OS=Homo sapiens GN=HNRNPAB PE=1 SV=2 |

|  | **Query** | **Observed** | **Mr(expt)** | **Mr(calc)** | **Delta** | **Miss** | **Score** | **Expect** | **Rank** | **Peptide** |
| --- | --- | --- | --- | --- | --- | --- | --- | --- | --- | --- |
|  | 1219 | **586.2977** | **585.2905** | **585.3486** | **-0.0581** | **1** | **11** | **16** | **1** | **K.KDPVK.K** |
|  | 1234 | 587.3912 | 586.3839 | 585.3486 | 1.0353 | 1 | 18 | 4.3 | 6 | K.DPVKK.I |
|  | 3440 | **441.7240** | **881.4335** | **881.4467** | **-0.0132** | **1** | **11** | **11** | **6** | **K.EHRLDGR.V** |
|  | 4939 | 495.2779 | 988.5413 | 989.5294 | -0.9881 | 1 | 11 | 15 | 10 | K.KFHTVSGSK.C 4935 |
|  | 7302 | 583.7759 | 1165.5372 | 1166.5642 | -1.0270 | 0 | 20 | 1.3 | 2 | K.FGEVVDCTIK.M 7323 |
|  | 13260 | **750.3423** | **1498.6700** | **1498.6801** | **-0.0100** | **0** | **53** | **0.00058** | **1** | **K.EVYQQQQYGSGGR.G** |

| **749.** | P04075\|ALDOA_HUMAN    **Mass:** 39851    **Score:** 53     **Queries matched:** 19   **emPAI:** 0.31 |
| --- | --- |
|  | Fructose-bisphosphate aldolase A OS=Homo sapiens GN=ALDOA PE=1 SV=2 |

|  | **Query** | **Observed** | **Mr(expt)** | **Mr(calc)** | **Delta** | **Miss** | **Score** | **Expect** | **Rank** | **Peptide** |
| --- | --- | --- | --- | --- | --- | --- | --- | --- | --- | --- |
|  | 1218 | 585.3762 | 584.3689 | 583.3693 | 0.9995 | 0 | 26 | 0.37 | 1 | R.IVAPGK.G |
|  | 1921 | 762.3217 | 761.3144 | 762.4640 | -1.1495 | 0 | 7 | 41 | 1 | K.VLAAVYK.A |
|  | 2177 | **401.2422** | **800.4698** | **800.4756** | **-0.0057** | **0** | **27** | **0.48** | **1** | **R.ALQASALK.A** 2176 |
|  | 2801 | 422.7581 | 843.5017 | 843.5178 | -0.0161 | 1 | 5 | 63 | 4 | K.SKGGVVGIK.V 2786 |
|  | 3057 | 861.4448 | 860.4375 | 860.4691 | -0.0315 | 1 | 17 | 4.5 | 6 | K.WRCVLK.I |
|  | 5683 | **522.7839** | **1043.5533** | **1043.5611** | **-0.0078** | **0** | **20** | **1.7** | **1** | **R.QLLLTADDR.V** |
|  | 6854 | 566.2819 | 1130.5493 | 1131.5706 | -1.0213 | 0 | 21 | 1.4 | 2 | R.ALANSLACQGK.Y 6871 |
|  | 10610 | **671.8544** | **1341.6943** | **1341.7041** | **-0.0098** | **0** | **8** | **22** | **2** | **K.ADDGRPFPQVIK.S** |
|  | 12229 | **717.8618** | **1433.7091** | **1433.7191** | **-0.0100** | **0** | **19** | **1.6** | **1** | **M.PYQYPALTPEQK.K** |
|  | 15482 | **823.9026** | **1645.7907** | **1645.8019** | **-0.0112** | **1** | **13** | **5.8** | **1** | **R.LQSIGTENTEENRR.F** |
|  | 15908 | **838.9232** | **1675.8319** | **1674.8399** | **0.9920** | **0** | **44** | **0.0043** | **1** | **K.FSHEEIAMATVTALR.R** |
|  | 18950 | 633.0118 | 1896.0135 | 1897.0520 | -1.0385 | 1 | 1 | 84 | 9 | R.IVAPGKGILAADESTGSIAK.R |
|  | 21295 | 703.4303 | 2107.2691 | 2106.0891 | 1.1799 | 0 | 1 | 64 | 7 | K.IGEHTPSALAIMENANVLAR.Y |
|  | 22806 | 1137.0597 | 2272.1048 | 2271.1343 | 0.9705 | 0 | 34 | 0.03 | 2 | K.GVVPLAGTNGETTTQGLDGLSER.C 22807 |
|  | 27891 | 627.1171 | 3130.5490 | 3130.5692 | -0.0202 | 0 | 25 | 0.16 | 1 | K.ALSDHHIYLEGTLLKPNMVTPGHACTQK.F |

| **750.** | P06396\|GELS_HUMAN    **Mass:** 86043    **Score:** 53     **Queries matched:** 16   **emPAI:** 0.09 |
| --- | --- |
|  | Gelsolin OS=Homo sapiens GN=GSN PE=1 SV=1 |

|  | **Query** | **Observed** | **Mr(expt)** | **Mr(calc)** | **Delta** | **Miss** | **Score** | **Expect** | **Rank** | **Peptide** |
| --- | --- | --- | --- | --- | --- | --- | --- | --- | --- | --- |
|  | 2722 | **838.7401** | **837.7328** | **838.4007** | **-0.6679** | **0** | **13** | **4.5** | **1** | **R.LFACSNK.I** |
|  | 2874 | 425.2186 | 848.4227 | 849.4709 | -1.0481 | 1 | 7 | 39 | 3 | K.KGGVASGFK.H 2907 |
|  | 3442 | **441.7294** | **881.4442** | **881.4494** | **-0.0053** | **0** | **7** | **30** | **5** | **K.TASDFITK.M** |
|  | 3518 | 444.2483 | 886.4820 | 886.4872 | -0.0052 | 0 | 53 | 0.00096 | 1 | K.TGAQELLR.V |
|  | 4642 | **484.3207** | **966.6268** | **966.4705** | **0.1563** | **1** | **14** | **5** | **1** | **K.KMDAHPPR.L** |
|  | 6129 | 538.2823 | 1074.5500 | 1073.5101 | 1.0399 | 0 | 5 | 51 | 9 | R.EGGQTAPASTR.L |
|  | 14102 | **778.9182** | **1555.8219** | **1555.7815** | **0.0404** | **1** | **2** | **86** | **1** | **R.AATASRGASQAGAPQGR.V** |
|  | 15759 | **556.3207** | **1665.9404** | **1664.7740** | **1.1663** | **1** | **0** | **1e+002** | **10** | **K.DSQEEEKTEALTSAK.R** |
|  | 16557 | 862.3929 | 1722.7713 | 1721.8301 | 0.9412 | 0 | 11 | 8.1 | 1 | R.EVQGFESATFLGYFK.S |
|  | 18015 | **915.4801** | **1828.9456** | **1828.9571** | **-0.0114** | **0** | **30** | **0.11** | **1** | **K.QTQVSVLPEGGETPLFK.Q** |
|  | 20660 | **683.6874** | **2048.0403** | **2047.0634** | **0.9769** | **0** | **3** | **43** | **2** | **K.EPAHLMSLFGGKPMIIYK.G** |
|  | 26636 | 1434.2376 | 2866.4605 | 2866.4535 | 0.0071 | 1 | 3 | 27 | 3 | K.LYKVSNGAGTMSVSLVADENPFAQGALK.S |
|  | 28311 | 646.7335 | 3228.6309 | 3229.4458 | -0.8149 | 0 | 0 | 41 | 7 | K.QGFEPPSFVGWFLGWDDDYWSVDPLDR.A |
|  | 28826 | **843.6882** | **3370.7236** | **3370.8152** | **-0.0916** | **1** | **2** | **25** | **1** | **-.MAPHRPAPALLCALSLALCALSLPVRAATASR.G** |
|  | 29131 | **1158.4939** | **3472.4599** | **3471.5936** | **0.8663** | **1** | **2** | **19** | **3** | **R.VPFDAATLHTSTAMAAQHGMDDDGTGQKQIWR.I** |

| **751.** | P61247\|RS3A_HUMAN    **Mass:** 30154    **Score:** 53     **Queries matched:** 20   **emPAI:** 0.27 |
| --- | --- |
|  | 40S ribosomal protein S3a OS=Homo sapiens GN=RPS3A PE=1 SV=2 |

|  | **Query** | **Observed** | **Mr(expt)** | **Mr(calc)** | **Delta** | **Miss** | **Score** | **Expect** | **Rank** | **Peptide** |
| --- | --- | --- | --- | --- | --- | --- | --- | --- | --- | --- |
|  | 1247 | **590.3190** | **589.3118** | **588.3595** | **0.9522** | **0** | **18** | **4.4** | **1** | **K.TLVTR.T** |
|  | 1377 | **618.0686** | **617.0613** | **617.3934** | **-0.3321** | **1** | **3** | **1.2e+002** | **6** | **K.VKMLK.K** |
|  | 2769 | 421.7283 | 841.4420 | 841.4909 | -0.0489 | 0 | 6 | 39 | 4 | K.LIPDSIGK.D |
|  | 3864 | **456.2081** | **910.4017** | **910.4074** | **-0.0057** | **0** | **25** | **0.42** | **1** | **K.MMEIMTR.E** |
|  | 3990 | **460.7313** | **919.4480** | **918.5175** | **0.9306** | **1** | **8** | **29** | **2** | **K.KVVDPFSK.K** |
|  | 5139 | **501.7736** | **1001.5327** | **1001.5393** | **-0.0066** | **0** | **28** | **0.27** | **1** | **K.LITEDVQGK.N** |
|  | 5275 | 507.2815 | 1012.5484 | 1013.4344 | -0.8860 | 1 | 13 | 8.4 | 7 | R.DKMCSMVK.K |
|  | 5624 | **520.2770** | **1038.5394** | **1038.5024** | **0.0370** | **1** | **(4)** | **68** | **10** | **K.KMMEIMTR.E** |
|  | 5829 | **528.7550** | **1055.4954** | **1054.4973** | **0.9981** | **1** | **8** | **27** | **7** | **K.KMMEIMTR.E** 5828 |
|  | 6048 | **536.2175** | **1070.4204** | **1070.4922** | **-0.0718** | **1** | **(4)** | **70** | **5** | **K.KMMEIMTR.E** |
|  | 8244 | 609.3006 | 1216.5866 | 1216.5949 | -0.0082 | 0 | 53 | 0.00081 | 1 | K.TSYAQHQQVR.Q |
|  | 10310 | 663.8644 | 1325.7143 | 1326.7395 | -1.0251 | 1 | 4 | 54 | 9 | K.LIPDSIGKDIEK.A |
|  | 10665 | **449.2339** | **1344.6798** | **1344.6898** | **-0.0101** | **1** | **6** | **34** | **5** | **R.KTSYAQHQQVR.Q** 10663 10669 10670 |
|  | 14408 | **526.9063** | **1577.6969** | **1577.7079** | **-0.0110** | **0** | **9** | **15** | **2** | **K.NCLTNFHGMDLTR.D** |
|  | 15899 | **559.3124** | **1674.9155** | **1673.7897** | **1.1258** | **1** | **2** | **76** | **4** | **K.VERADGYEPPVQESV.-** |
|  | 16304 | **568.9522** | **1703.8348** | **1703.8454** | **-0.0106** | **0** | **6** | **27** | **1** | **K.ACQSIYPLHDVFVR.K** |

| **752.** | P62244\|RS15A_HUMAN    **Mass:** 14944    **Score:** 53     **Queries matched:** 17   **emPAI:** 0.59 |
| --- | --- |
|  | 40S ribosomal protein S15a OS=Homo sapiens GN=RPS15A PE=1 SV=2 |

|  | **Query** | **Observed** | **Mr(expt)** | **Mr(calc)** | **Delta** | **Miss** | **Score** | **Expect** | **Rank** | **Peptide** |
| --- | --- | --- | --- | --- | --- | --- | --- | --- | --- | --- |
|  | 2003 | **395.2072** | **788.3998** | **787.4011** | **0.9987** | **0** | **28** | **0.35** | **1** | **K.CGVISPR.F** 1991 |
|  | 3240 | **435.2323** | **868.4500** | **868.4551** | **-0.0050** | **0** | **11** | **12** | **1** | **R.FLTVMMK.H** |
|  | 3279 | **436.2689** | **870.5232** | **870.5287** | **-0.0055** | **0** | **54** | **0.00071** | **1** | **K.IVVNLTGR.L** 3274 |
|  | 3493 | **443.2239** | **884.4333** | **884.4500** | **-0.0167** | **0** | **(7)** | **34** | **10** | **R.FLTVMMK.H** |
|  | 4739 | 487.7668 | 973.5191 | 973.5266 | -0.0075 | 0 | 18 | 3.2 | 2 | R.MNVLADALK.S 4762 |
|  | 4955 | 495.7687 | 989.5229 | 989.5215 | 0.0014 | 0 | (18) | 3.1 | 6 | R.MNVLADALK.S |
|  | 8480 | **615.3779** | **1228.7413** | **1228.6962** | **0.0452** | **1** | **0** | **1.4e+002** | **9** | **M.VRMNVLADALK.S** |
|  | 11190 | **459.7732** | **1376.2978** | **1375.7316** | **0.5663** | **1** | **8** | **13** | **1** | **-.MVRMNVLADALK.S** |
|  | 11464 | **464.7221** | **1391.1446** | **1391.7265** | **-0.5819** | **1** | **(1)** | **65** | **6** | **-.MVRMNVLADALK.S** |
|  | 16251 | **567.6015** | **1699.7827** | **1699.7954** | **-0.0128** | **0** | **27** | **0.2** | **1** | **K.HGYIGEFEIIDDHR.A** |
|  | 22535 | **746.3909** | **2236.1510** | **2237.0787** | **-0.9277** | **0** | **2** | **53** | **4** | **R.QFGFIVLTTSAGIMDHEEAR.R** |
|  | 24945 | **638.3276** | **2549.2815** | **2550.2399** | **-0.9585** | **1** | **5** | **20** | **7** | **R.FLTVMMKHGYIGEFEIIDDHR.A** 24944 24957 |

| **753.** | O96000\|NDUBA_HUMAN    **Mass:** 21048    **Score:** 53     **Queries matched:** 8   **emPAI:** 0.40 |
| --- | --- |
|  | NADH dehydrogenase [ubiquinone] 1 beta subcomplex subunit 10 OS=Homo sapiens GN=NDUFB10 PE=1 SV=3 |

|  | **Query** | **Observed** | **Mr(expt)** | **Mr(calc)** | **Delta** | **Miss** | **Score** | **Expect** | **Rank** | **Peptide** |
| --- | --- | --- | --- | --- | --- | --- | --- | --- | --- | --- |
|  | 7474 | **589.8022** | **1177.5899** | **1177.5979** | **-0.0080** | **0** | **42** | **0.011** | **1** | **K.EVEQFTQVAK.A** |
|  | 8492 | 615.7869 | 1229.5592 | 1229.5676 | -0.0085 | 0 | 35 | 0.045 | 1 | R.YQDLGAYSSAR.K |
|  | 11267 | **691.3076** | **1380.6007** | **1380.6092** | **-0.0085** | **0** | **5** | **44** | **2** | **R.EGQNYQQNCIK.E** |
|  | 14916 | **538.2962** | **1611.8668** | **1612.9301** | **-1.0633** | **0** | **14** | **4.6** | **1** | **K.AFDLIVDRPVTLVR.E** 14903 14931 |
|  | 17750 | 604.6277 | 1810.8612 | 1810.8850 | -0.0237 | 1 | 2 | 71 | 10 | K.EVEQFTQVAKAYQDR.Y |
|  | 18524 | 621.9509 | 1862.8310 | 1862.8547 | -0.0237 | 1 | 1 | 79 | 5 | K.AYQDRYQDLGAYSSAR.K |

| **754.** | Q58FF3\|ENPLL_HUMAN    **Mass:** 46343    **Score:** 53     **Queries matched:** 12   **emPAI:** 0.17 |
| --- | --- |
|  | Putative endoplasmin-like protein OS=Homo sapiens GN=HSP90B2P PE=5 SV=1 |

|  | **Query** | **Observed** | **Mr(expt)** | **Mr(calc)** | **Delta** | **Miss** | **Score** | **Expect** | **Rank** | **Peptide** |
| --- | --- | --- | --- | --- | --- | --- | --- | --- | --- | --- |
|  | 1727 | 706.3829 | 705.3757 | 705.3810 | -0.0053 | 0 | 34 | 0.087 | 1 | R.FQNVAK.E 1733 |
|  | 3333 | 438.2392 | 874.4638 | 875.4501 | -0.9863 | 0 | 22 | 1.5 | 5 | K.TFEINPR.H |
|  | 5169 | 502.7756 | 1003.5367 | 1003.5451 | -0.0084 | 1 | 8 | 26 | 1 | K.KTFEINPR.H |
|  | 6960 | 570.2896 | 1138.5647 | 1138.5731 | -0.0084 | 0 | 51 | 0.0012 | 1 | K.LGVIEDHSNR.T |
|  | 7315 | 584.2983 | 1166.5820 | 1167.4601 | -0.8781 | 0 | 1 | 1.1e+002 | 4 | K.EMDVGTDEEK.Q |
|  | 9363 | **425.9001** | **1274.6785** | **1274.7387** | **-0.0602** | **0** | **13** | **7.1** | **3** | **K.SILFVPTFVPR.G** 9362 9387 |
|  | 11794 | 705.8180 | 1409.6215 | 1410.5820 | -0.9605 | 1 | 5 | 43 | 7 | K.DKEMDVGTDEEK.Q 11795 |
|  | 17072 | **587.3183** | **1758.9331** | **1759.7910** | **-0.8579** | **1** | **1** | **94** | **6** | **R.CVFITDDFRDTMPK.N** |

| **755.** | O75746\|CMC1_HUMAN    **Mass:** 75114    **Score:** 52     **Queries matched:** 16   **emPAI:** 0.10 |
| --- | --- |
|  | Calcium-binding mitochondrial carrier protein Aralar1 OS=Homo sapiens GN=SLC25A12 PE=1 SV=2 |

|  | **Query** | **Observed** | **Mr(expt)** | **Mr(calc)** | **Delta** | **Miss** | **Score** | **Expect** | **Rank** | **Peptide** |
| --- | --- | --- | --- | --- | --- | --- | --- | --- | --- | --- |
|  | 1549 | 656.4037 | 655.3965 | 656.3969 | -1.0005 | 0 | 1 | 1e+002 | 8 | R.LQVAAR.A |
|  | 3363 | **439.2455** | **876.4763** | **876.4930** | **-0.0166** | **1** | **9** | **23** | **5** | **K.GTAARVFR.S** 3351 |
|  | 4033 | 462.2850 | 922.5554 | 921.4854 | 1.0699 | 1 | 21 | 1.3 | 1 | K.GAKACFLR.D |
|  | 4593 | 482.2637 | 962.5128 | 962.5185 | -0.0057 | 0 | 46 | 0.0048 | 1 | K.LTVNDFVR.D |
|  | 7103 | 576.2759 | 1150.5372 | 1150.5447 | -0.0075 | 0 | 31 | 0.12 | 1 | R.YEGFFGLYR.G |
|  | 9346 | 425.7343 | 1274.1811 | 1274.7347 | -0.5536 | 1 | 3 | 44 | 10 | K.AIKLTVNDFVR.D |
|  | 10469 | 667.9018 | 1333.7890 | 1333.7969 | -0.0079 | 0 | 2 | 85 | 3 | R.GLIPQLIGVAPEK.A 10462 |
|  | 15475 | 549.3360 | 1644.9862 | 1646.0130 | -1.0269 | 1 | 4 | 38 | 3 | R.GLIPQLIGVAPEKAIK.L |
|  | 16303 | 568.9247 | 1703.7524 | 1704.8584 | -1.1060 | 0 | 1 | 85 | 10 | R.IADLPPANPDHIGGYR.L |
|  | 18650 | 625.3312 | 1872.9719 | 1872.8961 | 0.0757 | 0 | 5 | 33 | 7 | K.SGMISGLDFSDIMVTIR.S |
|  | 19180 | **638.9917** | **1913.9533** | **1914.8928** | **-0.9395** | **1** | **7** | **18** | **1** | **R.MQNQRGSGSVVGELMYK.N** |
|  | 19348 | **643.6884** | **1928.0432** | **1926.9338** | **1.1094** | **0** | **1** | **81** | **4** | **R.DIPFSAIYFPVYAHCK.L** |
|  | 20909 | 519.2744 | 2073.0685 | 2072.0282 | 1.0403 | 1 | 8 | 15 | 3 | K.SKSGMISGLDFSDIMVTIR.S |
|  | 30451 | **1357.3273** | **4068.9600** | **4069.9534** | **-0.9934** | **1** | **2** | **16** | **1** | **K.SGNGEVTFENVKEIFGQTIIHHHIPFNWDCEFIR.L** |

| **756.** | Q9UI47\|CTNA3_HUMAN    **Mass:** 100772   **Score:** 52     **Queries matched:** 21   **emPAI:** 0.04 |
| --- | --- |
|  | Catenin alpha-3 OS=Homo sapiens GN=CTNNA3 PE=1 SV=2 |

|  | **Query** | **Observed** | **Mr(expt)** | **Mr(calc)** | **Delta** | **Miss** | **Score** | **Expect** | **Rank** | **Peptide** |
| --- | --- | --- | --- | --- | --- | --- | --- | --- | --- | --- |
|  | 1083 | **557.4534** | **556.4461** | **557.3285** | **-0.8824** | **0** | **5** | **44** | **1** | **R.AAGAIR.G** |
|  | 2227 | 804.4412 | 803.4339 | 803.4388 | -0.0050 | 1 | 7 | 41 | 10 | R.KESEALK.V |
|  | 2480 | 412.7308 | 823.4471 | 824.4253 | -0.9782 | 1 | 7 | 27 | 6 | R.DLHRER.I |
|  | 4078 | 464.2452 | 926.4759 | 927.3976 | -0.9218 | 0 | 0 | 1.5e+002 | 10 | R.CSVMMIR.T |
|  | 6049 | **536.2253** | **1070.4361** | **1071.4841** | **-1.0480** | **1** | **6** | **45** | **10** | **K.NTMEMYKR.T** |
|  | 8045 | 604.3278 | 1206.6411 | 1206.7197 | -0.0786 | 1 | 4 | 62 | 2 | K.IIRIQSPAGPR.H |
|  | 8233 | 608.8356 | 1215.6567 | 1215.6645 | -0.0078 | 0 | 52 | 0.00099 | 1 | K.NLMNAVVQTVK.M |
|  | 8552 | 617.3415 | 1232.6684 | 1231.6595 | 1.0090 | 0 | (3) | 77 | 4 | K.NLMNAVVQTVK.M |
|  | 9060 | 421.0560 | 1260.1463 | 1260.6197 | -0.4735 | 0 | 10 | 11 | 3 | K.DELTASLEEVR.K |
|  | 12368 | **722.9022** | **1443.7898** | **1444.6690** | **-0.8792** | **1** | **2** | **91** | **3** | **K.SQAVKNTMEMYK.R** |
|  | 12374 | **723.1811** | **1444.3476** | **1444.6690** | **-0.3214** | **1** | **(0)** | **85** | **9** | **K.SQAVKNTMEMYK.R** |
|  | 13384 | 754.8680 | 1507.7215 | 1508.6963 | -0.9748 | 0 | 5 | 40 | 4 | R.SNTLNIALDNMCK.K 13383 |
|  | 13431 | 504.5908 | 1510.7505 | 1509.7827 | 0.9678 | 1 | 14 | 4.5 | 1 | K.ELENLDYLAFKR.Q |
|  | 17527 | **897.4616** | **1792.9087** | **1793.8400** | **-0.9313** | **1** | **1** | **86** | **3** | **K.ERSNTLNIALDNMCK.K** |
|  | 18181 | **614.5689** | **1840.6847** | **1841.6949** | **-1.0101** | **0** | **(0)** | **79** | **8** | **K.NMCMIMMEMTDFTR.G** |
|  | 18206 | **615.0425** | **1842.1058** | **1841.6949** | **0.4109** | **0** | **1** | **79** | **3** | **K.NMCMIMMEMTDFTR.G** |
|  | 21378 | **705.2353** | **2112.6840** | **2113.0911** | **-0.4071** | **1** | **1** | **53** | **4** | **K.NLMNAVVQTVKMSYIASTK.I** |
|  | 23801 | **803.3599** | **2407.0578** | **2406.2134** | **0.8443** | **1** | **2** | **46** | **2** | **R.LVEVANLACSMSTNEDGIKIVK.I** |
|  | 25681 | 671.0435 | 2680.1450 | 2679.4735 | 0.6715 | 0 | 3 | 25 | 4 | K.AIIDHVSDSFLDTTVPLLVLIEAAK.N |
|  | 30464 | **817.0032** | **4079.9798** | **4079.8368** | **0.1429** | **1** | **3** | **13** | **7** | **K.LDAEIEIWDDTSNDIIVLAKNMCMIMMEMTDFTR.G** |

| **757.** | P14406\|CX7A2_HUMAN    **Mass:** 9390     **Score:** 52     **Queries matched:** 3   **emPAI:** 0.43 |
| --- | --- |
|  | Cytochrome c oxidase polypeptide 7A2, mitochondrial OS=Homo sapiens GN=COX7A2 PE=1 SV=1 |

|  | **Query** | **Observed** | **Mr(expt)** | **Mr(calc)** | **Delta** | **Miss** | **Score** | **Expect** | **Rank** | **Peptide** |
| --- | --- | --- | --- | --- | --- | --- | --- | --- | --- | --- |
|  | 5559 | **517.7817** | **1033.5488** | **1033.5556** | **-0.0068** | **0** | **52** | **0.0011** | **1** | **K.GGVADALLYR.A** |
|  | 15085 | 811.9131 | 1621.8117 | 1621.8239 | -0.0122 | 0 | 4 | 46 | 8 | K.LFQEDDEIPLYLK.G |
|  | 24449 | **1244.1613** | **2486.3079** | **2486.3131** | **-0.0051** | **0** | **9** | **8.3** | **1** | **R.ATMILTVGGTAYAIYELAVASFPK.K** |

| **758.** | P12074\|CX6A1_HUMAN    **Mass:** 12147    **Score:** 52     **Queries matched:** 14   **emPAI:** 1.34 |
| --- | --- |
|  | Cytochrome c oxidase subunit 6A1, mitochondrial OS=Homo sapiens GN=COX6A1 PE=1 SV=4 |

|  | **Query** | **Observed** | **Mr(expt)** | **Mr(calc)** | **Delta** | **Miss** | **Score** | **Expect** | **Rank** | **Peptide** |
| --- | --- | --- | --- | --- | --- | --- | --- | --- | --- | --- |
|  | 6356 | **546.7843** | **1091.5540** | **1090.5805** | **0.9736** | **0** | **10** | **16** | **1** | **-.MAVVGVSSVSR.L** 6354 |
|  | 6551 | 554.2609 | 1106.5072 | 1106.5754 | -0.0682 | 0 | (8) | 24 | 1 | -.MAVVGVSSVSR.L |
|  | 22299 | **738.0256** | **2211.0549** | **2211.0722** | **-0.0173** | **0** | **18** | **1.4** | **1** | **K.SHHGEHERPEFIAYPHLR.I** 22300 22301 22302 |
|  | 22920 | **1142.1401** | **2282.2657** | **2282.2749** | **-0.0091** | **0** | **41** | **0.0055** | **1** | **K.TLTFFVALPGVAVSMLNVYLK.S** 22921 |
|  | 28626 | **1106.1799** | **3315.5180** | **3315.5374** | **-0.0194** | **0** | **20** | **0.43** | **1** | **R.TKPFPWGDGNHTLFHNPHVNPLPTGYEDE.-** 28623 28624 28627 |
|  | 29446 | **897.4266** | **3585.6772** | **3584.7226** | **0.9546** | **1** | **3** | **19** | **1** | **R.IRTKPFPWGDGNHTLFHNPHVNPLPTGYEDE.-** |

| **759.** | Q8WWI5\|CTL1_HUMAN    **Mass:** 74793    **Score:** 52     **Queries matched:** 19   **emPAI:** 0.05 |
| --- | --- |
|  | Choline transporter-like protein 1 OS=Homo sapiens GN=SLC44A1 PE=1 SV=1 |

|  | **Query** | **Observed** | **Mr(expt)** | **Mr(calc)** | **Delta** | **Miss** | **Score** | **Expect** | **Rank** | **Peptide** |
| --- | --- | --- | --- | --- | --- | --- | --- | --- | --- | --- |
|  | 2037 | **395.7062** | **789.3978** | **789.4055** | **-0.0077** | **0** | **15** | **7.1** | **10** | **K.SSVLCPK.L** 2049 |
|  | 4448 | **476.7792** | **951.5438** | **950.5107** | **1.0331** | **0** | **8** | **26** | **3** | **R.LISGVMTSK.E** |
|  | 7975 | **402.2437** | **1203.7092** | **1202.5900** | **1.1192** | **0** | **10** | **16** | **4** | **K.SVALCVAACPR.Q** |
|  | 8698 | **620.8119** | **1239.6092** | **1238.5965** | **1.0127** | **0** | **5** | **41** | **5** | **K.VLMEFVENSR.K** |
|  | 9258 | 635.7968 | 1269.5791 | 1269.7557 | -0.1767 | 1 | 5 | 49 | 5 | R.LIRYHLGTVAK.G |
|  | 9596 | **430.2997** | **1287.8773** | **1287.7034** | **0.1739** | **1** | **7** | **27** | **5** | **R.QELKTLSDVQK.F** |
|  | 10764 | 676.8104 | 1351.6062 | 1350.6965 | 0.9096 | 1 | 6 | 33 | 4 | K.VLMEFVENSRK.A |
|  | 11378 | 463.1317 | 1386.3734 | 1386.5504 | -0.1770 | 0 | 3 | 49 | 4 | M.GCCSSASSAAQSSK.R 11377 |
|  | 11505 | 465.2715 | 1392.7925 | 1391.7305 | 1.0620 | 0 | (1) | 95 | 7 | R.MILMYIHSQLK.G |
|  | 11762 | **705.3025** | **1408.5904** | **1407.7254** | **0.8650** | **0** | **5** | **37** | **1** | **R.MILMYIHSQLK.G** |
|  | 13548 | **507.2339** | **1518.6800** | **1517.5909** | **1.0892** | **0** | **1** | **1e+002** | **10** | **-.MGCCSSASSAAQSSK.R** |
|  | 15671 | **830.8803** | **1659.7461** | **1659.7563** | **-0.0102** | **0** | **52** | **0.00073** | **1** | **R.LVSGYDSYGNICGQK.N** |
|  | 15846 | **558.5414** | **1672.6025** | **1673.6920** | **-1.0895** | **1** | **11** | **7.9** | **1** | **-.MGCCSSASSAAQSSKR.E** |
|  | 17046 | **879.4670** | **1756.9194** | **1757.9684** | **-1.0490** | **1** | **4** | **47** | **4** | **K.IPRMILMYIHSQLK.G** |
|  | 19143 | **638.3029** | **1911.8869** | **1910.9268** | **0.9601** | **1** | **1** | **80** | **4** | **K.NTKLEAIPNSGMDHTQR.K** |
|  | 20153 | 668.7888 | 2003.3446 | 2002.9418 | 0.4028 | 1 | 10 | 7 | 1 | R.LVSGYDSYGNICGQKNTK.L |
|  | 26003 | **909.4987** | **2725.4743** | **2726.5882** | **-1.1139** | **0** | **3** | **29** | **4** | **R.ALLIYAISATVFTVILFLIMLVMR.K** |

| **760.** | Q9Y624\|JAM1_HUMAN    **Mass:** 32962    **Score:** 52     **Queries matched:** 9   **emPAI:** 0.11 |
| --- | --- |
|  | Junctional adhesion molecule A OS=Homo sapiens GN=F11R PE=1 SV=1 |

|  | **Query** | **Observed** | **Mr(expt)** | **Mr(calc)** | **Delta** | **Miss** | **Score** | **Expect** | **Rank** | **Peptide** |
| --- | --- | --- | --- | --- | --- | --- | --- | --- | --- | --- |
|  | 3869 | **456.2626** | **910.5106** | **909.4378** | **1.0728** | **0** | **0** | **1.3e+002** | **5** | **R.LVCYNNK.I** |
|  | 5370 | **510.7738** | **1019.5331** | **1019.5400** | **-0.0069** | **0** | **11** | **15** | **2** | **K.VIYSQPSAR.S** |
|  | 6052 | **536.2746** | **1070.5346** | **1070.5430** | **-0.0084** | **0** | **11** | **14** | **1** | **K.DGIVMPTNPK.S** |
|  | 6313 | **544.8287** | **1087.6428** | **1086.5379** | **1.1048** | **0** | **(3)** | **82** | **5** | **K.DGIVMPTNPK.S** |
|  | 11021 | **684.8095** | **1367.6045** | **1366.6299** | **0.9745** | **0** | **13** | **7** | **1** | **R.NGYGTPMTSNAVR.M** |
|  | 12155 | **715.8170** | **1429.6195** | **1430.7188** | **-1.0993** | **1** | **7** | **25** | **4** | **K.DGIVMPTNPKSTR.A** |
|  | 15757 | **556.3116** | **1665.9129** | **1665.9454** | **-0.0325** | **1** | **1** | **81** | **6** | **R.VTFLPTGITFKSVTR.E** |
|  | 23056 | **1151.5115** | **2301.0084** | **2301.0260** | **-0.0176** | **0** | **52** | **0.00047** | **1** | **R.AVLTCSEQDGSPPSEYTWFK.D** |
|  | 23591 | **791.7954** | **2372.3644** | **2372.3791** | **-0.0147** | **0** | **17** | **1.1** | **1** | **K.LIVLVPPSKPTVNIPSSATIGNR.A** |

| **761.** | P01908\|HA21_HUMAN    **Mass:** 28316    **Score:** 52     **Queries matched:** 4   **emPAI:** 0.13 |
| --- | --- |
|  | HLA class II histocompatibility antigen, DQ(1) alpha chain OS=Homo sapiens PE=2 SV=2 |

|  | **Query** | **Observed** | **Mr(expt)** | **Mr(calc)** | **Delta** | **Miss** | **Score** | **Expect** | **Rank** | **Peptide** |
| --- | --- | --- | --- | --- | --- | --- | --- | --- | --- | --- |
|  | 3713 | 451.2454 | 900.4762 | 901.5094 | -1.0331 | 1 | 15 | 5.9 | 4 | R.GLRSVGASR.H 3711 |
|  | 12245 | 479.2544 | 1434.7413 | 1434.7507 | -0.0095 | 0 | 52 | 0.00091 | 1 | K.VEHWGLDEPLLK.H |
|  | 20493 | 679.3719 | 2035.0940 | 2033.9656 | 1.1284 | 0 | 1 | 77 | 8 | K.ISYLTFLPSADEIYDCK.V |

|  | **Proteins matching the same set of peptides:** |
| --- | --- |

|  | P04226\|HA22_HUMAN    **Mass:** 28147    **Score:** 52     **Queries matched:** 4 |
| --- | --- |
|  | HLA class II histocompatibility antigen, DQ(2) alpha chain OS=Homo sapiens GN=HLA-DQA1 PE=2 SV=1 |

|  | **Proteins matching a subset of these peptides:** |
| --- | --- |

|  | P04225\|HA24_HUMAN    **Mass:** 26020    **Score:** 52     **Queries matched:** 3 |
| --- | --- |
|  | HLA class II histocompatibility antigen, DQ(4) alpha chain (Fragment) OS=Homo sapiens GN=HLA-DQA1 PE=1 SV=1 |

|  | P01906\|HA26_HUMAN    **Mass:** 28243    **Score:** 52     **Queries matched:** 2 |
| --- | --- |
|  | HLA class II histocompatibility antigen, DQ(6) alpha chain OS=Homo sapiens GN=HLA-DQA2 PE=2 SV=2 |

| **762.** | P26373\|RL13_HUMAN    **Mass:** 24304    **Score:** 52     **Queries matched:** 5   **emPAI:** 0.16 |
| --- | --- |
|  | 60S ribosomal protein L13 OS=Homo sapiens GN=RPL13 PE=1 SV=4 |

|  | **Query** | **Observed** | **Mr(expt)** | **Mr(calc)** | **Delta** | **Miss** | **Score** | **Expect** | **Rank** | **Peptide** |
| --- | --- | --- | --- | --- | --- | --- | --- | --- | --- | --- |
|  | 1159 | **573.3321** | **572.3249** | **572.3394** | **-0.0145** | **1** | **9** | **35** | **8** | **R.QAKAR.R** |
|  | 1414 | 628.3623 | 627.3550 | 626.3751 | 0.9799 | 0 | 10 | 14 | 3 | R.KPSAPK.K 1412 |
|  | 5941 | **532.2631** | **1062.5117** | **1062.5193** | **-0.0076** | **1** | **22** | **0.99** | **1** | **K.KGDSSAEELK.L** |
|  | 8524 | **616.8111** | **1231.6076** | **1231.6157** | **-0.0080** | **0** | **52** | **0.0011** | **1** | **K.STESLQANVQR.L** |

| **763.** | P53618\|COPB_HUMAN    **Mass:** 108214   **Score:** 51     **Queries matched:** 18   **emPAI:** 0.03 |
| --- | --- |
|  | Coatomer subunit beta OS=Homo sapiens GN=COPB1 PE=1 SV=3 |

|  | **Query** | **Observed** | **Mr(expt)** | **Mr(calc)** | **Delta** | **Miss** | **Score** | **Expect** | **Rank** | **Peptide** |
| --- | --- | --- | --- | --- | --- | --- | --- | --- | --- | --- |
|  | 966 | **531.2998** | **530.2925** | **530.3064** | **-0.0139** | **0** | **19** | **4.1** | **10** | **K.SSLPK.K** |
|  | 2238 | **805.3824** | **804.3751** | **804.3613** | **0.0138** | **0** | **10** | **21** | **2** | **K.ESDNNVK.L** |
|  | 6755 | 562.2991 | 1122.5836 | 1121.5387 | 1.0449 | 0 | 6 | 37 | 2 | K.SQGMALSLGDK.I |
|  | 8942 | **628.3186** | **1254.6226** | **1255.6772** | **-1.0545** | **1** | **5** | **42** | **10** | **K.EAADPLASKLNK.V** |
|  | 9021 | **629.8527** | **1257.6908** | **1256.7526** | **0.9382** | **1** | **2** | **85** | **3** | **K.KVIIMILNGEK.L** |
|  | 10056 | **438.7222** | **1313.1447** | **1313.7377** | **-0.5930** | **0** | **7** | **18** | **4** | **R.VLQDLVMDILR.V** |
|  | 10214 | 441.2467 | 1320.7183 | 1320.6707 | 0.0475 | 1 | 5 | 42 | 6 | R.AKSQGMALSLGDK.I |
|  | 10508 | 446.2690 | 1335.7852 | 1335.6340 | 0.1512 | 0 | 5 | 40 | 8 | K.EDIQSVMTEIR.R |
|  | 13242 | 500.2324 | 1497.6753 | 1496.6394 | 1.0359 | 0 | 2 | 81 | 6 | R.QMWAEFEWENK.V |
|  | 13937 | **774.2358** | **1546.4571** | **1545.7392** | **0.7180** | **1** | **2** | **50** | **5** | **K.ECRQSLSHMLSAK.L** |
|  | 16353 | **570.2683** | **1707.7831** | **1706.7496** | **1.0335** | **1** | **5** | **33** | **6** | **K.TNNVSEHEDTDKYR.Q** |
|  | 16857 | **582.3157** | **1743.9254** | **1744.8665** | **-0.9411** | **1** | **4** | **43** | **1** | **R.QSLSHMLSAKLEEEK.L** |
|  | 17515 | **896.9828** | **1791.9510** | **1791.9618** | **-0.0108** | **0** | **51** | **0.00081** | **1** | **K.YEAAGTLVTLSSAPTAIK.A** |
|  | 22631 | **564.0087** | **2252.0056** | **2252.3364** | **-0.3308** | **1** | **1** | **57** | **7** | **K.VIIMILNGEKLPGLLMTIIR.F** |
|  | 22843 | **759.4930** | **2275.4571** | **2275.1089** | **0.3482** | **1** | **1** | **48** | **1** | **K.TTPDGRLLHEMILVCDAYR.K** |
|  | 23879 | 605.3137 | 2417.2255 | 2417.4232 | -0.1977 | 1 | 5 | 23 | 7 | K.LPGLLMTIIRFVLPLQDHTIK.K |
|  | 24624 | **837.1752** | **2508.5039** | **2508.1599** | **0.3439** | **1** | **1** | **39** | **1** | **K.CLTPEKALSGYCGFMAANLYAR.S** |
|  | 26815 | 1448.2643 | 2894.5140 | 2893.3725 | 1.1415 | 0 | 1 | 47 | 8 | M.TAAENVCYTLINVPMDSEPPSEISLK.N |

| **764.** | P09960\|LKHA4_HUMAN    **Mass:** 69868    **Score:** 51     **Queries matched:** 11   **emPAI:** 0.05 |
| --- | --- |
|  | Leukotriene A-4 hydrolase OS=Homo sapiens GN=LTA4H PE=1 SV=2 |

|  | **Query** | **Observed** | **Mr(expt)** | **Mr(calc)** | **Delta** | **Miss** | **Score** | **Expect** | **Rank** | **Peptide** |
| --- | --- | --- | --- | --- | --- | --- | --- | --- | --- | --- |
|  | 1333 | 609.3198 | 608.3125 | 608.3170 | -0.0045 | 0 | 2 | 79 | 9 | K.AYVEK.F |
|  | 8045 | 604.3278 | 1206.6411 | 1206.6496 | -0.0085 | 0 | 2 | 86 | 5 | K.LTYTAEVSVPK.E |
|  | 12346 | 722.3130 | 1442.6114 | 1441.6321 | 0.9793 | 1 | 12 | 8.2 | 10 | R.DGETPDPEDPSRK.I |
|  | 13823 | 770.8496 | 1539.6845 | 1539.7729 | -0.0883 | 1 | 11 | 9.8 | 3 | K.HLHLRCSVDFTR.R 13824 |
|  | 16036 | **843.4706** | **1684.9266** | **1683.9407** | **0.9859** | **1** | **18** | **1.9** | **1** | **K.DLTIEKVVINGQEVK.Y** |
|  | 16623 | **577.1246** | **1728.3521** | **1728.8903** | **-0.5382** | **1** | **2** | **43** | **2** | **K.ASMHPVTAMLVGKDLK.V** |
|  | 21375 | 705.0485 | 2112.1237 | 2113.0010 | -0.8773 | 1 | 3 | 40 | 6 | K.RMQEVYNFNAINNSEIR.F |
|  | 23304 | **1169.5825** | **2337.1505** | **2337.1628** | **-0.0123** | **0** | **51** | **0.00055** | **1** | **K.LVVDLTDIDPDVAYSSVPYEK.G** |
|  | 23805 | **803.4327** | **2407.2764** | **2406.2292** | **1.0472** | **0** | **6** | **18** | **5** | **K.SLSNVIAHEISHSWTGNLVTNK.T** |
|  | 28736 | 1116.9060 | 3347.6962 | 3346.6932 | 1.0030 | 1 | 3 | 23 | 5 | K.NQEIVIEISFETSPKSSALQWLTPEQTSGK.E |

| **765.** | P05783\|K1C18_HUMAN    **Mass:** 48029    **Score:** 51     **Queries matched:** 10   **emPAI:** 0.08 |
| --- | --- |
|  | Keratin, type I cytoskeletal 18 OS=Homo sapiens GN=KRT18 PE=1 SV=2 |

|  | **Query** | **Observed** | **Mr(expt)** | **Mr(calc)** | **Delta** | **Miss** | **Score** | **Expect** | **Rank** | **Peptide** |
| --- | --- | --- | --- | --- | --- | --- | --- | --- | --- | --- |
|  | 2714 | 419.7217 | 837.4289 | 836.4392 | 0.9897 | 0 | 7 | 29 | 1 | R.LASYLDR.V |
|  | 9668 | 431.2528 | 1290.7367 | 1291.7136 | -0.9769 | 1 | 10 | 15 | 1 | K.VKLEAEIATYR.R |
|  | 10892 | **680.3571** | **1358.6997** | **1359.7180** | **-1.0183** | **1** | **11** | **11** | **1** | **K.SQDLAKIMADIR.A** |
|  | 18774 | **628.9840** | **1883.9300** | **1883.0000** | **0.9300** | **0** | **1** | **86** | **2** | **K.GLQAQIASSGLTVEVDAPK.S** |
|  | 22878 | **760.0369** | **2277.0890** | **2276.0889** | **1.0000** | **0** | **2** | **49** | **3** | **R.GGMGSGGLATGIAGGLAGMGGIQNEK.E** |
|  | 24737 | 632.0470 | 2524.1589 | 2525.3337 | -1.1748 | 1 | 2 | 39 | 6 | K.GLQAQIASSGLTVEVDAPKSQDLAK.I |
|  | 25593 | **890.7991** | **2669.3756** | **2669.3846** | **-0.0091** | **0** | **51** | **0.00047** | **1** | **R.YALQMEQLNGILLHLESELAQTR.A** |
|  | 26651 | **957.4561** | **2869.3465** | **2870.3651** | **-1.0186** | **1** | **1** | **41** | **5** | **R.STSFRGGMGSGGLATGIAGGLAGMGGIQNEK.E** |
|  | 28818 | **842.8946** | **3367.5493** | **3366.5602** | **0.9890** | **1** | **(3)** | **21** | **4** | **R.GGMGSGGLATGIAGGLAGMGGIQNEKETMQSLNDR.L** |
|  | 28862 | **846.6155** | **3382.4331** | **3382.5552** | **-0.1221** | **1** | **4** | **15** | **3** | **R.GGMGSGGLATGIAGGLAGMGGIQNEKETMQSLNDR.L** |

| **766.** | P62081\|RS7_HUMAN    **Mass:** 22113    **Score:** 51     **Queries matched:** 14   **emPAI:** 0.38 |
| --- | --- |
|  | 40S ribosomal protein S7 OS=Homo sapiens GN=RPS7 PE=1 SV=1 |

|  | **Query** | **Observed** | **Mr(expt)** | **Mr(calc)** | **Delta** | **Miss** | **Score** | **Expect** | **Rank** | **Peptide** |
| --- | --- | --- | --- | --- | --- | --- | --- | --- | --- | --- |
|  | 1894 | 757.3978 | 756.3905 | 756.3476 | 0.0429 | 0 | 13 | 9.6 | 4 | -.MFSSSAK.I |
|  | 2501 | 413.2401 | 824.4657 | 823.5280 | 0.9377 | 0 | 3 | 64 | 4 | R.ILPKPTR.K |
|  | 3727 | 451.2683 | 900.5219 | 900.5392 | -0.0173 | 1 | 6 | 50 | 5 | K.LDGSRLIK.V |
|  | 5481 | **515.2631** | **1028.5117** | **1028.5179** | **-0.0062** | **0** | **8** | **28** | **8** | **K.VETFSGVYK.K** |
|  | 7183 | **578.9360** | **1155.8574** | **1156.6128** | **-0.7554** | **1** | **1** | **94** | **4** | **K.VETFSGVYKK.L** |
|  | 10326 | **443.0397** | **1326.0972** | **1326.7619** | **-0.6647** | **1** | **6** | **23** | **1** | **K.AQLRELNITAAK.E** |
|  | 20942 | **693.3630** | **2077.0671** | **2077.0229** | **0.0443** | **1** | **1** | **70** | **3** | **K.AQQNNVEHKVETFSGVYK.K** |
|  | 23565 | 789.7613 | 2366.2620 | 2366.2733 | -0.0113 | 0 | 40 | 0.008 | 1 | R.TLTAVHDAILEDLVFPSEIVGK.R 23552 23562 23564 23566 |
|  | 28684 | 833.4213 | 3329.6562 | 3329.6700 | -0.0138 | 0 | 13 | 1.9 | 1 | K.IVKPNGEKPDEFESGISQALLELEMNSDLK.A |
|  | 30486 | 1022.2587 | 4085.0058 | 4084.0020 | 1.0038 | 1 | 2 | 16 | 3 | -.MFSSSAKIVKPNGEKPDEFESGISQALLELEMNSDLK.A |

| **767.** | P60468\|SC61B_HUMAN    **Mass:** 10025    **Score:** 51     **Queries matched:** 4   **emPAI:** 0.40 |
| --- | --- |
|  | Protein transport protein Sec61 subunit beta OS=Homo sapiens GN=SEC61B PE=1 SV=2 |

|  | **Query** | **Observed** | **Mr(expt)** | **Mr(calc)** | **Delta** | **Miss** | **Score** | **Expect** | **Rank** | **Peptide** |
| --- | --- | --- | --- | --- | --- | --- | --- | --- | --- | --- |
|  | 6814 | **564.7822** | **1127.5498** | **1128.6363** | **-1.0865** | **1** | **1** | **1.2e+002** | **8** | **K.AVAARAAGSTVR.Q** |
|  | 11066 | **685.8319** | **1369.6493** | **1369.6586** | **-0.0094** | **0** | **51** | **0.001** | **1** | **M.PGPTPSGTNVGSSGR.S** 11082 11086 |

| **768.** | P46059\|S15A1_HUMAN    **Mass:** 79382    **Score:** 51     **Queries matched:** 17   **emPAI:** 0.20 |
| --- | --- |
|  | Solute carrier family 15 member 1 OS=Homo sapiens GN=SLC15A1 PE=2 SV=1 |

|  | **Query** | **Observed** | **Mr(expt)** | **Mr(calc)** | **Delta** | **Miss** | **Score** | **Expect** | **Rank** | **Peptide** |
| --- | --- | --- | --- | --- | --- | --- | --- | --- | --- | --- |
|  | 5468 | **514.7685** | **1027.5224** | **1027.5298** | **-0.0074** | **0** | **7** | **32** | **9** | **K.DGLNQKPEK.G** |
|  | 5832 | **528.7634** | **1055.5123** | **1055.5182** | **-0.0059** | **0** | **36** | **0.044** | **1** | **R.VQQCGIHSK.Q** |
|  | 6686 | 1118.4879 | 1117.4806 | 1118.5906 | -1.1100 | 0 | (4) | 69 | 8 | K.FKPQGNIMGK.V |
|  | 6910 | 568.2799 | 1134.5453 | 1134.5856 | -0.0403 | 0 | 4 | 58 | 10 | K.FKPQGNIMGK.V |
|  | 8367 | **612.3044** | **1222.5943** | **1222.6016** | **-0.0073** | **0** | **27** | **0.31** | **1** | **R.WTLQATTMSGK.I** 8368 |
|  | 8688 | 620.7758 | 1239.5370 | 1239.6149 | -0.0779 | 1 | 9 | 16 | 7 | K.REHWLDWAK.E |
|  | 9135 | **422.0044** | **1262.9913** | **1262.6805** | **0.3108** | **1** | **6** | **25** | **10** | **K.KFKPQGNIMGK.V** |
|  | 10563 | **670.8522** | **1339.6898** | **1340.6513** | **-0.9615** | **1** | **5** | **43** | **7** | **R.EHWLDWAKEK.Y** |
|  | 12156 | 715.8171 | 1429.6196 | 1429.6296 | -0.0100 | 0 | 31 | 0.11 | 1 | K.SNPYFMSGANSQK.Q 12155 |
|  | 17658 | **602.3291** | **1803.9655** | **1803.9784** | **-0.0130** | **0** | **22** | **0.67** | **1** | **R.HTLLVWAPNHYQVVK.D** |
|  | 18008 | **915.4541** | **1828.8936** | **1829.9233** | **-1.0297** | **0** | **2** | **70** | **3** | **R.FVNTFNELITITMSGK.V** |
|  | 27223 | 744.5941 | 2974.3471 | 2973.6150 | 0.7321 | 1 | 0 | 50 | 3 | R.NRFFSIFYLAINAGSLLSTIITPMLR.V |
|  | 29866 | 1242.6325 | 3724.8755 | 3724.9837 | -0.1082 | 1 | 1 | 28 | 5 | R.FFSIFYLAINAGSLLSTIITPMLRVQQCGIHSK.Q |
|  | 30240 | 1300.5900 | 3898.7481 | 3897.8773 | 0.8707 | 0 | 1 | 25 | 5 | K.VLNIGNNTMNISLPGEMVTLGPMSQTNAFMTFDVNK.L |
|  | 31536 | 1735.2394 | 5202.6963 | 5201.7834 | 0.9129 | 1 | 4 | 5.9 | 1 | R.WTLQATTMSGKIGALEIQPDQMQTVNAILIVIMVPIFDAVLYPLIAK.C |

| **769.** | Q99828\|CIB1_HUMAN    **Mass:** 21861    **Score:** 51     **Queries matched:** 3   **emPAI:** 0.38 |
| --- | --- |
|  | Calcium and integrin-binding protein 1 OS=Homo sapiens GN=CIB1 PE=1 SV=4 |

|  | **Query** | **Observed** | **Mr(expt)** | **Mr(calc)** | **Delta** | **Miss** | **Score** | **Expect** | **Rank** | **Peptide** |
| --- | --- | --- | --- | --- | --- | --- | --- | --- | --- | --- |
|  | 12670 | **732.3381** | **1462.6616** | **1462.6722** | **-0.0106** | **0** | **37** | **0.028** | **1** | **R.LVNCLTGEGEDTR.L** 12658 |
|  | 16425 | 856.9824 | 1711.9502 | 1710.9556 | 0.9946 | 0 | 40 | 0.011 | 1 | R.AQVPFEQILSLPELK.A |

| **770.** | P16219\|ACADS_HUMAN    **Mass:** 44611    **Score:** 51     **Queries matched:** 23   **emPAI:** 0.08 |
| --- | --- |
|  | Short-chain specific acyl-CoA dehydrogenase, mitochondrial OS=Homo sapiens GN=ACADS PE=1 SV=1 |

|  | **Query** | **Observed** | **Mr(expt)** | **Mr(calc)** | **Delta** | **Miss** | **Score** | **Expect** | **Rank** | **Peptide** |
| --- | --- | --- | --- | --- | --- | --- | --- | --- | --- | --- |
|  | 972 | **533.3079** | **532.3006** | **532.3043** | **-0.0036** | **0** | **16** | **7.5** | **1** | **R.AAMLK.D** |
|  | 4196 | **468.7524** | **935.4903** | **934.4946** | **0.9957** | **0** | **(12)** | **11** | **2** | **R.MAFGAPLTK.L** 4187 |
|  | 4414 | 476.1965 | 950.3784 | 950.4895 | -0.1111 | 0 | 12 | 9.9 | 4 | R.MAFGAPLTK.L 4433 4438 |
|  | 8409 | **409.2312** | **1224.6717** | **1223.7098** | **0.9619** | **1** | **9** | **17** | **2** | **M.AAALLARASGPAR.R** 8373 |
|  | 8537 | **411.7192** | **1232.1359** | **1232.6951** | **-0.5592** | **1** | **10** | **9.4** | **1** | **K.KPFIKEAAMAK.L** |
|  | 8866 | **625.8041** | **1249.5936** | **1249.6013** | **-0.0077** | **0** | **10** | **13** | **1** | **K.DSILGEPGMGFK.I** |
|  | 9366 | **425.9004** | **1274.6794** | **1275.6492** | **-0.9698** | **0** | **10** | **16** | **9** | **K.LADMALALESAR.L** 9361 |
|  | 11113 | **458.2819** | **1371.8239** | **1370.7452** | **1.0787** | **1** | **8** | **21** | **8** | **-.MAAALLARASGPAR.R** 11115 11119 11120 |
|  | 12763 | **735.3330** | **1468.6515** | **1468.6616** | **-0.0102** | **0** | **8** | **21** | **1** | **R.GSSTANLIFEDCR.I** |
|  | 13809 | **769.8831** | **1537.7517** | **1537.7624** | **-0.0107** | **0** | **51** | **0.0011** | **1** | **R.ITEIYEGTSEIQR.L** |
|  | 14782 | 802.9174 | 1603.8202 | 1603.8280 | -0.0078 | 1 | 12 | 7.9 | 2 | R.IPKDSILGEPGMGFK.I |
|  | 17586 | 900.0057 | 1797.9969 | 1798.0063 | -0.0094 | 0 | 18 | 1.4 | 1 | K.GISAFLVPMPTPGLTLGK.K |
|  | 17791 | **605.7412** | **1814.2018** | **1814.0012** | **0.2006** | **0** | **(11)** | **6** | **2** | **K.GISAFLVPMPTPGLTLGK.K** |
|  | 28166 | **1062.8641** | **3185.5706** | **3185.5849** | **-0.0143** | **0** | **11** | **4.1** | **1** | **K.LAASEAATAISHQAIQILGGMGYVTEMPAER.H** |
|  | 29909 | 938.7129 | 3750.8225 | 3750.8491 | -0.0267 | 1 | 5 | 12 | 3 | K.IAMQTLDMGRIGIASQALGIAQTALDCAVNYAENR.M |

| **771.** | P13010\|KU86_HUMAN    **Mass:** 83222    **Score:** 50     **Queries matched:** 19   **emPAI:** 0.09 |
| --- | --- |
|  | ATP-dependent DNA helicase 2 subunit 2 OS=Homo sapiens GN=XRCC5 PE=1 SV=3 |

|  | **Query** | **Observed** | **Mr(expt)** | **Mr(calc)** | **Delta** | **Miss** | **Score** | **Expect** | **Rank** | **Peptide** |
| --- | --- | --- | --- | --- | --- | --- | --- | --- | --- | --- |
|  | 1267 | **596.9543** | **595.9470** | **595.3442** | **0.6028** | **0** | **8** | **11** | **1** | **K.IPNPR.F** 1270 |
|  | 1438 | 632.3682 | 631.3609 | 631.3177 | 0.0432 | 0 | 8 | 50 | 1 | K.EDIQK.E |
|  | 4694 | 486.2706 | 970.5267 | 971.5764 | -1.0496 | 1 | 14 | 6.8 | 8 | K.SILQERVK.K |
|  | 6072 | **536.7814** | **1071.5483** | **1072.6240** | **-1.0757** | **0** | **13** | **9** | **5** | **R.LTIGSNLSIR.I** 6073 |
|  | 6591 | 555.7626 | 1109.5106 | 1108.6029 | 0.9077 | 0 | 30 | 0.13 | 2 | R.LGGHGPSFPLK.G |
|  | 7345 | 585.2987 | 1168.5829 | 1168.5434 | 0.0395 | 1 | 3 | 75 | 10 | K.VDEEQMKYK.S |
|  | 11214 | 689.8416 | 1377.6687 | 1376.7565 | 0.9122 | 0 | 7 | 27 | 7 | R.ANPQVGVAFPHIK.H |
|  | 13087 | 745.3881 | 1488.7617 | 1489.7361 | -0.9744 | 1 | 7 | 27 | 3 | K.IERHSIHWPCR.L |
|  | 13748 | **511.9525** | **1532.8355** | **1532.8576** | **-0.0221** | **1** | **4** | **48** | **9** | **K.RANPQVGVAFPHIK.H** |
|  | 23205 | **774.7253** | **2321.1542** | **2320.1620** | **0.9922** | **0** | **16** | **1.8** | **1** | **K.YAPTEAQLNAVDALIDSMSLAK.K** |
|  | 23299 | **779.8893** | **2336.6460** | **2336.1569** | **0.4891** | **0** | **(7)** | **10** | **1** | **K.YAPTEAQLNAVDALIDSMSLAK.K** |
|  | 25409 | **660.0715** | **2636.2568** | **2635.2033** | **1.0535** | **0** | **6** | **16** | **3** | **K.MVMISLEGEDGLDEIYSFSESLR.K** |
|  | 25976 | **908.4654** | **2722.3743** | **2722.3847** | **-0.0104** | **0** | **37** | **0.013** | **1** | **R.DDEAAAVALSSLIHALDDLDMVAIVR.Y** |
|  | 26835 | **967.7814** | **2900.3225** | **2900.3386** | **-0.0161** | **1** | **35** | **0.018** | **1** | **K.ETVYCLNDDDETEVLKEDIIQGFR.Y** |
|  | 28628 | **1106.1826** | **3315.5260** | **3315.5394** | **-0.0133** | **0** | **17** | **0.96** | **1** | **K.ASFEEASNQLINHIEQFLDTNETPYFMK.S** |
|  | 28863 | 1128.5354 | 3382.5844 | 3383.6200 | -1.0356 | 1 | 6 | 11 | 3 | R.SGNKAAVVLCMDVGFTMSNSIPGIESPFEQAK.K 28864 |

| **772.** | Q13838\|UAP56_HUMAN    **Mass:** 49416    **Score:** 50     **Queries matched:** 11   **emPAI:** 0.16 |
| --- | --- |
|  | Spliceosome RNA helicase BAT1 OS=Homo sapiens GN=BAT1 PE=1 SV=1 |

|  | **Query** | **Observed** | **Mr(expt)** | **Mr(calc)** | **Delta** | **Miss** | **Score** | **Expect** | **Rank** | **Peptide** |
| --- | --- | --- | --- | --- | --- | --- | --- | --- | --- | --- |
|  | 3046 | **430.8869** | **859.7592** | **859.4651** | **0.2941** | **1** | **1** | **1.1e+002** | **8** | **K.KDEEVLK.K** |
|  | 3807 | **454.2416** | **906.4686** | **905.4607** | **1.0079** | **0** | **4** | **63** | **6** | **R.DVQEIFR.M** |
|  | 5774 | 526.2625 | 1050.5105 | 1050.4838 | 0.0267 | 0 | 15 | 4.9 | 6 | K.MLEQLDMR.R |
|  | 5777 | 526.2768 | 1050.5390 | 1050.4838 | 0.0552 | 0 | (4) | 64 | 10 | K.MLEQLDMR.R |
|  | 8997 | 629.3116 | 1256.6086 | 1257.6097 | -1.0011 | 0 | 12 | 9.1 | 5 | K.QVMMFSATLSK.E |
|  | 9036 | 630.3244 | 1258.6342 | 1258.6095 | 0.0248 | 1 | 2 | 86 | 4 | R.YQQFKDFQR.R |
|  | 9782 | **649.3514** | **1296.6883** | **1295.6259** | **1.0625** | **0** | **31** | **0.11** | **1** | **K.GSYVSIHSSGFR.D** |
|  | 15449 | **548.9408** | **1643.8006** | **1644.7930** | **-0.9924** | **1** | **5** | **38** | **4** | **R.DVQEIFRMTPHEK.Q** |
|  | 18217 | **922.4462** | **1842.8779** | **1843.8008** | **-0.9229** | **0** | **8** | **18** | **1** | **K.FMQDPMEIFVDDETK.L** |
|  | 19497 | **648.3085** | **1941.9036** | **1943.0887** | **-1.1851** | **1** | **0** | **84** | **7** | **K.NCPHIVVGTPGRILALAR.N** |
|  | 23045 | **767.3428** | **2299.0065** | **2299.0215** | **-0.0150** | **0** | **40** | **0.0073** | **1** | **R.VNIAFNYDMPEDSDTYLHR.V** |

| **773.** | O75964\|ATP5L_HUMAN    **Mass:** 11421    **Score:** 50     **Queries matched:** 7   **emPAI:** 0.35 |
| --- | --- |
|  | ATP synthase subunit g, mitochondrial OS=Homo sapiens GN=ATP5L PE=1 SV=3 |

|  | **Query** | **Observed** | **Mr(expt)** | **Mr(calc)** | **Delta** | **Miss** | **Score** | **Expect** | **Rank** | **Peptide** |
| --- | --- | --- | --- | --- | --- | --- | --- | --- | --- | --- |
|  | 1564 | 659.4040 | 658.3968 | 658.4014 | -0.0046 | 0 | 26 | 0.73 | 1 | R.AIQSLK.K 1558 |
|  | 7098 | 575.8344 | 1149.6543 | 1150.5982 | -0.9440 | 0 | 6 | 38 | 9 | K.IVNSAQTGSFK.Q 7095 7111 |
|  | 9431 | 640.3520 | 1278.6894 | 1278.6932 | -0.0038 | 1 | 50 | 0.0013 | 1 | K.KIVNSAQTGSFK.Q 9427 |

| **774.** | O15484\|CAN5_HUMAN    **Mass:** 74092    **Score:** 50     **Queries matched:** 9   **emPAI:** 0.10 |
| --- | --- |
|  | Calpain-5 OS=Homo sapiens GN=CAPN5 PE=2 SV=2 |

|  | **Query** | **Observed** | **Mr(expt)** | **Mr(calc)** | **Delta** | **Miss** | **Score** | **Expect** | **Rank** | **Peptide** |
| --- | --- | --- | --- | --- | --- | --- | --- | --- | --- | --- |
|  | 2814 | **423.2552** | **844.4958** | **844.5018** | **-0.0060** | **0** | **11** | **19** | **7** | **R.GGLISASIK.A** |
|  | 6326 | **545.3003** | **1088.5860** | **1087.5411** | **1.0450** | **0** | **41** | **0.014** | **1** | **K.GHAYAVTDVR.K** |
|  | 10619 | 672.3144 | 1342.6143 | 1342.6564 | -0.0421 | 1 | 7 | 25 | 3 | R.QYRMHSLQHK.A |
|  | 10960 | **682.8516** | **1363.6886** | **1363.6983** | **-0.0098** | **0** | **21** | **1** | **1** | **K.DSPTGANSYVIIK.C** |
|  | 17276 | **888.4296** | **1774.8447** | **1774.8566** | **-0.0119** | **0** | **35** | **0.036** | **1** | **K.DTFFQNPQYIFEVK.K** 17277 |
|  | 18507 | 621.3006 | 1860.8800 | 1859.9775 | 0.9025 | 1 | 4 | 39 | 4 | R.GGLISASIKAVTAADMEAR.L 18503 |
|  | 19639 | **652.3132** | **1953.9177** | **1952.9149** | **1.0027** | **1** | **5** | **31** | **2** | **K.DSPTGANSYVIIKCEGDK.V** |

| **775.** | Q6P1A2\|MBOA5_HUMAN    **Mass:** 56511    **Score:** 50     **Queries matched:** 6   **emPAI:** 0.07 |
| --- | --- |
|  | Lysophospholipid acyltransferase 5 OS=Homo sapiens GN=LPCAT3 PE=1 SV=1 |

|  | **Query** | **Observed** | **Mr(expt)** | **Mr(calc)** | **Delta** | **Miss** | **Score** | **Expect** | **Rank** | **Peptide** |
| --- | --- | --- | --- | --- | --- | --- | --- | --- | --- | --- |
|  | 8361 | 408.2347 | 1221.6824 | 1220.7605 | 0.9219 | 1 | 7 | 32 | 10 | K.IPNSIIPALKR.L |
|  | 9481 | **428.2591** | **1281.7554** | **1280.7340** | **1.0214** | **0** | **1** | **94** | **9** | **K.LVQGELIDIPGK.I** |
|  | 19408 | 645.3266 | 1932.9580 | 1932.0913 | 0.8667 | 0 | 1 | 81 | 5 | R.LIISIFLGYPFALFYR.H |
|  | 23241 | 776.8925 | 2327.6556 | 2327.3828 | 0.2728 | 1 | 11 | 4.1 | 1 | K.LVQGELIDIPGKIPNSIIPALK.R |
|  | 25276 | **871.4264** | **2611.2573** | **2611.2766** | **-0.0192** | **1** | **50** | **0.00067** | **1** | **K.LIGLAVDYFDGGKDQNSLSSEQQK.Y** 25277 |

| **776.** | P08754\|GNAI3_HUMAN    **Mass:** 41076    **Score:** 50     **Queries matched:** 14   **emPAI:** 0.19 |
| --- | --- |
|  | Guanine nucleotide-binding protein G(k) subunit alpha OS=Homo sapiens GN=GNAI3 PE=1 SV=3 |

|  | **Query** | **Observed** | **Mr(expt)** | **Mr(calc)** | **Delta** | **Miss** | **Score** | **Expect** | **Rank** | **Peptide** |
| --- | --- | --- | --- | --- | --- | --- | --- | --- | --- | --- |
|  | 3372 | 439.7372 | 877.4599 | 877.4294 | 0.0306 | 0 | 11 | 16 | 2 | K.IDFGEAAR.A |
|  | 5850 | 529.3127 | 1056.6108 | 1056.6179 | -0.0071 | 0 | 26 | 0.46 | 1 | K.LLLLGAGESGK.S 5844 5849 |
|  | 6424 | 549.2708 | 1096.5269 | 1095.4768 | 1.0502 | 0 | 6 | 41 | 10 | R.DGGVQACFSR.S |
|  | 6591 | 555.7626 | 1109.5106 | 1109.5175 | -0.0069 | 0 | 37 | 0.028 | 1 | K.LFDSICNNK.W |
|  | 9442 | 640.7906 | 1279.5666 | 1280.5932 | -1.0265 | 1 | 2 | 95 | 10 | K.MFDVGGQRSER.K |
|  | 11258 | 460.9072 | 1379.6998 | 1379.7085 | -0.0088 | 0 | 19 | 1.7 | 2 | K.TTGIVETHFTFK.D |
|  | 14509 | 529.2706 | 1584.7899 | 1584.9450 | -0.1552 | 1 | 3 | 61 | 4 | K.LLLLGAGESGKSTIVK.Q |
|  | 14527 | 793.8569 | 1585.6992 | 1585.7083 | -0.0091 | 0 | 13 | 6 | 3 | K.EIYTHFTCATDTK.N |
|  | 16365 | 570.3245 | 1707.9516 | 1706.9243 | 1.0272 | 0 | 1 | 82 | 10 | K.NVQFVFDAVTDVIIK.N |
|  | 18805 | 629.6392 | 1885.8957 | 1884.8168 | 1.0788 | 1 | 5 | 31 | 4 | R.MHESMKLFDSICNNK.W |
|  | 24645 | **838.9614** | **2513.8625** | **2514.3516** | **-0.4891** | **1** | **0** | **48** | **2** | **R.QLFVLAGSAEEGVMTPELAGVIKR.L** |
|  | 30100 | **1268.2459** | **3801.7157** | **3802.6767** | **-0.9609** | **0** | **38** | **0.0053** | **1** | **R.SPLTICYPEYTGSNTYEEAAAYIQCQFEDLNR.R** |

| **777.** | P78310\|CXAR_HUMAN    **Mass:** 40575    **Score:** 49     **Queries matched:** 9   **emPAI:** 0.09 |
| --- | --- |
|  | Coxsackievirus and adenovirus receptor OS=Homo sapiens GN=CXADR PE=1 SV=1 |

|  | **Query** | **Observed** | **Mr(expt)** | **Mr(calc)** | **Delta** | **Miss** | **Score** | **Expect** | **Rank** | **Peptide** |
| --- | --- | --- | --- | --- | --- | --- | --- | --- | --- | --- |
|  | 751 | **490.0970** | **489.0897** | **489.2435** | **-0.1537** | **0** | **2** | **2.1e+002** | **4** | **K.DGSIV.-** |
|  | 1640 | 677.4423 | 676.4350 | 676.3392 | 0.0959 | 0 | 7 | 46 | 1 | K.LSDSQK.M |
|  | 5715 | 524.2645 | 1046.5145 | 1046.5179 | -0.0034 | 0 | 8 | 30 | 9 | R.VGSDQCLLR.L |
|  | 5910 | **531.2659** | **1060.5172** | **1059.5349** | **0.9823** | **0** | **7** | **40** | **2** | **R.VHFTSNDLK.S** |
|  | 10632 | **672.3990** | **1342.7835** | **1341.7148** | **1.0687** | **0** | **5** | **46** | **9** | **R.MGAIPVMIPAQSK.D** |
|  | 13165 | 498.5652 | 1492.6737 | 1492.7330 | -0.0594 | 0 | 3 | 65 | 7 | R.SLSITTPEEMIEK.A |
|  | 18259 | **615.9964** | **1844.9674** | **1845.8455** | **-0.8781** | **1** | **3** | **53** | **7** | **R.CYVDGSEEIGSDFKIK.C** |
|  | 24994 | 853.0930 | 2556.2571 | 2556.2451 | 0.0119 | 1 | 5 | 23 | 2 | K.LSDSQKMPTSWLAEMTSSVISVK.N |
|  | 25941 | 1357.1660 | 2712.3175 | 2712.3283 | -0.0108 | 0 | 49 | 0.0007 | 1 | K.FTLSPEDQGPLDIEWLISPADNQK.V |

| **778.** | P57105\|SYJ2B_HUMAN    **Mass:** 15918    **Score:** 49     **Queries matched:** 4   **emPAI:** 0.25 |
| --- | --- |
|  | Synaptojanin-2-binding protein OS=Homo sapiens GN=SYNJ2BP PE=1 SV=2 |

|  | **Query** | **Observed** | **Mr(expt)** | **Mr(calc)** | **Delta** | **Miss** | **Score** | **Expect** | **Rank** | **Peptide** |
| --- | --- | --- | --- | --- | --- | --- | --- | --- | --- | --- |
|  | 6294 | 544.3039 | 1086.5932 | 1085.6081 | 0.9852 | 0 | 1 | 1.4e+002 | 7 | K.ILSVNGQDLK.N |
|  | 8199 | **608.3079** | **1214.6013** | **1213.6415** | **0.9598** | **1** | **3** | **73** | **3** | **R.IKENGAAALDGR.L** |
|  | 15437 | **548.6401** | **1642.8984** | **1642.7910** | **0.1074** | **1** | **1** | **95** | **6** | **K.ENGAAALDGRLQEGDK.I** |
|  | 16137 | **847.4304** | **1692.8463** | **1692.8570** | **-0.0107** | **0** | **49** | **0.0013** | **1** | **R.VDYLVTEEEINLTR.G** |

| **779.** | Q9ULC6\|PADI1_HUMAN    **Mass:** 75416    **Score:** 49     **Queries matched:** 9   **emPAI:** 0.05 |
| --- | --- |
|  | Protein-arginine deiminase type-1 OS=Homo sapiens GN=PADI1 PE=1 SV=2 |

|  | **Query** | **Observed** | **Mr(expt)** | **Mr(calc)** | **Delta** | **Miss** | **Score** | **Expect** | **Rank** | **Peptide** |
| --- | --- | --- | --- | --- | --- | --- | --- | --- | --- | --- |
|  | 2487 | **825.0748** | **824.0675** | **824.4140** | **-0.3466** | **0** | **4** | **36** | **2** | **R.DNLHAQK.C** |
|  | 2584 | 415.7156 | 829.4167 | 830.4545 | -1.0378 | 1 | 12 | 13 | 8 | R.QMARAVR.N |
|  | 4253 | 940.4562 | 939.4490 | 939.4298 | 0.0192 | 0 | 49 | 0.0018 | 2 | R.GGNSLSDYK.Q |
|  | 13467 | **505.5968** | **1513.7686** | **1512.6732** | **1.0953** | **0** | **4** | **51** | **3** | **K.EEGYGEAAQFDGLK.H** |
|  | 14348 | 525.2634 | 1572.7683 | 1572.7355 | 0.0328 | 1 | 0 | 1.2e+002 | 9 | R.VFCARGGNSLSDYK.Q |
|  | 19090 | **636.6578** | **1906.9517** | **1906.8983** | **0.0534** | **0** | **3** | **56** | **1** | **R.WPLDTDADMVVSVGTASK.E** |
|  | 21582 | 712.6641 | 2134.9704 | 2134.0365 | 0.9339 | 1 | 3 | 39 | 8 | K.ARWPLDTDADMVVSVGTASK.E |
|  | 22260 | **736.4299** | **2206.2680** | **2207.0431** | **-0.7752** | **0** | **3** | **41** | **1** | **K.NFYAEAFFPDMVNMVVLGK.Y** |
|  | 24352 | 825.7382 | 2474.1928 | 2473.1505 | 1.0423 | 1 | 4 | 28 | 4 | R.VMDTHGSNEKFLEDMSYLTLK.A |

| **780.** | Q9BUP3\|HTAI2_HUMAN    **Mass:** 27329    **Score:** 49     **Queries matched:** 15   **emPAI:** 0.30 |
| --- | --- |
|  | Oxidoreductase HTATIP2 OS=Homo sapiens GN=HTATIP2 PE=1 SV=1 |

|  | **Query** | **Observed** | **Mr(expt)** | **Mr(calc)** | **Delta** | **Miss** | **Score** | **Expect** | **Rank** | **Peptide** |
| --- | --- | --- | --- | --- | --- | --- | --- | --- | --- | --- |
|  | 1742 | **709.3942** | **708.3869** | **708.3919** | **-0.0050** | **0** | **38** | **0.025** | **1** | **K.AHGSLKP.-** |
|  | 2256 | **403.7085** | **805.4024** | **805.4082** | **-0.0058** | **0** | **24** | **0.73** | **1** | **K.AGAEGFVR.V** 2236 |
|  | 2845 | 848.4490 | 847.4418 | 847.4287 | 0.0131 | 0 | 8 | 32 | 2 | M.AETEALSK.L |
|  | 4952 | **495.7555** | **989.4964** | **990.5247** | **-1.0283** | **1** | **3** | **96** | **2** | **R.GKAGAEGFVR.V** |
|  | 8817 | **624.3143** | **1246.6141** | **1246.6227** | **-0.0086** | **1** | **41** | **0.012** | **1** | **R.DKQMELLENK.A** 8813 |
|  | 9473 | 641.8085 | 1281.6024 | 1281.6142 | -0.0118 | 0 | 5 | 43 | 2 | K.FFGSLPDSWAR.G |
|  | 10194 | 660.8223 | 1319.6300 | 1319.6357 | -0.0058 | 0 | 16 | 3.5 | 2 | K.NVNQEVVDFEK.L 10214 |
|  | 14289 | **523.9142** | **1568.7207** | **1568.8198** | **-0.0991** | **1** | **2** | **79** | **9** | **K.GADKSSNFLYLQVK.G** |
|  | 14462 | **527.9418** | **1580.8037** | **1580.8133** | **-0.0097** | **0** | **2** | **70** | **3** | **R.YSVFRPGVLLCDR.Q** 14453 |
|  | 16971 | 585.2940 | 1752.8600 | 1753.9032 | -1.0432 | 1 | 8 | 19 | 5 | K.QMELLENKAIHDLGK.A |
|  | 24478 | **830.8803** | **2489.6191** | **2489.0740** | **0.5451** | **0** | **1** | **42** | **3** | **K.LDDYASAFQGHDVGFCCLGTTR.G** |

| **781.** | P53007\|TXTP_HUMAN    **Mass:** 34333    **Score:** 49     **Queries matched:** 14   **emPAI:** 0.11 |
| --- | --- |
|  | Tricarboxylate transport protein, mitochondrial OS=Homo sapiens GN=SLC25A1 PE=1 SV=2 |

|  | **Query** | **Observed** | **Mr(expt)** | **Mr(calc)** | **Delta** | **Miss** | **Score** | **Expect** | **Rank** | **Peptide** |
| --- | --- | --- | --- | --- | --- | --- | --- | --- | --- | --- |
|  | 2420 | 409.7211 | 817.4277 | 818.4188 | -0.9911 | 0 | 5 | 70 | 5 | R.GFFHGVR.E |
|  | 4087 | 464.7339 | 927.4532 | 928.4436 | -0.9904 | 0 | 5 | 56 | 8 | R.MQGLEAHK.Y 4083 4099 |
|  | 5111 | 500.7648 | 999.5151 | 999.5212 | -0.0061 | 0 | 25 | 0.56 | 1 | R.FFVMTSLR.N |
|  | 5137 | **501.7613** | **1001.5080** | **1001.5142** | **-0.0061** | **0** | **10** | **18** | **4** | **K.TQLQLDER.S** |
|  | 9489 | **642.3182** | **1282.6219** | **1282.6306** | **-0.0087** | **0** | **29** | **0.16** | **1** | **K.FIHDQTSPNPK.Y** 9506 9515 |
|  | 13712 | **766.5336** | **1531.0526** | **1530.6748** | **0.3778** | **0** | **4** | **36** | **7** | **R.FGMFEFLSNHMR.D** |
|  | 17695 | **603.3295** | **1806.9666** | **1805.9887** | **0.9779** | **1** | **6** | **29** | **2** | **R.EQGLKGTYQGLTATVLK.Q** |
|  | 19542 | 649.3320 | 1944.9743 | 1943.9135 | 1.0608 | 1 | 2 | 58 | 6 | K.AAVRFGMFEFLSNHMR.D |
|  | 29177 | **1165.9440** | **3494.8101** | **3494.8232** | **-0.0131** | **0** | **49** | **0.00048** | **1** | **R.GDNPNKPMNPLITGVFGAIAGAASVFGNTPLDVIK.T** 29179 |

| **782.** | Q15005\|SPCS2_HUMAN    **Mass:** 25272    **Score:** 49     **Queries matched:** 2   **emPAI:** 0.32 |
| --- | --- |
|  | Signal peptidase complex subunit 2 OS=Homo sapiens GN=SPCS2 PE=1 SV=3 |

|  | **Query** | **Observed** | **Mr(expt)** | **Mr(calc)** | **Delta** | **Miss** | **Score** | **Expect** | **Rank** | **Peptide** |
| --- | --- | --- | --- | --- | --- | --- | --- | --- | --- | --- |
|  | 18455 | **929.3586** | **1856.7027** | **1856.7126** | **-0.0099** | **0** | **46** | **0.0019** | **1** | **R.SGGSGGCSGAGGASNCGTGSGR.S** |
|  | 22315 | **738.6721** | **2212.9945** | **2213.0099** | **-0.0154** | **0** | **20** | **0.72** | **1** | **K.FFDHSGTLVMDAYEPEISR.L** |

| **783.** | Q30154\|2B51_HUMAN    **Mass:** 30322    **Score:** 48     **Queries matched:** 7   **emPAI:** 0.27 |
| --- | --- |
|  | HLA class II histocompatibility antigen, DRB5 beta chain OS=Homo sapiens GN=HLA-DRB5 PE=1 SV=1 |

|  | **Query** | **Observed** | **Mr(expt)** | **Mr(calc)** | **Delta** | **Miss** | **Score** | **Expect** | **Rank** | **Peptide** |
| --- | --- | --- | --- | --- | --- | --- | --- | --- | --- | --- |
|  | 1828 | 734.3819 | 733.3746 | 733.3242 | 0.0504 | 0 | 7 | 46 | 10 | R.NSQEEK.A |
|  | 12015 | **474.9214** | **1421.7424** | **1422.7363** | **-0.9939** | **1** | **1** | **1.1e+002** | **9** | **M.VCLKLPGGSYMAK.L** |
|  | 13168 | **747.3551** | **1492.6956** | **1492.7059** | **-0.0103** | **0** | **28** | **0.18** | **1** | **R.HNYGVGESFTVQR.R** |
|  | 25552 | **887.7537** | **2660.2392** | **2660.2541** | **-0.0149** | **0** | **37** | **0.014** | **1** | **R.SGEVYTCQVEHPSVTSPLTVEWR.A** |
|  | 27883 | 1044.1863 | 3129.5370 | 3130.6882 | -1.1512 | 1 | 3 | 22 | 1 | K.LPGGSYMAKLTVTLMVLSSPLALAGDTRPR.F 27887 |
|  | 29683 | **733.3729** | **3661.8282** | **3662.8250** | **-0.9968** | **1** | **10** | **3.7** | **1** | **R.NSQEEKAGVVSTGLIQNGDWTFQTLVMLETVPR.S** |

| **784.** | P04229\|2B11_HUMAN    **Mass:** 30294    **Score:** 48     **Queries matched:** 5   **emPAI:** 0.27 |
| --- | --- |
|  | HLA class II histocompatibility antigen, DRB1-1 beta chain OS=Homo sapiens GN=HLA-DRB1 PE=1 SV=2 |

|  | **Query** | **Observed** | **Mr(expt)** | **Mr(calc)** | **Delta** | **Miss** | **Score** | **Expect** | **Rank** | **Peptide** |
| --- | --- | --- | --- | --- | --- | --- | --- | --- | --- | --- |
|  | 12354 | 722.4034 | 1442.7922 | 1442.6037 | 0.1885 | 0 | 5 | 40 | 7 | K.FECHFFNGTER.V |
|  | 13168 | 747.3551 | 1492.6956 | 1492.7059 | -0.0103 | 0 | 28 | 0.18 | 1 | R.HNYGVGESFTVQR.R |
|  | 23552 | 789.4271 | 2365.2595 | 2365.0281 | 0.2315 | 1 | 0 | 68 | 8 | R.CIYNQEESVRFDSDVGEYR.A |
|  | 25552 | 887.7537 | 2660.2392 | 2660.2541 | -0.0149 | 0 | 37 | 0.014 | 1 | R.SGEVYTCQVEHPSVTSPLTVEWR.A |
|  | 28539 | **821.1301** | **3280.4914** | **3279.7366** | **0.7548** | **1** | **0** | **43** | **3** | **R.SESAQSKMLSGVGGFVLGLLFLGAGLFIYFR.N** |

| **785.** | P20039\|2B1B_HUMAN    **Mass:** 30483    **Score:** 48     **Queries matched:** 5   **emPAI:** 0.26 |
| --- | --- |
|  | HLA class II histocompatibility antigen, DRB1-11 beta chain OS=Homo sapiens GN=HLA-DRB1 PE=2 SV=1 |

|  | **Query** | **Observed** | **Mr(expt)** | **Mr(calc)** | **Delta** | **Miss** | **Score** | **Expect** | **Rank** | **Peptide** |
| --- | --- | --- | --- | --- | --- | --- | --- | --- | --- | --- |
|  | 1686 | **694.3577** | **693.3504** | **693.3302** | **0.0202** | **0** | **4** | **63** | **9** | **-.MVCLR.L** |
|  | 13168 | 747.3551 | 1492.6956 | 1492.7059 | -0.0103 | 0 | 28 | 0.18 | 1 | R.HNYGVGESFTVQR.R |
|  | 25552 | 887.7537 | 2660.2392 | 2660.2541 | -0.0149 | 0 | 37 | 0.014 | 1 | R.SGEVYTCQVEHPSVTSPLTVEWR.A |
|  | 28539 | 821.1301 | 3280.4914 | 3279.7366 | 0.7548 | 1 | 0 | 43 | 3 | R.SESAQSKMLSGVGGFVLGLLFLGAGLFIYFR.N |
|  | 29986 | **944.2311** | **3772.8955** | **3773.9487** | **-1.0532** | **1** | **1** | **25** | **2** | **-.MVCLRLPGGSCMAVLTVTLMVLSSPLALAGDTRPR.F** |

| **786.** | Q15717\|ELAV1_HUMAN    **Mass:** 36240    **Score:** 48     **Queries matched:** 3   **emPAI:** 0.10 |
| --- | --- |
|  | ELAV-like protein 1 OS=Homo sapiens GN=ELAVL1 PE=1 SV=2 |

|  | **Query** | **Observed** | **Mr(expt)** | **Mr(calc)** | **Delta** | **Miss** | **Score** | **Expect** | **Rank** | **Peptide** |
| --- | --- | --- | --- | --- | --- | --- | --- | --- | --- | --- |
|  | 5072 | **499.3705** | **996.7265** | **997.4175** | **-0.6910** | **0** | **3** | **61** | **10** | **K.DVEDMFSR.F** |
|  | 7644 | **594.8283** | **1187.6419** | **1187.6510** | **-0.0091** | **0** | **48** | **0.0023** | **1** | **R.VLVDQTTGLSR.G** |
|  | 15016 | **540.2307** | **1617.6703** | **1618.6967** | **-1.0264** | **1** | **3** | **62** | **6** | **R.TMTQKDVEDMFSR.F** |

| **787.** | Q9UDY2\|ZO2_HUMAN    **Mass:** 134118   **Score:** 48     **Queries matched:** 25   **emPAI:** 0.03 |
| --- | --- |
|  | Tight junction protein ZO-2 OS=Homo sapiens GN=TJP2 PE=1 SV=1 |

|  | **Query** | **Observed** | **Mr(expt)** | **Mr(calc)** | **Delta** | **Miss** | **Score** | **Expect** | **Rank** | **Peptide** |
| --- | --- | --- | --- | --- | --- | --- | --- | --- | --- | --- |
|  | 1067 | **552.2740** | **551.2668** | **550.2751** | **0.9917** | **0** | **5** | **60** | **6** | **R.EAGFK.R** |
|  | 1424 | **631.3115** | **630.3042** | **629.4112** | **0.8930** | **1** | **25** | **1.1** | **1** | **R.KLIEK.S** |
|  | 2587 | **415.7271** | **829.4395** | **830.4610** | **-1.0215** | **1** | **0** | **2.1e+002** | **10** | **K.KGDSVGLR.L** |
|  | 8620 | 619.2733 | 1236.5321 | 1236.7376 | -0.2056 | 0 | 2 | 80 | 9 | R.GRPGPIGVLLMK.S |
|  | 9978 | **437.2403** | **1308.6990** | **1308.4598** | **0.2391** | **0** | **10** | **15** | **4** | **K.MEGMDDDPEDR.M** 9986 |
|  | 10239 | **661.8369** | **1321.6593** | **1320.6860** | **0.9733** | **1** | **8** | **22** | **1** | **R.LSRMGATPTPFK.S** 10216 |
|  | 10302 | **663.8314** | **1325.6482** | **1324.4547** | **1.1934** | **0** | **(1)** | **1.1e+002** | **7** | **K.MEGMDDDPEDR.M** |
|  | 10381 | **665.7252** | **1329.4359** | **1330.5500** | **-1.1141** | **0** | **11** | **8.1** | **1** | **R.AFEVMDEFDGR.S** |
|  | 10435 | 667.3052 | 1332.5959 | 1332.6092 | -0.0132 | 0 | 7 | 32 | 9 | R.MQELQEAQNAR.I |
|  | 10442 | 667.3448 | 1332.6751 | 1331.6252 | 1.0500 | 0 | 48 | 0.0022 | 1 | R.AEQMASVQNAQR.D 10401 |
|  | 12857 | **738.4399** | **1474.8652** | **1474.6576** | **0.2076** | **1** | **4** | **54** | **2** | **K.TQNKEESYDFSK.S** 12846 |
|  | 14540 | **794.3797** | **1586.7448** | **1586.7536** | **-0.0088** | **0** | **2** | **74** | **3** | **K.SNPSAVAGNETPGASTK.G** |
|  | 15352 | **546.7836** | **1637.3289** | **1636.7726** | **0.5562** | **0** | **6** | **21** | **3** | **K.INGTVTENMSLTDAR.K** 15327 |
|  | 16403 | 856.4598 | 1710.9051 | 1710.9417 | -0.0365 | 1 | 2 | 72 | 7 | K.LGNWLAVRIGNELEK.G 16395 |
|  | 16658 | 866.4455 | 1730.8764 | 1729.8529 | 1.0235 | 1 | 2 | 67 | 7 | R.LQRMQELQEAQNAR.I |
|  | 17201 | 885.9467 | 1769.8789 | 1770.9702 | -1.0913 | 0 | 1 | 82 | 7 | K.RPVVLFGPIADIAMEK.L |
|  | 17921 | **608.9722** | **1823.8949** | **1822.9577** | **0.9371** | **1** | **1** | **91** | **2** | **R.ANEEYGLRLGSQIFVK.E** |
|  | 23884 | 807.0318 | 2418.0736 | 2418.2101 | -0.1365 | 1 | 4 | 25 | 4 | R.MGATPTPFKSTGDIAGTVVPETNK.E |
|  | 30479 | 1361.3560 | 4081.0461 | 4082.0655 | -1.0194 | 1 | 2 | 15 | 8 | K.LQLVVLRDSQQTLINIPSLNDSDSEIEDISEIESNR.S |

| **788.** | P49821\|NDUV1_HUMAN    **Mass:** 51469    **Score:** 48     **Queries matched:** 17   **emPAI:** 0.32 |
| --- | --- |
|  | NADH dehydrogenase [ubiquinone] flavoprotein 1, mitochondrial OS=Homo sapiens GN=NDUFV1 PE=1 SV=4 |

|  | **Query** | **Observed** | **Mr(expt)** | **Mr(calc)** | **Delta** | **Miss** | **Score** | **Expect** | **Rank** | **Peptide** |
| --- | --- | --- | --- | --- | --- | --- | --- | --- | --- | --- |
|  | 827 | 506.2578 | 505.2506 | 504.2478 | 1.0027 | 0 | 7 | 53 | 2 | R.AMGAR.A |
|  | 911 | **521.3043** | **520.2970** | **520.2428** | **0.0542** | **0** | **(6)** | **64** | **3** | **R.AMGAR.A** |
|  | 1221 | 586.3630 | 585.3557 | 584.3394 | 1.0163 | 0 | 8 | 28 | 4 | K.QGKPR.L |
|  | 1919 | 760.4263 | 759.4190 | 759.4603 | -0.0413 | 1 | 5 | 74 | 3 | R.LKGSLSR.G |
|  | 3780 | **453.2468** | **904.4790** | **903.4814** | **0.9976** | **0** | **19** | **2.6** | **1** | **R.GGAGFPTGLK.W** |
|  | 4867 | **492.2659** | **982.5173** | **982.5236** | **-0.0063** | **0** | **19** | **2.1** | **1** | **R.IFTNLYGR.H** |
|  | 6695 | **559.8016** | **1117.5887** | **1116.6040** | **0.9847** | **1** | **4** | **68** | **10** | **R.GRGGAGFPTGLK.W** |
|  | 8140 | 404.8731 | 1211.5975 | 1211.6047 | -0.0072 | 0 | 8 | 20 | 6 | R.HFRPELEER.M |
|  | 11441 | **696.2624** | **1390.5102** | **1390.5177** | **-0.0074** | **0** | **7** | **25** | **1** | **K.HESCGQCTPCR.E** |
|  | 13974 | 517.2592 | 1548.7558 | 1548.7507 | 0.0051 | 0 | 11 | 11 | 1 | K.WSFMNKPSDGRPK.Y |
|  | 15550 | **826.3800** | **1650.7454** | **1650.7559** | **-0.0105** | **0** | **18** | **2** | **1** | **K.YLVVNADEGEPGTCK.D** |
|  | 16391 | **855.9205** | **1709.8264** | **1709.8373** | **-0.0109** | **0** | **33** | **0.055** | **1** | **R.GEFYNEASNLQVAIR.E** |
|  | 17431 | **596.3003** | **1785.8792** | **1785.8897** | **-0.0105** | **0** | **25** | **0.36** | **1** | **R.GDARPAEIDSLWEISK.Q** |
|  | 19273 | **641.6306** | **1921.8700** | **1921.8840** | **-0.0140** | **1** | **12** | **6.3** | **1** | **K.YLVVNADEGEPGTCKDR.E** |
|  | 25282 | **1307.6958** | **2613.3770** | **2613.3915** | **-0.0145** | **0** | **37** | **0.013** | **1** | **K.HAGGVTGGWDNLLAVIPGGSSTPLIPK.S** |
|  | 25409 | 660.0715 | 2636.2568 | 2636.2727 | -0.0159 | 0 | 2 | 37 | 8 | K.LFNISGHVNHPCTVEEEMSVPLK.E |
|  | 28947 | **1133.9131** | **3398.7174** | **3398.7367** | **-0.0193** | **0** | **25** | **0.12** | **1** | **R.LKPPFPADVGVFGCPTTVANVETVAVSPTICR.R** |

| **789.** | O15143\|ARC1B_HUMAN    **Mass:** 41722    **Score:** 48     **Queries matched:** 8   **emPAI:** 0.09 |
| --- | --- |
|  | Actin-related protein 2/3 complex subunit 1B OS=Homo sapiens GN=ARPC1B PE=1 SV=3 |

|  | **Query** | **Observed** | **Mr(expt)** | **Mr(calc)** | **Delta** | **Miss** | **Score** | **Expect** | **Rank** | **Peptide** |
| --- | --- | --- | --- | --- | --- | --- | --- | --- | --- | --- |
|  | 2747 | **421.0560** | **840.0975** | **840.4745** | **-0.3770** | **0** | **6** | **23** | **9** | **R.IFSAYIK.E** |
|  | 9379 | 638.8055 | 1275.5964 | 1274.6830 | 0.9134 | 0 | 4 | 58 | 3 | K.NSVSQISVLSGGK.A |
|  | 14460 | **791.4025** | **1580.7905** | **1581.7787** | **-0.9882** | **0** | **10** | **10** | **1** | **K.EVEERPAPTPWGSK.M** |
|  | 15025 | 540.2929 | 1617.8567 | 1618.7852 | -0.9285 | 1 | 0 | 1.2e+002 | 8 | R.WAPNENKFAVGSGSR.V |
|  | 15191 | **814.8922** | **1627.7697** | **1627.7802** | **-0.0104** | **0** | **48** | **0.0018** | **1** | **K.ASSEGGTAAGAGLDSLHK.N** 15200 |
|  | 18634 | 625.0079 | 1872.0018 | 1872.8676 | -0.8658 | 0 | 4 | 38 | 4 | R.VAWVSHDSTVCLADADK.K |
|  | 26861 | **969.7744** | **2906.3012** | **2907.2911** | **-0.9899** | **1** | **6** | **13** | **2** | **K.CSQFCTTGMDGGMSIWDVKSLESALK.D** |

| **790.** | Q9H0X4\|ITFG3_HUMAN    **Mass:** 60249    **Score:** 48     **Queries matched:** 12   **emPAI:** 0.20 |
| --- | --- |
|  | Protein ITFG3 OS=Homo sapiens GN=ITFG3 PE=1 SV=1 |

|  | **Query** | **Observed** | **Mr(expt)** | **Mr(calc)** | **Delta** | **Miss** | **Score** | **Expect** | **Rank** | **Peptide** |
| --- | --- | --- | --- | --- | --- | --- | --- | --- | --- | --- |
|  | 1333 | 609.3198 | 608.3125 | 608.3170 | -0.0045 | 0 | 9 | 18 | 1 | K.GLYEK.V |
|  | 2858 | 424.7370 | 847.4595 | 848.4392 | -0.9797 | 0 | 7 | 38 | 6 | K.VTGSGGPFK.S 2857 |
|  | 5484 | 515.2797 | 1028.5449 | 1027.5774 | 0.9675 | 1 | 13 | 7.6 | 6 | K.VRDLVPSSR.V |
|  | 6951 | 569.8256 | 1137.6366 | 1137.6434 | -0.0068 | 0 | 41 | 0.011 | 1 | R.IQDVLFLYK.N |
|  | 6967 | 570.3373 | 1138.6601 | 1139.4955 | -0.8354 | 0 | 4 | 60 | 6 | K.NTNSSNNFSR.S |
|  | 7896 | 601.3029 | 1200.5913 | 1200.6098 | -0.0185 | 1 | 8 | 27 | 3 | R.KSQENLGNPSK.N |
|  | 10062 | **657.8126** | **1313.6106** | **1313.6212** | **-0.0106** | **0** | **31** | **0.12** | **1** | **R.LGEGGPDSDQAIR.D** 10071 |
|  | 12321 | **481.2430** | **1440.7072** | **1440.6957** | **0.0115** | **1** | **3** | **62** | **8** | **K.NEDNVKSAPPQSR.L** |
|  | 13244 | 500.2561 | 1497.7465 | 1497.7551 | -0.0086 | 0 | 14 | 4.8 | 1 | R.HSLYMFHPTLPR.V |
|  | 20377 | **676.3311** | **2025.9713** | **2025.9868** | **-0.0155** | **0** | **16** | **2** | **1** | **R.EEVSGHLYSGSTGHQIGLR.G** |

| **791.** | P62249\|RS16_HUMAN    **Mass:** 16549    **Score:** 48     **Queries matched:** 8   **emPAI:** 0.23 |
| --- | --- |
|  | 40S ribosomal protein S16 OS=Homo sapiens GN=RPS16 PE=1 SV=2 |

|  | **Query** | **Observed** | **Mr(expt)** | **Mr(calc)** | **Delta** | **Miss** | **Score** | **Expect** | **Rank** | **Peptide** |
| --- | --- | --- | --- | --- | --- | --- | --- | --- | --- | --- |
|  | 4238 | 470.2394 | 938.4643 | 938.4709 | -0.0066 | 1 | 7 | 27 | 4 | K.YVDEASKK.E |
|  | 4524 | **479.7398** | **957.4651** | **957.4702** | **-0.0051** | **0** | **16** | **4.8** | **1** | **K.TATAVAHCK.R** 4525 4526 |
|  | 11691 | 703.3827 | 1404.7508 | 1404.7612 | -0.0104 | 1 | 48 | 0.0021 | 1 | K.EIKDILIQYDR.T |
|  | 12072 | **476.1974** | **1425.5703** | **1425.7398** | **-0.1695** | **0** | **1** | **1e+002** | **9** | **K.VNGRPLEMIEPR.T** |
|  | 12737 | 734.4150 | 1466.8154 | 1467.8310 | -1.0156 | 1 | 0 | 1.1e+002 | 10 | R.VKGGGHVAQIYAIR.Q |
|  | 19020 | **634.6910** | **1901.0511** | **1900.0418** | **1.0093** | **1** | **0** | **96** | **2** | **K.DILIQYDRTLLVADPR.R** |

| **792.** | Q9Y2R5\|RT17_HUMAN    **Mass:** 14607    **Score:** 48     **Queries matched:** 2   **emPAI:** 0.27 |
| --- | --- |
|  | 28S ribosomal protein S17, mitochondrial OS=Homo sapiens GN=MRPS17 PE=1 SV=1 |

|  | **Query** | **Observed** | **Mr(expt)** | **Mr(calc)** | **Delta** | **Miss** | **Score** | **Expect** | **Rank** | **Peptide** |
| --- | --- | --- | --- | --- | --- | --- | --- | --- | --- | --- |
|  | 6101 | 537.3100 | 1072.6054 | 1072.6532 | -0.0478 | 0 | 12 | 12 | 4 | R.LVLDPYLLK.Y |
|  | 27706 | **1027.1819** | **3078.5238** | **3078.5431** | **-0.0193** | **0** | **48** | **0.00079** | **1** | **K.VIDPVTGKPCAGTTYLESPLSSETTQLSK.N** |

| **793.** | O43280\|TREA_HUMAN    **Mass:** 66925    **Score:** 47     **Queries matched:** 11   **emPAI:** 0.11 |
| --- | --- |
|  | Trehalase OS=Homo sapiens GN=TREH PE=1 SV=2 |

|  | **Query** | **Observed** | **Mr(expt)** | **Mr(calc)** | **Delta** | **Miss** | **Score** | **Expect** | **Rank** | **Peptide** |
| --- | --- | --- | --- | --- | --- | --- | --- | --- | --- | --- |
|  | 4776 | **488.7736** | **975.5327** | **974.4682** | **1.0645** | **0** | **9** | **24** | **7** | **R.DHNHSIPR.E** |
|  | 9263 | 635.8424 | 1269.6701 | 1268.5422 | 1.1280 | 0 | 11 | 10 | 4 | K.AGAESGWDFSSR.W |
|  | 9691 | 647.2989 | 1292.5833 | 1292.6799 | -0.0965 | 0 | 10 | 13 | 10 | K.GMLQNFLDLVK.T 9686 |
|  | 10518 | **446.5721** | **1336.6945** | **1336.7040** | **-0.0095** | **0** | **15** | **4.8** | **3** | **R.AWAGQLHQLWK.K** |
|  | 10526 | 446.7448 | 1337.2127 | 1337.6761 | -0.4635 | 0 | 11 | 8 | 2 | K.MKPEVLSHPER.F |
|  | 14447 | **790.9258** | **1579.8371** | **1579.8471** | **-0.0099** | **0** | **40** | **0.011** | **1** | **R.WLIGGPNPNSLSGIR.T** |
|  | 23850 | **806.0396** | **2415.0970** | **2414.2481** | **0.8489** | **1** | **0** | **72** | **3** | **K.YLEDNRILTYQYGIPTSLQK.T** |
|  | 25079 | **1286.0876** | **2570.1607** | **2570.1788** | **-0.0180** | **0** | **32** | **0.036** | **1** | **R.EFYPSNLTPLWAGCFSDPGVADK.A** |
|  | 28401 | **1082.2765** | **3243.8076** | **3244.8297** | **-1.0221** | **1** | **1** | **31** | **4** | **R.LTSGAKLAFLEPHCLAATLLPSLLLSLLPW.-** |
|  | 30977 | **905.8620** | **4524.2736** | **4525.1612** | **-0.8876** | **1** | **2** | **12** | **2** | **R.FVEFYYWDSYWVMEGLLLSEMAETVKGMLQNFLDLVK.T** |

| **794.** | P26641\|EF1G_HUMAN    **Mass:** 50429    **Score:** 47     **Queries matched:** 7   **emPAI:** 0.07 |
| --- | --- |
|  | Elongation factor 1-gamma OS=Homo sapiens GN=EEF1G PE=1 SV=3 |

|  | **Query** | **Observed** | **Mr(expt)** | **Mr(calc)** | **Delta** | **Miss** | **Score** | **Expect** | **Rank** | **Peptide** |
| --- | --- | --- | --- | --- | --- | --- | --- | --- | --- | --- |
|  | 4757 | 488.2789 | 974.5433 | 974.5185 | 0.0248 | 0 | 9 | 22 | 3 | K.QVLEPSFR.Q 4745 4755 |
|  | 6689 | 559.7864 | 1117.5582 | 1117.6747 | -0.1165 | 0 | 8 | 30 | 4 | R.ILGLLDAYLK.T |
|  | 10684 | 449.7417 | 1346.2033 | 1346.7306 | -0.5273 | 0 | 1 | 81 | 9 | K.ALIAAQYSGAQVR.V |
|  | 13835 | **514.2695** | **1539.7866** | **1540.7310** | **-0.9444** | **0** | **4** | **54** | **8** | **M.AAGTLYTYPENWR.A** |
|  | 25731 | **1344.1167** | **2686.2188** | **2686.2340** | **-0.0151** | **0** | **47** | **0.0012** | **1** | **R.GQELAFPLSPDWQVDYESYTWR.K** |

| **795.** | Q9P035\|PTAD1_HUMAN    **Mass:** 43360    **Score:** 47     **Queries matched:** 3   **emPAI:** 0.09 |
| --- | --- |
|  | Protein tyrosine phosphatase-like protein PTPLAD1 OS=Homo sapiens GN=PTPLAD1 PE=1 SV=2 |

|  | **Query** | **Observed** | **Mr(expt)** | **Mr(calc)** | **Delta** | **Miss** | **Score** | **Expect** | **Rank** | **Peptide** |
| --- | --- | --- | --- | --- | --- | --- | --- | --- | --- | --- |
|  | 4106 | **465.2441** | **928.4736** | **928.5342** | **-0.0606** | **0** | **9** | **26** | **10** | **R.QVNITVQK.K** |
|  | 13923 | **516.2758** | **1545.8056** | **1544.7682** | **1.0374** | **0** | **8** | **21** | **10** | **R.LESEGSPETLTNLR.K** |
|  | 25491 | **884.4467** | **2650.3182** | **2650.3319** | **-0.0137** | **0** | **47** | **0.0012** | **1** | **K.GDNVYEFHLEFLDLVKPEPVYK.L** |

| **796.** | O15372\|EIF3H_HUMAN    **Mass:** 40076    **Score:** 47     **Queries matched:** 6   **emPAI:** 0.09 |
| --- | --- |
|  | Eukaryotic translation initiation factor 3 subunit H OS=Homo sapiens GN=EIF3H PE=1 SV=1 |

|  | **Query** | **Observed** | **Mr(expt)** | **Mr(calc)** | **Delta** | **Miss** | **Score** | **Expect** | **Rank** | **Peptide** |
| --- | --- | --- | --- | --- | --- | --- | --- | --- | --- | --- |
|  | 8124 | **606.3233** | **1210.6320** | **1210.7285** | **-0.0965** | **0** | **4** | **56** | **7** | **K.QVQIDGLVVLK.I** 8132 |
|  | 8573 | **618.2659** | **1234.5173** | **1233.6461** | **0.8712** | **1** | **6** | **39** | **3** | **R.LTPKLMEVCK.E** |
|  | 15168 | **814.3683** | **1626.7221** | **1626.7333** | **-0.0111** | **0** | **47** | **0.0022** | **1** | **K.EGTGSTATSSSSTAGAAGK.G** |
|  | 16585 | **863.4397** | **1724.8648** | **1724.8919** | **-0.0271** | **0** | **3** | **58** | **7** | **K.NSHLINVLMWELEK.K** |
|  | 30006 | **1261.8696** | **3782.5871** | **3783.5474** | **-0.9603** | **0** | **1** | **24** | **1** | **R.LEITNCFPFPQHTEDDADFDEVQYQMEMMR.S** |

| **797.** | Q01814\|AT2B2_HUMAN    **Mass:** 137987   **Score:** 47     **Queries matched:** 38   **emPAI:** 0.03 |
| --- | --- |
|  | Plasma membrane calcium-transporting ATPase 2 OS=Homo sapiens GN=ATP2B2 PE=1 SV=2 |

|  | **Query** | **Observed** | **Mr(expt)** | **Mr(calc)** | **Delta** | **Miss** | **Score** | **Expect** | **Rank** | **Peptide** |
| --- | --- | --- | --- | --- | --- | --- | --- | --- | --- | --- |
|  | 863 | 514.8105 | 513.8032 | 514.3479 | -0.5446 | 0 | 14 | 4.7 | 4 | R.AIAIK.C |
|  | 3733 | **451.7188** | **901.4231** | **901.4981** | **-0.0750** | **1** | **15** | **6.9** | **6** | **K.EAGRLTQK.E** |
|  | 3974 | 460.2462 | 918.4779 | 918.5076 | -0.0296 | 0 | 8 | 31 | 3 | R.GQILWFR.G |
|  | 4886 | 493.2556 | 984.4965 | 984.5029 | -0.0063 | 0 | 8 | 27 | 3 | K.VYTFNSVR.K |
|  | 7709 | 596.3003 | 1190.5861 | 1190.5714 | 0.0148 | 0 | 11 | 13 | 8 | R.MVTGDNINTAR.A 7708 |
|  | 7849 | 400.2375 | 1197.6906 | 1197.6942 | -0.0035 | 1 | 8 | 21 | 3 | R.GLNRIQTQIR.V |
|  | 9053 | **421.0011** | **1259.9814** | **1259.6002** | **0.3812** | **0** | **6** | **23** | **8** | **K.VIEPMACDGLR.T** 9029 9048 9089 |
|  | 9401 | **639.3203** | **1276.6261** | **1275.5951** | **1.0309** | **0** | **(3)** | **69** | **2** | **K.VIEPMACDGLR.T** |
|  | 10090 | **439.2196** | **1314.6369** | **1313.5558** | **1.0811** | **0** | **5** | **42** | **6** | **K.ETYGDTEAICR.R** 10103 |
|  | 10155 | 440.2385 | 1317.6936 | 1318.7608 | -1.0673 | 1 | 6 | 35 | 8 | K.ILPPEKEGALPR.Q |
|  | 11151 | 687.8447 | 1373.6749 | 1373.6544 | 0.0205 | 1 | 8 | 22 | 2 | K.CCKILNGAGEPR.V |
|  | 11697 | **469.2846** | **1404.8321** | **1403.6901** | **1.1420** | **1** | **14** | **5.5** | **1** | **K.KVIEPMACDGLR.T** |
|  | 11929 | 473.3052 | 1416.8937 | 1415.7925 | 1.1012 | 1 | 4 | 51 | 5 | R.KQIFGQNFIPPK.K |
|  | 12117 | 714.8848 | 1427.7550 | 1427.8361 | -0.0811 | 1 | 13 | 7.6 | 4 | R.KPYGRNKPLISR.T 12144 |
|  | 12914 | 740.3799 | 1478.7452 | 1477.6831 | 1.0621 | 1 | 0 | 1.1e+002 | 3 | K.MQDGNVDASQSKAK.Q |
|  | 13722 | **766.8674** | **1531.7203** | **1532.6963** | **-0.9760** | **0** | **2** | **79** | **8** | **K.QQDGAAAMEMQPLK.S** 13742 |
|  | 15416 | 821.4199 | 1640.8253 | 1640.8370 | -0.0117 | 0 | 47 | 0.0022 | 1 | R.QVVAVTGDGTNDGPALK.K 15417 15418 |
|  | 15732 | 832.8961 | 1663.7776 | 1662.8060 | 0.9715 | 1 | 11 | 8.6 | 1 | K.IDESSLTGESDQVRK.S 15733 |
|  | 16278 | **568.2784** | **1701.8135** | **1702.9036** | **-1.0901** | **1** | **3** | **57** | **7** | **R.MVTGDNINTARAIAIK.C** |
|  | 16669 | 866.9659 | 1731.9172 | 1731.8284 | 0.0889 | 1 | 9 | 15 | 3 | K.AKQQDGAAAMEMQPLK.S |
|  | 17918 | **608.8705** | **1823.5898** | **1824.7407** | **-1.1509** | **1** | **12** | **4.7** | **3** | **-.MGDMTNSDFYSKNQR.N** |
|  | 19648 | 652.6575 | 1954.9508 | 1954.7785 | 0.1722 | 0 | 5 | 27 | 10 | R.NESSHGGEFGCTMEELR.S |
|  | 25342 | 875.7799 | 2624.3177 | 2625.2630 | -0.9453 | 0 | 0 | 64 | 5 | K.GDGLQLPAADGAAASNAADSANASLVNGK.M |
|  | 25531 | **887.1135** | **2658.3186** | **2659.4506** | **-1.1320** | **1** | **4** | **21** | **1** | **K.TMELLINAIAINSAYTTKILPPEK.E** |
|  | 26379 | 936.4367 | 2806.2883 | 2806.3848 | -0.0964 | 1 | 0 | 57 | 5 | R.MTVVQAYVGDVHYKEIPDPSSINTK.T |
|  | 26717 | 960.5004 | 2878.4793 | 2878.4634 | 0.0159 | 0 | 2 | 41 | 5 | R.MLVTAVGVNSQTGIIFTLLGAGGEEEEK.K |
|  | 28050 | 1055.5427 | 3163.6063 | 3163.6322 | -0.0259 | 1 | 5 | 15 | 2 | K.EIPDPSSINTKTMELLINAIAINSAYTTK.I |
|  | 30487 | 681.8417 | 4085.0068 | 4085.1548 | -0.1480 | 1 | 2 | 17 | 1 | R.NVYDSISKFLQFQLTVNVVAVIVAFTGACITQDSPLK.A |

| **798.** | Q58FG1\|HS904_HUMAN    **Mass:** 47796    **Score:** 47     **Queries matched:** 14   **emPAI:** 0.08 |
| --- | --- |
|  | Putative heat shock protein HSP 90-alpha A4 OS=Homo sapiens GN=HSP90AA4P PE=5 SV=1 |

|  | **Query** | **Observed** | **Mr(expt)** | **Mr(calc)** | **Delta** | **Miss** | **Score** | **Expect** | **Rank** | **Peptide** |
| --- | --- | --- | --- | --- | --- | --- | --- | --- | --- | --- |
|  | 1572 | 661.3661 | 660.3588 | 659.4218 | 0.9370 | 0 | 10 | 26 | 1 | K.VTVITK.H |
|  | 1868 | 747.4567 | 746.4494 | 746.4538 | -0.0044 | 0 | 23 | 1 | 1 | K.TLVSVTK.E |
|  | 4347 | 473.7424 | 945.4703 | 945.5429 | -0.0726 | 1 | 10 | 18 | 9 | R.IMKAQALR.D |
|  | 5218 | 504.5847 | 1007.1548 | 1006.4641 | 0.6907 | 0 | 0 | 1e+002 | 7 | -.MESLTDPSK.L |
|  | 9867 | **434.9200** | **1301.7383** | **1301.5921** | **0.1461** | **1** | **7** | **31** | **4** | **R.DNSTTGYMAAKK.H** |
|  | 10086 | **439.2101** | **1314.6086** | **1314.5663** | **0.0423** | **0** | **12** | **9.8** | **2** | **K.STYGWTANMER.I** |
|  | 10716 | 675.3210 | 1348.6275 | 1347.6572 | 0.9704 | 0 | 1 | 1.2e+002 | 10 | K.HFSVEGQLEFR.A |
|  | 10726 | 675.3670 | 1348.7194 | 1348.7272 | -0.0078 | 0 | 47 | 0.0031 | 1 | R.TLTIVDTGIGMTK.A |
|  | 13366 | **503.1062** | **1506.2969** | **1506.7236** | **-0.4267** | **1** | **(1)** | **64** | **8** | **-.MESLTDPSKLDSGK.E** |
|  | 13613 | 508.9797 | 1523.9172 | 1522.7185 | 1.1987 | 1 | 4 | 51 | 5 | -.MESLTDPSKLDSGK.E |
|  | 16173 | 848.8961 | 1695.7776 | 1696.8202 | -1.0427 | 1 | 6 | 25 | 3 | K.AQALRDNSTTGYMAAK.K |
|  | 19397 | **644.8454** | **1931.5144** | **1931.0000** | **0.5144** | **1** | **2** | **43** | **9** | **R.GVVDSEDLPLNIFRETK.D** |
|  | 25318 | 874.0991 | 2619.2754 | 2618.4201 | 0.8553 | 1 | 3 | 32 | 4 | R.TLTIVDTGIGMTKADLINNLGTITK.S |
|  | 30106 | **952.4792** | **3805.8877** | **3804.8267** | **1.0610** | **1** | **4** | **12** | **1** | **K.SETKVFMEVLQAGADISMIGQFSVGFYSAYSVAEK.V** |

| **799.** | Q96G23\|LASS2_HUMAN    **Mass:** 44961    **Score:** 47     **Queries matched:** 5   **emPAI:** 0.08 |
| --- | --- |
|  | LAG1 longevity assurance homolog 2 OS=Homo sapiens GN=LASS2 PE=1 SV=1 |

|  | **Query** | **Observed** | **Mr(expt)** | **Mr(calc)** | **Delta** | **Miss** | **Score** | **Expect** | **Rank** | **Peptide** |
| --- | --- | --- | --- | --- | --- | --- | --- | --- | --- | --- |
|  | 6075 | 536.7871 | 1071.5595 | 1071.5924 | -0.0329 | 0 | 8 | 29 | 3 | K.QVEVELLSR.Q |
|  | 16872 | 582.9638 | 1745.8696 | 1744.8784 | 0.9912 | 0 | 6 | 25 | 3 | R.APPNATLEHFYLTSGK.Q 16839 16841 |
|  | 18329 | **925.9287** | **1849.8429** | **1849.8498** | **-0.0069** | **0** | **47** | **0.0022** | **1** | **-.MLQTLYDYFWWER.L** |

| **800.** | Q53TN4\|CYBR1_HUMAN    **Mass:** 31735    **Score:** 47     **Queries matched:** 3   **emPAI:** 0.25 |
| --- | --- |
|  | Cytochrome b reductase 1 OS=Homo sapiens GN=CYBRD1 PE=1 SV=1 |

|  | **Query** | **Observed** | **Mr(expt)** | **Mr(calc)** | **Delta** | **Miss** | **Score** | **Expect** | **Rank** | **Peptide** |
| --- | --- | --- | --- | --- | --- | --- | --- | --- | --- | --- |
|  | 6274 | 543.7767 | 1085.5388 | 1085.5465 | -0.0077 | 0 | 31 | 0.12 | 1 | R.NLALDEAGQR.S |
|  | 8727 | **414.5603** | **1240.6592** | **1241.6476** | **-0.9885** | **1** | **5** | **50** | **8** | **K.RNLALDEAGQR.S** |
|  | 11081 | **686.2823** | **1370.5501** | **1370.5595** | **-0.0093** | **0** | **42** | **0.0083** | **1** | **R.GSMPAYSGNNMDK.S** |

| **801.** | P50440\|GATM_HUMAN    **Mass:** 48938    **Score:** 47     **Queries matched:** 18   **emPAI:** 0.16 |
| --- | --- |
|  | Glycine amidinotransferase, mitochondrial OS=Homo sapiens GN=GATM PE=1 SV=1 |

|  | **Query** | **Observed** | **Mr(expt)** | **Mr(calc)** | **Delta** | **Miss** | **Score** | **Expect** | **Rank** | **Peptide** |
| --- | --- | --- | --- | --- | --- | --- | --- | --- | --- | --- |
|  | 3273 | **436.2221** | **870.4295** | **870.4348** | **-0.0052** | **0** | **9** | **20** | **2** | **R.HLAPDYR.V** |
|  | 3408 | **440.7405** | **879.4663** | **879.3392** | **0.1271** | **0** | **11** | **12** | **3** | **R.NSCAADDK.A** |
|  | 9442 | 640.7906 | 1279.5666 | 1279.7210 | -0.1543 | 1 | 9 | 17 | 5 | K.MFEKLGITTIK.V 9419 |
|  | 9740 | **648.3520** | **1294.6895** | **1295.7159** | **-1.0263** | **1** | **(1)** | **1e+002** | **4** | **K.MFEKLGITTIK.V** |
|  | 10609 | **671.8511** | **1341.6877** | **1341.6962** | **-0.0085** | **0** | **22** | **0.79** | **1** | **R.VMVDANEVPIQK.M** 10597 |
|  | 13234 | **749.4139** | **1496.8133** | **1497.7973** | **-0.9840** | **1** | **0** | **1.2e+002** | **9** | **K.RVMVDANEVPIQK.M** |
|  | 13535 | 759.8252 | 1517.6358 | 1518.7422 | -1.1063 | 0 | 2 | 79 | 5 | K.AVAEIEEMCNILK.T 13547 |
|  | 15009 | **809.3991** | **1616.7837** | **1615.7512** | **1.0325** | **1** | **9** | **17** | **1** | **R.NSCAADDKATEPLPK.D** |
|  | 16049 | **422.4880** | **1685.9231** | **1686.7559** | **-0.8329** | **0** | **2** | **64** | **9** | **K.TPDFESTGLYSAMPR.D** |
|  | 19805 | **493.2395** | **1968.9288** | **1968.0429** | **0.8859** | **1** | **1** | **69** | **4** | **K.TEGVTVRRPDPIDWSLK.Y** |
|  | 20534 | **1020.0483** | **2038.0821** | **2038.0921** | **-0.0100** | **0** | **4** | **37** | **1** | **R.DILIVVGNEIIEAPMAWR.S** |
|  | 20819 | **1032.9662** | **2063.9178** | **2063.9299** | **-0.0121** | **0** | **46** | **0.0019** | **1** | **K.FVTTEFEPCFDAADFIR.A** |
|  | 23530 | **1182.5382** | **2363.0619** | **2363.0740** | **-0.0121** | **0** | **19** | **0.8** | **1** | **K.DCPVSSYNEWDPLEEVIVGR.A** |
|  | 25618 | **892.1263** | **2673.3570** | **2672.3672** | **0.9898** | **0** | **1** | **45** | **4** | **K.AGWTIITPPTPIIPDDHPLWMSSK.W** |
|  | 28741 | 838.4119 | 3349.6184 | 3348.5721 | 1.0463 | 1 | 1 | 35 | 10 | R.GAKWTTAPKPTMADELYNQDYPIHSVEDR.H |

| **802.** | P22307\|NLTP_HUMAN    **Mass:** 59640    **Score:** 47     **Queries matched:** 22   **emPAI:** 0.13 |
| --- | --- |
|  | Non-specific lipid-transfer protein OS=Homo sapiens GN=SCP2 PE=1 SV=2 |

|  | **Query** | **Observed** | **Mr(expt)** | **Mr(calc)** | **Delta** | **Miss** | **Score** | **Expect** | **Rank** | **Peptide** |
| --- | --- | --- | --- | --- | --- | --- | --- | --- | --- | --- |
|  | 1681 | **691.4092** | **690.4020** | **690.4064** | **-0.0045** | **0** | **21** | **1.8** | **2** | **K.ANLVFK.E** |
|  | 3995 | 460.7516 | 919.4886 | 919.5161 | -0.0275 | 1 | 13 | 9.1 | 7 | K.MSKGSLGIK.F 3994 4000 |
|  | 4424 | 476.2581 | 950.5016 | 950.5549 | -0.0533 | 0 | 14 | 7 | 3 | K.HVDLLINK.Y 4442 |
|  | 5567 | **518.2664** | **1034.5183** | **1034.5253** | **-0.0070** | **0** | **10** | **19** | **1** | **K.ITGNMGLAMK.L** |
|  | 7859 | 600.2753 | 1198.5361 | 1198.5441 | -0.0080 | 0 | 3 | 75 | 3 | K.MGFPEAASSFR.T |
|  | 8075 | **605.3129** | **1208.6112** | **1207.6747** | **0.9364** | **1** | **4** | **54** | **3** | **R.RVFVVGVGMTK.F** |
|  | 8926 | **627.7964** | **1253.5782** | **1253.5863** | **-0.0081** | **0** | **18** | **2.3** | **1** | **K.MNPQSAFFQGK.L** |
|  | 9258 | 635.7968 | 1269.5791 | 1269.5812 | -0.0021 | 0 | (3) | 72 | 9 | K.MNPQSAFFQGK.L 9244 |
|  | 9696 | 647.3106 | 1292.6065 | 1291.6992 | 0.9074 | 1 | 14 | 5.4 | 2 | K.LKITGNMGLAMK.L |
|  | 10259 | 662.3679 | 1322.7213 | 1322.7306 | -0.0093 | 0 | 37 | 0.024 | 1 | K.LQNLQLQPGNAK.L |
|  | 10497 | 668.8057 | 1335.5969 | 1334.6718 | 0.9251 | 1 | 26 | 0.34 | 2 | K.KLEEEGEQFVK.K |
|  | 10574 | **671.1193** | **1340.2240** | **1340.6217** | **-0.3977** | **1** | **(0)** | **87** | **7** | **K.MVGFDMSKEAAR.K** |
|  | 10849 | **679.3251** | **1356.6357** | **1356.6166** | **0.0191** | **1** | **4** | **62** | **4** | **K.MVGFDMSKEAAR.K** |
|  | 12251 | **718.3838** | **1434.7530** | **1435.8147** | **-1.0617** | **1** | **1** | **1.1e+002** | **5** | **K.LQNLQLQPGNAKL.-** |
|  | 13431 | 504.5908 | 1510.7505 | 1510.7602 | -0.0098 | 1 | 9 | 17 | 7 | K.MNPQSAFFQGKLK.I |
|  | 18913 | **631.9780** | **1892.9123** | **1892.9243** | **-0.0121** | **0** | **32** | **0.067** | **1** | **K.YGLSAHPVAPQMFGYAGK.E** |
|  | 20775 | **1030.5084** | **2059.0023** | **2059.9265** | **-0.9242** | **0** | **20** | **0.96** | **1** | **K.ADCTITMADSDFLALMTGK.M** |
|  | 25745 | **896.7293** | **2687.1661** | **2687.1809** | **-0.0148** | **0** | **14** | **2.6** | **1** | **K.HSVNNPYSQFQDEYSLDEVMASK.E** |

| **803.** | Q9Y3B3\|TMED7_HUMAN    **Mass:** 25498    **Score:** 47     **Queries matched:** 6   **emPAI:** 0.15 |
| --- | --- |
|  | Transmembrane emp24 domain-containing protein 7 OS=Homo sapiens GN=TMED7 PE=1 SV=2 |

|  | **Query** | **Observed** | **Mr(expt)** | **Mr(calc)** | **Delta** | **Miss** | **Score** | **Expect** | **Rank** | **Peptide** |
| --- | --- | --- | --- | --- | --- | --- | --- | --- | --- | --- |
|  | 5911 | 531.2668 | 1060.5190 | 1060.5261 | -0.0071 | 1 | 32 | 0.12 | 1 | R.SRAEDLNTR.V |
|  | 9162 | **633.3127** | **1264.6109** | **1264.6200** | **-0.0091** | **0** | **47** | **0.003** | **1** | **K.SVIDYQTHFR.L** |
|  | 12581 | 729.8711 | 1457.7276 | 1458.6449 | -0.9173 | 0 | 5 | 43 | 10 | K.QCFYEDIAQGTK.C 12590 |
|  | 20892 | 1037.0143 | 2072.0140 | 2073.0234 | -1.0094 | 0 | 1 | 74 | 8 | R.VSALTQMESACVSIHEALK.S |
|  | 22347 | **739.0245** | **2214.0516** | **2213.9841** | **0.0676** | **1** | **1** | **60** | **2** | **K.NGTYKFCFSNEFSTFTHK.T** |

| **804.** | Q9NRV9\|HEBP1_HUMAN    **Mass:** 21198    **Score:** 47     **Queries matched:** 5   **emPAI:** 0.39 |
| --- | --- |
|  | Heme-binding protein 1 OS=Homo sapiens GN=HEBP1 PE=1 SV=1 |

|  | **Query** | **Observed** | **Mr(expt)** | **Mr(calc)** | **Delta** | **Miss** | **Score** | **Expect** | **Rank** | **Peptide** |
| --- | --- | --- | --- | --- | --- | --- | --- | --- | --- | --- |
|  | 17473 | 597.3122 | 1788.9147 | 1788.9258 | -0.0110 | 0 | 49 | 0.0013 | 1 | K.FATVEVTDKPVDEALR.E |
|  | 21590 | **712.9836** | **2135.9289** | **2135.9445** | **-0.0156** | **0** | **17** | **1.5** | **1** | **R.GDIYFCTGYDPPMKPYGR.R** |
|  | 24513 | **1247.5751** | **2493.1356** | **2494.2236** | **-1.0880** | **0** | **27** | **0.14** | **1** | **K.GIGMGMTVPISFAVFPNEDGSLQK.K** 24528 |
|  | 26216 | 924.1555 | 2769.4446 | 2770.2908 | -0.8463 | 1 | 3 | 29 | 3 | R.EGITVYSMQFGGYAKEADYVAQATR.L |

| **805.** | P49326\|FMO5_HUMAN    **Mass:** 60638    **Score:** 46     **Queries matched:** 15   **emPAI:** 0.13 |
| --- | --- |
|  | Dimethylaniline monooxygenase [N-oxide-forming] 5 OS=Homo sapiens GN=FMO5 PE=2 SV=2 |

|  | **Query** | **Observed** | **Mr(expt)** | **Mr(calc)** | **Delta** | **Miss** | **Score** | **Expect** | **Rank** | **Peptide** |
| --- | --- | --- | --- | --- | --- | --- | --- | --- | --- | --- |
|  | 1495 | **645.5403** | **644.5331** | **643.4017** | **1.1314** | **1** | **4** | **1.2e+002** | **2** | **K.VKGNVK.E** |
|  | 1939 | 764.4496 | 763.4423 | 763.4116 | 0.0307 | 0 | 34 | 0.078 | 1 | K.EFDLLK.Y |
|  | 7378 | **586.3885** | **1170.7624** | **1169.5942** | **1.1683** | **1** | **0** | **1.3e+002** | **5** | **R.VQGPGKWDGAR.K** |
|  | 7776 | **598.2908** | **1194.5670** | **1195.6601** | **-1.0931** | **1** | **11** | **12** | **1** | **K.EFDLLKYIR.F** |
|  | 8757 | 415.2274 | 1242.6604 | 1243.7071 | -1.0466 | 1 | 3 | 74 | 4 | R.KPLMTRVVER.S |
|  | 8976 | **628.6353** | **1255.2561** | **1254.5881** | **0.6680** | **1** | **3** | **46** | **7** | **R.DYKNPEGFTGK.R** 8971 |
|  | 9111 | **421.5654** | **1261.6745** | **1261.5530** | **0.1215** | **0** | **12** | **10** | **5** | **R.SSSMTSTMTIGK.F** |
|  | 14247 | 783.8884 | 1565.7622 | 1566.7269 | -0.9647 | 0 | 8 | 19 | 4 | K.TLPSQSEMMAEISK.A |
|  | 14353 | 525.2928 | 1572.8565 | 1571.7104 | 1.1462 | 0 | 7 | 23 | 4 | K.EFTETAAIFEDGSR.E |
|  | 15476 | **549.3433** | **1645.0081** | **1644.7784** | **0.2297** | **0** | **4** | **42** | **8** | **R.VGDYGYPADVLFSSR.L** |
|  | 16096 | **845.9230** | **1689.8314** | **1689.8434** | **-0.0120** | **0** | **39** | **0.013** | **1** | **R.ALSQHPTLNDDLPNR.I** 16095 |
|  | 16629 | **577.2983** | **1728.8730** | **1728.8386** | **0.0344** | **1** | **3** | **55** | **9** | **R.VVERSSSMTSTMTIGK.F** |
|  | 17148 | **589.3520** | **1765.0341** | **1765.9517** | **-0.9176** | **0** | **3** | **55** | **2** | **K.FMLALAFFAIIIAYF.-** |

| **806.** | P47985\|UCRI_HUMAN    **Mass:** 29934    **Score:** 46     **Queries matched:** 8   **emPAI:** 0.27 |
| --- | --- |
|  | Cytochrome b-c1 complex subunit Rieske, mitochondrial OS=Homo sapiens GN=UQCRFS1 PE=1 SV=2 |

|  | **Query** | **Observed** | **Mr(expt)** | **Mr(calc)** | **Delta** | **Miss** | **Score** | **Expect** | **Rank** | **Peptide** |
| --- | --- | --- | --- | --- | --- | --- | --- | --- | --- | --- |
|  | 3006 | **429.7289** | **857.4432** | **857.4494** | **-0.0062** | **0** | **10** | **23** | **7** | **K.LSDIPEGK.N** |
|  | 4350 | **473.7481** | **945.4817** | **945.4879** | **-0.0062** | **0** | **18** | **3.4** | **1** | **R.ESLSGQAVR.R** |
|  | 5899 | **530.7999** | **1059.5853** | **1059.5924** | **-0.0071** | **1** | **25** | **0.57** | **1** | **R.RLEVLDSTK.S** |
|  | 14945 | **538.9460** | **1613.8163** | **1613.8260** | **-0.0097** | **0** | **28** | **0.21** | **1** | **K.EIEQEAAVELSQLR.D** |
|  | 21319 | **1055.5418** | **2109.0689** | **2109.0776** | **-0.0086** | **0** | **4** | **33** | **1** | **K.NAVTQFVSSMSASADVLALAK.I** |
|  | 25165 | **864.4233** | **2590.2482** | **2590.2623** | **-0.0141** | **1** | **35** | **0.021** | **1** | **K.EIEQEAAVELSQLRDPQHDLDR.V** |
|  | 25441 | **1320.7499** | **2639.4852** | **2639.5010** | **-0.0158** | **0** | **34** | **0.019** | **1** | **R.GVAGALRPLVQATVPATPEQPVLDLK.R** |
|  | 31145 | **1564.3574** | **4690.0504** | **4690.1403** | **-0.0899** | **0** | **1** | **12** | **3** | **K.KPEWVILIGVCTHLGCVPIANAGDFGGYYCPCHGSHYDASGR.I** |

| **807.** | Q92604\|LGAT1_HUMAN    **Mass:** 43289    **Score:** 46     **Queries matched:** 12   **emPAI:** 0.09 |
| --- | --- |
|  | Acyl-CoA:lysophosphatidylglycerol acyltransferase 1 OS=Homo sapiens GN=LPGAT1 PE=2 SV=1 |

|  | **Query** | **Observed** | **Mr(expt)** | **Mr(calc)** | **Delta** | **Miss** | **Score** | **Expect** | **Rank** | **Peptide** |
| --- | --- | --- | --- | --- | --- | --- | --- | --- | --- | --- |
|  | 3297 | **437.0528** | **872.0910** | **872.3988** | **-0.3078** | **0** | **10** | **14** | **9** | **K.NGSPAGGDAK.E** |
|  | 4317 | **472.7312** | **943.4479** | **944.4464** | **-0.9985** | **0** | **12** | **12** | **2** | **K.HLENNYR.S** 4329 |
|  | 6439 | **549.7974** | **1097.5802** | **1097.6808** | **-0.1006** | **1** | **16** | **4** | **1** | **R.DQQLLLLKK.H** |
|  | 8132 | 606.3773 | 1210.7400 | 1209.7445 | 0.9955 | 0 | 13 | 6.3 | 1 | K.IILNALVAQQK.N |
|  | 8599 | **618.7944** | **1235.5742** | **1236.6727** | **-1.0986** | **0** | **6** | **35** | **2** | **R.KPTVTHVHYR.I** |
|  | 14155 | **520.9579** | **1559.8518** | **1560.8817** | **-1.0298** | **1** | **4** | **43** | **8** | **K.WIVLFPEGGFLRK.R** |
|  | 15028 | **809.9424** | **1617.8702** | **1617.8766** | **-0.0064** | **0** | **35** | **0.038** | **1** | **K.GLQWIIDTTIAYPK.A** 15027 |
|  | 15588 | 551.9940 | 1652.9602 | 1653.9777 | -1.0175 | 1 | 14 | 4.5 | 3 | R.SGATKIILNALVAQQK.N |
|  | 19593 | 650.6653 | 1948.9740 | 1949.9566 | -0.9826 | 0 | 4 | 38 | 4 | K.GLVVAQMMWLMDHIFK.Y |
|  | 26810 | **965.2853** | **2892.8342** | **2892.5399** | **0.2943** | **1** | **2** | **27** | **6** | **K.AEPIDIQTWILGYRKPTVTHVHYR.I** |

| **808.** | O15145\|ARPC3_HUMAN    **Mass:** 20761    **Score:** 46     **Queries matched:** 9   **emPAI:** 0.18 |
| --- | --- |
|  | Actin-related protein 2/3 complex subunit 3 OS=Homo sapiens GN=ARPC3 PE=1 SV=3 |

|  | **Query** | **Observed** | **Mr(expt)** | **Mr(calc)** | **Delta** | **Miss** | **Score** | **Expect** | **Rank** | **Peptide** |
| --- | --- | --- | --- | --- | --- | --- | --- | --- | --- | --- |
|  | 1492 | 645.3882 | 644.3810 | 644.3130 | 0.0680 | 0 | 13 | 15 | 4 | K.SLSGPGQ.- |
|  | 7526 | **592.2958** | **1182.5771** | **1181.5804** | **0.9967** | **1** | **1** | **1.2e+002** | **3** | **K.WWTCFVKR.Q** |
|  | 8126 | **404.5615** | **1210.6626** | **1209.7267** | **0.9358** | **0** | **8** | **22** | **8** | **K.LIGNMALLPIR.S** |
|  | 8883 | **417.7312** | **1250.1718** | **1250.5891** | **-0.4173** | **1** | **8** | **16** | **5** | **K.NYEIKNEADR.T** |
|  | 9982 | **655.3839** | **1308.7533** | **1308.6132** | **0.1400** | **1** | **2** | **81** | **5** | **R.QFMNKSLSGPGQ.-** |
|  | 14607 | 796.3759 | 1590.7371 | 1590.7453 | -0.0082 | 0 | 46 | 0.003 | 1 | K.DTDIVDEAIYYFK.A |
|  | 18483 | **620.3726** | **1858.0960** | **1857.0321** | **1.0639** | **1** | **2** | **53** | **5** | **R.TLIYITLYISECLKK.L** |
|  | 25503 | **885.4083** | **2653.2030** | **2652.3767** | **0.8262** | **1** | **(0)** | **59** | **1** | **M.PAYHSSLMDPDTKLIGNMALLPIR.S** |
|  | 25583 | 667.8334 | 2667.3047 | 2668.3716 | -1.0670 | 1 | 1 | 54 | 5 | M.PAYHSSLMDPDTKLIGNMALLPIR.S |

| **809.** | Q96SQ9\|CP2S1_HUMAN    **Mass:** 56009    **Score:** 46     **Queries matched:** 18   **emPAI:** 0.21 |
| --- | --- |
|  | Cytochrome P450 2S1 OS=Homo sapiens GN=CYP2S1 PE=2 SV=2 |

|  | **Query** | **Observed** | **Mr(expt)** | **Mr(calc)** | **Delta** | **Miss** | **Score** | **Expect** | **Rank** | **Peptide** |
| --- | --- | --- | --- | --- | --- | --- | --- | --- | --- | --- |
|  | 4815 | **490.2843** | **978.5540** | **978.5684** | **-0.0144** | **1** | **5** | **54** | **10** | **R.KFTMLALR.D** |
|  | 7473 | 589.7730 | 1177.5313 | 1178.7209 | -1.1896 | 0 | (6) | 39 | 8 | R.LLALVPMGIPR.T |
|  | 7776 | 598.2908 | 1194.5670 | 1194.7158 | -0.1488 | 0 | 7 | 30 | 3 | R.LLALVPMGIPR.T |
|  | 9259 | **635.8184** | **1269.6223** | **1269.6313** | **-0.0090** | **0** | **33** | **0.07** | **1** | **R.ELGAGQAPSLGDR.T** |
|  | 12489 | 726.8422 | 1451.6698 | 1451.7629 | -0.0931 | 1 | 6 | 28 | 5 | K.FTMLALRDLGMGK.R |
|  | 12994 | **742.5356** | **1483.0566** | **1483.7527** | **-0.6961** | **1** | **(1)** | **73** | **4** | **K.FTMLALRDLGMGK.R** |
|  | 14993 | 808.8926 | 1615.7707 | 1616.7835 | -1.0127 | 1 | 1 | 91 | 8 | R.FSYEDKEFQAVVR.A |
|  | 16432 | **571.8131** | **1712.4175** | **1711.9841** | **0.4334** | **1** | **3** | **42** | **1** | **R.LLALVPMGIPRTLMR.T** |
|  | 19135 | **637.9810** | **1910.9211** | **1910.9446** | **-0.0235** | **1** | **3** | **45** | **2** | **R.EELNRELGAGQAPSLGDR.T** |
|  | 26063 | **913.0729** | **2736.1968** | **2737.3269** | **-1.1301** | **1** | **0** | **57** | **6** | **R.DLVDAFLLKMAQEEQNPGTEFTNK.N** |
|  | 26166 | **919.4899** | **2755.4480** | **2755.4585** | **-0.0105** | **0** | **20** | **0.63** | **1** | **R.GYTLPQGTEVFPLLGSILHDPNIFK.H** 26164 |
|  | 27942 | **787.9472** | **3147.7597** | **3147.7743** | **-0.0146** | **0** | **31** | **0.029** | **1** | **R.GHLPPGPTPLPLLGNLLQLRPGALYSGLMR.L** |
|  | 28821 | 1124.5665 | 3370.6778 | 3369.7712 | 0.9066 | 0 | (0) | 40 | 4 | K.NMLMTVIYLLFAGTMTVSTTVGYTLLLLMK.Y |
|  | 28879 | 1129.2028 | 3384.5865 | 3385.7661 | -1.1797 | 0 | 5 | 13 | 3 | K.NMLMTVIYLLFAGTMTVSTTVGYTLLLLMK.Y 28880 |
|  | 28891 | **1130.1854** | **3387.5344** | **3388.7027** | **-1.1683** | **0** | **0** | **38** | **1** | **R.AAGGTLLGVSSQGGQTYEMFSWFLRPLPGPHK.Q** |
|  | 30517 | **1369.7548** | **4106.2425** | **4106.1732** | **0.0692** | **1** | **0** | **19** | **5** | **K.NMLMTVIYLLFAGTMTVSTTVGYTLLLLMKYPHVQK.W** |

| **810.** | Q9UBX3\|DIC_HUMAN    **Mass:** 31718    **Score:** 46     **Queries matched:** 10   **emPAI:** 0.25 |
| --- | --- |
|  | Mitochondrial dicarboxylate carrier OS=Homo sapiens GN=SLC25A10 PE=1 SV=2 |

|  | **Query** | **Observed** | **Mr(expt)** | **Mr(calc)** | **Delta** | **Miss** | **Score** | **Expect** | **Rank** | **Peptide** |
| --- | --- | --- | --- | --- | --- | --- | --- | --- | --- | --- |
|  | 1332 | 608.3361 | 607.3288 | 607.2999 | 0.0289 | 0 | 12 | 11 | 6 | R.LMNSK.G |
|  | 1698 | **699.0814** | **698.0741** | **697.3871** | **0.6870** | **0** | **1** | **73** | **2** | **K.LPQGQR.R** |
|  | 1726 | **706.3505** | **705.3432** | **705.4173** | **-0.0742** | **1** | **6** | **56** | **10** | **R.KNFGIK.V** |
|  | 2329 | **406.2184** | **810.4223** | **810.3728** | **0.0495** | **0** | **9** | **18** | **1** | **R.MTGMALR.V** |
|  | 2955 | **854.9699** | **853.9626** | **853.4882** | **0.4744** | **1** | **5** | **30** | **2** | **K.LPQGQRR.N** |
|  | 9488 | **428.5477** | **1282.6214** | **1282.6452** | **-0.0238** | **1** | **7** | **28** | **3** | **R.RLFSGATMASSR.G** |
|  | 11873 | **472.2789** | **1413.8150** | **1412.7194** | **1.0955** | **1** | **5** | **43** | **6** | **R.MQNDVKLPQGQR.R** |
|  | 23041 | **1149.6390** | **2297.2635** | **2297.2744** | **-0.0108** | **0** | **48** | **0.0012** | **1** | **K.VLLGSVSGLAGGFVGTPADLVNVR.M** 23040 |
|  | 24544 | **833.0490** | **2496.1252** | **2496.1389** | **-0.0137** | **0** | **22** | **0.42** | **1** | **R.WYFGGLASCGAACCTHPLDLLK.V** |

| **811.** | Q2M2H8\|MGAL2_HUMAN    **Mass:** 55624    **Score:** 46     **Queries matched:** 6   **emPAI:** 0.14 |
| --- | --- |
|  | Putative maltase-glucoamylase-like protein LOC93432 OS=Homo sapiens PE=2 SV=2 |

|  | **Query** | **Observed** | **Mr(expt)** | **Mr(calc)** | **Delta** | **Miss** | **Score** | **Expect** | **Rank** | **Peptide** |
| --- | --- | --- | --- | --- | --- | --- | --- | --- | --- | --- |
|  | 1249 | 590.3257 | 589.3185 | 588.3231 | 0.9953 | 0 | 9 | 35 | 1 | K.EVVSR.N |
|  | 8828 | **624.7706** | **1247.5266** | **1247.6947** | **-0.1682** | **0** | **8** | **25** | **8** | **K.YLIIMNPGISK.N** |
|  | 18328 | **925.8928** | **1849.7711** | **1849.7822** | **-0.0112** | **0** | **33** | **0.053** | **1** | **R.IDCTPDQEVTEDICR.W** 18329 |
|  | 24795 | 1268.6466 | 2535.2787 | 2536.3690 | -1.0903 | 0 | 36 | 0.018 | 1 | R.VLLDTSIGPLQFAQQYLQLSFR.L 24794 |

| **812.** | Q969H8\|CS010_HUMAN    **Mass:** 18897    **Score:** 46     **Queries matched:** 3   **emPAI:** 0.20 |
| --- | --- |
|  | UPF0556 protein C19orf10 OS=Homo sapiens GN=C19orf10 PE=1 SV=1 |

|  | **Query** | **Observed** | **Mr(expt)** | **Mr(calc)** | **Delta** | **Miss** | **Score** | **Expect** | **Rank** | **Peptide** |
| --- | --- | --- | --- | --- | --- | --- | --- | --- | --- | --- |
|  | 7355 | 585.7771 | 1169.5396 | 1169.7383 | -0.1987 | 1 | 12 | 10 | 6 | K.AELSKLVIVAK.A |
|  | 16919 | 875.9356 | 1749.8565 | 1749.8672 | -0.0107 | 1 | 46 | 0.003 | 1 | R.ESDVPLKTEEFEVTK.T 16918 |

| **813.** | Q8N0Y7\|PGAM4_HUMAN    **Mass:** 28930    **Score:** 46     **Queries matched:** 8   **emPAI:** 0.45 |
| --- | --- |
|  | Probable phosphoglycerate mutase 4 OS=Homo sapiens GN=PGAM4 PE=1 SV=1 |

|  | **Query** | **Observed** | **Mr(expt)** | **Mr(calc)** | **Delta** | **Miss** | **Score** | **Expect** | **Rank** | **Peptide** |
| --- | --- | --- | --- | --- | --- | --- | --- | --- | --- | --- |
|  | 1250 | 590.3295 | 589.3222 | 589.3071 | 0.0151 | 0 | 16 | 6.9 | 3 | K.AETAAK.H |
|  | 1357 | 614.1743 | 613.1670 | 612.4323 | 0.7348 | 0 | 3 | 62 | 1 | K.LVLIR.H |
|  | 5875 | 530.2792 | 1058.5438 | 1058.5509 | -0.0071 | 0 | 37 | 0.041 | 1 | R.HYGGLTGLNK.A 5874 |
|  | 15483 | **823.9180** | **1645.8214** | **1646.9580** | **-1.1366** | **1** | **1** | **92** | **6** | **K.RVLIAAHGNSLQGIAK.H** |
|  | 16025 | 842.9556 | 1683.8967 | 1682.9032 | 0.9935 | 0 | 37 | 0.024 | 1 | R.ALPFWNEEIVPQIK.E |
|  | 21822 | 719.3879 | 2155.1418 | 2155.1500 | -0.0082 | 0 | 32 | 0.059 | 1 | R.TLWTVLDAIDQMWLPVVR.T |
|  | 22730 | **566.3278** | **2261.2822** | **2262.1388** | **-0.8566** | **0** | **2** | **43** | **3** | **K.NLKPIKPMQFLGDEETVCK.A** |

| **814.** | P60903\|S10AA_HUMAN    **Mass:** 11310    **Score:** 45     **Queries matched:** 3   **emPAI:** 0.83 |
| --- | --- |
|  | Protein S100-A10 OS=Homo sapiens GN=S100A10 PE=1 SV=2 |

|  | **Query** | **Observed** | **Mr(expt)** | **Mr(calc)** | **Delta** | **Miss** | **Score** | **Expect** | **Rank** | **Peptide** |
| --- | --- | --- | --- | --- | --- | --- | --- | --- | --- | --- |
|  | 19565 | 973.9928 | 1945.9710 | 1945.9785 | -0.0075 | 1 | 31 | 0.084 | 1 | K.EFPGFLENQKDPLAVDK.I |
|  | 20986 | 521.4748 | 2081.8703 | 2081.8831 | -0.0128 | 0 | 40 | 0.0078 | 1 | M.PSQMEHAMETMMFTFHK.F |
|  | 21168 | 700.3493 | 2098.0261 | 2097.8781 | 0.1480 | 0 | (1) | 75 | 9 | M.PSQMEHAMETMMFTFHK.F |

| **815.** | Q9P2E9\|RRBP1_HUMAN    **Mass:** 152780   **Score:** 45     **Queries matched:** 28   **emPAI:** 0.02 |
| --- | --- |
|  | Ribosome-binding protein 1 OS=Homo sapiens GN=RRBP1 PE=1 SV=4 |

|  | **Query** | **Observed** | **Mr(expt)** | **Mr(calc)** | **Delta** | **Miss** | **Score** | **Expect** | **Rank** | **Peptide** |
| --- | --- | --- | --- | --- | --- | --- | --- | --- | --- | --- |
|  | 1494 | 645.3884 | 644.3812 | 643.3653 | 1.0158 | 0 | 16 | 6.4 | 6 | K.EVAGLR.Q 1487 |
|  | 1785 | **720.3475** | **719.3402** | **718.3861** | **0.9541** | **1** | **8** | **31** | **4** | **R.EKDTVK.K** |
|  | 2804 | 423.0523 | 844.0901 | 844.4654 | -0.3753 | 1 | 16 | 3.7 | 2 | K.LASSPKDK.K |
|  | 2995 | **429.1944** | **856.3742** | **855.4450** | **0.9292** | **0** | **6** | **52** | **2** | **K.GEGAPIQGK.K** |
|  | 3084 | 431.7525 | 861.4904 | 860.3876 | 1.1028 | 0 | 5 | 67 | 9 | K.AEGSPSEGK.K |
|  | 3407 | 440.7394 | 879.4643 | 878.4167 | 1.0475 | 0 | 2 | 1.2e+002 | 8 | R.QQLSEMK.S |
|  | 3878 | **456.8827** | **911.7508** | **912.5280** | **-0.7772** | **0** | **6** | **26** | **6** | **K.AAILETAPK.E** |
|  | 4104 | 465.2366 | 928.4586 | 928.4978 | -0.0392 | 1 | 11 | 14 | 5 | K.KEEKPNGK.I |
|  | 4882 | 492.7916 | 983.5686 | 983.5400 | 0.0287 | 1 | 14 | 6.6 | 4 | K.GEGAPIQGKK.A |
|  | 5288 | 507.7976 | 1013.5807 | 1014.5094 | -0.9287 | 1 | 13 | 8.7 | 7 | K.AEGSPNQGKK.A |
|  | 5449 | 514.2695 | 1026.5244 | 1026.5206 | 0.0038 | 1 | 4 | 58 | 7 | K.AEGAPNQGRK.A |
|  | 5523 | 516.3124 | 1030.6103 | 1029.5455 | 1.0649 | 0 | 8 | 26 | 6 | K.QSDELALVR.Q 5498 |
|  | 5670 | **522.2686** | **1042.5227** | **1043.5359** | **-1.0132** | **1** | **8** | **29** | **6** | **K.AEGAQNQAKK.V** |
|  | 6478 | 551.3149 | 1100.6153 | 1099.5985 | 1.0168 | 0 | 7 | 33 | 6 | R.LQQENSILR.D |
|  | 7345 | 585.2987 | 1168.5829 | 1167.6037 | 0.9792 | 0 | 8 | 24 | 2 | K.AGIIQDTWHK.A |
|  | 8543 | 617.2654 | 1232.5162 | 1232.6360 | -0.1198 | 1 | 6 | 39 | 2 | K.LQSSEAEVRSK.C |
|  | 8705 | **414.2689** | **1239.7848** | **1239.7187** | **0.0662** | **1** | **3** | **60** | **5** | **K.ATQKGDPVAILK.R** |
|  | 10501 | **446.2269** | **1335.6589** | **1336.7425** | **-1.0836** | **0** | **4** | **56** | **7** | **K.EVPMVVVPPVGAK.G** |
|  | 10866 | **679.8045** | **1357.5944** | **1357.7089** | **-0.1144** | **0** | **45** | **0.0044** | **1** | **K.LLATEQEDAAVAK.S** |
|  | 10894 | **680.8054** | **1359.5962** | **1359.6994** | **-0.1032** | **1** | **2** | **1e+002** | **6** | **K.LKGELESSDQVR.E** |
|  | 11797 | 705.8332 | 1409.6518 | 1409.6422 | 0.0096 | 0 | 24 | 0.51 | 2 | K.ETSYEEALANQR.K |
|  | 12049 | **712.9973** | **1423.9799** | **1422.8082** | **1.1717** | **1** | **2** | **56** | **2** | **R.VTVKHLEEIVEK.L** |
|  | 12840 | 492.2463 | 1473.7170 | 1474.7878 | -1.0708 | 1 | 8 | 20 | 3 | K.LLSLTQAKEESEK.Q |
|  | 13864 | 514.9320 | 1541.7742 | 1542.8253 | -1.0511 | 0 | 11 | 10 | 7 | R.QLLLESQSQLDAAK.S 13897 |
|  | 21957 | 724.3757 | 2170.1054 | 2171.0454 | -0.9401 | 1 | 3 | 46 | 3 | K.TESASVQGRNTDVAQSPEAPK.Q |

| **816.** | O14907\|TX1B3_HUMAN    **Mass:** 13726    **Score:** 45     **Queries matched:** 3   **emPAI:** 0.29 |
| --- | --- |
|  | Tax1-binding protein 3 OS=Homo sapiens GN=TAX1BP3 PE=1 SV=2 |

|  | **Query** | **Observed** | **Mr(expt)** | **Mr(calc)** | **Delta** | **Miss** | **Score** | **Expect** | **Rank** | **Peptide** |
| --- | --- | --- | --- | --- | --- | --- | --- | --- | --- | --- |
|  | 15401 | **820.9226** | **1639.8307** | **1639.8417** | **-0.0110** | **0** | **45** | **0.0035** | **1** | **R.VSEGGPAEIAGLQIGDK.I** |
|  | 15459 | **823.3236** | **1644.6327** | **1644.8658** | **-0.2331** | **0** | **1** | **69** | **2** | **-.MSYIPGQPVTAVVQR.V** |
|  | 22878 | 760.0369 | 2277.0890 | 2276.0500 | 1.0389 | 1 | 1 | 68 | 5 | K.IMQVNGWDMTMVTHDQARK.R |

| **817.** | Q96HR9\|REEP6_HUMAN    **Mass:** 20891    **Score:** 45     **Queries matched:** 4   **emPAI:** 0.40 |
| --- | --- |
|  | Receptor expression-enhancing protein 6 OS=Homo sapiens GN=REEP6 PE=1 SV=1 |

|  | **Query** | **Observed** | **Mr(expt)** | **Mr(calc)** | **Delta** | **Miss** | **Score** | **Expect** | **Rank** | **Peptide** |
| --- | --- | --- | --- | --- | --- | --- | --- | --- | --- | --- |
|  | 4531 | **479.7663** | **957.5181** | **957.5243** | **-0.0062** | **0** | **44** | **0.0065** | **1** | **R.ALDAAAGITR.N** |
|  | 8373 | 612.3361 | 1222.6576 | 1222.6670 | -0.0094 | 0 | 28 | 0.25 | 1 | R.NVKPSQTPQPK.D |
|  | 10823 | 678.3283 | 1354.6421 | 1355.7660 | -1.1239 | 0 | 1 | 1.1e+002 | 8 | R.NLVTEVLGALEAK.T |
|  | 18596 | 935.4807 | 1868.9469 | 1870.0411 | -1.0943 | 1 | 3 | 52 | 10 | R.NLVTEVLGALEAKTGVEK.R |

| **818.** | P11234\|RALB_HUMAN    **Mass:** 23508    **Score:** 45     **Queries matched:** 9   **emPAI:** 0.16 |
| --- | --- |
|  | Ras-related protein Ral-B OS=Homo sapiens GN=RALB PE=1 SV=1 |

|  | **Query** | **Observed** | **Mr(expt)** | **Mr(calc)** | **Delta** | **Miss** | **Score** | **Expect** | **Rank** | **Peptide** |
| --- | --- | --- | --- | --- | --- | --- | --- | --- | --- | --- |
|  | 2403 | 409.2221 | 816.4296 | 817.4181 | -0.9885 | 1 | 16 | 5.8 | 1 | R.VKAEEDK.I |
|  | 3215 | **434.7449** | **867.4752** | **866.3804** | **1.0949** | **1** | **12** | **8.4** | **3** | **K.MSENKDK.N** |
|  | 3771 | **452.9024** | **903.7902** | **903.4410** | **0.3493** | **1** | **6** | **34** | **4** | **K.SDLEERR.Q** |
|  | 5158 | 1003.5538 | 1002.5466 | 1002.5532 | -0.0067 | 0 | 45 | 0.0059 | 1 | K.VIMVGSGGVGK.S |
|  | 6991 | **571.3050** | **1140.5954** | **1141.6091** | **-1.0137** | **1** | **2** | **99** | **7** | **R.QVPVEEARSK.A** |
|  | 13620 | 509.2384 | 1524.6935 | 1523.8559 | 0.8376 | 1 | 0 | 1.1e+002 | 8 | K.AEEDKIPLLVVGNK.S |
|  | 19472 | **647.5110** | **1939.5111** | **1940.0513** | **-0.5402** | **1** | **4** | **26** | **2** | **K.GQSSLALHKVIMVGSGGVGK.S** |
|  | 23956 | **1213.5518** | **2425.0890** | **2425.1035** | **-0.0146** | **0** | **14** | **3** | **1** | **K.SALTLQFMYDEFVEDYEPTK.A** |
|  | 24070 | **611.0365** | **2440.1169** | **2441.0985** | **-0.9816** | **0** | **(1)** | **62** | **9** | **K.SALTLQFMYDEFVEDYEPTK.A** |

| **819.** | P11233\|RALA_HUMAN    **Mass:** 23723    **Score:** 45     **Queries matched:** 7   **emPAI:** 0.16 |
| --- | --- |
|  | Ras-related protein Ral-A OS=Homo sapiens GN=RALA PE=1 SV=1 |

|  | **Query** | **Observed** | **Mr(expt)** | **Mr(calc)** | **Delta** | **Miss** | **Score** | **Expect** | **Rank** | **Peptide** |
| --- | --- | --- | --- | --- | --- | --- | --- | --- | --- | --- |
|  | 4651 | **484.7402** | **967.4659** | **966.5247** | **0.9413** | **0** | **22** | **0.89** | **2** | **K.GQNSLALHK.V** |
|  | 5158 | 1003.5538 | 1002.5466 | 1002.5532 | -0.0067 | 0 | 45 | 0.0059 | 1 | K.VIMVGSGGVGK.S |
|  | 15366 | 820.3582 | 1638.7019 | 1637.7685 | 0.9334 | 0 | 1 | 80 | 5 | R.AEQWNVNYVETSAK.T |
|  | 23956 | 1213.5518 | 2425.0890 | 2425.1035 | -0.0146 | 0 | 14 | 3 | 1 | K.SALTLQFMYDEFVEDYEPTK.A |
|  | 24070 | 611.0365 | 2440.1169 | 2441.0985 | -0.9816 | 0 | (1) | 62 | 9 | K.SALTLQFMYDEFVEDYEPTK.A |
|  | 28672 | 1109.9121 | 3326.7145 | 3326.5588 | 0.1557 | 1 | 3 | 24 | 2 | R.SGEGFLCVFSITEMESFAATADFREQILR.V |
|  | 28868 | **1128.7787** | **3383.3142** | **3382.4911** | **0.8232** | **1** | **0** | **27** | **1** | **R.DNYFRSGEGFLCVFSITEMESFAATADFR.E** |

| **820.** | Q9Y376\|CAB39_HUMAN    **Mass:** 40015    **Score:** 45     **Queries matched:** 10   **emPAI:** 0.09 |
| --- | --- |
|  | Calcium-binding protein 39 OS=Homo sapiens GN=CAB39 PE=1 SV=1 |

|  | **Query** | **Observed** | **Mr(expt)** | **Mr(calc)** | **Delta** | **Miss** | **Score** | **Expect** | **Rank** | **Peptide** |
| --- | --- | --- | --- | --- | --- | --- | --- | --- | --- | --- |
|  | 1681 | 691.4092 | 690.4020 | 690.3734 | 0.0285 | 0 | 16 | 5.7 | 7 | K.NLVAMK.E |
|  | 4968 | **496.2505** | **990.4865** | **990.4957** | **-0.0091** | **0** | **7** | **38** | **6** | **R.HNFTIMTK.Y** |
|  | 6198 | **541.2368** | **1080.4591** | **1080.5927** | **-0.1337** | **1** | **4** | **59** | **1** | **K.SHKSPADIVK.N** |
|  | 7134 | **577.1310** | **1152.2474** | **1152.7118** | **-0.4644** | **0** | **0** | **98** | **10** | **K.TQPILDILLK.N** |
|  | 9108 | **631.8309** | **1261.6472** | **1260.6747** | **0.9725** | **1** | **4** | **56** | **5** | **K.NLKESMAVLEK.Q** |
|  | 9390 | **638.8335** | **1275.6524** | **1276.6696** | **-1.0172** | **1** | **(3)** | **68** | **4** | **K.NLKESMAVLEK.Q** |
|  | 17111 | **882.4448** | **1762.8751** | **1762.8719** | **0.0032** | **0** | **45** | **0.0037** | **1** | **K.IILWSEQFYDFFR.Y** |
|  | 18131 | **613.3158** | **1836.9256** | **1837.8702** | **-0.9447** | **0** | **0** | **1e+002** | **3** | **K.GYESPEIALNCGIMLR.E** |
|  | 18480 | **620.3398** | **1857.9977** | **1857.8632** | **0.1345** | **1** | **7** | **20** | **1** | **R.TEDEQFNDEKTYLVK.Q** |
|  | 19724 | **981.9256** | **1961.8366** | **1962.0794** | **-0.2427** | **1** | **2** | **59** | **8** | **K.YISKPENLKLMMNLLR.D** |

| **821.** | Q8WU67\|ABHD3_HUMAN    **Mass:** 46492    **Score:** 45     **Queries matched:** 5   **emPAI:** 0.08 |
| --- | --- |
|  | Abhydrolase domain-containing protein 3 OS=Homo sapiens GN=ABHD3 PE=2 SV=2 |

|  | **Query** | **Observed** | **Mr(expt)** | **Mr(calc)** | **Delta** | **Miss** | **Score** | **Expect** | **Rank** | **Peptide** |
| --- | --- | --- | --- | --- | --- | --- | --- | --- | --- | --- |
|  | 5321 | 509.2376 | 1016.4605 | 1017.5389 | -1.0784 | 1 | 6 | 44 | 5 | M.QRLAMDLR.M |
|  | 7076 | **575.2405** | **1148.4664** | **1148.5794** | **-0.1130** | **1** | **(6)** | **41** | **9** | **-.MQRLAMDLR.M** |
|  | 7084 | 575.3068 | 1148.5991 | 1148.5794 | 0.0197 | 1 | 8 | 27 | 9 | -.MQRLAMDLR.M |
|  | 20247 | **672.3990** | **2014.1753** | **2015.0371** | **-0.8618** | **1** | **2** | **47** | **7** | **R.CVVFNNRGVAGENLLTPR.T** |
|  | 27183 | **989.5135** | **2965.5186** | **2964.5267** | **0.9920** | **0** | **45** | **0.0018** | **1** | **K.SVGIPVLCLNSVDDVFSPSHAIPIETAK.Q** |

| **822.** | Q9HCU5\|PREB_HUMAN    **Mass:** 46010    **Score:** 44     **Queries matched:** 5   **emPAI:** 0.08 |
| --- | --- |
|  | Prolactin regulatory element-binding protein OS=Homo sapiens GN=PREB PE=1 SV=2 |

|  | **Query** | **Observed** | **Mr(expt)** | **Mr(calc)** | **Delta** | **Miss** | **Score** | **Expect** | **Rank** | **Peptide** |
| --- | --- | --- | --- | --- | --- | --- | --- | --- | --- | --- |
|  | 7568 | 593.1536 | 1184.2926 | 1184.5687 | -0.2761 | 0 | 7 | 21 | 4 | R.FQAHQQQGNK.A 7575 7594 |
|  | 14248 | **522.9301** | **1565.7685** | **1565.7798** | **-0.0113** | **0** | **44** | **0.0043** | **1** | **R.LSASLLHSHDTETR.A** |
|  | 29800 | **1236.1365** | **3705.3876** | **3704.8264** | **0.5612** | **1** | **4** | **9.4** | **1** | **R.ATMNLALAGDILAAGQDAHCQLLRFQAHQQQGNK.A** |

| **823.** | P46977\|STT3A_HUMAN    **Mass:** 81104    **Score:** 44     **Queries matched:** 12   **emPAI:** 0.09 |
| --- | --- |
|  | Dolichyl-diphosphooligosaccharide--protein glycosyltransferase subunit STT3A OS=Homo sapiens GN=STT3A PE=1 SV=2 |

|  | **Query** | **Observed** | **Mr(expt)** | **Mr(calc)** | **Delta** | **Miss** | **Score** | **Expect** | **Rank** | **Peptide** |
| --- | --- | --- | --- | --- | --- | --- | --- | --- | --- | --- |
|  | 4052 | **463.2396** | **924.4647** | **924.4705** | **-0.0058** | **0** | **7** | **28** | **7** | **R.IIFDDFR.E** |
|  | 5311 | **508.7630** | **1015.5115** | **1014.5321** | **0.9794** | **1** | **11** | **15** | **1** | **-.MTKFGFLR.L** |
|  | 7107 | 576.2840 | 1150.5534 | 1149.5336 | 1.0199 | 0 | 40 | 0.016 | 1 | R.VGQAMASTEEK.A 7108 |
|  | 7582 | 593.3292 | 1184.6439 | 1184.6513 | -0.0074 | 1 | 16 | 3.7 | 1 | K.NLDISRPDKK.S |
|  | 13123 | **746.3181** | **1490.6217** | **1490.6314** | **-0.0097** | **0** | **17** | **2.6** | **1** | **K.ENDYYTPTGEFR.V** |
|  | 13603 | **508.6058** | **1522.7955** | **1522.7524** | **0.0431** | **0** | **6** | **34** | **4** | **R.EGSPVLLNCLMYK.M** |
|  | 23296 | 779.7624 | 2336.2653 | 2335.3185 | 0.9469 | 1 | 5 | 26 | 5 | K.QDTLLKLLILSMAAVLSFSTR.L |
|  | 27583 | 1015.5240 | 3043.5501 | 3044.4621 | -0.9120 | 1 | 7 | 10 | 2 | R.TILVDNNTWNNTHISRVGQAMASTEEK.A |
|  | 27748 | **773.6265** | **3090.4767** | **3090.4934** | **-0.0167** | **1** | **27** | **0.11** | **1** | **R.NAEIGNKDFELDVLEEAYTTEHWLVR.I** |
|  | 31426 | **1248.5154** | **4990.0324** | **4989.4465** | **0.5859** | **1** | **1** | **9.8** | **6** | **K.DAGAGLLAAAMIAVVPGYISRSVAGSYDNEGIAIFCMLLTYYMWIK.A** |
|  | 31692 | 1380.3986 | 5517.5651 | 5517.7153 | -0.1501 | 1 | 0 | 8.8 | 4 | K.QQDSTYPIKNEVASGMILVMAFFLITYTFHSTWVTSEAYSSPSIVLSAR.G |

| **824.** | P04233\|HG2A_HUMAN    **Mass:** 33950    **Score:** 44     **Queries matched:** 7   **emPAI:** 0.11 |
| --- | --- |
|  | HLA class II histocompatibility antigen gamma chain OS=Homo sapiens GN=CD74 PE=1 SV=3 |

|  | **Query** | **Observed** | **Mr(expt)** | **Mr(calc)** | **Delta** | **Miss** | **Score** | **Expect** | **Rank** | **Peptide** |
| --- | --- | --- | --- | --- | --- | --- | --- | --- | --- | --- |
|  | 3972 | **460.2448** | **918.4750** | **918.4559** | **0.0191** | **0** | **9** | **22** | **6** | **K.GSFPENLR.H** |
|  | 14718 | 800.4070 | 1598.7995 | 1598.8086 | -0.0091 | 0 | 15 | 3.3 | 1 | R.DLISNNEQLPMLGR.R |
|  | 15195 | **814.9465** | **1627.8785** | **1627.8893** | **-0.0108** | **0** | **44** | **0.0047** | **1** | **K.LTVTSQNLQLENLR.M** |
|  | 25345 | 876.0883 | 2625.2430 | 2625.3151 | -0.0721 | 0 | (2) | 37 | 2 | R.MATPLLMQALPMGALPQGPMQNATK.Y |
|  | 25635 | **892.4615** | **2674.3628** | **2673.2998** | **1.0630** | **0** | **5** | **21** | **1** | **R.MATPLLMQALPMGALPQGPMQNATK.Y** |
|  | 27164 | 987.4298 | 2959.2676 | 2960.4414 | -1.1738 | 1 | 2 | 28 | 4 | K.MRMATPLLMQALPMGALPQGPMQNATK.Y 27165 |

| **825.** | B1AK53\|ESPN_HUMAN    **Mass:** 92588    **Score:** 44     **Queries matched:** 14   **emPAI:** 0.04 |
| --- | --- |
|  | Espin OS=Homo sapiens GN=ESPN PE=1 SV=1 |

|  | **Query** | **Observed** | **Mr(expt)** | **Mr(calc)** | **Delta** | **Miss** | **Score** | **Expect** | **Rank** | **Peptide** |
| --- | --- | --- | --- | --- | --- | --- | --- | --- | --- | --- |
|  | 2229 | 402.8872 | 803.7599 | 803.4436 | 0.3163 | 1 | 7 | 27 | 2 | K.RQVMVR.K |
|  | 3867 | 456.2522 | 910.4898 | 911.4236 | -0.9338 | 0 | 6 | 38 | 7 | R.TLGYDESK.L |
|  | 6034 | **535.7840** | **1069.5534** | **1069.5880** | **-0.0345** | **0** | **5** | **49** | **6** | **M.ALEQALQAAR.Q** |
|  | 6786 | **563.7526** | **1125.4907** | **1124.5873** | **0.9034** | **1** | **4** | **55** | **9** | **R.AGCPRLGPAAR.G** |
|  | 7502 | 590.8102 | 1179.6059 | 1180.6088 | -1.0029 | 1 | 8 | 26 | 8 | K.LRTLGYDESK.L |
|  | 8260 | 406.6999 | 1217.0778 | 1216.6234 | 0.4545 | 0 | 4 | 45 | 7 | -.MALEQALQAAR.Q |
|  | 10639 | **672.7792** | **1343.5438** | **1344.5728** | **-1.0290** | **0** | **1** | **1.1e+002** | **3** | **K.ELSSCDGHDGLR.R** |
|  | 17537 | **897.9569** | **1793.8993** | **1793.9384** | **-0.0391** | **1** | **4** | **39** | **1** | **R.VQDKDNSGATVLHLAAR.F** |
|  | 20995 | **1042.4815** | **2082.9483** | **2082.9602** | **-0.0118** | **0** | **44** | **0.0036** | **1** | **K.SFNMMSPTGDNSELLAEIK.A** |
|  | 21171 | 700.4199 | 2098.2379 | 2098.9551 | -0.7171 | 0 | (2) | 43 | 4 | K.SFNMMSPTGDNSELLAEIK.A |
|  | 23583 | **791.1289** | **2370.3649** | **2371.1035** | **-0.7386** | **1** | **5** | **21** | **1** | **K.SFNMMSPTGDNSELLAEIKAGK.S** |
|  | 29637 | **1217.9586** | **3650.8540** | **3649.7889** | **1.0652** | **0** | **2** | **20** | **6** | **R.FGHPEVVNWLLHHGGGDPTAATDMGALPIHYAAAK.G** |
|  | 30244 | **1302.3267** | **3903.9582** | **3902.8390** | **1.1191** | **0** | **(0)** | **30** | **5** | **R.AHDGMTPLHAAAQMGHSPVIVWLVSCTDVSLSEQDK.D** |
|  | 30262 | 980.4788 | 3917.8862 | 3918.8339 | -0.9477 | 0 | 2 | 22 | 3 | R.AHDGMTPLHAAAQMGHSPVIVWLVSCTDVSLSEQDK.D |

| **826.** | P40227\|TCPZ_HUMAN    **Mass:** 58444    **Score:** 44     **Queries matched:** 19   **emPAI:** 0.06 |
| --- | --- |
|  | T-complex protein 1 subunit zeta OS=Homo sapiens GN=CCT6A PE=1 SV=3 |

|  | **Query** | **Observed** | **Mr(expt)** | **Mr(calc)** | **Delta** | **Miss** | **Score** | **Expect** | **Rank** | **Peptide** |
| --- | --- | --- | --- | --- | --- | --- | --- | --- | --- | --- |
|  | 894 | **518.2894** | **517.2822** | **518.2886** | **-1.0065** | **0** | **13** | **18** | **9** | **-.MAAVK.T** |
|  | 1412 | 628.2590 | 627.2517 | 626.2846 | 0.9671 | 0 | 5 | 43 | 9 | R.HPDMK.K |
|  | 1688 | **696.1807** | **695.1735** | **694.4126** | **0.7609** | **0** | **8** | **17** | **8** | **K.HKPSVK.G** |
|  | 3809 | 454.2528 | 906.4911 | 907.4069 | -0.9159 | 1 | 18 | 2.6 | 4 | R.KVCGDSDK.G 3830 3835 |
|  | 3916 | **458.2526** | **914.4906** | **915.5025** | **-1.0120** | **0** | **14** | **7** | **6** | **R.ETLIDVAR.T** |
|  | 4276 | 471.3338 | 940.6530 | 940.5705 | 0.0825 | 1 | 16 | 3.5 | 3 | M.AAVKTLNPK.A |
|  | 5206 | **504.2742** | **1006.5338** | **1005.5165** | **1.0173** | **0** | **6** | **42** | **8** | **K.MLVSGAGDIK.L** |
|  | 6445 | **550.2551** | **1098.4957** | **1097.6193** | **0.8764** | **1** | **0** | **1.5e+002** | **9** | **K.TLNPKAEVAR.A** |
|  | 8934 | 628.2241 | 1254.4337 | 1254.7044 | -0.2707 | 0 | 4 | 47 | 3 | R.AQAALAVNISAAR.G |
|  | 10506 | **446.2580** | **1335.7523** | **1334.7081** | **1.0441** | **1** | **3** | **62** | **8** | **R.IITEGFEAAKEK.A** |
|  | 12306 | **480.7983** | **1439.3732** | **1438.7160** | **0.6572** | **1** | **5** | **26** | **4** | **K.GTMKMLVSGAGDIK.L** |
|  | 12660 | 488.3048 | 1461.8926 | 1462.7086 | -0.8160 | 1 | 8 | 19 | 3 | R.EMDRETLIDVAR.T |
|  | 17097 | 881.8866 | 1761.7586 | 1760.9309 | 0.8278 | 0 | 29 | 0.13 | 2 | K.VLAQNSGFDLQETLVK.I 17096 |
|  | 22249 | 736.0800 | 2205.2181 | 2205.2256 | -0.0076 | 0 | 18 | 1.3 | 1 | K.VHAELADVLTEAVVDSILAIK.K |
|  | 23154 | **772.4196** | **2314.2369** | **2314.2103** | **0.0265** | **0** | **44** | **0.003** | **1** | **K.DGNVLLHEMQIQHPTASLIAK.V** |
|  | 24823 | **848.8728** | **2543.5966** | **2543.3330** | **0.2635** | **0** | **2** | **32** | **2** | **K.VATAQDDITGDGTTSNVLIIGELLK.Q** |

| **827.** | Q6UWP2\|DHR11_HUMAN    **Mass:** 28689    **Score:** 44     **Queries matched:** 6   **emPAI:** 0.13 |
| --- | --- |
|  | Dehydrogenase/reductase SDR family member 11 OS=Homo sapiens GN=DHRS11 PE=1 SV=1 |

|  | **Query** | **Observed** | **Mr(expt)** | **Mr(calc)** | **Delta** | **Miss** | **Score** | **Expect** | **Rank** | **Peptide** |
| --- | --- | --- | --- | --- | --- | --- | --- | --- | --- | --- |
|  | 2969 | **856.4105** | **855.4032** | **855.5178** | **-0.1146** | **0** | **3** | **92** | **7** | **R.ALVQQGLK.V** |
|  | 9915 | **435.7173** | **1304.1300** | **1304.6230** | **-0.4930** | **1** | **9** | **12** | **1** | **-.MARPGMERWR.D** |
|  | 12979 | 495.2238 | 1482.6495 | 1482.8518 | -0.2023 | 0 | 8 | 18 | 3 | R.LALVTGASGGIGAAVAR.A |
|  | 16981 | **877.9373** | **1753.8601** | **1753.8709** | **-0.0108** | **0** | **6** | **26** | **1** | **R.ATCISPGVVETQFAFK.L** |
|  | 16983 | **585.6392** | **1753.8958** | **1753.9799** | **-0.0840** | **1** | **3** | **51** | **3** | **R.DRLALVTGASGGIGAAVAR.A** |
|  | 18705 | **626.9631** | **1877.8674** | **1877.8802** | **-0.0129** | **0** | **44** | **0.0042** | **1** | **R.NVDDGHIININSMSGHR.V** |

| **828.** | P13640\|MT1G_HUMAN    **Mass:** 7277     **Score:** 44     **Queries matched:** 2   **emPAI:** 0.57 |
| --- | --- |
|  | Metallothionein-1G OS=Homo sapiens GN=MT1G PE=1 SV=2 |

|  | **Query** | **Observed** | **Mr(expt)** | **Mr(calc)** | **Delta** | **Miss** | **Score** | **Expect** | **Rank** | **Peptide** |
| --- | --- | --- | --- | --- | --- | --- | --- | --- | --- | --- |
|  | 12379 | **723.2534** | **1444.4923** | **1444.5026** | **-0.0103** | **0** | **44** | **0.0045** | **1** | **K.SCCSCCPVGCAK.C** |
|  | 25212 | 520.4521 | 2597.2241 | 2596.9566 | 0.2675 | 1 | 3 | 32 | 9 | -.MDPNCSCAAAGVSCTCASSCKCK.E |

|  | **Proteins matching a subset of these peptides:** |
| --- | --- |

|  | Q8N339\|MT1M_HUMAN    **Mass:** 7246     **Score:** 44     **Queries matched:** 1 |
| --- | --- |
|  | Metallothionein-1M OS=Homo sapiens GN=MT1M PE=2 SV=2 |

|  | P04732\|MT1E_HUMAN    **Mass:** 7150     **Score:** 44     **Queries matched:** 1 |
| --- | --- |
|  | Metallothionein-1E OS=Homo sapiens GN=MT1E PE=1 SV=1 |

|  | P80297\|MT1X_HUMAN    **Mass:** 7204     **Score:** 44     **Queries matched:** 1 |
| --- | --- |
|  | Metallothionein-1X OS=Homo sapiens GN=MT1X PE=1 SV=1 |

| **829.** | P02795\|MT2_HUMAN    **Mass:** 7178     **Score:** 44     **Queries matched:** 2   **emPAI:** 0.58 |
| --- | --- |
|  | Metallothionein-2 OS=Homo sapiens GN=MT2A PE=1 SV=1 |

|  | **Query** | **Observed** | **Mr(expt)** | **Mr(calc)** | **Delta** | **Miss** | **Score** | **Expect** | **Rank** | **Peptide** |
| --- | --- | --- | --- | --- | --- | --- | --- | --- | --- | --- |
|  | 712 | 478.2912 | 477.2840 | 476.2231 | 1.0609 | 0 | 1 | 1.4e+002 | 1 | K.GASDK.C |
|  | 12379 | 723.2534 | 1444.4923 | 1444.5026 | -0.0103 | 0 | 44 | 0.0045 | 1 | K.SCCSCCPVGCAK.C |

| **830.** | Q9H0U3\|MAGT1_HUMAN    **Mass:** 38411    **Score:** 44     **Queries matched:** 10   **emPAI:** 0.10 |
| --- | --- |
|  | Magnesium transporter protein 1 OS=Homo sapiens GN=MAGT1 PE=1 SV=1 |

|  | **Query** | **Observed** | **Mr(expt)** | **Mr(calc)** | **Delta** | **Miss** | **Score** | **Expect** | **Rank** | **Peptide** |
| --- | --- | --- | --- | --- | --- | --- | --- | --- | --- | --- |
|  | 2088 | **397.4031** | **792.7916** | **792.3622** | **0.4294** | **0** | **13** | **6.4** | **8** | **R.QCVVCK.Q** |
|  | 7587 | **593.6136** | **1185.2126** | **1184.6448** | **0.5678** | **1** | **4** | **41** | **9** | **K.RPVIRMNGDK.F** |
|  | 8602 | **618.8162** | **1235.6178** | **1235.6258** | **-0.0081** | **1** | **44** | **0.0063** | **1** | **K.RGDTYELQVR.G** 8632 8634 |
|  | 9538 | 643.3019 | 1284.5893 | 1284.6285 | -0.0391 | 1 | 9 | 19 | 2 | R.RSNMEFLFNK.T |
|  | 9848 | 651.8033 | 1301.5920 | 1300.6234 | 0.9686 | 1 | (4) | 58 | 5 | R.RSNMEFLFNK.T |
|  | 16326 | **853.4764** | **1704.9382** | **1705.8060** | **-0.8678** | **0** | **12** | **6.1** | **1** | **K.QADEEFQILANSWR.Y** |
|  | 17699 | **603.3407** | **1807.0003** | **1806.9451** | **0.0552** | **0** | **1** | **90** | **9** | **R.NYSVIVMFTALQLHR.Q** |
|  | 21000 | **695.3500** | **2083.0281** | **2082.9966** | **0.0316** | **1** | **5** | **26** | **1** | **K.EMVLSEKVSQLMEWTNK.R** |

| **831.** | P13796\|PLSL_HUMAN    **Mass:** 70815    **Score:** 43     **Queries matched:** 15   **emPAI:** 0.05 |
| --- | --- |
|  | Plastin-2 OS=Homo sapiens GN=LCP1 PE=1 SV=5 |

|  | **Query** | **Observed** | **Mr(expt)** | **Mr(calc)** | **Delta** | **Miss** | **Score** | **Expect** | **Rank** | **Peptide** |
| --- | --- | --- | --- | --- | --- | --- | --- | --- | --- | --- |
|  | 1020 | 545.3256 | 544.3183 | 543.3493 | 0.9690 | 1 | 17 | 7 | 1 | R.KIGAR.V |
|  | 4299 | 472.2808 | 942.5471 | 941.5334 | 1.0137 | 0 | 5 | 53 | 7 | R.VNKPPYPK.L |
|  | 6020 | 535.3128 | 1068.6111 | 1068.6179 | -0.0068 | 0 | 9 | 21 | 6 | K.LSPEELLLR.W |
|  | 11024 | **456.8827** | **1367.6264** | **1366.7027** | **0.9238** | **1** | **3** | **61** | **8** | **K.LNNAKYAISMAR.K** |
|  | 11769 | 705.3333 | 1408.6521 | 1408.6656 | -0.0136 | 0 | 7 | 22 | 1 | K.LENCNYAVELGK.N |
|  | 11964 | 474.2282 | 1419.6628 | 1418.6430 | 1.0198 | 0 | 7 | 27 | 10 | K.MVMTVFACLMGK.G |
|  | 13791 | 769.3817 | 1536.7489 | 1536.7606 | -0.0117 | 1 | 43 | 0.0054 | 1 | K.KLENCNYAVELGK.N 13793 |
|  | 16722 | 868.4882 | 1734.9619 | 1734.7999 | 0.1619 | 1 | 10 | 12 | 2 | K.MVMTVFACLMGKGMK.R |
|  | 17047 | **586.6556** | **1756.9451** | **1755.7590** | **1.1861** | **1** | **5** | **34** | **1** | **-.MARGSVSDEEMMELR.E** |
|  | 17615 | **601.3303** | **1800.9691** | **1800.9998** | **-0.0307** | **1** | **2** | **61** | **7** | **R.GNPKLNLAFIANLFNR.Y** |
|  | 18877 | **631.6099** | **1891.8079** | **1892.8422** | **-1.0342** | **0** | **1** | **84** | **5** | **R.EITENLMATGDLDQDGR.I** |
|  | 21653 | 715.4202 | 2143.2389 | 2143.1194 | 0.1195 | 1 | 3 | 36 | 4 | R.NEALIALLREGESLEDLMK.L |
|  | 29606 | **1217.6097** | **3649.8074** | **3650.8191** | **-1.0117** | **1** | **0** | **34** | **5** | **R.NWMNSLGVNPRVNHLYSDLSDALVIFQLYEK.I** |
|  | 29647 | **1826.4380** | **3650.8614** | **3649.9137** | **0.9477** | **1** | **1** | **27** | **2** | **K.KLTPFTIQENLNLALNSASAIGCHVVNIGAEDLK.E** |

| **832.** | Q9HBL7\|CI046_HUMAN    **Mass:** 17190    **Score:** 43     **Queries matched:** 10   **emPAI:** 0.23 |
| --- | --- |
|  | Transmembrane protein C9orf46 OS=Homo sapiens GN=C9orf46 PE=2 SV=1 |

|  | **Query** | **Observed** | **Mr(expt)** | **Mr(calc)** | **Delta** | **Miss** | **Score** | **Expect** | **Rank** | **Peptide** |
| --- | --- | --- | --- | --- | --- | --- | --- | --- | --- | --- |
|  | 5685 | **522.7955** | **1043.5765** | **1042.4576** | **1.1189** | **0** | **3** | **88** | **5** | **K.EFMLMNAR.L** 5673 |
|  | 6910 | 568.2799 | 1134.5453 | 1134.5525 | -0.0073 | 0 | 12 | 9.2 | 3 | R.QLIMQSEMR.E |
|  | 7126 | **576.8106** | **1151.6067** | **1150.5474** | **1.0592** | **0** | **(7)** | **30** | **10** | **R.QLIMQSEMR.E** |
|  | 7359 | 585.8415 | 1169.6685 | 1169.5638 | 0.1047 | 0 | 11 | 13 | 3 | R.GMITFESIEK.A 7360 |
|  | 11576 | 699.8549 | 1397.6952 | 1396.7020 | 0.9931 | 1 | 4 | 58 | 7 | R.GMITFESIEKAR.K |
|  | 13139 | **746.8583** | **1491.7020** | **1491.7126** | **-0.0106** | **1** | **43** | **0.006** | **1** | **R.MKGEAEDILETEK.S** |
|  | 13578 | 761.3937 | 1520.7728 | 1520.7003 | 0.0725 | 1 | 7 | 25 | 6 | M.GFIFSKSMNESMK.N 13579 |

| **833.** | P54886\|P5CS_HUMAN    **Mass:** 87989    **Score:** 43     **Queries matched:** 16   **emPAI:** 0.04 |
| --- | --- |
|  | Delta-1-pyrroline-5-carboxylate synthetase OS=Homo sapiens GN=ALDH18A1 PE=1 SV=2 |

|  | **Query** | **Observed** | **Mr(expt)** | **Mr(calc)** | **Delta** | **Miss** | **Score** | **Expect** | **Rank** | **Peptide** |
| --- | --- | --- | --- | --- | --- | --- | --- | --- | --- | --- |
|  | 2179 | **401.2844** | **800.5542** | **801.4709** | **-0.9167** | **0** | **25** | **0.68** | **1** | **K.LGSAVVTR.G** |
|  | 3392 | 440.2630 | 878.5114 | 878.3990 | 0.1124 | 0 | 6 | 44 | 8 | R.VGMGGMEAK.V |
|  | 5736 | 525.1028 | 1048.1910 | 1048.4495 | -0.2585 | 0 | 4 | 43 | 3 | R.EEVEDLCR.L |
|  | 6957 | **570.2683** | **1138.5221** | **1137.5271** | **0.9950** | **1** | **11** | **12** | **2** | **K.SRVGMGGMEAK.V** 6950 6965 |
|  | 9694 | 431.8719 | 1292.5938 | 1292.6725 | -0.0786 | 0 | 16 | 3.5 | 1 | R.FGLGAEVGISTSR.I |
|  | 12320 | 481.2417 | 1440.7034 | 1439.7153 | 0.9881 | 0 | 4 | 53 | 7 | R.EMMLVTSGAVAFGK.Q 12319 |
|  | 14163 | 781.3863 | 1560.7580 | 1559.7726 | 0.9855 | 1 | 4 | 50 | 5 | R.SGGRMLATLEPEQR.A |
|  | 15675 | **554.2714** | **1659.7924** | **1660.9624** | **-1.1700** | **1** | **3** | **57** | **5** | **R.IHARGPVGLEGLLTTK.W** |
|  | 16426 | 571.6674 | 1711.9804 | 1712.9607 | -0.9803 | 1 | 2 | 70 | 9 | K.MIDLIIPRGSSQLVR.D |
|  | 16787 | **580.9911** | **1739.9514** | **1739.8699** | **0.0816** | **1** | **2** | **61** | **3** | **R.EMMLVTSGAVAFGKQR.L** |
|  | 20405 | **1015.0120** | **2028.0094** | **2028.0204** | **-0.0111** | **0** | **43** | **0.0042** | **1** | **K.LIDIFYPGDQQSVTFGTK.S** |
|  | 24290 | **822.7385** | **2465.1936** | **2464.2057** | **0.9879** | **0** | **0** | **72** | **4** | **K.VGTFFSEVKPAGPTVEQQGEMAR.S** |
|  | 30675 | **1401.6065** | **4201.7975** | **4201.1325** | **0.6650** | **1** | **5** | **6.5** | **1** | **R.MNIVPIVNTNDAVVPPAEPNSDLQGVNVISVKDNDSLAAR.L** |

| **834.** | Q15800\|ERG25_HUMAN    **Mass:** 35592    **Score:** 43     **Queries matched:** 5   **emPAI:** 0.11 |
| --- | --- |
|  | C-4 methylsterol oxidase OS=Homo sapiens GN=SC4MOL PE=1 SV=1 |

|  | **Query** | **Observed** | **Mr(expt)** | **Mr(calc)** | **Delta** | **Miss** | **Score** | **Expect** | **Rank** | **Peptide** |
| --- | --- | --- | --- | --- | --- | --- | --- | --- | --- | --- |
|  | 12608 | 487.2953 | 1458.8641 | 1459.6732 | -0.8090 | 0 | 4 | 57 | 7 | K.DKPETWENQWK.C |
|  | 15522 | 825.3702 | 1648.7259 | 1648.7369 | -0.0110 | 0 | 43 | 0.0052 | 1 | R.IFGTDSQYNAYNEK.R 15510 15527 |
|  | 17995 | **610.3176** | **1827.9311** | **1828.9108** | **-0.9797** | **1** | **4** | **39** | **7** | **K.IQKDKPETWENQWK.C** |

| **835.** | Q58FF8\|H90B2_HUMAN    **Mass:** 44492    **Score:** 43     **Queries matched:** 11   **emPAI:** 0.08 |
| --- | --- |
|  | Putative heat shock protein HSP 90-beta 2 OS=Homo sapiens GN=HSP90AB2P PE=1 SV=2 |

|  | **Query** | **Observed** | **Mr(expt)** | **Mr(calc)** | **Delta** | **Miss** | **Score** | **Expect** | **Rank** | **Peptide** |
| --- | --- | --- | --- | --- | --- | --- | --- | --- | --- | --- |
|  | 2060 | 396.7319 | 791.4493 | 791.4211 | 0.0282 | 1 | 31 | 0.16 | 1 | R.MKEIQK.S 2061 |
|  | 5748 | **525.2686** | **1048.5227** | **1049.5175** | **-0.9949** | **0** | **2** | **1e+002** | **9** | **K.VEMVTVSNR.L** |
|  | 7104 | 576.2781 | 1150.5416 | 1150.5506 | -0.0090 | 0 | 12 | 9.1 | 1 | K.YIDQEELNK.T |
|  | 7239 | 581.2903 | 1160.5661 | 1159.5761 | 0.9900 | 0 | 20 | 1.8 | 1 | K.SIYYITGESK.E |
|  | 7559 | **592.7662** | **1183.5179** | **1183.5543** | **-0.0364** | **0** | **6** | **37** | **10** | **-.MPEEVHLGEK.E** 7568 |
|  | 8741 | 621.8521 | 1241.6897 | 1241.6979 | -0.0083 | 0 | 43 | 0.0066 | 1 | K.ADLINNLGTIAK.F 8742 8756 |
|  | 14052 | **518.6271** | **1552.8594** | **1552.7232** | **0.1363** | **1** | **3** | **56** | **6** | **R.YFSVEEYVSRMK.E** |

| **836.** | Q14568\|HS902_HUMAN    **Mass:** 39454    **Score:** 43     **Queries matched:** 8   **emPAI:** 0.10 |
| --- | --- |
|  | Putative heat shock protein HSP 90-alpha A2 OS=Homo sapiens GN=HSP90AA2 PE=1 SV=2 |

|  | **Query** | **Observed** | **Mr(expt)** | **Mr(calc)** | **Delta** | **Miss** | **Score** | **Expect** | **Rank** | **Peptide** |
| --- | --- | --- | --- | --- | --- | --- | --- | --- | --- | --- |
|  | 1572 | 661.3661 | 660.3588 | 659.4218 | 0.9370 | 0 | 10 | 26 | 1 | K.VTVITK.H |
|  | 7104 | 576.2781 | 1150.5416 | 1150.5506 | -0.0090 | 0 | 12 | 9.1 | 1 | K.YIDQEELNK.T |
|  | 8741 | 621.8521 | 1241.6897 | 1241.6979 | -0.0083 | 0 | 43 | 0.0066 | 1 | K.ADLINNLGTIAK.S 8742 8756 |
|  | 9659 | 646.3174 | 1290.6202 | 1290.6303 | -0.0101 | 0 | 18 | 2.3 | 1 | R.ELISNSSDALDK.I |
|  | 10716 | 675.3210 | 1348.6275 | 1347.6572 | 0.9704 | 0 | 1 | 1.2e+002 | 10 | K.HFSVEGQLEFR.A |
|  | 27161 | **987.3959** | **2959.1658** | **2959.5536** | **-0.3878** | **1** | **0** | **42** | **2** | **K.QDQTLTIVDTGIGMTKADLINNLGTIAK.S** |

| **837.** | Q9BVK6\|TMED9_HUMAN    **Mass:** 27374    **Score:** 43     **Queries matched:** 5   **emPAI:** 0.14 |
| --- | --- |
|  | Transmembrane emp24 domain-containing protein 9 OS=Homo sapiens GN=TMED9 PE=1 SV=2 |

|  | **Query** | **Observed** | **Mr(expt)** | **Mr(calc)** | **Delta** | **Miss** | **Score** | **Expect** | **Rank** | **Peptide** |
| --- | --- | --- | --- | --- | --- | --- | --- | --- | --- | --- |
|  | 1309 | 604.3009 | 603.2936 | 602.2548 | 1.0389 | 0 | 7 | 56 | 7 | K.DPEDK.V |
|  | 6454 | **550.3312** | **1098.6478** | **1097.5692** | **1.0786** | **0** | **9** | **21** | **2** | **K.FSLFAGGMLR.V** |
|  | 6493 | 551.7999 | 1101.5853 | 1100.6189 | 0.9664 | 1 | 12 | 10 | 2 | K.DKLSELQLR.V |
|  | 10591 | 671.3674 | 1340.7202 | 1340.7300 | -0.0098 | 0 | 43 | 0.0067 | 1 | R.QLVEQVEQIQK.E |
|  | 21256 | **702.8624** | **2105.5653** | **2106.2096** | **-0.6443** | **0** | **5** | **22** | **1** | **-.MAVELGVLLVRPRPGTGLGR.V** |

| **838.** | Q15366\|PCBP2_HUMAN    **Mass:** 38955    **Score:** 43     **Queries matched:** 12   **emPAI:** 0.10 |
| --- | --- |
|  | Poly(rC)-binding protein 2 OS=Homo sapiens GN=PCBP2 PE=1 SV=1 |

|  | **Query** | **Observed** | **Mr(expt)** | **Mr(calc)** | **Delta** | **Miss** | **Score** | **Expect** | **Rank** | **Peptide** |
| --- | --- | --- | --- | --- | --- | --- | --- | --- | --- | --- |
|  | 2185 | **401.2846** | **800.5547** | **801.4596** | **-0.9049** | **0** | **9** | **26** | **5** | **K.EVGSIIGK.K** |
|  | 3377 | 439.7605 | 877.5064 | 877.4327 | 0.0736 | 0 | 5 | 64 | 5 | R.QMSGAQIK.I |
|  | 4055 | **463.2443** | **924.4741** | **923.4786** | **0.9955** | **0** | **4** | **56** | **7** | **K.AFAMIIDK.L** |
|  | 5492 | **515.3279** | **1028.6412** | **1027.5774** | **1.0638** | **1** | **12** | **11** | **3** | **R.QGAKINEIR.Q** 5456 |
|  | 9579 | **644.7967** | **1287.5789** | **1287.5877** | **-0.0088** | **0** | **43** | **0.0074** | **1** | **R.INISEGNCPER.I** |
|  | 12095 | **714.3405** | **1426.6665** | **1427.7806** | **-1.1142** | **0** | **10** | **12** | **2** | **R.LVVPASQCGSLIGK.G** |
|  | 12947 | 741.3606 | 1480.7066 | 1480.8436 | -0.1369 | 1 | 5 | 36 | 4 | R.LLMHGKEVGSIIGK.K 12959 |
|  | 18015 | 915.4801 | 1828.9456 | 1828.9427 | 0.0030 | 0 | 1 | 84 | 4 | K.QICVVMLETLSQSPPK.G |
|  | 19230 | 640.3184 | 1917.9333 | 1916.8646 | 1.0686 | 1 | 2 | 72 | 3 | R.EESGARINISEGNCPER.I |
|  | 24396 | **828.4389** | **2482.2949** | **2483.3240** | **-1.0291** | **1** | **2** | **40** | **2** | **-.MDTGVIEGGLNVTLTIRLLMHGK.E** |

| **839.** | P57721\|PCBP3_HUMAN    **Mass:** 36201    **Score:** 43     **Queries matched:** 12   **emPAI:** 0.10 |
| --- | --- |
|  | Poly(rC)-binding protein 3 OS=Homo sapiens GN=PCBP3 PE=1 SV=1 |

|  | **Query** | **Observed** | **Mr(expt)** | **Mr(calc)** | **Delta** | **Miss** | **Score** | **Expect** | **Rank** | **Peptide** |
| --- | --- | --- | --- | --- | --- | --- | --- | --- | --- | --- |
|  | 2185 | 401.2846 | 800.5547 | 801.4596 | -0.9049 | 0 | 9 | 26 | 5 | K.EVGSIIGK.K |
|  | 3377 | 439.7605 | 877.5064 | 877.4327 | 0.0736 | 0 | 5 | 64 | 5 | R.QMSGAQIK.I |
|  | 9579 | 644.7967 | 1287.5789 | 1287.5877 | -0.0088 | 0 | 43 | 0.0074 | 1 | R.INISEGNCPER.I |
|  | 11922 | **473.2566** | **1416.7479** | **1415.7153** | **1.0326** | **0** | **7** | **26** | **3** | **K.QICVVMLESPPK.G** 11899 |
|  | 12095 | 714.3405 | 1426.6665 | 1427.7806 | -1.1142 | 0 | 10 | 12 | 2 | R.LVVPASQCGSLIGK.G |
|  | 12947 | 741.3606 | 1480.7066 | 1480.8436 | -0.1369 | 1 | 5 | 36 | 4 | R.LLMHGKEVGSIIGK.K 12959 |
|  | 17043 | 879.4592 | 1756.9039 | 1756.9506 | -0.0467 | 1 | 3 | 55 | 3 | R.LVVPASQCGSLIGKGGSK.I |
|  | 19230 | 640.3184 | 1917.9333 | 1916.8646 | 1.0686 | 1 | 2 | 72 | 3 | R.EESGARINISEGNCPER.I |
|  | 20713 | **685.3329** | **2052.9770** | **2053.1354** | **-0.1584** | **1** | **1** | **75** | **4** | **K.VSEGGLNVTLTIRLLMHGK.E** |
|  | 28776 | 841.4371 | 3361.7194 | 3362.7755 | -1.0561 | 1 | 3 | 22 | 3 | R.QITITGTPANISLAQYLINARLTSEVTGMGTL.- |

| **840.** | Q15365\|PCBP1_HUMAN    **Mass:** 37987    **Score:** 43     **Queries matched:** 13   **emPAI:** 0.10 |
| --- | --- |
|  | Poly(rC)-binding protein 1 OS=Homo sapiens GN=PCBP1 PE=1 SV=2 |

|  | **Query** | **Observed** | **Mr(expt)** | **Mr(calc)** | **Delta** | **Miss** | **Score** | **Expect** | **Rank** | **Peptide** |
| --- | --- | --- | --- | --- | --- | --- | --- | --- | --- | --- |
|  | 2185 | 401.2846 | 800.5547 | 801.4596 | -0.9049 | 0 | 9 | 26 | 5 | K.EVGSIIGK.K |
|  | 3377 | 439.7605 | 877.5064 | 877.4327 | 0.0736 | 0 | 5 | 64 | 5 | R.QMSGAQIK.I |
|  | 4055 | 463.2443 | 924.4741 | 923.4786 | 0.9955 | 0 | 4 | 56 | 7 | K.AFAMIIDK.L |
|  | 9579 | 644.7967 | 1287.5789 | 1287.5877 | -0.0088 | 0 | 43 | 0.0074 | 1 | R.INISEGNCPER.I |
|  | 11401 | **694.5180** | **1387.0215** | **1387.8075** | **-0.7860** | **0** | **0** | **92** | **10** | **R.IITLTGPTNAIFK.A** |
|  | 12317 | **721.3496** | **1440.6847** | **1441.7963** | **-1.1116** | **0** | **1** | **94** | **4** | **R.LVVPATQCGSLIGK.G** |
|  | 12947 | 741.3606 | 1480.7066 | 1480.8436 | -0.1369 | 1 | 5 | 36 | 4 | R.LLMHGKEVGSIIGK.K 12959 |
|  | 18228 | **615.3787** | **1843.1142** | **1843.9648** | **-0.8507** | **1** | **4** | **37** | **9** | **R.LVVPATQCGSLIGKGGCK.I** |
|  | 19230 | 640.3184 | 1917.9333 | 1916.8646 | 1.0686 | 1 | 2 | 72 | 3 | R.EESGARINISEGNCPER.I |
|  | 24421 | 829.7594 | 2486.2564 | 2487.2825 | -1.0262 | 1 | 2 | 41 | 2 | -.MDAGVTESGLNVTLTIRLLMHGK.E |
|  | 24476 | 830.7631 | 2489.2674 | 2490.1705 | -0.9032 | 0 | 3 | 39 | 2 | R.VMTIPYQPMPASSPVICAGGQDR.C |
|  | 28845 | **845.4277** | **3377.6818** | **3377.6959** | **-0.0141** | **1** | **8** | **7** | **1** | **K.AFAMIIDKLEEDINSSMTNSTAASRPPVTLR.L** |

| **841.** | O14979\|HNRDL_HUMAN    **Mass:** 46580    **Score:** 43     **Queries matched:** 10   **emPAI:** 0.08 |
| --- | --- |
|  | Heterogeneous nuclear ribonucleoprotein D-like OS=Homo sapiens GN=HNRPDL PE=1 SV=3 |

|  | **Query** | **Observed** | **Mr(expt)** | **Mr(calc)** | **Delta** | **Miss** | **Score** | **Expect** | **Rank** | **Peptide** |
| --- | --- | --- | --- | --- | --- | --- | --- | --- | --- | --- |
|  | 3548 | **445.5668** | **889.1190** | **888.4454** | **0.6736** | **0** | **3** | **66** | **9** | **R.YHQIGSGK.C** |
|  | 4130 | **466.2268** | **930.4391** | **929.4791** | **0.9599** | **1** | **5** | **66** | **4** | **R.GAAAGGRGGTR.G** |
|  | 5040 | 498.7649 | 995.5153 | 994.5811 | 0.9342 | 1 | 16 | 3.9 | 4 | K.VLELKEHK.L |
|  | 5086 | 998.5455 | 997.5382 | 997.5808 | -0.0426 | 1 | 8 | 26 | 6 | K.LDGKLIDPK.R |
|  | 7302 | 583.7759 | 1165.5372 | 1166.5642 | -1.0270 | 0 | 20 | 1.3 | 2 | R.FGEVVDCTIK.T 7323 |
|  | 13009 | **495.7555** | **1484.2446** | **1484.7334** | **-0.4888** | **1** | **1** | **75** | **5** | **K.MFIGGLSWDTSKK.D** |
|  | 13556 | **507.2891** | **1518.8454** | **1519.8722** | **-1.0268** | **0** | **0** | **1.2e+002** | **9** | **R.QLAPLLPSLAPSSAR.Q** |
|  | 16324 | 853.4551 | 1704.8956 | 1704.8570 | 0.0386 | 0 | 7 | 21 | 5 | K.VFVGGLSPDTSEEQIK.E |
|  | 30229 | **1297.2859** | **3888.8358** | **3888.8655** | **-0.0297** | **1** | **43** | **0.0016** | **1** | **K.VFVGGLSPDTSEEQIKEYFGAFGEIENIELPMDTK.T** |

| **842.** | P36873\|PP1G_HUMAN    **Mass:** 37701    **Score:** 43     **Queries matched:** 6   **emPAI:** 0.10 |
| --- | --- |
|  | Serine/threonine-protein phosphatase PP1-gamma catalytic subunit OS=Homo sapiens GN=PPP1CC PE=1 SV=1 |

|  | **Query** | **Observed** | **Mr(expt)** | **Mr(calc)** | **Delta** | **Miss** | **Score** | **Expect** | **Rank** | **Peptide** |
| --- | --- | --- | --- | --- | --- | --- | --- | --- | --- | --- |
|  | 3587 | **446.7578** | **891.5011** | **891.4848** | **0.0163** | **1** | **8** | **28** | **10** | **R.GMITKQAK.K** |
|  | 7232 | **581.2639** | **1160.5131** | **1159.5258** | **0.9873** | **0** | **10** | **18** | **3** | **K.DVLGWGENDR.G** |
|  | 10470 | 667.9755 | 1333.9365 | 1332.7626 | 1.1739 | 0 | 4 | 39 | 1 | K.KPNATRPVTPPR.G |
|  | 12300 | 480.6037 | 1438.7892 | 1438.7972 | -0.0081 | 1 | 43 | 0.0066 | 1 | K.IKYPENFFLLR.G |
|  | 14934 | 538.7795 | 1613.3168 | 1612.8784 | 0.4384 | 1 | 5 | 25 | 1 | M.ADLDKLNIDSIIQR.L |
|  | 18530 | 621.9794 | 1862.9165 | 1863.0513 | -0.1348 | 1 | 11 | 8.5 | 2 | K.KPNATRPVTPPRGMITK.Q |

| **843.** | P62140\|PP1B_HUMAN    **Mass:** 37961    **Score:** 43     **Queries matched:** 5   **emPAI:** 0.10 |
| --- | --- |
|  | Serine/threonine-protein phosphatase PP1-beta catalytic subunit OS=Homo sapiens GN=PPP1CB PE=1 SV=3 |

|  | **Query** | **Observed** | **Mr(expt)** | **Mr(calc)** | **Delta** | **Miss** | **Score** | **Expect** | **Rank** | **Peptide** |
| --- | --- | --- | --- | --- | --- | --- | --- | --- | --- | --- |
|  | 7702 | 596.2888 | 1190.5630 | 1190.5965 | -0.0335 | 0 | 4 | 60 | 5 | K.IVQMTEAEVR.G |
|  | 11626 | **468.2030** | **1401.5871** | **1401.7100** | **-0.1229** | **0** | **9** | **16** | **3** | **M.ADGELNVDSLITR.L** 11641 |
|  | 12300 | 480.6037 | 1438.7892 | 1438.7972 | -0.0081 | 1 | 43 | 0.0066 | 1 | K.IKYPENFFLLR.G |
|  | 20230 | **671.5046** | **2011.4919** | **2012.0902** | **-0.5983** | **1** | **0** | **58** | **7** | **M.ADGELNVDSLITRLLEVR.G** |

| **844.** | P62136\|PP1A_HUMAN    **Mass:** 38229    **Score:** 43     **Queries matched:** 2   **emPAI:** 0.10 |
| --- | --- |
|  | Serine/threonine-protein phosphatase PP1-alpha catalytic subunit OS=Homo sapiens GN=PPP1CA PE=1 SV=1 |

|  | **Query** | **Observed** | **Mr(expt)** | **Mr(calc)** | **Delta** | **Miss** | **Score** | **Expect** | **Rank** | **Peptide** |
| --- | --- | --- | --- | --- | --- | --- | --- | --- | --- | --- |
|  | 12300 | 480.6037 | 1438.7892 | 1438.7972 | -0.0081 | 1 | 43 | 0.0066 | 1 | K.IKYPENFFLLR.G |
|  | 13935 | 516.3547 | 1546.0424 | 1545.7998 | 0.2426 | 1 | 0 | 79 | 9 | M.SDSEKLNLDSIIGR.L |

| **845.** | P08237\|K6PF_HUMAN    **Mass:** 85984    **Score:** 43     **Queries matched:** 18   **emPAI:** 0.04 |
| --- | --- |
|  | 6-phosphofructokinase, muscle type OS=Homo sapiens GN=PFKM PE=1 SV=2 |

|  | **Query** | **Observed** | **Mr(expt)** | **Mr(calc)** | **Delta** | **Miss** | **Score** | **Expect** | **Rank** | **Peptide** |
| --- | --- | --- | --- | --- | --- | --- | --- | --- | --- | --- |
|  | 1027 | 546.2509 | 545.2436 | 544.3333 | 0.9104 | 0 | 7 | 72 | 5 | R.ILGSR.M |
|  | 2448 | **411.2324** | **820.4503** | **819.4603** | **0.9901** | **0** | **9** | **25** | **6** | **R.VGIFTGAR.V** |
|  | 3128 | **432.3089** | **862.6032** | **861.4668** | **1.1364** | **1** | **4** | **77** | **8** | **R.LSETRTR.G** |
|  | 4879 | 492.7398 | 983.4650 | 982.4906 | 0.9744 | 1 | 4 | 57 | 8 | R.NFATKMGAK.A |
|  | 5779 | 526.2806 | 1050.5467 | 1050.5532 | -0.0065 | 0 | 43 | 0.0081 | 1 | R.TFVLEVMGR.H |
|  | 7297 | **583.7281** | **1165.4416** | **1164.5783** | **0.8633** | **1** | **5** | **46** | **6** | **K.AMNWMSGKIK.E** |
|  | 7882 | **600.8184** | **1199.6223** | **1200.6350** | **-1.0127** | **0** | **2** | **99** | **2** | **K.NGKPITSEDIK.N** |
|  | 10987 | 683.3678 | 1364.7210 | 1364.7299 | -0.0089 | 1 | 5 | 42 | 7 | K.KSFEQISANITK.F |
|  | 11337 | 693.3088 | 1384.6030 | 1384.6735 | -0.0705 | 1 | 5 | 41 | 4 | K.DQTDFEHRIPK.E |
|  | 17261 | 592.2831 | 1773.8276 | 1774.8784 | -1.0508 | 1 | 1 | 90 | 9 | -.MTHEEHHAAKTLGIGK.A |
|  | 17610 | **601.3029** | **1800.8868** | **1800.9152** | **-0.0285** | **0** | **3** | **55** | **6** | **K.AIAVLTSGGDAQGMNAAVR.A** |
|  | 18181 | 614.5689 | 1840.6847 | 1839.8686 | 0.8161 | 0 | 3 | 45 | 2 | K.NVLGHMQQGGSPTPFDR.N |
|  | 19168 | 638.6595 | 1912.9566 | 1912.9677 | -0.0110 | 0 | 9 | 12 | 5 | R.IMEIVDAITTTAQSHQR.T |
|  | 21936 | 723.3474 | 2167.0202 | 2167.0626 | -0.0424 | 0 | 3 | 38 | 3 | K.SGSHTVAVMNVGAPAAGMNAAVR.S |
|  | 22196 | **734.3701** | **2200.0885** | **2199.0525** | **1.0361** | **0** | **(0)** | **79** | **2** | **K.SGSHTVAVMNVGAPAAGMNAAVR.S** |
|  | 23858 | **806.5664** | **2416.6774** | **2417.1546** | **-0.4772** | **1** | **0** | **49** | **2** | **K.NVLGHMQQGGSPTPFDRNFATK.M** |
|  | 28857 | **846.1389** | **3380.5263** | **3380.7922** | **-0.2659** | **1** | **2** | **23** | **1** | **K.LLAHVRPPVSKSGSHTVAVMNVGAPAAGMNAAVR.S** |
|  | 29498 | **603.3030** | **3613.7742** | **3613.8266** | **-0.0524** | **1** | **1** | **29** | **7** | **R.ILGSRMGVEAVMALLEGTPDTPACVVSLSGNQAVR.L** |

| **846.** | P63000\|RAC1_HUMAN    **Mass:** 21835    **Score:** 43     **Queries matched:** 11   **emPAI:** 0.18 |
| --- | --- |
|  | Ras-related C3 botulinum toxin substrate 1 OS=Homo sapiens GN=RAC1 PE=1 SV=1 |

|  | **Query** | **Observed** | **Mr(expt)** | **Mr(calc)** | **Delta** | **Miss** | **Score** | **Expect** | **Rank** | **Peptide** |
| --- | --- | --- | --- | --- | --- | --- | --- | --- | --- | --- |
|  | 4826 | **490.7530** | **979.4914** | **979.5525** | **-0.0610** | **0** | **10** | **15** | **4** | **R.AVLCPPPVK.K** 4830 |
|  | 8709 | 621.2919 | 1240.5693 | 1239.5918 | 0.9776 | 0 | 43 | 0.0077 | 1 | K.YLECSALTQR.G 8718 |
|  | 14535 | **529.6168** | **1585.8286** | **1585.8399** | **-0.0113** | **0** | **23** | **0.6** | **1** | **R.HHCPNTPIILVGTK.L** 14527 |
|  | 15266 | 544.9023 | 1631.6850 | 1630.9116 | 0.7734 | 1 | 3 | 58 | 7 | K.KLTPITYPQGLAMAK.E 15221 |
|  | 15272 | **408.9297** | **1631.6895** | **1630.8535** | **0.8360** | **1** | **8** | **19** | **9** | **-.MQAIKCVVVGDGAVGK.T** 15245 |
|  | 15482 | 823.9026 | 1645.7907 | 1646.8484 | -1.0577 | 1 | (3) | 57 | 8 | -.MQAIKCVVVGDGAVGK.T |

| **847.** | P60763\|RAC3_HUMAN    **Mass:** 21764    **Score:** 43     **Queries matched:** 9   **emPAI:** 0.18 |
| --- | --- |
|  | Ras-related C3 botulinum toxin substrate 3 OS=Homo sapiens GN=RAC3 PE=1 SV=1 |

|  | **Query** | **Observed** | **Mr(expt)** | **Mr(calc)** | **Delta** | **Miss** | **Score** | **Expect** | **Rank** | **Peptide** |
| --- | --- | --- | --- | --- | --- | --- | --- | --- | --- | --- |
|  | 3752 | 452.2244 | 902.4343 | 901.4981 | 0.9362 | 1 | 10 | 21 | 9 | K.DTIERLR.D |
|  | 4826 | 490.7530 | 979.4914 | 979.5525 | -0.0610 | 0 | 10 | 15 | 4 | R.AVLCPPPVK.K 4830 |
|  | 8709 | 621.2919 | 1240.5693 | 1239.5918 | 0.9776 | 0 | 43 | 0.0077 | 1 | K.YLECSALTQR.G 8718 |
|  | 15272 | 408.9297 | 1631.6895 | 1630.8535 | 0.8360 | 1 | 8 | 19 | 9 | -.MQAIKCVVVGDGAVGK.T 15245 |
|  | 15482 | 823.9026 | 1645.7907 | 1646.8484 | -1.0577 | 1 | (3) | 57 | 8 | -.MQAIKCVVVGDGAVGK.T |
|  | 18384 | **618.8524** | **1853.5353** | **1852.9353** | **0.6000** | **1** | **2** | **48** | **4** | **R.EIGSVKYLECSALTQR.G** |

| **848.** | P15153\|RAC2_HUMAN    **Mass:** 21814    **Score:** 43     **Queries matched:** 7   **emPAI:** 0.18 |
| --- | --- |
|  | Ras-related C3 botulinum toxin substrate 2 OS=Homo sapiens GN=RAC2 PE=1 SV=1 |

|  | **Query** | **Observed** | **Mr(expt)** | **Mr(calc)** | **Delta** | **Miss** | **Score** | **Expect** | **Rank** | **Peptide** |
| --- | --- | --- | --- | --- | --- | --- | --- | --- | --- | --- |
|  | 5539 | 517.2714 | 1032.5282 | 1031.5552 | 0.9729 | 1 | 5 | 63 | 9 | R.AKWFPEVR.H |
|  | 8709 | 621.2919 | 1240.5693 | 1239.5918 | 0.9776 | 0 | 43 | 0.0077 | 1 | K.YLECSALTQR.G 8718 |
|  | 12524 | 728.3299 | 1454.6452 | 1454.8497 | -0.2044 | 0 | 9 | 17 | 8 | K.LAPITYPQGLALAK.E |
|  | 15272 | 408.9297 | 1631.6895 | 1630.8535 | 0.8360 | 1 | 8 | 19 | 9 | -.MQAIKCVVVGDGAVGK.T 15245 |
|  | 15482 | 823.9026 | 1645.7907 | 1646.8484 | -1.0577 | 1 | (3) | 57 | 8 | -.MQAIKCVVVGDGAVGK.T |

| **849.** | P16520\|GBB3_HUMAN    **Mass:** 38166    **Score:** 42     **Queries matched:** 14   **emPAI:** 0.21 |
| --- | --- |
|  | Guanine nucleotide-binding protein G(I)/G(S)/G(T) subunit beta-3 OS=Homo sapiens GN=GNB3 PE=1 SV=1 |

|  | **Query** | **Observed** | **Mr(expt)** | **Mr(calc)** | **Delta** | **Miss** | **Score** | **Expect** | **Rank** | **Peptide** |
| --- | --- | --- | --- | --- | --- | --- | --- | --- | --- | --- |
|  | 2250 | 805.4991 | 804.4918 | 804.4970 | -0.0052 | 0 | 42 | 0.012 | 1 | K.VHAIPLR.S 2218 2233 2237 2244 2251 2258 |
|  | 5329 | 509.2790 | 1016.5434 | 1016.5502 | -0.0068 | 0 | 30 | 0.17 | 1 | K.LLVSASQDGK.L 5328 |
|  | 10548 | 670.3294 | 1338.6443 | 1337.6074 | 1.0369 | 0 | 2 | 84 | 8 | K.IYAMHWATDSK.L |
|  | 16298 | 568.7846 | 1703.3320 | 1703.8148 | -0.4828 | 1 | 4 | 34 | 4 | M.GEMEQLRQEAEQLK.K |
|  | 18337 | **617.8169** | **1850.4288** | **1850.8502** | **-0.4214** | **1** | **2** | **46** | **6** | **-.MGEMEQLRQEAEQLK.K** |
|  | 23284 | 1168.5649 | 2335.1153 | 2336.1471 | -1.0317 | 1 | 8 | 12 | 2 | K.IYAMHWATDSKLLVSASQDGK.L 23285 |

| **850.** | O60664\|M6PBP_HUMAN    **Mass:** 47189    **Score:** 42     **Queries matched:** 10   **emPAI:** 0.08 |
| --- | --- |
|  | Mannose-6-phosphate receptor-binding protein 1 OS=Homo sapiens GN=M6PRBP1 PE=1 SV=2 |

|  | **Query** | **Observed** | **Mr(expt)** | **Mr(calc)** | **Delta** | **Miss** | **Score** | **Expect** | **Rank** | **Peptide** |
| --- | --- | --- | --- | --- | --- | --- | --- | --- | --- | --- |
|  | 5500 | **515.7661** | **1029.5177** | **1029.5931** | **-0.0754** | **1** | **14** | **7.6** | **2** | **R.LGSLSERLR.Q** |
|  | 5829 | 528.7550 | 1055.4954 | 1055.5036 | -0.0082 | 0 | 14 | 6.1 | 1 | R.QEQSYFVR.L 5828 |
|  | 9643 | **645.8396** | **1289.6646** | **1288.7238** | **0.9408** | **1** | **8** | **24** | **1** | **K.VLADTKELVSSK.V** 9596 |
|  | 13661 | 764.4358 | 1526.8571 | 1526.8668 | -0.0096 | 0 | 20 | 1.1 | 2 | R.TLTAAAVSGAQPILSK.L |
|  | 18096 | **917.9631** | **1833.9117** | **1833.9221** | **-0.0104** | **0** | **42** | **0.0069** | **1** | **R.IATSLDGFDVASVQQQR.Q** |
|  | 18365 | **618.3343** | **1851.9812** | **1851.9248** | **0.0564** | **1** | **4** | **44** | **1** | **K.ELVSSKVSGAQEMVSSAK.D** 18338 |
|  | 28117 | **794.9008** | **3175.5742** | **3175.4875** | **0.0866** | **1** | **0** | **44** | **5** | **R.VASMPLISSTCDMVSAAYASTKESYPHIK.T** |

| **851.** | Q93050\|VPP1_HUMAN    **Mass:** 97148    **Score:** 42     **Queries matched:** 12   **emPAI:** 0.12 |
| --- | --- |
|  | V-type proton ATPase 116 kDa subunit a isoform 1 OS=Homo sapiens GN=ATP6V0A1 PE=1 SV=3 |

|  | **Query** | **Observed** | **Mr(expt)** | **Mr(calc)** | **Delta** | **Miss** | **Score** | **Expect** | **Rank** | **Peptide** |
| --- | --- | --- | --- | --- | --- | --- | --- | --- | --- | --- |
|  | 1707 | **700.4304** | **699.4231** | **699.4279** | **-0.0048** | **0** | **8** | **34** | **6** | **R.VLQAAAK.N** |
|  | 2719 | **419.7763** | **837.5379** | **838.2949** | **-0.7570** | **0** | **15** | **4.9** | **2** | **R.CEEMDR.K** |
|  | 4660 | **485.2237** | **968.4328** | **968.5331** | **-0.1003** | **0** | **6** | **33** | **10** | **K.LTFLNSFK.M** |
|  | 4837 | **491.2581** | **980.5016** | **979.4393** | **1.0623** | **0** | **23** | **0.68** | **1** | **K.EMASGVNTR.I** |
|  | 5708 | **523.7940** | **1045.5735** | **1044.6080** | **0.9655** | **0** | **29** | **0.21** | **1** | **R.LGFVAGVINR.E** |
|  | 6230 | **542.1294** | **1082.2442** | **1082.6560** | **-0.4118** | **1** | **9** | **11** | **4** | **R.VLQAAAKNIR.V** |
|  | 9100 | 631.8076 | 1261.6007 | 1261.7030 | -0.1023 | 1 | 5 | 53 | 8 | K.RNFLELTELK.F |
|  | 12011 | **711.8331** | **1421.6517** | **1421.6609** | **-0.0092** | **0** | **45** | **0.0042** | **1** | **R.MQTNQTPPTYNK.T** |
|  | 24596 | **835.7916** | **2504.3529** | **2505.2396** | **-0.8868** | **0** | **1** | **52** | **3** | **K.CLIAEVWCPVTDLDSIQFALR.R** |
|  | 26200 | **922.4430** | **2764.3071** | **2763.3362** | **0.9709** | **0** | **1** | **44** | **1** | **R.YIILLMGVFSMYTGLIYNDCFSK.S** |
|  | 27218 | 992.1266 | 2973.3579 | 2973.4250 | -0.0671 | 1 | 2 | 32 | 10 | R.GTEHSGSTVPSILNRMQTNQTPPTYNK.T |
|  | 31317 | **1220.5714** | **4878.2565** | **4878.4780** | **-0.2215** | **1** | **0** | **14** | **1** | **R.EINPAPYTIITFPFLFAVMFGDFGHGILMTLFAVWMVLRESR.I** |

| **852.** | Q10588\|BST1_HUMAN    **Mass:** 36328    **Score:** 42     **Queries matched:** 5   **emPAI:** 0.10 |
| --- | --- |
|  | ADP-ribosyl cyclase 2 OS=Homo sapiens GN=BST1 PE=1 SV=2 |

|  | **Query** | **Observed** | **Mr(expt)** | **Mr(calc)** | **Delta** | **Miss** | **Score** | **Expect** | **Rank** | **Peptide** |
| --- | --- | --- | --- | --- | --- | --- | --- | --- | --- | --- |
|  | 3901 | 457.7659 | 913.5172 | 912.5029 | 1.0144 | 0 | 13 | 8.1 | 1 | R.ALLSPEQR.N |
|  | 5388 | 511.7034 | 1021.3922 | 1021.4433 | -0.0511 | 0 | (4) | 69 | 3 | -.MAAQGCAASR.L |
|  | 5598 | 519.2594 | 1036.5042 | 1037.4382 | -0.9340 | 0 | 7 | 34 | 3 | -.MAAQGCAASR.L |
|  | 13853 | **771.3810** | **1540.7475** | **1540.7562** | **-0.0087** | **0** | **42** | **0.0077** | **1** | **K.GFFADYEIPNLQK.E** |
|  | 27583 | 1015.5240 | 3043.5501 | 3043.4453 | 0.1048 | 1 | 3 | 27 | 7 | R.IEIWVMHEIGGPNVESCGEGSMKVLEK.R |

| **853.** | P31146\|COR1A_HUMAN    **Mass:** 51678    **Score:** 42     **Queries matched:** 6   **emPAI:** 0.07 |
| --- | --- |
|  | Coronin-1A OS=Homo sapiens GN=CORO1A PE=1 SV=4 |

|  | **Query** | **Observed** | **Mr(expt)** | **Mr(calc)** | **Delta** | **Miss** | **Score** | **Expect** | **Rank** | **Peptide** |
| --- | --- | --- | --- | --- | --- | --- | --- | --- | --- | --- |
|  | 3613 | **448.2135** | **894.4124** | **893.4971** | **0.9154** | **0** | **12** | **9** | **1** | **K.ILTTGFSR.M** |
|  | 4701 | **486.5134** | **971.0123** | **970.4364** | **0.5759** | **1** | **3** | **56** | **9** | **R.GMGYMPKR.G** |
|  | 10644 | 672.8370 | 1343.6594 | 1344.6642 | -1.0048 | 1 | 2 | 79 | 7 | R.RCEPIAMTVPR.K |
|  | 12908 | 740.3431 | 1478.6716 | 1478.7188 | -0.0471 | 1 | 3 | 58 | 5 | R.MSERQVALWDTK.H |
|  | 13315 | **752.4139** | **1502.8132** | **1501.7009** | **1.1124** | **0** | **12** | **8.1** | **1** | **R.AAPEASGTPSSDAVSR.L** |
|  | 30171 | **1282.9706** | **3845.8899** | **3845.9074** | **-0.0175** | **0** | **42** | **0.0021** | **1** | **K.HLEEPLSLQELDTSSGVLLPFFDPDTNIVYLCGK.G** |

| **854.** | P10412\|H14_HUMAN    **Mass:** 21852    **Score:** 42     **Queries matched:** 10   **emPAI:** 0.38 |
| --- | --- |
|  | Histone H1.4 OS=Homo sapiens GN=HIST1H1E PE=1 SV=2 |

|  | **Query** | **Observed** | **Mr(expt)** | **Mr(calc)** | **Delta** | **Miss** | **Score** | **Expect** | **Rank** | **Peptide** |
| --- | --- | --- | --- | --- | --- | --- | --- | --- | --- | --- |
|  | 1477 | 642.7714 | 641.7641 | 641.3860 | 0.3781 | 0 | 15 | 3.2 | 2 | K.KPAGAAK.K |
|  | 1762 | **714.3370** | **713.3298** | **712.4595** | **0.8702** | **1** | **8** | **27** | **8** | **K.AKAAKPK.K** |
|  | 2322 | 811.3892 | 810.3819 | 810.3872 | -0.0053 | 0 | 27 | 0.28 | 1 | K.GTGASGSFK.L |
|  | 2814 | 423.2552 | 844.4958 | 843.4814 | 1.0144 | 1 | 12 | 13 | 4 | K.KATGAATPK.K |
|  | 2815 | **423.2557** | **844.4968** | **844.5018** | **-0.0050** | **0** | **41** | **0.016** | **1** | **R.SGVSLAALK.K** |
|  | 4728 | **487.3022** | **972.5899** | **972.5968** | **-0.0069** | **1** | **18** | **2.9** | **2** | **R.SGVSLAALKK.A** |
|  | 4870 | 492.2818 | 982.5490 | 982.5923 | -0.0433 | 1 | 9 | 21 | 4 | K.AKKPAAAAGAK.K |
|  | 6557 | 554.2840 | 1106.5534 | 1106.5608 | -0.0073 | 0 | 11 | 14 | 2 | K.ALAAAGYDVEK.N |
|  | 8587 | 618.3439 | 1234.6732 | 1234.6557 | 0.0175 | 1 | 2 | 92 | 6 | K.KALAAAGYDVEK.N |
|  | 13800 | 513.2672 | 1536.7796 | 1537.8100 | -1.0304 | 1 | 1 | 1e+002 | 8 | K.GTLVQTKGTGASGSFK.L |

| **855.** | P16403\|H12_HUMAN    **Mass:** 21352    **Score:** 42     **Queries matched:** 12   **emPAI:** 0.39 |
| --- | --- |
|  | Histone H1.2 OS=Homo sapiens GN=HIST1H1C PE=1 SV=2 |

|  | **Query** | **Observed** | **Mr(expt)** | **Mr(calc)** | **Delta** | **Miss** | **Score** | **Expect** | **Rank** | **Peptide** |
| --- | --- | --- | --- | --- | --- | --- | --- | --- | --- | --- |
|  | 1502 | **647.5560** | **646.5487** | **645.3809** | **1.1678** | **1** | **2** | **1.4e+002** | **4** | **K.AAKSAAK.A** |
|  | 1559 | **658.7995** | **657.7922** | **657.3810** | **0.4113** | **0** | **9** | **22** | **9** | **K.AGGTKPK.K** |
|  | 2168 | **400.7689** | **799.5232** | **799.4552** | **0.0680** | **1** | **11** | **15** | **2** | **K.KAAGGATPK.K** |
|  | 2322 | 811.3892 | 810.3819 | 810.3872 | -0.0053 | 0 | 27 | 0.28 | 1 | K.GTGASGSFK.L |
|  | 2815 | 423.2557 | 844.4968 | 844.5018 | -0.0050 | 0 | 41 | 0.016 | 1 | R.SGVSLAALK.K |
|  | 4728 | 487.3022 | 972.5899 | 972.5968 | -0.0069 | 1 | 18 | 2.9 | 2 | R.SGVSLAALKK.A |
|  | 5393 | 512.2444 | 1022.4742 | 1022.6600 | -0.1858 | 1 | 8 | 24 | 9 | K.KPVGAAKKPK.K 5417 |
|  | 6557 | 554.2840 | 1106.5534 | 1106.5608 | -0.0073 | 0 | 11 | 14 | 2 | K.ALAAAGYDVEK.N |
|  | 8587 | 618.3439 | 1234.6732 | 1234.6557 | 0.0175 | 1 | 2 | 92 | 6 | K.KALAAAGYDVEK.N |
|  | 9980 | 437.2477 | 1308.7213 | 1308.7878 | -0.0664 | 1 | 6 | 33 | 8 | K.AGGTKPKKPVGAAK.K |
|  | 13800 | 513.2672 | 1536.7796 | 1537.8100 | -1.0304 | 1 | 1 | 1e+002 | 8 | K.GTLVQTKGTGASGSFK.L |

| **856.** | P16402\|H13_HUMAN    **Mass:** 22336    **Score:** 42     **Queries matched:** 14   **emPAI:** 0.37 |
| --- | --- |
|  | Histone H1.3 OS=Homo sapiens GN=HIST1H1D PE=1 SV=2 |

|  | **Query** | **Observed** | **Mr(expt)** | **Mr(calc)** | **Delta** | **Miss** | **Score** | **Expect** | **Rank** | **Peptide** |
| --- | --- | --- | --- | --- | --- | --- | --- | --- | --- | --- |
|  | 1477 | 642.7714 | 641.7641 | 641.3860 | 0.3781 | 0 | 15 | 3.2 | 2 | R.KPAGAAK.K |
|  | 2131 | **399.2687** | **796.5229** | **797.4871** | **-0.9643** | **1** | **2** | **88** | **5** | **K.KAGAAKPR.K** |
|  | 2322 | 811.3892 | 810.3819 | 810.3872 | -0.0053 | 0 | 27 | 0.28 | 1 | K.GTGASGSFK.L |
|  | 2771 | **421.7556** | **841.4965** | **841.5021** | **-0.0056** | **1** | **13** | **7.4** | **2** | **K.VAGAATPKK.S** 2772 2782 |
|  | 2815 | 423.2557 | 844.4968 | 844.5018 | -0.0050 | 0 | 41 | 0.016 | 1 | R.SGVSLAALK.K |
|  | 4725 | **487.2957** | **972.5769** | **971.5400** | **1.0369** | **1** | **13** | **10** | **2** | **K.KAASGEGKPK.A** 4684 4732 |
|  | 4728 | 487.3022 | 972.5899 | 972.5968 | -0.0069 | 1 | 18 | 2.9 | 2 | R.SGVSLAALKK.A |
|  | 6557 | 554.2840 | 1106.5534 | 1106.5608 | -0.0073 | 0 | 11 | 14 | 2 | K.ALAAAGYDVEK.N |
|  | 8587 | 618.3439 | 1234.6732 | 1234.6557 | 0.0175 | 1 | 2 | 92 | 6 | K.KALAAAGYDVEK.N |
|  | 13800 | 513.2672 | 1536.7796 | 1537.8100 | -1.0304 | 1 | 1 | 1e+002 | 8 | K.GTLVQTKGTGASGSFK.L |

| **857.** | Q969Q0\|RL36L_HUMAN    **Mass:** 12746    **Score:** 42     **Queries matched:** 9   **emPAI:** 0.71 |
| --- | --- |
|  | 60S ribosomal protein L36a-like OS=Homo sapiens GN=RPL36AL PE=1 SV=3 |

|  | **Query** | **Observed** | **Mr(expt)** | **Mr(calc)** | **Delta** | **Miss** | **Score** | **Expect** | **Rank** | **Peptide** |
| --- | --- | --- | --- | --- | --- | --- | --- | --- | --- | --- |
|  | 1042 | 547.2689 | 546.2616 | 547.3329 | -1.0714 | 1 | 7 | 63 | 8 | K.AKTTK.K |
|  | 1418 | **629.3939** | **628.3866** | **627.4432** | **0.9434** | **1** | **3** | **1.1e+002** | **3** | **K.KIVLR.L** |
|  | 3734 | **451.7189** | **901.4232** | **901.4294** | **-0.0061** | **0** | **37** | **0.042** | **1** | **K.HFELGGDK.K** 3733 |
|  | 4558 | 480.7983 | 959.5821 | 959.5222 | 0.0599 | 1 | 11 | 16 | 6 | -.MVNVPKTR.R |
|  | 5507 | 516.2606 | 1030.5067 | 1029.5243 | 0.9823 | 1 | 4 | 66 | 6 | K.HFELGGDKK.R |
|  | 6390 | **547.7793** | **1093.5440** | **1093.5516** | **-0.0076** | **1** | **39** | **0.02** | **1** | **K.GKDSLYAQGR.R** |
|  | 7458 | **589.3288** | **1176.6430** | **1175.5063** | **1.1367** | **0** | **6** | **41** | **2** | **R.LECVEPNCR.S** |
|  | 9140 | 632.8073 | 1263.6001 | 1264.6676 | -1.0676 | 1 | 4 | 60 | 8 | K.HQPHKVTQYK.K |

|  | **Proteins matching a subset of these peptides:** |
| --- | --- |

|  | P83881\|RL36A_HUMAN    **Mass:** 12718    **Score:** 36     **Queries matched:** 8 |
| --- | --- |
|  | 60S ribosomal protein L36a OS=Homo sapiens GN=RPL36A PE=1 SV=2 |

| **858.** | Q9Y3I0\|CV028_HUMAN    **Mass:** 55688    **Score:** 42     **Queries matched:** 12   **emPAI:** 0.07 |
| --- | --- |
|  | UPF0027 protein C22orf28 OS=Homo sapiens GN=C22orf28 PE=1 SV=1 |

|  | **Query** | **Observed** | **Mr(expt)** | **Mr(calc)** | **Delta** | **Miss** | **Score** | **Expect** | **Rank** | **Peptide** |
| --- | --- | --- | --- | --- | --- | --- | --- | --- | --- | --- |
|  | 1042 | 547.2689 | 546.2616 | 547.3078 | -1.0462 | 1 | 6 | 78 | 9 | R.KGSTR.A |
|  | 2574 | 415.2402 | 828.4658 | 827.4323 | 1.0335 | 1 | 8 | 35 | 8 | K.KMGIDHK.G 2523 |
|  | 4768 | **488.3566** | **974.6986** | **974.5219** | **0.1768** | **0** | **1** | **1.2e+002** | **10** | **K.LADMGIAIR.V** |
|  | 10365 | 443.7359 | 1328.1860 | 1328.6870 | -0.5011 | 1 | 8 | 14 | 5 | R.MLQADPNKVSAR.A |
|  | 10637 | **448.7297** | **1343.1674** | **1343.6569** | **-0.4895** | **0** | **4** | **38** | **8** | **R.TNLDESDVQPVK.E** |
|  | 10681 | 673.7961 | 1345.5777 | 1345.6231 | -0.0454 | 0 | 10 | 15 | 1 | K.GQVCVMIHSGSR.G 10686 |
|  | 12568 | **486.7997** | **1457.3774** | **1456.8072** | **0.5702** | **1** | **2** | **51** | **4** | **K.LADMGIAIRVASPK.L** |
|  | 15380 | 820.4205 | 1638.8264 | 1638.8399 | -0.0135 | 0 | 42 | 0.008 | 1 | R.GLGHQVATDALVAMEK.A |
|  | 19809 | 985.5160 | 1969.0175 | 1969.0125 | 0.0051 | 1 | 1 | 78 | 4 | R.GLGHQVATDALVAMEKAMK.R |
|  | 28728 | 837.4211 | 3345.6552 | 3344.7041 | 0.9511 | 1 | 6 | 12 | 2 | R.GLPQLGTLGAGNHYAEIQVVDEIFNEYAAKK.M |

| **859.** | Q9NUU6\|F105A_HUMAN    **Mass:** 42568    **Score:** 41     **Queries matched:** 7   **emPAI:** 0.09 |
| --- | --- |
|  | Protein FAM105A OS=Homo sapiens GN=FAM105A PE=2 SV=1 |

|  | **Query** | **Observed** | **Mr(expt)** | **Mr(calc)** | **Delta** | **Miss** | **Score** | **Expect** | **Rank** | **Peptide** |
| --- | --- | --- | --- | --- | --- | --- | --- | --- | --- | --- |
|  | 2612 | **416.3038** | **830.5930** | **830.5014** | **0.0916** | **0** | **9** | **24** | **4** | **K.VIPSLFR.L** |
|  | 3173 | **433.4526** | **864.8906** | **865.3930** | **-0.5024** | **0** | **13** | **5.6** | **5** | **R.DNYDALR.S** |
|  | 4544 | **480.2782** | **958.5419** | **958.5964** | **-0.0545** | **1** | **7** | **35** | **10** | **K.KVIPSLFR.L** |
|  | 8876 | 417.7125 | 1250.1156 | 1250.6044 | -0.4887 | 0 | 14 | 4.1 | 1 | K.TQWTEFNGIR.D 8909 |
|  | 22914 | **761.4379** | **2281.2920** | **2281.1048** | **0.1871** | **1** | **2** | **48** | **8** | **R.NLSVEAEVDLLSYCAREWK.G** |
|  | 23066 | 1152.0767 | 2302.1388 | 2301.0888 | 1.0499 | 0 | 41 | 0.0054 | 2 | K.LLFSQGCNWIQQYSFGPEK.Y |

| **860.** | P61626\|LYSC_HUMAN    **Mass:** 16982    **Score:** 41     **Queries matched:** 7   **emPAI:** 0.23 |
| --- | --- |
|  | Lysozyme C OS=Homo sapiens GN=LYZ PE=1 SV=1 |

|  | **Query** | **Observed** | **Mr(expt)** | **Mr(calc)** | **Delta** | **Miss** | **Score** | **Expect** | **Rank** | **Peptide** |
| --- | --- | --- | --- | --- | --- | --- | --- | --- | --- | --- |
|  | 2540 | 414.7127 | 827.4107 | 826.3643 | 1.0464 | 0 | 5 | 52 | 4 | R.LGMDGYR.G |
|  | 3854 | **455.7317** | **909.4488** | **909.4015** | **0.0474** | **0** | **8** | **21** | **5** | **R.QYVQGCGV.-** 3829 |
|  | 4854 | 491.7846 | 981.5545 | 980.4312 | 1.1234 | 0 | 15 | 4.8 | 1 | R.ATNYNAGDR.S 4842 |
|  | 5261 | 506.7239 | 1011.4333 | 1011.4410 | -0.0077 | 0 | 23 | 0.82 | 1 | K.WESGYNTR.A |
|  | 26968 | 976.4583 | 2926.3529 | 2926.3735 | -0.0206 | 0 | 41 | 0.004 | 1 | K.TPGAVNACHLSCSALLQDNIADAVACAK.R |

| **861.** | P20339\|RAB5A_HUMAN    **Mass:** 23872    **Score:** 41     **Queries matched:** 6   **emPAI:** 0.16 |
| --- | --- |
|  | Ras-related protein Rab-5A OS=Homo sapiens GN=RAB5A PE=1 SV=2 |

|  | **Query** | **Observed** | **Mr(expt)** | **Mr(calc)** | **Delta** | **Miss** | **Score** | **Expect** | **Rank** | **Peptide** |
| --- | --- | --- | --- | --- | --- | --- | --- | --- | --- | --- |
|  | 10066 | 657.8320 | 1313.6494 | 1312.6623 | 0.9871 | 0 | 11 | 13 | 3 | R.GVDLTEPTQPTR.N |
|  | 10772 | 676.8751 | 1351.7357 | 1350.6204 | 1.1153 | 0 | 41 | 0.01 | 1 | K.FEIWDTAGQER.Y |
|  | 14491 | **528.7803** | **1583.3190** | **1583.7687** | **-0.4497** | **0** | **7** | **16** | **2** | **K.TSMNVNEIFMAIAK.K** |
|  | 16770 | 580.6484 | 1738.9235 | 1740.0509 | -1.1274 | 1 | 5 | 34 | 3 | K.LVLLGESAVGKSSLVLR.F |
|  | 20337 | **675.0051** | **2021.9936** | **2023.1062** | **-1.1126** | **1** | **4** | **36** | **1** | **R.QASPNIVIALSGNKADLANK.R** |
|  | 25782 | **899.0867** | **2694.2384** | **2695.1959** | **-0.9576** | **0** | **7** | **13** | **1** | **R.AVDFQEAQSYADDNSLLFMETSAK.T** |

| **862.** | P61020\|RAB5B_HUMAN    **Mass:** 23920    **Score:** 41     **Queries matched:** 6   **emPAI:** 0.16 |
| --- | --- |
|  | Ras-related protein Rab-5B OS=Homo sapiens GN=RAB5B PE=1 SV=1 |

|  | **Query** | **Observed** | **Mr(expt)** | **Mr(calc)** | **Delta** | **Miss** | **Score** | **Expect** | **Rank** | **Peptide** |
| --- | --- | --- | --- | --- | --- | --- | --- | --- | --- | --- |
|  | 5888 | 530.7101 | 1059.4056 | 1058.5873 | 0.8183 | 1 | 12 | 9.7 | 5 | K.TWVKELQR.Q |
|  | 10772 | 676.8751 | 1351.7357 | 1350.6204 | 1.1153 | 0 | 41 | 0.01 | 1 | K.FEIWDTAGQER.Y |
|  | 15150 | 813.4143 | 1624.8141 | 1624.7917 | 0.0223 | 1 | 2 | 67 | 8 | R.SRGVDLHEQSQQNK.S |
|  | 16770 | 580.6484 | 1738.9235 | 1740.0509 | -1.1274 | 1 | 5 | 34 | 3 | K.LVLLGESAVGKSSLVLR.F |
|  | 20275 | 673.3554 | 2017.0442 | 2017.0163 | 0.0279 | 1 | 2 | 56 | 7 | R.STARPNGQPQASKICQFK.L |
|  | 20838 | 689.6697 | 2065.9874 | 2066.0433 | -0.0559 | 0 | 1 | 65 | 7 | R.GAQAAIVVYDITNQETFAR.A |

| **863.** | P22223\|CADH3_HUMAN    **Mass:** 91875    **Score:** 41     **Queries matched:** 8   **emPAI:** 0.04 |
| --- | --- |
|  | Cadherin-3 OS=Homo sapiens GN=CDH3 PE=1 SV=2 |

|  | **Query** | **Observed** | **Mr(expt)** | **Mr(calc)** | **Delta** | **Miss** | **Score** | **Expect** | **Rank** | **Peptide** |
| --- | --- | --- | --- | --- | --- | --- | --- | --- | --- | --- |
|  | 1448 | 634.3263 | 633.3190 | 633.3082 | 0.0108 | 1 | 4 | 88 | 8 | K.DRDTK.I |
|  | 9257 | 635.7624 | 1269.5102 | 1269.5183 | -0.0081 | 1 | 41 | 0.011 | 1 | K.KLADMYGGGEDD.- |
|  | 9746 | **432.8770** | **1295.6093** | **1296.6561** | **-1.0468** | **0** | **5** | **39** | **3** | **K.EPLLLPEDDTR.D** |
|  | 10075 | **438.9158** | **1313.7256** | **1314.6714** | **-0.9458** | **0** | **6** | **38** | **7** | **R.QITICNQSPVR.Q** |
|  | 11262 | 461.0073 | 1380.0000 | 1380.6609 | -0.6608 | 0 | 6 | 25 | 1 | K.DPHDLMFTIHR.S |
|  | 14053 | **777.4454** | **1552.8763** | **1553.8566** | **-0.9802** | **0** | **2** | **75** | **3** | **K.ETGWLLLNKPLDR.E** |
|  | 20993 | **1042.2189** | **2082.4232** | **2083.0069** | **-0.5837** | **0** | **0** | **55** | **1** | **R.EAEVTLEAGGAEQEPGQALGK.V** |
|  | 31927 | 1897.2565 | 5688.7476 | 5688.8226 | -0.0751 | 1 | 3 | 5 | 1 | R.NNIYEVMVLAMDNGSPPTTGTGTLLLTLIDVNDHGPVPEPRQITICNQSPVR.Q |

| **864.** | P62988\|UBIQ_HUMAN    **Mass:** 8560     **Score:** 41     **Queries matched:** 5   **emPAI:** 0.48 |
[truncated: 6,608,819 more chars]
