# Supplementary material for: Dataset from proteomic analysis of human liver, lung, kidney and intestine microsomes
Source: Data Brief. 2018 Mar 30;18:831–4. doi: 10.1016/j.dib.2018.03.124 (PMC5996730; doi:10.1016/j.dib.2018.03.124)
Supplement: Supplementary file 4 — Supplementary material [file mmc4.doc]

Mascot Search Results

**User	: zhihong**

**Email	: peng_zhihong@hotmail.com**

**Search title	: kidney**

**MS data file	: kidney 2.mgf**

**Database	: SwissProt 101509 (510076 sequences; 179409349 residues)**

**Taxonomy	: Homo sapiens (human) (20335 sequences)**

**Timestamp	: 8 May 2013 at 18:19:19 GMT**

**Warning	: Invalid value (18+) specified for the "CHARGE" parameter**

**Warning	: Invalid value () specified for the "CHARGE" parameter**

**Warning	: Invalid value (9+) specified for the "CHARGE" parameter**

**Warning	: Invalid value () specified for the "CHARGE" parameter**

**Warning	: Invalid value (9+) specified for the "CHARGE" parameter**

**Warning	: Invalid value () specified for the "CHARGE" parameter**

**Warning	: Invalid value (10+) specified for the "CHARGE" parameter**

**Warning	: Invalid value () specified for the "CHARGE" parameter**

**Warning	: Invalid value (10+) specified for the "CHARGE" parameter**

**Warning	: Invalid value () specified for the "CHARGE" parameter**

**Warning	: Error 61 has been detected 114 times and only the first 10 messages have been output**

**Enzyme	: Trypsin**

**Fixed modifications	: Carbamidomethyl (C)**

**Variable modifications : Oxidation (M)**

**Mass values	: Monoisotopic**

**Protein Mass	: Unrestricted**

**Peptide Mass Tolerance : ± 1.2 Da**

**Fragment Mass Tolerance: ± 0.6 Da**

**Max Missed Cleavages	: 1**

**Instrument type	: Default**

**Number of queries	: 32510**

| **Protein hits           :** | **P06576\|ATPB_HUMAN** | ATP synthase subunit beta, mitochondrial OS=Homo sapiens GN=ATP5B PE=1 SV=3 |
| --- | --- | --- |
|  | **P05023\|AT1A1_HUMAN** | Sodium/potassium-transporting ATPase subunit alpha-1 OS=Homo sapiens GN=ATP1A1 PE=1 SV=1 |
|  | **P15144\|AMPN_HUMAN** | Aminopeptidase N OS=Homo sapiens GN=ANPEP PE=1 SV=4 |
|  | **P98164\|LRP2_HUMAN** | Low-density lipoprotein receptor-related protein 2 OS=Homo sapiens GN=LRP2 PE=1 SV=3 |
|  | **P68371\|TBB2C_HUMAN** | Tubulin beta-2C chain OS=Homo sapiens GN=TUBB2C PE=1 SV=1 |
|  | **P60709\|ACTB_HUMAN** | Actin, cytoplasmic 1 OS=Homo sapiens GN=ACTB PE=1 SV=1 |
|  | **P63261\|ACTG_HUMAN** | Actin, cytoplasmic 2 OS=Homo sapiens GN=ACTG1 PE=1 SV=1 |
|  | **P07437\|TBB5_HUMAN** | Tubulin beta chain OS=Homo sapiens GN=TUBB PE=1 SV=2 |
|  | **P25705\|ATPA_HUMAN** | ATP synthase subunit alpha, mitochondrial OS=Homo sapiens GN=ATP5A1 PE=1 SV=1 |
|  | **Q00610\|CLH1_HUMAN** | Clathrin heavy chain 1 OS=Homo sapiens GN=CLTC PE=1 SV=5 |
|  | **P04350\|TBB4_HUMAN** | Tubulin beta-4 chain OS=Homo sapiens GN=TUBB4 PE=1 SV=2 |
|  | **P68032\|ACTC_HUMAN** | Actin, alpha cardiac muscle 1 OS=Homo sapiens GN=ACTC1 PE=1 SV=1 |
|  | **Q9BVA1\|TBB2B_HUMAN** | Tubulin beta-2B chain OS=Homo sapiens GN=TUBB2B PE=1 SV=1 |
|  | **P62736\|ACTA_HUMAN** | Actin, aortic smooth muscle OS=Homo sapiens GN=ACTA2 PE=1 SV=1 |
|  | **P63267\|ACTH_HUMAN** | Actin, gamma-enteric smooth muscle OS=Homo sapiens GN=ACTG2 PE=1 SV=1 |
|  | **P35579\|MYH9_HUMAN** | Myosin-9 OS=Homo sapiens GN=MYH9 PE=1 SV=4 |
|  | **P13637\|AT1A3_HUMAN** | Sodium/potassium-transporting ATPase subunit alpha-3 OS=Homo sapiens GN=ATP1A3 PE=1 SV=3 |
|  | **P50993\|AT1A2_HUMAN** | Sodium/potassium-transporting ATPase subunit alpha-2 OS=Homo sapiens GN=ATP1A2 PE=1 SV=1 |
|  | **P68133\|ACTS_HUMAN** | Actin, alpha skeletal muscle OS=Homo sapiens GN=ACTA1 PE=1 SV=1 |
|  | **P68366\|TBA4A_HUMAN** | Tubulin alpha-4A chain OS=Homo sapiens GN=TUBA4A PE=1 SV=1 |
|  | **P31040\|DHSA_HUMAN** | Succinate dehydrogenase [ubiquinone] flavoprotein subunit, mitochondrial OS=Homo sapiens GN=SDHA PE=1 SV=2 |
|  | **O60494\|CUBN_HUMAN** | Cubilin OS=Homo sapiens GN=CUBN PE=1 SV=4 |
|  | **P31930\|QCR1_HUMAN** | Cytochrome b-c1 complex subunit 1, mitochondrial OS=Homo sapiens GN=UQCRC1 PE=1 SV=3 |
|  | **Q71U36\|TBA1A_HUMAN** | Tubulin alpha-1A chain OS=Homo sapiens GN=TUBA1A PE=1 SV=1 |
|  | **P40939\|ECHA_HUMAN** | Trifunctional enzyme subunit alpha, mitochondrial OS=Homo sapiens GN=HADHA PE=1 SV=2 |
|  | **Q14764\|MVP_HUMAN** | Major vault protein OS=Homo sapiens GN=MVP PE=1 SV=4 |
|  | **P68363\|TBA1B_HUMAN** | Tubulin alpha-1B chain OS=Homo sapiens GN=TUBA1B PE=1 SV=1 |
|  | **Q9BQE3\|TBA1C_HUMAN** | Tubulin alpha-1C chain OS=Homo sapiens GN=TUBA1C PE=1 SV=1 |
|  | **P08473\|NEP_HUMAN** | Neprilysin OS=Homo sapiens GN=MME PE=1 SV=2 |
|  | **Q6S8J3\|POTEE_HUMAN** | POTE ankyrin domain family member E OS=Homo sapiens GN=POTEE PE=1 SV=3 |
|  | **P11021\|GRP78_HUMAN** | 78 kDa glucose-regulated protein OS=Homo sapiens GN=HSPA5 PE=1 SV=2 |
|  | **P02768\|ALBU_HUMAN** | Serum albumin OS=Homo sapiens GN=ALB PE=1 SV=2 |
|  | **P14625\|ENPL_HUMAN** | Endoplasmin OS=Homo sapiens GN=HSP90B1 PE=1 SV=1 |
|  | **P04406\|G3P_HUMAN** | Glyceraldehyde-3-phosphate dehydrogenase OS=Homo sapiens GN=GAPDH PE=1 SV=3 |
|  | **P68871\|HBB_HUMAN** | Hemoglobin subunit beta OS=Homo sapiens GN=HBB PE=1 SV=2 |
|  | **P11142\|HSP7C_HUMAN** | Heat shock cognate 71 kDa protein OS=Homo sapiens GN=HSPA8 PE=1 SV=1 |
|  | **P55084\|ECHB_HUMAN** | Trifunctional enzyme subunit beta, mitochondrial OS=Homo sapiens GN=HADHB PE=1 SV=3 |
|  | **Q9NY65\|TBA8_HUMAN** | Tubulin alpha-8 chain OS=Homo sapiens GN=TUBA8 PE=1 SV=1 |
|  | **O75309\|CAD16_HUMAN** | Cadherin-16 OS=Homo sapiens GN=CDH16 PE=1 SV=1 |
|  | **O95831\|AIFM1_HUMAN** | Apoptosis-inducing factor 1, mitochondrial OS=Homo sapiens GN=AIFM1 PE=1 SV=1 |
|  | **Q13509\|TBB3_HUMAN** | Tubulin beta-3 chain OS=Homo sapiens GN=TUBB3 PE=1 SV=2 |
|  | **P05141\|ADT2_HUMAN** | ADP/ATP translocase 2 OS=Homo sapiens GN=SLC25A5 PE=1 SV=6 |
|  | **P33121\|ACSL1_HUMAN** | Long-chain-fatty-acid--CoA ligase 1 OS=Homo sapiens GN=ACSL1 PE=1 SV=1 |
|  | **P30049\|ATPD_HUMAN** | ATP synthase subunit delta, mitochondrial OS=Homo sapiens GN=ATP5D PE=1 SV=2 |
|  | **P38606\|VATA_HUMAN** | V-type proton ATPase catalytic subunit A OS=Homo sapiens GN=ATP6V1A PE=1 SV=2 |
|  | **Q13733\|AT1A4_HUMAN** | Sodium/potassium-transporting ATPase subunit alpha-4 OS=Homo sapiens GN=ATP1A4 PE=1 SV=3 |
|  | **Q3ZCM7\|TBB8_HUMAN** | Tubulin beta-8 chain OS=Homo sapiens GN=TUBB8 PE=1 SV=2 |
|  | **P04792\|HSPB1_HUMAN** | Heat shock protein beta-1 OS=Homo sapiens GN=HSPB1 PE=1 SV=2 |
|  | **P22695\|QCR2_HUMAN** | Cytochrome b-c1 complex subunit 2, mitochondrial OS=Homo sapiens GN=UQCRC2 PE=1 SV=3 |
|  | **P29972\|AQP1_HUMAN** | Aquaporin-1 OS=Homo sapiens GN=AQP1 PE=1 SV=3 |
|  | **P21796\|VDAC1_HUMAN** | Voltage-dependent anion-selective channel protein 1 OS=Homo sapiens GN=VDAC1 PE=1 SV=2 |
|  | **P00167\|CYB5_HUMAN** | Cytochrome b5 OS=Homo sapiens GN=CYB5A PE=1 SV=2 |
|  | **P20674\|COX5A_HUMAN** | Cytochrome c oxidase subunit 5A, mitochondrial OS=Homo sapiens GN=COX5A PE=1 SV=2 |
|  | **Q99867\|TBB4Q_HUMAN** | Putative tubulin beta-4q chain OS=Homo sapiens GN=TUBB4Q PE=5 SV=1 |
|  | **P08107\|HSP71_HUMAN** | Heat shock 70 kDa protein 1 OS=Homo sapiens GN=HSPA1A PE=1 SV=5 |
|  | **P10809\|CH60_HUMAN** | 60 kDa heat shock protein, mitochondrial OS=Homo sapiens GN=HSPD1 PE=1 SV=2 |
|  | **A6NNZ2\|TBB8B_HUMAN** | Tubulin beta-8 chain B OS=Homo sapiens PE=1 SV=1 |
|  | **Q16853\|AOC3_HUMAN** | Membrane primary amine oxidase OS=Homo sapiens GN=AOC3 PE=1 SV=3 |
|  | **O60656\|UD19_HUMAN** | UDP-glucuronosyltransferase 1-9 OS=Homo sapiens GN=UGT1A9 PE=1 SV=1 |
|  | **Q01082\|SPTB2_HUMAN** | Spectrin beta chain, brain 1 OS=Homo sapiens GN=SPTBN1 PE=1 SV=2 |
|  | **Q02928\|CP4AB_HUMAN** | Cytochrome P450 4A11 OS=Homo sapiens GN=CYP4A11 PE=1 SV=1 |
|  | **P27797\|CALR_HUMAN** | Calreticulin OS=Homo sapiens GN=CALR PE=1 SV=1 |
|  | **P08670\|VIME_HUMAN** | Vimentin OS=Homo sapiens GN=VIM PE=1 SV=4 |
|  | **Q13813\|SPTA2_HUMAN** | Spectrin alpha chain, brain OS=Homo sapiens GN=SPTAN1 PE=1 SV=3 |
|  | **P54819\|KAD2_HUMAN** | Adenylate kinase 2, mitochondrial OS=Homo sapiens GN=AK2 PE=1 SV=2 |
|  | **P16435\|NCPR_HUMAN** | NADPH--cytochrome P450 reductase OS=Homo sapiens GN=POR PE=1 SV=2 |
|  | **P02511\|CRYAB_HUMAN** | Alpha-crystallin B chain OS=Homo sapiens GN=CRYAB PE=1 SV=2 |
|  | **O60814\|H2B1K_HUMAN** | Histone H2B type 1-K OS=Homo sapiens GN=HIST1H2BK PE=1 SV=3 |
|  | **P57053\|H2BFS_HUMAN** | Histone H2B type F-S OS=Homo sapiens GN=H2BFS PE=1 SV=2 |
|  | **Q5QNW6\|H2B2F_HUMAN** | Histone H2B type 2-F OS=Homo sapiens GN=HIST2H2BF PE=1 SV=3 |
|  | **P36269\|GGT5_HUMAN** | Gamma-glutamyltransferase 5 OS=Homo sapiens GN=GGT5 PE=1 SV=2 |
|  | **P35232\|PHB_HUMAN** | Prohibitin OS=Homo sapiens GN=PHB PE=1 SV=1 |
|  | **P80365\|DHI2_HUMAN** | Corticosteroid 11-beta-dehydrogenase isozyme 2 OS=Homo sapiens GN=HSD11B2 PE=1 SV=2 |
|  | **P08195\|4F2_HUMAN** | 4F2 cell-surface antigen heavy chain OS=Homo sapiens GN=SLC3A2 PE=1 SV=3 |
|  | **Q13423\|NNTM_HUMAN** | NAD(P) transhydrogenase, mitochondrial OS=Homo sapiens GN=NNT PE=1 SV=3 |
|  | **Q16778\|H2B2E_HUMAN** | Histone H2B type 2-E OS=Homo sapiens GN=HIST2H2BE PE=1 SV=3 |
|  | **P23527\|H2B1O_HUMAN** | Histone H2B type 1-O OS=Homo sapiens GN=HIST1H2BO PE=1 SV=3 |
|  | **Q8N257\|H2B3B_HUMAN** | Histone H2B type 3-B OS=Homo sapiens GN=HIST3H2BB PE=1 SV=3 |
|  | **Q16134\|ETFD_HUMAN** | Electron transfer flavoprotein-ubiquinone oxidoreductase, mitochondrial OS=Homo sapiens GN=ETFDH PE=1 SV=1 |
|  | **O43451\|MGA_HUMAN** | Maltase-glucoamylase, intestinal OS=Homo sapiens GN=MGAM PE=1 SV=5 |
|  | **P21281\|VATB2_HUMAN** | V-type proton ATPase subunit B, brain isoform OS=Homo sapiens GN=ATP6V1B2 PE=1 SV=3 |
|  | **P12236\|ADT3_HUMAN** | ADP/ATP translocase 3 OS=Homo sapiens GN=SLC25A6 PE=1 SV=4 |
|  | **Q562R1\|ACTBL_HUMAN** | Beta-actin-like protein 2 OS=Homo sapiens GN=ACTBL2 PE=1 SV=2 |
|  | **P01876\|IGHA1_HUMAN** | Ig alpha-1 chain C region OS=Homo sapiens GN=IGHA1 PE=1 SV=2 |
|  | **P05026\|AT1B1_HUMAN** | Sodium/potassium-transporting ATPase subunit beta-1 OS=Homo sapiens GN=ATP1B1 PE=1 SV=1 |
|  | **P18859\|ATP5J_HUMAN** | ATP synthase-coupling factor 6, mitochondrial OS=Homo sapiens GN=ATP5J PE=1 SV=1 |
|  | **Q5VTE0\|EF1A3_HUMAN** | Putative elongation factor 1-alpha-like 3 OS=Homo sapiens GN=EEF1AL3 PE=5 SV=1 |
|  | **P01834\|IGKC_HUMAN** | Ig kappa chain C region OS=Homo sapiens GN=IGKC PE=1 SV=1 |
|  | **P07911\|UROM_HUMAN** | Uromodulin OS=Homo sapiens GN=UMOD PE=1 SV=1 |
|  | **P30040\|ERP29_HUMAN** | Endoplasmic reticulum protein ERp29 OS=Homo sapiens GN=ERP29 PE=1 SV=4 |
|  | **P16444\|DPEP1_HUMAN** | Dipeptidase 1 OS=Homo sapiens GN=DPEP1 PE=1 SV=3 |
|  | **P01877\|IGHA2_HUMAN** | Ig alpha-2 chain C region OS=Homo sapiens GN=IGHA2 PE=1 SV=3 |
|  | **P08758\|ANXA5_HUMAN** | Annexin A5 OS=Homo sapiens GN=ANXA5 PE=1 SV=2 |
|  | **P30086\|PEBP1_HUMAN** | Phosphatidylethanolamine-binding protein 1 OS=Homo sapiens GN=PEBP1 PE=1 SV=3 |
|  | **P35555\|FBN1_HUMAN** | Fibrillin-1 OS=Homo sapiens GN=FBN1 PE=1 SV=2 |
|  | **P30101\|PDIA3_HUMAN** | Protein disulfide-isomerase A3 OS=Homo sapiens GN=PDIA3 PE=1 SV=4 |
|  | **P02679\|FIBG_HUMAN** | Fibrinogen gamma chain OS=Homo sapiens GN=FGG PE=1 SV=3 |
|  | **P28331\|NDUS1_HUMAN** | NADH-ubiquinone oxidoreductase 75 kDa subunit, mitochondrial OS=Homo sapiens GN=NDUFS1 PE=1 SV=3 |
|  | **A5A3E0\|POTEF_HUMAN** | POTE ankyrin domain family member F OS=Homo sapiens GN=POTEF PE=1 SV=2 |
|  | **Q5TCH4\|CP4AM_HUMAN** | Cytochrome P450 4A22 OS=Homo sapiens GN=CYP4A22 PE=1 SV=1 |
|  | **P19440\|GGT1_HUMAN** | Gamma-glutamyltranspeptidase 1 OS=Homo sapiens GN=GGT1 PE=1 SV=2 |
|  | **P60033\|CD81_HUMAN** | CD81 antigen OS=Homo sapiens GN=CD81 PE=1 SV=1 |
|  | **P27487\|DPP4_HUMAN** | Dipeptidyl peptidase 4 OS=Homo sapiens GN=DPP4 PE=1 SV=2 |
|  | **P34931\|HS71L_HUMAN** | Heat shock 70 kDa protein 1L OS=Homo sapiens GN=HSPA1L PE=1 SV=2 |
|  | **O95182\|NDUA7_HUMAN** | NADH dehydrogenase [ubiquinone] 1 alpha subcomplex subunit 7 OS=Homo sapiens GN=NDUFA7 PE=1 SV=3 |
|  | **P12821\|ACE_HUMAN** | Angiotensin-converting enzyme OS=Homo sapiens GN=ACE PE=1 SV=1 |
|  | **P14854\|CX6B1_HUMAN** | Cytochrome c oxidase subunit 6B1 OS=Homo sapiens GN=COX6B1 PE=1 SV=2 |
|  | **P50440\|GATM_HUMAN** | Glycine amidinotransferase, mitochondrial OS=Homo sapiens GN=GATM PE=1 SV=1 |
|  | **P24539\|AT5F1_HUMAN** | ATP synthase subunit b, mitochondrial OS=Homo sapiens GN=ATP5F1 PE=1 SV=2 |
|  | **Q9HAW9\|UD18_HUMAN** | UDP-glucuronosyltransferase 1-8 OS=Homo sapiens GN=UGT1A8 PE=1 SV=1 |
|  | **P21397\|AOFA_HUMAN** | Amine oxidase [flavin-containing] A OS=Homo sapiens GN=MAOA PE=1 SV=1 |
|  | **P02730\|B3AT_HUMAN** | Band 3 anion transport protein OS=Homo sapiens GN=SLC4A1 PE=1 SV=3 |
|  | **Q9HAW7\|UD17_HUMAN** | UDP-glucuronosyltransferase 1-7 OS=Homo sapiens GN=UGT1A7 PE=1 SV=2 |
|  | **P12235\|ADT1_HUMAN** | ADP/ATP translocase 1 OS=Homo sapiens GN=SLC25A4 PE=1 SV=4 |
|  | **P62873\|GBB1_HUMAN** | Guanine nucleotide-binding protein G(I)/G(S)/G(T) subunit beta-1 OS=Homo sapiens GN=GNB1 PE=1 SV=3 |
|  | **P60660\|MYL6_HUMAN** | Myosin light polypeptide 6 OS=Homo sapiens GN=MYL6 PE=1 SV=2 |
|  | **P45880\|VDAC2_HUMAN** | Voltage-dependent anion-selective channel protein 2 OS=Homo sapiens GN=VDAC2 PE=1 SV=2 |
|  | **P53675\|CLH2_HUMAN** | Clathrin heavy chain 2 OS=Homo sapiens GN=CLTCL1 PE=1 SV=2 |
|  | **P27635\|RL10_HUMAN** | 60S ribosomal protein L10 OS=Homo sapiens GN=RPL10 PE=1 SV=4 |
|  | **P02735\|SAA_HUMAN** | Serum amyloid A protein OS=Homo sapiens GN=SAA1 PE=1 SV=2 |
|  | **P05556\|ITB1_HUMAN** | Integrin beta-1 OS=Homo sapiens GN=ITGB1 PE=1 SV=2 |
|  | **P19224\|UD16_HUMAN** | UDP-glucuronosyltransferase 1-6 OS=Homo sapiens GN=UGT1A6 PE=1 SV=2 |
|  | **P35504\|UD15_HUMAN** | UDP-glucuronosyltransferase 1-5 OS=Homo sapiens GN=UGT1A5 PE=1 SV=1 |
|  | **P22310\|UD14_HUMAN** | UDP-glucuronosyltransferase 1-4 OS=Homo sapiens GN=UGT1A4 PE=1 SV=1 |
|  | **P35503\|UD13_HUMAN** | UDP-glucuronosyltransferase 1-3 OS=Homo sapiens GN=UGT1A3 PE=1 SV=1 |
|  | **P22309\|UD11_HUMAN** | UDP-glucuronosyltransferase 1-1 OS=Homo sapiens GN=UGT1A1 PE=1 SV=1 |
|  | **P35613\|BASI_HUMAN** | Basigin OS=Homo sapiens GN=BSG PE=1 SV=2 |
|  | **P05387\|RLA2_HUMAN** | 60S acidic ribosomal protein P2 OS=Homo sapiens GN=RPLP2 PE=1 SV=1 |
|  | **Q9BYX7\|ACTBM_HUMAN** | Beta-actin-like protein 3 OS=Homo sapiens GN=ACTBL3 PE=1 SV=1 |
|  | **P04004\|VTNC_HUMAN** | Vitronectin OS=Homo sapiens GN=VTN PE=1 SV=1 |
|  | **Q99536\|VAT1_HUMAN** | Synaptic vesicle membrane protein VAT-1 homolog OS=Homo sapiens GN=VAT1 PE=1 SV=2 |
|  | **P08238\|HS90B_HUMAN** | Heat shock protein HSP 90-beta OS=Homo sapiens GN=HSP90AB1 PE=1 SV=4 |
|  | **Q6FI13\|H2A2A_HUMAN** | Histone H2A type 2-A OS=Homo sapiens GN=HIST2H2AA3 PE=1 SV=3 |
|  | **Q8N2G8\|GHDC_HUMAN** | GH3 domain-containing protein OS=Homo sapiens GN=GHDC PE=1 SV=2 |
|  | **Q92597\|NDRG1_HUMAN** | Protein NDRG1 OS=Homo sapiens GN=NDRG1 PE=1 SV=1 |
|  | **P07339\|CATD_HUMAN** | Cathepsin D OS=Homo sapiens GN=CTSD PE=1 SV=1 |
|  | **P07900\|HS90A_HUMAN** | Heat shock protein HSP 90-alpha OS=Homo sapiens GN=HSP90AA1 PE=1 SV=5 |
|  | **P06396\|GELS_HUMAN** | Gelsolin OS=Homo sapiens GN=GSN PE=1 SV=1 |
|  | **P27338\|AOFB_HUMAN** | Amine oxidase [flavin-containing] B OS=Homo sapiens GN=MAOB PE=1 SV=3 |
|  | **O14828\|SCAM3_HUMAN** | Secretory carrier-associated membrane protein 3 OS=Homo sapiens GN=SCAMP3 PE=1 SV=3 |
|  | **Q9BSJ8\|ESYT1_HUMAN** | Extended synaptotagmin-1 OS=Homo sapiens GN=ESYT1 PE=1 SV=1 |
|  | **P02749\|APOH_HUMAN** | Beta-2-glycoprotein 1 OS=Homo sapiens GN=APOH PE=1 SV=3 |
|  | **P36268\|GGT2_HUMAN** | Gamma-glutamyltranspeptidase 2 OS=Homo sapiens GN=GGT2 PE=1 SV=3 |
|  | **Q9NQC3\|RTN4_HUMAN** | Reticulon-4 OS=Homo sapiens GN=RTN4 PE=1 SV=2 |
|  | **P21926\|CD9_HUMAN** | CD9 antigen OS=Homo sapiens GN=CD9 PE=1 SV=4 |
|  | **O15127\|SCAM2_HUMAN** | Secretory carrier-associated membrane protein 2 OS=Homo sapiens GN=SCAMP2 PE=1 SV=2 |
|  | **P62820\|RAB1A_HUMAN** | Ras-related protein Rab-1A OS=Homo sapiens GN=RAB1A PE=1 SV=3 |
|  | **P69905\|HBA_HUMAN** | Hemoglobin subunit alpha OS=Homo sapiens GN=HBA1 PE=1 SV=2 |
|  | **A1L0T0\|ILVBL_HUMAN** | Acetolactate synthase-like protein OS=Homo sapiens GN=ILVBL PE=1 SV=2 |
|  | **P12111\|CO6A3_HUMAN** | Collagen alpha-3(VI) chain OS=Homo sapiens GN=COL6A3 PE=1 SV=4 |
|  | **P61006\|RAB8A_HUMAN** | Ras-related protein Rab-8A OS=Homo sapiens GN=RAB8A PE=1 SV=1 |
|  | **O14975\|S27A2_HUMAN** | Very long-chain acyl-CoA synthetase OS=Homo sapiens GN=SLC27A2 PE=1 SV=1 |
|  | **P35749\|MYH11_HUMAN** | Myosin-11 OS=Homo sapiens GN=MYH11 PE=1 SV=3 |
|  | **Q9BUF5\|TBB6_HUMAN** | Tubulin beta-6 chain OS=Homo sapiens GN=TUBB6 PE=1 SV=1 |
|  | **P10606\|COX5B_HUMAN** | Cytochrome c oxidase subunit 5B, mitochondrial OS=Homo sapiens GN=COX5B PE=1 SV=2 |
|  | **P36542\|ATPG_HUMAN** | ATP synthase subunit gamma, mitochondrial OS=Homo sapiens GN=ATP5C1 PE=1 SV=1 |
|  | **Q6DD88\|ATLA3_HUMAN** | Atlastin-3 OS=Homo sapiens GN=ATL3 PE=1 SV=1 |
|  | **Q9H0U4\|RAB1B_HUMAN** | Ras-related protein Rab-1B OS=Homo sapiens GN=RAB1B PE=1 SV=1 |
|  | **Q8N339\|MT1M_HUMAN** | Metallothionein-1M OS=Homo sapiens GN=MT1M PE=2 SV=2 |
|  | **O95299\|NDUAA_HUMAN** | NADH dehydrogenase [ubiquinone] 1 alpha subcomplex subunit 10, mitochondrial OS=Homo sapiens GN=NDUFA10 PE=1 SV=1 |
|  | **P00441\|SODC_HUMAN** | Superoxide dismutase [Cu-Zn] OS=Homo sapiens GN=SOD1 PE=1 SV=2 |
|  | **P51687\|SUOX_HUMAN** | Sulfite oxidase, mitochondrial OS=Homo sapiens GN=SUOX PE=1 SV=2 |
|  | **A6NGU5\|GGT3_HUMAN** | Putative gamma-glutamyltranspeptidase 3 OS=Homo sapiens GN=GGT3P PE=5 SV=2 |
|  | **Q96A33\|CCD47_HUMAN** | Coiled-coil domain-containing protein 47 OS=Homo sapiens GN=CCDC47 PE=1 SV=1 |
|  | **P15313\|VATB1_HUMAN** | V-type proton ATPase subunit B, kidney isoform OS=Homo sapiens GN=ATP6V1B1 PE=1 SV=3 |
|  | **P13073\|COX41_HUMAN** | Cytochrome c oxidase subunit 4 isoform 1, mitochondrial OS=Homo sapiens GN=COX4I1 PE=1 SV=1 |
|  | **O00264\|PGRC1_HUMAN** | Membrane-associated progesterone receptor component 1 OS=Homo sapiens GN=PGRMC1 PE=1 SV=3 |
|  | **P30050\|RL12_HUMAN** | 60S ribosomal protein L12 OS=Homo sapiens GN=RPL12 PE=1 SV=1 |
|  | **Q02338\|BDH_HUMAN** | D-beta-hydroxybutyrate dehydrogenase, mitochondrial OS=Homo sapiens GN=BDH1 PE=1 SV=3 |
|  | **O00217\|NDUS8_HUMAN** | NADH dehydrogenase [ubiquinone] iron-sulfur protein 8, mitochondrial OS=Homo sapiens GN=NDUFS8 PE=1 SV=1 |
|  | **Q96KR1\|ZFR_HUMAN** | Zinc finger RNA-binding protein OS=Homo sapiens GN=ZFR PE=1 SV=2 |
|  | **P84098\|RL19_HUMAN** | 60S ribosomal protein L19 OS=Homo sapiens GN=RPL19 PE=1 SV=1 |
|  | **P52272\|HNRPM_HUMAN** | Heterogeneous nuclear ribonucleoprotein M OS=Homo sapiens GN=HNRNPM PE=1 SV=3 |
|  | **P01009\|A1AT_HUMAN** | Alpha-1-antitrypsin OS=Homo sapiens GN=SERPINA1 PE=1 SV=3 |
|  | **P61421\|VA0D1_HUMAN** | V-type proton ATPase subunit d 1 OS=Homo sapiens GN=ATP6V0D1 PE=1 SV=1 |
|  | **P56199\|ITA1_HUMAN** | Integrin alpha-1 OS=Homo sapiens GN=ITGA1 PE=1 SV=2 |
|  | **P20648\|ATP4A_HUMAN** | Potassium-transporting ATPase alpha chain 1 OS=Homo sapiens GN=ATP4A PE=2 SV=5 |
|  | **Q99623\|PHB2_HUMAN** | Prohibitin-2 OS=Homo sapiens GN=PHB2 PE=1 SV=2 |
|  | **P55072\|TERA_HUMAN** | Transitional endoplasmic reticulum ATPase OS=Homo sapiens GN=VCP PE=1 SV=4 |
|  | **O43895\|XPP2_HUMAN** | Xaa-Pro aminopeptidase 2 OS=Homo sapiens GN=XPNPEP2 PE=1 SV=3 |
|  | **Q9NZ45\|CISD1_HUMAN** | CDGSH iron sulfur domain-containing protein 1 OS=Homo sapiens GN=CISD1 PE=1 SV=1 |
|  | **P12109\|CO6A1_HUMAN** | Collagen alpha-1(VI) chain OS=Homo sapiens GN=COL6A1 PE=1 SV=3 |
|  | **Q9Y5K8\|VATD_HUMAN** | V-type proton ATPase subunit D OS=Homo sapiens GN=ATP6V1D PE=1 SV=1 |
|  | **P15311\|EZRI_HUMAN** | Ezrin OS=Homo sapiens GN=EZR PE=1 SV=4 |
|  | **P39023\|RL3_HUMAN** | 60S ribosomal protein L3 OS=Homo sapiens GN=RPL3 PE=1 SV=2 |
|  | **O95292\|VAPB_HUMAN** | Vesicle-associated membrane protein-associated protein B/C OS=Homo sapiens GN=VAPB PE=1 SV=3 |
|  | **P49821\|NDUV1_HUMAN** | NADH dehydrogenase [ubiquinone] flavoprotein 1, mitochondrial OS=Homo sapiens GN=NDUFV1 PE=1 SV=4 |
|  | **O75306\|NDUS2_HUMAN** | NADH dehydrogenase [ubiquinone] iron-sulfur protein 2, mitochondrial OS=Homo sapiens GN=NDUFS2 PE=1 SV=2 |
|  | **Q9HCC0\|MCCB_HUMAN** | Methylcrotonoyl-CoA carboxylase beta chain, mitochondrial OS=Homo sapiens GN=MCCC2 PE=1 SV=1 |
|  | **P02545\|LMNA_HUMAN** | Lamin-A/C OS=Homo sapiens GN=LMNA PE=1 SV=1 |
|  | **P10909\|CLUS_HUMAN** | Clusterin OS=Homo sapiens GN=CLU PE=1 SV=1 |
|  | **Q9UJZ1\|STML2_HUMAN** | Stomatin-like protein 2 OS=Homo sapiens GN=STOML2 PE=1 SV=1 |
|  | **P09110\|THIK_HUMAN** | 3-ketoacyl-CoA thiolase, peroxisomal OS=Homo sapiens GN=ACAA1 PE=1 SV=2 |
|  | **P30041\|PRDX6_HUMAN** | Peroxiredoxin-6 OS=Homo sapiens GN=PRDX6 PE=1 SV=3 |
|  | **P09497\|CLCB_HUMAN** | Clathrin light chain B OS=Homo sapiens GN=CLTB PE=1 SV=1 |
|  | **P54652\|HSP72_HUMAN** | Heat shock-related 70 kDa protein 2 OS=Homo sapiens GN=HSPA2 PE=1 SV=1 |
|  | **Q15005\|SPCS2_HUMAN** | Signal peptidase complex subunit 2 OS=Homo sapiens GN=SPCS2 PE=1 SV=3 |
|  | **P04843\|RPN1_HUMAN** | Dolichyl-diphosphooligosaccharide--protein glycosyltransferase subunit 1 OS=Homo sapiens GN=RPN1 PE=1 SV=1 |
|  | **P62241\|RS8_HUMAN** | 40S ribosomal protein S8 OS=Homo sapiens GN=RPS8 PE=1 SV=2 |
|  | **P02647\|APOA1_HUMAN** | Apolipoprotein A-I OS=Homo sapiens GN=APOA1 PE=1 SV=1 |
|  | **Q9BPW8\|NIPS1_HUMAN** | Protein NipSnap homolog 1 OS=Homo sapiens GN=NIPSNAP1 PE=1 SV=1 |
|  | **P07195\|LDHB_HUMAN** | L-lactate dehydrogenase B chain OS=Homo sapiens GN=LDHB PE=1 SV=2 |
|  | **Q01740\|FMO1_HUMAN** | Dimethylaniline monooxygenase [N-oxide-forming] 1 OS=Homo sapiens GN=FMO1 PE=2 SV=3 |
|  | **P61106\|RAB14_HUMAN** | Ras-related protein Rab-14 OS=Homo sapiens GN=RAB14 PE=1 SV=4 |
|  | **P02748\|CO9_HUMAN** | Complement component C9 OS=Homo sapiens GN=C9 PE=1 SV=2 |
|  | **P48047\|ATPO_HUMAN** | ATP synthase subunit O, mitochondrial OS=Homo sapiens GN=ATP5O PE=1 SV=1 |
|  | **P50895\|BCAM_HUMAN** | Basal cell adhesion molecule OS=Homo sapiens GN=BCAM PE=1 SV=2 |
|  | **Q9GZP9\|DERL2_HUMAN** | Derlin-2 OS=Homo sapiens GN=DERL2 PE=1 SV=1 |
|  | **P11233\|RALA_HUMAN** | Ras-related protein Ral-A OS=Homo sapiens GN=RALA PE=1 SV=1 |
|  | **Q8NE62\|CHDH_HUMAN** | Choline dehydrogenase, mitochondrial OS=Homo sapiens GN=CHDH PE=2 SV=2 |
|  | **O15400\|STX7_HUMAN** | Syntaxin-7 OS=Homo sapiens GN=STX7 PE=1 SV=4 |
|  | **P14314\|GLU2B_HUMAN** | Glucosidase 2 subunit beta OS=Homo sapiens GN=PRKCSH PE=1 SV=2 |
|  | **P62841\|RS15_HUMAN** | 40S ribosomal protein S15 OS=Homo sapiens GN=RPS15 PE=1 SV=2 |
|  | **P60174\|TPIS_HUMAN** | Triosephosphate isomerase OS=Homo sapiens GN=TPI1 PE=1 SV=2 |
|  | **P36543\|VATE1_HUMAN** | V-type proton ATPase subunit E 1 OS=Homo sapiens GN=ATP6V1E1 PE=1 SV=1 |
|  | **P05388\|RLA0_HUMAN** | 60S acidic ribosomal protein P0 OS=Homo sapiens GN=RPLP0 PE=1 SV=1 |
|  | **P62917\|RL8_HUMAN** | 60S ribosomal protein L8 OS=Homo sapiens GN=RPL8 PE=1 SV=2 |
|  | **P62158\|CALM_HUMAN** | Calmodulin OS=Homo sapiens GN=CALM1 PE=1 SV=2 |
|  | **Q07075\|AMPE_HUMAN** | Glutamyl aminopeptidase OS=Homo sapiens GN=ENPEP PE=1 SV=2 |
|  | **Q9UL25\|RAB21_HUMAN** | Ras-related protein Rab-21 OS=Homo sapiens GN=RAB21 PE=1 SV=3 |
|  | **Q9UJS0\|CMC2_HUMAN** | Calcium-binding mitochondrial carrier protein Aralar2 OS=Homo sapiens GN=SLC25A13 PE=1 SV=2 |
|  | **Q00325\|MPCP_HUMAN** | Phosphate carrier protein, mitochondrial OS=Homo sapiens GN=SLC25A3 PE=1 SV=2 |
|  | **O60716\|CTND1_HUMAN** | Catenin delta-1 OS=Homo sapiens GN=CTNND1 PE=1 SV=1 |
|  | **Q53GQ0\|DHB12_HUMAN** | Estradiol 17-beta-dehydrogenase 12 OS=Homo sapiens GN=HSD17B12 PE=1 SV=2 |
|  | **Q92959\|SO2A1_HUMAN** | Solute carrier organic anion transporter family member 2A1 OS=Homo sapiens GN=SLCO2A1 PE=1 SV=2 |
|  | **Q7Z406\|MYH14_HUMAN** | Myosin-14 OS=Homo sapiens GN=MYH14 PE=1 SV=1 |
|  | **P21283\|VATC1_HUMAN** | V-type proton ATPase subunit C 1 OS=Homo sapiens GN=ATP6V1C1 PE=1 SV=4 |
|  | **Q9H8H3\|MET7A_HUMAN** | Methyltransferase-like protein 7A OS=Homo sapiens GN=METTL7A PE=1 SV=1 |
|  | **Q9NTJ5\|SAC1_HUMAN** | Phosphatidylinositide phosphatase SAC1 OS=Homo sapiens GN=SACM1L PE=1 SV=2 |
|  | **P21912\|DHSB_HUMAN** | Succinate dehydrogenase [ubiquinone] iron-sulfur subunit, mitochondrial OS=Homo sapiens GN=SDHB PE=1 SV=3 |
|  | **P16422\|EPCAM_HUMAN** | Epithelial cell adhesion molecule OS=Homo sapiens GN=EPCAM PE=1 SV=2 |
|  | **P51572\|BAP31_HUMAN** | B-cell receptor-associated protein 31 OS=Homo sapiens GN=BCAP31 PE=1 SV=3 |
|  | **P17568\|NDUB7_HUMAN** | NADH dehydrogenase [ubiquinone] 1 beta subcomplex subunit 7 OS=Homo sapiens GN=NDUFB7 PE=1 SV=4 |
|  | **Q9NZZ3\|CHMP5_HUMAN** | Charged multivesicular body protein 5 OS=Homo sapiens GN=CHMP5 PE=1 SV=1 |
|  | **P07237\|PDIA1_HUMAN** | Protein disulfide-isomerase OS=Homo sapiens GN=P4HB PE=1 SV=3 |
|  | **Q92928\|RAB1C_HUMAN** | Putative Ras-related protein Rab-1C OS=Homo sapiens GN=RAB1C PE=5 SV=2 |
|  | **Q969Z3\|MOSC2_HUMAN** | MOSC domain-containing protein 2, mitochondrial OS=Homo sapiens GN=MOSC2 PE=1 SV=1 |
|  | **P17066\|HSP76_HUMAN** | Heat shock 70 kDa protein 6 OS=Homo sapiens GN=HSPA6 PE=1 SV=2 |
|  | **P48741\|HSP77_HUMAN** | Putative heat shock 70 kDa protein 7 OS=Homo sapiens GN=HSPA7 PE=5 SV=2 |
|  | **Q16718\|NDUA5_HUMAN** | NADH dehydrogenase [ubiquinone] 1 alpha subcomplex subunit 5 OS=Homo sapiens GN=NDUFA5 PE=1 SV=3 |
|  | **P02675\|FIBB_HUMAN** | Fibrinogen beta chain OS=Homo sapiens GN=FGB PE=1 SV=2 |
|  | **P09211\|GSTP1_HUMAN** | Glutathione S-transferase P OS=Homo sapiens GN=GSTP1 PE=1 SV=2 |
|  | **Q16864\|VATF_HUMAN** | V-type proton ATPase subunit F OS=Homo sapiens GN=ATP6V1F PE=1 SV=2 |
|  | **O75947\|ATP5H_HUMAN** | ATP synthase subunit d, mitochondrial OS=Homo sapiens GN=ATP5H PE=1 SV=3 |
|  | **P05091\|ALDH2_HUMAN** | Aldehyde dehydrogenase, mitochondrial OS=Homo sapiens GN=ALDH2 PE=1 SV=2 |
|  | **P0C0S8\|H2A1_HUMAN** | Histone H2A type 1 OS=Homo sapiens GN=HIST1H2AG PE=1 SV=2 |
|  | **P27824\|CALX_HUMAN** | Calnexin OS=Homo sapiens GN=CANX PE=1 SV=2 |
|  | **Q15365\|PCBP1_HUMAN** | Poly(rC)-binding protein 1 OS=Homo sapiens GN=PCBP1 PE=1 SV=2 |
|  | **P06756\|ITAV_HUMAN** | Integrin alpha-V OS=Homo sapiens GN=ITGAV PE=1 SV=2 |
|  | **P02671\|FIBA_HUMAN** | Fibrinogen alpha chain OS=Homo sapiens GN=FGA PE=1 SV=2 |
|  | **P31943\|HNRH1_HUMAN** | Heterogeneous nuclear ribonucleoprotein H OS=Homo sapiens GN=HNRNPH1 PE=1 SV=4 |
|  | **P55795\|HNRH2_HUMAN** | Heterogeneous nuclear ribonucleoprotein H2 OS=Homo sapiens GN=HNRNPH2 PE=1 SV=1 |
|  | **P14927\|QCR7_HUMAN** | Cytochrome b-c1 complex subunit 7 OS=Homo sapiens GN=UQCRB PE=1 SV=2 |
|  | **P19404\|NDUV2_HUMAN** | NADH dehydrogenase [ubiquinone] flavoprotein 2, mitochondrial OS=Homo sapiens GN=NDUFV2 PE=1 SV=2 |
|  | **P21810\|PGS1_HUMAN** | Biglycan OS=Homo sapiens GN=BGN PE=1 SV=2 |
|  | **P51648\|AL3A2_HUMAN** | Fatty aldehyde dehydrogenase OS=Homo sapiens GN=ALDH3A2 PE=1 SV=1 |
|  | **P00395\|COX1_HUMAN** | Cytochrome c oxidase subunit 1 OS=Homo sapiens GN=MT-CO1 PE=1 SV=1 |
|  | **O00483\|NDUA4_HUMAN** | NADH dehydrogenase [ubiquinone] 1 alpha subcomplex subunit 4 OS=Homo sapiens GN=NDUFA4 PE=1 SV=1 |
|  | **P00387\|NB5R3_HUMAN** | NADH-cytochrome b5 reductase 3 OS=Homo sapiens GN=CYB5R3 PE=1 SV=3 |
|  | **Q02252\|MMSA_HUMAN** | Methylmalonate-semialdehyde dehydrogenase [acylating], mitochondrial OS=Homo sapiens GN=ALDH6A1 PE=1 SV=2 |
|  | **O75380\|NDUS6_HUMAN** | NADH dehydrogenase [ubiquinone] iron-sulfur protein 6, mitochondrial OS=Homo sapiens GN=NDUFS6 PE=1 SV=1 |
|  | **P04040\|CATA_HUMAN** | Catalase OS=Homo sapiens GN=CAT PE=1 SV=3 |
|  | **O14817\|TSN4_HUMAN** | Tetraspanin-4 OS=Homo sapiens GN=TSPAN4 PE=1 SV=1 |
|  | **P05062\|ALDOB_HUMAN** | Fructose-bisphosphate aldolase B OS=Homo sapiens GN=ALDOB PE=1 SV=2 |
|  | **P61247\|RS3A_HUMAN** | 40S ribosomal protein S3a OS=Homo sapiens GN=RPS3A PE=1 SV=2 |
|  | **P17858\|K6PL_HUMAN** | 6-phosphofructokinase, liver type OS=Homo sapiens GN=PFKL PE=1 SV=6 |
|  | **Q12955\|ANK3_HUMAN** | Ankyrin-3 OS=Homo sapiens GN=ANK3 PE=1 SV=3 |
|  | **P04003\|C4BPA_HUMAN** | C4b-binding protein alpha chain OS=Homo sapiens GN=C4BPA PE=1 SV=2 |
|  | **O75396\|SC22B_HUMAN** | Vesicle-trafficking protein SEC22b OS=Homo sapiens GN=SEC22B PE=1 SV=3 |
|  | **Q02978\|M2OM_HUMAN** | Mitochondrial 2-oxoglutarate/malate carrier protein OS=Homo sapiens GN=SLC25A11 PE=1 SV=3 |
|  | **O96000\|NDUBA_HUMAN** | NADH dehydrogenase [ubiquinone] 1 beta subcomplex subunit 10 OS=Homo sapiens GN=NDUFB10 PE=1 SV=3 |
|  | **P20039\|2B1B_HUMAN** | HLA class II histocompatibility antigen, DRB1-11 beta chain OS=Homo sapiens GN=HLA-DRB1 PE=2 SV=1 |
|  | **Q30154\|2B51_HUMAN** | HLA class II histocompatibility antigen, DRB5 beta chain OS=Homo sapiens GN=HLA-DRB5 PE=1 SV=1 |
|  | **P04229\|2B11_HUMAN** | HLA class II histocompatibility antigen, DRB1-1 beta chain OS=Homo sapiens GN=HLA-DRB1 PE=1 SV=2 |
|  | **Q9UBX3\|DIC_HUMAN** | Mitochondrial dicarboxylate carrier OS=Homo sapiens GN=SLC25A10 PE=1 SV=2 |
|  | **Q56VL3\|OCAD2_HUMAN** | OCIA domain-containing protein 2 OS=Homo sapiens GN=OCIAD2 PE=1 SV=1 |
|  | **P63104\|1433Z_HUMAN** | 14-3-3 protein zeta/delta OS=Homo sapiens GN=YWHAZ PE=1 SV=1 |
|  | **P46776\|RL27A_HUMAN** | 60S ribosomal protein L27a OS=Homo sapiens GN=RPL27A PE=1 SV=2 |
|  | **O43707\|ACTN4_HUMAN** | Alpha-actinin-4 OS=Homo sapiens GN=ACTN4 PE=1 SV=2 |
|  | **P05090\|APOD_HUMAN** | Apolipoprotein D OS=Homo sapiens GN=APOD PE=1 SV=1 |
|  | **P09622\|DLDH_HUMAN** | Dihydrolipoyl dehydrogenase, mitochondrial OS=Homo sapiens GN=DLD PE=1 SV=1 |
|  | **Q9UI12\|VATH_HUMAN** | V-type proton ATPase subunit H OS=Homo sapiens GN=ATP6V1H PE=1 SV=1 |
|  | **Q12846\|STX4_HUMAN** | Syntaxin-4 OS=Homo sapiens GN=STX4 PE=1 SV=2 |
|  | **P54707\|AT12A_HUMAN** | Potassium-transporting ATPase alpha chain 2 OS=Homo sapiens GN=ATP12A PE=2 SV=3 |
|  | **Q03154\|ACY1_HUMAN** | Aminoacylase-1 OS=Homo sapiens GN=ACY1 PE=1 SV=1 |
|  | **P53007\|TXTP_HUMAN** | Tricarboxylate transport protein, mitochondrial OS=Homo sapiens GN=SLC25A1 PE=1 SV=2 |
|  | **Q15084\|PDIA6_HUMAN** | Protein disulfide-isomerase A6 OS=Homo sapiens GN=PDIA6 PE=1 SV=1 |
|  | **P30479\|1B41_HUMAN** | HLA class I histocompatibility antigen, B-41 alpha chain OS=Homo sapiens GN=HLA-B PE=1 SV=1 |
|  | **P49411\|EFTU_HUMAN** | Elongation factor Tu, mitochondrial OS=Homo sapiens GN=TUFM PE=1 SV=2 |
|  | **P30508\|1C12_HUMAN** | HLA class I histocompatibility antigen, Cw-12 alpha chain OS=Homo sapiens GN=HLA-C PE=1 SV=2 |
|  | **P26373\|RL13_HUMAN** | 60S ribosomal protein L13 OS=Homo sapiens GN=RPL13 PE=1 SV=4 |
|  | **P52943\|CRIP2_HUMAN** | Cysteine-rich protein 2 OS=Homo sapiens GN=CRIP2 PE=1 SV=1 |
|  | **Q15366\|PCBP2_HUMAN** | Poly(rC)-binding protein 2 OS=Homo sapiens GN=PCBP2 PE=1 SV=1 |
|  | **P57721\|PCBP3_HUMAN** | Poly(rC)-binding protein 3 OS=Homo sapiens GN=PCBP3 PE=1 SV=1 |
|  | **Q9UM54\|MYO6_HUMAN** | Myosin-VI OS=Homo sapiens GN=MYO6 PE=1 SV=4 |
|  | **P54920\|SNAA_HUMAN** | Alpha-soluble NSF attachment protein OS=Homo sapiens GN=NAPA PE=1 SV=3 |
|  | **Q6UX53\|MET7B_HUMAN** | Methyltransferase-like protein 7B OS=Homo sapiens GN=METTL7B PE=2 SV=2 |
|  | **Q96GC9\|TMM49_HUMAN** | Transmembrane protein 49 OS=Homo sapiens GN=TMEM49 PE=2 SV=1 |
|  | **P10620\|MGST1_HUMAN** | Microsomal glutathione S-transferase 1 OS=Homo sapiens GN=MGST1 PE=1 SV=1 |
|  | **P08263\|GSTA1_HUMAN** | Glutathione S-transferase A1 OS=Homo sapiens GN=GSTA1 PE=1 SV=3 |
|  | **P09210\|GSTA2_HUMAN** | Glutathione S-transferase A2 OS=Homo sapiens GN=GSTA2 PE=1 SV=4 |
|  | **P78329\|CP4F2_HUMAN** | Leukotriene-B(4) omega-hydroxylase 1 OS=Homo sapiens GN=CYP4F2 PE=1 SV=1 |
|  | **P07099\|HYEP_HUMAN** | Epoxide hydrolase 1 OS=Homo sapiens GN=EPHX1 PE=1 SV=1 |
|  | **O75964\|ATP5L_HUMAN** | ATP synthase subunit g, mitochondrial OS=Homo sapiens GN=ATP5L PE=1 SV=3 |
|  | **Q6RW13\|ATRAP_HUMAN** | Type-1 angiotensin II receptor-associated protein OS=Homo sapiens GN=AGTRAP PE=1 SV=1 |
|  | **P39060\|COIA1_HUMAN** | Collagen alpha-1(XVIII) chain OS=Homo sapiens GN=COL18A1 PE=1 SV=5 |
|  | **P04222\|1C03_HUMAN** | HLA class I histocompatibility antigen, Cw-3 alpha chain OS=Homo sapiens GN=HLA-C PE=1 SV=2 |
|  | **O14880\|MGST3_HUMAN** | Microsomal glutathione S-transferase 3 OS=Homo sapiens GN=MGST3 PE=1 SV=1 |
|  | **P21695\|GPDA_HUMAN** | Glycerol-3-phosphate dehydrogenase [NAD+], cytoplasmic OS=Homo sapiens GN=GPD1 PE=1 SV=4 |
|  | **P38646\|GRP75_HUMAN** | Stress-70 protein, mitochondrial OS=Homo sapiens GN=HSPA9 PE=1 SV=2 |
|  | **P01833\|PIGR_HUMAN** | Polymeric immunoglobulin receptor OS=Homo sapiens GN=PIGR PE=1 SV=4 |
|  | **P35222\|CTNB1_HUMAN** | Catenin beta-1 OS=Homo sapiens GN=CTNNB1 PE=1 SV=1 |
|  | **P61224\|RAP1B_HUMAN** | Ras-related protein Rap-1b OS=Homo sapiens GN=RAP1B PE=1 SV=1 |
|  | **A6NIZ1\|RP1BL_HUMAN** | Ras-related protein Rap-1b-like protein OS=Homo sapiens PE=2 SV=1 |
|  | **Q99653\|CHP1_HUMAN** | Calcium-binding protein p22 OS=Homo sapiens GN=CHP PE=1 SV=3 |
|  | **Q05639\|EF1A2_HUMAN** | Elongation factor 1-alpha 2 OS=Homo sapiens GN=EEF1A2 PE=1 SV=1 |
|  | **Q9BYF1\|ACE2_HUMAN** | Angiotensin-converting enzyme 2 OS=Homo sapiens GN=ACE2 PE=1 SV=2 |
|  | **P62834\|RAP1A_HUMAN** | Ras-related protein Rap-1A OS=Homo sapiens GN=RAP1A PE=1 SV=1 |
|  | **P50416\|CPT1A_HUMAN** | Carnitine O-palmitoyltransferase 1, liver isoform OS=Homo sapiens GN=CPT1A PE=1 SV=2 |
|  | **P62937\|PPIA_HUMAN** | Peptidyl-prolyl cis-trans isomerase A OS=Homo sapiens GN=PPIA PE=1 SV=2 |
|  | **Q07000\|1C15_HUMAN** | HLA class I histocompatibility antigen, Cw-15 alpha chain OS=Homo sapiens GN=HLA-C PE=1 SV=1 |
|  | **P30501\|1C02_HUMAN** | HLA class I histocompatibility antigen, Cw-2 alpha chain OS=Homo sapiens GN=HLA-C PE=1 SV=1 |
|  | **P10321\|1C07_HUMAN** | HLA class I histocompatibility antigen, Cw-7 alpha chain OS=Homo sapiens GN=HLA-C PE=1 SV=3 |
|  | **Q31612\|1B73_HUMAN** | HLA class I histocompatibility antigen, B-73 alpha chain OS=Homo sapiens GN=HLA-B PE=1 SV=1 |
|  | **O15031\|PLXB2_HUMAN** | Plexin-B2 OS=Homo sapiens GN=PLXNB2 PE=1 SV=3 |
|  | **Q8WWT9\|S13A3_HUMAN** | Solute carrier family 13 member 3 OS=Homo sapiens GN=SLC13A3 PE=2 SV=1 |
|  | **P02452\|CO1A1_HUMAN** | Collagen alpha-1(I) chain OS=Homo sapiens GN=COL1A1 PE=1 SV=4 |
|  | **Q8NHW5\|RLA0L_HUMAN** | 60S acidic ribosomal protein P0-like OS=Homo sapiens PE=2 SV=1 |
|  | **P30519\|HMOX2_HUMAN** | Heme oxygenase 2 OS=Homo sapiens GN=HMOX2 PE=1 SV=2 |
|  | **O95202\|LETM1_HUMAN** | LETM1 and EF-hand domain-containing protein 1, mitochondrial OS=Homo sapiens GN=LETM1 PE=1 SV=1 |
|  | **O14745\|NHRF1_HUMAN** | Na(+)/H(+) exchange regulatory cofactor NHE-RF1 OS=Homo sapiens GN=SLC9A3R1 PE=1 SV=4 |
|  | **Q93050\|VPP1_HUMAN** | V-type proton ATPase 116 kDa subunit a isoform 1 OS=Homo sapiens GN=ATP6V0A1 PE=1 SV=3 |
|  | **P60468\|SC61B_HUMAN** | Protein transport protein Sec61 subunit beta OS=Homo sapiens GN=SEC61B PE=1 SV=2 |
|  | **Q5RI15\|FA36A_HUMAN** | Protein FAM36A OS=Homo sapiens GN=FAM36A PE=1 SV=2 |
|  | **Q8WW52\|F151A_HUMAN** | Protein FAM151A OS=Homo sapiens GN=FAM151A PE=2 SV=2 |
|  | **O14672\|ADA10_HUMAN** | Disintegrin and metalloproteinase domain-containing protein 10 OS=Homo sapiens GN=ADAM10 PE=1 SV=1 |
|  | **P30460\|1B08_HUMAN** | HLA class I histocompatibility antigen, B-8 alpha chain OS=Homo sapiens GN=HLA-B PE=1 SV=1 |
|  | **P01889\|1B07_HUMAN** | HLA class I histocompatibility antigen, B-7 alpha chain OS=Homo sapiens GN=HLA-B PE=1 SV=3 |
|  | **P16284\|PECA1_HUMAN** | Platelet endothelial cell adhesion molecule OS=Homo sapiens GN=PECAM1 PE=1 SV=1 |
|  | **Q13228\|SBP1_HUMAN** | Selenium-binding protein 1 OS=Homo sapiens GN=SELENBP1 PE=1 SV=2 |
|  | **P08574\|CY1_HUMAN** | Cytochrome c1, heme protein, mitochondrial OS=Homo sapiens GN=CYC1 PE=1 SV=2 |
|  | **Q9UI09\|NDUAC_HUMAN** | NADH dehydrogenase [ubiquinone] 1 alpha subcomplex subunit 12 OS=Homo sapiens GN=NDUFA12 PE=1 SV=1 |
|  | **P06744\|G6PI_HUMAN** | Glucose-6-phosphate isomerase OS=Homo sapiens GN=GPI PE=1 SV=4 |
|  | **Q8TCZ2\|C99L2_HUMAN** | CD99 antigen-like protein 2 OS=Homo sapiens GN=CD99L2 PE=2 SV=1 |
|  | **P05362\|ICAM1_HUMAN** | Intercellular adhesion molecule 1 OS=Homo sapiens GN=ICAM1 PE=1 SV=2 |
|  | **Q9BUR5\|APOO_HUMAN** | Apolipoprotein O OS=Homo sapiens GN=APOO PE=1 SV=1 |
|  | **Q9UF12\|PROD2_HUMAN** | Probable proline dehydrogenase 2 OS=Homo sapiens GN=PRODH2 PE=2 SV=1 |
|  | **P01861\|IGHG4_HUMAN** | Ig gamma-4 chain C region OS=Homo sapiens GN=IGHG4 PE=1 SV=1 |
|  | **P61019\|RAB2A_HUMAN** | Ras-related protein Rab-2A OS=Homo sapiens GN=RAB2A PE=1 SV=1 |
|  | **P07355\|ANXA2_HUMAN** | Annexin A2 OS=Homo sapiens GN=ANXA2 PE=1 SV=2 |
|  | **P13010\|KU86_HUMAN** | ATP-dependent DNA helicase 2 subunit 2 OS=Homo sapiens GN=XRCC5 PE=1 SV=3 |
|  | **Q9Y2B0\|CNPY2_HUMAN** | Protein canopy homolog 2 OS=Homo sapiens GN=CNPY2 PE=1 SV=1 |
|  | **Q10567\|AP1B1_HUMAN** | AP-1 complex subunit beta-1 OS=Homo sapiens GN=AP1B1 PE=1 SV=1 |
|  | **Q14697\|GANAB_HUMAN** | Neutral alpha-glucosidase AB OS=Homo sapiens GN=GANAB PE=1 SV=3 |
|  | **P23396\|RS3_HUMAN** | 40S ribosomal protein S3 OS=Homo sapiens GN=RPS3 PE=1 SV=2 |
|  | **P35558\|PCKGC_HUMAN** | Phosphoenolpyruvate carboxykinase, cytosolic [GTP] OS=Homo sapiens GN=PCK1 PE=1 SV=3 |
|  | **Q15836\|VAMP3_HUMAN** | Vesicle-associated membrane protein 3 OS=Homo sapiens GN=VAMP3 PE=1 SV=3 |
|  | **P27105\|STOM_HUMAN** | Erythrocyte band 7 integral membrane protein OS=Homo sapiens GN=STOM PE=1 SV=3 |
|  | **Q9NYQ3\|HAOX2_HUMAN** | Hydroxyacid oxidase 2 OS=Homo sapiens GN=HAO2 PE=2 SV=1 |
|  | **P16157\|ANK1_HUMAN** | Ankyrin-1 OS=Homo sapiens GN=ANK1 PE=1 SV=3 |
|  | **P46777\|RL5_HUMAN** | 60S ribosomal protein L5 OS=Homo sapiens GN=RPL5 PE=1 SV=3 |
|  | **P63027\|VAMP2_HUMAN** | Vesicle-associated membrane protein 2 OS=Homo sapiens GN=VAMP2 PE=1 SV=2 |
|  | **P63010\|AP2B1_HUMAN** | AP-2 complex subunit beta OS=Homo sapiens GN=AP2B1 PE=1 SV=1 |
|  | **Q13621\|S12A1_HUMAN** | Solute carrier family 12 member 1 OS=Homo sapiens GN=SLC12A1 PE=1 SV=2 |
|  | **P21980\|TGM2_HUMAN** | Protein-glutamine gamma-glutamyltransferase 2 OS=Homo sapiens GN=TGM2 PE=1 SV=2 |
|  | **Q16891\|IMMT_HUMAN** | Mitochondrial inner membrane protein OS=Homo sapiens GN=IMMT PE=1 SV=1 |
|  | **P0C7P4\|UCRIL_HUMAN** | Cytochrome b-c1 complex subunit Rieske-like protein 1 OS=Homo sapiens GN=UQCRFSL1 PE=2 SV=1 |
|  | **P47985\|UCRI_HUMAN** | Cytochrome b-c1 complex subunit Rieske, mitochondrial OS=Homo sapiens GN=UQCRFS1 PE=1 SV=2 |
|  | **P02042\|HBD_HUMAN** | Hemoglobin subunit delta OS=Homo sapiens GN=HBD PE=1 SV=2 |
|  | **Q04826\|1B40_HUMAN** | HLA class I histocompatibility antigen, B-40 alpha chain OS=Homo sapiens GN=HLA-B PE=1 SV=1 |
|  | **P16402\|H13_HUMAN** | Histone H1.3 OS=Homo sapiens GN=HIST1H1D PE=1 SV=2 |
|  | **P16403\|H12_HUMAN** | Histone H1.2 OS=Homo sapiens GN=HIST1H1C PE=1 SV=2 |
|  | **P10412\|H14_HUMAN** | Histone H1.4 OS=Homo sapiens GN=HIST1H1E PE=1 SV=2 |
|  | **P22492\|H1T_HUMAN** | Histone H1t OS=Homo sapiens GN=HIST1H1T PE=1 SV=4 |
|  | **Q02539\|H11_HUMAN** | Histone H1.1 OS=Homo sapiens GN=HIST1H1A PE=1 SV=3 |
|  | **Q14508\|WFDC2_HUMAN** | WAP four-disulfide core domain protein 2 OS=Homo sapiens GN=WFDC2 PE=1 SV=2 |
|  | **P62491\|RB11A_HUMAN** | Ras-related protein Rab-11A OS=Homo sapiens GN=RAB11A PE=1 SV=3 |
|  | **O43570\|CAH12_HUMAN** | Carbonic anhydrase 12 OS=Homo sapiens GN=CA12 PE=1 SV=1 |
|  | **P14174\|MIF_HUMAN** | Macrophage migration inhibitory factor OS=Homo sapiens GN=MIF PE=1 SV=4 |
|  | **P13667\|PDIA4_HUMAN** | Protein disulfide-isomerase A4 OS=Homo sapiens GN=PDIA4 PE=1 SV=2 |
|  | **P01842\|LAC_HUMAN** | Ig lambda chain C regions OS=Homo sapiens GN=IGLC1 PE=1 SV=1 |
|  | **Q14240\|IF4A2_HUMAN** | Eukaryotic initiation factor 4A-II OS=Homo sapiens GN=EIF4A2 PE=1 SV=2 |
|  | **P38919\|IF4A3_HUMAN** | Eukaryotic initiation factor 4A-III OS=Homo sapiens GN=EIF4A3 PE=1 SV=4 |
|  | **P60842\|IF4A1_HUMAN** | Eukaryotic initiation factor 4A-I OS=Homo sapiens GN=EIF4A1 PE=1 SV=1 |
|  | **P46782\|RS5_HUMAN** | 40S ribosomal protein S5 OS=Homo sapiens GN=RPS5 PE=1 SV=4 |
|  | **Q07065\|CKAP4_HUMAN** | Cytoskeleton-associated protein 4 OS=Homo sapiens GN=CKAP4 PE=1 SV=2 |
|  | **P62879\|GBB2_HUMAN** | Guanine nucleotide-binding protein G(I)/G(S)/G(T) subunit beta-2 OS=Homo sapiens GN=GNB2 PE=1 SV=3 |
|  | **P62701\|RS4X_HUMAN** | 40S ribosomal protein S4, X isoform OS=Homo sapiens GN=RPS4X PE=1 SV=2 |
|  | **P30464\|1B15_HUMAN** | HLA class I histocompatibility antigen, B-15 alpha chain OS=Homo sapiens GN=HLA-B PE=1 SV=2 |
|  | **P14550\|AK1A1_HUMAN** | Alcohol dehydrogenase [NADP+] OS=Homo sapiens GN=AKR1A1 PE=1 SV=3 |
|  | **P12956\|KU70_HUMAN** | ATP-dependent DNA helicase 2 subunit 1 OS=Homo sapiens GN=XRCC6 PE=1 SV=2 |
|  | **P16615\|AT2A2_HUMAN** | Sarcoplasmic/endoplasmic reticulum calcium ATPase 2 OS=Homo sapiens GN=ATP2A2 PE=1 SV=1 |
|  | **P24752\|THIL_HUMAN** | Acetyl-CoA acetyltransferase, mitochondrial OS=Homo sapiens GN=ACAT1 PE=1 SV=1 |
|  | **Q9HBG4\|VPP4_HUMAN** | V-type proton ATPase 116 kDa subunit a isoform 4 OS=Homo sapiens GN=ATP6V0A4 PE=1 SV=1 |
|  | **P02774\|VTDB_HUMAN** | Vitamin D-binding protein OS=Homo sapiens GN=GC PE=1 SV=1 |
|  | **Q9Y4P3\|TBL2_HUMAN** | Transducin beta-like protein 2 OS=Homo sapiens GN=TBL2 PE=1 SV=1 |
|  | **Q8NBJ7\|SUMF2_HUMAN** | Sulfatase-modifying factor 2 OS=Homo sapiens GN=SUMF2 PE=1 SV=1 |
|  | **P51149\|RAB7A_HUMAN** | Ras-related protein Rab-7a OS=Homo sapiens GN=RAB7A PE=1 SV=1 |
|  | **P02760\|AMBP_HUMAN** | Protein AMBP OS=Homo sapiens GN=AMBP PE=1 SV=1 |
|  | **Q29865\|1C18_HUMAN** | HLA class I histocompatibility antigen, Cw-18 alpha chain OS=Homo sapiens GN=HLA-C PE=2 SV=1 |
|  | **P30499\|1C01_HUMAN** | HLA class I histocompatibility antigen, Cw-1 alpha chain OS=Homo sapiens GN=HLA-C PE=2 SV=1 |
|  | **P37108\|SRP14_HUMAN** | Signal recognition particle 14 kDa protein OS=Homo sapiens GN=SRP14 PE=1 SV=2 |
|  | **Q01813\|K6PP_HUMAN** | 6-phosphofructokinase type C OS=Homo sapiens GN=PFKP PE=1 SV=2 |
|  | **P01911\|2B1E_HUMAN** | HLA class II histocompatibility antigen, DRB1-15 beta chain OS=Homo sapiens GN=HLA-DRB1 PE=1 SV=2 |
|  | **P56181\|NDUV3_HUMAN** | NADH dehydrogenase [ubiquinone] flavoprotein 3, mitochondrial OS=Homo sapiens GN=NDUFV3 PE=2 SV=2 |
|  | **P30457\|1A66_HUMAN** | HLA class I histocompatibility antigen, A-66 alpha chain OS=Homo sapiens GN=HLA-A PE=1 SV=1 |
|  | **P18462\|1A25_HUMAN** | HLA class I histocompatibility antigen, A-25 alpha chain OS=Homo sapiens GN=HLA-A PE=2 SV=1 |
|  | **P30456\|1A43_HUMAN** | HLA class I histocompatibility antigen, A-43 alpha chain OS=Homo sapiens GN=HLA-A PE=2 SV=1 |
|  | **P98187\|CP4F8_HUMAN** | Cytochrome P450 4F8 OS=Homo sapiens GN=CYP4F8 PE=1 SV=1 |
|  | **Q08477\|CP4F3_HUMAN** | Leukotriene-B(4) omega-hydroxylase 2 OS=Homo sapiens GN=CYP4F3 PE=2 SV=2 |
|  | **O15229\|KMO_HUMAN** | Kynurenine 3-monooxygenase OS=Homo sapiens GN=KMO PE=1 SV=2 |
|  | **Q13011\|ECH1_HUMAN** | Delta(3,5)-Delta(2,4)-dienoyl-CoA isomerase, mitochondrial OS=Homo sapiens GN=ECH1 PE=1 SV=2 |
|  | **Q00765\|REEP5_HUMAN** | Receptor expression-enhancing protein 5 OS=Homo sapiens GN=REEP5 PE=1 SV=3 |
|  | **P13987\|CD59_HUMAN** | CD59 glycoprotein OS=Homo sapiens GN=CD59 PE=1 SV=1 |
|  | **P80723\|BASP1_HUMAN** | Brain acid soluble protein 1 OS=Homo sapiens GN=BASP1 PE=1 SV=2 |
|  | **Q9UHE5\|NAT8_HUMAN** | Probable N-acetyltransferase 8 OS=Homo sapiens GN=NAT8 PE=2 SV=2 |
|  | **Q9NRZ7\|PLCC_HUMAN** | 1-acyl-sn-glycerol-3-phosphate acyltransferase gamma OS=Homo sapiens GN=AGPAT3 PE=2 SV=1 |
|  | **P04206\|KV307_HUMAN** | Ig kappa chain V-III region GOL OS=Homo sapiens PE=1 SV=1 |
|  | **P01620\|KV302_HUMAN** | Ig kappa chain V-III region SIE OS=Homo sapiens PE=1 SV=1 |
|  | **P01619\|KV301_HUMAN** | Ig kappa chain V-III region B6 OS=Homo sapiens PE=1 SV=1 |
|  | **Q9UBI6\|GBG12_HUMAN** | Guanine nucleotide-binding protein G(I)/G(S)/G(O) subunit gamma-12 OS=Homo sapiens GN=GNG12 PE=1 SV=3 |
|  | **Q9Y624\|JAM1_HUMAN** | Junctional adhesion molecule A OS=Homo sapiens GN=F11R PE=1 SV=1 |
|  | **P62888\|RL30_HUMAN** | 60S ribosomal protein L30 OS=Homo sapiens GN=RPL30 PE=1 SV=2 |
|  | **P11279\|LAMP1_HUMAN** | Lysosome-associated membrane glycoprotein 1 OS=Homo sapiens GN=LAMP1 PE=1 SV=3 |
|  | **P01024\|CO3_HUMAN** | Complement C3 OS=Homo sapiens GN=C3 PE=1 SV=2 |
|  | **Q7Z3B1\|NEGR1_HUMAN** | Neuronal growth regulator 1 OS=Homo sapiens GN=NEGR1 PE=1 SV=3 |
|  | **P07919\|QCR6_HUMAN** | Cytochrome b-c1 complex subunit 6, mitochondrial OS=Homo sapiens GN=UQCRH PE=1 SV=2 |
|  | **P56134\|ATPK_HUMAN** | ATP synthase subunit f, mitochondrial OS=Homo sapiens GN=ATP5J2 PE=1 SV=3 |
|  | **P14649\|MYL6B_HUMAN** | Myosin light chain 6B OS=Homo sapiens GN=MYL6B PE=1 SV=1 |
|  | **P31946\|1433B_HUMAN** | 14-3-3 protein beta/alpha OS=Homo sapiens GN=YWHAB PE=1 SV=3 |
|  | **O43795\|MYO1B_HUMAN** | Myosin-Ib OS=Homo sapiens GN=MYO1B PE=1 SV=3 |
|  | **Q9H9B4\|SFXN1_HUMAN** | Sideroflexin-1 OS=Homo sapiens GN=SFXN1 PE=1 SV=4 |
|  | **P11277\|SPTB1_HUMAN** | Spectrin beta chain, erythrocyte OS=Homo sapiens GN=SPTB PE=1 SV=5 |
|  | **P01857\|IGHG1_HUMAN** | Ig gamma-1 chain C region OS=Homo sapiens GN=IGHG1 PE=1 SV=1 |
|  | **O60884\|DNJA2_HUMAN** | DnaJ homolog subfamily A member 2 OS=Homo sapiens GN=DNAJA2 PE=1 SV=1 |
|  | **P04899\|GNAI2_HUMAN** | Guanine nucleotide-binding protein G(i), alpha-2 subunit OS=Homo sapiens GN=GNAI2 PE=1 SV=3 |
|  | **O94760\|DDAH1_HUMAN** | N(G),N(G)-dimethylarginine dimethylaminohydrolase 1 OS=Homo sapiens GN=DDAH1 PE=1 SV=3 |
|  | **P67809\|YBOX1_HUMAN** | Nuclease-sensitive element-binding protein 1 OS=Homo sapiens GN=YBX1 PE=1 SV=3 |
|  | **P16662\|UD2B7_HUMAN** | UDP-glucuronosyltransferase 2B7 OS=Homo sapiens GN=UGT2B7 PE=1 SV=1 |
|  | **P61981\|1433G_HUMAN** | 14-3-3 protein gamma OS=Homo sapiens GN=YWHAG PE=1 SV=2 |
|  | **P49368\|TCPG_HUMAN** | T-complex protein 1 subunit gamma OS=Homo sapiens GN=CCT3 PE=1 SV=4 |
|  | **Q9Y6C9\|MTCH2_HUMAN** | Mitochondrial carrier homolog 2 OS=Homo sapiens GN=MTCH2 PE=1 SV=1 |
|  | **Q13409\|DC1I2_HUMAN** | Cytoplasmic dynein 1 intermediate chain 2 OS=Homo sapiens GN=DYNC1I2 PE=1 SV=3 |
|  | **P30475\|1B39_HUMAN** | HLA class I histocompatibility antigen, B-39 alpha chain OS=Homo sapiens GN=HLA-B PE=2 SV=1 |
|  | **P30466\|1B18_HUMAN** | HLA class I histocompatibility antigen, B-18 alpha chain OS=Homo sapiens GN=HLA-B PE=1 SV=1 |
|  | **P61026\|RAB10_HUMAN** | Ras-related protein Rab-10 OS=Homo sapiens GN=RAB10 PE=1 SV=1 |
|  | **Q9P0I2\|TM111_HUMAN** | Transmembrane protein 111 OS=Homo sapiens GN=TMEM111 PE=1 SV=3 |
|  | **A6NKZ8\|YI016_HUMAN** | Putative tubulin beta chain-like protein ENSP00000290377 OS=Homo sapiens PE=5 SV=2 |
|  | **P10451\|OSTP_HUMAN** | Osteopontin OS=Homo sapiens GN=SPP1 PE=1 SV=1 |
|  | **P52597\|HNRPF_HUMAN** | Heterogeneous nuclear ribonucleoprotein F OS=Homo sapiens GN=HNRNPF PE=1 SV=3 |
|  | **O94973\|AP2A2_HUMAN** | AP-2 complex subunit alpha-2 OS=Homo sapiens GN=AP2A2 PE=1 SV=2 |
|  | **P04439\|1A03_HUMAN** | HLA class I histocompatibility antigen, A-3 alpha chain OS=Homo sapiens GN=HLA-A PE=1 SV=2 |
|  | **O95782\|AP2A1_HUMAN** | AP-2 complex subunit alpha-1 OS=Homo sapiens GN=AP2A1 PE=1 SV=2 |
|  | **Q9Y394\|DHRS7_HUMAN** | Dehydrogenase/reductase SDR family member 7 OS=Homo sapiens GN=DHRS7 PE=1 SV=1 |
|  | **P12110\|CO6A2_HUMAN** | Collagen alpha-2(VI) chain OS=Homo sapiens GN=COL6A2 PE=1 SV=4 |
|  | **Q07020\|RL18_HUMAN** | 60S ribosomal protein L18 OS=Homo sapiens GN=RPL18 PE=1 SV=2 |
|  | **P05186\|PPBT_HUMAN** | Alkaline phosphatase, tissue-nonspecific isozyme OS=Homo sapiens GN=ALPL PE=1 SV=4 |
|  | **P15954\|COX7C_HUMAN** | Cytochrome c oxidase subunit 7C, mitochondrial OS=Homo sapiens GN=COX7C PE=1 SV=1 |
|  | **Q12931\|TRAP1_HUMAN** | Heat shock protein 75 kDa, mitochondrial OS=Homo sapiens GN=TRAP1 PE=1 SV=3 |
|  | **O94826\|TOM70_HUMAN** | Mitochondrial import receptor subunit TOM70 OS=Homo sapiens GN=TOMM70A PE=1 SV=1 |
|  | **O75489\|NDUS3_HUMAN** | NADH dehydrogenase [ubiquinone] iron-sulfur protein 3, mitochondrial OS=Homo sapiens GN=NDUFS3 PE=1 SV=1 |
|  | **P07357\|CO8A_HUMAN** | Complement component C8 alpha chain OS=Homo sapiens GN=C8A PE=1 SV=2 |
|  | **O95168\|NDUB4_HUMAN** | NADH dehydrogenase [ubiquinone] 1 beta subcomplex subunit 4 OS=Homo sapiens GN=NDUFB4 PE=1 SV=3 |
|  | **O75381\|PEX14_HUMAN** | Peroxisomal membrane protein PEX14 OS=Homo sapiens GN=PEX14 PE=1 SV=1 |
|  | **P35606\|COPB2_HUMAN** | Coatomer subunit beta' OS=Homo sapiens GN=COPB2 PE=1 SV=2 |
|  | **P20073\|ANXA7_HUMAN** | Annexin A7 OS=Homo sapiens GN=ANXA7 PE=1 SV=3 |
|  | **P06133\|UD2B4_HUMAN** | UDP-glucuronosyltransferase 2B4 OS=Homo sapiens GN=UGT2B4 PE=1 SV=2 |
|  | **O00330\|ODPX_HUMAN** | Pyruvate dehydrogenase protein X component, mitochondrial OS=Homo sapiens GN=PDHX PE=1 SV=3 |
|  | **P04156\|PRIO_HUMAN** | Major prion protein OS=Homo sapiens GN=PRNP PE=1 SV=1 |
|  | **P04217\|A1BG_HUMAN** | Alpha-1B-glycoprotein OS=Homo sapiens GN=A1BG PE=1 SV=3 |
|  | **Q8WUD1\|RAB2B_HUMAN** | Ras-related protein Rab-2B OS=Homo sapiens GN=RAB2B PE=1 SV=1 |
|  | **P62910\|RL32_HUMAN** | 60S ribosomal protein L32 OS=Homo sapiens GN=RPL32 PE=1 SV=2 |
|  | **Q5VT66\|MOSC1_HUMAN** | MOSC domain-containing protein 1, mitochondrial OS=Homo sapiens GN=MOSC1 PE=1 SV=1 |
|  | **P50914\|RL14_HUMAN** | 60S ribosomal protein L14 OS=Homo sapiens GN=RPL14 PE=1 SV=4 |
|  | **Q9UGT4\|SUSD2_HUMAN** | Sushi domain-containing protein 2 OS=Homo sapiens GN=SUSD2 PE=1 SV=1 |
|  | **Q8TC12\|RDH11_HUMAN** | Retinol dehydrogenase 11 OS=Homo sapiens GN=RDH11 PE=1 SV=2 |
|  | **P00338\|LDHA_HUMAN** | L-lactate dehydrogenase A chain OS=Homo sapiens GN=LDHA PE=1 SV=2 |
|  | **P07864\|LDHC_HUMAN** | L-lactate dehydrogenase C chain OS=Homo sapiens GN=LDHC PE=2 SV=4 |
|  | **Q6ZMR3\|LDH6A_HUMAN** | L-lactate dehydrogenase A-like 6A OS=Homo sapiens GN=LDHAL6A PE=2 SV=1 |
|  | **P30481\|1B44_HUMAN** | HLA class I histocompatibility antigen, B-44 alpha chain OS=Homo sapiens GN=HLA-B PE=1 SV=1 |
|  | **P18124\|RL7_HUMAN** | 60S ribosomal protein L7 OS=Homo sapiens GN=RPL7 PE=1 SV=1 |
|  | **P28799\|GRN_HUMAN** | Granulins OS=Homo sapiens GN=GRN PE=1 SV=2 |
|  | **O14548\|COX7R_HUMAN** | Cytochrome c oxidase subunit 7A-related protein, mitochondrial OS=Homo sapiens GN=COX7A2L PE=1 SV=2 |
|  | **P11498\|PYC_HUMAN** | Pyruvate carboxylase, mitochondrial OS=Homo sapiens GN=PC PE=1 SV=2 |
|  | **P80294\|MT1H_HUMAN** | Metallothionein-1H OS=Homo sapiens GN=MT1H PE=1 SV=1 |
|  | **Q96S66\|CLCC1_HUMAN** | Chloride channel CLIC-like protein 1 OS=Homo sapiens GN=CLCC1 PE=1 SV=1 |
|  | **Q9Y5L4\|TIM13_HUMAN** | Mitochondrial import inner membrane translocase subunit Tim13 OS=Homo sapiens GN=TIMM13 PE=1 SV=1 |
|  | **P08865\|RSSA_HUMAN** | 40S ribosomal protein SA OS=Homo sapiens GN=RPSA PE=1 SV=4 |
|  | **P99999\|CYC_HUMAN** | Cytochrome c OS=Homo sapiens GN=CYCS PE=1 SV=2 |
|  | **P51153\|RAB13_HUMAN** | Ras-related protein Rab-13 OS=Homo sapiens GN=RAB13 PE=1 SV=1 |
|  | **O43181\|NDUS4_HUMAN** | NADH dehydrogenase [ubiquinone] iron-sulfur protein 4, mitochondrial OS=Homo sapiens GN=NDUFS4 PE=1 SV=1 |
|  | **Q29960\|1C16_HUMAN** | HLA class I histocompatibility antigen, Cw-16 alpha chain OS=Homo sapiens GN=HLA-C PE=2 SV=1 |
|  | **Q95604\|1C17_HUMAN** | HLA class I histocompatibility antigen, Cw-17 alpha chain OS=Homo sapiens GN=HLA-C PE=1 SV=1 |
|  | **P31639\|SC5A2_HUMAN** | Sodium/glucose cotransporter 2 OS=Homo sapiens GN=SLC5A2 PE=1 SV=1 |
|  | **P09601\|HMOX1_HUMAN** | Heme oxygenase 1 OS=Homo sapiens GN=HMOX1 PE=1 SV=1 |
|  | **O15126\|SCAM1_HUMAN** | Secretory carrier-associated membrane protein 1 OS=Homo sapiens GN=SCAMP1 PE=1 SV=2 |
|  | **P23229\|ITA6_HUMAN** | Integrin alpha-6 OS=Homo sapiens GN=ITGA6 PE=1 SV=4 |
|  | **P55145\|MANF_HUMAN** | Mesencephalic astrocyte-derived neurotrophic factor OS=Homo sapiens GN=MANF PE=1 SV=2 |
|  | **P50454\|SERPH_HUMAN** | Serpin H1 OS=Homo sapiens GN=SERPINH1 PE=1 SV=2 |
|  | **P09669\|COX6C_HUMAN** | Cytochrome c oxidase subunit 6C OS=Homo sapiens GN=COX6C PE=1 SV=2 |
|  | **P23284\|PPIB_HUMAN** | Peptidyl-prolyl cis-trans isomerase B OS=Homo sapiens GN=PPIB PE=1 SV=2 |
|  | **O75348\|VATG1_HUMAN** | V-type proton ATPase subunit G 1 OS=Homo sapiens GN=ATP6V1G1 PE=1 SV=3 |
|  | **Q9Y512\|SAM50_HUMAN** | Sorting and assembly machinery component 50 homolog OS=Homo sapiens GN=SAMM50 PE=1 SV=3 |
|  | **O95470\|SGPL1_HUMAN** | Sphingosine-1-phosphate lyase 1 OS=Homo sapiens GN=SGPL1 PE=1 SV=3 |
|  | **P13761\|2B17_HUMAN** | HLA class II histocompatibility antigen, DRB1-7 beta chain OS=Homo sapiens GN=HLA-DRB1 PE=1 SV=1 |
|  | **Q9Y536\|PAL4B_HUMAN** | Peptidylprolyl cis-trans isomerase A-like 4B OS=Homo sapiens GN=PPIAL4B PE=1 SV=1 |
|  | **P09327\|VILI_HUMAN** | Villin-1 OS=Homo sapiens GN=VIL1 PE=1 SV=4 |
|  | **Q8TBA6\|GOGA5_HUMAN** | Golgin subfamily A member 5 OS=Homo sapiens GN=GOLGA5 PE=1 SV=2 |
|  | **Q6UW02\|CP20A_HUMAN** | Cytochrome P450 20A1 OS=Homo sapiens GN=CYP20A1 PE=1 SV=1 |
|  | **P00966\|ASSY_HUMAN** | Argininosuccinate synthase OS=Homo sapiens GN=ASS1 PE=1 SV=2 |
|  | **Q08426\|ECHP_HUMAN** | Peroxisomal bifunctional enzyme OS=Homo sapiens GN=EHHADH PE=1 SV=3 |
|  | **Q8N5K1\|CISD2_HUMAN** | CDGSH iron sulfur domain-containing protein 2 OS=Homo sapiens GN=CISD2 PE=1 SV=1 |
|  | **Q9BS26\|ERP44_HUMAN** | Endoplasmic reticulum resident protein ERp44 OS=Homo sapiens GN=ERP44 PE=1 SV=1 |
|  | **P51970\|NDUA8_HUMAN** | NADH dehydrogenase [ubiquinone] 1 alpha subcomplex subunit 8 OS=Homo sapiens GN=NDUFA8 PE=1 SV=3 |
|  | **P62847\|RS24_HUMAN** | 40S ribosomal protein S24 OS=Homo sapiens GN=RPS24 PE=1 SV=1 |
|  | **O15173\|PGRC2_HUMAN** | Membrane-associated progesterone receptor component 2 OS=Homo sapiens GN=PGRMC2 PE=1 SV=1 |
|  | **P10319\|1B58_HUMAN** | HLA class I histocompatibility antigen, B-58 alpha chain OS=Homo sapiens GN=HLA-B PE=2 SV=1 |
|  | **P18465\|1B57_HUMAN** | HLA class I histocompatibility antigen, B-57 alpha chain OS=Homo sapiens GN=HLA-B PE=1 SV=1 |
|  | **P30685\|1B35_HUMAN** | HLA class I histocompatibility antigen, B-35 alpha chain OS=Homo sapiens GN=HLA-B PE=1 SV=1 |
|  | **P05106\|ITB3_HUMAN** | Integrin beta-3 OS=Homo sapiens GN=ITGB3 PE=1 SV=2 |
|  | **P13760\|2B14_HUMAN** | HLA class II histocompatibility antigen, DRB1-4 beta chain OS=Homo sapiens GN=HLA-DRB1 PE=1 SV=1 |
|  | **P01913\|2B32_HUMAN** | HLA class II histocompatibility antigen, DRB3-2 beta chain OS=Homo sapiens GN=HLA-DRB3 PE=2 SV=3 |
|  | **P79483\|2B31_HUMAN** | HLA class II histocompatibility antigen, DRB3-1 beta chain OS=Homo sapiens GN=HLA-DRB3 PE=1 SV=1 |
|  | **P36578\|RL4_HUMAN** | 60S ribosomal protein L4 OS=Homo sapiens GN=RPL4 PE=1 SV=5 |
|  | **P07910\|HNRPC_HUMAN** | Heterogeneous nuclear ribonucleoproteins C1/C2 OS=Homo sapiens GN=HNRNPC PE=1 SV=4 |
|  | **P09758\|TACD2_HUMAN** | Tumor-associated calcium signal transducer 2 OS=Homo sapiens GN=TACSTD2 PE=1 SV=3 |
|  | **A6NHL2\|TBAL3_HUMAN** | Tubulin alpha chain-like 3 OS=Homo sapiens GN=TUBAL3 PE=1 SV=2 |
|  | **Q93088\|BHMT1_HUMAN** | Betaine--homocysteine S-methyltransferase 1 OS=Homo sapiens GN=BHMT PE=1 SV=2 |
|  | **Q9NVJ2\|ARL8B_HUMAN** | ADP-ribosylation factor-like protein 8B OS=Homo sapiens GN=ARL8B PE=1 SV=1 |
|  | **Q13740\|CD166_HUMAN** | CD166 antigen OS=Homo sapiens GN=ALCAM PE=1 SV=2 |
|  | **P61769\|B2MG_HUMAN** | Beta-2-microglobulin OS=Homo sapiens GN=B2M PE=1 SV=1 |
|  | **Q9Y6N5\|SQRD_HUMAN** | Sulfide:quinone oxidoreductase, mitochondrial OS=Homo sapiens GN=SQRDL PE=1 SV=1 |
|  | **Q3LXA3\|DHAK_HUMAN** | Bifunctional ATP-dependent dihydroxyacetone kinase/FAD-AMP lyase (cyclizing) OS=Homo sapiens GN=DAK PE=1 SV=1 |
|  | **Q02218\|ODO1_HUMAN** | 2-oxoglutarate dehydrogenase E1 component, mitochondrial OS=Homo sapiens GN=OGDH PE=1 SV=3 |
|  | **P04733\|MT1F_HUMAN** | Metallothionein-1F OS=Homo sapiens GN=MT1F PE=1 SV=1 |
|  | **P35580\|MYH10_HUMAN** | Myosin-10 OS=Homo sapiens GN=MYH10 PE=1 SV=3 |
|  | **P21333\|FLNA_HUMAN** | Filamin-A OS=Homo sapiens GN=FLNA PE=1 SV=4 |
|  | **Q6NUK1\|SCMC1_HUMAN** | Calcium-binding mitochondrial carrier protein SCaMC-1 OS=Homo sapiens GN=SLC25A24 PE=1 SV=2 |
|  | **Q1EHB4\|SC5AC_HUMAN** | Sodium-coupled monocarboxylate transporter 2 OS=Homo sapiens GN=SLC5A12 PE=2 SV=2 |
|  | **P56385\|ATP5I_HUMAN** | ATP synthase subunit e, mitochondrial OS=Homo sapiens GN=ATP5I PE=1 SV=2 |
|  | **Q9Y5J7\|TIM9_HUMAN** | Mitochondrial import inner membrane translocase subunit Tim9 OS=Homo sapiens GN=TIMM9 PE=1 SV=1 |
|  | **P02549\|SPTA1_HUMAN** | Spectrin alpha chain, erythrocyte OS=Homo sapiens GN=SPTA1 PE=1 SV=4 |
|  | **P02790\|HEMO_HUMAN** | Hemopexin OS=Homo sapiens GN=HPX PE=1 SV=2 |
|  | **P07197\|NFM_HUMAN** | Neurofilament medium polypeptide OS=Homo sapiens GN=NEFM PE=1 SV=2 |
|  | **Q16352\|AINX_HUMAN** | Alpha-internexin OS=Homo sapiens GN=INA PE=1 SV=2 |
|  | **P17661\|DESM_HUMAN** | Desmin OS=Homo sapiens GN=DES PE=1 SV=3 |
|  | **O00291\|HIP1_HUMAN** | Huntingtin-interacting protein 1 OS=Homo sapiens GN=HIP1 PE=1 SV=5 |
|  | **P16188\|1A30_HUMAN** | HLA class I histocompatibility antigen, A-30 alpha chain OS=Homo sapiens GN=HLA-A PE=1 SV=2 |
|  | **P01891\|1A68_HUMAN** | HLA class I histocompatibility antigen, A-68 alpha chain OS=Homo sapiens GN=HLA-A PE=1 SV=4 |
|  | **P10316\|1A69_HUMAN** | HLA class I histocompatibility antigen, A-69 alpha chain OS=Homo sapiens GN=HLA-A PE=1 SV=2 |
|  | **P30512\|1A29_HUMAN** | HLA class I histocompatibility antigen, A-29 alpha chain OS=Homo sapiens GN=HLA-A PE=2 SV=2 |
|  | **P05534\|1A24_HUMAN** | HLA class I histocompatibility antigen, A-24 alpha chain OS=Homo sapiens GN=HLA-A PE=1 SV=2 |
|  | **P10314\|1A32_HUMAN** | HLA class I histocompatibility antigen, A-32 alpha chain OS=Homo sapiens GN=HLA-A PE=2 SV=2 |
|  | **P01892\|1A02_HUMAN** | HLA class I histocompatibility antigen, A-2 alpha chain OS=Homo sapiens GN=HLA-A PE=1 SV=1 |
|  | **P30447\|1A23_HUMAN** | HLA class I histocompatibility antigen, A-23 alpha chain OS=Homo sapiens GN=HLA-A PE=1 SV=1 |
|  | **P30459\|1A74_HUMAN** | HLA class I histocompatibility antigen, A-74 alpha chain OS=Homo sapiens GN=HLA-A PE=1 SV=1 |
|  | **P30443\|1A01_HUMAN** | HLA class I histocompatibility antigen, A-1 alpha chain OS=Homo sapiens GN=HLA-A PE=1 SV=1 |
|  | **Q13113\|PDZ1I_HUMAN** | PDZK1-interacting protein 1 OS=Homo sapiens GN=PDZK1IP1 PE=1 SV=1 |
|  | **P26885\|FKBP2_HUMAN** | FK506-binding protein 2 OS=Homo sapiens GN=FKBP2 PE=1 SV=2 |
|  | **P20711\|DDC_HUMAN** | Aromatic-L-amino-acid decarboxylase OS=Homo sapiens GN=DDC PE=1 SV=1 |
|  | **P98160\|PGBM_HUMAN** | Basement membrane-specific heparan sulfate proteoglycan core protein OS=Homo sapiens GN=HSPG2 PE=1 SV=3 |
|  | **O14949\|QCR8_HUMAN** | Cytochrome b-c1 complex subunit 8 OS=Homo sapiens GN=UQCRQ PE=1 SV=4 |
|  | **P09496\|CLCA_HUMAN** | Clathrin light chain A OS=Homo sapiens GN=CLTA PE=1 SV=1 |
|  | **P34896\|GLYC_HUMAN** | Serine hydroxymethyltransferase, cytosolic OS=Homo sapiens GN=SHMT1 PE=1 SV=1 |
|  | **Q7Z7H5\|TMED4_HUMAN** | Transmembrane emp24 domain-containing protein 4 OS=Homo sapiens GN=TMED4 PE=1 SV=1 |
|  | **Q9BVK6\|TMED9_HUMAN** | Transmembrane emp24 domain-containing protein 9 OS=Homo sapiens GN=TMED9 PE=1 SV=2 |
|  | **O14773\|TPP1_HUMAN** | Tripeptidyl-peptidase 1 OS=Homo sapiens GN=TPP1 PE=1 SV=2 |
|  | **Q9H330\|CI005_HUMAN** | Transmembrane protein C9orf5 OS=Homo sapiens GN=C9orf5 PE=1 SV=2 |
|  | **P47914\|RL29_HUMAN** | 60S ribosomal protein L29 OS=Homo sapiens GN=RPL29 PE=1 SV=2 |
|  | **Q9P0J0\|NDUAD_HUMAN** | NADH dehydrogenase [ubiquinone] 1 alpha subcomplex subunit 13 OS=Homo sapiens GN=NDUFA13 PE=1 SV=3 |
|  | **Q9NS69\|TOM22_HUMAN** | Mitochondrial import receptor subunit TOM22 homolog OS=Homo sapiens GN=TOMM22 PE=1 SV=3 |
|  | **P14618\|KPYM_HUMAN** | Pyruvate kinase isozymes M1/M2 OS=Homo sapiens GN=PKM2 PE=1 SV=4 |
|  | **O15372\|EIF3H_HUMAN** | Eukaryotic translation initiation factor 3 subunit H OS=Homo sapiens GN=EIF3H PE=1 SV=1 |
|  | **P08134\|RHOC_HUMAN** | Rho-related GTP-binding protein RhoC OS=Homo sapiens GN=RHOC PE=1 SV=1 |
|  | **P61586\|RHOA_HUMAN** | Transforming protein RhoA OS=Homo sapiens GN=RHOA PE=1 SV=1 |
|  | **P05164\|PERM_HUMAN** | Myeloperoxidase OS=Homo sapiens GN=MPO PE=1 SV=1 |
|  | **Q96NB2\|SFXN2_HUMAN** | Sideroflexin-2 OS=Homo sapiens GN=SFXN2 PE=1 SV=2 |
|  | **Q8NBX0\|SCPDH_HUMAN** | Probable saccharopine dehydrogenase OS=Homo sapiens GN=SCCPDH PE=1 SV=1 |
|  | **Q14624\|ITIH4_HUMAN** | Inter-alpha-trypsin inhibitor heavy chain H4 OS=Homo sapiens GN=ITIH4 PE=1 SV=4 |
|  | **Q7Z6K2\|TPISL_HUMAN** | Putative triosephosphate isomerase-like protein LOC286016 OS=Homo sapiens PE=1 SV=2 |
|  | **P09525\|ANXA4_HUMAN** | Annexin A4 OS=Homo sapiens GN=ANXA4 PE=1 SV=4 |
|  | **O00748\|EST2_HUMAN** | Carboxylesterase 2 OS=Homo sapiens GN=CES2 PE=1 SV=1 |
|  | **A6NMY6\|AXA2L_HUMAN** | Putative annexin A2-like protein OS=Homo sapiens GN=ANXA2P2 PE=5 SV=2 |
|  | **O43464\|HTRA2_HUMAN** | Serine protease HTRA2, mitochondrial OS=Homo sapiens GN=HTRA2 PE=1 SV=2 |
|  | **Q9Y4W6\|AFG32_HUMAN** | AFG3-like protein 2 OS=Homo sapiens GN=AFG3L2 PE=1 SV=2 |
|  | **P27348\|1433T_HUMAN** | 14-3-3 protein theta OS=Homo sapiens GN=YWHAQ PE=1 SV=1 |
|  | **P30485\|1B47_HUMAN** | HLA class I histocompatibility antigen, B-47 alpha chain OS=Homo sapiens GN=HLA-B PE=2 SV=1 |
|  | **P62280\|RS11_HUMAN** | 40S ribosomal protein S11 OS=Homo sapiens GN=RPS11 PE=1 SV=3 |
|  | **Q96KP4\|CNDP2_HUMAN** | Cytosolic non-specific dipeptidase OS=Homo sapiens GN=CNDP2 PE=1 SV=2 |
|  | **O15260\|SURF4_HUMAN** | Surfeit locus protein 4 OS=Homo sapiens GN=SURF4 PE=1 SV=3 |
|  | **P01859\|IGHG2_HUMAN** | Ig gamma-2 chain C region OS=Homo sapiens GN=IGHG2 PE=1 SV=2 |
|  | **Q14677\|EPN4_HUMAN** | Clathrin interactor 1 OS=Homo sapiens GN=CLINT1 PE=1 SV=1 |
|  | **P02655\|APOC2_HUMAN** | Apolipoprotein C-II OS=Homo sapiens GN=APOC2 PE=1 SV=1 |
|  | **Q14165\|MLEC_HUMAN** | Malectin OS=Homo sapiens GN=MLEC PE=1 SV=1 |
|  | **P08559\|ODPA_HUMAN** | Pyruvate dehydrogenase E1 component subunit alpha, somatic form, mitochondrial OS=Homo sapiens GN=PDHA1 PE=1 SV=3 |
|  | **Q08380\|LG3BP_HUMAN** | Galectin-3-binding protein OS=Homo sapiens GN=LGALS3BP PE=1 SV=1 |
|  | **P56556\|NDUA6_HUMAN** | NADH dehydrogenase [ubiquinone] 1 alpha subcomplex subunit 6 OS=Homo sapiens GN=NDUFA6 PE=1 SV=2 |
|  | **Q8N4Q1\|MIA40_HUMAN** | Mitochondrial intermembrane space import and assembly protein 40 OS=Homo sapiens GN=CHCHD4 PE=1 SV=1 |
|  | **Q96BM9\|ARL8A_HUMAN** | ADP-ribosylation factor-like protein 8A OS=Homo sapiens GN=ARL8A PE=1 SV=1 |
|  | **O43169\|CYB5B_HUMAN** | Cytochrome b5 type B OS=Homo sapiens GN=CYB5B PE=1 SV=2 |
|  | **P18463\|1B37_HUMAN** | HLA class I histocompatibility antigen, B-37 alpha chain OS=Homo sapiens GN=HLA-B PE=2 SV=1 |
|  | **Q8IYT4\|KATL2_HUMAN** | Katanin p60 ATPase-containing subunit A-like 2 OS=Homo sapiens GN=KATNAL2 PE=2 SV=3 |
|  | **Q9NX47\|MARH5_HUMAN** | E3 ubiquitin-protein ligase MARCH5 OS=Homo sapiens GN=MARCH5 PE=1 SV=1 |
|  | **Q9BWM7\|SFXN3_HUMAN** | Sideroflexin-3 OS=Homo sapiens GN=SFXN3 PE=2 SV=1 |
|  | **P61916\|NPC2_HUMAN** | Epididymal secretory protein E1 OS=Homo sapiens GN=NPC2 PE=1 SV=1 |
|  | **O00159\|MYO1C_HUMAN** | Myosin-Ic OS=Homo sapiens GN=MYO1C PE=1 SV=3 |
|  | **P62277\|RS13_HUMAN** | 40S ribosomal protein S13 OS=Homo sapiens GN=RPS13 PE=1 SV=2 |
|  | **O43920\|NDUS5_HUMAN** | NADH dehydrogenase [ubiquinone] iron-sulfur protein 5 OS=Homo sapiens GN=NDUFS5 PE=1 SV=3 |
|  | **Q9Y6M9\|NDUB9_HUMAN** | NADH dehydrogenase [ubiquinone] 1 beta subcomplex subunit 9 OS=Homo sapiens GN=NDUFB9 PE=1 SV=3 |
|  | **Q9Y679\|AUP1_HUMAN** | Ancient ubiquitous protein 1 OS=Homo sapiens GN=AUP1 PE=1 SV=1 |
|  | **Q16822\|PCKGM_HUMAN** | Phosphoenolpyruvate carboxykinase [GTP], mitochondrial OS=Homo sapiens GN=PCK2 PE=1 SV=2 |
|  | **O00476\|NPT4_HUMAN** | Sodium-dependent phosphate transport protein 4 OS=Homo sapiens GN=SLC17A3 PE=2 SV=2 |
|  | **Q9BXJ8\|T120A_HUMAN** | Transmembrane protein 120A OS=Homo sapiens GN=TMEM120A PE=2 SV=1 |
|  | **P62424\|RL7A_HUMAN** | 60S ribosomal protein L7a OS=Homo sapiens GN=RPL7A PE=1 SV=2 |
|  | **P53367\|ARFP1_HUMAN** | Arfaptin-1 OS=Homo sapiens GN=ARFIP1 PE=1 SV=2 |
|  | **Q9UHQ4\|BAP29_HUMAN** | B-cell receptor-associated protein 29 OS=Homo sapiens GN=BCAP29 PE=1 SV=2 |
|  | **P22307\|NLTP_HUMAN** | Non-specific lipid-transfer protein OS=Homo sapiens GN=SCP2 PE=1 SV=2 |
|  | **Q09160\|1A80_HUMAN** | HLA class I histocompatibility antigen, A-80 alpha chain OS=Homo sapiens GN=HLA-A PE=2 SV=1 |
|  | **P01893\|HLAH_HUMAN** | Putative HLA class I histocompatibility antigen, alpha chain H OS=Homo sapiens GN=HLA-H PE=5 SV=2 |
|  | **P22059\|OSBP1_HUMAN** | Oxysterol-binding protein 1 OS=Homo sapiens GN=OSBP PE=1 SV=1 |
|  | **P46779\|RL28_HUMAN** | 60S ribosomal protein L28 OS=Homo sapiens GN=RPL28 PE=1 SV=3 |
|  | **Q9UBC0\|HNF6_HUMAN** | Hepatocyte nuclear factor 6 OS=Homo sapiens GN=ONECUT1 PE=2 SV=1 |
|  | **P11234\|RALB_HUMAN** | Ras-related protein Ral-B OS=Homo sapiens GN=RALB PE=1 SV=1 |
|  | **O75438\|NDUB1_HUMAN** | NADH dehydrogenase [ubiquinone] 1 beta subcomplex subunit 1 OS=Homo sapiens GN=NDUFB1 PE=1 SV=1 |
|  | **Q9NX63\|CHCH3_HUMAN** | Coiled-coil-helix-coiled-coil-helix domain-containing protein 3, mitochondrial OS=Homo sapiens GN=CHCHD3 PE=1 SV=1 |
|  | **Q6UWM9\|UD2A3_HUMAN** | UDP-glucuronosyltransferase 2A3 OS=Homo sapiens GN=UGT2A3 PE=2 SV=1 |
|  | **Q9BXJ7\|AMNLS_HUMAN** | Protein amnionless OS=Homo sapiens GN=AMN PE=1 SV=1 |
|  | **P40926\|MDHM_HUMAN** | Malate dehydrogenase, mitochondrial OS=Homo sapiens GN=MDH2 PE=1 SV=3 |
|  | **P02649\|APOE_HUMAN** | Apolipoprotein E OS=Homo sapiens GN=APOE PE=1 SV=1 |
|  | **Q71UM5\|RS27L_HUMAN** | 40S ribosomal protein S27-like protein OS=Homo sapiens GN=RPS27L PE=1 SV=3 |
|  | **P03928\|ATP8_HUMAN** | ATP synthase protein 8 OS=Homo sapiens GN=MT-ATP8 PE=1 SV=1 |
|  | **Q6P1M0\|S27A4_HUMAN** | Long-chain fatty acid transport protein 4 OS=Homo sapiens GN=SLC27A4 PE=1 SV=1 |
|  | **P07858\|CATB_HUMAN** | Cathepsin B OS=Homo sapiens GN=CTSB PE=1 SV=3 |
|  | **P30085\|KCY_HUMAN** | UMP-CMP kinase OS=Homo sapiens GN=CMPK1 PE=1 SV=3 |
|  | **Q9HCK1\|ZDBF2_HUMAN** | DBF4-type zinc finger-containing protein 2 OS=Homo sapiens GN=ZDBF2 PE=1 SV=3 |
|  | **P14406\|CX7A2_HUMAN** | Cytochrome c oxidase polypeptide 7A2, mitochondrial OS=Homo sapiens GN=COX7A2 PE=1 SV=1 |
|  | **Q9H4M9\|EHD1_HUMAN** | EH domain-containing protein 1 OS=Homo sapiens GN=EHD1 PE=1 SV=2 |
|  | **P08183\|MDR1_HUMAN** | Multidrug resistance protein 1 OS=Homo sapiens GN=ABCB1 PE=1 SV=3 |
|  | **P21439\|MDR3_HUMAN** | Multidrug resistance protein 3 OS=Homo sapiens GN=ABCB4 PE=1 SV=2 |
|  | **Q9BV40\|VAMP8_HUMAN** | Vesicle-associated membrane protein 8 OS=Homo sapiens GN=VAMP8 PE=1 SV=1 |
|  | **P06753\|TPM3_HUMAN** | Tropomyosin alpha-3 chain OS=Homo sapiens GN=TPM3 PE=1 SV=1 |
|  | **P07951\|TPM2_HUMAN** | Tropomyosin beta chain OS=Homo sapiens GN=TPM2 PE=1 SV=1 |
|  | **P09493\|TPM1_HUMAN** | Tropomyosin alpha-1 chain OS=Homo sapiens GN=TPM1 PE=1 SV=2 |
|  | **O43175\|SERA_HUMAN** | D-3-phosphoglycerate dehydrogenase OS=Homo sapiens GN=PHGDH PE=1 SV=4 |
|  | **P19022\|CADH2_HUMAN** | Cadherin-2 OS=Homo sapiens GN=CDH2 PE=1 SV=4 |
|  | **P50402\|EMD_HUMAN** | Emerin OS=Homo sapiens GN=EMD PE=1 SV=1 |
|  | **P13639\|EF2_HUMAN** | Elongation factor 2 OS=Homo sapiens GN=EEF2 PE=1 SV=4 |
|  | **P10515\|ODP2_HUMAN** | Dihydrolipoyllysine-residue acetyltransferase component of pyruvate dehydrogenase complex, mitochondrial OS=Homo sapiens GN=DLAT PE=1 SV=3 |
|  | **Q6ZQN7\|SO4C1_HUMAN** | Solute carrier organic anion transporter family member 4C1 OS=Homo sapiens GN=SLCO4C1 PE=1 SV=1 |
|  | **Q96AG4\|LRC59_HUMAN** | Leucine-rich repeat-containing protein 59 OS=Homo sapiens GN=LRRC59 PE=1 SV=1 |
|  | **O94919\|ENDD1_HUMAN** | Endonuclease domain-containing 1 protein OS=Homo sapiens GN=ENDOD1 PE=1 SV=2 |
|  | **P08708\|RS17_HUMAN** | 40S ribosomal protein S17 OS=Homo sapiens GN=RPS17 PE=1 SV=2 |
|  | **P54289\|CA2D1_HUMAN** | Voltage-dependent calcium channel subunit alpha-2/delta-1 OS=Homo sapiens GN=CACNA2D1 PE=1 SV=2 |
|  | **O00299\|CLIC1_HUMAN** | Chloride intracellular channel protein 1 OS=Homo sapiens GN=CLIC1 PE=1 SV=4 |
|  | **Q9Y696\|CLIC4_HUMAN** | Chloride intracellular channel protein 4 OS=Homo sapiens GN=CLIC4 PE=1 SV=4 |
|  | **P05787\|K2C8_HUMAN** | Keratin, type II cytoskeletal 8 OS=Homo sapiens GN=KRT8 PE=1 SV=7 |
|  | **P35221\|CTNA1_HUMAN** | Catenin alpha-1 OS=Homo sapiens GN=CTNNA1 PE=1 SV=1 |
|  | **Q02543\|RL18A_HUMAN** | 60S ribosomal protein L18a OS=Homo sapiens GN=RPL18A PE=1 SV=2 |
|  | **P67936\|TPM4_HUMAN** | Tropomyosin alpha-4 chain OS=Homo sapiens GN=TPM4 PE=1 SV=3 |
|  | **P30038\|AL4A1_HUMAN** | Delta-1-pyrroline-5-carboxylate dehydrogenase, mitochondrial OS=Homo sapiens GN=ALDH4A1 PE=1 SV=3 |
|  | **P05783\|K1C18_HUMAN** | Keratin, type I cytoskeletal 18 OS=Homo sapiens GN=KRT18 PE=1 SV=2 |
|  | **Q96HR9\|REEP6_HUMAN** | Receptor expression-enhancing protein 6 OS=Homo sapiens GN=REEP6 PE=1 SV=1 |
|  | **Q9NX20\|RM16_HUMAN** | 39S ribosomal protein L16, mitochondrial OS=Homo sapiens GN=MRPL16 PE=1 SV=1 |
|  | **P05386\|RLA1_HUMAN** | 60S acidic ribosomal protein P1 OS=Homo sapiens GN=RPLP1 PE=1 SV=1 |
|  | **Q8IVB4\|SL9A9_HUMAN** | Sodium/hydrogen exchanger 9 OS=Homo sapiens GN=SLC9A9 PE=1 SV=1 |
|  | **P13929\|ENOB_HUMAN** | Beta-enolase OS=Homo sapiens GN=ENO3 PE=1 SV=4 |
|  | **Q15043\|S39AE_HUMAN** | Zinc transporter ZIP14 OS=Homo sapiens GN=SLC39A14 PE=1 SV=2 |
|  | **Q92820\|GGH_HUMAN** | Gamma-glutamyl hydrolase OS=Homo sapiens GN=GGH PE=1 SV=2 |
|  | **Q8WXE0\|CSKI2_HUMAN** | Caskin-2 OS=Homo sapiens GN=CASKIN2 PE=1 SV=1 |
|  | **Q9NZ01\|TECR_HUMAN** | Trans-2,3-enoyl-CoA reductase OS=Homo sapiens GN=TECR PE=1 SV=1 |
|  | **O43678\|NDUA2_HUMAN** | NADH dehydrogenase [ubiquinone] 1 alpha subcomplex subunit 2 OS=Homo sapiens GN=NDUFA2 PE=1 SV=3 |
|  | **P62906\|RL10A_HUMAN** | 60S ribosomal protein L10a OS=Homo sapiens GN=RPL10A PE=1 SV=2 |
|  | **P84095\|RHOG_HUMAN** | Rho-related GTP-binding protein RhoG OS=Homo sapiens GN=RHOG PE=1 SV=1 |
|  | **O75865\|TPC6A_HUMAN** | Trafficking protein particle complex subunit 6A OS=Homo sapiens GN=TRAPPC6A PE=1 SV=2 |
|  | **P51148\|RAB5C_HUMAN** | Ras-related protein Rab-5C OS=Homo sapiens GN=RAB5C PE=1 SV=2 |
|  | **P13498\|CY24A_HUMAN** | Cytochrome b-245 light chain OS=Homo sapiens GN=CYBA PE=1 SV=2 |
|  | **O43399\|TPD54_HUMAN** | Tumor protein D54 OS=Homo sapiens GN=TPD52L2 PE=1 SV=2 |
|  | **Q9BQB6\|VKOR1_HUMAN** | Vitamin K epoxide reductase complex subunit 1 OS=Homo sapiens GN=VKORC1 PE=1 SV=1 |
|  | **P26232\|CTNA2_HUMAN** | Catenin alpha-2 OS=Homo sapiens GN=CTNNA2 PE=1 SV=5 |
|  | **P53990\|IST1_HUMAN** | IST1 homolog OS=Homo sapiens GN=KIAA0174 PE=1 SV=1 |
|  | **Q2M3M2\|SC5A9_HUMAN** | Sodium/glucose cotransporter 4 OS=Homo sapiens GN=SLC5A9 PE=2 SV=2 |
|  | **Q9NVH1\|DJC11_HUMAN** | DnaJ homolog subfamily C member 11 OS=Homo sapiens GN=DNAJC11 PE=1 SV=2 |
|  | **Q8TF72\|SHRM3_HUMAN** | Protein Shroom3 OS=Homo sapiens GN=SHROOM3 PE=1 SV=1 |
|  | **Q13724\|MOGS_HUMAN** | Mannosyl-oligosaccharide glucosidase OS=Homo sapiens GN=MOGS PE=1 SV=5 |
|  | **Q14103\|HNRPD_HUMAN** | Heterogeneous nuclear ribonucleoprotein D0 OS=Homo sapiens GN=HNRNPD PE=1 SV=1 |
|  | **P02753\|RET4_HUMAN** | Retinol-binding protein 4 OS=Homo sapiens GN=RBP4 PE=1 SV=3 |
|  | **Q5JTJ3\|CA031_HUMAN** | Uncharacterized protein C1orf31 OS=Homo sapiens GN=C1orf31 PE=2 SV=1 |
|  | **P32119\|PRDX2_HUMAN** | Peroxiredoxin-2 OS=Homo sapiens GN=PRDX2 PE=1 SV=5 |
|  | **Q8ND61\|CC020_HUMAN** | Uncharacterized protein C3orf20 OS=Homo sapiens GN=C3orf20 PE=2 SV=2 |
|  | **Q8WUY1\|CH055_HUMAN** | UPF0670 protein C8orf55 OS=Homo sapiens GN=C8orf55 PE=1 SV=2 |
|  | **Q96N66\|MBOA7_HUMAN** | Lysophospholipid acyltransferase 7 OS=Homo sapiens GN=MBOAT7 PE=1 SV=2 |
|  | **Q13510\|ASAH1_HUMAN** | Acid ceramidase OS=Homo sapiens GN=ASAH1 PE=1 SV=5 |
|  | **Q15436\|SC23A_HUMAN** | Protein transport protein Sec23A OS=Homo sapiens GN=SEC23A PE=1 SV=2 |
|  | **P09382\|LEG1_HUMAN** | Galectin-1 OS=Homo sapiens GN=LGALS1 PE=1 SV=2 |
|  | **P54710\|ATNG_HUMAN** | Sodium/potassium-transporting ATPase gamma chain OS=Homo sapiens GN=FXYD2 PE=1 SV=3 |
|  | **P62753\|RS6_HUMAN** | 40S ribosomal protein S6 OS=Homo sapiens GN=RPS6 PE=1 SV=1 |
|  | **Q9Y2L6\|FRM4B_HUMAN** | FERM domain-containing protein 4B OS=Homo sapiens GN=FRMD4B PE=1 SV=2 |
|  | **Q9ULD0\|OGDHL_HUMAN** | 2-oxoglutarate dehydrogenase E1 component-like, mitochondrial OS=Homo sapiens GN=OGDHL PE=2 SV=3 |
|  | **Q9NP72\|RAB18_HUMAN** | Ras-related protein Rab-18 OS=Homo sapiens GN=RAB18 PE=1 SV=1 |
|  | **Q9UBQ7\|GRHPR_HUMAN** | Glyoxylate reductase/hydroxypyruvate reductase OS=Homo sapiens GN=GRHPR PE=1 SV=1 |
|  | **P16152\|CBR1_HUMAN** | Carbonyl reductase [NADPH] 1 OS=Homo sapiens GN=CBR1 PE=1 SV=3 |
|  | **O95169\|NDUB8_HUMAN** | NADH dehydrogenase [ubiquinone] 1 beta subcomplex subunit 8, mitochondrial OS=Homo sapiens GN=NDUFB8 PE=1 SV=1 |
|  | **P06703\|S10A6_HUMAN** | Protein S100-A6 OS=Homo sapiens GN=S100A6 PE=1 SV=1 |
|  | **Q9H0C2\|ADT4_HUMAN** | ADP/ATP translocase 4 OS=Homo sapiens GN=SLC25A31 PE=1 SV=1 |
|  | **Q9Y3D6\|FIS1_HUMAN** | Mitochondrial fission 1 protein OS=Homo sapiens GN=FIS1 PE=1 SV=2 |
|  | **Q9Y320\|TMX2_HUMAN** | Thioredoxin-related transmembrane protein 2 OS=Homo sapiens GN=TMX2 PE=1 SV=1 |
|  | **P50053\|KHK_HUMAN** | Ketohexokinase OS=Homo sapiens GN=KHK PE=1 SV=1 |
|  | **P01011\|AACT_HUMAN** | Alpha-1-antichymotrypsin OS=Homo sapiens GN=SERPINA3 PE=1 SV=2 |
|  | **P21964\|COMT_HUMAN** | Catechol O-methyltransferase OS=Homo sapiens GN=COMT PE=1 SV=2 |
|  | **O75954\|TSN9_HUMAN** | Tetraspanin-9 OS=Homo sapiens GN=TSPAN9 PE=1 SV=1 |
|  | **Q7Z4W1\|DCXR_HUMAN** | L-xylulose reductase OS=Homo sapiens GN=DCXR PE=1 SV=2 |
|  | **Q9H0J4\|QRIC2_HUMAN** | Glutamine-rich protein 2 OS=Homo sapiens GN=QRICH2 PE=1 SV=1 |
|  | **Q9H299\|SH3L3_HUMAN** | SH3 domain-binding glutamic acid-rich-like protein 3 OS=Homo sapiens GN=SH3BGRL3 PE=1 SV=1 |
|  | **P00738\|HPT_HUMAN** | Haptoglobin OS=Homo sapiens GN=HP PE=1 SV=1 |
|  | **P07305\|H10_HUMAN** | Histone H1.0 OS=Homo sapiens GN=H1F0 PE=1 SV=3 |
|  | **O60361\|NDK8_HUMAN** | Putative nucleoside diphosphate kinase OS=Homo sapiens GN=NME2P1 PE=5 SV=1 |
|  | **P15531\|NDKA_HUMAN** | Nucleoside diphosphate kinase A OS=Homo sapiens GN=NME1 PE=1 SV=1 |
|  | **O43747\|AP1G1_HUMAN** | AP-1 complex subunit gamma-1 OS=Homo sapiens GN=AP1G1 PE=1 SV=5 |
|  | **O00338\|ST1C2_HUMAN** | Sulfotransferase 1C2 OS=Homo sapiens GN=SULT1C2 PE=1 SV=1 |
|  | **P98082\|DAB2_HUMAN** | Disabled homolog 2 OS=Homo sapiens GN=DAB2 PE=1 SV=3 |
|  | **P53999\|TCP4_HUMAN** | Activated RNA polymerase II transcriptional coactivator p15 OS=Homo sapiens GN=SUB1 PE=1 SV=3 |
|  | **P30483\|1B45_HUMAN** | HLA class I histocompatibility antigen, B-45 alpha chain OS=Homo sapiens GN=HLA-B PE=2 SV=1 |
|  | **Q8NBS9\|TXND5_HUMAN** | Thioredoxin domain-containing protein 5 OS=Homo sapiens GN=TXNDC5 PE=1 SV=2 |
|  | **P51571\|SSRD_HUMAN** | Translocon-associated protein subunit delta OS=Homo sapiens GN=SSR4 PE=1 SV=1 |
|  | **Q9H553\|ALG2_HUMAN** | Alpha-1,3-mannosyltransferase ALG2 OS=Homo sapiens GN=ALG2 PE=2 SV=1 |
|  | **Q15286\|RAB35_HUMAN** | Ras-related protein Rab-35 OS=Homo sapiens GN=RAB35 PE=1 SV=1 |
|  | **Q96L21\|RL10L_HUMAN** | 60S ribosomal protein L10-like OS=Homo sapiens GN=RPL10L PE=1 SV=3 |
|  | **Q6UWE0\|LRSM1_HUMAN** | E3 ubiquitin-protein ligase LRSAM1 OS=Homo sapiens GN=LRSAM1 PE=1 SV=1 |
|  | **P16070\|CD44_HUMAN** | CD44 antigen OS=Homo sapiens GN=CD44 PE=1 SV=2 |
|  | **P22303\|ACES_HUMAN** | Acetylcholinesterase OS=Homo sapiens GN=ACHE PE=1 SV=1 |
|  | **Q5XG92\|EST8_HUMAN** | Carboxylesterase 8 OS=Homo sapiens GN=CES8 PE=2 SV=2 |
|  | **Q9H078\|CLPB_HUMAN** | Caseinolytic peptidase B protein homolog OS=Homo sapiens GN=CLPB PE=1 SV=1 |
|  | **P62263\|RS14_HUMAN** | 40S ribosomal protein S14 OS=Homo sapiens GN=RPS14 PE=1 SV=3 |
|  | **P10768\|ESTD_HUMAN** | S-formylglutathione hydrolase OS=Homo sapiens GN=ESD PE=1 SV=2 |
|  | **P61978\|HNRPK_HUMAN** | Heterogeneous nuclear ribonucleoprotein K OS=Homo sapiens GN=HNRNPK PE=1 SV=1 |
|  | **P17252\|KPCA_HUMAN** | Protein kinase C alpha type OS=Homo sapiens GN=PRKCA PE=1 SV=3 |
|  | **Q9NZM1\|MYOF_HUMAN** | Myoferlin OS=Homo sapiens GN=MYOF PE=1 SV=1 |
|  | **P78357\|CNTP1_HUMAN** | Contactin-associated protein 1 OS=Homo sapiens GN=CNTNAP1 PE=1 SV=1 |
|  | **Q9NQE9\|HINT3_HUMAN** | Histidine triad nucleotide-binding protein 3 OS=Homo sapiens GN=HINT3 PE=1 SV=1 |
|  | **Q06136\|KDSR_HUMAN** | 3-ketodihydrosphingosine reductase OS=Homo sapiens GN=KDSR PE=1 SV=1 |
|  | **P08727\|K1C19_HUMAN** | Keratin, type I cytoskeletal 19 OS=Homo sapiens GN=KRT19 PE=1 SV=3 |
|  | **Q9HCU5\|PREB_HUMAN** | Prolactin regulatory element-binding protein OS=Homo sapiens GN=PREB PE=1 SV=2 |
|  | **P62988\|UBIQ_HUMAN** | Ubiquitin OS=Homo sapiens GN=RPS27A PE=1 SV=1 |
|  | **P62913\|RL11_HUMAN** | 60S ribosomal protein L11 OS=Homo sapiens GN=RPL11 PE=1 SV=2 |
|  | **Q8N8Y2\|VA0D2_HUMAN** | V-type proton ATPase subunit d 2 OS=Homo sapiens GN=ATP6V0D2 PE=2 SV=1 |
|  | **Q96A26\|F162A_HUMAN** | UPF0389 protein FAM162A OS=Homo sapiens GN=FAM162A PE=1 SV=2 |
|  | **P07602\|SAP_HUMAN** | Proactivator polypeptide OS=Homo sapiens GN=PSAP PE=1 SV=2 |
|  | **A0PJK1\|SC5AA_HUMAN** | Sodium/glucose cotransporter 5 OS=Homo sapiens GN=SLC5A10 PE=2 SV=2 |
|  | **Q9Y639\|NPTN_HUMAN** | Neuroplastin OS=Homo sapiens GN=NPTN PE=1 SV=1 |
|  | **Q9HCG7\|GBA2_HUMAN** | Non-lysosomal glucosylceramidase OS=Homo sapiens GN=GBA2 PE=1 SV=2 |
|  | **O15269\|SPTC1_HUMAN** | Serine palmitoyltransferase 1 OS=Homo sapiens GN=SPTLC1 PE=1 SV=1 |
|  | **O95954\|FTCD_HUMAN** | Formimidoyltransferase-cyclodeaminase OS=Homo sapiens GN=FTCD PE=1 SV=2 |
|  | **P27482\|CALL3_HUMAN** | Calmodulin-like protein 3 OS=Homo sapiens GN=CALML3 PE=1 SV=2 |
|  | **P00558\|PGK1_HUMAN** | Phosphoglycerate kinase 1 OS=Homo sapiens GN=PGK1 PE=1 SV=3 |
|  | **O75880\|SCO1_HUMAN** | Protein SCO1 homolog, mitochondrial OS=Homo sapiens GN=SCO1 PE=1 SV=1 |

Top of Form

Select Summary Report

|  | Peptide Summary Select Summary (protein hits) Select Summary (unassigned) Export Search Results |  | Help |
| --- | --- | --- | --- |
|  | Significance threshold p< | Max. number of hits |  |
|  | Standard scoring  MudPIT scoring | Ions score or expect cut-off | Show sub-sets |
|  | Show pop-ups  Suppress pop-ups | Sort unassigned Decreasing Score Increasing query / Mr Decreasing Intensity | Require bold red |

Bottom of Form

Top of Form

| **1.** | P06576\|ATPB_HUMAN    **Mass:** 56525    **Score:** 3003   **Queries matched:** 172   **emPAI:** 14.57 |
| --- | --- |
|  | ATP synthase subunit beta, mitochondrial OS=Homo sapiens GN=ATP5B PE=1 SV=3 |

|  | **Query** | **Observed** | **Mr(expt)** | **Mr(calc)** | **Delta** | **Miss** | **Score** | **Expect** | **Rank** | **Peptide** |
| --- | --- | --- | --- | --- | --- | --- | --- | --- | --- | --- |
|  | 2868 | **899.5137** | **898.5064** | **898.5124** | **-0.0060** | **0** | **50** | **0.0014** | **1** | **K.VLDSGAPIK.I** 2869 |
|  | 3908 | **488.2819** | **974.5493** | **974.5549** | **-0.0056** | **0** | **54** | **0.0007** | **1** | **K.IGLFGGAGVGK.T** 3894 3897 3914 |
|  | 4777 | **520.8363** | **1039.6580** | **1039.6641** | **-0.0061** | **1** | **25** | **0.45** | **1** | **K.LVPLKETIK.G** 4776 |
|  | 5346 | **543.7535** | **1085.4924** | **1085.4989** | **-0.0065** | **1** | **40** | **0.018** | **1** | **K.ADKLAEEHSS.-** 5341 5343 5344 5345 5347 5348 |
|  | 8274 | **631.8202** | **1261.6258** | **1261.6336** | **-0.0078** | **0** | **56** | **0.00039** | **1** | **R.TIAMDGTEGLVR.G** 8268 |
|  | 8580 | **639.8178** | **1277.6211** | **1277.6286** | **-0.0075** | **0** | **(34)** | **0.052** | **1** | **R.TIAMDGTEGLVR.G** 8581 |
|  | 9588 | **665.8242** | **1329.6338** | **1329.7656** | **-0.1319** | **1** | **5** | **49** | **4** | **K.VVDLLAPYAKGGK.I** |
|  | 10553 | **693.3547** | **1384.6948** | **1384.7020** | **-0.0072** | **0** | **62** | **8.2e-005** | **1** | **R.IMNVIGEPIDER.G** |
|  | 10826 | **701.3518** | **1400.6891** | **1400.6969** | **-0.0079** | **0** | **(20)** | **1.4** | **1** | **R.IMNVIGEPIDER.G** |
|  | 10906 | **703.8406** | **1405.6666** | **1405.6739** | **-0.0073** | **0** | **67** | **2.5e-005** | **1** | **K.AHGGYSVFAGVGER.T** 10905 |
|  | 11403 | **718.3772** | **1434.7398** | **1434.7467** | **-0.0068** | **0** | **64** | **5.2e-005** | **1** | **R.FTQAGSEVSALLGR.I** 11402 |
|  | 11753 | **729.4208** | **1456.8271** | **1456.8323** | **-0.0052** | **0** | **59** | **0.00018** | **1** | **K.TVLIMELINNVAK.A** 11752 11776 |
|  | 13665 | **788.4072** | **1574.7999** | **1574.8086** | **-0.0087** | **1** | **64** | **4.6e-005** | **1** | **R.TIAMDGTEGLVRGQK.V** |
|  | 14083 | **801.4078** | **1600.8011** | **1600.8031** | **-0.0020** | **0** | **23** | **0.55** | **1** | **K.VALVYGQMNEPPGAR.A** 14084 14085 14086 14096 |
|  | 14330 | **809.4058** | **1616.7971** | **1616.7981** | **-0.0010** | **0** | **(7)** | **23** | **2** | **K.VALVYGQMNEPPGAR.A** |
|  | 14871 | **825.9569** | **1649.8991** | **1649.9101** | **-0.0109** | **0** | **63** | **5e-005** | **1** | **R.LVLEVAQHLGESTVR.T** 14853 14857 14859 14872 14874 14885 |
|  | 15115 | **556.6692** | **1666.9857** | **1665.9202** | **1.0655** | **1** | **4** | **44** | **6** | **R.ARVALTGLTVAEYFR.D** |
|  | 16178 | **872.3890** | **1742.7634** | **1742.9614** | **-0.1980** | **1** | **1** | **82** | **6** | **-.MLGFVGRVAAAPASGALR.R** |
|  | 16266 | **438.2285** | **1748.8847** | **1747.8489** | **1.0358** | **1** | **5** | **37** | **10** | **R.DYAAQTSPSPKAGAATGR.I** |
|  | 16653 | **890.9800** | **1779.9454** | **1779.9553** | **-0.0099** | **1** | **47** | **0.002** | **1** | **R.IMNVIGEPIDERGPIK.T** 16651 16652 16654 |
|  | 17061 | **908.4326** | **1814.8506** | **1814.8621** | **-0.0116** | **0** | **85** | **3.5e-007** | **1** | **R.IMDPNIVGSEHYDVAR.G** 17059 17060 17062 17063 17065 |
|  | 18360 | **960.0455** | **1918.0765** | **1918.0888** | **-0.0123** | **1** | **62** | **5.7e-005** | **1** | **K.VLDSGAPIKIPVGPETLGR.I** 18361 18362 18363 18365 18366 |
|  | 18409 | **961.4828** | **1920.9511** | **1920.9581** | **-0.0070** | **0** | **123** | **5.2e-011** | **1** | **R.DQEGQDVLLFIDNIFR.F** 18408 |
|  | 19171 | **995.0160** | **1988.0174** | **1987.0262** | **0.9912** | **0** | **53** | **0.00048** | **1** | **R.AIAELGIYPAVDPLDSTSR.I** 19160 19162 19163 |
|  | 19525 | **1012.0038** | **2021.9930** | **2022.0033** | **-0.0103** | **0** | **107** | **1.6e-009** | **1** | **R.FLSQPFQVAEVFTGHMGK.L** 19523 19524 19526 19528 19531 19532 19534 19545 19548 |
|  | 19674 | **679.3424** | **2035.0052** | **2036.1014** | **-1.0962** | **1** | **25** | **0.27** | **1** | **R.ETRLVLEVAQHLGESTVR.T** |
|  | 19935 | **1030.9950** | **2059.9754** | **2059.9884** | **-0.0130** | **0** | **73** | **4.7e-006** | **1** | **R.EGNDLYHEMIESGVINLK.D** 19936 |
|  | 20505 | **531.0005** | **2119.9731** | **2119.0368** | **0.9363** | **1** | **5** | **30** | **10** | **K.DATSKVALVYGQMNEPPGAR.A** |
|  | 21473 | **743.3704** | **2227.0893** | **2227.1055** | **-0.0163** | **1** | **15** | **2.6** | **1** | **R.IMDPNIVGSEHYDVARGVQK.I** 21474 |
|  | 21782 | **1133.5400** | **2265.0655** | **2265.0770** | **-0.0114** | **0** | **77** | **1.5e-006** | **1** | **R.IPSAVGYQPTLATDMGTMQER.I** 21780 21781 |
|  | 22167 | **1159.5676** | **2317.1207** | **2317.1372** | **-0.0165** | **1** | **98** | **1.2e-008** | **1** | **R.TREGNDLYHEMIESGVINLK.D** 22168 22169 22170 |
|  | 23846 | **855.0780** | **2562.2122** | **2562.2271** | **-0.0150** | **1** | **52** | **0.00046** | **1** | **R.EGNDLYHEMIESGVINLKDATSK.V** |
|  | 24465 | **1338.1903** | **2674.3661** | **2674.3735** | **-0.0074** | **1** | **111** | **5.2e-010** | **1** | **K.SLQDIIAILGMDELSEEDKLTVSR.A** 24412 24413 24415 24417 24418 24419 24421 24423 24424 24425 24426 24427 24430 24432 24433 24435 24436 24437 24438 24440 24441 24446 24447 24448 24450 24451 24453 24455 24458 24459 24461 24463 24466 |
|  | 24549 | **897.7940** | **2690.3600** | **2690.3684** | **-0.0084** | **1** | **(29)** | **0.072** | **1** | **K.SLQDIIAILGMDELSEEDKLTVSR.A** |
|  | 26243 | **758.6727** | **3030.6618** | **3030.6754** | **-0.0136** | **0** | **27** | **0.092** | **1** | **R.IVAVIGAVVDVQFDEGLPPILNALEVQGR.E** 26234 26236 26248 26249 26260 26263 26271 |
|  | 26324 | **1015.5038** | **3043.4895** | **3043.5034** | **-0.0139** | **0** | **60** | **4.6e-005** | **1** | **K.QFAPIHAEAPEFMEMSVEQEILVTGIK.V** 26322 26323 26325 26326 26327 26336 26337 |
|  | 27366 | **819.1646** | **3272.6293** | **3272.6461** | **-0.0168** | **1** | **6** | **12** | **1** | **K.TKQFAPIHAEAPEFMEMSVEQEILVTGIK.V** |
|  | 27623 | **1114.9127** | **3341.7163** | **3341.7296** | **-0.0133** | **1** | **71** | **3.5e-006** | **1** | **R.VALTGLTVAEYFRDQEGQDVLLFIDNIFR.F** 27624 27626 27627 27629 |
|  | 27671 | **1675.8156** | **3349.6165** | **3349.6329** | **-0.0163** | **0** | **60** | **4.2e-005** | **1** | **K.GFQQILAGEYDHLPEQAFYMVGPIEEAVAK.A** 27667 27668 27670 27672 27675 |
|  | 28788 | **916.9520** | **3663.7788** | **3663.7919** | **-0.0131** | **1** | **15** | **1.2** | **1** | **K.GFQQILAGEYDHLPEQAFYMVGPIEEAVAKADK.L** 28784 28785 28787 |
|  | 28832 | **1228.2730** | **3681.7970** | **3681.8131** | **-0.0161** | **1** | **104** | **1.5e-009** | **1** | **R.FTQAGSEVSALLGRIPSAVGYQPTLATDMGTMQER.I** 28833 28834 |
|  | 28932 | **1238.9613** | **3713.8621** | **3713.8789** | **-0.0168** | **0** | **71** | **2.9e-006** | **1** | **K.GSITSVQAIYVPADDLTDPAPATTFAHLDATTVLSR.A** 28930 28931 |
|  | 29288 | **1281.6578** | **3841.9517** | **3841.9738** | **-0.0221** | **1** | **94** | **1.4e-008** | **1** | **K.KGSITSVQAIYVPADDLTDPAPATTFAHLDATTVLSR.A** 29287 29289 29292 |
|  | 29963 | **1372.0435** | **4113.1086** | **4113.1206** | **-0.0121** | **1** | **28** | **0.043** | **1** | **K.QFAPIHAEAPEFMEMSVEQEILVTGIKVVDLLAPYAK.G** 29961 29962 |

| **2.** | P05023\|AT1A1_HUMAN    **Mass:** 114135   **Score:** 2333   **Queries matched:** 119   **emPAI:** 2.35 |
| --- | --- |
|  | Sodium/potassium-transporting ATPase subunit alpha-1 OS=Homo sapiens GN=ATP1A1 PE=1 SV=1 |

|  | **Query** | **Observed** | **Mr(expt)** | **Mr(calc)** | **Delta** | **Miss** | **Score** | **Expect** | **Rank** | **Peptide** |
| --- | --- | --- | --- | --- | --- | --- | --- | --- | --- | --- |
|  | 1630 | **406.1985** | **810.3824** | **810.3872** | **-0.0047** | **0** | **47** | **0.0029** | **1** | **K.YGTDLSR.G** |
|  | 2341 | **862.4400** | **861.4327** | **861.4378** | **-0.0051** | **0** | **33** | **0.1** | **1** | **R.IISANGCK.V** 2342 |
|  | 3421 | **470.2366** | **938.4586** | **938.4821** | **-0.0235** | **1** | **10** | **13** | **3** | **R.KYGTDLSR.G** 3435 |
|  | 3682 | **480.7050** | **959.3954** | **959.4018** | **-0.0065** | **0** | **16** | **4.4** | **1** | **K.EVSMDDHK.L** |
|  | 3885 | **487.7630** | **973.5114** | **973.5192** | **-0.0079** | **1** | **12** | **12** | **2** | **K.TDKLVNER.L** |
|  | 3934 | **489.7632** | **977.5119** | **978.5135** | **-1.0015** | **0** | **16** | **4.7** | **1** | **R.GIVVYTGDR.T** |
|  | 3991 | **491.7669** | **981.5193** | **981.5243** | **-0.0050** | **0** | **11** | **11** | **1** | **K.LSLDELHR.K** |
|  | 4440 | **507.7697** | **1013.5249** | **1013.5328** | **-0.0079** | **0** | **51** | **0.0012** | **1** | **R.CSSILLHGK.E** |
|  | 5329 | **543.2707** | **1084.5268** | **1084.5335** | **-0.0067** | **0** | **43** | **0.0079** | **1** | **K.ACVVHGSDLK.D** 5328 |
|  | 5359 | **544.2969** | **1086.5792** | **1086.5856** | **-0.0063** | **0** | **37** | **0.032** | **1** | **K.LIIVEGCQR.Q** |
|  | 5621 | **553.3001** | **1104.5855** | **1104.5927** | **-0.0072** | **0** | **38** | **0.025** | **1** | **K.TSATWLALSR.I** |
|  | 7503 | **613.3393** | **1224.6640** | **1224.5301** | **0.1339** | **0** | **7** | **29** | **8** | **K.CIELCCGSVK.E** |
|  | 7615 | **616.3265** | **1230.6384** | **1230.6456** | **-0.0071** | **0** | **73** | **7.2e-006** | **1** | **R.AVAGDASESALLK.C** 7608 7613 |
|  | 7718 | **618.8525** | **1235.6904** | **1235.6986** | **-0.0082** | **0** | **55** | **0.00049** | **1** | **R.LNIPVSQVNPR.D** 7706 7717 7726 |
|  | 8260 | **631.3400** | **1260.6655** | **1260.6714** | **-0.0059** | **0** | **46** | **0.0036** | **1** | **K.IVEIPFNSTNK.Y** 8261 8276 |
|  | 8394 | **634.8575** | **1267.7004** | **1267.7071** | **-0.0067** | **0** | **55** | **0.00044** | **1** | **K.NMVPQQALVIR.N** 8371 8393 |
|  | 8693 | **642.8540** | **1283.6934** | **1283.7020** | **-0.0085** | **0** | **(16)** | **3.4** | **1** | **K.NMVPQQALVIR.N** |
|  | 10497 | **691.3746** | **1380.7347** | **1380.7435** | **-0.0088** | **0** | **72** | **9.3e-006** | **1** | **K.VIMVTGDHPITAK.A** 10473 10499 |
|  | 11153 | **711.3447** | **1420.6749** | **1420.6834** | **-0.0085** | **1** | **13** | **6.7** | **2** | **K.YEPAAVSEQGDKK.G** |
|  | 12321 | **745.4360** | **1488.8575** | **1489.7446** | **-0.8871** | **0** | **5** | **39** | **5** | **K.ADIGVAMGIAGSDVSK.Q** |
|  | 12852 | **508.6018** | **1522.7837** | **1522.7926** | **-0.0089** | **1** | **(3)** | **63** | **4** | **R.GIVVYTGDRTVMGR.I** |
|  | 13019 | **768.8573** | **1535.7000** | **1535.7103** | **-0.0103** | **1** | **52** | **0.00086** | **1** | **R.DKYEPAAVSEQGDK.K** 13018 |
|  | 13100 | **514.2953** | **1539.8642** | **1538.7875** | **1.0767** | **1** | **5** | **43** | **3** | **R.GIVVYTGDRTVMGR.I** 13066 13069 |
|  | 13823 | **792.9365** | **1583.8584** | **1583.8671** | **-0.0087** | **0** | **40** | **0.011** | **1** | **R.AVFQANQENLPILK.R** |
|  | 14358 | **810.3731** | **1618.7317** | **1618.7435** | **-0.0118** | **0** | **63** | **6.3e-005** | **1** | **K.VDNSSLTGESEPQTR.S** 14360 14361 |
|  | 14604 | **545.3375** | **1632.9906** | **1633.8345** | **-0.8440** | **1** | **4** | **43** | **8** | **K.KADIGVAMGIAGSDVSK.Q** 14603 |
|  | 15405 | **843.8936** | **1685.7725** | **1685.7832** | **-0.0106** | **0** | **64** | **5e-005** | **1** | **R.NIAFFSTNCVEGTAR.G** 15411 |
|  | 16150 | **870.9860** | **1739.9575** | **1739.9682** | **-0.0107** | **1** | **75** | **3.3e-006** | **1** | **R.AVFQANQENLPILKR.A** 16151 16152 |
|  | 17016 | **906.4746** | **1810.9345** | **1810.9425** | **-0.0080** | **0** | **83** | **5.3e-007** | **1** | **R.QGAIVAVTGDGVNDSPALK.K** 17014 17015 17031 |
|  | 17263 | **915.4605** | **1828.9063** | **1828.9167** | **-0.0103** | **0** | **99** | **1.3e-008** | **1** | **K.GVGIISEGNETVEDIAAR.L** 17258 17259 17260 17262 17264 17272 |
|  | 17277 | **915.9743** | **1829.9340** | **1829.9445** | **-0.0104** | **0** | **93** | **5.7e-008** | **1** | **K.MSINAEEVVVGDLVEVK.G** 17278 17279 17282 17284 17288 17289 17291 17292 17293 17295 17296 17297 17304 |
|  | 17382 | **613.3414** | **1837.0023** | **1837.0132** | **-0.0109** | **1** | **45** | **0.0034** | **1** | **R.SAGIKVIMVTGDHPITAK.A** |
|  | 18447 | **962.9559** | **1923.8972** | **1923.9095** | **-0.0123** | **0** | **64** | **3.4e-005** | **1** | **K.NLEAVETLGSTSTICSDK.T** |
|  | 18630 | **970.5211** | **1939.0276** | **1939.0375** | **-0.0099** | **1** | **70** | **8.9e-006** | **1** | **R.QGAIVAVTGDGVNDSPALKK.A** 18628 18629 |
|  | 19349 | **669.0273** | **2004.0602** | **2003.0696** | **0.9906** | **1** | **15** | **3** | **1** | **K.IMESFKNMVPQQALVIR.N** |
|  | 21324 | **738.3924** | **2212.1554** | **2212.1699** | **-0.0145** | **1** | **54** | **0.00029** | **1** | **K.AIAKGVGIISEGNETVEDIAAR.L** |
|  | 21495 | **1116.0300** | **2230.0455** | **2231.1104** | **-1.0649** | **1** | **2** | **49** | **3** | **K.MSINAEEVVVGDLVEVKGGDR.I** |
|  | 23182 | **1232.5776** | **2463.1407** | **2462.1707** | **0.9700** | **1** | **73** | **4e-006** | **1** | **R.IISANGCKVDNSSLTGESEPQTR.S** 23167 23176 23181 23184 23185 |
|  | 23257 | **825.3972** | **2473.1698** | **2473.1829** | **-0.0130** | **1** | **54** | **0.00029** | **1** | **K.ACVVHGSDLKDMTSEQLDDILK.Y** 23255 23256 23258 |
|  | 23276 | **826.4325** | **2476.2757** | **2476.2896** | **-0.0140** | **1** | **52** | **0.00043** | **1** | **K.YQLSIHKNPNTSEPQHLLVMK.G** 23275 |
|  | 23369 | **623.5826** | **2490.3015** | **2489.1778** | **1.1237** | **1** | **(0)** | **63** | **5** | **K.ACVVHGSDLKDMTSEQLDDILK.Y** |
|  | 25121 | **1398.6792** | **2795.3438** | **2795.3607** | **-0.0169** | **1** | **123** | **3.5e-011** | **1** | **K.NLEAVETLGSTSTICSDKTGTLTQNR.M** 25117 25118 25119 25122 |
|  | 25303 | **1417.6917** | **2833.3687** | **2833.3770** | **-0.0082** | **1** | **56** | **0.00015** | **1** | **K.EQPLDEELKDAFQNAYLELGGLGER.V** 25298 25299 25302 |
|  | 26349 | **1016.5390** | **3046.5952** | **3046.6047** | **-0.0095** | **1** | **55** | **0.00015** | **1** | **K.GVGIISEGNETVEDIAARLNIPVSQVNPR.D** |
|  | 26695 | **1040.8096** | **3119.4069** | **3119.4279** | **-0.0211** | **1** | **63** | **2.1e-005** | **1** | **K.VDNSSLTGESEPQTRSPDFTNENPLETR.N** |
|  | 27236 | **1080.4883** | **3238.4430** | **3237.4132** | **1.0298** | **0** | **51** | **0.00037** | **1** | **R.MTVAHMWFDNQIHEADTTENQSGVSFDK.T** 27233 |
|  | 30293 | **1082.0004** | **4323.9724** | **4323.9954** | **-0.0230** | **1** | **69** | **2.9e-006** | **1** | **R.MTVAHMWFDNQIHEADTTENQSGVSFDKTSATWLALSR.I** 30292 30294 30295 30298 30302 30305 |
|  | 30329 | **869.0017** | **4339.9719** | **4339.9903** | **-0.0184** | **1** | **(6)** | **5.6** | **1** | **R.MTVAHMWFDNQIHEADTTENQSGVSFDKTSATWLALSR.I** |
|  | 31491 | **1318.6256** | **5270.4733** | **5270.4869** | **-0.0135** | **0** | **10** | **1.2** | **1** | **R.VLGFCHLFLPDEQFPEGFQFDTDDVNFPIDNLCFVGLISMIDPPR.A** |

| **3.** | P15144\|AMPN_HUMAN    **Mass:** 109870   **Score:** 2052   **Queries matched:** 111   **emPAI:** 2.40 |
| --- | --- |
|  | Aminopeptidase N OS=Homo sapiens GN=ANPEP PE=1 SV=4 |

|  | **Query** | **Observed** | **Mr(expt)** | **Mr(calc)** | **Delta** | **Miss** | **Score** | **Expect** | **Rank** | **Peptide** |
| --- | --- | --- | --- | --- | --- | --- | --- | --- | --- | --- |
|  | 2719 | **443.7558** | **885.4971** | **885.5032** | **-0.0061** | **0** | **42** | **0.011** | **1** | **K.IQTQLQR.D** |
|  | 2901 | **901.4937** | **900.4864** | **900.4916** | **-0.0052** | **0** | **25** | **0.59** | **1** | **R.ALEQALEK.T** 2899 2900 |
|  | 3023 | **455.7180** | **909.4214** | **909.4266** | **-0.0052** | **0** | **11** | **12** | **1** | **R.SEVYGPMK.N** |
|  | 4445 | **507.7977** | **1013.5809** | **1013.5869** | **-0.0060** | **0** | **6** | **42** | **9** | **R.SIQLPTTVR.D** |
|  | 4447 | **507.8030** | **1013.5913** | **1013.5982** | **-0.0068** | **1** | **53** | **0.00089** | **1** | **R.KIQTQLQR.D** 4426 4430 4446 |
|  | 5313 | **542.7365** | **1083.4585** | **1083.4655** | **-0.0070** | **0** | **39** | **0.019** | **1** | **R.SEYMEGNVR.K** 5312 |
|  | 5563 | **551.3149** | **1100.6152** | **1099.4604** | **1.1548** | **0** | **(8)** | **27** | **6** | **R.SEYMEGNVR.K** |
|  | 5840 | **561.3033** | **1120.5920** | **1120.5989** | **-0.0069** | **0** | **2** | **1.1e+002** | **6** | **R.DHSAIPVINR.A** |
|  | 6421 | **585.2893** | **1168.5639** | **1168.5724** | **-0.0085** | **0** | **8** | **22** | **3** | **R.GVGGSQPPDIDK.T** |
|  | 6734 | **397.2019** | **1188.5840** | **1187.6047** | **0.9793** | **0** | **7** | **33** | **6** | **K.LNYTLSQGHR.V** |
|  | 7030 | **601.8234** | **1201.6323** | **1201.6376** | **-0.0053** | **0** | **48** | **0.0025** | **1** | **K.DLTALSNMLPK.G** 6991 7027 7028 7029 |
|  | 7222 | **606.7834** | **1211.5522** | **1211.5604** | **-0.0082** | **1** | **8** | **23** | **1** | **R.SEYMEGNVRK.V** |
|  | 7360 | **610.2816** | **1218.5486** | **1217.6326** | **0.9160** | **0** | **(9)** | **21** | **1** | **K.DLTALSNMLPK.G** |
|  | 7469 | **612.7636** | **1223.5127** | **1223.5207** | **-0.0080** | **0** | **63** | **7.1e-005** | **1** | **R.VNYDEENWR.K** 7468 |
|  | 7503 | 613.3393 | 1224.6640 | 1224.6714 | -0.0074 | 0 | 38 | 0.022 | 1 | K.EATDVIIIHSK.K 7502 7504 |
|  | 8158 | **419.9058** | **1256.6956** | **1256.7030** | **-0.0074** | **0** | **22** | **0.89** | **1** | **K.QVTPLFIHFR.N** |
|  | 9046 | **434.8811** | **1301.6214** | **1302.6932** | **-1.0718** | **0** | **12** | **8.2** | **2** | **R.LPNTLKPDSYR.V** 9039 9040 9041 9047 9048 9050 |
|  | 9555 | **664.7820** | **1327.5494** | **1327.5577** | **-0.0083** | **0** | **18** | **2.4** | **1** | **K.SFPCFDEPAMK.A** |
|  | 9966 | **676.8106** | **1351.6065** | **1351.6156** | **-0.0091** | **1** | **35** | **0.042** | **1** | **R.VNYDEENWRK.I** |
|  | 10121 | **681.3408** | **1360.6671** | **1360.6769** | **-0.0098** | **0** | **77** | **2.8e-006** | **1** | **K.VVATTQMQAADAR.K** 10139 |
|  | 10417 | **689.3389** | **1376.6632** | **1376.6718** | **-0.0086** | **0** | **(69)** | **1.9e-005** | **1** | **K.VVATTQMQAADAR.K** 10418 |
|  | 10560 | **462.6043** | **1384.7910** | **1384.7979** | **-0.0069** | **1** | **51** | **0.0012** | **1** | **K.KQVTPLFIHFR.N** |
|  | 10757 | **466.5449** | **1396.6130** | **1396.6219** | **-0.0089** | **1** | **7** | **27** | **1** | **K.KDNEETGFGSGTR.A** 10735 |
|  | 10847 | **701.8465** | **1401.6784** | **1401.6850** | **-0.0066** | **0** | **64** | **6.1e-005** | **1** | **R.MLSSFLSEDVFK.Q** 10846 |
|  | 11533 | **482.2656** | **1443.7749** | **1443.7834** | **-0.0085** | **0** | **32** | **0.076** | **1** | **R.VTLRPYLTPNDR.G** 11531 11532 |
|  | 11864 | **732.3635** | **1462.7124** | **1462.7205** | **-0.0081** | **0** | **29** | **0.17** | **1** | **R.QQQDYWLIDVR.A** |
|  | 11939 | **734.3915** | **1466.7685** | **1466.7769** | **-0.0084** | **0** | **28** | **0.18** | **1** | **R.YLSYTLNPDLIR.K** 11940 11943 |
|  | 12122 | **740.3739** | **1478.7332** | **1478.7405** | **-0.0073** | **0** | **59** | **0.00017** | **1** | **K.EVVLQWFTENSK.-** |
|  | 12310 | **745.3881** | **1488.7616** | **1488.7719** | **-0.0103** | **1** | **102** | **8.8e-009** | **1** | **R.KVVATTQMQAADAR.K** 12312 12314 |
|  | 12313 | **745.3884** | **1488.7622** | **1488.7719** | **-0.0097** | **1** | **65** | **3.9e-005** | **1** | **K.VVATTQMQAADARK.S** 12311 |
|  | 13611 | **786.8771** | **1571.7397** | **1571.7476** | **-0.0079** | **1** | **53** | **0.0007** | **1** | **K.LMFDRSEVYGPMK.N** 13612 |
|  | 14254 | **806.9215** | **1611.8285** | **1611.8369** | **-0.0084** | **0** | **90** | **1.1e-007** | **1** | **R.AQIINDAFNLASAHK.V** 14252 14253 14255 |
|  | 14417 | **811.9318** | **1621.8490** | **1621.8576** | **-0.0086** | **1** | **24** | **0.41** | **1** | **R.YRLPNTLKPDSYR.V** 14416 |
|  | 14759 | **822.7694** | **1643.5243** | **1642.8825** | **0.6418** | **1** | **1** | **61** | **7** | **R.SIQLPTTVRDIMNR.W** |
|  | 15933 | **862.9163** | **1723.8180** | **1723.8264** | **-0.0085** | **0** | **63** | **5e-005** | **1** | **R.ENSLLFDPLSSSSSNK.E** 15932 |
|  | 16782 | **896.4377** | **1790.8609** | **1790.8700** | **-0.0091** | **1** | **6** | **26** | **3** | **R.DGRQQQDYWLIDVR.A** |
|  | 17572 | **925.9621** | **1849.9096** | **1849.9210** | **-0.0114** | **1** | **49** | **0.0013** | **1** | **K.ENKEVVLQWFTENSK.-** |
|  | 18345 | **959.4778** | **1916.9410** | **1916.9520** | **-0.0110** | **1** | **76** | **2.6e-006** | **1** | **R.FSTEYELQQLEQFKK.D** 18346 18347 |
|  | 18395 | **960.9590** | **1919.9034** | **1919.9127** | **-0.0093** | **0** | **62** | **5.9e-005** | **1** | **R.QYMPWEAALSSLSYFK.L** |
|  | 19007 | **987.4669** | **1972.9193** | **1972.9326** | **-0.0133** | **0** | **60** | **8.6e-005** | **1** | **K.QWMENPNNNPIHPNLR.S** 19006 19008 19009 19010 |
|  | 19381 | **1005.4869** | **2008.9592** | **2008.9701** | **-0.0109** | **1** | **46** | **0.0023** | **1** | **R.ENSLLFDPLSSSSSNKER.V** |
|  | 21983 | **1147.0609** | **2292.1073** | **2292.1175** | **-0.0102** | **0** | **53** | **0.00037** | **1** | **K.LFNDYGGGSFSFSNLIQAVTR.R** 21984 |
|  | 23775 | **1277.5994** | **2553.1842** | **2553.1958** | **-0.0116** | **0** | **72** | **4e-006** | **1** | **K.SDQIGLPDFNAGAMENWGLVTYR.E** 23776 |
|  | 24530 | **1344.0529** | **2686.0912** | **2686.1017** | **-0.0105** | **0** | **138** | **9.6e-013** | **1** | **K.DSQYEMDSEFEGELADDLAGFYR.S** 24529 |
|  | 25589 | **1447.1243** | **2892.2340** | **2892.2449** | **-0.0110** | **0** | **98** | **8.2e-009** | **1** | **R.STVYCNAIAQGGEEEWDFAWEQFR.N** 25590 |
|  | 25724 | **974.1754** | **2919.5044** | **2919.5230** | **-0.0185** | **1** | **59** | **6.7e-005** | **1** | **R.GVGGSQPPDIDKTELVEPTEYLVVHLK.G** 25725 |
|  | 26931 | **1586.2014** | **3170.3883** | **3170.4026** | **-0.0143** | **1** | **77** | **9.9e-007** | **1** | **K.GSLVKDSQYEMDSEFEGELADDLAGFYR.S** 26925 26929 26930 26932 26933 26934 26935 26936 26937 26938 |
|  | 27216 | **1078.8829** | **3233.6270** | **3233.6357** | **-0.0088** | **0** | **10** | **4.1** | **1** | **K.QDATSTIISITNNVIGQGLVWDFVQSNWK.K** |
|  | 27720 | **1121.5802** | **3361.7188** | **3361.7307** | **-0.0119** | **1** | **62** | **2.8e-005** | **1** | **R.KQDATSTIISITNNVIGQGLVWDFVQSNWK.K** |
|  | 29337 | **1287.6409** | **3859.9008** | **3859.9189** | **-0.0182** | **0** | **74** | **1.3e-006** | **1** | **R.VMAVDALASSHPLSTPASEINTPAQISELFDAISYSK.G** 29338 |
|  | 29364 | **1292.9741** | **3875.9005** | **3875.9139** | **-0.0133** | **0** | **(63)** | **1.6e-005** | **1** | **R.VMAVDALASSHPLSTPASEINTPAQISELFDAISYSK.G** |
|  | 30205 | **1420.6714** | **4258.9923** | **4259.0117** | **-0.0193** | **1** | **79** | **2.9e-007** | **1** | **K.SDQIGLPDFNAGAMENWGLVTYRENSLLFDPLSSSSSNK.E** 30204 |

| **4.** | P98164\|LRP2_HUMAN    **Mass:** 540376   **Score:** 1863   **Queries matched:** 158   **emPAI:** 0.41 |
| --- | --- |
|  | Low-density lipoprotein receptor-related protein 2 OS=Homo sapiens GN=LRP2 PE=1 SV=3 |

|  | **Query** | **Observed** | **Mr(expt)** | **Mr(calc)** | **Delta** | **Miss** | **Score** | **Expect** | **Rank** | **Peptide** |
| --- | --- | --- | --- | --- | --- | --- | --- | --- | --- | --- |
|  | 721 | **519.1365** | **518.1293** | **518.2700** | **-0.1408** | **0** | **0** | **2.7e+002** | **3** | **R.DSAVK.V** |
|  | 767 | **536.3046** | **535.2973** | **534.3166** | **0.9807** | **0** | **3** | **1.1e+002** | **7** | **R.FGAIK.R** |
|  | 952 | **603.3414** | **602.3341** | **601.3799** | **0.9542** | **1** | **15** | **11** | **4** | **R.KDLVK.T** |
|  | 1287 | **783.4020** | **782.3948** | **782.3996** | **-0.0049** | **0** | **19** | **2.3** | **1** | **R.CISIYK.V** |
|  | 1593 | **404.2372** | **806.4599** | **806.4650** | **-0.0051** | **0** | **16** | **4.8** | **1** | **R.HIVVDPK.N** |
|  | 1653 | **813.4674** | **812.4601** | **812.4657** | **-0.0056** | **0** | **16** | **3.6** | **2** | **R.IFHQLR.Y** |
|  | 1720 | **409.7214** | **817.4282** | **816.4164** | **1.0118** | **0** | **18** | **3** | **1** | **K.VTVDMPR.H** |
|  | 1829 | **414.7518** | **827.4890** | **826.4297** | **1.0593** | **0** | **12** | **11** | **1** | **R.DNVPNLR.G** 1824 1830 |
|  | 1948 | **418.2641** | **834.5136** | **835.4010** | **-0.8874** | **0** | **9** | **23** | **3** | **K.CAPGYLR.E** |
|  | 2231 | **427.6758** | **853.3371** | **853.3422** | **-0.0051** | **0** | **12** | **9.3** | **1** | **R.ASMDGSMR.T** |
|  | 2398 | **865.3554** | **864.3481** | **864.4957** | **-0.1476** | **0** | **8** | **28** | **6** | **K.IYLVETK.V** |
|  | 2495 | **436.2302** | **870.4458** | **869.3371** | **1.1087** | **0** | **(12)** | **12** | **6** | **R.ASMDGSMR.T** |
|  | 2507 | **436.7375** | **871.4604** | **871.4664** | **-0.0061** | **0** | **37** | **0.043** | **1** | **R.GLQVYHR.R** 2489 |
|  | 2672 | **442.2688** | **882.5230** | **881.4719** | **1.0512** | **0** | **2** | **91** | **10** | **K.ETIINHR.L** |
|  | 2753 | **445.2699** | **888.5252** | **889.4617** | **-0.9365** | **1** | **6** | **50** | **9** | **R.VKTDGTNR.T** |
|  | 2826 | **448.9058** | **895.7970** | **896.5919** | **-0.7949** | **1** | **1** | **85** | **3** | **R.LGAIIRVR.K** |
|  | 3745 | **964.4787** | **963.4714** | **963.4774** | **-0.0059** | **0** | **31** | **0.15** | **1** | **R.NLYSTNPR.T** |
|  | 3980 | **491.5829** | **981.1513** | **980.4246** | **0.7267** | **1** | **6** | **26** | **9** | **R.FRCDNNR.C** |
|  | 4182 | **498.2946** | **994.5746** | **993.4516** | **1.1230** | **0** | **27** | **0.33** | **1** | **R.TDNDGLGFR.C** 4166 |
|  | 4527 | **511.2501** | **1020.4856** | **1020.4923** | **-0.0067** | **0** | **17** | **3.3** | **4** | **R.TCRPGQFR.C** |
|  | 4575 | **513.2419** | **1024.4693** | **1024.4760** | **-0.0067** | **1** | **32** | **0.099** | **1** | **R.AFMDGSNRK.D** 4574 |
|  | 4623 | **514.7877** | **1027.5608** | **1027.5675** | **-0.0068** | **1** | **11** | **12** | **2** | **R.GLQVYHRR.N** |
|  | 4909 | **526.2360** | **1050.4574** | **1050.5280** | **-0.0707** | **1** | **9** | **21** | **9** | **K.TNYRCIPK.W** |
|  | 5081 | **532.7950** | **1063.5755** | **1063.5814** | **-0.0059** | **1** | **23** | **0.79** | **1** | **R.KLYWLDAR.L** |
|  | 5528 | **550.2244** | **1098.4342** | **1098.4409** | **-0.0067** | **0** | **32** | **0.092** | **1** | **R.VCGCPYGMR.L** 5533 |
|  | 5530 | **550.2472** | **1098.4798** | **1098.4876** | **-0.0078** | **0** | **41** | **0.011** | **1** | **K.HCIVDNGER.C** 5522 5523 |
|  | 5656 | **554.2841** | **1106.5537** | **1107.6037** | **-1.0500** | **0** | **5** | **50** | **10** | **R.DLLIGDIHGR.S** |
|  | 5667 | **554.7871** | **1107.5597** | **1106.4451** | **1.1146** | **0** | **3** | **86** | **10** | **R.EWSCPESGR.C** |
|  | 5811 | **560.0837** | **1118.1529** | **1118.6118** | **-0.4588** | **1** | **9** | **14** | **2** | **K.SISVMRLADK.T** |
|  | 5907 | **564.8412** | **1127.6679** | **1126.6710** | **0.9970** | **0** | **39** | **0.022** | **1** | **R.QVIASDLIIR.H** |
|  | 6009 | **568.9748** | **1135.9350** | **1135.7189** | **0.2161** | **1** | **3** | **52** | **2** | **R.TVIARVPKPR.A** |
|  | 6211 | **576.7844** | **1151.5543** | **1151.5611** | **-0.0068** | **0** | **15** | **4.8** | **1** | **R.IFWSDATQGK.T** |
|  | 6247 | **577.7551** | **1153.4957** | **1152.5564** | **0.9394** | **0** | **8** | **24** | **8** | **R.AYIPNFESGR.N** |
|  | 7049 | **602.3245** | **1202.6345** | **1202.6408** | **-0.0063** | **0** | **37** | **0.032** | **1** | **R.TVVQYLNNPR.S** |
|  | 7190 | **605.8270** | **1209.6395** | **1208.5707** | **1.0688** | **0** | **12** | **9.3** | **1** | **K.IASANMDGTSVK.T** |
|  | 7521 | **409.5522** | **1225.6347** | **1224.6575** | **0.9773** | **1** | **10** | **15** | **3** | **K.TNKETIINHR.L** |
|  | 7530 | **614.2941** | **1226.5736** | **1226.5826** | **-0.0090** | **1** | **13** | **8.3** | **1** | **R.KHCIVDNGER.C** |
|  | 7651 | **617.2868** | **1232.5590** | **1232.6626** | **-0.1035** | **1** | **5** | **48** | **8** | **K.IERATLGGNFR.V** |
|  | 8256 | **631.2883** | **1260.5621** | **1260.5703** | **-0.0082** | **0** | **34** | **0.056** | **1** | **R.CVNQQCIPSR.W** 8257 8272 |
|  | 8821 | **645.8529** | **1289.6913** | **1288.6048** | **1.0865** | **0** | **3** | **75** | **1** | **K.IEHSTFDGLDR.R** |
|  | 9099 | **652.7490** | **1303.4834** | **1302.5081** | **0.9752** | **0** | **14** | **5.2** | **2** | **R.CGASSFTCSNGR.C** 9100 |
|  | 9223 | **656.6475** | **1311.2804** | **1310.6037** | **0.6767** | **1** | **2** | **58** | **10** | **K.LERAFMDGSNR.K** |
|  | 9238 | **657.3217** | **1312.6289** | **1312.6387** | **-0.0098** | **0** | **22** | **0.91** | **1** | **K.NHVCIPPYWK.C** 9260 |
|  | 9542 | **664.3072** | **1326.5999** | **1325.6040** | **0.9959** | **0** | **10** | **13** | **1** | **K.NLYWTDSHYK.S** 9540 |
|  | 9920 | **450.5866** | **1348.7380** | **1348.7463** | **-0.0083** | **0** | **30** | **0.17** | **1** | **R.VTLITENLGHPR.G** |
|  | 10097 | **680.3740** | **1358.7335** | **1358.7419** | **-0.0084** | **1** | **21** | **1.1** | **1** | **R.RTVVQYLNNPR.S** |
|  | 10550 | **693.3494** | **1384.6843** | **1383.7398** | **0.9445** | **0** | **2** | **91** | **8** | **K.DIGLSVVYYTVR.G** |
|  | 10583 | **694.3456** | **1386.6766** | **1386.6852** | **-0.0086** | **1** | **21** | **1.1** | **1** | **R.GVIERGNVDGTDR.M** |
|  | 10813 | **700.8737** | **1399.7329** | **1398.7579** | **0.9750** | **1** | **4** | **58** | **1** | **R.IRINGENSEVIR.Y** |
|  | 11047 | **708.2958** | **1414.5771** | **1414.5871** | **-0.0099** | **0** | **27** | **0.25** | **1** | **R.CGSGHCIPADWR.C** 11048 |
|  | 11219 | **713.4106** | **1424.8066** | **1424.8140** | **-0.0074** | **0** | **66** | **3.4e-005** | **1** | **K.TLVVNPWLTQVR.I** 11207 |
|  | 11424 | **719.3265** | **1436.6384** | **1436.6500** | **-0.0116** | **1** | **1** | **1.1e+002** | **4** | **K.CASGDKCIGVTNR.C** |
|  | 11557 | **482.5936** | **1444.7590** | **1444.7674** | **-0.0085** | **1** | **19** | **1.6** | **1** | **R.DVTIFDKQVQPR.S** |
|  | 11778 | **730.2618** | **1458.5091** | **1457.5915** | **0.9176** | **0** | **1** | **93** | **9** | **K.CDCAFGTLQSDGK.N** |
|  | 12271 | **744.3331** | **1486.6516** | **1486.6610** | **-0.0094** | **0** | **63** | **6.7e-005** | **1** | **R.TVMSLDYDSVSDR.I** |
|  | 12274 | **744.3496** | **1486.6845** | **1487.6351** | **-0.9506** | **0** | **8** | **20** | **5** | **K.CTFGFQLDTDER.H** |
|  | 13151 | **772.3833** | **1542.7520** | **1542.7619** | **-0.0099** | **0** | **23** | **0.67** | **1** | **R.YLFWADYGQRPK.I** |
|  | 13777 | **791.4277** | **1580.8409** | **1580.8522** | **-0.0113** | **0** | **47** | **0.0022** | **1** | **R.TQSIEVLTLHGDIR.Y** 13778 |
|  | 13793 | **528.2988** | **1581.8747** | **1580.7657** | **1.1090** | **0** | **2** | **75** | **5** | **K.QPIIFENPMYSAR.D** |
|  | 13979 | **532.3010** | **1593.8811** | **1593.8879** | **-0.0068** | **0** | **24** | **0.5** | **1** | **R.QHIIFGISLNPEVK.S** |
|  | 14428 | **812.3813** | **1622.7480** | **1622.7577** | **-0.0096** | **0** | **70** | **1.1e-005** | **1** | **K.LYWSDQGTDSGVPAK.I** 14427 14429 |
|  | 14649 | **546.3534** | **1636.0383** | **1636.7913** | **-0.7529** | **1** | **4** | **35** | **9** | **R.ASMDGSMRTVIVQDK.I** |
|  | 14806 | **549.9452** | **1646.8137** | **1647.6076** | **-0.7938** | **0** | **4** | **51** | **1** | **R.DCNATTEFMCNNR.R** |
|  | 15527 | **847.9244** | **1693.8343** | **1693.8423** | **-0.0080** | **0** | **12** | **7.4** | **1** | **R.QNSNIEPYLIFSNR.Y** |
|  | 15680 | **853.4180** | **1704.8214** | **1704.8294** | **-0.0080** | **0** | **72** | **6.8e-006** | **1** | **K.YVMQPDGIAVDWVGR.H** 15679 |
|  | 15806 | **572.5889** | **1714.7448** | **1714.7555** | **-0.0108** | **0** | **42** | **0.0072** | **1** | **R.HLCHCEEGYILER.G** 15800 15805 |
|  | 15863 | **860.4086** | **1718.8026** | **1718.8152** | **-0.0126** | **0** | **15** | **4** | **1** | **R.FDYIETVTYDGIQR.K** |
|  | 15875 | **574.2800** | **1719.8183** | **1720.8243** | **-1.0060** | **0** | **(7)** | **21** | **2** | **K.YVMQPDGIAVDWVGR.H** |
|  | 15895 | **861.8513** | **1721.6881** | **1721.6985** | **-0.0104** | **0** | **36** | **0.022** | **1** | **K.DCSDGSDELALCPQR.F** |
|  | 15999 | **577.1229** | **1728.3469** | **1727.8002** | **0.5467** | **1** | **3** | **36** | **3** | **R.RDPTPTYSATEDTFK.D** 15977 |
|  | 16494 | **884.9699** | **1767.9253** | **1767.9367** | **-0.0114** | **0** | **85** | **3.6e-007** | **1** | **K.VVQPIQVTVSENVDNK.N** |
|  | 16737 | **447.7468** | **1786.9582** | **1787.7138** | **-0.7555** | **1** | **2** | **61** | **10** | **R.DCNATTEFMCNNRR.C** |
|  | 16974 | **904.9394** | **1807.8642** | **1807.8741** | **-0.0099** | **1** | **59** | **0.00014** | **1** | **R.GKLYWSDQGTDSGVPAK.I** 16968 16975 |
|  | 17012 | **906.4644** | **1810.9142** | **1810.9247** | **-0.0105** | **0** | **27** | **0.22** | **1** | **R.CAAEGSSPLLLLPDNVR.I** |
|  | 17033 | **604.9820** | **1811.9241** | **1812.9060** | **-0.9818** | **1** | **7** | **22** | **1** | **K.NRYLFWADYGQRPK.I** 17029 |
|  | 17401 | **920.9568** | **1839.8990** | **1839.7266** | **0.1724** | **0** | **0** | **1e+002** | **10** | **R.FPDGAYCQATMFECK.N** |
|  | 18162 | **476.2240** | **1900.8669** | **1900.7794** | **0.0875** | **1** | **1** | **72** | **8** | **K.CPSGYTGKYCEMAFSK.G** |
|  | 18665 | **972.3834** | **1942.7522** | **1942.7648** | **-0.0126** | **0** | **58** | **0.00013** | **1** | **R.CMHGGNCYFDETDLPK.C** 18664 18666 |
|  | 18720 | **649.6732** | **1945.9977** | **1946.0084** | **-0.0108** | **1** | **3** | **52** | **1** | **K.LKYVMQPDGIAVDWVGR.H** |
|  | 18780 | **976.5222** | **1951.0298** | **1951.0415** | **-0.0117** | **0** | **29** | **0.13** | **1** | **K.WLISTDLDQPAAIAVNPK.L** |
|  | 19869 | **686.0075** | **2055.0007** | **2054.7802** | **0.2205** | **1** | **3** | **45** | **4** | **R.CSCDTGYMLESDGRTCK.V** |
|  | 19949 | **688.3188** | **2061.9345** | **2060.9428** | **0.9918** | **0** | **26** | **0.22** | **1** | **R.MNEHLLFWSDWGHHPR.I** |
|  | 19978 | **689.0030** | **2063.9871** | **2065.0302** | **-1.0431** | **1** | **(1)** | **67** | **3** | **K.QPIIFENPMYSARDSAVK.V** |
|  | 20130 | **521.2477** | **2080.9616** | **2081.0251** | **-0.0635** | **1** | **4** | **39** | **6** | **K.QPIIFENPMYSARDSAVK.V** |
|  | 20972 | **1087.4464** | **2172.8783** | **2172.8915** | **-0.0132** | **0** | **51** | **0.00055** | **1** | **R.TCVEFDDCQIWGICDQK.C** |
|  | 21368 | **740.0222** | **2217.0447** | **2216.0532** | **0.9915** | **0** | **16** | **2.1** | **1** | **R.GFSLELSDHSETMVPVAGQGR.N** |
|  | 21372 | **1109.9522** | **2217.8897** | **2217.9051** | **-0.0153** | **0** | **103** | **3.7e-009** | **1** | **R.TCVDIDECTEMPFVCSQK.C** 21373 |
|  | 21415 | **1112.0193** | **2222.0240** | **2221.0321** | **0.9919** | **0** | **82** | **5.2e-007** | **1** | **R.SGADLNMDIGVSGFGPETAIDR.S** 21401 |
|  | 21537 | **745.3893** | **2233.1460** | **2233.1994** | **-0.0534** | **0** | **1** | **70** | **2** | **R.VVFDSSIILTETIAIDWVGR.N** |
|  | 21775 | **755.7098** | **2264.1075** | **2264.1219** | **-0.0144** | **1** | **33** | **0.039** | **1** | **R.ANKYDGSGQIAMTTNLLSQPR.G** |
|  | 22062 | **768.3725** | **2302.0957** | **2302.1072** | **-0.0115** | **0** | **57** | **0.00016** | **1** | **R.GLALHPQYGYLYWADWGHR.A** |
|  | 22234 | **776.2278** | **2325.6615** | **2324.9725** | **0.6890** | **0** | **3** | **23** | **1** | **R.MLAQHCVDANNTFCFDNPR.G** |
|  | 22256 | **777.3840** | **2329.1301** | **2329.1451** | **-0.0150** | **0** | **32** | **0.044** | **1** | **R.SLHLDPENHSPPFQTINVER.T** |
|  | 22556 | **1184.6405** | **2367.2664** | **2367.2798** | **-0.0134** | **0** | **101** | **5.9e-009** | **1** | **K.TLIANDGTALGVGFPIGITVDPAR.G** 22557 |
|  | 23207 | **822.9286** | **2465.7639** | **2465.2809** | **0.4831** | **1** | **2** | **34** | **9** | **R.RIKPDGSSLMNIVTHGIGENGVR.G** |
|  | 23309 | **828.5468** | **2482.6186** | **2483.2791** | **-0.6605** | **0** | **8** | **8** | **1** | **R.SVVVHPFAGYLFFTDWFRPAK.I** |
|  | 23476 | **838.0034** | **2510.9883** | **2511.0036** | **-0.0153** | **0** | **0** | **49** | **2** | **R.TSCLDINECEQFGTCPQHCR.N** |
|  | 23539 | **841.3940** | **2521.1601** | **2521.1736** | **-0.0135** | **0** | **16** | **1.9** | **1** | **R.AIVLDPCQGYLYWADWDTHAK.I** |
|  | 23565 | **842.9636** | **2525.8689** | **2525.8867** | **-0.0178** | **0** | **16** | **1.2** | **1** | **R.WQCDGQNDCGDNSDEENCAPR.E** |
|  | 24034 | **866.7624** | **2597.2655** | **2597.2762** | **-0.0107** | **1** | **48** | **0.0011** | **1** | **R.GLAVDRSDGYVYWVDDSLDIIAR.I** |
|  | 24103 | **1307.6326** | **2613.2506** | **2613.2639** | **-0.0133** | **0** | **79** | **7.8e-007** | **1** | **R.GIAVDPTVGYLFFSDWESLSGEPK.L** 24102 |
|  | 24349 | **1332.6613** | **2663.3079** | **2663.3231** | **-0.0152** | **0** | **97** | **1.4e-008** | **1** | **R.NILVFEDLGWPTGLSIDYLNNDR.I** |
|  | 24380 | **891.0063** | **2669.9970** | **2670.0129** | **-0.0159** | **1** | **43** | **0.0023** | **1** | **K.WAVCNGVDDCRDNSDEQGCEER.T** |
|  | 26787 | **1570.1960** | **3138.3775** | **3138.3992** | **-0.0217** | **1** | **55** | **0.00014** | **1** | **R.SMAMSEDFVMEMGKQPIIFENPMYSAR.D** 26788 26789 |
|  | 26918 | **1585.7396** | **3169.4647** | **3170.3891** | **-0.9244** | **1** | **(0)** | **46** | **1** | **R.SMAMSEDFVMEMGKQPIIFENPMYSAR.D** |
|  | 27148 | **1074.4039** | **3220.1900** | **3220.2074** | **-0.0175** | **0** | **48** | **0.00051** | **1** | **K.CDGDDDCGDGSDEELHLCLDVPCNSPNR.F** |
|  | 27937 | **1141.0731** | **3420.1975** | **3420.2296** | **-0.0321** | **0** | **45** | **0.00082** | **1** | **R.CIPESWQCDTFNDCEDNSDEDSSHCASR.T** |
|  | 28015 | **1145.9081** | **3434.7024** | **3434.7180** | **-0.0156** | **1** | **73** | **1.8e-006** | **1** | **K.TLFTGNLEHLECVTLDIEEQKLYWAVTGR.G** 28013 |
|  | 28413 | **890.0872** | **3556.3195** | **3556.3449** | **-0.0254** | **0** | **8** | **4.7** | **1** | **R.CIPQHWYCDQETDCFDASDEPASCGHSER.T** |
|  | 28436 | **1189.0983** | **3564.2730** | **3564.2943** | **-0.0213** | **1** | **102** | **1.6e-009** | **1** | **R.WQCDGQNDCGDNSDEENCAPRECTESEFR.C** 28435 |
|  | 28628 | **1203.8910** | **3608.6511** | **3608.6698** | **-0.0186** | **1** | **40** | **0.0035** | **1** | **K.CDCAFGTLQSDGKNCAISTENFLIFALSNSLR.S** |
|  | 29543 | **1318.2936** | **3951.8589** | **3951.8737** | **-0.0148** | **1** | **54** | **0.00012** | **1** | **K.IESAWMNGEDRNILVFEDLGWPTGLSIDYLNNDR.I** 29551 29553 |
|  | 30121 | **1402.6439** | **4204.9099** | **4203.9484** | **0.9616** | **0** | **37** | **0.0042** | **1** | **K.SNDAMVPIAGIQNGLDVEFDDAEQYIYWVENPGEIHR.V** 30118 30122 |
|  | 30451 | **1471.6013** | **4411.7821** | **4411.8081** | **-0.0260** | **1** | **82** | **9.9e-008** | **1** | **K.GFTCECPDDFRTLQLSGSTYCMPMCSSTQFLCANNEK.C** 30452 |
|  | 30884 | **1184.3422** | **4733.3395** | **4733.3672** | **-0.0277** | **1** | **60** | **1.6e-005** | **1** | **K.VVQPIQVTVSENVDNKNYGSPINPSEIVPETNPTSPAADGTQVTK.W** 30882 30883 |
|  | 30976 | **1599.1024** | **4794.2854** | **4794.8139** | **-0.5285** | **1** | **1** | **11** | **2** | **K.CDNDNDCGDGSDEMESVCALHTCSPTAFTCANGRCVQYSYR.C** |

| **5.** | P68371\|TBB2C_HUMAN    **Mass:** 50255    **Score:** 1735   **Queries matched:** 96   **emPAI:** 9.63 |
| --- | --- |
|  | Tubulin beta-2C chain OS=Homo sapiens GN=TUBB2C PE=1 SV=1 |

|  | **Query** | **Observed** | **Mr(expt)** | **Mr(calc)** | **Delta** | **Miss** | **Score** | **Expect** | **Rank** | **Peptide** |
| --- | --- | --- | --- | --- | --- | --- | --- | --- | --- | --- |
|  | 1602 | **404.7373** | **807.4600** | **807.3399** | **0.1201** | **0** | **8** | **28** | **4** | **R.EEYPDR.I** |
|  | 4618 | **514.7597** | **1027.5048** | **1027.5121** | **-0.0072** | **0** | **25** | **0.57** | **1** | **K.TAVCDIPPR.G** 4619 4624 4627 4629 |
|  | 4763 | **520.2977** | **1038.5809** | **1038.5862** | **-0.0053** | **0** | **7** | **29** | **2** | **R.YLTVAAVFR.G** |
|  | 5095 | **533.2719** | **1064.5293** | **1064.4201** | **0.1091** | **0** | **(18)** | **2.3** | **1** | **K.NMMAACDPR.H** 5090 5102 |
|  | 5226 | **539.2660** | **1076.5175** | **1076.5250** | **-0.0075** | **1** | **25** | **0.53** | **1** | **K.IREEYPDR.I** 5225 |
|  | 5275 | **541.2110** | **1080.4074** | **1080.4151** | **-0.0076** | **0** | **26** | **0.36** | **1** | **K.NMMAACDPR.H** |
|  | 5276 | **541.2111** | **1080.4076** | **1080.4151** | **-0.0075** | **0** | **(25)** | **0.48** | **1** | **K.NMMAACDPR.H** |
|  | 6084 | **572.3180** | **1142.6215** | **1142.6270** | **-0.0055** | **0** | **17** | **3.3** | **1** | **K.LAVNMVPFPR.L** |
|  | 8765 | **430.2513** | **1287.7319** | **1286.7169** | **1.0150** | **1** | **4** | **55** | **3** | **R.KLAVNMVPFPR.L** |
|  | 9381 | **660.3513** | **1318.6880** | **1318.6955** | **-0.0075** | **0** | **69** | **2e-005** | **1** | **R.IMNTFSVVPSPK.V** 9382 |
|  | 9543 | **664.3431** | **1326.6716** | **1325.7126** | **0.9591** | **1** | **5** | **48** | **9** | **K.TAVCDIPPRGLK.M** |
|  | 9561 | **664.8240** | **1327.6335** | **1327.6408** | **-0.0073** | **0** | **61** | **0.0001** | **1** | **R.INVYYNEATGGK.Y** 9562 |
|  | 10550 | 693.3494 | 1384.6843 | 1384.6921 | -0.0078 | 1 | 30 | 0.13 | 1 | K.RISEQFTAMFR.R |
|  | 11576 | **723.8442** | **1445.6739** | **1445.6820** | **-0.0081** | **0** | **71** | **1.1e-005** | **1** | **K.EVDEQMLNVQNK.N** 11577 |
|  | 14084 | 801.4085 | 1600.8025 | 1600.8131 | -0.0106 | 0 | 63 | 6.1e-005 | 1 | R.AVLVDLEPGTMDSVR.S 14085 14086 14096 |
|  | 14330 | 809.4058 | 1616.7971 | 1616.8080 | -0.0109 | 0 | (21) | 0.95 | 1 | R.AVLVDLEPGTMDSVR.S |
|  | 14380 | **540.9477** | **1619.8212** | **1619.8283** | **-0.0070** | **0** | **48** | **0.0018** | **1** | **R.LHFFMPGFAPLTSR.G** 14371 14377 14378 14379 |
|  | 15480 | **846.4329** | **1690.8512** | **1690.8600** | **-0.0088** | **0** | **66** | **3e-005** | **1** | **R.ALTVPELTQQMFDAK.N** 15478 |
|  | 15553 | **848.9153** | **1695.8160** | **1695.8257** | **-0.0096** | **0** | **19** | **1.4** | **1** | **K.NSSYFVEWIPNNVK.T** 15562 15563 15564 |
|  | 17173 | **911.9601** | **1821.9057** | **1821.9156** | **-0.0098** | **0** | **61** | **8.7e-005** | **1** | **R.EIVHLQAGQCGNQIGAK.F** 17174 |
|  | 17447 | **922.4657** | **1842.9168** | **1842.9264** | **-0.0096** | **1** | **56** | **0.00029** | **1** | **R.INVYYNEATGGKYVPR.A** |
|  | 17663 | **929.4699** | **1856.9253** | **1856.9342** | **-0.0090** | **0** | **55** | **0.0003** | **1** | **K.MSATFIGNSTAIQELFK.R** |
|  | 18438 | **962.4478** | **1922.8809** | **1922.8900** | **-0.0090** | **1** | **91** | **8.1e-008** | **1** | **R.MSMKEVDEQMLNVQNK.N** |
|  | 18859 | **979.9888** | **1957.9631** | **1957.9745** | **-0.0114** | **0** | **86** | **2.2e-007** | **1** | **K.GHYTEGAELVDSVLDVVR.K** 18858 |
|  | 19427 | **1007.5205** | **2013.0265** | **2013.0353** | **-0.0089** | **1** | **43** | **0.005** | **2** | **K.MSATFIGNSTAIQELFKR.I** 19426 19429 |
|  | 20166 | **1044.0366** | **2086.0587** | **2086.0695** | **-0.0108** | **1** | **114** | **3.4e-010** | **1** | **K.GHYTEGAELVDSVLDVVRK.E** 20162 20163 20167 20168 20169 |
|  | 20402 | **1055.5299** | **2109.0453** | **2109.0571** | **-0.0119** | **1** | **94** | **3.6e-008** | **1** | **-.MREIVHLQAGQCGNQIGAK.F** 20399 20400 20401 20403 20410 |
|  | 20534 | **532.2908** | **2125.1342** | **2125.0521** | **0.0822** | **1** | **(5)** | **26** | **4** | **-.MREIVHLQAGQCGNQIGAK.F** |
|  | 24649 | **1354.6668** | **2707.3189** | **2707.3310** | **-0.0120** | **0** | **100** | **5.8e-009** | **1** | **K.LTTPTYGDLNHLVSATMSGVTTCLR.F** 24651 |
|  | 25038 | **929.4656** | **2785.3749** | **2785.2543** | **0.1206** | **1** | **1** | **45** | **4** | **R.ALTVPELTQQMFDAKNMMAACDPR.H** |
|  | 25134 | **1399.6674** | **2797.3202** | **2797.3361** | **-0.0160** | **0** | **80** | **5.8e-007** | **1** | **R.SGPFGQIFRPDNFVFGQSGAGNNWAK.G** 25133 25135 25137 25138 25139 25144 25145 25147 25148 |
|  | 26680 | **1039.4745** | **3115.4016** | **3115.4159** | **-0.0143** | **0** | **67** | **1e-005** | **1** | **K.FWEVISDEHGIDPTGTYHGDSDLQLER.I** 26674 26676 26677 26679 |
|  | 27484 | **1656.2628** | **3310.5111** | **3310.5268** | **-0.0158** | **0** | **72** | **2.8e-006** | **1** | **K.EAESCDCLQGFQLTHSLGGGTGSGMGTLLISK.I** 27480 27481 27482 27483 |
|  | 27558 | **1109.8416** | **3326.5028** | **3326.5218** | **-0.0189** | **0** | **(3)** | **24** | **2** | **K.EAESCDCLQGFQLTHSLGGGTGSGMGTLLISK.I** 27556 |
|  | 28039 | **1147.2087** | **3438.6044** | **3438.6218** | **-0.0174** | **1** | **115** | **1.3e-010** | **1** | **R.KEAESCDCLQGFQLTHSLGGGTGSGMGTLLISK.I** 28036 28037 |
|  | 28070 | **1152.8376** | **3455.4911** | **3454.6167** | **0.8744** | **1** | **(1)** | **31** | **10** | **R.KEAESCDCLQGFQLTHSLGGGTGSGMGTLLISK.I** |
|  | 30696 | **1531.7006** | **4592.0799** | **4592.0999** | **-0.0201** | **0** | **53** | **8.6e-005** | **1** | **K.VSDTVVEPYNATLSVHQLVENTDETYCIDNEALYDICFR.T** 30694 30698 30701 |

| **6.** | P60709\|ACTB_HUMAN    **Mass:** 42052    **Score:** 1734   **Queries matched:** 94   **emPAI:** 6.75 |
| --- | --- |
|  | Actin, cytoplasmic 1 OS=Homo sapiens GN=ACTB PE=1 SV=1 |

|  | **Query** | **Observed** | **Mr(expt)** | **Mr(calc)** | **Delta** | **Miss** | **Score** | **Expect** | **Rank** | **Peptide** |
| --- | --- | --- | --- | --- | --- | --- | --- | --- | --- | --- |
|  | 1475 | **800.5302** | **799.5229** | **799.5280** | **-0.0051** | **1** | **14** | **8.2** | **3** | **K.RGILTLK.Y** |
|  | 4224 | **499.7444** | **997.4741** | **997.4790** | **-0.0049** | **0** | **10** | **15** | **4** | **R.DLTDYLMK.I** |
|  | 4734 | **518.8262** | **1035.6379** | **1035.6440** | **-0.0061** | **1** | **21** | **1.4** | **1** | **K.IKIIAPPER.K** |
|  | 5957 | **566.7640** | **1131.5134** | **1131.5197** | **-0.0062** | **0** | **51** | **0.0015** | **1** | **R.GYSFTTTAER.E** |
|  | 6341 | **581.3092** | **1160.6038** | **1160.6111** | **-0.0072** | **0** | **36** | **0.042** | **1** | **K.EITALAPSTMK.I** 6340 |
|  | 6445 | **586.2852** | **1170.5558** | **1170.5638** | **-0.0080** | **0** | **48** | **0.0025** | **1** | **R.HQGVMVGMGQK.D** 6444 6446 6447 6460 |
|  | 6691 | **594.2826** | **1186.5507** | **1186.5587** | **-0.0080** | **0** | **(40)** | **0.017** | **1** | **R.HQGVMVGMGQK.D** 6679 6693 6696 |
|  | 6692 | **594.2828** | **1186.5511** | **1186.5587** | **-0.0076** | **0** | **(42)** | **0.01** | **1** | **R.HQGVMVGMGQK.D** 6695 |
|  | 6934 | **599.7607** | **1197.5068** | **1197.5150** | **-0.0081** | **0** | **47** | **0.0031** | **1** | **K.DSYVGDEAQSK.R** |
|  | 6943 | **599.8527** | **1197.6908** | **1197.6982** | **-0.0075** | **0** | **52** | **0.00092** | **1** | **R.AVFPSIVGRPR.H** 6939 6944 6945 6946 |
|  | 7039 | **602.2794** | **1202.5442** | **1202.5536** | **-0.0095** | **0** | **(21)** | **1.3** | **1** | **R.HQGVMVGMGQK.D** |
|  | 10002 | **677.8109** | **1353.6073** | **1353.6161** | **-0.0088** | **1** | **60** | **0.00014** | **1** | **K.DSYVGDEAQSKR.G** 9990 10003 10004 10005 10006 10024 10031 |
|  | 12723 | **505.9181** | **1514.7325** | **1514.7419** | **-0.0093** | **0** | **40** | **0.014** | **1** | **K.IWHHTFYNELR.V** 12724 12725 |
|  | 12739 | **758.8506** | **1515.6866** | **1515.6954** | **-0.0087** | **0** | **42** | **0.0081** | **1** | **K.QEYDESGPSIVHR.K** 12738 |
|  | 13248 | **774.9052** | **1547.7957** | **1547.8051** | **-0.0094** | **1** | **53** | **0.0006** | **1** | **R.MQKEITALAPSTMK.I** 13247 13249 13250 |
|  | 13497 | **522.2708** | **1563.7904** | **1563.8000** | **-0.0096** | **1** | **(22)** | **0.73** | **1** | **R.MQKEITALAPSTMK.I** |
|  | 14437 | **541.9493** | **1622.8262** | **1622.8338** | **-0.0076** | **1** | **25** | **0.33** | **1** | **R.LDLAGRDLTDYLMK.I** |
|  | 14536 | **815.4099** | **1628.8053** | **1628.8158** | **-0.0106** | **1** | **21** | **0.9** | **1** | **R.GYSFTTTAEREIVR.D** |
|  | 16191 | **872.9021** | **1743.7896** | **1743.8792** | **-0.0895** | **1** | **3** | **60** | **4** | **K.ILTERGYSFTTTAER.E** |
|  | 16767 | **895.9441** | **1789.8736** | **1789.8846** | **-0.0110** | **0** | **66** | **2.5e-005** | **1** | **K.SYELPDGQVITIGNER.F** 16766 16769 16770 16771 16772 |
|  | 18474 | **642.6770** | **1925.0092** | **1925.8169** | **-0.8077** | **0** | **3** | **50** | **6** | **-.MDDDIAALVVDNGSGMCK.A** 18473 |
|  | 18797 | **977.5299** | **1953.0451** | **1953.0571** | **-0.0120** | **0** | **76** | **2.2e-006** | **1** | **R.VAPEEHPVLLTEAPLNPK.A** 18798 18799 18800 18812 |
|  | 21347 | **1108.0330** | **2214.0514** | **2214.0627** | **-0.0113** | **0** | **101** | **6.8e-009** | **1** | **K.DLYANTVLSGGTTMYPGIADR.M** 21343 21344 21345 21348 |
|  | 21495 | 1116.0300 | 2230.0455 | 2230.0576 | -0.0121 | 0 | (98) | 1.4e-008 | 1 | K.DLYANTVLSGGTTMYPGIADR.M |
|  | 22348 | **1172.0800** | **2342.1454** | **2342.1576** | **-0.0123** | **1** | **97** | **1.3e-008** | **1** | **R.KDLYANTVLSGGTTMYPGIADR.M** 22347 22350 |
|  | 22410 | **1176.0343** | **2350.0540** | **2350.0682** | **-0.0141** | **1** | **96** | **1.8e-008** | **1** | **R.HQGVMVGMGQKDSYVGDEAQSK.R** 22409 22411 |
|  | 22544 | **789.4450** | **2365.3132** | **2366.2464** | **-0.9332** | **1** | **4** | **30** | **1** | **R.AVFPSIVGRPRHQGVMVGMGQK.D** |
|  | 22547 | **789.6902** | **2366.0487** | **2366.0631** | **-0.0144** | **1** | **(6)** | **20** | **1** | **R.HQGVMVGMGQKDSYVGDEAQSK.R** |
|  | 23722 | **1275.5837** | **2549.1529** | **2549.1665** | **-0.0136** | **0** | **112** | **3.9e-010** | **1** | **K.LCYVALDFEQEMATAASSSSLEK.S** 23723 23724 23725 23729 23737 23744 23745 23746 |
|  | 24842 | **550.0686** | **2745.3066** | **2745.4200** | **-0.1134** | **1** | **5** | **22** | **3** | **R.KYSVWIGGSILASLSTFQQMWISK.Q** |
|  | 25170 | **936.4366** | **2806.2879** | **2806.3041** | **-0.0162** | **1** | **36** | **0.013** | **1** | **K.EKLCYVALDFEQEMATAASSSSLEK.S** 25171 |
|  | 27001 | **1061.8700** | **3182.5882** | **3182.6071** | **-0.0189** | **0** | **60** | **4.7e-005** | **1** | **R.TTGIVMDSGDGVTHTVPIYEGYALPHAILR.L** 27002 |
|  | 28328 | **1178.8758** | **3533.6057** | **3533.6240** | **-0.0183** | **1** | **47** | **0.0007** | **1** | **R.FRCPEALFQPSFLGMESCGIHETTFNSIMK.C** 28330 28331 28341 |
|  | 28366 | **1181.9708** | **3542.8907** | **3541.7295** | **1.1612** | **1** | **1** | **30** | **3** | **R.EKMTQIMFETFNTPAMYVAIQAVLSLYASGR.T** |
|  | 28386 | **888.4067** | **3549.5979** | **3549.6189** | **-0.0211** | **1** | **(4)** | **13** | **1** | **R.FRCPEALFQPSFLGMESCGIHETTFNSIMK.C** |
|  | 30286 | **1441.3481** | **4321.0226** | **4321.0406** | **-0.0180** | **1** | **129** | **2.7e-012** | **1** | **K.LCYVALDFEQEMATAASSSSLEKSYELPDGQVITIGNER.F** 30289 |

| **7.** | P63261\|ACTG_HUMAN    **Mass:** 42108    **Score:** 1734   **Queries matched:** 93   **emPAI:** 6.75 |
| --- | --- |
|  | Actin, cytoplasmic 2 OS=Homo sapiens GN=ACTG1 PE=1 SV=1 |

|  | **Query** | **Observed** | **Mr(expt)** | **Mr(calc)** | **Delta** | **Miss** | **Score** | **Expect** | **Rank** | **Peptide** |
| --- | --- | --- | --- | --- | --- | --- | --- | --- | --- | --- |
|  | 1475 | 800.5302 | 799.5229 | 799.5280 | -0.0051 | 1 | 14 | 8.2 | 3 | K.RGILTLK.Y |
|  | 4224 | 499.7444 | 997.4741 | 997.4790 | -0.0049 | 0 | 10 | 15 | 4 | R.DLTDYLMK.I |
|  | 4734 | 518.8262 | 1035.6379 | 1035.6440 | -0.0061 | 1 | 21 | 1.4 | 1 | K.IKIIAPPER.K |
|  | 5957 | 566.7640 | 1131.5134 | 1131.5197 | -0.0062 | 0 | 51 | 0.0015 | 1 | R.GYSFTTTAER.E |
|  | 6341 | 581.3092 | 1160.6038 | 1160.6111 | -0.0072 | 0 | 36 | 0.042 | 1 | K.EITALAPSTMK.I 6340 |
|  | 6445 | 586.2852 | 1170.5558 | 1170.5638 | -0.0080 | 0 | 48 | 0.0025 | 1 | R.HQGVMVGMGQK.D 6444 6446 6447 6460 |
|  | 6691 | 594.2826 | 1186.5507 | 1186.5587 | -0.0080 | 0 | (40) | 0.017 | 1 | R.HQGVMVGMGQK.D 6679 6693 6696 |
|  | 6692 | 594.2828 | 1186.5511 | 1186.5587 | -0.0076 | 0 | (42) | 0.01 | 1 | R.HQGVMVGMGQK.D 6695 |
|  | 6934 | 599.7607 | 1197.5068 | 1197.5150 | -0.0081 | 0 | 47 | 0.0031 | 1 | K.DSYVGDEAQSK.R |
|  | 6943 | 599.8527 | 1197.6908 | 1197.6982 | -0.0075 | 0 | 52 | 0.00092 | 1 | R.AVFPSIVGRPR.H 6939 6944 6945 6946 |
|  | 7039 | 602.2794 | 1202.5442 | 1202.5536 | -0.0095 | 0 | (21) | 1.3 | 1 | R.HQGVMVGMGQK.D |
|  | 10002 | 677.8109 | 1353.6073 | 1353.6161 | -0.0088 | 1 | 60 | 0.00014 | 1 | K.DSYVGDEAQSKR.G 9990 10003 10004 10005 10006 10024 10031 |
|  | 12723 | 505.9181 | 1514.7325 | 1514.7419 | -0.0093 | 0 | 40 | 0.014 | 1 | K.IWHHTFYNELR.V 12724 12725 |
|  | 12739 | 758.8506 | 1515.6866 | 1515.6954 | -0.0087 | 0 | 42 | 0.0081 | 1 | K.QEYDESGPSIVHR.K 12738 |
|  | 13248 | 774.9052 | 1547.7957 | 1547.8051 | -0.0094 | 1 | 53 | 0.0006 | 1 | R.MQKEITALAPSTMK.I 13247 13249 13250 |
|  | 13497 | 522.2708 | 1563.7904 | 1563.8000 | -0.0096 | 1 | (22) | 0.73 | 1 | R.MQKEITALAPSTMK.I |
|  | 14437 | 541.9493 | 1622.8262 | 1622.8338 | -0.0076 | 1 | 25 | 0.33 | 1 | R.LDLAGRDLTDYLMK.I |
|  | 14536 | 815.4099 | 1628.8053 | 1628.8158 | -0.0106 | 1 | 21 | 0.9 | 1 | R.GYSFTTTAEREIVR.D |
|  | 16191 | 872.9021 | 1743.7896 | 1743.8792 | -0.0895 | 1 | 3 | 60 | 4 | K.ILTERGYSFTTTAER.E |
|  | 16767 | 895.9441 | 1789.8736 | 1789.8846 | -0.0110 | 0 | 66 | 2.5e-005 | 1 | K.SYELPDGQVITIGNER.F 16766 16769 16770 16771 16772 |
|  | 18797 | 977.5299 | 1953.0451 | 1953.0571 | -0.0120 | 0 | 76 | 2.2e-006 | 1 | R.VAPEEHPVLLTEAPLNPK.A 18798 18799 18800 18812 |
|  | 19086 | **661.4286** | **1981.2641** | **1981.8795** | **-0.6153** | **0** | **0** | **64** | **10** | **-.MEEEIAALVIDNGSGMCK.A** |
|  | 21347 | 1108.0330 | 2214.0514 | 2214.0627 | -0.0113 | 0 | 101 | 6.8e-009 | 1 | K.DLYANTVLSGGTTMYPGIADR.M 21343 21344 21345 21348 |
|  | 21495 | 1116.0300 | 2230.0455 | 2230.0576 | -0.0121 | 0 | (98) | 1.4e-008 | 1 | K.DLYANTVLSGGTTMYPGIADR.M |
|  | 22348 | 1172.0800 | 2342.1454 | 2342.1576 | -0.0123 | 1 | 97 | 1.3e-008 | 1 | R.KDLYANTVLSGGTTMYPGIADR.M 22347 22350 |
|  | 22410 | 1176.0343 | 2350.0540 | 2350.0682 | -0.0141 | 1 | 96 | 1.8e-008 | 1 | R.HQGVMVGMGQKDSYVGDEAQSK.R 22409 22411 |
|  | 22544 | 789.4450 | 2365.3132 | 2366.2464 | -0.9332 | 1 | 4 | 30 | 1 | R.AVFPSIVGRPRHQGVMVGMGQK.D |
|  | 22547 | 789.6902 | 2366.0487 | 2366.0631 | -0.0144 | 1 | (6) | 20 | 1 | R.HQGVMVGMGQKDSYVGDEAQSK.R |
|  | 23722 | 1275.5837 | 2549.1529 | 2549.1665 | -0.0136 | 0 | 112 | 3.9e-010 | 1 | K.LCYVALDFEQEMATAASSSSLEK.S 23723 23724 23725 23729 23737 23744 23745 23746 |
|  | 24842 | 550.0686 | 2745.3066 | 2745.4200 | -0.1134 | 1 | 5 | 22 | 3 | R.KYSVWIGGSILASLSTFQQMWISK.Q |
|  | 25170 | 936.4366 | 2806.2879 | 2806.3041 | -0.0162 | 1 | 36 | 0.013 | 1 | K.EKLCYVALDFEQEMATAASSSSLEK.S 25171 |
|  | 27001 | 1061.8700 | 3182.5882 | 3182.6071 | -0.0189 | 0 | 60 | 4.7e-005 | 1 | R.TTGIVMDSGDGVTHTVPIYEGYALPHAILR.L 27002 |
|  | 28328 | 1178.8758 | 3533.6057 | 3533.6240 | -0.0183 | 1 | 47 | 0.0007 | 1 | R.FRCPEALFQPSFLGMESCGIHETTFNSIMK.C 28330 28331 28341 |
|  | 28366 | 1181.9708 | 3542.8907 | 3541.7295 | 1.1612 | 1 | 1 | 30 | 3 | R.EKMTQIMFETFNTPAMYVAIQAVLSLYASGR.T |
|  | 28386 | 888.4067 | 3549.5979 | 3549.6189 | -0.0211 | 1 | (4) | 13 | 1 | R.FRCPEALFQPSFLGMESCGIHETTFNSIMK.C |
|  | 30286 | 1441.3481 | 4321.0226 | 4321.0406 | -0.0180 | 1 | 129 | 2.7e-012 | 1 | K.LCYVALDFEQEMATAASSSSLEKSYELPDGQVITIGNER.F 30289 |

| **8.** | P07437\|TBB5_HUMAN    **Mass:** 50095    **Score:** 1694   **Queries matched:** 93   **emPAI:** 7.65 |
| --- | --- |
|  | Tubulin beta chain OS=Homo sapiens GN=TUBB PE=1 SV=2 |

|  | **Query** | **Observed** | **Mr(expt)** | **Mr(calc)** | **Delta** | **Miss** | **Score** | **Expect** | **Rank** | **Peptide** |
| --- | --- | --- | --- | --- | --- | --- | --- | --- | --- | --- |
|  | 1602 | 404.7373 | 807.4600 | 807.3399 | 0.1201 | 0 | 8 | 28 | 4 | R.EEYPDR.I |
|  | 4618 | 514.7597 | 1027.5048 | 1027.5121 | -0.0072 | 0 | 25 | 0.57 | 1 | K.TAVCDIPPR.G 4619 4624 4627 4629 |
|  | 4763 | 520.2977 | 1038.5809 | 1038.5862 | -0.0053 | 0 | 7 | 29 | 2 | R.YLTVAAVFR.G |
|  | 5095 | 533.2719 | 1064.5293 | 1064.4201 | 0.1091 | 0 | (18) | 2.3 | 1 | K.NMMAACDPR.H 5090 5102 |
|  | 5226 | 539.2660 | 1076.5175 | 1076.5250 | -0.0075 | 1 | 25 | 0.53 | 1 | K.IREEYPDR.I 5225 |
|  | 5275 | 541.2110 | 1080.4074 | 1080.4151 | -0.0076 | 0 | 26 | 0.36 | 1 | K.NMMAACDPR.H |
|  | 5276 | 541.2111 | 1080.4076 | 1080.4151 | -0.0075 | 0 | (25) | 0.48 | 1 | K.NMMAACDPR.H |
|  | 6084 | 572.3180 | 1142.6215 | 1142.6270 | -0.0055 | 0 | 17 | 3.3 | 1 | K.LAVNMVPFPR.L |
|  | 8765 | 430.2513 | 1287.7319 | 1286.7169 | 1.0150 | 1 | 4 | 55 | 3 | R.KLAVNMVPFPR.L |
|  | 9016 | **651.3185** | **1300.6224** | **1300.6299** | **-0.0075** | **0** | **64** | **5.4e-005** | **1** | **R.ISVYYNEATGGK.Y** 9017 |
|  | 9381 | 660.3513 | 1318.6880 | 1318.6955 | -0.0075 | 0 | 69 | 2e-005 | 1 | R.IMNTFSVVPSPK.V 9382 |
|  | 9543 | 664.3431 | 1326.6716 | 1325.7126 | 0.9591 | 1 | 5 | 48 | 9 | K.TAVCDIPPRGLK.M |
|  | 10550 | 693.3494 | 1384.6843 | 1384.6921 | -0.0078 | 1 | 30 | 0.13 | 1 | K.RISEQFTAMFR.R |
|  | 11576 | 723.8442 | 1445.6739 | 1445.6820 | -0.0081 | 0 | 71 | 1.1e-005 | 1 | K.EVDEQMLNVQNK.N 11577 |
|  | 14299 | **808.4167** | **1614.8189** | **1614.8287** | **-0.0098** | **0** | **44** | **0.0046** | **1** | **R.AILVDLEPGTMDSVR.S** 14300 14310 |
|  | 14380 | 540.9477 | 1619.8212 | 1619.8283 | -0.0070 | 0 | 48 | 0.0018 | 1 | R.LHFFMPGFAPLTSR.G 14371 14377 14378 14379 |
|  | 14564 | **816.4130** | **1630.8114** | **1630.8236** | **-0.0123** | **0** | **(21)** | **1.1** | **1** | **R.AILVDLEPGTMDSVR.S** 14576 |
|  | 15009 | **830.4469** | **1658.8792** | **1658.8879** | **-0.0087** | **0** | **54** | **0.00041** | **1** | **R.ALTVPELTQQVFDAK.N** 15007 15008 |
|  | 15553 | 848.9153 | 1695.8160 | 1695.8257 | -0.0096 | 0 | 19 | 1.4 | 1 | K.NSSYFVEWIPNNVK.T 15562 15563 15564 |
|  | 17088 | **908.9598** | **1815.9051** | **1815.9155** | **-0.0104** | **1** | **71** | **9.2e-006** | **1** | **R.ISVYYNEATGGKYVPR.A** 17089 |
|  | 17173 | 911.9601 | 1821.9057 | 1821.9156 | -0.0098 | 0 | 61 | 8.7e-005 | 1 | R.EIVHIQAGQCGNQIGAK.F 17174 |
|  | 17811 | **624.2341** | **1869.6806** | **1868.9706** | **0.7099** | **0** | **6** | **19** | **1** | **K.MAVTFIGNSTAIQELFK.R** 17803 |
|  | 18438 | 962.4478 | 1922.8809 | 1922.8900 | -0.0090 | 1 | 91 | 8.1e-008 | 1 | R.MSMKEVDEQMLNVQNK.N |
|  | 18859 | 979.9888 | 1957.9631 | 1957.9745 | -0.0114 | 0 | 86 | 2.2e-007 | 1 | K.GHYTEGAELVDSVLDVVR.K 18858 |
|  | 20166 | 1044.0366 | 2086.0587 | 2086.0695 | -0.0108 | 1 | 114 | 3.4e-010 | 1 | K.GHYTEGAELVDSVLDVVRK.E 20162 20163 20167 20168 20169 |
|  | 20402 | 1055.5299 | 2109.0453 | 2109.0571 | -0.0119 | 1 | 94 | 3.6e-008 | 1 | -.MREIVHIQAGQCGNQIGAK.F 20399 20400 20401 20403 20410 |
|  | 20534 | 532.2908 | 2125.1342 | 2125.0521 | 0.0822 | 1 | (5) | 26 | 4 | -.MREIVHIQAGQCGNQIGAK.F |
|  | 24649 | 1354.6668 | 2707.3189 | 2707.3310 | -0.0120 | 0 | 100 | 5.8e-009 | 1 | K.LTTPTYGDLNHLVSATMSGVTTCLR.F 24651 |
|  | 25134 | 1399.6674 | 2797.3202 | 2797.3361 | -0.0160 | 0 | 80 | 5.8e-007 | 1 | R.SGPFGQIFRPDNFVFGQSGAGNNWAK.G 25133 25135 25137 25138 25139 25144 25145 25147 25148 |
|  | 26634 | **1034.8009** | **3101.3809** | **3101.4003** | **-0.0194** | **0** | **53** | **0.00023** | **1** | **K.FWEVISDEHGIDPTGTYHGDSDLQLDR.I** 26633 26635 |
|  | 27484 | 1656.2628 | 3310.5111 | 3310.5268 | -0.0158 | 0 | 72 | 2.8e-006 | 1 | K.EAESCDCLQGFQLTHSLGGGTGSGMGTLLISK.I 27480 27481 27482 27483 |
|  | 27558 | 1109.8416 | 3326.5028 | 3326.5218 | -0.0189 | 0 | (3) | 24 | 2 | K.EAESCDCLQGFQLTHSLGGGTGSGMGTLLISK.I 27556 |
|  | 28039 | 1147.2087 | 3438.6044 | 3438.6218 | -0.0174 | 1 | 115 | 1.3e-010 | 1 | R.KEAESCDCLQGFQLTHSLGGGTGSGMGTLLISK.I 28036 28037 |
|  | 28070 | 1152.8376 | 3455.4911 | 3454.6167 | 0.8744 | 1 | (1) | 31 | 10 | R.KEAESCDCLQGFQLTHSLGGGTGSGMGTLLISK.I |
|  | 30696 | 1531.7006 | 4592.0799 | 4592.0999 | -0.0201 | 0 | 53 | 8.6e-005 | 1 | K.VSDTVVEPYNATLSVHQLVENTDETYCIDNEALYDICFR.T 30694 30698 30701 |

| **9.** | P25705\|ATPA_HUMAN    **Mass:** 59828    **Score:** 1688   **Queries matched:** 108   **emPAI:** 4.75 |
| --- | --- |
|  | ATP synthase subunit alpha, mitochondrial OS=Homo sapiens GN=ATP5A1 PE=1 SV=1 |

|  | **Query** | **Observed** | **Mr(expt)** | **Mr(calc)** | **Delta** | **Miss** | **Score** | **Expect** | **Rank** | **Peptide** |
| --- | --- | --- | --- | --- | --- | --- | --- | --- | --- | --- |
|  | 1314 | **395.2130** | **788.4114** | **788.4141** | **-0.0027** | **0** | **33** | **0.13** | **1** | **R.VGSAAQTR.A** 1310 1315 |
|  | 1675 | **408.2321** | **814.4496** | **814.4548** | **-0.0053** | **0** | **20** | **2.2** | **1** | **R.ELIIGDR.Q** |
|  | 2310 | **430.7521** | **859.4896** | **859.4949** | **-0.0053** | **0** | **27** | **0.42** | **1** | **R.QMSLLLR.R** |
|  | 2373 | **432.7076** | **863.4007** | **863.4059** | **-0.0051** | **0** | **18** | **2.7** | **1** | **R.LTDADAMK.Y** |
|  | 2778 | **446.7455** | **891.4764** | **891.4814** | **-0.0050** | **0** | **43** | **0.0091** | **1** | **K.LELAQYR.E** 2784 |
|  | 3049 | **456.7217** | **911.4289** | **910.3668** | **1.0620** | **0** | **9** | **20** | **10** | **R.FNDGSDEK.K** 3051 |
|  | 3117 | **458.3047** | **914.5949** | **914.5437** | **0.0512** | **1** | **5** | **56** | **9** | **K.LEPSKITK.F** |
|  | 4257 | **500.7903** | **999.5660** | **999.5713** | **-0.0053** | **0** | **48** | **0.0025** | **1** | **R.VLSIGDGIAR.V** 4256 4269 4285 |
|  | 4593 | **513.6124** | **1025.2103** | **1025.5869** | **-0.3766** | **0** | **8** | **15** | **2** | **K.AVDSLVPIGR.G** |
|  | 5244 | **540.2373** | **1078.4600** | **1079.5467** | **-1.0867** | **1** | **10** | **15** | **2** | **R.AMKQVAGTMK.L** 5243 5245 |
|  | 6465 | **586.8079** | **1171.6012** | **1170.6245** | **0.9767** | **0** | **14** | **7.1** | **1** | **R.VVDALGNAIDGK.G** |
|  | 7577 | **615.3420** | **1228.6694** | **1228.6775** | **-0.0081** | **1** | **14** | **6.2** | **2** | **R.ELIIGDRQTGK.T** 7578 |
|  | 7995 | **625.3043** | **1248.5941** | **1247.5993** | **0.9948** | **1** | **27** | **0.3** | **1** | **R.ADGKISEQSDAK.L** |
|  | 9324 | **658.8704** | **1315.7263** | **1315.7347** | **-0.0084** | **0** | **59** | **0.00018** | **1** | **K.TSIAIDTIINQK.R** 9325 |
|  | 9559 | **664.8080** | **1327.6015** | **1326.7006** | **0.9010** | **0** | **39** | **0.018** | **1** | **K.LYCIYVAIGQK.R** 9558 |
|  | 10080 | **679.8723** | **1357.7301** | **1357.7388** | **-0.0087** | **1** | **53** | **0.00076** | **1** | **R.ISVREPMQTGIK.A** 10079 10081 10082 |
|  | 10231 | **456.5940** | **1366.7601** | **1366.7681** | **-0.0080** | **1** | **0** | **1.3e+002** | **8** | **K.AVDSLVPIGRGQR.E** |
|  | 10367 | **458.9160** | **1373.7262** | **1373.7337** | **-0.0074** | **1** | **(10)** | **13** | **5** | **R.ISVREPMQTGIK.A** |
|  | 11183 | **712.3361** | **1422.6577** | **1422.6660** | **-0.0083** | **0** | **85** | **4.5e-007** | **1** | **K.TGTAEMSSILEER.I** 11182 |
|  | 11446 | **719.9236** | **1437.8326** | **1437.8416** | **-0.0090** | **0** | **48** | **0.0022** | **1** | **K.GIRPAINVGLSVSR.V** 11429 11447 |
|  | 12019 | **491.6164** | **1471.8274** | **1471.8358** | **-0.0084** | **1** | **20** | **1.3** | **1** | **K.TSIAIDTIINQKR.F** 12018 |
|  | 13267 | **517.2944** | **1548.8615** | **1547.8606** | **1.0009** | **1** | **5** | **42** | **7** | **K.QAVAYRQMSLLLR.R** |
|  | 13302 | **776.4214** | **1550.8282** | **1550.8344** | **-0.0062** | **1** | **64** | **5.3e-005** | **1** | **K.LKEIVTNFLAGFEA.-** 13298 13300 13309 |
|  | 13658 | **788.3921** | **1574.7696** | **1574.7788** | **-0.0092** | **0** | **96** | **2.9e-008** | **1** | **R.ILGADTSVDLEETGR.V** 13642 13646 13656 13657 13661 13670 13676 |
|  | 14180 | **804.4269** | **1606.8393** | **1606.8501** | **-0.0108** | **1** | **31** | **0.11** | **1** | **K.QVAGTMKLELAQYR.E** |
|  | 14454 | **542.2728** | **1623.7965** | **1622.8450** | **0.9515** | **1** | **(5)** | **38** | **4** | **K.QVAGTMKLELAQYR.E** |
|  | 14458 | **812.9440** | **1623.8735** | **1623.8832** | **-0.0097** | **0** | **58** | **0.00017** | **1** | **R.TGAIVDVPVGEELLGR.V** 14457 14459 |
|  | 15102 | **834.3953** | **1666.7761** | **1666.7872** | **-0.0111** | **0** | **90** | **1.1e-007** | **1** | **R.NVQAEEMVEFSSGLK.G** 15103 15106 15107 15121 |
|  | 15741 | **855.9672** | **1709.9198** | **1709.9312** | **-0.0114** | **1** | **60** | **0.00011** | **1** | **R.VVDALGNAIDGKGPIGSK.T** 15743 15756 15757 15760 |
|  | 16658 | **890.9951** | **1779.9756** | **1779.9843** | **-0.0087** | **1** | **59** | **0.00012** | **1** | **K.RTGAIVDVPVGEELLGR.V** 16659 |
|  | 20338 | **1052.5176** | **2103.0206** | **2103.0307** | **-0.0101** | **0** | **85** | **2.9e-007** | **1** | **K.GMSLNLEPDNVGVVVFGNDK.L** 20335 20336 20351 |
|  | 20483 | **706.6904** | **2117.0495** | **2116.0602** | **0.9892** | **1** | **13** | **4.7** | **1** | **R.RPPGREAYPGDVFYLHSR.L** 20475 |
|  | 20493 | **707.0501** | **2118.1283** | **2119.0256** | **-0.8973** | **0** | **(9)** | **10** | **1** | **K.GMSLNLEPDNVGVVVFGNDK.L** |
|  | 22101 | **1155.0787** | **2308.1429** | **2308.1521** | **-0.0092** | **0** | **107** | **1.6e-009** | **1** | **K.QGQYSPMAIEEQVAVIYAGVR.G** 22100 |
|  | 22299 | **1169.5826** | **2337.1507** | **2337.1601** | **-0.0094** | **0** | **140** | **7.6e-013** | **1** | **R.EVAAFAQFGSDLDAATQQLLSR.G** 22298 22301 |
|  | 23141 | **1229.6465** | **2457.2784** | **2457.2937** | **-0.0153** | **1** | **91** | **5.3e-008** | **1** | **K.GMSLNLEPDNVGVVVFGNDKLIK.E** 23142 23143 23144 23145 |
|  | 23261 | **825.4332** | **2473.2777** | **2473.2887** | **-0.0110** | **1** | **(15)** | **2.1** | **1** | **K.GMSLNLEPDNVGVVVFGNDKLIK.E** 23258 |
|  | 23810 | **1279.1709** | **2556.3272** | **2556.3395** | **-0.0123** | **1** | **31** | **0.056** | **1** | **R.ILGADTSVDLEETGRVLSIGDGIAR.V** 23808 23811 23812 23815 23816 23823 23825 |
|  | 24991 | **926.5035** | **2776.4888** | **2776.4971** | **-0.0083** | **1** | **47** | **0.0011** | **1** | **R.TGAIVDVPVGEELLGRVVDALGNAIDGK.G** |
|  | 25526 | **962.4658** | **2884.3755** | **2884.4429** | **-0.0674** | **1** | **2** | **36** | **3** | **K.QGQYSPMAIEEQVAVIYAGVRGYLDK.L** 25527 25528 |
|  | 27793 | **1694.7891** | **3387.5636** | **3387.5791** | **-0.0156** | **0** | **33** | **0.02** | **1** | **K.YTIVVSATASDAAPLQYLAPYSGCSMGEYFR.D** |
|  | 29054 | **1251.6033** | **3751.7880** | **3751.8073** | **-0.0193** | **1** | **28** | **0.058** | **1** | **R.NVQAEEMVEFSSGLKGMSLNLEPDNVGVVVFGNDK.L** |
|  | 30154 | **1411.9923** | **4232.9551** | **4232.9744** | **-0.0193** | **1** | **49** | **0.00035** | **1** | **R.LTDADAMKYTIVVSATASDAAPLQYLAPYSGCSMGEYFR.D** 30153 30156 |
|  | 31307 | **1277.3928** | **5105.5422** | **5105.5471** | **-0.0050** | **0** | **26** | **0.033** | **1** | **K.MNDAFGGGSLTALPVIETQAGDVSAYIPTNVISITDGQIFLETELFYK.G** 31303 |

| **10.** | Q00610\|CLH1_HUMAN    **Mass:** 193260   **Score:** 1621   **Queries matched:** 109   **emPAI:** 1.09 |
| --- | --- |
|  | Clathrin heavy chain 1 OS=Homo sapiens GN=CLTC PE=1 SV=5 |

|  | **Query** | **Observed** | **Mr(expt)** | **Mr(calc)** | **Delta** | **Miss** | **Score** | **Expect** | **Rank** | **Peptide** |
| --- | --- | --- | --- | --- | --- | --- | --- | --- | --- | --- |
|  | 1007 | **625.1442** | **624.1369** | **624.3119** | **-0.1750** | **0** | **7** | **18** | **3** | **K.DSLYK.D** |
|  | 1163 | **698.3130** | **697.3058** | **697.3105** | **-0.0047** | **0** | **20** | **1.2** | **1** | **K.MYDAAK.L** |
|  | 1446 | **399.7127** | **797.4107** | **796.3901** | **1.0206** | **0** | **7** | **31** | **5** | **K.YLQMAR.K** |
|  | 1804 | **413.5520** | **825.0894** | **825.5436** | **-0.4542** | **0** | **2** | **61** | **3** | **K.NLILVVR.G** |
|  | 1826 | **414.7136** | **827.4126** | **827.4177** | **-0.0051** | **0** | **30** | **0.2** | **1** | **R.AVNYFSK.V** |
|  | 2154 | **425.2187** | **848.4228** | **848.4756** | **-0.0528** | **1** | **21** | **1.7** | **1** | **K.NFLKEAK.L** |
|  | 2219 | **853.4179** | **852.4106** | **852.4164** | **-0.0057** | **0** | **42** | **0.01** | **1** | **K.YIQAACK.T** 2217 |
|  | 2710 | **443.2060** | **884.3974** | **884.4028** | **-0.0054** | **0** | **34** | **0.068** | **1** | **R.AYEFAER.C** |
|  | 3242 | **462.7316** | **923.4486** | **923.4535** | **-0.0048** | **0** | **16** | **3.6** | **1** | **R.VMEYINR.L** |
|  | 3665 | **479.7664** | **957.5182** | **956.5477** | **0.9705** | **0** | **7** | **34** | **5** | **-.MAQILPIR.F** |
|  | 4818 | **522.3002** | **1042.5859** | **1042.4356** | **0.1503** | **0** | **14** | **6.6** | **1** | **R.ENPYYDSR.V** |
|  | 5009 | **530.2895** | **1058.5645** | **1058.5720** | **-0.0075** | **1** | **15** | **5.4** | **2** | **K.TGQIKEVER.I** 5010 5014 5017 |
|  | 5144 | **535.7750** | **1069.5355** | **1068.5386** | **0.9969** | **0** | **24** | **0.59** | **1** | **R.AHIAQLCEK.A** |
|  | 5175 | **536.8125** | **1071.6104** | **1070.6124** | **0.9980** | **0** | **26** | **0.44** | **1** | **K.HDVVFLITK.Y** |
|  | 6418 | **585.2261** | **1168.4377** | **1168.4455** | **-0.0078** | **0** | **10** | **12** | **2** | **R.ESNCYDPER.V** |
|  | 7644 | **411.7181** | **1232.1324** | **1231.6408** | **0.4916** | **1** | **6** | **29** | **9** | **K.VDKLDASESLR.K** |
|  | 7711 | **618.8332** | **1235.6518** | **1235.6584** | **-0.0065** | **0** | **32** | **0.083** | **1** | **K.TLQIFNIEMK.S** |
|  | 9108 | **652.8290** | **1303.6434** | **1303.6520** | **-0.0086** | **0** | **20** | **1.3** | **1** | **R.NNLAGAEELFAR.K** 9122 |
|  | 9258 | **438.7480** | **1313.2221** | **1312.7139** | **0.5082** | **1** | **22** | **0.55** | **1** | **R.LEKHELIEFR.R** |
|  | 9649 | **667.8168** | **1333.6191** | **1333.6262** | **-0.0071** | **0** | **60** | **0.00014** | **1** | **K.IYIDSNNNPER.F** 9647 |
|  | 9699 | **669.3437** | **1336.6728** | **1336.6809** | **-0.0081** | **0** | **64** | **5.4e-005** | **1** | **R.VVGAMQLYSVDR.K** 9683 |
|  | 9963 | **676.4227** | **1350.8308** | **1350.8387** | **-0.0079** | **1** | **9** | **15** | **1** | **R.LKLLLPWLEAR.I** |
|  | 9978 | **451.7483** | **1352.2230** | **1352.6758** | **-0.4529** | **0** | **(4)** | **33** | **2** | **R.VVGAMQLYSVDR.K** |
|  | 9988 | **677.3536** | **1352.6927** | **1352.7010** | **-0.0082** | **1** | **29** | **0.17** | **1** | **K.GMVKEAIDSYIK.A** |
|  | 10254 | **456.9014** | **1367.6824** | **1367.6906** | **-0.0082** | **0** | **35** | **0.042** | **1** | **K.NNRPSEGPLQTR.L** 10253 |
|  | 10690 | **697.3577** | **1392.7009** | **1392.5690** | **0.1320** | **1** | **14** | **5.2** | **2** | **R.CYDEKMYDAAK.L** |
|  | 10732 | **466.2073** | **1395.6000** | **1395.6089** | **-0.0088** | **1** | **11** | **11** | **1** | **R.ESNCYDPERVK.N** |
|  | 10845 | **701.8386** | **1401.6627** | **1401.6711** | **-0.0084** | **0** | **46** | **0.0032** | **1** | **R.CNEPAVWSQLAK.A** |
|  | 11052 | **708.3633** | **1414.7120** | **1414.7205** | **-0.0085** | **0** | **63** | **6.3e-005** | **1** | **K.IVLDNSVFSEHR.N** 11066 |
|  | 11374 | **717.3997** | **1432.7849** | **1432.7926** | **-0.0077** | **0** | **78** | **2e-006** | **1** | **K.SVDPTLALSVYLR.A** 11375 |
|  | 11784 | **730.3471** | **1458.6797** | **1458.6891** | **-0.0095** | **1** | **39** | **0.019** | **1** | **R.FLRENPYYDSR.V** 11785 11787 |
|  | 11893 | **733.3904** | **1464.7663** | **1464.7759** | **-0.0096** | **1** | **1** | **94** | **1** | **R.VVGAMQLYSVDRK.V** |
|  | 11930 | **489.9078** | **1466.7015** | **1466.7803** | **-0.0788** | **1** | **3** | **59** | **7** | **K.TLQIFNIEMKSK.M** |
|  | 12462 | **750.4156** | **1498.8166** | **1498.8256** | **-0.0090** | **0** | **67** | **2.5e-005** | **1** | **K.WLLLTGISAQQNR.V** |
|  | 12525 | **501.9192** | **1502.7357** | **1502.7452** | **-0.0096** | **1** | **20** | **1.2** | **1** | **K.MREHLELFWSR.V** |
|  | 12774 | **759.8740** | **1517.7334** | **1517.7409** | **-0.0075** | **0** | **48** | **0.0022** | **1** | **R.HSSLAGCQIINYR.T** |
|  | 13289 | **776.3757** | **1550.7368** | **1550.7464** | **-0.0096** | **0** | **62** | **7.3e-005** | **1** | **R.GQFSTDELVAEVEK.R** |
|  | 14157 | **536.2795** | **1605.8166** | **1606.8871** | **-1.0705** | **1** | **1** | **83** | **7** | **K.KVGYTPDWIFLLR.N** |
|  | 14193 | **536.9269** | **1607.7588** | **1607.8671** | **-0.1083** | **1** | **1** | **92** | **1** | **K.YIEIYVQKVNPSR.L** |
|  | 14256 | **806.9225** | **1611.8304** | **1611.8396** | **-0.0091** | **0** | **57** | **0.00023** | **1** | **R.ESYVETELIFALAK.T** 14269 |
|  | 14381 | **540.9481** | **1619.8224** | **1619.8307** | **-0.0084** | **1** | **57** | **0.00025** | **1** | **R.ALEHFTDLYDIKR.A** |
|  | 14546 | **815.8922** | **1629.7699** | **1629.7787** | **-0.0088** | **0** | **66** | **3.4e-005** | **1** | **K.FNALFAQGNYSEAAK.V** |
|  | 15534 | **565.7885** | **1694.3437** | **1695.0043** | **-0.6606** | **1** | **3** | **33** | **3** | **R.NLQNLLILTAIKADR.T** |
|  | 15704 | **854.4262** | **1706.8377** | **1706.8475** | **-0.0098** | **1** | **89** | **1.3e-007** | **1** | **R.GQFSTDELVAEVEKR.N** |
|  | 15822 | **858.9332** | **1715.8519** | **1715.8631** | **-0.0112** | **0** | **68** | **1.7e-005** | **1** | **K.VSQPIEGHAASFAQFK.M** 15823 |
|  | 16377 | **879.9392** | **1757.8637** | **1757.8736** | **-0.0099** | **1** | **60** | **9.9e-005** | **1** | **R.KFNALFAQGNYSEAAK.V** 16376 16378 |
|  | 16580 | **888.9145** | **1775.8144** | **1775.8260** | **-0.0116** | **0** | **91** | **8e-008** | **1** | **R.IHEGCEEPATHNALAK.I** 16578 16579 16607 |
|  | 18653 | **971.9548** | **1941.8950** | **1941.9068** | **-0.0118** | **0** | **91** | **7.2e-008** | **1** | **R.TSIDAYDNFDNISLAQR.L** 18652 |
|  | 18691 | **973.0242** | **1944.0339** | **1944.0469** | **-0.0130** | **0** | **59** | **0.00011** | **1** | **K.LHIIEVGTPPTGNQPFPK.K** 18692 |
|  | 18721 | **974.0072** | **1945.9998** | **1946.0070** | **-0.0072** | **0** | **36** | **0.026** | **1** | **K.AFMTADLPNELIELLEK.I** |
|  | 19116 | **992.9721** | **1983.9297** | **1983.9409** | **-0.0112** | **0** | **68** | **1.5e-005** | **1** | **R.HNIMDFAMPYFIQVMK.E** |
|  | 19864 | **685.9481** | **2054.8224** | **2054.0394** | **0.7830** | **0** | **6** | **21** | **1** | **R.GYFEELITMLEAALGLER.A** |
|  | 20279 | **1049.5444** | **2097.0743** | **2097.1616** | **-0.0873** | **1** | **3** | **39** | **2** | **R.RPISADSAIMNPASKVIALK.A** 20278 20283 |
|  | 21737 | **754.4019** | **2260.1839** | **2259.1827** | **1.0012** | **1** | **2** | **53** | **9** | **R.AMLSANIRQNLQICVQVASK.Y** |
|  | 21888 | **760.0505** | **2277.1298** | **2276.1324** | **0.9974** | **1** | **24** | **0.31** | **1** | **K.DTELAEELLQWFLQEEKR.E** |
|  | 22137 | **771.7129** | **2312.1170** | **2312.1284** | **-0.0114** | **1** | **7** | **15** | **1** | **R.TSIDAYDNFDNISLAQRLEK.H** |
|  | 22435 | **785.4174** | **2353.2303** | **2352.2438** | **0.9865** | **0** | **28** | **0.12** | **1** | **R.ISGETIFVTAPHEATAGIIGVNR.K** |
|  | 22443 | **1178.0710** | **2354.1275** | **2354.1390** | **-0.0114** | **0** | **129** | **8.4e-012** | **1** | **K.SVNESLNNLFITEEDYQALR.T** 22441 22442 22444 |
|  | 23035 | **815.1030** | **2442.2871** | **2442.3020** | **-0.0149** | **1** | **5** | **25** | **2** | **R.GQAGGKLHIIEVGTPPTGNQPFPK.K** |
|  | 23089 | **818.0851** | **2451.2334** | **2451.2428** | **-0.0093** | **1** | **16** | **1.7** | **1** | **R.EKVGEQAQVVIIDMNDPSNPIR.R** |
|  | 23187 | **822.0732** | **2463.1979** | **2463.2142** | **-0.0163** | **0** | **10** | **7.7** | **1** | **R.LAELEEFINGPNNAHIQQVGDR.C** |
|  | 24136 | **873.7616** | **2618.2630** | **2618.2735** | **-0.0105** | **1** | **39** | **0.0077** | **1** | **R.HNIMDFAMPYFIQVMKEYLTK.V** |
|  | 24292 | **884.7047** | **2651.0923** | **2650.2634** | **0.8290** | **1** | **(0)** | **54** | **5** | **R.HNIMDFAMPYFIQVMKEYLTK.V** |
|  | 24812 | **914.0795** | **2739.2166** | **2739.2334** | **-0.0168** | **0** | **49** | **0.00079** | **1** | **K.ADDPSSYMEVVQAANTSGNWEELVK.Y** |
|  | 25313 | **946.1368** | **2835.3885** | **2834.4059** | **0.9826** | **1** | **60** | **5.7e-005** | **1** | **K.TNRLAELEEFINGPNNAHIQQVGDR.C** |
|  | 26271 | 1011.2318 | 3030.6734 | 3030.3528 | 0.3207 | 1 | 4 | 19 | 4 | K.YEEYDNAIITMMNHPTDAWKEGQFK.D 26240 |
|  | 26739 | **782.8807** | **3127.4936** | **3127.5041** | **-0.0105** | **0** | **53** | **0.00022** | **1** | **R.LLEMNLMHAPQVADAILGNQMFTHYDR.A** |
|  | 26809 | **786.8795** | **3143.4890** | **3143.4991** | **-0.0101** | **0** | **(25)** | **0.15** | **1** | **R.LLEMNLMHAPQVADAILGNQMFTHYDR.A** |
|  | 26961 | **1058.8507** | **3173.5303** | **3173.5492** | **-0.0189** | **0** | **2** | **27** | **2** | **K.WISLNTVALVTDNAVYHWSMEGESQPVK.M** |
|  | 27011 | **1063.2271** | **3186.6593** | **3186.6714** | **-0.0120** | **0** | **50** | **0.00047** | **1** | **R.FQSVPAQPGQTSPLLQYFGILLDQGQLNK.Y** |
|  | 28191 | **1166.5825** | **3496.7257** | **3496.7449** | **-0.0192** | **0** | **22** | **0.24** | **1** | **R.AVVHTHLLNPEWLVNYFGSLSVEDSLECLR.A** |
|  | 28769 | **1220.5679** | **3658.6818** | **3658.6984** | **-0.0167** | **1** | **73** | **1.6e-006** | **1** | **K.EAIDSYIKADDPSSYMEVVQAANTSGNWEELVK.Y** 28770 |
|  | 29003 | **934.2253** | **3732.8723** | **3732.8927** | **-0.0204** | **1** | **8** | **5** | **1** | **K.VGEQAQVVIIDMNDPSNPIRRPISADSAIMNPASK.V** |
|  | 29608 | **1326.3138** | **3975.9197** | **3975.9352** | **-0.0155** | **1** | **27** | **0.066** | **1** | **R.VMEYINRLDNYDAPDIANIAISNELFEEAFAIFR.K** |
|  | 30073 | **836.6100** | **4178.0136** | **4178.0322** | **-0.0186** | **1** | **2** | **15** | **1** | **R.LLEMNLMHAPQVADAILGNQMFTHYDRAHIAQLCEK.A** |
|  | 31227 | **1004.1218** | **5015.5725** | **5015.5967** | **-0.0243** | **1** | **5** | **4** | **1** | **R.FQSVPAQPGQTSPLLQYFGILLDQGQLNKYESLELCRPVLQQGR.K** 31230 31232 |
|  | 31720 | **1382.4266** | **5525.6775** | **5525.7085** | **-0.0310** | **0** | **42** | **0.00075** | **1** | **R.ISPDQGQQFAQMLVQDEEPLADITQIVDVFMEYNLIQQCTAFLLDALK.N** 31719 |
|  | 32031 | **1152.3329** | **5756.6281** | **5757.6960** | **-1.0679** | **0** | **5** | **2.7** | **1** | **K.EEEQATETQPIVYGQPQLMLTAGPSVAVPPQAPFGYGYTAPPYGQPQPGFGYSM.-** |

| **11.** | P04350\|TBB4_HUMAN    **Mass:** 50010    **Score:** 1432   **Queries matched:** 81   **emPAI:** 5.49 |
| --- | --- |
|  | Tubulin beta-4 chain OS=Homo sapiens GN=TUBB4 PE=1 SV=2 |

|  | **Query** | **Observed** | **Mr(expt)** | **Mr(calc)** | **Delta** | **Miss** | **Score** | **Expect** | **Rank** | **Peptide** |
| --- | --- | --- | --- | --- | --- | --- | --- | --- | --- | --- |
|  | 4618 | 514.7597 | 1027.5048 | 1027.5121 | -0.0072 | 0 | 25 | 0.57 | 1 | K.TAVCDIPPR.G 4619 4624 4627 4629 |
|  | 4763 | 520.2977 | 1038.5809 | 1038.5862 | -0.0053 | 0 | 7 | 29 | 2 | R.YLTVAAVFR.G |
|  | 5095 | 533.2719 | 1064.5293 | 1064.4201 | 0.1091 | 0 | (18) | 2.3 | 1 | K.NMMAACDPR.H 5090 5102 |
|  | 5275 | 541.2110 | 1080.4074 | 1080.4151 | -0.0076 | 0 | 26 | 0.36 | 1 | K.NMMAACDPR.H |
|  | 5276 | 541.2111 | 1080.4076 | 1080.4151 | -0.0075 | 0 | (25) | 0.48 | 1 | K.NMMAACDPR.H |
|  | 6084 | 572.3180 | 1142.6215 | 1142.6270 | -0.0055 | 0 | 17 | 3.3 | 1 | K.LAVNMVPFPR.L |
|  | 8765 | 430.2513 | 1287.7319 | 1286.7169 | 1.0150 | 1 | 4 | 55 | 3 | R.KLAVNMVPFPR.L |
|  | 9381 | 660.3513 | 1318.6880 | 1318.6955 | -0.0075 | 0 | 69 | 2e-005 | 1 | R.IMNTFSVVPSPK.V 9382 |
|  | 9543 | 664.3431 | 1326.6716 | 1325.7126 | 0.9591 | 1 | 5 | 48 | 9 | K.TAVCDIPPRGLK.M |
|  | 10550 | 693.3494 | 1384.6843 | 1384.6921 | -0.0078 | 1 | 30 | 0.13 | 1 | K.RISEQFTAMFR.R |
|  | 14084 | 801.4085 | 1600.8025 | 1600.8131 | -0.0106 | 0 | 63 | 6.1e-005 | 1 | R.AVLVDLEPGTMDSVR.S 14085 14086 14096 |
|  | 14330 | 809.4058 | 1616.7971 | 1616.8080 | -0.0109 | 0 | (21) | 0.95 | 1 | R.AVLVDLEPGTMDSVR.S |
|  | 14380 | 540.9477 | 1619.8212 | 1619.8283 | -0.0070 | 0 | 48 | 0.0018 | 1 | R.LHFFMPGFAPLTSR.G 14371 14377 14378 14379 |
|  | 15480 | 846.4329 | 1690.8512 | 1690.8600 | -0.0088 | 0 | 66 | 3e-005 | 1 | R.ALTVPELTQQMFDAK.N 15478 |
|  | 15553 | 848.9153 | 1695.8160 | 1695.8257 | -0.0096 | 0 | 19 | 1.4 | 1 | K.NSSYFVEWIPNNVK.T 15562 15563 15564 |
|  | 17173 | 911.9601 | 1821.9057 | 1821.9156 | -0.0098 | 0 | 61 | 8.7e-005 | 1 | R.EIVHLQAGQCGNQIGAK.F 17174 |
|  | 17255 | **915.4393** | **1828.8640** | **1828.8744** | **-0.0104** | **0** | **70** | **1e-005** | **1** | **R.INVYYNEATGGNYVPR.A** |
|  | 17663 | 929.4699 | 1856.9253 | 1856.9342 | -0.0090 | 0 | 55 | 0.0003 | 1 | K.MAATFIGNSTAIQELFK.R |
|  | 18347 | 639.9880 | 1916.9423 | 1916.8529 | 0.0894 | 1 | 4 | 43 | 10 | R.MSMKEVDEQMLSVQSK.N |
|  | 19427 | 1007.5205 | 2013.0265 | 2013.0353 | -0.0089 | 1 | 49 | 0.0013 | 1 | K.MAATFIGNSTAIQELFKR.I 19426 19429 |
|  | 20402 | 1055.5299 | 2109.0453 | 2109.0571 | -0.0119 | 1 | 94 | 3.6e-008 | 1 | -.MREIVHLQAGQCGNQIGAK.F 20399 20400 20401 20403 20410 |
|  | 20534 | 532.2908 | 2125.1342 | 2125.0521 | 0.0822 | 1 | (5) | 26 | 4 | -.MREIVHLQAGQCGNQIGAK.F |
|  | 24649 | 1354.6668 | 2707.3189 | 2707.3310 | -0.0120 | 0 | 100 | 5.8e-009 | 1 | K.LTTPTYGDLNHLVSATMSGVTTCLR.F 24651 |
|  | 25038 | 929.4656 | 2785.3749 | 2785.2543 | 0.1206 | 1 | 1 | 45 | 4 | R.ALTVPELTQQMFDAKNMMAACDPR.H |
|  | 25134 | 1399.6674 | 2797.3202 | 2797.3361 | -0.0160 | 0 | 80 | 5.8e-007 | 1 | R.SGPFGQIFRPDNFVFGQSGAGNNWAK.G 25133 25135 25137 25138 25139 25144 25145 25147 25148 |
|  | 26680 | 1039.4745 | 3115.4016 | 3115.4159 | -0.0143 | 0 | 67 | 1e-005 | 1 | K.FWEVISDEHGIDPTGTYHGDSDLQLER.I 26674 26676 26677 26679 |
|  | 27484 | 1656.2628 | 3310.5111 | 3310.5268 | -0.0158 | 0 | 72 | 2.8e-006 | 1 | K.EAESCDCLQGFQLTHSLGGGTGSGMGTLLISK.I 27480 27481 27482 27483 |
|  | 27558 | 1109.8416 | 3326.5028 | 3326.5218 | -0.0189 | 0 | (3) | 24 | 2 | K.EAESCDCLQGFQLTHSLGGGTGSGMGTLLISK.I 27556 |
|  | 28039 | 1147.2087 | 3438.6044 | 3438.6218 | -0.0174 | 1 | 115 | 1.3e-010 | 1 | R.KEAESCDCLQGFQLTHSLGGGTGSGMGTLLISK.I 28036 28037 |
|  | 28070 | 1152.8376 | 3455.4911 | 3454.6167 | 0.8744 | 1 | (1) | 31 | 10 | R.KEAESCDCLQGFQLTHSLGGGTGSGMGTLLISK.I |
|  | 30696 | 1531.7006 | 4592.0799 | 4592.0999 | -0.0201 | 0 | 53 | 8.6e-005 | 1 | K.VSDTVVEPYNATLSVHQLVENTDETYCIDNEALYDICFR.T 30694 30698 30701 |

| **12.** | P68032\|ACTC_HUMAN    **Mass:** 42334    **Score:** 1249   **Queries matched:** 82   **emPAI:** 5.47 |
| --- | --- |
|  | Actin, alpha cardiac muscle 1 OS=Homo sapiens GN=ACTC1 PE=1 SV=1 |

|  | **Query** | **Observed** | **Mr(expt)** | **Mr(calc)** | **Delta** | **Miss** | **Score** | **Expect** | **Rank** | **Peptide** |
| --- | --- | --- | --- | --- | --- | --- | --- | --- | --- | --- |
|  | 1475 | 800.5302 | 799.5229 | 799.5280 | -0.0051 | 1 | 14 | 8.2 | 3 | K.RGILTLK.Y |
|  | 4224 | 499.7444 | 997.4741 | 997.4790 | -0.0049 | 0 | 10 | 15 | 4 | R.DLTDYLMK.I |
|  | 4734 | 518.8262 | 1035.6379 | 1035.6440 | -0.0061 | 1 | 21 | 1.4 | 1 | K.IKIIAPPER.K |
|  | 5932 | **565.7742** | **1129.5339** | **1129.5404** | **-0.0065** | **0** | **4** | **72** | **2** | **R.GYSFVTTAER.E** |
|  | 6341 | 581.3092 | 1160.6038 | 1160.6111 | -0.0072 | 0 | 36 | 0.042 | 1 | K.EITALAPSTMK.I 6340 |
|  | 6445 | 586.2852 | 1170.5558 | 1170.5638 | -0.0080 | 0 | 48 | 0.0025 | 1 | R.HQGVMVGMGQK.D 6444 6446 6447 6460 |
|  | 6691 | 594.2826 | 1186.5507 | 1186.5587 | -0.0080 | 0 | (40) | 0.017 | 1 | R.HQGVMVGMGQK.D 6679 6693 6696 |
|  | 6692 | 594.2828 | 1186.5511 | 1186.5587 | -0.0076 | 0 | (42) | 0.01 | 1 | R.HQGVMVGMGQK.D 6695 |
|  | 6934 | 599.7607 | 1197.5068 | 1197.5150 | -0.0081 | 0 | 47 | 0.0031 | 1 | K.DSYVGDEAQSK.R |
|  | 6943 | 599.8527 | 1197.6908 | 1197.6982 | -0.0075 | 0 | 52 | 0.00092 | 1 | R.AVFPSIVGRPR.H 6939 6944 6945 6946 |
|  | 7039 | 602.2794 | 1202.5442 | 1202.5536 | -0.0095 | 0 | (21) | 1.3 | 1 | R.HQGVMVGMGQK.D |
|  | 10002 | 677.8109 | 1353.6073 | 1353.6161 | -0.0088 | 1 | 60 | 0.00014 | 1 | K.DSYVGDEAQSKR.G 9990 10003 10004 10005 10006 10024 10031 |
|  | 12455 | **750.3372** | **1498.6599** | **1499.7005** | **-1.0406** | **0** | **12** | **8** | **2** | **K.QEYDEAGPSIVHR.K** 12456 12471 |
|  | 12723 | 505.9181 | 1514.7325 | 1514.7419 | -0.0093 | 0 | 40 | 0.014 | 1 | K.IWHHTFYNELR.V 12724 12725 |
|  | 13248 | 774.9052 | 1547.7957 | 1547.8051 | -0.0094 | 1 | 53 | 0.0006 | 1 | R.MQKEITALAPSTMK.I 13247 13249 13250 |
|  | 13497 | 522.2708 | 1563.7904 | 1563.8000 | -0.0096 | 1 | (22) | 0.73 | 1 | R.MQKEITALAPSTMK.I |
|  | 14437 | 541.9493 | 1622.8262 | 1622.8338 | -0.0076 | 1 | 25 | 0.33 | 1 | R.LDLAGRDLTDYLMK.I |
|  | 14496 | **814.3845** | **1626.7544** | **1627.7954** | **-1.0410** | **1** | **16** | **3.2** | **2** | **K.QEYDEAGPSIVHRK.C** |
|  | 16767 | 895.9441 | 1789.8736 | 1789.8846 | -0.0110 | 0 | 66 | 2.5e-005 | 1 | K.SYELPDGQVITIGNER.F 16766 16769 16770 16771 16772 |
|  | 18819 | **978.5196** | **1955.0246** | **1955.0364** | **-0.0117** | **0** | **41** | **0.0076** | **1** | **R.VAPEEHPTLLTEAPLNPK.A** 18812 18820 |
|  | 21470 | **1114.5292** | **2227.0438** | **2227.0579** | **-0.0141** | **0** | **75** | **2.4e-006** | **1** | **K.DLYANNVLSGGTTMYPGIADR.M** 21471 21472 |
|  | 21602 | **561.5441** | **2242.1472** | **2243.0528** | **-0.9057** | **0** | **(4)** | **30** | **6** | **K.DLYANNVLSGGTTMYPGIADR.M** |
|  | 22410 | 1176.0343 | 2350.0540 | 2350.0682 | -0.0141 | 1 | 96 | 1.8e-008 | 1 | R.HQGVMVGMGQKDSYVGDEAQSK.R 22409 22411 |
|  | 22460 | **1178.5765** | **2355.1385** | **2355.1529** | **-0.0144** | **1** | **76** | **1.8e-006** | **1** | **R.KDLYANNVLSGGTTMYPGIADR.M** 22459 |
|  | 22544 | 789.4450 | 2365.3132 | 2366.2464 | -0.9332 | 1 | 4 | 30 | 1 | R.AVFPSIVGRPRHQGVMVGMGQK.D |
|  | 22547 | 789.6902 | 2366.0487 | 2366.0631 | -0.0144 | 1 | (6) | 20 | 1 | R.HQGVMVGMGQKDSYVGDEAQSK.R |
|  | 23627 | **1268.5759** | **2535.1373** | **2535.1509** | **-0.0136** | **0** | **120** | **6.5e-011** | **1** | **K.LCYVALDFENEMATAASSSSLEK.S** 23626 |
|  | 23753 | **1276.5885** | **2551.1624** | **2551.1458** | **0.0167** | **0** | **(45)** | **0.002** | **1** | **K.LCYVALDFENEMATAASSSSLEK.S** 23746 23754 23767 |
|  | 23957 | **862.7755** | **2585.3047** | **2585.3199** | **-0.0152** | **1** | **25** | **0.2** | **1** | **R.GILTLKYPIEHGIITNWDDMEK.I** |
|  | 24194 | **527.2899** | **2631.4129** | **2630.2469** | **1.1661** | **1** | **0** | **55** | **7** | **K.DLYANNVLSGGTTMYPGIADRMQK.E** |
|  | 24842 | 550.0686 | 2745.3066 | 2745.4200 | -0.1134 | 1 | 5 | 22 | 3 | R.KYSVWIGGSILASLSTFQQMWISK.Q |
|  | 25075 | **931.7656** | **2792.2751** | **2792.2884** | **-0.0133** | **1** | **26** | **0.15** | **1** | **K.EKLCYVALDFENEMATAASSSSLEK.S** |
|  | 25186 | **937.0732** | **2808.1977** | **2808.2833** | **-0.0856** | **1** | **(3)** | **24** | **1** | **K.EKLCYVALDFENEMATAASSSSLEK.S** |
|  | 27039 | **1066.2020** | **3195.5843** | **3195.6023** | **-0.0180** | **0** | **71** | **4.1e-006** | **1** | **R.TTGIVLDSGDGVTHNVPIYEGYALPHAIMR.L** 27040 |
|  | 27082 | **1069.0851** | **3204.2334** | **3203.4613** | **0.7721** | **0** | **3** | **19** | **1** | **R.CPETLFQPSFIGMESAGIHETTYNSIMK.C** |
|  | 27356 | **817.9298** | **3267.6901** | **3266.6178** | **1.0724** | **0** | **1** | **36** | **2** | **K.MTQIMFETFNVPAMYVAIQAVLSLYASGR.T** |
|  | 28174 | **1164.5455** | **3490.6148** | **3490.6360** | **-0.0212** | **1** | **66** | **8.5e-006** | **1** | **R.FRCPETLFQPSFIGMESAGIHETTYNSIMK.C** 28175 |

| **13.** | Q9BVA1\|TBB2B_HUMAN    **Mass:** 50377    **Score:** 1243   **Queries matched:** 85   **emPAI:** 4.97 |
| --- | --- |
|  | Tubulin beta-2B chain OS=Homo sapiens GN=TUBB2B PE=1 SV=1 |

|  | **Query** | **Observed** | **Mr(expt)** | **Mr(calc)** | **Delta** | **Miss** | **Score** | **Expect** | **Rank** | **Peptide** |
| --- | --- | --- | --- | --- | --- | --- | --- | --- | --- | --- |
|  | 1602 | 404.7373 | 807.4600 | 807.3399 | 0.1201 | 0 | 8 | 28 | 4 | R.EEYPDR.I |
|  | 4618 | 514.7597 | 1027.5048 | 1027.5121 | -0.0072 | 0 | 25 | 0.57 | 1 | K.TAVCDIPPR.G 4619 4624 4627 4629 |
|  | 5095 | 533.2719 | 1064.5293 | 1064.4201 | 0.1091 | 0 | (18) | 2.3 | 1 | K.NMMAACDPR.H 5090 5102 |
|  | 5226 | 539.2660 | 1076.5175 | 1076.5250 | -0.0075 | 1 | 25 | 0.53 | 1 | K.IREEYPDR.I 5225 |
|  | 5275 | 541.2110 | 1080.4074 | 1080.4151 | -0.0076 | 0 | 26 | 0.36 | 1 | K.NMMAACDPR.H |
|  | 5276 | 541.2111 | 1080.4076 | 1080.4151 | -0.0075 | 0 | (25) | 0.48 | 1 | K.NMMAACDPR.H |
|  | 6084 | 572.3180 | 1142.6215 | 1142.6270 | -0.0055 | 0 | 17 | 3.3 | 1 | K.LAVNMVPFPR.L |
|  | 8765 | 430.2513 | 1287.7319 | 1286.7169 | 1.0150 | 1 | 4 | 55 | 3 | R.KLAVNMVPFPR.L |
|  | 9543 | 664.3431 | 1326.6716 | 1325.7126 | 0.9591 | 1 | 5 | 48 | 9 | K.TAVCDIPPRGLK.M |
|  | 10232 | **684.3992** | **1366.7839** | **1366.6625** | **0.1214** | **0** | **7** | **25** | **1** | **R.IMNTFSVMPSPK.V** 10241 10242 10243 |
|  | 10507 | **691.9351** | **1381.8557** | **1382.6574** | **-0.8017** | **0** | **(2)** | **87** | **3** | **R.IMNTFSVMPSPK.V** |
|  | 10550 | 693.3494 | 1384.6843 | 1384.6921 | -0.0078 | 1 | 30 | 0.13 | 1 | K.RISEQFTAMFR.R |
|  | 11576 | 723.8442 | 1445.6739 | 1445.6820 | -0.0081 | 0 | 71 | 1.1e-005 | 1 | K.EVDEQMLNVQNK.N 11577 |
|  | 14299 | 808.4167 | 1614.8189 | 1614.8287 | -0.0098 | 0 | 44 | 0.0046 | 1 | R.AILVDLEPGTMDSVR.S 14300 14310 |
|  | 14380 | 540.9477 | 1619.8212 | 1619.8283 | -0.0070 | 0 | 48 | 0.0018 | 1 | R.LHFFMPGFAPLTSR.G 14371 14377 14378 14379 |
|  | 14564 | 816.4130 | 1630.8114 | 1630.8236 | -0.0123 | 0 | (21) | 1.1 | 1 | R.AILVDLEPGTMDSVR.S 14576 |
|  | 15553 | 848.9153 | 1695.8160 | 1695.8257 | -0.0096 | 0 | 19 | 1.4 | 1 | K.NSSYFVEWIPNNVK.T 15562 15563 15564 |
|  | 15939 | **575.6318** | **1723.8737** | **1722.8498** | **1.0239** | **0** | **4** | **46** | **7** | **R.ALTVPELTQQMFDSK.N** |
|  | 17173 | 911.9601 | 1821.9057 | 1821.9156 | -0.0098 | 0 | 61 | 8.7e-005 | 1 | R.EIVHIQAGQCGNQIGAK.F 17174 |
|  | 17663 | 929.4699 | 1856.9253 | 1856.9342 | -0.0090 | 0 | 55 | 0.0003 | 1 | K.MSATFIGNSTAIQELFK.R |
|  | 18438 | 962.4478 | 1922.8809 | 1922.8900 | -0.0090 | 1 | 91 | 8.1e-008 | 1 | R.MSMKEVDEQMLNVQNK.N |
|  | 18859 | 979.9888 | 1957.9631 | 1957.9745 | -0.0114 | 0 | 86 | 2.2e-007 | 1 | K.GHYTEGAELVDSVLDVVR.K 18858 |
|  | 19427 | 1007.5205 | 2013.0265 | 2013.0353 | -0.0089 | 1 | 43 | 0.005 | 2 | K.MSATFIGNSTAIQELFKR.I 19426 19429 |
|  | 20166 | 1044.0366 | 2086.0587 | 2086.0695 | -0.0108 | 1 | 114 | 3.4e-010 | 1 | K.GHYTEGAELVDSVLDVVRK.E 20162 20163 20167 20168 20169 |
|  | 20402 | 1055.5299 | 2109.0453 | 2109.0571 | -0.0119 | 1 | 94 | 3.6e-008 | 1 | -.MREIVHIQAGQCGNQIGAK.F 20399 20400 20401 20403 20410 |
|  | 20534 | 532.2908 | 2125.1342 | 2125.0521 | 0.0822 | 1 | (5) | 26 | 4 | -.MREIVHIQAGQCGNQIGAK.F |
|  | 24649 | 1354.6668 | 2707.3189 | 2707.3310 | -0.0120 | 0 | 100 | 5.8e-009 | 1 | K.LTTPTYGDLNHLVSATMSGVTTCLR.F 24651 |
|  | 25038 | 929.4656 | 2785.3749 | 2785.2543 | 0.1206 | 1 | 2 | 36 | 1 | R.ALTVPELTQQMFDSKNMMAACDPR.H 25040 |
|  | 25134 | 1399.6674 | 2797.3202 | 2797.3361 | -0.0160 | 0 | 80 | 5.8e-007 | 1 | R.SGPFGQIFRPDNFVFGQSGAGNNWAK.G 25133 25135 25137 25138 25139 25144 25145 25147 25148 |
|  | 26633 | 1034.8005 | 3101.3798 | 3101.4003 | -0.0205 | 0 | 45 | 0.0015 | 1 | K.FWEVISDEHGIDPTGSYHGDSDLQLER.I 26634 26635 |
|  | 27558 | 1109.8416 | 3326.5028 | 3326.5218 | -0.0189 | 0 | 65 | 1.5e-005 | 1 | K.ESESCDCLQGFQLTHSLGGGTGSGMGTLLISK.I 27557 |
|  | 28067 | **864.6573** | **3454.6003** | **3454.6167** | **-0.0164** | **1** | **9** | **5.1** | **1** | **R.KESESCDCLQGFQLTHSLGGGTGSGMGTLLISK.I** |
|  | 30696 | 1531.7006 | 4592.0799 | 4592.0999 | -0.0201 | 0 | 53 | 8.6e-005 | 1 | K.VSDTVVEPYNATLSVHQLVENTDETYCIDNEALYDICFR.T 30694 30698 30701 |

|  | **Proteins matching a subset of these peptides:** |
| --- | --- |

|  | Q13885\|TBB2A_HUMAN    **Mass:** 50274    **Score:** 1153   **Queries matched:** 81 |
| --- | --- |
|  | Tubulin beta-2A chain OS=Homo sapiens GN=TUBB2A PE=1 SV=1 |

| **14.** | P62736\|ACTA_HUMAN    **Mass:** 42381    **Score:** 1219   **Queries matched:** 80   **emPAI:** 4.92 |
| --- | --- |
|  | Actin, aortic smooth muscle OS=Homo sapiens GN=ACTA2 PE=1 SV=1 |

|  | **Query** | **Observed** | **Mr(expt)** | **Mr(calc)** | **Delta** | **Miss** | **Score** | **Expect** | **Rank** | **Peptide** |
| --- | --- | --- | --- | --- | --- | --- | --- | --- | --- | --- |
|  | 1475 | 800.5302 | 799.5229 | 799.5280 | -0.0051 | 1 | 14 | 8.2 | 3 | K.RGILTLK.Y |
|  | 4224 | 499.7444 | 997.4741 | 997.4790 | -0.0049 | 0 | 10 | 15 | 4 | R.DLTDYLMK.I |
|  | 4734 | 518.8262 | 1035.6379 | 1035.6440 | -0.0061 | 1 | 21 | 1.4 | 1 | K.IKIIAPPER.K |
|  | 5932 | 565.7742 | 1129.5339 | 1129.5404 | -0.0065 | 0 | 4 | 72 | 2 | R.GYSFVTTAER.E |
|  | 6341 | 581.3092 | 1160.6038 | 1160.6111 | -0.0072 | 0 | 36 | 0.042 | 1 | K.EITALAPSTMK.I 6340 |
|  | 6445 | 586.2852 | 1170.5558 | 1170.5638 | -0.0080 | 0 | 48 | 0.0025 | 1 | R.HQGVMVGMGQK.D 6444 6446 6447 6460 |
|  | 6691 | 594.2826 | 1186.5507 | 1186.5587 | -0.0080 | 0 | (40) | 0.017 | 1 | R.HQGVMVGMGQK.D 6679 6693 6696 |
|  | 6692 | 594.2828 | 1186.5511 | 1186.5587 | -0.0076 | 0 | (42) | 0.01 | 1 | R.HQGVMVGMGQK.D 6695 |
|  | 6934 | 599.7607 | 1197.5068 | 1197.5150 | -0.0081 | 0 | 47 | 0.0031 | 1 | K.DSYVGDEAQSK.R |
|  | 6943 | 599.8527 | 1197.6908 | 1197.6982 | -0.0075 | 0 | 52 | 0.00092 | 1 | R.AVFPSIVGRPR.H 6939 6944 6945 6946 |
|  | 7039 | 602.2794 | 1202.5442 | 1202.5536 | -0.0095 | 0 | (21) | 1.3 | 1 | R.HQGVMVGMGQK.D |
|  | 10002 | 677.8109 | 1353.6073 | 1353.6161 | -0.0088 | 1 | 60 | 0.00014 | 1 | K.DSYVGDEAQSKR.G 9990 10003 10004 10005 10006 10024 10031 |
|  | 12455 | 750.3372 | 1498.6599 | 1499.7005 | -1.0406 | 0 | 12 | 8 | 2 | K.QEYDEAGPSIVHR.K 12456 12471 |
|  | 12488 | **501.2467** | **1500.7182** | **1500.7262** | **-0.0080** | **0** | **26** | **0.3** | **1** | **K.IWHHSFYNELR.V** |
|  | 13248 | 774.9052 | 1547.7957 | 1547.8051 | -0.0094 | 1 | 53 | 0.0006 | 1 | R.MQKEITALAPSTMK.I 13247 13249 13250 |
|  | 13497 | 522.2708 | 1563.7904 | 1563.8000 | -0.0096 | 1 | (22) | 0.73 | 1 | R.MQKEITALAPSTMK.I |
|  | 14437 | 541.9493 | 1622.8262 | 1622.8338 | -0.0076 | 1 | 25 | 0.33 | 1 | R.LDLAGRDLTDYLMK.I |
|  | 14496 | 814.3845 | 1626.7544 | 1627.7954 | -1.0410 | 1 | 16 | 3.2 | 2 | K.QEYDEAGPSIVHRK.C |
|  | 16767 | 895.9441 | 1789.8736 | 1789.8846 | -0.0110 | 0 | 66 | 2.5e-005 | 1 | K.SYELPDGQVITIGNER.F 16766 16769 16770 16771 16772 |
|  | 18819 | 978.5196 | 1955.0246 | 1955.0364 | -0.0117 | 0 | 41 | 0.0076 | 1 | R.VAPEEHPTLLTEAPLNPK.A 18812 18820 |
|  | 21470 | 1114.5292 | 2227.0438 | 2227.0579 | -0.0141 | 0 | 75 | 2.4e-006 | 1 | K.DLYANNVLSGGTTMYPGIADR.M 21471 21472 |
|  | 21602 | 561.5441 | 2242.1472 | 2243.0528 | -0.9057 | 0 | (4) | 30 | 6 | K.DLYANNVLSGGTTMYPGIADR.M |
|  | 22410 | 1176.0343 | 2350.0540 | 2350.0682 | -0.0141 | 1 | 96 | 1.8e-008 | 1 | R.HQGVMVGMGQKDSYVGDEAQSK.R 22409 22411 |
|  | 22460 | 1178.5765 | 2355.1385 | 2355.1529 | -0.0144 | 1 | 76 | 1.8e-006 | 1 | R.KDLYANNVLSGGTTMYPGIADR.M 22459 |
|  | 22544 | 789.4450 | 2365.3132 | 2366.2464 | -0.9332 | 1 | 4 | 30 | 1 | R.AVFPSIVGRPRHQGVMVGMGQK.D |
|  | 22547 | 789.6902 | 2366.0487 | 2366.0631 | -0.0144 | 1 | (6) | 20 | 1 | R.HQGVMVGMGQKDSYVGDEAQSK.R |
|  | 23627 | 1268.5759 | 2535.1373 | 2535.1509 | -0.0136 | 0 | 120 | 6.5e-011 | 1 | K.LCYVALDFENEMATAASSSSLEK.S 23626 |
|  | 23753 | 1276.5885 | 2551.1624 | 2551.1458 | 0.0167 | 0 | (45) | 0.002 | 1 | K.LCYVALDFENEMATAASSSSLEK.S 23746 23754 23767 |
|  | 23957 | 862.7755 | 2585.3047 | 2585.3199 | -0.0152 | 1 | 25 | 0.2 | 1 | R.GILTLKYPIEHGIITNWDDMEK.I |
|  | 24194 | 527.2899 | 2631.4129 | 2630.2469 | 1.1661 | 1 | 0 | 55 | 7 | K.DLYANNVLSGGTTMYPGIADRMQK.E |
|  | 24842 | 550.0686 | 2745.3066 | 2745.4200 | -0.1134 | 1 | 5 | 22 | 3 | R.KYSVWIGGSILASLSTFQQMWISK.Q |
|  | 25075 | 931.7656 | 2792.2751 | 2792.2884 | -0.0133 | 1 | 26 | 0.15 | 1 | K.EKLCYVALDFENEMATAASSSSLEK.S |
|  | 25186 | 937.0732 | 2808.1977 | 2808.2833 | -0.0856 | 1 | (3) | 24 | 1 | K.EKLCYVALDFENEMATAASSSSLEK.S |
|  | 27039 | 1066.2020 | 3195.5843 | 3195.6023 | -0.0180 | 0 | 71 | 4.1e-006 | 1 | R.TTGIVLDSGDGVTHNVPIYEGYALPHAIMR.L 27040 |
|  | 27082 | 1069.0851 | 3204.2334 | 3203.4613 | 0.7721 | 0 | 3 | 19 | 1 | R.CPETLFQPSFIGMESAGIHETTYNSIMK.C |
|  | 27356 | 817.9298 | 3267.6901 | 3266.6178 | 1.0724 | 0 | 1 | 36 | 2 | K.MTQIMFETFNVPAMYVAIQAVLSLYASGR.T |
|  | 28174 | 1164.5455 | 3490.6148 | 3490.6360 | -0.0212 | 1 | 66 | 8.5e-006 | 1 | R.FRCPETLFQPSFIGMESAGIHETTYNSIMK.C 28175 |

| **15.** | P63267\|ACTH_HUMAN    **Mass:** 42249    **Score:** 1219   **Queries matched:** 77   **emPAI:** 4.95 |
| --- | --- |
|  | Actin, gamma-enteric smooth muscle OS=Homo sapiens GN=ACTG2 PE=1 SV=1 |

|  | **Query** | **Observed** | **Mr(expt)** | **Mr(calc)** | **Delta** | **Miss** | **Score** | **Expect** | **Rank** | **Peptide** |
| --- | --- | --- | --- | --- | --- | --- | --- | --- | --- | --- |
|  | 1475 | 800.5302 | 799.5229 | 799.5280 | -0.0051 | 1 | 14 | 8.2 | 3 | K.RGILTLK.Y |
|  | 4224 | 499.7444 | 997.4741 | 997.4790 | -0.0049 | 0 | 10 | 15 | 4 | R.DLTDYLMK.I |
|  | 4734 | 518.8262 | 1035.6379 | 1035.6440 | -0.0061 | 1 | 21 | 1.4 | 1 | K.IKIIAPPER.K |
|  | 5932 | 565.7742 | 1129.5339 | 1129.5404 | -0.0065 | 0 | 4 | 72 | 2 | R.GYSFVTTAER.E |
|  | 6341 | 581.3092 | 1160.6038 | 1160.6111 | -0.0072 | 0 | 36 | 0.042 | 1 | K.EITALAPSTMK.I 6340 |
|  | 6445 | 586.2852 | 1170.5558 | 1170.5638 | -0.0080 | 0 | 48 | 0.0025 | 1 | R.HQGVMVGMGQK.D 6444 6446 6447 6460 |
|  | 6691 | 594.2826 | 1186.5507 | 1186.5587 | -0.0080 | 0 | (40) | 0.017 | 1 | R.HQGVMVGMGQK.D 6679 6693 6696 |
|  | 6692 | 594.2828 | 1186.5511 | 1186.5587 | -0.0076 | 0 | (42) | 0.01 | 1 | R.HQGVMVGMGQK.D 6695 |
|  | 6934 | 599.7607 | 1197.5068 | 1197.5150 | -0.0081 | 0 | 47 | 0.0031 | 1 | K.DSYVGDEAQSK.R |
|  | 6943 | 599.8527 | 1197.6908 | 1197.6982 | -0.0075 | 0 | 52 | 0.00092 | 1 | R.AVFPSIVGRPR.H 6939 6944 6945 6946 |
|  | 7039 | 602.2794 | 1202.5442 | 1202.5536 | -0.0095 | 0 | (21) | 1.3 | 1 | R.HQGVMVGMGQK.D |
|  | 10002 | 677.8109 | 1353.6073 | 1353.6161 | -0.0088 | 1 | 60 | 0.00014 | 1 | K.DSYVGDEAQSKR.G 9990 10003 10004 10005 10006 10024 10031 |
|  | 12488 | 501.2467 | 1500.7182 | 1500.7262 | -0.0080 | 0 | 26 | 0.3 | 1 | K.IWHHSFYNELR.V |
|  | 13248 | 774.9052 | 1547.7957 | 1547.8051 | -0.0094 | 1 | 53 | 0.0006 | 1 | R.MQKEITALAPSTMK.I 13247 13249 13250 |
|  | 13497 | 522.2708 | 1563.7904 | 1563.8000 | -0.0096 | 1 | (22) | 0.73 | 1 | R.MQKEITALAPSTMK.I |
|  | 14437 | 541.9493 | 1622.8262 | 1622.8338 | -0.0076 | 1 | 25 | 0.33 | 1 | R.LDLAGRDLTDYLMK.I |
|  | 16767 | 895.9441 | 1789.8736 | 1789.8846 | -0.0110 | 0 | 66 | 2.5e-005 | 1 | K.SYELPDGQVITIGNER.F 16766 16769 16770 16771 16772 |
|  | 18819 | 978.5196 | 1955.0246 | 1955.0364 | -0.0117 | 0 | 41 | 0.0076 | 1 | R.VAPEEHPTLLTEAPLNPK.A 18812 18820 |
|  | 19753 | **681.8496** | **2042.5268** | **2041.8391** | **0.6877** | **0** | **6** | **17** | **1** | **M.CEEETTALVCDNGSGLCK.A** 19751 |
|  | 21470 | 1114.5292 | 2227.0438 | 2227.0579 | -0.0141 | 0 | 75 | 2.4e-006 | 1 | K.DLYANNVLSGGTTMYPGIADR.M 21471 21472 |
|  | 21602 | 561.5441 | 2242.1472 | 2243.0528 | -0.9057 | 0 | (4) | 30 | 6 | K.DLYANNVLSGGTTMYPGIADR.M |
|  | 22410 | 1176.0343 | 2350.0540 | 2350.0682 | -0.0141 | 1 | 96 | 1.8e-008 | 1 | R.HQGVMVGMGQKDSYVGDEAQSK.R 22409 22411 |
|  | 22460 | 1178.5765 | 2355.1385 | 2355.1529 | -0.0144 | 1 | 76 | 1.8e-006 | 1 | R.KDLYANNVLSGGTTMYPGIADR.M 22459 |
|  | 22544 | 789.4450 | 2365.3132 | 2366.2464 | -0.9332 | 1 | 4 | 30 | 1 | R.AVFPSIVGRPRHQGVMVGMGQK.D |
|  | 22547 | 789.6902 | 2366.0487 | 2366.0631 | -0.0144 | 1 | (6) | 20 | 1 | R.HQGVMVGMGQKDSYVGDEAQSK.R |
|  | 23627 | 1268.5759 | 2535.1373 | 2535.1509 | -0.0136 | 0 | 120 | 6.5e-011 | 1 | K.LCYVALDFENEMATAASSSSLEK.S 23626 |
|  | 23753 | 1276.5885 | 2551.1624 | 2551.1458 | 0.0167 | 0 | (45) | 0.002 | 1 | K.LCYVALDFENEMATAASSSSLEK.S 23746 23754 23767 |
|  | 23957 | 862.7755 | 2585.3047 | 2585.3199 | -0.0152 | 1 | 25 | 0.2 | 1 | R.GILTLKYPIEHGIITNWDDMEK.I |
|  | 24194 | 527.2899 | 2631.4129 | 2630.2469 | 1.1661 | 1 | 0 | 55 | 7 | K.DLYANNVLSGGTTMYPGIADRMQK.E |
|  | 25075 | 931.7656 | 2792.2751 | 2792.2884 | -0.0133 | 1 | 26 | 0.15 | 1 | K.EKLCYVALDFENEMATAASSSSLEK.S |
|  | 25186 | 937.0732 | 2808.1977 | 2808.2833 | -0.0856 | 1 | (3) | 24 | 1 | K.EKLCYVALDFENEMATAASSSSLEK.S |
|  | 27039 | 1066.2020 | 3195.5843 | 3195.6023 | -0.0180 | 0 | 71 | 4.1e-006 | 1 | R.TTGIVLDSGDGVTHNVPIYEGYALPHAIMR.L 27040 |
|  | 27082 | 1069.0851 | 3204.2334 | 3203.4613 | 0.7721 | 0 | 3 | 19 | 1 | R.CPETLFQPSFIGMESAGIHETTYNSIMK.C |
|  | 27356 | 817.9298 | 3267.6901 | 3266.6178 | 1.0724 | 0 | 1 | 36 | 2 | K.MTQIMFETFNVPAMYVAIQAVLSLYASGR.T |
|  | 28174 | 1164.5455 | 3490.6148 | 3490.6360 | -0.0212 | 1 | 66 | 8.5e-006 | 1 | R.FRCPETLFQPSFIGMESAGIHETTYNSIMK.C 28175 |

| **16.** | P35579\|MYH9_HUMAN    **Mass:** 227646   **Score:** 1208   **Queries matched:** 112   **emPAI:** 0.45 |
| --- | --- |
|  | Myosin-9 OS=Homo sapiens GN=MYH9 PE=1 SV=4 |

|  | **Query** | **Observed** | **Mr(expt)** | **Mr(calc)** | **Delta** | **Miss** | **Score** | **Expect** | **Rank** | **Peptide** |
| --- | --- | --- | --- | --- | --- | --- | --- | --- | --- | --- |
|  | 716 | **519.1362** | **518.1289** | **517.2608** | **0.8681** | **0** | **13** | **15** | **1** | **R.ANASR.R** |
|  | 751 | **533.0582** | **532.0509** | **531.3017** | **0.7492** | **0** | **9** | **30** | **1** | **R.IGQSK.V** 752 753 755 756 |
|  | 871 | **572.9955** | **571.9882** | **571.3806** | **0.6076** | **1** | **9** | **24** | **2** | **R.IKVGR.D** |
|  | 927 | **591.3232** | **590.3159** | **589.3257** | **0.9902** | **0** | **13** | **11** | **4** | **K.MQLAK.K** |
|  | 1609 | **405.1959** | **808.3773** | **807.4491** | **0.9282** | **0** | **19** | **2.1** | **1** | **R.TVGQLYK.E** |
|  | 2123 | **424.0561** | **846.0977** | **845.4793** | **0.6184** | **1** | **6** | **37** | **8** | **R.KLQAQMK.D** |
|  | 2369 | **432.2584** | **862.5022** | **861.4014** | **1.1007** | **0** | **9** | **25** | **1** | **-.MAQQAADK.Y** 2348 |
|  | 2777 | **446.7454** | **891.4762** | **891.4735** | **0.0026** | **1** | **8** | **33** | **5** | **K.KLEMDLK.D** |
|  | 2840 | **449.2418** | **896.4690** | **897.5508** | **-1.0819** | **1** | **2** | **1e+002** | **10** | **K.QLVRQVR.E** 2837 |
|  | 2996 | **454.2270** | **906.4394** | **907.4803** | **-1.0409** | **0** | **7** | **34** | **2** | **K.FVSELWK.D** |
|  | 3677 | **480.2771** | **958.5397** | **958.5083** | **0.0314** | **1** | **12** | **13** | **10** | **K.VNKDDIQK.M** |
|  | 3865 | **487.2953** | **972.5761** | **971.5287** | **1.0474** | **0** | **10** | **17** | **2** | **R.EQEVNILK.K** |
|  | 3873 | **487.3027** | **972.5909** | **971.5400** | **1.0509** | **1** | **10** | **20** | **10** | **R.DEAIKQLR.K** |
|  | 4062 | **494.2553** | **986.4961** | **985.5556** | **0.9405** | **1** | **7** | **33** | **10** | **K.GALALEEKR.R** 4067 |
|  | 4338 | **503.5118** | **1005.0089** | **1004.5185** | **0.4904** | **1** | **1** | **90** | **5** | **K.QRSMAVAAR.K** |
|  | 4392 | **505.7688** | **1009.5230** | **1010.4048** | **-0.8818** | **0** | **4** | **59** | **7** | **R.QEEEMMAK.E** 4405 |
|  | 4705 | **517.7596** | **1033.5047** | **1033.5114** | **-0.0067** | **0** | **19** | **2.1** | **2** | **K.LQEMEGTVK.S** |
|  | 4774 | **520.8052** | **1039.5958** | **1038.6550** | **0.9409** | **0** | **5** | **50** | **3** | **K.VKPLLQVSR.Q** |
|  | 4791 | **521.3040** | **1040.5935** | **1040.6495** | **-0.0560** | **1** | **6** | **35** | **9** | **R.WLVLRINK.A** |
|  | 4840 | **523.2856** | **1044.5567** | **1044.5637** | **-0.0070** | **0** | **4** | **63** | **1** | **R.LEVNLQAMK.A** |
|  | 5169 | **536.3107** | **1070.6068** | **1070.6124** | **-0.0056** | **1** | **2** | **1.1e+002** | **5** | **K.KLVWVPSDK.S** |
|  | 5595 | **552.2996** | **1102.5846** | **1102.5917** | **-0.0071** | **1** | **22** | **1.1** | **1** | **K.RQQQLTAMK.V** |
|  | 5775 | **559.0590** | **1116.1035** | **1115.5710** | **0.5325** | **0** | **6** | **27** | **4** | **R.DLGEELEALK.T** |
|  | 5808 | **559.8027** | **1117.5908** | **1118.5866** | **-0.9958** | **1** | **(3)** | **82** | **9** | **K.RQQQLTAMK.V** 5822 |
|  | 6252 | **577.7812** | **1153.5478** | **1153.5550** | **-0.0072** | **0** | **31** | **0.1** | **1** | **K.VMQEQGTHPK.F** 6251 |
|  | 6849 | **597.8362** | **1193.6579** | **1193.6655** | **-0.0076** | **1** | **18** | **2.6** | **1** | **K.YKASITALEAK.I** |
|  | 7393 | **610.8260** | **1219.6374** | **1219.6448** | **-0.0074** | **1** | **30** | **0.14** | **1** | **K.KFDQLLAEEK.T** |
|  | 7400 | **407.7223** | **1220.1451** | **1219.5754** | **0.5697** | **0** | **12** | **6.4** | **1** | **R.ALEQQVEEMK.T** |
|  | 7702 | **618.3408** | **1234.6671** | **1234.4950** | **0.1721** | **0** | **11** | **12** | **6** | **K.GAGDGSDEEVDGK.A** |
|  | 8206 | **630.3782** | **1258.7419** | **1258.7132** | **0.0287** | **1** | **6** | **35** | **6** | **K.LQLEKVTTEAK.L** |
|  | 8263 | **631.3405** | **1260.6665** | **1260.6748** | **-0.0083** | **1** | **35** | **0.049** | **1** | **K.VKLQEMEGTVK.S** 8264 |
|  | 8342 | **422.7274** | **1265.1603** | **1265.5081** | **-0.3478** | **0** | **9** | **12** | **1** | **R.EMEAELEDER.K** |
|  | 8517 | **637.8337** | **1273.6528** | **1273.6918** | **-0.0390** | **0** | **7** | **26** | **6** | **R.YEILTPNSIPK.G** 8511 |
|  | 8545 | **426.2688** | **1275.7847** | **1276.6697** | **-0.8850** | **1** | **(6)** | **36** | **8** | **K.VKLQEMEGTVK.S** |
|  | 8611 | **640.7838** | **1279.5529** | **1279.5835** | **-0.0306** | **1** | **(5)** | **39** | **9** | **K.LQAQMKDCMR.E** |
|  | 8788 | **645.3424** | **1288.6701** | **1288.7173** | **-0.0471** | **1** | **9** | **16** | **6** | **K.EQLAKLMATLR.N** |
|  | 8917 | **432.8774** | **1295.6104** | **1295.5784** | **0.0320** | **1** | **7** | **24** | **4** | **K.LQAQMKDCMR.E** |
|  | 9234 | **657.1148** | **1312.2151** | **1311.5733** | **0.6417** | **1** | **(4)** | **37** | **2** | **K.LQAQMKDCMR.E** |
|  | 9369 | **440.2517** | **1317.7333** | **1317.7405** | **-0.0072** | **0** | **11** | **11** | **1** | **K.LDPHLVLDQLR.C** |
|  | 9811 | **448.7283** | **1343.1630** | **1342.6187** | **0.5443** | **0** | **6** | **22** | **5** | **K.HEAMITDLEER.L** |
|  | 10769 | **466.7442** | **1397.2107** | **1396.6697** | **0.5410** | **1** | **15** | **3** | **1** | **K.VDYKADEWLMK.N** |
|  | 11007 | **706.9049** | **1411.7951** | **1411.8035** | **-0.0083** | **1** | **69** | **1.7e-005** | **1** | **K.KVEAQLQELQVK.F** 11008 |
|  | 11817 | **730.8790** | **1459.7435** | **1459.7340** | **0.0095** | **1** | **2** | **80** | **5** | **R.VEEEAAQKNMALK.K** |
|  | 12111 | **493.6023** | **1477.7850** | **1477.7929** | **-0.0079** | **0** | **15** | **3.8** | **1** | **K.VIQYLAYVASSHK.S** |
|  | 12939 | **765.3975** | **1528.7804** | **1529.7573** | **-0.9769** | **0** | **4** | **48** | **5** | **K.IAQLEEQLDNETK.E** |
|  | 13508 | **783.3367** | **1564.6588** | **1564.6675** | **-0.0087** | **0** | **13** | **5.1** | **1** | **R.ELEDATETADAMNR.E** |
|  | 13650 | **525.6359** | **1573.8858** | **1573.8940** | **-0.0083** | **1** | **10** | **11** | **1** | **K.AGKLDPHLVLDQLR.C** |
|  | 13720 | **526.9389** | **1577.7949** | **1578.8365** | **-1.0417** | **1** | **0** | **1.1e+002** | **7** | **R.AGVLAHLEEERDLK.I** |
|  | 13918 | **795.8720** | **1589.7295** | **1590.6944** | **-0.9649** | **0** | **81** | **1e-006** | **1** | **R.NTDQASMPDNTAAQK.V** 13934 |
|  | 14286 | **539.0003** | **1613.9791** | **1613.9869** | **-0.0078** | **1** | **11** | **8.7** | **1** | **R.VISGVLQLGNIVFKK.E** |
|  | 14297 | **808.4091** | **1614.8037** | **1614.8109** | **-0.0072** | **0** | **26** | **0.33** | **1** | **R.IMGIPEEEQMGLLR.V** |
|  | 14575 | **544.9342** | **1631.7808** | **1630.8058** | **0.9749** | **0** | **(5)** | **38** | **2** | **R.IMGIPEEEQMGLLR.V** |
|  | 14790 | **412.4516** | **1645.7774** | **1645.8722** | **-0.0948** | **1** | **7** | **22** | **2** | **K.LMATLRNTNPNFVR.C** |
|  | 14791 | **823.9021** | **1645.7896** | **1645.7981** | **-0.0084** | **1** | **39** | **0.014** | **1** | **R.ALEEAMEQKAELER.L** |
|  | 14835 | **550.2949** | **1647.8628** | **1646.8008** | **1.0620** | **0** | **(12)** | **8.4** | **1** | **R.IMGIPEEEQMGLLR.V** |
|  | 15007 | 830.4467 | 1658.8788 | 1658.8661 | 0.0127 | 1 | 6 | 27 | 2 | K.ALSLARALEEAMEQK.A 15008 15009 |
|  | 15045 | **831.8992** | **1661.7839** | **1661.8671** | **-0.0832** | **1** | **(1)** | **98** | **10** | **K.LMATLRNTNPNFVR.C** |
|  | 15185 | **836.9212** | **1671.8278** | **1671.8369** | **-0.0090** | **0** | **55** | **0.00038** | **1** | **K.NFINNPLAQADWAAK.K** |
|  | 15593 | **567.2902** | **1698.8488** | **1699.7281** | **-0.8792** | **1** | **3** | **57** | **2** | **R.TEMEDLMSSKDDVGK.S** |
|  | 15965 | **863.9725** | **1725.9305** | **1725.9413** | **-0.0108** | **0** | **86** | **2.8e-007** | **1** | **R.QLLQANPILEAFGNAK.T** 15966 |
|  | 16279 | **876.4261** | **1750.8376** | **1750.8481** | **-0.0105** | **0** | **46** | **0.0025** | **1** | **R.LTEMETLQSQLMAEK.L** 16294 |
|  | 17271 | **610.9539** | **1829.8399** | **1829.8503** | **-0.0104** | **1** | **51** | **0.00084** | **1** | **R.QLEEAEEEAQRANASR.R** |
|  | 17593 | **618.2819** | **1851.8239** | **1851.8342** | **-0.0103** | **1** | **9** | **14** | **1** | **K.MEDSVGCLETAEEVKR.K** |
|  | 17783 | **934.9547** | **1867.8947** | **1867.9051** | **-0.0103** | **1** | **92** | **5.7e-008** | **1** | **R.IAEFTTNLTEEEEKSK.S** |
|  | 17798 | **935.4818** | **1868.9491** | **1868.9592** | **-0.0101** | **0** | **84** | **3.5e-007** | **1** | **K.ANLQIDQINTDLNLER.S** |
|  | 17914 | **470.2449** | **1876.9504** | **1875.8381** | **1.1123** | **1** | **2** | **65** | **8** | **K.ERNTDQASMPDNTAAQK.V** |
|  | 18471 | **963.4701** | **1924.9256** | **1924.9604** | **-0.0348** | **1** | **2** | **60** | **3** | **R.YEILTPNSIPKGFMDGK.Q** |
|  | 18706 | **973.5020** | **1944.9895** | **1945.0004** | **-0.0109** | **0** | **131** | **7.7e-012** | **1** | **K.LQVELDNVTGLLSQSDSK.S** 18705 |
|  | 19238 | **665.6724** | **1993.9953** | **1995.0021** | **-1.0068** | **1** | **33** | **0.046** | **1** | **K.HSQAVEELAEQLEQTKR.V** |
|  | 19431 | **672.3058** | **2013.8957** | **2013.0014** | **0.8943** | **1** | **3** | **42** | **2** | **K.EEELQAALARVEEEAAQK.N** |
|  | 19469 | **1009.5310** | **2017.0475** | **2017.0554** | **-0.0079** | **0** | **90** | **9.4e-008** | **1** | **R.IIGLDQVAGMSETALPGAFK.T** |
|  | 19741 | **1021.9885** | **2041.9624** | **2041.9738** | **-0.0114** | **1** | **85** | **3.2e-007** | **1** | **K.TLEEEAKTHEAQIQEMR.Q** 19727 19739 19740 |
|  | 19799 | **1024.9786** | **2047.9427** | **2047.9554** | **-0.0126** | **0** | **73** | **4.5e-006** | **1** | **K.SMEAEMIQLQEELAAAER.A** |
|  | 20779 | **718.3787** | **2152.1143** | **2152.1020** | **0.0123** | **1** | **6** | **23** | **2** | **K.QACVLMIKALELDSNLYR.I** |
|  | 21963 | **764.0477** | **2289.1214** | **2289.1344** | **-0.0130** | **1** | **11** | **6.4** | **1** | **K.LKSMEAEMIQLQEELAAAER.A** |
|  | 22274 | **1167.0242** | **2332.0338** | **2332.0488** | **-0.0150** | **0** | **85** | **2.5e-007** | **1** | **K.MQQNIQELEEQLEEEESAR.Q** |
|  | 22677 | **1193.6090** | **2385.2035** | **2384.2263** | **0.9771** | **0** | **4** | **28** | **2** | **R.INFDVNGYIVGANIETYLLEK.S** |
|  | 23161 | **821.0514** | **2460.1323** | **2460.1438** | **-0.0114** | **1** | **17** | **1.4** | **1** | **K.KMQQNIQELEEQLEEEESAR.Q** |
|  | 23237 | **1236.5819** | **2471.1493** | **2471.1663** | **-0.0170** | **0** | **86** | **1.6e-007** | **1** | **R.IAQLEEELEEEQGNTELINDR.L** 23229 23236 23238 |
|  | 23376 | **1247.0840** | **2492.1534** | **2492.1667** | **-0.0132** | **0** | **33** | **0.038** | **1** | **K.DFSALESQLQDTQELLQEENR.Q** |
|  | 23530 | **630.8016** | **2519.1775** | **2518.2737** | **0.9037** | **1** | **1** | **57** | **9** | **K.DVDRIIGLDQVAGMSETALPGAFK.T** |
|  | 23981 | **863.7368** | **2588.1885** | **2588.2023** | **-0.0139** | **1** | **19** | **0.78** | **1** | **K.MQQNIQELEEQLEEEESARQK.L** |
|  | 24671 | **905.1182** | **2712.3329** | **2712.3453** | **-0.0124** | **1** | **1** | **52** | **2** | **R.IAQLEEELEEEQGNTELINDRLK.K** |
|  | 26131 | **1004.4562** | **3010.3469** | **3009.3783** | **0.9686** | **1** | **25** | **0.15** | **1** | **R.FLSNGHVTIPGQQDKDMFQETMEAMR.I** 26127 |
|  | 26161 | **1006.4943** | **3016.4610** | **3016.4724** | **-0.0114** | **1** | **21** | **0.47** | **1** | **R.DLGEELEALKTELEDTLDSTAAQQELR.S** |
|  | 26811 | **786.8869** | **3143.5185** | **3143.5306** | **-0.0121** | **1** | **4** | **22** | **1** | **R.NTDQASMPDNTAAQKVSHLLGINVTDFTR.G** |

| **17.** | P13637\|AT1A3_HUMAN    **Mass:** 113102   **Score:** 1117   **Queries matched:** 54   **emPAI:** 0.57 |
| --- | --- |
|  | Sodium/potassium-transporting ATPase subunit alpha-3 OS=Homo sapiens GN=ATP1A3 PE=1 SV=3 |

|  | **Query** | **Observed** | **Mr(expt)** | **Mr(calc)** | **Delta** | **Miss** | **Score** | **Expect** | **Rank** | **Peptide** |
| --- | --- | --- | --- | --- | --- | --- | --- | --- | --- | --- |
|  | 3682 | 480.7050 | 959.3954 | 959.4382 | -0.0428 | 0 | 14 | 7.7 | 4 | K.EVAMTEHK.M |
|  | 3885 | 487.7630 | 973.5114 | 973.5192 | -0.0079 | 1 | 12 | 12 | 2 | R.TDKLVNER.L |
|  | 5359 | 544.2969 | 1086.5792 | 1086.5856 | -0.0063 | 0 | 37 | 0.032 | 1 | K.LIIVEGCQR.Q |
|  | 7718 | 618.8525 | 1235.6904 | 1235.6986 | -0.0082 | 0 | 55 | 0.00049 | 1 | R.LNIPVSQVNPR.D 7706 7717 7726 |
|  | 8394 | 634.8575 | 1267.7004 | 1267.7071 | -0.0067 | 0 | 55 | 0.00044 | 1 | K.NMVPQQALVIR.E 8371 8393 |
|  | 8563 | **639.4437** | **1276.8729** | **1275.7009** | **1.1720** | **1** | **0** | **1.1e+002** | **6** | **R.YLLVMKGAPER.I** |
|  | 8693 | 642.8540 | 1283.6934 | 1283.7020 | -0.0085 | 0 | (16) | 3.4 | 1 | K.NMVPQQALVIR.E |
|  | 10497 | 691.3746 | 1380.7347 | 1380.7435 | -0.0088 | 0 | 72 | 9.3e-006 | 1 | K.VIMVTGDHPITAK.A 10473 10499 |
|  | 11348 | **478.2394** | **1431.6965** | **1430.7365** | **0.9600** | **1** | **5** | **47** | **7** | **K.RDVAGDASESALLK.C** |
|  | 12321 | 745.4360 | 1488.8575 | 1489.7446 | -0.8871 | 0 | 5 | 39 | 5 | K.ADIGVAMGIAGSDVSK.Q |
|  | 14358 | 810.3731 | 1618.7317 | 1618.7435 | -0.0118 | 0 | 63 | 6.3e-005 | 1 | K.VDNSSLTGESEPQTR.S 14360 14361 |
|  | 14604 | 545.3375 | 1632.9906 | 1633.8345 | -0.8440 | 1 | 4 | 43 | 8 | K.KADIGVAMGIAGSDVSK.Q 14603 |
|  | 17016 | 906.4746 | 1810.9345 | 1810.9425 | -0.0080 | 0 | 83 | 5.3e-007 | 1 | R.QGAIVAVTGDGVNDSPALK.K 17014 17015 17031 |
|  | 17263 | 915.4605 | 1828.9063 | 1828.9167 | -0.0103 | 0 | 99 | 1.3e-008 | 1 | K.GVGIISEGNETVEDIAAR.L 17258 17259 17260 17262 17264 17272 17282 17292 17295 17296 |
|  | 17382 | 613.3414 | 1837.0023 | 1837.0132 | -0.0109 | 1 | 45 | 0.0034 | 1 | R.SAGIKVIMVTGDHPITAK.A |
|  | 18447 | 962.9559 | 1923.8972 | 1923.9095 | -0.0123 | 0 | 64 | 3.4e-005 | 1 | K.NLEAVETLGSTSTICSDK.T |
|  | 18630 | 970.5211 | 1939.0276 | 1939.0375 | -0.0099 | 1 | 70 | 8.9e-006 | 1 | R.QGAIVAVTGDGVNDSPALKK.A 18628 18629 |
|  | 18758 | **650.7988** | **1949.3745** | **1949.8645** | **-0.4900** | **1** | **3** | **31** | **2** | **K.EVAMTEHKMSVEEVCR.K** |
|  | 19349 | 669.0273 | 2004.0602 | 2003.0696 | 0.9906 | 1 | 15 | 3 | 1 | K.IMESFKNMVPQQALVIR.E |
|  | 21324 | 738.3924 | 2212.1554 | 2212.1699 | -0.0145 | 1 | 54 | 0.00029 | 1 | K.AIAKGVGIISEGNETVEDIAAR.L |
|  | 22272 | **583.8065** | **2331.1967** | **2330.1675** | **1.0292** | **1** | **3** | **34** | **6** | **R.EGEKMQVNAEEVVVGDLVEIK.G** |
|  | 25121 | 1398.6792 | 2795.3438 | 2795.3607 | -0.0169 | 1 | 123 | 3.5e-011 | 1 | K.NLEAVETLGSTSTICSDKTGTLTQNR.M 25117 25118 25119 25122 |
|  | 26349 | 1016.5390 | 3046.5952 | 3046.6047 | -0.0095 | 1 | 55 | 0.00015 | 1 | K.GVGIISEGNETVEDIAARLNIPVSQVNPR.D |
|  | 29950 | **1371.3488** | **4111.0244** | **4111.8277** | **-0.8032** | **1** | **8** | **3.8** | **6** | **K.TGTLTQNRMTVAHMWFDNQIHEADTTEDQSGTSFDK.S** 29952 |

| **18.** | P50993\|AT1A2_HUMAN    **Mass:** 113505   **Score:** 1050   **Queries matched:** 60   **emPAI:** 0.52 |
| --- | --- |
|  | Sodium/potassium-transporting ATPase subunit alpha-2 OS=Homo sapiens GN=ATP1A2 PE=1 SV=1 |

|  | **Query** | **Observed** | **Mr(expt)** | **Mr(calc)** | **Delta** | **Miss** | **Score** | **Expect** | **Rank** | **Peptide** |
| --- | --- | --- | --- | --- | --- | --- | --- | --- | --- | --- |
|  | 3682 | 480.7050 | 959.3954 | 959.4018 | -0.0065 | 0 | 12 | 14 | 8 | K.EVAMDDHK.L |
|  | 4310 | **502.2953** | **1002.5760** | **1002.5233** | **0.0527** | **1** | **3** | **89** | **3** | **K.EKELDELK.K** |
|  | 4689 | **517.2581** | **1032.5017** | **1031.5247** | **0.9770** | **0** | **3** | **91** | **4** | **K.AGQENISVSK.R** 4699 |
|  | 5003 | **530.2474** | **1058.4802** | **1057.5114** | **0.9688** | **1** | **9** | **24** | **1** | **K.SSKIMDSFK.N** |
|  | 5329 | 543.2707 | 1084.5268 | 1084.5335 | -0.0067 | 0 | 43 | 0.0079 | 1 | K.ACVVHGSDLK.D 5328 |
|  | 5359 | 544.2969 | 1086.5792 | 1086.5856 | -0.0063 | 0 | 37 | 0.032 | 1 | K.LIIVEGCQR.Q |
|  | 6490 | **587.7923** | **1173.5700** | **1173.6254** | **-0.0554** | **1** | **13** | **8.2** | **2** | **K.RSPTWTALSR.I** |
|  | 6707 | **396.7318** | **1187.1737** | **1187.6258** | **-0.4521** | **1** | **7** | **20** | **4** | **K.AGQENISVSKR.D** |
|  | 8394 | 634.8575 | 1267.7004 | 1267.7071 | -0.0067 | 0 | 55 | 0.00044 | 1 | K.NMVPQQALVIR.E 8371 8393 |
|  | 8693 | 642.8540 | 1283.6934 | 1283.7020 | -0.0085 | 0 | (16) | 3.4 | 1 | K.NMVPQQALVIR.E |
|  | 10497 | 691.3746 | 1380.7347 | 1380.7435 | -0.0088 | 0 | 72 | 9.3e-006 | 1 | K.VIMVTGDHPITAK.A 10473 10499 |
|  | 11735 | **486.2650** | **1455.7732** | **1454.6565** | **1.1167** | **0** | **10** | **14** | **2** | **K.FDTDELNFPTEK.L** |
|  | 13743 | **527.2895** | **1578.8468** | **1579.7478** | **-0.9010** | **1** | **8** | **18** | **2** | **R.EYSPAATTAENGGGKK.K** 13737 |
|  | 14358 | 810.3731 | 1618.7317 | 1618.7435 | -0.0118 | 0 | 63 | 6.3e-005 | 1 | K.VDNSSLTGESEPQTR.S 14360 14361 |
|  | 16231 | **874.4340** | **1746.8535** | **1745.9458** | **0.9077** | **1** | **5** | **36** | **2** | **R.TVMGRIATLASGLEVGR.T** 16205 16228 |
|  | 16799 | **598.6442** | **1792.9109** | **1792.8340** | **0.0769** | **1** | **2** | **79** | **5** | **R.GAGREYSPAATTAENGGGK.K** |
|  | 17016 | 906.4746 | 1810.9345 | 1810.9425 | -0.0080 | 0 | 83 | 5.3e-007 | 1 | R.QGAIVAVTGDGVNDSPALK.K 17014 17015 17031 |
|  | 17263 | 915.4605 | 1828.9063 | 1828.9167 | -0.0103 | 0 | 99 | 1.3e-008 | 1 | K.GVGIISEGNETVEDIAAR.L 17258 17259 17260 17262 17264 17272 17282 17292 17295 17296 |
|  | 17382 | 613.3414 | 1837.0023 | 1837.0132 | -0.0109 | 1 | 45 | 0.0034 | 1 | R.SAGIKVIMVTGDHPITAK.A |
|  | 17476 | **615.6299** | **1843.8678** | **1842.8782** | **0.9897** | **1** | **4** | **42** | **3** | **K.EVAMDDHKLSLDELGR.K** 17473 |
|  | 18447 | 962.9559 | 1923.8972 | 1923.9095 | -0.0123 | 0 | 64 | 3.4e-005 | 1 | K.NLEAVETLGSTSTICSDK.T |
|  | 18630 | 970.5211 | 1939.0276 | 1939.0375 | -0.0099 | 1 | 70 | 8.9e-006 | 1 | R.QGAIVAVTGDGVNDSPALKK.A 18628 18629 |
|  | 19349 | 669.0273 | 2004.0602 | 2005.0489 | -0.9887 | 1 | 4 | 39 | 8 | K.IMDSFKNMVPQQALVIR.E |
|  | 21126 | **730.6738** | **2188.9997** | **2189.9357** | **-0.9361** | **0** | **1** | **72** | **3** | **K.EMQDAFQNAYMELGGLGER.V** |
|  | 21324 | 738.3924 | 2212.1554 | 2212.1699 | -0.0145 | 1 | 54 | 0.00029 | 1 | K.AIAKGVGIISEGNETVEDIAAR.L |
|  | 23325 | **829.7198** | **2486.1377** | **2487.1985** | **-1.0608** | **1** | **1** | **57** | **6** | **K.ACVVHGSDLKDMTSEQLDEILK.N** |
|  | 23425 | **834.7603** | **2501.2591** | **2501.1816** | **0.0775** | **1** | **8** | **12** | **2** | **R.IISSHGCKVDNSSLTGESEPQTR.S** |
|  | 25121 | 1398.6792 | 2795.3438 | 2795.3607 | -0.0169 | 1 | 123 | 3.5e-011 | 1 | K.NLEAVETLGSTSTICSDKTGTLTQNR.M 25117 25118 25119 25122 |
|  | 25553 | **722.3512** | **2885.3757** | **2885.3211** | **0.0546** | **1** | **5** | **19** | **6** | **K.EIPLDKEMQDAFQNAYMELGGLGER.V** |

| **19.** | P68133\|ACTS_HUMAN    **Mass:** 42366    **Score:** 1046   **Queries matched:** 75   **emPAI:** 4.00 |
| --- | --- |
|  | Actin, alpha skeletal muscle OS=Homo sapiens GN=ACTA1 PE=1 SV=1 |

|  | **Query** | **Observed** | **Mr(expt)** | **Mr(calc)** | **Delta** | **Miss** | **Score** | **Expect** | **Rank** | **Peptide** |
| --- | --- | --- | --- | --- | --- | --- | --- | --- | --- | --- |
|  | 1475 | 800.5302 | 799.5229 | 799.5280 | -0.0051 | 1 | 14 | 8.2 | 3 | K.RGILTLK.Y |
|  | 4224 | 499.7444 | 997.4741 | 997.4790 | -0.0049 | 0 | 10 | 15 | 4 | R.DLTDYLMK.I |
|  | 4734 | 518.8262 | 1035.6379 | 1035.6440 | -0.0061 | 1 | 21 | 1.4 | 1 | K.IKIIAPPER.K |
|  | 5932 | 565.7742 | 1129.5339 | 1129.5404 | -0.0065 | 0 | 4 | 72 | 2 | R.GYSFVTTAER.E |
|  | 6341 | 581.3092 | 1160.6038 | 1160.6111 | -0.0072 | 0 | 36 | 0.042 | 1 | K.EITALAPSTMK.I 6340 |
|  | 6445 | 586.2852 | 1170.5558 | 1170.5638 | -0.0080 | 0 | 48 | 0.0025 | 1 | R.HQGVMVGMGQK.D 6444 6446 6447 6460 |
|  | 6691 | 594.2826 | 1186.5507 | 1186.5587 | -0.0080 | 0 | (40) | 0.017 | 1 | R.HQGVMVGMGQK.D 6679 6693 6696 |
|  | 6692 | 594.2828 | 1186.5511 | 1186.5587 | -0.0076 | 0 | (42) | 0.01 | 1 | R.HQGVMVGMGQK.D 6695 |
|  | 6934 | 599.7607 | 1197.5068 | 1197.5150 | -0.0081 | 0 | 47 | 0.0031 | 1 | K.DSYVGDEAQSK.R |
|  | 6943 | 599.8527 | 1197.6908 | 1197.6982 | -0.0075 | 0 | 52 | 0.00092 | 1 | R.AVFPSIVGRPR.H 6939 6944 6945 6946 |
|  | 7039 | 602.2794 | 1202.5442 | 1202.5536 | -0.0095 | 0 | (21) | 1.3 | 1 | R.HQGVMVGMGQK.D |
|  | 10002 | 677.8109 | 1353.6073 | 1353.6161 | -0.0088 | 1 | 60 | 0.00014 | 1 | K.DSYVGDEAQSKR.G 9990 10003 10004 10005 10006 10024 10031 |
|  | 12455 | 750.3372 | 1498.6599 | 1499.7005 | -1.0406 | 0 | 12 | 8 | 2 | K.QEYDEAGPSIVHR.K 12456 12471 |
|  | 12723 | 505.9181 | 1514.7325 | 1514.7419 | -0.0093 | 0 | 40 | 0.014 | 1 | K.IWHHTFYNELR.V 12724 12725 |
|  | 13248 | 774.9052 | 1547.7957 | 1547.8051 | -0.0094 | 1 | 53 | 0.0006 | 1 | R.MQKEITALAPSTMK.I 13247 13249 13250 |
|  | 13497 | 522.2708 | 1563.7904 | 1563.8000 | -0.0096 | 1 | (22) | 0.73 | 1 | R.MQKEITALAPSTMK.I |
|  | 14437 | 541.9493 | 1622.8262 | 1622.8338 | -0.0076 | 1 | 25 | 0.33 | 1 | R.LDLAGRDLTDYLMK.I |
|  | 14496 | 814.3845 | 1626.7544 | 1627.7954 | -1.0410 | 1 | 16 | 3.2 | 2 | K.QEYDEAGPSIVHRK.C |
|  | 16767 | 895.9441 | 1789.8736 | 1789.8846 | -0.0110 | 0 | 66 | 2.5e-005 | 1 | K.SYELPDGQVITIGNER.F 16766 16769 16770 16771 16772 |
|  | 18819 | 978.5196 | 1955.0246 | 1955.0364 | -0.0117 | 0 | 41 | 0.0076 | 1 | R.VAPEEHPTLLTEAPLNPK.A 18812 18820 |
|  | 22410 | 1176.0343 | 2350.0540 | 2350.0682 | -0.0141 | 1 | 96 | 1.8e-008 | 1 | R.HQGVMVGMGQKDSYVGDEAQSK.R 22409 22411 |
|  | 22544 | 789.4450 | 2365.3132 | 2366.2464 | -0.9332 | 1 | 4 | 30 | 1 | R.AVFPSIVGRPRHQGVMVGMGQK.D |
|  | 22547 | 789.6902 | 2366.0487 | 2366.0631 | -0.0144 | 1 | (6) | 20 | 1 | R.HQGVMVGMGQKDSYVGDEAQSK.R |
|  | 23627 | 1268.5759 | 2535.1373 | 2535.1509 | -0.0136 | 0 | 120 | 6.5e-011 | 1 | K.LCYVALDFENEMATAASSSSLEK.S 23626 |
|  | 23753 | 1276.5885 | 2551.1624 | 2551.1458 | 0.0167 | 0 | (45) | 0.002 | 1 | K.LCYVALDFENEMATAASSSSLEK.S 23746 23754 23767 |
|  | 23957 | 862.7755 | 2585.3047 | 2585.3199 | -0.0152 | 1 | 25 | 0.2 | 1 | R.GILTLKYPIEHGIITNWDDMEK.I |
|  | 24353 | **888.8173** | **2663.4300** | **2664.1982** | **-0.7682** | **1** | **2** | **41** | **1** | **K.DLYANNVMSGGTTMYPGIADRMQK.E** |
|  | 25075 | 931.7656 | 2792.2751 | 2792.2884 | -0.0133 | 1 | 26 | 0.15 | 1 | K.EKLCYVALDFENEMATAASSSSLEK.S |
|  | 25186 | 937.0732 | 2808.1977 | 2808.2833 | -0.0856 | 1 | (3) | 24 | 1 | K.EKLCYVALDFENEMATAASSSSLEK.S |
|  | 27039 | 1066.2020 | 3195.5843 | 3195.6023 | -0.0180 | 0 | 71 | 4.1e-006 | 1 | R.TTGIVLDSGDGVTHNVPIYEGYALPHAIMR.L 27040 |
|  | 27082 | 1069.0851 | 3204.2334 | 3203.4613 | 0.7721 | 0 | 3 | 19 | 1 | R.CPETLFQPSFIGMESAGIHETTYNSIMK.C |
|  | 27356 | 817.9298 | 3267.6901 | 3266.6178 | 1.0724 | 0 | 1 | 36 | 2 | K.MTQIMFETFNVPAMYVAIQAVLSLYASGR.T |
|  | 28174 | 1164.5455 | 3490.6148 | 3490.6360 | -0.0212 | 1 | 66 | 8.5e-006 | 1 | R.FRCPETLFQPSFIGMESAGIHETTYNSIMK.C 28175 |

| **20.** | P68366\|TBA4A_HUMAN    **Mass:** 50634    **Score:** 1014   **Queries matched:** 51   **emPAI:** 3.46 |
| --- | --- |
|  | Tubulin alpha-4A chain OS=Homo sapiens GN=TUBA4A PE=1 SV=1 |

|  | **Query** | **Observed** | **Mr(expt)** | **Mr(calc)** | **Delta** | **Miss** | **Score** | **Expect** | **Rank** | **Peptide** |
| --- | --- | --- | --- | --- | --- | --- | --- | --- | --- | --- |
|  | 2728 | **444.2173** | **886.4200** | **886.4259** | **-0.0058** | **0** | **41** | **0.017** | **1** | **K.FDLMYAK.R** |
|  | 3028 | **455.7546** | **909.4947** | **908.4967** | **0.9979** | **1** | **22** | **1** | **1** | **R.LSVDYGKK.S** |
|  | 4037 | **493.2849** | **984.5551** | **984.5604** | **-0.0052** | **0** | **67** | **3.4e-005** | **1** | **K.DVNAAIAAIK.T** 4038 |
|  | 7994 | **625.2763** | **1248.5379** | **1248.5453** | **-0.0074** | **0** | **36** | **0.035** | **1** | **K.YMACCLLYR.G** |
|  | 10474 | **690.8484** | **1379.6822** | **1379.6907** | **-0.0085** | **1** | **16** | **3.6** | **1** | **R.LDHKFDLMYAK.R** |
|  | 13817 | **792.8756** | **1583.7367** | **1583.7443** | **-0.0076** | **0** | **48** | **0.0018** | **1** | **R.SIQFVDWCPTGFK.V** 13815 |
|  | 15812 | **858.4589** | **1714.9033** | **1714.9142** | **-0.0109** | **0** | **78** | **1.7e-006** | **1** | **R.AVFVDLEPTVIDEIR.N** 15813 |
|  | 15854 | **859.9388** | **1717.8631** | **1717.8747** | **-0.0116** | **0** | **51** | **0.00085** | **1** | **R.NLDIERPTYTNLNR.L** 15855 |
|  | 16144 | **580.9411** | **1739.8015** | **1739.8454** | **-0.0439** | **1** | **2** | **72** | **3** | **K.RSIQFVDWCPTGFK.V** |
|  | 16352 | **878.9811** | **1755.9476** | **1755.9559** | **-0.0083** | **0** | **41** | **0.009** | **1** | **R.IHFPLATYAPVISAEK.A** 16351 16353 |
|  | 17194 | **912.9911** | **1823.9676** | **1823.9782** | **-0.0105** | **0** | **46** | **0.0028** | **1** | **K.VGINYQPPTVVPGGDLAK.V** 17192 17193 17195 17209 |
|  | 17205 | **609.3275** | **1824.9607** | **1824.9734** | **-0.0127** | **1** | **40** | **0.01** | **1** | **R.GHYTIGKEIIDPVLDR.I** 17204 17218 |
|  | 17476 | 615.6299 | 1843.8678 | 1843.8783 | -0.0105 | 1 | 19 | 1.2 | 1 | K.YMACCLLYRGDVVPK.D 17477 |
|  | 17690 | **620.7950** | **1859.3631** | **1859.8732** | **-0.5101** | **1** | **(3)** | **39** | **5** | **K.YMACCLLYRGDVVPK.D** |
|  | 17729 | **932.9498** | **1863.8851** | **1863.8971** | **-0.0120** | **0** | **85** | **3.7e-007** | **1** | **R.AVCMLSNTTAIAEAWAR.L** 17730 |
|  | 20012 | **1034.4465** | **2066.8785** | **2066.8892** | **-0.0107** | **0** | **123** | **4.4e-011** | **1** | **K.TIGGGDDSFTTFFCETGAGK.H** 20011 |
|  | 22254 | **777.3408** | **2329.0006** | **2329.0110** | **-0.0103** | **0** | **39** | **0.0091** | **1** | **R.AFVHWYVGEGMEEGEFSEAR.E** |
|  | 22800 | **1205.1028** | **2408.1910** | **2408.2012** | **-0.0102** | **0** | **123** | **3.6e-011** | **1** | **R.FDGALNVDLTEFQTNLVPYPR.I** 22799 22801 |
|  | 22841 | **1208.0984** | **2414.1822** | **2414.1978** | **-0.0156** | **1** | **46** | **0.0019** | **1** | **R.QLFHPEQLITGKEDAANNYAR.G** 22842 22844 |
|  | 24859 | **1375.6443** | **2749.2740** | **2749.2840** | **-0.0099** | **0** | **74** | **2.5e-006** | **1** | **K.AYHEQLSVAEITNACFEPANQMVK.C** 24857 24862 |
|  | 27012 | **797.6893** | **3186.7282** | **3186.7360** | **-0.0078** | **1** | **5** | **15** | **2** | **R.NLDIERPTYTNLNRLISQIVSSITASLR.F** |
|  | 27136 | **805.1072** | **3216.3996** | **3216.4168** | **-0.0172** | **1** | **0** | **42** | **1** | **R.AFVHWYVGEGMEEGEFSEAREDMAALEK.D** |
|  | 27811 | **1130.9064** | **3389.6973** | **3389.7119** | **-0.0146** | **1** | **25** | **0.12** | **1** | **R.SIQFVDWCPTGFKVGINYQPPTVVPGGDLAK.V** |
|  | 27941 | **856.4022** | **3421.5798** | **3421.6072** | **-0.0274** | **0** | **2** | **28** | **1** | **K.LSDQCTGLQGFLVFHSFGGGTGSGFTSLLMER.L** |
|  | 28336 | **884.4282** | **3533.6835** | **3533.7072** | **-0.0237** | **1** | **8** | **5.7** | **1** | **R.KLSDQCTGLQGFLVFHSFGGGTGSGFTSLLMER.L** 28337 |
|  | 30522 | **1119.0217** | **4472.0578** | **4471.9865** | **0.0713** | **1** | **1** | **18** | **6** | **M.RECISVHVGQAGVQMGNACWELYCLEHGIQPDGQMPSDK.T** |
|  | 31994 | **1419.1687** | **5672.6457** | **5672.6902** | **-0.0445** | **1** | **7** | **1.9** | **1** | **K.LEFSIYPAPQVSTAVVEPYNSILTTHTTLEHSDCAFMVDNEAIYDICRR.N** 31995 31997 |

| **21.** | P31040\|DHSA_HUMAN    **Mass:** 73672    **Score:** 952    **Queries matched:** 66   **emPAI:** 2.41 |
| --- | --- |
|  | Succinate dehydrogenase [ubiquinone] flavoprotein subunit, mitochondrial OS=Homo sapiens GN=SDHA PE=1 SV=2 |

|  | **Query** | **Observed** | **Mr(expt)** | **Mr(calc)** | **Delta** | **Miss** | **Score** | **Expect** | **Rank** | **Peptide** |
| --- | --- | --- | --- | --- | --- | --- | --- | --- | --- | --- |
|  | 1065 | **648.3666** | **647.3593** | **647.3642** | **-0.0049** | **0** | **26** | **0.59** | **1** | **R.YAPVAK.D** 1067 1068 1069 |
|  | 2156 | **425.2263** | **848.4380** | **848.4426** | **-0.0046** | **0** | **34** | **0.085** | **1** | **R.SMTLEIR.E** 2155 |
|  | 3431 | **470.7280** | **939.4414** | **939.3361** | **0.1053** | **0** | **21** | **1.2** | **1** | **R.CCCVADR.T** 3440 3443 |
|  | 4114 | **495.7604** | **989.5062** | **988.4396** | **1.0666** | **1** | **7** | **36** | **6** | **R.EGRGCGPEK.D** |
|  | 5461 | **547.7797** | **1093.5449** | **1093.5516** | **-0.0067** | **0** | **55** | **0.00049** | **1** | **K.NTVVATGGYGR.T** 5463 |
|  | 5679 | **555.2794** | **1108.5442** | **1108.5513** | **-0.0071** | **1** | **43** | **0.0079** | **1** | **R.TEDGKIYQR.A** 5680 5682 5683 5691 |
|  | 5850 | **561.6075** | **1121.2004** | **1120.5302** | **0.6702** | **0** | **1** | **85** | **9** | **R.GFHFTVDGNK.R** |
|  | 5971 | **567.2814** | **1132.5482** | **1132.5547** | **-0.0065** | **0** | **61** | **0.00012** | **1** | **R.VGSVLQEGCGK.I** 5969 5970 |
|  | 6044 | **570.3215** | **1138.6285** | **1138.6135** | **0.0150** | **1** | **0** | **1.3e+002** | **4** | **R.AFGGQSLKFGK.G** |
|  | 6780 | **595.8196** | **1189.6247** | **1189.6455** | **-0.0208** | **1** | **5** | **54** | **7** | **R.YAPVAKDLASR.D** |
|  | 8536 | **638.8267** | **1275.6389** | **1275.6459** | **-0.0070** | **0** | **26** | **0.41** | **1** | **K.HTLSYVDVGTGK.V** |
|  | 8871 | **647.3450** | **1292.6754** | **1292.6837** | **-0.0083** | **1** | **38** | **0.023** | **1** | **R.AKNTVVATGGYGR.T** 8866 8889 |
|  | 9572 | **665.3374** | **1328.6602** | **1328.6684** | **-0.0082** | **0** | **38** | **0.023** | **1** | **R.GEGGILINSQGER.F** 9591 |
|  | 10011 | **452.2433** | **1353.7080** | **1353.7153** | **-0.0073** | **0** | **6** | **34** | **2** | **R.TGHSLLHTLYGR.S** 10031 |
|  | 10875 | **702.8731** | **1403.7317** | **1403.7409** | **-0.0092** | **1** | **69** | **1.8e-005** | **1** | **R.KHTLSYVDVGTGK.V** 10873 10874 |
|  | 11834 | **731.3719** | **1460.7292** | **1460.7657** | **-0.0365** | **1** | **5** | **44** | **2** | **R.VGSVLQEGCGKISK.L** |
|  | 12034 | **737.4216** | **1472.8286** | **1472.8351** | **-0.0065** | **0** | **83** | **5.9e-007** | **1** | **R.LGANSLLDLVVFGR.A** 12038 |
|  | 13069 | 513.7733 | 1538.2981 | 1538.7875 | -0.4894 | 0 | 2 | 45 | 9 | R.GVIALCIEDGSIHR.I |
|  | 16651 | 594.3219 | 1779.9439 | 1778.8556 | 1.0883 | 1 | 5 | 33 | 5 | R.LSMQKSMQNHAAVFR.V |
|  | 17009 | **906.4390** | **1810.8635** | **1810.8737** | **-0.0102** | **0** | **65** | **3.1e-005** | **1** | **R.IDEYDYSKPIQGQQK.K** 17007 17008 |
|  | 20008 | **1034.0234** | **2066.0323** | **2066.0432** | **-0.0109** | **1** | **55** | **0.00029** | **1** | **K.VRIDEYDYSKPIQGQQK.K** 20007 |
|  | 21768 | **1132.9922** | **2263.9698** | **2263.9838** | **-0.0140** | **0** | **93** | **3.6e-008** | **1** | **R.TYFSCTSAHTSTGDGTAMITR.A** 21767 21769 |
|  | 22458 | **786.0529** | **2355.1368** | **2355.1933** | **-0.0565** | **0** | **0** | **75** | **3** | **K.EPIPVLPTVHYNMGGIPTNYK.G** |
|  | 25102 | **699.6026** | **2794.3813** | **2794.3973** | **-0.0160** | **1** | **25** | **0.19** | **1** | **R.GCGPEKDHVYLQLHHLPPEQLATR.L** |
|  | 25689 | **1458.2239** | **2914.4332** | **2914.4461** | **-0.0129** | **0** | **92** | **3.1e-008** | **1** | **K.VSDSISAQYPVVDHEFDAVVVGAGGAGLR.A** 25686 25688 |
|  | 25820 | **981.1823** | **2940.5251** | **2940.5379** | **-0.0128** | **1** | **37** | **0.01** | **1** | **K.VTLEYRPVIDKTLNEADCATVPPAIR.S** |
|  | 25872 | **984.7808** | **2951.3206** | **2950.3563** | **0.9644** | **0** | **38** | **0.0073** | **1** | **R.HVNGQDQIVPGLYACGEAACASVHGANR.L** 25873 |
|  | 27725 | **1122.8560** | **3365.5461** | **3365.5632** | **-0.0171** | **0** | **53** | **0.0002** | **1** | **R.AGLPCQDLEFVQFHPTGIYGAGCLITEGCR.G** 27724 |
|  | 28392 | **1184.9186** | **3551.7339** | **3551.7462** | **-0.0123** | **0** | **64** | **1.5e-005** | **1** | **R.GMVWNTDLVETLELQNLMLCALQTIYGAEAR.K** |
|  | 29163 | **1266.5719** | **3796.6939** | **3796.7137** | **-0.0199** | **0** | **65** | **1e-005** | **1** | **K.GSDWLGDQDAIHYMTEQAPAAVVELENYGMPFSR.T** 29166 |
|  | 30439 | **1102.5441** | **4406.1472** | **4405.1808** | **0.9664** | **1** | **9** | **2.5** | **1** | **R.HVNGQDQIVPGLYACGEAACASVHGANRLGANSLLDLVVFGR.A** |
|  | 30826 | **1559.7413** | **4676.2022** | **4676.2210** | **-0.0189** | **1** | **59** | **2.4e-005** | **1** | **R.AGLPCQDLEFVQFHPTGIYGAGCLITEGCRGEGGILINSQGER.F** |
|  | 31077 | **1625.4031** | **4873.1874** | **4873.2217** | **-0.0343** | **1** | **45** | **0.00041** | **1** | **R.WHFYDTVKGSDWLGDQDAIHYMTEQAPAAVVELENYGMPFSR.T** 31078 31079 31081 |

| **22.** | O60494\|CUBN_HUMAN    **Mass:** 407202   **Score:** 908    **Queries matched:** 62   **emPAI:** 0.16 |
| --- | --- |
|  | Cubilin OS=Homo sapiens GN=CUBN PE=1 SV=4 |

|  | **Query** | **Observed** | **Mr(expt)** | **Mr(calc)** | **Delta** | **Miss** | **Score** | **Expect** | **Rank** | **Peptide** |
| --- | --- | --- | --- | --- | --- | --- | --- | --- | --- | --- |
|  | 1247 | **762.3516** | **761.3443** | **760.3967** | **0.9477** | **0** | **5** | **64** | **9** | **K.IDASVEK.A** |
|  | 1921 | **417.2436** | **832.4726** | **832.4337** | **0.0389** | **1** | **9** | **23** | **7** | **R.LARTCGR.E** |
|  | 2492 | **436.2219** | **870.4293** | **869.4395** | **0.9898** | **1** | **13** | **9.6** | **1** | **R.GFKAEYR.Q** |
|  | 3350 | **466.9101** | **931.8056** | **932.5113** | **-0.7057** | **1** | **3** | **63** | **1** | **K.TEMGKVLR.I** |
|  | 3598 | **477.7309** | **953.4472** | **954.5247** | **-1.0775** | **0** | **6** | **33** | **4** | **R.SINLQQPR.M** |
|  | 5681 | **555.2796** | **1108.5446** | **1108.5513** | **-0.0066** | **1** | **33** | **0.074** | **1** | **K.FKSDQIDTR.S** |
|  | 6190 | **576.2883** | **1150.5621** | **1149.6142** | **0.9479** | **1** | **2** | **90** | **7** | **R.FKTDLSINGR.G** |
|  | 7545 | **614.7445** | **1227.4743** | **1227.4826** | **-0.0083** | **0** | **18** | **2.3** | **1** | **K.YDDCEGGSVAR.C** |
|  | 7723 | **619.2812** | **1236.5478** | **1236.5557** | **-0.0079** | **0** | **16** | **3.6** | **1** | **R.SNTDCSWVIR.V** |
|  | 8093 | **418.9010** | **1253.6811** | **1254.6278** | **-0.9467** | **0** | **5** | **47** | **10** | **K.SSNNSMLLVFK.T** 8121 |
|  | 8902 | **648.2838** | **1294.5529** | **1294.5612** | **-0.0082** | **0** | **48** | **0.0021** | **1** | **K.SSSTENHGFMAK.F** 8912 |
|  | 9309 | **439.3740** | **1315.1002** | **1314.6820** | **0.4183** | **0** | **2** | **63** | **7** | **R.DGPLYQDPLLGK.F** |
|  | 9720 | **447.0295** | **1338.0668** | **1337.6252** | **0.4416** | **0** | **7** | **17** | **2** | **K.SSHGSAFELEFK.D** |
|  | 9799 | **672.3278** | **1342.6411** | **1342.6518** | **-0.0107** | **0** | **20** | **1.2** | **1** | **R.FVTDGSVTASGFR.L** |
|  | 10300 | **686.3504** | **1370.6862** | **1370.6943** | **-0.0080** | **0** | **16** | **3.7** | **1** | **K.IFGNDNIVGTHGK.V** |
|  | 10760 | **466.5793** | **1396.7162** | **1396.7575** | **-0.0413** | **1** | **8** | **21** | **10** | **K.LRIYDGPSIHAR.L** 10758 |
|  | 11461 | **480.6250** | **1438.8532** | **1438.8620** | **-0.0088** | **1** | **14** | **5.4** | **1** | **K.LQVLLLTDGVGRR.E** |
|  | 12341 | **497.9123** | **1490.7151** | **1490.7266** | **-0.0115** | **1** | **27** | **0.27** | **1** | **R.FRSDNSPTHVGFK.A** |
|  | 16472 | **883.4620** | **1764.9095** | **1764.9159** | **-0.0063** | **0** | **59** | **0.00014** | **1** | **R.GNLVFLTGSAQNIEFR.T** 16471 |
|  | 17366 | **919.4199** | **1836.8253** | **1836.8353** | **-0.0100** | **0** | **104** | **4.5e-009** | **1** | **R.FISDGSGSGTGFQATFMK.I** 17367 |
|  | 17417 | **614.6245** | **1840.8515** | **1840.8625** | **-0.0110** | **0** | **7** | **19** | **3** | **K.LNDEDLSECLHQIQK.N** |
|  | 17483 | **922.9912** | **1843.9679** | **1843.9792** | **-0.0113** | **0** | **76** | **2.8e-006** | **1** | **R.EQLANPIVSSGNSLFLR.F** 17484 |
|  | 17940 | **627.6549** | **1879.9429** | **1880.9454** | **-1.0025** | **1** | **4** | **39** | **2** | **R.EIPGPIRSTGEYMFIR.F** |
|  | 18587 | **968.9689** | **1935.9232** | **1935.9360** | **-0.0128** | **0** | **54** | **0.00037** | **1** | **R.AVYQVACGDELTGEGVIR.S** 18588 |
|  | 19220 | **997.0035** | **1991.9924** | **1992.0429** | **-0.0505** | **0** | **0** | **85** | **9** | **R.VPGQSGVVESIGHPTLPYR.D** |
|  | 19582 | **1014.0023** | **2025.9900** | **2025.9983** | **-0.0083** | **0** | **18** | **1.6** | **2** | **K.FCTTFSVPPLQTTGPFAR.I** 19592 |
|  | 20348 | **1052.9653** | **2103.9161** | **2102.9440** | **0.9722** | **0** | **49** | **0.0011** | **1** | **R.NGGSPESPIIGQYCGNSNPR.T** 20349 |
|  | 20480 | **706.4456** | **2116.3149** | **2117.1116** | **-0.7968** | **0** | **0** | **61** | **2** | **K.GSAIGLPQNISSQIYQLNSK.L** |
|  | 20512 | **708.3226** | **2121.9461** | **2121.9578** | **-0.0118** | **0** | **48** | **0.0013** | **1** | **R.CNFDVLEIYGGPDFHSPR.I** |
|  | 20933 | **1085.5010** | **2168.9874** | **2169.0008** | **-0.0134** | **0** | **88** | **1.2e-007** | **1** | **R.YCGNTIPDSIDTSSNTAVVR.F** |
|  | 21333 | **1107.5132** | **2213.0118** | **2212.0226** | **0.9893** | **0** | **39** | **0.011** | **1** | **R.TGDAPVFLFSPGWPDSYSNR.V** |
|  | 21394 | **1111.0271** | **2220.0396** | **2220.0521** | **-0.0125** | **0** | **52** | **0.00049** | **1** | **R.WETQQPECGGILTGPYGSIK.S** |
|  | 21971 | **1146.5189** | **2291.0233** | **2291.0351** | **-0.0118** | **0** | **106** | **1.7e-009** | **1** | **K.FCGTSLPSSQWSSGEVMYLR.F** 21972 |
|  | 22188 | **774.1159** | **2319.3259** | **2318.1801** | **1.1458** | **1** | **20** | **0.62** | **1** | **R.TCGREQLANPIVSSGNSLFLR.F** |
|  | 22365 | **782.3727** | **2344.0962** | **2344.1079** | **-0.0117** | **1** | **49** | **0.00088** | **1** | **R.ILEMDIEEIQNCYYDKLR.I** 22364 |
|  | 22741 | **1199.5892** | **2397.1639** | **2397.1787** | **-0.0148** | **0** | **58** | **0.00012** | **1** | **K.YCGTLLPNPVFSQNNELYLR.F** 22742 |
|  | 23301 | **828.4048** | **2482.1925** | **2482.1733** | **0.0192** | **1** | **7** | **14** | **1** | **R.GRYCGTDMPHPITSFSSALTLR.F** |
|  | 25758 | **733.0975** | **2928.3610** | **2929.5219** | **-1.1609** | **1** | **2** | **32** | **3** | **R.YCGVDLPPPGSTTSSKLQVLLLTDGVGR.R** |
|  | 26091 | **1001.1470** | **3000.4193** | **3000.4366** | **-0.0174** | **0** | **41** | **0.0045** | **1** | **R.IHFHSDSQISDQGFHITYLTSPSDLR.C** |
|  | 26597 | **774.1644** | **3092.6284** | **3092.4597** | **0.1687** | **1** | **2** | **31** | **2** | **R.CNFDVLEIYGGPDFHSPRIAQLCTQR.S** |
|  | 28627 | **1203.8557** | **3608.5453** | **3608.6532** | **-0.1079** | **0** | **2** | **21** | **7** | **R.GFNASWQAVTGGCGGIFQAPSGEIHSPNYPSPYR.S** |
|  | 28872 | **1233.1083** | **3696.3030** | **3696.7110** | **-0.4080** | **1** | **0** | **22** | **1** | **R.VLLNFTDFDLEPQDSCIMAYDGLSSTMSRLAR.T** |
|  | 29045 | **938.6713** | **3750.6559** | **3750.6791** | **-0.0232** | **1** | **81** | **2.5e-007** | **1** | **R.LRFESSMEECGGDLQGSIGTFTSPNYPNPNPHGR.I** 29044 |
|  | 29291 | **961.4959** | **3841.9545** | **3842.7246** | **-0.7701** | **1** | **9** | **4.1** | **1** | **K.GFQMQWFVYGCGGELSGATGSFSSPGFPNRYPPNK.E** |
|  | 29698 | **1005.9819** | **4019.8986** | **4019.6997** | **0.1989** | **1** | **3** | **13** | **1** | **K.MSFRQTLGPQQGCGGYLTGSNNTFASPDSDSNGMYDK.N** |
|  | 30177 | **849.2039** | **4240.9832** | **4241.8266** | **-0.8434** | **1** | **1** | **18** | **1** | **R.FESSMEECGGDLQGSIGTFTSPNYPNPNPHGRICEWR.I** |
|  | 31349 | **1711.5590** | **5131.6551** | **5131.3354** | **0.3196** | **1** | **1** | **8.3** | **8** | **K.FSYRIISCGGVFNFSSGIITSPAYSYADYPNDMHCLYTITVSDDK.V** |
|  | 31667 | **1367.6679** | **5466.6423** | **5466.6831** | **-0.0408** | **1** | **1** | **9** | **1** | **K.VWAGTEEVDKALLATGCGNVAPGPVITPSNTFTAVFQSQEAPAQGFSASFVSR.C** |
|  | 32012 | **1139.1656** | **5690.7919** | **5690.5679** | **0.2240** | **1** | **5** | **2.7** | **1** | **R.NGPDICSPPLGPPGGNGHFCGSHASSTLFTSDNQMFVQFISDHSNEGQGFKIK.Y** |

| **23.** | P31930\|QCR1_HUMAN    **Mass:** 53297    **Score:** 879    **Queries matched:** 43   **emPAI:** 1.95 |
| --- | --- |
|  | Cytochrome b-c1 complex subunit 1, mitochondrial OS=Homo sapiens GN=UQCRC1 PE=1 SV=3 |

|  | **Query** | **Observed** | **Mr(expt)** | **Mr(calc)** | **Delta** | **Miss** | **Score** | **Expect** | **Rank** | **Peptide** |
| --- | --- | --- | --- | --- | --- | --- | --- | --- | --- | --- |
|  | 1612 | **809.4102** | **808.4029** | **808.4079** | **-0.0050** | **0** | **19** | **2.2** | **1** | **R.FTGSEIR.H** 1604 |
|  | 1616 | **405.2275** | **808.4405** | **808.4443** | **-0.0038** | **0** | **35** | **0.052** | **1** | **R.SLLTYGR.R** |
|  | 4562 | **512.7553** | **1023.4959** | **1023.5025** | **-0.0066** | **0** | **14** | **6.6** | **1** | **R.EHTAYYIK.A** 4591 |
|  | 4991 | **529.3794** | **1056.7442** | **1057.5768** | **-0.8325** | **0** | **3** | **83** | **4** | **R.IAEVDASVVR.E** |
|  | 6860 | **598.2648** | **1194.5151** | **1193.7244** | **0.7907** | **1** | **3** | **70** | **7** | **R.SPALLRTPALR.S** |
|  | 7520 | **409.5522** | **1225.6347** | **1225.4703** | **0.1644** | **0** | **6** | **34** | **2** | **R.EMQENDASMR.D** |
|  | 8142 | **628.8375** | **1255.6604** | **1255.6673** | **-0.0069** | **1** | **29** | **0.17** | **1** | **R.RIPLAEWESR.I** 8105 8143 |
|  | 9451 | **662.3095** | **1322.6045** | **1322.6136** | **-0.0091** | **0** | **70** | **1.3e-005** | **1** | **R.LCTSATESEVAR.G** |
|  | 11465 | **720.8495** | **1439.6844** | **1439.6932** | **-0.0088** | **0** | **26** | **0.32** | **1** | **R.ADLTEYLSTHYK.A** |
|  | 15072 | **832.8838** | **1663.7530** | **1663.7624** | **-0.0094** | **0** | **74** | **5.1e-006** | **1** | **K.EVESMGAHLNAYSTR.E** |
|  | 15270 | **838.9250** | **1675.8354** | **1674.8611** | **0.9744** | **1** | **3** | **55** | **5** | **R.IAEVDASVVREICSK.Y** |
|  | 16453 | **588.9654** | **1763.8743** | **1763.8842** | **-0.0099** | **1** | **27** | **0.25** | **1** | **R.ADLTEYLSTHYKAPR.M** 16454 16466 |
|  | 16823 | **599.3477** | **1795.0212** | **1795.9104** | **-0.8893** | **1** | **2** | **67** | **9** | **K.LSRADLTEYLSTHYK.A** |
|  | 19600 | **676.9858** | **2027.9355** | **2027.9453** | **-0.0098** | **1** | **1** | **67** | **4** | **R.MKIDDMMFVLQGQWMR.L** |
|  | 19846 | **1027.4955** | **2052.9764** | **2052.9899** | **-0.0135** | **0** | **96** | **1.9e-008** | **1** | **R.NALVSHLDGTTPVCEDIGR.S** 19845 |
|  | 20216 | **1046.5575** | **2091.1004** | **2091.1146** | **-0.0142** | **0** | **95** | **3e-008** | **1** | **R.MVLAAAGGVEHQQLLDLAQK.H** 20215 20217 20218 |
|  | 22431 | **1177.5760** | **2353.1375** | **2353.1525** | **-0.0150** | **0** | **70** | **7.9e-006** | **1** | **K.HLGGIPWTYAEDAVPTLTPCR.F** 22432 |
|  | 22963 | **811.5511** | **2431.6314** | **2431.3005** | **0.3309** | **1** | **0** | **44** | **4** | **K.APRMVLAAAGGVEHQQLLDLAQK.H** |
|  | 24121 | **873.0929** | **2616.2569** | **2616.2714** | **-0.0145** | **1** | **79** | **8.6e-007** | **1** | **K.NRPGSALEKEVESMGAHLNAYSTR.E** 24120 24122 |
|  | 24610 | **1351.6237** | **2701.2328** | **2701.2482** | **-0.0155** | **0** | **125** | **2.1e-011** | **1** | **K.YIYDQCPAVAGYGPIEQLPDYNR.I** 24609 24612 |
|  | 24639 | **677.3545** | **2705.3889** | **2705.4025** | **-0.0136** | **0** | **3** | **31** | **3** | **R.STATFAQALQFVPETQVSLLDNGLR.V** |
|  | 25942 | **991.1461** | **2970.4165** | **2970.4334** | **-0.0169** | **1** | **96** | **1.2e-008** | **1** | **K.YIYDQCPAVAGYGPIEQLPDYNRIR.S** |
|  | 26751 | **1044.1867** | **3129.5381** | **3129.5520** | **-0.0138** | **0** | **11** | **3.6** | **1** | **R.DVVFNYLHATAFQGTPLAQAVEGPSENVR.K** |
|  | 30285 | **1441.3414** | **4321.0025** | **4321.0168** | **-0.0143** | **1** | **48** | **0.00033** | **1** | **R.EMQENDASMRDVVFNYLHATAFQGTPLAQAVEGPSENVR.K** 30283 |
|  | 30472 | **1107.5669** | **4426.2385** | **4426.2566** | **-0.0181** | **1** | **12** | **1.3** | **1** | **R.MVLAAAGGVEHQQLLDLAQKHLGGIPWTYAEDAVPTLTPCR.F** 30474 |
|  | 32165 | **1227.8129** | **6134.0280** | **6133.0293** | **0.9986** | **1** | **26** | **0.015** | **1** | **R.HRDDALPFAHVAIAVEGPGWASPDNVALQVANAIIGHYDCTYGGGVHLSSPLASGAVANK.L** |

| **24.** | Q71U36\|TBA1A_HUMAN    **Mass:** 50788    **Score:** 874    **Queries matched:** 52   **emPAI:** 3.43 |
| --- | --- |
|  | Tubulin alpha-1A chain OS=Homo sapiens GN=TUBA1A PE=1 SV=1 |

|  | **Query** | **Observed** | **Mr(expt)** | **Mr(calc)** | **Delta** | **Miss** | **Score** | **Expect** | **Rank** | **Peptide** |
| --- | --- | --- | --- | --- | --- | --- | --- | --- | --- | --- |
|  | 2728 | 444.2173 | 886.4200 | 886.4259 | -0.0058 | 0 | 41 | 0.017 | 1 | K.FDLMYAK.R |
|  | 3028 | 455.7546 | 909.4947 | 908.4967 | 0.9979 | 1 | 22 | 1 | 1 | R.LSVDYGKK.S |
|  | 5333 | **543.3108** | **1084.6071** | **1084.6128** | **-0.0057** | **0** | **13** | **7.8** | **1** | **K.EIIDLVLDR.I** |
|  | 7994 | 625.2763 | 1248.5379 | 1248.5453 | -0.0074 | 0 | 36 | 0.035 | 1 | K.YMACCLLYR.G |
|  | 10016 | **452.2676** | **1353.7808** | **1353.7980** | **-0.0172** | **1** | **6** | **34** | **3** | **K.EIIDLVLDRIR.K** |
|  | 10474 | 690.8484 | 1379.6822 | 1379.6907 | -0.0085 | 1 | 16 | 3.6 | 1 | R.LDHKFDLMYAK.R |
|  | 11755 | **729.4343** | **1456.8540** | **1456.8613** | **-0.0073** | **0** | **48** | **0.0021** | **1** | **R.LIGQIVSSITASLR.F** 11759 11769 |
|  | 14038 | **799.8832** | **1597.7518** | **1597.7599** | **-0.0081** | **0** | **58** | **0.00018** | **1** | **R.TIQFVDWCPTGFK.V** |
|  | 14224 | **805.9542** | **1609.8938** | **1609.9039** | **-0.0101** | **1** | **26** | **0.27** | **1** | **R.GDVVPKDVNAAIATIK.T** 14225 |
|  | 15621 | **851.4518** | **1700.8890** | **1700.8985** | **-0.0095** | **0** | **49** | **0.0014** | **1** | **R.AVFVDLEPTVIDEVR.T** 15623 15632 |
|  | 15854 | 859.9388 | 1717.8631 | 1717.8747 | -0.0116 | 0 | 51 | 0.00085 | 1 | R.NLDIERPTYTNLNR.L 15855 |
|  | 16352 | 878.9811 | 1755.9476 | 1755.9559 | -0.0083 | 0 | 41 | 0.009 | 1 | R.IHFPLATYAPVISAEK.A 16351 16353 |
|  | 17194 | 912.9911 | 1823.9676 | 1823.9782 | -0.0105 | 0 | 46 | 0.0028 | 1 | K.VGINYQPPTVVPGGDLAK.V 17192 17193 17195 17209 |
|  | 17476 | 615.6299 | 1843.8678 | 1843.8783 | -0.0105 | 1 | 19 | 1.2 | 1 | K.YMACCLLYRGDVVPK.D 17477 |
|  | 17690 | 620.7950 | 1859.3631 | 1859.8732 | -0.5101 | 1 | (3) | 39 | 5 | K.YMACCLLYRGDVVPK.D |
|  | 17729 | 932.9498 | 1863.8851 | 1863.8971 | -0.0120 | 0 | 85 | 3.7e-007 | 1 | R.AVCMLSNTTAIAEAWAR.L 17730 |
|  | 19368 | **1004.4435** | **2006.8725** | **2006.8858** | **-0.0133** | **0** | **85** | **3e-007** | **1** | **K.TIGGGDDSFNTFFSETGAGK.H** 19369 19370 19371 |
|  | 22254 | 777.3408 | 2329.0006 | 2329.0110 | -0.0103 | 0 | 39 | 0.0091 | 1 | R.AFVHWYVGEGMEEGEFSEAR.E |
|  | 22800 | 1205.1028 | 2408.1910 | 2408.2012 | -0.0102 | 0 | 123 | 3.6e-011 | 1 | R.FDGALNVDLTEFQTNLVPYPR.I 22799 22801 |
|  | 22841 | 1208.0984 | 2414.1822 | 2414.1978 | -0.0156 | 1 | 46 | 0.0019 | 1 | R.QLFHPEQLITGKEDAANNYAR.G 22842 22844 |
|  | 23398 | **625.0456** | **2496.1533** | **2496.1670** | **-0.0138** | **1** | **6** | **15** | **7** | **K.TIGGGDDSFNTFFSETGAGKHVPR.A** 23401 |
|  | 24859 | 1375.6443 | 2749.2740 | 2749.2840 | -0.0099 | 0 | 74 | 2.5e-006 | 1 | K.AYHEQLSVAEITNACFEPANQMVK.C 24857 24862 |
|  | 27136 | 805.1072 | 3216.3996 | 3216.4168 | -0.0172 | 1 | 0 | 42 | 1 | R.AFVHWYVGEGMEEGEFSEAREDMAALEK.D |
|  | 27872 | **1135.5789** | **3403.7148** | **3403.7275** | **-0.0128** | **1** | **42** | **0.0029** | **1** | **R.TIQFVDWCPTGFKVGINYQPPTVVPGGDLAK.V** |
|  | 30249 | **1075.2472** | **4296.9597** | **4295.9497** | **1.0100** | **0** | **5** | **6.9** | **1** | **R.ECISIHVGQAGVQIGNACWELYCLEHGIQPDGQMPSDK.T** |
|  | 31994 | 1419.1687 | 5672.6457 | 5672.6902 | -0.0445 | 1 | 7 | 1.9 | 1 | K.LEFSIYPAPQVSTAVVEPYNSILTTHTTLEHSDCAFMVDNEAIYDICRR.N 31995 31997 |

|  | **Proteins matching a subset of these peptides:** |
| --- | --- |

|  | Q13748\|TBA3C_HUMAN    **Mass:** 50612    **Score:** 805    **Queries matched:** 39 |
| --- | --- |
|  | Tubulin alpha-3C/D chain OS=Homo sapiens GN=TUBA3C PE=1 SV=3 |

|  | Q6PEY2\|TBA3E_HUMAN    **Mass:** 50568    **Score:** 747    **Queries matched:** 33 |
| --- | --- |
|  | Tubulin alpha-3E chain OS=Homo sapiens GN=TUBA3E PE=1 SV=2 |

| **25.** | P40939\|ECHA_HUMAN    **Mass:** 83688    **Score:** 872    **Queries matched:** 71   **emPAI:** 2.67 |
| --- | --- |
|  | Trifunctional enzyme subunit alpha, mitochondrial OS=Homo sapiens GN=HADHA PE=1 SV=2 |

|  | **Query** | **Observed** | **Mr(expt)** | **Mr(calc)** | **Delta** | **Miss** | **Score** | **Expect** | **Rank** | **Peptide** |
| --- | --- | --- | --- | --- | --- | --- | --- | --- | --- | --- |
|  | 1192 | **715.4053** | **714.3981** | **714.4025** | **-0.0044** | **0** | **27** | **0.37** | **1** | **K.GDVAVVR.I** |
|  | 1772 | **823.4262** | **822.4189** | **822.4236** | **-0.0046** | **0** | **15** | **4.6** | **1** | **K.ALTSFER.D** |
|  | 2427 | **868.4459** | **867.4386** | **867.4450** | **-0.0064** | **0** | **25** | **0.5** | **1** | **K.HVAEDLGK.V** 2428 |
|  | 3302 | **465.2361** | **928.4577** | **928.4654** | **-0.0077** | **1** | **42** | **0.013** | **1** | **K.KYEAAYGK.Q** |
|  | 3334 | **466.2505** | **930.4864** | **930.4923** | **-0.0059** | **0** | **12** | **11** | **1** | **R.THINYGVK.G** 3329 |
|  | 3439 | **471.0711** | **940.1277** | **939.4736** | **0.6541** | **0** | **3** | **49** | **4** | **K.FGELVMTK.E** |
|  | 4227 | **499.7759** | **997.5373** | **997.5444** | **-0.0071** | **0** | **42** | **0.0097** | **1** | **R.ILQEGVDPK.K** |
|  | 4425 | **507.2347** | **1012.4549** | **1012.4614** | **-0.0066** | **0** | **27** | **0.32** | **1** | **K.DGPGFYTTR.C** |
|  | 4772 | **520.7711** | **1039.5277** | **1039.5338** | **-0.0062** | **0** | **36** | **0.04** | **1** | **R.FVDLYGAQK.I** |
|  | 5806 | **559.7940** | **1117.5735** | **1117.5979** | **-0.0244** | **0** | **7** | **32** | **8** | **K.DTSASAVAVGLK.Q** |
|  | 6396 | **583.9808** | **1165.9470** | **1165.6091** | **0.3379** | **0** | **5** | **27** | **3** | **R.NFTGSSALLTR.T** |
|  | 7299 | **608.7369** | **1215.4593** | **1214.6917** | **0.7676** | **1** | **8** | **22** | **3** | **M.VACRAIGILSR.F** |
|  | 9368 | **659.8655** | **1317.7164** | **1317.7214** | **-0.0050** | **0** | **59** | **0.00019** | **1** | **K.MQLLEIITTEK.T** |
|  | 9865 | **449.7144** | **1346.1213** | **1345.7322** | **0.3891** | **1** | **6** | **23** | **9** | **-.MVACRAIGILSR.F** |
|  | 10669 | **696.8481** | **1391.6817** | **1391.6908** | **-0.0090** | **0** | **31** | **0.11** | **1** | **K.VIGMHYFSPVDK.M** |
|  | 10784 | **467.0989** | **1398.2749** | **1397.7377** | **0.5372** | **0** | **7** | **18** | **5** | **K.LTAYAMTIPFVR.Q** |
|  | 10926 | **470.2355** | **1407.6848** | **1407.6857** | **-0.0009** | **0** | **(10)** | **12** | **4** | **K.VIGMHYFSPVDK.M** |
|  | 10984 | **706.3164** | **1410.6181** | **1410.6263** | **-0.0081** | **0** | **22** | **0.71** | **1** | **K.SEVSSDEDIQFR.L** |
|  | 11333 | **716.3886** | **1430.7627** | **1430.7729** | **-0.0102** | **1** | **44** | **0.0053** | **1** | **K.DTSASAVAVGLKQGK.V** |
|  | 11407 | **479.2629** | **1434.7669** | **1433.7726** | **0.9943** | **1** | **21** | **1.1** | **1** | **K.TSKDTSASAVAVGLK.Q** |
|  | 11726 | **486.2537** | **1455.7392** | **1455.7470** | **-0.0078** | **1** | **28** | **0.23** | **1** | **K.HVAEDLGKVFGER.F** |
|  | 12049 | **737.8795** | **1473.7445** | **1474.7456** | **-1.0011** | **1** | **12** | **7.6** | **2** | **K.SGKGFYIYQEGVK.R** 12057 |
|  | 12308 | **745.3835** | **1488.7524** | **1488.7581** | **-0.0057** | **0** | **43** | **0.0063** | **1** | **K.ALMGLYHGQVLCK.K** |
|  | 12829 | **761.3733** | **1520.7321** | **1520.7392** | **-0.0070** | **0** | **35** | **0.044** | **1** | **K.DLNSDMDSILASLK.L** 12821 |
|  | 13449 | **781.3949** | **1560.7752** | **1560.7826** | **-0.0074** | **0** | **91** | **9.4e-008** | **1** | **K.MVGVPAALDMMLTGR.S** |
|  | 13698 | **789.3985** | **1576.7824** | **1576.7919** | **-0.0095** | **0** | **68** | **2e-005** | **1** | **R.FGGGNPELLTQMVSK.G** 13699 |
|  | 14332 | **539.9548** | **1616.8425** | **1616.8531** | **-0.0106** | **1** | **4** | **53** | **3** | **K.ALMGLYHGQVLCKK.N** |
|  | 14486 | **813.9318** | **1625.8491** | **1625.8552** | **-0.0061** | **0** | **70** | **1.1e-005** | **1** | **R.TIEYLEEVAITFAK.G** 14485 |
|  | 14516 | **543.6221** | **1627.8446** | **1626.8842** | **0.9604** | **1** | **24** | **0.45** | **1** | **R.THINYGVKGDVAVVR.I** |
|  | 14549 | **815.9181** | **1629.8217** | **1629.8322** | **-0.0105** | **0** | **59** | **0.00016** | **1** | **K.TLQEVTQLSQEAQR.I** 14550 14553 14562 |
|  | 15078 | **555.6475** | **1663.9207** | **1663.9298** | **-0.0090** | **1** | **21** | **0.96** | **1** | **K.VIIVVKDGPGFYTTR.C** |
|  | 15319 | **560.6210** | **1678.8413** | **1678.8488** | **-0.0075** | **0** | **29** | **0.15** | **1** | **K.ADMVIEAVFEDLSLK.H** |
|  | 16774 | **895.9686** | **1789.9226** | **1789.9323** | **-0.0097** | **1** | **44** | **0.0047** | **1** | **K.DATLTALDRGQQQVFK.G** |
|  | 17430 | **921.9076** | **1841.8007** | **1841.8102** | **-0.0094** | **0** | **86** | **2.7e-007** | **1** | **K.TGIEQGSDAGYLCESQK.F** 17428 17429 |
|  | 18474 | 642.6770 | 1925.0092 | 1924.0492 | 0.9600 | 1 | 1 | 67 | 10 | K.GLVEKLTAYAMTIPFVR.Q |
|  | 18638 | **970.9283** | **1939.8421** | **1939.9210** | **-0.0789** | **0** | **3** | **45** | **3** | **K.QFTPCQLLADHANSPNK.K** |
|  | 18738 | **487.9948** | **1947.9499** | **1946.9992** | **0.9507** | **1** | **1** | **87** | **7** | **R.LPKMVGVPAALDMMLTGR.S** |
|  | 19516 | **1011.5072** | **2020.9998** | **2020.1139** | **0.8859** | **0** | **3** | **50** | **2** | **K.HLAILGAGLMGAGIAQVSVDK.G** 19508 |
|  | 19940 | **1031.4844** | **2060.9542** | **2060.9691** | **-0.0149** | **0** | **82** | **6.5e-007** | **1** | **R.DSIFSNLTGQLDYQGFEK.A** |
|  | 20369 | **703.0374** | **2106.0904** | **2107.0885** | **-0.9981** | **1** | **(2)** | **58** | **2** | **R.FGGGNPELLTQMVSKGFLGR.K** |
|  | 20518 | **708.3751** | **2122.1035** | **2123.0834** | **-0.9798** | **1** | **6** | **22** | **4** | **R.FGGGNPELLTQMVSKGFLGR.K** |
|  | 21044 | **1091.0375** | **2180.0604** | **2181.0889** | **-1.0284** | **1** | **2** | **59** | **7** | **K.VFGERFGGGNPELLTQMVSK.G** |
|  | 21855 | **758.7391** | **2273.1954** | **2273.2089** | **-0.0136** | **0** | **55** | **0.00022** | **1** | **K.MGLVDQLVEPLGPGLKPPEER.T** 21856 |
|  | 22176 | **773.6805** | **2318.0196** | **2317.1480** | **0.8716** | **1** | **1** | **54** | **8** | **R.CLAPMMSEVIRILQEGVDPK.K** |
|  | 22523 | **788.7499** | **2363.2278** | **2362.3042** | **0.9236** | **1** | **0** | **64** | **6** | **K.DVKHLAILGAGLMGAGIAQVSVDK.G** |
|  | 22759 | **601.3301** | **2401.2915** | **2401.3039** | **-0.0124** | **1** | **18** | **1.1** | **1** | **K.KMGLVDQLVEPLGPGLKPPEER.T** |
|  | 22886 | **1210.5586** | **2419.1026** | **2419.1114** | **-0.0088** | **0** | **87** | **1.4e-007** | **1** | **K.ELHSEFSEVMNEIWASDQIR.S** 22884 22885 |
|  | 23999 | **1297.6520** | **2593.2894** | **2593.3066** | **-0.0172** | **0** | **77** | **1.4e-006** | **1** | **R.SAVLISSKPGCFIAGADINMLAACK.T** 24001 24003 |
|  | 24853 | **916.7613** | **2747.2622** | **2747.2782** | **-0.0160** | **1** | **38** | **0.0091** | **1** | **K.TGIEQGSDAGYLCESQKFGELVMTK.E** 24852 |
|  | 25316 | **1418.7472** | **2835.4798** | **2835.4906** | **-0.0108** | **1** | **51** | **0.00046** | **1** | **K.KLDSLTTSFGFPVGAATLVDEVGVDVAK.H** |
|  | 27211 | **809.1415** | **3232.5371** | **3233.6900** | **-1.1530** | **1** | **1** | **38** | **4** | **K.STKPIVAAINGSCLGGGLEVAISCQYRIATK.D** |
|  | 29919 | **1369.6704** | **4105.9894** | **4106.0104** | **-0.0210** | **0** | **20** | **0.28** | **1** | **R.FVNEAVMCLQEGILATPAEGDIGAVFGLGFPPCLGGPFR.F** |
|  | 31364 | **1029.7428** | **5143.6776** | **5143.5286** | **0.1490** | **1** | **1** | **10** | **5** | **R.FVNEAVMCLQEGILATPAEGDIGAVFGLGFPPCLGGPFRFVDLYGAQK.I** |

| **26.** | Q14764\|MVP_HUMAN    **Mass:** 99551    **Score:** 835    **Queries matched:** 56   **emPAI:** 0.86 |
| --- | --- |
|  | Major vault protein OS=Homo sapiens GN=MVP PE=1 SV=4 |

|  | **Query** | **Observed** | **Mr(expt)** | **Mr(calc)** | **Delta** | **Miss** | **Score** | **Expect** | **Rank** | **Peptide** |
| --- | --- | --- | --- | --- | --- | --- | --- | --- | --- | --- |
|  | 1012 | **628.3618** | **627.3545** | **627.3592** | **-0.0047** | **0** | **37** | **0.027** | **1** | **R.VEVGPK.T** |
|  | 1474 | **400.7633** | **799.5120** | **798.4422** | **1.0698** | **0** | **6** | **45** | **1** | **R.MVTVPPR.H** |
|  | 2221 | **427.2272** | **852.4399** | **852.4453** | **-0.0054** | **0** | **41** | **0.013** | **1** | **R.HADLEIR.L** |
|  | 2293 | **430.2271** | **858.4397** | **858.4447** | **-0.0050** | **0** | **8** | **34** | **2** | **K.VEVVEER.Q** |
|  | 2629 | **441.2615** | **880.5084** | **880.5130** | **-0.0046** | **0** | **27** | **0.32** | **1** | **K.SLQPLAPR.N** 2628 2663 |
|  | 4147 | **496.7715** | **991.5285** | **991.5338** | **-0.0053** | **0** | **22** | **1** | **1** | **R.ELELVYAR.A** |
|  | 4462 | **508.5110** | **1015.0074** | **1015.5549** | **-0.5475** | **0** | **3** | **65** | **3** | **R.AQLELEVSK.A** |
|  | 6844 | **597.7919** | **1193.5692** | **1193.5789** | **-0.0097** | **1** | **21** | **1.3** | **1** | **K.TYIRQDNER.V** 6860 |
|  | 8766 | **644.8854** | **1287.7563** | **1287.6670** | **0.0893** | **1** | **6** | **35** | **4** | **R.QKILDQSEAEK.A** |
|  | 8904 | **648.3113** | **1294.6080** | **1295.7197** | **-1.1117** | **1** | **0** | **1.3e+002** | **2** | **K.DTAKSLQPLAPR.N** |
|  | 9067 | **652.3098** | **1302.6049** | **1302.6139** | **-0.0090** | **0** | **22** | **0.88** | **1** | **R.HYCTVANPVSR.D** |
|  | 9364 | **659.8362** | **1317.6579** | **1318.6551** | **-0.9972** | **0** | **2** | **87** | **2** | **K.QMTEAIGPSTIR.D** |
|  | 9701 | **669.3650** | **1336.7154** | **1336.7238** | **-0.0084** | **0** | **10** | **15** | **1** | **K.ELPPGVEELLNK.G** |
|  | 10211 | **456.2293** | **1365.6661** | **1365.6749** | **-0.0088** | **1** | **14** | **4.8** | **1** | **K.HEAQRLEQEAR.G** |
|  | 11694 | **727.8490** | **1453.6834** | **1453.6912** | **-0.0077** | **0** | **18** | **2** | **1** | **K.LFSVPDFVGDACK.A** |
|  | 12263 | **743.9048** | **1485.7950** | **1485.6834** | **0.1116** | **0** | **3** | **60** | **4** | **R.ALQPLEEGEDEEK.V** |
|  | 13139 | **771.9053** | **1541.7960** | **1541.8049** | **-0.0089** | **0** | **86** | **2.8e-007** | **1** | **K.AQALAIETEAELQR.V** |
|  | 13997 | **399.7197** | **1594.8498** | **1593.8185** | **1.0314** | **1** | **4** | **44** | **7** | **K.FKQMTEAIGPSTIR.D** |
|  | 14517 | **814.9298** | **1627.8449** | **1627.8529** | **-0.0080** | **1** | **16** | **3** | **1** | **R.AEAARIEGEGSVLQAK.L** 14534 |
|  | 16131 | **870.4697** | **1738.9248** | **1739.0193** | **-0.0945** | **1** | **5** | **32** | **2** | **R.KEVEVVEIIQATIIR.Q** |
|  | 16698 | **892.4942** | **1782.9738** | **1782.9839** | **-0.0101** | **1** | **68** | **1.8e-005** | **1** | **K.LKAQALAIETEAELQR.V** 16699 |
|  | 18525 | **965.9857** | **1929.9569** | **1929.9684** | **-0.0115** | **0** | **88** | **1.7e-007** | **1** | **R.QAIPLDENEGIYVQDVK.T** 18526 18530 |
|  | 19364 | **669.6806** | **2006.0200** | **2007.0081** | **-0.9882** | **0** | **1** | **76** | **2** | **K.ELLELEALSMAVESTGTAK.A** |
|  | 20616 | **712.6989** | **2135.0749** | **2135.0912** | **-0.0163** | **1** | **43** | **0.004** | **1** | **R.VVSYRVPHNAAVQVYDYR.E** 20618 |
|  | 20755 | **718.0314** | **2151.0725** | **2151.0861** | **-0.0137** | **0** | **36** | **0.021** | **1** | **R.IPPYHYIHVLDQNSNVSR.V** |
|  | 22973 | **609.3071** | **2433.1992** | **2433.2111** | **-0.0119** | **1** | **5** | **21** | **2** | **R.TAVFGFETSEAKGPDGMALPRPR.D** |
|  | 23388 | **832.4276** | **2494.2608** | **2494.2775** | **-0.0167** | **1** | **6** | **18** | **1** | **R.SVQLAIEITTNSQEAAAKHEAQR.L** |
|  | 23674 | **848.4348** | **2542.2824** | **2541.2931** | **0.9893** | **1** | **6** | **17** | **1** | **K.QMTEAIGPSTIRDLAVAGPEMQVK.L** |
|  | 24912 | **553.0905** | **2760.4159** | **2760.4347** | **-0.0189** | **1** | **6** | **16** | **1** | **R.IPPYHYIHVLDQNSNVSRVEVGPK.T** 24913 |
|  | 25200 | **938.1381** | **2811.3924** | **2811.4039** | **-0.0116** | **0** | **59** | **7.2e-005** | **1** | **R.DQAVFPQNGLVVSSVDVQSVEPVDQR.T** |
|  | 25552 | **962.7980** | **2885.3723** | **2885.3944** | **-0.0221** | **1** | **9** | **7.6** | **1** | **R.ALQPLEEGEDEEKVSHQAGDHWLIR.G** 25551 |
|  | 26694 | **1040.5269** | **3118.5588** | **3118.5796** | **-0.0208** | **0** | **72** | **2.9e-006** | **1** | **R.VASGPSPGEGISPQSAQAPQAPGDNHVVPVLR.-** 26693 |
|  | 27375 | **1637.8373** | **3273.6600** | **3273.6704** | **-0.0104** | **0** | **89** | **5.5e-008** | **1** | **K.STLITDGSTPINLFNTAFGLLGMGPEGQPLGR.R** 27372 27374 |
|  | 27382 | **819.9167** | **3275.6377** | **3274.6807** | **0.9570** | **1** | **19** | **0.55** | **1** | **R.RVASGPSPGEGISPQSAQAPQAPGDNHVVPVLR.-** |
|  | 27593 | **1667.8639** | **3333.7132** | **3333.7245** | **-0.0113** | **0** | **106** | **1e-009** | **1** | **K.SFFLQPGEQLEQGIQDVYVLSEQQGLLLR.A** 27585 27590 27591 |
|  | 27980 | **1144.2595** | **3429.7567** | **3429.7715** | **-0.0148** | **1** | **71** | **3e-006** | **1** | **K.STLITDGSTPINLFNTAFGLLGMGPEGQPLGRR.V** |
|  | 28803 | **918.0102** | **3668.0117** | **3668.0229** | **-0.0112** | **1** | **13** | **1.5** | **1** | **R.LAQDPFPLYPGEVLEKDITPLQVVLPNTALHLK.A** 28801 28802 |
|  | 28949 | **1859.9784** | **3717.9422** | **3717.9645** | **-0.0222** | **0** | **1** | **25** | **7** | **R.VTGEEWLVTTVGAYLPAVFEEVLDLVDAVILTEK.T** |

| **27.** | P68363\|TBA1B_HUMAN    **Mass:** 50804    **Score:** 816    **Queries matched:** 52   **emPAI:** 3.12 |
| --- | --- |
|  | Tubulin alpha-1B chain OS=Homo sapiens GN=TUBA1B PE=1 SV=1 |

|  | **Query** | **Observed** | **Mr(expt)** | **Mr(calc)** | **Delta** | **Miss** | **Score** | **Expect** | **Rank** | **Peptide** |
| --- | --- | --- | --- | --- | --- | --- | --- | --- | --- | --- |
|  | 2728 | 444.2173 | 886.4200 | 886.4259 | -0.0058 | 0 | 41 | 0.017 | 1 | K.FDLMYAK.R |
|  | 3028 | 455.7546 | 909.4947 | 908.4967 | 0.9979 | 1 | 22 | 1 | 1 | R.LSVDYGKK.S |
|  | 5333 | 543.3108 | 1084.6071 | 1084.6128 | -0.0057 | 0 | 13 | 7.8 | 1 | K.EIIDLVLDR.I |
|  | 7994 | 625.2763 | 1248.5379 | 1248.5453 | -0.0074 | 0 | 36 | 0.035 | 1 | K.YMACCLLYR.G |
|  | 10016 | 452.2676 | 1353.7808 | 1353.7980 | -0.0172 | 1 | 6 | 34 | 3 | K.EIIDLVLDRIR.K |
|  | 10474 | 690.8484 | 1379.6822 | 1379.6907 | -0.0085 | 1 | 16 | 3.6 | 1 | R.LDHKFDLMYAK.R |
|  | 13817 | 792.8756 | 1583.7367 | 1583.7443 | -0.0076 | 0 | 48 | 0.0018 | 1 | R.SIQFVDWCPTGFK.V 13815 |
|  | 14224 | 805.9542 | 1609.8938 | 1609.9039 | -0.0101 | 1 | 26 | 0.27 | 1 | R.GDVVPKDVNAAIATIK.T 14225 |
|  | 15621 | 851.4518 | 1700.8890 | 1700.8985 | -0.0095 | 0 | 49 | 0.0014 | 1 | R.AVFVDLEPTVIDEVR.T 15623 15632 |
|  | 15854 | 859.9388 | 1717.8631 | 1717.8747 | -0.0116 | 0 | 51 | 0.00085 | 1 | R.NLDIERPTYTNLNR.L 15855 |
|  | 16144 | 580.9411 | 1739.8015 | 1739.8454 | -0.0439 | 1 | 2 | 72 | 3 | K.RSIQFVDWCPTGFK.V |
|  | 16352 | 878.9811 | 1755.9476 | 1755.9559 | -0.0083 | 0 | 41 | 0.009 | 1 | R.IHFPLATYAPVISAEK.A 16351 16353 |
|  | 17194 | 912.9911 | 1823.9676 | 1823.9782 | -0.0105 | 0 | 46 | 0.0028 | 1 | K.VGINYQPPTVVPGGDLAK.V 17192 17193 17195 17209 |
|  | 17476 | 615.6299 | 1843.8678 | 1843.8783 | -0.0105 | 1 | 19 | 1.2 | 1 | K.YMACCLLYRGDVVPK.D 17477 |
|  | 17690 | 620.7950 | 1859.3631 | 1859.8732 | -0.5101 | 1 | (3) | 39 | 5 | K.YMACCLLYRGDVVPK.D |
|  | 17729 | 932.9498 | 1863.8851 | 1863.8971 | -0.0120 | 0 | 85 | 3.7e-007 | 1 | R.AVCMLSNTTAIAEAWAR.L 17730 |
|  | 19368 | 1004.4435 | 2006.8725 | 2006.8858 | -0.0133 | 0 | 85 | 3e-007 | 1 | K.TIGGGDDSFNTFFSETGAGK.H 19369 19370 19371 |
|  | 22254 | 777.3408 | 2329.0006 | 2329.0110 | -0.0103 | 0 | 39 | 0.0091 | 1 | R.AFVHWYVGEGMEEGEFSEAR.E |
|  | 22800 | 1205.1028 | 2408.1910 | 2408.2012 | -0.0102 | 0 | 123 | 3.6e-011 | 1 | R.FDGALNVDLTEFQTNLVPYPR.I 22799 22801 |
|  | 22841 | 1208.0984 | 2414.1822 | 2414.1978 | -0.0156 | 1 | 46 | 0.0019 | 1 | R.QLFHPEQLITGKEDAANNYAR.G 22842 22844 |
|  | 23398 | 625.0456 | 2496.1533 | 2496.1670 | -0.0138 | 1 | 6 | 15 | 7 | K.TIGGGDDSFNTFFSETGAGKHVPR.A 23401 |
|  | 24859 | 1375.6443 | 2749.2740 | 2749.2840 | -0.0099 | 0 | 74 | 2.5e-006 | 1 | K.AYHEQLSVAEITNACFEPANQMVK.C 24857 24862 |
|  | 27012 | 797.6893 | 3186.7282 | 3186.7360 | -0.0078 | 1 | 5 | 15 | 2 | R.NLDIERPTYTNLNRLISQIVSSITASLR.F |
|  | 27136 | 805.1072 | 3216.3996 | 3216.4168 | -0.0172 | 1 | 0 | 42 | 1 | R.AFVHWYVGEGMEEGEFSEAREDMAALEK.D |
|  | 27811 | 1130.9064 | 3389.6973 | 3389.7119 | -0.0146 | 1 | 25 | 0.12 | 1 | R.SIQFVDWCPTGFKVGINYQPPTVVPGGDLAK.V |
|  | 30249 | 1075.2472 | 4296.9597 | 4295.9497 | 1.0100 | 0 | 5 | 6.9 | 1 | R.ECISIHVGQAGVQIGNACWELYCLEHGIQPDGQMPSDK.T |
|  | 31994 | 1419.1687 | 5672.6457 | 5672.6902 | -0.0445 | 1 | 7 | 1.9 | 1 | K.LEFSIYPAPQVSTAVVEPYNSILTTHTTLEHSDCAFMVDNEAIYDICRR.N 31995 31997 |

| **28.** | Q9BQE3\|TBA1C_HUMAN    **Mass:** 50548    **Score:** 777    **Queries matched:** 50   **emPAI:** 2.88 |
| --- | --- |
|  | Tubulin alpha-1C chain OS=Homo sapiens GN=TUBA1C PE=1 SV=1 |

|  | **Query** | **Observed** | **Mr(expt)** | **Mr(calc)** | **Delta** | **Miss** | **Score** | **Expect** | **Rank** | **Peptide** |
| --- | --- | --- | --- | --- | --- | --- | --- | --- | --- | --- |
|  | 2728 | 444.2173 | 886.4200 | 886.4259 | -0.0058 | 0 | 41 | 0.017 | 1 | K.FDLMYAK.R |
|  | 3028 | 455.7546 | 909.4947 | 908.4967 | 0.9979 | 1 | 22 | 1 | 1 | R.LSVDYGKK.S |
|  | 5333 | 543.3108 | 1084.6071 | 1084.6128 | -0.0057 | 0 | 13 | 7.8 | 1 | K.EIIDLVLDR.I |
|  | 7994 | 625.2763 | 1248.5379 | 1248.5453 | -0.0074 | 0 | 36 | 0.035 | 1 | K.YMACCLLYR.G |
|  | 10016 | 452.2676 | 1353.7808 | 1353.7980 | -0.0172 | 1 | 6 | 34 | 3 | K.EIIDLVLDRIR.K |
|  | 10474 | 690.8484 | 1379.6822 | 1379.6907 | -0.0085 | 1 | 16 | 3.6 | 1 | R.LDHKFDLMYAK.R |
|  | 14038 | 799.8832 | 1597.7518 | 1597.7599 | -0.0081 | 0 | 58 | 0.00018 | 1 | R.TIQFVDWCPTGFK.V |
|  | 14224 | 805.9542 | 1609.8938 | 1609.9039 | -0.0101 | 1 | 26 | 0.27 | 1 | R.GDVVPKDVNAAIATIK.T 14225 |
|  | 15621 | 851.4518 | 1700.8890 | 1700.8985 | -0.0095 | 0 | 49 | 0.0014 | 1 | R.AVFVDLEPTVIDEVR.T 15623 15632 |
|  | 15854 | 859.9388 | 1717.8631 | 1717.8747 | -0.0116 | 0 | 51 | 0.00085 | 1 | R.NLDIERPTYTNLNR.L 15855 |
|  | 16352 | 878.9811 | 1755.9476 | 1755.9559 | -0.0083 | 0 | 41 | 0.009 | 1 | R.IHFPLATYAPVISAEK.A 16351 16353 |
|  | 17194 | 912.9911 | 1823.9676 | 1823.9782 | -0.0105 | 0 | 46 | 0.0028 | 1 | K.VGINYQPPTVVPGGDLAK.V 17192 17193 17195 17209 |
|  | 17476 | 615.6299 | 1843.8678 | 1843.8783 | -0.0105 | 1 | 19 | 1.2 | 1 | K.YMACCLLYRGDVVPK.D 17477 |
|  | 17568 | **925.9430** | **1849.8715** | **1849.8815** | **-0.0099** | **0** | **85** | **3.6e-007** | **1** | **R.AVCMLSNTTAVAEAWAR.L** 17569 |
|  | 17690 | 620.7950 | 1859.3631 | 1859.8732 | -0.5101 | 1 | (3) | 39 | 5 | K.YMACCLLYRGDVVPK.D |
|  | 19368 | 1004.4435 | 2006.8725 | 2006.8858 | -0.0133 | 0 | 85 | 3e-007 | 1 | K.TIGGGDDSFNTFFSETGAGK.H 19369 19370 19371 |
|  | 22254 | 777.3408 | 2329.0006 | 2329.0110 | -0.0103 | 0 | 39 | 0.0091 | 1 | R.AFVHWYVGEGMEEGEFSEAR.E |
|  | 22800 | 1205.1028 | 2408.1910 | 2408.2012 | -0.0102 | 0 | 123 | 3.6e-011 | 1 | R.FDGALNVDLTEFQTNLVPYPR.I 22799 22801 |
|  | 22841 | 1208.0984 | 2414.1822 | 2414.1978 | -0.0156 | 1 | 46 | 0.0019 | 1 | R.QLFHPEQLITGKEDAANNYAR.G 22842 22844 |
|  | 23398 | 625.0456 | 2496.1533 | 2496.1670 | -0.0138 | 1 | 6 | 15 | 7 | K.TIGGGDDSFNTFFSETGAGKHVPR.A 23401 |
|  | 24925 | **922.1032** | **2763.2876** | **2763.2996** | **-0.0120** | **0** | **33** | **0.031** | **1** | **K.AYHEQLTVAEITNACFEPANQMVK.C** |
|  | 27012 | 797.6893 | 3186.7282 | 3186.7360 | -0.0078 | 1 | 5 | 15 | 2 | R.NLDIERPTYTNLNRLISQIVSSITASLR.F |
|  | 27136 | 805.1072 | 3216.3996 | 3216.4168 | -0.0172 | 1 | 0 | 42 | 1 | R.AFVHWYVGEGMEEGEFSEAREDMAALEK.D |
|  | 27468 | **1103.5216** | **3307.5430** | **3307.5060** | **0.0370** | **1** | **2** | **30** | **7** | **K.AYHEQLTVAEITNACFEPANQMVKCDPR.H** |
|  | 27872 | 1135.5789 | 3403.7148 | 3403.7275 | -0.0128 | 1 | 42 | 0.0029 | 1 | R.TIQFVDWCPTGFKVGINYQPPTVVPGGDLAK.V |
|  | 30249 | 1075.2472 | 4296.9597 | 4295.9497 | 1.0100 | 0 | 5 | 6.9 | 1 | R.ECISIHVGQAGVQIGNACWELYCLEHGIQPDGQMPSDK.T |
|  | 30604 | **1130.3251** | **4517.2712** | **4517.2399** | **0.0313** | **1** | **1** | **15** | **1** | **R.IHFPLATYAPVISAEKAYHEQLTVAEITNACFEPANQMVK.C** |
|  | 31994 | 1419.1687 | 5672.6457 | 5672.6902 | -0.0445 | 1 | 7 | 1.9 | 1 | K.LEFSIYPAPQVSTAVVEPYNSILTTHTTLEHSDCAFMVDNEAIYDICRR.N 31995 31997 |

| **29.** | P08473\|NEP_HUMAN    **Mass:** 86144    **Score:** 759    **Queries matched:** 62   **emPAI:** 1.32 |
| --- | --- |
|  | Neprilysin OS=Homo sapiens GN=MME PE=1 SV=2 |

|  | **Query** | **Observed** | **Mr(expt)** | **Mr(calc)** | **Delta** | **Miss** | **Score** | **Expect** | **Rank** | **Peptide** |
| --- | --- | --- | --- | --- | --- | --- | --- | --- | --- | --- |
|  | 1473 | **400.7506** | **799.4866** | **799.4915** | **-0.0049** | **1** | **44** | **0.0076** | **1** | **K.ALAIKER.I** |
|  | 2863 | **899.4567** | **898.4495** | **898.4548** | **-0.0054** | **0** | **21** | **1.2** | **1** | **R.AYQNYIK.K** 2864 |
|  | 4207 | **499.2557** | **996.4968** | **996.5029** | **-0.0061** | **0** | **34** | **0.053** | **1** | **R.YGNFDILR.D** |
|  | 4291 | **501.7614** | **1001.5082** | **1001.5142** | **-0.0060** | **0** | **24** | **0.78** | **1** | **R.NVIPETSSR.Y** 4290 4298 |
|  | 4293 | **501.7733** | **1001.5321** | **1001.5393** | **-0.0072** | **0** | **23** | **0.91** | **1** | **K.TEDIVAVQK.A** |
|  | 4416 | **506.7368** | **1011.4591** | **1011.4661** | **-0.0071** | **0** | **21** | **1.1** | **1** | **K.YGASWTAEK.A** |
|  | 5691 | 555.7623 | 1109.5100 | 1109.5175 | -0.0075 | 1 | 17 | 3 | 1 | K.NSYMNPEKK.C 5680 5683 5699 5701 5706 5708 |
|  | 5913 | **565.2694** | **1128.5243** | **1128.5312** | **-0.0070** | **0** | **14** | **7.5** | **1** | **K.TDVHSPGNFR.I** 5936 |
|  | 6287 | **579.3210** | **1156.6274** | **1157.6153** | **-0.9879** | **1** | **5** | **57** | **9** | **K.RNVIPETSSR.Y** |
|  | 8373 | **634.3364** | **1266.6583** | **1266.6642** | **-0.0059** | **0** | **67** | **2.8e-005** | **1** | **R.FIMDLVSSLSR.T** 8360 8374 |
|  | 8689 | **642.8233** | **1283.6320** | **1283.6397** | **-0.0077** | **0** | **32** | **0.08** | **1** | **R.LYVEAAFAGESK.H** |
|  | 8867 | **647.3145** | **1292.6145** | **1291.7248** | **0.8896** | **0** | **71** | **1.2e-005** | **1** | **K.HVVEDLIAQIR.E** 8840 |
|  | 11619 | **725.3417** | **1448.6688** | **1448.6783** | **-0.0095** | **0** | **45** | **0.0038** | **1** | **R.IGYPDDIVSNDNK.L** |
|  | 11721 | **728.8530** | **1455.6914** | **1455.6994** | **-0.0080** | **0** | **52** | **0.00087** | **1** | **K.ALYGTTSETATWR.R** |
|  | 12926 | **510.2675** | **1527.7807** | **1527.7906** | **-0.0099** | **0** | **3** | **67** | **9** | **K.NSVNHVIHIDQPR.L** |
|  | 14245 | **806.9026** | **1611.7906** | **1611.8005** | **-0.0099** | **1** | **8** | **21** | **1** | **K.ALYGTTSETATWRR.C** |
|  | 14933 | **552.5847** | **1654.7322** | **1654.7885** | **-0.0564** | **1** | **2** | **79** | **7** | **K.YSARDLQNLMSWR.F** |
|  | 15122 | **834.8603** | **1667.7060** | **1667.7144** | **-0.0084** | **0** | **64** | **4.2e-005** | **1** | **R.CANYVNGNMENAVGR.L** |
|  | 17143 | **455.9815** | **1819.8969** | **1818.8669** | **1.0300** | **0** | **2** | **77** | **10** | **K.SESQMDITDINTPKPK.K** |
|  | 19152 | **497.7428** | **1986.9420** | **1987.9884** | **-1.0465** | **1** | **9** | **12** | **1** | **M.GKSESQMDITDINTPKPK.K** |
|  | 19622 | **1015.9574** | **2029.9002** | **2029.9125** | **-0.0123** | **0** | **92** | **5.2e-008** | **1** | **R.LIQNMDATTEPCTDFFK.Y** 19621 19623 19624 19631 |
|  | 20104 | **1040.4934** | **2078.9723** | **2078.9844** | **-0.0121** | **0** | **84** | **3.7e-007** | **1** | **R.IIGTLQNSAEFSEAFHCR.K** 20103 20105 |
|  | 20612 | **712.6562** | **2134.9467** | **2135.0238** | **-0.0771** | **1** | **3** | **46** | **4** | **-.MGKSESQMDITDINTPKPK.K** |
|  | 20750 | **538.5455** | **2150.1528** | **2151.1661** | **-1.0133** | **1** | **2** | **52** | **6** | **K.NSVNHVIHIDQPRLGLPSR.D** |
|  | 20793 | **1078.0118** | **2154.0091** | **2154.0191** | **-0.0100** | **0** | **113** | **3.8e-010** | **1** | **R.EVFIQTLDDLTWMDAETK.K** 20791 20792 20794 20800 20806 |
|  | 20854 | **1080.0377** | **2158.0609** | **2158.0735** | **-0.0126** | **0** | **36** | **0.023** | **1** | **K.LLPDIYGWPVATENWEQK.Y** 20853 20855 |
|  | 21271 | **736.6960** | **2207.0663** | **2207.0793** | **-0.0130** | **1** | **11** | **5.9** | **1** | **R.IIGTLQNSAEFSEAFHCRK.N** |
|  | 21824 | **757.7012** | **2270.0819** | **2270.0968** | **-0.0149** | **1** | **41** | **0.0065** | **1** | **K.VDKDEWISGAAVVNAFYSSGR.N** 21832 |
|  | 21924 | **761.7104** | **2282.1093** | **2282.1140** | **-0.0047** | **1** | **9** | **10** | **1** | **R.EVFIQTLDDLTWMDAETKK.R** |
|  | 23415 | **834.0505** | **2499.1298** | **2499.1455** | **-0.0157** | **1** | **29** | **0.077** | **1** | **R.NFNKDGDLVDWWTQQSASNFK.E** |
|  | 23826 | **853.4559** | **2557.3460** | **2557.3540** | **-0.0080** | **1** | **28** | **0.11** | **1** | **R.LYVEAAFAGESKHVVEDLIAQIR.E** |
|  | 25362 | **948.8398** | **2843.4975** | **2842.5090** | **0.9885** | **1** | **35** | **0.019** | **1** | **K.VLINLFVGTDDKNSVNHVIHIDQPR.L** 25353 |
|  | 26351 | **1016.8276** | **3047.4609** | **3047.4763** | **-0.0154** | **1** | **45** | **0.0017** | **1** | **K.LNNEYLELNYKEDEYFENIIQNLK.F** |
|  | 26846 | **1051.5127** | **3151.5163** | **3151.5291** | **-0.0128** | **1** | **32** | **0.034** | **1** | **K.LLPDIYGWPVATENWEQKYGASWTAEK.A** |

| **30.** | Q6S8J3\|POTEE_HUMAN    **Mass:** 122882   **Score:** 757    **Queries matched:** 58   **emPAI:** 0.27 |
| --- | --- |
|  | POTE ankyrin domain family member E OS=Homo sapiens GN=POTEE PE=1 SV=3 |

|  | **Query** | **Observed** | **Mr(expt)** | **Mr(calc)** | **Delta** | **Miss** | **Score** | **Expect** | **Rank** | **Peptide** |
| --- | --- | --- | --- | --- | --- | --- | --- | --- | --- | --- |
|  | 1475 | 800.5302 | 799.5229 | 799.5280 | -0.0051 | 1 | 14 | 8.2 | 3 | K.RGILTLK.Y |
|  | 3029 | **455.7555** | **909.4965** | **908.3797** | **1.1168** | **0** | **4** | **59** | **10** | **R.EVEEEMK.K** |
|  | 6416 | **585.0891** | **1168.1635** | **1167.5264** | **0.6372** | **0** | **(9)** | **12** | **1** | **K.MNSELSLSCK.K** 6408 |
|  | 6646 | **593.1548** | **1184.2950** | **1183.5213** | **0.7737** | **0** | **16** | **2.5** | **1** | **K.MNSELSLSCK.K** 6659 |
|  | 6943 | 599.8527 | 1197.6908 | 1197.6982 | -0.0075 | 0 | 52 | 0.00092 | 1 | R.AVFPSIVGRPR.Q 6939 6944 6945 6946 |
|  | 7426 | **611.7866** | **1221.5587** | **1222.6445** | **-1.0858** | **1** | **5** | **44** | **5** | **K.YLEDIESVKK.K** |
|  | 7488 | **613.3124** | **1224.6102** | **1224.5986** | **0.0116** | **1** | **0** | **1.3e+002** | **6** | **K.ESYVGKEAQSK.R** |
|  | 7633 | **616.8056** | **1231.5966** | **1230.5815** | **1.0151** | **1** | **3** | **82** | **9** | **R.GYRFTTMAER.E** |
|  | 8306 | **422.2385** | **1263.6936** | **1263.5159** | **0.1777** | **0** | **5** | **50** | **9** | **R.QQGMMGGMHQK.E** 8334 |
|  | 9091 | **435.2689** | **1302.7848** | **1302.5485** | **0.2362** | **1** | **8** | **22** | **3** | **R.CFPCYRESGK.S** |
|  | 9379 | **660.3484** | **1318.6823** | **1318.6728** | **0.0095** | **1** | **1** | **1.1e+002** | **8** | **K.LTSEEESQRLK.G** |
|  | 11560 | **482.5971** | **1444.7696** | **1445.7734** | **-1.0038** | **1** | **15** | **4.2** | **2** | **K.EIAALAPSMMKIR.I** |
|  | 12299 | **497.2400** | **1488.6982** | **1489.6103** | **-0.9121** | **0** | **8** | **23** | **5** | **K.SNVGASGDHDDSAMK.T** |
|  | 12440 | **500.2620** | **1497.7643** | **1498.6683** | **-0.9040** | **0** | **0** | **1.1e+002** | **9** | **R.ELENFMAIEEMK.K** |
|  | 12723 | 505.9181 | 1514.7325 | 1514.7419 | -0.0093 | 0 | 40 | 0.014 | 1 | K.IWHHTFYNELR.V 12724 12725 |
|  | 12739 | 758.8506 | 1515.6866 | 1515.6954 | -0.0087 | 0 | 42 | 0.0081 | 1 | K.QEYDESGPSIVHR.K 12738 |
|  | 13248 | 774.9052 | 1547.7957 | 1547.7873 | 0.0084 | 1 | 21 | 0.93 | 2 | R.MQKEIAALAPSMMK.I 13249 |
|  | 13508 | 783.3367 | 1564.6588 | 1564.7477 | -0.0889 | 0 | 3 | 56 | 4 | -.MVVEVDSMPAASSVK.K |
|  | 13997 | 399.7197 | 1594.8498 | 1595.7721 | -0.9223 | 1 | (12) | 6.5 | 1 | R.MQKEIAALAPSMMK.I 13994 |
|  | 14848 | **825.3959** | **1648.7773** | **1648.8494** | **-0.0721** | **1** | **2** | **69** | **5** | **R.LDLAGRELPDYLMK.I** |
|  | 15697 | **569.6187** | **1705.8343** | **1706.8582** | **-1.0239** | **1** | **0** | **1e+002** | **10** | **R.EEIAMLRLELDTMK.H** |
|  | 16767 | 895.9441 | 1789.8736 | 1789.8846 | -0.0110 | 0 | 66 | 2.5e-005 | 1 | K.SYELPDGQVITIGNER.F 16766 16769 16770 16771 16772 |
|  | 17810 | **624.2331** | **1869.6774** | **1870.6943** | **-1.0168** | **1** | **(3)** | **39** | **2** | **K.WCCHCFPCCRGSGK.S** 17811 |
|  | 18087 | **632.3237** | **1893.9492** | **1894.8488** | **-0.8996** | **1** | **2** | **58** | **4** | **R.QQGMMGGMHQKESYVGK.E** |
|  | 19198 | **664.3737** | **1990.0993** | **1989.9677** | **0.1317** | **1** | **0** | **78** | **5** | **K.QMLKISSENSNPEQELK.L** |
|  | 23722 | 1275.5837 | 2549.1529 | 2549.1665 | -0.0136 | 0 | 112 | 3.9e-010 | 1 | K.LCYVALDFEQEMATAASSSSLEK.S 23723 23724 23725 23729 23737 23744 23745 23746 |
|  | 25170 | 936.4366 | 2806.2879 | 2806.3041 | -0.0162 | 1 | 36 | 0.013 | 1 | K.EKLCYVALDFEQEMATAASSSSLEK.S 25171 |
|  | 26215 | **1010.1639** | **3027.4700** | **3028.4751** | **-1.0051** | **1** | **4** | **20** | **2** | **K.KHGSTHVGFPENLTNGATAGNGDDGLIPPR.K** |
|  | 29159 | **1265.9736** | **3794.8991** | **3795.7390** | **-0.8399** | **1** | **1** | **29** | **4** | **K.ALQLNELTMDDDTAVLVIDNGSGMCKAGFAGDDAPR.A** |
|  | 30286 | 1441.3481 | 4321.0226 | 4321.0406 | -0.0180 | 1 | 129 | 2.7e-012 | 1 | K.LCYVALDFEQEMATAASSSSLEKSYELPDGQVITIGNER.F 30289 |

| **31.** | P11021\|GRP78_HUMAN    **Mass:** 72402    **Score:** 751    **Queries matched:** 49   **emPAI:** 1.34 |
| --- | --- |
|  | 78 kDa glucose-regulated protein OS=Homo sapiens GN=HSPA5 PE=1 SV=2 |

|  | **Query** | **Observed** | **Mr(expt)** | **Mr(calc)** | **Delta** | **Miss** | **Score** | **Expect** | **Rank** | **Peptide** |
| --- | --- | --- | --- | --- | --- | --- | --- | --- | --- | --- |
|  | 1021 | **631.3716** | **630.3644** | **630.3701** | **-0.0057** | **1** | **7** | **58** | **10** | **K.EKLGGK.L** |
|  | 1134 | **687.3989** | **686.3916** | **686.3963** | **-0.0047** | **0** | **23** | **1.2** | **3** | **R.LIGDAAK.N** 1136 |
|  | 1205 | **728.4621** | **727.4548** | **727.4592** | **-0.0044** | **0** | **35** | **0.061** | **1** | **K.IQQLVK.E** |
|  | 4212 | **499.2596** | **996.5047** | **996.5101** | **-0.0054** | **0** | **48** | **0.0023** | **1** | **R.ALSSQHQAR.I** 4225 |
|  | 5113 | **534.2675** | **1066.5205** | **1065.6910** | **0.8295** | **1** | **3** | **76** | **9** | **R.IPKIQQLVK.E** |
|  | 5198 | **537.7771** | **1073.5396** | **1073.5465** | **-0.0069** | **0** | **30** | **0.17** | **1** | **K.ITITNDQNR.L** |
|  | 7185 | **605.7926** | **1209.5706** | **1209.5778** | **-0.0072** | **1** | **13** | **6.8** | **1** | **K.EFFNGKEPSR.G** 7175 7176 |
|  | 7551 | **614.8137** | **1227.6128** | **1227.6207** | **-0.0080** | **0** | **44** | **0.0057** | **1** | **R.VEIIANDQGNR.I** |
|  | 7644 | 411.7181 | 1232.1324 | 1232.6183 | -0.4859 | 0 | 8 | 15 | 4 | K.DAGTIAGLNVMR.I |
|  | 7800 | **620.8118** | **1239.6091** | **1239.6169** | **-0.0078** | **1** | **20** | **1.3** | **1** | **K.MKETAEAYLGK.K** |
|  | 9237 | **657.3098** | **1312.6051** | **1312.6122** | **-0.0071** | **0** | **17** | **2.9** | **1** | **K.FEELNMDLFR.S** |
|  | 9314 | **658.8184** | **1315.6223** | **1315.6295** | **-0.0073** | **0** | **7** | **27** | **2** | **R.NELESYAYSLK.N** |
|  | 9316 | **658.8448** | **1315.6750** | **1315.6844** | **-0.0094** | **1** | **38** | **0.022** | **1** | **K.NKITITNDQNR.L** 9333 |
|  | 11305 | **715.8451** | **1429.6757** | **1429.6838** | **-0.0080** | **0** | **66** | **3.5e-005** | **1** | **R.TWNDPSVQQDIK.F** 11306 |
|  | 11817 | 730.8790 | 1459.7435 | 1459.7518 | -0.0083 | 0 | 9 | 17 | 1 | K.SDIDEIVLVGGSTR.I 11811 11818 |
|  | 12667 | **756.8759** | **1511.7371** | **1511.7442** | **-0.0071** | **1** | **59** | **0.00017** | **1** | **R.AKFEELNMDLFR.S** |
|  | 13026 | **768.8987** | **1535.7828** | **1535.7905** | **-0.0077** | **0** | **54** | **0.00046** | **1** | **K.TFAPEEISAMVLTK.M** |
|  | 13307 | **518.0089** | **1551.0047** | **1551.7854** | **-0.7807** | **0** | **(3)** | **48** | **1** | **K.TFAPEEISAMVLTK.M** |
|  | 13378 | **778.8876** | **1555.7606** | **1554.7862** | **0.9743** | **1** | **24** | **0.48** | **1** | **K.NGRVEIIANDQGNR.I** 13377 |
|  | 13521 | **783.8890** | **1565.7634** | **1565.7726** | **-0.0092** | **0** | **41** | **0.01** | **1** | **R.ITPSYVAFTPEGER.L** 13520 |
|  | 15003 | **830.4456** | **1658.8766** | **1658.8879** | **-0.0113** | **0** | **79** | **1.4e-006** | **1** | **R.IINEPTAAAIAYGLDK.R** 15005 |
|  | 15280 | **839.4020** | **1676.7894** | **1676.8006** | **-0.0112** | **0** | **25** | **0.35** | **1** | **K.NQLTSNPENTVFDAK.R** |
|  | 15383 | **421.9748** | **1683.8701** | **1685.0273** | **-1.1572** | **1** | **3** | **53** | **8** | **M.KLSLVAAMLLLLSAAR.A** |
|  | 17070 | **908.4958** | **1814.9770** | **1814.9890** | **-0.0120** | **1** | **78** | **1.7e-006** | **1** | **R.IINEPTAAAIAYGLDKR.E** 17069 17071 |
|  | 17328 | **917.4527** | **1832.8908** | **1832.9017** | **-0.0108** | **1** | **73** | **5.3e-006** | **1** | **K.NQLTSNPENTVFDAKR.L** |
|  | 17361 | **918.9651** | **1835.9157** | **1835.9265** | **-0.0108** | **0** | **78** | **1.7e-006** | **1** | **K.SQIFSTASDNQPTVTIK.V** 17362 |
|  | 17788 | **623.6606** | **1867.9599** | **1868.9745** | **-1.0145** | **1** | **1** | **76** | **3** | **R.LIGRTWNDPSVQQDIK.F** |
|  | 18010 | **944.4836** | **1886.9526** | **1886.9639** | **-0.0113** | **0** | **37** | **0.019** | **1** | **K.VTHAVVTVPAYFNDAQR.Q** 18011 |
|  | 19739 | 511.4976 | 2041.9611 | 2041.0440 | 0.9172 | 1 | 1 | 72 | 3 | K.ITITNDQNRLTPEEIER.M |
|  | 20895 | **1082.9936** | **2163.9727** | **2163.9848** | **-0.0120** | **0** | **107** | **1.8e-009** | **1** | **R.IEIESFYEGEDFSETLTR.A** |
|  | 20989 | **1088.4929** | **2174.9713** | **2174.9855** | **-0.0142** | **1** | **103** | **3.8e-009** | **1** | **K.LYGSAGPPPTGEEDTAEKDEL.-** 20988 |
|  | 22377 | **782.7315** | **2345.1727** | **2345.1863** | **-0.0136** | **1** | **5** | **25** | **1** | **R.LIGDAAKNQLTSNPENTVFDAK.R** |
|  | 24988 | **926.4111** | **2776.2116** | **2775.3828** | **0.8288** | **1** | **2** | **34** | **4** | **R.VEIIANDQGNRITPSYVAFTPEGER.L** |

| **32.** | P02768\|ALBU_HUMAN    **Mass:** 71317    **Score:** 740    **Queries matched:** 68   **emPAI:** 3.36 |
| --- | --- |
|  | Serum albumin OS=Homo sapiens GN=ALB PE=1 SV=2 |

|  | **Query** | **Observed** | **Mr(expt)** | **Mr(calc)** | **Delta** | **Miss** | **Score** | **Expect** | **Rank** | **Peptide** |
| --- | --- | --- | --- | --- | --- | --- | --- | --- | --- | --- |
|  | 1330 | **395.2366** | **788.4587** | **788.4644** | **-0.0057** | **0** | **35** | **0.076** | **1** | **K.LVTDLTK.V** |
|  | 2623 | **441.2180** | **880.4214** | **879.4338** | **0.9876** | **0** | **4** | **57** | **4** | **K.AEFAEVSK.L** |
|  | 2791 | **447.2289** | **892.4433** | **891.4297** | **1.0136** | **1** | **8** | **30** | **9** | **R.DEGKASSAK.Q** |
|  | 3277 | **464.2477** | **926.4808** | **926.4861** | **-0.0053** | **0** | **19** | **2.2** | **1** | **K.YLYEIAR.R** 3276 |
|  | 3365 | **467.2590** | **932.5034** | **932.5113** | **-0.0079** | **0** | **10** | **19** | **2** | **K.LCTVATLR.E** |
|  | 3524 | **474.2676** | **946.5207** | **946.5270** | **-0.0063** | **1** | **19** | **2.2** | **1** | **R.LKCASLQK.F** 3525 |
|  | 3700 | **481.2478** | **960.4810** | **959.5552** | **0.9258** | **0** | **11** | **16** | **7** | **K.FQNALLVR.Y** |
|  | 4269 | 501.2606 | 1000.5067 | 999.5964 | 0.9103 | 0 | 2 | 1.2e+002 | 8 | K.QTALVELVK.H |
|  | 4433 | **507.3006** | **1012.5867** | **1012.5917** | **-0.0050** | **0** | **27** | **0.33** | **1** | **K.LVAASQAALGL.-** |
|  | 4482 | **509.2688** | **1016.5230** | **1016.5291** | **-0.0061** | **0** | **22** | **1.1** | **1** | **K.SLHTLFGDK.L** 4496 |
|  | 5197 | **537.7714** | **1073.5282** | **1073.5353** | **-0.0071** | **1** | **12** | **11** | **3** | **K.LDELRDEGK.A** |
|  | 5908 | **564.8498** | **1127.6850** | **1127.6914** | **-0.0064** | **1** | **36** | **0.042** | **1** | **K.KQTALVELVK.H** |
|  | 6933 | **599.7232** | **1197.4319** | **1196.5495** | **0.8823** | **1** | **7** | **24** | **8** | **K.DVCKNYAEAK.D** |
|  | 6956 | **600.2591** | **1198.5036** | **1197.5336** | **0.9701** | **1** | **3** | **79** | **4** | **K.ETCFAEEGKK.L** |
|  | 7515 | **409.5375** | **1225.5907** | **1225.5979** | **-0.0072** | **1** | **25** | **0.5** | **1** | **R.FKDLGEENFK.A** 7532 |
|  | 9780 | **671.8174** | **1341.6202** | **1341.6275** | **-0.0073** | **0** | **46** | **0.0038** | **1** | **K.AVMDDFAAFVEK.C** |
|  | 11383 | **717.7659** | **1433.5173** | **1433.5261** | **-0.0088** | **0** | **41** | **0.0083** | **1** | **R.ETYGEMADCCAK.Q** |
|  | 11510 | **722.3203** | **1442.6261** | **1442.6347** | **-0.0087** | **0** | **40** | **0.012** | **1** | **K.YICENQDSISSK.L** 11508 11509 11520 |
|  | 11943 | 489.9493 | 1466.8262 | 1466.8358 | -0.0096 | 1 | 39 | 0.015 | 1 | R.RHPDYSVVLLLR.L 11929 11939 |
|  | 12434 | **749.7883** | **1497.5620** | **1497.5712** | **-0.0092** | **0** | **72** | **6.7e-006** | **1** | **K.TCVADESAENCDK.S** |
|  | 13205 | **773.8973** | **1545.7801** | **1545.7894** | **-0.0093** | **1** | **46** | **0.0032** | **1** | **K.LKECCEKPLLEK.S** 13206 |
|  | 13293 | **776.3925** | **1550.7704** | **1551.5905** | **-0.8201** | **0** | **40** | **0.012** | **1** | **K.CCAAADPHECYAK.V** |
|  | 14695 | **820.4665** | **1638.9184** | **1638.9305** | **-0.0121** | **1** | **75** | **3.4e-006** | **1** | **K.KVPQVSTPTLVEVSR.N** 14694 14696 14697 14698 |
|  | 14868 | **825.9465** | **1649.8785** | **1649.8876** | **-0.0091** | **1** | **61** | **8.4e-005** | **1** | **K.AEFAEVSKLVTDLTK.V** |
|  | 15040 | **831.8604** | **1661.7063** | **1661.7178** | **-0.0115** | **1** | **57** | **0.00024** | **1** | **R.YKAAFTECCQAADK.A** 15041 |
|  | 17401 | 920.9568 | 1839.8990 | 1839.9077 | -0.0086 | 1 | 60 | 0.00011 | 1 | K.EQLKAVMDDFAAFVEK.C |
|  | 18135 | **475.4928** | **1897.9422** | **1897.9879** | **-0.0457** | **1** | **4** | **35** | **9** | **R.HPYFYAPELLFFAKR.Y** |
|  | 18137 | **633.6664** | **1897.9773** | **1897.9879** | **-0.0106** | **1** | **12** | **6.8** | **1** | **R.RHPYFYAPELLFFAK.R** |
|  | 18552 | **966.5170** | **1931.0194** | **1931.0299** | **-0.0105** | **1** | **73** | **5.1e-006** | **1** | **K.SLHTLFGDKLCTVATLR.E** 18550 18551 |
|  | 19769 | **1023.0454** | **2044.0763** | **2044.0881** | **-0.0118** | **0** | **70** | **9.5e-006** | **1** | **K.VFDEFKPLVEEPQNLIK.Q** |
|  | 20154 | **696.2799** | **2085.8179** | **2085.8303** | **-0.0124** | **0** | **25** | **0.22** | **1** | **K.VHTECCHGDLLECADDR.A** |
|  | 20179 | **523.0380** | **2088.1227** | **2088.8187** | **-0.6959** | **1** | **11** | **7** | **1** | **R.ETYGEMADCCAKQEPER.N** |
|  | 22045 | **767.3693** | **2299.0861** | **2299.0983** | **-0.0122** | **1** | **7** | **16** | **1** | **K.NYAEAKDVFLGMFLYEYAR.R** |
|  | 22395 | **783.6781** | **2348.0125** | **2348.0269** | **-0.0144** | **1** | **30** | **0.074** | **1** | **K.LCTVATLRETYGEMADCCAK.Q** |
|  | 23360 | **830.7640** | **2489.2701** | **2489.2777** | **-0.0076** | **0** | **27** | **0.15** | **1** | **K.ALVLIAFAQYLQQCPFEDHVK.L** 23358 |
|  | 23945 | **862.3729** | **2584.0969** | **2584.1105** | **-0.0135** | **1** | **41** | **0.0052** | **1** | **K.VHTECCHGDLLECADDRADLAK.Y** |
|  | 24039 | **867.0999** | **2598.2779** | **2598.2900** | **-0.0121** | **1** | **38** | **0.0098** | **1** | **K.QNCELFEQLGEYKFQNALLVR.Y** |
|  | 24283 | **884.0877** | **2649.2411** | **2649.2567** | **-0.0156** | **0** | **5** | **22** | **1** | **R.LVRPEVDVMCTAFHDNEETFLK.K** |
|  | 24996 | **695.3401** | **2777.3315** | **2777.3517** | **-0.0202** | **1** | **39** | **0.0078** | **1** | **R.LVRPEVDVMCTAFHDNEETFLKK.Y** 24997 24999 |
|  | 25954 | **992.1146** | **2973.3220** | **2973.3372** | **-0.0151** | **0** | **51** | **0.00036** | **1** | **K.SHCIAEVENDEMPADLPSLAADFVESK.D** 25955 25958 25959 |
|  | 26226 | **758.3539** | **3029.3867** | **3029.4045** | **-0.0179** | **1** | **4** | **19** | **1** | **K.CCTESLVNRRPCFSALEVDETYVPK.E** |
|  | 27940 | **1711.5590** | **3421.1034** | **3421.7016** | **-0.5983** | **1** | **0** | **29** | **4** | **K.DLGEENFKALVLIAFAQYLQQCPFEDHVK.L** |
|  | 28126 | **869.8747** | **3475.4697** | **3475.5582** | **-0.0885** | **1** | **4** | **13** | **1** | **K.SHCIAEVENDEMPADLPSLAADFVESKDVCK.N** |
|  | 28667 | **1207.6290** | **3619.8653** | **3619.8749** | **-0.0096** | **1** | **15** | **0.95** | **1** | **K.ALVLIAFAQYLQQCPFEDHVKLVNEVTEFAK.T** 28663 |

| **33.** | P14625\|ENPL_HUMAN    **Mass:** 92696    **Score:** 731    **Queries matched:** 38   **emPAI:** 1.02 |
| --- | --- |
|  | Endoplasmin OS=Homo sapiens GN=HSP90B1 PE=1 SV=1 |

|  | **Query** | **Observed** | **Mr(expt)** | **Mr(calc)** | **Delta** | **Miss** | **Score** | **Expect** | **Rank** | **Peptide** |
| --- | --- | --- | --- | --- | --- | --- | --- | --- | --- | --- |
|  | 1908 | **417.2362** | **832.4578** | **832.4589** | **-0.0011** | **1** | **13** | **11** | **1** | **R.IERMLR.L** |
|  | 2362 | **432.2333** | **862.4520** | **863.4786** | **-1.0266** | **1** | **4** | **72** | **1** | **K.TLDMIKK.I** |
|  | 2563 | **438.7300** | **875.4454** | **875.4501** | **-0.0047** | **0** | **11** | **16** | **1** | **K.TFEINPR.H** |
|  | 3987 | **491.7427** | **981.4708** | **981.4767** | **-0.0059** | **0** | **23** | **0.69** | **1** | **K.SGTSEFLNK.M** |
|  | 5322 | **542.8798** | **1083.7450** | **1084.5764** | **-0.8314** | **0** | **2** | **76** | **4** | **R.LSLNIDPDAK.V** |
|  | 5821 | **560.3265** | **1118.6384** | **1118.6084** | **0.0300** | **1** | **18** | **2.7** | **8** | **R.FQNVAKEGVK.F** |
|  | 10760 | 466.5793 | 1396.7162 | 1395.7219 | 0.9944 | 1 | 9 | 18 | 8 | K.LGVIEDHSNRTR.L |
|  | 12246 | **743.3758** | **1484.7370** | **1484.7471** | **-0.0101** | **0** | **84** | **5.6e-007** | **1** | **K.GVVDSDDLPLNVSR.E** 12247 |
|  | 12690 | **505.2619** | **1512.7640** | **1512.7719** | **-0.0079** | **0** | **33** | **0.061** | **1** | **K.NLLHVTDTGVGMTR.E** 12674 12687 |
|  | 13177 | **772.9123** | **1543.8101** | **1543.8205** | **-0.0104** | **1** | **82** | **7.3e-007** | **1** | **R.ELISNASDALDKIR.L** 13178 |
|  | 13352 | **519.1348** | **1554.3827** | **1554.7678** | **-0.3851** | **1** | **0** | **79** | **10** | **R.SGYLLPDTKAYGDR.I** |
|  | 13988 | **798.3594** | **1594.7042** | **1594.7160** | **-0.0118** | **0** | **63** | **6.1e-005** | **1** | **R.VFITDDFHDMMPK.Y** 14009 |
|  | 16277 | **584.5441** | **1750.6106** | **1750.8171** | **-0.2065** | **1** | **23** | **0.46** | **1** | **R.RVFITDDFHDMMPK.Y** 16292 |
|  | 17925 | **939.9705** | **1877.9265** | **1877.9352** | **-0.0087** | **0** | **70** | **9.6e-006** | **1** | **K.YSQFINFPIYVWSSK.T** |
|  | 19778 | **1023.5278** | **2045.0410** | **2045.0528** | **-0.0118** | **0** | **101** | **6.7e-009** | **1** | **R.LISLTDENALSGNEELTVK.I** |
|  | 20421 | **1056.5533** | **2111.0921** | **2111.1045** | **-0.0124** | **1** | **46** | **0.0021** | **1** | **K.NLLHVTDTGVGMTREELVK.N** 20420 20422 |
|  | 20518 | 708.3751 | 2122.1035 | 2122.9099 | -0.8064 | 1 | 3 | 45 | 8 | K.TETVEEPMEEEEAAKEEK.E |
|  | 20625 | **1069.0077** | **2136.0008** | **2136.0123** | **-0.0115** | **1** | **71** | **6.9e-006** | **1** | **K.AQAYQTGKDISTNYYASQK.K** 20624 |
|  | 21644 | **750.7267** | **2249.1582** | **2249.1692** | **-0.0110** | **1** | **41** | **0.0058** | **1** | **K.YLNFVKGVVDSDDLPLNVSR.E** |
|  | 21726 | **754.0219** | **2259.0439** | **2259.0556** | **-0.0117** | **0** | **65** | **2.3e-005** | **1** | **R.FQSSHHPTDITSLDQYVER.M** 21724 21725 |
|  | 22453 | **1178.5309** | **2355.0472** | **2355.0623** | **-0.0151** | **0** | **92** | **4.1e-008** | **1** | **R.LTESPCALVASQYGWSGNMER.I** 22454 |
|  | 23093 | **818.1046** | **2451.2918** | **2451.2996** | **-0.0078** | **1** | **6** | **17** | **1** | **R.GTTITLVLKEEASDYLELDTIK.N** |
|  | 23663 | **848.0986** | **2541.2739** | **2541.2838** | **-0.0099** | **0** | **19** | **0.87** | **1** | **K.TVWDWELMNDIKPIWQRPSK.E** |
|  | 26642 | **1035.4982** | **3103.4727** | **3103.4848** | **-0.0122** | **1** | **32** | **0.028** | **1** | **K.GYEVIYLTEPVDEYCIQALPEFDGKR.F** |
|  | 29556 | **1318.6470** | **3952.9191** | **3951.9452** | **0.9739** | **1** | **84** | **1.3e-007** | **1** | **K.MTEAQEDGQSTSELIGQFGVGFYSAFLVADKVIVTSK.H** 29544 |

| **34.** | P04406\|G3P_HUMAN    **Mass:** 36201    **Score:** 648    **Queries matched:** 29   **emPAI:** 1.69 |
| --- | --- |
|  | Glyceraldehyde-3-phosphate dehydrogenase OS=Homo sapiens GN=GAPDH PE=1 SV=3 |

|  | **Query** | **Observed** | **Mr(expt)** | **Mr(calc)** | **Delta** | **Miss** | **Score** | **Expect** | **Rank** | **Peptide** |
| --- | --- | --- | --- | --- | --- | --- | --- | --- | --- | --- |
|  | 1541 | **402.7052** | **803.3958** | **804.4243** | **-1.0285** | **0** | **42** | **0.013** | **1** | **K.VGVNGFGR.I** 1554 |
|  | 5100 | **533.2951** | **1064.5756** | **1064.5839** | **-0.0083** | **1** | **21** | **1.2** | **1** | **K.AGAHLQGGAKR.V** |
|  | 7008 | **401.2425** | **1200.7056** | **1200.5995** | **0.1061** | **0** | **9** | **19** | **1** | **R.VVDLMAHMASK.E** |
|  | 7314 | **406.2100** | **1215.6081** | **1216.5944** | **-0.9864** | **0** | **(3)** | **79** | **7** | **R.VVDLMAHMASK.E** |
|  | 9588 | 665.8242 | 1329.6338 | 1329.6421 | -0.0083 | 1 | 64 | 6.2e-005 | 1 | R.VVDLMAHMASKE.- 9580 9587 9592 9593 9594 |
|  | 16131 | 870.4697 | 1738.9248 | 1738.9326 | -0.0078 | 1 | 32 | 0.066 | 1 | R.DGRGALQNIIPASTGAAK.A 16115 |
|  | 16440 | **882.4001** | **1762.7856** | **1762.7951** | **-0.0094** | **0** | **81** | **8.2e-007** | **1** | **K.LISWYDNEFGYSNR.V** 16439 |
|  | 17330 | **917.4583** | **1832.9021** | **1832.9124** | **-0.0104** | **0** | **91** | **8.4e-008** | **1** | **K.IISNASCTTNCLAPLAK.V** 17329 17331 17339 |
|  | 21873 | **1139.0164** | **2276.0182** | **2276.0307** | **-0.0126** | **0** | **111** | **6.2e-010** | **1** | **K.WGDAGAEYVVESTGVFTTMEK.A** 21872 |
|  | 23513 | **840.0731** | **2517.1975** | **2517.2097** | **-0.0122** | **1** | **12** | **4.1** | **1** | **K.IKWGDAGAEYVVESTGVFTTMEK.A** |
|  | 24006 | **865.7871** | **2594.3393** | **2594.3527** | **-0.0134** | **0** | **79** | **7.7e-007** | **1** | **K.VIHDNFGIVEGLMTTVHAITATQK.T** 24007 24009 |
|  | 24096 | **871.4537** | **2611.3392** | **2610.3476** | **0.9916** | **0** | **(1)** | **52** | **8** | **K.VIHDNFGIVEGLMTTVHAITATQK.T** |
|  | 27468 | 1103.5216 | 3307.5430 | 3307.5570 | -0.0140 | 0 | 38 | 0.0067 | 1 | K.VDIVAINDPFIDLNYMVYMFQYDSTHGK.F |
|  | 29718 | **1009.9735** | **4035.8647** | **4035.8875** | **-0.0228** | **0** | **53** | **0.00014** | **1** | **K.GILGYTEHQVVSSDFNSDTHSSTFDAGAGIALNDHFVK.L** 29717 |

| **35.** | P68871\|HBB_HUMAN    **Mass:** 16102    **Score:** 626    **Queries matched:** 29   **emPAI:** 7.70 |
| --- | --- |
|  | Hemoglobin subunit beta OS=Homo sapiens GN=HBB PE=1 SV=2 |

|  | **Query** | **Observed** | **Mr(expt)** | **Mr(calc)** | **Delta** | **Miss** | **Score** | **Expect** | **Rank** | **Peptide** |
| --- | --- | --- | --- | --- | --- | --- | --- | --- | --- | --- |
|  | 3347 | **466.7608** | **931.5070** | **931.5127** | **-0.0057** | **0** | **51** | **0.0016** | **1** | **K.SAVTALWGK.V** |
|  | 3582 | **476.7556** | **951.4965** | **951.5025** | **-0.0060** | **0** | **27** | **0.33** | **1** | **M.VHLTPEEK.S** |
|  | 8529 | **638.3271** | **1274.6396** | **1273.7183** | **0.9213** | **0** | **22** | **0.82** | **1** | **R.LLVVYPWTQR.F** 8523 8530 |
|  | 9266 | **657.8401** | **1313.6656** | **1313.6575** | **0.0081** | **0** | **57** | **0.00028** | **1** | **K.VNVDEVGGEALGR.L** 9256 9298 |
|  | 10437 | **689.8496** | **1377.6847** | **1377.6929** | **-0.0082** | **0** | **4** | **53** | **5** | **K.EFTPPVQAAYQK.V** |
|  | 11626 | **725.3976** | **1448.7807** | **1448.7888** | **-0.0081** | **1** | **62** | **7.8e-005** | **1** | **K.VVAGVANALAHKYH.-** 11613 11627 |
|  | 15154 | **835.4435** | **1668.8725** | **1668.8835** | **-0.0110** | **0** | **50** | **0.0011** | **1** | **K.VLGAFSDGLAHLDNLK.G** 15169 |
|  | 19905 | **1029.9730** | **2057.9315** | **2057.9405** | **-0.0090** | **0** | **98** | **1.4e-008** | **1** | **R.FFESFGDLSTPDAVMGNPK.V** 19900 19901 19902 19903 19904 19906 |
|  | 20062 | **1037.9703** | **2073.9261** | **2073.9354** | **-0.0093** | **0** | **(73)** | **4.1e-006** | **1** | **R.FFESFGDLSTPDAVMGNPK.V** 20061 |
|  | 21477 | **1114.5792** | **2227.1439** | **2227.1597** | **-0.0158** | **1** | **99** | **9.4e-009** | **1** | **K.SAVTALWGKVNVDEVGGEALGR.L** 21476 |
|  | 23954 | **862.7461** | **2585.2166** | **2585.2333** | **-0.0167** | **1** | **45** | **0.0021** | **1** | **K.GTFATLSELHCDKLHVDPENFR.L** |
|  | 26779 | **784.9211** | **3135.6555** | **3135.6692** | **-0.0137** | **1** | **13** | **2.2** | **1** | **R.LLGNVLVCVLAHHFGKEFTPPVQAAYQK.V** 26778 |
|  | 27490 | **1105.5547** | **3313.6422** | **3313.6482** | **-0.0060** | **1** | **14** | **1.7** | **1** | **R.LLVVYPWTQRFFESFGDLSTPDAVMGNPK.V** |

| **36.** | P11142\|HSP7C_HUMAN    **Mass:** 71082    **Score:** 603    **Queries matched:** 52   **emPAI:** 1.63 |
| --- | --- |
|  | Heat shock cognate 71 kDa protein OS=Homo sapiens GN=HSPA8 PE=1 SV=1 |

|  | **Query** | **Observed** | **Mr(expt)** | **Mr(calc)** | **Delta** | **Miss** | **Score** | **Expect** | **Rank** | **Peptide** |
| --- | --- | --- | --- | --- | --- | --- | --- | --- | --- | --- |
|  | 1134 | 687.3989 | 686.3916 | 686.3963 | -0.0047 | 0 | 23 | 1.2 | 3 | R.LIGDAAK.N 1136 |
|  | 1146 | **689.3898** | **688.3826** | **688.3755** | **0.0070** | **1** | **11** | **20** | **8** | **R.DAKLDK.S** |
|  | 1363 | **396.7318** | **791.4491** | **790.3709** | **1.0783** | **0** | **8** | **31** | **8** | **K.ATVEDEK.L** |
|  | 2193 | **426.2319** | **850.4492** | **849.3902** | **1.0591** | **0** | **5** | **53** | **6** | **R.MVQEAEK.Y** |
|  | 2541 | **437.7172** | **873.4198** | **874.4144** | **-0.9947** | **1** | **10** | **23** | **6** | **K.AEDEKQR.D** |
|  | 4487 | **509.2846** | **1016.5547** | **1016.5614** | **-0.0068** | **1** | **31** | **0.15** | **1** | **K.ITITNDKGR.L** 4494 |
|  | 6580 | **591.3058** | **1180.5970** | **1179.6135** | **0.9835** | **1** | **10** | **14** | **1** | **K.VQVEYKGETK.S** |
|  | 6966 | **600.3375** | **1198.6604** | **1198.6670** | **-0.0066** | **0** | **47** | **0.0029** | **1** | **K.DAGTIAGLNVLR.I** |
|  | 7551 | 614.8137 | 1227.6128 | 1227.6207 | -0.0080 | 0 | 44 | 0.0057 | 1 | K.VEIIANDQGNR.T |
|  | 7695 | **618.3115** | **1234.6084** | **1234.6169** | **-0.0085** | **0** | **29** | **0.19** | **1** | **R.MVNHFIAEFK.R** |
|  | 8050 | **626.8079** | **1251.6013** | **1250.6118** | **0.9895** | **0** | **(2)** | **87** | **1** | **R.MVNHFIAEFK.R** |
|  | 8052 | **626.8307** | **1251.6469** | **1251.6533** | **-0.0063** | **1** | **39** | **0.02** | **1** | **K.MKEIAEAYLGK.T** |
|  | 8084 | **627.7838** | **1253.5529** | **1253.5598** | **-0.0069** | **0** | **54** | **0.00062** | **1** | **R.FDDAVVQSDMK.H** |
|  | 8394 | 634.8575 | 1267.7004 | 1267.6482 | 0.0522 | 1 | (14) | 5.9 | 8 | K.MKEIAEAYLGK.T 8387 |
|  | 8431 | **424.0559** | **1269.1459** | **1269.5547** | **-0.4088** | **0** | **(6)** | **21** | **2** | **R.FDDAVVQSDMK.H** 8430 8438 |
|  | 12100 | **739.4137** | **1476.8128** | **1475.6926** | **1.1203** | **1** | **2** | **87** | **5** | **K.EDIERMVQEAEK.Y** |
|  | 12141 | **494.2534** | **1479.7385** | **1479.7470** | **-0.0085** | **1** | **38** | **0.022** | **1** | **R.ARFEELNADLFR.G** |
|  | 12167 | **741.4027** | **1480.7909** | **1480.7998** | **-0.0089** | **0** | **65** | **3.5e-005** | **1** | **K.SQIHDIVLVGGSTR.I** 12168 |
|  | 12274 | 744.3496 | 1486.6845 | 1486.6940 | -0.0095 | 0 | 49 | 0.0015 | 1 | R.TTPSYVAFTDTER.L |
|  | 13509 | **783.3471** | **1564.6797** | **1564.8249** | **-0.1453** | **1** | **7** | **25** | **2** | **K.LLQDFFNGKELNK.S** |
|  | 14310 | 808.8925 | 1615.7705 | 1615.7804 | -0.0099 | 0 | 37 | 0.023 | 1 | K.SFYPEEVSSMVLTK.M |
|  | 14848 | 825.3959 | 1648.7773 | 1648.7879 | -0.0106 | 0 | 59 | 0.00015 | 1 | K.NQVAMNPTNTVFDAK.R 14849 |
|  | 14910 | **551.9457** | **1652.8152** | **1652.8246** | **-0.0094** | **0** | **35** | **0.035** | **1** | **K.HWPFMVVNDAGRPK.V** |
|  | 15003 | 830.4456 | 1658.8766 | 1658.8879 | -0.0113 | 0 | 79 | 1.4e-006 | 1 | R.IINEPTAAAIAYGLDK.K 15005 |
|  | 15166 | **557.6285** | **1669.8638** | **1668.8195** | **1.0443** | **0** | **(1)** | **92** | **6** | **K.HWPFMVVNDAGRPK.V** |
|  | 15471 | **846.3610** | **1690.7074** | **1690.7183** | **-0.0110** | **0** | **27** | **0.21** | **1** | **K.STAGDTHLGGEDFDNR.M** 15472 |
|  | 16547 | **886.9491** | **1771.8836** | **1772.9131** | **-1.0294** | **1** | **3** | **55** | **3** | **K.ILDKCNEIINWLDK.N** 16546 |
|  | 16740 | **894.4932** | **1786.9719** | **1786.9828** | **-0.0109** | **1** | **38** | **0.017** | **1** | **R.IINEPTAAAIAYGLDKK.V** |
|  | 16934 | **903.4471** | **1804.8797** | **1804.8890** | **-0.0093** | **1** | **63** | **5e-005** | **1** | **K.NQVAMNPTNTVFDAKR.L** 16932 16933 |
|  | 17110 | **909.9404** | **1817.8663** | **1819.0316** | **-1.1653** | **1** | **1** | **87** | **3** | **K.SQIHDIVLVGGSTRIPK.I** 17111 |
|  | 17381 | **919.5043** | **1836.9941** | **1837.0058** | **-0.0117** | **1** | **68** | **1.5e-005** | **1** | **K.LDKSQIHDIVLVGGSTR.I** |
|  | 17872 | **625.6607** | **1873.9603** | **1874.9158** | **-0.9555** | **1** | **2** | **59** | **4** | **K.SFYPEEVSSMVLTKMK.E** |
|  | 19078 | **991.4960** | **1980.9775** | **1980.9905** | **-0.0130** | **0** | **23** | **0.48** | **1** | **K.TVTNAVVTVPAYFNDSQR.Q** 19079 |
|  | 24970 | **1387.6599** | **2773.3053** | **2773.3195** | **-0.0143** | **0** | **85** | **1.8e-007** | **1** | **K.QTQTFTTYSDNQPGVLIQVYEGER.A** 24969 24971 |
|  | 26845 | **1051.5122** | **3151.5148** | **3152.5513** | **-1.0365** | **1** | **4** | **21** | **7** | **K.RTLSSSTQASIEIDSLYEGIDFYTSITR.A** |
|  | 28307 | **1177.2451** | **3528.7135** | **3528.7373** | **-0.0237** | **1** | **53** | **0.00017** | **1** | **R.NTTIPTKQTQTFTTYSDNQPGVLIQVYEGER.A** |

| **37.** | P55084\|ECHB_HUMAN    **Mass:** 51547    **Score:** 601    **Queries matched:** 45   **emPAI:** 1.66 |
| --- | --- |
|  | Trifunctional enzyme subunit beta, mitochondrial OS=Homo sapiens GN=HADHB PE=1 SV=3 |

|  | **Query** | **Observed** | **Mr(expt)** | **Mr(calc)** | **Delta** | **Miss** | **Score** | **Expect** | **Rank** | **Peptide** |
| --- | --- | --- | --- | --- | --- | --- | --- | --- | --- | --- |
|  | 1288 | **785.4459** | **784.4386** | **784.4443** | **-0.0056** | **0** | **12** | **10** | **2** | **R.AAPAVQTK.T** |
|  | 2100 | **423.2332** | **844.4517** | **844.4589** | **-0.0071** | **0** | **10** | **23** | **7** | **R.LVMAAANR.L** |
|  | 2115 | **423.7387** | **845.4629** | **845.4680** | **-0.0051** | **0** | **24** | **0.84** | **1** | **K.LMLDLNK.A** |
|  | 2197 | **426.2743** | **850.5340** | **851.5116** | **-0.9777** | **0** | **9** | **21** | **4** | **K.VGLPPLEK.F** |
|  | 3042 | **456.2520** | **910.4893** | **911.5552** | **-1.0659** | **0** | **19** | **1.6** | **2** | **K.TLAKPNIR.N** 3057 |
|  | 3740 | **482.7264** | **963.4382** | **962.5913** | **0.8469** | **1** | **8** | **28** | **8** | **R.LSLISKFR.F** 3748 |
|  | 3936 | **489.7728** | **977.5310** | **977.5368** | **-0.0058** | **0** | **27** | **0.32** | **1** | **K.ALAMGYKPK.A** |
|  | 4112 | **495.7566** | **989.4987** | **989.5579** | **-0.0592** | **1** | **16** | **4.2** | **4** | **R.KLMLDLNK.A** 4127 4130 |
|  | 6000 | **568.7789** | **1135.5432** | **1135.5509** | **-0.0077** | **0** | **51** | **0.0011** | **1** | **R.LEQDEYALR.S** 6001 |
|  | 7375 | **610.3341** | **1218.6537** | **1218.6754** | **-0.0217** | **1** | **12** | **9.3** | **3** | **K.SMGQRLSLISK.F** 7376 |
|  | 7569 | **615.2754** | **1228.5362** | **1228.5434** | **-0.0072** | **0** | **16** | **4.2** | **1** | **R.DFMYVSQDPK.D** |
|  | 8159 | **629.3574** | **1256.7002** | **1255.7037** | **0.9965** | **1** | **6** | **41** | **9** | **K.NLPTASKWALR.F** |
|  | 13187 | **773.3831** | **1544.7516** | **1544.7617** | **-0.0101** | **0** | **56** | **0.00032** | **1** | **K.DNGIRPSSLEQMAK.L** |
|  | 15492 | **846.8391** | **1691.6635** | **1691.6708** | **-0.0073** | **0** | **38** | **0.015** | **1** | **K.AMDSDWFAENYMGR.K** |
|  | 15508 | **847.4106** | **1692.8067** | **1692.8431** | **-0.0364** | **1** | **5** | **37** | **5** | **K.TSNVAREAALGAGFSDK.T** |
|  | 17136 | **910.8858** | **1819.7570** | **1819.7658** | **-0.0087** | **1** | **84** | **4.5e-007** | **1** | **K.AMDSDWFAENYMGRK.T** |
|  | 17588 | **926.5054** | **1850.9963** | **1851.0030** | **-0.0067** | **0** | **44** | **0.004** | **1** | **K.EVVDYIIFGTVIQEVK.T** |
|  | 20598 | **1067.9905** | **2133.9664** | **2133.9803** | **-0.0139** | **0** | **108** | **1.5e-009** | **1** | **K.FNNWGGSLSLGHPFGATGCR.L** 20599 20600 |
|  | 21888 | 760.0505 | 2277.1298 | 2277.1463 | -0.0166 | 1 | 5 | 26 | 5 | R.TPFLLSGTSYKDLMPHDLAR.A |
|  | 22173 | **580.2921** | **2317.1391** | **2316.2372** | **0.9019** | **1** | **5** | **23** | **2** | **R.FSIRPLSCSSQLRAAPAVQTK.T** |
|  | 22527 | **1182.6517** | **2363.2889** | **2363.2988** | **-0.0099** | **1** | **81** | **4.8e-007** | **1** | **R.TSVPKEVVDYIIFGTVIQEVK.T** |
|  | 24169 | **1314.1493** | **2626.2840** | **2626.2989** | **-0.0149** | **1** | **55** | **0.00017** | **1** | **R.DFMYVSQDPKDQLLLGPTYATPK.V** 24170 |
|  | 24627 | **1353.1360** | **2704.2574** | **2704.2737** | **-0.0163** | **0** | **89** | **7.4e-008** | **1** | **K.EGGQYGLVAACAAGGQGHAMIVEAYPK.-** 24628 |
|  | 25542 | **962.7940** | **2885.3600** | **2885.3694** | **-0.0094** | **0** | **49** | **0.00078** | **1** | **K.AGLTMNDIDAFEFHEAFSGQILANFK.A** 25535 25537 25545 25558 |
|  | 26804 | **786.6240** | **3142.4667** | **3142.4818** | **-0.0151** | **1** | **13** | **2.1** | **1** | **K.FRFNFLAPELPAVSEFSTSETMGHSADR.L** |
|  | 27698 | **839.6687** | **3354.6457** | **3354.6594** | **-0.0137** | **1** | **2** | **29** | **4** | **K.VLEKAGLTMNDIDAFEFHEAFSGQILANFK.A** |
|  | 28459 | **1191.6113** | **3571.8122** | **3571.8306** | **-0.0184** | **0** | **17** | **0.78** | **1** | **K.LKPAFIKPYGTVTAANSSFLTDGASAMLIMAEEK.A** 28461 |
|  | 28994 | **1243.2727** | **3726.7963** | **3725.8148** | **0.9815** | **1** | **4** | **12** | **3** | **R.FNFLAPELPAVSEFSTSETMGHSADRLAAAFAVSR.L** |
|  | 31347 | **642.4502** | **5131.5434** | **5130.5715** | **0.9719** | **1** | **(1)** | **8.5** | **1** | **K.DNGIRPSSLEQMAKLKPAFIKPYGTVTAANSSFLTDGASAMLIMAEEK.A** |
|  | 31368 | **1716.6179** | **5146.8319** | **5146.5664** | **0.2655** | **1** | **2** | **5.6** | **2** | **K.DNGIRPSSLEQMAKLKPAFIKPYGTVTAANSSFLTDGASAMLIMAEEK.A** |

| **38.** | Q9NY65\|TBA8_HUMAN    **Mass:** 50746    **Score:** 581    **Queries matched:** 24   **emPAI:** 1.03 |
| --- | --- |
|  | Tubulin alpha-8 chain OS=Homo sapiens GN=TUBA8 PE=1 SV=1 |

|  | **Query** | **Observed** | **Mr(expt)** | **Mr(calc)** | **Delta** | **Miss** | **Score** | **Expect** | **Rank** | **Peptide** |
| --- | --- | --- | --- | --- | --- | --- | --- | --- | --- | --- |
|  | 2728 | 444.2173 | 886.4200 | 886.4259 | -0.0058 | 0 | 41 | 0.017 | 1 | K.FDLMYAK.R |
|  | 7835 | **414.6910** | **1241.0511** | **1241.7343** | **-0.6832** | **1** | **2** | **55** | **5** | **K.DVNVAIAAIKTK.R** |
|  | 10474 | 690.8484 | 1379.6822 | 1379.6907 | -0.0085 | 1 | 16 | 3.6 | 1 | R.LDHKFDLMYAK.R |
|  | 14038 | 799.8832 | 1597.7518 | 1597.7599 | -0.0081 | 0 | 58 | 0.00018 | 1 | R.TIQFVDWCPTGFK.V |
|  | 15623 | 851.4520 | 1700.8895 | 1700.8655 | 0.0240 | 0 | 10 | 11 | 2 | R.AVMIDLEPTVVDEVR.A 15621 |
|  | 15854 | 859.9388 | 1717.8631 | 1717.8747 | -0.0116 | 0 | 51 | 0.00085 | 1 | R.NLDIERPTYTNLNR.L 15855 |
|  | 17194 | 912.9911 | 1823.9676 | 1823.9782 | -0.0105 | 0 | 46 | 0.0028 | 1 | K.VGINYQPPTVVPGGDLAK.V 17192 17193 17195 17209 |
|  | 17729 | 932.9498 | 1863.8851 | 1863.8971 | -0.0120 | 0 | 85 | 3.7e-007 | 1 | R.AVCMLSNTTAIAEAWAR.L 17730 |
|  | 22254 | 777.3408 | 2329.0006 | 2329.0110 | -0.0103 | 0 | 39 | 0.0091 | 1 | R.AFVHWYVGEGMEEGEFSEAR.E |
|  | 22800 | 1205.1028 | 2408.1910 | 2408.2012 | -0.0102 | 0 | 123 | 3.6e-011 | 1 | R.FDGALNVDLTEFQTNLVPYPR.I 22799 22801 |
|  | 22841 | 1208.0984 | 2414.1822 | 2414.1978 | -0.0156 | 1 | 46 | 0.0019 | 1 | R.QLFHPEQLITGKEDAANNYAR.G 22842 22844 |
|  | 27012 | 797.6893 | 3186.7282 | 3186.7360 | -0.0078 | 1 | 5 | 15 | 2 | R.NLDIERPTYTNLNRLISQIVSSITASLR.F |
|  | 27872 | 1135.5789 | 3403.7148 | 3403.7275 | -0.0128 | 1 | 42 | 0.0029 | 1 | R.TIQFVDWCPTGFKVGINYQPPTVVPGGDLAK.V |

| **39.** | O75309\|CAD16_HUMAN    **Mass:** 90380    **Score:** 541    **Queries matched:** 34   **emPAI:** 0.69 |
| --- | --- |
|  | Cadherin-16 OS=Homo sapiens GN=CDH16 PE=1 SV=1 |

|  | **Query** | **Observed** | **Mr(expt)** | **Mr(calc)** | **Delta** | **Miss** | **Score** | **Expect** | **Rank** | **Peptide** |
| --- | --- | --- | --- | --- | --- | --- | --- | --- | --- | --- |
|  | 4167 | **497.7524** | **993.4903** | **993.4953** | **-0.0051** | **0** | **51** | **0.0011** | **1** | **R.LMDFAIER.G** 4154 4172 4178 |
|  | 5429 | **546.3238** | **1090.6330** | **1090.6386** | **-0.0056** | **0** | **9** | **21** | **1** | **R.TYQLLVQVK.D** |
|  | 6511 | **588.3679** | **1174.7212** | **1173.5262** | **1.1950** | **0** | **12** | **10** | **2** | **R.DEPGTANSDLR.F** |
|  | 8618 | **640.8531** | **1279.6916** | **1280.6248** | **-0.9332** | **0** | **2** | **93** | **4** | **K.DPDQPADSVPLK.A** |
|  | 11579 | **723.8525** | **1445.6904** | **1445.6998** | **-0.0094** | **0** | **17** | **2.7** | **1** | **R.EGAEGQIVLSGDSGK.A** |
|  | 17317 | **916.9740** | **1831.9334** | **1831.9428** | **-0.0093** | **1** | **57** | **0.0002** | **1** | **R.ELDREAQAEYLLQVR.A** |
|  | 18836 | **979.0352** | **1956.0558** | **1956.0681** | **-0.0123** | **0** | **9** | **11** | **1** | **R.AFQVDPTSGSVTLGVLPLR.A** 18826 |
|  | 19544 | **1012.4863** | **2022.9580** | **2022.9721** | **-0.0141** | **0** | **9** | **11** | **1** | **K.ATEGPFAMDPDSGFLLVTR.A** |
|  | 21386 | **1110.5896** | **2219.1646** | **2219.1772** | **-0.0126** | **0** | **49** | **0.001** | **1** | **K.APPAPALTLAPVPSQYLCTPR.Q** 21385 21387 |
|  | 21455 | **1113.5863** | **2225.1580** | **2225.1692** | **-0.0111** | **0** | **80** | **8.2e-007** | **1** | **K.NLSYEAAPSHEVVVVVQSVAK.L** |
|  | 22791 | **1205.0613** | **2408.1080** | **2408.1947** | **-0.0867** | **0** | **14** | **3.1** | **2** | **R.FHILSQAPAQPSPDMFQLEPR.L** 22792 |
|  | 23796 | **639.7959** | **2555.1545** | **2555.4146** | **-0.2601** | **1** | **4** | **29** | **8** | **K.LVGPGPGPGATATVTVLVERVMPPPK.L** |
|  | 25773 | **733.6164** | **2930.4365** | **2930.4522** | **-0.0158** | **1** | **50** | **0.00059** | **1** | **R.GTRPGIPFLFLEASDRDEPGTANSDLR.F** 25771 25772 |
|  | 26362 | **1017.4616** | **3049.3628** | **3049.3876** | **-0.0248** | **1** | **56** | **0.00011** | **1** | **R.LMDFAIERGDTEGTFGLDWEPDSGHVR.L** 26361 |
|  | 27596 | **1112.5216** | **3334.5430** | **3334.5589** | **-0.0159** | **0** | **35** | **0.014** | **1** | **R.LSAEDADAPGSPNSHVVYQLLSPEPEDGVEGR.A** 27595 |
|  | 28054 | **1151.2217** | **3450.6432** | **3450.6613** | **-0.0181** | **1** | **96** | **9.9e-009** | **1** | **R.EGAEGQIVLSGDSGKATEGPFAMDPDSGFLLVTR.A** 28053 |
|  | 28370 | **1182.8862** | **3545.6369** | **3545.6547** | **-0.0178** | **0** | **63** | **1.5e-005** | **1** | **K.FSGEVHTAQSLQGAQPGDTYTVLVEAQDTDEPR.L** |
|  | 28737 | **911.9522** | **3643.7797** | **3643.8020** | **-0.0222** | **1** | **14** | **1.5** | **1** | **R.QDHGLIVSGPSKDPDLASGHGPYSFTLGPNPTVQR.D** |
|  | 29508 | **1313.3411** | **3937.0014** | **3937.0183** | **-0.0169** | **1** | **32** | **0.021** | **1** | **R.VMPPPKLDQESYEASVPISAPAGSFLLTIQPSDPISR.T** 29509 |
|  | 31137 | **1231.6168** | **4922.4382** | **4922.4727** | **-0.0345** | **1** | **45** | **0.00053** | **1** | **K.FSGEVHTAQSLQGAQPGDTYTVLVEAQDTDEPRLSASAPLVIHFLK.A** 31138 |

| **40.** | O95831\|AIFM1_HUMAN    **Mass:** 67144    **Score:** 541    **Queries matched:** 53   **emPAI:** 1.94 |
| --- | --- |
|  | Apoptosis-inducing factor 1, mitochondrial OS=Homo sapiens GN=AIFM1 PE=1 SV=1 |

|  | **Query** | **Observed** | **Mr(expt)** | **Mr(calc)** | **Delta** | **Miss** | **Score** | **Expect** | **Rank** | **Peptide** |
| --- | --- | --- | --- | --- | --- | --- | --- | --- | --- | --- |
|  | 1688 | **408.7296** | **815.4446** | **816.4453** | **-1.0007** | **1** | **16** | **5.7** | **8** | **R.AGAEVKSR.T** |
|  | 1854 | **415.2284** | **828.4422** | **827.4865** | **0.9557** | **0** | **6** | **52** | **5** | **K.VVQLDVR.D** 1843 |
|  | 2726 | **444.2141** | **886.4136** | **886.4185** | **-0.0049** | **0** | **34** | **0.09** | **1** | **K.LFNIHED.-** |
|  | 2881 | **900.4841** | **899.4768** | **899.4825** | **-0.0057** | **0** | **15** | **5.3** | **1** | **R.VNAELQAR.S** |
|  | 4837 | **523.2736** | **1044.5326** | **1044.5386** | **-0.0061** | **0** | **18** | **2.7** | **1** | **K.CLIATGGTPR.S** |
|  | 5630 | **553.7713** | **1105.5280** | **1105.5364** | **-0.0083** | **0** | **57** | **0.00038** | **1** | **K.SATEQSGTGIR.S** 5631 5632 5633 5634 5635 5653 |
|  | 6077 | **571.8215** | **1141.6284** | **1141.6343** | **-0.0059** | **0** | **31** | **0.14** | **1** | **R.ISGLGLTPEQK.Q** 6076 |
|  | 8364 | **634.3079** | **1266.6012** | **1266.6092** | **-0.0080** | **0** | **62** | **8.5e-005** | **1** | **K.LNDGSQITYEK.C** 8365 |
|  | 9321 | **658.8577** | **1315.7009** | **1315.7095** | **-0.0086** | **1** | **72** | **8.8e-006** | **1** | **R.SLSAIDRAGAEVK.S** 9320 |
|  | 9783 | **448.2228** | **1341.6464** | **1341.6538** | **-0.0074** | **0** | **35** | **0.041** | **1** | **R.VEHHDHAVVSGR.L** |
|  | 9964 | **451.2844** | **1350.8313** | **1350.6900** | **0.1412** | **1** | **2** | **82** | **4** | **-.MFRCGGLAAGALK.Q** |
|  | 11057 | **472.5913** | **1414.7521** | **1414.7602** | **-0.0081** | **1** | **21** | **1.1** | **1** | **K.VVQLDVRDNMVK.L** |
|  | 11635 | **725.8419** | **1449.6692** | **1449.6776** | **-0.0084** | **0** | **35** | **0.042** | **1** | **K.ELWFSDDPNVTK.T** 11636 |
|  | 12205 | **742.3320** | **1482.6495** | **1482.6586** | **-0.0091** | **0** | **28** | **0.19** | **1** | **K.DGEQHEDLNEVAK.L** |
|  | 12439 | **500.2561** | **1497.7466** | **1497.7549** | **-0.0084** | **1** | **41** | **0.0099** | **1** | **R.RVEHHDHAVVSGR.L** 12438 |
|  | 13616 | **786.9197** | **1571.8248** | **1571.8341** | **-0.0093** | **0** | **67** | **2.3e-005** | **1** | **K.VMPNAIVQSVGVSSGK.L** 13609 |
|  | 14432 | **812.4036** | **1622.7926** | **1622.8014** | **-0.0088** | **0** | **40** | **0.012** | **1** | **K.ILPEYLSNWTMEK.V** |
|  | 14696 | 820.4670 | 1638.9195 | 1638.7963 | 0.1232 | 0 | (12) | 7 | 2 | K.ILPEYLSNWTMEK.V 14694 14695 |
|  | 15009 | 830.4469 | 1658.8792 | 1657.8159 | 1.0634 | 1 | 1 | 88 | 6 | K.KAALSASEGEEVPQDK.A |
|  | 15168 | **835.9421** | **1669.8697** | **1670.9355** | **-1.0658** | **1** | **2** | **81** | **10** | **K.ARALGTEVIQLFPEK.G** |
|  | 17251 | **915.4251** | **1828.8357** | **1828.8454** | **-0.0097** | **0** | **32** | **0.072** | **1** | **R.SNIWVAGDAACFYDIK.L** |
|  | 17373 | **613.3099** | **1836.9078** | **1836.9217** | **-0.0139** | **1** | **25** | **0.34** | **1** | **K.IIKDGEQHEDLNEVAK.L** |
|  | 18547 | **966.5117** | **1931.0089** | **1931.0186** | **-0.0097** | **1** | **53** | **0.00052** | **1** | **R.ALGTEVIQLFPEKGNMGK.I** 18546 18548 18549 |
|  | 18737 | **650.2888** | **1947.8446** | **1947.0135** | **0.8311** | **1** | **(2)** | **60** | **3** | **R.ALGTEVIQLFPEKGNMGK.I** |
|  | 19210 | **996.5280** | **1991.0413** | **1991.0510** | **-0.0096** | **0** | **91** | **7.9e-008** | **1** | **K.SITIIGGGFLGSELACALGR.K** |
|  | 21063 | **1092.0870** | **2182.1595** | **2182.1707** | **-0.0112** | **0** | **62** | **5.5e-005** | **1** | **R.VLIVSEDPELPYMRPPLSK.E** 21064 21065 |
|  | 21515 | **744.7417** | **2231.2033** | **2231.2161** | **-0.0129** | **1** | **62** | **5.2e-005** | **1** | **R.KVETDHIVAAVGLEPNVELAK.T** |
|  | 22280 | **1167.5316** | **2333.0487** | **2334.2419** | **-1.1932** | **1** | **0** | **69** | **9** | **R.LPGNLFQRWHVPLELQMTR.Q** |
|  | 27215 | **1078.8821** | **3233.6244** | **3233.6397** | **-0.0153** | **0** | **6** | **11** | **1** | **R.SIYFQPPSFYVSAQDLPHIENGGVAVLTGK.K** |
|  | 31222 | **1671.8026** | **5012.3860** | **5011.4201** | **0.9659** | **0** | **31** | **0.01** | **1** | **R.LAGENMTGAAKPYWHQSMFWSDLGPDVGYEAIGLVDSSLPTVGVFAK.A** 31223 |
|  | 31241 | **1257.8560** | **5027.3947** | **5027.4150** | **-0.0203** | **0** | **(19)** | **0.19** | **1** | **R.LAGENMTGAAKPYWHQSMFWSDLGPDVGYEAIGLVDSSLPTVGVFAK.A** |
|  | 31242 | **1257.8590** | **5027.4069** | **5027.4150** | **-0.0081** | **0** | **(11)** | **1** | **1** | **R.LAGENMTGAAKPYWHQSMFWSDLGPDVGYEAIGLVDSSLPTVGVFAK.A** |

| **41.** | Q13509\|TBB3_HUMAN    **Mass:** 50856    **Score:** 539    **Queries matched:** 43   **emPAI:** 1.51 |
| --- | --- |
|  | Tubulin beta-3 chain OS=Homo sapiens GN=TUBB3 PE=1 SV=2 |

|  | **Query** | **Observed** | **Mr(expt)** | **Mr(calc)** | **Delta** | **Miss** | **Score** | **Expect** | **Rank** | **Peptide** |
| --- | --- | --- | --- | --- | --- | --- | --- | --- | --- | --- |
|  | 1602 | 404.7373 | 807.4600 | 807.3399 | 0.1201 | 0 | 8 | 28 | 4 | R.EEYPDR.I |
|  | 5095 | 533.2719 | 1064.5293 | 1064.4201 | 0.1091 | 0 | (18) | 2.3 | 1 | K.NMMAACDPR.H 5090 5102 |
|  | 5275 | 541.2110 | 1080.4074 | 1080.4151 | -0.0076 | 0 | 26 | 0.36 | 1 | K.NMMAACDPR.H |
|  | 5276 | 541.2111 | 1080.4076 | 1080.4151 | -0.0075 | 0 | (25) | 0.48 | 1 | K.NMMAACDPR.H |
|  | 6084 | 572.3180 | 1142.6215 | 1142.6270 | -0.0055 | 0 | 17 | 3.3 | 1 | K.LAVNMVPFPR.L |
|  | 8765 | 430.2513 | 1287.7319 | 1286.7169 | 1.0150 | 1 | 4 | 55 | 3 | R.KLAVNMVPFPR.L |
|  | 9381 | 660.3513 | 1318.6880 | 1318.6955 | -0.0075 | 0 | 69 | 2e-005 | 1 | R.IMNTFSVVPSPK.V 9382 |
|  | 10550 | 693.3494 | 1384.6843 | 1384.6921 | -0.0078 | 1 | 30 | 0.13 | 1 | K.RISEQFTAMFR.R |
|  | 14299 | 808.4167 | 1614.8189 | 1614.8287 | -0.0098 | 0 | 44 | 0.0046 | 1 | R.AILVDLEPGTMDSVR.S 14300 14310 |
|  | 14378 | 810.9174 | 1619.8202 | 1619.8283 | -0.0081 | 0 | 16 | 2.7 | 2 | R.LHFFMPGFAPLTAR.G 14377 |
|  | 14564 | 816.4130 | 1630.8114 | 1630.8236 | -0.0123 | 0 | (21) | 1.1 | 1 | R.AILVDLEPGTMDSVR.S 14576 |
|  | 15480 | 846.4329 | 1690.8512 | 1690.8600 | -0.0088 | 0 | 66 | 3e-005 | 1 | R.ALTVPELTQQMFDAK.N 15478 |
|  | 15553 | 848.9153 | 1695.8160 | 1695.8257 | -0.0096 | 0 | 19 | 1.4 | 1 | K.NSSYFVEWIPNNVK.V 15562 15563 15564 |
|  | 17173 | 911.9601 | 1821.9057 | 1821.9156 | -0.0098 | 0 | 61 | 8.7e-005 | 1 | R.EIVHIQAGQCGNQIGAK.F 17174 |
|  | 17969 | **628.6407** | **1882.9002** | **1882.8838** | **0.0164** | **1** | **0** | **92** | **7** | **R.MSMKEVDEQMLAIQSK.N** |
|  | 18859 | 979.9888 | 1957.9631 | 1957.9745 | -0.0114 | 0 | 86 | 2.2e-007 | 1 | K.GHYTEGAELVDSVLDVVR.K 18858 |
|  | 20166 | 1044.0366 | 2086.0587 | 2086.0695 | -0.0108 | 1 | 114 | 3.4e-010 | 1 | K.GHYTEGAELVDSVLDVVRK.E 20162 20163 20167 20168 20169 |
|  | 20402 | 1055.5299 | 2109.0453 | 2109.0571 | -0.0119 | 1 | 94 | 3.6e-008 | 1 | -.MREIVHIQAGQCGNQIGAK.F 20399 20400 20401 20403 20410 |
|  | 20534 | 532.2908 | 2125.1342 | 2125.0521 | 0.0822 | 1 | (5) | 26 | 4 | -.MREIVHIQAGQCGNQIGAK.F |
|  | 25038 | 929.4656 | 2785.3749 | 2785.2543 | 0.1206 | 1 | 1 | 45 | 4 | R.ALTVPELTQQMFDAKNMMAACDPR.H |

| **42.** | P05141\|ADT2_HUMAN    **Mass:** 33102    **Score:** 519    **Queries matched:** 47   **emPAI:** 4.05 |
| --- | --- |
|  | ADP/ATP translocase 2 OS=Homo sapiens GN=SLC25A5 PE=1 SV=6 |

|  | **Query** | **Observed** | **Mr(expt)** | **Mr(calc)** | **Delta** | **Miss** | **Score** | **Expect** | **Rank** | **Peptide** |
| --- | --- | --- | --- | --- | --- | --- | --- | --- | --- | --- |
|  | 2024 | **420.6771** | **839.3397** | **839.3452** | **-0.0055** | **0** | **25** | **0.43** | **1** | **R.MMMQSGR.K** 2026 2042 |
|  | 2254 | **428.7456** | **855.4767** | **855.4814** | **-0.0047** | **0** | **36** | **0.043** | **1** | **K.TAVAPIER.V** 2274 |
|  | 2255 | **428.7509** | **855.4873** | **855.4926** | **-0.0053** | **0** | **31** | **0.12** | **1** | **R.GNLANVIR.Y** |
|  | 2267 | **429.0874** | **856.1602** | **855.3401** | **0.8201** | **0** | **(14)** | **4.8** | **1** | **R.MMMQSGR.K** 2269 |
|  | 2271 | **429.2248** | **856.4350** | **855.3401** | **1.0949** | **0** | **(13)** | **9** | **1** | **R.MMMQSGR.K** |
|  | 2272 | **429.2307** | **856.4469** | **855.3401** | **1.1068** | **0** | **(13)** | **9.6** | **3** | **R.MMMQSGR.K** |
|  | 2332 | **431.2261** | **860.4376** | **860.4426** | **-0.0049** | **0** | **41** | **0.016** | **1** | **R.GLGDCLVK.I** 2322 2333 |
|  | 3325 | **465.7691** | **929.5236** | **929.5294** | **-0.0058** | **1** | **14** | **8.1** | **3** | **R.TRLAADVGK.A** |
|  | 3385 | **468.2345** | **934.4544** | **934.4621** | **-0.0077** | **1** | **13** | **8.7** | **8** | **K.AGAEREFR.G** |
|  | 3784 | **484.7235** | **967.4325** | **967.4401** | **-0.0077** | **1** | **29** | **0.18** | **1** | **R.MMMQSGRK.G** |
|  | 4190 | **498.7267** | **995.4387** | **995.4463** | **-0.0075** | **1** | **22** | **1** | **1** | **R.RMMMQSGR.K** 4193 |
|  | 4763 | 520.2977 | 1038.5809 | 1039.5008 | -0.9199 | 0 | 1 | 1.2e+002 | 9 | -.MTDAAVSFAK.D |
|  | 5311 | **542.5944** | **1083.1743** | **1082.6448** | **0.5295** | **1** | **9** | **11** | **3** | **K.TAVAPIERVK.L** |
|  | 5835 | **561.2869** | **1120.5593** | **1120.5665** | **-0.0072** | **0** | **35** | **0.047** | **1** | **K.EQGVLSFWR.G** 5834 |
|  | 6005 | **568.8392** | **1135.6639** | **1135.6713** | **-0.0074** | **0** | **25** | **0.49** | **1** | **K.LLLQVQHASK.Q** 6000 |
|  | 7375 | 610.3341 | 1218.6537 | 1218.6608 | -0.0072 | 0 | 77 | 3.1e-006 | 1 | K.DFLAGGVAAAISK.T 7376 7377 |
|  | 10149 | **681.8502** | **1361.6859** | **1362.8347** | **-1.1488** | **1** | **66** | **3.3e-005** | **1** | **R.VKLLLQVQHASK.Q** 10159 10170 10171 |
|  | 15436 | **844.8689** | **1687.7232** | **1687.7334** | **-0.0102** | **0** | **64** | **4.2e-005** | **1** | **K.GTDIMYTGTLDCWR.K** 15435 |
|  | 16130 | **870.4696** | **1738.9246** | **1738.9328** | **-0.0081** | **1** | **38** | **0.017** | **1** | **R.GMGGAFVLVLYDEIKK.Y** |
|  | 17076 | **908.9162** | **1815.8178** | **1815.8284** | **-0.0106** | **1** | **78** | **1.6e-006** | **1** | **R.KGTDIMYTGTLDCWR.K** 17075 17093 |
|  | 17077 | **908.9170** | **1815.8194** | **1815.8284** | **-0.0090** | **1** | **43** | **0.0049** | **1** | **K.GTDIMYTGTLDCWRK.I** 17078 |
|  | 25112 | **932.7831** | **2795.3274** | **2795.3377** | **-0.0103** | **0** | **51** | **0.00045** | **1** | **R.YFAGNLASGGAAGATSLCFVYPLDFAR.T** 25108 25109 25110 25113 25114 25115 |
|  | 28238 | **879.4016** | **3513.5771** | **3513.6928** | **-0.1157** | **1** | **1** | **30** | **6** | **R.TQFWRYFAGNLASGGAAGATSLCFVYPLDFAR.T** |

| **43.** | P33121\|ACSL1_HUMAN    **Mass:** 78919    **Score:** 505    **Queries matched:** 47   **emPAI:** 0.99 |
| --- | --- |
|  | Long-chain-fatty-acid--CoA ligase 1 OS=Homo sapiens GN=ACSL1 PE=1 SV=1 |

|  | **Query** | **Observed** | **Mr(expt)** | **Mr(calc)** | **Delta** | **Miss** | **Score** | **Expect** | **Rank** | **Peptide** |
| --- | --- | --- | --- | --- | --- | --- | --- | --- | --- | --- |
|  | 1346 | **396.1872** | **790.3599** | **790.3643** | **-0.0044** | **0** | **44** | **0.009** | **1** | **K.AMEDLGR.A** |
|  | 2354 | **432.2156** | **862.4167** | **862.4470** | **-0.0303** | **0** | **7** | **44** | **8** | **R.LLMDDLK.V** |
|  | 2449 | **434.8811** | **867.7476** | **868.5242** | **-0.7767** | **1** | **11** | **6.9** | **1** | **K.AKRPELR.N** 2435 |
|  | 2891 | **451.0397** | **900.0649** | **899.4613** | **0.6036** | **0** | **9** | **15** | **9** | **M.QAHELFR.Y** |
|  | 4459 | **508.2895** | **1014.5644** | **1013.5757** | **0.9887** | **0** | **25** | **0.57** | **1** | **K.LLLEGVENK.L** 4443 |
|  | 4879 | **524.6259** | **1047.2373** | **1046.4967** | **0.7406** | **0** | **2** | **82** | **10** | **-.MQAHELFR.Y** |
|  | 4931 | **527.2718** | **1052.5290** | **1051.5451** | **0.9839** | **0** | **36** | **0.037** | **1** | **K.IGFFQGDIR.L** |
|  | 5003 | 530.2474 | 1058.4802 | 1057.5992 | 0.8810 | 1 | 5 | 66 | 7 | K.VQSSLGGRVR.L |
|  | 5256 | **540.7662** | **1079.5178** | **1079.5546** | **-0.0368** | **1** | **5** | **43** | **9** | **R.LLNRMFDR.I** |
|  | 5407 | **545.7416** | **1089.4687** | **1089.5852** | **-0.1165** | **1** | **7** | **35** | **5** | **K.KAILEDMVR.L** |
|  | 5849 | **561.5532** | **1121.0919** | **1121.5869** | **-0.4951** | **0** | **2** | **72** | **6** | **K.GPNVFQGYLK.D** |
|  | 7791 | **620.3020** | **1238.5894** | **1238.5965** | **-0.0071** | **0** | **49** | **0.0017** | **1** | **R.NIVSDCSAFVK.A** 7766 7792 |
|  | 7972 | **624.3313** | **1246.6480** | **1246.6557** | **-0.0077** | **0** | **22** | **0.88** | **1** | **K.DSGLKPFEQVK.G** |
|  | 9947 | **451.2287** | **1350.6643** | **1350.8024** | **-0.1381** | **0** | **4** | **58** | **4** | **K.VLQPTVFPVVPR.L** |
|  | 10507 | 691.9351 | 1381.8557 | 1380.7137 | 1.1420 | 1 | 14 | 5.5 | 1 | R.SQIDDLYSTIKV.- |
|  | 11766 | **729.8548** | **1457.6950** | **1457.7045** | **-0.0095** | **0** | **64** | **5.7e-005** | **1** | **R.GIQVSNNGPCLGSR.K** 11783 11786 11788 |
|  | 12060 | **738.3826** | **1474.7507** | **1473.8079** | **0.9428** | **0** | **19** | **1.9** | **1** | **K.AELSLVFVDKPEK.A** 12061 12062 |
|  | 12685 | **505.2525** | **1512.7356** | **1512.7296** | **0.0060** | **1** | **6** | **33** | **8** | **-.MQAHELFRYFR.M** |
|  | 13195 | **773.4310** | **1544.8475** | **1544.8562** | **-0.0087** | **1** | **54** | **0.00055** | **1** | **R.LGKDSGLKPFEQVK.G** 13194 13196 |
|  | 16211 | **873.4541** | **1744.8936** | **1744.9029** | **-0.0093** | **0** | **93** | **5.2e-008** | **1** | **K.QVAELSECIGSALIQK.G** |
|  | 17508 | **924.0220** | **1846.0294** | **1846.0387** | **-0.0093** | **0** | **16** | **2.7** | **1** | **R.LMVTGAAPVSATVLTFLR.A** |
|  | 22016 | **1149.1061** | **2296.1976** | **2296.2137** | **-0.0161** | **0** | **68** | **1.1e-005** | **1** | **K.GITLHPELFSIDNGLLTPTMK.A** 22004 22017 22018 22026 |
|  | 22692 | **597.7418** | **2386.9379** | **2387.0331** | **-0.0951** | **0** | **1** | **47** | **6** | **K.LVDVEEMNYMAAEGEGEVCVK.G** |
|  | 24507 | **894.4691** | **2680.3854** | **2680.4007** | **-0.0153** | **1** | **55** | **0.00019** | **1** | **R.RKPKPPAPEDLAVICFTSGTTGNPK.G** |
|  | 26398 | **1528.7190** | **3055.4234** | **3055.4339** | **-0.0104** | **0** | **93** | **2.8e-008** | **1** | **R.SALLDSDEPLVYFYDDVTTLYEGFQR.G** 26399 26400 |
|  | 27114 | **1071.5151** | **3211.5236** | **3211.5350** | **-0.0114** | **1** | **21** | **0.36** | **1** | **R.RSALLDSDEPLVYFYDDVTTLYEGFQR.G** |
|  | 30126 | **1052.7832** | **4207.1037** | **4208.0017** | **-0.8980** | **1** | **1** | **21** | **2** | **R.NIVSDCSAFVKATENTVNPCPDDTLISFLPLAHMFER.V** |
|  | 30913 | **1188.2579** | **4749.0026** | **4749.0348** | **-0.0322** | **0** | **32** | **0.0081** | **1** | **R.AALGCQFYEGYGQTECTAGCCLTMPGDWTAGHVGAPMPCNLIK.L** 30914 |
|  | 31343 | **1282.8492** | **5127.3679** | **5128.5613** | **-1.1935** | **0** | **1** | **11** | **4** | **R.TLPTNTLMGFGAFAALTTFWYATRPKPLKPPCDLSMQSVEVAGSGGAR.R** |

| **44.** | P30049\|ATPD_HUMAN    **Mass:** 17479    **Score:** 504    **Queries matched:** 35   **emPAI:** 1.23 |
| --- | --- |
|  | ATP synthase subunit delta, mitochondrial OS=Homo sapiens GN=ATP5D PE=1 SV=2 |

|  | **Query** | **Observed** | **Mr(expt)** | **Mr(calc)** | **Delta** | **Miss** | **Score** | **Expect** | **Rank** | **Peptide** |
| --- | --- | --- | --- | --- | --- | --- | --- | --- | --- | --- |
|  | 4049 | **493.7588** | **985.5031** | **985.5444** | **-0.0413** | **0** | **20** | **1.6** | **1** | **R.IEANEALVK.A** 4044 |
|  | 20666 | **1071.5538** | **2141.0931** | **2141.1076** | **-0.0145** | **1** | **47** | **0.0018** | **1** | **K.AQAELVGTADEATRAEIQIR.I** 20662 20667 20669 20670 20671 20672 20679 20682 20691 20692 |
|  | 28539 | **1195.6014** | **3583.7825** | **3583.7967** | **-0.0142** | **0** | **69** | **4.6e-006** | **1** | **K.YFVSSGSIAVNADSSVQLLAEEAVTLDMLDLGAAK.A** 28526 28527 28531 28532 28533 28534 28535 28536 28537 28538 28540 28541 28542 28543 28544 28547 28557 28562 |
|  | 29915 | **1369.4249** | **4105.2530** | **4106.2529** | **-0.9999** | **0** | **26** | **0.052** | **1** | **R.QVDVPTLTGAFGILAAHVPTLQVLRPGLVVVHAEDGTTSK.Y** 29925 |
|  | 30034 | **1386.0953** | **4155.2642** | **4155.0933** | **0.1709** | **1** | **2** | **14** | **6** | **K.YFVSSGSIAVNADSSVQLLAEEAVTLDMLDLGAAKANLEK.A** |

| **45.** | P38606\|VATA_HUMAN    **Mass:** 68660    **Score:** 492    **Queries matched:** 33   **emPAI:** 1.20 |
| --- | --- |
|  | V-type proton ATPase catalytic subunit A OS=Homo sapiens GN=ATP6V1A PE=1 SV=2 |

|  | **Query** | **Observed** | **Mr(expt)** | **Mr(calc)** | **Delta** | **Miss** | **Score** | **Expect** | **Rank** | **Peptide** |
| --- | --- | --- | --- | --- | --- | --- | --- | --- | --- | --- |
|  | 2919 | **451.7537** | **901.4928** | **901.4981** | **-0.0054** | **0** | **55** | **0.0006** | **1** | **R.GVNVSALSR.D** 2918 |
|  | 3710 | **481.7766** | **961.5387** | **961.5444** | **-0.0057** | **0** | **33** | **0.097** | **1** | **K.TVISQSLSK.Y** |
|  | 4432 | **507.3004** | **1012.5862** | **1011.5647** | **1.0215** | **1** | **3** | **73** | **6** | **K.IMLPPRNR.G** |
|  | 5627 | **553.6619** | **1105.3092** | **1104.5274** | **0.7818** | **0** | **13** | **6.6** | **1** | **R.EHMGDILYK.L** |
|  | 8590 | **427.1157** | **1278.3254** | **1277.6591** | **0.6663** | **0** | **3** | **44** | **7** | **K.FTMVQVWPVR.Q** |
|  | 9169 | **436.9112** | **1307.7117** | **1307.7197** | **-0.0080** | **0** | **29** | **0.17** | **1** | **R.VGHSELVGEIIR.L** 9194 |
|  | 9288 | **658.3444** | **1314.6743** | **1315.7361** | **-1.0618** | **0** | **2** | **95** | **1** | **K.LPANHPLLTGQR.V** |
|  | 12731 | **758.3963** | **1514.7780** | **1514.7875** | **-0.0094** | **0** | **49** | **0.0016** | **1** | **R.TALVANTSNMPVAAR.E** |
|  | 13700 | **789.4044** | **1576.7943** | **1576.8038** | **-0.0095** | **0** | **6** | **28** | **1** | **K.HFPSVNWLISYSK.Y** 13692 |
|  | 14899 | **551.5927** | **1651.7563** | **1650.8109** | **0.9454** | **1** | **2** | **73** | **5** | **R.EHMGDILYKLSSMK.F** |
|  | 15412 | **844.3420** | **1686.6694** | **1686.6800** | **-0.0106** | **0** | **68** | **1.7e-005** | **1** | **R.DMGYHVSMMADSTSR.W** 15428 |
|  | 15413 | **563.2596** | **1686.7569** | **1686.8835** | **-0.1266** | **1** | **1** | **85** | **4** | **K.RTALVANTSNMPVAAR.E** |
|  | 16869 | **900.9116** | **1799.8087** | **1799.8148** | **-0.0061** | **0** | **63** | **4.8e-005** | **1** | **K.SDYAQLLEDMQNAFR.S** |
|  | 17120 | **910.4267** | **1818.8388** | **1818.8467** | **-0.0078** | **0** | **75** | **3.4e-006** | **1** | **K.TVGMLSNMIAFYDMAR.R** 17135 |
|  | 20690 | **1072.0234** | **2142.0323** | **2142.0422** | **-0.0099** | **1** | **35** | **0.027** | **1** | **R.ALDEYYDKHFTEFVPLR.T** 20688 |
|  | 20858 | **720.3795** | **2158.1167** | **2158.1270** | **-0.0103** | **0** | **17** | **1.6** | **1** | **R.VGSHITGGDIYGIVSENSLIK.H** |
|  | 21678 | **1127.6074** | **2253.2003** | **2253.2103** | **-0.0101** | **1** | **121** | **6e-011** | **1** | **K.AKEILQEEEDLAEIVQLVGK.A** |
|  | 21680 | **752.0928** | **2253.2567** | **2253.2706** | **-0.0139** | **1** | **25** | **0.23** | **1** | **R.QVRPVTEKLPANHPLLTGQR.V** 21681 |
|  | 22990 | **1218.5994** | **2435.1842** | **2435.1978** | **-0.0136** | **0** | **56** | **0.00015** | **1** | **R.VLDALFPCVQGGTTAIPGAFGCGK.T** 22991 |
|  | 26926 | **1057.5266** | **3169.5580** | **3169.5707** | **-0.0127** | **1** | **0** | **46** | **9** | **R.GTVTYIAPPGNYDTSDVVLELEFEGVKEK.F** |
|  | 29206 | **1272.9899** | **3815.9478** | **3815.9622** | **-0.0145** | **1** | **49** | **0.00044** | **1** | **R.EGSVSIVGAVSPPGGDFSDPVTSATLGIVQVFWGLDKK.L** 29207 |
|  | 29572 | **1319.0018** | **3953.9837** | **3954.0045** | **-0.0208** | **1** | **56** | **7.7e-005** | **1** | **R.VGHSELVGEIIRLEGDMATIQVYEETSGVSVGDPVLR.T** |
|  | 30010 | **1381.3986** | **4141.1739** | **4141.1882** | **-0.0143** | **0** | **52** | **0.00013** | **1** | **R.TGKPLSVELGPGIMGAIFDGIQRPLSDISSQTQSIYIPR.G** 30005 |

| **46.** | Q13733\|AT1A4_HUMAN    **Mass:** 115119   **Score:** 485    **Queries matched:** 22   **emPAI:** 0.25 |
| --- | --- |
|  | Sodium/potassium-transporting ATPase subunit alpha-4 OS=Homo sapiens GN=ATP1A4 PE=1 SV=3 |

|  | **Query** | **Observed** | **Mr(expt)** | **Mr(calc)** | **Delta** | **Miss** | **Score** | **Expect** | **Rank** | **Peptide** |
| --- | --- | --- | --- | --- | --- | --- | --- | --- | --- | --- |
|  | 5359 | 544.2969 | 1086.5792 | 1086.5856 | -0.0063 | 0 | 37 | 0.032 | 1 | K.LIIVEGCQR.L |
|  | 7546 | **410.1950** | **1227.5630** | **1228.5547** | **-0.9916** | **0** | **10** | **16** | **1** | **K.SSDTWFMLAR.I** |
|  | 8394 | 634.8575 | 1267.7004 | 1267.7071 | -0.0067 | 0 | 55 | 0.00044 | 1 | K.NMVPQQALVIR.G 8371 8393 |
|  | 8693 | 642.8540 | 1283.6934 | 1283.7020 | -0.0085 | 0 | (16) | 3.4 | 1 | K.NMVPQQALVIR.G |
|  | 9404 | **661.3586** | **1320.7027** | **1319.6694** | **1.0333** | **1** | **5** | **46** | **3** | **K.GTVAPHDQSPRR.R** |
|  | 9462 | **662.3832** | **1322.7519** | **1321.6561** | **1.0958** | **1** | **4** | **50** | **5** | **R.NSLFQQGMRNK.V** |
|  | 10399 | **459.3275** | **1374.9605** | **1375.7130** | **-0.7524** | **0** | **4** | **37** | **7** | **R.GIVIATGDSTVMGR.I** |
|  | 10497 | 691.3746 | 1380.7347 | 1380.7435 | -0.0088 | 0 | 72 | 9.3e-006 | 1 | K.VIMVTGDHPITAK.A 10473 10499 |
|  | 14697 | 547.3140 | 1638.9201 | 1638.8875 | 0.0325 | 1 | 7 | 25 | 5 | K.NMVPQQALVIRGGEK.M |
|  | 17382 | 613.3414 | 1837.0023 | 1837.0132 | -0.0109 | 1 | 45 | 0.0034 | 1 | R.SAGIKVIMVTGDHPITAK.A |
|  | 18447 | 962.9559 | 1923.8972 | 1923.9095 | -0.0123 | 0 | 64 | 3.4e-005 | 1 | K.NLEAVETLGSTSTICSDK.T |
|  | 19349 | 669.0273 | 2004.0602 | 2003.0696 | 0.9906 | 1 | 15 | 3 | 1 | K.IMESFKNMVPQQALVIR.G |
|  | 25121 | 1398.6792 | 2795.3438 | 2795.3607 | -0.0169 | 1 | 123 | 3.5e-011 | 1 | K.NLEAVETLGSTSTICSDKTGTLTQNR.M 25117 25118 25119 25122 |
|  | 27563 | **666.7321** | **3328.6239** | **3329.4567** | **-0.8328** | **1** | **2** | **25** | **1** | **R.MTVAHMWFDMTVYEADTTEEQTGKTFTK.S** |

| **47.** | Q3ZCM7\|TBB8_HUMAN    **Mass:** 50257    **Score:** 452    **Queries matched:** 39   **emPAI:** 1.05 |
| --- | --- |
|  | Tubulin beta-8 chain OS=Homo sapiens GN=TUBB8 PE=1 SV=2 |

|  | **Query** | **Observed** | **Mr(expt)** | **Mr(calc)** | **Delta** | **Miss** | **Score** | **Expect** | **Rank** | **Peptide** |
| --- | --- | --- | --- | --- | --- | --- | --- | --- | --- | --- |
|  | 1602 | 404.7373 | 807.4600 | 807.3399 | 0.1201 | 0 | 8 | 28 | 4 | R.EEYPDR.I |
|  | 4597 | **513.7761** | **1025.5377** | **1024.5705** | **0.9671** | **0** | **1** | **1.1e+002** | **6** | **R.YLTAAAIFR.G** |
|  | 4618 | 514.7597 | 1027.5048 | 1027.5121 | -0.0072 | 0 | 25 | 0.57 | 1 | K.TAVCDIPPR.G 4619 4624 4627 4629 |
|  | 5095 | 533.2719 | 1064.5293 | 1064.4201 | 0.1091 | 0 | (18) | 2.3 | 1 | K.NMMAACDPR.H 5090 5102 |
|  | 5226 | 539.2660 | 1076.5175 | 1076.5250 | -0.0075 | 1 | 25 | 0.53 | 1 | K.IREEYPDR.I 5225 |
|  | 5275 | 541.2110 | 1080.4074 | 1080.4151 | -0.0076 | 0 | 26 | 0.36 | 1 | K.NMMAACDPR.H |
|  | 5276 | 541.2111 | 1080.4076 | 1080.4151 | -0.0075 | 0 | (25) | 0.48 | 1 | K.NMMAACDPR.H |
|  | 6084 | 572.3180 | 1142.6215 | 1142.6270 | -0.0055 | 0 | 17 | 3.3 | 1 | K.LAVNMVPFPR.L |
|  | 8765 | 430.2513 | 1287.7319 | 1286.7169 | 1.0150 | 1 | 4 | 55 | 3 | R.KLAVNMVPFPR.L |
|  | 9543 | 664.3431 | 1326.6716 | 1325.7126 | 0.9591 | 1 | 5 | 48 | 9 | K.TAVCDIPPRGLK.M |
|  | 14084 | 801.4085 | 1600.8025 | 1600.8131 | -0.0106 | 0 | 63 | 6.1e-005 | 1 | R.AVLVDLEPGTMDSVR.S 14085 14086 14096 |
|  | 14330 | 809.4058 | 1616.7971 | 1616.8080 | -0.0109 | 0 | (21) | 0.95 | 1 | R.AVLVDLEPGTMDSVR.S |
|  | 14380 | 540.9477 | 1619.8212 | 1619.8283 | -0.0070 | 0 | 48 | 0.0018 | 1 | R.LHFFMPGFAPLTSR.G 14371 14377 14378 14379 |
|  | 19132 | **497.2602** | **1985.0118** | **1984.0524** | **0.9594** | **1** | **1** | **73** | **4** | **M.REIVLTQIGQCGNQIGAK.F** |
|  | 21198 | **734.0298** | **2199.0675** | **2198.1405** | **0.9270** | **1** | **0** | **82** | **9** | **R.GLKMSATFIGNNTAIQELFK.R** |
|  | 27484 | 1656.2628 | 3310.5111 | 3310.5268 | -0.0158 | 0 | 72 | 2.8e-006 | 1 | K.EAESCDCLQGFQLTHSLGGGTGSGMGTLLLSK.I 27480 27481 27482 27483 |
|  | 27558 | 1109.8416 | 3326.5028 | 3326.5218 | -0.0189 | 0 | (3) | 24 | 2 | K.EAESCDCLQGFQLTHSLGGGTGSGMGTLLLSK.I 27556 |
|  | 28039 | 1147.2087 | 3438.6044 | 3438.6218 | -0.0174 | 1 | 115 | 1.3e-010 | 1 | R.KEAESCDCLQGFQLTHSLGGGTGSGMGTLLLSK.I 28036 28037 |

| **48.** | P04792\|HSPB1_HUMAN    **Mass:** 22826    **Score:** 445    **Queries matched:** 31   **emPAI:** 4.47 |
| --- | --- |
|  | Heat shock protein beta-1 OS=Homo sapiens GN=HSPB1 PE=1 SV=2 |

|  | **Query** | **Observed** | **Mr(expt)** | **Mr(calc)** | **Delta** | **Miss** | **Score** | **Expect** | **Rank** | **Peptide** |
| --- | --- | --- | --- | --- | --- | --- | --- | --- | --- | --- |
|  | 4070 | **494.3056** | **986.5966** | **986.6025** | **-0.0059** | **1** | **23** | **0.95** | **1** | **R.RVPFSLLR.G** |
|  | 5202 | **537.8199** | **1073.6253** | **1074.5669** | **-0.9416** | **0** | **38** | **0.031** | **1** | **R.QLSSGVSEIR.H** 5218 |
|  | 6125 | **573.4061** | **1144.7976** | **1145.6292** | **-0.8316** | **1** | **4** | **50** | **2** | **K.TKDGVVEITGK.H** 6117 |
|  | 6365 | **582.3107** | **1162.6068** | **1162.6135** | **-0.0067** | **0** | **57** | **0.0003** | **1** | **R.LFDQAFGLPR.L** 6364 |
|  | 14748 | **822.4100** | **1642.8054** | **1642.8162** | **-0.0108** | **1** | **57** | **0.00022** | **1** | **R.AQLGGPEAAKSDETAAK.-** 14749 14768 |
|  | 14933 | 552.5847 | 1654.7322 | 1654.7448 | -0.0126 | 1 | 39 | 0.014 | 1 | K.HEERQDEHGYISR.C |
|  | 16691 | **892.4600** | **1782.9055** | **1782.9152** | **-0.0097** | **0** | **62** | **6.4e-005** | **1** | **R.VSLDVNHFAPDELTVK.T** 16688 16689 16690 16692 |
|  | 18213 | **953.4946** | **1904.9747** | **1904.9843** | **-0.0096** | **0** | **56** | **0.00024** | **1** | **K.LATQSNEITIPVTFESR.A** 18210 18211 18220 18225 |
|  | 26614 | **1549.7762** | **3097.5379** | **3097.5529** | **-0.0150** | **0** | **55** | **0.00015** | **1** | **K.YTLPPGVDPTQVSSSLSPEGTLTVEAPMPK.L** 26613 |
|  | 27187 | **1076.2183** | **3225.6330** | **3225.6479** | **-0.0150** | **1** | **53** | **0.00019** | **1** | **R.KYTLPPGVDPTQVSSSLSPEGTLTVEAPMPK.L** 27182 27183 27184 27185 27186 |
|  | 31442 | **1310.4093** | **5237.6081** | **5236.6563** | **0.9518** | **1** | **29** | **0.016** | **1** | **R.LFDQAFGLPRLPEEWSQWLGGSSWPGYVRPLPPAAIESPAVAAPAYSR.A** 31440 |

| **49.** | P22695\|QCR2_HUMAN    **Mass:** 48584    **Score:** 434    **Queries matched:** 56   **emPAI:** 2.52 |
| --- | --- |
|  | Cytochrome b-c1 complex subunit 2, mitochondrial OS=Homo sapiens GN=UQCRC2 PE=1 SV=3 |

|  | **Query** | **Observed** | **Mr(expt)** | **Mr(calc)** | **Delta** | **Miss** | **Score** | **Expect** | **Rank** | **Peptide** |
| --- | --- | --- | --- | --- | --- | --- | --- | --- | --- | --- |
|  | 1230 | **747.4310** | **746.4238** | **746.4286** | **-0.0049** | **0** | **15** | **7.3** | **1** | **K.LSVTATR.E** |
|  | 1241 | **759.4308** | **758.4236** | **758.4286** | **-0.0051** | **0** | **15** | **7.8** | **1** | **R.GGLGLSGAK.A** |
|  | 4938 | **527.3029** | **1052.5913** | **1051.6066** | **0.9847** | **1** | **2** | **87** | **6** | **R.FYSLKVAPK.V** |
|  | 5551 | **550.8139** | **1099.6132** | **1099.6349** | **-0.0217** | **1** | **9** | **20** | **10** | **K.ITRGIEAVGGK.L** |
|  | 8290 | **632.3567** | **1262.6988** | **1262.6731** | **0.0257** | **1** | **5** | **47** | **5** | **R.GGLGLSGAKANYR.G** |
|  | 9499 | **663.8521** | **1325.6897** | **1325.6979** | **-0.0083** | **0** | **33** | **0.075** | **1** | **R.WEVADLQPQLK.I** 9500 |
|  | 10542 | **693.3078** | **1384.6010** | **1384.6115** | **-0.0104** | **0** | **14** | **5.8** | **1** | **R.ENMAYTVECLR.G** |
|  | 11370 | **478.5802** | **1432.7189** | **1433.8428** | **-1.1240** | **0** | **4** | **55** | **10** | **R.MALIGLGVSHPVLK.Q** |
|  | 11640 | **725.8685** | **1449.7225** | **1449.7324** | **-0.0099** | **0** | **11** | **11** | **1** | **R.GSNTTSHLHQAVAK.A** 11631 11632 11633 11641 11643 |
|  | 12191 | **741.9024** | **1481.7902** | **1481.7990** | **-0.0088** | **1** | **25** | **0.42** | **1** | **R.RWEVADLQPQLK.I** 12192 12212 |
|  | 14160 | **803.9181** | **1605.8216** | **1605.8335** | **-0.0119** | **1** | **45** | **0.0037** | **1** | **K.RGSNTTSHLHQAVAK.A** 14139 14140 14141 14144 14147 14158 14159 |
|  | 14551 | **815.9185** | **1629.8225** | **1629.8322** | **-0.0097** | **0** | **67** | **2.3e-005** | **1** | **K.TIAQGNLSNTDVQAAK.N** 14552 14568 |
|  | 15084 | **833.4103** | **1664.8060** | **1664.8158** | **-0.0098** | **0** | **63** | **5.2e-005** | **1** | **R.YEDFSNLGTTHLLR.L** |
|  | 15364 | **561.6389** | **1681.8949** | **1681.9039** | **-0.0090** | **1** | **20** | **1** | **1** | **R.WEVADLQPQLKIDK.A** |
|  | 16206 | **873.4077** | **1744.8009** | **1744.8090** | **-0.0082** | **0** | **25** | **0.35** | **1** | **K.SMAASGNLGHTPFVDEL.-** 16205 |
|  | 19671 | **679.3283** | **2034.9631** | **2036.0075** | **-1.0444** | **1** | **15** | **3.1** | **1** | **K.AGSRYEDFSNLGTTHLLR.L** |
|  | 20899 | **722.3483** | **2164.0232** | **2164.0338** | **-0.0106** | **0** | **34** | **0.038** | **1** | **K.VTSEELHYFVQNHFTSAR.M** |
|  | 21907 | **760.7219** | **2279.1438** | **2279.1508** | **-0.0070** | **0** | **18** | **1.2** | **1** | **R.GDVDILMEFLLNVTTAPEFR.R** |
|  | 22404 | **588.3036** | **2349.1853** | **2349.1978** | **-0.0125** | **0** | **13** | **3.7** | **1** | **K.AVAFQNPQTHVIENLHAAAYR.N** 22405 |
|  | 23177 | **821.7468** | **2462.2187** | **2462.2343** | **-0.0156** | **1** | **67** | **1.4e-005** | **1** | **R.IGKVTSEELHYFVQNHFTSAR.M** 23181 23182 |
|  | 23399 | **833.0701** | **2496.1886** | **2496.2860** | **-0.0974** | **1** | **3** | **35** | **3** | **R.YEDFSNLGTTHLLRLTSSLTTK.G** |
|  | 24639 | 677.3545 | 2705.3889 | 2705.4037 | -0.0149 | 1 | 37 | 0.011 | 1 | K.IDKAVAFQNPQTHVIENLHAAAYR.N 24640 24641 |
|  | 29215 | **1273.9943** | **3818.9610** | **3818.9843** | **-0.0233** | **1** | **41** | **0.0023** | **1** | **K.ATAAPAGAPPQPQDLEFTKLPNGLVIASLENYSPVSR.I** 29220 29222 |
|  | 29276 | **1280.9537** | **3839.8394** | **3839.8530** | **-0.0136** | **0** | **29** | **0.044** | **1** | **K.ATQQPFDVSAFNASYSDSGLFGIYTISQATAAGDVIK.A** |
|  | 29871 | **1022.2601** | **4085.0112** | **4084.0403** | **0.9709** | **0** | **24** | **0.11** | **1** | **R.EQNGDSLVHAAFVAESAVAGSAEANAFSVLQHVLGAGPHVK.R** 29865 |
|  | 30718 | **920.2666** | **4596.2966** | **4596.3109** | **-0.0143** | **1** | **18** | **0.28** | **1** | **R.GGEIREQNGDSLVHAAFVAESAVAGSAEANAFSVLQHVLGAGPHVK.R** |
|  | 30740 | **1539.0846** | **4614.2319** | **4614.2554** | **-0.0235** | **1** | **88** | **3.1e-008** | **1** | **K.ATQQPFDVSAFNASYSDSGLFGIYTISQATAAGDVIKAAYNQVK.T** 30741 30743 |
|  | 31473 | **1315.8820** | **5259.4987** | **5259.5301** | **-0.0314** | **0** | **9** | **1.4** | **1** | **K.AGYLMSVESSECFLEEVGSQALVAGSYMPPSTVLQQIDSVANADIINAAK.K** |

| **50.** | P29972\|AQP1_HUMAN    **Mass:** 28736    **Score:** 433    **Queries matched:** 12   **emPAI:** 0.86 |
| --- | --- |
|  | Aquaporin-1 OS=Homo sapiens GN=AQP1 PE=1 SV=3 |

|  | **Query** | **Observed** | **Mr(expt)** | **Mr(calc)** | **Delta** | **Miss** | **Score** | **Expect** | **Rank** | **Peptide** |
| --- | --- | --- | --- | --- | --- | --- | --- | --- | --- | --- |
|  | 2008 | **420.2217** | **838.4287** | **839.4211** | **-0.9923** | **1** | **5** | **52** | **7** | **-.MASEFKK.K** |
|  | 4522 | **511.2483** | **1020.4820** | **1019.5247** | **0.9572** | **1** | **7** | **32** | **6** | **R.SSDLTDRVK.V** |
|  | 22123 | **1156.5124** | **2311.0103** | **2311.0240** | **-0.0137** | **0** | **128** | **1.1e-011** | **1** | **K.VWTSGQVEEYDLDADDINSR.V** 22121 22122 22135 |
|  | 23647 | **847.0646** | **2538.1719** | **2538.1874** | **-0.0155** | **1** | **40** | **0.0074** | **1** | **R.VKVWTSGQVEEYDLDADDINSR.V** |
|  | 26180 | **1008.8074** | **3023.4003** | **3023.4182** | **-0.0179** | **1** | **78** | **7.7e-007** | **1** | **K.VWTSGQVEEYDLDADDINSRVEMKPK.-** 26177 26181 26182 |
|  | 27321 | **1088.2422** | **3261.7047** | **3261.7180** | **-0.0132** | **0** | **50** | **0.0004** | **1** | **R.DLGGSAPLAIGLSVALGHLLAIDYTGCGINPAR.S** |

| **51.** | P21796\|VDAC1_HUMAN    **Mass:** 30868    **Score:** 430    **Queries matched:** 25   **emPAI:** 1.52 |
| --- | --- |
|  | Voltage-dependent anion-selective channel protein 1 OS=Homo sapiens GN=VDAC1 PE=1 SV=2 |

|  | **Query** | **Observed** | **Mr(expt)** | **Mr(calc)** | **Delta** | **Miss** | **Score** | **Expect** | **Rank** | **Peptide** |
| --- | --- | --- | --- | --- | --- | --- | --- | --- | --- | --- |
|  | 2234 | **854.4710** | **853.4637** | **853.4698** | **-0.0061** | **0** | **25** | **0.5** | **1** | **K.GYGFGLIK.L** 2235 |
|  | 4655 | **515.8078** | **1029.6010** | **1029.6070** | **-0.0059** | **0** | **12** | **12** | **3** | **K.LTLSALLDGK.N** |
|  | 10365 | **687.8278** | **1373.6411** | **1373.6503** | **-0.0092** | **0** | **27** | **0.31** | **1** | **R.WTEYGLTFTEK.W** 10393 |
|  | 10808 | **700.8342** | **1399.6538** | **1399.6620** | **-0.0082** | **0** | **35** | **0.046** | **1** | **K.LTFDSSFSPNTGK.K** |
|  | 12924 | **510.2564** | **1527.7475** | **1527.7569** | **-0.0094** | **1** | **6** | **33** | **1** | **K.LTFDSSFSPNTGKK.N** |
|  | 15508 | 847.4106 | 1692.8067 | 1692.8148 | -0.0080 | 1 | 85 | 3.6e-007 | 1 | K.YRWTEYGLTFTEK.W |
|  | 15923 | **575.3360** | **1722.9862** | **1723.9006** | **-0.9144** | **1** | **2** | **63** | **9** | **K.NVNAGGHKLGLGLEFQA.-** |
|  | 20333 | **1052.0890** | **2102.1634** | **2102.1735** | **-0.0101** | **0** | **25** | **0.27** | **1** | **K.VNNSSLIGLGYTQTLKPGIK.L** 20330 20331 20332 20334 |
|  | 20865 | **1080.4911** | **2158.9676** | **2158.9776** | **-0.0099** | **0** | **39** | **0.011** | **1** | **R.EHINLGCDMDFDIAGPSIR.G** |
|  | 20994 | **1088.5239** | **2175.0333** | **2175.0444** | **-0.0111** | **0** | **138** | **1.5e-012** | **1** | **K.WNTDNTLGTEITVEDQLAR.G** 20995 |
|  | 21112 | **730.3411** | **2188.0016** | **2188.0131** | **-0.0116** | **1** | **19** | **1.1** | **1** | **K.TKSENGLEFTSSGSANTETTK.V** 21125 |
|  | 22870 | **1209.5874** | **2417.1602** | **2417.1725** | **-0.0123** | **0** | **61** | **6e-005** | **1** | **R.GALVLGYEGWLAGYQMNFETAK.S** 22869 |
|  | 24040 | **867.3964** | **2599.1673** | **2599.1827** | **-0.0154** | **0** | **58** | **0.00011** | **1** | **K.TDEFQLHTNVNDGTEFGGSIYQK.V** |
|  | 24479 | **893.0765** | **2676.2076** | **2676.3006** | **-0.0930** | **1** | **0** | **57** | **7** | **R.GALVLGYEGWLAGYQMNFETAKSR.V** |
|  | 28196 | **1749.7119** | **3497.4093** | **3497.7864** | **-0.3772** | **1** | **1** | **25** | **10** | **K.YQIDPDACFSAKVNNSSLIGLGYTQTLKPGIK.L** |
|  | 29153 | **949.4482** | **3793.7636** | **3793.7860** | **-0.0224** | **1** | **3** | **15** | **2** | **R.VTQSNFAVGYKTDEFQLHTNVNDGTEFGGSIYQK.V** |

| **52.** | P00167\|CYB5_HUMAN    **Mass:** 15321    **Score:** 412    **Queries matched:** 28   **emPAI:** 11.12 |
| --- | --- |
|  | Cytochrome b5 OS=Homo sapiens GN=CYB5A PE=1 SV=2 |

|  | **Query** | **Observed** | **Mr(expt)** | **Mr(calc)** | **Delta** | **Miss** | **Score** | **Expect** | **Rank** | **Peptide** |
| --- | --- | --- | --- | --- | --- | --- | --- | --- | --- | --- |
|  | 5984 | **1134.6343** | **1133.6270** | **1133.6346** | **-0.0075** | **0** | **39** | **0.021** | **1** | **K.STWLILHHK.V** 5985 5986 |
|  | 12638 | **756.3736** | **1510.7326** | **1510.7416** | **-0.0089** | **0** | **40** | **0.011** | **1** | **K.FLEEHPGGEEVLR.E** 12641 12663 |
|  | 14660 | **819.4307** | **1636.8468** | **1636.8573** | **-0.0105** | **0** | **38** | **0.019** | **1** | **K.TFIIGELHPDDRPK.L** 14659 14661 14662 14663 14669 |
|  | 16755 | **597.2971** | **1788.8694** | **1788.8795** | **-0.0101** | **1** | **2** | **74** | **8** | **K.YYTLEEIQKHNHSK.S** |
|  | 20426 | **705.0252** | **2112.0538** | **2112.0674** | **-0.0136** | **1** | **9** | **11** | **1** | **R.EMSKTFIIGELHPDDRPK.L** |
|  | 21249 | **1103.4620** | **2204.9095** | **2204.9207** | **-0.0111** | **0** | **114** | **3.2e-010** | **1** | **R.EQAGGDATENFEDVGHSTDAR.E** 21246 21247 21248 21259 |
|  | 21512 | **744.7133** | **2231.1181** | **2230.1270** | **0.9911** | **1** | **13** | **4.4** | **1** | **K.VYDLTKFLEEHPGGEEVLR.E** |
|  | 28879 | **740.5337** | **3697.6321** | **3697.6517** | **-0.0196** | **1** | **32** | **0.021** | **1** | **K.FLEEHPGGEEVLREQAGGDATENFEDVGHSTDAR.E** 28875 28876 28877 28878 28880 |
|  | 29991 | **1377.7223** | **4130.1450** | **4130.1550** | **-0.0100** | **0** | **81** | **1.8e-007** | **1** | **K.LNKPPETLITTIDSSSSWWTNWVIPAISAVAVALMYR.L** 29992 |

| **53.** | P20674\|COX5A_HUMAN    **Mass:** 16923    **Score:** 410    **Queries matched:** 29   **emPAI:** 6.92 |
| --- | --- |
|  | Cytochrome c oxidase subunit 5A, mitochondrial OS=Homo sapiens GN=COX5A PE=1 SV=2 |

|  | **Query** | **Observed** | **Mr(expt)** | **Mr(calc)** | **Delta** | **Miss** | **Score** | **Expect** | **Rank** | **Peptide** |
| --- | --- | --- | --- | --- | --- | --- | --- | --- | --- | --- |
|  | 1267 | **771.4673** | **770.4600** | **770.4650** | **-0.0050** | **0** | **33** | **0.098** | **1** | **K.IIDAALR.A** |
|  | 2151 | **425.0480** | **848.0814** | **848.4174** | **-0.3360** | **0** | **14** | **5.3** | **1** | **R.CAVAATTR.A** |
|  | 3472 | **472.2886** | **942.5627** | **942.5750** | **-0.0122** | **1** | **37** | **0.031** | **1** | **R.ILEVVKDK.A** |
|  | 4543 | **511.7637** | **1021.5128** | **1021.5193** | **-0.0064** | **0** | **55** | **0.00051** | **1** | **R.LNDFASTVR.I** 4554 |
|  | 6543 | **589.8135** | **1177.6124** | **1177.6204** | **-0.0080** | **1** | **50** | **0.0015** | **1** | **R.RLNDFASTVR.I** 6535 6549 |
|  | 15273 | **838.9260** | **1675.8375** | **1675.8491** | **-0.0116** | **0** | **93** | **6.2e-008** | **1** | **K.GINTLVTYDMVPEPK.I** 15239 15274 15275 15276 15283 15286 |
|  | 17131 | **607.5634** | **1819.6682** | **1819.9390** | **-0.2708** | **1** | **0** | **92** | **9** | **R.KGINTLVTYDMVPEPK.I** |
|  | 19836 | **685.0074** | **2052.0003** | **2052.0105** | **-0.0102** | **0** | **8** | **15** | **1** | **R.WVTYFNKPDIDAWELR.K** 19850 |
|  | 21047 | **1091.0530** | **2180.0914** | **2180.1055** | **-0.0140** | **1** | **69** | **1e-005** | **1** | **R.WVTYFNKPDIDAWELRK.G** 21035 21046 21048 21049 21051 |
|  | 27968 | **1714.9060** | **3427.7975** | **3427.8126** | **-0.0151** | **1** | **31** | **0.029** | **1** | **K.EIYPYVIQELRPTLNELGISTPEELGLDKV.-** 27967 27969 27970 27972 |

| **54.** | Q99867\|TBB4Q_HUMAN    **Mass:** 48917    **Score:** 408    **Queries matched:** 27   **emPAI:** 0.67 |
| --- | --- |
|  | Putative tubulin beta-4q chain OS=Homo sapiens GN=TUBB4Q PE=5 SV=1 |

|  | **Query** | **Observed** | **Mr(expt)** | **Mr(calc)** | **Delta** | **Miss** | **Score** | **Expect** | **Rank** | **Peptide** |
| --- | --- | --- | --- | --- | --- | --- | --- | --- | --- | --- |
|  | 1602 | 404.7373 | 807.4600 | 807.3399 | 0.1201 | 0 | 8 | 28 | 4 | R.EEYPDR.I |
|  | 5226 | 539.2660 | 1076.5175 | 1076.5250 | -0.0075 | 1 | 25 | 0.53 | 1 | K.IREEYPDR.I 5225 |
|  | 6084 | 572.3180 | 1142.6215 | 1142.6270 | -0.0055 | 0 | 17 | 3.3 | 1 | K.LAVNMVPFPR.L |
|  | 8765 | 430.2513 | 1287.7319 | 1286.7169 | 1.0150 | 1 | 4 | 55 | 3 | R.KLAVNMVPFPR.L |
|  | 14084 | 801.4085 | 1600.8025 | 1600.8131 | -0.0106 | 0 | 63 | 6.1e-005 | 1 | R.AVLVDLEPGTMDSVR.S 14085 14086 14096 |
|  | 14330 | 809.4058 | 1616.7971 | 1616.8080 | -0.0109 | 0 | (21) | 0.95 | 1 | R.AVLVDLEPGTMDSVR.S |
|  | 17460 | **615.3414** | **1843.0025** | **1843.8332** | **-0.8307** | **0** | **7** | **20** | **3** | **R.YTEGAELMESVMDVVR.K** 17464 |
|  | 19185 | **664.0126** | **1989.0159** | **1987.9231** | **1.0928** | **1** | **1** | **81** | **1** | **R.YTEGAELMESVMDVVRK.E** |
|  | 19724 | **511.2419** | **2040.9384** | **2040.9608** | **-0.0224** | **1** | **3** | **42** | **5** | **K.GRYTEGAELMESVMDVVR.K** |
|  | 19875 | **686.2848** | **2055.8325** | **2056.9558** | **-1.1232** | **1** | **(1)** | **61** | **5** | **K.GRYTEGAELMESVMDVVR.K** |
|  | 22470 | **472.2487** | **2356.2071** | **2355.1385** | **1.0686** | **1** | **7** | **15** | **3** | **R.ALTVAELTQQMFDAKNMMAAR.D** |
|  | 22471 | **472.2513** | **2356.2200** | **2355.1385** | **1.0815** | **1** | **(4)** | **33** | **2** | **R.ALTVAELTQQMFDAKNMMAAR.D** |
|  | 27484 | 1656.2628 | 3310.5111 | 3310.5268 | -0.0158 | 0 | 72 | 2.8e-006 | 1 | K.EAESCDCLQGFQLTHSLGGGTGSGMGTLLLSK.I 27480 27481 27482 27483 |
|  | 27558 | 1109.8416 | 3326.5028 | 3326.5218 | -0.0189 | 0 | (3) | 24 | 2 | K.EAESCDCLQGFQLTHSLGGGTGSGMGTLLLSK.I 27556 |
|  | 28039 | 1147.2087 | 3438.6044 | 3438.6218 | -0.0174 | 1 | 115 | 1.3e-010 | 1 | R.KEAESCDCLQGFQLTHSLGGGTGSGMGTLLLSK.I 28036 28037 |

| **55.** | P08107\|HSP71_HUMAN    **Mass:** 70294    **Score:** 407    **Queries matched:** 40   **emPAI:** 1.16 |
| --- | --- |
|  | Heat shock 70 kDa protein 1 OS=Homo sapiens GN=HSPA1A PE=1 SV=5 |

|  | **Query** | **Observed** | **Mr(expt)** | **Mr(calc)** | **Delta** | **Miss** | **Score** | **Expect** | **Rank** | **Peptide** |
| --- | --- | --- | --- | --- | --- | --- | --- | --- | --- | --- |
|  | 1134 | 687.3989 | 686.3916 | 686.3963 | -0.0047 | 0 | 23 | 1.2 | 3 | R.LIGDAAK.N 1136 |
|  | 1146 | 689.3898 | 688.3826 | 688.3755 | 0.0070 | 1 | 11 | 20 | 8 | R.DAKLDK.A |
|  | 2193 | 426.2319 | 850.4492 | 849.3902 | 1.0591 | 0 | 5 | 53 | 6 | R.MVQEAEK.Y |
|  | 4487 | 509.2846 | 1016.5547 | 1016.5614 | -0.0068 | 1 | 31 | 0.15 | 1 | K.ITITNDKGR.L 4494 |
|  | 5977 | **567.3701** | **1132.7257** | **1131.5771** | **1.1485** | **1** | **1** | **1.3e+002** | **3** | **K.SAVEDEGLKGK.I** |
|  | 6034 | **569.8051** | **1137.5955** | **1137.6030** | **-0.0074** | **1** | **10** | **14** | **1** | **K.VQVSYKGETK.A** |
|  | 7427 | **611.7890** | **1221.5634** | **1221.5700** | **-0.0065** | **0** | **49** | **0.0018** | **1** | **K.FGDPVVQSDMK.H** |
|  | 7551 | 614.8137 | 1227.6128 | 1227.6207 | -0.0080 | 0 | 44 | 0.0057 | 1 | K.VEIIANDQGNR.T |
|  | 8276 | 421.5560 | 1261.6462 | 1260.6503 | 0.9959 | 0 | 17 | 2.9 | 1 | R.LVNHFVEEFK.R |
|  | 9939 | **676.1088** | **1350.2030** | **1349.6649** | **0.5380** | **1** | **33** | **0.051** | **1** | **R.KFGDPVVQSDMK.H** |
|  | 11900 | **733.4047** | **1464.7948** | **1464.8049** | **-0.0101** | **0** | **46** | **0.0031** | **1** | **K.AQIHDLVLVGGSTR.I** 11908 |
|  | 12274 | 744.3496 | 1486.6845 | 1486.6940 | -0.0095 | 0 | 49 | 0.0015 | 1 | R.TTPSYVAFTDTER.L |
|  | 13130 | **771.8685** | **1541.7224** | **1541.7296** | **-0.0073** | **1** | **49** | **0.0016** | **1** | **R.ARFEELCSDLFR.S** 13128 13129 |
|  | 14469 | **542.5991** | **1624.7755** | **1624.9260** | **-0.1505** | **1** | **7** | **23** | **1** | **R.QATKDAGVIAGLNVLR.I** |
|  | 14978 | **829.9235** | **1657.8324** | **1657.8424** | **-0.0100** | **0** | **67** | **2.5e-005** | **1** | **K.NQVALNPQNTVFDAK.R** 14979 |
|  | 15235 | **838.3636** | **1674.7126** | **1674.7234** | **-0.0108** | **0** | **42** | **0.0064** | **1** | **K.ATAGDTHLGGEDFDNR.L** 15233 15236 |
|  | 15330 | **560.9514** | **1679.8324** | **1679.8420** | **-0.0096** | **0** | **46** | **0.0026** | **1** | **K.HWPFQVINDGDKPK.V** 15329 |
|  | 15415 | **563.2714** | **1686.7924** | **1687.8239** | **-1.0315** | **1** | **3** | **56** | **8** | **R.VSAKNALESYAFNMK.S** 15440 |
|  | 17054 | **605.6510** | **1813.9312** | **1813.9435** | **-0.0123** | **1** | **32** | **0.062** | **1** | **K.NQVALNPQNTVFDAKR.L** 17047 17055 |
|  | 17175 | **608.3256** | **1821.9549** | **1821.0108** | **0.9440** | **1** | **12** | **6.6** | **2** | **K.LDKAQIHDLVLVGGSTR.I** |
|  | 22177 | **580.5344** | **2318.1083** | **2319.1416** | **-1.0333** | **0** | **6** | **19** | **2** | **K.SINPDEAVAYGAAVQAAILMGDK.S** 22193 |
|  | 25030 | **1393.6780** | **2785.3414** | **2785.3559** | **-0.0145** | **0** | **73** | **2.8e-006** | **1** | **K.QTQIFTTYSDNQPGVLIQVYEGER.A** |
|  | 27034 | **799.6585** | **3194.6047** | **3195.7312** | **-1.1265** | **0** | **0** | **46** | **5** | **K.SENVQDLLLLDVAPLSLGLETAGGVMTALIK.R** |
|  | 28122 | **1158.9291** | **3473.7654** | **3473.7362** | **0.0293** | **1** | **1** | **32** | **6** | **K.AAAIGIDLGTTYSCVGVFQHGKVEIIANDQGNR.T** |
|  | 28299 | **1176.5861** | **3526.7364** | **3526.7580** | **-0.0216** | **1** | **70** | **4.1e-006** | **1** | **R.NSTIPTKQTQIFTTYSDNQPGVLIQVYEGER.A** 28300 28301 |

| **56.** | P10809\|CH60_HUMAN    **Mass:** 61187    **Score:** 407    **Queries matched:** 38   **emPAI:** 0.43 |
| --- | --- |
|  | 60 kDa heat shock protein, mitochondrial OS=Homo sapiens GN=HSPD1 PE=1 SV=2 |

|  | **Query** | **Observed** | **Mr(expt)** | **Mr(calc)** | **Delta** | **Miss** | **Score** | **Expect** | **Rank** | **Peptide** |
| --- | --- | --- | --- | --- | --- | --- | --- | --- | --- | --- |
|  | 669 | **503.1051** | **502.0979** | **501.2911** | **0.8068** | **0** | **2** | **2.7e+002** | **4** | **K.NIGAK.L** |
|  | 1145 | **689.3886** | **688.3813** | **688.3756** | **0.0057** | **0** | **9** | **30** | **7** | **K.DGVTVAK.S** |
|  | 1364 | **396.7319** | **791.4492** | **792.4494** | **-1.0002** | **1** | **7** | **39** | **4** | **K.YKNIGAK.L** |
|  | 2889 | **900.5095** | **899.5022** | **900.5280** | **-1.0258** | **0** | **9** | **22** | **1** | **K.LSDGVAVLK.V** |
|  | 3682 | 480.7050 | 959.3954 | 959.5036 | -0.1082 | 0 | 12 | 11 | 6 | R.VTDALNATR.A |
|  | 3951 | **490.2652** | **978.5159** | **977.4930** | **1.0229** | **1** | **7** | **32** | **9** | **K.DVKFGADAR.A** 3945 |
|  | 4395 | **505.7870** | **1009.5595** | **1010.5621** | **-1.0026** | **1** | **4** | **57** | **5** | **K.GANPVEIRR.G** |
|  | 4501 | **509.7994** | **1017.5841** | **1016.5866** | **0.9975** | **1** | **40** | **0.019** | **1** | **K.VTKDGVTVAK.S** 4479 |
|  | 5955 | **566.7239** | **1131.4333** | **1131.6587** | **-0.2253** | **1** | **15** | **5.3** | **5** | **-.MLRLPTVFR.Q** |
|  | 6610 | **395.2131** | **1182.6175** | **1182.6720** | **-0.0546** | **1** | **7** | **28** | **8** | **K.ISKGANPVEIR.R** |
|  | 7289 | **608.3346** | **1214.6546** | **1214.6507** | **0.0040** | **0** | **4** | **58** | **6** | **K.NAGVEGSLIVEK.I** 7291 |
|  | 9824 | **672.8571** | **1343.6996** | **1343.7085** | **-0.0089** | **0** | **42** | **0.0083** | **1** | **R.TVIIEQSWGSPK.V** |
|  | 12536 | **752.8776** | **1503.7406** | **1503.7490** | **-0.0085** | **0** | **81** | **1e-006** | **1** | **K.TLNDELEIIEGMK.F** |
|  | 15202 | **837.3611** | **1672.7076** | **1673.6129** | **-0.9053** | **0** | **(1)** | **94** | **4** | **K.DPGMGAMGGMGGGMGGGMF.-** |
|  | 15678 | **569.2788** | **1704.8146** | **1705.6027** | **-0.7881** | **0** | **(2)** | **72** | **7** | **K.DPGMGAMGGMGGGMGGGMF.-** |
|  | 15689 | **569.3045** | **1704.8917** | **1705.6027** | **-0.7111** | **0** | **19** | **1.4** | **1** | **K.DPGMGAMGGMGGGMGGGMF.-** 15695 |
|  | 15899 | **574.9500** | **1721.8282** | **1721.5977** | **0.2305** | **0** | **(1)** | **83** | **8** | **K.DPGMGAMGGMGGGMGGGMF.-** |
|  | 15902 | **575.0478** | **1722.1215** | **1721.5977** | **0.5239** | **0** | **(4)** | **32** | **1** | **K.DPGMGAMGGMGGGMGGGMF.-** |
|  | 15967 | **576.3315** | **1725.9728** | **1725.9526** | **0.0202** | **1** | **6** | **27** | **7** | **K.VGLQVVAVKAPGFGDNR.K** |
|  | 16104 | **580.0609** | **1737.1609** | **1737.5926** | **-0.4317** | **0** | **(1)** | **57** | **5** | **K.DPGMGAMGGMGGGMGGGMF.-** |
|  | 16312 | **585.3022** | **1752.8847** | **1753.5875** | **-0.7028** | **0** | **(2)** | **75** | **1** | **K.DPGMGAMGGMGGGMGGGMF.-** |
|  | 18361 | 960.0455 | 1918.0765 | 1918.0636 | 0.0129 | 0 | 1 | 82 | 7 | K.ISSIQSIVPALEIANAHR.K |
|  | 20213 | **697.8986** | **2090.6740** | **2091.7829** | **-1.1088** | **1** | **(0)** | **59** | **7** | **K.EEKDPGMGAMGGMGGGMGGGMF.-** |
|  | 20231 | **698.3538** | **2092.0395** | **2091.7829** | **0.2566** | **1** | **(0)** | **83** | **10** | **K.EEKDPGMGAMGGMGGGMGGGMF.-** |
|  | 20376 | **703.3306** | **2106.9699** | **2107.7778** | **-0.8079** | **1** | **(4)** | **40** | **8** | **K.EEKDPGMGAMGGMGGGMGGGMF.-** |
|  | 20380 | **527.7675** | **2107.0410** | **2107.7778** | **-0.7368** | **1** | **7** | **19** | **1** | **K.EEKDPGMGAMGGMGGGMGGGMF.-** |
|  | 20390 | **703.6530** | **2107.9370** | **2107.7778** | **0.1592** | **1** | **(1)** | **63** | **6** | **K.EEKDPGMGAMGGMGGGMGGGMF.-** |
|  | 20430 | **1057.0700** | **2112.1253** | **2112.1323** | **-0.0069** | **0** | **73** | **4.4e-006** | **1** | **R.ALMLQGVDLLADAVAVTMGPK.G** 20429 |
|  | 20477 | **706.3833** | **2116.1281** | **2115.1059** | **1.0221** | **1** | **0** | **83** | **6** | **K.LSDGVAVLKVGGTSDVEVNEK.K** |
|  | 22006 | **766.0545** | **2295.1417** | **2294.1529** | **0.9888** | **1** | **5** | **22** | **2** | **R.IQEIIEQLDVTTSEYEKEK.L** |
|  | 23830 | **1280.6195** | **2559.2245** | **2559.2413** | **-0.0168** | **0** | **148** | **1.1e-013** | **1** | **K.LVQDVANNTNEEAGDGTTTATVLAR.S** 23832 |
|  | 24836 | **915.8198** | **2744.4376** | **2745.4194** | **-0.9817** | **1** | **2** | **40** | **1** | **K.FGADARALMLQGVDLLADAVAVTMGPK.G** |

| **57.** | A6NNZ2\|TBB8B_HUMAN    **Mass:** 50168    **Score:** 385    **Queries matched:** 35   **emPAI:** 0.65 |
| --- | --- |
|  | Tubulin beta-8 chain B OS=Homo sapiens PE=1 SV=1 |

|  | **Query** | **Observed** | **Mr(expt)** | **Mr(calc)** | **Delta** | **Miss** | **Score** | **Expect** | **Rank** | **Peptide** |
| --- | --- | --- | --- | --- | --- | --- | --- | --- | --- | --- |
|  | 1602 | 404.7373 | 807.4600 | 807.3399 | 0.1201 | 0 | 8 | 28 | 4 | R.EEYPDR.I |
|  | 4618 | 514.7597 | 1027.5048 | 1027.5121 | -0.0072 | 0 | 25 | 0.57 | 1 | K.TAVCDIPPR.G 4619 4624 4627 4629 |
|  | 5095 | 533.2719 | 1064.5293 | 1064.4201 | 0.1091 | 0 | (18) | 2.3 | 1 | K.NMMAACDPR.H 5090 5102 |
|  | 5226 | 539.2660 | 1076.5175 | 1076.5250 | -0.0075 | 1 | 25 | 0.53 | 1 | K.IREEYPDR.I 5225 |
|  | 5275 | 541.2110 | 1080.4074 | 1080.4151 | -0.0076 | 0 | 26 | 0.36 | 1 | K.NMMAACDPR.H |
|  | 5276 | 541.2111 | 1080.4076 | 1080.4151 | -0.0075 | 0 | (25) | 0.48 | 1 | K.NMMAACDPR.H |
|  | 6084 | 572.3180 | 1142.6215 | 1142.6270 | -0.0055 | 0 | 17 | 3.3 | 1 | K.LAVNMVPFPR.L |
|  | 8765 | 430.2513 | 1287.7319 | 1286.7169 | 1.0150 | 1 | 4 | 55 | 3 | R.KLAVNMVPFPR.L |
|  | 9543 | 664.3431 | 1326.6716 | 1325.7126 | 0.9591 | 1 | 5 | 48 | 9 | K.TAVCDIPPRGLK.M |
|  | 10915 | **469.8835** | **1406.6287** | **1406.7129** | **-0.0842** | **0** | **7** | **24** | **7** | **R.HGCYLTVAAIFR.G** |
|  | 14380 | 540.9477 | 1619.8212 | 1619.8283 | -0.0070 | 0 | 48 | 0.0018 | 1 | R.LHFFMPGFAPLTSR.G 14371 14377 14378 14379 |
|  | 16851 | **600.2947** | **1797.8624** | **1797.8455** | **0.0169** | **0** | **2** | **71** | **2** | **R.YTEGAELTESVMDVVR.K** |
|  | 27132 | **1072.8164** | **3215.4274** | **3215.4726** | **-0.0452** | **0** | **1** | **39** | **1** | **K.MSATFIGNNAAIQELFTCVSEQFTAMFR.R** |
|  | 27484 | 1656.2628 | 3310.5111 | 3310.5268 | -0.0158 | 0 | 72 | 2.8e-006 | 1 | K.EAESCDCLQGFQLTHSLGGGTGSGMGTLLISK.I 27480 27481 27482 27483 |
|  | 27558 | 1109.8416 | 3326.5028 | 3326.5218 | -0.0189 | 0 | (3) | 24 | 2 | K.EAESCDCLQGFQLTHSLGGGTGSGMGTLLISK.I 27556 |
|  | 28039 | 1147.2087 | 3438.6044 | 3438.6218 | -0.0174 | 1 | 115 | 1.3e-010 | 1 | R.KEAESCDCLQGFQLTHSLGGGTGSGMGTLLISK.I 28036 28037 |
|  | 28070 | 1152.8376 | 3455.4911 | 3454.6167 | 0.8744 | 1 | (1) | 31 | 10 | R.KEAESCDCLQGFQLTHSLGGGTGSGMGTLLISK.I |

| **58.** | Q16853\|AOC3_HUMAN    **Mass:** 85138    **Score:** 383    **Queries matched:** 18   **emPAI:** 0.35 |
| --- | --- |
|  | Membrane primary amine oxidase OS=Homo sapiens GN=AOC3 PE=1 SV=3 |

|  | **Query** | **Observed** | **Mr(expt)** | **Mr(calc)** | **Delta** | **Miss** | **Score** | **Expect** | **Rank** | **Peptide** |
| --- | --- | --- | --- | --- | --- | --- | --- | --- | --- | --- |
|  | 1286 | **780.3949** | **779.3876** | **779.3926** | **-0.0050** | **0** | **17** | **4** | **1** | **R.FSVQGSR.V** |
|  | 2194 | **426.2322** | **850.4499** | **850.4549** | **-0.0050** | **0** | **6** | **46** | **3** | **K.YTTPLTR.G** |
|  | 5593 | **552.2946** | **1102.5747** | **1102.5805** | **-0.0058** | **0** | **48** | **0.0029** | **1** | **R.NLVTMTTAPR.G** |
|  | 9318 | **658.8546** | **1315.6947** | **1315.7030** | **-0.0084** | **1** | **37** | **0.03** | **1** | **R.GRNLVTMTTAPR.G** 9312 9319 |
|  | 16699 | 892.4945 | 1782.9743 | 1783.9733 | -0.9990 | 1 | 5 | 34 | 2 | R.GSPPPAREALAIVFFGR.Q |
|  | 17925 | 939.9705 | 1877.9265 | 1877.8982 | 0.0283 | 1 | 1 | 90 | 7 | R.YVDGGFGMGKYTTPLTR.G |
|  | 20190 | **1045.5463** | **2089.0780** | **2089.0877** | **-0.0098** | **1** | **114** | **3.2e-010** | **1** | **R.KLLEMEEQAAFLVGSATPR.Y** 20188 20189 |
|  | 24131 | **873.4348** | **2617.2826** | **2616.3489** | **0.9337** | **1** | **0** | **59** | **4** | **R.VASSLWTFSFGLGAFSGPRIFDVR.F** |
|  | 24657 | **903.7681** | **2708.2824** | **2708.2945** | **-0.0121** | **0** | **39** | **0.0081** | **1** | **R.GVDCPYLATYVDWHFLLESQAPK.T** 24658 |
|  | 25988 | **993.8470** | **2978.5191** | **2977.5331** | **0.9860** | **0** | **40** | **0.0052** | **1** | **R.LGPGLVDAAQARPSDNCVFSVELQLPPK.A** 25984 |
|  | 29231 | **1274.8876** | **3821.6409** | **3821.6621** | **-0.0212** | **0** | **77** | **5.7e-007** | **1** | **R.GDQDAGACEVNPLACLPQAAACAPDLPAFSHGGFSHN.-** 29230 |

| **59.** | O60656\|UD19_HUMAN    **Mass:** 60757    **Score:** 382    **Queries matched:** 39   **emPAI:** 1.30 |
| --- | --- |
|  | UDP-glucuronosyltransferase 1-9 OS=Homo sapiens GN=UGT1A9 PE=1 SV=1 |

|  | **Query** | **Observed** | **Mr(expt)** | **Mr(calc)** | **Delta** | **Miss** | **Score** | **Expect** | **Rank** | **Peptide** |
| --- | --- | --- | --- | --- | --- | --- | --- | --- | --- | --- |
|  | 1086 | **659.3677** | **658.3605** | **658.3650** | **-0.0045** | **0** | **25** | **0.78** | **1** | **K.AVINDK.S** |
|  | 1259 | **765.1535** | **764.1462** | **763.4480** | **0.6983** | **0** | **9** | **17** | **1** | **K.LVEYLK.E** 1258 |
|  | 2796 | **447.3454** | **892.6762** | **891.5429** | **1.1333** | **1** | **2** | **80** | **7** | **K.KLVEYLK.E** |
|  | 3687 | **960.5128** | **959.5055** | **959.5110** | **-0.0055** | **0** | **41** | **0.014** | **1** | **K.AMAIADALGK.I** |
|  | 4770 | **520.7600** | **1039.5055** | **1039.5120** | **-0.0066** | **1** | **39** | **0.017** | **1** | **K.SYKENIMR.L** 4771 |
|  | 5402 | **545.3372** | **1088.6598** | **1087.6059** | **1.0538** | **1** | **8** | **30** | **3** | **K.KAMAIADALGK.I** |
|  | 9788 | **671.8537** | **1341.6928** | **1341.7003** | **-0.0074** | **0** | **47** | **0.0026** | **1** | **R.ILLGFSDAMTFK.E** 9789 |
|  | 9791 | **448.2527** | **1341.7364** | **1342.7027** | **-0.9663** | **1** | **11** | **9.9** | **2** | **K.ENIMRLSSLHK.D** |
|  | 13477 | **782.3588** | **1562.7031** | **1562.7100** | **-0.0069** | **0** | **25** | **0.43** | **1** | **K.TYSTSYTLEDLDR.E** |
|  | 15287 | **839.4249** | **1676.8353** | **1676.8457** | **-0.0104** | **0** | **30** | **0.13** | **1** | **K.WLPQNDLLGHPMTR.A** 15288 15289 |
|  | 16746 | **596.9661** | **1787.8766** | **1787.8851** | **-0.0086** | **0** | **24** | **0.44** | **1** | **K.LLVVPMDGSHWFTMR.S** |
|  | 18411 | **961.4955** | **1920.9764** | **1920.9880** | **-0.0116** | **0** | **9** | **12** | **1** | **R.GHEVVVVMPEVSWQLGR.S** |
|  | 18806 | **652.0336** | **1953.0789** | **1953.0870** | **-0.0081** | **1** | **48** | **0.0013** | **1** | **K.AMAIADALGKIPQTVLWR.Y** 18804 18807 |
|  | 18958 | **493.2440** | **1968.9467** | **1969.0819** | **-0.1351** | **1** | **(7)** | **19** | **1** | **K.AMAIADALGKIPQTVLWR.Y** |
|  | 21226 | **735.3732** | **2203.0977** | **2203.1042** | **-0.0065** | **0** | **64** | **3.7e-005** | **1** | **K.GAGVTLNVLEMTSEDLENALK.A** |
|  | 21382 | **740.5198** | **2218.5377** | **2219.0991** | **-0.5614** | **0** | **(3)** | **28** | **5** | **K.GAGVTLNVLEMTSEDLENALK.A** 21379 |
|  | 22175 | **773.4023** | **2317.1850** | **2317.1929** | **-0.0079** | **0** | **29** | **0.1** | **1** | **K.DRPVEPLDLAVFWVEFVMR.H** |
|  | 22356 | **1172.5570** | **2343.0995** | **2343.1093** | **-0.0098** | **0** | **41** | **0.0059** | **1** | **K.ESSFDAVFLDPFDNCGLIVAK.Y** 22355 |
|  | 22378 | **1173.6072** | **2345.1998** | **2346.1865** | **-0.9867** | **1** | **1** | **66** | **6** | **K.LLVVPMDGSHWFTMRSVVEK.L** |
|  | 23589 | **1265.6078** | **2529.2010** | **2529.2144** | **-0.0134** | **0** | **95** | **2e-008** | **1** | **R.GILCHYLEEGAQCPAPLSYVPR.I** 23588 |
|  | 25357 | **948.8234** | **2843.4485** | **2843.4586** | **-0.0101** | **1** | **63** | **3.1e-005** | **1** | **K.GAGVTLNVLEMTSEDLENALKAVINDK.S** 25358 |
|  | 27764 | **1125.9159** | **3374.7258** | **3374.7398** | **-0.0139** | **0** | **19** | **0.48** | **1** | **K.NALEIASEILQTPVTEYDLYSHTSIWLLR.T** |
|  | 29241 | **957.1843** | **3824.7080** | **3823.7289** | **0.9791** | **0** | **28** | **0.046** | **1** | **R.AFITHAGSHGVYESICNGVPMVMMPLFGDQMDNAK.R** 29234 29240 |
|  | 29624 | **996.2063** | **3980.7961** | **3979.8300** | **0.9661** | **1** | **31** | **0.022** | **1** | **R.AFITHAGSHGVYESICNGVPMVMMPLFGDQMDNAKR.M** 29622 29623 |

| **60.** | Q01082\|SPTB2_HUMAN    **Mass:** 275237   **Score:** 380    **Queries matched:** 86   **emPAI:** 0.22 |
| --- | --- |
|  | Spectrin beta chain, brain 1 OS=Homo sapiens GN=SPTBN1 PE=1 SV=2 |

|  | **Query** | **Observed** | **Mr(expt)** | **Mr(calc)** | **Delta** | **Miss** | **Score** | **Expect** | **Rank** | **Peptide** |
| --- | --- | --- | --- | --- | --- | --- | --- | --- | --- | --- |
|  | 1082 | **658.3363** | **657.3290** | **657.3962** | **-0.0672** | **1** | **9** | **26** | **1** | **K.KHVFK.L** |
|  | 1147 | **689.4147** | **688.4075** | **688.4119** | **-0.0045** | **0** | **19** | **3.3** | **1** | **R.LTVQTK.F** |
|  | 1527 | **402.2436** | **802.4726** | **801.3844** | **1.0882** | **0** | **13** | **11** | **2** | **R.FFSMVR.D** |
|  | 1719 | **409.7095** | **817.4044** | **816.4341** | **0.9703** | **0** | **16** | **4.9** | **2** | **K.EVAELTR.L** |
|  | 2089 | **422.7637** | **843.5129** | **844.5130** | **-1.0001** | **1** | **4** | **75** | **4** | **K.RLTVQTK.F** |
|  | 2198 | **426.2762** | **850.5378** | **849.4378** | **1.1000** | **1** | **1** | **1.3e+002** | **9** | **R.KLTGMER.D** |
|  | 2648 | **441.2962** | **880.5778** | **879.3822** | **1.1957** | **0** | **4** | **64** | **5** | **K.STDEVDSK.R** |
|  | 3056 | **456.7821** | **911.5496** | **911.5553** | **-0.0056** | **0** | **27** | **0.31** | **1** | **R.VQAVVAVAR.E** 3059 |
|  | 3664 | **479.7662** | **957.5178** | **957.5243** | **-0.0065** | **0** | **10** | **17** | **8** | **K.INAVVETGR.R** 3646 3667 3669 |
|  | 3984 | **491.7083** | **981.4019** | **981.5144** | **-0.1125** | **0** | **8** | **25** | **4** | **K.WVNSHLAR.V** |
|  | 4105 | **495.5741** | **989.1336** | **988.4574** | **0.6762** | **0** | **9** | **14** | **1** | **R.DTGNIGQER.V** |
|  | 4280 | **501.3014** | **1000.5883** | **999.6077** | **0.9806** | **1** | **6** | **51** | **8** | **K.LLQLTEKR.K** |
|  | 5481 | **548.3079** | **1094.6012** | **1094.5720** | **0.0292** | **0** | **5** | **51** | **10** | **R.RPPSPEPSTK.V** |
|  | 5668 | **554.7991** | **1107.5837** | **1107.5924** | **-0.0087** | **1** | **32** | **0.11** | **1** | **R.YKEVAELTR.L** |
|  | 5758 | **558.3108** | **1114.6071** | **1113.6142** | **0.9930** | **1** | **6** | **43** | **3** | **R.QEKLEQLAR.R** |
|  | 6354 | **582.2665** | **1162.5185** | **1161.5414** | **0.9771** | **0** | **4** | **62** | **10** | **R.ETWLSENQR.L** |
|  | 7498 | **409.2278** | **1224.6614** | **1225.7142** | **-1.0528** | **1** | **2** | **86** | **6** | **R.ELALRNELIR.Q** |
|  | 7825 | **414.5602** | **1240.6588** | **1240.6928** | **-0.0340** | **0** | **5** | **45** | **5** | **R.LILEVHQFSR.D** |
|  | 8000 | **625.3336** | **1248.6526** | **1249.5873** | **-0.9348** | **0** | **(6)** | **38** | **2** | **R.QLMHSGHPSEK.E** |
|  | 8375 | **634.3374** | **1266.6602** | **1265.5823** | **1.0780** | **0** | **9** | **16** | **6** | **R.QLMHSGHPSEK.E** |
|  | 8560 | **426.5762** | **1276.7068** | **1277.7013** | **-0.9945** | **1** | **2** | **83** | **9** | **K.DLTSVMRLLSK.H** |
|  | 9203 | **655.8163** | **1309.6180** | **1309.6262** | **-0.0082** | **0** | **8** | **24** | **1** | **R.ALVADSHPESER.I** |
|  | 9641 | **667.3453** | **1332.6761** | **1333.5833** | **-0.9072** | **1** | **2** | **83** | **3** | **K.HRAFEDEMSGR.S** |
|  | 9676 | **446.1889** | **1335.5449** | **1334.6612** | **0.8837** | **1** | **1** | **95** | **7** | **K.NRETASELLMR.L** |
|  | 9845 | **449.2413** | **1344.7022** | **1344.7183** | **-0.0162** | **1** | **13** | **7.7** | **1** | **R.QRLEMNLGLQK.I** |
|  | 10646 | **695.8518** | **1389.6889** | **1389.6962** | **-0.0073** | **0** | **27** | **0.28** | **1** | **K.FMELLEPLNER.K** 10647 |
|  | 11408 | **479.2663** | **1434.7771** | **1435.6839** | **-0.9069** | **0** | **2** | **78** | **9** | **R.DLMLWMEDVIR.Q** |
|  | 11622 | **725.3687** | **1448.7229** | **1448.6857** | **0.0372** | **0** | **6** | **35** | **5** | **R.LAEISDVWEEMK.T** |
|  | 12229 | **495.5741** | **1483.7004** | **1483.7466** | **-0.0463** | **1** | **2** | **84** | **9** | **K.WVNSHLARVSCR.I** |
|  | 12360 | **498.2545** | **1491.7416** | **1491.7066** | **0.0349** | **1** | **5** | **40** | **6** | **R.EFARDTGNIGQER.V** |
|  | 12449 | **500.2819** | **1497.8240** | **1496.6817** | **1.1423** | **0** | **2** | **82** | **7** | **K.AELFTQSCADLDK.W** |
|  | 12742 | **506.2477** | **1515.7212** | **1515.7317** | **-0.0105** | **1** | **28** | **0.18** | **1** | **R.ELEAENYHDIKR.I** |
|  | 12749 | **506.2773** | **1515.8101** | **1515.8694** | **-0.0593** | **1** | **8** | **19** | **1** | **R.MLIKLLEVLSGER.L** |
|  | 12943 | **765.8265** | **1529.6384** | **1529.6490** | **-0.0106** | **0** | **67** | **2.3e-005** | **1** | **K.MLTAQDMSYDEAR.N** |
|  | 13171 | **515.3519** | **1543.0338** | **1541.8664** | **1.1673** | **1** | **6** | **24** | **3** | **R.DLVAIEAKLSDLQK.E** |
|  | 13258 | **517.2546** | **1548.7419** | **1548.7388** | **0.0031** | **1** | **(3)** | **61** | **4** | **K.KQQMLENQMEVR.K** 13271 |
|  | 13265 | **517.2774** | **1548.8104** | **1548.7388** | **0.0716** | **1** | **4** | **45** | **7** | **K.KQQMLENQMEVR.K** |
|  | 13385 | **778.9229** | **1555.8311** | **1556.7947** | **-0.9635** | **0** | **4** | **44** | **8** | **R.EQWANLEQLSAIR.K** 13397 |
|  | 13640 | **525.3108** | **1572.9105** | **1573.8980** | **-0.9875** | **1** | **4** | **45** | **9** | **R.LWEYLLELLRAR.R** |
|  | 14369 | **540.8147** | **1619.4223** | **1619.8089** | **-0.3867** | **1** | **4** | **33** | **3** | **R.QLMHSGHPSEKEIK.A** |
|  | 14687 | **547.2811** | **1638.8214** | **1637.7798** | **1.0416** | **1** | **4** | **46** | **6** | **K.SAATWDERFSALER.L** |
|  | 14880 | **826.3666** | **1650.7187** | **1650.7307** | **-0.0120** | **0** | **21** | **0.97** | **1** | **R.FESLEPEMNNQASR.V** |
|  | 15134 | **556.9746** | **1667.9020** | **1666.7257** | **1.1763** | **0** | **(6)** | **27** | **2** | **R.FESLEPEMNNQASR.V** |
|  | 15713 | **854.8968** | **1707.7791** | **1707.7886** | **-0.0095** | **0** | **85** | **3.1e-007** | **1** | **R.TQETPSAQMEGFLNR.K** 15722 |
|  | 15906 | **575.2787** | **1722.8144** | **1723.7835** | **-0.9691** | **0** | **(2)** | **75** | **4** | **R.TQETPSAQMEGFLNR.K** 15945 15946 |
|  | 16004 | **865.3839** | **1728.7533** | **1728.7606** | **-0.0073** | **0** | **27** | **0.22** | **1** | **K.FFWEMAEEEGWIR.E** |
|  | 16565 | **592.2885** | **1773.8435** | **1773.8533** | **-0.0098** | **1** | **12** | **6.9** | **1** | **K.KHEAIETDIAAYEER.V** |
|  | 16614 | **889.4451** | **1776.8756** | **1776.8853** | **-0.0098** | **0** | **51** | **0.00091** | **1** | **R.SQNIVTDSSSLSAEAIR.Q** 16613 |
|  | 16854 | **600.3331** | **1797.9776** | **1798.8883** | **-0.9108** | **0** | **1** | **74** | **7** | **R.DVSSVELLMNNHQGIK.A** |
|  | 16862 | **900.4411** | **1798.8676** | **1798.8771** | **-0.0095** | **1** | **94** | **4.3e-008** | **1** | **K.ILSSDDYGKDLTSVMR.L** |
|  | 17333 | **611.9830** | **1832.9271** | **1831.7604** | **1.1667** | **1** | **1** | **88** | **8** | **K.KQEDFMTTMDANEEK.I** |
|  | 19115 | **662.3152** | **1983.9239** | **1983.8514** | **0.0726** | **0** | **(3)** | **48** | **4** | **R.MAETVDTSEMVNGATEQR.T** |
|  | 19302 | **667.6652** | **1999.9738** | **1999.8463** | **0.1275** | **0** | **9** | **11** | **1** | **R.MAETVDTSEMVNGATEQR.T** |
|  | 19516 | 1011.5072 | 2020.9998 | 2021.0106 | -0.0107 | 0 | 70 | 8.2e-006 | 1 | R.LVSQDNFGFDLPAVEAATK.K |
|  | 19822 | **684.3317** | **2049.9734** | **2049.9855** | **-0.0121** | **0** | **20** | **1** | **1** | **K.IVSSSDVGHDEYSTQSLVK.K** |
|  | 20474 | **706.3507** | **2116.0303** | **2117.0827** | **-1.0524** | **1** | **1** | **65** | **5** | **K.LTGLHKMWEVLESTTQTK.A** |
|  | 20719 | **716.3745** | **2146.1017** | **2145.0736** | **1.0281** | **0** | **2** | **49** | **4** | **K.VIESTQDLGNDLAGVMALQR.K** |
|  | 21027 | **727.0298** | **2178.0675** | **2178.0804** | **-0.0129** | **1** | **30** | **0.083** | **1** | **K.IVSSSDVGHDEYSTQSLVKK.H** |
|  | 21095 | **729.3946** | **2185.1621** | **2185.0361** | **0.1260** | **1** | **3** | **43** | **3** | **R.LFDANKAELFTQSCADLDK.W** |
|  | 21828 | **1136.5414** | **2271.0682** | **2269.9918** | **1.0764** | **0** | **2** | **56** | **5** | **R.VAHMEFCYQELCQLAAER.R** |
|  | 21848 | **758.3882** | **2272.1429** | **2273.1824** | **-1.0395** | **1** | **1** | **64** | **4** | **K.IEKEGMQLISEKPETEAVVK.E** |
|  | 22351 | **586.5641** | **2342.2273** | **2342.0934** | **0.1338** | **1** | **4** | **31** | **9** | **K.EQRVHLENMGSHDIVDGNHR.L** |
|  | 22500 | **787.4108** | **2359.2105** | **2358.2213** | **0.9892** | **1** | **2** | **47** | **3** | **K.TKVIESTQDLGNDLAGVMALQR.K** 22485 22489 |
|  | 23312 | **828.7487** | **2483.2243** | **2482.2353** | **0.9891** | **0** | **35** | **0.022** | **1** | **K.SNAHYNLQNAFNLAEQHLGLTK.L** |
|  | 23651 | **847.4932** | **2539.4579** | **2539.0964** | **0.3615** | **1** | **3** | **33** | **1** | **K.MRDMGEMVTQGQTDAQYMFLR.Q** 23645 |
|  | 23876 | **1285.9949** | **2569.9752** | **2571.0862** | **-1.1110** | **1** | **(1)** | **38** | **3** | **K.MRDMGEMVTQGQTDAQYMFLR.Q** |
|  | 24626 | **677.0552** | **2704.1916** | **2704.2075** | **-0.0159** | **1** | **20** | **0.62** | **1** | **R.SGHFEQAIKEGEDMIAEEHFGSEK.I** 24639 |
|  | 26083 | **1000.8228** | **2999.4464** | **2999.4586** | **-0.0122** | **0** | **76** | **1.3e-006** | **1** | **R.LEEASLLHQFQADADDIDAWMLDILK.I** 26084 |
|  | 26986 | **530.9245** | **3179.5033** | **3180.4713** | **-0.9680** | **1** | **4** | **21** | **6** | **R.LEMNLGLQKIFQEMLYIMDWMDEMK.V** |
|  | 28950 | **1240.3249** | **3717.9530** | **3718.6799** | **-0.7269** | **1** | **2** | **20** | **1** | **K.GEQVSQNGLPAEQGSPRMAETVDTSEMVNGATEQR.T** |

| **61.** | Q02928\|CP4AB_HUMAN    **Mass:** 59766    **Score:** 375    **Queries matched:** 22   **emPAI:** 0.62 |
| --- | --- |
|  | Cytochrome P450 4A11 OS=Homo sapiens GN=CYP4A11 PE=1 SV=1 |

|  | **Query** | **Observed** | **Mr(expt)** | **Mr(calc)** | **Delta** | **Miss** | **Score** | **Expect** | **Rank** | **Peptide** |
| --- | --- | --- | --- | --- | --- | --- | --- | --- | --- | --- |
|  | 1180 | **709.1945** | **708.1872** | **708.4031** | **-0.2158** | **0** | **8** | **22** | **5** | **K.NGIHLR.L** |
|  | 3339 | **466.7362** | **931.4578** | **930.5134** | **0.9444** | **0** | **12** | **12** | **6** | **M.SVSVLSPSR.L** |
|  | 5244 | 540.2373 | 1078.4600 | 1077.5488 | 0.9112 | 0 | 9 | 21 | 5 | -.MSVSVLSPSR.L |
|  | 5671 | **554.9564** | **1107.8982** | **1108.5070** | **-0.6089** | **0** | **8** | **15** | **1** | **K.MENGSILSDK.D** |
|  | 8442 | **636.1109** | **1270.2072** | **1270.5904** | **-0.3831** | **0** | **5** | **28** | **1** | **R.VQLYDPDYMK.V** |
|  | 10975 | **705.9180** | **1409.8215** | **1409.8282** | **-0.0067** | **0** | **76** | **3.4e-006** | **1** | **R.HLDFLDILLLAK.M** 10974 |
|  | 12095 | **493.2463** | **1476.7171** | **1476.7242** | **-0.0071** | **1** | **8** | **21** | **2** | **K.MENGSILSDKDLR.A** |
|  | 17215 | **913.5181** | **1825.0217** | **1824.9444** | **0.0773** | **1** | **2** | **61** | **5** | **R.VQLYDPDYMKVILGR.S** |
|  | 18351 | **959.4839** | **1916.9532** | **1916.9639** | **-0.0107** | **0** | **85** | **3.5e-007** | **1** | **R.ACQLAHQHTDQVIQLR.K** 18348 18350 18352 |
|  | 20928 | **1085.0455** | **2168.0765** | **2168.0862** | **-0.0097** | **0** | **99** | **1.1e-008** | **1** | **R.NSQSYIQAISDLNNLVFSR.V** 20929 |
|  | 24216 | **660.0918** | **2636.3381** | **2636.3495** | **-0.0114** | **0** | **12** | **4.5** | **1** | **R.MLTPAFHYDILKPYVGLMADSVR.V** |
|  | 27239 | **1620.3160** | **3238.6175** | **3238.6593** | **-0.0417** | **1** | **3** | **25** | **7** | **R.MLTPAFHYDILKPYVGLMADSVRVMLDK.W** |
|  | 27976 | **858.4207** | **3429.6535** | **3429.6677** | **-0.0142** | **0** | **5** | **12** | **1** | **K.ALQQFPCPPSHWLFGHIQELQQDQELQR.I** |
|  | 28818 | **919.9271** | **3675.6791** | **3675.6942** | **-0.0151** | **1** | **36** | **0.0084** | **1** | **R.CREEIHSLLGDGASITWNHLDQMPYTTMCIK.E** 28817 28819 |
|  | 29036 | **1249.2980** | **3744.8721** | **3744.8817** | **-0.0096** | **1** | **96** | **8e-009** | **1** | **R.VMLDKWEELLGQDSPLEVFQHVSLMTLDTIMK.C** |

| **62.** | P27797\|CALR_HUMAN    **Mass:** 48283    **Score:** 372    **Queries matched:** 27   **emPAI:** 0.96 |
| --- | --- |
|  | Calreticulin OS=Homo sapiens GN=CALR PE=1 SV=1 |

|  | **Query** | **Observed** | **Mr(expt)** | **Mr(calc)** | **Delta** | **Miss** | **Score** | **Expect** | **Rank** | **Peptide** |
| --- | --- | --- | --- | --- | --- | --- | --- | --- | --- | --- |
|  | 1094 | **662.3465** | **661.3392** | **661.3435** | **-0.0043** | **0** | **22** | **1.3** | **1** | **R.WIESK.H** |
|  | 4143 | **496.7348** | **991.4551** | **991.4611** | **-0.0060** | **0** | **16** | **4.6** | **1** | **R.QIDNPDYK.G** |
|  | 5321 | **542.8240** | **1083.6334** | **1083.6400** | **-0.0066** | **1** | **12** | **9.2** | **1** | **K.NVLINKDIR.C** |
|  | 10964 | **705.8135** | **1409.6125** | **1409.6212** | **-0.0086** | **0** | **45** | **0.0037** | **1** | **K.EQFLDGDGWTSR.W** 10965 |
|  | 12081 | **738.8259** | **1475.6373** | **1475.6463** | **-0.0090** | **0** | **40** | **0.014** | **1** | **K.HEQNIDCGGGYVK.L** 12082 |
|  | 16871 | **900.9181** | **1799.8216** | **1799.8326** | **-0.0110** | **1** | **58** | **0.00017** | **1** | **K.IKDPDASKPEDWDER.A** 16870 16872 |
|  | 23517 | **840.3975** | **2518.1706** | **2518.1864** | **-0.0158** | **1** | **38** | **0.0099** | **1** | **K.IDNSQVESGSLEDDWDFLPPKK.I** 23518 |
|  | 24905 | **690.8168** | **2759.2380** | **2759.2562** | **-0.0182** | **0** | **16** | **1.5** | **1** | **K.IDDPTDSKPEDWDKPEHIPDPDAK.K** |
|  | 25401 | **714.8481** | **2855.3635** | **2855.3800** | **-0.0165** | **1** | **0** | **52** | **2** | **R.CKDDEFTHLYTLIVRPDNTYEVK.I** |
|  | 25900 | **740.5998** | **2958.3701** | **2958.3883** | **-0.0182** | **1** | **13** | **2.5** | **1** | **R.AKIDDPTDSKPEDWDKPEHIPDPDAK.K** |
|  | 27346 | **1634.7444** | **3267.4742** | **3267.4884** | **-0.0142** | **0** | **95** | **1.5e-008** | **1** | **K.SGTIFDNFLITNDEAYAEEFGNETWGVTK.A** 27342 27344 27350 |
|  | 28070 | 1152.8376 | 3455.4911 | 3455.5108 | -0.0197 | 0 | 28 | 0.057 | 1 | K.LFPNSLDQTDMHGDSEYNIMFGPDICGPGTK.K 28071 |
|  | 30021 | **1383.6617** | **4147.9634** | **4147.9844** | **-0.0210** | **0** | **88** | **3.9e-008** | **1** | **K.GTWIHPEIDNPEYSPDPSIYAYDNFGVLGLDLWQVK.S** 30022 |
|  | 31336 | **1281.3622** | **5121.4196** | **5121.4349** | **-0.0152** | **1** | **27** | **0.03** | **1** | **R.QIDNPDYKGTWIHPEIDNPEYSPDPSIYAYDNFGVLGLDLWQVK.S** 31334 31335 31337 |

| **63.** | P08670\|VIME_HUMAN    **Mass:** 53676    **Score:** 372    **Queries matched:** 43   **emPAI:** 1.56 |
| --- | --- |
|  | Vimentin OS=Homo sapiens GN=VIM PE=1 SV=4 |

|  | **Query** | **Observed** | **Mr(expt)** | **Mr(calc)** | **Delta** | **Miss** | **Score** | **Expect** | **Rank** | **Peptide** |
| --- | --- | --- | --- | --- | --- | --- | --- | --- | --- | --- |
|  | 721 | 519.1365 | 518.1293 | 518.2813 | -0.1520 | 0 | 0 | 2.7e+002 | 5 | R.SSAVR.L |
|  | 2975 | **453.7348** | **905.4550** | **905.4607** | **-0.0056** | **0** | **27** | **0.35** | **1** | **R.FLEQQNK.I** |
|  | 4552 | **512.2476** | **1022.4806** | **1022.5033** | **-0.0227** | **0** | **23** | **0.83** | **1** | **R.QQYESVAAK.N** 4569 |
|  | 4641 | **515.3267** | **1028.6388** | **1027.5047** | **1.1341** | **1** | **7** | **33** | **8** | **R.SVSSSSYRR.M** |
|  | 5026 | **530.7892** | **1059.5638** | **1059.5560** | **0.0078** | **1** | **6** | **43** | **10** | **R.KLLEGEESR.I** |
|  | 5851 | **561.7703** | **1121.5260** | **1120.5764** | **0.9496** | **0** | **3** | **77** | **5** | **R.EYQDLLNVK.M** |
|  | 6488 | **587.3843** | **1172.7540** | **1172.7129** | **0.0411** | **1** | **10** | **16** | **1** | **R.TLLIKTVETR.D** 6489 6491 |
|  | 7299 | 608.7369 | 1215.4593 | 1215.6208 | -0.1614 | 1 | 11 | 12 | 1 | R.RQVDQLTNDK.A 7327 |
|  | 8737 | **644.3320** | **1286.6494** | **1286.6579** | **-0.0085** | **1** | **40** | **0.016** | **1** | **R.QVDQLTNDKAR.V** |
|  | 9157 | **654.3775** | **1306.7404** | **1307.6469** | **-0.9065** | **1** | **1** | **1.1e+002** | **2** | **K.SKFADLSEAANR.N** |
|  | 9178 | **655.3026** | **1308.5907** | **1308.5986** | **-0.0079** | **0** | **19** | **1.8** | **1** | **K.NLQEAEEWYK.S** |
|  | 9884 | **450.1946** | **1347.5619** | **1346.6248** | **0.9371** | **1** | **6** | **42** | **5** | **-.MSTRSVSSSSYR.R** |
|  | 10124 | **681.3578** | **1360.7010** | **1360.6769** | **0.0242** | **1** | **6** | **39** | **9** | **R.DNLAEDIMRLR.E** |
|  | 11272 | **714.8560** | **1427.6975** | **1427.7045** | **-0.0070** | **0** | **53** | **0.00073** | **1** | **R.SLYASSPGGVYATR.S** |
|  | 12404 | **499.2844** | **1494.8313** | **1493.7045** | **1.1268** | **0** | **11** | **9.1** | **1** | **R.MFGGPGTASRPSSSR.S** 12381 |
|  | 12662 | **504.9078** | **1511.7015** | **1512.6878** | **-0.9863** | **1** | **10** | **13** | **4** | **K.SRLGDLYEEEMR.E** |
|  | 12914 | **764.4131** | **1526.8116** | **1526.8205** | **-0.0089** | **1** | **52** | **0.0008** | **1** | **R.HLREYQDLLNVK.M** 12915 |
|  | 12989 | **767.4257** | **1532.8369** | **1532.8450** | **-0.0081** | **1** | **66** | **3.4e-005** | **1** | **R.KVESLQEEIAFLK.K** 12988 12998 |
|  | 13084 | **770.4545** | **1538.8945** | **1538.9032** | **-0.0086** | **1** | **60** | **0.00012** | **1** | **K.ILLAELEQLKGQGK.S** 13083 |
|  | 13574 | **524.2632** | **1569.7679** | **1569.8878** | **-0.1200** | **0** | **9** | **18** | **4** | **R.ISLPLPNFSSLNLR.E** 13578 |
|  | 14882 | **551.2537** | **1650.7393** | **1649.8056** | **0.9337** | **1** | **15** | **3.6** | **1** | **R.RMFGGPGTASRPSSSR.S** |
|  | 15442 | **844.9128** | **1687.8111** | **1687.8199** | **-0.0088** | **1** | **24** | **0.41** | **1** | **R.VEVERDNLAEDIMR.L** |
|  | 16061 | **867.9061** | **1733.7976** | **1733.8076** | **-0.0100** | **1** | **56** | **0.00026** | **1** | **R.LQDEIQNMKEEMAR.H** 16060 |
|  | 17356 | **918.8977** | **1835.7809** | **1835.7922** | **-0.0113** | **0** | **39** | **0.012** | **1** | **R.DGQVINETSQHHDDLE.-** 17365 |
|  | 21100 | **1093.9812** | **2185.9478** | **2185.9586** | **-0.0107** | **0** | **81** | **6.1e-007** | **1** | **R.EMEENFAVEAANYQDTIGR.L** 21099 |
|  | 21228 | **551.7886** | **2203.1252** | **2201.9535** | **1.1717** | **0** | **(11)** | **6.2** | **2** | **R.EMEENFAVEAANYQDTIGR.L** |
|  | 23400 | **833.0868** | **2496.2385** | **2496.2496** | **-0.0111** | **1** | **35** | **0.02** | **1** | **R.LLQDSVDFSLADAINTEFKNTR.T** |
|  | 29475 | **981.5033** | **3921.9841** | **3922.0072** | **-0.0231** | **0** | **67** | **5.7e-006** | **1** | **K.LHEEEIQELQAQIQEQHVQIDVDVSKPDLTAALR.D** 29476 29477 |

| **64.** | Q13813\|SPTA2_HUMAN    **Mass:** 285163   **Score:** 370    **Queries matched:** 70   **emPAI:** 0.12 |
| --- | --- |
|  | Spectrin alpha chain, brain OS=Homo sapiens GN=SPTAN1 PE=1 SV=3 |

|  | **Query** | **Observed** | **Mr(expt)** | **Mr(calc)** | **Delta** | **Miss** | **Score** | **Expect** | **Rank** | **Peptide** |
| --- | --- | --- | --- | --- | --- | --- | --- | --- | --- | --- |
|  | 1486 | **401.2130** | **800.4115** | **801.3981** | **-0.9865** | **0** | **9** | **25** | **10** | **K.EEIQQR.L** |
|  | 1730 | **410.2248** | **818.4350** | **817.4294** | **1.0057** | **0** | **12** | **15** | **3** | **K.EAGSVSLR.M** |
|  | 2092 | **423.0527** | **844.0909** | **844.3828** | **-0.2918** | **0** | **8** | **24** | **6** | **R.EAHDAFR.S** |
|  | 2518 | **437.0529** | **872.0912** | **871.4399** | **0.6513** | **0** | **14** | **4.9** | **1** | **K.DPTNLQGK.L** |
|  | 2586 | **439.2582** | **876.5019** | **877.3712** | **-0.8693** | **0** | **6** | **46** | **2** | **R.EQAQSCR.Q** |
|  | 2856 | **450.1946** | **898.3746** | **897.5760** | **0.7986** | **1** | **6** | **37** | **8** | **R.LKGLALQR.Q** |
|  | 3097 | **458.2327** | **914.4509** | **913.5345** | **0.9164** | **1** | **12** | **12** | **1** | **K.KQEALVAR.Y** 3118 |
|  | 3488 | **473.0806** | **944.1466** | **943.4723** | **0.6743** | **0** | **11** | **11** | **1** | **K.LDPAQSASR.E** 3499 |
|  | 3618 | **478.2694** | **954.5243** | **953.5042** | **1.0201** | **1** | **8** | **22** | **1** | **R.AKLNESHR.L** |
|  | 3805 | **485.7269** | **969.4392** | **969.5468** | **-0.1075** | **1** | **6** | **35** | **6** | **K.QLAAARGQR.L** |
|  | 4051 | **493.8027** | **985.5909** | **985.5556** | **0.0353** | **0** | **1** | **1.4e+002** | **10** | **R.IDGITIQAR.Q** |
|  | 4468 | **508.7820** | **1015.5494** | **1014.5458** | **1.0036** | **0** | **30** | **0.2** | **1** | **R.DLTGVQNLR.K** |
|  | 5175 | 536.8125 | 1071.6104 | 1071.5924 | 0.0181 | 1 | 0 | 1.8e+002 | 9 | R.IDALEKAGQK.L |
|  | 5186 | **537.2811** | **1072.5477** | **1072.5513** | **-0.0035** | **0** | **28** | **0.32** | **1** | **R.SLQQLAEER.S** |
|  | 5326 | **543.2408** | **1084.4671** | **1085.6080** | **-1.1409** | **0** | **13** | **8.2** | **2** | **K.DLASVNNLLK.K** |
|  | 6681 | **396.2218** | **1185.6437** | **1186.6306** | **-0.9869** | **1** | **4** | **67** | **9** | **K.VSDLEKAAAQR.K** |
|  | 7074 | **402.2434** | **1203.7084** | **1203.6070** | **0.1014** | **0** | **20** | **1.5** | **2** | **R.DLMSWINGIR.G** |
|  | 7306 | **406.1849** | **1215.5330** | **1214.6255** | **0.9075** | **1** | **7** | **34** | **7** | **R.EKEPIAASTNR.G** |
|  | 7486 | **613.2998** | **1224.5850** | **1224.5696** | **0.0154** | **0** | **0** | **1.3e+002** | **7** | **R.DLTSWVTEMK.A** |
|  | 8777 | **645.3041** | **1288.5936** | **1287.7034** | **0.8902** | **0** | **6** | **39** | **5** | **K.GDILTLLNSTNK.D** |
|  | 9079 | **652.3339** | **1302.6533** | **1302.6602** | **-0.0069** | **0** | **58** | **0.00021** | **1** | **R.GVIDMGNSLIER.G** 9078 9093 |
|  | 9470 | **442.2339** | **1323.6798** | **1323.6895** | **-0.0097** | **0** | **20** | **1.5** | **1** | **R.SQLLGSAHEVQR.F** 9473 |
|  | 9854 | **673.3661** | **1344.7176** | **1343.7309** | **0.9867** | **1** | **0** | **1.4e+002** | **8** | **R.WSQLLANSAARK.K** |
|  | 10134 | **454.7478** | **1361.2217** | **1360.7173** | **0.5044** | **1** | **7** | **19** | **1** | **R.QWELLLEKMR.E** |
|  | 10235 | **456.7217** | **1367.1433** | **1367.5663** | **-0.4230** | **0** | **7** | **18** | **6** | **R.DTEQVDNWMSK.Q** |
|  | 11771 | **486.9202** | **1457.7387** | **1457.7474** | **-0.0087** | **1** | **4** | **55** | **7** | **K.VLETAEDIQERR.Q** |
|  | 12008 | **736.8578** | **1471.7010** | **1472.7583** | **-1.0573** | **1** | **8** | **18** | **3** | **R.QEQIDNQTRITK.E** |
|  | 12047 | **492.2554** | **1473.7444** | **1474.7086** | **-0.9642** | **0** | **10** | **13** | **5** | **R.VNDVCTNGQDLIK.K** |
|  | 12393 | **748.3713** | **1494.7281** | **1494.7361** | **-0.0080** | **0** | **42** | **0.0073** | **1** | **R.MQHNLEQQIQAR.N** 12392 |
|  | 13395 | **779.3887** | **1556.7628** | **1555.7855** | **0.9773** | **0** | **20** | **1.4** | **2** | **K.HQALQAEIAGHEPR.I** |
|  | 13885 | **530.2528** | **1587.7366** | **1586.7437** | **0.9929** | **0** | **16** | **3.4** | **1** | **K.HQAFEAELSANQSR.I** |
|  | 14363 | **810.3936** | **1618.7725** | **1618.7376** | **0.0350** | **0** | **3** | **66** | **5** | **R.QFQDAGHFDAENIK.K** 14364 |
|  | 15383 | 421.9748 | 1683.8701 | 1683.8805 | -0.0104 | 1 | 10 | 12 | 1 | K.KHQALQAEIAGHEPR.I |
|  | 15912 | **575.2844** | **1722.8313** | **1722.8424** | **-0.0112** | **0** | **31** | **0.079** | **1** | **R.LIQSHPESAEDLQEK.C** 15913 |
|  | 16113 | **580.3052** | **1737.8939** | **1738.7257** | **-0.8318** | **0** | **3** | **56** | **9** | **K.FEEFQTDMAAHEER.V** |
|  | 16728 | **894.4236** | **1786.8326** | **1785.9148** | **0.9178** | **0** | **2** | **74** | **9** | **K.QVEELYHSLLELGEK.R** |
|  | 17332 | **917.4602** | **1832.9059** | **1833.0221** | **-0.1162** | **1** | **0** | **1e+002** | **10** | **R.LQQSHPLSATQIQVKR.E** |
|  | 17449 | **922.4813** | **1842.9480** | **1843.9237** | **-0.9757** | **0** | **7** | **23** | **2** | **K.IEDLGAAMEEALILDNK.Y** 17482 |
|  | 17673 | **929.9727** | **1857.9309** | **1857.9432** | **-0.0123** | **1** | **94** | **4.1e-008** | **1** | **R.DLAALGDKVNSLGETAER.L** 17672 |
|  | 17706 | **621.3565** | **1861.0475** | **1859.9186** | **1.1289** | **0** | **(1)** | **70** | **10** | **K.IEDLGAAMEEALILDNK.Y** |
|  | 17976 | **942.5206** | **1883.0267** | **1882.8706** | **0.1561** | **0** | **1** | **73** | **3** | **R.DGHVSLQEYMAFMISR.E** |
|  | 18774 | **976.4661** | **1950.9176** | **1950.9283** | **-0.0107** | **0** | **108** | **1.7e-009** | **1** | **R.SSLSSAQADFNQLAELDR.Q** 18775 |
|  | 18991 | **657.8702** | **1970.5887** | **1971.0578** | **-0.4690** | **0** | **0** | **64** | **6** | **K.IAALQAFADQLIAAGHYAK.G** |
|  | 19586 | **676.6343** | **2026.8810** | **2025.8924** | **0.9886** | **0** | **0** | **85** | **1** | **R.EQADYCVSHMKPYVDGK.G** |
|  | 19950 | **1031.9905** | **2061.9664** | **2061.9751** | **-0.0087** | **1** | **19** | **1.2** | **1** | **R.NTTGVTEEALKEFSMMFK.H** |
|  | 20547 | **710.0280** | **2127.0622** | **2126.0855** | **0.9768** | **0** | **3** | **42** | **4** | **K.ALINADELASDVAGAEALLDR.H** 20546 |
|  | 21279 | **737.0485** | **2208.1236** | **2208.1348** | **-0.0112** | **0** | **3** | **42** | **1** | **K.MTLVASEDYGDTLAAIQGLLK.K** |
|  | 21499 | **558.5363** | **2230.1162** | **2230.0688** | **0.0474** | **1** | **7** | **17** | **3** | **K.AVTQKGNAMVEEGHFAAEDVK.A** |
|  | 22250 | **777.0331** | **2328.0774** | **2327.1254** | **0.9520** | **0** | **7** | **16** | **1** | **K.NQALNTDNYGHDLASVQALQR.K** |
|  | 22333 | **781.3958** | **2341.1654** | **2341.0719** | **0.0936** | **1** | **6** | **21** | **1** | **K.LVQYLRECEDVMDWINDK.E** 22327 |
|  | 22525 | **788.7682** | **2363.2827** | **2364.1049** | **-0.8222** | **1** | **0** | **64** | **2** | **R.GVIDMGNSLIERGACAGSEDAVK.A** |
|  | 22933 | **809.4191** | **2425.2354** | **2425.2489** | **-0.0135** | **1** | **16** | **1.7** | **1** | **R.ALSSEGKPYVTKEELYQNLTR.E** |
|  | 24877 | **918.4507** | **2752.3304** | **2752.3443** | **-0.0139** | **1** | **11** | **5** | **1** | **R.QQVAPTDDETGKELVLALYDYQEK.S** |
|  | 29016 | **1247.6139** | **3739.8198** | **3739.8428** | **-0.0229** | **1** | **21** | **0.25** | **1** | **R.EANELQQWINEKEAALTSEEVGADLEQVEVLQK.K** |
|  | 29101 | **1257.2507** | **3768.7304** | **3767.7328** | **0.9976** | **1** | **2** | **18** | **3** | **K.DINKVAEDLESEGLMAEEVQAVQQQEVYGMMPR.D** |
|  | 29430 | **650.8373** | **3898.9800** | **3899.8081** | **-0.8281** | **1** | **1** | **25** | **4** | **R.ECEDVMDWINDKEAIVTSEELGQDLEHVEVLQK.K** |
|  | 31319 | **852.7034** | **5110.1766** | **5111.3515** | **-1.1749** | **1** | **1** | **8.4** | **2** | **R.SLGYDLPMVEEGEPDPEFEAILDTVDPNRDGHVSLQEYMAFMISR.E** |

| **65.** | P54819\|KAD2_HUMAN    **Mass:** 26689    **Score:** 369    **Queries matched:** 31   **emPAI:** 3.95 |
| --- | --- |
|  | Adenylate kinase 2, mitochondrial OS=Homo sapiens GN=AK2 PE=1 SV=2 |

|  | **Query** | **Observed** | **Mr(expt)** | **Mr(calc)** | **Delta** | **Miss** | **Score** | **Expect** | **Rank** | **Peptide** |
| --- | --- | --- | --- | --- | --- | --- | --- | --- | --- | --- |
|  | 963 | **607.3883** | **606.3810** | **606.3853** | **-0.0043** | **0** | **9** | **22** | **4** | **R.LIHPK.S** |
|  | 3880 | **974.4920** | **973.4847** | **973.4902** | **-0.0055** | **0** | **35** | **0.062** | **1** | **K.NLETPLCK.N** 3879 |
|  | 3967 | **490.7954** | **979.5762** | **978.5862** | **0.9900** | **0** | **19** | **1.8** | **1** | **R.AVLLGPPGAGK.G** 3954 |
|  | 4894 | **1049.5225** | **1048.5152** | **1048.5223** | **-0.0071** | **0** | **46** | **0.0041** | **1** | **R.AMVASGSELGK.K** 4893 |
|  | 5995 | **568.2955** | **1134.5765** | **1134.5822** | **-0.0057** | **0** | **22** | **1** | **1** | **K.NGFLLDGFPR.T** |
|  | 9431 | **661.4575** | **1320.9004** | **1321.5894** | **-0.6890** | **0** | **4** | **38** | **1** | **R.QAEMLDDLMEK.R** 9432 |
|  | 12351 | **746.4309** | **1490.8473** | **1490.7994** | **0.0479** | **1** | **2** | **74** | **3** | **K.NGFLLDGFPRTVR.Q** |
|  | 12402 | **499.2636** | **1494.7688** | **1493.6854** | **1.0834** | **1** | **6** | **29** | **1** | **R.QAEMLDDLMEKR.K** |
|  | 13907 | **795.4143** | **1588.8139** | **1588.9049** | **-0.0910** | **1** | **27** | **0.27** | **1** | **R.AVLLGPPGAGKGTQAPR.L** 13891 13892 13895 13912 |
|  | 14272 | **538.6476** | **1612.9209** | **1612.8130** | **0.1079** | **1** | **9** | **15** | **3** | **K.EPMKDDITGEPLIR.R** |
|  | 14522 | **543.7547** | **1628.2423** | **1628.8080** | **-0.5657** | **1** | **(1)** | **67** | **6** | **K.EPMKDDITGEPLIR.R** |
|  | 17091 | **909.0027** | **1815.9909** | **1815.9982** | **-0.0073** | **0** | **40** | **0.01** | **1** | **K.LDSVIEFSIPDSLLIR.R** |
|  | 19260 | **999.0032** | **1995.9918** | **1996.0054** | **-0.0136** | **0** | **72** | **6.5e-006** | **1** | **R.LQAYHTQTTPLIEYYR.K** 19261 |
|  | 20067 | **1038.0714** | **2074.1283** | **2073.1357** | **0.9925** | **1** | **65** | **2.6e-005** | **1** | **K.EKLDSVIEFSIPDSLLIR.R** |
|  | 21016 | **726.7020** | **2177.0843** | **2177.0959** | **-0.0117** | **1** | **21** | **0.73** | **1** | **K.ATMDAGKLVSDEMVVELIEK.N** |
|  | 21161 | **731.7203** | **2192.1392** | **2193.0909** | **-0.9517** | **1** | **(4)** | **34** | **7** | **K.ATMDAGKLVSDEMVVELIEK.N** 21160 |
|  | 21786 | **756.0655** | **2265.1746** | **2265.1906** | **-0.0159** | **1** | **29** | **0.1** | **1** | **K.IRLQAYHTQTTPLIEYYR.K** |
|  | 23691 | **1273.1636** | **2544.3126** | **2544.3224** | **-0.0098** | **0** | **85** | **2.3e-007** | **1** | **R.GIHSAIDASQTPDVVFASILAAFSK.A** 23690 23693 |
|  | 24605 | **676.1108** | **2700.4143** | **2700.4235** | **-0.0093** | **1** | **50** | **0.00059** | **1** | **K.RGIHSAIDASQTPDVVFASILAAFSK.A** |

| **66.** | P16435\|NCPR_HUMAN    **Mass:** 77097    **Score:** 361    **Queries matched:** 29   **emPAI:** 0.39 |
| --- | --- |
|  | NADPH--cytochrome P450 reductase OS=Homo sapiens GN=POR PE=1 SV=2 |

|  | **Query** | **Observed** | **Mr(expt)** | **Mr(calc)** | **Delta** | **Miss** | **Score** | **Expect** | **Rank** | **Peptide** |
| --- | --- | --- | --- | --- | --- | --- | --- | --- | --- | --- |
|  | 3090 | **457.7535** | **913.4925** | **913.4981** | **-0.0056** | **0** | **24** | **0.66** | **1** | **R.LEQLGAQR.I** |
|  | 3137 | **458.9160** | **915.8175** | **915.4926** | **0.3248** | **0** | **6** | **34** | **9** | **K.GVATNWLR.A** 3146 |
|  | 3453 | **471.7097** | **941.4049** | **941.4099** | **-0.0050** | **0** | **20** | **1.4** | **1** | **K.VYMGEMGR.L** |
|  | 3495 | **473.2566** | **944.4987** | **944.5039** | **-0.0052** | **1** | **20** | **2.1** | **1** | **R.KLNQGTER.H** |
|  | 4318 | **502.7875** | **1003.5604** | **1003.5662** | **-0.0058** | **0** | **37** | **0.035** | **1** | **K.IQTLTSSVR.E** 4326 |
|  | 7299 | 608.7369 | 1215.4593 | 1215.5520 | -0.0927 | 1 | 3 | 69 | 7 | R.RSDEDYLYR.E |
|  | 8473 | **424.9105** | **1271.7096** | **1270.7146** | **0.9950** | **1** | **23** | **0.76** | **1** | **R.INKGVATNWLR.A** |
|  | 12021 | **491.7432** | **1472.2077** | **1472.7623** | **-0.5546** | **1** | **1** | **62** | **9** | **R.LQARYYSIASSSK.V** |
|  | 13049 | **513.2577** | **1536.7514** | **1536.7606** | **-0.0092** | **0** | **32** | **0.081** | **1** | **R.HLMHLELDISDSK.I** |
|  | 16941 | **602.7509** | **1805.2309** | **1805.9458** | **-0.7148** | **1** | **4** | **30** | **7** | **R.HLMHLELDISDSKIR.Y** |
|  | 17000 | **604.3373** | **1809.9900** | **1809.9472** | **0.0428** | **1** | **2** | **57** | **2** | **K.IQTLTSSVRESSFVEK.M** |
|  | 17079 | **606.2806** | **1815.8199** | **1814.9097** | **0.9102** | **1** | **5** | **32** | **2** | **K.EPAGENGGRALVPMFVR.K** |
|  | 20470 | **1059.0111** | **2116.0077** | **2116.0225** | **-0.0149** | **0** | **101** | **6.7e-009** | **1** | **R.NIIVFYGSQTGTAEEFANR.L** 20471 |
|  | 20571 | **711.0145** | **2130.0218** | **2130.0357** | **-0.0139** | **1** | **4** | **35** | **1** | **K.FAVFGLGNKTYEHFNAMGK.Y** |
|  | 21354 | **554.5460** | **2214.1547** | **2214.0627** | **0.0921** | **1** | **4** | **33** | **6** | **K.MASSSGEGKELYLSWVVEAR.R** |
|  | 22292 | **468.2292** | **2336.1094** | **2335.1590** | **0.9504** | **1** | **8** | **14** | **1** | **K.LNQGTERHLMHLELDISDSK.I** |
|  | 23107 | **1227.5703** | **2453.1261** | **2453.1387** | **-0.0126** | **0** | **134** | **2.9e-012** | **1** | **R.IFELGLGDDDGNLEEDFITWR.E** 23103 23108 23112 |
|  | 23146 | **820.1054** | **2457.2944** | **2457.3090** | **-0.0146** | **0** | **14** | **2.7** | **1** | **K.ATTPVIMVGPGTGVAPFIGFIQER.A** 23147 23148 |
|  | 23923 | **861.1061** | **2580.2966** | **2580.3071** | **-0.0105** | **1** | **52** | **0.00037** | **1** | **R.TNVLYELAQYASEPSEQELLRK.M** |
|  | 24678 | **906.1226** | **2715.3460** | **2715.3616** | **-0.0156** | **1** | **27** | **0.13** | **1** | **K.IRYESGDHVAVYPANDSALVNQLGK.I** |
|  | 29816 | **1358.0181** | **4071.0324** | **4071.0476** | **-0.0152** | **1** | **52** | **0.00015** | **1** | **R.TALTYYLDITNPPRTNVLYELAQYASEPSEQELLR.K** |

| **67.** | P02511\|CRYAB_HUMAN    **Mass:** 20146    **Score:** 361    **Queries matched:** 39   **emPAI:** 3.83 |
| --- | --- |
|  | Alpha-crystallin B chain OS=Homo sapiens GN=CRYAB PE=1 SV=2 |

|  | **Query** | **Observed** | **Mr(expt)** | **Mr(calc)** | **Delta** | **Miss** | **Score** | **Expect** | **Rank** | **Peptide** |
| --- | --- | --- | --- | --- | --- | --- | --- | --- | --- | --- |
|  | 2882 | **450.7459** | **899.4773** | **899.4825** | **-0.0051** | **1** | **34** | **0.064** | **1** | **R.KQVSGPER.T** 2857 2858 2859 2860 2879 2880 2885 2887 2888 2891 2892 2893 |
|  | 3214 | **461.2532** | **920.4919** | **920.4967** | **-0.0048** | **0** | **35** | **0.055** | **1** | **R.FSVNLDVK.H** |
|  | 4047 | **493.7478** | **985.4811** | **985.4869** | **-0.0058** | **0** | **29** | **0.24** | **1** | **K.HFSPEELK.V** 4046 |
|  | 6067 | **571.3415** | **1140.6684** | **1139.6186** | **1.0498** | **0** | **5** | **47** | **5** | **R.EEKPAVTAAPK.K** |
|  | 6380 | **583.3291** | **1164.6436** | **1164.6503** | **-0.0066** | **0** | **39** | **0.018** | **1** | **K.VLGDVIEVHGK.H** |
|  | 6794 | **596.3054** | **1190.5962** | **1191.6248** | **-1.0286** | **1** | **3** | **76** | **5** | **K.DRFSVNLDVK.H** |
|  | 7248 | **405.2212** | **1212.6417** | **1212.6503** | **-0.0086** | **1** | **32** | **0.091** | **1** | **K.HFSPEELKVK.V** 7232 7246 7247 |
|  | 8395 | **634.8591** | **1267.7036** | **1267.7136** | **-0.0100** | **1** | **18** | **2.3** | **1** | **R.EEKPAVTAAPKK.-** 8366 8424 8425 |
|  | 12698 | **505.2765** | **1512.8076** | **1511.6715** | **1.1362** | **0** | **6** | **28** | **3** | **R.APSWFDTGLSEMR.L** 12673 |
|  | 14682 | **547.2535** | **1638.7386** | **1638.7499** | **-0.0113** | **1** | **40** | **0.012** | **1** | **K.HEERQDEHGFISR.E** |
|  | 15826 | **572.9688** | **1715.8844** | **1715.8955** | **-0.0111** | **1** | **13** | **5.5** | **1** | **K.VLGDVIEVHGKHEER.Q** |
|  | 17159 | **911.5196** | **1821.0246** | **1821.0360** | **-0.0113** | **1** | **19** | **1.4** | **1** | **R.TIPITREEKPAVTAAPK.K** |
|  | 25824 | **981.8476** | **2942.5210** | **2942.5349** | **-0.0140** | **1** | **57** | **0.00011** | **1** | **K.YRIPADVDPLTITSSLSSDGVLTVNGPR.K** 25833 25835 |
|  | 29668 | **1335.3417** | **4003.0032** | **4003.0196** | **-0.0165** | **0** | **124** | **1e-011** | **1** | **R.LFDQFFGEHLLESDLFPTSTSLSPFYLRPPSFLR.A** 29667 29669 29670 |

| **68.** | O60814\|H2B1K_HUMAN    **Mass:** 13882    **Score:** 359    **Queries matched:** 29   **emPAI:** 1.71 |
| --- | --- |
|  | Histone H2B type 1-K OS=Homo sapiens GN=HIST1H2BK PE=1 SV=3 |

|  | **Query** | **Observed** | **Mr(expt)** | **Mr(calc)** | **Delta** | **Miss** | **Score** | **Expect** | **Rank** | **Peptide** |
| --- | --- | --- | --- | --- | --- | --- | --- | --- | --- | --- |
|  | 865 | **571.1000** | **570.0928** | **569.3173** | **0.7755** | **0** | **12** | **5.5** | **1** | **K.SAPAPK.K** 864 |
|  | 1172 | **704.5136** | **703.5063** | **702.3660** | **1.1402** | **0** | **11** | **19** | **1** | **R.IAGEASR.L** |
|  | 1844 | **415.2156** | **828.4167** | **827.4137** | **1.0029** | **0** | **23** | **0.95** | **1** | **K.HAVSEGTK.A** 1838 1839 1840 1841 1843 1855 |
|  | 5438 | **546.8013** | **1091.5881** | **1091.5975** | **-0.0094** | **1** | **27** | **0.31** | **2** | **M.PEPAKSAPAPK.K** |
|  | 6014 | **569.2734** | **1136.5322** | **1136.5390** | **-0.0068** | **0** | **13** | **7.1** | **1** | **K.ESYSVYVYK.V** |
|  | 6412 | **584.7980** | **1167.5814** | **1167.5884** | **-0.0070** | **0** | **25** | **0.5** | **1** | **K.QVHPDTGISSK.A** |
|  | 7805 | **620.8442** | **1239.6739** | **1238.6329** | **1.0411** | **1** | **4** | **55** | **7** | **-.MPEPAKSAPAPK.K** |
|  | 8333 | **633.3204** | **1264.6263** | **1264.6339** | **-0.0076** | **1** | **31** | **0.1** | **1** | **R.KESYSVYVYK.V** 8332 |
|  | 11304 | **715.8343** | **1429.6541** | **1428.7473** | **0.9068** | **1** | **9** | **16** | **3** | **R.IAGEASRLAHYNK.R** 11287 |
|  | 12604 | **754.9198** | **1507.8250** | **1507.8358** | **-0.0108** | **1** | **54** | **0.00046** | **1** | **K.VLKQVHPDTGISSK.A** |
|  | 16180 | **872.4089** | **1742.8032** | **1742.8120** | **-0.0088** | **0** | **99** | **1.3e-008** | **1** | **K.AMGIMNSFVNDIFER.I** 16168 16179 16181 16183 16191 |
|  | 16389 | **880.4067** | **1758.7988** | **1758.8069** | **-0.0081** | **0** | **(54)** | **0.00038** | **1** | **K.AMGIMNSFVNDIFER.I** 16378 |
|  | 16443 | **441.7535** | **1762.9851** | **1761.9989** | **0.9862** | **1** | **4** | **39** | **2** | **R.LLLPGELAKHAVSEGTK.A** |
|  | 23151 | **820.3754** | **2458.1045** | **2459.1573** | **-1.0528** | **1** | **2** | **48** | **2** | **K.AMGIMNSFVNDIFERIAGEASR.L** |

|  | **Proteins matching the same set of peptides:** |
| --- | --- |

|  | P62807\|H2B1C_HUMAN    **Mass:** 13811    **Score:** 359    **Queries matched:** 29 |
| --- | --- |
|  | Histone H2B type 1-C/E/F/G/I OS=Homo sapiens GN=HIST1H2BC PE=1 SV=3 |

| **69.** | P57053\|H2BFS_HUMAN    **Mass:** 13936    **Score:** 359    **Queries matched:** 28   **emPAI:** 1.69 |
| --- | --- |
|  | Histone H2B type F-S OS=Homo sapiens GN=H2BFS PE=1 SV=2 |

|  | **Query** | **Observed** | **Mr(expt)** | **Mr(calc)** | **Delta** | **Miss** | **Score** | **Expect** | **Rank** | **Peptide** |
| --- | --- | --- | --- | --- | --- | --- | --- | --- | --- | --- |
|  | 865 | 571.1000 | 570.0928 | 569.3173 | 0.7755 | 0 | 12 | 5.5 | 1 | K.SAPAPK.K 864 |
|  | 1172 | 704.5136 | 703.5063 | 702.3660 | 1.1402 | 0 | 11 | 19 | 1 | R.IAGEASR.L |
|  | 1844 | 415.2156 | 828.4167 | 827.4137 | 1.0029 | 0 | 23 | 0.95 | 1 | K.HAVSEGTK.A 1838 1839 1840 1841 1843 1855 |
|  | 5438 | 546.8013 | 1091.5881 | 1091.5975 | -0.0094 | 1 | 27 | 0.31 | 2 | M.PEPAKSAPAPK.K |
|  | 6014 | 569.2734 | 1136.5322 | 1136.5390 | -0.0068 | 0 | 13 | 7.1 | 1 | K.ESYSVYVYK.V |
|  | 6412 | 584.7980 | 1167.5814 | 1167.5884 | -0.0070 | 0 | 25 | 0.5 | 1 | K.QVHPDTGISSK.A |
|  | 7805 | 620.8442 | 1239.6739 | 1238.6329 | 1.0411 | 1 | 4 | 55 | 7 | -.MPEPAKSAPAPK.K |
|  | 8333 | 633.3204 | 1264.6263 | 1264.6339 | -0.0076 | 1 | 31 | 0.1 | 1 | R.KESYSVYVYK.V 8332 |
|  | 11698 | **727.8725** | **1453.7304** | **1454.7630** | **-1.0325** | **1** | **4** | **51** | **4** | **R.IAGEASRLPHYNK.R** |
|  | 12604 | 754.9198 | 1507.8250 | 1507.8358 | -0.0108 | 1 | 54 | 0.00046 | 1 | K.VLKQVHPDTGISSK.A |
|  | 16180 | 872.4089 | 1742.8032 | 1742.8120 | -0.0088 | 0 | 99 | 1.3e-008 | 1 | K.AMGIMNSFVNDIFER.I 16168 16179 16181 16183 16191 |
|  | 16389 | 880.4067 | 1758.7988 | 1758.8069 | -0.0081 | 0 | (54) | 0.00038 | 1 | K.AMGIMNSFVNDIFER.I 16378 |
|  | 16443 | 441.7535 | 1762.9851 | 1761.9989 | 0.9862 | 1 | 4 | 39 | 2 | R.LLLPGELAKHAVSEGTK.A |
|  | 23151 | 820.3754 | 2458.1045 | 2459.1573 | -1.0528 | 1 | 2 | 48 | 2 | K.AMGIMNSFVNDIFERIAGEASR.L |

| **70.** | Q5QNW6\|H2B2F_HUMAN    **Mass:** 13912    **Score:** 359    **Queries matched:** 30   **emPAI:** 1.71 |
| --- | --- |
|  | Histone H2B type 2-F OS=Homo sapiens GN=HIST2H2BF PE=1 SV=3 |

|  | **Query** | **Observed** | **Mr(expt)** | **Mr(calc)** | **Delta** | **Miss** | **Score** | **Expect** | **Rank** | **Peptide** |
| --- | --- | --- | --- | --- | --- | --- | --- | --- | --- | --- |
|  | 865 | 571.1000 | 570.0928 | 569.3173 | 0.7755 | 0 | 12 | 5.5 | 1 | K.SAPAPK.K 864 |
|  | 1172 | 704.5136 | 703.5063 | 702.3660 | 1.1402 | 0 | 11 | 19 | 1 | R.IAGEASR.L |
|  | 1844 | 415.2156 | 828.4167 | 827.4137 | 1.0029 | 0 | 23 | 0.95 | 1 | K.HAVSEGTK.A 1838 1839 1840 1841 1843 1855 |
|  | 4020 | **492.7686** | **983.5227** | **983.5287** | **-0.0061** | **1** | **13** | **8.5** | **1** | **K.AVTKYTSSK.-** |
|  | 6014 | 569.2734 | 1136.5322 | 1136.5390 | -0.0068 | 0 | 13 | 7.1 | 1 | K.ESYSVYVYK.V |
|  | 6412 | 584.7980 | 1167.5814 | 1167.5884 | -0.0070 | 0 | 25 | 0.5 | 1 | K.QVHPDTGISSK.A |
|  | 7166 | **605.3293** | **1208.6440** | **1208.6223** | **0.0217** | **1** | **(1)** | **1.1e+002** | **1** | **-.MPDPAKSAPAPK.K** |
|  | 7483 | **613.2940** | **1224.5733** | **1224.6172** | **-0.0439** | **1** | **10** | **15** | **9** | **-.MPDPAKSAPAPK.K** |
|  | 8333 | 633.3204 | 1264.6263 | 1264.6339 | -0.0076 | 1 | 31 | 0.1 | 1 | R.KESYSVYVYK.V 8332 |
|  | 11304 | 715.8343 | 1429.6541 | 1428.7473 | 0.9068 | 1 | 9 | 16 | 3 | R.IAGEASRLAHYNK.R 11287 |
|  | 12604 | 754.9198 | 1507.8250 | 1507.8358 | -0.0108 | 1 | 54 | 0.00046 | 1 | K.VLKQVHPDTGISSK.A |
|  | 16180 | 872.4089 | 1742.8032 | 1742.8120 | -0.0088 | 0 | 99 | 1.3e-008 | 1 | K.AMGIMNSFVNDIFER.I 16168 16179 16181 16183 16191 |
|  | 16389 | 880.4067 | 1758.7988 | 1758.8069 | -0.0081 | 0 | (54) | 0.00038 | 1 | K.AMGIMNSFVNDIFER.I 16378 |
|  | 16443 | 441.7535 | 1762.9851 | 1761.9989 | 0.9862 | 1 | 4 | 39 | 2 | R.LLLPGELAKHAVSEGTK.A |
|  | 23151 | 820.3754 | 2458.1045 | 2459.1573 | -1.0528 | 1 | 2 | 48 | 2 | K.AMGIMNSFVNDIFERIAGEASR.L |

|  | **Proteins matching the same set of peptides:** |
| --- | --- |

|  | Q93079\|H2B1H_HUMAN    **Mass:** 13884    **Score:** 359    **Queries matched:** 30 |
| --- | --- |
|  | Histone H2B type 1-H OS=Homo sapiens GN=HIST1H2BH PE=1 SV=3 |

|  | **Proteins matching a subset of these peptides:** |
| --- | --- |

|  | P58876\|H2B1D_HUMAN    **Mass:** 13928    **Score:** 359    **Queries matched:** 28 |
| --- | --- |
|  | Histone H2B type 1-D OS=Homo sapiens GN=HIST1H2BD PE=1 SV=2 |

|  | Q99877\|H2B1N_HUMAN    **Mass:** 13914    **Score:** 359    **Queries matched:** 28 |
| --- | --- |
|  | Histone H2B type 1-N OS=Homo sapiens GN=HIST1H2BN PE=1 SV=3 |

|  | Q99879\|H2B1M_HUMAN    **Mass:** 13981    **Score:** 359    **Queries matched:** 26 |
| --- | --- |
|  | Histone H2B type 1-M OS=Homo sapiens GN=HIST1H2BM PE=1 SV=3 |

|  | Q99880\|H2B1L_HUMAN    **Mass:** 13944    **Score:** 359    **Queries matched:** 24 |
| --- | --- |
|  | Histone H2B type 1-L OS=Homo sapiens GN=HIST1H2BL PE=1 SV=3 |

| **71.** | P36269\|GGT5_HUMAN    **Mass:** 62736    **Score:** 357    **Queries matched:** 27   **emPAI:** 0.50 |
| --- | --- |
|  | Gamma-glutamyltransferase 5 OS=Homo sapiens GN=GGT5 PE=1 SV=2 |

|  | **Query** | **Observed** | **Mr(expt)** | **Mr(calc)** | **Delta** | **Miss** | **Score** | **Expect** | **Rank** | **Peptide** |
| --- | --- | --- | --- | --- | --- | --- | --- | --- | --- | --- |
|  | 1497 | **401.2847** | **800.5549** | **799.4552** | **1.0997** | **0** | **6** | **51** | **5** | **K.VEVINAR.E** |
|  | 2873 | **450.2788** | **898.5431** | **898.4773** | **0.0657** | **1** | **3** | **69** | **7** | **R.WRLGDPR.S** |
|  | 5426 | **546.3164** | **1090.6181** | **1090.6247** | **-0.0066** | **0** | **16** | **4.5** | **1** | **R.GGHVVAPVLSR.F** |
|  | 7661 | **617.3408** | **1232.6670** | **1231.6482** | **1.0188** | **0** | **3** | **77** | **5** | **R.LGQMLVEDIAK.E** |
|  | 9056 | **651.8453** | **1301.6760** | **1301.6827** | **-0.0067** | **0** | **19** | **1.9** | **1** | **K.EGSQLTLQDLAK.F** |
|  | 9066 | **652.3011** | **1302.5876** | **1303.7612** | **-1.1736** | **0** | **5** | **47** | **4** | **R.AAIAAPILHVNSK.G** |
|  | 11660 | **484.6020** | **1450.7841** | **1450.7932** | **-0.0091** | **0** | **13** | **7** | **1** | **R.VNVYHHLVETLK.F** |
|  | 14537 | **543.9442** | **1628.8108** | **1629.7529** | **-0.9421** | **1** | **1** | **1e+002** | **6** | **R.CPRGSGTTPSPVSGDR.V** |
|  | 15475 | **564.6174** | **1690.8305** | **1690.8362** | **-0.0057** | **1** | **3** | **50** | **3** | **R.VGGAPGRCWPPVPGER.S** |
|  | 16551 | **886.9597** | **1771.9049** | **1771.9138** | **-0.0089** | **0** | **100** | **1.1e-008** | **1** | **R.TGIILNNELLDLCER.C** 16550 16562 |
|  | 17415 | **921.4044** | **1840.7942** | **1840.8050** | **-0.0108** | **0** | **81** | **7.9e-007** | **1** | **K.GCVEYEPNFSQEVQR.G** 17416 |
|  | 17475 | **922.9374** | **1843.8602** | **1842.9873** | **0.8729** | **1** | **3** | **57** | **3** | **R.SPSSMVPSILINKAQGSK.L** 17474 |
|  | 18790 | **977.0501** | **1952.0855** | **1952.0956** | **-0.0100** | **0** | **51** | **0.00075** | **1** | **R.FLHNSILRPSLQASTLR.Q** 18789 18791 18794 |
|  | 20451 | **1058.0576** | **2114.1007** | **2113.1817** | **0.9190** | **0** | **4** | **35** | **1** | **K.LVIGGAGGELIISAVAQAIMSK.L** |
|  | 20479 | **530.0563** | **2116.1963** | **2116.2058** | **-0.0095** | **1** | **0** | **78** | **7** | **R.HGRLPWAQLFQPTIALLR.G** |
|  | 23418 | **834.1120** | **2499.3142** | **2499.3254** | **-0.0112** | **1** | **68** | **1e-005** | **1** | **R.LGQMLVEDIAKEGSQLTLQDLAK.F** 23408 |
|  | 28719 | **1213.9523** | **3638.8350** | **3638.8515** | **-0.0165** | **0** | **48** | **0.0006** | **1** | **R.ETVPASHAPSLLDQCAQALPLGTGAQWIGVPGELR.G** 28718 |
|  | 29805 | **1017.7964** | **4067.1565** | **4067.1660** | **-0.0095** | **0** | **6** | **5.8** | **1** | **K.FQPEVVDALEVPLGDYTLYSPPPPAGGAILSFILNVLR.G** |

| **72.** | P35232\|PHB_HUMAN    **Mass:** 29843    **Score:** 356    **Queries matched:** 22   **emPAI:** 1.93 |
| --- | --- |
|  | Prohibitin OS=Homo sapiens GN=PHB PE=1 SV=1 |

|  | **Query** | **Observed** | **Mr(expt)** | **Mr(calc)** | **Delta** | **Miss** | **Score** | **Expect** | **Rank** | **Peptide** |
| --- | --- | --- | --- | --- | --- | --- | --- | --- | --- | --- |
|  | 4894 | 1049.5225 | 1048.5152 | 1048.5917 | -0.0765 | 1 | 9 | 23 | 2 | M.AAKVFESIGK.F |
|  | 6880 | **399.3449** | **1195.0129** | **1195.6271** | **-0.6142** | **1** | **10** | **10** | **5** | **-.MAAKVFESIGK.F** |
|  | 7099 | **603.3263** | **1204.6380** | **1204.6452** | **-0.0071** | **1** | **32** | **0.1** | **1** | **R.FVVEKAEQQK.K** |
|  | 8705 | **643.3292** | **1284.6439** | **1284.6534** | **-0.0095** | **1** | **30** | **0.15** | **1** | **K.QVAQQEAERAR.F** |
|  | 10740 | **698.9202** | **1395.8259** | **1395.8350** | **-0.0091** | **0** | **37** | **0.023** | **1** | **R.ILFRPVASQLPR.I** 10741 |
|  | 11524 | **722.8293** | **1443.6440** | **1443.6518** | **-0.0078** | **0** | **33** | **0.071** | **1** | **R.IFTSIGEDYDER.V** |
|  | 14161 | **803.9190** | **1605.8235** | **1605.8362** | **-0.0127** | **1** | **88** | **1.6e-007** | **1** | **R.KLEAAEDIAYQLSR.S** |
|  | 14776 | **549.2738** | **1644.7996** | **1645.8271** | **-1.0275** | **1** | **7** | **23** | **1** | **R.ELVSRQVSDDLTER.A** |
|  | 20126 | **1041.0842** | **2080.1539** | **2080.1640** | **-0.0101** | **1** | **103** | **4.3e-009** | **1** | **R.NVPVITGSKDLQNVNITLR.I** 20125 |
|  | 20280 | **1049.5809** | **2097.1473** | **2097.1582** | **-0.0109** | **1** | **51** | **0.00069** | **1** | **R.SRNITYLPAGQSVLLQLPQ.-** 20281 20290 |
|  | 20528 | **709.3404** | **2124.9993** | **2125.1742** | **-0.1749** | **1** | **2** | **51** | **6** | **K.AAELIANSLATAGDGLIELRK.L** |
|  | 26306 | **760.9061** | **3039.5954** | **3039.6087** | **-0.0133** | **1** | **58** | **7.9e-005** | **1** | **K.AAIISAEGDSKAAELIANSLATAGDGLIELR.K** 26310 |
|  | 26707 | **1041.8737** | **3122.5992** | **3122.6176** | **-0.0184** | **1** | **12** | **2.9** | **1** | **R.AATFGLILDDVSLTHLTFGKEFTEAVEAK.Q** |
|  | 26711 | **1041.8817** | **3122.6233** | **3122.6376** | **-0.0143** | **0** | **42** | **0.0033** | **1** | **R.GVQDIVVGEGTHFLIPWVQKPIIFDCR.S** 26712 26713 |
|  | 27959 | **857.4577** | **3425.8017** | **3425.8071** | **-0.0054** | **1** | **38** | **0.0067** | **1** | **R.FRGVQDIVVGEGTHFLIPWVQKPIIFDCR.S** |

| **73.** | P80365\|DHI2_HUMAN    **Mass:** 44612    **Score:** 355    **Queries matched:** 22   **emPAI:** 0.76 |
| --- | --- |
|  | Corticosteroid 11-beta-dehydrogenase isozyme 2 OS=Homo sapiens GN=HSD11B2 PE=1 SV=2 |

|  | **Query** | **Observed** | **Mr(expt)** | **Mr(calc)** | **Delta** | **Miss** | **Score** | **Expect** | **Rank** | **Peptide** |
| --- | --- | --- | --- | --- | --- | --- | --- | --- | --- | --- |
|  | 2301 | **860.4210** | **859.4137** | **859.4188** | **-0.0051** | **0** | **21** | **1.7** | **1** | **R.NVGQWEK.R** 2303 |
|  | 6150 | **574.8075** | **1147.6004** | **1147.6060** | **-0.0055** | **0** | **10** | **15** | **1** | **K.VSIIQPGCFK.T** |
|  | 13869 | **793.9274** | **1585.8402** | **1585.8498** | **-0.0096** | **0** | **73** | **5.7e-006** | **1** | **R.LLQMDLTKPGDISR.V** 13850 |
|  | 14614 | **545.7737** | **1634.2992** | **1634.8392** | **-0.5399** | **0** | **6** | **20** | **4** | **R.FLQAFFISHCLPR.A** |
|  | 15877 | **574.3029** | **1719.8869** | **1719.8978** | **-0.0109** | **1** | **12** | **7.1** | **1** | **K.VSIIQPGCFKTESVR.N** |
|  | 16308 | **877.4380** | **1752.8614** | **1752.8716** | **-0.0102** | **1** | **52** | **0.00072** | **1** | **R.AVLITGCDSGFGKETAK.K** |
|  | 18275 | **637.8214** | **1910.4424** | **1911.0829** | **-0.6405** | **0** | **3** | **33** | **3** | **K.QLLLANLPQELLQAYGK.D** |
|  | 18778 | **651.3422** | **1951.0048** | **1951.0217** | **-0.0168** | **1** | **4** | **39** | **6** | **M.ERWPWPSGGAWLLVAAR.A** 18786 |
|  | 20038 | **691.3362** | **2070.9867** | **2070.2353** | **0.7514** | **0** | **1** | **61** | **4** | **R.LLPPPAALAVLAAAGWIALSR.L** |
|  | 22319 | **1171.0576** | **2340.1007** | **2340.1130** | **-0.0123** | **0** | **89** | **8.7e-008** | **1** | **R.IVTVGSPAGDMPYPCLGAYGTSK.A** 22331 |
|  | 23556 | **842.1074** | **2523.3004** | **2523.3229** | **-0.0225** | **1** | **3** | **34** | **4** | **R.SCMEVNFFGALELTKGLLPLLR.S** |
|  | 23781 | **852.0811** | **2553.2215** | **2553.2356** | **-0.0141** | **1** | **15** | **2.2** | **1** | **R.GRIVTVGSPAGDMPYPCLGAYGTSK.A** |
|  | 24405 | **1337.7216** | **2673.4286** | **2673.4411** | **-0.0125** | **1** | **85** | **1.9e-007** | **1** | **K.KLDSMGFTVLATVLELNSPGAIELR.T** 24407 |
|  | 26286 | **1518.2539** | **3034.4933** | **3034.5108** | **-0.0176** | **0** | **45** | **0.0017** | **1** | **R.ALQPGQPGTTPPQDAAQDPNLSPGPSPAVAR.-** 26285 |
|  | 28006 | **1145.5684** | **3433.6833** | **3433.7015** | **-0.0182** | **0** | **65** | **1.3e-005** | **1** | **K.AHTTSTGLWGLVNNAGHNEVVADAELSPVATFR.S** 28007 |

| **74.** | P08195\|4F2_HUMAN    **Mass:** 68180    **Score:** 354    **Queries matched:** 34   **emPAI:** 0.70 |
| --- | --- |
|  | 4F2 cell-surface antigen heavy chain OS=Homo sapiens GN=SLC3A2 PE=1 SV=3 |

|  | **Query** | **Observed** | **Mr(expt)** | **Mr(calc)** | **Delta** | **Miss** | **Score** | **Expect** | **Rank** | **Peptide** |
| --- | --- | --- | --- | --- | --- | --- | --- | --- | --- | --- |
|  | 1210 | **732.2933** | **731.2861** | **730.3861** | **0.9000** | **0** | **18** | **3.8** | **1** | **R.DIENLK.D** |
|  | 3366 | **467.2951** | **932.5756** | **932.5807** | **-0.0051** | **0** | **34** | **0.071** | **1** | **K.GLVLGPIHK.N** |
|  | 4014 | **492.7144** | **983.4143** | **983.4209** | **-0.0066** | **0** | **34** | **0.057** | **1** | **R.HWDQNER.F** 4015 4016 4017 4018 4019 4028 4029 |
|  | 6532 | **589.2984** | **1176.5822** | **1175.6662** | **0.9160** | **0** | **10** | **18** | **2** | **K.LEPHEGLLLR.F** |
|  | 7910 | **623.2983** | **1244.5820** | **1244.5884** | **-0.0064** | **0** | **10** | **15** | **2** | **K.VAEDEAEAAAAAK.F** 7934 |
|  | 8307 | **422.2404** | **1263.6993** | **1263.7074** | **-0.0081** | **1** | **7** | **29** | **2** | **K.FTGLSKEELLK.V** |
|  | 10304 | **686.4188** | **1370.8230** | **1370.8286** | **-0.0056** | **0** | **45** | **0.0045** | **1** | **R.LLTSFLPAQLLR.L** |
|  | 11093 | **473.2864** | **1416.8375** | **1416.8452** | **-0.0078** | **1** | **28** | **0.21** | **1** | **R.LKLEPHEGLLLR.F** 11098 |
|  | 12260 | **743.8863** | **1485.7581** | **1485.7674** | **-0.0093** | **1** | **63** | **6.5e-005** | **1** | **K.IKVAEDEAEAAAAAK.F** |
|  | 12557 | **753.3790** | **1504.7434** | **1504.7522** | **-0.0088** | **0** | **55** | **0.00038** | **1** | **K.GQSEDPGSLLSLFR.R** 12558 |
|  | 15943 | **575.7982** | **1724.3727** | **1724.9250** | **-0.5523** | **1** | **5** | **26** | **5** | **K.LEPHEGLLLRFPYAA.-** |
|  | 16511 | **885.4150** | **1768.8154** | **1768.8268** | **-0.0114** | **0** | **45** | **0.0036** | **1** | **R.GENSWFSTQVDTVATK.V** |
|  | 17592 | **926.7769** | **1851.5393** | **1851.9764** | **-0.4371** | **0** | **3** | **31** | **1** | **-.MELQPPEASIAVVSIPR.Q** |
|  | 17762 | **933.9891** | **1865.9636** | **1865.9748** | **-0.0112** | **0** | **76** | **2.9e-006** | **1** | **R.IGDLQAFQGHGAGNLAGLK.G** 17759 17760 17761 |
|  | 19037 | **659.3795** | **1975.1166** | **1974.9535** | **0.1631** | **0** | **2** | **50** | **4** | **K.DDVAQTDLLQIDPNFGSK.E** |
|  | 20108 | **694.0359** | **2079.0858** | **2079.0974** | **-0.0115** | **1** | **17** | **2** | **1** | **R.IGDLQAFQGHGAGNLAGLKGR.L** |
|  | 21517 | **558.8202** | **2231.2517** | **2231.1038** | **0.1479** | **1** | **2** | **53** | **6** | **K.QPMNAASGAAMSLAGAEKNGLVK.I** 21510 |
|  | 21977 | **764.7196** | **2291.1370** | **2291.1487** | **-0.0118** | **0** | **34** | **0.028** | **1** | **R.SLLHGDFHAFSAGPGLFSYIR.H** |
|  | 22111 | **1155.5945** | **2309.1744** | **2309.1863** | **-0.0119** | **1** | **5** | **24** | **1** | **K.ADLLLSTQPGREEGSPLELER.L** |
|  | 24728 | **682.3174** | **2725.2407** | **2725.5910** | **-0.3503** | **0** | **0** | **53** | **3** | **R.WALLLLFWLGWLGMLAGAVVIIVR.A** |

| **75.** | Q13423\|NNTM_HUMAN    **Mass:** 114564   **Score:** 350    **Queries matched:** 43   **emPAI:** 0.42 |
| --- | --- |
|  | NAD(P) transhydrogenase, mitochondrial OS=Homo sapiens GN=NNT PE=1 SV=3 |

|  | **Query** | **Observed** | **Mr(expt)** | **Mr(calc)** | **Delta** | **Miss** | **Score** | **Expect** | **Rank** | **Peptide** |
| --- | --- | --- | --- | --- | --- | --- | --- | --- | --- | --- |
|  | 2028 | **420.7607** | **839.5068** | **840.5069** | **-1.0001** | **0** | **11** | **12** | **9** | **K.QLTVGVPK.E** |
|  | 2971 | **453.7181** | **905.4216** | **905.4277** | **-0.0060** | **0** | **15** | **6.5** | **2** | **K.TCDALQAK.V** 2961 2972 |
|  | 3228 | **462.2209** | **922.4273** | **922.5600** | **-0.1327** | **0** | **1** | **1.3e+002** | **8** | **K.SAPLLLPGR.H** |
|  | 3237 | **923.5252** | **922.5179** | **922.5236** | **-0.0057** | **0** | **43** | **0.0074** | **1** | **K.NIPQGAPVK.Q** |
|  | 3374 | **467.7511** | **933.4876** | **933.4954** | **-0.0077** | **1** | **22** | **1.1** | **1** | **R.GTVVMKDGK.V** 3393 |
|  | 3427 | **470.3181** | **938.6215** | **938.3882** | **0.2333** | **0** | **6** | **34** | **6** | **K.FSDDHYR.V** |
|  | 3798 | **485.2893** | **968.5641** | **968.5695** | **-0.0054** | **0** | **8** | **25** | **3** | **K.VIFPAPTPK.N** |
|  | 4087 | **495.0604** | **988.1062** | **988.5077** | **-0.4014** | **0** | **6** | **29** | **2** | **K.TVAELEAEK.A** |
|  | 4210 | **499.2592** | **996.5038** | **995.4633** | **1.0404** | **0** | **6** | **38** | **7** | **K.EFIEAEMK.L** |
|  | 4707 | **517.7644** | **1033.5142** | **1033.5226** | **-0.0084** | **1** | **40** | **0.017** | **1** | **K.KTCDALQAK.V** |
|  | 5058 | **532.2762** | **1062.5378** | **1062.5458** | **-0.0080** | **1** | **12** | **11** | **4** | **K.EIFQNEKR.V** |
|  | 5175 | 536.8125 | 1071.6104 | 1071.6176 | -0.0071 | 0 | 14 | 7.4 | 2 | K.EVLASDLVVK.V |
|  | 6254 | **577.8218** | **1153.6290** | **1153.6356** | **-0.0066** | **0** | **33** | **0.067** | **1** | **K.AVVLAANHFGR.F** |
|  | 7594 | **615.8257** | **1229.6368** | **1229.6438** | **-0.0070** | **0** | **33** | **0.077** | **1** | **K.TTVLAMDQVPR.V** |
|  | 8435 | **424.2059** | **1269.5960** | **1268.7129** | **0.8831** | **1** | **11** | **12** | **2** | **K.DGKVIFPAPTPK.N** |
|  | 9501 | **663.8527** | **1325.6908** | **1326.7871** | **-1.0963** | **1** | **9** | **18** | **2** | **K.EVLASDLVVKVR.A** 9503 |
|  | 11292 | **715.3715** | **1428.7284** | **1428.7361** | **-0.0078** | **0** | **49** | **0.0018** | **1** | **K.GITHIGYTDLPSR.M** |
|  | 12530 | **501.9467** | **1502.8182** | **1502.6632** | **0.1550** | **1** | **4** | **49** | **3** | **K.EMSKEFIEAEMK.L** |
|  | 12865 | **762.9545** | **1523.8945** | **1523.9035** | **-0.0090** | **0** | **108** | **2e-009** | **1** | **K.ILIVGGGVAGLASAGAAK.S** 12860 |
|  | 13713 | **789.8874** | **1577.7602** | **1577.7686** | **-0.0083** | **0** | **65** | **3.8e-005** | **1** | **K.QGFNVVVESGAGEASK.F** 13698 13710 13729 13748 |
|  | 14746 | **822.3967** | **1642.7789** | **1641.8032** | **0.9757** | **0** | **(1)** | **86** | **6** | **R.MATQASTLYSNNITK.L** |
|  | 14880 | 826.3666 | 1650.7187 | 1651.8313 | -1.1126 | 1 | 0 | 1.1e+002 | 8 | K.APVLFNKEMIESMK.E |
|  | 14969 | **829.5090** | **1657.0035** | **1657.7981** | **-0.7946** | **0** | **9** | **11** | **2** | **R.MATQASTLYSNNITK.L** |
|  | 16603 | **593.2668** | **1776.7787** | **1776.9080** | **-0.1293** | **0** | **4** | **39** | **4** | **R.EANSIIITPGYGLCAAK.A** |
|  | 19430 | **672.0834** | **2013.2283** | **2012.0612** | **1.1671** | **1** | **0** | **71** | **5** | **R.MATQASTLYSNNITKLLK.A** |
|  | 19926 | **687.3776** | **2059.1110** | **2059.1248** | **-0.0138** | **1** | **47** | **0.0017** | **1** | **K.VRAPMVNPTLGVHEADLLK.T** |
|  | 20044 | **1037.0151** | **2072.0157** | **2072.0248** | **-0.0091** | **0** | **111** | **6.1e-010** | **1** | **R.VTIAQGYDALSSMANIAGYK.A** |
|  | 20070 | **415.9797** | **2074.8623** | **2075.1197** | **-0.2574** | **1** | **(6)** | **19** | **2** | **K.VRAPMVNPTLGVHEADLLK.T** |
|  | 20184 | **697.3389** | **2088.9950** | **2088.0197** | **0.9752** | **0** | **(2)** | **61** | **5** | **R.VTIAQGYDALSSMANIAGYK.A** |
|  | 22282 | **778.7152** | **2333.1238** | **2333.1387** | **-0.0149** | **1** | **26** | **0.2** | **1** | **K.SLGAEPLEVDLKESGEGQGGYAK.E** |
|  | 25256 | **942.4841** | **2824.4306** | **2824.4469** | **-0.0164** | **0** | **39** | **0.008** | **1** | **R.SLGVGYAAVDNPIFYKPNTAMLLGDAK.K** |
|  | 29120 | **945.9496** | **3779.7692** | **3778.8433** | **0.9259** | **1** | **1** | **26** | **4** | **K.EMIESMKEGSVVVDLAAEAGGNFETTKPGELYIHK.G** |
|  | 30437 | **630.3223** | **4405.2050** | **4405.4151** | **-0.2101** | **0** | **2** | **14** | **2** | **R.LGNALGMIGVAGGLAATLGVLKPGPELLAQMSGAMALGGTIGLTIAK.R** |
|  | 30654 | **1137.5399** | **4546.1306** | **4545.5212** | **0.6093** | **1** | **2** | **13** | **10** | **R.LGNALGMIGVAGGLAATLGVLKPGPELLAQMSGAMALGGTIGLTIAKR.I** |

| **76.** | Q16778\|H2B2E_HUMAN    **Mass:** 13912    **Score:** 349    **Queries matched:** 28   **emPAI:** 1.11 |
| --- | --- |
|  | Histone H2B type 2-E OS=Homo sapiens GN=HIST2H2BE PE=1 SV=3 |

|  | **Query** | **Observed** | **Mr(expt)** | **Mr(calc)** | **Delta** | **Miss** | **Score** | **Expect** | **Rank** | **Peptide** |
| --- | --- | --- | --- | --- | --- | --- | --- | --- | --- | --- |
|  | 865 | 571.1000 | 570.0928 | 569.3173 | 0.7755 | 0 | 12 | 5.5 | 1 | K.SAPAPK.K 864 |
|  | 1172 | 704.5136 | 703.5063 | 702.3660 | 1.1402 | 0 | 11 | 19 | 1 | R.IAGEASR.L |
|  | 1844 | 415.2156 | 828.4167 | 827.4137 | 1.0029 | 0 | 23 | 0.95 | 1 | K.HAVSEGTK.A 1838 1839 1840 1841 1843 1855 |
|  | 4020 | 492.7686 | 983.5227 | 983.5287 | -0.0061 | 1 | 13 | 8.5 | 1 | K.AVTKYTSSK.- |
|  | 5438 | 546.8013 | 1091.5881 | 1091.5975 | -0.0094 | 1 | 27 | 0.31 | 2 | M.PEPAKSAPAPK.K |
|  | 6412 | 584.7980 | 1167.5814 | 1167.5884 | -0.0070 | 0 | 25 | 0.5 | 1 | K.QVHPDTGISSK.A |
|  | 7805 | 620.8442 | 1239.6739 | 1238.6329 | 1.0411 | 1 | 4 | 55 | 7 | -.MPEPAKSAPAPK.K |
|  | 8597 | **640.3269** | **1278.6392** | **1278.6496** | **-0.0103** | **1** | **16** | **3.6** | **2** | **R.KESYSIYVYK.V** |
|  | 11304 | 715.8343 | 1429.6541 | 1428.7473 | 0.9068 | 1 | 9 | 16 | 3 | R.IAGEASRLAHYNK.R 11287 |
|  | 12604 | 754.9198 | 1507.8250 | 1507.8358 | -0.0108 | 1 | 54 | 0.00046 | 1 | K.VLKQVHPDTGISSK.A |
|  | 16180 | 872.4089 | 1742.8032 | 1742.8120 | -0.0088 | 0 | 99 | 1.3e-008 | 1 | K.AMGIMNSFVNDIFER.I 16168 16179 16181 16183 16191 |
|  | 16389 | 880.4067 | 1758.7988 | 1758.8069 | -0.0081 | 0 | (54) | 0.00038 | 1 | K.AMGIMNSFVNDIFER.I 16378 |
|  | 16443 | 441.7535 | 1762.9851 | 1761.9989 | 0.9862 | 1 | 4 | 39 | 2 | R.LLLPGELAKHAVSEGTK.A |
|  | 23151 | 820.3754 | 2458.1045 | 2459.1573 | -1.0528 | 1 | 2 | 48 | 2 | K.AMGIMNSFVNDIFERIAGEASR.L |

|  | **Proteins matching a subset of these peptides:** |
| --- | --- |

|  | P06899\|H2B1J_HUMAN    **Mass:** 13896    **Score:** 349    **Queries matched:** 27 |
| --- | --- |
|  | Histone H2B type 1-J OS=Homo sapiens GN=HIST1H2BJ PE=1 SV=3 |

|  | P33778\|H2B1B_HUMAN    **Mass:** 13942    **Score:** 349    **Queries matched:** 26 |
| --- | --- |
|  | Histone H2B type 1-B OS=Homo sapiens GN=HIST1H2BB PE=1 SV=2 |

| **77.** | P23527\|H2B1O_HUMAN    **Mass:** 13898    **Score:** 349    **Queries matched:** 28   **emPAI:** 1.11 |
| --- | --- |
|  | Histone H2B type 1-O OS=Homo sapiens GN=HIST1H2BO PE=1 SV=3 |

|  | **Query** | **Observed** | **Mr(expt)** | **Mr(calc)** | **Delta** | **Miss** | **Score** | **Expect** | **Rank** | **Peptide** |
| --- | --- | --- | --- | --- | --- | --- | --- | --- | --- | --- |
|  | 865 | 571.1000 | 570.0928 | 569.3173 | 0.7755 | 0 | 12 | 5.5 | 1 | K.SAPAPK.K 864 |
|  | 1172 | 704.5136 | 703.5063 | 702.3660 | 1.1402 | 0 | 11 | 19 | 1 | R.IAGEASR.L |
|  | 1844 | 415.2156 | 828.4167 | 827.4137 | 1.0029 | 0 | 23 | 0.95 | 1 | K.HAVSEGTK.A 1838 1839 1840 1841 1843 1855 |
|  | 4020 | 492.7686 | 983.5227 | 983.5287 | -0.0061 | 1 | 13 | 8.5 | 1 | K.AVTKYTSSK.- |
|  | 6412 | 584.7980 | 1167.5814 | 1167.5884 | -0.0070 | 0 | 25 | 0.5 | 1 | K.QVHPDTGISSK.A |
|  | 7166 | 605.3293 | 1208.6440 | 1208.6223 | 0.0217 | 1 | (1) | 1.1e+002 | 1 | -.MPDPAKSAPAPK.K |
|  | 7483 | 613.2940 | 1224.5733 | 1224.6172 | -0.0439 | 1 | 10 | 15 | 9 | -.MPDPAKSAPAPK.K |
|  | 8597 | 640.3269 | 1278.6392 | 1278.6496 | -0.0103 | 1 | 16 | 3.6 | 2 | R.KESYSIYVYK.V |
|  | 11304 | 715.8343 | 1429.6541 | 1428.7473 | 0.9068 | 1 | 9 | 16 | 3 | R.IAGEASRLAHYNK.R 11287 |
|  | 12604 | 754.9198 | 1507.8250 | 1507.8358 | -0.0108 | 1 | 54 | 0.00046 | 1 | K.VLKQVHPDTGISSK.A |
|  | 16180 | 872.4089 | 1742.8032 | 1742.8120 | -0.0088 | 0 | 99 | 1.3e-008 | 1 | K.AMGIMNSFVNDIFER.I 16168 16179 16181 16183 16191 |
|  | 16389 | 880.4067 | 1758.7988 | 1758.8069 | -0.0081 | 0 | (54) | 0.00038 | 1 | K.AMGIMNSFVNDIFER.I 16378 |
|  | 16443 | 441.7535 | 1762.9851 | 1761.9989 | 0.9862 | 1 | 4 | 39 | 2 | R.LLLPGELAKHAVSEGTK.A |
|  | 23151 | 820.3754 | 2458.1045 | 2459.1573 | -1.0528 | 1 | 2 | 48 | 2 | K.AMGIMNSFVNDIFERIAGEASR.L |

| **78.** | Q8N257\|H2B3B_HUMAN    **Mass:** 13900    **Score:** 349    **Queries matched:** 24   **emPAI:** 1.11 |
| --- | --- |
|  | Histone H2B type 3-B OS=Homo sapiens GN=HIST3H2BB PE=1 SV=3 |

|  | **Query** | **Observed** | **Mr(expt)** | **Mr(calc)** | **Delta** | **Miss** | **Score** | **Expect** | **Rank** | **Peptide** |
| --- | --- | --- | --- | --- | --- | --- | --- | --- | --- | --- |
|  | 865 | 571.1000 | 570.0928 | 569.3173 | 0.7755 | 0 | 12 | 5.5 | 1 | K.SAPAPK.K 864 |
|  | 1524 | **402.2430** | **802.4715** | **801.4345** | **1.0371** | **0** | **4** | **88** | **9** | **R.EVQTAVR.L** |
|  | 1844 | 415.2156 | 828.4167 | 827.4137 | 1.0029 | 0 | 23 | 0.95 | 1 | K.HAVSEGTK.A 1838 1839 1840 1841 1843 1855 |
|  | 4020 | 492.7686 | 983.5227 | 983.5287 | -0.0061 | 1 | 13 | 8.5 | 1 | K.AVTKYTSSK.- |
|  | 6412 | 584.7980 | 1167.5814 | 1167.5884 | -0.0070 | 0 | 25 | 0.5 | 1 | K.QVHPDTGISSK.A |
|  | 7488 | 613.3124 | 1224.6102 | 1224.6172 | -0.0070 | 1 | 1 | 1.2e+002 | 3 | -.MPDPSKSAPAPK.K |
|  | 8597 | 640.3269 | 1278.6392 | 1278.6496 | -0.0103 | 1 | 16 | 3.6 | 2 | R.KESYSIYVYK.V |
|  | 12604 | 754.9198 | 1507.8250 | 1507.8358 | -0.0108 | 1 | 54 | 0.00046 | 1 | K.VLKQVHPDTGISSK.A |
|  | 16180 | 872.4089 | 1742.8032 | 1742.8120 | -0.0088 | 0 | 99 | 1.3e-008 | 1 | K.AMGIMNSFVNDIFER.I 16168 16179 16181 16183 16191 |
|  | 16389 | 880.4067 | 1758.7988 | 1758.8069 | -0.0081 | 0 | (54) | 0.00038 | 1 | K.AMGIMNSFVNDIFER.I 16378 |
|  | 16443 | 441.7535 | 1762.9851 | 1761.9989 | 0.9862 | 1 | 4 | 39 | 2 | R.LLLPGELAKHAVSEGTK.A |

| **79.** | Q16134\|ETFD_HUMAN    **Mass:** 69262    **Score:** 347    **Queries matched:** 22   **emPAI:** 0.52 |
| --- | --- |
|  | Electron transfer flavoprotein-ubiquinone oxidoreductase, mitochondrial OS=Homo sapiens GN=ETFDH PE=1 SV=1 |

|  | **Query** | **Observed** | **Mr(expt)** | **Mr(calc)** | **Delta** | **Miss** | **Score** | **Expect** | **Rank** | **Peptide** |
| --- | --- | --- | --- | --- | --- | --- | --- | --- | --- | --- |
|  | 1592 | **404.2316** | **806.4486** | **806.4538** | **-0.0051** | **0** | **25** | **0.6** | **1** | **R.FGILTEK.Y** |
|  | 4517 | **510.7291** | **1019.4436** | **1019.4494** | **-0.0059** | **0** | **11** | **16** | **1** | **R.WEGVNMER.F** |
|  | 5754 | **558.3032** | **1114.5919** | **1114.5982** | **-0.0063** | **0** | **18** | **2.3** | **1** | **K.GIATNDVGIQK.D** 5765 |
|  | 7477 | **409.1558** | **1224.4455** | **1225.6277** | **-1.1823** | **1** | **1** | **1e+002** | **7** | **R.GMEPWTLKHK.G** |
|  | 8303 | **632.8343** | **1263.6541** | **1263.6612** | **-0.0070** | **0** | **34** | **0.056** | **1** | **R.ITTHYTIYPR.D** 8304 |
|  | 8844 | **646.8149** | **1291.6152** | **1290.6204** | **0.9948** | **0** | **45** | **0.0046** | **1** | **R.NLSIYDGPEQR.F** 8828 |
|  | 8878 | **432.1801** | **1293.5184** | **1293.6466** | **-0.1282** | **0** | **6** | **34** | **4** | **R.VDHTVGWPLDR.H** |
|  | 12227 | **742.8536** | **1483.6927** | **1483.7024** | **-0.0097** | **0** | **57** | **0.00026** | **1** | **R.LQINAQNCVHCK.T** 12225 12226 |
|  | 13782 | **528.2706** | **1581.7901** | **1582.8315** | **-1.0414** | **1** | **2** | **73** | **5** | **K.GIATNDVGIQKDGAPK.A** |
|  | 19896 | **1029.5408** | **2057.0670** | **2057.0793** | **-0.0123** | **1** | **57** | **0.00018** | **1** | **K.GAPLNTPVTEDRFGILTEK.Y** |
|  | 20291 | **1050.0543** | **2098.0941** | **2098.1059** | **-0.0118** | **0** | **65** | **3e-005** | **1** | **R.FAEEADVVIVGAGPAGLSAAVR.L** |
|  | 20975 | **1087.4988** | **2172.9830** | **2172.9939** | **-0.0109** | **0** | **76** | **2e-006** | **1** | **R.FCPAGVYEFVPVEQGDGFR.L** 20967 |
|  | 21640 | **1125.5789** | **2249.1432** | **2249.1539** | **-0.0107** | **0** | **97** | **1.4e-008** | **1** | **K.SGILAAESIFNQLTSENLQSK.T** |
|  | 25482 | **1438.6454** | **2875.2762** | **2875.2905** | **-0.0143** | **1** | **48** | **0.00084** | **1** | **K.TCDIKDPSQNINWVVPEGGGGPAYNGM.-** |
|  | 28712 | **1819.9807** | **3637.9469** | **3638.6857** | **-0.7389** | **1** | **0** | **31** | **8** | **R.FCPAGVYEFVPVEQGDGFRLQINAQNCVHCK.T** |
|  | 29524 | **986.9811** | **3943.8955** | **3943.9091** | **-0.0136** | **0** | **5** | **10** | **1** | **R.LGHLVSWMGEQAEALGVEVYPGYAAAEVLFHDDGSVK.G** |

| **80.** | O43451\|MGA_HUMAN    **Mass:** 211031   **Score:** 339    **Queries matched:** 60   **emPAI:** 0.19 |
| --- | --- |
|  | Maltase-glucoamylase, intestinal OS=Homo sapiens GN=MGAM PE=1 SV=5 |

|  | **Query** | **Observed** | **Mr(expt)** | **Mr(calc)** | **Delta** | **Miss** | **Score** | **Expect** | **Rank** | **Peptide** |
| --- | --- | --- | --- | --- | --- | --- | --- | --- | --- | --- |
|  | 770 | **537.2993** | **536.2920** | **536.2264** | **0.0655** | **0** | **8** | **31** | **3** | **R.GSDMK.I** 769 |
|  | 1180 | 709.1945 | 708.1872 | 707.4442 | 0.7430 | 0 | 12 | 9.5 | 1 | K.IGLHLR.G |
|  | 1597 | **404.6917** | **807.3689** | **807.3731** | **-0.0042** | **1** | **20** | **1.6** | **1** | **R.MKADGMR.V** 1607 |
|  | 1995 | **838.3995** | **837.3923** | **837.3981** | **-0.0058** | **0** | **9** | **21** | **1** | **R.STFPSSGR.W** |
|  | 3049 | 456.7217 | 911.4289 | 911.4573 | -0.0285 | 0 | 9 | 17 | 7 | R.NHNTIGTR.R 3053 |
|  | 3695 | **481.1519** | **960.2892** | **960.4625** | **-0.1733** | **0** | **3** | **78** | **10** | **K.LTDQTNNR.F** |
|  | 7633 | 616.8056 | 1231.5966 | 1231.6026 | -0.0060 | 0 | 28 | 0.23 | 1 | R.AYVAFPDFFR.N |
|  | 8248 | **421.0573** | **1260.1501** | **1260.5445** | **-0.3944** | **0** | **7** | **19** | **3** | **R.YEYGTLDNMR.E** 8227 8233 8234 8243 8245 8246 |
|  | 8642 | **641.8226** | **1281.6306** | **1281.6387** | **-0.0081** | **0** | **7** | **27** | **2** | **R.INCIPDQPPTK.A** |
|  | 8759 | **430.2271** | **1287.6595** | **1288.6333** | **-0.9738** | **1** | **9** | **17** | **5** | **K.QKVEMELPGDK.I** 8822 |
|  | 9185 | **437.2276** | **1308.6611** | **1307.6734** | **0.9876** | **1** | **10** | **13** | **6** | **R.NARNVTAYFPR.A** 9158 9175 9187 9192 |
|  | 10481 | **461.0080** | **1380.0022** | **1379.7384** | **0.2638** | **1** | **4** | **42** | **10** | **K.FAGFPALINRMK.A** 10469 |
|  | 12007 | **736.8500** | **1471.6854** | **1470.7831** | **0.9023** | **1** | **0** | **1.3e+002** | **4** | **R.SFILTRSTFAGSGK.F** |
|  | 13030 | **512.9690** | **1535.8851** | **1536.8875** | **-1.0024** | **1** | **4** | **50** | **4** | **R.KNPLGLIIALDENK.E** |
|  | 13496 | **782.9016** | **1563.7887** | **1563.6929** | **0.0958** | **0** | **29** | **0.14** | **1** | **R.DLEWHTWGMFSR.D** |
|  | 13976 | **532.2883** | **1593.8430** | **1594.8943** | **-1.0514** | **0** | **11** | **9.2** | **1** | **K.TLPAPLDHINLHVR.G** |
|  | 14617 | **818.3709** | **1634.7271** | **1634.7365** | **-0.0094** | **0** | **78** | **1.8e-006** | **1** | **R.WYDYYTGVDINAR.G** 14618 |
|  | 14941 | **552.6314** | **1654.8724** | **1654.8793** | **-0.0069** | **0** | **3** | **53** | **4** | **R.YTLLPYLYTLMHK.A** |
|  | 16194 | **872.9058** | **1743.7970** | **1743.8063** | **-0.0094** | **0** | **71** | **8.4e-006** | **1** | **R.EIEELYNNPQNPER.S** 16193 |
|  | 16833 | **449.9724** | **1795.8605** | **1795.8893** | **-0.0288** | **1** | **9** | **12** | **2** | **R.LPSKYLYGFGETEHR.S** |
|  | 17145 | **910.9661** | **1819.9176** | **1818.9264** | **0.9911** | **0** | **11** | **10** | **1** | **K.SSVYANAFPSTPVNPLR.L** |
|  | 18079 | **632.0326** | **1893.0761** | **1891.8912** | **1.1849** | **0** | **2** | **66** | **2** | **K.DQDPASFGADSLLLNSSR.H** |
|  | 19832 | **1026.5316** | **2051.0487** | **2051.0588** | **-0.0101** | **0** | **60** | **9.3e-005** | **1** | **R.GGYILPWQEPALNTHLSR.Q** |
|  | 21636 | **750.6721** | **2248.9944** | **2249.0059** | **-0.0115** | **0** | **29** | **0.082** | **1** | **R.AAQLPYDVQHADIDYMDER.R** |
|  | 21784 | **1133.5482** | **2265.0819** | **2265.0954** | **-0.0135** | **1** | **61** | **6.6e-005** | **1** | **R.GVEDDVFIKYPNDGDIVWGK.V** 21783 |
|  | 22153 | **772.3984** | **2314.1733** | **2315.0892** | **-0.9159** | **0** | **9** | **10** | **1** | **K.QYDIHNLYGYSMAVATAEAAK.T** |
|  | 22396 | **1175.0402** | **2348.0658** | **2348.0783** | **-0.0126** | **0** | **81** | **6.1e-007** | **1** | **K.VMAYVPDAVWYDYETGSQVR.W** |
|  | 22829 | **805.3790** | **2413.1151** | **2414.2845** | **-1.1695** | **0** | **2** | **47** | **8** | **R.VILILDPAISGNETQPYPAFTR.G** |
|  | 22948 | **810.6991** | **2429.0755** | **2430.2656** | **-1.1901** | **1** | **4** | **30** | **2** | **R.QDPVSWDVAFVNISRTVLQTR.Y** |
|  | 23013 | **814.4034** | **2440.1883** | **2441.2200** | **-1.0317** | **1** | **0** | **73** | **6** | **K.NHSYHVEGNLVNTNAGFTARLK.N** |
|  | 26096 | **1001.4488** | **3001.3245** | **3000.3568** | **0.9677** | **1** | **34** | **0.022** | **1** | **R.SLKFDGMWIDMNEPSSFVNGAVSPGCR.D** 26095 26097 |
|  | 29031 | **624.7673** | **3742.5603** | **3741.7378** | **0.8225** | **1** | **0** | **24** | **3** | **K.TLCMDAVQHWGKQYDIHNLYGYSMAVATAEAAK.T** |
|  | 30625 | **756.3817** | **4532.2466** | **4533.0035** | **-0.7569** | **1** | **2** | **13** | **4** | **K.FDGMWIDMNEPSSFVNGAVSPGCRDASLNHPPYMPHLESR.D** |
|  | 30792 | **1160.8108** | **4639.2141** | **4639.2152** | **-0.0011** | **0** | **10** | **1.9** | **1** | **K.NSYGVHPYYMGLEEDGSAHGVLLLNSNAMDVTFQPLPALTYR.T** |
|  | 30989 | **1205.1322** | **4816.4997** | **4816.5229** | **-0.0232** | **0** | **22** | **0.088** | **1** | **K.AHTEGVTVVRPLLHEFVSDQVTWDIDSQFLLGPAFLVSPVLER.N** 30990 |
|  | 31352 | **1284.1167** | **5132.4377** | **5132.4509** | **-0.0132** | **0** | **12** | **0.77** | **1** | **K.TLCMESQQILPDGSLVQHYNVHNLYGWSQTRPTYEAVQEVTGQR.G** 31351 31354 |
|  | 31664 | **1821.1442** | **5460.4107** | **5459.4245** | **0.9862** | **1** | **5** | **3** | **6** | **K.SIIGMMEFSLFGISYTGADICGFFQDAEYEMCVRWMQLGAFYPFSR.N** |

| **81.** | P21281\|VATB2_HUMAN    **Mass:** 56807    **Score:** 335    **Queries matched:** 51   **emPAI:** 0.89 |
| --- | --- |
|  | V-type proton ATPase subunit B, brain isoform OS=Homo sapiens GN=ATP6V1B2 PE=1 SV=3 |

|  | **Query** | **Observed** | **Mr(expt)** | **Mr(calc)** | **Delta** | **Miss** | **Score** | **Expect** | **Rank** | **Peptide** |
| --- | --- | --- | --- | --- | --- | --- | --- | --- | --- | --- |
|  | 1029 | **633.3801** | **632.3728** | **631.3289** | **1.0439** | **0** | **3** | **1.5e+002** | **9** | **R.EVSAAR.E** |
|  | 2147 | **424.8947** | **847.7749** | **847.4520** | **0.3229** | **1** | **(3)** | **66** | **6** | **-.MALRAMR.G** |
|  | 2545 | **437.7423** | **873.4700** | **872.4716** | **0.9984** | **0** | **31** | **0.15** | **1** | **R.EQALAVSR.N** 2506 2528 |
|  | 2575 | **439.2322** | **876.4499** | **876.4453** | **0.0046** | **0** | **18** | **3.2** | **2** | **R.NYLSQPR.L** 2573 |
|  | 2639 | **441.2957** | **880.5768** | **879.4418** | **1.1350** | **1** | **6** | **41** | **3** | **-.MALRAMR.G** |
|  | 4883 | **525.1025** | **1048.1904** | **1048.5335** | **-0.3431** | **1** | **(9)** | **14** | **2** | **K.SAIGEGMTRK.D** |
|  | 5095 | 533.2719 | 1064.5293 | 1064.5284 | 0.0008 | 1 | 10 | 15 | 2 | K.SAIGEGMTRK.D 5075 5085 5089 5097 5102 |
|  | 5410 | **545.7871** | **1089.5597** | **1089.5666** | **-0.0069** | **0** | **47** | **0.0039** | **1** | **R.SGQVLEVSGSK.A** 5411 |
|  | 5598 | **552.3103** | **1102.6060** | **1103.5281** | **-0.9221** | **0** | **3** | **81** | **10** | **R.TPVSEDMLGR.V** |
|  | 6733 | **397.2011** | **1188.5813** | **1188.6251** | **-0.0438** | **0** | **12** | **8.9** | **2** | **R.VFNGSGKPIDR.G** 6765 |
|  | 7963 | **624.2767** | **1246.5388** | **1245.6677** | **0.8711** | **1** | **0** | **1.4e+002** | **7** | **K.RSGQVLEVSGSK.A** |
|  | 8980 | **433.9043** | **1298.6910** | **1298.6579** | **0.0331** | **1** | **4** | **51** | **8** | **R.EVSAAREEVPGR.R** |
|  | 11228 | **713.8490** | **1425.6834** | **1425.6922** | **-0.0088** | **1** | **41** | **0.01** | **1** | **K.KTSCEFTGDILR.T** |
|  | 12811 | **760.8964** | **1519.7782** | **1519.7882** | **-0.0101** | **0** | **65** | **3.9e-005** | **1** | **K.AVVQVFEGTSGIDAK.K** |
|  | 14010 | **798.9553** | **1595.8960** | **1595.9035** | **-0.0075** | **0** | **55** | **0.00038** | **1** | **R.QIYPPINVLPSLSR.L** 14011 |
|  | 15466 | **845.9571** | **1689.8997** | **1689.9090** | **-0.0093** | **0** | **61** | **8.9e-005** | **1** | **R.TVFETLDIGWQLLR.I** |
|  | 18106 | **948.9407** | **1895.8668** | **1895.8764** | **-0.0096** | **0** | **99** | **1.2e-008** | **1** | **R.GFPGYMYTDLATIYER.A** 18107 |
|  | 18281 | **638.0178** | **1911.0315** | **1911.8713** | **-0.8398** | **0** | **(2)** | **56** | **2** | **R.GFPGYMYTDLATIYER.A** |
|  | 18351 | 959.4839 | 1916.9532 | 1917.0030 | -0.0497 | 0 | 3 | 49 | 4 | K.HVLVILTDMSSYAEALR.E 18350 |
|  | 19307 | **1001.0363** | **2000.0579** | **1999.0851** | **0.9729** | **0** | **7** | **20** | **1** | **R.GIVNGAAPELPVPTGGPAVGAR.E** |
|  | 19789 | **512.7938** | **2047.1459** | **2047.1579** | **-0.0119** | **1** | **9** | **9.8** | **1** | **K.TVSGVNGPLVILDHVKFPR.Y** |
|  | 19834 | **684.8318** | **2051.4735** | **2051.9735** | **-0.4999** | **1** | **4** | **22** | **1** | **R.KDHADVSNQLYACYAIGK.D** |
|  | 22793 | **1205.0864** | **2408.1583** | **2408.1715** | **-0.0132** | **0** | **38** | **0.012** | **1** | **R.IYPEEMIQTGISAIDGMNSIAR.G** 22794 |
|  | 23369 | 623.5826 | 2490.3015 | 2490.3165 | -0.0151 | 1 | 10 | 6.9 | 1 | R.GQKIPIFSAAGLPHNEIAAQICR.Q |
|  | 27945 | **1141.8505** | **3422.5296** | **3421.5595** | **0.9701** | **1** | **33** | **0.021** | **1** | **R.FFKSDFEENGSMDNVCLFLNLANDPTIER.I** |
|  | 28716 | **910.7125** | **3638.8210** | **3638.8338** | **-0.0128** | **1** | **16** | **0.85** | **1** | **R.VFNGSGKPIDRGPVVLAEDFLDIMGQPINPQCR.I** 28727 28728 28729 |
|  | 29945 | **1371.0210** | **4110.0412** | **4110.0620** | **-0.0208** | **0** | **27** | **0.047** | **1** | **R.NGSITQIPILTMPNDDITHPIPDLTGYITEGQIYVDR.Q** 29944 29946 29948 29949 29950 29952 |
|  | 30923 | **1190.3148** | **4757.2302** | **4758.4075** | **-1.1774** | **1** | **1** | **12** | **2** | **R.NGSITQIPILTMPNDDITHPIPDLTGYITEGQIYVDRQLHNR.Q** |

| **82.** | P12236\|ADT3_HUMAN    **Mass:** 33073    **Score:** 334    **Queries matched:** 47   **emPAI:** 3.53 |
| --- | --- |
|  | ADP/ATP translocase 3 OS=Homo sapiens GN=SLC25A6 PE=1 SV=4 |

|  | **Query** | **Observed** | **Mr(expt)** | **Mr(calc)** | **Delta** | **Miss** | **Score** | **Expect** | **Rank** | **Peptide** |
| --- | --- | --- | --- | --- | --- | --- | --- | --- | --- | --- |
|  | 803 | **549.3108** | **548.3036** | **548.2554** | **0.0481** | **0** | **3** | **1.1e+002** | **2** | **K.SGTER.E** |
|  | 2024 | 420.6771 | 839.3397 | 839.3452 | -0.0055 | 0 | 25 | 0.43 | 1 | R.MMMQSGR.K 2026 2042 |
|  | 2254 | 428.7456 | 855.4767 | 855.4814 | -0.0047 | 0 | 36 | 0.043 | 1 | K.TAVAPIER.V 2274 |
|  | 2255 | 428.7509 | 855.4873 | 855.4926 | -0.0053 | 0 | 31 | 0.12 | 1 | R.GNLANVIR.Y |
|  | 2267 | 429.0874 | 856.1602 | 855.3401 | 0.8201 | 0 | (14) | 4.8 | 1 | R.MMMQSGR.K 2269 |
|  | 2271 | 429.2248 | 856.4350 | 855.3401 | 1.0949 | 0 | (13) | 9 | 1 | R.MMMQSGR.K |
|  | 2272 | 429.2307 | 856.4469 | 855.3401 | 1.1068 | 0 | (13) | 9.6 | 3 | R.MMMQSGR.K |
|  | 2332 | 431.2261 | 860.4376 | 860.4426 | -0.0049 | 0 | 41 | 0.016 | 1 | R.GLGDCLVK.I 2322 2333 |
|  | 2552 | **438.2198** | **874.4250** | **873.4246** | **1.0004** | **0** | **11** | **18** | **2** | **K.HTQFWR.Y** |
|  | 3200 | **461.0072** | **919.9999** | **920.4716** | **-0.4717** | **1** | **2** | **69** | **9** | **K.IFRDEGGK.A** |
|  | 3325 | 465.7691 | 929.5236 | 929.5294 | -0.0058 | 1 | 14 | 8.1 | 3 | R.TRLAADVGK.S |
|  | 3784 | 484.7235 | 967.4325 | 967.4401 | -0.0077 | 1 | 29 | 0.18 | 1 | R.MMMQSGRK.G |
|  | 4190 | 498.7267 | 995.4387 | 995.4463 | -0.0075 | 1 | 22 | 1 | 1 | R.RMMMQSGR.K 4193 |
|  | 5311 | 542.5944 | 1083.1743 | 1082.6448 | 0.5295 | 1 | 9 | 11 | 3 | K.TAVAPIERVK.L |
|  | 5835 | 561.2869 | 1120.5593 | 1120.5665 | -0.0072 | 0 | 35 | 0.047 | 1 | K.EQGVLSFWR.G 5834 |
|  | 6005 | 568.8392 | 1135.6639 | 1135.6713 | -0.0074 | 0 | 25 | 0.49 | 1 | K.LLLQVQHASK.Q 6000 |
|  | 7662 | **617.3424** | **1232.6703** | **1232.6765** | **-0.0062** | **0** | **87** | **2.8e-007** | **1** | **K.DFLAGGIAAAISK.T** 7661 |
|  | 9944 | **451.2267** | **1350.6582** | **1349.7126** | **0.9457** | **1** | **5** | **42** | **8** | **K.QYKGIVDCIVR.I** |
|  | 10149 | 681.8502 | 1361.6859 | 1362.8347 | -1.1488 | 1 | 66 | 3.3e-005 | 1 | R.VKLLLQVQHASK.Q 10159 10170 10171 |
|  | 14774 | **823.3579** | **1644.7013** | **1643.7072** | **0.9940** | **0** | **3** | **57** | **5** | **K.GADIMYTGTVDCWR.K** 14763 |
|  | 16130 | 870.4696 | 1738.9246 | 1738.9328 | -0.0081 | 1 | 38 | 0.017 | 1 | R.GMGGAFVLVLYDELKK.V |
|  | 16542 | **886.9032** | **1771.7918** | **1771.8022** | **-0.0103** | **1** | **56** | **0.00027** | **1** | **R.KGADIMYTGTVDCWR.K** 16540 16541 |
|  | 21294 | **737.3701** | **2209.0885** | **2208.1790** | **0.9095** | **1** | **2** | **47** | **2** | **M.TEQAISFAKDFLAGGIAAAISK.T** |
|  | 25112 | 932.7831 | 2795.3274 | 2795.3377 | -0.0103 | 0 | 51 | 0.00045 | 1 | R.YFAGNLASGGAAGATSLCFVYPLDFAR.T 25108 25109 25110 25113 25114 25115 |
|  | 27513 | **830.3661** | **3317.4355** | **3316.7391** | **0.6964** | **1** | **4** | **17** | **10** | **K.NTHIVVSWMIAQTVTAVAGVVSYPFDTVRR.R** |

| **83.** | Q562R1\|ACTBL_HUMAN    **Mass:** 42318    **Score:** 332    **Queries matched:** 30   **emPAI:** 0.29 |
| --- | --- |
|  | Beta-actin-like protein 2 OS=Homo sapiens GN=ACTBL2 PE=1 SV=2 |

|  | **Query** | **Observed** | **Mr(expt)** | **Mr(calc)** | **Delta** | **Miss** | **Score** | **Expect** | **Rank** | **Peptide** |
| --- | --- | --- | --- | --- | --- | --- | --- | --- | --- | --- |
|  | 4224 | 499.7444 | 997.4741 | 997.4790 | -0.0049 | 0 | 10 | 15 | 4 | R.DLTDYLMK.I |
|  | 4734 | 518.8262 | 1035.6379 | 1035.6440 | -0.0061 | 1 | 21 | 1.4 | 1 | K.IKIIAPPER.K |
|  | 6445 | 586.2852 | 1170.5558 | 1170.5638 | -0.0080 | 0 | 48 | 0.0025 | 1 | R.HQGVMVGMGQK.D 6444 6446 6447 6460 |
|  | 6691 | 594.2826 | 1186.5507 | 1186.5587 | -0.0080 | 0 | (40) | 0.017 | 1 | R.HQGVMVGMGQK.D 6679 6693 6696 |
|  | 6692 | 594.2828 | 1186.5511 | 1186.5587 | -0.0076 | 0 | (42) | 0.01 | 1 | R.HQGVMVGMGQK.D 6695 |
|  | 7039 | 602.2794 | 1202.5442 | 1202.5536 | -0.0095 | 0 | (21) | 1.3 | 1 | R.HQGVMVGMGQK.D |
|  | 14437 | 541.9493 | 1622.8262 | 1622.8338 | -0.0076 | 1 | 25 | 0.33 | 1 | R.LDLAGRDLTDYLMK.I |
|  | 16767 | 895.9441 | 1789.8736 | 1789.8846 | -0.0110 | 0 | 66 | 2.5e-005 | 1 | R.SYELPDGQVITIGNER.F 16766 16769 16770 16771 16772 |
|  | 18745 | **650.5480** | **1948.6221** | **1947.8673** | **0.7548** | **0** | **0** | **63** | **6** | **K.YPIEHGVVTNWDDMEK.I** |
|  | 18800 | 652.0226 | 1953.0461 | 1953.0571 | -0.0110 | 0 | 30 | 0.1 | 2 | R.VAPDEHPILLTEAPLNPK.I 18797 18798 18799 18812 |
|  | 19867 | **1028.4718** | **2054.9290** | **2055.8911** | **-0.9621** | **0** | **0** | **78** | **5** | **-.MTDNELSALVVDNGSGMCK.A** |
|  | 24842 | 550.0686 | 2745.3066 | 2745.4200 | -0.1134 | 1 | 5 | 22 | 3 | R.KYSVWIGGSILASLSTFQQMWISK.Q |
|  | 26858 | **1578.8817** | **3155.7489** | **3155.4944** | **0.2545** | **0** | **1** | **30** | **4** | **R.CPEAIFQPSFLGIESSGIHETTFNSIMK.C** |

| **84.** | P01876\|IGHA1_HUMAN    **Mass:** 38486    **Score:** 331    **Queries matched:** 16   **emPAI:** 1.54 |
| --- | --- |
|  | Ig alpha-1 chain C region OS=Homo sapiens GN=IGHA1 PE=1 SV=2 |

|  | **Query** | **Observed** | **Mr(expt)** | **Mr(calc)** | **Delta** | **Miss** | **Score** | **Expect** | **Rank** | **Peptide** |
| --- | --- | --- | --- | --- | --- | --- | --- | --- | --- | --- |
|  | 2819 | **448.7325** | **895.4504** | **895.4552** | **-0.0048** | **0** | **14** | **5.7** | **2** | **K.YLTWASR.Q** |
|  | 3435 | 470.7423 | 939.4701 | 939.4774 | -0.0073 | 0 | 11 | 13 | 2 | K.SAVQGPPER.D |
|  | 5793 | **559.3184** | **1116.6223** | **1116.6292** | **-0.0069** | **1** | **51** | **0.0014** | **1** | **R.GFSPKDVLVR.W** |
|  | 10385 | **688.3092** | **1374.6038** | **1374.6126** | **-0.0087** | **0** | **56** | **0.00033** | **1** | **K.TFTCTAAYPESK.T** |
|  | 11987 | **735.8840** | **1469.7535** | **1469.7626** | **-0.0091** | **1** | **40** | **0.012** | **1** | **R.WLQGSQELPREK.Y** 11988 |
|  | 13107 | **771.3334** | **1540.6522** | **1539.7206** | **0.9316** | **0** | **4** | **52** | **9** | **R.DASGVTFTWTPSSGK.S** |
|  | 17353 | **918.4741** | **1834.9336** | **1834.9425** | **-0.0089** | **0** | **97** | **2.4e-008** | **1** | **R.QEPSQGTTTFAVTSILR.V** 17352 |
|  | 22294 | **779.7119** | **2336.1139** | **2336.1293** | **-0.0154** | **1** | **28** | **0.11** | **1** | **K.KGDTFSCMVGHEALPLAFTQK.T** |
|  | 23994 | **865.0431** | **2592.1074** | **2592.1196** | **-0.0121** | **0** | **45** | **0.0018** | **1** | **R.DLCGCYSVSSVLPGCAEPWNHGK.T** |
|  | 28235 | **1172.1954** | **3513.5645** | **3513.5864** | **-0.0219** | **1** | **48** | **0.0005** | **1** | **K.SAVQGPPERDLCGCYSVSSVLPGCAEPWNHGK.T** 28236 |
|  | 28475 | **1191.9585** | **3572.8537** | **3572.8661** | **-0.0124** | **0** | **80** | **4e-007** | **1** | **K.SGNTFRPEVHLLPPPSEELALNELVTLTCLAR.G** 28476 28479 |

| **85.** | P05026\|AT1B1_HUMAN    **Mass:** 35438    **Score:** 328    **Queries matched:** 35   **emPAI:** 0.83 |
| --- | --- |
|  | Sodium/potassium-transporting ATPase subunit beta-1 OS=Homo sapiens GN=ATP1B1 PE=1 SV=1 |

|  | **Query** | **Observed** | **Mr(expt)** | **Mr(calc)** | **Delta** | **Miss** | **Score** | **Expect** | **Rank** | **Peptide** |
| --- | --- | --- | --- | --- | --- | --- | --- | --- | --- | --- |
|  | 934 | **599.0203** | **598.0130** | **597.3850** | **0.6281** | **0** | **2** | **45** | **6** | **K.LLQPK.Y** |
|  | 2376 | **432.7421** | **863.4696** | **862.4185** | **1.0511** | **1** | **14** | **7.8** | **1** | **K.EEGSWKK.F** 2354 |
|  | 3243 | **462.7337** | **923.4529** | **922.4548** | **0.9981** | **0** | **10** | **14** | **6** | **K.FIWNSEK.K** |
|  | 3935 | **489.7652** | **977.5158** | **976.5593** | **0.9565** | **1** | **5** | **55** | **3** | **R.FDVKIEVK.S** |
|  | 7309 | **406.1856** | **1215.5350** | **1215.5381** | **-0.0031** | **1** | **14** | **6.5** | **1** | **R.GDFNHERGER.K** 7302 7307 7310 |
|  | 9073 | **652.3312** | **1302.6478** | **1302.6568** | **-0.0090** | **0** | **20** | **1.4** | **1** | **K.TEISFRPNDPK.S** 9074 9075 |
|  | 9504 | **663.8531** | **1325.6916** | **1325.6979** | **-0.0063** | **0** | **63** | **7e-005** | **1** | **K.SYEAYVLNIVR.F** 9501 9503 9514 |
|  | 9523 | **443.0385** | **1326.0938** | **1325.6173** | **0.4765** | **0** | **3** | **44** | **7** | **K.NESLETYPVMK.Y** |
|  | 11481 | **481.2425** | **1440.7056** | **1439.8282** | **0.8774** | **1** | **2** | **84** | **10** | **K.EGKPCIIIKLNR.V** |
|  | 12302 | **745.3725** | **1488.7304** | **1488.7395** | **-0.0091** | **0** | **36** | **0.03** | **1** | **K.YNPNVLPVQCTGK.R** 12303 |
|  | 12321 | 745.4360 | 1488.8575 | 1488.8664 | -0.0089 | 0 | 49 | 0.0015 | 1 | R.VAPPGLTQIPQIQK.T 12320 12322 |
|  | 12480 | **751.3435** | **1500.6725** | **1500.6844** | **-0.0120** | **1** | **54** | **0.00047** | **1** | **K.AYGENIGYSEKDR.F** 12481 |
|  | 14780 | **823.4229** | **1644.8311** | **1644.8406** | **-0.0095** | **1** | **49** | **0.0016** | **1** | **K.YNPNVLPVQCTGKR.D** 14778 14779 |
|  | 17607 | **927.3772** | **1852.7398** | **1852.7495** | **-0.0097** | **0** | **7** | **18** | **1** | **R.DDMIFEDCGDVPSEPK.E** |
|  | 24092 | **653.5893** | **2610.3281** | **2610.3442** | **-0.0161** | **1** | **19** | **0.84** | **1** | **K.TEISFRPNDPKSYEAYVLNIVR.F** 24093 24096 |
|  | 25270 | **1415.1742** | **2828.3338** | **2828.3486** | **-0.0148** | **0** | **88** | **9.9e-008** | **1** | **K.VGNVEYFGLGNSPGFPLQYYPYYGK.L** 25271 25272 |

| **86.** | P18859\|ATP5J_HUMAN    **Mass:** 12580    **Score:** 328    **Queries matched:** 16   **emPAI:** 5.80 |
| --- | --- |
|  | ATP synthase-coupling factor 6, mitochondrial OS=Homo sapiens GN=ATP5J PE=1 SV=1 |

|  | **Query** | **Observed** | **Mr(expt)** | **Mr(calc)** | **Delta** | **Miss** | **Score** | **Expect** | **Rank** | **Peptide** |
| --- | --- | --- | --- | --- | --- | --- | --- | --- | --- | --- |
|  | 2459 | **435.1989** | **868.3832** | **867.4926** | **0.8906** | **0** | **7** | **26** | **4** | **R.SAVSVHLR.R** |
|  | 5434 | **546.7660** | **1091.5175** | **1091.6274** | **-0.1098** | **1** | **11** | **14** | **4** | **-.MILQRLFR.F** |
|  | 15270 | 838.9250 | 1675.8354 | 1675.8457 | -0.0103 | 1 | 46 | 0.0026 | 1 | K.FEDPKFEVIEKPQA.- 15247 15271 15290 |
|  | 16243 | **874.8882** | **1747.7618** | **1747.7698** | **-0.0080** | **0** | **33** | **0.052** | **1** | **K.QMFGNADMNTFPTFK.F** 16241 16242 16244 |
|  | 19345 | **501.9878** | **2003.9221** | **2004.9438** | **-1.0217** | **1** | **2** | **63** | **3** | **K.LKQMFGNADMNTFPTFK.F** |
|  | 20114 | **1040.9691** | **2079.9237** | **2079.9345** | **-0.0108** | **0** | **120** | **9.1e-011** | **1** | **R.QTSGGPVDASSEYQQELER.E** 20115 |
|  | 21556 | **746.3487** | **2236.0242** | **2236.0356** | **-0.0114** | **1** | **43** | **0.0037** | **1** | **K.RQTSGGPVDASSEYQQELER.E** |
|  | 24030 | **1299.6112** | **2597.2079** | **2597.2245** | **-0.0167** | **1** | **52** | **0.00036** | **1** | **R.QTSGGPVDASSEYQQELERELFK.L** 24031 |

| **87.** | Q5VTE0\|EF1A3_HUMAN    **Mass:** 50495    **Score:** 320    **Queries matched:** 31   **emPAI:** 0.90 |
| --- | --- |
|  | Putative elongation factor 1-alpha-like 3 OS=Homo sapiens GN=EEF1AL3 PE=5 SV=1 |

|  | **Query** | **Observed** | **Mr(expt)** | **Mr(calc)** | **Delta** | **Miss** | **Score** | **Expect** | **Rank** | **Peptide** |
| --- | --- | --- | --- | --- | --- | --- | --- | --- | --- | --- |
|  | 2462 | **435.2236** | **868.4327** | **869.5334** | **-1.1007** | **0** | **14** | **6** | **2** | **K.QLIVGVNK.M** 2491 |
|  | 2801 | **447.7473** | **893.4800** | **893.4858** | **-0.0058** | **1** | **13** | **8.6** | **3** | **R.TIEKFEK.E** 2802 |
|  | 3093 | **457.7845** | **913.5544** | **913.5597** | **-0.0053** | **0** | **48** | **0.0029** | **1** | **R.QTVAVGVIK.A** |
|  | 4586 | **513.3055** | **1024.5965** | **1024.6030** | **-0.0064** | **0** | **68** | **2.4e-005** | **1** | **K.IGGIGTVPVGR.V** 4587 4596 |
|  | 9262 | **657.8151** | **1313.6157** | **1312.6816** | **0.9341** | **1** | **6** | **33** | **2** | **K.GSFKYAWVLDK.L** |
|  | 10870 | **468.9110** | **1403.7112** | **1403.7197** | **-0.0085** | **0** | **30** | **0.12** | **1** | **K.YYVTIIDAPGHR.D** 10871 |
|  | 23492 | **1258.1892** | **2514.3639** | **2514.3768** | **-0.0129** | **0** | **60** | **5.8e-005** | **1** | **R.VETGVLKPGMVVTFAPVNVTTEVK.S** 23491 23493 |
|  | 24550 | **897.8085** | **2690.4038** | **2689.4552** | **0.9486** | **1** | **2** | **37** | **2** | **K.THINIVVIGHVDSGKSTTTGHLIYK.C** 24548 |
|  | 26057 | **999.1331** | **2994.3775** | **2994.3926** | **-0.0150** | **0** | **37** | **0.011** | **1** | **K.SGDAAIVDMVPGKPMCVESFSDYPPLGR.F** |
|  | 26201 | **1009.8564** | **3026.5473** | **3026.3824** | **0.1649** | **0** | **(2)** | **31** | **2** | **K.SGDAAIVDMVPGKPMCVESFSDYPPLGR.F** |
|  | 28281 | **881.2468** | **3520.9580** | **3520.9692** | **-0.0112** | **1** | **7** | **5.8** | **1** | **K.IGGIGTVPVGRVETGVLKPGMVVTFAPVNVTTEVK.S** |
|  | 28444 | **1189.8899** | **3566.6478** | **3566.6639** | **-0.0161** | **0** | **30** | **0.035** | **1** | **K.IGYNPDTVAFVPISGWNGDNMLEPSANMPWFK.G** 28445 28448 |
|  | 28520 | **1195.5659** | **3583.6759** | **3582.6588** | **1.0171** | **0** | **(12)** | **2.5** | **1** | **K.IGYNPDTVAFVPISGWNGDNMLEPSANMPWFK.G** |
|  | 28868 | **1232.5868** | **3694.7385** | **3694.7589** | **-0.0203** | **1** | **42** | **0.0022** | **1** | **K.KIGYNPDTVAFVPISGWNGDNMLEPSANMPWFK.G** 28869 |
|  | 28920 | **928.7244** | **3710.8686** | **3710.7538** | **0.1148** | **1** | **(1)** | **28** | **8** | **K.KIGYNPDTVAFVPISGWNGDNMLEPSANMPWFK.G** |
|  | 29512 | **1313.6204** | **3937.8393** | **3937.8596** | **-0.0204** | **1** | **76** | **7.9e-007** | **1** | **K.IGYNPDTVAFVPISGWNGDNMLEPSANMPWFKGWK.V** 29511 29514 |
|  | 29613 | **1326.6969** | **3977.0689** | **3977.0932** | **-0.0243** | **1** | **39** | **0.0033** | **1** | **K.DGNASGTTLLEALDCILPPTRPTDKPLRLPLQDVYK.I** |
|  | 31235 | **1004.6422** | **5018.1747** | **5018.4340** | **-0.2593** | **1** | **1** | **11** | **4** | **K.NDPPMEAAGFTAQVIILNHPGQISAGYAPVLDCHMAHIACKFAELK.E** |

|  | **Proteins matching a subset of these peptides:** |
| --- | --- |

|  | P68104\|EF1A1_HUMAN    **Mass:** 50451    **Score:** 320    **Queries matched:** 30 |
| --- | --- |
|  | Elongation factor 1-alpha 1 OS=Homo sapiens GN=EEF1A1 PE=1 SV=1 |

| **88.** | P01834\|IGKC_HUMAN    **Mass:** 11773    **Score:** 318    **Queries matched:** 12   **emPAI:** 3.28 |
| --- | --- |
|  | Ig kappa chain C region OS=Homo sapiens GN=IGKC PE=1 SV=1 |

|  | **Query** | **Observed** | **Mr(expt)** | **Mr(calc)** | **Delta** | **Miss** | **Score** | **Expect** | **Rank** | **Peptide** |
| --- | --- | --- | --- | --- | --- | --- | --- | --- | --- | --- |
|  | 2456 | **869.3510** | **868.3437** | **868.3497** | **-0.0060** | **1** | **10** | **13** | **1** | **K.SFNRGEC.-** |
|  | 2754 | **445.3122** | **888.6098** | **887.4865** | **1.1233** | **1** | **9** | **25** | **5** | **R.EAKVQWK.V** 2753 |
|  | 16842 | **899.4457** | **1796.8769** | **1796.8880** | **-0.0110** | **0** | **43** | **0.0054** | **1** | **K.SGTASVVCLLNNFYPR.E** 16843 |
|  | 17882 | **938.4619** | **1874.9093** | **1874.9197** | **-0.0104** | **0** | **69** | **1.3e-005** | **1** | **K.VYACEVTHQGLSSPVTK.S** 17881 |
|  | 20611 | **1068.4801** | **2134.9456** | **2134.9614** | **-0.0158** | **0** | **100** | **7.5e-009** | **1** | **K.VDNALQSGNSQESVTEQDSK.D** 20610 |
|  | 28659 | **1207.2351** | **3618.6835** | **3618.7020** | **-0.0185** | **1** | **66** | **9.8e-006** | **1** | **K.VDNALQSGNSQESVTEQDSKDSTYSLSSTLTLSK.A** 28660 28662 |

| **89.** | P07911\|UROM_HUMAN    **Mass:** 72451    **Score:** 317    **Queries matched:** 41   **emPAI:** 0.65 |
| --- | --- |
|  | Uromodulin OS=Homo sapiens GN=UMOD PE=1 SV=1 |

|  | **Query** | **Observed** | **Mr(expt)** | **Mr(calc)** | **Delta** | **Miss** | **Score** | **Expect** | **Rank** | **Peptide** |
| --- | --- | --- | --- | --- | --- | --- | --- | --- | --- | --- |
|  | 1351 | **396.2092** | **790.4038** | **790.4086** | **-0.0048** | **0** | **38** | **0.03** | **1** | **R.FVGQGGAR.M** 1349 1352 1360 1363 |
|  | 1693 | **408.7357** | **815.4569** | **816.4454** | **-0.9885** | **0** | **8** | **37** | **4** | **K.GVQATVSR.A** |
|  | 3095 | **458.2303** | **914.4461** | **913.4480** | **0.9981** | **0** | **26** | **0.42** | **1** | **R.FSVQMFR.F** 3085 |
|  | 3488 | 473.0806 | 944.1466 | 944.5403 | -0.3937 | 1 | 8 | 21 | 5 | R.KGVQATVSR.A |
|  | 3536 | **474.7378** | **947.4610** | **947.4672** | **-0.0063** | **0** | **32** | **0.12** | **1** | **R.SGSVIDQSR.V** |
|  | 3985 | **491.7322** | **981.4498** | **981.4556** | **-0.0058** | **0** | **10** | **16** | **2** | **R.TLDEYWR.S** |
|  | 3995 | **491.8035** | **981.5925** | **981.5971** | **-0.0046** | **0** | **20** | **1.6** | **1** | **R.VLNLGPITR.K** 3977 |
|  | 4739 | **519.2194** | **1036.4243** | **1036.4317** | **-0.0075** | **0** | **19** | **1.8** | **1** | **R.LECGANDMK.V** 4747 |
|  | 4786 | **521.2746** | **1040.5346** | **1039.5121** | **1.0225** | **0** | **4** | **58** | **10** | **R.VGGTGMFTVR.M** |
|  | 5774 | **558.8332** | **1115.6518** | **1116.5274** | **-0.8755** | **0** | **10** | **15** | **6** | **K.VFMYLSDSR.C** |
|  | 5918 | **565.3005** | **1128.5865** | **1128.5928** | **-0.0062** | **0** | **17** | **3.5** | **1** | **R.DWVSVVTPAR.D** |
|  | 5967 | **567.2761** | **1132.5377** | **1132.5223** | **0.0154** | **0** | **(5)** | **52** | **6** | **K.VFMYLSDSR.C** |
|  | 6779 | **595.8184** | **1189.6223** | **1190.5788** | **-0.9565** | **0** | **1** | **1.2e+002** | **5** | **R.MAETCVPVLR.C** |
|  | 8025 | **417.5519** | **1249.6338** | **1250.6367** | **-1.0029** | **1** | **10** | **14** | **8** | **R.FRSGSVIDQSR.V** |
|  | 11310 | **715.8671** | **1429.7197** | **1428.7759** | **0.9438** | **0** | **10** | **14** | **6** | **K.TALQPMVSALNIR.V** |
|  | 12825 | **761.3694** | **1520.7242** | **1520.7327** | **-0.0085** | **1** | **51** | **0.0011** | **1** | **R.LECGANDMKVSLGK.C** 12812 |
|  | 13025 | **512.9348** | **1535.7824** | **1536.7276** | **-0.9452** | **1** | **(4)** | **46** | **2** | **R.LECGANDMKVSLGK.C** |
|  | 15673 | **853.3978** | **1704.7810** | **1704.7915** | **-0.0105** | **0** | **68** | **1.9e-005** | **1** | **R.DSTIQVVENGESSQGR.F** 15682 15692 15693 |
|  | 16089 | **868.8518** | **1735.6891** | **1735.6995** | **-0.0105** | **0** | **108** | **1.7e-009** | **1** | **R.STEYGEGYACDTDLR.G** 16090 |
|  | 17404 | **920.9588** | **1839.9030** | **1840.0604** | **-0.1574** | **1** | **2** | **60** | **10** | **K.VSLKTALQPMVSALNIR.V** |
|  | 18903 | **655.3398** | **1962.9975** | **1962.9768** | **0.0207** | **1** | **2** | **54** | **2** | **R.FVGQGGARMAETCVPVLR.C** |
|  | 22038 | **1149.9830** | **2297.9515** | **2297.9648** | **-0.0132** | **1** | **33** | **0.037** | **1** | **R.STEYGEGYACDTDLRGWYR.F** 22036 22037 |
|  | 27509 | **1106.2719** | **3315.7937** | **3315.5978** | **0.1959** | **0** | **0** | **37** | **6** | **-.MGQPSLTWMLMVVVASWFITTAATDTSEAR.W** |
|  | 29631 | **996.6429** | **3982.5427** | **3982.5645** | **-0.0218** | **0** | **56** | **6e-005** | **1** | **R.GDGWHCECSPGSCGPGLDCVPEGDALVCADPCQAHR.T** 29629 29630 |
|  | 32481 | **1241.8068** | **8685.5964** | **8685.6439** | **-0.0475** | **1** | **1** | **1.3** | **1** | **R.LSPGLGCTDVDECAEPGLSHCHALATCVNVVGSYLCVCPAGYRGDGWHCECSPGSCGPGLDCVPEGDALVCADPCQAHR.T** |

| **90.** | P30040\|ERP29_HUMAN    **Mass:** 29032    **Score:** 316    **Queries matched:** 10   **emPAI:** 0.44 |
| --- | --- |
|  | Endoplasmic reticulum protein ERp29 OS=Homo sapiens GN=ERP29 PE=1 SV=4 |

|  | **Query** | **Observed** | **Mr(expt)** | **Mr(calc)** | **Delta** | **Miss** | **Score** | **Expect** | **Rank** | **Peptide** |
| --- | --- | --- | --- | --- | --- | --- | --- | --- | --- | --- |
|  | 8451 | **636.3373** | **1270.6600** | **1269.6274** | **1.0326** | **1** | **13** | **7.8** | **2** | **K.LNMELSEKYK.L** |
|  | 14190 | **804.8762** | **1607.7379** | **1607.7468** | **-0.0089** | **0** | **13** | **6.1** | **1** | **R.DGDFENPVPYTGAVK.V** |
|  | 15711 | **854.8908** | **1707.7669** | **1707.7774** | **-0.0104** | **0** | **80** | **1.1e-006** | **1** | **K.ILDQGEDFPASEMTR.I** 15712 |
|  | 15912 | 575.2844 | 1722.8313 | 1723.7723 | -0.9411 | 0 | (12) | 6.5 | 3 | K.ILDQGEDFPASEMTR.I |
|  | 17324 | **459.2269** | **1832.8784** | **1832.9349** | **-0.0565** | **1** | **6** | **27** | **6** | **K.FVLVKFDTQYPYGEK.Q** |
|  | 20646 | **713.7012** | **2138.0817** | **2137.0184** | **1.0633** | **1** | **4** | **30** | **1** | **K.IMGKILDQGEDFPASEMTR.I** |
|  | 23935 | **1292.1345** | **2582.2545** | **2582.2662** | **-0.0117** | **0** | **99** | **8.2e-009** | **1** | **K.GQGVYLGMPGCLPVYDALAGEFIR.A** 23936 |
|  | 27904 | **1138.2085** | **3411.6037** | **3411.6239** | **-0.0202** | **1** | **89** | **4.7e-008** | **1** | **R.LAENSASSDDLLVAEVGISDYGDKLNMELSEK.Y** |

| **91.** | P16444\|DPEP1_HUMAN    **Mass:** 46101    **Score:** 315    **Queries matched:** 21   **emPAI:** 0.87 |
| --- | --- |
|  | Dipeptidase 1 OS=Homo sapiens GN=DPEP1 PE=1 SV=3 |

|  | **Query** | **Observed** | **Mr(expt)** | **Mr(calc)** | **Delta** | **Miss** | **Score** | **Expect** | **Rank** | **Peptide** |
| --- | --- | --- | --- | --- | --- | --- | --- | --- | --- | --- |
|  | 1329 | **395.2339** | **788.4532** | **787.4552** | **0.9980** | **0** | **13** | **11** | **1** | **K.ATLQLSR.A** |
|  | 7533 | **409.8743** | **1226.6012** | **1226.6078** | **-0.0066** | **0** | **29** | **0.21** | **1** | **R.TLEQMDVVHR.M** |
|  | 8885 | **647.8124** | **1293.6102** | **1292.6150** | **0.9952** | **0** | **49** | **0.0018** | **1** | **R.AVGFGGDFDGVPR.V** |
|  | 10091 | **453.7826** | **1358.3260** | **1357.7717** | **0.5543** | **1** | **8** | **16** | **3** | **K.YPDLIAELLRR.N** 10078 |
|  | 17340 | **917.9509** | **1833.8872** | **1833.8971** | **-0.0099** | **0** | **76** | **2.6e-006** | **1** | **R.MYPETFLYVTSSAGIR.Q** 17326 |
|  | 17566 | **617.4323** | **1849.2751** | **1849.8920** | **-0.6169** | **0** | **(1)** | **55** | **4** | **R.MYPETFLYVTSSAGIR.Q** |
|  | 18157 | **634.3469** | **1900.0189** | **1898.9850** | **1.0339** | **1** | **2** | **63** | **1** | **R.NWTEAEVKGALADNLLR.V** |
|  | 21014 | **1089.5232** | **2177.0318** | **2178.2194** | **-1.1876** | **1** | **6** | **22** | **2** | **K.ELNRLGVLIDLAHVSVATMK.A** |
|  | 21751 | **1132.0165** | **2262.0184** | **2261.0212** | **0.9972** | **0** | **57** | **0.00016** | **1** | **R.AGFVGGQFWSVYTPCDTQNK.D** |
|  | 24016 | **1298.6106** | **2595.2066** | **2595.2176** | **-0.0110** | **0** | **79** | **7.5e-007** | **1** | **R.DSPVIDGHNDLPWQLLDMFNNR.L** 24015 24018 |
|  | 26068 | **999.8184** | **2996.4334** | **2995.4433** | **0.9902** | **1** | **29** | **0.069** | **1** | **R.IMRDSPVIDGHNDLPWQLLDMFNNR.L** |
|  | 26198 | **1009.8284** | **3026.4633** | **3027.4331** | **-0.9698** | **1** | **(0)** | **50** | **3** | **R.IMRDSPVIDGHNDLPWQLLDMFNNR.L** |
|  | 27401 | **1095.5226** | **3283.5459** | **3283.5667** | **-0.0208** | **0** | **18** | **0.69** | **1** | **R.VFEAVEQASNLTQAPEEEPIPLDQLGGSCR.T** |
|  | 29833 | **1359.9550** | **4076.8431** | **4076.8599** | **-0.0169** | **0** | **64** | **1e-005** | **1** | **R.YLTLTHSCNTPWADNWLVDTGDSEPQSQGLSPFGQR.V** 29832 29834 29835 |

| **92.** | P01877\|IGHA2_HUMAN    **Mass:** 37301    **Score:** 310    **Queries matched:** 13   **emPAI:** 0.96 |
| --- | --- |
|  | Ig alpha-2 chain C region OS=Homo sapiens GN=IGHA2 PE=1 SV=3 |

|  | **Query** | **Observed** | **Mr(expt)** | **Mr(calc)** | **Delta** | **Miss** | **Score** | **Expect** | **Rank** | **Peptide** |
| --- | --- | --- | --- | --- | --- | --- | --- | --- | --- | --- |
|  | 2819 | 448.7325 | 895.4504 | 895.4552 | -0.0048 | 0 | 14 | 5.7 | 2 | K.YLTWASR.Q |
|  | 3435 | 470.7423 | 939.4701 | 939.4774 | -0.0073 | 0 | 11 | 13 | 2 | K.SAVQGPPER.D |
|  | 5793 | 559.3184 | 1116.6223 | 1116.6292 | -0.0069 | 1 | 51 | 0.0014 | 1 | R.GFSPKDVLVR.W |
|  | 11987 | 735.8840 | 1469.7535 | 1469.7626 | -0.0091 | 1 | 40 | 0.012 | 1 | R.WLQGSQELPREK.Y 11988 |
|  | 12659 | **756.8470** | **1511.6795** | **1511.6893** | **-0.0097** | **0** | **70** | **1.4e-005** | **1** | **R.DASGATFTWTPSSGK.S** 12658 |
|  | 17353 | 918.4741 | 1834.9336 | 1834.9425 | -0.0089 | 0 | 97 | 2.4e-008 | 1 | R.QEPSQGTTTFAVTSILR.V 17352 |
|  | 22294 | 779.7119 | 2336.1139 | 2336.1293 | -0.0154 | 1 | 28 | 0.11 | 1 | K.KGDTFSCMVGHEALPLAFTQK.T |
|  | 28475 | 1191.9585 | 3572.8537 | 3572.8661 | -0.0124 | 0 | 80 | 4e-007 | 1 | K.SGNTFRPEVHLLPPPSEELALNELVTLTCLAR.G 28476 28479 |

| **93.** | P08758\|ANXA5_HUMAN    **Mass:** 35971    **Score:** 304    **Queries matched:** 9   **emPAI:** 0.35 |
| --- | --- |
|  | Annexin A5 OS=Homo sapiens GN=ANXA5 PE=1 SV=2 |

|  | **Query** | **Observed** | **Mr(expt)** | **Mr(calc)** | **Delta** | **Miss** | **Score** | **Expect** | **Rank** | **Peptide** |
| --- | --- | --- | --- | --- | --- | --- | --- | --- | --- | --- |
|  | 4279 | **501.3005** | **1000.5863** | **1000.5917** | **-0.0053** | **0** | **45** | **0.0059** | **1** | **K.VLTEIIASR.T** |
|  | 10849 | **468.2346** | **1401.6820** | **1401.7326** | **-0.0506** | **1** | **4** | **54** | **5** | **R.KNFATSLYSMIK.G** |
|  | 13250 | 516.9396 | 1547.7969 | 1546.8905 | 0.9064 | 1 | 11 | 8.9 | 3 | K.FEKLIVALMKPSR.L |
|  | 15507 | **565.2754** | **1692.8043** | **1693.8821** | **-1.0778** | **1** | **1** | **86** | **4** | **R.VMVSRSEIDLFNIR.K** |
|  | 15663 | **852.9505** | **1703.8864** | **1703.8941** | **-0.0077** | **0** | **116** | **2.6e-010** | **1** | **K.GLGTDEESILTLLTSR.S** 15652 15662 |
|  | 29159 | 1265.9736 | 3794.8991 | 3794.9149 | -0.0158 | 1 | 102 | 1.9e-009 | 1 | R.MLVVLLQANRDPDAGIDEAQVEQDAQALFQAGELK.W 29158 |

| **94.** | P30086\|PEBP1_HUMAN    **Mass:** 21158    **Score:** 303    **Queries matched:** 13   **emPAI:** 1.30 |
| --- | --- |
|  | Phosphatidylethanolamine-binding protein 1 OS=Homo sapiens GN=PEBP1 PE=1 SV=3 |

|  | **Query** | **Observed** | **Mr(expt)** | **Mr(calc)** | **Delta** | **Miss** | **Score** | **Expect** | **Rank** | **Peptide** |
| --- | --- | --- | --- | --- | --- | --- | --- | --- | --- | --- |
|  | 2712 | **443.2709** | **884.5272** | **884.5331** | **-0.0059** | **0** | **23** | **0.85** | **1** | **K.VLTPTQVK.N** |
|  | 5605 | **552.7736** | **1103.5327** | **1102.5077** | **1.0250** | **0** | **17** | **3.5** | **2** | **K.CDEPILSNR.S** |
|  | 13437 | **780.9127** | **1559.8109** | **1559.8195** | **-0.0086** | **0** | **34** | **0.052** | **1** | **K.LYTLVLTDPDAPSR.K** |
|  | 14574 | **816.8974** | **1631.7802** | **1631.7903** | **-0.0101** | **0** | **70** | **1.3e-005** | **1** | **K.NRPTSISWDGLDSGK.L** |
|  | 18748 | **975.4703** | **1948.9260** | **1948.9378** | **-0.0118** | **0** | **84** | **3.6e-007** | **1** | **K.GNDISSGTVLSDYVGSGPPK.G** 18747 |
|  | 25084 | **931.8020** | **2792.3842** | **2792.3956** | **-0.0114** | **1** | **38** | **0.0094** | **1** | **R.YVWLVYEQDRPLKCDEPILSNR.S** 25083 25085 |
|  | 28115 | **1157.9033** | **3470.6881** | **3470.7106** | **-0.0225** | **0** | **82** | **2.4e-007** | **1** | **K.WSGPLSLQEVDEQPQHPLHVTYAGAAVDELGK.V** 28116 |
|  | 29947 | **823.0178** | **4110.0524** | **4110.0698** | **-0.0174** | **1** | **8** | **4.5** | **1** | **M.PVDLSKWSGPLSLQEVDEQPQHPLHVTYAGAAVDELGK.V** |
|  | 30202 | **852.4692** | **4257.3095** | **4257.1052** | **0.2043** | **1** | **2** | **13** | **2** | **-.MPVDLSKWSGPLSLQEVDEQPQHPLHVTYAGAAVDELGK.V** |

| **95.** | P35555\|FBN1_HUMAN    **Mass:** 332667   **Score:** 302    **Queries matched:** 56   **emPAI:** 0.14 |
| --- | --- |
|  | Fibrillin-1 OS=Homo sapiens GN=FBN1 PE=1 SV=2 |

|  | **Query** | **Observed** | **Mr(expt)** | **Mr(calc)** | **Delta** | **Miss** | **Score** | **Expect** | **Rank** | **Peptide** |
| --- | --- | --- | --- | --- | --- | --- | --- | --- | --- | --- |
|  | 1696 | **408.7381** | **815.4617** | **815.4654** | **-0.0037** | **0** | **15** | **7.4** | **1** | **R.GFIPNIR.T** 1703 |
|  | 1698 | **408.8883** | **815.7620** | **816.4341** | **-0.6721** | **0** | **8** | **23** | **4** | **K.LNEVSQK.C** |
|  | 1753 | **411.2350** | **820.4554** | **820.3861** | **0.0693** | **1** | **6** | **48** | **5** | **K.CSSPKSR.N** |
|  | 2141 | **424.7283** | **847.4421** | **846.4712** | **0.9709** | **1** | **10** | **20** | **7** | **K.INGYPKR.G** |
|  | 4560 | **512.7167** | **1023.4189** | **1023.4266** | **-0.0077** | **0** | **11** | **12** | **4** | **R.GSYHCICK.T** |
|  | 4903 | **525.7612** | **1049.5079** | **1049.5142** | **-0.0063** | **0** | **31** | **0.13** | **1** | **K.GYILQEDGR.S** |
|  | 4946 | **528.2473** | **1054.4801** | **1054.4324** | **0.0476** | **0** | **6** | **40** | **8** | **R.CDCKPGYR.F** |
|  | 5304 | **542.2840** | **1082.5534** | **1081.5339** | **1.0196** | **1** | **0** | **1.4e+002** | **8** | **K.CRNTIGSFK.C** |
|  | 5538 | **550.2949** | **1098.5752** | **1099.3481** | **-0.7730** | **0** | **7** | **31** | **1** | **K.SECCCDGGR.G** |
|  | 6280 | **579.2676** | **1156.5207** | **1156.3882** | **0.1325** | **0** | **1** | **1.2e+002** | **8** | **K.MQCCCDAGR.C** |
|  | 9932 | **450.8886** | **1349.6441** | **1349.6510** | **-0.0069** | **0** | **27** | **0.31** | **1** | **R.SGNCYLDIRPR.G** |
|  | 10237 | **456.7337** | **1367.1794** | **1366.6010** | **0.5784** | **0** | **4** | **36** | **8** | **R.NTPGSFVCTCPK.G** |
|  | 11117 | **473.9305** | **1418.7698** | **1417.7752** | **0.9946** | **0** | **16** | **3.1** | **2** | **R.GQCIKPLFGAVTK.S** |
|  | 11291 | **477.2497** | **1428.7272** | **1429.5602** | **-0.8330** | **0** | **4** | **50** | **8** | **K.NTEGSYQCSCPK.G** |
|  | 11532 | 722.8946 | 1443.7747 | 1442.5806 | 1.1942 | 0 | 17 | 2.8 | 2 | R.CECEIGYELDR.S |
|  | 11843 | **487.9714** | **1460.8924** | **1461.6275** | **-0.7351** | **0** | **4** | **55** | **2** | **R.IGQGHCVSGMGMGR.G** |
|  | 12169 | **494.6055** | **1480.7947** | **1480.5745** | **0.2202** | **1** | **4** | **53** | **2** | **K.HDCTEKQMECK.N** |
|  | 13316 | **776.8461** | **1551.6776** | **1551.6875** | **-0.0099** | **0** | **67** | **2.5e-005** | **1** | **R.YEDEECTLPIAGR.H** 13317 |
|  | 13334 | **777.3243** | **1552.6341** | **1552.6433** | **-0.0091** | **0** | **51** | **0.00091** | **1** | **K.CQCPSGMTLDATGR.I** |
|  | 13649 | **525.6300** | **1573.8682** | **1573.8763** | **-0.0081** | **1** | **7** | **24** | **1** | **K.RGQCIKPLFGAVTK.S** |
|  | 13820 | **792.8986** | **1583.7827** | **1583.7912** | **-0.0085** | **0** | **19** | **1.4** | **1** | **K.TLPGGNQCIVPICR.H** |
|  | 14191 | **536.9229** | **1607.7469** | **1607.7548** | **-0.0079** | **0** | **6** | **33** | **6** | **K.CICKPGFQLASDGR.Y** |
|  | 14340 | **809.4761** | **1616.9376** | **1617.6374** | **-0.6998** | **0** | **6** | **28** | **2** | **K.GSFICHCDMGYSGK.K** |
|  | 14346 | **809.8765** | **1617.7385** | **1617.7491** | **-0.0106** | **0** | **61** | **9.4e-005** | **1** | **R.TCVDINECLLEPR.K** 14347 |
|  | 14674 | **547.0314** | **1638.0725** | **1638.6549** | **-0.5824** | **1** | **1** | **67** | **8** | **R.NPVTKSECCCDGGR.G** |
|  | 16646 | **890.8809** | **1779.7473** | **1778.7741** | **0.9732** | **0** | **22** | **0.59** | **1** | **R.GDNGDTACSNEIGVGVSK.A** |
|  | 17128 | **607.3401** | **1818.9984** | **1818.0363** | **0.9621** | **0** | **2** | **75** | **6** | **R.ILELLPALTTLTNHNR.Y** |
|  | 17683 | **930.4283** | **1858.8421** | **1858.8528** | **-0.0107** | **0** | **52** | **0.00062** | **1** | **R.CWSPGVTVAPEMCPIR.A** |
|  | 18171 | **634.9325** | **1901.7757** | **1901.8798** | **-0.1041** | **1** | **0** | **88** | **10** | **K.DINECKMIPSLCTHGK.C** |
|  | 18174 | **951.9318** | **1901.8490** | **1901.8586** | **-0.0097** | **0** | **47** | **0.002** | **1** | **K.NLIGTYMCICGPGYQR.R** 18183 |
|  | 19180 | **663.9810** | **1988.9212** | **1987.8550** | **1.0662** | **1** | **1** | **64** | **9** | **R.GSFKCQCPSGMTLDATGR.I** 19159 |
|  | 19601 | **677.0201** | **2028.0384** | **2028.7823** | **-0.7439** | **0** | **1** | **77** | **1** | **K.TNDDQTMCLDINECER.D** |
|  | 19669 | **679.2764** | **2034.8073** | **2034.8194** | **-0.0121** | **0** | **22** | **0.47** | **1** | **K.TGCTDINECEIGAHNCGK.H** |
|  | 20199 | **523.4661** | **2089.8354** | **2089.8492** | **-0.0138** | **0** | **12** | **4.7** | **1** | **R.AWGHPCEMCPAQPHPCR.R** |
|  | 21365 | **739.9681** | **2216.8826** | **2215.9119** | **0.9707** | **1** | **9** | **9.3** | **1** | **R.YCKDINECETPGICMNGR.C** |
|  | 22216 | **775.0513** | **2322.1320** | **2323.0541** | **-0.9222** | **1** | **1** | **63** | **3** | **K.CQCPSGMTLDATGRICLDIR.L** |
|  | 22656 | **795.3519** | **2383.0338** | **2383.0494** | **-0.0157** | **1** | **32** | **0.051** | **1** | **R.IKGTQCEDIDECEVFPGVCK.N** |
|  | 24292 | 884.7047 | 2651.0923 | 2651.1428 | -0.0505 | 1 | 0 | 52 | 3 | R.CINTDGSFHCVCNAGFHVTRDGK.N |
|  | 24495 | **671.0839** | **2680.3063** | **2680.2084** | **0.0979** | **1** | **0** | **56** | **6** | **R.CWSPGVTVAPEMCPIRATEDFNK.L** |
|  | 24520 | **537.8537** | **2684.2321** | **2685.2383** | **-1.0062** | **0** | **13** | **3.4** | **1** | **R.TGPCFTVISNQMCQGQLSGIVCTK.T** |
|  | 25225 | **940.0870** | **2817.2393** | **2817.1575** | **0.0818** | **1** | **4** | **21** | **3** | **K.DINECETPGICMNGRCVNTDGSYR.C** |
|  | 26142 | **1005.1826** | **3012.5259** | **3013.2254** | **-0.6996** | **1** | **1** | **39** | **8** | **K.MQCCCDAGRCWSPGVTVAPEMCPIR.A** |
|  | 26594 | **1031.7273** | **3092.1600** | **3092.1789** | **-0.0189** | **1** | **3** | **16** | **1** | **R.GQCVNTPGDFECKCDEGYESGFMMMK.N** |
|  | 26786 | **785.5994** | **3138.3684** | **3139.2636** | **-0.8952** | **1** | **8** | **6.7** | **1** | **K.ASCCCSLGKAWGTPCEMCPAVNTSEYK.I** |
|  | 26814 | **629.8844** | **3144.3856** | **3144.3522** | **0.0335** | **1** | **1** | **33** | **4** | **K.CAPGTCQNLDGSYRCICPPGYSLQNEK.C** |
|  | 26868 | **632.7269** | **3158.5983** | **3158.2304** | **0.3679** | **0** | **1** | **41** | **2** | **R.HSCGDGFCSRPNMCTCPSGQIAPSCGSR.S** |
|  | 27736 | **1123.8800** | **3368.6182** | **3369.4510** | **-0.8328** | **1** | **1** | **32** | **4** | **R.QICPYGSGIIVGPDDSAVDMDECKEPDVCK.H** |
|  | 28088 | **1154.1748** | **3459.5026** | **3459.5204** | **-0.0178** | **0** | **58** | **5.9e-005** | **1** | **K.TGYTPDITGTSCVDLNECNQAPKPCNFICK.N** |
|  | 29386 | **1294.6283** | **3880.8631** | **3880.8798** | **-0.0167** | **0** | **32** | **0.022** | **1** | **K.ILCPGGEGFRPNPITVILEDIDECQELPGLCQGGK.C** 29385 |
|  | 29518 | **789.5170** | **3942.5488** | **3942.5253** | **0.0235** | **1** | **1** | **21** | **4** | **K.CSCSPGWIGDGIKCTDLDECSNGTHMCSQHADCK.N** |

| **96.** | P30101\|PDIA3_HUMAN    **Mass:** 57146    **Score:** 301    **Queries matched:** 28   **emPAI:** 0.88 |
| --- | --- |
|  | Protein disulfide-isomerase A3 OS=Homo sapiens GN=PDIA3 PE=1 SV=4 |

|  | **Query** | **Observed** | **Mr(expt)** | **Mr(calc)** | **Delta** | **Miss** | **Score** | **Expect** | **Rank** | **Peptide** |
| --- | --- | --- | --- | --- | --- | --- | --- | --- | --- | --- |
|  | 939 | **601.1233** | **600.1161** | **599.3279** | **0.7882** | **0** | **3** | **66** | **1** | **K.NLEPK.Y** |
|  | 2425 | **434.7185** | **867.4224** | **868.5018** | **-1.0794** | **0** | **1** | **1.1e+002** | **8** | **K.DPNIVIAK.M** |
|  | 2587 | **439.2582** | **876.5019** | **876.4817** | **0.0202** | **0** | **10** | **20** | **7** | **K.LNFAVASR.K** |
|  | 3757 | **483.2539** | **964.4932** | **963.4994** | **0.9938** | **1** | **12** | **12** | **6** | **R.NRVMMVAK.K** |
|  | 5316 | **542.7838** | **1083.5531** | **1083.5601** | **-0.0070** | **0** | **35** | **0.053** | **1** | **K.YGVSGYPTLK.I** |
|  | 6793 | **596.3007** | **1190.5869** | **1190.5931** | **-0.0062** | **0** | **68** | **2.5e-005** | **1** | **R.LAPEYEAAATR.L** |
|  | 6950 | **400.2437** | **1197.7092** | **1196.7128** | **0.9964** | **1** | **32** | **0.089** | **1** | **K.LSKDPNIVIAK.M** 6923 |
|  | 7729 | **619.3185** | **1236.6225** | **1235.5054** | **1.1171** | **0** | **12** | **8.9** | **1** | **R.DGEEAGAYDGPR.T** |
|  | 9759 | **671.3424** | **1340.6701** | **1340.6765** | **-0.0063** | **0** | **5** | **40** | **1** | **R.GFPTIYFSPANK.K** |
|  | 9874 | **674.3497** | **1346.6848** | **1346.6942** | **-0.0094** | **1** | **51** | **0.0012** | **1** | **K.RLAPEYEAAATR.L** |
|  | 10250 | **684.8317** | **1367.6489** | **1367.6569** | **-0.0080** | **0** | **7** | **25** | **2** | **K.SEPIPESNDGPVK.V** |
|  | 10749 | **699.2887** | **1396.5628** | **1396.5711** | **-0.0083** | **0** | **64** | **5.4e-005** | **1** | **K.VDCTANTNTCNK.Y** 10750 10753 10754 10769 |
|  | 12005 | **491.5634** | **1471.6685** | **1471.6766** | **-0.0080** | **1** | **5** | **37** | **6** | **K.FVMQEEFSRDGK.A** |
|  | 13737 | 790.4149 | 1578.8152 | 1578.8253 | -0.0101 | 0 | 39 | 0.016 | 1 | R.EATNPPVIQEEKPK.K 13738 |
|  | 14896 | **826.8819** | **1651.7492** | **1651.7590** | **-0.0098** | **1** | **67** | **2.1e-005** | **1** | **K.IFRDGEEAGAYDGPR.T** 14890 14897 |
|  | 15068 | **832.8773** | **1663.7401** | **1663.7512** | **-0.0111** | **0** | **70** | **1.2e-005** | **1** | **K.MDATANDVPSPYEVR.G** 15069 |
|  | 23174 | **821.7094** | **2462.1064** | **2462.1206** | **-0.0142** | **1** | **9** | **9.1** | **1** | **K.VDCTANTNTCNKYGVSGYPTLK.I** |
|  | 24620 | **901.8001** | **2702.3785** | **2702.3915** | **-0.0130** | **1** | **33** | **0.03** | **1** | **R.KTFSHELSDFGLESTAGEIPVVAIR.T** |
|  | 25374 | **712.5956** | **2846.3532** | **2846.3731** | **-0.0199** | **1** | **9** | **6.3** | **1** | **K.FIQENIFGICPHMTEDNKDLIQGK.D** |

| **97.** | P02679\|FIBG_HUMAN    **Mass:** 52106    **Score:** 290    **Queries matched:** 17   **emPAI:** 0.32 |
| --- | --- |
|  | Fibrinogen gamma chain OS=Homo sapiens GN=FGG PE=1 SV=3 |

|  | **Query** | **Observed** | **Mr(expt)** | **Mr(calc)** | **Delta** | **Miss** | **Score** | **Expect** | **Rank** | **Peptide** |
| --- | --- | --- | --- | --- | --- | --- | --- | --- | --- | --- |
|  | 2067 | **422.0606** | **842.1067** | **842.5225** | **-0.4159** | **1** | **9** | **14** | **1** | **K.IVNLKEK.V** 2064 2086 |
|  | 2620 | **440.9027** | **879.7909** | **879.4338** | **0.3571** | **0** | **9** | **13** | **4** | **R.LDGSVDFK.K** |
|  | 3019 | **455.2536** | **908.4926** | **908.4347** | **0.0580** | **0** | **9** | **20** | **1** | **K.MLEEIMK.Y** |
|  | 4616 | **514.7528** | **1027.4911** | **1028.4862** | **-0.9951** | **0** | **2** | **1e+002** | **9** | **-.MSWSLHPR.N** |
|  | 4706 | **517.7635** | **1033.5124** | **1033.5192** | **-0.0068** | **1** | **33** | **0.076** | **1** | **K.VGPEADKYR.L** |
|  | 4739 | 519.2194 | 1036.4243 | 1036.5296 | -0.1054 | 1 | 4 | 56 | 9 | R.KMLEEIMK.Y |
|  | 5785 | **559.2842** | **1116.5539** | **1116.5200** | **0.0340** | **0** | **6** | **44** | **2** | **R.VELEDWNGR.T** |
|  | 12683 | **757.3635** | **1512.7124** | **1512.7208** | **-0.0085** | **0** | **77** | **2.6e-006** | **1** | **R.YLQEIYNSNNQK.I** |
|  | 19416 | **671.6309** | **2011.8708** | **2010.9007** | **0.9701** | **0** | **13** | **4.4** | **1** | **K.CHAGHLNGVYYQGGTYSK.A** |
|  | 21517 | 558.8202 | 2231.2517 | 2232.0157 | -0.7641 | 1 | 2 | 47 | 4 | R.VELEDWNGRTSTADYAMFK.V |
|  | 22533 | **789.0462** | **2364.1168** | **2365.1657** | **-1.0489** | **1** | **42** | **0.0051** | **1** | **K.MLEEIMKYEASILTHDSSIR.Y** 22540 |
|  | 22856 | **1209.0363** | **2416.0579** | **2416.0716** | **-0.0136** | **0** | **123** | **3.2e-011** | **1** | **R.FGSYCPTTCGIADFLSTYQTK.V** 22855 |
|  | 23531 | **840.7546** | **2519.2421** | **2519.2577** | **-0.0156** | **0** | **14** | **3.2** | **1** | **K.AIQLTYNPDESSKPNMIDAATLK.S** |

| **98.** | P28331\|NDUS1_HUMAN    **Mass:** 80443    **Score:** 289    **Queries matched:** 37   **emPAI:** 0.57 |
| --- | --- |
|  | NADH-ubiquinone oxidoreductase 75 kDa subunit, mitochondrial OS=Homo sapiens GN=NDUFS1 PE=1 SV=3 |

|  | **Query** | **Observed** | **Mr(expt)** | **Mr(calc)** | **Delta** | **Miss** | **Score** | **Expect** | **Rank** | **Peptide** |
| --- | --- | --- | --- | --- | --- | --- | --- | --- | --- | --- |
|  | 1496 | **401.2846** | **800.5547** | **799.4374** | **1.1173** | **0** | **16** | **5.8** | **10** | **K.VGMQIPR.F** |
|  | 2082 | **422.7274** | **843.4402** | **844.4477** | **-1.0074** | **0** | **(4)** | **88** | **6** | **R.LTEPMVR.N** |
|  | 2339 | **431.7036** | **861.3925** | **860.4426** | **0.9500** | **0** | **17** | **4.5** | **2** | **R.LTEPMVR.N** 2304 2313 2314 |
|  | 2560 | **438.7189** | **875.4232** | **875.4283** | **-0.0051** | **0** | **2** | **1.1e+002** | **9** | **R.LSVAGNCR.M** |
|  | 3048 | **456.6940** | **911.3734** | **910.3756** | **0.9979** | **0** | **12** | **9.8** | **4** | **R.FCYHER.L** 3038 |
|  | 3670 | **480.2413** | **958.4680** | **958.5236** | **-0.0555** | **1** | **10** | **18** | **2** | **R.EDWKIIR.A** |
|  | 3967 | 490.7954 | 979.5762 | 979.5815 | -0.0053 | 0 | 14 | 6.1 | 3 | K.VAVTPPGLAR.E |
|  | 5277 | **541.2493** | **1080.4841** | **1080.4910** | **-0.0069** | **0** | **13** | **6.8** | **1** | **R.MTSGVTGDWK.V** |
|  | 6336 | **581.2954** | **1160.5763** | **1160.5826** | **-0.0063** | **0** | **28** | **0.27** | **2** | **K.GWNILTNSEK.S** |
|  | 7160 | **403.7399** | **1208.1980** | **1207.5763** | **0.6216** | **0** | **5** | **27** | **9** | **K.VVAACAMPVMK.G** |
|  | 8642 | 641.8226 | 1281.6306 | 1281.7292 | -0.0986 | 1 | 27 | 0.26 | 1 | R.AVEDKNIGPLVK.T |
|  | 10193 | **683.3632** | **1364.7119** | **1365.6711** | **-0.9592** | **1** | **2** | **87** | **1** | **K.IRMTSGVTGDWK.V** |
|  | 11996 | **491.2541** | **1470.7404** | **1471.7713** | **-1.0310** | **1** | **2** | **75** | **8** | **K.APKVVAACAMPVMK.G** |
|  | 13066 | 513.6124 | 1537.8155 | 1537.8253 | -0.0098 | 1 | 8 | 17 | 2 | K.VAVTPPGLAREDWK.I |
|  | 13430 | **780.8592** | **1559.7039** | **1559.7137** | **-0.0098** | **0** | **4** | **52** | **5** | **K.AVTEGAQAVEEPSIC.-** |
|  | 15791 | **572.3043** | **1713.8911** | **1712.9607** | **0.9304** | **1** | **4** | **44** | **4** | **K.EAKKPMVVLGSSALQR.N** |
|  | 17394 | **920.4960** | **1838.9774** | **1838.9890** | **-0.0117** | **0** | **60** | **0.00011** | **1** | **K.ILQDIASGSHPFSQVLK.E** |
|  | 18173 | **951.9312** | **1901.8479** | **1901.8577** | **-0.0099** | **1** | **89** | **1.1e-007** | **1** | **R.MHEDINEEWISDKTR.F** 18172 |
|  | 19045 | **659.7991** | **1976.3754** | **1975.9608** | **0.4146** | **1** | **2** | **37** | **6** | **R.MTSGVTGDWKVMNILHR.I** |
|  | 20233 | **1047.0271** | **2092.0396** | **2092.0510** | **-0.0114** | **0** | **47** | **0.0017** | **1** | **R.ALSEIAGMTLPYDTLDQVR.N** 20232 20236 |
|  | 20302 | **1050.6189** | **2099.2232** | **2099.2354** | **-0.0122** | **0** | **20** | **0.82** | **1** | **K.LVNQQLLADPLVPPQLTIK.D** |
|  | 20651 | **713.9758** | **2138.9055** | **2140.0259** | **-1.1204** | **1** | **0** | **79** | **5** | **R.ILPRMHEDINEEWISDK.T** |
|  | 21185 | **733.3650** | **2197.0731** | **2198.2423** | **-1.1691** | **1** | **4** | **33** | **2** | **R.IASQVAALDLGYKPGVEAIRK.N** |
|  | 24902 | **920.4761** | **2758.4064** | **2757.3796** | **1.0268** | **1** | **2** | **37** | **2** | **K.GLLTYTSWEDALSRVAGMLQSFQGK.D** |
|  | 25829 | **982.1315** | **2943.3726** | **2943.3920** | **-0.0194** | **1** | **36** | **0.014** | **1** | **R.FASEIAGVDDLGTTGRGNDMQVGTYIEK.M** |
|  | 27189 | **1076.5281** | **3226.5624** | **3226.5757** | **-0.0133** | **0** | **22** | **0.29** | **1** | **K.DCFIIYQGHHGDVGAPIADVILPGAAYTEK.S** |
|  | 27496 | **1105.9225** | **3314.7456** | **3314.7584** | **-0.0128** | **1** | **27** | **0.083** | **1** | **K.LVNQQLLADPLVPPQLTIKDFYMTDSISR.A** 27495 |
|  | 30252 | **1433.6000** | **4297.7781** | **4297.7942** | **-0.0161** | **0** | **50** | **0.0002** | **1** | **R.EGVMEFLLANHPLDCPICDQGGECDLQDQSMMFGNDR.S** 30250 |

| **99.** | A5A3E0\|POTEF_HUMAN    **Mass:** 123020   **Score:** 286    **Queries matched:** 41   **emPAI:** 0.16 |
| --- | --- |
|  | POTE ankyrin domain family member F OS=Homo sapiens GN=POTEF PE=1 SV=2 |

|  | **Query** | **Observed** | **Mr(expt)** | **Mr(calc)** | **Delta** | **Miss** | **Score** | **Expect** | **Rank** | **Peptide** |
| --- | --- | --- | --- | --- | --- | --- | --- | --- | --- | --- |
|  | 1475 | 800.5302 | 799.5229 | 799.5280 | -0.0051 | 1 | 14 | 8.2 | 3 | K.RGILTLK.Y |
|  | 3029 | 455.7555 | 909.4965 | 908.3797 | 1.1168 | 0 | 4 | 59 | 10 | R.EVEEEMK.K |
|  | 4072 | **494.5778** | **987.1410** | **987.5138** | **-0.3727** | **0** | **13** | **5.7** | **1** | **K.ILTEHGYR.F** |
|  | 6416 | 585.0891 | 1168.1635 | 1167.5264 | 0.6372 | 0 | (9) | 12 | 1 | K.MNSELSLSCK.K 6408 |
|  | 6646 | 593.1548 | 1184.2950 | 1183.5213 | 0.7737 | 0 | 16 | 2.5 | 1 | K.MNSELSLSCK.K 6659 |
|  | 6919 | **599.3468** | **1196.6790** | **1196.6149** | **0.0641** | **0** | **3** | **73** | **10** | **K.DILHENSTLR.E** |
|  | 6943 | 599.8527 | 1197.6908 | 1197.6982 | -0.0075 | 0 | 52 | 0.00092 | 1 | R.AVFPSIVGRPR.Q 6939 6944 6945 6946 |
|  | 7426 | 611.7866 | 1221.5587 | 1222.6445 | -1.0858 | 1 | 5 | 44 | 5 | K.YLEDIESVKK.R |
|  | 7488 | 613.3124 | 1224.6102 | 1224.5986 | 0.0116 | 1 | 0 | 1.3e+002 | 6 | K.ESYVGKEAQSK.R |
|  | 8306 | 422.2385 | 1263.6936 | 1263.5159 | 0.1777 | 0 | 5 | 50 | 9 | R.QQGMMGGMHQK.E 8334 |
|  | 9379 | 660.3484 | 1318.6823 | 1318.6728 | 0.0095 | 1 | 1 | 1.1e+002 | 8 | K.LTSEEESQRLK.G |
|  | 11560 | 482.5971 | 1444.7696 | 1445.7734 | -1.0038 | 1 | 15 | 4.2 | 2 | K.EIAALAPSMMKIR.I |
|  | 12299 | 497.2400 | 1488.6982 | 1489.6103 | -0.9121 | 0 | 8 | 23 | 5 | K.SNVGASGDHDDSAMK.T |
|  | 12440 | 500.2620 | 1497.7643 | 1498.6683 | -0.9040 | 0 | 0 | 1.1e+002 | 9 | R.ELENFMAIEEMK.K |
|  | 12723 | 505.9181 | 1514.7325 | 1514.7419 | -0.0093 | 0 | 40 | 0.014 | 1 | K.IWHHTFYNELR.V 12724 12725 |
|  | 12739 | 758.8506 | 1515.6866 | 1515.6954 | -0.0087 | 0 | 42 | 0.0081 | 1 | K.QEYDESGPSIVHR.K 12738 |
|  | 13248 | 774.9052 | 1547.7957 | 1547.7873 | 0.0084 | 1 | 21 | 0.93 | 2 | R.MQKEIAALAPSMMK.I 13249 |
|  | 13508 | 783.3367 | 1564.6588 | 1564.7477 | -0.0889 | 0 | 3 | 56 | 4 | -.MVVEVDSMPAASSVK.K |
|  | 13997 | 399.7197 | 1594.8498 | 1595.7721 | -0.9223 | 1 | (12) | 6.5 | 1 | R.MQKEIAALAPSMMK.I 13994 |
|  | 14848 | 825.3959 | 1648.7773 | 1648.8494 | -0.0721 | 1 | 2 | 69 | 5 | R.LDLAGRELPDYLMK.I |
|  | 15697 | 569.6187 | 1705.8343 | 1706.8582 | -1.0239 | 1 | 0 | 1e+002 | 10 | R.EEIAMLRLELDTMK.H |
|  | 16767 | 895.9441 | 1789.8736 | 1789.8846 | -0.0110 | 0 | 66 | 2.5e-005 | 1 | K.SYELPDGQVITIGNER.F 16766 16769 16770 16771 16772 |
|  | 18087 | 632.3237 | 1893.9492 | 1894.8488 | -0.8996 | 1 | 2 | 58 | 4 | R.QQGMMGGMHQKESYVGK.E |
|  | 29159 | 1265.9736 | 3794.8991 | 3795.7390 | -0.8399 | 1 | 1 | 29 | 4 | K.ALQLNELTMDDDTAVLVIDNGSGMCKAGFAGDDAPR.A |

| **100.** | Q5TCH4\|CP4AM_HUMAN    **Mass:** 59721    **Score:** 283    **Queries matched:** 20   **emPAI:** 0.35 |
| --- | --- |
|  | Cytochrome P450 4A22 OS=Homo sapiens GN=CYP4A22 PE=1 SV=1 |

|  | **Query** | **Observed** | **Mr(expt)** | **Mr(calc)** | **Delta** | **Miss** | **Score** | **Expect** | **Rank** | **Peptide** |
| --- | --- | --- | --- | --- | --- | --- | --- | --- | --- | --- |
|  | 1180 | 709.1945 | 708.1872 | 708.4031 | -0.2158 | 0 | 8 | 22 | 5 | K.NGIHLR.L |
|  | 1664 | **407.7530** | **813.4915** | **812.4578** | **1.0337** | **0** | **5** | **55** | **5** | **R.IPIPMAR.L** |
|  | 3339 | 466.7362 | 931.4578 | 930.5134 | 0.9444 | 0 | 12 | 12 | 6 | M.SVSVLSPSR.R |
|  | 4412 | **506.2938** | **1010.5731** | **1011.6553** | **-1.0822** | **1** | **5** | **42** | **3** | **K.VARALTLLR.F** |
|  | 5244 | 540.2373 | 1078.4600 | 1077.5488 | 0.9112 | 0 | 9 | 21 | 5 | -.MSVSVLSPSR.R |
|  | 5671 | 554.9564 | 1107.8982 | 1108.5070 | -0.6089 | 0 | 8 | 15 | 1 | K.MENGSILSDK.D |
|  | 8442 | 636.1109 | 1270.2072 | 1270.5904 | -0.3831 | 0 | 5 | 28 | 1 | R.VQLYDPDYMK.V |
|  | 9444 | **661.8292** | **1321.6439** | **1320.6972** | **0.9467** | **1** | **5** | **43** | **1** | **K.QFAMNQLKVAR.A** |
|  | 10975 | 705.9180 | 1409.8215 | 1409.8282 | -0.0067 | 0 | 76 | 3.4e-006 | 1 | R.HLDFLDILLLAK.M 10974 |
|  | 12095 | 493.2463 | 1476.7171 | 1476.7242 | -0.0071 | 1 | 8 | 21 | 2 | K.MENGSILSDKDLR.A |
|  | 17215 | 913.5181 | 1825.0217 | 1824.9444 | 0.0773 | 1 | 2 | 61 | 5 | R.VQLYDPDYMKVILGR.S |
|  | 17746 | **933.4198** | **1864.8250** | **1865.0233** | **-0.1983** | **1** | **4** | **42** | **3** | **R.FELLPDPTRIPIPMAR.L** |
|  | 18351 | 959.4839 | 1916.9532 | 1916.9639 | -0.0107 | 0 | 85 | 3.5e-007 | 1 | R.ACQLAHQHTDQVIQLR.K 18348 18350 18352 |
|  | 24062 | **651.8266** | **2603.2773** | **2603.3240** | **-0.0467** | **0** | **4** | **24** | **3** | **R.MLTPAFHNDILKPYVGLMADSVR.V** |
|  | 27165 | **806.3997** | **3221.5695** | **3221.6287** | **-0.0592** | **1** | **1** | **35** | **2** | **R.MLTPAFHNDILKPYVGLMADSVRVMLDK.W** |
|  | 29036 | 1249.2980 | 3744.8721 | 3744.8817 | -0.0096 | 1 | 96 | 8e-009 | 1 | R.VMLDKWEELLGQDSPLEVFQHVSLMTLDTIMK.S |

| **101.** | P19440\|GGT1_HUMAN    **Mass:** 61714    **Score:** 282    **Queries matched:** 23   **emPAI:** 0.42 |
| --- | --- |
|  | Gamma-glutamyltranspeptidase 1 OS=Homo sapiens GN=GGT1 PE=1 SV=2 |

|  | **Query** | **Observed** | **Mr(expt)** | **Mr(calc)** | **Delta** | **Miss** | **Score** | **Expect** | **Rank** | **Peptide** |
| --- | --- | --- | --- | --- | --- | --- | --- | --- | --- | --- |
|  | 1219 | **734.9947** | **733.9874** | **733.4123** | **0.5752** | **0** | **3** | **70** | **1** | **R.IVEAFR.F** |
|  | 2238 | **428.2274** | **854.4402** | **855.4562** | **-1.0161** | **1** | **7** | **33** | **3** | **K.RAVEEPR.L** |
|  | 2278 | **429.6938** | **857.3730** | **857.4719** | **-0.0989** | **1** | **3** | **1.1e+002** | **3** | **K.VLREGER.L** |
|  | 3839 | **486.7559** | **971.4972** | **972.4777** | **-0.9805** | **0** | **33** | **0.097** | **1** | **R.GYELAHQR.H** 3876 |
|  | 4124 | **496.2474** | **990.4802** | **991.5464** | **-1.0662** | **1** | **9** | **25** | **7** | **R.HGRLPWAR.L** |
|  | 4801 | **521.9270** | **1041.8394** | **1041.5930** | **0.2464** | **1** | **2** | **67** | **4** | **K.GLAAALENKR.T** |
|  | 5067 | **532.2894** | **1062.5643** | **1062.5710** | **-0.0067** | **0** | **34** | **0.062** | **1** | **K.FVDVTEVVR.N** 5080 |
|  | 6469 | **586.8398** | **1171.6650** | **1171.6713** | **-0.0063** | **0** | **49** | **0.0019** | **1** | **R.LFQPSIQLAR.Q** |
|  | 9270 | **657.8625** | **1313.7104** | **1313.7132** | **-0.0028** | **1** | **12** | **9.2** | **5** | **R.IVEAFRFAYAK.R** 9300 |
|  | 10828 | **701.3660** | **1400.7175** | **1400.7259** | **-0.0084** | **0** | **87** | **2.7e-007** | **1** | **R.NIDQAVTAALETR.H** |
|  | 11313 | **477.6096** | **1429.8071** | **1429.6660** | **0.1411** | **0** | **23** | **0.71** | **1** | **R.NMTSEFFAAQLR.A** 11322 |
|  | 17904 | **939.4615** | **1876.9084** | **1876.9176** | **-0.0091** | **0** | **69** | **1.2e-005** | **1** | **R.TVIEQQPVLCEVFCR.D** 17903 17905 17924 |
|  | 18376 | **960.4720** | **1918.9295** | **1918.9384** | **-0.0089** | **0** | **124** | **3.7e-011** | **1** | **K.DIQAAGGIVTAEDLNNYR.A** |
|  | 20391 | **703.7077** | **2108.1013** | **2108.1127** | **-0.0114** | **1** | **14** | **3.7** | **1** | **K.GGLSVAVPGEIRGYELAHQR.H** |
|  | 20732 | **717.0189** | **2148.0348** | **2148.0456** | **-0.0109** | **1** | **12** | **6** | **1** | **R.TVIEQQPVLCEVFCRDR.K** |
|  | 30516 | **745.3474** | **4466.0404** | **4465.2192** | **0.8212** | **1** | **0** | **18** | **5** | **R.DALRDGGSAVDAAIAALLCVGLMNAHSMGIGGGLFLTIYNSTTR.K** |

| **102.** | P60033\|CD81_HUMAN    **Mass:** 26476    **Score:** 281    **Queries matched:** 4   **emPAI:** 0.49 |
| --- | --- |
|  | CD81 antigen OS=Homo sapiens GN=CD81 PE=1 SV=1 |

|  | **Query** | **Observed** | **Mr(expt)** | **Mr(calc)** | **Delta** | **Miss** | **Score** | **Expect** | **Rank** | **Peptide** |
| --- | --- | --- | --- | --- | --- | --- | --- | --- | --- | --- |
|  | 21665 | **1127.0182** | **2252.0218** | **2252.0345** | **-0.0127** | **0** | **119** | **1.1e-010** | **1** | **K.QFYDQALQQAVVDDDANNAK.A** 21664 |
|  | 23661 | **1271.6050** | **2541.1954** | **2541.2091** | **-0.0137** | **0** | **95** | **2.1e-008** | **1** | **K.TFHETLDCCGSSTLTALTTSVLK.N** 23660 |

| **103.** | P27487\|DPP4_HUMAN    **Mass:** 88907    **Score:** 277    **Queries matched:** 31   **emPAI:** 0.39 |
| --- | --- |
|  | Dipeptidyl peptidase 4 OS=Homo sapiens GN=DPP4 PE=1 SV=2 |

|  | **Query** | **Observed** | **Mr(expt)** | **Mr(calc)** | **Delta** | **Miss** | **Score** | **Expect** | **Rank** | **Peptide** |
| --- | --- | --- | --- | --- | --- | --- | --- | --- | --- | --- |
|  | 2253 | **428.7454** | **855.4763** | **855.4814** | **-0.0051** | **0** | **34** | **0.062** | **1** | **K.AGAVNPTVK.F** |
|  | 3762 | **483.7412** | **965.4679** | **965.4753** | **-0.0074** | **1** | **31** | **0.13** | **1** | **K.MGFVDNKR.I** |
|  | 4394 | **505.7836** | **1009.5526** | **1009.5603** | **-0.0077** | **1** | **18** | **2.2** | **2** | **K.IMHAINRR.L** 4378 4393 |
|  | 5257 | **540.7663** | **1079.5180** | **1079.5247** | **-0.0067** | **0** | **38** | **0.024** | **1** | **K.IISNEEGYR.H** 5256 |
|  | 5453 | **547.2985** | **1092.5824** | **1091.5546** | **1.0278** | **1** | **11** | **12** | **3** | **K.GMPGGRNLYK.I** 5448 5454 |
|  | 7915 | **623.3360** | **1244.6574** | **1244.6652** | **-0.0078** | **1** | **16** | **4.1** | **1** | **R.KTYTLTDYLK.N** |
|  | 12091 | **739.3378** | **1476.6611** | **1476.6701** | **-0.0090** | **0** | **16** | **3.3** | **1** | **K.VTCLSCELNPER.C** |
|  | 12236 | **743.0601** | **1484.1057** | **1484.7875** | **-0.6818** | **1** | **2** | **59** | **1** | **R.NLYKIQLSDYTK.V** |
|  | 12621 | **755.8237** | **1509.6329** | **1509.6412** | **-0.0083** | **0** | **39** | **0.016** | **1** | **R.WEYYDSVYTER.Y** |
|  | 12764 | **506.6164** | **1516.8273** | **1516.7093** | **0.1180** | **0** | **4** | **47** | **8** | **R.QHIEMSTTGWVGR.F** |
|  | 13696 | **789.3892** | **1576.7639** | **1576.7733** | **-0.0094** | **0** | **69** | **1.4e-005** | **1** | **R.LGTFEVEDQIEAAR.Q** 13695 13702 |
|  | 16555 | **887.4087** | **1772.8028** | **1773.9413** | **-1.1385** | **1** | **5** | **33** | **1** | **K.IEPNLPSYRITWTGK.E** |
|  | 17488 | **615.9598** | **1844.8577** | **1844.8693** | **-0.0116** | **1** | **20** | **1.1** | **1** | **R.HSYTASYDIYDLNKR.Q** |
|  | 19725 | **681.3273** | **2040.9602** | **2040.9694** | **-0.0092** | **0** | **9** | **11** | **1** | **R.FRPSEPHFTLDGNSFYK.I** |
|  | 20869 | **720.7198** | **2159.1375** | **2159.0072** | **0.1304** | **1** | **5** | **25** | **3** | **K.QWRHSYTASYDIYDLNK.R** |
|  | 22322 | **1171.0904** | **2340.1663** | **2340.1750** | **-0.0086** | **0** | **97** | **1.6e-008** | **1** | **R.LNWATYLASTENIIVASFDGR.G** 22323 22324 |
|  | 22753 | **800.9798** | **2399.9176** | **2399.5495** | **0.3681** | **0** | **0** | **57** | **1** | **K.VLLGLLGAAALVTIITVPVVLLNK.G** 22755 |
|  | 27979 | **858.4270** | **3429.6789** | **3429.6953** | **-0.0164** | **1** | **43** | **0.0019** | **1** | **R.AENFKQVEYLLIHGTADDNVHFQQSAQISK.A** 27977 27978 |
|  | 31941 | **1871.7090** | **5612.1051** | **5611.7604** | **0.3448** | **1** | **2** | **5.9** | **2** | **K.AGAVNPTVKFFVVNTDSLSSVTNATSIQITAPASMLIGDHYLCDVTWATQER.I** |

| **104.** | P34931\|HS71L_HUMAN    **Mass:** 70730    **Score:** 276    **Queries matched:** 21   **emPAI:** 0.58 |
| --- | --- |
|  | Heat shock 70 kDa protein 1L OS=Homo sapiens GN=HSPA1L PE=1 SV=2 |

|  | **Query** | **Observed** | **Mr(expt)** | **Mr(calc)** | **Delta** | **Miss** | **Score** | **Expect** | **Rank** | **Peptide** |
| --- | --- | --- | --- | --- | --- | --- | --- | --- | --- | --- |
|  | 1134 | 687.3989 | 686.3916 | 686.3963 | -0.0047 | 0 | 23 | 1.2 | 3 | R.LIGDAAK.N 1136 |
|  | 4487 | 509.2846 | 1016.5547 | 1016.5614 | -0.0068 | 1 | 31 | 0.15 | 1 | K.ITITNDKGR.L 4494 |
|  | 5884 | **563.6105** | **1125.2065** | **1124.5614** | **0.6451** | **0** | **0** | **88** | **7** | **R.LLQDYFNGR.D** |
|  | 7551 | 614.8137 | 1227.6128 | 1227.6207 | -0.0080 | 0 | 44 | 0.0057 | 1 | K.VEIIANDQGNR.T |
|  | 11900 | 733.4047 | 1464.7948 | 1464.8413 | -0.0465 | 1 | 46 | 0.0031 | 1 | K.AKIHDIVLVGGSTR.I 11908 |
|  | 12274 | 744.3496 | 1486.6845 | 1486.6940 | -0.0095 | 0 | 49 | 0.0015 | 1 | R.TTPSYVAFTDTER.L |
|  | 14469 | 542.5991 | 1624.7755 | 1624.9260 | -0.1505 | 1 | 7 | 23 | 1 | R.QATKDAGVIAGLNVLR.I |
|  | 15003 | 830.4456 | 1658.8766 | 1658.8879 | -0.0113 | 0 | 79 | 1.4e-006 | 1 | R.IINEPTAAAIAYGLDK.G 15005 |
|  | 15235 | 838.3636 | 1674.7126 | 1674.7234 | -0.0108 | 0 | 42 | 0.0064 | 1 | K.ATAGDTHLGGEDFDNR.L 15233 15236 |
|  | 22177 | 580.5344 | 2318.1083 | 2319.1416 | -1.0333 | 0 | 6 | 19 | 2 | K.SINPDEAVAYGAAVQAAILMGDK.S 22193 |
|  | 25030 | 1393.6780 | 2785.3414 | 2785.3559 | -0.0145 | 0 | 73 | 2.8e-006 | 1 | K.QTQIFTTYSDNQPGVLIQVYEGER.A |
|  | 28299 | 1176.5861 | 3526.7364 | 3526.7580 | -0.0216 | 1 | 70 | 4.1e-006 | 1 | R.NSTIPTKQTQIFTTYSDNQPGVLIQVYEGER.A 28300 28301 |

| **105.** | O95182\|NDUA7_HUMAN    **Mass:** 12601    **Score:** 274    **Queries matched:** 10   **emPAI:** 1.26 |
| --- | --- |
|  | NADH dehydrogenase [ubiquinone] 1 alpha subcomplex subunit 7 OS=Homo sapiens GN=NDUFA7 PE=1 SV=3 |

|  | **Query** | **Observed** | **Mr(expt)** | **Mr(calc)** | **Delta** | **Miss** | **Score** | **Expect** | **Rank** | **Peptide** |
| --- | --- | --- | --- | --- | --- | --- | --- | --- | --- | --- |
|  | 3229 | **462.2474** | **922.4803** | **922.4872** | **-0.0069** | **1** | **21** | **1.2** | **1** | **R.YQEISKR.T** |
|  | 4545 | **511.7945** | **1021.5744** | **1020.6331** | **0.9412** | **1** | **4** | **63** | **3** | **K.KAVTPAPPIK.R** |
|  | 4899 | **525.3237** | **1048.6328** | **1048.6393** | **-0.0065** | **1** | **16** | **4.1** | **1** | **K.AVTPAPPIKR.W** |
|  | 12165 | **741.3789** | **1480.7431** | **1480.7535** | **-0.0103** | **1** | **50** | **0.0013** | **1** | **R.LRNWASGHDLQGK.L** |
|  | 15811 | **858.4573** | **1714.9001** | **1714.9101** | **-0.0100** | **0** | **92** | **6.6e-008** | **1** | **K.ALVSGKPAESSAVAATEK.K** 15809 15810 |
|  | 17456 | **922.5034** | **1842.9923** | **1843.0051** | **-0.0128** | **1** | **94** | **4.5e-008** | **1** | **K.ALVSGKPAESSAVAATEKK.A** 17455 |
|  | 19352 | **669.2872** | **2004.8399** | **2004.9840** | **-0.1441** | **1** | **1** | **65** | **6** | **K.LPVGPSHKLSNNYYCTR.D** |

| **106.** | P12821\|ACE_HUMAN    **Mass:** 150418   **Score:** 271    **Queries matched:** 31   **emPAI:** 0.21 |
| --- | --- |
|  | Angiotensin-converting enzyme OS=Homo sapiens GN=ACE PE=1 SV=1 |

|  | **Query** | **Observed** | **Mr(expt)** | **Mr(calc)** | **Delta** | **Miss** | **Score** | **Expect** | **Rank** | **Peptide** |
| --- | --- | --- | --- | --- | --- | --- | --- | --- | --- | --- |
|  | 1560 | **403.2280** | **804.4414** | **804.4024** | **0.0390** | **1** | **10** | **23** | **2** | **-.MGAASGRR.G** |
|  | 2409 | **433.7320** | **865.4494** | **865.4545** | **-0.0051** | **0** | **16** | **4.4** | **1** | **R.VFDGSITK.E** 2418 |
|  | 2725 | **444.0429** | **886.0713** | **886.4872** | **-0.4159** | **1** | **3** | **72** | **10** | **K.KVQDLER.A** |
|  | 3084 | **457.7071** | **913.3996** | **912.4011** | **0.9985** | **0** | **23** | **0.96** | **1** | **K.CDIYQSK.E** 3070 |
|  | 4453 | **508.2502** | **1014.4858** | **1013.4778** | **1.0080** | **0** | **5** | **54** | **8** | **R.SEGPLPDSGR.V** |
|  | 5320 | **542.8189** | **1083.6233** | **1083.6288** | **-0.0055** | **0** | **42** | **0.011** | **1** | **R.TLGSANLPLAK.R** |
|  | 6522 | **588.8190** | **1175.6234** | **1175.6298** | **-0.0064** | **0** | **58** | **0.00026** | **1** | **K.YVELINQAAR.L** 6502 |
|  | 6788 | **397.8583** | **1190.5532** | **1190.6077** | **-0.0545** | **1** | **12** | **9.8** | **1** | **K.EAGQRLATAMK.L** |
|  | 6799 | **596.3179** | **1190.6212** | **1189.5914** | **1.0298** | **0** | **5** | **55** | **4** | **K.YQGICPPVTR.N** |
|  | 9326 | **658.8707** | **1315.7268** | **1314.7085** | **1.0183** | **0** | **6** | **38** | **2** | **K.FHIPSSVPYIR.Y** |
|  | 10137 | **681.8134** | **1361.6123** | **1361.6193** | **-0.0070** | **0** | **28** | **0.22** | **1** | **R.YNFDWWYLR.T** |
|  | 11479 | **481.2419** | **1440.7038** | **1439.6827** | **1.0211** | **0** | **3** | **62** | **5** | **R.QQYNALLSNMSR.I** |
|  | 12859 | **762.8419** | **1523.6693** | **1523.6793** | **-0.0100** | **0** | **32** | **0.079** | **1** | **K.ENYNQEWWSLR.L** |
|  | 13660 | **788.3926** | **1574.7707** | **1575.8369** | **-1.0662** | **1** | **5** | **42** | **2** | **K.FDVNQLQNTTIKR.I** 13681 |
|  | 14015 | **533.1882** | **1596.5429** | **1595.7838** | **0.7591** | **1** | **2** | **49** | **7** | **K.RQQYNALLSNMSR.I** 14014 |
|  | 14042 | **799.9226** | **1597.8305** | **1597.8385** | **-0.0080** | **0** | **45** | **0.0035** | **1** | **K.DMVGLDALDAQPLLK.Y** 14043 |
|  | 15559 | **849.3834** | **1696.7523** | **1696.7614** | **-0.0091** | **0** | **7** | **22** | **1** | **R.SMYETPSLEQDLER.L** |
|  | 15766 | **571.5717** | **1711.6932** | **1712.7563** | **-1.0632** | **0** | **(6)** | **23** | **2** | **R.SMYETPSLEQDLER.L** |
|  | 21134 | **1096.0366** | **2190.0587** | **2190.0705** | **-0.0118** | **1** | **97** | **1.8e-008** | **1** | **R.RQEEAALLSQEFAEAWGQK.A** 21135 |
|  | 22103 | **770.3929** | **2308.1568** | **2308.1699** | **-0.0131** | **0** | **11** | **6.1** | **1** | **R.EGANPGFHEAIGDVLALSVSTPK.H** |
|  | 22584 | **791.3778** | **2371.1116** | **2371.1233** | **-0.0117** | **1** | **15** | **2.1** | **1** | **R.VFDGSITKENYNQEWWSLR.L** |
|  | 24562 | **674.0761** | **2692.2751** | **2692.2915** | **-0.0164** | **0** | **26** | **0.15** | **1** | **K.HLHSLNLLSSEGGSDEHDINFLMK.M** |
|  | 27386 | **1093.2532** | **3276.7377** | **3276.7540** | **-0.0163** | **1** | **81** | **3.2e-007** | **1** | **K.VLQAGSSRPWQEVLKDMVGLDALDAQPLLK.Y** |
|  | 27527 | **1108.1624** | **3321.4652** | **3322.5210** | **-1.0557** | **0** | **1** | **29** | **4** | **K.QCTTVNLEDLVVAHHEMGHIQYFMQYK.D** |

| **107.** | P14854\|CX6B1_HUMAN    **Mass:** 10414    **Score:** 269    **Queries matched:** 18   **emPAI:** 6.04 |
| --- | --- |
|  | Cytochrome c oxidase subunit 6B1 OS=Homo sapiens GN=COX6B1 PE=1 SV=2 |

|  | **Query** | **Observed** | **Mr(expt)** | **Mr(calc)** | **Delta** | **Miss** | **Score** | **Expect** | **Rank** | **Peptide** |
| --- | --- | --- | --- | --- | --- | --- | --- | --- | --- | --- |
|  | 3597 | **477.7235** | **953.4325** | **952.4470** | **0.9855** | **1** | **3** | **66** | **7** | **R.CQKAMTAK.G** |
|  | 7208 | **606.3098** | **1210.6049** | **1210.5573** | **0.0476** | **1** | **9** | **18** | **6** | **-.MAEDMETKIK.N** |
|  | 7226 | **404.8845** | **1211.6316** | **1210.5573** | **1.0743** | **1** | **(9)** | **19** | **5** | **-.MAEDMETKIK.N** |
|  | 11969 | **735.3232** | **1468.6318** | **1468.6405** | **-0.0087** | **0** | **42** | **0.0073** | **1** | **K.GGDISVCEWYQR.V** 11967 11970 |
|  | 13314 | **776.8367** | **1551.6589** | **1551.6677** | **-0.0088** | **0** | **56** | **0.00027** | **1** | **R.NCWQNYLDFHR.C** 13315 13336 |
|  | 16627 | **889.9270** | **1777.8394** | **1777.8496** | **-0.0101** | **1** | **29** | **0.12** | **1** | **K.TAPFDSRFPNQNQTR.N** 16623 16625 16626 |
|  | 21815 | **1135.5068** | **2268.9991** | **2269.0110** | **-0.0119** | **0** | **99** | **1e-008** | **1** | **R.VYQSLCPTSWVTDWDEQR.A** 21814 21822 |
|  | 23647 | 847.0646 | 2538.1719 | 2537.1407 | 1.0312 | 1 | 1 | 62 | 3 | R.FPNQNQTRNCWQNYLDFHR.C |
|  | 26406 | **1019.8014** | **3056.3823** | **3056.3975** | **-0.0151** | **1** | **27** | **0.094** | **1** | **R.VYQSLCPTSWVTDWDEQRAEGTFPGK.I** |

| **108.** | P50440\|GATM_HUMAN    **Mass:** 48938    **Score:** 269    **Queries matched:** 19   **emPAI:** 0.55 |
| --- | --- |
|  | Glycine amidinotransferase, mitochondrial OS=Homo sapiens GN=GATM PE=1 SV=1 |

|  | **Query** | **Observed** | **Mr(expt)** | **Mr(calc)** | **Delta** | **Miss** | **Score** | **Expect** | **Rank** | **Peptide** |
| --- | --- | --- | --- | --- | --- | --- | --- | --- | --- | --- |
|  | 2492 | 436.2219 | 870.4293 | 870.4348 | -0.0055 | 0 | 10 | 17 | 2 | R.HLAPDYR.V |
|  | 7538 | **409.8881** | **1226.6425** | **1226.7710** | **-0.1285** | **1** | **10** | **14** | **8** | **K.LGITTIKVNIR.N** |
|  | 13645 | **787.8925** | **1573.7705** | **1573.7810** | **-0.0105** | **0** | **32** | **0.088** | **1** | **R.AENACVPPFTIEVK.A** 13643 13644 13646 |
|  | 15407 | **562.9549** | **1685.8429** | **1686.7559** | **-0.9131** | **0** | **4** | **50** | **3** | **K.TPDFESTGLYSAMPR.D** |
|  | 18893 | **654.9766** | **1961.9080** | **1961.9193** | **-0.0113** | **1** | **18** | **1.5** | **1** | **K.YKTPDFESTGLYSAMPR.D** |
|  | 18949 | **657.1148** | **1968.3226** | **1968.0429** | **0.2797** | **1** | **1** | **48** | **6** | **K.TEGVTVRRPDPIDWSLK.Y** |
|  | 19711 | **1020.5505** | **2039.0865** | **2038.0921** | **0.9944** | **0** | **29** | **0.1** | **1** | **R.DILIVVGNEIIEAPMAWR.S** 19701 |
|  | 19974 | **1032.9668** | **2063.9190** | **2063.9299** | **-0.0109** | **0** | **26** | **0.23** | **1** | **K.FVTTEFEPCFDAADFIR.A** 19973 |
|  | 22530 | **1183.0403** | **2364.0660** | **2363.0740** | **0.9920** | **0** | **84** | **2.5e-007** | **1** | **K.DCPVSSYNEWDPLEEVIVGR.A** 22517 |
|  | 24397 | **891.7929** | **2672.3569** | **2672.3672** | **-0.0103** | **0** | **5** | **21** | **1** | **K.AGWTIITPPTPIIPDDHPLWMSSK.W** 24396 |
|  | 26507 | **1026.4760** | **3076.4060** | **3076.4236** | **-0.0176** | **0** | **82** | **3.1e-007** | **1** | **K.WTTAPKPTMADELYNQDYPIHSVEDR.H** 26506 |

| **109.** | P24539\|AT5F1_HUMAN    **Mass:** 28947    **Score:** 267    **Queries matched:** 20   **emPAI:** 0.63 |
| --- | --- |
|  | ATP synthase subunit b, mitochondrial OS=Homo sapiens GN=ATP5F1 PE=1 SV=2 |

|  | **Query** | **Observed** | **Mr(expt)** | **Mr(calc)** | **Delta** | **Miss** | **Score** | **Expect** | **Rank** | **Peptide** |
| --- | --- | --- | --- | --- | --- | --- | --- | --- | --- | --- |
|  | 2748 | **445.2175** | **888.4204** | **887.4535** | **0.9670** | **1** | **2** | **1.2e+002** | **1** | **K.KAQAQPVM.-** |
|  | 4980 | **529.3051** | **1056.5955** | **1056.6040** | **-0.0084** | **1** | **36** | **0.043** | **1** | **K.SQQALVQKR.H** 4964 4979 4981 |
|  | 5227 | **539.2737** | **1076.5328** | **1076.5403** | **-0.0075** | **0** | **21** | **1.4** | **1** | **R.HYLFDVQR.N** 5228 |
|  | 7655 | **617.3075** | **1232.6004** | **1232.6414** | **-0.0410** | **1** | **5** | **53** | **1** | **K.RHYLFDVQR.N** |
|  | 8265 | **631.3580** | **1260.7015** | **1260.6747** | **0.0268** | **1** | **2** | **93** | **1** | **K.ETIAKCIADLK.L** |
|  | 10514 | **692.3604** | **1382.7061** | **1382.7154** | **-0.0092** | **0** | **56** | **0.00032** | **1** | **K.HVVQSISTQQEK.E** 10517 10518 |
|  | 15310 | **560.3451** | **1678.0134** | **1677.8079** | **0.2055** | **1** | **2** | **60** | **7** | **R.LDYHISVQNMMRR.K** |
|  | 15311 | **560.3553** | **1678.0440** | **1678.8460** | **-0.8020** | **1** | **4** | **36** | **4** | **R.NNIAMALEVTYRER.L** |
|  | 16725 | **596.3195** | **1785.9367** | **1785.0182** | **0.9185** | **1** | **2** | **63** | **10** | **-.MLSRVVLSAAATAAPSLK.N** |
|  | 18475 | **963.5120** | **1925.0094** | **1925.0218** | **-0.0124** | **1** | **88** | **1.4e-007** | **1** | **K.HVVQSISTQQEKETIAK.C** 18473 18474 18476 |
|  | 24574 | **674.6005** | **2694.3727** | **2693.5228** | **0.8499** | **1** | **1** | **51** | **2** | **R.VVLSAAATAAPSLKNAAFLGPGVLQATR.T** |

| **110.** | Q9HAW9\|UD18_HUMAN    **Mass:** 60444    **Score:** 266    **Queries matched:** 33   **emPAI:** 1.05 |
| --- | --- |
|  | UDP-glucuronosyltransferase 1-8 OS=Homo sapiens GN=UGT1A8 PE=1 SV=1 |

|  | **Query** | **Observed** | **Mr(expt)** | **Mr(calc)** | **Delta** | **Miss** | **Score** | **Expect** | **Rank** | **Peptide** |
| --- | --- | --- | --- | --- | --- | --- | --- | --- | --- | --- |
|  | 1086 | 659.3677 | 658.3605 | 658.3650 | -0.0045 | 0 | 25 | 0.78 | 1 | K.AVINDK.S |
|  | 1259 | 765.1535 | 764.1462 | 763.4480 | 0.6983 | 0 | 9 | 17 | 1 | K.LVEYLK.E 1258 |
|  | 2617 | **440.7277** | **879.4408** | **878.4610** | **0.9798** | **1** | **20** | **1.7** | **1** | **R.SLFNDRK.L** |
|  | 2796 | 447.3454 | 892.6762 | 891.5429 | 1.1333 | 1 | 2 | 80 | 7 | R.KLVEYLK.E |
|  | 3687 | 960.5128 | 959.5055 | 959.5110 | -0.0055 | 0 | 41 | 0.014 | 1 | K.AMAIADALGK.I |
|  | 4770 | 520.7600 | 1039.5055 | 1039.5120 | -0.0066 | 1 | 39 | 0.017 | 1 | K.SYKENIMR.L 4771 |
|  | 5402 | 545.3372 | 1088.6598 | 1087.6059 | 1.0538 | 1 | 8 | 30 | 3 | K.KAMAIADALGK.I |
|  | 9788 | 671.8537 | 1341.6928 | 1341.7003 | -0.0074 | 0 | 47 | 0.0026 | 1 | R.ILLGFSDAMTFK.E 9789 |
|  | 9791 | 448.2527 | 1341.7364 | 1342.7027 | -0.9663 | 1 | 11 | 9.9 | 2 | K.ENIMRLSSLHK.D |
|  | 13477 | 782.3588 | 1562.7031 | 1562.7100 | -0.0069 | 0 | 25 | 0.43 | 1 | K.TYSTSYTLEDLDR.E |
|  | 15287 | 839.4249 | 1676.8353 | 1676.8457 | -0.0104 | 0 | 30 | 0.13 | 1 | K.WLPQNDLLGHPMTR.A 15288 15289 |
|  | 17437 | **615.0034** | **1841.9883** | **1840.8566** | **1.1316** | **1** | **1** | **92** | **7** | **R.EFMDFADAQWKAQVR.S** |
|  | 18806 | 652.0336 | 1953.0789 | 1953.0870 | -0.0081 | 1 | 48 | 0.0013 | 1 | K.AMAIADALGKIPQTVLWR.Y 18804 18807 |
|  | 18958 | 493.2440 | 1968.9467 | 1969.0819 | -0.1351 | 1 | (7) | 19 | 1 | K.AMAIADALGKIPQTVLWR.Y |
|  | 21226 | 735.3732 | 2203.0977 | 2203.1042 | -0.0065 | 0 | 64 | 3.7e-005 | 1 | K.GAGVTLNVLEMTSEDLENALK.A |
|  | 21382 | 740.5198 | 2218.5377 | 2219.0991 | -0.5614 | 0 | (3) | 28 | 5 | K.GAGVTLNVLEMTSEDLENALK.A 21379 |
|  | 22175 | 773.4023 | 2317.1850 | 2317.1929 | -0.0079 | 0 | 29 | 0.1 | 1 | K.DRPVEPLDLAVFWVEFVMR.H |
|  | 25357 | 948.8234 | 2843.4485 | 2843.4586 | -0.0101 | 1 | 63 | 3.1e-005 | 1 | K.GAGVTLNVLEMTSEDLENALKAVINDK.S 25358 |
|  | 29241 | 957.1843 | 3824.7080 | 3823.7289 | 0.9791 | 0 | 28 | 0.046 | 1 | R.AFITHAGSHGVYESICNGVPMVMMPLFGDQMDNAK.R 29234 29240 |
|  | 29624 | 996.2063 | 3980.7961 | 3979.8300 | 0.9661 | 1 | 31 | 0.022 | 1 | R.AFITHAGSHGVYESICNGVPMVMMPLFGDQMDNAKR.M 29622 29623 |

|  | **Proteins matching a subset of these peptides:** |
| --- | --- |

|  | Q9HAW8\|UD110_HUMAN    **Mass:** 60568    **Score:** 243    **Queries matched:** 29 |
| --- | --- |
|  | UDP-glucuronosyltransferase 1-10 OS=Homo sapiens GN=UGT1A10 PE=1 SV=1 |

| **111.** | P21397\|AOFA_HUMAN    **Mass:** 60157    **Score:** 263    **Queries matched:** 37   **emPAI:** 0.94 |
| --- | --- |
|  | Amine oxidase [flavin-containing] A OS=Homo sapiens GN=MAOA PE=1 SV=1 |

|  | **Query** | **Observed** | **Mr(expt)** | **Mr(calc)** | **Delta** | **Miss** | **Score** | **Expect** | **Rank** | **Peptide** |
| --- | --- | --- | --- | --- | --- | --- | --- | --- | --- | --- |
|  | 1308 | **395.2123** | **788.4101** | **788.4908** | **-0.0808** | **1** | **12** | **16** | **3** | **K.YKLLPR.S** 1339 1343 |
|  | 1841 | 415.2008 | 828.3870 | 828.4705 | -0.0835 | 0 | 4 | 80 | 9 | R.EVLNGLGK.V |
|  | 2095 | **423.2104** | **844.4063** | **843.4888** | **0.9175** | **0** | **18** | **3.4** | **3** | **R.LPMGAVIK.C** |
|  | 2817 | **448.7283** | **895.4420** | **895.4473** | **-0.0053** | **0** | **30** | **0.16** | **1** | **K.ICELYAK.V** |
|  | 3048 | 456.6940 | 911.3734 | 910.3387 | 1.0347 | 0 | 13 | 7.4 | 3 | K.CMMYYK.E |
|  | 4567 | **512.7747** | **1023.5348** | **1023.5423** | **-0.0075** | **1** | **27** | **0.31** | **1** | **K.KICELYAK.V** 4568 4569 |
|  | 5837 | **561.2955** | **1120.5765** | **1121.5466** | **-0.9700** | **0** | **73** | **7.4e-006** | **1** | **K.FVGGSGQVSER.I** 5856 |
|  | 5947 | **566.3046** | **1130.5946** | **1130.6005** | **-0.0060** | **0** | **40** | **0.016** | **1** | **R.IMDLLGDQVK.L** |
|  | 6663 | **593.3077** | **1184.6008** | **1184.6077** | **-0.0069** | **0** | **46** | **0.0042** | **1** | **R.IFFAGTETATK.W** |
|  | 7152 | **604.8002** | **1207.5859** | **1206.5993** | **0.9866** | **0** | **9** | **19** | **1** | **R.IFSVTNGGQER.K** |
|  | 8025 | 417.5519 | 1249.6338 | 1249.6415 | -0.0077 | 1 | 48 | 0.0023 | 1 | R.KFVGGSGQVSER.I 8013 8024 8033 |
|  | 8620 | **427.5725** | **1279.6955** | **1279.7023** | **-0.0068** | **1** | **7** | **27** | **2** | **R.LSKELGIETYK.V** |
|  | 8709 | **429.2270** | **1284.6591** | **1285.7242** | **-1.0650** | **1** | **4** | **62** | **6** | **R.EVLNGLGKVTEK.D** |
|  | 10179 | **455.5837** | **1363.7292** | **1363.7360** | **-0.0068** | **0** | **12** | **8.5** | **1** | **K.IHFRPELPAER.N** |
|  | 13107 | 771.3334 | 1540.6522 | 1540.6616 | -0.0094 | 0 | 70 | 1.2e-005 | 1 | K.WSGYMEGAVEAGER.A 13095 |
|  | 13910 | **795.4351** | **1588.8557** | **1587.6560** | **1.1997** | **1** | **2** | **80** | **3** | **K.CMMYYKEAFWK.K** |
|  | 14660 | 819.4307 | 1636.8468 | 1635.8468 | 1.0000 | 1 | 4 | 51 | 3 | K.ELGIETYKVNVSER.L |
|  | 15031 | **831.4725** | **1660.9304** | **1660.9400** | **-0.0096** | **0** | **39** | **0.014** | **1** | **K.LLTEYGVSVLVLEAR.D** |
|  | 15419 | **844.4253** | **1686.8361** | **1686.8465** | **-0.0103** | **1** | **1** | **86** | **4** | **K.VTEKDIWVQEPESK.D** |
|  | 17349 | **612.6438** | **1834.9096** | **1834.9213** | **-0.0118** | **0** | **6** | **28** | **1** | **K.VLGSQEALHPVHYEEK.N** |
|  | 21127 | **730.6741** | **2189.0006** | **2189.0138** | **-0.0132** | **0** | **44** | **0.0034** | **1** | **R.NEHVDYVDVGGAYVGPTQNR.I** 21126 |
|  | 21650 | **563.5494** | **2250.1684** | **2250.1315** | **0.0370** | **1** | **3** | **35** | **6** | **K.FVGGSGQVSERIMDLLGDQVK.L** |
|  | 25249 | **942.1224** | **2823.3455** | **2823.3576** | **-0.0121** | **1** | **78** | **8.8e-007** | **1** | **R.TYTIRNEHVDYVDVGGAYVGPTQNR.I** |
|  | 25255 | **565.8729** | **2824.3279** | **2823.6150** | **0.7129** | **1** | **3** | **26** | **5** | **R.NLPSVSGLLKIIGFSTSVTALGFVLYK.Y** |
|  | 25525 | **962.2511** | **2883.7315** | **2884.4753** | **-0.7438** | **1** | **0** | **39** | **1** | **M.ENQEKASIAGHMFDVVVIGGGISGLSAAK.L** |
|  | 26251 | **758.6734** | **3030.6645** | **3031.5107** | **-0.8462** | **1** | **0** | **43** | **2** | **-.MENQEKASIAGHMFDVVVIGGGISGLSAAK.L** |
|  | 27617 | **669.3184** | **3341.5557** | **3341.5735** | **-0.0178** | **0** | **13** | **2.1** | **1** | **K.LNHPVTHVDQSSDNIIIETLNHEHYECK.Y** |

| **112.** | P02730\|B3AT_HUMAN    **Mass:** 102013   **Score:** 261    **Queries matched:** 30   **emPAI:** 0.38 |
| --- | --- |
|  | Band 3 anion transport protein OS=Homo sapiens GN=SLC4A1 PE=1 SV=3 |

|  | **Query** | **Observed** | **Mr(expt)** | **Mr(calc)** | **Delta** | **Miss** | **Score** | **Expect** | **Rank** | **Peptide** |
| --- | --- | --- | --- | --- | --- | --- | --- | --- | --- | --- |
|  | 3803 | **485.4900** | **968.9654** | **969.6375** | **-0.6721** | **0** | **6** | **27** | **4** | **R.VLLPLIFR.N** |
|  | 4941 | **527.7492** | **1053.4839** | **1053.4913** | **-0.0074** | **0** | **40** | **0.017** | **1** | **R.IDAYMAQSR.G** |
|  | 6926 | **599.3837** | **1196.7529** | **1196.7492** | **0.0037** | **1** | **3** | **68** | **5** | **R.EELLRALLLK.H** |
|  | 9544 | **664.3605** | **1326.7065** | **1327.6918** | **-0.9853** | **0** | **3** | **75** | **1** | **R.SVTHANALTVMGK.A** |
|  | 11909 | **489.2753** | **1464.8040** | **1465.8140** | **-1.0101** | **0** | **12** | **8.5** | **1** | **K.IPPDSEATLVLVGR.A** 11894 11905 |
|  | 12005 | 491.5634 | 1471.6685 | 1471.7242 | -0.0556 | 1 | 5 | 40 | 8 | R.VFRIDAYMAQSR.G |
|  | 12328 | **745.8998** | **1489.7850** | **1489.7929** | **-0.0079** | **0** | **66** | **3.6e-005** | **1** | **R.ADFLEQPVLGFVR.L** 12301 12329 |
|  | 14115 | **802.8785** | **1603.7425** | **1603.7518** | **-0.0093** | **0** | **45** | **0.0042** | **1** | **R.YQSSPAKPDSSFYK.G** 14116 |
|  | 16412 | **587.9553** | **1760.8440** | **1759.8529** | **0.9910** | **1** | **7** | **22** | **2** | **R.RYQSSPAKPDSSFYK.G** 16406 |
|  | 17892 | **939.0170** | **1876.0195** | **1876.0305** | **-0.0110** | **0** | **54** | **0.0004** | **1** | **R.LQEAAELEAVELPVPIR.F** 17893 17899 |
|  | 19737 | **511.2808** | **2041.0942** | **2041.1069** | **-0.0127** | **0** | **82** | **6.1e-007** | **1** | **K.HSHAGELEALGGVKPAVLTR.S** |
|  | 21542 | **745.7123** | **2234.1152** | **2234.1252** | **-0.0101** | **1** | **28** | **0.14** | **1** | **K.VYVELQELVMDEKNQELR.W** |
|  | 21580 | **747.3889** | **2239.1449** | **2240.2578** | **-1.1128** | **0** | **2** | **48** | **3** | **R.MHLFTGIQIICLAVLWVVK.S** |
|  | 21939 | **762.7435** | **2285.2088** | **2285.2208** | **-0.0121** | **0** | **45** | **0.0023** | **1** | **R.FLFVLLGPEAPHIDYTQLGR.A** |
|  | 23214 | **823.4145** | **2467.2218** | **2466.2503** | **0.9715** | **0** | **8** | **13** | **1** | **K.GLDLNGGPDDPLQQTGQLFGGLVR.D** |
|  | 24639 | 677.3545 | 2705.3889 | 2705.5877 | -0.1989 | 1 | 4 | 27 | 2 | K.EQRISGLLVAVLVGLSILMEPILSR.I |
|  | 28782 | **1832.5205** | **3663.0265** | **3661.9187** | **1.1078** | **1** | **5** | **8.8** | **2** | **K.MVKGSGFHLDLLLVVGMGGVAALFGMPWLSATTVR.S** |
|  | 29974 | **1030.7295** | **4118.8889** | **4118.9128** | **-0.0239** | **0** | **29** | **0.029** | **1** | **R.SGDPSQPLLPQHSSLETQLFCEQGDGGTEGHSPSGILEK.I** |
|  | 30013 | **1036.8131** | **4143.2233** | **4143.2408** | **-0.0175** | **1** | **23** | **0.11** | **1** | **R.LQEAAELEAVELPVPIRFLFVLLGPEAPHIDYTQLGR.A** |
|  | 30721 | **657.7637** | **4597.2948** | **4597.4012** | **-0.1064** | **1** | **2** | **10** | **1** | **K.GSGFHLDLLLVVGMGGVAALFGMPWLSATTVRSVTHANALTVMGK.A** |
|  | 31605 | **1794.4332** | **5380.2779** | **5380.8041** | **-0.5262** | **1** | **2** | **5.4** | **5** | **K.IFQDHPLQKTYNYNVLMVPKPQGPLPNTALLSLVLMAGTFFFAMMLR.K** |
|  | 31879 | **1114.3435** | **5566.6812** | **5566.7162** | **-0.0351** | **1** | **6** | **3** | **1** | **R.SGDPSQPLLPQHSSLETQLFCEQGDGGTEGHSPSGILEKIPPDSEATLVLVGR.A** |

| **113.** | Q9HAW7\|UD17_HUMAN    **Mass:** 60578    **Score:** 260    **Queries matched:** 33   **emPAI:** 0.93 |
| --- | --- |
|  | UDP-glucuronosyltransferase 1-7 OS=Homo sapiens GN=UGT1A7 PE=1 SV=2 |

|  | **Query** | **Observed** | **Mr(expt)** | **Mr(calc)** | **Delta** | **Miss** | **Score** | **Expect** | **Rank** | **Peptide** |
| --- | --- | --- | --- | --- | --- | --- | --- | --- | --- | --- |
|  | 1086 | 659.3677 | 658.3605 | 658.3650 | -0.0045 | 0 | 25 | 0.78 | 1 | K.AVINDK.S |
|  | 1259 | 765.1535 | 764.1462 | 763.4480 | 0.6983 | 0 | 9 | 17 | 1 | K.LVEYLK.E 1258 |
|  | 2617 | 440.7277 | 879.4408 | 878.4610 | 0.9798 | 1 | 20 | 1.7 | 1 | R.SLFNDRK.L |
|  | 2796 | 447.3454 | 892.6762 | 891.5429 | 1.1333 | 1 | 2 | 80 | 7 | R.KLVEYLK.E |
|  | 3687 | 960.5128 | 959.5055 | 959.5110 | -0.0055 | 0 | 41 | 0.014 | 1 | K.AMAIADALGK.I |
|  | 4770 | 520.7600 | 1039.5055 | 1039.5120 | -0.0066 | 1 | 39 | 0.017 | 1 | K.SYKENIMR.L 4771 |
|  | 5402 | 545.3372 | 1088.6598 | 1087.6059 | 1.0538 | 1 | 8 | 30 | 3 | K.KAMAIADALGK.I |
|  | 9788 | 671.8537 | 1341.6928 | 1341.7003 | -0.0074 | 0 | 47 | 0.0026 | 1 | R.LLLGFSDAMTFK.E 9789 |
|  | 9791 | 448.2527 | 1341.7364 | 1342.7027 | -0.9663 | 1 | 11 | 9.9 | 2 | K.ENIMRLSSLHK.D |
|  | 15287 | 839.4249 | 1676.8353 | 1676.8457 | -0.0104 | 0 | 30 | 0.13 | 1 | K.WLPQNDLLGHPMTR.A 15288 15289 |
|  | 18411 | 961.4955 | 1920.9764 | 1920.9880 | -0.0116 | 0 | 9 | 12 | 1 | R.GHEVVVVMPEVSWQLGR.S |
|  | 18806 | 652.0336 | 1953.0789 | 1953.0870 | -0.0081 | 1 | 48 | 0.0013 | 1 | K.AMAIADALGKIPQTVLWR.Y 18804 18807 |
|  | 18958 | 493.2440 | 1968.9467 | 1969.0819 | -0.1351 | 1 | (7) | 19 | 1 | K.AMAIADALGKIPQTVLWR.Y |
|  | 21226 | 735.3732 | 2203.0977 | 2203.1042 | -0.0065 | 0 | 64 | 3.7e-005 | 1 | K.GAGVTLNVLEMTSEDLENALK.A |
|  | 21382 | 740.5198 | 2218.5377 | 2219.0991 | -0.5614 | 0 | (3) | 28 | 5 | K.GAGVTLNVLEMTSEDLENALK.A 21379 |
|  | 22175 | 773.4023 | 2317.1850 | 2317.1929 | -0.0079 | 0 | 29 | 0.1 | 1 | K.DRPVEPLDLAVFWVEFVMR.H |
|  | 22590 | **594.0574** | **2372.2004** | **2373.1021** | **-0.9018** | **0** | **8** | **12** | **5** | **K.ESCFDAVFLDPFDACGLIVAK.Y** |
|  | 25357 | 948.8234 | 2843.4485 | 2843.4586 | -0.0101 | 1 | 63 | 3.1e-005 | 1 | K.GAGVTLNVLEMTSEDLENALKAVINDK.S 25358 |
|  | 29241 | 957.1843 | 3824.7080 | 3823.7289 | 0.9791 | 0 | 28 | 0.046 | 1 | R.AFITHAGSHGVYESICNGVPMVMMPLFGDQMDNAK.R 29234 29240 |
|  | 29624 | 996.2063 | 3980.7961 | 3979.8300 | 0.9661 | 1 | 31 | 0.022 | 1 | R.AFITHAGSHGVYESICNGVPMVMMPLFGDQMDNAKR.M 29622 29623 |

| **114.** | P12235\|ADT1_HUMAN    **Mass:** 33271    **Score:** 256    **Queries matched:** 35   **emPAI:** 2.25 |
| --- | --- |
|  | ADP/ATP translocase 1 OS=Homo sapiens GN=SLC25A4 PE=1 SV=4 |

|  | **Query** | **Observed** | **Mr(expt)** | **Mr(calc)** | **Delta** | **Miss** | **Score** | **Expect** | **Rank** | **Peptide** |
| --- | --- | --- | --- | --- | --- | --- | --- | --- | --- | --- |
|  | 2024 | 420.6771 | 839.3397 | 839.3452 | -0.0055 | 0 | 25 | 0.43 | 1 | R.MMMQSGR.K 2026 2042 |
|  | 2254 | 428.7456 | 855.4767 | 855.4814 | -0.0047 | 0 | 36 | 0.043 | 1 | K.TAVAPIER.V 2274 |
|  | 2255 | 428.7509 | 855.4873 | 855.4926 | -0.0053 | 0 | 31 | 0.12 | 1 | R.GNLANVIR.Y |
|  | 2267 | 429.0874 | 856.1602 | 855.3401 | 0.8201 | 0 | (14) | 4.8 | 1 | R.MMMQSGR.K 2269 |
|  | 2271 | 429.2248 | 856.4350 | 855.3401 | 1.0949 | 0 | (13) | 9 | 1 | R.MMMQSGR.K |
|  | 2272 | 429.2307 | 856.4469 | 855.3401 | 1.1068 | 0 | (13) | 9.6 | 3 | R.MMMQSGR.K |
|  | 3325 | 465.7691 | 929.5236 | 929.5294 | -0.0058 | 1 | 14 | 8.1 | 3 | R.TRLAADVGK.G |
|  | 3784 | 484.7235 | 967.4325 | 967.4401 | -0.0077 | 1 | 29 | 0.18 | 1 | R.MMMQSGRK.G |
|  | 4190 | 498.7267 | 995.4387 | 995.4463 | -0.0075 | 1 | 22 | 1 | 1 | R.RMMMQSGR.K 4193 |
|  | 4318 | 502.7875 | 1003.5604 | 1003.5451 | 0.0153 | 0 | 8 | 27 | 8 | K.QLFLGGVDR.H |
|  | 5311 | 542.5944 | 1083.1743 | 1082.6448 | 0.5295 | 1 | 9 | 11 | 3 | K.TAVAPIERVK.L |
|  | 6005 | 568.8392 | 1135.6639 | 1135.6713 | -0.0074 | 0 | 25 | 0.49 | 1 | K.LLLQVQHASK.Q 6000 |
|  | 10149 | 681.8502 | 1361.6859 | 1362.8347 | -1.1488 | 1 | 66 | 3.3e-005 | 1 | R.VKLLLQVQHASK.Q 10159 10170 10171 |
|  | 14774 | 823.3579 | 1644.7013 | 1643.7072 | 0.9940 | 0 | 3 | 57 | 5 | K.GADIMYTGTVDCWR.K 14763 |
|  | 16130 | 870.4696 | 1738.9246 | 1738.9328 | -0.0081 | 1 | 38 | 0.017 | 1 | R.GMGGAFVLVLYDEIKK.Y |
|  | 16542 | 886.9032 | 1771.7918 | 1771.8022 | -0.0103 | 1 | 56 | 0.00027 | 1 | R.KGADIMYTGTVDCWR.K 16540 16541 |
|  | 25112 | 932.7831 | 2795.3274 | 2795.3377 | -0.0103 | 0 | 51 | 0.00045 | 1 | R.YFAGNLASGGAAGATSLCFVYPLDFAR.T 25108 25109 25110 25113 25114 25115 |

| **115.** | P62873\|GBB1_HUMAN    **Mass:** 38151    **Score:** 256    **Queries matched:** 28   **emPAI:** 0.76 |
| --- | --- |
|  | Guanine nucleotide-binding protein G(I)/G(S)/G(T) subunit beta-1 OS=Homo sapiens GN=GNB1 PE=1 SV=3 |

|  | **Query** | **Observed** | **Mr(expt)** | **Mr(calc)** | **Delta** | **Miss** | **Score** | **Expect** | **Rank** | **Peptide** |
| --- | --- | --- | --- | --- | --- | --- | --- | --- | --- | --- |
|  | 1563 | **403.2297** | **804.4449** | **804.4970** | **-0.0521** | **0** | **12** | **14** | **2** | **K.VHAIPLR.S** 1546 1549 |
|  | 4394 | 505.7836 | 1009.5526 | 1008.5101 | 1.0425 | 0 | 20 | 1.6 | 1 | R.AGVLAGHDNR.V 4371 4377 4378 4379 4393 |
|  | 4485 | **509.2794** | **1016.5442** | **1016.5502** | **-0.0060** | **0** | **7** | **40** | **2** | **R.LLVSASQDGK.L** |
|  | 7484 | **613.2940** | **1224.5733** | **1224.5809** | **-0.0075** | **0** | **70** | **1.5e-005** | **1** | **R.LFVSGACDASAK.L** 7483 |
|  | 9662 | **668.3080** | **1334.6015** | **1335.6030** | **-1.0015** | **0** | **8** | **24** | **8** | **K.IYAMHWGTDSR.L** |
|  | 9937 | **451.0399** | **1350.0979** | **1350.6752** | **-0.5773** | **1** | **9** | **12** | **5** | **K.ADRAGVLAGHDNR.V** |
|  | 9977 | **451.7476** | **1352.2209** | **1351.5979** | **0.6230** | **0** | **(4)** | **37** | **2** | **K.IYAMHWGTDSR.L** |
|  | 9986 | **677.3525** | **1352.6905** | **1352.6976** | **-0.0071** | **0** | **19** | **1.7** | **1** | **K.LIIWDSYTTNK.V** |
|  | 12846 | **762.3356** | **1522.6566** | **1522.6657** | **-0.0091** | **0** | **58** | **0.00019** | **1** | **R.ELAGHTGYLSCCR.F** |
|  | 17740 | **622.3517** | **1864.0332** | **1864.8672** | **-0.8340** | **1** | **2** | **68** | **5** | **R.VSRELAGHTGYLSCCR.F** |
|  | 19443 | **1008.4898** | **2014.9649** | **2014.9742** | **-0.0092** | **0** | **97** | **1.7e-008** | **1** | **K.ACADATLSQITNNIDPVGR.I** |
|  | 22847 | **1208.5555** | **2415.0965** | **2415.1087** | **-0.0122** | **0** | **84** | **2.9e-007** | **1** | **R.VSCLGVTDDGMAVATGSWDSFLK.I** 22848 |
|  | 22965 | **487.4543** | **2432.2352** | **2431.1036** | **1.1316** | **0** | **(5)** | **24** | **3** | **R.VSCLGVTDDGMAVATGSWDSFLK.I** |
|  | 23219 | **823.7302** | **2468.1687** | **2468.1794** | **-0.0108** | **1** | **20** | **0.73** | **1** | **R.LLLAGYDDFNCNVWDALKADR.A** |
|  | 23834 | **854.4025** | **2560.1856** | **2559.2533** | **0.9322** | **1** | **2** | **40** | **1** | **K.ACADATLSQITNNIDPVGRIQMR.T** |
|  | 26243 | 758.6727 | 3030.6618 | 3031.4015 | -0.7397 | 1 | 1 | 34 | 4 | R.ADQELMTYSHDNIICGITSVSFSKSGR.L |
|  | 27707 | **1120.9186** | **3359.7339** | **3359.6166** | **0.1173** | **1** | **1** | **30** | **5** | **R.LFDLRADQELMTYSHDNIICGITSVSFSK.S** 27708 |
|  | 27943 | **856.4415** | **3421.7368** | **3421.6031** | **0.1336** | **1** | **1** | **36** | **4** | **R.AGVLAGHDNRVSCLGVTDDGMAVATGSWDSFLK.I** |

| **116.** | P60660\|MYL6_HUMAN    **Mass:** 17090    **Score:** 255    **Queries matched:** 16   **emPAI:** 3.18 |
| --- | --- |
|  | Myosin light polypeptide 6 OS=Homo sapiens GN=MYL6 PE=1 SV=2 |

|  | **Query** | **Observed** | **Mr(expt)** | **Mr(calc)** | **Delta** | **Miss** | **Score** | **Expect** | **Rank** | **Peptide** |
| --- | --- | --- | --- | --- | --- | --- | --- | --- | --- | --- |
|  | 1011 | **627.8264** | **626.8191** | **626.3752** | **0.4440** | **0** | **34** | **0.033** | **1** | **K.VLGNPK.S** |
|  | 4166 | 497.7301 | 993.4457 | 994.5811 | -1.1354 | 0 | 35 | 0.05 | 1 | R.HVLVTLGEK.M 4182 |
|  | 9756 | **671.3142** | **1340.6139** | **1340.6217** | **-0.0078** | **0** | **61** | **0.00011** | **1** | **K.ILYSQCGDVMR.A** |
|  | 10012 | **677.8652** | **1353.7158** | **1353.7252** | **-0.0094** | **0** | **38** | **0.024** | **1** | **R.ALGQNPTNAEVLK.V** 10033 |
|  | 10074 | **453.5691** | **1357.6856** | **1356.6166** | **1.0690** | **0** | **(4)** | **65** | **4** | **K.ILYSQCGDVMR.A** |
|  | 11308 | **715.8647** | **1429.7149** | **1429.7235** | **-0.0085** | **1** | **64** | **5.2e-005** | **1** | **K.VLGNPKSDEMNVK.V** |
|  | 15907 | **575.2788** | **1722.8146** | **1721.8407** | **0.9739** | **1** | **20** | **1.2** | **1** | **R.VFDKEGNGTVMGAEIR.H** 15908 15909 |
|  | 16716 | **893.9094** | **1785.8042** | **1785.8169** | **-0.0128** | **1** | **86** | **2.7e-007** | **1** | **K.NKDQGTYEDYVEGLR.V** 16717 |
|  | 16873 | **600.9497** | **1799.8271** | **1798.8342** | **0.9929** | **1** | **14** | **4.6** | **1** | **R.TGDGKILYSQCGDVMR.A** |
|  | 18013 | **944.5015** | **1886.9884** | **1886.9964** | **-0.0081** | **0** | **48** | **0.0016** | **1** | **K.VLDFEHFLPMLQTVAK.N** |
|  | 26816 | **1049.1615** | **3144.4627** | **3144.3474** | **0.1152** | **0** | **1** | **42** | **4** | **K.MTEEEVEMLVAGHEDSNGCINYEAFVR.H** |

| **117.** | P45880\|VDAC2_HUMAN    **Mass:** 32060    **Score:** 253    **Queries matched:** 16   **emPAI:** 0.74 |
| --- | --- |
|  | Voltage-dependent anion-selective channel protein 2 OS=Homo sapiens GN=VDAC2 PE=1 SV=2 |

|  | **Query** | **Observed** | **Mr(expt)** | **Mr(calc)** | **Delta** | **Miss** | **Score** | **Expect** | **Rank** | **Peptide** |
| --- | --- | --- | --- | --- | --- | --- | --- | --- | --- | --- |
|  | 3445 | **471.2526** | **940.4906** | **939.4563** | **1.0343** | **0** | **5** | **53** | **3** | **R.NNFAVGYR.T** 3433 3439 |
|  | 8589 | **427.1154** | **1278.3243** | **1278.7336** | **-0.4093** | **1** | **5** | **30** | **5** | **K.GFGFGLVKLDVK.T** |
|  | 8869 | **647.3341** | **1292.6535** | **1292.6612** | **-0.0077** | **0** | **22** | **1** | **1** | **K.YQLDPTASISAK.V** |
|  | 11270 | **714.8496** | **1427.6845** | **1427.6933** | **-0.0087** | **0** | **3** | **67** | **1** | **K.LTFDTTFSPNTGK.K** |
|  | 11491 | **481.2638** | **1440.7695** | **1440.7766** | **-0.0071** | **1** | **21** | **1.1** | **1** | **R.DIFNKGFGFGLVK.L** |
|  | 13380 | **778.8965** | **1555.7784** | **1555.7882** | **-0.0098** | **1** | **56** | **0.00033** | **1** | **K.LTFDTTFSPNTGKK.S** 13381 |
|  | 20332 | 1052.0752 | 2102.1358 | 2102.1484 | -0.0126 | 0 | 68 | 1.4e-005 | 1 | K.VNNSSLIGVGYTQTLRPGVK.L 20330 20331 |
|  | 23520 | **1260.1010** | **2518.1873** | **2518.2010** | **-0.0136** | **0** | **120** | **6.5e-011** | **1** | **K.WNTDNTLGTEIAIEDQICQGLK.L** |
|  | 23574 | **843.3891** | **2527.1455** | **2527.1616** | **-0.0161** | **0** | **43** | **0.0032** | **1** | **R.TGDFQLHTNVNDGTEFGGSIYQK.V** |
|  | 24796 | **913.0820** | **2736.2243** | **2736.2396** | **-0.0154** | **1** | **57** | **0.00012** | **1** | **K.SCSGVEFSTSGSSNTDTGKVTGTLETK.Y** |
|  | 30908 | **950.0516** | **4745.2218** | **4746.1142** | **-0.8924** | **1** | **2** | **11** | **1** | **R.ECINLGCDVDFDFAGPAIHGSAVFGYEGWLAGYQMTFDSAKSK.L** |

| **118.** | P53675\|CLH2_HUMAN    **Mass:** 189020   **Score:** 251    **Queries matched:** 42   **emPAI:** 0.19 |
| --- | --- |
|  | Clathrin heavy chain 2 OS=Homo sapiens GN=CLTCL1 PE=1 SV=2 |

|  | **Query** | **Observed** | **Mr(expt)** | **Mr(calc)** | **Delta** | **Miss** | **Score** | **Expect** | **Rank** | **Peptide** |
| --- | --- | --- | --- | --- | --- | --- | --- | --- | --- | --- |
|  | 874 | **572.9959** | **571.9886** | **572.2918** | **-0.3032** | **0** | **13** | **8.6** | **1** | **R.NPTGGK.L** |
|  | 1401 | **398.2106** | **794.4066** | **795.4967** | **-1.0901** | **0** | **15** | **4.7** | **1** | **M.AQILPVR.F** 1433 |
|  | 2154 | 425.2187 | 848.4228 | 848.4756 | -0.0528 | 1 | 21 | 1.7 | 1 | K.NFLKEAK.L |
|  | 2219 | 853.4179 | 852.4106 | 852.4164 | -0.0057 | 0 | 42 | 0.01 | 1 | K.YIQAACK.T 2217 |
|  | 2710 | 443.2060 | 884.3974 | 884.4028 | -0.0054 | 0 | 34 | 0.068 | 1 | R.AYEFAER.C |
|  | 3748 | 482.8958 | 963.7771 | 964.4978 | -0.7206 | 0 | 5 | 34 | 8 | K.EAINSYIR.G 3753 |
|  | 5009 | 530.2895 | 1058.5645 | 1058.5720 | -0.0075 | 1 | 15 | 5.4 | 2 | K.TGQIKEVER.I 5010 5014 5017 |
|  | 5144 | 535.7750 | 1069.5355 | 1068.5386 | 0.9969 | 0 | 24 | 0.59 | 1 | R.AHIAQLCEK.A |
|  | 5296 | **541.9092** | **1081.8038** | **1081.6608** | **0.1431** | **1** | **1** | **87** | **4** | **K.VAASAPKGILR.T** |
|  | 7711 | 618.8332 | 1235.6518 | 1235.6584 | -0.0065 | 0 | 32 | 0.083 | 1 | K.TLQIFNIEMK.S |
|  | 9699 | 669.3437 | 1336.6728 | 1336.6809 | -0.0081 | 0 | 64 | 5.4e-005 | 1 | R.VVGAMQLYSVDR.K 9683 |
|  | 9978 | 451.7483 | 1352.2230 | 1352.6758 | -0.4529 | 0 | (4) | 33 | 2 | R.VVGAMQLYSVDR.K |
|  | 11052 | 708.3633 | 1414.7120 | 1414.7205 | -0.0085 | 0 | 63 | 6.3e-005 | 1 | K.IVLDNSVFSEHR.N 11066 |
|  | 11893 | 733.3904 | 1464.7663 | 1464.7759 | -0.0096 | 1 | 1 | 94 | 1 | R.VVGAMQLYSVDRK.V |
|  | 11930 | 489.9078 | 1466.7015 | 1466.7803 | -0.0788 | 1 | 3 | 59 | 7 | K.TLQIFNIEMKSK.M |
|  | 12401 | **499.2604** | **1494.7593** | **1494.7752** | **-0.0159** | **0** | **2** | **71** | **10** | **K.TTDPMLALSVYLR.A** |
|  | 13289 | 776.3757 | 1550.7368 | 1550.7464 | -0.0096 | 0 | 62 | 7.3e-005 | 1 | R.GQFSTDELVAEVEK.R |
|  | 13583 | **524.2962** | **1569.8668** | **1569.6664** | **0.2004** | **1** | **6** | **31** | **5** | **R.ICRESSCYNPER.V** |
|  | 14157 | 536.2795 | 1605.8166 | 1606.8871 | -1.0705 | 1 | 1 | 83 | 7 | K.KVGYTPDWIFLLR.G |
|  | 14193 | 536.9269 | 1607.7588 | 1607.8671 | -0.1083 | 1 | 1 | 92 | 1 | R.YIEIYVQKVNPSR.T |
|  | 15534 | 565.7885 | 1694.3437 | 1695.0043 | -0.6606 | 1 | 3 | 33 | 3 | R.NLQNLLILTAIKADR.T |
|  | 15704 | 854.4262 | 1706.8377 | 1706.8475 | -0.0098 | 1 | 89 | 1.3e-007 | 1 | R.GQFSTDELVAEVEKR.N |
|  | 18721 | 974.0072 | 1945.9998 | 1946.0070 | -0.0072 | 0 | 36 | 0.026 | 1 | K.AFMTADLPNELIELLEK.I |
|  | 18901 | **655.3359** | **1962.9858** | **1961.9856** | **1.0003** | **0** | **(0)** | **93** | **7** | **R.HNLVDLAMPYFIQVMR.E** |
|  | 19057 | **495.4786** | **1977.8855** | **1977.9805** | **-0.0950** | **0** | **6** | **27** | **1** | **R.HNLVDLAMPYFIQVMR.E** |
|  | 21083 | **1093.5175** | **2185.0204** | **2186.0711** | **-1.0508** | **0** | **4** | **36** | **1** | **K.VGEQAQVTIIDMSDPMAPIR.R** |
|  | 23047 | **611.8062** | **2443.1957** | **2443.2087** | **-0.0129** | **1** | **2** | **51** | **1** | **R.EKVGEQAQVTIIDMSDPMAPIR.R** |
|  | 23866 | **856.7257** | **2567.1553** | **2567.3265** | **-0.1712** | **1** | **1** | **51** | **1** | **K.VNPSRTPAVIGGLLDVDCSEEVIK.H** |
|  | 24204 | **659.8084** | **2635.2043** | **2634.3152** | **0.8891** | **1** | **2** | **37** | **7** | **K.KAVDVFFPPEAQNDFPVAMQIGAK.H** |
|  | 29013 | **1246.9882** | **3737.9427** | **3738.8743** | **-0.9316** | **1** | **0** | **32** | **10** | **K.VGEQAQVTIIDMSDPMAPIRRPISAESAIMNPASK.V** |
|  | 30565 | **899.2342** | **4491.1349** | **4491.2580** | **-0.1231** | **1** | **1** | **18** | **4** | **K.NNRPAEGLLQTWLLEMNLVHAPQVADAILGNKMFTHYDR.A** |
|  | 30788 | **774.2023** | **4639.1703** | **4639.1862** | **-0.0159** | **1** | **4** | **6.7** | **2** | **K.AHTMAEEVIFWKWVSVNTVALVTETAVYHWSMEGDSQPMK.M** |
|  | 30811 | **1164.5927** | **4654.3415** | **4655.1811** | **-0.8396** | **1** | **(1)** | **15** | **6** | **K.AHTMAEEVIFWKWVSVNTVALVTETAVYHWSMEGDSQPMK.M** |
|  | 31737 | **924.4777** | **5540.8223** | **5539.7565** | **1.0659** | **1** | **0** | **9.5** | **1** | **K.ISPEQGLQFSRMLVQDEEPLANISQIVDIFMENSLIQQCTSFLLDALK.N** |

| **119.** | P27635\|RL10_HUMAN    **Mass:** 25044    **Score:** 249    **Queries matched:** 12   **emPAI:** 0.76 |
| --- | --- |
|  | 60S ribosomal protein L10 OS=Homo sapiens GN=RPL10 PE=1 SV=4 |

|  | **Query** | **Observed** | **Mr(expt)** | **Mr(calc)** | **Delta** | **Miss** | **Score** | **Expect** | **Rank** | **Peptide** |
| --- | --- | --- | --- | --- | --- | --- | --- | --- | --- | --- |
|  | 2227 | **427.4372** | **852.8599** | **852.5181** | **0.3418** | **1** | **5** | **32** | **2** | **R.QKIHISK.K** |
|  | 3964 | **490.7628** | **979.5110** | **979.4215** | **0.0894** | **0** | **23** | **0.75** | **1** | **K.MLSCAGADR.L** 3945 |
|  | 4216 | **499.2638** | **996.5131** | **995.4164** | **1.0967** | **0** | **(12)** | **9.5** | **2** | **K.MLSCAGADR.L** 4202 |
|  | 5763 | **558.3566** | **1114.6986** | **1113.5965** | **1.1021** | **1** | **1** | **1.3e+002** | **5** | **K.RLIPDGCGVK.Y** |
|  | 8050 | 626.8079 | 1251.6013 | 1251.7122 | -0.1109 | 0 | 0 | 1.3e+002 | 4 | R.VHIGQVIMSIR.T |
|  | 13173 | **772.8275** | **1543.6405** | **1543.6501** | **-0.0096** | **0** | **89** | **1.3e-007** | **1** | **K.FNADEFEDMVAEK.R** |
|  | 15599 | **567.5879** | **1699.7420** | **1699.7512** | **-0.0091** | **1** | **33** | **0.056** | **1** | **K.FNADEFEDMVAEKR.L** |
|  | 26534 | **1028.1252** | **3081.3539** | **3081.3695** | **-0.0156** | **0** | **64** | **1.7e-005** | **1** | **K.VDEFPLCGHMVSDEYEQLSSEALEAAR.I** 26535 |
|  | 27393 | **1094.5038** | **3280.4895** | **3280.5016** | **-0.0121** | **1** | **108** | **7.2e-010** | **1** | **K.AKVDEFPLCGHMVSDEYEQLSSEALEAAR.I** |

| **120.** | P02735\|SAA_HUMAN    **Mass:** 13581    **Score:** 249    **Queries matched:** 11   **emPAI:** 1.15 |
| --- | --- |
|  | Serum amyloid A protein OS=Homo sapiens GN=SAA1 PE=1 SV=2 |

|  | **Query** | **Observed** | **Mr(expt)** | **Mr(calc)** | **Delta** | **Miss** | **Score** | **Expect** | **Rank** | **Peptide** |
| --- | --- | --- | --- | --- | --- | --- | --- | --- | --- | --- |
|  | 1226 | **742.3131** | **741.3058** | **741.3116** | **-0.0057** | **0** | **6** | **37** | **1** | **R.AYSDMR.E** |
|  | 4214 | **499.2604** | **996.5062** | **995.4560** | **1.0502** | **0** | **6** | **33** | **3** | **R.EANYIGSDK.Y** |
|  | 13276 | **775.8636** | **1549.7126** | **1549.7202** | **-0.0075** | **0** | **48** | **0.0019** | **1** | **R.SFFSFLGEAFDGAR.D** 13287 |
|  | 14249 | **806.9078** | **1611.8010** | **1611.8117** | **-0.0107** | **1** | **47** | **0.0025** | **1** | **K.RGPGGVWAAEAISDAR.E** 14236 14250 14251 |
|  | 17156 | **456.2537** | **1820.9857** | **1820.0230** | **0.9626** | **0** | **2** | **67** | **9** | **K.LLTGLVFCSLVLGVSSR.S** |
|  | 21011 | **1089.4779** | **2176.9413** | **2176.9562** | **-0.0150** | **0** | **112** | **5.6e-010** | **1** | **R.FFGHGAEDSLADQAANEWGR.S** 21012 |

| **121.** | P05556\|ITB1_HUMAN    **Mass:** 91664    **Score:** 246    **Queries matched:** 21   **emPAI:** 0.43 |
| --- | --- |
|  | Integrin beta-1 OS=Homo sapiens GN=ITGB1 PE=1 SV=2 |

|  | **Query** | **Observed** | **Mr(expt)** | **Mr(calc)** | **Delta** | **Miss** | **Score** | **Expect** | **Rank** | **Peptide** |
| --- | --- | --- | --- | --- | --- | --- | --- | --- | --- | --- |
|  | 4006 | **492.2609** | **982.5072** | **982.5124** | **-0.0052** | **0** | **14** | **5.8** | **1** | **R.IGFGSFVEK.T** |
|  | 5285 | **541.7215** | **1081.4284** | **1081.4355** | **-0.0070** | **0** | **30** | **0.14** | **1** | **R.GICECGVCK.C** |
|  | 5666 | **554.7863** | **1107.5581** | **1106.5608** | **0.9973** | **0** | **15** | **5** | **2** | **R.SGEPQTFTLK.F** |
|  | 7426 | 611.7866 | 1221.5587 | 1221.5666 | -0.0079 | 0 | 32 | 0.096 | 1 | K.WDTGENPIYK.S |
|  | 8349 | **633.7911** | **1265.5676** | **1265.5744** | **-0.0068** | **0** | **34** | **0.055** | **1** | **K.SLGTDLMNEMR.R** |
|  | 9677 | **668.7985** | **1335.5824** | **1334.6071** | **0.9753** | **0** | **8** | **23** | **6** | **R.SNGLICGGNGVCK.C** |
|  | 10403 | **459.5867** | **1375.7384** | **1375.7460** | **-0.0076** | **1** | **8** | **20** | **2** | **R.LRSGEPQTFTLK.F** |
|  | 11455 | **720.3523** | **1438.6900** | **1437.6704** | **1.0196** | **1** | **5** | **37** | **2** | **K.SLGTDLMNEMRR.I** |
|  | 13016 | **768.7638** | **1535.5130** | **1535.5228** | **-0.0098** | **0** | **49** | **0.0013** | **1** | **K.FCECDNFNCDR.S** |
|  | 17479 | **615.6448** | **1843.9127** | **1843.9251** | **-0.0124** | **1** | **12** | **6.8** | **1** | **R.DKLPQPVQPDPVSHCK.E** 17458 |
|  | 18877 | **981.0337** | **1960.0528** | **1960.0629** | **-0.0101** | **1** | **82** | **5.7e-007** | **1** | **K.NVLSLTNKGEVFNELVGK.Q** |
|  | 19482 | **1010.0772** | **2018.1399** | **2018.1524** | **-0.0125** | **0** | **54** | **0.00035** | **1** | **K.LKPEDITQIQPQQLVLR.L** |
|  | 22618 | **793.2811** | **2376.8214** | **2376.8351** | **-0.0138** | **0** | **32** | **0.03** | **1** | **R.HCECSTDEVNSEDMDAYCR.K** |
|  | 23226 | **1235.6238** | **2469.2330** | **2469.2427** | **-0.0097** | **0** | **69** | **9.3e-006** | **1** | **K.LSENNIQTIFAVTEEFQPVYK.E** 23225 |
|  | 26421 | **1021.1478** | **3060.4217** | **3060.2327** | **0.1890** | **1** | **1** | **45** | **7** | **K.CTDPKFQGQTCEMCQTCLGVCAEHK.E** |
|  | 27379 | **819.8309** | **3275.2946** | **3275.3168** | **-0.0221** | **1** | **32** | **0.021** | **1** | **K.FQGQTCEMCQTCLGVCAEHKECVQCR.A** 27380 |
|  | 29191 | **1270.6359** | **3808.8858** | **3808.7998** | **0.0860** | **0** | **9** | **4.2** | **1** | **K.IRPLGFTEEVEVILQYICECECQSEGIPESPK.C** |
|  | 30070 | **836.3939** | **4176.9332** | **4176.9496** | **-0.0164** | **0** | **6** | **6.2** | **2** | **K.LGGIVLPNDGQCHLENNMYTMSHYYDYPSIAHLVQK.L** |

| **122.** | P19224\|UD16_HUMAN    **Mass:** 61338    **Score:** 243    **Queries matched:** 28   **emPAI:** 0.80 |
| --- | --- |
|  | UDP-glucuronosyltransferase 1-6 OS=Homo sapiens GN=UGT1A6 PE=1 SV=2 |

|  | **Query** | **Observed** | **Mr(expt)** | **Mr(calc)** | **Delta** | **Miss** | **Score** | **Expect** | **Rank** | **Peptide** |
| --- | --- | --- | --- | --- | --- | --- | --- | --- | --- | --- |
|  | 1086 | 659.3677 | 658.3605 | 658.3650 | -0.0045 | 0 | 25 | 0.78 | 1 | K.AVINDK.S |
|  | 2531 | **437.2521** | **872.4896** | **871.5280** | **0.9616** | **0** | **29** | **0.25** | **2** | **K.VSVWLLR.Y** 2515 |
|  | 3687 | 960.5128 | 959.5055 | 959.5110 | -0.0055 | 0 | 41 | 0.014 | 1 | K.AMAIADALGK.I |
|  | 4770 | 520.7600 | 1039.5055 | 1039.5120 | -0.0066 | 1 | 39 | 0.017 | 1 | K.SYKENIMR.L 4771 |
|  | 5402 | 545.3372 | 1088.6598 | 1087.6059 | 1.0538 | 1 | 8 | 30 | 3 | K.KAMAIADALGK.I |
|  | 8600 | **427.2313** | **1278.6720** | **1277.6979** | **0.9741** | **1** | **5** | **47** | **10** | **K.YEELASAVLKR.D** |
|  | 9791 | 448.2527 | 1341.7364 | 1342.7027 | -0.9663 | 1 | 11 | 9.9 | 2 | K.ENIMRLSSLHK.D |
|  | 15287 | 839.4249 | 1676.8353 | 1676.8457 | -0.0104 | 0 | 30 | 0.13 | 1 | K.WLPQNDLLGHPMTR.A 15288 15289 |
|  | 18806 | 652.0336 | 1953.0789 | 1953.0870 | -0.0081 | 1 | 48 | 0.0013 | 1 | K.AMAIADALGKIPQTVLWR.Y 18804 18807 |
|  | 18958 | 493.2440 | 1968.9467 | 1969.0819 | -0.1351 | 1 | (7) | 19 | 1 | K.AMAIADALGKIPQTVLWR.Y |
|  | 21226 | 735.3732 | 2203.0977 | 2203.1042 | -0.0065 | 0 | 64 | 3.7e-005 | 1 | K.GAGVTLNVLEMTSEDLENALK.A |
|  | 21382 | 740.5198 | 2218.5377 | 2219.0991 | -0.5614 | 0 | (3) | 28 | 5 | K.GAGVTLNVLEMTSEDLENALK.A 21379 |
|  | 22175 | 773.4023 | 2317.1850 | 2317.1929 | -0.0079 | 0 | 29 | 0.1 | 1 | K.DRPVEPLDLAVFWVEFVMR.H |
|  | 25357 | 948.8234 | 2843.4485 | 2843.4586 | -0.0101 | 1 | 63 | 3.1e-005 | 1 | K.GAGVTLNVLEMTSEDLENALKAVINDK.S 25358 |
|  | 29241 | 957.1843 | 3824.7080 | 3823.7289 | 0.9791 | 0 | 28 | 0.046 | 1 | R.AFITHAGSHGVYESICNGVPMVMMPLFGDQMDNAK.R 29234 29240 |
|  | 29624 | 996.2063 | 3980.7961 | 3979.8300 | 0.9661 | 1 | 31 | 0.022 | 1 | R.AFITHAGSHGVYESICNGVPMVMMPLFGDQMDNAKR.M 29622 29623 |

| **123.** | P35504\|UD15_HUMAN    **Mass:** 60602    **Score:** 243    **Queries matched:** 34   **emPAI:** 0.81 |
| --- | --- |
|  | UDP-glucuronosyltransferase 1-5 OS=Homo sapiens GN=UGT1A5 PE=1 SV=1 |

|  | **Query** | **Observed** | **Mr(expt)** | **Mr(calc)** | **Delta** | **Miss** | **Score** | **Expect** | **Rank** | **Peptide** |
| --- | --- | --- | --- | --- | --- | --- | --- | --- | --- | --- |
|  | 1086 | 659.3677 | 658.3605 | 658.3650 | -0.0045 | 0 | 25 | 0.78 | 1 | K.AVINDK.S |
|  | 3687 | 960.5128 | 959.5055 | 959.5110 | -0.0055 | 0 | 41 | 0.014 | 1 | K.AMAIADALGK.I |
|  | 4770 | 520.7600 | 1039.5055 | 1039.5120 | -0.0066 | 1 | 39 | 0.017 | 1 | K.SYKENIMR.L 4771 |
|  | 5271 | **541.1182** | **1080.2219** | **1079.5836** | **0.6384** | **1** | **1** | **84** | **6** | **R.EALRDLHAR.G** |
|  | 5402 | 545.3372 | 1088.6598 | 1087.6059 | 1.0538 | 1 | 8 | 30 | 3 | K.KAMAIADALGK.I |
|  | 9791 | 448.2527 | 1341.7364 | 1342.7027 | -0.9663 | 1 | 11 | 9.9 | 2 | K.ENIMRLSSLHK.D |
|  | 13941 | **531.3212** | **1590.9417** | **1590.7680** | **0.1737** | **0** | **4** | **44** | **6** | **R.MAIMNNMSLIIHR.S** |
|  | 15287 | 839.4249 | 1676.8353 | 1676.8457 | -0.0104 | 0 | 30 | 0.13 | 1 | K.WLPQNDLLGHPMTR.A 15288 15289 |
|  | 15808 | **429.7259** | **1714.8746** | **1714.8793** | **-0.0047** | **1** | **13** | **5.4** | **2** | **R.RMAIMNNMSLIIHR.S** |
|  | 15831 | **572.9943** | **1715.9611** | **1714.8793** | **1.0819** | **1** | **(4)** | **41** | **3** | **R.RMAIMNNMSLIIHR.S** 15790 |
|  | 16020 | **865.8720** | **1729.7295** | **1730.8742** | **-1.1447** | **1** | **(6)** | **28** | **2** | **R.RMAIMNNMSLIIHR.S** |
|  | 16224 | **583.0347** | **1746.0824** | **1746.8691** | **-0.7867** | **1** | **(7)** | **19** | **3** | **R.RMAIMNNMSLIIHR.S** |
|  | 18363 | 640.3666 | 1918.0781 | 1918.9935 | -0.9154 | 1 | 6 | 24 | 3 | R.LLTTNSDHMTFLQRVK.N |
|  | 18806 | 652.0336 | 1953.0789 | 1953.0870 | -0.0081 | 1 | 48 | 0.0013 | 1 | K.AMAIADALGKIPQTVLWR.Y 18804 18807 |
|  | 18958 | 493.2440 | 1968.9467 | 1969.0819 | -0.1351 | 1 | (7) | 19 | 1 | K.AMAIADALGKIPQTVLWR.Y |
|  | 21226 | 735.3732 | 2203.0977 | 2203.1042 | -0.0065 | 0 | 64 | 3.7e-005 | 1 | K.GAGVTLNVLEMTSEDLENALK.A |
|  | 21382 | 740.5198 | 2218.5377 | 2219.0991 | -0.5614 | 0 | (3) | 28 | 5 | K.GAGVTLNVLEMTSEDLENALK.A 21379 |
|  | 22175 | 773.4023 | 2317.1850 | 2317.1929 | -0.0079 | 0 | 29 | 0.1 | 1 | K.DRPVEPLDLAVFWVEFVMR.H |
|  | 25357 | 948.8234 | 2843.4485 | 2843.4586 | -0.0101 | 1 | 63 | 3.1e-005 | 1 | K.GAGVTLNVLEMTSEDLENALKAVINDK.S 25358 |
|  | 26602 | **516.7648** | **3094.5453** | **3093.5707** | **0.9746** | **1** | **2** | **31** | **5** | **R.MAIMNNMSLIIHRSCVELLHNEALIR.H** |
|  | 29241 | 957.1843 | 3824.7080 | 3823.7289 | 0.9791 | 0 | 28 | 0.046 | 1 | R.AFITHAGSHGVYESICNGVPMVMMPLFGDQMDNAK.R 29234 29240 |
|  | 29624 | 996.2063 | 3980.7961 | 3979.8300 | 0.9661 | 1 | 31 | 0.022 | 1 | R.AFITHAGSHGVYESICNGVPMVMMPLFGDQMDNAKR.M 29622 29623 |

| **124.** | P22310\|UD14_HUMAN    **Mass:** 60613    **Score:** 243    **Queries matched:** 30   **emPAI:** 0.81 |
| --- | --- |
|  | UDP-glucuronosyltransferase 1-4 OS=Homo sapiens GN=UGT1A4 PE=1 SV=1 |

|  | **Query** | **Observed** | **Mr(expt)** | **Mr(calc)** | **Delta** | **Miss** | **Score** | **Expect** | **Rank** | **Peptide** |
| --- | --- | --- | --- | --- | --- | --- | --- | --- | --- | --- |
|  | 1086 | 659.3677 | 658.3605 | 658.3650 | -0.0045 | 0 | 25 | 0.78 | 1 | K.AVINDK.S |
|  | 3687 | 960.5128 | 959.5055 | 959.5110 | -0.0055 | 0 | 41 | 0.014 | 1 | K.AMAIADALGK.I |
|  | 4770 | 520.7600 | 1039.5055 | 1039.5120 | -0.0066 | 1 | 39 | 0.017 | 1 | K.SYKENIMR.L 4771 |
|  | 5402 | 545.3372 | 1088.6598 | 1087.6059 | 1.0538 | 1 | 8 | 30 | 3 | K.KAMAIADALGK.I |
|  | 5652 | **554.2758** | **1106.5370** | **1105.6720** | **0.8650** | **1** | **1** | **1.4e+002** | **6** | **M.ARGLQVPLPR.L** |
|  | 9791 | 448.2527 | 1341.7364 | 1342.7027 | -0.9663 | 1 | 11 | 9.9 | 2 | K.ENIMRLSSLHK.D |
|  | 10774 | **699.7831** | **1397.5516** | **1397.7496** | **-0.1980** | **0** | **3** | **61** | **5** | **K.YLSIPAVFFWR.Y** |
|  | 15287 | 839.4249 | 1676.8353 | 1676.8457 | -0.0104 | 0 | 30 | 0.13 | 1 | K.WLPQNDLLGHPMTR.A 15288 15289 |
|  | 18363 | 640.3666 | 1918.0781 | 1918.9935 | -0.9154 | 1 | 6 | 24 | 3 | K.LLTTNSDHMTFLQRVK.N |
|  | 18806 | 652.0336 | 1953.0789 | 1953.0870 | -0.0081 | 1 | 48 | 0.0013 | 1 | K.AMAIADALGKIPQTVLWR.Y 18804 18807 |
|  | 18958 | 493.2440 | 1968.9467 | 1969.0819 | -0.1351 | 1 | (7) | 19 | 1 | K.AMAIADALGKIPQTVLWR.Y |
|  | 19264 | **666.3565** | **1996.0475** | **1995.1404** | **0.9071** | **0** | **5** | **28** | **3** | **R.LATGLLLLLSVQPWAESGK.V** |
|  | 21226 | 735.3732 | 2203.0977 | 2203.1042 | -0.0065 | 0 | 64 | 3.7e-005 | 1 | K.GAGVTLNVLEMTSEDLENALK.A |
|  | 21382 | 740.5198 | 2218.5377 | 2219.0991 | -0.5614 | 0 | (3) | 28 | 5 | K.GAGVTLNVLEMTSEDLENALK.A 21379 |
|  | 22175 | 773.4023 | 2317.1850 | 2317.1929 | -0.0079 | 0 | 29 | 0.1 | 1 | K.DRPVEPLDLAVFWVEFVMR.H |
|  | 25357 | 948.8234 | 2843.4485 | 2843.4586 | -0.0101 | 1 | 63 | 3.1e-005 | 1 | K.GAGVTLNVLEMTSEDLENALKAVINDK.S 25358 |
|  | 25401 | 714.8481 | 2855.3635 | 2855.6637 | -0.3002 | 1 | 0 | 51 | 1 | R.GLQVPLPRLATGLLLLLSVQPWAESGK.V |
|  | 29241 | 957.1843 | 3824.7080 | 3823.7289 | 0.9791 | 0 | 28 | 0.046 | 1 | R.AFITHAGSHGVYESICNGVPMVMMPLFGDQMDNAK.R 29234 29240 |
|  | 29624 | 996.2063 | 3980.7961 | 3979.8300 | 0.9661 | 1 | 31 | 0.022 | 1 | R.AFITHAGSHGVYESICNGVPMVMMPLFGDQMDNAKR.M 29622 29623 |

| **125.** | P35503\|UD13_HUMAN    **Mass:** 60869    **Score:** 243    **Queries matched:** 30   **emPAI:** 0.81 |
| --- | --- |
|  | UDP-glucuronosyltransferase 1-3 OS=Homo sapiens GN=UGT1A3 PE=1 SV=1 |

|  | **Query** | **Observed** | **Mr(expt)** | **Mr(calc)** | **Delta** | **Miss** | **Score** | **Expect** | **Rank** | **Peptide** |
| --- | --- | --- | --- | --- | --- | --- | --- | --- | --- | --- |
|  | 1086 | 659.3677 | 658.3605 | 658.3650 | -0.0045 | 0 | 25 | 0.78 | 1 | K.AVINDK.S |
|  | 3687 | 960.5128 | 959.5055 | 959.5110 | -0.0055 | 0 | 41 | 0.014 | 1 | K.AMAIADALGK.I |
|  | 4770 | 520.7600 | 1039.5055 | 1039.5120 | -0.0066 | 1 | 39 | 0.017 | 1 | K.SYKENIMR.L 4771 |
|  | 5402 | 545.3372 | 1088.6598 | 1087.6059 | 1.0538 | 1 | 8 | 30 | 3 | K.KAMAIADALGK.I |
|  | 9791 | 448.2527 | 1341.7364 | 1342.7027 | -0.9663 | 1 | 11 | 9.9 | 2 | K.ENIMRLSSLHK.D |
|  | 15287 | 839.4249 | 1676.8353 | 1676.8457 | -0.0104 | 0 | 30 | 0.13 | 1 | K.WLPQNDLLGHPMTR.A 15288 15289 |
|  | 15361 | **561.6353** | **1681.8841** | **1681.7738** | **0.1103** | **0** | **0** | **1e+002** | **8** | **R.SMAMLNNMSLVYHR.S** |
|  | 15916 | **575.2949** | **1722.8628** | **1723.9080** | **-1.0452** | **0** | **5** | **33** | **1** | **K.VLVVPIDGSHWLSMR.E** |
|  | 15967 | 576.3315 | 1725.9728 | 1725.7814 | 0.1913 | 0 | 5 | 34 | 8 | R.LLTTNSDHMTFMQR.V |
|  | 18806 | 652.0336 | 1953.0789 | 1953.0870 | -0.0081 | 1 | 48 | 0.0013 | 1 | K.AMAIADALGKIPQTVLWR.Y 18804 18807 |
|  | 18958 | 493.2440 | 1968.9467 | 1969.0819 | -0.1351 | 1 | (7) | 19 | 1 | K.AMAIADALGKIPQTVLWR.Y |
|  | 21226 | 735.3732 | 2203.0977 | 2203.1042 | -0.0065 | 0 | 64 | 3.7e-005 | 1 | K.GAGVTLNVLEMTSEDLENALK.A |
|  | 21382 | 740.5198 | 2218.5377 | 2219.0991 | -0.5614 | 0 | (3) | 28 | 5 | K.GAGVTLNVLEMTSEDLENALK.A 21379 |
|  | 22175 | 773.4023 | 2317.1850 | 2317.1929 | -0.0079 | 0 | 29 | 0.1 | 1 | K.DRPVEPLDLAVFWVEFVMR.H |
|  | 25357 | 948.8234 | 2843.4485 | 2843.4586 | -0.0101 | 1 | 63 | 3.1e-005 | 1 | K.GAGVTLNVLEMTSEDLENALKAVINDK.S 25358 |
|  | 26156 | **754.8655** | **3015.4328** | **3015.3864** | **0.0464** | **0** | **5** | **17** | **1** | **R.GDFVMDYPRPIMPNMVFIGGINCANR.K** |
|  | 26236 | 1011.2276 | 3030.6610 | 3031.3813 | -0.7203 | 0 | (1) | 39 | 5 | R.GDFVMDYPRPIMPNMVFIGGINCANR.K |
|  | 29241 | 957.1843 | 3824.7080 | 3823.7289 | 0.9791 | 0 | 28 | 0.046 | 1 | R.AFITHAGSHGVYESICNGVPMVMMPLFGDQMDNAK.R 29234 29240 |
|  | 29624 | 996.2063 | 3980.7961 | 3979.8300 | 0.9661 | 1 | 31 | 0.022 | 1 | R.AFITHAGSHGVYESICNGVPMVMMPLFGDQMDNAKR.M 29622 29623 |

| **126.** | P22309\|UD11_HUMAN    **Mass:** 60180    **Score:** 243    **Queries matched:** 29   **emPAI:** 0.82 |
| --- | --- |
|  | UDP-glucuronosyltransferase 1-1 OS=Homo sapiens GN=UGT1A1 PE=1 SV=1 |

|  | **Query** | **Observed** | **Mr(expt)** | **Mr(calc)** | **Delta** | **Miss** | **Score** | **Expect** | **Rank** | **Peptide** |
| --- | --- | --- | --- | --- | --- | --- | --- | --- | --- | --- |
|  | 1086 | 659.3677 | 658.3605 | 658.3650 | -0.0045 | 0 | 25 | 0.78 | 1 | K.AVINDK.S |
|  | 3687 | 960.5128 | 959.5055 | 959.5110 | -0.0055 | 0 | 41 | 0.014 | 1 | K.AMAIADALGK.I |
|  | 4770 | 520.7600 | 1039.5055 | 1039.5120 | -0.0066 | 1 | 39 | 0.017 | 1 | K.SYKENIMR.L 4771 |
|  | 5402 | 545.3372 | 1088.6598 | 1087.6059 | 1.0538 | 1 | 8 | 30 | 3 | K.KAMAIADALGK.I |
|  | 9791 | 448.2527 | 1341.7364 | 1342.7027 | -0.9663 | 1 | 11 | 9.9 | 2 | K.ENIMRLSSLHK.D |
|  | 15287 | 839.4249 | 1676.8353 | 1676.8457 | -0.0104 | 0 | 30 | 0.13 | 1 | K.WLPQNDLLGHPMTR.A 15288 15289 |
|  | 16283 | **584.6289** | **1750.8649** | **1751.9570** | **-1.0921** | **0** | **2** | **77** | **5** | **R.GHEIVVLAPDASLYIR.D** |
|  | 18169 | **634.8320** | **1901.4743** | **1901.9676** | **-0.4933** | **1** | **1** | **60** | **6** | **R.DGAFYTLKTYPVPFQR.E** |
|  | 18494 | **643.3171** | **1926.9294** | **1925.9451** | **0.9843** | **1** | **1** | **80** | **5** | **K.KDSAMLLSGCSHLLHNK.E** |
|  | 18806 | 652.0336 | 1953.0789 | 1953.0870 | -0.0081 | 1 | 48 | 0.0013 | 1 | K.AMAIADALGKIPQTVLWR.Y 18804 18807 |
|  | 18958 | 493.2440 | 1968.9467 | 1969.0819 | -0.1351 | 1 | (7) | 19 | 1 | K.AMAIADALGKIPQTVLWR.Y |
|  | 21226 | 735.3732 | 2203.0977 | 2203.1042 | -0.0065 | 0 | 64 | 3.7e-005 | 1 | K.GAGVTLNVLEMTSEDLENALK.A |
|  | 21382 | 740.5198 | 2218.5377 | 2219.0991 | -0.5614 | 0 | (3) | 28 | 5 | K.GAGVTLNVLEMTSEDLENALK.A 21379 |
|  | 22175 | 773.4023 | 2317.1850 | 2317.1929 | -0.0079 | 0 | 29 | 0.1 | 1 | K.DRPVEPLDLAVFWVEFVMR.H |
|  | 25357 | 948.8234 | 2843.4485 | 2843.4586 | -0.0101 | 1 | 63 | 3.1e-005 | 1 | K.GAGVTLNVLEMTSEDLENALKAVINDK.S 25358 |
|  | 29241 | 957.1843 | 3824.7080 | 3823.7289 | 0.9791 | 0 | 28 | 0.046 | 1 | R.AFITHAGSHGVYESICNGVPMVMMPLFGDQMDNAK.R 29234 29240 |
|  | 29624 | 996.2063 | 3980.7961 | 3979.8300 | 0.9661 | 1 | 31 | 0.022 | 1 | R.AFITHAGSHGVYESICNGVPMVMMPLFGDQMDNAKR.M 29622 29623 |
|  | 31562 | **1776.8149** | **5327.4230** | **5327.6675** | **-0.2445** | **1** | **2** | **6.8** | **3** | **K.NMLIAFSQNFLCDVVYSPYATLASEFLQREVTVQDLLSSASVWLFR.S** |

| **127.** | P35613\|BASI_HUMAN    **Mass:** 42573    **Score:** 238    **Queries matched:** 14   **emPAI:** 0.66 |
| --- | --- |
|  | Basigin OS=Homo sapiens GN=BSG PE=1 SV=2 |

|  | **Query** | **Observed** | **Mr(expt)** | **Mr(calc)** | **Delta** | **Miss** | **Score** | **Expect** | **Rank** | **Peptide** |
| --- | --- | --- | --- | --- | --- | --- | --- | --- | --- | --- |
|  | 10973 | **470.9339** | **1409.7799** | **1409.7878** | **-0.0080** | **1** | **26** | **0.33** | **1** | **K.GGVVLKEDALPGQK.T** 10984 |
|  | 15074 | **832.8973** | **1663.7800** | **1663.7882** | **-0.0082** | **0** | **28** | **0.17** | **1** | **K.SESVPPVTDWAWYK.I** 15073 |
|  | 16504 | **590.3344** | **1767.9814** | **1767.7945** | **0.1869** | **1** | **6** | **24** | **1** | **K.ITDSEDKALMNGSESR.F** 16502 |
|  | 16908 | **902.9086** | **1803.8027** | **1803.8131** | **-0.0104** | **0** | **71** | **8.8e-006** | **1** | **K.SSEHINEGETAMLVCK.S** 16909 16910 |
|  | 20456 | **1058.4860** | **2114.9574** | **2114.9691** | **-0.0117** | **0** | **85** | **2.9e-007** | **1** | **R.SELHIENLNMEADPGQYR.C** 20454 20455 |
|  | 29696 | **1005.9633** | **4019.8242** | **4019.8459** | **-0.0217** | **1** | **46** | **0.00059** | **1** | **K.TEFKVDSDDQWGEYSCVFLPEPMGTANIQLHGPPR.V** |
|  | 30268 | **1078.4840** | **4309.9069** | **4308.9730** | **0.9339** | **1** | **0** | **18** | **8** | **R.VHIHATYHQHAASTISIDTLVEEDTGTYECRASNDPDR.N** |

| **128.** | P05387\|RLA2_HUMAN    **Mass:** 11658    **Score:** 238    **Queries matched:** 13   **emPAI:** 2.23 |
| --- | --- |
|  | 60S acidic ribosomal protein P2 OS=Homo sapiens GN=RPLP2 PE=1 SV=1 |

|  | **Query** | **Observed** | **Mr(expt)** | **Mr(calc)** | **Delta** | **Miss** | **Score** | **Expect** | **Rank** | **Peptide** |
| --- | --- | --- | --- | --- | --- | --- | --- | --- | --- | --- |
|  | 2296 | **859.5027** | **858.4954** | **858.4811** | **0.0144** | **0** | **16** | **5.2** | **2** | **K.VISELNGK.N** |
|  | 8144 | **628.8420** | **1255.6695** | **1255.6772** | **-0.0077** | **0** | **78** | **2.5e-006** | **1** | **K.NIEDVIAQGIGK.L** 8156 |
|  | 16547 | 886.9491 | 1771.8836 | 1771.8952 | -0.0115 | 1 | 67 | 2.1e-005 | 1 | K.ILDSVGIEADDDRLNK.V 16546 |
|  | 17786 | **934.9862** | **1867.9579** | **1867.9679** | **-0.0101** | **0** | **54** | **0.00039** | **1** | **R.YVASYLLAALGGNSSPSAK.D** 17796 17797 |
|  | 20800 | 1078.0408 | 2154.0670 | 2155.1095 | -1.0425 | 1 | 5 | 25 | 7 | -.MRYVASYLLAALGGNSSPSAK.D |
|  | 21442 | **742.3946** | **2224.1619** | **2224.1739** | **-0.0120** | **1** | **16** | **2.3** | **1** | **R.YVASYLLAALGGNSSPSAKDIK.K** |
|  | 24977 | **1387.7113** | **2773.4080** | **2773.4246** | **-0.0165** | **0** | **48** | **0.00087** | **1** | **K.LASVPAGGAVAVSAAPGSAAPAAGSAPAAAEEK.K** 24976 24978 |

| **129.** | Q9BYX7\|ACTBM_HUMAN    **Mass:** 42331    **Score:** 236    **Queries matched:** 22   **emPAI:** 0.40 |
| --- | --- |
|  | Beta-actin-like protein 3 OS=Homo sapiens GN=ACTBL3 PE=1 SV=1 |

|  | **Query** | **Observed** | **Mr(expt)** | **Mr(calc)** | **Delta** | **Miss** | **Score** | **Expect** | **Rank** | **Peptide** |
| --- | --- | --- | --- | --- | --- | --- | --- | --- | --- | --- |
|  | 6742 | **397.2191** | **1188.6355** | **1188.6424** | **-0.0068** | **0** | **8** | **28** | **1** | **K.EITALAPSIMK.I** |
|  | 7488 | 613.3124 | 1224.6102 | 1224.5986 | 0.0116 | 1 | 0 | 1.3e+002 | 6 | K.ESYVGKEAQSK.R |
|  | 9568 | **443.7562** | **1328.2469** | **1328.5424** | **-0.2955** | **0** | **(9)** | **13** | **5** | **R.HQGMMEGMHQK.E** |
|  | 9582 | **444.0429** | **1329.1070** | **1328.5424** | **0.5646** | **0** | **13** | **5.1** | **1** | **R.HQGMMEGMHQK.E** |
|  | 10110 | **680.8521** | **1359.6897** | **1360.5322** | **-0.8426** | **0** | **(2)** | **1e+002** | **6** | **R.HQGMMEGMHQK.E** 10142 |
|  | 11317 | **477.7309** | **1430.1708** | **1429.8214** | **0.3494** | **1** | **2** | **63** | **6** | **K.EITALAPSIMKIK.I** |
|  | 12723 | 505.9181 | 1514.7325 | 1514.7419 | -0.0093 | 0 | 40 | 0.014 | 1 | K.IWHHTFYNELR.V 12724 12725 |
|  | 12739 | 758.8506 | 1515.6866 | 1515.6954 | -0.0087 | 0 | 42 | 0.0081 | 1 | K.QEYDESGPSIVHR.K 12738 |
|  | 16767 | 895.9441 | 1789.8736 | 1789.8846 | -0.0110 | 0 | 66 | 2.5e-005 | 1 | K.SYELPDGQVITIGNER.F 16766 16769 16770 16771 16772 |
|  | 19216 | **664.8218** | **1991.4435** | **1991.8652** | **-0.4217** | **1** | **2** | **40** | **5** | **R.HQGMMEGMHQKESYVGK.E** |
|  | 24228 | **660.5363** | **2638.1162** | **2637.2277** | **0.8885** | **1** | **0** | **54** | **6** | **R.GMLTLKYPMEHGIITNWDDMEK.I** |
|  | 25099 | **932.4548** | **2794.3427** | **2794.2347** | **0.1080** | **1** | **1** | **49** | **3** | **K.EKLCYVALDSEQEMAMAASSSSVEK.S** |
|  | 28031 | **1719.2308** | **3436.4471** | **3437.6180** | **-1.1709** | **1** | **0** | **31** | **7** | **K.AGFAGDDAPQAVFPSIVGRPRHQGMMEGMHQK.E** |

| **130.** | P04004\|VTNC_HUMAN    **Mass:** 55069    **Score:** 236    **Queries matched:** 16   **emPAI:** 0.48 |
| --- | --- |
|  | Vitronectin OS=Homo sapiens GN=VTN PE=1 SV=1 |

|  | **Query** | **Observed** | **Mr(expt)** | **Mr(calc)** | **Delta** | **Miss** | **Score** | **Expect** | **Rank** | **Peptide** |
| --- | --- | --- | --- | --- | --- | --- | --- | --- | --- | --- |
|  | 6910 | **599.2780** | **1196.5413** | **1196.5496** | **-0.0082** | **1** | **32** | **0.092** | **1** | **R.CTEGFNVDKK.C** 6936 |
|  | 9101 | **652.7725** | **1303.5305** | **1303.5390** | **-0.0086** | **0** | **40** | **0.013** | **1** | **R.GQYCYELDEK.A** |
|  | 9265 | **438.8953** | **1313.6641** | **1313.6728** | **-0.0087** | **1** | **20** | **1.5** | **1** | **R.RVDTVDPPYPR.S** |
|  | 11160 | **711.8262** | **1421.6378** | **1421.6463** | **-0.0085** | **0** | **53** | **0.00066** | **1** | **R.FEDGVLDPDYPR.N** |
|  | 11607 | **724.8793** | **1447.7441** | **1447.7532** | **-0.0091** | **1** | **8** | **20** | **1** | **R.TSAGTRQPQFISR.D** |
|  | 12531 | **752.4166** | **1502.8187** | **1502.8279** | **-0.0092** | **0** | **13** | **5.9** | **1** | **R.IYISGMAPRPSLAK.K** 12530 |
|  | 14793 | **823.9079** | **1645.8012** | **1645.8100** | **-0.0088** | **0** | **39** | **0.016** | **1** | **R.DVWGIEGPIDAAFTR.I** |
|  | 15090 | **833.8871** | **1665.7596** | **1665.7682** | **-0.0086** | **0** | **55** | **0.00037** | **1** | **R.DWHGVPGQVDAAMAGR.I** 15089 15092 |
|  | 15161 | **835.9095** | **1669.8044** | **1668.8082** | **0.9962** | **0** | **12** | **7.2** | **1** | **R.SIAQYWLGCPAPGHL.-** |
|  | 22679 | **597.3286** | **2385.2853** | **2385.1390** | **0.1464** | **1** | **0** | **68** | **9** | **R.DSWEDIFELLFWGRTSAGTR.Q** |
|  | 23988 | **864.4063** | **2590.1971** | **2589.1920** | **1.0051** | **0** | **1** | **47** | **3** | **R.MDWLVPATCEPIQSVFFFSGDK.Y** |
|  | 28142 | **1161.4481** | **3481.3225** | **3481.3448** | **-0.0223** | **0** | **104** | **1.2e-009** | **1** | **K.CQCDELCSYYQSCCTDYTAECKPQVTR.G** |

| **131.** | Q99536\|VAT1_HUMAN    **Mass:** 42122    **Score:** 235    **Queries matched:** 17   **emPAI:** 0.53 |
| --- | --- |
|  | Synaptic vesicle membrane protein VAT-1 homolog OS=Homo sapiens GN=VAT1 PE=1 SV=2 |

|  | **Query** | **Observed** | **Mr(expt)** | **Mr(calc)** | **Delta** | **Miss** | **Score** | **Expect** | **Rank** | **Peptide** |
| --- | --- | --- | --- | --- | --- | --- | --- | --- | --- | --- |
|  | 3508 | **473.3245** | **944.6344** | **943.5385** | **1.0959** | **1** | **7** | **40** | **6** | **K.RNLMALAR.T** |
|  | 3671 | **480.2413** | **958.4681** | **959.5334** | **-1.0653** | **1** | **(5)** | **57** | **4** | **K.RNLMALAR.T** |
|  | 5828 | **560.7731** | **1119.5316** | **1119.6110** | **-0.0794** | **0** | **24** | **0.69** | **1** | **K.GYNLLKPMGK.V** |
|  | 11869 | **732.3957** | **1462.7768** | **1462.7854** | **-0.0086** | **0** | **(2)** | **91** | **8** | **K.VVTYGMANLLTGPK.R** |
|  | 12139 | **494.2391** | **1479.6955** | **1478.7803** | **0.9152** | **0** | **3** | **62** | **7** | **K.VVTYGMANLLTGPK.R** |
|  | 13383 | **519.6066** | **1555.7979** | **1555.8069** | **-0.0090** | **1** | **24** | **0.47** | **1** | **R.CLVLTGFGGYDKVK.L** |
|  | 13644 | 787.8821 | 1573.7497 | 1573.7658 | -0.0161 | 0 | 64 | 5e-005 | 1 | K.GVDIVMDPLGGSDTAK.G 13643 |
|  | 13931 | **531.2559** | **1590.7459** | **1589.7607** | **0.9852** | **0** | **(10)** | **11** | **1** | **K.GVDIVMDPLGGSDTAK.G** 13915 13926 |
|  | 14194 | **402.9471** | **1607.7592** | **1606.8072** | **0.9520** | **1** | **6** | **33** | **7** | **R.LRACGLNFADLMAR.Q** |
|  | 18488 | **964.0410** | **1926.0675** | **1926.0799** | **-0.0124** | **0** | **51** | **0.00077** | **1** | **K.LQSRPAAPPAPGPGQLTLR.L** |
|  | 24474 | **892.7922** | **2675.3547** | **2675.3663** | **-0.0115** | **1** | **34** | **0.022** | **1** | **K.GVDIVMDPLGGSDTAKGYNLLKPMGK.V** |
|  | 24562 | 674.0761 | 2692.2751 | 2691.3612 | 0.9139 | 1 | (3) | 32 | 8 | K.GVDIVMDPLGGSDTAKGYNLLKPMGK.V |
|  | 27267 | **1083.8982** | **3248.6727** | **3248.6864** | **-0.0136** | **1** | **95** | **1.3e-008** | **1** | **R.QGLYDRLPPLPVTPGMEGAGVVIAVGEGVSDR.K** 27266 |

| **132.** | P08238\|HS90B_HUMAN    **Mass:** 83554    **Score:** 235    **Queries matched:** 24   **emPAI:** 0.35 |
| --- | --- |
|  | Heat shock protein HSP 90-beta OS=Homo sapiens GN=HSP90AB1 PE=1 SV=4 |

|  | **Query** | **Observed** | **Mr(expt)** | **Mr(calc)** | **Delta** | **Miss** | **Score** | **Expect** | **Rank** | **Peptide** |
| --- | --- | --- | --- | --- | --- | --- | --- | --- | --- | --- |
|  | 1207 | **731.1756** | **730.1683** | **729.4385** | **0.7299** | **0** | **5** | **57** | **1** | **R.LSELLR.Y** |
|  | 1625 | **405.6919** | **809.3692** | **809.3742** | **-0.0049** | **0** | **23** | **0.73** | **1** | **K.FENLCK.L** |
|  | 2028 | 420.7607 | 839.5068 | 838.4735 | 1.0333 | 1 | 16 | 3.9 | 2 | R.IYRMIK.L |
|  | 2766 | **446.2213** | **890.4281** | **890.4174** | **0.0107** | **0** | **8** | **28** | **3** | **K.FYEAFSK.N** |
|  | 3511 | **473.7746** | **945.5347** | **945.5429** | **-0.0082** | **1** | **7** | **42** | **6** | **R.IMKAQALR.D** |
|  | 6175 | **575.7858** | **1149.5571** | **1150.5506** | **-0.9935** | **0** | **17** | **3.1** | **1** | **K.YIDQEELNK.T** |
|  | 7845 | **621.8527** | **1241.6908** | **1241.6979** | **-0.0072** | **0** | **22** | **0.9** | **1** | **K.ADLINNLGTIAK.S** 7870 |
|  | 12693 | **757.3920** | **1512.7694** | **1512.7784** | **-0.0090** | **0** | **66** | **3.1e-005** | **1** | **R.GVVDSEDLPLNISR.E** 12656 |
|  | 12907 | **764.3709** | **1526.7271** | **1526.7365** | **-0.0094** | **0** | **57** | **0.00026** | **1** | **K.SLTNDWEDHLAVK.H** 12908 12909 |
|  | 13177 | 772.9123 | 1543.8101 | 1543.8205 | -0.0104 | 1 | 82 | 7.3e-007 | 1 | R.ELISNASDALDKIR.Y 13178 |
|  | 16695 | **595.3192** | **1782.9358** | **1781.9424** | **0.9934** | **0** | **42** | **0.0073** | **1** | **K.HLEINPDHPIVETLR.Q** 16676 |
|  | 18271 | **478.5140** | **1910.0268** | **1910.0374** | **-0.0106** | **1** | **13** | **4.4** | **1** | **K.KHLEINPDHPIVETLR.Q** |
|  | 22709 | **797.7279** | **2390.1619** | **2390.1754** | **-0.0135** | **1** | **27** | **0.16** | **1** | **K.SIYYITGESKEQVANSAFVER.V** |
|  | 23889 | **858.4749** | **2572.4029** | **2572.4146** | **-0.0117** | **1** | **37** | **0.012** | **1** | **R.TLTLVDTGIGMTKADLINNLGTIAK.S** |
|  | 27409 | **822.4361** | **3285.7153** | **3285.7245** | **-0.0092** | **1** | **24** | **0.19** | **1** | **K.AVKDLVVLLFETALLSSGFSLEDPQTHSNR.I** |
|  | 27503 | **829.8867** | **3315.5178** | **3315.5832** | **-0.0654** | **0** | **0** | **41** | **6** | **K.AFMEALQAGADISMIGQFGVGFYSAYLVAEK.V** |
|  | 28813 | **1225.1722** | **3672.4949** | **3672.7844** | **-0.2895** | **1** | **1** | **21** | **5** | **K.SGTKAFMEALQAGADISMIGQFGVGFYSAYLVAEK.V** |
|  | 30031 | **831.3789** | **4151.8582** | **4150.8536** | **1.0046** | **1** | **2** | **16** | **9** | **K.LGLGIDEDEVAAEEPNAAVPDEIPPLEGDEDASRMEEVD.-** |

| **133.** | Q6FI13\|H2A2A_HUMAN    **Mass:** 14087    **Score:** 234    **Queries matched:** 9   **emPAI:** 0.63 |
| --- | --- |
|  | Histone H2A type 2-A OS=Homo sapiens GN=HIST2H2AA3 PE=1 SV=3 |

|  | **Query** | **Observed** | **Mr(expt)** | **Mr(calc)** | **Delta** | **Miss** | **Score** | **Expect** | **Rank** | **Peptide** |
| --- | --- | --- | --- | --- | --- | --- | --- | --- | --- | --- |
|  | 2185 | **425.7640** | **849.5135** | **849.5184** | **-0.0049** | **0** | **27** | **0.35** | **1** | **R.HLQLAIR.N** 2184 |
|  | 2337 | **431.2445** | **860.4744** | **860.3875** | **0.0869** | **0** | **7** | **42** | **4** | **R.NDEELNK.L** |
|  | 3484 | **472.7663** | **943.5180** | **943.5240** | **-0.0060** | **0** | **40** | **0.019** | **1** | **R.AGLQFPVGR.V** 3470 |
|  | 18534 | **966.0835** | **1930.1524** | **1930.1615** | **-0.0091** | **0** | **19** | **1.1** | **1** | **K.VTIAQGGVLPNIQAVLLPK.K** |
|  | 25782 | **1467.2699** | **2932.5252** | **2932.5368** | **-0.0115** | **0** | **124** | **1.9e-011** | **1** | **R.VGAGAPVYMAAVLEYLTAEILELAGNAAR.D** 25783 25784 |

|  | **Proteins matching the same set of peptides:** |
| --- | --- |

|  | Q16777\|H2A2C_HUMAN    **Mass:** 13980    **Score:** 234    **Queries matched:** 9 |
| --- | --- |
|  | Histone H2A type 2-C OS=Homo sapiens GN=HIST2H2AC PE=1 SV=4 |

| **134.** | Q8N2G8\|GHDC_HUMAN    **Mass:** 58058    **Score:** 233    **Queries matched:** 10   **emPAI:** 0.21 |
| --- | --- |
|  | GH3 domain-containing protein OS=Homo sapiens GN=GHDC PE=1 SV=2 |

|  | **Query** | **Observed** | **Mr(expt)** | **Mr(calc)** | **Delta** | **Miss** | **Score** | **Expect** | **Rank** | **Peptide** |
| --- | --- | --- | --- | --- | --- | --- | --- | --- | --- | --- |
|  | 2729 | **444.2376** | **886.4607** | **886.4661** | **-0.0054** | **0** | **14** | **8.9** | **1** | **R.AVGQWAGAK.L** |
|  | 9634 | **667.3006** | **1332.5866** | **1331.7099** | **0.8768** | **1** | **3** | **76** | **5** | **K.SLRFWGSVGPAR.V** |
|  | 11301 | **715.8123** | **1429.6100** | **1430.7630** | **-1.1530** | **1** | **4** | **50** | **4** | **K.AYPEVLAQGRTAR.V** |
|  | 11594 | **483.2463** | **1446.7170** | **1446.7262** | **-0.0092** | **1** | **13** | **7.4** | **1** | **R.HRHLAQCLQER.V** |
|  | 22248 | **776.7457** | **2327.2152** | **2327.5146** | **-0.2994** | **0** | **2** | **50** | **1** | **-.MLLWPLLLLLLLLPTLALLR.Q** |
|  | 23534 | **841.0871** | **2520.2395** | **2519.2504** | **0.9891** | **1** | **28** | **0.12** | **1** | **R.LDQTLSVRGEDIGEDLFSEALGR.A** |
|  | 28967 | **1240.9801** | **3719.9185** | **3719.9258** | **-0.0073** | **0** | **7** | **6.7** | **1** | **R.ALEAGTAVELLDVFLGLETDGEELAGAIAAGNPGAPLR.E** |
|  | 29675 | **1336.0253** | **4005.0540** | **4005.0694** | **-0.0155** | **1** | **149** | **3.3e-014** | **1** | **R.ALEAGTAVELLDVFLGLETDGEELAGAIAAGNPGAPLRER.A** 29674 |
|  | 29699 | **1341.0017** | **4019.9833** | **4019.9923** | **-0.0090** | **0** | **52** | **0.00018** | **1** | **K.ASQTQQEDSGEQPLPPTSNQDLGEASLQATLLGLAALNK.A** |

| **135.** | Q92597\|NDRG1_HUMAN    **Mass:** 43264    **Score:** 225    **Queries matched:** 12   **emPAI:** 0.51 |
| --- | --- |
|  | Protein NDRG1 OS=Homo sapiens GN=NDRG1 PE=1 SV=1 |

|  | **Query** | **Observed** | **Mr(expt)** | **Mr(calc)** | **Delta** | **Miss** | **Score** | **Expect** | **Rank** | **Peptide** |
| --- | --- | --- | --- | --- | --- | --- | --- | --- | --- | --- |
|  | 5798 | **559.3414** | **1116.6682** | **1116.5272** | **0.1410** | **1** | **3** | **89** | **7** | **R.SRSHTSEGTR.S** |
|  | 13762 | **790.9226** | **1579.8305** | **1579.8392** | **-0.0087** | **0** | **25** | **0.4** | **1** | **K.SIIGMGTGAGAYILTR.F** |
|  | 13774 | **791.3893** | **1580.7640** | **1580.7754** | **-0.0114** | **1** | **68** | **1.9e-005** | **1** | **R.SRTASGSSVTSLDGTR.S** |
|  | 13985 | **399.6766** | **1594.6772** | **1595.8341** | **-1.1569** | **0** | **(10)** | **12** | **4** | **K.SIIGMGTGAGAYILTR.F** |
|  | 17199 | **913.4019** | **1824.7893** | **1824.7997** | **-0.0104** | **0** | **87** | **1.9e-007** | **1** | **K.YFVQGMGYMPSASMTR.L** 17198 17216 |
|  | 22599 | **792.3721** | **2374.0944** | **2375.1102** | **-1.0158** | **0** | **36** | **0.016** | **1** | **R.SHTSEGAHLDITPNSGAAGNSAGPK.S** 22600 22608 |
|  | 29218 | **764.9703** | **3819.8153** | **3818.8396** | **0.9757** | **1** | **2** | **22** | **5** | **K.ISGWTQALPDMVVSHLFGKEEMQSNVEVVHTYR.Q** |
|  | 30263 | **1077.0293** | **4304.0881** | **4304.1127** | **-0.0246** | **0** | **17** | **0.5** | **1** | **R.DLEIERPMPGTHTVTLQCPALLVVGDSSPAVDAVVECNSK.L** |

| **136.** | P07339\|CATD_HUMAN    **Mass:** 45037    **Score:** 223    **Queries matched:** 11   **emPAI:** 0.38 |
| --- | --- |
|  | Cathepsin D OS=Homo sapiens GN=CTSD PE=1 SV=1 |

|  | **Query** | **Observed** | **Mr(expt)** | **Mr(calc)** | **Delta** | **Miss** | **Score** | **Expect** | **Rank** | **Peptide** |
| --- | --- | --- | --- | --- | --- | --- | --- | --- | --- | --- |
|  | 2611 | **440.2479** | **878.4812** | **878.4498** | **0.0314** | **0** | **23** | **0.83** | **1** | **R.QVFGEATK.Q** |
|  | 7794 | **620.3106** | **1238.6067** | **1238.6118** | **-0.0051** | **0** | **62** | **8.7e-005** | **1** | **K.FDGILGMAYPR.I** 7793 |
|  | 8826 | **646.3075** | **1290.6004** | **1290.6092** | **-0.0087** | **1** | **33** | **0.065** | **1** | **K.YNSDKSSTYVK.N** 8827 |
|  | 19169 | **995.0068** | **1987.9990** | **1988.0111** | **-0.0121** | **0** | **(11)** | **7.1** | **2** | **K.AIGAVPLIQGEYMIPCEK.V** |
|  | 19358 | **1003.5087** | **2005.0028** | **2004.0060** | **0.9968** | **0** | **23** | **0.47** | **1** | **K.AIGAVPLIQGEYMIPCEK.V** |
|  | 22283 | **584.3043** | **2333.1879** | **2333.0879** | **0.1000** | **0** | **0** | **72** | **7** | **K.EGCEAIVDTGTSLMVGPVDEVR.E** |
|  | 26144 | **1005.2183** | **3012.6330** | **3011.6439** | **0.9890** | **1** | **2** | **33** | **1** | **K.AIGAVPLIQGEYMIPCEKVSTLPAITLK.L** |
|  | 27738 | **1124.2074** | **3369.6004** | **3369.6187** | **-0.0184** | **1** | **95** | **1.2e-008** | **1** | **K.LVDQNIFSFYLSRDPDAQPGGELMLGGTDSK.Y** 27739 |

| **137.** | P07900\|HS90A_HUMAN    **Mass:** 85006    **Score:** 223    **Queries matched:** 35   **emPAI:** 0.41 |
| --- | --- |
|  | Heat shock protein HSP 90-alpha OS=Homo sapiens GN=HSP90AA1 PE=1 SV=5 |

|  | **Query** | **Observed** | **Mr(expt)** | **Mr(calc)** | **Delta** | **Miss** | **Score** | **Expect** | **Rank** | **Peptide** |
| --- | --- | --- | --- | --- | --- | --- | --- | --- | --- | --- |
|  | 1207 | 731.1756 | 730.1683 | 729.4385 | 0.7299 | 0 | 5 | 57 | 1 | K.LSELLR.Y |
|  | 1625 | 405.6919 | 809.3692 | 809.3742 | -0.0049 | 0 | 23 | 0.73 | 1 | K.FENLCK.I |
|  | 2028 | 420.7607 | 839.5068 | 838.4735 | 1.0333 | 1 | 16 | 3.9 | 2 | R.IYRMIK.L |
|  | 3511 | 473.7746 | 945.5347 | 945.5429 | -0.0082 | 1 | 7 | 42 | 6 | R.IMKAQALR.D |
|  | 3744 | **482.7392** | **963.4639** | **962.4127** | **1.0512** | **0** | **24** | **0.68** | **1** | **R.TDTGEPMGR.G** |
|  | 6175 | 575.7858 | 1149.5571 | 1150.5506 | -0.9935 | 0 | 17 | 3.1 | 1 | K.YIDQEELNK.T |
|  | 6711 | **594.7511** | **1187.4876** | **1187.4951** | **-0.0074** | **0** | **57** | **0.00029** | **1** | **R.DNSTMGYMAAK.K** |
|  | 7359 | **610.2755** | **1218.5365** | **1219.4849** | **-0.9484** | **0** | **(2)** | **98** | **10** | **R.DNSTMGYMAAK.K** |
|  | 7845 | 621.8527 | 1241.6908 | 1241.6979 | -0.0072 | 0 | 22 | 0.9 | 1 | K.ADLINNLGTIAK.S 7870 |
|  | 9349 | **659.3793** | **1316.7440** | **1315.5900** | **1.1540** | **1** | **(1)** | **1.1e+002** | **8** | **R.DNSTMGYMAAKK.H** |
|  | 9887 | **450.2158** | **1347.6255** | **1347.5799** | **0.0457** | **1** | **9** | **22** | **5** | **R.DNSTMGYMAAKK.H** 9886 |
|  | 12693 | 757.3920 | 1512.7694 | 1512.7784 | -0.0090 | 0 | 66 | 3.1e-005 | 1 | R.GVVDSEDLPLNISR.E 12656 |
|  | 12907 | 764.3709 | 1526.7271 | 1526.7365 | -0.0094 | 0 | 57 | 0.00026 | 1 | K.SLTNDWEDHLAVK.H 12908 12909 |
|  | 13423 | **780.4182** | **1558.8219** | **1559.8155** | **-0.9936** | **1** | **8** | **22** | **3** | **R.ELISNSSDALDKIR.Y** |
|  | 16386 | **587.1135** | **1758.3186** | **1758.8029** | **-0.4843** | **1** | **1** | **63** | **4** | **K.AQALRDNSTMGYMAAK.K** |
|  | 18318 | **479.5118** | **1914.0180** | **1914.0323** | **-0.0143** | **1** | **15** | **2.8** | **1** | **K.KHLEINPDHSIIETLR.Q** |
|  | 18740 | **650.3556** | **1948.0451** | **1949.0105** | **-0.9654** | **1** | **0** | **88** | **6** | **K.EIFLRELISNSSDALDK.I** |
|  | 21611 | **749.3248** | **2244.9527** | **2244.9667** | **-0.0141** | **1** | **24** | **0.27** | **1** | **R.YYTSASGDEMVSLKDYCTR.M** |
|  | 22196 | **581.0389** | **2320.1264** | **2320.0077** | **0.1187** | **1** | **2** | **46** | **9** | **K.ESEDKPEIEDVGSDEEEEKK.D** |
|  | 22839 | **1208.0853** | **2414.1561** | **2414.1650** | **-0.0089** | **0** | **2** | **47** | **2** | **R.VFIMDNCEELIPEYLNFIR.G** |
|  | 23013 | 814.4034 | 2440.1883 | 2440.2023 | -0.0140 | 1 | 59 | 0.0001 | 1 | K.HIYYITGETKDQVANSAFVER.L 23012 |
|  | 23889 | 858.4749 | 2572.4029 | 2572.4146 | -0.0117 | 1 | 37 | 0.012 | 1 | R.TLTIVDTGIGMTKADLINNLGTIAK.S |
|  | 23900 | **859.7589** | **2576.2547** | **2576.2655** | **-0.0107** | **0** | **5** | **23** | **1** | **K.HGLEVIYMIEPIDEYCVQQLK.E** |
|  | 25295 | **945.4613** | **2833.3621** | **2833.3271** | **0.0350** | **1** | **1** | **43** | **1** | **R.LVTSPCCIVTSTYGWTANMERIMK.A** |
|  | 26907 | **1056.8495** | **3167.5266** | **3166.5355** | **0.9912** | **1** | **40** | **0.0048** | **1** | **K.HGLEVIYMIEPIDEYCVQQLKEFEGK.T** 26893 |
|  | 27503 | 829.8867 | 3315.5178 | 3315.5832 | -0.0654 | 0 | 0 | 41 | 6 | K.AFMEALQAGADISMIGQFGVGFYSAYLVAEK.V |
|  | 28426 | **1782.2015** | **3562.3885** | **3562.5265** | **-0.1379** | **0** | **1** | **24** | **1** | **K.LGLGIDEDDPTADDTSAAVTEEMPPLEGDDDTSR.M** |
|  | 28813 | 1225.1722 | 3672.4949 | 3672.7844 | -0.2895 | 1 | 1 | 21 | 5 | K.SGTKAFMEALQAGADISMIGQFGVGFYSAYLVAEK.V |

| **138.** | P06396\|GELS_HUMAN    **Mass:** 86043    **Score:** 222    **Queries matched:** 22   **emPAI:** 0.23 |
| --- | --- |
|  | Gelsolin OS=Homo sapiens GN=GSN PE=1 SV=1 |

|  | **Query** | **Observed** | **Mr(expt)** | **Mr(calc)** | **Delta** | **Miss** | **Score** | **Expect** | **Rank** | **Peptide** |
| --- | --- | --- | --- | --- | --- | --- | --- | --- | --- | --- |
|  | 1779 | **412.5438** | **823.0731** | **822.3806** | **0.6924** | **0** | **(4)** | **39** | **1** | **K.MDAHPPR.L** |
|  | 2001 | **419.8801** | **837.7456** | **838.3756** | **-0.6299** | **0** | **7** | **19** | **1** | **K.MDAHPPR.L** |
|  | 2350 | **863.3760** | **862.3687** | **863.3508** | **-0.9821** | **0** | **14** | **8.7** | **2** | **K.DSQEEEK.T** |
|  | 2661 | **441.7294** | **881.4442** | **881.4494** | **-0.0053** | **0** | **21** | **1.1** | **1** | **K.TASDFITK.M** |
|  | 5205 | **538.2461** | **1074.4776** | **1073.5101** | **0.9675** | **0** | **6** | **44** | **5** | **R.EGGQTAPASTR.L** |
|  | 5234 | **539.7585** | **1077.5024** | **1077.5091** | **-0.0067** | **0** | **10** | **15** | **1** | **R.YIETDPANR.D** |
|  | 8534 | **638.3577** | **1274.7009** | **1274.7095** | **-0.0086** | **0** | **15** | **4.5** | **1** | **K.HVVPNEVVVQR.L** |
|  | 9379 | 660.3484 | 1318.6823 | 1318.6881 | -0.0057 | 0 | 53 | 0.00069 | 1 | K.AGALNSNDAFVLK.T |
|  | 9912 | **675.3216** | **1348.6286** | **1348.6371** | **-0.0085** | **1** | **3** | **79** | **8** | **R.YIETDPANRDR.R** |
|  | 15897 | **861.9178** | **1721.8210** | **1721.8301** | **-0.0091** | **0** | **1** | **80** | **4** | **R.EVQGFESATFLGYFK.S** |
|  | 17265 | **915.4799** | **1828.9452** | **1828.9571** | **-0.0119** | **0** | **20** | **0.93** | **1** | **K.QTQVSVLPEGGETPLFK.Q** |
|  | 19807 | **513.0358** | **2048.1142** | **2047.0634** | **1.0508** | **0** | **0** | **88** | **10** | **K.EPAHLMSLFGGKPMIIYK.G** |
|  | 21830 | **1136.5421** | **2271.0697** | **2271.0808** | **-0.0111** | **0** | **90** | **8.3e-008** | **1** | **R.AQPVQVAEGSEPDGFWEALGGK.A** 21827 |
|  | 23450 | **502.2358** | **2506.1425** | **2505.2872** | **0.8553** | **1** | **2** | **43** | **9** | **K.EPAHLMSLFGGKPMIIYKGGTSR.E** |
|  | 23676 | **509.4809** | **2542.3683** | **2542.3804** | **-0.0121** | **1** | **6** | **15** | **1** | **R.VVQGKEPAHLMSLFGGKPMIIYK.G** |
|  | 24640 | 542.0851 | 2705.3893 | 2705.3660 | 0.0233 | 1 | 2 | 39 | 10 | K.TPSAAYLWVGTGASEAEKTGAQELLR.V |
|  | 25347 | **711.5946** | **2842.3493** | **2842.3634** | **-0.0141** | **1** | **7** | **12** | **1** | **K.NWRDPDQTDGLGLSYLSSHIANVER.V** |
|  | 26418 | **1020.8660** | **3059.5761** | **3059.5896** | **-0.0135** | **1** | **26** | **0.12** | **1** | **R.VEKFDLVPVPTNLYGDFFTGDAYVILK.T** |
|  | 27850 | **1133.8835** | **3398.6288** | **3398.6459** | **-0.0171** | **1** | **61** | **3.5e-005** | **1** | **R.IEGSNKVPVDPATYGQFYGGDSYIILYNYR.H** 27851 |
|  | 29576 | **792.3896** | **3956.9116** | **3956.9425** | **-0.0310** | **1** | **9** | **3.5** | **1** | **R.VHVSEEGTEPEAMLQVLGPKPALPAGTEDTAKEDAANR.K** |

| **139.** | P27338\|AOFB_HUMAN    **Mass:** 59238    **Score:** 222    **Queries matched:** 36   **emPAI:** 1.08 |
| --- | --- |
|  | Amine oxidase [flavin-containing] B OS=Homo sapiens GN=MAOB PE=1 SV=3 |

|  | **Query** | **Observed** | **Mr(expt)** | **Mr(calc)** | **Delta** | **Miss** | **Score** | **Expect** | **Rank** | **Peptide** |
| --- | --- | --- | --- | --- | --- | --- | --- | --- | --- | --- |
|  | 2817 | 448.7283 | 895.4420 | 895.4473 | -0.0053 | 0 | 30 | 0.16 | 1 | K.LCELYAK.V |
|  | 3346 | **466.7442** | **931.4738** | **931.4797** | **-0.0059** | **0** | **24** | **0.7** | **1** | **R.IMDLLGDR.V** |
|  | 3993 | **491.7901** | **981.5657** | **981.5720** | **-0.0063** | **1** | **22** | **0.92** | **1** | **R.VLRQPVDR.I** |
|  | 4557 | **512.2792** | **1022.5439** | **1022.5509** | **-0.0069** | **1** | **10** | **16** | **1** | **R.TYTLRNQK.V** |
|  | 4567 | 512.7747 | 1023.5348 | 1023.5423 | -0.0075 | 1 | 27 | 0.31 | 1 | K.KLCELYAK.V 4568 4569 |
|  | 5837 | 561.2955 | 1120.5765 | 1121.5466 | -0.9700 | 0 | 73 | 7.4e-006 | 1 | K.FVGGSGQVSER.I 5856 |
|  | 6518 | **588.7928** | **1175.5710** | **1174.5942** | **0.9768** | **0** | **13** | **8.2** | **2** | **R.IISTTNGGQER.K** |
|  | 7250 | **405.2267** | **1212.6583** | **1211.6444** | **1.0139** | **1** | **2** | **83** | **7** | **R.AAREILHAMGK.I** |
|  | 8025 | 417.5519 | 1249.6338 | 1249.6415 | -0.0077 | 1 | 48 | 0.0023 | 1 | R.KFVGGSGQVSER.I 8013 8024 8033 |
|  | 9971 | **676.8484** | **1351.6823** | **1351.6893** | **-0.0070** | **0** | **36** | **0.037** | **1** | **K.IHFNPPLPMMR.N** 9970 |
|  | 12509 | **501.5002** | **1501.4787** | **1501.8253** | **-0.3465** | **0** | **8** | **15** | **2** | **K.LERPVIYIDQTR.E** 12525 |
|  | 13306 | **776.4329** | **1550.8513** | **1550.6745** | **0.1768** | **0** | **1** | **1.1e+002** | **4** | **K.APLAEEWDNMTMK.E** |
|  | 13447 | **521.2584** | **1560.7533** | **1559.7595** | **0.9938** | **1** | **10** | **13** | **3** | **K.CIVYYKEPFWR.K** |
|  | 14605 | **545.3377** | **1632.9913** | **1633.9151** | **-0.9238** | **0** | **11** | **9** | **1** | **K.LLHDSGLNVVVLEAR.D** 14588 14589 |
|  | 15309 | **560.3327** | **1677.9763** | **1677.8573** | **0.1189** | **1** | **10** | **11** | **2** | **K.ELGLETYKVNEVER.L** |
|  | 17595 | **926.9757** | **1851.9369** | **1851.9479** | **-0.0111** | **1** | **68** | **1.5e-005** | **1** | **K.VKYVDLGGSYVGPTQNR.I** 17596 17597 |
|  | 18354 | **959.9603** | **1917.9061** | **1917.9142** | **-0.0081** | **0** | **55** | **0.0003** | **1** | **R.ENVLVETLNHEMYEAK.Y** 18355 |
|  | 24779 | **911.7427** | **2732.2064** | **2732.2177** | **-0.0113** | **0** | **26** | **0.14** | **1** | **R.IYFAGTETATHWSGYMEGAVEAGER.A** 24765 24778 |
|  | 29907 | **1027.2607** | **4105.0139** | **4105.0354** | **-0.0215** | **1** | **16** | **0.66** | **1** | **R.EILHAMGKIPEDEIWQSEPESVDVPAQPITTTFLER.H** 29918 |
|  | 31147 | **1236.0698** | **4940.2502** | **4940.2738** | **-0.0236** | **1** | **23** | **0.063** | **1** | **K.VLGSLEALEPVHYEEKNWCEEQYSGGCYTTYFPPGILTQYGR.V** |

| **140.** | O14828\|SCAM3_HUMAN    **Mass:** 38661    **Score:** 221    **Queries matched:** 9   **emPAI:** 0.45 |
| --- | --- |
|  | Secretory carrier-associated membrane protein 3 OS=Homo sapiens GN=SCAMP3 PE=1 SV=3 |

|  | **Query** | **Observed** | **Mr(expt)** | **Mr(calc)** | **Delta** | **Miss** | **Score** | **Expect** | **Rank** | **Peptide** |
| --- | --- | --- | --- | --- | --- | --- | --- | --- | --- | --- |
|  | 2878 | **450.7347** | **899.4548** | **898.5124** | **0.9424** | **1** | **31** | **0.15** | **1** | **R.KLSPTEPK.N** |
|  | 9472 | **662.8477** | **1323.6809** | **1323.6895** | **-0.0086** | **0** | **29** | **0.16** | **1** | **R.ELQHAALGGTATR.Q** |
|  | 14203 | **537.2821** | **1608.8245** | **1608.8332** | **-0.0087** | **1** | **18** | **2** | **1** | **R.ERELQHAALGGTATR.Q** |
|  | 15477 | **846.4233** | **1690.8321** | **1690.8427** | **-0.0106** | **0** | **35** | **0.033** | **1** | **K.AQQEFAAGVFSNPAVR.T** 15490 |
|  | 19448 | **1008.9914** | **2015.9682** | **2015.9799** | **-0.0117** | **0** | **122** | **5.4e-011** | **1** | **K.NYGSYSTQASAAAATAELLK.K** 19447 |
|  | 25568 | **578.6925** | **2888.4261** | **2887.6894** | **0.7367** | **0** | **1** | **46** | **6** | **K.GNTAVSVLMLLVALLFTGIAVLGIVMLK.R** |
|  | 28824 | **736.5612** | **3677.7697** | **3678.7233** | **-0.9536** | **1** | **1** | **31** | **1** | **-.MAQSRDGGNPFAEPSELDNPFQDPAVIQHRPSR.Q** |

| **141.** | Q9BSJ8\|ESYT1_HUMAN    **Mass:** 123293   **Score:** 217    **Queries matched:** 27   **emPAI:** 0.19 |
| --- | --- |
|  | Extended synaptotagmin-1 OS=Homo sapiens GN=ESYT1 PE=1 SV=1 |

|  | **Query** | **Observed** | **Mr(expt)** | **Mr(calc)** | **Delta** | **Miss** | **Score** | **Expect** | **Rank** | **Peptide** |
| --- | --- | --- | --- | --- | --- | --- | --- | --- | --- | --- |
|  | 1331 | **395.2466** | **788.4787** | **787.4803** | **0.9983** | **1** | **16** | **5.7** | **6** | **R.KLDVSVK.S** |
|  | 2775 | **446.7311** | **891.4477** | **892.4654** | **-1.0178** | **1** | **4** | **78** | **3** | **K.GKSDPYVK.L** |
|  | 4392 | 505.7688 | 1009.5230 | 1009.5491 | -0.0261 | 0 | 5 | 49 | 2 | K.GMQLHGVLR.V 4395 |
|  | 5430 | **546.3534** | **1090.6922** | **1091.5247** | **-0.8325** | **0** | **8** | **25** | **1** | **R.TLSPEFNER.F** 5452 |
|  | 7286 | **405.8886** | **1214.6438** | **1215.5302** | **-0.8864** | **1** | **8** | **23** | **10** | **K.SNSSFMSRER.E** |
|  | 10501 | **461.2591** | **1380.7554** | **1380.7660** | **-0.0106** | **1** | **2** | **86** | **9** | **K.AGVKGMQLHGVLR.V** |
|  | 10760 | 466.5793 | 1396.7162 | 1395.7769 | 0.9393 | 1 | 11 | 11 | 1 | K.LVSIVHGCRSLR.Q |
|  | 10848 | **701.8474** | **1401.6803** | **1401.6987** | **-0.0184** | **0** | **12** | **8.8** | **1** | **R.QLLDDEEQLTAK.T** |
|  | 12256 | **496.2530** | **1485.7372** | **1486.7456** | **-1.0084** | **1** | **2** | **75** | **4** | **K.LTLWYYSEERK.L** |
|  | 12565 | **502.9239** | **1505.7499** | **1504.7423** | **1.0076** | **0** | **6** | **27** | **3** | **R.GSNPHLQTFTFTR.V** |
|  | 13953 | **398.9468** | **1591.7579** | **1590.8518** | **0.9061** | **0** | **5** | **40** | **8** | **R.WFTLSSGQGQVLLR.A** |
|  | 15768 | **571.6213** | **1711.8420** | **1711.8530** | **-0.0109** | **0** | **34** | **0.05** | **1** | **K.HLSPYATLTVGDSSHK.T** |
|  | 15789 | **857.9478** | **1713.8809** | **1713.8897** | **-0.0088** | **0** | **100** | **1.2e-008** | **1** | **K.VQLDLAETDLSQGVAR.W** |
|  | 16746 | 596.9661 | 1787.8766 | 1788.0509 | -0.1744 | 0 | 8 | 15 | 5 | R.LLVPLVPDLQDVAQLR.S |
|  | 17238 | **914.9131** | **1827.8117** | **1827.8250** | **-0.0133** | **0** | **21** | **0.71** | **1** | **K.AVYSTNCPVWEEAFR.F** 17240 |
|  | 20381 | **1054.5320** | **2107.0494** | **2107.0586** | **-0.0092** | **0** | **66** | **2.2e-005** | **1** | **K.TISQTSAPVWDESASFLIR.K** |
|  | 20623 | **1069.0016** | **2135.9886** | **2136.1215** | **-0.1329** | **1** | **4** | **36** | **1** | **R.QNGRDPPDPYVSLLLLPDK.N** |
|  | 22311 | **780.6106** | **2338.8100** | **2338.3736** | **0.4363** | **1** | **1** | **47** | **4** | **R.LLVPLVPDLQDVAQLRSPLPR.G** |
|  | 22469 | **590.0590** | **2356.2070** | **2357.1533** | **-0.9463** | **1** | **1** | **66** | **7** | **K.GNKEPNPMVQLSIQDVTQESK.A** |
|  | 22721 | **798.7201** | **2393.1384** | **2393.1499** | **-0.0115** | **1** | **23** | **0.34** | **1** | **R.FFLQDPQSQELDVQVKDDSR.A** |
|  | 23840 | **1281.6534** | **2561.2923** | **2561.3013** | **-0.0090** | **0** | **63** | **3.4e-005** | **1** | **R.LTTVLNSGFLDEWLTLEDVPSGR.L** 23839 |
|  | 24488 | **893.4918** | **2677.4536** | **2677.4650** | **-0.0114** | **0** | **10** | **6** | **1** | **R.LTPRPTAAELEEVLQVNSLIQTQK.S** |
|  | 30625 | 756.3817 | 4532.2466 | 4533.2957 | -1.0491 | 0 | 1 | 14 | 5 | R.RPTLDINWTGMTNLLDIPGLSSLSDTMIMDSIAAFLVLPNR.L |

| **142.** | P02749\|APOH_HUMAN    **Mass:** 39584    **Score:** 215    **Queries matched:** 21   **emPAI:** 0.57 |
| --- | --- |
|  | Beta-2-glycoprotein 1 OS=Homo sapiens GN=APOH PE=1 SV=3 |

|  | **Query** | **Observed** | **Mr(expt)** | **Mr(calc)** | **Delta** | **Miss** | **Score** | **Expect** | **Rank** | **Peptide** |
| --- | --- | --- | --- | --- | --- | --- | --- | --- | --- | --- |
|  | 4144 | **496.7468** | **991.4791** | **991.4281** | **0.0510** | **0** | **24** | **0.74** | **1** | **K.TDASDVKPC.-** 4140 4141 4162 |
|  | 4544 | **511.7638** | **1021.5130** | **1021.5193** | **-0.0063** | **0** | **12** | **11** | **1** | **K.ATVVYQGER.V** |
|  | 12516 | **751.8883** | **1501.7621** | **1501.7711** | **-0.0091** | **0** | **44** | **0.0047** | **1** | **R.VCPFAGILENGAVR.Y** 12482 12505 |
|  | 16539 | **886.8086** | **1771.6026** | **1771.9695** | **-0.3669** | **0** | **20** | **0.87** | **1** | **K.FICPLTGLWPINTLK.C** |
|  | 21207 | **734.3922** | **2200.1548** | **2199.2238** | **0.9310** | **0** | **3** | **42** | **3** | **M.ISPVLILFSSFLCHVAIAGR.T** |
|  | 21330 | **738.6686** | **2212.9839** | **2212.9980** | **-0.0141** | **1** | **10** | **7.4** | **1** | **K.KCSYTEDAQCIDGTIEVPK.C** 21329 |
|  | 22651 | **1192.0427** | **2382.0709** | **2382.0838** | **-0.0129** | **0** | **90** | **8.3e-008** | **1** | **K.TFYEPGEEITYSCKPGYVSR.G** 22650 22652 |
|  | 22674 | **795.9992** | **2384.9758** | **2384.9889** | **-0.0131** | **0** | **25** | **0.22** | **1** | **K.ATFGCHDGYSLDGPEEIECTK.L** |
|  | 24176 | **877.0387** | **2628.0943** | **2628.1108** | **-0.0166** | **1** | **23** | **0.25** | **1** | **K.DKATFGCHDGYSLDGPEEIECTK.L** 24175 |
|  | 25234 | **940.8206** | **2819.4399** | **2818.4550** | **0.9848** | **0** | **5** | **20** | **1** | **K.WSPELPVCAPIICPPPSIPTFATLR.V** |
|  | 28288 | **1175.2473** | **3522.7201** | **3522.7350** | **-0.0149** | **1** | **54** | **0.00016** | **1** | **K.CTEEGKWSPELPVCAPIICPPPSIPTFATLR.V** 28290 |

| **143.** | P36268\|GGT2_HUMAN    **Mass:** 62074    **Score:** 210    **Queries matched:** 18   **emPAI:** 0.34 |
| --- | --- |
|  | Gamma-glutamyltranspeptidase 2 OS=Homo sapiens GN=GGT2 PE=1 SV=3 |

|  | **Query** | **Observed** | **Mr(expt)** | **Mr(calc)** | **Delta** | **Miss** | **Score** | **Expect** | **Rank** | **Peptide** |
| --- | --- | --- | --- | --- | --- | --- | --- | --- | --- | --- |
|  | 1219 | 734.9947 | 733.9874 | 733.4123 | 0.5752 | 0 | 3 | 70 | 1 | R.IVEAFR.F |
|  | 2238 | 428.2274 | 854.4402 | 855.4562 | -1.0161 | 1 | 7 | 33 | 3 | K.RAVEEPR.L |
|  | 2278 | 429.6938 | 857.3730 | 857.4719 | -0.0989 | 1 | 3 | 1.1e+002 | 3 | K.VLREGER.L |
|  | 3839 | 486.7559 | 971.4972 | 972.4777 | -0.9805 | 0 | 33 | 0.097 | 1 | R.GYELAHQR.H 3876 |
|  | 4124 | 496.2474 | 990.4802 | 991.5464 | -1.0662 | 1 | 9 | 25 | 7 | R.HGRLPWAR.L |
|  | 5067 | 532.2894 | 1062.5643 | 1062.5710 | -0.0067 | 0 | 34 | 0.062 | 1 | K.FVDVTEVVR.N 5080 |
|  | 5139 | **535.3054** | **1068.5962** | **1069.5880** | **-0.9918** | **1** | **10** | **16** | **8** | **R.EGERLTLPR.L** |
|  | 6469 | 586.8398 | 1171.6650 | 1171.6713 | -0.0063 | 0 | 49 | 0.0019 | 1 | R.LFQPSIQLAR.Q |
|  | 9270 | 657.8625 | 1313.7104 | 1313.7132 | -0.0028 | 1 | 12 | 9.2 | 5 | R.IVEAFRFAYAK.R 9300 |
|  | 10828 | 701.3660 | 1400.7175 | 1400.7259 | -0.0084 | 0 | 87 | 2.7e-007 | 1 | R.NIDQAVTAALETR.H |
|  | 11313 | 477.6096 | 1429.8071 | 1429.6660 | 0.1411 | 0 | 23 | 0.71 | 1 | R.NMTSEFFAAQLR.S 11322 |
|  | 18376 | 960.4720 | 1918.9295 | 1918.9384 | -0.0089 | 0 | 124 | 3.7e-011 | 1 | K.DIQAAGGIVTAEDLNNYR.A |
|  | 20391 | 703.7077 | 2108.1013 | 2108.1127 | -0.0114 | 1 | 14 | 3.7 | 1 | K.GGLSVAVPGEIRGYELAHQR.H |
|  | 29851 | **817.2436** | **4081.1816** | **4081.0323** | **0.1493** | **0** | **3** | **11** | **1** | **R.DGGSAVDAAIAALLCVGLMNAHSMGIGVGLFLTIYNSTTGK.A** |

| **144.** | Q9NQC3\|RTN4_HUMAN    **Mass:** 130250   **Score:** 208    **Queries matched:** 18   **emPAI:** 0.15 |
| --- | --- |
|  | Reticulon-4 OS=Homo sapiens GN=RTN4 PE=1 SV=2 |

|  | **Query** | **Observed** | **Mr(expt)** | **Mr(calc)** | **Delta** | **Miss** | **Score** | **Expect** | **Rank** | **Peptide** |
| --- | --- | --- | --- | --- | --- | --- | --- | --- | --- | --- |
|  | 934 | 599.0203 | 598.0130 | 598.3438 | -0.3308 | 0 | 4 | 32 | 2 | K.NIQPK.V |
|  | 1024 | **632.3677** | **631.3605** | **630.4064** | **0.9540** | **1** | **18** | **5** | **2** | **K.KVSGIK.E** |
|  | 2572 | **439.2216** | **876.4286** | **875.4535** | **0.9751** | **1** | **10** | **18** | **4** | **K.NVKDAMAK.I** |
|  | 3485 | **472.7888** | **943.5630** | **943.4148** | **0.1482** | **0** | **15** | **6.1** | **4** | **K.SDEGHPFR.A** |
|  | 4158 | **497.2498** | **992.4850** | **993.4437** | **-0.9587** | **0** | **8** | **25** | **10** | **R.SSAENMDLK.E** |
|  | 8456 | **424.5792** | **1270.7157** | **1270.7034** | **0.0123** | **0** | **12** | **8.4** | **2** | **R.GPLPAAPPVAPER.Q** |
|  | 11437 | **719.8186** | **1437.6226** | **1438.6246** | **-1.0019** | **1** | **6** | **31** | **3** | **K.DSKEDSDMLAAGGK.I** |
|  | 13479 | **782.3776** | **1562.7407** | **1562.7511** | **-0.0104** | **0** | **71** | **9.1e-006** | **1** | **K.YSNSALGHVNCTIK.E** |
|  | 14178 | **804.4133** | **1606.8121** | **1606.8216** | **-0.0095** | **0** | **84** | **5.3e-007** | **1** | **R.HQAQIDHYLGLANK.N** 14148 14179 |
|  | 14646 | **546.3203** | **1635.9391** | **1635.8104** | **0.1287** | **1** | **5** | **37** | **3** | **K.EDRSPSAIFSAELSK.T** |
|  | 16964 | **904.4648** | **1806.9151** | **1806.9251** | **-0.0099** | **0** | **20** | **1.1** | **1** | **R.AYLESEVAISEELVQK.Y** |
|  | 17377 | **613.3323** | **1836.9752** | **1837.0309** | **-0.0557** | **1** | **4** | **45** | **3** | **K.AESAVIVANPREEIIVK.N** |
|  | 18739 | **488.0117** | **1948.0175** | **1948.0279** | **-0.0103** | **1** | **15** | **3.1** | **1** | **R.HQAQIDHYLGLANKNVK.D** |
|  | 19388 | **503.2792** | **2009.0876** | **2008.9259** | **0.1617** | **1** | **4** | **38** | **9** | **K.EDSDMLAAGGKIESNLESK.V** |
|  | 29076 | **627.5077** | **3759.0028** | **3758.8890** | **0.1138** | **0** | **1** | **27** | **3** | **K.EQPGNTISAGQEDFPSVLLETAASLPSLSPLSAASFK.E** |
|  | 30098 | **1396.2384** | **4185.6934** | **4185.7192** | **-0.0259** | **1** | **97** | **4.1e-009** | **1** | **K.YQFVREPEDEEEEEEEEEEDEDEDLEELEVLER.K** |

| **145.** | P21926\|CD9_HUMAN    **Mass:** 25969    **Score:** 205    **Queries matched:** 9   **emPAI:** 0.51 |
| --- | --- |
|  | CD9 antigen OS=Homo sapiens GN=CD9 PE=1 SV=4 |

|  | **Query** | **Observed** | **Mr(expt)** | **Mr(calc)** | **Delta** | **Miss** | **Score** | **Expect** | **Rank** | **Peptide** |
| --- | --- | --- | --- | --- | --- | --- | --- | --- | --- | --- |
|  | 6558 | **590.3312** | **1178.6478** | **1178.6547** | **-0.0069** | **1** | **44** | **0.0064** | **1** | **K.KDVLETFTVK.S** |
|  | 25076 | **1397.1529** | **2792.2913** | **2792.3084** | **-0.0171** | **0** | **97** | **1.1e-008** | **1** | **K.AIHYALNCCGLAGGVEQFISDICPK.K** 25077 25079 25080 25081 25089 |
|  | 27546 | **832.0873** | **3324.3203** | **3324.7446** | **-0.4243** | **0** | **3** | **17** | **1** | **K.FHIIGAVGIGIAVVMIFGMIFSMILCCAIR.R** |
|  | 29876 | **1023.2242** | **4088.8679** | **4089.0786** | **-0.2108** | **1** | **1** | **20** | **2** | **K.EVFDNKFHIIGAVGIGIAVVMIFGMIFSMILCCAIR.R** |

| **146.** | O15127\|SCAM2_HUMAN    **Mass:** 37082    **Score:** 203    **Queries matched:** 7   **emPAI:** 0.21 |
| --- | --- |
|  | Secretory carrier-associated membrane protein 2 OS=Homo sapiens GN=SCAMP2 PE=1 SV=2 |

|  | **Query** | **Observed** | **Mr(expt)** | **Mr(calc)** | **Delta** | **Miss** | **Score** | **Expect** | **Rank** | **Peptide** |
| --- | --- | --- | --- | --- | --- | --- | --- | --- | --- | --- |
|  | 1707 | **409.2280** | **816.4414** | **815.4501** | **0.9914** | **1** | **9** | **30** | **5** | **R.KAAELER.K** |
|  | 3334 | 466.2505 | 930.4864 | 929.5195 | 0.9669 | 1 | 5 | 56 | 4 | R.VHSLYRR.T |
|  | 10708 | **465.5766** | **1393.7080** | **1392.7473** | **0.9606** | **0** | **5** | **47** | **8** | **R.ELQNTVANLHVR.Q** |
|  | 15307 | **560.3010** | **1677.8813** | **1677.8910** | **-0.0098** | **1** | **42** | **0.008** | **1** | **K.ERELQNTVANLHVR.Q** 15308 |
|  | 21235 | **1103.0087** | **2204.0028** | **2204.0134** | **-0.0106** | **0** | **106** | **2.3e-009** | **1** | **R.TGASFQQAQEEFSQGIFSSR.T** 21234 |

| **147.** | P62820\|RAB1A_HUMAN    **Mass:** 22891    **Score:** 202    **Queries matched:** 31   **emPAI:** 1.17 |
| --- | --- |
|  | Ras-related protein Rab-1A OS=Homo sapiens GN=RAB1A PE=1 SV=3 |

|  | **Query** | **Observed** | **Mr(expt)** | **Mr(calc)** | **Delta** | **Miss** | **Score** | **Expect** | **Rank** | **Peptide** |
| --- | --- | --- | --- | --- | --- | --- | --- | --- | --- | --- |
|  | 4106 | **495.6060** | **989.1975** | **989.4818** | **-0.2843** | **0** | **4** | **45** | **8** | **R.TITSSYYR.G** |
|  | 4569 | 512.7840 | 1023.5534 | 1023.5601 | -0.0066 | 1 | 24 | 0.62 | 1 | K.KVVDYTTAK.E 4552 4583 4589 |
|  | 4847 | **523.7471** | **1045.4796** | **1045.4862** | **-0.0066** | **0** | **41** | **0.015** | **1** | **R.MGPGATAGGAEK.S** 4848 4849 4850 4856 4872 |
|  | 5171 | **536.3210** | **1070.6274** | **1070.6336** | **-0.0062** | **0** | **21** | **1.3** | **1** | **K.LLLIGDSGVGK.S** |
|  | 7056 | **602.3575** | **1202.7004** | **1201.5873** | **1.1131** | **1** | **37** | **0.032** | **1** | **K.RMGPGATAGGAEK.S** |
|  | 7381 | **407.2626** | **1218.7661** | **1217.5823** | **1.1838** | **1** | **(5)** | **52** | **7** | **K.RMGPGATAGGAEK.S** 7357 |
|  | 9315 | **658.8296** | **1315.6446** | **1315.6521** | **-0.0074** | **0** | **18** | **2.1** | **1** | **K.LQIWDTAGQER.F** 9284 |
|  | 12042 | **737.8648** | **1473.7151** | **1473.7246** | **-0.0095** | **1** | **51** | **0.001** | **1** | **R.MGPGATAGGAEKSNVK.I** 12043 12051 |
|  | 12328 | 745.8998 | 1489.7850 | 1489.7195 | 0.0655 | 1 | (9) | 15 | 5 | R.MGPGATAGGAEKSNVK.I 12329 |
|  | 14723 | **547.8219** | **1640.4439** | **1639.6898** | **0.7541** | **0** | **4** | **30** | **6** | **-.MSSMNPEYDYLFK.L** 14720 |
|  | 14973 | **829.6645** | **1657.3144** | **1657.8788** | **-0.5643** | **1** | **3** | **37** | **1** | **K.TIKLQIWDTAGQER.F** |
|  | 15938 | **862.9366** | **1723.8587** | **1723.8669** | **-0.0081** | **0** | **16** | **2.8** | **2** | **K.EFADSLGIPFLETSAK.N** |
|  | 17979 | **942.9399** | **1883.8653** | **1883.8757** | **-0.0104** | **0** | **66** | **2.3e-005** | **1** | **K.NATNVEQSFMTMAAEIK.K** 17978 17980 |
|  | 20967 | 1087.0000 | 2171.9854 | 2170.9947 | 0.9908 | 0 | 69 | 1.1e-005 | 1 | R.FADDTYTESYISTIGVDFK.I 20961 |

| **148.** | P69905\|HBA_HUMAN    **Mass:** 15305    **Score:** 201    **Queries matched:** 17   **emPAI:** 3.89 |
| --- | --- |
|  | Hemoglobin subunit alpha OS=Homo sapiens GN=HBA1 PE=1 SV=2 |

|  | **Query** | **Observed** | **Mr(expt)** | **Mr(calc)** | **Delta** | **Miss** | **Score** | **Expect** | **Rank** | **Peptide** |
| --- | --- | --- | --- | --- | --- | --- | --- | --- | --- | --- |
|  | 5163 | **536.2778** | **1070.5411** | **1070.5471** | **-0.0060** | **0** | **28** | **0.26** | **1** | **R.MFLSFPTTK.T** |
|  | 6452 | **586.3336** | **1170.6527** | **1170.6608** | **-0.0081** | **1** | **45** | **0.0047** | **1** | **M.VLSPADKTNVK.A** 6453 6454 |
|  | 12934 | **765.3659** | **1528.7173** | **1528.7270** | **-0.0097** | **0** | **71** | **1.1e-005** | **1** | **K.VGAHAGEYGAEALER.M** 12933 12935 12937 |
|  | 17320 | **917.4442** | **1832.8737** | **1832.8846** | **-0.0109** | **0** | **61** | **8.3e-005** | **1** | **K.TYFPHFDLSHGSAQVK.G** 17313 17321 17322 |
|  | 19743 | **681.6692** | **2041.9857** | **2041.9969** | **-0.0112** | **1** | **42** | **0.0063** | **1** | **K.AAWGKVGAHAGEYGAEALER.M** |
|  | 26063 | **749.8735** | **2995.4651** | **2995.4821** | **-0.0171** | **0** | **16** | **1.4** | **1** | **K.VADALTNAVAHVDDMPNALSALSDLHAHK.L** |
|  | 26716 | **781.8967** | **3123.5576** | **3123.5771** | **-0.0195** | **1** | **42** | **0.0031** | **1** | **K.KVADALTNAVAHVDDMPNALSALSDLHAHK.L** 26717 |
|  | 30202 | 852.4692 | 4257.3095 | 4257.3235 | -0.0140 | 1 | 13 | 1 | 1 | K.LLSHCLLVTLAAHLPAEFTPAVHASLDKFLASVSTVLTSK.Y |

| **149.** | A1L0T0\|ILVBL_HUMAN    **Mass:** 68452    **Score:** 197    **Queries matched:** 26   **emPAI:** 0.53 |
| --- | --- |
|  | Acetolactate synthase-like protein OS=Homo sapiens GN=ILVBL PE=1 SV=2 |

|  | **Query** | **Observed** | **Mr(expt)** | **Mr(calc)** | **Delta** | **Miss** | **Score** | **Expect** | **Rank** | **Peptide** |
| --- | --- | --- | --- | --- | --- | --- | --- | --- | --- | --- |
|  | 773 | **538.3419** | **537.3346** | **538.2976** | **-0.9629** | **0** | **5** | **45** | **1** | **R.AHGVR.F** |
|  | 3681 | **480.5604** | **959.1062** | **959.4560** | **-0.3498** | **0** | **4** | **55** | **9** | **R.ENEDQVVK.V** |
|  | 5262 | **540.7848** | **1079.5550** | **1079.5248** | **0.0303** | **1** | **15** | **5.1** | **2** | **R.TDFRDGSIAV.-** 5258 |
|  | 5852 | **561.8007** | **1121.5868** | **1121.5941** | **-0.0074** | **0** | **34** | **0.063** | **1** | **R.HGGENVAAVLR.A** |
|  | 8087 | **627.8002** | **1253.5859** | **1253.5935** | **-0.0076** | **0** | **46** | **0.0039** | **1** | **K.VLHDAQQQCR.D** |
|  | 10599 | **694.7256** | **1387.4367** | **1387.7936** | **-0.3568** | **0** | **5** | **28** | **1** | **R.DGHPVVVNILIGR.T** |
|  | 11904 | **489.2746** | **1464.8020** | **1464.8776** | **-0.0757** | **1** | **3** | **57** | **8** | **R.VLSHSSKIIIVNR.N** |
|  | 12534 | **502.2398** | **1503.6974** | **1503.7140** | **-0.0166** | **0** | **3** | **64** | **5** | **R.HEVTAVFAADAMAR.L** |
|  | 13008 | **768.3356** | **1534.6567** | **1534.8328** | **-0.1761** | **1** | **2** | **81** | **6** | **K.ASVRHGGENVAAVLR.A** |
|  | 14055 | **800.4336** | **1598.8527** | **1598.8628** | **-0.0100** | **1** | **21** | **0.89** | **1** | **R.GLLLSRENEDQVVK.V** |
|  | 16252 | **874.9405** | **1747.8664** | **1747.8750** | **-0.0085** | **0** | **38** | **0.018** | **1** | **R.AAVETLGVPCFLGGMAR.G** 16253 |
|  | 19334 | **668.6276** | **2002.8610** | **2004.0469** | **-1.1859** | **0** | **4** | **33** | **1** | **R.WLDPGAFGTLGVGAGFALGAK.L** |
|  | 20070 | 415.9797 | 2074.8623 | 2074.0266 | 0.8357 | 1 | 2 | 51 | 10 | R.VVDTRHEVTAVFAADAMAR.L |
|  | 21073 | **1092.6090** | **2183.2035** | **2183.2162** | **-0.0127** | **0** | **53** | **0.00036** | **1** | **R.LSGTVGVAAVTAGPGLTNTVTAVK.N** 21072 |
|  | 21451 | **1113.5561** | **2225.0977** | **2225.1117** | **-0.0139** | **0** | **57** | **0.00018** | **1** | **K.LVEGLQGQTWAPDWVEELR.E** 21452 |
|  | 22550 | **1184.1395** | **2366.2645** | **2366.2740** | **-0.0095** | **0** | **40** | **0.0071** | **1** | **K.NAQMAQSPILLLGGAASTLLQNR.G** 22549 |
|  | 24581 | **899.7749** | **2696.3029** | **2696.3194** | **-0.0166** | **1** | **19** | **0.84** | **1** | **K.LVEGLQGQTWAPDWVEELREADR.Q** |
|  | 30287 | **1081.2631** | **4321.0231** | **4321.1877** | **-0.1646** | **1** | **1** | **18** | **2** | **R.AAMAAAQSGTPGPVFVELPVDVLYPYFMVQKEMVPAKPPK.G** |
|  | 31054 | **1215.3900** | **4857.5309** | **4857.5488** | **-0.0178** | **0** | **49** | **0.00018** | **1** | **K.AAMPVAQHLNPVQVLQLVEETLPDNSILVVDGGDFVGTAAHLVQPR.G** 31055 31056 |

| **150.** | P12111\|CO6A3_HUMAN    **Mass:** 345163   **Score:** 194    **Queries matched:** 57   **emPAI:** 0.04 |
| --- | --- |
|  | Collagen alpha-3(VI) chain OS=Homo sapiens GN=COL6A3 PE=1 SV=4 |

|  | **Query** | **Observed** | **Mr(expt)** | **Mr(calc)** | **Delta** | **Miss** | **Score** | **Expect** | **Rank** | **Peptide** |
| --- | --- | --- | --- | --- | --- | --- | --- | --- | --- | --- |
|  | 494 | **447.1147** | **446.1074** | **446.2237** | **-0.1163** | **0** | **3** | **1.6e+002** | **4** | **R.AGGSR.V** |
|  | 661 | **501.0781** | **500.0709** | **499.2503** | **0.8206** | **0** | **6** | **44** | **2** | **K.GNPGR.R** 660 |
|  | 1254 | **763.1150** | **762.1077** | **762.3885** | **-0.2808** | **1** | **7** | **28** | **10** | **K.NGGFGRR.G** |
|  | 1504 | **401.2854** | **800.5562** | **799.4552** | **1.1010** | **0** | **8** | **34** | **5** | **K.VDAILNR.I** |
|  | 1807 | **413.6150** | **825.2155** | **824.4756** | **0.7399** | **0** | **9** | **14** | **2** | **R.GPIGSIGPK.G** 1800 1809 |
|  | 2675 | **442.4728** | **882.9311** | **882.4784** | **0.4527** | **1** | **3** | **47** | **8** | **K.GGIGNRGPR.G** |
|  | 2819 | 448.7325 | 895.4504 | 896.5120 | -1.0616 | 0 | 12 | 10 | 4 | K.YIAYLVR.Q 2850 |
|  | 2859 | 450.2158 | 898.4170 | 899.4825 | -1.0655 | 0 | 10 | 16 | 8 | K.QDVVNAVR.Q 2857 2858 |
|  | 4161 | **497.2769** | **992.5392** | **992.4927** | **0.0465** | **0** | **14** | **6.2** | **2** | **R.DSFQEVLR.F** |
|  | 4656 | **515.9562** | **1029.8978** | **1030.6288** | **-0.7310** | **1** | **1** | **97** | **3** | **R.GVKVFAVGVR.N** |
|  | 4740 | **519.2558** | **1036.4970** | **1036.5301** | **-0.0331** | **0** | **7** | **33** | **6** | **K.IASNSATAFR.V** |
|  | 5137 | **535.2867** | **1068.5588** | **1068.5927** | **-0.0339** | **0** | **17** | **2.8** | **7** | **R.SQAPVLDAIR.R** |
|  | 5165 | **536.2842** | **1070.5539** | **1069.5040** | **1.0499** | **0** | **12** | **12** | **4** | **R.DGVPGGPGETGK.N** 5168 |
|  | 6256 | **577.9376** | **1153.8607** | **1153.5801** | **0.2806** | **0** | **10** | **9.3** | **1** | **R.SSIMAFAIGNK.G** |
|  | 7245 | **607.3161** | **1212.6176** | **1213.7394** | **-1.1217** | **1** | **7** | **27** | **1** | **K.IKNLQVALTSK.Q** |
|  | 7764 | **413.5473** | **1237.6200** | **1237.6455** | **-0.0256** | **0** | **3** | **71** | **10** | **K.VAVFFSNTPTR.A** |
|  | 9891 | **450.2324** | **1347.6753** | **1348.6735** | **-0.9983** | **0** | **5** | **49** | **7** | **K.SVHNGAPAPVSGEK.D** |
|  | 9893 | **450.2430** | **1347.7072** | **1347.7146** | **-0.0074** | **1** | **4** | **57** | **10** | **K.GFESKVDAILNR.I** |
|  | 10888 | **469.2281** | **1404.6624** | **1404.7249** | **-0.0625** | **0** | **5** | **45** | **3** | **K.DVVFLLDGSEGVR.S** |
|  | 12015 | **491.5986** | **1471.7739** | **1470.7791** | **0.9948** | **1** | **8** | **18** | **1** | **R.VVESLDVGQDRVR.V** |
|  | 12215 | **495.2797** | **1482.8174** | **1482.7137** | **0.1038** | **0** | **2** | **72** | **7** | **K.QQSLETAMSFVAR.N** |
|  | 12950 | **765.8888** | **1529.7630** | **1529.7726** | **-0.0095** | **0** | **27** | **0.24** | **1** | **R.SQHPYVLTEDTLK.V** |
|  | 13201 | **773.8661** | **1545.7177** | **1546.7661** | **-1.0483** | **0** | **1** | **96** | **10** | **R.GDSIDQCALIQSIK.D** |
|  | 14026 | **533.2764** | **1596.8075** | **1595.8056** | **1.0019** | **0** | **3** | **53** | **5** | **K.QASVFSFGLGAQAASR.A** 13996 |
|  | 14340 | 809.4761 | 1616.9376 | 1616.9436 | -0.0060 | 0 | 46 | 0.0027 | 1 | R.AAPLQGMLPGLLAPLR.T |
|  | 15409 | **843.9844** | **1685.9542** | **1684.9611** | **0.9931** | **0** | **59** | **0.00014** | **1** | **K.LLTPITTLTSEQIQK.L** 15401 15407 |
|  | 15505 | **565.2737** | **1692.7994** | **1693.8093** | **-1.0099** | **1** | **0** | **1e+002** | **5** | **K.STELNEEPLMRFGR.L** |
|  | 16158 | **871.4537** | **1740.8928** | **1742.0203** | **-1.1275** | **1** | **1** | **77** | **1** | **R.LQPVLQPLPSPGVGGKR.D** |
|  | 16160 | **581.3101** | **1740.9084** | **1741.0138** | **-0.1055** | **0** | **2** | **67** | **8** | **R.IEDGVLQFLVLLVAGR.S** |
|  | 16758 | **597.3010** | **1788.8813** | **1789.8880** | **-1.0067** | **1** | **3** | **55** | **4** | **R.GDSIDQCALIQSIKDK.C** |
|  | 17246 | **610.3344** | **1827.9814** | **1826.8734** | **1.1081** | **1** | **4** | **38** | **6** | **R.QLDMSPDPKASQHFAR.V** |
|  | 17891 | **938.9646** | **1875.9146** | **1874.9487** | **0.9660** | **1** | **1** | **71** | **7** | **K.EFVQRVVESLDVGQDR.V** |
|  | 18338 | **639.6502** | **1915.9288** | **1915.0011** | **0.9277** | **1** | **9** | **13** | **1** | **K.SVEDAQDVSLALTQRGVK.V** |
|  | 18908 | **655.3458** | **1963.0155** | **1962.9258** | **0.0896** | **1** | **0** | **88** | **7** | **K.QCDWFQGDQPTKNLVK.F** |
|  | 19159 | 994.5074 | 1987.0003 | 1987.0091 | -0.0088 | 0 | 102 | 5.4e-009 | 1 | R.VGVVQFSNDVFPEFYLK.T 19170 |
|  | 19813 | **410.8384** | **2049.1558** | **2047.9997** | **1.1561** | **0** | **6** | **20** | **8** | **R.DFVMNLVNSLDIGNDNIR.V** |
|  | 21146 | **439.0324** | **2190.1254** | **2191.1637** | **-1.0383** | **0** | **1** | **67** | **10** | **R.DIVFLVDGSSALGLANFNAIR.D** |
|  | 21618 | **749.4173** | **2245.2301** | **2246.1259** | **-0.8959** | **1** | **1** | **56** | **8** | **K.VNIKEVYTFASEPNDVFFK.L** |
|  | 21924 | 761.7104 | 2282.1093 | 2283.1607 | -1.0514 | 1 | 5 | 27 | 5 | K.VEFLLNAHSSKDEVQNAVQR.L |
|  | 23247 | **825.0473** | **2472.1201** | **2473.2377** | **-1.1176** | **0** | **2** | **43** | **1** | **R.VGLVQFSDTPVTEFSLNTYQTK.S** |
|  | 23552 | **631.5983** | **2522.3642** | **2522.1999** | **0.1643** | **1** | **3** | **30** | **3** | **R.VGVVQFSDEPRTMFSLDTYSTK.A** |
|  | 23859 | **856.0904** | **2565.2493** | **2565.2646** | **-0.0152** | **1** | **3** | **30** | **1** | **K.ACNLDVILGFDGSRDQNVFVAQK.G** |
|  | 25341 | **711.3438** | **2841.3461** | **2841.5236** | **-0.1775** | **1** | **0** | **56** | **8** | **R.VPQIAFVITGGKSVEDAQDVSLALTQR.G** |
|  | 26011 | **746.8493** | **2983.3681** | **2984.3220** | **-0.9539** | **1** | **2** | **35** | **1** | **R.GYPGDEGGPGERGPPGVNGTQGFQGCPGQR.G** |
|  | 27541 | **831.9328** | **3323.7021** | **3323.5578** | **0.1443** | **1** | **4** | **16** | **1** | **R.AAGSDVDIDMAFILDSAETTTLFQFNEMKK.Y** |
|  | 30522 | 1119.0217 | 4472.0578 | 4471.0243 | 1.0335 | 1 | 3 | 11 | 3 | R.DLTDFLENVLTCHVCLDICNIDPSCGFGSWRPSFRDR.R |

| **151.** | P61006\|RAB8A_HUMAN    **Mass:** 23824    **Score:** 194    **Queries matched:** 19   **emPAI:** 0.81 |
| --- | --- |
|  | Ras-related protein Rab-8A OS=Homo sapiens GN=RAB8A PE=1 SV=1 |

|  | **Query** | **Observed** | **Mr(expt)** | **Mr(calc)** | **Delta** | **Miss** | **Score** | **Expect** | **Rank** | **Peptide** |
| --- | --- | --- | --- | --- | --- | --- | --- | --- | --- | --- |
|  | 1863 | **830.4077** | **829.4004** | **828.3687** | **1.0317** | **0** | **24** | **0.87** | **1** | **K.FMETSAK.A** 1832 1833 |
|  | 2994 | **454.2086** | **906.4027** | **905.4025** | **1.0002** | **1** | **10** | **16** | **4** | **K.CDVNDKR.Q** |
|  | 5171 | 536.3210 | 1070.6274 | 1070.6336 | -0.0062 | 0 | 21 | 1.3 | 1 | K.LLLIGDSGVGK.T |
|  | 9315 | 658.8296 | 1315.6446 | 1315.6521 | -0.0074 | 0 | 18 | 2.1 | 1 | K.LQIWDTAGQER.F 9284 |
|  | 9755 | **671.3141** | **1340.6136** | **1340.6208** | **-0.0072** | **0** | **35** | **0.04** | **1** | **R.NIEEHASADVEK.M** |
|  | 11028 | **707.8455** | **1413.6765** | **1413.6848** | **-0.0083** | **0** | **61** | **0.0001** | **1** | **K.LEGNSPQGSNQGVK.I** |
|  | 13133 | **771.8918** | **1541.7691** | **1541.7798** | **-0.0106** | **1** | **70** | **1.2e-005** | **1** | **K.KLEGNSPQGSNQGVK.I** 13134 13135 13166 13169 |
|  | 13736 | **790.4102** | **1578.8059** | **1578.8154** | **-0.0095** | **0** | **78** | **1.7e-006** | **1** | **K.ANINVENAFFTLAR.D** |
|  | 15395 | **562.6180** | **1684.8321** | **1685.8004** | **-0.9683** | **0** | **12** | **6.5** | **1** | **R.GAMGIMLVYDITNEK.S** |
|  | 19263 | **666.3415** | **1996.0028** | **1996.9887** | **-0.9859** | **1** | **4** | **37** | **3** | **R.NIEEHASADVEKMILGNK.C** |
|  | 22598 | **792.3673** | **2374.0801** | **2373.1787** | **0.9014** | **1** | **6** | **16** | **2** | **K.FMETSAKANINVENAFFTLAR.D** |
|  | 25693 | **729.6279** | **2914.4824** | **2914.4212** | **0.0612** | **1** | **1** | **40** | **2** | **K.TCVLFRFSEDAFNSTFISTIGIDFK.I** |

| **152.** | O14975\|S27A2_HUMAN    **Mass:** 71066    **Score:** 193    **Queries matched:** 26   **emPAI:** 0.29 |
| --- | --- |
|  | Very long-chain acyl-CoA synthetase OS=Homo sapiens GN=SLC27A2 PE=1 SV=1 |

|  | **Query** | **Observed** | **Mr(expt)** | **Mr(calc)** | **Delta** | **Miss** | **Score** | **Expect** | **Rank** | **Peptide** |
| --- | --- | --- | --- | --- | --- | --- | --- | --- | --- | --- |
|  | 5109 | **534.2562** | **1066.4978** | **1067.6563** | **-1.1586** | **1** | **3** | **82** | **7** | **K.VRLALGNGLR.G** |
|  | 7702 | 618.3408 | 1234.6671 | 1233.5448 | 1.1223 | 1 | 43 | 0.008 | 1 | K.MKENHEFDGK.K |
|  | 9698 | **669.3274** | **1336.6402** | **1335.6241** | **1.0161** | **1** | **4** | **60** | **9** | **R.DENGYCVRVPK.G** |
|  | 9810 | **448.7047** | **1343.0921** | **1343.6721** | **-0.5800** | **1** | **2** | **63** | **8** | **K.KDDVSIYYVSR.T** |
|  | 10540 | **462.2646** | **1383.7719** | **1382.7823** | **0.9896** | **0** | **8** | **24** | **5** | **R.QTPHKPFLLFR.D** |
|  | 11236 | **476.2801** | **1425.8186** | **1425.7841** | **0.0345** | **1** | **10** | **12** | **2** | **R.LALGNGLRGDVWR.Q** 11223 11224 |
|  | 12146 | **740.8898** | **1479.7650** | **1479.7722** | **-0.0072** | **0** | **1** | **92** | **1** | **K.ITQLTPFNGYAGAK.A** |
|  | 13369 | **778.4565** | **1554.8985** | **1554.7105** | **0.1880** | **0** | **14** | **6.5** | **1** | **K.LGCAMACLNYNIR.A** |
|  | 13475 | **521.7606** | **1562.2599** | **1562.8014** | **-0.5416** | **0** | **3** | **42** | **10** | **K.MTLVEEGFNPAVIK.D** 13483 |
|  | 15006 | **553.9667** | **1658.8784** | **1657.8788** | **0.9996** | **1** | **2** | **70** | **4** | **R.IQDTIEITGTFKHR.K** |
|  | 15457 | **564.2536** | **1689.7390** | **1690.8964** | **-1.1574** | **1** | **4** | **47** | **4** | **R.KMTLVEEGFNPAVIK.D** |
|  | 16329 | **585.9416** | **1754.8029** | **1753.8426** | **0.9604** | **1** | **4** | **43** | **6** | **K.LGCAMACLNYNIRAK.S** 16314 |
|  | 17490 | **923.4381** | **1844.8617** | **1844.8688** | **-0.0072** | **0** | **55** | **0.00036** | **1** | **K.MYVPMTEDIYNAISAK.T** 17491 |
|  | 17705 | **621.3559** | **1861.0459** | **1860.8637** | **0.1821** | **0** | **(5)** | **32** | **4** | **K.MYVPMTEDIYNAISAK.T** |
|  | 22277 | **1167.0923** | **2332.1700** | **2332.1839** | **-0.0138** | **0** | **89** | **9.6e-008** | **1** | **R.SEVTFSTPALYIYTSGTTGLPK.A** 22278 |
|  | 26291 | **1013.1433** | **3036.4079** | **3036.4200** | **-0.0121** | **1** | **33** | **0.022** | **1** | **R.TSNTDGIDSFLDKVDEVSTEPIPESWR.S** |
|  | 26332 | **762.1060** | **3044.3950** | **3044.4127** | **-0.0177** | **1** | **27** | **0.09** | **1** | **K.KGDLYFNSGDLLMVDHENFIYFHDR.V** |
|  | 28632 | **1805.8231** | **3609.6317** | **3608.8768** | **0.7548** | **1** | **1** | **26** | **8** | **K.SLLHCFQCCGAKVLLVSPELQAAVEEILPSLK.K** |
|  | 29046 | **938.7059** | **3750.7944** | **3750.8126** | **-0.0182** | **0** | **5** | **11** | **1** | **K.GENVATTEVADTVGLVDFVQEVNVYGVHVPDHEGR.I** |
|  | 30629 | **1512.6941** | **4535.0604** | **4534.4565** | **0.6040** | **1** | **0** | **17** | **8** | **M.LSAIYTVLAGLLFLPLLVNLCCPYFFQDIGYFLKVAAVGR.R** |

| **153.** | P35749\|MYH11_HUMAN    **Mass:** 228054   **Score:** 193    **Queries matched:** 73   **emPAI:** 0.03 |
| --- | --- |
|  | Myosin-11 OS=Homo sapiens GN=MYH11 PE=1 SV=3 |

|  | **Query** | **Observed** | **Mr(expt)** | **Mr(calc)** | **Delta** | **Miss** | **Score** | **Expect** | **Rank** | **Peptide** |
| --- | --- | --- | --- | --- | --- | --- | --- | --- | --- | --- |
|  | 751 | 533.0582 | 532.0509 | 531.3017 | 0.7492 | 0 | 9 | 30 | 1 | R.IGQSK.I 752 753 755 756 |
|  | 757 | **533.1888** | **532.1816** | **531.2877** | **0.8938** | **1** | **7** | **64** | **1** | **R.RSGGR.R** |
|  | 871 | 572.9955 | 571.9882 | 571.3806 | 0.6076 | 1 | 9 | 24 | 2 | R.IKVGR.D |
|  | 882 | **576.3053** | **575.2980** | **575.2663** | **0.0317** | **0** | **8** | **43** | **9** | **K.NESAR.Q** 886 |
|  | 927 | 591.3232 | 590.3159 | 589.3257 | 0.9902 | 0 | 13 | 11 | 4 | K.MQLAK.K |
|  | 1507 | **401.2857** | **800.5568** | **801.4596** | **-0.9028** | **1** | **11** | **16** | **2** | **K.KLEGDLK.D** 1504 |
|  | 1609 | 405.1959 | 808.3773 | 807.4491 | 0.9282 | 0 | 19 | 2.1 | 1 | R.TVGQLYK.E |
|  | 2018 | **420.3173** | **838.6200** | **839.4613** | **-0.8414** | **1** | **2** | **75** | **5** | **K.ALDKTHR.Q** |
|  | 2123 | 424.0561 | 846.0977 | 845.4793 | 0.6184 | 1 | 6 | 37 | 8 | R.KLQAQMK.D |
|  | 2881 | 900.4841 | 899.4768 | 899.5189 | -0.0421 | 1 | 10 | 19 | 5 | K.VGRDVVQK.A |
|  | 3129 | **458.7838** | **915.5530** | **915.4774** | **0.0757** | **1** | **12** | **13** | **8** | **R.ARAELNDK.V** |
|  | 3417 | **469.8835** | **937.7525** | **937.5022** | **0.2503** | **0** | **5** | **35** | **3** | **R.IVFQEFR.Q** 3403 3412 |
|  | 4840 | 523.2856 | 1044.5567 | 1044.5637 | -0.0070 | 0 | 0 | 1.7e+002 | 9 | R.LEVNMQALK.G |
|  | 4858 | **524.2432** | **1046.4719** | **1045.5152** | **0.9567** | **1** | **16** | **4.8** | **4** | **R.ELEDARASR.D** 4860 |
|  | 5582 | **551.9453** | **1101.8761** | **1101.6030** | **0.2731** | **1** | **0** | **1.1e+002** | **5** | **K.VTVGKDDIQK.M** |
|  | 5595 | 552.2996 | 1102.5846 | 1102.5917 | -0.0071 | 1 | 22 | 1.1 | 1 | K.RQQQLTAMK.V |
|  | 5775 | 559.0590 | 1116.1035 | 1115.5710 | 0.5325 | 0 | 6 | 27 | 4 | R.DLGEELEALK.T |
|  | 5808 | 559.8027 | 1117.5908 | 1118.5866 | -0.9958 | 1 | (3) | 82 | 9 | K.RQQQLTAMK.V 5822 |
|  | 7029 | 601.8234 | 1201.6322 | 1201.6706 | -0.0385 | 0 | 5 | 55 | 9 | R.YEILAANAIPK.G 6991 |
|  | 7275 | **405.7425** | **1214.2057** | **1213.5509** | **0.6548** | **0** | **3** | **59** | **8** | **R.SHEAQVQEMR.Q** |
|  | 7393 | 610.8260 | 1219.6374 | 1219.6448 | -0.0074 | 1 | 30 | 0.14 | 1 | R.KFDQLLAEEK.N |
|  | 7400 | 407.7223 | 1220.1451 | 1219.6296 | 0.5156 | 1 | 7 | 19 | 10 | K.KDTSITGELEK.Q 7362 |
|  | 7587 | **615.3779** | **1228.7412** | **1228.7027** | **0.0385** | **1** | **4** | **66** | **6** | **K.LQLEKVTAEAK.I** |
|  | 8588 | **427.0750** | **1278.2032** | **1277.6000** | **0.6032** | **1** | **7** | **19** | **6** | **K.DFQRELEDAR.A** |
|  | 8774 | **645.2997** | **1288.5848** | **1288.7173** | **-0.1325** | **1** | **5** | **51** | **3** | **K.EQLGKLMTTLR.N** |
|  | 8812 | **430.8870** | **1289.6392** | **1290.6456** | **-1.0063** | **0** | **13** | **6.8** | **1** | **K.EQADFAVEALAK.A** 8805 8811 |
|  | 10279 | **685.8251** | **1369.6357** | **1369.6183** | **0.0174** | **1** | **3** | **70** | **4** | **K.MAEQYKEQAEK.G** |
|  | 12225 | 495.5715 | 1483.6927 | 1483.8246 | -0.1319 | 1 | 5 | 41 | 8 | K.LKEILLQVEDER.K |
|  | 12296 | **497.2352** | **1488.6839** | **1489.7260** | **-1.0421** | **0** | **5** | **44** | **3** | **K.EELAEELASSLSGR.N** |
|  | 12422 | **499.9376** | **1496.7909** | **1495.7262** | **1.0647** | **1** | **(0)** | **1.1e+002** | **7** | **K.MLKAEMEDLVSSK.D** |
|  | 12640 | **504.5852** | **1510.7339** | **1511.7211** | **-0.9872** | **1** | **6** | **34** | **10** | **K.MLKAEMEDLVSSK.D** 12641 |
|  | 13596 | **524.6171** | **1570.8296** | **1570.7306** | **0.0990** | **0** | **1** | **1e+002** | **8** | **K.ITDVIMAFQAMCR.G** 13610 |
|  | 13918 | 795.8720 | 1589.7295 | 1590.6944 | -0.9649 | 0 | 81 | 1e-006 | 1 | R.NTDQASMPDNTAAQK.V 13934 |
|  | 14630 | **546.0035** | **1634.9886** | **1635.7992** | **-0.8106** | **0** | **1** | **80** | **7** | **K.LQDFASTVEALEEGK.K** |
|  | 15042 | **554.9216** | **1661.7429** | **1661.8018** | **-0.0589** | **0** | **3** | **63** | **6** | **K.VCHLMGINVTDFTR.S** |
|  | 15048 | **831.9439** | **1661.8733** | **1661.8559** | **0.0174** | **1** | **3** | **52** | **4** | **R.LEVNMQALKGQFER.D** |
|  | 15471 | 846.3610 | 1690.7074 | 1690.7720 | -0.0646 | 0 | 0 | 96 | 6 | K.LEDEILVMDDQNNK.L |
|  | 15826 | 572.9688 | 1715.8844 | 1715.9206 | -0.0362 | 1 | 4 | 44 | 4 | K.ALELDPNLYRIGQSK.I |
|  | 15965 | 863.9725 | 1725.9305 | 1725.9413 | -0.0108 | 0 | 86 | 2.8e-007 | 1 | K.QLLQANPILEAFGNAK.T 15966 |
|  | 16283 | 584.6289 | 1750.8649 | 1750.8964 | -0.0315 | 1 | 1 | 84 | 9 | K.HLPIYSEKIVDMYK.G |
|  | 16725 | 596.3195 | 1785.9367 | 1786.9114 | -0.9747 | 1 | 5 | 38 | 5 | K.NFINSPVAQADWAAKR.L |
|  | 17248 | **610.3379** | **1827.9920** | **1828.8551** | **-0.8631** | **1** | **1** | **74** | **5** | **R.LEEEEDRGQQLQAER.K** |
|  | 17345 | **612.3072** | **1833.8999** | **1833.9124** | **-0.0124** | **1** | **1** | **85** | **1** | **R.TFHIFYYMIAGAKEK.M** |
|  | 17372 | **613.2998** | **1836.8776** | **1836.9444** | **-0.0668** | **1** | **3** | **51** | **7** | **R.YEILAANAIPKGFMDGK.Q** |
|  | 17914 | 470.2449 | 1876.9504 | 1875.8381 | 1.1123 | 1 | 2 | 65 | 8 | K.ERNTDQASMPDNTAAQK.V |
|  | 18665 | 972.3834 | 1942.7522 | 1942.9315 | -0.1793 | 1 | 0 | 70 | 4 | R.DLKITDVIMAFQAMCR.G |
|  | 18740 | 650.3556 | 1948.0451 | 1948.9225 | -0.8774 | 0 | 3 | 50 | 3 | K.TELEDTLDSTATQQELR.A |
|  | 18898 | **655.3026** | **1962.8860** | **1962.9204** | **-0.0344** | **0** | **15** | **2.9** | **1** | **K.LQNEVESVTGMLNEAEGK.A** |
|  | 20590 | **534.0056** | **2131.9934** | **2131.0377** | **0.9557** | **1** | **5** | **31** | **7** | **K.ITDVIMAFQAMCRGYLAR.K** |
|  | 21520 | **744.8460** | **2231.5160** | **2231.1168** | **0.3992** | **1** | **0** | **54** | **10** | **K.LLEERISDLTTNLAEEEEK.A** |
|  | 23207 | 822.9286 | 2465.7639 | 2466.0526 | -0.2886 | 0 | 7 | 11 | 1 | R.IAQLEEELEEEQGNMEAMSDR.V |
|  | 23450 | 502.2358 | 2506.1425 | 2507.1849 | -1.0424 | 0 | 4 | 25 | 4 | K.NLLQEQLQAETELYAEAEEMR.V |
|  | 24104 | **872.1188** | **2613.3345** | **2614.3642** | **-1.0298** | **1** | **2** | **42** | **4** | **R.INFDVTGYIVGANIETYLLEKSR.A** |
|  | 24264 | **1324.1637** | **2646.3128** | **2646.2319** | **0.0810** | **1** | **0** | **61** | **3** | **R.HEMPPHIYAIADTAYRSMLQDR.E** |
|  | 24701 | **545.0704** | **2720.3155** | **2721.2221** | **-0.9066** | **1** | **1** | **51** | **10** | **R.IAQLEEELEEEQGNMEAMSDRVR.K** |

| **154.** | Q9BUF5\|TBB6_HUMAN    **Mass:** 50281    **Score:** 192    **Queries matched:** 34   **emPAI:** 0.65 |
| --- | --- |
|  | Tubulin beta-6 chain OS=Homo sapiens GN=TUBB6 PE=1 SV=1 |

|  | **Query** | **Observed** | **Mr(expt)** | **Mr(calc)** | **Delta** | **Miss** | **Score** | **Expect** | **Rank** | **Peptide** |
| --- | --- | --- | --- | --- | --- | --- | --- | --- | --- | --- |
|  | 5095 | 533.2719 | 1064.5293 | 1064.4201 | 0.1091 | 0 | (18) | 2.3 | 1 | R.NMMAACDPR.H 5090 5102 |
|  | 5275 | 541.2110 | 1080.4074 | 1080.4151 | -0.0076 | 0 | 26 | 0.36 | 1 | R.NMMAACDPR.H |
|  | 5276 | 541.2111 | 1080.4076 | 1080.4151 | -0.0075 | 0 | (25) | 0.48 | 1 | R.NMMAACDPR.H |
|  | 6084 | 572.3180 | 1142.6215 | 1142.6270 | -0.0055 | 0 | 17 | 3.3 | 1 | K.LAVNMVPFPR.L |
|  | 8765 | 430.2513 | 1287.7319 | 1286.7169 | 1.0150 | 1 | 4 | 55 | 3 | R.KLAVNMVPFPR.L |
|  | 10232 | 684.3992 | 1366.7839 | 1366.6625 | 0.1214 | 0 | 7 | 25 | 1 | R.IMNTFSVMPSPK.V 10241 10242 10243 |
|  | 10290 | **457.7286** | **1370.1639** | **1370.6765** | **-0.5126** | **1** | **1** | **71** | **10** | **R.ISEQFSAMFRR.K** |
|  | 10507 | 691.9351 | 1381.8557 | 1382.6574 | -0.8017 | 0 | (2) | 87 | 3 | R.IMNTFSVMPSPK.V |
|  | 14380 | 540.9477 | 1619.8212 | 1619.8283 | -0.0070 | 0 | 48 | 0.0018 | 1 | R.LHFFMPGFAPLTSR.G 14371 14377 14378 14379 |
|  | 15553 | 848.9153 | 1695.8160 | 1695.8257 | -0.0096 | 0 | 19 | 1.4 | 1 | K.NSSYFVEWIPNNVK.V 15562 15563 15564 |
|  | 17663 | 929.4699 | 1856.9253 | 1856.9342 | -0.0090 | 0 | 55 | 0.0003 | 1 | K.MASTFIGNSTAIQELFK.R |
|  | 19427 | 1007.5205 | 2013.0265 | 2013.0353 | -0.0089 | 1 | 34 | 0.033 | 3 | K.MASTFIGNSTAIQELFKR.I 19426 19429 |
|  | 19525 | 1012.0038 | 2021.9930 | 2020.9631 | 1.0299 | 1 | 5 | 29 | 4 | R.GPMSMKEVDEQMLAIQSK.N |
|  | 20344 | **526.7992** | **2103.1677** | **2104.0623** | **-0.8946** | **1** | **3** | **40** | **3** | **K.YVPRAALVDLEPGTMDSVR.S** |
|  | 20678 | **714.7081** | **2141.1024** | **2139.9969** | **1.1055** | **1** | **1** | **72** | **1** | **R.EEFPDRIMNTFSVMPSPK.V** |
|  | 29087 | **941.4768** | **3761.8779** | **3761.9047** | **-0.0268** | **1** | **4** | **12** | **2** | **K.LTTPTYGDLNHLVSATMSGVTTSLRFPGQLNADLR.K** |
|  | 30696 | 1531.7006 | 4592.0799 | 4592.0999 | -0.0201 | 0 | 53 | 8.6e-005 | 1 | K.VSDTVVEPYNATLSVHQLVENTDETYCIDNEALYDICFR.T 30694 30698 30701 |

| **155.** | P10606\|COX5B_HUMAN    **Mass:** 13915    **Score:** 191    **Queries matched:** 18   **emPAI:** 0.65 |
| --- | --- |
|  | Cytochrome c oxidase subunit 5B, mitochondrial OS=Homo sapiens GN=COX5B PE=1 SV=2 |

|  | **Query** | **Observed** | **Mr(expt)** | **Mr(calc)** | **Delta** | **Miss** | **Score** | **Expect** | **Rank** | **Peptide** |
| --- | --- | --- | --- | --- | --- | --- | --- | --- | --- | --- |
|  | 2966 | **453.2609** | **904.5072** | **904.5130** | **-0.0058** | **0** | **11** | **16** | **3** | **K.LVPQQLAH.-** |
|  | 4809 | **522.2661** | **1042.5177** | **1043.5294** | **-1.0118** | **1** | **5** | **59** | **6** | **R.ARGPSGAAAMR.S** |
|  | 8093 | 418.9010 | 1253.6811 | 1254.7156 | -1.0346 | 1 | 10 | 14 | 2 | R.GAGTLAAQALRAR.G 8102 8107 |
|  | 9242 | **657.3262** | **1312.6379** | **1313.7343** | **-1.0964** | **1** | **56** | **0.00033** | **1** | **K.KGLDPYNVLAPK.G** 9251 9262 9263 9272 9274 |
|  | 10945 | **705.3403** | **1408.6661** | **1409.8466** | **-1.1805** | **1** | **0** | **1.1e+002** | **8** | **R.LLRGAGTLAAQALR.A** |
|  | 11954 | **734.8867** | **1467.7589** | **1467.7681** | **-0.0092** | **1** | **7** | **27** | **2** | **R.EDPNLVPSISNKR.I** |
|  | 19203 | **996.4456** | **1990.8767** | **1990.8902** | **-0.0135** | **0** | **91** | **7.1e-008** | **1** | **R.SMASGGGVPTDEEQATGLER.E** 19201 19202 |
|  | 19370 | 669.9649 | 2006.8729 | 2006.8851 | -0.0122 | 0 | (2) | 53 | 2 | R.SMASGGGVPTDEEQATGLER.E |
|  | 23377 | **831.7256** | **2492.1549** | **2491.1665** | **0.9885** | **0** | **5** | **21** | **1** | **R.IVGCICEEDNTSVVWFWLHK.G** |

| **156.** | P36542\|ATPG_HUMAN    **Mass:** 33032    **Score:** 191    **Queries matched:** 22   **emPAI:** 0.72 |
| --- | --- |
|  | ATP synthase subunit gamma, mitochondrial OS=Homo sapiens GN=ATP5C1 PE=1 SV=1 |

|  | **Query** | **Observed** | **Mr(expt)** | **Mr(calc)** | **Delta** | **Miss** | **Score** | **Expect** | **Rank** | **Peptide** |
| --- | --- | --- | --- | --- | --- | --- | --- | --- | --- | --- |
|  | 1119 | **677.3607** | **676.3534** | **676.3578** | **-0.0043** | **0** | **13** | **9.9** | **5** | **R.NMATLK.D** |
|  | 2373 | 432.7076 | 863.4007 | 863.4865 | -0.0858 | 0 | 9 | 26 | 9 | K.LTLTFNR.T |
|  | 3131 | **458.8804** | **915.7463** | **915.5502** | **0.1962** | **1** | **1** | **1.1e+002** | **10** | **R.TRQAVITK.E** |
|  | 3572 | **476.2687** | **950.5228** | **951.4881** | **-0.9653** | **1** | **3** | **73** | **7** | **K.SMKMVAAAK.Y** |
|  | 3593 | **477.3170** | **952.6194** | **951.4881** | **1.1312** | **1** | **(0)** | **1.4e+002** | **10** | **K.SMKMVAAAK.Y** |
|  | 3774 | **484.2484** | **966.4822** | **967.4103** | **-0.9281** | **0** | **9** | **20** | **1** | **R.MTAMDNASK.N** |
|  | 3838 | **486.7493** | **971.4841** | **971.4924** | **-0.0082** | **1** | **15** | **5.2** | **1** | **K.ADIKGPEDK.K** |
|  | 4041 | **493.2991** | **984.5837** | **983.4052** | **1.1785** | **0** | **(3)** | **86** | **3** | **R.MTAMDNASK.N** 4009 |
|  | 4870 | **524.2845** | **1046.5545** | **1046.5608** | **-0.0062** | **0** | **33** | **0.081** | **1** | **K.SEVATLTAAGK.E** |
|  | 5139 | 535.3054 | 1068.5962 | 1068.6039 | -0.0078 | 1 | 15 | 5.4 | 4 | R.AERELKPAR.I |
|  | 5493 | **548.8060** | **1095.5974** | **1095.6037** | **-0.0063** | **0** | **33** | **0.066** | **1** | **K.HLLIGVSSDR.G** |
|  | 7246 | 607.3187 | 1212.6229 | 1212.6285 | -0.0056 | 0 | 69 | 1.7e-005 | 1 | R.GLCGAIHSSIAK.Q 7247 |
|  | 7468 | 612.7634 | 1223.5122 | 1223.6986 | -0.1864 | 1 | 9 | 18 | 3 | K.KHLLIGVSSDR.G 7469 |
|  | 8851 | **646.8319** | **1291.6493** | **1291.6561** | **-0.0068** | **0** | **22** | **0.89** | **1** | **R.THSDQFLVAFK.E** |
|  | 9509 | **663.8647** | **1325.7148** | **1325.7231** | **-0.0082** | **0** | **69** | **1.8e-005** | **1** | **R.IYGLGSLALYEK.A** 9489 9490 |
|  | 17838 | **624.9412** | **1871.8018** | **1871.8063** | **-0.0045** | **1** | **7** | **21** | **4** | **R.MTAMDNASKNASEMIDK.L** |
|  | 27742 | **1124.5680** | **3370.6821** | **3370.6973** | **-0.0151** | **0** | **67** | **7.3e-006** | **1** | **R.KPPTFGDASVIALELLNSGYEFDEGSIIFNK.F** |

| **157.** | Q6DD88\|ATLA3_HUMAN    **Mass:** 60960    **Score:** 188    **Queries matched:** 9   **emPAI:** 0.06 |
| --- | --- |
|  | Atlastin-3 OS=Homo sapiens GN=ATL3 PE=1 SV=1 |

|  | **Query** | **Observed** | **Mr(expt)** | **Mr(calc)** | **Delta** | **Miss** | **Score** | **Expect** | **Rank** | **Peptide** |
| --- | --- | --- | --- | --- | --- | --- | --- | --- | --- | --- |
|  | 6510 | **588.3678** | **1174.7210** | **1173.5812** | **1.1398** | **0** | **4** | **67** | **9** | **R.DAVVGRPSMDK.K** |
|  | 9055 | **651.8405** | **1301.6665** | **1301.6762** | **-0.0097** | **1** | **4** | **60** | **8** | **R.DAVVGRPSMDKK.A** |
|  | 10333 | **686.9948** | **1371.9751** | **1372.7245** | **-0.7494** | **1** | **2** | **57** | **3** | **-.MLSPQRVAAAASR.G** |
|  | 10551 | **693.3539** | **1384.6932** | **1384.7173** | **-0.0241** | **1** | **0** | **1.3e+002** | **4** | **K.VTCRGLLEYFK.A** |
|  | 17316 | **916.9567** | **1831.8988** | **1831.9097** | **-0.0110** | **0** | **120** | **1e-010** | **1** | **K.SMLQATAEANNLAAAASAK.D** 17315 17332 |
|  | 26726 | **1563.6362** | **3125.2579** | **3125.5451** | **-0.2872** | **1** | **4** | **13** | **1** | **K.IYQGEDLPHPKSMLQATAEANNLAAAASAK.D** |
|  | 31144 | **1645.4795** | **4933.4166** | **4934.2935** | **-0.8769** | **1** | **2** | **9.7** | **7** | **K.SMLQATAEANNLAAAASAKDIYYNNMEEVCGGEKPYLSPDILEEK.H** |

| **158.** | Q9H0U4\|RAB1B_HUMAN    **Mass:** 22328    **Score:** 187    **Queries matched:** 22   **emPAI:** 0.61 |
| --- | --- |
|  | Ras-related protein Rab-1B OS=Homo sapiens GN=RAB1B PE=1 SV=1 |

|  | **Query** | **Observed** | **Mr(expt)** | **Mr(calc)** | **Delta** | **Miss** | **Score** | **Expect** | **Rank** | **Peptide** |
| --- | --- | --- | --- | --- | --- | --- | --- | --- | --- | --- |
|  | 3907 | **488.2804** | **974.5463** | **974.5397** | **0.0066** | **1** | **5** | **62** | **5** | **K.KVVDNTTAK.E** |
|  | 4106 | 495.6060 | 989.1975 | 989.4818 | -0.2843 | 0 | 4 | 45 | 8 | R.TITSSYYR.G |
|  | 5171 | 536.3210 | 1070.6274 | 1070.6336 | -0.0062 | 0 | 21 | 1.3 | 1 | K.LLLIGDSGVGK.S |
|  | 8731 | **644.2775** | **1286.5404** | **1287.7398** | **-1.1994** | **1** | **5** | **47** | **4** | **K.LLVGNKSDLTTK.K** 8767 |
|  | 9315 | 658.8296 | 1315.6446 | 1315.6521 | -0.0074 | 0 | 18 | 2.1 | 1 | K.LQIWDTAGQER.F 9284 |
|  | 11479 | 481.2419 | 1440.7038 | 1440.7143 | -0.0105 | 0 | 13 | 6.1 | 1 | R.MGPGAASGGERPNLK.I 11481 11484 |
|  | 11738 | **486.3488** | **1456.0246** | **1456.7092** | **-0.6846** | **0** | **(5)** | **27** | **4** | **R.MGPGAASGGERPNLK.I** |
|  | 14973 | 829.6645 | 1657.3144 | 1657.8788 | -0.5643 | 1 | 3 | 37 | 1 | K.TIKLQIWDTAGQER.F |
|  | 15938 | 862.9366 | 1723.8587 | 1723.8669 | -0.0081 | 0 | 16 | 2.8 | 2 | K.EFADSLGIPFLETSAK.N |
|  | 17781 | **934.9435** | **1867.8725** | **1867.8808** | **-0.0083** | **0** | **63** | **4.5e-005** | **1** | **K.NATNVEQAFMTMAAEIK.K** 17803 |
|  | 17979 | 942.9399 | 1883.8653 | 1883.8757 | -0.0104 | 0 | (56) | 0.00026 | 2 | K.NATNVEQAFMTMAAEIK.K 17978 17980 |
|  | 18155 | **951.0157** | **1900.0168** | **1899.8706** | **0.1462** | **0** | **(3)** | **49** | **9** | **K.NATNVEQAFMTMAAEIK.K** 18154 |
|  | 20967 | 1087.0000 | 2171.9854 | 2170.9947 | 0.9908 | 0 | 69 | 1.1e-005 | 1 | R.FADDTYTESYISTIGVDFK.I 20961 |

| **159.** | Q8N339\|MT1M_HUMAN    **Mass:** 7246     **Score:** 187    **Queries matched:** 8   **emPAI:** 2.92 |
| --- | --- |
|  | Metallothionein-1M OS=Homo sapiens GN=MT1M PE=2 SV=2 |

|  | **Query** | **Observed** | **Mr(expt)** | **Mr(calc)** | **Delta** | **Miss** | **Score** | **Expect** | **Rank** | **Peptide** |
| --- | --- | --- | --- | --- | --- | --- | --- | --- | --- | --- |
|  | 11544 | **723.2539** | **1444.4933** | **1444.5026** | **-0.0093** | **0** | **53** | **0.00053** | **1** | **K.SCCSCCPVGCAK.C** 11542 11545 11546 11547 11548 |
|  | 13627 | **787.3009** | **1572.5872** | **1572.5976** | **-0.0103** | **1** | **61** | **9.1e-005** | **1** | **K.KSCCSCCPVGCAK.C** 13626 |

|  | **Proteins matching the same set of peptides:** |
| --- | --- |

|  | P02795\|MT2_HUMAN    **Mass:** 7178     **Score:** 187    **Queries matched:** 8 |
| --- | --- |
|  | Metallothionein-2 OS=Homo sapiens GN=MT2A PE=1 SV=1 |

|  | P04732\|MT1E_HUMAN    **Mass:** 7150     **Score:** 187    **Queries matched:** 8 |
| --- | --- |
|  | Metallothionein-1E OS=Homo sapiens GN=MT1E PE=1 SV=1 |

|  | P13640\|MT1G_HUMAN    **Mass:** 7277     **Score:** 187    **Queries matched:** 8 |
| --- | --- |
|  | Metallothionein-1G OS=Homo sapiens GN=MT1G PE=1 SV=2 |

|  | P80297\|MT1X_HUMAN    **Mass:** 7204     **Score:** 187    **Queries matched:** 8 |
| --- | --- |
|  | Metallothionein-1X OS=Homo sapiens GN=MT1X PE=1 SV=1 |

| **160.** | O95299\|NDUAA_HUMAN    **Mass:** 41067    **Score:** 186    **Queries matched:** 16   **emPAI:** 0.42 |
| --- | --- |
|  | NADH dehydrogenase [ubiquinone] 1 alpha subcomplex subunit 10, mitochondrial OS=Homo sapiens GN=NDUFA10 PE=1 SV=1 |

|  | **Query** | **Observed** | **Mr(expt)** | **Mr(calc)** | **Delta** | **Miss** | **Score** | **Expect** | **Rank** | **Peptide** |
| --- | --- | --- | --- | --- | --- | --- | --- | --- | --- | --- |
|  | 1460 | **799.3966** | **798.3893** | **798.4501** | **-0.0607** | **0** | **5** | **49** | **4** | **R.VLHQFR.E** |
|  | 1659 | **407.6878** | **813.3611** | **812.3487** | **1.0124** | **0** | **4** | **71** | **8** | **K.GDPHEMK.I** |
|  | 2900 | 451.2503 | 900.4861 | 900.5280 | -0.0419 | 1 | 10 | 22 | 7 | K.LAKEIAEK.L 2915 |
|  | 3073 | **457.2330** | **912.4514** | **911.3733** | **1.0781** | **0** | **5** | **47** | **10** | **R.SNDGNSYR.L** |
|  | 5289 | **541.7638** | **1081.5130** | **1080.5161** | **0.9969** | **0** | **10** | **15** | **5** | **K.TFLPEMSEK.C** |
|  | 7156 | **605.0363** | **1208.0579** | **1208.6111** | **-0.5531** | **1** | **0** | **88** | **6** | **K.KTFLPEMSEK.C** |
|  | 8425 | 423.9114 | 1268.7122 | 1269.6612 | -0.9490 | 1 | 6 | 32 | 3 | R.VRGIHSSVQCK.L |
|  | 8539 | **638.8282** | **1275.6418** | **1275.6493** | **-0.0075** | **0** | **51** | **0.0011** | **1** | **R.VITVDGNICTGK.G** 8538 |
|  | 9563 | **664.8397** | **1327.6649** | **1328.5885** | **-0.9236** | **0** | **15** | **5.1** | **2** | **K.YSPGYNTEVGDK.W** |
|  | 12797 | **760.3931** | **1518.7716** | **1518.7824** | **-0.0109** | **1** | **57** | **0.00025** | **1** | **R.SRVITVDGNICTGK.G** 12778 |
|  | 19868 | **1028.5072** | **2054.9998** | **2055.0102** | **-0.0103** | **1** | **69** | **1.1e-005** | **1** | **K.YSPGYNTEVGDKWIWLK.-** 19870 |
|  | 21108 | **730.0149** | **2187.0228** | **2187.0340** | **-0.0112** | **1** | **27** | **0.18** | **1** | **K.TFLPEMSEKCEVLQYSAR.E** |

| **161.** | P00441\|SODC_HUMAN    **Mass:** 16154    **Score:** 185    **Queries matched:** 9   **emPAI:** 1.36 |
| --- | --- |
|  | Superoxide dismutase [Cu-Zn] OS=Homo sapiens GN=SOD1 PE=1 SV=2 |

|  | **Query** | **Observed** | **Mr(expt)** | **Mr(calc)** | **Delta** | **Miss** | **Score** | **Expect** | **Rank** | **Peptide** |
| --- | --- | --- | --- | --- | --- | --- | --- | --- | --- | --- |
|  | 5829 | **560.7819** | **1119.5492** | **1119.6144** | **-0.0652** | **1** | **12** | **10** | **5** | **-.MATKAVCVLK.G** |
|  | 21329 | 738.6683 | 2212.9830 | 2212.1124 | 0.8706 | 1 | 3 | 41 | 5 | K.GDGPVQGIINFEQKESNGPVK.V 21330 |
|  | 28270 | **1173.8722** | **3518.5947** | **3518.6175** | **-0.0227** | **0** | **78** | **6.2e-007** | **1** | **K.GLTEGLHGFHVHEFGDNTAGCTSAGPHFNPLSR.K** 28272 |
|  | 28960 | **930.9544** | **3719.7885** | **3719.8061** | **-0.0176** | **1** | **61** | **2.6e-005** | **1** | **R.HVGDLGNVTADKDGVADVSIEDSVISLSGDHCIIGR.T** 28958 28959 28964 |

| **162.** | P51687\|SUOX_HUMAN    **Mass:** 60759    **Score:** 184    **Queries matched:** 16   **emPAI:** 0.13 |
| --- | --- |
|  | Sulfite oxidase, mitochondrial OS=Homo sapiens GN=SUOX PE=1 SV=2 |

|  | **Query** | **Observed** | **Mr(expt)** | **Mr(calc)** | **Delta** | **Miss** | **Score** | **Expect** | **Rank** | **Peptide** |
| --- | --- | --- | --- | --- | --- | --- | --- | --- | --- | --- |
|  | 4474 | **508.9801** | **1015.9456** | **1015.5451** | **0.4006** | **1** | **6** | **29** | **3** | **K.EVKGLEWR.T** 4473 |
|  | 4484 | **509.2752** | **1016.5358** | **1015.5597** | **0.9761** | **1** | **8** | **27** | **4** | **R.LQQACRLK.S** |
|  | 4776 | 520.8357 | 1039.6568 | 1038.5359 | 1.1209 | 0 | 6 | 41 | 9 | R.GVLSNAWHR.V |
|  | 6840 | **597.6312** | **1193.2479** | **1193.5962** | **-0.3483** | **1** | **1** | **74** | **1** | **R.SEMTQVKEVK.G** |
|  | 9337 | **659.2846** | **1316.5547** | **1315.8089** | **0.7458** | **1** | **6** | **40** | **1** | **R.VVVPGVVGARHVK.W** |
|  | 13860 | **529.5776** | **1585.7111** | **1586.8304** | **-1.1194** | **0** | **2** | **87** | **9** | **R.VDVSLDGGLTWQVAK.L** |
|  | 16032 | **577.9376** | **1730.7910** | **1730.8012** | **-0.0102** | **0** | **10** | **9.9** | **3** | **R.VSVQPEESYSHWQR.R** 16023 |
|  | 19589 | **1014.4808** | **2026.9471** | **2026.1211** | **0.8260** | **1** | **2** | **62** | **3** | **R.AVIRVDVSLDGGLTWQVAK.L** |
|  | 21597 | **1122.0521** | **2242.0897** | **2242.1019** | **-0.0122** | **0** | **58** | **0.00014** | **1** | **K.AVDDGYNVQPDTVAPIWNLR.G** 21598 |
|  | 22359 | **782.0448** | **2343.1126** | **2343.1239** | **-0.0113** | **0** | **3** | **34** | **1** | **R.AMDPEAEVLLAYEMNGQPLPR.D** |
|  | 28200 | **1167.9254** | **3500.7544** | **3500.7728** | **-0.0184** | **0** | **71** | **2.7e-006** | **1** | **K.VNSQRPFNAEPPPELLTENYITPNPIFFTR.N** 28201 |
|  | 29758 | **810.6375** | **4048.1512** | **4047.1006** | **1.0505** | **1** | **1** | **19** | **7** | **R.HPALKVNSQRPFNAEPPPELLTENYITPNPIFFTR.N** |

| **163.** | A6NGU5\|GGT3_HUMAN    **Mass:** 61919    **Score:** 183    **Queries matched:** 22   **emPAI:** 0.34 |
| --- | --- |
|  | Putative gamma-glutamyltranspeptidase 3 OS=Homo sapiens GN=GGT3P PE=5 SV=2 |

|  | **Query** | **Observed** | **Mr(expt)** | **Mr(calc)** | **Delta** | **Miss** | **Score** | **Expect** | **Rank** | **Peptide** |
| --- | --- | --- | --- | --- | --- | --- | --- | --- | --- | --- |
|  | 1219 | 734.9947 | 733.9874 | 733.4123 | 0.5752 | 0 | 3 | 70 | 1 | R.IVEAFR.F |
|  | 2238 | 428.2274 | 854.4402 | 855.4562 | -1.0161 | 1 | 7 | 33 | 3 | K.RAVEEPR.L |
|  | 2278 | 429.6938 | 857.3730 | 857.4719 | -0.0989 | 1 | 3 | 1.1e+002 | 3 | K.VLREGER.L |
|  | 3839 | 486.7559 | 971.4972 | 972.4777 | -0.9805 | 0 | 33 | 0.097 | 1 | R.GYELAHQR.H 3876 |
|  | 4124 | 496.2474 | 990.4802 | 991.5464 | -1.0662 | 1 | 9 | 25 | 7 | R.HGRLPWAR.L |
|  | 5067 | 532.2894 | 1062.5643 | 1062.5710 | -0.0067 | 0 | 34 | 0.062 | 1 | K.FVDVTEVVR.N 5080 |
|  | 5139 | 535.3054 | 1068.5962 | 1069.5880 | -0.9918 | 1 | 10 | 16 | 8 | R.EGERLTLPR.L |
|  | 6469 | 586.8398 | 1171.6650 | 1171.6713 | -0.0063 | 0 | 49 | 0.0019 | 1 | R.LFQPSIQLAR.Q |
|  | 9270 | 657.8625 | 1313.7104 | 1313.7132 | -0.0028 | 1 | 12 | 9.2 | 5 | R.IVEAFRFAYAK.R 9300 |
|  | 10828 | 701.3660 | 1400.7175 | 1400.7259 | -0.0084 | 0 | 87 | 2.7e-007 | 1 | R.NIDQAVTAALETR.H |
|  | 11313 | 477.6096 | 1429.8071 | 1429.6660 | 0.1411 | 0 | 23 | 0.71 | 1 | R.NMTSEFFAAQLR.S 11322 |
|  | 17904 | 939.4615 | 1876.9084 | 1876.9176 | -0.0091 | 0 | 69 | 1.2e-005 | 1 | R.TVIEQQPVLCEVFCR.D 17903 17905 17924 |
|  | 20391 | 703.7077 | 2108.1013 | 2108.1127 | -0.0114 | 1 | 14 | 3.7 | 1 | K.GGLSVAVPGEIRGYELAHQR.H |
|  | 20732 | 717.0189 | 2148.0348 | 2148.0456 | -0.0109 | 1 | 12 | 6 | 1 | R.TVIEQQPVLCEVFCRDR.K |
|  | 29803 | **1356.7242** | **4067.1509** | **4068.0119** | **-0.8610** | **0** | **6** | **6.8** | **2** | **R.DGGSAVDAAIAALLCVGLMNAHSMGIGVGLFLTIYNSTTR.K** |

| **164.** | Q96A33\|CCD47_HUMAN    **Mass:** 56123    **Score:** 183    **Queries matched:** 21   **emPAI:** 0.29 |
| --- | --- |
|  | Coiled-coil domain-containing protein 47 OS=Homo sapiens GN=CCDC47 PE=1 SV=1 |

|  | **Query** | **Observed** | **Mr(expt)** | **Mr(calc)** | **Delta** | **Miss** | **Score** | **Expect** | **Rank** | **Peptide** |
| --- | --- | --- | --- | --- | --- | --- | --- | --- | --- | --- |
|  | 1306 | **395.2119** | **788.4093** | **788.3777** | **0.0317** | **0** | **4** | **97** | **6** | **R.QEAAQSR.R** |
|  | 3655 | **479.2744** | **956.5343** | **956.5403** | **-0.0060** | **1** | **49** | **0.0023** | **1** | **R.RLEEAALR.R** |
|  | 5636 | **553.7758** | **1105.5370** | **1104.5927** | **0.9442** | **1** | **5** | **50** | **9** | **R.ARVEENFLK.L** |
|  | 6750 | **397.2313** | **1188.6720** | **1187.5856** | **1.0863** | **0** | **13** | **8.9** | **4** | **K.IMQEEGQPLK.L** |
|  | 10600 | **694.7437** | **1387.4729** | **1387.6401** | **-0.1673** | **1** | **9** | **13** | **1** | **R.IMNEEDPEKQR.R** |
|  | 12853 | **508.6026** | **1522.7861** | **1522.7964** | **-0.0104** | **1** | **22** | **0.73** | **1** | **K.LTHVQRQEAAQSR.R** |
|  | 14074 | **534.3038** | **1599.8897** | **1599.8018** | **0.0878** | **1** | **0** | **1.1e+002** | **8** | **K.NSRLAQAWFNTHR.E** |
|  | 16697 | **595.3232** | **1782.9477** | **1781.8990** | **1.0487** | **1** | **1** | **84** | **5** | **R.VCCEGMLIQLRFLK.R** |
|  | 17630 | **928.4791** | **1854.9436** | **1854.9516** | **-0.0080** | **0** | **33** | **0.048** | **1** | **R.TLLFTFNVPGSGNTYPK.D** 17631 |
|  | 19954 | **516.5148** | **2062.0300** | **2062.8976** | **-0.8676** | **0** | **4** | **36** | **2** | **K.VTMNDEDMDTYVFAVGTR.K** 19957 |
|  | 20296 | **700.6284** | **2098.8633** | **2097.9711** | **0.8922** | **1** | **5** | **22** | **3** | **R.LQKEMQDLSEFCSDKPK.S** |
|  | 22329 | **781.3588** | **2341.0545** | **2342.1571** | **-1.1027** | **1** | **0** | **69** | **7** | **K.DMEALLPLMNMVIYSIDKAK.K** |
|  | 23569 | **632.5491** | **2526.1674** | **2525.2133** | **0.9541** | **1** | **3** | **37** | **2** | **R.ELLESNFTLVGDDGTNKEATSTGK.L** |
|  | 23885 | **1287.0892** | **2572.1639** | **2572.1747** | **-0.0108** | **0** | **87** | **1.1e-007** | **1** | **K.YGLPDSLAILSEMGEVTDGMMDTK.M** 23884 23886 |
|  | 23977 | **1294.6536** | **2587.2926** | **2588.1696** | **-0.8770** | **0** | **(0)** | **66** | **5** | **K.YGLPDSLAILSEMGEVTDGMMDTK.M** |
|  | 25719 | **730.8526** | **2919.3813** | **2919.4014** | **-0.0201** | **1** | **13** | **3** | **1** | **K.MVHFLTHYADKIESVHFSDQFSGPK.I** 25721 |

| **165.** | P15313\|VATB1_HUMAN    **Mass:** 57196    **Score:** 181    **Queries matched:** 33   **emPAI:** 0.13 |
| --- | --- |
|  | V-type proton ATPase subunit B, kidney isoform OS=Homo sapiens GN=ATP6V1B1 PE=1 SV=3 |

|  | **Query** | **Observed** | **Mr(expt)** | **Mr(calc)** | **Delta** | **Miss** | **Score** | **Expect** | **Rank** | **Peptide** |
| --- | --- | --- | --- | --- | --- | --- | --- | --- | --- | --- |
|  | 1029 | 633.3801 | 632.3728 | 631.3289 | 1.0439 | 0 | 3 | 1.5e+002 | 9 | R.EVSAAR.E |
|  | 3843 | **486.7781** | **971.5415** | **970.4654** | **1.0761** | **0** | **5** | **56** | **6** | **R.EHMQAVTR.N** 3813 |
|  | 4883 | 525.1025 | 1048.1904 | 1048.5335 | -0.3431 | 1 | (9) | 14 | 2 | K.SAIGEGMTRK.D |
|  | 5095 | 533.2719 | 1064.5293 | 1064.5284 | 0.0008 | 1 | 10 | 15 | 2 | K.SAIGEGMTRK.D 5075 5085 5089 5097 5102 |
|  | 5401 | **545.3364** | **1088.6582** | **1087.5873** | **1.0708** | **0** | **9** | **21** | **1** | **R.SGQVLEVAGTK.A** |
|  | 5598 | 552.3103 | 1102.6060 | 1103.5281 | -0.9221 | 0 | 3 | 81 | 10 | R.TPVSEDMLGR.V |
|  | 6327 | **580.9122** | **1159.8098** | **1160.6190** | **-0.8092** | **0** | **1** | **1e+002** | **4** | **R.VFNGSGKPIDK.G** |
|  | 8980 | 433.9043 | 1298.6910 | 1298.6579 | 0.0331 | 1 | 4 | 51 | 8 | R.EVSAAREEVPGR.R |
|  | 11414 | **479.5697** | **1435.6872** | **1436.7300** | **-1.0428** | **0** | **1** | **1.1e+002** | **4** | **R.IPQAVIDEFYSR.E** |
|  | 12930 | **510.2885** | **1527.8438** | **1527.8079** | **0.0359** | **0** | **4** | **45** | **7** | **R.TVCSVNGPLVVLDR.V** 12936 |
|  | 14010 | 798.9553 | 1595.8960 | 1595.9035 | -0.0075 | 0 | 55 | 0.00038 | 1 | R.QIYPPINVLPSLSR.L 14011 |
|  | 18106 | 948.9407 | 1895.8668 | 1895.8764 | -0.0096 | 0 | 99 | 1.2e-008 | 1 | R.GFPGYMYTDLATIYER.A 18107 |
|  | 18281 | 638.0178 | 1911.0315 | 1911.8713 | -0.8398 | 0 | (2) | 56 | 2 | R.GFPGYMYTDLATIYER.A |
|  | 18351 | 959.4839 | 1916.9532 | 1917.0030 | -0.0497 | 0 | 3 | 49 | 4 | K.HVLVILTDMSSYAEALR.E 18350 |
|  | 23369 | 623.5826 | 2490.3015 | 2490.3165 | -0.0151 | 1 | 10 | 6.9 | 1 | R.GQKIPIFSAAGLPHNEIAAQICR.Q |
|  | 23371 | **623.8174** | **2491.2407** | **2492.2291** | **-0.9884** | **0** | **7** | **14** | **1** | **R.IYPEEMIQTGISPIDVMNSIAR.G** |
|  | 27171 | **1075.4965** | **3223.4676** | **3223.5107** | **-0.0431** | **1** | **2** | **29** | **1** | **K.AVLDYHDDNFAIVFAAMGVNMETARFFK.S** |
|  | 27500 | **829.8864** | **3315.5165** | **3314.5376** | **0.9789** | **1** | **(0)** | **41** | **2** | **-.MAMEIDSRPGGLPGSSCNLGAAREHMQAVTR.N** |
|  | 27571 | **833.8956** | **3331.5534** | **3330.5326** | **1.0209** | **1** | **(1)** | **33** | **6** | **-.MAMEIDSRPGGLPGSSCNLGAAREHMQAVTR.N** |
|  | 27573 | **833.9150** | **3331.6311** | **3330.5326** | **1.0985** | **1** | **5** | **13** | **1** | **-.MAMEIDSRPGGLPGSSCNLGAAREHMQAVTR.N** |
|  | 29726 | **1346.6838** | **4037.0297** | **4037.0456** | **-0.0159** | **0** | **17** | **0.55** | **1** | **R.GGSITQIPILTMPNDDITHPIPDLTGFITEGQIYVDR.Q** 29727 29728 |

| **166.** | P13073\|COX41_HUMAN    **Mass:** 19621    **Score:** 181    **Queries matched:** 18   **emPAI:** 2.50 |
| --- | --- |
|  | Cytochrome c oxidase subunit 4 isoform 1, mitochondrial OS=Homo sapiens GN=COX4I1 PE=1 SV=1 |

|  | **Query** | **Observed** | **Mr(expt)** | **Mr(calc)** | **Delta** | **Miss** | **Score** | **Expect** | **Rank** | **Peptide** |
| --- | --- | --- | --- | --- | --- | --- | --- | --- | --- | --- |
|  | 3987 | 491.7427 | 981.4708 | 982.4178 | -0.9470 | 0 | 14 | 6.2 | 3 | K.ESFAEMNR.G |
|  | 4081 | **494.7911** | **987.5677** | **988.5342** | **-0.9664** | **1** | **10** | **17** | **1** | **K.EWVAKQTK.R** 4117 |
|  | 4598 | **513.7977** | **1025.5808** | **1025.5869** | **-0.0061** | **0** | **50** | **0.0016** | **1** | **K.VNPIQGLASK.W** 4599 |
|  | 6168 | **575.4109** | **1148.8073** | **1147.6132** | **1.1942** | **1** | **0** | **1.1e+002** | **10** | **K.RAISTSVCVR.A** |
|  | 7822 | **414.5567** | **1240.6483** | **1240.6564** | **-0.0081** | **0** | **33** | **0.076** | **1** | **R.DHPLPEVAHVK.H** 7820 7821 |
|  | 8515 | **637.8214** | **1273.6283** | **1273.5761** | **0.0522** | **1** | **5** | **48** | **3** | **K.FKESFAEMNR.G** |
|  | 11302 | **715.8128** | **1429.6111** | **1429.6184** | **-0.0073** | **0** | **45** | **0.0043** | **1** | **K.SEDFSLPAYMDR.R** 11301 |
|  | 13859 | **793.8622** | **1585.7098** | **1585.7195** | **-0.0097** | **1** | **35** | **0.035** | **1** | **K.SEDFSLPAYMDRR.D** 13858 13860 |
|  | 18150 | **950.9537** | **1899.8929** | **1899.9036** | **-0.0107** | **1** | **84** | **4.4e-007** | **1** | **K.ASWSSLSMDEKVELYR.I** 18149 18151 |

| **167.** | O00264\|PGRC1_HUMAN    **Mass:** 21772    **Score:** 178    **Queries matched:** 16   **emPAI:** 1.25 |
| --- | --- |
|  | Membrane-associated progesterone receptor component 1 OS=Homo sapiens GN=PGRMC1 PE=1 SV=3 |

|  | **Query** | **Observed** | **Mr(expt)** | **Mr(calc)** | **Delta** | **Miss** | **Score** | **Expect** | **Rank** | **Peptide** |
| --- | --- | --- | --- | --- | --- | --- | --- | --- | --- | --- |
|  | 2296 | 859.5027 | 858.4954 | 858.4997 | -0.0043 | 0 | 37 | 0.045 | 1 | R.ILMAINGK.V |
|  | 3357 | **467.2219** | **932.4292** | **932.4352** | **-0.0060** | **0** | **36** | **0.048** | **1** | **R.FDGVQDPR.I** 3358 |
|  | 3537 | **474.7401** | **947.4655** | **947.4712** | **-0.0057** | **0** | **16** | **4.6** | **1** | **R.DFTPAELR.R** 3538 |
|  | 4563 | **512.7567** | **1023.4989** | **1023.5059** | **-0.0071** | **0** | **16** | **4.1** | **1** | **R.GLATFCLDK.E** |
|  | 5387 | **545.2722** | **1088.5299** | **1088.5363** | **-0.0064** | **1** | **11** | **13** | **3** | **R.RFDGVQDPR.I** 5408 |
|  | 5618 | **553.2795** | **1104.5445** | **1103.5723** | **0.9722** | **1** | **11** | **14** | **1** | **R.DFTPAELRR.F** |
|  | 5620 | **553.2914** | **1104.5683** | **1103.5723** | **0.9960** | **1** | **22** | **1.2** | **1** | **R.RDFTPAELR.R** |
|  | 12741 | **758.8593** | **1515.7041** | **1515.7147** | **-0.0106** | **0** | **45** | **0.0041** | **1** | **K.FYGPEGPYGVFAGR.D** |
|  | 14765 | **822.9075** | **1643.8004** | **1643.8096** | **-0.0092** | **1** | **71** | **8.1e-006** | **1** | **R.KFYGPEGPYGVFAGR.D** 14751 14766 |
|  | 16755 | 597.2971 | 1788.8694 | 1788.9192 | -0.0499 | 1 | 3 | 54 | 3 | R.FDGVQDPRILMAINGK.V |
|  | 28675 | **1208.5503** | **3622.6290** | **3622.6475** | **-0.0184** | **1** | **63** | **1.9e-005** | **1** | **K.EALKDEYDDLSDLTAAQQETLSDWESQFTFK.Y** |

| **168.** | P30050\|RL12_HUMAN    **Mass:** 17979    **Score:** 177    **Queries matched:** 4   **emPAI:** 1.18 |
| --- | --- |
|  | 60S ribosomal protein L12 OS=Homo sapiens GN=RPL12 PE=1 SV=1 |

|  | **Query** | **Observed** | **Mr(expt)** | **Mr(calc)** | **Delta** | **Miss** | **Score** | **Expect** | **Rank** | **Peptide** |
| --- | --- | --- | --- | --- | --- | --- | --- | --- | --- | --- |
|  | 2632 | **441.2739** | **880.5333** | **880.5382** | **-0.0049** | **0** | **33** | **0.071** | **1** | **K.IGPLGLSPK.K** |
|  | 11099 | **709.8465** | **1417.6784** | **1417.6871** | **-0.0087** | **0** | **85** | **4.8e-007** | **1** | **R.CTGGEVGATSALAPK.I** |
|  | 15396 | **843.4288** | **1684.8430** | **1684.8533** | **-0.0103** | **0** | **100** | **1.1e-008** | **1** | **K.HSGNITFDEIVNIAR.Q** |
|  | 22762 | **802.0715** | **2403.1926** | **2403.2064** | **-0.0138** | **1** | **33** | **0.04** | **1** | **R.ELSGTIKEILGTAQSVGCNVDGR.H** |

| **169.** | Q02338\|BDH_HUMAN    **Mass:** 38532    **Score:** 176    **Queries matched:** 9   **emPAI:** 0.20 |
| --- | --- |
|  | D-beta-hydroxybutyrate dehydrogenase, mitochondrial OS=Homo sapiens GN=BDH1 PE=1 SV=3 |

|  | **Query** | **Observed** | **Mr(expt)** | **Mr(calc)** | **Delta** | **Miss** | **Score** | **Expect** | **Rank** | **Peptide** |
| --- | --- | --- | --- | --- | --- | --- | --- | --- | --- | --- |
|  | 9362 | **659.8167** | **1317.6189** | **1316.6684** | **0.9505** | **1** | **3** | **69** | **8** | **K.ELDSLNSDRLR.T** |
|  | 11054 | **708.3675** | **1414.7204** | **1414.7278** | **-0.0074** | **1** | **20** | **1.2** | **1** | **K.KMWEELPEVVR.K** |
|  | 11317 | 477.7309 | 1430.1708 | 1430.7228 | -0.5520 | 1 | 1 | 71 | 10 | K.MWEELPEVVRK.D |
|  | 16009 | **433.2310** | **1728.8949** | **1727.8553** | **1.0396** | **0** | **5** | **33** | **6** | **K.AVLVTGCDSGFGFSLAK.H** |
|  | 20358 | **702.4100** | **2104.2081** | **2105.0471** | **-0.8391** | **0** | **3** | **44** | **3** | **R.MQIMTHLPGAISDMIYIR.-** |
|  | 24551 | **1346.2098** | **2690.4051** | **2690.4167** | **-0.0115** | **0** | **66** | **1.6e-005** | **1** | **K.VSVVEPGNFIAATSLYSPESIQAIAK.K** |
|  | 27576 | **1111.8412** | **3332.5017** | **3332.5177** | **-0.0160** | **0** | **83** | **2.3e-007** | **1** | **K.METYCSSGSTDTSPVIDAVTHALTATTPYTR.Y** 27577 |
|  | 28944 | **1858.8318** | **3715.6490** | **3715.7600** | **-0.1110** | **1** | **0** | **29** | **2** | **R.YHPMDYYWWLRMQIMTHLPGAISDMIYIR.-** |

| **170.** | O00217\|NDUS8_HUMAN    **Mass:** 24203    **Score:** 174    **Queries matched:** 18   **emPAI:** 0.55 |
| --- | --- |
|  | NADH dehydrogenase [ubiquinone] iron-sulfur protein 8, mitochondrial OS=Homo sapiens GN=NDUFS8 PE=1 SV=1 |

|  | **Query** | **Observed** | **Mr(expt)** | **Mr(calc)** | **Delta** | **Miss** | **Score** | **Expect** | **Rank** | **Peptide** |
| --- | --- | --- | --- | --- | --- | --- | --- | --- | --- | --- |
|  | 1981 | **419.3138** | **836.6129** | **836.3664** | **0.2465** | **0** | **3** | **65** | **3** | **R.YPSGEER.C** |
|  | 4034 | **493.2668** | **984.5191** | **984.5253** | **-0.0062** | **1** | **16** | **4.5** | **1** | **R.FRGEHALR.R** 4033 |
|  | 4153 | **497.2381** | **992.4616** | **992.4675** | **-0.0059** | **1** | **25** | **0.51** | **1** | **R.RYPSGEER.C** 4140 4154 4155 4156 |
|  | 6545 | **589.8295** | **1177.6444** | **1177.6495** | **-0.0051** | **0** | **39** | **0.022** | **1** | **R.TLLWTELFR.G** |
|  | 7860 | **622.2992** | **1242.5838** | **1242.5914** | **-0.0076** | **1** | **10** | **16** | **2** | **R.TTRYDIDMTK.C** |
|  | 8177 | **420.2217** | **1257.6431** | **1256.6587** | **0.9844** | **0** | **5** | **43** | **4** | **R.GLGMTLSYLFR.E** |
|  | 8181 | **629.8414** | **1257.6682** | **1258.5863** | **-0.9182** | **1** | **(9)** | **20** | **7** | **R.TTRYDIDMTK.C** |
|  | 12470 | **750.8073** | **1499.6001** | **1499.6095** | **-0.0094** | **0** | **48** | **0.002** | **1** | **K.YVNMQDPEMDMK.S** 12486 |
|  | 19728 | **1021.5007** | **2040.9868** | **2040.9972** | **-0.0104** | **0** | **97** | **1.6e-008** | **1** | **K.LCEAICPAQAITIEAEPR.A** 19746 |
|  | 22858 | **806.3708** | **2416.0905** | **2416.2977** | **-0.2072** | **1** | **3** | **36** | **2** | **R.TLLWTELFRGLGMTLSYLFR.E** |
|  | 23704 | **849.7659** | **2546.2758** | **2546.2879** | **-0.0121** | **1** | **14** | **2.8** | **1** | **R.GLGMTLSYLFREPATINYPFEK.G** |

| **171.** | Q96KR1\|ZFR_HUMAN    **Mass:** 118079   **Score:** 174    **Queries matched:** 20   **emPAI:** 0.03 |
| --- | --- |
|  | Zinc finger RNA-binding protein OS=Homo sapiens GN=ZFR PE=1 SV=2 |

|  | **Query** | **Observed** | **Mr(expt)** | **Mr(calc)** | **Delta** | **Miss** | **Score** | **Expect** | **Rank** | **Peptide** |
| --- | --- | --- | --- | --- | --- | --- | --- | --- | --- | --- |
|  | 1223 | **739.4416** | **738.4344** | **737.4548** | **0.9796** | **1** | **5** | **48** | **10** | **K.HQKVVK.L** |
|  | 4766 | **520.3379** | **1038.6612** | **1037.4699** | **1.1913** | **0** | **4** | **61** | **5** | **R.EGDVTSGMVK.D** |
|  | 4792 | **521.3046** | **1040.5947** | **1041.5468** | **-0.9521** | **1** | **5** | **50** | **10** | **R.FNIHNNRK.R** |
|  | 5191 | **537.2976** | **1072.5805** | **1072.5811** | **-0.0006** | **1** | **6** | **55** | **5** | **R.ILRDLCQR.V** |
|  | 5368 | **544.4226** | **1086.8307** | **1087.5696** | **-0.7389** | **0** | **3** | **58** | **1** | **K.VSAVPTNMAAK.K** |
|  | 6591 | **395.0792** | **1182.2157** | **1181.6040** | **0.6116** | **1** | **7** | **18** | **4** | **K.DPPDVLDRQK.C** |
|  | 7627 | **616.5414** | **1231.0682** | **1231.6594** | **-0.5912** | **1** | **0** | **1e+002** | **7** | **K.VSAVPTNMAAKK.T** |
|  | 7845 | 621.8527 | 1241.6908 | 1242.6092 | -0.9184 | 0 | 11 | 12 | 2 | K.LVSDSLSEHEK.N |
|  | 12006 | **736.8492** | **1471.6838** | **1471.8432** | **-0.1594** | **0** | **7** | **25** | **3** | **K.MQVTITLTSPIIR.E** |
|  | 12638 | 756.3736 | 1510.7326 | 1510.5895 | 0.1431 | 0 | 5 | 35 | 7 | R.MEEEQHHWDDR.R |
|  | 12894 | **763.8662** | **1525.7179** | **1526.5844** | **-0.8666** | **0** | **(1)** | **94** | **8** | **R.MEEEQHHWDDR.R** |
|  | 13358 | **778.3768** | **1554.7390** | **1554.8253** | **-0.0863** | **1** | **0** | **1.2e+002** | **5** | **R.ALKLVSDSLSEHEK.N** |
|  | 13550 | **523.6246** | **1567.8519** | **1568.7253** | **-0.8734** | **1** | **3** | **68** | **6** | **R.RPDSSDDRYVMTK.H** |
|  | 15358 | **841.8800** | **1681.7454** | **1681.8119** | **-0.0664** | **0** | **2** | **62** | **3** | **K.GLTTTGNSSLNSTSNTK.V** |
|  | 15967 | 576.3315 | 1725.9728 | 1725.0083 | 0.9645 | 1 | 16 | 2.7 | 1 | R.ANGLQSCVIIIRILR.D |
|  | 17668 | **929.9322** | **1857.8499** | **1857.8606** | **-0.0106** | **0** | **106** | **2.9e-009** | **1** | **K.AGYSQGATQYTQAQQTR.Q** 17669 17676 |
|  | 18503 | **643.6030** | **1927.7873** | **1928.9150** | **-1.1277** | **1** | **2** | **57** | **2** | **R.EGDVTSGMVKDPPDVLDR.Q** |
|  | 19640 | **1016.5432** | **2031.0717** | **2031.0836** | **-0.0119** | **1** | **5** | **28** | **1** | **K.WFQARANGLQSCVIIIR.I** |

| **172.** | P84098\|RL19_HUMAN    **Mass:** 23565    **Score:** 172    **Queries matched:** 13   **emPAI:** 0.35 |
| --- | --- |
|  | 60S ribosomal protein L19 OS=Homo sapiens GN=RPL19 PE=1 SV=1 |

|  | **Query** | **Observed** | **Mr(expt)** | **Mr(calc)** | **Delta** | **Miss** | **Score** | **Expect** | **Rank** | **Peptide** |
| --- | --- | --- | --- | --- | --- | --- | --- | --- | --- | --- |
|  | 878 | **574.9915** | **573.9842** | **573.3486** | **0.6357** | **1** | **6** | **58** | **1** | **K.LKADK.A** |
|  | 1420 | **399.2146** | **796.4147** | **797.4330** | **-1.0183** | **1** | **14** | **5.4** | **1** | **R.HMGIGKR.K** 1423 1445 1453 1467 |
|  | 2912 | **451.2798** | **900.5450** | **900.5505** | **-0.0054** | **1** | **14** | **7.2** | **5** | **K.RLASSVLR.C** |
|  | 4063 | **494.2593** | **986.5041** | **985.5192** | **0.9849** | **0** | **43** | **0.0093** | **1** | **K.LLADQAEAR.R** |
|  | 5750 | **558.2845** | **1114.5545** | **1113.6142** | **0.9404** | **1** | **4** | **67** | **5** | **K.KLLADQAEAR.R** |
|  | 6083 | **572.3156** | **1142.6167** | **1141.6203** | **0.9963** | **1** | **20** | **1.5** | **1** | **K.LLADQAEARR.S** |
|  | 6840 | 597.6312 | 1193.2479 | 1192.5733 | 0.6746 | 1 | 0 | 90 | 3 | R.MPEKVTWMR.R |
|  | 18655 | **971.9598** | **1941.9051** | **1941.9180** | **-0.0129** | **0** | **98** | **1.4e-008** | **1** | **K.VWLDPNETNEIANANSR.Q** 18654 |

| **173.** | P52272\|HNRPM_HUMAN    **Mass:** 77749    **Score:** 172    **Queries matched:** 83   **emPAI:** 0.26 |
| --- | --- |
|  | Heterogeneous nuclear ribonucleoprotein M OS=Homo sapiens GN=HNRNPM PE=1 SV=3 |

|  | **Query** | **Observed** | **Mr(expt)** | **Mr(calc)** | **Delta** | **Miss** | **Score** | **Expect** | **Rank** | **Peptide** |
| --- | --- | --- | --- | --- | --- | --- | --- | --- | --- | --- |
|  | 955 | **605.0369** | **604.0296** | **604.3003** | **-0.2707** | **0** | **9** | **25** | **7** | **R.MGLER.M** |
|  | 1232 | **749.3560** | **748.3487** | **748.3538** | **-0.0051** | **0** | **13** | **10** | **1** | **R.MGSGIER.M** |
|  | 1314 | 395.2130 | 788.4114 | 788.4028 | 0.0086 | 0 | 20 | 2.2 | 3 | R.VGSEIER.M |
|  | 1345 | **395.8697** | **789.7249** | **789.3803** | **0.3446** | **0** | **12** | **9.8** | **5** | **R.LGGAGMER.M** |
|  | 2424 | **434.5419** | **867.0693** | **867.3579** | **-0.2886** | **0** | **6** | **24** | **5** | **R.MGQTMER.I** |
|  | 2475 | **435.2793** | **868.5441** | **867.3579** | **1.1862** | **0** | **(4)** | **51** | **7** | **R.MGQTMER.I** |
|  | 2942 | **452.7162** | **903.4179** | **903.4232** | **-0.0053** | **0** | **20** | **2.1** | **1** | **R.MGANNLER.M** 2954 |
|  | 3174 | **460.2486** | **918.4826** | **919.4181** | **-0.9355** | **0** | **(4)** | **80** | **6** | **R.MGANNLER.M** |
|  | 3607 | **478.1928** | **954.3710** | **955.4004** | **-1.0294** | **0** | **(2)** | **86** | **9** | **R.FGSGMNMGR.I** |
|  | 3826 | **486.2642** | **970.5139** | **971.3953** | **-0.8814** | **0** | **12** | **10** | **1** | **R.FGSGMNMGR.I** 3813 3818 3870 |
|  | 3828 | **486.2650** | **970.5154** | **971.3953** | **-0.8799** | **0** | **(11)** | **14** | **5** | **R.FGSGMNMGR.I** 3822 3829 3857 |
|  | 4097 | **495.2732** | **988.5318** | **987.3902** | **1.1415** | **0** | **(3)** | **89** | **7** | **R.FGSGMNMGR.I** |
|  | 5573 | **551.7481** | **1101.4815** | **1100.4855** | **0.9960** | **0** | **59** | **0.0002** | **1** | **R.MGAGLGHGMDR.V** |
|  | 5575 | **551.7842** | **1101.5538** | **1102.5506** | **-0.9968** | **1** | **6** | **45** | **6** | **R.ADILEDKDGK.S** |
|  | 5784 | **559.2799** | **1116.5451** | **1116.4805** | **0.0647** | **0** | **(8)** | **28** | **10** | **R.MGAGLGHGMDR.V** 5772 |
|  | 5888 | **563.7894** | **1125.5642** | **1124.5107** | **1.0535** | **0** | **33** | **0.077** | **1** | **R.MGAGMGFGLER.M** |
|  | 6050 | **570.7856** | **1139.5566** | **1140.5056** | **-0.9490** | **0** | **(6)** | **39** | **4** | **R.MGAGMGFGLER.M** |
|  | 6267 | **578.7943** | **1155.5741** | **1155.5199** | **0.0542** | **1** | **(1)** | **1.3e+002** | **8** | **R.MMNGMKLSGR.E** |
|  | 6292 | **579.3281** | **1156.6417** | **1155.5199** | **1.1219** | **1** | **10** | **17** | **1** | **R.MMNGMKLSGR.E** 6261 6276 6286 |
|  | 6458 | **586.4145** | **1170.8144** | **1171.5148** | **-0.7003** | **1** | **(6)** | **35** | **5** | **R.MMNGMKLSGR.E** |
|  | 6715 | **594.7933** | **1187.5720** | **1187.5791** | **-0.0071** | **0** | **16** | **4.2** | **1** | **R.MVPAGMGAGLER.M** |
|  | 7104 | **603.3675** | **1204.7204** | **1203.5740** | **1.1464** | **0** | **(6)** | **43** | **4** | **R.MVPAGMGAGLER.M** 7095 7096 |
|  | 7167 | **403.9092** | **1208.7057** | **1208.6224** | **0.0834** | **0** | **4** | **54** | **7** | **K.EVFSMAGVVVR.A** |
|  | 7367 | **610.3215** | **1218.6285** | **1219.5689** | **-0.9404** | **0** | **(9)** | **21** | **1** | **R.MVPAGMGAGLER.M** |
|  | 8430 | 424.0557 | 1269.1452 | 1269.7404 | -0.5953 | 1 | 12 | 6.3 | 1 | R.INEILSNALKR.G |
|  | 10788 | **700.3214** | **1398.6283** | **1398.6020** | **0.0262** | **0** | **2** | **82** | **5** | **R.MGLAMGGGGGASFDR.A** |
|  | 11264 | **714.8367** | **1427.6588** | **1426.7061** | **0.9527** | **0** | **(14)** | **5.8** | **2** | **R.MGPAMGPALGAGIER.M** 11238 |
|  | 11394 | **718.3406** | **1434.6666** | **1433.8065** | **0.8601** | **1** | **(1)** | **1e+002** | **6** | **K.LKEVFSMAGVVVR.A** |
|  | 11476 | **481.2383** | **1440.6930** | **1439.6497** | **1.0433** | **1** | **2** | **82** | **5** | **R.MGSVERMGSGIER.M** |
|  | 11522 | **482.2153** | **1443.6241** | **1442.7010** | **0.9231** | **0** | **16** | **3.5** | **1** | **R.MGPAMGPALGAGIER.M** |
|  | 11639 | **484.2470** | **1449.7191** | **1449.8014** | **-0.0823** | **1** | **3** | **61** | **9** | **K.LKEVFSMAGVVVR.A** |
|  | 12297 | **497.2377** | **1488.6912** | **1488.7177** | **-0.0265** | **1** | **(3)** | **58** | **9** | **R.MGSGVERMGPAIER.M** |
|  | 12395 | **499.2557** | **1494.7452** | **1495.6945** | **-0.9493** | **1** | **10** | **13** | **4** | **R.MGLVMDRMGSVER.M** |
|  | 12571 | **503.1042** | **1506.2908** | **1505.7078** | **0.5830** | **1** | **0** | **75** | **7** | **R.MGANNLERMGLER.M** |
|  | 12731 | 758.3963 | 1514.7780 | 1515.7286 | -0.9506 | 1 | 6 | 34 | 9 | R.MGPGIDRLGGAGMER.M 12734 |
|  | 12827 | **507.9174** | **1520.7303** | **1520.7075** | **0.0228** | **1** | **8** | **19** | **1** | **R.MGSGVERMGPAIER.M** |
|  | 13265 | 517.2774 | 1548.8104 | 1547.7184 | 1.0919 | 1 | (5) | 37 | 5 | R.MGPGIDRLGGAGMER.M 13227 |
|  | 13540 | **523.2768** | **1566.8085** | **1565.7290** | **1.0795** | **1** | **2** | **79** | **9** | **R.MGQTMERIGSGVER.M** |
|  | 13624 | **787.1291** | **1572.2436** | **1571.8090** | **0.4347** | **1** | **2** | **62** | **4** | **R.MAAPIDRVGQTIER.M** |
|  | 13957 | **531.7524** | **1592.2353** | **1592.7473** | **-0.5120** | **1** | **4** | **34** | **2** | **R.MGPAIERMGLSMER.M** |
|  | 14266 | **538.5942** | **1612.7607** | **1612.7701** | **-0.0094** | **0** | **46** | **0.0029** | **1** | **R.MGPLGLDHMASSIER.M** 14265 |
|  | 14536 | 815.4099 | 1628.8053 | 1628.7650 | 0.0402 | 0 | (6) | 31 | 4 | R.MGPLGLDHMASSIER.M |
|  | 14789 | **549.4796** | **1645.4170** | **1644.7600** | **0.6570** | **0** | **(2)** | **45** | **4** | **R.MGPLGLDHMASSIER.M** |
|  | 14844 | **550.3313** | **1647.9721** | **1646.8185** | **1.1536** | **0** | **3** | **56** | **9** | **-.MAAGVEAAAEVAATEIK.M** |
|  | 15782 | **857.8394** | **1713.6642** | **1713.6732** | **-0.0090** | **0** | **51** | **0.0007** | **1** | **K.MGGMEGPFGGGMENMGR.F** |
|  | 16021 | **865.9331** | **1729.8517** | **1729.6681** | **0.1836** | **0** | **(4)** | **42** | **2** | **K.MGGMEGPFGGGMENMGR.F** |
|  | 16022 | **865.9334** | **1729.8523** | **1729.6681** | **0.1842** | **0** | **(0)** | **1e+002** | **4** | **K.MGGMEGPFGGGMENMGR.F** |
|  | 17842 | **624.9734** | **1871.8985** | **1870.8778** | **1.0207** | **1** | **10** | **9.8** | **1** | **R.MGAGLGHGMDRVGSEIER.M** |
|  | 18005 | **629.9392** | **1886.7956** | **1887.8502** | **-1.0546** | **1** | **2** | **64** | **3** | **R.LGGAGMERMGAGLGHGMDR.V** |
|  | 18280 | **478.7589** | **1911.0065** | **1910.8801** | **0.1264** | **1** | **3** | **55** | **10** | **R.MGAGMGFGLERMAAPIDR.V** |
|  | 19359 | **669.3635** | **2005.0686** | **2005.9206** | **-0.8520** | **1** | **0** | **87** | **5** | **R.MVPAGMGAGLERMGPVMDR.M** |
|  | 19392 | **1005.5635** | **2009.1124** | **2009.0027** | **0.1097** | **1** | **0** | **81** | **2** | **K.EKVGEVTYVELLMDAEGK.S** |
|  | 19663 | **678.9858** | **2033.9357** | **2033.9457** | **-0.0100** | **0** | **22** | **0.58** | **1** | **R.GNFGGSFAGSFGGAGGHAPGVAR.K** 19660 |
|  | 21014 | 1089.5232 | 2177.0318 | 2177.0432 | -0.0113 | 0 | 63 | 4.3e-005 | 1 | K.GIGMGNIGPAGMGMEGIGFGINK.M |
|  | 24760 | **911.1312** | **2730.3717** | **2731.0376** | **-0.6659** | **1** | **4** | **26** | **1** | **K.MGGMEGPFGGGMENMGRFGSGMNMGR.I** |
|  | 25409 | **953.0931** | **2856.2576** | **2855.2823** | **0.9753** | **1** | **4** | **20** | **2** | **R.MGPAMGPALGAGIERMGLAMGGGGGASFDR.A** 25408 |
|  | 29441 | **977.2210** | **3904.8549** | **3904.6956** | **0.1593** | **1** | **3** | **15** | **1** | **K.GIGMGNIGPAGMGMEGIGFGINKMGGMEGPFGGGMENMGR.F** |
|  | 29444 | **1302.9625** | **3905.8657** | **3904.6956** | **1.1701** | **1** | **(0)** | **30** | **1** | **K.GIGMGNIGPAGMGMEGIGFGINKMGGMEGPFGGGMENMGR.F** |
|  | 29543 | 1318.2936 | 3951.8589 | 3952.6803 | -0.8214 | 1 | (3) | 15 | 4 | K.GIGMGNIGPAGMGMEGIGFGINKMGGMEGPFGGGMENMGR.F |
|  | 29563 | **989.2723** | **3953.0603** | **3952.6803** | **0.3799** | **1** | **(2)** | **16** | **2** | **K.GIGMGNIGPAGMGMEGIGFGINKMGGMEGPFGGGMENMGR.F** |
|  | 30137 | **1409.4891** | **4225.4456** | **4226.1109** | **-0.6653** | **0** | **2** | **9.9** | **3** | **K.VMATTGGMGMGPGGPGMITIPPSILNNPNIPNEIIHALQAGR.L** |
|  | 30179 | **707.8577** | **4241.1024** | **4242.1058** | **-1.0034** | **0** | **(1)** | **20** | **1** | **K.VMATTGGMGMGPGGPGMITIPPSILNNPNIPNEIIHALQAGR.L** |
|  | 30864 | **1179.8722** | **4715.4597** | **4716.3318** | **-0.8722** | **1** | **(0)** | **15** | **5** | **R.AMQKVMATTGGMGMGPGGPGMITIPPSILNNPNIPNEIIHALQAGR.L** |
|  | 30885 | **1578.7926** | **4733.3560** | **4732.3267** | **1.0292** | **1** | **1** | **16** | **3** | **R.AMQKVMATTGGMGMGPGGPGMITIPPSILNNPNIPNEIIHALQAGR.L** |

| **174.** | P01009\|A1AT_HUMAN    **Mass:** 46878    **Score:** 172    **Queries matched:** 20   **emPAI:** 0.47 |
| --- | --- |
|  | Alpha-1-antitrypsin OS=Homo sapiens GN=SERPINA1 PE=1 SV=3 |

|  | **Query** | **Observed** | **Mr(expt)** | **Mr(calc)** | **Delta** | **Miss** | **Score** | **Expect** | **Rank** | **Peptide** |
| --- | --- | --- | --- | --- | --- | --- | --- | --- | --- | --- |
|  | 1288 | 785.4459 | 784.4386 | 784.4443 | -0.0057 | 0 | 20 | 1.7 | 1 | K.VVNPTQK.- |
|  | 2752 | **445.2545** | **888.4945** | **887.4964** | **0.9982** | **0** | **30** | **0.2** | **1** | **K.AVLTIDEK.G** |
|  | 4349 | **504.2380** | **1006.4615** | **1007.5876** | **-1.1261** | **1** | **8** | **28** | **1** | **R.RSASLHLPK.L** |
|  | 4367 | **504.9023** | **1007.7901** | **1007.4924** | **0.2978** | **0** | **6** | **30** | **5** | **K.QINDYVEK.G** |
|  | 4461 | **508.3079** | **1014.6012** | **1014.6073** | **-0.0061** | **0** | **39** | **0.025** | **1** | **K.SVLGQLGITK.V** |
|  | 5236 | **539.7641** | **1077.5136** | **1077.5203** | **-0.0066** | **1** | **7** | **37** | **10** | **K.FLENEDRR.S** |
|  | 7072 | **402.2401** | **1203.6984** | **1203.7049** | **-0.0065** | **1** | **11** | **13** | **8** | **K.KLSSWVLLMK.Y** |
|  | 7390 | **610.8025** | **1219.5904** | **1219.6998** | **-0.1094** | **1** | **(10)** | **16** | **3** | **K.KLSSWVLLMK.Y** |
|  | 7965 | **416.5365** | **1246.5877** | **1246.5951** | **-0.0074** | **0** | **10** | **15** | **4** | **R.LGMFNIQHCK.K** |
|  | 16632 | **593.9233** | **1778.7480** | **1778.7609** | **-0.0129** | **0** | **17** | **2.1** | **1** | **K.TDTSHHDQDHPTFNK.I** |
|  | 17332 | 917.4602 | 1832.9059 | 1832.9156 | -0.0097 | 0 | 91 | 9e-008 | 1 | K.VFSNGADLSGVTEEAPLK.L 17330 17342 17343 |
|  | 17632 | **619.3281** | **1854.9624** | **1854.9702** | **-0.0079** | **0** | **35** | **0.034** | **1** | **K.FNKPFVFLMIEQNTK.S** |
|  | 20885 | **1082.0553** | **2162.0960** | **2161.1266** | **0.9694** | **1** | **52** | **0.00049** | **1** | **K.VFSNGADLSGVTEEAPLKLSK.A** 20880 20884 20886 |
|  | 26877 | **791.1658** | **3160.6342** | **3159.6083** | **1.0259** | **1** | **5** | **16** | **1** | **K.AVLTIDEKGTEAAGAMFLEAIPMSIPPEVK.F** |

| **175.** | P61421\|VA0D1_HUMAN    **Mass:** 40759    **Score:** 171    **Queries matched:** 15   **emPAI:** 0.55 |
| --- | --- |
|  | V-type proton ATPase subunit d 1 OS=Homo sapiens GN=ATP6V0D1 PE=1 SV=1 |

|  | **Query** | **Observed** | **Mr(expt)** | **Mr(calc)** | **Delta** | **Miss** | **Score** | **Expect** | **Rank** | **Peptide** |
| --- | --- | --- | --- | --- | --- | --- | --- | --- | --- | --- |
|  | 1416 | **398.8770** | **795.7393** | **795.3949** | **0.3444** | **0** | **9** | **13** | **2** | **K.MVVEFR.H** |
|  | 2254 | 428.7456 | 855.4767 | 855.5065 | -0.0299 | 0 | 16 | 4.4 | 4 | R.SIAELVPK.C |
|  | 3720 | **482.2153** | **962.4160** | **961.4651** | **0.9510** | **1** | **5** | **56** | **6** | **K.LKEQECR.N** |
|  | 4177 | **498.2095** | **994.4044** | **993.5171** | **0.8873** | **0** | **4** | **61** | **5** | **K.IDNYIPIF.-** |
|  | 7595 | **615.8417** | **1229.6688** | **1229.6768** | **-0.0080** | **0** | **31** | **0.12** | **1** | **R.LYPEGLAQLAR.A** |
|  | 9107 | **652.8237** | **1303.6328** | **1303.6408** | **-0.0080** | **0** | **69** | **1.6e-005** | **1** | **K.LLFEGAGSNPGDK.T** 9083 9084 |
|  | 21287 | **1105.4922** | **2208.9698** | **2208.9851** | **-0.0153** | **1** | **63** | **4.3e-005** | **1** | **R.ADDYEQVKNVADYYPEYK.L** 21286 21288 21289 |
|  | 23969 | **863.3949** | **2587.1629** | **2587.1757** | **-0.0128** | **0** | **1** | **49** | **3** | **K.FCTLLGGTTADAMCPILEFEADR.R** |
|  | 31640 | **1359.9302** | **5435.6916** | **5434.6879** | **1.0038** | **1** | **37** | **0.002** | **1** | **K.AGVLSQADYLNLVQCETLEDLKLHLQSTDYGNFLANEASPLTVSVIDDR.L** |
|  | 32293 | **1704.2952** | **6813.1516** | **6814.2590** | **-1.1075** | **1** | **1** | **3.5** | **1** | **K.CHPLGSFEQMEAVNIAQTPAELYNAILVDTPLAAFFQDCISEQDLDEMNIEIIRNTLYK.A** |

| **176.** | P56199\|ITA1_HUMAN    **Mass:** 132304   **Score:** 169    **Queries matched:** 24   **emPAI:** 0.06 |
| --- | --- |
|  | Integrin alpha-1 OS=Homo sapiens GN=ITGA1 PE=1 SV=2 |

|  | **Query** | **Observed** | **Mr(expt)** | **Mr(calc)** | **Delta** | **Miss** | **Score** | **Expect** | **Rank** | **Peptide** |
| --- | --- | --- | --- | --- | --- | --- | --- | --- | --- | --- |
|  | 1590 | **404.2242** | **806.4338** | **805.3640** | **1.0698** | **0** | **7** | **34** | **3** | **R.MEDGNIK.I** |
|  | 2193 | 426.2319 | 850.4492 | 849.3651 | 1.0842 | 1 | 4 | 64 | 8 | K.DCGNKEK.C |
|  | 3006 | **454.7041** | **907.3937** | **906.5175** | **0.8762** | **0** | **13** | **9.2** | **1** | **K.FNVSLTVK.N** |
|  | 5193 | **537.3182** | **1072.6218** | **1071.5924** | **1.0294** | **1** | **5** | **68** | **8** | **R.IPSGGDGKTLK.F** |
|  | 7370 | **610.3311** | **1218.6477** | **1217.5095** | **1.1382** | **0** | **5** | **51** | **4** | **R.QHNSCTTENK.N** 7339 |
|  | 8035 | **626.3068** | **1250.5991** | **1250.6078** | **-0.0087** | **1** | **24** | **0.54** | **2** | **R.TGDVYKCPVGR.G** |
|  | 8880 | **647.7958** | **1293.5771** | **1292.6394** | **0.9377** | **0** | **1** | **99** | **4** | **R.QTMTALGIDTAR.K** |
|  | 10875 | 702.8731 | 1403.7317 | 1404.7395 | -1.0078 | 1 | 2 | 89 | 9 | R.QTMTALGIDTARK.E 10874 |
|  | 11884 | **488.9267** | **1463.7584** | **1462.7263** | **1.0320** | **0** | **6** | **35** | **9** | **R.SENASLVLSSSNQK.R** |
|  | 12771 | **759.8414** | **1517.6683** | **1517.6780** | **-0.0097** | **0** | **57** | **0.00025** | **1** | **K.VIQDCEDENIQR.F** |
|  | 15931 | **575.6013** | **1723.7821** | **1723.7948** | **-0.0127** | **0** | **3** | **50** | **2** | **K.VMVIVTDGESHDNHR.L** |
|  | 17377 | 613.3323 | 1836.9752 | 1835.8948 | 1.0804 | 1 | 22 | 0.74 | 1 | K.KVMVIVTDGESHDNHR.L 17368 |
|  | 17600 | **618.3351** | **1851.9836** | **1851.8898** | **0.0938** | **1** | **(1)** | **76** | **7** | **K.KVMVIVTDGESHDNHR.L** |
|  | 19561 | **506.9845** | **2023.9090** | **2023.9211** | **-0.0120** | **1** | **7** | **20** | **1** | **K.HSFYMLDKHDFQDSVR.I** |
|  | 19716 | **511.0177** | **2040.0417** | **2040.1125** | **-0.0708** | **0** | **1** | **66** | **9** | **R.ARPGVAVACCWLLTVVLR.C** |
|  | 24084 | **652.8301** | **2607.2912** | **2608.3782** | **-1.0870** | **1** | **6** | **19** | **2** | **R.GESLPCVKLDLPVNTSIPNVTEVK.E** |
|  | 24129 | **873.1431** | **2616.4076** | **2617.4574** | **-1.0498** | **1** | **2** | **38** | **3** | **M.APRPRARPGVAVACCWLLTVVLR.C** |
|  | 28237 | **703.7220** | **3513.5739** | **3514.1281** | **-0.5542** | **1** | **3** | **17** | **2** | **K.DGLPGRVPLWVILLSAFAGLLLLMLLILALWK.I** |
|  | 28407 | **889.7120** | **3554.8191** | **3553.6937** | **1.1254** | **0** | **2** | **23** | **3** | **K.FFGQSIHGEMDLNGDGLTDVTIGGLGGAALFWSR.D** |
|  | 30353 | **1454.6870** | **4361.0392** | **4361.0661** | **-0.0269** | **1** | **82** | **1.4e-007** | **1** | **K.NSMTFSGPVEDMFGYTVQQYENEEGKWVLIGSPLVGQPK.N** 30354 |

| **177.** | P20648\|ATP4A_HUMAN    **Mass:** 115756   **Score:** 168    **Queries matched:** 18   **emPAI:** 0.06 |
| --- | --- |
|  | Potassium-transporting ATPase alpha chain 1 OS=Homo sapiens GN=ATP4A PE=2 SV=5 |

|  | **Query** | **Observed** | **Mr(expt)** | **Mr(calc)** | **Delta** | **Miss** | **Score** | **Expect** | **Rank** | **Peptide** |
| --- | --- | --- | --- | --- | --- | --- | --- | --- | --- | --- |
|  | 1907 | **417.2335** | **832.4524** | **832.4337** | **0.0187** | **1** | **8** | **34** | **6** | **K.CRTAGIR.V** |
|  | 2309 | **430.7468** | **859.4791** | **859.4586** | **0.0206** | **0** | **16** | **6** | **2** | **R.ILAAQGCK.V** |
|  | 7008 | 401.2425 | 1200.7056 | 1199.6087 | 1.0968 | 0 | 5 | 49 | 10 | R.LSAFQQGFFR.N |
|  | 8032 | **417.7261** | **1250.1565** | **1249.6965** | **0.4600** | **1** | **10** | **10** | **1** | **R.HLLVMKGAPER.V** |
|  | 9729 | **447.2377** | **1338.6914** | **1339.6666** | **-0.9753** | **0** | **6** | **32** | **7** | **K.AESDIMHLRPR.N** |
|  | 10497 | 691.3746 | 1380.7347 | 1380.7435 | -0.0088 | 0 | 72 | 9.3e-006 | 1 | R.VIMVTGDHPITAK.A 10473 10499 |
|  | 12228 | **742.8571** | **1483.6996** | **1484.8562** | **-1.1566** | **1** | **4** | **51** | **9** | **K.RIVIGDASETALLK.F** |
|  | 14358 | 810.3731 | 1618.7317 | 1618.7435 | -0.0118 | 0 | 63 | 6.3e-005 | 1 | K.VDNSSLTGESEPQTR.S 14360 14361 |
|  | 16824 | **599.3484** | **1795.0235** | **1795.9680** | **-0.9445** | **0** | **1** | **85** | **6** | **R.LGAIVAVTGDGVNDSPALK.K** |
|  | 21117 | **730.3809** | **2188.1208** | **2187.9506** | **0.1701** | **1** | **10** | **8.2** | **1** | **K.LGVRCCPGSWWDQELYY.-** |
|  | 22195 | **465.0324** | **2320.1257** | **2319.1529** | **0.9728** | **1** | **4** | **31** | **6** | **K.FQINADQLVVGDLVEMKGGDR.V** |
|  | 27124 | **1072.1433** | **3213.4081** | **3213.7256** | **-0.3175** | **0** | **0** | **39** | **4** | **R.AMVFFMAIVVAYVPEGLLATVTVCLSLTAK.R** |
|  | 28659 | 1207.2351 | 3618.6835 | 3619.6462 | -0.9627 | 0 | 7 | 7.9 | 2 | K.DYPPGYAFDVEAMNFPSSGLCFAGLVSMIDPPR.A |
|  | 30202 | 852.4692 | 4257.3095 | 4258.2615 | -0.9519 | 0 | 1 | 16 | 4 | K.TPIAIEIEHFVDIIAGLAILFGATFFIVAMCIGYTFLR.A |

| **178.** | Q99623\|PHB2_HUMAN    **Mass:** 33276    **Score:** 168    **Queries matched:** 18   **emPAI:** 0.11 |
| --- | --- |
|  | Prohibitin-2 OS=Homo sapiens GN=PHB2 PE=1 SV=2 |

|  | **Query** | **Observed** | **Mr(expt)** | **Mr(calc)** | **Delta** | **Miss** | **Score** | **Expect** | **Rank** | **Peptide** |
| --- | --- | --- | --- | --- | --- | --- | --- | --- | --- | --- |
|  | 749 | **531.2849** | **530.2776** | **530.2813** | **-0.0036** | **0** | **14** | **14** | **4** | **K.DLAGR.L** |
|  | 1929 | **834.4673** | **833.4600** | **833.4647** | **-0.0047** | **0** | **25** | **0.58** | **2** | **R.AQFLVEK.A** 1944 |
|  | 2141 | 424.7283 | 847.4421 | 847.4473 | -0.0052 | 0 | 17 | 3.9 | 2 | K.MLGEALSK.N 2140 2144 |
|  | 2928 | **452.2403** | **902.4660** | **903.5025** | **-1.0366** | **1** | **8** | **31** | **7** | **R.GSDSLIKGK.K** |
|  | 4167 | 497.7524 | 993.4903 | 993.4767 | 0.0136 | 0 | 10 | 16 | 10 | R.LGLDYEER.V |
|  | 7072 | 402.2401 | 1203.6984 | 1203.6281 | 0.0703 | 0 | 11 | 12 | 6 | K.DLQMVNISLR.V 7096 |
|  | 8680 | **642.6005** | **1283.1864** | **1283.7020** | **-0.5156** | **1** | **1** | **78** | **8** | **R.LPAGPRGMGTALK.L** |
|  | 18025 | **630.3243** | **1887.9510** | **1887.9625** | **-0.0115** | **0** | **24** | **0.38** | **1** | **R.VLSRPNAQELPSMYQR.L** 18046 |
|  | 19736 | **681.3711** | **2041.0916** | **2040.0415** | **1.0501** | **0** | **4** | **35** | **2** | **K.DFSLILDDVAITELSFSR.E** 19731 |
|  | 21439 | **1113.0707** | **2224.1268** | **2224.1375** | **-0.0107** | **0** | **119** | **1e-010** | **1** | **R.IYLTADNLVLNLQDESFTR.G** 21432 21440 |

| **179.** | P55072\|TERA_HUMAN    **Mass:** 89950    **Score:** 167    **Queries matched:** 24   **emPAI:** 0.13 |
| --- | --- |
|  | Transitional endoplasmic reticulum ATPase OS=Homo sapiens GN=VCP PE=1 SV=4 |

|  | **Query** | **Observed** | **Mr(expt)** | **Mr(calc)** | **Delta** | **Miss** | **Score** | **Expect** | **Rank** | **Peptide** |
| --- | --- | --- | --- | --- | --- | --- | --- | --- | --- | --- |
|  | 1814 | **414.2092** | **826.4038** | **827.5229** | **-1.1190** | **1** | **2** | **98** | **4** | **R.KQLAQIK.E** |
|  | 1815 | **414.2275** | **826.4405** | **826.3933** | **0.0472** | **0** | **3** | **71** | **6** | **K.THGEVER.R** |
|  | 3720 | 482.2153 | 962.4160 | 961.4716 | 0.9444 | 0 | 4 | 69 | 9 | R.ESIESEIR.R |
|  | 4053 | **493.8937** | **985.7728** | **985.5266** | **0.2462** | **0** | **6** | **35** | **2** | **K.EMVELPLR.H** |
|  | 4702 | **517.7184** | **1033.4222** | **1033.4287** | **-0.0065** | **0** | **13** | **9.4** | **2** | **R.DHFEEAMR.F** |
|  | 4913 | **526.2629** | **1050.5112** | **1050.5168** | **-0.0056** | **0** | **54** | **0.00059** | **1** | **K.MDELQLFR.G** |
|  | 5817 | **560.2936** | **1118.5727** | **1117.5727** | **1.0000** | **1** | **3** | **90** | **5** | **R.ESIESEIRR.E** |
|  | 6270 | **578.8370** | **1155.6595** | **1154.6158** | **1.0437** | **1** | **9** | **19** | **1** | **K.FLKFGMTPSK.G** |
|  | 6467 | **586.8339** | **1171.6533** | **1171.6601** | **-0.0068** | **0** | **46** | **0.0042** | **1** | **R.GILLYGPPGTGK.T** |
|  | 8781 | **645.3296** | **1288.6446** | **1287.7034** | **0.9412** | **1** | **5** | **47** | **2** | **K.GDDLSTAILKQK.N** |
|  | 9560 | **664.8218** | **1327.6290** | **1328.6837** | **-1.0547** | **0** | **1** | **1.2e+002** | **6** | **R.WALSQSNPSALR.E** |
|  | 9806 | **448.5816** | **1342.7231** | **1342.6338** | **0.0893** | **1** | **3** | **70** | **3** | **K.ARGGNIGDGGGAADR.V** |
|  | 12772 | **506.9066** | **1517.6979** | **1516.6980** | **0.9998** | **0** | **1** | **1e+002** | **9** | **K.YEMFAQTLQQSR.G** |
|  | 15532 | **565.7611** | **1694.2615** | **1694.9178** | **-0.6563** | **1** | **6** | **20** | **2** | **K.EMVELPLRHPALFK.A** |
|  | 16864 | **900.4473** | **1798.8801** | **1798.7978** | **0.0823** | **0** | **1** | **80** | **6** | **K.MTNGFSGADLTEICQR.A** |
|  | 17396 | **920.8135** | **1839.6124** | **1839.7867** | **-0.1742** | **0** | **0** | **73** | **1** | **R.EAVCIVLSDDTCSDEK.I** |
|  | 17401 | 920.9568 | 1839.8990 | 1838.9118 | 0.9873 | 1 | 1 | 92 | 8 | R.VINQILTEMDGMSTKK.N |
|  | 20943 | **1085.5598** | **2169.1051** | **2169.1165** | **-0.0114** | **0** | **96** | **1.9e-008** | **1** | **R.LIVDEAINEDNSVVSLSQPK.M** 20941 20946 |
|  | 21319 | **738.0550** | **2211.1431** | **2211.1575** | **-0.0144** | **1** | **4** | **36** | **2** | **R.ELQELVQYPVEHPDKFLK.F** |
|  | 27486 | **828.8873** | **3311.5200** | **3311.6496** | **-0.1296** | **1** | **1** | **33** | **4** | **R.AVANETGAFFFLINGPEIMSKLAGESESNLR.K** |
|  | 29172 | **760.9934** | **3799.9307** | **3799.8799** | **0.0508** | **1** | **0** | **30** | **1** | **K.LADDVDLEQVANETHGHVGADLAALCSEAALQAIRK.K** |
|  | 29648 | **799.2310** | **3991.1184** | **3990.1295** | **0.9889** | **0** | **4** | **11** | **3** | **R.IHVLPIDDTVEGITGNLFEVYLKPYFLEAYRPIR.K** |

| **180.** | O43895\|XPP2_HUMAN    **Mass:** 76090    **Score:** 167    **Queries matched:** 14   **emPAI:** 0.15 |
| --- | --- |
|  | Xaa-Pro aminopeptidase 2 OS=Homo sapiens GN=XPNPEP2 PE=1 SV=3 |

|  | **Query** | **Observed** | **Mr(expt)** | **Mr(calc)** | **Delta** | **Miss** | **Score** | **Expect** | **Rank** | **Peptide** |
| --- | --- | --- | --- | --- | --- | --- | --- | --- | --- | --- |
|  | 1423 | 399.2147 | 796.4149 | 797.4395 | -1.0247 | 0 | 12 | 7.8 | 7 | K.VGPELQR.R 1457 |
|  | 3618 | 478.2694 | 954.5243 | 953.5406 | 0.9837 | 1 | 6 | 34 | 5 | K.VGPELQRR.Q |
|  | 4648 | **515.3519** | **1028.6892** | **1029.5818** | **-0.8926** | **1** | **17** | **3.2** | **1** | **K.NSKEQALLK.A** |
|  | 5987 | **567.8213** | **1133.6280** | **1134.5815** | **-0.9535** | **1** | **0** | **1.6e+002** | **5** | **K.VSGVRSQMQK.H** |
|  | 12245 | **743.3654** | **1484.7162** | **1484.7255** | **-0.0093** | **0** | **50** | **0.0012** | **1** | **K.LVTDTYSPVMMTK.A** 12259 |
|  | 16677 | **446.4907** | **1781.9335** | **1782.9260** | **-0.9924** | **1** | **9** | **15** | **3** | **K.LVTDTYSPVMMTKAVK.N** |
|  | 17074 | **606.2447** | **1815.7122** | **1814.9158** | **0.7964** | **1** | **(2)** | **51** | **7** | **K.LVTDTYSPVMMTKAVK.N** |
|  | 21066 | **728.4253** | **2182.2542** | **2182.0041** | **0.2501** | **1** | **8** | **11** | **1** | **K.GMFTSIEPGYYKDGEFGIR.L** |
|  | 23873 | **1285.6223** | **2569.2301** | **2569.2377** | **-0.0076** | **0** | **98** | **1e-008** | **1** | **R.ASDIPYNPFFYSYTLLTDSSIR.L** |
|  | 25385 | **951.1022** | **2850.2847** | **2850.3018** | **-0.0171** | **1** | **71** | **4.5e-006** | **1** | **R.KLSSDEMYLLDSGGQYWDGTTDITR.T** |
|  | 31143 | **1645.4074** | **4933.2002** | **4933.5178** | **-0.3176** | **1** | **2** | **7.5** | **4** | **K.EVGTTPIVTWLLTEIPAGGRVGFDPFLLSIDTWESYDLALQGSNR.Q** |
|  | 32165 | 1227.8129 | 6134.0280 | 6133.9030 | 0.1249 | 1 | 3 | 3.1 | 2 | R.NCSTNPPYLPVTVVNTTMSLTALRQQMQTQNLSAYIIPGTDAHMNEYIGQHDER.R |

| **181.** | Q9NZ45\|CISD1_HUMAN    **Mass:** 12362    **Score:** 166    **Queries matched:** 6   **emPAI:** 2.05 |
| --- | --- |
|  | CDGSH iron sulfur domain-containing protein 1 OS=Homo sapiens GN=CISD1 PE=1 SV=1 |

|  | **Query** | **Observed** | **Mr(expt)** | **Mr(calc)** | **Delta** | **Miss** | **Score** | **Expect** | **Rank** | **Peptide** |
| --- | --- | --- | --- | --- | --- | --- | --- | --- | --- | --- |
|  | 5117 | **534.3024** | **1066.5902** | **1066.5957** | **-0.0055** | **0** | **13** | **7.3** | **2** | **K.AMINLHIQK.D** |
|  | 12296 | 497.2352 | 1488.6839 | 1488.6919 | -0.0080 | 0 | 33 | 0.072 | 1 | K.IVHAFDMEDLGDK.A |
|  | 12831 | **761.4096** | **1520.8047** | **1520.8133** | **-0.0086** | **1** | **39** | **0.015** | **1** | **K.AMINLHIQKDNPK.I** |
|  | 14625 | **818.4159** | **1634.8172** | **1634.8264** | **-0.0092** | **0** | **91** | **8.4e-008** | **1** | **K.HNEETGDNVGPLIIK.K** 14623 |
|  | 20634 | **713.3453** | **2137.0142** | **2137.9925** | **-0.9783** | **1** | **30** | **0.096** | **1** | **K.IVHAFDMEDLGDKAVYCR.C** |

| **182.** | P12109\|CO6A1_HUMAN    **Mass:** 109602   **Score:** 165    **Queries matched:** 18   **emPAI:** 0.07 |
| --- | --- |
|  | Collagen alpha-1(VI) chain OS=Homo sapiens GN=COL6A1 PE=1 SV=3 |

|  | **Query** | **Observed** | **Mr(expt)** | **Mr(calc)** | **Delta** | **Miss** | **Score** | **Expect** | **Rank** | **Peptide** |
| --- | --- | --- | --- | --- | --- | --- | --- | --- | --- | --- |
|  | 752 | 533.0582 | 532.0509 | 532.2428 | -0.1919 | 0 | 5 | 71 | 9 | R.MPGGR.D |
|  | 2628 | 441.2615 | 880.5084 | 881.4468 | -0.9384 | 1 | 8 | 25 | 7 | R.GGPGERGPR.G |
|  | 3353 | **467.0989** | **932.1833** | **931.3970** | **0.7863** | **1** | **10** | **13** | **6** | **R.YYRCDR.N** |
|  | 6458 | 586.4145 | 1170.8144 | 1170.6608 | 0.1536 | 1 | 9 | 14 | 3 | R.NVQELKEAIK.S |
|  | 8790 | **430.5642** | **1288.6708** | **1288.6775** | **-0.0067** | **0** | **10** | **15** | **3** | **R.LSIIATDHTYR.R** |
|  | 13361 | **519.2670** | **1554.7791** | **1554.7903** | **-0.0112** | **1** | **5** | **42** | **1** | **R.FLTAGRTDPAHDVR.V** |
|  | 13760 | **527.6164** | **1579.8273** | **1579.8358** | **-0.0085** | **0** | **7** | **21** | **1** | **K.VFSVAITPDHLEPR.L** |
|  | 14589 | 545.2770 | 1632.8091 | 1631.7726 | 1.0365 | 0 | (2) | 81 | 4 | R.GAPGPAGPPGDPGLMGER.G |
|  | 14850 | **550.6042** | **1648.7909** | **1647.7675** | **1.0234** | **0** | **3** | **53** | **1** | **R.GAPGPAGPPGDPGLMGER.G** 14805 |
|  | 18545 | **966.5005** | **1930.9864** | **1931.0000** | **-0.0136** | **0** | **22** | **0.7** | **1** | **R.LLLFSDGNSQGATPAAIEK.A** |
|  | 20052 | **692.0260** | **2073.0562** | **2074.0735** | **-1.0173** | **1** | **0** | **79** | **1** | **K.ENKYLIVVTDGHPLEGYK.E** |
|  | 20472 | **706.3474** | **2116.0204** | **2116.0048** | **0.0156** | **0** | **2** | **62** | **3** | **K.SLQWMAGGTFTGEALQYTR.D** |
|  | 22581 | **593.5700** | **2370.2509** | **2371.1227** | **-0.8717** | **1** | **3** | **36** | **1** | **K.GEAGDPGRPGDLGPVGYQGMKGEK.G** |
|  | 26958 | **794.1384** | **3172.5244** | **3172.5387** | **-0.0143** | **1** | **2** | **31** | **4** | **K.YLIVVTDGHPLEGYKEPCGGLEDAVNEAK.H** |
|  | 29599 | **1324.5979** | **3970.7719** | **3969.7853** | **0.9866** | **1** | **103** | **1.2e-009** | **1** | **R.DAEEAISQTIDTIVDMIKNNVEQVCCSFECQPAR.G** 29595 |
|  | 30828 | **1170.3079** | **4677.2023** | **4677.9949** | **-0.7925** | **1** | **2** | **13** | **1** | **K.GEPGADGEAGRPGSSGPSGDEGQPGEPGPPGEKGEAGDEGNPGPDGAPGER.G** |

| **183.** | Q9Y5K8\|VATD_HUMAN    **Mass:** 28245    **Score:** 165    **Queries matched:** 12   **emPAI:** 0.29 |
| --- | --- |
|  | V-type proton ATPase subunit D OS=Homo sapiens GN=ATP6V1D PE=1 SV=1 |

|  | **Query** | **Observed** | **Mr(expt)** | **Mr(calc)** | **Delta** | **Miss** | **Score** | **Expect** | **Rank** | **Peptide** |
| --- | --- | --- | --- | --- | --- | --- | --- | --- | --- | --- |
|  | 1756 | **411.2401** | **820.4657** | **821.4139** | **-0.9482** | **0** | **7** | **41** | **6** | **R.MAQTIMK.A** |
|  | 2336 | **431.2423** | **860.4700** | **860.4756** | **-0.0056** | **0** | **12** | **14** | **3** | **R.IEIFPSR.M** |
|  | 3989 | **491.7516** | **981.4886** | **981.4446** | **0.0441** | **0** | **4** | **65** | **5** | **K.MLMGEVMR.E** |
|  | 4893 | 525.2640 | 1048.5135 | 1048.5521 | -0.0386 | 1 | 11 | 14 | 8 | R.MAQTIMKAR.L |
|  | 5090 | 533.2144 | 1064.4142 | 1064.5470 | -0.1329 | 1 | (1) | 1.1e+002 | 9 | R.MAQTIMKAR.L |
|  | 14037 | **799.8719** | **1597.7292** | **1597.7878** | **-0.0585** | **1** | **3** | **54** | **2** | **K.IIETKMLMGEVMR.E** |
|  | 14903 | **551.7842** | **1652.3307** | **1652.8621** | **-0.5313** | **0** | **6** | **22** | **1** | **R.AAGEVLEPANLLAEEK.D** |
|  | 15889 | **574.6374** | **1720.8905** | **1720.8995** | **-0.0090** | **1** | **7** | **23** | **3** | **R.TLAYIITELDERER.E** |
|  | 16157 | **871.4370** | **1740.8593** | **1740.8683** | **-0.0089** | **0** | **110** | **9.8e-010** | **1** | **K.FTAGDFSTTVIQNVNK.A** 16156 |
|  | 23486 | **1257.5902** | **2513.1659** | **2514.2377** | **-1.0718** | **1** | **33** | **0.035** | **1** | **R.AAGEVLEPANLLAEEKDEDLLFE.-** 23490 |

| **184.** | P15311\|EZRI_HUMAN    **Mass:** 69484    **Score:** 164    **Queries matched:** 19   **emPAI:** 0.17 |
| --- | --- |
|  | Ezrin OS=Homo sapiens GN=EZR PE=1 SV=4 |

|  | **Query** | **Observed** | **Mr(expt)** | **Mr(calc)** | **Delta** | **Miss** | **Score** | **Expect** | **Rank** | **Peptide** |
| --- | --- | --- | --- | --- | --- | --- | --- | --- | --- | --- |
|  | 1580 | **404.0306** | **806.0467** | **805.4004** | **0.6463** | **0** | **8** | **22** | **4** | **K.EELMLR.L** |
|  | 4017 | 492.7148 | 983.4151 | 982.4212 | 0.9939 | 1 | 3 | 75 | 7 | R.EKEQMMR.E 4015 |
|  | 4062 | 494.2553 | 986.4961 | 986.5032 | -0.0071 | 0 | 11 | 14 | 3 | R.ALQLEEER.K |
|  | 5765 | 558.7153 | 1115.4160 | 1115.5822 | -0.1662 | 1 | 9 | 21 | 1 | R.AKEAQDDLVK.T |
|  | 5829 | 560.7819 | 1119.5492 | 1119.5560 | -0.0068 | 1 | 23 | 0.88 | 1 | K.QRIDEFEAL.- |
|  | 6127 | **573.5405** | **1145.0664** | **1144.6022** | **0.4642** | **1** | **5** | **33** | **1** | **R.LEADRMAALR.A** |
|  | 6494 | **588.0978** | **1174.1810** | **1174.5996** | **-0.4186** | **0** | **2** | **73** | **4** | **R.IQVWHAEHR.G** |
|  | 10665 | **696.5037** | **1390.9928** | **1390.6841** | **0.3087** | **1** | **0** | **87** | **3** | **K.EVHKSGYLSSER.L** |
|  | 12305 | **745.3792** | **1488.7439** | **1487.7766** | **0.9673** | **1** | **3** | **60** | **7** | **R.RKPDTIEVQQMK.A** |
|  | 12372 | **747.3445** | **1492.6745** | **1492.6841** | **-0.0096** | **0** | **68** | **2e-005** | **1** | **R.THNDIIHNENMR.Q** 12373 |
|  | 12569 | **753.8884** | **1505.7623** | **1504.7998** | **0.9625** | **1** | **4** | **45** | **6** | **K.SGYLSSERLIPQR.V** 12568 |
|  | 14884 | **826.4081** | **1650.8016** | **1650.8100** | **-0.0084** | **0** | **85** | **3.5e-007** | **1** | **K.SQEQLAAELAEYTAK.I** 14885 |
|  | 15076 | **555.6294** | **1663.8663** | **1664.7801** | **-0.9138** | **1** | **1** | **93** | **5** | **K.RTHNDIIHNENMR.Q** |
|  | 22973 | 609.3071 | 2433.1992 | 2433.2387 | -0.0395 | 1 | 2 | 50 | 8 | R.QAVDQIKSQEQLAAELAEYTAK.I |
|  | 26090 | **751.0930** | **3000.3427** | **3000.5412** | **-0.1985** | **1** | **1** | **44** | **7** | **-.MPKPINVRVTTMDAELEFAIQPNTTGK.Q** |

| **185.** | P39023\|RL3_HUMAN    **Mass:** 46365    **Score:** 163    **Queries matched:** 20   **emPAI:** 0.26 |
| --- | --- |
|  | 60S ribosomal protein L3 OS=Homo sapiens GN=RPL3 PE=1 SV=2 |

|  | **Query** | **Observed** | **Mr(expt)** | **Mr(calc)** | **Delta** | **Miss** | **Score** | **Expect** | **Rank** | **Peptide** |
| --- | --- | --- | --- | --- | --- | --- | --- | --- | --- | --- |
|  | 1624 | **810.2764** | **809.2692** | **810.4123** | **-1.1432** | **0** | **18** | **2** | **1** | **K.FIDTTSK.F** 1644 |
|  | 2063 | **421.9068** | **841.7991** | **841.4004** | **0.3987** | **1** | **2** | **72** | **3** | **K.DFSSMKK.Y** |
|  | 2419 | **434.2338** | **866.4530** | **866.4610** | **-0.0080** | **1** | **10** | **17** | **4** | **K.GYKGVTSR.W** |
|  | 2591 | **439.3740** | **876.7335** | **876.3614** | **0.3721** | **0** | **3** | **59** | **2** | **K.WQDEDGK.K** |
|  | 2904 | **451.2627** | **900.5109** | **899.4647** | **1.0462** | **0** | **10** | **18** | **6** | **K.AGMTHIVR.E** |
|  | 3152 | **459.1706** | **916.3266** | **915.5753** | **0.7513** | **1** | **1** | **1.6e+002** | **5** | **R.KSLLVQTK.R** |
|  | 3597 | 477.7235 | 953.4325 | 954.5069 | -1.0744 | 0 | 16 | 3.8 | 1 | R.VIAHTQMR.L |
|  | 4023 | **492.7734** | **983.5323** | **982.5349** | **0.9974** | **0** | **13** | **7.8** | **1** | **R.HGSLGFLPR.K** |
|  | 4927 | **526.9556** | **1051.8967** | **1052.5264** | **-0.6297** | **1** | **2** | **57** | **6** | **R.AGQKGYHHR.T** |
|  | 6818 | **398.2280** | **1191.6621** | **1190.6659** | **0.9962** | **1** | **9** | **18** | **3** | **K.IGQGYLIKDGK.L** |
|  | 7575 | **615.3414** | **1228.6683** | **1227.6571** | **1.0112** | **1** | **4** | **67** | **10** | **R.EVDRPGSKVNK.K** |
|  | 9777 | **671.7872** | **1341.5599** | **1341.5684** | **-0.0085** | **0** | **37** | **0.026** | **1** | **K.NNASTDYDLSDK.S** 9776 |
|  | 10203 | **456.0271** | **1365.0596** | **1364.6837** | **0.3758** | **1** | **2** | **60** | **7** | **K.FIDTTSKFGHGR.F** |
|  | 15550 | **566.2613** | **1695.7620** | **1695.8315** | **-0.0695** | **1** | **5** | **32** | **7** | **K.LIKNNASTDYDLSDK.S** |
|  | 16495 | **590.3162** | **1767.9268** | **1767.9050** | **0.0218** | **1** | **1** | **92** | **5** | **K.AGMTHIVREVDRPGSK.V** |
|  | 24527 | **1343.6836** | **2685.3526** | **2685.3684** | **-0.0158** | **0** | **105** | **1.9e-009** | **1** | **R.LEQQVPVNQVFGQDEMIDVIGVTK.G** |
|  | 25943 | **991.1733** | **2970.4982** | **2970.5121** | **-0.0139** | **1** | **67** | **1.2e-005** | **1** | **R.ERLEQQVPVNQVFGQDEMIDVIGVTK.G** |
|  | 26131 | 1004.4562 | 3010.3469 | 3009.6461 | 0.7008 | 1 | 1 | 44 | 4 | K.KEVVEAVTIVETPPMVVVGIVGYVETPR.G |

| **186.** | O95292\|VAPB_HUMAN    **Mass:** 27439    **Score:** 163    **Queries matched:** 15   **emPAI:** 0.68 |
| --- | --- |
|  | Vesicle-associated membrane protein-associated protein B/C OS=Homo sapiens GN=VAPB PE=1 SV=3 |

|  | **Query** | **Observed** | **Mr(expt)** | **Mr(calc)** | **Delta** | **Miss** | **Score** | **Expect** | **Rank** | **Peptide** |
| --- | --- | --- | --- | --- | --- | --- | --- | --- | --- | --- |
|  | 1846 | **829.4465** | **828.4393** | **828.4454** | **-0.0061** | **0** | **20** | **2.1** | **1** | **R.LQGEVQR.L** |
|  | 3435 | 470.7423 | 939.4701 | 938.4201 | 1.0499 | 1 | 7 | 27 | 9 | K.KVMEECK.R |
|  | 3576 | **476.2801** | **950.5457** | **950.4314** | **0.1144** | **1** | **12** | **10** | **1** | **K.VMEECKR.L** |
|  | 8843 | **646.7986** | **1291.5827** | **1290.6820** | **0.9008** | **0** | **7** | **28** | **5** | **R.GPFTDVVTTNLK.L** |
|  | 13977 | **797.9290** | **1593.8434** | **1593.8515** | **-0.0081** | **1** | **57** | **0.00024** | **1** | **K.FRGPFTDVVTTNLK.L** |
|  | 14838 | **824.9435** | **1647.8725** | **1647.8832** | **-0.0106** | **0** | **57** | **0.00021** | **1** | **K.VEQVLSLEPQHELK.F** |
|  | 15254 | **838.4728** | **1674.9310** | **1674.9404** | **-0.0093** | **1** | **43** | **0.0061** | **1** | **K.IISTTASKTETPIVSK.S** 15255 |
|  | 15596 | **567.3325** | **1698.9757** | **1697.9312** | **1.0445** | **1** | **6** | **23** | **3** | **R.KTVQSNSPISALAPTGK.E** |
|  | 17044 | **605.3048** | **1812.8924** | **1813.1845** | **-0.2921** | **0** | **1** | **84** | **8** | **R.LLALVVLFFIVGVIIGK.I** |
|  | 22190 | **1161.0129** | **2320.0113** | **2320.0214** | **-0.0101** | **0** | **49** | **0.00081** | **1** | **K.FMVQSMFAPTDTSDMEAVWK.E** 22189 |
|  | 22425 | **785.0544** | **2352.1415** | **2352.0113** | **0.1302** | **0** | **(1)** | **66** | **3** | **K.FMVQSMFAPTDTSDMEAVWK.E** |
|  | 28681 | **907.6618** | **3626.6181** | **3627.5765** | **-0.9584** | **1** | **1** | **28** | **3** | **K.FMVQSMFAPTDTSDMEAVWKEAKPEDLMDSK.L** |
|  | 28983 | **1242.3042** | **3723.8908** | **3724.7977** | **-0.9070** | **1** | **2** | **21** | **1** | **R.RYCVRPNSGIIDAGASINVSVMLQPFDYDPNEK.S** |

| **187.** | P49821\|NDUV1_HUMAN    **Mass:** 51469    **Score:** 163    **Queries matched:** 28   **emPAI:** 0.52 |
| --- | --- |
|  | NADH dehydrogenase [ubiquinone] flavoprotein 1, mitochondrial OS=Homo sapiens GN=NDUFV1 PE=1 SV=4 |

|  | **Query** | **Observed** | **Mr(expt)** | **Mr(calc)** | **Delta** | **Miss** | **Score** | **Expect** | **Rank** | **Peptide** |
| --- | --- | --- | --- | --- | --- | --- | --- | --- | --- | --- |
|  | 1095 | **662.3670** | **661.3597** | **661.3647** | **-0.0049** | **0** | **23** | **1.1** | **1** | **R.STDIVK.A** |
|  | 4007 | **492.2663** | **982.5180** | **982.5236** | **-0.0056** | **0** | **28** | **0.24** | **1** | **R.IFTNLYGR.H** |
|  | 4947 | **528.2539** | **1054.4933** | **1054.4985** | **-0.0052** | **0** | **37** | **0.029** | **1** | **R.GGTWFAGFGR.E** 4962 |
|  | 5191 | 537.2976 | 1072.5805 | 1072.5699 | 0.0106 | 0 | 4 | 76 | 9 | K.LLEGCLVGGR.A |
|  | 5789 | **559.3055** | **1116.5964** | **1116.6040** | **-0.0076** | **1** | **1** | **1.3e+002** | **5** | **R.GRGGAGFPTGLK.W** |
|  | 7224 | **404.8730** | **1211.5973** | **1211.6047** | **-0.0074** | **0** | **19** | **1.9** | **1** | **R.HFRPELEER.M** 7205 7225 |
|  | 10173 | **455.3684** | **1363.0834** | **1363.7096** | **-0.6262** | **1** | **5** | **29** | **1** | **R.VSVRFSGDTTAPK.K** |
|  | 10324 | **458.2637** | **1371.7694** | **1370.7128** | **1.0565** | **1** | **3** | **72** | **7** | **R.AMGARAAYIYIR.G** |
|  | 11657 | **484.5764** | **1450.7073** | **1450.6697** | **0.0376** | **1** | **1** | **93** | **8** | **R.EGVDWMNKVMAR.F** |
|  | 13258 | 517.2546 | 1548.7419 | 1548.7507 | -0.0088 | 0 | 19 | 1.6 | 1 | K.WSFMNKPSDGRPK.Y 13257 |
|  | 14743 | **548.3264** | **1641.9574** | **1642.7998** | **-0.8423** | **1** | **8** | **19** | **2** | **R.HFRPELEERMQR.F** |
|  | 14883 | **826.3815** | **1650.7484** | **1650.7559** | **-0.0075** | **0** | **30** | **0.11** | **1** | **K.YLVVNADEGEPGTCK.D** |
|  | 15737 | **855.9208** | **1709.8271** | **1709.8373** | **-0.0101** | **0** | **99** | **1.4e-008** | **1** | **R.GEFYNEASNLQVAIR.E** 15736 |
|  | 15915 | **862.4316** | **1722.8486** | **1722.9920** | **-0.1434** | **1** | **38** | **0.016** | **1** | **K.EILLKGPDWILGEIK.T** |
|  | 22539 | **789.3614** | **2365.0623** | **2365.9297** | **-0.8673** | **1** | **1** | **63** | **4** | **K.HESCGQCTPCREGVDWMNK.V** |
|  | 23170 | **821.4206** | **2461.2399** | **2461.2536** | **-0.0137** | **0** | **2** | **53** | **4** | **K.QIEGHTICALGDGAAWPVQGLIR.H** |
|  | 24108 | **872.1343** | **2613.3812** | **2613.3915** | **-0.0103** | **0** | **5** | **20** | **1** | **K.HAGGVTGGWDNLLAVIPGGSSTPLIPK.S** |
|  | 24827 | **914.7911** | **2741.3516** | **2741.3660** | **-0.0144** | **1** | **6** | **16** | **1** | **R.GEFYNEASNLQVAIREAYEAGLIGK.N** |
|  | 27183 | 1076.2164 | 3225.6275 | 3225.7398 | -0.1123 | 1 | 3 | 22 | 2 | K.ELIEKHAGGVTGGWDNLLAVIPGGSSTPLIPK.S |
|  | 27854 | **1133.9132** | **3398.7178** | **3398.7367** | **-0.0189** | **0** | **14** | **1.7** | **1** | **R.LKPPFPADVGVFGCPTTVANVETVAVSPTICR.R** 27853 |
|  | 28406 | **889.7114** | **3554.8163** | **3554.8378** | **-0.0215** | **1** | **21** | **0.31** | **1** | **R.LKPPFPADVGVFGCPTTVANVETVAVSPTICRR.G** 28407 |

| **188.** | O75306\|NDUS2_HUMAN    **Mass:** 52911    **Score:** 162    **Queries matched:** 18   **emPAI:** 0.31 |
| --- | --- |
|  | NADH dehydrogenase [ubiquinone] iron-sulfur protein 2, mitochondrial OS=Homo sapiens GN=NDUFS2 PE=1 SV=2 |

|  | **Query** | **Observed** | **Mr(expt)** | **Mr(calc)** | **Delta** | **Miss** | **Score** | **Expect** | **Rank** | **Peptide** |
| --- | --- | --- | --- | --- | --- | --- | --- | --- | --- | --- |
|  | 4521 | **511.2228** | **1020.4309** | **1020.4375** | **-0.0065** | **0** | **34** | **0.07** | **1** | **K.MFEFYER.V** |
|  | 4660 | **516.2646** | **1030.5147** | **1030.5196** | **-0.0048** | **0** | **8** | **27** | **5** | **R.GSGIQWDLR.K** |
|  | 4950 | **528.2761** | **1054.5376** | **1054.5659** | **-0.0283** | **1** | **6** | **44** | **8** | **K.VDDAKVSPPK.R** |
|  | 5210 | **538.2946** | **1074.5746** | **1074.6662** | **-0.0916** | **0** | **6** | **43** | **5** | **K.LLNIRPPPR.A** |
|  | 6317 | **580.3115** | **1158.6084** | **1158.6145** | **-0.0062** | **1** | **23** | **0.91** | **1** | **R.GSGIQWDLRK.T** |
|  | 6915 | **599.2979** | **1196.5813** | **1195.6349** | **0.9463** | **0** | **7** | **28** | **10** | **K.APGFAHLAGLDK.M** |
|  | 7315 | **406.2101** | **1215.6084** | **1216.6135** | **-1.0051** | **0** | **2** | **90** | **10** | **K.CDPHIGLLHR.G** |
|  | 8283 | **632.3210** | **1262.6274** | **1262.6363** | **-0.0089** | **0** | **5** | **43** | **1** | **R.LVMELSGEMVR.K** |
|  | 9956 | **451.2632** | **1350.7679** | **1349.7892** | **0.9787** | **0** | **2** | **87** | **9** | **R.GVAAQVLRPGAGVR.L** |
|  | 12355 | **498.2093** | **1491.6062** | **1490.6976** | **0.9086** | **1** | **8** | **22** | **5** | **K.MFEFYERVSGAR.M** |
|  | 20076 | **1038.5367** | **2075.0589** | **2075.0647** | **-0.0058** | **1** | **22** | **0.59** | **1** | **K.NFSLRLDELEELLTNNR.I** |
|  | 21342 | **1108.0156** | **2214.0167** | **2213.0277** | **0.9890** | **0** | **73** | **4.2e-006** | **1** | **K.TQPYDVYDQVEFDVPVGSR.G** 21334 |
|  | 21790 | **756.3740** | **2266.1002** | **2265.9956** | **0.1047** | **0** | **1** | **56** | **1** | **R.LDYVSMMCNEQAYSLAVEK.L** |
|  | 23087 | **1226.5657** | **2451.1168** | **2451.1318** | **-0.0150** | **0** | **84** | **2.5e-007** | **1** | **R.QWQPDVEWAQQFGGAVMYPSK.E** |
|  | 23875 | **643.3275** | **2569.2809** | **2569.2965** | **-0.0156** | **1** | **0** | **60** | **8** | **K.ETAHWKPPPWNDVDPPKDTIVK.N** |
|  | 26959 | **794.1444** | **3172.5485** | **3171.5634** | **0.9852** | **0** | **12** | **3.1** | **1** | **R.MHAAYIRPGGVHQDLPLGLMDDIYQFSK.N** 26949 |

| **189.** | Q9HCC0\|MCCB_HUMAN    **Mass:** 61808    **Score:** 161    **Queries matched:** 15   **emPAI:** 0.26 |
| --- | --- |
|  | Methylcrotonoyl-CoA carboxylase beta chain, mitochondrial OS=Homo sapiens GN=MCCC2 PE=1 SV=1 |

|  | **Query** | **Observed** | **Mr(expt)** | **Mr(calc)** | **Delta** | **Miss** | **Score** | **Expect** | **Rank** | **Peptide** |
| --- | --- | --- | --- | --- | --- | --- | --- | --- | --- | --- |
|  | 841 | **561.3206** | **560.3133** | **559.3078** | **1.0055** | **1** | **10** | **30** | **1** | **R.AREGK.Q** |
|  | 5042 | **531.3220** | **1060.6295** | **1059.5131** | **1.1164** | **0** | **9** | **23** | **4** | **R.AQEIAMQNR.L** |
|  | 5217 | **538.7799** | **1075.5453** | **1075.5080** | **0.0373** | **0** | **(7)** | **31** | **10** | **R.AQEIAMQNR.L** |
|  | 6814 | **596.8210** | **1191.6274** | **1190.6408** | **0.9866** | **1** | **6** | **40** | **9** | **R.SFDVREVIAR.I** 6789 |
|  | 11759 | 486.7781 | 1457.3123 | 1456.7568 | 0.5555 | 1 | 5 | 29 | 4 | K.QLRAQEIAMQNR.L |
|  | 11960 | **490.3153** | **1467.9240** | **1467.8813** | **0.0427** | **1** | **0** | **97** | **10** | **R.KQGTIFLAGPPLVK.A** |
|  | 12006 | 736.8492 | 1471.6838 | 1471.6943 | -0.0105 | 0 | 51 | 0.00094 | 1 | R.VWDDGIIDPADTR.L |
|  | 15268 | **838.8762** | **1675.7379** | **1675.7478** | **-0.0099** | **1** | **50** | **0.0012** | **1** | **K.KFEEEGNPYYSSAR.V** 15235 |
|  | 15974 | **864.4446** | **1726.8746** | **1727.9439** | **-1.0693** | **1** | **6** | **27** | **4** | **-.MWAVLRLALRPCAR.A** |
|  | 16786 | **896.9265** | **1791.8385** | **1792.9182** | **-1.0797** | **0** | **1** | **85** | **10** | **R.LPCIYLVDSGGAYLPR.Q** |
|  | 16896 | **901.9825** | **1801.9505** | **1801.9608** | **-0.0102** | **0** | **56** | **0.00026** | **1** | **R.ISVMGGEQAANVLATITK.D** 16895 |
|  | 30501 | **1484.0714** | **4449.1924** | **4449.2128** | **-0.0204** | **1** | **73** | **1.1e-006** | **1** | **R.ERIDNLIDPGSPFLELSQFAGYQLYDNEEVPGGGIITGIGR.V** |

| **190.** | P02545\|LMNA_HUMAN    **Mass:** 74380    **Score:** 161    **Queries matched:** 34   **emPAI:** 0.41 |
| --- | --- |
|  | Lamin-A/C OS=Homo sapiens GN=LMNA PE=1 SV=1 |

|  | **Query** | **Observed** | **Mr(expt)** | **Mr(calc)** | **Delta** | **Miss** | **Score** | **Expect** | **Rank** | **Peptide** |
| --- | --- | --- | --- | --- | --- | --- | --- | --- | --- | --- |
|  | 2317 | **430.8863** | **859.7580** | **860.4967** | **-0.7387** | **1** | **4** | **56** | **4** | **R.KTLDSVAK.E** 2315 |
|  | 3856 | **487.2599** | **972.5052** | **973.4716** | **-0.9664** | **0** | **10** | **21** | **2** | **K.LLEGEEER.L** |
|  | 4822 | **522.7444** | **1043.4742** | **1042.5407** | **0.9335** | **0** | **1** | **1.5e+002** | **8** | **K.EGDLIAAQAR.L** |
|  | 5391 | **545.2769** | **1088.5392** | **1088.5462** | **-0.0070** | **0** | **36** | **0.049** | **1** | **R.SLETENAGLR.L** |
|  | 5448 | 547.2808 | 1092.5471 | 1092.5564 | -0.0093 | 0 | 9 | 21 | 3 | R.SYLLGNSSPR.T 5463 |
|  | 6461 | **586.7958** | **1171.5771** | **1170.6357** | **0.9415** | **1** | **5** | **56** | **8** | **K.KEGDLIAAQAR.L** |
|  | 6727 | **595.2176** | **1188.4206** | **1187.6081** | **0.8126** | **1** | **6** | **37** | **1** | **K.QLQDEMLRR.V** |
|  | 7078 | **402.2436** | **1203.7089** | **1202.5891** | **1.1198** | **1** | **8** | **24** | **9** | **R.DLEDSLARER.D** |
|  | 8832 | **431.2315** | **1290.6726** | **1290.6204** | **0.0522** | **0** | **4** | **53** | **8** | **R.QNGDDPLLTYR.F** |
|  | 9402 | **661.3475** | **1320.6804** | **1319.6503** | **1.0301** | **1** | **2** | **95** | **6** | **R.VDAENRLQTMK.E** |
|  | 10063 | **453.2318** | **1356.6735** | **1357.7313** | **-1.0578** | **1** | **9** | **18** | **2** | **R.SLETENAGLRLR.I** 10089 |
|  | 10094 | **680.3427** | **1358.6709** | **1358.6790** | **-0.0081** | **0** | **46** | **0.0038** | **1** | **R.SGAQASSTPLSPTR.I** 10111 |
|  | 10155 | **682.3076** | **1362.6007** | **1362.6099** | **-0.0092** | **0** | **54** | **0.00058** | **1** | **K.AQNTWGCGNSLR.T** |
|  | 10343 | **687.3213** | **1372.6280** | **1373.6211** | **-0.9931** | **0** | **5** | **41** | **4** | **R.AQHEDQVEQYK.K** |
|  | 11093 | 473.2864 | 1416.8375 | 1416.7572 | 0.0803 | 1 | 7 | 28 | 7 | R.LRITESEEVVSR.E |
|  | 12345 | **746.3760** | **1490.7375** | **1490.7399** | **-0.0023** | **0** | **43** | **0.0072** | **1** | **R.TALINSTGEEVAMR.K** |
|  | 12511 | **501.5754** | **1501.7043** | **1501.7161** | **-0.0118** | **1** | **38** | **0.023** | **1** | **R.AQHEDQVEQYKK.E** 12510 |
|  | 12799 | **507.2819** | **1518.8239** | **1517.7070** | **1.1169** | **0** | **8** | **19** | **5** | **R.ASSHSSQTQGGGSVTK.K** 12772 |
|  | 13516 | **522.7768** | **1565.3085** | **1565.7434** | **-0.4349** | **0** | **3** | **38** | **9** | **R.SVGGSGGGSFGDNLVTR.S** |
|  | 14163 | **536.2838** | **1605.8294** | **1604.8046** | **1.0248** | **1** | **3** | **57** | **5** | **R.VAVEEVDEEGKFVR.L** |
|  | 14779 | 549.2842 | 1644.8307 | 1645.8020 | -0.9713 | 1 | 8 | 19 | 5 | R.ASSHSSQTQGGGSVTKK.R |
|  | 15595 | **850.4842** | **1698.9538** | **1698.9628** | **-0.0090** | **1** | **24** | **0.42** | **1** | **R.IRIDSLSAQLSQLQK.Q** 15590 |
|  | 16295 | **876.9296** | **1751.8446** | **1751.8550** | **-0.0105** | **0** | **47** | **0.0023** | **1** | **R.NSNLVGAAHEELQQSR.I** 16283 16296 |
|  | 16604 | **593.2670** | **1776.7791** | **1777.8846** | **-1.1055** | **1** | **8** | **18** | **2** | **R.EVSGIKAAYEAELGDAR.K** 16640 |

| **191.** | P10909\|CLUS_HUMAN    **Mass:** 53031    **Score:** 160    **Queries matched:** 19   **emPAI:** 0.23 |
| --- | --- |
|  | Clusterin OS=Homo sapiens GN=CLU PE=1 SV=1 |

|  | **Query** | **Observed** | **Mr(expt)** | **Mr(calc)** | **Delta** | **Miss** | **Score** | **Expect** | **Rank** | **Peptide** |
| --- | --- | --- | --- | --- | --- | --- | --- | --- | --- | --- |
|  | 3003 | **454.2631** | **906.5116** | **906.5174** | **-0.0059** | **1** | **44** | **0.0076** | **1** | **R.KYNELLK.S** 3001 |
|  | 3424 | **470.2632** | **938.5119** | **939.3790** | **-0.8671** | **1** | **6** | **35** | **4** | **R.MKDQCDK.C** |
|  | 3519 | **474.2496** | **946.4846** | **946.4356** | **0.0490** | **0** | **9** | **23** | **8** | **K.EDALNETR.E** |
|  | 3598 | 477.7309 | 953.4472 | 953.4528 | -0.0056 | 0 | 15 | 5 | 1 | K.FMETVAEK.A |
|  | 5205 | 538.2461 | 1074.4776 | 1074.5305 | -0.0529 | 1 | 7 | 35 | 3 | K.KEDALNETR.E |
|  | 6145 | **574.3270** | **1146.6395** | **1146.5928** | **0.0467** | **1** | **4** | **68** | **5** | **R.VCRSGSGLVGR.Q** |
|  | 7636 | **616.8237** | **1231.6329** | **1231.6408** | **-0.0079** | **1** | **15** | **4.8** | **1** | **K.TLIEKTNEER.K** 7618 |
|  | 7935 | **416.2150** | **1245.6231** | **1244.6976** | **0.9255** | **1** | **8** | **25** | **8** | **R.KTLLSNLEEAK.K** 7936 |
|  | 8756 | **644.8184** | **1287.6222** | **1287.6306** | **-0.0085** | **0** | **10** | **14** | **1** | **R.ELDESLQVAER.L** |
|  | 11981 | **735.8249** | **1469.6353** | **1470.7507** | **-1.1153** | **1** | **2** | **73** | **3** | **K.YNELLKSYQWK.M** |
|  | 14968 | **553.3313** | **1656.9721** | **1657.8635** | **-0.8914** | **1** | **3** | **53** | **5** | **R.ELDESLQVAERLTR.K** |
|  | 16423 | **881.9122** | **1761.8098** | **1761.8203** | **-0.0105** | **0** | **46** | **0.0026** | **1** | **R.EILSVDCSTNNPSQAK.L** 16424 |
|  | 22144 | **1157.5857** | **2313.1568** | **2313.1700** | **-0.0132** | **0** | **69** | **9.9e-006** | **1** | **R.VTTVASHTSDSDVPSGVTEVVVK.L** 22145 |
|  | 30297 | **865.8026** | **4323.9767** | **4324.1607** | **-0.1840** | **1** | **0** | **21** | **2** | **M.MKTLLLFVGLLLTWESGQVLGDQTVSDNELQEMSNQGSK.Y** |

| **192.** | Q9UJZ1\|STML2_HUMAN    **Mass:** 38624    **Score:** 160    **Queries matched:** 14   **emPAI:** 0.20 |
| --- | --- |
|  | Stomatin-like protein 2 OS=Homo sapiens GN=STOML2 PE=1 SV=1 |

|  | **Query** | **Observed** | **Mr(expt)** | **Mr(calc)** | **Delta** | **Miss** | **Score** | **Expect** | **Rank** | **Peptide** |
| --- | --- | --- | --- | --- | --- | --- | --- | --- | --- | --- |
|  | 1492 | **401.2304** | **800.4463** | **799.4916** | **0.9547** | **0** | **9** | **27** | **4** | **R.GTGALLLR.G** 1497 |
|  | 5048 | **531.7534** | **1061.4922** | **1061.5353** | **-0.0431** | **0** | **7** | **33** | **10** | **R.ATVLESEGTR.E** |
|  | 7368 | **407.2168** | **1218.6286** | **1217.6364** | **0.9922** | **1** | **4** | **58** | **6** | **K.RATVLESEGTR.E** 7352 |
|  | 9690 | **669.2903** | **1336.5661** | **1336.5751** | **-0.0090** | **0** | **12** | **8.5** | **1** | **K.ESMQMQVEAER.R** |
|  | 10231 | 456.5940 | 1366.7601 | 1365.7405 | 1.0196 | 1 | 3 | 60 | 1 | R.YEIKDIHVPPR.V |
|  | 18041 | **945.4534** | **1888.8923** | **1888.9014** | **-0.0091** | **1** | **91** | **8.8e-008** | **1** | **R.DVQGTDASLDEELDRVK.M** 18042 18060 |
|  | 19429 | 1007.5378 | 2013.0611 | 2013.0684 | -0.0072 | 0 | 27 | 0.18 | 1 | R.NTVVLFVPQQEAWVVER.M |
|  | 22067 | **1152.5547** | **2303.0948** | **2302.1012** | **0.9937** | **0** | **5** | **22** | **1** | **R.ESLNASIVDAINQAADCWGIR.C** |
|  | 26454 | **1022.7636** | **3065.2688** | **3065.5049** | **-0.2361** | **0** | **3** | **19** | **1** | **K.DSNTILLPSNPGDVTSMVAQAMGVYGALTK.A** |
|  | 26508 | **1026.4865** | **3076.4375** | **3076.4521** | **-0.0146** | **1** | **24** | **0.2** | **1** | **R.IMDPYKASYGVEDPEYAVTQLAQTTMR.S** |

| **193.** | P09110\|THIK_HUMAN    **Mass:** 44834    **Score:** 155    **Queries matched:** 12   **emPAI:** 0.17 |
| --- | --- |
|  | 3-ketoacyl-CoA thiolase, peroxisomal OS=Homo sapiens GN=ACAA1 PE=1 SV=2 |

|  | **Query** | **Observed** | **Mr(expt)** | **Mr(calc)** | **Delta** | **Miss** | **Score** | **Expect** | **Rank** | **Peptide** |
| --- | --- | --- | --- | --- | --- | --- | --- | --- | --- | --- |
|  | 1172 | 704.5136 | 703.5063 | 702.4428 | 1.0634 | 0 | 11 | 21 | 6 | K.LKPAFK.K |
|  | 3120 | **458.3054** | **914.5963** | **914.4570** | **0.1393** | **0** | **13** | **9.5** | **7** | **R.GNPGNITSR.L** 3089 |
|  | 9523 | 443.0385 | 1326.0938 | 1325.8031 | 0.2907 | 1 | 4 | 39 | 6 | R.QVITLLNELKR.R 9539 |
|  | 12378 | **747.3781** | **1492.7417** | **1492.7522** | **-0.0105** | **1** | **55** | **0.00044** | **1** | **R.EKQDTFALASQQK.A** |
|  | 15217 | **558.7917** | **1673.3534** | **1673.8519** | **-0.4985** | **0** | **3** | **36** | **1** | **R.QCSSGLQAVASIAGGIR.N** |
|  | 16911 | **902.9272** | **1803.8398** | **1803.8495** | **-0.0097** | **0** | **17** | **2.3** | **1** | **R.DCLIPMGITSENVAER.F** |
|  | 17144 | **910.9560** | **1819.8974** | **1819.8444** | **0.0530** | **0** | **(3)** | **63** | **7** | **R.DCLIPMGITSENVAER.F** |
|  | 20878 | **1081.5322** | **2161.0499** | **2161.0611** | **-0.0112** | **0** | **127** | **1.8e-011** | **1** | **K.DGSTTAGNSSQVSDGAAAILLAR.R** |
|  | 21281 | **442.6329** | **2208.1280** | **2208.1348** | **-0.0068** | **1** | **2** | **52** | **9** | **R.GGFKDTTPDELLSAVMTAVLK.D** |
|  | 21534 | **745.3784** | **2233.1134** | **2233.1260** | **-0.0126** | **0** | **13** | **4.3** | **1** | **R.SITVTQDEGIRPSTTMEGLAK.L** |

| **194.** | P30041\|PRDX6_HUMAN    **Mass:** 25133    **Score:** 155    **Queries matched:** 11   **emPAI:** 0.15 |
| --- | --- |
|  | Peroxiredoxin-6 OS=Homo sapiens GN=PRDX6 PE=1 SV=3 |

|  | **Query** | **Observed** | **Mr(expt)** | **Mr(calc)** | **Delta** | **Miss** | **Score** | **Expect** | **Rank** | **Peptide** |
| --- | --- | --- | --- | --- | --- | --- | --- | --- | --- | --- |
|  | 3119 | **458.3051** | **914.5956** | **914.4862** | **0.1094** | **0** | **6** | **48** | **2** | **R.VATPVDWK.D** |
|  | 3337 | **466.5793** | **931.1441** | **930.5287** | **0.6155** | **1** | **12** | **7.6** | **4** | **K.LAPEFAKR.N** |
|  | 5822 | 560.3327 | 1118.6508 | 1118.5390 | 0.1118 | 1 | 5 | 47 | 2 | K.DEKGMPVTAR.V 5817 |
|  | 10719 | **698.3278** | **1394.6410** | **1394.6500** | **-0.0091** | **0** | **20** | **1.3** | **1** | **R.DFTPVCTTELGR.A** |
|  | 13768 | **791.3476** | **1580.6806** | **1581.6617** | **-0.9810** | **0** | **14** | **4.8** | **2** | **K.DINAYNCEEPTEK.L** |
|  | 18145 | **634.2648** | **1899.7725** | **1899.9863** | **-0.2138** | **1** | **3** | **47** | **5** | **R.ELAILLGMLDPAEKDEK.G** 18152 |
|  | 19620 | **677.6404** | **2029.8995** | **2029.9799** | **-0.0804** | **0** | **1** | **77** | **2** | **R.FHDFLGDSWGILFSHPR.D** |
|  | 20279 | 1049.5444 | 2097.0743 | 2097.0855 | -0.0112 | 0 | 106 | 2.2e-009 | 1 | M.PGGLLLGDVAPNFEANTTVGR.I 20278 |

| **195.** | P09497\|CLCB_HUMAN    **Mass:** 25289    **Score:** 152    **Queries matched:** 7   **emPAI:** 0.15 |
| --- | --- |
|  | Clathrin light chain B OS=Homo sapiens GN=CLTB PE=1 SV=1 |

|  | **Query** | **Observed** | **Mr(expt)** | **Mr(calc)** | **Delta** | **Miss** | **Score** | **Expect** | **Rank** | **Peptide** |
| --- | --- | --- | --- | --- | --- | --- | --- | --- | --- | --- |
|  | 3518 | **474.2295** | **946.4444** | **946.4508** | **-0.0064** | **0** | **13** | **10** | **2** | **K.VTEQEWR.E** 3520 |
|  | 3883 | **487.7586** | **973.5026** | **973.5080** | **-0.0054** | **0** | **15** | **6.2** | **4** | **R.LQELDAASK.V** |
|  | 5173 | **536.7808** | **1071.5471** | **1071.5560** | **-0.0089** | **0** | **13** | **9.1** | **1** | **R.LTQEPESIR.K** 5189 |
|  | 18177 | **951.9763** | **1901.9380** | **1901.9483** | **-0.0103** | **1** | **90** | **1.1e-007** | **1** | **R.LQELDAASKVTEQEWR.E** 18176 |

| **196.** | P54652\|HSP72_HUMAN    **Mass:** 70263    **Score:** 152    **Queries matched:** 27   **emPAI:** 0.36 |
| --- | --- |
|  | Heat shock-related 70 kDa protein 2 OS=Homo sapiens GN=HSPA2 PE=1 SV=1 |

|  | **Query** | **Observed** | **Mr(expt)** | **Mr(calc)** | **Delta** | **Miss** | **Score** | **Expect** | **Rank** | **Peptide** |
| --- | --- | --- | --- | --- | --- | --- | --- | --- | --- | --- |
|  | 1134 | 687.3989 | 686.3916 | 686.3963 | -0.0047 | 0 | 23 | 1.2 | 3 | R.LIGDAAK.N 1136 |
|  | 1146 | 689.3898 | 688.3826 | 688.3755 | 0.0070 | 1 | 11 | 20 | 8 | R.DAKLDK.G |
|  | 1268 | **772.3564** | **771.3491** | **770.4286** | **0.9205** | **1** | **21** | **1.6** | **2** | **K.KDIGPNK.R** |
|  | 4487 | 509.2846 | 1016.5547 | 1016.5614 | -0.0068 | 1 | 31 | 0.15 | 1 | K.ITITNDKGR.L 4494 |
|  | 6430 | **585.3319** | **1168.6493** | **1168.5546** | **0.0946** | **1** | **7** | **27** | **1** | **R.MVQEAERYK.S** |
|  | 6580 | 591.3058 | 1180.5970 | 1179.6135 | 0.9835 | 1 | 10 | 14 | 1 | K.VQVEYKGETK.T |
|  | 7551 | 614.8137 | 1227.6128 | 1227.6207 | -0.0080 | 0 | 44 | 0.0057 | 1 | K.VEIIANDQGNR.T |
|  | 8732 | **644.2858** | **1286.5571** | **1285.5496** | **1.0075** | **0** | **2** | **96** | **5** | **K.FEDATVQSDMK.H** |
|  | 9513 | **663.9353** | **1325.8560** | **1324.6696** | **1.1864** | **1** | **3** | **58** | **1** | **K.MKEIAEAYLGGK.V** |
|  | 11733 | **486.2642** | **1455.7708** | **1455.8046** | **-0.0337** | **0** | **8** | **23** | **3** | **K.GQIQEIVLVGGSTR.I** |
|  | 12100 | 739.4137 | 1476.8128 | 1475.6674 | 1.1454 | 1 | 1 | 1e+002 | 10 | K.DDIDRMVQEAER.Y |
|  | 12141 | 494.2534 | 1479.7385 | 1479.7470 | -0.0085 | 1 | 38 | 0.022 | 1 | R.ARFEELNADLFR.G |
|  | 12274 | 744.3496 | 1486.6845 | 1486.6940 | -0.0095 | 0 | 49 | 0.0015 | 1 | R.TTPSYVAFTDTER.L |
|  | 13509 | 783.3471 | 1564.6797 | 1564.8249 | -0.1453 | 1 | 7 | 25 | 2 | K.LLQDFFNGKELNK.S |
|  | 14770 | **548.9617** | **1643.8634** | **1643.8117** | **0.0517** | **0** | **0** | **1.1e+002** | **3** | **K.TFFPEEISSMVLTK.M** |
|  | 15003 | 830.4456 | 1658.8766 | 1658.8879 | -0.0113 | 0 | 79 | 1.4e-006 | 1 | R.IINEPTAAAIAYGLDK.K 15005 |
|  | 15471 | 846.3610 | 1690.7074 | 1690.7183 | -0.0110 | 0 | 27 | 0.21 | 1 | K.STAGDTHLGGEDFDNR.M 15472 |
|  | 16740 | 894.4932 | 1786.9719 | 1786.9828 | -0.0109 | 1 | 38 | 0.017 | 1 | R.IINEPTAAAIAYGLDKK.G |
|  | 18386 | **640.7838** | **1919.3294** | **1918.9420** | **0.3874** | **1** | **3** | **35** | **1** | **K.TFFPEEISSMVLTKMK.E** 18400 |
|  | 19913 | **687.0319** | **2058.0738** | **2059.0184** | **-0.9446** | **1** | **3** | **48** | **2** | **K.GETKTFFPEEISSMVLTK.M** |
|  | 22200 | **774.3956** | **2320.1651** | **2319.0252** | **1.1399** | **0** | **6** | **17** | **4** | **K.LYQGGPGGGSGGGGSGASGGPTIEEVD.-** |
|  | 26003 | **597.3143** | **2981.5353** | **2982.4346** | **-0.8993** | **0** | **0** | **50** | **4** | **R.TLSSSTQASIEIDSLYEGVDFYTSITR.A** |

| **197.** | Q15005\|SPCS2_HUMAN    **Mass:** 25272    **Score:** 151    **Queries matched:** 9   **emPAI:** 0.15 |
| --- | --- |
|  | Signal peptidase complex subunit 2 OS=Homo sapiens GN=SPCS2 PE=1 SV=3 |

|  | **Query** | **Observed** | **Mr(expt)** | **Mr(calc)** | **Delta** | **Miss** | **Score** | **Expect** | **Rank** | **Peptide** |
| --- | --- | --- | --- | --- | --- | --- | --- | --- | --- | --- |
|  | 1505 | **401.2854** | **800.5563** | **799.4300** | **1.1263** | **0** | **16** | **5.9** | **2** | **M.AAAAVQGGR.S** 1504 1508 |
|  | 5824 | **560.3553** | **1118.6960** | **1117.5768** | **1.1193** | **1** | **11** | **14** | **3** | **K.IDKWDGSAVK.N** |
|  | 6948 | **400.2377** | **1197.6914** | **1198.6822** | **-0.9909** | **1** | **11** | **13** | **1** | **K.EKSIFLVAHR.K** |
|  | 8649 | **428.2586** | **1281.7540** | **1281.6353** | **0.1187** | **0** | **10** | **12** | **2** | **K.YVENFGLIDGR.L** |
|  | 17653 | **929.3588** | **1856.7030** | **1856.7126** | **-0.0097** | **0** | **89** | **9.8e-008** | **1** | **R.SGGSGGCSGAGGASNCGTGSGR.S** 17654 |
|  | 25040 | 929.4662 | 2785.3769 | 2785.1675 | 0.2094 | 1 | 0 | 56 | 5 | -.MAAAAVQGGRSGGSGGCSGAGGASNCGTGSGR.S |

| **198.** | P04843\|RPN1_HUMAN    **Mass:** 68641    **Score:** 151    **Queries matched:** 27   **emPAI:** 0.23 |
| --- | --- |
|  | Dolichyl-diphosphooligosaccharide--protein glycosyltransferase subunit 1 OS=Homo sapiens GN=RPN1 PE=1 SV=1 |

|  | **Query** | **Observed** | **Mr(expt)** | **Mr(calc)** | **Delta** | **Miss** | **Score** | **Expect** | **Rank** | **Peptide** |
| --- | --- | --- | --- | --- | --- | --- | --- | --- | --- | --- |
|  | 2039 | **841.1056** | **840.0983** | **839.4025** | **0.6958** | **0** | **20** | **0.88** | **1** | **R.NVESYTK.L** 2036 |
|  | 2040 | **421.0565** | **840.0984** | **839.5116** | **0.5867** | **0** | **4** | **41** | **8** | **K.IILPEGAK.N** |
|  | 4476 | **509.2376** | **1016.4606** | **1016.4676** | **-0.0070** | **0** | **33** | **0.1** | **1** | **R.HFDETVNR.Y** 4472 4475 4477 |
|  | 5052 | **531.7896** | **1061.5647** | **1061.5717** | **-0.0070** | **1** | **14** | **6.2** | **1** | **R.DISTLNSGKK.S** 5063 |
|  | 7576 | **615.3419** | **1228.6692** | **1228.6775** | **-0.0084** | **1** | **73** | **8.4e-006** | **1** | **K.SAVEAERLVAGK.L** |
|  | 8865 | **647.3042** | **1292.5938** | **1292.6031** | **-0.0092** | **1** | **13** | **6.9** | **1** | **R.LKTEGSDLCDR.V** 8866 |
|  | 9021 | **651.3703** | **1300.7260** | **1300.7350** | **-0.0090** | **0** | **58** | **0.0002** | **1** | **K.ALTSEIALLQSR.L** 8993 9022 |
|  | 9202 | **437.5446** | **1309.6121** | **1310.6143** | **-1.0022** | **0** | **12** | **8.7** | **3** | **R.SEDLLDYGPFR.D** |
|  | 12154 | **494.3223** | **1479.9450** | **1479.7722** | **0.1728** | **0** | **3** | **59** | **4** | **K.TILPAAAQDVYYR.D** |
|  | 12616 | **755.3893** | **1508.7641** | **1508.7722** | **-0.0081** | **1** | **46** | **0.0035** | **1** | **K.DTYIENEKLISGK.R** 12615 |
|  | 12982 | **511.9262** | **1532.7567** | **1532.7834** | **-0.0267** | **1** | **8** | **18** | **3** | **R.LDFSITKDPAAEAR.M** |
|  | 13167 | **515.3269** | **1542.9589** | **1541.9140** | **1.0448** | **1** | **5** | **34** | **5** | **K.ALTSEIALLQSRLK.T** |
|  | 13866 | **793.9190** | **1585.8233** | **1586.8702** | **-1.0468** | **0** | **7** | **27** | **6** | **K.VACITEQVLTLVNK.R** |
|  | 14914 | **551.9654** | **1652.8743** | **1652.8846** | **-0.0103** | **0** | **38** | **0.02** | **1** | **K.VTAEVVLAHLGGGSTSR.A** 14913 |
|  | 15004 | **830.4462** | **1658.8778** | **1658.8879** | **-0.0101** | **0** | **1** | **83** | **1** | **R.ATSFLLALEPELEAR.L** |
|  | 22226 | **775.4099** | **2323.2077** | **2324.2277** | **-1.0200** | **1** | **2** | **44** | **8** | **K.KNLVEQHIQDIVVHYTFNK.V** |
|  | 25334 | **568.8644** | **2839.2855** | **2839.3428** | **-0.0573** | **1** | **3** | **30** | **1** | **K.QFVVFEGNHYFYSPYPTKTQTMR.V** |

| **199.** | P62241\|RS8_HUMAN    **Mass:** 24475    **Score:** 150    **Queries matched:** 13   **emPAI:** 1.06 |
| --- | --- |
|  | 40S ribosomal protein S8 OS=Homo sapiens GN=RPS8 PE=1 SV=2 |

|  | **Query** | **Observed** | **Mr(expt)** | **Mr(calc)** | **Delta** | **Miss** | **Score** | **Expect** | **Rank** | **Peptide** |
| --- | --- | --- | --- | --- | --- | --- | --- | --- | --- | --- |
|  | 2650 | **441.2963** | **880.5780** | **879.5403** | **1.0378** | **1** | **8** | **25** | **4** | **R.IHTVRVR.G** |
|  | 7390 | 610.8025 | 1219.5904 | 1218.6357 | 0.9548 | 0 | 32 | 0.11 | 1 | K.YELGRPAANTK.I 7370 7386 |
|  | 9266 | 657.8401 | 1313.6656 | 1313.6714 | -0.0058 | 0 | 9 | 17 | 8 | K.LTPEEEEILNK.K |
|  | 9879 | **449.9148** | **1346.7225** | **1346.7306** | **-0.0081** | **1** | **6** | **35** | **4** | **R.KYELGRPAANTK.I** |
|  | 12568 | 753.8882 | 1505.7619 | 1505.7726 | -0.0106 | 0 | 55 | 0.00035 | 1 | K.ISSLLEEQFQQGK.L 12569 |
|  | 13410 | **779.8962** | **1557.7778** | **1557.7868** | **-0.0090** | **0** | **14** | **4.5** | **1** | **K.LLACIASRPGQCGR.A** |
|  | 15856 | **859.9524** | **1717.8902** | **1717.8999** | **-0.0096** | **0** | **60** | **0.00012** | **1** | **R.IIDVVYNASNNELVR.T** 15857 |
|  | 18163 | **951.4633** | **1900.9120** | **1900.9570** | **-0.0451** | **1** | **30** | **0.098** | **1** | **R.ADGYVLEGKELEFYLR.K** 18178 |

| **200.** | P02647\|APOA1_HUMAN    **Mass:** 30759    **Score:** 149    **Queries matched:** 10   **emPAI:** 0.12 |
| --- | --- |
|  | Apolipoprotein A-I OS=Homo sapiens GN=APOA1 PE=1 SV=1 |

|  | **Query** | **Observed** | **Mr(expt)** | **Mr(calc)** | **Delta** | **Miss** | **Score** | **Expect** | **Rank** | **Peptide** |
| --- | --- | --- | --- | --- | --- | --- | --- | --- | --- | --- |
|  | 2822 | **448.7417** | **895.4689** | **895.4763** | **-0.0074** | **0** | **13** | **7.2** | **1** | **K.LHELQEK.L** |
|  | 4662 | **1031.5243** | **1030.5170** | **1030.5117** | **0.0053** | **0** | **2** | **1e+002** | **5** | **K.LSPLGEEMR.D** |
|  | 8980 | 433.9043 | 1298.6910 | 1298.5601 | 0.1309 | 0 | 5 | 46 | 4 | K.WQEEMELYR.Q |
|  | 9052 | **651.8234** | **1301.6323** | **1300.6411** | **0.9912** | **0** | **11** | **12** | **1** | **R.THLAPYSDELR.Q** |
|  | 11835 | **731.3832** | **1460.7519** | **1461.8443** | **-1.0924** | **1** | **3** | **69** | **4** | **R.VKDLATVYVDVLK.D** |
|  | 13841 | **529.2708** | **1584.7904** | **1584.8008** | **-0.0104** | **1** | **19** | **1.4** | **1** | **R.THLAPYSDELRQR.L** |
|  | 14242 | **806.8915** | **1611.7684** | **1611.7781** | **-0.0097** | **0** | **102** | **7.9e-009** | **1** | **K.LLDNWDSVTSTFSK.L** 14243 |
|  | 18789 | 977.0501 | 1952.0855 | 1951.8602 | 0.2254 | 0 | 2 | 57 | 7 | R.HFWQQDEPPQSPWDR.V |
|  | 21048 | 546.0308 | 2180.0942 | 2179.0235 | 1.0706 | 1 | 2 | 49 | 8 | R.HFWQQDEPPQSPWDRVK.D |

| **201.** | Q9BPW8\|NIPS1_HUMAN    **Mass:** 33460    **Score:** 149    **Queries matched:** 12   **emPAI:** 0.53 |
| --- | --- |
|  | Protein NipSnap homolog 1 OS=Homo sapiens GN=NIPSNAP1 PE=1 SV=1 |

|  | **Query** | **Observed** | **Mr(expt)** | **Mr(calc)** | **Delta** | **Miss** | **Score** | **Expect** | **Rank** | **Peptide** |
| --- | --- | --- | --- | --- | --- | --- | --- | --- | --- | --- |
|  | 3990 | **491.7600** | **981.5053** | **981.5131** | **-0.0077** | **1** | **19** | **1.8** | **1** | **K.KETSNLYK.I** |
|  | 5530 | 550.2472 | 1098.4798 | 1098.6033 | -0.1235 | 1 | 9 | 21 | 2 | R.KDAHSTLLSK.K |
|  | 8068 | **627.2694** | **1252.5243** | **1251.7624** | **0.7618** | **1** | **2** | **90** | **4** | **R.IMIPLKISPLQ.-** |
|  | 8362 | **634.3013** | **1266.5881** | **1267.7574** | **-1.1692** | **1** | **(1)** | **1e+002** | **6** | **R.IMIPLKISPLQ.-** |
|  | 13916 | **795.8365** | **1589.6584** | **1589.6676** | **-0.0092** | **0** | **8** | **20** | **1** | **R.FSGGYPALMDCMNK.L** |
|  | 15739 | **855.9241** | **1709.8337** | **1709.8413** | **-0.0076** | **0** | **42** | **0.0063** | **1** | **R.GWDENVYYTVPLVR.H** 15738 |
|  | 16544 | **591.6312** | **1771.8719** | **1771.8828** | **-0.0109** | **0** | **21** | **0.78** | **1** | **K.LKPGTMIEWGNNWAR.A** 16545 |
|  | 17510 | **616.5414** | **1846.6023** | **1846.8416** | **-0.2393** | **1** | **4** | **31** | **4** | **R.FSGGYPALMDCMNKLK.N** |
|  | 25057 | **930.4832** | **2788.4276** | **2788.4435** | **-0.0159** | **0** | **50** | **0.00057** | **1** | **K.IQFHNVKPEYLDAYNSLTEAVLPK.L** |
|  | 28894 | **1234.5608** | **3700.6605** | **3700.6794** | **-0.0188** | **0** | **78** | **5.4e-007** | **1** | **K.LHLDEDYPCSLVGNWNTWYGEQDQAVHLWR.F** |

| **202.** | P07195\|LDHB_HUMAN    **Mass:** 36900    **Score:** 148    **Queries matched:** 17   **emPAI:** 0.79 |
| --- | --- |
|  | L-lactate dehydrogenase B chain OS=Homo sapiens GN=LDHB PE=1 SV=2 |

|  | **Query** | **Observed** | **Mr(expt)** | **Mr(calc)** | **Delta** | **Miss** | **Score** | **Expect** | **Rank** | **Peptide** |
| --- | --- | --- | --- | --- | --- | --- | --- | --- | --- | --- |
|  | 978 | **615.0428** | **614.0355** | **613.3799** | **0.6556** | **0** | **5** | **41** | **4** | **K.LSGLPK.H** |
|  | 3079 | **457.2924** | **912.5703** | **912.5757** | **-0.0053** | **0** | **55** | **0.00049** | **1** | **K.IVVVTAGVR.Q** |
|  | 3677 | 480.2771 | 958.5397 | 958.5447 | -0.0050 | 0 | 42 | 0.011 | 1 | R.GLTSVINQK.L |
|  | 4409 | **506.2830** | **1010.5514** | **1010.5583** | **-0.0069** | **0** | **10** | **16** | **2** | **R.IHPVSTMVK.G** |
|  | 6533 | **589.2996** | **1176.5846** | **1175.5822** | **1.0023** | **0** | **8** | **26** | **3** | **K.SADTLWDIQK.D** |
|  | 7983 | **624.7997** | **1247.5849** | **1247.5928** | **-0.0079** | **0** | **63** | **8.1e-005** | **1** | **R.VIGSGCNLDSAR.F** |
|  | 8395 | 634.8591 | 1267.7036 | 1266.6530 | 1.0506 | 0 | 8 | 23 | 10 | K.MVVESAYEVIK.L |
|  | 12625 | **755.8861** | **1509.7577** | **1509.7675** | **-0.0098** | **1** | **68** | **2.1e-005** | **1** | **K.IVADKDYSVTANSK.I** 12626 |
|  | 12872 | **763.3508** | **1524.6870** | **1523.8269** | **0.8601** | **1** | **3** | **57** | **2** | **K.MVVESAYEVIKLK.G** |
|  | 13113 | **514.5881** | **1540.7424** | **1540.7529** | **-0.0105** | **1** | **27** | **0.22** | **1** | **K.HRVIGSGCNLDSAR.F** |
|  | 13282 | **517.7407** | **1550.2002** | **1550.7624** | **-0.5622** | **1** | **7** | **17** | **3** | **R.VIGSGCNLDSARFR.Y** 13295 |
|  | 14536 | 815.4099 | 1628.8053 | 1628.8509 | -0.0456 | 0 | 2 | 77 | 9 | K.SLADELALVDVLEDK.L |
|  | 15530 | **847.9460** | **1693.8775** | **1693.8886** | **-0.0111** | **0** | **18** | **1.6** | **1** | **K.LIAPVAEEEATVPNNK.I** |
|  | 21183 | **733.3610** | **2197.0612** | **2196.1136** | **0.9476** | **0** | **4** | **34** | **2** | **K.GYTNWAIGLSVADLIESMLK.N** |
|  | 22004 | 766.0504 | 2295.1294 | 2295.1391 | -0.0097 | 0 | 10 | 6.9 | 1 | K.GMYGIENEVFLSLPCILNAR.G |

| **203.** | Q01740\|FMO1_HUMAN    **Mass:** 60899    **Score:** 148    **Queries matched:** 19   **emPAI:** 0.34 |
| --- | --- |
|  | Dimethylaniline monooxygenase [N-oxide-forming] 1 OS=Homo sapiens GN=FMO1 PE=2 SV=3 |

|  | **Query** | **Observed** | **Mr(expt)** | **Mr(calc)** | **Delta** | **Miss** | **Score** | **Expect** | **Rank** | **Peptide** |
| --- | --- | --- | --- | --- | --- | --- | --- | --- | --- | --- |
|  | 872 | **572.9957** | **571.9884** | **571.3330** | **0.6554** | **0** | **7** | **40** | **5** | **R.LTGPGK.W** |
|  | 3231 | **462.2488** | **922.4830** | **921.4954** | **0.9876** | **1** | **16** | **3.8** | **2** | **K.TKVCSVTK.C** |
|  | 3677 | 480.2771 | 958.5397 | 958.5964 | -0.0567 | 0 | 15 | 5.6 | 3 | K.VFIRPSIK.E |
|  | 4508 | **510.2498** | **1018.4850** | **1017.4880** | **0.9970** | **0** | **56** | **0.00043** | **1** | **R.SDDLGGLWR.F** |
|  | 5599 | **552.3104** | **1102.6063** | **1102.5043** | **0.1020** | **0** | **15** | **6** | **3** | **R.FTEHVEEGR.A** |
|  | 8037 | **626.3096** | **1250.6046** | **1250.6118** | **-0.0072** | **0** | **21** | **1.1** | **1** | **K.MYANHFDLLK.H** |
|  | 12442 | **500.2792** | **1497.8158** | **1496.9039** | **0.9119** | **1** | **3** | **67** | **8** | **K.RVAIVGAGVSGLASIK.C** 12439 |
|  | 16043 | **578.2918** | **1731.8536** | **1732.8103** | **-0.9567** | **1** | **3** | **53** | **3** | **K.WEGARNAIMTQWDR.T** |
|  | 16383 | **879.9681** | **1757.9216** | **1757.9312** | **-0.0096** | **1** | **75** | **3.6e-006** | **1** | **R.TQLKEFVLNDELPGR.I** 16382 |
|  | 16859 | **900.3683** | **1798.7221** | **1798.7324** | **-0.0103** | **0** | **34** | **0.036** | **1** | **K.CCLEEGLEPTCFER.S** |
|  | 17746 | 933.4198 | 1864.8250 | 1863.8324 | 0.9926 | 0 | 40 | 0.01 | 1 | R.IFDSGYPWDMVFMTR.F 17719 |
|  | 20911 | **723.0353** | **2166.0842** | **2166.0970** | **-0.0128** | **1** | **36** | **0.021** | **1** | **R.KINNWLNHANYGLIPEDR.T** |
|  | 22119 | **771.0658** | **2310.1756** | **2310.1889** | **-0.0134** | **0** | **4** | **33** | **1** | **R.VLVIGMGNSGTDIAVEASHLAEK.V** |
|  | 28575 | **898.4412** | **3589.7355** | **3590.0058** | **-0.2703** | **0** | **1** | **26** | **3** | **K.YIFPAHLQKPTLAIIGLIKPLGSMIPTGETQAR.W** |
|  | 28822 | **920.1992** | **3676.7675** | **3677.7786** | **-1.0111** | **0** | **0** | **33** | **5** | **K.QESAIFDAVMVCTGFLTNPYLPLDSFPGINAFK.G** |
|  | 29107 | **943.7449** | **3770.9504** | **3770.9666** | **-0.0162** | **1** | **7** | **6.5** | **1** | **R.VLVIGMGNSGTDIAVEASHLAEKVFLSTTGGGWVISR.I** |

| **204.** | P61106\|RAB14_HUMAN    **Mass:** 24110    **Score:** 148    **Queries matched:** 19   **emPAI:** 1.41 |
| --- | --- |
|  | Ras-related protein Rab-14 OS=Homo sapiens GN=RAB14 PE=1 SV=4 |

|  | **Query** | **Observed** | **Mr(expt)** | **Mr(calc)** | **Delta** | **Miss** | **Score** | **Expect** | **Rank** | **Peptide** |
| --- | --- | --- | --- | --- | --- | --- | --- | --- | --- | --- |
|  | 1232 | 749.3560 | 748.3487 | 748.4344 | -0.0857 | 1 | 5 | 59 | 9 | R.FRAVTR.S |
|  | 2519 | **437.1247** | **872.2348** | **872.4967** | **-0.2620** | **0** | **4** | **66** | **9** | **R.IIEVSGQK.I** |
|  | 9315 | 658.8296 | 1315.6446 | 1315.6521 | -0.0074 | 0 | 18 | 2.1 | 1 | K.LQIWDTAGQER.F 9284 |
|  | 9982 | **451.8851** | **1352.6334** | **1352.6758** | **-0.0424** | **0** | **2** | **85** | **8** | **R.GAAGALMVYDITR.R** 9987 |
|  | 14394 | **811.4017** | **1620.7889** | **1620.7995** | **-0.0106** | **1** | **50** | **0.0011** | **1** | **K.TGENVEDAFLEAAKK.I** 14395 |
|  | 14443 | **812.4704** | **1622.9262** | **1622.9355** | **-0.0093** | **0** | **20** | **1.1** | **1** | **R.NLTNPNTVIILIGNK.A** 14439 |
|  | 14864 | **825.8923** | **1649.7700** | **1649.7798** | **-0.0098** | **0** | **38** | **0.017** | **1** | **R.STYNHLSSWLTDAR.N** |
|  | 16107 | **869.8798** | **1737.7451** | **1736.8217** | **0.9234** | **1** | **3** | **49** | **3** | **K.ADLEAQRDVTYEEAK.Q** |
|  | 16959 | **603.2905** | **1806.8496** | **1805.8809** | **0.9687** | **1** | **0** | **1.1e+002** | **6** | **R.RSTYNHLSSWLTDAR.N** |
|  | 18229 | **636.3737** | **1906.0992** | **1905.9407** | **0.1585** | **1** | **(2)** | **52** | **3** | **R.SYYRGAAGALMVYDITR.R** |
|  | 18412 | **641.3333** | **1920.9779** | **1921.9356** | **-0.9577** | **1** | **3** | **48** | **1** | **R.SYYRGAAGALMVYDITR.R** |
|  | 26839 | **1050.8501** | **3149.5285** | **3149.5490** | **-0.0205** | **0** | **61** | **4e-005** | **1** | **K.IYQNIQDGSLDLNAAESGVQHKPSAPQGGR.L** 26840 |
|  | 27391 | **820.4136** | **3277.6255** | **3277.6439** | **-0.0185** | **1** | **32** | **0.028** | **1** | **K.KIYQNIQDGSLDLNAAESGVQHKPSAPQGGR.L** 27388 |

| **205.** | P02748\|CO9_HUMAN    **Mass:** 64615    **Score:** 147    **Queries matched:** 16   **emPAI:** 0.32 |
| --- | --- |
|  | Complement component C9 OS=Homo sapiens GN=C9 PE=1 SV=2 |

|  | **Query** | **Observed** | **Mr(expt)** | **Mr(calc)** | **Delta** | **Miss** | **Score** | **Expect** | **Rank** | **Peptide** |
| --- | --- | --- | --- | --- | --- | --- | --- | --- | --- | --- |
|  | 2803 | **447.7739** | **893.5332** | **892.3709** | **1.1623** | **0** | **1** | **1.4e+002** | **7** | **R.CTDAVGDR.R** |
|  | 4663 | **516.2685** | **1030.5224** | **1030.5295** | **-0.0070** | **0** | **35** | **0.062** | **1** | **R.VVEESELAR.T** |
|  | 4866 | **524.2740** | **1046.5335** | **1046.5583** | **-0.0247** | **1** | **2** | **1e+002** | **9** | **K.EKMFLHVK.G** |
|  | 6382 | **583.3528** | **1164.6910** | **1164.6139** | **0.0771** | **0** | **0** | **1.4e+002** | **9** | **K.TSNFNAAISLK.F** |
|  | 7848 | **621.8734** | **1241.7323** | **1241.7383** | **-0.0061** | **0** | **44** | **0.005** | **1** | **K.LSPIYNLVPVK.M** 7849 |
|  | 9053 | **651.8319** | **1301.6493** | **1301.6575** | **-0.0082** | **1** | **26** | **0.34** | **1** | **R.DRVVEESELAR.T** |
|  | 11712 | **728.3550** | **1454.6955** | **1454.7041** | **-0.0086** | **0** | **60** | **0.00014** | **1** | **R.AIEDYINEFSVR.K** |
|  | 11948 | **490.1571** | **1467.4496** | **1467.7470** | **-0.2974** | **1** | **2** | **49** | **9** | **R.SRSIEVFGQFNGK.R** |
|  | 12518 | **501.6375** | **1501.8907** | **1500.8738** | **1.0169** | **1** | **4** | **50** | **3** | **K.LSPIYNLVPVKMK.N** |
|  | 13132 | **514.9185** | **1541.7336** | **1542.7929** | **-1.0594** | **0** | **4** | **45** | **8** | **K.ISEGLPALEFPNEK.-** |
|  | 13692 | 526.4296 | 1576.2671 | 1575.8409 | 0.4262 | 0 | 1 | 70 | 2 | R.RPWNVASLIYETK.G |
|  | 13805 | **528.6038** | **1582.7895** | **1582.7991** | **-0.0096** | **1** | **7** | **22** | **2** | **R.AIEDYINEFSVRK.C** |
|  | 18364 | **640.3668** | **1918.0785** | **1917.8859** | **0.1925** | **1** | **4** | **36** | **1** | **R.KCHTCQNGGTVILMDGK.C** |
|  | 25055 | **930.4795** | **2788.4166** | **2788.4283** | **-0.0117** | **0** | **83** | **3.1e-007** | **1** | **R.GTVIDVTDFVNWASSINDAPVLISQK.L** |
|  | 26930 | 1057.8032 | 3170.3878 | 3170.6976 | -0.3097 | 1 | 0 | 43 | 2 | K.LLRGTVIDVTDFVNWASSINDAPVLISQK.L |

| **206.** | P48047\|ATPO_HUMAN    **Mass:** 23377    **Score:** 147    **Queries matched:** 23   **emPAI:** 1.13 |
| --- | --- |
|  | ATP synthase subunit O, mitochondrial OS=Homo sapiens GN=ATP5O PE=1 SV=1 |

|  | **Query** | **Observed** | **Mr(expt)** | **Mr(calc)** | **Delta** | **Miss** | **Score** | **Expect** | **Rank** | **Peptide** |
| --- | --- | --- | --- | --- | --- | --- | --- | --- | --- | --- |
|  | 2057 | **841.4069** | **840.3996** | **840.4051** | **-0.0055** | **0** | **21** | **1.3** | **1** | **K.YVDMSVK.T** 2058 |
|  | 2343 | **431.7351** | **861.4557** | **860.4603** | **0.9954** | **0** | **7** | **37** | **6** | **K.SLNDITAK.E** |
|  | 3291 | **464.7634** | **927.5122** | **927.5138** | **-0.0016** | **0** | **5** | **57** | **1** | **M.AAPAVSGLSR.Q** |
|  | 4582 | **513.2816** | **1024.5487** | **1024.6281** | **-0.0794** | **1** | **10** | **16** | **1** | **R.VAQILKEPK.V** |
|  | 4999 | **529.7929** | **1057.5711** | **1058.5542** | **-0.9831** | **0** | **7** | **36** | **8** | **-.MAAPAVSGLSR.Q** |
|  | 5358 | **544.2967** | **1086.5788** | **1085.5427** | **1.0361** | **1** | **1** | **1.5e+002** | **6** | **K.YVDMSVKTK.I** |
|  | 5374 | **544.8158** | **1087.6170** | **1087.6237** | **-0.0067** | **1** | **6** | **50** | **8** | **K.VKSLNDITAK.E** 5362 |
|  | 5622 | **553.3001** | **1104.5855** | **1105.6132** | **-1.0276** | **0** | **6** | **43** | **2** | **K.SFLSQGQVLK.L** |
|  | 6127 | 573.5405 | 1145.0664 | 1145.6040 | -0.5376 | 1 | 2 | 65 | 3 | K.SLNDITAKER.F |
|  | 8181 | 629.8414 | 1257.6682 | 1257.6751 | -0.0069 | 0 | 37 | 0.032 | 1 | K.TDPSILGGMIVR.I 8180 |
|  | 8390 | **423.5546** | **1267.6420** | **1267.6482** | **-0.0062** | **1** | **10** | **15** | **2** | **R.IGEKYVDMSVK.T** |
|  | 12726 | **505.9219** | **1514.7440** | **1513.8504** | **0.8936** | **1** | **4** | **48** | **7** | **K.EPKVAASVLNPYVK.R** |
|  | 13795 | **791.9520** | **1581.8895** | **1581.8991** | **-0.0096** | **0** | **16** | **2.7** | **1** | **K.LVRPPVQVYGIEGR.Y** 13796 13811 |
|  | 17867 | **937.5007** | **1872.9868** | **1872.0105** | **0.9763** | **0** | **56** | **0.00025** | **1** | **R.FSPLTTNLINLLAENGR.L** 17851 |
|  | 19828 | **684.6674** | **2050.9804** | **2050.9928** | **-0.0124** | **0** | **24** | **0.37** | **1** | **R.LSNTQGVVSAFSTMMSVHR.G** |
|  | 22171 | **1159.5682** | **2317.1219** | **2317.1359** | **-0.0140** | **0** | **75** | **2.6e-006** | **1** | **R.GEVPCTVTSASPLEEATLSELK.T** |
|  | 30333 | **1088.5391** | **4350.1271** | **4350.1182** | **0.0090** | **1** | **1** | **16** | **1** | **R.LSNTQGVVSAFSTMMSVHRGEVPCTVTSASPLEEATLSELK.T** |

| **207.** | P50895\|BCAM_HUMAN    **Mass:** 68161    **Score:** 147    **Queries matched:** 27   **emPAI:** 0.30 |
| --- | --- |
|  | Basal cell adhesion molecule OS=Homo sapiens GN=BCAM PE=1 SV=2 |

|  | **Query** | **Observed** | **Mr(expt)** | **Mr(calc)** | **Delta** | **Miss** | **Score** | **Expect** | **Rank** | **Peptide** |
| --- | --- | --- | --- | --- | --- | --- | --- | --- | --- | --- |
|  | 4851 | **523.7609** | **1045.5072** | **1045.5152** | **-0.0080** | **0** | **71** | **1.3e-005** | **1** | **R.AGAAGTAEATAR.L** 4848 4850 4862 4868 4871 4872 4873 4874 |
|  | 7797 | **620.3568** | **1238.6989** | **1238.7057** | **-0.0067** | **0** | **24** | **0.54** | **1** | **R.LSVPPLVEVMR.G** |
|  | 8969 | **433.7402** | **1298.1989** | **1298.6830** | **-0.4842** | **0** | **2** | **64** | **7** | **R.LVLAEAQVGDER.D** |
|  | 11190 | **475.2390** | **1422.6951** | **1423.8221** | **-1.1270** | **1** | **3** | **64** | **6** | **R.LSVPPLVEVMRGK.S** |
|  | 11357 | **478.2694** | **1431.7865** | **1431.7218** | **0.0646** | **1** | **7** | **29** | **4** | **M.EPPDAPAQARGAPR.L** |
|  | 11816 | **730.8500** | **1459.6855** | **1459.7168** | **-0.0313** | **1** | **6** | **34** | **2** | **R.GRSPPYQLDSQGR.L** 11813 |
|  | 13533 | **784.3556** | **1566.6967** | **1566.7049** | **-0.0082** | **0** | **65** | **3.6e-005** | **1** | **R.VEDYDAADDVQLSK.T** |
|  | 13757 | **395.9571** | **1579.7993** | **1578.7572** | **1.0420** | **1** | **4** | **47** | **6** | **-.MEPPDAPAQARGAPR.L** |
|  | 14726 | **821.3676** | **1640.7207** | **1640.7325** | **-0.0118** | **1** | **53** | **0.0006** | **1** | **R.DGISCEASNPHGNKR.H** |
|  | 16274 | **584.3721** | **1750.0946** | **1750.9002** | **-0.8057** | **0** | **6** | **23** | **2** | **K.LSWSQLGGSPAEPIPGR.Q** |
|  | 18068 | **631.3734** | **1891.0984** | **1892.0731** | **-0.9747** | **1** | **2** | **55** | **3** | **R.EASGLLSLTSTLYLRLR.K** |
|  | 19506 | **674.3447** | **2020.0124** | **2020.9272** | **-0.9149** | **1** | **12** | **5.8** | **1** | **K.ADGSWREGDEVTLICSAR.G** |
|  | 20337 | **1052.5175** | **2103.0204** | **2102.9725** | **0.0479** | **0** | **1** | **71** | **2** | **R.LASAEMQGSELQVTMHDTR.G** |
|  | 20621 | **712.9969** | **2135.9688** | **2134.9623** | **1.0065** | **0** | **(0)** | **77** | **7** | **R.LASAEMQGSELQVTMHDTR.G** |
|  | 21427 | **742.1926** | **2223.5561** | **2223.0664** | **0.4897** | **1** | **(3)** | **31** | **9** | **R.LEVPVEMNPEGYMTSRTVR.E** |
|  | 21590 | **449.0419** | **2240.1733** | **2239.0613** | **1.1120** | **1** | **3** | **43** | **6** | **R.LEVPVEMNPEGYMTSRTVR.E** |
|  | 24032 | **866.7442** | **2597.2108** | **2596.2228** | **0.9880** | **1** | **32** | **0.043** | **1** | **R.EGDTVQLLCRGDGSPSPEYTLFR.L** |
|  | 27701 | **840.3763** | **3357.4763** | **3358.5848** | **-1.1085** | **1** | **2** | **26** | **4** | **R.EKGAPPPGEPGLSHSGSEQPEQTGLLMGGASGGAR.G** |

| **208.** | Q9GZP9\|DERL2_HUMAN    **Mass:** 27663    **Score:** 146    **Queries matched:** 5   **emPAI:** 0.14 |
| --- | --- |
|  | Derlin-2 OS=Homo sapiens GN=DERL2 PE=1 SV=1 |

|  | **Query** | **Observed** | **Mr(expt)** | **Mr(calc)** | **Delta** | **Miss** | **Score** | **Expect** | **Rank** | **Peptide** |
| --- | --- | --- | --- | --- | --- | --- | --- | --- | --- | --- |
|  | 2423 | **434.4995** | **866.9845** | **867.4272** | **-0.4428** | **0** | **9** | **12** | **6** | **-.MAYQSLR.L** |
|  | 3783 | **484.5764** | **967.1382** | **967.4433** | **-0.3051** | **0** | **6** | **27** | **1** | **R.MLEEGSFR.G** |
|  | 6903 | **399.7148** | **1196.1225** | **1196.5608** | **-0.4383** | **1** | **13** | **5.1** | **1** | **R.MLEEGSFRGR.T** |
|  | 27755 | **1125.5118** | **3373.5137** | **3373.5276** | **-0.0139** | **0** | **95** | **1.4e-008** | **1** | **K.AIFDTPDEDPNYNPLPEERPGGFAWGEGQR.L** 27754 |

| **209.** | P11233\|RALA_HUMAN    **Mass:** 23723    **Score:** 144    **Queries matched:** 8   **emPAI:** 0.35 |
| --- | --- |
|  | Ras-related protein Ral-A OS=Homo sapiens GN=RALA PE=1 SV=1 |

|  | **Query** | **Observed** | **Mr(expt)** | **Mr(calc)** | **Delta** | **Miss** | **Score** | **Expect** | **Rank** | **Peptide** |
| --- | --- | --- | --- | --- | --- | --- | --- | --- | --- | --- |
|  | 790 | **546.2806** | **545.2733** | **545.2809** | **-0.0076** | **0** | **3** | **1.9e+002** | **5** | **R.ANVDK.V** |
|  | 2761 | **445.8225** | **889.6304** | **888.4552** | **1.1752** | **0** | **3** | **1e+002** | **4** | **R.QVSVEEAK.N** |
|  | 4306 | **502.2809** | **1002.5472** | **1002.5532** | **-0.0060** | **0** | **22** | **1** | **1** | **K.VIMVGSGGVGK.S** |
|  | 11698 | 727.8725 | 1453.7304 | 1453.7388 | -0.0083 | 1 | 43 | 0.0067 | 1 | R.ANVDKVFFDLMR.E 11696 |
|  | 14669 | 819.8868 | 1637.7590 | 1637.7685 | -0.0095 | 0 | 21 | 0.92 | 2 | R.AEQWNVNYVETSAK.T |
|  | 18245 | **954.9577** | **1907.9008** | **1907.9126** | **-0.0117** | **1** | **86** | **2.3e-007** | **1** | **K.NRAEQWNVNYVETSAK.T** 18262 |

| **210.** | Q8NE62\|CHDH_HUMAN    **Mass:** 66058    **Score:** 143    **Queries matched:** 26   **emPAI:** 0.31 |
| --- | --- |
|  | Choline dehydrogenase, mitochondrial OS=Homo sapiens GN=CHDH PE=2 SV=2 |

|  | **Query** | **Observed** | **Mr(expt)** | **Mr(calc)** | **Delta** | **Miss** | **Score** | **Expect** | **Rank** | **Peptide** |
| --- | --- | --- | --- | --- | --- | --- | --- | --- | --- | --- |
|  | 1751 | **411.2268** | **820.4391** | **820.4443** | **-0.0052** | **0** | **13** | **8.7** | **1** | **R.VLFEGTR.A** |
|  | 3072 | **457.2264** | **912.4382** | **913.4658** | **-1.0276** | **0** | **7** | **28** | **3** | **K.GQPALWDK.D** 3112 |
|  | 3324 | **465.7687** | **929.5229** | **929.4454** | **0.0775** | **0** | **16** | **5.1** | **2** | **R.LTEDPAER.V** |
|  | 3428 | **470.3305** | **938.6465** | **938.5800** | **0.0664** | **0** | **6** | **30** | **6** | **R.VLLLEAGPK.D** 3427 |
|  | 3783 | 484.5764 | 967.1382 | 966.5723 | 0.5659 | 0 | 5 | 29 | 2 | R.GLGRPGALAR.G |
|  | 3906 | **488.2800** | **974.5455** | **974.5032** | **0.0423** | **0** | **13** | **9.9** | **1** | **K.AEAETLVSR.V** |
|  | 11347 | **716.8538** | **1431.6931** | **1430.7729** | **0.9202** | **1** | **38** | **0.022** | **1** | **R.TNLKAEAETLVSR.V** |
|  | 13685 | **526.2939** | **1575.8598** | **1575.8409** | **0.0189** | **1** | **6** | **28** | **1** | **R.EIFAQEALAPFRGK.E** |
|  | 13816 | **528.9033** | **1583.6881** | **1583.6980** | **-0.0098** | **0** | **7** | **26** | **1** | **R.GWDYAHCLPYFR.K** |
|  | 13984 | **798.3451** | **1594.6756** | **1594.6868** | **-0.0112** | **1** | **42** | **0.0077** | **1** | **R.AKADSAYHPSCTCK.M** 13971 13972 13983 13986 13987 |
|  | 14094 | **534.9304** | **1601.7694** | **1601.7773** | **-0.0078** | **0** | **24** | **0.52** | **1** | **R.WSAACAYLHPALSR.T** |
|  | 14732 | **548.2458** | **1641.7155** | **1642.9155** | **-1.1999** | **1** | **4** | **45** | **3** | **K.DVPVYKPRTLATQR.-** |
|  | 16585 | **592.9555** | **1775.8447** | **1776.9370** | **-1.0923** | **1** | **3** | **50** | **1** | **K.AEAETLVSRVLFEGTR.A** |
|  | 17435 | **614.9851** | **1841.9333** | **1841.9869** | **-0.0536** | **1** | **7** | **23** | **9** | **-.MWCLLRGLGRPGALAR.G** |
|  | 17773 | **623.3360** | **1866.9862** | **1867.9945** | **-1.0083** | **1** | **10** | **8.9** | **1** | **K.GQPALWDKDVPVYKPR.T** |
|  | 23977 | 1294.6536 | 2587.2926 | 2587.3059 | -0.0134 | 0 | 67 | 1.4e-005 | 1 | R.VVDASIMPSMVSGNLNAPTIMIAEK.A 23978 |
|  | 30616 | **647.8982** | **4528.2364** | **4528.2612** | **-0.0248** | **1** | **4** | **8.2** | **7** | **R.SQPGVPHPDIQFHFLPSQVIDHGRVPTQQEAYQVHVGPMR.G** 30618 |

| **211.** | O15400\|STX7_HUMAN    **Mass:** 29911    **Score:** 142    **Queries matched:** 12   **emPAI:** 0.61 |
| --- | --- |
|  | Syntaxin-7 OS=Homo sapiens GN=STX7 PE=1 SV=4 |

|  | **Query** | **Observed** | **Mr(expt)** | **Mr(calc)** | **Delta** | **Miss** | **Score** | **Expect** | **Rank** | **Peptide** |
| --- | --- | --- | --- | --- | --- | --- | --- | --- | --- | --- |
|  | 2831 | **449.2130** | **896.4115** | **895.4651** | **0.9465** | **1** | **6** | **34** | **7** | **K.ETDKYIK.E** |
|  | 5451 | **547.2826** | **1092.5507** | **1092.5564** | **-0.0056** | **0** | **50** | **0.0014** | **1** | **K.QQYTNQLAK.E** 5450 |
|  | 14041 | **799.9207** | **1597.8269** | **1597.8352** | **-0.0083** | **0** | **49** | **0.0015** | **1** | **R.LVAEFTTSLTNFQK.V** |
|  | 15126 | **834.9261** | **1667.8376** | **1667.8479** | **-0.0102** | **0** | **54** | **0.00047** | **1** | **R.TLNQLGTPQDSPELR.Q** |
|  | 15504 | **847.4067** | **1692.7989** | **1693.8233** | **-1.0243** | **0** | **4** | **47** | **3** | **R.QLEADIMDINEIFK.D** |
|  | 16440 | 882.4001 | 1762.7856 | 1762.8308 | -0.0452 | 0 | 1 | 90 | 6 | -.MSYTPGVGGDPAQLAQR.I |
|  | 22681 | **796.3671** | **2386.0794** | **2386.2241** | **-0.1447** | **1** | **3** | **40** | **3** | **M.SYTPGVGGDPAQLAQRISSNIQK.I** |
|  | 26551 | **1543.7218** | **3085.4290** | **3085.4476** | **-0.0186** | **0** | **50** | **0.00042** | **1** | **R.NLVSWESQTQPQVQVQDEEITEDDLR.L** |
|  | 29759 | **810.6386** | **4048.1567** | **4048.8855** | **-0.7288** | **0** | **1** | **19** | **2** | **K.DLGMMIHEQGDVIDSIEANVENAEVHVQQANQQLSR.A** |
|  | 32003 | **1136.3461** | **5676.6940** | **5676.7134** | **-0.0194** | **1** | **12** | **0.69** | **1** | **R.QLEADIMDINEIFKDLGMMIHEQGDVIDSIEANVENAEVHVQQANQQLSR.A** 32006 |

| **212.** | P14314\|GLU2B_HUMAN    **Mass:** 60357    **Score:** 142    **Queries matched:** 12   **emPAI:** 0.20 |
| --- | --- |
|  | Glucosidase 2 subunit beta OS=Homo sapiens GN=PRKCSH PE=1 SV=2 |

|  | **Query** | **Observed** | **Mr(expt)** | **Mr(calc)** | **Delta** | **Miss** | **Score** | **Expect** | **Rank** | **Peptide** |
| --- | --- | --- | --- | --- | --- | --- | --- | --- | --- | --- |
|  | 4247 | **500.3103** | **998.6060** | **998.6124** | **-0.0064** | **1** | **17** | **3.5** | **1** | **K.LIELQAGKK.S** |
|  | 4523 | **511.2494** | **1020.4842** | **1021.4828** | **-0.9986** | **1** | **10** | **18** | **3** | **R.NKFEEAER.S** 4524 |
|  | 6301 | **579.8079** | **1157.6013** | **1157.6080** | **-0.0067** | **0** | **51** | **0.0013** | **1** | **K.LWEEQLAAAK.A** |
|  | 7364 | **610.2992** | **1218.5838** | **1218.5914** | **-0.0076** | **0** | **53** | **0.00083** | **1** | **K.SLEDQVEMLR.T** |
|  | 9289 | **439.2322** | **1314.6749** | **1314.6779** | **-0.0030** | **1** | **10** | **16** | **5** | **R.TVKEEAEKPER.E** |
|  | 10155 | 682.3076 | 1362.6007 | 1362.6813 | -0.0806 | 1 | 5 | 38 | 7 | K.KSLEDQVEMLR.T |
|  | 11607 | 724.8793 | 1447.7441 | 1447.6943 | 0.0498 | 0 | 1 | 1.1e+002 | 4 | K.AQQEQELAADAFK.E |
|  | 13225 | **774.3956** | **1546.7767** | **1546.8025** | **-0.0258** | **1** | **3** | **56** | **1** | **K.SLEDQVEMLRTVK.E** |
|  | 13313 | **776.8289** | **1551.6432** | **1551.6525** | **-0.0093** | **0** | **28** | **0.21** | **1** | **K.YEQGTGCWQGPNR.S** |
|  | 27438 | **1100.0896** | **3297.2470** | **3297.2625** | **-0.0155** | **1** | **87** | **6.3e-008** | **1** | **R.VNDGVCDCCDGTDEYNSGVICENTCKEK.G** |
|  | 31862 | **927.8110** | **5560.8226** | **5560.4232** | **0.3994** | **0** | **0** | **10** | **1** | **K.ELDDDMDGTVSVTELQTHPELDTDGDGALSEAEAQALLSGDTQTDATSFYDR.V** |

| **213.** | P62841\|RS15_HUMAN    **Mass:** 17029    **Score:** 141    **Queries matched:** 11   **emPAI:** 0.85 |
| --- | --- |
|  | 40S ribosomal protein S15 OS=Homo sapiens GN=RPS15 PE=1 SV=2 |

|  | **Query** | **Observed** | **Mr(expt)** | **Mr(calc)** | **Delta** | **Miss** | **Score** | **Expect** | **Rank** | **Peptide** |
| --- | --- | --- | --- | --- | --- | --- | --- | --- | --- | --- |
|  | 9987 | 677.3527 | 1352.6909 | 1352.7010 | -0.0101 | 0 | 1 | 1.1e+002 | 10 | K.EAPPMEKPEVVK.T |
|  | 10275 | **685.3951** | **1368.7756** | **1368.6959** | **0.0797** | **0** | **(1)** | **1.1e+002** | **5** | **K.EAPPMEKPEVVK.T** |
|  | 19838 | **685.0245** | **2052.0518** | **2052.0094** | **0.0424** | **0** | **(2)** | **62** | **4** | **R.DMIILPEMVGSMVGVYNGK.T** |
|  | 20024 | **690.3485** | **2068.0237** | **2068.0043** | **0.0194** | **0** | **2** | **54** | **1** | **R.DMIILPEMVGSMVGVYNGK.T** |
|  | 23973 | **1294.6172** | **2587.2198** | **2587.2298** | **-0.0100** | **0** | **92** | **3.9e-008** | **1** | **R.GVDLDQLLDMSYEQLMQLYSAR.Q** 23971 23972 |
|  | 26908 | **792.9104** | **3167.6125** | **3167.6365** | **-0.0240** | **0** | **33** | **0.022** | **1** | **K.TFNQVEIKPEMIGHYLGEFSITYKPVK.H** 26893 |
|  | 26944 | **635.1161** | **3170.5441** | **3170.5052** | **0.0388** | **1** | **3** | **25** | **4** | **K.FTYRGVDLDQLLDMSYEQLMQLYSAR.Q** |
|  | 31485 | **1756.1051** | **5265.2935** | **5265.6150** | **-0.3215** | **1** | **3** | **5** | **3** | **R.DMIILPEMVGSMVGVYNGKTFNQVEIKPEMIGHYLGEFSITYKPVK.H** |

| **214.** | P60174\|TPIS_HUMAN    **Mass:** 26938    **Score:** 140    **Queries matched:** 15   **emPAI:** 1.20 |
| --- | --- |
|  | Triosephosphate isomerase OS=Homo sapiens GN=TPI1 PE=1 SV=2 |

|  | **Query** | **Observed** | **Mr(expt)** | **Mr(calc)** | **Delta** | **Miss** | **Score** | **Expect** | **Rank** | **Peptide** |
| --- | --- | --- | --- | --- | --- | --- | --- | --- | --- | --- |
|  | 3622 | **478.2987** | **954.5829** | **953.4760** | **1.1069** | **0** | **1** | **1.3e+002** | **8** | **K.FFVGGNWK.M** |
|  | 7680 | **617.8008** | **1233.5871** | **1233.5949** | **-0.0078** | **0** | **37** | **0.027** | **1** | **K.SNVSDAVAQSTR.I** |
|  | 8518 | **637.8367** | **1273.6589** | **1273.6666** | **-0.0077** | **1** | **50** | **0.0016** | **1** | **K.VIADNVKDWSK.V** |
|  | 9509 | 663.8647 | 1325.7148 | 1325.6649 | 0.0499 | 0 | 5 | 44 | 5 | R.IIYGGSVTGATCK.E |
|  | 11023 | **471.9498** | **1412.8275** | **1411.6819** | **1.1456** | **1** | **3** | **65** | **6** | **K.FFVGGNWKMNGR.K** |
|  | 11767 | **729.8607** | **1457.7069** | **1457.7151** | **-0.0082** | **0** | **52** | **0.00094** | **1** | **R.HVFGESDELIGQK.V** |
|  | 11915 | **733.8604** | **1465.7061** | **1465.7161** | **-0.0100** | **0** | **54** | **0.00048** | **1** | **K.TATPQQAQEVHEK.L** |
|  | 13163 | **515.3266** | **1542.9580** | **1541.8777** | **1.0803** | **1** | **7** | **21** | **3** | **R.KQSLGELIGTLNAAK.V** |
|  | 14098 | **801.9440** | **1601.8734** | **1601.8817** | **-0.0083** | **0** | **37** | **0.027** | **1** | **K.VVLAYEPVWAIGTGK.T** |
|  | 14412 | **811.9040** | **1621.7935** | **1620.8181** | **0.9754** | **0** | **22** | **0.72** | **1** | **K.VTNGAFTGEISPGMIK.D** 14411 |
|  | 15850 | **573.3974** | **1717.1704** | **1717.8747** | **-0.7044** | **1** | **3** | **40** | **1** | **R.GWLKSNVSDAVAQSTR.I** |
|  | 22195 | 465.0324 | 2320.1257 | 2320.2209 | -0.0952 | 1 | 2 | 47 | 9 | K.VAHALAEGLGVIACIGEKLDER.E |
|  | 26225 | **1010.5285** | **3028.5637** | **3028.5757** | **-0.0120** | **0** | **4** | **21** | **3** | **K.ELASQPDVDGFLVGGASLKPEFVDIINAK.Q** 26210 |

| **215.** | P36543\|VATE1_HUMAN    **Mass:** 26186    **Score:** 139    **Queries matched:** 19   **emPAI:** 0.72 |
| --- | --- |
|  | V-type proton ATPase subunit E 1 OS=Homo sapiens GN=ATP6V1E1 PE=1 SV=1 |

|  | **Query** | **Observed** | **Mr(expt)** | **Mr(calc)** | **Delta** | **Miss** | **Score** | **Expect** | **Rank** | **Peptide** |
| --- | --- | --- | --- | --- | --- | --- | --- | --- | --- | --- |
|  | 2972 | 453.7183 | 905.4220 | 904.4614 | 0.9606 | 0 | 29 | 0.22 | 1 | K.VSNTLESR.L 2961 2971 |
|  | 3641 | **479.2556** | **956.4966** | **956.5516** | **-0.0550** | **1** | **18** | **2.7** | **2** | **K.GRLVQTQR.L** 3652 |
|  | 3890 | **487.7846** | **973.5545** | **973.5597** | **-0.0051** | **1** | **9** | **24** | **8** | **R.KQDFPLVK.A** |
|  | 4112 | 495.7566 | 989.4987 | 989.5043 | -0.0056 | 0 | 48 | 0.003 | 1 | R.GALFGANANR.K 4127 |
|  | 4146 | **496.7513** | **991.4881** | **990.4368** | **1.0513** | **0** | **11** | **13** | **5** | **K.IMEYYEK.K** |
|  | 5809 | **559.8031** | **1117.5916** | **1117.5992** | **-0.0076** | **1** | **50** | **0.0015** | **1** | **R.GALFGANANRK.F** 5810 |
|  | 6135 | **573.8237** | **1145.6329** | **1145.6404** | **-0.0075** | **1** | **24** | **0.6** | **1** | **K.IKVSNTLESR.L** |
|  | 11387 | **478.9080** | **1433.7022** | **1432.7278** | **0.9744** | **1** | **10** | **12** | **4** | **K.KIQMSNLMNQAR.L** |
|  | 11610 | **483.7363** | **1448.1871** | **1448.7228** | **-0.5357** | **1** | **(3)** | **44** | **3** | **K.KIQMSNLMNQAR.L** |
|  | 11907 | **489.2750** | **1464.8031** | **1464.7177** | **0.0854** | **1** | **(1)** | **87** | **9** | **K.KIQMSNLMNQAR.L** |
|  | 13866 | 793.9190 | 1585.8233 | 1585.8311 | -0.0078 | 1 | 83 | 6.4e-007 | 1 | R.ARDDLITDLLNEAK.Q |
|  | 13978 | **532.2897** | **1593.8472** | **1592.6963** | **1.1509** | **0** | **3** | **59** | **9** | **K.HMMAFIEQEANEK.A** |
|  | 22259 | **777.4392** | **2329.2956** | **2329.3045** | **-0.0089** | **0** | **2** | **40** | **3** | **R.YQVLLDGLVLQGLYQLLEPR.M** |
|  | 22281 | **778.6915** | **2333.0528** | **2333.0667** | **-0.0139** | **1** | **16** | **1.9** | **1** | **K.HMMAFIEQEANEKAEEIDAK.A** |

| **216.** | P05388\|RLA0_HUMAN    **Mass:** 34423    **Score:** 138    **Queries matched:** 16   **emPAI:** 0.52 |
| --- | --- |
|  | 60S acidic ribosomal protein P0 OS=Homo sapiens GN=RPLP0 PE=1 SV=1 |

|  | **Query** | **Observed** | **Mr(expt)** | **Mr(calc)** | **Delta** | **Miss** | **Score** | **Expect** | **Rank** | **Peptide** |
| --- | --- | --- | --- | --- | --- | --- | --- | --- | --- | --- |
|  | 1542 | **402.7128** | **803.4110** | **802.4119** | **0.9990** | **0** | **9** | **29** | **8** | **K.QMQQIR.M** |
|  | 3787 | **484.7611** | **967.5075** | **967.5127** | **-0.0052** | **0** | **13** | **7.3** | **1** | **R.GNVGFVFTK.E** |
|  | 7414 | **611.3104** | **1220.6063** | **1220.6149** | **-0.0086** | **0** | **53** | **0.00072** | **1** | **R.GHLENNPALEK.L** 7413 7428 |
|  | 8357 | **423.0529** | **1266.1368** | **1265.6074** | **0.5294** | **0** | **0** | **83** | **4** | **K.CFIVGADNVGSK.Q** |
|  | 9244 | **657.3550** | **1312.6954** | **1312.7027** | **-0.0073** | **0** | **14** | **5.4** | **1** | **K.TSFFQALGITTK.I** |
|  | 9450 | **441.7436** | **1322.2089** | **1321.6595** | **0.5495** | **1** | **7** | **17** | **2** | **K.QMQQIRMSLR.G** |
|  | 10216 | **456.2520** | **1365.7340** | **1365.6931** | **0.0410** | **1** | **9** | **18** | **1** | **K.AVVLMGKNTMMR.K** |
|  | 18097 | **948.4982** | **1894.9819** | **1894.9928** | **-0.0109** | **0** | **67** | **2.1e-005** | **1** | **R.VLALSVETDYTFPLAEK.V** |
|  | 21032 | **1090.5472** | **2179.0799** | **2179.0943** | **-0.0144** | **0** | **41** | **0.0063** | **1** | **R.AGAIAPCEVTVPAQNTGLGPEK.T** 21033 21034 |
|  | 24870 | **918.1547** | **2751.4423** | **2751.4595** | **-0.0172** | **0** | **18** | **1** | **1** | **K.AFLADPSAFVAAAPVAAATTAAPAAAAAPAK.V** |
|  | 28122 | 1158.9291 | 3473.7654 | 3473.7865 | -0.0211 | 1 | 7 | 8.2 | 2 | R.AGAIAPCEVTVPAQNTGLGPEKTSFFQALGITTK.I |
|  | 32006 | 1420.4434 | 5677.7443 | 5678.8454 | -1.1011 | 1 | 9 | 1.1 | 1 | K.TGDKVGASEATLLNMLNISPFSFGLVIQQVFDNGSIYNPEVLDITEETLHSR.F |

| **217.** | P62917\|RL8_HUMAN    **Mass:** 28235    **Score:** 138    **Queries matched:** 14   **emPAI:** 0.29 |
| --- | --- |
|  | 60S ribosomal protein L8 OS=Homo sapiens GN=RPL8 PE=1 SV=2 |

|  | **Query** | **Observed** | **Mr(expt)** | **Mr(calc)** | **Delta** | **Miss** | **Score** | **Expect** | **Rank** | **Peptide** |
| --- | --- | --- | --- | --- | --- | --- | --- | --- | --- | --- |
|  | 665 | **502.3719** | **501.3646** | **501.3023** | **0.0623** | **1** | **8** | **63** | **3** | **R.KGAAR.L** 677 |
|  | 824 | **556.3412** | **555.3340** | **555.3380** | **-0.0041** | **0** | **21** | **0.98** | **1** | **R.GAPLAK.V** |
|  | 1211 | **732.3198** | **731.3125** | **731.3173** | **-0.0048** | **0** | **7** | **52** | **2** | **R.NCWPR.V** |
|  | 1585 | **807.3976** | **806.3903** | **806.3923** | **-0.0020** | **0** | **5** | **55** | **7** | **R.AVDFAER.H** |
|  | 1824 | 414.6910 | 827.3674 | 826.5388 | 0.8286 | 1 | 5 | 60 | 9 | R.KVGLIAAR.R |
|  | 3226 | **462.1591** | **922.3037** | **921.4668** | **0.8369** | **0** | **18** | **2.2** | **1** | **K.DIIHDPGR.G** 3219 |
|  | 4972 | **529.2372** | **1056.4599** | **1056.6444** | **-0.1845** | **1** | **1** | **1.3e+002** | **7** | **R.GAPLAKVVFR.D** |
|  | 9383 | **660.3525** | **1318.6904** | **1318.7357** | **-0.0453** | **1** | **16** | **3.8** | **1** | **K.GIVKDIIHDPGR.G** |
|  | 15126 | 834.9261 | 1667.8376 | 1667.9431 | -0.1055 | 1 | 2 | 71 | 7 | K.VISSANRAVVGVVAGGGR.I |
|  | 15439 | **844.9105** | **1687.8065** | **1687.8165** | **-0.0101** | **0** | **99** | **1.4e-008** | **1** | **R.ASGNYATVISHNPETK.K** 15440 |
|  | 27338 | **1089.8706** | **3266.5900** | **3266.6098** | **-0.0198** | **0** | **20** | **0.42** | **1** | **K.AQLNIGNVLPVGTMPEGTIVCCLEEKPGDR.G** |

| **218.** | P62158\|CALM_HUMAN    **Mass:** 16827    **Score:** 137    **Queries matched:** 9   **emPAI:** 0.23 |
| --- | --- |
|  | Calmodulin OS=Homo sapiens GN=CALM1 PE=1 SV=2 |

|  | **Query** | **Observed** | **Mr(expt)** | **Mr(calc)** | **Delta** | **Miss** | **Score** | **Expect** | **Rank** | **Peptide** |
| --- | --- | --- | --- | --- | --- | --- | --- | --- | --- | --- |
|  | 1552 | **805.4176** | **804.4103** | **804.4164** | **-0.0060** | **0** | **21** | **1.9** | **1** | **K.ELGTVMR.S** |
|  | 15721 | **570.3539** | **1708.0400** | **1708.8302** | **-0.7902** | **1** | **4** | **35** | **3** | **K.DGDGTITTKELGTVMR.S** |
|  | 16325 | **877.9334** | **1753.8521** | **1753.8635** | **-0.0113** | **1** | **87** | **2.2e-007** | **1** | **R.VFDKDGNGYISAAELR.H** 16324 16332 |
|  | 29815 | **1357.6117** | **4069.8132** | **4068.8391** | **0.9742** | **0** | **9** | **3.2** | **1** | **R.SLGQNPTEAELQDMINEVDADGNGTIDFPEFLTMMAR.K** 29809 29814 |
|  | 31097 | **978.4472** | **4887.1996** | **4887.2347** | **-0.0351** | **1** | **2** | **8.8** | **1** | **K.ELGTVMRSLGQNPTEAELQDMINEVDADGNGTIDFPEFLTMMAR.K** |

| **219.** | Q07075\|AMPE_HUMAN    **Mass:** 109717   **Score:** 137    **Queries matched:** 23   **emPAI:** 0.26 |
| --- | --- |
|  | Glutamyl aminopeptidase OS=Homo sapiens GN=ENPEP PE=1 SV=2 |

|  | **Query** | **Observed** | **Mr(expt)** | **Mr(calc)** | **Delta** | **Miss** | **Score** | **Expect** | **Rank** | **Peptide** |
| --- | --- | --- | --- | --- | --- | --- | --- | --- | --- | --- |
|  | 3138 | **458.9210** | **915.8274** | **915.4814** | **0.3460** | **0** | **6** | **33** | **3** | **K.NNIEWLK.Q** |
|  | 3792 | **485.2455** | **968.4765** | **967.4797** | **0.9968** | **0** | **1** | **1.1e+002** | **9** | **R.SSVLGFACK.M** |
|  | 4252 | **500.7501** | **999.4856** | **999.5654** | **-0.0798** | **0** | **4** | **68** | **5** | **R.YLWLHLR.E** |
|  | 5226 | 539.2660 | 1076.5175 | 1077.5819 | -1.0643 | 0 | 7 | 32 | 5 | K.TQDVFTVIR.Y |
|  | 6646 | 593.1548 | 1184.2950 | 1183.5291 | 0.7659 | 1 | 9 | 15 | 2 | -.MNFAEREGSK.R |
|  | 7346 | **406.6951** | **1217.0635** | **1217.6040** | **-0.5405** | **0** | **4** | **45** | **2** | **K.HTAEYAANITK.S** |
|  | 7617 | **616.3339** | **1230.6533** | **1230.6608** | **-0.0075** | **0** | **33** | **0.075** | **1** | **K.ATYTISITHPK.E** |
|  | 8927 | **432.8836** | **1295.6291** | **1294.6227** | **1.0064** | **0** | **1** | **99** | **3** | **K.EYGALSNMPVAK.E** |
|  | 10243 | 684.8124 | 1367.6103 | 1367.6180 | -0.0077 | 1 | 13 | 6.5 | 1 | K.SFPCFDEPNKK.A |
|  | 10985 | **706.3400** | **1410.6654** | **1410.6739** | **-0.0085** | **0** | **33** | **0.058** | **1** | **K.SIVATDHEPTDAR.K** |
|  | 13078 | **513.9268** | **1538.7585** | **1538.7689** | **-0.0104** | **1** | **11** | **11** | **1** | **K.SIVATDHEPTDARK.S** 13065 |
|  | 13888 | **530.2739** | **1587.8000** | **1588.9188** | **-1.1188** | **1** | **1** | **1e+002** | **9** | **R.AQLLDYKVALNLTK.Y** |
|  | 15283 | 839.4133 | 1676.8121 | 1676.8232 | -0.0111 | 0 | 38 | 0.02 | 1 | R.MLEDWIKPENFQK.G 15284 |
|  | 16813 | **599.2784** | **1794.8135** | **1794.8536** | **-0.0401** | **1** | **4** | **44** | **2** | **K.NAKTSDFWAALEEASR.L** |
|  | 18167 | **951.5145** | **1901.0144** | **1901.0258** | **-0.0114** | **0** | **37** | **0.019** | **1** | **R.ISNSGKPLTIYVQPEQK.H** |
|  | 20142 | **1042.5203** | **2083.0260** | **2083.0374** | **-0.0115** | **0** | **59** | **0.00011** | **1** | **R.ANPSQPPSDLGYTWNIPVK.W** |
|  | 20304 | **700.8892** | **2099.6457** | **2099.1011** | **0.5446** | **1** | **3** | **32** | **9** | **K.YPQAGAGEKPREQVLETVK.N** |
|  | 21508 | **744.7116** | **2231.1130** | **2231.1222** | **-0.0092** | **0** | **32** | **0.049** | **1** | **R.VNYEVATWDSIATALSLNHK.T** 21511 |
|  | 22679 | 597.3286 | 2385.2853 | 2385.4294 | -0.1441 | 0 | 3 | 41 | 2 | K.HVAILCAVVVGVGLIVGLAVGLTR.S |
|  | 31665 | **1093.3329** | **5461.6281** | **5461.6414** | **-0.0133** | **1** | **0** | **10** | **7** | **R.VISAVTYIISMFEDDKELYPMIEEYFQGQVKPIADSLGWNDAGDHVTK.L** |

| **220.** | Q9UL25\|RAB21_HUMAN    **Mass:** 24731    **Score:** 136    **Queries matched:** 13   **emPAI:** 0.33 |
| --- | --- |
|  | Ras-related protein Rab-21 OS=Homo sapiens GN=RAB21 PE=1 SV=3 |

|  | **Query** | **Observed** | **Mr(expt)** | **Mr(calc)** | **Delta** | **Miss** | **Score** | **Expect** | **Rank** | **Peptide** |
| --- | --- | --- | --- | --- | --- | --- | --- | --- | --- | --- |
|  | 5164 | **536.2838** | **1070.5529** | **1070.5217** | **0.0312** | **0** | **2** | **1.1e+002** | **8** | **M.AAAGGGGGGAAAAGR.A** 5152 |
|  | 6787 | **596.2836** | **1190.5527** | **1190.5601** | **-0.0074** | **0** | **55** | **0.00048** | **1** | **R.MIETAQVDER.A** 6803 |
|  | 6997 | **401.2130** | **1200.6173** | **1201.5622** | **-0.9449** | **0** | **12** | **9.1** | **1** | **-.MAAAGGGGGGAAAAGR.A** |
|  | 7339 | 609.3283 | 1216.6419 | 1217.5571 | -0.9152 | 0 | (5) | 50 | 2 | -.MAAAGGGGGGAAAAGR.A |
|  | 7585 | **410.5723** | **1228.6952** | **1229.6113** | **-0.9161** | **1** | **8** | **24** | **3** | **R.AKGNGSSQPGTAR.R** |
|  | 12022 | **491.7516** | **1472.2329** | **1471.7419** | **0.4910** | **0** | **5** | **25** | **3** | **R.VNLAIWDTAGQER.F** |
|  | 15962 | **576.3133** | **1725.9180** | **1725.9124** | **0.0057** | **1** | **2** | **64** | **6** | **R.AYSFKVVLLGEGCVGK.T** |
|  | 16442 | **441.7426** | **1762.9412** | **1763.8732** | **-0.9320** | **1** | **6** | **26** | **2** | **R.KMLGNEICLCIVGNK.I** |
|  | 16912 | **902.9334** | **1803.8521** | **1803.8639** | **-0.0117** | **0** | **105** | **3.3e-009** | **1** | **R.HVSIQEAESYAESVGAK.H** |
|  | 20203 | **418.9932** | **2089.9294** | **2089.0076** | **0.9219** | **1** | **4** | **36** | **2** | **K.ERHVSIQEAESYAESVGAK.H** 20180 |

| **221.** | Q9UJS0\|CMC2_HUMAN    **Mass:** 74528    **Score:** 135    **Queries matched:** 18   **emPAI:** 0.05 |
| --- | --- |
|  | Calcium-binding mitochondrial carrier protein Aralar2 OS=Homo sapiens GN=SLC25A13 PE=1 SV=2 |

|  | **Query** | **Observed** | **Mr(expt)** | **Mr(calc)** | **Delta** | **Miss** | **Score** | **Expect** | **Rank** | **Peptide** |
| --- | --- | --- | --- | --- | --- | --- | --- | --- | --- | --- |
|  | 292 | **432.2366** | **431.2293** | **430.2288** | **1.0005** | **0** | **6** | **1.2e+002** | **2** | **K.GAGAR.V** 290 |
|  | 1856 | **415.2296** | **828.4446** | **829.5022** | **-1.0575** | **0** | **12** | **13** | **3** | **R.VSALSVVR.D** |
|  | 3451 | **471.2712** | **940.5278** | **940.5341** | **-0.0063** | **1** | **5** | **49** | **9** | **K.ILREEGPK.A** |
|  | 5991 | **568.2477** | **1134.4808** | **1134.6146** | **-0.1338** | **1** | **3** | **80** | **6** | **R.TGRVTAIDFR.D** |
|  | 7831 | **621.3520** | **1240.6895** | **1240.6776** | **0.0120** | **0** | **10** | **14** | **1** | **R.LQVAGEITTGPR.V** |
|  | 8539 | 638.8282 | 1275.6418 | 1274.7347 | 0.9072 | 1 | 3 | 68 | 9 | K.AIKLTVNDFVR.D |
|  | 9641 | 667.3453 | 1332.6761 | 1333.6224 | -0.9463 | 0 | 0 | 1.4e+002 | 5 | R.STGSFVGELMYK.N |
|  | 9643 | **445.2545** | **1332.7418** | **1333.7969** | **-1.0551** | **0** | **10** | **16** | **2** | **R.GLLPQLLGVAPEK.A** |
|  | 12582 | **754.3795** | **1506.7445** | **1505.7514** | **0.9931** | **0** | **15** | **3.7** | **1** | **R.YLNIFGESQPNPK.T** |
|  | 15235 | 838.3636 | 1674.7126 | 1675.7301 | -1.0174 | 0 | 1 | 96 | 9 | K.NGEFFMSPNDFVTR.Y |
|  | 19194 | **498.5226** | **1990.0615** | **1989.0279** | **1.0335** | **0** | **5** | **28** | **10** | **K.ASGDSARPVLLQVAESAYR.F** |
|  | 20356 | **1053.0815** | **2104.1485** | **2104.1569** | **-0.0083** | **0** | **93** | **4.3e-008** | **1** | **R.FGLGSVAGAVGATAVYPIDLVK.T** 20357 |
|  | 21523 | **744.9944** | **2231.9613** | **2231.9868** | **-0.0254** | **1** | **1** | **58** | **10** | **R.STGSFVGELMYKNSFDCFK.K** |
|  | 25077 | 931.7722 | 2792.2947 | 2793.4160 | -1.1213 | 1 | 7 | 12 | 2 | K.FGQVTPMEVDILFQLADLYEPRGR.M |
|  | 25161 | **702.0934** | **2804.3447** | **2805.4813** | **-1.1367** | **1** | **1** | **51** | **9** | **R.INLPAPNPDHVGGYKLAVATFAGIENK.F** |
|  | 25483 | **576.1027** | **2875.4769** | **2875.4967** | **-0.0198** | **1** | **3** | **27** | **7** | **R.YLNIFGESQPNPKTVELLSGVVDQTK.D** |

| **222.** | Q00325\|MPCP_HUMAN    **Mass:** 40525    **Score:** 134    **Queries matched:** 27   **emPAI:** 0.42 |
| --- | --- |
|  | Phosphate carrier protein, mitochondrial OS=Homo sapiens GN=SLC25A3 PE=1 SV=2 |

|  | **Query** | **Observed** | **Mr(expt)** | **Mr(calc)** | **Delta** | **Miss** | **Score** | **Expect** | **Rank** | **Peptide** |
| --- | --- | --- | --- | --- | --- | --- | --- | --- | --- | --- |
|  | 1781 | **412.7310** | **823.4475** | **822.4487** | **0.9988** | **0** | **9** | **20** | **1** | **R.TVEALYK.F** |
|  | 2097 | **845.4138** | **844.4065** | **844.4113** | **-0.0048** | **0** | **32** | **0.12** | **2** | **R.MQVDPQK.Y** 2095 2096 |
|  | 3308 | **465.2524** | **928.4903** | **928.4953** | **-0.0050** | **0** | **25** | **0.58** | **1** | **K.GVAPLWMR.Q** |
|  | 3372 | **467.7320** | **933.4494** | **933.5436** | **-0.0942** | **1** | **12** | **13** | **5** | **R.LGFKGVWK.G** 3370 |
|  | 4203 | **499.2066** | **996.3986** | **996.4950** | **-0.0963** | **1** | **7** | **31** | **5** | **K.MYKEEGLK.A** |
|  | 4443 | 507.7919 | 1013.5692 | 1012.4899 | 1.0793 | 1 | (6) | 46 | 8 | K.MYKEEGLK.A |
|  | 4491 | **509.3033** | **1016.5921** | **1016.5978** | **-0.0058** | **1** | **40** | **0.017** | **1** | **K.GSSASLVLKR.L** |
|  | 4626 | **514.8005** | **1027.5864** | **1026.4878** | **1.0986** | **0** | **4** | **67** | **3** | **R.QIPYTMMK.F** |
|  | 5132 | **535.2617** | **1068.5089** | **1069.5152** | **-1.0064** | **0** | **6** | **41** | **4** | **R.SSSPGPTGQPR.R** |
|  | 5811 | 560.0837 | 1118.1529 | 1117.6343 | 0.5187 | 1 | 6 | 27 | 5 | K.EKGSSASLVLK.R 5825 |
|  | 6219 | **576.8180** | **1151.6215** | **1151.5645** | **0.0571** | **1** | **2** | **1e+002** | **10** | **R.MQVDPQKYK.G** |
|  | 10124 | 681.3578 | 1360.7010 | 1360.7099 | -0.0089 | 0 | 54 | 0.00066 | 1 | R.IQTQPGYANTLR.D 10123 |
|  | 16125 | **870.4473** | **1738.8801** | **1737.9050** | **0.9751** | **1** | **36** | **0.03** | **1** | **K.GIFNGFSVTLKEDGVR.G** 16119 16124 |
|  | 17449 | 922.4813 | 1842.9480 | 1842.9588 | -0.0108 | 1 | 49 | 0.0013 | 1 | R.IQTQPGYANTLRDAAPK.M 17450 |
|  | 18605 | **646.8149** | **1937.4228** | **1936.9725** | **0.4503** | **1** | **0** | **62** | **9** | **K.GVAPLWMRQIPYTMMK.F** |
|  | 24414 | **669.5965** | **2674.3569** | **2674.3200** | **0.0369** | **0** | **1** | **46** | **9** | **R.TSLYLAASASAEFFADIALAPMEAAK.V** 24459 |
|  | 25083 | 699.1028 | 2792.3823 | 2792.4975 | -0.1153 | 1 | 3 | 32 | 10 | R.IIMIGTLTALQWFIYDSVKVYFR.L |
|  | 25776 | **733.6197** | **2930.4499** | **2929.4895** | **0.9604** | **1** | **3** | **28** | **1** | **R.TSLYLAASASAEFFADIALAPMEAAKVR.I** |

| **223.** | O60716\|CTND1_HUMAN    **Mass:** 108674   **Score:** 134    **Queries matched:** 25   **emPAI:** 0.18 |
| --- | --- |
|  | Catenin delta-1 OS=Homo sapiens GN=CTNND1 PE=1 SV=1 |

|  | **Query** | **Observed** | **Mr(expt)** | **Mr(calc)** | **Delta** | **Miss** | **Score** | **Expect** | **Rank** | **Peptide** |
| --- | --- | --- | --- | --- | --- | --- | --- | --- | --- | --- |
|  | 2900 | 451.2503 | 900.4861 | 901.4617 | -0.9756 | 1 | 12 | 13 | 2 | R.ALEEERR.H |
|  | 3422 | **470.2450** | **938.4754** | **938.4934** | **-0.0179** | **0** | **1** | **1.3e+002** | **6** | **R.HVSAQLER.V** |
|  | 3806 | **485.7402** | **969.4658** | **970.5923** | **-1.1266** | **1** | **7** | **27** | **7** | **R.VVKAASGALR.N** 3837 |
|  | 4434 | **507.3269** | **1012.6393** | **1011.6076** | **1.0317** | **1** | **0** | **1.5e+002** | **7** | **K.ELRKPLEK.E** |
|  | 5190 | **537.2972** | **1072.5799** | **1072.5876** | **-0.0077** | **1** | **9** | **24** | **1** | **K.LRETQGIEK.L** |
|  | 5559 | **551.2607** | **1100.5068** | **1099.5444** | **0.9624** | **0** | **6** | **43** | **8** | **K.NCDGVPALVR.L** |
|  | 5740 | **557.3988** | **1112.7830** | **1112.6666** | **0.1165** | **1** | **3** | **68** | **10** | **K.LVLINKSGNR.S** |
|  | 6835 | **398.6289** | **1192.8650** | **1193.6629** | **-0.7979** | **1** | **3** | **55** | **3** | **R.HVSAQLERVR.V** |
|  | 13816 | 528.9033 | 1583.6881 | 1584.7467 | -1.0585 | 1 | 2 | 69 | 9 | R.YRPSMEGYRAPSR.Q |
|  | 14092 | **534.9073** | **1601.7002** | **1601.7110** | **-0.0108** | **0** | **21** | **1** | **1** | **R.FHPEPYGLEDDQR.S** |
|  | 14314 | **808.9350** | **1615.8554** | **1614.8439** | **1.0115** | **0** | **6** | **29** | **2** | **R.QPELPEVIAMLGFR.L** |
|  | 14900 | **826.9294** | **1651.8442** | **1651.8529** | **-0.0087** | **0** | **73** | **5.8e-006** | **1** | **K.ALSAIADLLTNEHER.V** |
|  | 15376 | **562.2653** | **1683.7740** | **1684.8308** | **-1.0569** | **0** | **4** | **41** | **5** | **K.KPIEDPANDTVDFPK.R** |
|  | 17306 | **611.3214** | **1830.9424** | **1831.0356** | **-0.0932** | **1** | **3** | **55** | **2** | **R.AAALVLQTIWGYKELR.K** |
|  | 17412 | **921.3464** | **1840.6783** | **1840.9319** | **-0.2536** | **1** | **0** | **74** | **1** | **K.KPIEDPANDTVDFPKR.T** |
|  | 19021 | **658.9442** | **1973.8108** | **1972.8187** | **0.9921** | **0** | **24** | **0.31** | **1** | **R.HYEDGYPGGSDNYGSLSR.V** |
|  | 19886 | **686.4151** | **2056.2235** | **2055.0531** | **1.1704** | **1** | **2** | **44** | **3** | **R.VRVSPQDANPLMANGTLTR.R** |
|  | 20726 | **1074.4135** | **2146.8123** | **2146.8282** | **-0.0158** | **0** | **23** | **0.36** | **1** | **R.SMGYDDLDYGMMSDYGTAR.R** |
|  | 22597 | **792.3614** | **2374.0623** | **2374.0760** | **-0.0137** | **0** | **32** | **0.043** | **1** | **R.YQEAAPNVANNTGPHAASCFGAK.K** |
|  | 23265 | **826.0723** | **2475.1952** | **2474.1999** | **0.9953** | **0** | **19** | **0.99** | **1** | **R.DMDLTEVITGTLWNLSSHDSIK.M** |
|  | 23642 | **846.9177** | **2537.7312** | **2537.3027** | **0.4285** | **1** | **0** | **46** | **3** | **K.FSDLKLNGPQDHSHLLYSTIPR.M** |
|  | 28425 | **1187.8750** | **3560.6032** | **3559.7941** | **0.8091** | **1** | **3** | **16** | **1** | **K.TVTTRTVQPVAMGPDGLPVDASSVSNNYIQTLGR.D** |
|  | 29860 | **1021.5177** | **4082.0417** | **4081.8508** | **0.1909** | **0** | **1** | **19** | **1** | **K.GTTPLMQDEGQESLEEELDVLVLDDEGGQVSYPSMQK.I** |
|  | 30011 | **1382.0282** | **4143.0628** | **4143.0760** | **-0.0132** | **1** | **63** | **1.4e-005** | **1** | **K.NLPGGQQNSSWNFSEDTVISILNTINEVIAENLEAAKK.L** |

| **224.** | Q53GQ0\|DHB12_HUMAN    **Mass:** 34416    **Score:** 132    **Queries matched:** 10   **emPAI:** 0.23 |
| --- | --- |
|  | Estradiol 17-beta-dehydrogenase 12 OS=Homo sapiens GN=HSD17B12 PE=1 SV=2 |

|  | **Query** | **Observed** | **Mr(expt)** | **Mr(calc)** | **Delta** | **Miss** | **Score** | **Expect** | **Rank** | **Peptide** |
| --- | --- | --- | --- | --- | --- | --- | --- | --- | --- | --- |
|  | 7170 | **605.5350** | **1209.0554** | **1208.6005** | **0.4548** | **1** | **1** | **76** | **2** | **K.IVMNMNKSTR.A** |
|  | 11615 | **483.7787** | **1448.3142** | **1447.7891** | **0.5252** | **1** | **6** | **21** | **3** | **K.KMININILSVCK.M** |
|  | 14944 | **828.4605** | **1654.9063** | **1653.9131** | **0.9933** | **0** | **61** | **9.8e-005** | **1** | **K.GVFVQSVLPYFVATK.L** 14929 |
|  | 17232 | **914.4811** | **1826.9476** | **1826.9302** | **0.0174** | **1** | **3** | **46** | **5** | **R.TIAVDFASEDIYDKIK.T** |
|  | 17637 | **464.7688** | **1855.0460** | **1855.0567** | **-0.0107** | **1** | **10** | **9.5** | **1** | **K.IRKPTLDKPSPETFVK.S** 17638 |
|  | 23434 | **1252.1910** | **2502.3675** | **2502.3767** | **-0.0092** | **0** | **94** | **2.6e-008** | **1** | **K.GAILNISSGSGMLPVPLLTIYSATK.T** 23435 |
|  | 28443 | **1189.8648** | **3566.5724** | **3565.8499** | **0.7226** | **1** | **0** | **31** | **3** | **R.TNGYLIHALMGSIISNLPSWIYLKIVMNMNK.S** |

| **225.** | Q92959\|SO2A1_HUMAN    **Mass:** 71367    **Score:** 132    **Queries matched:** 9   **emPAI:** 0.05 |
| --- | --- |
|  | Solute carrier organic anion transporter family member 2A1 OS=Homo sapiens GN=SLCO2A1 PE=1 SV=2 |

|  | **Query** | **Observed** | **Mr(expt)** | **Mr(calc)** | **Delta** | **Miss** | **Score** | **Expect** | **Rank** | **Peptide** |
| --- | --- | --- | --- | --- | --- | --- | --- | --- | --- | --- |
|  | 4716 | **518.2639** | **1034.5131** | **1033.5920** | **0.9211** | **1** | **10** | **18** | **8** | **R.GSLVDFIKR.F** |
|  | 6179 | **575.8826** | **1149.7507** | **1150.5917** | **-0.8410** | **1** | **4** | **58** | **1** | **R.CARSVFGNIK.V** 6175 |
|  | 8351 | **633.7951** | **1265.5756** | **1265.5848** | **-0.0091** | **0** | **81** | **1.2e-006** | **1** | **K.LGASQGSDTSTSR.A** 8350 |
|  | 10472 | **460.8786** | **1379.6141** | **1380.7588** | **-1.1447** | **0** | **4** | **60** | **8** | **K.SFAIGVQFLLMR.L** |
|  | 18412 | 641.3333 | 1920.9779 | 1920.9575 | 0.0204 | 1 | 1 | 77 | 3 | -.MGLLPKLGASQGSDTSTSR.A |
|  | 28262 | **1172.9504** | **3515.8295** | **3514.8468** | **0.9826** | **1** | **2** | **22** | **4** | **K.QYGTSAAYANFLIGAVNLPAAALGMLFGGILMKR.F** |
|  | 29417 | **973.9706** | **3891.8535** | **3892.0307** | **-0.1772** | **1** | **5** | **9.1** | **3** | **K.FLEKQYGTSAAYANFLIGAVNLPAAALGMLFGGILMK.R** |

| **226.** | Q7Z406\|MYH14_HUMAN    **Mass:** 228889   **Score:** 131    **Queries matched:** 46   **emPAI:** 0.02 |
| --- | --- |
|  | Myosin-14 OS=Homo sapiens GN=MYH14 PE=1 SV=1 |

|  | **Query** | **Observed** | **Mr(expt)** | **Mr(calc)** | **Delta** | **Miss** | **Score** | **Expect** | **Rank** | **Peptide** |
| --- | --- | --- | --- | --- | --- | --- | --- | --- | --- | --- |
|  | 871 | 572.9955 | 571.9882 | 571.3806 | 0.6076 | 1 | 9 | 24 | 2 | R.IKVGR.D |
|  | 1609 | 405.1959 | 808.3773 | 807.4491 | 0.9282 | 0 | 19 | 2.1 | 1 | R.TVGQLYK.E |
|  | 1688 | 408.7296 | 815.4446 | 814.5025 | 0.9422 | 1 | 18 | 3.5 | 5 | R.AKLALGSR.V |
|  | 2270 | **429.2163** | **856.4180** | **855.5178** | **0.9002** | **0** | **7** | **37** | **9** | **R.LVLDQLR.C** |
|  | 2626 | **441.2470** | **880.4793** | **879.4306** | **1.0487** | **1** | **8** | **27** | **5** | **R.KMQAQMK.E** |
|  | 3141 | **459.0118** | **916.0091** | **916.4250** | **-0.4159** | **0** | **12** | **9.1** | **1** | **R.LGEEDAGAR.A** |
|  | 3866 | **487.2955** | **972.5764** | **971.5400** | **1.0364** | **1** | **10** | **20** | **6** | **K.EEAVKQLR.K** 3865 |
|  | 4837 | 523.2736 | 1044.5326 | 1044.6179 | -0.0853 | 1 | 10 | 19 | 4 | R.QLVSTLEKK.Q |
|  | 5196 | **537.7714** | **1073.5282** | **1074.5669** | **-1.0387** | **0** | **9** | **24** | **2** | **R.AELSSLQTAR.Q** |
|  | 5756 | **558.3104** | **1114.6062** | **1115.6121** | **-1.0059** | **1** | **6** | **44** | **4** | **M.AAVTMSVPGRK.A** |
|  | 5761 | **558.3287** | **1114.6428** | **1113.6003** | **1.0425** | **1** | **10** | **18** | **2** | **K.RQQQQSALR.V** |
|  | 6517 | **588.7927** | **1175.5709** | **1176.6503** | **-1.0794** | **0** | **9** | **20** | **1** | **R.LLGLGVTDFSR.A** |
|  | 7393 | 610.8260 | 1219.6374 | 1219.6448 | -0.0074 | 1 | 30 | 0.14 | 1 | R.KFDQLLAEEK.A |
|  | 8206 | 630.3782 | 1258.7419 | 1258.7132 | 0.0287 | 1 | 6 | 35 | 6 | K.LQLEKVTTEAK.M |
|  | 8474 | **424.9337** | **1271.7792** | **1272.6608** | **-0.8816** | **1** | **9** | **19** | **8** | **R.VRAMEAEAAGLR.E** |
|  | 10073 | **453.5623** | **1357.6650** | **1356.6997** | **0.9653** | **1** | **5** | **44** | **5** | **R.AEDEGGARAQLLK.S** |
|  | 10494 | **461.2430** | **1380.7070** | **1379.7409** | **0.9662** | **1** | **5** | **43** | **2** | **R.TVGQLYKESLSR.L** |
|  | 12283 | **744.4406** | **1486.8665** | **1485.8151** | **1.0515** | **1** | **6** | **33** | **1** | **K.TRLALEAEVSELR.A** |
|  | 12298 | **497.2381** | **1488.6925** | **1487.7627** | **0.9298** | **1** | **3** | **60** | **5** | **R.QQQQSALRVMQR.N** |
|  | 12572 | **503.1043** | **1506.2911** | **1505.8276** | **0.4635** | **0** | **7** | **18** | **1** | **R.MVSAVLQFGNIALK.R** |
|  | 12802 | **507.3270** | **1518.9591** | **1519.7300** | **-0.7709** | **1** | **7** | **21** | **1** | **K.AQMASAGQGKEEAVK.Q** |
|  | 13134 | 771.8920 | 1541.7694 | 1541.8565 | -0.0871 | 1 | 4 | 45 | 8 | R.QRYEILTPNAIPK.G |
|  | 13166 | 515.3269 | 1542.9589 | 1543.7478 | -0.7889 | 0 | 4 | 42 | 10 | K.LAQAEEQLEQETR.E |
|  | 13257 | 517.2545 | 1548.7416 | 1549.7923 | -1.0507 | 0 | 2 | 75 | 7 | R.LMATLSNTNPSFVR.C |
|  | 13544 | **784.4622** | **1566.9098** | **1565.7872** | **1.1226** | **0** | **(2)** | **77** | **2** | **R.LMATLSNTNPSFVR.C** |
|  | 13655 | **525.9266** | **1574.7579** | **1573.8199** | **0.9380** | **1** | **4** | **54** | **3** | **R.AELEALLSSKDDVGK.S** |
|  | 13932 | **531.2673** | **1590.7802** | **1590.8075** | **-0.0274** | **0** | **1** | **89** | **2** | **K.MIQALELDPNLYR.V** |
|  | 14940 | **828.4402** | **1654.8659** | **1654.8638** | **0.0021** | **1** | **6** | **26** | **2** | **R.QDEVLQARAQELQK.V** |
|  | 15948 | **863.3517** | **1724.6888** | **1724.8807** | **-0.1919** | **0** | **6** | **25** | **1** | **K.QLPIYTEAIVEMYR.G** |
|  | 15965 | 863.9725 | 1725.9305 | 1725.9413 | -0.0108 | 0 | 86 | 2.8e-007 | 1 | R.QLLQANPILEAFGNAK.T 15966 |
|  | 16898 | **601.8234** | **1802.4483** | **1801.8918** | **0.5564** | **1** | **5** | **24** | **4** | **R.AELSSLQTARQEGEQR.R** |
|  | 17184 | **608.6363** | **1822.8872** | **1823.8955** | **-1.0083** | **0** | **0** | **98** | **4** | **R.HEVPPHVYAVTEGAYR.S** |
|  | 17437 | 615.0034 | 1841.9883 | 1840.9279 | 1.0604 | 1 | 2 | 71 | 4 | R.EVGELQGRVAQLEEER.A 17407 |
|  | 18263 | **637.3085** | **1908.9038** | **1909.9971** | **-1.0934** | **1** | **0** | **91** | **5** | **K.QLPIYTEAIVEMYRGK.K** |
|  | 18811 | **652.3367** | **1953.9884** | **1954.9133** | **-0.9250** | **0** | **2** | **63** | **1** | **R.GPSAGGGPGSGTSPQVEWTAR.R** |
|  | 18949 | 657.1148 | 1968.3226 | 1967.9272 | 0.3954 | 0 | 1 | 52 | 8 | R.ASISYGSNMRPQSQTWR.D |
|  | 19180 | 663.9810 | 1988.9212 | 1987.9884 | 0.9328 | 1 | 1 | 64 | 8 | K.LEGELEELKAQMASAGQGK.E |
|  | 20225 | **523.8046** | **2091.1894** | **2090.0830** | **1.1064** | **1** | **3** | **45** | **8** | **K.MIQALELDPNLYRVGQSK.I** |
|  | 21538 | **745.4088** | **2233.2046** | **2233.9869** | **-0.7823** | **1** | **0** | **76** | **2** | **R.QAQQDRDEMADEVANGNLSK.A** |
|  | 25772 | 733.6164 | 2930.4365 | 2930.3981 | 0.0384 | 1 | 0 | 53 | 10 | K.ADLLLEPCSHYRFLTNGPSSSPGQER.E |
|  | 25929 | **495.2610** | **2965.5223** | **2964.4763** | **1.0460** | **1** | **2** | **34** | **6** | **K.NMDPLNDNVAALLHQSTDRLTAEIWK.D** |
|  | 29858 | **681.3463** | **4082.0342** | **4081.0559** | **0.9783** | **1** | **3** | **14** | **7** | **K.APPRPGPVPEAAQPFLFTPRGPSAGGGPGSGTSPQVEWTAR.R** 29847 |

| **227.** | P21283\|VATC1_HUMAN    **Mass:** 44085    **Score:** 131    **Queries matched:** 7   **emPAI:** 0.08 |
| --- | --- |
|  | V-type proton ATPase subunit C 1 OS=Homo sapiens GN=ATP6V1C1 PE=1 SV=4 |

|  | **Query** | **Observed** | **Mr(expt)** | **Mr(calc)** | **Delta** | **Miss** | **Score** | **Expect** | **Rank** | **Peptide** |
| --- | --- | --- | --- | --- | --- | --- | --- | --- | --- | --- |
|  | 2127 | **424.2183** | **846.4220** | **845.4647** | **0.9573** | **0** | **7** | **38** | **6** | **K.FNIPDLK.V** |
|  | 3672 | **480.2556** | **958.4967** | **958.5560** | **-0.0593** | **1** | **2** | **1.2e+002** | **8** | **R.KNAGSLLTR.S** |
|  | 5275 | 541.2110 | 1080.4074 | 1079.4706 | 0.9369 | 0 | 16 | 3.7 | 4 | K.TCQQTWEK.L |
|  | 11393 | **479.0831** | **1434.2274** | **1434.7177** | **-0.4903** | **0** | **3** | **41** | **7** | **K.QYETLAEMVVPR.S** |
|  | 12241 | **495.7736** | **1484.2989** | **1483.6864** | **0.6125** | **0** | **1** | **67** | **8** | **K.VAQYMADVLEDSK.D** |
|  | 22383 | **1174.0403** | **2346.0660** | **2346.0798** | **-0.0138** | **0** | **90** | **7.2e-008** | **1** | **R.SSNVLSEDQDSYLCNVTLFR.K** 22382 |

| **228.** | Q9H8H3\|MET7A_HUMAN    **Mass:** 28814    **Score:** 131    **Queries matched:** 14   **emPAI:** 1.10 |
| --- | --- |
|  | Methyltransferase-like protein 7A OS=Homo sapiens GN=METTL7A PE=1 SV=1 |

|  | **Query** | **Observed** | **Mr(expt)** | **Mr(calc)** | **Delta** | **Miss** | **Score** | **Expect** | **Rank** | **Peptide** |
| --- | --- | --- | --- | --- | --- | --- | --- | --- | --- | --- |
|  | 1857 | **415.2452** | **828.4759** | **828.4242** | **0.0516** | **0** | **31** | **0.17** | **1** | **R.HLQFER.F** |
|  | 4483 | **509.2704** | **1016.5263** | **1017.5243** | **-0.9980** | **1** | **10** | **19** | **10** | **R.ESWKALER.A** |
|  | 6197 | **576.3315** | **1150.6485** | **1150.6420** | **0.0065** | **0** | **7** | **31** | **8** | **-.MELTIFILR.L** |
|  | 11364 | **717.3356** | **1432.6567** | **1432.6657** | **-0.0090** | **0** | **37** | **0.027** | **1** | **R.VTCIDPNPNFEK.F** 11365 |
|  | 12460 | **500.5923** | **1498.7550** | **1498.7640** | **-0.0090** | **1** | **9** | **14** | **3** | **K.SIAENRHLQFER.F** |
|  | 13513 | **783.3987** | **1564.7829** | **1564.7919** | **-0.0090** | **0** | **61** | **8.5e-005** | **1** | **K.LSLLEVGCGTGANFK.F** |
|  | 14431 | **812.4006** | **1622.7866** | **1622.7940** | **-0.0074** | **0** | **72** | **6.8e-006** | **1** | **R.ELFSNLQEFAGPSGK.L** |
|  | 18575 | **645.6690** | **1933.9852** | **1933.9972** | **-0.0120** | **1** | **20** | **0.94** | **1** | **R.VTCIDPNPNFEKFLIK.S** |
|  | 24689 | **544.5004** | **2717.4655** | **2717.4805** | **-0.0151** | **0** | **23** | **0.26** | **1** | **K.LQHIQAPLSWELVRPHIYGYAVK.-** 24690 |
|  | 25904 | **592.7364** | **2958.6459** | **2958.6596** | **-0.0137** | **1** | **35** | **0.016** | **1** | **K.LKLQHIQAPLSWELVRPHIYGYAVK.-** 25902 25903 |

| **229.** | Q9NTJ5\|SAC1_HUMAN    **Mass:** 67437    **Score:** 130    **Queries matched:** 11   **emPAI:** 0.11 |
| --- | --- |
|  | Phosphatidylinositide phosphatase SAC1 OS=Homo sapiens GN=SACM1L PE=1 SV=2 |

|  | **Query** | **Observed** | **Mr(expt)** | **Mr(calc)** | **Delta** | **Miss** | **Score** | **Expect** | **Rank** | **Peptide** |
| --- | --- | --- | --- | --- | --- | --- | --- | --- | --- | --- |
|  | 3478 | **472.3464** | **942.6782** | **942.5247** | **0.1535** | **0** | **5** | **49** | **3** | **R.SLQAQLQR.L** |
|  | 4848 | 523.7473 | 1045.4801 | 1045.6019 | -0.1219 | 0 | 9 | 24 | 4 | R.VSTEVTLAVK.K |
|  | 6490 | 587.7923 | 1173.5700 | 1173.6969 | -0.1268 | 1 | 11 | 13 | 5 | R.VSTEVTLAVKK.D |
|  | 9116 | **435.7714** | **1304.2923** | **1304.5567** | **-0.2644** | **0** | **4** | **40** | **7** | **K.NAWADNANACAK.Q** |
|  | 16569 | **592.3422** | **1774.0048** | **1774.8495** | **-0.8446** | **0** | **1** | **75** | **9** | **R.THLGLIMDGWNSMIR.Y** |
|  | 17938 | **940.9504** | **1879.8862** | **1879.8985** | **-0.0123** | **0** | **57** | **0.00018** | **1** | **R.LSNTSPEFQEMSLLER.A** 17939 |
|  | 18520 | **644.2775** | **1929.8106** | **1930.9506** | **-1.1400** | **1** | **5** | **27** | **1** | **K.RTHLGLIMDGWNSMIR.Y** 18541 |
|  | 18658 | **648.3229** | **1941.9468** | **1943.0186** | **-1.0718** | **1** | **1** | **68** | **3** | **-.MATAAYEQLKLHITPEK.F** |
|  | 29401 | **1295.3152** | **3882.9237** | **3882.9350** | **-0.0112** | **0** | **68** | **4.7e-006** | **1** | **R.LSILLDQVAEMQDELSYFLVDSAGQVVANQEGVFR.S** |

| **230.** | P21912\|DHSB_HUMAN    **Mass:** 32407    **Score:** 130    **Queries matched:** 37   **emPAI:** 2.01 |
| --- | --- |
|  | Succinate dehydrogenase [ubiquinone] iron-sulfur subunit, mitochondrial OS=Homo sapiens GN=SDHB PE=1 SV=3 |

|  | **Query** | **Observed** | **Mr(expt)** | **Mr(calc)** | **Delta** | **Miss** | **Score** | **Expect** | **Rank** | **Peptide** |
| --- | --- | --- | --- | --- | --- | --- | --- | --- | --- | --- |
|  | 910 | **585.3315** | **584.3243** | **584.3282** | **-0.0039** | **0** | **6** | **30** | **3** | **K.GLNPGK.A** |
|  | 1241 | 759.4308 | 758.4236 | 759.3295 | -0.9059 | 0 | 5 | 78 | 9 | K.MMATYK.E |
|  | 1271 | **772.4882** | **771.4809** | **771.4854** | **-0.0045** | **1** | **37** | **0.045** | **1** | **K.AIAEIKK.M** |
|  | 1425 | **399.2272** | **796.4399** | **796.4595** | **-0.0196** | **1** | **10** | **13** | **2** | **K.KFAIYR.W** |
|  | 2889 | 900.5095 | 899.5022 | 898.5600 | 0.9423 | 0 | 4 | 66 | 4 | M.AAVVALSLR.R |
|  | 3860 | **487.2718** | **972.5291** | **972.5352** | **-0.0061** | **1** | **23** | **0.91** | **1** | **R.RIDTNLNK.V** |
|  | 4266 | **501.2402** | **1000.4658** | **1000.4721** | **-0.0063** | **1** | **23** | **1** | **1** | **K.MMATYKEK.K** |
|  | 5158 | **536.2432** | **1070.4718** | **1070.5543** | **-0.0825** | **1** | **9** | **24** | **7** | **R.TCPKGLNPGK.A** |
|  | 5962 | **566.7898** | **1131.5650** | **1130.6295** | **0.9355** | **1** | **10** | **18** | **3** | **R.IDTNLNKVSK.I** |
|  | 6033 | **569.7949** | **1137.5753** | **1137.5818** | **-0.0066** | **0** | **34** | **0.063** | **1** | **K.LQDPFSLYR.C** 6016 6047 |
|  | 6805 | **596.7506** | **1191.4867** | **1191.4947** | **-0.0081** | **0** | **29** | **0.18** | **1** | **R.CHTIMNCTR.T** |
|  | 7059 | **602.3914** | **1202.7682** | **1201.6965** | **1.0717** | **1** | **0** | **1.5e+002** | **4** | **-.MAAVVALSLRR.R** |
|  | 7318 | **608.8210** | **1215.6274** | **1215.6355** | **-0.0081** | **0** | **50** | **0.0015** | **1** | **K.CGPMVLDALIK.I** |
|  | 7613 | 616.3264 | 1230.6381 | 1231.6305 | -0.9923 | 0 | (8) | 27 | 2 | K.CGPMVLDALIK.I |
|  | 8879 | **432.1822** | **1293.5247** | **1292.6360** | **0.8886** | **0** | **10** | **14** | **2** | **K.QQYLQSIEER.E** 8876 |
|  | 9710 | **446.7766** | **1337.3080** | **1336.6735** | **0.6345** | **1** | **7** | **17** | **1** | **K.NEVDSTLTFRR.S** 9693 |
|  | 11175 | **711.8782** | **1421.7419** | **1421.7514** | **-0.0095** | **1** | **41** | **0.011** | **1** | **K.IKNEVDSTLTFR.R** 11176 |
|  | 11752 | 729.4208 | 1456.8271 | 1456.8146 | 0.0126 | 1 | 19 | 1.6 | 4 | K.CGPMVLDALIKIK.N 11753 |
|  | 12041 | **737.8569** | **1473.6993** | **1472.8095** | **0.8898** | **1** | **(3)** | **69** | **1** | **K.CGPMVLDALIKIK.N** |
|  | 15184 | **836.9105** | **1671.8065** | **1671.8144** | **-0.0079** | **0** | **44** | **0.0046** | **1** | **K.DLVPDLSNFYAQYK.S** |
|  | 15589 | **850.3679** | **1698.7212** | **1698.7308** | **-0.0096** | **1** | **32** | **0.062** | **1** | **R.WMIDSRDDFTEER.L** 15588 |
|  | 21102 | **729.7433** | **2186.2080** | **2185.0812** | **1.1268** | **1** | **6** | **20** | **2** | **K.YLGPAVLMQAYRWMIDSR.D** |
|  | 21698 | **1128.9702** | **2255.9259** | **2254.9551** | **0.9707** | **0** | **31** | **0.061** | **1** | **R.EGICGSCAMNINGGNTLACTR.R** |
|  | 23325 | 829.7198 | 2486.1377 | 2486.1536 | -0.0159 | 1 | 5 | 20 | 2 | R.WDPDKAGDKPHMQTYEVDLNK.C |
|  | 24328 | **887.3694** | **2659.0863** | **2658.1189** | **0.9674** | **1** | **14** | **1.8** | **1** | **R.SCREGICGSCAMNINGGNTLACTR.R** |
|  | 26201 | 1009.8564 | 3026.5473 | 3026.5616 | -0.0142 | 1 | 34 | 0.023 | 1 | K.IYPLPHMYVIKDLVPDLSNFYAQYK.S 26200 26202 26203 26205 |

| **231.** | P16422\|EPCAM_HUMAN    **Mass:** 35594    **Score:** 130    **Queries matched:** 11   **emPAI:** 0.22 |
| --- | --- |
|  | Epithelial cell adhesion molecule OS=Homo sapiens GN=EPCAM PE=1 SV=2 |

|  | **Query** | **Observed** | **Mr(expt)** | **Mr(calc)** | **Delta** | **Miss** | **Score** | **Expect** | **Rank** | **Peptide** |
| --- | --- | --- | --- | --- | --- | --- | --- | --- | --- | --- |
|  | 2970 | **453.7180** | **905.4215** | **904.3531** | **1.0684** | **0** | **4** | **68** | **4** | **K.EMGEMHR.E** |
|  | 3210 | **461.2338** | **920.4531** | **920.3480** | **0.1051** | **0** | **(2)** | **1.2e+002** | **7** | **K.EMGEMHR.E** |
|  | 4687 | **517.2425** | **1032.4704** | **1031.5400** | **0.9305** | **1** | **4** | **70** | **3** | **K.GESLFHSKK.M** |
|  | 9595 | **444.2560** | **1329.7461** | **1329.6169** | **0.1292** | **1** | **(4)** | **58** | **5** | **K.AEIKEMGEMHR.E** |
|  | 9624 | **445.0340** | **1332.0803** | **1331.5598** | **0.5205** | **1** | **7** | **18** | **1** | **K.EMGEMHRELNA.-** |
|  | 9865 | 449.7144 | 1346.1213 | 1345.6118 | 0.5095 | 1 | 10 | 11 | 4 | K.AEIKEMGEMHR.E |
|  | 10163 | **682.3501** | **1362.6856** | **1362.7143** | **-0.0287** | **1** | **4** | **50** | **3** | **K.EITTRYQLDPK.F** |
|  | 18637 | **970.9283** | **1939.8421** | **1939.8550** | **-0.0129** | **0** | **66** | **2.3e-005** | **1** | **R.QCQCTSVGAQNTVICSK.L** 18638 |
|  | 21354 | 554.5460 | 2214.1547 | 2214.9391 | -0.7844 | 1 | 3 | 36 | 7 | K.AKQCNGTSMCWCVNTAGVR.R |
|  | 24873 | **918.4116** | **2752.2130** | **2752.2286** | **-0.0156** | **0** | **43** | **0.0028** | **1** | **R.AKPEGALQNNDGLYDPDCDESGLFK.A** |

| **232.** | P51572\|BAP31_HUMAN    **Mass:** 28031    **Score:** 129    **Queries matched:** 21   **emPAI:** 1.14 |
| --- | --- |
|  | B-cell receptor-associated protein 31 OS=Homo sapiens GN=BCAP31 PE=1 SV=3 |

|  | **Query** | **Observed** | **Mr(expt)** | **Mr(calc)** | **Delta** | **Miss** | **Score** | **Expect** | **Rank** | **Peptide** |
| --- | --- | --- | --- | --- | --- | --- | --- | --- | --- | --- |
|  | 2012 | **420.2320** | **838.4495** | **838.4548** | **-0.0053** | **0** | **9** | **17** | **6** | **R.LLEEHAK.L** 2011 |
|  | 2932 | **452.2678** | **902.5211** | **901.5233** | **0.9978** | **1** | **29** | **0.27** | **1** | **R.SLKADLQK.L** |
|  | 4661 | **516.2653** | **1030.5160** | **1030.5229** | **-0.0070** | **0** | **49** | **0.002** | **1** | **K.AENQVLAMR.K** 4662 |
|  | 4876 | **524.3037** | **1046.5929** | **1046.5179** | **0.0750** | **0** | **(9)** | **21** | **1** | **K.AENQVLAMR.K** |
|  | 5816 | **560.2860** | **1118.5575** | **1118.5567** | **0.0008** | **1** | **5** | **50** | **2** | **K.KQAESASEAAK.K** |
|  | 6093 | **572.7830** | **1143.5515** | **1143.5594** | **-0.0079** | **0** | **36** | **0.046** | **1** | **K.LQAAVDGPMDK.K** 6109 |
|  | 6649 | **593.1554** | **1184.2962** | **1184.5019** | **-0.2057** | **0** | **0** | **99** | **7** | **K.YMEENDQLK.K** |
|  | 8738 | **644.3322** | **1286.6499** | **1287.6493** | **-0.9994** | **1** | **15** | **4.9** | **1** | **K.LQAAVDGPMDKK.E** 8741 8766 |
|  | 8942 | **649.3035** | **1296.5925** | **1296.6020** | **-0.0095** | **1** | **28** | **0.19** | **1** | **K.YMEENDQLKK.G** 8943 |
|  | 10848 | 701.8474 | 1401.6803 | 1401.6888 | -0.0085 | 1 | 8 | 22 | 2 | K.EYDRLLEEHAK.L |
|  | 11075 | **473.2273** | **1416.6600** | **1416.7395** | **-0.0795** | **1** | **2** | **77** | **3** | **K.LEKAENQVLAMR.K** |
|  | 15786 | **857.9291** | **1713.8437** | **1713.8533** | **-0.0096** | **1** | **50** | **0.0011** | **1** | **K.LDVGNAEVKLEEENR.S** 15785 15787 15788 |

| **233.** | P17568\|NDUB7_HUMAN    **Mass:** 16619    **Score:** 128    **Queries matched:** 18   **emPAI:** 0.87 |
| --- | --- |
|  | NADH dehydrogenase [ubiquinone] 1 beta subcomplex subunit 7 OS=Homo sapiens GN=NDUFB7 PE=1 SV=4 |

|  | **Query** | **Observed** | **Mr(expt)** | **Mr(calc)** | **Delta** | **Miss** | **Score** | **Expect** | **Rank** | **Peptide** |
| --- | --- | --- | --- | --- | --- | --- | --- | --- | --- | --- |
|  | 1468 | **400.5567** | **799.0989** | **798.4170** | **0.6819** | **0** | **2** | **77** | **2** | **-.MGAHLVR.R** |
|  | 6654 | **593.2825** | **1184.5504** | **1183.5556** | **0.9947** | **0** | **31** | **0.12** | **1** | **R.DYCAHHLIR.L** 6636 |
|  | 9335 | **439.8378** | **1316.4916** | **1316.4993** | **-0.0077** | **0** | **10** | **14** | **1** | **R.HDWDYCEHR.D** |
|  | 14232 | **806.3768** | **1610.7390** | **1610.7511** | **-0.0121** | **1** | **16** | **2.9** | **1** | **R.DSFPNFLACKQER.H** 14235 14236 14241 |
|  | 16826 | **599.3721** | **1795.0946** | **1795.7903** | **-0.6957** | **0** | **6** | **20** | **1** | **R.EMVATQQEMMDAQLR.L** |
|  | 17235 | **610.1804** | **1827.5194** | **1827.7801** | **-0.2607** | **0** | **(3)** | **35** | **3** | **R.EMVATQQEMMDAQLR.L** |
|  | 19997 | **689.3164** | **2064.9272** | **2064.9390** | **-0.0118** | **1** | **37** | **0.016** | **1** | **K.EREMVATQQEMMDAQLR.L** |
|  | 20446 | **529.4995** | **2113.9687** | **2112.9238** | **1.0449** | **1** | **(1)** | **73** | **4** | **K.EREMVATQQEMMDAQLR.L** |
|  | 22093 | **770.0568** | **2307.1485** | **2306.1181** | **1.0304** | **1** | **(0)** | **79** | **6** | **R.EMVATQQEMMDAQLRLQLR.D** |
|  | 22094 | **770.0622** | **2307.1647** | **2306.1181** | **1.0467** | **1** | **(1)** | **65** | **3** | **R.EMVATQQEMMDAQLRLQLR.D** |
|  | 22215 | **775.0491** | **2322.1254** | **2322.1130** | **0.0124** | **1** | **(0)** | **71** | **6** | **R.EMVATQQEMMDAQLRLQLR.D** |
|  | 22224 | **465.6314** | **2323.1205** | **2322.1130** | **1.0075** | **1** | **4** | **34** | **7** | **R.EMVATQQEMMDAQLRLQLR.D** |
|  | 26455 | **1022.8180** | **3065.4321** | **3065.4481** | **-0.0159** | **1** | **69** | **6.6e-006** | **1** | **R.YLGDASVEPDPLQMPTFPPDYGFPERK.E** 26457 |

| **234.** | Q9NZZ3\|CHMP5_HUMAN    **Mass:** 24612    **Score:** 128    **Queries matched:** 4   **emPAI:** 0.54 |
| --- | --- |
|  | Charged multivesicular body protein 5 OS=Homo sapiens GN=CHMP5 PE=1 SV=1 |

|  | **Query** | **Observed** | **Mr(expt)** | **Mr(calc)** | **Delta** | **Miss** | **Score** | **Expect** | **Rank** | **Peptide** |
| --- | --- | --- | --- | --- | --- | --- | --- | --- | --- | --- |
|  | 15393 | **843.4069** | **1684.7993** | **1684.8091** | **-0.0098** | **0** | **60** | **0.00011** | **1** | **K.APPPSLTDCIGTVDSR.A** |
|  | 22254 | 777.3408 | 2329.0006 | 2328.1267 | 0.8739 | 1 | 6 | 16 | 3 | K.APPPSLTDCIGTVDSRAESIDK.K |
|  | 25042 | **929.7751** | **2786.3034** | **2786.3181** | **-0.0146** | **1** | **36** | **0.013** | **1** | **R.DNLAQQSFNMEQANYTIQSLKDTK.T** |
|  | 27021 | **1064.4785** | **3190.4137** | **3190.4281** | **-0.0144** | **0** | **70** | **4.4e-006** | **1** | **K.IDQIEDLQDQLEDMMEDANEIQEALSR.S** |

| **235.** | P07237\|PDIA1_HUMAN    **Mass:** 57480    **Score:** 127    **Queries matched:** 22   **emPAI:** 0.29 |
| --- | --- |
|  | Protein disulfide-isomerase OS=Homo sapiens GN=P4HB PE=1 SV=3 |

|  | **Query** | **Observed** | **Mr(expt)** | **Mr(calc)** | **Delta** | **Miss** | **Score** | **Expect** | **Rank** | **Peptide** |
| --- | --- | --- | --- | --- | --- | --- | --- | --- | --- | --- |
|  | 2343 | 431.7351 | 861.4557 | 861.4596 | -0.0039 | 0 | 14 | 7.3 | 2 | K.ALAPEYAK.A |
|  | 3032 | **455.7563** | **909.4981** | **909.4345** | **0.0636** | **0** | **8** | **24** | **6** | **K.FFPASADR.T** |
|  | 3696 | **481.1525** | **960.2904** | **961.4440** | **-1.1535** | **0** | **8** | **26** | **7** | **R.ITEFCHR.F** 3693 3711 |
|  | 4093 | **495.2354** | **988.4563** | **987.4807** | **0.9756** | **1** | **9** | **27** | **6** | **K.KEECPAVR.L** |
|  | 4295 | **501.7791** | **1001.5437** | **1001.5505** | **-0.0069** | **1** | **29** | **0.22** | **3** | **K.LKAEGSEIR.L** |
|  | 7429 | **408.2145** | **1221.6217** | **1221.6162** | **0.0055** | **0** | **8** | **25** | **3** | **R.LITLEEEMTK.Y** |
|  | 7755 | **619.8086** | **1237.6027** | **1238.6044** | **-1.0016** | **1** | **8** | **24** | **7** | **K.FFRNGDTASPK.E** |
|  | 8863 | **647.2992** | **1292.5838** | **1292.5918** | **-0.0080** | **0** | **58** | **0.00022** | **1** | **K.MDSTANEVEAVK.V** 8864 |
|  | 11654 | **726.3489** | **1450.6832** | **1450.6939** | **-0.0107** | **0** | **28** | **0.22** | **1** | **K.YKPESEELTAER.I** 11653 11662 |
|  | 16009 | 433.2310 | 1728.8949 | 1728.9046 | -0.0098 | 1 | 8 | 16 | 3 | K.LGETYKDHENIVIAK.M |
|  | 16647 | **890.9155** | **1779.8164** | **1779.8275** | **-0.0111** | **0** | **67** | **2.1e-005** | **1** | **K.VDATEESDLAQQYGVR.G** |
|  | 21222 | **735.3707** | **2203.0904** | **2202.0991** | **0.9913** | **1** | **8** | **13** | **2** | **K.MDSTANEVEAVKVHSFPTLK.F** |
|  | 21373 | 1109.9528 | 2217.8910 | 2218.0940 | -0.2030 | 1 | (5) | 20 | 2 | K.MDSTANEVEAVKVHSFPTLK.F |
|  | 22877 | **605.3138** | **2417.2260** | **2417.2413** | **-0.0153** | **1** | **43** | **0.0033** | **1** | **K.IKPHLMSQELPEDWDKQPVK.V** |
|  | 24660 | **542.6998** | **2708.4625** | **2708.4537** | **0.0087** | **1** | **4** | **26** | **5** | **K.HNQLPLVIEFTEQTAPKIFGGEIK.T** |
|  | 25031 | **558.0818** | **2785.3726** | **2785.3396** | **0.0330** | **1** | **4** | **23** | **6** | **K.YLLVEFYAPWCGHCKALAPEYAK.A** |
|  | 29094 | **1256.2858** | **3765.8355** | **3765.8473** | **-0.0118** | **1** | **14** | **1.3** | **1** | **R.TGPAATTLPDGAAAESLVESSEVAVIGFFKDVESDSAK.Q** |

| **236.** | Q92928\|RAB1C_HUMAN    **Mass:** 22231    **Score:** 126    **Queries matched:** 22   **emPAI:** 0.61 |
| --- | --- |
|  | Putative Ras-related protein Rab-1C OS=Homo sapiens GN=RAB1C PE=5 SV=2 |

|  | **Query** | **Observed** | **Mr(expt)** | **Mr(calc)** | **Delta** | **Miss** | **Score** | **Expect** | **Rank** | **Peptide** |
| --- | --- | --- | --- | --- | --- | --- | --- | --- | --- | --- |
|  | 3907 | 488.2804 | 974.5463 | 974.5397 | 0.0066 | 1 | 5 | 62 | 5 | K.KVVDNTTAK.E |
|  | 5171 | 536.3210 | 1070.6274 | 1070.6336 | -0.0062 | 0 | 21 | 1.3 | 1 | K.LLLIGDSGVGK.S |
|  | 8731 | 644.2775 | 1286.5404 | 1287.7398 | -1.1994 | 1 | 5 | 47 | 4 | K.LLVGNKSDLTTK.K 8767 |
|  | 9315 | 658.8296 | 1315.6446 | 1315.6521 | -0.0074 | 0 | 18 | 2.1 | 1 | K.LQIWDTAGQER.F 9284 |
|  | 10079 | 679.8721 | 1357.7296 | 1357.7817 | -0.0521 | 1 | 34 | 0.056 | 2 | K.IQTIELDGKTIK.L 10080 10081 10082 |
|  | 13836 | **397.1942** | **1584.7479** | **1584.7678** | **-0.0200** | **0** | **7** | **22** | **1** | **K.QMGPGAASGGERPNLK.I** 13823 13865 |
|  | 14973 | 829.6645 | 1657.3144 | 1657.8788 | -0.5643 | 1 | 3 | 37 | 1 | K.TIKLQIWDTAGQER.F |
|  | 15938 | 862.9366 | 1723.8587 | 1723.8669 | -0.0081 | 0 | 16 | 2.8 | 2 | K.EFADSLGIPFLETSAK.N |
|  | 17781 | 934.9435 | 1867.8725 | 1867.8808 | -0.0083 | 0 | 63 | 4.5e-005 | 1 | K.NATNVEQAFMTMAAEIK.K 17803 |
|  | 17979 | 942.9399 | 1883.8653 | 1883.8757 | -0.0104 | 0 | (56) | 0.00026 | 2 | K.NATNVEQAFMTMAAEIK.K 17978 17980 |
|  | 18155 | 951.0157 | 1900.0168 | 1899.8706 | 0.1462 | 0 | (3) | 49 | 9 | K.NATNVEQAFMTMAAEIK.K 18154 |

| **237.** | Q969Z3\|MOSC2_HUMAN    **Mass:** 38569    **Score:** 126    **Queries matched:** 23   **emPAI:** 0.59 |
| --- | --- |
|  | MOSC domain-containing protein 2, mitochondrial OS=Homo sapiens GN=MOSC2 PE=1 SV=1 |

|  | **Query** | **Observed** | **Mr(expt)** | **Mr(calc)** | **Delta** | **Miss** | **Score** | **Expect** | **Rank** | **Peptide** |
| --- | --- | --- | --- | --- | --- | --- | --- | --- | --- | --- |
|  | 1088 | **660.3627** | **659.3554** | **659.3238** | **0.0316** | **0** | **13** | **12** | **3** | **K.QPSSNK.L** |
|  | 2587 | 439.2582 | 876.5019 | 876.4310 | 0.0710 | 1 | 9 | 24 | 8 | K.KVMACPR.C |
|  | 3162 | **459.7736** | **917.5326** | **917.5375** | **-0.0049** | **0** | **31** | **0.17** | **1** | **K.LWIYPVK.S** 3154 |
|  | 3462 | **472.2480** | **942.4814** | **941.5546** | **0.9269** | **1** | **13** | **8.6** | **2** | **R.KQPLDTLK.S** 3459 |
|  | 3468 | **472.2792** | **942.5439** | **942.5498** | **-0.0059** | **0** | **31** | **0.13** | **1** | **R.LQQVGTVAK.L** |
|  | 4449 | **507.9174** | **1013.8202** | **1014.4553** | **-0.6350** | **0** | **1** | **91** | **2** | **K.EDGHMVTAR.Q** |
|  | 4923 | **526.7471** | **1051.4797** | **1050.5168** | **0.9629** | **1** | **9** | **17** | **2** | **R.VGDPVYRMV.-** |
|  | 4932 | **527.2894** | **1052.5643** | **1052.4920** | **0.0723** | **0** | **7** | **31** | **6** | **-.MGASSSSALAR.L** |
|  | 5684 | **555.2832** | **1108.5518** | **1108.5587** | **-0.0068** | **0** | **21** | **1** | **1** | **R.LVQFETNMK.G** |
|  | 9711 | **669.6826** | **1337.3506** | **1337.6762** | **-0.3256** | **1** | **2** | **63** | **3** | **R.LVQFETNMKGR.T** |
|  | 12733 | **758.4045** | **1514.7945** | **1514.8021** | **-0.0075** | **0** | **39** | **0.017** | **1** | **K.LSPLFGIYYSVEK.I** |
|  | 13124 | **514.7601** | **1541.2586** | **1540.7052** | **0.5533** | **1** | **1** | **59** | **9** | **K.EDGHMVTARQEPR.L** |
|  | 13676 | 788.8721 | 1575.7297 | 1575.7385 | -0.0088 | 0 | 63 | 5.6e-005 | 1 | K.GVPVSEAECTAMGLR.S |
|  | 13963 | **399.2196** | **1592.8495** | **1591.7334** | **1.1160** | **0** | **(9)** | **14** | **2** | **K.GVPVSEAECTAMGLR.S** |
|  | 15225 | **837.9169** | **1673.8192** | **1673.8295** | **-0.0103** | **0** | **32** | **0.081** | **1** | **R.CILTTVDPDTGVIDR.K** |
|  | 18951 | **985.4375** | **1968.8604** | **1969.9779** | **-1.1174** | **1** | **1** | **73** | **7** | **R.APDMDQLVLPSKQPSSNK.L** |
|  | 22771 | **1203.6489** | **2405.2833** | **2404.1549** | **1.1284** | **1** | **1** | **59** | **4** | **K.VMACPRCILTTVDPDTGVIDR.K** |
|  | 29192 | **1270.6366** | **3808.8880** | **3808.9056** | **-0.0176** | **0** | **46** | **0.00084** | **1** | **K.LLPTLDQNFQVAYPDYCPLLIMTDASLVDLNTR.M** |
|  | 29513 | **1313.6968** | **3938.0685** | **3937.0005** | **1.0680** | **1** | **43** | **0.0015** | **1** | **R.KLLPTLDQNFQVAYPDYCPLLIMTDASLVDLNTR.M** 29503 29506 |

| **238.** | P17066\|HSP76_HUMAN    **Mass:** 71440    **Score:** 126    **Queries matched:** 21   **emPAI:** 0.35 |
| --- | --- |
|  | Heat shock 70 kDa protein 6 OS=Homo sapiens GN=HSPA6 PE=1 SV=2 |

|  | **Query** | **Observed** | **Mr(expt)** | **Mr(calc)** | **Delta** | **Miss** | **Score** | **Expect** | **Rank** | **Peptide** |
| --- | --- | --- | --- | --- | --- | --- | --- | --- | --- | --- |
|  | 1146 | 689.3898 | 688.3826 | 688.3755 | 0.0070 | 1 | 11 | 20 | 8 | R.DAKLDK.A |
|  | 4487 | 509.2846 | 1016.5547 | 1016.5614 | -0.0068 | 1 | 31 | 0.15 | 1 | K.ITITNDKGR.L 4494 |
|  | 6415 | **584.9444** | **1167.8742** | **1168.6564** | **-0.7822** | **0** | **2** | **66** | **5** | **K.DAGAIAGLNVLR.I** |
|  | 7551 | 614.8137 | 1227.6128 | 1227.6207 | -0.0080 | 0 | 44 | 0.0057 | 1 | R.VEILANDQGNR.T |
|  | 12274 | 744.3496 | 1486.6845 | 1486.6940 | -0.0095 | 0 | 49 | 0.0015 | 1 | R.TTPSYVAFTDTER.L |
|  | 13130 | 771.8685 | 1541.7224 | 1541.7296 | -0.0073 | 1 | 49 | 0.0016 | 1 | R.ARFEELCSDLFR.S 13128 13129 |
|  | 13509 | 783.3471 | 1564.6797 | 1564.8249 | -0.1453 | 1 | 7 | 25 | 2 | K.LLQDFFNGKELNK.S |
|  | 13769 | **527.9224** | **1580.7453** | **1579.7916** | **0.9537** | **1** | **3** | **61** | **2** | **K.MKETAEAYLGQPVK.H** |
|  | 15235 | 838.3636 | 1674.7126 | 1674.7234 | -0.0108 | 0 | 42 | 0.0064 | 1 | K.ATAGDTHLGGEDFDNR.L 15233 15236 |
|  | 15311 | 560.3553 | 1678.0440 | 1678.8678 | -0.8238 | 0 | 4 | 37 | 7 | R.EVLAWLEHNQLAEK.E |
|  | 16496 | **590.3210** | **1767.9413** | **1767.9016** | **0.0397** | **1** | **8** | **17** | **1** | **K.SQAALNPHNTVFDAKR.L** |
|  | 18024 | **630.3235** | **1887.9488** | **1888.9314** | **-0.9826** | **1** | **1** | **83** | **7** | **K.TFYPEEISSMVLSKMK.E** 18038 |
|  | 18394 | **640.9750** | **1919.9033** | **1920.9213** | **-1.0180** | **1** | **(1)** | **84** | **3** | **K.TFYPEEISSMVLSKMK.E** |
|  | 26370 | **763.6301** | **3050.4914** | **3051.6890** | **-1.1976** | **0** | **6** | **12** | **1** | **K.VQDLLLLDVAPLSLGLETAGGVMTTLIQR.N** |
|  | 29113 | **1889.1348** | **3776.2550** | **3777.0962** | **-0.8412** | **1** | **3** | **11** | **1** | **K.VQDLLLLDVAPLSLGLETAGGVMTTLIQRNATIPTK.Q** |

| **239.** | P48741\|HSP77_HUMAN    **Mass:** 40448    **Score:** 126    **Queries matched:** 17   **emPAI:** 0.70 |
| --- | --- |
|  | Putative heat shock 70 kDa protein 7 OS=Homo sapiens GN=HSPA7 PE=5 SV=2 |

|  | **Query** | **Observed** | **Mr(expt)** | **Mr(calc)** | **Delta** | **Miss** | **Score** | **Expect** | **Rank** | **Peptide** |
| --- | --- | --- | --- | --- | --- | --- | --- | --- | --- | --- |
|  | 1146 | 689.3898 | 688.3826 | 688.3755 | 0.0070 | 1 | 11 | 20 | 8 | R.DAKLDK.A |
|  | 1691 | **408.7300** | **815.4454** | **814.4548** | **0.9906** | **0** | **13** | **11** | **8** | **K.DAGAIAGLK.V** |
|  | 7551 | 614.8137 | 1227.6128 | 1227.6207 | -0.0080 | 0 | 44 | 0.0057 | 1 | R.VEILANDQGNR.T |
|  | 12274 | 744.3496 | 1486.6845 | 1486.6940 | -0.0095 | 0 | 49 | 0.0015 | 1 | R.TTPSYVAFTDTER.L |
|  | 13130 | 771.8685 | 1541.7224 | 1541.7296 | -0.0073 | 1 | 49 | 0.0016 | 1 | R.ARFEELCSDLFR.S 13128 13129 |
|  | 13769 | 527.9224 | 1580.7453 | 1579.7916 | 0.9537 | 1 | 3 | 61 | 2 | K.MKETAEAYLGQPVK.H |
|  | 14003 | **532.7570** | **1595.2491** | **1594.8256** | **0.4234** | **0** | **6** | **21** | **1** | **K.HWPFQVVSEGGKPK.V** |
|  | 15235 | 838.3636 | 1674.7126 | 1674.7234 | -0.0108 | 0 | 42 | 0.0064 | 1 | K.ATAGDTHLGGEDFDNR.L 15233 15236 |
|  | 16496 | 590.3210 | 1767.9413 | 1767.9016 | 0.0397 | 1 | 8 | 17 | 1 | K.SQAALNPHNTVFDAKR.L |
|  | 18024 | 630.3235 | 1887.9488 | 1888.9314 | -0.9826 | 1 | 1 | 83 | 7 | K.TFYPEEISSMVLSKMK.E 18038 |
|  | 18394 | 640.9750 | 1919.9033 | 1920.9213 | -1.0180 | 1 | (1) | 84 | 3 | K.TFYPEEISSMVLSKMK.E |
|  | 18985 | **657.8015** | **1970.3825** | **1970.0836** | **0.2989** | **0** | **1** | **52** | **10** | **K.VLPIINEATAAAIAYGLDR.R** |

| **240.** | Q16718\|NDUA5_HUMAN    **Mass:** 13507    **Score:** 126    **Queries matched:** 7   **emPAI:** 0.66 |
| --- | --- |
|  | NADH dehydrogenase [ubiquinone] 1 alpha subcomplex subunit 5 OS=Homo sapiens GN=NDUFA5 PE=1 SV=3 |

|  | **Query** | **Observed** | **Mr(expt)** | **Mr(calc)** | **Delta** | **Miss** | **Score** | **Expect** | **Rank** | **Peptide** |
| --- | --- | --- | --- | --- | --- | --- | --- | --- | --- | --- |
|  | 1248 | **762.3770** | **761.3697** | **761.4469** | **-0.0772** | **1** | **13** | **11** | **4** | **-.MAGVLKK.T** |
|  | 5874 | **563.2711** | **1124.5276** | **1124.5349** | **-0.0074** | **0** | **16** | **3.5** | **1** | **K.YTEQITNEK.L** |
|  | 15935 | **862.9353** | **1723.8560** | **1723.8676** | **-0.0115** | **0** | **87** | **2.2e-007** | **1** | **K.TTGLVGLAVCNTPHER.L** |
|  | 17359 | **918.9570** | **1835.8995** | **1835.9094** | **-0.0099** | **0** | **33** | **0.064** | **1** | **K.LWEPLVEEPPADQWK.W** |
|  | 21528 | **1117.0645** | **2232.1143** | **2232.1255** | **-0.0112** | **1** | **13** | **4.1** | **1** | **K.LWEPLVEEPPADQWKWPI.-** 21526 |
|  | 25952 | **744.1433** | **2972.5441** | **2972.5567** | **-0.0125** | **1** | **65** | **1.6e-005** | **1** | **K.KLEDQLQGGQLEEVILQAEHELNLAR.K** |

| **241.** | P02675\|FIBB_HUMAN    **Mass:** 56577    **Score:** 125    **Queries matched:** 23   **emPAI:** 0.38 |
| --- | --- |
|  | Fibrinogen beta chain OS=Homo sapiens GN=FGB PE=1 SV=2 |

|  | **Query** | **Observed** | **Mr(expt)** | **Mr(calc)** | **Delta** | **Miss** | **Score** | **Expect** | **Rank** | **Peptide** |
| --- | --- | --- | --- | --- | --- | --- | --- | --- | --- | --- |
|  | 3960 | **490.7223** | **979.4300** | **979.4359** | **-0.0059** | **0** | **37** | **0.03** | **1** | **R.QDGSVDFGR.K** 3962 |
|  | 4679 | **516.7813** | **1031.5479** | **1031.5553** | **-0.0073** | **0** | **15** | **6.1** | **1** | **K.IRPFFPQQ.-** 4680 |
|  | 7782 | **620.2589** | **1238.5033** | **1238.5105** | **-0.0072** | **0** | **12** | **8.4** | **2** | **K.EDGGGWWYNR.C** 7781 |
|  | 13125 | **514.7706** | **1541.2901** | **1540.7530** | **0.5370** | **1** | **2** | **53** | **3** | **K.MSMKIRPFFPQQ.-** |
|  | 13746 | **527.2980** | **1578.8721** | **1579.9888** | **-1.1167** | **0** | **2** | **72** | **8** | **K.HLLLLLLCVFLVK.S** |
|  | 15121 | 834.8553 | 1667.6960 | 1667.7079 | -0.0118 | 0 | 18 | 1.6 | 1 | R.YYWGGQYTWDMAK.H |
|  | 17349 | 612.6438 | 1834.9096 | 1835.7971 | -0.8875 | 0 | 3 | 59 | 5 | R.TMTIHNGMFFSTYDR.D |
|  | 19194 | 498.5226 | 1990.0615 | 1990.9380 | -0.8765 | 1 | 8 | 14 | 3 | R.MGPTELLIEMEDWKGDK.V |
|  | 20823 | **1078.9716** | **2155.9286** | **2155.9415** | **-0.0130** | **0** | **67** | **1.5e-005** | **1** | **R.VYCDMNTENGGWTVIQNR.Q** 20838 20839 |
|  | 21700 | **753.0264** | **2256.0573** | **2256.0732** | **-0.0159** | **0** | **41** | **0.0058** | **1** | **K.GGETSEMYLIQPDSSVKPYR.V** 21701 |
|  | 21840 | **758.2191** | **2271.6355** | **2272.0681** | **-0.4326** | **0** | **(1)** | **39** | **5** | **K.GGETSEMYLIQPDSSVKPYR.V** 21835 |
|  | 22553 | **790.0555** | **2367.1446** | **2367.1607** | **-0.0161** | **1** | **11** | **5.8** | **1** | **K.AHYGGFTVQNEANKYQISVNK.Y** |
|  | 22665 | **795.7243** | **2384.1511** | **2384.1682** | **-0.0171** | **1** | **30** | **0.075** | **1** | **R.KGGETSEMYLIQPDSSVKPYR.V** |
|  | 23310 | **828.7316** | **2483.1731** | **2483.1903** | **-0.0173** | **1** | **6** | **20** | **2** | **K.NYCGLPGEYWLGNDKISQLTR.M** |
|  | 26892 | **1056.4941** | **3166.4606** | **3166.4764** | **-0.0158** | **0** | **14** | **2** | **1** | **R.NSVDELNNNVEAVSQTSSSSFQYMYLLK.D** |
|  | 29863 | **1021.7532** | **4082.9838** | **4083.0088** | **-0.0250** | **1** | **19** | **0.31** | **1** | **R.KAPDAGGCLHADPDLGVLCPTGCQLQEALLQQERPIR.N** |

| **242.** | P09211\|GSTP1_HUMAN    **Mass:** 23569    **Score:** 125    **Queries matched:** 6   **emPAI:** 0.16 |
| --- | --- |
|  | Glutathione S-transferase P OS=Homo sapiens GN=GSTP1 PE=1 SV=2 |

|  | **Query** | **Observed** | **Mr(expt)** | **Mr(calc)** | **Delta** | **Miss** | **Score** | **Expect** | **Rank** | **Peptide** |
| --- | --- | --- | --- | --- | --- | --- | --- | --- | --- | --- |
|  | 1543 | **402.7246** | **803.4345** | **802.4232** | **1.0114** | **1** | **16** | **6** | **2** | **R.GRCAALR.M** |
|  | 8843 | 646.7986 | 1291.5827 | 1291.6231 | -0.0403 | 0 | 5 | 41 | 6 | R.MLLADQGQSWK.E |
|  | 17972 | **942.4725** | **1882.9304** | **1882.9425** | **-0.0121** | **0** | **125** | **3.2e-011** | **1** | **K.FQDGDLTLYQSNTILR.H** 17955 |
|  | 20457 | **529.7466** | **2114.9575** | **2115.9743** | **-1.0168** | **0** | **8** | **15** | **4** | **K.DQQEAALVDMVNDGVEDLR.C** |
|  | 26090 | 751.0930 | 3000.3427 | 3000.5015 | -0.1588 | 1 | 4 | 22 | 2 | K.ASCLYGQLPKFQDGDLTLYQSNTILR.H |

| **243.** | Q16864\|VATF_HUMAN    **Mass:** 13362    **Score:** 125    **Queries matched:** 8   **emPAI:** 1.17 |
| --- | --- |
|  | V-type proton ATPase subunit F OS=Homo sapiens GN=ATP6V1F PE=1 SV=2 |

|  | **Query** | **Observed** | **Mr(expt)** | **Mr(calc)** | **Delta** | **Miss** | **Score** | **Expect** | **Rank** | **Peptide** |
| --- | --- | --- | --- | --- | --- | --- | --- | --- | --- | --- |
|  | 4756 | **520.2442** | **1038.4738** | **1038.4804** | **-0.0066** | **0** | **46** | **0.0039** | **1** | **R.GMFTAEDLR.-** |
|  | 4963 | **528.7667** | **1055.5189** | **1054.4753** | **1.0435** | **0** | **(0)** | **1.5e+002** | **8** | **R.GMFTAEDLR.-** |
|  | 9975 | **677.0559** | **1352.0973** | **1351.7361** | **0.3612** | **1** | **3** | **46** | **2** | **K.NRHPNFLVVEK.D** |
|  | 23050 | **1222.6613** | **2443.3079** | **2443.3210** | **-0.0131** | **0** | **100** | **6.9e-009** | **1** | **K.LIAVIGDEDTVTGFLLGGIGELNK.N** |
|  | 25923 | **593.9061** | **2964.4940** | **2964.5093** | **-0.0154** | **1** | **25** | **0.16** | **1** | **R.HALDAHQQSIPAVLEIPSKEHPYDAAK.D** 25921 25922 25929 |

| **244.** | O75947\|ATP5H_HUMAN    **Mass:** 18537    **Score:** 123    **Queries matched:** 16   **emPAI:** 1.13 |
| --- | --- |
|  | ATP synthase subunit d, mitochondrial OS=Homo sapiens GN=ATP5H PE=1 SV=3 |

|  | **Query** | **Observed** | **Mr(expt)** | **Mr(calc)** | **Delta** | **Miss** | **Score** | **Expect** | **Rank** | **Peptide** |
| --- | --- | --- | --- | --- | --- | --- | --- | --- | --- | --- |
|  | 1399 | **398.0502** | **794.0858** | **794.3666** | **-0.2808** | **1** | **3** | **51** | **6** | **K.EMEKMK.N** |
|  | 4360 | **504.3061** | **1006.5977** | **1006.5447** | **0.0530** | **1** | **4** | **74** | **7** | **K.ARIVEYEK.E** |
|  | 5445 | **547.2641** | **1092.5136** | **1092.5200** | **-0.0063** | **0** | **29** | **0.19** | **1** | **K.SWNETLTSR.L** |
|  | 8944 | **649.3164** | **1296.6181** | **1296.6271** | **-0.0090** | **1** | **35** | **0.047** | **1** | **R.IVEYEKEMEK.M** |
|  | 15378 | **842.9223** | **1683.8300** | **1683.8409** | **-0.0108** | **1** | **33** | **0.055** | **1** | **K.KYPYWPHQPIENL.-** 15369 15379 15380 15381 |
|  | 17845 | **936.9914** | **1871.9682** | **1871.9781** | **-0.0099** | **0** | **38** | **0.015** | **1** | **K.TIDWVAFAEIIPQNQK.A** 17856 17858 |
|  | 23193 | **1233.0946** | **2464.1746** | **2464.1832** | **-0.0085** | **0** | **93** | **3.4e-008** | **1** | **K.NLIPFDQMTIEDLNEAFPETK.L** |
|  | 24710 | **908.7751** | **2723.3034** | **2723.3186** | **-0.0152** | **1** | **14** | **2.5** | **1** | **K.MKNLIPFDQMTIEDLNEAFPETK.L** |
|  | 25238 | **941.1331** | **2820.3774** | **2820.3891** | **-0.0118** | **1** | **4** | **26** | **3** | **K.NLIPFDQMTIEDLNEAFPETKLDK.K** 25237 |

| **245.** | P05091\|ALDH2_HUMAN    **Mass:** 56859    **Score:** 123    **Queries matched:** 10   **emPAI:** 0.14 |
| --- | --- |
|  | Aldehyde dehydrogenase, mitochondrial OS=Homo sapiens GN=ALDH2 PE=1 SV=2 |

|  | **Query** | **Observed** | **Mr(expt)** | **Mr(calc)** | **Delta** | **Miss** | **Score** | **Expect** | **Rank** | **Peptide** |
| --- | --- | --- | --- | --- | --- | --- | --- | --- | --- | --- |
|  | 3850 | **487.2308** | **972.4471** | **971.5764** | **0.8707** | **1** | **7** | **35** | **8** | **K.RVTLELGGK.S** |
|  | 5972 | **567.2878** | **1132.5610** | **1131.5825** | **0.9785** | **0** | **2** | **1.1e+002** | **9** | **R.AAFQLGSPWR.R** |
|  | 10533 | **462.2393** | **1383.6960** | **1384.7748** | **-1.0788** | **0** | **2** | **91** | **6** | **K.LGPALATGNVVVMK.V** |
|  | 12348 | **746.4072** | **1490.7999** | **1490.8093** | **-0.0094** | **1** | **26** | **0.29** | **1** | **K.ILGYINTGKQEGAK.L** |
|  | 12912 | **764.3888** | **1526.7630** | **1526.7729** | **-0.0099** | **0** | **91** | **1e-007** | **1** | **R.ANNSTYGLAAAVFTK.D** |
|  | 16750 | **895.4122** | **1788.8099** | **1788.8206** | **-0.0107** | **0** | **56** | **0.00025** | **1** | **R.TFVQEDIYDEFVER.S** |
|  | 19473 | **673.3739** | **2017.0999** | **2015.9741** | **1.1257** | **1** | **3** | **46** | **7** | **K.YHGKTIPIDGDFFSYTR.H** |
|  | 21142 | **548.5286** | **2190.0854** | **2190.0263** | **0.0591** | **1** | **1** | **73** | **10** | **K.MSGSGRELGEYGLQAYTEVK.T** |
|  | 23074 | **817.3951** | **2449.1636** | **2449.1762** | **-0.0126** | **1** | **30** | **0.073** | **1** | **R.VVGNPFDSKTEQGPQVDETQFK.K** |
|  | 24698 | **907.4627** | **2719.3663** | **2719.3767** | **-0.0104** | **0** | **18** | **0.91** | **1** | **R.HEPVGVCGQIIPWNFPLLMQAWK.L** |

| **246.** | P0C0S8\|H2A1_HUMAN    **Mass:** 14083    **Score:** 123    **Queries matched:** 9   **emPAI:** 1.09 |
| --- | --- |
|  | Histone H2A type 1 OS=Homo sapiens GN=HIST1H2AG PE=1 SV=2 |

|  | **Query** | **Observed** | **Mr(expt)** | **Mr(calc)** | **Delta** | **Miss** | **Score** | **Expect** | **Rank** | **Peptide** |
| --- | --- | --- | --- | --- | --- | --- | --- | --- | --- | --- |
|  | 2185 | 425.7640 | 849.5135 | 849.5184 | -0.0049 | 0 | 27 | 0.35 | 1 | R.HLQLAIR.N 2184 |
|  | 2337 | 431.2445 | 860.4744 | 860.3875 | 0.0869 | 0 | 7 | 42 | 4 | R.NDEELNK.L |
|  | 3484 | 472.7663 | 943.5180 | 943.5240 | -0.0060 | 0 | 40 | 0.019 | 1 | R.AGLQFPVGR.V 3470 |
|  | 18534 | 966.0835 | 1930.1524 | 1930.1615 | -0.0091 | 0 | 19 | 1.1 | 1 | K.VTIAQGGVLPNIQAVLLPK.K |
|  | 25694 | **1458.2902** | **2914.5658** | **2914.5804** | **-0.0146** | **0** | **107** | **9.7e-010** | **1** | **R.VGAGAPVYLAAVLEYLTAEILELAGNAAR.D** 25695 25696 |

|  | **Proteins matching the same set of peptides:** |
| --- | --- |

|  | Q7L7L0\|H2A3_HUMAN    **Mass:** 14113    **Score:** 123    **Queries matched:** 9 |
| --- | --- |
|  | Histone H2A type 3 OS=Homo sapiens GN=HIST3H2A PE=1 SV=3 |

|  | Q9BTM1\|H2AJ_HUMAN    **Mass:** 14011    **Score:** 123    **Queries matched:** 9 |
| --- | --- |
|  | Histone H2A.J OS=Homo sapiens GN=H2AFJ PE=1 SV=1 |

|  | Q96KK5\|H2A1H_HUMAN    **Mass:** 13898    **Score:** 123    **Queries matched:** 9 |
| --- | --- |
|  | Histone H2A type 1-H OS=Homo sapiens GN=HIST1H2AH PE=1 SV=3 |

|  | P04908\|H2A1B_HUMAN    **Mass:** 14127    **Score:** 123    **Queries matched:** 9 |
| --- | --- |
|  | Histone H2A type 1-B/E OS=Homo sapiens GN=HIST1H2AB PE=1 SV=2 |

|  | P20671\|H2A1D_HUMAN    **Mass:** 14099    **Score:** 123    **Queries matched:** 9 |
| --- | --- |
|  | Histone H2A type 1-D OS=Homo sapiens GN=HIST1H2AD PE=1 SV=2 |

|  | Q93077\|H2A1C_HUMAN    **Mass:** 14097    **Score:** 123    **Queries matched:** 9 |
| --- | --- |
|  | Histone H2A type 1-C OS=Homo sapiens GN=HIST1H2AC PE=1 SV=3 |

|  | Q99878\|H2A1J_HUMAN    **Mass:** 13928    **Score:** 123    **Queries matched:** 9 |
| --- | --- |
|  | Histone H2A type 1-J OS=Homo sapiens GN=HIST1H2AJ PE=1 SV=3 |

|  | **Proteins matching a subset of these peptides:** |
| --- | --- |

|  | P16104\|H2AX_HUMAN    **Mass:** 15135    **Score:** 123    **Queries matched:** 8 |
| --- | --- |
|  | Histone H2A.x OS=Homo sapiens GN=H2AFX PE=1 SV=2 |

|  | Q8IUE6\|H2A2B_HUMAN    **Mass:** 13987    **Score:** 123    **Queries matched:** 6 |
| --- | --- |
|  | Histone H2A type 2-B OS=Homo sapiens GN=HIST2H2AB PE=1 SV=3 |

| **247.** | P27824\|CALX_HUMAN    **Mass:** 67982    **Score:** 121    **Queries matched:** 20   **emPAI:** 0.24 |
| --- | --- |
|  | Calnexin OS=Homo sapiens GN=CANX PE=1 SV=2 |

|  | **Query** | **Observed** | **Mr(expt)** | **Mr(calc)** | **Delta** | **Miss** | **Score** | **Expect** | **Rank** | **Peptide** |
| --- | --- | --- | --- | --- | --- | --- | --- | --- | --- | --- |
|  | 1652 | **407.2266** | **812.4386** | **813.4344** | **-0.9959** | **0** | **1** | **1.3e+002** | **6** | **K.RPDADLK.T** |
|  | 1904 | **417.2193** | **832.4241** | **831.4755** | **0.9486** | **0** | **4** | **79** | **2** | **K.LHFIFR.H** |
|  | 2025 | **420.6775** | **839.3404** | **838.4072** | **0.9331** | **0** | **9** | **18** | **2** | **K.TGIYEEK.H** |
|  | 3712 | **481.7776** | **961.5407** | **961.5457** | **-0.0050** | **1** | **20** | **1.9** | **1** | **R.AKHHAISAK.L** 3711 |
|  | 4305 | **502.2679** | **1002.5211** | **1001.4301** | **1.0910** | **0** | **9** | **24** | **7** | **R.EIEDPEDR.K** |
|  | 5032 | **531.2673** | **1060.5201** | **1060.5917** | **-0.0716** | **0** | **4** | **70** | **2** | **R.GTLSGWILSK.A** |
|  | 5372 | **544.7613** | **1087.5081** | **1087.5145** | **-0.0064** | **0** | **54** | **0.00075** | **1** | **K.AEEDEILNR.S** |
|  | 6154 | **574.9918** | **1147.9690** | **1148.5197** | **-0.5507** | **1** | **3** | **54** | **3** | **K.KDDTDDEIAK.Y** |
|  | 9031 | **434.7329** | **1301.1769** | **1300.7173** | **0.4596** | **1** | **10** | **9.1** | **3** | **K.LPGDKGLVLMSR.A** 8995 9020 |
|  | 9186 | **655.3398** | **1308.6650** | **1309.5860** | **-0.9210** | **1** | **2** | **95** | **4** | **K.WEVEEMKESK.L** |
|  | 11271 | **714.8528** | **1427.6911** | **1427.7004** | **-0.0093** | **1** | **6** | **38** | **2** | **K.AEEDEILNRSPR.N** |
|  | 12291 | **744.8549** | **1487.6953** | **1487.7045** | **-0.0092** | **0** | **44** | **0.0046** | **1** | **R.IVDDWANDGWGLK.K** |
|  | 14313 | **808.9028** | **1615.7910** | **1615.7995** | **-0.0085** | **1** | **51** | **0.001** | **1** | **R.IVDDWANDGWGLKK.A** 14311 14312 |
|  | 28899 | **926.2271** | **3700.8793** | **3700.8963** | **-0.0170** | **0** | **4** | **13** | **1** | **K.LNKPFLFDTKPLIVQYEVNFQNGIECGGAYVK.L** |
|  | 30485 | **1479.7445** | **4436.2117** | **4436.2322** | **-0.0205** | **0** | **32** | **0.012** | **1** | **K.THLYTLILNPDNSFEILVDQSVVNSGNLLNDMTPPVNPSR.E** |

| **248.** | Q15365\|PCBP1_HUMAN    **Mass:** 37987    **Score:** 121    **Queries matched:** 11   **emPAI:** 0.21 |
| --- | --- |
|  | Poly(rC)-binding protein 1 OS=Homo sapiens GN=PCBP1 PE=1 SV=2 |

|  | **Query** | **Observed** | **Mr(expt)** | **Mr(calc)** | **Delta** | **Miss** | **Score** | **Expect** | **Rank** | **Peptide** |
| --- | --- | --- | --- | --- | --- | --- | --- | --- | --- | --- |
|  | 3323 | **465.7680** | **929.5215** | **929.5546** | **-0.0331** | **1** | **16** | **5.1** | **5** | **K.EVGSIIGKK.G** |
|  | 8769 | **430.2997** | **1287.8773** | **1287.5877** | **0.2896** | **0** | **5** | **41** | **1** | **R.INISEGNCPER.I** |
|  | 10612 | **694.9070** | **1387.7995** | **1387.8075** | **-0.0080** | **0** | **45** | **0.0039** | **1** | **R.IITLTGPTNAIFK.A** |
|  | 11501 | **721.9012** | **1441.7878** | **1441.7963** | **-0.0085** | **0** | **5** | **44** | **1** | **R.LVVPATQCGSLIGK.G** |
|  | 12426 | **499.9808** | **1496.9206** | **1496.8385** | **0.0821** | **1** | **5** | **31** | **3** | **R.LLMHGKEVGSIIGK.K** 12418 |
|  | 15190 | **836.9459** | **1671.8773** | **1671.8866** | **-0.0093** | **0** | **4** | **44** | **1** | **R.AITIAGVPQSVTECVK.Q** |
|  | 17845 | 936.9914 | 1871.9682 | 1872.9476 | -0.9793 | 1 | 9 | 11 | 4 | R.QGANINEIRQMSGAQIK.I |
|  | 20183 | **1045.4883** | **2088.9620** | **2088.9746** | **-0.0126** | **0** | **71** | **7.4e-006** | **1** | **R.ESTGAQVQVAGDMLPNSTER.A** 20182 |
|  | 22231 | **582.2912** | **2325.1357** | **2324.2522** | **0.8835** | **1** | **2** | **49** | **1** | **M.DAGVTESGLNVTLTIRLLMHGK.E** |

| **249.** | P06756\|ITAV_HUMAN    **Mass:** 117048   **Score:** 120    **Queries matched:** 21   **emPAI:** 0.06 |
| --- | --- |
|  | Integrin alpha-V OS=Homo sapiens GN=ITGAV PE=1 SV=2 |

|  | **Query** | **Observed** | **Mr(expt)** | **Mr(calc)** | **Delta** | **Miss** | **Score** | **Expect** | **Rank** | **Peptide** |
| --- | --- | --- | --- | --- | --- | --- | --- | --- | --- | --- |
|  | 2449 | 434.8811 | 867.7476 | 868.4807 | -0.7331 | 0 | 5 | 28 | 10 | R.ALFLYSR.S |
|  | 2537 | **437.5446** | **873.0747** | **873.4643** | **-0.3896** | **1** | **11** | **11** | **3** | **-.MAFPPRR.R** 2524 2542 2551 |
|  | 2768 | **446.2583** | **890.5021** | **889.4592** | **1.0429** | **1** | **(10)** | **18** | **1** | **-.MAFPPRR.R** |
|  | 4259 | **500.8024** | **999.5903** | **998.5873** | **1.0030** | **0** | **28** | **0.28** | **1** | **K.AGTQLLAGLR.F** |
|  | 4863 | **524.2600** | **1046.5055** | **1045.4789** | **1.0266** | **0** | **3** | **98** | **4** | **K.NDTVAGQGER.D** |
|  | 9741 | **670.8052** | **1339.5959** | **1339.6296** | **-0.0336** | **1** | **0** | **1.3e+002** | **5** | **R.DYAKDDPLEFK.S** |
|  | 11276 | **476.9414** | **1427.8023** | **1428.7006** | **-0.8983** | **1** | **6** | **30** | **2** | **K.VSCFNVRFCLK.A** |
|  | 12370 | **747.1406** | **1492.2667** | **1492.7456** | **-0.4789** | **1** | **1** | **79** | **1** | **R.NNEALARLSCAFK.T** |
|  | 13624 | 787.1291 | 1572.2436 | 1572.8763 | -0.6327 | 0 | 16 | 2 | 1 | K.IYIGDDNPLTLIVK.A |
|  | 14631 | **546.0280** | **1635.0620** | **1633.9953** | **1.0667** | **0** | **2** | **51** | **4** | **R.GLPLLLSGLLLPLCR.A** |
|  | 17736 | **622.3218** | **1863.9435** | **1863.8785** | **0.0650** | **1** | **0** | **96** | **4** | **K.QEREPVGTCFLQDGTK.T** |
|  | 19270 | **999.5183** | **1997.0221** | **1997.0330** | **-0.0110** | **0** | **69** | **1.2e-005** | **1** | **R.STGLNAVPSQILEGQWAAR.S** 19271 |
|  | 20863 | **720.3992** | **2158.1759** | **2159.1336** | **-0.9578** | **1** | **2** | **53** | **3** | **K.SAILYVKSLLWTETFMNK.E** |
|  | 21384 | **555.7886** | **2219.1255** | **2220.0786** | **-0.9531** | **1** | **1** | **65** | **10** | **R.NNGPSSFSKAMLHLQWPYK.Y** |
|  | 25069 | **931.1564** | **2790.4475** | **2790.4592** | **-0.0118** | **0** | **22** | **0.4** | **1** | **R.VLLGGPGSFYWQGQLISDQVAEIVSK.Y** |
|  | 27468 | 1103.5216 | 3307.5430 | 3307.7717 | -0.2287 | 1 | 3 | 24 | 4 | K.VDLAVLAAVEIRGVSSPDHVFLPIPNWEHK.E |
|  | 30692 | **1530.3927** | **4588.1563** | **4588.1937** | **-0.0375** | **1** | **1** | **17** | **9** | **R.AFNLDVDSPAEYSGPEGSYFGFAVDFFVPSASSRMFLLVGAPK.A** |

| **250.** | P02671\|FIBA_HUMAN    **Mass:** 95656    **Score:** 119    **Queries matched:** 25   **emPAI:** 0.16 |
| --- | --- |
|  | Fibrinogen alpha chain OS=Homo sapiens GN=FGA PE=1 SV=2 |

|  | **Query** | **Observed** | **Mr(expt)** | **Mr(calc)** | **Delta** | **Miss** | **Score** | **Expect** | **Rank** | **Peptide** |
| --- | --- | --- | --- | --- | --- | --- | --- | --- | --- | --- |
|  | 3767 | **484.0637** | **966.1129** | **966.4155** | **-0.3026** | **0** | **9** | **12** | **2** | **R.GDFSSANNR.D** 3761 |
|  | 3825 | **486.2641** | **970.5136** | **970.5196** | **-0.0060** | **1** | **15** | **5.3** | **3** | **K.DLLPSRDR.Q** |
|  | 3931 | **489.2999** | **976.5852** | **976.4872** | **0.0980** | **1** | **7** | **41** | **3** | **R.GSCSRALAR.E** |
|  | 5049 | **531.7539** | **1061.4933** | **1061.4998** | **-0.0065** | **0** | **9** | **20** | **3** | **K.ALTDMPQMR.M** |
|  | 5233 | **539.7563** | **1077.4980** | **1077.4947** | **0.0033** | **0** | **(7)** | **30** | **8** | **K.ALTDMPQMR.M** |
|  | 6764 | **595.5643** | **1189.1140** | **1188.6285** | **0.4855** | **1** | **2** | **62** | **7** | **K.NVRAQLVDMK.R** |
|  | 11499 | **721.8947** | **1441.7749** | **1440.7799** | **0.9950** | **0** | **11** | **9.4** | **1** | **K.MKPVPDLVPGNFK.S** |
|  | 11765 | **729.8481** | **1457.6816** | **1456.7748** | **0.9068** | **0** | **(1)** | **98** | **4** | **K.MKPVPDLVPGNFK.S** |
|  | 12063 | **492.6004** | **1474.7794** | **1474.8732** | **-0.0938** | **1** | **4** | **51** | **3** | **K.VQHIQLLQKNVR.A** |
|  | 12489 | **751.3703** | **1500.7260** | **1500.7354** | **-0.0094** | **0** | **35** | **0.044** | **1** | **R.MELERPGGNEITR.G** |
|  | 13607 | **786.8375** | **1571.6605** | **1571.6700** | **-0.0095** | **0** | **42** | **0.0081** | **1** | **R.GGSTSYGTGSETESPR.N** 13606 |
|  | 13655 | 525.9266 | 1574.7579 | 1574.9508 | -0.1929 | 1 | 3 | 63 | 4 | K.VIEKVQHIQLLQK.N |
|  | 14773 | **549.2125** | **1644.6157** | **1644.8141** | **-0.1984** | **0** | **1** | **77** | **10** | **K.DSHSLTTNIMEILR.G** |
|  | 16020 | 865.8720 | 1729.7295 | 1729.7404 | -0.0109 | 1 | 19 | 1.3 | 1 | R.GDFSSANNRDNTYNR.V 16003 16019 |
|  | 19139 | **663.0047** | **1985.9923** | **1985.0000** | **0.9923** | **1** | **43** | **0.0051** | **1** | **K.NNKDSHSLTTNIMEILR.G** |
|  | 19302 | 667.6652 | 1999.9738 | 2000.9949 | -1.0211 | 1 | (8) | 15 | 2 | K.NNKDSHSLTTNIMEILR.G |
|  | 21771 | **1133.0261** | **2264.0377** | **2264.0460** | **-0.0083** | **0** | **46** | **0.0021** | **1** | **K.TFPGFFSPMLGEFVSETESR.G** 21772 |
|  | 28409 | **1186.1508** | **3555.4305** | **3555.7491** | **-0.3186** | **1** | **3** | **14** | **4** | **M.FSMRIVCLVLSVVGTAWTADSGEGDFLAEGGGVR.G** |
|  | 28426 | 1782.2015 | 3562.3885 | 3562.6973 | -0.3088 | 1 | 1 | 25 | 2 | K.IFSVYCDQETSLGGWLLIQQRMDGSLNFNR.T |
|  | 32162 | **1022.6232** | **6129.6954** | **6129.7272** | **-0.0318** | **1** | **2** | **4.5** | **1** | **R.GSAGHWTSESSVSGSTGQWHSESGSFRPDSPGSGNARPNNPDWGTFEEVSGNVSPGTRR.E** |

| **251.** | P31943\|HNRH1_HUMAN    **Mass:** 49484    **Score:** 119    **Queries matched:** 12   **emPAI:** 0.08 |
| --- | --- |
|  | Heterogeneous nuclear ribonucleoprotein H OS=Homo sapiens GN=HNRNPH1 PE=1 SV=4 |

|  | **Query** | **Observed** | **Mr(expt)** | **Mr(calc)** | **Delta** | **Miss** | **Score** | **Expect** | **Rank** | **Peptide** |
| --- | --- | --- | --- | --- | --- | --- | --- | --- | --- | --- |
|  | 3228 | 462.2209 | 922.4273 | 922.4443 | -0.0170 | 1 | 3 | 86 | 4 | R.GAGFERMR.R |
|  | 5429 | 546.3238 | 1090.6330 | 1091.5724 | -0.9393 | 0 | 3 | 93 | 9 | R.VHIEIGPDGR.V |
|  | 9451 | 662.3095 | 1322.6045 | 1322.6541 | -0.0496 | 0 | 28 | 0.24 | 2 | M.MLGTEGGEGFVVK.V |
|  | 9737 | **447.2723** | **1338.7951** | **1339.6633** | **-0.8682** | **1** | **13** | **7.3** | **1** | **R.AEVRTHYDPPR.K** |
|  | 13963 | 399.2196 | 1592.8495 | 1593.8185 | -0.9690 | 1 | 4 | 43 | 8 | M.MLGTEGGEGFVVKVR.G |
|  | 17419 | **921.4446** | **1840.8746** | **1840.8843** | **-0.0097** | **0** | **84** | **4e-007** | **1** | **R.STGEAFVQFASQEIAEK.A** 17418 |
|  | 18792 | **651.8234** | **1952.4485** | **1952.9453** | **-0.4968** | **1** | **2** | **42** | **1** | **K.HTGPNSPDTANDGFVRLR.G** |
|  | 19258 | **998.9877** | **1995.9608** | **1995.9690** | **-0.0082** | **0** | **3** | **49** | **2** | **R.ATENDIYNFFSPLNPVR.V** |
|  | 24201 | **527.8900** | **2634.4137** | **2635.2860** | **-0.8723** | **1** | **0** | **56** | **8** | **K.LMAMQRPGPYDRPGAGRGYNSIGR.G** |
|  | 27338 | 1089.8706 | 3266.5900 | 3266.5514 | 0.0386 | 1 | 1 | 39 | 8 | R.VHIEIGPDGRVTGEADVEFATHEDAVAAMSK.D 27355 |

| **252.** | P55795\|HNRH2_HUMAN    **Mass:** 49517    **Score:** 119    **Queries matched:** 16   **emPAI:** 0.08 |
| --- | --- |
|  | Heterogeneous nuclear ribonucleoprotein H2 OS=Homo sapiens GN=HNRNPH2 PE=1 SV=1 |

|  | **Query** | **Observed** | **Mr(expt)** | **Mr(calc)** | **Delta** | **Miss** | **Score** | **Expect** | **Rank** | **Peptide** |
| --- | --- | --- | --- | --- | --- | --- | --- | --- | --- | --- |
|  | 3228 | 462.2209 | 922.4273 | 922.4443 | -0.0170 | 1 | 3 | 86 | 4 | R.GAGFERMR.R |
|  | 3374 | 467.7511 | 933.4876 | 932.5444 | 0.9433 | 1 | 12 | 12 | 4 | R.EGFVVKVR.G |
|  | 3431 | 470.7280 | 939.4414 | 939.4154 | 0.0261 | 0 | 14 | 6 | 2 | -.MMLSTEGR.E 3442 3443 3450 |
|  | 3437 | **470.7484** | **939.4823** | **939.4154** | **0.0670** | **0** | **(12)** | **9.4** | **3** | **-.MMLSTEGR.E** 3441 |
|  | 5429 | 546.3238 | 1090.6330 | 1091.5724 | -0.9393 | 0 | 3 | 93 | 9 | R.VHIEIGPDGR.V |
|  | 9737 | 447.2723 | 1338.7951 | 1339.6633 | -0.8682 | 1 | 13 | 7.3 | 1 | R.AEVRTHYDPPR.K |
|  | 14475 | **542.7587** | **1625.2544** | **1624.8685** | **0.3858** | **1** | **4** | **31** | **1** | **K.IQNGTSGIRFIYTR.E** |
|  | 17419 | 921.4446 | 1840.8746 | 1840.8843 | -0.0097 | 0 | 84 | 4e-007 | 1 | R.STGEAFVQFASQEIAEK.A 17418 |
|  | 18792 | 651.8234 | 1952.4485 | 1952.9453 | -0.4968 | 1 | 2 | 42 | 1 | K.HTGPNSPDTANDGFVRLR.G |
|  | 24201 | 527.8900 | 2634.4137 | 2635.2860 | -0.8723 | 1 | 0 | 56 | 8 | K.LMAMQRPGPYDRPGAGRGYNSIGR.G |
|  | 25808 | **980.4695** | **2938.3868** | **2937.4583** | **0.9285** | **0** | **2** | **37** | **2** | **K.EEIVQFFSGLEIVPNGMTLPVDFQGR.S** |

| **253.** | P14927\|QCR7_HUMAN    **Mass:** 13522    **Score:** 119    **Queries matched:** 15   **emPAI:** 2.58 |
| --- | --- |
|  | Cytochrome b-c1 complex subunit 7 OS=Homo sapiens GN=UQCRB PE=1 SV=2 |

|  | **Query** | **Observed** | **Mr(expt)** | **Mr(calc)** | **Delta** | **Miss** | **Score** | **Expect** | **Rank** | **Peptide** |
| --- | --- | --- | --- | --- | --- | --- | --- | --- | --- | --- |
|  | 2731 | **444.2559** | **886.4972** | **886.5025** | **-0.0053** | **1** | **13** | **9.9** | **4** | **K.WLDGIRK.W** |
|  | 5983 | **567.8044** | **1133.5943** | **1133.5863** | **0.0081** | **1** | **14** | **7.2** | **5** | **-.MAGKQAVSASGK.W** 5967 |
|  | 6190 | 576.2883 | 1150.5621 | 1149.5812 | 0.9809 | 1 | (10) | 16 | 2 | -.MAGKQAVSASGK.W |
|  | 7650 | **617.2842** | **1232.5538** | **1232.5614** | **-0.0076** | **0** | **34** | **0.058** | **1** | **K.WYYNAAGFNK.L** 7677 |
|  | 8780 | **645.3296** | **1288.6446** | **1288.6524** | **-0.0077** | **1** | **43** | **0.0075** | **1** | **R.RLPENLYNDR.M** 8778 8782 |
|  | 10118 | **681.3311** | **1360.6475** | **1360.6564** | **-0.0088** | **1** | **34** | **0.056** | **1** | **R.KWYYNAAGFNK.L** |
|  | 12518 | 501.6375 | 1501.8907 | 1501.8980 | -0.0073 | 1 | 22 | 0.74 | 1 | R.ALDLNLKHQILPK.E |
|  | 16993 | **604.2792** | **1809.8159** | **1809.8268** | **-0.0109** | **1** | **6** | **23** | **1** | **R.DDTIYEDEDVKEAIR.R** |
|  | 17116 | **607.2084** | **1818.6033** | **1818.8875** | **-0.2842** | **1** | **1** | **61** | **5** | **K.WYYNAAGFNKLGLMR.D** |
|  | 22791 | 1205.0613 | 2408.1080 | 2408.1212 | -0.0132 | 1 | 66 | 1.8e-005 | 1 | K.EQWTKYEEENFYLEPYLK.E 22792 |

| **254.** | P19404\|NDUV2_HUMAN    **Mass:** 27659    **Score:** 119    **Queries matched:** 10   **emPAI:** 0.47 |
| --- | --- |
|  | NADH dehydrogenase [ubiquinone] flavoprotein 2, mitochondrial OS=Homo sapiens GN=NDUFV2 PE=1 SV=2 |

|  | **Query** | **Observed** | **Mr(expt)** | **Mr(calc)** | **Delta** | **Miss** | **Score** | **Expect** | **Rank** | **Peptide** |
| --- | --- | --- | --- | --- | --- | --- | --- | --- | --- | --- |
|  | 6666 | **593.3621** | **1184.7096** | **1184.6124** | **0.0971** | **1** | **1** | **1.2e+002** | **4** | **-.MFFSAALRAR.A** |
|  | 7316 | **608.8156** | **1215.6165** | **1215.6234** | **-0.0069** | **0** | **53** | **0.00078** | **1** | **K.DIEEIIDELK.A** |
|  | 7335 | **609.3185** | **1216.6224** | **1216.6299** | **-0.0075** | **0** | **34** | **0.057** | **1** | **R.NSDSILEAIQK.K** |
|  | 8069 | **627.2766** | **1252.5385** | **1253.6802** | **-1.1416** | **0** | **7** | **30** | **3** | **K.VAEVLQVPPMR.V** |
|  | 9853 | **449.2459** | **1344.7159** | **1344.7249** | **-0.0090** | **1** | **10** | **15** | **2** | **R.NSDSILEAIQKK.L** |
|  | 13614 | **524.9218** | **1571.7436** | **1572.7831** | **-1.0395** | **0** | **0** | **1.2e+002** | **7** | **K.TVMQNGAGGALFVHR.D** |
|  | 16634 | **593.9457** | **1778.8154** | **1778.8266** | **-0.0112** | **0** | **2** | **70** | **8** | **K.YHIQVCTTTPCMLR.N** |
|  | 24401 | **1337.6549** | **2673.2953** | **2673.3109** | **-0.0156** | **1** | **55** | **0.00017** | **1** | **R.FSCEPAGGLTSLTEPPKGPGFGVQAGL.-** 24402 |
|  | 28956 | **1240.8503** | **3719.5292** | **3718.7569** | **0.7723** | **0** | **2** | **16** | **6** | **K.LFTLIEVECLGACVNAPMVQINDNYYEDLTAK.D** |

| **255.** | P21810\|PGS1_HUMAN    **Mass:** 42027    **Score:** 118    **Queries matched:** 12   **emPAI:** 0.19 |
| --- | --- |
|  | Biglycan OS=Homo sapiens GN=BGN PE=1 SV=2 |

|  | **Query** | **Observed** | **Mr(expt)** | **Mr(calc)** | **Delta** | **Miss** | **Score** | **Expect** | **Rank** | **Peptide** |
| --- | --- | --- | --- | --- | --- | --- | --- | --- | --- | --- |
|  | 1857 | 415.2452 | 828.4759 | 828.4494 | 0.0265 | 1 | 17 | 4.4 | 3 | R.YSKLYR.L |
|  | 3227 | **462.2133** | **922.4120** | **922.5097** | **-0.0977** | **1** | **6** | **39** | **10** | **R.IHDNRIR.K** 3245 |
|  | 5806 | 559.7940 | 1117.5735 | 1117.5802 | -0.0067 | 0 | 43 | 0.009 | 1 | R.VVQCSDLGLK.S 5784 |
|  | 6948 | 400.2377 | 1197.6914 | 1196.6666 | 1.0248 | 1 | 5 | 44 | 7 | K.IHEKAFSPLR.K |
|  | 13133 | 771.8918 | 1541.7691 | 1541.7007 | 0.0684 | 0 | 5 | 37 | 10 | K.VGVNDFCPMGFGVK.R 13134 |
|  | 13958 | **531.7616** | **1592.2630** | **1592.8232** | **-0.5602** | **0** | **3** | **44** | **8** | **R.MIENGSLSFLPTLR.E** |
|  | 23797 | **639.7960** | **2555.1550** | **2556.3006** | **-1.1456** | **1** | **3** | **37** | **7** | **R.MIENGSLSFLPTLRELHLDNNK.L** |
|  | 28081 | **1153.1992** | **3456.5758** | **3456.5900** | **-0.0142** | **1** | **101** | **2.9e-009** | **1** | **R.NMNCIEMGGNPLENSGFEPGAFDGLKLNYLR.I** |
|  | 31941 | 1871.7090 | 5612.1051 | 5612.3136 | -0.2084 | 0 | 6 | 2.2 | 1 | R.GFWDFTLDDGPFMMNDEEASGADTSGVLDPDSVTPTYSAMCPFGCHCHLR.V |

| **256.** | P51648\|AL3A2_HUMAN    **Mass:** 55269    **Score:** 118    **Queries matched:** 22   **emPAI:** 0.30 |
| --- | --- |
|  | Fatty aldehyde dehydrogenase OS=Homo sapiens GN=ALDH3A2 PE=1 SV=1 |

|  | **Query** | **Observed** | **Mr(expt)** | **Mr(calc)** | **Delta** | **Miss** | **Score** | **Expect** | **Rank** | **Peptide** |
| --- | --- | --- | --- | --- | --- | --- | --- | --- | --- | --- |
|  | 1375 | **397.2119** | **792.4092** | **791.3847** | **1.0245** | **0** | **7** | **35** | **10** | **-.MELEVR.R** |
|  | 3203 | **461.0081** | **920.0016** | **919.4399** | **0.5616** | **0** | **4** | **42** | **6** | **R.YPPNSQSK.V** 3204 |
|  | 4260 | **500.8029** | **999.5913** | **999.5964** | **-0.0051** | **0** | **24** | **0.64** | **1** | **R.ILSLLEGQK.I** |
|  | 6729 | **595.2795** | **1188.5444** | **1188.6251** | **-0.0807** | **1** | **12** | **10** | **3** | **K.LRYPPNSQSK.V** 6772 |
|  | 8308 | **632.8630** | **1263.7114** | **1263.7187** | **-0.0073** | **0** | **9** | **17** | **1** | **K.HLTPVTLELGGK.S** |
|  | 11332 | **716.3860** | **1430.7574** | **1430.7657** | **-0.0083** | **0** | **48** | **0.0024** | **1** | **R.YIAPTVLTDVDPK.T** |
|  | 11366 | **717.3505** | **1432.6864** | **1432.6946** | **-0.0083** | **0** | **32** | **0.076** | **1** | **K.NVDEAINFINER.E** |
|  | 13568 | **524.2197** | **1569.6374** | **1568.7624** | **0.8750** | **0** | **17** | **2.2** | **1** | **R.FDHIFYTGNTAVGK.I** |
|  | 15361 | 561.6353 | 1681.8841 | 1681.8940 | -0.0099 | 0 | 2 | 74 | 5 | R.EKPLALYVFSHNHK.L |
|  | 17841 | **468.9819** | **1871.8985** | **1871.9101** | **-0.0116** | **0** | **9** | **11** | **1** | **K.HSFDTFSHQRPCLLK.S** 17826 |
|  | 20400 | 704.0222 | 2109.0448 | 2109.3541 | -0.3092 | 1 | 4 | 39 | 6 | K.EKLGLLLLTFLGIVAAVLVK.A |
|  | 24491 | **893.7833** | **2678.3282** | **2678.3439** | **-0.0158** | **1** | **10** | **6.3** | **1** | **K.IAFGGETDEATRYIAPTVLTDVDPK.T** |
|  | 27376 | **1092.4996** | **3274.4771** | **3274.4919** | **-0.0149** | **0** | **28** | **0.06** | **1** | **K.YMNCGQTCIAPDYILCEASLQNQIVWK.I** |
|  | 27534 | **1108.5906** | **3322.7499** | **3322.7635** | **-0.0136** | **1** | **65** | **1.3e-005** | **1** | **K.VMQEEIFGPILPIVPVKNVDEAINFINER.E** 27533 27535 |
|  | 29790 | **1016.2132** | **4060.8237** | **4060.8394** | **-0.0157** | **0** | **12** | **1.7** | **1** | **R.MIDETSSGGVTGNDVIMHFTLNSFPFGGVGSSGMGAYHGK.H** |
|  | 30220 | **854.0212** | **4265.0698** | **4264.9253** | **0.1445** | **1** | **1** | **18** | **1** | **K.RMIDETSSGGVTGNDVIMHFTLNSFPFGGVGSSGMGAYHGK.H** |
|  | 32113 | **1193.3811** | **5961.8691** | **5962.7237** | **-0.8546** | **1** | **4** | **2.8** | **2** | **R.MIDETSSGGVTGNDVIMHFTLNSFPFGGVGSSGMGAYHGKHSFDTFSHQRPCLLK.S** |

| **257.** | P00395\|COX1_HUMAN    **Mass:** 57060    **Score:** 118    **Queries matched:** 10   **emPAI:** 0.21 |
| --- | --- |
|  | Cytochrome c oxidase subunit 1 OS=Homo sapiens GN=MT-CO1 PE=1 SV=1 |

|  | **Query** | **Observed** | **Mr(expt)** | **Mr(calc)** | **Delta** | **Miss** | **Score** | **Expect** | **Rank** | **Peptide** |
| --- | --- | --- | --- | --- | --- | --- | --- | --- | --- | --- |
|  | 12811 | 760.8964 | 1519.7782 | 1520.7524 | -0.9743 | 1 | 5 | 44 | 5 | M.FADRWLFSTNHK.D |
|  | 16703 | **595.5643** | **1783.6710** | **1782.9590** | **0.7120** | **0** | **7** | **17** | **6** | **R.AYFTSATMIIAIPTGVK.V** |
|  | 29135 | **1262.5789** | **3784.7148** | **3784.7325** | **-0.0178** | **0** | **74** | **1.3e-006** | **1** | **K.VLMVEEPSMNLEWLYGCPPPYHTFEEPVYMK.S** 29138 |
|  | 29173 | **1267.9136** | **3800.7189** | **3800.7275** | **-0.0086** | **0** | **(40)** | **0.0031** | **1** | **K.VLMVEEPSMNLEWLYGCPPPYHTFEEPVYMK.S** |
|  | 29356 | **1291.5900** | **3871.7481** | **3871.7646** | **-0.0165** | **1** | **28** | **0.047** | **1** | **K.VLMVEEPSMNLEWLYGCPPPYHTFEEPVYMKS.-** 29355 29357 |
|  | 30564 | **899.0403** | **4490.1653** | **4490.0166** | **0.1488** | **1** | **2** | **13** | **1** | **K.KEPFGYMGMVWAMMSIGFLGFIVWAHHMFTVGMDVDTR.A** 30562 |

| **258.** | O00483\|NDUA4_HUMAN    **Mass:** 9421     **Score:** 118    **Queries matched:** 9   **emPAI:** 1.92 |
| --- | --- |
|  | NADH dehydrogenase [ubiquinone] 1 alpha subcomplex subunit 4 OS=Homo sapiens GN=NDUFA4 PE=1 SV=1 |

|  | **Query** | **Observed** | **Mr(expt)** | **Mr(calc)** | **Delta** | **Miss** | **Score** | **Expect** | **Rank** | **Peptide** |
| --- | --- | --- | --- | --- | --- | --- | --- | --- | --- | --- |
|  | 3370 | 467.7319 | 933.4493 | 933.4556 | -0.0063 | 0 | 65 | 5.6e-005 | 1 | K.LGPNDQYK.F 3371 3372 |
|  | 4223 | **499.7353** | **997.4561** | **997.4617** | **-0.0056** | **0** | **3** | **79** | **8** | **R.NNPEPWNK.L** |
|  | 7410 | **611.2893** | **1220.5641** | **1220.5713** | **-0.0073** | **0** | **52** | **0.00099** | **1** | **K.FYSVNVDYSK.L** 7388 7404 7412 |
|  | 20627 | **713.0081** | **2136.0025** | **2136.0164** | **-0.0138** | **1** | **2** | **49** | **2** | **K.LGPNDQYKFYSVNVDYSK.L** |

| **259.** | P00387\|NB5R3_HUMAN    **Mass:** 34441    **Score:** 117    **Queries matched:** 14   **emPAI:** 0.23 |
| --- | --- |
|  | NADH-cytochrome b5 reductase 3 OS=Homo sapiens GN=CYB5R3 PE=1 SV=3 |

|  | **Query** | **Observed** | **Mr(expt)** | **Mr(calc)** | **Delta** | **Miss** | **Score** | **Expect** | **Rank** | **Peptide** |
| --- | --- | --- | --- | --- | --- | --- | --- | --- | --- | --- |
|  | 1166 | **699.4105** | **698.4032** | **698.4075** | **-0.0043** | **0** | **11** | **13** | **8** | **K.SNPIIR.T** |
|  | 5881 | **563.3185** | **1124.6224** | **1125.5891** | **-0.9666** | **1** | **9** | **18** | **9** | **R.EIISHDTRR.F** 5896 |
|  | 11942 | **734.3944** | **1466.7743** | **1466.7841** | **-0.0099** | **1** | **14** | **5.2** | **1** | **R.LIDREIISHDTR.R** |
|  | 18155 | 951.0157 | 1900.0168 | 1900.0274 | -0.0106 | 0 | 55 | 0.00033 | 1 | K.SVGMIAGGTGITPMLQVIR.A 18154 |
|  | 20122 | **694.3472** | **2080.0199** | **2078.9289** | **1.0910** | **0** | **4** | **39** | **1** | **K.MSQYLESMQIGDTIEFR.G** |
|  | 21615 | **562.3004** | **2245.1726** | **2244.2334** | **0.9392** | **1** | **5** | **24** | **3** | **R.TVKSVGMIAGGTGITPMLQVIR.A** |
|  | 25225 | 940.0870 | 2817.2393 | 2816.5602 | 0.6791 | 1 | 3 | 26 | 6 | R.FRFALPSPQHILGLPVGQHIYLSAR.I |
|  | 25976 | **744.6447** | **2974.5497** | **2974.5652** | **-0.0154** | **1** | **14** | **2.2** | **1** | **R.IDGNLVVRPYTPISSDDDKGFVDLVIK.V** 25975 |
|  | 26828 | **1049.8490** | **3146.5252** | **3146.5416** | **-0.0165** | **1** | **65** | **1.4e-005** | **1** | **K.MSQYLESMQIGDTIEFRGPSGLLVYQGK.G** |
|  | 27959 | 857.4577 | 3425.8017 | 3425.8219 | -0.0202 | 1 | 3 | 21 | 3 | -.MGAQLSTLGHMVLFPVWFLYSLLMKLFQR.S |
|  | 30343 | **1089.7689** | **4355.0466** | **4355.0636** | **-0.0170** | **0** | **5** | **5.9** | **1** | **R.DHLPPPEEEPLVLMCGPPPMIQYACLPNLDHVGHPTER.C** |

| **260.** | Q02252\|MMSA_HUMAN    **Mass:** 58259    **Score:** 117    **Queries matched:** 23   **emPAI:** 0.06 |
| --- | --- |
|  | Methylmalonate-semialdehyde dehydrogenase [acylating], mitochondrial OS=Homo sapiens GN=ALDH6A1 PE=1 SV=2 |

|  | **Query** | **Observed** | **Mr(expt)** | **Mr(calc)** | **Delta** | **Miss** | **Score** | **Expect** | **Rank** | **Peptide** |
| --- | --- | --- | --- | --- | --- | --- | --- | --- | --- | --- |
|  | 3884 | **487.7598** | **973.5051** | **973.5127** | **-0.0076** | **1** | **14** | **8.3** | **2** | **K.RVQANMGAK.N** |
|  | 4023 | 492.7734 | 983.5323 | 983.4712 | 0.0610 | 0 | 9 | 19 | 2 | K.AGEYIFER.G |
|  | 4269 | 501.2606 | 1000.5067 | 1000.6281 | -0.1214 | 1 | 3 | 86 | 5 | R.ILQVSSKVK.S |
|  | 4653 | **515.7771** | **1029.5396** | **1029.5455** | **-0.0058** | **0** | **8** | **26** | **2** | **K.EGASILLDGR.K** |
|  | 4993 | **529.3798** | **1056.7451** | **1056.6113** | **0.1337** | **0** | **5** | **53** | **10** | **-.MAALLAAAAVR.A** 4985 |
|  | 7416 | **611.3214** | **1220.6283** | **1220.6553** | **-0.0271** | **0** | **3** | **84** | **5** | **K.WLPELVEHAK.N** |
|  | 9452 | **441.8762** | **1322.6067** | **1321.6118** | **0.9949** | **1** | **10** | **14** | **10** | **K.AEMDAAIASCKR.A** |
|  | 9724 | **447.2100** | **1338.6081** | **1337.6067** | **1.0013** | **1** | **(8)** | **23** | **6** | **K.AEMDAAIASCKR.A** |
|  | 11617 | **725.3401** | **1448.6657** | **1448.7367** | **-0.0710** | **0** | **(4)** | **54** | **10** | **R.CMALSTAVLVGEAK.K** |
|  | 11875 | **732.8453** | **1463.6761** | **1464.7316** | **-1.0555** | **0** | **11** | **11** | **3** | **R.CMALSTAVLVGEAK.K** 11912 |
|  | 14125 | **535.6227** | **1603.8464** | **1603.8834** | **-0.0370** | **1** | **10** | **12** | **2** | **K.WLPELVEHAKNLR.V** |
|  | 16937 | **452.2407** | **1804.9337** | **1805.8651** | **-0.9315** | **1** | **11** | **9.4** | **2** | **R.VPQATKAEMDAAIASCK.R** |
|  | 17063 | 908.4327 | 1814.8509 | 1815.9227 | -1.0718 | 0 | 7 | 20 | 7 | K.ENTLNQLVGAAFGAAGQR.C |
|  | 18888 | **981.5122** | **1961.0099** | **1961.0218** | **-0.0119** | **0** | **77** | **2e-006** | **1** | **R.VNAGDQPGADLGPLITPQAK.E** 18890 |
|  | 19998 | **689.3206** | **2064.9399** | **2064.0388** | **0.9010** | **1** | **1** | **69** | **6** | **K.SDKWIDIHNPATNEVIGR.V** |
|  | 25769 | **977.8148** | **2930.4225** | **2929.4466** | **0.9758** | **1** | **2** | **34** | **9** | **K.VKGYENGNFVGPTIISNVKPNMTCYK.E** |
|  | 27170 | **1075.4231** | **3223.2475** | **3223.7216** | **-0.4742** | **1** | **0** | **29** | **4** | **R.KYAHLVDVGQVGVNVPIPVPLPMFSFTGSR.S** |
|  | 30610 | **755.3908** | **4526.3012** | **4527.1970** | **-0.8958** | **0** | **3** | **10** | **1** | **R.LPLGVCAGIAPFNFPAMIPLWMFPMAMVCGNTFLMKPSER.V** |
|  | 31774 | **794.8419** | **5556.8421** | **5556.7499** | **0.0922** | **1** | **1** | **6.3** | **2** | **R.LPLGVCAGIAPFNFPAMIPLWMFPMAMVCGNTFLMKPSERVPGATMLLAK.L** |
|  | 31923 | **931.9654** | **5585.7487** | **5586.6302** | **-0.8815** | **1** | **0** | **9.2** | **1** | **K.DMDLYSYRLPLGVCAGIAPFNFPAMIPLWMFPMAMVCGNTFLMKPSER.V** |

| **261.** | O75380\|NDUS6_HUMAN    **Mass:** 14045    **Score:** 117    **Queries matched:** 10   **emPAI:** 2.41 |
| --- | --- |
|  | NADH dehydrogenase [ubiquinone] iron-sulfur protein 6, mitochondrial OS=Homo sapiens GN=NDUFS6 PE=1 SV=1 |

|  | **Query** | **Observed** | **Mr(expt)** | **Mr(calc)** | **Delta** | **Miss** | **Score** | **Expect** | **Rank** | **Peptide** |
| --- | --- | --- | --- | --- | --- | --- | --- | --- | --- | --- |
|  | 1197 | **717.4090** | **716.4017** | **716.3705** | **0.0313** | **0** | **3** | **1.2e+002** | **5** | **R.VSPTGEK.V** |
|  | 3285 | **464.2947** | **926.5748** | **926.4102** | **0.1646** | **0** | **13** | **7.9** | **3** | **M.AAAMTFCR.L** |
|  | 7433 | **611.8340** | **1221.6534** | **1221.6605** | **-0.0071** | **1** | **39** | **0.019** | **1** | **K.VYINLDKETK.T** |
|  | 10927 | **704.8497** | **1407.6848** | **1407.6929** | **-0.0081** | **0** | **48** | **0.0022** | **1** | **R.VIACDGGGGALGHPK.V** 10945 |
|  | 11111 | **710.3066** | **1418.5987** | **1418.6071** | **-0.0084** | **0** | **48** | **0.002** | **1** | **K.TGTCGYCGLQFR.Q** 11110 |
|  | 15551 | **566.2651** | **1695.7734** | **1695.7853** | **-0.0119** | **1** | **30** | **0.12** | **1** | **K.VTHTGQVYDDKDYR.R** 15552 |
|  | 24897 | **920.1301** | **2757.3686** | **2757.3821** | **-0.0135** | **1** | **36** | **0.015** | **1** | **R.QKEVNENFAIDLIAEQPVSEVETR.V** |

| **262.** | P04040\|CATA_HUMAN    **Mass:** 59947    **Score:** 117    **Queries matched:** 23   **emPAI:** 0.35 |
| --- | --- |
|  | Catalase OS=Homo sapiens GN=CAT PE=1 SV=3 |

|  | **Query** | **Observed** | **Mr(expt)** | **Mr(calc)** | **Delta** | **Miss** | **Score** | **Expect** | **Rank** | **Peptide** |
| --- | --- | --- | --- | --- | --- | --- | --- | --- | --- | --- |
|  | 3796 | **485.2603** | **968.5060** | **967.5815** | **0.9245** | **0** | **7** | **27** | **10** | **K.LNVITVGPR.G** 3782 |
|  | 3896 | **487.9712** | **973.9278** | **973.4828** | **0.4450** | **0** | **2** | **79** | **7** | **K.NLSVEDAAR.L** |
|  | 6254 | 577.8218 | 1153.6290 | 1153.5914 | 0.0377 | 0 | 5 | 50 | 4 | R.LCENIAGHLK.D |
|  | 8891 | **432.2203** | **1293.6389** | **1293.5561** | **0.0829** | **0** | **8** | **22** | **3** | **R.HMNGYGSHTFK.L** |
|  | 9714 | **669.8503** | **1337.6861** | **1336.6445** | **1.0416** | **0** | **4** | **50** | **6** | **K.LVNANGEAVYCK.F** 9699 |
|  | 12162 | **741.3680** | **1480.7215** | **1480.7310** | **-0.0095** | **0** | **34** | **0.053** | **1** | **R.AFYVNVLNEEQR.K** |
|  | 12197 | **495.0605** | **1482.1596** | **1482.6951** | **-0.5354** | **0** | **4** | **33** | **4** | **R.FSTVAGESGSADTVR.D** 12196 |
|  | 12374 | **747.3480** | **1492.6814** | **1492.6906** | **-0.0093** | **0** | **44** | **0.0054** | **1** | **R.FNTANDDNVTQVR.A** |
|  | 14021 | **533.2535** | **1596.7386** | **1595.9399** | **0.7987** | **1** | **5** | **36** | **1** | **K.DYPLIPVGKLVLNR.N** 13996 |
|  | 14333 | **405.2181** | **1616.8433** | **1615.8165** | **1.0268** | **1** | **9** | **15** | **2** | **K.TDQGIKNLSVEDAAR.L** 14308 |
|  | 17015 | 906.4746 | 1810.9345 | 1811.9377 | -1.0032 | 1 | 0 | 96 | 4 | R.AAQKADVLTTGAGNPVGDK.L |
|  | 17584 | **617.9621** | **1850.8645** | **1850.8759** | **-0.0114** | **1** | **25** | **0.35** | **1** | **R.FSTVAGESGSADTVRDPR.G** 17583 |
|  | 19399 | **671.1026** | **2010.2860** | **2009.9279** | **0.3581** | **1** | **2** | **40** | **2** | **R.GIPDGHRHMNGYGSHTFK.L** |
|  | 27992 | **858.8884** | **3431.5246** | **3431.6213** | **-0.0967** | **0** | **0** | **35** | **4** | **K.YPSWTFYIQVMTFNQAETFPFNPFDLTK.V** |
|  | 29106 | **1257.9562** | **3770.8467** | **3770.8614** | **-0.0147** | **1** | **63** | **1.7e-005** | **1** | **K.LVLNRNPVNYFAEVEQIAFDPSNMPPGIEASPDK.M** |
|  | 30798 | **1161.5625** | **4642.2209** | **4641.9860** | **0.2349** | **0** | **3** | **8.9** | **2** | **R.DGPMCMQDNQGGAPNYYPNSFGAPEQQPSALEHSIQYSGEVR.R** |
|  | 30962 | **1196.5225** | **4782.0607** | **4782.0922** | **-0.0314** | **1** | **38** | **0.0021** | **1** | **R.DGPMCMQDNQGGAPNYYPNSFGAPEQQPSALEHSIQYSGEVRR.F** |

| **263.** | O14817\|TSN4_HUMAN    **Mass:** 26841    **Score:** 117    **Queries matched:** 3   **emPAI:** 0.14 |
| --- | --- |
|  | Tetraspanin-4 OS=Homo sapiens GN=TSPAN4 PE=1 SV=1 |

|  | **Query** | **Observed** | **Mr(expt)** | **Mr(calc)** | **Delta** | **Miss** | **Score** | **Expect** | **Rank** | **Peptide** |
| --- | --- | --- | --- | --- | --- | --- | --- | --- | --- | --- |
|  | 8012 | **417.2435** | **1248.7086** | **1248.6462** | **0.0624** | **1** | **8** | **22** | **2** | **K.IDRYAQQDLK.K** |
|  | 24908 | **921.1359** | **2760.3858** | **2760.3984** | **-0.0126** | **0** | **79** | **7.6e-007** | **1** | **K.GLHLYGTQGNVGLTNAWSIIQTDFR.C** 24909 |

| **264.** | P05062\|ALDOB_HUMAN    **Mass:** 39961    **Score:** 117    **Queries matched:** 31   **emPAI:** 0.71 |
| --- | --- |
|  | Fructose-bisphosphate aldolase B OS=Homo sapiens GN=ALDOB PE=1 SV=2 |

|  | **Query** | **Observed** | **Mr(expt)** | **Mr(calc)** | **Delta** | **Miss** | **Score** | **Expect** | **Rank** | **Peptide** |
| --- | --- | --- | --- | --- | --- | --- | --- | --- | --- | --- |
|  | 1249 | **762.4305** | **761.4233** | **762.4640** | **-1.0407** | **0** | **12** | **15** | **2** | **K.VLAAVYK.A** |
|  | 3468 | 472.2792 | 942.5439 | 941.5910 | 0.9530 | 1 | 18 | 2.6 | 3 | K.EKGIVVGIK.L |
|  | 3733 | **482.7175** | **963.4205** | **963.4266** | **-0.0061** | **0** | **44** | **0.0074** | **1** | **R.AMANCQAAK.G** 3730 3732 3734 3736 3738 |
|  | 5053 | **531.8575** | **1061.7005** | **1060.5553** | **1.1452** | **0** | **1** | **1.2e+002** | **3** | **R.FPALTQEQK.K** |
|  | 5155 | **536.1630** | **1070.3114** | **1069.4750** | **0.8364** | **0** | **1** | **90** | **2** | **K.EATQEAFMK.R** |
|  | 5258 | 540.7666 | 1079.5186 | 1078.5196 | 0.9990 | 1 | 13 | 7.1 | 1 | K.DGVDFGKWR.A |
|  | 5816 | 560.2860 | 1118.5575 | 1119.5277 | -0.9702 | 1 | 7 | 31 | 1 | K.RAMANCQAAK.G |
|  | 7501 | **409.2285** | **1224.6636** | **1225.5761** | **-0.9125** | **1** | **6** | **39** | **7** | **K.EATQEAFMKR.A** |
|  | 7800 | 620.8118 | 1239.6091 | 1240.6412 | -1.0321 | 0 | 8 | 20 | 3 | K.LDQGGAPLAGTNK.E 7833 |
|  | 11793 | **730.3810** | **1458.7474** | **1457.7725** | **0.9749** | **0** | **31** | **0.11** | **1** | **K.ELSEIAQSIVANGK.G** |
|  | 12326 | **745.8620** | **1489.7094** | **1489.7195** | **-0.0100** | **0** | **77** | **2.5e-006** | **1** | **K.GILAADESVGTMGNR.L** |
|  | 12573 | **503.1047** | **1506.2923** | **1505.7144** | **0.5779** | **0** | **(4)** | **33** | **3** | **K.GILAADESVGTMGNR.L** 12571 12575 |
|  | 18200 | **635.4981** | **1903.4723** | **1902.9581** | **0.5142** | **1** | **4** | **29** | **3** | **K.GILAADESVGTMGNRLQR.I** |
|  | 18325 | **479.7540** | **1914.9868** | **1914.9986** | **-0.0118** | **1** | **9** | **13** | **2** | **K.KYTPEQVAMATVTALHR.T** |
|  | 18541 | 644.5974 | 1930.7702 | 1930.9935 | -0.2232 | 1 | (3) | 49 | 7 | K.KYTPEQVAMATVTALHR.T |
|  | 20694 | **715.0536** | **2142.1391** | **2141.0535** | **1.0857** | **0** | **1** | **76** | **8** | **R.IADQCPSSLAIQENANALAR.Y** |
|  | 26041 | **748.3761** | **2989.4753** | **2989.4902** | **-0.0149** | **0** | **29** | **0.071** | **1** | **K.ALNDHHVYLEGTLLKPNMVTAGHACTK.K** 26039 |
|  | 29769 | **1014.2241** | **4052.8674** | **4051.8932** | **0.9742** | **0** | **19** | **0.29** | **1** | **R.YASICQQNGLVPIVEPEVIPDGDHDLEHCQYVTEK.V** 29765 29768 |
|  | 29848 | **1361.0204** | **4080.0393** | **4080.0522** | **-0.0129** | **0** | **29** | **0.034** | **1** | **R.TVPAAVPGICFLSGGMSEEDATLNLNAINLCPLPKPWK.L** 29847 |

| **265.** | P61247\|RS3A_HUMAN    **Mass:** 30154    **Score:** 116    **Queries matched:** 16   **emPAI:** 0.42 |
| --- | --- |
|  | 40S ribosomal protein S3a OS=Homo sapiens GN=RPS3A PE=1 SV=2 |

|  | **Query** | **Observed** | **Mr(expt)** | **Mr(calc)** | **Delta** | **Miss** | **Score** | **Expect** | **Rank** | **Peptide** |
| --- | --- | --- | --- | --- | --- | --- | --- | --- | --- | --- |
|  | 1496 | 401.2846 | 800.5547 | 799.4413 | 1.1135 | 1 | 20 | 2 | 3 | K.RNNQIR.K |
|  | 3154 | 459.2442 | 916.4738 | 915.5138 | 0.9600 | 1 | 11 | 17 | 1 | K.IASDGLKGR.V |
|  | 4953 | **528.2852** | **1054.5558** | **1054.4973** | **0.0585** | **1** | **7** | **32** | **1** | **K.KMMEIMTR.E** |
|  | 7331 | **609.3001** | **1216.5855** | **1216.5949** | **-0.0093** | **0** | **69** | **2.1e-005** | **1** | **K.TSYAQHQQVR.Q** |
|  | 7653 | **617.3027** | **1232.5908** | **1232.5997** | **-0.0089** | **1** | **45** | **0.0049** | **1** | **K.ATGDETGAKVER.A** 7645 7670 |
|  | 9509 | 663.8647 | 1325.7148 | 1326.7395 | -1.0246 | 1 | 5 | 44 | 6 | K.LIPDSIGKDIEK.A |
|  | 9601 | **444.5442** | **1330.6107** | **1330.6187** | **-0.0080** | **0** | **8** | **25** | **7** | **K.LMELHGEGSSSGK.A** |
|  | 9837 | **449.2133** | **1344.6182** | **1344.6898** | **-0.0716** | **1** | **6** | **33** | **7** | **R.KTSYAQHQQVR.Q** 9822 |
|  | 12732 | **758.3998** | **1514.7851** | **1514.7940** | **-0.0089** | **1** | **56** | **0.00034** | **1** | **R.EVQTNDLKEVVNK.L** |
|  | 15682 | 569.2869 | 1704.8388 | 1703.8454 | 0.9934 | 0 | 27 | 0.23 | 1 | K.ACQSIYPLHDVFVR.K |
|  | 17628 | **619.3200** | **1854.9382** | **1853.8685** | **1.0697** | **1** | **6** | **27** | **4** | **K.MMEIMTREVQTNDLK.E** |
|  | 18422 | **481.5254** | **1922.0726** | **1920.9251** | **1.1475** | **1** | **10** | **8.9** | **4** | **K.FELGKLMELHGEGSSSGK.A** |
|  | 20107 | **694.0248** | **2079.0527** | **2079.0637** | **-0.0110** | **1** | **10** | **9** | **1** | **R.VFEVSLADLQNDEVAFRK.F** |

| **266.** | P17858\|K6PL_HUMAN    **Mass:** 85762    **Score:** 113    **Queries matched:** 22   **emPAI:** 0.09 |
| --- | --- |
|  | 6-phosphofructokinase, liver type OS=Homo sapiens GN=PFKL PE=1 SV=6 |

|  | **Query** | **Observed** | **Mr(expt)** | **Mr(calc)** | **Delta** | **Miss** | **Score** | **Expect** | **Rank** | **Peptide** |
| --- | --- | --- | --- | --- | --- | --- | --- | --- | --- | --- |
|  | 1180 | 709.1945 | 708.1872 | 707.3602 | 0.8270 | 0 | 7 | 28 | 6 | R.LGFDTR.V |
|  | 2564 | **438.7480** | **875.4814** | **875.4422** | **0.0391** | **0** | **9** | **28** | **4** | **-.MAAVDLEK.L** |
|  | 2991 | **453.8384** | **905.6623** | **906.4196** | **-0.7573** | **0** | **8** | **25** | **5** | **R.GGTPSAFDR.I** |
|  | 3075 | **457.2397** | **912.4649** | **913.4215** | **-0.9566** | **1** | **5** | **51** | **4** | **R.TLSMDKGF.-** |
|  | 3167 | **460.0161** | **918.0177** | **918.3832** | **-0.3654** | **0** | **3** | **67** | **8** | **K.DTDFEHR.M** |
|  | 3292 | **464.7792** | **927.5439** | **927.5501** | **-0.0063** | **1** | **4** | **69** | **9** | **R.GLVLRNEK.C** |
|  | 5349 | **543.7613** | **1085.5081** | **1085.5175** | **-0.0094** | **0** | **9** | **23** | **2** | **K.VNVEHMTEK.M** |
|  | 8167 | **420.1982** | **1257.5726** | **1256.6724** | **0.9002** | **0** | **22** | **0.84** | **1** | **K.GQLESIVENIR.I** |
|  | 8266 | **631.3586** | **1260.7026** | **1261.6852** | **-0.9826** | **1** | **9** | **18** | **4** | **K.AMLWLSEKLR.E** |
|  | 8632 | **641.3083** | **1280.6020** | **1280.6911** | **-0.0891** | **1** | **2** | **85** | **5** | **R.AVTRMGIYVGAK.V** |
|  | 8767 | 430.2594 | 1287.7565 | 1287.7663 | -0.0098 | 1 | 19 | 1.7 | 1 | K.LLAHQKPPKEK.S 8766 |
|  | 8788 | 645.3424 | 1288.6701 | 1288.7391 | -0.0689 | 1 | 4 | 52 | 9 | K.AVAFSPVTELKK.D |
|  | 10132 | **454.7214** | **1361.1424** | **1360.6479** | **0.4945** | **1** | **4** | **35** | **3** | **K.VNVEHMTEKMK.T** |
|  | 10196 | **455.9706** | **1364.8900** | **1364.6502** | **0.2398** | **0** | **3** | **52** | **8** | **R.LPLMECVQMTK.E** |
|  | 13453 | **781.4008** | **1560.7871** | **1560.7970** | **-0.0099** | **0** | **60** | **0.00012** | **1** | **R.VFANAPDSACVIGLK.K** |
|  | 16731 | **894.4530** | **1786.8914** | **1786.8996** | **-0.0081** | **0** | **76** | **2.5e-006** | **1** | **K.AIGVLTSGGDAQGMNAAVR.A** |
|  | 16890 | **601.6373** | **1801.8900** | **1802.8945** | **-1.0045** | **0** | **(0)** | **99** | **9** | **K.AIGVLTSGGDAQGMNAAVR.A** |
|  | 20702 | **1072.5612** | **2143.1078** | **2143.1208** | **-0.0130** | **0** | **24** | **0.34** | **1** | **K.SNFSLAILNVGAPAAGMNAAVR.S** |
|  | 27046 | **640.3002** | **3196.4645** | **3195.4920** | **0.9725** | **0** | **3** | **22** | **10** | **K.MGMEAVMALLEATPDTPACVVTLSGNQSVR.L** 27042 |
|  | 31615 | **1079.1222** | **5390.5746** | **5390.5978** | **-0.0233** | **1** | **3** | **5.1** | **1** | **R.VFIVETMGGYCGYLATVTGIAVGADAAYVFEDPFNIHDLKVNVEHMTEK.M** |

| **267.** | Q12955\|ANK3_HUMAN    **Mass:** 482394   **Score:** 113    **Queries matched:** 92   **emPAI:** 0.02 |
| --- | --- |
|  | Ankyrin-3 OS=Homo sapiens GN=ANK3 PE=1 SV=3 |

|  | **Query** | **Observed** | **Mr(expt)** | **Mr(calc)** | **Delta** | **Miss** | **Score** | **Expect** | **Rank** | **Peptide** |
| --- | --- | --- | --- | --- | --- | --- | --- | --- | --- | --- |
|  | 951 | **603.3413** | **602.3340** | **602.3275** | **0.0065** | **0** | **23** | **1.5** | **2** | **K.EDLVK.V** |
|  | 1762 | **411.7164** | **821.4182** | **822.3766** | **-0.9584** | **1** | **4** | **67** | **9** | **R.GGSMRGSR.H** |
|  | 1871 | **415.7500** | **829.4854** | **829.4658** | **0.0196** | **0** | **0** | **2e+002** | **10** | **R.VTGGLLDR.L** |
|  | 1878 | **416.2296** | **830.4447** | **829.3462** | **1.0985** | **0** | **7** | **49** | **2** | **K.MFEMTR.S** 1863 |
|  | 1983 | **419.3138** | **836.6131** | **835.5643** | **1.0487** | **1** | **2** | **83** | **10** | **R.IIIPPRK.C** |
|  | 2121 | **424.0557** | **846.0968** | **845.3412** | **0.7556** | **0** | **(3)** | **79** | **8** | **K.MFEMTR.S** |
|  | 2714 | **443.4206** | **884.8266** | **884.5443** | **0.2823** | **1** | **0** | **1.1e+002** | **3** | **K.LLLDRGAK.I** |
|  | 3288 | **464.7326** | **927.4506** | **927.4774** | **-0.0268** | **0** | **10** | **18** | **10** | **R.TSPADGKPR.L** |
|  | 3831 | **486.2934** | **970.5723** | **971.4858** | **-0.9135** | **0** | **1** | **1.2e+002** | **2** | **-.MAHAASQLK.K** |
|  | 3995 | 491.8035 | 981.5925 | 981.4549 | 0.1376 | 1 | 7 | 31 | 5 | K.QRTEMSSK.A |
|  | 4213 | **499.2597** | **996.5049** | **996.5314** | **-0.0265** | **1** | **7** | **26** | **6** | **K.DFMVLKTK.D** |
|  | 4216 | 499.2638 | 996.5131 | 995.4672 | 1.0460 | 0 | 8 | 22 | 7 | K.SDANASYLR.A |
|  | 4409 | 506.2830 | 1010.5514 | 1010.4193 | 0.1321 | 0 | 10 | 15 | 1 | R.GDDEVFDSK.S 4392 |
|  | 4677 | **516.7416** | **1031.4686** | **1030.5196** | **0.9490** | **0** | **7** | **38** | **6** | **K.EQHLTFTR.E** |
|  | 4855 | **524.2197** | **1046.4249** | **1045.5404** | **0.8845** | **1** | **0** | **1.7e+002** | **6** | **R.EANVDAATKK.G** |
|  | 4910 | **526.2372** | **1050.4598** | **1050.5610** | **-0.1012** | **1** | **3** | **85** | **3** | **K.KHHYLPEK.E** |
|  | 5037 | **531.3004** | **1060.5863** | **1061.5361** | **-0.9499** | **1** | **10** | **18** | **1** | **K.LKMPPASMR.T** |
|  | 5877 | **563.2822** | **1124.5499** | **1123.5621** | **0.9878** | **1** | **5** | **46** | **2** | **K.KSDANASYLR.A** |
|  | 5961 | **566.7801** | **1131.5456** | **1132.6492** | **-1.1036** | **0** | **15** | **5.2** | **1** | **K.SSLFLAPSALK.L** |
|  | 6370 | **582.6303** | **1163.2461** | **1162.5288** | **0.7172** | **0** | **11** | **8.3** | **1** | **K.MNDPVESSLR.C** |
|  | 6408 | 584.7654 | 1167.5163 | 1166.5469 | 0.9694 | 0 | 3 | 67 | 7 | R.QHGPDGQGFPK.A |
|  | 6409 | **584.7834** | **1167.5522** | **1168.5910** | **-1.0388** | **1** | **11** | **12** | **1** | **R.SFRTMSSPIK.T** |
|  | 6489 | 587.4213 | 1172.8281 | 1173.6605 | -0.8324 | 0 | 5 | 38 | 3 | K.LLTNGSLSEIK.E |
|  | 6693 | 594.2828 | 1186.5511 | 1187.6259 | -1.0747 | 1 | 3 | 76 | 7 | R.GGQPSVTTKSAR.D |
|  | 6768 | **397.4026** | **1189.1860** | **1188.5189** | **0.6672** | **0** | **(6)** | **27** | **8** | **K.MGISASTMTMK.K** |
|  | 6864 | **399.2146** | **1194.6221** | **1193.5313** | **1.0908** | **0** | **9** | **17** | **6** | **R.TWESSGATNNK.S** 6851 6869 |
|  | 7078 | 402.2436 | 1203.7089 | 1204.5138 | -0.8048 | 0 | 17 | 3.2 | 1 | K.MGISASTMTMK.K 7076 7084 |
|  | 7711 | 618.8332 | 1235.6518 | 1235.6431 | 0.0087 | 0 | 6 | 34 | 9 | K.ITMASSLSSPVK.Q |
|  | 7778 | **413.6150** | **1237.8232** | **1238.6142** | **-0.7910** | **1** | **2** | **64** | **10** | **K.AASEKDYNLTK.V** |
|  | 7785 | **620.2960** | **1238.5774** | **1238.6039** | **-0.0265** | **0** | **6** | **37** | **6** | **R.ELICVPYMAK.F** |
|  | 8991 | **650.7919** | **1299.5692** | **1300.6189** | **-1.0497** | **1** | **(4)** | **54** | **7** | **K.MGISASTMTMKK.E** |
|  | 9040 | 434.8810 | 1301.6211 | 1300.6189 | 1.0022 | 1 | (4) | 57 | 3 | K.MGISASTMTMKK.E |
|  | 9324 | 658.8704 | 1315.7263 | 1316.6138 | -0.8875 | 1 | 7 | 28 | 10 | K.MGISASTMTMKK.E 9323 |
|  | 9390 | **440.9027** | **1319.6864** | **1320.6885** | **-1.0021** | **1** | **7** | **27** | **6** | **K.STVEAKGSISQSK.A** |
|  | 9395 | **661.3018** | **1320.5891** | **1319.6721** | **0.9170** | **0** | **47** | **0.003** | **1** | **R.YLVQDGAQVEAK.A** |
|  | 9644 | **667.3861** | **1332.7577** | **1333.7089** | **-0.9512** | **0** | **1** | **1.3e+002** | **10** | **K.NATTDALTSVLTK.I** |
|  | 9818 | **448.9049** | **1343.6930** | **1343.6568** | **0.0361** | **1** | **7** | **28** | **10** | **K.EVDDEQKEKPK.A** |
|  | 9847 | **673.3598** | **1344.7050** | **1344.6456** | **0.0595** | **1** | **1** | **1.2e+002** | **10** | **K.ACATQKQGQPEK.G** |
|  | 10776 | **699.8519** | **1397.6893** | **1396.6405** | **1.0488** | **1** | **1** | **98** | **3** | **K.NEFMSVTERER.K** |
|  | 10875 | 702.8731 | 1403.7317 | 1403.6140 | 0.1177 | 0 | 4 | 59 | 2 | K.DTFPPNHMSNTK.A |
|  | 10897 | **469.3106** | **1404.9099** | **1405.7201** | **-0.8102** | **1** | **4** | **39** | **8** | **K.QYEKEIQQGGVK.K** 10910 |
|  | 11593 | **483.2422** | **1446.7047** | **1447.6687** | **-0.9640** | **0** | **5** | **40** | **10** | **R.IEETMSVHDIMK.A** |
|  | 11987 | 735.8840 | 1469.7535 | 1469.7514 | 0.0021 | 1 | 15 | 4.1 | 3 | R.DPSKELAGLFEHK.S |
|  | 12257 | **743.8760** | **1485.7374** | **1486.7780** | **-1.0406** | **0** | **2** | **81** | **8** | **K.VTVLHYSGNVSSPK.H** |
|  | 12271 | 744.3331 | 1486.6516 | 1486.7126 | -0.0610 | 0 | 4 | 46 | 2 | K.SNINMYSSSLPFK.S |
|  | 13421 | **780.4057** | **1558.7968** | **1558.7740** | **0.0229** | **1** | **11** | **8.9** | **5** | **K.TVQETRGTFYTTR.Q** |
|  | 13893 | **530.3057** | **1587.8952** | **1588.6940** | **-0.7988** | **0** | **3** | **61** | **7** | **R.MVYHSPPGGEGASER.I** 13914 |
|  | 14253 | 538.2832 | 1611.8278 | 1611.8034 | 0.0244 | 1 | 2 | 75 | 10 | R.TPIKMGISASTMTMK.K |
|  | 14378 | 810.9174 | 1619.8202 | 1619.9247 | -0.1045 | 1 | 2 | 84 | 10 | R.VGLQAQPVPDEIVKK.I |
|  | 14618 | 818.3710 | 1634.7275 | 1633.8523 | 0.8752 | 1 | 0 | 1.1e+002 | 4 | R.DGKNATTDALTSVLTK.I |
|  | 14924 | **552.2900** | **1653.8483** | **1652.8192** | **1.0291** | **0** | **4** | **47** | **10** | **K.LANPPPMVEGEGLASR.L** |
|  | 15178 | **557.9672** | **1670.8797** | **1671.9407** | **-1.0611** | **1** | **6** | **29** | **5** | **K.SVVSPVKSAVDVISSAK.I** |
|  | 15641 | **852.4569** | **1702.8991** | **1703.8227** | **-0.9235** | **1** | **4** | **48** | **8** | **R.ENHIGHPESKSVDQK.N** |
|  | 16342 | **586.2946** | **1755.8620** | **1754.7628** | **1.0992** | **1** | **1** | **94** | **10** | **K.KMQSELSDEEESTSR.N** |
|  | 16406 | 587.6206 | 1759.8400 | 1759.8159 | 0.0241 | 1 | 3 | 56 | 1 | R.EMPNDVSKDSNQRPK.N |
|  | 16443 | 441.7535 | 1762.9851 | 1761.9546 | 1.0305 | 1 | 3 | 56 | 6 | K.LLTNGSLSEIKEMTVK.S |
|  | 16550 | 886.9597 | 1771.9049 | 1772.8428 | -0.9379 | 1 | 7 | 20 | 4 | K.VGPDEDKPPSKSSSSEK.T |
|  | 16626 | 593.6204 | 1777.8393 | 1777.9686 | -0.1294 | 0 | 8 | 16 | 3 | K.GNTALHIASLAGQAEVVK.V 16634 |
|  | 17422 | **921.5041** | **1840.9936** | **1841.0411** | **-0.0474** | **1** | **59** | **0.00013** | **1** | **K.ILGNKATFSPIVTVEPR.R** |
|  | 18006 | **629.9689** | **1886.8848** | **1887.0209** | **-0.1361** | **1** | **2** | **67** | **4** | **K.LIIEETKPCVPVSMKK.M** |
|  | 18334 | **639.4045** | **1915.1918** | **1915.9058** | **-0.7140** | **1** | **3** | **34** | **1** | **R.VTGGLLDRLDDSPDQCR.D** |
|  | 18436 | **641.9646** | **1922.8720** | **1923.0174** | **-0.1454** | **1** | **5** | **31** | **3** | **R.QQAAASQSPSLPERVQVK.A** |
|  | 18725 | **649.8530** | **1946.5371** | **1946.0408** | **0.4963** | **0** | **1** | **57** | **1** | **K.GLPQTAVCNLNITLPAHK.K** |
|  | 18898 | 655.3026 | 1962.8860 | 1963.0448 | -0.1588 | 1 | 8 | 14 | 2 | R.VENPNSLISQSFMLLKK.W 18919 |
|  | 18903 | 655.3398 | 1962.9975 | 1963.0163 | -0.0188 | 1 | 2 | 61 | 3 | K.ELAGLFEHKSAVSPDVHK.S |
|  | 20885 | 1082.0553 | 2162.0960 | 2162.0161 | 0.0799 | 1 | 3 | 48 | 3 | K.QTTVEETAVEREMPNDVSK.D |
|  | 21574 | **747.0452** | **2238.1137** | **2238.9443** | **-0.8306** | **0** | **(3)** | **40** | **9** | **K.MNVPETMNEVLDMSDDEVR.K** |
|  | 21698 | 1128.9702 | 2255.9259 | 2254.9392 | 0.9867 | 0 | 6 | 17 | 2 | K.MNVPETMNEVLDMSDDEVR.K |
|  | 21784 | 1133.5482 | 2265.0819 | 2266.0899 | -1.0080 | 0 | 7 | 15 | 2 | K.NQMDIATTLLEYGADANAVTR.Q |
|  | 21927 | **762.0497** | **2283.1274** | **2282.0849** | **1.0425** | **0** | **(6)** | **18** | **1** | **K.NQMDIATTLLEYGADANAVTR.Q** |
|  | 23165 | **616.1323** | **2460.5002** | **2461.2673** | **-0.7671** | **1** | **3** | **30** | **3** | **R.NANVNLSNKSGLTPLHLAAQEDR.V** |
|  | 24552 | **673.6241** | **2690.4672** | **2690.4742** | **-0.0070** | **1** | **4** | **23** | **1** | **K.SSLFLAPSALKLSTPSSLSSSQEILK.D** |
|  | 24676 | **679.8221** | **2715.2595** | **2716.3088** | **-1.0493** | **1** | **5** | **21** | **1** | **R.SSITMTPPASPKSNINMYSSSLPFK.S** |
|  | 24869 | **918.1489** | **2751.4248** | **2751.4241** | **0.0007** | **0** | **58** | **9.5e-005** | **1** | **R.LVEMGPAGAQFLGPVIVEIPHFGSMR.G** 24879 |
|  | 24939 | **923.4208** | **2767.2407** | **2767.4190** | **-0.1783** | **0** | **(2)** | **41** | **2** | **R.LVEMGPAGAQFLGPVIVEIPHFGSMR.G** |
|  | 28476 | 894.2208 | 3572.8542 | 3571.7729 | 1.0813 | 0 | 2 | 21 | 2 | K.VLVTNGANVNAQSQNGFTPLYMAAQENHLEVVK.F |
|  | 29149 | **1264.9010** | **3791.6812** | **3791.7587** | **-0.0775** | **0** | **0** | **28** | **1** | **K.IEAVMTSCQGLENETITMISNTANSQMGVRPHEK.H** |
|  | 30750 | **1155.8003** | **4619.1721** | **4618.2922** | **0.8799** | **0** | **3** | **10** | **3** | **K.HGASIQAVTESGLTPIHVAAFMGHVNIVSQLMHHGASPNTTNVR.G** |
|  | 31000 | **965.8307** | **4824.1174** | **4823.1675** | **0.9499** | **1** | **1** | **11** | **5** | **K.HDFQKDNFNNNNNLDSSTIQTDNIMSNIVLTEHSAPTCTTEK.D** |
|  | 31544 | **1330.1834** | **5316.7043** | **5315.7361** | **0.9682** | **1** | **0** | **12** | **2** | **K.GNTALHIASLAGQAEVVKVLVTNGANVNAQSQNGFTPLYMAAQENHLEVVK.F** |

| **268.** | P04003\|C4BPA_HUMAN    **Mass:** 69042    **Score:** 112    **Queries matched:** 14   **emPAI:** 0.05 |
| --- | --- |
|  | C4b-binding protein alpha chain OS=Homo sapiens GN=C4BPA PE=1 SV=2 |

|  | **Query** | **Observed** | **Mr(expt)** | **Mr(calc)** | **Delta** | **Miss** | **Score** | **Expect** | **Rank** | **Peptide** |
| --- | --- | --- | --- | --- | --- | --- | --- | --- | --- | --- |
|  | 2002 | **419.9058** | **837.7971** | **837.4457** | **0.3514** | **0** | **6** | **26** | **9** | **K.TPSGALHR.K** |
|  | 2222 | **427.2313** | **852.4480** | **852.4415** | **0.0065** | **0** | **7** | **30** | **8** | **K.MALEVYK.L** 2203 2225 |
|  | 3993 | 491.7901 | 981.5657 | 981.5066 | 0.0591 | 0 | 16 | 4 | 7 | K.CKPPPDIR.N 3970 |
|  | 8929 | **432.8839** | **1295.6298** | **1296.7051** | **-1.0753** | **1** | **6** | **34** | **2** | **M.HPPKTPSGALHR.K** 8951 |
|  | 10677 | **465.0194** | **1392.0364** | **1391.7172** | **0.3192** | **1** | **2** | **57** | **9** | **K.MAAWPFSRLWK.V** |
|  | 15690 | **853.5090** | **1705.0034** | **1705.8610** | **-0.8576** | **0** | **10** | **10** | **1** | **R.GVGWSHPLPQCEIVK.C** |
|  | 19242 | **998.4179** | **1994.8211** | **1994.8325** | **-0.0113** | **0** | **69** | **1.2e-005** | **1** | **R.WTPYQGCEALCCPEPK.L** 19243 |
|  | 21496 | **744.3662** | **2230.0768** | **2231.0351** | **-0.9583** | **0** | **2** | **57** | **4** | **R.CHPGYKPTTDEPTTVICQK.N** |
|  | 30674 | **1523.4081** | **4567.2024** | **4567.3780** | **-0.1756** | **0** | **0** | **18** | **3** | **K.VSDPILFQMTLIAALLPAVLGNCGPPPTLSFAAPMDITLTETR.F** |

| **269.** | O75396\|SC22B_HUMAN    **Mass:** 24896    **Score:** 112    **Queries matched:** 19   **emPAI:** 1.04 |
| --- | --- |
|  | Vesicle-trafficking protein SEC22b OS=Homo sapiens GN=SEC22B PE=1 SV=3 |

|  | **Query** | **Observed** | **Mr(expt)** | **Mr(calc)** | **Delta** | **Miss** | **Score** | **Expect** | **Rank** | **Peptide** |
| --- | --- | --- | --- | --- | --- | --- | --- | --- | --- | --- |
|  | 3128 | **458.7833** | **915.5520** | **915.5575** | **-0.0055** | **0** | **42** | **0.013** | **1** | **M.VLLTMIAR.V** |
|  | 3363 | **467.2509** | **932.4872** | **932.4927** | **-0.0054** | **0** | **18** | **2.7** | **1** | **K.ANNLSSLSK.K** |
|  | 3504 | **473.2879** | **944.5612** | **943.4723** | **1.0890** | **0** | **12** | **11** | **4** | **K.LNEQSPTR.C** |
|  | 4120 | **495.7820** | **989.5494** | **989.5029** | **0.0465** | **0** | **18** | **2.7** | **1** | **R.GEALSALDSK.A** 4110 4119 |
|  | 4864 | **524.2632** | **1046.5119** | **1046.5980** | **-0.0861** | **0** | **15** | **5.8** | **8** | **-.MVLLTMIAR.V** |
|  | 5077 | **532.7766** | **1063.5385** | **1062.5929** | **0.9456** | **0** | **(13)** | **8.9** | **1** | **-.MVLLTMIAR.V** |
|  | 5174 | **536.7872** | **1071.5599** | **1071.5672** | **-0.0073** | **1** | **48** | **0.0027** | **1** | **R.KLNEQSPTR.C** 5176 |
|  | 5238 | **539.7797** | **1077.5448** | **1078.5879** | **-1.0431** | **0** | **(2)** | **1e+002** | **7** | **-.MVLLTMIAR.V** |
|  | 6046 | **570.3570** | **1138.6994** | **1137.5600** | **1.1394** | **1** | **2** | **91** | **6** | **R.QDAKYLNMR.S** |
|  | 11031 | **707.8861** | **1413.7577** | **1413.7650** | **-0.0073** | **0** | **38** | **0.023** | **1** | **R.IMVANIEEVLQR.G** |
|  | 11321 | **477.7710** | **1430.2913** | **1429.7599** | **0.5314** | **0** | **(4)** | **35** | **7** | **R.IMVANIEEVLQR.G** |
|  | 13864 | **793.9063** | **1585.7979** | **1585.8060** | **-0.0080** | **0** | **64** | **4.8e-005** | **1** | **R.NLGSINTELQDVQR.I** |
|  | 19170 | 663.6787 | 1988.0143 | 1988.9109 | -0.8966 | 0 | 6 | 22 | 2 | R.VADGLPLAASMQEDEQSGR.D |
|  | 26097 | 1001.4492 | 3001.3257 | 3001.5035 | -0.1778 | 1 | (1) | 38 | 4 | -.MVLLTMIARVADGLPLAASMQEDEQSGR.D |
|  | 26166 | **1510.1079** | **3018.2013** | **3017.4984** | **0.7029** | **1** | **3** | **19** | **3** | **-.MVLLTMIARVADGLPLAASMQEDEQSGR.D** |
|  | 26881 | **1055.1648** | **3162.4725** | **3162.4887** | **-0.0162** | **1** | **32** | **0.031** | **1** | **R.VADGLPLAASMQEDEQSGRDLQQYQSQAK.Q** |

| **270.** | Q02978\|M2OM_HUMAN    **Mass:** 34211    **Score:** 112    **Queries matched:** 21   **emPAI:** 0.37 |
| --- | --- |
|  | Mitochondrial 2-oxoglutarate/malate carrier protein OS=Homo sapiens GN=SLC25A11 PE=1 SV=3 |

|  | **Query** | **Observed** | **Mr(expt)** | **Mr(calc)** | **Delta** | **Miss** | **Score** | **Expect** | **Rank** | **Peptide** |
| --- | --- | --- | --- | --- | --- | --- | --- | --- | --- | --- |
|  | 1091 | **661.3405** | **660.3332** | **660.3377** | **-0.0045** | **0** | **7** | **49** | **1** | **R.IQNMR.M** |
|  | 3511 | 473.7746 | 945.5347 | 945.5396 | -0.0049 | 0 | 16 | 4.9 | 1 | K.NVFNALIR.I |
|  | 6790 | **397.8683** | **1190.5831** | **1189.5873** | **0.9958** | **1** | **3** | **72** | **1** | **K.NRMQLSGEGAK.T** |
|  | 6828 | **597.3035** | **1192.5924** | **1192.5870** | **0.0054** | **1** | **13** | **8.3** | **1** | **R.MQLSGEGAKTR.E** |
|  | 7129 | **604.3295** | **1206.6444** | **1205.5822** | **1.0621** | **1** | **(3)** | **77** | **1** | **K.NRMQLSGEGAK.T** |
|  | 9602 | **666.3271** | **1330.6396** | **1329.6459** | **0.9937** | **1** | **11** | **12** | **1** | **R.MTADGRLPADQR.R** |
|  | 9872 | **674.3260** | **1346.6374** | **1345.6408** | **0.9966** | **1** | **(5)** | **50** | **1** | **R.MTADGRLPADQR.R** |
|  | 10787 | **467.1008** | **1398.2806** | **1398.7215** | **-0.4410** | **0** | **13** | **4.8** | **2** | **M.AATASAGAGGIDGKPR.T** 10777 |
|  | 12033 | **491.9496** | **1472.8270** | **1471.8147** | **1.0123** | **1** | **6** | **32** | **6** | **R.ITREEGVLTLWR.G** |
|  | 12958 | **511.2505** | **1530.7296** | **1529.7620** | **0.9675** | **0** | **4** | **54** | **8** | **-.MAATASAGAGGIDGKPR.T** 12961 |
|  | 14627 | **818.4342** | **1634.8538** | **1634.8628** | **-0.0089** | **0** | **71** | **9.4e-006** | **1** | **R.AVVVNAAQLASYSQSK.Q** |
|  | 16172 | **871.9678** | **1741.9211** | **1742.9679** | **-1.0468** | **1** | **3** | **47** | **10** | **K.TSFHALTSILKAEGLR.G** |
|  | 19335 | **668.6372** | **2002.8898** | **2003.9921** | **-1.1023** | **1** | **0** | **92** | **1** | **R.EEGVLTLWRGCIPTMAR.A** |
|  | 21910 | **760.7504** | **2279.2295** | **2279.2388** | **-0.0094** | **0** | **53** | **0.00042** | **1** | **K.FLFGGLAGMGATVFVQPLDLVK.N** 21909 21913 |
|  | 25726 | **730.8845** | **2919.5090** | **2919.4947** | **0.0143** | **1** | **0** | **54** | **10** | **K.AVIGMTAGATGAFVGTPAEVALIRMTADGR.L** |
|  | 28718 | 910.7158 | 3638.8342 | 3639.9699 | -1.1357 | 1 | 7 | 6.6 | 2 | R.LTGADGTPPGFLLKAVIGMTAGATGAFVGTPAEVALIR.M 28719 |

| **271.** | O96000\|NDUBA_HUMAN    **Mass:** 21048    **Score:** 111    **Queries matched:** 8   **emPAI:** 1.31 |
| --- | --- |
|  | NADH dehydrogenase [ubiquinone] 1 beta subcomplex subunit 10 OS=Homo sapiens GN=NDUFB10 PE=1 SV=3 |

|  | **Query** | **Observed** | **Mr(expt)** | **Mr(calc)** | **Delta** | **Miss** | **Score** | **Expect** | **Rank** | **Peptide** |
| --- | --- | --- | --- | --- | --- | --- | --- | --- | --- | --- |
|  | 6541 | **589.8029** | **1177.5912** | **1177.5979** | **-0.0067** | **0** | **41** | **0.012** | **1** | **K.EVEQFTQVAK.A** 6526 |
|  | 10486 | **691.3077** | **1380.6008** | **1380.6092** | **-0.0084** | **0** | **18** | **1.9** | **1** | **R.EGQNYQQNCIK.E** |
|  | 14272 | 538.6476 | 1612.9209 | 1612.9301 | -0.0092 | 0 | 43 | 0.0061 | 1 | K.AFDLIVDRPVTLVR.E |
|  | 17716 | **932.4294** | **1862.8443** | **1862.8547** | **-0.0104** | **1** | **43** | **0.0049** | **1** | **K.AYQDRYQDLGAYSSAR.K** 17715 17717 |
|  | 23657 | **847.7351** | **2540.1833** | **2540.1965** | **-0.0132** | **1** | **30** | **0.067** | **1** | **R.EGQNYQQNCIKEVEQFTQVAK.A** |

| **272.** | P20039\|2B1B_HUMAN    **Mass:** 30483    **Score:** 110    **Queries matched:** 9   **emPAI:** 0.26 |
| --- | --- |
|  | HLA class II histocompatibility antigen, DRB1-11 beta chain OS=Homo sapiens GN=HLA-DRB1 PE=2 SV=1 |

|  | **Query** | **Observed** | **Mr(expt)** | **Mr(calc)** | **Delta** | **Miss** | **Score** | **Expect** | **Rank** | **Peptide** |
| --- | --- | --- | --- | --- | --- | --- | --- | --- | --- | --- |
|  | 1045 | **637.2899** | **636.2826** | **635.3867** | **0.8959** | **1** | **8** | **29** | **5** | **R.RVHPK.V** |
|  | 1541 | 402.7052 | 803.3958 | 804.4606 | -1.0648 | 1 | 12 | 14 | 6 | R.VRFLDR.Y |
|  | 7401 | **407.7233** | **1220.1482** | **1220.6374** | **-0.4892** | **1** | **7** | **19** | **3** | **R.NQKGHSGLQPR.G** |
|  | 10966 | **705.8158** | **1409.6170** | **1409.6252** | **-0.0081** | **0** | **7** | **27** | **1** | **R.YFYNQEEYVR.F** |
|  | 12375 | **747.3555** | **1492.6965** | **1492.7059** | **-0.0094** | **0** | **56** | **0.00035** | **1** | **R.HNYGVGESFTVQR.R** |
|  | 24338 | **887.7534** | **2660.2383** | **2660.2541** | **-0.0158** | **0** | **76** | **1.5e-006** | **1** | **R.SGEVYTCQVEHPSVTSPLTVEWR.A** 24337 |
|  | 28687 | **1210.0779** | **3627.2118** | **3626.9133** | **0.2985** | **1** | **3** | **13** | **1** | **M.VCLRLPGGSCMAVLTVTLMVLSSPLALAGDTRPR.F** |
|  | 29029 | **1872.2601** | **3742.5057** | **3741.9588** | **0.5469** | **1** | **6** | **5.5** | **1** | **-.MVCLRLPGGSCMAVLTVTLMVLSSPLALAGDTRPR.F** |

| **273.** | Q30154\|2B51_HUMAN    **Mass:** 30322    **Score:** 110    **Queries matched:** 8   **emPAI:** 0.27 |
| --- | --- |
|  | HLA class II histocompatibility antigen, DRB5 beta chain OS=Homo sapiens GN=HLA-DRB5 PE=1 SV=1 |

|  | **Query** | **Observed** | **Mr(expt)** | **Mr(calc)** | **Delta** | **Miss** | **Score** | **Expect** | **Rank** | **Peptide** |
| --- | --- | --- | --- | --- | --- | --- | --- | --- | --- | --- |
|  | 12375 | 747.3555 | 1492.6965 | 1492.7059 | -0.0094 | 0 | 56 | 0.00035 | 1 | R.HNYGVGESFTVQR.R |
|  | 13569 | **524.2421** | **1569.7046** | **1569.7717** | **-0.0672** | **1** | **16** | **3.1** | **1** | **-.MVCLKLPGGSYMAK.L** |
|  | 15799 | **572.3365** | **1713.9878** | **1713.7682** | **0.2196** | **1** | **9** | **13** | **1** | **K.YECHFFNGTERVR.F** |
|  | 23623 | **1268.2090** | **2534.4034** | **2534.4011** | **0.0023** | **0** | **4** | **23** | **2** | **K.MLSGVGGFVLGLLFLGAGLFIYFK.N** |
|  | 24338 | 887.7534 | 2660.2383 | 2660.2541 | -0.0158 | 0 | 76 | 1.5e-006 | 1 | R.SGEVYTCQVEHPSVTSPLTVEWR.A 24337 |
|  | 26762 | **783.8526** | **3131.3813** | **3130.6882** | **0.6930** | **1** | **(0)** | **41** | **7** | **K.LPGGSYMAKLTVTLMVLSSPLALAGDTRPR.F** |
|  | 26833 | **630.4966** | **3147.4468** | **3146.6832** | **0.7637** | **1** | **1** | **33** | **3** | **K.LPGGSYMAKLTVTLMVLSSPLALAGDTRPR.F** |

| **274.** | P04229\|2B11_HUMAN    **Mass:** 30294    **Score:** 110    **Queries matched:** 4   **emPAI:** 0.27 |
| --- | --- |
|  | HLA class II histocompatibility antigen, DRB1-1 beta chain OS=Homo sapiens GN=HLA-DRB1 PE=1 SV=2 |

|  | **Query** | **Observed** | **Mr(expt)** | **Mr(calc)** | **Delta** | **Miss** | **Score** | **Expect** | **Rank** | **Peptide** |
| --- | --- | --- | --- | --- | --- | --- | --- | --- | --- | --- |
|  | 12375 | 747.3555 | 1492.6965 | 1492.7059 | -0.0094 | 0 | 56 | 0.00035 | 1 | R.HNYGVGESFTVQR.R |
|  | 22545 | **789.4672** | **2365.3798** | **2365.0281** | **0.3518** | **1** | **2** | **36** | **2** | **R.CIYNQEESVRFDSDVGEYR.A** |
|  | 24338 | 887.7534 | 2660.2383 | 2660.2541 | -0.0158 | 0 | 76 | 1.5e-006 | 1 | R.SGEVYTCQVEHPSVTSPLTVEWR.A 24337 |

| **275.** | Q9UBX3\|DIC_HUMAN    **Mass:** 31718    **Score:** 110    **Queries matched:** 10   **emPAI:** 0.25 |
| --- | --- |
|  | Mitochondrial dicarboxylate carrier OS=Homo sapiens GN=SLC25A10 PE=1 SV=2 |

|  | **Query** | **Observed** | **Mr(expt)** | **Mr(calc)** | **Delta** | **Miss** | **Score** | **Expect** | **Rank** | **Peptide** |
| --- | --- | --- | --- | --- | --- | --- | --- | --- | --- | --- |
|  | 6895 | **598.8024** | **1195.5903** | **1195.5986** | **-0.0083** | **0** | **21** | **1.2** | **1** | **K.GSQGPLPFHEK.V** |
|  | 8673 | **642.3658** | **1282.7171** | **1282.6452** | **0.0719** | **1** | **(2)** | **89** | **2** | **R.RLFSGATMASSR.G** |
|  | 8846 | **646.8182** | **1291.6219** | **1291.6309** | **-0.0090** | **0** | **30** | **0.13** | **1** | **R.NYAHALDGLYR.V** 8869 |
|  | 8962 | **433.5725** | **1297.6956** | **1298.6401** | **-0.9445** | **1** | **5** | **46** | **6** | **R.RLFSGATMASSR.G** 8980 |
|  | 11615 | 483.7787 | 1448.3142 | 1447.7320 | 0.5822 | 1 | 4 | 34 | 7 | R.RNYAHALDGLYR.V |
|  | 21810 | **757.0245** | **2268.0516** | **2268.0667** | **-0.0151** | **1** | **9** | **9.8** | **1** | **R.LMNSKGEYQGVFHCAVETAK.L** |
|  | 22032 | **1149.6399** | **2297.2652** | **2297.2744** | **-0.0091** | **0** | **101** | **6.4e-009** | **1** | **K.VLLGSVSGLAGGFVGTPADLVNVR.M** |
|  | 23397 | **833.0486** | **2496.1239** | **2496.1389** | **-0.0149** | **0** | **14** | **2.6** | **1** | **R.WYFGGLASCGAACCTHPLDLLK.V** |

| **276.** | Q56VL3\|OCAD2_HUMAN    **Mass:** 17285    **Score:** 110    **Queries matched:** 10   **emPAI:** 1.24 |
| --- | --- |
|  | OCIA domain-containing protein 2 OS=Homo sapiens GN=OCIAD2 PE=1 SV=1 |

|  | **Query** | **Observed** | **Mr(expt)** | **Mr(calc)** | **Delta** | **Miss** | **Score** | **Expect** | **Rank** | **Peptide** |
| --- | --- | --- | --- | --- | --- | --- | --- | --- | --- | --- |
|  | 4769 | **520.7508** | **1039.4870** | **1039.4948** | **-0.0078** | **0** | **19** | **2** | **1** | **R.GAGFGPQHNR.H** |
|  | 7777 | **413.6149** | **1237.8230** | **1237.5880** | **0.2349** | **0** | **7** | **24** | **4** | **K.FHFFEDQLR.G** |
|  | 9909 | **675.2831** | **1348.5516** | **1348.5574** | **-0.0058** | **0** | **49** | **0.0018** | **1** | **R.HCLLTCEECK.I** |
|  | 10788 | 700.3214 | 1398.6283 | 1398.6375 | -0.0093 | 1 | 43 | 0.0072 | 1 | K.HGLSEKGDSQPSAS.- 10789 |
|  | 13043 | **513.2482** | **1536.7229** | **1536.7321** | **-0.0092** | **1** | **39** | **0.016** | **1** | **R.GNQDKDAHFPPPSK.Q** 13041 13042 13047 |
|  | 13917 | **530.9164** | **1589.7275** | **1589.7364** | **-0.0089** | **1** | **27** | **0.24** | **1** | **R.HCLLTCEECKIK.H** |

| **277.** | P63104\|1433Z_HUMAN    **Mass:** 27899    **Score:** 110    **Queries matched:** 11   **emPAI:** 0.29 |
| --- | --- |
|  | 14-3-3 protein zeta/delta OS=Homo sapiens GN=YWHAZ PE=1 SV=1 |

|  | **Query** | **Observed** | **Mr(expt)** | **Mr(calc)** | **Delta** | **Miss** | **Score** | **Expect** | **Rank** | **Peptide** |
| --- | --- | --- | --- | --- | --- | --- | --- | --- | --- | --- |
|  | 6742 | 397.2191 | 1188.6355 | 1188.6536 | -0.0181 | 0 | 7 | 35 | 7 | K.DSTLIMQLLR.D |
|  | 11108 | **710.2915** | **1418.5684** | **1417.7123** | **0.8562** | **0** | **8** | **20** | **1** | **R.DICNDVLSLLEK.F** |
|  | 13241 | **774.8558** | **1547.6971** | **1547.7063** | **-0.0092** | **0** | **63** | **5.5e-005** | **1** | **K.SVTEQGAELSNEER.N** 13242 13247 |
|  | 20585 | **427.2421** | **2131.1740** | **2130.9844** | **0.1895** | **0** | **14** | **3.1** | **2** | **K.TAFDEAIAELDTLSEESYK.D** |
|  | 24196 | **659.3366** | **2633.3173** | **2633.1043** | **0.2130** | **1** | **2** | **47** | **9** | **R.YDDMAACMKSVTEQGAELSNEER.N** |
|  | 27448 | **1101.5464** | **3301.6173** | **3301.6275** | **-0.0102** | **1** | **74** | **1.8e-006** | **1** | **K.TAFDEAIAELDTLSEESYKDSTLIMQLLR.D** 27446 27449 27450 |

| **278.** | P46776\|RL27A_HUMAN    **Mass:** 16665    **Score:** 110    **Queries matched:** 6   **emPAI:** 0.23 |
| --- | --- |
|  | 60S ribosomal protein L27a OS=Homo sapiens GN=RPL27A PE=1 SV=2 |

|  | **Query** | **Observed** | **Mr(expt)** | **Mr(calc)** | **Delta** | **Miss** | **Score** | **Expect** | **Rank** | **Peptide** |
| --- | --- | --- | --- | --- | --- | --- | --- | --- | --- | --- |
|  | 7611 | **411.2143** | **1230.6210** | **1231.6561** | **-1.0351** | **0** | **7** | **29** | **5** | **K.LWTLVSEQTR.V** |
|  | 9994 | **677.3918** | **1352.7690** | **1352.7776** | **-0.0086** | **1** | **7** | **26** | **1** | **K.NKTGAAPIIDVVR.S** |
|  | 10531 | **462.2209** | **1383.6409** | **1382.6805** | **0.9604** | **1** | **6** | **32** | **4** | **K.YHPGYFGKVGMK.H** |
|  | 13838 | **529.2613** | **1584.7622** | **1584.7725** | **-0.0103** | **1** | **12** | **8.4** | **1** | **R.INFDKYHPGYFGK.V** |
|  | 24210 | **1318.6543** | **2635.2940** | **2635.3064** | **-0.0124** | **1** | **110** | **7.1e-010** | **1** | **R.NQSFCPTVNLDKLWTLVSEQTR.V** 24211 |

| **279.** | O43707\|ACTN4_HUMAN    **Mass:** 105245   **Score:** 109    **Queries matched:** 21   **emPAI:** 0.11 |
| --- | --- |
|  | Alpha-actinin-4 OS=Homo sapiens GN=ACTN4 PE=1 SV=2 |

|  | **Query** | **Observed** | **Mr(expt)** | **Mr(calc)** | **Delta** | **Miss** | **Score** | **Expect** | **Rank** | **Peptide** |
| --- | --- | --- | --- | --- | --- | --- | --- | --- | --- | --- |
|  | 2368 | **432.2536** | **862.4926** | **863.4752** | **-0.9827** | **0** | **9** | **24** | **5** | **K.ALDFIASK.G** |
|  | 2872 | **450.2786** | **898.5427** | **899.4647** | **-0.9220** | **1** | **10** | **15** | **2** | **K.EAMLKHR.D** |
|  | 6760 | **595.4190** | **1188.8233** | **1188.6979** | **0.1255** | **1** | **2** | **78** | **7** | **R.VHKPPKVQEK.C** |
|  | 8994 | **434.2019** | **1299.5840** | **1298.6619** | **0.9221** | **0** | **34** | **0.055** | **1** | **R.HRPELIEYDK.L** |
|  | 9039 | 434.8809 | 1301.6208 | 1300.5982 | 1.0226 | 0 | 6 | 38 | 6 | K.HTNYTMEHIR.V 9046 |
|  | 10263 | **457.0744** | **1368.2013** | **1367.6140** | **0.5873** | **0** | **5** | **31** | **4** | **K.GISQEQMQEFR.A** |
|  | 10313 | **458.2329** | **1371.6768** | **1371.7795** | **-0.1027** | **0** | **7** | **26** | **5** | **K.LMLLLEVISGER.L** 10320 10324 |
|  | 11134 | **474.2892** | **1419.8458** | **1420.6986** | **-0.8528** | **0** | **8** | **20** | **3** | **K.GYEEWLLNEIR.R** |
|  | 13243 | **516.9233** | **1547.7480** | **1547.7580** | **-0.0100** | **1** | **28** | **0.21** | **1** | **K.HRDYETATLSDIK.A** |
|  | 16786 | 896.9265 | 1791.8385 | 1791.8502 | -0.0117 | 0 | 52 | 0.0007 | 1 | R.MAPYQGPDAVPGALDYK.S |
|  | 17316 | 916.9567 | 1831.8988 | 1831.8622 | 0.0366 | 0 | 4 | 37 | 3 | K.MLDAEDIVNTARPDEK.A |
|  | 18305 | **479.4833** | **1913.9041** | **1913.9166** | **-0.0125** | **1** | **6** | **22** | **5** | **K.ICDQWDALGSLTHSRR.E** |
|  | 18378 | **960.5003** | **1918.9861** | **1919.0000** | **-0.0139** | **0** | **78** | **1.5e-006** | **1** | **K.LSGSNPYTTVTPQIINSK.W** |
|  | 19931 | **687.6537** | **2059.9394** | **2059.9520** | **-0.0126** | **0** | **9** | **11** | **1** | **K.VLAVNQENEHLMEDYEK.L** |
|  | 25639 | **969.4655** | **2905.3747** | **2904.3889** | **0.9858** | **0** | **3** | **25** | **1** | **R.VEQIAAIAQELNELDYYDSHNVNTR.C** |
|  | 25894 | **592.5001** | **2957.4642** | **2958.5922** | **-1.1280** | **1** | **0** | **47** | **10** | **K.LVSIGAEEIVDGNAKMTLGMIWTIILR.F** |
|  | 30183 | **1062.7695** | **4247.0490** | **4246.6600** | **0.3890** | **0** | **7** | **4.9** | **2** | **-.MVDYHAANQSYQYGPSSAGNGAGGGGSMGDYMAQEDDWDR.D** |
|  | 30546 | **1494.1882** | **4479.5429** | **4479.1251** | **0.4178** | **1** | **0** | **18** | **10** | **R.IMSLVDPNHSGLVTFQAFIDFMSRETTDTDTADQVIASFK.V** |

| **280.** | P05090\|APOD_HUMAN    **Mass:** 21547    **Score:** 109    **Queries matched:** 5   **emPAI:** 0.63 |
| --- | --- |
|  | Apolipoprotein D OS=Homo sapiens GN=APOD PE=1 SV=1 |

|  | **Query** | **Observed** | **Mr(expt)** | **Mr(calc)** | **Delta** | **Miss** | **Score** | **Expect** | **Rank** | **Peptide** |
| --- | --- | --- | --- | --- | --- | --- | --- | --- | --- | --- |
|  | 2500 | **436.2510** | **870.4875** | **870.4923** | **-0.0048** | **0** | **42** | **0.0099** | **1** | **K.VLNQELR.A** |
|  | 7600 | **616.0477** | **1230.0809** | **1229.6615** | **0.4194** | **0** | **6** | **25** | **1** | **K.NILTSNNIDVK.K** |
|  | 8843 | 646.7986 | 1291.5827 | 1291.5901 | -0.0073 | 0 | 57 | 0.00027 | 1 | K.MTVTDQVNCPK.L |
|  | 11127 | **710.8454** | **1419.6762** | **1419.6850** | **-0.0088** | **1** | **71** | **1.1e-005** | **1** | **K.KMTVTDQVNCPK.L** |
|  | 17969 | 628.6407 | 1882.9002 | 1881.9261 | 0.9742 | 1 | 10 | 10 | 1 | R.WYEIEKIPTTFENGR.C |

| **281.** | P09622\|DLDH_HUMAN    **Mass:** 54686    **Score:** 107    **Queries matched:** 14   **emPAI:** 0.14 |
| --- | --- |
|  | Dihydrolipoyl dehydrogenase, mitochondrial OS=Homo sapiens GN=DLD PE=1 SV=1 |

|  | **Query** | **Observed** | **Mr(expt)** | **Mr(calc)** | **Delta** | **Miss** | **Score** | **Expect** | **Rank** | **Peptide** |
| --- | --- | --- | --- | --- | --- | --- | --- | --- | --- | --- |
|  | 4210 | 499.2592 | 996.5038 | 995.5222 | 0.9816 | 1 | 6 | 37 | 6 | R.VYCSLAKR.G |
|  | 4909 | 526.2360 | 1050.4574 | 1050.4652 | -0.0078 | 0 | 28 | 0.22 | 1 | K.TNADTDGMVK.I |
|  | 6646 | 593.1548 | 1184.2950 | 1183.5577 | 0.7373 | 1 | 7 | 24 | 3 | K.MMEQKSTAVK.A |
|  | 8034 | **626.3032** | **1250.5918** | **1249.5972** | **0.9945** | **1** | **23** | **0.7** | **1** | **R.AKTNADTDGMVK.I** |
|  | 11934 | **489.9104** | **1466.7093** | **1467.7358** | **-1.0265** | **1** | **9** | **16** | **2** | **R.EANLAASFGKSINF.-** |
|  | 12862 | **508.9141** | **1523.7204** | **1523.7303** | **-0.0100** | **0** | **18** | **2.1** | **1** | **R.VCHAHPTLSEAFR.E** |
|  | 13541 | **784.4155** | **1566.8165** | **1566.8253** | **-0.0088** | **0** | **78** | **2e-006** | **1** | **K.NLGLEELGIELDPR.G** 13542 |
|  | 13921 | **795.9156** | **1589.8166** | **1589.8083** | **0.0083** | **1** | **2** | **84** | **6** | **K.TNADTDGMVKILGQK.S** |
|  | 14274 | **538.7799** | **1613.3179** | **1612.9301** | **0.3878** | **1** | **2** | **46** | **4** | **K.STAVKALTGGIAHLFK.Q** |
|  | 17112 | **606.9691** | **1817.8855** | **1818.8604** | **-0.9749** | **0** | **2** | **65** | **4** | **K.NETLGGTCLNVGCIPSK.A** |
|  | 19140 | **663.0128** | **1986.0165** | **1985.0768** | **0.9396** | **0** | **9** | **12** | **1** | **K.MVVIGAGVIGVELGSVWQR.L** |
|  | 27157 | **806.3658** | **3221.4340** | **3221.4771** | **-0.0430** | **1** | **4** | **18** | **3** | **K.ALLNNSHYYHMAHGTDFASRGIEMSEVR.L** |
|  | 27689 | **1118.8818** | **3353.6237** | **3353.6384** | **-0.0147** | **0** | **21** | **0.33** | **1** | **R.VLGAHILGPGAGEMVNEAALALEYGASCEDIAR.V** |

| **282.** | Q9UI12\|VATH_HUMAN    **Mass:** 56417    **Score:** 106    **Queries matched:** 17   **emPAI:** 0.21 |
| --- | --- |
|  | V-type proton ATPase subunit H OS=Homo sapiens GN=ATP6V1H PE=1 SV=1 |

|  | **Query** | **Observed** | **Mr(expt)** | **Mr(calc)** | **Delta** | **Miss** | **Score** | **Expect** | **Rank** | **Peptide** |
| --- | --- | --- | --- | --- | --- | --- | --- | --- | --- | --- |
|  | 2076 | **422.2480** | **842.4815** | **842.4862** | **-0.0046** | **0** | **15** | **6.4** | **2** | **R.VIEQLGGK.Q** |
|  | 4516 | **510.2950** | **1018.5755** | **1018.5811** | **-0.0056** | **0** | **32** | **0.11** | **1** | **R.YNALLAVQK.L** |
|  | 5023 | **530.7550** | **1059.4954** | **1059.6036** | **-0.1082** | **1** | **10** | **19** | **3** | **K.TQLSSQKLR.G** |
|  | 8742 | **429.9149** | **1286.7229** | **1287.6030** | **-0.8801** | **0** | **10** | **14** | **1** | **R.QDPFTVHMAAR.I** 8760 8765 8783 |
|  | 11248 | **714.3607** | **1426.7068** | **1426.7164** | **-0.0097** | **0** | **40** | **0.013** | **1** | **K.QLQSEQPQTAAAR.S** |
|  | 14435 | **541.9463** | **1622.8170** | **1621.8075** | **1.0095** | **0** | **(3)** | **63** | **6** | **K.NTAWPYFLPMLNR.Q** |
|  | 14687 | 547.2811 | 1638.8214 | 1637.8024 | 1.0190 | 0 | 3 | 62 | 10 | K.NTAWPYFLPMLNR.Q |
|  | 15513 | **565.3033** | **1692.8880** | **1693.8895** | **-1.0015** | **1** | **4** | **47** | **7** | **R.QEYALAMIQCKVLK.Q** |
|  | 16138 | **870.8558** | **1739.6971** | **1739.8334** | **-0.1363** | **1** | **5** | **30** | **1** | **R.ETRQEYALAMIQCK.V** |
|  | 17300 | **916.4349** | **1830.8553** | **1830.0138** | **0.8415** | **0** | **3** | **55** | **1** | **R.YNIIPVLSDILQESVK.E** |
|  | 20589 | **1066.9917** | **2131.9688** | **2131.9797** | **-0.0109** | **0** | **88** | **1.5e-007** | **1** | **K.LGESVQDLSSFDEYSSELK.S** |
|  | 24278 | **883.5852** | **2647.7338** | **2647.2780** | **0.4558** | **1** | **0** | **43** | **3** | **K.LAAWGKELMEGSDLNYYFNWIK.T** |
|  | 25212 | **704.5862** | **2814.3156** | **2813.3919** | **0.9238** | **1** | **1** | **47** | **7** | **K.LMVHNWEYLGKQLQSEQPQTAAAR.S** |
|  | 27281 | **1085.5035** | **3253.4888** | **3252.4678** | **1.0210** | **1** | **2** | **32** | **2** | **K.VNWQSYLQGQMISAEDCEFIQRFEMK.R** |

| **283.** | Q12846\|STX4_HUMAN    **Mass:** 34273    **Score:** 106    **Queries matched:** 4   **emPAI:** 0.23 |
| --- | --- |
|  | Syntaxin-4 OS=Homo sapiens GN=STX4 PE=1 SV=2 |

|  | **Query** | **Observed** | **Mr(expt)** | **Mr(calc)** | **Delta** | **Miss** | **Score** | **Expect** | **Rank** | **Peptide** |
| --- | --- | --- | --- | --- | --- | --- | --- | --- | --- | --- |
|  | 2497 | **436.2508** | **870.4871** | **870.5035** | **-0.0164** | **1** | **4** | **67** | **4** | **K.QLGREIR.L** |
|  | 11348 | 478.2394 | 1431.6965 | 1430.5918 | 1.1047 | 1 | 7 | 29 | 3 | K.CNSMQSEYREK.N |
|  | 21257 | **736.3410** | **2206.0012** | **2206.0138** | **-0.0126** | **1** | **20** | **0.84** | **1** | **K.AIEPQKEEADENYNSVNTR.M** |
|  | 29635 | **1328.6385** | **3982.8938** | **3982.9140** | **-0.0201** | **1** | **109** | **3.5e-010** | **1** | **K.ITNAGMVSDEELEQMLDSGQSEVFVSNILKDTQVTR.Q** |

| **284.** | P54707\|AT12A_HUMAN    **Mass:** 116292   **Score:** 106    **Queries matched:** 16   **emPAI:** 0.10 |
| --- | --- |
|  | Potassium-transporting ATPase alpha chain 2 OS=Homo sapiens GN=ATP12A PE=2 SV=3 |

|  | **Query** | **Observed** | **Mr(expt)** | **Mr(calc)** | **Delta** | **Miss** | **Score** | **Expect** | **Rank** | **Peptide** |
| --- | --- | --- | --- | --- | --- | --- | --- | --- | --- | --- |
|  | 4492 | **509.3054** | **1016.5962** | **1017.5277** | **-0.9315** | **1** | **3** | **1e+002** | **9** | **K.NNCLELKK.K** |
|  | 5359 | 544.2969 | 1086.5792 | 1086.5856 | -0.0063 | 0 | 37 | 0.032 | 1 | K.LIIVEGCQR.Q |
|  | 7051 | **602.3365** | **1202.6585** | **1201.7070** | **0.9515** | **1** | **3** | **72** | **3** | **K.QTPEIVKFLK.Q** |
|  | 7168 | **605.3679** | **1208.7213** | **1208.6302** | **0.0911** | **0** | **2** | **88** | **6** | **R.NSIFQQGLFR.N** |
|  | 10497 | 691.3746 | 1380.7347 | 1380.7435 | -0.0088 | 0 | 72 | 9.3e-006 | 1 | K.VIMVTGDHPITAK.A 10473 10499 |
|  | 10809 | **700.8373** | **1399.6601** | **1399.7823** | **-0.1222** | **1** | **5** | **43** | **4** | **R.TLINLRVEWEK.D** |
|  | 11054 | 708.3675 | 1414.7204 | 1414.8031 | -0.0827 | 1 | 7 | 24 | 5 | K.KAVIGDASETALLK.F |
|  | 11841 | **487.9712** | **1460.8917** | **1459.7341** | **1.1576** | **0** | **(2)** | **88** | **6** | **K.ADIGIAMGIAGSDAAK.N** |
|  | 12099 | **493.2780** | **1476.8121** | **1475.7290** | **1.0831** | **0** | **4** | **50** | **5** | **K.ADIGIAMGIAGSDAAK.N** |
|  | 15094 | **556.2880** | **1665.8421** | **1665.9050** | **-0.0629** | **0** | **1** | **96** | **8** | **R.TIIGHIASLASGVGNEK.T** |
|  | 16040 | **866.8754** | **1731.7362** | **1730.9025** | **0.8337** | **1** | **2** | **65** | **2** | **R.AEFKPGQENVPIMKK.A** |
|  | 17382 | 613.3414 | 1837.0023 | 1837.0132 | -0.0109 | 1 | 45 | 0.0034 | 1 | R.SAGIKVIMVTGDHPITAK.A |
|  | 17913 | **470.2367** | **1876.9178** | **1877.9986** | **-1.0808** | **1** | **5** | **33** | **3** | **K.TPEIYSVELSGTKDIVK.T** |
|  | 19176 | **663.8647** | **1988.5722** | **1987.8912** | **0.6811** | **1** | **0** | **56** | **3** | **K.DYVNDLKDSYGQEWTR.Y** |

| **285.** | Q03154\|ACY1_HUMAN    **Mass:** 46084    **Score:** 106    **Queries matched:** 14   **emPAI:** 0.17 |
| --- | --- |
|  | Aminoacylase-1 OS=Homo sapiens GN=ACY1 PE=1 SV=1 |

|  | **Query** | **Observed** | **Mr(expt)** | **Mr(calc)** | **Delta** | **Miss** | **Score** | **Expect** | **Rank** | **Peptide** |
| --- | --- | --- | --- | --- | --- | --- | --- | --- | --- | --- |
|  | 5981 | **567.7711** | **1133.5277** | **1133.5928** | **-0.0651** | **0** | **8** | **25** | **3** | **K.EGSVTSVNLTK.L** |
|  | 8531 | **425.8973** | **1274.6701** | **1274.7347** | **-0.0645** | **1** | **4** | **56** | **3** | **K.VVNSILAFREK.E** |
|  | 9596 | **444.2685** | **1329.7836** | **1330.6630** | **-0.8793** | **0** | **1** | **1.2e+002** | **10** | **R.TPVLLHDHDER.L** |
|  | 10739 | **466.2611** | **1395.7616** | **1395.8350** | **-0.0734** | **1** | **3** | **65** | **9** | **K.LHKVVNSILAFR.E** |
|  | 11313 | 477.6096 | 1429.8071 | 1430.7340 | -0.9269 | 0 | 3 | 71 | 7 | R.AVGVPALGFSPMNR.T |
|  | 17725 | **466.7409** | **1862.9344** | **1862.9825** | **-0.0481** | **1** | **9** | **12** | **4** | **R.YIRAVGVPALGFSPMNR.T** |
|  | 21611 | 749.3248 | 2244.9527 | 2244.0885 | 0.8642 | 0 | 1 | 56 | 4 | K.LEGGVAYNVIPATMSASFDFR.V |
|  | 22866 | **806.7155** | **2417.1245** | **2417.1389** | **-0.0143** | **1** | **13** | **4** | **1** | **R.VCKDMNLTLEPEIMPAATDNR.Y** |
|  | 23365 | **1246.0874** | **2490.1602** | **2490.1703** | **-0.0101** | **0** | **65** | **2.1e-005** | **1** | **R.AGFALDEGIANPTDAFTVFYSER.S** 23363 |
|  | 23584 | **843.7147** | **2528.1223** | **2528.1332** | **-0.0108** | **0** | **38** | **0.011** | **1** | **K.WMHPQVTPTDDSNPWWAAFSR.V** |
|  | 27707 | 1120.9186 | 3359.7339 | 3359.6708 | 0.0632 | 1 | 1 | 33 | 6 | K.EGSVTSVNLTKLEGGVAYNVIPATMSASFDFR.V |
|  | 28600 | **900.4305** | **3597.6931** | **3597.7086** | **-0.0155** | **1** | **13** | **1.7** | **1** | **R.VAPDVDFKAFEEQLQSWCQAAGEGVTLEFAQK.W** 28602 |

| **286.** | P53007\|TXTP_HUMAN    **Mass:** 34333    **Score:** 105    **Queries matched:** 16   **emPAI:** 0.68 |
| --- | --- |
|  | Tricarboxylate transport protein, mitochondrial OS=Homo sapiens GN=SLC25A1 PE=1 SV=2 |

|  | **Query** | **Observed** | **Mr(expt)** | **Mr(calc)** | **Delta** | **Miss** | **Score** | **Expect** | **Rank** | **Peptide** |
| --- | --- | --- | --- | --- | --- | --- | --- | --- | --- | --- |
|  | 744 | **529.3420** | **528.3347** | **528.3020** | **0.0327** | **0** | **15** | **6.6** | **1** | **K.GTVPR.L** |
|  | 1278 | **776.3671** | **775.3598** | **775.3647** | **-0.0049** | **0** | **22** | **1.6** | **1** | **R.GIGDCVR.Q** |
|  | 4305 | 502.2679 | 1002.5211 | 1001.5142 | 1.0070 | 0 | 21 | 1.3 | 1 | K.TQLQLDER.S |
|  | 4755 | **520.2441** | **1038.4737** | **1037.5658** | **0.9079** | **1** | **8** | **22** | **3** | **K.AFYKGTVPR.L** |
|  | 7691 | **617.8523** | **1233.6900** | **1233.6969** | **-0.0068** | **0** | **45** | **0.005** | **1** | **R.GLSSLLYGSIPK.A** |
|  | 8665 | **428.5481** | **1282.6225** | **1282.6306** | **-0.0081** | **0** | **33** | **0.073** | **1** | **K.FIHDQTSPNPK.Y** |
|  | 12062 | 738.3839 | 1474.7531 | 1474.7602 | -0.0071 | 1 | 32 | 0.085 | 1 | R.NTWDCGLQILKK.E 12060 |
|  | 12946 | **765.8717** | **1529.7288** | **1530.6748** | **-0.9459** | **0** | **(2)** | **86** | **7** | **R.FGMFEFLSNHMR.D** |
|  | 12972 | **766.8982** | **1531.7818** | **1530.6748** | **1.1070** | **0** | **4** | **51** | **4** | **R.FGMFEFLSNHMR.D** |
|  | 23032 | **1222.1360** | **2442.2574** | **2442.2685** | **-0.0111** | **0** | **67** | **1.4e-005** | **1** | **R.GLLCGLGAGVAEAVVVVCPMETIK.V** 23030 23031 |
|  | 28187 | **1165.9440** | **3494.8101** | **3494.8232** | **-0.0131** | **0** | **25** | **0.11** | **1** | **R.GDNPNKPMNPLITGVFGAIAGAASVFGNTPLDVIK.T** |
|  | 29095 | **942.7313** | **3766.8962** | **3767.9669** | **-1.0707** | **1** | **3** | **16** | **4** | **R.GDNPNKPMNPLITGVFGAIAGAASVFGNTPLDVIKTR.M** |
|  | 29992 | 1378.0597 | 4131.1572 | 4130.1048 | 1.0525 | 1 | 1 | 19 | 5 | R.NWYRGDNPNKPMNPLITGVFGAIAGAASVFGNTPLDVIK.T |

| **287.** | Q15084\|PDIA6_HUMAN    **Mass:** 48490    **Score:** 104    **Queries matched:** 13   **emPAI:** 0.35 |
| --- | --- |
|  | Protein disulfide-isomerase A6 OS=Homo sapiens GN=PDIA6 PE=1 SV=1 |

|  | **Query** | **Observed** | **Mr(expt)** | **Mr(calc)** | **Delta** | **Miss** | **Score** | **Expect** | **Rank** | **Peptide** |
| --- | --- | --- | --- | --- | --- | --- | --- | --- | --- | --- |
|  | 1098 | **665.3569** | **664.3496** | **664.3544** | **-0.0048** | **0** | **9** | **26** | **6** | **K.IFGSNK.N** |
|  | 1114 | **676.3571** | **675.3498** | **675.3551** | **-0.0054** | **0** | **18** | **3.8** | **1** | **R.SDIVSR.A** |
|  | 1269 | **772.3785** | **771.3713** | **772.4119** | **-1.0407** | **0** | **2** | **1.2e+002** | **8** | **R.LTPEWK.K** |
|  | 6786 | **596.2752** | **1190.5357** | **1190.5428** | **-0.0071** | **0** | **18** | **2.5** | **1** | **K.NRPEDYQGGR.T** 6816 |
|  | 7319 | **608.8212** | **1215.6278** | **1214.6507** | **0.9771** | **1** | **7** | **34** | **1** | **K.DVVKVGAVDADK.H** |
|  | 10570 | **693.8794** | **1385.7442** | **1385.7514** | **-0.0072** | **0** | **10** | **13** | **1** | **R.TGEAIVDAALSALR.Q** |
|  | 15112 | **834.4002** | **1666.7857** | **1666.7951** | **-0.0094** | **1** | **42** | **0.0067** | **1** | **K.IFQKGESPVDYDGGR.T** |
|  | 19302 | 667.6652 | 1999.9738 | 1999.9850 | -0.0112 | 1 | 6 | 25 | 4 | K.NLEPEWAAAASEVKEQTK.G |
|  | 23924 | **861.1062** | **2580.2968** | **2580.3085** | **-0.0117** | **1** | **46** | **0.0017** | **1** | **K.VGAVDADKHHSLGGQYGVQGFPTIK.I** |
|  | 24218 | **879.7904** | **2636.3494** | **2636.3585** | **-0.0091** | **0** | **31** | **0.059** | **1** | **R.ALDLFSDNAPPPELLEIINEDIAK.R** 24217 |
|  | 25086 | **931.8239** | **2792.4499** | **2792.4596** | **-0.0097** | **1** | **29** | **0.08** | **1** | **R.ALDLFSDNAPPPELLEIINEDIAKR.T** |

| **288.** | P30479\|1B41_HUMAN    **Mass:** 40856    **Score:** 104    **Queries matched:** 6   **emPAI:** 0.30 |
| --- | --- |
|  | HLA class I histocompatibility antigen, B-41 alpha chain OS=Homo sapiens GN=HLA-B PE=1 SV=1 |

|  | **Query** | **Observed** | **Mr(expt)** | **Mr(calc)** | **Delta** | **Miss** | **Score** | **Expect** | **Rank** | **Peptide** |
| --- | --- | --- | --- | --- | --- | --- | --- | --- | --- | --- |
|  | 7513 | **613.7462** | **1225.4779** | **1225.4856** | **-0.0077** | **0** | **54** | **0.00053** | **1** | **R.MYGCDVGPDGR.L** 7482 |
|  | 12793 | **760.3734** | **1518.7323** | **1518.7427** | **-0.0104** | **0** | **73** | **7.3e-006** | **1** | **R.SWTAADTAAQITQR.K** |
|  | 14460 | **406.9821** | **1623.8995** | **1623.7498** | **0.1497** | **1** | **4** | **43** | **10** | **R.MYGCDVGPDGRLLR.G** |
|  | 23135 | **492.2564** | **2456.2458** | **2456.3097** | **-0.0639** | **0** | **2** | **47** | **5** | **R.TVLLLLSAALALTETWAGSHSMR.Y** |
|  | 24792 | **912.7573** | **2735.2500** | **2735.2635** | **-0.0135** | **1** | **34** | **0.023** | **1** | **R.DGEDQTQDTELVETRPAGDRTFQK.W** |

| **289.** | P49411\|EFTU_HUMAN    **Mass:** 49852    **Score:** 103    **Queries matched:** 16   **emPAI:** 0.54 |
| --- | --- |
|  | Elongation factor Tu, mitochondrial OS=Homo sapiens GN=TUFM PE=1 SV=2 |

|  | **Query** | **Observed** | **Mr(expt)** | **Mr(calc)** | **Delta** | **Miss** | **Score** | **Expect** | **Rank** | **Peptide** |
| --- | --- | --- | --- | --- | --- | --- | --- | --- | --- | --- |
|  | 1677 | **815.4574** | **814.4501** | **814.4548** | **-0.0047** | **0** | **28** | **0.29** | **1** | **K.ILAEGGGAK.F** 1681 |
|  | 3180 | **460.2736** | **918.5327** | **918.5386** | **-0.0058** | **0** | **19** | **2.3** | **1** | **K.TTLTAAITK.I** |
|  | 5533 | 550.2700 | 1098.5255 | 1098.5597 | -0.0342 | 0 | 6 | 39 | 2 | R.ELLTEFGYK.G |
|  | 8258 | **631.3301** | **1260.6456** | **1260.6537** | **-0.0080** | **0** | **33** | **0.069** | **1** | **R.TVVTGIEMFHK.S** |
|  | 13142 | **771.9262** | **1541.8377** | **1541.8453** | **-0.0076** | **0** | **17** | **2.3** | **1** | **K.LLDAVDTYIPVPAR.D** |
|  | 14753 | **548.7426** | **1643.2058** | **1643.8817** | **-0.6759** | **1** | **1** | **57** | **9** | **K.NIRTVVTGIEMFHK.S** |
|  | 15211 | **837.4286** | **1672.8426** | **1672.8533** | **-0.0106** | **0** | **52** | **0.00068** | **1** | **R.GITINAAHVEYSTAAR.H** 15210 |
|  | 15287 | 839.4249 | 1676.8353 | 1676.8733 | -0.0380 | 1 | 3 | 57 | 8 | K.VEAQVYILSKEEGGR.H |
|  | 16523 | **590.9224** | **1769.7454** | **1769.7580** | **-0.0126** | **0** | **26** | **0.29** | **1** | **R.HYAHTDCPGHADYVK.N** |
|  | 16758 | 597.3010 | 1788.8813 | 1790.0349 | -1.1536 | 1 | 2 | 65 | 5 | R.RGLVMVKPGSIKPHQK.V |
|  | 16979 | **904.9644** | **1807.9143** | **1808.9282** | **-1.0139** | **0** | **1** | **93** | **7** | **R.DKPHVNVGTIGHVDHGK.T** |
|  | 21756 | **1132.0906** | **2262.1666** | **2262.1777** | **-0.0111** | **1** | **54** | **0.00034** | **1** | **R.TIGTGLVTNTLAMTEEEKNIK.W** 21755 |
|  | 25196 | **703.5927** | **2810.3415** | **2811.3718** | **-1.0303** | **0** | **3** | **31** | **2** | **K.NMITGTAPLDGCILVVAANDGPMPQTR.E** |

| **290.** | P30508\|1C12_HUMAN    **Mass:** 41316    **Score:** 103    **Queries matched:** 8   **emPAI:** 0.42 |
| --- | --- |
|  | HLA class I histocompatibility antigen, Cw-12 alpha chain OS=Homo sapiens GN=HLA-C PE=1 SV=2 |

|  | **Query** | **Observed** | **Mr(expt)** | **Mr(calc)** | **Delta** | **Miss** | **Score** | **Expect** | **Rank** | **Peptide** |
| --- | --- | --- | --- | --- | --- | --- | --- | --- | --- | --- |
|  | 3749 | **483.2167** | **964.4188** | **964.4250** | **-0.0062** | **0** | **35** | **0.057** | **1** | **R.FDSDAASPR.G** |
|  | 9948 | **676.3410** | **1350.6674** | **1349.6939** | **0.9736** | **1** | **14** | **5.9** | **1** | **R.YLENGKETLQR.A** 9957 |
|  | 12793 | 760.3734 | 1518.7323 | 1518.7427 | -0.0104 | 0 | 73 | 7.3e-006 | 1 | R.SWTAADTAAQITQR.K |
|  | 14538 | **815.4131** | **1628.8117** | **1628.8199** | **-0.0081** | **0** | **28** | **0.17** | **1** | **R.FIAVGYVDDTQFVR.F** |
|  | 14674 | 547.0314 | 1638.0725 | 1637.7654 | 0.3071 | 1 | 2 | 52 | 6 | R.MYGCDLGPDGRLLR.G |
|  | 17358 | **612.9700** | **1835.8883** | **1835.9027** | **-0.0144** | **0** | **3** | **52** | **2** | **K.THVTHHPVSDHEATLR.C** |
|  | 24215 | **879.7303** | **2636.1690** | **2636.1838** | **-0.0148** | **0** | **43** | **0.003** | **1** | **R.DGEDQTQDTELVETRPAGDGTFQK.W** |

|  | **Proteins matching a subset of these peptides:** |
| --- | --- |

|  | Q29963\|1C06_HUMAN    **Mass:** 41399    **Score:** 94     **Queries matched:** 6 |
| --- | --- |
|  | HLA class I histocompatibility antigen, Cw-6 alpha chain OS=Homo sapiens GN=HLA-C PE=1 SV=2 |

|  | P30505\|1C08_HUMAN    **Mass:** 41203    **Score:** 87     **Queries matched:** 4 |
| --- | --- |
|  | HLA class I histocompatibility antigen, Cw-8 alpha chain OS=Homo sapiens GN=HLA-C PE=2 SV=1 |

|  | P30504\|1C04_HUMAN    **Mass:** 41368    **Score:** 84     **Queries matched:** 6 |
| --- | --- |
|  | HLA class I histocompatibility antigen, Cw-4 alpha chain OS=Homo sapiens GN=HLA-C PE=1 SV=1 |

|  | P30510\|1C14_HUMAN    **Mass:** 41268    **Score:** 84     **Queries matched:** 6 |
| --- | --- |
|  | HLA class I histocompatibility antigen, Cw-14 alpha chain OS=Homo sapiens GN=HLA-C PE=2 SV=2 |

| **291.** | P26373\|RL13_HUMAN    **Mass:** 24304    **Score:** 103    **Queries matched:** 15   **emPAI:** 0.16 |
| --- | --- |
|  | 60S ribosomal protein L13 OS=Homo sapiens GN=RPL13 PE=1 SV=4 |

|  | **Query** | **Observed** | **Mr(expt)** | **Mr(calc)** | **Delta** | **Miss** | **Score** | **Expect** | **Rank** | **Peptide** |
| --- | --- | --- | --- | --- | --- | --- | --- | --- | --- | --- |
|  | 1011 | 627.8264 | 626.8191 | 626.3751 | 0.4440 | 0 | 19 | 1.1 | 2 | R.KPSAPK.K |
|  | 2122 | **424.0559** | **846.0973** | **846.4334** | **-0.3362** | **0** | **5** | **42** | **4** | **R.VITEEEK.N** |
|  | 3633 | **478.9037** | **955.7929** | **956.5291** | **-0.7362** | **0** | **12** | **7.4** | **1** | **R.TIGISVDPR.R** 3634 3646 3649 3650 3663 |
|  | 3874 | **487.3107** | **972.6068** | **972.6120** | **-0.0052** | **1** | **20** | **1.8** | **1** | **R.SKLILFPR.K** |
|  | 10216 | 456.2520 | 1365.7340 | 1365.8496 | -0.1156 | 1 | 5 | 39 | 3 | K.LILFPRKPSAPK.K |
|  | 12049 | 737.8795 | 1473.7445 | 1473.7535 | -0.0091 | 1 | 73 | 7.2e-006 | 1 | R.NKSTESLQANVQR.L 12027 12047 |
|  | 16340 | **439.7734** | **1755.0644** | **1755.0744** | **-0.0099** | **1** | **19** | **1.2** | **1** | **R.RIAPRPASGPIRPIVR.C** |
|  | 18010 | 944.4836 | 1886.9526 | 1886.0448 | 0.9078 | 1 | 1 | 79 | 4 | K.LATQLTGPVMPVRNVYK.K |

| **292.** | P52943\|CRIP2_HUMAN    **Mass:** 23276    **Score:** 102    **Queries matched:** 4   **emPAI:** 0.16 |
| --- | --- |
|  | Cysteine-rich protein 2 OS=Homo sapiens GN=CRIP2 PE=1 SV=1 |

|  | **Query** | **Observed** | **Mr(expt)** | **Mr(calc)** | **Delta** | **Miss** | **Score** | **Expect** | **Rank** | **Peptide** |
| --- | --- | --- | --- | --- | --- | --- | --- | --- | --- | --- |
|  | 1621 | **405.5355** | **809.0564** | **808.3320** | **0.7244** | **1** | **5** | **30** | **8** | **R.CERCGK.T** |
|  | 11276 | 476.9414 | 1427.8023 | 1427.7660 | 0.0363 | 1 | 1 | 98 | 9 | K.TVYFAEKVSSLGK.D |
|  | 26706 | **781.6244** | **3122.4685** | **3122.4855** | **-0.0170** | **0** | **5** | **14** | **1** | **K.TLTPGGHAEHDGKPFCHKPCYATLFGPK.G** |
|  | 27106 | **1070.8922** | **3209.6548** | **3209.6721** | **-0.0173** | **0** | **102** | **2.5e-009** | **1** | **K.GVNIGGAGSYIYEKPLAEGPQVTGPIEVPAAR.A** |

| **293.** | Q15366\|PCBP2_HUMAN    **Mass:** 38955    **Score:** 102    **Queries matched:** 14   **emPAI:** 0.10 |
| --- | --- |
|  | Poly(rC)-binding protein 2 OS=Homo sapiens GN=PCBP2 PE=1 SV=1 |

|  | **Query** | **Observed** | **Mr(expt)** | **Mr(calc)** | **Delta** | **Miss** | **Score** | **Expect** | **Rank** | **Peptide** |
| --- | --- | --- | --- | --- | --- | --- | --- | --- | --- | --- |
|  | 1057 | **645.3534** | **644.3462** | **643.3653** | **0.9809** | **0** | **14** | **11** | **5** | **K.INEIR.Q** |
|  | 3323 | 465.7680 | 929.5215 | 929.5546 | -0.0331 | 1 | 16 | 5.1 | 5 | K.EVGSIIGKK.G |
|  | 4643 | **515.3269** | **1028.6392** | **1027.5774** | **1.0618** | **1** | **11** | **14** | **3** | **R.QGAKINEIR.Q** 4606 4638 4640 |
|  | 8769 | 430.2997 | 1287.8773 | 1287.5877 | 0.2896 | 0 | 5 | 41 | 1 | R.INISEGNCPER.I |
|  | 12426 | 499.9808 | 1496.9206 | 1496.8385 | 0.0821 | 1 | 5 | 31 | 3 | R.LLMHGKEVGSIIGK.K 12418 |
|  | 17273 | **610.9777** | **1829.9113** | **1828.9427** | **0.9686** | **0** | **7** | **23** | **7** | **K.QICVVMLETLSQSPPK.G** |
|  | 20183 | 1045.4883 | 2088.9620 | 2088.9746 | -0.0126 | 0 | 71 | 7.4e-006 | 1 | R.ESTGAQVQVAGDMLPNSTER.A 20182 |
|  | 21626 | **749.9367** | **2246.7883** | **2247.2701** | **-0.4818** | **1** | **1** | **41** | **6** | **R.IITLAGPTNAIFKAFAMIIDK.L** |
|  | 27851 | 1133.8840 | 3398.6303 | 3397.5820 | 1.0483 | 0 | 2 | 28 | 3 | K.LHQLAMQQSHFPMTHGNTGFSGIESSSPEVK.G |

| **294.** | P57721\|PCBP3_HUMAN    **Mass:** 36201    **Score:** 102    **Queries matched:** 9   **emPAI:** 0.10 |
| --- | --- |
|  | Poly(rC)-binding protein 3 OS=Homo sapiens GN=PCBP3 PE=1 SV=1 |

|  | **Query** | **Observed** | **Mr(expt)** | **Mr(calc)** | **Delta** | **Miss** | **Score** | **Expect** | **Rank** | **Peptide** |
| --- | --- | --- | --- | --- | --- | --- | --- | --- | --- | --- |
|  | 1057 | 645.3534 | 644.3462 | 643.3653 | 0.9809 | 0 | 14 | 11 | 5 | K.INEIR.Q |
|  | 3323 | 465.7680 | 929.5215 | 929.5546 | -0.0331 | 1 | 16 | 5.1 | 5 | K.EVGSIIGKK.G |
|  | 8769 | 430.2997 | 1287.8773 | 1287.5877 | 0.2896 | 0 | 5 | 41 | 1 | R.INISEGNCPER.I |
|  | 12426 | 499.9808 | 1496.9206 | 1496.8385 | 0.0821 | 1 | 5 | 31 | 3 | R.LLMHGKEVGSIIGK.K 12418 |
|  | 17524 | **616.9612** | **1847.8617** | **1848.9615** | **-1.0998** | **1** | **6** | **26** | **1** | **-.MESKVSEGGLNVTLTIR.L** |
|  | 19842 | **685.2001** | **2052.5784** | **2053.1354** | **-0.5570** | **1** | **3** | **26** | **3** | **K.VSEGGLNVTLTIRLLMHGK.E** |
|  | 20183 | 1045.4883 | 2088.9620 | 2088.9746 | -0.0126 | 0 | 71 | 7.4e-006 | 1 | R.ESTGAQVQVAGDMLPNSTER.A 20182 |

| **295.** | Q9UM54\|MYO6_HUMAN    **Mass:** 150965   **Score:** 101    **Queries matched:** 36   **emPAI:** 0.05 |
| --- | --- |
|  | Myosin-VI OS=Homo sapiens GN=MYO6 PE=1 SV=4 |

|  | **Query** | **Observed** | **Mr(expt)** | **Mr(calc)** | **Delta** | **Miss** | **Score** | **Expect** | **Rank** | **Peptide** |
| --- | --- | --- | --- | --- | --- | --- | --- | --- | --- | --- |
|  | 2355 | **432.2157** | **862.4168** | **863.4752** | **-1.0584** | **1** | **11** | **15** | **4** | **R.KSPEYLK.A** |
|  | 2778 | 446.7455 | 891.4764 | 891.4484 | 0.0280 | 1 | 10 | 18 | 8 | K.LEMEAKR.K |
|  | 3381 | **468.2172** | **934.4199** | **934.4252** | **-0.0053** | **0** | **(4)** | **74** | **6** | **K.MTPEQMAK.E** |
|  | 3568 | **476.2402** | **950.4658** | **950.4201** | **0.0457** | **0** | **11** | **13** | **6** | **K.MTPEQMAK.E** |
|  | 3937 | **489.7732** | **977.5319** | **977.5216** | **0.0103** | **0** | **6** | **41** | **6** | **R.VMLTTAGGTK.G** 3935 |
|  | 4171 | **497.8008** | **993.5871** | **993.5165** | **0.0706** | **0** | **(4)** | **68** | **10** | **R.VMLTTAGGTK.G** |
|  | 7896 | **622.8297** | **1243.6449** | **1243.6520** | **-0.0072** | **1** | **12** | **10** | **1** | **K.ETDKQILQNR.K** |
|  | 8723 | **643.8096** | **1285.6046** | **1286.6214** | **-1.0169** | **1** | **7** | **30** | **4** | **K.QQEEEAERLR.R** |
|  | 9521 | **443.0382** | **1326.0928** | **1325.8031** | **0.2897** | **1** | **10** | **9** | **3** | **K.TQLNLLLDKLR.S** 9539 9543 |
|  | 9877 | **674.3592** | **1346.7038** | **1346.7115** | **-0.0077** | **0** | **7** | **27** | **3** | **K.NLEISIDTLMAK.I** 9862 9899 |
|  | 11887 | **733.3359** | **1464.6572** | **1465.7083** | **-1.0510** | **0** | **2** | **75** | **5** | **K.MSQSIIVSGESGAGK.T** |
|  | 13753 | **527.5957** | **1579.7653** | **1580.7902** | **-1.0249** | **1** | **(2)** | **65** | **5** | **K.IKSTMMTQEQIQK.E** |
|  | 14018 | **799.3640** | **1596.7133** | **1596.7851** | **-0.0718** | **1** | **7** | **21** | **5** | **K.IKSTMMTQEQIQK.E** |
|  | 14133 | **803.4209** | **1604.8272** | **1603.8855** | **0.9418** | **1** | **1** | **95** | **8** | **K.NLEISIDTLMAKIK.S** |
|  | 14178 | 804.4133 | 1606.8121 | 1607.8784 | -1.0663 | 0 | 6 | 29 | 8 | K.SLGTRPPHVFAIADK.A |
|  | 15616 | **567.9211** | **1700.7416** | **1701.7805** | **-1.0389** | **0** | **5** | **36** | **3** | **K.EEESQQQAVLEQER.R** |
|  | 15843 | **573.3160** | **1716.9261** | **1715.9491** | **0.9770** | **1** | **1** | **81** | **8** | **K.QIKNLEISIDTLMAK.I** |
|  | 15867 | **573.9687** | **1718.8842** | **1718.8080** | **0.0763** | **1** | **(4)** | **51** | **2** | **K.NDGTRPKMTPEQMAK.E** |
|  | 16063 | **434.4696** | **1733.8493** | **1734.8029** | **-0.9536** | **1** | **7** | **20** | **6** | **K.NDGTRPKMTPEQMAK.E** |
|  | 16788 | **598.2952** | **1791.8639** | **1790.9713** | **0.8926** | **0** | **2** | **71** | **4** | **R.QARPTYATAMLQSLLK.-** |
|  | 17718 | **466.7230** | **1862.8627** | **1863.9843** | **-1.1216** | **1** | **7** | **18** | **3** | **K.SSVVGGFVSHYLLEKSR.I** |
|  | 17999 | **629.6372** | **1885.8898** | **1885.8992** | **-0.0094** | **1** | **49** | **0.0012** | **1** | **K.AGSMKDPLLDDHGDFIR.M** |
|  | 18159 | **634.3546** | **1900.0420** | **1901.0444** | **-1.0024** | **0** | **3** | **53** | **4** | **R.QMELHPDKPPILLVAGK.D** |
|  | 18394 | 640.9750 | 1919.9033 | 1920.9833 | -1.0800 | 0 | 2 | 69 | 2 | K.TFLALINQVFPAEEDSK.K |
|  | 18709 | **649.3421** | **1945.0045** | **1945.8583** | **-0.8539** | **1** | **1** | **74** | **5** | **K.MTPEQMAKEMSEFLSR.G** |
|  | 19555 | **675.3532** | **2023.0376** | **2022.9892** | **0.0485** | **1** | **7** | **18** | **2** | **K.MSQSIIVSGESGAGKTENTK.F** |
|  | 20142 | 1042.5203 | 2083.0260 | 2084.0803 | -1.0544 | 1 | 0 | 83 | 5 | R.DALAKTVYSHLFDHVVNR.V |
|  | 20666 | 1071.5538 | 2141.0931 | 2142.0804 | -0.9873 | 0 | 6 | 24 | 8 | R.IAQSEAELISDEAQADLALR.R |
|  | 24251 | **882.1006** | **2643.2799** | **2643.2963** | **-0.0163** | **0** | **7** | **12** | **1** | **R.VVAGVLHLGNIDFEEAGSTSGGCNLK.N** |
|  | 25422 | **573.0917** | **2860.4220** | **2860.3780** | **0.0440** | **0** | **3** | **27** | **1** | **K.SVTDYDFAPFLNNSPQQNPAAQIPAR.Q** |
|  | 27442 | **1100.5369** | **3298.5888** | **3298.5993** | **-0.0106** | **1** | **73** | **2.1e-006** | **1** | **R.YLTESYGTGQDIDDRIVEANPLLEAFGNAK.T** |

| **296.** | P54920\|SNAA_HUMAN    **Mass:** 33667    **Score:** 100    **Queries matched:** 16   **emPAI:** 0.53 |
| --- | --- |
|  | Alpha-soluble NSF attachment protein OS=Homo sapiens GN=NAPA PE=1 SV=3 |

|  | **Query** | **Observed** | **Mr(expt)** | **Mr(calc)** | **Delta** | **Miss** | **Score** | **Expect** | **Rank** | **Peptide** |
| --- | --- | --- | --- | --- | --- | --- | --- | --- | --- | --- |
|  | 4551 | **512.2393** | **1022.4640** | **1021.4312** | **1.0328** | **0** | **5** | **46** | **10** | **K.GEESNSSANK.C** 4528 |
|  | 4611 | **514.2958** | **1026.5771** | **1026.4990** | **0.0781** | **1** | **4** | **68** | **7** | **R.AANMFKMAK.N** |
|  | 4813 | **522.2915** | **1042.5684** | **1042.4939** | **0.0745** | **1** | **(2)** | **1.1e+002** | **2** | **R.AANMFKMAK.N** |
|  | 6511 | 588.3679 | 1174.7212 | 1174.5466 | 0.1746 | 0 | 24 | 0.69 | 1 | K.TIQGDEEDLR.- |
|  | 8071 | **627.2917** | **1252.5688** | **1252.5757** | **-0.0069** | **0** | **40** | **0.014** | **1** | **K.IEEACEIYAR.A** 8055 |
|  | 11375 | 717.4002 | 1432.7859 | 1432.6439 | 0.1420 | 0 | 2 | 93 | 10 | K.ADPQEAINCLMR.A |
|  | 14425 | **812.3587** | **1622.7028** | **1622.7148** | **-0.0119** | **0** | **72** | **7.9e-006** | **1** | **K.HDAATCFVDAGNAFK.K** |
|  | 18521 | **644.2858** | **1929.8357** | **1930.8917** | **-1.0560** | **1** | **5** | **30** | **3** | **K.IEEACEIYARAANMFK.M** |
|  | 23029 | **815.0652** | **2442.1737** | **2442.1862** | **-0.0125** | **0** | **11** | **6.8** | **1** | **K.NWSAAGNAFCQAAQLHLQLQSK.H** 23040 |
|  | 23836 | **854.7128** | **2561.1167** | **2561.1306** | **-0.0139** | **1** | **28** | **0.11** | **1** | **K.AIAHYEQSADYYKGEESNSSANK.C** |
|  | 24036 | **650.3504** | **2597.3725** | **2598.1876** | **-0.8151** | **1** | **1** | **53** | **5** | **K.ADPQEAINCLMRAIEIYTDMGR.F** |
|  | 25823 | **981.4572** | **2941.3498** | **2940.3624** | **0.9874** | **1** | **32** | **0.032** | **1** | **K.LLEAHEEQNVDSYTESVKEYDSISR.L** 25817 |

| **297.** | Q6UX53\|MET7B_HUMAN    **Mass:** 28269    **Score:** 100    **Queries matched:** 12   **emPAI:** 0.29 |
| --- | --- |
|  | Methyltransferase-like protein 7B OS=Homo sapiens GN=METTL7B PE=2 SV=2 |

|  | **Query** | **Observed** | **Mr(expt)** | **Mr(calc)** | **Delta** | **Miss** | **Score** | **Expect** | **Rank** | **Peptide** |
| --- | --- | --- | --- | --- | --- | --- | --- | --- | --- | --- |
|  | 2512 | **436.7692** | **871.5238** | **870.5287** | **0.9951** | **1** | **12** | **14** | **6** | **R.KVLQEVR.R** 2489 |
|  | 5828 | 560.7731 | 1119.5316 | 1119.5383 | -0.0067 | 0 | 17 | 3 | 2 | R.FVVAPGEDMR.Q |
|  | 7228 | **405.0345** | **1212.0816** | **1211.5968** | **0.4848** | **1** | **5** | **31** | **4** | **K.FLTKSMAENR.H** 7227 |
|  | 7688 | **412.2278** | **1233.6617** | **1233.6692** | **-0.0075** | **0** | **12** | **9.1** | **1** | **K.WLPVGPHIMGK.A** |
|  | 12924 | 510.2564 | 1527.7475 | 1528.8000 | -1.0525 | 0 | 2 | 82 | 10 | K.SYFPYLMAVLTPK.S |
|  | 12978 | **511.7638** | **1532.2695** | **1532.7154** | **-0.4459** | **1** | **4** | **35** | **7** | **K.SMAENRHLQYER.F** |
|  | 18707 | **649.3373** | **1944.9902** | **1944.9768** | **0.0134** | **1** | **3** | **53** | **5** | **R.VTCLDPNPHFEKFLTK.S** |
|  | 21673 | **1127.5183** | **2253.0221** | **2253.0372** | **-0.0151** | **1** | **99** | **1e-008** | **1** | **R.ETWKDLENAQFSEIQMER.Q** 21674 21679 |

| **298.** | Q96GC9\|TMM49_HUMAN    **Mass:** 46607    **Score:** 100    **Queries matched:** 7   **emPAI:** 0.08 |
| --- | --- |
|  | Transmembrane protein 49 OS=Homo sapiens GN=TMEM49 PE=2 SV=1 |

|  | **Query** | **Observed** | **Mr(expt)** | **Mr(calc)** | **Delta** | **Miss** | **Score** | **Expect** | **Rank** | **Peptide** |
| --- | --- | --- | --- | --- | --- | --- | --- | --- | --- | --- |
|  | 1098 | 665.3569 | 664.3496 | 664.2850 | 0.0646 | 0 | 5 | 59 | 9 | -.MAENGK.N |
|  | 6789 | 596.2986 | 1190.5827 | 1190.5098 | 0.0729 | 1 | 2 | 94 | 5 | M.AENGKNCDQR.R |
|  | 7927 | **623.3940** | **1244.7734** | **1243.6520** | **1.1214** | **1** | **6** | **41** | **1** | **R.IQQRLNSEEK.T** |
|  | 22582 | **791.1037** | **2370.2893** | **2370.0659** | **0.2234** | **1** | **2** | **41** | **1** | **R.VAMNKEHHNGNFTDPSSVNEK.K** |
|  | 24534 | **896.4579** | **2686.3520** | **2687.2261** | **-0.8740** | **1** | **0** | **60** | **1** | **K.LHHKSEMGTPQGENWLSWMFEK.L** |
|  | 28087 | **1153.8148** | **3458.4226** | **3458.4368** | **-0.0142** | **0** | **98** | **5.5e-009** | **1** | **R.LSGAEPDDEEYQEFEEMLEHAESAQDFASR.A** 28086 |

| **299.** | P10620\|MGST1_HUMAN    **Mass:** 17644    **Score:** 100    **Queries matched:** 10   **emPAI:** 0.81 |
| --- | --- |
|  | Microsomal glutathione S-transferase 1 OS=Homo sapiens GN=MGST1 PE=1 SV=1 |

|  | **Query** | **Observed** | **Mr(expt)** | **Mr(calc)** | **Delta** | **Miss** | **Score** | **Expect** | **Rank** | **Peptide** |
| --- | --- | --- | --- | --- | --- | --- | --- | --- | --- | --- |
|  | 11163 | **711.8297** | **1421.6447** | **1421.6505** | **-0.0058** | **0** | **60** | **0.00014** | **1** | **K.MMLMSTATAFYR.L** |
|  | 13771 | **791.3852** | **1580.7558** | **1580.7657** | **-0.0099** | **1** | **44** | **0.0043** | **1** | **R.KVFANPEDCVAFGK.G** 13772 |
|  | 16989 | **603.9994** | **1808.9763** | **1807.8783** | **1.0980** | **1** | **6** | **29** | **5** | **K.MMLMSTATAFYRLTR.K** |
|  | 18786 | 651.6403 | 1951.8991 | 1951.9098 | -0.0107 | 1 | 1 | 68 | 2 | K.VFANPEDCVAFGKGENAK.K |
|  | 25181 | **936.7976** | **2807.3710** | **2806.3809** | **0.9901** | **0** | **30** | **0.056** | **1** | **M.VDLTQVMDDEVFMAFASYATIILSK.M** 25174 25182 |
|  | 30416 | **1098.5513** | **4390.1760** | **4389.0461** | **1.1299** | **1** | **(1)** | **17** | **2** | **-.MVDLTQVMDDEVFMAFASYATIILSKMMLMSTATAFYR.L** |
|  | 30437 | 630.3223 | 4405.2050 | 4405.0410 | 0.1639 | 1 | 3 | 10 | 1 | -.MVDLTQVMDDEVFMAFASYATIILSKMMLMSTATAFYR.L |

| **300.** | P08263\|GSTA1_HUMAN    **Mass:** 25672    **Score:** 99     **Queries matched:** 17   **emPAI:** 0.51 |
| --- | --- |
|  | Glutathione S-transferase A1 OS=Homo sapiens GN=GSTA1 PE=1 SV=3 |

|  | **Query** | **Observed** | **Mr(expt)** | **Mr(calc)** | **Delta** | **Miss** | **Score** | **Expect** | **Rank** | **Peptide** |
| --- | --- | --- | --- | --- | --- | --- | --- | --- | --- | --- |
|  | 2093 | **423.0527** | **844.0909** | **843.4160** | **0.6749** | **0** | **13** | **7.8** | **5** | **R.KPPMDEK.S** |
|  | 2505 | **436.6971** | **871.3797** | **870.5538** | **0.8259** | **1** | **11** | **15** | **2** | **K.DAKLALIK.E** |
|  | 2899 | 451.2457 | 900.4768 | 900.4818 | -0.0049 | 0 | 25 | 0.62 | 2 | K.FLQPGSPR.K 2917 |
|  | 4634 | **515.2918** | **1028.5691** | **1028.5767** | **-0.0076** | **1** | **13** | **8.8** | **2** | **K.KFLQPGSPR.K** 4618 4635 |
|  | 4867 | **524.2759** | **1046.5373** | **1045.5404** | **0.9970** | **1** | **21** | **1.4** | **1** | **K.SAEDLDKLR.N** |
|  | 6461 | 586.7958 | 1171.5771 | 1170.5822 | 0.9950 | 1 | 18 | 2.7 | 1 | K.NRYFPAFEK.V 6448 |
|  | 7328 | **609.2950** | **1216.5754** | **1216.5836** | **-0.0082** | **0** | **36** | **0.045** | **1** | **K.SHGQDYLVGNK.L** |
|  | 13376 | **519.5918** | **1555.7536** | **1556.8311** | **-1.0775** | **1** | **3** | **57** | **4** | **K.VLKSHGQDYLVGNK.L** |
|  | 13634 | **525.2712** | **1572.7917** | **1572.8008** | **-0.0091** | **1** | **26** | **0.29** | **1** | **K.SHGQDYLVGNKLSR.A** |
|  | 14379 | 540.9475 | 1619.8207 | 1619.8242 | -0.0035 | 1 | 19 | 1.7 | 1 | -.MAEKPKLHYFNAR.G 14386 |
|  | 21341 | **1108.0104** | **2214.0062** | **2214.0159** | **-0.0097** | **0** | **87** | **1.8e-007** | **1** | **R.NDGYLMFQQVPMVEIDGMK.L** |
|  | 27967 | 1143.6063 | 3427.7971 | 3426.7488 | 1.0483 | 1 | 0 | 33 | 7 | K.ERALIDMYIEGIADLGEMILLLPVCPPEEK.D |

| **301.** | P09210\|GSTA2_HUMAN    **Mass:** 25648    **Score:** 99     **Queries matched:** 15   **emPAI:** 0.51 |
| --- | --- |
|  | Glutathione S-transferase A2 OS=Homo sapiens GN=GSTA2 PE=1 SV=4 |

|  | **Query** | **Observed** | **Mr(expt)** | **Mr(calc)** | **Delta** | **Miss** | **Score** | **Expect** | **Rank** | **Peptide** |
| --- | --- | --- | --- | --- | --- | --- | --- | --- | --- | --- |
|  | 2093 | 423.0527 | 844.0909 | 843.4160 | 0.6749 | 0 | 13 | 7.8 | 5 | R.KPPMDEK.S |
|  | 2138 | **424.7193** | **847.4240** | **847.4334** | **-0.0093** | **1** | **12** | **14** | **5** | **R.GRMESIR.W** |
|  | 2899 | 451.2457 | 900.4768 | 900.4818 | -0.0049 | 0 | 25 | 0.62 | 2 | K.FLQPGSPR.K 2917 |
|  | 4634 | 515.2918 | 1028.5691 | 1028.5767 | -0.0076 | 1 | 13 | 8.8 | 2 | K.KFLQPGSPR.K 4618 4635 |
|  | 4867 | 524.2759 | 1046.5373 | 1045.5404 | 0.9970 | 1 | 21 | 1.4 | 1 | K.SAEDLDKLR.N |
|  | 6461 | 586.7958 | 1171.5771 | 1170.5822 | 0.9950 | 1 | 18 | 2.7 | 1 | K.NRYFPAFEK.V 6448 |
|  | 7328 | 609.2950 | 1216.5754 | 1216.5836 | -0.0082 | 0 | 36 | 0.045 | 1 | K.SHGQDYLVGNK.L |
|  | 13376 | 519.5918 | 1555.7536 | 1556.8311 | -1.0775 | 1 | 3 | 57 | 4 | K.VLKSHGQDYLVGNK.L |
|  | 13634 | 525.2712 | 1572.7917 | 1572.8008 | -0.0091 | 1 | 26 | 0.29 | 1 | K.SHGQDYLVGNKLSR.A |
|  | 14107 | **535.2618** | **1602.7637** | **1601.8347** | **0.9289** | **1** | **5** | **40** | **4** | **-.MAEKPKLHYSNIR.G** |
|  | 21341 | 1108.0104 | 2214.0062 | 2214.0159 | -0.0097 | 0 | 87 | 1.8e-007 | 1 | R.NDGYLMFQQVPMVEIDGMK.L |

| **302.** | P78329\|CP4F2_HUMAN    **Mass:** 60442    **Score:** 99     **Queries matched:** 13   **emPAI:** 0.13 |
| --- | --- |
|  | Leukotriene-B(4) omega-hydroxylase 1 OS=Homo sapiens GN=CYP4F2 PE=1 SV=1 |

|  | **Query** | **Observed** | **Mr(expt)** | **Mr(calc)** | **Delta** | **Miss** | **Score** | **Expect** | **Rank** | **Peptide** |
| --- | --- | --- | --- | --- | --- | --- | --- | --- | --- | --- |
|  | 6077 | 571.8215 | 1141.6284 | 1140.6502 | 0.9781 | 0 | 15 | 5.2 | 3 | R.SVINASAAIAPK.D 6060 |
|  | 6421 | 585.2893 | 1168.5639 | 1169.6128 | -1.0488 | 1 | 5 | 41 | 5 | R.LRCFPQPPR.R |
|  | 9918 | **675.3584** | **1348.7022** | **1348.7099** | **-0.0077** | **0** | **42** | **0.0091** | **1** | **R.HVTQDIVLPDGR.V** |
|  | 10851 | **701.8612** | **1401.7079** | **1401.7074** | **0.0004** | **0** | **15** | **4.1** | **1** | **K.IFNESVNIMHAK.W** |
|  | 13137 | **771.8940** | **1541.7735** | **1541.7838** | **-0.0103** | **0** | **56** | **0.00031** | **1** | **R.LVHDFTDAVIQER.R** 13136 13138 |
|  | 21381 | **740.5195** | **2218.5368** | **2219.1595** | **-0.6227** | **0** | **4** | **25** | **6** | **K.VWMGPISPLLSLCHPDIIR.S** |
|  | 22532 | **789.0400** | **2364.0983** | **2363.0324** | **1.0659** | **1** | **1** | **51** | **2** | **R.RNWFWGHQGMVNPTEEGMR.V** |
|  | 23390 | **832.4546** | **2494.3419** | **2494.3110** | **0.0310** | **1** | **1** | **49** | **6** | **R.NCIGQTFAMAEMKVVLALTLLR.F** |
|  | 27551 | **665.9396** | **3324.6618** | **3325.8043** | **-1.1424** | **1** | **6** | **13** | **1** | **K.VWMGPISPLLSLCHPDIIRSVINASAAIAPK.D** |
|  | 29280 | **769.2078** | **3841.0024** | **3840.7941** | **0.2083** | **1** | **2** | **23** | **1** | **K.LSDEDIRAEADTFMFEGHDTTASGLSWVLYHLAK.H** |

| **303.** | P07099\|HYEP_HUMAN    **Mass:** 53143    **Score:** 99     **Queries matched:** 19   **emPAI:** 0.50 |
| --- | --- |
|  | Epoxide hydrolase 1 OS=Homo sapiens GN=EPHX1 PE=1 SV=1 |

|  | **Query** | **Observed** | **Mr(expt)** | **Mr(calc)** | **Delta** | **Miss** | **Score** | **Expect** | **Rank** | **Peptide** |
| --- | --- | --- | --- | --- | --- | --- | --- | --- | --- | --- |
|  | 4131 | **496.2788** | **990.5431** | **990.5498** | **-0.0068** | **1** | **38** | **0.027** | **1** | **K.FLSVLERQ.-** 4116 |
|  | 4387 | **505.3157** | **1008.6168** | **1008.6219** | **-0.0051** | **0** | **5** | **48** | **3** | **K.IIPLLTDPK.N** |
|  | 4903 | 525.7612 | 1049.5079 | 1049.5618 | -0.0539 | 1 | 8 | 24 | 4 | K.KGFNSVATAR.I |
|  | 5945 | **566.2938** | **1130.5731** | **1130.5794** | **-0.0063** | **0** | **16** | **4.3** | **1** | **K.LISYSYMVR.G** |
|  | 12021 | 491.7432 | 1472.2077 | 1472.7776 | -0.5699 | 1 | 5 | 29 | 3 | K.GFNSVATARIFYK.L |
|  | 16006 | **865.4158** | **1728.8171** | **1728.8294** | **-0.0123** | **1** | **32** | **0.069** | **1** | **R.FYKENLGQGWMTQK.H** |
|  | 19294 | **667.3310** | **1998.9711** | **1998.9799** | **-0.0088** | **0** | **41** | **0.0079** | **1** | **R.GGHFAAFEEPELLAQDIR.K** |
|  | 20168 | 696.3605 | 2086.0596 | 2084.9803 | 1.0793 | 1 | 3 | 49 | 7 | R.DKEETLPLEDGWWGPGTR.S |
|  | 20547 | 710.0280 | 2127.0622 | 2127.0749 | -0.0126 | 1 | 46 | 0.0023 | 1 | R.GGHFAAFEEPELLAQDIRK.F 20548 20552 |
|  | 21908 | **760.7277** | **2279.1612** | **2278.1674** | **0.9937** | **0** | **4** | **29** | **3** | **K.VYVPTGFSAFPFELLHTPEK.W** |
|  | 23783 | **639.3218** | **2553.2580** | **2553.2978** | **-0.0398** | **1** | **3** | **33** | **5** | **R.MKVYVPTGFSAFPFELLHTPEK.W** |
|  | 25097 | **932.4410** | **2794.3013** | **2794.3158** | **-0.0145** | **1** | **38** | **0.011** | **1** | **R.EDDSIRPFKVETSDEEIHDLHQR.I** 25098 |
|  | 26428 | **1021.4979** | **3061.4719** | **3062.4080** | **-0.9361** | **0** | **24** | **0.22** | **1** | **K.NHGLSDEHVFEVICPSIPGYGFSEASSK.K** |
|  | 28431 | **891.9349** | **3563.7107** | **3563.7276** | **-0.0169** | **0** | **17** | **0.81** | **1** | **R.ESGYMHIQCTKPDTVGSALNDSPVGLAAYILEK.F** |
|  | 30570 | **1498.7113** | **4493.1121** | **4492.1753** | **0.9368** | **1** | **3** | **11** | **1** | **K.VFYSLMRESGYMHIQCTKPDTVGSALNDSPVGLAAYILEK.F** |

| **304.** | O75964\|ATP5L_HUMAN    **Mass:** 11421    **Score:** 99     **Queries matched:** 4   **emPAI:** 0.82 |
| --- | --- |
|  | ATP synthase subunit g, mitochondrial OS=Homo sapiens GN=ATP5L PE=1 SV=3 |

|  | **Query** | **Observed** | **Mr(expt)** | **Mr(calc)** | **Delta** | **Miss** | **Score** | **Expect** | **Rank** | **Peptide** |
| --- | --- | --- | --- | --- | --- | --- | --- | --- | --- | --- |
|  | 2775 | 446.7311 | 891.4477 | 891.4814 | -0.0337 | 1 | 1 | 1.4e+002 | 8 | K.RGIIGYDV.- |
|  | 6193 | **576.3029** | **1150.5912** | **1150.5982** | **-0.0071** | **0** | **59** | **0.00019** | **1** | **K.IVNSAQTGSFK.Q** |
|  | 9953 | **676.3724** | **1350.7302** | **1349.7125** | **1.0177** | **1** | **4** | **61** | **4** | **-.MAQFVRNLVEK.T** |
|  | 20954 | **724.4063** | **2170.1971** | **2170.2110** | **-0.0139** | **1** | **67** | **1.8e-005** | **1** | **R.NLVEKTPALVNAAVTYSKPR.L** |

| **305.** | Q6RW13\|ATRAP_HUMAN    **Mass:** 17579    **Score:** 98     **Queries matched:** 4   **emPAI:** 0.22 |
| --- | --- |
|  | Type-1 angiotensin II receptor-associated protein OS=Homo sapiens GN=AGTRAP PE=1 SV=1 |

|  | **Query** | **Observed** | **Mr(expt)** | **Mr(calc)** | **Delta** | **Miss** | **Score** | **Expect** | **Rank** | **Peptide** |
| --- | --- | --- | --- | --- | --- | --- | --- | --- | --- | --- |
|  | 4455 | **508.2737** | **1014.5329** | **1013.5579** | **0.9749** | **0** | **21** | **1.3** | **2** | **-.MELPAVNLK.V** |
|  | 21974 | **1146.5353** | **2291.0560** | **2291.0706** | **-0.0146** | **0** | **63** | **4.1e-005** | **1** | **R.SAYQTIDSAEAPADPFAVPEGR.S** 21967 21973 |

| **306.** | P39060\|COIA1_HUMAN    **Mass:** 179389   **Score:** 98     **Queries matched:** 19   **emPAI:** 0.02 |
| --- | --- |
|  | Collagen alpha-1(XVIII) chain OS=Homo sapiens GN=COL18A1 PE=1 SV=5 |

|  | **Query** | **Observed** | **Mr(expt)** | **Mr(calc)** | **Delta** | **Miss** | **Score** | **Expect** | **Rank** | **Peptide** |
| --- | --- | --- | --- | --- | --- | --- | --- | --- | --- | --- |
|  | 539 | **459.0126** | **458.0053** | **458.2601** | **-0.2548** | **0** | **3** | **90** | **1** | **R.LGGGR.L** 534 536 537 |
|  | 8620 | 427.5725 | 1279.6955 | 1280.6473 | -0.9518 | 1 | 4 | 57 | 8 | R.EGIAGPQGPKGDR.G |
|  | 9547 | **664.3733** | **1326.7321** | **1326.5775** | **0.1546** | **0** | **8** | **20** | **1** | **R.GADFQCFQQAR.A** |
|  | 9616 | **666.8664** | **1331.7182** | **1331.6946** | **0.0236** | **1** | **0** | **1.4e+002** | **8** | **K.GQKGEPGVPGPPGR.A** |
|  | 10224 | **684.1998** | **1366.3851** | **1366.6490** | **-0.2639** | **1** | **1** | **72** | **1** | **K.SVWHGSDPNGRR.L** |
|  | 14095 | **801.9031** | **1601.7916** | **1601.8009** | **-0.0093** | **0** | **98** | **2.1e-008** | **1** | **R.TEAPSATGQASSLLGGR.L** 14094 |
|  | 15342 | **561.2783** | **1680.8131** | **1681.8577** | **-1.0445** | **0** | **3** | **53** | **4** | **R.EGPPGFPGLPGPPGPPGR.E** |
|  | 17082 | **606.2936** | **1815.8591** | **1816.8857** | **-1.0266** | **0** | **5** | **36** | **4** | **R.GQPGPPGPQGPPGIGYEGR.Q** |
|  | 18515 | **483.2289** | **1928.8865** | **1928.9646** | **-0.0781** | **0** | **6** | **24** | **3** | **R.QGPPGPPGPPGPPSFPGPHR.Q** |
|  | 19712 | **680.7403** | **2039.1991** | **2040.0276** | **-0.8285** | **1** | **3** | **39** | **1** | **K.GEPGSIFSPDGGALGPAQKGAK.G** |
|  | 20740 | **717.4250** | **2149.2531** | **2148.1229** | **1.1302** | **1** | **5** | **23** | **1** | **R.GPRGFPGPPGPPGVPGLPGEPGR.F** |
|  | 23374 | **831.4725** | **2491.3956** | **2492.3322** | **-0.9366** | **1** | **7** | **13** | **1** | **R.DFQPVLHLVALNSPLSGGMRGIR.G** |
|  | 26983 | **636.7587** | **3178.7573** | **3178.6346** | **0.1226** | **1** | **1** | **33** | **9** | **R.QTISVPGPPGPPGPPGPPGTMGASSGVRLWATR.Q** |
|  | 29943 | **1028.4458** | **4109.7541** | **4109.0980** | **0.6561** | **0** | **1** | **17** | **1** | **R.AGPPGSPCLPGPPGLPCPVSPLGPAGPALQTVPGPQGPPGPPGR.D** 29947 |

| **307.** | P04222\|1C03_HUMAN    **Mass:** 41234    **Score:** 98     **Queries matched:** 6   **emPAI:** 0.30 |
| --- | --- |
|  | HLA class I histocompatibility antigen, Cw-3 alpha chain OS=Homo sapiens GN=HLA-C PE=1 SV=2 |

|  | **Query** | **Observed** | **Mr(expt)** | **Mr(calc)** | **Delta** | **Miss** | **Score** | **Expect** | **Rank** | **Peptide** |
| --- | --- | --- | --- | --- | --- | --- | --- | --- | --- | --- |
|  | 3749 | 483.2167 | 964.4188 | 964.4250 | -0.0062 | 0 | 35 | 0.057 | 1 | R.FDSDAASPR.G |
|  | 7513 | 613.7462 | 1225.4779 | 1225.4856 | -0.0077 | 0 | 54 | 0.00053 | 1 | R.MYGCDVGPDGR.L 7482 |
|  | 12793 | 760.3734 | 1518.7323 | 1518.7427 | -0.0104 | 0 | 73 | 7.3e-006 | 1 | R.SWTAADTAAQITQR.K |
|  | 14460 | 406.9821 | 1623.8995 | 1623.7498 | 0.1497 | 1 | 4 | 43 | 10 | R.MYGCDVGPDGRLLR.G |
|  | 17358 | 612.9700 | 1835.8883 | 1835.9027 | -0.0144 | 0 | 3 | 52 | 2 | K.THVTHHPVSDHEATLR.C |

| **308.** | O14880\|MGST3_HUMAN    **Mass:** 16734    **Score:** 98     **Queries matched:** 6   **emPAI:** 1.30 |
| --- | --- |
|  | Microsomal glutathione S-transferase 3 OS=Homo sapiens GN=MGST3 PE=1 SV=1 |

|  | **Query** | **Observed** | **Mr(expt)** | **Mr(calc)** | **Delta** | **Miss** | **Score** | **Expect** | **Rank** | **Peptide** |
| --- | --- | --- | --- | --- | --- | --- | --- | --- | --- | --- |
|  | 1170 | **702.3730** | **701.3657** | **701.3708** | **-0.0051** | **0** | **33** | **0.14** | **1** | **K.SGLGSGPK.C** |
|  | 24513 | **895.0761** | **2682.2065** | **2682.2206** | **-0.0141** | **0** | **11** | **4.9** | **1** | **K.VEYPIMYSTDPENGHIFNCIQR.A** |
|  | 24887 | **919.1673** | **2754.4801** | **2754.4891** | **-0.0090** | **0** | **44** | **0.0023** | **1** | **R.GALGSIALLGLVGTTVCSAFQHLGWVK.S** |
|  | 25957 | **992.1283** | **2973.3631** | **2973.3789** | **-0.0159** | **1** | **59** | **6.4e-005** | **1** | **K.YKVEYPIMYSTDPENGHIFNCIQR.A** 25956 25972 |

| **309.** | P21695\|GPDA_HUMAN    **Mass:** 38171    **Score:** 98     **Queries matched:** 8   **emPAI:** 0.33 |
| --- | --- |
|  | Glycerol-3-phosphate dehydrogenase [NAD+], cytoplasmic OS=Homo sapiens GN=GPD1 PE=1 SV=4 |

|  | **Query** | **Observed** | **Mr(expt)** | **Mr(calc)** | **Delta** | **Miss** | **Score** | **Expect** | **Rank** | **Peptide** |
| --- | --- | --- | --- | --- | --- | --- | --- | --- | --- | --- |
|  | 2767 | **446.2530** | **890.4915** | **890.4974** | **-0.0059** | **1** | **7** | **42** | **8** | **R.KVAEAFAR.T** |
|  | 4459 | 508.2895 | 1014.5644 | 1014.5709 | -0.0065 | 0 | 18 | 2.7 | 2 | K.LISEVIGER.L |
|  | 13384 | **778.9080** | **1555.8015** | **1555.8107** | **-0.0092** | **0** | **66** | **2.8e-005** | **1** | **K.IVGGNAAQLAQFDPR.V** |
|  | 15096 | **556.3052** | **1665.8939** | **1665.9050** | **-0.0111** | **1** | **34** | **0.044** | **1** | **K.KLTEIINTQHENVK.Y** |
|  | 20322 | **701.4899** | **2101.4480** | **2101.0626** | **0.3854** | **1** | **3** | **28** | **1** | **K.DPAQGQLLKELMQTPNFR.I** |
|  | 24506 | **671.1032** | **2680.3837** | **2680.1621** | **0.2216** | **0** | **3** | **33** | **8** | **K.VCYEGQPVGEFIHCLQNHPEHM.-** |
|  | 26951 | **635.3176** | **3171.5515** | **3172.6359** | **-1.0844** | **1** | **0** | **46** | **6** | **K.LISEVIGERLGIPMSVLMGANIASEVADEK.F** |
|  | 27896 | **1137.6246** | **3409.8521** | **3409.8650** | **-0.0129** | **0** | **40** | **0.0035** | **1** | **K.LPPNVVAVPDVVQAAEDADILIFVVPHQFIGK.I** |

| **310.** | P38646\|GRP75_HUMAN    **Mass:** 73920    **Score:** 97     **Queries matched:** 40   **emPAI:** 0.28 |
| --- | --- |
|  | Stress-70 protein, mitochondrial OS=Homo sapiens GN=HSPA9 PE=1 SV=2 |

|  | **Query** | **Observed** | **Mr(expt)** | **Mr(calc)** | **Delta** | **Miss** | **Score** | **Expect** | **Rank** | **Peptide** |
| --- | --- | --- | --- | --- | --- | --- | --- | --- | --- | --- |
|  | 943 | **601.3628** | **600.3556** | **600.3595** | **-0.0039** | **0** | **24** | **1.1** | **1** | **R.ELLAR.K** |
|  | 1082 | 658.3363 | 657.3290 | 657.3268 | 0.0022 | 0 | 9 | 26 | 1 | K.HLNMK.L |
|  | 1285 | **780.3941** | **779.3868** | **779.3120** | **0.0749** | **0** | **11** | **14** | **1** | **R.EMAGDNK.L** |
|  | 2956 | **453.2259** | **904.4372** | **903.4960** | **0.9412** | **1** | **18** | **3** | **1** | **K.MRELLAR.K** 2954 |
|  | 3662 | **479.7482** | **957.4818** | **957.4879** | **-0.0061** | **0** | **62** | **0.00011** | **1** | **K.VLENAEGAR.T** |
|  | 3686 | **480.7455** | **959.4765** | **959.5440** | **-0.0675** | **1** | **8** | **32** | **10** | **K.DIKNVPFK.I** |
|  | 3857 | 487.2624 | 972.5102 | 972.5175 | -0.0073 | 1 | 24 | 0.77 | 1 | R.RTIAPCQK.A |
|  | 3937 | 489.7732 | 977.5319 | 978.4328 | -0.9009 | 0 | 9 | 22 | 3 | K.DDIENMVK.N |
|  | 3947 | **490.2372** | **978.4599** | **977.4488** | **1.0112** | **0** | **13** | **7.6** | **2** | **K.AMQDAEVSK.S** |
|  | 5808 | 559.8027 | 1117.5908 | 1117.5662 | 0.0246 | 1 | 10 | 16 | 2 | K.DNMALQRVR.E |
|  | 5839 | **561.3032** | **1120.5919** | **1119.5818** | **1.0101** | **1** | **3** | **79** | **1** | **-.MISASRAAAAR.L** |
|  | 6147 | **574.6171** | **1147.2196** | **1147.5469** | **-0.3273** | **1** | **6** | **27** | **2** | **R.KDSETGENIR.Q** |
|  | 6629 | **395.4237** | **1183.2492** | **1183.6785** | **-0.4293** | **1** | **0** | **89** | **9** | **R.AAAARLVGAAASR.G** |
|  | 8803 | **430.8862** | **1289.6367** | **1289.6728** | **-0.0361** | **0** | **9** | **16** | **4** | **K.VQQTVQDLFGR.A** 8823 |
|  | 8944 | 649.3164 | 1296.6181 | 1296.7262 | -0.1081 | 1 | 7 | 30 | 8 | R.LVGAAASRGPTAAR.H 8940 |
|  | 10129 | **681.3711** | **1360.7277** | **1360.7351** | **-0.0073** | **0** | **45** | **0.0052** | **1** | **R.AQFEGIVTDLIR.R** |
|  | 11158 | **474.7592** | **1421.2557** | **1420.6504** | **0.6054** | **1** | **9** | **11** | **5** | **K.DDIENMVKNAEK.Y** |
|  | 11591 | **483.2276** | **1446.6610** | **1445.7548** | **0.9062** | **0** | **(8)** | **19** | **3** | **K.SDIGEVILVGGMTR.M** 11568 |
|  | 11832 | **487.9094** | **1460.7063** | **1461.7497** | **-1.0435** | **0** | **10** | **15** | **1** | **K.SDIGEVILVGGMTR.M** 11841 |
|  | 13339 | **777.4192** | **1552.8238** | **1552.8323** | **-0.0085** | **0** | **34** | **0.05** | **1** | **K.LYSPSQIGAFVLMK.M** |
|  | 13952 | **398.9462** | **1591.7558** | **1591.7664** | **-0.0106** | **1** | **15** | **3.4** | **1** | **K.MKETAENYLGHTAK.N** |
|  | 14428 | 812.3813 | 1622.7480 | 1622.7020 | 0.0460 | 1 | 6 | 29 | 5 | R.EGSGSSGTGEQKEDQK.E |
|  | 15035 | **554.7863** | **1661.3371** | **1661.8559** | **-0.5188** | **1** | **6** | **17** | **5** | **R.MPKVQQTVQDLFGR.A** |
|  | 15213 | **558.6339** | **1672.8797** | **1672.8897** | **-0.0100** | **1** | **25** | **0.39** | **1** | **K.VQQTVQDLFGRAPSK.A** 15227 |
|  | 15526 | **847.9227** | **1693.8308** | **1693.8424** | **-0.0116** | **0** | **24** | **0.4** | **1** | **K.NAVITVPAYFNDSQR.Q** |
|  | 16883 | **601.3001** | **1800.8785** | **1801.9430** | **-1.0645** | **1** | **3** | **51** | **3** | **K.SDIGEVILVGGMTRMPK.V** |
|  | 16974 | 904.9394 | 1807.8642 | 1807.8952 | -0.0310 | 0 | 0 | 1.1e+002 | 5 | K.SQVFSTAADGQTQVEIK.V |
|  | 17239 | **610.2816** | **1827.8229** | **1827.9627** | **-0.1398** | **1** | **3** | **48** | **6** | **K.LYSPSQIGAFVLMKMK.E** |
|  | 20682 | 714.8411 | 2141.5016 | 2141.0423 | 0.4593 | 1 | 3 | 26 | 2 | K.ERVEAVNMAEGIIHDTETK.M |
|  | 21548 | **559.7432** | **2234.9435** | **2236.0522** | **-1.1087** | **1** | **1** | **57** | **7** | **R.GPTAARHQDSWNGLSHEAFR.L** |
|  | 22770 | **802.7338** | **2405.1797** | **2405.1930** | **-0.0134** | **1** | **4** | **31** | **1** | **K.AMQDAEVSKSDIGEVILVGGMTR.M** |
|  | 22875 | **806.7402** | **2417.1987** | **2417.2108** | **-0.0121** | **1** | **10** | **7.9** | **1** | **R.EQQIVIQSSGGLSKDDIENMVK.N** |
|  | 23777 | **639.3082** | **2553.2036** | **2552.1774** | **1.0261** | **1** | **3** | **35** | **1** | **R.VEAVNMAEGIIHDTETKMEEFK.D** |
|  | 25579 | **964.4597** | **2890.3572** | **2891.4276** | **-1.0704** | **1** | **1** | **45** | **8** | **R.ASNGDAWVEAHGKLYSPSQIGAFVLMK.M** |

| **311.** | P01833\|PIGR_HUMAN    **Mass:** 84429    **Score:** 97     **Queries matched:** 21   **emPAI:** 0.09 |
| --- | --- |
|  | Polymeric immunoglobulin receptor OS=Homo sapiens GN=PIGR PE=1 SV=4 |

|  | **Query** | **Observed** | **Mr(expt)** | **Mr(calc)** | **Delta** | **Miss** | **Score** | **Expect** | **Rank** | **Peptide** |
| --- | --- | --- | --- | --- | --- | --- | --- | --- | --- | --- |
|  | 1378 | **397.2209** | **792.4272** | **792.4130** | **0.0142** | **0** | **3** | **85** | **1** | **K.VLDSGFR.E** |
|  | 1708 | **409.2280** | **816.4415** | **815.3661** | **1.0754** | **0** | **13** | **11** | **2** | **K.ADAAPDEK.V** 1687 |
|  | 2100 | 423.2332 | 844.4517 | 845.4355 | -0.9838 | 1 | 21 | 1.8 | 1 | K.TENAQKR.K |
|  | 2558 | **438.5746** | **875.1347** | **875.4283** | **-0.2936** | **0** | **9** | **19** | **8** | **K.CGLGINSR.G** |
|  | 2914 | **451.7317** | **901.4488** | **902.4722** | **-1.0234** | **1** | **20** | **2** | **3** | **K.RAPAFEGR.I** 2945 2946 |
|  | 7220 | **606.7573** | **1211.5000** | **1210.5037** | **0.9963** | **0** | **16** | **3.2** | **1** | **K.AFVNCDENSR.L** |
|  | 7548 | **614.8049** | **1227.5952** | **1227.7551** | **-0.1599** | **0** | **13** | **7** | **2** | **R.LVSLTLNLVTR.A** |
|  | 8003 | **417.2426** | **1248.7060** | **1249.6667** | **-0.9607** | **0** | **9** | **17** | **2** | **K.DGSFSVVITGLR.K** |
|  | 10419 | **459.8965** | **1376.6675** | **1377.7616** | **-1.0941** | **1** | **3** | **77** | **4** | **K.DGSFSVVITGLRK.E** |
|  | 10765 | **699.3983** | **1396.7821** | **1396.7926** | **-0.0105** | **0** | **8** | **19** | **5** | **K.VPGNVTAVLGETLK.V** |
|  | 10847 | 701.8465 | 1401.6784 | 1400.5878 | 1.0906 | 0 | 7 | 25 | 3 | R.TDISMSDFENSR.E |
|  | 14964 | **553.2795** | **1656.8168** | **1656.8029** | **0.0139** | **0** | **2** | **74** | **6** | **R.GGCITLISSEGYVSSK.Y** |
|  | 15112 | 834.4002 | 1666.7857 | 1667.8995 | -1.1137 | 1 | 5 | 32 | 2 | R.APAFEGRILLNPQDK.D |
|  | 16948 | **602.9820** | **1805.9241** | **1806.7843** | **-0.8601** | **1** | **1** | **92** | **4** | **R.SYRTDISMSDFENSR.E** |
|  | 19006 | 658.6464 | 1972.9174 | 1973.8730 | -0.9556 | 0 | 1 | 82 | 6 | R.DAGFYWCLTNGDTLWR.T |
|  | 20947 | **724.3224** | **2169.9453** | **2169.9597** | **-0.0143** | **1** | **48** | **0.0013** | **1** | **R.LSDAGQYLCQAGDDSNSNKK.N** |
|  | 26987 | **455.2221** | **3179.5038** | **3180.3625** | **-0.8587** | **1** | **1** | **43** | **4** | **K.WNNTGCQALPSQDEGPSKAFVNCDENSR.L** |
|  | 28692 | **1210.9075** | **3629.7006** | **3629.7209** | **-0.0203** | **0** | **70** | **3.6e-006** | **1** | **R.YLCGAHSDGQLQEGSPIQAWQLFVNEESTIPR.S** |

| **312.** | P35222\|CTNB1_HUMAN    **Mass:** 86069    **Score:** 96     **Queries matched:** 17   **emPAI:** 0.13 |
| --- | --- |
|  | Catenin beta-1 OS=Homo sapiens GN=CTNNB1 PE=1 SV=1 |

|  | **Query** | **Observed** | **Mr(expt)** | **Mr(calc)** | **Delta** | **Miss** | **Score** | **Expect** | **Rank** | **Peptide** |
| --- | --- | --- | --- | --- | --- | --- | --- | --- | --- | --- |
|  | 2057 | 841.4069 | 840.3996 | 840.5069 | -0.1073 | 0 | 4 | 57 | 7 | K.SGGIPALVK.M |
|  | 7324 | **608.9091** | **1215.8037** | **1215.7339** | **0.0698** | **1** | **1** | **1.1e+002** | **1** | **K.LLWTTSRVLK.V** |
|  | 7542 | **614.3514** | **1226.6882** | **1226.6805** | **0.0077** | **1** | **13** | **8.3** | **1** | **K.AAVMVHQLSKK.E** |
|  | 9057 | **651.8491** | **1301.6837** | **1301.6914** | **-0.0077** | **0** | **13** | **7** | **1** | **R.LVQNCLWTLR.N** |
|  | 10452 | **690.3102** | **1378.6058** | **1378.6147** | **-0.0089** | **0** | **57** | **0.00029** | **1** | **R.TMQNTNDVETAR.C** |
|  | 10512 | **692.3234** | **1382.6323** | **1382.6361** | **-0.0038** | **0** | **26** | **0.34** | **1** | **R.HQEAEMAQNAVR.L** |
|  | 10796 | **700.3498** | **1398.6850** | **1398.6310** | **0.0540** | **0** | **(6)** | **31** | **1** | **R.HQEAEMAQNAVR.L** |
|  | 11600 | **483.2548** | **1446.7425** | **1445.7561** | **0.9864** | **0** | **3** | **67** | **4** | **R.NLALCPANHAPLR.E** |
|  | 12180 | **494.7922** | **1481.3547** | **1480.7674** | **0.5873** | **0** | **8** | **14** | **4** | **R.NEGVATYAAAVLFR.M** 12144 |
|  | 13982 | **798.3180** | **1594.6215** | **1593.7677** | **0.8538** | **0** | **4** | **39** | **1** | **K.MMVCQVGGIEALVR.T** |
|  | 14454 | 542.2728 | 1623.7965 | 1623.7675 | 0.0290 | 0 | 3 | 55 | 7 | R.TSMGGTQQQFVEGVR.M |
|  | 20140 | **695.3207** | **2082.9404** | **2082.8730** | **0.0674** | **0** | **0** | **88** | **1** | **-.MATQADLMELDMAMEPDR.K** |
|  | 21316 | **738.0308** | **2211.0706** | **2210.9680** | **0.1027** | **1** | **3** | **38** | **3** | **-.MATQADLMELDMAMEPDRK.A** |
|  | 24694 | **680.8129** | **2719.2224** | **2718.2959** | **0.9265** | **1** | **4** | **23** | **4** | **R.NEGVATYAAAVLFRMSEDKPQDYK.K** |
|  | 26463 | **767.6603** | **3066.6120** | **3066.6212** | **-0.0092** | **0** | **8** | **7.2** | **1** | **K.MLGSPVDSVLFYAITTLHNLLLHQEGAK.M** |
|  | 27074 | **1068.1625** | **3201.4656** | **3201.4859** | **-0.0203** | **0** | **55** | **0.00017** | **1** | **R.AAMFPETLDEGMQIPSTQFDAAHPTNVQR.L** |

| **313.** | P61224\|RAP1B_HUMAN    **Mass:** 21040    **Score:** 96     **Queries matched:** 5   **emPAI:** 0.40 |
| --- | --- |
|  | Ras-related protein Rap-1b OS=Homo sapiens GN=RAP1B PE=1 SV=1 |

|  | **Query** | **Observed** | **Mr(expt)** | **Mr(calc)** | **Delta** | **Miss** | **Score** | **Expect** | **Rank** | **Peptide** |
| --- | --- | --- | --- | --- | --- | --- | --- | --- | --- | --- |
|  | 10562 | **462.7337** | **1385.1794** | **1385.6463** | **-0.4669** | **1** | **5** | **33** | **10** | **K.YDPTIEDSYRK.Q** |
|  | 13346 | **777.8488** | **1553.6829** | **1553.6933** | **-0.0103** | **0** | **65** | **4.2e-005** | **1** | **R.QWNNCAFLESSAK.S** |
|  | 15068 | 832.8773 | 1663.7401 | 1664.9138 | -1.1737 | 0 | 8 | 18 | 4 | K.SALTVQFVQGIFVEK.Y 15073 |
|  | 15729 | **855.4597** | **1708.9049** | **1708.9148** | **-0.0099** | **1** | **57** | **0.0002** | **1** | **K.SKINVNEIFYDLVR.Q** |

| **314.** | A6NIZ1\|RP1BL_HUMAN    **Mass:** 21140    **Score:** 96     **Queries matched:** 5   **emPAI:** 0.40 |
| --- | --- |
|  | Ras-related protein Rap-1b-like protein OS=Homo sapiens PE=2 SV=1 |

|  | **Query** | **Observed** | **Mr(expt)** | **Mr(calc)** | **Delta** | **Miss** | **Score** | **Expect** | **Rank** | **Peptide** |
| --- | --- | --- | --- | --- | --- | --- | --- | --- | --- | --- |
|  | 5324 | **543.0407** | **1084.0667** | **1083.6764** | **0.3903** | **1** | **2** | **70** | **6** | **K.LVVLGSRGVGK.S** |
|  | 13346 | 777.8488 | 1553.6829 | 1553.6933 | -0.0103 | 0 | 65 | 4.2e-005 | 1 | R.QWNNCAFLESSAK.S |
|  | 15068 | 832.8773 | 1663.7401 | 1664.9138 | -1.1737 | 0 | 8 | 18 | 4 | K.SALTVQFVQGIFVEK.Y 15073 |
|  | 15729 | 855.4597 | 1708.9049 | 1708.9148 | -0.0099 | 1 | 57 | 0.0002 | 1 | K.SKINVNEIFYDLVR.Q |

| **315.** | Q99653\|CHP1_HUMAN    **Mass:** 22442    **Score:** 95     **Queries matched:** 7   **emPAI:** 0.37 |
| --- | --- |
|  | Calcium-binding protein p22 OS=Homo sapiens GN=CHP PE=1 SV=3 |

|  | **Query** | **Observed** | **Mr(expt)** | **Mr(calc)** | **Delta** | **Miss** | **Score** | **Expect** | **Rank** | **Peptide** |
| --- | --- | --- | --- | --- | --- | --- | --- | --- | --- | --- |
|  | 6703 | **594.3351** | **1186.6557** | **1185.6605** | **0.9952** | **1** | **54** | **0.00069** | **1** | **K.VLEKVDVEQK.M** |
|  | 6910 | 599.2780 | 1196.5413 | 1196.5786 | -0.0372 | 0 | 6 | 40 | 9 | K.DVNGPEPLNSR.S |
|  | 12905 | **764.3090** | **1526.6034** | **1525.7485** | **0.8549** | **1** | **1** | **89** | **4** | **K.DVNGPEPLNSRSNK.L** |
|  | 13559 | **523.9351** | **1568.7834** | **1568.7947** | **-0.0113** | **0** | **9** | **17** | **2** | **R.TLAHFRPIEDNEK.S** |
|  | 16672 | **594.7933** | **1781.3580** | **1780.8856** | **0.4723** | **1** | **8** | **11** | **5** | **K.ETGFSHSQITRLYSR.F** |
|  | 19107 | **661.9908** | **1982.9507** | **1982.0221** | **0.9286** | **1** | **3** | **49** | **2** | **R.EDFQRIPELAINPLGDR.I** |
|  | 21203 | **1101.0101** | **2200.0057** | **2200.0172** | **-0.0115** | **0** | **73** | **4.6e-006** | **1** | **R.TIQEADQDGDSAISFTEFVK.V** |

| **316.** | Q05639\|EF1A2_HUMAN    **Mass:** 50780    **Score:** 95     **Queries matched:** 16   **emPAI:** 0.15 |
| --- | --- |
|  | Elongation factor 1-alpha 2 OS=Homo sapiens GN=EEF1A2 PE=1 SV=1 |

|  | **Query** | **Observed** | **Mr(expt)** | **Mr(calc)** | **Delta** | **Miss** | **Score** | **Expect** | **Rank** | **Peptide** |
| --- | --- | --- | --- | --- | --- | --- | --- | --- | --- | --- |
|  | 2462 | 435.2236 | 868.4327 | 869.5334 | -1.1007 | 0 | 14 | 6 | 2 | K.QLIVGVNK.M 2491 |
|  | 2801 | 447.7473 | 893.4800 | 893.4858 | -0.0058 | 1 | 13 | 8.6 | 3 | R.TIEKFEK.E 2802 |
|  | 3093 | 457.7845 | 913.5544 | 913.5597 | -0.0053 | 0 | 48 | 0.0029 | 1 | R.QTVAVGVIK.N |
|  | 4586 | 513.3055 | 1024.5965 | 1024.6030 | -0.0064 | 0 | 68 | 2.4e-005 | 1 | K.IGGIGTVPVGR.V 4587 4596 |
|  | 8162 | **629.3940** | **1256.7734** | **1256.5231** | **0.2504** | **0** | **2** | **84** | **4** | **K.MDSTEPAYSEK.R** |
|  | 9262 | 657.8151 | 1313.6157 | 1312.6816 | 0.9341 | 1 | 6 | 33 | 2 | K.GSFKYAWVLDK.L |
|  | 20386 | **703.3942** | **2107.1607** | **2108.0459** | **-0.8853** | **1** | **2** | **50** | **7** | **K.QLIVGVNKMDSTEPAYSEK.R** |
|  | 23873 | 1285.6223 | 2569.2301 | 2570.4142 | -1.1841 | 0 | 1 | 52 | 6 | R.VETGILRPGMVVTFAPVNITTEVK.S |
|  | 24550 | 897.8085 | 2690.4038 | 2689.4552 | 0.9486 | 1 | 2 | 37 | 2 | K.THINIVVIGHVDSGKSTTTGHLIYK.C 24548 |
|  | 25903 | 494.1146 | 2958.6439 | 2959.5978 | -0.9539 | 0 | 2 | 35 | 8 | K.EGNASGVSLLEALDTILPPTRPTDKPLR.L |
|  | 29589 | **794.5792** | **3967.8597** | **3968.9171** | **-1.0574** | **1** | **1** | **22** | **4** | **K.IGYNPATVPFVPISGWHGDNMLEPSPNMPWFKGWK.V** |

| **317.** | Q9BYF1\|ACE2_HUMAN    **Mass:** 92860    **Score:** 94     **Queries matched:** 17   **emPAI:** 0.08 |
| --- | --- |
|  | Angiotensin-converting enzyme 2 OS=Homo sapiens GN=ACE2 PE=1 SV=2 |

|  | **Query** | **Observed** | **Mr(expt)** | **Mr(calc)** | **Delta** | **Miss** | **Score** | **Expect** | **Rank** | **Peptide** |
| --- | --- | --- | --- | --- | --- | --- | --- | --- | --- | --- |
|  | 959 | **605.3281** | **604.3209** | **604.3068** | **0.0141** | **0** | **10** | **25** | **7** | **R.TEVEK.A** |
|  | 1260 | **765.3946** | **764.3874** | **764.3925** | **-0.0051** | **0** | **19** | **2.2** | **1** | **R.ILMCTK.V** |
|  | 2897 | **451.2267** | **900.4388** | **899.4171** | **1.0217** | **0** | **11** | **15** | **5** | **R.SSVAYAMR.Q** 2871 2908 |
|  | 3063 | **456.8827** | **911.7509** | **912.5029** | **-0.7520** | **0** | **1** | **89** | **6** | **K.NVSDIIPR.T** |
|  | 8439 | **635.8417** | **1269.6689** | **1269.6758** | **-0.0068** | **0** | **7** | **28** | **2** | **R.ISFNFFVTAPK.N** |
|  | 12453 | **500.3528** | **1498.0366** | **1498.7991** | **-0.7625** | **1** | **1** | **72** | **5** | **K.NVSDIIPRTEVEK.A** |
|  | 15528 | **565.6199** | **1693.8380** | **1692.8505** | **0.9875** | **1** | **2** | **71** | **8** | **K.VKNQMILFGEEDVR.V** |
|  | 16348 | **878.9564** | **1755.8982** | **1755.9077** | **-0.0095** | **0** | **76** | **3e-006** | **1** | **R.LNTILNTMSTIYSTGK.V** |
|  | 18283 | **638.0192** | **1911.0359** | **1911.0465** | **-0.0107** | **1** | **44** | **0.0039** | **1** | **R.LGKSEPWTLALENVVGAK.N** |
|  | 20899 | 722.3483 | 2164.0232 | 2164.1681 | -0.1449 | 1 | 0 | 80 | 4 | R.ISFNFFVTAPKNVSDIIPR.T |
|  | 22386 | **587.5533** | **2346.1843** | **2345.0957** | **1.0885** | **0** | **2** | **52** | **5** | **R.NGANEGFHEAVGEIMSLSAATPK.H** |
|  | 26834 | **630.5064** | **3147.4957** | **3146.5504** | **0.9453** | **1** | **2** | **30** | **1** | **R.AKLMNAYPSYISPIGCLPAHLLGDMWGR.F** |
|  | 28278 | **881.1614** | **3520.6164** | **3520.6366** | **-0.0202** | **0** | **7** | **6.8** | **1** | **K.VTMDDFLTAHHEMGHIQYDMAYAAQPFLLR.N** |
|  | 28344 | **1179.5664** | **3535.6774** | **3536.6315** | **-0.9541** | **0** | **(1)** | **27** | **7** | **K.VTMDDFLTAHHEMGHIQYDMAYAAQPFLLR.N** |
|  | 30253 | **860.6018** | **4297.9724** | **4299.0084** | **-1.0360** | **1** | **6** | **5.8** | **1** | **R.ILMCTKVTMDDFLTAHHEMGHIQYDMAYAAQPFLLR.N** |

| **318.** | P62834\|RAP1A_HUMAN    **Mass:** 21316    **Score:** 94     **Queries matched:** 6   **emPAI:** 0.39 |
| --- | --- |
|  | Ras-related protein Rap-1A OS=Homo sapiens GN=RAP1A PE=1 SV=1 |

|  | **Query** | **Observed** | **Mr(expt)** | **Mr(calc)** | **Delta** | **Miss** | **Score** | **Expect** | **Rank** | **Peptide** |
| --- | --- | --- | --- | --- | --- | --- | --- | --- | --- | --- |
|  | 10562 | 462.7337 | 1385.1794 | 1385.6463 | -0.4669 | 1 | 5 | 33 | 10 | K.YDPTIEDSYRK.Q |
|  | 14061 | **800.8427** | **1599.6707** | **1599.6810** | **-0.0102** | **0** | **58** | **0.00017** | **1** | **R.QWCNCAFLESSAK.S** |
|  | 15068 | 832.8773 | 1663.7401 | 1664.9138 | -1.1737 | 0 | 8 | 18 | 4 | K.SALTVQFVQGIFVEK.Y 15073 |
|  | 15729 | 855.4597 | 1708.9049 | 1708.9148 | -0.0099 | 1 | 57 | 0.0002 | 1 | K.SKINVNEIFYDLVR.Q |
|  | 17087 | **606.3038** | **1815.8897** | **1814.8080** | **1.0817** | **1** | **6** | **28** | **3** | **R.QWCNCAFLESSAKSK.I** |

| **319.** | P50416\|CPT1A_HUMAN    **Mass:** 88995    **Score:** 94     **Queries matched:** 26   **emPAI:** 0.18 |
| --- | --- |
|  | Carnitine O-palmitoyltransferase 1, liver isoform OS=Homo sapiens GN=CPT1A PE=1 SV=2 |

|  | **Query** | **Observed** | **Mr(expt)** | **Mr(calc)** | **Delta** | **Miss** | **Score** | **Expect** | **Rank** | **Peptide** |
| --- | --- | --- | --- | --- | --- | --- | --- | --- | --- | --- |
|  | 1781 | 412.7310 | 823.4475 | 824.4504 | -1.0029 | 0 | 4 | 59 | 4 | R.LSHEALR.Q |
|  | 2620 | 440.9027 | 879.7909 | 879.4347 | 0.3563 | 0 | 12 | 6.9 | 1 | K.IWMGMVK.I 2653 |
|  | 2747 | **445.1189** | **888.2232** | **888.4552** | **-0.2320** | **0** | **12** | **10** | **2** | **K.QSLDAVEK.A** |
|  | 4595 | **513.7418** | **1025.4690** | **1025.6121** | **-0.1431** | **0** | **7** | **31** | **5** | **K.IDPSLGIIAK.I** |
|  | 5243 | 540.2306 | 1078.4467 | 1078.4535 | -0.0068 | 0 | 31 | 0.14 | 1 | R.EMEQQMQR.I |
|  | 5943 | **566.2613** | **1130.5080** | **1130.5931** | **-0.0851** | **1** | **13** | **7.4** | **1** | **K.NKQSLDAVEK.A** |
|  | 6381 | **583.3411** | **1164.6677** | **1163.6195** | **1.0482** | **1** | **3** | **76** | **4** | **R.ATKIWMGMVK.I** |
|  | 6755 | **595.4188** | **1188.8231** | **1188.5022** | **0.3209** | **1** | **4** | **52** | **2** | **R.CYDRWFDK.S** |
|  | 8045 | **626.4199** | **1250.8253** | **1249.6853** | **1.1400** | **0** | **1** | **1e+002** | **7** | **R.YLQSVRPLMK.E** |
|  | 8088 | **418.8696** | **1253.5870** | **1252.6676** | **0.9193** | **1** | **9** | **20** | **5** | **R.DSKHIVVYHR.G** |
|  | 10044 | **678.8442** | **1355.6738** | **1355.6834** | **-0.0095** | **0** | **14** | **5.8** | **1** | **K.GDINPNIPYPTR.L** |
|  | 10115 | **681.2720** | **1360.5294** | **1360.5388** | **-0.0094** | **0** | **23** | **0.7** | **1** | **R.SCTTESCDFVR.A** |
|  | 10462 | **460.5864** | **1378.7374** | **1377.6057** | **1.1317** | **0** | **(5)** | **44** | **9** | **K.FCLTYEASMTR.L** |
|  | 10705 | **697.7873** | **1393.5601** | **1393.6006** | **-0.0405** | **0** | **6** | **35** | **2** | **K.FCLTYEASMTR.L** 10684 |
|  | 12896 | **763.8717** | **1525.7288** | **1525.7372** | **-0.0084** | **0** | **55** | **0.00038** | **1** | **R.ILDNTSEPQPGEAR.L** 12895 |
|  | 17206 | **609.3283** | **1824.9629** | **1824.7845** | **0.1785** | **1** | **3** | **48** | **5** | **K.DMGKFCLTYEASMTR.L** |
|  | 17211 | **609.3320** | **1824.9743** | **1824.7845** | **0.1898** | **1** | **(2)** | **69** | **7** | **K.DMGKFCLTYEASMTR.L** |
|  | 17864 | **625.3336** | **1872.9789** | **1871.8883** | **1.0906** | **1** | **(2)** | **62** | **10** | **K.HQHMYRLAMTGSGIDR.H** |
|  | 18021 | **944.8499** | **1887.6853** | **1887.8832** | **-0.1979** | **1** | **4** | **31** | **1** | **K.HQHMYRLAMTGSGIDR.H** |
|  | 18974 | **657.4001** | **1969.1784** | **1970.0030** | **-0.8246** | **1** | **0** | **71** | **8** | **K.EAMTDIITLFGLSSNSKK.-** |
|  | 28934 | **1239.2488** | **3714.7245** | **3714.8784** | **-0.1538** | **1** | **0** | **32** | **3** | **R.TLETANCMSSQTKNVVSGVLFGTGLWVALIVTMR.Y** 28944 |
|  | 30465 | **1474.7098** | **4421.1077** | **4421.1315** | **-0.0238** | **0** | **54** | **7.7e-005** | **1** | **R.LQWDIPGECQEVIETSLNTANLLANDVDFHSFPFVAFGK.G** |

| **320.** | P62937\|PPIA_HUMAN    **Mass:** 18229    **Score:** 94     **Queries matched:** 12   **emPAI:** 0.78 |
| --- | --- |
|  | Peptidyl-prolyl cis-trans isomerase A OS=Homo sapiens GN=PPIA PE=1 SV=2 |

|  | **Query** | **Observed** | **Mr(expt)** | **Mr(calc)** | **Delta** | **Miss** | **Score** | **Expect** | **Rank** | **Peptide** |
| --- | --- | --- | --- | --- | --- | --- | --- | --- | --- | --- |
|  | 1135 | **687.3989** | **686.3917** | **685.3912** | **1.0005** | **0** | **13** | **10** | **4** | **K.HVVFGK.V** |
|  | 9194 | 437.2521 | 1308.7344 | 1309.5642 | -0.8298 | 0 | 7 | 25 | 6 | K.EGMNIVEAMER.F |
|  | 12554 | **753.3715** | **1504.7285** | **1504.7377** | **-0.0093** | **1** | **50** | **0.0013** | **1** | **K.VKEGMNIVEAMER.F** 12556 |
|  | 12805 | **760.8289** | **1519.6433** | **1520.7327** | **-1.0894** | **1** | **(3)** | **65** | **6** | **K.VKEGMNIVEAMER.F** |
|  | 12807 | **507.5618** | **1519.6636** | **1520.7327** | **-1.0691** | **1** | **(9)** | **17** | **2** | **K.VKEGMNIVEAMER.F** |
|  | 14036 | **799.8714** | **1597.7282** | **1597.7381** | **-0.0099** | **0** | **58** | **0.00017** | **1** | **R.IIPGFMCQGGDFTR.H** 14037 |
|  | 17273 | 610.9777 | 1829.9113 | 1830.9039 | -0.9926 | 1 | 7 | 22 | 6 | K.SIYGEKFEDENFILK.H |
|  | 18704 | **649.3361** | **1944.9865** | **1944.9946** | **-0.0080** | **0** | **33** | **0.049** | **1** | **M.VNPTVFFDIAVDGEPLGR.V** |
|  | 21771 | 1133.0261 | 2264.0377 | 2265.0419 | -1.0043 | 1 | 3 | 39 | 5 | R.IIPGFMCQGGDFTRHNGTGGK.S |
|  | 25071 | **931.4359** | **2791.2859** | **2790.3218** | **0.9641** | **0** | **12** | **3.9** | **1** | **K.HTGPGILSMANAGPNTNGSQFFICTAK.T** |

| **321.** | Q07000\|1C15_HUMAN    **Mass:** 41293    **Score:** 94     **Queries matched:** 9   **emPAI:** 0.30 |
| --- | --- |
|  | HLA class I histocompatibility antigen, Cw-15 alpha chain OS=Homo sapiens GN=HLA-C PE=1 SV=1 |

|  | **Query** | **Observed** | **Mr(expt)** | **Mr(calc)** | **Delta** | **Miss** | **Score** | **Expect** | **Rank** | **Peptide** |
| --- | --- | --- | --- | --- | --- | --- | --- | --- | --- | --- |
|  | 3749 | 483.2167 | 964.4188 | 964.4250 | -0.0062 | 0 | 35 | 0.057 | 1 | R.FDSDAASPR.G |
|  | 7000 | **401.2285** | **1200.6638** | **1199.6371** | **1.0268** | **1** | **14** | **5.9** | **1** | **R.QAQTDRVNLR.K** |
|  | 9948 | 676.3410 | 1350.6674 | 1349.6939 | 0.9736 | 1 | 14 | 5.9 | 1 | R.YLENGKETLQR.A 9957 |
|  | 12793 | 760.3734 | 1518.7323 | 1518.7427 | -0.0104 | 0 | 73 | 7.3e-006 | 1 | R.SWTAADTAAQITQR.K |
|  | 14674 | 547.0314 | 1638.0725 | 1637.7654 | 0.3071 | 1 | 2 | 52 | 6 | R.MYGCDLGPDGRLLR.G |
|  | 17358 | 612.9700 | 1835.8883 | 1835.9027 | -0.0144 | 0 | 3 | 52 | 2 | K.THVTHHPVSDHEATLR.C |
|  | 24215 | 879.7303 | 2636.1690 | 2636.1838 | -0.0148 | 0 | 43 | 0.003 | 1 | R.DGEDQTQDTELVETRPAGDGTFQK.W |
|  | 25835 | 982.1769 | 2943.5090 | 2943.3028 | 0.2062 | 1 | 1 | 46 | 9 | R.GYYNQSEAGSHIIQRMYGCDLGPDGR.L |

| **322.** | P30501\|1C02_HUMAN    **Mass:** 41468    **Score:** 94     **Queries matched:** 7   **emPAI:** 0.30 |
| --- | --- |
|  | HLA class I histocompatibility antigen, Cw-2 alpha chain OS=Homo sapiens GN=HLA-C PE=1 SV=1 |

|  | **Query** | **Observed** | **Mr(expt)** | **Mr(calc)** | **Delta** | **Miss** | **Score** | **Expect** | **Rank** | **Peptide** |
| --- | --- | --- | --- | --- | --- | --- | --- | --- | --- | --- |
|  | 3749 | 483.2167 | 964.4188 | 964.4250 | -0.0062 | 0 | 35 | 0.057 | 1 | R.FDSDAASPR.G |
|  | 7000 | 401.2285 | 1200.6638 | 1199.6371 | 1.0268 | 1 | 14 | 5.9 | 1 | R.QAQTDRVNLR.K |
|  | 12793 | 760.3734 | 1518.7323 | 1518.7427 | -0.0104 | 0 | 73 | 7.3e-006 | 1 | R.SWTAADTAAQITQR.K |
|  | 14674 | 547.0314 | 1638.0725 | 1637.7654 | 0.3071 | 1 | 2 | 52 | 6 | R.MYGCDLGPDGRLLR.G |
|  | 17358 | 612.9700 | 1835.8883 | 1835.9027 | -0.0144 | 0 | 3 | 52 | 2 | K.THVTHHPVSDHEATLR.C |
|  | 24215 | 879.7303 | 2636.1690 | 2636.1838 | -0.0148 | 0 | 43 | 0.003 | 1 | R.DGEDQTQDTELVETRPAGDGTFQK.W |
|  | 27011 | 1063.2271 | 3186.6593 | 3187.6192 | -0.9598 | 1 | 3 | 26 | 3 | R.VMEPRTLILLLSGALALTETWACSHSMR.Y |

| **323.** | P10321\|1C07_HUMAN    **Mass:** 41136    **Score:** 94     **Queries matched:** 8   **emPAI:** 0.30 |
| --- | --- |
|  | HLA class I histocompatibility antigen, Cw-7 alpha chain OS=Homo sapiens GN=HLA-C PE=1 SV=3 |

|  | **Query** | **Observed** | **Mr(expt)** | **Mr(calc)** | **Delta** | **Miss** | **Score** | **Expect** | **Rank** | **Peptide** |
| --- | --- | --- | --- | --- | --- | --- | --- | --- | --- | --- |
|  | 3749 | 483.2167 | 964.4188 | 964.4250 | -0.0062 | 0 | 35 | 0.057 | 1 | R.FDSDAASPR.G |
|  | 9948 | 676.3410 | 1350.6674 | 1349.6939 | 0.9736 | 1 | 14 | 5.9 | 1 | R.YLENGKETLQR.A 9957 |
|  | 11244 | **714.3414** | **1426.6683** | **1425.7365** | **0.9318** | **0** | **3** | **60** | **3** | **K.WAAVVVPSGQEQR.Y** |
|  | 12793 | 760.3734 | 1518.7323 | 1518.7427 | -0.0104 | 0 | 73 | 7.3e-006 | 1 | R.SWTAADTAAQITQR.K |
|  | 13213 | **516.2897** | **1545.8474** | **1545.7392** | **0.1082** | **1** | **5** | **41** | **6** | **R.MSGCDLGPDGRLLR.G** |
|  | 13471 | **521.6091** | **1561.8056** | **1561.7341** | **0.0715** | **1** | **(2)** | **82** | **3** | **R.MSGCDLGPDGRLLR.G** |
|  | 24215 | 879.7303 | 2636.1690 | 2636.1838 | -0.0148 | 0 | 43 | 0.003 | 1 | R.DGEDQTQDTELVETRPAGDGTFQK.W |

| **324.** | Q31612\|1B73_HUMAN    **Mass:** 40809    **Score:** 94     **Queries matched:** 10   **emPAI:** 0.30 |
| --- | --- |
|  | HLA class I histocompatibility antigen, B-73 alpha chain OS=Homo sapiens GN=HLA-B PE=1 SV=1 |

|  | **Query** | **Observed** | **Mr(expt)** | **Mr(calc)** | **Delta** | **Miss** | **Score** | **Expect** | **Rank** | **Peptide** |
| --- | --- | --- | --- | --- | --- | --- | --- | --- | --- | --- |
|  | 1255 | **763.1151** | **762.1078** | **762.3694** | **-0.2616** | **0** | **7** | **33** | **7** | **R.NTQICK.A** 1256 1257 |
|  | 3749 | 483.2167 | 964.4188 | 964.4250 | -0.0062 | 0 | 35 | 0.057 | 1 | R.FDSDAASPR.E |
|  | 11244 | 714.3414 | 1426.6683 | 1425.7365 | 0.9318 | 0 | 3 | 60 | 3 | K.WAAVVVPSGQEQR.Y |
|  | 12793 | 760.3734 | 1518.7323 | 1518.7427 | -0.0104 | 0 | 73 | 7.3e-006 | 1 | R.SWTAADTAAQITQR.K |
|  | 23135 | 492.2564 | 2456.2458 | 2456.3097 | -0.0639 | 0 | 2 | 47 | 5 | R.TVLLLLSAALALTETWAGSHSMR.Y |
|  | 24215 | 879.7303 | 2636.1690 | 2636.1838 | -0.0148 | 0 | 43 | 0.003 | 1 | R.DGEDQTQDTELVETRPAGDGTFQK.W |
|  | 27235 | **648.6937** | **3238.4319** | **3238.7392** | **-0.3073** | **1** | **3** | **24** | **5** | **-.MLVMAPRTVLLLLSAALALTETWAGSHSMR.Y** |
|  | 27288 | **651.9504** | **3254.7158** | **3254.7341** | **-0.0183** | **1** | **(1)** | **39** | **4** | **-.MLVMAPRTVLLLLSAALALTETWAGSHSMR.Y** |

| **325.** | O15031\|PLXB2_HUMAN    **Mass:** 207734   **Score:** 93     **Queries matched:** 36   **emPAI:** 0.04 |
| --- | --- |
|  | Plexin-B2 OS=Homo sapiens GN=PLXNB2 PE=1 SV=3 |

|  | **Query** | **Observed** | **Mr(expt)** | **Mr(calc)** | **Delta** | **Miss** | **Score** | **Expect** | **Rank** | **Peptide** |
| --- | --- | --- | --- | --- | --- | --- | --- | --- | --- | --- |
|  | 882 | 576.3053 | 575.2980 | 575.2915 | 0.0066 | 0 | 8 | 37 | 3 | K.NIDSK.L |
|  | 1143 | **689.3318** | **688.3245** | **688.3755** | **-0.0510** | **0** | **5** | **77** | **4** | **K.NLDTVK.G** |
|  | 1503 | **401.2853** | **800.5561** | **799.4552** | **1.1009** | **0** | **5** | **64** | **5** | **K.TNSLPLR.F** |
|  | 2023 | **420.5760** | **839.1374** | **838.4548** | **0.6826** | **0** | **9** | **12** | **2** | **K.LDIPEPR.R** 2000 2001 |
|  | 2739 | **444.7422** | **887.4698** | **886.5058** | **0.9640** | **1** | **6** | **58** | **5** | **K.NPKLMLR.R** |
|  | 3894 | 487.9707 | 973.9268 | 974.5145 | -0.5877 | 1 | 2 | 78 | 9 | R.LLDRTDSR.E |
|  | 4409 | 506.2830 | 1010.5514 | 1010.5033 | 0.0481 | 1 | 10 | 16 | 3 | K.SYGKNIDSK.L |
|  | 6372 | **582.7817** | **1163.5489** | **1162.6346** | **0.9143** | **1** | **8** | **24** | **5** | **K.EKIIDQVYR.G** |
|  | 6838 | **398.7113** | **1193.1122** | **1193.5598** | **-0.4477** | **1** | **10** | **8.8** | **1** | **K.DGDKDVMITGK.L** |
|  | 8507 | **637.8061** | **1273.5976** | **1272.6172** | **0.9804** | **1** | **3** | **75** | **7** | **K.MVEDYYKGIR.Q** |
|  | 9298 | 658.3602 | 1314.7059 | 1314.7330 | -0.0271 | 1 | 3 | 82 | 8 | R.VTLNGVPCKVTK.F |
|  | 10693 | **465.2420** | **1392.7043** | **1393.7102** | **-1.0060** | **1** | **5** | **44** | **1** | **R.AFEPLRSFASGGR.S** 10692 10712 |
|  | 11035 | **472.2832** | **1413.8279** | **1412.7300** | **1.0979** | **0** | **7** | **28** | **3** | **R.DTTHNLPEFIVK.F** |
|  | 11088 | **473.2796** | **1416.8169** | **1417.7248** | **-0.9080** | **1** | **8** | **20** | **3** | **K.RVNTLMHYNVR.D** |
|  | 11888 | **489.2268** | **1464.6585** | **1465.7136** | **-1.0551** | **0** | **2** | **72** | **4** | **K.LHVTLYNCSFGR.S** |
|  | 12024 | **491.7851** | **1472.3334** | **1472.7664** | **-0.4330** | **1** | **0** | **78** | **10** | **K.TYTDRVFFLPSK.D** |
|  | 14410 | **811.8915** | **1621.7685** | **1621.7770** | **-0.0084** | **0** | **100** | **1e-008** | **1** | **K.AMTLQEAEAFVGAER.C** |
|  | 14491 | **543.0407** | **1626.1001** | **1625.8988** | **0.2013** | **1** | **6** | **19** | **3** | **R.LQQIAAALENKVTDL.-** 14468 |
|  | 18940 | **656.8490** | **1967.5252** | **1967.9185** | **-0.3933** | **0** | **3** | **26** | **2** | **K.VGVSQQPEDSQQDLPGER.H** |
|  | 20852 | **720.3523** | **2158.0350** | **2157.9857** | **0.0494** | **1** | **10** | **9.7** | **1** | **K.AMTLQEAEAFVGAERCTMK.T** |
|  | 21959 | **573.0519** | **2288.1787** | **2289.3685** | **-1.1898** | **0** | **3** | **40** | **7** | **M.ALQLWALTLLGLLGAGASLRPR.K** |
|  | 22838 | **805.4772** | **2413.4099** | **2413.1341** | **0.2758** | **1** | **6** | **14** | **1** | **K.VFRLPVQECLSYPTCTQCR.D** |
|  | 23411 | **833.7535** | **2498.2388** | **2498.2540** | **-0.0152** | **1** | **15** | **2.3** | **1** | **K.VYLTPDGTSSEYDSILVEINKR.V** |
|  | 23740 | **638.3208** | **2549.2541** | **2548.5039** | **0.7502** | **1** | **11** | **5.2** | **1** | **-.MALQLWALTLLGLLGAGASLRPRK.L** |
|  | 28299 | 1176.5861 | 3526.7364 | 3527.0064 | -0.2700 | 1 | 0 | 35 | 9 | R.VSDVPLSLILPLVIVPMVVVIAVSVYCYWRK.S |
|  | 28842 | **922.4091** | **3685.6071** | **3685.8298** | **-0.2226** | **0** | **0** | **28** | **7** | **R.AQGEVQLTVSPLPALSEEDELLCLFGESPPHPAR.V** |
|  | 29291 | 961.4959 | 3841.9545 | 3841.9309 | 0.0237 | 1 | 1 | 27 | 2 | R.RAQGEVQLTVSPLPALSEEDELLCLFGESPPHPAR.V |
|  | 29417 | 973.9706 | 3891.8535 | 3891.8645 | -0.0111 | 1 | 19 | 0.37 | 1 | K.AGYLSTNTQQFVAAFEDGPYVFFVFNQQDKHPAR.N |
|  | 30105 | **1400.2283** | **4197.6630** | **4196.9606** | **0.7024** | **1** | **1** | **14** | **2** | **R.AHMEDSCPQFLGPSPLVIPMNHETDVNFQGKNLDTVK.G** |
|  | 30204 | 1420.6709 | 4258.9909 | 4259.0933 | -0.1024 | 1 | 2 | 13 | 4 | K.FGAQLQCVTGPQATRGQMLLEVSYGGSPVPNPGIFFTYR.E |
|  | 30796 | **1161.3043** | **4641.1882** | **4642.2544** | **-1.0662** | **1** | **3** | **8.4** | **1** | **R.QMVQVSDQDMNTHLAEISRAHTDSLNTLVALHQLYQYTQK.Y** |

| **326.** | Q8WWT9\|S13A3_HUMAN    **Mass:** 67197    **Score:** 93     **Queries matched:** 5   **emPAI:** 0.06 |
| --- | --- |
|  | Solute carrier family 13 member 3 OS=Homo sapiens GN=SLC13A3 PE=2 SV=1 |

|  | **Query** | **Observed** | **Mr(expt)** | **Mr(calc)** | **Delta** | **Miss** | **Score** | **Expect** | **Rank** | **Peptide** |
| --- | --- | --- | --- | --- | --- | --- | --- | --- | --- | --- |
|  | 1185 | **711.0927** | **710.0854** | **710.2871** | **-0.2017** | **0** | **4** | **39** | **3** | **K.EDEYR.R** |
|  | 15019 | **830.8871** | **1659.7596** | **1659.7700** | **-0.0104** | **1** | **93** | **5.8e-008** | **1** | **R.KDPSQESEENTAAVR.R** |
|  | 30421 | **879.9874** | **4394.9005** | **4394.2472** | **0.6533** | **1** | **0** | **18** | **1** | **R.LILGMMVTTSFLSMWLSNTASTAMMLPIANAILKSLFGQK.E** |
|  | 31100 | **979.4502** | **4892.2146** | **4891.6321** | **0.5826** | **1** | **1** | **12** | **1** | **K.EGRCLFVILLMAVYWCTEALPLSVTALLPIVLFPFMGILPSNK.V** |
|  | 31139 | **1233.0222** | **4928.0598** | **4927.5831** | **0.4767** | **1** | **0** | **12** | **2** | **K.ILMLVGVQPARLILGMMVTTSFLSMWLSNTASTAMMLPIANAILK.S** |

| **327.** | P02452\|CO1A1_HUMAN    **Mass:** 139853   **Score:** 93     **Queries matched:** 40   **emPAI:** 0.05 |
| --- | --- |
|  | Collagen alpha-1(I) chain OS=Homo sapiens GN=COL1A1 PE=1 SV=4 |

|  | **Query** | **Observed** | **Mr(expt)** | **Mr(calc)** | **Delta** | **Miss** | **Score** | **Expect** | **Rank** | **Peptide** |
| --- | --- | --- | --- | --- | --- | --- | --- | --- | --- | --- |
|  | 4620 | **514.7601** | **1027.5057** | **1027.4683** | **0.0374** | **0** | **6** | **41** | **1** | **R.GANGAPGNDGAK.G** 4608 |
|  | 5054 | **531.9184** | **1061.8222** | **1062.5683** | **-0.7460** | **0** | **2** | **77** | **6** | **R.GRPGAPGPAGAR.G** 5076 |
|  | 5750 | 558.2845 | 1114.5545 | 1114.5367 | 0.0178 | 1 | 6 | 39 | 2 | K.GADGSPGKDGVR.G |
|  | 6327 | 580.9122 | 1159.8098 | 1159.6350 | 0.1748 | 0 | 1 | 1e+002 | 4 | R.GVPGPPGAVGPAGK.D |
|  | 6335 | **581.2865** | **1160.5584** | **1160.5649** | **-0.0064** | **0** | **2** | **1.1e+002** | **2** | **R.GQAGVMGFPGPK.G** |
|  | 6713 | **396.8441** | **1187.5104** | **1186.6306** | **0.8799** | **0** | **10** | **17** | **3** | **K.SLSQQIENIR.S** 6709 |
|  | 8962 | 433.5725 | 1297.6956 | 1296.6058 | 1.0898 | 0 | 4 | 53 | 7 | K.GESGPSGPAGPTGAR.G |
|  | 9675 | **446.0377** | **1335.0912** | **1334.5747** | **0.5165** | **1** | **1** | **78** | **4** | **R.DLKMCHSDWK.S** |
|  | 11257 | **476.7288** | **1427.1646** | **1426.6953** | **0.4693** | **0** | **5** | **32** | **3** | **R.GSAGPPGATGFPGAAGR.V** 11244 |
|  | 11625 | **725.3864** | **1448.7583** | **1447.7718** | **0.9865** | **1** | **1** | **97** | **1** | **R.GLPGTAGLPGMKGHR.G** |
|  | 13064 | **513.4946** | **1537.4619** | **1536.7797** | **0.6822** | **0** | **5** | **28** | **3** | **K.GANGAPGIAGAPGFPGAR.G** |
|  | 13316 | 776.8461 | 1551.6776 | 1552.7668 | -1.0892 | 0 | (0) | 1.2e+002 | 9 | K.STGGISVPGPMGPSGPR.G |
|  | 13422 | **520.6078** | **1558.8017** | **1557.8151** | **0.9866** | **0** | **4** | **53** | **2** | **R.GLTGPIGPPGPAGAPGDK.G** |
|  | 13566 | **785.6344** | **1569.2542** | **1568.7617** | **0.4925** | **0** | **3** | **44** | **1** | **K.STGGISVPGPMGPSGPR.G** |
|  | 14703 | **547.3433** | **1639.0080** | **1637.8162** | **1.1918** | **1** | **1** | **73** | **7** | **R.GERGFPGLPGPSGEPGK.Q** |
|  | 14707 | **547.6074** | **1639.8003** | **1638.8114** | **0.9888** | **1** | **11** | **9.7** | **1** | **R.GFPGADGVAGPKGPAGER.G** 14675 14677 |
|  | 15518 | **565.3266** | **1692.9580** | **1693.7478** | **-0.7899** | **0** | **(5)** | **31** | **3** | **K.GEPGSPGENGAPGQMGPR.G** |
|  | 15749 | **571.2786** | **1710.8141** | **1709.7428** | **1.0713** | **0** | **9** | **14** | **3** | **K.GEPGSPGENGAPGQMGPR.G** |
|  | 17250 | **610.5522** | **1828.6347** | **1827.8938** | **0.7410** | **1** | **1** | **63** | **8** | **R.GQAGVMGFPGPKGAAGEPGK.A** |
|  | 18542 | **644.6480** | **1930.9222** | **1931.0047** | **-0.0825** | **1** | **2** | **60** | **3** | **R.GPPGPQGARGLPGTAGLPGMK.G** 18529 |
|  | 18886 | **981.4868** | **1960.9591** | **1960.9715** | **-0.0124** | **1** | **73** | **5.4e-006** | **1** | **K.SGDRGETGPAGPAGPVGPVGAR.G** 18885 |
|  | 20501 | **530.7684** | **2119.0444** | **2120.0651** | **-1.0207** | **0** | **5** | **29** | **10** | **R.GETGPAGPPGAPGAPGAPGPVGPAGK.S** |
|  | 20600 | 1067.9911 | 2133.9676 | 2135.0760 | -1.1084 | 1 | 2 | 50 | 3 | R.VGPPGPSGNAGPPGPPGPAGKEGGK.G |
|  | 23416 | **834.0932** | **2499.2578** | **2499.2143** | **0.0435** | **0** | **2** | **45** | **2** | **R.GNDGATGAAGPPGPTGPAGPPGFPGAVGAK.G** 23408 |
|  | 26777 | **628.1065** | **3135.4962** | **3134.5469** | **0.9493** | **1** | **1** | **40** | **6** | **K.TGPPGPAGQDGRPGPPGPPGARGQAGVMGFPGPK.G** |
|  | 26809 | 786.8795 | 3143.4890 | 3143.4439 | 0.0451 | 1 | 3 | 26 | 3 | R.GANGAPGNDGAKGDAGAPGAPGSQGAPGLQGMPGER.G |
|  | 28334 | **884.4097** | **3533.6096** | **3532.7084** | **0.9012** | **1** | **2** | **22** | **1** | **R.GFSGLQGPPGPPGSPGEQGPSGASGPAGPRGPPGSAGAPGK.D** 28332 28335 |
|  | 29342 | **773.0307** | **3860.1171** | **3859.8959** | **0.2212** | **1** | **5** | **8.6** | **2** | **R.GRTGDAGPVGPPGPPGPPGPPGPPSAGFDFSFLPQPPQEK.A** |
|  | 31503 | **1057.8914** | **5284.4204** | **5285.5881** | **-1.1677** | **1** | **1** | **8.1** | **4** | **R.DGIPGQPGLPGPPGPPGPPGPPGLGGNFAPQLSYGYDEKSTGGISVPGPMGPSGPR.G** |

| **328.** | Q8NHW5\|RLA0L_HUMAN    **Mass:** 34514    **Score:** 93     **Queries matched:** 17   **emPAI:** 0.36 |
| --- | --- |
|  | 60S acidic ribosomal protein P0-like OS=Homo sapiens PE=2 SV=1 |

|  | **Query** | **Observed** | **Mr(expt)** | **Mr(calc)** | **Delta** | **Miss** | **Score** | **Expect** | **Rank** | **Peptide** |
| --- | --- | --- | --- | --- | --- | --- | --- | --- | --- | --- |
|  | 1542 | 402.7128 | 803.4110 | 802.4119 | 0.9990 | 0 | 9 | 29 | 8 | K.QMQQIR.M |
|  | 3521 | **474.2633** | **946.5120** | **945.5681** | **0.9439** | **1** | **5** | **62** | **5** | **R.GKVVVLMGK.N** |
|  | 7414 | 611.3104 | 1220.6063 | 1220.6149 | -0.0086 | 0 | 53 | 0.00072 | 1 | R.GHLENNPALEK.L 7413 7428 |
|  | 8357 | 423.0529 | 1266.1368 | 1265.6074 | 0.5294 | 0 | 0 | 83 | 4 | K.CFIVGADNVGSK.Q |
|  | 9244 | 657.3550 | 1312.6954 | 1312.7027 | -0.0073 | 0 | 14 | 5.4 | 1 | K.TSFFQALGITTK.I |
|  | 9450 | 441.7436 | 1322.2089 | 1321.6595 | 0.5495 | 1 | 7 | 17 | 2 | K.QMQQIRMSLR.G |
|  | 10696 | **465.2555** | **1392.7446** | **1393.7244** | **-0.9797** | **1** | **(4)** | **60** | **4** | **K.VVVLMGKNTMMR.K** |
|  | 10714 | **465.7636** | **1394.2690** | **1393.7244** | **0.5446** | **1** | **(2)** | **60** | **8** | **K.VVVLMGKNTMMR.K** |
|  | 10992 | **471.2526** | **1410.7359** | **1409.7193** | **1.0166** | **1** | **(2)** | **80** | **10** | **K.VVVLMGKNTMMR.K** |
|  | 11234 | **476.2729** | **1425.7970** | **1425.7142** | **0.0828** | **1** | **7** | **27** | **10** | **K.VVVLMGKNTMMR.K** |
|  | 21032 | 1090.5472 | 2179.0799 | 2179.0943 | -0.0144 | 0 | 41 | 0.0063 | 1 | R.AGAIAPCEVTVPAQNTGLGPEK.T 21033 21034 |
|  | 27159 | **806.3901** | **3221.5315** | **3222.6924** | **-1.1610** | **1** | **0** | **44** | **1** | **K.AFLADPSAFVAAAPVAADTTAAPAAAAAPAKVEAK.E** |
|  | 28122 | 1158.9291 | 3473.7654 | 3473.7865 | -0.0211 | 1 | 7 | 8.2 | 2 | R.AGAIAPCEVTVPAQNTGLGPEKTSFFQALGITTK.I |

| **329.** | P30519\|HMOX2_HUMAN    **Mass:** 36181    **Score:** 93     **Queries matched:** 14   **emPAI:** 0.10 |
| --- | --- |
|  | Heme oxygenase 2 OS=Homo sapiens GN=HMOX2 PE=1 SV=2 |

|  | **Query** | **Observed** | **Mr(expt)** | **Mr(calc)** | **Delta** | **Miss** | **Score** | **Expect** | **Rank** | **Peptide** |
| --- | --- | --- | --- | --- | --- | --- | --- | --- | --- | --- |
|  | 1147 | 689.4147 | 688.4075 | 688.4119 | -0.0045 | 1 | 11 | 22 | 10 | R.KEALTK.D |
|  | 1289 | **786.1808** | **785.1736** | **784.4919** | **0.6817** | **1** | **5** | **44** | **2** | **K.VAQRALK.L** |
|  | 8595 | **640.3173** | **1278.6201** | **1277.6471** | **0.9729** | **1** | **(5)** | **42** | **2** | **R.MNALDLNMKTK.E** |
|  | 8863 | 647.2992 | 1292.5838 | 1293.6421 | -1.0582 | 1 | 17 | 3.2 | 2 | R.MNALDLNMKTK.E |
|  | 9174 | **437.0529** | **1308.1368** | **1307.6326** | **0.5043** | **1** | **1** | **82** | **10** | **R.ARMNALDLNMK.T** |
|  | 11516 | **722.3865** | **1442.7585** | **1442.6678** | **0.0907** | **0** | **1** | **98** | **7** | **R.ETLEDGFPVHDGK.G** |
|  | 15493 | **846.8583** | **1691.7021** | **1692.8174** | **-1.1153** | **1** | **3** | **47** | **5** | **K.ENQMRMADLSELLK.E** |
|  | 24948 | **1386.0953** | **2770.1761** | **2770.4191** | **-0.2429** | **1** | **0** | **48** | **4** | **K.YVERIHYIGQNEPELLVAHAYTR.Y** |
|  | 25841 | **982.4904** | **2944.4493** | **2944.4607** | **-0.0114** | **1** | **57** | **0.00011** | **1** | **R.ALKLPSTGEGTQFYLFENVDNAQQFK.Q** 25840 |
|  | 27071 | **1067.8513** | **3200.5321** | **3200.5448** | **-0.0126** | **1** | **14** | **2.1** | **1** | **R.IVEEANKAFEYNMQIFNELDQAGSTLAR.E** 27072 |
|  | 27554 | **832.4207** | **3325.6535** | **3324.8130** | **0.8405** | **1** | **(2)** | **29** | **4** | **R.TAMAVLRKPSLQFILAAGVALAAGLLAWYYM.-** |
|  | 27617 | 669.3184 | 3341.5557 | 3340.8079 | 0.7478 | 1 | 2 | 25 | 4 | R.TAMAVLRKPSLQFILAAGVALAAGLLAWYYM.- |

| **330.** | O95202\|LETM1_HUMAN    **Mass:** 83986    **Score:** 92     **Queries matched:** 18   **emPAI:** 0.19 |
| --- | --- |
|  | LETM1 and EF-hand domain-containing protein 1, mitochondrial OS=Homo sapiens GN=LETM1 PE=1 SV=1 |

|  | **Query** | **Observed** | **Mr(expt)** | **Mr(calc)** | **Delta** | **Miss** | **Score** | **Expect** | **Rank** | **Peptide** |
| --- | --- | --- | --- | --- | --- | --- | --- | --- | --- | --- |
|  | 2789 | **447.2100** | **892.4054** | **893.4429** | **-1.0375** | **0** | **9** | **23** | **1** | **R.ICADLFR.L** 2811 |
|  | 3962 | 490.7507 | 979.4868 | 978.4460 | 1.0408 | 0 | 7 | 31 | 2 | K.HYYHGFR.L |
|  | 4488 | **509.2853** | **1016.5559** | **1016.4968** | **0.0592** | **0** | **3** | **1e+002** | **6** | **K.DFSVFFQK.I** |
|  | 4526 | **511.2500** | **1020.4854** | **1019.5135** | **0.9719** | **1** | **2** | **1e+002** | **6** | **K.ELSKTGEEK.Y** |
|  | 7097 | **402.5364** | **1204.5874** | **1203.6030** | **0.9844** | **1** | **11** | **11** | **8** | **R.GMRALGVTEDR.L** |
|  | 7119 | **403.0463** | **1206.1170** | **1205.6373** | **0.4797** | **1** | **6** | **26** | **4** | **-.MASILLRSCR.G** |
|  | 9313 | **658.8158** | **1315.6170** | **1315.6256** | **-0.0085** | **0** | **38** | **0.022** | **1** | **K.VAEVEGEQVDNK.A** |
|  | 15056 | **555.2794** | **1662.8164** | **1661.8334** | **0.9830** | **0** | **(5)** | **41** | **8** | **R.AMYLPDTLSPADQLK.S** |
|  | 15321 | **560.6440** | **1678.9101** | **1677.8284** | **1.0818** | **0** | **5** | **34** | **3** | **R.AMYLPDTLSPADQLK.S** |
|  | 15748 | **571.2780** | **1710.8122** | **1710.9767** | **-0.1645** | **1** | **4** | **50** | **9** | **K.VNIDDLVKVIELVDK.E** |
|  | 16487 | **884.9415** | **1767.8685** | **1768.9247** | **-1.0562** | **0** | **6** | **25** | **2** | **K.LEEGGPVYSPPAEVVVK.K** |
|  | 17094 | **606.6343** | **1816.8810** | **1815.8897** | **0.9913** | **1** | **27** | **0.19** | **1** | **K.IRETGERPSNEEIMR.F** |
|  | 17800 | **623.9934** | **1868.9584** | **1867.9390** | **1.0194** | **0** | **0** | **88** | **7** | **K.LFPNMLPSTFETQSLK.E** |
|  | 17827 | **624.6387** | **1870.8942** | **1870.9272** | **-0.0330** | **1** | **6** | **26** | **2** | **K.EAQVKVAEVEGEQVDNK.A** |
|  | 23017 | **1221.1169** | **2440.2193** | **2439.2501** | **0.9692** | **0** | **65** | **2.2e-005** | **1** | **K.LAPANGMPTGENVISVAELINAMK.Q** 23018 |
|  | 30192 | **1064.7168** | **4254.8381** | **4254.9820** | **-0.1439** | **1** | **5** | **5.6** | **1** | **R.GSPGDPAHLSCASTLGLRNCLNVPFGCCTPIHPVYTSSR.G** |

| **331.** | O14745\|NHRF1_HUMAN    **Mass:** 39130    **Score:** 92     **Queries matched:** 8   **emPAI:** 0.10 |
| --- | --- |
|  | Na(+)/H(+) exchange regulatory cofactor NHE-RF1 OS=Homo sapiens GN=SLC9A3R1 PE=1 SV=4 |

|  | **Query** | **Observed** | **Mr(expt)** | **Mr(calc)** | **Delta** | **Miss** | **Score** | **Expect** | **Rank** | **Peptide** |
| --- | --- | --- | --- | --- | --- | --- | --- | --- | --- | --- |
|  | 4596 | 513.7733 | 1025.5321 | 1025.5393 | -0.0072 | 0 | 11 | 11 | 1 | R.LVEPGSPAEK.A 4587 |
|  | 9665 | **668.3129** | **1334.6113** | **1333.6370** | **0.9743** | **0** | **18** | **2.2** | **1** | **R.IVEVNGVCMEGK.Q** |
|  | 12577 | **503.2268** | **1506.6587** | **1505.7263** | **0.9324** | **1** | **5** | **36** | **1** | **K.KGPSGYGFNLHSDK.S** |
|  | 14482 | **813.9275** | **1625.8404** | **1625.8512** | **-0.0108** | **0** | **58** | **0.00019** | **1** | **R.LLVVDPETDEQLQK.L** 14483 |
|  | 24514 | **537.5048** | **2682.4877** | **2682.2694** | **0.2183** | **0** | **7** | **11** | **2** | **K.QDSTAPSSTSSSDPILDFNISLAMAK.E** |
|  | 26253 | **1011.2288** | **3030.6646** | **3030.3914** | **0.2732** | **1** | **5** | **15** | **1** | **R.AQEAPGQAEPPAAAEVQGAGNENEPREADK.S** |

| **332.** | Q93050\|VPP1_HUMAN    **Mass:** 97148    **Score:** 92     **Queries matched:** 30   **emPAI:** 0.21 |
| --- | --- |
|  | V-type proton ATPase 116 kDa subunit a isoform 1 OS=Homo sapiens GN=ATP6V0A1 PE=1 SV=3 |

|  | **Query** | **Observed** | **Mr(expt)** | **Mr(calc)** | **Delta** | **Miss** | **Score** | **Expect** | **Rank** | **Peptide** |
| --- | --- | --- | --- | --- | --- | --- | --- | --- | --- | --- |
|  | 1168 | **700.4304** | **699.4231** | **699.4279** | **-0.0048** | **0** | **26** | **0.55** | **1** | **R.VLQAAAK.N** |
|  | 2767 | 446.2530 | 890.4915 | 890.4974 | -0.0059 | 1 | 14 | 7.8 | 2 | R.KFVNEVR.R |
|  | 3195 | **460.7605** | **919.5064** | **919.5127** | **-0.0063** | **1** | **36** | **0.044** | **1** | **R.FVEKEIR.K** |
|  | 3961 | **490.7387** | **979.4629** | **979.4393** | **0.0236** | **0** | **7** | **32** | **5** | **K.EMASGVNTR.I** |
|  | 5438 | 546.8013 | 1091.5881 | 1091.5393 | 0.0488 | 1 | 28 | 0.24 | 1 | R.KEMASGVNTR.I 5421 5441 5442 5453 5454 |
|  | 5662 | **554.3207** | **1106.6268** | **1107.5342** | **-0.9074** | **1** | **(8)** | **27** | **4** | **R.KEMASGVNTR.I** 5669 |
|  | 6642 | **395.7425** | **1184.2058** | **1183.6462** | **0.5596** | **0** | **9** | **14** | **7** | **K.HLGTLNFGGIR.V** |
|  | 9309 | 439.3740 | 1315.1002 | 1315.6521 | -0.5518 | 0 | 1 | 78 | 10 | R.DLNPDVNVFQR.K |
|  | 9600 | **666.0188** | **1330.0230** | **1329.7517** | **0.2713** | **1** | **2** | **61** | **2** | **R.LGFVAGVINRER.I** |
|  | 11164 | **711.8329** | **1421.6512** | **1421.6609** | **-0.0097** | **0** | **37** | **0.026** | **1** | **R.MQTNQTPPTYNK.T** 11165 |
|  | 11459 | **720.3992** | **1438.7839** | **1437.6558** | **1.1281** | **0** | **(9)** | **17** | **1** | **R.MQTNQTPPTYNK.T** 11437 |
|  | 13223 | **774.3574** | **1546.7002** | **1546.7086** | **-0.0084** | **0** | **13** | **5.8** | **1** | **R.ASLYPCPETPQER.K** |
|  | 13349 | **777.8923** | **1553.7700** | **1553.7798** | **-0.0098** | **0** | **56** | **0.0003** | **1** | **R.GTEHSGSTVPSILNR.M** |
|  | 14643 | **546.2999** | **1635.8778** | **1634.9395** | **0.9382** | **1** | **3** | **62** | **5** | **R.NFLELTELKFILR.K** |
|  | 19259 | **998.9880** | **1995.9615** | **1995.9724** | **-0.0109** | **0** | **41** | **0.0071** | **1** | **K.ANIPIMDTGENPEVPFPR.D** |
|  | 20541 | **709.7010** | **2126.0811** | **2126.0175** | **0.0637** | **1** | **6** | **21** | **2** | **R.IDDLQMVLNQTEDHRQR.V** |
|  | 20795 | **719.0131** | **2154.0175** | **2153.0277** | **0.9899** | **0** | **19** | **1.2** | **1** | **R.QAEIENPLEDPVTGDYVHK.S** |
|  | 21024 | **545.4877** | **2177.9218** | **2179.0891** | **-1.1673** | **1** | **8** | **13** | **3** | **K.FYSGTGFKFLPFSFEHIR.E** |
|  | 26786 | 785.5994 | 3138.3684 | 3137.6996 | 0.6688 | 1 | 3 | 21 | 5 | K.MKMSVILGIIHMLFGVSLSLFNHIYFK.K |
|  | 27561 | **1110.5059** | **3328.4958** | **3328.5115** | **-0.0158** | **1** | **26** | **0.095** | **1** | **R.KTQQFFDEMADPDLLEESSSLLEPSEMGR.G** |
|  | 27638 | **1115.8661** | **3344.5764** | **3344.5064** | **0.0700** | **1** | **(3)** | **24** | **3** | **R.KTQQFFDEMADPDLLEESSSLLEPSEMGR.G** |
|  | 30212 | **1066.2686** | **4261.0451** | **4261.9873** | **-0.9422** | **1** | **2** | **14** | **2** | **K.NENEMFSTVFSGRYIILLMGVFSMYTGLIYNDCFSK.S** |

| **333.** | P60468\|SC61B_HUMAN    **Mass:** 10025    **Score:** 90     **Queries matched:** 8   **emPAI:** 0.97 |
| --- | --- |
|  | Protein transport protein Sec61 subunit beta OS=Homo sapiens GN=SEC61B PE=1 SV=2 |

|  | **Query** | **Observed** | **Mr(expt)** | **Mr(calc)** | **Delta** | **Miss** | **Score** | **Expect** | **Rank** | **Peptide** |
| --- | --- | --- | --- | --- | --- | --- | --- | --- | --- | --- |
|  | 5865 | **562.7579** | **1123.5013** | **1123.5081** | **-0.0067** | **0** | **48** | **0.0025** | **1** | **R.TTSAGTGGMWR.F** 5864 |
|  | 5995 | 568.2955 | 1134.5765 | 1135.5152 | -0.9387 | 1 | 2 | 93 | 7 | K.NASCGTRSAGR.T |
|  | 6058 | **571.2839** | **1140.5532** | **1139.5030** | **1.0502** | **0** | **(5)** | **54** | **1** | **R.TTSAGTGGMWR.F** |
|  | 10280 | **685.8321** | **1369.6496** | **1369.6586** | **-0.0090** | **0** | **56** | **0.00034** | **1** | **M.PGPTPSGTNVGSSGR.S** |
|  | 21753 | **755.0492** | **2262.1257** | **2261.0423** | **1.0834** | **1** | **4** | **30** | **1** | **R.TTSAGTGGMWRFYTEDSPGLK.V** |
|  | 21896 | **760.3245** | **2277.9516** | **2277.0372** | **0.9143** | **1** | **(3)** | **33** | **2** | **R.TTSAGTGGMWRFYTEDSPGLK.V** |
|  | 24869 | 918.1489 | 2751.4248 | 2752.5576 | -1.1329 | 0 | 0 | 55 | 5 | K.VGPVPVLVMSLLFIASVFMLHIWGK.Y |

| **334.** | Q5RI15\|FA36A_HUMAN    **Mass:** 13511    **Score:** 90     **Queries matched:** 3   **emPAI:** 0.29 |
| --- | --- |
|  | Protein FAM36A OS=Homo sapiens GN=FAM36A PE=1 SV=2 |

|  | **Query** | **Observed** | **Mr(expt)** | **Mr(calc)** | **Delta** | **Miss** | **Score** | **Expect** | **Rank** | **Peptide** |
| --- | --- | --- | --- | --- | --- | --- | --- | --- | --- | --- |
|  | 1875 | **416.0282** | **830.0418** | **829.3678** | **0.6740** | **1** | **2** | **1e+002** | **7** | **R.KHNGSSSN.-** |
|  | 14118 | **802.9045** | **1603.7945** | **1603.8028** | **-0.0083** | **0** | **74** | **4.4e-006** | **1** | **K.LLGFLDVENTPCAR.H** 14119 |

| **335.** | Q8WW52\|F151A_HUMAN    **Mass:** 64329    **Score:** 90     **Queries matched:** 7   **emPAI:** 0.06 |
| --- | --- |
|  | Protein FAM151A OS=Homo sapiens GN=FAM151A PE=2 SV=2 |

|  | **Query** | **Observed** | **Mr(expt)** | **Mr(calc)** | **Delta** | **Miss** | **Score** | **Expect** | **Rank** | **Peptide** |
| --- | --- | --- | --- | --- | --- | --- | --- | --- | --- | --- |
|  | 2727 | **444.2154** | **886.4162** | **885.5032** | **0.9130** | **0** | **0** | **1.9e+002** | **3** | **K.QLALNATR.K** |
|  | 6258 | **578.2918** | **1154.5691** | **1155.6724** | **-1.1033** | **1** | **6** | **41** | **1** | **R.TALLAARAVDR.T** |
|  | 8894 | **432.2332** | **1293.6776** | **1293.6864** | **-0.0087** | **1** | **10** | **15** | **7** | **R.VTFPVRSSMVR.A** |
|  | 18616 | **647.0004** | **1937.9793** | **1937.0306** | **0.9487** | **1** | **3** | **47** | **4** | **K.MHELVGGVPQRVTFPVR.S** |
|  | 23653 | **847.5826** | **2539.7259** | **2539.3143** | **0.4116** | **1** | **4** | **18** | **1** | **R.ATVTVEHNPAGGDYASVRTALLAAR.A** |
|  | 23806 | **1279.1331** | **2556.2516** | **2556.2570** | **-0.0054** | **0** | **90** | **7.1e-008** | **1** | **R.YSLTLWQAASDPMSVEDLLYVR.D** |
|  | 29356 | 1291.5900 | 3871.7481 | 3871.9706 | -0.2226 | 0 | 1 | 25 | 8 | R.KPMYYTGGSLIPLLQLPGDDGLNVEWLVPDVQGSGK.T |

| **336.** | O14672\|ADA10_HUMAN    **Mass:** 86140    **Score:** 89     **Queries matched:** 26   **emPAI:** 0.13 |
| --- | --- |
|  | Disintegrin and metalloproteinase domain-containing protein 10 OS=Homo sapiens GN=ADAM10 PE=1 SV=1 |

|  | **Query** | **Observed** | **Mr(expt)** | **Mr(calc)** | **Delta** | **Miss** | **Score** | **Expect** | **Rank** | **Peptide** |
| --- | --- | --- | --- | --- | --- | --- | --- | --- | --- | --- |
|  | 2925 | **452.2097** | **902.4048** | **902.4862** | **-0.0813** | **0** | **6** | **54** | **7** | **R.FPNIGVEK.F** |
|  | 3587 | **477.1947** | **952.3748** | **951.4675** | **0.9073** | **0** | **5** | **43** | **3** | **R.LDFHAHGR.H** |
|  | 4587 | 513.3060 | 1024.5974 | 1025.5506 | -0.9532 | 0 | 4 | 59 | 10 | R.LVDADGPLAR.L |
|  | 7921 | **415.9131** | **1244.7175** | **1245.5448** | **-0.8273** | **0** | **9** | **21** | **2** | **K.ENGNYIMYAR.A** |
|  | 9551 | **443.2710** | **1326.7913** | **1325.6398** | **1.1515** | **0** | **8** | **20** | **4** | **K.ICSVHTPSSNPK.L** 9506 9526 9531 9546 |
|  | 9852 | **449.2457** | **1344.7152** | **1343.6357** | **1.0794** | **1** | **4** | **56** | **3** | **K.RDTSLFSDEFK.V** |
|  | 12474 | **500.9519** | **1499.8338** | **1499.8433** | **-0.0095** | **1** | **24** | **0.55** | **1** | **R.RRPPQPIQQPQR.Q** |
|  | 14131 | **803.3835** | **1604.7524** | **1604.7617** | **-0.0093** | **0** | **36** | **0.028** | **1** | **R.TITLQPGSPCNDFR.G** 14125 |
|  | 14561 | **544.3379** | **1629.9918** | **1628.8198** | **1.1720** | **1** | **12** | **7.6** | **1** | **R.GGTFYVEPAERYIK.D** |
|  | 16509 | **885.3779** | **1768.7412** | **1768.7509** | **-0.0097** | **0** | **75** | **2.9e-006** | **1** | **K.MDPSTCASTGSVQWSR.H** |
|  | 16909 | 602.2750 | 1803.8031 | 1804.8050 | -1.0019 | 1 | 2 | 75 | 5 | K.ENGNYIMYARATSGDK.L |
|  | 18127 | **949.4373** | **1896.8600** | **1896.8458** | **0.0141** | **1** | **0** | **96** | **3** | **K.KMDPSTCASTGSVQWSR.H** |
|  | 22266 | **583.5835** | **2330.3049** | **2330.2457** | **0.0592** | **0** | **4** | **28** | **3** | **R.VLILLLSWAAGMGGQYGNPLNK.Y** 22265 |
|  | 25897 | **1480.1733** | **2958.3321** | **2958.6187** | **-0.2866** | **1** | **4** | **22** | **1** | **-.MVLLRVLILLLSWAAGMGGQYGNPLNK.Y** 25900 25901 |
|  | 29403 | **1295.6328** | **3883.8766** | **3883.5059** | **0.3707** | **1** | **0** | **27** | **10** | **K.RNNCFVESGQPICGNGMVEQGEECDCGYSDQCK.D** |
|  | 30626 | **1512.6925** | **4535.0557** | **4535.3863** | **-0.3306** | **1** | **2** | **12** | **2** | **K.KAIFSPELYENIAEWIVAHWWAVLLMGIALIMLMAGFIK.I** |
|  | 31427 | **1306.8402** | **5223.3317** | **5223.3503** | **-0.0185** | **1** | **21** | **0.093** | **1** | **K.FLELNSEQNHDDYCLAYVFTDRDFDDGVLGLAWVGAPSGSSGGICEK.S** 31429 |

| **337.** | P30460\|1B08_HUMAN    **Mass:** 40648    **Score:** 89     **Queries matched:** 8   **emPAI:** 0.30 |
| --- | --- |
|  | HLA class I histocompatibility antigen, B-8 alpha chain OS=Homo sapiens GN=HLA-B PE=1 SV=1 |

|  | **Query** | **Observed** | **Mr(expt)** | **Mr(calc)** | **Delta** | **Miss** | **Score** | **Expect** | **Rank** | **Peptide** |
| --- | --- | --- | --- | --- | --- | --- | --- | --- | --- | --- |
|  | 3749 | 483.2167 | 964.4188 | 964.4250 | -0.0062 | 0 | 35 | 0.057 | 1 | R.FDSDAASPR.E |
|  | 9015 | **434.5419** | **1300.6040** | **1299.6030** | **1.0010** | **0** | **8** | **24** | **10** | **R.YFDTAMSRPGR.G** |
|  | 12793 | 760.3734 | 1518.7323 | 1518.7427 | -0.0104 | 0 | 73 | 7.3e-006 | 1 | R.SWTAADTAAQITQR.K |
|  | 16109 | **580.2962** | **1737.8668** | **1738.8209** | **-0.9542** | **1** | **2** | **63** | **7** | **R.YFDTAMSRPGRGEPR.F** |
|  | 23135 | 492.2564 | 2456.2458 | 2456.3097 | -0.0639 | 0 | 2 | 47 | 5 | R.TVLLLLSAALALTETWAGSHSMR.Y |
|  | 24792 | 912.7573 | 2735.2500 | 2735.2635 | -0.0135 | 1 | 34 | 0.023 | 1 | R.DGEDQTQDTELVETRPAGDRTFQK.W |
|  | 27235 | 648.6937 | 3238.4319 | 3238.7392 | -0.3073 | 1 | 3 | 24 | 5 | -.MLVMAPRTVLLLLSAALALTETWAGSHSMR.Y |
|  | 27288 | 651.9504 | 3254.7158 | 3254.7341 | -0.0183 | 1 | (1) | 39 | 4 | -.MLVMAPRTVLLLLSAALALTETWAGSHSMR.Y |

|  | **Proteins matching a subset of these peptides:** |
| --- | --- |

|  | P30480\|1B42_HUMAN    **Mass:** 40650    **Score:** 89     **Queries matched:** 6 |
| --- | --- |
|  | HLA class I histocompatibility antigen, B-42 alpha chain OS=Homo sapiens GN=HLA-B PE=1 SV=1 |

| **338.** | P01889\|1B07_HUMAN    **Mass:** 40777    **Score:** 89     **Queries matched:** 7   **emPAI:** 0.30 |
| --- | --- |
|  | HLA class I histocompatibility antigen, B-7 alpha chain OS=Homo sapiens GN=HLA-B PE=1 SV=3 |

|  | **Query** | **Observed** | **Mr(expt)** | **Mr(calc)** | **Delta** | **Miss** | **Score** | **Expect** | **Rank** | **Peptide** |
| --- | --- | --- | --- | --- | --- | --- | --- | --- | --- | --- |
|  | 3266 | **463.7024** | **925.3902** | **924.5029** | **0.8874** | **1** | **4** | **51** | **2** | **K.LERADPPK.T** |
|  | 3749 | 483.2167 | 964.4188 | 964.4250 | -0.0062 | 0 | 35 | 0.057 | 1 | R.FDSDAASPR.E |
|  | 12793 | 760.3734 | 1518.7323 | 1518.7427 | -0.0104 | 0 | 73 | 7.3e-006 | 1 | R.SWTAADTAAQITQR.K |
|  | 23135 | 492.2564 | 2456.2458 | 2456.3097 | -0.0639 | 0 | 2 | 47 | 5 | R.TVLLLLSAALALTETWAGSHSMR.Y |
|  | 24792 | 912.7573 | 2735.2500 | 2735.2635 | -0.0135 | 1 | 34 | 0.023 | 1 | R.DGEDQTQDTELVETRPAGDRTFQK.W |
|  | 27235 | 648.6937 | 3238.4319 | 3238.7392 | -0.3073 | 1 | 3 | 24 | 5 | -.MLVMAPRTVLLLLSAALALTETWAGSHSMR.Y |
|  | 27288 | 651.9504 | 3254.7158 | 3254.7341 | -0.0183 | 1 | (1) | 39 | 4 | -.MLVMAPRTVLLLLSAALALTETWAGSHSMR.Y |

| **339.** | P16284\|PECA1_HUMAN    **Mass:** 83396    **Score:** 89     **Queries matched:** 12   **emPAI:** 0.04 |
| --- | --- |
|  | Platelet endothelial cell adhesion molecule OS=Homo sapiens GN=PECAM1 PE=1 SV=1 |

|  | **Query** | **Observed** | **Mr(expt)** | **Mr(calc)** | **Delta** | **Miss** | **Score** | **Expect** | **Rank** | **Peptide** |
| --- | --- | --- | --- | --- | --- | --- | --- | --- | --- | --- |
|  | 3464 | **472.2734** | **942.5322** | **941.5294** | **1.0028** | **0** | **6** | **45** | **4** | **K.EAIQGGIVR.V** |
|  | 7092 | **603.0800** | **1204.1455** | **1203.6281** | **0.5173** | **1** | **4** | **40** | **2** | **K.CTVIVNNKEK.T** |
|  | 8918 | **648.8180** | **1295.6214** | **1296.6244** | **-1.0030** | **0** | **3** | **64** | **1** | **R.NHAMKPINDNK.E** |
|  | 11318 | **477.7424** | **1430.2055** | **1430.6752** | **-0.4697** | **0** | **7** | **18** | **2** | **K.DDVLFYNISSMK.S** |
|  | 15561 | **849.4054** | **1696.7962** | **1696.9611** | **-0.1648** | **0** | **64** | **4.4e-005** | **1** | **K.VIAPVDEVQISILSSK.V** 15560 15566 |
|  | 17988 | **629.3206** | **1884.9400** | **1885.9091** | **-0.9691** | **1** | **1** | **72** | **2** | **K.IASKSDSGTYICTAGIDK.V** |
|  | 19952 | **688.3499** | **2062.0279** | **2061.8958** | **0.1321** | **0** | **1** | **69** | **3** | **K.AVYSVMAMVEHSGNYTCK.V** |
|  | 20524 | **708.8615** | **2123.5627** | **2124.1136** | **-0.5509** | **1** | **2** | **35** | **2** | **R.CESISGTLPISYQLLKTSK.V** |
|  | 23922 | **861.1053** | **2580.2940** | **2579.3020** | **0.9920** | **1** | **1** | **56** | **1** | **R.DQNFVILEFPVEEQDRVLSFR.C** |
|  | 31488 | **879.0975** | **5268.5412** | **5269.5774** | **-1.0362** | **1** | **1** | **9.1** | **1** | **R.WAQGATMWLGVLLTLLLCSSLEGQENSFTINSVDMKSLPDWTVQNGK.N** |

| **340.** | Q13228\|SBP1_HUMAN    **Mass:** 52928    **Score:** 89     **Queries matched:** 10   **emPAI:** 0.07 |
| --- | --- |
|  | Selenium-binding protein 1 OS=Homo sapiens GN=SELENBP1 PE=1 SV=2 |

|  | **Query** | **Observed** | **Mr(expt)** | **Mr(calc)** | **Delta** | **Miss** | **Score** | **Expect** | **Rank** | **Peptide** |
| --- | --- | --- | --- | --- | --- | --- | --- | --- | --- | --- |
|  | 5312 | 542.7364 | 1083.4582 | 1083.6652 | -0.2069 | 0 | 3 | 74 | 4 | K.LVLPSLISSR.I |
|  | 7645 | 411.7246 | 1232.1518 | 1232.6401 | -0.4883 | 0 | 1 | 88 | 10 | R.IYVVDVGSEPR.A |
|  | 9023 | **434.5829** | **1300.7269** | **1301.7092** | **-0.9822** | **0** | **5** | **48** | **7** | **K.EPLGPALAHELR.Y** |
|  | 12944 | **510.9116** | **1529.7130** | **1528.7919** | **0.9211** | **0** | **2** | **82** | **4** | **R.VAGGPQMIQLSLDGK.R** |
|  | 18209 | **953.4521** | **1904.8897** | **1904.9004** | **-0.0106** | **0** | **91** | **8.4e-008** | **1** | **R.NTGTEAPDYLATVDVDPK.S** 18208 18215 |
|  | 19825 | **684.5778** | **2050.7116** | **2050.9023** | **-0.1907** | **1** | **3** | **31** | **2** | **K.CGNCGPGYSTPLEAMKGPR.E** |
|  | 23755 | **851.4162** | **2551.2268** | **2551.2992** | **-0.0725** | **1** | **4** | **26** | **3** | **K.QFYPDLIREGSVMLQVDVDTVK.G** |
|  | 30738 | **1154.1007** | **4612.3737** | **4613.1975** | **-0.8238** | **1** | **2** | **11** | **6** | **R.HNVMISTEWAAPNVLRDGFNPADVEAGLYGSHLYVWDWQR.H** |

| **341.** | P08574\|CY1_HUMAN    **Mass:** 35709    **Score:** 88     **Queries matched:** 14   **emPAI:** 0.22 |
| --- | --- |
|  | Cytochrome c1, heme protein, mitochondrial OS=Homo sapiens GN=CYC1 PE=1 SV=2 |

|  | **Query** | **Observed** | **Mr(expt)** | **Mr(calc)** | **Delta** | **Miss** | **Score** | **Expect** | **Rank** | **Peptide** |
| --- | --- | --- | --- | --- | --- | --- | --- | --- | --- | --- |
|  | 3864 | **487.2799** | **972.5453** | **971.5916** | **0.9537** | **1** | **10** | **21** | **2** | **R.KLAYRPPK.-** |
|  | 12509 | 501.5002 | 1501.4787 | 1500.8260 | 0.6527 | 1 | 4 | 42 | 6 | R.TPQAVALSSKSGLSR.G |
|  | 12824 | **761.3619** | **1520.7093** | **1519.6977** | **1.0116** | **0** | **2** | **83** | **7** | **R.HLVGVCYTEDEAK.E** |
|  | 13063 | **513.3363** | **1536.9871** | **1536.8558** | **0.1312** | **0** | **2** | **55** | **8** | **R.GLLCSARPGQLPLR.T** |
|  | 13347 | **777.8889** | **1553.7633** | **1554.8664** | **-1.1031** | **1** | **3** | **66** | **9** | **-.MAAAAASLRGVVLGPR.G** |
|  | 18131 | **633.3339** | **1896.9799** | **1898.0153** | **-1.0354** | **0** | **3** | **48** | **7** | **K.MLMMMALLVPLVYTIK.R** |
|  | 18307 | **957.9674** | **1913.9203** | **1913.9312** | **-0.0109** | **0** | **47** | **0.0018** | **1** | **K.LFDYFPKPYPNSEAAR.A** 18306 18308 18309 |
|  | 18331 | **639.3673** | **1915.0799** | **1914.0102** | **1.0697** | **0** | **(1)** | **73** | **1** | **K.MLMMMALLVPLVYTIK.R** |
|  | 24502 | **894.4642** | **2680.3709** | **2679.1792** | **1.1916** | **0** | **6** | **15** | **1** | **K.ELAAEVEVQDGPNEDGEMFMRPGK.L** 24500 |
|  | 31519 | **1768.1763** | **5301.5070** | **5301.5025** | **0.0045** | **1** | **2** | **8.1** | **9** | **R.EGLYFNPYFPGQAIAMAPPIYTDVLEFDDGTPATMSQIAKDVCTFLR.W** |

| **342.** | Q9UI09\|NDUAC_HUMAN    **Mass:** 17104    **Score:** 88     **Queries matched:** 4   **emPAI:** 0.50 |
| --- | --- |
|  | NADH dehydrogenase [ubiquinone] 1 alpha subcomplex subunit 12 OS=Homo sapiens GN=NDUFA12 PE=1 SV=1 |

|  | **Query** | **Observed** | **Mr(expt)** | **Mr(calc)** | **Delta** | **Miss** | **Score** | **Expect** | **Rank** | **Peptide** |
| --- | --- | --- | --- | --- | --- | --- | --- | --- | --- | --- |
|  | 11780 | **730.3428** | **1458.6710** | **1457.7554** | **0.9155** | **0** | **22** | **0.87** | **1** | **K.IQEWIPPSTPYK.-** |
|  | 18874 | **980.9719** | **1959.9292** | **1958.9374** | **0.9917** | **0** | **57** | **0.00017** | **1** | **K.FNVTGTPEQYVPYSTTR.K** 18866 |
|  | 20005 | **689.6783** | **2066.0130** | **2066.0255** | **-0.0125** | **0** | **22** | **0.52** | **1** | **R.WLHSMTDDPPTTKPLTAR.K** |

| **343.** | P06744\|G6PI_HUMAN    **Mass:** 63335    **Score:** 88     **Queries matched:** 16   **emPAI:** 0.06 |
| --- | --- |
|  | Glucose-6-phosphate isomerase OS=Homo sapiens GN=GPI PE=1 SV=4 |

|  | **Query** | **Observed** | **Mr(expt)** | **Mr(calc)** | **Delta** | **Miss** | **Score** | **Expect** | **Rank** | **Peptide** |
| --- | --- | --- | --- | --- | --- | --- | --- | --- | --- | --- |
|  | 961 | **607.2069** | **606.1996** | **606.3853** | **-0.1857** | **0** | **4** | **78** | **1** | **R.LLPHK.V** |
|  | 2095 | 423.2104 | 844.4063 | 843.4814 | 0.9250 | 1 | 15 | 7.2 | 6 | R.KELQAAGK.S |
|  | 2106 | **423.4552** | **844.8959** | **844.4515** | **0.4444** | **1** | **6** | **41** | **5** | **K.SRGVEAAR.E** |
|  | 7350 | **609.8235** | **1217.6325** | **1217.6404** | **-0.0079** | **0** | **14** | **6.8** | **1** | **K.HFVALSTNTTK.V** |
|  | 10862 | **702.3447** | **1402.6748** | **1401.7174** | **0.9574** | **1** | **6** | **38** | **6** | **K.DVMPEVNKVLDK.M** 10833 |
|  | 13161 | **515.3265** | **1542.9576** | **1541.7685** | **1.1891** | **1** | **12** | **7.2** | **2** | **K.ELQAAGKSPEDLER.L** 13144 |
|  | 13629 | **787.3494** | **1572.6843** | **1573.7195** | **-1.0352** | **1** | **2** | **76** | **6** | **R.MFNGEKINYTEGR.A** |
|  | 14090 | **534.8102** | **1601.4089** | **1601.8202** | **-0.4113** | **0** | **11** | **6.7** | **1** | **R.VWYVSNIDGTHIAK.T** 14083 |
|  | 15647 | **852.4762** | **1702.9378** | **1702.9440** | **-0.0061** | **0** | **57** | **0.00023** | **1** | **K.ILLANFLAQTEALMR.G** 15646 |
|  | 15866 | **573.9545** | **1718.8416** | **1718.9389** | **-0.0973** | **0** | **(4)** | **48** | **6** | **K.ILLANFLAQTEALMR.G** |
|  | 17278 | 915.9745 | 1829.9344 | 1831.0091 | -1.0747 | 0 | 12 | 6.2 | 3 | K.TLAQLNPESSLFIIASK.T |
|  | 18041 | 945.4534 | 1888.8923 | 1888.0604 | 0.8319 | 1 | 1 | 81 | 6 | K.ILLANFLAQTEALMRGK.S |

| **344.** | Q8TCZ2\|C99L2_HUMAN    **Mass:** 28140    **Score:** 88     **Queries matched:** 13   **emPAI:** 0.13 |
| --- | --- |
|  | CD99 antigen-like protein 2 OS=Homo sapiens GN=CD99L2 PE=2 SV=1 |

|  | **Query** | **Observed** | **Mr(expt)** | **Mr(calc)** | **Delta** | **Miss** | **Score** | **Expect** | **Rank** | **Peptide** |
| --- | --- | --- | --- | --- | --- | --- | --- | --- | --- | --- |
|  | 2010 | **420.2298** | **838.4451** | **839.5090** | **-1.0638** | **1** | **9** | **18** | **2** | **R.RKPGIGGR.E** 2008 2048 2053 |
|  | 8780 | 645.3296 | 1288.6446 | 1288.6888 | -0.0442 | 1 | 8 | 22 | 10 | R.RKPIAGGGGFSDK.D 8778 8782 |
|  | 10301 | **457.9087** | **1370.7042** | **1371.6895** | **-0.9854** | **1** | **5** | **41** | **1** | **R.ERWNHVTTTTK.R** |
|  | 15606 | **850.9415** | **1699.8685** | **1699.8087** | **0.0598** | **0** | **1** | **97** | **6** | **K.GENLEAVVCEEPQVK.Y** |
|  | 20071 | **692.6392** | **2074.8957** | **2074.0119** | **0.8837** | **0** | **3** | **40** | **1** | **K.YSTLHTQSAEPPPPPEPAR.I** |
|  | 30230 | **1425.6953** | **4274.0641** | **4274.0875** | **-0.0234** | **1** | **62** | **1.4e-005** | **1** | **R.YGSNDDPGSGMVAEPGTIAGVASALAMALIGAVSSYISYQQKK.F** 30229 |
|  | 30243 | **1073.2526** | **4288.9811** | **4290.0824** | **-1.1013** | **1** | **(5)** | **7.1** | **1** | **R.YGSNDDPGSGMVAEPGTIAGVASALAMALIGAVSSYISYQQKK.F** |

| **345.** | P05362\|ICAM1_HUMAN    **Mass:** 58587    **Score:** 87     **Queries matched:** 16   **emPAI:** 0.20 |
| --- | --- |
|  | Intercellular adhesion molecule 1 OS=Homo sapiens GN=ICAM1 PE=1 SV=2 |

|  | **Query** | **Observed** | **Mr(expt)** | **Mr(calc)** | **Delta** | **Miss** | **Score** | **Expect** | **Rank** | **Peptide** |
| --- | --- | --- | --- | --- | --- | --- | --- | --- | --- | --- |
|  | 3106 | **458.2457** | **914.4769** | **915.4886** | **-1.0117** | **1** | **15** | **5.9** | **10** | **K.NQTRELR.V** |
|  | 5256 | 540.7662 | 1079.5178 | 1079.6590 | -0.1412 | 0 | 15 | 4.6 | 2 | K.LLGIETPLPK.K 5257 5274 5281 |
|  | 6218 | **576.8155** | **1151.6164** | **1152.6615** | **-1.0450** | **1** | **6** | **36** | **3** | **K.KELLLPGNNR.K** |
|  | 9725 | **670.3331** | **1338.6516** | **1338.6602** | **-0.0086** | **0** | **6** | **38** | **1** | **K.GTPMKPNTQATPP.-** |
|  | 10031 | 678.3373 | 1354.6601 | 1354.6551 | 0.0050 | 0 | (1) | 1e+002 | 6 | K.GTPMKPNTQATPP.- |
|  | 11617 | 725.3401 | 1448.6657 | 1448.6743 | -0.0086 | 0 | 72 | 8.2e-006 | 1 | K.ASVSVTAEDEGTQR.L |
|  | 14346 | 809.8765 | 1617.7385 | 1616.9362 | 0.8023 | 1 | 2 | 77 | 8 | R.AKVTLNGVPAQPLGPR.A |
|  | 15537 | **848.3824** | **1694.7503** | **1694.7604** | **-0.0101** | **0** | **46** | **0.003** | **1** | **R.GGSVLVTCSTSCDQPK.L** |
|  | 20192 | **697.3674** | **2089.0803** | **2089.0878** | **-0.0075** | **1** | **25** | **0.29** | **1** | **K.CLKDGTFPLPIGESVTVTR.D** |
|  | 25668 | **729.1012** | **2912.3757** | **2913.5429** | **-1.1672** | **1** | **4** | **26** | **1** | **K.TFLTVYWTPERVELAPLPSWQPVGK.N** |
|  | 27217 | **809.4155** | **3233.6328** | **3234.3904** | **-0.7576** | **1** | **4** | **17** | **2** | **R.KVYELSNVQEDSQPMCYSNCPDGQSTAK.T** |
|  | 29595 | 1324.2653 | 3969.7740 | 3969.7509 | 0.0231 | 1 | 7 | 5 | 2 | R.LDERDCPGNWTWPENSQQTPMCQAWGNPLPELK.C 29599 |

| **346.** | Q9BUR5\|APOO_HUMAN    **Mass:** 22385    **Score:** 87     **Queries matched:** 3   **emPAI:** 0.37 |
| --- | --- |
|  | Apolipoprotein O OS=Homo sapiens GN=APOO PE=1 SV=1 |

|  | **Query** | **Observed** | **Mr(expt)** | **Mr(calc)** | **Delta** | **Miss** | **Score** | **Expect** | **Rank** | **Peptide** |
| --- | --- | --- | --- | --- | --- | --- | --- | --- | --- | --- |
|  | 8785 | **645.3342** | **1288.6539** | **1288.6623** | **-0.0084** | **0** | **62** | **8.8e-005** | **1** | **R.SQLEESISQLR.H** |
|  | 17074 | 606.2447 | 1815.7122 | 1815.0618 | 0.6504 | 1 | 2 | 60 | 10 | K.VIQRSVGPASLSLLTFK.V |
|  | 24079 | **869.7094** | **2606.1064** | **2606.1206** | **-0.0142** | **0** | **55** | **0.00018** | **1** | **R.HYCEPYTTWCQETYSQTKPK.M** |

| **347.** | Q9UF12\|PROD2_HUMAN    **Mass:** 59689    **Score:** 87     **Queries matched:** 14   **emPAI:** 0.27 |
| --- | --- |
|  | Probable proline dehydrogenase 2 OS=Homo sapiens GN=PRODH2 PE=2 SV=1 |

|  | **Query** | **Observed** | **Mr(expt)** | **Mr(calc)** | **Delta** | **Miss** | **Score** | **Expect** | **Rank** | **Peptide** |
| --- | --- | --- | --- | --- | --- | --- | --- | --- | --- | --- |
|  | 2145 | **848.4788** | **847.4715** | **847.4763** | **-0.0048** | **0** | **4** | **81** | **1** | **K.VTALTSTR.L** |
|  | 3438 | **470.7668** | **939.5191** | **938.5410** | **0.9781** | **1** | **3** | **80** | **8** | **R.ASLSRLHR.V** |
|  | 4159 | **497.2558** | **992.4971** | **992.5039** | **-0.0068** | **1** | **8** | **29** | **4** | **K.DTFERLGR.D** 4164 |
|  | 5492 | **548.7914** | **1095.5683** | **1094.5581** | **1.0103** | **1** | **6** | **33** | **4** | **R.LGRDAEAAHR.A** 5494 |
|  | 9571 | **443.8895** | **1328.6468** | **1327.6956** | **0.9511** | **1** | **5** | **42** | **10** | **R.AQENRSVLQGAR.R** |
|  | 10778 | **699.8713** | **1397.7280** | **1397.8354** | **-0.1074** | **1** | **7** | **25** | **7** | **K.GTGELTRALLVLR.L** |
|  | 16834 | **898.9910** | **1795.9675** | **1795.9753** | **-0.0078** | **0** | **49** | **0.0012** | **1** | **R.GLLEPPSLAEASLMQLK.V** |
|  | 20201 | **523.4854** | **2089.9125** | **2089.9244** | **-0.0119** | **0** | **41** | **0.0064** | **1** | **R.HGPMCHLMVASHNEESVR.Q** 20200 |
|  | 22658 | **795.4077** | **2383.2013** | **2383.1049** | **0.0964** | **1** | **1** | **66** | **4** | **K.SGEAWYEGNLGAMLRCVDLSR.G** |
|  | 23612 | **634.3088** | **2533.2063** | **2534.1577** | **-0.9514** | **1** | **3** | **35** | **4** | **R.HGPMCHLMVASHNEESVRQATK.R** |
|  | 25008 | **927.7461** | **2780.2166** | **2780.2348** | **-0.0181** | **0** | **39** | **0.0075** | **1** | **R.AVAQLHGMEDPTQPDYEATSQSYSR.C** |

| **348.** | P01861\|IGHG4_HUMAN    **Mass:** 36431    **Score:** 86     **Queries matched:** 8   **emPAI:** 0.34 |
| --- | --- |
|  | Ig gamma-4 chain C region OS=Homo sapiens GN=IGHG4 PE=1 SV=1 |

|  | **Query** | **Observed** | **Mr(expt)** | **Mr(calc)** | **Delta** | **Miss** | **Score** | **Expect** | **Rank** | **Peptide** |
| --- | --- | --- | --- | --- | --- | --- | --- | --- | --- | --- |
|  | 6343 | **581.3152** | **1160.6159** | **1160.6223** | **-0.0064** | **0** | **29** | **0.21** | **1** | **K.NQVSLTCLVK.G** |
|  | 8728 | **429.7405** | **1286.1995** | **1286.6442** | **-0.4446** | **0** | **6** | **25** | **5** | **K.GPSVFPLAPCSR.S** |
|  | 11189 | **712.3544** | **1422.6942** | **1422.7024** | **-0.0083** | **0** | **40** | **0.012** | **1** | **R.STSESTAALGCLVK.D** 11170 |
|  | 15075 | **555.6051** | **1663.7935** | **1663.7624** | **0.0311** | **0** | **2** | **79** | **10** | **K.TYTCNVDHKPSNTK.V** |
|  | 23683 | **1272.5614** | **2543.1082** | **2543.1241** | **-0.0158** | **0** | **49** | **0.00079** | **1** | **K.GFYPSDIAVEWESNGQPENNYK.T** 23688 |
|  | 32275 | **1341.2495** | **6701.2112** | **6701.2913** | **-0.0801** | **1** | **10** | **0.64** | **1** | **K.DYFPEPVTVSWNSGALTSGVHTFPAVLQSSGLYSLSSVVTVPSSSLGTKTYTCNVDHKPSNTK.V** |

| **349.** | P61019\|RAB2A_HUMAN    **Mass:** 23702    **Score:** 86     **Queries matched:** 7   **emPAI:** 0.35 |
| --- | --- |
|  | Ras-related protein Rab-2A OS=Homo sapiens GN=RAB2A PE=1 SV=1 |

|  | **Query** | **Observed** | **Mr(expt)** | **Mr(calc)** | **Delta** | **Miss** | **Score** | **Expect** | **Rank** | **Peptide** |
| --- | --- | --- | --- | --- | --- | --- | --- | --- | --- | --- |
|  | 4743 | **519.2856** | **1036.5566** | **1035.4985** | **1.0581** | **1** | **12** | **10** | **3** | **K.KEEGEAFAR.E** |
|  | 9366 | **440.2390** | **1317.6952** | **1318.7245** | **-1.0293** | **0** | **12** | **10** | **2** | **R.GAAGALLVYDITR.R** |
|  | 10142 | 681.8351 | 1361.6556 | 1361.6649 | -0.0093 | 0 | 10 | 14 | 1 | R.EHGLIFMETSAK.T |
|  | 12044 | **492.2528** | **1473.7365** | **1474.8256** | **-1.0891** | **1** | **6** | **34** | **5** | **R.GAAGALLVYDITRR.D** |
|  | 13279 | **775.8789** | **1549.7431** | **1549.7525** | **-0.0094** | **0** | **64** | **4.9e-005** | **1** | **K.LQIWDTAGQESFR.S** 13258 |
|  | 16407 | **880.9396** | **1759.8646** | **1759.8741** | **-0.0094** | **0** | **46** | **0.003** | **1** | **K.IQEGVFDINNEANGIK.I** |

| **350.** | P07355\|ANXA2_HUMAN    **Mass:** 38808    **Score:** 86     **Queries matched:** 18   **emPAI:** 0.20 |
| --- | --- |
|  | Annexin A2 OS=Homo sapiens GN=ANXA2 PE=1 SV=2 |

|  | **Query** | **Observed** | **Mr(expt)** | **Mr(calc)** | **Delta** | **Miss** | **Score** | **Expect** | **Rank** | **Peptide** |
| --- | --- | --- | --- | --- | --- | --- | --- | --- | --- | --- |
|  | 2302 | **430.7141** | **859.4137** | **858.5174** | **0.8963** | **1** | **5** | **69** | **5** | **K.KELASALK.S** |
|  | 2535 | **437.2709** | **872.5273** | **872.5517** | **-0.0244** | **1** | **6** | **48** | **9** | **R.KLMVALAK.G** |
|  | 5375 | **544.8180** | **1087.6214** | **1086.5921** | **1.0294** | **0** | **2** | **1e+002** | **6** | **R.DALNIETAIK.T** |
|  | 5423 | **546.2999** | **1090.5852** | **1089.5852** | **1.0000** | **1** | **1** | **1.3e+002** | **7** | **R.SEVDMLKIR.S** |
|  | 5570 | **551.5013** | **1100.9881** | **1101.6692** | **-0.6811** | **1** | **4** | **48** | **2** | **K.VLIRIMVSR.S** |
|  | 6471 | **586.9057** | **1171.7968** | **1171.5431** | **0.2538** | **1** | **3** | **68** | **3** | **K.EMYKTDLEK.D** 6473 6483 |
|  | 7306 | 406.1849 | 1215.5330 | 1216.5944 | -1.0614 | 0 | 8 | 23 | 4 | -.MSTVHEILCK.L 7307 7348 |
|  | 7894 | **622.8109** | **1243.6073** | **1243.6156** | **-0.0084** | **0** | **49** | **0.0019** | **1** | **R.TNQELQEINR.V** 7865 |
|  | 11181 | **712.3343** | **1422.6540** | **1422.7211** | **-0.0670** | **1** | **19** | **1.7** | **2** | **R.IMVSRSEVDMLK.I** 11200 |
|  | 13140 | **771.9239** | **1541.8332** | **1541.8413** | **-0.0081** | **0** | **68** | **2e-005** | **1** | **K.GVDEVTIVNILTNR.S** |
|  | 16589 | **445.0015** | **1775.9767** | **1776.8564** | **-0.8796** | **0** | **8** | **16** | **7** | **K.GLGTDEDSLIEIICSR.T** |
|  | 17403 | **614.3082** | **1839.9027** | **1839.9665** | **-0.0638** | **1** | **5** | **33** | **3** | **K.WISIMTERSVPHLQK.V** |

| **351.** | P13010\|KU86_HUMAN    **Mass:** 83222    **Score:** 85     **Queries matched:** 20   **emPAI:** 0.19 |
| --- | --- |
|  | ATP-dependent DNA helicase 2 subunit 2 OS=Homo sapiens GN=XRCC5 PE=1 SV=3 |

|  | **Query** | **Observed** | **Mr(expt)** | **Mr(calc)** | **Delta** | **Miss** | **Score** | **Expect** | **Rank** | **Peptide** |
| --- | --- | --- | --- | --- | --- | --- | --- | --- | --- | --- |
|  | 1244 | **760.4507** | **759.4435** | **759.4126** | **0.0308** | **1** | **6** | **68** | **2** | **K.KEDIQK.E** |
|  | 1270 | **772.4516** | **771.4443** | **771.4490** | **-0.0047** | **0** | **31** | **0.18** | **1** | **K.SQIPLSK.I** |
|  | 2607 | **879.0596** | **878.0523** | **877.3851** | **0.6672** | **0** | **1** | **1.1e+002** | **3** | **K.VDEEQMK.Y** |
|  | 5201 | **537.8038** | **1073.5931** | **1072.6240** | **0.9691** | **0** | **12** | **12** | **2** | **R.LTIGSNLSIR.I** |
|  | 8474 | 424.9337 | 1271.7792 | 1271.7853 | -0.0060 | 1 | 14 | 6 | 2 | K.IKTLFPLIEAK.K |
|  | 8825 | **431.1936** | **1290.5590** | **1290.7295** | **-0.1706** | **1** | **6** | **36** | **4** | **R.IAAYKSILQER.V** |
|  | 14500 | **543.2748** | **1626.8027** | **1626.8705** | **-0.0678** | **1** | **6** | **28** | **3** | **R.FFMGNQVLKVFAAR.D** 14491 14494 |
|  | 18312 | **638.9857** | **1913.9352** | **1913.9444** | **-0.0093** | **0** | **22** | **0.57** | **1** | **R.HLMLPDFDLLEDIESK.I** |
|  | 19150 | **663.3079** | **1986.9018** | **1986.9245** | **-0.0227** | **1** | **1** | **71** | **9** | **R.YGSDIVPFSKVDEEQMK.Y** |
|  | 20719 | 716.3745 | 2146.1017 | 2146.1218 | -0.0201 | 1 | 2 | 59 | 6 | R.HSIHWPCRLTIGSNLSIR.I |
|  | 22199 | **774.3917** | **2320.1532** | **2320.1620** | **-0.0089** | **0** | **18** | **1.1** | **1** | **K.YAPTEAQLNAVDALIDSMSLAK.K** |
|  | 23076 | **817.4245** | **2449.2517** | **2448.2570** | **0.9947** | **1** | **32** | **0.048** | **1** | **K.KYAPTEAQLNAVDALIDSMSLAK.K** |
|  | 23464 | **1255.0627** | **2508.1109** | **2508.1214** | **-0.0105** | **0** | **42** | **0.0046** | **1** | **K.DKPSGDTAAVFEEGGDVDDLLDMI.-** 23463 |
|  | 24706 | **908.4662** | **2722.3769** | **2722.3847** | **-0.0078** | **0** | **30** | **0.054** | **1** | **R.DDEAAAVALSSLIHALDDLDMVAIVR.Y** |
|  | 26447 | **767.1104** | **3064.4123** | **3064.4587** | **-0.0464** | **1** | **0** | **44** | **8** | **K.FLAPKDKPSGDTAAVFEEGGDVDDLLDMI.-** |
|  | 29792 | **1016.2469** | **4060.9585** | **4060.9767** | **-0.0182** | **1** | **8** | **3.9** | **1** | **R.QVFAENKDEIALVLFGTDGTDNPLSGGDQYQNITVHR.H** |
|  | 29834 | 1020.2181 | 4076.8432 | 4075.8931 | 0.9501 | 1 | 4 | 10 | 2 | K.ASFEEASNQLINHIEQFLDTNETPYFMKSIDCIR.A |

| **352.** | Q9Y2B0\|CNPY2_HUMAN    **Mass:** 20981    **Score:** 85     **Queries matched:** 8   **emPAI:** 0.40 |
| --- | --- |
|  | Protein canopy homolog 2 OS=Homo sapiens GN=CNPY2 PE=1 SV=1 |

|  | **Query** | **Observed** | **Mr(expt)** | **Mr(calc)** | **Delta** | **Miss** | **Score** | **Expect** | **Rank** | **Peptide** |
| --- | --- | --- | --- | --- | --- | --- | --- | --- | --- | --- |
|  | 4878 | **524.6221** | **1047.2297** | **1047.5448** | **-0.3151** | **0** | **8** | **20** | **5** | **R.IDSDISGTLK.F** |
|  | 5306 | **542.2974** | **1082.5803** | **1082.5543** | **0.0260** | **1** | **4** | **59** | **1** | **K.KTIQMGSFR.I** |
|  | 7063 | **602.7509** | **1203.4873** | **1202.4921** | **0.9952** | **0** | **46** | **0.0042** | **1** | **R.SQDLHCGACR.A** |
|  | 11102 | **709.8899** | **1417.7652** | **1416.6845** | **1.0808** | **0** | **4** | **56** | **5** | **R.NGESSELDLQGIR.I** |
|  | 15475 | 564.6174 | 1690.8305 | 1689.7780 | 1.0524 | 1 | 2 | 70 | 6 | R.MKEYGEQIDPSTHR.K |
|  | 16021 | 865.9331 | 1729.8517 | 1729.8635 | -0.0118 | 0 | 67 | 2.2e-005 | 1 | R.INPDGSQSVVEVPYAR.S 16022 |
|  | 28359 | **886.1721** | **3540.6591** | **3540.6494** | **0.0098** | **1** | **3** | **20** | **1** | **R.IDSDISGTLKFACESIVEEYEDELIEFFSR.E** |

| **353.** | Q10567\|AP1B1_HUMAN    **Mass:** 105452   **Score:** 85     **Queries matched:** 17   **emPAI:** 0.11 |
| --- | --- |
|  | AP-1 complex subunit beta-1 OS=Homo sapiens GN=AP1B1 PE=1 SV=1 |

|  | **Query** | **Observed** | **Mr(expt)** | **Mr(calc)** | **Delta** | **Miss** | **Score** | **Expect** | **Rank** | **Peptide** |
| --- | --- | --- | --- | --- | --- | --- | --- | --- | --- | --- |
|  | 1282 | **777.0353** | **776.0281** | **775.4262** | **0.6019** | **0** | **8** | **22** | **3** | **K.LDIMIR.L** 1279 1281 |
|  | 4155 | 497.2400 | 992.4655 | 991.4393 | 1.0262 | 0 | 9 | 21 | 9 | R.EAQSICER.V |
|  | 4443 | 507.7919 | 1013.5692 | 1013.5757 | -0.0065 | 0 | 7 | 36 | 6 | R.LLSTDPVAAK.E |
|  | 7145 | **604.6749** | **1207.3353** | **1206.6608** | **0.6745** | **0** | **6** | **29** | **4** | **K.LQSSNIFTVAK.R** |
|  | 8585 | **427.0655** | **1278.1747** | **1278.6932** | **-0.5185** | **1** | **6** | **22** | **1** | **K.AKGLEISGTFTR.Q** 8607 |
|  | 10173 | 455.3684 | 1363.0834 | 1362.7619 | 0.3215 | 1 | 0 | 87 | 6 | K.LQSSNIFTVAKR.N |
|  | 10627 | **695.3228** | **1388.6309** | **1387.6913** | **0.9396** | **1** | **3** | **64** | **1** | **K.VLMKFMEMLSK.D** 10621 |
|  | 11505 | **481.7335** | **1442.1786** | **1441.6846** | **0.4940** | **1** | **3** | **44** | **4** | **K.MDRQMFLATWK.D** |
|  | 12861 | **762.8671** | **1523.7197** | **1523.7290** | **-0.0093** | **0** | **51** | **0.0011** | **1** | **R.NVEGQDMLYQSLK.L** |
|  | 29105 | **1257.6710** | **3769.9912** | **3770.0077** | **-0.0164** | **0** | **42** | **0.0019** | **1** | **R.NSFGLAPAAPLQVHAPLSPNQTVEISLPLSTVGSVMK.M** |
|  | 29490 | **1310.5469** | **3928.6188** | **3928.8499** | **-0.2310** | **1** | **3** | **11** | **1** | **K.YESVIATLCENLDSLDEPEARAAMIWIVGEYAER.I** |
|  | 30101 | **1397.6534** | **4189.9385** | **4189.0848** | **0.8537** | **0** | **2** | **16** | **2** | **K.DLISDSNPMVVANAVAALSEIAESHPSSNLLDLNPQSINK.L** |
|  | 32210 | **1265.6387** | **6323.1570** | **6323.1907** | **-0.0337** | **1** | **34** | **0.0024** | **1** | **K.LHDINAQLVEDQGFLDTLKDLISDSNPMVVANAVAALSEIAESHPSSNLLDLNPQSINK.L** |

| **354.** | Q14697\|GANAB_HUMAN    **Mass:** 107263   **Score:** 85     **Queries matched:** 18   **emPAI:** 0.07 |
| --- | --- |
|  | Neutral alpha-glucosidase AB OS=Homo sapiens GN=GANAB PE=1 SV=3 |

|  | **Query** | **Observed** | **Mr(expt)** | **Mr(calc)** | **Delta** | **Miss** | **Score** | **Expect** | **Rank** | **Peptide** |
| --- | --- | --- | --- | --- | --- | --- | --- | --- | --- | --- |
|  | 3323 | 465.7680 | 929.5215 | 929.5116 | 0.0098 | 0 | 15 | 5.9 | 6 | -.MAAVAAVAAR.R |
|  | 3516 | **474.2284** | **946.4423** | **945.5066** | **0.9357** | **0** | **(7)** | **35** | **7** | **-.MAAVAAVAAR.R** 3530 |
|  | 5624 | **553.3313** | **1104.6480** | **1103.6703** | **0.9778** | **0** | **5** | **59** | **2** | **K.LVAIVDPHIK.V** |
|  | 6112 | **573.3215** | **1144.6284** | **1144.6353** | **-0.0069** | **0** | **22** | **1** | **1** | **R.SIRPGLSPYR.A** |
|  | 7641 | **616.9612** | **1231.9078** | **1231.7652** | **0.1426** | **1** | **5** | **37** | **3** | **R.KLVAIVDPHIK.V** |
|  | 13559 | 523.9351 | 1568.7834 | 1567.6750 | 1.1083 | 0 | 14 | 5.2 | 1 | K.DPAEGDGAQPEETPR.D |
|  | 13966 | **797.7782** | **1593.5418** | **1592.7235** | **0.8184** | **0** | **16** | **2** | **1** | **R.WYQMGAYQPFFR.A** |
|  | 15078 | 555.6475 | 1663.9207 | 1663.0396 | 0.8811 | 0 | 14 | 4.3 | 2 | R.VVIIGAGKPAAVVLQTK.G |
|  | 15334 | **560.9827** | **1679.9264** | **1679.9359** | **-0.0095** | **1** | **21** | **0.89** | **1** | **R.YRVPDVLVADPPIAR.L** |
|  | 18489 | **964.4227** | **1926.8308** | **1926.8452** | **-0.0144** | **0** | **88** | **1.4e-007** | **1** | **K.MMDYLQGSGETPQTDVR.W** |
|  | 26128 | **1004.1385** | **3009.3936** | **3009.4145** | **-0.0208** | **0** | **24** | **0.18** | **1** | **R.FSFSGNTLVSSSADPEGHFETPIWIER.V** |
|  | 28739 | **912.4670** | **3645.8391** | **3645.8442** | **-0.0052** | **0** | **14** | **1.3** | **1** | **R.LYNLDVFQYELYNPMALYGSVPVLLAHNPHR.D** |
|  | 29550 | **791.5729** | **3952.8283** | **3952.8513** | **-0.0229** | **0** | **18** | **0.47** | **1** | **K.THSDSKPYGPMSVGLDFSLPGMEHVYGIPEHADNLR.L** 29545 29552 |
|  | 29589 | 794.5792 | 3967.8597 | 3968.8462 | -0.9865 | 0 | (5) | 8.6 | 2 | K.THSDSKPYGPMSVGLDFSLPGMEHVYGIPEHADNLR.L |
|  | 32338 | **1449.7234** | **7243.5806** | **7243.5706** | **0.0099** | **0** | **14** | **0.18** | **1** | **R.EGIPVMRPLWVQYPQDVTTFNIDDQYLLGDALLVHPVSDSGAHGVQVYLPGQGEVWYDIQSYQK.H** |

| **355.** | P23396\|RS3_HUMAN    **Mass:** 26842    **Score:** 85     **Queries matched:** 18   **emPAI:** 0.30 |
| --- | --- |
|  | 40S ribosomal protein S3 OS=Homo sapiens GN=RPS3 PE=1 SV=2 |

|  | **Query** | **Observed** | **Mr(expt)** | **Mr(calc)** | **Delta** | **Miss** | **Score** | **Expect** | **Rank** | **Peptide** |
| --- | --- | --- | --- | --- | --- | --- | --- | --- | --- | --- |
|  | 1429 | **399.2328** | **796.4510** | **797.5123** | **-1.0613** | **0** | **4** | **59** | **6** | **K.LLGGLAVR.R** |
|  | 2660 | **441.7206** | **881.4265** | **881.4317** | **-0.0051** | **0** | **15** | **4.4** | **2** | **R.FIMESGAK.G** |
|  | 2735 | **444.5442** | **887.0738** | **886.5124** | **0.5614** | **0** | **2** | **91** | **4** | **R.ELTAVVQK.R** |
|  | 5440 | **547.0314** | **1092.0483** | **1091.5611** | **0.4872** | **0** | **5** | **32** | **3** | **K.AELNEFLTR.E** |
|  | 6291 | **579.3238** | **1156.6330** | **1155.6975** | **0.9355** | **1** | **6** | **40** | **4** | **R.IRELTAVVQK.R** 6260 |
|  | 7077 | **402.2436** | **1203.7089** | **1202.6442** | **1.0647** | **1** | **6** | **36** | **9** | **K.GCEVVVSGKLR.G** |
|  | 11181 | 712.3343 | 1422.6540 | 1422.6627 | -0.0086 | 0 | 63 | 6.1e-005 | 1 | R.ELAEDGYSGVEVR.V 11200 |
|  | 11986 | **735.8839** | **1469.7533** | **1469.7613** | **-0.0081** | **0** | **28** | **0.24** | **1** | **K.DEILPTTPISEQK.G** |
|  | 13613 | **786.8781** | **1571.7415** | **1571.7508** | **-0.0092** | **0** | **14** | **5.6** | **1** | **R.FGFPEGSVELYAEK.V** |
|  | 13887 | **530.2739** | **1587.8000** | **1588.7919** | **-0.9920** | **0** | **3** | **67** | **3** | **K.GGKPEPPAMPQPVPTA.-** |
|  | 15818 | **572.8071** | **1715.3996** | **1714.9148** | **0.4848** | **1** | **2** | **42** | **7** | **K.VATRGLCAIAQAESLR.Y** |
|  | 17052 | **605.5483** | **1813.6232** | **1812.8750** | **0.7482** | **1** | **4** | **32** | **5** | **R.FIMESGAKGCEVVVSGK.L** 17043 |
|  | 18142 | **475.7448** | **1898.9499** | **1898.0836** | **0.8662** | **1** | **10** | **9** | **1** | **R.TEIIILATRTQNVLGEK.G** |
|  | 23212 | **823.3972** | **2467.1697** | **2467.1842** | **-0.0146** | **0** | **26** | **0.18** | **1** | **K.FVDGLMIHSGDPVNYYVDTAVR.H** |
|  | 26635 | 776.3528 | 3101.3823 | 3101.5757 | -0.1934 | 1 | 5 | 16 | 3 | K.FVDGLMIHSGDPVNYYVDTAVRHVLLR.Q |

| **356.** | P35558\|PCKGC_HUMAN    **Mass:** 69948    **Score:** 85     **Queries matched:** 15   **emPAI:** 0.23 |
| --- | --- |
|  | Phosphoenolpyruvate carboxykinase, cytosolic [GTP] OS=Homo sapiens GN=PCK1 PE=1 SV=3 |

|  | **Query** | **Observed** | **Mr(expt)** | **Mr(calc)** | **Delta** | **Miss** | **Score** | **Expect** | **Rank** | **Peptide** |
| --- | --- | --- | --- | --- | --- | --- | --- | --- | --- | --- |
|  | 1349 | 396.2091 | 790.4037 | 791.4323 | -1.0287 | 1 | 6 | 47 | 10 | R.MASRLAK.E |
|  | 3453 | 471.7097 | 941.4049 | 942.4672 | -1.0623 | 0 | 11 | 12 | 2 | K.FDAQGHLR.A |
|  | 8105 | 628.3065 | 1254.5984 | 1254.6067 | -0.0083 | 0 | 6 | 41 | 6 | K.YLAAAFPSACGK.T |
|  | 10509 | **461.7323** | **1382.1750** | **1382.7016** | **-0.5266** | **1** | **5** | **32** | **10** | **K.KYLAAAFPSACGK.T** |
|  | 10868 | **702.8265** | **1403.6384** | **1402.7126** | **0.9258** | **0** | **2** | **88** | **1** | **R.LLGQMEEEGILR.R** 10855 |
|  | 16271 | **875.9515** | **1749.8884** | **1749.8971** | **-0.0087** | **0** | **45** | **0.0036** | **1** | **K.IGIELTDSPYVVASMR.I** |
|  | 16468 | **589.3030** | **1764.8871** | **1765.8920** | **-1.0049** | **0** | **(3)** | **52** | **6** | **K.IGIELTDSPYVVASMR.I** |
|  | 16915 | **902.9583** | **1803.9019** | **1803.9156** | **-0.0136** | **0** | **37** | **0.022** | **1** | **R.AINPENGFFGVAPGTSVK.T** 16936 |
|  | 20296 | 700.6284 | 2098.8633 | 2098.8763 | -0.0130 | 1 | 45 | 0.0024 | 1 | K.NKEWSSEDGEPCAHPNSR.F 20295 |
|  | 22189 | 1161.0129 | 2320.0113 | 2319.0969 | 0.9144 | 0 | 2 | 44 | 3 | K.IIMHDPFAMRPFFGYNFGK.Y 22190 |
|  | 25275 | **943.8065** | **2828.3977** | **2828.5508** | **-0.1531** | **1** | **0** | **60** | **2** | **M.PPQLQNGLNLSAKVVQGSLDSLPQAVR.E** |

| **357.** | Q15836\|VAMP3_HUMAN    **Mass:** 11359    **Score:** 84     **Queries matched:** 4   **emPAI:** 0.83 |
| --- | --- |
|  | Vesicle-associated membrane protein 3 OS=Homo sapiens GN=VAMP3 PE=1 SV=3 |

|  | **Query** | **Observed** | **Mr(expt)** | **Mr(calc)** | **Delta** | **Miss** | **Score** | **Expect** | **Rank** | **Peptide** |
| --- | --- | --- | --- | --- | --- | --- | --- | --- | --- | --- |
|  | 2114 | **423.7295** | **845.4444** | **846.4083** | **-0.9639** | **0** | **10** | **21** | **2** | **K.LSELDDR.A** |
|  | 15069 | 832.8787 | 1663.7429 | 1664.8006 | -1.0577 | 0 | 18 | 1.7 | 2 | R.ADALQAGASQFETSAAK.L |
|  | 18322 | **958.4751** | **1914.9356** | **1914.9469** | **-0.0113** | **0** | **53** | **0.00044** | **1** | **R.LQQTQNQVDEVVDIMR.V** |
|  | 23382 | **1247.5988** | **2493.1829** | **2493.1983** | **-0.0153** | **1** | **56** | **0.00016** | **1** | **K.LSELDDRADALQAGASQFETSAAK.L** |

| **358.** | P27105\|STOM_HUMAN    **Mass:** 31882    **Score:** 84     **Queries matched:** 13   **emPAI:** 0.75 |
| --- | --- |
|  | Erythrocyte band 7 integral membrane protein OS=Homo sapiens GN=STOM PE=1 SV=3 |

|  | **Query** | **Observed** | **Mr(expt)** | **Mr(calc)** | **Delta** | **Miss** | **Score** | **Expect** | **Rank** | **Peptide** |
| --- | --- | --- | --- | --- | --- | --- | --- | --- | --- | --- |
|  | 1022 | **631.3733** | **630.3660** | **630.3701** | **-0.0041** | **0** | **25** | **0.98** | **1** | **R.NVLGTK.N** |
|  | 3115 | **458.2821** | **914.5496** | **914.5549** | **-0.0053** | **0** | **40** | **0.018** | **1** | **R.LLAQTTLR.N** 3129 |
|  | 4329 | **503.2523** | **1004.4900** | **1005.4549** | **-0.9649** | **0** | **46** | **0.0041** | **1** | **R.AMAAEAEASR.E** |
|  | 7982 | **624.7928** | **1247.5710** | **1246.5975** | **0.9735** | **0** | **6** | **36** | **3** | **K.VIAAEGEMNASR.A** |
|  | 9953 | 676.3724 | 1350.7302 | 1350.7395 | -0.0092 | 0 | 23 | 0.73 | 1 | R.YLQTLTTIAAEK.N |
|  | 10427 | **460.0151** | **1377.0234** | **1377.6306** | **-0.6072** | **1** | **7** | **20** | **7** | **R.AMAAEAEASREAR.A** |
|  | 11580 | **723.8673** | **1445.7199** | **1445.7296** | **-0.0097** | **1** | **32** | **0.08** | **1** | **R.AKVIAAEGEMNASR.A** 11581 |
|  | 14952 | **414.9648** | **1655.8303** | **1656.9159** | **-1.0856** | **1** | **6** | **28** | **6** | **R.NVLGTKNLSQILSDR.E** |
|  | 18517 | **965.4977** | **1928.9809** | **1928.9915** | **-0.0106** | **0** | **51** | **0.00075** | **1** | **R.VQNATLAVANITNADSATR.L** |
|  | 19594 | **676.7061** | **2027.0963** | **2027.1085** | **-0.0121** | **1** | **3** | **42** | **1** | **R.ALKEASMVITESPAALQLR.Y** |
|  | 20665 | **714.6998** | **2141.0777** | **2142.1759** | **-1.0982** | **0** | **4** | **33** | **2** | **K.NSTIVFPLPIDMLQGIIGAK.H** |

| **359.** | Q9NYQ3\|HAOX2_HUMAN    **Mass:** 39384    **Score:** 84     **Queries matched:** 8   **emPAI:** 0.20 |
| --- | --- |
|  | Hydroxyacid oxidase 2 OS=Homo sapiens GN=HAO2 PE=2 SV=1 |

|  | **Query** | **Observed** | **Mr(expt)** | **Mr(calc)** | **Delta** | **Miss** | **Score** | **Expect** | **Rank** | **Peptide** |
| --- | --- | --- | --- | --- | --- | --- | --- | --- | --- | --- |
|  | 2372 | **432.5885** | **863.1624** | **862.4661** | **0.6963** | **0** | **2** | **91** | **7** | **R.NLVQFSR.L** |
|  | 7574 | **615.3361** | **1228.6576** | **1228.6663** | **-0.0087** | **0** | **36** | **0.041** | **1** | **R.NLTLTDLQSPK.K** 7584 |
|  | 9969 | **676.8397** | **1351.6649** | **1351.6732** | **-0.0083** | **1** | **13** | **7.6** | **1** | **R.YLRDVSEVDTR.T** |
|  | 12290 | **744.8460** | **1487.6773** | **1486.7753** | **0.9020** | **0** | **2** | **87** | **6** | **K.HNVQGIIVSNHGGR.Q** |
|  | 16118 | **580.3207** | **1737.9404** | **1738.8122** | **-0.8718** | **1** | **1** | **88** | **9** | **K.STRDFIEGGADDSITR.D** |
|  | 28736 | **1215.5719** | **3643.6939** | **3643.7109** | **-0.0171** | **0** | **48** | **0.00058** | **1** | **R.TTIQGEEISAPICIAPTGFHCLVWPDGEMSTAR.A** 28735 |

| **360.** | P16157\|ANK1_HUMAN    **Mass:** 207334   **Score:** 84     **Queries matched:** 38   **emPAI:** 0.07 |
| --- | --- |
|  | Ankyrin-1 OS=Homo sapiens GN=ANK1 PE=1 SV=3 |

|  | **Query** | **Observed** | **Mr(expt)** | **Mr(calc)** | **Delta** | **Miss** | **Score** | **Expect** | **Rank** | **Peptide** |
| --- | --- | --- | --- | --- | --- | --- | --- | --- | --- | --- |
|  | 1322 | **395.2199** | **788.4252** | **787.4374** | **0.9878** | **0** | **9** | **29** | **9** | **R.GNVIMVR.L** |
|  | 1762 | 411.7164 | 821.4182 | 822.3766 | -0.9584 | 1 | 4 | 67 | 9 | R.GGSMRGSR.H |
|  | 2453 | **434.8816** | **867.7486** | **867.3723** | **0.3763** | **0** | **2** | **61** | **4** | **K.DHTSTPNP.-** |
|  | 3079 | 457.2924 | 912.5703 | 912.5579 | 0.0125 | 1 | 10 | 17 | 9 | R.LAMPVKVR.D |
|  | 3460 | **471.9977** | **941.9809** | **941.5545** | **0.4264** | **0** | **6** | **29** | **1** | **R.VAELLLER.D** |
|  | 3680 | **480.3261** | **958.6377** | **959.5335** | **-0.8958** | **1** | **5** | **63** | **5** | **R.RGNVIMVR.L** |
|  | 3828 | 486.2650 | 970.5154 | 971.4535 | -0.9381 | 0 | 20 | 1.8 | 1 | -.MPYSVGFR.E 3826 3827 3857 3868 |
|  | 5055 | **532.2594** | **1062.5042** | **1061.5506** | **0.9537** | **0** | **9** | **20** | **5** | **R.EPGGSLSFLR.K** 5063 5068 |
|  | 5279 | **541.2656** | **1080.5167** | **1079.5247** | **0.9920** | **0** | **10** | **14** | **1** | **R.EADAATSFLR.A** 5253 |
|  | 5349 | 543.7613 | 1085.5081 | 1085.5288 | -0.0206 | 0 | 18 | 2.9 | 1 | K.HGVMVDATTR.M 5340 |
|  | 6491 | 587.8085 | 1173.6024 | 1174.6809 | -1.0785 | 1 | 6 | 41 | 4 | K.EIILETTTKK.G |
|  | 10427 | 460.0151 | 1377.0234 | 1377.7000 | -0.6766 | 1 | 5 | 30 | 9 | R.EADAATSFLRAAR.S |
|  | 10537 | **462.2530** | **1383.7373** | **1382.7306** | **1.0067** | **0** | **7** | **27** | **5** | **K.FLLQHQADVNAK.T** |
|  | 12190 | **494.9264** | **1481.7574** | **1481.7045** | **0.0529** | **1** | **5** | **37** | **4** | **K.AKDDQTPLHCAAR.I** 12214 |
|  | 12574 | **503.1049** | **1506.2930** | **1506.7427** | **-0.4496** | **1** | **3** | **42** | **8** | **R.DSSREPGGSLSFLR.K** |
|  | 13393 | **779.3526** | **1556.6906** | **1556.7543** | **-0.0636** | **1** | **4** | **47** | **7** | **R.RQQGQEEQVQEAK.N** |
|  | 14126 | **535.6354** | **1603.8843** | **1603.8933** | **-0.0090** | **0** | **30** | **0.12** | **1** | **R.ISEILLDHGAPIQAK.T** 14131 14133 |
|  | 14236 | 806.4173 | 1610.8200 | 1611.8733 | -1.0532 | 1 | 6 | 28 | 8 | K.FLLQHQADVNAKTK.L |
|  | 14576 | 544.9410 | 1631.8011 | 1631.8049 | -0.0038 | 1 | 5 | 35 | 1 | R.GEIVNMLEGSGRQSR.N |
|  | 15110 | **834.3977** | **1666.7809** | **1665.8573** | **0.9235** | **0** | **1** | **80** | **10** | **K.LSTPPPLAEEEGLASR.I** |
|  | 16809 | **897.9767** | **1793.9388** | **1793.9523** | **-0.0135** | **0** | **36** | **0.029** | **1** | **R.TAAVLLQNDPNPDVLSK.T** |
|  | 17422 | 921.5041 | 1840.9936 | 1841.0047 | -0.0111 | 0 | 59 | 0.00013 | 1 | K.LLGNQATFSPIVTVEPR.R |
|  | 20093 | **693.3623** | **2077.0651** | **2077.0738** | **-0.0088** | **1** | **5** | **31** | **2** | **R.GASPNVSNVKVETPLHMAAR.A** |
|  | 23046 | **815.4054** | **2443.1944** | **2443.2087** | **-0.0143** | **1** | **27** | **0.16** | **1** | **R.EGHVETVLALLEKEASQACMTK.K** |
|  | 23340 | **830.0671** | **2487.1794** | **2487.3294** | **-0.1500** | **1** | **0** | **66** | **3** | **R.DIEVLEGMSLFAELSGNLVPVKK.A** |
|  | 23810 | 1279.1709 | 2556.3272 | 2555.1597 | 1.1676 | 1 | 0 | 61 | 10 | K.RQDDATGAGQDSENEVSLVSGHQR.G |
|  | 26043 | **748.3937** | **2989.5459** | **2990.3803** | **-0.8345** | **1** | **3** | **26** | **1** | **K.LVYANECANFTTNVSARFWLSDCPR.T** |

| **361.** | P46777\|RL5_HUMAN    **Mass:** 34569    **Score:** 84     **Queries matched:** 11   **emPAI:** 0.36 |
| --- | --- |
|  | 60S ribosomal protein L5 OS=Homo sapiens GN=RPL5 PE=1 SV=3 |

|  | **Query** | **Observed** | **Mr(expt)** | **Mr(calc)** | **Delta** | **Miss** | **Score** | **Expect** | **Rank** | **Peptide** |
| --- | --- | --- | --- | --- | --- | --- | --- | --- | --- | --- |
|  | 5933 | **565.7885** | **1129.5625** | **1128.5676** | **0.9949** | **1** | **31** | **0.15** | **1** | **K.EFNAEVHRK.H** |
|  | 6653 | **593.2758** | **1184.5371** | **1184.5462** | **-0.0091** | **1** | **27** | **0.31** | **1** | **K.RFPGYDSESK.E** |
|  | 7714 | **618.8470** | **1235.6794** | **1235.6874** | **-0.0080** | **1** | **11** | **11** | **1** | **R.TTTGNKVFGALK.G** |
|  | 9714 | 669.8503 | 1337.6861 | 1337.6939 | -0.0078 | 0 | 54 | 0.00052 | 1 | K.GAVDGGLSIPHSTK.R 9715 |
|  | 11384 | **717.8300** | **1433.6454** | **1433.6544** | **-0.0090** | **0** | **38** | **0.019** | **1** | **K.HIMGQNVADYMR.Y** 11396 |
|  | 13274 | **517.3312** | **1548.9717** | **1548.6653** | **0.3064** | **1** | **4** | **43** | **10** | **R.YLMEEDEDAYKK.Q** |
|  | 13642 | 787.8256 | 1573.6366 | 1573.6462 | -0.0097 | 0 | 12 | 7.2 | 2 | K.NSVTPDMMEEMYK.K |
|  | 15627 | **851.8726** | **1701.7307** | **1701.7412** | **-0.0105** | **1** | **43** | **0.0053** | **1** | **K.NSVTPDMMEEMYKK.A** |
|  | 18786 | 651.6403 | 1951.8991 | 1953.0684 | -1.1692 | 1 | 0 | 91 | 5 | K.VFGALKGAVDGGLSIPHSTK.R |

| **362.** | P63027\|VAMP2_HUMAN    **Mass:** 12698    **Score:** 84     **Queries matched:** 4   **emPAI:** 0.72 |
| --- | --- |
|  | Vesicle-associated membrane protein 2 OS=Homo sapiens GN=VAMP2 PE=1 SV=2 |

|  | **Query** | **Observed** | **Mr(expt)** | **Mr(calc)** | **Delta** | **Miss** | **Score** | **Expect** | **Rank** | **Peptide** |
| --- | --- | --- | --- | --- | --- | --- | --- | --- | --- | --- |
|  | 2114 | 423.7295 | 845.4444 | 846.4083 | -0.9639 | 0 | 10 | 21 | 2 | K.LSELDDR.A |
|  | 15069 | 832.8787 | 1663.7429 | 1664.8006 | -1.0577 | 0 | 18 | 1.7 | 2 | R.ADALQAGASQFETSAAK.L |
|  | 17843 | **936.9724** | **1871.9303** | **1871.9411** | **-0.0108** | **0** | **59** | **0.00014** | **1** | **R.LQQTQAQVDEVVDIMR.V** |
|  | 23382 | 1247.5988 | 2493.1829 | 2493.1983 | -0.0153 | 1 | 56 | 0.00016 | 1 | K.LSELDDRADALQAGASQFETSAAK.L |

| **363.** | P63010\|AP2B1_HUMAN    **Mass:** 105398   **Score:** 83     **Queries matched:** 17   **emPAI:** 0.07 |
| --- | --- |
|  | AP-2 complex subunit beta OS=Homo sapiens GN=AP2B1 PE=1 SV=1 |

|  | **Query** | **Observed** | **Mr(expt)** | **Mr(calc)** | **Delta** | **Miss** | **Score** | **Expect** | **Rank** | **Peptide** |
| --- | --- | --- | --- | --- | --- | --- | --- | --- | --- | --- |
|  | 1282 | 777.0353 | 776.0281 | 775.4262 | 0.6019 | 0 | 8 | 22 | 3 | K.LDIMIR.L 1279 1281 |
|  | 4155 | 497.2400 | 992.4655 | 991.4393 | 1.0262 | 0 | 9 | 21 | 9 | R.EAQSICER.V |
|  | 4827 | **522.7972** | **1043.5799** | **1043.5863** | **-0.0063** | **0** | **12** | **10** | **4** | **R.LLSTDPVTAK.E** 4810 |
|  | 5285 | 541.7215 | 1081.4284 | 1081.6648 | -0.2363 | 1 | 2 | 93 | 3 | K.AVWLPAVKAK.G |
|  | 10105 | **680.8073** | **1359.6001** | **1359.6089** | **-0.0088** | **0** | **5** | **53** | **7** | **K.ECHLNADTVSSK.L** |
|  | 10617 | **463.7297** | **1388.1673** | **1388.6646** | **-0.4973** | **1** | **13** | **4.6** | **1** | **K.DSDYYNMLLKK.L** |
|  | 10802 | **467.2832** | **1398.8277** | **1397.8031** | **1.0246** | **0** | **1** | **95** | **9** | **K.LTNGIWILAELR.I** |
|  | 11523 | **722.8234** | **1443.6323** | **1443.7722** | **-0.1398** | **0** | **4** | **47** | **6** | **R.IQPGNPNYTLSLK.C** |
|  | 12861 | 762.8671 | 1523.7197 | 1523.7290 | -0.0093 | 0 | 51 | 0.0011 | 1 | R.NVEGQDMLYQSLK.L |
|  | 15128 | **417.9743** | **1667.8681** | **1668.7851** | **-0.9170** | **0** | **9** | **15** | **1** | **K.SQPDMAIMAVNSFVK.D** 15162 |
|  | 21769 | 1132.9922 | 2263.9698 | 2264.2780 | -0.3082 | 0 | 8 | 11 | 2 | K.LAPPLVTLLSGEPEVQYVALR.N |
|  | 29428 | **1300.3621** | **3898.0644** | **3898.0737** | **-0.0093** | **0** | **47** | **0.0006** | **1** | **K.NSFGVIPSTPLAIHTPLMPNQSIDVSLPLNTLGPVMK.M** 29427 |

| **364.** | Q13621\|S12A1_HUMAN    **Mass:** 122627   **Score:** 83     **Queries matched:** 26   **emPAI:** 0.09 |
| --- | --- |
|  | Solute carrier family 12 member 1 OS=Homo sapiens GN=SLC12A1 PE=1 SV=2 |

|  | **Query** | **Observed** | **Mr(expt)** | **Mr(calc)** | **Delta** | **Miss** | **Score** | **Expect** | **Rank** | **Peptide** |
| --- | --- | --- | --- | --- | --- | --- | --- | --- | --- | --- |
|  | 1046 | **637.3261** | **636.3188** | **635.3643** | **0.9545** | **0** | **18** | **2.7** | **1** | **R.IYVGGK.I** |
|  | 2863 | 899.4567 | 898.4495 | 898.3524 | 0.0970 | 0 | 5 | 54 | 8 | K.EMNSGMAK.K |
|  | 4279 | 501.3005 | 1000.5863 | 1000.5665 | 0.0198 | 0 | 18 | 3 | 4 | R.SLLQASGLGR.M |
|  | 4514 | **510.2885** | **1018.5625** | **1017.5706** | **0.9919** | **1** | **3** | **92** | **2** | **K.DLTTAEKLK.R** |
|  | 7766 | 413.5520 | 1237.6341 | 1237.6415 | -0.0074 | 0 | 9 | 17 | 1 | R.LNELLQEHSR.A |
|  | 8589 | 427.1154 | 1278.3243 | 1278.6357 | -0.3113 | 1 | 3 | 52 | 9 | R.GNHKNVLTFYS.- |
|  | 9847 | 673.3598 | 1344.7050 | 1344.7136 | -0.0086 | 1 | 49 | 0.0018 | 1 | K.ITDAELEAVKEK.S 9849 |
|  | 10798 | **467.2452** | **1398.7137** | **1398.6305** | **0.0832** | **1** | **1** | **1.1e+002** | **5** | **K.LCVKEMNSGMAK.K** |
|  | 11293 | **715.3729** | **1428.7312** | **1427.7845** | **0.9467** | **1** | **5** | **46** | **1** | **R.DGVRSLLQASGLGR.M** 11266 11297 |
|  | 13831 | **793.2664** | **1584.5183** | **1583.7943** | **0.7239** | **1** | **3** | **44** | **2** | **K.IEYYRNTGSISGPK.V** |
|  | 14221 | **403.4558** | **1609.7939** | **1608.6760** | **1.1179** | **0** | **11** | **8.9** | **4** | **K.ESDSMMVDPTNDIR.I** |
|  | 14715 | **547.6138** | **1639.8197** | **1640.6658** | **-0.8461** | **0** | **(6)** | **31** | **4** | **K.ESDSMMVDPTNDIR.I** |
|  | 15138 | **557.0177** | **1668.0313** | **1667.8350** | **0.1963** | **0** | **2** | **54** | **9** | **R.CMLNIWGVMLFIR.L** |
|  | 16223 | **582.9968** | **1745.9685** | **1745.9788** | **-0.0103** | **0** | **43** | **0.0058** | **1** | **K.VNRPSLLEIHEQLAK.N** 16222 |
|  | 20376 | 703.3306 | 2106.9699 | 2105.9800 | 0.9899 | 1 | 29 | 0.12 | 1 | K.KDGSINTSQSMHVGEFNQK.L |
|  | 21296 | **737.3934** | **2209.1585** | **2208.1734** | **0.9851** | **1** | **0** | **76** | **4** | **K.GVLVRCMLNIWGVMLFIR.L** |
|  | 21625 | **749.7383** | **2246.1930** | **2245.2616** | **0.9314** | **1** | **2** | **49** | **7** | **R.SLLQASGLGRMKPNTLVIGYK.K** |
|  | 23833 | **854.3907** | **2560.1502** | **2560.1652** | **-0.0150** | **0** | **38** | **0.0099** | **1** | **R.ISFRPGNQECYDNFLQSGETAK.T** |
|  | 26920 | **1057.4957** | **3169.4654** | **3168.3005** | **1.1648** | **0** | **1** | **38** | **7** | **R.DATGNMNDTIISGMNCNGSAACGLGYDFSR.C** |
|  | 27074 | 1068.1625 | 3201.4656 | 3200.2904 | 1.1753 | 0 | (1) | 41 | 6 | R.DATGNMNDTIISGMNCNGSAACGLGYDFSR.C |
|  | 29613 | 1326.6969 | 3977.0689 | 3977.7100 | -0.6411 | 0 | 1 | 20 | 7 | R.FQVSVINENHESSAAADDNTDPPHYEETSFGDEAQK.R |
|  | 32012 | 1139.1656 | 5690.7919 | 5690.8108 | -0.0189 | 1 | 2 | 5.8 | 3 | R.HEPCQYGLMNNFQVMSMVSGFGPLITAGIFSATLSSALASLVSAPKVFQALCK.D |

| **365.** | P21980\|TGM2_HUMAN    **Mass:** 78420    **Score:** 83     **Queries matched:** 14   **emPAI:** 0.15 |
| --- | --- |
|  | Protein-glutamine gamma-glutamyltransferase 2 OS=Homo sapiens GN=TGM2 PE=1 SV=2 |

|  | **Query** | **Observed** | **Mr(expt)** | **Mr(calc)** | **Delta** | **Miss** | **Score** | **Expect** | **Rank** | **Peptide** |
| --- | --- | --- | --- | --- | --- | --- | --- | --- | --- | --- |
|  | 3437 | 470.7484 | 939.4823 | 939.3790 | 0.1033 | 0 | 10 | 16 | 10 | K.EETGMAMR.I |
|  | 3445 | 471.2526 | 940.4906 | 939.3790 | 1.1116 | 0 | (4) | 63 | 5 | K.EETGMAMR.I |
|  | 3633 | 478.9037 | 955.7929 | 955.3739 | 0.4190 | 0 | (6) | 31 | 2 | K.EETGMAMR.I |
|  | 5394 | **545.3000** | **1088.5854** | **1088.5536** | **0.0319** | **0** | **5** | **52** | **8** | **-.MAEELVLER.C** |
|  | 7189 | **404.2191** | **1209.6354** | **1208.5641** | **1.0712** | **1** | **5** | **41** | **9** | **K.EETGMAMRIR.V** 7174 |
|  | 10767 | **466.7372** | **1397.1898** | **1396.6326** | **0.5572** | **1** | **6** | **24** | **4** | **K.LAEKEETGMAMR.I** |
|  | 10876 | **468.9182** | **1403.7326** | **1403.7417** | **-0.0091** | **0** | **7** | **29** | **5** | **R.MDLLPLHMGLHK.L** |
|  | 11139 | **474.3262** | **1419.9567** | **1419.7366** | **0.2200** | **0** | **(6)** | **25** | **4** | **R.MDLLPLHMGLHK.L** |
|  | 16874 | **600.9653** | **1799.8742** | **1799.0192** | **0.8549** | **0** | **2** | **73** | **1** | **R.ALLVEPVINSYLLAER.D** |
|  | 18560 | **966.9609** | **1931.9072** | **1931.9194** | **-0.0121** | **0** | **17** | **1.9** | **1** | **R.VVSGMVNCNDDQGVLLGR.W** |
|  | 23288 | **1241.0739** | **2480.1331** | **2480.1431** | **-0.0099** | **0** | **67** | **1.5e-005** | **1** | **R.WDNNYGDGVSPMSWIGSVDILR.R** |
|  | 24222 | **880.0844** | **2637.2314** | **2636.2442** | **0.9872** | **1** | **31** | **0.052** | **1** | **R.WDNNYGDGVSPMSWIGSVDILRR.W** |
|  | 30447 | **631.0184** | **4410.0777** | **4410.0468** | **0.0309** | **1** | **1** | **16** | **2** | **R.VVSGMVNCNDDQGVLLGRWDNNYGDGVSPMSWIGSVDILR.R** |

| **366.** | Q16891\|IMMT_HUMAN    **Mass:** 84026    **Score:** 82     **Queries matched:** 24   **emPAI:** 0.09 |
| --- | --- |
|  | Mitochondrial inner membrane protein OS=Homo sapiens GN=IMMT PE=1 SV=1 |

|  | **Query** | **Observed** | **Mr(expt)** | **Mr(calc)** | **Delta** | **Miss** | **Score** | **Expect** | **Rank** | **Peptide** |
| --- | --- | --- | --- | --- | --- | --- | --- | --- | --- | --- |
|  | 1244 | 760.4507 | 759.4435 | 759.4126 | 0.0308 | 0 | 4 | 95 | 9 | K.SVIENAK.K |
|  | 1545 | **402.7379** | **803.4612** | **802.4185** | **1.0428** | **0** | **17** | **4.6** | **3** | **K.SLEDALR.Q** |
|  | 1604 | 808.8525 | 807.8452 | 807.4127 | 0.4325 | 0 | 13 | 5.5 | 1 | R.AFDSAVAK.A |
|  | 3329 | 466.2273 | 930.4400 | 930.4770 | -0.0371 | 0 | 9 | 24 | 9 | K.VQAAQSEAK.V |
|  | 3757 | 483.2539 | 964.4932 | 963.5138 | 0.9794 | 1 | 15 | 5.6 | 3 | K.RAFDSAVAK.A |
|  | 4169 | **497.7791** | **993.5436** | **994.5005** | **-0.9569** | **0** | **7** | **33** | **7** | **K.ISSVSEVMK.E** |
|  | 4296 | **501.7794** | **1001.5442** | **1001.5505** | **-0.0063** | **1** | **31** | **0.13** | **1** | **R.TVEGALKER.R** |
|  | 4623 | 514.7877 | 1027.5608 | 1026.3746 | 1.1861 | 0 | 9 | 21 | 6 | R.DAMENEMR.T |
|  | 5011 | **530.2903** | **1058.5661** | **1058.5720** | **-0.0059** | **1** | **26** | **0.5** | **1** | **K.KVQAAQSEAK.V** |
|  | 5489 | **548.7872** | **1095.5598** | **1094.6236** | **0.9361** | **1** | **0** | **1.4e+002** | **8** | **R.FYAVQKLAR.R** |
|  | 7050 | **401.8859** | **1202.6358** | **1202.5891** | **0.0467** | **1** | **8** | **24** | **10** | **R.SEIQAEQDRK.I** |
|  | 7887 | **622.3709** | **1242.7273** | **1242.6819** | **0.0453** | **1** | **0** | **1.3e+002** | **8** | **R.KAVDEAADALLK.A** |
|  | 7895 | **622.8115** | **1243.6084** | **1244.5885** | **-0.9801** | **0** | **24** | **0.65** | **1** | **R.YSTSGSSGLTTGK.I** |
|  | 12110 | **493.6001** | **1477.7785** | **1477.7889** | **-0.0104** | **0** | **8** | **22** | **1** | **K.EVAGAKPHITAAEGK.L** |
|  | 12182 | **494.8830** | **1481.6273** | **1480.8249** | **0.8024** | **1** | **2** | **73** | **6** | **K.ATEKQHITLALEK.Q** |
|  | 12881 | **509.2666** | **1524.7779** | **1524.6660** | **0.1118** | **1** | **4** | **51** | **8** | **R.DAMENEMRTQLR.R** |
|  | 12913 | **764.4127** | **1526.8109** | **1526.8205** | **-0.0096** | **0** | **76** | **2.8e-006** | **1** | **K.VVSQYHELVVQAR.D** |
|  | 13808 | **792.4150** | **1582.8155** | **1582.8243** | **-0.0087** | **0** | **31** | **0.093** | **1** | **R.ELDSITPEVLPGWK.G** |
|  | 14165 | **536.2844** | **1605.8314** | **1605.8838** | **-0.0524** | **1** | **7** | **22** | **1** | **K.KEVAGAKPHITAAEGK.L** |
|  | 17109 | **606.9625** | **1817.8657** | **1817.8768** | **-0.0111** | **1** | **12** | **6.2** | **1** | **K.ALEHHRSEIQAEQDR.K** |
|  | 17952 | **628.2940** | **1881.8600** | **1880.8543** | **1.0057** | **0** | **3** | **54** | **7** | **R.ACQLSGVTAAAQSCLCGK.F** |
|  | 18726 | **649.8658** | **1946.5757** | **1947.1193** | **-0.5436** | **0** | **3** | **36** | **2** | **K.IAGAGLLFVGGGIGGTILYAK.W** |
|  | 19140 | 663.0128 | 1986.0165 | 1986.1012 | -0.0848 | 1 | 2 | 56 | 3 | K.LFEMVLGPAAYNVPLPKK.S |
|  | 23454 | **836.3951** | **2506.1634** | **2505.1805** | **0.9829** | **0** | **7** | **14** | **1** | **K.ANCSDNEFTQALTAAIPPESLTR.G** |

| **367.** | P0C7P4\|UCRIL_HUMAN    **Mass:** 31081    **Score:** 82     **Queries matched:** 13   **emPAI:** 0.41 |
| --- | --- |
|  | Cytochrome b-c1 complex subunit Rieske-like protein 1 OS=Homo sapiens GN=UQCRFSL1 PE=2 SV=1 |

|  | **Query** | **Observed** | **Mr(expt)** | **Mr(calc)** | **Delta** | **Miss** | **Score** | **Expect** | **Rank** | **Peptide** |
| --- | --- | --- | --- | --- | --- | --- | --- | --- | --- | --- |
|  | 670 | **503.1053** | **502.0980** | **502.2863** | **-0.1883** | **0** | **8** | **60** | **1** | **R.SVAAR.S** 666 |
|  | 2966 | 453.2609 | 904.5072 | 903.4913 | 1.0159 | 0 | 10 | 20 | 4 | R.LEVLDSTK.S |
|  | 3509 | **473.7482** | **945.4819** | **945.4879** | **-0.0060** | **0** | **31** | **0.17** | **1** | **R.ESLSGQAVR.R** |
|  | 4409 | 506.2830 | 1010.5514 | 1009.5266 | 1.0247 | 0 | 6 | 42 | 6 | -.MQQIYTVK.E |
|  | 5005 | **530.2739** | **1058.5333** | **1059.5924** | **-1.0591** | **1** | **11** | **14** | **2** | **R.RLEVLDSTK.S** |
|  | 7446 | **612.2604** | **1222.5063** | **1221.6102** | **0.8961** | **1** | **8** | **24** | **4** | **R.DPQHDLDRVK.K** |
|  | 8788 | 645.3424 | 1288.6701 | 1288.6776 | -0.0074 | 0 | 52 | 0.00094 | 1 | R.SGPFAPVLSATSR.G |
|  | 14258 | **538.2946** | **1611.8619** | **1612.8784** | **-1.0165** | **1** | **7** | **25** | **5** | **K.EIKQEAAVELSQLR.D** |
|  | 16542 | 886.9032 | 1771.7918 | 1772.9533 | -1.1615 | 1 | 2 | 62 | 3 | R.SVAARSGPFAPVLSATSR.G |
|  | 20397 | **1055.4800** | **2108.9454** | **2109.0776** | **-0.1322** | **0** | **4** | **36** | **1** | **K.NAVTQFVSSMSASADVLALAK.I** |
|  | 24234 | **1320.7502** | **2639.4859** | **2639.5010** | **-0.0151** | **0** | **41** | **0.0041** | **1** | **R.GVAGALRPLVQATVPATPEQPVLDLK.R** 24233 |

| **368.** | P47985\|UCRI_HUMAN    **Mass:** 29934    **Score:** 82     **Queries matched:** 11   **emPAI:** 0.43 |
| --- | --- |
|  | Cytochrome b-c1 complex subunit Rieske, mitochondrial OS=Homo sapiens GN=UQCRFS1 PE=1 SV=2 |

|  | **Query** | **Observed** | **Mr(expt)** | **Mr(calc)** | **Delta** | **Miss** | **Score** | **Expect** | **Rank** | **Peptide** |
| --- | --- | --- | --- | --- | --- | --- | --- | --- | --- | --- |
|  | 2966 | 453.2609 | 904.5072 | 903.4913 | 1.0159 | 0 | 10 | 20 | 4 | R.LEVLDSTK.S |
|  | 3509 | 473.7482 | 945.4819 | 945.4879 | -0.0060 | 0 | 31 | 0.17 | 1 | R.ESLSGQAVR.R |
|  | 5005 | 530.2739 | 1058.5333 | 1059.5924 | -1.0591 | 1 | 11 | 14 | 2 | R.RLEVLDSTK.S |
|  | 7446 | 612.2604 | 1222.5063 | 1221.6102 | 0.8961 | 1 | 8 | 24 | 4 | R.DPQHDLDRVK.K |
|  | 8788 | 645.3424 | 1288.6701 | 1288.6776 | -0.0074 | 0 | 52 | 0.00094 | 1 | R.SGPFAPVLSATSR.G |
|  | 14289 | **539.0960** | **1614.2660** | **1613.8260** | **0.4400** | **0** | **2** | **52** | **8** | **K.EIEQEAAVELSQLR.D** |
|  | 19804 | **683.7060** | **2048.0961** | **2049.0677** | **-0.9716** | **1** | **1** | **79** | **2** | **-.MLSVASRSGPFAPVLSATSR.G** |
|  | 20397 | 1055.4800 | 2108.9454 | 2109.0776 | -0.1322 | 0 | 4 | 36 | 1 | K.NAVTQFVSSMSASADVLALAK.I |
|  | 23901 | **1289.1376** | **2576.2606** | **2576.2720** | **-0.0114** | **0** | **5** | **22** | **1** | **R.LGPAPLNLEVPTYEFTSDDMVIVG.-** |
|  | 24234 | 1320.7502 | 2639.4859 | 2639.5010 | -0.0151 | 0 | 41 | 0.0041 | 1 | R.GVAGALRPLVQATVPATPEQPVLDLK.R 24233 |

| **369.** | P02042\|HBD_HUMAN    **Mass:** 16159    **Score:** 82     **Queries matched:** 11   **emPAI:** 0.91 |
| --- | --- |
|  | Hemoglobin subunit delta OS=Homo sapiens GN=HBD PE=1 SV=2 |

|  | **Query** | **Observed** | **Mr(expt)** | **Mr(calc)** | **Delta** | **Miss** | **Score** | **Expect** | **Rank** | **Peptide** |
| --- | --- | --- | --- | --- | --- | --- | --- | --- | --- | --- |
|  | 3582 | 476.7556 | 951.4965 | 951.5025 | -0.0060 | 0 | 27 | 0.33 | 1 | M.VHLTPEEK.T |
|  | 8529 | 638.3271 | 1274.6396 | 1273.7183 | 0.9213 | 0 | 22 | 0.82 | 1 | R.LLVVYPWTQR.F 8523 8530 |
|  | 11626 | 725.3976 | 1448.7807 | 1448.7888 | -0.0081 | 1 | 62 | 7.8e-005 | 1 | K.VVAGVANALAHKYH.- 11613 11627 |
|  | 15154 | 835.4435 | 1668.8725 | 1668.8835 | -0.0110 | 0 | 50 | 0.0011 | 1 | K.VLGAFSDGLAHLDNLK.G 15169 |
|  | 16550 | 886.9597 | 1771.9049 | 1772.0131 | -0.1082 | 1 | 4 | 44 | 8 | R.LLGNVLVCVLARNFGK.E 16562 |

| **370.** | Q04826\|1B40_HUMAN    **Mass:** 40822    **Score:** 81     **Queries matched:** 5   **emPAI:** 0.19 |
| --- | --- |
|  | HLA class I histocompatibility antigen, B-40 alpha chain OS=Homo sapiens GN=HLA-B PE=1 SV=1 |

|  | **Query** | **Observed** | **Mr(expt)** | **Mr(calc)** | **Delta** | **Miss** | **Score** | **Expect** | **Rank** | **Peptide** |
| --- | --- | --- | --- | --- | --- | --- | --- | --- | --- | --- |
|  | 9948 | 676.3410 | 1350.6674 | 1349.6939 | 0.9736 | 1 | 14 | 5.9 | 1 | R.YLENGKETLQR.A 9957 |
|  | 12793 | 760.3734 | 1518.7323 | 1518.7427 | -0.0104 | 0 | 73 | 7.3e-006 | 1 | R.SWTAADTAAQITQR.K |
|  | 23671 | **509.4508** | **2542.2177** | **2541.3414** | **0.8763** | **0** | **2** | **40** | **10** | **R.TLLLLLWGAVALTETWAGSHSMR.Y** |
|  | 24792 | 912.7573 | 2735.2500 | 2735.2635 | -0.0135 | 1 | 34 | 0.023 | 1 | R.DGEDQTQDTELVETRPAGDRTFQK.W |

| **371.** | P16402\|H13_HUMAN    **Mass:** 22336    **Score:** 81     **Queries matched:** 13   **emPAI:** 0.37 |
| --- | --- |
|  | Histone H1.3 OS=Homo sapiens GN=HIST1H1D PE=1 SV=2 |

|  | **Query** | **Observed** | **Mr(expt)** | **Mr(calc)** | **Delta** | **Miss** | **Score** | **Expect** | **Rank** | **Peptide** |
| --- | --- | --- | --- | --- | --- | --- | --- | --- | --- | --- |
|  | 3843 | 486.7781 | 971.5415 | 971.5764 | -0.0348 | 1 | 10 | 19 | 3 | K.KPATAAGTKK.V |
|  | 3866 | 487.2955 | 972.5764 | 971.5400 | 1.0364 | 1 | 12 | 11 | 1 | K.KAASGEGKPK.A 3845 3851 |
|  | 4200 | **498.9061** | **995.7976** | **994.6287** | **1.1689** | **1** | **1** | **75** | **6** | **R.KPAGAAKKPK.K** |
|  | 5656 | 554.2841 | 1106.5537 | 1106.5608 | -0.0071 | 0 | 50 | 0.0018 | 1 | K.ALAAAGYDVEK.N |
|  | 6944 | 599.8527 | 1197.6909 | 1197.6605 | 0.0304 | 0 | 21 | 1.3 | 1 | K.ASGPPVSELITK.A 6939 |
|  | 8864 | 647.2993 | 1292.5840 | 1292.7677 | -0.1837 | 1 | 14 | 5.8 | 3 | K.AGAAKPRKPAGAAK.K 8871 |
|  | 13049 | 513.2577 | 1536.7514 | 1537.8100 | -1.0586 | 1 | 4 | 50 | 6 | K.GTLVQTKGTGASGSFK.L |
|  | 13714 | **789.8922** | **1577.7697** | **1577.7797** | **-0.0100** | **1** | **53** | **0.00057** | **1** | **K.ALAAAGYDVEKNNSR.I** |
|  | 15902 | 575.0478 | 1722.1215 | 1722.8862 | -0.7646 | 0 | 2 | 57 | 4 | -.MSETAPLAPTIPAPAEK.T |

| **372.** | P16403\|H12_HUMAN    **Mass:** 21352    **Score:** 81     **Queries matched:** 11   **emPAI:** 0.39 |
| --- | --- |
|  | Histone H1.2 OS=Homo sapiens GN=HIST1H1C PE=1 SV=2 |

|  | **Query** | **Observed** | **Mr(expt)** | **Mr(calc)** | **Delta** | **Miss** | **Score** | **Expect** | **Rank** | **Peptide** |
| --- | --- | --- | --- | --- | --- | --- | --- | --- | --- | --- |
|  | 828 | **559.0961** | **558.0889** | **557.2922** | **0.7967** | **0** | **12** | **13** | **2** | **K.AGGTPR.K** |
|  | 1483 | **401.2126** | **800.4105** | **799.4552** | **0.9554** | **1** | **12** | **13** | **6** | **K.KAAGGATPK.K** 1479 1482 1501 |
|  | 4557 | 512.2792 | 1022.5439 | 1022.6600 | -0.1161 | 1 | 4 | 58 | 10 | K.KPVGAAKKPK.K |
|  | 5656 | 554.2841 | 1106.5537 | 1106.5608 | -0.0071 | 0 | 50 | 0.0018 | 1 | K.ALAAAGYDVEK.N |
|  | 6944 | 599.8527 | 1197.6909 | 1197.6605 | 0.0304 | 0 | 21 | 1.3 | 1 | K.ASGPPVSELITK.A 6939 |
|  | 13049 | 513.2577 | 1536.7514 | 1537.8100 | -1.0586 | 1 | 4 | 50 | 6 | K.GTLVQTKGTGASGSFK.L |
|  | 13714 | 789.8922 | 1577.7697 | 1577.7797 | -0.0100 | 1 | 53 | 0.00057 | 1 | K.ALAAAGYDVEKNNSR.I |

| **373.** | P10412\|H14_HUMAN    **Mass:** 21852    **Score:** 81     **Queries matched:** 9   **emPAI:** 0.38 |
| --- | --- |
|  | Histone H1.4 OS=Homo sapiens GN=HIST1H1E PE=1 SV=2 |

|  | **Query** | **Observed** | **Mr(expt)** | **Mr(calc)** | **Delta** | **Miss** | **Score** | **Expect** | **Rank** | **Peptide** |
| --- | --- | --- | --- | --- | --- | --- | --- | --- | --- | --- |
|  | 145 | **418.2642** | **417.2569** | **416.2383** | **1.0186** | **0** | **4** | **2e+002** | **5** | **K.AGAAK.A** |
|  | 2094 | **423.0529** | **844.0912** | **843.4814** | **0.6098** | **1** | **5** | **43** | **4** | **K.KATGAATPK.K** |
|  | 3058 | **456.8776** | **911.7406** | **911.5552** | **0.1854** | **1** | **0** | **98** | **5** | **K.KPAAAAGAKK.A** |
|  | 4200 | 498.9061 | 995.7976 | 994.6287 | 1.1689 | 1 | 1 | 75 | 6 | K.KPAGAAKKPK.K |
|  | 5656 | 554.2841 | 1106.5537 | 1106.5608 | -0.0071 | 0 | 50 | 0.0018 | 1 | K.ALAAAGYDVEK.N |
|  | 6944 | 599.8527 | 1197.6909 | 1197.6605 | 0.0304 | 0 | 21 | 1.3 | 1 | K.ASGPPVSELITK.A 6939 |
|  | 13049 | 513.2577 | 1536.7514 | 1537.8100 | -1.0586 | 1 | 4 | 50 | 6 | K.GTLVQTKGTGASGSFK.L |
|  | 13714 | 789.8922 | 1577.7697 | 1577.7797 | -0.0100 | 1 | 53 | 0.00057 | 1 | K.ALAAAGYDVEKNNSR.I |

| **374.** | P22492\|H1T_HUMAN    **Mass:** 22006    **Score:** 81     **Queries matched:** 9   **emPAI:** 0.38 |
| --- | --- |
|  | Histone H1t OS=Homo sapiens GN=HIST1H1T PE=1 SV=4 |

|  | **Query** | **Observed** | **Mr(expt)** | **Mr(calc)** | **Delta** | **Miss** | **Score** | **Expect** | **Rank** | **Peptide** |
| --- | --- | --- | --- | --- | --- | --- | --- | --- | --- | --- |
|  | 910 | 585.3315 | 584.3243 | 583.4057 | 0.9185 | 1 | 8 | 21 | 1 | K.KVIPK.S |
|  | 912 | **587.1049** | **586.0976** | **585.3850** | **0.7127** | **1** | **1** | **1.2e+002** | **6** | **K.LPTKK.R** |
|  | 5656 | 554.2841 | 1106.5537 | 1106.5608 | -0.0071 | 0 | 50 | 0.0018 | 1 | K.ALAAAGYDVEK.N |
|  | 7242 | **405.2088** | **1212.6045** | **1211.7098** | **0.8947** | **1** | **4** | **63** | **9** | **R.GRKPAGLISASR.K** |
|  | 9543 | 664.3431 | 1326.6716 | 1326.7983 | -0.1267 | 1 | 6 | 33 | 6 | K.SLVNKGILVQTR.G |
|  | 13714 | 789.8922 | 1577.7697 | 1577.7797 | -0.0100 | 1 | 53 | 0.00057 | 1 | K.ALAAAGYDVEKNNSR.I |
|  | 17189 | **608.8210** | **1823.4411** | **1822.8441** | **0.5970** | **0** | **3** | **38** | **6** | **-.MSETVPAASASAGVAAMEK.L** |
|  | 21625 | 749.7383 | 2246.1930 | 2246.1286 | 0.0644 | 1 | (2) | 46 | 6 | -.MSETVPAASASAGVAAMEKLPTK.K |
|  | 21754 | **566.5461** | **2262.1555** | **2262.1235** | **0.0319** | **1** | **4** | **33** | **2** | **-.MSETVPAASASAGVAAMEKLPTK.K** |

| **375.** | Q02539\|H11_HUMAN    **Mass:** 21829    **Score:** 81     **Queries matched:** 6   **emPAI:** 0.38 |
| --- | --- |
|  | Histone H1.1 OS=Homo sapiens GN=HIST1H1A PE=1 SV=3 |

|  | **Query** | **Observed** | **Mr(expt)** | **Mr(calc)** | **Delta** | **Miss** | **Score** | **Expect** | **Rank** | **Peptide** |
| --- | --- | --- | --- | --- | --- | --- | --- | --- | --- | --- |
|  | 3259 | **463.3012** | **924.5879** | **924.6120** | **-0.0241** | **1** | **2** | **81** | **10** | **K.KPKTVKPK.K** |
|  | 5656 | 554.2841 | 1106.5537 | 1106.5608 | -0.0071 | 0 | 50 | 0.0018 | 1 | K.ALAAAGYDVEK.N |
|  | 13049 | 513.2577 | 1536.7514 | 1537.8100 | -1.0586 | 1 | 4 | 50 | 6 | K.GTLVQTKGTGASGSFK.L |
|  | 13714 | 789.8922 | 1577.7697 | 1577.7797 | -0.0100 | 1 | 53 | 0.00057 | 1 | K.ALAAAGYDVEKNNSR.I |
|  | 19122 | **662.3834** | **1984.1284** | **1983.1000** | **1.0284** | **1** | **3** | **43** | **1** | **K.KKPAGPSVSELIVQAASSSK.E** |
|  | 20659 | **714.4099** | **2140.2079** | **2140.1488** | **0.0591** | **1** | **6** | **23** | **2** | **K.KPAGPSVSELIVQAASSSKER.G** |

| **376.** | Q14508\|WFDC2_HUMAN    **Mass:** 13953    **Score:** 81     **Queries matched:** 5   **emPAI:** 0.28 |
| --- | --- |
|  | WAP four-disulfide core domain protein 2 OS=Homo sapiens GN=WFDC2 PE=1 SV=2 |

|  | **Query** | **Observed** | **Mr(expt)** | **Mr(calc)** | **Delta** | **Miss** | **Score** | **Expect** | **Rank** | **Peptide** |
| --- | --- | --- | --- | --- | --- | --- | --- | --- | --- | --- |
|  | 764 | **535.1846** | **534.1774** | **534.2220** | **-0.0447** | **0** | **5** | **69** | **1** | **R.NGCGK.V** |
|  | 11451 | **720.3278** | **1438.6411** | **1438.6333** | **0.0077** | **1** | **7** | **28** | **7** | **R.NGCGKVSCVTPNF.-** |
|  | 15325 | **840.8372** | **1679.6598** | **1679.6702** | **-0.0104** | **0** | **68** | **1.4e-005** | **1** | **R.DQCQVDSQCPGQMK.C** 15326 |
|  | 27455 | **1102.2285** | **3303.6637** | **3302.8134** | **0.8503** | **1** | **1** | **33** | **4** | **-.MPACRLGPLAAALLLSLLLFGFTLVSGTGAEK.T** |

| **377.** | P62491\|RB11A_HUMAN    **Mass:** 24492    **Score:** 81     **Queries matched:** 12   **emPAI:** 0.33 |
| --- | --- |
|  | Ras-related protein Rab-11A OS=Homo sapiens GN=RAB11A PE=1 SV=3 |

|  | **Query** | **Observed** | **Mr(expt)** | **Mr(calc)** | **Delta** | **Miss** | **Score** | **Expect** | **Rank** | **Peptide** |
| --- | --- | --- | --- | --- | --- | --- | --- | --- | --- | --- |
|  | 1146 | 689.3898 | 688.3826 | 688.3868 | -0.0042 | 0 | 26 | 0.58 | 1 | K.SNLLSR.F |
|  | 1597 | 404.6917 | 807.3689 | 807.3657 | 0.0031 | 1 | 9 | 20 | 10 | K.QMSDRR.E 1607 |
|  | 4829 | **522.8053** | **1043.5960** | **1042.6023** | **0.9938** | **0** | **4** | **65** | **5** | **K.VVLIGDSGVGK.S** 4795 |
|  | 5262 | 540.7848 | 1079.5550 | 1079.5611 | -0.0061 | 0 | 38 | 0.023 | 1 | K.STIGVEFATR.S 5258 5261 |
|  | 10549 | **693.3447** | **1384.6748** | **1383.6783** | **0.9965** | **1** | **24** | **0.53** | **1** | **R.FTRNEFNLESK.S** |
|  | 14323 | **539.7637** | **1616.2692** | **1615.8318** | **0.4374** | **1** | **3** | **42** | **9** | **K.TIKAQIWDTAGQER.Y** |
|  | 19523 | 675.0045 | 2021.9916 | 2023.0520 | -1.0605 | 1 | 0 | 82 | 5 | K.ELRDHADSNIVIMLVGNK.S |
|  | 29040 | **1250.9585** | **3749.8537** | **3748.8835** | **0.9701** | **1** | **54** | **0.00014** | **1** | **R.AFAEKNGLSFIETSALDSTNVEAAFQTILTEIYR.I** |

| **378.** | O43570\|CAH12_HUMAN    **Mass:** 39711    **Score:** 81     **Queries matched:** 7   **emPAI:** 0.09 |
| --- | --- |
|  | Carbonic anhydrase 12 OS=Homo sapiens GN=CA12 PE=1 SV=1 |

|  | **Query** | **Observed** | **Mr(expt)** | **Mr(calc)** | **Delta** | **Miss** | **Score** | **Expect** | **Rank** | **Peptide** |
| --- | --- | --- | --- | --- | --- | --- | --- | --- | --- | --- |
|  | 1523 | **402.2401** | **802.4656** | **801.3657** | **1.0999** | **0** | **11** | **16** | **2** | **R.TAEYYR.Y** |
|  | 10812 | **467.5786** | **1399.7139** | **1398.7772** | **0.9367** | **1** | **2** | **87** | **3** | **K.IFSHLQHVKYK.G** |
|  | 15202 | 837.3611 | 1672.7076 | 1672.7158 | -0.0082 | 0 | 55 | 0.00039 | 1 | K.WTYFGPDGENSWSK.K 15201 |
|  | 22295 | **779.7335** | **2336.1787** | **2335.1848** | **0.9939** | **1** | **8** | **12** | **1** | **K.YKGQEAFVPGFNIEELLPER.T** |
|  | 22534 | **789.0714** | **2364.1924** | **2364.2049** | **-0.0125** | **1** | **3** | **40** | **1** | **R.YRGSLTTPPCNPTVLWTVFR.N** |
|  | 31592 | **1074.2756** | **5366.3418** | **5366.9543** | **-0.6125** | **1** | **0** | **8.5** | **1** | **K.FDERLVYTSFSQVQVCTAAGLSLGIILSLALAGILGICIVVVVSIWLFR.R** |

| **379.** | P14174\|MIF_HUMAN    **Mass:** 12639    **Score:** 81     **Queries matched:** 5   **emPAI:** 0.72 |
| --- | --- |
|  | Macrophage migration inhibitory factor OS=Homo sapiens GN=MIF PE=1 SV=4 |

|  | **Query** | **Observed** | **Mr(expt)** | **Mr(calc)** | **Delta** | **Miss** | **Score** | **Expect** | **Rank** | **Peptide** |
| --- | --- | --- | --- | --- | --- | --- | --- | --- | --- | --- |
|  | 4826 | **522.7946** | **1043.5746** | **1043.5797** | **-0.0052** | **0** | **66** | **4.1e-005** | **1** | **K.LLCGLLAER.L** |
|  | 8739 | **644.3444** | **1286.6743** | **1286.6805** | **-0.0062** | **0** | **45** | **0.005** | **1** | **M.PMFIVNTNVPR.A** 8757 |
|  | 32042 | **1162.3790** | **5806.8588** | **5806.8797** | **-0.0210** | **0** | **16** | **0.2** | **1** | **R.ASVPDGFLSELTQQLAQATGKPPQYIAVHVVPDQLMAFGGSSEPCALCSLHSIGK.I** 32041 |

| **380.** | P13667\|PDIA4_HUMAN    **Mass:** 73229    **Score:** 80     **Queries matched:** 13   **emPAI:** 0.16 |
| --- | --- |
|  | Protein disulfide-isomerase A4 OS=Homo sapiens GN=PDIA4 PE=1 SV=2 |

|  | **Query** | **Observed** | **Mr(expt)** | **Mr(calc)** | **Delta** | **Miss** | **Score** | **Expect** | **Rank** | **Peptide** |
| --- | --- | --- | --- | --- | --- | --- | --- | --- | --- | --- |
|  | 2655 | **441.3279** | **880.6413** | **881.5699** | **-0.9285** | **1** | **3** | **64** | **5** | **K.GPVKVVVGK.T** |
|  | 3939 | **489.8056** | **977.5966** | **977.6022** | **-0.0055** | **1** | **11** | **14** | **4** | **K.RSPPIPLAK.V** |
|  | 5252 | **540.4449** | **1078.8752** | **1078.6022** | **0.2730** | **1** | **1** | **87** | **4** | **K.DKDPPIPVAK.I** |
|  | 5290 | **541.7642** | **1081.5139** | **1080.4836** | **1.0303** | **0** | **4** | **67** | **3** | **K.GQAVDYEGSR.T** |
|  | 5888 | 563.7894 | 1125.5642 | 1125.5706 | -0.0064 | 0 | 28 | 0.21 | 2 | R.FDVSGYPTIK.I |
|  | 6780 | 595.8196 | 1189.6247 | 1189.6302 | -0.0055 | 0 | 65 | 5.2e-005 | 1 | K.IDATSASVLASR.F |
|  | 8259 | **631.3367** | **1260.6588** | **1260.6714** | **-0.0126** | **0** | **5** | **46** | **2** | **K.QLEPVYNSLAK.K** |
|  | 8646 | **641.8392** | **1281.6639** | **1281.6717** | **-0.0078** | **1** | **40** | **0.014** | **1** | **K.RFDVSGYPTLK.I** |
|  | 8753 | **430.2136** | **1287.6191** | **1288.6623** | **-1.0432** | **1** | **17** | **2.7** | **5** | **K.VDATAETDLAKR.F** |
|  | 8824 | **646.2787** | **1290.5428** | **1290.5510** | **-0.0082** | **0** | **22** | **0.8** | **1** | **K.MDATANDVPSDR.Y** |
|  | 17256 | **610.6297** | **1828.8673** | **1829.8142** | **-0.9469** | **1** | **1** | **88** | **10** | **K.KFAMEPEEFDSDTLR.E** |
|  | 19261 | 666.3381 | 1995.9924 | 1994.9019 | 1.0905 | 0 | 1 | 73 | 5 | K.DTVLLEFYAPWCGHCK.Q |
|  | 23289 | **827.7658** | **2480.2754** | **2480.2872** | **-0.0118** | **1** | **31** | **0.055** | **1** | **K.YGIVDYMIEQSGPPSKEILTLK.Q** |

| **381.** | P01842\|LAC_HUMAN    **Mass:** 11401    **Score:** 80     **Queries matched:** 10   **emPAI:** 0.82 |
| --- | --- |
|  | Ig lambda chain C regions OS=Homo sapiens GN=IGLC1 PE=1 SV=1 |

|  | **Query** | **Observed** | **Mr(expt)** | **Mr(calc)** | **Delta** | **Miss** | **Score** | **Expect** | **Rank** | **Peptide** |
| --- | --- | --- | --- | --- | --- | --- | --- | --- | --- | --- |
|  | 2399 | **865.3750** | **864.3677** | **863.3695** | **0.9982** | **0** | **16** | **4.3** | **1** | **K.TVAPTECS.-** |
|  | 4111 | **495.7566** | **989.4986** | **989.5029** | **-0.0044** | **0** | **15** | **5.8** | **3** | **K.AGVETTTPSK.Q** 4106 4109 |
|  | 13448 | **521.2612** | **1560.7617** | **1560.7744** | **-0.0127** | **1** | **16** | **3.2** | **1** | **K.AGVETTTPSKQSNNK.Y** |
|  | 15245 | **838.4278** | **1674.8410** | **1673.8472** | **0.9939** | **1** | **52** | **0.00075** | **1** | **K.ADSSPVKAGVETTTPSK.Q** 15227 |
|  | 15759 | **856.4830** | **1710.9514** | **1710.7519** | **0.1995** | **0** | **33** | **0.053** | **1** | **R.SYSCQVTHEGSTVEK.T** |
|  | 23803 | **853.0387** | **2556.0943** | **2556.1109** | **-0.0166** | **1** | **6** | **14** | **1** | **R.SYSCQVTHEGSTVEKTVAPTECS.-** 23806 |

| **382.** | Q14240\|IF4A2_HUMAN    **Mass:** 46601    **Score:** 80     **Queries matched:** 17   **emPAI:** 0.17 |
| --- | --- |
|  | Eukaryotic initiation factor 4A-II OS=Homo sapiens GN=EIF4A2 PE=1 SV=2 |

|  | **Query** | **Observed** | **Mr(expt)** | **Mr(calc)** | **Delta** | **Miss** | **Score** | **Expect** | **Rank** | **Peptide** |
| --- | --- | --- | --- | --- | --- | --- | --- | --- | --- | --- |
|  | 3267 | **463.7297** | **925.4449** | **925.3890** | **0.0559** | **0** | **4** | **60** | **9** | **M.SGGSADYNR.E** |
|  | 5183 | **537.2618** | **1072.5091** | **1072.4244** | **0.0848** | **0** | **5** | **57** | **4** | **-.MSGGSADYNR.E** |
|  | 5754 | 558.3032 | 1114.5919 | 1113.6758 | 0.9161 | 0 | 8 | 27 | 3 | R.VLITTDLLAR.G |
|  | 6950 | 400.2437 | 1197.7092 | 1197.6717 | 0.0375 | 0 | 16 | 3.6 | 2 | K.ETQALVLAPTR.E 6923 |
|  | 13479 | 782.3776 | 1562.7407 | 1562.7035 | 0.0372 | 0 | 12 | 7.5 | 2 | R.DFTVSALHGDMDQK.E |
|  | 13608 | **524.9099** | **1571.7077** | **1570.7007** | **1.0070** | **0** | **2** | **74** | **10** | **K.MFVLDEADEMLSR.G** |
|  | 17228 | **914.4659** | **1826.9173** | **1826.9315** | **-0.0142** | **0** | **76** | **2.8e-006** | **1** | **R.GIYAYGFEKPSAIQQR.A** 17227 |
|  | 19290 | **667.3006** | **1998.8800** | **1997.9590** | **0.9209** | **1** | **3** | **45** | **5** | **K.WIKMFVLDEADEMLSR.G** |
|  | 19905 | 1029.9730 | 2057.9315 | 2057.9411 | -0.0096 | 1 | 0 | 88 | 5 | K.MHARDFTVSALHGDMDQK.E |
|  | 20707 | **715.3938** | **2143.1596** | **2143.1273** | **0.0322** | **0** | **3** | **42** | **9** | **R.GIDVQQVSLVINYDLPTNR.E** |
|  | 21153 | **731.3832** | **2191.1279** | **2190.1215** | **1.0064** | **1** | **3** | **39** | **4** | **R.NEMQKLQAEAPHIVVGTPGR.V** 21144 |
|  | 22989 | **812.6999** | **2435.0778** | **2435.2744** | **-0.1965** | **1** | **5** | **19** | **4** | **K.LQAEAPHIVVGTPGRVFDMLNR.R** |
|  | 28206 | **701.7232** | **3503.5797** | **3504.4722** | **-0.8925** | **0** | **7** | **7.6** | **3** | **R.EHGGPEGMDPDGVIESNWNEIVDNFDDMNLK.E** |
|  | 30612 | **1510.1079** | **4527.3019** | **4526.8962** | **0.4057** | **1** | **5** | **5.8** | **4** | **-.MSGGSADYNREHGGPEGMDPDGVIESNWNEIVDNFDDMNLK.E** |

| **383.** | P38919\|IF4A3_HUMAN    **Mass:** 47126    **Score:** 80     **Queries matched:** 14   **emPAI:** 0.16 |
| --- | --- |
|  | Eukaryotic initiation factor 4A-III OS=Homo sapiens GN=EIF4A3 PE=1 SV=4 |

|  | **Query** | **Observed** | **Mr(expt)** | **Mr(calc)** | **Delta** | **Miss** | **Score** | **Expect** | **Rank** | **Peptide** |
| --- | --- | --- | --- | --- | --- | --- | --- | --- | --- | --- |
|  | 6788 | 397.8583 | 1190.5532 | 1189.5939 | 0.9593 | 0 | 7 | 32 | 8 | R.DVIAQSQSGTGK.T |
|  | 7253 | **607.3521** | **1212.6897** | **1211.6874** | **1.0023** | **0** | **1** | **1.1e+002** | **8** | **R.ETQALILAPTR.E** |
|  | 9987 | 677.3527 | 1352.6909 | 1352.6718 | 0.0191 | 1 | 14 | 5.4 | 1 | M.ATTATMATSGSARK.R |
|  | 10312 | **458.2328** | **1371.6767** | **1371.6122** | **0.0645** | **0** | **(7)** | **29** | **1** | **-.MATTATMATSGSAR.K** 10337 |
|  | 10331 | **458.3054** | **1371.8944** | **1371.6122** | **0.2822** | **0** | **7** | **22** | **6** | **-.MATTATMATSGSAR.K** |
|  | 10618 | **463.7411** | **1388.2014** | **1387.6072** | **0.5943** | **0** | **(2)** | **58** | **10** | **-.MATTATMATSGSAR.K** 10625 |
|  | 12463 | **500.7501** | **1499.2284** | **1499.7072** | **-0.4788** | **1** | **1** | **65** | **7** | **-.MATTATMATSGSARK.R** |
|  | 14353 | **540.3104** | **1617.9093** | **1616.8634** | **1.0458** | **1** | **4** | **48** | **8** | **R.SGASRVLISTDVWAR.G** |
|  | 17228 | 914.4659 | 1826.9173 | 1826.9315 | -0.0142 | 0 | 76 | 2.8e-006 | 1 | R.GIYAYGFEKPSAIQQR.A 17227 |
|  | 21496 | 744.3662 | 2230.0768 | 2230.1317 | -0.0549 | 1 | (1) | 73 | 5 | K.LDYGQHVVAGTPGRVFDMIR.R |
|  | 21614 | **562.2974** | **2245.1603** | **2246.1266** | **-0.9663** | **1** | **6** | **22** | **1** | **K.LDYGQHVVAGTPGRVFDMIR.R** |

| **384.** | P60842\|IF4A1_HUMAN    **Mass:** 46353    **Score:** 80     **Queries matched:** 9   **emPAI:** 0.17 |
| --- | --- |
|  | Eukaryotic initiation factor 4A-I OS=Homo sapiens GN=EIF4A1 PE=1 SV=1 |

|  | **Query** | **Observed** | **Mr(expt)** | **Mr(calc)** | **Delta** | **Miss** | **Score** | **Expect** | **Rank** | **Peptide** |
| --- | --- | --- | --- | --- | --- | --- | --- | --- | --- | --- |
|  | 5754 | 558.3032 | 1114.5919 | 1113.6758 | 0.9161 | 0 | 8 | 27 | 3 | R.VLITTDLLAR.G |
|  | 6427 | **585.3130** | **1168.6114** | **1168.5764** | **0.0350** | **0** | **4** | **55** | **8** | **K.DQIYDIFQK.L** |
|  | 13608 | 524.9099 | 1571.7077 | 1570.7007 | 1.0070 | 0 | 2 | 74 | 10 | K.MFVLDEADEMLSR.G |
|  | 14359 | **540.5850** | **1618.7331** | **1617.8661** | **0.8670** | **0** | **3** | **57** | **6** | **K.LQMEAPHIIVGTPGR.V** |
|  | 17228 | 914.4659 | 1826.9173 | 1826.9315 | -0.0142 | 0 | 76 | 2.8e-006 | 1 | R.GIYAYGFEKPSAIQQR.A 17227 |
|  | 18871 | **654.0151** | **1959.0234** | **1958.9481** | **0.0753** | **1** | **2** | **63** | **7** | **K.YIKMFVLDEADEMLSR.G** |
|  | 20225 | 523.8046 | 2091.1894 | 2091.8925 | -0.7031 | 1 | 5 | 24 | 3 | K.MHARDFTVSAMHGDMDQK.E |
|  | 20707 | 715.3938 | 2143.1596 | 2143.1273 | 0.0322 | 0 | 3 | 42 | 9 | R.GIDVQQVSLVINYDLPTNR.E |

| **385.** | P46782\|RS5_HUMAN    **Mass:** 23033    **Score:** 80     **Queries matched:** 7   **emPAI:** 0.36 |
| --- | --- |
|  | 40S ribosomal protein S5 OS=Homo sapiens GN=RPS5 PE=1 SV=4 |

|  | **Query** | **Observed** | **Mr(expt)** | **Mr(calc)** | **Delta** | **Miss** | **Score** | **Expect** | **Rank** | **Peptide** |
| --- | --- | --- | --- | --- | --- | --- | --- | --- | --- | --- |
|  | 3340 | **466.7369** | **931.4593** | **932.4675** | **-1.0082** | **1** | **25** | **0.54** | **1** | **R.EDSTRIGR.A** 3356 3368 |
|  | 12492 | **751.3975** | **1500.7804** | **1500.7871** | **-0.0067** | **0** | **42** | **0.008** | **1** | **R.VNQAIWLLCTGAR.E** |
|  | 14551 | 815.9185 | 1629.8225 | 1630.8236 | -1.0011 | 0 | 6 | 30 | 3 | K.TIAECLADELINAAK.G 14552 |
|  | 22214 | **1162.0690** | **2322.1234** | **2322.1380** | **-0.0146** | **0** | **66** | **2e-005** | **1** | **K.WSTDDVQINDISLQDYIAVK.E** |

| **386.** | Q07065\|CKAP4_HUMAN    **Mass:** 66097    **Score:** 80     **Queries matched:** 13   **emPAI:** 0.06 |
| --- | --- |
|  | Cytoskeleton-associated protein 4 OS=Homo sapiens GN=CKAP4 PE=1 SV=2 |

|  | **Query** | **Observed** | **Mr(expt)** | **Mr(calc)** | **Delta** | **Miss** | **Score** | **Expect** | **Rank** | **Peptide** |
| --- | --- | --- | --- | --- | --- | --- | --- | --- | --- | --- |
|  | 1343 | 395.7425 | 789.4705 | 790.3457 | -0.8751 | 0 | 5 | 72 | 9 | K.EAADTER.L |
|  | 5447 | **547.2684** | **1092.5223** | **1091.5281** | **0.9942** | **1** | **19** | **2.1** | **1** | **R.SQKEINDMK.A** |
|  | 5495 | **548.8366** | **1095.6587** | **1095.5673** | **0.0914** | **1** | **4** | **58** | **6** | **R.QLKSDSHGPK.E** |
|  | 6126 | **573.4061** | **1144.7976** | **1145.5928** | **-0.7952** | **0** | **0** | **1.2e+002** | **5** | **K.ASVSQVEADLK.M** |
|  | 7049 | 602.3245 | 1202.6345 | 1202.6506 | -0.0161 | 1 | 9 | 22 | 3 | K.EAVKEIQTSAK.S |
|  | 8406 | **635.0503** | **1268.0860** | **1268.6109** | **-0.5249** | **1** | **3** | **46** | **2** | **R.GSKGGHGAASPSEK.G** |
|  | 8979 | **433.8993** | **1298.6760** | **1299.6419** | **-0.9658** | **1** | **2** | **86** | **8** | **R.SSQHKQDLTEK.A** |
|  | 9162 | **436.8798** | **1307.6175** | **1307.5928** | **0.0246** | **1** | **8** | **24** | **6** | **K.SREWDMEALR.S** |
|  | 10133 | **454.7238** | **1361.1495** | **1360.6834** | **0.4662** | **1** | **2** | **64** | **10** | **K.AKVASLEESEGNK.Q** |
|  | 12943 | 765.8265 | 1529.6384 | 1529.7685 | -0.1301 | 1 | 7 | 24 | 3 | R.SEESVSRLPEEIR.R |
|  | 17625 | **619.3093** | **1854.9060** | **1854.9258** | **-0.0198** | **0** | **4** | **45** | **4** | **R.LQHVEDGVLSMQVASAR.Q** |
|  | 18204 | **952.9579** | **1903.9013** | **1903.9123** | **-0.0110** | **0** | **80** | **1e-006** | **1** | **R.LEGLGSSEADQDGLASTVR.S** |
|  | 20185 | **697.3541** | **2089.0406** | **2088.8978** | **0.1428** | **0** | **1** | **75** | **3** | **K.SSSSSSASAAAAAAAASSSASCSR.R** |

| **387.** | P62879\|GBB2_HUMAN    **Mass:** 38048    **Score:** 80     **Queries matched:** 22   **emPAI:** 0.21 |
| --- | --- |
|  | Guanine nucleotide-binding protein G(I)/G(S)/G(T) subunit beta-2 OS=Homo sapiens GN=GNB2 PE=1 SV=3 |

|  | **Query** | **Observed** | **Mr(expt)** | **Mr(calc)** | **Delta** | **Miss** | **Score** | **Expect** | **Rank** | **Peptide** |
| --- | --- | --- | --- | --- | --- | --- | --- | --- | --- | --- |
|  | 1563 | 403.2297 | 804.4449 | 804.4970 | -0.0521 | 0 | 12 | 14 | 2 | K.VHAIPLR.S 1546 1549 |
|  | 4394 | 505.7836 | 1009.5526 | 1008.5101 | 1.0425 | 0 | 20 | 1.6 | 1 | R.AGVLAGHDNR.V 4371 4377 4378 4379 4393 |
|  | 4485 | 509.2794 | 1016.5442 | 1016.5502 | -0.0060 | 0 | 7 | 40 | 2 | R.LLVSASQDGK.L |
|  | 8101 | **628.2996** | **1254.5846** | **1254.5914** | **-0.0069** | **0** | **3** | **67** | **8** | **R.TFVSGACDASIK.L** |
|  | 9662 | 668.3080 | 1334.6015 | 1335.6030 | -1.0015 | 0 | 8 | 24 | 8 | K.IYAMHWGTDSR.L |
|  | 9977 | 451.7476 | 1352.2209 | 1351.5979 | 0.6230 | 0 | (4) | 37 | 2 | K.IYAMHWGTDSR.L |
|  | 9986 | 677.3525 | 1352.6905 | 1352.6976 | -0.0071 | 0 | 19 | 1.7 | 1 | K.LIIWDSYTTNK.V |
|  | 18547 | 966.5117 | 1931.0089 | 1930.9419 | 0.0670 | 0 | 1 | 77 | 3 | K.ACGDSTLTQITAGLDPVGR.I |
|  | 20849 | **1079.9950** | **2157.9754** | **2157.9863** | **-0.0109** | **0** | **6** | **21** | **1** | **R.LLLAGYDDFNCNIWDAMK.G** |
|  | 22847 | 1208.5555 | 2415.0965 | 2415.1087 | -0.0122 | 0 | 84 | 2.9e-007 | 1 | R.VSCLGVTDDGMAVATGSWDSFLK.I 22848 |
|  | 22965 | 487.4543 | 2432.2352 | 2431.1036 | 1.1316 | 0 | (5) | 24 | 3 | R.VSCLGVTDDGMAVATGSWDSFLK.I |
|  | 23263 | **619.5761** | **2474.2751** | **2475.2210** | **-0.9459** | **1** | **0** | **65** | **5** | **K.ACGDSTLTQITAGLDPVGRIQMR.T** |
|  | 27943 | 856.4415 | 3421.7368 | 3421.6031 | 0.1336 | 1 | 1 | 36 | 4 | R.AGVLAGHDNRVSCLGVTDDGMAVATGSWDSFLK.I |
|  | 28898 | **1234.6220** | **3700.8440** | **3700.6783** | **0.1658** | **1** | **1** | **24** | **10** | **R.SSWVMTCAYAPSGNFVACGGLDNICSIYSLKTR.E** |

| **388.** | P62701\|RS4X_HUMAN    **Mass:** 29807    **Score:** 79     **Queries matched:** 11   **emPAI:** 0.13 |
| --- | --- |
|  | 40S ribosomal protein S4, X isoform OS=Homo sapiens GN=RPS4X PE=1 SV=2 |

|  | **Query** | **Observed** | **Mr(expt)** | **Mr(calc)** | **Delta** | **Miss** | **Score** | **Expect** | **Rank** | **Peptide** |
| --- | --- | --- | --- | --- | --- | --- | --- | --- | --- | --- |
|  | 1365 | **396.7506** | **791.4867** | **791.4905** | **-0.0039** | **1** | **18** | **3.4** | **1** | **R.KIFVGTK.G** 1362 1366 1371 1382 |
|  | 1878 | 416.2296 | 830.4447 | 830.4498 | -0.0050 | 0 | 3 | 1e+002 | 4 | R.LTIAEER.D |
|  | 5198 | 537.7771 | 1073.5396 | 1073.5717 | -0.0320 | 1 | 13 | 8.4 | 5 | R.LTIAEERDK.R |
|  | 13161 | 515.3265 | 1542.9576 | 1541.9116 | 1.0461 | 1 | 6 | 30 | 8 | K.LRECLPLIIFLR.N |
|  | 16675 | **891.9127** | **1781.8108** | **1781.8189** | **-0.0081** | **0** | **79** | **1.2e-006** | **1** | **K.FDTGNLCMVTGGANLGR.I** |
|  | 17706 | 621.3565 | 1861.0475 | 1860.0370 | 1.0105 | 1 | 3 | 54 | 4 | K.IFVGTKGIPHLVTHDAR.T |
|  | 23502 | **839.4458** | **2515.3156** | **2515.2200** | **0.0956** | **1** | **0** | **61** | **4** | **K.ITDFIKFDTGNLCMVTGGANLGR.I** |

| **389.** | P30464\|1B15_HUMAN    **Mass:** 40648    **Score:** 79     **Queries matched:** 9   **emPAI:** 0.42 |
| --- | --- |
|  | HLA class I histocompatibility antigen, B-15 alpha chain OS=Homo sapiens GN=HLA-B PE=1 SV=2 |

|  | **Query** | **Observed** | **Mr(expt)** | **Mr(calc)** | **Delta** | **Miss** | **Score** | **Expect** | **Rank** | **Peptide** |
| --- | --- | --- | --- | --- | --- | --- | --- | --- | --- | --- |
|  | 3749 | 483.2167 | 964.4188 | 964.4250 | -0.0062 | 0 | 35 | 0.057 | 1 | R.FDSDAASPR.M |
|  | 7513 | 613.7462 | 1225.4779 | 1225.4856 | -0.0077 | 0 | 54 | 0.00053 | 1 | R.MYGCDVGPDGR.L 7482 |
|  | 9948 | 676.3410 | 1350.6674 | 1349.6939 | 0.9736 | 1 | 14 | 5.9 | 1 | R.YLENGKETLQR.A 9957 |
|  | 11128 | **710.8602** | **1419.7058** | **1419.6565** | **0.0493** | **1** | **8** | **22** | **2** | **R.FDSDAASPRMAPR.A** |
|  | 14460 | 406.9821 | 1623.8995 | 1623.7498 | 0.1497 | 1 | 4 | 43 | 10 | R.MYGCDVGPDGRLLR.G |
|  | 14538 | 815.4131 | 1628.8117 | 1628.8199 | -0.0081 | 0 | 28 | 0.17 | 1 | R.FIAVGYVDDTQFVR.F |
|  | 24792 | 912.7573 | 2735.2500 | 2735.2635 | -0.0135 | 1 | 34 | 0.023 | 1 | R.DGEDQTQDTELVETRPAGDRTFQK.W |

|  | **Proteins matching the same set of peptides:** |
| --- | --- |

|  | P30484\|1B46_HUMAN    **Mass:** 40700    **Score:** 79     **Queries matched:** 9 |
| --- | --- |
|  | HLA class I histocompatibility antigen, B-46 alpha chain OS=Homo sapiens GN=HLA-B PE=2 SV=1 |

| **390.** | P14550\|AK1A1_HUMAN    **Mass:** 36892    **Score:** 79     **Queries matched:** 8   **emPAI:** 0.21 |
| --- | --- |
|  | Alcohol dehydrogenase [NADP+] OS=Homo sapiens GN=AKR1A1 PE=1 SV=3 |

|  | **Query** | **Observed** | **Mr(expt)** | **Mr(calc)** | **Delta** | **Miss** | **Score** | **Expect** | **Rank** | **Peptide** |
| --- | --- | --- | --- | --- | --- | --- | --- | --- | --- | --- |
|  | 5876 | **563.2817** | **1124.5489** | **1123.5986** | **0.9504** | **1** | **18** | **2.6** | **1** | **K.EDVGPGKAVPR.E** 5883 |
|  | 7712 | **412.8949** | **1235.6628** | **1234.6631** | **0.9996** | **0** | **9** | **19** | **3** | **R.YIVPMLTVDGK.R** |
|  | 8931 | **432.8840** | **1295.6302** | **1296.7190** | **-1.0888** | **1** | **3** | **66** | **8** | **K.AAVKYALSVGYR.H** |
|  | 10400 | **688.7741** | **1375.5337** | **1374.7507** | **0.7830** | **1** | **7** | **24** | **1** | **K.AVPREELFVTSK.L** |
|  | 12064 | **738.3975** | **1474.7804** | **1474.7892** | **-0.0088** | **0** | **69** | **1.6e-005** | **1** | **K.GLVQALGLSNFNSR.Q** |
|  | 13291 | **776.3818** | **1550.7491** | **1550.7576** | **-0.0085** | **0** | **32** | **0.08** | **1** | **R.GLEVTAYSPLGSSDR.A** |
|  | 13995 | **532.6235** | **1594.8488** | **1593.9103** | **0.9385** | **1** | **2** | **69** | **5** | **R.SPAQILLRWQVQR.K** |

| **391.** | P12956\|KU70_HUMAN    **Mass:** 70084    **Score:** 79     **Queries matched:** 30   **emPAI:** 0.23 |
| --- | --- |
|  | ATP-dependent DNA helicase 2 subunit 1 OS=Homo sapiens GN=XRCC6 PE=1 SV=2 |

|  | **Query** | **Observed** | **Mr(expt)** | **Mr(calc)** | **Delta** | **Miss** | **Score** | **Expect** | **Rank** | **Peptide** |
| --- | --- | --- | --- | --- | --- | --- | --- | --- | --- | --- |
|  | 1845 | **415.2170** | **828.4194** | **827.5229** | **0.8965** | **1** | **3** | **94** | **7** | **K.AIVEKLR.F** |
|  | 1929 | 834.4673 | 833.4600 | 833.4680 | -0.0080 | 1 | 25 | 0.58 | 1 | K.MKAIVEK.L 1944 |
|  | 1939 | **418.2383** | **834.4621** | **834.4673** | **-0.0052** | **0** | **17** | **3.5** | **1** | **K.FTVPMLK.E** |
|  | 2140 | 424.7261 | 847.4376 | 846.4381 | 0.9995 | 1 | 10 | 19 | 3 | K.VEAMNKR.L 2130 |
|  | 2377 | **432.7424** | **863.4702** | **862.4331** | **1.0372** | **1** | **(2)** | **1.1e+002** | **7** | **K.VEAMNKR.L** |
|  | 4331 | **503.2819** | **1004.5492** | **1004.5542** | **-0.0050** | **0** | **21** | **1.3** | **1** | **R.ILELDQFK.G** |
|  | 5199 | **537.7853** | **1073.5561** | **1072.5587** | **0.9975** | **0** | **20** | **2** | **1** | **K.IMATPEQVGK.M** |
|  | 6476 | **587.2661** | **1172.5177** | **1171.6812** | **0.8365** | **1** | **22** | **0.94** | **2** | **K.KQELLEALTK.H** |
|  | 9677 | 668.7985 | 1335.5824 | 1334.6765 | 0.9059 | 1 | 7 | 28 | 9 | K.EVAALCRYTPR.R |
|  | 10862 | 702.3447 | 1402.6748 | 1401.7326 | 0.9422 | 0 | 5 | 48 | 9 | R.DTGIFLDLMHLK.K |
|  | 13635 | **787.4096** | **1572.8047** | **1572.8147** | **-0.0101** | **0** | **61** | **9.8e-005** | **1** | **K.NIYVLQELDNPGAK.R** |
|  | 15476 | **423.7151** | **1690.8311** | **1690.8413** | **-0.0102** | **1** | **13** | **5.4** | **1** | **K.VEYSEEELKTHISK.G** |
|  | 16966 | **603.3197** | **1806.9373** | **1806.9476** | **-0.0103** | **1** | **21** | **0.95** | **1** | **R.TFNTSTGGLLLPSDTKR.S** 16961 |
|  | 17160 | **456.2635** | **1821.0250** | **1821.9005** | **-0.8755** | **1** | **9** | **15** | **3** | **K.MPFTEKIMATPEQVGK.M** |
|  | 17180 | **608.3444** | **1822.0113** | **1821.9005** | **0.1108** | **1** | **(1)** | **83** | **8** | **K.MPFTEKIMATPEQVGK.M** |
|  | 17369 | **919.4404** | **1836.8663** | **1837.8954** | **-1.0291** | **1** | **(4)** | **44** | **6** | **K.MPFTEKIMATPEQVGK.M** |
|  | 18167 | 951.5145 | 1901.0144 | 1901.8578 | -0.8434 | 0 | 4 | 44 | 3 | R.IMLFTNEDNPHGNDSAK.A |
|  | 19662 | **678.8442** | **2033.5107** | **2033.1094** | **0.4014** | **0** | **1** | **56** | **2** | **R.FDDPGLMLMGFKPLVLLK.K** |
|  | 20575 | **711.0915** | **2130.2528** | **2131.1354** | **-0.8826** | **0** | **10** | **7.5** | **1** | **K.IQVTPPGFQLVFLPFADDK.R** |
|  | 21517 | 558.8202 | 2231.2517 | 2232.0229 | -0.7713 | 1 | 5 | 26 | 1 | R.IMLFTNEDNPHGNDSAKASR.A 21520 |
|  | 22910 | **1212.1337** | **2422.2528** | **2422.2665** | **-0.0137** | **0** | **36** | **0.017** | **1** | **R.NLEALALDLMEPEQAVDLTLPK.V** |
|  | 23467 | **837.0848** | **2508.2327** | **2508.2424** | **-0.0097** | **1** | **31** | **0.062** | **1** | **R.LGSLVDEFKELVYPPDYNPEGK.V** 23470 |
|  | 25915 | **741.6086** | **2962.4055** | **2963.2064** | **-0.8009** | **1** | **4** | **24** | **2** | **K.TEGDEEAEEEQEENLEASGDYKYSGR.D** |
|  | 26662 | **623.3190** | **3111.5588** | **3110.5879** | **0.9709** | **1** | **1** | **35** | **7** | **R.NLEALALDLMEPEQAVDLTLPKVEAMNK.R** |
|  | 30555 | **1122.5230** | **4486.0627** | **4487.1110** | **-1.0483** | **1** | **0** | **20** | **2** | **R.DSLIFLVDASKAMFESQSEDELTPFDMSIQCIQSVYISK.I** |

| **392.** | P16615\|AT2A2_HUMAN    **Mass:** 116336   **Score:** 78     **Queries matched:** 22   **emPAI:** 0.10 |
| --- | --- |
|  | Sarcoplasmic/endoplasmic reticulum calcium ATPase 2 OS=Homo sapiens GN=ATP2A2 PE=1 SV=1 |

|  | **Query** | **Observed** | **Mr(expt)** | **Mr(calc)** | **Delta** | **Miss** | **Score** | **Expect** | **Rank** | **Peptide** |
| --- | --- | --- | --- | --- | --- | --- | --- | --- | --- | --- |
|  | 844 | **562.5030** | **561.4957** | **560.3533** | **1.1424** | **0** | **14** | **8.6** | **1** | **R.LTSIK.S** |
|  | 869 | **572.3472** | **571.3399** | **571.3442** | **-0.0043** | **0** | **12** | **12** | **4** | **K.NAIVR.S** |
|  | 5162 | **536.2756** | **1070.5367** | **1071.5560** | **-1.0193** | **0** | **2** | **1.2e+002** | **5** | **R.NAENAIEALK.E** |
|  | 6279 | **579.1780** | **1156.3415** | **1155.5924** | **0.7491** | **1** | **1** | **1e+002** | **8** | **K.KEFTLEFSR.D** |
|  | 6300 | **579.7953** | **1157.5761** | **1157.5975** | **-0.0214** | **1** | **6** | **46** | **1** | **R.CTHIRVGSTK.V** |
|  | 9119 | **653.3032** | **1304.5918** | **1304.5997** | **-0.0079** | **0** | **9** | **16** | **1** | **R.EFDELNPSAQR.D** |
|  | 9155 | **654.3419** | **1306.6692** | **1305.6963** | **0.9729** | **1** | **0** | **1.3e+002** | **5** | **R.VGSTKVPMTSGVK.Q** |
|  | 9732 | **670.3647** | **1338.7149** | **1338.6602** | **0.0548** | **0** | **2** | **97** | **2** | **K.NMLFSGTNIAAGK.A** |
|  | 10668 | **464.7792** | **1391.3158** | **1390.7126** | **0.6032** | **0** | **6** | **23** | **3** | **K.AEIGIAMGSGTAVAK.T** |
|  | 10945 | 705.3403 | 1408.6661 | 1407.6882 | 0.9779 | 0 | 7 | 27 | 3 | R.IGIFGQDEDVTSK.A |
|  | 12181 | **741.7845** | **1481.5545** | **1480.7596** | **0.7950** | **1** | **18** | **1.6** | **1** | **K.MNVFDTELKGLSK.I** |
|  | 12710 | **505.7688** | **1514.2846** | **1514.8239** | **-0.5393** | **1** | **13** | **4.6** | **2** | **R.QAGIRVIMITGDNK.G** |
|  | 12892 | **763.8536** | **1525.6926** | **1525.7017** | **-0.0091** | **0** | **43** | **0.0064** | **1** | **K.SMSVYCTPNKPSR.T** |
|  | 13631 | **525.2534** | **1572.7384** | **1573.8563** | **-1.1179** | **0** | **6** | **30** | **5** | **R.VDQSILTGESVSVIK.H** |
|  | 13719 | **526.9376** | **1577.7909** | **1577.7031** | **0.0877** | **0** | **4** | **48** | **3** | **R.EEMHLEDSANFIK.Y** |
|  | 16079 | **579.3202** | **1734.9389** | **1733.8042** | **1.1347** | **1** | **1** | **77** | **6** | **R.REEMHLEDSANFIK.Y** |
|  | 17171 | **911.8998** | **1821.7850** | **1821.9043** | **-0.1193** | **1** | **2** | **71** | **9** | **K.IMSVIREWGSGSDTLR.C** |
|  | 17333 | 611.9830 | 1832.9271 | 1833.9441 | -1.0170 | 1 | 3 | 54 | 4 | R.VIMITGDNKGTAVAICR.R |
|  | 17369 | 919.4404 | 1836.8663 | 1836.8755 | -0.0092 | 1 | 21 | 0.8 | 1 | K.AFTGREFDELNPSAQR.D |
|  | 20090 | **693.3447** | **2077.0122** | **2075.9438** | **1.0684** | **1** | **5** | **27** | **5** | **K.SMSVYCTPNKPSRTSMSK.M** |
|  | 24044 | **867.4526** | **2599.3359** | **2599.3494** | **-0.0134** | **1** | **58** | **0.00011** | **1** | **K.TVEEVLGHFGVNESTGLSLEQVKK.L** |
|  | 31550 | **1774.4956** | **5320.4650** | **5319.4430** | **1.0220** | **0** | **3** | **6.1** | **8** | **K.EDNPDFEGVDCAIFESPYPMTMALSVLVTIEMCNALNSLSENQSLLR.M** |

| **393.** | P24752\|THIL_HUMAN    **Mass:** 45456    **Score:** 78     **Queries matched:** 22   **emPAI:** 0.17 |
| --- | --- |
|  | Acetyl-CoA acetyltransferase, mitochondrial OS=Homo sapiens GN=ACAT1 PE=1 SV=1 |

|  | **Query** | **Observed** | **Mr(expt)** | **Mr(calc)** | **Delta** | **Miss** | **Score** | **Expect** | **Rank** | **Peptide** |
| --- | --- | --- | --- | --- | --- | --- | --- | --- | --- | --- |
|  | 2672 | 442.2688 | 882.5230 | 882.5287 | -0.0056 | 0 | 6 | 33 | 1 | R.LNVTPLAR.I |
|  | 3783 | 484.5764 | 967.1382 | 967.4247 | -0.2864 | 1 | 1 | 86 | 10 | K.EDEEYKR.V |
|  | 3854 | **487.2572** | **972.4999** | **972.5790** | **-0.0791** | **0** | **10** | **21** | **9** | **-.MAVLAALLR.S** |
|  | 3890 | 487.7846 | 973.5545 | 972.5604 | 0.9942 | 0 | 21 | 1.6 | 1 | K.EVVIVSATR.T |
|  | 6951 | **400.2644** | **1197.7715** | **1196.7353** | **1.0362** | **1** | **9** | **15** | **1** | **M.AVLAALLRSGAR.S** 6902 |
|  | 9376 | **660.2948** | **1318.5750** | **1317.5805** | **0.9945** | **0** | **30** | **0.14** | **1** | **K.IHMGSCAENTAK.K** |
|  | 9642 | **445.2455** | **1332.7147** | **1333.5755** | **-0.8608** | **0** | **(5)** | **47** | **7** | **K.IHMGSCAENTAK.K** |
|  | 10858 | **702.0634** | **1402.1123** | **1401.7868** | **0.3255** | **0** | **12** | **6.3** | **1** | **K.FGNEVIPVTVTVK.G** |
|  | 13174 | **772.8478** | **1543.6811** | **1543.6903** | **-0.0091** | **0** | **73** | **5.7e-006** | **1** | **R.NEQDAYAINSYTR.S** |
|  | 20457 | 529.7466 | 2114.9575 | 2115.1364 | -0.1790 | 1 | 5 | 25 | 7 | K.AAWEAGKFGNEVIPVTVTVK.G |
|  | 29121 | **945.9499** | **3779.7705** | **3779.6036** | **0.1668** | **0** | **1** | **26** | **3** | **K.AIMMASQSLMCGHQDVMVAGGMESMSNVPYVMNR.G** |
|  | 30576 | **750.4021** | **4496.3689** | **4496.9338** | **-0.5649** | **1** | **(1)** | **15** | **1** | **K.VCASGMKAIMMASQSLMCGHQDVMVAGGMESMSNVPYVMNR.G** |
|  | 30618 | 755.8807 | 4529.2404 | 4528.9237 | 0.3167 | 1 | (3) | 9.2 | 1 | K.VCASGMKAIMMASQSLMCGHQDVMVAGGMESMSNVPYVMNR.G |
|  | 30642 | **1137.0397** | **4544.1296** | **4544.9186** | **-0.7890** | **1** | **4** | **8.9** | **8** | **K.VCASGMKAIMMASQSLMCGHQDVMVAGGMESMSNVPYVMNR.G** |
|  | 30643 | **1137.0400** | **4544.1311** | **4544.9186** | **-0.7875** | **1** | **(2)** | **13** | **7** | **K.VCASGMKAIMMASQSLMCGHQDVMVAGGMESMSNVPYVMNR.G** |
|  | 30671 | **1141.0421** | **4560.1393** | **4560.9135** | **-0.7742** | **1** | **(3)** | **9.5** | **4** | **K.VCASGMKAIMMASQSLMCGHQDVMVAGGMESMSNVPYVMNR.G** |
|  | 30706 | **1149.5221** | **4594.0593** | **4592.9034** | **1.1559** | **1** | **(2)** | **12** | **1** | **K.VCASGMKAIMMASQSLMCGHQDVMVAGGMESMSNVPYVMNR.G** |
|  | 30734 | **769.2258** | **4609.3113** | **4610.0323** | **-0.7210** | **1** | **(1)** | **13** | **7** | **K.AIMMASQSLMCGHQDVMVAGGMESMSNVPYVMNRGSTPYGGVK.L** |
|  | 30765 | **1542.8727** | **4625.5962** | **4626.0272** | **-0.4310** | **1** | **5** | **4** | **4** | **K.AIMMASQSLMCGHQDVMVAGGMESMSNVPYVMNRGSTPYGGVK.L** |
|  | 30767 | **1157.8046** | **4627.1892** | **4626.0272** | **1.1620** | **1** | **(0)** | **16** | **4** | **K.AIMMASQSLMCGHQDVMVAGGMESMSNVPYVMNRGSTPYGGVK.L** |
|  | 30822 | **1169.6307** | **4674.4939** | **4674.0120** | **0.4819** | **1** | **(0)** | **14** | **5** | **K.AIMMASQSLMCGHQDVMVAGGMESMSNVPYVMNRGSTPYGGVK.L** |

| **394.** | Q9HBG4\|VPP4_HUMAN    **Mass:** 97207    **Score:** 78     **Queries matched:** 15   **emPAI:** 0.04 |
| --- | --- |
|  | V-type proton ATPase 116 kDa subunit a isoform 4 OS=Homo sapiens GN=ATP6V0A4 PE=1 SV=1 |

|  | **Query** | **Observed** | **Mr(expt)** | **Mr(calc)** | **Delta** | **Miss** | **Score** | **Expect** | **Rank** | **Peptide** |
| --- | --- | --- | --- | --- | --- | --- | --- | --- | --- | --- |
|  | 2767 | 446.2530 | 890.4915 | 890.4974 | -0.0059 | 1 | 14 | 7.8 | 2 | R.KFVNEVR.R |
|  | 16326 | **877.9452** | **1753.8758** | **1753.9210** | **-0.0452** | **0** | **1** | **84** | **4** | **R.LEDLITVITQTESHR.Q** |
|  | 17028 | **906.9418** | **1811.8690** | **1811.9013** | **-0.0323** | **0** | **3** | **48** | **8** | **K.LEGELQEANQNQQALK.Q** |
|  | 19048 | **659.8655** | **1976.5746** | **1977.0870** | **-0.5124** | **1** | **1** | **49** | **9** | **K.AVPAYMTGKLGFIAGVINR.E** |
|  | 19513 | **1011.4443** | **2020.8740** | **2020.8821** | **-0.0081** | **0** | **78** | **1.4e-006** | **1** | **R.IQEDATENIEGDSSSPSSR.S** |
|  | 22937 | **607.5749** | **2426.2707** | **2427.1331** | **-0.8624** | **0** | **2** | **42** | **8** | **R.ALEQGMELSGSSMAPIMTTVQSK.T** |
|  | 23053 | **612.0266** | **2444.0771** | **2443.1281** | **0.9490** | **0** | **(2)** | **46** | **6** | **R.ALEQGMELSGSSMAPIMTTVQSK.T** |
|  | 23949 | **862.4136** | **2584.2191** | **2583.2342** | **0.9849** | **1** | **1** | **52** | **2** | **K.RALEQGMELSGSSMAPIMTTVQSK.T** |
|  | 28202 | **1168.0124** | **3501.0155** | **3500.9080** | **0.1075** | **0** | **7** | **6.4** | **1** | **K.HQQEVQSFFVVMALISVPWMLLIKPFILR.A** |
|  | 29512 | 1313.6204 | 3937.8393 | 3937.8987 | -0.0595 | 1 | 3 | 15 | 4 | K.MKAVYHILNMCNIDVTQQCVIAEIWFPVADATR.I |
|  | 30448 | **1471.5964** | **4411.7675** | **4412.1023** | **-0.3348** | **1** | **3** | **6.6** | **1** | **-.MASVFRSEEMCLSQLFLQVEAAYCCVAELGELGLVQFK.D** 30449 |
|  | 31078 | 1219.3069 | 4873.1984 | 4872.4304 | 0.7680 | 1 | 6 | 3.7 | 1 | R.EINPAPYTIITFPFLFAVMFGDCGHGTVMLLAALWMILNERR.L 31079 31081 |

| **395.** | P02774\|VTDB_HUMAN    **Mass:** 54526    **Score:** 78     **Queries matched:** 10   **emPAI:** 0.14 |
| --- | --- |
|  | Vitamin D-binding protein OS=Homo sapiens GN=GC PE=1 SV=1 |

|  | **Query** | **Observed** | **Mr(expt)** | **Mr(calc)** | **Delta** | **Miss** | **Score** | **Expect** | **Rank** | **Peptide** |
| --- | --- | --- | --- | --- | --- | --- | --- | --- | --- | --- |
|  | 1760 | **411.6937** | **821.3729** | **821.4139** | **-0.0410** | **0** | **10** | **19** | **2** | **K.LCMAALK.H** 1763 |
|  | 5120 | **534.3447** | **1066.6748** | **1065.5706** | **1.1042** | **1** | **4** | **64** | **8** | **K.KELSSFIDK.G** |
|  | 6577 | **591.2753** | **1180.5360** | **1181.6656** | **-1.1296** | **1** | **0** | **1.4e+002** | **10** | **K.LPDATPKELAK.L** |
|  | 8074 | **627.3524** | **1252.6903** | **1253.7092** | **-1.0189** | **0** | **2** | **95** | **7** | **K.HLSLLTTLSNR.V** |
|  | 17893 | 939.0170 | 1876.0195 | 1876.1411 | -0.1215 | 1 | 1 | 81 | 5 | K.RVLVLLLAVAFGHALER.G |
|  | 20227 | **1046.9103** | **2091.8060** | **2091.8184** | **-0.0124** | **0** | **77** | **1.5e-006** | **1** | **K.SLGECCDVEDSTTCFNAK.G** |
|  | 21766 | **755.6464** | **2263.9174** | **2263.9297** | **-0.0122** | **0** | **19** | **0.78** | **1** | **K.SCESNSPFPVHPGTAECCTK.E** |
|  | 23505 | **839.6768** | **2516.0086** | **2517.0094** | **-1.0008** | **0** | **3** | **27** | **2** | **K.EVVSLTEACCAEGADPDCYDTR.T** |
|  | 24230 | **880.4564** | **2638.3473** | **2639.1852** | **-0.8380** | **1** | **3** | **35** | **1** | **K.SYLSMVGSCCTSASPTVCFLKER.L** |

| **396.** | Q9Y4P3\|TBL2_HUMAN    **Mass:** 50393    **Score:** 77     **Queries matched:** 11   **emPAI:** 0.07 |
| --- | --- |
|  | Transducin beta-like protein 2 OS=Homo sapiens GN=TBL2 PE=1 SV=1 |

|  | **Query** | **Observed** | **Mr(expt)** | **Mr(calc)** | **Delta** | **Miss** | **Score** | **Expect** | **Rank** | **Peptide** |
| --- | --- | --- | --- | --- | --- | --- | --- | --- | --- | --- |
|  | 918 | **588.3677** | **587.3605** | **587.3642** | **-0.0038** | **0** | **12** | **21** | **1** | **K.SLGALK.K** |
|  | 7181 | **605.5483** | **1209.0821** | **1208.5860** | **0.4962** | **1** | **0** | **94** | **4** | **R.MASVSKDGTWK.L** |
|  | 11546 | 723.2544 | 1444.4942 | 1444.6841 | -0.1899 | 1 | 1 | 88 | 3 | R.AGEERSGRPACQK.A |
|  | 12927 | **764.9162** | **1527.8178** | **1527.8257** | **-0.0078** | **0** | **77** | **2.2e-006** | **1** | **R.LQQQLTQAQETLK.S** |
|  | 18520 | 644.2775 | 1929.8106 | 1929.8745 | -0.0639 | 1 | 2 | 56 | 3 | K.REDGGYTFTATPEDFPK.K |
|  | 20330 | 1052.0748 | 2102.1351 | 2101.1544 | 0.9807 | 1 | 3 | 39 | 2 | K.EKPQQHNFTHRLLAAALK.S 20332 |
|  | 25559 | **963.2482** | **2886.7227** | **2887.5584** | **-0.8358** | **0** | **1** | **32** | **1** | **M.ELSQMSELMGLSVLLGLLALMATAAVAR.G** 25558 |
|  | 26374 | **509.4273** | **3050.5202** | **3050.5888** | **-0.0686** | **0** | **6** | **11** | **2** | **-.MELSQMSELMGLSVLLGLLALMATAAVAR.G** |
|  | 27919 | **854.9196** | **3415.6494** | **3415.8393** | **-0.1899** | **1** | **2** | **30** | **3** | **M.ELSQMSELMGLSVLLGLLALMATAAVARGWLR.A** |

| **397.** | Q8NBJ7\|SUMF2_HUMAN    **Mass:** 33950    **Score:** 77     **Queries matched:** 6   **emPAI:** 0.11 |
| --- | --- |
|  | Sulfatase-modifying factor 2 OS=Homo sapiens GN=SUMF2 PE=1 SV=1 |

|  | **Query** | **Observed** | **Mr(expt)** | **Mr(calc)** | **Delta** | **Miss** | **Score** | **Expect** | **Rank** | **Peptide** |
| --- | --- | --- | --- | --- | --- | --- | --- | --- | --- | --- |
|  | 3417 | 469.8835 | 937.7525 | 938.4934 | -0.7409 | 0 | 2 | 58 | 8 | R.QPAGPGSGIR.E |
|  | 13768 | 791.3476 | 1580.6806 | 1580.6889 | -0.0083 | 0 | 73 | 5.6e-006 | 1 | R.MGNTPDSASDNLGFR.C 13767 |
|  | 19863 | **685.9479** | **2054.8220** | **2053.9487** | **0.8733** | **1** | **1** | **55** | **3** | **R.VTTRMGNTPDSASDNLGFR.C** |
|  | 25080 | 931.7731 | 2792.2974 | 2791.2290 | 1.0684 | 1 | 3 | 34 | 2 | R.MGNTPDSASDNLGFRCAADAGRPPGEL.- 25077 |

| **398.** | P51149\|RAB7A_HUMAN    **Mass:** 23760    **Score:** 77     **Queries matched:** 7   **emPAI:** 0.35 |
| --- | --- |
|  | Ras-related protein Rab-7a OS=Homo sapiens GN=RAB7A PE=1 SV=1 |

|  | **Query** | **Observed** | **Mr(expt)** | **Mr(calc)** | **Delta** | **Miss** | **Score** | **Expect** | **Rank** | **Peptide** |
| --- | --- | --- | --- | --- | --- | --- | --- | --- | --- | --- |
|  | 4984 | **529.3129** | **1056.6113** | **1056.6179** | **-0.0066** | **0** | **38** | **0.026** | **1** | **K.VIILGDSGVGK.T** |
|  | 13906 | **530.6111** | **1588.8116** | **1588.8209** | **-0.0093** | **0** | **4** | **52** | **7** | **K.EAINVEQAFQTIAR.N** |
|  | 14805 | 824.4060 | 1646.7975 | 1646.8086 | -0.0112 | 0 | 66 | 3e-005 | 1 | R.LVTMQIWDTAGQER.F 14803 |
|  | 18920 | **982.9580** | **1963.9015** | **1963.9415** | **-0.0400** | **0** | **1** | **78** | **1** | **K.QETEVELYNEFPEPIK.L** |
|  | 21668 | **564.0392** | **2252.1276** | **2251.1882** | **0.9394** | **1** | **0** | **81** | **9** | **K.VIILGDSGVGKTSLMNQYVNK.K** |
|  | 23556 | 842.1074 | 2523.3004 | 2523.1734 | 0.1270 | 1 | 3 | 36 | 5 | K.EVMVDDRLVTMQIWDTAGQER.F |

| **399.** | P02760\|AMBP_HUMAN    **Mass:** 39886    **Score:** 77     **Queries matched:** 8   **emPAI:** 0.20 |
| --- | --- |
|  | Protein AMBP OS=Homo sapiens GN=AMBP PE=1 SV=1 |

|  | **Query** | **Observed** | **Mr(expt)** | **Mr(calc)** | **Delta** | **Miss** | **Score** | **Expect** | **Rank** | **Peptide** |
| --- | --- | --- | --- | --- | --- | --- | --- | --- | --- | --- |
|  | 4535 | **511.2660** | **1020.5175** | **1020.5240** | **-0.0065** | **0** | **46** | **0.0041** | **1** | **R.ETLLQDFR.V** |
|  | 7270 | **607.8446** | **1213.6746** | **1212.6649** | **1.0098** | **0** | **20** | **1.5** | **1** | **R.TVAACNLPIVR.G** 7251 7255 |
|  | 15727 | **855.4263** | **1708.8380** | **1707.8443** | **0.9937** | **0** | **57** | **0.00025** | **1** | **K.WYNLAIGSTCPWLK.K** |
|  | 20512 | 708.3226 | 2121.9461 | 2121.8224 | 0.1237 | 0 | 2 | 55 | 6 | K.EDSCQLGYSAGPCMGMTSR.Y |
|  | 30070 | 836.3939 | 4176.9332 | 4177.2027 | -0.2695 | 1 | 7 | 4.4 | 1 | -.MRSLGALLLLLSACLAVSAGPVPTPPDNIQVQENFNISR.I |
|  | 30268 | 1078.4840 | 4309.9069 | 4309.7367 | 0.1703 | 1 | 1 | 17 | 4 | R.YFYNGTSMACETFQYGGCMGNGNNFVTEKECLQTCR.T |

| **400.** | Q29865\|1C18_HUMAN    **Mass:** 41363    **Score:** 77     **Queries matched:** 6   **emPAI:** 0.19 |
| --- | --- |
|  | HLA class I histocompatibility antigen, Cw-18 alpha chain OS=Homo sapiens GN=HLA-C PE=2 SV=1 |

|  | **Query** | **Observed** | **Mr(expt)** | **Mr(calc)** | **Delta** | **Miss** | **Score** | **Expect** | **Rank** | **Peptide** |
| --- | --- | --- | --- | --- | --- | --- | --- | --- | --- | --- |
|  | 3749 | 483.2167 | 964.4188 | 964.4250 | -0.0062 | 0 | 35 | 0.057 | 1 | R.FDSDAASPR.G |
|  | 9948 | 676.3410 | 1350.6674 | 1349.6939 | 0.9736 | 1 | 14 | 5.9 | 1 | R.YLENGKETLQR.A 9957 |
|  | 12793 | 760.3734 | 1518.7323 | 1518.7427 | -0.0104 | 0 | 73 | 7.3e-006 | 1 | R.SWTAADTAAQITQR.K |
|  | 17358 | 612.9700 | 1835.8883 | 1835.9027 | -0.0144 | 0 | 3 | 52 | 2 | K.THVTHHPVSDHEATLR.C |
|  | 29171 | **760.7784** | **3798.8555** | **3798.2032** | **0.6523** | **0** | **2** | **23** | **4** | **R.WKPSSQPTIPIVGIVAGLAVLVVLAVLGAVVAVVMCR.R** |

| **401.** | P30499\|1C01_HUMAN    **Mass:** 41395    **Score:** 77     **Queries matched:** 5   **emPAI:** 0.19 |
| --- | --- |
|  | HLA class I histocompatibility antigen, Cw-1 alpha chain OS=Homo sapiens GN=HLA-C PE=2 SV=1 |

|  | **Query** | **Observed** | **Mr(expt)** | **Mr(calc)** | **Delta** | **Miss** | **Score** | **Expect** | **Rank** | **Peptide** |
| --- | --- | --- | --- | --- | --- | --- | --- | --- | --- | --- |
|  | 3749 | 483.2167 | 964.4188 | 964.4250 | -0.0062 | 0 | 35 | 0.057 | 1 | R.FDSDAASPR.G |
|  | 12078 | **492.8015** | **1475.3827** | **1474.6875** | **0.6952** | **0** | **5** | **28** | **3** | **K.WAAVMVPSGEEQR.Y** |
|  | 12793 | 760.3734 | 1518.7323 | 1518.7427 | -0.0104 | 0 | 73 | 7.3e-006 | 1 | R.SWTAADTAAQITQR.K |
|  | 17358 | 612.9700 | 1835.8883 | 1835.9027 | -0.0144 | 0 | 3 | 52 | 2 | K.THVTHHPVSDHEATLR.C |
|  | 26739 | 782.8807 | 3127.4936 | 3127.6232 | -0.1296 | 1 | 6 | 12 | 2 | R.VMEPRTLILLLSGALALTETWACSHSMK.Y |

| **402.** | P37108\|SRP14_HUMAN    **Mass:** 14675    **Score:** 76     **Queries matched:** 4   **emPAI:** 0.27 |
| --- | --- |
|  | Signal recognition particle 14 kDa protein OS=Homo sapiens GN=SRP14 PE=1 SV=2 |

|  | **Query** | **Observed** | **Mr(expt)** | **Mr(calc)** | **Delta** | **Miss** | **Score** | **Expect** | **Rank** | **Peptide** |
| --- | --- | --- | --- | --- | --- | --- | --- | --- | --- | --- |
|  | 1744 | **411.0965** | **820.1784** | **819.4702** | **0.7082** | **0** | **4** | **62** | **5** | **K.ISTVVSSK.E** |
|  | 7838 | **414.7479** | **1241.2218** | **1241.6227** | **-0.4009** | **0** | **6** | **25** | **4** | **K.FQMAYSNLLR.A** |
|  | 15294 | **839.4524** | **1676.8902** | **1676.8985** | **-0.0083** | **0** | **76** | **2.6e-006** | **1** | **M.VLLESEQFLTELTR.L** |
|  | 22318 | **781.0077** | **2340.0014** | **2340.2399** | **-0.2385** | **1** | **0** | **66** | **9** | **-.MVLLESEQFLTELTRLFQK.C** |

| **403.** | Q01813\|K6PP_HUMAN    **Mass:** 86454    **Score:** 76     **Queries matched:** 18   **emPAI:** 0.04 |
| --- | --- |
|  | 6-phosphofructokinase type C OS=Homo sapiens GN=PFKP PE=1 SV=2 |

|  | **Query** | **Observed** | **Mr(expt)** | **Mr(calc)** | **Delta** | **Miss** | **Score** | **Expect** | **Rank** | **Peptide** |
| --- | --- | --- | --- | --- | --- | --- | --- | --- | --- | --- |
|  | 919 | **588.3677** | **587.3605** | **587.3139** | **0.0465** | **1** | **2** | **1.8e+002** | **4** | **K.EARGR.G** |
|  | 1780 | **412.5605** | **823.1065** | **823.4301** | **-0.3235** | **0** | **5** | **30** | **2** | **R.VGIADGHR.M** |
|  | 2600 | **439.7613** | **877.5081** | **876.4851** | **1.0230** | **1** | **9** | **21** | **9** | **K.MKTTIQR.G** |
|  | 2991 | 453.8384 | 905.6623 | 906.4196 | -0.7573 | 0 | 8 | 25 | 5 | R.GGTPSAFDR.I |
|  | 4321 | **503.1042** | **1004.1939** | **1003.5563** | **0.6376** | **1** | **7** | **22** | **3** | **R.FQDAVRLR.G** 4323 |
|  | 6696 | 396.5248 | 1186.5527 | 1185.6652 | 0.8875 | 1 | 8 | 23 | 6 | R.LKAACNLLQR.G |
|  | 7756 | **413.5422** | **1237.6049** | **1236.6687** | **0.9362** | **1** | **4** | **61** | **9** | **R.SAVRVGIADGHR.M** |
|  | 8292 | **632.3866** | **1262.7586** | **1262.7169** | **0.0417** | **1** | **7** | **29** | **6** | **R.AVVRMGIYVGAK.V** |
|  | 16731 | 894.4530 | 1786.8914 | 1786.8996 | -0.0081 | 0 | 76 | 2.5e-006 | 1 | K.AIGVLTSGGDAQGMNAAVR.A |
|  | 16890 | 601.6373 | 1801.8900 | 1802.8945 | -1.0045 | 0 | (0) | 99 | 9 | K.AIGVLTSGGDAQGMNAAVR.A |
|  | 21097 | **729.4209** | **2185.2409** | **2185.0732** | **0.1677** | **0** | **0** | **69** | **9** | **K.TNCNVAVINVGAPAAGMNAAVR.S** |
|  | 23692 | **637.0856** | **2544.3134** | **2544.2973** | **0.0161** | **1** | **2** | **38** | **1** | **K.GQIKEIGWTDVGGWTGQGGSILGTK.R** |
|  | 24031 | 866.7440 | 2597.2101 | 2598.3119 | -1.1018 | 1 | 1 | 47 | 4 | K.TNCNVAVINVGAPAAGMNAAVRSAVR.V |
|  | 29665 | **1001.4568** | **4001.7981** | **4001.8678** | **-0.0697** | **0** | **2** | **19** | **8** | **R.VFIIETMGGYCGYLANMGGLAAGADAAYIFEEPFDIR.D** |
|  | 30062 | **1044.2499** | **4172.9704** | **4173.9638** | **-0.9934** | **1** | **5** | **8.5** | **1** | **R.RVFIIETMGGYCGYLANMGGLAAGADAAYIFEEPFDIR.D** |
|  | 30344 | **872.0174** | **4355.0506** | **4354.0740** | **0.9766** | **0** | **1** | **16** | **2** | **K.VYFIYEGYQGMVDGGSNIAEADWESVSSILQVGGTIIGSAR.C** 30340 |

| **404.** | P01911\|2B1E_HUMAN    **Mass:** 30289    **Score:** 76     **Queries matched:** 10   **emPAI:** 0.12 |
| --- | --- |
|  | HLA class II histocompatibility antigen, DRB1-15 beta chain OS=Homo sapiens GN=HLA-DRB1 PE=1 SV=2 |

|  | **Query** | **Observed** | **Mr(expt)** | **Mr(calc)** | **Delta** | **Miss** | **Score** | **Expect** | **Rank** | **Peptide** |
| --- | --- | --- | --- | --- | --- | --- | --- | --- | --- | --- |
|  | 1541 | 402.7052 | 803.3958 | 804.4606 | -1.0648 | 1 | 12 | 14 | 6 | R.VRFLDR.Y |
|  | 24338 | 887.7534 | 2660.2383 | 2660.2541 | -0.0158 | 0 | 76 | 1.5e-006 | 1 | R.SGEVYTCQVEHPSVTSPLTVEWR.A 24337 |
|  | 25992 | **994.1039** | **2979.2898** | **2979.4834** | **-0.1936** | **0** | **3** | **26** | **10** | **K.AGMVSTGLIQNGDWTFQTLVMLETVPR.S** |
|  | 26061 | **999.4920** | **2995.4542** | **2995.4784** | **-0.0242** | **0** | **(1)** | **44** | **3** | **K.AGMVSTGLIQNGDWTFQTLVMLETVPR.S** |
|  | 29092 | **753.9499** | **3764.7134** | **3763.9167** | **0.7967** | **1** | **2** | **23** | **4** | **-.MVCLKLPGGSCMTALTVTLMVLSSPLALSGDTRPR.F** |
|  | 29950 | 1371.3488 | 4111.0244 | 4111.0183 | 0.0061 | 1 | 12 | 1.9 | 2 | R.WFLNGQEEKAGMVSTGLIQNGDWTFQTLVMLETVPR.S 29944 29945 29949 |

| **405.** | P56181\|NDUV3_HUMAN    **Mass:** 11990    **Score:** 76     **Queries matched:** 4   **emPAI:** 0.33 |
| --- | --- |
|  | NADH dehydrogenase [ubiquinone] flavoprotein 3, mitochondrial OS=Homo sapiens GN=NDUFV3 PE=2 SV=2 |

|  | **Query** | **Observed** | **Mr(expt)** | **Mr(calc)** | **Delta** | **Miss** | **Score** | **Expect** | **Rank** | **Peptide** |
| --- | --- | --- | --- | --- | --- | --- | --- | --- | --- | --- |
|  | 2284 | **429.7405** | **857.4663** | **858.4018** | **-0.9354** | **0** | **8** | **36** | **10** | **R.MPQPSSGR.E** 2290 |
|  | 22133 | **771.4148** | **2311.2225** | **2311.1848** | **0.0377** | **1** | **7** | **14** | **1** | **K.QSPPKKPAPVPAEPFDNTTYK.N** |
|  | 23755 | 851.4162 | 2551.2268 | 2550.2390 | 0.9877 | 0 | 76 | 1.6e-006 | 1 | K.NLQHHDYSTYTFLDLNLELSK.F |

| **406.** | P30457\|1A66_HUMAN    **Mass:** 41341    **Score:** 76     **Queries matched:** 15   **emPAI:** 0.30 |
| --- | --- |
|  | HLA class I histocompatibility antigen, A-66 alpha chain OS=Homo sapiens GN=HLA-A PE=1 SV=1 |

|  | **Query** | **Observed** | **Mr(expt)** | **Mr(calc)** | **Delta** | **Miss** | **Score** | **Expect** | **Rank** | **Peptide** |
| --- | --- | --- | --- | --- | --- | --- | --- | --- | --- | --- |
|  | 4195 | **498.7497** | **995.4848** | **995.4308** | **0.0539** | **0** | **8** | **22** | **3** | **R.FDSDAASQR.M** 4211 |
|  | 7513 | 613.7462 | 1225.4779 | 1225.4856 | -0.0077 | 0 | 54 | 0.00053 | 1 | R.MYGCDVGPDGR.F 7482 |
|  | 9948 | 676.3410 | 1350.6674 | 1349.6939 | 0.9736 | 1 | 14 | 5.9 | 1 | R.YLENGKETLQR.T 9957 |
|  | 12866 | **508.9794** | **1523.9163** | **1524.6627** | **-0.7464** | **1** | **2** | **65** | **3** | **R.FDSDAASQRMEPR.A** |
|  | 13421 | 780.4057 | 1558.7968 | 1558.8063 | -0.0095 | 1 | 15 | 3.7 | 3 | K.AQSQTDRVDLGTLR.G 13420 |
|  | 13526 | **522.9525** | **1565.8355** | **1564.7304** | **1.1051** | **0** | **2** | **67** | **6** | **R.SWTAADMAAQITQR.K** |
|  | 14538 | 815.4131 | 1628.8117 | 1628.8199 | -0.0081 | 0 | 28 | 0.17 | 1 | R.FIAVGYVDDTQFVR.F |
|  | 17461 | **615.3420** | **1843.0041** | **1841.8591** | **1.1450** | **0** | **10** | **11** | **2** | **K.THMTHHAVSDHEATLR.C** |
|  | 24080 | **869.7491** | **2606.2254** | **2606.2402** | **-0.0147** | **1** | **15** | **1.9** | **1** | **R.FIAVGYVDDTQFVRFDSDAASQR.M** |
|  | 24215 | 879.7303 | 2636.1690 | 2636.1838 | -0.0148 | 0 | 43 | 0.003 | 1 | R.DGEDQTQDTELVETRPAGDGTFQK.W |
|  | 27203 | **647.1058** | **3230.4925** | **3229.6774** | **0.8151** | **1** | **4** | **20** | **1** | **-.MAVMAPRTLVLLLSGALALTQTWAGSHSMR.Y** |

|  | **Proteins matching a subset of these peptides:** |
| --- | --- |

|  | P30453\|1A34_HUMAN    **Mass:** 41314    **Score:** 76     **Queries matched:** 14 |
| --- | --- |
|  | HLA class I histocompatibility antigen, A-34 alpha chain OS=Homo sapiens GN=HLA-A PE=1 SV=1 |

|  | P30450\|1A26_HUMAN    **Mass:** 41321    **Score:** 76     **Queries matched:** 13 |
| --- | --- |
|  | HLA class I histocompatibility antigen, A-26 alpha chain OS=Homo sapiens GN=HLA-A PE=1 SV=2 |

| **407.** | P18462\|1A25_HUMAN    **Mass:** 41477    **Score:** 76     **Queries matched:** 14   **emPAI:** 0.30 |
| --- | --- |
|  | HLA class I histocompatibility antigen, A-25 alpha chain OS=Homo sapiens GN=HLA-A PE=2 SV=1 |

|  | **Query** | **Observed** | **Mr(expt)** | **Mr(calc)** | **Delta** | **Miss** | **Score** | **Expect** | **Rank** | **Peptide** |
| --- | --- | --- | --- | --- | --- | --- | --- | --- | --- | --- |
|  | 4195 | 498.7497 | 995.4848 | 995.4308 | 0.0539 | 0 | 8 | 22 | 3 | R.FDSDAASQR.M 4211 |
|  | 7513 | 613.7462 | 1225.4779 | 1225.4856 | -0.0077 | 0 | 54 | 0.00053 | 1 | R.MYGCDVGPDGR.F 7482 |
|  | 9004 | **434.2593** | **1299.7561** | **1298.6327** | **1.1234** | **1** | **3** | **71** | **8** | **K.AHSQTDRESLR.I** |
|  | 9948 | 676.3410 | 1350.6674 | 1349.6939 | 0.9736 | 1 | 14 | 5.9 | 1 | R.YLENGKETLQR.T 9957 |
|  | 12866 | 508.9794 | 1523.9163 | 1524.6627 | -0.7464 | 1 | 2 | 65 | 3 | R.FDSDAASQRMEPR.A |
|  | 13526 | 522.9525 | 1565.8355 | 1564.7304 | 1.1051 | 0 | 2 | 67 | 6 | R.SWTAADMAAQITQR.K |
|  | 14538 | 815.4131 | 1628.8117 | 1628.8199 | -0.0081 | 0 | 28 | 0.17 | 1 | R.FIAVGYVDDTQFVR.F |
|  | 17461 | 615.3420 | 1843.0041 | 1841.8591 | 1.1450 | 0 | 10 | 11 | 2 | K.THMTHHAVSDHEATLR.C |
|  | 24080 | 869.7491 | 2606.2254 | 2606.2402 | -0.0147 | 1 | 15 | 1.9 | 1 | R.FIAVGYVDDTQFVRFDSDAASQR.M |
|  | 24215 | 879.7303 | 2636.1690 | 2636.1838 | -0.0148 | 0 | 43 | 0.003 | 1 | R.DGEDQTQDTELVETRPAGDGTFQK.W |
|  | 27203 | 647.1058 | 3230.4925 | 3229.6774 | 0.8151 | 1 | 4 | 20 | 1 | -.MAVMAPRTLVLLLSGALALTQTWAGSHSMR.Y |

| **408.** | P30456\|1A43_HUMAN    **Mass:** 41292    **Score:** 76     **Queries matched:** 14   **emPAI:** 0.30 |
| --- | --- |
|  | HLA class I histocompatibility antigen, A-43 alpha chain OS=Homo sapiens GN=HLA-A PE=2 SV=1 |

|  | **Query** | **Observed** | **Mr(expt)** | **Mr(calc)** | **Delta** | **Miss** | **Score** | **Expect** | **Rank** | **Peptide** |
| --- | --- | --- | --- | --- | --- | --- | --- | --- | --- | --- |
|  | 4195 | 498.7497 | 995.4848 | 995.4308 | 0.0539 | 0 | 8 | 22 | 3 | R.FDSDAASQR.M 4211 |
|  | 7513 | 613.7462 | 1225.4779 | 1225.4856 | -0.0077 | 0 | 54 | 0.00053 | 1 | R.MYGCDVGPDGR.F 7482 |
|  | 9948 | 676.3410 | 1350.6674 | 1349.6939 | 0.9736 | 1 | 14 | 5.9 | 1 | R.YLENGKETLQR.T 9957 |
|  | 12866 | 508.9794 | 1523.9163 | 1524.6627 | -0.7464 | 1 | 2 | 65 | 3 | R.FDSDAASQRMEPR.A |
|  | 13526 | 522.9525 | 1565.8355 | 1564.7304 | 1.1051 | 0 | 2 | 67 | 6 | R.SWTAADMAAQITQR.K |
|  | 14538 | 815.4131 | 1628.8117 | 1628.8199 | -0.0081 | 0 | 28 | 0.17 | 1 | R.FIAVGYVDDTQFVR.F |
|  | 17461 | 615.3420 | 1843.0041 | 1841.8591 | 1.1450 | 0 | 10 | 11 | 2 | K.THMTHHAVSDHEATLR.C |
|  | 23144 | 820.1008 | 2457.2807 | 2458.1917 | -0.9110 | 1 | 3 | 40 | 8 | R.APWIEQEGPEYWDLQTRNVK.A |
|  | 24080 | 869.7491 | 2606.2254 | 2606.2402 | -0.0147 | 1 | 15 | 1.9 | 1 | R.FIAVGYVDDTQFVRFDSDAASQR.M |
|  | 24215 | 879.7303 | 2636.1690 | 2636.1838 | -0.0148 | 0 | 43 | 0.003 | 1 | R.DGEDQTQDTELVETRPAGDGTFQK.W |
|  | 27203 | 647.1058 | 3230.4925 | 3229.6774 | 0.8151 | 1 | 4 | 20 | 1 | -.MAVMAPRTLVLLLSGALALTQTWAGSHSMR.Y |

| **409.** | P98187\|CP4F8_HUMAN    **Mass:** 60640    **Score:** 75     **Queries matched:** 14   **emPAI:** 0.06 |
| --- | --- |
|  | Cytochrome P450 4F8 OS=Homo sapiens GN=CYP4F8 PE=1 SV=1 |

|  | **Query** | **Observed** | **Mr(expt)** | **Mr(calc)** | **Delta** | **Miss** | **Score** | **Expect** | **Rank** | **Peptide** |
| --- | --- | --- | --- | --- | --- | --- | --- | --- | --- | --- |
|  | 1812 | **827.3103** | **826.3030** | **826.3717** | **-0.0687** | **0** | **16** | **3.6** | **3** | **K.FAMAEMK.V** |
|  | 2082 | 422.7274 | 843.4402 | 842.3666 | 1.0736 | 0 | (3) | 98 | 10 | K.FAMAEMK.V |
|  | 3687 | 960.5128 | 959.5055 | 958.4872 | 1.0183 | 0 | 16 | 4.6 | 5 | R.AEDGLWLR.V |
|  | 3927 | **489.2752** | **976.5359** | **975.4509** | **1.0850** | **0** | **11** | **16** | **3** | **K.ELSDEDIR.A** |
|  | 3971 | **491.2371** | **980.4597** | **980.4940** | **-0.0343** | **1** | **10** | **14** | **8** | **K.RNNQFFR.Y** |
|  | 5608 | **552.8001** | **1103.5855** | **1103.5360** | **0.0496** | **1** | **18** | **2.6** | **1** | **R.FDPENAQKR.S** |
|  | 10707 | **697.8539** | **1393.6932** | **1392.6860** | **1.0072** | **0** | **6** | **36** | **8** | **R.SPMAFIPFSAGPR.N** |
|  | 11693 | **485.4900** | **1453.4481** | **1453.7565** | **-0.3084** | **1** | **17** | **1.9** | **2** | **R.AEDGLWLRVEPLG.-** |
|  | 13137 | 771.8940 | 1541.7735 | 1541.7838 | -0.0103 | 0 | 56 | 0.00031 | 1 | R.LVHDFTDAVIQER.R 13136 13138 |
|  | 13437 | 780.9127 | 1559.8109 | 1558.6942 | 1.1167 | 1 | 6 | 28 | 3 | R.NCIGQKFAMAEMK.V |
|  | 15878 | **574.3154** | **1719.9243** | **1720.8996** | **-0.9753** | **1** | **5** | **36** | **3** | **R.TLTSQGVDDFLQAKAK.S** |
|  | 17138 | **910.9202** | **1819.8258** | **1821.0256** | **-1.1998** | **1** | **9** | **15** | **1** | **K.FAMAEMKVVLALTLLR.F** |

| **410.** | Q08477\|CP4F3_HUMAN    **Mass:** 60663    **Score:** 75     **Queries matched:** 9   **emPAI:** 0.06 |
| --- | --- |
|  | Leukotriene-B(4) omega-hydroxylase 2 OS=Homo sapiens GN=CYP4F3 PE=2 SV=2 |

|  | **Query** | **Observed** | **Mr(expt)** | **Mr(calc)** | **Delta** | **Miss** | **Score** | **Expect** | **Rank** | **Peptide** |
| --- | --- | --- | --- | --- | --- | --- | --- | --- | --- | --- |
|  | 3991 | 491.7669 | 981.5193 | 982.6539 | -1.1346 | 0 | 7 | 33 | 5 | K.VVLGLTLLR.F 3997 |
|  | 4646 | **515.3273** | **1028.6400** | **1028.5226** | **0.1174** | **1** | **7** | **33** | **9** | **R.CFPQPPKR.N** |
|  | 10851 | 701.8612 | 1401.7079 | 1401.7074 | 0.0004 | 0 | 15 | 4.1 | 1 | K.IFNESVNIMHAK.W |
|  | 13137 | 771.8940 | 1541.7735 | 1541.7838 | -0.0103 | 0 | 56 | 0.00031 | 1 | R.LVHDFTDAVIQER.R 13136 13138 |
|  | 23200 | **822.7374** | **2465.1905** | **2466.2797** | **-1.0892** | **1** | **2** | **41** | **3** | **R.NCIGQAFAMAEMKVVLGLTLLR.F** |
|  | 29280 | 769.2078 | 3841.0024 | 3840.7941 | 0.2083 | 1 | 2 | 23 | 1 | K.LSDEDIRAEADTFMFEGHDTTASGLSWVLYHLAK.H |

| **411.** | O15229\|KMO_HUMAN    **Mass:** 56344    **Score:** 75     **Queries matched:** 13   **emPAI:** 0.14 |
| --- | --- |
|  | Kynurenine 3-monooxygenase OS=Homo sapiens GN=KMO PE=1 SV=2 |

|  | **Query** | **Observed** | **Mr(expt)** | **Mr(calc)** | **Delta** | **Miss** | **Score** | **Expect** | **Rank** | **Peptide** |
| --- | --- | --- | --- | --- | --- | --- | --- | --- | --- | --- |
|  | 2331 | **431.2192** | **860.4239** | **859.4651** | **0.9588** | **0** | **19** | **2.6** | **2** | **K.DLLTAAEK.Y** 2312 2314 |
|  | 3380 | **468.2029** | **934.3912** | **934.4542** | **-0.0630** | **0** | **3** | **97** | **10** | **-.MDSSVIQR.K** |
|  | 4255 | **500.7802** | **999.5458** | **999.5535** | **-0.0077** | **1** | **10** | **18** | **2** | **R.MIHSLSGKK.S** |
|  | 4374 | **505.2519** | **1008.4892** | **1009.5668** | **-1.0776** | **0** | **14** | **5.9** | **1** | **R.SINLALSHR.G** 4408 |
|  | 4926 | **526.7875** | **1051.5605** | **1051.5662** | **-0.0057** | **0** | **18** | **2.5** | **1** | **K.SQYILSVSR.E** |
|  | 12879 | **763.3944** | **1524.7743** | **1524.7824** | **-0.0082** | **0** | **48** | **0.0021** | **1** | **K.LLTSNDVVDFFQK.Y** |
|  | 13398 | **519.9478** | **1556.8214** | **1555.7197** | **1.1018** | **0** | **1** | **93** | **10** | **R.NTFMMIALPNMNK.S** |
|  | 15075 | 555.6051 | 1663.7935 | 1664.7386 | -0.9451 | 0 | 3 | 63 | 8 | K.CNPEEGMITVLGSDK.V |
|  | 19192 | **664.3431** | **1990.0074** | **1990.8996** | **-0.8921** | **0** | **3** | **43** | **8** | **K.NGDYAMEPNYLHIWPR.N** |
|  | 19301 | **1000.9501** | **1999.8856** | **1999.8980** | **-0.0124** | **0** | **53** | **0.00047** | **1** | **K.DVTCDLIVGCDGAYSTVR.S** |

| **412.** | Q13011\|ECH1_HUMAN    **Mass:** 36136    **Score:** 75     **Queries matched:** 9   **emPAI:** 0.10 |
| --- | --- |
|  | Delta(3,5)-Delta(2,4)-dienoyl-CoA isomerase, mitochondrial OS=Homo sapiens GN=ECH1 PE=1 SV=2 |

|  | **Query** | **Observed** | **Mr(expt)** | **Mr(calc)** | **Delta** | **Miss** | **Score** | **Expect** | **Rank** | **Peptide** |
| --- | --- | --- | --- | --- | --- | --- | --- | --- | --- | --- |
|  | 2546 | **437.7449** | **873.4752** | **874.4695** | **-0.9942** | **0** | **10** | **21** | **9** | **-.MAAGIVASR.R** |
|  | 11657 | 484.5764 | 1450.7073 | 1451.6748 | -0.9675 | 0 | (2) | 79 | 5 | K.MMADEALGSGLVSR.V |
|  | 11960 | 490.3153 | 1467.9240 | 1467.6698 | 0.2543 | 0 | 9 | 14 | 2 | K.MMADEALGSGLVSR.V |
|  | 13150 | **772.3779** | **1542.7413** | **1541.8049** | **0.9364** | **0** | **8** | **21** | **2** | **K.EVDVGLAADVGTLQR.L** |
|  | 14610 | **545.6360** | **1633.8861** | **1632.8947** | **0.9914** | **1** | **8** | **20** | **3** | **R.RLTGSNYPGLSISLR.L** |
|  | 16036 | **866.4689** | **1730.9232** | **1730.9315** | **-0.0083** | **0** | **50** | **0.0011** | **1** | **K.VIGNQSLVNELAFTAR.K** 16045 |
|  | 20417 | **704.6438** | **2110.9096** | **2111.9941** | **-1.0846** | **0** | **(1)** | **65** | **3** | **K.MFTAGIDLMDMASDILQPK.G** |
|  | 20437 | **705.3301** | **2112.9686** | **2111.9941** | **0.9745** | **0** | **1** | **63** | **6** | **K.MFTAGIDLMDMASDILQPK.G** |

| **413.** | Q00765\|REEP5_HUMAN    **Mass:** 21707    **Score:** 75     **Queries matched:** 19   **emPAI:** 0.38 |
| --- | --- |
|  | Receptor expression-enhancing protein 5 OS=Homo sapiens GN=REEP5 PE=1 SV=3 |

|  | **Query** | **Observed** | **Mr(expt)** | **Mr(calc)** | **Delta** | **Miss** | **Score** | **Expect** | **Rank** | **Peptide** |
| --- | --- | --- | --- | --- | --- | --- | --- | --- | --- | --- |
|  | 2138 | 424.7193 | 847.4240 | 847.4287 | -0.0046 | 0 | 23 | 1 | 1 | K.ETADAITK.E |
|  | 4049 | 493.7588 | 985.5031 | 986.5509 | -1.0478 | 1 | 15 | 6.1 | 3 | K.LEAKTGVNR.S |
|  | 4869 | **524.2833** | **1046.5521** | **1046.5608** | **-0.0087** | **1** | **45** | **0.0058** | **1** | **K.AKETADAITK.E** 4848 4849 4850 4857 4862 4871 4872 4873 4874 |
|  | 6308 | **580.2706** | **1158.5267** | **1158.5339** | **-0.0072** | **0** | **43** | **0.0085** | **1** | **K.HESQMDSVVK.D** 6307 |
|  | 6533 | 589.2996 | 1176.5846 | 1175.6033 | 0.9812 | 1 | 7 | 31 | 4 | K.ETADAITKEAK.K |
|  | 7004 | **401.2304** | **1200.6693** | **1200.6714** | **-0.0021** | **1** | **6** | **36** | **6** | **K.KATVNLLGEEK.K** |
|  | 7026 | **601.8084** | **1201.6021** | **1200.6714** | **0.9308** | **1** | **6** | **40** | **3** | **K.ATVNLLGEEKK.S** 7022 |
|  | 12966 | **511.3821** | **1531.1246** | **1530.7348** | **0.3898** | **1** | **0** | **78** | **7** | **K.HESQMDSVVKDLK.D** |

| **414.** | P13987\|CD59_HUMAN    **Mass:** 14795    **Score:** 75     **Queries matched:** 5   **emPAI:** 1.02 |
| --- | --- |
|  | CD59 glycoprotein OS=Homo sapiens GN=CD59 PE=1 SV=1 |

|  | **Query** | **Observed** | **Mr(expt)** | **Mr(calc)** | **Delta** | **Miss** | **Score** | **Expect** | **Rank** | **Peptide** |
| --- | --- | --- | --- | --- | --- | --- | --- | --- | --- | --- |
|  | 2777 | 446.7454 | 891.4762 | 891.4814 | -0.0052 | 0 | 28 | 0.3 | 1 | K.AGLQVYNK.C |
|  | 13072 | **770.3311** | **1538.6477** | **1538.6572** | **-0.0096** | **0** | **23** | **0.57** | **1** | **K.FEHCNFNDVTTR.L** |
|  | 14821 | **824.8723** | **1647.7299** | **1647.7385** | **-0.0085** | **1** | **47** | **0.0022** | **1** | **R.LRENELTYYCCK.K** 14822 |
|  | 18740 | 650.3556 | 1948.0451 | 1948.1186 | -0.0735 | 0 | 5 | 29 | 1 | K.TVLLLVTPFLAAAWSLHP.- |

| **415.** | P80723\|BASP1_HUMAN    **Mass:** 22680    **Score:** 75     **Queries matched:** 10   **emPAI:** 0.37 |
| --- | --- |
|  | Brain acid soluble protein 1 OS=Homo sapiens GN=BASP1 PE=1 SV=2 |

|  | **Query** | **Observed** | **Mr(expt)** | **Mr(calc)** | **Delta** | **Miss** | **Score** | **Expect** | **Rank** | **Peptide** |
| --- | --- | --- | --- | --- | --- | --- | --- | --- | --- | --- |
|  | 4104 | **495.5088** | **989.0030** | **988.4825** | **0.5205** | **1** | **13** | **6.5** | **3** | **K.EGEKDAAAAK.E** |
|  | 8776 | **645.3036** | **1288.5927** | **1288.5783** | **0.0145** | **0** | **0** | **1.3e+002** | **7** | **K.AEGAATEEEGTPK.E** |
|  | 10565 | **693.8365** | **1385.6584** | **1385.6674** | **-0.0090** | **0** | **39** | **0.016** | **1** | **K.ETPAATEAPSSTPK.A** |
|  | 11074 | **473.0817** | **1416.2233** | **1416.6732** | **-0.4499** | **1** | **2** | **60** | **9** | **K.KAEGAATEEEGTPK.E** |
|  | 16578 | 592.9453 | 1775.8141 | 1774.8486 | 0.9655 | 0 | 0 | 1e+002 | 9 | K.APEQEQAAPGPAAGGEAPK.A |
|  | 18559 | **644.9597** | **1931.8573** | **1931.8708** | **-0.0135** | **0** | **1** | **75** | **9** | **K.SDGAPASDSKPGSSEAAPSSK.E** |
|  | 22012 | **766.3789** | **2296.1149** | **2297.1287** | **-1.0138** | **1** | **3** | **40** | **3** | **K.AEPPKAPEQEQAAPGPAAGGEAPK.A** |
|  | 24206 | **879.4092** | **2635.2057** | **2635.2248** | **-0.0191** | **1** | **4** | **23** | **1** | **K.AAEAAAAPAESAAPAAGEEPSKEEGEPK.K** |
|  | 25584 | **964.8074** | **2891.4003** | **2891.4148** | **-0.0145** | **1** | **65** | **1.9e-005** | **1** | **K.AQGPAASAEEPKPVEAPAANSDQTVTVKE.-** 25591 |

| **416.** | Q9UHE5\|NAT8_HUMAN    **Mass:** 25831    **Score:** 75     **Queries matched:** 11   **emPAI:** 0.32 |
| --- | --- |
|  | Probable N-acetyltransferase 8 OS=Homo sapiens GN=NAT8 PE=2 SV=2 |

|  | **Query** | **Observed** | **Mr(expt)** | **Mr(calc)** | **Delta** | **Miss** | **Score** | **Expect** | **Rank** | **Peptide** |
| --- | --- | --- | --- | --- | --- | --- | --- | --- | --- | --- |
|  | 1237 | **754.3682** | **753.3610** | **753.3657** | **-0.0047** | **0** | **13** | **7.5** | **1** | **K.SYLSER.G** |
|  | 2692 | **443.0385** | **884.0625** | **883.4156** | **0.6469** | **0** | **4** | **40** | **5** | **-.MAPCHIR.K** |
|  | 3610 | **478.2394** | **954.4643** | **954.5974** | **-0.1331** | **1** | **5** | **49** | **10** | **R.QGIAKALVR.T** 3599 |
|  | 7065 | **602.8157** | **1203.6168** | **1202.5502** | **1.0666** | **0** | **7** | **29** | **2** | **R.GMAEHAPATFR.Q** |
|  | 14672 | **819.9434** | **1637.8723** | **1637.8811** | **-0.0088** | **0** | **61** | **8.4e-005** | **1** | **K.VVGMVGALPVDDPTLR.E** 14673 |
|  | 14937 | **828.4047** | **1654.7948** | **1653.8760** | **0.9187** | **0** | **(1)** | **95** | **10** | **K.VVGMVGALPVDDPTLR.E** |
|  | 17351 | **612.6480** | **1834.9222** | **1834.9326** | **-0.0104** | **1** | **40** | **0.011** | **1** | **K.YQESDRQWVVGLLSR.G** |
|  | 26532 | **1028.0925** | **3081.2558** | **3081.4675** | **-0.2117** | **0** | **2** | **28** | **8** | **R.DQGYSEVILDTGTIQLSAMALYQSMGFK.K** |
|  | 29386 | 1294.6283 | 3880.8631 | 3880.9379 | -0.0749 | 1 | 2 | 20 | 3 | R.TVLQFARDQGYSEVILDTGTIQLSAMALYQSMGFK.K |

| **417.** | Q9NRZ7\|PLCC_HUMAN    **Mass:** 43923    **Score:** 75     **Queries matched:** 7   **emPAI:** 0.18 |
| --- | --- |
|  | 1-acyl-sn-glycerol-3-phosphate acyltransferase gamma OS=Homo sapiens GN=AGPAT3 PE=2 SV=1 |

|  | **Query** | **Observed** | **Mr(expt)** | **Mr(calc)** | **Delta** | **Miss** | **Score** | **Expect** | **Rank** | **Peptide** |
| --- | --- | --- | --- | --- | --- | --- | --- | --- | --- | --- |
|  | 2969 | **453.5623** | **905.1100** | **904.4688** | **0.6412** | **0** | **5** | **38** | **3** | **R.VSMEVAAAK.G** |
|  | 5786 | **559.2891** | **1116.5636** | **1115.4883** | **1.0752** | **0** | **2** | **1.2e+002** | **2** | **K.GSSYGNQEFK.K** |
|  | 7277 | **608.2787** | **1214.5429** | **1213.6237** | **0.9192** | **1** | **6** | **36** | **1** | **K.HRVSMEVAAAK.G** |
|  | 7414 | 611.3104 | 1220.6063 | 1220.6037 | 0.0026 | 0 | 12 | 9.1 | 4 | K.DALQEIYNQK.G |
|  | 14732 | 548.2458 | 1641.7155 | 1642.8467 | -1.1312 | 1 | 2 | 77 | 9 | K.EAAQWLHKLYQEK.D |
|  | 17956 | **941.9754** | **1881.9362** | **1881.9472** | **-0.0109** | **1** | **68** | **1.7e-005** | **1** | **K.LYQEKDALQEIYNQK.G** 17957 |

| **418.** | P04206\|KV307_HUMAN    **Mass:** 11937    **Score:** 74     **Queries matched:** 5   **emPAI:** 0.33 |
| --- | --- |
|  | Ig kappa chain V-III region GOL OS=Homo sapiens PE=1 SV=1 |

|  | **Query** | **Observed** | **Mr(expt)** | **Mr(calc)** | **Delta** | **Miss** | **Score** | **Expect** | **Rank** | **Peptide** |
| --- | --- | --- | --- | --- | --- | --- | --- | --- | --- | --- |
|  | 10889 | **469.2335** | **1404.6786** | **1404.7507** | **-0.0722** | **1** | **4** | **55** | **4** | **R.ATLSCRAALLSSR.G** |
|  | 15912 | 575.2844 | 1722.8313 | 1722.8723 | -0.0410 | 1 | 19 | 1.5 | 2 | R.LLMYGASSRATGIPDR.F 15903 |
|  | 17975 | **942.5016** | **1882.9886** | **1883.0000** | **-0.0114** | **0** | **75** | **3e-006** | **1** | **-.EIVLTQSPGTLSLSPGER.A** 17974 |

|  | **Proteins matching a subset of these peptides:** |
| --- | --- |

|  | P01622\|KV304_HUMAN    **Mass:** 11895    **Score:** 74     **Queries matched:** 2 |
| --- | --- |
|  | Ig kappa chain V-III region Ti OS=Homo sapiens PE=1 SV=1 |

| **419.** | P01620\|KV302_HUMAN    **Mass:** 11882    **Score:** 74     **Queries matched:** 3   **emPAI:** 0.33 |
| --- | --- |
|  | Ig kappa chain V-III region SIE OS=Homo sapiens PE=1 SV=1 |

|  | **Query** | **Observed** | **Mr(expt)** | **Mr(calc)** | **Delta** | **Miss** | **Score** | **Expect** | **Rank** | **Peptide** |
| --- | --- | --- | --- | --- | --- | --- | --- | --- | --- | --- |
|  | 3967 | 490.7954 | 979.5762 | 978.5498 | 1.0264 | 0 | 7 | 30 | 6 | R.LLIYGASSR.A |
|  | 17975 | 942.5016 | 1882.9886 | 1883.0000 | -0.0114 | 0 | 75 | 3e-006 | 1 | -.EIVLTQSPGTLSLSPGER.A 17974 |

|  | **Proteins matching the same set of peptides:** |
| --- | --- |

|  | P01623\|KV305_HUMAN    **Mass:** 11853    **Score:** 74     **Queries matched:** 3 |
| --- | --- |
|  | Ig kappa chain V-III region WOL OS=Homo sapiens PE=1 SV=1 |

| **420.** | P01619\|KV301_HUMAN    **Score:** 74     **Queries matched:** 3 |
| --- | --- |
|  | Ig kappa chain V-III region B6 OS=Homo sapiens PE=1 SV=1 |

|  | **Query** | **Observed** | **Mr(expt)** | **Mr(calc)** | **Delta** | **Miss** | **Score** | **Expect** | **Rank** | **Peptide** |
| --- | --- | --- | --- | --- | --- | --- | --- | --- | --- | --- |
|  | 17975 | 942.5016 | 1882.9886 | 1883.0000 | -0.0114 | 0 | 75 | 3e-006 | 1 | -.EIVLTQSPGTLSLSPGER.A 17974 |
|  | 23676 | 509.4809 | 2542.3683 | 2542.3061 | 0.0622 | 1 | 2 | 37 | 9 | -.EIVLTESPGTLSLSPGERAALSCR.A |

| **421.** | Q9UBI6\|GBG12_HUMAN    **Mass:** 8115     **Score:** 74     **Queries matched:** 6   **emPAI:** 1.27 |
| --- | --- |
|  | Guanine nucleotide-binding protein G(I)/G(S)/G(O) subunit gamma-12 OS=Homo sapiens GN=GNG12 PE=1 SV=3 |

|  | **Query** | **Observed** | **Mr(expt)** | **Mr(calc)** | **Delta** | **Miss** | **Score** | **Expect** | **Rank** | **Peptide** |
| --- | --- | --- | --- | --- | --- | --- | --- | --- | --- | --- |
|  | 6136 | **573.9545** | **1145.8944** | **1145.5789** | **0.3155** | **0** | **9** | **12** | **1** | **K.TASTNNIAQAR.R** |
|  | 13979 | 532.3010 | 1593.8811 | 1594.7733 | -0.8922 | 1 | 6 | 30 | 8 | -.MSSKTASTNNIAQAR.R |
|  | 14684 | **820.4118** | **1638.8090** | **1638.6766** | **0.1324** | **0** | **75** | **3.3e-006** | **1** | **K.ASADLMSYCEEHAR.S** |
|  | 15976 | **864.4616** | **1726.9087** | **1726.9141** | **-0.0055** | **0** | **21** | **0.84** | **1** | **R.SDPLLIGIPTSENPFK.D** |
|  | 18958 | 493.2440 | 1968.9467 | 1968.8669 | 0.0798 | 1 | 2 | 55 | 10 | K.VSKASADLMSYCEEHAR.S |
|  | 18969 | **657.3626** | **1969.0660** | **1970.0360** | **-0.9700** | **1** | **4** | **36** | **7** | **R.SDPLLIGIPTSENPFKDK.K** |

| **422.** | Q9Y624\|JAM1_HUMAN    **Mass:** 32962    **Score:** 74     **Queries matched:** 6   **emPAI:** 0.11 |
| --- | --- |
|  | Junctional adhesion molecule A OS=Homo sapiens GN=F11R PE=1 SV=1 |

|  | **Query** | **Observed** | **Mr(expt)** | **Mr(calc)** | **Delta** | **Miss** | **Score** | **Expect** | **Rank** | **Peptide** |
| --- | --- | --- | --- | --- | --- | --- | --- | --- | --- | --- |
|  | 3437 | 470.7484 | 939.4823 | 938.4094 | 1.0729 | 0 | 13 | 7.3 | 2 | K.FDQGDTTR.L 3414 |
|  | 11352 | **478.2616** | **1431.7631** | **1430.7188** | **1.0443** | **1** | **5** | **46** | **7** | **K.DGIVMPTNPKSTR.A** |
|  | 22593 | **791.7971** | **2372.3695** | **2372.3791** | **-0.0096** | **0** | **2** | **41** | **3** | **K.LIVLVPPSKPTVNIPSSATIGNR.A** |
|  | 27687 | **1118.8540** | **3353.5402** | **3353.5584** | **-0.0183** | **1** | **74** | **1.6e-006** | **1** | **R.AVLTCSEQDGSPPSEYTWFKDGIVMPTNPK.S** |
|  | 31245 | **1259.3707** | **5033.4538** | **5032.2767** | **1.1771** | **1** | **1** | **12** | **3** | **R.AFSNSSYVLNPTTGELVFDPLSASDTGEYSCEARNGYGTPMTSNAVR.M** |

| **423.** | P62888\|RL30_HUMAN    **Mass:** 12947    **Score:** 74     **Queries matched:** 2   **emPAI:** 0.30 |
| --- | --- |
|  | 60S ribosomal protein L30 OS=Homo sapiens GN=RPL30 PE=1 SV=2 |

|  | **Query** | **Observed** | **Mr(expt)** | **Mr(calc)** | **Delta** | **Miss** | **Score** | **Expect** | **Rank** | **Peptide** |
| --- | --- | --- | --- | --- | --- | --- | --- | --- | --- | --- |
|  | 9359 | **659.7991** | **1317.5836** | **1316.6322** | **0.9514** | **0** | **1** | **1.2e+002** | **7** | **K.SEIEYYAMLAK.T** |
|  | 16363 | **879.4545** | **1756.8945** | **1756.9029** | **-0.0084** | **0** | **74** | **4.7e-006** | **1** | **R.VCTLAIIDPGDSDIIR.S** |

| **424.** | P11279\|LAMP1_HUMAN    **Mass:** 45367    **Score:** 73     **Queries matched:** 8   **emPAI:** 0.17 |
| --- | --- |
|  | Lysosome-associated membrane glycoprotein 1 OS=Homo sapiens GN=LAMP1 PE=1 SV=3 |

|  | **Query** | **Observed** | **Mr(expt)** | **Mr(calc)** | **Delta** | **Miss** | **Score** | **Expect** | **Rank** | **Peptide** |
| --- | --- | --- | --- | --- | --- | --- | --- | --- | --- | --- |
|  | 769 | 537.2986 | 536.2914 | 537.2217 | -0.9303 | 0 | 6 | 47 | 7 | R.SSCGK.E |
|  | 4691 | **517.2714** | **1032.5283** | **1032.5451** | **-0.0168** | **0** | **9** | **22** | **1** | **K.TVESITDIR.A** |
|  | 6192 | **576.3027** | **1150.5909** | **1150.5982** | **-0.0073** | **0** | **53** | **0.00075** | **1** | **R.ALQATVGNSYK.C** 6193 6215 |
|  | 16799 | 598.6442 | 1792.9109 | 1791.8825 | 1.0283 | 0 | 0 | 1e+002 | 7 | K.NMTFDLPSDATVVLNR.S |
|  | 22765 | **802.1030** | **2403.2873** | **2403.2951** | **-0.0078** | **1** | **46** | **0.0019** | **1** | **R.FFLQGIQLNTILPDARDPAFK.A** |
|  | 30680 | **1143.7842** | **4571.1076** | **4570.4431** | **0.6646** | **0** | **2** | **10** | **1** | **K.VEGGQFGSVEECLLDENSMLIPIAVGGALAGLVLIVLIAYLVGR.K** |

| **425.** | P01024\|CO3_HUMAN    **Mass:** 188569   **Score:** 73     **Queries matched:** 22   **emPAI:** 0.02 |
| --- | --- |
|  | Complement C3 OS=Homo sapiens GN=C3 PE=1 SV=2 |

|  | **Query** | **Observed** | **Mr(expt)** | **Mr(calc)** | **Delta** | **Miss** | **Score** | **Expect** | **Rank** | **Peptide** |
| --- | --- | --- | --- | --- | --- | --- | --- | --- | --- | --- |
|  | 2739 | 444.7422 | 887.4698 | 886.4508 | 1.0190 | 0 | 4 | 80 | 7 | R.NEQVEIR.A |
|  | 2875 | **450.2847** | **898.5548** | **897.4708** | **1.0840** | **0** | **8** | **26** | **5** | **R.AVLYNYR.Q** |
|  | 3243 | 462.7337 | 923.4529 | 922.5124 | 0.9405 | 1 | 9 | 17 | 7 | K.FLTTAKDK.N |
|  | 4606 | 514.2944 | 1026.5743 | 1026.6185 | -0.0442 | 1 | 8 | 25 | 3 | K.RQGALELIK.K |
|  | 4815 | **522.2996** | **1042.5847** | **1041.6182** | **0.9665** | **1** | **6** | **44** | **1** | **R.ISLPESLKR.I** 4814 |
|  | 6243 | **577.3420** | **1152.6694** | **1151.6009** | **1.0685** | **1** | **7** | **32** | **4** | **R.FISLGEACKK.V** |
|  | 6309 | **580.2780** | **1158.5413** | **1157.5968** | **0.9445** | **1** | **4** | **69** | **10** | **R.YISKYELDK.A** 6283 |
|  | 7869 | **415.2227** | **1242.6463** | **1241.6438** | **1.0025** | **0** | **7** | **30** | **1** | **R.QPVPGQQMTLK.I** |
|  | 10301 | 457.9087 | 1370.7042 | 1369.7242 | 0.9800 | 0 | 4 | 58 | 3 | K.TIYTPGSTVLYR.I 10272 |
|  | 15453 | **564.2318** | **1689.6734** | **1689.8872** | **-0.2138** | **1** | **(3)** | **50** | **3** | **K.GQGTLSVVTMYHAKAK.D** |
|  | 15632 | 851.9615 | 1701.9085 | 1700.9209 | 0.9876 | 1 | 9 | 13 | 2 | R.LPYSVVRNEQVEIR.A |
|  | 15693 | 853.8898 | 1705.7651 | 1705.8821 | -0.1170 | 1 | 10 | 10 | 1 | K.GQGTLSVVTMYHAKAK.D |
|  | 20830 | **1079.0406** | **2156.0667** | **2156.0783** | **-0.0116** | **0** | **73** | **4.5e-006** | **1** | **R.ILLQGTPVAQMTEDAVDAER.L** |
|  | 22744 | **800.0897** | **2397.2473** | **2397.2573** | **-0.0100** | **1** | **10** | **6.7** | **1** | **R.ILLQGTPVAQMTEDAVDAERLK.H** |
|  | 24771 | **683.8660** | **2731.4348** | **2732.4385** | **-1.0037** | **1** | **0** | **57** | **5** | **K.VEGTAFVIFGIQDGEQRISLPESLK.R** |
|  | 26584 | **619.1226** | **3090.5767** | **3090.4984** | **0.0784** | **1** | **0** | **48** | **5** | **K.TPKYFKPGMPFDLMVFVTNPDGSPAYR.V** |
|  | 28728 | 910.9589 | 3639.8066 | 3640.6695 | -0.8629 | 1 | 3 | 16 | 5 | R.GDQDATMSILDISMMTGFAPDTDDLKQLANGVDR.Y |
|  | 29507 | **788.4075** | **3937.0010** | **3936.1037** | **0.8973** | **1** | **4** | **13** | **1** | **R.EPGQDLVVLPLSITTDFIPSFRLVAYYTLIGASGQR.E** |
|  | 30198 | **1419.5358** | **4255.5855** | **4256.0592** | **-0.4737** | **1** | **2** | **10** | **2** | **R.LKHLIVTPSGCGEQNMIGMTPTVIAVHYLDETEQWEK.F** |

| **426.** | Q7Z3B1\|NEGR1_HUMAN    **Mass:** 39379    **Score:** 72     **Queries matched:** 6   **emPAI:** 0.10 |
| --- | --- |
|  | Neuronal growth regulator 1 OS=Homo sapiens GN=NEGR1 PE=1 SV=3 |

|  | **Query** | **Observed** | **Mr(expt)** | **Mr(calc)** | **Delta** | **Miss** | **Score** | **Expect** | **Rank** | **Peptide** |
| --- | --- | --- | --- | --- | --- | --- | --- | --- | --- | --- |
|  | 2288 | **429.9611** | **857.9076** | **858.4923** | **-0.5847** | **1** | **14** | **6.3** | **7** | **R.KGDTAVLR.C** 2305 |
|  | 4485 | 509.2794 | 1016.5442 | 1016.5978 | -0.0536 | 1 | 6 | 46 | 4 | R.VSISTLNKR.D |
|  | 14097 | **801.9128** | **1601.8111** | **1600.8573** | **0.9538** | **1** | **9** | **15** | **5** | **K.WSVDPRVSISTLNK.R** |
|  | 22847 | 1208.5555 | 2415.0965 | 2415.0285 | 0.0681 | 1 | 9 | 10 | 2 | R.DQAGEYECSAENDVSFPDVRK.V |
|  | 27228 | **1079.8236** | **3236.4490** | **3236.4681** | **-0.0191** | **0** | **72** | **2.6e-006** | **1** | **R.DYSLQIQNVDVTDDGPYTCSVQTQHTPR.T** |

| **427.** | P07919\|QCR6_HUMAN    **Mass:** 11017    **Score:** 72     **Queries matched:** 4   **emPAI:** 0.86 |
| --- | --- |
|  | Cytochrome b-c1 complex subunit 6, mitochondrial OS=Homo sapiens GN=UQCRH PE=1 SV=2 |

|  | **Query** | **Observed** | **Mr(expt)** | **Mr(calc)** | **Delta** | **Miss** | **Score** | **Expect** | **Rank** | **Peptide** |
| --- | --- | --- | --- | --- | --- | --- | --- | --- | --- | --- |
|  | 3369 | **467.7158** | **933.4171** | **933.4226** | **-0.0055** | **0** | **25** | **0.66** | **1** | **R.LELCDER.V** 3384 |
|  | 21547 | **745.9880** | **2234.9421** | **2234.9539** | **-0.0117** | **0** | **31** | **0.053** | **1** | **R.SHTEEDCTEELFDFLHAR.D** |
|  | 29761 | **1350.5911** | **4048.7514** | **4048.7776** | **-0.0263** | **1** | **62** | **1.5e-005** | **1** | **K.MLTESGDPEEEEEEEEELVDPLTTVREQCEQLEK.C** |

| **428.** | P56134\|ATPK_HUMAN    **Mass:** 11025    **Score:** 72     **Queries matched:** 4   **emPAI:** 0.36 |
| --- | --- |
|  | ATP synthase subunit f, mitochondrial OS=Homo sapiens GN=ATP5J2 PE=1 SV=3 |

|  | **Query** | **Observed** | **Mr(expt)** | **Mr(calc)** | **Delta** | **Miss** | **Score** | **Expect** | **Rank** | **Peptide** |
| --- | --- | --- | --- | --- | --- | --- | --- | --- | --- | --- |
|  | 9270 | 657.8625 | 1313.7104 | 1313.7166 | -0.0061 | 0 | 54 | 0.00057 | 1 | K.LGELPSWILMR.D 9269 |
|  | 11268 | **714.8449** | **1427.6753** | **1427.6834** | **-0.0081** | **0** | **3** | **71** | **10** | **R.DFSPSGIFGAFQR.G** |
|  | 15599 | 567.5879 | 1699.7420 | 1699.8273 | -0.0853 | 1 | 15 | 3.7 | 2 | -.MASVGECPAPVPVKDK.K |

| **429.** | P14649\|MYL6B_HUMAN    **Mass:** 22864    **Score:** 72     **Queries matched:** 8   **emPAI:** 0.59 |
| --- | --- |
|  | Myosin light chain 6B OS=Homo sapiens GN=MYL6B PE=1 SV=1 |

|  | **Query** | **Observed** | **Mr(expt)** | **Mr(calc)** | **Delta** | **Miss** | **Score** | **Expect** | **Rank** | **Peptide** |
| --- | --- | --- | --- | --- | --- | --- | --- | --- | --- | --- |
|  | 1011 | 627.8264 | 626.8191 | 626.3752 | 0.4440 | 0 | 34 | 0.033 | 1 | K.VLGNPK.S |
|  | 4218 | **499.2718** | **996.5291** | **996.5604** | **-0.0312** | **0** | **26** | **0.34** | **1** | **R.HVLTTLGEK.M** |
|  | 8543 | **426.2319** | **1275.6739** | **1275.6241** | **0.0498** | **1** | **11** | **11** | **2** | **K.EGNGKVMGAELR.H** |
|  | 9756 | 671.3142 | 1340.6139 | 1340.6217 | -0.0078 | 0 | 61 | 0.00011 | 1 | K.ILYSQCGDVMR.A |
|  | 10012 | 677.8652 | 1353.7158 | 1353.7252 | -0.0094 | 0 | 38 | 0.024 | 1 | R.ALGQNPTNAEVLK.V 10033 |
|  | 10074 | 453.5691 | 1357.6856 | 1356.6166 | 1.0690 | 0 | (4) | 65 | 4 | K.ILYSQCGDVMR.A |
|  | 16495 | 590.3162 | 1767.9268 | 1768.9505 | -1.0237 | 1 | 4 | 42 | 3 | K.VMGAELRHVLTTLGEK.M |

| **430.** | P31946\|1433B_HUMAN    **Mass:** 28179    **Score:** 72     **Queries matched:** 2   **emPAI:** 0.13 |
| --- | --- |
|  | 14-3-3 protein beta/alpha OS=Homo sapiens GN=YWHAB PE=1 SV=3 |

|  | **Query** | **Observed** | **Mr(expt)** | **Mr(calc)** | **Delta** | **Miss** | **Score** | **Expect** | **Rank** | **Peptide** |
| --- | --- | --- | --- | --- | --- | --- | --- | --- | --- | --- |
|  | 6742 | 397.2191 | 1188.6355 | 1188.6536 | -0.0181 | 0 | 7 | 35 | 7 | K.DSTLIMQLLR.D |
|  | 27564 | **1110.5494** | **3328.6265** | **3328.6384** | **-0.0119** | **1** | **72** | **3e-006** | **1** | **K.TAFDEAIAELDTLNEESYKDSTLIMQLLR.D** |

| **431.** | O43795\|MYO1B_HUMAN    **Mass:** 132928   **Score:** 72     **Queries matched:** 16   **emPAI:** 0.03 |
| --- | --- |
|  | Myosin-Ib OS=Homo sapiens GN=MYO1B PE=1 SV=3 |

|  | **Query** | **Observed** | **Mr(expt)** | **Mr(calc)** | **Delta** | **Miss** | **Score** | **Expect** | **Rank** | **Peptide** |
| --- | --- | --- | --- | --- | --- | --- | --- | --- | --- | --- |
|  | 921 | **589.3896** | **588.3824** | **587.3755** | **1.0069** | **1** | **9** | **44** | **1** | **K.SRVVK.Q** |
|  | 1730 | 410.2248 | 818.4350 | 819.4199 | -0.9848 | 1 | 12 | 14 | 2 | R.ANGKSTSR.I |
|  | 2008 | 420.2217 | 838.4287 | 839.4402 | -1.0115 | 1 | 5 | 43 | 5 | R.RAGYAFR.Q 2056 |
|  | 3045 | **456.2895** | **910.5644** | **909.4821** | **1.0823** | **1** | **11** | **12** | **1** | **K.FFRANAGK.K** |
|  | 7935 | 416.2150 | 1245.6231 | 1244.6434 | 0.9796 | 1 | 8 | 23 | 7 | K.NKMPSLSPIDK.N |
|  | 8970 | **650.1074** | **1298.2002** | **1297.7104** | **0.4898** | **1** | **6** | **26** | **3** | **R.IVQKYFLEMK.N** |
|  | 9263 | 657.8234 | 1313.6323 | 1313.7053 | -0.0730 | 1 | (2) | 95 | 10 | R.IVQKYFLEMK.N |
|  | 10887 | **469.2278** | **1404.6614** | **1403.7806** | **0.8808** | **1** | **9** | **18** | **2** | **K.ASVATLMKNLQTK.N** 10872 10898 |
|  | 17248 | 610.3379 | 1827.9920 | 1828.9063 | -0.9143 | 1 | 5 | 29 | 3 | K.TEASKLVMSYVAAVCGK.G |
|  | 20967 | 1087.0000 | 2171.9854 | 2171.0323 | 0.9531 | 0 | 4 | 33 | 3 | R.NFYELSPHIFALSDEAYR.S |
|  | 22826 | **1207.0950** | **2412.1754** | **2412.1849** | **-0.0095** | **0** | **72** | **5.2e-006** | **1** | **R.SGVEVLFNELEIPVEEYSFGR.S** |
|  | 24429 | **892.4614** | **2674.3623** | **2673.3506** | **1.0117** | **0** | **2** | **40** | **3** | **R.NAMQIVGFMDHEAESVLAVVAAVLK.L** |
|  | 26079 | **1000.4558** | **2998.3454** | **2997.5780** | **0.7674** | **1** | **5** | **17** | **1** | **R.TVRNAMQIVGFMDHEAESVLAVVAAVLK.L** |

| **432.** | Q9H9B4\|SFXN1_HUMAN    **Mass:** 35881    **Score:** 71     **Queries matched:** 20   **emPAI:** 0.35 |
| --- | --- |
|  | Sideroflexin-1 OS=Homo sapiens GN=SFXN1 PE=1 SV=4 |

|  | **Query** | **Observed** | **Mr(expt)** | **Mr(calc)** | **Delta** | **Miss** | **Score** | **Expect** | **Rank** | **Peptide** |
| --- | --- | --- | --- | --- | --- | --- | --- | --- | --- | --- |
|  | 2026 | 420.6775 | 839.3405 | 839.4541 | -0.1136 | 1 | 4 | 53 | 5 | R.VYFNKGL.- |
|  | 2298 | **430.5614** | **859.1082** | **859.4399** | **-0.3317** | **0** | **9** | **18** | **2** | **R.LGESANAAK.Q** |
|  | 2600 | 439.7613 | 877.5081 | 877.5134 | -0.0053 | 0 | 16 | 4.8 | 3 | K.HVSPLIGR.F |
|  | 3332 | **466.2390** | **930.4635** | **929.5083** | **0.9552** | **1** | **11** | **14** | **4** | **R.KIVHDYR.Q** |
|  | 5677 | **555.2689** | **1108.5233** | **1108.5302** | **-0.0069** | **0** | **46** | **0.0041** | **1** | **R.WDQSTFIGR.A** |
|  | 9105 | **435.5484** | **1303.6233** | **1303.6310** | **-0.0077** | **0** | **22** | **1** | **1** | **R.ANHFFTVTDPR.N** 9106 |
|  | 13754 | **790.8907** | **1579.7668** | **1579.7763** | **-0.0095** | **0** | **27** | **0.21** | **1** | **K.SSMSVTSLEAELQAK.I** |
|  | 15717 | **854.9508** | **1707.8871** | **1707.8944** | **-0.0073** | **0** | **44** | **0.0041** | **1** | **R.QGIVPPGLTENELWR.A** |
|  | 20225 | 523.8046 | 2091.1894 | 2090.0805 | 1.1089 | 0 | 10 | 8.9 | 1 | R.FVPFAAVAAANCINIPLMR.Q |
|  | 20978 | **725.3705** | **2173.0898** | **2173.1349** | **-0.0451** | **0** | **(4)** | **38** | **1** | **R.ILMAAPGMAIPPFIMNTLEK.K** |
|  | 21146 | 439.0324 | 2190.1254 | 2189.1298 | 0.9956 | 0 | 10 | 8.2 | 1 | R.ILMAAPGMAIPPFIMNTLEK.K |
|  | 22597 | 792.3614 | 2374.0623 | 2374.2402 | -0.1778 | 1 | 2 | 43 | 5 | R.FVPFAAVAAANCINIPLMRQR.E |
|  | 22667 | **597.0455** | **2384.1528** | **2385.0118** | **-0.8590** | **0** | **0** | **70** | **6** | **R.MSAQVPMNMTITGCMMTFYR.T** |
|  | 24532 | **896.4305** | **2686.2696** | **2685.3279** | **0.9417** | **1** | **2** | **45** | **6** | **K.SSMSVTSLEAELQAKIQESHPELR.R** |
|  | 26471 | **614.9201** | **3069.5641** | **3068.4270** | **1.1371** | **1** | **(1)** | **41** | **8** | **K.MILIGRMSAQVPMNMTITGCMMTFYR.T** |
|  | 26542 | **1028.7828** | **3083.3267** | **3084.4220** | **-1.0953** | **1** | **4** | **17** | **3** | **K.MILIGRMSAQVPMNMTITGCMMTFYR.T** |
|  | 26624 | **1034.4452** | **3100.3137** | **3100.4169** | **-0.1031** | **1** | **(2)** | **25** | **1** | **K.MILIGRMSAQVPMNMTITGCMMTFYR.T** |
|  | 26675 | **1039.4459** | **3115.3159** | **3116.4118** | **-1.0959** | **1** | **(4)** | **17** | **5** | **K.MILIGRMSAQVPMNMTITGCMMTFYR.T** |
|  | 27414 | **658.5375** | **3287.6510** | **3286.7491** | **0.9019** | **1** | **0** | **46** | **4** | **K.QAITQVVVSRILMAAPGMAIPPFIMNTLEK.K** |

| **433.** | P11277\|SPTB1_HUMAN    **Mass:** 247171   **Score:** 71     **Queries matched:** 60   **emPAI:** 0.05 |
| --- | --- |
|  | Spectrin beta chain, erythrocyte OS=Homo sapiens GN=SPTB PE=1 SV=5 |

|  | **Query** | **Observed** | **Mr(expt)** | **Mr(calc)** | **Delta** | **Miss** | **Score** | **Expect** | **Rank** | **Peptide** |
| --- | --- | --- | --- | --- | --- | --- | --- | --- | --- | --- |
|  | 1688 | 408.7296 | 815.4446 | 814.5025 | 0.9422 | 1 | 19 | 2.4 | 2 | R.AKLQISR.D |
|  | 2099 | **845.4414** | **844.4341** | **845.4243** | **-0.9901** | **1** | **4** | **79** | **2** | **K.DVAEERK.R** 2128 |
|  | 3160 | **459.3275** | **916.6403** | **917.4930** | **-0.8527** | **1** | **10** | **19** | **3** | **K.QLESSRAK.L** |
|  | 3297 | **465.0200** | **928.0254** | **928.5454** | **-0.5200** | **1** | **11** | **9.3** | **2** | **R.NIKQLASR.A** 3296 3299 3303 |
|  | 3667 | 479.7845 | 957.5544 | 957.4880 | 0.0664 | 0 | 12 | 10 | 1 | R.VEDQVNVR.K |
|  | 3695 | 481.1519 | 960.2892 | 959.4672 | 0.8220 | 0 | 10 | 18 | 1 | R.ETGAIGQER.V |
|  | 3841 | **486.7620** | **971.5095** | **970.5308** | **0.9787** | **1** | **13** | **9.5** | **3** | **R.EAAAGRLQR.L** |
|  | 3842 | **486.7742** | **971.5338** | **972.4988** | **-0.9650** | **1** | **16** | **4.7** | **8** | **K.EDIGQRQK.H** 3843 |
|  | 3984 | 491.7083 | 981.4019 | 981.5144 | -0.1125 | 0 | 8 | 25 | 4 | K.WVNSHLAR.V |
|  | 4038 | 493.2849 | 984.5551 | 985.5556 | -1.0005 | 0 | 8 | 24 | 10 | K.AQEASVLLR.D |
|  | 4111 | 495.7566 | 989.4986 | 989.5579 | -0.0594 | 1 | 16 | 4.7 | 2 | K.MKVLAVEGK.R |
|  | 5142 | **535.6227** | **1069.2309** | **1068.6291** | **0.6018** | **0** | **9** | **12** | **9** | **R.DLAGIIAIQR.K** |
|  | 5599 | 552.3104 | 1102.6063 | 1102.5982 | 0.0081 | 1 | 21 | 1.3 | 1 | K.VVESTKDLGR.D |
|  | 5752 | **558.3027** | **1114.5908** | **1113.5891** | **1.0017** | **1** | **15** | **5.3** | **1** | **K.RVEDQVNVR.K** |
|  | 5758 | 558.3108 | 1114.6071 | 1113.6142 | 0.9930 | 1 | 6 | 43 | 3 | R.QEKLEQLAR.R |
|  | 5937 | **565.8207** | **1129.6269** | **1128.6139** | **1.0131** | **1** | **3** | **77** | **7** | **K.EKVQLIEDR.H** |
|  | 6354 | 582.2665 | 1162.5185 | 1161.5414 | 0.9771 | 0 | 4 | 62 | 10 | R.ETWLSENQR.L |
|  | 6825 | **597.3032** | **1192.5918** | **1191.5342** | **1.0576** | **1** | **1** | **1.2e+002** | **10** | **K.EMNEKWEAR.W** 6822 |
|  | 6939 | 599.8332 | 1197.6518 | 1197.5891 | 0.0628 | 0 | 0 | 1.4e+002 | 10 | R.LGHLQSSWDR.L |
|  | 7093 | **402.4630** | **1204.3672** | **1203.6135** | **0.7537** | **0** | **10** | **11** | **2** | **R.LWDELQATTK.E** 7050 7075 |
|  | 7416 | 611.3214 | 1220.6283 | 1220.5898 | 0.0385 | 1 | 3 | 80 | 4 | R.QQRAVEDYGR.N |
|  | 7498 | 409.2278 | 1224.6614 | 1225.7142 | -1.0528 | 1 | 2 | 86 | 6 | R.ELALRNELIR.Q |
|  | 8186 | **629.9689** | **1257.9232** | **1257.6565** | **0.2667** | **1** | **3** | **52** | **2** | **R.DKVLSPVDSGNK.L** 8204 |
|  | 10580 | **463.2327** | **1386.6764** | **1386.6891** | **-0.0128** | **1** | **12** | **7.8** | **3** | **K.HYAGLKDVAEER.K** |
|  | 10840 | **468.2029** | **1401.5868** | **1402.6688** | **-1.0820** | **1** | **11** | **11** | **3** | **R.SDDPGKDLTSVNR.M** |
|  | 11408 | 479.2663 | 1434.7771 | 1435.6956 | -0.9186 | 0 | 23 | 0.62 | 1 | R.HNLEHAFNVAER.Q 11414 11425 |
|  | 12229 | 495.5741 | 1483.7004 | 1483.7466 | -0.0463 | 1 | 2 | 84 | 9 | K.WVNSHLARVSCR.I |
|  | 12293 | **496.9360** | **1487.7863** | **1488.8300** | **-1.0437** | **0** | **1** | **1e+002** | **7** | **K.GNLEVLLFTIQSR.M** |
|  | 12417 | **499.7615** | **1496.2627** | **1495.7154** | **0.5473** | **0** | **2** | **52** | **7** | **K.LLTSQDVSYDEAR.N** |
|  | 13201 | 773.8661 | 1545.7177 | 1545.7271 | -0.0093 | 0 | 54 | 0.00049 | 1 | R.LLSGEDVGQDEGATR.A 13200 |
|  | 13224 | **516.5956** | **1546.7651** | **1545.8072** | **0.9579** | **1** | **10** | **13** | **8** | **K.LMEADIAIQGDKVK.A** |
|  | 14011 | 798.9553 | 1595.8960 | 1596.8220 | -0.9260 | 1 | 6 | 31 | 6 | R.SSDLRLQTHADLNK.W |
|  | 14699 | **547.3154** | **1638.9243** | **1639.6607** | **-0.7364** | **0** | **2** | **71** | **6** | **R.VHNYCVDCEETSK.W** |
|  | 14914 | 551.9654 | 1652.8743 | 1653.9375 | -1.0632 | 0 | 7 | 23 | 3 | K.LLEVLSGEMLPKPTK.G 14938 |
|  | 16085 | **579.3362** | **1734.9869** | **1735.8563** | **-0.8694** | **0** | **2** | **64** | **10** | **K.NFSACLELGESLLQR.Q** |
|  | 16574 | **592.6476** | **1774.9209** | **1774.9326** | **-0.0117** | **0** | **19** | **1.5** | **1** | **R.AQGLLSAGHPEGEQIIR.L** |
|  | 18084 | **947.9747** | **1893.9348** | **1893.9472** | **-0.0124** | **0** | **7** | **20** | **1** | **R.LVAQDNFGYDLAAVEAAK.K** |
|  | 18317 | **479.5056** | **1913.9932** | **1914.9647** | **-0.9715** | **1** | **4** | **36** | **2** | **R.LLSGEDVGQDEGATRALGK.K** |
|  | 21027 | 727.0298 | 2178.0675 | 2179.0545 | -0.9870 | 1 | 3 | 40 | 4 | K.LVSDINRAWESLEEAEYR.R |
|  | 22351 | 586.5641 | 2342.2273 | 2342.0934 | 0.1338 | 1 | 4 | 31 | 9 | K.EQRVHLENMGSHDIVDGNHR.L |
|  | 22908 | **808.4122** | **2422.2149** | **2422.2274** | **-0.0126** | **0** | **2** | **48** | **1** | **K.TLMTQIDGVNLAANSLVESGHPR.S** |
|  | 22982 | **812.4058** | **2434.1956** | **2434.2757** | **-0.0801** | **1** | **1** | **52** | **5** | **K.DGLAFNALIHKHRPDLIDFDK.L** |
|  | 23741 | **638.3331** | **2549.3032** | **2550.2139** | **-0.9107** | **0** | **3** | **32** | **10** | **K.HQAFVAELASHEGWLENIDAEGK.Q** |
|  | 27084 | **1069.1768** | **3204.5085** | **3204.5251** | **-0.0167** | **0** | **20** | **0.51** | **1** | **R.DASVAEAWLIAQEPYLASGDFGHTVDSVEK.L** |
|  | 28486 | **1192.6265** | **3574.8576** | **3573.7443** | **1.1132** | **1** | **1** | **29** | **10** | **R.FDILDQEMKTLMTQIDGVNLAANSLVESGHPR.S** |
|  | 28948 | **930.1904** | **3716.7326** | **3716.7516** | **-0.0190** | **1** | **2** | **23** | **2** | **R.ETDDLEQWISEKELVASSPEMGQDFDHVTLLR.D** |
|  | 29318 | **1284.9637** | **3851.8694** | **3851.8927** | **-0.0233** | **1** | **39** | **0.0037** | **1** | **R.DLEDETLWVEERLPLAQSADYGTNLQTVQLFMK.K** |

| **434.** | P01857\|IGHG1_HUMAN    **Mass:** 36596    **Score:** 71     **Queries matched:** 15   **emPAI:** 0.34 |
| --- | --- |
|  | Ig gamma-1 chain C region OS=Homo sapiens GN=IGHG1 PE=1 SV=1 |

|  | **Query** | **Observed** | **Mr(expt)** | **Mr(calc)** | **Delta** | **Miss** | **Score** | **Expect** | **Rank** | **Peptide** |
| --- | --- | --- | --- | --- | --- | --- | --- | --- | --- | --- |
|  | 1294 | **395.0789** | **788.1432** | **787.4440** | **0.6993** | **0** | **6** | **39** | **4** | **K.SLSLSPGK.-** |
|  | 1998 | **838.4983** | **837.4911** | **837.4960** | **-0.0049** | **0** | **23** | **0.78** | **1** | **K.ALPAPIEK.T** |
|  | 6343 | 581.3152 | 1160.6159 | 1160.6223 | -0.0064 | 0 | 29 | 0.21 | 1 | K.NQVSLTCLVK.G |
|  | 6766 | **397.4023** | **1189.1852** | **1188.5047** | **0.6805** | **0** | **9** | **15** | **4** | **R.EEQYNSTYR.V** |
|  | 8384 | **634.6945** | **1267.3744** | **1266.7547** | **0.6197** | **1** | **4** | **39** | **1** | **K.ALPAPIEKTISK.A** |
|  | 9443 | **661.8124** | **1321.6102** | **1320.6708** | **0.9394** | **0** | **4** | **58** | **5** | **K.STSGGTAALGCLVK.D** |
|  | 13638 | **525.2925** | **1572.8556** | **1572.8512** | **0.0045** | **1** | **3** | **71** | **10** | **-.ASTKGPSVFPLAPSSK.S** |
|  | 15279 | **839.4006** | **1676.7866** | **1676.7947** | **-0.0081** | **0** | **26** | **0.28** | **1** | **K.FNWYVDGVEVHNAK.T** |
|  | 23343 | **622.8307** | **2487.2937** | **2488.2996** | **-1.0059** | **1** | **3** | **34** | **1** | **K.GPSVFPLAPSSKSTSGGTAALGCLVK.D** 23342 |
|  | 23683 | 1272.5614 | 2543.1082 | 2543.1241 | -0.0158 | 0 | 49 | 0.00079 | 1 | K.GFYPSDIAVEWESNGQPENNYK.T 23688 |
|  | 25940 | **990.8422** | **2969.5047** | **2970.4315** | **-0.9268** | **1** | **1** | **42** | **5** | **K.DTLMISRTPEVTCVVVDVSHEDPEVK.F** |
|  | 27588 | **834.4100** | **3333.6108** | **3333.6349** | **-0.0241** | **1** | **23** | **0.21** | **1** | **K.SCDKTHTCPPCPAPELLGGPSVFLFPPKPK.D** |
|  | 32280 | **1343.4620** | **6712.2738** | **6712.3072** | **-0.0334** | **0** | **9** | **0.73** | **1** | **K.DYFPEPVTVSWNSGALTSGVHTFPAVLQSSGLYSLSSVVTVPSSSLGTQTYICNVNHKPSNTK.V** |

| **435.** | O60884\|DNJA2_HUMAN    **Mass:** 46344    **Score:** 71     **Queries matched:** 5   **emPAI:** 0.08 |
| --- | --- |
|  | DnaJ homolog subfamily A member 2 OS=Homo sapiens GN=DNAJA2 PE=1 SV=1 |

|  | **Query** | **Observed** | **Mr(expt)** | **Mr(calc)** | **Delta** | **Miss** | **Score** | **Expect** | **Rank** | **Peptide** |
| --- | --- | --- | --- | --- | --- | --- | --- | --- | --- | --- |
|  | 784 | **544.3205** | **543.3132** | **543.3241** | **-0.0109** | **1** | **0** | **2.5e+002** | **10** | **R.GRGVR.I** |
|  | 3179 | **460.2687** | **918.5228** | **917.5546** | **0.9682** | **1** | **10** | **18** | **9** | **K.TTKLQLSK.N** |
|  | 9691 | **669.2967** | **1336.5789** | **1336.5864** | **-0.0074** | **0** | **56** | **0.00033** | **1** | **K.NVLCSACSGQGGK.S** 9692 |
|  | 21141 | **731.0326** | **2190.0761** | **2191.0289** | **-0.9528** | **1** | **1** | **69** | **6** | **R.GEDMMHPLKVSLEDLYNGK.T** |

| **436.** | P04899\|GNAI2_HUMAN    **Mass:** 40995    **Score:** 71     **Queries matched:** 15   **emPAI:** 0.19 |
| --- | --- |
|  | Guanine nucleotide-binding protein G(i), alpha-2 subunit OS=Homo sapiens GN=GNAI2 PE=1 SV=3 |

|  | **Query** | **Observed** | **Mr(expt)** | **Mr(calc)** | **Delta** | **Miss** | **Score** | **Expect** | **Rank** | **Peptide** |
| --- | --- | --- | --- | --- | --- | --- | --- | --- | --- | --- |
|  | 1285 | 780.3941 | 779.3868 | 779.3701 | 0.0167 | 0 | 7 | 35 | 4 | K.DLFEEK.I |
|  | 2743 | **445.0340** | **888.0535** | **888.4851** | **-0.4316** | **1** | **0** | **1.4e+002** | **7** | **K.MIDKNLR.E** |
|  | 3030 | **455.7558** | **909.4971** | **908.4175** | **1.0797** | **0** | **11** | **13** | **1** | **K.MFDVGGQR.S** |
|  | 5689 | **555.5287** | **1109.0428** | **1109.5175** | **-0.4747** | **0** | **6** | **26** | **3** | **K.LFDSICNNK.W** |
|  | 13848 | **529.3129** | **1584.9170** | **1584.9450** | **-0.0281** | **1** | **2** | **78** | **8** | **K.LLLLGAGESGKSTIVK.Q** 13830 |
|  | 14018 | 799.3640 | 1596.7133 | 1596.8552 | -0.1418 | 0 | 9 | 14 | 2 | K.WFTDTSIILFLNK.K 14019 |
|  | 16097 | **868.9806** | **1735.9466** | **1735.9542** | **-0.0076** | **0** | **57** | **0.00024** | **1** | **R.AVVYSNTIQSIMAIVK.A** |
|  | 18538 | **644.4310** | **1930.2711** | **1929.8779** | **0.3932** | **1** | **11** | **5.2** | **3** | **K.DTKEIYTHFTCATDTK.N** |
|  | 21070 | **728.7047** | **2183.0921** | **2183.1772** | **-0.0851** | **1** | **0** | **79** | **6** | **R.QYRAVVYSNTIQSIMAIVK.A** |
|  | 22181 | **774.0281** | **2319.0624** | **2319.0767** | **-0.0143** | **1** | **34** | **0.028** | **1** | **R.SREYQLNDSAAYYLNDLER.I** |
|  | 24138 | **873.7933** | **2618.3582** | **2617.3058** | **1.0524** | **0** | **1** | **55** | **4** | **R.QLFALSCTAEEQGVLPDDLSGVIR.R** |
|  | 24972 | **925.4690** | **2773.3851** | **2773.4069** | **-0.0217** | **1** | **13** | **2.9** | **1** | **R.QLFALSCTAEEQGVLPDDLSGVIRR.L** |
|  | 27453 | **826.8942** | **3303.5478** | **3303.5758** | **-0.0279** | **1** | **3** | **23** | **2** | **K.ITHSPLTICFPEYTGANKYDEAASYIQSK.F** |

| **437.** | O94760\|DDAH1_HUMAN    **Mass:** 31444    **Score:** 71     **Queries matched:** 11   **emPAI:** 0.12 |
| --- | --- |
|  | N(G),N(G)-dimethylarginine dimethylaminohydrolase 1 OS=Homo sapiens GN=DDAH1 PE=1 SV=3 |

|  | **Query** | **Observed** | **Mr(expt)** | **Mr(calc)** | **Delta** | **Miss** | **Score** | **Expect** | **Rank** | **Peptide** |
| --- | --- | --- | --- | --- | --- | --- | --- | --- | --- | --- |
|  | 1868 | **415.7399** | **829.4652** | **830.4875** | **-1.0223** | **0** | **2** | **1.3e+002** | **8** | **K.GHVLLHR.T** |
|  | 3067 | **457.0743** | **912.1341** | **911.4092** | **0.7249** | **1** | **10** | **11** | **1** | **R.KEVDMMK.E** 3068 |
|  | 6642 | 395.7425 | 1184.2058 | 1183.5920 | 0.6138 | 0 | 9 | 14 | 6 | -.MAGLGHPAAFGR.A |
|  | 11743 | **486.5891** | **1456.7456** | **1456.7279** | **0.0177** | **1** | **6** | **34** | **2** | **K.ALKIMQQMSDHR.Y** |
|  | 13494 | **522.2574** | **1563.7505** | **1562.8053** | **0.9453** | **1** | **5** | **38** | **4** | **R.TNQRGAEILADTFK.D** |
|  | 14768 | 548.9439 | 1643.8099 | 1643.7899 | 0.0200 | 0 | 3 | 54 | 6 | K.DHMLIPVSMSELEK.V |
|  | 15372 | **842.4551** | **1682.8957** | **1683.9056** | **-1.0099** | **1** | **4** | **44** | **6** | **R.AERQHQLYVGVLGSK.L** |
|  | 20789 | **1078.0048** | **2153.9950** | **2154.0085** | **-0.0135** | **0** | **71** | **7e-006** | **1** | **K.SFCSMAGPNLIAIGSSESAQK.A** |
|  | 24754 | **910.8122** | **2729.4147** | **2729.4276** | **-0.0129** | **1** | **10** | **6.2** | **1** | **R.GAEILADTFKDYAVSTVPVADGLHLK.S** |
|  | 27137 | **805.1450** | **3216.5507** | **3216.5903** | **-0.0395** | **1** | **0** | **44** | **5** | **K.DHMLIPVSMSELEKVDGLLTCCSVLINK.K** |

| **438.** | P67809\|YBOX1_HUMAN    **Mass:** 35903    **Score:** 71     **Queries matched:** 5   **emPAI:** 0.10 |
| --- | --- |
|  | Nuclease-sensitive element-binding protein 1 OS=Homo sapiens GN=YBX1 PE=1 SV=3 |

|  | **Query** | **Observed** | **Mr(expt)** | **Mr(calc)** | **Delta** | **Miss** | **Score** | **Expect** | **Rank** | **Peptide** |
| --- | --- | --- | --- | --- | --- | --- | --- | --- | --- | --- |
|  | 3869 | **487.2960** | **972.5774** | **972.4527** | **0.1247** | **0** | **(8)** | **31** | **10** | **R.FPPYYMR.R** |
|  | 4093 | 495.2354 | 988.4563 | 988.4477 | 0.0087 | 0 | 10 | 20 | 3 | R.FPPYYMR.R 4089 |
|  | 14743 | 548.3264 | 1641.9574 | 1641.8164 | 0.1410 | 1 | 3 | 60 | 9 | K.WFNVRNGYGFINR.N |
|  | 27294 | **1086.4635** | **3256.3687** | **3256.3889** | **-0.0202** | **1** | **71** | **3.3e-006** | **1** | **R.NYQQNYQNSESGEKNEGSESAPEGQAQQR.R** |

| **439.** | P16662\|UD2B7_HUMAN    **Mass:** 61168    **Score:** 71     **Queries matched:** 25   **emPAI:** 0.43 |
| --- | --- |
|  | UDP-glucuronosyltransferase 2B7 OS=Homo sapiens GN=UGT2B7 PE=1 SV=1 |

|  | **Query** | **Observed** | **Mr(expt)** | **Mr(calc)** | **Delta** | **Miss** | **Score** | **Expect** | **Rank** | **Peptide** |
| --- | --- | --- | --- | --- | --- | --- | --- | --- | --- | --- |
|  | 2531 | 437.2521 | 872.4896 | 871.4916 | 0.9980 | 0 | 32 | 0.15 | 1 | K.ADVWLIR.N 2515 |
|  | 3913 | **488.7533** | **975.4921** | **975.5171** | **-0.0250** | **1** | **9** | **25** | **7** | **K.ENVMKLSR.I** 3919 3925 |
|  | 11083 | **709.3779** | **1416.7413** | **1416.7514** | **-0.0101** | **0** | **10** | **14** | **2** | **K.WIPQNDLLGHPK.T** 11066 11084 |
|  | 11196 | **712.4131** | **1422.8116** | **1422.8194** | **-0.0078** | **0** | **58** | **0.00019** | **1** | **R.ANVIASALAQIPQK.V** 11195 |
|  | 11526 | **722.8627** | **1443.7108** | **1444.7787** | **-1.0679** | **0** | **22** | **0.92** | **1** | **R.IQHDQPVKPLDR.A** 11560 11561 |
|  | 13023 | **768.8854** | **1535.7563** | **1535.7653** | **-0.0090** | **1** | **30** | **0.13** | **1** | **R.VINDPSYKENVMK.L** 13022 |
|  | 13224 | 516.5956 | 1546.7651 | 1546.7740 | -0.0089 | 0 | 37 | 0.023 | 1 | R.FDGNKPDTLGLNTR.L |
|  | 16486 | **884.9379** | **1767.8613** | **1767.8713** | **-0.0100** | **0** | **19** | **1.2** | **1** | **R.VDFNTMSSTDLLNALK.R** 16485 |
|  | 18455 | **962.9883** | **1923.9621** | **1923.9724** | **-0.0103** | **1** | **30** | **0.098** | **1** | **R.VDFNTMSSTDLLNALKR.V** 18456 |
|  | 24749 | **683.3484** | **2729.3645** | **2730.3873** | **-1.0229** | **1** | **2** | **37** | **2** | **M.SVKWTSVILLIQLSFCFSSGNCGK.V** |
|  | 27075 | **1068.1681** | **3201.4824** | **3201.4974** | **-0.0149** | **0** | **14** | **1.7** | **1** | **K.HSGGFIFPPSYVPVVMSELTDQMTFMER.V** |
|  | 29844 | **1020.7543** | **4078.9882** | **4078.0233** | **0.9649** | **0** | **13** | **1.2** | **2** | **R.AFITHGGANGIYEAIYHGIPMVGIPLFADQPDNIAHMK.A** |
|  | 30283 | 1081.2565 | 4320.9968 | 4321.1565 | -0.1597 | 1 | 2 | 14 | 2 | R.AFITHGGANGIYEAIYHGIPMVGIPLFADQPDNIAHMKAR.G |
|  | 30285 | 1441.3414 | 4321.0025 | 4321.1565 | -0.1540 | 1 | (1) | 20 | 4 | R.AFITHGGANGIYEAIYHGIPMVGIPLFADQPDNIAHMKAR.G |

| **440.** | P61981\|1433G_HUMAN    **Mass:** 28456    **Score:** 70     **Queries matched:** 6   **emPAI:** 0.13 |
| --- | --- |
|  | 14-3-3 protein gamma OS=Homo sapiens GN=YWHAG PE=1 SV=2 |

|  | **Query** | **Observed** | **Mr(expt)** | **Mr(calc)** | **Delta** | **Miss** | **Score** | **Expect** | **Rank** | **Peptide** |
| --- | --- | --- | --- | --- | --- | --- | --- | --- | --- | --- |
|  | 5260 | **540.7843** | **1079.5540** | **1079.5499** | **0.0042** | **0** | **15** | **4.5** | **3** | **R.YLAEVATGEK.R** |
|  | 6742 | 397.2191 | 1188.6355 | 1188.6536 | -0.0181 | 0 | 7 | 35 | 7 | K.DSTLIMQLLR.D |
|  | 7712 | 412.8949 | 1235.6628 | 1235.6510 | 0.0118 | 1 | 9 | 18 | 2 | R.YLAEVATGEKR.A |
|  | 27446 | 1101.2057 | 3300.5952 | 3300.6071 | -0.0119 | 1 | 70 | 4.5e-006 | 1 | K.TAFDDAIAELDTLNEDSYKDSTLIMQLLR.D 27449 27450 |

| **441.** | P49368\|TCPG_HUMAN    **Mass:** 61066    **Score:** 70     **Queries matched:** 14   **emPAI:** 0.06 |
| --- | --- |
|  | T-complex protein 1 subunit gamma OS=Homo sapiens GN=CCT3 PE=1 SV=4 |

|  | **Query** | **Observed** | **Mr(expt)** | **Mr(calc)** | **Delta** | **Miss** | **Score** | **Expect** | **Rank** | **Peptide** |
| --- | --- | --- | --- | --- | --- | --- | --- | --- | --- | --- |
|  | 995 | **619.5248** | **618.5176** | **618.2721** | **0.2454** | **0** | **3** | **1.1e+002** | **1** | **K.TDNNR.I** |
|  | 1357 | **396.2098** | **790.4050** | **789.4497** | **0.9553** | **1** | **13** | **9.7** | **6** | **R.YIKNPR.I** 1354 |
|  | 4006 | 492.2609 | 982.5072 | 982.5084 | -0.0011 | 0 | 10 | 15 | 3 | R.IDDIVSGHK.K |
|  | 5935 | **565.8173** | **1129.6200** | **1128.6251** | **0.9949** | **1** | **65** | **5.3e-005** | **1** | **R.KVQSGNINAAK.T** 5923 |
|  | 6703 | 594.3351 | 1186.6557 | 1185.6540 | 1.0018 | 1 | 5 | 47 | 6 | K.DPKACTILLR.G |
|  | 6730 | **595.2797** | **1188.5448** | **1188.6649** | **-0.1201** | **1** | **0** | **1.5e+002** | **7** | **K.ACTILLRGASK.E** |
|  | 8594 | **427.2132** | **1278.6179** | **1278.7071** | **-0.0892** | **0** | **8** | **21** | **5** | **R.IVLLDSSLEYK.K** |
|  | 14804 | **549.9393** | **1646.7960** | **1645.9039** | **0.8921** | **1** | **2** | **79** | **8** | **K.NPRIVLLDSSLEYK.K** |
|  | 20719 | 716.3745 | 2146.1017 | 2146.0585 | 0.0432 | 0 | 2 | 53 | 5 | K.MLLDPMGGIVMTNDGNAILR.E |
|  | 23390 | 832.4546 | 2494.3419 | 2495.3231 | -0.9812 | 1 | 1 | 48 | 2 | R.IVSRPEELREDDVGTGAGLLEIK.K |
|  | 24133 | **873.4567** | **2617.3482** | **2616.3581** | **0.9901** | **0** | **5** | **20** | **2** | **R.NVLLDPQLVPGGGASEMAVAHALTEK.S** |
|  | 29305 | **642.1774** | **3847.0206** | **3847.9019** | **-0.8813** | **1** | **0** | **29** | **7** | **R.NLQDAMQVCRNVLLDPQLVPGGGASEMAVAHALTEK.S** |

| **442.** | Q9Y6C9\|MTCH2_HUMAN    **Mass:** 33936    **Score:** 70     **Queries matched:** 9   **emPAI:** 0.52 |
| --- | --- |
|  | Mitochondrial carrier homolog 2 OS=Homo sapiens GN=MTCH2 PE=1 SV=1 |

|  | **Query** | **Observed** | **Mr(expt)** | **Mr(calc)** | **Delta** | **Miss** | **Score** | **Expect** | **Rank** | **Peptide** |
| --- | --- | --- | --- | --- | --- | --- | --- | --- | --- | --- |
|  | 3674 | **480.2581** | **958.5017** | **958.3888** | **0.1128** | **0** | **16** | **4.6** | **1** | **K.TYCCDLK.M** |
|  | 5795 | **559.3242** | **1116.6339** | **1116.6404** | **-0.0065** | **1** | **34** | **0.067** | **1** | **R.RGLFTGLTPR.L** |
|  | 9506 | 663.8597 | 1325.7049 | 1325.7126 | -0.0077 | 0 | 54 | 0.0005 | 1 | R.LCSGVLGTVVHGK.V |
|  | 16061 | 867.9061 | 1733.7976 | 1733.8584 | -0.0608 | 1 | 5 | 31 | 6 | K.EVSSSFDHVIKETTR.E |
|  | 16593 | **593.1540** | **1776.4403** | **1776.0046** | **0.4357** | **0** | **6** | **19** | **4** | **R.SAATLITHPFHVITLR.S** 16602 |
|  | 22311 | 780.6106 | 2338.8100 | 2338.3161 | 0.4938 | 1 | 4 | 23 | 1 | K.VLIQVGYEPLPPTIGRNIFGR.Q |
|  | 22446 | **785.7197** | **2354.1374** | **2354.1502** | **-0.0129** | **1** | **28** | **0.1** | **1** | **K.VLQHYQESDKGEELGPGNVQK.E** 22445 |

| **443.** | Q13409\|DC1I2_HUMAN    **Mass:** 71811    **Score:** 70     **Queries matched:** 6   **emPAI:** 0.05 |
| --- | --- |
|  | Cytoplasmic dynein 1 intermediate chain 2 OS=Homo sapiens GN=DYNC1I2 PE=1 SV=3 |

|  | **Query** | **Observed** | **Mr(expt)** | **Mr(calc)** | **Delta** | **Miss** | **Score** | **Expect** | **Rank** | **Peptide** |
| --- | --- | --- | --- | --- | --- | --- | --- | --- | --- | --- |
|  | 5173 | 536.7808 | 1071.5471 | 1071.5713 | -0.0242 | 0 | 8 | 32 | 2 | K.ITQVDFPPR.E 5189 |
|  | 7909 | **415.7559** | **1244.2459** | **1244.6071** | **-0.3612** | **0** | **1** | **81** | **8** | **K.ETQTPVMAQPK.E** |
|  | 12239 | **495.7566** | **1484.2480** | **1484.7947** | **-0.5466** | **1** | **8** | **14** | **1** | **K.EGEIQAGAKLSLNR.Q** |
|  | 18764 | **975.9298** | **1949.8451** | **1949.8563** | **-0.0112** | **0** | **70** | **9.7e-006** | **1** | **K.SVSTPSEAGSQDSGDGAVGSR.T** |
|  | 22877 | 605.3138 | 2417.2260 | 2418.1348 | -0.9088 | 1 | 4 | 28 | 6 | K.KTTPEYVFHCQSAVMSATFAK.F |

| **444.** | P30475\|1B39_HUMAN    **Mass:** 40645    **Score:** 70     **Queries matched:** 13   **emPAI:** 0.30 |
| --- | --- |
|  | HLA class I histocompatibility antigen, B-39 alpha chain OS=Homo sapiens GN=HLA-B PE=2 SV=1 |

|  | **Query** | **Observed** | **Mr(expt)** | **Mr(calc)** | **Delta** | **Miss** | **Score** | **Expect** | **Rank** | **Peptide** |
| --- | --- | --- | --- | --- | --- | --- | --- | --- | --- | --- |
|  | 1255 | 763.1151 | 762.1078 | 762.3694 | -0.2616 | 0 | 7 | 33 | 7 | R.NTQICK.T 1256 1257 |
|  | 3749 | 483.2167 | 964.4188 | 964.4250 | -0.0062 | 0 | 35 | 0.057 | 1 | R.FDSDAASPR.E |
|  | 7513 | 613.7462 | 1225.4779 | 1225.4856 | -0.0077 | 0 | 54 | 0.00053 | 1 | R.MYGCDVGPDGR.L 7482 |
|  | 9948 | 676.3410 | 1350.6674 | 1349.6939 | 0.9736 | 1 | 14 | 5.9 | 1 | R.YLENGKETLQR.A 9957 |
|  | 14460 | 406.9821 | 1623.8995 | 1623.7498 | 0.1497 | 1 | 4 | 43 | 10 | R.MYGCDVGPDGRLLR.G |
|  | 23135 | 492.2564 | 2456.2458 | 2456.3097 | -0.0639 | 0 | 2 | 47 | 5 | R.TVLLLLSAALALTETWAGSHSMR.Y |
|  | 24792 | 912.7573 | 2735.2500 | 2735.2635 | -0.0135 | 1 | 34 | 0.023 | 1 | R.DGEDQTQDTELVETRPAGDRTFQK.W |
|  | 27235 | 648.6937 | 3238.4319 | 3238.7392 | -0.3073 | 1 | 3 | 24 | 5 | -.MLVMAPRTVLLLLSAALALTETWAGSHSMR.Y |
|  | 27288 | 651.9504 | 3254.7158 | 3254.7341 | -0.0183 | 1 | (1) | 39 | 4 | -.MLVMAPRTVLLLLSAALALTETWAGSHSMR.Y |

|  | **Proteins matching the same set of peptides:** |
| --- | --- |

|  | Q95365\|1B38_HUMAN    **Mass:** 40733    **Score:** 70     **Queries matched:** 13 |
| --- | --- |
|  | HLA class I histocompatibility antigen, B-38 alpha chain OS=Homo sapiens GN=HLA-B PE=2 SV=1 |

|  | **Proteins matching a subset of these peptides:** |
| --- | --- |

|  | Q29836\|1B67_HUMAN    **Mass:** 40602    **Score:** 70     **Queries matched:** 10 |
| --- | --- |
|  | HLA class I histocompatibility antigen, B-67 alpha chain OS=Homo sapiens GN=HLA-B PE=1 SV=1 |

| **445.** | P30466\|1B18_HUMAN    **Mass:** 40535    **Score:** 70     **Queries matched:** 7   **emPAI:** 0.30 |
| --- | --- |
|  | HLA class I histocompatibility antigen, B-18 alpha chain OS=Homo sapiens GN=HLA-B PE=1 SV=1 |

|  | **Query** | **Observed** | **Mr(expt)** | **Mr(calc)** | **Delta** | **Miss** | **Score** | **Expect** | **Rank** | **Peptide** |
| --- | --- | --- | --- | --- | --- | --- | --- | --- | --- | --- |
|  | 3749 | 483.2167 | 964.4188 | 964.4250 | -0.0062 | 0 | 35 | 0.057 | 1 | R.FDSDAASPR.T |
|  | 7513 | 613.7462 | 1225.4779 | 1225.4856 | -0.0077 | 0 | 54 | 0.00053 | 1 | R.MYGCDVGPDGR.L 7482 |
|  | 11588 | **483.2167** | **1446.6282** | **1447.6692** | **-1.0410** | **1** | **13** | **7.2** | **1** | **R.FDSDAASPRTEPR.A** |
|  | 14460 | 406.9821 | 1623.8995 | 1623.7498 | 0.1497 | 1 | 4 | 43 | 10 | R.MYGCDVGPDGRLLR.G |
|  | 23671 | 509.4508 | 2542.2177 | 2541.3414 | 0.8763 | 0 | 2 | 40 | 10 | R.TLLLLLWGAVALTETWAGSHSMR.Y |
|  | 24792 | 912.7573 | 2735.2500 | 2735.2635 | -0.0135 | 1 | 34 | 0.023 | 1 | R.DGEDQTQDTELVETRPAGDRTFQK.W |

| **446.** | P61026\|RAB10_HUMAN    **Mass:** 22755    **Score:** 69     **Queries matched:** 8   **emPAI:** 0.36 |
| --- | --- |
|  | Ras-related protein Rab-10 OS=Homo sapiens GN=RAB10 PE=1 SV=1 |

|  | **Query** | **Observed** | **Mr(expt)** | **Mr(calc)** | **Delta** | **Miss** | **Score** | **Expect** | **Rank** | **Peptide** |
| --- | --- | --- | --- | --- | --- | --- | --- | --- | --- | --- |
|  | 1863 | 830.4077 | 829.4004 | 828.4018 | 0.9987 | 0 | 24 | 0.87 | 1 | R.FFETSAK.A |
|  | 4441 | **507.7781** | **1013.5416** | **1014.6073** | **-1.0657** | **1** | **6** | **43** | **7** | **K.IKTVELQGK.K** |
|  | 5171 | 536.3210 | 1070.6274 | 1070.6336 | -0.0062 | 0 | 21 | 1.3 | 1 | K.LLLIGDSGVGK.T |
|  | 8266 | 631.3586 | 1260.7026 | 1260.7078 | -0.0052 | 0 | 34 | 0.067 | 1 | K.AFLTLAEDILR.K |
|  | 9315 | 658.8296 | 1315.6446 | 1315.6521 | -0.0074 | 0 | 18 | 2.1 | 1 | K.LQIWDTAGQER.F 9284 |
|  | 11464 | **720.8162** | **1439.6179** | **1439.6277** | **-0.0098** | **0** | **61** | **8.8e-005** | **1** | **R.NIDEHANEDVER.M** |
|  | 13771 | 791.3852 | 1580.7558 | 1581.7895 | -1.0336 | 0 | 0 | 1.1e+002 | 7 | R.GAMGIMLVYDITNGK.S |

| **447.** | Q9P0I2\|TM111_HUMAN    **Mass:** 29932    **Score:** 69     **Queries matched:** 10   **emPAI:** 0.13 |
| --- | --- |
|  | Transmembrane protein 111 OS=Homo sapiens GN=TMEM111 PE=1 SV=3 |

|  | **Query** | **Observed** | **Mr(expt)** | **Mr(calc)** | **Delta** | **Miss** | **Score** | **Expect** | **Rank** | **Peptide** |
| --- | --- | --- | --- | --- | --- | --- | --- | --- | --- | --- |
|  | 9028 | **434.7211** | **1301.1415** | **1301.6979** | **-0.5565** | **0** | **6** | **23** | **10** | **R.HYVSILLQSDK.K** |
|  | 16171 | **436.4738** | **1741.8662** | **1742.9527** | **-1.0865** | **1** | **7** | **23** | **10** | **K.KLTQEQVSDSQVLIR.S** |
|  | 19558 | **675.3777** | **2023.1112** | **2021.9182** | **1.1930** | **0** | **3** | **47** | **2** | **K.VVPPSPMTDPTMLTDMMK.G** |
|  | 19876 | **1028.9302** | **2055.8458** | **2055.8556** | **-0.0098** | **0** | **69** | **9e-006** | **1** | **R.MMQEQMTGAAMAMPADTNK.A** |
|  | 20745 | **538.5182** | **2150.0439** | **2150.0132** | **0.0307** | **1** | **7** | **16** | **4** | **R.KVVPPSPMTDPTMLTDMMK.G** |
|  | 20916 | **723.1944** | **2166.5614** | **2166.0081** | **0.5533** | **1** | **(2)** | **33** | **1** | **R.KVVPPSPMTDPTMLTDMMK.G** |
|  | 20919 | **723.3225** | **2166.9457** | **2166.0081** | **0.9376** | **1** | **(3)** | **42** | **1** | **R.KVVPPSPMTDPTMLTDMMK.G** |
|  | 23080 | **490.8772** | **2449.3495** | **2450.0408** | **-0.6913** | **1** | **3** | **31** | **8** | **R.MMQEQMTGAAMAMPADTNKAFK.T** |
|  | 29440 | **1302.3597** | **3904.0574** | **3903.7569** | **0.3005** | **1** | **1** | **24** | **7** | **R.SIYSLILGQDNAADQSRMMQEQMTGAAMAMPADTNK.A** |
|  | 31171 | **993.5190** | **4962.5585** | **4961.4108** | **1.1477** | **1** | **7** | **3** | **1** | **K.VVPPSPMTDPTMLTDMMKGNVTNVLPMILIGGWINMTFSGFVTTK.V** |

| **448.** | A6NKZ8\|YI016_HUMAN    **Mass:** 42204    **Score:** 69     **Queries matched:** 13   **emPAI:** 0.19 |
| --- | --- |
|  | Putative tubulin beta chain-like protein ENSP00000290377 OS=Homo sapiens PE=5 SV=2 |

|  | **Query** | **Observed** | **Mr(expt)** | **Mr(calc)** | **Delta** | **Miss** | **Score** | **Expect** | **Rank** | **Peptide** |
| --- | --- | --- | --- | --- | --- | --- | --- | --- | --- | --- |
|  | 1602 | 404.7373 | 807.4600 | 807.3399 | 0.1201 | 0 | 8 | 28 | 4 | R.EEYPDR.I |
|  | 5226 | 539.2660 | 1076.5175 | 1076.5250 | -0.0075 | 1 | 25 | 0.53 | 1 | K.IREEYPDR.I 5225 |
|  | 6084 | 572.3180 | 1142.6215 | 1142.6270 | -0.0055 | 0 | 17 | 3.3 | 1 | K.LAVNMVPFPR.L |
|  | 8765 | 430.2513 | 1287.7319 | 1286.7169 | 1.0150 | 1 | 4 | 55 | 3 | R.KLAVNMVPFPR.L |
|  | 14380 | 540.9477 | 1619.8212 | 1619.8283 | -0.0070 | 0 | 48 | 0.0018 | 1 | R.LHFFMPGFAPLTSR.G 14371 14377 14378 14379 |
|  | 16851 | 600.2947 | 1797.8624 | 1797.8455 | 0.0169 | 0 | 2 | 71 | 2 | R.YTEGAELTESVMDVVR.K |
|  | 25311 | **709.8330** | **2835.3029** | **2836.3504** | **-1.0475** | **0** | **6** | **14** | **1** | **R.SGPFGQVLRPDNFIFGQCGAGNNWAK.G** |
|  | 28156 | **1162.5587** | **3484.6543** | **3483.6625** | **0.9918** | **1** | **2** | **27** | **2** | **R.GLKMSASFIGNNAAIQELFTCVSEQFTAMFR.R** |

| **449.** | P10451\|OSTP_HUMAN    **Mass:** 35572    **Score:** 69     **Queries matched:** 4   **emPAI:** 0.11 |
| --- | --- |
|  | Osteopontin OS=Homo sapiens GN=SPP1 PE=1 SV=1 |

|  | **Query** | **Observed** | **Mr(expt)** | **Mr(calc)** | **Delta** | **Miss** | **Score** | **Expect** | **Rank** | **Peptide** |
| --- | --- | --- | --- | --- | --- | --- | --- | --- | --- | --- |
|  | 1521 | **402.2197** | **802.4248** | **801.4232** | **1.0016** | **0** | **12** | **16** | **7** | **K.QETLPSK.S** |
|  | 17616 | **927.9473** | **1853.8800** | **1853.8908** | **-0.0108** | **0** | **69** | **1.4e-005** | **1** | **K.AIPVAQDLNAPSDWDSR.G** |
|  | 23140 | **615.3158** | **2457.2341** | **2458.1208** | **-0.8867** | **1** | **1** | **57** | **9** | **K.ANDESNEHSDVIDSQELSKVSR.E** |
|  | 23741 | 638.3331 | 2549.3032 | 2548.1062 | 1.1970 | 1 | 9 | 8 | 1 | K.DSYETSQLDDQSAETHSHKQSR.L |

| **450.** | P52597\|HNRPF_HUMAN    **Mass:** 45985    **Score:** 68     **Queries matched:** 9   **emPAI:** 0.08 |
| --- | --- |
|  | Heterogeneous nuclear ribonucleoprotein F OS=Homo sapiens GN=HNRNPF PE=1 SV=3 |

|  | **Query** | **Observed** | **Mr(expt)** | **Mr(calc)** | **Delta** | **Miss** | **Score** | **Expect** | **Rank** | **Peptide** |
| --- | --- | --- | --- | --- | --- | --- | --- | --- | --- | --- |
|  | 5429 | 546.3238 | 1090.6330 | 1091.5724 | -0.9393 | 0 | 3 | 93 | 9 | R.VHIEIGPDGR.V |
|  | 9363 | **659.8223** | **1317.6301** | **1318.6591** | **-1.0290** | **0** | **2** | **1e+002** | **3** | **M.MLGPEGGEGFVVK.L** |
|  | 11901 | **489.2725** | **1464.7956** | **1465.6945** | **-0.8989** | **0** | **0** | **1.1e+002** | **9** | **-.MMLGPEGGEGFVVK.L** |
|  | 14324 | **539.7668** | **1616.2785** | **1615.6759** | **0.6026** | **0** | **4** | **36** | **4** | **R.DLSYCLSGMYDHR.Y** 14309 |
|  | 16064 | **578.9614** | **1733.8625** | **1734.8797** | **-1.0172** | **1** | **3** | **54** | **8** | **-.MMLGPEGGEGFVVKLR.G** |
|  | 17770 | **934.4707** | **1866.9268** | **1866.9363** | **-0.0095** | **0** | **68** | **1.5e-005** | **1** | **K.ITGEAFVQFASQELAEK.A** |
|  | 19258 | 998.9877 | 1995.9608 | 1995.9690 | -0.0082 | 0 | 3 | 49 | 2 | K.ATENDIYNFFSPLNPVR.V |
|  | 21556 | 746.3487 | 2236.0242 | 2236.1739 | -0.1497 | 1 | 3 | 36 | 5 | K.ITGEAFVQFASQELAEKALGK.H |

| **451.** | O94973\|AP2A2_HUMAN    **Mass:** 104807   **Score:** 68     **Queries matched:** 20   **emPAI:** 0.15 |
| --- | --- |
|  | AP-2 complex subunit alpha-2 OS=Homo sapiens GN=AP2A2 PE=1 SV=2 |

|  | **Query** | **Observed** | **Mr(expt)** | **Mr(calc)** | **Delta** | **Miss** | **Score** | **Expect** | **Rank** | **Peptide** |
| --- | --- | --- | --- | --- | --- | --- | --- | --- | --- | --- |
|  | 2697 | **443.0392** | **884.0637** | **883.4763** | **0.5874** | **1** | **9** | **12** | **1** | **K.AQEPPKSK.K** 2684 2688 2690 2696 2706 2708 |
|  | 3603 | **477.9149** | **953.8152** | **953.6022** | **0.2130** | **0** | **7** | **17** | **7** | **R.VIQIVINR.D** |
|  | 4346 | **503.8681** | **1005.7216** | **1004.5251** | **1.1966** | **1** | **5** | **40** | **9** | **R.TSKEAVSQR.L** |
|  | 5147 | **535.8153** | **1069.6161** | **1069.6243** | **-0.0083** | **1** | **26** | **0.43** | **1** | **R.INKELANIR.S** |
|  | 5172 | **536.7695** | **1071.5244** | **1071.5309** | **-0.0065** | **0** | **38** | **0.03** | **1** | **R.NADVELQQR.A** |
|  | 9182 | **437.2142** | **1308.6207** | **1307.6067** | **1.0140** | **0** | **7** | **26** | **1** | **R.EMAEAFAGEIPK.V** |
|  | 9779 | **671.8173** | **1341.6201** | **1341.6057** | **0.0144** | **0** | **15** | **4.1** | **1** | **R.AVDLLYAMCDR.S** |
|  | 14435 | 541.9463 | 1622.8170 | 1623.8944 | -1.0774 | 1 | 3 | 62 | 6 | K.THIETVINALKTER.D |
|  | 15046 | **554.9353** | **1661.7841** | **1662.8577** | **-1.0736** | **1** | **2** | **68** | **7** | **K.NPEEFKTSVSLAVSR.L** |
|  | 17527 | **924.9833** | **1847.9520** | **1846.9577** | **0.9943** | **0** | **22** | **0.65** | **1** | **K.VGGYILGEFGNLIAGDPR.S** |
|  | 18094 | **948.4726** | **1894.9306** | **1893.8356** | **1.0950** | **0** | **1** | **80** | **7** | **K.FFQPTEMASQDFFQR.W** |
|  | 19141 | **663.0148** | **1986.0227** | **1986.0323** | **-0.0096** | **1** | **28** | **0.16** | **1** | **R.WKQLSNPQQEVQNIFK.A** |
|  | 25495 | **960.5093** | **2878.5062** | **2878.5229** | **-0.0167** | **0** | **37** | **0.013** | **1** | **K.IIGFGSALLEEVDPNPANFVGAGIIHTK.T** |
|  | 28345 | **708.3209** | **3536.5682** | **3536.7948** | **-0.2266** | **1** | **1** | **23** | **2** | **K.YVCKLLFIFLLGHDIDFGHMEAVNLLSSNR.Y** |

| **452.** | P04439\|1A03_HUMAN    **Mass:** 41100    **Score:** 68     **Queries matched:** 15   **emPAI:** 0.30 |
| --- | --- |
|  | HLA class I histocompatibility antigen, A-3 alpha chain OS=Homo sapiens GN=HLA-A PE=1 SV=2 |

|  | **Query** | **Observed** | **Mr(expt)** | **Mr(calc)** | **Delta** | **Miss** | **Score** | **Expect** | **Rank** | **Peptide** |
| --- | --- | --- | --- | --- | --- | --- | --- | --- | --- | --- |
|  | 1644 | 406.7126 | 811.4107 | 810.4500 | 0.9606 | 1 | 4 | 56 | 10 | R.FLRGYR.Q |
|  | 4195 | 498.7497 | 995.4848 | 995.4308 | 0.0539 | 0 | 8 | 22 | 3 | R.FDSDAASQR.M 4211 |
|  | 9948 | 676.3410 | 1350.6674 | 1349.6939 | 0.9736 | 1 | 14 | 5.9 | 1 | R.YLENGKETLQR.T 9957 |
|  | 12185 | **741.8507** | **1481.6869** | **1481.6973** | **-0.0104** | **0** | **38** | **0.021** | **1** | **R.AYLDGTCVEWLR.R** |
|  | 12866 | 508.9794 | 1523.9163 | 1524.6627 | -0.7464 | 1 | 2 | 65 | 3 | R.FDSDAASQRMEPR.A |
|  | 13421 | 780.4057 | 1558.7968 | 1558.8063 | -0.0095 | 1 | 15 | 3.7 | 3 | K.AQSQTDRVDLGTLR.G 13420 |
|  | 13526 | 522.9525 | 1565.8355 | 1564.7668 | 1.0688 | 1 | 2 | 67 | 6 | R.SWTAADMAAQITKR.K |
|  | 14538 | 815.4131 | 1628.8117 | 1628.8199 | -0.0081 | 0 | 28 | 0.17 | 1 | R.FIAVGYVDDTQFVR.F |
|  | 17947 | **471.2618** | **1881.0179** | **1881.8904** | **-0.8725** | **0** | **11** | **8.5** | **2** | **K.THMTHHPISDHEATLR.C** |
|  | 24080 | 869.7491 | 2606.2254 | 2606.2402 | -0.0147 | 1 | 15 | 1.9 | 1 | R.FIAVGYVDDTQFVRFDSDAASQR.M |
|  | 24215 | 879.7303 | 2636.1690 | 2636.1838 | -0.0148 | 0 | 43 | 0.003 | 1 | R.DGEDQTQDTELVETRPAGDGTFQK.W |
|  | 29354 | **968.9431** | **3871.7434** | **3872.2114** | **-0.4680** | **1** | **2** | **17** | **1** | **R.WELSSQPTIPIVGIIAGLVLLGAVITGAVVAAVMWRR.K** |

| **453.** | O95782\|AP2A1_HUMAN    **Mass:** 108570   **Score:** 68     **Queries matched:** 21   **emPAI:** 0.11 |
| --- | --- |
|  | AP-2 complex subunit alpha-1 OS=Homo sapiens GN=AP2A1 PE=1 SV=2 |

|  | **Query** | **Observed** | **Mr(expt)** | **Mr(calc)** | **Delta** | **Miss** | **Score** | **Expect** | **Rank** | **Peptide** |
| --- | --- | --- | --- | --- | --- | --- | --- | --- | --- | --- |
|  | 2697 | 443.0392 | 884.0637 | 883.4763 | 0.5874 | 1 | 9 | 12 | 1 | K.AQEPPKSK.K 2684 2688 2690 2696 2706 2708 |
|  | 3878 | **487.7495** | **973.4843** | **974.5219** | **-1.0375** | **0** | **12** | **12** | **1** | **K.QIVSEMLR.Y** |
|  | 5147 | 535.8153 | 1069.6161 | 1069.6243 | -0.0083 | 1 | 26 | 0.43 | 1 | R.INKELANIR.S |
|  | 5172 | 536.7695 | 1071.5244 | 1071.5309 | -0.0065 | 0 | 38 | 0.03 | 1 | R.NADVELQQR.A |
|  | 6992 | **401.2084** | **1200.6035** | **1199.5895** | **1.0140** | **1** | **7** | **28** | **8** | **K.KGPGAGSALDDGR.R** 6995 |
|  | 7282 | **405.8763** | **1214.6069** | **1213.5979** | **1.0091** | **0** | **4** | **67** | **6** | **R.YLETADYAIR.E** |
|  | 11214 | **475.9131** | **1424.7176** | **1425.7616** | **-1.0440** | **0** | **2** | **82** | **9** | **R.SSPPVQFSLLHSK.F** |
|  | 14957 | **553.2515** | **1656.7328** | **1656.9199** | **-0.1871** | **1** | **9** | **13** | **2** | **K.QLSLPQQEAQKIFK.A** |
|  | 17527 | 924.9833 | 1847.9520 | 1846.9577 | 0.9943 | 0 | 22 | 0.65 | 1 | K.VGGYILGEFGNLIAGDPR.S |
|  | 17708 | **931.6624** | **1861.3103** | **1861.8458** | **-0.5355** | **0** | **52** | **0.0004** | **1** | **K.FFQPTEMAAQDFFQR.W** |
|  | 21745 | **566.2764** | **2261.0764** | **2262.1712** | **-1.0948** | **1** | **8** | **13** | **1** | **R.ILVAGDSMDSVKQSAALCLLR.L** |
|  | 25894 | 592.5001 | 2957.4642 | 2958.5313 | -1.0671 | 0 | 8 | 8.7 | 2 | K.LLFIFLLGHDIDFGHMEAVNLLSSNK.Y |
|  | 26989 | **795.8839** | **3179.5065** | **3179.5948** | **-0.0883** | **0** | **4** | **21** | **2** | **R.AVEYLTLSSVASTDVLATVLEEMPPFPER.E** 26984 |

| **454.** | Q9Y394\|DHRS7_HUMAN    **Mass:** 38673    **Score:** 68     **Queries matched:** 8   **emPAI:** 0.10 |
| --- | --- |
|  | Dehydrogenase/reductase SDR family member 7 OS=Homo sapiens GN=DHRS7 PE=1 SV=1 |

|  | **Query** | **Observed** | **Mr(expt)** | **Mr(calc)** | **Delta** | **Miss** | **Score** | **Expect** | **Rank** | **Peptide** |
| --- | --- | --- | --- | --- | --- | --- | --- | --- | --- | --- |
|  | 3416 | **469.7590** | **937.5035** | **937.5093** | **-0.0058** | **1** | **22** | **1** | **1** | **R.RVHELER.V** |
|  | 6496 | **588.2611** | **1174.5077** | **1174.5142** | **-0.0066** | **0** | **68** | **2.9e-005** | **1** | **K.SGVDADSSYFK.I** |
|  | 11083 | 709.3779 | 1416.7413 | 1415.7191 | 1.0222 | 0 | 18 | 2.3 | 1 | R.IDILVNNGGMSQR.S 11066 |
|  | 12084 | **492.9100** | **1475.7082** | **1474.6432** | **1.0650** | **0** | **3** | **68** | **8** | **R.SLCMDTSLDVYR.K** |
|  | 13476 | **782.3472** | **1562.6798** | **1562.7617** | **-0.0819** | **1** | **8** | **17** | **5** | **K.SGVDADSSYFKIFK.T** |
|  | 13482 | **782.3981** | **1562.7816** | **1562.8919** | **-0.1103** | **0** | **1** | **90** | **5** | **K.LIELNYLGTVSLTK.C** |
|  | 28454 | **1190.9452** | **3569.8137** | **3569.8287** | **-0.0150** | **0** | **7** | **6.8** | **1** | **R.TELATYPGIIVSNICPGPVQSNIVENSLAGEVTK.T** |

| **455.** | P12110\|CO6A2_HUMAN    **Mass:** 109709   **Score:** 68     **Queries matched:** 25   **emPAI:** 0.03 |
| --- | --- |
|  | Collagen alpha-2(VI) chain OS=Homo sapiens GN=COL6A2 PE=1 SV=4 |

|  | **Query** | **Observed** | **Mr(expt)** | **Mr(calc)** | **Delta** | **Miss** | **Score** | **Expect** | **Rank** | **Peptide** |
| --- | --- | --- | --- | --- | --- | --- | --- | --- | --- | --- |
|  | 777 | **541.1181** | **540.1108** | **539.2816** | **0.8292** | **0** | **7** | **17** | **3** | **K.GGPGPR.G** 776 778 780 |
|  | 1415 | **398.7114** | **795.4082** | **795.4351** | **-0.0269** | **1** | **10** | **16** | **3** | **K.GAKGGPGPR.G** 1432 |
|  | 1439 | **399.3459** | **796.6772** | **797.4759** | **-0.7987** | **1** | **2** | **57** | **4** | **R.KGAPGLAGK.N** |
|  | 2314 | 430.8862 | 859.7578 | 859.4035 | 0.3543 | 0 | 4 | 61 | 6 | R.DDDLNLR.A |
|  | 2629 | 441.2615 | 880.5084 | 880.4039 | 0.1045 | 0 | 9 | 19 | 6 | K.GDPGPEGPR.G |
|  | 4302 | **502.2421** | **1002.4696** | **1003.5563** | **-1.0867** | **1** | **6** | **42** | **10** | **R.FVEQVARR.L** |
|  | 4390 | **505.6847** | **1009.3548** | **1009.5193** | **-0.1645** | **0** | **2** | **79** | **4** | **R.GPQGALGEPGK.Q** |
|  | 5164 | 536.2838 | 1070.5529 | 1070.5105 | 0.0425 | 1 | 6 | 50 | 4 | R.RDDDPLNAR.V |
|  | 5201 | 537.8038 | 1073.5931 | 1073.5982 | -0.0051 | 0 | 19 | 2.4 | 1 | K.NFVINVVNR.L |
|  | 5972 | 567.2878 | 1132.5610 | 1131.5673 | 0.9937 | 1 | 2 | 1.1e+002 | 8 | R.GDFGLKGEPGR.K |
|  | 7270 | 607.8446 | 1213.6746 | 1213.6819 | -0.0072 | 0 | 9 | 21 | 3 | R.LFAVAPNQNLK.E |
|  | 8419 | **635.3295** | **1268.6445** | **1268.5858** | **0.0587** | **1** | **5** | **47** | **8** | **K.QGSRGDPGDAGPR.G** |
|  | 11771 | 486.9202 | 1457.7387 | 1458.7004 | -0.9617 | 0 | 3 | 76 | 9 | K.GDPGRPGFSYPGPR.G |
|  | 12027 | 737.3527 | 1472.6909 | 1471.6725 | 1.0184 | 1 | 3 | 59 | 6 | K.GAKGNMGEPGEPGQK.G |
|  | 12705 | **505.5605** | **1513.6598** | **1513.6693** | **-0.0095** | **1** | **22** | **0.78** | **1** | **K.VMKHEAYGECYK.V** |
|  | 13548 | **523.4698** | **1567.3877** | **1566.8518** | **0.5359** | **1** | **3** | **37** | **3** | **R.ASFIKNLQGISSFR.R** |
|  | 15825 | **572.9662** | **1715.8769** | **1715.8665** | **0.0104** | **1** | **10** | **11** | **1** | **K.VSCLEIPGPSGPKGYR.G** |
|  | 16184 | **872.4520** | **1742.8895** | **1742.8992** | **-0.0096** | **0** | **68** | **1.8e-005** | **1** | **K.NLEWIAGGTWTPSALK.F** |
|  | 17461 | 615.3420 | 1843.0041 | 1842.8240 | 0.1801 | 0 | 4 | 40 | 7 | R.GTFTDCALANMTEQIR.Q |
|  | 19092 | **661.6780** | **1982.0123** | **1982.9302** | **-0.9179** | **1** | **1** | **70** | **1** | **R.RGTFTDCALANMTEQIR.Q** |
|  | 28351 | **1180.4867** | **3538.4382** | **3537.6868** | **0.7515** | **1** | **1** | **22** | **2** | **K.HESENLYSIACDKPQQVRNMTLFSDLVAEK.F** |

| **456.** | Q07020\|RL18_HUMAN    **Mass:** 21735    **Score:** 67     **Queries matched:** 10   **emPAI:** 0.63 |
| --- | --- |
|  | 60S ribosomal protein L18 OS=Homo sapiens GN=RPL18 PE=1 SV=2 |

|  | **Query** | **Observed** | **Mr(expt)** | **Mr(calc)** | **Delta** | **Miss** | **Score** | **Expect** | **Rank** | **Peptide** |
| --- | --- | --- | --- | --- | --- | --- | --- | --- | --- | --- |
|  | 1651 | **407.2241** | **812.4337** | **812.4504** | **-0.0167** | **1** | **14** | **6.9** | **1** | **K.LPGRENK.T** |
|  | 5767 | **558.7917** | **1115.5689** | **1115.5757** | **-0.0068** | **0** | **27** | **0.37** | **1** | **K.GCGTVLLSGPR.K** 5795 |
|  | 7998 | **625.3148** | **1248.6150** | **1247.6510** | **0.9640** | **1** | **2** | **88** | **6** | **K.EPKSQDIYLR.L** |
|  | 9854 | 673.3661 | 1344.7176 | 1344.7249 | -0.0073 | 0 | 26 | 0.37 | 1 | K.TAVVVGTITDDVR.V |
|  | 10887 | 469.2278 | 1404.6614 | 1405.7678 | -1.1063 | 1 | 5 | 38 | 5 | R.RTNSTFNQVVLK.R 10889 |
|  | 11838 | **731.4013** | **1460.7879** | **1459.7922** | **0.9957** | **0** | **63** | **6.9e-005** | **1** | **K.ILTFDQLALDSPK.G** |
|  | 15847 | **573.3325** | **1716.9756** | **1715.9054** | **1.0702** | **1** | **5** | **37** | **4** | **R.ENKTAVVVGTITDDVR.V** |
|  | 16759 | **597.3032** | **1788.8877** | **1789.9621** | **-1.0744** | **1** | **7** | **21** | **2** | **R.LFMSRTNRPPLSLSR.M** |

| **457.** | P05186\|PPBT_HUMAN    **Mass:** 57611    **Score:** 67     **Queries matched:** 14   **emPAI:** 0.06 |
| --- | --- |
|  | Alkaline phosphatase, tissue-nonspecific isozyme OS=Homo sapiens GN=ALPL PE=1 SV=4 |

|  | **Query** | **Observed** | **Mr(expt)** | **Mr(calc)** | **Delta** | **Miss** | **Score** | **Expect** | **Rank** | **Peptide** |
| --- | --- | --- | --- | --- | --- | --- | --- | --- | --- | --- |
|  | 3015 | **455.2131** | **908.4116** | **907.5167** | **0.8949** | **0** | **2** | **91** | **2** | **K.FPFVALSK.T** |
|  | 7263 | **607.5637** | **1213.1128** | **1213.4986** | **-0.3859** | **0** | **2** | **56** | **4** | **K.TDVEYESDEK.A** |
|  | 8975 | **433.8810** | **1298.6213** | **1297.6085** | **1.0128** | **0** | **8** | **20** | **1** | **K.QALHEAVEMDR.A** |
|  | 9087 | **652.3692** | **1302.7238** | **1303.7096** | **-0.9857** | **1** | **14** | **5.7** | **1** | **K.DAGKSVGIVTTTR.V** 9088 9102 |
|  | 10120 | **681.3319** | **1360.6493** | **1360.6583** | **-0.0090** | **0** | **67** | **3e-005** | **1** | **K.ANEGTVGVSAATER.S** |
|  | 14134 | **803.4279** | **1604.8412** | **1603.7914** | **1.0498** | **1** | **2** | **78** | **2** | **K.ANEGTVGVSAATERSR.C** |
|  | 17352 | 918.4738 | 1834.9329 | 1834.8956 | 0.0374 | 1 | 8 | 17 | 3 | R.SRCNTTQGNEVTSILR.W 17353 |
|  | 21411 | **741.4091** | **2221.2055** | **2222.1848** | **-0.9793** | **1** | **3** | **35** | **9** | **K.KPFTAILYGNGPGYKVVGGER.E** |
|  | 21995 | **574.3001** | **2293.1713** | **2292.2334** | **0.9379** | **1** | **6** | **19** | **3** | **K.NVIMFLGDGMGVSTVTAARILK.G** |
|  | 22765 | 802.1030 | 2403.2873 | 2402.1835 | 1.1038 | 1 | 2 | 50 | 6 | K.DIAYQLMHNIRDIDVIMGGGR.K |
|  | 29143 | **1264.3982** | **3790.1727** | **3790.6376** | **-0.4649** | **1** | **4** | **11** | **1** | **R.VNHATPSAAYAHSADRDWYSDNEMPPEALSQGCK.D** |

| **458.** | P15954\|COX7C_HUMAN    **Mass:** 7298     **Score:** 67     **Queries matched:** 6   **emPAI:** 1.46 |
| --- | --- |
|  | Cytochrome c oxidase subunit 7C, mitochondrial OS=Homo sapiens GN=COX7C PE=1 SV=1 |

|  | **Query** | **Observed** | **Mr(expt)** | **Mr(calc)** | **Delta** | **Miss** | **Score** | **Expect** | **Rank** | **Peptide** |
| --- | --- | --- | --- | --- | --- | --- | --- | --- | --- | --- |
|  | 1223 | 739.4416 | 738.4344 | 738.4388 | -0.0044 | 1 | 21 | 1 | 1 | R.HQLLKT.- 1221 |
|  | 16199 | **872.9617** | **1743.9089** | **1744.9512** | **-1.0423** | **1** | **5** | **35** | **1** | **K.NLPFSVENKWSLLAK.M** |
|  | 19634 | **1016.4878** | **2030.9610** | **2030.9697** | **-0.0087** | **1** | **64** | **3.5e-005** | **1** | **R.SHYEEGPGKNLPFSVENK.W** 19632 |
|  | 24099 | **654.0955** | **2612.3527** | **2613.3600** | **-1.0073** | **1** | **1** | **49** | **4** | **K.MCLYFGSAFATPFLVVRHQLLK.T** |

| **459.** | Q12931\|TRAP1_HUMAN    **Mass:** 80345    **Score:** 66     **Queries matched:** 19   **emPAI:** 0.05 |
| --- | --- |
|  | Heat shock protein 75 kDa, mitochondrial OS=Homo sapiens GN=TRAP1 PE=1 SV=3 |

|  | **Query** | **Observed** | **Mr(expt)** | **Mr(calc)** | **Delta** | **Miss** | **Score** | **Expect** | **Rank** | **Peptide** |
| --- | --- | --- | --- | --- | --- | --- | --- | --- | --- | --- |
|  | 1865 | **830.4572** | **829.4499** | **828.5433** | **0.9067** | **1** | **19** | **2.7** | **3** | **R.KVLIQTK.A** |
|  | 5279 | 541.2656 | 1080.5167 | 1079.5360 | 0.9808 | 1 | 2 | 97 | 10 | K.EFSSEARVR.D |
|  | 6470 | **586.8399** | **1171.6652** | **1170.6608** | **1.0045** | **0** | **6** | **45** | **2** | **R.ELLQESALIR.K** |
|  | 7509 | **409.4392** | **1225.2957** | **1225.7295** | **-0.4338** | **1** | **5** | **29** | **4** | **R.ELRALLLWGR.R** |
|  | 8641 | **641.8185** | **1281.6224** | **1282.6128** | **-0.9904** | **0** | **3** | **64** | **4** | **R.NIYYLCAPNR.H** |
|  | 8815 | **645.8380** | **1289.6615** | **1288.6510** | **1.0105** | **0** | **8** | **23** | **4** | **R.ELISNASDALEK.L** 8776 8794 |
|  | 8881 | **647.8005** | **1293.5864** | **1294.5686** | **-0.9821** | **0** | **(0)** | **1.3e+002** | **7** | **K.ETEELMAWMR.N** |
|  | 9071 | **435.2183** | **1302.6330** | **1302.6667** | **-0.0337** | **0** | **11** | **13** | **6** | **R.EGIVTATEQEVK.E** |
|  | 9220 | **656.3625** | **1310.7104** | **1310.5635** | **0.1470** | **0** | **4** | **51** | **10** | **K.ETEELMAWMR.N** |
|  | 9785 | **671.8335** | **1341.6524** | **1341.7802** | **-0.1278** | **1** | **6** | **34** | **7** | **R.AMVGRLNELLVK.A** |
|  | 11460 | **480.6212** | **1438.8419** | **1438.8759** | **-0.0340** | **1** | **12** | **8.3** | **1** | **K.VLIQTKATDILPK.W** |
|  | 12584 | **754.3898** | **1506.7650** | **1505.8453** | **0.9197** | **1** | **1** | **1e+002** | **1** | **K.LLDIVARSLYSEK.E** |
|  | 12693 | 757.3920 | 1512.7694 | 1512.7784 | -0.0090 | 0 | 66 | 3.1e-005 | 1 | R.GVVDSEDIPLNLSR.E 12656 |
|  | 15416 | **563.2817** | **1686.8234** | **1686.9603** | **-0.1369** | **0** | **4** | **51** | **10** | **R.APALAAVPGGKPILCPR.R** |
|  | 17033 | 604.9820 | 1811.9241 | 1810.9070 | 1.0172 | 0 | 5 | 33 | 2 | R.LDTHPAMVTVLEMGAAR.H |
|  | 17312 | **611.3526** | **1831.0360** | **1831.9369** | **-0.9010** | **1** | **1** | **83** | **7** | **K.YSNFVSFPLYLNGRR.M** |

| **460.** | O94826\|TOM70_HUMAN    **Mass:** 68096    **Score:** 66     **Queries matched:** 16   **emPAI:** 0.17 |
| --- | --- |
|  | Mitochondrial import receptor subunit TOM70 OS=Homo sapiens GN=TOMM70A PE=1 SV=1 |

|  | **Query** | **Observed** | **Mr(expt)** | **Mr(calc)** | **Delta** | **Miss** | **Score** | **Expect** | **Rank** | **Peptide** |
| --- | --- | --- | --- | --- | --- | --- | --- | --- | --- | --- |
|  | 919 | 588.3677 | 587.3605 | 587.3139 | 0.0465 | 1 | 2 | 1.8e+002 | 4 | R.EARGR.G |
|  | 2303 | 430.7143 | 859.4141 | 859.4512 | -0.0371 | 1 | 10 | 21 | 7 | R.GRGDASGLK.R |
|  | 4039 | **493.2923** | **984.5701** | **984.5756** | **-0.0055** | **0** | **24** | **0.65** | **1** | **K.GLLQLQWK.Q** |
|  | 5540 | **550.3234** | **1098.6323** | **1099.6601** | **-1.0278** | **1** | **5** | **50** | **6** | **K.VISLKEANVK.L** |
|  | 8298 | **632.7989** | **1263.5833** | **1263.5918** | **-0.0084** | **1** | **28** | **0.22** | **1** | **K.WKEVAQDCTK.A** |
|  | 9221 | **656.3685** | **1310.7225** | **1310.7306** | **-0.0081** | **0** | **32** | **0.078** | **1** | **R.LRPESALAQAQK.C** |
|  | 10060 | **679.3283** | **1356.6421** | **1355.6470** | **0.9951** | **0** | **50** | **0.0014** | **1** | **K.NVDLSTFYQNR.A** |
|  | 11308 | 715.8647 | 1429.7149 | 1428.6377 | 1.0772 | 1 | 2 | 79 | 8 | R.GNMEKAIDMFNK.A |
|  | 11888 | 489.2268 | 1464.6585 | 1463.7806 | 0.8779 | 1 | 1 | 91 | 8 | K.AIDMFNKAINLAK.S |
|  | 13378 | 778.8876 | 1555.7606 | 1555.8181 | -0.0575 | 1 | (6) | 33 | 7 | K.NREPLMPSPQFIK.S |
|  | 13625 | **525.1025** | **1572.2856** | **1571.8130** | **0.4726** | **1** | **6** | **20** | **4** | **K.NREPLMPSPQFIK.S** |
|  | 17141 | **607.6252** | **1819.8537** | **1820.9342** | **-1.0805** | **1** | **2** | **79** | **4** | **K.EIDAEGKYMAEALLLR.A** |
|  | 19232 | **665.3826** | **1993.1261** | **1993.9343** | **-0.8082** | **0** | **3** | **43** | **4** | **K.SYFSSFTDDIISQPMLK.G** |
|  | 19658 | **678.7076** | **2033.1011** | **2034.0786** | **-0.9775** | **0** | **0** | **84** | **2** | **R.ATFYLLIGNANAAKPDLDK.V** |
|  | 23638 | **846.6123** | **2536.8151** | **2537.1791** | **-0.3640** | **1** | **1** | **39** | **7** | **K.CFALYRQAYTGNNSSQIQAAMK.G** |
|  | 24694 | 680.8129 | 2719.2224 | 2719.2404 | -0.0180 | 1 | 16 | 1.6 | 1 | K.SEMEMAHLYSLCDAAHAQTEVAKK.Y |

| **461.** | O75489\|NDUS3_HUMAN    **Mass:** 30337    **Score:** 66     **Queries matched:** 7   **emPAI:** 0.12 |
| --- | --- |
|  | NADH dehydrogenase [ubiquinone] iron-sulfur protein 3, mitochondrial OS=Homo sapiens GN=NDUFS3 PE=1 SV=1 |

|  | **Query** | **Observed** | **Mr(expt)** | **Mr(calc)** | **Delta** | **Miss** | **Score** | **Expect** | **Rank** | **Peptide** |
| --- | --- | --- | --- | --- | --- | --- | --- | --- | --- | --- |
|  | 3679 | **480.3062** | **958.5979** | **957.5607** | **1.0372** | **0** | **2** | **1.1e+002** | **2** | **R.GILGASALTR.G** |
|  | 3683 | **480.7268** | **959.4391** | **959.4461** | **-0.0070** | **0** | **12** | **12** | **7** | **R.DHTNAQFK.S** |
|  | 13290 | **517.9215** | **1550.7425** | **1550.7518** | **-0.0093** | **0** | **5** | **43** | **6** | **R.ILTDYGFEGHPFR.K** |
|  | 16145 | **870.9190** | **1739.8233** | **1739.8308** | **-0.0074** | **0** | **66** | **2.7e-005** | **1** | **K.FDLNSPWEAFPVYR.Q** 16146 |
|  | 21633 | **750.6220** | **2248.8442** | **2249.0840** | **-0.2398** | **1** | **1** | **47** | **6** | **R.EIWDMFGVFFANHPDLRR.I** 21632 |

| **462.** | P07357\|CO8A_HUMAN    **Mass:** 66832    **Score:** 66     **Queries matched:** 8   **emPAI:** 0.06 |
| --- | --- |
|  | Complement component C8 alpha chain OS=Homo sapiens GN=C8A PE=1 SV=2 |

|  | **Query** | **Observed** | **Mr(expt)** | **Mr(calc)** | **Delta** | **Miss** | **Score** | **Expect** | **Rank** | **Peptide** |
| --- | --- | --- | --- | --- | --- | --- | --- | --- | --- | --- |
|  | 4164 | 497.3162 | 992.6178 | 992.4961 | 0.1218 | 0 | 5 | 53 | 1 | K.MESLGITSR.D |
|  | 9257 | **438.7300** | **1313.1681** | **1312.6194** | **0.5487** | **0** | **7** | **18** | **3** | **K.INVGGGLSGDHCK.K** |
|  | 11475 | **481.1525** | **1440.4357** | **1440.7144** | **-0.2787** | **1** | **5** | **30** | **5** | **K.INVGGGLSGDHCKK.F** 11480 |
|  | 11940 | 734.3917 | 1466.7689 | 1467.6670 | -0.8981 | 1 | 8 | 18 | 5 | R.LYYGDDEKYFR.K |
|  | 15866 | 573.9545 | 1718.8416 | 1719.8098 | -0.9682 | 0 | 5 | 38 | 3 | R.LGSLGAACEQTQTEGAK.A |
|  | 27554 | 832.4207 | 3325.6535 | 3326.4855 | -0.8320 | 1 | 1 | 34 | 5 | R.KDDIMLDEGMLQSLMELPDQYNYGMYAK.F |
|  | 27994 | **1144.8513** | **3431.5321** | **3431.5545** | **-0.0223** | **1** | **66** | **1e-005** | **1** | **R.SLLQPNKFGGTICSGDIWDQASCSSSTTCVR.Q** |

| **463.** | O95168\|NDUB4_HUMAN    **Mass:** 15256    **Score:** 65     **Queries matched:** 4   **emPAI:** 0.26 |
| --- | --- |
|  | NADH dehydrogenase [ubiquinone] 1 beta subcomplex subunit 4 OS=Homo sapiens GN=NDUFB4 PE=1 SV=3 |

|  | **Query** | **Observed** | **Mr(expt)** | **Mr(calc)** | **Delta** | **Miss** | **Score** | **Expect** | **Rank** | **Peptide** |
| --- | --- | --- | --- | --- | --- | --- | --- | --- | --- | --- |
|  | 5483 | **548.3264** | **1094.6383** | **1094.6448** | **-0.0065** | **0** | **9** | **20** | **3** | **R.GLIENPALLR.W** |
|  | 8067 | **418.2641** | **1251.7705** | **1250.7459** | **1.0246** | **1** | **7** | **25** | **10** | **R.RGLIENPALLR.W** |
|  | 13753 | 527.5957 | 1579.7653 | 1579.7743 | -0.0090 | 1 | 3 | 59 | 4 | R.EYLLQYNDPNRR.G |
|  | 19774 | **1023.4990** | **2044.9835** | **2044.9953** | **-0.0118** | **0** | **65** | **2.7e-005** | **1** | **R.TLPETLDPAEYNISPETR.R** |

| **464.** | O75381\|PEX14_HUMAN    **Mass:** 41269    **Score:** 65     **Queries matched:** 6   **emPAI:** 0.09 |
| --- | --- |
|  | Peroxisomal membrane protein PEX14 OS=Homo sapiens GN=PEX14 PE=1 SV=1 |

|  | **Query** | **Observed** | **Mr(expt)** | **Mr(calc)** | **Delta** | **Miss** | **Score** | **Expect** | **Rank** | **Peptide** |
| --- | --- | --- | --- | --- | --- | --- | --- | --- | --- | --- |
|  | 5467 | **547.8596** | **1093.7046** | **1093.4597** | **0.2448** | **0** | **2** | **87** | **6** | **R.MEVQGEEEK.R** |
|  | 7838 | 414.7479 | 1241.2218 | 1241.7860 | -0.5642 | 1 | 5 | 29 | 8 | K.KYLLPLILGGR.E |
|  | 8005 | **417.2430** | **1248.7072** | **1249.5608** | **-0.8536** | **1** | **13** | **6.6** | **3** | **R.MEVQGEEEKR.E** |
|  | 15404 | **562.8013** | **1685.3822** | **1685.9464** | **-0.5643** | **1** | **7** | **15** | **3** | **R.EPLIATAVKFLQNSR.V** |
|  | 29329 | **1286.6736** | **3856.9989** | **3857.0092** | **-0.0103** | **0** | **65** | **9e-006** | **1** | **R.MEAGLSELSGSVAQTVTQLQTTLASVQELLIQQQQK.I** |
|  | 31350 | **1283.9254** | **5131.6726** | **5131.7074** | **-0.0349** | **1** | **2** | **7.4** | **2** | **R.MEAGLSELSGSVAQTVTQLQTTLASVQELLIQQQQKIQELAHELAAAK.A** |

| **465.** | P35606\|COPB2_HUMAN    **Mass:** 103278   **Score:** 65     **Queries matched:** 15   **emPAI:** 0.04 |
| --- | --- |
|  | Coatomer subunit beta' OS=Homo sapiens GN=COPB2 PE=1 SV=2 |

|  | **Query** | **Observed** | **Mr(expt)** | **Mr(calc)** | **Delta** | **Miss** | **Score** | **Expect** | **Rank** | **Peptide** |
| --- | --- | --- | --- | --- | --- | --- | --- | --- | --- | --- |
|  | 2078 | **422.2609** | **842.5072** | **842.4650** | **0.0422** | **0** | **5** | **64** | **1** | **R.VAHFLEK.Q** |
|  | 2240 | **428.2480** | **854.4814** | **853.5385** | **0.9429** | **1** | **11** | **12** | **2** | **M.PLRLDIK.R** |
|  | 6236 | **577.2982** | **1152.5818** | **1152.5887** | **-0.0069** | **0** | **50** | **0.0015** | **1** | **K.HSEVQQANLK.A** 6234 |
|  | 7010 | **401.2847** | **1200.8324** | **1200.6536** | **0.1787** | **0** | **12** | **7.4** | **1** | **K.VDACLELLIR.T** |
|  | 7697 | **618.3249** | **1234.6352** | **1234.6016** | **0.0336** | **0** | **4** | **62** | **1** | **K.TFEVCDLPVR.A** |
|  | 9128 | **435.8883** | **1304.6432** | **1305.7081** | **-1.0649** | **1** | **7** | **28** | **5** | **R.LVKIWDYQNK.T** |
|  | 9221 | 656.3685 | 1310.7225 | 1310.5118 | 0.2107 | 0 | 4 | 56 | 10 | R.EEPAMSMDANGK.I |
|  | 12800 | **507.3004** | **1518.8793** | **1519.6878** | **-0.8085** | **0** | **4** | **50** | **2** | **R.VHMFEAHSDYIR.C** |
|  | 13458 | **521.3040** | **1560.8902** | **1560.8222** | **0.0681** | **1** | **12** | **7** | **1** | **R.DFSMADKVLPTIPK.E** 13448 |
|  | 18215 | 636.0601 | 1905.1584 | 1905.8964 | -0.7381 | 1 | 2 | 57 | 8 | R.EEPAMSMDANGKIIWAK.H |
|  | 19111 | **496.7665** | **1983.0368** | **1983.9684** | **-0.9315** | **1** | **4** | **35** | **4** | **K.HSEVQQANLKAMGDAEIK.D** |
|  | 22976 | **812.0884** | **2433.2435** | **2434.2025** | **-0.9590** | **1** | **1** | **61** | **6** | **R.FVVVCGDGEYIIYTAMALRNK.S** |
|  | 26279 | **607.7408** | **3033.6678** | **3032.5414** | **1.1264** | **1** | **0** | **40** | **3** | **R.STAQQELDGKPASPTPVIVASHTANKEEK.S** |

| **466.** | P20073\|ANXA7_HUMAN    **Mass:** 52991    **Score:** 65     **Queries matched:** 17   **emPAI:** 0.07 |
| --- | --- |
|  | Annexin A7 OS=Homo sapiens GN=ANXA7 PE=1 SV=3 |

|  | **Query** | **Observed** | **Mr(expt)** | **Mr(calc)** | **Delta** | **Miss** | **Score** | **Expect** | **Rank** | **Peptide** |
| --- | --- | --- | --- | --- | --- | --- | --- | --- | --- | --- |
|  | 2740 | **444.7660** | **887.5175** | **887.4712** | **0.0463** | **1** | **6** | **53** | **8** | **R.DLEKDIR.S** |
|  | 2779 | **446.7622** | **891.5098** | **892.4403** | **-0.9305** | **0** | **2** | **1.1e+002** | **6** | **R.LYQAGEGR.L** |
|  | 4720 | **518.2897** | **1034.5649** | **1034.4417** | **0.1232** | **0** | **19** | **2** | **1** | **R.SDTSGHFER.L** 4716 |
|  | 4896 | **525.2760** | **1048.5374** | **1047.4767** | **1.0607** | **0** | **2** | **1.1e+002** | **2** | **K.AMQGAGTQER.V** |
|  | 6498 | **588.2725** | **1174.5304** | **1173.5311** | **0.9993** | **0** | **0** | **1.6e+002** | **7** | **K.QMFAQMYQK.T** |
|  | 6833 | **597.3229** | **1192.6313** | **1192.5693** | **0.0621** | **0** | **2** | **89** | **7** | **R.LLVSMCQGNR.D** |
|  | 7308 | **406.1855** | **1215.5348** | **1214.5819** | **0.9529** | **0** | **8** | **25** | **2** | **R.EFSGYVESGLK.T** |
|  | 8181 | 629.8414 | 1257.6682 | 1256.6837 | 0.9845 | 1 | 10 | 13 | 6 | R.TNQEIREIVR.C 8180 |
|  | 11070 | **472.9973** | **1415.9702** | **1415.6285** | **0.3417** | **1** | **(4)** | **36** | **6** | **R.ATMEAYSRMANR.D** |
|  | 11335 | **477.9283** | **1430.7631** | **1431.6234** | **-0.8603** | **1** | **13** | **7** | **3** | **R.ATMEAYSRMANR.D** |
|  | 13371 | **519.5651** | **1555.6734** | **1556.7141** | **-1.0407** | **0** | **1** | **88** | **6** | **K.TLGTMIAGDTSGDYR.R** 13389 |
|  | 14948 | **552.8012** | **1655.3818** | **1655.8089** | **-0.4271** | **1** | **5** | **24** | **1** | **R.SFPQLRATMEAYSR.M** |
|  | 15971 | **864.4044** | **1726.7943** | **1726.8018** | **-0.0075** | **0** | **65** | **3.3e-005** | **1** | **R.LGTDESCFNMILATR.S** |
|  | 26873 | **1581.2154** | **3160.4163** | **3161.5089** | **-1.0926** | **0** | **0** | **44** | **4** | **K.SELSGNMEELILALFMPPTYYDAWSLR.K** |

| **467.** | P06133\|UD2B4_HUMAN    **Mass:** 60872    **Score:** 65     **Queries matched:** 12   **emPAI:** 0.19 |
| --- | --- |
|  | UDP-glucuronosyltransferase 2B4 OS=Homo sapiens GN=UGT2B4 PE=1 SV=2 |

|  | **Query** | **Observed** | **Mr(expt)** | **Mr(calc)** | **Delta** | **Miss** | **Score** | **Expect** | **Rank** | **Peptide** |
| --- | --- | --- | --- | --- | --- | --- | --- | --- | --- | --- |
|  | 3553 | **475.2394** | **948.4642** | **947.4858** | **0.9784** | **1** | **16** | **4.5** | **4** | **K.ENAMKLSR.I** 3554 |
|  | 11083 | 709.3779 | 1416.7413 | 1416.7514 | -0.0101 | 0 | 10 | 14 | 2 | K.WIPQNDLLGHPK.T 11066 11084 |
|  | 11196 | 712.4131 | 1422.8116 | 1422.8558 | -0.0442 | 1 | 58 | 0.00019 | 1 | R.ANVIASALAKIPQK.V 11195 |
|  | 13224 | 516.5956 | 1546.7651 | 1546.7740 | -0.0089 | 0 | 37 | 0.023 | 1 | R.FDGNKPDTLGLNTR.L |
|  | 21259 | 736.3749 | 2206.1028 | 2206.0940 | 0.0088 | 0 | 4 | 32 | 7 | K.GAAVSLDFHTMSSTDLLNALK.T |
|  | 22710 | **797.7782** | **2390.3128** | **2389.2311** | **1.0816** | **1** | **2** | **44** | **2** | **K.AKGAAVSLDFHTMSSTDLLNALK.T** |
|  | 29844 | 1020.7543 | 4078.9882 | 4080.0026 | -1.0144 | 0 | 15 | 0.9 | 1 | R.AFITHGGANGIYEAIYHGIPMVGVPLFADQPDNIAHMK.A 29851 |

| **468.** | O00330\|ODPX_HUMAN    **Mass:** 54317    **Score:** 65     **Queries matched:** 18   **emPAI:** 0.07 |
| --- | --- |
|  | Pyruvate dehydrogenase protein X component, mitochondrial OS=Homo sapiens GN=PDHX PE=1 SV=3 |

|  | **Query** | **Observed** | **Mr(expt)** | **Mr(calc)** | **Delta** | **Miss** | **Score** | **Expect** | **Rank** | **Peptide** |
| --- | --- | --- | --- | --- | --- | --- | --- | --- | --- | --- |
|  | 952 | 603.3414 | 602.3341 | 601.3435 | 0.9906 | 0 | 15 | 11 | 4 | R.QDLVK.D |
|  | 3140 | **459.0113** | **916.0081** | **916.5243** | **-0.5161** | **1** | **8** | **23** | **4** | **R.FRLSPAAR.N** |
|  | 3226 | 462.1591 | 922.3037 | 921.5032 | 0.8005 | 0 | 7 | 28 | 9 | K.EHIPGTLR.F |
|  | 3330 | **466.2381** | **930.4617** | **931.4876** | **-1.0259** | **0** | **8** | **32** | **5** | **K.GALGWSVSR.G** 3338 |
|  | 4440 | 507.7697 | 1013.5249 | 1013.6233 | -0.0984 | 1 | 13 | 8.1 | 4 | K.LVQLKQTGK.I |
|  | 4493 | **509.3333** | **1016.6519** | **1016.5138** | **0.1381** | **0** | **2** | **1.3e+002** | **4** | **R.VVDDELATR.F** |
|  | 7196 | **606.0740** | **1210.1335** | **1210.7033** | **-0.5698** | **1** | **5** | **31** | **2** | **R.LSPAARNILEK.H** |
|  | 10758 | 699.3376 | 1396.6606 | 1396.6695 | -0.0089 | 0 | 65 | 4.4e-005 | 1 | K.HSLDASQGTATGPR.G |
|  | 11114 | **473.9099** | **1418.7080** | **1418.6547** | **0.0533** | **1** | **4** | **51** | **6** | **-.MAASWRLGCDPR.L** |
|  | 14537 | 543.9442 | 1628.8108 | 1629.7958 | -0.9850 | 1 | 0 | 1.1e+002 | 10 | K.LTEDEEGNAKLQQR.Q |
|  | 17700 | **931.4453** | **1860.8761** | **1861.9680** | **-1.0919** | **1** | **3** | **48** | **10** | **K.LQQRQLITVTMSSDSR.V** 17699 |
|  | 22168 | 1159.5679 | 2317.1212 | 2317.2061 | -0.0850 | 1 | 9 | 8.7 | 5 | K.ILMPSLSPTMEEGNIVKWLK.K 22167 22169 22170 |
|  | 23101 | **818.4102** | **2452.2088** | **2451.4352** | **0.7736** | **1** | **1** | **50** | **4** | **K.QLPFIDISVAVATDKGLLTPIIK.D** |

| **469.** | P04156\|PRIO_HUMAN    **Mass:** 27871    **Score:** 65     **Queries matched:** 1   **emPAI:** 0.14 |
| --- | --- |
|  | Major prion protein OS=Homo sapiens GN=PRNP PE=1 SV=1 |

|  | **Query** | **Observed** | **Mr(expt)** | **Mr(calc)** | **Delta** | **Miss** | **Score** | **Expect** | **Rank** | **Peptide** |
| --- | --- | --- | --- | --- | --- | --- | --- | --- | --- | --- |
|  | 13357 | **778.3608** | **1554.7070** | **1554.7170** | **-0.0100** | **0** | **65** | **4e-005** | **1** | **R.VVEQMCITQYER.E** |

| **470.** | P04217\|A1BG_HUMAN    **Mass:** 54809    **Score:** 65     **Queries matched:** 10   **emPAI:** 0.07 |
| --- | --- |
|  | Alpha-1B-glycoprotein OS=Homo sapiens GN=A1BG PE=1 SV=3 |

|  | **Query** | **Observed** | **Mr(expt)** | **Mr(calc)** | **Delta** | **Miss** | **Score** | **Expect** | **Rank** | **Peptide** |
| --- | --- | --- | --- | --- | --- | --- | --- | --- | --- | --- |
|  | 4067 | 494.2942 | 986.5738 | 985.5015 | 1.0723 | 0 | 5 | 62 | 9 | R.CLAPLEGAR.F |
|  | 7573 | **410.5489** | **1228.6249** | **1229.7608** | **-1.1359** | **1** | **10** | **16** | **3** | **R.GVLRGVTFLLR.R** 7623 |
|  | 8301 | **632.8259** | **1263.6373** | **1263.6459** | **-0.0086** | **0** | **65** | **5.1e-005** | **1** | **R.SGLSTGWTQLSK.L** |
|  | 10302 | **686.4143** | **1370.8139** | **1371.6895** | **-0.8756** | **0** | **30** | **0.13** | **1** | **K.HQFLLTGDTQGR.Y** 10319 10321 |
|  | 11719 | **486.2363** | **1455.6870** | **1455.7681** | **-0.0812** | **1** | **4** | **54** | **3** | **K.ELLVPRSSTSPDR.I** |
|  | 13609 | 786.8649 | 1571.7152 | 1571.8606 | -0.1454 | 1 | 1 | 1.1e+002 | 8 | R.CLAPLEGARFALVR.E |
|  | 24096 | 871.4537 | 2611.3392 | 2611.3078 | 0.0314 | 1 | 1 | 56 | 9 | R.TPGAAANLELIFVGPQHAGNYRCR.Y |

| **471.** | Q8WUD1\|RAB2B_HUMAN    **Mass:** 24427    **Score:** 64     **Queries matched:** 8   **emPAI:** 0.16 |
| --- | --- |
|  | Ras-related protein Rab-2B OS=Homo sapiens GN=RAB2B PE=1 SV=1 |

|  | **Query** | **Observed** | **Mr(expt)** | **Mr(calc)** | **Delta** | **Miss** | **Score** | **Expect** | **Rank** | **Peptide** |
| --- | --- | --- | --- | --- | --- | --- | --- | --- | --- | --- |
|  | 1351 | 396.2092 | 790.4038 | 791.3847 | -0.9809 | 0 | 10 | 23 | 9 | R.MVNIDGK.Q |
|  | 6103 | **572.9965** | **1143.9785** | **1144.6274** | **-0.6489** | **1** | **6** | **29** | **6** | **R.MVNIDGKQIK.L** |
|  | 9366 | 440.2390 | 1317.6952 | 1318.7245 | -1.0293 | 0 | 12 | 10 | 2 | R.GAAGALLVYDITR.R |
|  | 10142 | 681.8351 | 1361.6556 | 1361.6649 | -0.0093 | 0 | 10 | 14 | 1 | R.EHGLIFMETSAK.T |
|  | 12044 | 492.2528 | 1473.7365 | 1474.8256 | -1.0891 | 1 | 6 | 34 | 5 | R.GAAGALLVYDITRR.E |
|  | 13279 | 775.8789 | 1549.7431 | 1549.7525 | -0.0094 | 0 | 64 | 4.9e-005 | 1 | K.LQIWDTAGQESFR.S 13258 |
|  | 15232 | **559.0970** | **1674.2691** | **1673.8229** | **0.4462** | **0** | **3** | **41** | **8** | **R.QHSSSNMVIMLIGNK.S** |

| **472.** | P62910\|RL32_HUMAN    **Mass:** 15964    **Score:** 64     **Queries matched:** 6   **emPAI:** 0.24 |
| --- | --- |
|  | 60S ribosomal protein L32 OS=Homo sapiens GN=RPL32 PE=1 SV=2 |

|  | **Query** | **Observed** | **Mr(expt)** | **Mr(calc)** | **Delta** | **Miss** | **Score** | **Expect** | **Rank** | **Peptide** |
| --- | --- | --- | --- | --- | --- | --- | --- | --- | --- | --- |
|  | 3481 | **472.5334** | **943.0523** | **943.4698** | **-0.4175** | **0** | **3** | **56** | **5** | **K.HMLPSGFR.K** |
|  | 7459 | **612.3411** | **1222.6677** | **1222.7583** | **-0.0906** | **0** | **5** | **45** | **7** | **-.MAALRPLVKPK.I** |
|  | 11887 | 733.3359 | 1464.6572 | 1464.6667 | -0.0095 | 0 | 64 | 5.1e-005 | 1 | K.SYCAEIAHNVSSK.N 11888 11889 |
|  | 14366 | **540.7754** | **1619.3045** | **1618.8501** | **0.4544** | **1** | **11** | **5.9** | **1** | **K.GQILMPNIGYGSNKK.T** |

| **473.** | Q5VT66\|MOSC1_HUMAN    **Mass:** 37989    **Score:** 63     **Queries matched:** 5   **emPAI:** 0.10 |
| --- | --- |
|  | MOSC domain-containing protein 1, mitochondrial OS=Homo sapiens GN=MOSC1 PE=1 SV=1 |

|  | **Query** | **Observed** | **Mr(expt)** | **Mr(calc)** | **Delta** | **Miss** | **Score** | **Expect** | **Rank** | **Peptide** |
| --- | --- | --- | --- | --- | --- | --- | --- | --- | --- | --- |
|  | 2613 | **440.2704** | **878.5263** | **878.4215** | **0.1048** | **1** | **4** | **79** | **8** | **K.RVMACSR.C** |
|  | 4126 | **496.2530** | **990.4915** | **990.4916** | **-0.0001** | **0** | **1** | **1.3e+002** | **8** | **-.MGAAGSSALAR.F** |
|  | 11084 | 473.2547 | 1416.7424 | 1417.7401 | -0.9977 | 0 | 8 | 19 | 7 | R.LVHFEPHMRPR.R |
|  | 13676 | 788.8721 | 1575.7297 | 1575.7385 | -0.0088 | 0 | 63 | 5.6e-005 | 1 | K.GVPVSEAECTAMGLR.S |
|  | 13963 | 399.2196 | 1592.8495 | 1591.7334 | 1.1160 | 0 | (9) | 14 | 2 | K.GVPVSEAECTAMGLR.S |

| **474.** | P50914\|RL14_HUMAN    **Mass:** 23531    **Score:** 63     **Queries matched:** 9   **emPAI:** 0.35 |
| --- | --- |
|  | 60S ribosomal protein L14 OS=Homo sapiens GN=RPL14 PE=1 SV=4 |

|  | **Query** | **Observed** | **Mr(expt)** | **Mr(calc)** | **Delta** | **Miss** | **Score** | **Expect** | **Rank** | **Peptide** |
| --- | --- | --- | --- | --- | --- | --- | --- | --- | --- | --- |
|  | 1810 | **413.7769** | **825.5392** | **826.4661** | **-0.9269** | **1** | **2** | **97** | **3** | **K.GQKAPAQK.A** |
|  | 3576 | 476.2801 | 950.5457 | 950.4722 | 0.0735 | 0 | 6 | 43 | 5 | K.FPHSAHQK.Y 3564 |
|  | 4105 | 495.5741 | 989.1336 | 988.5243 | 0.6093 | 1 | 4 | 54 | 7 | K.WAATRWAK.K |
|  | 5002 | **530.2469** | **1058.4792** | **1058.4855** | **-0.0063** | **1** | **18** | **2.7** | **1** | **K.MTDFDRFK.V** |
|  | 7313 | **608.8092** | **1215.6038** | **1214.6078** | **0.9961** | **0** | **27** | **0.3** | **1** | **R.ALVDGPCTQVR.R** |
|  | 7657 | **617.3130** | **1232.6115** | **1231.6350** | **0.9766** | **0** | **3** | **70** | **2** | **R.VAYVSFGPHAGK.L** |
|  | 7958 | **624.1220** | **1246.2295** | **1245.6466** | **0.5829** | **1** | **3** | **55** | **3** | **K.ADINTKWAATR.W** |
|  | 10014 | **677.8849** | **1353.7552** | **1353.7616** | **-0.0064** | **0** | **60** | **0.00014** | **1** | **K.LVAIVDVIDQNR.A** |

| **475.** | Q9UGT4\|SUSD2_HUMAN    **Mass:** 91746    **Score:** 63     **Queries matched:** 9   **emPAI:** 0.13 |
| --- | --- |
|  | Sushi domain-containing protein 2 OS=Homo sapiens GN=SUSD2 PE=1 SV=1 |

|  | **Query** | **Observed** | **Mr(expt)** | **Mr(calc)** | **Delta** | **Miss** | **Score** | **Expect** | **Rank** | **Peptide** |
| --- | --- | --- | --- | --- | --- | --- | --- | --- | --- | --- |
|  | 1243 | **760.4258** | **759.4186** | **759.4239** | **-0.0053** | **0** | **9** | **31** | **2** | **R.SVQASLR.Y** |
|  | 1510 | **401.7172** | **801.4198** | **801.4245** | **-0.0047** | **0** | **10** | **20** | **3** | **R.NYRPPR.L** |
|  | 7703 | **412.7310** | **1235.1713** | **1235.6510** | **-0.4796** | **1** | **8** | **17** | **4** | **R.IIDSKNYAGQK.D** |
|  | 13445 | **521.2253** | **1560.6540** | **1561.8351** | **-1.1811** | **0** | **3** | **62** | **5** | **R.GEYVLLEAALTDLR.V** |
|  | 15399 | **422.2390** | **1684.9271** | **1683.7807** | **1.1463** | **1** | **8** | **19** | **3** | **K.VSMMEKSELVNETR.W** |
|  | 19304 | **1001.0096** | **2000.0047** | **2000.0175** | **-0.0127** | **0** | **11** | **8.4** | **1** | **R.GTGLTAVAVQEGNSDVVEVR.L** |
|  | 24814 | **914.4498** | **2740.3277** | **2739.3392** | **0.9885** | **0** | **32** | **0.039** | **1** | **K.HDPTFEPLFPSETTLNPSLAQEAAK.L** |
|  | 27137 | 805.1450 | 3216.5507 | 3215.5670 | 0.9838 | 1 | 22 | 0.29 | 1 | R.FKDSIQTLGHVDSSGQVHCVSPLLYESGR.I |
|  | 30057 | **1044.1998** | **4172.7702** | **4172.7978** | **-0.0276** | **1** | **41** | **0.0015** | **1** | **R.YGSGQQCCYTADGTQLLTADSSGGSTPDRGHDWGAPPFR.T** |

| **476.** | Q8TC12\|RDH11_HUMAN    **Mass:** 35763    **Score:** 63     **Queries matched:** 8   **emPAI:** 0.22 |
| --- | --- |
|  | Retinol dehydrogenase 11 OS=Homo sapiens GN=RDH11 PE=1 SV=2 |

|  | **Query** | **Observed** | **Mr(expt)** | **Mr(calc)** | **Delta** | **Miss** | **Score** | **Expect** | **Rank** | **Peptide** |
| --- | --- | --- | --- | --- | --- | --- | --- | --- | --- | --- |
|  | 3193 | **460.7569** | **919.4992** | **918.5022** | **0.9970** | **1** | **31** | **0.15** | **1** | **R.KLDLSDTK.S** 3166 3176 |
|  | 7290 | **608.3370** | **1214.6594** | **1213.7031** | **0.9563** | **0** | **60** | **0.00014** | **1** | **K.VVVVTGANTGIGK.E** |
|  | 7441 | **408.2505** | **1221.7296** | **1221.6254** | **0.1042** | **0** | **11** | **13** | **2** | **R.IHFHNLQGEK.F** |
|  | 10863 | **468.5772** | **1402.7098** | **1401.7841** | **0.9257** | **0** | **8** | **21** | **2** | **R.IVNVSSLAHHLGR.I** |
|  | 13868 | **793.9243** | **1585.8341** | **1585.8424** | **-0.0083** | **0** | **11** | **9.9** | **1** | **K.EIQTTTGNQQVLVR.K** |
|  | 14297 | 808.4091 | 1614.8037 | 1614.7964 | 0.0073 | 0 | 0 | 1.2e+002 | 10 | R.LWDVSCDLLGLPID.- |

| **477.** | P00338\|LDHA_HUMAN    **Mass:** 36950    **Score:** 63     **Queries matched:** 13   **emPAI:** 0.10 |
| --- | --- |
|  | L-lactate dehydrogenase A chain OS=Homo sapiens GN=LDHA PE=1 SV=2 |

|  | **Query** | **Observed** | **Mr(expt)** | **Mr(calc)** | **Delta** | **Miss** | **Score** | **Expect** | **Rank** | **Peptide** |
| --- | --- | --- | --- | --- | --- | --- | --- | --- | --- | --- |
|  | 3079 | 457.2924 | 912.5703 | 912.5756 | -0.0053 | 0 | 11 | 11 | 4 | K.LVIITAGAR.Q |
|  | 3305 | **465.2400** | **928.4654** | **928.5746** | **-0.1092** | **0** | **13** | **8.3** | **2** | **K.FIIPNVVK.Y** |
|  | 3877 | **487.7476** | **973.4807** | **972.4512** | **1.0295** | **0** | **23** | **0.87** | **1** | **K.EEQTPQNK.I** |
|  | 5996 | **568.3052** | **1134.5959** | **1133.5564** | **1.0395** | **0** | **9** | **19** | **5** | **K.VTLTSEEEAR.L** |
|  | 6414 | **584.9363** | **1167.8581** | **1166.6594** | **1.1987** | **1** | **(5)** | **36** | **2** | **R.RVHPVSTMIK.G** |
|  | 6625 | **395.4230** | **1183.2471** | **1182.6543** | **0.5928** | **1** | **5** | **33** | **9** | **R.RVHPVSTMIK.G** |
|  | 7983 | 624.7997 | 1247.5849 | 1247.5928 | -0.0079 | 0 | 63 | 8.1e-005 | 1 | R.VIGSGCNLDSAR.F |
|  | 13282 | 517.7407 | 1550.2002 | 1550.7624 | -0.5622 | 1 | 7 | 17 | 3 | R.VIGSGCNLDSARFR.Y 13295 |
|  | 18139 | **633.7911** | **1898.3514** | **1898.0248** | **0.3266** | **1** | **4** | **28** | **3** | **K.DLADELALVDVIEDKLK.G** |
|  | 19219 | **997.0035** | **1991.9924** | **1990.9605** | **1.0319** | **1** | **1** | **77** | **2** | **K.GEMMDLQHGSLFLRTPK.I** |
|  | 20421 | 1056.5533 | 2111.0921 | 2111.0609 | 0.0313 | 0 | 0 | 80 | 9 | K.GYTSWAIGLSVADLAESIMK.N |
|  | 25898 | **987.1183** | **2958.3332** | **2959.5181** | **-1.1849** | **1** | **0** | **53** | **3** | **K.EEQTPQNKITVVGVGAVGMACAISILMK.D** |

| **478.** | P07864\|LDHC_HUMAN    **Mass:** 36630    **Score:** 63     **Queries matched:** 10   **emPAI:** 0.10 |
| --- | --- |
|  | L-lactate dehydrogenase C chain OS=Homo sapiens GN=LDHC PE=2 SV=4 |

|  | **Query** | **Observed** | **Mr(expt)** | **Mr(calc)** | **Delta** | **Miss** | **Score** | **Expect** | **Rank** | **Peptide** |
| --- | --- | --- | --- | --- | --- | --- | --- | --- | --- | --- |
|  | 1217 | **734.3818** | **733.3745** | **734.3447** | **-0.9702** | **0** | **7** | **46** | **3** | **K.LGTDSDK.E** |
|  | 6414 | 584.9363 | 1167.8581 | 1168.6387 | -0.7805 | 1 | 3 | 50 | 4 | R.RVHPVSTMVK.G |
|  | 7983 | 624.7997 | 1247.5849 | 1247.5928 | -0.0079 | 0 | 63 | 8.1e-005 | 1 | R.VIGSGCNLDSAR.F |
|  | 9125 | **653.3255** | **1304.6364** | **1304.7010** | **-0.0645** | **1** | **3** | **63** | **8** | **-.MSTVKEQLIEK.L** |
|  | 10330 | **458.3051** | **1371.8934** | **1370.8068** | **1.0866** | **1** | **7** | **24** | **3** | **R.LALVQRNVAIMK.S** |
|  | 13282 | 517.7407 | 1550.2002 | 1550.7624 | -0.5622 | 1 | 7 | 17 | 3 | R.VIGSGCNLDSARFR.Y 13295 |
|  | 20958 | **724.5850** | **2170.7331** | **2171.0391** | **-0.3061** | **1** | **2** | **37** | **1** | **K.LKGEMMDLQHGSLFFSTSK.I** |
|  | 23472 | **837.4308** | **2509.2707** | **2508.3410** | **0.9297** | **1** | **4** | **28** | **1** | **K.GYTSWAIGLSVMDLVGSILKNLR.R** |
|  | 27594 | **1112.3098** | **3333.9076** | **3334.6822** | **-0.7746** | **1** | **1** | **27** | **4** | **K.LIEDDENSQCKITIVGTGAVGMACAISILLK.D** |

| **479.** | Q6ZMR3\|LDH6A_HUMAN    **Mass:** 36826    **Score:** 63     **Queries matched:** 8   **emPAI:** 0.10 |
| --- | --- |
|  | L-lactate dehydrogenase A-like 6A OS=Homo sapiens GN=LDHAL6A PE=2 SV=1 |

|  | **Query** | **Observed** | **Mr(expt)** | **Mr(calc)** | **Delta** | **Miss** | **Score** | **Expect** | **Rank** | **Peptide** |
| --- | --- | --- | --- | --- | --- | --- | --- | --- | --- | --- |
|  | 2566 | **438.7555** | **875.4964** | **874.4582** | **1.0381** | **0** | **2** | **1.1e+002** | **2** | **K.MPNIVSSK.D** |
|  | 7983 | 624.7997 | 1247.5849 | 1247.5928 | -0.0079 | 0 | 63 | 8.1e-005 | 1 | R.VIGSGCNLDSAR.F |
|  | 9626 | **667.1722** | **1332.3299** | **1332.6595** | **-0.3296** | **0** | **0** | **99** | **5** | **K.LTLEEEACLQK.S** |
|  | 13282 | 517.7407 | 1550.2002 | 1550.7624 | -0.5622 | 1 | 7 | 17 | 3 | R.VIGSGCNLDSARFR.Y 13295 |
|  | 13980 | **532.3011** | **1593.8814** | **1592.6963** | **1.1851** | **0** | **7** | **24** | **5** | **K.GETMDLQHGSPFMK.M** 13978 |
|  | 24921 | **921.5041** | **2761.4904** | **2762.4636** | **-0.9732** | **1** | **1** | **47** | **1** | **K.MPNIVSSKDYLVTANSNLVIITAGAR.Q** |

| **480.** | P30481\|1B44_HUMAN    **Mass:** 40798    **Score:** 62     **Queries matched:** 9   **emPAI:** 0.19 |
| --- | --- |
|  | HLA class I histocompatibility antigen, B-44 alpha chain OS=Homo sapiens GN=HLA-B PE=1 SV=1 |

|  | **Query** | **Observed** | **Mr(expt)** | **Mr(calc)** | **Delta** | **Miss** | **Score** | **Expect** | **Rank** | **Peptide** |
| --- | --- | --- | --- | --- | --- | --- | --- | --- | --- | --- |
|  | 3854 | 487.2572 | 972.4999 | 971.5512 | 0.9487 | 1 | 11 | 14 | 5 | R.ENLRTALR.Y |
|  | 7513 | 613.7462 | 1225.4779 | 1225.4856 | -0.0077 | 0 | 54 | 0.00053 | 1 | R.MYGCDVGPDGR.L 7482 |
|  | 9948 | 676.3410 | 1350.6674 | 1349.6939 | 0.9736 | 1 | 14 | 5.9 | 1 | R.YLENGKETLQR.A 9957 |
|  | 14460 | 406.9821 | 1623.8995 | 1623.7498 | 0.1497 | 1 | 4 | 43 | 10 | R.MYGCDVGPDGRLLR.G |
|  | 20385 | **527.7902** | **2107.1315** | **2107.0368** | **0.0948** | **1** | **4** | **37** | **9** | **R.VAEQDRAYLEGLCVESLR.R** |
|  | 23671 | 509.4508 | 2542.2177 | 2541.3414 | 0.8763 | 0 | 2 | 40 | 10 | R.TLLLLLWGAVALTETWAGSHSMR.Y |
|  | 24792 | 912.7573 | 2735.2500 | 2735.2635 | -0.0135 | 1 | 34 | 0.023 | 1 | R.DGEDQTQDTELVETRPAGDRTFQK.W |

| **481.** | P18124\|RL7_HUMAN    **Mass:** 29264    **Score:** 62     **Queries matched:** 12   **emPAI:** 0.28 |
| --- | --- |
|  | 60S ribosomal protein L7 OS=Homo sapiens GN=RPL7 PE=1 SV=1 |

|  | **Query** | **Observed** | **Mr(expt)** | **Mr(calc)** | **Delta** | **Miss** | **Score** | **Expect** | **Rank** | **Peptide** |
| --- | --- | --- | --- | --- | --- | --- | --- | --- | --- | --- |
|  | 826 | **558.3550** | **557.3477** | **558.3125** | **-0.9648** | **0** | **9** | **25** | **4** | **K.LSSPR.G** |
|  | 3758 | **483.2661** | **964.5177** | **964.5229** | **-0.0052** | **0** | **22** | **1** | **1** | **K.SVNELIYK.R** |
|  | 5476 | **548.2824** | **1094.5503** | **1095.5495** | **-0.9992** | **1** | **5** | **45** | **9** | **R.QMYRTEIR.M** |
|  | 6493 | **588.0930** | **1174.1714** | **1174.6492** | **-0.4778** | **1** | **2** | **65** | **6** | **K.LNKASINMLR.I** |
|  | 6824 | **597.3011** | **1192.5876** | **1191.5924** | **0.9952** | **0** | **1** | **1.1e+002** | **4** | **K.AGNFYVPAEPK.L** |
|  | 12284 | **496.6555** | **1486.9447** | **1487.7117** | **-0.7670** | **1** | **4** | **41** | **10** | **K.KTTHFVEGGDAGNR.E** |
|  | 13115 | **771.3987** | **1540.7829** | **1539.7874** | **0.9955** | **1** | **42** | **0.0089** | **1** | **R.FKEANNFLWPFK.L** |
|  | 15048 | 831.9439 | 1661.8733 | 1661.8817 | -0.0084 | 0 | 51 | 0.00087 | 1 | R.IVEPYIAWGYPNLK.S |
|  | 18054 | **473.4943** | **1889.9479** | **1891.0356** | **-1.0876** | **1** | **4** | **38** | **2** | **K.AGNFYVPAEPKLAFVIR.I** |
|  | 20457 | 529.7466 | 2114.9575 | 2114.9730 | -0.0155 | 1 | 15 | 2.9 | 1 | K.TTHFVEGGDAGNREDQINR.L 20458 |
|  | 23191 | **1233.0930** | **2464.1715** | **2463.2984** | **0.8731** | **1** | **1** | **52** | **2** | **K.ASINMLRIVEPYIAWGYPNLK.S** |

| **482.** | P28799\|GRN_HUMAN    **Mass:** 68518    **Score:** 62     **Queries matched:** 8   **emPAI:** 0.05 |
| --- | --- |
|  | Granulins OS=Homo sapiens GN=GRN PE=1 SV=2 |

|  | **Query** | **Observed** | **Mr(expt)** | **Mr(calc)** | **Delta** | **Miss** | **Score** | **Expect** | **Rank** | **Peptide** |
| --- | --- | --- | --- | --- | --- | --- | --- | --- | --- | --- |
|  | 907 | **585.0508** | **584.0435** | **583.3442** | **0.6993** | **0** | **1** | **56** | **4** | **K.LPAQR.T** |
|  | 11196 | 712.4131 | 1422.8116 | 1422.7653 | 0.0463 | 1 | 3 | 66 | 7 | R.CITPTGTHPLAKK.L |
|  | 11727 | **486.2601** | **1455.7586** | **1456.7708** | **-1.0122** | **1** | **16** | **3.1** | **1** | **R.GSEIVAGLEKMPAR.R** 11769 |
|  | 11873 | **488.7898** | **1463.3477** | **1462.6908** | **0.6568** | **0** | **5** | **27** | **3** | **R.AVALSSSVMCPDAR.S** |
|  | 12016 | **491.6006** | **1471.7799** | **1472.7657** | **-0.9858** | **1** | **(7)** | **27** | **2** | **R.GSEIVAGLEKMPAR.R** |
|  | 16991 | **905.8740** | **1809.7334** | **1809.7444** | **-0.0110** | **1** | **46** | **0.0025** | **1** | **R.SRCPDGSTCCELPSGK.Y** 16992 |

| **483.** | O14548\|COX7R_HUMAN    **Mass:** 12664    **Score:** 62     **Queries matched:** 4   **emPAI:** 0.31 |
| --- | --- |
|  | Cytochrome c oxidase subunit 7A-related protein, mitochondrial OS=Homo sapiens GN=COX7A2L PE=1 SV=2 |

|  | **Query** | **Observed** | **Mr(expt)** | **Mr(calc)** | **Delta** | **Miss** | **Score** | **Expect** | **Rank** | **Peptide** |
| --- | --- | --- | --- | --- | --- | --- | --- | --- | --- | --- |
|  | 3632 | **478.7807** | **955.5468** | **954.5498** | **0.9970** | **1** | **10** | **17** | **1** | **K.NKVPELQK.F** 3620 |
|  | 11112 | **710.3320** | **1418.6494** | **1418.6565** | **-0.0072** | **0** | **62** | **8.3e-005** | **1** | **K.LTSDSTVYDYAGK.N** |
|  | 12665 | **504.9180** | **1511.7323** | **1510.8184** | **0.9139** | **1** | **0** | **1.1e+002** | **6** | **K.FFQKADGVPVYLK.R** |

| **484.** | P11498\|PYC_HUMAN    **Mass:** 130293   **Score:** 62     **Queries matched:** 50   **emPAI:** 0.03 |
| --- | --- |
|  | Pyruvate carboxylase, mitochondrial OS=Homo sapiens GN=PC PE=1 SV=2 |

|  | **Query** | **Observed** | **Mr(expt)** | **Mr(calc)** | **Delta** | **Miss** | **Score** | **Expect** | **Rank** | **Peptide** |
| --- | --- | --- | --- | --- | --- | --- | --- | --- | --- | --- |
|  | 834 | **560.2997** | **559.2925** | **558.3741** | **0.9184** | **0** | **21** | **2.5** | **1** | **R.SILVK.D** |
|  | 1567 | **403.7051** | **805.3957** | **805.3752** | **0.0204** | **0** | **18** | **3.2** | **1** | **R.SGEGMGIR.L** 1584 |
|  | 1917 | **417.2434** | **832.4723** | **832.4589** | **0.0134** | **1** | **8** | **34** | **4** | **K.KVMVANR.G** |
|  | 2044 | **421.0566** | **840.0986** | **839.5229** | **0.5757** | **1** | **8** | **14** | **2** | **K.VLKDLPR.V** 2013 2040 |
|  | 2369 | 432.2584 | 862.5022 | 861.3981 | 1.1041 | 0 | 8 | 35 | 3 | R.EGPEGFAR.A |
|  | 3187 | **460.7259** | **919.4372** | **918.4593** | **0.9779** | **0** | **12** | **11** | **1** | **R.ACTELGIR.T** |
|  | 3199 | **460.8787** | **919.7428** | **920.4386** | **-0.6958** | **1** | **1** | **99** | **6** | **K.MGDKVEAR.A** |
|  | 3435 | 470.7423 | 939.4701 | 940.4225 | -0.9524 | 0 | 8 | 24 | 6 | K.EMHFHPK.A |
|  | 3598 | 477.7309 | 953.4472 | 954.4957 | -1.0485 | 0 | 8 | 21 | 2 | K.GQIGAPMPGK.V |
|  | 4015 | 492.7145 | 983.4144 | 982.4178 | 0.9966 | 0 | 4 | 59 | 4 | K.ENGMDVFR.V |
|  | 4854 | **523.8007** | **1045.5868** | **1045.6284** | **-0.0416** | **1** | **10** | **20** | **3** | **R.RLEYKPIK.K** |
|  | 5560 | **551.2899** | **1100.5652** | **1099.6026** | **0.9626** | **0** | **11** | **15** | **4** | **R.FIGPSPEVVR.K** 5559 |
|  | 5717 | **556.7854** | **1111.5562** | **1111.6965** | **-0.1402** | **1** | **3** | **76** | **2** | **K.VIDIKVVAGAK.V** |
|  | 9240 | **657.3262** | **1312.6378** | **1312.6809** | **-0.0431** | **1** | **3** | **69** | **5** | **K.DVKGQIGAPMPGK.V** |
|  | 9503 | 663.8531 | 1325.6916 | 1326.7004 | -1.0088 | 1 | 7 | 30 | 4 | R.RTSTAPAASPNVR.R 9501 9504 |
|  | 11896 | **489.2635** | **1464.7686** | **1465.7347** | **-0.9662** | **1** | **6** | **28** | **3** | **R.IEVFRSGEGMGIR.L** 11894 11928 |
|  | 12681 | **757.3455** | **1512.6764** | **1513.7711** | **-1.0948** | **0** | **3** | **60** | **7** | **R.NHPGLLLMDTTFR.D** |
|  | 12857 | **508.7952** | **1523.3638** | **1522.8541** | **0.5096** | **1** | **3** | **40** | **10** | **K.GQIGAPMPGKVIDIK.V** |
|  | 13219 | **516.3118** | **1545.9135** | **1546.7263** | **-0.8128** | **0** | **6** | **30** | **8** | **R.AEAEAQAEELSFPR.S** 13216 13217 13237 |
|  | 13295 | 517.9327 | 1550.7764 | 1550.7841 | -0.0078 | 1 | 3 | 67 | 6 | R.SFQPDTGRIEVFR.S |
|  | 13496 | 782.9016 | 1563.7887 | 1563.7966 | -0.0080 | 0 | 10 | 13 | 2 | K.AYVEANQMLGDLIK.V |
|  | 14077 | **534.3447** | **1600.0122** | **1598.8854** | **1.1268** | **0** | **4** | **41** | **5** | **R.ELIPNIPFQMLLR.G** |
|  | 15327 | **840.9034** | **1679.7923** | **1679.8222** | **-0.0299** | **1** | **5** | **35** | **3** | **K.METVVTSPMEGTVRK.V** |
|  | 16079 | 579.3202 | 1734.9389 | 1734.7995 | 0.1394 | 0 | 2 | 63 | 3 | R.TVAIYSEQDTGQMHR.Q |
|  | 16283 | 584.6289 | 1750.8649 | 1750.7944 | 0.0705 | 0 | (1) | 78 | 6 | R.TVAIYSEQDTGQMHR.Q |
|  | 16437 | **588.3950** | **1762.1632** | **1761.9274** | **0.2358** | **1** | **3** | **41** | **1** | **R.AGQRQVFFELNGQLR.S** |
|  | 17644 | **465.0237** | **1856.0657** | **1855.9727** | **0.0930** | **1** | **3** | **46** | **8** | **R.AVRNHPGLLLMDTTFR.D** |
|  | 17655 | **619.9091** | **1856.7055** | **1856.9308** | **-0.2253** | **0** | **4** | **31** | **2** | **R.AYSEALAAFGNGALFVEK.F** |
|  | 20107 | 694.0248 | 2079.0527 | 2079.0526 | 0.0000 | 0 | 0 | 87 | 5 | K.DMAGLLKPTACTMLVSSLR.D |
|  | 20248 | **699.0812** | **2094.2219** | **2095.0476** | **-0.8257** | **0** | **(0)** | **74** | **6** | **K.DMAGLLKPTACTMLVSSLR.D** |
|  | 20502 | **707.3682** | **2119.0827** | **2118.0494** | **1.0332** | **1** | **1** | **63** | **5** | **K.QVGYENAGTVEFLVDRHGK.H** |
|  | 21204 | **734.3807** | **2200.1204** | **2201.0865** | **-0.9661** | **1** | **3** | **43** | **2** | **K.VAKENNVDAVHPGYGFLSER.A** 21205 |
|  | 22359 | 782.0448 | 2343.1126 | 2344.2063 | -1.0937 | 0 | 3 | 40 | 2 | R.LDNASAFQGAVISPHYDSLLVK.V |
|  | 22401 | **588.2850** | **2349.1108** | **2350.1807** | **-1.0699** | **1** | **2** | **42** | **2** | **K.DMAGLLKPTACTMLVSSLRDR.F** |
|  | 22958 | **811.4139** | **2431.2198** | **2431.2325** | **-0.0127** | **0** | **62** | **4.6e-005** | **1** | **R.SVVEFLQGYIGVPHGGFPEPFR.S** 22959 |
|  | 27447 | **826.3690** | **3301.4467** | **3300.5761** | **0.8706** | **1** | **6** | **11** | **3** | **K.ELVDRHGEEVTPEDVLSAAMYPDVFAHFK.D** |
|  | 30980 | **960.4316** | **4797.1215** | **4798.1374** | **-1.0159** | **1** | **0** | **15** | **7** | **R.GLYAAFDCTATMKSGNSDVYENEIPGGQYTNLHFQAHSMGLGSK.F** |

| **485.** | P80294\|MT1H_HUMAN    **Mass:** 7175     **Score:** 62     **Queries matched:** 2   **emPAI:** 0.58 |
| --- | --- |
|  | Metallothionein-1H OS=Homo sapiens GN=MT1H PE=1 SV=1 |

|  | **Query** | **Observed** | **Mr(expt)** | **Mr(calc)** | **Delta** | **Miss** | **Score** | **Expect** | **Rank** | **Peptide** |
| --- | --- | --- | --- | --- | --- | --- | --- | --- | --- | --- |
|  | 11778 | 730.2618 | 1458.5091 | 1458.5183 | -0.0091 | 0 | 62 | 6.9e-005 | 1 | K.SCCSCCPLGCAK.C |
|  | 13860 | 529.5776 | 1585.7111 | 1586.6132 | -0.9021 | 1 | 1 | 88 | 10 | K.KSCCSCCPLGCAK.C |

| **486.** | Q96S66\|CLCC1_HUMAN    **Mass:** 62667    **Score:** 62     **Queries matched:** 3   **emPAI:** 0.06 |
| --- | --- |
|  | Chloride channel CLIC-like protein 1 OS=Homo sapiens GN=CLCC1 PE=1 SV=1 |

|  | **Query** | **Observed** | **Mr(expt)** | **Mr(calc)** | **Delta** | **Miss** | **Score** | **Expect** | **Rank** | **Peptide** |
| --- | --- | --- | --- | --- | --- | --- | --- | --- | --- | --- |
|  | 3873 | 487.3027 | 972.5909 | 972.5491 | 0.0418 | 0 | 13 | 9.4 | 2 | R.ETLLEIQK.F |
|  | 9385 | **660.6646** | **1319.3147** | **1318.6373** | **0.6774** | **1** | **2** | **67** | **7** | **K.MEPLNNVCAKK.M** |
|  | 10156 | **682.3138** | **1362.6130** | **1362.6238** | **-0.0108** | **0** | **62** | **9.1e-005** | **1** | **R.GQMGPTEQGPYAK.T** |

| **487.** | Q9Y5L4\|TIM13_HUMAN    **Mass:** 10721    **Score:** 61     **Queries matched:** 8   **emPAI:** 0.37 |
| --- | --- |
|  | Mitochondrial import inner membrane translocase subunit Tim13 OS=Homo sapiens GN=TIMM13 PE=1 SV=1 |

|  | **Query** | **Observed** | **Mr(expt)** | **Mr(calc)** | **Delta** | **Miss** | **Score** | **Expect** | **Rank** | **Peptide** |
| --- | --- | --- | --- | --- | --- | --- | --- | --- | --- | --- |
|  | 8160 | **629.3576** | **1256.7006** | **1257.6639** | **-0.9632** | **0** | **7** | **30** | **9** | **K.LDPGLIMEQVK.V** |
|  | 13325 | **776.9394** | **1551.8643** | **1551.8733** | **-0.0089** | **0** | **60** | **0.00013** | **1** | **K.VQIAVANAQELLQR.M** 13317 13326 |
|  | 13900 | **530.5879** | **1588.7418** | **1588.7515** | **-0.0097** | **0** | **15** | **4.1** | **2** | **K.CIGKPGGSLDNSEQK.C** |
|  | 19590 | **1014.4808** | **2026.9471** | **2027.0833** | **-0.1362** | **1** | **0** | **83** | **3** | **K.VQIAVANAQELLQRMTDK.C** |
|  | 22008 | **766.0745** | **2295.2017** | **2294.9363** | **0.2654** | **1** | **1** | **54** | **5** | **K.CIAMCMDRYMDAWNTVSR.A** |
|  | 24318 | **886.8086** | **2657.4039** | **2658.1328** | **-0.7289** | **1** | **1** | **46** | **2** | **K.CIGKPGGSLDNSEQKCIAMCMDR.Y** |

| **488.** | P08865\|RSSA_HUMAN    **Mass:** 32947    **Score:** 61     **Queries matched:** 5   **emPAI:** 0.11 |
| --- | --- |
|  | 40S ribosomal protein SA OS=Homo sapiens GN=RPSA PE=1 SV=4 |

|  | **Query** | **Observed** | **Mr(expt)** | **Mr(calc)** | **Delta** | **Miss** | **Score** | **Expect** | **Rank** | **Peptide** |
| --- | --- | --- | --- | --- | --- | --- | --- | --- | --- | --- |
|  | 8519 | **425.5763** | **1273.7072** | **1273.6514** | **0.0559** | **1** | **7** | **30** | **4** | **K.EEQAAAEKAVTK.E** |
|  | 16755 | 597.2971 | 1788.8694 | 1789.9131 | -1.0438 | 1 | 2 | 67 | 6 | M.SGALDVLQMKEEDVLK.F |
|  | 20549 | **710.0431** | **2127.1076** | **2127.0870** | **0.0206** | **1** | **0** | **79** | **10** | **K.GAHSVGLMWWMLAREVLR.M** |
|  | 22629 | **595.5656** | **2378.2333** | **2377.3692** | **0.8641** | **1** | **4** | **30** | **1** | **K.LLLAARAIVAIENPADVSVISSR.N** |
|  | 26061 | 999.4920 | 2995.4542 | 2995.4709 | -0.0167 | 0 | 61 | 4e-005 | 1 | R.ADHQPLTEASYVNLPTIALCNTDSPLR.Y |

| **489.** | P99999\|CYC_HUMAN    **Mass:** 11855    **Score:** 61     **Queries matched:** 5   **emPAI:** 0.78 |
| --- | --- |
|  | Cytochrome c OS=Homo sapiens GN=CYCS PE=1 SV=2 |

|  | **Query** | **Observed** | **Mr(expt)** | **Mr(calc)** | **Delta** | **Miss** | **Score** | **Expect** | **Rank** | **Peptide** |
| --- | --- | --- | --- | --- | --- | --- | --- | --- | --- | --- |
|  | 1396 | **397.8583** | **793.7021** | **794.4724** | **-0.7703** | **1** | **5** | **37** | **4** | **K.KIFIMK.C** |
|  | 11264 | 714.8367 | 1427.6588 | 1427.6681 | -0.0093 | 0 | 52 | 0.00079 | 1 | K.TGQAPGYSYTAANK.N |
|  | 13373 | **519.5913** | **1555.7521** | **1555.7630** | **-0.0109** | **1** | **33** | **0.056** | **1** | **R.KTGQAPGYSYTAANK.N** |
|  | 19543 | **675.3143** | **2022.9212** | **2022.9608** | **-0.0397** | **0** | **2** | **55** | **6** | **K.GIIWGEDTLMEYLENPK.K** |
|  | 21775 | 755.7098 | 2264.1075 | 2265.0987 | -0.9912 | 1 | 1 | 57 | 4 | K.NKGIIWGEDTLMEYLENPK.K |

| **490.** | P51153\|RAB13_HUMAN    **Mass:** 22988    **Score:** 61     **Queries matched:** 18   **emPAI:** 0.36 |
| --- | --- |
|  | Ras-related protein Rab-13 OS=Homo sapiens GN=RAB13 PE=1 SV=1 |

|  | **Query** | **Observed** | **Mr(expt)** | **Mr(calc)** | **Delta** | **Miss** | **Score** | **Expect** | **Rank** | **Peptide** |
| --- | --- | --- | --- | --- | --- | --- | --- | --- | --- | --- |
|  | 1863 | 830.4077 | 829.4004 | 828.4018 | 0.9987 | 0 | 24 | 0.87 | 1 | R.FFETSAK.S |
|  | 2746 | **445.1187** | **888.2227** | **888.4916** | **-0.2689** | **1** | **7** | **30** | **3** | **R.TVDIEGKK.I** 2760 |
|  | 3360 | **467.2221** | **932.4297** | **931.4359** | **0.9938** | **0** | **15** | **6.1** | **1** | **K.ENASAGVER.L** 3350 3352 3363 3364 |
|  | 3512 | **473.7821** | **945.5496** | **944.4927** | **1.0570** | **1** | **7** | **37** | **7** | **K.VQKEQADK.L** |
|  | 5171 | 536.3210 | 1070.6274 | 1070.6336 | -0.0062 | 0 | 21 | 1.3 | 1 | K.LLLIGDSGVGK.T |
|  | 7435 | **408.2275** | **1221.6607** | **1222.6168** | **-0.9561** | **1** | **3** | **84** | **8** | **-.MAKAYDHLFK.L** |
|  | 8218 | **630.8267** | **1259.6389** | **1259.6469** | **-0.0081** | **1** | **37** | **0.033** | **1** | **K.SIKENASAGVER.L** 8237 8238 |
|  | 12681 | 757.3455 | 1512.6764 | 1512.6878 | -0.0115 | 0 | 49 | 0.0014 | 1 | K.SSMNVDEAFSSLAR.D |
|  | 13177 | 772.9123 | 1543.8101 | 1542.8154 | 0.9947 | 1 | 9 | 17 | 9 | K.IKLQVWDTAGQER.F 13137 |
|  | 24084 | 652.8301 | 2607.2912 | 2606.3302 | 0.9610 | 1 | 1 | 54 | 8 | K.TITTAYYRGAMGIILVYDITDEK.S |

| **491.** | O43181\|NDUS4_HUMAN    **Mass:** 20095    **Score:** 61     **Queries matched:** 7   **emPAI:** 0.42 |
| --- | --- |
|  | NADH dehydrogenase [ubiquinone] iron-sulfur protein 4, mitochondrial OS=Homo sapiens GN=NDUFS4 PE=1 SV=1 |

|  | **Query** | **Observed** | **Mr(expt)** | **Mr(calc)** | **Delta** | **Miss** | **Score** | **Expect** | **Rank** | **Peptide** |
| --- | --- | --- | --- | --- | --- | --- | --- | --- | --- | --- |
|  | 1448 | **399.7148** | **797.4150** | **797.3378** | **0.0772** | **0** | **5** | **47** | **1** | **K.MEFDTR.E** |
|  | 2311 | **430.7571** | **859.4997** | **858.4824** | **1.0173** | **1** | **9** | **28** | **4** | **R.QTLWRR.R** |
|  | 7110 | **603.7752** | **1205.5359** | **1205.5459** | **-0.0100** | **0** | **21** | **1.4** | **1** | **R.NNMQSGVNNTK.K** |
|  | 8412 | **635.2696** | **1268.5246** | **1267.5469** | **0.9777** | **0** | **16** | **3.3** | **2** | **K.NGWSYDIEER.K** |
|  | 9666 | **668.3142** | **1334.6137** | **1333.6408** | **0.9729** | **1** | **5** | **46** | **5** | **R.NNMQSGVNNTKK.W** 9644 |
|  | 29108 | **1258.2634** | **3771.7685** | **3771.7800** | **-0.0116** | **1** | **61** | **2.8e-005** | **1** | **R.WENPLMGWASTADPLSNMVLTFSTKEDAVSFAEK.N** |

| **492.** | Q29960\|1C16_HUMAN    **Mass:** 41240    **Score:** 61     **Queries matched:** 9   **emPAI:** 0.30 |
| --- | --- |
|  | HLA class I histocompatibility antigen, Cw-16 alpha chain OS=Homo sapiens GN=HLA-C PE=2 SV=1 |

|  | **Query** | **Observed** | **Mr(expt)** | **Mr(calc)** | **Delta** | **Miss** | **Score** | **Expect** | **Rank** | **Peptide** |
| --- | --- | --- | --- | --- | --- | --- | --- | --- | --- | --- |
|  | 3749 | 483.2167 | 964.4188 | 964.4250 | -0.0062 | 0 | 35 | 0.057 | 1 | R.FDSDAASPR.G |
|  | 7868 | **622.3301** | **1242.6457** | **1242.6357** | **0.0101** | **0** | **1** | **1.3e+002** | **2** | **K.DYIALNEHLR.S** |
|  | 9276 | **438.9270** | **1313.7593** | **1314.6429** | **-0.8836** | **1** | **5** | **48** | **3** | **K.WEAARAAEQQR.A** |
|  | 9948 | 676.3410 | 1350.6674 | 1349.6939 | 0.9736 | 1 | 14 | 5.9 | 1 | R.YLENGKETLQR.A 9957 |
|  | 14417 | 811.9318 | 1621.8490 | 1620.7890 | 1.0600 | 1 | 1 | 1e+002 | 6 | R.SCTAADTAAQITQRK.W |
|  | 14538 | 815.4131 | 1628.8117 | 1628.8199 | -0.0081 | 0 | 28 | 0.17 | 1 | R.FIAVGYVDDTQFVR.F |
|  | 24215 | 879.7303 | 2636.1690 | 2636.1838 | -0.0148 | 0 | 43 | 0.003 | 1 | R.DGEDQTQDTELVETRPAGDGTFQK.W |
|  | 27706 | **1680.7860** | **3359.5575** | **3360.4677** | **-0.9102** | **1** | **1** | **30** | **9** | **R.NLRGYYNQSEAGSHTLQWMYGCDLGPDGR.L** |

| **493.** | Q95604\|1C17_HUMAN    **Mass:** 41612    **Score:** 61     **Queries matched:** 6   **emPAI:** 0.30 |
| --- | --- |
|  | HLA class I histocompatibility antigen, Cw-17 alpha chain OS=Homo sapiens GN=HLA-C PE=1 SV=1 |

|  | **Query** | **Observed** | **Mr(expt)** | **Mr(calc)** | **Delta** | **Miss** | **Score** | **Expect** | **Rank** | **Peptide** |
| --- | --- | --- | --- | --- | --- | --- | --- | --- | --- | --- |
|  | 3749 | 483.2167 | 964.4188 | 964.4250 | -0.0062 | 0 | 35 | 0.057 | 1 | R.FDSDAASPR.G |
|  | 11244 | 714.3414 | 1426.6683 | 1425.7365 | 0.9318 | 0 | 3 | 60 | 3 | K.WAAVVVPSGQEQR.Y |
|  | 14538 | 815.4131 | 1628.8117 | 1628.8199 | -0.0081 | 0 | 28 | 0.17 | 1 | R.FIAVGYVDDTQFVR.F |
|  | 14674 | 547.0314 | 1638.0725 | 1637.7654 | 0.3071 | 1 | 2 | 52 | 6 | R.MYGCDLGPDGRLLR.G |
|  | 17358 | 612.9700 | 1835.8883 | 1835.9027 | -0.0144 | 0 | 3 | 52 | 2 | K.THVTHHPVSDHEATLR.C |
|  | 24215 | 879.7303 | 2636.1690 | 2636.1838 | -0.0148 | 0 | 43 | 0.003 | 1 | R.DGEDQTQDTELVETRPAGDGTFQK.W |

| **494.** | P31639\|SC5A2_HUMAN    **Mass:** 73932    **Score:** 61     **Queries matched:** 16   **emPAI:** 0.05 |
| --- | --- |
|  | Sodium/glucose cotransporter 2 OS=Homo sapiens GN=SLC5A2 PE=1 SV=1 |

|  | **Query** | **Observed** | **Mr(expt)** | **Mr(calc)** | **Delta** | **Miss** | **Score** | **Expect** | **Rank** | **Peptide** |
| --- | --- | --- | --- | --- | --- | --- | --- | --- | --- | --- |
|  | 7523 | **409.5571** | **1225.6495** | **1226.6805** | **-1.0310** | **1** | **11** | **11** | **3** | **R.CLAGKSLTHIK.A** |
|  | 7787 | **413.8682** | **1238.5827** | **1238.7533** | **-0.1705** | **1** | **4** | **53** | **9** | **R.LVVKLMPNGLR.G** |
|  | 8555 | **639.3309** | **1276.6473** | **1276.7516** | **-0.1043** | **1** | **2** | **91** | **5** | **K.HLHRLVFSLR.H** |
|  | 11100 | **709.8554** | **1417.6963** | **1416.6521** | **1.0442** | **0** | **1** | **1.2e+002** | **5** | **R.LEDISEDPSWAR.V** |
|  | 11948 | 490.1571 | 1467.4496 | 1467.7583 | -0.3087 | 1 | 3 | 43 | 6 | R.TNRGTVGGYFLAGR.S |
|  | 13638 | 525.2925 | 1572.8556 | 1572.7532 | 0.1024 | 1 | 5 | 40 | 2 | R.RLEDISEDPSWAR.V |
|  | 15358 | 841.8800 | 1681.7454 | 1681.7552 | -0.0098 | 0 | 61 | 9e-005 | 1 | R.VCGTEVGCSNIAYPR.L 15357 |
|  | 15421 | **422.7207** | **1686.8538** | **1686.7155** | **0.1383** | **0** | **9** | **16** | **1** | **M.EEHTEAGSAPEMGAQK.A** |
|  | 16350 | **586.3228** | **1755.9464** | **1754.8592** | **1.0873** | **0** | **5** | **32** | **3** | **K.LTPMFLMVMPGMISR.I** |
|  | 16628 | **889.9501** | **1777.8857** | **1777.8959** | **-0.0102** | **0** | **15** | **3.4** | **1** | **R.GGVGSPPPLTQEEAAAAAR.R** |
|  | 28403 | **889.6727** | **3554.6618** | **3554.7897** | **-0.1278** | **0** | **3** | **20** | **2** | **R.GLMLAVMLAALMSSLASIFNSSSTLFTMDIYTR.L** 28404 |
|  | 28472 | **1786.9767** | **3571.9388** | **3570.7846** | **1.1542** | **0** | **(0)** | **30** | **7** | **R.GLMLAVMLAALMSSLASIFNSSSTLFTMDIYTR.L** |
|  | 28761 | **1828.1301** | **3654.2457** | **3654.7815** | **-0.5358** | **1** | **(0)** | **25** | **9** | **K.LTPMFLMVMPGMISRILYPDEVACVVPEVCR.R** |
|  | 28848 | **1844.4064** | **3686.7982** | **3686.7713** | **0.0269** | **1** | **1** | **28** | **4** | **K.LTPMFLMVMPGMISRILYPDEVACVVPEVCR.R** |

| **495.** | P09601\|HMOX1_HUMAN    **Mass:** 32798    **Score:** 61     **Queries matched:** 8   **emPAI:** 0.24 |
| --- | --- |
|  | Heme oxygenase 1 OS=Homo sapiens GN=HMOX1 PE=1 SV=1 |

|  | **Query** | **Observed** | **Mr(expt)** | **Mr(calc)** | **Delta** | **Miss** | **Score** | **Expect** | **Rank** | **Peptide** |
| --- | --- | --- | --- | --- | --- | --- | --- | --- | --- | --- |
|  | 687 | **513.0441** | **512.0368** | **512.3071** | **-0.2703** | **0** | **2** | **43** | **1** | **R.APGLR.Q** |
|  | 1430 | **399.2328** | **796.4510** | **796.4668** | **-0.0158** | **1** | **9** | **19** | **7** | **R.APGLRQR.A** |
|  | 3831 | 486.2934 | 970.5723 | 971.5400 | -0.9677 | 1 | 1 | 1.2e+002 | 3 | R.QRVIEEAK.T |
|  | 10437 | 689.8496 | 1377.6847 | 1376.7664 | 0.9183 | 1 | 27 | 0.3 | 1 | R.YLGDLSGGQVLKK.I |
|  | 14040 | **799.9054** | **1597.7962** | **1597.8060** | **-0.0097** | **1** | **58** | **0.0002** | **1** | **R.ASNKVQDSAPVETPR.G** 14039 |
|  | 14362 | **810.3817** | **1618.7487** | **1617.7973** | **0.9514** | **0** | **0** | **1.1e+002** | **5** | **R.WQEVIPYTPAMQR.Y** |
|  | 23499 | **839.4269** | **2515.2590** | **2516.1886** | **-0.9297** | **1** | **0** | **62** | **3** | **-.MERPQPDSMPQDLSEALKEATK.E** |

| **496.** | O15126\|SCAM1_HUMAN    **Mass:** 38295    **Score:** 60     **Queries matched:** 12   **emPAI:** 0.10 |
| --- | --- |
|  | Secretory carrier-associated membrane protein 1 OS=Homo sapiens GN=SCAMP1 PE=1 SV=2 |

|  | **Query** | **Observed** | **Mr(expt)** | **Mr(calc)** | **Delta** | **Miss** | **Score** | **Expect** | **Rank** | **Peptide** |
| --- | --- | --- | --- | --- | --- | --- | --- | --- | --- | --- |
|  | 1519 | **401.8859** | **801.7572** | **802.3821** | **-0.6248** | **0** | **8** | **25** | **8** | **R.QEELER.K** |
|  | 3676 | **480.2641** | **958.5136** | **958.4832** | **0.0304** | **1** | **17** | **3.6** | **6** | **K.RQEELER.K** |
|  | 9802 | **672.3393** | **1342.6640** | **1342.6412** | **0.0229** | **1** | **1** | **1.1e+002** | **3** | **R.EMQNLSQHGRK.N** |
|  | 10464 | **460.5882** | **1378.7428** | **1377.7728** | **0.9700** | **1** | **7** | **31** | **5** | **K.EHALAQAELLKR.Q** 10454 |
|  | 10967 | **705.8335** | **1409.6524** | **1409.6609** | **-0.0085** | **0** | **60** | **0.00013** | **1** | **K.AQQEFATGVMSNK.T** |
|  | 11217 | **475.9290** | **1424.7651** | **1425.6558** | **-0.8908** | **0** | **(4)** | **52** | **4** | **K.AQQEFATGVMSNK.T** 11225 |
|  | 12484 | **501.2422** | **1500.7047** | **1499.6899** | **1.0148** | **1** | **4** | **50** | **6** | **R.EREMQNLSQHGR.K** |
|  | 13527 | **783.9255** | **1565.8364** | **1564.7998** | **1.0366** | **1** | **5** | **36** | **4** | **K.VHGLYRTTGASFEK.A** |
|  | 28727 | 910.9581 | 3639.8032 | 3638.8589 | 0.9443 | 1 | 3 | 17 | 5 | R.TPPPGGVKMPNVPNTQPAIMKPTEEHPAYTQIAK.E 28728 |

| **497.** | P23229\|ITA6_HUMAN    **Mass:** 127751   **Score:** 60     **Queries matched:** 24   **emPAI:** 0.06 |
| --- | --- |
|  | Integrin alpha-6 OS=Homo sapiens GN=ITGA6 PE=1 SV=4 |

|  | **Query** | **Observed** | **Mr(expt)** | **Mr(calc)** | **Delta** | **Miss** | **Score** | **Expect** | **Rank** | **Peptide** |
| --- | --- | --- | --- | --- | --- | --- | --- | --- | --- | --- |
|  | 1082 | 658.3363 | 657.3290 | 657.2945 | 0.0345 | 0 | 9 | 26 | 1 | K.CGFFK.R |
|  | 2000 | 419.7763 | 837.5380 | 837.5436 | -0.0056 | 0 | 23 | 0.82 | 1 | R.LLLVGAPR.A 1978 1997 |
|  | 2737 | **444.7130** | **887.4114** | **887.4461** | **-0.0347** | **1** | **15** | **6.8** | **7** | **K.QIDDNRK.F** |
|  | 3292 | 464.7792 | 927.5439 | 927.5502 | -0.0063 | 1 | 4 | 69 | 9 | K.GIVRVEQK.N |
|  | 4086 | **495.0602** | **988.1059** | **988.5341** | **-0.4283** | **1** | **4** | **52** | **5** | **R.ELRAFPEK.Q** |
|  | 4946 | 528.2473 | 1054.4801 | 1053.6295 | 0.8506 | 0 | 9 | 22 | 2 | R.SRPVINIQK.T |
|  | 5660 | **554.3010** | **1106.5875** | **1106.5469** | **0.0406** | **1** | **5** | **56** | **10** | **K.QESRDIFGR.C** |
|  | 6223 | **576.9037** | **1151.7929** | **1151.5757** | **0.2172** | **0** | **1** | **96** | **9** | **R.CYVLSQNLR.I** |
|  | 7478 | **409.1558** | **1224.4455** | **1225.6163** | **-1.1708** | **1** | **4** | **55** | **7** | **R.QHVNTKQESR.D** |
|  | 8325 | **633.1579** | **1264.3012** | **1263.7411** | **0.5601** | **1** | **3** | **51** | **4** | **R.ANHSGAVVLLKR.D** |
|  | 8754 | **430.2140** | **1287.6201** | **1287.6493** | **-0.0291** | **1** | **2** | **86** | **5** | **K.GLEKVTCEPQK.E** |
|  | 8773 | **645.2981** | **1288.5816** | **1288.6524** | **-0.0707** | **1** | **3** | **79** | **10** | **R.VQFRNQGSEPK.Y** |
|  | 9236 | **657.3074** | **1312.6002** | **1312.6082** | **-0.0080** | **0** | **5** | **43** | **1** | **R.TGGLYSCDITAR.G** |
|  | 13085 | **514.0620** | **1539.1640** | **1538.8681** | **0.2959** | **1** | **3** | **48** | **2** | **R.WNNVKPIRLNGTK.D** |
|  | 15812 | 858.4589 | 1714.9033 | 1714.8737 | 0.0296 | 0 | 7 | 20 | 4 | K.LETTSNQDNLAPITAK.A 15813 |
|  | 17134 | **607.5637** | **1819.6692** | **1818.8207** | **0.8485** | **0** | **2** | **64** | **6** | **K.EDQWMGVTVQSQGPGGK.V** |
|  | 17676 | 620.3389 | 1857.9950 | 1857.8316 | 0.1634 | 0 | 6 | 27 | 5 | R.DGEVGGAVYVYMNQQGR.W |
|  | 19231 | **665.3710** | **1993.0911** | **1992.1004** | **0.9907** | **0** | **5** | **28** | **1** | **K.LRPIPITASVEIQEPSSR.R** |
|  | 19592 | 1014.5031 | 2026.9915 | 2026.0007 | 0.9908 | 0 | 34 | 0.038 | 1 | R.NSYPDVAVGSLSDSVTIFR.S 19582 |
|  | 29634 | **1328.6351** | **3982.8836** | **3982.9047** | **-0.0212** | **1** | **51** | **0.00021** | **1** | **K.GISPYFGYSIAGNMDLDRNSYPDVAVGSLSDSVTIFR.S** |

| **498.** | P55145\|MANF_HUMAN    **Mass:** 20700    **Score:** 60     **Queries matched:** 5   **emPAI:** 0.19 |
| --- | --- |
|  | Mesencephalic astrocyte-derived neurotrophic factor OS=Homo sapiens GN=MANF PE=1 SV=2 |

|  | **Query** | **Observed** | **Mr(expt)** | **Mr(calc)** | **Delta** | **Miss** | **Score** | **Expect** | **Rank** | **Peptide** |
| --- | --- | --- | --- | --- | --- | --- | --- | --- | --- | --- |
|  | 3365 | 467.2590 | 932.5034 | 933.5032 | -0.9997 | 1 | 10 | 17 | 1 | K.YAPKAASAR.T |
|  | 7731 | **619.3200** | **1236.6255** | **1235.5492** | **1.0762** | **0** | **3** | **76** | **10** | **K.ILDDWGETCK.G** |
|  | 13430 | 780.8592 | 1559.7039 | 1560.7130 | -1.0090 | 0 | 60 | 0.00013 | 1 | R.LCYYIGATDDAATK.I |
|  | 18439 | **481.7784** | **1923.0844** | **1923.0941** | **-0.0097** | **0** | **5** | **29** | **6** | **K.IINEVSKPLAHHIPVEK.I** |
|  | 19875 | 686.2848 | 2055.8325 | 2056.1139 | -0.2814 | 0 | 0 | 73 | 6 | -.MWATQGLAVALALSVLPGSR.A |

| **499.** | P50454\|SERPH_HUMAN    **Mass:** 46525    **Score:** 60     **Queries matched:** 10   **emPAI:** 0.08 |
| --- | --- |
|  | Serpin H1 OS=Homo sapiens GN=SERPINH1 PE=1 SV=2 |

|  | **Query** | **Observed** | **Mr(expt)** | **Mr(calc)** | **Delta** | **Miss** | **Score** | **Expect** | **Rank** | **Peptide** |
| --- | --- | --- | --- | --- | --- | --- | --- | --- | --- | --- |
|  | 3041 | **456.2427** | **910.4709** | **911.5552** | **-1.0843** | **1** | **2** | **1e+002** | **2** | **R.LVRPKGDK.M** |
|  | 3265 | **463.6054** | **925.1963** | **925.5960** | **-0.3998** | **1** | **2** | **64** | **4** | **K.KAVAISLPK.G** |
|  | 7046 | **602.3179** | **1202.6213** | **1201.4934** | **1.1279** | **0** | **3** | **81** | **7** | **K.QHYNCEHSK.I** |
|  | 8908 | **432.5885** | **1294.7436** | **1293.7115** | **1.0321** | **0** | **5** | **48** | **8** | **K.LQIVEMPLAHK.L** |
|  | 9468 | **662.8151** | **1323.6157** | **1324.6380** | **-1.0223** | **1** | **9** | **17** | **2** | **K.MVDNRGFMVTR.S** |
|  | 9806 | 448.5816 | 1342.7231 | 1342.5832 | 0.1399 | 0 | 3 | 71 | 5 | R.SYTVGVMMMHR.T |
|  | 14155 | **803.9030** | **1605.7915** | **1606.9042** | **-1.1127** | **1** | **5** | **36** | **2** | **K.KPAAAAAPGTAEKLSPK.A** |
|  | 14993 | **830.3995** | **1658.7844** | **1658.7941** | **-0.0096** | **0** | **60** | **0.00012** | **1** | **R.LYGPSSVSFADDFVR.S** |
|  | 17998 | **629.6195** | **1885.8365** | **1886.0738** | **-0.2373** | **1** | **1** | **83** | **6** | **R.DTQSGSLLFIGRLVRPK.G** |
|  | 19829 | **513.7574** | **2051.0005** | **2049.9257** | **1.0748** | **1** | **1** | **65** | **9** | **R.GFMVTRSYTVGVMMMHR.T** |

| **500.** | P09669\|COX6C_HUMAN    **Mass:** 8776     **Score:** 59     **Queries matched:** 2   **emPAI:** 0.46 |
| --- | --- |
|  | Cytochrome c oxidase subunit 6C OS=Homo sapiens GN=COX6C PE=1 SV=2 |

|  | **Query** | **Observed** | **Mr(expt)** | **Mr(calc)** | **Delta** | **Miss** | **Score** | **Expect** | **Rank** | **Peptide** |
| --- | --- | --- | --- | --- | --- | --- | --- | --- | --- | --- |
|  | 2158 | **425.2429** | **848.4713** | **848.4756** | **-0.0043** | **0** | **59** | **0.00023** | **1** | **K.AGIFQSVK.-** |
|  | 13952 | 398.9462 | 1591.7558 | 1591.6647 | 0.0912 | 1 | 4 | 45 | 5 | R.NYDVMKDFEEMR.K |

| **501.** | P23284\|PPIB_HUMAN    **Mass:** 23785    **Score:** 59     **Queries matched:** 12   **emPAI:** 0.16 |
| --- | --- |
|  | Peptidyl-prolyl cis-trans isomerase B OS=Homo sapiens GN=PPIB PE=1 SV=2 |

|  | **Query** | **Observed** | **Mr(expt)** | **Mr(calc)** | **Delta** | **Miss** | **Score** | **Expect** | **Rank** | **Peptide** |
| --- | --- | --- | --- | --- | --- | --- | --- | --- | --- | --- |
|  | 1135 | 687.3989 | 686.3917 | 685.3912 | 1.0005 | 0 | 13 | 10 | 4 | K.HVVFGK.V |
|  | 3204 | 461.0300 | 920.0454 | 919.4909 | 0.5545 | 1 | 1 | 85 | 10 | -.MLRLSER.N |
|  | 4673 | **516.3873** | **1030.7601** | **1030.5481** | **0.2120** | **0** | **3** | **72** | **10** | **K.VLEGMEVVR.K** |
|  | 7777 | 413.6149 | 1237.8230 | 1238.7023 | -0.8794 | 1 | 9 | 15 | 1 | K.VTVKVYFDLR.I 7771 |
|  | 7861 | **415.2059** | **1242.5958** | **1243.7176** | **-1.1218** | **0** | **5** | **50** | **9** | **K.IEVEKPFAIAK.E** |
|  | 12039 | **737.8437** | **1473.6728** | **1473.6823** | **-0.0095** | **0** | **59** | **0.00018** | **1** | **K.HYGPGWVSMANAGK.D** |
|  | 13597 | **524.6171** | **1570.8296** | **1570.8389** | **-0.0093** | **1** | **8** | **21** | **2** | **R.DKPLKDVIIADCGK.I** 13596 |
|  | 14093 | **534.9226** | **1601.7460** | **1600.7522** | **0.9939** | **1** | **8** | **21** | **5** | **K.SIYGERFPDENFK.L** 14083 |
|  | 16757 | **597.3010** | **1788.8811** | **1788.9622** | **-0.0811** | **1** | **5** | **35** | **4** | **K.TVPKTVDNFVALATGEK.G** |

| **502.** | O75348\|VATG1_HUMAN    **Mass:** 13863    **Score:** 59     **Queries matched:** 7   **emPAI:** 1.11 |
| --- | --- |
|  | V-type proton ATPase subunit G 1 OS=Homo sapiens GN=ATP6V1G1 PE=1 SV=3 |

|  | **Query** | **Observed** | **Mr(expt)** | **Mr(calc)** | **Delta** | **Miss** | **Score** | **Expect** | **Rank** | **Peptide** |
| --- | --- | --- | --- | --- | --- | --- | --- | --- | --- | --- |
|  | 3859 | **487.2710** | **972.5274** | **972.5352** | **-0.0078** | **1** | **37** | **0.036** | **1** | **K.AKEAAALGSR.G** 3868 3887 |
|  | 6463 | **586.8076** | **1171.6007** | **1171.6060** | **-0.0053** | **0** | **35** | **0.048** | **1** | **K.MTILQTYFR.Q** |
|  | 10187 | **683.3131** | **1364.6117** | **1364.6208** | **-0.0091** | **0** | **13** | **7.2** | **4** | **K.EEAQAEIEQYR.L** |
|  | 14230 | **806.3565** | **1610.6983** | **1610.7094** | **-0.0110** | **1** | **44** | **0.0048** | **1** | **R.GSCSTEVEKETQEK.M** 14231 |

| **503.** | Q9Y512\|SAM50_HUMAN    **Mass:** 52342    **Score:** 58     **Queries matched:** 10   **emPAI:** 0.07 |
| --- | --- |
|  | Sorting and assembly machinery component 50 homolog OS=Homo sapiens GN=SAMM50 PE=1 SV=3 |

|  | **Query** | **Observed** | **Mr(expt)** | **Mr(calc)** | **Delta** | **Miss** | **Score** | **Expect** | **Rank** | **Peptide** |
| --- | --- | --- | --- | --- | --- | --- | --- | --- | --- | --- |
|  | 5385 | **545.2647** | **1088.5149** | **1088.6012** | **-0.0863** | **1** | **5** | **63** | **6** | **K.AKNLIEVMR.K** |
|  | 13711 | **526.7515** | **1577.2328** | **1576.8111** | **0.4217** | **0** | **6** | **18** | **1** | **K.DVVVQHVHFDGLGR.T** |
|  | 16469 | **883.4509** | **1764.8873** | **1764.8968** | **-0.0095** | **1** | **58** | **0.00016** | **1** | **R.TKDDIIICEIGDVFK.A** |
|  | 16604 | 593.2670 | 1776.7791 | 1777.8748 | -1.0957 | 1 | 2 | 61 | 8 | K.VTGQFPWSSLRETDR.G |
|  | 20026 | **690.3580** | **2068.0523** | **2068.0088** | **0.0435** | **1** | **7** | **17** | **1** | **R.GMSAEYSFPIWKTSHTVK.W** |
|  | 21197 | **733.9954** | **2198.9644** | **2200.0504** | **-1.0860** | **0** | **(1)** | **63** | **3** | **R.LTGSYNTMVGNNEGSMVLGLK.L** 21215 |
|  | 21369 | **555.2892** | **2217.1276** | **2216.0453** | **1.0823** | **0** | **1** | **60** | **8** | **R.LTGSYNTMVGNNEGSMVLGLK.L** |
|  | 22473 | **590.0870** | **2356.3188** | **2356.1515** | **0.1673** | **1** | **(0)** | **66** | **5** | **R.RLTGSYNTMVGNNEGSMVLGLK.L** |
|  | 22475 | **786.7028** | **2357.0865** | **2356.1515** | **0.9349** | **1** | **2** | **44** | **1** | **R.RLTGSYNTMVGNNEGSMVLGLK.L** |

| **504.** | O95470\|SGPL1_HUMAN    **Mass:** 64053    **Score:** 58     **Queries matched:** 9   **emPAI:** 0.06 |
| --- | --- |
|  | Sphingosine-1-phosphate lyase 1 OS=Homo sapiens GN=SGPL1 PE=1 SV=3 |

|  | **Query** | **Observed** | **Mr(expt)** | **Mr(calc)** | **Delta** | **Miss** | **Score** | **Expect** | **Rank** | **Peptide** |
| --- | --- | --- | --- | --- | --- | --- | --- | --- | --- | --- |
|  | 1887 | **416.2590** | **830.5034** | **829.4844** | **1.0191** | **1** | **8** | **35** | **4** | **K.MPIIGRK.I** |
|  | 3666 | **479.7844** | **957.5542** | **956.5654** | **0.9887** | **1** | **8** | **31** | **5** | **R.KIEAEIVR.I** |
|  | 8181 | 629.8414 | 1257.6682 | 1257.6540 | 0.0142 | 1 | 8 | 23 | 8 | K.AASYFGMKIVR.V |
|  | 11350 | **478.2525** | **1431.7356** | **1432.7418** | **-1.0062** | **1** | **1** | **1.1e+002** | **7** | **R.VPLTKMMEVDVR.A** |
|  | 12263 | 743.9048 | 1485.7950 | 1485.8038 | -0.0088 | 0 | 58 | 0.00019 | 1 | K.ALPSQGLSSSAVLEK.L |
|  | 14116 | 535.5881 | 1603.7426 | 1604.6565 | -0.9140 | 0 | 16 | 3.3 | 1 | K.EYSSMDAFWQEGR.A 14115 |
|  | 14396 | **811.4142** | **1620.8138** | **1620.6515** | **0.1623** | **0** | **(0)** | **1.1e+002** | **5** | **K.EYSSMDAFWQEGR.A** |
|  | 19168 | **994.9828** | **1987.9511** | **1986.9503** | **1.0008** | **1** | **0** | **83** | **4** | **K.AKTTGMGAIYGMAQTTVDR.N** |

| **505.** | P13761\|2B17_HUMAN    **Mass:** 30145    **Score:** 58     **Queries matched:** 4   **emPAI:** 0.27 |
| --- | --- |
|  | HLA class II histocompatibility antigen, DRB1-7 beta chain OS=Homo sapiens GN=HLA-DRB1 PE=1 SV=1 |

|  | **Query** | **Observed** | **Mr(expt)** | **Mr(calc)** | **Delta** | **Miss** | **Score** | **Expect** | **Rank** | **Peptide** |
| --- | --- | --- | --- | --- | --- | --- | --- | --- | --- | --- |
|  | 3378 | **468.1884** | **934.3621** | **933.4338** | **0.9283** | **0** | **14** | **7.5** | **1** | **R.GQVDTVCR.H** |
|  | 5404 | **545.3375** | **1088.6604** | **1089.5349** | **-0.8746** | **1** | **6** | **50** | **3** | **R.RGQVDTVCR.H** |
|  | 10739 | 466.2611 | 1395.7616 | 1394.7670 | 0.9946 | 1 | 23 | 0.64 | 1 | R.RVHPEVTVYPAK.T |
|  | 12375 | 747.3555 | 1492.6965 | 1492.7059 | -0.0094 | 0 | 56 | 0.00035 | 1 | R.HNYGVGESFTVQR.R |

|  | **Proteins matching a subset of these peptides:** |
| --- | --- |

|  | Q9TQE0\|2B19_HUMAN    **Mass:** 30149    **Score:** 58     **Queries matched:** 2 |
| --- | --- |
|  | HLA class II histocompatibility antigen, DRB1-9 beta chain OS=Homo sapiens GN=HLA-DRB1 PE=2 SV=1 |

| **506.** | Q9Y536\|PAL4B_HUMAN    **Mass:** 18398    **Score:** 58     **Queries matched:** 7   **emPAI:** 0.21 |
| --- | --- |
|  | Peptidylprolyl cis-trans isomerase A-like 4B OS=Homo sapiens GN=PPIAL4B PE=1 SV=1 |

|  | **Query** | **Observed** | **Mr(expt)** | **Mr(calc)** | **Delta** | **Miss** | **Score** | **Expect** | **Rank** | **Peptide** |
| --- | --- | --- | --- | --- | --- | --- | --- | --- | --- | --- |
|  | 4522 | 511.2483 | 1020.4820 | 1020.4876 | -0.0056 | 0 | 9 | 24 | 1 | K.FDDENLIR.K |
|  | 13490 | **522.1323** | **1563.3751** | **1563.7504** | **-0.3752** | **0** | **0** | **78** | **5** | **R.VNIVEAMEHFGYR.N** |
|  | 14036 | 799.8714 | 1597.7282 | 1597.7381 | -0.0099 | 0 | 58 | 0.00017 | 1 | R.IIPGFMCQGGDFTR.H 14037 |
|  | 17578 | **617.6863** | **1850.0370** | **1848.8941** | **1.1429** | **1** | **(4)** | **37** | **1** | **K.ERVNIVEAMEHFGYR.N** |
|  | 17761 | 622.9951 | 1865.9634 | 1864.8890 | 1.0744 | 1 | 7 | 19 | 4 | K.ERVNIVEAMEHFGYR.N |
|  | 24862 | 917.7648 | 2750.2725 | 2750.2905 | -0.0180 | 0 | 1 | 43 | 5 | K.HTGSGILSMANAGPNTNGSQFFICAAK.T |

| **507.** | P09327\|VILI_HUMAN    **Mass:** 93093    **Score:** 58     **Queries matched:** 20   **emPAI:** 0.08 |
| --- | --- |
|  | Villin-1 OS=Homo sapiens GN=VIL1 PE=1 SV=4 |

|  | **Query** | **Observed** | **Mr(expt)** | **Mr(calc)** | **Delta** | **Miss** | **Score** | **Expect** | **Rank** | **Peptide** |
| --- | --- | --- | --- | --- | --- | --- | --- | --- | --- | --- |
|  | 4014 | 492.7144 | 983.4143 | 983.4672 | -0.0529 | 1 | 6 | 41 | 7 | K.HANEEEKK.A |
|  | 5684 | 555.2832 | 1108.5518 | 1109.6332 | -1.0814 | 0 | 7 | 31 | 8 | R.DPETPIIVVK.Q |
|  | 8868 | **431.8895** | **1292.6468** | **1293.6928** | **-1.0461** | **1** | **7** | **29** | **1** | **K.KAAATTAQEYLK.T** |
|  | 9037 | **434.8806** | **1301.6200** | **1301.6472** | **-0.0271** | **0** | **8** | **22** | **1** | **K.LMEVMNHVLGK.R** 9034 9036 9039 9044 |
|  | 10421 | **689.3554** | **1376.6963** | **1376.7048** | **-0.0086** | **0** | **41** | **0.012** | **1** | **R.LFQVQGTGANNTK.A** |
|  | 10528 | **462.1591** | **1383.4555** | **1382.6534** | **0.8022** | **1** | **4** | **41** | **8** | **R.EMAKMVADTISR.T** |
|  | 10872 | 468.9163 | 1403.7270 | 1402.7391 | 0.9879 | 1 | 6 | 34 | 7 | K.GAMSHALNFIKAK.Q |
|  | 11238 | 713.9177 | 1425.8208 | 1425.7584 | 0.0623 | 1 | 5 | 46 | 4 | K.LMEVMNHVLGKR.R |
|  | 11848 | **731.8690** | **1461.7234** | **1461.5839** | **0.1394** | **0** | **5** | **45** | **3** | **K.TQSCCYLWCGK.G** |
|  | 15285 | **839.4149** | **1676.8152** | **1676.8257** | **-0.0106** | **0** | **43** | **0.0053** | **1** | **R.TYVGVVDGENELASPK.L** |
|  | 15314 | **560.6055** | **1678.7948** | **1678.9505** | **-0.1558** | **1** | **0** | **1.1e+002** | **9** | **R.ELKAAVPDTVVEPALK.A** |
|  | 17992 | **629.3574** | **1885.0503** | **1884.9516** | **0.0986** | **0** | **2** | **56** | **6** | **K.FDATSMHVKPQVAAQQK.M** |
|  | 22965 | 487.4543 | 2432.2352 | 2433.1998 | -0.9646 | 1 | 3 | 39 | 7 | R.TEKQVVVEGQEPANFWMALGGK.A 22964 |
|  | 25212 | 704.5862 | 2814.3156 | 2813.3906 | 0.9251 | 1 | 1 | 50 | 8 | K.MVDDGSGEVQVWRIENLELVPVDSK.W |
|  | 28760 | **914.4899** | **3653.9306** | **3652.7546** | **1.1760** | **1** | **2** | **21** | **1** | **K.QYPPSTQVEVQNDGAESAVFQQLFQKWTASNR.T** |

| **508.** | Q8TBA6\|GOGA5_HUMAN    **Mass:** 82997    **Score:** 58     **Queries matched:** 19   **emPAI:** 0.04 |
| --- | --- |
|  | Golgin subfamily A member 5 OS=Homo sapiens GN=GOLGA5 PE=1 SV=2 |

|  | **Query** | **Observed** | **Mr(expt)** | **Mr(calc)** | **Delta** | **Miss** | **Score** | **Expect** | **Rank** | **Peptide** |
| --- | --- | --- | --- | --- | --- | --- | --- | --- | --- | --- |
|  | 1136 | 688.2893 | 687.2820 | 686.4326 | 0.8494 | 0 | 18 | 4 | 2 | K.LINSLK.E |
|  | 1889 | **416.2593** | **830.5040** | **829.4294** | **1.0747** | **0** | **7** | **42** | **4** | **K.AEDLLNR.V** 1887 |
|  | 4108 | **495.7520** | **989.4893** | **989.4665** | **0.0228** | **0** | **7** | **39** | **9** | **K.ETQEELNK.A** |
|  | 5367 | **544.3379** | **1086.6612** | **1087.5622** | **-0.9010** | **0** | **5** | **51** | **9** | **R.VDQGAATALSR.K** |
|  | 7120 | **604.2720** | **1206.5295** | **1205.5710** | **0.9585** | **1** | **2** | **87** | **7** | **K.EMQREEIQK.L** |
|  | 8520 | **637.8609** | **1273.7072** | **1272.6674** | **1.0399** | **0** | **1** | **1.1e+002** | **3** | **R.LQEADQLLSTR.T** |
|  | 8657 | **428.2842** | **1281.8309** | **1281.6122** | **0.2187** | **0** | **2** | **79** | **9** | **K.QTMLESLSTEK.N** |
|  | 9446 | **441.5628** | **1321.6667** | **1321.6990** | **-0.0323** | **1** | **6** | **35** | **9** | **R.KAASSIDQFSIR.L** |
|  | 9546 | 443.2513 | 1326.7321 | 1327.7095 | -0.9774 | 0 | 2 | 92 | 9 | R.QNLAEAITLAER.K |
|  | 11295 | **715.3764** | **1428.7382** | **1427.8096** | **0.9286** | **1** | **6** | **33** | **4** | **R.NQKATILAGTANVK.V** |
|  | 11460 | 480.6212 | 1438.8419 | 1437.7034 | 1.1385 | 1 | 3 | 59 | 8 | K.QMQSEFAARLNK.V |
|  | 11875 | 732.8453 | 1463.6761 | 1463.6852 | -0.0091 | 0 | 58 | 0.0002 | 1 | K.TLSNSSQSELENR.L |
|  | 17317 | 916.9740 | 1831.9334 | 1832.9421 | -1.0086 | 1 | 5 | 31 | 6 | M.SWFVDLAGKAEDLLNR.V |
|  | 23598 | **633.5631** | **2530.2233** | **2530.2081** | **0.0152** | **0** | **3** | **37** | **10** | **R.IMQDQSEGNSLQNQALQTLQER.L** |
|  | 24816 | **914.4539** | **2740.3398** | **2740.2133** | **0.1264** | **1** | **3** | **30** | **2** | **K.EGSGFEGLDSSTASSMELEELRHEK.E** |
|  | 26241 | **1011.2277** | **3030.6613** | **3030.2891** | **0.3723** | **0** | **6** | **10** | **1** | **R.LEQQMNSASGSSSNGSSINMSGIDNGEGTR.L** 26239 26249 |

| **509.** | Q6UW02\|CP20A_HUMAN    **Mass:** 52741    **Score:** 58     **Queries matched:** 6   **emPAI:** 0.15 |
| --- | --- |
|  | Cytochrome P450 20A1 OS=Homo sapiens GN=CYP20A1 PE=1 SV=1 |

|  | **Query** | **Observed** | **Mr(expt)** | **Mr(calc)** | **Delta** | **Miss** | **Score** | **Expect** | **Rank** | **Peptide** |
| --- | --- | --- | --- | --- | --- | --- | --- | --- | --- | --- |
|  | 1071 | **650.1066** | **649.0994** | **648.3377** | **0.7616** | **1** | **5** | **50** | **1** | **K.NMTRK.K** |
|  | 4202 | 499.1936 | 996.3726 | 995.4634 | 0.9093 | 0 | 4 | 56 | 7 | R.FDDELVMK.T |
|  | 11950 | **490.2372** | **1467.6899** | **1468.6980** | **-1.0081** | **1** | **5** | **40** | **8** | **K.GFLDGSLDKNMTR.K** |
|  | 12594 | **754.8261** | **1507.6377** | **1507.6474** | **-0.0097** | **0** | **52** | **0.00074** | **1** | **R.YQSGGGSVSENHMR.K** 12595 |
|  | 24405 | 1337.7216 | 2673.4286 | 2672.3810 | 1.0476 | 1 | 0 | 52 | 3 | K.LYEEINQVFGNGPVTPEKIEQLR.Y |

| **510.** | P00966\|ASSY_HUMAN    **Mass:** 46786    **Score:** 57     **Queries matched:** 17   **emPAI:** 0.26 |
| --- | --- |
|  | Argininosuccinate synthase OS=Homo sapiens GN=ASS1 PE=1 SV=2 |

|  | **Query** | **Observed** | **Mr(expt)** | **Mr(calc)** | **Delta** | **Miss** | **Score** | **Expect** | **Rank** | **Peptide** |
| --- | --- | --- | --- | --- | --- | --- | --- | --- | --- | --- |
|  | 974 | **612.3780** | **611.3707** | **610.3149** | **1.0558** | **0** | **1** | **95** | **1** | **R.FIGMK.S** |
|  | 1168 | 700.4304 | 699.4231 | 699.4643 | -0.0411 | 1 | 12 | 14 | 10 | K.ALKLGAK.K |
|  | 2005 | **420.2052** | **838.3959** | **837.4531** | **0.9428** | **1** | **6** | **36** | **5** | **R.FIGMKSR.G** |
|  | 2270 | 429.2163 | 856.4180 | 857.4607 | -1.0427 | 0 | 9 | 24 | 4 | R.IDIVENR.F 2280 2283 |
|  | 3197 | **460.7907** | **919.5668** | **918.4559** | **1.1109** | **0** | **9** | **21** | **2** | **K.YVSHGATGK.G** |
|  | 3830 | **486.2817** | **970.5489** | **970.5560** | **-0.0071** | **1** | **35** | **0.052** | **1** | **R.KQVEIAQR.E** |
|  | 3999 | **492.2262** | **982.4379** | **982.4429** | **-0.0050** | **0** | **16** | **4** | **1** | **R.NDLMEYAK.Q** |
|  | 9122 | 653.3202 | 1304.6258 | 1303.6521 | 0.9738 | 1 | 0 | 1.3e+002 | 6 | R.EGAKYVSHGATGK.G |
|  | 13360 | **778.3908** | **1554.7671** | **1554.7752** | **-0.0081** | **0** | **41** | **0.0087** | **1** | **R.FELSCYSLAPQIK.V** |
|  | 13906 | 530.6111 | 1588.8116 | 1589.8712 | -1.0595 | 0 | 12 | 7.8 | 1 | R.YLLGTSLARPCIAR.K |
|  | 15002 | **830.4445** | **1658.8745** | **1657.9879** | **0.8866** | **1** | **1** | **80** | **2** | **K.VQVSVLKGQVYILGR.E** |
|  | 15984 | **576.9037** | **1727.6892** | **1727.8842** | **-0.1950** | **1** | **1** | **73** | **7** | **K.NQAPPGLYTKTQDPAK.A** |
|  | 20541 | 709.7010 | 2126.0811 | 2126.0895 | -0.0084 | 1 | 37 | 0.016 | 1 | K.TQDPAKAPNTPDILEIEFK.K |
|  | 22440 | **785.7136** | **2354.1189** | **2353.1260** | **0.9928** | **0** | **22** | **0.47** | **1** | **K.DGTTHQTSLELFMYLNEVAGK.H** |
|  | 22598 | 792.3673 | 2374.0801 | 2373.0889 | 0.9912 | 0 | 3 | 37 | 3 | K.FAELVYTGFWHSPECEFVR.H |

| **511.** | Q08426\|ECHP_HUMAN    **Mass:** 80072    **Score:** 57     **Queries matched:** 9   **emPAI:** 0.05 |
| --- | --- |
|  | Peroxisomal bifunctional enzyme OS=Homo sapiens GN=EHHADH PE=1 SV=3 |

|  | **Query** | **Observed** | **Mr(expt)** | **Mr(calc)** | **Delta** | **Miss** | **Score** | **Expect** | **Rank** | **Peptide** |
| --- | --- | --- | --- | --- | --- | --- | --- | --- | --- | --- |
|  | 9964 | 451.2844 | 1350.8313 | 1350.7064 | 0.1248 | 1 | 1 | 1e+002 | 7 | K.MITSVLEKEASK.M |
|  | 10493 | **461.2425** | **1380.7056** | **1381.6779** | **-0.9723** | **0** | **20** | **1.3** | **1** | **K.GWYQYDKPLGR.I** |
|  | 11113 | **710.3568** | **1418.6989** | **1418.6942** | **0.0047** | **1** | **5** | **47** | **3** | **K.ANKWSTPSGASWK.T** 11123 |
|  | 12955 | **511.2483** | **1530.7230** | **1531.7743** | **-1.0513** | **1** | **7** | **25** | **3** | **R.FAQRVSDQPLESR.R** |
|  | 13930 | **796.3798** | **1590.7451** | **1590.7534** | **-0.0083** | **0** | **1** | **98** | **1** | **R.YCPIPDVLCELGR.F** |
|  | 20093 | 693.3623 | 2077.0651 | 2077.0653 | -0.0002 | 0 | 1 | 75 | 6 | K.ELGGVDLVIEAVFEEMSLK.K |
|  | 24920 | **691.3605** | **2761.4130** | **2760.5069** | **0.9061** | **1** | **1** | **46** | **10** | **K.TASARPVSSVGVVGLGTMGRGIVISFAR.A** |
|  | 29247 | **1276.2710** | **3825.7912** | **3825.8123** | **-0.0212** | **0** | **57** | **6e-005** | **1** | **R.MLNPYYNQAYFLLEEGSKPEEVDQVLEEFGFK.M** |

| **512.** | Q8N5K1\|CISD2_HUMAN    **Mass:** 15497    **Score:** 57     **Queries matched:** 5   **emPAI:** 0.25 |
| --- | --- |
|  | CDGSH iron sulfur domain-containing protein 2 OS=Homo sapiens GN=CISD2 PE=1 SV=1 |

|  | **Query** | **Observed** | **Mr(expt)** | **Mr(calc)** | **Delta** | **Miss** | **Score** | **Expect** | **Rank** | **Peptide** |
| --- | --- | --- | --- | --- | --- | --- | --- | --- | --- | --- |
|  | 1539 | **402.2882** | **802.5619** | **801.4596** | **1.1023** | **0** | **11** | **19** | **6** | **K.DSLINLK.I** |
|  | 7865 | 622.3218 | 1242.6290 | 1243.7322 | -1.1032 | 1 | 1 | 1.1e+002 | 6 | -.MVLESVARIVK.V |
|  | 8437 | **424.2189** | **1269.6348** | **1270.8013** | **-1.1665** | **1** | **4** | **56** | **6** | **R.IVKVQLPAYLK.R** |
|  | 14364 | 540.6269 | 1618.8590 | 1618.8679 | -0.0088 | 0 | 1 | 88 | 5 | K.HNELTGDNVGPLILK.K |
|  | 14995 | **830.4296** | **1658.8447** | **1658.8549** | **-0.0102** | **0** | **57** | **0.00022** | **1** | **K.VVNEINIEDLCLTK.A** |

| **513.** | Q9BS26\|ERP44_HUMAN    **Mass:** 47341    **Score:** 57     **Queries matched:** 5   **emPAI:** 0.16 |
| --- | --- |
|  | Endoplasmic reticulum resident protein ERp44 OS=Homo sapiens GN=ERP44 PE=1 SV=1 |

|  | **Query** | **Observed** | **Mr(expt)** | **Mr(calc)** | **Delta** | **Miss** | **Score** | **Expect** | **Rank** | **Peptide** |
| --- | --- | --- | --- | --- | --- | --- | --- | --- | --- | --- |
|  | 1952 | **418.6959** | **835.3773** | **834.4235** | **0.9537** | **0** | **7** | **38** | **10** | **K.LAPSEYR.Y** |
|  | 3559 | **475.5140** | **949.0134** | **948.5644** | **0.4490** | **1** | **8** | **20** | **4** | **R.ISKYPTLK.L** |
|  | 10150 | **681.8503** | **1361.6861** | **1360.7198** | **0.9663** | **1** | **4** | **55** | **1** | **R.DLAEITTLDRSK.R** |
|  | 11507 | **722.3129** | **1442.6112** | **1442.6209** | **-0.0097** | **0** | **64** | **5.1e-005** | **1** | **R.VDCDQHSDIAQR.Y** |
|  | 28387 | **888.4347** | **3549.7097** | **3549.7238** | **-0.0142** | **1** | **17** | **0.75** | **1** | **R.FSQMLHPIFEEASDVIKEEFPNENQVVFAR.V** |

| **514.** | P51970\|NDUA8_HUMAN    **Mass:** 20548    **Score:** 57     **Queries matched:** 5   **emPAI:** 0.41 |
| --- | --- |
|  | NADH dehydrogenase [ubiquinone] 1 alpha subcomplex subunit 8 OS=Homo sapiens GN=NDUFA8 PE=1 SV=3 |

|  | **Query** | **Observed** | **Mr(expt)** | **Mr(calc)** | **Delta** | **Miss** | **Score** | **Expect** | **Rank** | **Peptide** |
| --- | --- | --- | --- | --- | --- | --- | --- | --- | --- | --- |
|  | 2766 | 446.2213 | 890.4281 | 890.4327 | -0.0046 | 0 | 14 | 7.6 | 1 | R.FYFWTK.- |
|  | 10491 | **461.2327** | **1380.6764** | **1381.7176** | **-1.0413** | **1** | **2** | **87** | **9** | **K.LVNKCALDFFR.Q** |
|  | 11508 | 722.3192 | 1442.6237 | 1442.6322 | -0.0085 | 1 | 2 | 86 | 10 | K.EFMLCRWEEK.D |
|  | 19377 | **670.3762** | **2008.1067** | **2007.1140** | **0.9927** | **1** | **31** | **0.073** | **1** | **M.PGIVELPTLEELKVDEVK.I** |
|  | 23267 | **826.0856** | **2475.2351** | **2475.2468** | **-0.0117** | **1** | **44** | **0.0032** | **1** | **K.FDECVLDKLGWVRPDLGELSK.V** |

| **515.** | P62847\|RS24_HUMAN    **Mass:** 15413    **Score:** 57     **Queries matched:** 9   **emPAI:** 0.57 |
| --- | --- |
|  | 40S ribosomal protein S24 OS=Homo sapiens GN=RPS24 PE=1 SV=1 |

|  | **Query** | **Observed** | **Mr(expt)** | **Mr(calc)** | **Delta** | **Miss** | **Score** | **Expect** | **Rank** | **Peptide** |
| --- | --- | --- | --- | --- | --- | --- | --- | --- | --- | --- |
|  | 1412 | **398.7081** | **795.4016** | **795.4061** | **-0.0045** | **1** | **26** | **0.33** | **1** | **R.KFMTNR.L** |
|  | 1637 | **406.2527** | **810.4909** | **811.4010** | **-0.9102** | **1** | **(2)** | **93** | **10** | **R.KFMTNR.L** |
|  | 1729 | **410.2246** | **818.4346** | **817.4294** | **1.0052** | **0** | **12** | **14** | **4** | **M.NDTVTIR.T** |
|  | 3551 | **475.2381** | **948.4617** | **948.4699** | **-0.0082** | **0** | **1** | **1.3e+002** | **9** | **-.MNDTVTIR.T** |
|  | 4439 | **507.7580** | **1013.5014** | **1013.5869** | **-0.0855** | **1** | **7** | **32** | **8** | **K.ATVPKTEIR.E** |
|  | 7729 | 619.3185 | 1236.6225 | 1235.6696 | 0.9529 | 0 | 11 | 10 | 2 | K.QMVIDVLHPGK.A |
|  | 10778 | 699.8713 | 1397.7280 | 1397.7344 | -0.0064 | 0 | 51 | 0.0012 | 1 | K.TTPDVIFVFGFR.T |
|  | 15341 | **841.3878** | **1680.7610** | **1680.7705** | **-0.0096** | **0** | **36** | **0.026** | **1** | **K.TTGFGMIYDSLDYAK.K** |
|  | 22639 | **794.3796** | **2380.1169** | **2381.0998** | **-0.9829** | **1** | **2** | **46** | **2** | **R.THFGGGKTTGFGMIYDSLDYAK.K** |

| **516.** | O15173\|PGRC2_HUMAN    **Mass:** 23861    **Score:** 57     **Queries matched:** 6   **emPAI:** 0.16 |
| --- | --- |
|  | Membrane-associated progesterone receptor component 2 OS=Homo sapiens GN=PGRMC2 PE=1 SV=1 |

|  | **Query** | **Observed** | **Mr(expt)** | **Mr(calc)** | **Delta** | **Miss** | **Score** | **Expect** | **Rank** | **Peptide** |
| --- | --- | --- | --- | --- | --- | --- | --- | --- | --- | --- |
|  | 1203 | **726.3724** | **725.3652** | **724.3140** | **1.0512** | **0** | **6** | **32** | **2** | **R.QYDGSR.N** |
|  | 4352 | **1007.5104** | **1006.5032** | **1006.5083** | **-0.0052** | **0** | **12** | **10** | **1** | **R.DFSLEQLR.Q** |
|  | 4563 | 512.7567 | 1023.4989 | 1023.5059 | -0.0071 | 0 | 16 | 4.1 | 1 | R.GLATFCLDK.D |
|  | 12009 | **736.8652** | **1471.7158** | **1471.7248** | **-0.0091** | **0** | **57** | **0.00029** | **1** | **K.FYGPAGPYGIFAGR.D** |
|  | 15555 | **566.2974** | **1695.8704** | **1696.8380** | **-0.9676** | **0** | **1** | **84** | **9** | **R.GLGAGAGAGEESPATSLPR.M** |
|  | 18993 | **658.0335** | **1971.0787** | **1971.9684** | **-0.8897** | **1** | **6** | **27** | **3** | **R.GLGAGAGAGEESPATSLPRMK.K** |

| **517.** | P10319\|1B58_HUMAN    **Mass:** 40597    **Score:** 56     **Queries matched:** 10   **emPAI:** 0.30 |
| --- | --- |
|  | HLA class I histocompatibility antigen, B-58 alpha chain OS=Homo sapiens GN=HLA-B PE=2 SV=1 |

|  | **Query** | **Observed** | **Mr(expt)** | **Mr(calc)** | **Delta** | **Miss** | **Score** | **Expect** | **Rank** | **Peptide** |
| --- | --- | --- | --- | --- | --- | --- | --- | --- | --- | --- |
|  | 3749 | 483.2167 | 964.4188 | 964.4250 | -0.0062 | 0 | 35 | 0.057 | 1 | R.FDSDAASPR.T |
|  | 6427 | 585.3130 | 1168.6114 | 1168.5659 | 0.0456 | 1 | 12 | 8.3 | 1 | R.NMKASAQTYR.E |
|  | 9948 | 676.3410 | 1350.6674 | 1349.6939 | 0.9736 | 1 | 14 | 5.9 | 1 | R.YLENGKETLQR.A 9957 |
|  | 11588 | 483.2167 | 1446.6282 | 1447.6692 | -1.0410 | 1 | 13 | 7.2 | 1 | R.FDSDAASPRTEPR.A |
|  | 14538 | 815.4131 | 1628.8117 | 1628.8199 | -0.0081 | 0 | 28 | 0.17 | 1 | R.FIAVGYVDDTQFVR.F |
|  | 14674 | 547.0314 | 1638.0725 | 1637.7654 | 0.3071 | 1 | 2 | 52 | 6 | R.MYGCDLGPDGRLLR.G |
|  | 17358 | 612.9700 | 1835.8883 | 1835.9027 | -0.0144 | 0 | 3 | 52 | 2 | K.THVTHHPVSDHEATLR.C |
|  | 23688 | 1273.0597 | 2544.1048 | 2545.1510 | -1.0461 | 1 | 1 | 55 | 3 | R.TEPRAPWIEQEGPEYWDGETR.N |
|  | 24792 | 912.7573 | 2735.2500 | 2735.2635 | -0.0135 | 1 | 34 | 0.023 | 1 | R.DGEDQTQDTELVETRPAGDRTFQK.W |

|  | **Proteins matching a subset of these peptides:** |
| --- | --- |

|  | P30491\|1B53_HUMAN    **Mass:** 40755    **Score:** 56     **Queries matched:** 8 |
| --- | --- |
|  | HLA class I histocompatibility antigen, B-53 alpha chain OS=Homo sapiens GN=HLA-B PE=1 SV=1 |

|  | P18464\|1B51_HUMAN    **Mass:** 40826    **Score:** 56     **Queries matched:** 5 |
| --- | --- |
|  | HLA class I histocompatibility antigen, B-51 alpha chain OS=Homo sapiens GN=HLA-B PE=1 SV=1 |

|  | P30490\|1B52_HUMAN    **Mass:** 40781    **Score:** 56     **Queries matched:** 5 |
| --- | --- |
|  | HLA class I histocompatibility antigen, B-52 alpha chain OS=Homo sapiens GN=HLA-B PE=2 SV=1 |

|  | P30498\|1B78_HUMAN    **Mass:** 40738    **Score:** 56     **Queries matched:** 5 |
| --- | --- |
|  | HLA class I histocompatibility antigen, B-78 alpha chain OS=Homo sapiens GN=HLA-B PE=2 SV=1 |

| **518.** | P18465\|1B57_HUMAN    **Mass:** 40541    **Score:** 56     **Queries matched:** 7   **emPAI:** 0.30 |
| --- | --- |
|  | HLA class I histocompatibility antigen, B-57 alpha chain OS=Homo sapiens GN=HLA-B PE=1 SV=1 |

|  | **Query** | **Observed** | **Mr(expt)** | **Mr(calc)** | **Delta** | **Miss** | **Score** | **Expect** | **Rank** | **Peptide** |
| --- | --- | --- | --- | --- | --- | --- | --- | --- | --- | --- |
|  | 3749 | 483.2167 | 964.4188 | 964.4250 | -0.0062 | 0 | 35 | 0.057 | 1 | R.FDSDAASPR.M |
|  | 6427 | 585.3130 | 1168.6114 | 1168.5659 | 0.0456 | 1 | 12 | 8.3 | 1 | R.NMKASAQTYR.E |
|  | 9948 | 676.3410 | 1350.6674 | 1349.6939 | 0.9736 | 1 | 14 | 5.9 | 1 | R.YLENGKETLQR.A 9957 |
|  | 11128 | 710.8602 | 1419.7058 | 1419.6565 | 0.0493 | 1 | 8 | 22 | 2 | R.FDSDAASPRMAPR.A |
|  | 14538 | 815.4131 | 1628.8117 | 1628.8199 | -0.0081 | 0 | 28 | 0.17 | 1 | R.FIAVGYVDDTQFVR.F |
|  | 24792 | 912.7573 | 2735.2500 | 2735.2635 | -0.0135 | 1 | 34 | 0.023 | 1 | R.DGEDQTQDTELVETRPAGDRTFQK.W |

| **519.** | P30685\|1B35_HUMAN    **Mass:** 40715    **Score:** 56     **Queries matched:** 9   **emPAI:** 0.30 |
| --- | --- |
|  | HLA class I histocompatibility antigen, B-35 alpha chain OS=Homo sapiens GN=HLA-B PE=1 SV=1 |

|  | **Query** | **Observed** | **Mr(expt)** | **Mr(calc)** | **Delta** | **Miss** | **Score** | **Expect** | **Rank** | **Peptide** |
| --- | --- | --- | --- | --- | --- | --- | --- | --- | --- | --- |
|  | 3749 | 483.2167 | 964.4188 | 964.4250 | -0.0062 | 0 | 35 | 0.057 | 1 | R.FDSDAASPR.T |
|  | 9948 | 676.3410 | 1350.6674 | 1349.6939 | 0.9736 | 1 | 14 | 5.9 | 1 | R.YLENGKETLQR.A 9957 |
|  | 11588 | 483.2167 | 1446.6282 | 1447.6692 | -1.0410 | 1 | 13 | 7.2 | 1 | R.FDSDAASPRTEPR.A |
|  | 14538 | 815.4131 | 1628.8117 | 1628.8199 | -0.0081 | 0 | 28 | 0.17 | 1 | R.FIAVGYVDDTQFVR.F |
|  | 14674 | 547.0314 | 1638.0725 | 1637.7654 | 0.3071 | 1 | 2 | 52 | 6 | R.MYGCDLGPDGRLLR.G |
|  | 17358 | 612.9700 | 1835.8883 | 1835.9027 | -0.0144 | 0 | 3 | 52 | 2 | K.THVTHHPVSDHEATLR.C |
|  | 24792 | 912.7573 | 2735.2500 | 2735.2635 | -0.0135 | 1 | 34 | 0.023 | 1 | R.DGEDQTQDTELVETRPAGDRTFQK.W |
|  | 25835 | 982.1769 | 2943.5090 | 2943.3028 | 0.2062 | 1 | 1 | 46 | 9 | R.GYYNQSEAGSHIIQRMYGCDLGPDGR.L |

| **520.** | P05106\|ITB3_HUMAN    **Mass:** 90194    **Score:** 56     **Queries matched:** 10   **emPAI:** 0.04 |
| --- | --- |
|  | Integrin beta-3 OS=Homo sapiens GN=ITGB3 PE=1 SV=2 |

|  | **Query** | **Observed** | **Mr(expt)** | **Mr(calc)** | **Delta** | **Miss** | **Score** | **Expect** | **Rank** | **Peptide** |
| --- | --- | --- | --- | --- | --- | --- | --- | --- | --- | --- |
|  | 989 | **616.3986** | **615.3913** | **615.3591** | **0.0321** | **0** | **11** | **24** | **7** | **K.ENLLK.D** |
|  | 1896 | **416.2959** | **830.5773** | **830.4974** | **0.0799** | **1** | **14** | **7.9** | **1** | **R.KLTSNLR.I** |
|  | 2621 | **441.2115** | **880.4084** | **881.4719** | **-1.0635** | **0** | **3** | **79** | **7** | **K.THIALDGR.L** |
|  | 7426 | 611.7866 | 1221.5587 | 1220.5826 | 0.9761 | 0 | 6 | 42 | 4 | K.WDTANNPLYK.E |
|  | 8350 | 633.7947 | 1265.5749 | 1265.6503 | -0.0754 | 0 | 1 | 1.1e+002 | 10 | K.IGDTVSFSIEAK.V |
|  | 10322 | **458.2572** | **1371.7499** | **1370.6136** | **1.1362** | **1** | **3** | **65** | **10** | **K.DTGKDAVNCTYK.N** |
|  | 10876 | 468.9182 | 1403.7326 | 1402.6729 | 1.0598 | 0 | 9 | 15 | 2 | K.EATSTFTNITYR.G |
|  | 12972 | 766.8982 | 1531.7818 | 1530.7678 | 1.0140 | 0 | 17 | 2.3 | 1 | R.NDASHLLVFTTDAK.T |
|  | 18652 | 971.9538 | 1941.8930 | 1941.9540 | -0.0609 | 1 | 1 | 76 | 6 | K.SCMGLKIGDTVSFSIEAK.V |
|  | 24521 | **895.7805** | **2684.3196** | **2684.3366** | **-0.0170** | **1** | **56** | **0.00018** | **1** | **R.VLEDRPLSDKGSGDSSQVTQVSPQR.I** |

| **521.** | P13760\|2B14_HUMAN    **Mass:** 30435    **Score:** 56     **Queries matched:** 3   **emPAI:** 0.12 |
| --- | --- |
|  | HLA class II histocompatibility antigen, DRB1-4 beta chain OS=Homo sapiens GN=HLA-DRB1 PE=1 SV=1 |

|  | **Query** | **Observed** | **Mr(expt)** | **Mr(calc)** | **Delta** | **Miss** | **Score** | **Expect** | **Rank** | **Peptide** |
| --- | --- | --- | --- | --- | --- | --- | --- | --- | --- | --- |
|  | 1541 | 402.7052 | 803.3958 | 804.4606 | -1.0648 | 1 | 12 | 14 | 6 | R.VRFLDR.Y |
|  | 12375 | 747.3555 | 1492.6965 | 1492.7059 | -0.0094 | 0 | 56 | 0.00035 | 1 | R.HNYGVGESFTVQR.R |
|  | 28671 | **725.3559** | **3621.7431** | **3620.8551** | **0.8880** | **1** | **0** | **33** | **4** | **M.VCLKFPGGSCMAALTVTLMVLSSPLALAGDTRPR.F** |

| **522.** | P01913\|2B32_HUMAN    **Mass:** 30255    **Score:** 56     **Queries matched:** 7   **emPAI:** 0.12 |
| --- | --- |
|  | HLA class II histocompatibility antigen, DRB3-2 beta chain OS=Homo sapiens GN=HLA-DRB3 PE=2 SV=3 |

|  | **Query** | **Observed** | **Mr(expt)** | **Mr(calc)** | **Delta** | **Miss** | **Score** | **Expect** | **Rank** | **Peptide** |
| --- | --- | --- | --- | --- | --- | --- | --- | --- | --- | --- |
|  | 12375 | 747.3555 | 1492.6965 | 1492.7059 | -0.0094 | 0 | 56 | 0.00035 | 1 | R.HNYGVGESFTVQR.R |
|  | 20534 | 532.2908 | 2125.1342 | 2126.0255 | -0.8913 | 1 | 4 | 33 | 10 | R.FLELLKSECHFFNGTER.V |
|  | 23349 | **830.4445** | **2488.3117** | **2488.3757** | **-0.0640** | **1** | **1** | **51** | **3** | **M.VCLKLPGGSSLAALTVTLMVLSSR.L** |
|  | 24149 | **874.1476** | **2619.4211** | **2619.4162** | **0.0049** | **1** | **(1)** | **50** | **3** | **-.MVCLKLPGGSSLAALTVTLMVLSSR.L** |
|  | 24204 | 659.8084 | 2635.2043 | 2635.4111 | -0.2068 | 1 | 3 | 35 | 4 | -.MVCLKLPGGSSLAALTVTLMVLSSR.L 24207 24208 |

| **523.** | P79483\|2B31_HUMAN    **Mass:** 30228    **Score:** 56     **Queries matched:** 7   **emPAI:** 0.13 |
| --- | --- |
|  | HLA class II histocompatibility antigen, DRB3-1 beta chain OS=Homo sapiens GN=HLA-DRB3 PE=1 SV=1 |

|  | **Query** | **Observed** | **Mr(expt)** | **Mr(calc)** | **Delta** | **Miss** | **Score** | **Expect** | **Rank** | **Peptide** |
| --- | --- | --- | --- | --- | --- | --- | --- | --- | --- | --- |
|  | 12375 | 747.3555 | 1492.6965 | 1492.7059 | -0.0094 | 0 | 56 | 0.00035 | 1 | R.HNYGVGESFTVQR.R |
|  | 12621 | 755.8237 | 1509.6329 | 1510.6623 | -1.0294 | 1 | 1 | 88 | 4 | R.KSECHFFNGTER.V |
|  | 23349 | 830.4445 | 2488.3117 | 2488.3757 | -0.0640 | 1 | 1 | 51 | 3 | M.VCLKLPGGSSLAALTVTLMVLSSR.L |
|  | 24149 | 874.1476 | 2619.4211 | 2619.4162 | 0.0049 | 1 | (1) | 50 | 3 | -.MVCLKLPGGSSLAALTVTLMVLSSR.L |
|  | 24204 | 659.8084 | 2635.2043 | 2635.4111 | -0.2068 | 1 | 3 | 35 | 4 | -.MVCLKLPGGSSLAALTVTLMVLSSR.L 24207 24208 |

| **524.** | P36578\|RL4_HUMAN    **Mass:** 47953    **Score:** 56     **Queries matched:** 19   **emPAI:** 0.16 |
| --- | --- |
|  | 60S ribosomal protein L4 OS=Homo sapiens GN=RPL4 PE=1 SV=5 |

|  | **Query** | **Observed** | **Mr(expt)** | **Mr(calc)** | **Delta** | **Miss** | **Score** | **Expect** | **Rank** | **Peptide** |
| --- | --- | --- | --- | --- | --- | --- | --- | --- | --- | --- |
|  | 1173 | **705.3884** | **704.3812** | **704.3857** | **-0.0045** | **0** | **21** | **1.8** | **2** | **K.LNPYAK.T** 1174 |
|  | 3553 | 475.2394 | 948.4642 | 948.4698 | -0.0056 | 0 | 37 | 0.033 | 1 | K.MINTDLSR.I 3554 3555 |
|  | 3836 | **486.6005** | **971.1864** | **970.5672** | **0.6192** | **1** | **5** | **39** | **8** | **R.NTILRQAR.N** |
|  | 4082 | **494.7922** | **987.5698** | **987.5753** | **-0.0055** | **0** | **5** | **54** | **3** | **K.NVTLPAVFK.A** |
|  | 4448 | **507.8283** | **1013.6419** | **1013.6485** | **-0.0065** | **1** | **15** | **5.3** | **1** | **K.TKEAVLLLK.K** |
|  | 5308 | **542.3261** | **1082.6375** | **1082.6448** | **-0.0072** | **1** | **21** | **1.2** | **1** | **R.ILKSPEIQR.A** 5292 |
|  | 5717 | 556.7854 | 1111.5562 | 1110.6397 | 0.9166 | 1 | 1 | 1.2e+002 | 5 | K.AAATKKPAPEK.K |
|  | 6249 | **577.7577** | **1153.5008** | **1152.5750** | **0.9258** | **1** | **3** | **78** | **3** | **R.MFAPTKTWR.R** |
|  | 8611 | 640.7838 | 1279.5529 | 1279.6561 | -0.1031 | 1 | 20 | 1.3 | 1 | R.KLDELYGTWR.K |
|  | 8977 | **650.3214** | **1298.6281** | **1297.7354** | **0.8928** | **1** | **6** | **33** | **5** | **R.VDKAAAAAAALQAK.S** |
|  | 9368 | 659.8655 | 1317.7164 | 1318.7278 | -1.0114 | 1 | 12 | 9.8 | 4 | K.MINTDLSRILK.S 9388 |
|  | 16474 | **883.5852** | **1765.1559** | **1764.7533** | **0.4026** | **1** | **0** | **72** | **2** | **R.GGGTHRSGQGAFGNMCR.G** |
|  | 21941 | **762.7688** | **2285.2846** | **2286.1976** | **-0.9131** | **1** | **2** | **44** | **8** | **R.YAICSALAASALPALVMSKGHR.I** |
|  | 24681 | **906.4276** | **2716.2608** | **2715.2749** | **0.9859** | **1** | **51** | **0.0005** | **1** | **K.NNRQPYAVSELAGHQTSAESWGTGR.A** |

| **525.** | P07910\|HNRPC_HUMAN    **Mass:** 33707    **Score:** 55     **Queries matched:** 11   **emPAI:** 0.11 |
| --- | --- |
|  | Heterogeneous nuclear ribonucleoproteins C1/C2 OS=Homo sapiens GN=HNRNPC PE=1 SV=4 |

|  | **Query** | **Observed** | **Mr(expt)** | **Mr(calc)** | **Delta** | **Miss** | **Score** | **Expect** | **Rank** | **Peptide** |
| --- | --- | --- | --- | --- | --- | --- | --- | --- | --- | --- |
|  | 1208 | **731.1758** | **730.1685** | **730.4225** | **-0.2540** | **0** | **2** | **1.3e+002** | **8** | **K.ELTQIK.Q** |
|  | 2298 | 430.5614 | 859.1082 | 858.4447 | 0.6635 | 0 | 7 | 32 | 6 | K.GDDLQAIK.K |
|  | 2925 | 452.2097 | 902.4048 | 902.3956 | 0.0092 | 0 | 6 | 48 | 5 | R.MYSYPAR.V |
|  | 3011 | **454.8277** | **907.6408** | **908.2995** | **-0.6587** | **0** | **4** | **64** | **4** | **R.DSANGEDDS.-** |
|  | 3481 | 472.5334 | 943.0523 | 942.5651 | 0.4872 | 0 | 3 | 55 | 4 | R.VPPPPPIAR.A |
|  | 5252 | 540.4449 | 1078.8752 | 1078.4825 | 0.3927 | 1 | 1 | 86 | 3 | K.TDPRSMNSR.V |
|  | 7001 | **401.2286** | **1200.6640** | **1201.6051** | **-0.9411** | **1** | **6** | **43** | **9** | **M.ASNVTNKTDPR.S** |
|  | 9303 | **439.2722** | **1314.7948** | **1315.7864** | **-0.9915** | **0** | **5** | **49** | **4** | **R.VFIGNLNTLVVK.K** 9311 |
|  | 13336 | 518.5554 | 1552.6444 | 1552.6852 | -0.0408 | 1 | 10 | 11 | 4 | K.NDKSEEEQSSSSVK.K |
|  | 15365 | **841.9564** | **1681.8982** | **1681.9073** | **-0.0091** | **0** | **55** | **0.0003** | **1** | **R.MIAGQVLDINLAAEPK.V** |

| **526.** | P09758\|TACD2_HUMAN    **Mass:** 36371    **Score:** 55     **Queries matched:** 3   **emPAI:** 0.10 |
| --- | --- |
|  | Tumor-associated calcium signal transducer 2 OS=Homo sapiens GN=TACSTD2 PE=1 SV=3 |

|  | **Query** | **Observed** | **Mr(expt)** | **Mr(calc)** | **Delta** | **Miss** | **Score** | **Expect** | **Rank** | **Peptide** |
| --- | --- | --- | --- | --- | --- | --- | --- | --- | --- | --- |
|  | 3289 | **464.7557** | **927.4968** | **926.4821** | **1.0147** | **0** | **1** | **1.4e+002** | **9** | **R.GEPLQVER.T** |
|  | 7014 | **401.2850** | **1200.8331** | **1200.7091** | **0.1240** | **1** | **10** | **14** | **5** | **M.ARGPGLAPPPLR.L** |
|  | 14020 | **799.3765** | **1596.7384** | **1596.7487** | **-0.0104** | **0** | **55** | **0.00032** | **1** | **R.ALGSGMAVDCSTLTSK.C** |

| **527.** | A6NHL2\|TBAL3_HUMAN    **Mass:** 50675    **Score:** 55     **Queries matched:** 8   **emPAI:** 0.33 |
| --- | --- |
|  | Tubulin alpha chain-like 3 OS=Homo sapiens GN=TUBAL3 PE=1 SV=2 |

|  | **Query** | **Observed** | **Mr(expt)** | **Mr(calc)** | **Delta** | **Miss** | **Score** | **Expect** | **Rank** | **Peptide** |
| --- | --- | --- | --- | --- | --- | --- | --- | --- | --- | --- |
|  | 2728 | 444.2173 | 886.4200 | 886.4259 | -0.0058 | 0 | 41 | 0.017 | 1 | K.FDLMYAK.R |
|  | 7994 | 625.2763 | 1248.5379 | 1248.5453 | -0.0074 | 0 | 36 | 0.035 | 1 | K.YMACCLLYR.G |
|  | 10474 | 690.8484 | 1379.6822 | 1379.6907 | -0.0085 | 1 | 16 | 3.6 | 1 | R.LDHKFDLMYAK.R |
|  | 13729 | 527.2589 | 1578.7547 | 1577.8301 | 0.9246 | 0 | 3 | 65 | 9 | R.YSVGSEVIDLVLER.T |
|  | 17476 | 615.6299 | 1843.8678 | 1843.8783 | -0.0105 | 1 | 19 | 1.2 | 1 | K.YMACCLLYRGDVVPK.E 17477 |
|  | 17690 | 620.7950 | 1859.3631 | 1859.8732 | -0.5101 | 1 | (3) | 39 | 5 | K.YMACCLLYRGDVVPK.E |
|  | 23559 | **842.3738** | **2524.0995** | **2525.1857** | **-1.0862** | **1** | **3** | **31** | **1** | **R.SFGGGTGSGFTSLLMERLTGEYSR.K** |

| **528.** | Q93088\|BHMT1_HUMAN    **Mass:** 45426    **Score:** 54     **Queries matched:** 7   **emPAI:** 0.17 |
| --- | --- |
|  | Betaine--homocysteine S-methyltransferase 1 OS=Homo sapiens GN=BHMT PE=1 SV=2 |

|  | **Query** | **Observed** | **Mr(expt)** | **Mr(calc)** | **Delta** | **Miss** | **Score** | **Expect** | **Rank** | **Peptide** |
| --- | --- | --- | --- | --- | --- | --- | --- | --- | --- | --- |
|  | 4328 | **503.2522** | **1004.4898** | **1004.4961** | **-0.0063** | **0** | **30** | **0.17** | **1** | **K.GTAELMQQK.E** |
|  | 5782 | **559.2662** | **1116.5179** | **1116.5961** | **-0.0782** | **1** | **8** | **27** | **1** | **K.LMKEGLEAAR.L** 5801 |
|  | 9666 | 668.3142 | 1334.6137 | 1333.6990 | 0.9148 | 1 | 4 | 59 | 9 | K.LENRGNYVLEK.I |
|  | 12879 | 763.3944 | 1524.7743 | 1524.8374 | -0.0631 | 1 | 8 | 19 | 3 | K.VFLQQLEVFMKK.N |
|  | 15509 | **847.4310** | **1692.8474** | **1692.8570** | **-0.0096** | **1** | **50** | **0.001** | **1** | **K.EATTEQQLKELFEK.Q** |
|  | 18765 | **488.4877** | **1949.9216** | **1949.9319** | **-0.0103** | **0** | **10** | **9.7** | **1** | **K.HGSWGSGLDMHTKPWVR.A** |

| **529.** | Q9NVJ2\|ARL8B_HUMAN    **Mass:** 21753    **Score:** 54     **Queries matched:** 4   **emPAI:** 0.38 |
| --- | --- |
|  | ADP-ribosylation factor-like protein 8B OS=Homo sapiens GN=ARL8B PE=1 SV=1 |

|  | **Query** | **Observed** | **Mr(expt)** | **Mr(calc)** | **Delta** | **Miss** | **Score** | **Expect** | **Rank** | **Peptide** |
| --- | --- | --- | --- | --- | --- | --- | --- | --- | --- | --- |
|  | 4594 | **513.7285** | **1025.4425** | **1026.4705** | **-1.0280** | **1** | **11** | **11** | **7** | **R.FRSMWER.Y** |
|  | 4864 | 524.2632 | 1046.5119 | 1046.5179 | -0.0059 | 0 | 46 | 0.0043 | 1 | K.MNLSAIQDR.E |
|  | 14471 | **813.4363** | **1624.8581** | **1624.8672** | **-0.0090** | **1** | **38** | **0.02** | **1** | **R.DLPNALDEKQLIEK.M** |
|  | 16665 | **594.5333** | **1780.5780** | **1779.8825** | **0.6954** | **1** | **0** | **75** | **6** | **R.GVNAIVYMIDAADREK.I** |

| **530.** | Q13740\|CD166_HUMAN    **Mass:** 65745    **Score:** 54     **Queries matched:** 8   **emPAI:** 0.06 |
| --- | --- |
|  | CD166 antigen OS=Homo sapiens GN=ALCAM PE=1 SV=2 |

|  | **Query** | **Observed** | **Mr(expt)** | **Mr(calc)** | **Delta** | **Miss** | **Score** | **Expect** | **Rank** | **Peptide** |
| --- | --- | --- | --- | --- | --- | --- | --- | --- | --- | --- |
|  | 2362 | 432.2333 | 862.4520 | 862.4582 | -0.0062 | 1 | 1 | 1.5e+002 | 7 | K.CSLIDKK.S |
|  | 8108 | **419.2138** | **1254.6197** | **1255.6044** | **-0.9848** | **0** | **4** | **54** | **10** | **R.SSNTYTLTDVR.R** |
|  | 8569 | **639.4451** | **1276.8756** | **1276.6486** | **0.2270** | **0** | **2** | **64** | **2** | **R.LDVPQNLMFGK.W** |
|  | 9391 | **660.8675** | **1319.7204** | **1318.7496** | **0.9708** | **1** | **3** | **78** | **3** | **K.ALFLETEQLKK.L** |
|  | 15261 | **559.3412** | **1675.0019** | **1673.8407** | **1.1613** | **0** | **8** | **15** | **5** | **R.QIGDALPVSCTISASR.N** |
|  | 17340 | 917.9509 | 1833.8872 | 1833.0876 | 0.7996 | 1 | 2 | 77 | 3 | R.NGKVLHPLEGAVVIIFK.K |
|  | 27133 | **1072.8252** | **3215.4538** | **3215.4717** | **-0.0179** | **0** | **54** | **0.00017** | **1** | **R.SSPSFSSLHYQDAGNYVCETALQEVEGLK.K** |
|  | 29514 | 1313.9476 | 3938.8211 | 3937.8945 | 0.9266 | 1 | 5 | 8.7 | 2 | K.CLGNGNPPPEEFLFYLPGQPEGIRSSNTYTLTDVR.R |

| **531.** | P61769\|B2MG_HUMAN    **Mass:** 13820    **Score:** 54     **Queries matched:** 4   **emPAI:** 1.11 |
| --- | --- |
|  | Beta-2-microglobulin OS=Homo sapiens GN=B2M PE=1 SV=1 |

|  | **Query** | **Observed** | **Mr(expt)** | **Mr(calc)** | **Delta** | **Miss** | **Score** | **Expect** | **Rank** | **Peptide** |
| --- | --- | --- | --- | --- | --- | --- | --- | --- | --- | --- |
|  | 5856 | 1122.6193 | 1121.6120 | 1121.6193 | -0.0073 | 0 | 28 | 0.23 | 1 | R.VNHVTLSQPK.I 5837 |
|  | 23780 | **852.0768** | **2553.2085** | **2553.2210** | **-0.0125** | **0** | **44** | **0.0028** | **1** | **K.SNFLNCYVSGFHPSDIEVDLLK.N** 23779 |

| **532.** | Q9Y6N5\|SQRD_HUMAN    **Mass:** 50214    **Score:** 54     **Queries matched:** 17   **emPAI:** 0.15 |
| --- | --- |
|  | Sulfide:quinone oxidoreductase, mitochondrial OS=Homo sapiens GN=SQRDL PE=1 SV=1 |

|  | **Query** | **Observed** | **Mr(expt)** | **Mr(calc)** | **Delta** | **Miss** | **Score** | **Expect** | **Rank** | **Peptide** |
| --- | --- | --- | --- | --- | --- | --- | --- | --- | --- | --- |
|  | 6420 | **585.2795** | **1168.5444** | **1169.5936** | **-1.0492** | **1** | **(1)** | **1.1e+002** | **9** | **K.ERLSMYLMK.A** |
|  | 6994 | **401.2126** | **1200.6158** | **1201.5835** | **-0.9677** | **1** | **6** | **39** | **9** | **K.ERLSMYLMK.A** |
|  | 11816 | 730.8500 | 1459.6855 | 1459.8068 | -0.1213 | 1 | 0 | 1.3e+002 | 10 | R.TISVIMKNQTPTK.K |
|  | 11986 | 735.8839 | 1469.7533 | 1468.7522 | 1.0011 | 0 | 5 | 43 | 9 | K.VGAENVAIVEPSER.H |
|  | 12613 | **755.3784** | **1508.7422** | **1507.7195** | **1.0227** | **0** | **3** | **68** | **1** | **K.AEPLETFPFDQSK.E** |
|  | 13696 | 789.3892 | 1576.7639 | 1575.8256 | 0.9383 | 1 | 6 | 28 | 9 | K.KYADALQEIIQER.N |
|  | 14028 | **533.2864** | **1596.8373** | **1596.8471** | **-0.0098** | **1** | **4** | **45** | **5** | **R.KVGAENVAIVEPSER.H** |
|  | 15423 | **844.4464** | **1686.8781** | **1686.8882** | **-0.0101** | **0** | **46** | **0.0027** | **1** | **R.HFYQPIWTLVGAGAK.Q** |
|  | 19474 | **673.4200** | **2017.2383** | **2017.0051** | **0.2332** | **0** | **0** | **68** | **7** | **R.NHYEVLVLGGGSGGITMAAR.M** |
|  | 19709 | **1020.5307** | **2039.0468** | **2037.9942** | **1.0526** | **1** | **4** | **34** | **3** | **R.RYPNVFGIGDCTNLPTSK.T** |
|  | 20947 | 724.3224 | 2169.9453 | 2170.2037 | -0.2584 | 1 | 1 | 58 | 7 | K.ISYRYLIIALGIQLDYEK.I |
|  | 21561 | **746.3876** | **2236.1409** | **2236.1528** | **-0.0119** | **1** | **13** | **4** | **1** | **K.ALQDFKEGNAIFTFPNTPVK.C** |
|  | 21997 | **765.7120** | **2294.1143** | **2294.1291** | **-0.0148** | **1** | **28** | **0.12** | **1** | **K.TSPVADAAGWVDVDKETLQHR.R** 21999 |
|  | 23967 | **863.1003** | **2586.2790** | **2586.2894** | **-0.0103** | **1** | **3** | **34** | **1** | **R.VILAEFDYKAEPLETFPFDQSK.E** |
|  | 24597 | **900.4473** | **2698.3202** | **2698.2853** | **0.0348** | **1** | **0** | **56** | **7** | **R.LSMYLMKADLMPFLYWNMMLR.G** |
|  | 30188 | **1064.0272** | **4252.0798** | **4252.1072** | **-0.0274** | **1** | **24** | **0.083** | **1** | **R.ADKQEAVFENLDKPGETQVISYEMLHVTPPMSPPDVLK.T** |

| **533.** | Q3LXA3\|DHAK_HUMAN    **Mass:** 59282    **Score:** 54     **Queries matched:** 20   **emPAI:** 0.06 |
| --- | --- |
|  | Bifunctional ATP-dependent dihydroxyacetone kinase/FAD-AMP lyase (cyclizing) OS=Homo sapiens GN=DAK PE=1 SV=1 |

|  | **Query** | **Observed** | **Mr(expt)** | **Mr(calc)** | **Delta** | **Miss** | **Score** | **Expect** | **Rank** | **Peptide** |
| --- | --- | --- | --- | --- | --- | --- | --- | --- | --- | --- |
|  | 1898 | **416.3041** | **830.5935** | **830.4684** | **0.1252** | **0** | **14** | **8.8** | **1** | **R.MALVLER.V** |
|  | 2231 | 427.6758 | 853.3371 | 853.4294 | -0.0922 | 0 | 6 | 34 | 4 | R.ASYISSAR.L |
|  | 4070 | 494.3056 | 986.5966 | 986.5695 | 0.0271 | 1 | 13 | 8.4 | 6 | K.RMALVLER.V |
|  | 6658 | **593.2931** | **1184.5716** | **1184.6665** | **-0.0949** | **1** | **1** | **1.2e+002** | **3** | **R.AARAIQEWLK.E** |
|  | 13937 | **796.4392** | **1590.8637** | **1590.8729** | **-0.0092** | **0** | **54** | **0.00047** | **1** | **R.LEQPDPGAVAAAAILR.A** 13938 13953 |
|  | 14700 | **547.3184** | **1638.9333** | **1639.7736** | **-0.8404** | **1** | **3** | **59** | **6** | **K.NMEAGAGRASYISSAR.L** 14703 |
|  | 18637 | 970.9283 | 1939.8421 | 1939.9123 | -0.0702 | 0 | 0 | 89 | 3 | R.VAPAEPQEAPDSTAAGGSASK.R |
|  | 20266 | **699.6741** | **2096.0006** | **2096.0134** | **-0.0128** | **1** | **6** | **20** | **2** | **R.VAPAEPQEAPDSTAAGGSASKR.M** |
|  | 21077 | **728.8762** | **2183.6068** | **2183.0454** | **0.5614** | **1** | **1** | **43** | **10** | **R.SRVAPAEPQEAPDSTAAGGSASK.R** |
|  | 22334 | **781.3979** | **2341.1718** | **2341.1082** | **0.0636** | **1** | **1** | **58** | **4** | **K.TSLPAWSAAMDAGLEAMQKYGK.A** |
|  | 24441 | 892.4619 | 2674.3637 | 2673.3684 | 0.9954 | 1 | 9 | 7.9 | 2 | R.EQARAEGIPVEMVVIGDDSAFTVLK.K 24405 24414 24418 24425 24427 24446 |

| **534.** | Q02218\|ODO1_HUMAN    **Mass:** 117059   **Score:** 54     **Queries matched:** 21   **emPAI:** 0.13 |
| --- | --- |
|  | 2-oxoglutarate dehydrogenase E1 component, mitochondrial OS=Homo sapiens GN=OGDH PE=1 SV=3 |

|  | **Query** | **Observed** | **Mr(expt)** | **Mr(calc)** | **Delta** | **Miss** | **Score** | **Expect** | **Rank** | **Peptide** |
| --- | --- | --- | --- | --- | --- | --- | --- | --- | --- | --- |
|  | 4421 | **507.0752** | **1012.1358** | **1011.4985** | **0.6373** | **0** | **8** | **17** | **2** | **R.DPAAAPATGNK.K** |
|  | 5434 | 546.7660 | 1091.5175 | 1091.5247 | -0.0072 | 0 | 36 | 0.039 | 1 | R.SSPYPTDVAR.V |
|  | 5881 | 563.3185 | 1124.6224 | 1124.6302 | -0.0078 | 1 | 36 | 0.038 | 1 | K.KTHLTELQR.L 5892 |
|  | 7345 | **406.5653** | **1216.6742** | **1217.6265** | **-0.9523** | **0** | **3** | **85** | **5** | **K.TFSQNRPAAAR.T** |
|  | 7678 | **412.1865** | **1233.5376** | **1233.6111** | **-0.0735** | **1** | **7** | **32** | **3** | **-.MFHLRTCAAK.L** |
|  | 9619 | **444.9164** | **1331.7274** | **1331.6980** | **0.0294** | **1** | **5** | **42** | **5** | **K.ARDMVGQVAITR.I** |
|  | 12012 | **491.5839** | **1471.7298** | **1470.6878** | **1.0419** | **0** | **5** | **39** | **10** | **K.LGFYGLDESDLDK.V** |
|  | 12376 | **498.5765** | **1492.7075** | **1491.8409** | **0.8666** | **0** | **6** | **31** | **2** | **K.LVEDHLAVQSLIR.A** |
|  | 15012 | **553.9766** | **1658.9080** | **1658.9283** | **-0.0203** | **0** | **7** | **21** | **3** | **R.IEQLSPFPFDLLLK.E** |
|  | 16769 | 895.9442 | 1789.8739 | 1790.8080 | -0.9341 | 0 | 11 | 9.4 | 2 | K.SSENGVDYVIMGMPHR.G 16766 16771 16772 |
|  | 19408 | **1006.5067** | **2010.9989** | **2011.0123** | **-0.0134** | **0** | **20** | **0.92** | **1** | **R.NTNAGAPPGTAYQSPLPLSR.G** |
|  | 19716 | 511.0177 | 2040.0417 | 2040.0541 | -0.0124 | 1 | 11 | 7.6 | 1 | R.AKPVWYAGRDPAAAPATGNK.K |
|  | 20335 | 1052.5161 | 2103.0177 | 2104.0913 | -1.0736 | 0 | 8 | 12 | 2 | R.GSLAAVAHAQSLVEAQPNVDK.L |
|  | 20807 | **719.3468** | **2155.0186** | **2155.0276** | **-0.0090** | **0** | **21** | **0.63** | **1** | **K.HWLDSPWPGFFTLDGQPR.S** |
|  | 23439 | **835.4355** | **2503.2846** | **2503.1876** | **0.0971** | **0** | **7** | **14** | **1** | **R.VVNAPIFHVNSDDPEAVMYVCK.V** |
|  | 24096 | 871.4537 | 2611.3392 | 2610.1665 | 1.1727 | 1 | 1 | 48 | 7 | R.NGHNEMDEPMFTQPLMYKQIR.K |
|  | 31722 | **1383.1376** | **5528.5212** | **5528.5363** | **-0.0151** | **1** | **34** | **0.0035** | **1** | **R.FLQMCNDDPDVLPDLKEANFDINQLYDCNWVVVNCSTPGNFFHVLR.R** |

| **535.** | P04733\|MT1F_HUMAN    **Mass:** 7222     **Score:** 54     **Queries matched:** 1   **emPAI:** 0.58 |
| --- | --- |
|  | Metallothionein-1F OS=Homo sapiens GN=MT1F PE=1 SV=1 |

|  | **Query** | **Observed** | **Mr(expt)** | **Mr(calc)** | **Delta** | **Miss** | **Score** | **Expect** | **Rank** | **Peptide** |
| --- | --- | --- | --- | --- | --- | --- | --- | --- | --- | --- |
|  | 11826 | **731.2512** | **1460.4879** | **1460.4975** | **-0.0096** | **0** | **54** | **0.00043** | **1** | **K.SCCSCCPVGCSK.C** |

| **536.** | P35580\|MYH10_HUMAN    **Mass:** 229827   **Score:** 53     **Queries matched:** 80   **emPAI:** 0.08 |
| --- | --- |
|  | Myosin-10 OS=Homo sapiens GN=MYH10 PE=1 SV=3 |

|  | **Query** | **Observed** | **Mr(expt)** | **Mr(calc)** | **Delta** | **Miss** | **Score** | **Expect** | **Rank** | **Peptide** |
| --- | --- | --- | --- | --- | --- | --- | --- | --- | --- | --- |
|  | 716 | 519.1362 | 518.1289 | 517.2608 | 0.8681 | 0 | 13 | 15 | 1 | R.ANASR.R |
|  | 751 | 533.0582 | 532.0509 | 531.3017 | 0.7492 | 0 | 9 | 30 | 1 | R.IGQSK.I 752 753 755 756 |
|  | 871 | 572.9955 | 571.9882 | 571.3806 | 0.6076 | 1 | 9 | 24 | 2 | R.IKVGR.D |
|  | 921 | 589.3896 | 588.3824 | 587.3503 | 1.0320 | 1 | 6 | 79 | 7 | K.SRAVR.Q |
|  | 1029 | 633.3801 | 632.3728 | 631.3289 | 1.0439 | 0 | 3 | 1.3e+002 | 7 | R.DLQTR.D |
|  | 1086 | 659.3677 | 658.3605 | 658.4014 | -0.0409 | 0 | 21 | 2.2 | 3 | R.ALAVASK.K |
|  | 1168 | 700.4304 | 699.4231 | 699.4643 | -0.0411 | 1 | 18 | 3.4 | 4 | K.LQLAKK.E |
|  | 1609 | 405.1959 | 808.3773 | 807.4491 | 0.9282 | 0 | 19 | 2.1 | 1 | R.TVGQLYK.E |
|  | 2123 | 424.0561 | 846.0977 | 845.4793 | 0.6184 | 1 | 6 | 37 | 8 | R.KLQAQMK.D |
|  | 3417 | 469.8835 | 937.7525 | 937.5022 | 0.2503 | 0 | 5 | 35 | 3 | R.IVFQEFR.Q 3403 3412 |
|  | 3517 | **474.2290** | **946.4434** | **945.5243** | **0.9191** | **1** | **12** | **13** | **2** | **R.EVSTLKNR.L** |
|  | 4589 | 513.3106 | 1024.6067 | 1023.5349 | 1.0718 | 0 | 9 | 19 | 7 | K.HQQLLEEK.N |
|  | 4748 | **519.7731** | **1037.5316** | **1037.5393** | **-0.0077** | **1** | **21** | **1.4** | **1** | **K.ATDKTFVEK.L** |
|  | 5246 | **540.2562** | **1078.4978** | **1078.5012** | **-0.0034** | **1** | **5** | **55** | **5** | **K.QACERMIR.A** |
|  | 5453 | 547.2985 | 1092.5824 | 1091.5029 | 1.0795 | 1 | 9 | 19 | 5 | K.EQMEKANAR.M |
|  | 5568 | **551.3930** | **1100.7715** | **1100.5826** | **0.1889** | **1** | **7** | **26** | **2** | **K.REQEVAELK.K** |
|  | 6226 | **577.0380** | **1152.0614** | **1151.6298** | **0.4315** | **1** | **7** | **22** | **4** | **R.KHQQLLEEK.N** |
|  | 6561 | **590.3625** | **1178.7104** | **1178.6183** | **0.0922** | **1** | **2** | **88** | **3** | **R.LEEKALAYDK.L** |
|  | 7023 | **601.6528** | **1201.2911** | **1200.6714** | **0.6197** | **1** | **7** | **21** | **2** | **K.LQELEGAVKSK.F** |
|  | 7286 | 405.8886 | 1214.6438 | 1215.5956 | -0.9518 | 1 | 9 | 20 | 6 | K.SDNARQQLER.Q |
|  | 7393 | 610.8260 | 1219.6374 | 1219.6448 | -0.0074 | 1 | 30 | 0.14 | 1 | K.KFDQLLAEEK.S |
|  | 7587 | 615.3779 | 1228.7412 | 1228.7027 | 0.0385 | 1 | 4 | 66 | 6 | K.LQLEKVTAEAK.I |
|  | 8267 | **631.3734** | **1260.7323** | **1261.7064** | **-0.9741** | **1** | **1** | **1.3e+002** | **7** | **K.ESLTKLMATLR.N** |
|  | 8441 | **636.0601** | **1270.1056** | **1270.6266** | **-0.5210** | **1** | **4** | **36** | **4** | **M.AQRTGLEDPER.Y** |
|  | 8812 | 430.8870 | 1289.6392 | 1290.6456 | -1.0063 | 0 | 13 | 6.8 | 1 | K.EQADFAVEALAK.A 8805 8811 |
|  | 9223 | 656.6475 | 1311.2804 | 1310.5813 | 0.6991 | 0 | 10 | 8.4 | 1 | K.EIFMQVEDER.R 9202 |
|  | 9369 | 440.2517 | 1317.7333 | 1317.7405 | -0.0072 | 0 | 11 | 11 | 1 | K.LDPHLVLDQLR.C |
|  | 10167 | **682.3820** | **1362.7495** | **1363.6289** | **-0.8794** | **1** | **3** | **70** | **2** | **K.VEGELEEMERK.H** |
|  | 10769 | 466.7442 | 1397.2107 | 1396.6697 | 0.5410 | 1 | 15 | 3 | 1 | K.VDYKADEWLMK.N |
|  | 10837 | **468.0981** | **1401.2723** | **1401.6670** | **-0.3947** | **1** | **7** | **17** | **10** | **-.MAQRTGLEDPER.Y** 10839 |
|  | 11336 | **716.3894** | **1430.7642** | **1430.6161** | **0.1481** | **0** | **2** | **92** | **10** | **K.TSDVNETQPPQSE.-** |
|  | 12532 | **501.9470** | **1502.8191** | **1503.6801** | **-0.8610** | **1** | **4** | **51** | **5** | **R.DLQTRDEQNEEK.K** |
|  | 12791 | **760.3417** | **1518.6688** | **1518.6798** | **-0.0110** | **0** | **27** | **0.25** | **1** | **R.ELDDATEANEGLSR.E** 12798 12808 |
|  | 13134 | 771.8920 | 1541.7694 | 1541.8565 | -0.0871 | 1 | 4 | 45 | 8 | R.QRYEILTPNAIPK.G |
|  | 13323 | **518.2911** | **1551.8514** | **1551.7603** | **0.0911** | **1** | **1** | **1.1e+002** | **9** | **K.LKEIFMQVEDER.R** |
|  | 13650 | 525.6359 | 1573.8858 | 1573.8940 | -0.0083 | 1 | 10 | 11 | 1 | R.AGKLDPHLVLDQLR.C |
|  | 13677 | **526.2629** | **1575.7668** | **1576.7403** | **-0.9734** | **1** | **(3)** | **56** | **10** | **K.QTKVEGELEEMER.K** |
|  | 13720 | 526.9389 | 1577.7949 | 1578.8365 | -1.0417 | 1 | 0 | 1.1e+002 | 7 | R.AGVLAHLEEERDLK.I |
|  | 13919 | **795.8881** | **1589.7617** | **1589.7719** | **-0.0102** | **1** | **19** | **1.5** | **1** | **R.NKQEVMISDLEER.L** |
|  | 13975 | **532.2876** | **1593.8410** | **1592.7352** | **1.1058** | **1** | **7** | **26** | **5** | **K.QTKVEGELEEMER.K** |
|  | 14155 | 803.9030 | 1605.7915 | 1605.7668 | 0.0247 | 1 | (6) | 32 | 1 | R.NKQEVMISDLEER.L |
|  | 14275 | **807.8166** | **1613.6186** | **1614.7849** | **-1.1663** | **1** | **3** | **52** | **4** | **K.QVRELEAELEDER.K** |
|  | 14584 | **817.3723** | **1632.7301** | **1632.7413** | **-0.0113** | **0** | **28** | **0.18** | **1** | **R.NTDQASMPENTVAQK.L** |
|  | 14790 | 412.4516 | 1645.7774 | 1645.8722 | -0.0948 | 1 | 7 | 22 | 2 | K.LMATLRNTNPNFVR.C |
|  | 14814 | **550.2144** | **1647.6213** | **1648.7363** | **-1.1150** | **0** | **(8)** | **18** | **2** | **R.NTDQASMPENTVAQK.L** |
|  | 14937 | 828.4047 | 1654.7948 | 1655.7018 | -0.9071 | 1 | 7 | 22 | 1 | R.ADMEDLMSSKDDVGK.N |
|  | 15045 | 831.8992 | 1661.7839 | 1661.8671 | -0.0832 | 1 | (1) | 98 | 10 | K.LMATLRNTNPNFVR.C |
|  | 15261 | 559.3412 | 1675.0019 | 1675.8570 | -0.8550 | 0 | 6 | 27 | 7 | R.FLSNGYIPIPGQQDK.D 15235 |
|  | 15614 | **567.9007** | **1700.6803** | **1700.8468** | **-0.1665** | **1** | **1** | **77** | **5** | **R.QEEELQAKDEELLK.V** |
|  | 15755 | **571.3052** | **1710.8939** | **1709.8229** | **1.0710** | **1** | **4** | **46** | **2** | **R.LEVNMQAMKAQFER.D** |
|  | 15826 | 572.9688 | 1715.8844 | 1715.9206 | -0.0362 | 1 | 4 | 44 | 4 | R.ALELDPNLYRIGQSK.I |
|  | 15968 | **576.4088** | **1726.2044** | **1725.8178** | **0.3866** | **1** | **(0)** | **67** | **10** | **R.LEVNMQAMKAQFER.D** |
|  | 16117 | **435.4871** | **1737.9194** | **1737.8897** | **0.0297** | **1** | **12** | **6.3** | **2** | **K.KQELEEILHDLESR.V** |
|  | 16185 | **872.4724** | **1742.9301** | **1741.9362** | **0.9939** | **0** | **38** | **0.015** | **1** | **R.QLLQANPILESFGNAK.T** |
|  | 16335 | **878.4724** | **1754.9301** | **1753.8709** | **1.0593** | **0** | **1** | **99** | **3** | **K.NLPIYSENIIEMYR.G** |
|  | 17352 | 918.4738 | 1834.9329 | 1833.9029 | 1.0300 | 1 | 11 | 9.2 | 2 | K.KMEEEILLLEDQNSK.F |
|  | 18344 | **639.9692** | **1916.8857** | **1917.8850** | **-0.9993** | **1** | **1** | **85** | **10** | **K.ERNTDQASMPENTVAQK.L** |
|  | 18600 | **646.6534** | **1936.9383** | **1936.9490** | **-0.0107** | **0** | **4** | **41** | **3** | **R.LQQELDDLTVDLDHQR.Q** |
|  | 18728 | **487.7433** | **1946.9441** | **1945.9666** | **0.9775** | **1** | **7** | **19** | **2** | **K.MEIDLKDLEAQIEAANK.A** |
|  | 20358 | 702.4100 | 2104.2081 | 2103.0194 | 1.1886 | 0 | 3 | 43 | 2 | R.IVGLDQVTGMTETAFGSAYK.T |
|  | 21011 | 1089.4779 | 2176.9413 | 2176.0495 | 0.8918 | 1 | 1 | 70 | 4 | R.ELDDATEANEGLSREVSTLK.N |
|  | 22575 | **791.0314** | **2370.0725** | **2370.0250** | **0.0475** | **1** | **0** | **65** | **2** | **R.CMLQDREDQSILCTGESGAGK.T** |
|  | 24104 | 872.1188 | 2613.3345 | 2614.3642 | -1.0298 | 1 | 2 | 42 | 4 | R.INFDVTGYIVGANIETYLLEKSR.A |
|  | 24479 | 893.0765 | 2676.2076 | 2677.3017 | -1.0941 | 1 | 0 | 53 | 5 | K.NILAEQLQAETELFAEAEEMRAR.L |
|  | 24860 | **917.4497** | **2749.3273** | **2749.3406** | **-0.0133** | **1** | **19** | **0.71** | **1** | **K.FAKDAASLESQLQDTQELLQEETR.Q** |
|  | 26211 | **1010.1635** | **3027.4685** | **3028.4713** | **-1.0027** | **1** | **6** | **12** | **1** | **K.NMDPLNDNVATLLHQSSDRFVAELWK.D** |
|  | 26422 | **1021.1688** | **3060.4846** | **3060.4986** | **-0.0140** | **1** | **1** | **42** | **5** | **R.DLSEELEALKTELEDTLDTTAAQQELR.T** |

| **537.** | P21333\|FLNA_HUMAN    **Mass:** 283301   **Score:** 53     **Queries matched:** 54   **emPAI:** 0.03 |
| --- | --- |
|  | Filamin-A OS=Homo sapiens GN=FLNA PE=1 SV=4 |

|  | **Query** | **Observed** | **Mr(expt)** | **Mr(calc)** | **Delta** | **Miss** | **Score** | **Expect** | **Rank** | **Peptide** |
| --- | --- | --- | --- | --- | --- | --- | --- | --- | --- | --- |
|  | 2562 | **438.7263** | **875.4380** | **874.3967** | **1.0413** | **0** | **5** | **61** | **7** | **K.CSGPGLER.A** |
|  | 3141 | 459.0118 | 916.0091 | 916.3089 | -0.2998 | 0 | 5 | 40 | 8 | K.MSCMDNK.D |
|  | 4062 | 494.2553 | 986.4961 | 987.5349 | -1.0388 | 1 | 10 | 20 | 5 | K.KGEITGEVR.M 4101 |
|  | 5520 | **550.0256** | **1098.0367** | **1098.5669** | **-0.5302** | **0** | **1** | **86** | **1** | **K.GTVEPQLEAR.G** |
|  | 6118 | **573.4056** | **1144.7967** | **1144.6452** | **0.1515** | **1** | **2** | **86** | **6** | **K.GAGSGELKVTVK.G** |
|  | 6714 | **396.8594** | **1187.5565** | **1186.6558** | **0.9007** | **1** | **12** | **9.4** | **2** | **K.VSGLGEKVDVGK.D** 6717 |
|  | 6766 | 397.4023 | 1189.1852 | 1188.6867 | 0.4985 | 1 | 9 | 15 | 6 | K.VYGPGVAKTGLK.A 6737 |
|  | 6866 | **399.2156** | **1194.6249** | **1193.6768** | **0.9481** | **1** | **11** | **11** | **1** | **K.VLPTHDASKVK.A** |
|  | 7500 | **409.2280** | **1224.6623** | **1225.6455** | **-0.9833** | **0** | **7** | **26** | **6** | **R.AWGPGLEGGVVGK.S** 7489 |
|  | 7503 | 613.3393 | 1224.6640 | 1224.5622 | 0.1018 | 0 | 7 | 26 | 7 | R.EATTEFSVDAR.A |
|  | 7678 | 412.1865 | 1233.5376 | 1232.5642 | 0.9734 | 0 | 6 | 40 | 4 | K.CSGPGLSPGMVR.A 7645 |
|  | 9050 | 434.8813 | 1301.6220 | 1300.6564 | 0.9656 | 0 | 4 | 56 | 5 | K.FNGTHIPGSPFK.I |
|  | 9684 | **446.2693** | **1335.7862** | **1334.7419** | **1.0443** | **1** | **1** | **1e+002** | **6** | **R.ALTQTGGPHVKAR.V** |
|  | 11013 | **471.7638** | **1412.2695** | **1412.6473** | **-0.3778** | **0** | **5** | **27** | **1** | **K.WGDEHIPGSPYR.V** |
|  | 11388 | **717.8607** | **1433.7069** | **1433.7151** | **-0.0082** | **0** | **53** | **0.00074** | **1** | **R.ANLPQSFQVDTSK.A** |
|  | 11496 | **721.8477** | **1441.6809** | **1441.6910** | **-0.0101** | **0** | **28** | **0.22** | **1** | **R.AGQSAAGAAPGGGVDTR.D** |
|  | 11648 | **484.3824** | **1450.1254** | **1449.7511** | **0.3744** | **0** | **9** | **12** | **2** | **K.AGNNMLLVGVHGPR.T** 11647 |
|  | 11830 | **487.8884** | **1460.6434** | **1459.7276** | **0.9158** | **1** | **10** | **13** | **1** | **K.VKCSGPGLSPGMVR.A** 11812 11836 11841 |
|  | 12376 | 498.5765 | 1492.7075 | 1491.6810 | 1.0265 | 1 | 3 | 71 | 9 | R.VTGDDSMRMSHLK.V |
|  | 12482 | 501.2354 | 1500.6844 | 1499.7467 | 0.9377 | 0 | 5 | 37 | 10 | K.DAGEGGLSLAIEGPSK.A |
|  | 13910 | 795.4351 | 1588.8557 | 1587.7351 | 1.1206 | 0 | (1) | 93 | 4 | R.YAPSEAGLHEMDIR.Y |
|  | 14125 | 535.6227 | 1603.8464 | 1603.7300 | 0.1164 | 0 | 7 | 26 | 7 | R.YAPSEAGLHEMDIR.Y |
|  | 14887 | **551.3149** | **1650.9228** | **1651.8529** | **-0.9301** | **0** | **7** | **21** | **6** | **K.VTAQGPGLEPSGNIANK.T** |
|  | 15024 | **830.9251** | **1659.8357** | **1659.8403** | **-0.0046** | **1** | **1** | **82** | **5** | **K.ARAYGPGIEPTGNMVK.K** |
|  | 16344 | **878.9393** | **1755.8641** | **1755.8315** | **0.0326** | **1** | **1** | **92** | **8** | **K.SPFEVYVDKSQGDASK.V** |
|  | 16749 | **597.2772** | **1788.8098** | **1788.8213** | **-0.0115** | **0** | **18** | **1.5** | **1** | **K.ATCAPQHGAPGPGPADASK.V** |
|  | 18264 | **637.3106** | **1908.9098** | **1908.9105** | **-0.0007** | **0** | **3** | **52** | **3** | **R.EGPYSISVLYGDEEVPR.S** |
|  | 18593 | **646.3491** | **1936.0254** | **1937.0106** | **-0.9852** | **0** | **2** | **64** | **6** | **K.DAGEGLLAVQITDPEGKPK.K** |
|  | 22125 | **1156.5352** | **2311.0558** | **2312.0921** | **-1.0363** | **0** | **1** | **55** | **2** | **R.SAGQGEVLVYVEDPAGHQEEAK.V** 22126 |
|  | 22324 | 781.0630 | 2340.1673 | 2341.2166 | -1.0492 | 0 | 4 | 31 | 2 | K.ASGPGLNTTGVPASLPVEFTIDAK.D |
|  | 22677 | 1193.6090 | 2385.2035 | 2385.1159 | 0.0876 | 1 | 5 | 24 | 1 | R.KDGSCGVAYVVQEPGDYEVSVK.F |
|  | 22948 | 810.6991 | 2429.0755 | 2430.1081 | -1.0327 | 1 | 3 | 36 | 4 | R.AGQSAAGAAPGGGVDTRDAEMPATEK.D |
|  | 22952 | **608.3249** | **2429.2705** | **2430.1122** | **-0.8417** | **1** | **1** | **58** | **3** | **R.VANPSGNLTETYVQDRGDGMYK.V** |
|  | 23069 | **817.0430** | **2448.1071** | **2447.2405** | **0.8666** | **0** | **9** | **9.9** | **1** | **K.NGQHVASSPIPVVISQSEIGDASR.V** |
|  | 23620 | **634.5930** | **2534.3427** | **2535.1488** | **-0.8061** | **0** | **2** | **41** | **9** | **R.YWPQEAGEYAVHVLCNSEDIR.L** 23618 |
|  | 23955 | **518.0507** | **2585.2172** | **2586.2463** | **-1.0291** | **1** | **0** | **65** | **8** | **K.KTHIQDNHDGTYTVAYVPDVTGR.Y** |
|  | 24839 | **915.9743** | **2744.9011** | **2744.4708** | **0.4302** | **1** | **1** | **37** | **6** | **R.LTVSSLQESGLKVNQPASFAVSLNGAK.G** |
|  | 25570 | **723.1151** | **2888.4311** | **2887.3950** | **1.0361** | **0** | **2** | **34** | **6** | **K.TTYFEIFTAGAGTGEVEVVIQDPMGQK.G** |
|  | 26892 | 1056.4941 | 3166.4606 | 3167.4565 | -0.9959 | 1 | 0 | 45 | 4 | R.DAGYGGLSLSIEGPSKVDINTEDLEDGTCR.V |
|  | 28499 | **1194.2579** | **3579.7520** | **3578.6300** | **1.1219** | **0** | **6** | **9.5** | **1** | **K.DGSCSVEYIPYEAGTYSLNVTYGGHQVPGSPFK.V** |
|  | 29595 | 1324.2653 | 3969.7740 | 3970.9337 | -1.1598 | 1 | 3 | 13 | 3 | R.VSGQGLHEGHTFEPAEFIIDTRDAGYGGLSLSIEGPSK.V |
|  | 30278 | **1080.2575** | **4317.0007** | **4316.1636** | **0.8371** | **1** | **2** | **13** | **1** | **K.AIVDGNLKLILGLIWTLILHYSISMPMWDEEEDEEAK.K** |
|  | 32290 | **1129.5200** | **6771.0765** | **6772.1572** | **-1.0807** | **1** | **2** | **2.5** | **2** | **R.FTIDTKGAGTGGLGLTVEGPCEAQLECLDNGDGTCSVSYVPTEPGDYNINILFADTHIPGSPFK.A** |

| **538.** | Q6NUK1\|SCMC1_HUMAN    **Mass:** 53548    **Score:** 53     **Queries matched:** 5   **emPAI:** 0.07 |
| --- | --- |
|  | Calcium-binding mitochondrial carrier protein SCaMC-1 OS=Homo sapiens GN=SLC25A24 PE=1 SV=2 |

|  | **Query** | **Observed** | **Mr(expt)** | **Mr(calc)** | **Delta** | **Miss** | **Score** | **Expect** | **Rank** | **Peptide** |
| --- | --- | --- | --- | --- | --- | --- | --- | --- | --- | --- |
|  | 2950 | **453.0367** | **904.0589** | **903.4814** | **0.5775** | **0** | **8** | **22** | **4** | **K.EGIPGLYR.G** |
|  | 7243 | **607.3110** | **1212.6074** | **1211.6986** | **0.9088** | **0** | **7** | **31** | **1** | **R.QLLAGGIAGAVSR.T** |
|  | 10136 | **681.7994** | **1361.5842** | **1361.5922** | **-0.0080** | **0** | **53** | **0.00069** | **1** | **K.TGQYSGIYDCAK.K** |
|  | 22730 | **799.6871** | **2396.0394** | **2395.1262** | **0.9132** | **0** | **6** | **17** | **1** | **R.FISGSMAGATAQTFIYPMEVMK.T** |
|  | 23057 | **815.7460** | **2444.2161** | **2444.2416** | **-0.0255** | **1** | **1** | **61** | **8** | **R.DYFLFNPVTDIEEIIRFWK.H** |

| **539.** | Q1EHB4\|SC5AC_HUMAN    **Mass:** 68629    **Score:** 53     **Queries matched:** 4   **emPAI:** 0.05 |
| --- | --- |
|  | Sodium-coupled monocarboxylate transporter 2 OS=Homo sapiens GN=SLC5A12 PE=2 SV=2 |

|  | **Query** | **Observed** | **Mr(expt)** | **Mr(calc)** | **Delta** | **Miss** | **Score** | **Expect** | **Rank** | **Peptide** |
| --- | --- | --- | --- | --- | --- | --- | --- | --- | --- | --- |
|  | 11827 | **731.3049** | **1460.5953** | **1460.6031** | **-0.0077** | **0** | **7** | **24** | **1** | **K.SYNNMAFETTHF.-** |
|  | 15672 | **853.3663** | **1704.7180** | **1703.7250** | **0.9930** | **1** | **53** | **0.00052** | **1** | **K.DKSYNNMAFETTHF.-** |
|  | 21828 | 1136.5414 | 2271.0682 | 2271.2011 | -0.1329 | 1 | 1 | 60 | 9 | R.SGITSTYEYLQLRFNKPVR.Y |
|  | 32244 | **1311.7672** | **6553.7997** | **6553.2376** | **0.5621** | **1** | **1** | **4.4** | **1** | **K.LSTWISKGLCLLFGVMCTSMAVAASVMGGVVQASLSIHGMCGGPMLGLFSLGIVFPFVNWK.G** |

| **540.** | P56385\|ATP5I_HUMAN    **Mass:** 7928     **Score:** 53     **Queries matched:** 4   **emPAI:** 1.32 |
| --- | --- |
|  | ATP synthase subunit e, mitochondrial OS=Homo sapiens GN=ATP5I PE=1 SV=2 |

|  | **Query** | **Observed** | **Mr(expt)** | **Mr(calc)** | **Delta** | **Miss** | **Score** | **Expect** | **Rank** | **Peptide** |
| --- | --- | --- | --- | --- | --- | --- | --- | --- | --- | --- |
|  | 3591 | **477.2611** | **952.5075** | **952.5130** | **-0.0055** | **0** | **47** | **0.0029** | **1** | **R.YNYLKPR.A** |
|  | 6523 | **588.8678** | **1175.7210** | **1175.7278** | **-0.0067** | **0** | **32** | **0.092** | **1** | **M.VPPVQVSPLIK.L** 6514 |
|  | 14824 | **824.8940** | **1647.7735** | **1648.9698** | **-1.1963** | **1** | **0** | **1.1e+002** | **2** | **-.MVPPVQVSPLIKLGR.Y** |

| **541.** | Q9Y5J7\|TIM9_HUMAN    **Mass:** 10599    **Score:** 52     **Queries matched:** 4   **emPAI:** 0.38 |
| --- | --- |
|  | Mitochondrial import inner membrane translocase subunit Tim9 OS=Homo sapiens GN=TIMM9 PE=1 SV=1 |

|  | **Query** | **Observed** | **Mr(expt)** | **Mr(calc)** | **Delta** | **Miss** | **Score** | **Expect** | **Rank** | **Peptide** |
| --- | --- | --- | --- | --- | --- | --- | --- | --- | --- | --- |
|  | 4558 | **512.3534** | **1022.6922** | **1021.5161** | **1.1761** | **1** | **12** | **8.9** | **1** | **K.MTQRISMR.F** |
|  | 9847 | 673.3598 | 1344.7050 | 1345.6547 | -0.9497 | 0 | 4 | 54 | 3 | -.MAAQIPESDQIK.Q |
|  | 16756 | **895.4420** | **1788.8695** | **1788.8795** | **-0.0100** | **0** | **52** | **0.00062** | **1** | **R.FQEYHIQQNEALAAK.A** 16760 |

| **542.** | P02549\|SPTA1_HUMAN    **Mass:** 281023   **Score:** 52     **Queries matched:** 41   **emPAI:** 0.04 |
| --- | --- |
|  | Spectrin alpha chain, erythrocyte OS=Homo sapiens GN=SPTA1 PE=1 SV=4 |

|  | **Query** | **Observed** | **Mr(expt)** | **Mr(calc)** | **Delta** | **Miss** | **Score** | **Expect** | **Rank** | **Peptide** |
| --- | --- | --- | --- | --- | --- | --- | --- | --- | --- | --- |
|  | 1507 | 401.2857 | 800.5568 | 799.4664 | 1.0904 | 1 | 9 | 26 | 5 | R.DGLLARR.D |
|  | 1681 | 408.2505 | 814.4864 | 813.4708 | 1.0156 | 1 | 2 | 1.3e+002 | 8 | K.EPLATRK.K |
|  | 1704 | **409.2122** | **816.4098** | **816.4453** | **-0.0355** | **1** | **9** | **28** | **3** | **K.KLNEASR.Q** |
|  | 2903 | **451.2622** | **900.5098** | **900.5141** | **-0.0043** | **1** | **18** | **3.4** | **1** | **R.RQEVLTR.Y** |
|  | 3524 | 474.2676 | 946.5207 | 947.4746 | -0.9539 | 1 | 7 | 34 | 7 | K.ELCAKAEK.L |
|  | 4999 | 529.7929 | 1057.5711 | 1056.5676 | 1.0036 | 0 | 6 | 40 | 10 | K.ALSNAANLQR.F |
|  | 5374 | 544.8158 | 1087.6170 | 1087.5047 | 0.1124 | 0 | 4 | 74 | 10 | K.HFDENLTGR.L |
|  | 5750 | 558.2845 | 1114.5545 | 1113.5376 | 1.0170 | 1 | 7 | 32 | 1 | K.SYITKEDMK.Q |
|  | 5819 | **560.3005** | **1118.5864** | **1117.5662** | **1.0202** | **1** | **16** | **3.7** | **1** | **R.ARQESLVCR.F** |
|  | 6115 | **573.3939** | **1144.7733** | **1143.6440** | **1.1292** | **1** | **4** | **52** | **1** | **K.FYLFLSKAR.D** |
|  | 6942 | **400.2353** | **1197.6841** | **1198.6054** | **-0.9213** | **1** | **12** | **11** | **4** | **R.REEPGNITQR.Q** |
|  | 7192 | **404.2245** | **1209.6517** | **1208.5958** | **1.0559** | **0** | **7** | **25** | **5** | **K.SSLDSLEALMK.K** |
|  | 7238 | **607.3011** | **1212.5876** | **1212.5961** | **-0.0085** | **0** | **5** | **48** | **2** | **R.VMALYDFQAR.S** |
|  | 7360 | 610.2816 | 1218.5486 | 1217.6503 | 0.8983 | 1 | 2 | 96 | 9 | K.DIKGVSEETLK.E |
|  | 8196 | **630.2968** | **1258.5791** | **1258.6153** | **-0.0362** | **1** | **5** | **41** | **9** | **R.EQELQKEEAR.Q** |
|  | 8418 | **635.3217** | **1268.6289** | **1268.6361** | **-0.0072** | **0** | **35** | **0.04** | **1** | **K.HQSLEAEVQTK.S** |
|  | 10063 | 453.2318 | 1356.6735 | 1357.7354 | -1.0619 | 1 | 8 | 24 | 7 | K.RDVTEAIQWIK.E 10075 10084 |
|  | 10108 | **680.8449** | **1359.6753** | **1359.7245** | **-0.0492** | **1** | **7** | **34** | **6** | **K.EISSKIEALNEK.T** |
|  | 10874 | 702.8731 | 1403.7317 | 1402.6940 | 1.0377 | 1 | 4 | 55 | 2 | R.DDLDKAITAQEGK.I 10855 |
|  | 13869 | 793.9274 | 1585.8402 | 1586.7722 | -0.9321 | 0 | 4 | 50 | 7 | R.VQNVCAQGEDILNK.V |
|  | 13906 | 530.6111 | 1588.8116 | 1589.7144 | -0.9027 | 0 | 3 | 66 | 10 | K.MELNEAWEDLQGR.T |
|  | 14034 | **799.4619** | **1596.9091** | **1596.7380** | **0.1712** | **0** | **2** | **72** | **4** | **R.LSESHPDATEDLQR.Q** |
|  | 15704 | 854.4262 | 1706.8377 | 1707.7951 | -0.9574 | 0 | 2 | 75 | 6 | K.SSDEIENAFQALAEGK.S |
|  | 15921 | **575.3157** | **1722.9252** | **1722.9053** | **0.0199** | **0** | **2** | **62** | **3** | **K.VEAADHQGIVPAVYVR.R** |
|  | 16794 | **598.5643** | **1792.6710** | **1791.8713** | **0.7997** | **1** | **(1)** | **80** | **5** | **-.MEQFPKETVVESSGPK.V** |
|  | 16988 | **603.9882** | **1808.9428** | **1807.8662** | **1.0766** | **1** | **3** | **49** | **5** | **-.MEQFPKETVVESSGPK.V** |
|  | 17109 | 606.9625 | 1817.8657 | 1818.8570 | -0.9913 | 1 | 6 | 27 | 3 | K.MELNEAWEDLQGRTK.D |
|  | 17121 | **607.2900** | **1818.8483** | **1817.8195** | **1.0288** | **0** | **1** | **81** | **9** | **R.QYEQCLDFHLFYR.D** |
|  | 18484 | **482.4911** | **1925.9353** | **1925.0470** | **0.8883** | **1** | **2** | **56** | **5** | **R.DLVPLGDKVTILGETAER.L** |
|  | 19994 | **689.3085** | **2064.9036** | **2064.9171** | **-0.0135** | **0** | **32** | **0.055** | **1** | **K.TGQEMIEGGHYASDNVTTR.L** |
|  | 21618 | 749.4173 | 2245.2301 | 2246.2018 | -0.9718 | 1 | 3 | 40 | 5 | R.VILENIASHEPRIQEITER.G |
|  | 22701 | **797.4219** | **2389.2440** | **2389.1808** | **0.0632** | **1** | **2** | **45** | **1** | **K.CQALSAADPGSDLFSVQALQRR.H** |
|  | 22812 | **603.5360** | **2410.1149** | **2411.1968** | **-1.0819** | **1** | **3** | **34** | **2** | **K.EAIATSVELGEDWERTEVLHK.K** |
|  | 23064 | **816.4130** | **2446.2171** | **2446.1863** | **0.0307** | **1** | **22** | **0.42** | **1** | **R.EKEPIVDNTNYGADEEAAGALLK.K** |
|  | 24246 | **881.7540** | **2642.2403** | **2642.2970** | **-0.0567** | **1** | **0** | **60** | **5** | **R.VQNVCAQGEDILNKVLQEESQNK.E** |
|  | 24429 | 892.4614 | 2674.3623 | 2674.3450 | 0.0173 | 1 | 3 | 29 | 2 | K.QDTLDASLQSFQQERLPEITDLK.D |
|  | 27010 | **797.6395** | **3186.5290** | **3186.5482** | **-0.0192** | **1** | **2** | **30** | **2** | **R.RQNDLEANVQFQQYLADLHEAETWIR.E** |
|  | 29528 | **1315.8190** | **3944.4351** | **3944.8190** | **-0.3839** | **1** | **1** | **18** | **1** | **R.SEQVHGVINLGNSLIECSACDGNEEAMKEQLEQLK.E** |

| **543.** | P02790\|HEMO_HUMAN    **Mass:** 52385    **Score:** 52     **Queries matched:** 11   **emPAI:** 0.15 |
| --- | --- |
|  | Hemopexin OS=Homo sapiens GN=HPX PE=1 SV=2 |

|  | **Query** | **Observed** | **Mr(expt)** | **Mr(calc)** | **Delta** | **Miss** | **Score** | **Expect** | **Rank** | **Peptide** |
| --- | --- | --- | --- | --- | --- | --- | --- | --- | --- | --- |
|  | 7043 | **401.8779** | **1202.6120** | **1202.6044** | **0.0076** | **1** | **10** | **16** | **5** | **K.WDRELISER.W** |
|  | 7363 | **407.1997** | **1218.5774** | **1219.5986** | **-1.0212** | **0** | **9** | **22** | **2** | **K.NFPSPVDAAFR.Q** |
|  | 9313 | 658.8158 | 1315.6170 | 1316.6765 | -1.0594 | 1 | 1 | 1.3e+002 | 7 | K.GDKVWVYPPEK.K |
|  | 12656 | 756.8355 | 1511.6563 | 1511.6650 | -0.0086 | 1 | 15 | 3.9 | 1 | R.DVRDYFMPCPGR.G 12657 |
|  | 20415 | **704.4001** | **2110.1784** | **2108.9891** | **1.1894** | **1** | **0** | **78** | **8** | **R.YYCFQGNQFLRFDPVR.G** |
|  | 23403 | **833.4122** | **2497.2147** | **2497.2271** | **-0.0125** | **0** | **26** | **0.16** | **1** | **K.EVGTPHGIILDSVDAAFICPGSSR.L** |
|  | 27395 | **1094.8378** | **3281.4915** | **3281.5121** | **-0.0206** | **0** | **43** | **0.002** | **1** | **K.SLGPNSCSANGPGLYLIHGPNLYCYSDVEK.L** 27398 |
|  | 29122 | **945.9601** | **3779.8112** | **3778.8083** | **1.0029** | **1** | **14** | **1.3** | **1** | **K.SLGPNSCSANGPGLYLIHGPNLYCYSDVEKLNAAK.A** 29121 |

| **544.** | P07197\|NFM_HUMAN    **Mass:** 102444   **Score:** 52     **Queries matched:** 22   **emPAI:** 0.04 |
| --- | --- |
|  | Neurofilament medium polypeptide OS=Homo sapiens GN=NEFM PE=1 SV=2 |

|  | **Query** | **Observed** | **Mr(expt)** | **Mr(calc)** | **Delta** | **Miss** | **Score** | **Expect** | **Rank** | **Peptide** |
| --- | --- | --- | --- | --- | --- | --- | --- | --- | --- | --- |
|  | 1284 | **780.3431** | **779.3359** | **779.3450** | **-0.0091** | **0** | **0** | **1.8e+002** | **5** | **R.FEEEAR.L** |
|  | 1561 | **403.2284** | **804.4422** | **803.4025** | **1.0397** | **1** | **7** | **41** | **3** | **K.KEAAEEK.E** |
|  | 1876 | **416.2169** | **830.4192** | **829.4592** | **0.9600** | **1** | **12** | **15** | **3** | **K.RSMLAPR.L** |
|  | 2242 | **428.2584** | **854.5023** | **853.5022** | **1.0001** | **0** | **0** | **1.4e+002** | **7** | **K.VTSHAIVK.E** |
|  | 3293 | **464.9208** | **927.8271** | **927.5389** | **0.2882** | **1** | **1** | **91** | **10** | **K.VEKPEKAK.S** |
|  | 5851 | 561.7703 | 1121.5260 | 1120.5764 | 0.9496 | 0 | 3 | 77 | 5 | R.EYQDLLNVK.M |
|  | 6474 | **586.9147** | **1171.8149** | **1170.6244** | **1.1905** | **0** | **3** | **65** | **8** | **K.EIEAEIQALR.Q** |
|  | 6708 | **396.7319** | **1187.1738** | **1186.5652** | **0.6086** | **0** | **7** | **22** | **10** | **R.ATLEMVNHEK.A** |
|  | 6885 | **399.3459** | **1195.0158** | **1195.6448** | **-0.6290** | **1** | **8** | **14** | **1** | **K.SPVPKSPVEEK.G** |
|  | 7916 | **415.8938** | **1244.6595** | **1245.6928** | **-1.0334** | **1** | **7** | **30** | **9** | **K.TDISTALKEIR.S** |
|  | 12914 | 764.4131 | 1526.8116 | 1526.8205 | -0.0089 | 1 | 52 | 0.0008 | 1 | R.HLREYQDLLNVK.M 12915 |
|  | 12989 | 767.4257 | 1532.8369 | 1531.8358 | 1.0011 | 1 | 6 | 28 | 8 | K.KVQSLQDEVAFLR.S |
|  | 13527 | 783.9255 | 1565.8364 | 1564.6489 | 1.1875 | 1 | 4 | 47 | 5 | K.EKAGGEGGSEEEGSDK.G |
|  | 13885 | 530.2528 | 1587.7366 | 1586.8403 | 0.8963 | 1 | 5 | 44 | 8 | K.DIEEASLVKVELDK.K |
|  | 15813 | 858.4601 | 1714.9056 | 1715.7737 | -0.8681 | 1 | 3 | 52 | 8 | K.EEEPEAEEEEVAAKK.S |
|  | 16290 | **876.8754** | **1751.7362** | **1752.7286** | **-0.9924** | **1** | **7** | **22** | **2** | **K.SDQAEEGGSEKEGSSEK.E** |
|  | 17298 | **915.9776** | **1829.9406** | **1829.8544** | **0.0862** | **1** | **1** | **86** | **1** | **R.SQSWSRGSPSTVSSSYK.R** |
|  | 18139 | 633.7911 | 1898.3514 | 1899.0201 | -0.6686 | 1 | 1 | 52 | 8 | K.AESPVKEEAVAEVVTITK.S |
|  | 22133 | 771.4148 | 2311.2225 | 2310.1967 | 1.0258 | 1 | 2 | 53 | 4 | K.VHYLEQQNKEIEAEIQALR.Q |
|  | 29290 | **961.4954** | **3841.9526** | **3841.7952** | **0.1574** | **1** | **6** | **9.3** | **1** | **R.LAYSSAMLSSAESSLDFSQSSSLLNGGSGPGGDYKLSR.S** |
|  | 30006 | **1036.2991** | **4141.1672** | **4140.9256** | **0.2416** | **1** | **9** | **2.5** | **1** | **R.SMLAPRLAYSSAMLSSAESSLDFSQSSSLLNGGSGPGGDYK.L** |

| **545.** | Q16352\|AINX_HUMAN    **Mass:** 55528    **Score:** 52     **Queries matched:** 14   **emPAI:** 0.07 |
| --- | --- |
|  | Alpha-internexin OS=Homo sapiens GN=INA PE=1 SV=2 |

|  | **Query** | **Observed** | **Mr(expt)** | **Mr(calc)** | **Delta** | **Miss** | **Score** | **Expect** | **Rank** | **Peptide** |
| --- | --- | --- | --- | --- | --- | --- | --- | --- | --- | --- |
|  | 3583 | **476.7636** | **951.5127** | **951.4444** | **0.0683** | **1** | **9** | **22** | **4** | **R.NTKSEMAR.H** |
|  | 5851 | 561.7703 | 1121.5260 | 1120.5764 | 0.9496 | 0 | 3 | 77 | 5 | R.EYQDLLNVK.M |
|  | 5979 | **567.7622** | **1133.5097** | **1132.5625** | **0.9473** | **0** | **5** | **54** | **9** | **K.FANLNEQAAR.S** |
|  | 7916 | 415.8938 | 1244.6595 | 1245.6830 | -1.0235 | 1 | 11 | 12 | 2 | R.VGELFQRELR.D |
|  | 8705 | 643.3292 | 1284.6439 | 1284.6786 | -0.0347 | 1 | 19 | 1.9 | 4 | R.DGLAEEVQRLR.A |
|  | 10447 | **460.3102** | **1377.9088** | **1377.7252** | **0.1836** | **1** | **5** | **35** | **4** | **R.EIRAQYESLAAK.N** |
|  | 10791 | **467.2221** | **1398.6445** | **1399.7168** | **-1.0723** | **1** | **5** | **44** | **9** | **K.AQQRDVDGATLAR.L** 10800 10821 |
|  | 11633 | 484.0645 | 1449.1718 | 1448.7358 | 0.4360 | 1 | 5 | 28 | 1 | K.SNIEETTISSQKI.- |
|  | 12914 | 764.4131 | 1526.8116 | 1526.8205 | -0.0089 | 1 | 52 | 0.0008 | 1 | R.HLREYQDLLNVK.M 12915 |
|  | 14471 | 813.4363 | 1624.8581 | 1623.8329 | 1.0253 | 0 | 6 | 32 | 3 | R.RPPASDGLDLSQAAAR.T |
|  | 16759 | 597.3032 | 1788.8877 | 1789.9071 | -1.0194 | 1 | 3 | 58 | 7 | K.FANLNEQAARSTEAIR.A |

| **546.** | P17661\|DESM_HUMAN    **Mass:** 53560    **Score:** 52     **Queries matched:** 14   **emPAI:** 0.07 |
| --- | --- |
|  | Desmin OS=Homo sapiens GN=DES PE=1 SV=3 |

|  | **Query** | **Observed** | **Mr(expt)** | **Mr(calc)** | **Delta** | **Miss** | **Score** | **Expect** | **Rank** | **Peptide** |
| --- | --- | --- | --- | --- | --- | --- | --- | --- | --- | --- |
|  | 5026 | 530.7892 | 1059.5638 | 1059.5560 | 0.0078 | 1 | 6 | 43 | 10 | R.KLLEGEESR.I |
|  | 5851 | 561.7703 | 1121.5260 | 1120.5764 | 0.9496 | 0 | 3 | 77 | 5 | R.EYQDLLNVK.M |
|  | 6791 | **397.8684** | **1190.5834** | **1190.6693** | **-0.0859** | **1** | **6** | **37** | **8** | **K.TVMIKTIETR.D** |
|  | 8377 | **634.3546** | **1266.6947** | **1267.5720** | **-0.8774** | **0** | **2** | **85** | **10** | **K.NISEAEEWYK.S** |
|  | 9463 | **441.9246** | **1322.7521** | **1323.6023** | **-0.8502** | **1** | **8** | **22** | **2** | **K.GTNDSLMRQMR.E** |
|  | 9840 | **449.2298** | **1344.6677** | **1345.7062** | **-1.0386** | **1** | **7** | **28** | **1** | **R.TSGGAGGLGSLRASR.L** |
|  | 10442 | **460.2564** | **1377.7473** | **1377.7252** | **0.0221** | **1** | **10** | **13** | **1** | **R.DIRAQYETIAAK.N** 10439 |
|  | 10898 | 469.3273 | 1404.9601 | 1404.7500 | 0.2101 | 0 | 2 | 66 | 7 | R.IESLNEEIAFLK.K |
|  | 11041 | **472.3210** | **1413.9411** | **1413.6848** | **0.2563** | **1** | **2** | **64** | **10** | **R.AGFGSKGSSSSVTSR.V** |
|  | 11231 | **476.2549** | **1425.7430** | **1424.7333** | **1.0097** | **1** | **3** | **64** | **9** | **K.MALDVEIATYRK.L** |
|  | 12914 | 764.4131 | 1526.8116 | 1526.8205 | -0.0089 | 1 | 52 | 0.0008 | 1 | R.HLREYQDLLNVK.M 12915 |
|  | 14819 | **550.2502** | **1647.7287** | **1648.8937** | **-1.1649** | **0** | **2** | **70** | **5** | **R.INLPIQTYSALNFR.E** |

| **547.** | O00291\|HIP1_HUMAN    **Mass:** 117232   **Score:** 52     **Queries matched:** 16   **emPAI:** 0.03 |
| --- | --- |
|  | Huntingtin-interacting protein 1 OS=Homo sapiens GN=HIP1 PE=1 SV=5 |

|  | **Query** | **Observed** | **Mr(expt)** | **Mr(calc)** | **Delta** | **Miss** | **Score** | **Expect** | **Rank** | **Peptide** |
| --- | --- | --- | --- | --- | --- | --- | --- | --- | --- | --- |
|  | 1342 | **395.7424** | **789.4702** | **788.4028** | **1.0674** | **0** | **2** | **1.3e+002** | **3** | **R.EIVESGR.G** |
|  | 1928 | **417.7261** | **833.4376** | **832.4290** | **1.0086** | **0** | **9** | **23** | **2** | **R.SLSEIER.K** |
|  | 2088 | **422.7550** | **843.4954** | **842.4974** | **0.9981** | **1** | **5** | **62** | **3** | **R.QKLGELR.K** |
|  | 2761 | 445.8225 | 889.6304 | 889.4617 | 0.1687 | 1 | 2 | 1.3e+002 | 8 | K.SRAGDTGVK.L |
|  | 3442 | 471.2355 | 940.4564 | 940.4106 | 0.0458 | 1 | 8 | 22 | 4 | M.DRMASSMK.Q |
|  | 3644 | **479.2561** | **956.4977** | **957.4879** | **-0.9902** | **0** | **9** | **19** | **10** | **R.QAQVDLER.E** |
|  | 4065 | **494.2934** | **986.5722** | **985.4869** | **1.0854** | **0** | **6** | **43** | **8** | **R.SSNLQYFK.R** |
|  | 4210 | 499.2592 | 996.5038 | 996.5604 | -0.0566 | 0 | 6 | 35 | 5 | K.AIGEELLPR.G 4201 |
|  | 12360 | 498.2545 | 1491.7416 | 1492.7191 | -0.9776 | 0 | (4) | 55 | 10 | K.EMAATSAAIETATAR.I |
|  | 12625 | 755.8861 | 1509.7577 | 1508.7140 | 1.0436 | 0 | 6 | 29 | 4 | K.EMAATSAAIETATAR.I 12626 |
|  | 13420 | 520.6062 | 1558.7968 | 1558.7847 | 0.0121 | 1 | 3 | 60 | 7 | R.MASSMKQVPNPLPK.V |
|  | 13423 | 780.4182 | 1558.8219 | 1558.8315 | -0.0096 | 0 | 52 | 0.00085 | 1 | R.GVNQATAGVVASTISGK.S |
|  | 17952 | 628.2940 | 1881.8600 | 1881.8560 | 0.0040 | 1 | 3 | 52 | 6 | K.LASTEESMCQLAKDQR.K |
|  | 21024 | 545.4877 | 2177.9218 | 2178.0011 | -0.0793 | 1 | 4 | 31 | 10 | R.QQAADDCEFLRAELDELR.R |

| **548.** | P16188\|1A30_HUMAN    **Mass:** 41164    **Score:** 52     **Queries matched:** 15   **emPAI:** 0.19 |
| --- | --- |
|  | HLA class I histocompatibility antigen, A-30 alpha chain OS=Homo sapiens GN=HLA-A PE=1 SV=2 |

|  | **Query** | **Observed** | **Mr(expt)** | **Mr(calc)** | **Delta** | **Miss** | **Score** | **Expect** | **Rank** | **Peptide** |
| --- | --- | --- | --- | --- | --- | --- | --- | --- | --- | --- |
|  | 1530 | **402.2437** | **802.4729** | **801.4133** | **1.0596** | **0** | **12** | **15** | **5** | **R.WAEQLR.A** |
|  | 4195 | 498.7497 | 995.4848 | 995.4308 | 0.0539 | 0 | 8 | 22 | 3 | R.FDSDAASQR.M 4211 |
|  | 9948 | 676.3410 | 1350.6674 | 1349.6939 | 0.9736 | 1 | 14 | 5.9 | 1 | R.YLENGKETLQR.T 9957 |
|  | 12866 | 508.9794 | 1523.9163 | 1524.6627 | -0.7464 | 1 | 2 | 65 | 3 | R.FDSDAASQRMEPR.A |
|  | 13421 | 780.4057 | 1558.7968 | 1558.8063 | -0.0095 | 1 | 15 | 3.7 | 3 | K.AQSQTDRVDLGTLR.G 13420 |
|  | 13526 | 522.9525 | 1565.8355 | 1564.7304 | 1.1051 | 0 | 2 | 67 | 6 | R.SWTAADMAAQITQR.K |
|  | 14538 | 815.4131 | 1628.8117 | 1628.8199 | -0.0081 | 0 | 28 | 0.17 | 1 | R.FIAVGYVDDTQFVR.F |
|  | 17947 | 471.2618 | 1881.0179 | 1881.8904 | -0.8725 | 0 | 11 | 8.5 | 2 | K.THMTHHPISDHEATLR.C |
|  | 23192 | **822.3981** | **2464.1726** | **2464.3260** | **-0.1535** | **0** | **1** | **57** | **2** | **R.TLLLLLSGALALTHTWAGSHSMR.Y** |
|  | 24080 | 869.7491 | 2606.2254 | 2606.2402 | -0.0147 | 1 | 15 | 1.9 | 1 | R.FIAVGYVDDTQFVRFDSDAASQR.M |
|  | 24215 | 879.7303 | 2636.1690 | 2636.1838 | -0.0148 | 0 | 43 | 0.003 | 1 | R.DGEDQTQDTELVETRPAGDGTFQK.W |
|  | 29354 | 968.9431 | 3871.7434 | 3872.2114 | -0.4680 | 1 | 2 | 17 | 1 | R.WELSSQPTIPIVGIIAGLVLLGAVITGAVVAAVMWRR.K |

| **549.** | P01891\|1A68_HUMAN    **Mass:** 41168    **Score:** 52     **Queries matched:** 13   **emPAI:** 0.19 |
| --- | --- |
|  | HLA class I histocompatibility antigen, A-68 alpha chain OS=Homo sapiens GN=HLA-A PE=1 SV=4 |

|  | **Query** | **Observed** | **Mr(expt)** | **Mr(calc)** | **Delta** | **Miss** | **Score** | **Expect** | **Rank** | **Peptide** |
| --- | --- | --- | --- | --- | --- | --- | --- | --- | --- | --- |
|  | 1644 | 406.7126 | 811.4107 | 810.4500 | 0.9606 | 1 | 4 | 56 | 10 | R.FLRGYR.Q |
|  | 4195 | 498.7497 | 995.4848 | 995.4308 | 0.0539 | 0 | 8 | 22 | 3 | R.FDSDAASQR.M 4211 |
|  | 9948 | 676.3410 | 1350.6674 | 1349.6939 | 0.9736 | 1 | 14 | 5.9 | 1 | R.YLENGKETLQR.T 9957 |
|  | 12866 | 508.9794 | 1523.9163 | 1524.6627 | -0.7464 | 1 | 2 | 65 | 3 | R.FDSDAASQRMEPR.A |
|  | 13421 | 780.4057 | 1558.7968 | 1558.8063 | -0.0095 | 1 | 15 | 3.7 | 3 | K.AQSQTDRVDLGTLR.G 13420 |
|  | 14538 | 815.4131 | 1628.8117 | 1628.8199 | -0.0081 | 0 | 28 | 0.17 | 1 | R.FIAVGYVDDTQFVR.F |
|  | 17461 | 615.3420 | 1843.0041 | 1841.8591 | 1.1450 | 0 | 10 | 11 | 2 | K.THMTHHAVSDHEATLR.C |
|  | 24080 | 869.7491 | 2606.2254 | 2606.2402 | -0.0147 | 1 | 15 | 1.9 | 1 | R.FIAVGYVDDTQFVRFDSDAASQR.M |
|  | 24215 | 879.7303 | 2636.1690 | 2636.1838 | -0.0148 | 0 | 43 | 0.003 | 1 | R.DGEDQTQDTELVETRPAGDGTFQK.W |
|  | 27203 | 647.1058 | 3230.4925 | 3229.6774 | 0.8151 | 1 | 4 | 20 | 1 | -.MAVMAPRTLVLLLSGALALTQTWAGSHSMR.Y |

| **550.** | P10316\|1A69_HUMAN    **Mass:** 41236    **Score:** 52     **Queries matched:** 13   **emPAI:** 0.19 |
| --- | --- |
|  | HLA class I histocompatibility antigen, A-69 alpha chain OS=Homo sapiens GN=HLA-A PE=1 SV=2 |

|  | **Query** | **Observed** | **Mr(expt)** | **Mr(calc)** | **Delta** | **Miss** | **Score** | **Expect** | **Rank** | **Peptide** |
| --- | --- | --- | --- | --- | --- | --- | --- | --- | --- | --- |
|  | 4195 | 498.7497 | 995.4848 | 995.4308 | 0.0539 | 0 | 8 | 22 | 3 | R.FDSDAASQR.M 4211 |
|  | 9948 | 676.3410 | 1350.6674 | 1349.6939 | 0.9736 | 1 | 14 | 5.9 | 1 | R.YLENGKETLQR.T 9957 |
|  | 11244 | 714.3414 | 1426.6683 | 1425.7365 | 0.9318 | 0 | 3 | 60 | 3 | K.WAAVVVPSGQEQR.Y |
|  | 12866 | 508.9794 | 1523.9163 | 1524.6627 | -0.7464 | 1 | 2 | 65 | 3 | R.FDSDAASQRMEPR.A |
|  | 13421 | 780.4057 | 1558.7968 | 1558.8063 | -0.0095 | 1 | 15 | 3.7 | 3 | K.AQSQTDRVDLGTLR.G 13420 |
|  | 14538 | 815.4131 | 1628.8117 | 1628.8199 | -0.0081 | 0 | 28 | 0.17 | 1 | R.FIAVGYVDDTQFVR.F |
|  | 17461 | 615.3420 | 1843.0041 | 1841.8591 | 1.1450 | 0 | 10 | 11 | 2 | K.THMTHHAVSDHEATLR.C |
|  | 24080 | 869.7491 | 2606.2254 | 2606.2402 | -0.0147 | 1 | 15 | 1.9 | 1 | R.FIAVGYVDDTQFVRFDSDAASQR.M |
|  | 24215 | 879.7303 | 2636.1690 | 2636.1838 | -0.0148 | 0 | 43 | 0.003 | 1 | R.DGEDQTQDTELVETRPAGDGTFQK.W |
|  | 27203 | 647.1058 | 3230.4925 | 3229.6774 | 0.8151 | 1 | 4 | 20 | 1 | -.MAVMAPRTLVLLLSGALALTQTWAGSHSMR.Y |

| **551.** | P30512\|1A29_HUMAN    **Mass:** 41122    **Score:** 52     **Queries matched:** 13   **emPAI:** 0.19 |
| --- | --- |
|  | HLA class I histocompatibility antigen, A-29 alpha chain OS=Homo sapiens GN=HLA-A PE=2 SV=2 |

|  | **Query** | **Observed** | **Mr(expt)** | **Mr(calc)** | **Delta** | **Miss** | **Score** | **Expect** | **Rank** | **Peptide** |
| --- | --- | --- | --- | --- | --- | --- | --- | --- | --- | --- |
|  | 1644 | 406.7126 | 811.4107 | 810.4500 | 0.9606 | 1 | 4 | 56 | 10 | R.FLRGYR.Q |
|  | 4195 | 498.7497 | 995.4848 | 995.4308 | 0.0539 | 0 | 8 | 22 | 3 | R.FDSDAASQR.M 4211 |
|  | 9948 | 676.3410 | 1350.6674 | 1349.6939 | 0.9736 | 1 | 14 | 5.9 | 1 | R.YLENGKETLQR.T 9957 |
|  | 12866 | 508.9794 | 1523.9163 | 1524.6627 | -0.7464 | 1 | 2 | 65 | 3 | R.FDSDAASQRMEPR.A |
|  | 13526 | 522.9525 | 1565.8355 | 1564.7304 | 1.1051 | 0 | 2 | 67 | 6 | R.SWTAADMAAQITQR.K |
|  | 14538 | 815.4131 | 1628.8117 | 1628.8199 | -0.0081 | 0 | 28 | 0.17 | 1 | R.FIAVGYVDDTQFVR.F |
|  | 17461 | 615.3420 | 1843.0041 | 1841.8591 | 1.1450 | 0 | 10 | 11 | 2 | K.THMTHHAVSDHEATLR.C |
|  | 23144 | 820.1008 | 2457.2807 | 2458.1917 | -0.9110 | 1 | 3 | 40 | 8 | R.APWIEQEGPEYWDLQTRNVK.A |
|  | 24080 | 869.7491 | 2606.2254 | 2606.2402 | -0.0147 | 1 | 15 | 1.9 | 1 | R.FIAVGYVDDTQFVRFDSDAASQR.M |
|  | 24215 | 879.7303 | 2636.1690 | 2636.1838 | -0.0148 | 0 | 43 | 0.003 | 1 | R.DGEDQTQDTELVETRPAGDGTFQK.W |
|  | 26713 | 781.6638 | 3122.6259 | 3122.7096 | -0.0837 | 1 | 9 | 6.6 | 2 | M.AVMAPRTLLLLLLGALALTQTWAGSHSMR.Y |

|  | **Proteins matching a subset of these peptides:** |
| --- | --- |

|  | P16189\|1A31_HUMAN    **Mass:** 41263    **Score:** 52     **Queries matched:** 11 |
| --- | --- |
|  | HLA class I histocompatibility antigen, A-31 alpha chain OS=Homo sapiens GN=HLA-A PE=2 SV=2 |

|  | P16190\|1A33_HUMAN    **Mass:** 41151    **Score:** 52     **Queries matched:** 9 |
| --- | --- |
|  | HLA class I histocompatibility antigen, A-33 alpha chain OS=Homo sapiens GN=HLA-A PE=1 SV=3 |

| **552.** | P05534\|1A24_HUMAN    **Mass:** 40948    **Score:** 52     **Queries matched:** 13   **emPAI:** 0.19 |
| --- | --- |
|  | HLA class I histocompatibility antigen, A-24 alpha chain OS=Homo sapiens GN=HLA-A PE=1 SV=2 |

|  | **Query** | **Observed** | **Mr(expt)** | **Mr(calc)** | **Delta** | **Miss** | **Score** | **Expect** | **Rank** | **Peptide** |
| --- | --- | --- | --- | --- | --- | --- | --- | --- | --- | --- |
|  | 4195 | 498.7497 | 995.4848 | 995.4308 | 0.0539 | 0 | 8 | 22 | 3 | R.FDSDAASQR.M 4211 |
|  | 9948 | 676.3410 | 1350.6674 | 1349.6939 | 0.9736 | 1 | 14 | 5.9 | 1 | R.YLENGKETLQR.T 9957 |
|  | 12866 | 508.9794 | 1523.9163 | 1524.6627 | -0.7464 | 1 | 2 | 65 | 3 | R.FDSDAASQRMEPR.A |
|  | 13526 | 522.9525 | 1565.8355 | 1564.7668 | 1.0688 | 1 | 2 | 67 | 6 | R.SWTAADMAAQITKR.K |
|  | 14538 | 815.4131 | 1628.8117 | 1628.8199 | -0.0081 | 0 | 28 | 0.17 | 1 | R.FIAVGYVDDTQFVR.F |
|  | 17947 | 471.2618 | 1881.0179 | 1881.8904 | -0.8725 | 0 | 11 | 8.5 | 2 | K.THMTHHPISDHEATLR.C |
|  | 18437 | **641.9667** | **1922.8782** | **1921.9203** | **0.9578** | **1** | **9** | **13** | **1** | **K.EDLRSWTAADMAAQITK.R** |
|  | 22705 | **797.6228** | **2389.8466** | **2390.1066** | **-0.2601** | **1** | **1** | **42** | **3** | **R.APWIEQEGPEYWDEETGKVK.A** |
|  | 24080 | 869.7491 | 2606.2254 | 2606.2402 | -0.0147 | 1 | 15 | 1.9 | 1 | R.FIAVGYVDDTQFVRFDSDAASQR.M |
|  | 24215 | 879.7303 | 2636.1690 | 2636.1838 | -0.0148 | 0 | 43 | 0.003 | 1 | R.DGEDQTQDTELVETRPAGDGTFQK.W |
|  | 27203 | 647.1058 | 3230.4925 | 3229.6774 | 0.8151 | 1 | 4 | 20 | 1 | -.MAVMAPRTLVLLLSGALALTQTWAGSHSMR.Y |

| **553.** | P10314\|1A32_HUMAN    **Mass:** 41307    **Score:** 52     **Queries matched:** 12   **emPAI:** 0.19 |
| --- | --- |
|  | HLA class I histocompatibility antigen, A-32 alpha chain OS=Homo sapiens GN=HLA-A PE=2 SV=2 |

|  | **Query** | **Observed** | **Mr(expt)** | **Mr(calc)** | **Delta** | **Miss** | **Score** | **Expect** | **Rank** | **Peptide** |
| --- | --- | --- | --- | --- | --- | --- | --- | --- | --- | --- |
|  | 4195 | 498.7497 | 995.4848 | 995.4308 | 0.0539 | 0 | 8 | 22 | 3 | R.FDSDAASQR.M 4211 |
|  | 9004 | 434.2593 | 1299.7561 | 1298.6327 | 1.1234 | 1 | 3 | 71 | 8 | K.AHSQTDRESLR.I |
|  | 9948 | 676.3410 | 1350.6674 | 1349.6939 | 0.9736 | 1 | 14 | 5.9 | 1 | R.YLENGKETLQR.T 9957 |
|  | 12866 | 508.9794 | 1523.9163 | 1524.6627 | -0.7464 | 1 | 2 | 65 | 3 | R.FDSDAASQRMEPR.A |
|  | 13526 | 522.9525 | 1565.8355 | 1564.7304 | 1.1051 | 0 | 2 | 67 | 6 | R.SWTAADMAAQITQR.K |
|  | 14538 | 815.4131 | 1628.8117 | 1628.8199 | -0.0081 | 0 | 28 | 0.17 | 1 | R.FIAVGYVDDTQFVR.F |
|  | 17461 | 615.3420 | 1843.0041 | 1841.8591 | 1.1450 | 0 | 10 | 11 | 2 | K.THMTHHAVSDHEATLR.C |
|  | 24080 | 869.7491 | 2606.2254 | 2606.2402 | -0.0147 | 1 | 15 | 1.9 | 1 | R.FIAVGYVDDTQFVRFDSDAASQR.M |
|  | 24215 | 879.7303 | 2636.1690 | 2636.1838 | -0.0148 | 0 | 43 | 0.003 | 1 | R.DGEDQTQDTELVETRPAGDGTFQK.W |
|  | 26713 | 781.6638 | 3122.6259 | 3122.7096 | -0.0837 | 1 | 9 | 6.6 | 2 | M.AVMAPRTLLLLLLGALALTQTWAGSHSMR.Y |

| **554.** | P01892\|1A02_HUMAN    **Mass:** 41181    **Score:** 52     **Queries matched:** 12   **emPAI:** 0.19 |
| --- | --- |
|  | HLA class I histocompatibility antigen, A-2 alpha chain OS=Homo sapiens GN=HLA-A PE=1 SV=1 |

|  | **Query** | **Observed** | **Mr(expt)** | **Mr(calc)** | **Delta** | **Miss** | **Score** | **Expect** | **Rank** | **Peptide** |
| --- | --- | --- | --- | --- | --- | --- | --- | --- | --- | --- |
|  | 4195 | 498.7497 | 995.4848 | 995.4308 | 0.0539 | 0 | 8 | 22 | 3 | R.FDSDAASQR.M 4211 |
|  | 5062 | **532.2807** | **1062.5468** | **1062.5683** | **-0.0214** | **1** | **4** | **63** | **9** | **K.VKAHSQTHR.V** |
|  | 9948 | 676.3410 | 1350.6674 | 1349.6939 | 0.9736 | 1 | 14 | 5.9 | 1 | R.YLENGKETLQR.T 9957 |
|  | 11244 | 714.3414 | 1426.6683 | 1425.7365 | 0.9318 | 0 | 3 | 60 | 3 | K.WAAVVVPSGQEQR.Y |
|  | 12866 | 508.9794 | 1523.9163 | 1524.6627 | -0.7464 | 1 | 2 | 65 | 3 | R.FDSDAASQRMEPR.A |
|  | 14538 | 815.4131 | 1628.8117 | 1628.8199 | -0.0081 | 0 | 28 | 0.17 | 1 | R.FIAVGYVDDTQFVR.F |
|  | 17461 | 615.3420 | 1843.0041 | 1841.8591 | 1.1450 | 0 | 10 | 11 | 2 | K.THMTHHAVSDHEATLR.C |
|  | 24080 | 869.7491 | 2606.2254 | 2606.2402 | -0.0147 | 1 | 15 | 1.9 | 1 | R.FIAVGYVDDTQFVRFDSDAASQR.M |
|  | 24215 | 879.7303 | 2636.1690 | 2636.1838 | -0.0148 | 0 | 43 | 0.003 | 1 | R.DGEDQTQDTELVETRPAGDGTFQK.W |
|  | 27203 | 647.1058 | 3230.4925 | 3229.6774 | 0.8151 | 1 | 4 | 20 | 1 | -.MAVMAPRTLVLLLSGALALTQTWAGSHSMR.Y |

| **555.** | P30447\|1A23_HUMAN    **Mass:** 40992    **Score:** 52     **Queries matched:** 12   **emPAI:** 0.19 |
| --- | --- |
|  | HLA class I histocompatibility antigen, A-23 alpha chain OS=Homo sapiens GN=HLA-A PE=1 SV=1 |

|  | **Query** | **Observed** | **Mr(expt)** | **Mr(calc)** | **Delta** | **Miss** | **Score** | **Expect** | **Rank** | **Peptide** |
| --- | --- | --- | --- | --- | --- | --- | --- | --- | --- | --- |
|  | 4195 | 498.7497 | 995.4848 | 995.4308 | 0.0539 | 0 | 8 | 22 | 3 | R.FDSDAASQR.M 4211 |
|  | 9948 | 676.3410 | 1350.6674 | 1349.6939 | 0.9736 | 1 | 14 | 5.9 | 1 | R.YLENGKETLQR.T 9957 |
|  | 12866 | 508.9794 | 1523.9163 | 1524.6627 | -0.7464 | 1 | 2 | 65 | 3 | R.FDSDAASQRMEPR.A |
|  | 13526 | 522.9525 | 1565.8355 | 1564.7304 | 1.1051 | 0 | 2 | 67 | 6 | R.SWTAADMAAQITQR.K |
|  | 14538 | 815.4131 | 1628.8117 | 1628.8199 | -0.0081 | 0 | 28 | 0.17 | 1 | R.FIAVGYVDDTQFVR.F |
|  | 17947 | 471.2618 | 1881.0179 | 1881.8904 | -0.8725 | 0 | 11 | 8.5 | 2 | K.THMTHHPISDHEATLR.C |
|  | 22705 | 797.6228 | 2389.8466 | 2390.1066 | -0.2601 | 1 | 1 | 42 | 3 | R.APWIEQEGPEYWDEETGKVK.A |
|  | 24080 | 869.7491 | 2606.2254 | 2606.2402 | -0.0147 | 1 | 15 | 1.9 | 1 | R.FIAVGYVDDTQFVRFDSDAASQR.M |
|  | 24215 | 879.7303 | 2636.1690 | 2636.1838 | -0.0148 | 0 | 43 | 0.003 | 1 | R.DGEDQTQDTELVETRPAGDGTFQK.W |
|  | 27203 | 647.1058 | 3230.4925 | 3229.6774 | 0.8151 | 1 | 4 | 20 | 1 | -.MAVMAPRTLVLLLSGALALTQTWAGSHSMR.Y |

| **556.** | P30459\|1A74_HUMAN    **Mass:** 41150    **Score:** 52     **Queries matched:** 11   **emPAI:** 0.19 |
| --- | --- |
|  | HLA class I histocompatibility antigen, A-74 alpha chain OS=Homo sapiens GN=HLA-A PE=1 SV=1 |

|  | **Query** | **Observed** | **Mr(expt)** | **Mr(calc)** | **Delta** | **Miss** | **Score** | **Expect** | **Rank** | **Peptide** |
| --- | --- | --- | --- | --- | --- | --- | --- | --- | --- | --- |
|  | 4195 | 498.7497 | 995.4848 | 995.4308 | 0.0539 | 0 | 8 | 22 | 3 | R.FDSDAASQR.M 4211 |
|  | 9948 | 676.3410 | 1350.6674 | 1349.6939 | 0.9736 | 1 | 14 | 5.9 | 1 | R.YLENGKETLQR.T 9957 |
|  | 12866 | 508.9794 | 1523.9163 | 1524.6627 | -0.7464 | 1 | 2 | 65 | 3 | R.FDSDAASQRMEPR.A |
|  | 13526 | 522.9525 | 1565.8355 | 1564.7304 | 1.1051 | 0 | 2 | 67 | 6 | R.SWTAADMAAQITQR.K |
|  | 14538 | 815.4131 | 1628.8117 | 1628.8199 | -0.0081 | 0 | 28 | 0.17 | 1 | R.FIAVGYVDDTQFVR.F |
|  | 17461 | 615.3420 | 1843.0041 | 1841.8591 | 1.1450 | 0 | 10 | 11 | 2 | K.THMTHHAVSDHEATLR.C |
|  | 23104 | **818.4385** | **2452.2936** | **2451.3995** | **0.8941** | **1** | **1** | **58** | **2** | **R.TLLLLLLGALALTQTRAGSHSMR.Y** |
|  | 24080 | 869.7491 | 2606.2254 | 2606.2402 | -0.0147 | 1 | 15 | 1.9 | 1 | R.FIAVGYVDDTQFVRFDSDAASQR.M |
|  | 24215 | 879.7303 | 2636.1690 | 2636.1838 | -0.0148 | 0 | 43 | 0.003 | 1 | R.DGEDQTQDTELVETRPAGDGTFQK.W |

| **557.** | P30443\|1A01_HUMAN    **Mass:** 41105    **Score:** 52     **Queries matched:** 10   **emPAI:** 0.19 |
| --- | --- |
|  | HLA class I histocompatibility antigen, A-1 alpha chain OS=Homo sapiens GN=HLA-A PE=1 SV=1 |

|  | **Query** | **Observed** | **Mr(expt)** | **Mr(calc)** | **Delta** | **Miss** | **Score** | **Expect** | **Rank** | **Peptide** |
| --- | --- | --- | --- | --- | --- | --- | --- | --- | --- | --- |
|  | 1644 | 406.7126 | 811.4107 | 810.4500 | 0.9606 | 1 | 4 | 56 | 10 | R.FLRGYR.Q |
|  | 7072 | 402.2401 | 1203.6984 | 1202.5462 | 1.1522 | 1 | 11 | 11 | 5 | R.NMKAHSQTDR.A |
|  | 9948 | 676.3410 | 1350.6674 | 1349.6939 | 0.9736 | 1 | 14 | 5.9 | 1 | R.YLENGKETLQR.T 9957 |
|  | 12448 | **500.2805** | **1497.8197** | **1496.6565** | **1.1632** | **1** | **4** | **52** | **2** | **R.FDSDAASQKMEPR.A** |
|  | 13526 | 522.9525 | 1565.8355 | 1564.7668 | 1.0688 | 1 | 2 | 67 | 6 | R.SWTAADMAAQITKR.K |
|  | 14538 | 815.4131 | 1628.8117 | 1628.8199 | -0.0081 | 0 | 28 | 0.17 | 1 | R.FIAVGYVDDTQFVR.F |
|  | 17947 | 471.2618 | 1881.0179 | 1881.8904 | -0.8725 | 0 | 11 | 8.5 | 2 | K.THMTHHPISDHEATLR.C |
|  | 24215 | 879.7303 | 2636.1690 | 2636.1838 | -0.0148 | 0 | 43 | 0.003 | 1 | R.DGEDQTQDTELVETRPAGDGTFQK.W |
|  | 29354 | 968.9431 | 3871.7434 | 3872.2114 | -0.4680 | 1 | 2 | 17 | 1 | R.WELSSQPTIPIVGIIAGLVLLGAVITGAVVAAVMWRR.K |

|  | **Proteins matching the same set of peptides:** |
| --- | --- |

|  | P30455\|1A36_HUMAN    **Mass:** 41193    **Score:** 52     **Queries matched:** 10 |
| --- | --- |
|  | HLA class I histocompatibility antigen, A-36 alpha chain OS=Homo sapiens GN=HLA-A PE=1 SV=1 |

| **558.** | Q13113\|PDZ1I_HUMAN    **Mass:** 12333    **Score:** 51     **Queries matched:** 5   **emPAI:** 0.75 |
| --- | --- |
|  | PDZK1-interacting protein 1 OS=Homo sapiens GN=PDZK1IP1 PE=1 SV=1 |

|  | **Query** | **Observed** | **Mr(expt)** | **Mr(calc)** | **Delta** | **Miss** | **Score** | **Expect** | **Rank** | **Peptide** |
| --- | --- | --- | --- | --- | --- | --- | --- | --- | --- | --- |
|  | 4506 | **510.2315** | **1018.4483** | **1018.4542** | **-0.0058** | **0** | **25** | **0.54** | **1** | **R.YSSMAASFR.S** |
|  | 5002 | 530.2469 | 1058.4792 | 1058.5357 | -0.0564 | 0 | 3 | 97 | 7 | K.ADGVLVGTDGR.Y |
|  | 21217 | **734.9971** | **2201.9696** | **2201.9825** | **-0.0129** | **1** | **45** | **0.0025** | **1** | **R.SSEHENAYENVPEEEGKVR.S** 21218 21223 |

| **559.** | P26885\|FKBP2_HUMAN    **Mass:** 15810    **Score:** 51     **Queries matched:** 4   **emPAI:** 0.25 |
| --- | --- |
|  | FK506-binding protein 2 OS=Homo sapiens GN=FKBP2 PE=1 SV=2 |

|  | **Query** | **Observed** | **Mr(expt)** | **Mr(calc)** | **Delta** | **Miss** | **Score** | **Expect** | **Rank** | **Peptide** |
| --- | --- | --- | --- | --- | --- | --- | --- | --- | --- | --- |
|  | 592 | **470.2579** | **469.2507** | **468.2696** | **0.9810** | **0** | **10** | **7.1** | **1** | **R.GAPPK.I** |
|  | 13727 | **790.3422** | **1578.6698** | **1578.6807** | **-0.0109** | **0** | **51** | **0.00082** | **1** | **K.GWDQGLLGMCEGEK.R** |
|  | 13996 | 399.7196 | 1594.8495 | 1594.6756 | 0.1739 | 0 | (0) | 1.1e+002 | 10 | K.GWDQGLLGMCEGEK.R |
|  | 19665 | **679.0973** | **2034.2700** | **2035.1718** | **-0.9017** | **1** | **1** | **60** | **6** | **R.GAPPKIPGGATLVFEVELLK.I** |

| **560.** | P20711\|DDC_HUMAN    **Mass:** 54544    **Score:** 51     **Queries matched:** 11   **emPAI:** 0.07 |
| --- | --- |
|  | Aromatic-L-amino-acid decarboxylase OS=Homo sapiens GN=DDC PE=1 SV=1 |

|  | **Query** | **Observed** | **Mr(expt)** | **Mr(calc)** | **Delta** | **Miss** | **Score** | **Expect** | **Rank** | **Peptide** |
| --- | --- | --- | --- | --- | --- | --- | --- | --- | --- | --- |
|  | 2492 | 436.2219 | 870.4293 | 869.3701 | 1.0592 | 0 | 8 | 29 | 4 | -.MNASEFR.R |
|  | 9549 | **664.3985** | **1326.7824** | **1327.7208** | **-0.9383** | **1** | **5** | **48** | **1** | **K.GSNKVNEALLQR.I** |
|  | 11357 | 478.2694 | 1431.7865 | 1431.7795 | 0.0070 | 1 | 8 | 23 | 2 | K.MLELPKAFLNEK.A |
|  | 14825 | **824.8945** | **1647.7745** | **1647.7852** | **-0.0107** | **0** | **51** | **0.00087** | **1** | **K.LVAYSSDQAHSSVER.A** 14826 |
|  | 15606 | 850.9415 | 1699.8685 | 1699.8814 | -0.0129 | 0 | 15 | 3.4 | 2 | R.LQAASPELTQAAIMEK.L 15601 15602 |
|  | 18065 | **946.4726** | **1890.9306** | **1890.8128** | **0.1179** | **0** | **1** | **89** | **1** | **K.EMVDYVANYMEGIEGR.Q** |
|  | 20077 | **1038.8031** | **2075.5916** | **2075.9292** | **-0.3376** | **1** | **6** | **14** | **1** | **R.GKEMVDYVANYMEGIEGR.Q** |
|  | 21417 | **741.7845** | **2222.3318** | **2221.1888** | **1.1430** | **1** | **5** | **19** | **2** | **K.VIHRLQAASPELTQAAIMEK.L** |

| **561.** | P98160\|PGBM_HUMAN    **Mass:** 479221   **Score:** 51     **Queries matched:** 42   **emPAI:** 0.01 |
| --- | --- |
|  | Basement membrane-specific heparan sulfate proteoglycan core protein OS=Homo sapiens GN=HSPG2 PE=1 SV=3 |

|  | **Query** | **Observed** | **Mr(expt)** | **Mr(calc)** | **Delta** | **Miss** | **Score** | **Expect** | **Rank** | **Peptide** |
| --- | --- | --- | --- | --- | --- | --- | --- | --- | --- | --- |
|  | 1752 | **411.2288** | **820.4430** | **819.4100** | **1.0330** | **0** | **9** | **21** | **1** | **R.HQVHGSR.L** |
|  | 1861 | **415.5591** | **829.1037** | **828.4566** | **0.6471** | **1** | **6** | **33** | **1** | **K.RGGSLPSR.H** |
|  | 2374 | **432.7116** | **863.4087** | **862.4409** | **0.9677** | **1** | **5** | **56** | **5** | **R.SSARWTR.I** |
|  | 3955 | **490.3153** | **978.6160** | **978.5974** | **0.0186** | **0** | **11** | **14** | **4** | **R.LLAVTHGLR.A** |
|  | 4395 | 505.7870 | 1009.5595 | 1010.5145 | -0.9550 | 0 | 4 | 58 | 6 | K.EGGSLPPQAR.S |
|  | 4703 | **517.7304** | **1033.4463** | **1034.5985** | **-1.1522** | **1** | **12** | **11** | **4** | **R.LHNGKLPTR.A** |
|  | 6073 | **571.7742** | **1141.5338** | **1140.5234** | **1.0104** | **0** | **9** | **21** | **1** | **R.FDAGSGMATIR.H** |
|  | 6293 | **579.3355** | **1156.6563** | **1155.6261** | **1.0302** | **0** | **12** | **9.8** | **4** | **R.AVLHVHGGGGPR.V** |
|  | 6519 | **588.7935** | **1175.5724** | **1176.6503** | **-1.0779** | **0** | **4** | **66** | **3** | **R.YELGSGLAVLR.S** 6515 |
|  | 7148 | **604.7866** | **1207.5586** | **1206.5629** | **0.9957** | **0** | **6** | **40** | **4** | **R.ASYAQQPAESR.V** |
|  | 7595 | 615.8417 | 1229.6688 | 1230.5526 | -0.8838 | 1 | 12 | 9.9 | 2 | K.CKAGFFGDAMK.A |
|  | 8239 | **421.0566** | **1260.1479** | **1260.6245** | **-0.4766** | **1** | **1** | **80** | **9** | **R.GCVGEVSVNGKR.L** |
|  | 10591 | **694.3760** | **1386.7375** | **1387.6766** | **-0.9390** | **0** | **0** | **1.3e+002** | **9** | **K.AVTLECVSAGEPR.S** |
|  | 11722 | **728.8576** | **1455.7006** | **1456.7423** | **-1.0416** | **0** | **3** | **60** | **6** | **R.LSGSHSQGVAYPVR.I** |
|  | 15678 | 569.2788 | 1704.8146 | 1705.9614 | -1.1468 | 0 | 2 | 75 | 9 | R.IQVVVLSASDASPPPVK.I |
|  | 15802 | **572.3479** | **1714.0219** | **1714.7621** | **-0.7402** | **0** | **11** | **8.1** | **5** | **R.LYQASPADSGEYVCR.V** |
|  | 15946 | 575.8835 | 1724.6286 | 1725.7893 | -1.1607 | 0 | 5 | 27 | 1 | R.LHHVSPADSGEYVCR.V 15945 |
|  | 16207 | **873.4232** | **1744.8318** | **1744.8857** | **-0.0539** | **0** | **13** | **5.7** | **1** | **K.EGGQLPPGHSVQDGVLR.I** |
|  | 17018 | **604.6749** | **1811.0030** | **1809.8884** | **1.1146** | **0** | **3** | **47** | **5** | **R.EVSEAVVDTLESEYLK.I** |
|  | 17206 | 609.3283 | 1824.9629 | 1825.9761 | -1.0131 | 0 | 3 | 54 | 7 | R.GMVFGIPDGVLELVPQR.G |
|  | 17301 | **611.3047** | **1830.8922** | **1829.8625** | **1.0298** | **1** | **5** | **32** | **1** | **K.CRPVNQEIVRCDER.G** |
|  | 17303 | **611.3201** | **1830.9384** | **1831.8233** | **-0.8850** | **0** | **1** | **80** | **4** | **R.SDEFGCMPPQVVTPPR.E** |
|  | 17409 | **614.3461** | **1840.0166** | **1839.8863** | **0.1302** | **1** | **2** | **61** | **8** | **R.NELLHFERAAPEDSGR.Y** |
|  | 17454 | **615.3300** | **1842.9681** | **1841.9710** | **0.9971** | **0** | **(3)** | **58** | **6** | **R.GMVFGIPDGVLELVPQR.G** |
|  | 17800 | 623.9934 | 1868.9584 | 1867.9363 | 1.0221 | 1 | 5 | 34 | 3 | R.CSATGSPAPTIHWSKLR.S |
|  | 18570 | **645.3424** | **1933.0054** | **1934.1036** | **-1.0982** | **1** | **0** | **1e+002** | **9** | **-.MGWRAAGALLLALLLHGR.L** |
|  | 18859 | 979.9888 | 1957.9631 | 1958.0433 | -0.0802 | 0 | 5 | 31 | 2 | R.VVGSSGTQEASVLVTIQQR.L |
|  | 21602 | 561.5441 | 2242.1472 | 2242.1005 | 0.0467 | 1 | 9 | 9.5 | 2 | R.EFREVSEAVVDTLESEYLK.I |
|  | 22476 | **590.2820** | **2357.0991** | **2357.1104** | **-0.0113** | **0** | **6** | **19** | **4** | **K.MASVGLSDIAMDTTVTHATSHGR.A** |
|  | 23251 | **825.1334** | **2472.3784** | **2472.3839** | **-0.0055** | **0** | **51** | **0.00048** | **1** | **R.AELLQVLQSLEAVLIQTVYNTK.M** |
|  | 23748 | **638.5649** | **2550.2307** | **2550.1050** | **0.1257** | **1** | **2** | **42** | **2** | **R.LCNECADGSFHLSTRNPDGCLK.C** |
|  | 24480 | **670.0596** | **2676.2092** | **2677.3613** | **-1.1521** | **1** | **0** | **56** | **4** | **K.DFISLGLQDGHLVFRYQLGSGEAR.L** |
|  | 26624 | 1034.4452 | 3100.3137 | 3100.6128 | -0.2991 | 1 | 1 | 33 | 4 | R.ESIQASRGQTVTFTCVAIGVPTPIINWR.L |
|  | 27505 | **829.8878** | **3315.5219** | **3314.6830** | **0.8389** | **1** | **0** | **43** | **1** | **R.CSATGSPTPTLEWTGGPGGQLPAKAQIHGGILR.L** |
|  | 29672 | **801.6128** | **4003.0279** | **4003.9190** | **-0.8911** | **1** | **0** | **26** | **3** | **K.LDGSLPPDSRLENNMLMLPSVRPQDAGTYVCTATNR.Q** |
|  | 30503 | **1485.3867** | **4453.1383** | **4454.2540** | **-1.1157** | **1** | **1** | **17** | **10** | **K.LRYTLSYTAGPQGSPLSDPDVQITGNNIMLVASQPALQGPER.R** |
|  | 31138 | 985.7000 | 4923.4637 | 4922.4410 | 1.0227 | 1 | 0 | 14 | 7 | R.LSGSHSQGVAYPVRIESSSASLANGHTLDLNCLVASQAPHTITWYK.R |
|  | 31233 | **837.2031** | **5017.1747** | **5018.2288** | **-1.0541** | **1** | **1** | **9.6** | **1** | **R.WTHSYLSDDEDMLADSISGDDLGSGDLGSGDFQMVYFRALVNFTR.S** |
|  | 31477 | **1316.6316** | **5262.4973** | **5262.5251** | **-0.0278** | **1** | **10** | **1.2** | **1** | **R.LTGEFTVEPVPEGAQLSFGNFAQLGHESFYWQLPETYQGDKVAAYGGK.L** 31483 |

| **562.** | O14949\|QCR8_HUMAN    **Mass:** 9900     **Score:** 51     **Queries matched:** 8   **emPAI:** 0.98 |
| --- | --- |
|  | Cytochrome b-c1 complex subunit 8 OS=Homo sapiens GN=UQCRQ PE=1 SV=4 |

|  | **Query** | **Observed** | **Mr(expt)** | **Mr(calc)** | **Delta** | **Miss** | **Score** | **Expect** | **Rank** | **Peptide** |
| --- | --- | --- | --- | --- | --- | --- | --- | --- | --- | --- |
|  | 3247 | **463.0035** | **923.9924** | **923.5665** | **0.4259** | **1** | **4** | **42** | **4** | **K.GIPNVLRR.I** |
|  | 4881 | **524.7496** | **1047.4846** | **1048.5414** | **-1.0568** | **1** | **44** | **0.0071** | **1** | **M.GREFGNLTR.M** 4895 |
|  | 6173 | **575.7687** | **1149.5229** | **1148.5462** | **0.9768** | **1** | **20** | **1.4** | **1** | **R.KNPAAYENDK.-** 6154 6160 |
|  | 13468 | **781.8973** | **1561.7800** | **1561.7889** | **-0.0089** | **0** | **32** | **0.086** | **1** | **R.HVISYSLSPFEQR.A** 13469 |

| **563.** | P09496\|CLCA_HUMAN    **Mass:** 27174    **Score:** 51     **Queries matched:** 13   **emPAI:** 0.14 |
| --- | --- |
|  | Clathrin light chain A OS=Homo sapiens GN=CLTA PE=1 SV=1 |

|  | **Query** | **Observed** | **Mr(expt)** | **Mr(calc)** | **Delta** | **Miss** | **Score** | **Expect** | **Rank** | **Peptide** |
| --- | --- | --- | --- | --- | --- | --- | --- | --- | --- | --- |
|  | 1242 | **759.4935** | **758.4863** | **758.4902** | **-0.0039** | **0** | **20** | **2.4** | **1** | **R.SVLISLK.Q** |
|  | 2790 | **447.2102** | **892.4059** | **892.4113** | **-0.0054** | **0** | **24** | **0.8** | **1** | **R.LCDFNPK.S** 2789 |
|  | 3165 | **460.0151** | **918.0156** | **917.4606** | **0.5550** | **1** | **20** | **1.1** | **4** | **R.KQEAEWK.E** |
|  | 4076 | **494.7539** | **987.4932** | **987.4985** | **-0.0053** | **0** | **51** | **0.0014** | **1** | **R.LEALDANSR.K** 4059 |
|  | 4865 | **524.2678** | **1046.5211** | **1046.5032** | **0.0179** | **1** | **5** | **58** | **5** | **K.QEAEWKEK.A** |
|  | 4995 | **529.7744** | **1057.5341** | **1057.5404** | **-0.0062** | **0** | **28** | **0.26** | **1** | **R.LQSEPESIR.K** |
|  | 5787 | **559.2897** | **1116.5649** | **1115.5934** | **0.9715** | **1** | **4** | **74** | **1** | **R.LEALDANSRK.Q** |
|  | 6353 | **582.2631** | **1162.5116** | **1162.5189** | **-0.0073** | **1** | **22** | **1.1** | **2** | **K.WREEQMER.L** |
|  | 6693 | 594.2828 | 1186.5511 | 1185.6353 | 0.9158 | 1 | 8 | 26 | 3 | R.LQSEPESIRK.W |
|  | 10920 | **469.9113** | **1406.7119** | **1406.7194** | **-0.0074** | **1** | **19** | **1.7** | **1** | **K.AIKELEEWYAR.Q** 10921 |

| **564.** | P34896\|GLYC_HUMAN    **Mass:** 53619    **Score:** 51     **Queries matched:** 14   **emPAI:** 0.14 |
| --- | --- |
|  | Serine hydroxymethyltransferase, cytosolic OS=Homo sapiens GN=SHMT1 PE=1 SV=1 |

|  | **Query** | **Observed** | **Mr(expt)** | **Mr(calc)** | **Delta** | **Miss** | **Score** | **Expect** | **Rank** | **Peptide** |
| --- | --- | --- | --- | --- | --- | --- | --- | --- | --- | --- |
|  | 1027 | **633.3314** | **632.3241** | **632.3242** | **-0.0000** | **1** | **8** | **44** | **1** | **K.KESNR.Q** |
|  | 1313 | **395.2125** | **788.4105** | **787.4188** | **0.9917** | **1** | **10** | **22** | **4** | **K.ERLAGDK.Y** |
|  | 4948 | **528.2706** | **1054.5267** | **1055.4672** | **-0.9405** | **0** | **6** | **36** | **1** | **K.YSEGYPGQR.Y** |
|  | 7783 | **620.2945** | **1238.5744** | **1239.6281** | **-1.0537** | **0** | **9** | **17** | **2** | **K.LIIAGTSCYSR.N** |
|  | 10328 | **458.3047** | **1371.8923** | **1372.6670** | **-0.7747** | **0** | **17** | **2.7** | **1** | **K.VYQHQVVANCR.A** 10341 |
|  | 10592 | **463.2536** | **1386.7389** | **1387.7129** | **-0.9740** | **0** | **6** | **37** | **4** | **R.AVLEALGSCLNNK.Y** |
|  | 11709 | **728.3441** | **1454.6736** | **1454.8457** | **-0.1721** | **1** | **3** | **66** | **5** | **R.LGTPALTSRGLLEK.D** |
|  | 12539 | **502.2842** | **1503.8306** | **1502.8205** | **1.0101** | **1** | **5** | **40** | **8** | **R.LAGDKYQAAVQALR.E** |
|  | 18985 | 657.8015 | 1970.3825 | 1969.9567 | 0.4258 | 1 | 2 | 45 | 7 | K.DADLWSSHDKMLAQPLK.D |
|  | 22039 | **575.5415** | **2298.1369** | **2298.1137** | **0.0232** | **1** | **0** | **80** | **7** | **R.IMGLDLPDGGHLTHGFMTDKK.K** |
|  | 24272 | **1324.5892** | **2647.1639** | **2648.3380** | **-1.1741** | **1** | **2** | **36** | **2** | **K.VYQHQVVANCRALSEALTELGYK.I** |
|  | 28306 | **1177.2300** | **3528.6681** | **3528.6885** | **-0.0204** | **0** | **46** | **0.00092** | **1** | **K.LDPQCWGVNVQPYSGSPANFAVYTALVEPHGR.I** |
|  | 28923 | **1238.2463** | **3711.7172** | **3711.7402** | **-0.0231** | **1** | **29** | **0.04** | **1** | **K.ISATSIFFESMPYKVNPDTGYINYDQLEENAR.L** |

| **565.** | Q7Z7H5\|TMED4_HUMAN    **Mass:** 26097    **Score:** 51     **Queries matched:** 11   **emPAI:** 0.15 |
| --- | --- |
|  | Transmembrane emp24 domain-containing protein 4 OS=Homo sapiens GN=TMED4 PE=1 SV=1 |

|  | **Query** | **Observed** | **Mr(expt)** | **Mr(calc)** | **Delta** | **Miss** | **Score** | **Expect** | **Rank** | **Peptide** |
| --- | --- | --- | --- | --- | --- | --- | --- | --- | --- | --- |
|  | 1405 | **398.2280** | **794.4414** | **793.4156** | **1.0258** | **0** | **1** | **1.4e+002** | **2** | **R.MALFAGGK.L** |
|  | 1447 | **399.7128** | **797.4110** | **796.4555** | **0.9555** | **0** | **5** | **42** | **6** | **M.AGVGAGPLR.A** 1435 |
|  | 2010 | 420.2298 | 838.4451 | 837.3617 | 1.0834 | 0 | 5 | 46 | 8 | K.EQDYQR.Y |
|  | 5253 | 540.5850 | 1079.1554 | 1078.5957 | 0.5596 | 1 | 7 | 23 | 2 | R.MALFAGGKLR.V |
|  | 9803 | **448.5628** | **1342.6666** | **1342.6962** | **-0.0296** | **1** | **2** | **77** | **9** | **-.MAGVGAGPLRAMGR.Q** |
|  | 10076 | **679.8512** | **1357.6878** | **1358.6911** | **-1.0033** | **1** | **(1)** | **1.2e+002** | **1** | **-.MAGVGAGPLRAMGR.Q** 10077 10086 |
|  | 13555 | **523.8007** | **1568.3802** | **1567.8682** | **0.5120** | **1** | **1** | **60** | **4** | **R.ARQLLDQVEQIQK.E** |
|  | 20148 | **1043.4788** | **2084.9430** | **2084.9547** | **-0.0117** | **0** | **51** | **0.00069** | **1** | **R.CFIEEIPDETMVIGNYR.T** |

| **566.** | Q9BVK6\|TMED9_HUMAN    **Mass:** 27374    **Score:** 51     **Queries matched:** 3   **emPAI:** 0.14 |
| --- | --- |
|  | Transmembrane emp24 domain-containing protein 9 OS=Homo sapiens GN=TMED9 PE=1 SV=2 |

|  | **Query** | **Observed** | **Mr(expt)** | **Mr(calc)** | **Delta** | **Miss** | **Score** | **Expect** | **Rank** | **Peptide** |
| --- | --- | --- | --- | --- | --- | --- | --- | --- | --- | --- |
|  | 5763 | 558.3566 | 1114.6986 | 1113.5641 | 1.1345 | 0 | 1 | 1.1e+002 | 3 | K.FSLFAGGMLR.V |
|  | 20148 | 1043.4788 | 2084.9430 | 2084.9547 | -0.0117 | 0 | 51 | 0.00069 | 1 | K.CFIEEIPDETMVIGNYR.T |
|  | 28291 | **881.9316** | **3523.6972** | **3523.6766** | **0.0207** | **1** | **3** | **18** | **1** | **R.FTFTSHTPGEHQICLHSNSTKFSLFAGGMLR.V** |

| **567.** | O14773\|TPP1_HUMAN    **Mass:** 61723    **Score:** 51     **Queries matched:** 7   **emPAI:** 0.12 |
| --- | --- |
|  | Tripeptidyl-peptidase 1 OS=Homo sapiens GN=TPP1 PE=1 SV=2 |

|  | **Query** | **Observed** | **Mr(expt)** | **Mr(calc)** | **Delta** | **Miss** | **Score** | **Expect** | **Rank** | **Peptide** |
| --- | --- | --- | --- | --- | --- | --- | --- | --- | --- | --- |
|  | 2178 | **425.7345** | **849.4544** | **849.4266** | **0.0279** | **0** | **3** | **84** | **7** | **R.VNTELMK.A** |
|  | 7351 | **609.8261** | **1217.6375** | **1218.6390** | **-1.0015** | **1** | **1** | **1.1e+002** | **6** | **R.VNTELMKAAAR.G** |
|  | 10689 | **465.2400** | **1392.6981** | **1393.6044** | **-0.9064** | **1** | **5** | **43** | **8** | **K.CSYSPEPDQRR.T** 10691 |
|  | 13060 | **513.3104** | **1536.9094** | **1535.8936** | **1.0158** | **0** | **3** | **57** | **9** | **R.ILSGRPPLGFLNPR.L** |
|  | 14122 | **802.9080** | **1603.8015** | **1603.8107** | **-0.0092** | **0** | **45** | **0.0038** | **1** | **R.LYQQHGAGLFDVTR.G** 14121 |

| **568.** | Q9H330\|CI005_HUMAN    **Mass:** 101508   **Score:** 51     **Queries matched:** 12   **emPAI:** 0.04 |
| --- | --- |
|  | Transmembrane protein C9orf5 OS=Homo sapiens GN=C9orf5 PE=1 SV=2 |

|  | **Query** | **Observed** | **Mr(expt)** | **Mr(calc)** | **Delta** | **Miss** | **Score** | **Expect** | **Rank** | **Peptide** |
| --- | --- | --- | --- | --- | --- | --- | --- | --- | --- | --- |
|  | 3233 | **462.2530** | **922.4915** | **923.5301** | **-1.0385** | **1** | **4** | **58** | **5** | **K.GQKLHVSR.Q** |
|  | 5377 | **544.9342** | **1087.8538** | **1087.6138** | **0.2400** | **0** | **2** | **80** | **5** | **R.RPEIGTFLR.K** |
|  | 6056 | **571.2797** | **1140.5448** | **1140.5524** | **-0.0076** | **0** | **51** | **0.0012** | **1** | **R.AVGPSGGGGETPR.T** 6052 6055 |
|  | 7861 | 415.2059 | 1242.5958 | 1243.7149 | -1.1191 | 1 | 9 | 20 | 4 | R.RRPEIGTFLR.K 7888 |
|  | 8937 | **432.9128** | **1295.7166** | **1294.6418** | **1.0748** | **1** | **11** | **11** | **1** | **K.WNASTERYLR.A** |
|  | 9114 | **435.7389** | **1304.1949** | **1303.7500** | **0.4449** | **0** | **9** | **12** | **3** | **K.VDVVSSAVIFLR.K** |
|  | 12382 | **498.7319** | **1493.1739** | **1492.7747** | **0.3992** | **1** | **10** | **9.3** | **1** | **R.VPRAVGPSGGGGETPR.T** 12388 |
|  | 31406 | **1298.6229** | **5190.4626** | **5191.4739** | **-1.0113** | **1** | **0** | **13** | **9** | **K.QNGKESSGAELPGQVISMAASTLANLAISITGYESSSEDQPSTQPAEAVDR.G** |

| **569.** | P47914\|RL29_HUMAN    **Mass:** 17798    **Score:** 51     **Queries matched:** 9   **emPAI:** 0.22 |
| --- | --- |
|  | 60S ribosomal protein L29 OS=Homo sapiens GN=RPL29 PE=1 SV=2 |

|  | **Query** | **Observed** | **Mr(expt)** | **Mr(calc)** | **Delta** | **Miss** | **Score** | **Expect** | **Rank** | **Peptide** |
| --- | --- | --- | --- | --- | --- | --- | --- | --- | --- | --- |
|  | 1264 | **767.0089** | **766.0016** | **765.3956** | **0.6061** | **1** | **6** | **31** | **8** | **R.NMRFAK.K** |
|  | 1379 | **397.2288** | **792.4430** | **791.3596** | **1.0834** | **0** | **3** | **99** | **5** | **K.MQANNAK.A** |
|  | 2210 | **427.0750** | **852.1355** | **851.4436** | **0.6919** | **1** | **10** | **9.6** | **1** | **K.FLRNMR.F** |
|  | 2958 | **453.2351** | **904.4557** | **903.4596** | **0.9961** | **1** | **8** | **27** | **8** | **K.KMQANNAK.A** |
|  | 3077 | **457.2419** | **912.4693** | **911.5229** | **0.9464** | **0** | **5** | **49** | **9** | **R.LAYIAHPK.L** |
|  | 3425 | **470.2640** | **938.5133** | **937.5960** | **0.9173** | **1** | **4** | **66** | **1** | **K.EVKPKIPK.G** |
|  | 9192 | 437.2483 | 1308.7230 | 1308.6283 | 0.0947 | 1 | 8 | 24 | 7 | K.SKNHTTHNQSR.K |
|  | 10422 | **689.3733** | **1376.7321** | **1376.7412** | **-0.0090** | **0** | **51** | **0.0011** | **1** | **K.AQAAAPASVPAQAPK.R** |
|  | 12987 | **511.9516** | **1532.8329** | **1532.8423** | **-0.0094** | **1** | **18** | **2** | **1** | **K.AQAAAPASVPAQAPKR.T** |

| **570.** | Q9P0J0\|NDUAD_HUMAN    **Mass:** 16688    **Score:** 50     **Queries matched:** 9   **emPAI:** 0.52 |
| --- | --- |
|  | NADH dehydrogenase [ubiquinone] 1 alpha subcomplex subunit 13 OS=Homo sapiens GN=NDUFA13 PE=1 SV=3 |

|  | **Query** | **Observed** | **Mr(expt)** | **Mr(calc)** | **Delta** | **Miss** | **Score** | **Expect** | **Rank** | **Peptide** |
| --- | --- | --- | --- | --- | --- | --- | --- | --- | --- | --- |
|  | 5944 | **566.2897** | **1130.5649** | **1131.5673** | **-1.0024** | **0** | **47** | **0.0031** | **1** | **K.VGESVFHTTR.W** |
|  | 12675 | **756.9287** | **1511.8429** | **1511.8500** | **-0.0072** | **0** | **30** | **0.12** | **1** | **R.WVPPLIGELYGLR.T** |
|  | 14594 | **545.2971** | **1632.8695** | **1633.7446** | **-0.8751** | **0** | **6** | **30** | **2** | **K.QDMPPPGGYGPIDYK.R** |
|  | 14857 | 550.6260 | 1648.8563 | 1649.7396 | -0.8833 | 0 | (2) | 68 | 4 | K.QDMPPPGGYGPIDYK.R |
|  | 16967 | **603.3291** | **1806.9655** | **1805.8407** | **1.1248** | **1** | **13** | **6.3** | **1** | **K.QDMPPPGGYGPIDYKR.N** 16931 |
|  | 17890 | **626.3096** | **1875.9069** | **1876.9029** | **-0.9961** | **1** | **2** | **64** | **8** | **K.VKQDMPPPGGYGPIDYK.R** |
|  | 19946 | **1031.5306** | **2061.0467** | **2060.0645** | **0.9822** | **1** | **0** | **83** | **3** | **R.TLQMLRENLEEEAIIMK.D** |
|  | 24887 | 919.1673 | 2754.4801 | 2755.4189 | -0.9389 | 1 | 4 | 24 | 5 | R.RGLSGYSMLAIGIGTLIYGHWSIMK.W |

| **571.** | Q9NS69\|TOM22_HUMAN    **Mass:** 15512    **Score:** 50     **Queries matched:** 4   **emPAI:** 0.25 |
| --- | --- |
|  | Mitochondrial import receptor subunit TOM22 homolog OS=Homo sapiens GN=TOMM22 PE=1 SV=3 |

|  | **Query** | **Observed** | **Mr(expt)** | **Mr(calc)** | **Delta** | **Miss** | **Score** | **Expect** | **Rank** | **Peptide** |
| --- | --- | --- | --- | --- | --- | --- | --- | --- | --- | --- |
|  | 10434 | **689.8406** | **1377.6666** | **1377.6751** | **-0.0085** | **0** | **18** | **2.4** | **1** | **R.LWGLTEMFPER.V** |
|  | 13395 | 779.3887 | 1556.7628 | 1556.7729 | -0.0101 | 0 | 50 | 0.0013 | 1 | K.LQMEQQQQLQQR.Q |
|  | 14819 | 550.2502 | 1647.7287 | 1648.8395 | -1.1108 | 1 | 1 | 1e+002 | 8 | R.LWGLTEMFPERVR.S |
|  | 20413 | **704.3571** | **2110.0495** | **2109.0412** | **1.0083** | **0** | **2** | **55** | **7** | **-.MAAAVAAAGAGEPQSPDELLPK.G** |

| **572.** | P14618\|KPYM_HUMAN    **Mass:** 58470    **Score:** 50     **Queries matched:** 17   **emPAI:** 0.06 |
| --- | --- |
|  | Pyruvate kinase isozymes M1/M2 OS=Homo sapiens GN=PKM2 PE=1 SV=4 |

|  | **Query** | **Observed** | **Mr(expt)** | **Mr(calc)** | **Delta** | **Miss** | **Score** | **Expect** | **Rank** | **Peptide** |
| --- | --- | --- | --- | --- | --- | --- | --- | --- | --- | --- |
|  | 1106 | **674.3576** | **673.3503** | **672.4170** | **0.9333** | **1** | **11** | **21** | **5** | **R.KVLGEK.G** 1107 |
|  | 1196 | **717.4082** | **716.4009** | **716.4069** | **-0.0059** | **0** | **17** | **5.3** | **1** | **K.VVEVGSK.I** |
|  | 1210 | 732.2933 | 731.2861 | 730.3861 | 0.8999 | 0 | 18 | 3.8 | 1 | K.DIQDLK.F |
|  | 2692 | 443.0385 | 884.0625 | 883.4698 | 0.5927 | 0 | 4 | 39 | 4 | R.MQHLIAR.E |
|  | 6820 | **398.4886** | **1192.4439** | **1192.6373** | **-0.1934** | **1** | **7** | **31** | **4** | **R.SVETLKEMIK.S** 6839 |
|  | 7428 | 408.1999 | 1221.5778 | 1221.6288 | -0.0510 | 1 | 4 | 56 | 9 | R.VNFAMNVGKAR.G |
|  | 9469 | **662.8272** | **1323.6399** | **1324.6995** | **-1.0597** | **1** | **10** | **13** | **2** | **K.VFLAQKMMIGR.C** |
|  | 9737 | 447.2723 | 1338.7951 | 1339.7221 | -0.9270 | 1 | 6 | 33 | 6 | R.SAHQVARYRPR.A |
|  | 12506 | **501.3377** | **1500.9911** | **1501.8352** | **-0.8440** | **1** | **0** | **93** | **9** | **R.TGLIKGSGTAEVELK.K** |
|  | 16658 | 890.9951 | 1779.9756 | 1778.8687 | 1.1069 | 0 | 11 | 9.1 | 2 | K.GADFLVTEVENGGSLGSK.K |
|  | 17358 | 612.9700 | 1835.8883 | 1836.9040 | -1.0157 | 1 | 1 | 84 | 6 | R.RFDEILEASDGIMVAR.G |
|  | 17694 | **930.9505** | **1859.8864** | **1858.8924** | **0.9941** | **0** | **22** | **0.6** | **1** | **K.FGVEQDVDMVFASFIR.K** |
|  | 20985 | **1088.0561** | **2174.0977** | **2174.1107** | **-0.0129** | **0** | **50** | **0.00086** | **1** | **R.LAPITSDPTEATAVGAVEASFK.C** |
|  | 21742 | **754.6658** | **2260.9755** | **2262.1580** | **-1.1825** | **0** | **0** | **68** | **10** | **K.GDVVIVLTGWRPGSGFTNTMR.V** |
|  | 26157 | **754.9020** | **3015.5788** | **3016.5869** | **-1.0081** | **1** | **9** | **6** | **1** | **R.TATESFASDPILYRPVAVALDTKGPEIR.T** |

| **573.** | O15372\|EIF3H_HUMAN    **Mass:** 40076    **Score:** 50     **Queries matched:** 2   **emPAI:** 0.09 |
| --- | --- |
|  | Eukaryotic translation initiation factor 3 subunit H OS=Homo sapiens GN=EIF3H PE=1 SV=1 |

|  | **Query** | **Observed** | **Mr(expt)** | **Mr(calc)** | **Delta** | **Miss** | **Score** | **Expect** | **Rank** | **Peptide** |
| --- | --- | --- | --- | --- | --- | --- | --- | --- | --- | --- |
|  | 14495 | **814.3682** | **1626.7219** | **1626.7333** | **-0.0114** | **0** | **50** | **0.0012** | **1** | **K.EGTGSTATSSSSTAGAAGK.G** |
|  | 17008 | 906.4386 | 1810.8626 | 1811.8497 | -0.9871 | 1 | 0 | 94 | 9 | K.EGTGSTATSSSSTAGAAGKGK.G |

| **574.** | P08134\|RHOC_HUMAN    **Mass:** 22334    **Score:** 50     **Queries matched:** 5   **emPAI:** 0.37 |
| --- | --- |
|  | Rho-related GTP-binding protein RhoC OS=Homo sapiens GN=RHOC PE=1 SV=1 |

|  | **Query** | **Observed** | **Mr(expt)** | **Mr(calc)** | **Delta** | **Miss** | **Score** | **Expect** | **Rank** | **Peptide** |
| --- | --- | --- | --- | --- | --- | --- | --- | --- | --- | --- |
|  | 1051 | **643.3839** | **642.3766** | **642.3813** | **-0.0047** | **0** | **20** | **2** | **2** | **R.AGLQVR.K** |
|  | 3986 | **491.7341** | **981.4536** | **981.4590** | **-0.0053** | **0** | **38** | **0.022** | **1** | **R.EVFEMATR.A** |
|  | 6370 | 582.6303 | 1163.2461 | 1163.4989 | -0.2528 | 1 | 5 | 30 | 5 | R.SEEGRDMANR.I |
|  | 7292 | **608.3444** | **1214.6742** | **1215.6646** | **-0.9904** | **1** | **1** | **1.3e+002** | **4** | **K.KLVIVGDGACGK.T** |
|  | 16083 | **579.3238** | **1734.9495** | **1734.9603** | **-0.0108** | **1** | **35** | **0.038** | **1** | **K.HFCPNVPIILVGNKK.D** |

| **575.** | P61586\|RHOA_HUMAN    **Mass:** 22096    **Score:** 50     **Queries matched:** 6   **emPAI:** 0.38 |
| --- | --- |
|  | Transforming protein RhoA OS=Homo sapiens GN=RHOA PE=1 SV=1 |

|  | **Query** | **Observed** | **Mr(expt)** | **Mr(calc)** | **Delta** | **Miss** | **Score** | **Expect** | **Rank** | **Peptide** |
| --- | --- | --- | --- | --- | --- | --- | --- | --- | --- | --- |
|  | 3986 | 491.7341 | 981.4536 | 981.4590 | -0.0053 | 0 | 38 | 0.022 | 1 | R.EVFEMATR.A |
|  | 7292 | 608.3444 | 1214.6742 | 1215.6646 | -0.9904 | 1 | 1 | 1.3e+002 | 4 | K.KLVIVGDGACGK.T |
|  | 9642 | 445.2455 | 1332.7147 | 1332.5842 | 0.1305 | 0 | 8 | 24 | 4 | R.IGAFGYMECSAK.T |
|  | 13726 | **527.1554** | **1578.4444** | **1577.7218** | **0.7226** | **1** | **12** | **4.9** | **2** | **R.IGAFGYMECSAKTK.D** |
|  | 16083 | 579.3238 | 1734.9495 | 1734.9603 | -0.0108 | 1 | 35 | 0.038 | 1 | K.HFCPNVPIILVGNKK.D |
|  | 16340 | 439.7734 | 1755.0644 | 1754.8369 | 0.2275 | 1 | 3 | 52 | 4 | K.QEPVKPEEGRDMANR.I |

| **576.** | P05164\|PERM_HUMAN    **Mass:** 84784    **Score:** 50     **Queries matched:** 23   **emPAI:** 0.09 |
| --- | --- |
|  | Myeloperoxidase OS=Homo sapiens GN=MPO PE=1 SV=1 |

|  | **Query** | **Observed** | **Mr(expt)** | **Mr(calc)** | **Delta** | **Miss** | **Score** | **Expect** | **Rank** | **Peptide** |
| --- | --- | --- | --- | --- | --- | --- | --- | --- | --- | --- |
|  | 1564 | **403.2298** | **804.4450** | **803.4211** | **1.0239** | **0** | **11** | **15** | **3** | **R.GLMATPAK.L** |
|  | 3407 | **469.2734** | **936.5323** | **937.4691** | **-0.9369** | **0** | **11** | **14** | **1** | **R.LFEQVMR.I** |
|  | 3431 | 470.7280 | 939.4414 | 939.5178 | -0.0763 | 1 | 11 | 13 | 5 | R.KYLPTYR.S 3443 |
|  | 3491 | **473.0817** | **944.1488** | **943.5239** | **0.6249** | **0** | **7** | **27** | **4** | **R.NGFPVALAR.A** |
|  | 4378 | 505.2587 | 1008.5028 | 1008.5393 | -0.0365 | 0 | 8 | 26 | 8 | M.GVPFFSSLR.C |
|  | 4929 | **527.1554** | **1052.2962** | **1051.4570** | **0.8392** | **0** | **6** | **31** | **1** | **R.SYNDSVDPR.I** |
|  | 4996 | **529.7845** | **1057.5545** | **1057.5628** | **-0.0083** | **1** | **40** | **0.017** | **1** | **R.RSPTLGASNR.A** 4997 |
|  | 5283 | **541.3059** | **1080.5973** | **1081.4645** | **-0.8672** | **0** | **13** | **7.5** | **2** | **R.TITGMCNNR.R** |
|  | 9744 | **670.8677** | **1339.7209** | **1339.7282** | **-0.0073** | **0** | **2** | **83** | **1** | **R.IGLDLPALNMQR.S** |
|  | 10365 | 687.8278 | 1373.6411 | 1374.7368 | -1.0957 | 1 | 10 | 15 | 4 | R.SPTLGASNRAFVR.W |
|  | 11155 | **711.3641** | **1420.7137** | **1420.7232** | **-0.0095** | **0** | **33** | **0.065** | **1** | **R.IICDNTGITTVSK.N** |
|  | 11853 | **488.2797** | **1461.8172** | **1460.7697** | **1.0474** | **0** | **5** | **41** | **4** | **R.DYLPLVLGPTAMR.K** 11842 |
|  | 12350 | **746.4181** | **1490.8216** | **1490.8643** | **-0.0427** | **1** | **(1)** | **1.1e+002** | **1** | **R.KIVGAMVQIITYR.D** |
|  | 12587 | **503.2819** | **1506.8238** | **1506.8592** | **-0.0354** | **1** | **7** | **23** | **6** | **R.KIVGAMVQIITYR.D** |
|  | 17495 | **462.2476** | **1844.9613** | **1844.8693** | **0.0919** | **1** | **2** | **70** | **8** | **K.YLPTYRSYNDSVDPR.I** |
|  | 18826 | 978.9538 | 1955.8930 | 1956.0323 | -0.1392 | 1 | 1 | 65 | 5 | R.NLRNMSNQLGLLAVNQR.F |
|  | 19529 | **675.0054** | **2021.9945** | **2023.0098** | **-1.0153** | **1** | **1** | **76** | **9** | **R.YGHTLIQPFMFRLDNR.Y** 19524 |
|  | 22835 | **805.4141** | **2413.2204** | **2412.1525** | **1.0678** | **1** | **0** | **76** | **1** | **R.SSEMPELTSMHTLLLREHNR.L** |
|  | 26039 | 598.9019 | 2989.4729 | 2990.4664 | -0.9935 | 1 | 1 | 37 | 5 | R.IPCFLAGDTRSSEMPELTSMHTLLLR.E |

| **577.** | Q96NB2\|SFXN2_HUMAN    **Mass:** 36493    **Score:** 50     **Queries matched:** 20   **emPAI:** 0.48 |
| --- | --- |
|  | Sideroflexin-2 OS=Homo sapiens GN=SFXN2 PE=1 SV=2 |

|  | **Query** | **Observed** | **Mr(expt)** | **Mr(calc)** | **Delta** | **Miss** | **Score** | **Expect** | **Rank** | **Peptide** |
| --- | --- | --- | --- | --- | --- | --- | --- | --- | --- | --- |
|  | 959 | 605.3281 | 604.3209 | 604.3254 | -0.0046 | 0 | 12 | 14 | 5 | K.VMVEK.S |
|  | 1198 | **717.4092** | **716.4020** | **716.4068** | **-0.0049** | **0** | **24** | **1** | **1** | **K.LQDTIK.A** |
|  | 11681 | **485.2603** | **1452.7590** | **1453.8729** | **-1.1139** | **1** | **15** | **4** | **4** | **R.RAAAIGITQVVISR.I** |
|  | 12824 | 761.3619 | 1520.7093 | 1520.7187 | -0.0094 | 0 | 41 | 0.01 | 1 | K.YGELEPYVYFNK.G 12823 |
|  | 13315 | 518.2269 | 1551.6590 | 1550.7035 | 0.9555 | 0 | 3 | 60 | 7 | -.MEADLSGFNIDAPR.W |
|  | 13735 | **790.4049** | **1578.7953** | **1578.8042** | **-0.0089** | **1** | **29** | **0.16** | **1** | **R.TVFVSERELDWAK.V** 13734 |
|  | 14157 | 536.2795 | 1605.8166 | 1606.7627 | -0.9461 | 1 | 20 | 1.2 | 1 | K.KLYDSAFHPDTGEK.M |
|  | 15495 | **846.9177** | **1691.8208** | **1690.8242** | **0.9965** | **1** | **27** | **0.22** | **1** | **K.YGELEPYVYFNKGL.-** 15472 15474 |
|  | 17266 | **915.4896** | **1828.9646** | **1828.9757** | **-0.0111** | **0** | **25** | **0.3** | **1** | **R.MGVVPPGTQVEQLLYAK.K** 17267 |
|  | 18032 | **630.3775** | **1888.1107** | **1887.0032** | **1.1075** | **0** | **4** | **33** | **5** | **R.ITMSAPGMILLPVIMER.L** |
|  | 20196 | **697.4197** | **2089.2374** | **2088.1038** | **1.1336** | **1** | **5** | **21** | **4** | **K.SRMGVVPPGTQVEQLLYAK.K** |
|  | 22297 | **779.8970** | **2336.6691** | **2336.1520** | **0.5171** | **0** | **1** | **44** | **9** | **R.MSFQLPGGMIITGFMLQFYR.T** |
|  | 26181 | 756.8578 | 3023.4023 | 3022.5054 | 0.8969 | 1 | 1 | 37 | 10 | K.MNVIGRMSFQLPGGMIITGFMLQFYR.T |
|  | 26383 | **764.3635** | **3053.4247** | **3054.4952** | **-1.0705** | **1** | **(1)** | **36** | **1** | **K.MNVIGRMSFQLPGGMIITGFMLQFYR.T** |
|  | 26896 | **1056.8234** | **3167.4483** | **3166.7644** | **0.6839** | **1** | **2** | **29** | **1** | **R.AAAIGITQVVISRITMSAPGMILLPVIMER.L** |

| **578.** | Q8NBX0\|SCPDH_HUMAN    **Mass:** 47464    **Score:** 50     **Queries matched:** 4   **emPAI:** 0.08 |
| --- | --- |
|  | Probable saccharopine dehydrogenase OS=Homo sapiens GN=SCCPDH PE=1 SV=1 |

|  | **Query** | **Observed** | **Mr(expt)** | **Mr(calc)** | **Delta** | **Miss** | **Score** | **Expect** | **Rank** | **Peptide** |
| --- | --- | --- | --- | --- | --- | --- | --- | --- | --- | --- |
|  | 5702 | **556.2763** | **1110.5379** | **1111.6250** | **-1.0871** | **1** | **12** | **9.7** | **2** | **R.LPWAVAGRSR.E** |
|  | 14424 | **812.3583** | **1622.7021** | **1622.8683** | **-0.1662** | **0** | **2** | **77** | **3** | **K.LMFAGLFFLFFVR.F** |
|  | 14536 | 815.4099 | 1628.8053 | 1628.7869 | 0.0184 | 0 | 4 | 48 | 6 | K.GYSIPFMGSDVSVVR.R |
|  | 25940 | 990.8422 | 2969.5047 | 2969.5202 | -0.0155 | 0 | 50 | 0.00059 | 1 | K.LGRPTLSSEVGIIICDIANPASLDEMAK.Q |

| **579.** | Q14624\|ITIH4_HUMAN    **Mass:** 103521   **Score:** 50     **Queries matched:** 12   **emPAI:** 0.04 |
| --- | --- |
|  | Inter-alpha-trypsin inhibitor heavy chain H4 OS=Homo sapiens GN=ITIH4 PE=1 SV=4 |

|  | **Query** | **Observed** | **Mr(expt)** | **Mr(calc)** | **Delta** | **Miss** | **Score** | **Expect** | **Rank** | **Peptide** |
| --- | --- | --- | --- | --- | --- | --- | --- | --- | --- | --- |
|  | 2058 | 421.2073 | 840.4000 | 841.4334 | -1.0334 | 0 | 6 | 34 | 5 | K.YYLQGAK.I |
|  | 10700 | **697.4197** | **1392.8249** | **1393.7639** | **-0.9390** | **1** | **3** | **71** | **8** | **R.LLFKGSEMVVAGK.L** |
|  | 12240 | **495.7604** | **1484.2593** | **1484.8212** | **-0.5619** | **1** | **5** | **28** | **4** | **R.FAHTVVTSRVVNR.A** |
|  | 13010 | **768.4036** | **1534.7926** | **1534.8218** | **-0.0292** | **1** | **1** | **96** | **5** | **K.WKETLFSVMPGLK.M** 13003 |
|  | 16470 | **589.3094** | **1764.9063** | **1763.8512** | **1.0551** | **0** | **0** | **1e+002** | **3** | **R.NMEQFQVSVSVAPNAK.I** |
|  | 17549 | **617.3215** | **1848.9428** | **1849.9244** | **-0.9816** | **1** | **7** | **24** | **1** | **R.ANTVQEATFQMELPKK.A** |
|  | 17671 | **620.3001** | **1857.8785** | **1858.8879** | **-1.0094** | **1** | **(2)** | **73** | **9** | **K.ETLFSVMPGLKMTMDK.T** |
|  | 17690 | 620.7950 | 1859.3631 | 1858.8879 | 0.4752 | 1 | 2 | 44 | 9 | K.ETLFSVMPGLKMTMDK.T |
|  | 16939 | **903.4802** | **2707.4187** | **2707.3314** | **0.0872** | **1** | **3** | **78** | **2** | **R.HRQGPVNLLSDPEQGVEVTGQYER.E** |
|  | 27250 | **649.7227** | **3243.5772** | **3242.6111** | **0.9662** | **1** | **0** | **40** | **2** | **R.YSLFCLGFGFDVSYAFLEKLALDNGGLAR.R** |
|  | 31409 | **1300.6271** | **5198.4792** | **5197.4905** | **0.9888** | **1** | **50** | **0.00013** | **1** | **R.RIHEDSDSALQLQDFYQEVANPLLTAVTFEYPSNAVEEVTQNNFR.L** |

| **580.** | Q7Z6K2\|TPISL_HUMAN    **Mass:** 25016    **Score:** 50     **Queries matched:** 2   **emPAI:** 0.15 |
| --- | --- |
|  | Putative triosephosphate isomerase-like protein LOC286016 OS=Homo sapiens PE=1 SV=2 |

|  | **Query** | **Observed** | **Mr(expt)** | **Mr(calc)** | **Delta** | **Miss** | **Score** | **Expect** | **Rank** | **Peptide** |
| --- | --- | --- | --- | --- | --- | --- | --- | --- | --- | --- |
|  | 8518 | 637.8367 | 1273.6589 | 1273.6666 | -0.0077 | 1 | 50 | 0.0016 | 1 | K.VIADNVKDWSK.V |
|  | 14412 | 811.9040 | 1621.7935 | 1622.7974 | -1.0039 | 0 | 4 | 43 | 2 | K.VTNGAFTGEISPGMVK.D |

| **581.** | P09525\|ANXA4_HUMAN    **Mass:** 36088    **Score:** 49     **Queries matched:** 6   **emPAI:** 0.10 |
| --- | --- |
|  | Annexin A4 OS=Homo sapiens GN=ANXA4 PE=1 SV=4 |

|  | **Query** | **Observed** | **Mr(expt)** | **Mr(calc)** | **Delta** | **Miss** | **Score** | **Expect** | **Rank** | **Peptide** |
| --- | --- | --- | --- | --- | --- | --- | --- | --- | --- | --- |
|  | 10298 | **686.3316** | **1370.6486** | **1370.6579** | **-0.0092** | **0** | **49** | **0.0016** | **1** | **R.ISQTYQQQYGR.S** |
|  | 12825 | 761.3694 | 1520.7242 | 1519.7664 | 0.9578 | 1 | 9 | 16 | 5 | K.SMKGLGTDDNTLIR.V |
|  | 13041 | 513.2478 | 1536.7216 | 1535.7614 | 0.9602 | 1 | (3) | 59 | 4 | K.SMKGLGTDDNTLIR.V |
|  | 13778 | 527.9551 | 1580.8436 | 1580.7253 | 0.1183 | 0 | 5 | 40 | 8 | K.AASGFNAMEDAQTLR.K |
|  | 16421 | **588.2694** | **1761.7862** | **1761.9043** | **-0.1181** | **1** | **5** | **39** | **3** | **K.GLGTDDNTLIRVMVSR.A** |
|  | 19196 | **498.5251** | **1990.0711** | **1990.9816** | **-0.9105** | **1** | **6** | **21** | **6** | **R.AMKGAGTDEGCLIEILASR.T** |

| **582.** | O00748\|EST2_HUMAN    **Mass:** 62109    **Score:** 49     **Queries matched:** 9   **emPAI:** 0.26 |
| --- | --- |
|  | Carboxylesterase 2 OS=Homo sapiens GN=CES2 PE=1 SV=1 |

|  | **Query** | **Observed** | **Mr(expt)** | **Mr(calc)** | **Delta** | **Miss** | **Score** | **Expect** | **Rank** | **Peptide** |
| --- | --- | --- | --- | --- | --- | --- | --- | --- | --- | --- |
|  | 2158 | 425.2429 | 848.4713 | 848.4908 | -0.0196 | 1 | 22 | 1.4 | 9 | R.LQFWKK.A |
|  | 3273 | **464.2248** | **926.4351** | **926.4399** | **-0.0048** | **0** | **34** | **0.068** | **1** | **K.YWANFAR.N** |
|  | 8360 | 634.2889 | 1266.5632 | 1266.5728 | -0.0096 | 0 | 34 | 0.053 | 1 | K.FTEEEEQLSR.K |
|  | 8994 | 434.2019 | 1299.5840 | 1300.7390 | -1.1551 | 0 | 17 | 2.4 | 4 | K.EEILAINKPFK.M 9024 |
|  | 10815 | **700.8892** | **1399.7638** | **1398.7694** | **0.9944** | **0** | **32** | **0.082** | **1** | **K.MIPGVVDGVFLPR.H** |
|  | 12753 | **506.2925** | **1515.8558** | **1515.8660** | **-0.0102** | **1** | **30** | **0.13** | **1** | **K.SKEEILAINKPFK.M** |
|  | 13686 | **526.2947** | **1575.8624** | **1575.8733** | **-0.0109** | **0** | **30** | **0.13** | **1** | **R.TTHTGQVLGSLVHVK.G** |
|  | 16133 | **580.6495** | **1738.9266** | **1737.8336** | **1.0931** | **0** | **1** | **95** | **10** | **R.WVQQNIAHFGGNPDR.V** |

| **583.** | A6NMY6\|AXA2L_HUMAN    **Mass:** 38806    **Score:** 49     **Queries matched:** 18   **emPAI:** 0.10 |
| --- | --- |
|  | Putative annexin A2-like protein OS=Homo sapiens GN=ANXA2P2 PE=5 SV=2 |

|  | **Query** | **Observed** | **Mr(expt)** | **Mr(calc)** | **Delta** | **Miss** | **Score** | **Expect** | **Rank** | **Peptide** |
| --- | --- | --- | --- | --- | --- | --- | --- | --- | --- | --- |
|  | 2302 | 430.7141 | 859.4137 | 858.5174 | 0.8963 | 1 | 5 | 69 | 5 | K.KELASALK.S |
|  | 2535 | 437.2709 | 872.5273 | 872.5517 | -0.0244 | 1 | 6 | 48 | 9 | R.KLMVALAK.G |
|  | 5375 | 544.8180 | 1087.6214 | 1086.5921 | 1.0294 | 0 | 2 | 1e+002 | 6 | R.DALNIETAIK.T |
|  | 5423 | 546.2999 | 1090.5852 | 1089.5852 | 1.0000 | 1 | 1 | 1.3e+002 | 7 | R.SEVDMLKIR.S |
|  | 5570 | 551.5013 | 1100.9881 | 1101.6692 | -0.6811 | 1 | 4 | 48 | 2 | K.VLIRIMVSR.S |
|  | 6471 | 586.9057 | 1171.7968 | 1171.5431 | 0.2538 | 1 | 3 | 68 | 3 | K.EMYKTDLEK.D 6473 6483 |
|  | 7306 | 406.1849 | 1215.5330 | 1216.5944 | -1.0614 | 0 | 8 | 23 | 4 | -.MSTVHEILCK.L 7307 7348 |
|  | 7894 | 622.8109 | 1243.6073 | 1243.6156 | -0.0084 | 0 | 49 | 0.0019 | 1 | R.TNQELQEINR.V 7865 |
|  | 11181 | 712.3343 | 1422.6540 | 1422.7211 | -0.0670 | 1 | 19 | 1.7 | 2 | R.IMVSRSEVDMLK.I 11200 |
|  | 16589 | 445.0015 | 1775.9767 | 1776.8564 | -0.8796 | 0 | 8 | 16 | 7 | K.GLGTDEDSLIEIICSR.T |
|  | 17403 | 614.3082 | 1839.9027 | 1839.9665 | -0.0638 | 1 | 5 | 33 | 3 | K.WISIMTERSVPHLQK.V |
|  | 20433 | **705.1389** | **2112.3949** | **2112.0923** | **0.3026** | **1** | **1** | **45** | **1** | **K.GVDEVTIVNIVTNRDNAQR.Q** |

| **584.** | O43464\|HTRA2_HUMAN    **Mass:** 48868    **Score:** 49     **Queries matched:** 10   **emPAI:** 0.08 |
| --- | --- |
|  | Serine protease HTRA2, mitochondrial OS=Homo sapiens GN=HTRA2 PE=1 SV=2 |

|  | **Query** | **Observed** | **Mr(expt)** | **Mr(calc)** | **Delta** | **Miss** | **Score** | **Expect** | **Rank** | **Peptide** |
| --- | --- | --- | --- | --- | --- | --- | --- | --- | --- | --- |
|  | 838 | **561.0925** | **560.0853** | **560.2740** | **-0.1888** | **0** | **11** | **20** | **3** | **-.MAAPR.A** |
|  | 5258 | 540.7666 | 1079.5186 | 1078.5003 | 1.0183 | 0 | 6 | 38 | 8 | K.NSSSGISGSQR.R |
|  | 5421 | 546.2981 | 1090.5816 | 1091.6339 | -1.0522 | 0 | 7 | 31 | 9 | K.EPLPTLPLGR.S 5428 |
|  | 5795 | 559.3242 | 1116.6339 | 1116.5775 | 0.0564 | 0 | 10 | 16 | 7 | R.ALLTSGTSDPR.A |
|  | 6832 | **398.5500** | **1192.6282** | **1192.5432** | **0.0850** | **1** | **10** | **14** | **1** | **R.EASENSGTRSR.A** 6844 |
|  | 7145 | 604.6749 | 1207.3353 | 1206.5953 | 0.7400 | 1 | 6 | 29 | 4 | K.KNSSSGISGSQR.R |
|  | 11003 | **706.7797** | **1411.5448** | **1411.6983** | **-0.1536** | **0** | **4** | **45** | **5** | **R.SQYNFIADVVEK.T** |
|  | 26142 | 1005.1826 | 3012.5259 | 3012.5338 | -0.0080 | 0 | 49 | 0.00071 | 1 | R.AGLRPGDVILAIGEQMVQNAEDVYEAVR.T |

| **585.** | Q9Y4W6\|AFG32_HUMAN    **Mass:** 88984    **Score:** 49     **Queries matched:** 25   **emPAI:** 0.08 |
| --- | --- |
|  | AFG3-like protein 2 OS=Homo sapiens GN=AFG3L2 PE=1 SV=2 |

|  | **Query** | **Observed** | **Mr(expt)** | **Mr(calc)** | **Delta** | **Miss** | **Score** | **Expect** | **Rank** | **Peptide** |
| --- | --- | --- | --- | --- | --- | --- | --- | --- | --- | --- |
|  | 1211 | 732.3198 | 731.3125 | 731.3173 | -0.0048 | 0 | 7 | 52 | 2 | R.GGCWPR.G |
|  | 2076 | 422.2480 | 842.4815 | 842.5225 | -0.0410 | 1 | 12 | 11 | 4 | R.VIGGLEKK.T 2067 |
|  | 3340 | 466.7369 | 931.4593 | 932.4424 | -0.9831 | 1 | 25 | 0.63 | 2 | R.SSGGGGGGGGKR.G 3352 3354 3356 3361 3368 |
|  | 3702 | **481.2500** | **960.4854** | **959.5036** | **0.9818** | **0** | **4** | **71** | **3** | **K.ESKPAATTR.S** |
|  | 4785 | **521.2662** | **1040.5179** | **1040.5251** | **-0.0071** | **0** | **42** | **0.01** | **1** | **R.HLSDSINQK.H** |
|  | 5166 | **536.2844** | **1070.5543** | **1070.5608** | **-0.0065** | **0** | **8** | **26** | **2** | **K.TQVLQPEEK.K** |
|  | 5902 | **564.5554** | **1127.0962** | **1126.6386** | **0.4575** | **0** | **1** | **94** | **4** | **R.QIFIGPPDIK.G** |
|  | 5992 | **568.2508** | **1134.4870** | **1135.6098** | **-1.1228** | **1** | **4** | **70** | **3** | **R.FVTTQARASR.N** |
|  | 8202 | **630.3603** | **1258.7060** | **1259.6874** | **-0.9813** | **1** | **7** | **32** | **1** | **K.QYQDLGAKIPK.G** |
|  | 14000 | **399.7287** | **1594.8858** | **1593.9454** | **0.9404** | **1** | **3** | **60** | **7** | **K.GAILTGPPGTGKTLLAK.A** |
|  | 15394 | **843.4193** | **1684.8239** | **1683.8250** | **0.9989** | **1** | **3** | **53** | **1** | **R.TGRGMGGLFSVGETTAK.V** |
|  | 15737 | 855.9208 | 1709.8271 | 1710.7851 | -0.9580 | 1 | 0 | 1e+002 | 8 | K.EQLLDRMCMTLGGR.V |
|  | 18544 | **966.5005** | **1930.9864** | **1929.9982** | **0.9882** | **1** | **4** | **38** | **1** | **K.NAPCILFIDEIDAVGRK.R** 18545 |
|  | 19180 | 663.9810 | 1988.9212 | 1988.9271 | -0.0058 | 1 | 1 | 67 | 10 | R.MCMTLGGRVSEEIFFGR.I |
|  | 23406 | **833.7124** | **2498.1154** | **2498.2935** | **-0.1781** | **0** | **1** | **55** | **6** | **R.MFFLWTALFWGGVMFYLLLK.R** |
|  | 24679 | **906.1353** | **2715.3841** | **2715.4014** | **-0.0173** | **1** | **31** | **0.051** | **1** | **R.KLASLTPGFSGADVANVCNEAALIAAR.H** |
|  | 28635 | **1204.5597** | **3610.6572** | **3609.8209** | **0.8363** | **1** | **2** | **23** | **4** | **K.LASLTPGFSGADVANVCNEAALIAARHLSDSINQK.H** |
|  | 31624 | **1349.9337** | **5395.7058** | **5394.7267** | **0.9791** | **0** | **5** | **3.4** | **1** | **R.GNFGGQSEQENTLNQLLVEMDGFNTTTNVVILAGTNRPDILDPALLRPGR.F** |

| **586.** | P27348\|1433T_HUMAN    **Mass:** 28032    **Score:** 49     **Queries matched:** 4   **emPAI:** 0.13 |
| --- | --- |
|  | 14-3-3 protein theta OS=Homo sapiens GN=YWHAQ PE=1 SV=1 |

|  | **Query** | **Observed** | **Mr(expt)** | **Mr(calc)** | **Delta** | **Miss** | **Score** | **Expect** | **Rank** | **Peptide** |
| --- | --- | --- | --- | --- | --- | --- | --- | --- | --- | --- |
|  | 940 | **601.1234** | **600.1161** | **600.3344** | **-0.2182** | **0** | **4** | **53** | **4** | **K.NVVGGR.R** |
|  | 6742 | 397.2191 | 1188.6355 | 1188.6536 | -0.0181 | 0 | 7 | 35 | 7 | K.DSTLIMQLLR.D |
|  | 12970 | **766.8580** | **1531.7014** | **1531.7114** | **-0.0100** | **0** | **49** | **0.0017** | **1** | **K.AVTEQGAELSNEER.N** |
|  | 18897 | **655.2960** | **1962.8662** | **1962.8121** | **0.0541** | **1** | **1** | **68** | **3** | **K.LAEQAERYDDMATCMK.A** |

| **587.** | P30485\|1B47_HUMAN    **Mass:** 40888    **Score:** 48     **Queries matched:** 7   **emPAI:** 0.19 |
| --- | --- |
|  | HLA class I histocompatibility antigen, B-47 alpha chain OS=Homo sapiens GN=HLA-B PE=2 SV=1 |

|  | **Query** | **Observed** | **Mr(expt)** | **Mr(calc)** | **Delta** | **Miss** | **Score** | **Expect** | **Rank** | **Peptide** |
| --- | --- | --- | --- | --- | --- | --- | --- | --- | --- | --- |
|  | 7513 | 613.7462 | 1225.4779 | 1225.4856 | -0.0077 | 0 | 40 | 0.013 | 2 | R.MFGCDVGPDGR.L 7528 |
|  | 9948 | 676.3410 | 1350.6674 | 1349.6939 | 0.9736 | 1 | 14 | 5.9 | 1 | R.YLENGKETLQR.A 9957 |
|  | 23671 | 509.4508 | 2542.2177 | 2541.3414 | 0.8763 | 0 | 2 | 40 | 10 | R.TLLLLLWGAVALTETWAGSHSMR.Y |
|  | 24792 | 912.7573 | 2735.2500 | 2735.2635 | -0.0135 | 1 | 34 | 0.023 | 1 | R.DGEDQTQDTELVETRPAGDRTFQK.W |
|  | 29026 | **749.3204** | **3741.5658** | **3742.1332** | **-0.5674** | **1** | **0** | **27** | **5** | **R.WEPSSQSTVPIVGIVAGLAVLAVVVIGAVVAAVVCRR.K** |

| **588.** | P62280\|RS11_HUMAN    **Mass:** 18590    **Score:** 48     **Queries matched:** 6   **emPAI:** 0.21 |
| --- | --- |
|  | 40S ribosomal protein S11 OS=Homo sapiens GN=RPS11 PE=1 SV=3 |

|  | **Query** | **Observed** | **Mr(expt)** | **Mr(calc)** | **Delta** | **Miss** | **Score** | **Expect** | **Rank** | **Peptide** |
| --- | --- | --- | --- | --- | --- | --- | --- | --- | --- | --- |
|  | 4689 | 517.2581 | 1032.5017 | 1031.5910 | 0.9107 | 1 | 4 | 68 | 2 | K.MQRTIVIR.R |
|  | 5190 | 537.2972 | 1072.5799 | 1072.6128 | -0.0329 | 1 | 6 | 52 | 8 | R.VLLGETGKEK.L |
|  | 7017 | **401.2854** | **1200.8343** | **1201.6495** | **-0.8152** | **1** | **12** | **7.2** | **1** | **R.YYKNIGLGFK.T** |
|  | 10178 | **455.5757** | **1363.7054** | **1362.6173** | **1.0881** | **0** | **3** | **75** | **5** | **K.NMSVHLSPCFR.D** |
|  | 19130 | **993.5072** | **1984.9998** | **1985.0252** | **-0.0253** | **0** | **48** | **0.0013** | **1** | **R.DVQIGDIVTVGECRPLSK.T** 19129 |

| **589.** | Q96KP4\|CNDP2_HUMAN    **Mass:** 53187    **Score:** 48     **Queries matched:** 12   **emPAI:** 0.15 |
| --- | --- |
|  | Cytosolic non-specific dipeptidase OS=Homo sapiens GN=CNDP2 PE=1 SV=2 |

|  | **Query** | **Observed** | **Mr(expt)** | **Mr(calc)** | **Delta** | **Miss** | **Score** | **Expect** | **Rank** | **Peptide** |
| --- | --- | --- | --- | --- | --- | --- | --- | --- | --- | --- |
|  | 4197 | **498.7811** | **995.5476** | **994.5521** | **0.9955** | **0** | **9** | **18** | **1** | **-.MAALTTLFK.Y** |
|  | 5722 | **556.8333** | **1111.6521** | **1110.5305** | **1.1215** | **1** | **3** | **64** | **1** | **K.LYGRGSTDDK.G** |
|  | 7364 | 610.2992 | 1218.5838 | 1219.6053 | -1.0215 | 1 | 8 | 28 | 7 | R.RMMEVAAADVK.Q 7421 |
|  | 11557 | 482.5936 | 1444.7590 | 1444.8150 | -0.0561 | 1 | 9 | 17 | 4 | K.DVGAQILLHSHKK.D |
|  | 15422 | **563.2929** | **1686.8567** | **1685.9716** | **0.8851** | **0** | **5** | **37** | **3** | **K.LPDGSEIPLPPILLGR.L** |
|  | 16068 | **867.9446** | **1733.8746** | **1733.8836** | **-0.0090** | **0** | **19** | **1.3** | **1** | **R.EGGSIPVTLTFQEATGK.N** 16067 |
|  | 17897 | **626.4199** | **1876.2379** | **1876.9319** | **-0.6940** | **0** | **6** | **18** | **1** | **R.YPSLSLHGIEGAFSGSGAK.T** 17907 |
|  | 22280 | 1167.5316 | 2333.0487 | 2332.2348 | 0.8139 | 1 | 1 | 52 | 3 | R.LVPNMTPEVVGEQVTSYLTKK.F |
|  | 27512 | **1106.5248** | **3316.5525** | **3316.5711** | **-0.0185** | **0** | **48** | **0.00077** | **1** | **K.TVCIYGHLDVQPAALEDGWDSEPFTLVER.D** |

| **590.** | O15260\|SURF4_HUMAN    **Mass:** 30602    **Score:** 48     **Queries matched:** 7   **emPAI:** 0.12 |
| --- | --- |
|  | Surfeit locus protein 4 OS=Homo sapiens GN=SURF4 PE=1 SV=3 |

|  | **Query** | **Observed** | **Mr(expt)** | **Mr(calc)** | **Delta** | **Miss** | **Score** | **Expect** | **Rank** | **Peptide** |
| --- | --- | --- | --- | --- | --- | --- | --- | --- | --- | --- |
|  | 4020 | 492.7686 | 983.5227 | 982.5348 | 0.9879 | 0 | 6 | 40 | 2 | K.QYLPHVAR.L |
|  | 5904 | **564.7898** | **1127.5650** | **1127.5104** | **0.0547** | **0** | **2** | **1e+002** | **1** | **K.SMFAGVPTMR.E** |
|  | 12681 | 757.3455 | 1512.6764 | 1512.7065 | -0.0301 | 1 | (4) | 52 | 5 | R.SEGKSMFAGVPTMR.E |
|  | 12918 | **510.2452** | **1527.7136** | **1528.7014** | **-0.9878** | **1** | **6** | **33** | **4** | **R.SEGKSMFAGVPTMR.E** |
|  | 19599 | **1014.9552** | **2027.8958** | **2026.9055** | **0.9904** | **0** | **48** | **0.0014** | **1** | **M.GQNDLMGTAEDFADQFLR.V** 19587 |
|  | 20991 | **726.0031** | **2174.9875** | **2173.9409** | **1.0466** | **0** | **4** | **34** | **6** | **-.MGQNDLMGTAEDFADQFLR.V** |

| **591.** | P01859\|IGHG2_HUMAN    **Mass:** 36505    **Score:** 48     **Queries matched:** 7   **emPAI:** 0.22 |
| --- | --- |
|  | Ig gamma-2 chain C region OS=Homo sapiens GN=IGHG2 PE=1 SV=2 |

|  | **Query** | **Observed** | **Mr(expt)** | **Mr(calc)** | **Delta** | **Miss** | **Score** | **Expect** | **Rank** | **Peptide** |
| --- | --- | --- | --- | --- | --- | --- | --- | --- | --- | --- |
|  | 1294 | 395.0789 | 788.1432 | 787.4440 | 0.6993 | 0 | 6 | 39 | 4 | K.SLSLSPGK.- |
|  | 6343 | 581.3152 | 1160.6159 | 1160.6223 | -0.0064 | 0 | 29 | 0.21 | 1 | K.NQVSLTCLVK.G |
|  | 8728 | 429.7405 | 1286.1995 | 1286.6442 | -0.4446 | 0 | 6 | 25 | 5 | K.GPSVFPLAPCSR.S |
|  | 11189 | 712.3544 | 1422.6942 | 1422.7024 | -0.0083 | 0 | 40 | 0.012 | 1 | R.STSESTAALGCLVK.D 11170 |
|  | 18192 | **635.3273** | **1902.9600** | **1903.9349** | **-0.9750** | **1** | **2** | **68** | **7** | **R.EPQVYTLPPSREEMTK.N** 18201 |

| **592.** | Q14677\|EPN4_HUMAN    **Mass:** 68273    **Score:** 48     **Queries matched:** 5   **emPAI:** 0.17 |
| --- | --- |
|  | Clathrin interactor 1 OS=Homo sapiens GN=CLINT1 PE=1 SV=1 |

|  | **Query** | **Observed** | **Mr(expt)** | **Mr(calc)** | **Delta** | **Miss** | **Score** | **Expect** | **Rank** | **Peptide** |
| --- | --- | --- | --- | --- | --- | --- | --- | --- | --- | --- |
|  | 4902 | **525.7440** | **1049.4734** | **1049.4812** | **-0.0078** | **0** | **25** | **0.54** | **1** | **R.SQNTDMVQK.S** |
|  | 14200 | **805.4126** | **1608.8106** | **1608.8220** | **-0.0113** | **0** | **46** | **0.0028** | **1** | **K.HIHITQATETTTTR.H** 14219 |
|  | 20453 | **1058.4821** | **2114.9496** | **2114.9579** | **-0.0083** | **0** | **8** | **14** | **1** | **R.EATNDDPWGPSGQLMGEIAK.A** |
|  | 26904 | **792.8803** | **3167.4921** | **3167.5119** | **-0.0198** | **1** | **21** | **0.35** | **1** | **K.TIDLGAAAHYTGDKASPDQNASTHTPQSSVK.T** |

| **593.** | P02655\|APOC2_HUMAN    **Mass:** 11277    **Score:** 47     **Queries matched:** 1   **emPAI:** 0.35 |
| --- | --- |
|  | Apolipoprotein C-II OS=Homo sapiens GN=APOC2 PE=1 SV=1 |

|  | **Query** | **Observed** | **Mr(expt)** | **Mr(calc)** | **Delta** | **Miss** | **Score** | **Expect** | **Rank** | **Peptide** |
| --- | --- | --- | --- | --- | --- | --- | --- | --- | --- | --- |
|  | 23707 | **1274.6183** | **2547.2220** | **2547.2414** | **-0.0194** | **1** | **47** | **0.0013** | **1** | **K.STAAMSTYTGIFTDQVLSVLKGEE.-** |

| **594.** | Q14165\|MLEC_HUMAN    **Mass:** 32385    **Score:** 47     **Queries matched:** 4   **emPAI:** 0.12 |
| --- | --- |
|  | Malectin OS=Homo sapiens GN=MLEC PE=1 SV=1 |

|  | **Query** | **Observed** | **Mr(expt)** | **Mr(calc)** | **Delta** | **Miss** | **Score** | **Expect** | **Rank** | **Peptide** |
| --- | --- | --- | --- | --- | --- | --- | --- | --- | --- | --- |
|  | 6109 | 573.3106 | 1144.6067 | 1145.6557 | -1.0490 | 1 | 7 | 35 | 6 | K.LQPHPGLEKK.E |
|  | 13950 | **796.8766** | **1591.7386** | **1591.7478** | **-0.0092** | **0** | **47** | **0.0022** | **1** | **R.SNPEDQILYQTER.Y** |
|  | 15193 | **418.9849** | **1671.9103** | **1672.8970** | **-0.9867** | **0** | **6** | **27** | **10** | **-.MLGAWAVEGTAVALLR.L** |
|  | 24786 | **912.1160** | **2733.3263** | **2733.3425** | **-0.0162** | **1** | **13** | **3.1** | **1** | **R.YNEETFGYEVPIKEEGDYVLVLK.F** |

| **595.** | P08559\|ODPA_HUMAN    **Mass:** 43952    **Score:** 47     **Queries matched:** 18   **emPAI:** 0.09 |
| --- | --- |
|  | Pyruvate dehydrogenase E1 component subunit alpha, somatic form, mitochondrial OS=Homo sapiens GN=PDHA1 PE=1 SV=3 |

|  | **Query** | **Observed** | **Mr(expt)** | **Mr(calc)** | **Delta** | **Miss** | **Score** | **Expect** | **Rank** | **Peptide** |
| --- | --- | --- | --- | --- | --- | --- | --- | --- | --- | --- |
|  | 2545 | 437.7423 | 873.4700 | 874.5058 | -1.0358 | 1 | 11 | 16 | 4 | R.KMLAAVSR.V |
|  | 4240 | **500.2795** | **998.5443** | **998.4491** | **0.0952** | **0** | **10** | **17** | **1** | **R.YGMGTSVER.A** |
|  | 4636 | **515.2983** | **1028.5820** | **1029.5720** | **-0.9900** | **1** | **5** | **55** | **7** | **K.RGDFIPGLR.V** |
|  | 5933 | 565.7885 | 1129.5625 | 1128.6502 | 0.9122 | 1 | 17 | 3.6 | 2 | R.EILAELTGRK.G |
|  | 6311 | **580.3036** | **1158.5926** | **1158.5993** | **-0.0066** | **1** | **16** | **3.9** | **1** | **R.TREEIQEVR.S** |
|  | 6709 | 396.7506 | 1187.2300 | 1186.6380 | 0.5920 | 1 | 13 | 5.8 | 2 | K.SDPIMLLKDR.M |
|  | 6939 | 599.8332 | 1197.6518 | 1197.5271 | 0.1248 | 1 | 2 | 91 | 7 | K.GKGGSMHMYAK.N |
|  | 7019 | **401.2856** | **1200.8350** | **1199.6622** | **1.1728** | **0** | **13** | **7** | **1** | **R.VLSGASQKPASR.V** 7018 |
|  | 9682 | **446.2583** | **1335.7532** | **1335.6857** | **0.0675** | **0** | **6** | **32** | **3** | **K.GPILMELQTYR.Y** |
|  | 11615 | 483.7787 | 1448.3142 | 1448.7181 | -0.4038 | 0 | 1 | 70 | 10 | R.MVNSNLASVEELK.E |
|  | 13954 | **531.6171** | **1591.8296** | **1591.8392** | **-0.0096** | **1** | **4** | **49** | **4** | **R.SGKGPILMELQTYR.Y** |
|  | 15874 | **574.2662** | **1719.7769** | **1719.8461** | **-0.0692** | **1** | **5** | **38** | **1** | **K.DRMVNSNLASVEELK.E** 15879 |
|  | 17736 | 622.3218 | 1863.9435 | 1864.8275 | -0.8840 | 1 | 1 | 82 | 3 | R.YHGHSMSDPGVSYRTR.E |
|  | 18798 | 652.0225 | 1953.0457 | 1953.0419 | 0.0039 | 1 | 1 | 82 | 9 | R.LEEGPPVTTVLTREDGLK.Y |
|  | 27544 | **665.8670** | **3324.2986** | **3324.6206** | **-0.3220** | **1** | **0** | **30** | **3** | **K.GGSMHMYAKNFYGGNGIVGAQVPLGAGIALACK.Y** |
|  | 28324 | **884.3931** | **3533.5434** | **3533.5585** | **-0.0151** | **0** | **47** | **0.00067** | **1** | **R.GFCHLCDGQEACCVGLEAGINPTDHLITAYR.A** |

| **596.** | Q08380\|LG3BP_HUMAN    **Mass:** 66202    **Score:** 46     **Queries matched:** 7   **emPAI:** 0.06 |
| --- | --- |
|  | Galectin-3-binding protein OS=Homo sapiens GN=LGALS3BP PE=1 SV=1 |

|  | **Query** | **Observed** | **Mr(expt)** | **Mr(calc)** | **Delta** | **Miss** | **Score** | **Expect** | **Rank** | **Peptide** |
| --- | --- | --- | --- | --- | --- | --- | --- | --- | --- | --- |
|  | 884 | **577.1222** | **576.1149** | **577.2456** | **-1.1307** | **0** | **4** | **84** | **8** | **K.SGGSDR.T** |
|  | 7439 | **408.2467** | **1221.7184** | **1220.5456** | **1.1728** | **0** | **6** | **35** | **10** | **R.DAGVVCTNETR.S** |
|  | 13961 | **797.4067** | **1592.7989** | **1591.7842** | **1.0147** | **0** | **46** | **0.0027** | **1** | **R.ELSEALGQIFDSQR.G** |
|  | 20231 | 698.3538 | 2092.0395 | 2092.9228 | -0.8833 | 0 | (1) | 79 | 8 | K.EPGSNVTMSVDAECVPMVR.D |
|  | 20237 | **524.0565** | **2092.1967** | **2092.9228** | **-0.7260** | **0** | **(1)** | **59** | **2** | **K.EPGSNVTMSVDAECVPMVR.D** |
|  | 20391 | 703.7077 | 2108.1013 | 2108.9177 | -0.8164 | 0 | 2 | 53 | 8 | K.EPGSNVTMSVDAECVPMVR.D |
|  | 27575 | **667.3638** | **3331.7828** | **3332.6361** | **-0.8533** | **1** | **2** | **26** | **3** | **R.LFWVWLLVAGTQGVNDGDMRLADGGATNQGR.V** |

| **597.** | P56556\|NDUA6_HUMAN    **Mass:** 15127    **Score:** 46     **Queries matched:** 3   **emPAI:** 0.26 |
| --- | --- |
|  | NADH dehydrogenase [ubiquinone] 1 alpha subcomplex subunit 6 OS=Homo sapiens GN=NDUFA6 PE=1 SV=2 |

|  | **Query** | **Observed** | **Mr(expt)** | **Mr(calc)** | **Delta** | **Miss** | **Score** | **Expect** | **Rank** | **Peptide** |
| --- | --- | --- | --- | --- | --- | --- | --- | --- | --- | --- |
|  | 10095 | **453.8983** | **1358.6729** | **1357.6779** | **0.9950** | **0** | **12** | **9.9** | **2** | **R.FFHETEAPRPK.D** |
|  | 14169 | **536.3152** | **1605.9239** | **1606.7232** | **-0.7993** | **1** | **2** | **62** | **2** | **R.EMFMKNAHVTDPR.V** |
|  | 17771 | **934.4935** | **1866.9724** | **1866.9840** | **-0.0116** | **0** | **46** | **0.0023** | **1** | **R.EVPNTVHQFQLDITVK.M** |

| **598.** | Q8N4Q1\|MIA40_HUMAN    **Mass:** 16385    **Score:** 46     **Queries matched:** 2   **emPAI:** 0.53 |
| --- | --- |
|  | Mitochondrial intermembrane space import and assembly protein 40 OS=Homo sapiens GN=CHCHD4 PE=1 SV=1 |

|  | **Query** | **Observed** | **Mr(expt)** | **Mr(calc)** | **Delta** | **Miss** | **Score** | **Expect** | **Rank** | **Peptide** |
| --- | --- | --- | --- | --- | --- | --- | --- | --- | --- | --- |
|  | 24946 | **924.0616** | **2769.1631** | **2769.1799** | **-0.0168** | **1** | **47** | **0.001** | **1** | **K.SAFSCFHYSTEEIKGSDCVDQFR.A** |
|  | 32224 | **1288.7875** | **6438.9010** | **6438.9242** | **-0.0232** | **1** | **15** | **0.2** | **1** | **R.IIFVTKEDHETPSSAELVADDPNDPYEEHGLILPNGNINWNCPCLGGMASGPCGEQFK.S** |

| **599.** | Q96BM9\|ARL8A_HUMAN    **Mass:** 21630    **Score:** 46     **Queries matched:** 3   **emPAI:** 0.18 |
| --- | --- |
|  | ADP-ribosylation factor-like protein 8A OS=Homo sapiens GN=ARL8A PE=1 SV=1 |

|  | **Query** | **Observed** | **Mr(expt)** | **Mr(calc)** | **Delta** | **Miss** | **Score** | **Expect** | **Rank** | **Peptide** |
| --- | --- | --- | --- | --- | --- | --- | --- | --- | --- | --- |
|  | 1938 | **418.2348** | **834.4550** | **835.4626** | **-1.0076** | **0** | **6** | **51** | **8** | **-.MIALFNK.L** |
|  | 4594 | 513.7285 | 1025.4425 | 1026.4705 | -1.0280 | 1 | 11 | 11 | 7 | R.FRSMWER.Y |
|  | 4864 | 524.2632 | 1046.5119 | 1046.5179 | -0.0059 | 0 | 46 | 0.0043 | 1 | K.MNLSAIQDR.E |

| **600.** | O43169\|CYB5B_HUMAN    **Mass:** 16436    **Score:** 46     **Queries matched:** 3   **emPAI:** 0.53 |
| --- | --- |
|  | Cytochrome b5 type B OS=Homo sapiens GN=CYB5B PE=1 SV=2 |

|  | **Query** | **Observed** | **Mr(expt)** | **Mr(calc)** | **Delta** | **Miss** | **Score** | **Expect** | **Rank** | **Peptide** |
| --- | --- | --- | --- | --- | --- | --- | --- | --- | --- | --- |
|  | 13503 | **522.3016** | **1563.8829** | **1563.8885** | **-0.0056** | **1** | **30** | **0.12** | **1** | **R.NSLKELWLVIHGR.V** |
|  | 28682 | **907.6623** | **3626.6203** | **3626.6397** | **-0.0194** | **0** | **29** | **0.047** | **1** | **R.FLNEHPGGEEVLLEQAGVDASESFEDVGHSSDAR.E** 28681 |

| **601.** | P18463\|1B37_HUMAN    **Mass:** 40716    **Score:** 46     **Queries matched:** 8   **emPAI:** 0.19 |
| --- | --- |
|  | HLA class I histocompatibility antigen, B-37 alpha chain OS=Homo sapiens GN=HLA-B PE=2 SV=1 |

|  | **Query** | **Observed** | **Mr(expt)** | **Mr(calc)** | **Delta** | **Miss** | **Score** | **Expect** | **Rank** | **Peptide** |
| --- | --- | --- | --- | --- | --- | --- | --- | --- | --- | --- |
|  | 3749 | 483.2167 | 964.4188 | 964.4250 | -0.0062 | 0 | 35 | 0.057 | 1 | R.FDSDAASPR.T |
|  | 9948 | 676.3410 | 1350.6674 | 1349.6939 | 0.9736 | 1 | 14 | 5.9 | 1 | R.YLENGKETLQR.A 9957 |
|  | 11588 | 483.2167 | 1446.6282 | 1447.6692 | -1.0410 | 1 | 13 | 7.2 | 1 | R.FDSDAASPRTEPR.A |
|  | 12971 | **511.5793** | **1531.7160** | **1531.7235** | **-0.0075** | **1** | **4** | **45** | **8** | **R.MSGCDVGPDGRLLR.G** 12956 |
|  | 23671 | 509.4508 | 2542.2177 | 2541.3414 | 0.8763 | 0 | 2 | 40 | 10 | R.TLLLLLWGAVALTETWAGSHSMR.Y |
|  | 24792 | 912.7573 | 2735.2500 | 2735.2635 | -0.0135 | 1 | 34 | 0.023 | 1 | R.DGEDQTQDTELVETRPAGDRTFQK.W |

|  | **Proteins matching a subset of these peptides:** |
| --- | --- |

|  | P03989\|1B27_HUMAN    **Mass:** 40802    **Score:** 46     **Queries matched:** 5 |
| --- | --- |
|  | HLA class I histocompatibility antigen, B-27 alpha chain OS=Homo sapiens GN=HLA-B PE=1 SV=2 |

| **602.** | Q8IYT4\|KATL2_HUMAN    **Mass:** 61557    **Score:** 46     **Queries matched:** 8   **emPAI:** 0.06 |
| --- | --- |
|  | Katanin p60 ATPase-containing subunit A-like 2 OS=Homo sapiens GN=KATNAL2 PE=2 SV=3 |

|  | **Query** | **Observed** | **Mr(expt)** | **Mr(calc)** | **Delta** | **Miss** | **Score** | **Expect** | **Rank** | **Peptide** |
| --- | --- | --- | --- | --- | --- | --- | --- | --- | --- | --- |
|  | 2077 | **422.2572** | **842.4998** | **843.4814** | **-0.9816** | **0** | **9** | **25** | **3** | **R.ELAAVVSR.D** |
|  | 2139 | **424.7215** | **847.4284** | **846.4963** | **0.9321** | **0** | **9** | **28** | **8** | **R.VLFELAR.Y** |
|  | 3516 | 474.2284 | 946.4423 | 945.5284 | 0.9139 | 0 | 8 | 30 | 6 | K.EAVVYPIR.Y |
|  | 6467 | 586.8339 | 1171.6533 | 1171.6601 | -0.0068 | 0 | 46 | 0.0042 | 1 | K.GLLLYGPPGTGK.T |
|  | 7508 | **409.4391** | **1225.2955** | **1225.6455** | **-0.3500** | **0** | **1** | **82** | **8** | **R.DIYLHNPNIK.W** |
|  | 17387 | **613.7814** | **1838.3223** | **1837.9430** | **0.3793** | **0** | **10** | **7.4** | **1** | **R.LLKPLSAFIGMNSEMR.E** |
|  | 17774 | **623.3373** | **1866.9900** | **1867.9250** | **-0.9350** | **1** | **12** | **6.7** | **1** | **-.MELSYQTLKFTHQAR.E** |
|  | 24276 | **883.4509** | **2647.3310** | **2647.4189** | **-0.0880** | **1** | **0** | **63** | **7** | **R.LLKPLSAFIGMNSEMRELAAVVSR.D** |

| **603.** | Q9NX47\|MARH5_HUMAN    **Mass:** 31781    **Score:** 46     **Queries matched:** 4   **emPAI:** 0.12 |
| --- | --- |
|  | E3 ubiquitin-protein ligase MARCH5 OS=Homo sapiens GN=MARCH5 PE=1 SV=1 |

|  | **Query** | **Observed** | **Mr(expt)** | **Mr(calc)** | **Delta** | **Miss** | **Score** | **Expect** | **Rank** | **Peptide** |
| --- | --- | --- | --- | --- | --- | --- | --- | --- | --- | --- |
|  | 9263 | 657.8234 | 1313.6323 | 1313.6398 | -0.0075 | 0 | 46 | 0.004 | 1 | M.PDQALQQMLDR.S |
|  | 10324 | 458.2637 | 1371.7694 | 1371.7299 | 0.0395 | 1 | 4 | 62 | 6 | K.VYFKQQQYLR.Q |
|  | 10726 | **465.9283** | **1394.7631** | **1394.6976** | **0.0655** | **0** | **6** | **36** | **3** | **K.LMFSSVNSNLQR.T** 10739 |

| **604.** | Q9BWM7\|SFXN3_HUMAN    **Mass:** 35823    **Score:** 46     **Queries matched:** 11   **emPAI:** 0.10 |
| --- | --- |
|  | Sideroflexin-3 OS=Homo sapiens GN=SFXN3 PE=2 SV=1 |

|  | **Query** | **Observed** | **Mr(expt)** | **Mr(calc)** | **Delta** | **Miss** | **Score** | **Expect** | **Rank** | **Peptide** |
| --- | --- | --- | --- | --- | --- | --- | --- | --- | --- | --- |
|  | 2747 | 445.1189 | 888.2232 | 887.5341 | 0.6891 | 0 | 8 | 26 | 9 | K.HLPPLVGR.F |
|  | 3002 | **454.2630** | **906.5114** | **905.4719** | **1.0395** | **0** | **4** | **74** | **1** | **R.NIVQNYR.A** |
|  | 5677 | 555.2689 | 1108.5233 | 1108.5302 | -0.0069 | 0 | 46 | 0.0041 | 1 | R.WDQSTFLGR.A |
|  | 9344 | **439.8886** | **1316.6438** | **1316.7928** | **-0.1490** | **1** | **5** | **45** | **3** | **K.SLTKHLPPLVGR.F** |
|  | 14701 | **547.3253** | **1638.9540** | **1639.8239** | **-0.8700** | **0** | **8** | **16** | **5** | **-.MGELPLDINIQEPR.W** 14681 14686 14693 |
|  | 14740 | **821.9298** | **1641.8451** | **1640.8522** | **0.9928** | **0** | **12** | **6.6** | **1** | **R.AGVVTPGITEDQLWR.A** |
|  | 20225 | 523.8046 | 2091.1894 | 2090.0805 | 1.1089 | 0 | 10 | 8.9 | 1 | R.FVPFAAVAAANCINIPLMR.Q |
|  | 22597 | 792.3614 | 2374.0623 | 2374.2402 | -0.1778 | 1 | 2 | 43 | 5 | R.FVPFAAVAAANCINIPLMRQR.E |

| **605.** | P61916\|NPC2_HUMAN    **Mass:** 16902    **Score:** 45     **Queries matched:** 3   **emPAI:** 0.23 |
| --- | --- |
|  | Epididymal secretory protein E1 OS=Homo sapiens GN=NPC2 PE=1 SV=1 |

|  | **Query** | **Observed** | **Mr(expt)** | **Mr(calc)** | **Delta** | **Miss** | **Score** | **Expect** | **Rank** | **Peptide** |
| --- | --- | --- | --- | --- | --- | --- | --- | --- | --- | --- |
|  | 4465 | **508.7600** | **1015.5053** | **1015.5121** | **-0.0067** | **0** | **17** | **3.4** | **2** | **K.SGINCPIQK.D** |
|  | 17444 | **922.4322** | **1842.8499** | **1842.8604** | **-0.0105** | **0** | **45** | **0.003** | **1** | **K.EVNVSPCPTQPCQLSK.G** |
|  | 23057 | 815.7460 | 2444.2161 | 2444.2596 | -0.0436 | 0 | 0 | 66 | 10 | K.AVVHGILMGVPVPFPIPEPDGCK.S |

| **606.** | O00159\|MYO1C_HUMAN    **Mass:** 122503   **Score:** 45     **Queries matched:** 22   **emPAI:** 0.03 |
| --- | --- |
|  | Myosin-Ic OS=Homo sapiens GN=MYO1C PE=1 SV=3 |

|  | **Query** | **Observed** | **Mr(expt)** | **Mr(calc)** | **Delta** | **Miss** | **Score** | **Expect** | **Rank** | **Peptide** |
| --- | --- | --- | --- | --- | --- | --- | --- | --- | --- | --- |
|  | 529 | **459.0115** | **458.0043** | **458.2601** | **-0.2559** | **0** | **13** | **9** | **1** | **R.GGAVR.D** 528 549 |
|  | 839 | **561.0930** | **560.0857** | **559.3442** | **0.7415** | **1** | **5** | **76** | **3** | **R.KSLGR.G** |
|  | 1567 | 403.7051 | 805.3957 | 805.4181 | -0.0225 | 1 | 9 | 25 | 4 | R.SELSDKK.R |
|  | 2032 | **421.0011** | **839.9876** | **839.4402** | **0.5474** | **1** | **7** | **19** | **3** | **R.AGFAYRR.K** |
|  | 7085 | **402.2448** | **1203.7126** | **1202.6044** | **1.1082** | **1** | **7** | **30** | **10** | **K.KDNYPQSVPR.L** |
|  | 7466 | **408.7607** | **1223.2603** | **1223.6550** | **-0.3947** | **0** | **2** | **67** | **9** | **R.NPQSYLYLVK.G** |
|  | 9129 | **435.8884** | **1304.6434** | **1305.6347** | **-0.9913** | **1** | **12** | **8.7** | **1** | **K.GQCAKVSSINDK.S** 9131 |
|  | 9920 | 450.5866 | 1348.7380 | 1348.6769 | 0.0611 | 1 | (7) | 33 | 7 | R.VTMESALTARDR.V |
|  | 10207 | **456.1922** | **1365.5549** | **1364.6718** | **0.8831** | **1** | **8** | **19** | **1** | **R.VTMESALTARDR.V** |
|  | 14062 | **534.2503** | **1599.7291** | **1598.8416** | **0.8875** | **1** | **7** | **25** | **9** | **R.SISPEWKQQLQQK.A** |
|  | 14071 | **800.9357** | **1599.8568** | **1599.8620** | **-0.0052** | **0** | **26** | **0.3** | **1** | **R.LLQSNPVLEAFGNAK.T** |
|  | 15012 | 553.9766 | 1658.9080 | 1659.8402 | -0.9322 | 1 | 5 | 35 | 7 | K.EPAYVRCIKPNDAK.Q |
|  | 15525 | **847.9086** | **1693.8027** | **1693.8134** | **-0.0107** | **0** | **45** | **0.0031** | **1** | **R.LLQFYAETCPAPER.G** |
|  | 16306 | **585.2795** | **1752.8166** | **1752.9258** | **-0.1092** | **0** | **1** | **86** | **8** | **K.GDVVLQSDHVIETLTK.T** |
|  | 18222 | **636.3051** | **1905.8933** | **1905.0095** | **0.8838** | **1** | **9** | **13** | **1** | **R.DGTIDFTPGSELLITKAK.N** |
|  | 18284 | **956.5475** | **1911.0804** | **1910.0877** | **0.9927** | **0** | **14** | **3.7** | **1** | **R.VLQALGSEPIQYAVPVVK.Y** |
|  | 18984 | **493.5260** | **1970.0750** | **1970.0949** | **-0.0199** | **1** | **4** | **34** | **5** | **R.LLQSNPVLEAFGNAKTLR.N** |
|  | 22369 | **782.4291** | **2344.2656** | **2344.2791** | **-0.0135** | **1** | **14** | **2.7** | **1** | **R.VLQALGSEPIQYAVPVVKYDR.K** |
|  | 24444 | **669.5982** | **2674.3637** | **2674.4727** | **-0.1090** | **1** | **4** | **22** | **1** | **K.RPETVATQFKMSLLQLVEILQSK.E** |

| **607.** | P62277\|RS13_HUMAN    **Mass:** 17212    **Score:** 45     **Queries matched:** 15   **emPAI:** 0.23 |
| --- | --- |
|  | 40S ribosomal protein S13 OS=Homo sapiens GN=RPS13 PE=1 SV=2 |

|  | **Query** | **Observed** | **Mr(expt)** | **Mr(calc)** | **Delta** | **Miss** | **Score** | **Expect** | **Rank** | **Peptide** |
| --- | --- | --- | --- | --- | --- | --- | --- | --- | --- | --- |
|  | 695 | **515.3260** | **514.3187** | **514.3227** | **-0.0040** | **0** | **12** | **14** | **6** | **K.AVAVR.K** 693 698 |
|  | 1098 | 665.3569 | 664.3496 | 664.3544 | -0.0048 | 0 | 23 | 1 | 1 | R.FVTGNK.I |
|  | 3793 | **485.2469** | **968.4792** | **967.4836** | **0.9957** | **0** | **10** | **14** | **4** | **R.DSHGVAQVR.F** |
|  | 4285 | 501.3377 | 1000.6607 | 999.4742 | 1.1865 | 1 | 15 | 5.5 | 1 | -.MGRMHAPGK.G 4267 |
|  | 4877 | **524.3052** | **1046.5958** | **1046.6237** | **-0.0278** | **1** | **0** | **1.6e+002** | **6** | **R.FVTGNKILR.I** |
|  | 7935 | 416.2150 | 1245.6231 | 1246.6782 | -1.0551 | 1 | 8 | 25 | 9 | K.GLSQSALPYRR.S |
|  | 8057 | **418.2388** | **1251.6947** | **1252.7503** | **-1.0557** | **0** | **15** | **4.2** | **2** | **K.GLTPSQIGVILR.D** 8076 8093 |
|  | 10502 | **691.4261** | **1380.8376** | **1380.8453** | **-0.0076** | **1** | **45** | **0.0038** | **1** | **K.KGLTPSQIGVILR.D** |
|  | 11441 | **480.2497** | **1437.7274** | **1437.7351** | **-0.0077** | **1** | **10** | **13** | **1** | **K.LTSDDVKEQIYK.L** 11437 |

| **608.** | O43920\|NDUS5_HUMAN    **Mass:** 12737    **Score:** 45     **Queries matched:** 6   **emPAI:** 0.71 |
| --- | --- |
|  | NADH dehydrogenase [ubiquinone] iron-sulfur protein 5 OS=Homo sapiens GN=NDUFS5 PE=1 SV=3 |

|  | **Query** | **Observed** | **Mr(expt)** | **Mr(calc)** | **Delta** | **Miss** | **Score** | **Expect** | **Rank** | **Peptide** |
| --- | --- | --- | --- | --- | --- | --- | --- | --- | --- | --- |
|  | 989 | 616.3986 | 615.3913 | 615.3955 | -0.0043 | 1 | 12 | 20 | 1 | R.DKLIK.E |
|  | 1122 | **678.4758** | **677.4685** | **678.3483** | **-0.8798** | **1** | **7** | **38** | **4** | **R.QKTMR.R** |
|  | 4469 | **508.7952** | **1015.5758** | **1015.5815** | **-0.0056** | **1** | **35** | **0.055** | **1** | **M.PFLDIQKR.F** |
|  | 7116 | **403.0460** | **1206.1163** | **1205.5434** | **0.5730** | **1** | **4** | **37** | **9** | **K.MAGRCHAFEK.E** |
|  | 11622 | 725.3687 | 1448.7229 | 1448.7300 | -0.0071 | 0 | 6 | 31 | 4 | R.WLTIQSGEQPYK.M |
|  | 13594 | **786.3724** | **1570.7302** | **1570.7337** | **-0.0035** | **0** | **38** | **0.018** | **1** | **K.IEYDDFVECLLR.Q** |

| **609.** | Q9Y6M9\|NDUB9_HUMAN    **Mass:** 22045    **Score:** 45     **Queries matched:** 10   **emPAI:** 0.38 |
| --- | --- |
|  | NADH dehydrogenase [ubiquinone] 1 beta subcomplex subunit 9 OS=Homo sapiens GN=NDUFB9 PE=1 SV=3 |

|  | **Query** | **Observed** | **Mr(expt)** | **Mr(calc)** | **Delta** | **Miss** | **Score** | **Expect** | **Rank** | **Peptide** |
| --- | --- | --- | --- | --- | --- | --- | --- | --- | --- | --- |
|  | 5825 | 560.4135 | 1118.8123 | 1117.6165 | 1.1958 | 1 | 9 | 17 | 1 | K.DMAKATQLLK.E 5823 |
|  | 13422 | 520.6078 | 1558.8017 | 1559.8096 | -1.0079 | 0 | 3 | 70 | 4 | M.AFLASGPYLTHQQK.V |
|  | 18286 | **956.9708** | **1911.9271** | **1911.9366** | **-0.0095** | **1** | **15** | **3.1** | **1** | **K.ATQLLKEAEEEFWYR.Q** |
|  | 20535 | **709.3866** | **2125.1380** | **2125.1473** | **-0.0093** | **1** | **2** | **49** | **1** | **R.KEGDLPPLWWYIVTRPR.E** |
|  | 20990 | **1088.5007** | **2174.9869** | **2175.0021** | **-0.0152** | **0** | **33** | **0.039** | **1** | **R.QHPQPYIFPDSPGGTSYER.Y** 20991 20992 20993 |
|  | 23567 | **843.0227** | **2526.0463** | **2526.0620** | **-0.0157** | **1** | **11** | **4.2** | **1** | **R.YDCYKVPEWCLDDWHPSEK.A** |

| **610.** | Q9Y679\|AUP1_HUMAN    **Mass:** 53508    **Score:** 45     **Queries matched:** 6   **emPAI:** 0.07 |
| --- | --- |
|  | Ancient ubiquitous protein 1 OS=Homo sapiens GN=AUP1 PE=1 SV=1 |

|  | **Query** | **Observed** | **Mr(expt)** | **Mr(calc)** | **Delta** | **Miss** | **Score** | **Expect** | **Rank** | **Peptide** |
| --- | --- | --- | --- | --- | --- | --- | --- | --- | --- | --- |
|  | 1548 | **403.0460** | **804.0775** | **803.3773** | **0.7002** | **0** | **8** | **21** | **1** | **R.QEDSGLR.D** |
|  | 3845 | 486.8886 | 971.7627 | 972.3793 | -0.6166 | 0 | 3 | 73 | 2 | R.GFMEMNGR.G |
|  | 15720 | **570.3215** | **1707.9428** | **1706.9324** | **1.0103** | **1** | **6** | **29** | **4** | **R.FVVRTMCAVLGLVAR.Q** |
|  | 20682 | 714.8411 | 2141.5016 | 2142.1433 | -0.6417 | 1 | 3 | 31 | 3 | R.QLGEANEEFALRVQQLVAK.E |
|  | 25962 | **992.1842** | **2973.5308** | **2973.5448** | **-0.0140** | **1** | **45** | **0.0016** | **1** | **K.GTQSLPTASASKFPSSGPVTPQPTALTFAK.S** |
|  | 26868 | 632.7269 | 3158.5983 | 3158.6836 | -0.0854 | 1 | 2 | 29 | 1 | R.LRPQSAQSSFPPSPGPSPDVQLATLAQRVK.E |

| **611.** | Q16822\|PCKGM_HUMAN    **Mass:** 71447    **Score:** 44     **Queries matched:** 17   **emPAI:** 0.11 |
| --- | --- |
|  | Phosphoenolpyruvate carboxykinase [GTP], mitochondrial OS=Homo sapiens GN=PCK2 PE=1 SV=2 |

|  | **Query** | **Observed** | **Mr(expt)** | **Mr(calc)** | **Delta** | **Miss** | **Score** | **Expect** | **Rank** | **Peptide** |
| --- | --- | --- | --- | --- | --- | --- | --- | --- | --- | --- |
|  | 1181 | **711.0908** | **710.0835** | **709.3031** | **0.7804** | **0** | **3** | **42** | **3** | **R.FDSEGR.L** |
|  | 2557 | **438.5746** | **875.1346** | **875.3985** | **-0.2638** | **0** | **8** | **21** | **9** | **R.LEGEDSAR.E** |
|  | 3686 | 480.7455 | 959.4765 | 959.4461 | 0.0304 | 0 | 8 | 28 | 6 | R.DFVEHSAR.L 3673 |
|  | 4652 | **515.7770** | **1029.5395** | **1029.5455** | **-0.0059** | **0** | **18** | **2.8** | **1** | **K.EGALDLSGLR.A** |
|  | 5122 | **534.4467** | **1066.8789** | **1066.4614** | **0.4175** | **0** | **0** | **92** | **9** | **K.EPCAHPNSR.F** |
|  | 11635 | 725.8419 | 1449.6692 | 1449.6381 | 0.0311 | 0 | 11 | 11 | 2 | K.VECVGDDIAWMR.F 11623 11656 |
|  | 12828 | **507.9180** | **1520.7321** | **1521.6817** | **-0.9496** | **1** | **8** | **20** | **1** | **R.AVDERFPGCMQGR.T** |
|  | 14150 | **803.8705** | **1605.7264** | **1605.7358** | **-0.0094** | **0** | **43** | **0.0062** | **1** | **R.GQLGNWMSPADFQR.A** |
|  | 14420 | **541.7672** | **1622.2798** | **1621.7307** | **0.5491** | **0** | **(2)** | **51** | **5** | **R.GQLGNWMSPADFQR.A** |
|  | 14540 | **815.4413** | **1628.8680** | **1628.8774** | **-0.0094** | **0** | **26** | **0.3** | **1** | **R.LGTPVLQALGDGDFVK.C** |
|  | 18910 | **491.7618** | **1963.0180** | **1964.0942** | **-1.0762** | **1** | **8** | **15** | **2** | **R.ETPIGLVPKEGALDLSGLR.A** |
|  | 20803 | **719.0369** | **2154.0888** | **2153.0827** | **1.0061** | **0** | **7** | **18** | **1** | **R.DEGWLAEHMLILGITSPAGK.K** |
|  | 25731 | **731.1367** | **2920.5175** | **2919.3540** | **1.1635** | **1** | **0** | **52** | **5** | **R.FPGCMQGRTMYVLPFSMGPVGSPLSR.I** |
|  | 28779 | **1221.6191** | **3661.8356** | **3660.8354** | **1.0002** | **1** | **1** | **30** | **10** | **R.TMYVLPFSMGPVGSPLSRIGVQLTDSAYVVASMR.I** |

| **612.** | O00476\|NPT4_HUMAN    **Mass:** 46703    **Score:** 44     **Queries matched:** 5   **emPAI:** 0.08 |
| --- | --- |
|  | Sodium-dependent phosphate transport protein 4 OS=Homo sapiens GN=SLC17A3 PE=2 SV=2 |

|  | **Query** | **Observed** | **Mr(expt)** | **Mr(calc)** | **Delta** | **Miss** | **Score** | **Expect** | **Rank** | **Peptide** |
| --- | --- | --- | --- | --- | --- | --- | --- | --- | --- | --- |
|  | 9144 | **436.2237** | **1305.6492** | **1304.6758** | **0.9733** | **1** | **9** | **19** | **4** | **-.MATKTELSPTAR.E** |
|  | 14533 | **815.3936** | **1628.7725** | **1628.7828** | **-0.0103** | **0** | **39** | **0.014** | **1** | **K.NAQDMQVDETLIPR.K** 14534 |
|  | 14764 | **411.9527** | **1643.7816** | **1644.7777** | **-0.9961** | **0** | **(4)** | **46** | **10** | **K.NAQDMQVDETLIPR.K** |
|  | 16557 | **887.4111** | **1772.8077** | **1772.8727** | **-0.0650** | **1** | **0** | **1e+002** | **6** | **K.NAQDMQVDETLIPRK.V** |

| **613.** | Q9BXJ8\|T120A_HUMAN    **Mass:** 41040    **Score:** 44     **Queries matched:** 10   **emPAI:** 0.09 |
| --- | --- |
|  | Transmembrane protein 120A OS=Homo sapiens GN=TMEM120A PE=2 SV=1 |

|  | **Query** | **Observed** | **Mr(expt)** | **Mr(calc)** | **Delta** | **Miss** | **Score** | **Expect** | **Rank** | **Peptide** |
| --- | --- | --- | --- | --- | --- | --- | --- | --- | --- | --- |
|  | 5892 | 563.8602 | 1125.7059 | 1125.7121 | -0.0062 | 1 | 12 | 8.3 | 2 | R.LQELALALKK.C |
|  | 8866 | 647.3104 | 1292.6063 | 1292.6143 | -0.0080 | 0 | 44 | 0.0057 | 1 | K.LQNNCTSSITR.Q 8865 8871 8884 |
|  | 13280 | **517.6063** | **1549.7970** | **1548.7678** | **1.0291** | **1** | **2** | **76** | **7** | **K.LQNNCTSSITRQK.K** |
|  | 13297 | **517.9424** | **1550.8053** | **1549.7381** | **1.0672** | **0** | **5** | **36** | **9** | **-.MQPPPPGPLGDCLR.D** |
|  | 15405 | 843.8936 | 1685.7725 | 1685.8487 | -0.0761 | 1 | (1) | 84 | 3 | R.QGLFFDMEAYLPKK.N |
|  | 15640 | **426.7241** | **1702.8671** | **1701.8436** | **1.0235** | **1** | **1** | **85** | **8** | **R.QGLFFDMEAYLPKK.N** |
|  | 28559 | **1195.8931** | **3584.6574** | **3585.6695** | **-1.0122** | **1** | **1** | **27** | **3** | **M.QPPPPGPLGDCLRDWEDLQQDFQNIQETHR.L** |

| **614.** | P62424\|RL7A_HUMAN    **Mass:** 30148    **Score:** 44     **Queries matched:** 10   **emPAI:** 0.27 |
| --- | --- |
|  | 60S ribosomal protein L7a OS=Homo sapiens GN=RPL7A PE=1 SV=2 |

|  | **Query** | **Observed** | **Mr(expt)** | **Mr(calc)** | **Delta** | **Miss** | **Score** | **Expect** | **Rank** | **Peptide** |
| --- | --- | --- | --- | --- | --- | --- | --- | --- | --- | --- |
|  | 2195 | **426.2688** | **850.5231** | **850.5276** | **-0.0045** | **0** | **9** | **21** | **2** | **K.VAPAPAVVK.K** 2175 |
|  | 3955 | 490.3153 | 978.6160 | 978.6226 | -0.0066 | 1 | 14 | 6.4 | 2 | K.KVAPAPAVVK.K |
|  | 5078 | **532.7828** | **1063.5510** | **1063.5563** | **-0.0053** | **0** | **6** | **43** | **1** | **R.HWGGNVLGPK.S** |
|  | 6066 | **571.3164** | **1140.6181** | **1139.7026** | **0.9155** | **1** | **5** | **44** | **5** | **K.GALAKLVEAIR.T** |
|  | 7317 | **1216.6243** | **1215.6170** | **1215.6248** | **-0.0078** | **0** | **19** | **2.1** | **1** | **K.NFGIGQDIQPK.R** |
|  | 9863 | **673.8668** | **1345.7191** | **1344.7249** | **0.9942** | **0** | **47** | **0.0027** | **1** | **R.AGVNTVTTLVENK.K** |
|  | 11762 | **486.8886** | **1457.6441** | **1457.6535** | **-0.0094** | **1** | **17** | **2.4** | **1** | **R.TNYNDRYDEIR.R** |
|  | 13563 | **785.4174** | **1568.8202** | **1568.8311** | **-0.0109** | **0** | **19** | **1.4** | **1** | **K.VPPAINQFTQALDR.Q** |
|  | 20656 | **714.3524** | **2140.0353** | **2140.0470** | **-0.0118** | **1** | **20** | **0.96** | **1** | **K.TCTTVAFTQVNSEDKGALAK.L** |

| **615.** | P53367\|ARFP1_HUMAN    **Mass:** 41770    **Score:** 44     **Queries matched:** 3   **emPAI:** 0.09 |
| --- | --- |
|  | Arfaptin-1 OS=Homo sapiens GN=ARFIP1 PE=1 SV=2 |

|  | **Query** | **Observed** | **Mr(expt)** | **Mr(calc)** | **Delta** | **Miss** | **Score** | **Expect** | **Rank** | **Peptide** |
| --- | --- | --- | --- | --- | --- | --- | --- | --- | --- | --- |
|  | 3003 | 454.2631 | 906.5116 | 906.5174 | -0.0059 | 1 | 44 | 0.0076 | 1 | K.KYENILK.L 3001 |
|  | 8547 | **426.2743** | **1275.8010** | **1276.6663** | **-0.8654** | **0** | **13** | **7.5** | **1** | **R.QLGDAFADLSLK.S** |

| **616.** | Q9UHQ4\|BAP29_HUMAN    **Mass:** 28416    **Score:** 44     **Queries matched:** 11   **emPAI:** 0.13 |
| --- | --- |
|  | B-cell receptor-associated protein 29 OS=Homo sapiens GN=BCAP29 PE=1 SV=2 |

|  | **Query** | **Observed** | **Mr(expt)** | **Mr(calc)** | **Delta** | **Miss** | **Score** | **Expect** | **Rank** | **Peptide** |
| --- | --- | --- | --- | --- | --- | --- | --- | --- | --- | --- |
|  | 1243 | 760.4258 | 759.4186 | 758.4650 | 0.9536 | 1 | 9 | 30 | 1 | K.LKTELR.K |
|  | 2154 | 425.2187 | 848.4228 | 848.4603 | -0.0376 | 1 | 11 | 16 | 8 | R.KTSDALSK.A 2178 |
|  | 3003 | 454.2631 | 906.5116 | 907.4651 | -0.9535 | 0 | 44 | 0.0078 | 3 | K.EYDQLLK.E 3001 |
|  | 5091 | **533.2346** | **1064.4547** | **1064.4630** | **-0.0084** | **0** | **12** | **10** | **1** | **K.AQNDVMEMK.M** |
|  | 5151 | **536.1623** | **1070.3101** | **1069.4750** | **0.8352** | **1** | **2** | **75** | **2** | **K.KFMEENEK.L** |
|  | 6613 | **395.2170** | **1182.6293** | **1182.5590** | **0.0703** | **1** | **8** | **20** | **5** | **K.FMEENEKLK.R** |
|  | 15988 | **864.8699** | **1727.7253** | **1727.7277** | **-0.0024** | **1** | **1** | **79** | **6** | **K.AQNDVMEMKMQSER.L** |
|  | 19737 | 511.2808 | 2041.0942 | 2042.2907 | -1.1966 | 0 | 2 | 59 | 10 | K.AFLTIIILLIVLFLDAVR.E |
|  | 20793 | 1078.0118 | 2154.0091 | 2153.0324 | 0.9768 | 1 | 4 | 37 | 2 | K.SSTSRPDAYEHTQMKLFR.S |

| **617.** | P22307\|NLTP_HUMAN    **Mass:** 59640    **Score:** 43     **Queries matched:** 17   **emPAI:** 0.13 |
| --- | --- |
|  | Non-specific lipid-transfer protein OS=Homo sapiens GN=SCP2 PE=1 SV=2 |

|  | **Query** | **Observed** | **Mr(expt)** | **Mr(calc)** | **Delta** | **Miss** | **Score** | **Expect** | **Rank** | **Peptide** |
| --- | --- | --- | --- | --- | --- | --- | --- | --- | --- | --- |
|  | 2195 | 426.2688 | 850.5231 | 851.4905 | -0.9674 | 0 | 2 | 94 | 8 | K.IGGIFAFK.V |
|  | 8086 | **627.7959** | **1253.5772** | **1253.5863** | **-0.0090** | **0** | **25** | **0.47** | **1** | **K.MNPQSAFFQGK.L** |
|  | 8423 | **635.3491** | **1268.6836** | **1269.7081** | **-1.0245** | **0** | **8** | **24** | **4** | **K.WVINPSGGLISK.G** |
|  | 8457 | **636.3668** | **1270.7191** | **1269.5812** | **1.1379** | **0** | **(6)** | **40** | **4** | **K.MNPQSAFFQGK.L** |
|  | 9160 | **436.7711** | **1307.2915** | **1307.6941** | **-0.4026** | **1** | **5** | **28** | **2** | **K.LKITGNMGLAMK.L** 9197 |
|  | 9460 | **662.3582** | **1322.7017** | **1322.7306** | **-0.0289** | **0** | **41** | **0.011** | **1** | **K.LQNLQLQPGNAK.L** |
|  | 9677 | 668.7985 | 1335.5824 | 1334.6718 | 0.9106 | 1 | 28 | 0.23 | 1 | K.KLEEEGEQFVK.K |
|  | 9750 | **671.2244** | **1340.4342** | **1340.6217** | **-0.1875** | **1** | **(1)** | **77** | **5** | **K.MVGFDMSKEAAR.K** |
|  | 10069 | **679.6714** | **1357.3282** | **1356.6166** | **0.7116** | **1** | **(0)** | **90** | **10** | **K.MVGFDMSKEAAR.K** |
|  | 10077 | 679.8513 | 1357.6881 | 1356.6166 | 1.0715 | 1 | (0) | 1.4e+002 | 5 | K.MVGFDMSKEAAR.K |
|  | 10309 | **458.2303** | **1371.6691** | **1370.6938** | **0.9753** | **1** | **2** | **96** | **6** | **K.SIIKMVGFDMSK.E** |
|  | 10331 | 458.3054 | 1371.8944 | 1372.6115 | -0.7171 | 1 | 8 | 20 | 5 | K.MVGFDMSKEAAR.K 10338 |
|  | 14184 | **536.6387** | **1606.8944** | **1605.9090** | **0.9854** | **1** | **3** | **57** | **4** | **R.TIPTDKHVDLLINK.Y** |
|  | 27741 | **843.6381** | **3370.5231** | **3371.6418** | **-1.1186** | **0** | **0** | **35** | **8** | **K.EVFDFLTILQCCPTSDGAAAAILASEAFVQK.Y** |
|  | 32224 | 1288.7875 | 6438.9010 | 6439.0229 | -0.1219 | 1 | 0 | 6.1 | 2 | K.ALADAQIPYSAVDQACVGYVFGDSTCGQRAIYHSLGMTGIPIINVNNNCATGSTALFMAR.Q |

| **618.** | Q09160\|1A80_HUMAN    **Mass:** 41051    **Score:** 43     **Queries matched:** 12   **emPAI:** 0.09 |
| --- | --- |
|  | HLA class I histocompatibility antigen, A-80 alpha chain OS=Homo sapiens GN=HLA-A PE=2 SV=1 |

|  | **Query** | **Observed** | **Mr(expt)** | **Mr(calc)** | **Delta** | **Miss** | **Score** | **Expect** | **Rank** | **Peptide** |
| --- | --- | --- | --- | --- | --- | --- | --- | --- | --- | --- |
|  | 1133 | **687.3375** | **686.3302** | **685.3581** | **0.9721** | **0** | **9** | **27** | **2** | **M.AVMPPR.T** 1131 |
|  | 1487 | **401.2285** | **800.4425** | **800.4037** | **0.0389** | **0** | **10** | **20** | **6** | **-.MAVMPPR.T** |
|  | 1644 | 406.7126 | 811.4107 | 810.4500 | 0.9606 | 1 | 4 | 56 | 10 | R.FLRGYR.Q |
|  | 4423 | **507.2242** | **1012.4338** | **1012.5706** | **-0.1368** | **0** | **1** | **1.3e+002** | **5** | **K.WAAVVVPSGK.E** |
|  | 9948 | 676.3410 | 1350.6674 | 1349.6939 | 0.9736 | 1 | 14 | 5.9 | 1 | R.YLENGKETLQR.T 9957 |
|  | 13042 | 513.2479 | 1536.7218 | 1537.8073 | -1.0856 | 1 | 4 | 46 | 3 | K.AHSQTNRANLGTLR.G |
|  | 13526 | 522.9525 | 1565.8355 | 1564.7668 | 1.0688 | 1 | 2 | 67 | 6 | R.SWTAADMAAQITKR.K |
|  | 17947 | 471.2618 | 1881.0179 | 1881.8904 | -0.8725 | 0 | 11 | 8.5 | 2 | K.THMTHHPISDHEATLR.C |
|  | 21263 | **736.4193** | **2206.2359** | **2205.1113** | **1.1246** | **1** | **3** | **39** | **1** | **K.RYTCHVQHEGLPEPLTLR.W** |
|  | 24215 | 879.7303 | 2636.1690 | 2636.1838 | -0.0148 | 0 | 43 | 0.003 | 1 | R.DGEDQTQDTELVETRPAGDGTFQK.W |

| **619.** | P01893\|HLAH_HUMAN    **Mass:** 41052    **Score:** 43     **Queries matched:** 12   **emPAI:** 0.09 |
| --- | --- |
|  | Putative HLA class I histocompatibility antigen, alpha chain H OS=Homo sapiens GN=HLA-H PE=5 SV=2 |

|  | **Query** | **Observed** | **Mr(expt)** | **Mr(calc)** | **Delta** | **Miss** | **Score** | **Expect** | **Rank** | **Peptide** |
| --- | --- | --- | --- | --- | --- | --- | --- | --- | --- | --- |
|  | 1255 | 763.1151 | 762.1078 | 762.3694 | -0.2616 | 0 | 7 | 33 | 7 | R.NTQICK.A 1256 1257 |
|  | 4914 | **526.2745** | **1050.5344** | **1049.4930** | **1.0414** | **1** | **2** | **92** | **2** | **R.EGPKYWDR.N** |
|  | 9312 | 658.8048 | 1315.5951 | 1315.5979 | -0.0028 | 1 | 5 | 51 | 5 | R.EEPRAPWMER.E |
|  | 9948 | 676.3410 | 1350.6674 | 1349.6939 | 0.9736 | 1 | 14 | 5.9 | 1 | R.YLENGKETLQR.A 9957 |
|  | 13526 | 522.9525 | 1565.8355 | 1564.7668 | 1.0688 | 1 | 2 | 67 | 6 | R.SWTAADMAAQITKR.K |
|  | 17947 | 471.2618 | 1881.0179 | 1881.8904 | -0.8725 | 0 | 11 | 8.5 | 2 | K.THMTHHPISDHEATLR.C |
|  | 22259 | 777.4392 | 2329.2956 | 2330.0684 | -0.7728 | 1 | (0) | 62 | 7 | R.SHSMRYFYTTMSRPGAGEPR.F |
|  | 22388 | **587.5664** | **2346.2363** | **2346.0634** | **0.1729** | **1** | **8** | **12** | **1** | **R.SHSMRYFYTTMSRPGAGEPR.F** |
|  | 24215 | 879.7303 | 2636.1690 | 2636.1838 | -0.0148 | 0 | 43 | 0.003 | 1 | R.DGEDQTQDTELVETRPAGDGTFQK.W |

| **620.** | P22059\|OSBP1_HUMAN    **Mass:** 90220    **Score:** 43     **Queries matched:** 16   **emPAI:** 0.04 |
| --- | --- |
|  | Oxysterol-binding protein 1 OS=Homo sapiens GN=OSBP PE=1 SV=1 |

|  | **Query** | **Observed** | **Mr(expt)** | **Mr(calc)** | **Delta** | **Miss** | **Score** | **Expect** | **Rank** | **Peptide** |
| --- | --- | --- | --- | --- | --- | --- | --- | --- | --- | --- |
|  | 858 | **569.1239** | **568.1166** | **567.3380** | **0.7786** | **0** | **0** | **79** | **1** | **R.NPLPK.N** |
|  | 1601 | **404.7351** | **807.4556** | **806.3956** | **1.0600** | **0** | **10** | **16** | **7** | **-.MAATELR.G** 1588 |
|  | 6941 | **400.2278** | **1197.6617** | **1197.5486** | **0.1131** | **1** | **7** | **33** | **5** | **R.QRGHEAEESR.V** |
|  | 7650 | 617.2842 | 1232.5538 | 1232.6626 | -0.1087 | 1 | 0 | 1.4e+002 | 4 | K.QVNERATLFR.I |
|  | 8379 | **423.2555** | **1266.7447** | **1265.5856** | **1.1591** | **0** | **14** | **5.3** | **1** | **R.ITSNAMINACR.D** |
|  | 8640 | **641.8180** | **1281.6215** | **1280.7453** | **0.8763** | **0** | **0** | **1.2e+002** | **4** | **K.VTTTVHNIIVGK.L** |
|  | 9134 | **653.3575** | **1304.7005** | **1305.6387** | **-0.9382** | **0** | **4** | **59** | **10** | **R.DFLMLAQTHSK.K** |
|  | 11385 | **478.8892** | **1433.6457** | **1433.7337** | **-0.0880** | **1** | **4** | **49** | **10** | **R.DFLMLAQTHSKK.W** |
|  | 11502 | **721.9096** | **1441.8047** | **1441.8140** | **-0.0093** | **1** | **43** | **0.0064** | **1** | **R.IRLEETLEQLAK.Q** |
|  | 12228 | 742.8571 | 1483.6996 | 1483.7677 | -0.0682 | 1 | 11 | 10 | 4 | R.LRPDQRLMENGR.W |
|  | 12332 | **745.9880** | **1489.9614** | **1489.8100** | **0.1514** | **1** | **3** | **47** | **2** | **K.TELQNTLRTLSSK.V** |
|  | 16494 | 884.9699 | 1767.9253 | 1767.9479 | -0.0226 | 1 | 3 | 48 | 2 | K.HGTALQRSLSELESLK.L 16490 |
|  | 26135 | **753.6434** | **3010.5444** | **3011.3513** | **-0.8069** | **1** | **7** | **11** | **1** | **K.MLAESDESGDEESVSQTDKTELQNTLR.T** |
|  | 29744 | **1349.6344** | **4045.8814** | **4045.6234** | **0.2579** | **1** | **0** | **25** | **3** | **K.DQCCSGKGDMSDEDDENEFFDAPEIITMPENLGHK.R** |

| **621.** | P46779\|RL28_HUMAN    **Mass:** 15795    **Score:** 43     **Queries matched:** 7   **emPAI:** 0.25 |
| --- | --- |
|  | 60S ribosomal protein L28 OS=Homo sapiens GN=RPL28 PE=1 SV=3 |

|  | **Query** | **Observed** | **Mr(expt)** | **Mr(calc)** | **Delta** | **Miss** | **Score** | **Expect** | **Rank** | **Peptide** |
| --- | --- | --- | --- | --- | --- | --- | --- | --- | --- | --- |
|  | 2463 | **435.2250** | **868.4355** | **868.5858** | **-0.1503** | **1** | **10** | **15** | **5** | **K.GVVVVIKR.R** 2436 |
|  | 8686 | **642.8033** | **1283.5921** | **1283.7132** | **-0.1211** | **1** | **3** | **63** | **7** | **R.ATLSSIRHMIR.K** |
|  | 9901 | **450.2788** | **1347.8146** | **1348.7211** | **-0.9065** | **1** | **4** | **64** | **4** | **R.SGQRKPATSYVR.T** |
|  | 10366 | **687.8702** | **1373.7258** | **1372.6744** | **1.0514** | **0** | **9** | **20** | **1** | **-.MSAHLQWMVVR.N** |
|  | 12120 | **493.9085** | **1478.7035** | **1477.8253** | **0.8783** | **1** | **5** | **40** | **4** | **R.KPATSYVRTTINK.N** |
|  | 13021 | **768.8808** | **1535.7470** | **1535.7579** | **-0.0109** | **1** | **43** | **0.0062** | **1** | **R.NKQTYSTEPNNLK.A** |

| **622.** | Q9UBC0\|HNF6_HUMAN    **Mass:** 51219    **Score:** 43     **Queries matched:** 8   **emPAI:** 0.07 |
| --- | --- |
|  | Hepatocyte nuclear factor 6 OS=Homo sapiens GN=ONECUT1 PE=2 SV=1 |

|  | **Query** | **Observed** | **Mr(expt)** | **Mr(calc)** | **Delta** | **Miss** | **Score** | **Expect** | **Rank** | **Peptide** |
| --- | --- | --- | --- | --- | --- | --- | --- | --- | --- | --- |
|  | 2212 | **427.1154** | **852.2162** | **851.4250** | **0.7913** | **1** | **9** | **15** | **2** | **K.SGRETFR.R** |
|  | 2291 | **430.2136** | **858.4127** | **859.5127** | **-1.0999** | **1** | **43** | **0.011** | **1** | **R.ITTELKR.Y** |
|  | 9990 | 677.3607 | 1352.7069 | 1351.6918 | 1.0151 | 0 | 2 | 88 | 6 | R.LAGNVSGSFTLMR.D 9994 |
|  | 17175 | 608.3256 | 1821.9549 | 1820.9719 | 0.9829 | 1 | 7 | 24 | 4 | R.YSIPQAIFAQRVLCR.S |
|  | 18090 | **632.3567** | **1894.0482** | **1893.8978** | **0.1504** | **0** | **(0)** | **90** | **3** | **K.MLTPNGFEAHHPAMLGR.H** |
|  | 18280 | 478.7589 | 1911.0065 | 1909.8927 | 1.1138 | 0 | 3 | 47 | 9 | K.MLTPNGFEAHHPAMLGR.H |
|  | 24402 | 1337.6552 | 2673.2957 | 2674.2140 | -0.9183 | 0 | 0 | 56 | 5 | R.EPNPSVTGAQVSNGSNSGQMEEINTK.E |

| **623.** | P11234\|RALB_HUMAN    **Mass:** 23508    **Score:** 43     **Queries matched:** 10   **emPAI:** 0.16 |
| --- | --- |
|  | Ras-related protein Ral-B OS=Homo sapiens GN=RALB PE=1 SV=1 |

|  | **Query** | **Observed** | **Mr(expt)** | **Mr(calc)** | **Delta** | **Miss** | **Score** | **Expect** | **Rank** | **Peptide** |
| --- | --- | --- | --- | --- | --- | --- | --- | --- | --- | --- |
|  | 790 | 546.2806 | 545.2733 | 545.2809 | -0.0076 | 0 | 3 | 1.9e+002 | 5 | R.ANVDK.V |
|  | 1725 | **409.8881** | **817.7617** | **817.4181** | **0.3436** | **1** | **8** | **24** | **8** | **R.VKAEEDK.I** 1712 |
|  | 3446 | **471.2531** | **940.4917** | **939.5138** | **0.9779** | **0** | **6** | **40** | **4** | **K.GQSSLALHK.V** |
|  | 4306 | 502.2809 | 1002.5472 | 1002.5532 | -0.0060 | 0 | 22 | 1 | 1 | K.VIMVGSGGVGK.S |
|  | 5305 | **542.2966** | **1082.5786** | **1082.5832** | **-0.0046** | **1** | **4** | **56** | **2** | **R.RQVPVEEAR.S** |
|  | 11698 | 727.8725 | 1453.7304 | 1453.7388 | -0.0083 | 1 | 43 | 0.0067 | 1 | R.ANVDKVFFDLMR.E 11696 |
|  | 15344 | **561.2955** | **1680.8648** | **1680.9410** | **-0.0762** | **1** | **4** | **40** | **6** | **K.IPLLVVGNKSDLEER.R** 15327 |

| **624.** | O75438\|NDUB1_HUMAN    **Mass:** 7014     **Score:** 43     **Queries matched:** 2   **emPAI:** 1.55 |
| --- | --- |
|  | NADH dehydrogenase [ubiquinone] 1 beta subcomplex subunit 1 OS=Homo sapiens GN=NDUFB1 PE=1 SV=1 |

|  | **Query** | **Observed** | **Mr(expt)** | **Mr(calc)** | **Delta** | **Miss** | **Score** | **Expect** | **Rank** | **Peptide** |
| --- | --- | --- | --- | --- | --- | --- | --- | --- | --- | --- |
|  | 3603 | 477.9149 | 953.8152 | 953.6022 | 0.2130 | 0 | 38 | 0.016 | 1 | M.VNLLQIVR.D |
|  | 9839 | **673.3316** | **1344.6486** | **1344.6561** | **-0.0075** | **0** | **36** | **0.034** | **1** | **R.ELQPSEEVTWK.-** |

| **625.** | Q9NX63\|CHCH3_HUMAN    **Mass:** 26421    **Score:** 43     **Queries matched:** 11   **emPAI:** 0.31 |
| --- | --- |
|  | Coiled-coil-helix-coiled-coil-helix domain-containing protein 3, mitochondrial OS=Homo sapiens GN=CHCHD3 PE=1 SV=1 |

|  | **Query** | **Observed** | **Mr(expt)** | **Mr(calc)** | **Delta** | **Miss** | **Score** | **Expect** | **Rank** | **Peptide** |
| --- | --- | --- | --- | --- | --- | --- | --- | --- | --- | --- |
|  | 1320 | **395.2160** | **788.4174** | **787.3501** | **1.0674** | **0** | **7** | **46** | **3** | **R.SSEFYR.V** |
|  | 1629 | **406.1849** | **810.3553** | **809.3702** | **0.9852** | **0** | **8** | **23** | **2** | **-.MGGTTSTR.R** |
|  | 2204 | **426.7208** | **851.4271** | **851.4323** | **-0.0052** | **0** | **19** | **1.8** | **1** | **K.ILQCYR.E** |
|  | 3762 | 483.7412 | 965.4679 | 965.4713 | -0.0034 | 1 | 7 | 35 | 9 | -.MGGTTSTRR.V |
|  | 4194 | **498.7494** | **995.4842** | **995.4924** | **-0.0081** | **0** | **9** | **17** | **1** | **R.VTTEQYQK.A** |
|  | 6161 | **575.2844** | **1148.5542** | **1148.5422** | **0.0120** | **1** | **3** | **71** | **5** | **K.ESSPSGSKSQR.Y** |
|  | 10053 | **679.0973** | **1356.1800** | **1355.7408** | **0.4392** | **1** | **1** | **73** | **3** | **R.RVAEELALEQAK.K** |
|  | 12761 | **759.3513** | **1516.6880** | **1516.6980** | **-0.0100** | **0** | **38** | **0.019** | **1** | **R.YESHPVCADLQAK.I** |
|  | 13653 | **788.3577** | **1574.7009** | **1574.7100** | **-0.0091** | **0** | **26** | **0.32** | **1** | **R.YSGAYGASVSDEELK.R** 13687 |
|  | 15184 | 836.9105 | 1671.8065 | 1672.7991 | -0.9926 | 1 | 5 | 38 | 5 | K.RYESHPVCADLQAK.I |

| **626.** | Q6UWM9\|UD2A3_HUMAN    **Mass:** 60929    **Score:** 42     **Queries matched:** 15   **emPAI:** 0.06 |
| --- | --- |
|  | UDP-glucuronosyltransferase 2A3 OS=Homo sapiens GN=UGT2A3 PE=2 SV=1 |

|  | **Query** | **Observed** | **Mr(expt)** | **Mr(calc)** | **Delta** | **Miss** | **Score** | **Expect** | **Rank** | **Peptide** |
| --- | --- | --- | --- | --- | --- | --- | --- | --- | --- | --- |
|  | 1650 | **407.2032** | **812.3918** | **811.3898** | **1.0020** | **0** | **8** | **24** | **3** | **R.MTFLER.V** |
|  | 3913 | 488.7533 | 975.4921 | 975.4920 | 0.0001 | 1 | 9 | 25 | 7 | K.ENAMRLSR.I |
|  | 4832 | **523.0570** | **1044.0993** | **1043.5723** | **0.5270** | **0** | **4** | **43** | **2** | **K.KPSTLGANTR.L** |
|  | 7596 | **615.8493** | **1229.6840** | **1228.6888** | **0.9953** | **1** | **6** | **36** | **2** | **K.GKKPSTLGANTR.L** |
|  | 8511 | 425.5486 | 1273.6239 | 1272.5921 | 1.0318 | 0 | 13 | 7.5 | 1 | K.FEVVHMPQDR.T |
|  | 14280 | **807.8887** | **1613.7629** | **1613.7719** | **-0.0090** | **1** | **42** | **0.0067** | **1** | **R.TVITDSSYKENAMR.L** 14278 14279 |
|  | 16979 | 904.9644 | 1807.9143 | 1807.9257 | -0.0114 | 0 | 11 | 10 | 1 | R.LYDWIPQNDLLGHPK.T |
|  | 18821 | **652.8237** | **1955.4492** | **1954.8661** | **0.5831** | **1** | **3** | **32** | **6** | **K.MMCESFIYNQTLMKK.L** |
|  | 19404 | **671.3141** | **2010.9204** | **2010.0092** | **0.9113** | **1** | **2** | **56** | **4** | **K.GAAVEINFKTMTSEDLLR.A** |
|  | 26679 | 779.8575 | 3115.4011 | 3115.7393 | -0.3383 | 1 | 4 | 21 | 6 | K.VILEELIVRGHEVTVLTHSKPSLIDYR.K |
|  | 27519 | **1660.5739** | **3319.1331** | **3318.5602** | **0.5729** | **0** | **(3)** | **14** | **7** | **K.EMENFVQSSGEDGIVVFSLGSLFQNVTEEK.A** |
|  | 27599 | **834.9262** | **3335.6755** | **3334.5551** | **1.1204** | **0** | **3** | **22** | **1** | **K.EMENFVQSSGEDGIVVFSLGSLFQNVTEEK.A** |
|  | 30348 | **1090.2940** | **4357.1467** | **4357.1486** | **-0.0019** | **1** | **4** | **9.8** | **1** | **K.TKAFITHGGMNGIYEAIYHGVPMVGVPIFGDQLDNIAHMK.A** |

| **627.** | Q9BXJ7\|AMNLS_HUMAN    **Mass:** 48526    **Score:** 42     **Queries matched:** 5   **emPAI:** 0.08 |
| --- | --- |
|  | Protein amnionless OS=Homo sapiens GN=AMN PE=1 SV=1 |

|  | **Query** | **Observed** | **Mr(expt)** | **Mr(calc)** | **Delta** | **Miss** | **Score** | **Expect** | **Rank** | **Peptide** |
| --- | --- | --- | --- | --- | --- | --- | --- | --- | --- | --- |
|  | 14222 | **537.6054** | **1609.7942** | **1608.7420** | **1.0522** | **0** | **2** | **67** | **4** | **R.SGDEAPGLFFVDAER.V** |
|  | 17436 | **614.9998** | **1841.9776** | **1842.0437** | **-0.0661** | **0** | **2** | **62** | **3** | **R.VLLWLQLCALTQAVSK.L** |
|  | 22229 | **1163.5297** | **2325.0448** | **2325.0549** | **-0.0102** | **0** | **42** | **0.0043** | **1** | **K.AAADSTSHSYFVNPLFAGAEAEA.-** |
|  | 23926 | **861.1202** | **2580.3387** | **2580.3143** | **0.0244** | **1** | **4** | **28** | **2** | **R.EADTEIQVVLVENGPETGGAGRLAR.A** |
|  | 31986 | **1414.1741** | **5652.6672** | **5653.7128** | **-1.0456** | **1** | **2** | **6.6** | **1** | **K.MVSVLVQEGHAVSDMLLPLDGELVLASGAGFGVSDVGSHLDCGAGEPAVFRDSDR.F** |

| **628.** | P40926\|MDHM_HUMAN    **Mass:** 35937    **Score:** 42     **Queries matched:** 7   **emPAI:** 0.10 |
| --- | --- |
|  | Malate dehydrogenase, mitochondrial OS=Homo sapiens GN=MDH2 PE=1 SV=3 |

|  | **Query** | **Observed** | **Mr(expt)** | **Mr(calc)** | **Delta** | **Miss** | **Score** | **Expect** | **Rank** | **Peptide** |
| --- | --- | --- | --- | --- | --- | --- | --- | --- | --- | --- |
|  | 940 | 601.1234 | 600.1161 | 600.3595 | -0.2434 | 0 | 5 | 49 | 1 | K.NLGIGK.V |
|  | 1776 | **412.2280** | **822.4415** | **821.4283** | **1.0132** | **1** | **7** | **30** | **3** | **K.KGEDFVK.T** |
|  | 8500 | **425.2600** | **1272.7582** | **1271.7085** | **1.0497** | **1** | **4** | **53** | **9** | **R.IQEAGTEVVKAK.A** |
|  | 11973 | **490.5954** | **1468.7643** | **1469.6933** | **-0.9290** | **0** | **3** | **65** | **6** | **K.AGAGSATLSMAYAGAR.F** |
|  | 22535 | **789.0803** | **2364.2190** | **2364.2325** | **-0.0136** | **0** | **42** | **0.0043** | **1** | **R.LTLYDIAHTPGVAADLSHIETK.A** 22536 |
|  | 25188 | **937.1454** | **2808.4143** | **2808.4303** | **-0.0159** | **1** | **22** | **0.39** | **1** | **K.GYLGPEQLPDCLKGCDVVVIPAGVPR.K** |

| **629.** | P02649\|APOE_HUMAN    **Mass:** 36246    **Score:** 42     **Queries matched:** 16   **emPAI:** 0.22 |
| --- | --- |
|  | Apolipoprotein E OS=Homo sapiens GN=APOE PE=1 SV=1 |

|  | **Query** | **Observed** | **Mr(expt)** | **Mr(calc)** | **Delta** | **Miss** | **Score** | **Expect** | **Rank** | **Peptide** |
| --- | --- | --- | --- | --- | --- | --- | --- | --- | --- | --- |
|  | 3789 | **484.7764** | **967.5382** | **967.5451** | **-0.0069** | **0** | **20** | **1.6** | **1** | **R.LGPLVEQGR.V** |
|  | 5279 | 541.2656 | 1080.5167 | 1081.4644 | -0.9477 | 1 | (4) | 57 | 3 | R.ARMEEMGSR.T |
|  | 5537 | **550.2947** | **1098.5749** | **1097.4594** | **1.1156** | **1** | **10** | **16** | **1** | **R.ARMEEMGSR.T** 5507 5512 5539 |
|  | 5736 | **557.2740** | **1112.5335** | **1111.4750** | **1.0585** | **1** | **9** | **21** | **2** | **R.MEEMGSRTR.D** |
|  | 7269 | **405.5642** | **1213.6707** | **1213.6778** | **-0.0071** | **1** | **16** | **3.5** | **1** | **R.LSKELQAAQAR.L** |
|  | 9247 | **657.3583** | **1312.7020** | **1312.7099** | **-0.0079** | **1** | **28** | **0.2** | **1** | **R.AKLEEQAQQIR.L** |
|  | 9483 | **442.4876** | **1324.4410** | **1323.6414** | **0.7996** | **1** | **4** | **42** | **6** | **R.ALMDETMKELK.A** |
|  | 11034 | **472.2740** | **1413.8001** | **1413.7463** | **0.0537** | **1** | **10** | **12** | **5** | **R.LDEVKEQVAEVR.A** |
|  | 12427 | **500.0012** | **1496.9817** | **1496.7947** | **0.1870** | **0** | **1** | **76** | **10** | **R.AATVGSLAGQPLQER.A** |
|  | 14373 | **810.8975** | **1619.7805** | **1619.7904** | **-0.0099** | **0** | **33** | **0.056** | **1** | **K.VQAAVGTSAAPVPSDNH.-** 14374 |
|  | 20615 | **712.6914** | **2135.0524** | **2135.1635** | **-0.1111** | **1** | **0** | **83** | **1** | **-.MKVLWAALLVTFLAGCQAK.V** |
|  | 28636 | **1204.5599** | **3610.6580** | **3609.8103** | **0.8476** | **1** | **2** | **22** | **6** | **R.FWDYLRWVQTLSEQVQEELLSSQVTQELR.A** |

| **630.** | Q71UM5\|RS27L_HUMAN    **Mass:** 9813     **Score:** 42     **Queries matched:** 5   **emPAI:** 0.41 |
| --- | --- |
|  | 40S ribosomal protein S27-like protein OS=Homo sapiens GN=RPS27L PE=1 SV=3 |

|  | **Query** | **Observed** | **Mr(expt)** | **Mr(calc)** | **Delta** | **Miss** | **Score** | **Expect** | **Rank** | **Peptide** |
| --- | --- | --- | --- | --- | --- | --- | --- | --- | --- | --- |
|  | 3790 | **485.0677** | **968.1209** | **968.4386** | **-0.3177** | **0** | **2** | **59** | **9** | **R.LTEGCSFR.R** |
|  | 6884 | **399.3459** | **1195.0158** | **1195.5768** | **-0.5609** | **1** | **3** | **46** | **6** | **K.ARLTEGCSFR.R** |
|  | 12911 | **764.3746** | **1526.7346** | **1526.7439** | **-0.0093** | **0** | **42** | **0.0075** | **1** | **R.LVQSPNSYFMDVK.C** 12915 |
|  | 13144 | 515.0280 | 1542.0620 | 1542.7388 | -0.6768 | 0 | (4) | 36 | 3 | R.LVQSPNSYFMDVK.C |

|  | **Proteins matching the same set of peptides:** |
| --- | --- |

|  | P42677\|RS27_HUMAN    **Mass:** 9797     **Score:** 42     **Queries matched:** 5 |
| --- | --- |
|  | 40S ribosomal protein S27 OS=Homo sapiens GN=RPS27 PE=1 SV=3 |

| **631.** | P03928\|ATP8_HUMAN    **Mass:** 8043     **Score:** 42     **Queries matched:** 7   **emPAI:** 0.51 |
| --- | --- |
|  | ATP synthase protein 8 OS=Homo sapiens GN=MT-ATP8 PE=1 SV=1 |

|  | **Query** | **Observed** | **Mr(expt)** | **Mr(calc)** | **Delta** | **Miss** | **Score** | **Expect** | **Rank** | **Peptide** |
| --- | --- | --- | --- | --- | --- | --- | --- | --- | --- | --- |
|  | 7759 | **619.8098** | **1237.6051** | **1237.6125** | **-0.0074** | **0** | **37** | **0.028** | **1** | **K.ICSLHSLPPQS.-** 7757 7761 7762 |
|  | 11387 | 478.9080 | 1433.7022 | 1433.7125 | -0.0103 | 1 | 23 | 0.6 | 1 | K.MKNYNKPWEPK.W |
|  | 26326 | 1015.5042 | 3043.4906 | 3044.6694 | -1.1788 | 0 | 1 | 36 | 6 | M.PQLNTTVWPTMITPMLLTLFLITQLK.M 26324 |

| **632.** | Q6P1M0\|S27A4_HUMAN    **Mass:** 72930    **Score:** 42     **Queries matched:** 15   **emPAI:** 0.05 |
| --- | --- |
|  | Long-chain fatty acid transport protein 4 OS=Homo sapiens GN=SLC27A4 PE=1 SV=1 |

|  | **Query** | **Observed** | **Mr(expt)** | **Mr(calc)** | **Delta** | **Miss** | **Score** | **Expect** | **Rank** | **Peptide** |
| --- | --- | --- | --- | --- | --- | --- | --- | --- | --- | --- |
|  | 1748 | **821.1983** | **820.1910** | **819.4854** | **0.7056** | **1** | **5** | **44** | **8** | **K.IAKDVFK.K** |
|  | 4012 | **492.2899** | **982.5653** | **981.5971** | **0.9683** | **1** | **4** | **58** | **9** | **R.IIQKDPLR.R** |
|  | 5607 | **552.7767** | **1103.5389** | **1102.6022** | **0.9367** | **1** | **8** | **27** | **2** | **R.KEGFDPAIVK.D** |
|  | 6745 | **397.2219** | **1188.6439** | **1189.5954** | **-0.9515** | **0** | **12** | **9.5** | **1** | **R.MAALVYYGFR.M** |
|  | 7086 | **402.2672** | **1203.7798** | **1202.7023** | **1.0775** | **0** | **13** | **7** | **2** | **R.TVPILFASTVR.R** |
|  | 12393 | 748.3713 | 1494.7281 | 1495.7168 | -0.9886 | 1 | 6 | 31 | 10 | R.RFDGYLNQGANNK.K |
|  | 13066 | 513.6124 | 1537.8155 | 1538.8892 | -1.0738 | 1 | 6 | 30 | 4 | K.LGVEAALINTNLRR.D |
|  | 15862 | **860.3876** | **1718.7607** | **1718.7716** | **-0.0109** | **0** | **42** | **0.0073** | **1** | **R.AGMAAVASPTGNCDLER.F** |
|  | 16059 | **578.9324** | **1733.7753** | **1734.7665** | **-0.9912** | **0** | **(0)** | **1e+002** | **6** | **R.AGMAAVASPTGNCDLER.F** |
|  | 18618 | **647.0008** | **1937.9805** | **1937.9490** | **0.0316** | **1** | **4** | **42** | **2** | **R.EAENQHQVRMALGNGLR.Q** |
|  | 23755 | 851.4162 | 2551.2268 | 2550.2206 | 1.0061 | 1 | 1 | 52 | 8 | R.AGMAAVASPTGNCDLERFAQVLEK.E |
|  | 23783 | 639.3218 | 2553.2580 | 2553.2097 | 0.0483 | 0 | 1 | 53 | 7 | K.GDQAYLTGDVLVMDELGYLYFR.D |
|  | 25604 | **724.8406** | **2895.3332** | **2895.4411** | **-0.1079** | **1** | **0** | **48** | **9** | **R.GLASGDVAAIFMENRNEFVGLWLGMAK.L** |
|  | 27758 | **675.7553** | **3373.7402** | **3374.6201** | **-0.8799** | **1** | **5** | **14** | **1** | **R.QLDEYSSSVANFLQARGLASGDVAAIFMENR.N** |
|  | 31318 | **1278.1403** | **5108.5319** | **5108.4610** | **0.0710** | **0** | **1** | **10** | **1** | **R.ALVFGSEMASAICEVHASLDPSLSLFCSGSWEPGAVPPSTEHLDPLLK.D** |

| **633.** | P07858\|CATB_HUMAN    **Mass:** 38766    **Score:** 42     **Queries matched:** 9   **emPAI:** 0.10 |
| --- | --- |
|  | Cathepsin B OS=Homo sapiens GN=CTSB PE=1 SV=3 |

|  | **Query** | **Observed** | **Mr(expt)** | **Mr(calc)** | **Delta** | **Miss** | **Score** | **Expect** | **Rank** | **Peptide** |
| --- | --- | --- | --- | --- | --- | --- | --- | --- | --- | --- |
|  | 2565 | **438.7517** | **875.4889** | **875.4501** | **0.0388** | **0** | **2** | **1.2e+002** | **2** | **K.LPASFDAR.E** |
|  | 4203 | 499.2066 | 996.3986 | 997.4790 | -1.0804 | 0 | 13 | 7.4 | 1 | K.DIMAEIYK.N 4223 |
|  | 8723 | 643.8096 | 1285.6046 | 1285.6125 | -0.0079 | 0 | 19 | 1.6 | 1 | R.EQWPQCPTIK.E |
|  | 9261 | **657.8015** | **1313.5883** | **1313.5962** | **-0.0078** | **0** | **42** | **0.0097** | **1** | **K.ICEPGYSPTYK.Q** |
|  | 15392 | **843.3909** | **1684.7673** | **1684.7767** | **-0.0093** | **1** | **13** | **5.2** | **1** | **K.ICEPGYSPTYKQDK.H** |
|  | 18515 | 483.2289 | 1928.8865 | 1928.8985 | -0.0120 | 0 | 19 | 1.2 | 1 | K.SGVYQHVTGEMMGGHAIR.I |
|  | 29533 | **790.4053** | **3946.9903** | **3947.8611** | **-0.8708** | **1** | **7** | **5.2** | **1** | **K.NGPVEGAFSVYSDFLLYKSGVYQHVTGEMMGGHAIR.I** |
|  | 31234 | **1255.3983** | **5017.5642** | **5017.1082** | **0.4560** | **0** | **1** | **11** | **7** | **R.ICIHTNAHVSVEVSAEDLLTCCGSMCGDGCNGGYPAEAWNFWTR.K** |

| **634.** | P30085\|KCY_HUMAN    **Mass:** 22436    **Score:** 42     **Queries matched:** 12   **emPAI:** 0.17 |
| --- | --- |
|  | UMP-CMP kinase OS=Homo sapiens GN=CMPK1 PE=1 SV=3 |

|  | **Query** | **Observed** | **Mr(expt)** | **Mr(calc)** | **Delta** | **Miss** | **Score** | **Expect** | **Rank** | **Peptide** |
| --- | --- | --- | --- | --- | --- | --- | --- | --- | --- | --- |
|  | 3751 | **483.2346** | **964.4546** | **963.5178** | **0.9368** | **0** | **8** | **25** | **4** | **K.FLIDGFPR.N** 3741 3752 |
|  | 9692 | 669.2975 | 1336.5804 | 1336.5751 | 0.0053 | 0 | 42 | 0.0093 | 1 | R.EMDQTMAANAQK.N 9691 9705 |
|  | 10261 | **457.0741** | **1368.2006** | **1368.5649** | **-0.3644** | **0** | **(8)** | **15** | **2** | **R.EMDQTMAANAQK.N** 10277 |
|  | 12151 | **494.3054** | **1479.8943** | **1478.7518** | **1.1425** | **0** | **3** | **61** | **4** | **K.YGYTHLSAGELLR.D** |
|  | 13562 | **523.9439** | **1568.8097** | **1568.9113** | **-0.1015** | **0** | **7** | **25** | **1** | **-.MKPLVVFVLGGPGAGK.G** |
|  | 13716 | **789.9000** | **1577.7854** | **1578.7130** | **-0.9276** | **1** | **(2)** | **74** | **5** | **R.EMDQTMAANAQKNK.F** |
|  | 14248 | **403.9556** | **1611.7933** | **1610.7028** | **1.0905** | **1** | **10** | **12** | **1** | **R.EMDQTMAANAQKNK.F** |

| **635.** | Q9HCK1\|ZDBF2_HUMAN    **Mass:** 268589   **Score:** 42     **Queries matched:** 30   **emPAI:** 0.01 |
| --- | --- |
|  | DBF4-type zinc finger-containing protein 2 OS=Homo sapiens GN=ZDBF2 PE=1 SV=3 |

|  | **Query** | **Observed** | **Mr(expt)** | **Mr(calc)** | **Delta** | **Miss** | **Score** | **Expect** | **Rank** | **Peptide** |
| --- | --- | --- | --- | --- | --- | --- | --- | --- | --- | --- |
|  | 2163 | **425.2595** | **848.5044** | **848.4538** | **0.0506** | **1** | **6** | **53** | **3** | **R.MGTKGSLR.V** |
|  | 2249 | **428.6744** | **855.3341** | **854.3738** | **0.9603** | **1** | **4** | **56** | **7** | **R.ERMMTR.L** |
|  | 2671 | **442.2504** | **882.4863** | **882.5035** | **-0.0172** | **1** | **8** | **23** | **2** | **R.TTHRLQK.A** |
|  | 2969 | 453.5623 | 905.1100 | 904.4865 | 0.6235 | 1 | 3 | 60 | 6 | K.VSSDLKEK.N |
|  | 2980 | **453.7821** | **905.5497** | **906.4745** | **-0.9248** | **1** | **12** | **11** | **10** | **K.NYPVMKR.K** 2999 |
|  | 3024 | **455.7237** | **909.4328** | **910.4984** | **-1.0657** | **1** | **3** | **80** | **3** | **K.AHKEASLR.K** |
|  | 3318 | **465.7334** | **929.4523** | **929.4930** | **-0.0407** | **0** | **5** | **64** | **8** | **K.EISLQNAR.H** |
|  | 3413 | **469.7402** | **937.4658** | **936.4487** | **1.0171** | **0** | **10** | **15** | **6** | **K.TWSQIMR.E** |
|  | 4100 | **495.2889** | **988.5633** | **988.4648** | **0.0986** | **1** | **42** | **0.013** | **1** | **K.CDPNKVEK.Y** 4090 4091 4116 |
|  | 4622 | **514.7706** | **1027.5267** | **1027.5563** | **-0.0296** | **0** | **2** | **1e+002** | **7** | **R.HPPAERPPK.Q** |
|  | 5987 | 567.8213 | 1133.6280 | 1133.5387 | 0.0894 | 0 | 6 | 43 | 1 | K.ELNIDMEVR.S 5992 |
|  | 7481 | **613.2894** | **1224.5642** | **1223.6510** | **0.9132** | **1** | **8** | **23** | **2** | **K.IIRQEEDPPK.S** |
|  | 7650 | 617.2842 | 1232.5538 | 1232.6587 | -0.1049 | 1 | 3 | 84 | 3 | R.MNSDKLVLWK.D |
|  | 11770 | **729.8728** | **1457.7310** | **1457.7110** | **0.0200** | **1** | **5** | **47** | **1** | **K.EKNHDSQSSSVLK.V** 11773 |
|  | 13814 | **528.7678** | **1583.2815** | **1582.7522** | **0.5293** | **1** | **1** | **72** | **9** | **R.AYDLRSSSCLQQR.E** |
|  | 15386 | **562.3157** | **1683.9254** | **1684.8784** | **-0.9530** | **1** | **4** | **48** | **3** | **K.EVIQKEEYIHLER.K** |
|  | 16355 | **586.3339** | **1755.9799** | **1756.8704** | **-0.8905** | **1** | **1** | **81** | **2** | **K.NHDSQSSSVLKVDSVR.N** |
|  | 16815 | **599.2898** | **1794.8475** | **1795.8675** | **-1.0200** | **0** | **1** | **92** | **9** | **K.HVNLGNENHMYLEVK.N** |
|  | 17789 | **467.9996** | **1867.9692** | **1868.9745** | **-1.0053** | **0** | **0** | **91** | **10** | **K.LIHHPDVSVQSVADQPK.V** |
|  | 17833 | **624.8162** | **1871.4267** | **1870.9749** | **0.4518** | **0** | **1** | **55** | **6** | **K.SQEGTQEVSVRPSVIQK.L** |
|  | 21590 | 449.0419 | 2240.1733 | 2241.1965 | -1.0232 | 1 | 2 | 48 | 10 | K.SQEGTQEVSVRPSVIQKLEK.G |
|  | 21908 | 760.7277 | 2279.1612 | 2280.2590 | -1.0979 | 1 | 3 | 36 | 7 | K.LIHHPDVSVQSVADQPKVAIK.H |
|  | 22964 | 811.7513 | 2432.2322 | 2431.1723 | 1.0599 | 0 | 1 | 51 | 5 | K.ISSDCDDILHLVTNQSQMIVK.E |
|  | 28892 | **1234.1929** | **3699.5568** | **3698.6530** | **0.9038** | **1** | **2** | **16** | **1** | **R.SYDCSSSELTFDSDPPLLSVTEQSHLDAEGKER.H** |

| **636.** | P14406\|CX7A2_HUMAN    **Mass:** 9390     **Score:** 42     **Queries matched:** 4   **emPAI:** 1.04 |
| --- | --- |
|  | Cytochrome c oxidase polypeptide 7A2, mitochondrial OS=Homo sapiens GN=COX7A2 PE=1 SV=1 |

|  | **Query** | **Observed** | **Mr(expt)** | **Mr(calc)** | **Delta** | **Miss** | **Score** | **Expect** | **Rank** | **Peptide** |
| --- | --- | --- | --- | --- | --- | --- | --- | --- | --- | --- |
|  | 4709 | **517.7826** | **1033.5506** | **1033.5556** | **-0.0050** | **0** | **47** | **0.0033** | **1** | **K.GGVADALLYR.A** 4711 |
|  | 14413 | **811.9150** | **1621.8155** | **1621.8239** | **-0.0084** | **0** | **12** | **6.7** | **1** | **K.LFQEDDEIPLYLK.G** |
|  | 24225 | **880.1269** | **2637.3588** | **2637.3690** | **-0.0101** | **1** | **17** | **1.2** | **1** | **K.LFQEDDEIPLYLKGGVADALLYR.A** |

| **637.** | Q9H4M9\|EHD1_HUMAN    **Mass:** 60646    **Score:** 42     **Queries matched:** 11   **emPAI:** 0.06 |
| --- | --- |
|  | EH domain-containing protein 1 OS=Homo sapiens GN=EHD1 PE=1 SV=2 |

|  | **Query** | **Observed** | **Mr(expt)** | **Mr(calc)** | **Delta** | **Miss** | **Score** | **Expect** | **Rank** | **Peptide** |
| --- | --- | --- | --- | --- | --- | --- | --- | --- | --- | --- |
|  | 1047 | **638.3466** | **637.3393** | **636.3595** | **0.9798** | **0** | **8** | **22** | **3** | **K.TTFIR.H** |
|  | 2494 | **436.2239** | **870.4331** | **870.5287** | **-0.0955** | **1** | **10** | **19** | **5** | **K.LNDLIKR.A** |
|  | 4786 | 521.2746 | 1040.5346 | 1039.6026 | 0.9321 | 0 | 5 | 47 | 8 | K.IINTPEVVR.V |
|  | 5097 | 533.2767 | 1064.5389 | 1064.5325 | 0.0065 | 1 | 1 | 1.3e+002 | 6 | K.KEMPNVFGK.E |
|  | 9605 | **444.5844** | **1330.7315** | **1331.6503** | **-0.9188** | **0** | **8** | **24** | **5** | **K.ADQIETQQLMR.V** |
|  | 15707 | **570.0398** | **1707.0975** | **1705.9138** | **1.1838** | **1** | **4** | **37** | **4** | **K.LDISDEFSEVIKALK.N** |
|  | 21908 | 760.7277 | 2279.1612 | 2279.1984 | -0.0372 | 1 | 3 | 36 | 8 | K.MQELLQTQDFSKFQALKPK.L |
|  | 22888 | **605.7963** | **2419.1562** | **2419.2273** | **-0.0711** | **1** | **(3)** | **37** | **4** | **K.LLDTVDDMLANDIARLMVMVR.Q** |
|  | 22985 | **487.8577** | **2434.2523** | **2435.2222** | **-0.9700** | **1** | **5** | **22** | **6** | **K.LLDTVDDMLANDIARLMVMVR.Q** |
|  | 27363 | **1091.5533** | **3271.6382** | **3271.6548** | **-0.0166** | **0** | **42** | **0.0033** | **1** | **R.IGPEPTTDSFIAVMHGPTEGVVPGNALVVDPR.R** |
|  | 30768 | **1158.0346** | **4628.1091** | **4627.2840** | **0.8251** | **1** | **0** | **17** | **2** | **R.HLIEQDFPGMRIGPEPTTDSFIAVMHGPTEGVVPGNALVVDPR.R** |

| **638.** | P08183\|MDR1_HUMAN    **Mass:** 141788   **Score:** 42     **Queries matched:** 22   **emPAI:** 0.03 |
| --- | --- |
|  | Multidrug resistance protein 1 OS=Homo sapiens GN=ABCB1 PE=1 SV=3 |

|  | **Query** | **Observed** | **Mr(expt)** | **Mr(calc)** | **Delta** | **Miss** | **Score** | **Expect** | **Rank** | **Peptide** |
| --- | --- | --- | --- | --- | --- | --- | --- | --- | --- | --- |
|  | 840 | **561.2799** | **560.2726** | **559.3078** | **0.9648** | **0** | **4** | **1.1e+002** | **1** | **K.GAIGSR.L** |
|  | 883 | **576.3103** | **575.3030** | **574.3438** | **0.9592** | **0** | **15** | **9** | **1** | **R.SSLIR.K** |
|  | 3993 | 491.7901 | 981.5657 | 981.6447 | -0.0790 | 1 | 16 | 3.5 | 4 | R.IAIARALVR.N |
|  | 4169 | 497.7791 | 993.5436 | 994.5196 | -0.9760 | 0 | 11 | 12 | 1 | K.SGHKPDNIK.G |
|  | 4841 | **523.3081** | **1044.6017** | **1043.5975** | **1.0042** | **0** | **3** | **94** | **6** | **K.STVVQLLER.F** |
|  | 5662 | 554.3207 | 1106.6268 | 1107.5560 | -0.9292 | 1 | 8 | 28 | 5 | R.YNKNLEEAK.R |
|  | 7427 | 611.7890 | 1221.5634 | 1222.5387 | -0.9753 | 0 | 7 | 28 | 4 | R.ENVTMDEIEK.A |
|  | 8517 | 637.8337 | 1273.6528 | 1273.6626 | -0.0098 | 1 | 42 | 0.01 | 1 | K.VGDKGTQLSGGQK.Q |
|  | 8578 | **639.8052** | **1277.5959** | **1277.5670** | **0.0289** | **1** | **6** | **40** | **3** | **-.MDLEGDRNGGAK.K** |
|  | 10015 | **677.8852** | **1353.7558** | **1353.7690** | **-0.0131** | **0** | **3** | **63** | **2** | **K.ISAAHIIMIIEK.T** |
|  | 12131 | **740.5195** | **1479.0245** | **1478.7616** | **0.2629** | **0** | **4** | **37** | **3** | **K.IIDNKPSIDSYSK.S** |
|  | 12536 | 752.8776 | 1503.7406 | 1504.7443 | -1.0037 | 1 | 6 | 28 | 3 | R.ENVTMDEIEKAVK.E |
|  | 15031 | 831.4725 | 1660.9304 | 1660.8308 | 0.0996 | 0 | 3 | 62 | 3 | R.NADVIAGFDDGVIVEK.G |
|  | 19885 | **686.4143** | **2056.2209** | **2057.1520** | **-0.9311** | **1** | **3** | **36** | **3** | **K.ELLAYAKAGAVAEEVLAAIR.T** |
|  | 20403 | 528.2686 | 2109.0454 | 2109.0008 | 0.0446 | 1 | 11 | 6.9 | 3 | K.SEIDALEMSSNDSRSSLIR.K |
|  | 20601 | **712.3343** | **2133.9810** | **2134.3129** | **-0.3319** | **0** | **2** | **62** | **10** | **K.LTLVILAISPVLGLSAAVWAK.I** |
|  | 22896 | **606.0449** | **2420.1506** | **2420.1698** | **-0.0192** | **0** | **2** | **47** | **1** | **K.IGMFFQSMATFFTGFIVGFTR.G** |
|  | 30597 | **1505.1438** | **4512.4096** | **4513.2742** | **-0.8647** | **1** | **1** | **14** | **6** | **R.FGAYLVAHKLMSFEDVLLVFSAVVFGAMAVGQVSSFAPDYAK.A** |
|  | 31283 | **1272.0858** | **5084.3142** | **5084.5193** | **-0.2052** | **0** | **2** | **7.6** | **2** | **K.LYMVVGTLAAIIHGAGLPLMMLVFGEMTDIFANAGNLEDLMSNITNR.S** |
|  | 31285 | **848.6014** | **5085.5650** | **5084.5193** | **1.0457** | **0** | **(0)** | **14** | **1** | **K.LYMVVGTLAAIIHGAGLPLMMLVFGEMTDIFANAGNLEDLMSNITNR.S** |
|  | 31325 | **1280.1047** | **5116.3899** | **5116.5092** | **-0.1193** | **0** | **(1)** | **9.8** | **2** | **K.LYMVVGTLAAIIHGAGLPLMMLVFGEMTDIFANAGNLEDLMSNITNR.S** |
|  | 31550 | 1774.4956 | 5320.4650 | 5320.4620 | 0.0030 | 1 | 6 | 3.2 | 4 | R.SDINDTGFFMNLEEDMTRYAYYYSGIGAGVLVAAYIQVSFWCLAAGR.Q |

| **639.** | P21439\|MDR3_HUMAN    **Mass:** 142004   **Score:** 42     **Queries matched:** 22   **emPAI:** 0.03 |
| --- | --- |
|  | Multidrug resistance protein 3 OS=Homo sapiens GN=ABCB4 PE=1 SV=2 |

|  | **Query** | **Observed** | **Mr(expt)** | **Mr(calc)** | **Delta** | **Miss** | **Score** | **Expect** | **Rank** | **Peptide** |
| --- | --- | --- | --- | --- | --- | --- | --- | --- | --- | --- |
|  | 2063 | 421.9068 | 841.7991 | 841.5134 | 0.2858 | 1 | 0 | 1.1e+002 | 6 | K.LLAGNAKR.D |
|  | 3993 | 491.7901 | 981.5657 | 981.6447 | -0.0790 | 1 | 16 | 3.5 | 4 | R.IAIARALVR.N |
|  | 4658 | **516.2529** | **1030.4913** | **1031.5611** | **-1.0698** | **0** | **8** | **30** | **8** | **R.TVVSLTQER.K** |
|  | 4756 | 520.2442 | 1038.4738 | 1038.5247 | -0.0508 | 0 | 8 | 27 | 8 | R.NFNVNYLR.E |
|  | 4841 | 523.3081 | 1044.6017 | 1043.5975 | 1.0042 | 0 | 3 | 94 | 6 | K.STVVQLLER.F |
|  | 4847 | 523.7471 | 1045.4796 | 1045.5127 | -0.0331 | 1 | 9 | 22 | 7 | R.MAPNGWKSR.L |
|  | 5133 | **535.2618** | **1068.5091** | **1069.5412** | **-1.0321** | **1** | **7** | **28** | **10** | **R.SMAFKAMLR.Q** 5129 |
|  | 6194 | **576.3133** | **1150.6120** | **1149.5700** | **1.0421** | **1** | **3** | **70** | **8** | **R.GNVTMDEIKK.A** |
|  | 6991 | 601.3058 | 1200.5971 | 1201.6026 | -1.0054 | 1 | 5 | 47 | 3 | K.AATRMAPNGWK.S |
|  | 8517 | 637.8337 | 1273.6528 | 1273.6626 | -0.0098 | 1 | 42 | 0.01 | 1 | R.VGDKGTQLSGGQK.Q |
|  | 10673 | **464.9245** | **1391.7518** | **1392.8163** | **-1.0645** | **1** | **6** | **31** | **10** | **K.TVKMIGVLTLFR.Y** |
|  | 10857 | **468.3070** | **1401.8992** | **1401.7616** | **0.1376** | **1** | **0** | **1.1e+002** | **5** | **K.LPQKFDTLVGER.G** |
|  | 11806 | **487.2958** | **1458.8657** | **1458.7427** | **0.1230** | **0** | **10** | **14** | **2** | **R.LATDAAQVQGATGTR.L** |
|  | 13993 | **399.7130** | **1594.8228** | **1593.9454** | **0.8774** | **1** | **4** | **49** | **9** | **R.ANVPVLQGLSLEVKK.G** |
|  | 20556 | **710.3460** | **2128.0160** | **2129.0892** | **-1.0732** | **0** | **0** | **76** | **5** | **K.SLDVETDGLEANVPPVSFLK.V** |
|  | 21413 | **741.4465** | **2221.3176** | **2222.0604** | **-0.7428** | **0** | **0** | **58** | **3** | **K.NGTAWRPTSAEGDFELGISSK.Q** |
|  | 27181 | **807.4135** | **3225.6249** | **3226.5973** | **-0.9723** | **0** | **2** | **27** | **2** | **K.LFMSLGTIMAIAHGSGLPLMMIVFGEMTDK.F** |
|  | 27379 | 819.8309 | 3275.2946 | 3274.5820 | 0.7126 | 0 | (1) | 25 | 5 | K.LFMSLGTIMAIAHGSGLPLMMIVFGEMTDK.F 27378 |
|  | 30052 | **1042.4768** | **4165.8781** | **4164.9743** | **0.9039** | **1** | **3** | **13** | **1** | **R.YSDWQDKLFMSLGTIMAIAHGSGLPLMMIVFGEMTDK.F** |
|  | 32015 | **1899.1774** | **5694.5103** | **5694.8349** | **-0.3246** | **1** | **2** | **4.6** | **1** | **K.LFMSLGTIMAIAHGSGLPLMMIVFGEMTDKFVDTAGNFSFPVNFSLSLLNPGK.I** |

| **640.** | Q9BV40\|VAMP8_HUMAN    **Mass:** 11488    **Score:** 41     **Queries matched:** 11   **emPAI:** 1.45 |
| --- | --- |
|  | Vesicle-associated membrane protein 8 OS=Homo sapiens GN=VAMP8 PE=1 SV=1 |

|  | **Query** | **Observed** | **Mr(expt)** | **Mr(calc)** | **Delta** | **Miss** | **Score** | **Expect** | **Rank** | **Peptide** |
| --- | --- | --- | --- | --- | --- | --- | --- | --- | --- | --- |
|  | 5576 | **551.7872** | **1101.5598** | **1101.5666** | **-0.0068** | **0** | **24** | **0.64** | **1** | **R.NLQSEVEGVK.N** |
|  | 5605 | 552.7736 | 1103.5327 | 1103.5393 | -0.0066 | 0 | 29 | 0.24 | 1 | K.NIMTQNVER.I |
|  | 8023 | **417.5375** | **1249.5907** | **1250.4833** | **-0.8926** | **0** | **7** | **27** | **9** | **-.MEEASEGGGNDR.V** 8049 |
|  | 8381 | **634.6639** | **1267.3132** | **1266.4782** | **0.8350** | **0** | **(3)** | **47** | **1** | **-.MEEASEGGGNDR.V** |
|  | 10881 | **469.2196** | **1404.6371** | **1405.6361** | **-0.9990** | **0** | **6** | **31** | **7** | **K.TEDLEATSEHFK.T** 10898 |
|  | 12579 | **503.2523** | **1506.7350** | **1505.6528** | **1.0821** | **1** | **7** | **26** | **4** | **-.MEEASEGGGNDRVR.N** |
|  | 14823 | **550.2623** | **1647.7652** | **1647.7740** | **-0.0088** | **1** | **36** | **0.031** | **1** | **R.NKTEDLEATSEHFK.T** |
|  | 21110 | **730.0356** | **2187.0849** | **2187.0954** | **-0.0104** | **1** | **25** | **0.29** | **1** | **R.NLQSEVEGVKNIMTQNVER.I** 21109 |

| **641.** | P06753\|TPM3_HUMAN    **Mass:** 32856    **Score:** 41     **Queries matched:** 11   **emPAI:** 0.11 |
| --- | --- |
|  | Tropomyosin alpha-3 chain OS=Homo sapiens GN=TPM3 PE=1 SV=1 |

|  | **Query** | **Observed** | **Mr(expt)** | **Mr(calc)** | **Delta** | **Miss** | **Score** | **Expect** | **Rank** | **Peptide** |
| --- | --- | --- | --- | --- | --- | --- | --- | --- | --- | --- |
|  | 1397 | **397.8684** | **793.7223** | **793.4190** | **0.3033** | **1** | **3** | **53** | **6** | **K.KMQMLK.L** |
|  | 6145 | 574.3270 | 1146.6395 | 1146.5954 | 0.0441 | 0 | 3 | 79 | 6 | K.MELQEIQLK.E |
|  | 7867 | **622.3263** | **1242.6380** | **1242.6456** | **-0.0075** | **0** | **8** | **25** | **4** | **R.IQLVEEELDR.A** |
|  | 8410 | **635.2521** | **1268.4896** | **1267.6156** | **0.8740** | **1** | **9** | **17** | **4** | **K.EAKHIAEEADR.K** |
|  | 8697 | **429.0874** | **1284.2404** | **1283.7449** | **0.4955** | **1** | **9** | **12** | **5** | **R.KLVIIEGDLER.T** |
|  | 9329 | **439.7158** | **1316.1255** | **1315.6368** | **0.4888** | **0** | **11** | **7.2** | **1** | **K.AADAEAEVASLNR.R** |
|  | 10859 | **702.2108** | **1402.4071** | **1402.7126** | **-0.3055** | **1** | **(3)** | **54** | **1** | **K.QLEDELAAMQKK.L** |
|  | 11118 | **710.3982** | **1418.7818** | **1418.7075** | **0.0743** | **1** | **11** | **12** | **4** | **K.QLEDELAAMQKK.L** 11132 |
|  | 12027 | 737.3527 | 1472.6909 | 1471.7379 | 0.9530 | 1 | 2 | 78 | 9 | K.AADAEAEVASLNRR.I |
|  | 15974 | 864.4446 | 1726.8746 | 1726.8849 | -0.0103 | 1 | 41 | 0.0079 | 1 | R.IQLVEEELDRAQER.L |

| **642.** | P07951\|TPM2_HUMAN    **Mass:** 32945    **Score:** 41     **Queries matched:** 8   **emPAI:** 0.11 |
| --- | --- |
|  | Tropomyosin beta chain OS=Homo sapiens GN=TPM2 PE=1 SV=1 |

|  | **Query** | **Observed** | **Mr(expt)** | **Mr(calc)** | **Delta** | **Miss** | **Score** | **Expect** | **Rank** | **Peptide** |
| --- | --- | --- | --- | --- | --- | --- | --- | --- | --- | --- |
|  | 1397 | 397.8684 | 793.7223 | 793.4190 | 0.3033 | 1 | 3 | 53 | 6 | K.KMQMLK.L |
|  | 7867 | 622.3263 | 1242.6380 | 1242.6456 | -0.0075 | 0 | 8 | 25 | 4 | R.IQLVEEELDR.A |
|  | 9052 | 651.8234 | 1301.6323 | 1301.6463 | -0.0139 | 1 | 5 | 42 | 7 | K.EAQEKLEQAEK.K 9077 |
|  | 11813 | 487.3586 | 1459.0539 | 1459.7267 | -0.6727 | 1 | 4 | 40 | 6 | K.KATDAEADVASLNR.R |
|  | 12615 | 503.9286 | 1508.7640 | 1508.7214 | 0.0426 | 1 | 8 | 21 | 3 | K.MELQEMQLKEAK.H |
|  | 15191 | **558.3032** | **1671.8878** | **1670.8839** | **1.0040** | **1** | **1** | **90** | **10** | **K.LVILEGELERSEER.A** |
|  | 15974 | 864.4446 | 1726.8746 | 1726.8849 | -0.0103 | 1 | 41 | 0.0079 | 1 | R.IQLVEEELDRAQER.L |

| **643.** | P09493\|TPM1_HUMAN    **Mass:** 32746    **Score:** 41     **Queries matched:** 7   **emPAI:** 0.12 |
| --- | --- |
|  | Tropomyosin alpha-1 chain OS=Homo sapiens GN=TPM1 PE=1 SV=2 |

|  | **Query** | **Observed** | **Mr(expt)** | **Mr(calc)** | **Delta** | **Miss** | **Score** | **Expect** | **Rank** | **Peptide** |
| --- | --- | --- | --- | --- | --- | --- | --- | --- | --- | --- |
|  | 1397 | 397.8684 | 793.7223 | 793.4190 | 0.3033 | 1 | 3 | 53 | 6 | K.KMQMLK.L |
|  | 6145 | 574.3270 | 1146.6395 | 1146.5954 | 0.0441 | 0 | 3 | 79 | 6 | K.MEIQEIQLK.E |
|  | 7867 | 622.3263 | 1242.6380 | 1242.6456 | -0.0075 | 0 | 8 | 25 | 4 | R.IQLVEEELDR.A |
|  | 8085 | **627.7909** | **1253.5673** | **1253.6000** | **-0.0326** | **1** | **3** | **71** | **2** | **K.EAKHIAEDADR.K** |
|  | 11813 | 487.3586 | 1459.0539 | 1459.7267 | -0.6727 | 1 | 4 | 40 | 6 | K.KATDAEADVASLNR.R |
|  | 15191 | 558.3032 | 1671.8878 | 1670.8839 | 1.0040 | 1 | 2 | 72 | 7 | K.LVIIESDLERAEER.A |
|  | 15974 | 864.4446 | 1726.8746 | 1726.8849 | -0.0103 | 1 | 41 | 0.0079 | 1 | R.IQLVEEELDRAQER.L |

| **644.** | O43175\|SERA_HUMAN    **Mass:** 57356    **Score:** 41     **Queries matched:** 16   **emPAI:** 0.13 |
| --- | --- |
|  | D-3-phosphoglycerate dehydrogenase OS=Homo sapiens GN=PHGDH PE=1 SV=4 |

|  | **Query** | **Observed** | **Mr(expt)** | **Mr(calc)** | **Delta** | **Miss** | **Score** | **Expect** | **Rank** | **Peptide** |
| --- | --- | --- | --- | --- | --- | --- | --- | --- | --- | --- |
|  | 1763 | 411.7181 | 821.4216 | 821.4218 | -0.0002 | 0 | 11 | 14 | 1 | -.MAFANLR.K |
|  | 2002 | 419.9058 | 837.7971 | 837.4167 | 0.3804 | 0 | (7) | 19 | 5 | -.MAFANLR.K |
|  | 4030 | **493.2460** | **984.4774** | **985.5960** | **-1.1186** | **0** | **4** | **60** | **4** | **R.DLPLLLFR.T** |
|  | 6038 | **570.2509** | **1138.4871** | **1139.5645** | **-1.0773** | **1** | **2** | **82** | **4** | **K.KFMGTELNGK.T** |
|  | 10654 | **464.2634** | **1389.7684** | **1389.6922** | **0.0762** | **1** | **4** | **59** | **7** | **R.QIPQATASMKDGK.W** |
|  | 12246 | 743.3758 | 1484.7370 | 1485.7787 | -1.0417 | 1 | 10 | 15 | 3 | K.EASKQADVNLVNAK.L |
|  | 13448 | 521.2612 | 1560.7617 | 1561.7592 | -0.9976 | 1 | 5 | 40 | 3 | R.KVLISDSLDPCCR.K |
|  | 14295 | **539.2616** | **1614.7630** | **1615.8166** | **-1.0536** | **1** | **4** | **48** | **4** | **R.AGTGVDNVDLEAATRK.G** |
|  | 16695 | 595.3192 | 1782.9358 | 1781.9999 | 0.9359 | 1 | 1 | 78 | 9 | K.VTADVINAAEKLQVVGR.A |
|  | 17793 | **623.6690** | **1867.9850** | **1868.0367** | **-0.0517** | **1** | **10** | **8.8** | **1** | **K.ILQDGGLQVVEKQNLSK.E** |
|  | 17906 | **939.4634** | **1876.9122** | **1876.0240** | **0.8882** | **1** | **5** | **32** | **1** | **K.FMGTELNGKTLGILGLGR.I** |
|  | 19785 | **683.3408** | **2047.0005** | **2047.0157** | **-0.0152** | **0** | **41** | **0.0074** | **1** | **R.ALVDHENVISCPHLGASTK.E** 19786 |
|  | 23126 | **819.3791** | **2455.1154** | **2456.2580** | **-1.1426** | **1** | **1** | **59** | **7** | **K.QNLSKEELIAELQDCEGLIVR.S** |
|  | 26786 | 785.5994 | 3138.3684 | 3137.5164 | 0.8520 | 1 | 2 | 25 | 9 | R.KGILVMNTPNGNSLSAAELTCGMIMCLAR.Q |
|  | 29847 | 1361.0194 | 4080.0364 | 4080.9597 | -0.9233 | 1 | 8 | 4.6 | 2 | K.GILVMNTPNGNSLSAAELTCGMIMCLARQIPQATASMK.D |

| **645.** | P19022\|CADH2_HUMAN    **Mass:** 100203   **Score:** 41     **Queries matched:** 15   **emPAI:** 0.04 |
| --- | --- |
|  | Cadherin-2 OS=Homo sapiens GN=CDH2 PE=1 SV=4 |

|  | **Query** | **Observed** | **Mr(expt)** | **Mr(calc)** | **Delta** | **Miss** | **Score** | **Expect** | **Rank** | **Peptide** |
| --- | --- | --- | --- | --- | --- | --- | --- | --- | --- | --- |
|  | 1360 | 396.2218 | 790.4291 | 789.4133 | 1.0158 | 0 | 6 | 49 | 5 | R.NWTITR.L |
|  | 1786 | **413.2033** | **824.3921** | **825.3439** | **-0.9518** | **0** | **9** | **19** | **2** | **K.FSNCNGK.R** |
|  | 1907 | 417.2335 | 832.4524 | 833.4256 | -0.9732 | 0 | 8 | 34 | 5 | K.HSGHLQR.Q |
|  | 5077 | 532.7766 | 1063.5385 | 1062.5426 | 0.9959 | 1 | 7 | 31 | 2 | -.MCRIAGALR.T 5050 |
|  | 8429 | **635.4982** | **1268.9819** | **1268.5707** | **0.4112** | **0** | **7** | **21** | **2** | **K.VDEDGMVYAVR.S** |
|  | 9837 | 449.2133 | 1344.6182 | 1343.6656 | 0.9526 | 1 | 6 | 35 | 9 | R.YMQQNIRYTK.L |
|  | 12017 | **491.6033** | **1471.7882** | **1472.8351** | **-1.0469** | **1** | **3** | **58** | **6** | **R.LNGDFAQLNLKIK.F** |
|  | 12639 | **756.3740** | **1510.7335** | **1511.7508** | **-1.0173** | **0** | **3** | **68** | **9** | **K.TGFPEDVYSAVLSK.D** |
|  | 14271 | **807.4672** | **1612.9199** | **1611.8144** | **1.1055** | **1** | **3** | **63** | **3** | **K.FLIYAQDKETQEK.W** |
|  | 14682 | 547.2535 | 1638.7386 | 1638.8577 | -0.1191 | 1 | 1 | 98 | 7 | R.QAKQLLIDPEDDVR.D |
|  | 16025 | **865.9742** | **1729.9339** | **1729.9437** | **-0.0097** | **0** | **6** | **29** | **1** | **R.MFVLTVAAENQVPLAK.G** |
|  | 19376 | **670.3673** | **2008.0801** | **2008.9901** | **-0.9100** | **1** | **3** | **47** | **2** | **R.RMDERPIHAEPQYPVR.S** |
|  | 28611 | **901.6556** | **3602.5932** | **3601.8893** | **0.7039** | **1** | **0** | **30** | **6** | **K.GIQHPPQSTATVSVTVIDVNENPYFAPNPKIIR.Q** |
|  | 29716 | **1009.7876** | **4035.1213** | **4035.1429** | **-0.0216** | **1** | **41** | **0.002** | **1** | **R.YSVTGPGADQPPTGIFIINPISGQLSVTKPLDREQIAR.F** |

| **646.** | P50402\|EMD_HUMAN    **Mass:** 29033    **Score:** 41     **Queries matched:** 9   **emPAI:** 0.13 |
| --- | --- |
|  | Emerin OS=Homo sapiens GN=EMD PE=1 SV=1 |

|  | **Query** | **Observed** | **Mr(expt)** | **Mr(calc)** | **Delta** | **Miss** | **Score** | **Expect** | **Rank** | **Peptide** |
| --- | --- | --- | --- | --- | --- | --- | --- | --- | --- | --- |
|  | 2248 | **428.5833** | **855.1521** | **854.4722** | **0.6799** | **0** | **8** | **15** | **1** | **R.AIRPENR.A** 2249 |
|  | 4414 | **506.3164** | **1010.6183** | **1010.5733** | **0.0450** | **1** | **15** | **4.2** | **2** | **R.RAIRPENR.A** 4394 |
|  | 6054 | **571.2780** | **1140.5415** | **1139.4805** | **1.0610** | **0** | **6** | **34** | **4** | **R.GDADMYDLPK.K** 6055 |
|  | 7267 | **405.5587** | **1213.6544** | **1212.6139** | **1.0406** | **1** | **7** | **28** | **8** | **K.KIFEYETQR.R** |
|  | 8022 | **625.7819** | **1249.5493** | **1249.5575** | **-0.0082** | **0** | **41** | **0.012** | **1** | **R.TYGEPESAGPSR.A** 8002 |

| **647.** | P13639\|EF2_HUMAN    **Mass:** 96246    **Score:** 41     **Queries matched:** 18   **emPAI:** 0.04 |
| --- | --- |
|  | Elongation factor 2 OS=Homo sapiens GN=EEF2 PE=1 SV=4 |

|  | **Query** | **Observed** | **Mr(expt)** | **Mr(calc)** | **Delta** | **Miss** | **Score** | **Expect** | **Rank** | **Peptide** |
| --- | --- | --- | --- | --- | --- | --- | --- | --- | --- | --- |
|  | 2814 | **448.5710** | **895.1274** | **895.4143** | **-0.2869** | **1** | **1** | **79** | **1** | **K.VEDMMKK.L** |
|  | 3066 | **457.0741** | **912.1337** | **911.4092** | **0.7245** | **1** | **10** | **11** | **2** | **K.KVEDMMK.K** 3067 3068 |
|  | 3804 | **485.5794** | **969.1442** | **968.5403** | **0.6039** | **0** | **9** | **12** | **4** | **R.GGGQIIPTAR.R** |
|  | 4960 | **528.5098** | **1055.0051** | **1054.5191** | **0.4860** | **0** | **13** | **5.2** | **1** | **K.GPLMMYISK.M** |
|  | 5069 | **532.2897** | **1062.5648** | **1062.5709** | **-0.0062** | **0** | **2** | **1.1e+002** | **7** | **K.GVQYLNEIK.D** |
|  | 5420 | **546.2926** | **1090.5706** | **1090.5771** | **-0.0065** | **0** | **11** | **13** | **1** | **M.VNFTVDQIR.A** 5418 |
|  | 5757 | **558.3104** | **1114.6062** | **1115.5935** | **-0.9873** | **1** | **2** | **98** | **5** | **K.GEVSARQELK.Q** |
|  | 6015 | **569.2766** | **1136.5385** | **1137.5091** | **-0.9705** | **0** | **3** | **79** | **4** | **K.YEWDVAEAR.K** |
|  | 8522 | **637.8630** | **1273.7115** | **1272.6139** | **1.0977** | **1** | **2** | **92** | **5** | **R.YFDPANGKFSK.S** |
|  | 13036 | **513.0793** | **1536.2162** | **1535.6820** | **0.5342** | **1** | **2** | **51** | **10** | **K.EGALCEENMRGVR.F** |
|  | 16627 | 889.9270 | 1777.8394 | 1776.9053 | 0.9341 | 1 | 3 | 59 | 7 | K.ANIRNMSVIAHVDHGK.S |
|  | 16864 | 900.4473 | 1798.8801 | 1798.8890 | -0.0089 | 0 | 41 | 0.0084 | 1 | K.AYLPVNESFGFTADLR.S |
|  | 25152 | **934.3117** | **2799.9133** | **2800.4032** | **-0.4899** | **0** | **1** | **36** | **1** | **K.DGAGFLINLIDSPGHVDFSSEVTAALR.V** |
|  | 26686 | **780.3835** | **3117.5051** | **3116.6402** | **0.8648** | **1** | **(1)** | **44** | **9** | **R.LMEPIYLVEIQCPEQVVGGIYGVLNRK.R** |
|  | 26766 | **783.9121** | **3131.6191** | **3132.6351** | **-1.0161** | **1** | **1** | **36** | **4** | **R.LMEPIYLVEIQCPEQVVGGIYGVLNRK.R** |

| **648.** | P10515\|ODP2_HUMAN    **Mass:** 69466    **Score:** 41     **Queries matched:** 17   **emPAI:** 0.05 |
| --- | --- |
|  | Dihydrolipoyllysine-residue acetyltransferase component of pyruvate dehydrogenase complex, mitochondrial OS=Homo sapiens GN=DLAT PE=1 SV=3 |

|  | **Query** | **Observed** | **Mr(expt)** | **Mr(calc)** | **Delta** | **Miss** | **Score** | **Expect** | **Rank** | **Peptide** |
| --- | --- | --- | --- | --- | --- | --- | --- | --- | --- | --- |
|  | 841 | 561.3206 | 560.3133 | 559.3078 | 1.0055 | 1 | 10 | 30 | 1 | K.AREGK.L |
|  | 1638 | **406.2585** | **810.5025** | **810.4460** | **0.0565** | **1** | **10** | **15** | **1** | **R.SGPAPARR.N** |
|  | 2532 | **437.2530** | **872.4915** | **872.4967** | **-0.0052** | **0** | **41** | **0.017** | **1** | **K.GIDLTQVK.G** |
|  | 4093 | 495.2354 | 988.4563 | 987.6117 | 0.8446 | 1 | 9 | 23 | 5 | R.VFVSPLAKK.L |
|  | 4185 | **498.3368** | **994.6590** | **993.4637** | **1.1953** | **1** | **8** | **21** | **9** | **-.MWRVCAR.R** |
|  | 6002 | **568.7850** | **1135.5554** | **1134.5921** | **0.9633** | **1** | **8** | **25** | **7** | **K.KDIDSFVPSK.V** |
|  | 6462 | **586.7995** | **1171.5844** | **1172.6699** | **-1.0855** | **1** | **1** | **1.2e+002** | **10** | **R.VIAQRLMQSK.Q** |
|  | 7712 | 412.8949 | 1235.6628 | 1235.6835 | -0.0207 | 0 | 8 | 25 | 5 | K.YLEKPITMLL.- 7706 |
|  | 9416 | **441.2957** | **1320.8653** | **1321.8194** | **-0.9540** | **1** | **14** | **4.8** | **1** | **R.LLLQLLGSPGRR.Y** 9428 |
|  | 11420 | **479.7574** | **1436.2504** | **1435.8623** | **0.3881** | **1** | **2** | **51** | **8** | **R.NRLLLQLLGSPGR.R** |
|  | 11809 | **487.2967** | **1458.8682** | **1459.6878** | **-0.8196** | **0** | **6** | **31** | **5** | **R.ALCGWTPSSGATPR.N** 11828 11830 |
|  | 14116 | 535.5881 | 1603.7426 | 1603.7664 | -0.0239 | 0 | 1 | 87 | 7 | K.VPEANSSWMDTVIR.Q |
|  | 16277 | 584.5441 | 1750.6106 | 1749.7590 | 0.8516 | 0 | 0 | 76 | 7 | K.ATVGFESLEECYMAK.I |

| **649.** | Q6ZQN7\|SO4C1_HUMAN    **Mass:** 80379    **Score:** 41     **Queries matched:** 14   **emPAI:** 0.05 |
| --- | --- |
|  | Solute carrier organic anion transporter family member 4C1 OS=Homo sapiens GN=SLCO4C1 PE=1 SV=1 |

|  | **Query** | **Observed** | **Mr(expt)** | **Mr(calc)** | **Delta** | **Miss** | **Score** | **Expect** | **Rank** | **Peptide** |
| --- | --- | --- | --- | --- | --- | --- | --- | --- | --- | --- |
|  | 793 | **546.3980** | **545.3907** | **544.3221** | **1.0687** | **1** | **1** | **3e+002** | **10** | **K.IVKEG.-** |
|  | 7441 | 408.2505 | 1221.7296 | 1220.6513 | 1.0783 | 0 | 7 | 29 | 5 | K.HLPGTAEIQAGK.T |
|  | 9876 | **674.3584** | **1346.7022** | **1346.7744** | **-0.0722** | **0** | **3** | **84** | **3** | **R.SLALGIQFMVLR.L** 9870 |
|  | 12421 | **499.9362** | **1496.7869** | **1497.7134** | **-0.9264** | **0** | **7** | **28** | **4** | **K.LGSLFEDTCVTTR.N** 12442 |
|  | 14573 | **544.8181** | **1631.4325** | **1630.9341** | **0.4984** | **1** | **5** | **29** | **2** | **R.QRSLALGIQFMVLR.L** |
|  | 16442 | 441.7426 | 1762.9412 | 1763.8577 | -0.9165 | 0 | 1 | 91 | 7 | K.SPEPSLPSAPPNVSEEK.L |
|  | 18227 | **636.3521** | **1906.0345** | **1906.9384** | **-0.9039** | **0** | **6** | **26** | **3** | **R.ENSQPQELQKPQEPQK.S** |
|  | 21325 | **738.3975** | **2212.1706** | **2212.1642** | **0.0063** | **0** | **2** | **56** | **4** | **K.FALFTSGVALTLSFVFMYAK.C** |
|  | 24543 | **897.4589** | **2689.3549** | **2688.3215** | **1.0334** | **1** | **6** | **17** | **1** | **K.HLPGTAEIQAGKTSQAHQSNSNADVK.F** |
|  | 27307 | **1086.7942** | **3257.3607** | **3257.3794** | **-0.0187** | **0** | **41** | **0.0031** | **1** | **R.SYYYPVCGDGVQYFSPCFAGCSNPVAHR.K** |
|  | 29092 | 753.9499 | 3764.7134 | 3764.0553 | 0.6581 | 0 | 2 | 22 | 2 | K.FIENQFGLTSSFAATLGGAVLIPGAALGQILGGFLVSK.F |
|  | 30625 | 756.3817 | 4532.2466 | 4531.2969 | 0.9497 | 1 | 2 | 12 | 3 | K.MAHMLVAISVTCKVITMFFNGFAIFLYKPPPSATDVSFHK.E |

| **650.** | Q96AG4\|LRC59_HUMAN    **Mass:** 35308    **Score:** 41     **Queries matched:** 10   **emPAI:** 0.22 |
| --- | --- |
|  | Leucine-rich repeat-containing protein 59 OS=Homo sapiens GN=LRRC59 PE=1 SV=1 |

|  | **Query** | **Observed** | **Mr(expt)** | **Mr(calc)** | **Delta** | **Miss** | **Score** | **Expect** | **Rank** | **Peptide** |
| --- | --- | --- | --- | --- | --- | --- | --- | --- | --- | --- |
|  | 1884 | **416.2589** | **830.5033** | **830.4246** | **0.0787** | **1** | **8** | **35** | **8** | **R.AKEAQER.E** |
|  | 2288 | 429.9611 | 857.9076 | 858.4671 | -0.5596 | 1 | 13 | 8.2 | 9 | K.AGSKGGNLR.D |
|  | 6094 | **572.8054** | **1143.5963** | **1143.6037** | **-0.0074** | **0** | **8** | **26** | **1** | **K.LQQLPADFGR.L** |
|  | 10568 | **693.8735** | **1385.7324** | **1385.7415** | **-0.0091** | **1** | **38** | **0.02** | **1** | **K.NKLQQLPADFGR.L** 10562 |
|  | 12990 | **511.9602** | **1532.8588** | **1532.8674** | **-0.0086** | **0** | **23** | **0.62** | **1** | **R.LVNLQHLDLLNNK.L** |
|  | 15147 | **557.2740** | **1668.8003** | **1667.8413** | **0.9590** | **1** | **5** | **40** | **4** | **K.VLQHMKAVQADQER.E** |
|  | 16083 | 579.3238 | 1734.9495 | 1735.9509 | -1.0013 | 1 | 3 | 58 | 4 | K.WLDLKDNPLDPVLAK.V |
|  | 17652 | **619.8449** | **1856.5129** | **1856.0077** | **0.5052** | **1** | **2** | **48** | **5** | **K.ELAALPKATILDLSCNK.L** |
|  | 23933 | **646.3344** | **2581.3085** | **2581.5546** | **-0.2460** | **1** | **0** | **59** | **5** | **R.SWAVLKLLLLLLLFGVAGGLVACR.V** |

| **651.** | O94919\|ENDD1_HUMAN    **Mass:** 55723    **Score:** 41     **Queries matched:** 8   **emPAI:** 0.07 |
| --- | --- |
|  | Endonuclease domain-containing 1 protein OS=Homo sapiens GN=ENDOD1 PE=1 SV=2 |

|  | **Query** | **Observed** | **Mr(expt)** | **Mr(calc)** | **Delta** | **Miss** | **Score** | **Expect** | **Rank** | **Peptide** |
| --- | --- | --- | --- | --- | --- | --- | --- | --- | --- | --- |
|  | 2013 | 420.2476 | 838.4806 | 839.5705 | -1.0899 | 1 | 6 | 37 | 4 | K.VIRALLR.I 2006 |
|  | 3192 | **460.7554** | **919.4963** | **920.4563** | **-0.9600** | **0** | **9** | **23** | **3** | **K.SSSPLSSTR.S** |
|  | 7137 | **403.2280** | **1206.6622** | **1205.6265** | **1.0357** | **0** | **10** | **15** | **2** | **R.APRPAPGGAEQR.W** 7119 |
|  | 13821 | **792.9101** | **1583.8056** | **1583.8155** | **-0.0098** | **0** | **41** | **0.01** | **1** | **K.ILEVVNQIQDEER.M** |
|  | 18885 | 654.6603 | 1960.9590 | 1962.0058 | -1.0468 | 1 | 3 | 47 | 3 | K.RSTLLPPEASEGSSSFLGK.L |
|  | 22061 | **576.5311** | **2302.0954** | **2302.2620** | **-0.1665** | **1** | **2** | **46** | **10** | **-.MGTARWLALGSLFALAGLLEGR.L** |

| **652.** | P08708\|RS17_HUMAN    **Mass:** 15597    **Score:** 40     **Queries matched:** 2   **emPAI:** 0.25 |
| --- | --- |
|  | 40S ribosomal protein S17 OS=Homo sapiens GN=RPS17 PE=1 SV=2 |

|  | **Query** | **Observed** | **Mr(expt)** | **Mr(calc)** | **Delta** | **Miss** | **Score** | **Expect** | **Rank** | **Peptide** |
| --- | --- | --- | --- | --- | --- | --- | --- | --- | --- | --- |
|  | 6989 | **601.3032** | **1200.5918** | **1200.6000** | **-0.0082** | **1** | **40** | **0.015** | **1** | **R.LGNDFHTNKR.V** |
|  | 10573 | **462.9240** | **1385.7501** | **1385.7588** | **-0.0087** | **1** | **5** | **46** | **8** | **R.VCEEIAIIPSKK.L** |

| **653.** | P54289\|CA2D1_HUMAN    **Mass:** 124246   **Score:** 40     **Queries matched:** 22   **emPAI:** 0.06 |
| --- | --- |
|  | Voltage-dependent calcium channel subunit alpha-2/delta-1 OS=Homo sapiens GN=CACNA2D1 PE=1 SV=2 |

|  | **Query** | **Observed** | **Mr(expt)** | **Mr(calc)** | **Delta** | **Miss** | **Score** | **Expect** | **Rank** | **Peptide** |
| --- | --- | --- | --- | --- | --- | --- | --- | --- | --- | --- |
|  | 2790 | 447.2102 | 892.4059 | 892.4654 | -0.0595 | 0 | 12 | 11 | 3 | K.IDLYDVR.R 2799 |
|  | 8200 | **630.3472** | **1258.6798** | **1258.6881** | **-0.0083** | **1** | **36** | **0.033** | **1** | **K.AKLEETITQAR.S** |
|  | 8696 | **429.0871** | **1284.2396** | **1284.7401** | **-0.5006** | **1** | **16** | **2.3** | **1** | **K.VLKDAVNNITAK.G** 8709 |
|  | 8824 | 646.2787 | 1290.5428 | 1290.7045 | -0.1616 | 1 | 12 | 7.9 | 2 | K.ARFVVTDGGITR.V |
|  | 9890 | **450.2317** | **1347.6732** | **1348.5904** | **-0.9171** | **0** | **6** | **39** | **4** | **R.GPIQWMACENK.G** 9912 |
|  | 10557 | **693.3815** | **1384.7484** | **1384.6293** | **0.1191** | **1** | **3** | **72** | **1** | **K.MIDGESGEKTFR.T** |
|  | 13863 | **529.5988** | **1585.7744** | **1584.7314** | **1.0430** | **0** | **1** | **99** | **10** | **K.TPNNPSCNADLINR.V** |
|  | 16095 | **579.6543** | **1735.9411** | **1734.7488** | **1.1923** | **1** | **7** | **22** | **4** | **K.TSIRDPCAGPVCDCK.R** |
|  | 18865 | **653.8610** | **1958.5612** | **1958.0394** | **0.5218** | **0** | **0** | **58** | **9** | **K.NQLILGVMGVDVSLEDIK.R** |
|  | 20452 | **1058.0579** | **2114.1012** | **2114.1405** | **-0.0394** | **1** | **7** | **20** | **2** | **K.NQLILGVMGVDVSLEDIKR.L** 20451 |
|  | 20459 | **1058.4995** | **2114.9845** | **2114.9976** | **-0.0132** | **0** | **23** | **0.46** | **1** | **R.LLIQAEQTSDGPNPCDMVK.Q** |
|  | 20573 | **711.0911** | **2130.2516** | **2130.9926** | **-0.7410** | **0** | **(2)** | **48** | **2** | **R.LLIQAEQTSDGPNPCDMVK.Q** |
|  | 21510 | 744.7122 | 2231.1148 | 2231.1620 | -0.0472 | 0 | 3 | 40 | 2 | R.INTQEYLDVLGRPMVLAGDK.A |
|  | 23400 | 833.0868 | 2496.2385 | 2496.2101 | 0.0285 | 1 | 3 | 32 | 4 | R.LLIQAEQTSDGPNPCDMVKQPR.Y |
|  | 25248 | **706.8431** | **2823.3435** | **2822.2347** | **1.1087** | **1** | **3** | **32** | **3** | **R.VYPKEAGENWQENPETYEDSFYK.R** |
|  | 30124 | **1052.2773** | **4205.0803** | **4206.1996** | **-1.1194** | **1** | **2** | **15** | **2** | **-.MAAGCLLALTLTLFQSLLIGPSSEEPFPSAVTIKSWVDK.M** |
|  | 30562 | 1123.3068 | 4489.1979 | 4489.0439 | 0.1541 | 0 | 0 | 21 | 2 | R.TSVSEMLETLSDDDFVNVASFNSNAQDVSCFQHLVQANVR.N |
|  | 31072 | **1218.8081** | **4871.2033** | **4871.3131** | **-0.1097** | **1** | **1** | **13** | **2** | **K.LIRTSVSEMLETLSDDDFVNVASFNSNAQDVSCFQHLVQANVR.N** |

| **654.** | O00299\|CLIC1_HUMAN    **Mass:** 27248    **Score:** 40     **Queries matched:** 7   **emPAI:** 0.14 |
| --- | --- |
|  | Chloride intracellular channel protein 1 OS=Homo sapiens GN=CLIC1 PE=1 SV=4 |

|  | **Query** | **Observed** | **Mr(expt)** | **Mr(calc)** | **Delta** | **Miss** | **Score** | **Expect** | **Rank** | **Peptide** |
| --- | --- | --- | --- | --- | --- | --- | --- | --- | --- | --- |
|  | 10920 | 469.9113 | 1406.7119 | 1405.7215 | 0.9905 | 1 | 8 | 23 | 3 | R.GVHRYLSNAYAR.E |
|  | 12282 | **496.6289** | **1486.8648** | **1485.7841** | **1.0807** | **1** | **6** | **33** | **4** | **R.GFTIPEAFRGVHR.Y** |
|  | 13479 | 782.3776 | 1562.7407 | 1562.7650 | -0.0243 | 0 | 6 | 31 | 5 | -.MAEEQPQVELFVK.A |
|  | 19326 | **668.3496** | **2002.0268** | **2002.0007** | **0.0261** | **1** | **10** | **9.8** | **2** | **M.AEEQPQVELFVKAGSDGAK.I** |
|  | 19935 | 1030.9950 | 2059.9754 | 2060.0612 | -0.0858 | 1 | 0 | 89 | 9 | R.KFLDGNELTLADCNLLPK.L |
|  | 26696 | **1040.8267** | **3119.4582** | **3119.4782** | **-0.0201** | **1** | **40** | **0.0044** | **1** | **K.VLDNYLTSPLPEEVDETSAEDEGVSQRK.F** |
|  | 26813 | **787.1035** | **3144.3850** | **3145.5754** | **-1.1904** | **1** | **4** | **17** | **2** | **R.TETVQKLCPGGQLPFLLYGTEVHTDTNK.I** |

| **655.** | Q9Y696\|CLIC4_HUMAN    **Mass:** 28982    **Score:** 40     **Queries matched:** 9   **emPAI:** 0.28 |
| --- | --- |
|  | Chloride intracellular channel protein 4 OS=Homo sapiens GN=CLIC4 PE=1 SV=4 |

|  | **Query** | **Observed** | **Mr(expt)** | **Mr(calc)** | **Delta** | **Miss** | **Score** | **Expect** | **Rank** | **Peptide** |
| --- | --- | --- | --- | --- | --- | --- | --- | --- | --- | --- |
|  | 964 | **609.4042** | **608.3969** | **608.4010** | **-0.0041** | **0** | **19** | **1.6** | **1** | **K.LHIVK.V** |
|  | 2774 | **446.7183** | **891.4220** | **891.4273** | **-0.0053** | **0** | **8** | **28** | **4** | **K.EMTGIWR.Y** |
|  | 2877 | **450.6637** | **899.3129** | **899.5804** | **-0.2675** | **1** | **1** | **1.2e+002** | **1** | **R.GLLKTLQK.L** |
|  | 10546 | **693.3376** | **1384.6606** | **1384.6694** | **-0.0088** | **0** | **25** | **0.45** | **1** | **K.NSRPEANEALER.G** |
|  | 14152 | **536.2701** | **1605.7886** | **1605.7973** | **-0.0087** | **1** | **38** | **0.016** | **1** | **R.NFDIPKEMTGIWR.Y** |
|  | 15490 | 564.8412 | 1691.5019 | 1690.8269 | 0.6750 | 1 | 6 | 21 | 8 | -.MALSMPLNGLKEEDK.E |
|  | 15705 | **569.9538** | **1706.8396** | **1706.8219** | **0.0177** | **1** | **(4)** | **42** | **4** | **-.MALSMPLNGLKEEDK.E** |
|  | 18675 | **972.5256** | **1943.0367** | **1941.9618** | **1.0749** | **1** | **0** | **86** | **9** | **K.LSPKHPESNTAGMDIFAK.F** |
|  | 23799 | **852.7872** | **2555.3399** | **2556.3080** | **-0.9681** | **1** | **1** | **59** | **8** | **K.FLDGNEMTLADCNLLPKLHIVK.V** |

| **656.** | P05787\|K2C8_HUMAN    **Mass:** 53671    **Score:** 40     **Queries matched:** 18   **emPAI:** 0.14 |
| --- | --- |
|  | Keratin, type II cytoskeletal 8 OS=Homo sapiens GN=KRT8 PE=1 SV=7 |

|  | **Query** | **Observed** | **Mr(expt)** | **Mr(calc)** | **Delta** | **Miss** | **Score** | **Expect** | **Rank** | **Peptide** |
| --- | --- | --- | --- | --- | --- | --- | --- | --- | --- | --- |
|  | 1046 | 637.3261 | 636.3188 | 635.2697 | 1.0491 | 0 | 14 | 6.4 | 3 | K.QDMAR.Q |
|  | 2975 | 453.7348 | 905.4550 | 905.4607 | -0.0056 | 0 | 27 | 0.35 | 1 | R.FLEQQNK.M |
|  | 5026 | 530.7892 | 1059.5638 | 1059.5560 | 0.0078 | 1 | 6 | 43 | 10 | R.KLLEGEESR.L |
|  | 5246 | 540.2562 | 1078.4978 | 1078.5043 | -0.0065 | 0 | 24 | 0.6 | 1 | K.AQYEDIANR.S |
|  | 7016 | **401.2853** | **1200.8341** | **1200.6462** | **0.1879** | **1** | **6** | **30** | **7** | **R.RQLETLGQEK.L** |
|  | 7167 | 403.9092 | 1208.7057 | 1207.5867 | 1.1191 | 1 | 4 | 59 | 9 | R.TKTEISEMNR.N |
|  | 7575 | 615.3414 | 1228.6683 | 1227.5441 | 1.1242 | 1 | 6 | 38 | 5 | K.KDVDEAYMNK.V |
|  | 8738 | 644.3322 | 1286.6499 | 1285.7241 | 0.9257 | 1 | 9 | 20 | 2 | R.QLETLGQEKLK.L |
|  | 9673 | **445.9172** | **1334.7298** | **1335.6592** | **-0.9294** | **0** | **2** | **90** | **8** | **R.SLDMDSIIAEVK.A** |
|  | 9791 | 448.2527 | 1341.7364 | 1340.7412 | 0.9952 | 1 | 5 | 43 | 4 | R.LQAEIEGLKGQR.A |
|  | 9856 | **449.2583** | **1344.7531** | **1343.6681** | **1.0850** | **0** | **8** | **25** | **2** | **R.ASLEAAIADAEQR.G** |
|  | 10749 | 699.2887 | 1396.5628 | 1396.7350 | -0.1722 | 1 | 10 | 12 | 3 | K.TLNNKFASFIDK.V |
|  | 12593 | **503.5118** | **1507.5134** | **1506.6806** | **0.8328** | **0** | **5** | **28** | **3** | **R.LESGMQNMSIHTK.T** |
|  | 19806 | **683.7118** | **2048.1135** | **2049.0452** | **-0.9317** | **1** | **6** | **25** | **1** | **K.LKLEAELGNMQGLVEDFK.N** |
|  | 22811 | **603.5333** | **2410.1042** | **2411.2406** | **-1.1364** | **1** | **1** | **58** | **8** | **R.EYQELMNVKLALDIEIATYR.K** |
|  | 26907 | 1056.8495 | 3167.5266 | 3168.5244 | -0.9978 | 1 | 1 | 43 | 10 | R.QLYEEEIRELQSQISDTSVVLSMDNSR.S |
|  | 29482 | **1309.3473** | **3925.0200** | **3924.0415** | **0.9785** | **0** | **38** | **0.0046** | **1** | **R.GGLGGGYGGASGMGGITAVTVNQSLLSPLVLEVDPNIQAVR.T** |
|  | 30842 | **1563.6362** | **4687.8869** | **4687.4028** | **0.4840** | **1** | **4** | **5.2** | **4** | **R.VGSSNFRGGLGGGYGGASGMGGITAVTVNQSLLSPLVLEVDPNIQAVR.T** |

| **657.** | P35221\|CTNA1_HUMAN    **Mass:** 100693   **Score:** 40     **Queries matched:** 25   **emPAI:** 0.07 |
| --- | --- |
|  | Catenin alpha-1 OS=Homo sapiens GN=CTNNA1 PE=1 SV=1 |

|  | **Query** | **Observed** | **Mr(expt)** | **Mr(calc)** | **Delta** | **Miss** | **Score** | **Expect** | **Rank** | **Peptide** |
| --- | --- | --- | --- | --- | --- | --- | --- | --- | --- | --- |
|  | 2130 | 424.2369 | 846.4593 | 845.5157 | 0.9436 | 1 | 8 | 32 | 2 | R.KAVLMIR.T |
|  | 3124 | **458.7470** | **915.4795** | **916.4614** | **-0.9819** | **1** | **2** | **1.2e+002** | **7** | **K.NGNEKEVK.E** |
|  | 4344 | **503.7607** | **1005.5068** | **1004.5138** | **0.9930** | **0** | **7** | **38** | **9** | **K.NTSDVISAAK.K** |
|  | 4654 | **515.7863** | **1029.5579** | **1029.5641** | **-0.0061** | **0** | **3** | **88** | **9** | **K.LLSNTVMPR.F** |
|  | 4688 | **517.2532** | **1032.4919** | **1032.5352** | **-0.0433** | **0** | **6** | **42** | **8** | **R.FRPSLEER.L** |
|  | 7671 | **617.4320** | **1232.8495** | **1231.6595** | **1.1900** | **0** | **4** | **46** | **2** | **K.NLMNAVVQTVK.A** |
|  | 8141 | **628.8337** | **1255.6528** | **1255.6594** | **-0.0066** | **0** | **32** | **0.099** | **1** | **R.AIMAQLPQEQK.A** |
|  | 8413 | **635.2757** | **1268.5368** | **1267.6561** | **0.8808** | **1** | **5** | **39** | **2** | **K.EVKEYAQVFR.E** |
|  | 9351 | **659.3795** | **1316.7444** | **1316.6870** | **0.0573** | **1** | **3** | **75** | **6** | **R.DQMAAARGILQK.N** |
|  | 9380 | **660.3510** | **1318.6873** | **1317.6524** | **1.0349** | **1** | **9** | **19** | **5** | **K.ERSDALNSAIDK.M** |
|  | 9514 | 663.9355 | 1325.8564 | 1325.6259 | 0.2306 | 1 | 2 | 68 | 6 | K.DVGHRDQMAAAR.G |
|  | 9794 | **448.2621** | **1341.7645** | **1341.6208** | **0.1438** | **1** | **(2)** | **89** | **3** | **K.DVGHRDQMAAAR.G** |
|  | 10454 | 460.5629 | 1378.6669 | 1379.7370 | -1.0701 | 0 | 6 | 32 | 3 | R.LLILADMADVYK.L |
|  | 10929 | **704.8818** | **1407.7491** | **1408.6868** | **-0.9376** | **1** | **2** | **73** | **2** | **R.SDALNSAIDKMTK.K** |
|  | 12972 | 766.8982 | 1531.7818 | 1531.7882 | -0.0064 | 0 | 1 | 90 | 7 | K.QIIVDPLSFSEER.F |
|  | 17110 | 909.9404 | 1817.8663 | 1817.8755 | -0.0092 | 0 | 37 | 0.021 | 1 | R.TSVQTEDDQLIAGQSAR.A 17111 |
|  | 17938 | 940.9504 | 1879.8862 | 1880.8972 | -1.0110 | 0 | 14 | 4.4 | 2 | R.LESIISGAALMADSSCTR.D |
|  | 18271 | 478.5140 | 1910.0268 | 1908.9363 | 1.0905 | 1 | 1 | 72 | 8 | R.QALQDLLSEYMGNAGRK.E 18268 |
|  | 18877 | 981.0337 | 1960.0528 | 1961.0105 | -0.9577 | 1 | 3 | 43 | 4 | K.ESQFLKEELVAAVEDVR.K |
|  | 22107 | **770.6984** | **2309.0735** | **2309.0886** | **-0.0151** | **0** | **22** | **0.51** | **1** | **R.VIHVVTSEMDNYEPGVYTEK.V** 22108 |
|  | 23159 | **820.7467** | **2459.2183** | **2458.2315** | **0.9868** | **0** | **14** | **3** | **1** | **K.NVPILYTASQACLQHPDVAAYK.A** |
|  | 25199 | **703.8503** | **2811.3723** | **2812.5004** | **-1.1282** | **1** | **2** | **38** | **3** | **K.VKAEVQNLGGELVVSGVDSAMSLIQAAK.N** |

| **658.** | Q02543\|RL18A_HUMAN    **Mass:** 21034    **Score:** 40     **Queries matched:** 14   **emPAI:** 0.18 |
| --- | --- |
|  | 60S ribosomal protein L18a OS=Homo sapiens GN=RPL18A PE=1 SV=2 |

|  | **Query** | **Observed** | **Mr(expt)** | **Mr(calc)** | **Delta** | **Miss** | **Score** | **Expect** | **Rank** | **Peptide** |
| --- | --- | --- | --- | --- | --- | --- | --- | --- | --- | --- |
|  | 808 | **550.3306** | **549.3234** | **548.2377** | **1.0857** | **0** | **14** | **9.2** | **1** | **R.DMGAR.H** 803 |
|  | 853 | **566.1292** | **565.1219** | **564.2326** | **0.8893** | **0** | **(6)** | **34** | **3** | **R.DMGAR.H** |
|  | 949 | **603.2903** | **602.2830** | **603.3340** | **-1.0510** | **0** | **0** | **3e+002** | **6** | **K.ASGTLR.E** |
|  | 2114 | 423.7295 | 845.4444 | 845.4494 | -0.0050 | 0 | 18 | 3.1 | 1 | K.VEEIAASK.C |
|  | 2363 | **432.2387** | **862.4629** | **862.4695** | **-0.0066** | **1** | **23** | **1** | **2** | **-.MKASGTLR.E** |
|  | 3279 | **464.2555** | **926.4964** | **926.5007** | **-0.0043** | **0** | **7** | **33** | **2** | **R.AHSIQIMK.V** |
|  | 9721 | **447.0296** | **1338.0670** | **1337.7568** | **0.3102** | **1** | **2** | **58** | **7** | **R.IFAPNHVVAKSR.F** |
|  | 10497 | 691.3746 | 1380.7347 | 1381.7652 | -1.0305 | 1 | 2 | 77 | 6 | R.MRIFAPNHVVAK.S |
|  | 11709 | 728.3441 | 1454.6736 | 1454.6824 | -0.0088 | 0 | 35 | 0.038 | 1 | R.DLTTAGAVTQCYR.D 11708 11736 |
|  | 16327 | **439.4898** | **1753.9300** | **1753.9396** | **-0.0096** | **1** | **4** | **43** | **5** | **R.AHSIQIMKVEEIAASK.C** 16337 |

| **659.** | P67936\|TPM4_HUMAN    **Mass:** 28619    **Score:** 40     **Queries matched:** 7   **emPAI:** 0.28 |
| --- | --- |
|  | Tropomyosin alpha-4 chain OS=Homo sapiens GN=TPM4 PE=1 SV=3 |

|  | **Query** | **Observed** | **Mr(expt)** | **Mr(calc)** | **Delta** | **Miss** | **Score** | **Expect** | **Rank** | **Peptide** |
| --- | --- | --- | --- | --- | --- | --- | --- | --- | --- | --- |
|  | 6142 | **574.3029** | **1146.5913** | **1147.5907** | **-0.9994** | **0** | **3** | **88** | **2** | **-.MAGLNSLEAVK.R** |
|  | 7867 | 622.3263 | 1242.6380 | 1242.6456 | -0.0075 | 0 | 8 | 25 | 4 | R.IQLVEEELDR.A |
|  | 8410 | 635.2521 | 1268.4896 | 1267.6156 | 0.8740 | 1 | 9 | 17 | 4 | K.EAKHIAEEADR.K |
|  | 8753 | 430.2136 | 1287.6191 | 1287.6969 | -0.0777 | 1 | 17 | 3.1 | 6 | -.MAGLNSLEAVKR.K |
|  | 12615 | 503.9286 | 1508.7640 | 1508.7214 | 0.0426 | 1 | 8 | 21 | 3 | K.MEIQEMQLKEAK.H |
|  | 15974 | 864.4446 | 1726.8746 | 1726.8849 | -0.0103 | 1 | 41 | 0.0079 | 1 | R.IQLVEEELDRAQER.L |
|  | 21806 | **756.7114** | **2267.1125** | **2267.1254** | **-0.0129** | **1** | **18** | **1.3** | **1** | **K.IQALQQQADEAEDRAQGLQR.E** |

| **660.** | P30038\|AL4A1_HUMAN    **Mass:** 62137    **Score:** 39     **Queries matched:** 11   **emPAI:** 0.12 |
| --- | --- |
|  | Delta-1-pyrroline-5-carboxylate dehydrogenase, mitochondrial OS=Homo sapiens GN=ALDH4A1 PE=1 SV=3 |

|  | **Query** | **Observed** | **Mr(expt)** | **Mr(calc)** | **Delta** | **Miss** | **Score** | **Expect** | **Rank** | **Peptide** |
| --- | --- | --- | --- | --- | --- | --- | --- | --- | --- | --- |
|  | 1481 | **401.2084** | **800.4023** | **799.4915** | **0.9108** | **1** | **7** | **41** | **7** | **R.RAEILAK.T** |
|  | 2713 | **443.2710** | **884.5275** | **884.5079** | **0.0196** | **0** | **9** | **19** | **3** | **K.AIEAALAAR.K** |
|  | 4362 | **504.3074** | **1006.6002** | **1005.6447** | **0.9555** | **1** | **2** | **1.1e+002** | **10** | **M.LLPAPALRR.A** |
|  | 5385 | 545.2647 | 1088.5149 | 1088.5397 | -0.0248 | 1 | 3 | 87 | 8 | K.AADMLSGPRR.A |
|  | 9824 | 672.8571 | 1343.6996 | 1344.7435 | -1.0439 | 1 | 11 | 9.9 | 2 | R.AEILAKTMVGQGK.T |
|  | 10132 | 454.7214 | 1361.1424 | 1360.7384 | 0.4040 | 1 | (0) | 94 | 10 | R.AEILAKTMVGQGK.T |
|  | 11457 | **720.3889** | **1438.7633** | **1439.8459** | **-1.0827** | **1** | **1** | **1e+002** | **4** | **K.SLLNKAIEAALAAR.K** |
|  | 11833 | **731.3711** | **1460.7277** | **1460.7372** | **-0.0095** | **0** | **39** | **0.018** | **1** | **K.STGSIVGQQPFGGAR.A** 11841 |
|  | 12138 | **494.2384** | **1479.6934** | **1479.8238** | **-0.1304** | **0** | **22** | **0.82** | **1** | **R.LYVPHSLWPQIK.G** |
|  | 13325 | 776.9394 | 1551.8643 | 1552.8950 | -1.0307 | 1 | 9 | 16 | 2 | R.RALLSRPWTGAGLR.W |

| **661.** | P05783\|K1C18_HUMAN    **Mass:** 48029    **Score:** 39     **Queries matched:** 17   **emPAI:** 0.16 |
| --- | --- |
|  | Keratin, type I cytoskeletal 18 OS=Homo sapiens GN=KRT18 PE=1 SV=2 |

|  | **Query** | **Observed** | **Mr(expt)** | **Mr(calc)** | **Delta** | **Miss** | **Score** | **Expect** | **Rank** | **Peptide** |
| --- | --- | --- | --- | --- | --- | --- | --- | --- | --- | --- |
|  | 3761 | 483.7363 | 965.4580 | 964.4614 | 0.9967 | 0 | 9 | 21 | 1 | R.AQYDELAR.K 3751 |
|  | 4322 | **503.1043** | **1004.1941** | **1003.5046** | **0.6894** | **1** | **10** | **12** | **1** | **R.SLETENRR.L** |
|  | 4790 | **521.3032** | **1040.5918** | **1040.5978** | **-0.0061** | **0** | **33** | **0.074** | **1** | **R.IVLQIDNAR.L** |
|  | 4867 | 524.2759 | 1046.5373 | 1045.5404 | 0.9969 | 0 | 18 | 2.5 | 2 | K.VIDDTNITR.L |
|  | 5434 | 546.7660 | 1091.5175 | 1091.6087 | -0.0912 | 1 | 10 | 15 | 5 | R.LASYLDRVR.S |
|  | 5682 | 555.2799 | 1108.5453 | 1108.4819 | 0.0634 | 0 | 7 | 28 | 4 | K.ETMQSLNDR.L |
|  | 7923 | **415.9140** | **1244.7202** | **1243.6884** | **1.0318** | **1** | **8** | **23** | **4** | **R.NLKASLENSLR.E** |
|  | 8088 | 418.8696 | 1253.5870 | 1254.6278 | -1.0408 | 1 | 8 | 24 | 7 | R.VKYETELAMR.Q |
|  | 10423 | **689.3973** | **1376.7801** | **1375.7129** | **1.0672** | **1** | **3** | **62** | **5** | **K.SQDLAKIMADIR.A** |
|  | 12574 | 503.1049 | 1506.2930 | 1505.7395 | 0.5535 | 0 | 9 | 9.2 | 1 | R.TVQSLEIDLDSMR.N |
|  | 15886 | **861.4329** | **1720.8513** | **1720.8573** | **-0.0060** | **1** | **4** | **49** | **7** | **R.DWSHYFKIIEDLR.A** |
|  | 18504 | **643.6033** | **1927.7882** | **1926.9105** | **0.8777** | **1** | **3** | **40** | **1** | **K.ETMQSLNDRLASYLDR.V** |
|  | 21746 | **754.7169** | **2261.1288** | **2260.0940** | **1.0347** | **0** | **1** | **63** | **1** | **R.GGMGSGGLATGIAGGLAGMGGIQNEK.E** |
|  | 22339 | **586.5245** | **2342.0691** | **2341.2502** | **0.8188** | **1** | **0** | **74** | **7** | **R.AQIFANTVDNARIVLQIDNAR.L** |
|  | 27597 | **1112.5237** | **3334.5492** | **3334.5704** | **-0.0212** | **1** | **33** | **0.023** | **1** | **R.GGMGSGGLATGIAGGLAGMGGIQNEKETMQSLNDR.L** |
|  | 27680 | **671.0757** | **3350.3420** | **3350.5653** | **-0.2233** | **1** | **(1)** | **25** | **4** | **R.GGMGSGGLATGIAGGLAGMGGIQNEKETMQSLNDR.L** |

| **662.** | Q96HR9\|REEP6_HUMAN    **Mass:** 20891    **Score:** 39     **Queries matched:** 8   **emPAI:** 0.18 |
| --- | --- |
|  | Receptor expression-enhancing protein 6 OS=Homo sapiens GN=REEP6 PE=1 SV=1 |

|  | **Query** | **Observed** | **Mr(expt)** | **Mr(calc)** | **Delta** | **Miss** | **Score** | **Expect** | **Rank** | **Peptide** |
| --- | --- | --- | --- | --- | --- | --- | --- | --- | --- | --- |
|  | 3201 | **461.0075** | **920.0003** | **920.4386** | **-0.4382** | **0** | **9** | **14** | **3** | **R.IMNDLSGR.A** |
|  | 7452 | **612.3361** | **1222.6577** | **1222.6670** | **-0.0093** | **0** | **35** | **0.046** | **1** | **R.NVKPSQTPQPK.D** 7453 |
|  | 9779 | 671.8173 | 1341.6201 | 1340.6949 | 0.9252 | 1 | 1 | 1.1e+002 | 7 | R.QRVEHFLEQR.N |
|  | 10045 | **452.8989** | **1355.6748** | **1355.7660** | **-0.0913** | **0** | **3** | **77** | **7** | **R.NLVTEVLGALEAK.T** |
|  | 11907 | 489.2750 | 1464.8031 | 1465.7889 | -0.9858 | 1 | 4 | 48 | 5 | R.NVKPSQTPQPKDK.- 11896 |
|  | 17827 | 624.6387 | 1870.8942 | 1870.0411 | 0.8531 | 1 | 3 | 53 | 6 | R.NLVTEVLGALEAKTGVEK.R |

| **663.** | Q9NX20\|RM16_HUMAN    **Mass:** 28488    **Score:** 39     **Queries matched:** 12   **emPAI:** 0.13 |
| --- | --- |
|  | 39S ribosomal protein L16, mitochondrial OS=Homo sapiens GN=MRPL16 PE=1 SV=1 |

|  | **Query** | **Observed** | **Mr(expt)** | **Mr(calc)** | **Delta** | **Miss** | **Score** | **Expect** | **Rank** | **Peptide** |
| --- | --- | --- | --- | --- | --- | --- | --- | --- | --- | --- |
|  | 1062 | **647.5555** | **646.5483** | **645.3850** | **1.1633** | **0** | **3** | **1e+002** | **4** | **K.LPFAAK.A** |
|  | 2187 | **425.9054** | **849.7963** | **849.4378** | **0.3585** | **1** | **4** | **45** | **3** | **R.GTLEKMR.K** |
|  | 2338 | **431.2610** | **860.5074** | **859.4586** | **1.0489** | **0** | **13** | **12** | **3** | **R.LVVEMGGR.C** 2328 |
|  | 2560 | 438.7189 | 875.4232 | 875.4535 | -0.0303 | 0 | (3) | 92 | 4 | R.LVVEMGGR.C |
|  | 3677 | 480.2771 | 958.5397 | 959.5400 | -1.0003 | 1 | 13 | 8.9 | 5 | K.AVSRGTLEK.M |
|  | 5210 | 538.2946 | 1074.5746 | 1074.5855 | -0.0110 | 0 | 11 | 13 | 1 | R.IATANMLGIR.K |
|  | 6467 | 586.8339 | 1171.6533 | 1171.6237 | 0.0296 | 0 | 39 | 0.02 | 3 | K.VLSPYDLTHK.G 6452 6453 |
|  | 6507 | **588.3308** | **1174.6471** | **1173.6176** | **1.0295** | **1** | **6** | **38** | **6** | **R.LTINRSMDPK.N** |
|  | 30192 | 1064.7168 | 4254.8381 | 4255.0255 | -0.1874 | 1 | 1 | 17 | 6 | R.GPSTEATEFTEGNFAILALGGGYLHWGHFEMMRLTINR.S |

| **664.** | P05386\|RLA1_HUMAN    **Mass:** 11621    **Score:** 39     **Queries matched:** 2   **emPAI:** 0.34 |
| --- | --- |
|  | 60S acidic ribosomal protein P1 OS=Homo sapiens GN=RPLP1 PE=1 SV=1 |

|  | **Query** | **Observed** | **Mr(expt)** | **Mr(calc)** | **Delta** | **Miss** | **Score** | **Expect** | **Rank** | **Peptide** |
| --- | --- | --- | --- | --- | --- | --- | --- | --- | --- | --- |
|  | 22455 | **786.0318** | **2355.0736** | **2354.3150** | **0.7585** | **1** | **2** | **52** | **4** | **K.INALIKAAGVNVEPFWPGLFAK.A** |
|  | 29188 | **1270.3077** | **3807.9014** | **3807.9213** | **-0.0199** | **0** | **39** | **0.004** | **1** | **K.ALANVNIGSLICNVGAGGPAPAAGAAPAGGPAPSTAAAPAEEK.K** |

| **665.** | Q8IVB4\|SL9A9_HUMAN    **Mass:** 73088    **Score:** 39     **Queries matched:** 5   **emPAI:** 0.05 |
| --- | --- |
|  | Sodium/hydrogen exchanger 9 OS=Homo sapiens GN=SLC9A9 PE=1 SV=1 |

|  | **Query** | **Observed** | **Mr(expt)** | **Mr(calc)** | **Delta** | **Miss** | **Score** | **Expect** | **Rank** | **Peptide** |
| --- | --- | --- | --- | --- | --- | --- | --- | --- | --- | --- |
|  | 4295 | 501.7791 | 1001.5437 | 1001.6022 | -0.0585 | 0 | 39 | 0.024 | 1 | R.GAIAFALAIR.N |
|  | 4554 | 512.2559 | 1022.4973 | 1022.4815 | 0.0158 | 1 | 9 | 19 | 7 | K.NMTKAESAR.L |
|  | 18092 | **632.3866** | **1894.1380** | **1893.8841** | **0.2539** | **0** | **4** | **36** | **2** | **K.IPWNFQHMMMFSGLR.G** |
|  | 29055 | **938.9807** | **3751.8937** | **3750.9381** | **0.9557** | **0** | **0** | **30** | **2** | **K.QMMFTTTLLLVFFTVWVFGGGTTPMLTWLQIR.V** |
|  | 30749 | **1155.7975** | **4619.1609** | **4618.3246** | **0.8362** | **1** | **4** | **6.6** | **3** | **K.ENPNAFDAAAFFQSVGNFLGIFAGSFAMGSAYAIITALLTKFTK.L** |

| **666.** | P13929\|ENOB_HUMAN    **Mass:** 47244    **Score:** 39     **Queries matched:** 15   **emPAI:** 0.08 |
| --- | --- |
|  | Beta-enolase OS=Homo sapiens GN=ENO3 PE=1 SV=4 |

|  | **Query** | **Observed** | **Mr(expt)** | **Mr(calc)** | **Delta** | **Miss** | **Score** | **Expect** | **Rank** | **Peptide** |
| --- | --- | --- | --- | --- | --- | --- | --- | --- | --- | --- |
|  | 1790 | **413.2429** | **824.4712** | **823.4010** | **1.0701** | **0** | **5** | **42** | **7** | **K.YNQLMR.I** |
|  | 3738 | 482.7193 | 963.4241 | 963.5324 | -0.1083 | 1 | 12 | 12 | 6 | M.AMQKIFAR.E 3730 3731 3732 3736 3737 3739 |
|  | 4494 | 509.6205 | 1017.2264 | 1016.5502 | 0.6762 | 1 | 2 | 80 | 9 | K.KLSVADQEK.V |
|  | 5718 | **556.7855** | **1111.5564** | **1110.5678** | **0.9886** | **1** | **5** | **46** | **6** | **-.MAMQKIFAR.E** |
|  | 6215 | 576.7943 | 1151.5739 | 1151.6121 | -0.0381 | 1 | 4 | 63 | 3 | R.LAKYNQLMR.I |
|  | 16058 | **578.9089** | **1733.7050** | **1733.9498** | **-0.2448** | **1** | **0** | **92** | **6** | **K.SKFGANAILGVSLAVCK.A** |
|  | 20374 | **703.2097** | **2106.6073** | **2107.1062** | **-0.4988** | **1** | **2** | **39** | **4** | **R.FRAAVPSGASTGIYEALELR.D** |
|  | 26583 | **1031.1874** | **3090.5403** | **3090.5145** | **0.0258** | **1** | **39** | **0.0069** | **1** | **K.YGKDATNVGDEGGFAPNILENNEALELLK.T** 26582 |

| **667.** | Q15043\|S39AE_HUMAN    **Mass:** 54902    **Score:** 39     **Queries matched:** 2   **emPAI:** 0.07 |
| --- | --- |
|  | Zinc transporter ZIP14 OS=Homo sapiens GN=SLC39A14 PE=1 SV=2 |

|  | **Query** | **Observed** | **Mr(expt)** | **Mr(calc)** | **Delta** | **Miss** | **Score** | **Expect** | **Rank** | **Peptide** |
| --- | --- | --- | --- | --- | --- | --- | --- | --- | --- | --- |
|  | 22193 | 1161.0686 | 2320.1226 | 2320.1369 | -0.0143 | 0 | 39 | 0.01 | 1 | R.IGSSELQEFCPTILQQLDSR.A |
|  | 22792 | 803.7107 | 2408.1102 | 2409.0689 | -0.9587 | 0 | 1 | 59 | 5 | K.LQNGDLDHMIPQHCSSELDGK.A |

| **668.** | Q92820\|GGH_HUMAN    **Mass:** 36340    **Score:** 39     **Queries matched:** 6   **emPAI:** 0.10 |
| --- | --- |
|  | Gamma-glutamyl hydrolase OS=Homo sapiens GN=GGH PE=1 SV=2 |

|  | **Query** | **Observed** | **Mr(expt)** | **Mr(calc)** | **Delta** | **Miss** | **Score** | **Expect** | **Rank** | **Peptide** |
| --- | --- | --- | --- | --- | --- | --- | --- | --- | --- | --- |
|  | 2672 | 442.2688 | 882.5230 | 882.4382 | 0.0849 | 1 | 3 | 66 | 4 | K.VMKNYGR.Y |
|  | 6059 | **571.2844** | **1140.5542** | **1139.7100** | **0.8442** | **0** | **8** | **25** | **2** | **K.KPIIGILMQK.C** |
|  | 9616 | 666.8664 | 1331.7182 | 1332.6271 | -0.9089 | 0 | 0 | 1.3e+002 | 6 | K.IEFISTMEGYK.Y |
|  | 9678 | **668.8057** | **1335.5969** | **1334.6942** | **0.9027** | **0** | **39** | **0.019** | **1** | **K.NLDGISHAPNAVK.T** |
|  | 12224 | **742.8462** | **1483.6778** | **1483.7671** | **-0.0893** | **1** | **1** | **1e+002** | **6** | **K.KFFNVLTTNTDGK.I** |
|  | 23106 | **818.5812** | **2452.7219** | **2452.3002** | **0.4217** | **1** | **2** | **32** | **5** | **K.DYEILFKSINGILFPGGSVDLR.R** |

| **669.** | Q8WXE0\|CSKI2_HUMAN    **Mass:** 127204   **Score:** 38     **Queries matched:** 14   **emPAI:** 0.03 |
| --- | --- |
|  | Caskin-2 OS=Homo sapiens GN=CASKIN2 PE=1 SV=1 |

|  | **Query** | **Observed** | **Mr(expt)** | **Mr(calc)** | **Delta** | **Miss** | **Score** | **Expect** | **Rank** | **Peptide** |
| --- | --- | --- | --- | --- | --- | --- | --- | --- | --- | --- |
|  | 3840 | **486.7566** | **971.4986** | **971.5764** | **-0.0778** | **1** | **38** | **0.026** | **1** | **R.EASGILKVR.A** |
|  | 6444 | 586.2845 | 1170.5544 | 1169.5677 | 0.9867 | 0 | 1 | 1.2e+002 | 5 | R.VGLSPDSPAGDR.N |
|  | 7553 | **614.8389** | **1227.6633** | **1226.7346** | **0.9287** | **1** | **11** | **13** | **4** | **K.QLLREASGILK.V** |
|  | 7774 | **619.9095** | **1237.8045** | **1236.6462** | **1.1583** | **0** | **8** | **19** | **2** | **R.NLPEGTERPPK.L** 7726 |
|  | 8505 | **425.4512** | **1273.3319** | **1273.6375** | **-0.3056** | **1** | **6** | **23** | **3** | **R.SPSQESIGARSR.G** 8501 8525 |
|  | 8576 | **426.8695** | **1277.5868** | **1277.6074** | **-0.0206** | **0** | **4** | **63** | **8** | **K.TPLDLACEFGR.L** |
|  | 8704 | **643.3197** | **1284.6248** | **1285.6626** | **-1.0378** | **0** | **0** | **1.3e+002** | **10** | **R.GLLQGEALSEGGR.R** |
|  | 16911 | 902.9272 | 1803.8398 | 1804.8843 | -1.0445 | 0 | 6 | 26 | 4 | R.GSSGEGLPFAEEGNLTIK.Q |
|  | 20850 | **1079.9959** | **2157.9771** | **2157.1066** | **0.8706** | **1** | **2** | **60** | **1** | **K.RLSSVSGPSPEPPPLDGSPGPK.E** |
|  | 29636 | **797.7877** | **3983.9019** | **3985.0771** | **-1.1752** | **1** | **3** | **14** | **1** | **R.WNGETEPPAAPAALLKVPGAGTAPKPVSVACTQLAFSGPK.L** |
|  | 29880 | **1023.3101** | **4089.2111** | **4090.1375** | **-0.9263** | **0** | **3** | **11** | **7** | **R.LNVNYQDADGFSALHHAALGGSLELIALLLEAQATVDIK.D** |

| **670.** | Q9NZ01\|TECR_HUMAN    **Mass:** 36410    **Score:** 38     **Queries matched:** 11   **emPAI:** 0.10 |
| --- | --- |
|  | Trans-2,3-enoyl-CoA reductase OS=Homo sapiens GN=TECR PE=1 SV=1 |

|  | **Query** | **Observed** | **Mr(expt)** | **Mr(calc)** | **Delta** | **Miss** | **Score** | **Expect** | **Rank** | **Peptide** |
| --- | --- | --- | --- | --- | --- | --- | --- | --- | --- | --- |
|  | 2078 | 422.2609 | 842.5072 | 842.4610 | 0.0462 | 0 | 4 | 66 | 6 | R.DLRPAGSK.T |
|  | 3464 | 472.2734 | 942.5322 | 942.5612 | -0.0290 | 0 | 4 | 76 | 8 | R.MPIIPFLL.- |
|  | 4836 | **523.2634** | **1044.5123** | **1044.5175** | **-0.0052** | **0** | **0** | **1.6e+002** | **2** | **R.FSHGTMPLR.N** |
|  | 7318 | 608.8210 | 1215.6274 | 1215.6135 | 0.0139 | 0 | 8 | 28 | 8 | K.HYEVEILDAK.T |
|  | 8671 | **642.3522** | **1282.6898** | **1282.7510** | **-0.0612** | **1** | **1** | **1.1e+002** | **7** | **K.RLLETLFVHR.F** |
|  | 9825 | **448.9075** | **1343.7008** | **1343.7085** | **-0.0077** | **1** | **2** | **92** | **9** | **M.KHYEVEILDAK.T** |
|  | 12058 | **738.3774** | **1474.7403** | **1474.7490** | **-0.0086** | **1** | **38** | **0.02** | **1** | **-.MKHYEVEILDAK.T** 12059 |
|  | 13476 | 782.3472 | 1562.6798 | 1562.8028 | -0.1230 | 1 | 8 | 17 | 5 | R.FSHGTMPLRNIFK.N 13469 |
|  | 27192 | **1076.6246** | **3226.8521** | **3226.7471** | **0.1050** | **1** | **3** | **17** | **4** | **K.LALAIFVICQLGNFSIHMALRDLRPAGSK.T** |

| **671.** | O43678\|NDUA2_HUMAN    **Mass:** 11029    **Score:** 38     **Queries matched:** 4   **emPAI:** 0.36 |
| --- | --- |
|  | NADH dehydrogenase [ubiquinone] 1 alpha subcomplex subunit 2 OS=Homo sapiens GN=NDUFA2 PE=1 SV=3 |

|  | **Query** | **Observed** | **Mr(expt)** | **Mr(calc)** | **Delta** | **Miss** | **Score** | **Expect** | **Rank** | **Peptide** |
| --- | --- | --- | --- | --- | --- | --- | --- | --- | --- | --- |
|  | 4617 | **514.7534** | **1027.4922** | **1028.5727** | **-1.0805** | **1** | **8** | **28** | **2** | **M.AAAAASRGVGAK.L** |
|  | 5844 | **561.3345** | **1120.6545** | **1120.6604** | **-0.0059** | **0** | **38** | **0.026** | **1** | **K.ANPDLPILIR.E** |
|  | 6346 | **581.3484** | **1160.6823** | **1159.6131** | **1.0692** | **1** | **5** | **47** | **6** | **-.MAAAAASRGVGAK.L** |
|  | 6538 | **589.3249** | **1176.6352** | **1175.6081** | **1.0272** | **1** | **(4)** | **67** | **2** | **-.MAAAAASRGVGAK.L** |

| **672.** | P62906\|RL10A_HUMAN    **Mass:** 24987    **Score:** 38     **Queries matched:** 10   **emPAI:** 0.15 |
| --- | --- |
|  | 60S ribosomal protein L10a OS=Homo sapiens GN=RPL10A PE=1 SV=2 |

|  | **Query** | **Observed** | **Mr(expt)** | **Mr(calc)** | **Delta** | **Miss** | **Score** | **Expect** | **Rank** | **Peptide** |
| --- | --- | --- | --- | --- | --- | --- | --- | --- | --- | --- |
|  | 2960 | **453.2353** | **904.4560** | **903.4596** | **0.9963** | **0** | **6** | **50** | **10** | **K.STMGKPQR.L** |
|  | 3773 | **484.2481** | **966.4816** | **965.4818** | **0.9998** | **0** | **7** | **26** | **5** | **R.DTLYEAVR.E** 3765 3770 |
|  | 4510 | **510.2564** | **1018.4983** | **1017.5706** | **0.9277** | **1** | **10** | **18** | **2** | **K.VDEVKSTIK.F** |
|  | 9191 | **437.2480** | **1308.7221** | **1307.6834** | **1.0387** | **1** | **10** | **15** | **3** | **K.VSRDTLYEAVR.E** |
|  | 12234 | **742.8995** | **1483.7845** | **1483.7922** | **-0.0077** | **1** | **38** | **0.021** | **1** | **K.KYDAFLASESLIK.Q** |
|  | 13205 | 773.8973 | 1545.7801 | 1546.8970 | -1.1169 | 1 | 6 | 31 | 10 | R.KFLETVELQISLK.N |
|  | 13981 | **798.3175** | **1594.6204** | **1594.8389** | **-0.2184** | **1** | **4** | **45** | **3** | **K.AVDIPHMDIEALKK.L** |
|  | 18070 | **631.6099** | **1891.8078** | **1891.8193** | **-0.0116** | **0** | **10** | **10** | **1** | **K.FSVCVLGDQQHCDEAK.A** |

| **673.** | P84095\|RHOG_HUMAN    **Mass:** 21751    **Score:** 38     **Queries matched:** 7   **emPAI:** 0.18 |
| --- | --- |
|  | Rho-related GTP-binding protein RhoG OS=Homo sapiens GN=RHOG PE=1 SV=1 |

|  | **Query** | **Observed** | **Mr(expt)** | **Mr(calc)** | **Delta** | **Miss** | **Score** | **Expect** | **Rank** | **Peptide** |
| --- | --- | --- | --- | --- | --- | --- | --- | --- | --- | --- |
|  | 3631 | **478.7634** | **955.5122** | **955.5199** | **-0.0077** | **1** | **4** | **58** | **3** | **R.AQPDTLRR.L** |
|  | 6645 | **593.1540** | **1184.2935** | **1183.6309** | **0.6626** | **1** | **9** | **12** | **4** | **K.DLRAQPDTLR.R** 6596 |
|  | 12615 | 503.9286 | 1508.7640 | 1509.7133 | -0.9493 | 0 | 6 | 33 | 6 | R.YLECSALQQDGVK.E |
|  | 15050 | **555.1699** | **1662.4878** | **1662.8433** | **-0.3556** | **1** | **2** | **44** | **4** | **-.MQSIKCVVVGDGAVGK.T** |
|  | 15619 | **567.9610** | **1700.8612** | **1700.8266** | **0.0346** | **0** | **1** | **81** | **5** | **K.TCLLICYTTNAFPK.E** |
|  | 19361 | **669.3682** | **2005.0829** | **2005.0956** | **-0.0127** | **1** | **38** | **0.015** | **1** | **R.LKEQGQAPITPQQGQALAK.Q** |

| **674.** | O75865\|TPC6A_HUMAN    **Mass:** 17764    **Score:** 38     **Queries matched:** 8   **emPAI:** 0.22 |
| --- | --- |
|  | Trafficking protein particle complex subunit 6A OS=Homo sapiens GN=TRAPPC6A PE=1 SV=2 |

|  | **Query** | **Observed** | **Mr(expt)** | **Mr(calc)** | **Delta** | **Miss** | **Score** | **Expect** | **Rank** | **Peptide** |
| --- | --- | --- | --- | --- | --- | --- | --- | --- | --- | --- |
|  | 2096 | 423.2105 | 844.4065 | 844.4542 | -0.0477 | 0 | 38 | 0.039 | 1 | R.EELDVLK.F 2095 2097 |
|  | 6845 | **398.8644** | **1193.5712** | **1194.6833** | **-1.1121** | **1** | **10** | **14** | **3** | **R.VGQALGERLPR.E** |
|  | 9778 | **448.2138** | **1341.6195** | **1341.6421** | **-0.0226** | **0** | **4** | **56** | **9** | **K.MSLSVLEGMGFR.V** |
|  | 10705 | 697.7873 | 1393.5601 | 1392.7323 | 0.8279 | 1 | 0 | 1.2e+002 | 10 | R.EELDVLKFLCK.D |
|  | 17580 | **617.8008** | **1850.3807** | **1850.9349** | **-0.5542** | **1** | **1** | **60** | **5** | **K.DLWVAVFQKQMDSLR.T** |
|  | 20940 | **724.0345** | **2169.0818** | **2168.0718** | **1.0100** | **1** | **3** | **42** | **1** | **K.MSLSVLEGMGFRVGQALGER.L** |

| **675.** | P51148\|RAB5C_HUMAN    **Mass:** 23696    **Score:** 37     **Queries matched:** 13   **emPAI:** 0.16 |
| --- | --- |
|  | Ras-related protein Rab-5C OS=Homo sapiens GN=RAB5C PE=1 SV=2 |

|  | **Query** | **Observed** | **Mr(expt)** | **Mr(calc)** | **Delta** | **Miss** | **Score** | **Expect** | **Rank** | **Peptide** |
| --- | --- | --- | --- | --- | --- | --- | --- | --- | --- | --- |
|  | 5172 | 536.7695 | 1071.5244 | 1071.5825 | -0.0581 | 1 | 12 | 11 | 3 | K.NWVKELQR.Q |
|  | 7202 | **606.2876** | **1210.5606** | **1210.5690** | **-0.0084** | **0** | **32** | **0.081** | **1** | **K.NEPQNATGAPGR.N** 7182 7201 7204 7206 |
|  | 9015 | 434.5419 | 1300.6040 | 1299.6070 | 0.9970 | 0 | 25 | 0.41 | 1 | R.YHSLAPMYYR.G 8996 |
|  | 13569 | 524.2421 | 1569.7046 | 1568.7655 | 0.9391 | 1 | 10 | 14 | 2 | R.NRGVDLQENNPASR.S 13581 13585 |
|  | 15112 | 834.4002 | 1666.7857 | 1667.8274 | -1.0416 | 1 | 3 | 51 | 4 | -.MAGRGGAARPNGPAAGNK.I 15127 |

| **676.** | P13498\|CY24A_HUMAN    **Mass:** 21059    **Score:** 37     **Queries matched:** 3   **emPAI:** 0.18 |
| --- | --- |
|  | Cytochrome b-245 light chain OS=Homo sapiens GN=CYBA PE=1 SV=2 |

|  | **Query** | **Observed** | **Mr(expt)** | **Mr(calc)** | **Delta** | **Miss** | **Score** | **Expect** | **Rank** | **Peptide** |
| --- | --- | --- | --- | --- | --- | --- | --- | --- | --- | --- |
|  | 1769 | **412.2079** | **822.4012** | **823.3858** | **-0.9846** | **1** | **10** | **16** | **1** | **K.KGSTMER.W** |
|  | 8984 | **650.3886** | **1298.7627** | **1299.6758** | **-0.9131** | **1** | **7** | **27** | **10** | **R.WGQKHMTAVVK.L** |
|  | 26133 | **1004.5101** | **3010.5086** | **3010.5247** | **-0.0162** | **1** | **37** | **0.0097** | **1** | **R.KKPSEEEAAAAAGGPPGGPQVNPIPVTDEVV.-** |

| **677.** | O43399\|TPD54_HUMAN    **Mass:** 22281    **Score:** 37     **Queries matched:** 6   **emPAI:** 0.17 |
| --- | --- |
|  | Tumor protein D54 OS=Homo sapiens GN=TPD52L2 PE=1 SV=2 |

|  | **Query** | **Observed** | **Mr(expt)** | **Mr(calc)** | **Delta** | **Miss** | **Score** | **Expect** | **Rank** | **Peptide** |
| --- | --- | --- | --- | --- | --- | --- | --- | --- | --- | --- |
|  | 6678 | **396.2158** | **1185.6255** | **1185.7081** | **-0.0826** | **1** | **9** | **23** | **4** | **R.RLGLSTLGELK.Q** |
|  | 6775 | **595.8002** | **1189.5859** | **1189.5939** | **-0.0080** | **0** | **37** | **0.029** | **1** | **K.TQETLSQAGQK.T** |
|  | 9394 | **661.2988** | **1320.5831** | **1319.7045** | **0.8786** | **0** | **8** | **20** | **1** | **K.TSAALSTVGSAISR.K** 9391 |
|  | 14097 | 801.9128 | 1601.8111 | 1602.7308 | -0.9196 | 0 | 8 | 21 | 9 | -.MDSAGQDINLNSPNK.G |
|  | 20341 | **1052.5366** | **2103.0587** | **2103.0497** | **0.0089** | **1** | **6** | **24** | **2** | **K.QNLSRSWHDVQVSSAYVK.T** |

| **678.** | Q9BQB6\|VKOR1_HUMAN    **Mass:** 18622    **Score:** 37     **Queries matched:** 1   **emPAI:** 0.21 |
| --- | --- |
|  | Vitamin K epoxide reductase complex subunit 1 OS=Homo sapiens GN=VKORC1 PE=1 SV=1 |

|  | **Query** | **Observed** | **Mr(expt)** | **Mr(calc)** | **Delta** | **Miss** | **Score** | **Expect** | **Rank** | **Peptide** |
| --- | --- | --- | --- | --- | --- | --- | --- | --- | --- | --- |
|  | 10943 | **705.3254** | **1408.6362** | **1408.6439** | **-0.0077** | **0** | **37** | **0.023** | **1** | **R.ALCDVGTAISCSR.V** |

| **679.** | P26232\|CTNA2_HUMAN    **Mass:** 106045   **Score:** 37     **Queries matched:** 17   **emPAI:** 0.03 |
| --- | --- |
|  | Catenin alpha-2 OS=Homo sapiens GN=CTNNA2 PE=1 SV=5 |

|  | **Query** | **Observed** | **Mr(expt)** | **Mr(calc)** | **Delta** | **Miss** | **Score** | **Expect** | **Rank** | **Peptide** |
| --- | --- | --- | --- | --- | --- | --- | --- | --- | --- | --- |
|  | 2130 | 424.2369 | 846.4593 | 845.5157 | 0.9436 | 1 | 8 | 32 | 2 | R.KAVLMIR.T |
|  | 2178 | 425.7345 | 849.4544 | 848.4174 | 1.0370 | 1 | 13 | 9.9 | 1 | R.KQGETMR.I |
|  | 2380 | **432.8830** | **863.7514** | **864.4123** | **-0.6610** | **1** | **(10)** | **13** | **9** | **R.KQGETMR.I** |
|  | 2414 | **433.8875** | **865.7605** | **865.4406** | **0.3199** | **0** | **1** | **84** | **10** | **R.HPDVAATR.A** |
|  | 4688 | 517.2532 | 1032.4919 | 1032.5352 | -0.0433 | 0 | 6 | 42 | 8 | R.FRPSLEER.L |
|  | 6338 | **581.3088** | **1160.6030** | **1159.6197** | **0.9833** | **1** | **4** | **61** | **9** | **K.NTSDVINAAKK.I** |
|  | 8141 | 628.8337 | 1255.6528 | 1256.6434 | -0.9906 | 0 | 27 | 0.27 | 2 | R.AIMAQLPQEEK.A |
|  | 8413 | 635.2757 | 1268.5368 | 1267.6561 | 0.8808 | 1 | 5 | 39 | 2 | K.EVKEYAQVFR.E |
|  | 9343 | **659.3265** | **1316.6384** | **1315.7459** | **0.8925** | **1** | **5** | **42** | **2** | **K.SLEIRTLTVER.L** |
|  | 11355 | **478.2665** | **1431.7777** | **1430.7439** | **1.0338** | **1** | **10** | **13** | **9** | **K.GDPLNIAIDKMTK.K** |
|  | 14251 | 806.9085 | 1611.8025 | 1611.7086 | 0.0938 | 0 | 5 | 35 | 4 | R.IASSEFADDPCSSVK.R |
|  | 17110 | 909.9404 | 1817.8663 | 1817.8755 | -0.0092 | 0 | 37 | 0.021 | 1 | R.TSVQTEDDQLIAGQSAR.A 17111 |
|  | 17938 | 940.9504 | 1879.8862 | 1880.8972 | -1.0110 | 0 | 14 | 4.4 | 2 | R.LESIISGAALMADSSCTR.D |
|  | 19261 | 666.3381 | 1995.9924 | 1996.0418 | -0.0494 | 1 | 0 | 88 | 8 | K.YQKVYGTAAVNSPVVSWK.M |
|  | 20136 | **1042.0034** | **2081.9923** | **2083.1062** | **-1.1139** | **1** | **1** | **66** | **1** | **K.KAHVLAASVEQATQNFLEK.G** |
|  | 22254 | 777.3408 | 2329.0006 | 2329.9346 | -0.9340 | 0 | 3 | 38 | 5 | R.TPEELEDDSDFEQEDYDVR.S |

| **680.** | P53990\|IST1_HUMAN    **Mass:** 39897    **Score:** 37     **Queries matched:** 13   **emPAI:** 0.09 |
| --- | --- |
|  | IST1 homolog OS=Homo sapiens GN=KIAA0174 PE=1 SV=1 |

|  | **Query** | **Observed** | **Mr(expt)** | **Mr(calc)** | **Delta** | **Miss** | **Score** | **Expect** | **Rank** | **Peptide** |
| --- | --- | --- | --- | --- | --- | --- | --- | --- | --- | --- |
|  | 394 | **443.0393** | **442.0320** | **442.2288** | **-0.1968** | **0** | **5** | **12** | **1** | **K.GGPGR.G** 396 |
|  | 2159 | **425.2552** | **848.4959** | **848.5007** | **-0.0048** | **0** | **20** | **1.8** | **2** | **R.YLIEIAK.N** |
|  | 4483 | 509.2704 | 1016.5263 | 1016.5325 | -0.0061 | 0 | 28 | 0.3 | 1 | K.IVADQLCAK.Y |
|  | 4920 | **526.3691** | **1050.7236** | **1049.5393** | **1.1843** | **0** | **6** | **32** | **1** | **K.EIADYLAAGK.D** |
|  | 5486 | **548.7426** | **1095.4705** | **1095.6876** | **-0.2171** | **1** | **4** | **52** | **8** | **R.VNLRLVINR.L** |
|  | 5784 | 559.2799 | 1116.5451 | 1116.5524 | -0.0072 | 0 | 37 | 0.032 | 1 | R.TNQIGTVNDR.L |
|  | 10196 | 455.9706 | 1364.8900 | 1364.7486 | 0.1414 | 1 | 8 | 19 | 2 | R.LMHKLSVEAPPK.I 10216 |
|  | 11622 | 725.3687 | 1448.7229 | 1449.8555 | -1.1326 | 1 | 5 | 40 | 6 | K.LSVEAPPKILVER.Y 11638 |
|  | 13201 | 773.8661 | 1545.7177 | 1545.7682 | -0.0504 | 1 | 2 | 72 | 8 | K.LCRTNQIGTVNDR.L |
|  | 26138 | **753.8767** | **3011.4777** | **3011.5815** | **-0.1037** | **1** | **1** | **47** | **2** | **K.ELDSGLAESVSTLIWAAPRLQSEVAELK.I** |

| **681.** | Q2M3M2\|SC5A9_HUMAN    **Mass:** 74880    **Score:** 37     **Queries matched:** 11   **emPAI:** 0.05 |
| --- | --- |
|  | Sodium/glucose cotransporter 4 OS=Homo sapiens GN=SLC5A9 PE=2 SV=2 |

|  | **Query** | **Observed** | **Mr(expt)** | **Mr(calc)** | **Delta** | **Miss** | **Score** | **Expect** | **Rank** | **Peptide** |
| --- | --- | --- | --- | --- | --- | --- | --- | --- | --- | --- |
|  | 5923 | 565.3159 | 1128.6172 | 1127.6298 | 0.9873 | 1 | 10 | 18 | 5 | R.SLSAKSLSHAK.G 5927 |
|  | 8294 | **422.0406** | **1263.1000** | **1263.6129** | **-0.5129** | **0** | **3** | **44** | **3** | **K.STEQELMVVGR.V** 8293 |
|  | 14113 | **535.3206** | **1602.9400** | **1601.7831** | **1.1569** | **1** | **1** | **1e+002** | **6** | **M.SKELAAMGPGASGDGVR.T** |
|  | 16460 | **883.4076** | **1764.8007** | **1764.8135** | **-0.0127** | **1** | **0** | **1e+002** | **7** | **-.MSKELAAMGPGASGDGVR.T** |
|  | 16506 | **590.3625** | **1768.0656** | **1766.9286** | **1.1371** | **0** | **(1)** | **64** | **10** | **K.ILPMFFIVMPGMISR.A** |
|  | 16697 | 595.3232 | 1782.9477 | 1782.9235 | 0.0242 | 0 | 3 | 50 | 2 | K.ILPMFFIVMPGMISR.A |
|  | 18986 | **657.8234** | **1970.4485** | **1969.8914** | **0.5571** | **0** | **0** | **59** | **3** | **R.MILEFSYPAPACGEVDR.R** |
|  | 19041 | **988.9389** | **1975.8632** | **1975.8768** | **-0.0136** | **0** | **37** | **0.018** | **1** | **R.ALFPDEVGCVDPDVCQR.I** |
|  | 32338 | 1449.7234 | 7243.5806 | 7244.7051 | -1.1245 | 0 | 0 | 4 | 2 | R.SMSWWPIGASLMSSNVGSGLFIGLAGTGAAGGLAVGGFEWNATWLLLALGWVFVPVYIAAGVVTMPQYLK.K |

| **682.** | Q9NVH1\|DJC11_HUMAN    **Mass:** 63524    **Score:** 37     **Queries matched:** 15   **emPAI:** 0.06 |
| --- | --- |
|  | DnaJ homolog subfamily C member 11 OS=Homo sapiens GN=DNAJC11 PE=1 SV=2 |

|  | **Query** | **Observed** | **Mr(expt)** | **Mr(calc)** | **Delta** | **Miss** | **Score** | **Expect** | **Rank** | **Peptide** |
| --- | --- | --- | --- | --- | --- | --- | --- | --- | --- | --- |
|  | 941 | **601.1237** | **600.1164** | **599.3391** | **0.7773** | **0** | **2** | **89** | **2** | **R.NLTPR.C** |
|  | 4513 | **510.2877** | **1018.5608** | **1017.5389** | **1.0219** | **1** | **11** | **14** | **3** | **R.LMQESVRR.I** |
|  | 5660 | 554.3010 | 1106.5875 | 1105.5516 | 1.0359 | 1 | 8 | 25 | 3 | R.EEFERLQR.E |
|  | 9788 | 671.8537 | 1341.6928 | 1341.8496 | -0.1567 | 1 | 10 | 13 | 4 | R.LIIKPYLRAQK.E 9789 |
|  | 12110 | 493.6001 | 1477.7785 | 1477.7347 | 0.0437 | 0 | 3 | 59 | 5 | R.WGIQSAMNTSIVR.D 12093 12094 12104 |
|  | 15108 | **834.3969** | **1666.7793** | **1665.8872** | **0.8920** | **0** | **15** | **3.7** | **1** | **R.GVLHQVMVLDSEALR.I** |
|  | 15458 | **564.2564** | **1689.7474** | **1689.7603** | **-0.0129** | **0** | **5** | **37** | **2** | **R.CFVTTNCALQFSSR.G** |
|  | 16912 | 902.9334 | 1803.8521 | 1804.0207 | -0.1686 | 0 | 4 | 41 | 2 | R.HSVLGAAVSVGVPQGVSLK.V |
|  | 23356 | **623.2887** | **2489.1257** | **2488.3261** | **0.7996** | **1** | **1** | **50** | **4** | **K.VLYQFRGVLHQVMVLDSEALR.I** 23343 |
|  | 29422 | **1299.6000** | **3895.7781** | **3894.7960** | **0.9822** | **1** | **37** | **0.0062** | **1** | **K.GTISVGVDATDLFDRYDEEYEDVSGSSFPQIEINK.M** |

| **683.** | Q8TF72\|SHRM3_HUMAN    **Mass:** 218125   **Score:** 37     **Queries matched:** 41   **emPAI:** 0.02 |
| --- | --- |
|  | Protein Shroom3 OS=Homo sapiens GN=SHROOM3 PE=1 SV=1 |

|  | **Query** | **Observed** | **Mr(expt)** | **Mr(calc)** | **Delta** | **Miss** | **Score** | **Expect** | **Rank** | **Peptide** |
| --- | --- | --- | --- | --- | --- | --- | --- | --- | --- | --- |
|  | 888 | **577.1248** | **576.1175** | **575.2663** | **0.8512** | **0** | **7** | **37** | **7** | **K.DAQSR.V** |
|  | 1278 | 776.3671 | 775.3598 | 775.3712 | -0.0114 | 0 | 9 | 28 | 4 | R.SSPATADK.R |
|  | 1545 | 402.7379 | 803.4612 | 802.4548 | 1.0064 | 0 | 17 | 4.6 | 3 | R.ISESVLR.D |
|  | 2067 | 422.0606 | 842.1067 | 842.4861 | -0.3795 | 1 | 5 | 39 | 9 | R.LTPEQKK.R |
|  | 2545 | 437.7423 | 873.4700 | 872.5331 | 0.9369 | 1 | 10 | 22 | 6 | R.KEAVSLVK.G 2542 |
|  | 2637 | **441.2956** | **880.5766** | **881.4606** | **-0.8840** | **1** | **3** | **68** | **8** | **R.SSLYEKR.K** 2640 |
|  | 3221 | **462.1436** | **922.2725** | **923.4573** | **-1.1848** | **1** | **4** | **47** | **2** | **R.SSSFAGGRR.L** |
|  | 3344 | **466.7404** | **931.4663** | **931.4723** | **-0.0060** | **1** | **37** | **0.038** | **1** | **R.SSPATADKR.Q** 3330 3343 3353 |
|  | 5693 | **555.7939** | **1109.5732** | **1108.5625** | **1.0107** | **0** | **3** | **68** | **9** | **K.ILAGQHEDAR.E** |
|  | 6099 | **572.9713** | **1143.9281** | **1143.5924** | **0.3356** | **1** | **1** | **86** | **1** | **R.YVKTVYDTR.R** |
|  | 7688 | 412.2278 | 1233.6617 | 1233.6751 | -0.0134 | 1 | 6 | 33 | 6 | K.MKSTLLIEQR.K 7663 |
|  | 8165 | **629.7687** | **1257.5228** | **1257.6928** | **-0.1700** | **1** | **1** | **1.1e+002** | **3** | **R.DVNLLKENSVK.R** |
|  | 8474 | 424.9337 | 1271.7792 | 1271.7561 | 0.0231 | 1 | 9 | 17 | 7 | R.ISLRISESVLR.D |
|  | 9218 | **437.8867** | **1310.6382** | **1309.5681** | **1.0701** | **1** | **12** | **7.8** | **3** | **R.ERSGSMDNTSAR.G** |
|  | 9541 | **664.3031** | **1326.5916** | **1325.5630** | **1.0287** | **1** | **(7)** | **25** | **6** | **R.ERSGSMDNTSAR.G** |
|  | 10132 | 454.7214 | 1361.1424 | 1360.6848 | 0.4576 | 0 | 3 | 49 | 5 | R.SNGHTLTQPPGPR.G |
|  | 10705 | 697.7873 | 1393.5601 | 1393.6296 | -0.0694 | 0 | 8 | 22 | 1 | K.ESGYIAPQGACNK.M |
|  | 11038 | **472.2926** | **1413.8561** | **1414.7351** | **-0.8790** | **1** | **9** | **17** | **4** | **R.GGLLEGMRQADIR.Y** |
|  | 11114 | 473.9099 | 1418.7080 | 1418.7783 | -0.0703 | 1 | 2 | 81 | 8 | K.RVSLPQWPPPSR.A |
|  | 11118 | 710.3982 | 1418.7818 | 1418.6790 | 0.1028 | 0 | 10 | 14 | 7 | R.ERPSSWSSLDQK.R |
|  | 11192 | **475.2600** | **1422.7580** | **1421.6820** | **1.0760** | **1** | **5** | **45** | **7** | **R.AGKSMSAEDLLER.S** |
|  | 11223 | 476.1921 | 1425.5544 | 1425.6632 | -0.1088 | 0 | 8 | 19 | 2 | K.TTMDLMEGLFPR.D |
|  | 11920 | **489.6055** | **1465.7947** | **1464.6779** | **1.1167** | **1** | **8** | **18** | **2** | **R.NGMRFPESSVADR.R** |
|  | 12646 | **504.6291** | **1510.8656** | **1509.9355** | **0.9302** | **1** | **11** | **9.6** | **1** | **K.VVNLLLSLSGRLAR.V** |
|  | 12851 | **508.5992** | **1522.7758** | **1521.7787** | **0.9971** | **1** | **7** | **25** | **2** | **R.EGLGQSLSGNFGKTK.S** 12823 12843 |
|  | 13684 | **526.2911** | **1575.8516** | **1574.8304** | **1.0211** | **0** | **2** | **68** | **9** | **R.QDVLLGQDSGFGLVK.D** |
|  | 14878 | **551.0200** | **1650.0382** | **1650.8473** | **-0.8091** | **1** | **4** | **38** | **7** | **R.LKTTMDLMEGLFPR.D** |
|  | 15321 | 560.6440 | 1678.9101 | 1678.8023 | 0.1078 | 1 | 5 | 37 | 4 | R.SPEASASASPHTPRER.H |
|  | 16841 | **599.9551** | **1796.8434** | **1797.8870** | **-1.0436** | **1** | **0** | **90** | **9** | **R.HLTPPQGNSPHSNERK.S** |
|  | 17376 | **613.3301** | **1836.9686** | **1835.9352** | **1.0334** | **0** | **1** | **81** | **1** | **R.GQEEMLLLFHHLTPR.W** |
|  | 17608 | **464.2140** | **1852.8267** | **1853.8360** | **-1.0092** | **1** | **17** | **2.2** | **1** | **R.SGSMDNTSARGGLLEGMR.Q** |
|  | 20840 | **720.0015** | **2156.9828** | **2157.0661** | **-0.0834** | **1** | **1** | **73** | **3** | **R.LARVENVLSGLGEDASNEER.S** |
|  | 30942 | **795.3931** | **4766.3151** | **4766.4591** | **-0.1440** | **1** | **1** | **14** | **6** | **K.CLLESLPSDFIPKAGALALPPNLTSEPIPAGGCTFSGIFPTLTSPL.-** |

| **684.** | Q13724\|MOGS_HUMAN    **Mass:** 92032    **Score:** 37     **Queries matched:** 7   **emPAI:** 0.04 |
| --- | --- |
|  | Mannosyl-oligosaccharide glucosidase OS=Homo sapiens GN=MOGS PE=1 SV=5 |

|  | **Query** | **Observed** | **Mr(expt)** | **Mr(calc)** | **Delta** | **Miss** | **Score** | **Expect** | **Rank** | **Peptide** |
| --- | --- | --- | --- | --- | --- | --- | --- | --- | --- | --- |
|  | 394 | 443.0393 | 442.0320 | 442.2288 | -0.1968 | 0 | 5 | 12 | 1 | R.GGPGR.R 396 |
|  | 911 | **585.3682** | **584.3610** | **583.3806** | **0.9804** | **1** | **8** | **22** | **1** | **R.KALPR.L** |
|  | 3594 | **477.3566** | **952.6986** | **953.5406** | **-0.8420** | **1** | **6** | **32** | **8** | **R.RAVPAEGVR.T** |
|  | 17998 | 629.6195 | 1885.8365 | 1885.0058 | 0.8307 | 0 | 3 | 52 | 3 | R.GPSGQGQGQFLIQQVTLK.I |
|  | 23186 | **616.8018** | **2463.1782** | **2464.2441** | **-1.0659** | **1** | **1** | **52** | **5** | **R.SFFPRGFLWDEGFHQLVVQR.W** |
|  | 29440 | 1302.3597 | 3904.0574 | 3904.0762 | -0.0188 | 1 | 37 | 0.0059 | 1 | R.VTVEPQDSGTSALPLVSLFFYVVTDGKEVLLPEVGAK.G |

| **685.** | Q14103\|HNRPD_HUMAN    **Mass:** 38581    **Score:** 37     **Queries matched:** 5   **emPAI:** 0.10 |
| --- | --- |
|  | Heterogeneous nuclear ribonucleoprotein D0 OS=Homo sapiens GN=HNRNPD PE=1 SV=1 |

|  | **Query** | **Observed** | **Mr(expt)** | **Mr(calc)** | **Delta** | **Miss** | **Score** | **Expect** | **Rank** | **Peptide** |
| --- | --- | --- | --- | --- | --- | --- | --- | --- | --- | --- |
|  | 3797 | **485.2684** | **968.5222** | **969.4628** | **-0.9406** | **0** | **2** | **83** | **5** | **R.HSEAATAQR.E** |
|  | 11758 | **486.7620** | **1457.2642** | **1456.5815** | **0.6828** | **0** | **5** | **27** | **1** | **K.NEEDEGHSNSSPR.H** |
|  | 12292 | **744.8786** | **1487.7426** | **1487.7508** | **-0.0081** | **0** | **37** | **0.027** | **1** | **K.IFVGGLSPDTPEEK.I** |
|  | 16363 | 879.4545 | 1756.8945 | 1756.9359 | -0.0414 | 1 | 16 | 2.5 | 2 | K.IFVGGLSPDTPEEKIR.E |
|  | 18498 | **643.3731** | **1927.0973** | **1926.9186** | **0.1787** | **1** | **1** | **65** | **5** | **R.EEWKMFIGGLSWDTTK.K** |

| **686.** | P02753\|RET4_HUMAN    **Mass:** 23337    **Score:** 37     **Queries matched:** 4   **emPAI:** 0.16 |
| --- | --- |
|  | Retinol-binding protein 4 OS=Homo sapiens GN=RBP4 PE=1 SV=3 |

|  | **Query** | **Observed** | **Mr(expt)** | **Mr(calc)** | **Delta** | **Miss** | **Score** | **Expect** | **Rank** | **Peptide** |
| --- | --- | --- | --- | --- | --- | --- | --- | --- | --- | --- |
|  | 9087 | 652.3692 | 1302.7238 | 1302.6139 | 0.1099 | 0 | 7 | 31 | 6 | R.LIVHNGYCDGR.S |
|  | 14468 | 542.5949 | 1624.7629 | 1624.9453 | -0.1824 | 0 | 4 | 44 | 9 | K.WVWALLLLAALGSGR.A |
|  | 19976 | **1032.9838** | **2063.9530** | **2063.9623** | **-0.0093** | **0** | **37** | **0.018** | **1** | **R.LLNLDGTCADSYSFVFSR.D** |
|  | 25442 | **1434.2028** | **2866.3910** | **2866.3266** | **0.0644** | **1** | **2** | **32** | **1** | **R.VRLLNNWDVCADMVGTFTDTEDPAK.F** |

| **687.** | Q5JTJ3\|CA031_HUMAN    **Mass:** 14449    **Score:** 37     **Queries matched:** 4   **emPAI:** 0.27 |
| --- | --- |
|  | Uncharacterized protein C1orf31 OS=Homo sapiens GN=C1orf31 PE=2 SV=1 |

|  | **Query** | **Observed** | **Mr(expt)** | **Mr(calc)** | **Delta** | **Miss** | **Score** | **Expect** | **Rank** | **Peptide** |
| --- | --- | --- | --- | --- | --- | --- | --- | --- | --- | --- |
|  | 4738 | **519.1361** | **1036.2577** | **1036.5665** | **-0.3089** | **0** | **3** | **55** | **1** | **M.GPGGPLLSPSR.G** |
|  | 13099 | **514.2953** | **1539.8640** | **1540.7045** | **-0.8405** | **0** | **3** | **64** | **6** | **K.FEAGQFEPSETTAK.S** |
|  | 13802 | **792.3561** | **1582.6976** | **1582.7086** | **-0.0110** | **0** | **37** | **0.024** | **1** | **R.SSFESSCPQQWIK.Y** |
|  | 28876 | 925.4144 | 3697.6286 | 3697.7804 | -0.1517 | 1 | 1 | 27 | 5 | K.TGWHSNRLLGDCGPHTPVSTALSFIAVGMAAPSMK.E |

| **688.** | P32119\|PRDX2_HUMAN    **Mass:** 22049    **Score:** 37     **Queries matched:** 5   **emPAI:** 0.17 |
| --- | --- |
|  | Peroxiredoxin-2 OS=Homo sapiens GN=PRDX2 PE=1 SV=5 |

|  | **Query** | **Observed** | **Mr(expt)** | **Mr(calc)** | **Delta** | **Miss** | **Score** | **Expect** | **Rank** | **Peptide** |
| --- | --- | --- | --- | --- | --- | --- | --- | --- | --- | --- |
|  | 6549 | 590.3057 | 1178.5968 | 1178.6295 | -0.0327 | 1 | 5 | 53 | 10 | R.RLSEDYGVLK.T |
|  | 17710 | **621.6907** | **1862.0502** | **1862.0625** | **-0.0123** | **1** | **4** | **38** | **1** | **R.KEGGLGPLNIPLLADVTR.R** 17705 |
|  | 18517 | 965.4977 | 1928.9809 | 1927.9527 | 1.0282 | 1 | 37 | 0.021 | 2 | R.LSEDYGVLKTDEGIAYR.G |
|  | 18541 | 644.5974 | 1930.7702 | 1931.0517 | -0.2814 | 1 | 1 | 66 | 10 | R.IGKPAPDFKATAVVDGAFK.E |

| **689.** | Q8ND61\|CC020_HUMAN    **Mass:** 102228   **Score:** 37     **Queries matched:** 10   **emPAI:** 0.04 |
| --- | --- |
|  | Uncharacterized protein C3orf20 OS=Homo sapiens GN=C3orf20 PE=2 SV=2 |

|  | **Query** | **Observed** | **Mr(expt)** | **Mr(calc)** | **Delta** | **Miss** | **Score** | **Expect** | **Rank** | **Peptide** |
| --- | --- | --- | --- | --- | --- | --- | --- | --- | --- | --- |
|  | 1271 | 772.4882 | 771.4809 | 771.4854 | -0.0045 | 1 | 37 | 0.045 | 1 | R.ALAEIKK.R |
|  | 4711 | 517.9327 | 1033.8509 | 1033.5590 | 0.2920 | 1 | 3 | 55 | 5 | K.MSRALAEIK.K |
|  | 12233 | **495.6020** | **1483.7841** | **1484.6752** | **-0.8910** | **0** | **2** | **75** | **3** | **K.EMAFNCLISTAGR.S** |
|  | 13422 | 520.6078 | 1558.8017 | 1558.8211 | -0.0194 | 0 | 2 | 76 | 5 | K.LLMICQNAGISVPK.G |
|  | 17371 | **613.2973** | **1836.8701** | **1835.9153** | **0.9548** | **0** | **2** | **73** | **3** | **K.FSVPNSVLSLEDSESVK.K** |
|  | 19998 | 689.3206 | 2064.9399 | 2065.0006 | -0.0607 | 1 | (1) | 62 | 3 | K.MKAMVESMSVGANPLDITR.R |
|  | 20123 | **521.0284** | **2080.0847** | **2080.9955** | **-0.9109** | **1** | **2** | **63** | **9** | **K.MKAMVESMSVGANPLDITR.R** |
|  | 20287 | **700.3498** | **2098.0275** | **2096.9904** | **1.0371** | **1** | **(0)** | **80** | **1** | **K.MKAMVESMSVGANPLDITR.R** |
|  | 21474 | 557.7803 | 2227.0922 | 2226.1653 | 0.9269 | 1 | 2 | 57 | 10 | K.NSVVQGMILMFAGGKLIFGGR.V |
|  | 29062 | **751.9688** | **3754.8077** | **3754.8600** | **-0.0523** | **1** | **2** | **23** | **5** | **R.GRTITCLFNDIPGFSLLALFNTEGQGCVHYNLK.T** |

| **690.** | Q8WUY1\|CH055_HUMAN    **Mass:** 24135    **Score:** 37     **Queries matched:** 4   **emPAI:** 0.16 |
| --- | --- |
|  | UPF0670 protein C8orf55 OS=Homo sapiens GN=C8orf55 PE=1 SV=2 |

|  | **Query** | **Observed** | **Mr(expt)** | **Mr(calc)** | **Delta** | **Miss** | **Score** | **Expect** | **Rank** | **Peptide** |
| --- | --- | --- | --- | --- | --- | --- | --- | --- | --- | --- |
|  | 1860 | **415.2661** | **828.5175** | **827.4687** | **1.0488** | **0** | **8** | **32** | **8** | **R.LPCAVLR.A** |
|  | 4297 | **501.7824** | **1001.5503** | **1001.5546** | **-0.0042** | **0** | **5** | **53** | **7** | **R.LLEPFEVR.T** |
|  | 9177 | **655.2960** | **1308.5775** | **1308.5867** | **-0.0093** | **1** | **37** | **0.03** | **1** | **R.MESGLSDVTKDQ.-** |
|  | 9505 | **442.9065** | **1325.6976** | **1324.5817** | **1.1159** | **1** | **(5)** | **46** | **1** | **R.MESGLSDVTKDQ.-** |

| **691.** | Q96N66\|MBOA7_HUMAN    **Mass:** 53415    **Score:** 36     **Queries matched:** 6   **emPAI:** 0.07 |
| --- | --- |
|  | Lysophospholipid acyltransferase 7 OS=Homo sapiens GN=MBOAT7 PE=1 SV=2 |

|  | **Query** | **Observed** | **Mr(expt)** | **Mr(calc)** | **Delta** | **Miss** | **Score** | **Expect** | **Rank** | **Peptide** |
| --- | --- | --- | --- | --- | --- | --- | --- | --- | --- | --- |
|  | 2245 | **428.2660** | **854.5175** | **855.3796** | **-0.8622** | **0** | **12** | **8.7** | **4** | **K.EMASGFSK.G** |
|  | 2533 | **437.2534** | **872.4923** | **871.3746** | **1.1178** | **0** | **(10)** | **21** | **8** | **K.EMASGFSK.G** |
|  | 13543 | **523.2820** | **1566.8243** | **1565.8105** | **1.0138** | **0** | **5** | **36** | **9** | **R.LFYMIPVFFAFR.M** |
|  | 15313 | **840.4017** | **1678.7888** | **1678.7985** | **-0.0097** | **0** | **36** | **0.025** | **1** | **R.AGGGPTLQCPPPSSPEK.A** |
|  | 24346 | **888.4570** | **2662.3493** | **2662.2712** | **0.0780** | **0** | **1** | **49** | **6** | **R.FYVAWIAAECGCIAAGFGAYPVAAK.A** |
|  | 25387 | **571.1212** | **2850.5697** | **2849.6108** | **0.9589** | **1** | **9** | **5.6** | **1** | **R.RAWPAPLFGLLFLLSSHLFPLEAVR.E** |

| **692.** | Q13510\|ASAH1_HUMAN    **Mass:** 45087    **Score:** 36     **Queries matched:** 9   **emPAI:** 0.17 |
| --- | --- |
|  | Acid ceramidase OS=Homo sapiens GN=ASAH1 PE=1 SV=5 |

|  | **Query** | **Observed** | **Mr(expt)** | **Mr(calc)** | **Delta** | **Miss** | **Score** | **Expect** | **Rank** | **Peptide** |
| --- | --- | --- | --- | --- | --- | --- | --- | --- | --- | --- |
|  | 3147 | **459.0344** | **916.0543** | **916.5355** | **-0.4812** | **1** | **10** | **14** | **2** | **K.KGHLIHGR.N** 3144 3152 |
|  | 7513 | 613.7462 | 1225.4779 | 1224.5775 | 0.9004 | 0 | 3 | 67 | 5 | K.STYPPSGPTYR.G |
|  | 7849 | 621.8735 | 1241.7324 | 1242.6179 | -0.8855 | 1 | 11 | 12 | 2 | K.RWHELMLDK.A |
|  | 8641 | 641.8185 | 1281.6224 | 1280.6136 | 1.0088 | 0 | 22 | 0.77 | 1 | K.ESLDVYELDAK.Q |
|  | 9798 | **672.3181** | **1342.6217** | **1342.6306** | **-0.0089** | **0** | **10** | **14** | **1** | **R.WYVVQTNYDR.W** |
|  | 10479 | **461.0072** | **1379.9999** | **1380.7224** | **-0.7225** | **1** | **1** | **84** | **9** | **K.KDVMWIGFLTR.T** |
|  | 14969 | 829.5090 | 1657.0035 | 1656.8049 | 0.1986 | 1 | 37 | 0.022 | 1 | R.WYVVQTNYDRWK.H |

| **693.** | Q15436\|SC23A_HUMAN    **Mass:** 87018    **Score:** 36     **Queries matched:** 20   **emPAI:** 0.04 |
| --- | --- |
|  | Protein transport protein Sec23A OS=Homo sapiens GN=SEC23A PE=1 SV=2 |

|  | **Query** | **Observed** | **Mr(expt)** | **Mr(calc)** | **Delta** | **Miss** | **Score** | **Expect** | **Rank** | **Peptide** |
| --- | --- | --- | --- | --- | --- | --- | --- | --- | --- | --- |
|  | 5019 | **530.3453** | **1058.6761** | **1057.6066** | **1.0695** | **1** | **5** | **52** | **7** | **R.QLIRLCQK.F** |
|  | 9555 | 664.7820 | 1327.5494 | 1327.6157 | -0.0663 | 1 | 8 | 20 | 9 | R.SWHDIDKDNAK.Y |
|  | 9646 | **667.8067** | **1333.5988** | **1332.6058** | **0.9930** | **0** | **36** | **0.033** | **1** | **R.YIDTEHGGSQAR.F** 9634 |
|  | 10854 | **468.2648** | **1401.7726** | **1400.8102** | **0.9624** | **0** | **9** | **16** | **3** | **R.MVVPVAALFTPLK.E** |
|  | 11005 | **706.8132** | **1411.6118** | **1411.7130** | **-0.1012** | **1** | **6** | **30** | **2** | **K.MGFGGTLEIKTSR.E** |
|  | 11601 | **724.3789** | **1446.7433** | **1446.7289** | **0.0143** | **0** | **3** | **74** | **7** | **R.AVLNPLCQVDYR.A** |
|  | 14081 | **801.3800** | **1600.7454** | **1601.7542** | **-1.0087** | **0** | **2** | **73** | **5** | **R.MVQVHELGCEGISK.S** 14108 |
|  | 14899 | 551.5927 | 1651.7563 | 1650.6620 | 1.0943 | 0 | 2 | 69 | 4 | K.SGYQDMPEYENFR.H |
|  | 15288 | 839.4249 | 1676.8353 | 1675.8814 | 0.9539 | 1 | 2 | 69 | 10 | K.DLSAKQLQEMLGLSK.V |
|  | 16649 | **594.3112** | **1779.9117** | **1778.7570** | **1.1547** | **1** | **2** | **61** | **10** | **R.KSGYQDMPEYENFR.H** |
|  | 18488 | 964.0410 | 1926.0675 | 1926.8968 | -0.8293 | 1 | 2 | 61 | 3 | K.DMHGQFKMGFGGTLEIK.T |
|  | 21693 | **1128.5677** | **2255.1209** | **2254.0875** | **1.0335** | **1** | **1** | **68** | **1** | **R.MVQVHELGCEGISKSYVFR.G** |
|  | 24747 | **683.3276** | **2729.2812** | **2730.3907** | **-1.1095** | **1** | **(0)** | **60** | **5** | **R.IMMFIGGPATQGPGMVVGDELKTPIR.S** |
|  | 24751 | **683.3589** | **2729.4065** | **2730.3907** | **-0.9843** | **1** | **(0)** | **62** | **4** | **R.IMMFIGGPATQGPGMVVGDELKTPIR.S** |
|  | 24758 | **683.5785** | **2730.2851** | **2730.3907** | **-0.1056** | **1** | **1** | **45** | **10** | **R.IMMFIGGPATQGPGMVVGDELKTPIR.S** 24754 |
|  | 29737 | **809.8400** | **4044.1635** | **4044.0556** | **0.1078** | **1** | **1** | **21** | **1** | **K.ESMQMSLSLLPPTALVGLITFGRMVQVHELGCEGISK.S** |
|  | 30056 | **835.0021** | **4169.9743** | **4169.9939** | **-0.0196** | **1** | **10** | **2.6** | **2** | **K.VNPSQTHNNMYAWGQESGAPILTDDVSLQVFMDHLKK.L** |

| **694.** | P09382\|LEG1_HUMAN    **Mass:** 15048    **Score:** 36     **Queries matched:** 3   **emPAI:** 0.26 |
| --- | --- |
|  | Galectin-1 OS=Homo sapiens GN=LGALS1 PE=1 SV=2 |

|  | **Query** | **Observed** | **Mr(expt)** | **Mr(calc)** | **Delta** | **Miss** | **Score** | **Expect** | **Rank** | **Peptide** |
| --- | --- | --- | --- | --- | --- | --- | --- | --- | --- | --- |
|  | 4788 | **521.2838** | **1040.5529** | **1040.5614** | **-0.0085** | **1** | **17** | **3.4** | **1** | **R.VRGEVAPDAK.S** 4791 |
|  | 14801 | **824.3759** | **1646.7373** | **1646.7471** | **-0.0098** | **0** | **36** | **0.028** | **1** | **R.FNAHGDANTIVCNSK.D** |

| **695.** | P54710\|ATNG_HUMAN    **Mass:** 7336     **Score:** 36     **Queries matched:** 9   **emPAI:** 0.57 |
| --- | --- |
|  | Sodium/potassium-transporting ATPase gamma chain OS=Homo sapiens GN=FXYD2 PE=1 SV=3 |

|  | **Query** | **Observed** | **Mr(expt)** | **Mr(calc)** | **Delta** | **Miss** | **Score** | **Expect** | **Rank** | **Peptide** |
| --- | --- | --- | --- | --- | --- | --- | --- | --- | --- | --- |
|  | 5649 | **554.2606** | **1106.5066** | **1105.5074** | **0.9992** | **0** | **36** | **0.039** | **1** | **M.TGLSMDGGGSPK.G** 5629 |
|  | 7760 | **619.8098** | **1237.6051** | **1236.5479** | **1.0572** | **0** | **(5)** | **42** | **2** | **-.MTGLSMDGGGSPK.G** 7735 |
|  | 8085 | 627.7909 | 1253.5673 | 1252.5428 | 1.0246 | 0 | (3) | 66 | 1 | -.MTGLSMDGGGSPK.G 8079 |
|  | 8412 | 635.2696 | 1268.5246 | 1268.5377 | -0.0131 | 0 | 5 | 39 | 9 | -.MTGLSMDGGGSPK.G 8433 |
|  | 20823 | 1078.9716 | 2155.9286 | 2156.3085 | -0.3799 | 0 | 2 | 51 | 6 | R.NGGLIFAGLAFIVGLLILLSR.R |

| **696.** | P62753\|RS6_HUMAN    **Mass:** 28834    **Score:** 36     **Queries matched:** 11   **emPAI:** 0.13 |
| --- | --- |
|  | 40S ribosomal protein S6 OS=Homo sapiens GN=RPS6 PE=1 SV=1 |

|  | **Query** | **Observed** | **Mr(expt)** | **Mr(calc)** | **Delta** | **Miss** | **Score** | **Expect** | **Rank** | **Peptide** |
| --- | --- | --- | --- | --- | --- | --- | --- | --- | --- | --- |
|  | 1099 | **665.3577** | **664.3505** | **663.3704** | **0.9800** | **0** | **22** | **1.2** | **1** | **R.QYVVR.K** |
|  | 2158 | 425.2429 | 848.4713 | 848.5120 | -0.0407 | 1 | 36 | 0.047 | 2 | R.KLFNLSK.E |
|  | 3043 | **456.2540** | **910.4934** | **909.4079** | **1.0855** | **0** | **1** | **1.2e+002** | **1** | **K.EEAAEYAK.L** |
|  | 5756 | 558.3104 | 1114.6062 | 1115.5822 | -0.9761 | 1 | 6 | 43 | 3 | K.LIEVDDERK.L |
|  | 6200 | **576.7761** | **1151.5376** | **1151.5458** | **-0.0082** | **1** | **12** | **9.2** | **1** | **K.NKEEAAEYAK.L** |
|  | 8300 | **632.8221** | **1263.6296** | **1263.6572** | **-0.0275** | **1** | **1** | **1.1e+002** | **8** | **K.GYVVRISGGNDK.Q** |
|  | 9671 | **445.8993** | **1334.6760** | **1334.6830** | **-0.0071** | **1** | **7** | **25** | **4** | **K.LFNLSKEDDVR.Q** |
|  | 9685 | **446.2929** | **1335.8568** | **1334.6653** | **1.1915** | **0** | **4** | **52** | **8** | **K.LNISFPATGCQK.L** 9674 |
|  | 13595 | **786.3859** | **1570.7572** | **1570.8038** | **-0.0467** | **1** | **7** | **25** | **1** | **K.QGFPMKQGVLTHGR.V** |
|  | 14627 | 818.4342 | 1634.8538 | 1635.7450 | -0.8912 | 0 | 2 | 73 | 4 | R.MATEVAADALGEEWK.G |

| **697.** | Q9Y2L6\|FRM4B_HUMAN    **Score:** 36     **Queries matched:** 12 |
| --- | --- |
|  | FERM domain-containing protein 4B OS=Homo sapiens GN=FRMD4B PE=1 SV=2 |

|  | **Query** | **Observed** | **Mr(expt)** | **Mr(calc)** | **Delta** | **Miss** | **Score** | **Expect** | **Rank** | **Peptide** |
| --- | --- | --- | --- | --- | --- | --- | --- | --- | --- | --- |
|  | 1138 | **688.3848** | **687.3776** | **686.4439** | **0.9337** | **1** | **12** | **17** | **2** | **K.IKGLTR.G** |
|  | 2158 | 425.2429 | 848.4713 | 848.4908 | -0.0196 | 1 | 36 | 0.047 | 2 | R.KLFQWK.Q |
|  | 3579 | **476.7288** | **951.4431** | **952.4726** | **-1.0296** | **0** | **8** | **25** | **2** | **R.SSSVPHSPR.I** 3589 |
|  | 6905 | **399.7247** | **1196.1524** | **1196.6302** | **-0.4778** | **1** | **4** | **39** | **10** | **K.KFAVEVHDPR.R** |
|  | 7437 | **408.2323** | **1221.6752** | **1221.7445** | **-0.0693** | **1** | **4** | **54** | **5** | **R.ILPPKSLGIER.I** |
|  | 8133 | **419.3140** | **1254.9202** | **1254.5476** | **0.3725** | **1** | **4** | **46** | **8** | **K.GDYTSDENARK.D** |
|  | 14302 | **539.2825** | **1614.8258** | **1615.8682** | **-1.0424** | **1** | **2** | **85** | **6** | **K.GIGQYDIQDKVKPR.K** |
|  | 16826 | 599.3721 | 1795.0946 | 1794.9740 | 0.1205 | 1 | 0 | 76 | 7 | K.EYPLNIGEKPPQVRR.R |
|  | 18052 | **630.9823** | **1889.9251** | **1889.9265** | **-0.0014** | **1** | **2** | **64** | **2** | **K.KQNVSTSNSGSMPNLAQK.D** |
|  | 21636 | 750.6721 | 2248.9944 | 2249.0706 | -0.0762 | 1 | 1 | 60 | 7 | K.QNVSTSNSGSMPNLAQKDSLR.N |
|  | 25343 | **569.2775** | **2841.3510** | **2842.4728** | **-1.1218** | **1** | **1** | **44** | **2** | **R.GQAVVQYMKIVEALPTYGVHYYAVK.D** |

| **698.** | Q9ULD0\|OGDHL_HUMAN    **Mass:** 115264   **Score:** 36     **Queries matched:** 8   **emPAI:** 0.03 |
| --- | --- |
|  | 2-oxoglutarate dehydrogenase E1 component-like, mitochondrial OS=Homo sapiens GN=OGDHL PE=2 SV=3 |

|  | **Query** | **Observed** | **Mr(expt)** | **Mr(calc)** | **Delta** | **Miss** | **Score** | **Expect** | **Rank** | **Peptide** |
| --- | --- | --- | --- | --- | --- | --- | --- | --- | --- | --- |
|  | 5434 | 546.7660 | 1091.5175 | 1091.5247 | -0.0072 | 0 | 36 | 0.039 | 1 | R.SSPYPTDVAR.V |
|  | 12376 | 498.5765 | 1492.7075 | 1491.8409 | 0.8666 | 0 | 6 | 31 | 2 | K.LVEDHLAVQSLIR.A |
|  | 20141 | **695.3234** | **2082.9483** | **2082.8849** | **0.0633** | **0** | **6** | **23** | **3** | **R.GHNEMDEPMFTQPLMYK.Q** |
|  | 20801 | **539.5265** | **2154.0769** | **2153.9800** | **0.0968** | **1** | **5** | **24** | **3** | **R.HPEAKSSFDQMVSGTSFQR.V** |
|  | 21443 | **742.4014** | **2224.1823** | **2222.9911** | **1.1912** | **1** | **3** | **43** | **5** | **R.RGHNEMDEPMFTQPLMYK.Q** |
|  | 21691 | **752.7014** | **2255.0822** | **2254.9809** | **0.1013** | **1** | **(0)** | **75** | **1** | **R.RGHNEMDEPMFTQPLMYK.Q** |
|  | 25325 | **1419.7101** | **2837.4056** | **2836.3861** | **1.0195** | **0** | **3** | **29** | **2** | **R.HNGIVLLLPHGMEGMGPEHSSARPER.F** |
|  | 27706 | 1680.7860 | 3359.5575 | 3358.5209 | 1.0365 | 0 | 1 | 30 | 8 | R.LENTYCQHIGLEFMFINDVEQCQWIR.Q |

| **699.** | Q9NP72\|RAB18_HUMAN    **Mass:** 23248    **Score:** 36     **Queries matched:** 5   **emPAI:** 0.16 |
| --- | --- |
|  | Ras-related protein Rab-18 OS=Homo sapiens GN=RAB18 PE=1 SV=1 |

|  | **Query** | **Observed** | **Mr(expt)** | **Mr(calc)** | **Delta** | **Miss** | **Score** | **Expect** | **Rank** | **Peptide** |
| --- | --- | --- | --- | --- | --- | --- | --- | --- | --- | --- |
|  | 5334 | **543.3289** | **1084.6433** | **1084.6492** | **-0.0059** | **0** | **22** | **1** | **2** | **K.ILIIGESGVGK.S** |
|  | 7995 | 625.3043 | 1248.5941 | 1248.6172 | -0.0231 | 0 | 3 | 74 | 8 | K.HSMLFIEASAK.T |
|  | 16345 | **878.9416** | **1755.8686** | **1755.8791** | **-0.0105** | **0** | **29** | **0.15** | **1** | **K.IIQTPGLWESENQNK.G** 16346 |
|  | 21614 | 562.2974 | 2245.1603 | 2246.2079 | -1.0476 | 1 | 6 | 22 | 2 | -.MDEDVLTTLKILIIGESGVGK.S |

| **700.** | Q9UBQ7\|GRHPR_HUMAN    **Mass:** 36045    **Score:** 36     **Queries matched:** 4   **emPAI:** 0.22 |
| --- | --- |
|  | Glyoxylate reductase/hydroxypyruvate reductase OS=Homo sapiens GN=GRHPR PE=1 SV=1 |

|  | **Query** | **Observed** | **Mr(expt)** | **Mr(calc)** | **Delta** | **Miss** | **Score** | **Expect** | **Rank** | **Peptide** |
| --- | --- | --- | --- | --- | --- | --- | --- | --- | --- | --- |
|  | 4605 | **514.2699** | **1026.5252** | **1026.5597** | **-0.0344** | **0** | **4** | **65** | **4** | **R.LPEAIEEVK.N** |
|  | 15353 | **561.3566** | **1681.0479** | **1680.9464** | **0.1015** | **1** | **3** | **41** | **4** | **R.LKPFGVQRFLYTGR.Q** |
|  | 16782 | 896.4377 | 1790.8609 | 1791.8639 | -1.0030 | 0 | 30 | 0.1 | 1 | R.GDVVNQDDLYQALASGK.I |
|  | 23962 | **863.0529** | **2586.1369** | **2586.1544** | **-0.0174** | **1** | **28** | **0.11** | **1** | **R.AADCEVEQWDSDEPIPAKELER.G** |

| **701.** | P16152\|CBR1_HUMAN    **Mass:** 30641    **Score:** 36     **Queries matched:** 4   **emPAI:** 0.26 |
| --- | --- |
|  | Carbonyl reductase [NADPH] 1 OS=Homo sapiens GN=CBR1 PE=1 SV=3 |

|  | **Query** | **Observed** | **Mr(expt)** | **Mr(calc)** | **Delta** | **Miss** | **Score** | **Expect** | **Rank** | **Peptide** |
| --- | --- | --- | --- | --- | --- | --- | --- | --- | --- | --- |
|  | 11769 | 729.8665 | 1457.7185 | 1457.7272 | -0.0087 | 0 | 32 | 0.091 | 1 | K.ILLNACCPGWVR.T |
|  | 12797 | 760.3931 | 1518.7716 | 1517.8599 | 0.9116 | 1 | 2 | 84 | 4 | R.VVNVSSIMSVRALK.S |
|  | 14052 | **533.9396** | **1598.7969** | **1598.8053** | **-0.0084** | **0** | **31** | **0.088** | **1** | **R.FHQLDIDDLQSIR.A** |
|  | 14818 | **550.2242** | **1647.6509** | **1647.8944** | **-0.2435** | **1** | **4** | **44** | **8** | **R.LFSGDVVLTARDVTR.G** |

| **702.** | O95169\|NDUB8_HUMAN    **Mass:** 21865    **Score:** 36     **Queries matched:** 17   **emPAI:** 0.38 |
| --- | --- |
|  | NADH dehydrogenase [ubiquinone] 1 beta subcomplex subunit 8, mitochondrial OS=Homo sapiens GN=NDUFB8 PE=1 SV=1 |

|  | **Query** | **Observed** | **Mr(expt)** | **Mr(calc)** | **Delta** | **Miss** | **Score** | **Expect** | **Rank** | **Peptide** |
| --- | --- | --- | --- | --- | --- | --- | --- | --- | --- | --- |
|  | 289 | **430.8875** | **429.8802** | **430.2540** | **-0.3738** | **0** | **4** | **84** | **1** | **R.AAAAK.K** 269 |
|  | 801 | **548.2583** | **547.2510** | **546.2948** | **0.9562** | **0** | **16** | **7.5** | **7** | **-.MAVAR.A** |
|  | 2539 | **437.5658** | **873.1170** | **872.4538** | **0.6632** | **0** | **10** | **13** | **9** | **R.NVMPLGAR.T** |
|  | 5245 | 540.2496 | 1078.4846 | 1078.4906 | -0.0060 | 0 | 9 | 22 | 1 | K.DMFPGPYPR.T 5253 |
|  | 5484 | **548.3817** | **1094.7489** | **1094.4855** | **0.2633** | **0** | **(1)** | **1.1e+002** | **3** | **K.DMFPGPYPR.T** |
|  | 12008 | 736.8578 | 1471.7010 | 1471.7095 | -0.0085 | 0 | 12 | 8.2 | 1 | K.QYPYNNLYLER.G 12011 |
|  | 14558 | **544.3167** | **1629.9283** | **1628.8127** | **1.1156** | **1** | **0** | **1.2e+002** | **6** | **R.NVMPLGARTASHMTK.D** |
|  | 16307 | **585.2944** | **1752.8613** | **1753.9774** | **-1.1161** | **1** | **8** | **17** | **1** | **-.MAVARAGVLGVQWLQR.A** |
|  | 16523 | 590.9224 | 1769.7454 | 1769.9723 | -0.2269 | 1 | (6) | 27 | 3 | -.MAVARAGVLGVQWLQR.A 16528 |
|  | 17584 | 617.9621 | 1850.8645 | 1850.8444 | 0.0201 | 1 | 3 | 55 | 4 | R.TASHMTKDMFPGPYPR.T |
|  | 20284 | **700.3138** | **2097.9197** | **2097.9301** | **-0.0105** | **0** | **15** | **2.6** | **1** | **R.LNWGEPMHWHLDMYNR.N** |
|  | 20826 | **719.6636** | **2155.9691** | **2155.9824** | **-0.0133** | **1** | **36** | **0.022** | **1** | **R.SQHERDPWYSWDQPGLR.L** |
|  | 21297 | **1105.5933** | **2209.1720** | **2208.8834** | **0.2886** | **0** | **11** | **6.2** | **1** | **R.VEDYEPYPDDGMGYGDYPK.L** |

| **703.** | P06703\|S10A6_HUMAN    **Mass:** 10230    **Score:** 36     **Queries matched:** 5   **emPAI:** 0.39 |
| --- | --- |
|  | Protein S100-A6 OS=Homo sapiens GN=S100A6 PE=1 SV=1 |

|  | **Query** | **Observed** | **Mr(expt)** | **Mr(calc)** | **Delta** | **Miss** | **Score** | **Expect** | **Rank** | **Peptide** |
| --- | --- | --- | --- | --- | --- | --- | --- | --- | --- | --- |
|  | 3053 | 456.7410 | 911.4674 | 910.4144 | 1.0530 | 1 | 5 | 44 | 9 | K.YSGREGDK.H 3037 3051 |
|  | 3106 | 458.2457 | 914.4769 | 914.4821 | -0.0052 | 0 | 36 | 0.047 | 1 | K.LQDAEIAR.L 3121 |

| **704.** | Q9H0C2\|ADT4_HUMAN    **Mass:** 35285    **Score:** 36     **Queries matched:** 12   **emPAI:** 0.11 |
| --- | --- |
|  | ADP/ATP translocase 4 OS=Homo sapiens GN=SLC25A31 PE=1 SV=1 |

|  | **Query** | **Observed** | **Mr(expt)** | **Mr(calc)** | **Delta** | **Miss** | **Score** | **Expect** | **Rank** | **Peptide** |
| --- | --- | --- | --- | --- | --- | --- | --- | --- | --- | --- |
|  | 2254 | 428.7456 | 855.4767 | 855.4814 | -0.0047 | 0 | 36 | 0.043 | 1 | K.TAVAPIER.V 2274 |
|  | 2255 | 428.7509 | 855.4873 | 855.4926 | -0.0053 | 0 | 31 | 0.12 | 1 | R.GNLANVIR.Y |
|  | 4427 | **507.2642** | **1012.5138** | **1011.4187** | **1.0951** | **0** | **3** | **83** | **4** | **R.MMMQSGEAK.R** |
|  | 5311 | 542.5944 | 1083.1743 | 1082.6448 | 0.5295 | 1 | 9 | 11 | 3 | K.TAVAPIERVK.L |
|  | 6636 | 395.5236 | 1183.5491 | 1183.5148 | 0.0344 | 1 | 7 | 33 | 8 | R.MMMQSGEAKR.Q |
|  | 7338 | **609.3204** | **1216.6263** | **1215.5046** | **1.1217** | **1** | **4** | **65** | **5** | **R.RMMMQSGEAK.R** |
|  | 7412 | 611.3063 | 1220.5980 | 1220.5713 | 0.0266 | 0 | 4 | 61 | 9 | R.ASYFGAYDTVK.G |
|  | 9296 | **439.2363** | **1314.6872** | **1313.6802** | **1.0070** | **1** | **2** | **84** | **6** | **K.YKQLFMSGVNK.E** |
|  | 11305 | 715.8451 | 1429.6757 | 1430.7670 | -1.0913 | 1 | 12 | 8.8 | 3 | K.IKEFFHIDIGGR.- 11306 |
|  | 13215 | **516.2967** | **1545.8684** | **1545.9130** | **-0.0446** | **1** | **0** | **1.2e+002** | **10** | **R.GTGGALVLVLYDKIK.E** |

| **705.** | Q9Y3D6\|FIS1_HUMAN    **Mass:** 16984    **Score:** 36     **Queries matched:** 7   **emPAI:** 0.23 |
| --- | --- |
|  | Mitochondrial fission 1 protein OS=Homo sapiens GN=FIS1 PE=1 SV=2 |

|  | **Query** | **Observed** | **Mr(expt)** | **Mr(calc)** | **Delta** | **Miss** | **Score** | **Expect** | **Rank** | **Peptide** |
| --- | --- | --- | --- | --- | --- | --- | --- | --- | --- | --- |
|  | 1911 | **417.2430** | **832.4715** | **832.4039** | **0.0676** | **1** | **11** | **18** | **4** | **K.GSKEEQR.D** |
|  | 7796 | **620.3389** | **1238.6633** | **1237.6302** | **1.0331** | **1** | **12** | **8.2** | **1** | **K.FQSEKAAGSVSK.S** |
|  | 11470 | **720.8719** | **1439.7292** | **1439.7368** | **-0.0076** | **0** | **36** | **0.036** | **1** | **R.GLLQTEPQNNQAK.E** |
|  | 15150 | **557.2925** | **1668.8558** | **1669.9138** | **-1.0580** | **0** | **7** | **23** | **2** | **M.EAVLNELVSVEDLLK.F** |
|  | 16883 | 601.3001 | 1800.8785 | 1800.9543 | -0.0758 | 0 | 4 | 44 | 2 | -.MEAVLNELVSVEDLLK.F |
|  | 17076 | 908.9162 | 1815.8178 | 1816.9492 | -1.1313 | 0 | (1) | 84 | 7 | -.MEAVLNELVSVEDLLK.F |
|  | 25256 | 942.4841 | 2824.4306 | 2824.5918 | -0.1613 | 1 | 1 | 43 | 4 | K.DGLVGMAIVGGMALGVAGLAGLIGLAVSKSK.S |

| **706.** | Q9Y320\|TMX2_HUMAN    **Mass:** 34358    **Score:** 35     **Queries matched:** 4   **emPAI:** 0.11 |
| --- | --- |
|  | Thioredoxin-related transmembrane protein 2 OS=Homo sapiens GN=TMX2 PE=1 SV=1 |

|  | **Query** | **Observed** | **Mr(expt)** | **Mr(calc)** | **Delta** | **Miss** | **Score** | **Expect** | **Rank** | **Peptide** |
| --- | --- | --- | --- | --- | --- | --- | --- | --- | --- | --- |
|  | 9500 | 663.8526 | 1325.6906 | 1324.6411 | 1.0495 | 0 | 35 | 0.039 | 1 | R.EFNLNELYQR.A |
|  | 10216 | 456.2520 | 1365.7340 | 1366.6841 | -0.9501 | 1 | 8 | 20 | 2 | K.VDVGRYTDVSTR.Y |
|  | 13756 | **527.6041** | **1579.7904** | **1580.9654** | **-1.1750** | **0** | **4** | **52** | **8** | **M.AVLAPLIALVYSVPR.L** |
|  | 16724 | **596.3113** | **1785.9120** | **1784.9126** | **0.9994** | **0** | **7** | **23** | **6** | **R.EVEILMFLSAIVMMK.N** |

| **707.** | P50053\|KHK_HUMAN    **Mass:** 33109    **Score:** 35     **Queries matched:** 7   **emPAI:** 0.11 |
| --- | --- |
|  | Ketohexokinase OS=Homo sapiens GN=KHK PE=1 SV=1 |

|  | **Query** | **Observed** | **Mr(expt)** | **Mr(calc)** | **Delta** | **Miss** | **Score** | **Expect** | **Rank** | **Peptide** |
| --- | --- | --- | --- | --- | --- | --- | --- | --- | --- | --- |
|  | 2195 | 426.2688 | 850.5231 | 849.4596 | 1.0635 | 0 | 3 | 82 | 6 | K.VDLTQFK.W |
|  | 9182 | 437.2142 | 1308.6207 | 1307.6245 | 0.9962 | 0 | 1 | 1.1e+002 | 9 | R.SLPDVSATDFEK.V |
|  | 9215 | **437.7630** | **1310.2673** | **1310.7670** | **-0.4998** | **1** | **6** | **24** | **5** | **K.IRVSVEVEKPR.E** |
|  | 12983 | **767.3870** | **1532.7594** | **1532.7695** | **-0.0102** | **1** | **35** | **0.035** | **1** | **R.IDAHNTRQPPEQK.I** 13000 |
|  | 21517 | 558.8202 | 2231.2517 | 2232.0950 | -0.8434 | 1 | 2 | 46 | 3 | R.TILYYDRSLPDVSATDFEK.V |
|  | 29511 | 1313.6175 | 3937.8308 | 3938.8060 | -0.9752 | 0 | 5 | 8.7 | 2 | R.GGNASNSCTVLSLLGAPCAFMGSMAPGHVADFVLDDLR.R |

| **708.** | P01011\|AACT_HUMAN    **Mass:** 47792    **Score:** 35     **Queries matched:** 12   **emPAI:** 0.08 |
| --- | --- |
|  | Alpha-1-antichymotrypsin OS=Homo sapiens GN=SERPINA3 PE=1 SV=2 |

|  | **Query** | **Observed** | **Mr(expt)** | **Mr(calc)** | **Delta** | **Miss** | **Score** | **Expect** | **Rank** | **Peptide** |
| --- | --- | --- | --- | --- | --- | --- | --- | --- | --- | --- |
|  | 1025 | **633.1579** | **632.1506** | **631.3402** | **0.8105** | **1** | **0** | **2.3e+002** | **2** | **K.NGTRGK.I** |
|  | 5023 | 530.7550 | 1059.4954 | 1060.5804 | -1.0850 | 0 | 8 | 27 | 4 | R.EIGELYLPK.F 5030 |
|  | 5464 | **547.8162** | **1093.6179** | **1093.6244** | **-0.0065** | **0** | **35** | **0.043** | **1** | **R.NLAVSQVVHK.A** |
|  | 7294 | **608.3649** | **1214.7152** | **1214.7234** | **-0.0082** | **0** | **2** | **1e+002** | **2** | **K.ITLLSALVETR.T** |
|  | 14840 | **550.3234** | **1647.9485** | **1647.8099** | **0.1386** | **0** | **5** | **39** | **8** | **K.MEEVEAMLLPETLK.R** |
|  | 15397 | **562.6341** | **1684.8804** | **1684.0247** | **0.8558** | **1** | **9** | **15** | **3** | **K.ITLLSALVETRTIVR.F** |
|  | 20266 | 699.6741 | 2096.0006 | 2095.0006 | 1.0000 | 0 | 4 | 40 | 8 | K.DLDSQTMMVLVNYIFFK.A |
|  | 21888 | 760.0505 | 2277.1298 | 2278.1378 | -1.0080 | 1 | 4 | 29 | 7 | K.DLDSQTMMVLVNYIFFKAK.W |
|  | 22672 | **795.8720** | **2384.5942** | **2385.2312** | **-0.6370** | **1** | **4** | **21** | **1** | **K.KWVMVPMMSLHHLTIPYFR.D** 22685 |
|  | 24409 | **669.5156** | **2674.0334** | **2674.3651** | **-0.3317** | **0** | **1** | **39** | **3** | **R.FNRPFLMIIVPTDTQNIFFMSK.V** |

| **709.** | P21964\|COMT_HUMAN    **Mass:** 30474    **Score:** 35     **Queries matched:** 9   **emPAI:** 0.12 |
| --- | --- |
|  | Catechol O-methyltransferase OS=Homo sapiens GN=COMT PE=1 SV=2 |

|  | **Query** | **Observed** | **Mr(expt)** | **Mr(calc)** | **Delta** | **Miss** | **Score** | **Expect** | **Rank** | **Peptide** |
| --- | --- | --- | --- | --- | --- | --- | --- | --- | --- | --- |
|  | 5353 | **543.9442** | **1085.8739** | **1086.5968** | **-0.7229** | **1** | **4** | **45** | **2** | **R.MARLLSPGAR.L** 5348 |
|  | 5679 | 555.2794 | 1108.5442 | 1108.5587 | -0.0145 | 1 | 13 | 7.7 | 3 | R.MVDFAGVKDK.V 5680 |
|  | 6123 | **573.4061** | **1144.7976** | **1145.5717** | **-0.7741** | **1** | **3** | **70** | **2** | **K.AIYKGPGSEAGP.-** |
|  | 15340 | **561.2531** | **1680.7373** | **1679.9822** | **0.7551** | **0** | **0** | **1e+002** | **7** | **K.VTLVVGASQDIIPQLK.K** |
|  | 17232 | 914.4811 | 1826.9476 | 1826.9560 | -0.0084 | 0 | 35 | 0.03 | 1 | R.LITIEINPDCAAITQR.M |
|  | 18117 | **633.1570** | **1896.4493** | **1895.8764** | **0.5729** | **0** | **3** | **38** | **1** | **K.YDVDTLDMVFLDHWK.D** |
|  | 18435 | **962.4426** | **1922.8707** | **1923.1041** | **-0.2334** | **1** | **1** | **75** | **5** | **K.DKVTLVVGASQDIIPQLK.K** |

| **710.** | O75954\|TSN9_HUMAN    **Mass:** 27559    **Score:** 35     **Queries matched:** 4   **emPAI:** 0.14 |
| --- | --- |
|  | Tetraspanin-9 OS=Homo sapiens GN=TSPAN9 PE=1 SV=1 |

|  | **Query** | **Observed** | **Mr(expt)** | **Mr(calc)** | **Delta** | **Miss** | **Score** | **Expect** | **Rank** | **Peptide** |
| --- | --- | --- | --- | --- | --- | --- | --- | --- | --- | --- |
|  | 10375 | **459.0125** | **1374.0155** | **1373.4581** | **0.5574** | **0** | **7** | **21** | **6** | **R.CCMENSQGCGR.N** 10374 |
|  | 15606 | 850.9415 | 1699.8685 | 1698.8941 | 0.9745 | 0 | 35 | 0.033 | 1 | K.EGLLLYHTENNVGLK.N |
|  | 19887 | **686.4406** | **2056.2998** | **2055.1000** | **1.1998** | **1** | **2** | **37** | **3** | **K.DLKEGLLLYHTENNVGLK.N** |

| **711.** | Q7Z4W1\|DCXR_HUMAN    **Mass:** 26182    **Score:** 35     **Queries matched:** 14   **emPAI:** 0.15 |
| --- | --- |
|  | L-xylulose reductase OS=Homo sapiens GN=DCXR PE=1 SV=2 |

|  | **Query** | **Observed** | **Mr(expt)** | **Mr(calc)** | **Delta** | **Miss** | **Score** | **Expect** | **Rank** | **Peptide** |
| --- | --- | --- | --- | --- | --- | --- | --- | --- | --- | --- |
|  | 737 | **528.2407** | **527.2334** | **526.3479** | **0.8855** | **0** | **12** | **9.2** | **2** | **R.IPLGK.F** |
|  | 1572 | **403.7399** | **805.4653** | **804.4494** | **1.0159** | **0** | **7** | **35** | **8** | **M.ELFLAGR.R** |
|  | 2157 | **425.2267** | **848.4387** | **848.4538** | **-0.0150** | **1** | **8** | **29** | **2** | **K.AKTMLNR.I** |
|  | 2378 | **432.7480** | **863.4815** | **863.4501** | **0.0314** | **0** | **11** | **16** | **9** | **R.SFEVNLR.A** |
|  | 2398 | 865.3554 | 864.3481 | 863.4422 | 0.9058 | 0 | 14 | 7 | 1 | K.GALDMLTK.V |
|  | 3586 | **477.1944** | **952.3743** | **951.4848** | **0.8895** | **0** | **3** | **63** | **9** | **-.MELFLAGR.R** |
|  | 5418 | 546.2847 | 1090.5548 | 1091.5910 | -1.0362 | 1 | 4 | 67 | 10 | -.MELFLAGRR.V |
|  | 5795 | 559.3242 | 1116.6339 | 1116.5775 | 0.0564 | 0 | 10 | 18 | 8 | R.TQADLDSLVR.E 5793 |
|  | 6581 | **591.3187** | **1180.6228** | **1180.6313** | **-0.0085** | **0** | **35** | **0.045** | **1** | **R.GTVQALHATGAR.V** |
|  | 6622 | **395.2466** | **1182.7180** | **1182.7085** | **0.0096** | **0** | **8** | **22** | **6** | **R.AVIQVSQIVAR.G** |
|  | 12185 | 741.8507 | 1481.6869 | 1481.7263 | -0.0394 | 1 | 2 | 74 | 6 | K.EAFDRSFEVNLR.A |
|  | 20928 | 1085.0455 | 2168.0765 | 2168.1484 | -0.0719 | 1 | 1 | 73 | 4 | R.GLIARGVPGAIVNVSSQCSQR.A |
|  | 23425 | 834.7603 | 2501.2591 | 2501.1679 | 0.0912 | 0 | 5 | 22 | 3 | R.VNAVNPTVVMTSMGQATWSDPHK.A |

| **712.** | Q9H0J4\|QRIC2_HUMAN    **Mass:** 181228   **Score:** 35     **Queries matched:** 25   **emPAI:** 0.02 |
| --- | --- |
|  | Glutamine-rich protein 2 OS=Homo sapiens GN=QRICH2 PE=1 SV=1 |

|  | **Query** | **Observed** | **Mr(expt)** | **Mr(calc)** | **Delta** | **Miss** | **Score** | **Expect** | **Rank** | **Peptide** |
| --- | --- | --- | --- | --- | --- | --- | --- | --- | --- | --- |
|  | 3344 | 466.7404 | 931.4663 | 931.5451 | -0.0788 | 1 | 35 | 0.058 | 2 | K.SALATKVSR.V 3343 |
|  | 3597 | 477.7235 | 953.4325 | 952.5164 | 0.9161 | 0 | 2 | 86 | 9 | R.MLPPSVPGR.D |
|  | 4562 | 512.7553 | 1023.4959 | 1023.5825 | -0.0866 | 0 | 4 | 59 | 6 | R.GLVQPGAVQR.G |
|  | 5132 | 535.2617 | 1068.5089 | 1067.5724 | 0.9365 | 0 | 5 | 45 | 8 | R.GLVQPGVDQR.G |
|  | 5494 | 548.8254 | 1095.6362 | 1095.6400 | -0.0038 | 0 | 4 | 53 | 8 | R.SLVQPGIVQR.G |
|  | 6688 | **594.2336** | **1186.4526** | **1186.6492** | **-0.1966** | **1** | **4** | **57** | **5** | **R.MDTRLPGILR.K** 6689 |
|  | 6870 | **399.2328** | **1194.6765** | **1194.5009** | **0.1756** | **0** | **8** | **24** | **6** | **R.SMEPLGMDQR.G** |
|  | 7120 | 604.2720 | 1206.5295 | 1205.6842 | 0.8453 | 0 | 2 | 92 | 9 | K.IQFLLAQMVK.R |
|  | 7833 | 621.3565 | 1240.6983 | 1241.5962 | -0.8978 | 0 | 2 | 80 | 3 | R.EHLEMEIDVK.A |
|  | 9182 | 437.2142 | 1308.6207 | 1309.6851 | -1.0644 | 1 | 1 | 1.1e+002 | 8 | K.VPSGTGRQQQPR.A |
|  | 10673 | 464.9245 | 1391.7518 | 1390.7205 | 1.0313 | 1 | 19 | 1.8 | 1 | K.DREQFLELVSR.K 10674 |
|  | 11264 | 714.8367 | 1427.6588 | 1428.6555 | -0.9967 | 0 | 5 | 43 | 10 | R.ASQAGSETLMGFSK.H |
|  | 11627 | 483.9344 | 1448.7814 | 1448.7082 | 0.0732 | 0 | 5 | 42 | 6 | R.GLVQPGMDQYGLR.Q |
|  | 12372 | 747.3445 | 1492.6745 | 1493.7218 | -1.0472 | 1 | 5 | 42 | 4 | K.MLDRLLTEMDNK.L |
|  | 13526 | 522.9525 | 1565.8355 | 1566.8617 | -1.0262 | 1 | 2 | 76 | 9 | R.LELDPVKQLLEDR.W |
|  | 16052 | **578.6285** | **1732.8638** | **1732.8202** | **0.0436** | **0** | **3** | **56** | **3** | **R.ERPPLYQADEAAAMR.R** |
|  | 16502 | 590.3298 | 1767.9677 | 1766.7716 | 1.1961 | 1 | 2 | 59 | 10 | K.MSGQEQDWQKMLDR.L |
|  | 16736 | **596.6581** | **1786.9524** | **1786.9914** | **-0.0390** | **1** | **1** | **77** | **10** | **R.RNARPGPVQQDLPLAR.D** |
|  | 22942 | **607.8166** | **2427.2375** | **2426.1565** | **1.0810** | **0** | **(0)** | **67** | **3** | **R.MIIESMLTSSSTLLSMSMAPHK.A** |
|  | 23050 | 1222.6613 | 2443.3079 | 2442.1514 | 1.1565 | 0 | 3 | 35 | 4 | R.MIIESMLTSSSTLLSMSMAPHK.A |
|  | 28513 | **896.7056** | **3582.7934** | **3581.6225** | **1.1709** | **1** | **2** | **25** | **1** | **R.VQFDATTEQLNHMMQELVAKMSGQEQDWQK.M** |
|  | 30314 | **1082.7380** | **4326.9231** | **4328.1028** | **-1.1798** | **1** | **0** | **20** | **1** | **R.RGLEPTGMNQPGLVPASTYPHGVVPLSMGQLGVPPPEMDDR.E** |

| **713.** | Q9H299\|SH3L3_HUMAN    **Mass:** 10488    **Score:** 35     **Queries matched:** 6   **emPAI:** 0.38 |
| --- | --- |
|  | SH3 domain-binding glutamic acid-rich-like protein 3 OS=Homo sapiens GN=SH3BGRL3 PE=1 SV=1 |

|  | **Query** | **Observed** | **Mr(expt)** | **Mr(calc)** | **Delta** | **Miss** | **Score** | **Expect** | **Rank** | **Peptide** |
| --- | --- | --- | --- | --- | --- | --- | --- | --- | --- | --- |
|  | 4974 | **529.2634** | **1056.5122** | **1055.5248** | **0.9874** | **0** | **13** | **8.8** | **1** | **R.VYSTSVTGSR.E** 4968 |
|  | 7006 | **401.2324** | **1200.6755** | **1200.5921** | **0.0834** | **1** | **9** | **19** | **4** | **R.DEMRALAGNPK.A** |
|  | 7332 | **609.3069** | **1216.5992** | **1216.5870** | **0.0122** | **1** | **(3)** | **84** | **2** | **R.DEMRALAGNPK.A** |
|  | 22088 | **769.7137** | **2306.1192** | **2306.1325** | **-0.0133** | **1** | **35** | **0.024** | **1** | **R.IQYQLVDISQDNALRDEMR.A** 22086 |

| **714.** | P00738\|HPT_HUMAN    **Mass:** 45861    **Score:** 35     **Queries matched:** 6   **emPAI:** 0.08 |
| --- | --- |
|  | Haptoglobin OS=Homo sapiens GN=HP PE=1 SV=1 |

|  | **Query** | **Observed** | **Mr(expt)** | **Mr(calc)** | **Delta** | **Miss** | **Score** | **Expect** | **Rank** | **Peptide** |
| --- | --- | --- | --- | --- | --- | --- | --- | --- | --- | --- |
|  | 6131 | **573.7709** | **1145.5272** | **1145.5353** | **-0.0081** | **0** | **35** | **0.051** | **1** | **R.HYEGSTVPEK.K** |
|  | 7046 | 602.3179 | 1202.6213 | 1202.6295 | -0.0082 | 0 | 14 | 7 | 1 | K.VTSIQDWVQK.T |
|  | 8509 | **425.5482** | **1273.6227** | **1273.6302** | **-0.0076** | **1** | **18** | **2.2** | **1** | **R.HYEGSTVPEKK.T** 8508 8510 |
|  | 21120 | **730.4200** | **2188.2383** | **2187.0453** | **1.1930** | **0** | **5** | **26** | **1** | **K.SPVGVQPILNEHTFCAGMSK.Y** |

| **715.** | P07305\|H10_HUMAN    **Mass:** 20850    **Score:** 35     **Queries matched:** 9   **emPAI:** 0.40 |
| --- | --- |
|  | Histone H1.0 OS=Homo sapiens GN=H1F0 PE=1 SV=3 |

|  | **Query** | **Observed** | **Mr(expt)** | **Mr(calc)** | **Delta** | **Miss** | **Score** | **Expect** | **Rank** | **Peptide** |
| --- | --- | --- | --- | --- | --- | --- | --- | --- | --- | --- |
|  | 1966 | **419.2119** | **836.4093** | **836.4141** | **-0.0047** | **0** | **23** | **0.92** | **1** | **K.GVGASGSFR.L** 1969 1984 |
|  | 2457 | **435.1798** | **868.3451** | **867.5542** | **0.7910** | **1** | **5** | **40** | **4** | **K.KPKATPVK.K** |
|  | 6867 | **399.2325** | **1194.6757** | **1193.6153** | **1.0604** | **1** | **10** | **16** | **3** | **K.QTKGVGASGSFR.L** |
|  | 11452 | **720.3300** | **1438.6455** | **1437.7173** | **0.9282** | **0** | **20** | **1.2** | **1** | **K.YSDMIVAAIQAEK.N** |
|  | 13655 | 525.9266 | 1574.7579 | 1574.7689 | -0.0110 | 1 | 33 | 0.058 | 1 | K.SHYKVGENADSQIK.L |
|  | 14110 | **535.2823** | **1602.8252** | **1603.7988** | **-0.9735** | **1** | **4** | **48** | **4** | **-.MTENSTSAPAAKPKR.A** 14131 |

| **716.** | O60361\|NDK8_HUMAN    **Mass:** 15690    **Score:** 35     **Queries matched:** 5   **emPAI:** 0.25 |
| --- | --- |
|  | Putative nucleoside diphosphate kinase OS=Homo sapiens GN=NME2P1 PE=5 SV=1 |

|  | **Query** | **Observed** | **Mr(expt)** | **Mr(calc)** | **Delta** | **Miss** | **Score** | **Expect** | **Rank** | **Peptide** |
| --- | --- | --- | --- | --- | --- | --- | --- | --- | --- | --- |
|  | 3145 | **459.0125** | **916.0103** | **915.4814** | **0.5289** | **0** | **4** | **50** | **7** | **K.QHYIDLK.D** |
|  | 4925 | **526.7515** | **1051.4885** | **1050.4917** | **0.9968** | **0** | **35** | **0.048** | **1** | **R.GDFCIQVGR.N** |
|  | 5135 | **535.2823** | **1068.5501** | **1068.5564** | **-0.0062** | **0** | **14** | **6.8** | **1** | **R.NIIHGSDSVK.S** |
|  | 8285 | **632.3311** | **1262.6475** | **1261.6886** | **0.9589** | **1** | **4** | **53** | **1** | **-.MQCGLVGKIIK.R** |
|  | 16868 | **600.9431** | **1799.8075** | **1800.9040** | **-1.0965** | **0** | **8** | **16** | **1** | **R.VMLGETNPADSKPGTIR.G** |

|  | **Proteins matching a subset of these peptides:** |
| --- | --- |

|  | P22392\|NDKB_HUMAN    **Mass:** 17401    **Score:** 35     **Queries matched:** 4 |
| --- | --- |
|  | Nucleoside diphosphate kinase B OS=Homo sapiens GN=NME2 PE=1 SV=1 |

| **717.** | P15531\|NDKA_HUMAN    **Mass:** 17309    **Score:** 35     **Queries matched:** 4   **emPAI:** 0.22 |
| --- | --- |
|  | Nucleoside diphosphate kinase A OS=Homo sapiens GN=NME1 PE=1 SV=1 |

|  | **Query** | **Observed** | **Mr(expt)** | **Mr(calc)** | **Delta** | **Miss** | **Score** | **Expect** | **Rank** | **Peptide** |
| --- | --- | --- | --- | --- | --- | --- | --- | --- | --- | --- |
|  | 4925 | 526.7515 | 1051.4885 | 1050.4917 | 0.9968 | 0 | 35 | 0.048 | 1 | R.GDFCIQVGR.N |
|  | 16868 | 600.9431 | 1799.8075 | 1800.9040 | -1.0965 | 0 | 8 | 16 | 1 | R.VMLGETNPADSKPGTIR.G |
|  | 23053 | 612.0266 | 2444.0771 | 2445.2297 | -1.1526 | 1 | 8 | 12 | 1 | K.YMHSGPVVAMVWEGLNVVKTGR.V |
|  | 23167 | 616.2835 | 2461.1047 | 2461.2246 | -0.1199 | 1 | (6) | 17 | 2 | K.YMHSGPVVAMVWEGLNVVKTGR.V |

| **718.** | O43747\|AP1G1_HUMAN    **Mass:** 92148    **Score:** 35     **Queries matched:** 18   **emPAI:** 0.04 |
| --- | --- |
|  | AP-1 complex subunit gamma-1 OS=Homo sapiens GN=AP1G1 PE=1 SV=5 |

|  | **Query** | **Observed** | **Mr(expt)** | **Mr(calc)** | **Delta** | **Miss** | **Score** | **Expect** | **Rank** | **Peptide** |
| --- | --- | --- | --- | --- | --- | --- | --- | --- | --- | --- |
|  | 2181 | **425.7403** | **849.4661** | **848.3963** | **1.0698** | **1** | **11** | **15** | **1** | **K.KYDHMR.S** 2186 |
|  | 5108 | **534.2503** | **1066.4861** | **1065.5819** | **0.9042** | **0** | **6** | **43** | **7** | **R.VLTTAGSYVR.D** 5093 5112 |
|  | 6912 | **599.2883** | **1196.5621** | **1195.5768** | **0.9853** | **1** | **6** | **41** | **1** | **K.ECAAIRSSFR.E** |
|  | 7940 | **416.2372** | **1245.6898** | **1245.6829** | **0.0069** | **1** | **8** | **27** | **9** | **R.FLLNNDKNIR.Y** |
|  | 8410 | 635.2521 | 1268.4896 | 1267.6269 | 0.8627 | 0 | 35 | 0.041 | 1 | K.TVQTDHNAVQR.H |
|  | 9969 | 676.8397 | 1351.6649 | 1351.6442 | 0.0207 | 0 | 1 | 1e+002 | 10 | K.ADCASGIFLAAEK.Y 9989 |
|  | 12150 | **494.2935** | **1479.8587** | **1478.7803** | **1.0785** | **0** | **4** | **46** | **5** | **R.IGYLGAMLLLDER.Q** |
|  | 12259 | 496.2584 | 1485.7535 | 1486.7272 | -0.9737 | 0 | 3 | 70 | 4 | R.QDVHLLMTNCIK.N |
|  | 13026 | 768.8987 | 1535.7828 | 1536.7568 | -0.9740 | 0 | 2 | 73 | 9 | K.VPELMEMFLPATK.N |
|  | 15685 | **569.2943** | **1704.8611** | **1704.8968** | **-0.0357** | **1** | **3** | **49** | **9** | **R.STIVDCLKDLDVSIK.R** 15675 |
|  | 20482 | **530.2679** | **2117.0427** | **2118.1044** | **-1.0618** | **1** | **2** | **51** | **7** | **R.WHIDTIMRVLTTAGSYVR.D** |
|  | 24340 | **533.2514** | **2661.2206** | **2660.2905** | **0.9302** | **0** | **1** | **44** | **3** | **K.NLIMSGYSPEHDVSGISDPFLQVR.I** |
|  | 25164 | **702.1215** | **2804.4567** | **2805.4265** | **-0.9698** | **1** | **2** | **39** | **3** | **K.NLLNEKNHGVLHTSVVLLTEMCER.S** |

| **719.** | O00338\|ST1C2_HUMAN    **Mass:** 35142    **Score:** 35     **Queries matched:** 7   **emPAI:** 0.23 |
| --- | --- |
|  | Sulfotransferase 1C2 OS=Homo sapiens GN=SULT1C2 PE=1 SV=1 |

|  | **Query** | **Observed** | **Mr(expt)** | **Mr(calc)** | **Delta** | **Miss** | **Score** | **Expect** | **Rank** | **Peptide** |
| --- | --- | --- | --- | --- | --- | --- | --- | --- | --- | --- |
|  | 1015 | **630.3420** | **629.3348** | **628.4272** | **0.9076** | **1** | **4** | **1e+002** | **3** | **K.QIKLK.E** |
|  | 2242 | 428.2584 | 854.5023 | 855.3983 | -0.8960 | 0 | 2 | 90 | 2 | K.VMQFMGK.K |
|  | 5841 | **561.3046** | **1120.5946** | **1119.5165** | **1.0781** | **1** | **31** | **0.12** | **1** | **K.MKENPMTNR.S** |
|  | 10513 | **692.3457** | **1382.6768** | **1382.6864** | **-0.0095** | **0** | **(1)** | **1e+002** | **1** | **K.SILDQSISSFMR.K** |
|  | 10801 | **467.2590** | **1398.7552** | **1398.6813** | **0.0739** | **0** | **7** | **27** | **2** | **K.SILDQSISSFMR.K** |
|  | 18859 | 979.9888 | 1957.9631 | 1957.9395 | 0.0236 | 1 | 2 | 59 | 8 | K.GTVGDWKNHFTVAQNER.F |
|  | 30183 | 1062.7695 | 4247.0490 | 4246.1508 | 0.8983 | 1 | 28 | 0.036 | 1 | K.LKEVEGTLLQPATVDNWSQIQSFEAKPDDLLICTYPK.A |

| **720.** | P98082\|DAB2_HUMAN    **Mass:** 82511    **Score:** 35     **Queries matched:** 20   **emPAI:** 0.04 |
| --- | --- |
|  | Disabled homolog 2 OS=Homo sapiens GN=DAB2 PE=1 SV=3 |

|  | **Query** | **Observed** | **Mr(expt)** | **Mr(calc)** | **Delta** | **Miss** | **Score** | **Expect** | **Rank** | **Peptide** |
| --- | --- | --- | --- | --- | --- | --- | --- | --- | --- | --- |
|  | 592 | 470.2579 | 469.2507 | 468.2696 | 0.9810 | 0 | 10 | 7.1 | 1 | R.AGPPK.D |
|  | 3061 | **456.8826** | **911.7507** | **911.4422** | **0.3085** | **1** | **9** | **12** | **1** | **K.DVKEMFK.D** 3075 |
|  | 5819 | 560.3005 | 1118.5864 | 1117.6244 | 0.9620 | 0 | 8 | 27 | 6 | K.INEPPKPAPR.Q 5786 5811 |
|  | 7796 | 620.3389 | 1238.6633 | 1237.6707 | 0.9926 | 0 | 6 | 37 | 10 | K.DLFQVIYNVK.K |
|  | 8502 | **637.3879** | **1272.7613** | **1272.5148** | **0.2465** | **1** | **(2)** | **92** | **9** | **R.GDKMSQDSMMK.L** |
|  | 8518 | 637.8367 | 1273.6589 | 1272.5148 | 1.1441 | 1 | (4) | 54 | 10 | R.GDKMSQDSMMK.L |
|  | 8786 | **430.5614** | **1288.6623** | **1288.5097** | **0.1525** | **1** | **10** | **13** | **5** | **R.GDKMSQDSMMK.L** 8775 |
|  | 9141 | **653.8010** | **1305.5874** | **1304.5047** | **1.0827** | **1** | **(0)** | **1.3e+002** | **3** | **R.GDKMSQDSMMK.L** |
|  | 12202 | **742.1916** | **1482.3687** | **1482.7678** | **-0.3991** | **1** | **2** | **55** | **3** | **K.LIGIDDVPDARGDK.M** |
|  | 19441 | **672.6484** | **2014.9233** | **2014.9360** | **-0.0127** | **0** | **7** | **17** | **1** | **R.AFGYVCGGEGQHQFFAIK.T** |
|  | 21226 | 735.3732 | 2203.0977 | 2204.0994 | -1.0017 | 1 | 1 | 62 | 6 | K.AVENGSEALMILDDQTNKLK.S |
|  | 22423 | **785.0348** | **2352.0825** | **2352.0982** | **-0.0157** | **0** | **18** | **1.1** | **1** | **K.VGIPQENADHDDFDANQLLNK.I** |
|  | 24129 | 873.1431 | 2616.4076 | 2616.4163 | -0.0087 | 1 | 35 | 0.021 | 1 | K.TGQQAEPLVVDLKDLFQVIYNVK.K |
|  | 24149 | 874.1476 | 2619.4211 | 2620.2537 | -0.8326 | 1 | 2 | 40 | 2 | K.IEEASKAVENGSEALMILDDQTNK.L |
|  | 26899 | **792.8708** | **3167.4541** | **3167.3778** | **0.0762** | **1** | **1** | **37** | **3** | **K.DSFGSSQASVASSQPVSSEMYRDPFGNPFA.-** |
|  | 30277 | **1439.5313** | **4315.5719** | **4314.9941** | **0.5778** | **1** | **4** | **6.3** | **2** | **K.GEQTSSGTLSAFASYFNSKVGIPQENADHDDFDANQLLNK.I** |

| **721.** | P53999\|TCP4_HUMAN    **Mass:** 14386    **Score:** 34     **Queries matched:** 6   **emPAI:** 0.27 |
| --- | --- |
|  | Activated RNA polymerase II transcriptional coactivator p15 OS=Homo sapiens GN=SUB1 PE=1 SV=3 |

|  | **Query** | **Observed** | **Mr(expt)** | **Mr(calc)** | **Delta** | **Miss** | **Score** | **Expect** | **Rank** | **Peptide** |
| --- | --- | --- | --- | --- | --- | --- | --- | --- | --- | --- |
|  | 4183 | **498.3030** | **994.5913** | **994.5811** | **0.0102** | **0** | **3** | **79** | **1** | **K.QVAPEKPVK.K** |
|  | 16160 | 581.3101 | 1740.9084 | 1739.7283 | 1.1800 | 0 | 3 | 49 | 4 | R.EYWMDPEGEMKPGR.K |
|  | 17054 | 605.6510 | 1813.9312 | 1813.7701 | 0.1610 | 0 | 4 | 44 | 8 | K.ELVSSSSSGSDSDSEVDK.K 17055 |
|  | 24818 | **914.4589** | **2740.3548** | **2740.3668** | **-0.0120** | **1** | **34** | **0.022** | **1** | **K.GISLNPEQWSQLKEQISDIDDAVR.K** 24821 |

| **722.** | P30483\|1B45_HUMAN    **Mass:** 40674    **Score:** 34     **Queries matched:** 7   **emPAI:** 0.09 |
| --- | --- |
|  | HLA class I histocompatibility antigen, B-45 alpha chain OS=Homo sapiens GN=HLA-B PE=2 SV=1 |

|  | **Query** | **Observed** | **Mr(expt)** | **Mr(calc)** | **Delta** | **Miss** | **Score** | **Expect** | **Rank** | **Peptide** |
| --- | --- | --- | --- | --- | --- | --- | --- | --- | --- | --- |
|  | 5925 | **565.3266** | **1128.6386** | **1127.5247** | **1.1139** | **0** | **3** | **81** | **8** | **R.GYNQLAYDGK.D** |
|  | 9948 | 676.3410 | 1350.6674 | 1349.6939 | 0.9736 | 1 | 14 | 5.9 | 1 | R.YLENGKETLQR.A 9957 |
|  | 14674 | 547.0314 | 1638.0725 | 1637.7654 | 0.3071 | 1 | 2 | 52 | 6 | R.MYGCDLGPDGRLLR.G |
|  | 20385 | 527.7902 | 2107.1315 | 2107.0368 | 0.0948 | 1 | 4 | 37 | 9 | R.VAEQDRAYLEGLCVESLR.R |
|  | 23135 | 492.2564 | 2456.2458 | 2456.3097 | -0.0639 | 0 | 2 | 47 | 5 | R.TVLLLLSAALALTETWAGSHSMR.Y |
|  | 24792 | 912.7573 | 2735.2500 | 2735.2635 | -0.0135 | 1 | 34 | 0.023 | 1 | R.DGEDQTQDTELVETRPAGDRTFQK.W |

|  | **Proteins matching a subset of these peptides:** |
| --- | --- |

|  | P30487\|1B49_HUMAN    **Mass:** 40841    **Score:** 34     **Queries matched:** 6 |
| --- | --- |
|  | HLA class I histocompatibility antigen, B-49 alpha chain OS=Homo sapiens GN=HLA-B PE=2 SV=2 |

|  | P30488\|1B50_HUMAN    **Mass:** 40801    **Score:** 34     **Queries matched:** 6 |
| --- | --- |
|  | HLA class I histocompatibility antigen, B-50 alpha chain OS=Homo sapiens GN=HLA-B PE=2 SV=1 |

| **723.** | Q8NBS9\|TXND5_HUMAN    **Mass:** 48283    **Score:** 34     **Queries matched:** 11   **emPAI:** 0.08 |
| --- | --- |
|  | Thioredoxin domain-containing protein 5 OS=Homo sapiens GN=TXNDC5 PE=1 SV=2 |

|  | **Query** | **Observed** | **Mr(expt)** | **Mr(calc)** | **Delta** | **Miss** | **Score** | **Expect** | **Rank** | **Peptide** |
| --- | --- | --- | --- | --- | --- | --- | --- | --- | --- | --- |
|  | 1349 | 396.2091 | 790.4037 | 791.4541 | -1.0505 | 0 | 12 | 12 | 2 | R.FVLSQAK.D |
|  | 1489 | **401.2290** | **800.4435** | **799.4123** | **1.0312** | **0** | **5** | **67** | **4** | **-.MPARPGR.L** |
|  | 1836 | **414.9418** | **827.8691** | **827.3886** | **0.4805** | **0** | **4** | **51** | **2** | **K.VSEHSGGR.D** |
|  | 5796 | **559.3405** | **1116.6665** | **1115.6339** | **1.0326** | **0** | **11** | **13** | **3** | **K.LFKPGQEAVK.Y** |
|  | 12994 | **512.2502** | **1533.7289** | **1532.7181** | **1.0108** | **1** | **5** | **38** | **5** | **K.YNSMEDAKVYVAK.V** |
|  | 13834 | **529.2372** | **1584.6899** | **1583.8824** | **0.8075** | **1** | **3** | **56** | **4** | **K.YSVRGYPTLLLFR.G** |
|  | 16410 | **587.9227** | **1760.7464** | **1760.7570** | **-0.0107** | **0** | **34** | **0.038** | **1** | **K.VDCTAHSDVCSAQGVR.G** |
|  | 16590 | **445.0059** | **1775.9946** | **1774.9981** | **0.9964** | **1** | **5** | **38** | **3** | **R.GYPTLKLFKPGQEAVK.Y** |
|  | 17745 | **622.5694** | **1864.6864** | **1863.9326** | **0.7538** | **1** | **7** | **18** | **6** | **R.DLESLREYVESQLQR.T** |
|  | 21160 | 731.7200 | 2192.1381 | 2192.1477 | -0.0096 | 0 | 3 | 41 | 1 | K.ALAPTWEQLALGLEHSETVK.I |
|  | 29011 | **748.5717** | **3737.8222** | **3738.6530** | **-0.8308** | **0** | **1** | **28** | **2** | **K.HLYTADMFTHGIQSAAHFVMFFAPWCGHCQR.L** |

| **724.** | P51571\|SSRD_HUMAN    **Mass:** 19158    **Score:** 34     **Queries matched:** 8   **emPAI:** 0.20 |
| --- | --- |
|  | Translocon-associated protein subunit delta OS=Homo sapiens GN=SSR4 PE=1 SV=1 |

|  | **Query** | **Observed** | **Mr(expt)** | **Mr(calc)** | **Delta** | **Miss** | **Score** | **Expect** | **Rank** | **Peptide** |
| --- | --- | --- | --- | --- | --- | --- | --- | --- | --- | --- |
|  | 10078 | 453.5709 | 1357.6910 | 1358.7055 | -1.0145 | 1 | 11 | 12 | 1 | K.QFPVTRGQDVGR.Y 10084 10085 10088 10091 |
|  | 10183 | **455.7546** | **1364.2420** | **1364.6758** | **-0.4338** | **0** | **7** | **19** | **4** | **R.VQNMALYADVGGK.Q** |
|  | 10479 | 461.0072 | 1379.9999 | 1380.6707 | -0.6709 | 0 | (1) | 78 | 8 | R.VQNMALYADVGGK.Q |
|  | 10884 | **703.3313** | **1404.6480** | **1404.6561** | **-0.0081** | **0** | **34** | **0.049** | **1** | **R.FFDEESYSLLR.K** |

| **725.** | Q9H553\|ALG2_HUMAN    **Mass:** 47746    **Score:** 34     **Queries matched:** 5   **emPAI:** 0.08 |
| --- | --- |
|  | Alpha-1,3-mannosyltransferase ALG2 OS=Homo sapiens GN=ALG2 PE=2 SV=1 |

|  | **Query** | **Observed** | **Mr(expt)** | **Mr(calc)** | **Delta** | **Miss** | **Score** | **Expect** | **Rank** | **Peptide** |
| --- | --- | --- | --- | --- | --- | --- | --- | --- | --- | --- |
|  | 3910 | **488.3163** | **974.6179** | **973.4440** | **1.1740** | **0** | **9** | **27** | **9** | **R.CAGDWLPR.G** |
|  | 7951 | **416.2597** | **1245.7574** | **1246.6054** | **-0.8480** | **1** | **3** | **69** | **6** | **R.GRLTSQDWER.V** |
|  | 9588 | 665.8242 | 1329.6338 | 1329.7074 | -0.0737 | 1 | 3 | 68 | 6 | R.EPSLKATMGLAGR.A |
|  | 12290 | 744.8460 | 1487.6773 | 1486.7092 | 0.9681 | 0 | 34 | 0.05 | 1 | K.FSPEAFTEQLYR.Y |
|  | 12502 | **501.3056** | **1500.8949** | **1499.7620** | **1.1329** | **0** | **4** | **52** | **9** | **R.VLENVEHYQELK.K** |

| **726.** | Q15286\|RAB35_HUMAN    **Mass:** 23296    **Score:** 34     **Queries matched:** 4   **emPAI:** 0.16 |
| --- | --- |
|  | Ras-related protein Rab-35 OS=Homo sapiens GN=RAB35 PE=1 SV=1 |

|  | **Query** | **Observed** | **Mr(expt)** | **Mr(calc)** | **Delta** | **Miss** | **Score** | **Expect** | **Rank** | **Peptide** |
| --- | --- | --- | --- | --- | --- | --- | --- | --- | --- | --- |
|  | 5171 | 536.3210 | 1070.6274 | 1070.6336 | -0.0062 | 0 | 21 | 1.3 | 1 | K.LLIIGDSGVGK.S |
|  | 9315 | 658.8296 | 1315.6446 | 1315.6521 | -0.0074 | 0 | 18 | 2.1 | 1 | K.LQIWDTAGQER.F 9284 |
|  | 9785 | 671.8335 | 1341.6524 | 1341.6637 | -0.0112 | 0 | 34 | 0.055 | 1 | K.QQQQQQNDVVK.L |

| **727.** | Q96L21\|RL10L_HUMAN    **Mass:** 24959    **Score:** 34     **Queries matched:** 9   **emPAI:** 0.15 |
| --- | --- |
|  | 60S ribosomal protein L10-like OS=Homo sapiens GN=RPL10L PE=1 SV=3 |

|  | **Query** | **Observed** | **Mr(expt)** | **Mr(calc)** | **Delta** | **Miss** | **Score** | **Expect** | **Rank** | **Peptide** |
| --- | --- | --- | --- | --- | --- | --- | --- | --- | --- | --- |
|  | 2227 | 427.4372 | 852.8599 | 852.5181 | 0.3418 | 1 | 5 | 32 | 2 | R.QKIHISK.K |
|  | 3964 | 490.7628 | 979.5110 | 979.4215 | 0.0894 | 0 | 23 | 0.75 | 1 | K.MLSCAGADR.L 3945 |
|  | 4216 | 499.2638 | 996.5131 | 995.4164 | 1.0967 | 0 | (12) | 9.5 | 2 | K.MLSCAGADR.L 4202 |
|  | 8050 | 626.8079 | 1251.6013 | 1251.7122 | -0.1109 | 0 | 0 | 1.3e+002 | 4 | R.VHIGQVIMSIR.T |
|  | 11356 | **478.2666** | **1431.7780** | **1430.6024** | **1.1756** | **0** | **1** | **1e+002** | **6** | **K.FNADEFEDMVAK.K** |
|  | 13173 | 772.8275 | 1543.6405 | 1542.7024 | 0.9380 | 1 | 34 | 0.05 | 2 | K.FNADEFEDMVAKK.C |
|  | 19822 | 684.3317 | 2049.9734 | 2049.9142 | 0.0591 | 1 | 2 | 57 | 7 | K.WGFTKFNADEFEDMVAK.K |

| **728.** | Q6UWE0\|LRSM1_HUMAN    **Mass:** 84567    **Score:** 34     **Queries matched:** 10   **emPAI:** 0.04 |
| --- | --- |
|  | E3 ubiquitin-protein ligase LRSAM1 OS=Homo sapiens GN=LRSAM1 PE=1 SV=1 |

|  | **Query** | **Observed** | **Mr(expt)** | **Mr(calc)** | **Delta** | **Miss** | **Score** | **Expect** | **Rank** | **Peptide** |
| --- | --- | --- | --- | --- | --- | --- | --- | --- | --- | --- |
|  | 1700 | **409.0727** | **816.1309** | **815.4137** | **0.7173** | **0** | **4** | **54** | **6** | **R.KPSEEAR.K** |
|  | 4256 | 500.7896 | 999.5647 | 998.5331 | 1.0316 | 0 | 7 | 31 | 9 | R.NQLMQLPR.S |
|  | 4832 | 523.0570 | 1044.0993 | 1044.5703 | -0.4709 | 0 | 4 | 43 | 1 | R.EILTELEAK.S |
|  | 6233 | **577.2977** | **1152.5808** | **1152.7230** | **-0.1422** | **0** | **11** | **12** | **5** | **R.LLNQKPLSLK.L** |
|  | 12914 | 764.4131 | 1526.8116 | 1527.8620 | -1.0504 | 0 | 34 | 0.052 | 2 | R.SIGNLTQLQTLNVK.D 12915 |
|  | 16879 | **601.2902** | **1800.8488** | **1800.9330** | **-0.0841** | **1** | **3** | **53** | **5** | **R.LQEQLKQTEQNISSR.I** |
|  | 18261 | **637.3049** | **1908.8930** | **1908.9325** | **-0.0395** | **0** | **5** | **34** | **1** | **R.TLEMLSLDASAMVYPPR.E** |
|  | 18477 | **482.2632** | **1925.0237** | **1923.8917** | **1.1320** | **0** | **4** | **41** | **5** | **R.MEQLMSITQEETESLR.R** |
|  | 20126 | 1041.0842 | 2080.1539 | 2079.9928 | 0.1611 | 1 | 3 | 47 | 4 | R.MEQLMSITQEETESLRR.R |

| **729.** | P16070\|CD44_HUMAN    **Mass:** 82017    **Score:** 34     **Queries matched:** 14   **emPAI:** 0.05 |
| --- | --- |
|  | CD44 antigen OS=Homo sapiens GN=CD44 PE=1 SV=2 |

|  | **Query** | **Observed** | **Mr(expt)** | **Mr(calc)** | **Delta** | **Miss** | **Score** | **Expect** | **Rank** | **Peptide** |
| --- | --- | --- | --- | --- | --- | --- | --- | --- | --- | --- |
|  | 3694 | **481.1516** | **960.2887** | **960.4698** | **-0.1811** | **0** | **6** | **39** | **1** | **R.NLQNVDMK.I** |
|  | 4796 | **521.7657** | **1041.5168** | **1041.5243** | **-0.0075** | **1** | **34** | **0.07** | **1** | **R.YVQKGEYR.T** |
|  | 10587 | **463.2447** | **1386.7122** | **1385.7456** | **0.9666** | **0** | **7** | **30** | **7** | **R.YGFIEGHVVIPR.I** 10564 10575 |
|  | 11087 | **473.2795** | **1416.8166** | **1415.6641** | **1.1525** | **0** | **4** | **59** | **2** | **K.DHPTTSTLTSSNR.N** |
|  | 14061 | 800.8427 | 1599.6707 | 1599.7273 | -0.0565 | 0 | 6 | 26 | 2 | K.AFNSTLPTMAQMEK.A |
|  | 17668 | 929.9322 | 1857.8499 | 1858.7527 | -0.9028 | 0 | 3 | 50 | 3 | K.ESSETPDQFMTADETR.N |
|  | 20468 | **706.1022** | **2115.2849** | **2114.9941** | **0.2908** | **1** | **5** | **23** | **1** | **K.DHPTTSTLTSSNRNDVTGGR.R** 20448 20466 |
|  | 22329 | 781.3588 | 2341.0545 | 2341.1168 | -0.0623 | 1 | 1 | 64 | 5 | K.RQETWDWFSWLFLPSESK.N |
|  | 24694 | 680.8129 | 2719.2224 | 2718.2815 | 0.9409 | 1 | 3 | 27 | 5 | K.AFNSTLPTMAQMEKALSIGFETCR.Y |
|  | 28495 | **1193.9120** | **3578.7141** | **3578.6927** | **0.0214** | **1** | **1** | **29** | **8** | **R.AFDHTKQNQDWTQWNPSHSNPEVLLQTTTR.M** |

| **730.** | P22303\|ACES_HUMAN    **Mass:** 68209    **Score:** 34     **Queries matched:** 10   **emPAI:** 0.05 |
| --- | --- |
|  | Acetylcholinesterase OS=Homo sapiens GN=ACHE PE=1 SV=1 |

|  | **Query** | **Observed** | **Mr(expt)** | **Mr(calc)** | **Delta** | **Miss** | **Score** | **Expect** | **Rank** | **Peptide** |
| --- | --- | --- | --- | --- | --- | --- | --- | --- | --- | --- |
|  | 830 | **559.0965** | **558.0892** | **557.3398** | **0.7495** | **1** | **11** | **17** | **5** | **R.GGRLR.G** |
|  | 2219 | 853.4179 | 852.4106 | 853.3817 | -0.9711 | 0 | 6 | 37 | 4 | R.NYTAEEK.I |
|  | 3273 | 464.2248 | 926.4351 | 926.4399 | -0.0048 | 0 | 34 | 0.068 | 1 | R.YWANFAR.T |
|  | 7503 | 613.3393 | 1224.6640 | 1224.5735 | 0.0906 | 1 | 7 | 31 | 9 | R.TGDPNEPRDPK.A |
|  | 7756 | 413.5422 | 1237.6049 | 1238.5543 | -0.9494 | 0 | 10 | 13 | 1 | R.WSSYMVHWK.N |
|  | 8828 | 646.3137 | 1290.6129 | 1290.7085 | -0.0956 | 0 | 5 | 43 | 4 | R.VGAFGFLALPGSR.E 8837 |
|  | 22572 | **790.7974** | **2369.3704** | **2370.2518** | **-0.8814** | **1** | **2** | **38** | **5** | **R.TVLVSMNYRVGAFGFLALPGSR.E** |
|  | 24292 | 884.7047 | 2651.0923 | 2650.2551 | 0.8372 | 1 | 2 | 36 | 1 | K.DEGSYFLVYGAPGFSKDNESLISR.A |
|  | 30778 | **1158.5310** | **4630.0949** | **4629.5833** | **0.5117** | **1** | **11** | **1.3** | **1** | **M.RPPQCLLHTPSLASPLLLLLLWLLGGGVGAEGREDAELLVTVR.G** |

| **731.** | Q5XG92\|EST8_HUMAN    **Mass:** 63887    **Score:** 34     **Queries matched:** 6   **emPAI:** 0.06 |
| --- | --- |
|  | Carboxylesterase 8 OS=Homo sapiens GN=CES8 PE=2 SV=2 |

|  | **Query** | **Observed** | **Mr(expt)** | **Mr(calc)** | **Delta** | **Miss** | **Score** | **Expect** | **Rank** | **Peptide** |
| --- | --- | --- | --- | --- | --- | --- | --- | --- | --- | --- |
|  | 1606 | **405.0414** | **808.0683** | **808.3902** | **-0.3218** | **0** | **2** | **70** | **1** | **K.MLWSTR.T** |
|  | 3273 | 464.2248 | 926.4351 | 926.4399 | -0.0048 | 0 | 34 | 0.068 | 1 | K.YWANFAR.T |
|  | 10235 | 456.7217 | 1367.1433 | 1367.7132 | -0.5699 | 1 | 7 | 17 | 5 | K.MRFLQLNFQR.D |
|  | 10480 | **461.0075** | **1380.0005** | **1380.7071** | **-0.7066** | **1** | **4** | **42** | **5** | **K.ETITKMLWSTR.T** |
|  | 17502 | **923.9553** | **1845.8960** | **1844.9065** | **0.9894** | **1** | **3** | **53** | **3** | **K.ALSLQMMKYWANFAR.T** |
|  | 26114 | **752.6050** | **3006.3908** | **3005.5112** | **0.8797** | **1** | **1** | **45** | **3** | **-.MRWILCWSLTLCLMAQTALGALHTK.R** |

| **732.** | Q9H078\|CLPB_HUMAN    **Mass:** 79193    **Score:** 34     **Queries matched:** 22   **emPAI:** 0.05 |
| --- | --- |
|  | Caseinolytic peptidase B protein homolog OS=Homo sapiens GN=CLPB PE=1 SV=1 |

|  | **Query** | **Observed** | **Mr(expt)** | **Mr(calc)** | **Delta** | **Miss** | **Score** | **Expect** | **Rank** | **Peptide** |
| --- | --- | --- | --- | --- | --- | --- | --- | --- | --- | --- |
|  | 842 | **562.3155** | **561.3082** | **560.3533** | **0.9549** | **0** | **9** | **33** | **7** | **K.ITISK.N** |
|  | 3298 | **465.0205** | **928.0264** | **928.4978** | **-0.4713** | **0** | **5** | **39** | **3** | **K.DAALLEAAR.A** |
|  | 4880 | **524.7422** | **1047.4698** | **1047.4767** | **-0.0069** | **0** | **34** | **0.074** | **1** | **R.ANNMQEVSR.L** |
|  | 5033 | **531.2727** | **1060.5309** | **1059.6223** | **0.9086** | **1** | **7** | **34** | **4** | **-.MLGSLVLRR.K** |
|  | 5202 | 537.8199 | 1073.6253 | 1073.5903 | 0.0351 | 1 | 10 | 20 | 6 | R.EGEVMKLLR.T |
|  | 5428 | 546.3209 | 1090.6272 | 1089.5852 | 1.0420 | 1 | (7) | 31 | 3 | R.EGEVMKLLR.T 5385 5390 5412 |
|  | 8744 | **644.3932** | **1286.7719** | **1287.7299** | **-0.9579** | **1** | **4** | **52** | **10** | **R.LDIRAPLHPEK.V** 8766 |
|  | 11310 | 715.8671 | 1429.7197 | 1430.7704 | -1.0507 | 0 | 10 | 15 | 9 | R.LGWTALMVAAINR.N 11309 |
|  | 13393 | 779.3526 | 1556.6906 | 1556.7947 | -0.1041 | 0 | 7 | 27 | 2 | R.NVTTGSLGEPQWLR.V |
|  | 15722 | 570.3570 | 1708.0491 | 1707.9355 | 0.1137 | 1 | 7 | 20 | 5 | K.HRLGWTALMVAAINR.N |
|  | 16390 | **587.2890** | **1758.8452** | **1757.8961** | **0.9490** | **1** | **0** | **1e+002** | **7** | **R.HITNQWTSALEFRR.W** |
|  | 18669 | **972.4834** | **1942.9522** | **1943.0575** | **-0.1052** | **1** | **3** | **52** | **1** | **R.IAENLGDVQISDKITISK.N** |
|  | 19000 | **658.5075** | **1972.5007** | **1971.9691** | **0.5316** | **0** | **3** | **28** | **1** | **K.FIGSPPGYVGHEEGGQLTK.K** |
|  | 20885 | 1082.0553 | 2162.0960 | 2161.0433 | 1.0527 | 1 | 10 | 7.8 | 2 | R.ANNMQEVSRLLSEGADVNAK.H |
|  | 21567 | **560.0665** | **2236.2368** | **2236.1097** | **0.1270** | **1** | **4** | **33** | **6** | **R.GHGGASGRNVTTGSLGEPQWLR.V** |
|  | 24007 | 649.5928 | 2594.3420 | 2593.2489 | 1.0931 | 0 | 4 | 25 | 4 | K.ENGWYDEEHPLVFLFLGSSGIGK.T |
|  | 26709 | **781.6579** | **3122.6025** | **3121.5040** | **1.0985** | **1** | **2** | **30** | **5** | **K.CLAAATWGRLPGPEETLPGQDSWNGVPSR.A** |

| **733.** | P62263\|RS14_HUMAN    **Mass:** 16434    **Score:** 34     **Queries matched:** 4   **emPAI:** 0.24 |
| --- | --- |
|  | 40S ribosomal protein S14 OS=Homo sapiens GN=RPS14 PE=1 SV=3 |

|  | **Query** | **Observed** | **Mr(expt)** | **Mr(calc)** | **Delta** | **Miss** | **Score** | **Expect** | **Rank** | **Peptide** |
| --- | --- | --- | --- | --- | --- | --- | --- | --- | --- | --- |
|  | 11272 | 714.8560 | 1427.6975 | 1428.7096 | -1.0122 | 0 | 1 | 1.1e+002 | 10 | R.IEDVTPIPSDSTR.R |
|  | 13850 | 529.3183 | 1584.9331 | 1584.8107 | 0.1223 | 1 | 5 | 34 | 5 | R.IEDVTPIPSDSTRR.K 13846 |
|  | 21778 | **756.0198** | **2265.0377** | **2264.0491** | **0.9886** | **1** | **34** | **0.034** | **1** | **K.ADRDESSPYAAMLAAQDVAQR.C** |

| **734.** | P10768\|ESTD_HUMAN    **Mass:** 31956    **Score:** 34     **Queries matched:** 6   **emPAI:** 0.25 |
| --- | --- |
|  | S-formylglutathione hydrolase OS=Homo sapiens GN=ESD PE=1 SV=2 |

|  | **Query** | **Observed** | **Mr(expt)** | **Mr(calc)** | **Delta** | **Miss** | **Score** | **Expect** | **Rank** | **Peptide** |
| --- | --- | --- | --- | --- | --- | --- | --- | --- | --- | --- |
|  | 7185 | 605.7926 | 1209.5706 | 1208.6627 | 0.9079 | 1 | 6 | 35 | 4 | K.MKFAVYLPPK.A |
|  | 22093 | 770.0568 | 2307.1485 | 2307.1244 | 0.0241 | 0 | 20 | 0.78 | 1 | K.SGYHQSASEHGLVVIAPDTSPR.G |
|  | 22829 | 805.3790 | 2413.1151 | 2414.1657 | -1.0506 | 1 | 3 | 37 | 5 | R.MSIFGHSMGGHGALICALKNPGK.Y 22846 |
|  | 27309 | **815.4166** | **3257.6371** | **3257.5815** | **0.0556** | **1** | **1** | **35** | **2** | **K.TNYRMYSYVTEELPQLINANFPVDPQR.M** |
|  | 30000 | **1380.3444** | **4138.0113** | **4137.0252** | **0.9860** | **1** | **33** | **0.015** | **1** | **K.SYPGSQLDILIDQGKDDQFLLDGQLLPDNFIAACTEK.K** |

| **735.** | P61978\|HNRPK_HUMAN    **Mass:** 51230    **Score:** 34     **Queries matched:** 16   **emPAI:** 0.07 |
| --- | --- |
|  | Heterogeneous nuclear ribonucleoprotein K OS=Homo sapiens GN=HNRNPK PE=1 SV=1 |

|  | **Query** | **Observed** | **Mr(expt)** | **Mr(calc)** | **Delta** | **Miss** | **Score** | **Expect** | **Rank** | **Peptide** |
| --- | --- | --- | --- | --- | --- | --- | --- | --- | --- | --- |
|  | 4219 | **499.2954** | **996.5762** | **997.3811** | **-0.8049** | **0** | **9** | **18** | **4** | **R.DYDDMSPR.R** 4241 |
|  | 4598 | 513.7977 | 1025.5808 | 1026.5723 | -0.9915 | 1 | 13 | 7.2 | 4 | R.RGPPPPPPGR.G 4599 |
|  | 5843 | **561.3310** | **1120.6474** | **1121.5023** | **-0.8548** | **0** | **7** | **34** | **6** | **R.NTDEMVELR.I** |
|  | 6723 | **396.9795** | **1187.9167** | **1187.6346** | **0.2821** | **1** | **2** | **62** | **6** | **R.RGRPVGFPMR.G** |
|  | 8182 | **420.2320** | **1257.6743** | **1258.5677** | **-0.8934** | **0** | **7** | **27** | **8** | **K.IDEPLEGSEDR.I** 8171 |
|  | 8450 | **424.5569** | **1270.6487** | **1270.6993** | **-0.0506** | **1** | **3** | **72** | **7** | **K.DLAGSIIGKGGQR.I** |
|  | 12363 | **498.3030** | **1491.8870** | **1491.7551** | **0.1319** | **1** | **(5)** | **36** | **5** | **R.MPPGRGGRPMPPSR.R** |
|  | 12846 | 762.3356 | 1522.6566 | 1523.7449 | -1.0883 | 1 | 6 | 31 | 4 | R.MPPGRGGRPMPPSR.R |
|  | 15794 | **572.3085** | **1713.9036** | **1713.9764** | **-0.0728** | **0** | **5** | **39** | **8** | **R.ILSISADIETIGEILK.K** |
|  | 16082 | **579.3211** | **1734.9415** | **1734.7995** | **0.1420** | **1** | **2** | **65** | **9** | **K.RPAEDMEEEQAFKR.S** |
|  | 16936 | 903.4520 | 1804.8894 | 1803.9400 | 0.9494 | 1 | 2 | 62 | 4 | R.NTDEMVELRILLQSK.N |
|  | 18342 | **959.0153** | **1916.0160** | **1916.0255** | **-0.0095** | **0** | **34** | **0.044** | **1** | **R.GSYGDLGGPIITTQVTIPK.D** |
|  | 27412 | **1096.8058** | **3287.3955** | **3286.3284** | **1.0671** | **1** | **0** | **35** | **3** | **K.GRAQPYDPNFYDETYDYGGFTMMFDDR.R** |

| **736.** | P17252\|KPCA_HUMAN    **Mass:** 77855    **Score:** 33     **Queries matched:** 15   **emPAI:** 0.05 |
| --- | --- |
|  | Protein kinase C alpha type OS=Homo sapiens GN=PRKCA PE=1 SV=3 |

|  | **Query** | **Observed** | **Mr(expt)** | **Mr(calc)** | **Delta** | **Miss** | **Score** | **Expect** | **Rank** | **Peptide** |
| --- | --- | --- | --- | --- | --- | --- | --- | --- | --- | --- |
|  | 1081 | **657.4253** | **656.4181** | **655.3653** | **1.0528** | **0** | **12** | **11** | **2** | **K.LGPAGNK.V** |
|  | 2152 | **425.0480** | **848.0814** | **847.4586** | **0.6229** | **1** | **9** | **16** | **6** | **K.VMLADRK.G** |
|  | 7477 | 409.1558 | 1224.4455 | 1223.5605 | 0.8850 | 1 | 1 | 1.1e+002 | 9 | K.VCGKGAENFDK.F |
|  | 9367 | **659.8589** | **1317.7032** | **1317.6353** | **0.0679** | **0** | **8** | **25** | **5** | **R.LSVEIWDWDR.T** |
|  | 14458 | 812.9440 | 1623.8735 | 1623.9058 | -0.0323 | 1 | 4 | 46 | 5 | R.VKLTDFNFLMVLGK.G |
|  | 15572 | **849.9056** | **1697.7966** | **1697.8049** | **-0.0083** | **0** | **33** | **0.05** | **1** | **R.STLNPQWNESFTFK.L** |
|  | 16593 | 593.1540 | 1776.4403 | 1776.8063 | -0.3659 | 0 | (4) | 33 | 10 | R.NDFMGSLSFGVSELMK.M |
|  | 16626 | 593.6204 | 1777.8393 | 1776.8063 | 1.0330 | 0 | 4 | 39 | 10 | R.NDFMGSLSFGVSELMK.M |
|  | 16757 | 597.3010 | 1788.8811 | 1787.8764 | 1.0047 | 0 | 2 | 62 | 8 | K.NLIPMDPNGLSDPYVK.L |
|  | 16788 | 598.2952 | 1791.8639 | 1792.8012 | -0.9373 | 0 | (0) | 1.1e+002 | 9 | R.NDFMGSLSFGVSELMK.M |
|  | 17441 | **615.2460** | **1842.7163** | **1841.9193** | **0.7970** | **1** | **2** | **58** | **7** | **R.DLKLDNVMLDSEGHIK.I** 17464 |
|  | 17729 | 932.9498 | 1863.8851 | 1862.8395 | 1.0456 | 0 | 1 | 93 | 10 | M.ADVFPGNDSTASQDVANR.F |
|  | 21573 | **746.6937** | **2237.0592** | **2237.0461** | **0.0130** | **1** | **4** | **30** | **5** | **M.ADVFPGNDSTASQDVANRFAR.K** |
|  | 26295 | **1013.4594** | **3037.3562** | **3036.3658** | **0.9904** | **0** | **7** | **9.2** | **1** | **K.LLNQEEGEYYNVPIPEGDEEGNMELR.Q** |

| **737.** | Q9NZM1\|MYOF_HUMAN    **Mass:** 236100   **Score:** 33     **Queries matched:** 43   **emPAI:** 0.02 |
| --- | --- |
|  | Myoferlin OS=Homo sapiens GN=MYOF PE=1 SV=1 |

|  | **Query** | **Observed** | **Mr(expt)** | **Mr(calc)** | **Delta** | **Miss** | **Score** | **Expect** | **Rank** | **Peptide** |
| --- | --- | --- | --- | --- | --- | --- | --- | --- | --- | --- |
|  | 740 | **528.4092** | **527.4019** | **528.2656** | **-0.8637** | **0** | **2** | **1e+002** | **7** | **K.NNGPK.V** |
|  | 1856 | 415.2296 | 828.4446 | 827.4977 | 0.9469 | 1 | 12 | 14 | 4 | R.VRVIEGR.Q |
|  | 1952 | 418.6959 | 835.3773 | 834.5439 | 0.8334 | 0 | 9 | 22 | 4 | R.LALHILR.T |
|  | 2783 | **446.7822** | **891.5498** | **890.4094** | **1.1405** | **0** | **3** | **1.1e+002** | **9** | **K.DLTGDQSR.S** |
|  | 3341 | **466.7370** | **931.4594** | **931.4876** | **-0.0282** | **0** | **21** | **1.6** | **1** | **K.WGTSGLVGR.H** 3340 3352 3354 3356 |
|  | 3488 | 473.0806 | 944.1466 | 943.5048 | 0.6418 | 0 | 8 | 22 | 10 | R.LDMIPDLK.A |
|  | 3865 | 487.2953 | 972.5761 | 971.4672 | 1.1089 | 0 | 9 | 26 | 10 | K.EADERPAGK.G |
|  | 4486 | **509.2844** | **1016.5543** | **1017.5244** | **-0.9700** | **1** | **10** | **17** | **4** | **R.HKFSDVTGK.I** |
|  | 5433 | **546.7595** | **1091.5045** | **1092.5247** | **-1.0202** | **0** | **9** | **22** | **9** | **K.VHVCGQTHR.T** |
|  | 6187 | **576.2841** | **1150.5537** | **1149.5626** | **0.9911** | **0** | **2** | **86** | **2** | **K.DLTQTASSTAR.A** |
|  | 6358 | **582.2737** | **1162.5329** | **1162.5540** | **-0.0210** | **0** | **10** | **16** | **2** | **K.LNSEMDITPK.L** |
|  | 7428 | 408.1999 | 1221.5778 | 1221.6830 | -0.1051 | 1 | 5 | 49 | 7 | K.NNGPKVPVELR.V |
|  | 7548 | 614.8049 | 1227.5952 | 1228.6776 | -1.0824 | 0 | 17 | 3.5 | 1 | K.ANVTVLDTQIR.K 7555 |
|  | 9369 | 440.2517 | 1317.7333 | 1317.6850 | 0.0483 | 0 | 5 | 48 | 10 | K.VEMTLEILNEK.E |
|  | 9574 | **665.3710** | **1328.7274** | **1327.7500** | **0.9774** | **0** | **1** | **1.2e+002** | **2** | **R.VNIWLGLSAVEK.K** |
|  | 9651 | **445.5534** | **1333.6383** | **1333.6799** | **-0.0416** | **0** | **(4)** | **56** | **8** | **K.VEMTLEILNEK.E** |
|  | 9924 | **450.5954** | **1348.7644** | **1349.6735** | **-0.9091** | **1** | **3** | **82** | **8** | **K.VHVCGQTHRTR.I** |
|  | 10443 | **460.2630** | **1377.7673** | **1378.6405** | **-0.8732** | **0** | **8** | **24** | **2** | **K.ISVYDYDTFTR.D** |
|  | 10838 | **468.1884** | **1401.5432** | **1401.6888** | **-0.1456** | **0** | **7** | **23** | **6** | **K.GFPQPILSEDGSR.I** |
|  | 11059 | **708.3987** | **1414.7829** | **1415.7442** | **-0.9613** | **0** | **6** | **34** | **5** | **R.LDAVNTLLAMAER.L** |
|  | 12016 | 491.6006 | 1471.7799 | 1470.6912 | 1.0887 | 0 | 2 | 82 | 7 | K.SITGEEMSDIYVK.G |
|  | 12110 | 493.6001 | 1477.7785 | 1478.7365 | -0.9580 | 1 | 3 | 64 | 6 | K.DLTGDQSRSLPYK.L |
|  | 14000 | 399.7287 | 1594.8858 | 1594.6987 | 0.1871 | 1 | 4 | 51 | 5 | K.GWWPCYAEKDGAR.V |
|  | 14532 | **815.3777** | **1628.7409** | **1629.8210** | **-1.0800** | **1** | **12** | **8.2** | **3** | **R.DEKVGETIIDLENR.F** |
|  | 15174 | **836.3951** | **1670.7756** | **1669.8934** | **0.8822** | **1** | **1** | **1e+002** | **1** | **R.KPVVGQCTIERLDR.F** |
|  | 17491 | 923.4381 | 1844.8617 | 1844.8105 | 0.0512 | 0 | 8 | 17 | 2 | R.EYTGFPDPYDELNTGK.G 17490 |
|  | 17935 | **627.3592** | **1879.0557** | **1878.0799** | **0.9758** | **1** | **1** | **79** | **6** | **R.VIEGRQLSGNNIRPVVK.V** |
|  | 20541 | 709.7010 | 2126.0811 | 2126.0491 | 0.0320 | 0 | 4 | 35 | 6 | R.DNDSDDVESNLLLPAGIALR.W |
|  | 21433 | **742.3652** | **2224.0737** | **2224.0834** | **-0.0097** | **1** | **2** | **59** | **3** | **K.VIMELFDNDQVGKDEFLGR.S** |
|  | 23467 | 837.0848 | 2508.2327 | 2508.2801 | -0.0474 | 1 | 1 | 59 | 10 | K.GWEYGITIPPDHKPKSWVAAEK.M |
|  | 23891 | **858.7038** | **2573.0896** | **2573.2692** | **-0.1796** | **0** | **1** | **44** | **1** | **K.LMQLTEEPQNSMPDIIIWMIR.G** |
|  | 24095 | **871.3915** | **2611.1528** | **2611.1674** | **-0.0146** | **0** | **33** | **0.028** | **1** | **R.SLLTEADAGHTEFTDEVYQNESR.Y** |
|  | 24663 | **904.0410** | **2709.1012** | **2708.4149** | **0.6863** | **1** | **0** | **47** | **5** | **K.LQMWVDVFPKSLGPPGPPFNITPR.K** |
|  | 25237 | 941.1328 | 2820.3766 | 2819.4826 | 0.8940 | 1 | 3 | 29 | 2 | K.LLWHPVMNGDKACGDVLVTAELILR.G |
|  | 25455 | **575.0711** | **2870.3192** | **2871.3030** | **-0.9839** | **1** | **2** | **35** | **10** | **R.ADCLMGEFKIDVGFVYDEPGHAVMR.K** |
|  | 25603 | **724.8391** | **2895.3271** | **2894.2932** | **1.0339** | **0** | **0** | **48** | **3** | **K.FNSFAEGTFTVFAEMYENQALMFGK.W** |
|  | 28667 | 1207.6290 | 3619.8653 | 3618.7664 | 1.0988 | 1 | 4 | 12 | 2 | R.KYSLSAVFHSATMLQDVGEAIQFEVSIGNYGNK.F |

| **738.** | P78357\|CNTP1_HUMAN    **Mass:** 158220   **Score:** 33     **Queries matched:** 14   **emPAI:** 0.02 |
| --- | --- |
|  | Contactin-associated protein 1 OS=Homo sapiens GN=CNTNAP1 PE=1 SV=1 |

|  | **Query** | **Observed** | **Mr(expt)** | **Mr(calc)** | **Delta** | **Miss** | **Score** | **Expect** | **Rank** | **Peptide** |
| --- | --- | --- | --- | --- | --- | --- | --- | --- | --- | --- |
|  | 3108 | **458.2534** | **914.4922** | **913.3600** | **1.1322** | **0** | **4** | **70** | **10** | **K.ESCEAYR.L** |
|  | 3803 | 485.4900 | 968.9654 | 969.5607 | -0.5953 | 0 | 33 | 0.045 | 1 | R.VNIADLAVR.R 3817 |
|  | 4937 | **527.3020** | **1052.5894** | **1052.5767** | **0.0128** | **1** | **10** | **14** | **4** | **K.KLQFAAGYR.L** |
|  | 5688 | **555.3578** | **1108.7011** | **1108.5778** | **0.1234** | **0** | **8** | **25** | **5** | **R.LHGISGWSPR.I** |
|  | 5689 | 555.5287 | 1109.0428 | 1109.5506 | -0.5077 | 0 | 9 | 13 | 1 | R.FILNGDFER.L |
|  | 10431 | **460.0398** | **1377.0975** | **1377.4860** | **-0.3886** | **0** | **5** | **29** | **6** | **R.CSPNMCEHDGR.C** |
|  | 13852 | **529.3419** | **1585.0038** | **1584.8147** | **0.1890** | **0** | **2** | **68** | **10** | **R.SLGASSYYSLLTAPR.F** |
|  | 15772 | **571.7925** | **1712.3556** | **1711.8695** | **0.4861** | **1** | **10** | **7.7** | **1** | **R.HDLHFHFTARYIR.I** |
|  | 15923 | 575.3360 | 1722.9862 | 1723.9410 | -0.9548 | 1 | 7 | 22 | 5 | R.FRTWDLTGLLLFSR.L |
|  | 22788 | **1204.9688** | **2407.9229** | **2406.9595** | **0.9634** | **0** | **3** | **27** | **1** | **R.CYQSWDDFICYCELTGYK.G** |
|  | 26793 | **1047.4403** | **3139.2991** | **3139.5649** | **-0.2658** | **1** | **2** | **25** | **7** | **R.LSGKTSGNFTIDPDGSGPLKPFVVYCDIR.E** |
|  | 28981 | **1242.2815** | **3723.8226** | **3722.9169** | **0.9057** | **1** | **2** | **20** | **1** | **R.YQLGTSPYVYQLTTRPVTDGQPHSINITRVYR.N** |
|  | 29029 | 1872.2601 | 3742.5057 | 3742.7654 | -0.2597 | 1 | 2 | 16 | 8 | R.WDCHSNQTAFHGCMELLKVDGQLVNLTLVEGR.R |

| **739.** | Q9NQE9\|HINT3_HUMAN    **Mass:** 20804    **Score:** 33     **Queries matched:** 4   **emPAI:** 0.18 |
| --- | --- |
|  | Histidine triad nucleotide-binding protein 3 OS=Homo sapiens GN=HINT3 PE=1 SV=1 |

|  | **Query** | **Observed** | **Mr(expt)** | **Mr(calc)** | **Delta** | **Miss** | **Score** | **Expect** | **Rank** | **Peptide** |
| --- | --- | --- | --- | --- | --- | --- | --- | --- | --- | --- |
|  | 2690 | 443.0382 | 884.0619 | 883.4446 | 0.6172 | 1 | 5 | 35 | 4 | K.KHIGNCR.T |
|  | 15443 | **422.9901** | **1687.9315** | **1687.9410** | **-0.0095** | **0** | **33** | **0.053** | **1** | **K.DIKPAATHHYLVVPK.K** |
|  | 15449 | **563.9598** | **1688.8577** | **1687.8815** | **0.9762** | **1** | **11** | **8.9** | **2** | **R.KDQVELVENMVTVGK.T** |
|  | 15672 | 853.3663 | 1704.7180 | 1703.8764 | 0.8416 | 1 | (0) | 94 | 6 | R.KDQVELVENMVTVGK.T |

| **740.** | Q06136\|KDSR_HUMAN    **Mass:** 36563    **Score:** 33     **Queries matched:** 6   **emPAI:** 0.10 |
| --- | --- |
|  | 3-ketodihydrosphingosine reductase OS=Homo sapiens GN=KDSR PE=1 SV=1 |

|  | **Query** | **Observed** | **Mr(expt)** | **Mr(calc)** | **Delta** | **Miss** | **Score** | **Expect** | **Rank** | **Peptide** |
| --- | --- | --- | --- | --- | --- | --- | --- | --- | --- | --- |
|  | 2094 | 423.0529 | 844.0912 | 843.4814 | 0.6098 | 0 | 4 | 55 | 9 | R.TKPLETR.L |
|  | 2637 | 441.2956 | 880.5766 | 880.3830 | 0.1937 | 1 | 3 | 67 | 7 | R.RCMMQR.E |
|  | 10324 | 458.2637 | 1371.7694 | 1370.6354 | 1.1340 | 0 | 6 | 39 | 2 | K.FEDLEVSTFER.L |
|  | 14292 | **539.1592** | **1614.4557** | **1614.8076** | **-0.3518** | **0** | **11** | **6.2** | **1** | **R.LMSINYLGSVYPSR.A** |
|  | 16761 | **895.4665** | **1788.9185** | **1788.9291** | **-0.0106** | **0** | **33** | **0.051** | **1** | **R.LISETTSVCKPEQVAK.Q** |
|  | 17575 | **617.6457** | **1849.9152** | **1849.8736** | **0.0416** | **0** | **5** | **35** | **6** | **K.LGPVDMLVNCAGMAVSGK.F** |

| **741.** | P08727\|K1C19_HUMAN    **Mass:** 44065    **Score:** 33     **Queries matched:** 7   **emPAI:** 0.08 |
| --- | --- |
|  | Keratin, type I cytoskeletal 19 OS=Homo sapiens GN=KRT19 PE=1 SV=3 |

|  | **Query** | **Observed** | **Mr(expt)** | **Mr(calc)** | **Delta** | **Miss** | **Score** | **Expect** | **Rank** | **Peptide** |
| --- | --- | --- | --- | --- | --- | --- | --- | --- | --- | --- |
|  | 2389 | **432.8838** | **863.7530** | **864.4592** | **-0.7063** | **0** | **0** | **1.1e+002** | **8** | **K.EELAYLK.K** |
|  | 4790 | 521.3032 | 1040.5918 | 1040.5978 | -0.0061 | 0 | 33 | 0.074 | 1 | R.IVLQIDNAR.L |
|  | 7074 | 402.2434 | 1203.7084 | 1203.5918 | 0.1167 | 0 | 20 | 1.5 | 2 | R.MSVEADINGLR.R |
|  | 9988 | 677.3536 | 1352.6927 | 1353.5983 | -0.9056 | 0 | 8 | 20 | 2 | R.SQYEVMAEQNR.K |
|  | 10400 | 688.7741 | 1375.5337 | 1375.6878 | -0.1541 | 1 | 1 | 1e+002 | 7 | R.MSVEADINGLRR.V |
|  | 19200 | **664.3985** | **1990.1737** | **1989.0564** | **1.1172** | **1** | **1** | **62** | **7** | **R.RTLQGLEIELQSQLSMK.A** |
|  | 21478 | **743.4095** | **2227.2066** | **2227.0651** | **0.1415** | **1** | **1** | **67** | **2** | **R.TEELNREVAGHTEQLQMSR.S** |

| **742.** | Q9HCU5\|PREB_HUMAN    **Mass:** 46010    **Score:** 33     **Queries matched:** 11   **emPAI:** 0.08 |
| --- | --- |
|  | Prolactin regulatory element-binding protein OS=Homo sapiens GN=PREB PE=1 SV=2 |

|  | **Query** | **Observed** | **Mr(expt)** | **Mr(calc)** | **Delta** | **Miss** | **Score** | **Expect** | **Rank** | **Peptide** |
| --- | --- | --- | --- | --- | --- | --- | --- | --- | --- | --- |
|  | 3319 | **465.7392** | **929.4638** | **928.4726** | **0.9912** | **1** | **8** | **33** | **7** | **K.AGSKEQGPR.Q** 3296 |
|  | 4257 | 500.7903 | 999.5660 | 999.6077 | -0.0417 | 1 | 10 | 17 | 8 | K.LVTVGRDLK.A 4285 |
|  | 6652 | **593.2670** | **1184.5194** | **1184.5687** | **-0.0493** | **0** | **1** | **1.4e+002** | **5** | **R.FQAHQQQGNK.A** |
|  | 12461 | **500.6127** | **1498.8164** | **1498.6834** | **0.1329** | **0** | **4** | **47** | **6** | **K.CGAETQHEGLELR.V** |
|  | 12685 | 505.2525 | 1512.7356 | 1512.7433 | -0.0077 | 1 | 23 | 0.62 | 1 | R.FQAHQQQGNKAEK.A |
|  | 21867 | **759.3828** | **2275.1266** | **2275.1808** | **-0.0542** | **1** | **7** | **17** | **1** | **K.AHEGEIEDLALGPDGKLVTVGR.D** |
|  | 22099 | **770.3665** | **2308.0777** | **2308.0907** | **-0.0129** | **0** | **3** | **42** | **3** | **K.VVCFNHDNTLLATGGTDGYVR.V** |
|  | 23425 | 834.7603 | 2501.2591 | 2500.2685 | 0.9906 | 1 | 33 | 0.034 | 1 | R.LRQPPPCYLTAWDGSNFLPLR.T |
|  | 23542 | **841.4420** | **2521.3041** | **2522.2733** | **-0.9693** | **0** | **18** | **1.2** | **1** | **R.ATMNLALAGDILAAGQDAHCQLLR.F** |

| **743.** | P62988\|UBIQ_HUMAN    **Mass:** 8560     **Score:** 33     **Queries matched:** 6   **emPAI:** 0.48 |
| --- | --- |
|  | Ubiquitin OS=Homo sapiens GN=RPS27A PE=1 SV=1 |

|  | **Query** | **Observed** | **Mr(expt)** | **Mr(calc)** | **Delta** | **Miss** | **Score** | **Expect** | **Rank** | **Peptide** |
| --- | --- | --- | --- | --- | --- | --- | --- | --- | --- | --- |
|  | 5119 | **534.3110** | **1066.6075** | **1066.6135** | **-0.0060** | **0** | **3** | **73** | **8** | **K.ESTLHLVLR.L** |
|  | 8310 | **632.8682** | **1263.7218** | **1264.7213** | **-0.9996** | **1** | **11** | **10** | **5** | **-.MQIFVKTLTGK.T** |
|  | 16634 | 593.9457 | 1778.8154 | 1778.8799 | -0.0645 | 1 | 2 | 63 | 7 | K.QLEDGRTLSDYNIQK.E |
|  | 16735 | **894.4629** | **1786.9112** | **1786.9200** | **-0.0088** | **0** | **33** | **0.053** | **1** | **K.TITLEVEPSDTIENVK.A** 16734 |
|  | 19139 | 663.0047 | 1985.9923 | 1986.0521 | -0.0598 | 1 | 5 | 27 | 6 | K.TITLEVEPSDTIENVKAK.I |

| **744.** | P62913\|RL11_HUMAN    **Mass:** 20468    **Score:** 33     **Queries matched:** 7   **emPAI:** 0.19 |
| --- | --- |
|  | 60S ribosomal protein L11 OS=Homo sapiens GN=RPL11 PE=1 SV=2 |

|  | **Query** | **Observed** | **Mr(expt)** | **Mr(calc)** | **Delta** | **Miss** | **Score** | **Expect** | **Rank** | **Peptide** |
| --- | --- | --- | --- | --- | --- | --- | --- | --- | --- | --- |
|  | 2994 | 454.2086 | 906.4027 | 905.3913 | 1.0114 | 0 | 10 | 16 | 4 | -.MAQDQGEK.E |
|  | 3723 | **482.2461** | **962.4776** | **962.4855** | **-0.0079** | **1** | **21** | **1.3** | **1** | **R.ISKEEAMR.W** |
|  | 5368 | 544.4226 | 1086.8307 | 1086.5920 | 0.2386 | 1 | 1 | 1.1e+002 | 7 | R.GAKAEEILEK.G |
|  | 13211 | **773.9224** | **1545.8303** | **1545.8403** | **-0.0100** | **0** | **33** | **0.062** | **1** | **K.VLEQLTGQTPVFSK.A** |
|  | 13253 | **517.0737** | **1548.1992** | **1548.6660** | **-0.4669** | **1** | **7** | **16** | **2** | **-.MAQDQGEKENPMR.E** 13259 |
|  | 15500 | **564.9714** | **1691.8923** | **1691.9035** | **-0.0112** | **1** | **29** | **0.14** | **1** | **R.WFQQKYDGIILPGK.-** |

| **745.** | Q8N8Y2\|VA0D2_HUMAN    **Mass:** 40913    **Score:** 33     **Queries matched:** 2   **emPAI:** 0.09 |
| --- | --- |
|  | V-type proton ATPase subunit d 2 OS=Homo sapiens GN=ATP6V0D2 PE=2 SV=1 |

|  | **Query** | **Observed** | **Mr(expt)** | **Mr(calc)** | **Delta** | **Miss** | **Score** | **Expect** | **Rank** | **Peptide** |
| --- | --- | --- | --- | --- | --- | --- | --- | --- | --- | --- |
|  | 10945 | 705.3403 | 1408.6661 | 1407.6704 | 0.9957 | 0 | 33 | 0.068 | 1 | R.LLAQAEDFDQMK.N |
|  | 24799 | **548.2850** | **2736.3885** | **2737.2298** | **-0.8414** | **1** | **3** | **33** | **2** | **K.FCKNHGDVTAEVMCPILEFEADR.R** |

| **746.** | Q96A26\|F162A_HUMAN    **Mass:** 17559    **Score:** 33     **Queries matched:** 5   **emPAI:** 0.22 |
| --- | --- |
|  | UPF0389 protein FAM162A OS=Homo sapiens GN=FAM162A PE=1 SV=2 |

|  | **Query** | **Observed** | **Mr(expt)** | **Mr(calc)** | **Delta** | **Miss** | **Score** | **Expect** | **Rank** | **Peptide** |
| --- | --- | --- | --- | --- | --- | --- | --- | --- | --- | --- |
|  | 1965 | **419.2045** | **836.3945** | **835.4222** | **0.9723** | **0** | **15** | **5.1** | **7** | **-.MGSLSGLR.L** 1942 |
|  | 3191 | **460.7480** | **919.4815** | **918.4844** | **0.9971** | **1** | **33** | **0.098** | **1** | **R.LKEEAAMK.A** |
|  | 3844 | **486.8000** | **971.5855** | **971.5916** | **-0.0061** | **1** | **20** | **1.9** | **1** | **K.KILIWSGR.F** |
|  | 23310 | 828.7316 | 2483.1731 | 2484.3558 | -1.1827 | 1 | 2 | 42 | 6 | K.ISYLMIALTVVGCIFMVIEGKK.A |

| **747.** | P07602\|SAP_HUMAN    **Mass:** 59899    **Score:** 33     **Queries matched:** 10   **emPAI:** 0.20 |
| --- | --- |
|  | Proactivator polypeptide OS=Homo sapiens GN=PSAP PE=1 SV=2 |

|  | **Query** | **Observed** | **Mr(expt)** | **Mr(calc)** | **Delta** | **Miss** | **Score** | **Expect** | **Rank** | **Peptide** |
| --- | --- | --- | --- | --- | --- | --- | --- | --- | --- | --- |
|  | 2361 | **432.2203** | **862.4259** | **862.4405** | **-0.0145** | **1** | **6** | **56** | **6** | **K.MCSKLPK.S** |
|  | 8240 | **421.0566** | **1260.1479** | **1259.6254** | **0.5225** | **0** | **10** | **11** | **1** | **K.EMPMQTLVPAK.V** |
|  | 14779 | 549.2842 | 1644.8307 | 1644.8579 | -0.0272 | 1 | 6 | 26 | 8 | K.EMPMQTLVPAKVASK.N |
|  | 15298 | **560.2656** | **1677.7751** | **1677.8872** | **-0.1121** | **0** | **5** | **40** | **2** | **K.IGACPSAHKPLLGTEK.C** |
|  | 23025 | **1221.6191** | **2441.2237** | **2442.1204** | **-0.8966** | **0** | **29** | **0.091** | **1** | **K.NYISQYSEIAIQMMMHMQPK.E** 23028 23038 |
|  | 26954 | **793.9108** | **3171.6140** | **3170.6032** | **1.0108** | **0** | **19** | **0.66** | **1** | **K.IPELDMTEVVAPFMANIPLLLYPQDGPR.S** |
|  | 28112 | **1157.5649** | **3469.6730** | **3469.6859** | **-0.0130** | **0** | **20** | **0.42** | **1** | **K.QCDQFVAEYEPVLIEILVEVMDPSFVCLK.I** |
|  | 29267 | **768.5686** | **3837.8066** | **3837.9685** | **-0.1619** | **1** | **1** | **27** | **1** | **K.QLESNKIPELDMTEVVAPFMANIPLLLYPQDGPR.S** |

| **748.** | A0PJK1\|SC5AA_HUMAN    **Mass:** 64928    **Score:** 33     **Queries matched:** 7   **emPAI:** 0.06 |
| --- | --- |
|  | Sodium/glucose cotransporter 5 OS=Homo sapiens GN=SLC5A10 PE=2 SV=2 |

|  | **Query** | **Observed** | **Mr(expt)** | **Mr(calc)** | **Delta** | **Miss** | **Score** | **Expect** | **Rank** | **Peptide** |
| --- | --- | --- | --- | --- | --- | --- | --- | --- | --- | --- |
|  | 3815 | **486.2525** | **970.4905** | **969.5468** | **0.9437** | **1** | **3** | **92** | **7** | **R.LRPRSGER.E** |
|  | 7585 | 410.5723 | 1228.6952 | 1227.6935 | 1.0016 | 1 | 9 | 19 | 2 | R.SGERELLLVGR.L 7535 |
|  | 9075 | 435.2236 | 1302.6491 | 1302.7039 | -0.0549 | 0 | 5 | 50 | 4 | K.LVMELMPIGLR.G |
|  | 14008 | **798.8561** | **1595.6976** | **1595.7072** | **-0.0096** | **0** | **33** | **0.061** | **1** | **R.ACGAEVGCSNIAYPK.L** |
|  | 15705 | 569.9538 | 1706.8396 | 1706.8591 | -0.0196 | 0 | 4 | 46 | 5 | K.MLPMGLIIMPGMISR.A 15720 |

| **749.** | Q9Y639\|NPTN_HUMAN    **Mass:** 31500    **Score:** 33     **Queries matched:** 4   **emPAI:** 0.12 |
| --- | --- |
|  | Neuroplastin OS=Homo sapiens GN=NPTN PE=1 SV=1 |

|  | **Query** | **Observed** | **Mr(expt)** | **Mr(calc)** | **Delta** | **Miss** | **Score** | **Expect** | **Rank** | **Peptide** |
| --- | --- | --- | --- | --- | --- | --- | --- | --- | --- | --- |
|  | 4261 | **500.9519** | **999.8892** | **999.4080** | **0.4812** | **0** | **7** | **23** | **1** | **K.NASNMEYR.I** 4280 |
|  | 12696 | **505.2710** | **1512.7912** | **1511.7310** | **1.0602** | **0** | **3** | **61** | **2** | **K.SVGYPHPDWIWR.K** |
|  | 16564 | **887.8667** | **1773.7188** | **1773.7298** | **-0.0109** | **1** | **33** | **0.055** | **1** | **R.SENKNEGQDATMYCK.S** |

| **750.** | Q9HCG7\|GBA2_HUMAN    **Mass:** 105892   **Score:** 32     **Queries matched:** 14   **emPAI:** 0.03 |
| --- | --- |
|  | Non-lysosomal glucosylceramidase OS=Homo sapiens GN=GBA2 PE=1 SV=2 |

|  | **Query** | **Observed** | **Mr(expt)** | **Mr(calc)** | **Delta** | **Miss** | **Score** | **Expect** | **Rank** | **Peptide** |
| --- | --- | --- | --- | --- | --- | --- | --- | --- | --- | --- |
|  | 7105 | **603.3778** | **1204.7411** | **1203.6282** | **1.1129** | **0** | **7** | **27** | **6** | **K.GVGIAGAVCVSSK.L** |
|  | 7197 | **404.4139** | **1210.2198** | **1210.6090** | **-0.3892** | **0** | **5** | **30** | **7** | **R.YLMSGVMAPVK.R** |
|  | 11193 | **475.2674** | **1422.7803** | **1423.6707** | **-0.8904** | **0** | **4** | **47** | **6** | **K.AMGYQVPPFGWR.I** |
|  | 11848 | 731.8690 | 1461.7234 | 1461.7399 | -0.0165 | 1 | 0 | 1.2e+002 | 9 | K.QGTGLRTGPMFGPK.E |
|  | 12503 | **501.3109** | **1500.9108** | **1501.7963** | **-0.8855** | **0** | **13** | **6.4** | **1** | **K.TPFIDMINSVPLR.Q** |
|  | 13706 | **526.6196** | **1576.8371** | **1577.8348** | **-0.9977** | **1** | **5** | **35** | **5** | **R.TVIADQFTVCLRR.E** |
|  | 14426 | **541.9092** | **1622.7057** | **1621.7121** | **0.9936** | **0** | **16** | **2.9** | **1** | **R.YYNYDSSSRPQSR.S** |
|  | 14532 | 815.3777 | 1628.7409 | 1629.8912 | -1.1503 | 1 | 7 | 22 | 7 | K.KTPFIDMINSVPLR.Q |
|  | 14950 | **828.8748** | **1655.7351** | **1655.7436** | **-0.0085** | **0** | **32** | **0.064** | **1** | **R.SVMSDQCAGQWFLK.A** |
|  | 17213 | **609.3401** | **1824.9984** | **1824.7919** | **0.2066** | **0** | **(1)** | **78** | **4** | **K.DMWPVCLAVMESEMK.F** |
|  | 17503 | **616.3108** | **1845.9107** | **1845.9883** | **-0.0776** | **1** | **2** | **72** | **7** | **K.RKPFQANNVSLSNMIK.H** |
|  | 17663 | 929.4699 | 1856.9253 | 1856.7817 | 0.1436 | 0 | 4 | 45 | 5 | K.DMWPVCLAVMESEMK.F |
|  | 27441 | **825.6397** | **3298.5297** | **3299.6067** | **-1.0770** | **0** | **1** | **35** | **2** | **R.ALQTIFELNVQAFAGGAMGAVNGMQPHGVPDK.S** |
|  | 29449 | **1304.6115** | **3910.8125** | **3910.6932** | **0.1193** | **1** | **0** | **30** | **5** | **M.GTQDPGNMGTGVPASEQISCAKEDPQVYCPEETGGTK.D** |

| **751.** | O15269\|SPTC1_HUMAN    **Mass:** 53281    **Score:** 32     **Queries matched:** 7   **emPAI:** 0.07 |
| --- | --- |
|  | Serine palmitoyltransferase 1 OS=Homo sapiens GN=SPTLC1 PE=1 SV=1 |

|  | **Query** | **Observed** | **Mr(expt)** | **Mr(calc)** | **Delta** | **Miss** | **Score** | **Expect** | **Rank** | **Peptide** |
| --- | --- | --- | --- | --- | --- | --- | --- | --- | --- | --- |
|  | 2300 | **430.7058** | **859.3971** | **858.4923** | **0.9048** | **0** | **7** | **48** | **3** | **R.SIALTQAR.Y** |
|  | 3180 | 460.2736 | 918.5327 | 918.5022 | 0.0306 | 1 | 10 | 17 | 7 | R.SDLTVKEK.E |
|  | 3407 | 469.2734 | 936.5323 | 936.5029 | 0.0294 | 1 | 7 | 29 | 8 | K.TYKLQER.S |
|  | 11031 | 707.8861 | 1413.7577 | 1412.8239 | 0.9338 | 1 | 6 | 34 | 6 | R.AASTIKEVAQAVLL.- |
|  | 12282 | 496.6289 | 1486.8648 | 1487.7191 | -0.8543 | 1 | 3 | 66 | 9 | K.LFKHNDMADLER.L |
|  | 12773 | **759.8687** | **1517.7227** | **1517.7330** | **-0.0103** | **0** | **32** | **0.074** | **1** | **R.LLQEIVDQCMNR.S** |
|  | 23057 | 815.7460 | 2444.2161 | 2443.2608 | 0.9553 | 1 | 3 | 37 | 3 | K.DHPALNYNIVSGPPSHKTVVNGK.E |

| **752.** | O95954\|FTCD_HUMAN    **Mass:** 59574    **Score:** 32     **Queries matched:** 15   **emPAI:** 0.06 |
| --- | --- |
|  | Formimidoyltransferase-cyclodeaminase OS=Homo sapiens GN=FTCD PE=1 SV=2 |

|  | **Query** | **Observed** | **Mr(expt)** | **Mr(calc)** | **Delta** | **Miss** | **Score** | **Expect** | **Rank** | **Peptide** |
| --- | --- | --- | --- | --- | --- | --- | --- | --- | --- | --- |
|  | 868 | **571.3161** | **570.3088** | **571.2714** | **-0.9626** | **0** | **2** | **98** | **6** | **K.DQPGR.L** |
|  | 1219 | 734.9947 | 733.9874 | 733.3792 | 0.6082 | 0 | 3 | 70 | 1 | R.LIDMSR.H |
|  | 1700 | 409.0727 | 816.1309 | 816.4817 | -0.3508 | 1 | 4 | 64 | 10 | R.GLGSKSLR.A |
|  | 2153 | **425.2120** | **848.4094** | **849.3585** | **-0.9491** | **0** | **5** | **56** | **3** | **R.CGNLACR.S** |
|  | 5735 | **557.2731** | **1112.5317** | **1113.6254** | **-1.0937** | **1** | **0** | **1.4e+002** | **6** | **R.TAALQEGLRR.A** |
|  | 7832 | **621.3559** | **1240.6972** | **1241.5710** | **-0.8738** | **0** | **3** | **63** | **8** | **R.QFQSLDTTMR.R** |
|  | 10488 | **461.2237** | **1380.6492** | **1381.6772** | **-1.0280** | **1** | **7** | **29** | **2** | **R.QFQSLDTTMRR.L** |
|  | 12314 | 497.2614 | 1488.7625 | 1489.7422 | -0.9797 | 0 | 9 | 18 | 8 | R.MGALDVCPFIPVR.G |
|  | 13599 | **524.6221** | **1570.8446** | **1569.8515** | **0.9931** | **1** | **10** | **12** | **1** | **R.IIEYLVPERGPER.G** |
|  | 15923 | 575.3360 | 1722.9862 | 1723.7909 | -0.8048 | 0 | 5 | 31 | 6 | -.MSQLVECVPNFSEGK.N |
|  | 18242 | **636.8220** | **1907.4441** | **1906.9676** | **0.4764** | **0** | **1** | **48** | **1** | **R.LAEELDVPVYLYGEAAR.M** |
|  | 21633 | 750.6220 | 2248.8442 | 2249.1151 | -0.2709 | 0 | 5 | 19 | 2 | R.TVYTFVGPPECVVEGALNAAR.V 21636 |
|  | 23623 | 1268.2090 | 2534.4034 | 2534.4108 | -0.0074 | 0 | 32 | 0.036 | 1 | R.AVSVPLTLAETVASLWPALQELAR.C |
|  | 31143 | 1645.4074 | 4933.2002 | 4932.3580 | 0.8422 | 1 | 0 | 12 | 9 | -.MSQLVECVPNFSEGKNQEVIDAISGAITQTPGCVLLDVDAGPSTNR.T |

| **753.** | P27482\|CALL3_HUMAN    **Mass:** 16937    **Score:** 32     **Queries matched:** 8   **emPAI:** 0.23 |
| --- | --- |
|  | Calmodulin-like protein 3 OS=Homo sapiens GN=CALML3 PE=1 SV=2 |

|  | **Query** | **Observed** | **Mr(expt)** | **Mr(calc)** | **Delta** | **Miss** | **Score** | **Expect** | **Rank** | **Peptide** |
| --- | --- | --- | --- | --- | --- | --- | --- | --- | --- | --- |
|  | 1552 | 805.4176 | 804.4103 | 804.4164 | -0.0060 | 0 | 21 | 1.9 | 1 | R.ELGTVMR.S |
|  | 4163 | **497.2933** | **992.5720** | **993.4186** | **-0.8465** | **0** | **1** | **1.5e+002** | **2** | **K.DGDGCITTR.E** |
|  | 13041 | 513.2478 | 1536.7216 | 1536.7308 | -0.0092 | 0 | 0 | 1.1e+002 | 8 | M.ADQLTEEQVTEFK.E |
|  | 15908 | 862.4147 | 1722.8148 | 1723.8530 | -1.0382 | 1 | 32 | 0.066 | 1 | R.VFDKDGNGFVSAAELR.H 15907 15909 |
|  | 25436 | **717.1083** | **2864.4040** | **2865.2078** | **-0.8038** | **1** | **(5)** | **20** | **1** | **R.DMMSEIDRDGNGTVDFPEFLGMMAR.K** |
|  | 25609 | **966.4111** | **2896.2116** | **2897.1976** | **-0.9861** | **1** | **6** | **11** | **1** | **R.DMMSEIDRDGNGTVDFPEFLGMMAR.K** |

| **754.** | P00558\|PGK1_HUMAN    **Mass:** 44985    **Score:** 32     **Queries matched:** 24   **emPAI:** 0.08 |
| --- | --- |
|  | Phosphoglycerate kinase 1 OS=Homo sapiens GN=PGK1 PE=1 SV=3 |

|  | **Query** | **Observed** | **Mr(expt)** | **Mr(calc)** | **Delta** | **Miss** | **Score** | **Expect** | **Rank** | **Peptide** |
| --- | --- | --- | --- | --- | --- | --- | --- | --- | --- | --- |
|  | 2431 | **434.7322** | **867.4498** | **866.4684** | **0.9814** | **1** | **10** | **14** | **5** | **K.AGGFLMKK.E** |
|  | 3265 | 463.6054 | 925.1963 | 925.5960 | -0.3998 | 1 | 0 | 92 | 6 | R.IKAAVPSIK.F |
|  | 5571 | **551.5913** | **1101.1681** | **1100.5323** | **0.6358** | **0** | **5** | **34** | **2** | **K.NNQITNNQR.I** |
|  | 7981 | **624.7523** | **1247.4901** | **1248.6747** | **-1.1847** | **1** | **14** | **5.9** | **2** | **-.MSLSNKLTLDK.L** |
|  | 10253 | 456.9011 | 1367.6815 | 1366.7027 | 0.9788 | 0 | 1 | 1.2e+002 | 10 | R.AHSSMVGVNLPQK.A |
|  | 10518 | 692.3695 | 1382.7243 | 1382.6976 | 0.0267 | 0 | (0) | 1.3e+002 | 8 | R.AHSSMVGVNLPQK.A |
|  | 11627 | 483.9344 | 1448.7814 | 1449.7472 | -0.9658 | 1 | 8 | 22 | 4 | R.VVMRVDFNVPMK.N 11648 |
|  | 13921 | 795.9156 | 1589.8166 | 1589.8235 | -0.0069 | 1 | 4 | 52 | 4 | K.AAVPSIKFCLDNGAK.S |
|  | 14591 | **545.2830** | **1632.8271** | **1633.7849** | **-0.9578** | **0** | **8** | **19** | **1** | **K.LGDVYVNDAFGTAHR.A** |
|  | 16203 | **873.0958** | **1744.1770** | **1744.9975** | **-0.8205** | **1** | **1** | **54** | **1** | **K.YSLEPVAVELKSLLGK.D** |
|  | 16322 | **877.8915** | **1753.7685** | **1753.7798** | **-0.0112** | **0** | **16** | **2.7** | **1** | **R.GCITIIGGGDTATCCAK.W** |
|  | 16492 | **884.9677** | **1767.9207** | **1767.9883** | **-0.0675** | **0** | **15** | **3.5** | **1** | **K.ALESPERPFLAILGGAK.V** |
|  | 19537 | **675.0138** | **2022.0195** | **2022.0310** | **-0.0114** | **1** | **1** | **73** | **8** | **K.ITLPVDFVTADKFDENAK.T** |
|  | 19633 | **508.7470** | **2030.9587** | **2030.9956** | **-0.0369** | **1** | **2** | **52** | **7** | **R.VDFNVPMKNNQITNNQR.I** |
|  | 19995 | **689.3122** | **2064.9147** | **2066.0289** | **-1.1142** | **0** | **2** | **48** | **4** | **K.SVVLMSHLGRPDGVPMPDK.Y** |
|  | 22092 | **577.7865** | **2307.1169** | **2306.1828** | **0.9341** | **1** | **(2)** | **52** | **3** | **K.VLNNMEIGTSLFDEEGAKIVK.D** |
|  | 22226 | 775.4099 | 2323.2077 | 2322.1777 | 1.0300 | 1 | 8 | 12 | 3 | K.VLNNMEIGTSLFDEEGAKIVK.D |
|  | 23487 | **838.7486** | **2513.2240** | **2513.2398** | **-0.0158** | **1** | **7** | **14** | **1** | **K.WNTEDKVSHVSTGGGASLELLEGK.V** |
|  | 24067 | **869.0801** | **2604.2186** | **2604.2312** | **-0.0126** | **1** | **32** | **0.039** | **1** | **K.TGQATVASGIPAGWMGLDCGPESSKK.Y** |
|  | 27038 | **799.8826** | **3195.5014** | **3196.4441** | **-0.9427** | **1** | **3** | **23** | **8** | **K.FDENAKTGQATVASGIPAGWMGLDCGPESSK.K** 27053 |
|  | 27325 | **816.6816** | **3262.6972** | **3262.7094** | **-0.0122** | **1** | **17** | **0.82** | **1** | **K.SVVLMSHLGRPDGVPMPDKYSLEPVAVELK.S** 27324 |

| **755.** | O75880\|SCO1_HUMAN    **Mass:** 34021    **Score:** 32     **Queries matched:** 9   **emPAI:** 0.11 |
| --- | --- |
|  | Protein SCO1 homolog, mitochondrial OS=Homo sapiens GN=SCO1 PE=1 SV=1 |

|  | **Query** | **Observed** | **Mr(expt)** | **Mr(calc)** | **Delta** | **Miss** | **Score** | **Expect** | **Rank** | **Peptide** |
| --- | --- | --- | --- | --- | --- | --- | --- | --- | --- | --- |
|  | 5559 | 551.2607 | 1100.5068 | 1101.6039 | -1.0970 | 0 | 8 | 26 | 1 | -.MAMLVLVPGR.V 5565 5572 5599 |
|  | 5780 | **559.2545** | **1116.4944** | **1117.5988** | **-1.1044** | **0** | **(4)** | **72** | **5** | **-.MAMLVLVPGR.V** |
|  | 11191 | **475.2590** | **1422.7553** | **1421.6622** | **1.0930** | **1** | **6** | **33** | **8** | **R.QFCARQAEAWR.A** |
|  | 15447 | **563.8415** | **1688.5028** | **1687.8464** | **0.6564** | **0** | **3** | **38** | **3** | **K.GEIAASIATHMRPYR.K** |
|  | 16172 | 871.9678 | 1741.9211 | 1741.9323 | -0.0112 | 1 | 32 | 0.061 | 1 | K.LVGLTGTREEVDQVAR.A |
|  | 20485 | **530.2887** | **2117.1257** | **2117.1382** | **-0.0125** | **0** | **12** | **6.1** | **1** | **R.HIGKPLLGGPFSLTTHTGER.K** |

Bottom of Form

**Mascot:**  http://www.matrixscience.com/

Top scoring peptide matches to query 145
File7043 Spectrum2424 scans: 1880
Score greater than 17 indicates homology
Score greater than 39 indicates identity

**Score	Expect	Delta Hit Protein	Peptide**
 8.5	63	1.0437	AGGGR
 8.5	63	1.0437	GAGGR
 4.6 1.6e+002	1.0185	K.GVGGK.L
 4.0 1.8e+002	1.0437	GGGAR
 3.5	2e+002	1.0186 373	P10412|H14_HUMAN K.AGAAK.A
 3.3 2.1e+002	1.0186	GAAAK
 2.9 2.3e+002	1.0185	R.GGGVK.A

Top scoring peptide matches to query 269
File7043 Spectrum1755 scans: 1325
Score greater than 14 indicates homology
Score greater than 36 indicates identity

**Score	Expect	Delta Hit Protein	Peptide**
 1.4 1.5e+002	-0.3747 702	O95169|NDUB8_HUMAN AAAAK

Top scoring peptide matches to query 289
File7043 Spectrum153 scans: 248
Score greater than 17 indicates homology
Score greater than 36 indicates identity

**Score	Expect	Delta Hit Protein	Peptide**
 4.0	84	-0.3738 702	O95169|NDUB8_HUMAN AAAAK

Top scoring peptide matches to query 290
File7043 Spectrum16408 scans: 13597
Score greater than 20 indicates homology
Score greater than 38 indicates identity

**Score	Expect	Delta Hit Protein	Peptide**
 12.6	20	0.5165	AGAGR
 12.3	21	0.5165	AGGAR
 6.7	77	0.4913	R.VGGAK.V
 6.7	77	0.5277	K.VGGAQ.-
 5.2 1.1e+002	0.5165	GAAGR
 4.8 1.2e+002	0.5165 221	Q9UJS0|CMC2_HUMAN GAGAR
 0.7	3e+002	0.4913	AGVGK

Top scoring peptide matches to query 292
File7043 Spectrum28735 scans: 25549
Score greater than 18 indicates homology
Score greater than 39 indicates identity

**Score	Expect	Delta Hit Protein	Peptide**
 6.9	85	1.0005	GGAAR
 5.6 1.2e+002	1.0005	AGGAR
 5.6 1.2e+002	1.0005 221	Q9UJS0|CMC2_HUMAN GAGAR

Top scoring peptide matches to query 394
File7043 Spectrum28428 scans: 25290
Score greater than 17 indicates homology
Score greater than 28 indicates identity

**Score	Expect	Delta Hit Protein	Peptide**
 5.1	12	-0.1968 680+ P53990|IST1_HUMAN GGPGR
 4.8	13	-0.1968	GGGPR

Top scoring peptide matches to query 396
File7043 Spectrum29374 scans: 26072
Score greater than 13 indicates homology
Score greater than 28 indicates identity

**Score	Expect	Delta Hit Protein	Peptide**
 0.9	32	-0.1968 680+ P53990|IST1_HUMAN GGPGR

Top scoring peptide matches to query 494
File7043 Spectrum30387 scans: 26891
Score greater than 18 indicates homology
Score greater than 37 indicates identity

**Score	Expect	Delta Hit Protein	Peptide**
 8.9	40	-0.1163	AGSGR
 5.4	91	-0.1163	GAGSR
 5.4	91	-0.1163	GASGR
 2.9 1.6e+002	-0.1163 150	P12111|CO6A3_HUMAN AGGSR

Top scoring peptide matches to query 528
File7043 Spectrum3214 scans: 2491
Score greater than 21 indicates homology
Score greater than 35 indicates identity

**Score	Expect	Delta Hit Protein	Peptide**
 8.3	28	-0.2560 606	O00159|MYO1C_HUMAN R.GGAVR.D
 7.7	32	-0.2447	K.GGTPK.S
 6.7	41	-0.2560	GGVAR
 6.0	47	-0.2811	R.GGVVK.S
 5.3	57	-0.2560	GGLGR
 1.6 1.3e+002	-0.2560	GGGLR
 0.7 1.6e+002	-0.2560	GLGGR
 0.7 1.6e+002	-0.2560	R.AGGVR.G

Top scoring peptide matches to query 529
File7043 Spectrum3397 scans: 2634
Score greater than 22 indicates homology
Score greater than 35 indicates identity

**Score	Expect	Delta Hit Protein	Peptide**
 13.3	9	-0.2559 606	O00159|MYO1C_HUMAN R.GGAVR.D
 8.6	27	-0.2559	GGVAR
 8.2	29	-0.2559	GGGLR
 8.0	30	-0.2810	R.GGVVK.S
 3.1	93	-0.2559	GGLGR

Top scoring peptide matches to query 534
File7043 Spectrum2636 scans: 2035
Score greater than 14 indicates homology
Score greater than 35 indicates identity

**Score	Expect	Delta Hit Protein	Peptide**
 2.0 1.2e+002	-0.2550	R.AVGGR.R
 2.0 1.2e+002	-0.2550	GLGGR
 2.0 1.2e+002	-0.2550	IGGGR
 2.0 1.2e+002	-0.2550 306	P39060|COIA1_HUMAN LGGGR
 2.0 1.2e+002	-0.2550	VAGGR

Top scoring peptide matches to query 536
File7043 Spectrum2382 scans: 1846
Score greater than 13 indicates homology
Score greater than 35 indicates identity

**Score	Expect	Delta Hit Protein	Peptide**
 0.4 1.7e+002	-0.2550	R.AVGGR.R
 0.4 1.7e+002	-0.2550	GLGGR
 0.4 1.7e+002	-0.2550	IGGGR
 0.4 1.7e+002	-0.2550 306	P39060|COIA1_HUMAN LGGGR
 0.4 1.7e+002	-0.2550	VAGGR

Top scoring peptide matches to query 537
File7043 Spectrum1728 scans: 1304
Score greater than 15 indicates homology
Score greater than 35 indicates identity

**Score	Expect	Delta Hit Protein	Peptide**
 2.9	97	-0.2549	R.AVGGR.R
 2.9	97	-0.2549	GLGGR
 2.9	97	-0.2549	IGGGR
 2.9	97	-0.2549 306	P39060|COIA1_HUMAN LGGGR
 2.9	97	-0.2549	VAGGR

Top scoring peptide matches to query 539
File7043 Spectrum1888 scans: 1439
Score greater than 16 indicates homology
Score greater than 35 indicates identity

**Score	Expect	Delta Hit Protein	Peptide**
 3.3	90	-0.2548	R.AVGGR.R
 3.3	90	-0.2548	GLGGR
 3.3	90	-0.2548	IGGGR
 3.3	90	-0.2548 306	P39060|COIA1_HUMAN LGGGR
 3.3	90	-0.2548	VAGGR
 3.0	95	-0.2548	GAVGR
 3.0	95	-0.2548	GGLGR
 3.0	95	-0.2548	M.GVAGR.N
 3.0	95	-0.2548	R.VGAGR.R

Top scoring peptide matches to query 549
File7043 Spectrum5388 scans: 4203
Score greater than 17 indicates homology
Score greater than 38 indicates identity

**Score	Expect	Delta Hit Protein	Peptide**
 4.8 1.2e+002	-0.0678	K.GAAIK.D
 4.8 1.2e+002	-0.0678	GAALK
 3.9 1.5e+002	-0.0427	GGGLR
 2.7 1.9e+002	-0.0315	K.GGTPK.S
 2.5	2e+002	-0.0427 606	O00159|MYO1C_HUMAN R.GGAVR.D
 2.5	2e+002	-0.0427	GGLGR
 2.5	2e+002	-0.0427	GGVAR
 2.5	2e+002	-0.0427	GLGGR

Top scoring peptide matches to query 592
File7043 Spectrum18490 scans: 15555
Score greater than 23 indicates homology
Score greater than 31 indicates identity

**Score	Expect	Delta Hit Protein	Peptide**
 10.4	7.1	0.9810 720	P98082|DAB2_HUMAN R.AGPPK.D
 10.4	7.1	0.9810 559	P26885|FKBP2_HUMAN GAPPK

Top scoring peptide matches to query 660
File7043 Spectrum2347 scans: 1817
Score greater than 19 indicates homology
Score greater than 35 indicates identity

**Score	Expect	Delta Hit Protein	Peptide**
 7.5	28	0.8203	K.GGGGPR.S
 6.1	39	0.8203	K.NGGPR.N
 4.3	60	-0.1637	DGGPR
 4.3	60	-0.1637	DGPGR
 2.1	99	0.8203	R.GGNPR.K
 1.4 1.2e+002	0.8203 150	P12111|CO6A3_HUMAN K.GNPGR.R

Top scoring peptide matches to query 661
File7043 Spectrum2018 scans: 1545
Score greater than 19 indicates homology
Score greater than 35 indicates identity

**Score	Expect	Delta Hit Protein	Peptide**
 12.6	8.7	0.8205	GGPGGR
 5.6	44	0.8206 150	P12111|CO6A3_HUMAN K.GNPGR.R
 5.5	45	0.8206	R.GGNPR.K
 5.4	45	-0.1635	DGPGR
 5.1	49	0.8206	R.NPGGR.G
 5.0	51	0.8205	K.GGGGPR.S
 4.7	54	0.8206	K.GPNGR.D
 3.8	65	0.8205	R.GPGGGR.I

Top scoring peptide matches to query 665
File7043 Spectrum29454 scans: 26138
Score greater than 20 indicates homology
Score greater than 38 indicates identity

**Score	Expect	Delta Hit Protein	Peptide**
 8.2	59	-0.9217	GSALR
 8.2	59	-0.9217	SGALR
 7.9	63	0.0987	ANAAR
 7.9	63	0.0623	GKAAR
 7.9	63	0.0623 217	P62917|RL8_HUMAN KGAAR
 7.9	63	0.0987	QGAAR
 7.8	65	1.0575	AAAIR
 7.8	65	1.0575	AAALR
 7.4	72	0.0987	GAGAAR
 2.8 2.1e+002	-0.8854	DAAAR

Top scoring peptide matches to query 666
File7043 Spectrum29626 scans: 26286
Score greater than 15 indicates homology
Score greater than 38 indicates identity

**Score	Expect	Delta Hit Protein	Peptide**
 7.2	75	-0.8852	R.AAADR.D
 7.2	75	-0.8852	R.AADAR.D
 7.2	75	-0.8852	AAEGR
 7.2	75	-0.8852	AAGER
 7.2	75	-0.9216	AASVR
 7.2	75	-0.9216	AAVSR
 2.4 2.3e+002	-0.8852	DAAAR
 2.4 2.3e+002	-0.8852	EGAAR
 2.4 2.3e+002	-0.8852	R.GEAAR.R
 2.4 2.3e+002	-0.9216 367	P0C7P4|UCRIL_HUMAN R.SVAAR.S

Top scoring peptide matches to query 669
File7043 Spectrum2412 scans: 1870
Score greater than 18 indicates homology
Score greater than 38 indicates identity

**Score	Expect	Delta Hit Protein	Peptide**
 5.8	1e+002	0.8207	GGGRR
 4.8 1.3e+002	0.8068	R.NGLAK.L
 4.2 1.4e+002	-0.1885	R.GGLTR.F
 1.5 2.7e+002	0.8068	56	P10809|CH60_HUMAN K.NIGAK.L

Top scoring peptide matches to query 670
File7043 Spectrum30303 scans: 26821
Score greater than 16 indicates homology
Score greater than 38 indicates identity

**Score	Expect	Delta Hit Protein	Peptide**
 8.1	60	-0.1883 367	P0C7P4|UCRIL_HUMAN R.SVAAR.S
 8.1	60	-0.2135	SVAVK
 3.2 1.8e+002	0.7957	GKAAR
 3.2 1.8e+002	0.8069	R.GQAVK.E
 2.5 2.1e+002	0.8321	GAGAAR
 1.6 2.6e+002	-0.1883	R.SGIAR.V
 1.6 2.6e+002	-0.1883	R.SGLAR.H

Top scoring peptide matches to query 677
File7043 Spectrum29068 scans: 25821
Score greater than 16 indicates homology
Score greater than 39 indicates identity

**Score	Expect	Delta Hit Protein	Peptide**
 3.6 1.7e+002	1.0079 217	P62917|RL8_HUMAN KGAAR
 3.6 1.7e+002	1.0443	QGAAR
 3.5 1.8e+002	1.0443	AGQAR
 3.5 1.8e+002	1.0443	M.QAGAR.F
 3.5 1.8e+002	0.0238	K.GGTLR.G
 3.3 1.9e+002	0.0603	AGEAR
 3.3 1.9e+002	0.0603	R.GAEAR.A
 2.9	2e+002	1.0443	GAGAAR
 2.9 2.1e+002	0.0603	R.EAGAR.G
 2.9 2.1e+002	0.0603	EGAAR

Top scoring peptide matches to query 687
File7043 Spectrum2278 scans: 1762
Score greater than 14 indicates homology
Score greater than 31 indicates identity

**Score	Expect	Delta Hit Protein	Peptide**
 1.7	43	-0.2703	M.AGPLR.A
 1.7	43	-0.2703	R.APGIR.S
 1.7	43	-0.2703 495	P09601|HMOX1_HUMAN APGLR
 1.7	43	-0.2703	R.GAPIR.R
 1.7	43	-0.2703	R.GPAIR.A
 1.7	43	-0.2703	GPALR

Top scoring peptide matches to query 693
File7043 Spectrum2004 scans: 1532
Score greater than 13 indicates homology
Score greater than 34 indicates identity

**Score	Expect	Delta Hit Protein	Peptide**
 1.4	95	-0.2095	GGEPR
 1.4	95	-0.2823	K.GGILR.K
 1.4	95	-0.2823	GGLLR
 1.4	95	-0.2095	R.GGPER.E
 0.5 1.2e+002	-0.2823	K.AAVVR.L
 0.5 1.2e+002	-0.2823	K.AGLVR.K
 0.5 1.2e+002	-0.2823	ALGVR
 0.5 1.2e+002	-0.2823 607	P62277|RS13_HUMAN AVAVR
 0.5 1.2e+002	-0.2823	GAIVR
 0.5 1.2e+002	-0.2823	R.GALVR.G

Top scoring peptide matches to query 695
File7043 Spectrum3818 scans: 2970
Score greater than 26 indicates homology
Score greater than 36 indicates identity

**Score	Expect	Delta Hit Protein	Peptide**
 13.4	10	-0.0040	R.LAGVR.G
 13.4	10	-0.0040	R.LGAVR.S
 13.1	11	-0.9629	GGQVR
 13.1	11	-0.9629	NGAVR
 12.6	12	-0.9741	RGGAR
 11.9	14	-0.0040	K.AAVVR.L
 11.9	14	-0.0040	K.AGLVR.K
 11.9	14	-0.0040	ALGVR
 11.9	14	-0.0040 607	P62277|RS13_HUMAN AVAVR
 11.9	14	-0.0040	GAIVR

Top scoring peptide matches to query 698
File7043 Spectrum3406 scans: 2642
Score greater than 25 indicates homology
Score greater than 36 indicates identity

**Score	Expect	Delta Hit Protein	Peptide**
 16.6	4.9	-0.0037	K.AGLVR.K
 16.6	4.9	-0.0037	GAIVR
 16.6	4.9	-0.0037	R.GALVR.G
 11.4	16	-0.0037	R.LAGVR.G
 11.4	16	-0.0037	R.LGAVR.S
 10.1	22	-0.0037	K.AAVVR.L
 10.1	22	-0.0037	ALGVR
 10.1	22	-0.0037 607	P62277|RS13_HUMAN AVAVR
 10.1	22	-0.0037	K.GIAVR.G
 10.1	22	0.0327	R.GPSVR.L

Top scoring peptide matches to query 716
File7043 Spectrum29412 scans: 26107
Score greater than 25 indicates homology
Score greater than 37 indicates identity

**Score	Expect	Delta Hit Protein	Peptide**
 13.0	15	0.8681	16+ P35579|MYH9_HUMAN ANASR
 13.0	15	0.8680	K.ANTGR.K
 11.9	19	0.8680	AGGSAR
 9.4	33	-0.1160	R.DAASR.R
 9.4	33	-0.1160	R.DAGTR.G
 8.9	37	0.8681	R.NAASR.L
 8.9	37	0.8680	K.NAGTR.T
 8.9	37	0.8681	NASAR
 8.9	37	0.8680	R.NATGR.G
 8.1	45	0.8680	GGAASR

Top scoring peptide matches to query 721
File7043 Spectrum2166 scans: 1670
Score greater than 14 indicates homology
Score greater than 37 indicates identity

**Score	Expect	Delta Hit Protein	Peptide**
 4.6	1e+002	0.8909	R.LPSDS.-
 1.4 2.1e+002	-0.1156	GGTER
 0.3 2.7e+002	-0.1156	DSAAR
 0.3 2.7e+002	-0.1408	4	P98164|LRP2_HUMAN DSAVK
 0.3 2.7e+002	-0.1520	63	P08670|VIME_HUMAN SSAVR
 0.0 2.9e+002	-0.1156	DGTAR

Top scoring peptide matches to query 737
File7043 Spectrum28536 scans: 25387
Score greater than 24 indicates homology
Score greater than 34 indicates identity

**Score	Expect	Delta Hit Protein	Peptide**
 12.1	8.9	-0.0733	R.NPLGK.L
 12.0	9.2	0.8855 711	Q7Z4W1|DCXR_HUMAN IPLGK
 12.0	9.2	0.8855	K.LPIGK.R
 12.0	9.2	0.8855	R.LPLGK.I
 10.5	13	0.8855	R.ILPGK.S
 10.5	13	0.8855	K.LIPGK.N
 10.5	13	0.8855	LLPGK
 7.6	25	-0.0733	R.NIPGK.V
 7.1	29	-1.0574	K.DPLGK.D
 4.9	47	-0.0733	NLGPK

Top scoring peptide matches to query 740
File7043 Spectrum29243 scans: 25967
Score greater than 15 indicates homology
Score greater than 34 indicates identity

**Score	Expect	Delta Hit Protein	Peptide**
 2.6	79	1.1156	SAPPR
 2.6	79	1.1156	SPAPR
 2.3	85	0.1203	R.AGQPR.L
 2.3	85	0.0839	AKGPR
 2.3	85	0.1203	K.AQGPR.G
 1.9	95	-0.8637	K.THGSK.D
 1.5	1e+002	-0.8637 737	Q9NZM1|MYOF_HUMAN K.NNGPK.V
 1.5	1e+002	1.1407	K.SAGHR.L
 1.5	1e+002	1.1407	R.SHGAR.S
 1.5	1e+002	1.1156	R.SPPAR.S

Top scoring peptide matches to query 744
File7043 Spectrum3176 scans: 2460
Score greater than 27 indicates homology
Score greater than 36 indicates identity

**Score	Expect	Delta Hit Protein	Peptide**
 15.0	6.6	0.0691	K.AADPR.F
 15.0	6.6	0.0691	K.ADAPR.E
 15.0	6.6	0.0691	R.AEGPR.E
 15.0	6.6	0.0327	K.ASVPR.E
 15.0	6.6	0.0691	K.DAAPR.A
 15.0	6.6	0.0327	R.GISPR.A
 15.0	6.6	0.0327	GLSPR
 15.0	6.6	0.0327	GSIPR
 15.0	6.6	0.0327 286	P53007|TXTP_HUMAN K.GTVPR.L
 15.0	6.6	0.0327	SAVPR

Top scoring peptide matches to query 749
File7043 Spectrum7729 scans: 6062
Score greater than 29 indicates homology
Score greater than 38 indicates identity

**Score	Expect	Delta Hit Protein	Peptide**
 21.8	2.3	0.0328	K.DGSPR.E
 21.8	2.3	1.0168	K.NSGPR.N
 15.3	10	0.0328	R.DSPGR.E
 13.9	14	-0.0036 178	Q99623|PHB2_HUMAN DLAGR
 12.9	18	-0.0148	R.NKGGR.K
 12.5	20	0.0141	MPAGR
 12.4	20	-0.0036	DVAAR
 11.4	25	0.0215	R.GGQGGR.W
 10.3	32	-0.0036	DAIGR
 10.3	32	-0.0036	DALGR

Top scoring peptide matches to query 751
File7043 Spectrum1930 scans: 1473
Score greater than 17 indicates homology
Score greater than 36 indicates identity

**Score	Expect	Delta Hit Protein	Peptide**
 9.1	30	0.7492	16+ P35579|MYH9_HUMAN IGQSK
 9.1	30	0.7129	K.IGSKK.I
 9.1	30	0.7492	K.IGSQK.K
 9.1	30	0.7129	K.LGKSK.I
 9.1	30	0.7129	K.LGSKK.V
 9.1	30	0.7492	R.LGSQK.F
 4.5	88	-0.2460	R.NGKSK.W
 4.5	88	-0.2460	NGSKK
 2.2 1.5e+002	-1.1936	R.DGASGK.D
 2.2 1.5e+002	-1.1936	R.DGQSK.E

Top scoring peptide matches to query 752
File7043 Spectrum2091 scans: 1608
Score greater than 24 indicates homology
Score greater than 36 indicates identity

**Score	Expect	Delta Hit Protein	Peptide**
 14.0	9.8	-0.1732	K.DEGGR.Q
 11.1	19	0.8108	R.GGEGGR.A
 8.9	32	0.7492	16+ P35579|MYH9_HUMAN IGQSK
 8.9	32	0.7129	K.IGSKK.I
 8.9	32	0.7492	K.IGSQK.K
 8.9	32	0.7129	K.LGKSK.I
 8.9	32	0.7129	K.LGSKK.V
 8.9	32	0.7492	R.LGSQK.F
 5.4	71	-0.1732	R.EDGGR.Q
 5.4	71	-0.1919 182	P12109|CO6A1_HUMAN MPGGR

Top scoring peptide matches to query 753
File7043 Spectrum1770 scans: 1338
Score greater than 17 indicates homology
Score greater than 36 indicates identity

**Score	Expect	Delta Hit Protein	Peptide**
 9.1	31	0.7494	16+ P35579|MYH9_HUMAN IGQSK
 9.1	31	0.7130	K.IGSKK.I
 9.1	31	0.7494	K.IGSQK.K
 9.1	31	0.7130	K.LGKSK.I
 9.1	31	0.7130	K.LGSKK.V
 9.1	31	0.7494	R.LGSQK.F
 4.4	89	-0.2459	R.NGKSK.W
 4.4	89	-0.2459	NGSKK
 2.5 1.4e+002	-1.1935	R.DGASGK.D
 2.5 1.4e+002	-1.1935	R.DGQSK.E

Top scoring peptide matches to query 755
File7043 Spectrum2257 scans: 1744
Score greater than 17 indicates homology
Score greater than 37 indicates identity

**Score	Expect	Delta Hit Protein	Peptide**
 8.6	40	0.8796	16+ P35579|MYH9_HUMAN IGQSK
 8.6	40	0.8432	K.IGSKK.I
 8.6	40	0.8796	K.IGSQK.K
 8.6	40	0.8432	K.LGKSK.I
 8.6	40	0.8432	K.LGSKK.V
 8.6	40	0.8796	R.LGSQK.F
 4.1 1.1e+002	-0.1157	R.NGKSK.W
 3.9 1.2e+002	-0.1157	NGSKK
 2.1 1.8e+002	-1.0633	R.DGASGK.D
 2.1 1.8e+002	-1.0997	DGKSK

Top scoring peptide matches to query 756
File7043 Spectrum2426 scans: 1882
Score greater than 17 indicates homology
Score greater than 37 indicates identity

**Score	Expect	Delta Hit Protein	Peptide**
 8.5	41	0.8797	16+ P35579|MYH9_HUMAN IGQSK
 8.5	41	0.8434	K.IGSKK.I
 8.5	41	0.8797	K.IGSQK.K
 8.5	41	0.8434	K.LGKSK.I
 8.5	41	0.8434	K.LGSKK.V
 8.5	41	0.8797	R.LGSQK.F
 4.1 1.1e+002	0.9413	R.GGEGGR.A
 4.0 1.2e+002	-0.0679	SSPDK
 4.0 1.2e+002	-0.0791	SSPSR
 3.9 1.2e+002	-0.0428	K.DEGGR.Q

Top scoring peptide matches to query 757
File7043 Spectrum479 scans: 460
Score greater than 18 indicates homology
Score greater than 37 indicates identity

**Score	Expect	Delta Hit Protein	Peptide**
 6.6	64	0.8938 153	P35749|MYH11_HUMAN RSGGR
 5.1	90	-1.0742	R.TGSNR.F
 3.8 1.2e+002	-0.0426	K.GGDER.G
 2.8 1.5e+002	-0.0976	R.VAGMR.A
 2.6 1.6e+002	-1.0742	K.ASSNR.S
 2.6 1.6e+002	-1.0742	R.SASNR.L
 2.3 1.7e+002	-0.0426	K.DEGGR.Q
 2.1 1.8e+002	0.9415	R.DAGNR.E
 2.0 1.8e+002	0.9414	K.DQGGR.T
 1.9 1.9e+002	-0.1153	R.VATSR.I

Top scoring peptide matches to query 764
File7043 Spectrum2319 scans: 1794
Score greater than 18 indicates homology
Score greater than 36 indicates identity

**Score	Expect	Delta Hit Protein	Peptide**
 5.3	69	-0.0447 376	Q14508|WFDC2_HUMAN R.NGCGK.V
 5.1	72	0.9142	R.IGCGK.G
 5.1	72	0.9142	LGCGK

Top scoring peptide matches to query 767
File7043 Spectrum4971 scans: 3879
Score greater than 18 indicates homology
Score greater than 36 indicates identity

**Score	Expect	Delta Hit Protein	Peptide**
 11.4	17	1.0575	R.DSGTR.R
 5.3	69	0.9960	R.SSTIK.I
 5.3	69	0.9960	R.SSTLK.R
 5.0	74	0.0371	R.SSNTK.Y
 4.0	93	0.0371	R.SSGGTK.M
 3.6	1e+002	1.0389	K.GVGMR.L
 3.5 1.1e+002	0.9807	4	P98164|LRP2_HUMAN R.FGAIK.R
 3.5 1.1e+002	0.9807	R.FGALK.Y
 3.5 1.1e+002	1.0137	K.MGALK.V
 3.3 1.1e+002	-0.0145	R.FKGGK.G

Top scoring peptide matches to query 769
File7043 Spectrum4041 scans: 3159
Score greater than 20 indicates homology
Score greater than 35 indicates identity

**Score	Expect	Delta Hit Protein	Peptide**
 11.3	13	-0.0045	K.GGYLK.G
 11.3	13	-0.0045	YGGLK
 6.7	39	0.0472	SSESK
 6.7	39	0.0472	R.SSSEK.K
 6.7	39	0.0472	R.SSTDK.L
 6.2	44	-0.9633	R.GGYGGK.M
 5.9	47	-0.9303 424	P11279|LAMP1_HUMAN R.SSCGK.E
 3.4	83	0.0649	DSGMK
 3.4	83	0.0463	MAGMK
 3.4	84	0.0649	80	O43451|MGA_HUMAN	R.GSDMK.I

Top scoring peptide matches to query 770
File7043 Spectrum4205 scans: 3285
Score greater than 21 indicates homology
Score greater than 35 indicates identity

**Score	Expect	Delta Hit Protein	Peptide**
 12.8	9.6	-0.0039	K.GGYLK.G
 12.8	9.6	-0.0039	YGGLK
 7.7	31	0.0655	DSGMK
 7.7	31	0.0655	80	O43451|MGA_HUMAN R.GSDMK.I
 7.7	31	0.0469	MAGMK
 7.7	31	0.0655	R.SGDMK.D
 4.3	68	0.0292	K.TMSAK.E
 4.1	72	0.0543	R.GGMTR.N
 3.1	90	0.0213	K.FGASR.A
 3.1	90	0.0543	MAGSR

Top scoring peptide matches to query 773
File7043 Spectrum4300 scans: 3359
Score greater than 18 indicates homology
Score greater than 34 indicates identity

**Score	Expect	Delta Hit Protein	Peptide**
 5.2	45	-0.9629 149	A1L0T0|ILVBL_HUMAN AHGVR

Top scoring peptide matches to query 776
File7043 Spectrum1486 scans: 1135
Score greater than 17 indicates homology
Score greater than 32 indicates identity

**Score	Expect	Delta Hit Protein	Peptide**
 8.8	11	-0.1299	R.GGDHR.V
 4.5	30	-0.2279	K.IPGVR.A
 4.5	30	-0.2279	R.IPVGR.Y
 4.5	30	-0.2279	K.LPGVR.T
 2.6	47	0.8541	R.GGGHGR.G
 2.6	47	0.8289	GGGPPR
 2.6	47	0.8289 455	P12110|CO6A2_HUMAN K.GGPGPR.G
 2.6	47	0.8289	K.GGPPGR.G

Top scoring peptide matches to query 777
File7043 Spectrum2274 scans: 1758
Score greater than 21 indicates homology
Score greater than 32 indicates identity

**Score	Expect	Delta Hit Protein	Peptide**
 9.2	10	-0.1296	R.GGDHR.V
 7.9	14	0.8292	R.NPGPR.A
 6.9	17	0.8292 455	P12110|CO6A2_HUMAN K.GGPGPR.G
 6.9	17	0.8292	K.GGPPGR.G
 6.0	21	-0.1548	DPGPR
 6.0	21	-0.1548	DPPGR
 5.3	25	0.8544	R.GGGHGR.G
 5.3	25	0.8292	GGGPPR

Top scoring peptide matches to query 778
File7043 Spectrum1729 scans: 1305
Score greater than 19 indicates homology
Score greater than 32 indicates identity

**Score	Expect	Delta Hit Protein	Peptide**
 10.2	8.2	-0.1296	R.GGDHR.V
 6.5	19	0.8544	R.GGGHGR.G
 6.5	19	0.8292	GGGPPR
 3.1	41	0.8292 455	P12110|CO6A2_HUMAN K.GGPGPR.G
 3.1	41	0.8292	K.GGPPGR.G

Top scoring peptide matches to query 780
File7043 Spectrum1891 scans: 1441
Score greater than 16 indicates homology
Score greater than 32 indicates identity

**Score	Expect	Delta Hit Protein	Peptide**
 4.7	29	0.8294 455	P12110|CO6A2_HUMAN K.GGPGPR.G
 4.7	29	0.8294	K.GGPPGR.G
 3.1	42	0.8546	R.GGGHGR.G
 3.1	42	0.8294	GGGPPR
 0.4	77	-0.1294	R.GGDHR.V

Top scoring peptide matches to query 784
File7043 Spectrum3514 scans: 2723
Score greater than 19 indicates homology
Score greater than 37 indicates identity

**Score	Expect	Delta Hit Protein	Peptide**
 12.6	15	1.0320	DPGVR
 6.0	70	1.0432	K.DPSPK.T
 4.5	98	1.0320	DPVGR
 4.5	1e+002	0.9592	R.IVGVR.Y
 4.5	1e+002	0.9592	K.LVGVR.E
 4.5	1e+002	0.9592	R.VLGVR.R
 3.7 1.2e+002	0.0116	R.KDGPK.R
 2.7 1.5e+002	-0.9837	R.VDGVR.E
 1.4	2e+002	-0.9473	R.DRSPA.-
 0.4 2.5e+002	-0.0109 435	O60884|DNJA2_HUMAN GRGVR

Top scoring peptide matches to query 790
File7043 Spectrum29445 scans: 26132
Score greater than 16 indicates homology
Score greater than 38 indicates identity

**Score	Expect	Delta Hit Protein	Peptide**
 3.2 1.8e+002	0.0176	K.ANGER.R
 3.0 1.9e+002	0.0175	R.AGGEGR.D
 3.0 1.9e+002	0.0176	ANEGR
 3.0 1.9e+002	-0.0440	ANTLK
 2.9 1.9e+002	-0.0440	ANLTK
 2.9 1.9e+002	-0.0188	K.ANSVR.N
 2.9 1.9e+002	-0.0076 209+ P11233|RALA_HUMAN R.ANVDK.V
 2.7	2e+002	-0.0076	M.ASPSGK.G
 2.0 2.3e+002	-1.0466	R.VLGMK.S

Top scoring peptide matches to query 793
File7043 Spectrum29636 scans: 26294
Score greater than 19 indicates homology
Score greater than 38 indicates identity

**Score	Expect	Delta Hit Protein	Peptide**
 7.0	74	1.1302	K.ADSPR.T
 7.0	74	1.1302	R.DRSPA.-
 7.0	74	1.1302	EGSPR
 7.0	74	1.0938	K.VSSPR.N
 6.5	82	1.1302	K.DPTGR.K
 6.5	82	1.0574	R.LVTGR.G
 6.5	82	1.0574	VLTGR
 6.2	90	1.0938	SSVPR
 2.2 2.2e+002	0.1350	R.AADGGR.L
 0.9	3e+002	1.0687 649	Q6ZQN7|SO4C1_HUMAN K.IVKEG.-

Top scoring peptide matches to query 801
File7043 Spectrum3393 scans: 2629
Score greater than 29 indicates homology
Score greater than 37 indicates identity

**Score	Expect	Delta Hit Protein	Peptide**
 18.4	4.1	0.9562	R.IGMAR.M
 18.4	4.1	0.9385	ISTAR
 18.4	4.1	0.9385	R.ITSAR.K
 18.4	4.1	0.9385	R.LSTAR.L
 18.4	4.1	0.9385	R.LTSAR.C
 15.7	7.5	0.9385	TTVAR
 15.7	7.5	0.9562	AMVAR
 15.7	7.5	0.9749	R.DSVAR.I
 15.7	7.5	0.9562 702	O95169|NDUB8_HUMAN MAVAR
 13.6	12	-0.0204	NTSAR

Top scoring peptide matches to query 803
File7043 Spectrum5012 scans: 3915
Score greater than 16 indicates homology
Score greater than 36 indicates identity

**Score	Expect	Delta Hit Protein	Peptide**
 6.4	50	1.0499	K.CVGGR.G
 2.8 1.1e+002	0.0482	SASER
 2.8 1.1e+002	0.0481	82	P12236|ADT3_HUMAN K.SGTER.E
 2.8 1.1e+002	0.0659 658	Q02543|RL18A_HUMAN DMGAR
 2.7 1.2e+002	0.0482	SSEAR
 2.5 1.2e+002	0.0230	SSEVK
 2.4 1.2e+002	0.0043	R.VMGVK.C
 1.4 1.6e+002	1.0499	K.AGMNR.V
 1.4 1.6e+002	1.0685	K.DGSNR.L
 1.4 1.6e+002	1.0685	R.GDSGGR.D

Top scoring peptide matches to query 808
File7043 Spectrum4301 scans: 3360
Score greater than 26 indicates homology
Score greater than 36 indicates identity

**Score	Expect	Delta Hit Protein	Peptide**
 13.8	9.2	1.0857 658	Q02543|RL18A_HUMAN DMGAR
 13.7	9.4	1.0428	SSEVK
 12.8	11	1.0241	R.VMGVK.C
 12.2	13	1.0241	R.LGMTK.E
 12.0	14	1.0679	SSEAR
 10.5	19	1.0163	LGGFR
 7.6	38	1.0605	SSPMK
 7.6	38	1.0493	K.MTAAR.I
 6.6	48	1.0163	LGFGR
 5.9	56	1.0606	AMAEK

Top scoring peptide matches to query 824
File7043 Spectrum3280 scans: 2545
Score greater than 28 indicates homology
Score greater than 34 indicates identity

**Score	Expect	Delta Hit Protein	Peptide**
 21.2	0.98	-0.0041 217	P62917|RL8_HUMAN GAPLAK
 14.6	4.4	-0.0404	K.IPKAK.D
 14.6	4.4	-0.0404	R.LPKAK.L
 14.4	4.6	-0.0404	K.IPAKK.E
 14.4	4.6	-0.0041	R.IPAQK.K
 14.4	4.6	-0.0404	LPAKK
 14.4	4.6	-0.0041	K.LPAQK.T
 13.9	5.2	-0.0041	K.AGLPAK.A
 13.9	5.2	-0.0404	KIPAK
 13.9	5.2	-0.0041	R.QLPAK.W

Top scoring peptide matches to query 826
File7043 Spectrum3348 scans: 2593
Score greater than 23 indicates homology
Score greater than 36 indicates identity

**Score	Expect	Delta Hit Protein	Peptide**
 11.3	16	0.0192	R.GAALAR.E
 9.9	22	1.0508	K.EGVPR.F
 9.9	22	1.0508	EVGPR
 9.4	25	-0.9648	ISSPR
 9.4	25	-0.9648 481	P18124|RL7_HUMAN LSSPR
 6.4	50	-0.9471	MGVPR
 4.7	75	0.9780	KPKGK
 4.7	75	1.0144	K.KPQGK.Q
 4.7	75	1.0144	K.QPKGK.H
 4.4	78	0.0556	K.AGASPR.S

Top scoring peptide matches to query 828
File7043 Spectrum3047 scans: 2355
Score greater than 24 indicates homology
Score greater than 35 indicates identity

**Score	Expect	Delta Hit Protein	Peptide**
 11.7	13	0.7603	R.LGVGGR.T
 11.6	13	0.7967	K.AGASPR.S
 11.6	13	0.7967 372	P16403|H12_HUMAN K.AGGTPR.K
 11.6	13	0.7967	AGSAPR
 11.6	13	0.7603	K.KASPR.A
 11.6	13	0.7603	KGTPR
 11.6	13	0.7603	R.KSAPR.R
 11.6	13	0.7603	K.KTGPR.C
 11.6	13	0.7967	K.QASPR.D
 11.6	13	0.7967	K.QGTPR.A

Top scoring peptide matches to query 830
File7043 Spectrum2847 scans: 2192
Score greater than 24 indicates homology
Score greater than 35 indicates identity

**Score	Expect	Delta Hit Protein	Peptide**
 12.7	10	0.7607	GGVAVR
 12.6	11	0.7607	R.NVGIR.E
 10.7	16	0.7607	R.NIGVR.R
 10.7	16	0.7607	K.NLGVR.D
 10.5	17	0.7607	K.GGGVIR.V
 10.5	17	0.7495	GGRIR
 10.5	17	0.7495 730	P22303|ACES_HUMAN GGRLR
 10.5	17	0.7607	NGVIR
 7.2	36	0.7971	K.NTPAR.V
 7.0	38	0.7495	GRVAR

Top scoring peptide matches to query 834
File7043 Spectrum4736 scans: 3696
Score greater than 32 indicates homology
Score greater than 38 indicates identity

**Score	Expect	Delta Hit Protein	Peptide**
 21.1	2.5	0.9548	K.AELVK.L
 21.1	2.5	0.9548	K.EAIVK.F
 21.1	2.5	0.9548	EALVK
 21.1	2.5	0.9184	R.ISIVK.S
 21.1	2.5	0.9184	ISLVK
 21.1	2.5	0.9184 484	P11498|PYC_HUMAN R.SILVK.D
 21.1	2.5	0.9184	K.SLIVK.Q
 21.1	2.5	0.9184	K.SLLVK.A
 21.1	2.5	0.9184	R.TVIVK.G
 21.1	2.5	0.9184	TVLVK

Top scoring peptide matches to query 838
File7043 Spectrum2543 scans: 1968
Score greater than 26 indicates homology
Score greater than 37 indicates identity

**Score	Expect	Delta Hit Protein	Peptide**
 16.0	6.4	0.8138	NSSPR
 12.1	16	-0.1702	DPSSR
 11.2	20	-0.1888 584	O43464|HTRA2_HUMAN MAAPR
 9.6	28	0.8138	R.GGAEAR.G
 9.6	28	0.7775	R.GGALSR.V
 9.6	28	0.8138	R.GGEAAR.L
 9.6	28	0.7774	R.GGGLTR.Y
 9.6	28	0.8026	GGRDR
 9.6	28	0.7775	K.NALSR.T
 9.6	28	0.7775	R.NASLR.M

Top scoring peptide matches to query 839
File7043 Spectrum2145 scans: 1653
Score greater than 18 indicates homology
Score greater than 37 indicates identity

**Score	Expect	Delta Hit Protein	Peptide**
 5.9	66	0.8143	K.NDVGR.S
 5.9	66	0.7779	K.NLTGR.L
 5.3	76	0.7415	R.KSIGR.Q
 5.3	76	0.7415 606	O00159|MYO1C_HUMAN KSLGR
 5.2	77	0.7779	K.AGVTGR.R
 5.2	77	0.7779	K.GAISGR.S
 5.2	77	0.7779	KEAGR
 5.2	77	0.7415	K.KLSGR.L
 5.2	77	0.7415	R.KVTGR.S
 5.2	77	0.8143	QAEGR

Top scoring peptide matches to query 840
File7043 Spectrum30176 scans: 26722
Score greater than 15 indicates homology
Score greater than 37 indicates identity

**Score	Expect	Delta Hit Protein	Peptide**
 4.3 1.1e+002	0.9648	R.AVAGSR.S
 4.3 1.1e+002	0.9648 638	P08183|MDR1_HUMAN K.GAIGSR.L
 4.3 1.1e+002	0.9648	R.GALGSR.C
 4.3 1.1e+002	1.0012	GSPGSR
 4.3 1.1e+002	0.9285	R.KIGSR.S
 4.3 1.1e+002	0.9285	KLGSR
 4.3 1.1e+002	0.9648	LQGSR
 4.3 1.1e+002	0.9648	R.QIGSR.S
 4.3 1.1e+002	0.9648	QLGSR
 2.2 1.8e+002	0.9648	K.GAISGR.S

Top scoring peptide matches to query 841
File7043 Spectrum5007 scans: 3911
Score greater than 22 indicates homology
Score greater than 37 indicates identity

**Score	Expect	Delta Hit Protein	Peptide**
 10.0	30	1.0055	R.ARADK.Q
 10.0	30	1.0055	K.ARDAK.F
 10.0	30	1.0055 189+ Q9HCC0|MCCB_HUMAN AREGK
 10.0	30	1.0055	R.ARGEK.V
 10.0	30	0.9691	R.ARSVK.L
 10.0	30	0.9803	R.AVGVSK.Q
 10.0	30	1.0015	K.GAVWK.A
 10.0	30	1.0167	R.GGIGEK.G
 10.0	30	1.0167	K.GVAGEK.G
 10.0	30	1.0167	R.INDAK.C

Top scoring peptide matches to query 842
File7043 Spectrum3223 scans: 2498
Score greater than 24 indicates homology
Score greater than 37 indicates identity

**Score	Expect	Delta Hit Protein	Peptide**
 12.7	16	0.9912	R.DVISK.F
 12.7	16	0.9912	DVLSK
 10.9	23	-0.0040	NTISK
 10.7	25	0.0211	R.NTATR.M
 10.7	25	0.0575	NTDGR
 10.7	25	0.0211	R.NTTAR.A
 9.5	33	1.0164	DVATR
 9.5	33	1.0528	K.DVDGR.T
 9.5	33	1.0164	R.DVTAR.D
 9.5	33	0.9549 732	Q9H078|CLPB_HUMAN ITISK

Top scoring peptide matches to query 844
File7043 Spectrum4415 scans: 3447
Score greater than 24 indicates homology
Score greater than 36 indicates identity

**Score	Expect	Delta Hit Protein	Peptide**
 14.1	8.6	1.1424	R.ITSIK.E
 14.1	8.6	1.1424 392	P16615|AT2A2_HUMAN LTSIK
 14.1	8.6	1.1424	LTSLK
 11.1	17	1.1601	K.LMGIK.S
 11.0	17	1.1675	R.LTTAR.R
 10.3	21	1.1424	R.ISTLK.D
 10.3	21	1.1424	R.LSTIK.N
 10.3	21	1.1424	LSTLK
 10.2	21	1.1787	DVSLK
 10.2	21	1.1424	R.TLSIK.E

Top scoring peptide matches to query 853
File7043 Spectrum5168 scans: 4032
Score greater than 19 indicates homology
Score greater than 34 indicates identity

**Score	Expect	Delta Hit Protein	Peptide**
 6.2	33	0.8563	R.DAGFR.V
 6.2	33	0.8893	R.DAGMR.R
 6.1	34	0.8563	R.DGFAR.I
 6.1	34	0.8893 658	Q02543|RL18A_HUMAN DMGAR
 6.0	35	0.8530	MATAR
 6.0	35	0.8530	MSTAR
 4.2	53	0.8716	K.SSESR.R
 4.2	53	0.8716	SSSER
 3.5	62	0.8563	DAFGR
 3.4	64	0.8893	R.DMGSR.D

Top scoring peptide matches to query 858
File7043 Spectrum2221 scans: 1716
Score greater than 13 indicates homology
Score greater than 32 indicates identity

**Score	Expect	Delta Hit Protein	Peptide**
 0.3	79	0.7786	NLPPK
 0.3	79	0.7786 620	P22059|OSBP1_HUMAN R.NPLPK.N
 0.3	80	0.7674	M.PRAPK.R

Top scoring peptide matches to query 864
File7043 Spectrum1480 scans: 1129
Score greater than 17 indicates homology
Score greater than 32 indicates identity

**Score	Expect	Delta Hit Protein	Peptide**
 10.1	8.6	0.7384	K.NIVPK.L
 10.1	8.6	0.7384	NLVPK
 3.7	38	-1.1867	K.CPAPK.C
 3.7	38	0.7748	68+ O60814|H2B1K_HUMAN K.SAPAPK.K
 2.5	50	0.7748	K.GAPTPK.R
 2.1	55	0.7384	K.GIGVPK.W

Top scoring peptide matches to query 865
File7043 Spectrum2360 scans: 1828
Score greater than 20 indicates homology
Score greater than 32 indicates identity

**Score	Expect	Delta Hit Protein	Peptide**
 12.0	5.5	0.7755	68+ O60814|H2B1K_HUMAN K.SAPAPK.K
 6.9	18	0.7642	R.KHASK.V
 6.9	18	0.8006	K.QHASK.L
 6.5	20	-1.1860	K.CPAPK.C
 5.3	26	0.7755	K.GAPTPK.R
 5.3	26	0.7391	K.KPTPK.E
 3.5	40	0.7279	K.RVAPK.R
 0.7	75	0.8118	K.NDPPK.R
 0.7	75	0.7391	K.NIVPK.L
 0.7	75	0.7391	NLVPK

Top scoring peptide matches to query 868
File7043 Spectrum3099 scans: 2397
Score greater than 18 indicates homology
Score greater than 34 indicates identity

**Score	Expect	Delta Hit Protein	Peptide**
 6.2	38	0.9915	K.GAPTPK.R
 6.2	38	0.9551	K.KPTPK.E
 5.3	46	0.9551	K.VIPNK.V
 3.9	64	0.9551	KTPPK
 2.1	97	-1.0241	TLNPK
 2.0	98	-0.9990	R.AATPGR.K
 2.0	98	-0.9626	K.AGDPGR.D
 2.0	98	-0.9626 752	O95954|FTCD_HUMAN DQPGR
 2.0	98	-0.9626	ENPGR
 2.0	98	-0.9626	K.GDAPGR.Y

Top scoring peptide matches to query 869
File7043 Spectrum3517 scans: 2725
Score greater than 26 indicates homology
Score greater than 36 indicates identity

**Score	Expect	Delta Hit Protein	Peptide**
 15.2	6.6	-0.0043	K.INAVR.A
 14.5	7.8	-0.0043	R.INGIR.L
 14.5	7.8	-0.0043	LNGIR
 12.4	12	-0.0043	K.ANIVR.S
 12.4	12	-0.0043	K.GQLVR.S
 12.4	12	-0.0407	KGIVR
 12.4	12	-0.0407	KGLVR
 12.4	12	-0.0043 392	P16615|AT2A2_HUMAN K.NAIVR.S
 12.4	12	-0.0043	K.QGIVR.F
 12.4	12	-0.0043	QGLVR

Top scoring peptide matches to query 871
File7043 Spectrum2055 scans: 1580
Score greater than 21 indicates homology
Score greater than 35 indicates identity

**Score	Expect	Delta Hit Protein	Peptide**
 8.8	24	0.6692	PRGSR
 8.8	24	0.6440	K.IGLGGR.G
 8.8	24	0.6440	K.IGLNR.L
 8.8	24	0.6076	16+ P35579|MYH9_HUMAN R.IKVGR.D
 8.8	24	0.6440	K.ILGNR.V
 8.8	24	0.6440	K.ILNGR.Q
 8.8	24	0.6440	K.INAVR.A
 8.8	24	0.6440	R.INGIR.L
 8.8	24	0.6328	K.IRAGR.S
 8.8	24	0.6328	R.IRGAR.L

Top scoring peptide matches to query 872
File7043 Spectrum2542 scans: 1967
Score greater than 20 indicates homology
Score greater than 35 indicates identity

**Score	Expect	Delta Hit Protein	Peptide**
 7.8	30	0.6442	R.INGIR.L
 7.8	30	0.6442	LNGIR
 6.9	37	0.6442	GVAGLR
 6.9	37	0.6330	RAGLR
 6.5	40	0.6554 203	Q01740|FMO1_HUMAN R.LTGPGK.W
 5.9	47	-0.3398	DLGLR
 5.7	49	0.6078	K.KVGIR.E
 5.7	49	0.6442	R.QVGLR.L
 5.6	50	0.6442	K.GAVGIR.F
 5.5	52	0.7170	R.DNAPR.T

Top scoring peptide matches to query 874
File7043 Spectrum2541 scans: 1965
Score greater than 25 indicates homology
Score greater than 35 indicates identity

**Score	Expect	Delta Hit Protein	Peptide**
 13.3	8.6	-0.3032 118	P53675|CLH2_HUMAN R.NPTGGK.L
 11.4	13	-0.3396	K.NPKSK.G
 11.1	14	0.6193	K.IPKSK.R
 11.1	14	0.6557	K.LPAGSK.K
 11.1	14	0.6193	K.LPKSK.Q
 11.1	14	0.6557	K.LPQSK.V
 10.4	17	0.6193	K.IKSPK.D
 10.4	17	0.6557	R.IQSPK.R
 10.4	17	0.6193	R.ISKPK.T
 10.4	17	0.6557	ISQPK

Top scoring peptide matches to query 878
File7043 Spectrum896 scans: 741
Score greater than 18 indicates homology
Score greater than 36 indicates identity

**Score	Expect	Delta Hit Protein	Peptide**
 5.5	58	0.6357	IKDAK
 5.5	58	0.6720	IQADK
 5.5	58	0.6720	K.LGADAK.I
 5.5	58	0.6357 172	P84098|RL19_HUMAN LKADK
 5.5	58	0.6720	R.LQDAK.T
 5.4	60	0.5993	K.IKSVK.E
 5.4	60	0.6356	R.IQSVK.T
 5.4	60	0.5993	LKSVK
 5.4	60	0.6356	LQSVK
 4.7	70	0.7149	K.GGMGPR.M

Top scoring peptide matches to query 882
File7043 Spectrum4065 scans: 3183
Score greater than 22 indicates homology
Score greater than 37 indicates identity

**Score	Expect	Delta Hit Protein	Peptide**
 9.6	28	0.0066	K.SSPASK.D
 8.6	35	0.0317	K.GGEASR.V
 8.3	37	0.0066 325	O15031|PLXB2_HUMAN NIDSK
 8.3	37	0.0066	K.NLDSK.V
 8.2	39	0.0317	K.NNNSK.W
 8.0	40	0.0066	R.SSPSAK.G
 8.0	41	0.0065	K.DIGGSK.A
 8.0	41	0.0066	DLNSK
 7.8	43	0.0317 153	P35749|MYH11_HUMAN NESAR
 7.4	47	-0.0233	R.ARAMK.I

Top scoring peptide matches to query 883
File7043 Spectrum6295 scans: 4922
Score greater than 27 indicates homology
Score greater than 37 indicates identity

**Score	Expect	Delta Hit Protein	Peptide**
 14.5	9	0.9592 638	P08183|MDR1_HUMAN SSLIR
 14.5	9	0.9592	SSLLR
 13.3	12	0.9518	AMLLK
 13.3	12	0.9704	DSILK
 13.3	12	0.9704	DSLIK
 13.3	12	0.9704	DSLLK
 13.3	12	0.9518	R.MALIK.D
 13.3	12	0.9518	MALLK
 13.3	12	0.9704	K.SDILK.D
 13.3	12	0.9704	SDLIK

Top scoring peptide matches to query 884
File7043 Spectrum16 scans: 24
Score greater than 17 indicates homology
Score greater than 36 indicates identity

**Score	Expect	Delta Hit Protein	Peptide**
 6.9	41	-1.1307	K.SSNDR.D
 4.3	74	0.8122	SAKDR
 4.3	74	0.8486	K.SQADR.E
 3.8	83	0.8122	K.AKSDR.I
 3.8	83	0.8486	R.ANTDR.A
 3.8	83	0.8122	ASKDR
 3.8	83	0.8486	K.ASQDR.K
 3.7	84	-1.1307 596	Q08380|LG3BP_HUMAN K.SGGSDR.T
 3.7	84	-1.1307	K.SNSDR.E

Top scoring peptide matches to query 886
File7043 Spectrum1725 scans: 1300
Score greater than 13 indicates homology
Score greater than 36 indicates identity

**Score	Expect	Delta Hit Protein	Peptide**
 5.7	54	0.8063	K.LVGCK.S
 5.5	56	-0.1414	K.SMLNP.-
 0.3 1.9e+002	0.8249	K.NDTVK.Y
 0.3 1.9e+002	0.8501 153	P35749|MYH11_HUMAN NESAR
 0.3 1.9e+002	0.8501	NSEAR
 0.3 1.9e+002	0.8249	NSEVK

Top scoring peptide matches to query 888
File7043 Spectrum32038 scans: 28074
Score greater than 20 indicates homology
Score greater than 36 indicates identity

**Score	Expect	Delta Hit Protein	Peptide**
 9.5	22	0.7784	K.KTATR.V
 9.5	22	0.8260	DLSNK
 9.4	23	0.8260	R.EVSNK.I
 7.3	37	0.8511	K.DGAGTR.V
 7.3	37	0.8260	K.DIGGSK.A
 7.3	37	0.8260	DLNSK
 7.3	37	0.8148	R.DAKSR.Q
 7.3	37	0.8512 683	Q8TF72|SHRM3_HUMAN DAQSR
 7.3	37	0.8148	K.DASKR.G
 7.3	37	0.8512	K.DASQR.R

Top scoring peptide matches to query 907
File7043 Spectrum1766 scans: 1334
Score greater than 14 indicates homology
Score greater than 31 indicates identity

**Score	Expect	Delta Hit Protein	Peptide**
 2.5	42	0.7609	K.NHGTR.E
 2.5	42	0.7357	R.NHSVK.K
 2.5	42	0.7609	K.NHTGR.S
 1.2	56	0.6993	R.IAPQR.A
 1.2	56	0.6630	IPAKR
 1.2	56	0.6993	R.LAPAGR.V
 1.2	56	0.6630	LAPKR
 1.2	56	0.6630	LPAKR
 1.2	56	0.6993 482	P28799|GRN_HUMAN K.LPAQR.T
 0.5	66	-0.2232	DHTGR

Top scoring peptide matches to query 910
File7043 Spectrum3083 scans: 2383
Score greater than 19 indicates homology
Score greater than 34 indicates identity

**Score	Expect	Delta Hit Protein	Peptide**
 7.9	21	0.9185 374	P22492|H1T_HUMAN KVIPK
 7.9	21	0.9185	K.KVLPK.C
 6.5	30	-0.0039 230	P21912|DHSB_HUMAN K.GLNPGK.A
 6.1	32	1.0277	GEPGPK
 3.8	55	1.0164	R.GGPTPR.N
 2.9	68	1.0165	K.KDHGK.E
 2.7	71	1.0124	K.GWPPK.Y
 2.0	83	-0.0515	K.KRPGK.L
 2.0	83	-0.0152	QRPGK
 1.5	94	-0.0291	K.EVIPK.E

Top scoring peptide matches to query 911
File7043 Spectrum6220 scans: 4860
Score greater than 20 indicates homology
Score greater than 34 indicates identity

**Score	Expect	Delta Hit Protein	Peptide**
 7.8	22	0.9804	AKLPR
 7.8	22	1.0168	K.AQLPR.R
 7.8	22	0.9804 684	Q13724|MOGS_HUMAN KALPR
 7.8	22	1.0168	R.QALPR.A
 7.2	25	0.0328	R.AELPR.I
 7.2	25	0.0328	K.EALPR.E
 7.2	25	-0.0036	ISIPR
 7.2	25	-0.0036	R.LSIPR.V
 7.2	25	-0.0036	LSLPR
 7.2	25	-0.0036	R.SLIPR.Y

Top scoring peptide matches to query 912
File7043 Spectrum1879 scans: 1430
Score greater than 16 indicates homology
Score greater than 34 indicates identity

**Score	Expect	Delta Hit Protein	Peptide**
 6.8	31	0.7491	M.IPASAK.A
 6.8	31	0.7491	K.LAPSAK.N
 6.8	31	0.7490	R.LPAGTK.V
 6.8	31	0.7491	K.LPASAK.G
 3.6	66	-0.2098	K.DTVPR.A
 0.9 1.2e+002	0.7490	ITPQK
 0.9 1.2e+002	0.7126	K.IVVQK.Y
 0.9 1.2e+002	0.7127 374	P22492|H1T_HUMAN LPTKK
 0.9 1.2e+002	0.7127	K.LTPKK.D
 0.9 1.2e+002	0.7490	R.LTPQK.K

Top scoring peptide matches to query 918
File7043 Spectrum4989 scans: 3894
Score greater than 23 indicates homology
Score greater than 37 indicates identity

**Score	Expect	Delta Hit Protein	Peptide**
 11.7	21	-0.0038 396	Q9Y4P3|TBL2_HUMAN K.SLGALK.K
 10.4	28	-0.0038	AEKIK
 10.4	28	-0.0038	AEKLK
 10.4	28	0.0326	AEQLK
 10.4	28	-0.0038	K.EAKIK.E
 10.4	28	-0.0038	K.EAKLK.E
 10.4	28	0.0326	EAQIK
 10.4	28	0.0326	EAQLK
 10.4	28	-0.0402	R.ISKLK.D
 10.4	28	-0.0038	ISQIK

Top scoring peptide matches to query 919
File7043 Spectrum4994 scans: 3899
Score greater than 16 indicates homology
Score greater than 37 indicates identity

**Score	Expect	Delta Hit Protein	Peptide**
 9.8	32	0.0578	R.EAAAAR.E
 3.4 1.4e+002	0.0578	AEAAAR
 3.4 1.4e+002	0.0214	R.SLAAAR.R
 2.4 1.8e+002	0.0465 403+ Q01813|K6PP_HUMAN EARGR
 1.8 2.1e+002	1.0054	R.KAVNR.K
 1.4 2.3e+002	1.0054	K.AKAAAR.Q
 1.2 2.3e+002	1.0418	R.QVAGGR.G
 0.7 2.6e+002	1.0305	QARGR
 0.6 2.7e+002	0.9802	R.KAAGLK.K

Top scoring peptide matches to query 921
File7043 Spectrum29659 scans: 26312
Score greater than 20 indicates homology
Score greater than 38 indicates identity

**Score	Expect	Delta Hit Protein	Peptide**
 8.7	44	1.0320	SRVAR
 8.7	44	1.0069 431	O43795|MYO1B_HUMAN K.SRVVK.Q
 6.7	69	1.0433	K.KAQNK.E
 6.7	69	1.0433	M.QAKNK.D
 6.6	71	0.0229	R.VTKNK.L
 6.6	71	0.0593	EAKNK
 6.1	79	1.0320 536	P35580|MYH10_HUMAN SRAVR
 6.1	79	1.0320	SRLGR
 6.1	79	1.0433	SRPTK
 6.1	79	1.0433	R.SRTPK.T

Top scoring peptide matches to query 927
File7043 Spectrum3949 scans: 3080
Score greater than 25 indicates homology
Score greater than 36 indicates identity

**Score	Expect	Delta Hit Protein	Peptide**
 14.3	8.8	0.9538	KLMAK
 14.2	8.9	0.9538	R.KMLAK.R
 14.2	8.9	0.9902	K.QMLAK.N
 13.4	11	0.9724	R.ASTLAK.F
 13.4	11	0.9901	K.CVLAK.R
 13.4	11	0.9538	-.MKLAK.T
 13.4	11	0.9902	16+ P35579|MYH9_HUMAN MQLAK
 12.1	15	0.9901	R.CVALK.L
 12.1	15	0.9538	R.KMAIK.L
 12.1	15	0.9538	R.KMALK.Y

Top scoring peptide matches to query 934
File7043 Spectrum2193 scans: 1693
Score greater than 16 indicates homology
Score greater than 31 indicates identity

**Score	Expect	Delta Hit Protein	Peptide**
 5.1	23	-0.3057	R.NVPNR.Y
 3.6	32	-0.3308 144	Q9NQC3|RTN4_HUMAN K.NIQPK.V
 3.6	32	-0.3672	R.NLKPK.R
 3.6	32	-0.3308	K.NLQPK.I
 3.1	36	0.7260	R.DPPNR.V
 2.1	45	0.5917	ILKPK
 2.1	45	0.6281	K.ILQPK.L
 2.1	45	0.5917	LIKPK
 2.1	45	0.6281	85	P05026|AT1B1_HUMAN LLQPK

Top scoring peptide matches to query 939
File7043 Spectrum2288 scans: 1770
Score greater than 16 indicates homology
Score greater than 34 indicates identity

**Score	Expect	Delta Hit Protein	Peptide**
 3.4	66	0.7770	R.NKGGPK.W
 3.4	66	0.7882	96	P30101|PDIA3_HUMAN NLEPK
 3.1	71	0.7406	K.LRSPK.S
 3.1	71	0.7770	R.RLSAPG.-
 3.1	71	0.7406	RLSPK
 2.5	82	-0.1958	R.DELPK.L
 2.5	82	-0.2070	K.DNKPK.I
 2.2	87	-0.1859	K.YHGPK.A
 2.1	90	-0.2183	K.GRGSPK.E
 2.1	90	-0.2070	K.QAASPK.G

Top scoring peptide matches to query 940
File7043 Spectrum1803 scans: 1367
Score greater than 17 indicates homology
Score greater than 34 indicates identity

**Score	Expect	Delta Hit Protein	Peptide**
 4.7	49	-0.2434 628	P40926|MDHM_HUMAN K.NLGIGK.V
 4.7	49	-0.2434	R.NLGLGK.K
 4.5	51	0.7294	LVRGR
 4.4	53	-0.2295	R.GGVRGR.G
 4.4	53	-0.2182 586	P27348|1433T_HUMAN K.NVVGGR.R
 1.2 1.1e+002	0.7155	R.NIILK.L
 1.2 1.1e+002	0.7155	K.NILIK.K
 1.2 1.1e+002	0.7155	NILLK
 1.2 1.1e+002	0.7155	NLILK
 1.2 1.1e+002	0.7155	NLLIK

Top scoring peptide matches to query 941
File7043 Spectrum2486 scans: 1923
Score greater than 15 indicates homology
Score greater than 34 indicates identity

**Score	Expect	Delta Hit Protein	Peptide**
 7.6	25	-0.1452	K.DSSHR.A
 2.1	89	0.7773	K.NITPR.K
 2.1	89	0.7773 682	Q9NVH1|DJC11_HUMAN R.NLTPR.C
 2.1	89	0.7409	R.NLVVR.D
 2.1	89	0.8137	NPDVR
 2.1	89	-0.1744	K.YFSGK.N
 1.9	94	0.7773	K.NLPTR.G
 1.9	94	0.7773	K.NTPIR.T
 1.9	94	0.7773	R.NTPLR.M
 1.9	94	0.7409	NVVLR

Top scoring peptide matches to query 943
File7043 Spectrum5771 scans: 4509
Score greater than 36 indicates homology
Score greater than 37 indicates identity

**Score	Expect	Delta Hit Protein	Peptide**
 23.8	1.1	-0.0039	K.EIIAR.G
 23.8	1.1	-0.0039	EILAR
 23.8	1.1	-0.0039	ELIAR
 23.8	1.1	-0.0039 310	P38646|GRP75_HUMAN ELLAR
 22.1	1.6	0.0212	R.AGNLAR.R
 22.1	1.6	-0.0151	R.KNIAR.D
 22.1	1.6	-0.0151	R.KNLAR.M
 22.1	1.6	0.0212	R.QGGLAR.R
 22.1	1.6	0.0212	K.QNLAR.L
 19.1	3.1	-0.0291	K.EIIVK.S

Top scoring peptide matches to query 949
File7043 Spectrum29945 scans: 26543
Score greater than 18 indicates homology
Score greater than 37 indicates identity

**Score	Expect	Delta Hit Protein	Peptide**
 7.3	58	-1.0398	K.ELTGGK.V
 4.8	1e+002	-1.0398	R.EGTIGK.K
 4.8	1e+002	-1.0398	K.EGTLGK.T
 4.4 1.1e+002	-1.0146	R.AQSNGK.W
 0.6 2.7e+002	-1.0146	R.ANGQSK.G
 0.2	3e+002	-1.0510	R.AGSTLR.N
 0.2	3e+002	-1.0332	K.ANMLR.T
 0.2	3e+002	-1.0510 658	Q02543|RL18A_HUMAN K.ASGTLR.E

Top scoring peptide matches to query 951
File7043 Spectrum4067 scans: 3185
Score greater than 37 indicates identity

**Score	Expect	Delta Hit Protein	Peptide**
 26.3	0.74	0.0065	EDVLK
 23.1	1.5	0.0793	K.EDDPK.A
 23.1	1.5	0.1157	R.EDDPQ.-
 23.1	1.5	0.0065 267	Q12955|ANK3_HUMAN K.EDLVK.V
 23.1	1.5	0.0793	R.EDPDK.G
 18.4	4.5	-0.0299	K.EITIK.K
 18.4	4.5	-0.0299	EITLK
 18.4	4.5	-0.0299	ELTLK
 17.5	5.6	1.0157	KDPSR
 17.5	5.6	1.0633	R.QDPDK.Q

Top scoring peptide matches to query 952
File7043 Spectrum4080 scans: 3195
Score greater than 28 indicates homology
Score greater than 37 indicates identity

**Score	Expect	Delta Hit Protein	Peptide**
 17.1	6.2	1.0083	R.AGMPVK.D
 17.0	6.3	0.9906	R.KEPTK.E
 16.9	6.4	0.9906	R.GISPTK.G
 14.6	11	0.9542	4	P98164|LRP2_HUMAN KDLVK
 14.6	11	0.9906 468	O00330|ODPX_HUMAN QDLVK
 14.6	11	0.9906	K.AGDVIK.V
 14.6	11	0.9542	K.KDVIK.M
 14.6	11	0.9542	K.KDVLK.H
 14.6	11	0.9906	QDVLK
 13.2	15	0.9542	R.AVLATK.T

Top scoring peptide matches to query 955
File7043 Spectrum2134 scans: 1644
Score greater than 27 indicates homology
Score greater than 35 indicates identity

**Score	Expect	Delta Hit Protein	Peptide**
 14.2	6.7	0.7167	R.NAFPR.F
 13.4	8.1	-0.2343	-.MSDPR.Q
 13.4	8.1	-0.2707	-.MTTPR.N
 11.4	13	0.7497	-.MGSGPR.G
 11.4	13	0.7497	MSNPR
 8.8	23	-0.2455	R.GMGGAGR.G
 8.5	25	-0.2707 173	P52272|HNRPM_HUMAN R.MGLER.M
 7.5	31	-0.2269	R.DNSNR.D
 7.5	31	-0.2269	K.DSNNR.I
 7.4	32	-0.2157	DEEGR

Top scoring peptide matches to query 959
File7043 Spectrum3798 scans: 2953
Score greater than 26 indicates homology
Score greater than 36 indicates identity

**Score	Expect	Delta Hit Protein	Peptide**
 14.0	10	1.0232	R.DNVTR.Y
 13.8	10	0.9868	R.GATVTR.G
 13.6	11	1.0345	DNVEK
 12.7	14	1.0596	R.GGDEAR.A
 12.4	14	-0.0046 577	Q96NB2|SFXN2_HUMAN K.VMVEK.S
 11.6	17	0.0028	R.DLTTR.L
 10.0	25	0.0504	K.DDVEK.N
 10.0	25	0.0141	K.ETVEK.Y
 10.0	25	0.0141 317	Q9BYF1|ACE2_HUMAN R.TEVEK.A
 9.4	28	1.0232	K.GEAATR.A

Top scoring peptide matches to query 961
File7043 Spectrum1882 scans: 1433
Score greater than 15 indicates homology
Score greater than 35 indicates identity

**Score	Expect	Delta Hit Protein	Peptide**
 3.9	78	-0.1129	K.EPPHK.V
 3.9	78	-0.1857	R.LIPHK.G
 3.9	78	-0.1857 343	P06744|G6PI_HUMAN R.LLPHK.V
 2.1 1.2e+002	-0.0500	R.DTEDK.L
 2.1 1.2e+002	-0.0500	ESEDK
 2.1 1.2e+002	-0.0500	SEEDK
 2.1 1.2e+002	-0.0500	R.TDEDK.A

Top scoring peptide matches to query 963
File7043 Spectrum3309 scans: 2564
Score greater than 23 indicates homology
Score greater than 35 indicates identity

**Score	Expect	Delta Hit Protein	Peptide**
 12.0	12	0.0797	K.AYEPK.L
 12.0	12	0.0797	K.FSEPK.E
 12.0	12	0.1127	-.MSEPK.A
 9.4	22	-0.0043	R.IIHPK.T
 9.4	22	-0.0043	R.ILHPK.F
 9.4	22	-0.0043	65	P54819|KAD2_HUMAN LIHPK
 9.4	22	-0.0043	R.LLHPK.D
 7.0	38	1.0637	R.QPFSK.W
 6.5	43	-0.0043	K.HIIPK.G
 6.5	43	-0.0043	HLIPK

Top scoring peptide matches to query 964
File7043 Spectrum5280 scans: 4116
Score greater than 30 indicates homology
Score greater than 34 indicates identity

**Score	Expect	Delta Hit Protein	Peptide**
 19.2	1.6	-0.0041	R.IHIVK.K
 19.2	1.6	-0.0041 655	Q9Y696|CLIC4_HUMAN LHIVK
 19.2	1.6	-0.0041	K.LHLVK.G
 16.7	2.9	-0.9629	K.NHLVK.E
 14.2	5.2	-0.9629	K.IHNVK.I
 14.0	5.5	-0.0041	R.HILVK.F
 14.0	5.5	-0.0041	R.HLLVK.V
 14.0	5.5	0.0435	K.YSLVK.N
 13.3	6.3	0.0211	R.LHLAR.R
 13.2	6.5	-0.9629	R.HLNVK.S

Top scoring peptide matches to query 974
File7043 Spectrum3730 scans: 2898
Score greater than 13 indicates homology
Score greater than 33 indicates identity

**Score	Expect	Delta Hit Protein	Peptide**
 0.6	95	1.0558 510	P00966|ASSY_HUMAN R.FIGMK.S
 0.6	95	1.0228	K.LFGFK.E
 0.6	95	1.1253	R.MEGMK.G
 0.6	95	1.0922	K.YPGMK.K

Top scoring peptide matches to query 978
File7043 Spectrum1730 scans: 1306
Score greater than 18 indicates homology
Score greater than 33 indicates identity

**Score	Expect	Delta Hit Protein	Peptide**
 8.0	19	0.7423	R.GGGGGGPR.L
 4.8	40	0.6444	K.NKKPK.S
 4.8	40	0.6808	NQKPK
 4.8	41	0.6556 202	P07195|LDHB_HUMAN K.LSGLPK.H
 4.1	48	-0.2417	K.DNPNR.G
 3.5	55	-0.3032	DQKPK
 3.5	55	-0.2669	R.DQQPK.A
 1.2	94	-0.1685	R.MSTNM.-

Top scoring peptide matches to query 989
File7043 Spectrum3563 scans: 2760
Score greater than 24 indicates homology
Score greater than 37 indicates identity

**Score	Expect	Delta Hit Protein	Peptide**
 11.7	20	-0.0043	K.DKILK.L
 11.7	20	-0.0043 608	O43920|NDUS5_HUMAN R.DKLIK.E
 11.7	20	-0.0043	DKLLK
 11.7	20	0.0321	R.DQIIK.K
 11.7	20	0.0321	DQLIK
 11.7	20	0.0321	R.DQLLK.R
 11.0	24	0.0321	K.ENIIK.V
 11.0	24	0.0321	R.ENILK.T
 11.0	24	0.0321	R.ENLIK.Q
 11.0	24	0.0321 520	P05106|ITB3_HUMAN ENLLK

Top scoring peptide matches to query 995
File7043 Spectrum29383 scans: 26080
Score greater than 15 indicates homology
Score greater than 36 indicates identity

**Score	Expect	Delta Hit Protein	Peptide**
 2.9 1.1e+002	0.2454 441	P49368|TCPG_HUMAN K.TDNNR.I
 2.5 1.2e+002	0.2050	K.YSPPR.D
 0.9 1.8e+002	1.1679	K.SKNAAK.T
 0.9 1.8e+002	1.1931	SKNGGR
 0.4	2e+002	0.2090	K.DRSNK.A
 0.4	2e+002	0.1839	K.GLTSNK.S
 0.3 2.1e+002	-0.7936	K.KCNAK.D

Top scoring peptide matches to query 1007
File7043 Spectrum3816 scans: 2968
Score greater than 15 indicates homology
Score greater than 32 indicates identity

**Score	Expect	Delta Hit Protein	Peptide**
 7.5	17	-0.1386	K.DSEFK.Q
 7.5	17	-0.1056	R.DSEMK.E
 7.4	18	-0.1750	10	Q00610|CLH1_HUMAN K.DSLYK.D
 1.7	66	-0.2266	K.AIFFK.N
 1.7	66	-0.2266	R.ALFFK.V
 1.7	66	-0.1936	R.ALFMK.Q
 1.7	66	-0.1936	R.LAFMK.G
 1.7	66	-0.1606	K.LAMMK.K
 1.7	66	-0.1572	R.SPFMK.F

Top scoring peptide matches to query 1011
File7043 Spectrum3886 scans: 3027
Score greater than 32 indicates identity

**Score	Expect	Delta Hit Protein	Peptide**
 34.3	0.033	0.4440 116+ P60660|MYL6_HUMAN K.VLGNPK.S
 18.8	1.1	0.4440 291	P26373|RL13_HUMAN R.KPSAPK.K
 14.8	2.9	0.4440	K.VGINPK.I
 13.5	3.9	0.5168	K.DPNGPK.W
 11.1	6.7	0.4440	R.KTPGPK.D
 10.3	8.1	0.4076	R.KPLAAK.N
 10.0	8.8	0.4440	R.QIAAPK.A
 9.6	9.6	0.4328	R.QRVPK.V
 8.9	11	0.4440	R.ALQPAK.L
 8.6	12	0.4328	K.IRNPK.G

Top scoring peptide matches to query 1012
File7043 Spectrum4234 scans: 3310
Score greater than 34 indicates identity

**Score	Expect	Delta Hit Protein	Peptide**
 37.5	0.027	-0.0047	26	Q14764|MVP_HUMAN R.VEVGPK.T
 21.3	1.1	-0.0047	K.DIVGPK.G
 21.3	1.1	-0.0047	R.EVVGPK.V
 13.0	7.4	-0.0159	K.KAQGPK.G
 10.5	13	0.9569	ARRPK
 10.5	13	0.9681	R.AVGRPK.G
 10.5	13	0.9681	R.NLRPK.F
 10.5	13	0.9569	R.RARPK.F
 10.5	13	0.9681	R.VQRPK.E
 9.7	16	-0.9999	R.ISQGPK.E

Top scoring peptide matches to query 1015
File7043 Spectrum4854 scans: 3786
Score greater than 19 indicates homology
Score greater than 37 indicates identity

**Score	Expect	Delta Hit Protein	Peptide**
 7.8	45	0.9803	K.QIGALQ.-
 5.6	73	0.9440	R.KLSGPK.G
 4.1	1e+002	0.9076	KIQLK
 4.1	1e+002	0.9076	R.KLQLK.T
 4.1	1e+002	0.9076	R.QIKIK.T
 4.1	1e+002	0.9076 719	O00338|ST1C2_HUMAN K.QIKLK.E
 4.1	1e+002	0.9440	R.QIQIK.G
 4.1	1e+002	0.9076	R.QLKIK.E
 4.1	1e+002	0.9076	QLKLK
 4.1	1e+002	0.9440	K.QLQIK.L

Top scoring peptide matches to query 1021
File7043 Spectrum4053 scans: 3170
Score greater than 26 indicates homology
Score greater than 38 indicates identity

**Score	Expect	Delta Hit Protein	Peptide**
 16.0	8.1	-0.0169	R.LRSGAK.A
 12.8	17	0.9896	R.LIAEGK.A
 12.8	17	0.9896	R.LLAEGK.K
 11.8	21	0.0154	R.WAGVAK.G
 10.9	26	1.0259	R.EPSGIK.N
 10.9	26	0.9532	K.ILSGLK.S
 10.7	28	0.9532	K.IISLGK.K
 10.7	28	0.9532	R.LISIGK.W
 8.3	48	1.0259	K.EPSAVK.K
 7.5	58	-0.0057	31	P11021|GRP78_HUMAN K.EKLGGK.L

Top scoring peptide matches to query 1022
File7043 Spectrum4915 scans: 3835
Score greater than 37 indicates homology
Score greater than 38 indicates identity

**Score	Expect	Delta Hit Protein	Peptide**
 25.1	0.98	-0.0041 358	P27105|STOM_HUMAN R.NVLGTK.N
 24.0	1.3	-0.0040	K.AAALGTK.I
 23.2	1.5	-0.0041	K.IVNGTK.R
 18.6	4.5	-0.0041	K.LNVGTK.K
 14.8	11	-0.0153	K.ARVGTK.S
 14.8	11	0.0647	K.YYGTK.D
 14.2	12	0.9548	K.VIIGTK.T
 13.6	14	-0.0153	R.GRLSAK.E
 13.6	14	-0.0041	K.NVITGK.S
 13.6	14	-0.0041	R.VNLTGK.A

Top scoring peptide matches to query 1024
File7043 Spectrum3349 scans: 2594
Score greater than 27 indicates homology
Score greater than 37 indicates identity

**Score	Expect	Delta Hit Protein	Peptide**
 17.8	4.8	0.9540	R.KVSAVK.I
 17.7	5	0.9540 144	Q9NQC3|RTN4_HUMAN K.KVSGIK.E
 13.1	14	1.0744	EEPEK
 12.9	15	0.9904	R.KVAGEK.I
 12.9	15	0.9904	QVKEK
 12.9	15	1.0268	QVQEK
 12.4	17	0.9792	R.ARSGLK.I
 12.0	19	1.0268	LNQEK
 11.9	19	1.0744	K.EEEPK.I
 11.6	20	1.0744	K.EPEEK.A

Top scoring peptide matches to query 1025
File7043 Spectrum654 scans: 577
Score greater than 13 indicates homology
Score greater than 37 indicates identity

**Score	Expect	Delta Hit Protein	Peptide**
 0.6 2.2e+002	0.7965	K.ILDSGK.I
 0.4 2.3e+002	0.8105 708	P01011|AACT_HUMAN K.NGTRGK.I

Top scoring peptide matches to query 1027
File7043 Spectrum6344 scans: 4958
Score greater than 21 indicates homology
Score greater than 37 indicates identity

**Score	Expect	Delta Hit Protein	Peptide**
 8.1	44	-0.0000	R.EKSNR.S
 8.1	44	-0.0000 564	P34896|GLYC_HUMAN K.KESNR.Q
 8.1	44	-0.0113	R.TRSNR.V
 7.6	50	0.0363	R.EAGSGGR.E
 5.8	76	0.0112	K.GLDSNK.N
 5.8	76	0.9588	R.GVTSLR.F
 5.8	76	0.0112	R.LDGSNK.S
 5.8	76	0.0112	R.RESEI.-
 5.8	76	0.9476	K.RTSLR.D
 5.8	76	-0.0041	WVSNK

Top scoring peptide matches to query 1029
File7043 Spectrum2818 scans: 2170
Score greater than 17 indicates homology
Score greater than 37 indicates identity

**Score	Expect	Delta Hit Protein	Peptide**
 8.9	37	0.0558	YPPEK
 8.7	39	1.0690	R.QSNGAR.L
 4.0 1.2e+002	1.0187	K.EGLSVK.E
 4.0 1.2e+002	1.0187	R.EIGSVK.Y
 3.6 1.3e+002	1.0439	R.EVAGTR.V
 3.5 1.3e+002	1.0075	R.EVKTR.E
 3.5 1.3e+002	1.0075	DLKTR
 3.5 1.3e+002	1.0439 536	P35580|MYH10_HUMAN DLQTR
 2.8 1.5e+002	1.0439	81+ P21281|VATB2_HUMAN R.EVSAAR.E
 2.8 1.5e+002	1.0439	ESGLAR

Top scoring peptide matches to query 1045
File7043 Spectrum4883 scans: 3808
Score greater than 21 indicates homology
Score greater than 35 indicates identity

**Score	Expect	Delta Hit Protein	Peptide**
 11.6	11	0.9183	K.DKVFK.D
 11.6	11	0.9547	R.DQVFK.V
 11.6	11	0.9725	K.FPNMK.I
 11.6	11	0.9691	K.IMNMK.E
 7.6	29	0.8959	HVRPK
 7.6	29	0.9911	K.NYDPK.V
 7.6	29	0.8959 272	P20039|2B1B_HUMAN R.RVHPK.V
 7.6	29	0.8819	K.VPVPPK.S
 7.6	29	0.8819	R.VVPPPK.K
 5.0	52	1.0241	K.DQMDK.E

Top scoring peptide matches to query 1046
File7043 Spectrum5137 scans: 4011
Score greater than 28 indicates homology
Score greater than 35 indicates identity

**Score	Expect	Delta Hit Protein	Peptide**
 17.8	2.7	0.9545 364	Q13621|S12A1_HUMAN R.IYVGGK.I
 14.4	5.9	1.0239	R.LMDNK.Q
 14.1	6.4	1.0491 656	P05787|K2C8_HUMAN QDMAR
 13.5	7.3	0.9909	R.FEVNK.K
 13.4	7.5	1.0491	R.EMGAGR.Q
 12.9	8.3	0.9797	KDFAR
 12.9	8.3	1.0161	K.QDFAR.R
 12.6	8.9	0.9181	R.IFTKK.L
 12.6	8.9	0.9545	K.IFTQK.S
 12.6	8.9	0.9545	R.LFTQK.L

Top scoring peptide matches to query 1047
File7043 Spectrum5376 scans: 4195
Score greater than 23 indicates homology
Score greater than 34 indicates identity

**Score	Expect	Delta Hit Protein	Peptide**
 10.8	11	1.0492	K.DSMIR.K
 9.8	14	1.0128	-.MTTLR.A
 7.7	22	0.9798 637	Q9H4M9|EHD1_HUMAN TTFIR
 6.0	33	0.9910	K.EFTIK.K
 6.0	33	1.0240	R.EMTLK.I
 5.5	37	0.9546	R.IYTLK.E
 5.5	37	0.9546	LYTLK
 5.5	37	1.0240	-.METLK.D
 5.5	37	0.9546	YITLK
 5.5	37	0.9546	K.YLTLK.V

Top scoring peptide matches to query 1051
File7043 Spectrum5886 scans: 4599
Score greater than 33 indicates homology
Score greater than 36 indicates identity

**Score	Expect	Delta Hit Protein	Peptide**
 20.6	1.8	-1.0251	R.KLQAGK.K
 20.0	2	-0.0047	K.AGIQVR.H
 20.0	2	-0.0047 574	P08134|RHOC_HUMAN R.AGLQVR.K
 20.0	2	-0.0410	QLKVR
 20.0	2	-0.0047	QLQVR
 19.8	2.1	-0.0410	IQKVR
 19.8	2.1	-0.0410	K.LKQVR.N
 19.5	2.3	-0.9887	R.GGLVNGK.G
 18.8	2.7	-0.0047	R.IAGGAVR.D
 15.9	5.3	-0.0662	R.GVIKVK.G

Top scoring peptide matches to query 1057
File7043 Spectrum5138 scans: 4012
Score greater than 27 indicates homology
Score greater than 37 indicates identity

**Score	Expect	Delta Hit Protein	Peptide**
 16.8	6	0.9809	EVAGLR
 16.8	6	0.9445	K.EVKIR.E
 16.8	6	0.9445	R.EVKLR.D
 16.8	6	0.9809	EVQLR
 14.0	11	0.9809	K.IGGELR.I
 14.0	11	0.9809 293+ Q15366|PCBP2_HUMAN INEIR
 14.0	11	0.9809	LNELR
 11.1	22	0.9809	R.ENIIR.Y
 11.1	22	0.9809	K.ENILR.A
 11.1	22	0.9809	K.ENLIR.E

Top scoring peptide matches to query 1062
File7043 Spectrum30369 scans: 26876
Score greater than 16 indicates homology
Score greater than 36 indicates identity

**Score	Expect	Delta Hit Protein	Peptide**
 4.9	70	0.2448	K.DKDGGR.K
 4.9	70	0.2812	K.DQDGGR.K
 3.3	1e+002	1.1673	K.GKVQSK.L
 3.2	1e+002	1.1633 663	Q9NX20|RM16_HUMAN K.LPFAAK.A
 3.2	1e+002	1.1884	K.LPFNR.S

Top scoring peptide matches to query 1065
File7043 Spectrum4141 scans: 3236
Score greater than 36 indicates identity

**Score	Expect	Delta Hit Protein	Peptide**
 26.0	0.59	-0.0049	21	P31040|DHSA_HUMAN R.YAPVAK.D
 21.5	1.6	-0.0050	K.FSPVAK.K
 21.5	1.6	0.0532	K.NNMAAK.R
 18.8	3.1	0.0532	K.GCVGGAK.S
 16.2	5.6	0.0784	R.CGAGGAR.G
 13.4	11	0.0168	K.MGGKGAK.G
 12.3	14	-0.9420	-.MAAGQR.E
 12.2	14	0.0103	R.ALTESK.E
 12.2	14	0.0467	K.GDLESK.N
 12.2	14	0.0831	R.LDGESQ.-

Top scoring peptide matches to query 1067
File7043 Spectrum4167 scans: 3255
Score greater than 31 indicates homology
Score greater than 36 indicates identity

**Score	Expect	Delta Hit Protein	Peptide**
 19.3	2.8	0.8882	K.NNMAAK.R
 17.5	4.3	0.8816	R.IDGTDK.A
 17.5	4.3	0.9180	R.LDGESQ.-
 17.5	4.3	0.8817	R.LDGSEK.V
 15.3	7.2	0.9133	R.CGAGGAR.G
 14.6	8.4	0.8300	21	P31040|DHSA_HUMAN R.YAPVAK.D
 9.5	27	-1.0985	-.MPGAMK.I
 9.4	27	0.8882	K.GCVGGAK.S
 7.2	46	0.8300	K.FSPVAK.K
 7.1	47	-0.1071	-.MAAGQR.E

Top scoring peptide matches to query 1068
File7043 Spectrum3967 scans: 3096
Score greater than 30 indicates homology
Score greater than 36 indicates identity

**Score	Expect	Delta Hit Protein	Peptide**
 17.4	4.4	0.9180	R.LDGESQ.-
 16.2	5.7	0.8300	21	P31040|DHSA_HUMAN R.YAPVAK.D
 14.8	8	0.8882	K.NNMAAK.R
 14.2	9	0.8300	K.FSPVAK.K
 13.2	11	0.8994	-.MPSDAK.D
 10.3	22	0.8340	R.KSQSAK.S
 10.1	23	0.8882	K.GCVGGAK.S
 8.7	33	-0.1071	K.MAGGAAR.M
 8.1	37	0.8704	R.REESK.K
 8.1	38	-0.1289	K.FSPNGK.Y

Top scoring peptide matches to query 1069
File7043 Spectrum3483 scans: 2701
Score greater than 25 indicates homology
Score greater than 36 indicates identity

**Score	Expect	Delta Hit Protein	Peptide**
 15.4	6.9	0.8886	K.NNMAAK.R
 11.6	17	0.8304	K.FSPVAK.K
 11.3	18	0.8634	-.MEAAVK.E
 10.2	23	0.8158	R.RMVVK.M
 10.0	24	0.8708	K.GGNSSVK.L
 9.5	27	0.8304	21	P31040|DHSA_HUMAN R.YAPVAK.D
 9.1	29	0.8634	-.MEAVAK.F
 8.2	36	0.7940	R.FVALAK.Q
 7.2	46	0.8304	R.APYVAK.E
 7.2	46	0.8270	R.IMVAAK.N

Top scoring peptide matches to query 1071
File7043 Spectrum2489 scans: 1925
Score greater than 14 indicates homology
Score greater than 35 indicates identity

**Score	Expect	Delta Hit Protein	Peptide**
 5.2	50	0.7616 509	Q6UW02|CP20A_HUMAN K.NMTRK.K
 1.2 1.3e+002	-0.1748	K.DDLCK.D
 1.2 1.3e+002	-0.1496	R.DNGGCK.Q
 1.2 1.3e+002	-0.1748	K.DVECK.F

Top scoring peptide matches to query 1081
File7043 Spectrum6594 scans: 5156
Score greater than 24 indicates homology
Score greater than 35 indicates identity

**Score	Expect	Delta Hit Protein	Peptide**
 12.7	9.4	0.0939	K.SHAAGSK.Q
 12.1	11	1.0528 736	P17252|KPCA_HUMAN K.LGPAGNK.V
 11.2	13	0.0324	R.KIPGDK.C
 11.2	13	0.0687	R.QIPGDK.Y
 11.2	13	0.0687	K.QLPGDK.R
 8.1	27	-0.0152	K.IGAKIR.W
 6.9	36	0.1190	R.NHSGSR.T
 6.5	39	0.0575	K.GLPQSR.L
 6.3	40	0.0211	R.KPLSGR.T
 6.2	42	0.1190	R.HSGGGSR.D

Top scoring peptide matches to query 1082
File7043 Spectrum5853 scans: 4573
Score greater than 20 indicates homology
Score greater than 36 indicates identity

**Score	Expect	Delta Hit Protein	Peptide**
 9.3	26	0.0345 497	P23229|ITA6_HUMAN K.CGFFK.R
 9.3	26	-0.0308	K.GIGHFK.R
 9.3	26	0.0022 310	P38646|GRP75_HUMAN K.HLNMK.L
 9.3	26	-0.0308	K.IHNFK.V
 9.3	26	-0.0672	60	Q01082|SPTB2_HUMAN KHVFK
 9.3	26	-0.0342	R.KVHMK.D
 9.3	26	0.0022	R.LHNMK.L
 9.3	26	0.1006	MGCMK
 9.3	26	0.0022	M.NIHMK.R
 9.3	26	-0.0308	R.NLHFK.N

Top scoring peptide matches to query 1086
File7043 Spectrum3533 scans: 2738
Score greater than 34 indicates homology
Score greater than 37 indicates identity

**Score	Expect	Delta Hit Protein	Peptide**
 25.3	0.78	-0.0045	59+ O60656|UD19_HUMAN K.AVINDK.S
 25.3	0.78	-0.0045	R.LGLNDK.V
 20.7	2.2	-0.0409 536	P35580|MYH10_HUMAN R.ALAVASK.K
 20.0	2.6	-0.0270	R.RVAASR.T
 19.4	3	-0.0521	K.KVVASR.Q
 18.7	3.5	-0.0157	R.VALNSR.E
 15.3	7.8	-0.0045	K.ASPSGLK.S
 14.5	9.2	-0.0409	K.GLVKDK.I
 14.5	9.2	-0.0045	R.GLVQDK.T
 13.9	11	-0.0409	K.INVVSK.C

Top scoring peptide matches to query 1088
File7043 Spectrum6789 scans: 5311
Score greater than 28 indicates homology
Score greater than 37 indicates identity

**Score	Expect	Delta Hit Protein	Peptide**
 19.0	3.3	0.0428	R.DTAEPK.W
 14.2	10	-0.0048	R.QAINSK.M
 13.4	12	0.0316 237	Q969Z3|MOSC2_HUMAN K.QPSSNK.L
 11.9	17	0.0792	R.DDGPEK.Q
 10.6	23	-0.0300	R.EASLLK.G
 10.4	24	0.0064	K.ESSLPK.S
 10.3	25	0.0428	K.ESEAPK.T
 9.2	32	0.0064	K.VTSPEK.A
 8.9	34	0.0203	R.QAGQTR.T
 8.5	37	1.0156	R.QPSATR.L

Top scoring peptide matches to query 1091
File7043 Spectrum3152 scans: 2440
Score greater than 20 indicates homology
Score greater than 37 indicates identity

**Score	Expect	Delta Hit Protein	Peptide**
 7.2	49	-0.0409	IKNMR
 7.2	49	-0.0045 270	Q02978|M2OM_HUMAN R.IQNMR.M
 6.8	55	0.9907	K.KEMPR.S
 5.8	68	0.9366	R.KINTGK.N
 5.5	74	0.9366	R.LKNTGK.R
 5.4	75	1.0093	R.AGELDR.A
 5.4	75	1.0093	R.AGEVER.D
 5.4	75	0.9730	R.KEDLR.Q
 5.4	75	0.9730	KELDR
 5.4	75	0.9730	KEQQK

Top scoring peptide matches to query 1094
File7043 Spectrum5870 scans: 4587
Score greater than 32 indicates homology
Score greater than 36 indicates identity

**Score	Expect	Delta Hit Protein	Peptide**
 22.5	1.3	-0.0043	62	P27797|CALR_HUMAN R.WIESK.H
 18.2	3.6	-0.0003	K.QGDKSK.E
 17.9	3.9	0.0361	K.GNQESK.K
 15.0	7.4	-0.0003	K.ADNKSK.I
 12.7	13	0.9950	K.ANIESK.R
 12.6	13	-0.0115	K.AGGSRSK.R
 12.3	14	-0.0003	R.DAKNSK.T
 12.3	14	-0.0366	R.VSKNSK.Y
 12.3	14	0.0209	K.WAGNSK.A
 12.3	14	0.0209	WQNSK

Top scoring peptide matches to query 1095
File7043 Spectrum4169 scans: 3257
Score greater than 33 indicates homology
Score greater than 36 indicates identity

**Score	Expect	Delta Hit Protein	Peptide**
 23.5	1.1	-0.0678	K.FRIVK.L
 23.5	1.1	-0.0049 187	P49821|NDUV1_HUMAN R.STDIVK.A
 19.0	3	-0.0235	R.SIMLAK.K
 19.0	3	-0.0235	K.SLMLAK.A
 18.3	3.5	0.0016	GMVLAR
 18.3	3.5	0.0016	-.MATLAR.L
 18.3	3.5	-0.0096	MRLAR
 18.3	3.5	-0.0426	RFLAR
 18.2	3.6	0.0202	M.SSAIER.K
 18.0	3.7	0.0315	R.AESEVK.K

Top scoring peptide matches to query 1098
File7043 Spectrum3532 scans: 2737
Score greater than 27 indicates homology
Score greater than 36 indicates identity

**Score	Expect	Delta Hit Protein	Peptide**
 22.9	1	-0.0048 607	P62277|RS13_HUMAN R.FVTGNK.I
 13.3	9.5	0.0646	-.MDTGNK.T
 11.0	16	-0.0048	R.FLGNSK.S
 10.0	20	0.0646	-.MDANSK.D
 9.2	25	0.9870	-.MLGTVK.M
 8.9	26	-0.0048 287	Q15084|PDIA6_HUMAN IFGSNK
 7.2	39	0.9870	R.MVATVK.Q
 7.2	39	1.0057	K.TSETVK.Y
 5.4	59	0.0646 298	Q96GC9|TMM49_HUMAN -.MAENGK.N
 5.0	65	-0.0776	K.KPPVPK.H

Top scoring peptide matches to query 1099
File7043 Spectrum5169 scans: 4033
Score greater than 32 indicates homology
Score greater than 36 indicates identity

**Score	Expect	Delta Hit Protein	Peptide**
 22.2	1.2	0.9800 696	P62753|RS6_HUMAN QYVVR
 18.2	3.1	1.0308	K.QMMVR.D
 17.1	4	0.9437	K.YKVVR.D
 14.9	6.5	0.9800	R.FGSVVR.R
 14.9	6.5	1.0131	-.MSGVVR.T
 11.7	14	0.9185	R.KYILK.F
 11.7	14	0.9185	KYLIK
 11.7	14	0.9185	KYLLK
 11.7	14	0.9549	K.QYLIK.H
 11.7	14	0.9549	R.QYLLK.K

Top scoring peptide matches to query 1106
File7043 Spectrum5712 scans: 4463
Score greater than 25 indicates homology
Score greater than 37 indicates identity

**Score	Expect	Delta Hit Protein	Peptide**
 13.4	12	0.9697	K.KVDPSK.F
 11.7	18	0.9472	R.GRAITR.G
 11.2	20	0.9221	K.KVGLTR.C
 11.2	20	0.9585	K.NVALTR.S
 11.1	21	0.9333 572	P14618|KPYM_HUMAN KVLGEK
 11.0	21	0.9948	R.LNGDVR.L
 10.9	22	0.9697	R.NLLGEK.G
 10.5	24	0.9697	K.LINGEK.A
 10.3	25	0.9697	K.NVALEK.V
 10.1	26	0.9333	K.AAVVVSK.S

Top scoring peptide matches to query 1107
File7043 Spectrum4190 scans: 3275
Score greater than 19 indicates homology
Score greater than 37 indicates identity

**Score	Expect	Delta Hit Protein	Peptide**
 10.3	25	0.9533	R.KVAVEK.F
 5.9	69	0.9897	R.KTEAPK.G
 5.8	70	0.9897	K.KTAPEK.S
 5.6	73	1.0439	-.MAGGPPK.A
 5.3	78	0.0057	K.EVTTPK.E
 5.3	80	0.9785	K.KVNGQK.F
 5.0	84	1.0585	K.YFSEK.E
 5.0	86	0.9897	R.NLAVEK.A
 4.5	94	0.9533 572	P14618|KPYM_HUMAN KVLGEK
 3.6 1.2e+002	0.9421	K.KVGLTR.C

Top scoring peptide matches to query 1114
File7043 Spectrum4094 scans: 3205
Score greater than 29 indicates homology
Score greater than 36 indicates identity

**Score	Expect	Delta Hit Protein	Peptide**
 17.9	3.8	-0.0054 287	Q15084|PDIA6_HUMAN R.SDIVSR.A
 17.9	3.8	-0.0418	K.TTIVSR.L
 15.6	6.4	0.0674	R.EDAEGR.L
 15.2	7	0.0674	K.DEAGER.D
 14.8	7.8	-0.0054	R.SVEVSR.T
 14.4	8.4	-0.0127	-.MALVDK.H
 14.2	8.8	0.0236	-.MPAVDK.L
 13.8	9.7	-0.0206	R.FPGISR.S
 13.7	9.8	-0.0054	K.DTVVSR.S
 13.7	9.8	0.0124	-.MSPVSR.V

Top scoring peptide matches to query 1119
File7043 Spectrum4809 scans: 3753
Score greater than 28 indicates homology
Score greater than 36 indicates identity

**Score	Expect	Delta Hit Protein	Peptide**
 22.0	1.4	-0.0043	R.MQSALK.T
 22.0	1.4	-0.0221	K.TASSAIK.G
 13.7	9.5	-0.0122	K.YGGKPR.K
 13.7	9.5	0.0242	K.YGGQPR.R
 13.5	9.9	-0.0043	K.KMGVDK.I
 13.5	9.9	-0.0585	K.KTSTLK.H
 13.5	9.9	-0.0043 156	P36542|ATPG_HUMAN R.NMATLK.D
 13.5	9.9	-0.0221	K.QTSTLK.Q
 13.5	9.9	-0.0585	R.STKTLK.V
 13.5	9.9	-0.0585	R.TSKTIK.R

Top scoring peptide matches to query 1122
File7043 Spectrum29564 scans: 26236
Score greater than 20 indicates homology
Score greater than 35 indicates identity

**Score	Expect	Delta Hit Protein	Peptide**
 7.3	36	-0.8401	R.DQGGFR.E
 7.3	36	-0.8070	K.NDAGMR.E
 7.3	36	-0.8401	K.QGDGFR.A
 7.1	38	-0.9128	K.AKGTFR.E
 7.1	38	-0.9128	R.GKAFTR.G
 7.1	38	-0.9128	K.QKFTR.A
 7.1	38	-0.8798 608	O43920|NDUS5_HUMAN R.QKTMR.R
 7.1	38	-0.8434	K.QQMTR.S
 5.1	61	-0.8434	K.GDRGMK.G
 5.1	61	-0.8686	R.GIASGMK.Y

Top scoring peptide matches to query 1131
File7043 Spectrum28110 scans: 24960
Score greater than 19 indicates homology
Score greater than 36 indicates identity

**Score	Expect	Delta Hit Protein	Peptide**
 9.2	25	0.0468	R.VLSLVR.S
 9.2	25	0.1195	R.VLSPDR.G
 5.6	57	1.1036	R.ALPQTR.T
 5.6	57	1.0784	K.APIKEK.K
 5.6	57	1.0672	R.APLKTR.D
 5.5	58	1.0784	K.IAPKEK.G
 2.4 1.2e+002	0.1261	K.VHMKR.C
 2.4 1.2e+002	0.1373 618	Q09160|1A80_HUMAN M.AVMPPR.T
 2.4 1.2e+002	0.1261	R.HMVKR.A
 2.4 1.2e+002	0.1373	-.MAVPPR.G

Top scoring peptide matches to query 1133
File7043 Spectrum4964 scans: 3873
Score greater than 23 indicates homology
Score greater than 36 indicates identity

**Score	Expect	Delta Hit Protein	Peptide**
 12.3	14	0.9543	K.VPSAQGK.L
 9.3	27	0.9721 618	Q09160|1A80_HUMAN M.AVMPPR.T
 9.2	28	0.9543	R.DVSLPR.G
 9.2	28	0.9179	K.ITSLPR.A
 8.5	33	-0.0410	K.AGVLDGR.F
 8.5	33	-0.0410	K.IQVDGR.T
 8.5	33	-0.0522	R.IRADGR.S
 8.5	33	-0.0522	R.LARDGR.R
 8.5	33	-0.0410	R.QVLDGR.Q
 8.5	33	-0.0522	R.RLADGR.T

Top scoring peptide matches to query 1134
File7043 Spectrum5330 scans: 4158
Score greater than 36 indicates identity

**Score	Expect	Delta Hit Protein	Peptide**
 26.5	0.52	-0.0047	K.AVDAALK.K
 26.5	0.52	-0.0159	R.REAALK.E
 22.8	1.2	-0.0047	31+ P11021|GRP78_HUMAN R.LIGDAAK.N
 19.2	2.8	-0.0523	R.ILRSAK.L
 19.2	2.8	-0.0523	K.LLRSAK.N
 19.2	2.8	-0.0047	R.QDLLAK.C
 19.1	2.8	-0.0047	R.DQIALK.N
 19.1	2.8	-0.0047	R.ENLALK.E
 19.1	2.8	-0.0411	K.KDIAIK.Y
 19.0	2.9	-0.0159	R.REALAK.H

Top scoring peptide matches to query 1135
File7043 Spectrum5339 scans: 4165
Score greater than 27 indicates homology
Score greater than 36 indicates identity

**Score	Expect	Delta Hit Protein	Peptide**
 17.3	4.3	-0.0046	R.DQIALK.N
 17.3	4.3	-0.0046	R.ENLALK.E
 17.3	4.3	-0.0410	K.KDIAIK.Y
 13.5	10	1.0005 320+ P62937|PPIA_HUMAN HVVFGK
 12.7	12	-0.0046	R.ENALLK.S
 12.5	13	-0.0046	K.AVDAALK.K
 12.5	13	-0.0158	R.REAALK.E
 12.5	13	0.0569	R.EPSAAGR.L
 12.4	13	-0.0410	K.DKALIK.F
 12.4	13	-0.0410	R.DKALLK.R

Top scoring peptide matches to query 1136
File7043 Spectrum4764 scans: 3718
Score greater than 31 indicates homology
Score greater than 37 indicates identity

**Score	Expect	Delta Hit Protein	Peptide**
 18.8	3.4	0.8858	31+ P11021|GRP78_HUMAN R.LIGDAAK.N
 18.1	4	0.8494	K.ILNSLK.K
 18.1	4	0.8494 508	Q8TBA6|GOGA5_HUMAN K.LINSLK.E
 17.8	4.2	0.8858	R.ILNEAK.K
 17.8	4.2	0.8858	K.LLNEAK.M
 16.8	5.4	0.8382	K.IRASLK.R
 16.6	5.7	0.8382	R.RLASLK.T
 16.6	5.7	0.8745	R.SRPSIK.R
 16.4	5.9	0.8494	R.LIGGISK.G
 16.1	6.3	0.8745	R.RIAEAK.G

Top scoring peptide matches to query 1138
File7043 Spectrum5844 scans: 4565
Score greater than 19 indicates homology
Score greater than 37 indicates identity

**Score	Expect	Delta Hit Protein	Peptide**
 11.9	17	0.9700	R.IQGTLR.G
 11.9	17	0.9337 697	Q9Y2L6|FRM4B_HUMAN K.IKGLTR.G
 5.5	72	0.9701	M.ISSKPR.L
 5.5	72	1.0064	R.LSSAGPR.S
 5.5	73	0.9952	IRENR
 5.5	73	0.9952	LRENR
 4.4	94	0.9813	R.ISPKDK.S
 4.1	1e+002	0.9337	K.LSAKIR.L
 4.1	1e+002	0.9337	R.LKSALR.K
 4.1	1e+002	0.9701	R.IKNAGGK.V

Top scoring peptide matches to query 1143
File7043 Spectrum3368 scans: 2609
Score greater than 18 indicates homology
Score greater than 37 indicates identity

**Score	Expect	Delta Hit Protein	Peptide**
 9.9	26	0.9330	R.NLGSVAK.V
 9.8	27	0.9330	K.KVNEAK.R
 9.8	27	0.9694	K.VQNEAK.T
 5.2	77	-0.0510	K.LNDLSK.I
 5.2	77	-0.0510	R.LNDSLK.E
 5.2	77	-0.0510	R.NLDLSK.F
 5.2	77	-0.0510 325	O15031|PLXB2_HUMAN K.NLDTVK.G
 5.2	77	-0.0510	NLDVTK
 5.2	77	-0.0510	R.QVDSLK.Q
 4.9	82	0.9582	M.ARNAEK.A

Top scoring peptide matches to query 1145
File7043 Spectrum4135 scans: 3231
Score greater than 26 indicates homology
Score greater than 37 indicates identity

**Score	Expect	Delta Hit Protein	Peptide**
 15.7	6.9	-0.0055	K.RDSLAK.T
 12.9	13	-0.9718	-.MAISPR.S
 12.4	15	0.0486	R.GCLSPR.A
 12.0	16	0.0057	R.SSPSLAK.I
 11.4	18	1.0261	R.EISSPR.I
 10.8	21	1.0261	K.TDISPR.E
 9.3	30	0.0057	56	P10809|CH60_HUMAN K.DGVTVAK.S
 8.9	33	1.0261	K.LESSPR.K
 8.9	33	1.0261	K.SLESPR.E
 8.4	37	-0.0307	R.ASILTGK.Y

Top scoring peptide matches to query 1146
File7043 Spectrum7147 scans: 5590
Score greater than 36 indicates homology
Score greater than 37 indicates identity

**Score	Expect	Delta Hit Protein	Peptide**
 26.4	0.58	-0.0042 377	P62491|RB11A_HUMAN K.SNLLSR.F
 25.1	0.78	-0.0042	R.NSLLSR.H
 22.5	1.4	0.0070	R.NSLIDK.Q
 19.0	3.2	-0.0294	K.ASAILSK.Q
 19.0	3.2	0.0070	R.NDLISK.E
 19.0	3.2	-0.0294	R.TQIISK.K
 14.6	8.8	-0.0154	R.RSALSR.E
 11.1	20	-0.0042	K.DAKISR.L
 11.1	20	0.0070	36+ P11142|HSP7C_HUMAN DAKLDK
 11.1	20	0.0322	EQGLSR

Top scoring peptide matches to query 1147
File7043 Spectrum4818 scans: 3759
Score greater than 28 indicates homology
Score greater than 37 indicates identity

**Score	Expect	Delta Hit Protein	Peptide**
 18.9	3.3	-0.0045	60	Q01082|SPTB2_HUMAN R.LTVQTK.F
 14.3	9.5	0.0319	R.LEAQTK.G
 13.2	12	-0.0045	LTKAEK
 13.0	13	-0.0045	K.AVGTITK.L
 12.9	13	0.0319	NLDVTK
 12.9	13	0.0207	K.RAVDTK.K
 12.8	14	0.0133	K.NLMALK.K
 12.8	14	0.0133	K.QVMAIK.S
 12.8	14	0.0133	K.QVMALK.D
 10.7	22	-0.0045 329	P30519|HMOX2_HUMAN R.KEALTK.D

Top scoring peptide matches to query 1163
File7043 Spectrum3468 scans: 2690
Score greater than 22 indicates homology
Score greater than 34 indicates identity

**Score	Expect	Delta Hit Protein	Peptide**
 20.4	1.2	-0.0047	K.MFDAAK.S
 20.4	1.2	-0.0047	10	Q00610|CLH1_HUMAN K.MYDAAK.L
 8.2	20	-0.0047	R.MDFAAK.L
 7.9	22	-0.0047	R.MDYAAK.L
 5.8	35	-0.0225	K.ATDTYK.L
 5.8	35	-0.0047	K.DLCYK.D
 5.8	35	0.0139	K.DSGEYK.A
 5.8	35	-0.0238	K.HHNYK.E
 5.8	35	-0.0411	K.IMQYK.Y
 5.8	35	-0.0589	R.ITSSYK.I

Top scoring peptide matches to query 1166
File7043 Spectrum6909 scans: 5405
Score greater than 27 indicates homology
Score greater than 34 indicates identity

**Score	Expect	Delta Hit Protein	Peptide**
 15.9	4	-0.0519	R.ARALIR.E
 15.9	4	-0.0771	K.KVAIIR.T
 15.9	4	-0.0519	R.RAAILR.R
 15.9	4	-0.0519	R.RAALLR.A
 15.9	4	-0.0771	K.VKALLR.D
 13.2	7.5	-1.0359	R.VKALNR.Q
 11.3	12	-0.0407	K.VAGALLR.A
 10.7	13	-0.0043 259	P00387|NB5R3_HUMAN K.SNPIIR.T
 10.6	13	-0.9996	K.AGGILNR.F
 10.6	13	-0.9996	R.ALNINR.M

Top scoring peptide matches to query 1168
File7043 Spectrum3799 scans: 2954
Score greater than 32 indicates homology
Score greater than 35 indicates identity

**Score	Expect	Delta Hit Protein	Peptide**
 25.6	0.55	-0.0048 332	Q93050|VPP1_HUMAN R.VLQAAAK.N
 18.1	3.1	-0.0048	K.QILQAK.R
 18.1	3.1	-0.0048	K.QLLQAK.A
 17.7	3.4	-0.0411	K.IKLAQK.S
 17.7	3.4	-0.0411	R.KLLAQK.Y
 17.7	3.4	-0.0411 536	P35580|MYH10_HUMAN K.LQLAKK.E
 17.7	3.4	-0.0411	K.QLIAKK.I
 15.2	6	-0.0411	VIKVNK
 11.6	14	0.0204	K.VLQNAR.G
 11.6	14	-0.0411 510	P00966|ASSY_HUMAN ALKLGAK

Top scoring peptide matches to query 1170
File7043 Spectrum3700 scans: 2873
Score greater than 27 indicates homology
Score greater than 37 indicates identity

**Score	Expect	Delta Hit Protein	Peptide**
 32.7	0.14	-0.0051 308	O14880|MGST3_HUMAN K.SGLGSGPK.C
 12.8	13	1.0153	R.GPSAGGQK.L
 12.7	14	0.9426	R.KQGIQK.G
 9.1	31	0.9426	K.KPSNKK.K
 8.9	33	0.9426	K.KIANQK.Q
 8.9	33	0.9426	R.KLANQK.R
 8.9	33	0.9789	R.QLANQK.Q
 8.6	35	0.9426	K.LQANKK.V
 8.6	35	0.9789	LQANQK
 8.2	38	0.9426	R.KLQANK.K

Top scoring peptide matches to query 1172
File7043 Spectrum29220 scans: 25949
Score greater than 23 indicates homology
Score greater than 36 indicates identity

**Score	Expect	Delta Hit Protein	Peptide**
 11.0	19	1.1402	68+ O60814|H2B1K_HUMAN R.IAGEASR.L
 11.0	19	1.1039	LKEASR
 10.7	20	1.0787	R.LKSDIK.N
 10.7	20	1.0787	K.LKSDLK.K
 10.7	20	1.0675	R.RSSIIK.D
 10.6	21	1.0634 193	P09110|THIK_HUMAN K.LKPAFK.K
 10.1	24	-0.8278	K.DKEADK.D
 10.1	24	-0.8026	K.DQEASR.G
 6.3	56	1.1151	R.NESLIK.S
 6.3	56	1.1151	K.NESLLK.S

Top scoring peptide matches to query 1173
File7043 Spectrum5282 scans: 4119
Score greater than 35 indicates homology
Score greater than 36 indicates identity

**Score	Expect	Delta Hit Protein	Peptide**
 22.5	1.2	-0.0443	K.MLAKVK.Q
 20.7	1.8	-0.0045 524	P36578|RL4_HUMAN K.LNPYAK.T
 20.4	2	-0.0409	K.LFNLAK.D
 20.2	2.1	-0.0079	K.MLNIAK.K
 18.9	2.8	-0.0522	K.ARFIAK.S
 14.1	8.3	0.0285	R.INMEAK.H
 13.7	9.1	-0.0079	K.QVMAIK.S
 13.7	9.1	-0.0079	K.QVMALK.D
 13.6	9.3	-0.0079	K.NLMALK.K
 13.6	9.3	-0.0522	R.RAFALK.I

Top scoring peptide matches to query 1174
File7043 Spectrum3766 scans: 2926
Score greater than 19 indicates homology
Score greater than 36 indicates identity

**Score	Expect	Delta Hit Protein	Peptide**
 8.9	26	0.9681 524	P36578|RL4_HUMAN K.LNPYAK.T
 5.9	52	0.9283	K.KMIGLK.A
 5.9	52	0.9283	K.KMLGLK.G
 4.8	67	0.9647	K.QMIAVK.I
 4.7	68	0.9317	K.FQVLAK.S
 4.6	70	0.9283	R.KLMGIK.S
 4.6	71	1.0197	R.DEDKAK.A
 4.6	71	1.0011	IMNEAK
 4.6	71	-0.0231	R.KNTSTR.V
 4.6	71	-0.0119	KTSNEK

Top scoring peptide matches to query 1180
File7043 Spectrum2517 scans: 1945
Score greater than 23 indicates homology
Score greater than 34 indicates identity

**Score	Expect	Delta Hit Protein	Peptide**
 11.8	9.5	0.7430	80	O43451|MGA_HUMAN	K.IGLHLR.G
 9.7	15	0.8270	K.IGNNYK.A
 9.6	16	0.8600	K.LGMESR.L
 9.6	16	0.7906	K.LGYISR.V
 8.1	22	-0.2158	61+ Q02928|CP4AB_HUMAN K.NGIHLR.L
 7.1	28	0.7906	K.IGLSYR.K
 7.1	28	0.8270	R.LGAEYR.L
 7.1	28	0.8270	R.LGDTFR.W
 7.1	28	0.8270 266	P17858|K6PL_HUMAN R.LGFDTR.V
 7.1	28	0.7832	R.LGLMFK.V

Top scoring peptide matches to query 1181
File7043 Spectrum3641 scans: 2824
Score greater than 16 indicates homology
Score greater than 32 indicates identity

**Score	Expect	Delta Hit Protein	Peptide**
 4.2	35	-0.2586	R.FLEMR.T
 4.2	35	-0.2256	MLEMR
 3.4	42	0.6712	K.ALHELK.L
 3.4	42	0.7770	K.ASTEMR.L
 3.4	42	0.7189	K.ASYEIK.K
 3.4	42	0.7804	R.DSGEFR.R
 3.4	42	0.7804 611	Q16822|PCKGM_HUMAN R.FDSEGR.L
 3.4	42	0.6712	K.HLAEIK.R
 3.4	42	0.7076	R.HPSELK.K
 3.4	42	0.6712	R.IHAEIK.N

Top scoring peptide matches to query 1185
File7043 Spectrum2570 scans: 1988
Score greater than 17 indicates homology
Score greater than 32 indicates identity

**Score	Expect	Delta Hit Protein	Peptide**
 4.5	32	0.7459	K.AGKFSNS.-
 4.4	33	0.7306	R.AGFPYR.E
 3.7	39	-0.2017 326	Q8WWT9|S13A3_HUMAN EDEYR
 3.3	43	-0.2970	R.ERPPGR.G

Top scoring peptide matches to query 1192
File7043 Spectrum5964 scans: 4661
Score greater than 30 indicates homology
Score greater than 36 indicates identity

**Score	Expect	Delta Hit Protein	Peptide**
 27.4	0.37	-0.0044	25	P40939|ECHA_HUMAN K.GDVAVVR.I
 16.0	5.1	-0.0044	R.DIAAAVR.F
 16.0	5.1	-0.0044	R.EVAAAVR.A
 14.2	7.7	-0.0118	K.LMGAPVK.L
 13.8	8.4	-0.0044	R.ATSIPAR.F
 12.6	11	-0.0044	K.LGEAAVR.A
 12.0	13	-0.0295	K.DGVALLK.V
 11.9	13	1.0412	R.GNRDPR.H
 10.4	19	-0.0044	K.TSLAPAR.A
 10.2	19	-0.0156	K.DGRIVR.G

Top scoring peptide matches to query 1196
File7043 Spectrum3998 scans: 3122
Score greater than 28 indicates homology
Score greater than 36 indicates identity

**Score	Expect	Delta Hit Protein	Peptide**
 16.6	5.3	-0.0059 572	P14618|KPYM_HUMAN K.VVEVGSK.I
 14.0	9.6	1.0509	R.QDAGTPK.F
 8.4	35	0.9781	R.KSGISPK.K
 7.8	40	1.0032	R.QDGRIK.E
 6.7	52	0.0305	R.TPEKDK.I
 6.3	56	1.0210	R.VCGRPK.A
 6.3	57	0.9781	K.LGSSKPK.E
 6.1	59	0.0305	K.DLVENK.F
 6.1	59	0.0305	R.DVLENK.L
 6.1	59	-0.0059	R.TLLENK.D

Top scoring peptide matches to query 1197
File7043 Spectrum3577 scans: 2771
Score greater than 19 indicates homology
Score greater than 36 indicates identity

**Score	Expect	Delta Hit Protein	Peptide**
 6.3	57	-0.9462	R.NSMAPAK.D
 5.8	63	0.0088	R.RTGGAQK.A
 4.9	79	1.0153	R.AQTAPTK.T
 4.9	79	1.0040	AQTIQR
 2.9 1.2e+002	-0.0051	K.SVPTVSK.G
 2.9 1.2e+002	0.0313 261	O75380|NDUS6_HUMAN R.VSPTGEK.V
 2.4 1.4e+002	0.0200	K.NLDTVR.A
 2.4 1.4e+002	-0.0065	K.RWTVR.L
 2.4 1.4e+002	0.0048	R.VGWTVR.R
 0.4 2.2e+002	-0.8847	R.HDSMGR.Y

Top scoring peptide matches to query 1198
File7043 Spectrum5851 scans: 4571
Score greater than 31 indicates homology
Score greater than 36 indicates identity

**Score	Expect	Delta Hit Protein	Peptide**
 23.7	1	-0.0413	K.LKDTLK.S
 23.7	1	-0.0049 577	Q96NB2|SFXN2_HUMAN K.LQDTIK.A
 16.8	5.1	-0.0525	K.RLSTLK.F
 13.4	11	-0.0412	K.LKSEIK.K
 13.4	11	-0.0049	R.LQSELK.T
 13.4	11	-0.0049	K.QISELK.I
 13.4	11	-0.0049	K.QLSELK.K
 13.2	12	0.0129	-.MAAAPLK.V
 12.4	14	0.0017	R.KLMPGR.I
 12.0	15	-1.0405	FLTPLK

Top scoring peptide matches to query 1203
File7043 Spectrum4052 scans: 3169
Score greater than 19 indicates homology
Score greater than 34 indicates identity

**Score	Expect	Delta Hit Protein	Peptide**
 6.6	28	0.9420	LPRPDK
 6.1	32	1.0512 516	O15173|PGRC2_HUMAN R.QYDGSR.N
 6.0	32	0.8692	K.LPIVRK.V
 5.9	33	1.0148	R.KDGYSR.N
 5.9	33	0.9896	K.KTFSDK.S
 5.9	33	0.9784	R.KTFSSR.K
 5.9	33	1.0148	R.QTSFSR.D
 5.7	35	0.9533	K.KSSIYK.T
 5.7	35	0.9896	R.QSSYLK.I
 5.5	36	1.0292	R.KMAMGR.K

Top scoring peptide matches to query 1205
File7043 Spectrum7347 scans: 5756
Score greater than 35 indicates identity

**Score	Expect	Delta Hit Protein	Peptide**
 34.6	0.061	-0.0044	31	P11021|GRP78_HUMAN K.IQQLVK.E
 34.6	0.061	-0.0408	K.LKQLVK.F
 23.8	0.73	-0.0408	R.LKVQLK.R
 22.6	0.96	-0.0044	K.SPGKVLK.R
 21.8	1.1	-0.0408	R.KQLVIK.E
 21.8	1.1	-0.0408	R.QKIVIK.-
 21.2	1.3	-0.0408	K.QIVKLK.G
 20.7	1.5	-0.0156	K.LQRLAK.D
 20.6	1.5	-0.0156	K.LKQLAR.Q
 19.9	1.8	0.0320	R.SGPVAGIK.T

Top scoring peptide matches to query 1207
File7043 Spectrum1431 scans: 1095
Score greater than 15 indicates homology
Score greater than 35 indicates identity

**Score	Expect	Delta Hit Protein	Peptide**
 5.2	57	0.8026	R.IESPER.T
 5.2	57	0.7298	K.ITDILR.T
 5.2	57	0.8026	LSEEPR
 5.2	57	0.7299 132+ P08238|HS90B_HUMAN LSELLR
 2.6	1e+002	-0.2654	K.LGKTNAK.A
 1.9 1.2e+002	-0.2191	K.GGWAGRK.I
 1.9 1.2e+002	-0.1827	NWQQR
 1.4 1.4e+002	-1.1338	K.DCGQPR.N
 1.4 1.4e+002	-1.1403	K.DSEPER.K

Top scoring peptide matches to query 1208
File7043 Spectrum1228 scans: 963
Score greater than 19 indicates homology
Score greater than 35 indicates identity

**Score	Expect	Delta Hit Protein	Peptide**
 8.9	24	0.7664	K.QTLPGSK.A
 5.7	51	0.7664	K.IASAPGSK.M
 5.2	57	0.7188	R.KLTNVR.I
 4.2	71	0.6936	R.IQTKIK.D
 4.2	71	0.6936	K.LKTQIK.H
 3.6	82	0.7300	K.KTIGSPK.R
 3.0	94	0.7300	K.LKPTGSK.V
 1.5 1.3e+002	-0.2540	K.EITQLK.H
 1.5 1.3e+002	-0.2904	K.ELTKLK.E
 1.5 1.3e+002	-0.2540 525	P07910|HNRPC_HUMAN ELTQIK

Top scoring peptide matches to query 1210
File7043 Spectrum3173 scans: 2457
Score greater than 24 indicates homology
Score greater than 36 indicates identity

**Score	Expect	Delta Hit Protein	Peptide**
 18.1	3.8	0.9000	74	P08195|4F2_HUMAN R.DIENLK.D
 18.1	3.8	0.8636	K.DIKDLK.A
 18.1	3.8	0.8999 572	P14618|KPYM_HUMAN K.DIQDLK.F
 18.1	3.8	0.8636	R.DIVSGLK.Y
 18.1	3.8	0.9000	K.DLENLK.A
 18.1	3.8	0.8636	K.DLKDLK.E
 10.5	22	0.8887	K.DKQAAAK.-
 8.5	35	0.8926	R.LEMLLP.-
 6.2	59	0.9251	R.DIAQER.M
 6.2	59	0.8999	R.DIEVQK.M

Top scoring peptide matches to query 1211
File7043 Spectrum6707 scans: 5244
Score greater than 20 indicates homology
Score greater than 36 indicates identity

**Score	Expect	Delta Hit Protein	Peptide**
 10.3	23	-0.0259	K.NCSVPR.E
 6.8	52	-0.0048 585	Q9Y4W6|AFG32_HUMAN R.GGCWPR.G
 6.8	52	-0.0048 217	P62917|RL8_HUMAN	R.NCWPR.V
 5.8	66	0.9693	R.CIEGPR.G
 5.8	66	0.9541	R.CLWPR.S
 4.3	92	-1.0892	K.EKTVEK.K
 3.1 1.2e+002	-0.0324	K.AAEEGQK.A
 3.1 1.2e+002	-0.1416	K.LSASVKK.E
 1.4 1.8e+002	-1.0641	K.GSQSVQK.F
 1.0 1.9e+002	-0.0371	K.RGCSPR.V

Top scoring peptide matches to query 1217
File7043 Spectrum3271 scans: 2538
Score greater than 22 indicates homology
Score greater than 36 indicates identity

**Score	Expect	Delta Hit Protein	Peptide**
 10.0	21	0.0350	K.SPYDPR.H
 9.1	26	0.0502	K.QNGDTSL.-
 6.7	46	0.0350	R.FAEDPR.Q
 6.7	46	-0.9702 478	P07864|LDHC_HUMAN K.LGTDSDK.E
 6.7	46	-0.0014	R.YALDPR.Q
 2.8 1.1e+002	-0.9966	R.GYKDPR.F
 2.8 1.1e+002	-0.9603	K.SFNDPR.L
 2.7 1.2e+002	-0.9789	-.MPAFNR.L
 2.5 1.2e+002	-1.0079	R.KNGFNR.H
 2.5 1.2e+002	-1.0330	K.VSLFNR.L

Top scoring peptide matches to query 1219
File7043 Spectrum1981 scans: 1514
Score greater than 14 indicates homology
Score greater than 34 indicates identity

**Score	Expect	Delta Hit Protein	Peptide**
 2.9	70	0.5752	R.INNVFK.D
 2.9	70	0.5752 101+ P19440|GGT1_HUMAN R.IVEAFR.F
 2.9	70	0.5752	R.LEVFAR.E
 2.9	70	0.6082 752	O95954|FTCD_HUMAN R.LIDMSR.H
 2.9	70	0.6082	R.LLDAMR.M
 2.9	70	0.5929	R.LMPAFR.T
 1.0 1.1e+002	0.5752	K.NLNFVK.G
 1.0 1.1e+002	0.6333	NLNMSR
 1.0 1.1e+002	0.6333	R.NNLAMR.A

Top scoring peptide matches to query 1221
File7043 Spectrum5316 scans: 4148
Score greater than 13 indicates homology
Score greater than 34 indicates identity

**Score	Expect	Delta Hit Protein	Peptide**
 5.6	43	0.9908	R.DVPGPPR.A
 5.3	46	0.9986	-.MLGSSVK.S
 0.4 1.4e+002	-1.0037	K.AHPWTK.V
 0.1 1.5e+002	-1.0613 458	P15954|COX7C_HUMAN R.HQLLKT.-
 0.1 1.5e+002	-1.0136	K.TYNITK.S

Top scoring peptide matches to query 1223
File7043 Spectrum5195 scans: 4053
Score greater than 31 indicates homology
Score greater than 34 indicates identity

**Score	Expect	Delta Hit Protein	Peptide**
 21.2	1	-0.0044 458	P15954|COX7C_HUMAN R.HQLLKT.-
 17.4	2.6	0.0940	R.MVNMTK.E
 16.4	3.2	1.0047	R.HQLKGR.N
 10.9	11	-0.0408	K.HKLITK.T
 8.6	19	1.0892	R.VMMDVK.F
 7.2	26	1.1395	K.CAMNAR.E
 7.0	28	1.0047	R.APPARAR.E
 6.7	30	1.1144	K.TMMPAR.Y
 5.4	40	1.1000	K.ENYNAK.C
 4.6	48	0.9796 171	Q96KR1|ZFR_HUMAN	K.HQKVVK.L

Top scoring peptide matches to query 1226
File7043 Spectrum4258 scans: 3328
Score greater than 18 indicates homology
Score greater than 34 indicates identity

**Score	Expect	Delta Hit Protein	Peptide**
 6.3	37	-0.0057 120	P02735|SAA_HUMAN R.AYSDMR.E
 5.3	46	-0.0500	K.HPADFR.D
 2.4	90	-0.0639	K.AVYDFK.A
 2.4	90	-0.0275	R.FEGDFK.L
 2.4	90	-0.1003	R.LYGVYK.V
 2.4	90	0.0199	R.SDMMMK.Y
 2.4	90	-0.0673	K.SMVYVK.V
 2.4	90	-0.0309	K.SVFDMK.H
 2.4	90	-0.0428	K.WFDFK.L
 2.1	97	0.9242	K.LHDAASK.L

Top scoring peptide matches to query 1230
File7043 Spectrum6828 scans: 5342
Score greater than 19 indicates homology
Score greater than 36 indicates identity

**Score	Expect	Delta Hit Protein	Peptide**
 14.9	7.3	-0.0049	49	P22695|QCR2_HUMAN K.LSVTATR.E
 6.3	53	0.0679	R.EEATAAR.G
 6.3	53	0.0791	R.EEGDLGK.A
 4.9	72	-0.0487	K.KLVMLK.E
 4.7	75	-0.0235	R.ALRMLK.L
 4.7	75	-0.0123	K.LVQMLK.D
 4.7	75	0.0605	R.NPEMLK.Q
 4.7	76	-0.0235	R.AIRIMK.F
 4.7	76	-0.0565	R.RLAIFK.D
 4.6	77	0.0605	R.MADGPLK.D

Top scoring peptide matches to query 1232
File7043 Spectrum4826 scans: 3764
Score greater than 25 indicates homology
Score greater than 35 indicates identity

**Score	Expect	Delta Hit Protein	Peptide**
 12.8	10	-0.0051 173	P52272|HNRPM_HUMAN R.MGSGIER.M
 11.7	13	-0.0857	R.FRLGTR.N
 9.1	24	0.9789	-.MSNPRK.R
 6.4	44	-0.0051	R.MALENR.L
 6.4	44	-0.0051	K.MALQDR.E
 6.4	44	-0.0527	-.MALRSR.F
 6.4	44	-0.0302	K.MSPTIGK.Q
 6.2	47	-0.0051	R.MDSPKR.A
 5.2	59	-0.0857 204	P61106|RAB14_HUMAN R.FRAVTR.S
 4.2	74	0.9790	R.NAEMRK.A

Top scoring peptide matches to query 1237
File7043 Spectrum6557 scans: 5128
Score greater than 24 indicates homology
Score greater than 34 indicates identity

**Score	Expect	Delta Hit Protein	Peptide**
 12.7	7.5	-0.0047 416	Q9UHE5|NAT8_HUMAN K.SYLSER.G
 10.8	12	-0.0524	R.HLLSER.S
 10.1	14	-0.0524	R.LHLSER.R
 10.1	14	-0.0047	K.YSLSER.L
 7.8	23	-0.0411	R.HILDEK.D
 7.8	23	-0.0411	K.HILEDK.N
 7.8	23	-0.0411	R.HLLDEK.R
 7.8	23	-0.0928	R.IHIPFK.G
 7.8	23	-0.0411	K.IHLDEK.S
 7.8	23	-0.0485	K.YSLIMK.V

Top scoring peptide matches to query 1241
File7043 Spectrum7641 scans: 5993
Score greater than 25 indicates homology
Score greater than 36 indicates identity

**Score	Expect	Delta Hit Protein	Peptide**
 14.9	7.8	-0.0051	49	P22695|QCR2_HUMAN R.GGLGLSGAK.A
 11.3	18	-0.0051	K.NVPSKSK.A
 10.2	23	0.0967	R.YMSQSK.H
 8.1	37	0.9749	R.IHVVYK.R
 7.6	42	-0.0415	R.VKGLTNK.K
 6.5	54	-0.0163	K.IRQSGAK.Q
 6.3	56	1.0589	R.YDGIYK.V
 6.2	57	-0.0163	R.RQISQK.A
 4.9	78	-0.9059 230	P21912|DHSB_HUMAN K.MMATYK.E
 4.0	96	0.0491	R.MEHSKK.Y

Top scoring peptide matches to query 1242
File7043 Spectrum13486 scans: 10917
Score greater than 29 indicates homology
Score greater than 36 indicates identity

**Score	Expect	Delta Hit Protein	Peptide**
 20.1	2.4	0.0325	R.EGILSLK.K
 20.1	2.4	-0.0039	K.SVIISIK.G
 20.1	2.4	-0.0039 563	P09496|CLCA_HUMAN R.SVLISLK.Q
 20.1	2.4	0.0172	K.WLISIK.D
 18.9	3.1	0.0026	R.IRVIMK.S
 15.0	7.6	0.0325	R.ALDLSLK.Y
 15.0	7.6	0.0325	K.ELGISIK.V
 15.0	7.6	0.0325	R.IGELSLK.L
 15.0	7.6	0.0325	K.TPTISLK.E
 9.8	25	0.0325	R.ADISIIK.G

Top scoring peptide matches to query 1243
File7043 Spectrum6109 scans: 4777
Score greater than 21 indicates homology
Score greater than 36 indicates identity

**Score	Expect	Delta Hit Protein	Peptide**
 9.2	30	0.9536 616	Q9UHQ4|BAP29_HUMAN K.LKTELR.K
 9.0	31	-0.0053 475	Q9UGT4|SUSD2_HUMAN R.SVQASLR.Y
 8.8	33	0.9899	R.IEAASLR.L
 7.6	43	-0.0053	K.QSVNKGK.Q
 6.4	57	0.0311	K.NSLQNGK.L
 6.0	62	1.0077	R.LNPMLR.K
 5.9	63	1.0077	R.IIMNPR.L
 5.5	70	-0.9893	DISGTLR
 5.5	70	-0.9893	K.DISSALR.V
 4.5	87	1.0151	R.NDLNKR.V

Top scoring peptide matches to query 1244
File7043 Spectrum4386 scans: 3423
Score greater than 18 indicates homology
Score greater than 36 indicates identity

**Score	Expect	Delta Hit Protein	Peptide**
 6.6	54	0.0308	K.GETKTPK.K
 5.6	68	0.0308	K.EKDIQK.K
 5.6	68	0.0308	K.EKDLQK.F
 5.6	68	0.0308	K.GSLDLQK.C
 5.6	68	-0.0056	K.IGSDLKK.H
 5.6	68	0.0308 351	P13010|KU86_HUMAN K.KEDIQK.E
 5.0	79	0.0196	R.RLGASEK.D
 4.6	86	0.0043	R.WARVTK.D
 4.1	95	0.0308 366	Q16891|IMMT_HUMAN K.SVIENAK.K
 4.1	96	-1.0525	FRLVVK

Top scoring peptide matches to query 1247
File7043 Spectrum2941 scans: 2268
Score greater than 25 indicates homology
Score greater than 36 indicates identity

**Score	Expect	Delta Hit Protein	Peptide**
 12.7	12	-0.0952	K.KSTALSR.T
 11.6	16	-0.0476	K.STAGALDK.K
 7.5	41	0.9477	K.ALDSLDK.A
 6.2	54	0.9477	R.DSLALDK.E
 6.2	54	0.9113	K.TTLALDK.Q
 6.0	56	0.9113	R.ATLTIDK.H
 5.8	60	-0.0377	K.FQQPSR.S
 5.7	61	0.9728	K.VESSPSR.S
 5.5	64	0.9477	R.DLASEVK.Y
 5.5	64	0.9477	22	O60494|CUBN_HUMAN K.IDASVEK.A

Top scoring peptide matches to query 1248
File7043 Spectrum6382 scans: 4987
Score greater than 28 indicates homology
Score greater than 36 indicates identity

**Score	Expect	Delta Hit Protein	Peptide**
 21.7	1.5	-0.0408	R.LCTLQK.K
 14.0	9	-0.0408	R.LTCIQK.G
 13.6	10	0.0142	K.GDSDLQK.A
 13.0	11	-0.0772 240	Q16718|NDUA5_HUMAN -.MAGVLKK.T
 12.2	14	-0.0044	R.DCVLQK.S
 10.7	19	-0.0408	R.LATAMAGK.T
 10.6	20	-0.0851	R.FARLQK.L
 10.6	20	-0.1102	R.FLNLKK.T
 10.6	20	-0.0408	K.MNIIQK.L
 10.6	20	-0.0408	K.MNLIQK.S

Top scoring peptide matches to query 1249
File7043 Spectrum9202 scans: 7251
Score greater than 25 indicates homology
Score greater than 36 indicates identity

**Score	Expect	Delta Hit Protein	Peptide**
 17.8	3.8	-1.0407	R.IQLVYK.N
 11.8	15	-1.0407 264	P05062|ALDOB_HUMAN K.VLAAVYK.A
 9.8	24	-1.0519	R.RLLAYK.Q
 6.0	57	-1.0189	K.RILMSK.K
 6.0	57	-1.0519	R.RLIFSK.R
 4.3	85	-1.0771	K.LKVLYK.L
 4.3	85	-1.0407	QLVIYK
 3.5	1e+002	1.0517	K.QEVGSGGK.H
 3.4	1e+002	-0.9791	K.KEPYAR.E
 3.2 1.1e+002	-1.0407	R.ILGVFSK.C

Top scoring peptide matches to query 1254
File7043 Spectrum1647 scans: 1240
Score greater than 32 indicates homology
Score greater than 34 indicates identity

**Score	Expect	Delta Hit Protein	Peptide**
 21.4	1.1	0.7336	K.IDINCK.Q
 21.4	1.1	0.7513	K.LMPNCK.W
 17.8	2.4	-0.2253	K.NVENCK.K
 8.0	23	0.6972	R.IEVVMR.E
 8.0	23	0.6972	R.LEVMVR.I
 8.0	24	0.6781	K.LGFGRGR.H
 8.0	24	0.6608	R.LGIKMGK.Q
 8.0	24	0.6859	M.LGSMARK.K
 7.8	25	0.6608	R.LKTLCK.D
 7.2	28	-0.2808 150	P12111|CO6A3_HUMAN K.NGGFGRR.G

Top scoring peptide matches to query 1255
File7043 Spectrum1216 scans: 955
Score greater than 33 indicates homology
Score greater than 34 indicates identity

**Score	Expect	Delta Hit Protein	Peptide**
 23.1	0.73	0.7336	K.IDINCK.Q
 23.1	0.73	0.7514	K.LMPNCK.W
 19.5	1.6	-0.2252	K.NVENCK.K
 9.3	17	0.6972	R.IEVVMR.E
 9.3	17	0.6972	R.LEVMVR.I
 8.8	20	0.6608	R.LKTLCK.D
 6.5	33	-0.2980	R.GGKTLCK.G
 6.5	33	-0.2365	K.NRDACK.T
 6.5	33	-0.2616 324+ Q31612|1B73_HUMAN NTQICK
 6.5	33	0.7085	R.IEEIMK.R

Top scoring peptide matches to query 1256
File7043 Spectrum588 scans: 531
Score greater than 17 indicates homology
Score greater than 34 indicates identity

**Score	Expect	Delta Hit Protein	Peptide**
 16.9	3	0.7343	K.IDINCK.Q
 16.9	3	0.7521	K.LMPNCK.W
 16.9	3	-0.2245	K.NVENCK.K
 4.0	59	0.6980	R.IEVVMR.E
 4.0	59	0.6980	R.LEVMVR.I
 3.8	62	-0.2973	R.GGKTLCK.G
 3.8	62	0.6616	R.LKTLCK.D
 3.8	62	-0.2357	K.NRDACK.T
 3.8	62	-0.2609 324+ Q31612|1B73_HUMAN NTQICK
 1.4 1.1e+002	-0.3150	R.LSTSSLR.H

Top scoring peptide matches to query 1257
File7043 Spectrum382 scans: 398
Score greater than 20 indicates homology
Score greater than 34 indicates identity

**Score	Expect	Delta Hit Protein	Peptide**
 9.3	17	0.7350	K.IDINCK.Q
 9.3	17	0.7528	K.LMPNCK.W
 7.2	28	-0.2238	K.NVENCK.K
 5.3	44	-0.2304	K.TEADAEK.T
 1.0 1.2e+002	-0.2966	R.GGKTLCK.G
 1.0 1.2e+002	0.6623	R.LKTLCK.D
 1.0 1.2e+002	-0.2351	K.NRDACK.T
 1.0 1.2e+002	-0.2602 324+ Q31612|1B73_HUMAN NTQICK

Top scoring peptide matches to query 1258
File7043 Spectrum1850 scans: 1405
Score greater than 20 indicates homology
Score greater than 34 indicates identity

**Score	Expect	Delta Hit Protein	Peptide**
 7.8	21	-0.3005	K.NNPRHK.S
 7.3	23	0.7251	R.IDIMEK.D
 7.3	23	0.6557	K.ILDLYK.H
 7.3	23	0.6557	R.LIDIYK.N
 7.3	23	0.6921	K.LLDFEK.K
 7.3	23	0.7139	R.LVETMR.F
 7.3	23	0.6557	59+ O60656|UD19_HUMAN K.LVEYLK.E
 7.3	24	0.7173	K.INFNEK.L
 7.3	24	0.7060	K.INFNTR.F
 7.3	24	0.7754	R.LGGSDCR.V

Top scoring peptide matches to query 1259
File7043 Spectrum2039 scans: 1564
Score greater than 20 indicates homology
Score greater than 34 indicates identity

**Score	Expect	Delta Hit Protein	Peptide**
 9.1	17	0.7677	R.IDIMEK.D
 9.1	17	0.6983	K.ILDLYK.H
 9.1	17	0.6983	R.LIDIYK.N
 9.1	17	0.7346	K.LLDFEK.K
 9.1	17	0.7564	R.LVETMR.F
 9.1	17	0.6983	59+ O60656|UD19_HUMAN K.LVEYLK.E
 6.9	28	-0.2355	R.GGDLFTR.L
 6.9	28	-0.2606	K.GGLDYLK.Q
 6.9	28	-0.2606	K.NDILYK.A
 6.9	28	-0.2242	R.NDLEFK.Y

Top scoring peptide matches to query 1260
File7043 Spectrum7568 scans: 5934
Score greater than 27 indicates homology
Score greater than 35 indicates identity

**Score	Expect	Delta Hit Protein	Peptide**
 19.1	2.2	-0.0051 317	Q9BYF1|ACE2_HUMAN R.ILMCTK.V
 13.5	7.9	-0.0381	K.LINFMK.M
 9.6	19	-0.0195	K.ILNDYK.M
 8.9	23	-0.0558	R.ILQYTK.E
 7.1	35	0.9394	K.YILVEK.K
 7.1	35	0.9282	R.YLLSIR.L
 6.9	36	1.0122	K.YPELDK.L
 6.7	38	-0.0195	R.KFDLDK.S
 6.5	40	0.0023	R.KMVESR.K
 5.5	50	-0.0783	K.LINHIR.V

Top scoring peptide matches to query 1264
File7043 Spectrum1881 scans: 1432
Score greater than 21 indicates homology
Score greater than 33 indicates identity

**Score	Expect	Delta Hit Protein	Peptide**
 10.7	10	0.5519	R.IDLLHR.M
 8.3	18	-0.4069	R.NDIIHR.T
 8.3	18	-0.4069	K.NDLLHR.V
 8.1	19	0.5658	R.LGHGRAR.Y
 8.0	19	0.5921	K.ILDFMK.D
 8.0	19	0.5921	R.IVEMFK.D
 8.0	19	0.5519	R.LIDHIR.N
 6.0	31	0.6061 569	P47914|RL29_HUMAN R.NMRFAK.K

Top scoring peptide matches to query 1267
File7043 Spectrum9993 scans: 7898
Score greater than 32 indicates homology
Score greater than 35 indicates identity

**Score	Expect	Delta Hit Protein	Peptide**
 32.5	0.098	-0.0050	53	P20674|COX5A_HUMAN K.IIDAALR.A
 17.9	2.8	0.0201	R.GVGQAAIR.G
 17.5	3.1	-0.0050	R.LGELALR.G
 17.2	3.3	-0.0050	R.DLALAIR.D
 17.2	3.3	-0.0302	K.LDLAVIK.I
 16.8	3.6	-0.0302	R.IVEGILK.R
 14.7	5.9	-0.0414	R.IVSAILR.A
 14.2	6.6	-0.9639	R.LNDAALR.A
 14.1	6.9	-0.0302	K.LLDLLGK.E
 13.2	8.4	-0.0302	K.LLTPSLK.S

Top scoring peptide matches to query 1268
File7043 Spectrum6790 scans: 5312
Score greater than 35 indicates homology
Score greater than 36 indicates identity

**Score	Expect	Delta Hit Protein	Peptide**
 23.0	1	0.9205	K.SLVNNPK.T
 21.1	1.6	0.9205 196	P54652|HSP72_HUMAN K.KDIGPNK.R
 16.0	5.2	0.9568	K.ENVQGPK.C
 14.2	7.8	0.9164	K.WLDLPK.A
 9.9	21	0.9205	K.ENKGVPK.Q
 9.8	21	0.9456	R.RADQGPK.T
 8.7	28	0.9416	K.HGYIPGK.N
 8.6	29	0.9205	K.NEPVGKK.T
 7.3	39	0.8841	R.SKSKPPK.R
 7.0	41	0.8980	K.SRPRQK.K

Top scoring peptide matches to query 1269
File7043 Spectrum9866 scans: 7792
Score greater than 19 indicates homology
Score greater than 36 indicates identity

**Score	Expect	Delta Hit Protein	Peptide**
 7.6	36	-0.1142	R.VNLLVSK.W
 6.5	46	-1.0407	K.TLEPWK.L
 5.6	57	-0.0414	K.VPDQSVK.R
 3.0	1e+002	0.8699	R.IVSAILR.A
 3.0	1e+002	0.0054	-.MSFIMK.L
 2.9	1e+002	0.0604	K.AEDYMK.T
 2.6 1.1e+002	-1.0189	R.LTQHMK.T
 2.3 1.2e+002	-1.0407 287	Q15084|PDIA6_HUMAN R.LTPEWK.K

Top scoring peptide matches to query 1270
File7043 Spectrum7881 scans: 6183
Score greater than 31 indicates homology
Score greater than 36 indicates identity

**Score	Expect	Delta Hit Protein	Peptide**
 30.6	0.18	-0.0047 351	P13010|KU86_HUMAN K.SQIPLSK.I
 16.2	4.9	-0.0047	R.QSLPSLK.D
 15.5	5.8	-0.0411	R.KTVLPSK.H
 14.4	7.5	-0.0047	R.ASAVIPSK.D
 12.8	11	0.0382	K.CHKLSK.A
 11.8	14	-0.9887	K.SELIPSK.V
 10.0	21	0.0204	K.EKVSPGR.T
 9.2	25	-0.0411	K.LSPLKSK.F
 8.9	26	-0.0775	R.SKILLAK.A
 8.9	26	-0.0775	K.SKLLLAK.E

Top scoring peptide matches to query 1271
File7043 Spectrum5062 scans: 3952
Score greater than 36 indicates identity

**Score	Expect	Delta Hit Protein	Peptide**
 36.6	0.045	-0.0045 230	P21912|DHSB_HUMAN K.AIAEIKK.M
 36.6	0.045	-0.0045 689	Q8ND61|CC020_HUMAN R.ALAEIKK.R
 23.4	0.95	0.0206	K.KQQLQK.S
 23.4	0.95	0.0206	R.QAGQIKK.L
 23.4	0.95	0.0570	QQQLQK
 20.9	1.7	-0.0409	K.LLSALKK.K
 20.9	1.7	-0.0045	R.ALLSLQK.F
 20.9	1.7	-0.0409	K.ALSLLKK.E
 20.9	1.7	-0.0045	K.IALSIQK.L
 16.4	4.8	0.0318	R.IGDVIQK.L

Top scoring peptide matches to query 1278
File7043 Spectrum6604 scans: 5164
Score greater than 23 indicates homology
Score greater than 36 indicates identity

**Score	Expect	Delta Hit Protein	Peptide**
 21.5	1.6	-0.0049 286	P53007|TXTP_HUMAN R.GIGDCVR.Q
 9.3	26	-0.0413	K.CIQSLR.S
 9.3	26	-0.0379	K.SWTNLR.L
 9.0	28	-0.0114 683	Q8TF72|SHRM3_HUMAN R.SSPATADK.R
 8.1	35	-0.0413	-.MNAAVVR.R
 7.3	41	-0.0300	R.LTSCPAK.D
 6.8	46	-1.0318	R.ASEEITK.M
 6.8	46	0.0064	M.DPMELR.N
 6.8	46	-0.0114	K.EETELR.F
 6.8	46	-0.0114	ETEELR

Top scoring peptide matches to query 1279
File7043 Spectrum2380 scans: 1844
Score greater than 29 indicates homology
Score greater than 34 indicates identity

**Score	Expect	Delta Hit Protein	Peptide**
 19.1	1.8	0.6620	R.IGNGQCK.N
 19.1	1.8	0.6005	R.LEVKCK.N
 15.5	4.1	-0.3736	R.NPMFIR.V
 7.1	28	0.6005	R.ELVKCK.K
 6.5	32	0.6443	K.INNSSNK.I
 6.5	32	0.5675	R.IVEIFR.E
 6.5	32	0.5675	R.IVELFR.N
 6.5	32	0.6005 353+ Q10567|AP1B1_HUMAN K.LDIMIR.L
 6.5	32	0.6369	K.LVEMER.E
 6.5	32	0.6005	R.LVEMLR.A

Top scoring peptide matches to query 1281
File7043 Spectrum2033 scans: 1558
Score greater than 23 indicates homology
Score greater than 34 indicates identity

**Score	Expect	Delta Hit Protein	Peptide**
 20.4	1.3	0.6627	R.IGNGQCK.N
 20.4	1.3	0.6012	R.LEVKCK.N
 8.9	19	0.5835	K.ITEAKSK.D
 7.8	24	0.6012	R.ELVKCK.K
 7.6	25	0.6264	R.IAERCK.N
 7.6	25	0.6376	R.LNELCK.I
 7.4	26	0.5682	R.IVEIFR.E
 7.4	26	0.5682	R.IVELFR.N
 7.4	27	0.6450	K.INNSSNK.I
 7.4	27	0.6012 353+ Q10567|AP1B1_HUMAN K.LDIMIR.L

Top scoring peptide matches to query 1282
File7043 Spectrum1713 scans: 1290
Score greater than 22 indicates homology
Score greater than 34 indicates identity

**Score	Expect	Delta Hit Protein	Peptide**
 21.8	0.95	0.6634	R.IGNGQCK.N
 21.8	0.95	0.6019	R.LEVKCK.N
 8.2	22	0.6457	K.INNSSNK.I
 8.2	22	0.5689	R.IVEIFR.E
 8.2	22	0.5689	R.IVELFR.N
 8.2	22	0.6019 353+ Q10567|AP1B1_HUMAN K.LDIMIR.L
 8.2	22	0.6383	K.LVEMER.E
 8.2	22	0.6019	R.LVEMLR.A
 7.3	27	0.6019	R.ELVKCK.K
 7.1	29	0.6270	R.IAERCK.N

Top scoring peptide matches to query 1284
File7043 Spectrum4121 scans: 3222
Score greater than 20 indicates homology
Score greater than 35 indicates identity

**Score	Expect	Delta Hit Protein	Peptide**
 10.7	15	-0.0059	K.AMGTMNR.Q
 6.5	40	-1.0845	R.SIVMFGK.G
 5.8	48	-1.0118	K.GMDFAPK.D
 0.6 1.6e+002	-0.0819	K.FTEKQK.Y
 0.0 1.8e+002	-0.0091 544	P07197|NFM_HUMAN R.FEEEAR.L

Top scoring peptide matches to query 1285
File7043 Spectrum10132 scans: 8012
Score greater than 22 indicates homology
Score greater than 35 indicates identity

**Score	Expect	Delta Hit Protein	Peptide**
 11.0	14	0.0749 310	P38646|GRP75_HUMAN R.EMAGDNK.L
 8.5	25	0.9280	K.YLKDIK.I
 8.1	28	0.0497	R.DLMEEK.F
 7.2	35	0.0167 436	P04899|GNAI2_HUMAN K.DLFEEK.I
 6.2	44	0.9895	GPSYSLR
 5.7	48	0.0055	R.DAAFTQK.K
 5.3	54	0.0497	R.MLDEGSL.-
 5.3	54	0.9974	R.EMKDLK.E
 5.3	54	0.9280	K.LYKDLK.N
 5.1	56	0.9742	R.GFTWLR.Y

Top scoring peptide matches to query 1286
File7043 Spectrum6466 scans: 5056
Score greater than 15 indicates homology
Score greater than 35 indicates identity

**Score	Expect	Delta Hit Protein	Peptide**
 16.6	4	-0.0050	58	Q16853|AOC3_HUMAN R.FSVQGSR.V
 1.3 1.3e+002	0.0644	-.MEGQSGR.C
 1.1 1.4e+002	0.0281	K.MGEKSGR.I
 0.2 1.7e+002	0.0570	K.MNSPMGK.S

Top scoring peptide matches to query 1287
File7043 Spectrum9408 scans: 7417
Score greater than 25 indicates homology
Score greater than 35 indicates identity

**Score	Expect	Delta Hit Protein	Peptide**
 18.5	2.3	-0.0049	4	P98164|LRP2_HUMAN R.CISIYK.V
 10.9	13	0.0203	R.GMAYSVR.V
 10.8	13	0.0137	K.DGTYTVK.F
 10.7	14	-0.0954	K.VPLVEVK.A
 10.0	16	-0.0339	K.ETPLPAR.R
 8.2	24	-0.9637	K.MNYNVK.E
 7.9	26	-0.0525	R.VPMIAPR.V
 7.7	28	-0.0637	K.VRHLMK.S
 7.1	31	-0.0703	K.TAHLITK.E
 6.2	39	0.0567	R.MYQDAR.L

Top scoring peptide matches to query 1288
File7043 Spectrum3992 scans: 3117
Score greater than 26 indicates homology
Score greater than 35 indicates identity

**Score	Expect	Delta Hit Protein	Peptide**
 20.0	1.7	-0.0057 174	P01009|A1AT_HUMAN K.VVNPTQK.-
 12.2	10	-0.0056	37	P55084|ECHB_HUMAN R.AAPAVQTK.T
 6.8	35	-0.0281	K.RRPQTK.L
 6.4	38	-0.0533	R.AAVALGRK.R
 5.4	48	-0.0420	R.VVNVINK.V
 4.1	65	0.0307	K.VVPNNDK.D
 3.9	68	0.0233	R.YMTVKK.R
 3.8	70	-0.0169	K.QSPRVAK.T
 2.9	86	-0.0533	K.SKPARVK.A
 2.6	92	-0.0056	K.QPLATQK.D

Top scoring peptide matches to query 1289
File7043 Spectrum97 scans: 205
Score greater than 18 indicates homology
Score greater than 34 indicates identity

**Score	Expect	Delta Hit Protein	Peptide**
 6.6	28	-0.2772	R.AVQRANK.H
 4.7	44	0.6817	R.NALRAIK.A
 4.7	44	0.6817 329	P30519|HMOX2_HUMAN K.VAQRALK.L
 3.9	52	0.6565	K.VAQKVLK.N
 0.5 1.2e+002	0.6994	R.IPMLRR.M
 0.2 1.2e+002	0.7470	K.TPMLPAR.N

Top scoring peptide matches to query 1294
File7043 Spectrum2005 scans: 1533
Score greater than 21 indicates homology
Score greater than 35 indicates identity

**Score	Expect	Delta Hit Protein	Peptide**
 9.2	19	0.6517	K.KTTVALR.D
 7.7	28	0.7357	KTEEPGK
 6.7	35	0.7244	R.GAGDLSLR.E
 6.2	39	0.6517	R.KITSALR.S
 6.2	39	0.6993 434+ P01857|IGHG1_HUMAN K.SLSLSPGK.-
 6.2	39	0.7608	R.SPTGSPSR.L
 5.5	46	0.6728	K.KFLGPAR.D
 5.0	51	0.6880	K.RGLEVSK.C
 4.7	56	0.7244	R.IEGASVGR.C
 4.6	56	0.7422	K.QMAVPSR.N

Top scoring peptide matches to query 1306
File7043 Spectrum1460 scans: 1116
Score greater than 18 indicates homology
Score greater than 36 indicates identity

**Score	Expect	Delta Hit Protein	Peptide**
 6.3	55	-0.9524	R.SSSGEPAR.E
 6.0	59	0.0204	R.GDARGASR.A
 5.6	65	-1.0186	K.QRMLAR.E
 4.7	80	0.0065	K.GDKVENK.V
 4.1	92	-1.0404	R.KGVPFSR.T
 3.8	97	0.0317	R.QAEAQSR.D
 3.8	97	0.0317 164	Q96A33|CCD47_HUMAN R.QEAAQSR.R
 3.5 1.1e+002	-0.0411	R.QLTGKSR.K
 3.2 1.1e+002	-0.0663	R.SILTISR.H
 2.6 1.3e+002	-0.0047	R.REELSR.Q

Top scoring peptide matches to query 1308
File7043 Spectrum2678 scans: 2067
Score greater than 25 indicates homology
Score greater than 36 indicates identity

**Score	Expect	Delta Hit Protein	Peptide**
 13.6	10	0.9549	K.ELKVGSR.E
 13.6	10	0.9800	R.ELQRSR.R
 11.8	16	0.0064	-.MCLLPR.G
 11.8	16	-0.0808 111	P21397|AOFA_HUMAN K.YKLLPR.S
 10.8	19	0.0072	K.ENGSVGVK.N
 9.3	28	1.0025	K.ESKAEPK.A
 8.9	30	0.0059	K.GWGSRAR.R
 8.9	30	-0.0152	K.SRGSNLR.V
 8.6	33	0.9614	R.RMLAAAR.N
 8.5	33	0.9298	K.ELSALKK.L

Top scoring peptide matches to query 1310
File7043 Spectrum1051 scans: 847
Score greater than 27 indicates homology
Score greater than 36 indicates identity

**Score	Expect	Delta Hit Protein	Peptide**
 15.6	6.5	0.0074	K.ENGSVGVK.N
 13.4	11	0.9662	K.EALVSAAK.L
 11.8	16	0.9663	K.ELAAEKK.R
 11.5	17	1.0026	K.EISSQPK.E
 10.9	19	-1.0177	R.LMASRGR.S
 10.8	19	0.9299	K.ELSALKK.L
 10.7	20	0.0060	K.GWGSRAR.R
 10.7	20	-0.0151	K.SRGSNLR.V
 10.2	22	0.9550	K.EATLAKR.F
 10.0	24	-0.0039	9	P25705|ATPA_HUMAN R.VGSAAQTR.A

Top scoring peptide matches to query 1313
File7043 Spectrum2498 scans: 1933
Score greater than 24 indicates homology
Score greater than 36 indicates identity

**Score	Expect	Delta Hit Protein	Peptide**
 15.7	6.4	0.9441	K.GVRTLSR.C
 11.0	19	0.9917	R.GSAPSKNK.R
 10.9	19	1.0281	K.EQEQVR.A
 10.3	22	0.9917 564	P34896|GLYC_HUMAN K.ERLAGDK.Y
 9.9	24	0.9037	R.FLKPRK.V
 9.6	26	1.0281	EGAENLR
 9.6	26	0.9917	K.EKENIR.Q
 9.6	26	0.9553	R.EKGSLVR.H
 9.6	26	1.0281	EQEVQR
 9.4	27	1.0029	K.EQAALEK.I

Top scoring peptide matches to query 1314
File7043 Spectrum241 scans: 308
Score greater than 34 indicates homology
Score greater than 36 indicates identity

**Score	Expect	Delta Hit Protein	Peptide**
 32.7	0.13	-0.0027	9	P25705|ATPA_HUMAN R.VGSAAQTR.A
 20.2	2.2	0.0086	K.ENGSVGVK.N
 20.2	2.2	0.0086 173	P52272|HNRPM_HUMAN R.VGSEIER.M
 14.9	7.7	-1.0231	R.VTQSSIR.T
 14.2	9	-0.9503	K.EQSAAER.C
 13.9	9.6	0.9562	K.ELKVGSR.E
 12.7	13	0.0627	-.MEGAEPR.A
 12.4	13	-0.9907	K.NFDIGPK.F
 12.4	13	-1.0230	K.EKASSLR.I
 11.8	16	-0.0101	R.VGTLPMR.L

Top scoring peptide matches to query 1315
File7043 Spectrum456 scans: 445
Score greater than 27 indicates homology
Score greater than 36 indicates identity

**Score	Expect	Delta Hit Protein	Peptide**
 14.5	8.3	1.0180	R.EQARER.E
 13.6	10	-0.0024	9	P25705|ATPA_HUMAN R.VGSAAQTR.A
 13.3	11	1.0041	R.EAESPKK.T
 12.8	12	-0.0177	R.QATRWK.Y
 12.4	14	0.9929	R.KEQAQGK.V
 12.4	14	-0.0024	R.QKEAASR.G
 12.4	14	1.0180	K.REQAER.L
 12.4	14	1.0180	K.RQEAER.K
 12.4	14	0.9816	R.RVGTAER.R
 12.1	15	0.9816	K.GQKGSVGR.H

Top scoring peptide matches to query 1320
File7043 Spectrum4308 scans: 3365
Score greater than 21 indicates homology
Score greater than 36 indicates identity

**Score	Expect	Delta Hit Protein	Peptide**
 11.2	18	0.9987	K.ENSLAVR.S
 8.2	36	0.9834	K.APGLGAFR.R
 7.1	46	1.0674 625	Q9NX63|CHCH3_HUMAN R.SSEFYR.V
 6.8	49	-1.0687	K.AIGVFKR.L
 5.8	62	1.0238	R.RSPSGER.R
 5.8	62	0.0510	R.EPDATTR.V
 5.8	62	1.0375	K.MRFYR.L
 5.4	68	0.9874	K.ARGAGATGK.D
 5.3	70	0.9987	K.DKEIQR.L
 5.3	70	1.0350	R.DQEIQR.L

Top scoring peptide matches to query 1322
File7043 Spectrum3519 scans: 2727
Score greater than 24 indicates homology
Score greater than 36 indicates identity

**Score	Expect	Delta Hit Protein	Peptide**
 11.4	17	1.0064	K.ANDTLVR.S
 10.7	20	-0.9915	K.QQMIVR.E
 10.6	20	-0.0656	R.AFALLVR.R
 10.6	21	-1.0027	R.RCSIVR.S
 10.4	21	0.9700	R.EKGSLVR.H
 10.4	22	1.0064	K.EAVSQVR.A
 10.2	22	-1.0027	K.ARGTMVR.W
 9.7	25	-0.9365	K.EDGTVNR.D
 9.1	29	0.9878 360	P16157|ANK1_HUMAN R.GNVIMVR.L
 8.9	30	-0.9252	EEEEVR

Top scoring peptide matches to query 1329
File7043 Spectrum8606 scans: 6761
Score greater than 25 indicates homology
Score greater than 36 indicates identity

**Score	Expect	Delta Hit Protein	Peptide**
 13.3	11	0.9980	91	P16444|DPEP1_HUMAN K.ATLQLSR.A
 12.8	12	-0.8973	K.EEEVER.L
 12.8	12	-0.9085	K.NESGVER.C
 12.8	12	-0.9635	R.QMRVEK.F
 12.8	12	-0.9700	K.SSEVQIK.Q
 11.9	15	-0.9523	-.MASKPEK.R
 11.1	18	-0.9085	R.SSSGEPAR.E
 10.8	20	-0.9449	R.ALSSAGER.A
 8.8	31	-0.9197	R.RQEDSR.V
 8.5	33	1.0231	R.ARLDASR.L

Top scoring peptide matches to query 1330
File7043 Spectrum9258 scans: 7295
Score greater than 36 indicates identity

**Score	Expect	Delta Hit Protein	Peptide**
 34.9	0.076	-0.0057	32	P02768|ALBU_HUMAN K.LVTDLTK.V
 26.6	0.51	-0.9757	K.RGSLETK.D
 23.1	1.1	-0.9832	K.LVCGLTK.R
 17.1	4.6	-0.0056	R.LVETSLK.G
 14.2	9	0.0672	K.IAEEEAK.A
 14.2	9	0.0308	R.IAELESK.T
 14.2	9	0.0308	K.IAESEIK.L
 14.2	9	0.0559	R.LAEAETR.A
 13.7	10	-0.0169	R.LVSSQKK.S
 12.3	14	0.0671	K.DPVTETK.N

Top scoring peptide matches to query 1331
File7043 Spectrum6824 scans: 5339
Score greater than 31 indicates homology
Score greater than 36 indicates identity

**Score	Expect	Delta Hit Protein	Peptide**
 23.1	1.1	0.0759	K.EISQANK.N
 23.1	1.1	0.0395	K.ELASKNK.E
 23.0	1.2	0.0242	K.KVGFNPK.K
 17.8	3.9	-0.9710	R.QRVYPK.C
 17.0	4.7	1.0599	K.REELNK.M
 16.2	5.7	0.9983 141	Q9BSJ8|ESYT1_HUMAN R.KLDVSVK.S
 16.0	5.9	0.0572	R.ELCLVR.A
 15.4	6.7	1.0486	K.GARTDLR.N
 15.4	6.7	1.0486	R.QRTLDR.L
 14.9	7.6	1.0122	K.GVRTLSR.C

Top scoring peptide matches to query 1339
File7043 Spectrum10724 scans: 8507
Score greater than 16 indicates homology
Score greater than 34 indicates identity

**Score	Expect	Delta Hit Protein	Peptide**
 4.5	54	0.5472	R.TFPRIR.R
 3.2	74	0.5914	K.TGVMLPR.V
 2.0	97	0.6026	K.MPTTPVK.A
 1.9	99	0.5849	K.EDTIIAK.D
 1.1 1.2e+002	0.6092	-.MCLLPR.G
 1.1 1.2e+002	0.5220 111	P21397|AOFA_HUMAN K.YKLLPR.S
 0.0 1.5e+002	-0.3713	R.SRGGGGGSR.G

Top scoring peptide matches to query 1342
File7043 Spectrum14266 scans: 11625
Score greater than 16 indicates homology
Score greater than 36 indicates identity

**Score	Expect	Delta Hit Protein	Peptide**
 4.2	84	1.1038	R.EPDATTR.V
 3.1 1.1e+002	-0.9305	K.VSINCAK.T
 2.2 1.3e+002	-0.8941	K.EDVAMAR.R
 2.2 1.3e+002	1.0674 547	O00291|HIP1_HUMAN EIVESGR
 2.0 1.4e+002	1.0627	-.MAAAGRGR.E
 1.8 1.5e+002	1.1427	K.YFSCGR.E
 0.7 1.9e+002	-0.8755	K.VEEGDSR.F
 0.4	2e+002	1.0158	K.ILPQYR.D
 0.3 2.1e+002	1.0926	M.AGAAAESGR.E

Top scoring peptide matches to query 1343
File7043 Spectrum11148 scans: 8864
Score greater than 22 indicates homology
Score greater than 36 indicates identity

**Score	Expect	Delta Hit Protein	Peptide**
 13.2	11	0.0175	K.AKVLGMR.R
 9.0	28	-0.9666	R.SLLVMGR.G
 8.3	33	0.9797 111	P21397|AOFA_HUMAN K.YKLLPR.S
 8.0	35	-0.9115	R.LSEESAR.L
 6.7	47	-0.9380	R.HGDPIPR.V
 5.9	57	-1.0247	R.LSAILFK.L
 5.9	57	-1.0247	K.LSALLFK.H
 5.1	69	0.1088	R.GVTADGDR.V
 4.9	72	-0.8751 386	Q07065|CKAP4_HUMAN K.EAADTER.L
 4.9	71	0.1089	R.GDSEQVR.Y

Top scoring peptide matches to query 1345
File7043 Spectrum11740 scans: 9377
Score greater than 26 indicates homology
Score greater than 34 indicates identity

**Score	Expect	Delta Hit Protein	Peptide**
 14.1	5.7	0.3996	R.EDGAAGDR.D
 12.8	7.7	0.3156	R.SSQQKGR.L
 12.2	8.8	0.3446	R.SSPCALR.L
 12.2	8.8	0.3156	R.SSTSRPR.Q
 11.7	9.8	0.3446 173	P52272|HNRPM_HUMAN R.LGGAGMER.M
 10.4	13	0.2904	K.TGLESKR.L
 10.4	13	0.3268	K.TLGESQR.T
 7.4	26	0.3810	R.MPENQR.L
 6.5	32	0.3116	K.SITWQR.L
 5.9	37	0.2752	R.LNFLQR.V

Top scoring peptide matches to query 1346
File7043 Spectrum7073 scans: 5531
Score greater than 36 indicates identity

**Score	Expect	Delta Hit Protein	Peptide**
 43.7	0.009	-0.0044	43	P33121|ACSL1_HUMAN K.AMEDLGR.A
 29.7	0.22	-0.0520	K.ACLSKGR.R
 23.7	0.88	-0.0011	K.DSWVER.L
 20.6	1.8	-0.0269	-.MASRGGGR.G
 20.4	1.9	0.0207	K.MANDQGR.H
 19.9	2.1	-0.0334	R.SSGQLSGR.S
 19.5	2.4	-0.0446	K.ASRASSGR.A
 18.8	2.7	-0.9997	K.MNSVEGR.K
 18.0	3.3	-0.0156	R.MAEERR.K
 16.9	4.3	0.0030	K.DTSGAGAGR.G

Top scoring peptide matches to query 1349
File7043 Spectrum4553 scans: 3552
Score greater than 26 indicates homology
Score greater than 36 indicates identity

**Score	Expect	Delta Hit Protein	Peptide**
 14.5	7.4	-0.0049	89	P07911|UROM_HUMAN R.FVGQGGAR.M
 12.4	12	-1.0505 723	Q8NBS9|TXND5_HUMAN R.FVLSQAK.D
 9.7	22	-0.9923	K.MVRNEK.R
 9.2	25	-0.0334	R.MVGKNVK.L
 8.2	32	-0.9777	R.QDFPGTK.L
 7.1	41	-1.0174	K.AMQSLVK.I
 7.0	42	1.0233	K.MPGQSVR.K
 6.9	43	-1.0868	R.KIYAAVK.I
 6.9	43	-1.0141	K.QFEAAVK.Q
 6.5	47	-1.0287 356	P35558|PCKGC_HUMAN R.MASRLAK.E

Top scoring peptide matches to query 1351
File7043 Spectrum4512 scans: 3524
Score greater than 36 indicates identity

**Score	Expect	Delta Hit Protein	Peptide**
 38.4	0.03	-0.0048	89	P07911|UROM_HUMAN R.FVGQGGAR.M
 25.8	0.55	-0.0333	R.MVGKNVK.L
 21.8	1.4	-0.0663	K.FVLKER.T
 15.1	6.4	0.9256	-.MVLGKVK.S
 12.5	12	-0.0299	K.FVALEGR.H
 11.9	13	0.9620	K.AGGMVLVK.G
 10.6	18	0.0647	R.DMGQQGR.Q
 10.5	19	0.0031	R.MVQELR.K
 9.5	23	-0.9809 471	Q8WUD1|RAB2B_HUMAN R.MVNIDGK.Q
 9.1	25	1.0235	R.MVPSNAR.F

Top scoring peptide matches to query 1352
File7043 Spectrum4499 scans: 3515
Score greater than 36 indicates identity

**Score	Expect	Delta Hit Protein	Peptide**
 33.5	0.093	-0.0046	89	P07911|UROM_HUMAN R.FVGQGGAR.M
 28.9	0.27	-0.0331	R.MVGKNVK.L
 18.3	3.1	-0.0661	K.FVLKER.T
 17.5	3.7	0.9621	K.AGGMVLVK.G
 17.1	4	0.9257	-.MVLGKVK.S
 16.9	4.3	1.0171	R.EDGGTLAK.G
 13.9	8.4	-0.9774	R.QDFPGTK.L
 13.4	9.5	-0.9444	MDLEER
 12.7	11	0.9179	K.FRQLVK.D
 12.7	11	0.9291	R.IGNFIVK.K

Top scoring peptide matches to query 1354
File7043 Spectrum4724 scans: 3687
Score greater than 26 indicates homology
Score greater than 36 indicates identity

**Score	Expect	Delta Hit Protein	Peptide**
 17.1	4	1.0603	R.EMQNPR.E
 13.0	10	-0.9665	R.DRAMQR.L
 13.0	10	0.9909	R.GYLAGPGR.E
 13.0	10	0.9764	R.LRAQMR.L
 13.0	10	0.9764	K.RIAQMR.Q
 10.7	18	0.9876	R.ERQVMK.K
 9.9	21	1.0062	R.EAAERSK.E
 9.2	25	0.9546 441	P49368|TCPG_HUMAN R.YIKNPR.I
 8.3	30	1.0174	K.EEQAVSK.E
 8.4	30	-0.9957	-.MFQPAAK.R

Top scoring peptide matches to query 1357
File7043 Spectrum851 scans: 710
Score greater than 27 indicates homology
Score greater than 36 indicates identity

**Score	Expect	Delta Hit Protein	Peptide**
 14.3	7.8	1.0611	R.EMQNPR.E
 13.8	8.8	0.9917	R.GYLAGPGR.E
 13.5	9.2	-0.9658	R.DRAMQR.L
 13.5	9.2	0.9771	R.LRAQMR.L
 13.5	9.2	0.9771	K.RIAQMR.Q
 13.3	9.7	-0.9910	R.ITQMQR.L
 13.3	9.7	0.9553 441	P49368|TCPG_HUMAN R.YIKNPR.I
 13.1	10	0.9883	R.VLQMQR.D
 10.4	19	0.9805	K.AGYRAPR.S
 10.2	20	-1.0644	K.RLFPFL.-

Top scoring peptide matches to query 1360
File7043 Spectrum10711 scans: 8498
Score greater than 21 indicates homology
Score greater than 36 indicates identity

**Score	Expect	Delta Hit Protein	Peptide**
 10.0	21	0.0205	89	P07911|UROM_HUMAN R.FVGQGGAR.M
 8.3	31	-0.0080	K.MSILGVR.S
 7.4	38	0.9947	KGSETLR
 7.4	38	0.0284	-.MATPSLR.G
 6.3	49	1.0158 645	P19022|CADH2_HUMAN NWTITR
 6.1	52	0.0205	K.FAQNVGR.N
 5.5	58	0.0093	R.KHDHVR.H
 5.2	63	0.0470	R.DSSVEVR.S
 4.8	69	0.0318	K.GDFPSLR.L
 4.7	70	-0.9920	R.KQEMLK.E

Top scoring peptide matches to query 1362
File7043 Spectrum4891 scans: 3815
Score greater than 23 indicates homology
Score greater than 35 indicates identity

**Score	Expect	Delta Hit Protein	Peptide**
 12.9	10	-1.1338	R.QYLTIR.Y
 12.6	11	-0.1749 388	P62701|RS4X_HUMAN R.KIFVGTK.G
 12.3	12	-0.1498	R.KVYNIR.G
 10.2	19	-1.0974	K.VQDYLR.R
 9.3	23	-1.0974	R.QYNIQK.K
 9.2	23	-1.0644	K.AGGMATTGK.E
 8.8	26	-1.1589	K.KYEIIK.R
 8.8	26	-1.1225	R.QYELLK.L
 8.3	29	-1.0974	R.KFDDIR.I
 8.3	29	-1.0830	K.KMMDLR.T

Top scoring peptide matches to query 1363
File7043 Spectrum616 scans: 549
Score greater than 27 indicates homology
Score greater than 35 indicates identity

**Score	Expect	Delta Hit Protein	Peptide**
 17.9	3.2	1.0405	89	P07911|UROM_HUMAN R.FVGQGGAR.M
 13.7	8.4	1.0120	R.MVGKNVK.L
 13.0	9.8	1.0484	K.MNQALAK.M
 10.5	18	-0.9382	R.MAPSIMK.S
 9.2	24	1.0882	K.EFNEPR.V
 8.7	26	0.0532	K.MVRNEK.R
 8.7	27	1.0518	R.KFDEPR.S
 8.0	31	1.0783	36	P11142|HSP7C_HUMAN K.ATVEDEK.L
 7.9	32	1.0372	R.QAMAKSR.R
 7.2	37	0.9902	R.DGVLFLK.L

Top scoring peptide matches to query 1364
File7043 Spectrum9991 scans: 7896
Score greater than 22 indicates homology
Score greater than 35 indicates identity

**Score	Expect	Delta Hit Protein	Peptide**
 15.9	5	-0.9638	K.YANNAIK.T
 9.0	25	-0.9274	R.FSPNSNK.S
 8.0	31	-0.9162	R.VGDDPYK.L
 7.0	39	0.0355	R.SSRDLSK.H
 7.0	39	-1.0002	56	P10809|CH60_HUMAN K.YKNIGAK.L
 6.2	47	1.0810	R.SSDGNRR.R
 6.1	48	-0.0022	K.HLPNRR.F
 6.0	50	-0.9195	-.MSEDLAK.Q
 5.9	50	-0.9638	K.SFIGQNK.S
 5.8	52	-0.9638	K.VEFSGVR.G

Top scoring peptide matches to query 1365
File7043 Spectrum6201 scans: 4847
Score greater than 28 indicates homology
Score greater than 35 indicates identity

**Score	Expect	Delta Hit Protein	Peptide**
 17.6	3.4	-0.0039 388	P62701|RS4X_HUMAN R.KIFVGTK.G
 15.1	6.1	-0.0038	R.KIYAAVK.I
 14.8	6.5	-0.9151	R.EIYPGSK.I
 14.8	6.5	-0.9515	K.EIYQIK.Q
 14.8	6.5	-0.9263	R.ELYAAAR.R
 14.8	6.5	-0.9375	R.ELYGRR.R
 10.2	19	-0.9297	-.MTASVLR.S
 10.2	19	0.0213	R.QLFRTK.S
 9.8	20	0.0656	K.QIASVMK.H
 9.2	24	0.1053	R.QDFPGTK.L

Top scoring peptide matches to query 1366
File7043 Spectrum4946 scans: 3861
Score greater than 26 indicates homology
Score greater than 34 indicates identity

**Score	Expect	Delta Hit Protein	Peptide**
 13.1	6.3	0.3083	-.MSGGGVIR.G
 12.4	7.3	0.3018	R.EGSLTGTK.D
 12.3	7.5	0.3230	R.QDFPGTK.L
 11.4	9.3	0.2832	R.KSSMPVK.I
 10.0	13	0.3005	K.ARNFER.V
 9.4	15	-0.7120	-.MTASVLR.S
 9.3	15	0.2138 388	P62701|RS4X_HUMAN R.KIFVGTK.G
 9.2	15	0.3196	K.AGVEAMSK.S
 8.9	17	0.2138	R.QYLLKK.K
 8.3	19	0.3084	R.KNGGMGTK.S

Top scoring peptide matches to query 1371
File7043 Spectrum4139 scans: 3234
Score greater than 25 indicates homology
Score greater than 35 indicates identity

**Score	Expect	Delta Hit Protein	Peptide**
 13.6	7.9	0.9916	-.MRVNEK.Y
 13.0	9.1	-0.0288	-.MTASVLR.S
 12.2	11	0.9916	K.MVRNEK.R
 10.2	17	0.0498	K.GWCGWK.G
 10.1	18	0.9916	R.KNGGMGTK.S
 9.9	18	0.8970 388	P62701|RS4X_HUMAN R.KIFVGTK.G
 8.2	28	1.0214	R.SSPEGSTK.D
 7.7	31	1.0062	R.QDFPGTK.L
 7.6	32	0.9851	R.EGSLTGTK.D
 7.1	35	0.0222	R.VGDDPYK.L

Top scoring peptide matches to query 1375
File7043 Spectrum2728 scans: 2103
Score greater than 24 indicates homology
Score greater than 35 indicates identity

**Score	Expect	Delta Hit Protein	Peptide**
 12.3	11	0.9802	K.AALFNTR.I
 10.5	16	1.0642	R.EFEDPR.D
 10.5	16	0.9915	R.EFEVLR.K
 9.0	23	1.0133	-.MRVNEK.Y
 8.8	24	1.0133	K.EMKAAAR.E
 7.6	31	-0.9734	R.MMEVLR.K
 7.5	32	1.0133	K.MVRNEK.R
 7.2	34	0.9690	R.FRNISR.I
 7.2	34	1.0384	K.MRNAER.A
 7.1	35	1.0245 256	P51648|AL3A2_HUMAN -.MELEVR.R

Top scoring peptide matches to query 1378
File7043 Spectrum14849 scans: 12157
Score greater than 14 indicates homology
Score greater than 35 indicates identity

**Score	Expect	Delta Hit Protein	Peptide**
 3.3	85	0.0142 311	P01833|PIGR_HUMAN K.VLDSGFR.E
 1.6 1.3e+002	1.0975	K.GDSQEEK.G
 1.5 1.3e+002	0.0870	K.HGSDYSK.D
 0.2 1.7e+002	0.1200	K.VDDCER.C

Top scoring peptide matches to query 1379
File7043 Spectrum3943 scans: 3076
Score greater than 17 indicates homology
Score greater than 35 indicates identity

**Score	Expect	Delta Hit Protein	Peptide**
 8.8	24	0.0300	K.DGSFIVR.Y
 8.8	24	0.0300	DGSFLVR
 4.1	71	-0.9145	R.VMGQMGR.S
 3.0	91	-0.9760	K.LMMLVR.E
 2.6	99	1.0470	K.MKAGGNAK.F
 2.6	99	1.0834 569	P47914|RL29_HUMAN K.MQANNAK.A
 2.6	1e+002	1.0219	K.DLGKTMK.D
 2.5	1e+002	0.0300	R.YDVGAIR.A
 2.5	1e+002	-0.0250	R.MKFLVR.R
 1.9 1.2e+002	-0.9588	R.CVRFGR.M

Top scoring peptide matches to query 1382
File7043 Spectrum1019 scans: 822
Score greater than 19 indicates homology
Score greater than 35 indicates identity

**Score	Expect	Delta Hit Protein	Peptide**
 13.4	8.2	1.0446	K.FPQLCK.F
 5.2	55	1.0487	R.EMNRVK.K
 4.8	59	1.0632	R.QDFPGTK.L
 4.8	60	1.0269	K.QPYEKK.A
 4.8	60	1.0633	K.QPYEQK.A
 4.7	62	0.9541 388	P62701|RS4X_HUMAN R.KIFVGTK.G
 4.7	62	0.9792	R.QLFRTK.S
 3.7	77	0.9905	R.KPGVYTK.V
 3.5	81	0.0646	R.ENMGKAK.E
 3.5	81	1.0487	K.QMETRK.R

Top scoring peptide matches to query 1396
File7043 Spectrum5256 scans: 4098
Score greater than 20 indicates homology
Score greater than 33 indicates identity

**Score	Expect	Delta Hit Protein	Peptide**
 9.9	11	0.3051	R.NTSTPFK.G
 7.4	20	-0.8033	K.KIFLFK.R
 7.3	20	-0.7339	-.MIAVSFK.C
 4.6	37	-0.7703 489	P99999|CYC_HUMAN K.KIFIMK.C
 4.3	40	0.2687	R.QTASIFK.Q
 4.2	41	0.2687	K.QLYKDK.E
 3.5	49	0.2099	R.IHRQLK.N
 3.4	49	0.3116	K.RMTWGK.V
 2.8	56	-0.7451	-.MALHVPK.A
 2.3	64	0.2575	K.SARSVFK.I

Top scoring peptide matches to query 1397
File7043 Spectrum815 scans: 686
Score greater than 17 indicates homology
Score greater than 33 indicates identity

**Score	Expect	Delta Hit Protein	Peptide**
 5.9	27	0.3140	K.EQFSKR.K
 5.6	29	0.2889	R.YGDALKK.C
 4.6	37	0.2889	R.QTASIFK.Q
 4.0	42	-0.6304	-.MEMGRR.I
 3.9	43	0.3722	R.SCRSER.S
 3.0	53	0.3219	K.KDSGMLK.Y
 3.0	53	0.3033 641+ P06753|TPM3_HUMAN K.KMQMLK.L
 3.0	54	-0.6006	K.AEMGSER.A
 2.9	54	-0.7064	K.IKYSER.V
 1.7	72	0.2855	K.TKISAMK.E

Top scoring peptide matches to query 1399
File7043 Spectrum2345 scans: 1816
Score greater than 18 indicates homology
Score greater than 33 indicates identity

**Score	Expect	Delta Hit Protein	Peptide**
 8.1	17	0.7218	K.ESAMVNK.I
 4.8	35	0.6854	K.KDSGMLK.Y
 4.4	39	-0.3502	-.MFEIKK.I
 4.3	40	-0.2808	K.KMMEEK.L
 3.5	48	0.7218	R.KMDEQK.K
 3.2	51	-0.2808 244	O75947|ATP5H_HUMAN K.EMEKMK.N
 2.6	59	0.6524	K.YDKQIK.F
 2.4	62	-0.3502	K.MLYQLK.V
 1.9	69	-0.3098	R.KMSSSGAK.D
 1.4	77	-0.3541	K.QHNLVGK.K

Top scoring peptide matches to query 1401
File7043 Spectrum11222 scans: 8924
Score greater than 21 indicates homology
Score greater than 34 indicates identity

**Score	Expect	Delta Hit Protein	Peptide**
 15.1	4.7	-1.0901 118	P53675|CLH2_HUMAN M.AQILPVR.F
 7.5	27	-1.0901	K.VVNIPVR.I
 3.7	65	-0.0697	K.LELKHR.I
 3.1	74	0.9620	K.VPISHNK.R
 3.0	77	-0.0445	R.LRNQHK.V
 2.9	78	-0.8938	R.QACMDR.D
 2.9	78	-0.9301	R.QAMSGMR.W
 2.9	78	-0.9301	R.QAMSGMR.W
 2.9	79	-0.0697	R.VLGHTLR.A
 2.7	82	-1.0901	R.AQPLLVR.A

Top scoring peptide matches to query 1405
File7043 Spectrum9864 scans: 7791
Score greater than 13 indicates homology
Score greater than 34 indicates identity

**Score	Expect	Delta Hit Protein	Peptide**
 2.0	97	-0.0138	K.VRAFFR.S
 0.5 1.4e+002	1.0258 565	Q7Z7H5|TMED4_HUMAN R.MALFAGGK.L

Top scoring peptide matches to query 1412
File7043 Spectrum3768 scans: 2928
Score greater than 31 indicates homology
Score greater than 34 indicates identity

**Score	Expect	Delta Hit Protein	Peptide**
 26.5	0.33	-0.0045 515	P62847|RS24_HUMAN R.KFMTNR.L
 16.7	3.2	0.0835	R.AGMDGSSR.F
 16.7	3.2	0.0649	K.KMECGR.M
 16.7	3.2	-0.0375	K.QFKAFR.R
 16.4	3.4	-0.0739	K.KPLPWR.S
 16.4	3.4	-0.0950	K.KPLSPVR.D
 16.4	3.4	-0.0950	R.QPLVIAR.L
 16.1	3.6	-0.9335	EFSGSDR
 15.8	3.9	-1.0790	M.EPLLLGR.G
 13.3	6.9	0.0285	K.MKMTNR.A

Top scoring peptide matches to query 1415
File7043 Spectrum5106 scans: 3985
Score greater than 23 indicates homology
Score greater than 34 indicates identity

**Score	Expect	Delta Hit Protein	Peptide**
 14.8	4.9	0.9571	R.GRGVGPPR.G
 14.6	5.2	0.0207	R.GESPPGPR.V
 9.6	16	-0.0269 455	P12110|CO6A2_HUMAN K.GAKGGPGPR.G
 7.0	29	0.0208	R.ENSPPPR.G
 6.6	32	0.0901	K.EGNSMSR.H
 6.5	33	-0.0156	R.HEIELR.M
 6.5	33	-0.0269	R.HELKNR.D
 6.5	33	-0.0017	R.RSHGSPR.R
 6.2	36	0.0134	-.MFTELR.S
 6.0	38	0.0095	K.QQNGPPR.K

Top scoring peptide matches to query 1416
File7043 Spectrum6597 scans: 5158
Score greater than 22 indicates homology
Score greater than 32 indicates identity

**Score	Expect	Delta Hit Protein	Peptide**
 16.0	2.4	0.2175	R.IKPLPTK.K
 8.7	13	0.3444 175	P61421|VA0D1_HUMAN K.MVVEFR.H
 8.1	15	0.3155	K.DQPPALR.R
 8.0	15	0.3081	R.IMNYKK.E
 8.0	15	0.3808	K.NPMYQK.E
 7.8	16	0.3523	R.LIMMEK.D
 7.8	16	0.3155	R.LEPPGAGR.G
 7.8	16	0.2427	R.LPLLGQR.Y
 6.2	23	0.2791	K.DPKPAIR.F
 6.1	24	0.2427	R.RLVPPSK.R

Top scoring peptide matches to query 1420
File7043 Spectrum1017 scans: 821
Score greater than 25 indicates homology
Score greater than 34 indicates identity

**Score	Expect	Delta Hit Protein	Peptide**
 14.1	5.4	-1.0183 172	P84098|RL19_HUMAN R.HMGIGKR.K
 13.5	6.1	-0.9707	R.CFTSKR.N
 13.3	6.6	-1.0149	K.HFNPKR.S
 13.3	6.6	-1.0183	HLMNKR
 11.1	11	-1.0109	M.AGGRGAPGR.G
 7.7	24	1.0780	R.NMTMQR.T
 7.2	26	1.0966	R.AGMDGSSR.F
 7.0	28	-0.0044	R.APAPGTAGR.G
 6.6	30	-1.0248	R.IPTSPAGR.F
 6.3	33	-0.0157	K.RHDLTR.L

Top scoring peptide matches to query 1423
File7043 Spectrum3481 scans: 2700
Score greater than 27 indicates homology
Score greater than 34 indicates identity

**Score	Expect	Delta Hit Protein	Peptide**
 17.3	2.6	-1.1338	K.AVLLVRK.F
 14.1	5.5	0.9910	K.GPVDVPGR.E
 14.0	5.6	-1.1338	K.LIGLVRK.A
 13.5	6.1	-1.0181 172	P84098|RL19_HUMAN R.HMGIGKR.K
 13.5	6.2	-0.9011	M.TMCSGAR.L
 12.8	7.4	-0.9743	K.RSEGGHR.G
 12.5	7.8	-1.0359	K.RIEPQR.Y
 12.5	7.8	-1.1086	K.RLLLQR.L
 12.5	7.8	-1.0359	R.RPELQR.V
 12.5	7.8	-1.0247 180	O43895|XPP2_HUMAN K.VGPELQR.R

Top scoring peptide matches to query 1425
File7043 Spectrum10757 scans: 8532
Score greater than 19 indicates homology
Score greater than 34 indicates identity

**Score	Expect	Delta Hit Protein	Peptide**
 10.9	11	0.0861	K.CEGIYR.V
 10.4	13	-0.0196 230	P21912|DHSB_HUMAN K.KFAIYR.W
 6.1	34	0.0861	-.MGSAFER.V
 5.2	42	0.0320	R.TTATTFR.A
 4.8	46	1.0776	R.SPEHNGR.G
 4.6	48	1.0524	R.SASQFTR.D
 3.5	62	0.1039	-.MGWTMR.L
 3.5	63	0.0464	-.MVKMTR.S
 3.4	63	0.0385	K.MATRYR.E
 3.4	64	0.0497	K.TCLTFR.F

Top scoring peptide matches to query 1429
File7043 Spectrum4820 scans: 3761
Score greater than 20 indicates homology
Score greater than 34 indicates identity

**Score	Expect	Delta Hit Protein	Peptide**
 9.7	15	0.0794	K.SYGLSDR.Y
 6.5	31	1.0046	HSRLQR
 6.3	32	-0.0232	K.HLKMLR.D
 5.0	44	1.0410	R.HSSGPRR.N
 4.4	51	0.0972	K.AVECYR.R
 3.8	59	-1.0613 355	P23396|RS3_HUMAN K.LLGGLAVR.R
 3.6	61	-0.9708	R.ELHLMR.N
 3.4	64	1.0700	K.AAHPNMR.T
 3.4	64	1.1320	R.AVCCMR.F
 3.4	64	-0.8617	K.AGDGCYR.R

Top scoring peptide matches to query 1430
File7043 Spectrum3707 scans: 2880
Score greater than 22 indicates homology
Score greater than 34 indicates identity

**Score	Expect	Delta Hit Protein	Peptide**
 9.6	15	-0.0409	R.KGLVPQR.C
 9.6	15	0.0244	K.SFLMKR.G
 8.8	18	-0.8650	M.TMCSGAR.L
 8.8	18	-0.9634	R.TVGHTQR.K
 8.8	18	0.9907	K.VEAPPKR.R
 8.8	18	1.1142	R.VSCMQR.K
 8.7	19	-0.0158 495	P09601|HMOX1_HUMAN R.APGLRQR.A
 8.7	19	1.0046	HSRLQR
 8.7	19	-1.0726	LLRLQR
 8.7	19	-1.0250	K.LLTPAQR.T

Top scoring peptide matches to query 1432
File7043 Spectrum8806 scans: 6922
Score greater than 20 indicates homology
Score greater than 34 indicates identity

**Score	Expect	Delta Hit Protein	Peptide**
 7.8	23	1.0199 455	P12110|CO6A2_HUMAN K.GAKGGPGPR.G
 6.8	29	0.0358	R.QVAHSGAK.A
 6.2	33	1.0562	K.QQNGPPR.K
 4.8	46	-0.9482	R.SPPGSTPR.L
 4.6	48	0.0359	R.KAAGDHAK.G
 4.6	49	-0.0621	K.KIQPLAK.A
 3.6	60	-1.0461	M.PAILVASK.M
 3.5	61	-0.0369	R.RGGPLVAK.E
 3.5	62	-0.9230	R.SGGGPPNGR.G
 3.3	66	-0.9845	K.KNQPPSK.A

Top scoring peptide matches to query 1433
File7043 Spectrum1644 scans: 1237
Score greater than 17 indicates homology
Score greater than 32 indicates identity

**Score	Expect	Delta Hit Protein	Peptide**
 8.2	15	0.3543	K.MASEGMR.F
 4.5	34	-0.7394	K.ATHVNTR.D
 4.2	37	0.3794	R.MCNNSR.F
 3.4	44	0.2849	R.MGIFSSR.G
 3.2	46	-0.8009	R.AAALLPSR.S
 3.2	46	-0.7645	K.APALSPSR.E
 3.2	46	-0.7394	K.ARPPDSR.L
 2.5	54	1.1784	R.LVPPSKR.R
 2.4	56	1.1784 118	P53675|CLH2_HUMAN M.AQILPVR.F
 2.4	56	1.1784	K.VVNIPVR.I

Top scoring peptide matches to query 1435
File7043 Spectrum32287 scans: 28284
Score greater than 17 indicates homology
Score greater than 32 indicates identity

**Score	Expect	Delta Hit Protein	Peptide**
 5.4	28	-0.8355	K.LNAVLIR.S
 4.7	33	-0.8970	K.LLAKLLK.S
 3.7	41	0.1849	R.KGLVPQR.C
 3.1	47	0.2465	K.RHDLTR.L
 3.0	49	-0.7739	K.KHSGTLR.G
 2.5	54	0.2577	R.HIEDKR.M
 2.5	54	0.2941	K.HLEDGAR.T
 2.5	54	0.2027	K.HLKMLR.D
 2.4	55	0.2213 565	Q7Z7H5|TMED4_HUMAN M.AGVGAGPLR.A
 1.9	62	0.2424	R.QHPLFR.E

Top scoring peptide matches to query 1439
File7043 Spectrum32335 scans: 28346
Score greater than 17 indicates homology
Score greater than 32 indicates identity

**Score	Expect	Delta Hit Protein	Peptide**
 6.5	22	0.3196	R.QGHGENR.Q
 4.0	38	0.2837	R.RLMMSK.E
 2.6	53	1.1442	K.KVIIPAR.M
 2.3	57	-0.7987 455	P12110|CO6A2_HUMAN R.KGAPGLAGK.N
 0.9	78	-0.7372	R.QAVPNGGR.F
 0.2	91	0.2983	R.GDLMAYK.V

Top scoring peptide matches to query 1445
File7043 Spectrum1217 scans: 956
Score greater than 24 indicates homology
Score greater than 34 indicates identity

**Score	Expect	Delta Hit Protein	Peptide**
 13.0	7.5	-1.0245	K.TPSPNKR.S
 10.6	13	-0.0769	R.GLSRPIR.R
 9.8	15	1.0050	R.GRAGSHGR.R
 8.0	23	-0.0228 172	P84098|RL19_HUMAN R.HMGIGKR.K
 8.0	24	-0.0042	K.GDHSKVR.I
 7.4	27	-1.0246	K.LTPQGQR.D
 6.8	31	-0.9630	R.GHNTQSR.V
 6.5	33	-1.0973	R.LAALQKR.R
 6.3	35	-0.0657	R.LATAPLGR.R
 6.2	36	-0.0657	R.GATALPLR.T

Top scoring peptide matches to query 1446
File7043 Spectrum2402 scans: 1862
Score greater than 20 indicates homology
Score greater than 34 indicates identity

**Score	Expect	Delta Hit Protein	Peptide**
 8.3	22	1.0206	R.LYQSMR.K
 7.9	24	0.9876	R.IYQFAR.L
 7.2	28	1.0206	M.PPGGGGPMK.D
 6.8	31	1.0462	R.QMIMMK.K
 6.8	31	1.0206	10	Q00610|CLH1_HUMAN K.YLQMAR.K
 4.7	50	-0.9223	K.YDMQSR.C
 4.7	50	-0.9223	K.YDMSGAR.L
 4.5	53	1.0318	R.KEDMFK.D
 4.4	54	-0.0903	R.KEIPLAK.D
 4.3	55	0.9301	R.TLTPPLR.R

Top scoring peptide matches to query 1447
File7043 Spectrum2235 scans: 1727
Score greater than 20 indicates homology
Score greater than 34 indicates identity

**Score	Expect	Delta Hit Protein	Peptide**
 11.1	12	-0.0074	K.KGGFSFR.F
 7.0	30	1.0209	K.EKFSMR.S
 7.0	30	1.0243	K.EQFFAR.L
 6.3	35	0.9807	M.RGAGPSPR.Q
 6.1	36	-0.0034	K.ATHVNTR.D
 5.5	42	0.9555 565	Q7Z7H5|TMED4_HUMAN M.AGVGAGPLR.A
 5.3	44	1.0573	QMEAFR
 5.2	45	1.0420	K.AFWAMR.R
 5.0	47	0.9192	R.KGNLPIR.S
 4.7	50	0.0256	K.KTCSFR.I

Top scoring peptide matches to query 1448
File7043 Spectrum6927 scans: 5420
Score greater than 17 indicates homology
Score greater than 34 indicates identity

**Score	Expect	Delta Hit Protein	Peptide**
 5.0	47	0.0772 491	O43181|NDUS4_HUMAN K.MEFDTR.E
 4.9	48	0.9555	R.IFKFSR.H
 4.9	48	0.9555	K.LFKFSR.E
 4.6	52	0.0772	-.MEFSER.K
 3.8	62	1.0579	K.LMMSQR.E
 2.7	80	0.0296	R.CFTSKR.N
 2.2	90	-0.9583	R.GHNTQSR.V
 2.2	90	0.0773	-.MEEYAR.E
 1.7	1e+002	1.0725	R.MEFENK.E
 1.5	1e+002	0.0772	-.MEFTDR.N

Top scoring peptide matches to query 1453
File7043 Spectrum837 scans: 701
Score greater than 23 indicates homology
Score greater than 34 indicates identity

**Score	Expect	Delta Hit Protein	Peptide**
 13.4	6.7	0.0439	R.GVAAPVER.A
 9.4	17	0.0327	R.EAGRIPR.T
 8.4	22	0.0690	K.IANNPGGR.E
 6.9	31	0.0504 172	P84098|RL19_HUMAN R.HMGIGKR.K
 6.5	33	-0.0037	R.GLSRPIR.R
 6.3	35	0.0578	R.RAQGPGGR.G
 6.0	37	0.0327	RQEPLR
 5.9	38	0.0075	R.GATALPLR.T
 5.8	40	1.0820	K.QMHLPR.S
 5.8	40	1.0279	K.QQVGIPR.S

Top scoring peptide matches to query 1457
File7043 Spectrum7726 scans: 6059
Score greater than 17 indicates homology
Score greater than 33 indicates identity

**Score	Expect	Delta Hit Protein	Peptide**
 5.3	30	0.6421	R.RHVACR.Y
 3.8	42	0.5992	R.RPELQR.V
 3.8	42	0.6104 180	O43895|XPP2_HUMAN K.VGPELQR.R
 3.7	43	0.5264	K.RLLLQR.L
 3.5	45	0.5628	R.RPEKLR.D
 3.4	46	0.5264	R.RLLQLR.A
 3.4	47	0.5992	R.RPEAVAR.I
 3.3	47	-0.3848	R.RLEEPR.T
 3.0	51	0.5516	RPTLRR
 2.5	56	0.5992	R.ERPAGLR.R

Top scoring peptide matches to query 1460
File7043 Spectrum5854 scans: 4574
Score greater than 22 indicates homology
Score greater than 35 indicates identity

**Score	Expect	Delta Hit Protein	Peptide**
 22.8	0.87	-0.0052	K.MIYASSK.D
 7.6	29	0.9862	R.RSSFSSK.D
 5.6	46	0.0087	K.RHPDMK.I
 5.3	49	-0.0607 160	O95299|NDUAA_HUMAN R.VLHQFR.E
 4.5	58	0.0273	R.GHATEER.G
 4.5	58	-0.0971	K.HKVFLR.F
 4.5	58	0.0338	R.HQCQAR.E
 4.5	58	-0.0607	K.QHVFLR.T
 4.4	60	0.0080	R.WYQFR.V
 3.9	69	0.0273	K.HSPESSR.G

Top scoring peptide matches to query 1467
File7043 Spectrum1424 scans: 1090
Score greater than 20 indicates homology
Score greater than 35 indicates identity

**Score	Expect	Delta Hit Protein	Peptide**
 8.8	22	1.0389	R.GATALPLR.T
 8.6	23	0.0760	K.KTTFFR.K
 7.0	33	-0.9404	R.QGQALGVK.R
 6.5	37	0.0648	K.AVVAFHR.G
 6.3	38	1.1182	K.MHAINGR.M
 5.9	43	1.0818 172	P84098|RL19_HUMAN R.HMGIGKR.K
 5.5	46	0.0324	R.LGRIGQR.K
 5.5	46	0.0324	R.VAARVQR.R
 5.5	47	-0.9265	R.GRGGGGAIR.V
 5.5	47	-0.9516	K.QNAALRK.E

Top scoring peptide matches to query 1468
File7043 Spectrum7188 scans: 5627
Score greater than 15 indicates homology
Score greater than 33 indicates identity

**Score	Expect	Delta Hit Protein	Peptide**
 2.3	72	0.6165	K.RQQLVR.K
 2.0	77	0.6819 233	P17568|NDUB7_HUMAN -.MGAHLVR.R
 1.8	81	0.7733	K.DDEHQR.R
 1.8	81	0.6489	R.FIVHQR.I
 1.8	81	0.7183	K.IMDHQR.I
 1.8	81	0.7183	R.LMDHQR.T
 1.8	81	0.7183	R.MVEHQR.V
 1.8	81	0.7434	K.NNMQHR.Y
 1.8	81	0.7369	R.TEEAHGR.E
 0.7	1e+002	0.7369	R.EQTEHR.G

Top scoring peptide matches to query 1473
File7043 Spectrum5759 scans: 4500
Score greater than 35 indicates identity

**Score	Expect	Delta Hit Protein	Peptide**
 44.1	0.0076	-0.0049	29	P08473|NEP_HUMAN K.ALAIKER.I
 31.0	0.15	-0.0049	R.ALAKIER.F
 24.6	0.68	-0.9526	R.LAALEER.L
 20.3	1.8	0.0314	R.AISPREK.I
 20.3	1.8	0.0314	R.ALLAEQR.D
 20.3	1.8	-0.0049	K.AIAAKQAK.K
 20.3	1.8	0.0314	R.AIAEQLR.A
 17.8	3.2	-0.9638	K.ALAGQSVR.I
 15.9	4.9	-0.9890	R.ALQGTIAK.N
 15.4	5.6	-0.9638	K.ANKAIER.N

Top scoring peptide matches to query 1474
File7043 Spectrum8857 scans: 6967
Score greater than 15 indicates homology
Score greater than 35 indicates identity

**Score	Expect	Delta Hit Protein	Peptide**
 6.3	45	1.0698	26	Q14764|MVP_HUMAN R.MVTVPPR.H
 6.2	46	1.0521	R.AGLQPVSK.W
 6.2	46	1.0157	R.KPLQVSK.I
 2.3 1.1e+002	1.1136	K.IHSAESR.H
 1.7 1.3e+002	0.0667	R.KHVGGFR.K
 1.4 1.4e+002	-0.9020	R.RESQGPK.K
 0.8 1.6e+002	1.0772	K.RQLPTGQ.-
 0.8 1.6e+002	1.0885	K.ETLSPPR.R
 0.4 1.8e+002	0.0456	R.RTSAIPR.A

Top scoring peptide matches to query 1475
File7043 Spectrum10044 scans: 7938
Score greater than 29 indicates homology
Score greater than 35 indicates identity

**Score	Expect	Delta Hit Protein	Peptide**
 18.3	2.9	0.0425	K.QLILEGK.D
 15.7	5.3	0.0313	R.AGLIITGR.T
 13.8	8.2	-0.0051	6+ P60709|ACTB_HUMAN K.RGILTLK.Y
 13.7	8.2	-0.9527	R.VINLTNK.K
 12.4	11	0.0062	K.KIIADIK.K
 12.4	11	0.0425	K.QILEGLK.Y
 11.5	14	0.0313	R.KLVVADR.E
 9.4	22	0.0062	K.AGLISLVK.M
 9.1	24	0.0888	K.QHPKYK.H
 9.1	24	0.1364	R.QSFQYK.T

Top scoring peptide matches to query 1479
File7043 Spectrum1821 scans: 1380
Score greater than 21 indicates homology
Score greater than 34 indicates identity

**Score	Expect	Delta Hit Protein	Peptide**
 8.9	18	0.6564 372	P16403|H12_HUMAN K.KAAGGATPK.K
 7.8	23	0.7179	R.KHSGTDR.T
 7.0	27	-0.2847	R.MAAQGAPR.F
 6.9	28	0.6703	R.GAAGAAARR.N
 6.9	29	-0.3501	R.TVAAGRAR.G
 4.9	45	0.7720	R.GEHNGMR.G
 4.7	47	-0.3681	K.TLPVFPK.G
 4.2	52	-0.3501	R.LGTQRAR.A
 3.6	61	0.6564	K.KADTLPR.V
 3.3	64	0.7543	K.GSHQSER.V

Top scoring peptide matches to query 1481
File7043 Spectrum5325 scans: 4155
Score greater than 22 indicates homology
Score greater than 36 indicates identity

**Score	Expect	Delta Hit Protein	Peptide**
 10.0	22	0.9471	K.VGDLGIAR.V
 9.4	25	-0.0481	K.GVAQGLTR.S
 9.2	26	1.0087	R.SHSSAGVR.Q
 9.2	26	0.9723	R.SSHVSKR.A
 8.3	32	0.9220	K.SPATLLAK.T
 7.6	37	0.9471	K.TTPTPRK.M
 7.2	41	0.9108 660	P30038|AL4A1_HUMAN R.RAEILAK.T
 6.5	48	-0.0732	R.AIDLNKK.G
 6.4	50	0.9359	R.RTSAIPR.A
 6.2	52	0.9610	R.QVGQGRR.S

Top scoring peptide matches to query 1482
File7043 Spectrum2205 scans: 1703
Score greater than 17 indicates homology
Score greater than 36 indicates identity

**Score	Expect	Delta Hit Protein	Peptide**
 6.8	46	0.9548 372	P16403|H12_HUMAN K.KAAGGATPK.K
 6.5	48	1.0163	R.KHSGTDR.T
 3.7	92	0.9759	K.QHPKYK.H
 2.9 1.1e+002	0.9646	R.KHVGGFR.K
 2.5 1.2e+002	0.9548	K.KADTLPR.V
 2.3 1.3e+002	0.0058	R.RHEGFR.Q
 2.3 1.3e+002	1.0163	K.EGADRPR.Q
 2.1 1.3e+002	0.9759	R.KHPFSGK.S
 1.5 1.5e+002	-0.9670	R.NTPWER.G
 1.2 1.6e+002	0.9687	R.GAAGAAARR.N

Top scoring peptide matches to query 1483
File7043 Spectrum145 scans: 242
Score greater than 27 indicates homology
Score greater than 36 indicates identity

**Score	Expect	Delta Hit Protein	Peptide**
 16.1	5.4	-0.0287	R.QTASVAPK.E
 16.0	5.4	0.9441	K.KGGASKPR.T
 15.6	6	0.9441	K.QNAALRK.E
 13.9	8.9	0.9918	R.IEASPQR.S
 12.8	11	0.9805	R.KQGSQPR.G
 12.2	13	0.9554 372	P16403|H12_HUMAN K.KAAGGATPK.K
 11.9	14	0.9554	K.LEAAIQR.S
 11.1	17	-1.0239	K.AAEEVKR.E
 10.7	19	0.9190	R.EIAALKR.N
 9.9	22	-0.0286	K.TSNLAAPK.S

Top scoring peptide matches to query 1486
File7043 Spectrum551 scans: 508
Score greater than 24 indicates homology
Score greater than 36 indicates identity

**Score	Expect	Delta Hit Protein	Peptide**
 16.4	4.9	1.0542	K.GDQGNPGR.T
 11.1	17	-0.0138	K.GEAGVRGR.A
 10.9	18	-0.0025	R.QKPSEGR.T
 10.9	18	-0.9865	QSSDLPR
 10.9	18	-0.0138	K.GEAVRGGR.R
 9.7	23	0.0338	R.EGTGQPGR.G
 9.7	24	-0.9614	R.EGAAGNQR.A
 9.7	24	-0.9865	R.EQELQR.Q
 9.6	24	-0.9865	QEELQR
 9.4	25	-0.9865	64	Q13813|SPTA2_HUMAN K.EEIQQR.L

Top scoring peptide matches to query 1487
File7043 Spectrum4124 scans: 3224
Score greater than 30 indicates homology
Score greater than 36 indicates identity

**Score	Expect	Delta Hit Protein	Peptide**
 26.7	0.46	1.0237	R.SNVDIPR.S
 16.1	5.3	-0.9555	QSSDLPR
 13.3	10	0.0761	K.DDADLPR.G
 13.3	10	-0.0079	K.LKGNDVR.I
 10.7	18	-0.0191	R.SGRAAALR.Q
 10.4	20	1.0415	K.HVMELR.Q
 10.4	20	-0.0517	-.MAVLVLR.L
 10.4	20	0.0389 618	Q09160|1A80_HUMAN -.MAVMPPR.T
 10.1	21	-1.0283	K.GSSALVLR.D
 9.6	24	0.0397	R.LGGGPSDAK.E

Top scoring peptide matches to query 1489
File7043 Spectrum9343 scans: 7367
Score greater than 20 indicates homology
Score greater than 36 indicates identity

**Score	Expect	Delta Hit Protein	Peptide**
 11.0	17	0.0506	K.FEGGVHR.V
 6.7	47	0.9519	R.KGQLVQK.E
 5.5	61	0.9771	R.QAGIQKR.K
 5.1	67	1.0312 723	Q8NBS9|TXND5_HUMAN -.MPARPGR.L
 5.0	69	0.9883	R.QGQALGVK.R
 4.2	82	0.9771	R.VKAAQAGR.Q
 4.1	84	-0.9117	R.QQGGCPR.G
 4.0	85	1.0247	R.ELQTPGR.A
 3.5	98	0.9982	R.FRPAGPR.A
 3.3	1e+002	1.0061	R.KAHGMLK.L

Top scoring peptide matches to query 1492
File7043 Spectrum3122 scans: 2417
Score greater than 22 indicates homology
Score greater than 36 indicates identity

**Score	Expect	Delta Hit Protein	Peptide**
 11.4	16	-1.0034	R.KAFPPSR.A
 10.8	18	1.0414	ASRSHSR
 10.8	18	1.0414	R.ASSRHSR.G
 9.0	27	0.9547 192	Q9UJZ1|STML2_HUMAN R.GTGALLLR.G
 8.5	31	0.0686	R.EGTGQPGR.G
 5.6	60	-0.0293	R.DTALLLR.D
 5.6	60	-0.0293	R.TDALLLR.L
 5.4	62	1.0850	R.NYFSGGR.G
 5.3	64	-0.0154	K.QRQTLR.K
 5.3	64	-0.0406	R.QVGKTLR.I

Top scoring peptide matches to query 1496
File7043 Spectrum3306 scans: 2562
Score greater than 36 indicates identity

**Score	Expect	Delta Hit Protein	Peptide**
 25.6	0.58	1.0632	K.RAALLEK.R
 23.1	1	1.0995	R.ITGLSPGR.S
 20.2	2	1.1135 265	P61247|RS3A_HUMAN K.RNNQIR.K
 20.2	2	1.1247	K.VGNNLQR.L
 19.8	2.3	1.0883	R.RDLLQR.L
 19.8	2.3	1.0995	K.VGDLGIAR.V
 18.6	2.9	-0.8620	-.MQASPIR.I
 16.4	4.9	1.0558	K.MAPLKIK.L
 16.2	5.1	1.0744	K.QVAIIEK.E
 15.7	5.8	1.1173	98	P28331|NDUS1_HUMAN K.VGMQIPR.F

Top scoring peptide matches to query 1497
File7043 Spectrum7465 scans: 5849
Score greater than 25 indicates homology
Score greater than 36 indicates identity

**Score	Expect	Delta Hit Protein	Peptide**
 12.6	12	0.0793	R.TGLLELR.A
 11.3	16	0.1732	K.TSYQFR.V
 7.2	40	1.1249	K.RTSSPPR.K
 7.0	43	0.1296	K.AAQERAR.Q
 6.2	51	1.0997	71	P36269|GGT5_HUMAN K.VEVINAR.E
 6.2	52	0.2136	R.TGSHEDR.M
 6.1	52	1.0633 192	Q9UJZ1|STML2_HUMAN R.GTGALLLR.G
 6.0	53	1.0633	K.GTALVLAR.L
 5.8	55	1.0885	R.RTSAIPR.A
 5.7	57	0.0932	R.TVAAGRAR.G

Top scoring peptide matches to query 1501
File7043 Spectrum5078 scans: 3965
Score greater than 21 indicates homology
Score greater than 36 indicates identity

**Score	Expect	Delta Hit Protein	Peptide**
 11.9	14	-0.8328	K.VHGNYGR.V
 8.1	33	-0.8427	R.INDGDLR.N
 7.9	34	1.1002 372	P16403|H12_HUMAN K.KAAGGATPK.K
 7.1	42	1.1002	R.KPVTEAR.S
 6.7	45	-0.9155	R.VQVTSLR.N
 6.4	49	0.1049	R.AVATNGLR.D
 6.2	51	-0.8651	R.ARAGGSQR.T
 5.8	55	1.1617	R.SAPAGGGGAR.A
 5.6	59	0.0533	K.FLRPLR.V
 5.5	61	-0.9518	K.VISTVKR.K

Top scoring peptide matches to query 1503
File7043 Spectrum748 scans: 640
Score greater than 21 indicates homology
Score greater than 36 indicates identity

**Score	Expect	Delta Hit Protein	Peptide**
 12.5	12	1.1009	K.KADTLPR.V
 8.2	33	-0.8420	K.ALAEENR.N
 6.4	48	-0.8671	K.ALAEEAAK.K
 5.6	59	-0.8169	R.SPQNSGGR.K
 5.2	64	1.1009 325	O15031|PLXB2_HUMAN K.TNSLPLR.F
 4.9	69	1.0897	R.RLDIAGR.D
 4.7	72	1.1624	K.ASVHSSGR.G
 4.5	75	-0.9148	R.ERVTVAK.L
 4.4	78	0.0692	R.QVGKTLR.I
 4.2	81	1.0645	R.RDLVIGK.C

Top scoring peptide matches to query 1504
File7043 Spectrum30402 scans: 26903
Score greater than 26 indicates homology
Score greater than 36 indicates identity

**Score	Expect	Delta Hit Protein	Peptide**
 14.8	7.1	1.1261	K.GRPGQTGK.E
 13.2	10	1.1262 197	Q15005|SPCS2_HUMAN M.AAAAVQGGR.S
 10.0	21	1.0395	R.VLQSLLK.V
 8.0	34	0.0806	R.GIVDKAAK.E
 7.9	34	1.1010 150	P12111|CO6A3_HUMAN K.VDAILNR.I
 7.7	36	1.1122	R.LVSQEPK.A
 7.7	36	1.0646	K.VDAVIRK.N
 7.4	38	-0.9034	K.IEKGDLK.K
 7.4	38	-0.9034 153	P35749|MYH11_HUMAN KLEGDLK
 7.1	41	1.1738	K.RGDADPVA.-

Top scoring peptide matches to query 1505
File7043 Spectrum30259 scans: 26787
Score greater than 26 indicates homology
Score greater than 36 indicates identity

**Score	Expect	Delta Hit Protein	Peptide**
 15.8	5.6	1.1124	R.LVSQEPK.A
 15.6	5.9	1.1263 197	Q15005|SPCS2_HUMAN M.AAAAVQGGR.S
 12.7	11	1.0647	M.IATGGLLR.I
 9.9	22	1.1263	R.GQGTGKPR.E
 9.3	25	1.1011	R.SPSAGILR.T
 8.0	34	-0.9397	R.VLSKDLK.Q
 7.8	36	-0.9509	R.VSTKVIR.T
 7.5	38	1.0647	GLGATLLR
 7.5	38	-0.8426	K.VMMHIR.N
 7.2	40	1.0899	K.GEVRAIR.Y

Top scoring peptide matches to query 1507
File7043 Spectrum30647 scans: 27115
Score greater than 23 indicates homology
Score greater than 36 indicates identity

**Score	Expect	Delta Hit Protein	Peptide**
 11.5	15	-0.8929	R.NVFGPIR.E
 11.3	16	-0.9028 153	P35749|MYH11_HUMAN KLEGDLK
 11.1	17	1.0904	R.DVAARIR.F
 9.9	22	1.0652	R.VEGVRLK.T
 9.1	26	1.0904 542	P02549|SPTA1_HUMAN R.DGLLARR.D
 9.0	27	-0.9028	K.IEKGDLK.K
 8.8	28	1.0652	R.ATLNLLR.R
 8.8	28	-0.9140	R.VAATTAIR.R
 8.7	29	1.0652	M.IATGGLLR.I
 8.2	32	1.0652	R.DGLKLVR.K

Top scoring peptide matches to query 1508
File7043 Spectrum30539 scans: 27018
Score greater than 26 indicates homology
Score greater than 36 indicates identity

**Score	Expect	Delta Hit Protein	Peptide**
 14.0	8.5	1.1270 197	Q15005|SPCS2_HUMAN M.AAAAVQGGR.S
 12.7	12	1.1158	R.GRGGGGAIR.V
 12.7	12	-0.8927	R.NVFGPIR.E
 8.3	31	1.1383	R.ESAPGALR.E
 8.2	32	0.0815	K.SKKPDVK.K
 8.0	34	0.0815	K.VNLNTIK.R
 7.1	42	-0.8774	R.SSVGSPLR.L
 6.9	44	1.0906	R.LDGRLAR.C
 6.7	45	-0.8596	-.MQASPIR.I
 6.6	46	0.1542	K.NVPSQEK.R

Top scoring peptide matches to query 1510
File7043 Spectrum3491 scans: 2707
Score greater than 23 indicates homology
Score greater than 36 indicates identity

**Score	Expect	Delta Hit Protein	Peptide**
 11.6	15	0.0357	R.RNNNER.V
 11.6	16	-0.0146	R.SSDKPIR.T
 10.4	20	-0.0047 475	Q9UGT4|SUSD2_HUMAN R.NYRPPR.L
 10.0	22	-0.0146	R.SSVGSPLR.L
 10.0	22	-0.0510	R.SSVLQLR.E
 9.8	23	-0.0047	R.YNRPPR.E
 9.8	23	0.9694	K.ANILTNR.Y
 9.7	24	0.0218	K.VGNEIDR.S
 9.0	28	0.0105	K.RDADGIR.D
 9.0	28	-0.0147	R.VGDGSVLR.T

Top scoring peptide matches to query 1519
File7043 Spectrum1597 scans: 1207
Score greater than 24 indicates homology
Score greater than 34 indicates identity

**Score	Expect	Delta Hit Protein	Peptide**
 14.4	5.5	-0.6877	K.KNAAPFR.S
 11.0	12	-0.6725	R.KNIGSER.Y
 11.0	12	-0.6361	R.QLNGSER.A
 8.5	21	0.3479	R.AGQKQDR.T
 8.3	22	-0.6547	R.QIAANMR.G
 8.2	23	0.2864	K.QLSSLVR.N
 8.1	24	-0.6361	R.QGAGATNGK.D
 7.7	25	-0.6612	K.KEELER.L
 7.7	25	-0.6248	R.QEEIER.Q
 7.7	25	-0.6248 496	O15126|SCAM1_HUMAN QEELER

Top scoring peptide matches to query 1521
File7043 Spectrum4074 scans: 3191
Score greater than 28 indicates homology
Score greater than 36 indicates identity

**Score	Expect	Delta Hit Protein	Peptide**
 17.4	4.1	0.9903	K.TQTLPSR.G
 14.6	8	0.9751	R.NVFGPIR.E
 14.4	8.3	0.9717	K.KPTGMIR.I
 13.8	9.5	0.9903	R.KESPSVR.S
 12.8	12	-0.0665	R.LTLSTLR.H
 11.7	16	0.9903	R.SSVGSPLR.L
 11.5	16	1.0016 449	P10451|OSTP_HUMAN K.QETLPSK.S
 11.1	18	0.9539	K.NVITSLR.A
 10.5	20	1.0267	R.GAEEQLR.S
 10.5	20	1.0267	R.QEAGELR.R

Top scoring peptide matches to query 1523
File7043 Spectrum17728 scans: 14834
Score greater than 25 indicates homology
Score greater than 36 indicates identity

**Score	Expect	Delta Hit Protein	Peptide**
 13.5	10	1.0199	R.TVGDVRR.A
 11.5	16	1.0999 378	O43570|CAH12_HUMAN R.TAEYYR.Y
 9.6	25	1.0312	R.KAVEEAR.H
 9.6	25	1.0424	R.QADDLLK.C
 9.1	28	0.0472	K.LSVENNK.T
 8.6	32	-0.9238	K.MRMGPGR.K
 8.5	32	-0.9456	-.MAGAWLR.W
 8.4	33	1.0312	K.AQTQLNK.K
 7.7	38	0.9948	R.TVDARLK.L
 7.6	40	0.9874	K.ALGGIIMK.I

Top scoring peptide matches to query 1524
File7043 Spectrum6761 scans: 5290
Score greater than 21 indicates homology
Score greater than 36 indicates identity

**Score	Expect	Delta Hit Protein	Peptide**
 11.5	16	0.1323	R.ADPQGCR.R
 8.1	36	1.0735	K.EQVAEAR.A
 8.1	36	1.0735	R.QEVSSPR.K
 6.4	53	1.0735	R.APTSEAAR.A
 6.3	54	-0.9244	R.DILACGR.G
 6.1	56	0.0054	R.ALRATSGK.R
 5.7	61	1.0622	EINDRR
 4.9	73	1.0007	R.GVLSALSR.F
 4.2	88	1.0371	78	Q8N257|H2B3B_HUMAN R.EVQTAVR.L
 4.0	92	0.0531	K.EEVASLR.A

Top scoring peptide matches to query 1527
File7043 Spectrum3489 scans: 2706
Score greater than 26 indicates homology
Score greater than 36 indicates identity

**Score	Expect	Delta Hit Protein	Peptide**
 14.0	9	1.0195	R.QVMLGVR.K
 13.3	11	1.0882	60	Q01082|SPTB2_HUMAN FFSMVR
 11.5	16	1.1246	R.FMFEGR.E
 11.5	16	1.1212	-.MFTMTR.A
 11.5	16	1.1424	-.MMFWR.K
 10.4	21	1.0381	R.SSVGSPLR.L
 9.8	24	0.0065	K.TAGTAKVR.E
 8.1	35	0.0065	K.TKSVNVR.E
 8.1	35	0.0680	K.TQERNR.C
 8.1	35	0.0680	R.TQQDRR.M

Top scoring peptide matches to query 1530
File7043 Spectrum6041 scans: 4718
Score greater than 28 indicates homology
Score greater than 36 indicates identity

**Score	Expect	Delta Hit Protein	Peptide**
 15.9	5.9	1.0232	R.WASGILR.H
 14.5	8.1	0.0358	-.MLSSPLR.V
 14.1	8.8	1.0384	R.SSVGSPLR.L
 12.9	12	1.0562	-.MQASPIR.I
 11.9	15	1.0748	R.DAAQELR.A
 11.9	15	1.1000	R.EGAAGNQR.A
 11.9	15	1.0748	R.GEAEALGR.E
 11.9	15	-0.9157	K.STAAQGNR.Q
 11.9	15	1.0596 548	P16188|1A30_HUMAN R.WAEQLR.A
 11.9	15	1.0596	R.WAQELR.Q

Top scoring peptide matches to query 1539
File7043 Spectrum29591 scans: 26261
Score greater than 28 indicates homology
Score greater than 36 indicates identity

**Score	Expect	Delta Hit Protein	Peptide**
 15.4	6.5	0.0707	R.SLTTLLR.A
 14.3	8.3	1.0976	K.MRGGLLR.T
 12.8	12	1.1639	K.NESSPIR.F
 12.4	13	1.1275	K.DQGITLR.K
 12.1	14	1.1122	R.NVFGPIR.E
 10.7	19	1.1023 512	Q8N5K1|CISD2_HUMAN K.DSLINLK.I
 10.6	20	0.1500	K.CQNLLR.H
 10.4	20	1.0799	R.GRTSILR.D
 9.9	23	1.1275	K.SLSPGVSR.D
 9.8	24	1.1275	K.SEALNIR.G

Top scoring peptide matches to query 1541
File7043 Spectrum9421 scans: 7426
Score greater than 31 indicates homology
Score greater than 36 indicates identity

**Score	Expect	Delta Hit Protein	Peptide**
 42.3	0.013	-1.0285	34	P04406|G3P_HUMAN K.VGVNGFGR.I
 16.0	5.4	-1.0244	R.VRSGSGSR.G
 14.2	8.1	-0.0114	K.DMAVRGR.L
 13.3	10	0.9336	K.EIMLGLK.R
 12.3	13	0.9773	R.DVTNGGIK.R
 11.8	14	-1.0648 272+ P20039|2B1B_HUMAN R.VRFLDR.Y
 11.8	14	-1.0504	R.VRMMLR.-
 11.8	14	-1.0318	K.VRVMER.D
 11.8	14	-1.0284	K.VRYPDR.I
 11.3	16	-1.0318	R.RVDMLR.D

Top scoring peptide matches to query 1542
File7043 Spectrum4687 scans: 3657
Score greater than 26 indicates homology
Score greater than 36 indicates identity

**Score	Expect	Delta Hit Protein	Peptide**
 15.9	5.5	-0.0577	K.TGKVMIR.I
 12.8	11	-0.0391	R.TASEKLR.N
 12.2	13	0.9925	K.VADTEIR.T
 11.8	14	0.0112	R.TRSGGGGGR.S
 10.5	19	0.9739	R.SSLMPLR.N
 9.7	23	-0.0326	R.TRQMIR.A
 9.1	27	0.9925	K.EEEKIR.E
 8.8	29	0.9990 216+ P05388|RLA0_HUMAN K.QMQQIR.M
 8.6	30	-0.0214	K.IMVVNGR.R
 8.6	30	0.0224	K.STAAQGNR.Q

Top scoring peptide matches to query 1543
File7043 Spectrum6273 scans: 4903
Score greater than 29 indicates homology
Score greater than 36 indicates identity

**Score	Expect	Delta Hit Protein	Peptide**
 25.1	0.67	-1.0108	R.GGTLSSKR.N
 15.5	6	1.0114 242	P09211|GSTP1_HUMAN R.GRCAALR.M
 15.0	6.9	-0.9567	-.MAGSGAGVR.C
 14.6	7.5	0.0096	R.ITRSGDR.D
 14.6	7.5	0.0096	R.LTSDRGR.A
 14.6	7.5	-0.0268	K.LTSRATR.R
 14.5	7.7	-0.0308	K.IQQFLR.A
 14.5	7.7	-0.0342	R.LKMSGLR.Y
 14.5	7.7	0.0022	R.LMQQLR.E
 14.5	7.7	-0.0672	LQKFLR

Top scoring peptide matches to query 1545
File7043 Spectrum10948 scans: 8693
Score greater than 30 indicates homology
Score greater than 36 indicates identity

**Score	Expect	Delta Hit Protein	Peptide**
 17.9	3.5	-0.8824	-.MPSGEER.R
 17.0	4.3	-0.9154	R.LDSWER.Q
 16.7	4.6	1.0064 683	Q8TF72|SHRM3_HUMAN R.ISESVLR.D
 16.7	4.6	1.0428	R.LSEADIR.G
 16.7	4.6	1.0428 366	Q16891|IMMT_HUMAN K.SLEDALR.Q
 16.0	5.4	-1.0609	R.LLPPPLR.H
 15.9	5.6	0.0839	R.EASPGSTR.R
 15.5	6.1	-0.9114	K.DSLGGNSR.T
 14.8	7.2	-0.9300	-.MAGSGAGVR.C
 14.5	7.7	-0.0042	K.IQQFLR.A

Top scoring peptide matches to query 1546
File7043 Spectrum5104 scans: 3984
Score greater than 22 indicates homology
Score greater than 34 indicates identity

**Score	Expect	Delta Hit Protein	Peptide**
 11.9	9.3	1.1995	R.FVIIRR.E
 8.7	19	-0.6991	R.LALDAMR.D
 5.8	38	-0.7797 115+ P62873|GBB1_HUMAN K.VHAIPLR.S
 5.7	39	-0.8049	R.LLPPPLR.H
 5.7	39	0.2923	K.RAANASSK.L
 4.5	52	0.3577	R.MNDAPTR.G
 4.4	52	1.1995	R.FVRILR.I
 4.0	58	0.3287	R.QASSQAGR.L
 3.2	69	0.2308	K.TLKLSSR.V
 2.6	80	-0.7467	K.LTMRLR.T

Top scoring peptide matches to query 1548
File7043 Spectrum4545 scans: 3547
Score greater than 19 indicates homology
Score greater than 34 indicates identity

**Score	Expect	Delta Hit Protein	Peptide**
 8.3	21	0.7002 610	Q9Y679|AUP1_HUMAN R.QEDSGLR.D
 6.0	36	0.6452	R.KEMQLR.Q
 5.7	39	0.6122	K.KYPQLR.N
 5.4	42	0.5758	K.KQIFLR.N
 5.4	42	0.6122	R.QQIFLR.V
 4.5	51	0.6261	R.HHNKLR.L
 3.9	58	-0.4082	K.ELKFLR.Q
 3.9	58	-0.3752	R.ELKMLR.T
 3.9	58	-0.3388	R.EQLMLR.A
 3.9	58	0.6630	K.KCPMIR.Y

Top scoring peptide matches to query 1549
File7043 Spectrum4290 scans: 3353
Score greater than 21 indicates homology
Score greater than 34 indicates identity

**Score	Expect	Delta Hit Protein	Peptide**
 9.4	17	0.7180	-.MASNPER.G
 7.7	25	0.6453	K.MALSGGLR.C
 7.6	25	-0.3574	-.MALAMLR.D
 7.6	25	-0.3387	-.MASDLIR.T
 7.2	27	0.6453	-.MALADKR.L
 7.0	28	-0.4194 115+ P62873|GBB1_HUMAN K.VHAIPLR.S
 6.8	30	-0.3201	R.LSDTVDR.L
 6.5	33	0.6453	-.MAAAATLR.L
 6.5	33	0.6664	-.MAGAWLR.W
 6.5	33	0.6300	-.MAKWLR.D

Top scoring peptide matches to query 1552
File7043 Spectrum8679 scans: 6820
Score greater than 25 indicates homology
Score greater than 36 indicates identity

**Score	Expect	Delta Hit Protein	Peptide**
 20.5	1.9	-0.0060 218+ P62158|CALM_HUMAN ELGTVMR
 11.2	16	0.0126	R.ELASTER.E
 10.9	17	0.9780	K.QLEKMR.K
 10.8	18	-0.0060	R.ECQKLK.S
 10.8	18	0.0602	R.EEEEAAK.K
 10.8	18	-0.0424	R.EIRMLK.Q
 10.8	18	0.0126	R.ESSNQLK.C
 10.5	19	-0.0312	R.GMLEVIK.L
 9.8	22	-0.0060	K.QECKIK.Y
 9.3	25	0.8834	K.KVIAVFK.D

Top scoring peptide matches to query 1554
File7043 Spectrum9451 scans: 7454
Score greater than 24 indicates homology
Score greater than 36 indicates identity

**Score	Expect	Delta Hit Protein	Peptide**
 15.4	6.1	0.0316	K.RFDPDR.T
 10.5	19	-1.0252	R.NFGTVLR.L
 10.0	21	0.9871	R.GLSCVLR.K
 9.8	22	-0.0160	R.GVFGGRGR.G
 9.4	24	-0.0048	34	P04406|G3P_HUMAN K.VGVNGFGR.I
 9.3	25	-0.0081	R.RNMLAGK.Q
 8.5	30	-1.0252	R.FNVAVTR.A
 8.5	30	-0.0663	K.IFVVATR.Q
 8.5	30	-0.0081	R.MERITR.K
 8.5	30	0.9759	K.QMLRTR.S

Top scoring peptide matches to query 1560
File7043 Spectrum2419 scans: 1875
Score greater than 23 indicates homology
Score greater than 36 indicates identity

**Score	Expect	Delta Hit Protein	Peptide**
 10.9	17	1.0641	EENEKR
 9.6	23	0.0390 106	P12821|ACE_HUMAN -.MGAASGRR.G
 9.6	23	0.0390	-.MGARQSR.A
 9.6	23	0.0390	-.MGARSGAR.G
 9.5	24	0.0139	-.MGGKSAVR.H
 8.2	32	0.0714	-.MAGGAWGR.L
 8.2	32	0.0390	-.MAGSRQR.G
 8.2	32	0.0251	R.MKELER.Q
 8.2	32	0.0350	K.MQKGWR.Q
 8.2	32	0.0251	-.MELEKR.E

Top scoring peptide matches to query 1561
File7043 Spectrum393 scans: 405
Score greater than 20 indicates homology
Score greater than 36 indicates identity

**Score	Expect	Delta Hit Protein	Peptide**
 7.5	38	0.9921	K.RTIAESK.K
 7.3	40	0.0081	K.SSKDQLK.E
 7.2	41	1.0397 544	P07197|NFM_HUMAN K.KEAAEEK.E
 6.6	46	0.0986	-.MEEPQR.A
 6.6	46	1.0397	R.QLEASEK.Q
 6.6	46	-1.0058	M.GVLMSKR.Q
 6.6	47	0.9986	R.RMLAAAR.N
 6.0	53	1.0350	R.QMTRPR.C
 5.9	54	1.0285	K.QENSAKK.F
 5.4	60	0.1415	R.MHADCR.T

Top scoring peptide matches to query 1563
File7043 Spectrum11718 scans: 9359
Score greater than 25 indicates homology
Score greater than 36 indicates identity

**Score	Expect	Delta Hit Protein	Peptide**
 15.4	6.1	-0.0521	K.IAVHPLR.E
 11.7	14	-1.0109	R.GHVQPIR.C
 11.7	14	-1.0109	R.GVQHPLR.G
 11.7	14	-1.0222	R.HGRAPLR.K
 11.7	14	0.9796	R.KSGFPIR.Y
 11.7	14	-1.0109	K.LGNHPIR.R
 11.7	14	-1.0109	K.LHNGPLR.N
 11.7	14	-0.0772	R.LLPPPLR.H
 11.7	14	-0.0772	M.PPLLPLR.L
 11.7	14	-0.0521 115+ P62873|GBB1_HUMAN K.VHAIPLR.S

Top scoring peptide matches to query 1564
File7043 Spectrum12615 scans: 10150
Score greater than 26 indicates homology
Score greater than 36 indicates identity

**Score	Expect	Delta Hit Protein	Peptide**
 14.6	7.3	0.0360	K.GNKTETR.F
 12.5	12	-1.0109	R.GVQHPLR.G
 11.5	15	1.0239 576	P05164|PERM_HUMAN R.GLMATPAK.L
 10.4	19	1.0126	R.GLSCVLR.K
 10.2	20	0.9511	R.KVAMVLK.E
 10.2	20	1.0126	K.MALSGGLR.C
 9.8	22	1.0061	R.EASTGVLK.D
 9.4	24	1.0677	R.EGKAEDR.G
 8.9	27	0.0538	R.ANMSQVR.Q
 8.9	27	0.0248	R.SSAASRAR.R

Top scoring peptide matches to query 1567
File7043 Spectrum6241 scans: 4877
Score greater than 25 indicates homology
Score greater than 35 indicates identity

**Score	Expect	Delta Hit Protein	Peptide**
 17.8	3.2	0.0204 484	P11498|PYC_HUMAN	R.SGEGMGIR.L
 11.0	16	0.9503	R.GGTLSSKR.N
 10.1	19	0.9212	K.LTPLAYK.Q
 8.9	25	-0.0225 606	O00159|MYO1C_HUMAN R.SELSDKK.R
 8.4	28	0.9867	R.SQLTSNR.Q
 7.6	34	0.0027	K.SQETSVR.T
 6.2	47	0.9728	K.LTSELDK.L
 5.4	57	0.0754	K.DTDQDGR.G
 5.2	60	-1.0330	K.SQLFNAK.N
 5.1	60	0.9793	K.LESMGIR.K

Top scoring peptide matches to query 1572
File7043 Spectrum12192 scans: 9781
Score greater than 27 indicates homology
Score greater than 35 indicates identity

**Score	Expect	Delta Hit Protein	Peptide**
 16.1	4.8	1.0523	K.ELPQYR.A
 14.0	7.8	1.0047	K.NQFLKR.L
 13.5	8.8	0.0571	R.NEPPPPR.N
 13.5	8.8	0.0571	K.VSGPQYR.L
 12.6	11	-0.9820	-.MLPPPPR.Q
 8.4	28	-0.9667	-.MLSSTLR.V
 8.2	30	1.0199	R.NKTSVTR.Q
 7.5	35	1.0159 711	Q7Z4W1|DCXR_HUMAN M.ELFLAGR.R
 7.5	35	1.0490	K.ELMIAGR.L
 6.7	41	1.0490	R.EIQIMR.D

Top scoring peptide matches to query 1580
File7043 Spectrum794 scans: 672
Score greater than 23 indicates homology
Score greater than 34 indicates identity

**Score	Expect	Delta Hit Protein	Peptide**
 14.1	5.1	0.6827	R.EMEELR.S
 9.6	14	0.6715	K.ENQMLR.R
 8.4	19	0.6238	R.LMASRGR.S
 7.7	22	0.6133	R.EELFLR.A
 7.7	22	0.6463 184	P15311|EZRI_HUMAN K.EELMLR.L
 7.5	23	0.6827	K.MEEEIR.Q
 6.3	30	0.6164	K.KMCVLR.L
 6.2	31	0.6892	-.MAPGMSGR.G
 6.2	31	0.6827	R.EEEMIR.H
 5.9	33	0.6133	R.FIEELR.T

Top scoring peptide matches to query 1584
File7043 Spectrum7074 scans: 5532
Score greater than 21 indicates homology
Score greater than 35 indicates identity

**Score	Expect	Delta Hit Protein	Peptide**
 15.6	5.1	-0.0423	R.TAYPSLR.L
 7.6	32	1.0111 484	P11498|PYC_HUMAN R.SGEGMGIR.L
 7.3	35	1.0297	K.GSTEEGAR.L
 7.2	36	0.9821	R.SASRETR.N
 7.2	36	0.9821	R.SSARETR.T
 6.8	39	-0.0899	K.LRYSLR.Q
 6.6	41	1.0904	K.SSCHCR.F
 5.2	56	0.9305	K.SQRFLR.V
 5.2	57	-1.0198	R.CGGFLVR.E
 5.1	58	0.0271	K.SMTPAER.E

Top scoring peptide matches to query 1585
File7043 Spectrum6846 scans: 5354
Score greater than 20 indicates homology
Score greater than 35 indicates identity

**Score	Expect	Delta Hit Protein	Peptide**
 9.0	24	0.0093	K.DGVPYEK.K
 8.4	27	-0.0496	R.RFSLER.S
 7.3	35	0.0012	R.AMGNMRK.T
 7.3	35	-0.0206	K.YPLMQR.M
 6.8	39	-0.0383	K.VFGSLER.G
 6.1	46	-0.0053	K.TALEMAR.T
 5.3	55	-0.0020 217	P62917|RL8_HUMAN R.AVDFAER.H
 5.3	55	0.0012	R.MMKNQR.V
 5.0	60	-0.0496	R.RFSELR.G
 4.8	62	-0.9754	R.MRSADGR.R

Top scoring peptide matches to query 1588
File7043 Spectrum7508 scans: 5882
Score greater than 26 indicates homology
Score greater than 35 indicates identity

**Score	Expect	Delta Hit Protein	Peptide**
 19.4	2.1	0.9677	K.LGNHPIR.R
 12.7	10	-0.9639	K.DSFGNIR.G
 12.7	10	1.0120	R.LSCASIR.V
 10.6	16	-1.0003	R.DGGYKLR.L
 9.6	20	0.0280 620	P22059|OSBP1_HUMAN -.MAATELR.G
 9.6	20	0.0457	-.MAPMGIR.L
 7.8	31	-0.9309	K.DSITCGR.L
 7.8	31	-0.9132	K.DCMGGIR.D
 7.8	31	-0.9527	K.DEEFIR.K
 7.8	31	-0.9639	R.DQYGGIR.S

Top scoring peptide matches to query 1590
File7043 Spectrum602 scans: 541
Score greater than 20 indicates homology
Score greater than 35 indicates identity

**Score	Expect	Delta Hit Protein	Peptide**
 8.1	29	1.0036	K.KMCVLR.L
 7.9	30	1.0763	-.MAPGMSGR.G
 7.4	34	1.0698 176	P56199|ITA1_HUMAN R.MEDGNIK.I
 7.0	37	0.0382	K.TMQAVNK.K
 6.3	44	1.0222	R.REVMGSK.E
 5.5	53	1.0698	R.EMEELR.S
 5.3	56	1.0334	R.EIEIMR.G
 5.3	56	1.0698	R.EIMEER.F
 5.3	56	1.0368	K.EIPEYR.K
 5.3	56	1.0586	M.ELCGATR.L

Top scoring peptide matches to query 1592
File7043 Spectrum12725 scans: 10235
Score greater than 35 indicates identity

**Score	Expect	Delta Hit Protein	Peptide**
 24.9	0.6	-0.0051	79	Q16134|ETFD_HUMAN R.FGILTEK.Y
 23.6	0.82	0.0894	R.MGLNDNK.A
 16.4	4.3	1.1024	R.GMPGMPGK.H
 15.4	5.3	1.0483	K.MGIGGLDK.E
 15.0	5.9	1.0119	K.TMIKEGK.N
 14.4	6.8	0.9789	R.LYVGGLGK.E
 14.4	6.8	0.9933	R.MKVMLGK.V
 14.4	6.8	1.0370	K.SMDRLGK.Q
 13.5	8.4	0.9788	K.AFVVSVGK.D
 13.5	8.4	1.0040	R.FVAERGK.H

Top scoring peptide matches to query 1593
File7043 Spectrum5764 scans: 4505
Score greater than 26 indicates homology
Score greater than 35 indicates identity

**Score	Expect	Delta Hit Protein	Peptide**
 15.9	4.8	-0.0051	4	P98164|LRP2_HUMAN R.HIVVDPK.N
 15.2	5.6	0.1119	-.MAETDPK.T
 15.2	5.6	0.1297	R.MMDADPK.W
 13.0	9.4	0.0966	K.MDPGFPK.L
 12.6	10	-0.0415	K.KVPAAPPK.K
 11.2	14	-0.9019	-.MPNTAMK.K
 10.9	15	-0.9891	K.QPIPEPK.G
 9.3	22	1.0670	K.STASAQNK.E
 9.2	22	-0.9164	R.FPTSDNK.W
 8.1	29	0.0821	R.KCMQPK.D

Top scoring peptide matches to query 1597
File7043 Spectrum3057 scans: 2363
Score greater than 32 indicates homology
Score greater than 35 indicates identity

**Score	Expect	Delta Hit Protein	Peptide**
 20.4	1.6	-0.0042	80	O43451|MGA_HUMAN	R.MKADGMR.V
 17.9	2.8	-0.0550	R.QHPDALK.F
 17.0	3.4	-0.0155	R.MQARMR.G
 14.6	6	-0.0914	K.KDHPALK.A
 14.5	6.2	-0.0802	K.KDSAFIK.K
 14.3	6.5	-0.0406	R.KMSVMGR.Q
 12.2	10	0.0103	K.QMWDTK.T
 11.0	14	0.9402	K.EAGYLVR.N
 9.5	19	-0.0624	K.CIGFAIK.N
 9.3	20	0.0031 377	P62491|RB11A_HUMAN K.QMSDRR.E

Top scoring peptide matches to query 1601
File7043 Spectrum5614 scans: 4388
Score greater than 29 indicates homology
Score greater than 35 indicates identity

**Score	Expect	Delta Hit Protein	Peptide**
 20.9	1.4	1.1076	-.MAETDPK.T
 15.7	4.7	1.0712	K.MLSSEPK.D
 12.1	11	1.0674	R.SSKSQDR.K
 10.7	15	-0.9597	R.FPVATMK.T
 10.7	15	1.0487	K.SSAVCRK.S
 10.6	15	1.0633	R.STSHTFK.N
 10.5	16	1.0600 620	P22059|OSBP1_HUMAN -.MAATELR.G
 10.4	16	0.9542	K.KVPAAPPK.K
 10.3	16	1.0777	-.MAPMGIR.L
 9.8	18	1.0600	K.MSSIVDR.D

Top scoring peptide matches to query 1602
File7043 Spectrum15468 scans: 12727
Score greater than 24 indicates homology
Score greater than 35 indicates identity

**Score	Expect	Delta Hit Protein	Peptide**
 12.1	11	0.1055	R.EMKNDR.I
 10.9	14	1.0314	R.KFELDR.H
 8.5	25	-0.8976	K.HSHASDR.F
 7.9	28	0.1201	5+ P68371|TBB2C_HUMAN R.EEYPDR.I
 7.8	29	0.1167	K.EMQQEK.Q
 7.8	29	-0.9592	K.HVSVPDR.I
 7.5	31	1.0314	R.KFIEDR.V
 7.5	31	1.0644	K.KMIEDR.S
 6.2	42	1.0314	R.KFQQEK.L
 6.1	43	0.0361	K.LYTAQGR.T

Top scoring peptide matches to query 1604
File7043 Spectrum7113 scans: 5564
Score greater than 22 indicates homology
Score greater than 33 indicates identity

**Score	Expect	Delta Hit Protein	Peptide**
 13.2	5.5	0.4325 366	Q16891|IMMT_HUMAN R.AFDSAVAK.A
 8.7	15	-0.5813	K.VMAGFLR.G
 7.1	22	-0.5627	23	P31930|QCR1_HUMAN FTGSEIR
 6.1	28	-0.5371	R.MAPSIMK.S
 5.9	29	-0.6143	R.FNLFLR.R
 5.9	29	-0.5991	R.FSVSTLR.N
 5.9	29	-0.5483	R.MLNMIR.Q
 5.9	29	-0.5926	-.MRAFLR.N
 5.9	29	-0.5813	M.ICTFLR.A
 5.8	30	0.4278	M.CGRFLR.R

Top scoring peptide matches to query 1606
File7043 Spectrum3754 scans: 2916
Score greater than 14 indicates homology
Score greater than 33 indicates identity

**Score	Expect	Delta Hit Protein	Peptide**
 2.1	70	-0.3218 731	Q5XG92|EST8_HUMAN K.MLWSTR.T
 1.6	79	0.6523	R.SLGEKMK.L
 1.0	90	0.6193	K.GYIGTLGK.N

Top scoring peptide matches to query 1607
File7043 Spectrum3197 scans: 2476
Score greater than 25 indicates homology
Score greater than 33 indicates identity

**Score	Expect	Delta Hit Protein	Peptide**
 16.0	2.8	0.6959	80	O43451|MGA_HUMAN	R.MKADGMR.V
 11.9	7.4	0.6595	R.KMSVMGR.Q
 10.1	11	0.6847	R.MQARMR.G
 9.0	14	0.7033 377	P62491|RB11A_HUMAN K.QMSDRR.E
 8.6	16	0.6451	K.VGQYVSR.A
 8.2	17	0.7331	K.DTDSSAGR.R
 8.0	18	0.6530	R.MKDSALK.V
 7.9	18	0.6451	K.LASFTGGR.Y
 7.9	18	0.6967	R.TASTGTDR.V
 7.7	19	0.6604	R.NSSTKSGK.V

Top scoring peptide matches to query 1609
File7043 Spectrum6015 scans: 4699
Score greater than 25 indicates homology
Score greater than 35 indicates identity

**Score	Expect	Delta Hit Protein	Peptide**
 18.9	2.1	0.9282	16+ P35579|MYH9_HUMAN R.TVGQLYK.E
 11.2	12	-1.0259	R.EKHPDGK.I
 11.2	12	-0.0605	R.GMPHVIR.I
 11.2	12	-0.0895	R.REHLVR.H
 11.2	12	-0.0895	R.REHVLR.H
 11.0	13	0.9752	K.MRSVSGR.R
 9.9	17	0.9283	K.DAAKLYK.K
 9.8	17	0.9283	K.ISANLYK.Q
 9.7	17	0.9613	R.SLGEKMK.L
 8.6	23	0.9686	R.TTTAASTR.R

Top scoring peptide matches to query 1612
File7043 Spectrum7084 scans: 5541
Score greater than 25 indicates homology
Score greater than 35 indicates identity

**Score	Expect	Delta Hit Protein	Peptide**
 18.7	2.2	-0.0050	23	P31930|QCR1_HUMAN FTGSEIR
 11.7	11	-0.0387	R.GGRGHGLR.G
 11.1	13	-0.0567	R.FNLFLR.R
 11.1	13	0.0094	R.MLNMIR.Q
 11.1	13	-0.0349	-.MRAFLR.N
 10.7	14	0.0345	R.RMESMR.K
 9.1	20	0.0206	R.MAPSIMK.S
 7.6	28	0.0314	R.FTADEAR.D
 6.5	36	0.0240	K.WESMLK.I
 6.5	37	-0.0124	K.FISSPMK.E

Top scoring peptide matches to query 1616
File7043 Spectrum11017 scans: 8745
Score greater than 35 indicates identity

**Score	Expect	Delta Hit Protein	Peptide**
 34.9	0.052	-0.0038	23	P31930|QCR1_HUMAN R.SLLTYGR.R
 28.0	0.26	-0.0289	K.SLLTTFK.K
 24.5	0.57	-0.0150	R.EALAHLR.N
 24.5	0.57	-0.0150	K.ISLHSPR.V
 18.2	2.4	0.0028	R.SLMFRR.G
 16.1	3.9	-1.0102	K.SLIGHQR.I
 15.2	4.9	0.0326	R.SLFTEGR.E
 13.9	6.7	-0.0401	K.EVLHALK.E
 13.5	7.2	0.0802	K.EALDQSF.-
 10.1	16	-0.9514	R.NSLDYAK.S

Top scoring peptide matches to query 1621
File7043 Spectrum4383 scans: 3421
Score greater than 20 indicates homology
Score greater than 33 indicates identity

**Score	Expect	Delta Hit Protein	Peptide**
 7.9	17	0.7027	K.CTWASGK.Q
 7.3	19	0.6928	K.MEVASEK.K
 7.0	21	-0.3177	K.QEQFMK.K
 6.7	22	0.6148	R.GAVHGRGR.G
 6.0	26	0.6234	LYTLDGK
 5.6	28	0.5757	R.LPPDVLR.W
 5.5	29	0.6928	K.MEGLESK.R
 5.4	30	0.7244 292	P52943|CRIP2_HUMAN CERCGK
 5.4	30	0.7244	CRECGK
 5.2	31	0.5784	K.APRPRGR.R

Top scoring peptide matches to query 1624
File7043 Spectrum6111 scans: 4779
Score greater than 22 indicates homology
Score greater than 34 indicates identity

**Score	Expect	Delta Hit Protein	Peptide**
 18.5	2	-1.1432 185	P39023|RL3_HUMAN K.FIDTTSK.F
 8.1	22	-0.1955	R.YSALKTK.H
 6.5	32	-0.0356	K.CVDCEK.S
 6.4	33	-0.2068	K.KGLHDIK.N
 5.9	37	-0.0832	R.DMKTCR.F
 5.3	42	-0.1162	R.YALQCR.F
 4.4	52	-1.1003	R.DFITCR.Y
 4.3	53	-0.1704	R.AHNIDLK.G
 4.3	53	-0.1414	KCFDLK
 4.3	53	-0.1084	R.KCMDLK.G

Top scoring peptide matches to query 1625
File7043 Spectrum7301 scans: 5722
Score greater than 29 indicates homology
Score greater than 34 indicates identity

**Score	Expect	Delta Hit Protein	Peptide**
 23.1	0.73	-0.0049 132+ P08238|HS90B_HUMAN FENLCK
 15.7	4	-1.0617	K.YSGILMK.D
 15.6	4.1	0.0580	K.MEEEEK.-
 15.3	4.4	0.9791	K.FQNLCK.F
 13.8	6.2	0.0315	-.MENWSK.D
 12.2	9	1.0420	K.MEEQEK.N
 12.2	9	1.0056	K.MEVASEK.K
 12.1	9.4	-0.0743	K.FEFIVR.R
 11.4	11	-0.0049	K.FEMDLR.N
 11.4	11	0.0355	-.MEGQSSR.G

Top scoring peptide matches to query 1629
File7043 Spectrum2664 scans: 2057
Score greater than 21 indicates homology
Score greater than 34 indicates identity

**Score	Expect	Delta Hit Protein	Peptide**
 9.9	14	-0.0907	R.NKLGHSR.A
 7.8	23	0.9852 625	Q9NX63|CHCH3_HUMAN -.MGGTTSTR.R
 6.4	32	1.0216	R.TSSDMNR.R
 4.1	54	1.0110	R.EFEEEK.V
 4.0	56	1.0328	R.EDSGSGMK.A
 3.6	61	1.0142	R.CTCELK.N
 2.9	71	-0.0538	-.MKMLTR.L
 2.9	71	0.9369	R.EFLHHK.E
 2.9	72	0.9747	EYEELK
 2.9	72	0.9747	K.EYELEK.Q

Top scoring peptide matches to query 1630
File7043 Spectrum6520 scans: 5099
Score greater than 34 indicates identity

**Score	Expect	Delta Hit Protein	Peptide**
 46.9	0.0029	-0.0047	2	P05023|AT1A1_HUMAN K.YGTDLSR.G
 18.9	1.8	0.0460	K.MNLDMR.K
 16.1	3.5	0.0712	-.MGGCAGSR.R
 15.6	3.8	-0.0022	K.YGVWMR.D
 13.8	5.9	-0.0047	R.AYSDLSR.A
 12.5	7.9	-0.1251	R.ALKLNPR.Y
 11.7	9.5	-0.0234	R.MALSFSR.F
 11.6	9.8	0.0130	-.MNIFDR.K
 11.2	10	-0.0200	K.YWSSLR.N
 11.1	11	-0.0233	R.YGKMANK.A

Top scoring peptide matches to query 1637
File7043 Spectrum13505 scans: 10936
Score greater than 18 indicates homology
Score greater than 34 indicates identity

**Score	Expect	Delta Hit Protein	Peptide**
 5.5	40	0.0561	R.GHTEVLR.L
 5.0	44	1.0626	R.NYVSSLK.R
 4.6	49	1.1055	R.CINFTR.V
 4.3	51	1.0947	K.CMIKMK.L
 3.8	58	1.0803	R.GAYGLAMK.V
 3.3	65	1.0652	R.GPGPGGGRR.A
 3.2	67	-0.8408	K.AMGTMNR.Q
 2.6	76	1.0691	R.TFKVCR.G
 2.2	83	1.0289	K.HIRTQR.N
 1.7	93	-0.9102 515	P62847|RS24_HUMAN R.KFMTNR.L

Top scoring peptide matches to query 1638
File7043 Spectrum7168 scans: 5612
Score greater than 21 indicates homology
Score greater than 34 indicates identity

**Score	Expect	Delta Hit Protein	Peptide**
 9.8	15	0.0565 648	P10515|ODP2_HUMAN R.SGPAPARR.N
 8.2	21	1.0517	R.SGLIQHR.R
 7.8	23	-0.9276	K.GGQQPLGR.A
 7.8	23	1.1687	K.EAGSMGSR.M
 6.5	31	1.0517	K.EQKLHR.V
 6.5	31	1.0517	R.QEKLHR.C
 6.5	31	1.0881	K.QELQHR.Q
 6.3	33	1.0881	R.VSASHGPR.K
 6.3	33	1.1422	R.CPGSHPR.V
 6.3	33	1.1534	R.CPGYGTR.T

Top scoring peptide matches to query 1644
File7043 Spectrum6117 scans: 4784
Score greater than 21 indicates homology
Score greater than 34 indicates identity

**Score	Expect	Delta Hit Protein	Peptide**
 8.6	20	0.9983 185	P39023|RL3_HUMAN K.FIDTTSK.F
 8.4	20	0.9797	K.MLYQLK.V
 7.5	25	1.0777	R.FENCNK.A
 7.4	26	1.0678	R.MESTDTK.R
 7.0	29	0.9104	K.FIYKIK.L
 6.0	35	1.0161	K.MIQFEK.E
 5.7	38	0.0031	K.VSQTYSK.N
 5.4	41	1.0678	R.EMTSSEK.G
 4.7	48	0.9937	R.MLRGYR.G
 4.0	56	0.9606 452+ P04439|1A03_HUMAN R.FLRGYR.Q

Top scoring peptide matches to query 1650
File7043 Spectrum4845 scans: 3779
Score greater than 22 indicates homology
Score greater than 34 indicates identity

**Score	Expect	Delta Hit Protein	Peptide**
 11.0	12	0.9730	R.EVSPPGAR.T
 9.3	18	0.9981	R.DLQSHGR.M
 8.1	24	1.0020 626	Q6UWM9|UD2A3_HUMAN R.MTFLER.V
 7.8	26	0.9981	R.NNAPGSPR.Y
 7.3	29	1.0197	R.FMVMER.L
 6.4	35	1.0424	-.MQTSSSR.S
 5.2	46	1.0020	-.MFLTER.N
 5.2	46	1.0714	-.MMETER.L
 5.2	47	1.0965	-.MSECGGR.G
 4.9	51	0.0213	R.DFPGSYK.H

Top scoring peptide matches to query 1651
File7043 Spectrum5182 scans: 4044
Score greater than 23 indicates homology
Score greater than 34 indicates identity

**Score	Expect	Delta Hit Protein	Peptide**
 13.5	6.9	-0.0167 456	Q07020|RL18_HUMAN K.LPGRENK.T
 10.1	15	0.0963	R.MEFENK.E
 9.2	19	0.9897	K.SSSYLKK.C
 8.8	20	0.0565	K.MVSSVMK.M
 8.5	22	0.0599	-.MLENYK.N
 7.8	26	0.9785	K.VHSTIQK.I
 7.3	29	-0.0572	K.FLVGHLK.T
 7.1	30	0.0122	K.VMEGIHK.A
 7.0	31	-0.0572	K.FVIGHLK.G
 6.8	32	0.0268	R.EYIGGFK.K

Top scoring peptide matches to query 1652
File7043 Spectrum9732 scans: 7678
Score greater than 16 indicates homology
Score greater than 34 indicates identity

**Score	Expect	Delta Hit Protein	Peptide**
 4.7	53	-0.0635	K.RLFHLK.H
 3.7	66	-0.0635	K.RLFLHK.T
 1.4 1.1e+002	0.0609	K.EATKNNH.-
 1.3 1.2e+002	1.0600	K.MFEELK.S
 1.2 1.2e+002	1.0892	-.MQTSSSR.S
 0.7 1.3e+002	-0.9959 247	P27824|CALX_HUMAN K.RPDADLK.T

Top scoring peptide matches to query 1653
File7043 Spectrum7212 scans: 5645
Score greater than 30 indicates homology
Score greater than 34 indicates identity

**Score	Expect	Delta Hit Protein	Peptide**
 19.1	1.9	0.0097	R.SSLKNHK.G
 16.4	3.6	-0.0420	K.FLHKLR.S
 16.4	3.6	-0.0056	4	P98164|LRP2_HUMAN R.IFHQLR.Y
 16.4	3.6	-0.0380	R.IRQQLR.F
 16.4	3.6	-0.0380	R.LQRQLR.D
 16.4	3.6	-0.0380	K.RIQQIR.M
 15.2	4.7	-0.0380	RQQILR
 14.6	5.4	-0.0380	K.ARLGQLR.G
 14.6	5.4	-0.0380	K.GRALQIR.D
 14.6	5.4	-0.0380	K.GRPSKLR.Q

Top scoring peptide matches to query 1659
File7043 Spectrum4000 scans: 3124
Score greater than 21 indicates homology
Score greater than 35 indicates identity

**Score	Expect	Delta Hit Protein	Peptide**
 9.7	19	-1.0033	K.MEEPGPR.F
 7.4	32	-1.1454	R.LFPVAIR.L
 6.2	43	0.9648	R.GHPVTMR.T
 5.7	48	-0.1461	K.IKPKNSK.Q
 5.4	51	1.0236	K.EYMENK.K
 4.9	58	-1.0284	K.MIYASSK.D
 4.2	67	-1.0435	K.GGQQGAAAR.E
 4.0	71	1.0124 160	O95299|NDUAA_HUMAN K.GDPHEMK.I
 3.6	76	-0.9920	-.MEYTGSK.Y
 3.6	78	-0.1461	K.IKGQGLAK.V

Top scoring peptide matches to query 1664
File7043 Spectrum9683 scans: 7641
Score greater than 21 indicates homology
Score greater than 35 indicates identity

**Score	Expect	Delta Hit Protein	Peptide**
 10.2	17	-0.9746	K.KGSPGLTR.R
 8.1	27	-0.0521	K.KAIILTR.E
 5.5	50	1.0523	K.ILPAEDR.A
 5.3	52	1.1064	K.KSFMER.S
 5.0	55	1.0337 100	Q5TCH4|CP4AM_HUMAN R.IPIPMAR.L
 5.0	55	1.0006	K.LPLPAFR.L
 4.6	61	1.0298	K.RPGGIGTR.E
 4.1	69	-0.9456	K.DLPAMLR.L
 3.9	72	0.0458	R.GAQIQAAR.E
 3.8	74	-0.9634	R.QQLGIEK.E

Top scoring peptide matches to query 1675
File7043 Spectrum10737 scans: 8517
Score greater than 32 indicates homology
Score greater than 36 indicates identity

**Score	Expect	Delta Hit Protein	Peptide**
 19.6	2.2	-0.0053	9	P25705|ATPA_HUMAN R.ELIIGDR.Q
 18.6	2.8	-0.0165	R.LQGGLTAR.R
 18.2	3	0.0199	R.GGGALLGDR.W
 15.8	5.4	1.0515	R.QPELGDR.K
 11.2	15	0.0562	R.GSNPLDGR.D
 10.5	18	0.0562	R.GPSQVGDR.L
 10.5	18	-0.0165	R.IKLGGDGR.T
 10.1	20	-0.0304	K.ELLLAEK.W
 10.1	20	-0.0668	R.ELLLLSK.A
 10.1	20	-0.0668	K.LEILTVK.F

Top scoring peptide matches to query 1677
File7043 Spectrum4999 scans: 3905
Score greater than 36 indicates identity

**Score	Expect	Delta Hit Protein	Peptide**
 28.4	0.29	-0.0047 289	P49411|EFTU_HUMAN K.ILAEGGGAK.F
 23.6	0.87	-0.0299	K.ILAEELK.E
 14.4	7.4	-0.9999	K.ARAELEK.R
 14.4	7.4	-0.9887	R.INAEIEK.Q
 14.4	7.4	-0.9887	K.NLAELEK.S
 11.0	16	0.0429	R.EAPEELK.Q
 10.7	17	-0.0047	K.LEQNALK.R
 10.1	20	-0.0411	R.AIVNSALK.L
 9.4	23	-0.0411	K.ILSLNKQ.-
 8.0	32	-0.0048	R.IVDVGGQK.S

Top scoring peptide matches to query 1681
File7043 Spectrum5645 scans: 4411
Score greater than 20 indicates homology
Score greater than 36 indicates identity

**Score	Expect	Delta Hit Protein	Peptide**
 9.4	23	1.0407	R.EPAVRSR.L
 7.4	37	1.0520	K.EPALTAGR.L
 4.8	67	1.0407	K.EPLGSRR.Q
 4.0	80	0.0316 289	P49411|EFTU_HUMAN K.ILAEGGGAK.F
 3.8	83	-0.8659	K.FMFDEK.L
 3.5	91	1.0697	K.MIVGNHK.D
 2.3 1.2e+002	-0.9888	K.DLISAGLK.E
 2.0 1.3e+002	1.0156 542	P02549|SPTA1_HUMAN K.EPLATRK.K
 1.9 1.3e+002	0.0931	K.GEAGPTGAR.G
 1.2 1.5e+002	-0.9273	R.SSEPQIR.R

Top scoring peptide matches to query 1687
File7043 Spectrum11965 scans: 9574
Score greater than 23 indicates homology
Score greater than 36 indicates identity

**Score	Expect	Delta Hit Protein	Peptide**
 10.8	17	-0.0361	K.KEPAPMK.S
 10.4	19	0.0189 311	P01833|PIGR_HUMAN K.ADAAPDEK.V
 9.5	24	0.9804	R.AQGQAGGAR.A
 8.2	32	0.8825	R.AQLISRK.T
 7.7	36	0.9553	M.KPPGSSSR.Q
 6.7	45	-0.9876	R.EEQQQR.Q
 6.4	49	0.9730	K.ISVHMGR.V
 6.3	49	0.9989	K.ASYEAFK.N
 6.1	51	-0.0400	K.ERDLQR.K
 6.0	52	0.8825	R.AQLLKSR.I

Top scoring peptide matches to query 1688
File7043 Spectrum6128 scans: 4790
Score greater than 33 indicates homology
Score greater than 36 indicates identity

**Score	Expect	Delta Hit Protein	Peptide**
 28.7	0.28	0.9422	R.AQLLKSR.I
 19.4	2.4	0.9422 433	P11277|SPTB1_HUMAN R.AKLQISR.D
 19.1	2.6	0.9422	R.KALSQLR.E
 17.9	3.4	0.9785	R.KAKPESR.L
 17.8	3.5	0.9422 226	Q7Z406|MYH14_HUMAN R.AKLALGSR.V
 17.6	3.6	1.0149	K.KAQESPR.G
 16.3	4.9	1.0149	R.AQKSEPR.E
 15.7	5.7	-1.0007	40	O95831|AIFM1_HUMAN R.AGAEVKSR.T
 12.9	11	0.0197	R.AQNIQSR.L
 12.8	11	0.9422	R.AKLSQIR.Q

Top scoring peptide matches to query 1691
File7043 Spectrum3385 scans: 2624
Score greater than 31 indicates homology
Score greater than 36 indicates identity

**Score	Expect	Delta Hit Protein	Peptide**
 28.5	0.3	0.9542	K.DLAKAVAK.Q
 17.0	4.1	0.9793	K.NGQKAAVK.I
 16.7	4.5	-0.9458	R.CVSQAPR.G
 16.4	4.8	0.9906	K.ENLAAGIK.Q
 15.5	5.9	1.0229	K.AIYSSFK.D
 14.8	6.9	0.9429	R.ITGRAGLK.E
 13.3	9.8	-0.9710	K.MVVTDPR.Y
 12.9	11	0.9906 239	P48741|HSP77_HUMAN K.DAGAIAGLK.V
 12.2	13	0.9681	K.QLRATAR.A
 12.1	13	0.9793	R.AVEKAAAR.H

Top scoring peptide matches to query 1693
File7043 Spectrum12483 scans: 10037
Score greater than 23 indicates homology
Score greater than 36 indicates identity

**Score	Expect	Delta Hit Protein	Peptide**
 11.6	15	-0.9997	R.TGIRQSR.L
 10.3	20	0.9908	R.LAQLQSR.M
 8.7	29	-0.9885	M.DALKSAGR.A
 7.5	37	-0.9885	89	P07911|UROM_HUMAN K.GVQATVSR.A
 7.5	37	-0.9633	R.RQATADR.D
 7.3	39	0.9722	VGKPMRK
 7.2	40	-1.0136	K.TILSEVR.F
 7.0	42	1.0119	K.RQWPTK.S
 6.8	44	1.0636	R.VGPNGESR.A
 6.3	49	1.0119	K.VGNALWR.R

Top scoring peptide matches to query 1696
File7043 Spectrum14719 scans: 12041
Score greater than 25 indicates homology
Score greater than 36 indicates identity

**Score	Expect	Delta Hit Protein	Peptide**
 14.5	7.4	-0.0037	95	P35555|FBN1_HUMAN R.GFIPNIR.T
 11.6	15	-0.9295	K.SSMHLAR.C
 9.2	25	0.0579	R.GHYGVQR.T
 8.7	28	-0.0037	K.FIPGLNR.S
 8.6	29	-0.9407	-.MGPGARGR.R
 8.4	30	-0.9585	K.SASRPGSR.A
 7.2	40	-0.9149	K.SSFHDPK.L
 6.4	48	0.0327	R.WGGLDLR.R
 6.3	49	0.0480	R.NSSIPNGK.L
 6.2	51	-0.9836	K.SAKENLR.D

Top scoring peptide matches to query 1698
File7043 Spectrum3956 scans: 3087
Score greater than 21 indicates homology
Score greater than 34 indicates identity

**Score	Expect	Delta Hit Protein	Peptide**
 11.4	10	-0.7020	R.NVRAVMK.R
 11.0	11	-0.7271	K.GKQILMK.D
 11.0	11	-0.6795	R.TDPLIMK.T
 8.0	23	-0.6721	95	P35555|FBN1_HUMAN K.LNEVSQK.C
 7.0	29	-0.6357	R.LNAENEK.D
 6.9	29	-0.6908	R.CPVVKSK.C
 6.5	32	-0.6721	R.LVDATNGK.E
 6.2	35	-0.6721	K.VASVAENK.G
 5.2	44	-0.7085	K.LSAQASLK.R
 5.2	44	-0.6543	R.NPMEAKK.R

Top scoring peptide matches to query 1700
File7043 Spectrum1687 scans: 1272
Score greater than 17 indicates homology
Score greater than 34 indicates identity

**Score	Expect	Delta Hit Protein	Peptide**
 5.0	46	0.6808	K.RADEVVK.Y
 4.5	52	0.7060	K.KPSQGSGR.R
 4.4	52	0.6656	R.QVIGPFR.R
 4.4	52	0.6696	R.RVSEGLR.D
 4.4	52	0.6696	R.RVSVAER.R
 4.3	54	0.6404	K.KPFTPVK.Y
 4.3	54	0.7173 728	Q6UWE0|LRSM1_HUMAN R.KPSEEAR.K
 4.1	56	-0.3144	K.KGESQLR.V
 3.8	61	0.7900	R.HDSSGEGK.R
 3.5	64	-0.3508 752	O95954|FTCD_HUMAN R.GLGSKSLR.A

Top scoring peptide matches to query 1703
File7043 Spectrum5060 scans: 3951
Score greater than 29 indicates homology
Score greater than 36 indicates identity

**Score	Expect	Delta Hit Protein	Peptide**
 17.6	3.7	0.9256	K.GTLAGEIR.D
 17.4	4	0.9467	R.GYPGPGLR.S
 15.5	6.1	-1.0835	K.CIRTLR.A
 15.5	6.1	-1.0835	R.CLRTIR.L
 15.3	6.4	0.9256	R.TTSPAALR.A
 14.2	8.3	0.9103	95	P35555|FBN1_HUMAN R.GFIPNIR.T
 13.5	9.8	-1.0835	R.VQMRLR.R
 11.8	14	-0.1246	R.IMRILR.I
 11.7	15	0.0031	K.DGDQNLR.D
 11.7	15	-1.0689	R.YVGNPLR.G

Top scoring peptide matches to query 1704
File7043 Spectrum4160 scans: 3250
Score greater than 22 indicates homology
Score greater than 36 indicates identity

**Score	Expect	Delta Hit Protein	Peptide**
 13.0	11	0.9710	K.QLLADEK.M
 12.4	13	-0.0607	K.QIALGTSK.L
 8.8	28	-0.0355 542	P02549|SPTA1_HUMAN K.KLNEASR.Q
 8.8	29	-0.0243	K.ENLAVGSK.I
 8.7	30	-0.0243	K.LKGENEK.I
 8.7	30	0.0121	R.LQGENEK.L
 8.4	31	-0.0607	K.LKDQVSK.D
 8.2	33	0.9233	K.KLLADTR.K
 7.8	36	-0.0104	K.AGGGKGAGSR.L
 7.2	42	0.0121	K.IQDAQDK.L

Top scoring peptide matches to query 1707
File7043 Spectrum1420 scans: 1088
Score greater than 26 indicates homology
Score greater than 36 indicates identity

**Score	Expect	Delta Hit Protein	Peptide**
 16.0	5.4	1.0165	K.AGADQRAK.N
 12.8	11	0.9550	R.AGAVKVGSK.V
 10.7	19	1.0455	K.KSHMGEK.Q
 8.9	28	0.9914	R.KAQSSAPK.V
 8.6	30	0.9914 146	O15127|SCAM2_HUMAN R.KAAELER.K
 8.2	33	0.9662	R.AKIEVEK.L
 8.2	33	0.9914	R.LAELAGSR.G
 8.1	34	0.0213	R.EAAQSRR.Q
 8.0	34	0.0213	R.RSAEAQR.G
 7.7	37	0.9662	R.QSIIDLK.E

Top scoring peptide matches to query 1708
File7043 Spectrum1209 scans: 951
Score greater than 26 indicates homology
Score greater than 36 indicates identity

**Score	Expect	Delta Hit Protein	Peptide**
 15.8	5.7	-0.9627	R.SRSPDTR.K
 12.9	11	1.0754 311	P01833|PIGR_HUMAN K.ADAAPDEK.V
 10.8	18	0.9438	R.ATRSLLR.E
 10.0	22	0.9914	M.AAAVLTDR.A
 9.4	25	1.0204	K.AAMPVEAK.C
 8.2	33	-0.9668	R.FDGVPQR.F
 7.3	40	1.0891	K.FMFDEK.L
 7.2	41	-1.0242	K.SVIKSER.I
 7.0	43	1.0278	R.ASELSPGR.S
 6.8	46	-0.9879	R.SLVQDTR.I

Top scoring peptide matches to query 1712
File7043 Spectrum3585 scans: 2778
Score greater than 18 indicates homology
Score greater than 36 indicates identity

**Score	Expect	Delta Hit Protein	Peptide**
 7.7	37	1.0440	K.KEPAPMK.S
 5.2	65	1.0037	R.ANSQLKR.Q
 3.6	94	0.0375	-.MAAQVAAR.E
 3.4	99	1.0037	K.KAGSQGLR.F
 3.4	1e+002	1.0037	M.AVSRLDR.L
 2.8 1.1e+002	0.0045	R.EPRLFR.L
 2.5 1.2e+002	1.0401	K.AGADQRAK.N
 2.4 1.2e+002	1.0150	K.AKAAAGEAK.V
 1.4 1.6e+002	-1.0523	K.KVGVIFR.L
 0.8 1.8e+002	-0.9531 623	P11234|RALB_HUMAN R.VKAEEDK.I

Top scoring peptide matches to query 1719
File7043 Spectrum2368 scans: 1835
Score greater than 29 indicates homology
Score greater than 36 indicates identity

**Score	Expect	Delta Hit Protein	Peptide**
 17.2	4	1.0496	R.EAGCPAGR.L
 16.3	4.9	0.9703	60	Q01082|SPTB2_HUMAN K.EVAELTR.L
 14.8	6.9	0.9703	R.SVALGQDK.V
 14.6	7.3	1.0683	R.DSESHSR.V
 13.6	9.1	0.9227	R.GTVTGVRK.D
 13.4	9.5	1.0067	R.EQGENIK.H
 12.0	13	-0.9483	R.MQCPQR.Q
 11.9	14	0.9663	R.EPFLPSK.E
 11.6	15	0.8976	R.KASLLSAK.S
 10.9	17	-0.0249	R.ASLRDEK.G

Top scoring peptide matches to query 1720
File7043 Spectrum10159 scans: 8035
Score greater than 30 indicates homology
Score greater than 36 indicates identity

**Score	Expect	Delta Hit Protein	Peptide**
 18.4	3	1.0118	4	P98164|LRP2_HUMAN K.VTVDMPR.H
 16.4	4.7	0.9828	K.AEVQSKR.K
 16.4	4.7	1.0192	R.AEKEGQR.L
 16.3	4.9	1.0734	R.EACGPAGR.T
 16.3	4.9	1.0734	R.EAGCPAGR.L
 15.4	6	1.0556	K.AEENAQR.L
 12.6	11	-0.9964	R.SLTAGSQR.K
 11.5	15	1.0192	K.ASASTPQR.R
 11.3	16	-0.0012	R.SLVQDTR.I
 10.8	17	-0.9964	R.DSLNSKR.R

Top scoring peptide matches to query 1725
File7043 Spectrum581 scans: 526
Score greater than 27 indicates homology
Score greater than 34 indicates identity

**Score	Expect	Delta Hit Protein	Peptide**
 16.0	3.5	0.3534	R.FDGVPQR.F
 13.3	6.6	-0.5910	R.MQCPQR.Q
 13.3	6.6	-0.6266	R.SGSSTPQR.S
 13.3	6.6	0.3348	M.WMTPKR.S
 9.4	16	0.3799	K.QEAATGLE.-
 9.3	17	0.3137	K.VAAIAMAR.I
 9.1	17	0.3501	K.DLGNLMR.T
 7.7	24	0.3436 623	P11234|RALB_HUMAN R.VKAEEDK.I
 7.5	25	-0.6452	K.IECNKR.H
 7.3	26	0.2773	M.KATVLMR.Q

Top scoring peptide matches to query 1729
File7043 Spectrum6480 scans: 5067
Score greater than 25 indicates homology
Score greater than 36 indicates identity

**Score	Expect	Delta Hit Protein	Peptide**
 13.2	10	0.9940	R.NNSSKLR.S
 13.1	10	0.9689	R.EVSSKLR.R
 13.1	10	1.0052	R.EVSSQLR.L
 11.9	14	1.0052 515	P62847|RS24_HUMAN M.NDTVTIR.T
 11.5	15	0.0828	R.EASGGDQR.E
 10.5	19	1.0053	R.EASRLDK.F
 9.9	21	0.0390	K.LGQMENK.T
 9.3	24	1.0053	R.EVSAKER.S
 8.9	27	-0.9376	K.NNDSSVGK.S
 8.1	32	0.0100	K.DNSSIRK.L

Top scoring peptide matches to query 1730
File7043 Spectrum6363 scans: 4974
Score greater than 25 indicates homology
Score greater than 36 indicates identity

**Score	Expect	Delta Hit Protein	Peptide**
 12.5	12	0.9870	R.GASAMLLR.D
 11.7	14	-0.9848 431	O43795|MYO1B_HUMAN R.ANGKSTSR.I
 11.5	15	1.0421	K.AESQDLR.H
 11.5	15	1.0057	64	Q13813|SPTA2_HUMAN K.EAGSVSLR.M
 11.5	15	1.0057	K.EASTAALR.S
 11.5	15	0.9693	R.LSKDSIR.T
 11.5	15	1.0057	R.LSSDQIR.R
 11.5	15	0.9693	K.TVSKDLR.N
 11.4	15	0.0030	R.SSLMPLR.N
 10.9	17	0.9870	K.AKAQLCK.I

Top scoring peptide matches to query 1744
File7043 Spectrum2328 scans: 1801
Score greater than 18 indicates homology
Score greater than 34 indicates identity

**Score	Expect	Delta Hit Protein	Peptide**
 5.7	37	-1.1731	K.DSSDSGVR.S
 4.8	46	-1.1731	K.DSETSQR.L
 4.1	54	-1.1918	K.LACSDTR.V
 3.7	59	-0.2845	-.MALLFAR.S
 3.6	62	0.7082 402	P37108|SRP14_HUMAN K.ISTVVSSK.E
 3.2	66	-0.2295	R.YLSGPER.A
 2.4	81	-0.2632	R.QHQRPR.W
 2.4	81	-0.2329	K.ECKVGTK.T
 2.3	83	0.7698	R.SLQESTR.F
 1.3	1e+002	0.7810	R.LSSEQEK.E

Top scoring peptide matches to query 1748
File7043 Spectrum1763 scans: 1332
Score greater than 21 indicates homology
Score greater than 34 indicates identity

**Score	Expect	Delta Hit Protein	Peptide**
 8.6	20	0.7420	K.LFENIGK.G
 8.3	21	0.7750	K.NEAIVMK.E
 7.7	25	0.7056	K.LVEKFGK.E
 7.7	25	0.7889	K.NARGMQK.H
 7.7	25	0.7671	R.NNLQFGK.T
 5.8	38	0.7274	K.IRMSGIK.G
 5.8	38	0.7056	K.LYNLGLK.G
 5.1	44	0.7056 632	Q6P1M0|S27A4_HUMAN K.IAKDVFK.K
 5.1	44	0.7308	K.LAKDFAR.H
 5.1	44	0.7056	R.LKADVFK.N

Top scoring peptide matches to query 1751
File7043 Spectrum9998 scans: 7902
Score greater than 24 indicates homology
Score greater than 35 indicates identity

**Score	Expect	Delta Hit Protein	Peptide**
 13.2	8.7	-0.0416	K.VIFSLSR.V
 13.2	8.7	-0.0052 210	Q8NE62|CHDH_HUMAN R.VLFEGTR.A
 11.1	14	-0.0528	R.VLSPLHR.G
 9.1	23	1.0482	K.VINMDGR.L
 8.6	26	-0.0165	R.VLDYRR.K
 8.0	30	1.0958	R.MENGEPK.G
 6.0	46	0.9788	K.GRLAFEK.M
 5.7	50	0.9900	R.VLNSYPK.R
 5.4	53	0.9424	R.VLPAPAPR.R
 4.6	64	-0.0052	K.VLYNANK.N

Top scoring peptide matches to query 1752
File7043 Spectrum5283 scans: 4120
Score greater than 21 indicates homology
Score greater than 35 indicates identity

**Score	Expect	Delta Hit Protein	Peptide**
 9.4	21	1.0218	R.HQRHSR.V
 9.4	21	1.0330 561	P98160|PGBM_HUMAN R.HQVHGSR.L
 9.2	22	0.0867	K.TEEEASR.G
 9.2	22	0.0278	R.TGSRTGSR.A
 9.2	22	-0.9636	K.CSSSLIR.H
 8.2	28	1.0409	R.DMRNIR.L
 8.2	28	1.0409	DMRNLR
 7.2	35	1.0343	R.LSADTASR.F
 7.0	37	0.0569	-.MAAAAQSR.V
 7.0	37	-0.8908	-.MASEDNR.V

Top scoring peptide matches to query 1753
File7043 Spectrum5288 scans: 4124
Score greater than 19 indicates homology
Score greater than 35 indicates identity

**Score	Expect	Delta Hit Protein	Peptide**
 7.3	34	0.0403	R.RTSSAGSR.G
 6.2	44	1.0282	-.MVLAQSR.V
 6.2	44	0.9951	K.VFIAQSR.S
 6.1	46	-0.9399	K.SLMAQEK.G
 5.8	48	0.0693	95	P35555|FBN1_HUMAN K.CSSPKSR.N
 5.7	49	0.0251	K.RFQQSR.R
 5.6	51	0.0507	R.MIRMDR.R
 5.6	51	0.0507	R.MLRMDR.R
 5.3	54	0.0918	-.MEAEVDK.L
 5.3	55	0.1057	R.MENGQSR.T

Top scoring peptide matches to query 1756
File7043 Spectrum431 scans: 428
Score greater than 24 indicates homology
Score greater than 35 indicates identity

**Score	Expect	Delta Hit Protein	Peptide**
 15.7	4.9	-0.9738	R.KSSTFPR.T
 10.4	17	1.0054	R.VGIFTGAR.V
 8.6	26	-0.9262	R.SGDATFPK.A
 7.6	32	0.0544	R.LTVDSMR.R
 7.4	33	1.0021	K.IRMSGIK.G
 6.5	41	-0.9482 183	Q9Y5K8|VATD_HUMAN R.MAQTIMK.A
 6.3	44	-0.9738	K.VDGFTRK.S
 6.2	45	0.0214	K.GVFNSGLK.G
 6.1	45	0.0684	R.RTGNMAR.V
 5.9	48	1.0385	K.ISLSNMR.I

Top scoring peptide matches to query 1760
File7043 Spectrum6460 scans: 5050
Score greater than 23 indicates homology
Score greater than 35 indicates identity

**Score	Expect	Delta Hit Protein	Peptide**
 13.7	7.2	0.9365	K.LVSEMVK.K
 9.6	19	-0.0410 395	P02774|VTDB_HUMAN K.LCMAALK.H
 9.1	21	0.9980	K.NASECLK.L
 8.3	25	-1.0540	R.NTSVMKK.G
 7.5	30	0.9035	K.EGLYLVK.A
 7.4	31	-0.0853	R.LCRFVK.E
 7.2	32	-1.1598	K.ILHSLLK.K
 6.3	39	-0.0455	DWFAKR
 6.2	41	-0.1104	-.MFGLKVK.K
 5.9	43	1.0418	R.EGSDSGNR.Q

Top scoring peptide matches to query 1762
File7043 Spectrum12489 scans: 10042
Score greater than 23 indicates homology
Score greater than 35 indicates identity

**Score	Expect	Delta Hit Protein	Peptide**
 12.6	9.4	-0.9326	R.GEPSYDR.L
 9.4	20	0.9950	R.SPYLWR.R
 9.4	20	-1.0054	R.SVYIDAR.D
 7.8	28	0.0303	R.RSSSEEK.L
 7.2	32	-1.0530	R.APPRASPK.R
 6.1	42	-0.0941	K.HLKLSPK.S
 4.3	64	-1.0682	R.WVHILR.R
 4.2	65	0.0263	R.SGDATFPK.A
 4.0	67	-0.9584 267+ Q12955|ANK3_HUMAN R.GGSMRGSR.H
 4.0	67	0.0229	K.VSAMSSPK.V

Top scoring peptide matches to query 1763
File7043 Spectrum3782 scans: 2941
Score greater than 20 indicates homology
Score greater than 35 indicates identity

**Score	Expect	Delta Hit Protein	Peptide**
 10.7	14	-0.0002 644	O43175|SERA_HUMAN -.MAFANLR.K
 10.1	17	1.0276	M.RGGGFGDR.D
 7.0	34	0.9917	K.MMGGKGLK.Q
 5.3	50	1.0314	R.VNGFPCK.N
 5.2	51	0.0077 395	P02774|VTDB_HUMAN K.LCMAALK.H
 5.1	53	1.0164	R.RTHHDR.A
 4.3	63	0.9409	R.GYSLLIR.G
 4.1	66	0.0185	K.NYDAALR.K
 4.0	68	-0.0179	R.ISGYNIR.H
 3.8	71	0.9409	R.LPEPPLR.G

Top scoring peptide matches to query 1769
File7043 Spectrum9671 scans: 7632
Score greater than 18 indicates homology
Score greater than 34 indicates identity

**Score	Expect	Delta Hit Protein	Peptide**
 9.8	16	-0.9846 676	P13498|CY24A_HUMAN K.KGSTMER.W
 5.3	46	1.0562	R.CGGGTQSR.K
 4.8	52	0.0470	K.DGTVEMR.G
 4.8	52	-0.0561	R.GHQRVAR.R
 4.8	52	-0.0013	K.WSVEFR.D
 4.8	52	-0.0376	K.WSVYIR.A
 4.5	55	-0.0224	K.IESTFAR.F
 4.1	61	0.0656	K.SDSEGNSK.D
 3.6	68	0.9616	R.AIFSTQR.Q
 3.2	75	1.0674	-.MADGGSER.A

Top scoring peptide matches to query 1772
File7043 Spectrum9516 scans: 7507
Score greater than 27 indicates homology
Score greater than 34 indicates identity

**Score	Expect	Delta Hit Protein	Peptide**
 15.4	4.6	-0.0046	25	P40939|ECHA_HUMAN K.ALTSFER.D
 13.7	6.6	-0.0410	R.SSFLTLR.D
 9.9	16	-0.0484	R.MLATLFK.D
 9.0	20	-0.0046	K.FSSLNQK.V
 8.7	21	0.0284	K.VSTQNMK.M
 8.5	22	-0.0159	R.GTGAFKSR.V
 8.4	23	-0.0233	-.MGVLTFR.D
 8.4	23	0.0317	R.TGEVDFR.E
 8.2	23	-0.0563	R.AGILFFR.I
 8.1	24	-0.0522	K.IALHNQK.Q

Top scoring peptide matches to query 1776
File7043 Spectrum3679 scans: 2854
Score greater than 21 indicates homology
Score greater than 34 indicates identity

**Score	Expect	Delta Hit Protein	Peptide**
 14.1	6.1	1.0132	R.EQATVFK.R
 7.5	27	-0.9265	R.MQGVSMR.D
 7.1	30	1.0132 628	P40926|MDHM_HUMAN K.KGEDFVK.T
 6.3	37	-0.9820	-.MQRPHR.R
 5.9	40	1.0350	K.KVSDSMR.T
 5.9	40	-0.9153	-.MPNTAMK.K
 5.9	40	0.9986	K.SRLTAMK.M
 5.9	40	1.0635	K.GNNGSAFR.V
 5.9	40	-0.9773	K.AYSTLNR.K
 5.6	43	0.9907	R.ERPAPPR.E

Top scoring peptide matches to query 1779
File7043 Spectrum14208 scans: 11571
Score greater than 16 indicates homology
Score greater than 32 indicates identity

**Score	Expect	Delta Hit Protein	Peptide**
 4.0	39	0.6924 138	P06396|GELS_HUMAN K.MDAHPPR.L
 3.7	42	-0.3201	R.EKMMAAK.R

Top scoring peptide matches to query 1780
File7043 Spectrum7979 scans: 6261
Score greater than 18 indicates homology
Score greater than 33 indicates identity

**Score	Expect	Delta Hit Protein	Peptide**
 6.2	24	-0.4214	K.IVVIAPGR.I
 5.3	30	-0.3235 403	Q01813|K6PP_HUMAN R.VGIADGHR.M
 3.4	46	0.6861	R.RVMVMR.R
 2.7	54	-0.3123	K.YGSDKVR.V
 2.4	58	0.6796	R.KNMVSTK.I
 2.3	59	-0.2429	R.QDTGTMR.A
 2.0	63	0.6895	R.HSPPMVR.S
 1.9	64	-0.3347	R.GAGGPRGPR.G
 1.8	67	0.6242	R.ELLRHR.D
 1.8	67	0.6242	M.LELRHR.G

Top scoring peptide matches to query 1781
File7043 Spectrum8841 scans: 6953
Score greater than 21 indicates homology
Score greater than 34 indicates identity

**Score	Expect	Delta Hit Protein	Peptide**
 8.7	20	0.9988 222	Q00325|MPCP_HUMAN R.TVEALYK.F
 7.7	25	-1.0756	K.LSPKPKR.A
 6.3	34	0.9651	K.ANHVVKR.D
 4.0	59	-0.9665	R.EAHLEAR.M
 4.0	59	-1.0029 319	P50416|CPT1A_HUMAN R.LSHEALR.Q
 4.0	59	-1.0029	R.SIHEALR.R
 4.0	59	-1.0393	K.SLHSIIR.H
 2.7	79	-0.9804	K.SLETTFK.N
 2.7	79	0.9876	K.TVKFNSK.T
 2.7	79	0.9988	K.TVTVEFK.H

Top scoring peptide matches to query 1786
File7043 Spectrum9417 scans: 7424
Score greater than 22 indicates homology
Score greater than 34 indicates identity

**Score	Expect	Delta Hit Protein	Peptide**
 9.2	17	0.9621	R.VYSGSRR.S
 8.7	19	-0.9518 645	P19022|CADH2_HUMAN K.FSNCNGK.R
 8.5	20	0.0322	-.MNFSGGGR.Q
 6.7	30	1.0613	K.SSEDSGSR.K
 6.5	31	0.9733	K.AFSEKSR.L
 5.9	36	1.1090	K.SNEGSDSE.-
 4.9	45	0.9733	R.SNIFTSR.K
 4.9	45	0.9733	R.SNLFTSR.K
 4.9	46	1.0129	-.MAMRSGR.H
 3.8	59	0.9985	R.DSFRGSR.S

Top scoring peptide matches to query 1790
File7043 Spectrum4624 scans: 3603
Score greater than 20 indicates homology
Score greater than 34 indicates identity

**Score	Expect	Delta Hit Protein	Peptide**
 11.5	10	-0.9381	K.AVHSQER.S
 6.8	29	0.0827	K.KMMDLR.T
 6.5	31	1.1404	K.SSEDSGSR.K
 6.4	32	1.0047	R.HLTAQVR.S
 5.5	40	1.0622	R.HFDHLR.A
 5.5	40	0.9432	R.KPTLPLR.L
 5.3	42	1.0701 666	P13929|ENOB_HUMAN K.YNQLMR.I
 5.3	42	-0.9309	K.EFFDLR.L
 5.1	44	1.0524	K.SSASFGIR.R
 5.0	44	1.0048	R.EPGLRPR.I

Top scoring peptide matches to query 1800
File7043 Spectrum475 scans: 458
Score greater than 19 indicates homology
Score greater than 34 indicates identity

**Score	Expect	Delta Hit Protein	Peptide**
 7.1	27	-0.8352	K.QDGESYK.N
 5.8	37	1.0740	K.RHELNR.Q
 4.5	49	1.1077	R.LYGDSAAK.I
 4.3	52	1.0488	R.HRELAAK.A
 2.3	82	0.0396 150	P12111|CO6A3_HUMAN R.GPIGSIGPK.G
 1.3	1e+002	1.0124	K.RLTIHGK.E
 1.1 1.1e+002	1.1692	K.QDGDSFR.M
 0.5 1.3e+002	0.0760	R.AGPATSPPK.T
 0.5 1.3e+002	1.0713	K.EPPSLPGK.E
 0.2 1.3e+002	1.0964	R.GPPGSQGPK.G

Top scoring peptide matches to query 1804
File7043 Spectrum9167 scans: 7223
Score greater than 15 indicates homology
Score greater than 32 indicates identity

**Score	Expect	Delta Hit Protein	Peptide**
 3.1	45	-0.3927	R.LPRDVAR.D
 2.8	49	0.6792	R.TVYGLCL.-
 1.8	61	-0.4542	10	Q00610|CLH1_HUMAN K.NLILVVR.G
 1.0	75	0.7560	-.MDGTETR.Q
 0.8	78	0.7706	K.NDIYGED.-
 0.7	79	0.7625	R.SGCVSCR.R

Top scoring peptide matches to query 1807
File7043 Spectrum676 scans: 591
Score greater than 22 indicates homology
Score greater than 33 indicates identity

**Score	Expect	Delta Hit Protein	Peptide**
 12.7	6	0.7650	R.GAPGIPGTR.G
 9.0	14	0.7399 150	P12111|CO6A3_HUMAN R.GPIGSIGPK.G
 4.5	39	0.7763	R.KDSPPPGK.V
 3.8	46	0.7287	K.SHILSIR.D
 3.6	48	0.8014	K.GDVGPAGPR.G
 2.1	67	0.8821	K.SEMSESR.Q
 2.0	70	0.7399	K.GIEKPPGK.N
 1.1	85	-0.2190	K.GKDPNAPK.R
 0.9	90	0.8742	SGGGYGGDR
 0.7	93	-1.1852	K.NIIHAEM.-

Top scoring peptide matches to query 1809
File7043 Spectrum20448 scans: 17418
Score greater than 20 indicates homology
Score greater than 34 indicates identity

**Score	Expect	Delta Hit Protein	Peptide**
 8.7	19	-1.0058	R.SILTEHK.R
 8.3	21	0.9735 150	P12111|CO6A3_HUMAN R.GPIGSIGPK.G
 6.7	31	-0.9074	K.KMMEEK.L
 5.3	42	1.0098	R.VVHDLDK.L
 5.0	45	0.9986	R.HEALLSR.G
 4.2	54	-1.0534	R.AVPGSIKR.T
 3.6	62	0.9834	R.WNLKHK.Y
 3.4	65	0.0358	K.YEHHLK.E
 3.3	66	0.0324	K.RMQFTK.A
 2.9	73	-1.0210	R.APVYLHK.M

Top scoring peptide matches to query 1810
File7043 Spectrum14862 scans: 12167
Score greater than 15 indicates homology
Score greater than 34 indicates identity

**Score	Expect	Delta Hit Protein	Peptide**
 3.7	59	-0.9157	R.GGLASTPPK.A
 2.1	85	0.0207	K.GKPLQRK.V
 1.6	97	-0.9269 474	P50914|RL14_HUMAN K.GQKAPAQK.A
 0.3 1.3e+002	1.0887	K.TQLAQHK.E

Top scoring peptide matches to query 1812
File7043 Spectrum3725 scans: 2892
Score greater than 30 indicates homology
Score greater than 34 indicates identity

**Score	Expect	Delta Hit Protein	Peptide**
 18.0	2.3	0.8976	R.GAYGLAMK.V
 17.2	2.7	0.9162	R.SFTAADSK.D
 16.0	3.6	-0.0687 409	P98187|CP4F8_HUMAN K.FAMAEMK.V
 15.7	3.8	-0.0977	R.MALSFSR.F
 14.1	5.6	0.9050	K.FSSSDRK.K
 11.2	11	0.9050	R.SSFSDRK.F
 10.3	13	-0.0501	-.MAEADFK.M
 8.5	20	0.8976	R.AYMNLAK.K
 8.5	20	0.8322	K.HKETLAK.M
 8.5	20	0.8645	R.WDPPLAK.C

Top scoring peptide matches to query 1814
File7043 Spectrum13445 scans: 10881
Score greater than 16 indicates homology
Score greater than 34 indicates identity

**Score	Expect	Delta Hit Protein	Peptide**
 3.8	64	-1.0867	R.LATWKPL.-
 2.9	79	-1.0939	K.KQINVAR.L
 2.3	92	-1.1190	K.KQILQAK.R
 2.0	98	-1.1190 179	P55072|TERA_HUMAN R.KQLAQIK.E
 1.1 1.2e+002	-0.9986	K.ADPPATEK.T
 0.2 1.5e+002	-1.0979	M.WAGLLLR.A

Top scoring peptide matches to query 1815
File7043 Spectrum7666 scans: 6011
Score greater than 20 indicates homology
Score greater than 34 indicates identity

**Score	Expect	Delta Hit Protein	Peptide**
 12.0	9.8	0.9584	R.TPPVTRR.R
 6.5	35	0.9948	R.SPQPNKR.H
 5.0	48	0.9585	R.TPPLSRR.K
 4.6	54	-0.9004	R.HEEEER.K
 4.0	61	0.9585	K.SLPPTRR.A
 3.4	71	0.0472 179	P55072|TERA_HUMAN K.THGEVER.R
 3.2	73	-0.0143	R.ELAELPR.S
 3.2	74	0.9585	R.SPRTLPR.S
 2.8	81	0.9948	R.TPPEARR.L
 2.5	87	0.0841	K.KMMEEK.L

Top scoring peptide matches to query 1824
File7043 Spectrum975 scans: 795
Score greater than 19 indicates homology
Score greater than 35 indicates identity

**Score	Expect	Delta Hit Protein	Peptide**
 7.3	33	0.8762	K.ILLSPER.V
 7.3	33	0.8398	R.LLLLAER.Y
 6.9	37	-1.1395	K.VAGLLTQK.L
 6.1	44	0.9377	4	P98164|LRP2_HUMAN R.DNVPNLR.G
 5.7	48	-0.0324	R.RGDSTHR.R
 5.6	49	-1.0780	R.NEGGVVVR.F
 5.3	53	0.9992	R.DRDHER.R
 5.1	55	0.9126	R.EPLAEIR.A
 4.7	60	0.8286 217	P62917|RL8_HUMAN R.KVGLIAAR.R
 3.7	76	0.8398	R.IILEALR.Q

Top scoring peptide matches to query 1826
File7043 Spectrum8947 scans: 7043
Score greater than 35 indicates identity

**Score	Expect	Delta Hit Protein	Peptide**
 29.6	0.2	-0.0051	10	Q00610|CLH1_HUMAN R.AVNYFSK.V
 21.4	1.3	-0.0487	R.AVNQAIGR.V
 17.0	3.5	-0.0375	K.LGNNPVSK.G
 13.7	7.6	-0.0640	R.IGAFRHK.L
 11.4	13	-0.0051	R.IGNSYFK.E
 11.2	14	-0.0375	K.AVAEPVSR.L
 11.1	14	-0.0487	K.AVQQVQR.L
 11.1	14	-0.0851	R.AVAVAKNR.K
 9.2	21	-0.0813	K.GLAMVPLK.F
 9.2	21	-0.0739	K.ANVGLNLK.F

Top scoring peptide matches to query 1829
File7043 Spectrum12587 scans: 10125
Score greater than 23 indicates homology
Score greater than 35 indicates identity

**Score	Expect	Delta Hit Protein	Peptide**
 12.1	11	1.0593	4	P98164|LRP2_HUMAN R.DNVPNLR.G
 9.3	21	-0.0338	R.EAKLVIR.W
 8.8	24	0.9502	K.VGVGKVIR.G
 6.6	39	0.9978	R.TEPIVLR.L
 5.8	47	0.9978	K.DKNILPK.V
 5.8	47	1.0342	K.DQNILPK.Y
 5.6	49	0.0026	R.VKTLPDR.T
 5.6	49	0.9614	K.VIEVVIR.S
 5.2	54	0.0455	K.KAIMHGR.G
 5.0	56	1.0481	K.RATHSAGK.R

Top scoring peptide matches to query 1830
File7043 Spectrum12604 scans: 10139
Score greater than 23 indicates homology
Score greater than 35 indicates identity

**Score	Expect	Delta Hit Protein	Peptide**
 15.1	5.5	-0.0045	R.MVVPAALK.V
 9.2	22	0.0571	K.MVEKHGK.G
 6.2	43	-0.9560	R.TTQLGPGR.F
 4.9	58	0.9506	K.KGLVALAR.Q
 4.6	62	-0.9382	K.KEMHLR.S
 3.9	72	-0.8906	K.MLDHEGK.E
 3.9	72	1.0233	K.QGPGLLSR.M
 3.8	74	1.0961	R.NDHGGTVK.K
 3.2	86	-0.8364	K.MNDFMR.M
 3.1	87	1.0597	4	P98164|LRP2_HUMAN R.DNVPNLR.G

Top scoring peptide matches to query 1832
File7043 Spectrum10915 scans: 8665
Score greater than 21 indicates homology
Score greater than 35 indicates identity

**Score	Expect	Delta Hit Protein	Peptide**
 10.8	15	1.1057	K.QLTMYR.V
 7.4	33	-0.9501	R.RIEQAGR.K
 7.4	33	-0.9501	K.RLEQQR.K
 7.4	33	-0.9501	R.RLQEGAR.K
 7.4	33	-0.9501	RLQEQR
 6.9	36	-0.9654	R.RLWGAAR.K
 6.9	37	0.0200	R.IPTLQTR.G
 6.2	43	0.0299	K.QLRHFK.F
 5.0	56	-0.8623 151	P61006|RAB8A_HUMAN K.FMETSAK.A
 4.6	61	-1.0004	K.KLEQALK.D

Top scoring peptide matches to query 1833
File7043 Spectrum10738 scans: 8519
Score greater than 25 indicates homology
Score greater than 35 indicates identity

**Score	Expect	Delta Hit Protein	Peptide**
 16.6	3.9	-0.8619 151	P61006|RAB8A_HUMAN K.FMETSAK.A
 11.4	13	0.0639	K.LIEYYK.N
 8.9	23	0.1398	R.AMMNAFK.E
 7.3	33	0.0203	R.KPIEVSR.E
 6.4	41	0.1585	R.YMNEQK.D
 6.1	44	-0.9749	R.AQLLAASR.E
 5.9	46	-0.0048	R.LLVELNK.I
 5.9	46	0.0679	R.EPVDLQK.K
 5.9	46	0.0679	K.EPVLQDK.R
 5.3	53	0.0745	K.GHQMLVK.M

Top scoring peptide matches to query 1836
File7043 Spectrum4368 scans: 3409
Score greater than 17 indicates homology
Score greater than 34 indicates identity

**Score	Expect	Delta Hit Protein	Peptide**
 13.6	5.6	0.3714	R.VARVDLR.K
 4.0	51	0.4805 723	Q8NBS9|TXND5_HUMAN K.VSEHSGGR.D
 2.4	75	0.4844	K.VSSMGFGK.D
 2.3	76	0.4078	R.QPTAKQR.V
 2.1	80	-0.5915	K.QYLHLR.A
 2.0	81	0.4078	R.ALGGVNAAR.R
 1.9	84	0.4554	R.ATPSPEAR.V
 0.7 1.1e+002	-0.6239	K.GAAVIRSR.E
 0.1 1.3e+002	0.4513	K.VFSFGSGK.D
 0.1 1.3e+002	-0.6239	R.AGLRLGSR.A

Top scoring peptide matches to query 1838
File7043 Spectrum1591 scans: 1204
Score greater than 21 indicates homology
Score greater than 34 indicates identity

**Score	Expect	Delta Hit Protein	Peptide**
 15.6	3.6	0.6481	K.HATNVMR.S
 7.5	23	0.6415	68+ O60814|H2B1K_HUMAN K.HAVSEGTK.A
 5.7	35	-0.3975	R.LLMPSPR.G
 5.5	36	0.6957	R.HADNMPK.K
 5.5	36	0.6593	K.HAMEVNK.H
 4.3	48	0.6892	K.QYDTSSK.T
 2.1	80	0.6845	R.QHMQER.R
 0.9	1e+002	0.6052	R.KELPGER.K
 0.8 1.1e+002	0.6052	K.ESLVPQR.H
 0.4 1.2e+002	0.6303	R.SGAGGPLGGR.G

Top scoring peptide matches to query 1839
File7043 Spectrum1949 scans: 1487
Score greater than 30 indicates homology
Score greater than 34 indicates identity

**Score	Expect	Delta Hit Protein	Peptide**
 20.7	1.1	0.6416	68+ O60814|H2B1K_HUMAN K.HAVSEGTK.A
 16.3	3	0.6481	K.HATNVMR.S
 7.4	24	0.6958	R.HADNMPK.K
 6.7	28	-0.3788	K.QPSLEQK.E
 6.0	32	0.6594	K.HAMEVNK.H
 5.6	35	0.5073	R.LLLIATGK.R
 5.5	36	-0.4516	K.VAGLLTQK.L
 4.0	51	0.6706	K.YSGNLMK.E
 3.1	62	0.5437	K.TPKLIEK.L
 3.1	63	0.6845	R.QHMQER.R

Top scoring peptide matches to query 1840
File7043 Spectrum309 scans: 352
Score greater than 18 indicates homology
Score greater than 34 indicates identity

**Score	Expect	Delta Hit Protein	Peptide**
 9.2	15	0.6426	68+ O60814|H2B1K_HUMAN K.HAVSEGTK.A
 5.3	38	-0.4142	K.LVGGTTPGK.G
 4.7	44	0.5698	K.LLTPQTR.L
 4.3	48	-0.3778	K.SSGPVPSAK.G
 3.9	53	-0.4506	R.TTVKVPGK.R
 3.9	53	0.6565	R.RGDSTHR.R
 3.6	56	0.6790	K.TFSSGSSR.T
 2.0	81	0.6426	R.TPTQEPR.E
 1.2	97	-0.3778	M.AEQLSPGK.A
 0.6 1.1e+002	0.5763	K.RVLMGPR.S

Top scoring peptide matches to query 1841
File7043 Spectrum2630 scans: 2031
Score greater than 21 indicates homology
Score greater than 36 indicates identity

**Score	Expect	Delta Hit Protein	Peptide**
 11.1	16	0.9798	K.HATNVMR.S
 8.2	31	0.9369	K.RAVPEEK.V
 5.4	60	0.9621	K.EGRQGPGK.Q
 4.8	68	0.9733	68+ O60814|H2B1K_HUMAN K.HAVSEGTK.A
 4.5	74	0.9183	R.INVPMVR.K
 4.4	75	1.0274	R.HADNMPK.K
 4.4	75	0.9944	QSFQYR
 4.2	78	0.9468	K.HQQLFR.N
 4.1	80	-0.0835 111	P21397|AOFA_HUMAN R.EVLNGLGK.V
 4.1	80	-0.9995	K.HGRGMEK.S

Top scoring peptide matches to query 1843
File7043 Spectrum2431 scans: 1887
Score greater than 22 indicates homology
Score greater than 36 indicates identity

**Score	Expect	Delta Hit Protein	Peptide**
 11.1	16	1.0020	68+ O60814|H2B1K_HUMAN K.HAVSEGTK.A
 8.8	27	-0.9708	K.HGRGMEK.S
 8.2	31	-0.9595	K.HQAAMEK.E
 7.9	34	1.0085	K.HATNVMR.S
 3.9	84	0.9470	R.INVPMVR.K
 2.6 1.1e+002	0.0613	R.QMIMMK.K
 2.2 1.2e+002	-0.0912	K.LIKEAAGK.S
 2.0 1.3e+002	0.8928	K.LIQSIVR.Q
 2.0 1.3e+002	0.8564	K.LLKTVVR.Q
 1.5 1.5e+002	0.9292	40	O95831|AIFM1_HUMAN K.VVQLDVR.D

Top scoring peptide matches to query 1844
File7043 Spectrum2267 scans: 1753
Score greater than 30 indicates homology
Score greater than 36 indicates identity

**Score	Expect	Delta Hit Protein	Peptide**
 23.4	0.95	1.0029	68+ O60814|H2B1K_HUMAN K.HAVSEGTK.A
 15.8	5.5	1.0095	K.HATNVMR.S
 8.3	31	1.0571	R.HADNMPK.K
 8.3	31	-0.9586	K.HNMADVK.E
 7.3	39	1.0207	K.HAMEVNK.H
 6.8	43	0.9666	K.NAIPQTGK.I
 6.7	44	-0.9698	K.HGRGMEK.S
 6.7	44	-0.9950	K.IPEGCVR.R
 6.2	50	-0.9585	K.HQAAMEK.E
 6.0	52	-0.9950	-.MDKHGVK.T

Top scoring peptide matches to query 1845
File7043 Spectrum7021 scans: 5492
Score greater than 20 indicates homology
Score greater than 36 indicates identity

**Score	Expect	Delta Hit Protein	Peptide**
 9.1	26	-0.9524	R.SYGGQYR.C
 6.7	44	0.9758	K.LKSHGMR.R
 5.1	64	-0.0082	R.IHGSMLR.A
 4.2	79	1.0268	R.GAYASFGR.D
 4.1	80	0.9693	R.AIGSVPER.S
 3.5	91	1.0412	K.MFKSCR.R
 3.4	94	0.8965 391	P12956|KU70_HUMAN K.AIVEKLR.F
 3.0	1e+002	-0.0333	R.LLMPSPR.G
 2.8 1.1e+002	1.1106	K.MNTMCR.S
 2.5 1.1e+002	0.9217	R.ALAERLR.L

Top scoring peptide matches to query 1846
File7043 Spectrum3907 scans: 3043
Score greater than 26 indicates homology
Score greater than 36 indicates identity

**Score	Expect	Delta Hit Protein	Peptide**
 19.8	2.1	-0.0061 186	O95292|VAPB_HUMAN R.LQGEVQR.L
 12.6	11	-0.0061	K.IQGDLAGR.A
 11.5	15	0.0051	K.LQGEAPSK.T
 8.4	30	-0.0577	K.LKWLNR.T
 8.4	30	-0.0789	R.LGSVAVKR.E
 6.4	47	-0.9901	R.QLGDLER.A
 6.2	50	-0.9901	K.LQGEIDR.C
 5.8	54	-1.0305	K.VEGVWLK.D
 5.4	59	-0.0676	K.LEAQLKK.A
 5.4	59	-0.0537	K.IQGRLSR.T

Top scoring peptide matches to query 1854
File7043 Spectrum721 scans: 621
Score greater than 22 indicates homology
Score greater than 36 indicates identity

**Score	Expect	Delta Hit Protein	Peptide**
 10.5	18	0.9808	R.QPTAKQR.V
 10.0	21	1.0350	K.HATNVMR.S
 9.3	24	-1.0123	K.KPTVEEK.A
 7.1	40	-1.0348	R.RPTTTVR.T
 6.0	52	0.9557	K.VLNLDVR.N
 6.0	52	0.9557	40	O95831|AIFM1_HUMAN K.VVQLDVR.D
 4.3	77	1.0574	K.VSSMGFGK.D
 4.1	79	0.9734	R.INVPMVR.K
 3.5	91	-0.9695	-.MDKHGVK.T
 3.5	91	-0.9946	K.TQPPLMK.R

Top scoring peptide matches to query 1855
File7043 Spectrum515 scans: 486
Score greater than 21 indicates homology
Score greater than 36 indicates identity

**Score	Expect	Delta Hit Protein	Peptide**
 8.7	28	1.0363	K.HATNVMR.S
 8.2	31	0.9934	R.SVLNNPGK.R
 7.6	35	-0.9027	K.QMMGGFK.E
 7.2	39	-0.9317	K.AEHAMQK.R
 6.1	51	-0.9430	K.HGRGMEK.S
 6.0	51	1.0913	R.HGGGGGDSGK.I
 5.8	54	-0.9317	K.HQAAMEK.E
 5.8	54	-0.0535	R.KLYIHR.V
 5.7	56	1.0298	68+ O60814|H2B1K_HUMAN K.HAVSEGTK.A
 5.5	57	0.0892	R.QMIMMK.K

Top scoring peptide matches to query 1856
File7043 Spectrum11153 scans: 8867
Score greater than 25 indicates homology
Score greater than 36 indicates identity

**Score	Expect	Delta Hit Protein	Peptide**
 13.9	8.4	-1.0576	K.TVGVIVSR.E
 12.0	13	-1.0324	R.RTVTPTR.K
 12.0	13	-1.0575 221	Q9UJS0|CMC2_HUMAN R.VSALSVVR.D
 11.6	14	0.9469 737	Q9NZM1|MYOF_HUMAN R.VRVIEGR.Q
 11.3	15	0.9469	R.VRTVPTR.K
 10.7	17	-0.0120	K.REAANLR.H
 10.6	18	0.9646	K.RVLMGPR.S
 10.2	19	-1.0324	K.RTEVGLR.I
 10.1	20	-0.0623	R.KSLGSLPK.V
 10.0	21	-1.0211	K.EQTLALR.K

Top scoring peptide matches to query 1857
File7043 Spectrum6775 scans: 5299
Score greater than 32 indicates homology
Score greater than 36 indicates identity

**Score	Expect	Delta Hit Protein	Peptide**
 30.8	0.17	0.0516 228	Q9H8H3|MET7A_HUMAN R.HLQFER.F
 17.2	3.9	0.0516	R.HLAADFR.H
 16.6	4.4	0.0847	K.LHQMER.E
 16.6	4.4	0.0265 255	P21810|PGS1_HUMAN R.YSKLYR.L
 15.8	5.5	-0.0211	K.HLPPLPR.T
 11.7	14	-0.0211	K.HKIYLR.K
 9.2	25	-0.0171	K.RLAQSVR.N
 8.7	27	0.0483	R.IHGSMLR.A
 8.7	27	0.0153	K.LHYQLR.N
 8.3	30	0.0442	R.HLMVWK.V

Top scoring peptide matches to query 1860
File7043 Spectrum17152 scans: 14294
Score greater than 24 indicates homology
Score greater than 36 indicates identity

**Score	Expect	Delta Hit Protein	Peptide**
 11.3	15	1.0562	R.TAQPQKR.E
 10.8	17	-0.9846	R.LVSISVGR.E
 9.3	24	1.0886	K.EGLPWAR.K
 9.0	26	1.1654	K.GDQSEHR.L
 9.0	26	1.1137	K.YHQPQR.A
 9.0	26	0.9834	R.LTRAVLR.D
 8.1	31	-1.0210	K.LTLSKIR.S
 8.1	32	1.0488 690	Q8WUY1|CH055_HUMAN R.LPCAVLR.A
 8.1	32	-0.9127	R.APMPMKR.G
 8.1	32	1.0562	R.LADPTRR.T

Top scoring peptide matches to query 1861
File7043 Spectrum5027 scans: 3926
Score greater than 18 indicates homology
Score greater than 34 indicates identity

**Score	Expect	Delta Hit Protein	Peptide**
 6.4	33	0.6471 561	P98160|PGBM_HUMAN K.RGGSLPSR.H
 4.7	50	0.7159	SFDAHPR
 1.9	95	0.6795	R.RYTAYR.Q
 0.8 1.2e+002	0.6359	K.RPSSARR.H
 0.7 1.2e+002	0.6649	-.MAAAGPRR.S
 0.5 1.3e+002	0.6510	R.EMPALIR.M
 0.5 1.3e+002	0.6874	EMPSLPR
 0.4 1.3e+002	0.6795	R.GGWGISPR.G
 0.1 1.4e+002	0.6696	R.EDLPSLR.K
 0.1 1.4e+002	0.6696	K.EDLPVTR.K

Top scoring peptide matches to query 1863
File7043 Spectrum8244 scans: 6473
Score greater than 36 indicates identity

**Score	Expect	Delta Hit Protein	Peptide**
 24.0	0.87	0.9987 446+ P61026|RAB10_HUMAN FFETSAK
 24.0	0.87	1.0317 151	P61006|RAB8A_HUMAN K.FMETSAK.A
 21.9	1.4	0.9953	K.YIMTSAK.L
 8.8	29	0.9801	K.FFEMKK.G
 6.2	52	0.9987	R.YLENYK.C
 4.4	80	0.0542 267	Q12955|ANK3_HUMAN K.MFEMTR.S
 3.4	99	1.0317	K.ATDMTFK.A
 3.2	1e+002	0.8936	R.KPSLVASK.L
 3.2	1e+002	0.8572	K.LKAIVASK.A
 2.2 1.3e+002	0.0728	R.GSCTYDK.V

Top scoring peptide matches to query 1865
File7043 Spectrum9388 scans: 7401
Score greater than 33 indicates homology
Score greater than 36 indicates identity

**Score	Expect	Delta Hit Protein	Peptide**
 22.2	1.3	0.9682	R.QVDIARK.R
 21.5	1.5	0.9390	K.YPPIVIK.S
 19.1	2.7	0.9067 459	Q12931|TRAP1_HUMAN R.KVLIQTK.A
 19.1	2.7	0.9682	R.QVLQVSR.Q
 19.0	2.7	-0.0158	K.EVEVAKR.N
 16.6	4.7	-0.0158	K.DILDKAR.A
 16.6	4.7	0.0206	K.DLLDQAR.S
 16.1	5.3	-0.0158	R.EVIINSR.F
 16.1	5.3	-0.0410	K.EVLTIQK.Q
 15.7	5.9	0.9794	K.QVNEVLK.W

Top scoring peptide matches to query 1868
File7043 Spectrum13349 scans: 10797
Score greater than 21 indicates homology
Score greater than 36 indicates identity

**Score	Expect	Delta Hit Protein	Peptide**
 9.6	24	1.0523	K.YKEAYR.K
 7.8	36	1.0338	R.GAGAGGRQR.G
 4.5	77	1.0410	R.GGWGISPR.G
 3.3	1e+002	1.0700	K.LCFSFR.L
 2.8 1.1e+002	0.9835	K.GQDLLKR.V
 2.5 1.2e+002	0.9934	R.AVHLPHR.A
 2.2 1.3e+002	-0.9382	R.DWLNQR.N
 2.1 1.3e+002	-0.9382	R.EYNLHR.V
 2.1 1.3e+002	-1.0223 437	O94760|DDAH1_HUMAN K.GHVLLHR.T
 2.1 1.3e+002	0.0999	K.HHFMSR.F

Top scoring peptide matches to query 1871
File7043 Spectrum8710 scans: 6845
Score greater than 18 indicates homology
Score greater than 36 indicates identity

**Score	Expect	Delta Hit Protein	Peptide**
 5.8	57	0.1063	K.GNAAGGGGGGR.S
 5.5	61	-0.9644	K.TDVILDR.K
 4.8	73	0.0626	R.IECPRR.L
 4.6	76	1.0288	K.VNTSPRR.K
 4.2	82	-0.9141	R.NQGQTAGR.A
 3.7	93	-0.9505	K.VSERAGGR.R
 2.3 1.3e+002	1.0466	R.CPGALRR.L
 1.8 1.4e+002	0.0448	R.EEGALRR.A
 0.9 1.8e+002	0.0196	K.DVKEALR.K
 0.5	2e+002	0.0196 267	Q12955|ANK3_HUMAN R.VTGGLLDR.L

Top scoring peptide matches to query 1875
File7043 Spectrum3028 scans: 2339
Score greater than 20 indicates homology
Score greater than 34 indicates identity

**Score	Expect	Delta Hit Protein	Peptide**
 10.4	13	-0.3141	R.DSYGSFR.E
 7.0	30	-0.2885	R.KMYGDMA.-
 4.1	58	0.5760	K.KPAQTASK.L
 4.0	59	0.6955	K.QMMGGFK.E
 3.0	75	0.6739	R.THADSSGR.L
 2.0	93	-0.4444	R.GLDALSKK.A
 1.6	1e+002	0.6740 334	Q5RI15|FA36A_HUMAN R.KHNGSSSN.-
 1.1 1.2e+002	0.5972	R.QPPLYGR.D
 0.9 1.2e+002	0.5760	R.KLLDEGR.S
 0.9 1.2e+002	0.5574	K.KMTVPVR.R

Top scoring peptide matches to query 1876
File7043 Spectrum3640 scans: 2823
Score greater than 26 indicates homology
Score greater than 36 indicates identity

**Score	Expect	Delta Hit Protein	Peptide**
 14.1	8.6	-1.0410	R.GLLNYPR.V
 13.2	11	-1.0556	M.AAVRMLR.T
 11.8	15	0.9600 544	P07197|NFM_HUMAN K.RSMLAPR.L
 11.7	15	0.9422	R.RTAVDLR.G
 11.4	16	-0.0207	R.GPPGPPGPR.G
 11.0	17	1.0215	R.RGGCPQR.C
 10.3	21	0.9422	K.RTEVGLR.I
 10.3	21	0.9422	R.RTGEVLR.I
 8.0	35	0.9348	R.CVIAVIR.H
 7.7	37	1.0287	K.QWMPPR.S

Top scoring peptide matches to query 1878
File7043 Spectrum8247 scans: 6476
Score greater than 20 indicates homology
Score greater than 36 indicates identity

**Score	Expect	Delta Hit Protein	Peptide**
 11.0	17	1.0039	K.MFYLLK.T
 6.6	49	1.0985 267	Q12955|ANK3_HUMAN K.MFEMTR.S
 5.2	67	-0.9832	R.FFSYLR.S
 3.4	1e+002	-0.0050 388	P62701|RS4X_HUMAN R.LTIAEER.D
 2.2 1.3e+002	-0.8669	R.CEHCAR.F
 2.2 1.3e+002	1.1428	R.MMMEEK.I
 2.1 1.4e+002	0.9790	R.TGEALLAR.V
 1.9 1.4e+002	0.9790	K.TSVPTGLR.L
 1.9 1.4e+002	0.9637	R.THGLFKK.L
 1.9 1.4e+002	0.9749	K.TPPPPPPK.T

Top scoring peptide matches to query 1884
File7043 Spectrum4584 scans: 3574
Score greater than 27 indicates homology
Score greater than 36 indicates identity

**Score	Expect	Delta Hit Protein	Peptide**
 15.8	5.9	0.9760	K.KLEVLTK.E
 15.8	5.9	1.0012	K.KVQNITK.E
 15.8	5.9	1.0124	K.QEVIITK.F
 13.3	10	1.0991	R.GAAAQAGER.E
 13.0	11	1.0012	K.RELALTK.E
 11.4	16	1.0012	R.ERLAITK.E
 9.8	23	-0.9239	R.ALLEACR.L
 8.0	35	0.0787	K.AKAEEQR.A
 8.0	35	0.0787 650	Q96AG4|LRC59_HUMAN R.AKEAQER.E
 8.0	35	0.0172	K.AQSLAITK.Q

Top scoring peptide matches to query 1887
File7043 Spectrum3441 scans: 2670
Score greater than 22 indicates homology
Score greater than 36 indicates identity

**Score	Expect	Delta Hit Protein	Peptide**
 10.1	21	0.0537	M.ASSDVKPK.S
 9.1	27	0.1040	R.GADRDAAR.N
 8.6	31	1.0918	K.MAPATAPR.R
 8.0	35	1.0191 504	O95470|SGPL1_HUMAN K.MPIIGRK.I
 7.8	37	0.0425	R.RDALSAAK.E
 7.5	39	0.0788	R.KDLNDAR.D
 7.4	40	1.0700	K.FPGDVAPK.T
 6.4	51	1.0840	R.QVWQNR.F
 6.0	55	0.0537	K.ETGALAAAK.V
 5.6	60	1.0741 508	Q8TBA6|GOGA5_HUMAN K.AEDLLNR.V

Top scoring peptide matches to query 1889
File7043 Spectrum3235 scans: 2508
Score greater than 24 indicates homology
Score greater than 36 indicates identity

**Score	Expect	Delta Hit Protein	Peptide**
 15.9	5.6	1.0495	R.SLDITPGK.N
 10.1	21	0.0430	K.VSVGGEKR.D
 7.2	42	1.0958	EIFHER
 7.2	42	1.0747 508	Q8TBA6|GOGA5_HUMAN K.AEDLLNR.V
 7.2	42	1.0270	K.LSDLRAR.L
 7.2	42	1.0019	R.LSDVLKR.K
 7.2	42	1.0270	R.SLDARLR.A
 7.2	42	1.0270	K.SLDLARR.A
 6.3	52	1.1362	R.THADSSGR.L
 6.0	56	0.1764	R.HCQACR.L

Top scoring peptide matches to query 1896
File7043 Spectrum2616 scans: 2022
Score greater than 25 indicates homology
Score greater than 36 indicates identity

**Score	Expect	Delta Hit Protein	Peptide**
 14.4	7.9	0.0799 520	P05106|ITB3_HUMAN R.KLTSNLR.I
 11.9	14	1.1327	R.AGLYPGPR.E
 10.1	21	1.1116	R.QLTAELR.E
 9.8	23	0.1664	R.FMKFSR.V
 9.8	23	0.1163	R.QITDKAR.E
 9.1	27	1.1843	R.GEEDPRK.Q
 7.7	37	1.0752	R.QIISTIR.T
 6.4	50	1.1293	R.QLMVPAR.D
 5.9	55	1.1657	R.IPMEGQR.D
 5.8	57	1.1116	K.EGVLEKR.S

Top scoring peptide matches to query 1898
File7043 Spectrum29206 scans: 25937
Score greater than 25 indicates homology
Score greater than 36 indicates identity

**Score	Expect	Delta Hit Protein	Peptide**
 13.8	8.8	0.1252 533	Q3LXA3|DHAK_HUMAN R.MALVLER.V
 13.6	9.3	1.1166	R.LERSGIR.T
 11.6	15	1.1278	R.GVELEKR.L
 11.6	15	1.1530	K.REIQER.D
 11.6	15	1.1530	K.RELQER.E
 11.6	15	1.1166	R.RELSLGR.K
 11.6	15	1.1530	R.RLEDAAR.Q
 11.6	15	1.1530	RLEEQR
 11.6	15	1.1530	RLEQER
 11.6	15	-0.8515	K.SAEITGVR.S

Top scoring peptide matches to query 1904
File7043 Spectrum6956 scans: 5444
Score greater than 17 indicates homology
Score greater than 36 indicates identity

**Score	Expect	Delta Hit Protein	Peptide**
 8.9	27	1.0366	K.DHYIER.G
 4.2	79	0.9486 247	P27824|CALX_HUMAN K.LHFIFR.H
 3.9	84	1.0002	R.LSFSHNK.I
 2.5 1.2e+002	0.9856	R.KGLQCAR.L
 0.2	2e+002	0.0492	K.DAAPACTK.K
 0.2	2e+002	0.9856	R.RPMTTAR.G

Top scoring peptide matches to query 1907
File7043 Spectrum29404 scans: 26100
Score greater than 24 indicates homology
Score greater than 36 indicates identity

**Score	Expect	Delta Hit Protein	Peptide**
 16.1	5.1	-0.9541	K.EMAAQIR.E
 10.1	20	1.0438	R.DDVSAGLR.G
 8.8	27	0.9962	K.RGTETLR.Q
 7.9	34	1.0616	R.NMAPDLR.Q
 7.9	34	-0.9732 645	P19022|CADH2_HUMAN K.HSGHLQR.Q
 7.8	34	0.0187 177	P20648|ATP4A_HUMAN K.CRTAGIR.V
 7.5	36	1.0074	K.ETEGKLR.N
 7.5	36	1.0252	K.VMADQLR.K
 7.5	37	-0.9905	-.MTGEKIR.S
 7.2	40	1.0099	K.MVWLQR.R

Top scoring peptide matches to query 1908
File7043 Spectrum13944 scans: 11322
Score greater than 23 indicates homology
Score greater than 36 indicates identity

**Score	Expect	Delta Hit Protein	Peptide**
 12.7	11	0.9829	M.AAVRMLR.T
 12.7	11	-0.0011	33	P14625|ENPL_HUMAN R.IERMLR.L
 12.7	11	-0.0341	R.LERFLR.A
 12.7	11	0.9829	R.LQRMIR.Y
 12.3	12	0.0313	R.VMEWLR.S
 12.0	13	0.0135	R.VPDSLFR.S
 9.5	23	-0.9665	K.DKTDSLR.E
 9.5	23	0.0102	K.ELMTALR.E
 9.5	23	-1.0181	K.KDVFGLR.R
 9.5	23	0.9248	R.KLFGVLR.L

Top scoring peptide matches to query 1911
File7043 Spectrum4364 scans: 3407
Score greater than 25 indicates homology
Score greater than 36 indicates identity

**Score	Expect	Delta Hit Protein	Peptide**
 14.2	7.8	1.0629	R.SALEEQR.L
 11.4	15	1.0516	K.RLESGDR.I
 10.9	17	-1.0157	R.QLAPHLR.V
 10.5	18	1.0265	R.AISEKER.M
 10.5	18	1.0880	R.DREQER.R
 10.5	18	-0.9164	K.EGSEKER.H
 10.5	18	-0.9277	R.GSEERTR.R
 10.5	18	0.0676 705	Q9Y3D6|FIS1_HUMAN K.GSKEEQR.D
 10.5	18	0.0676	R.KGSEGAER.L
 10.5	18	0.0676	R.KSGEEQR.S

Top scoring peptide matches to query 1917
File7043 Spectrum910 scans: 753
Score greater than 21 indicates homology
Score greater than 36 indicates identity

**Score	Expect	Delta Hit Protein	Peptide**
 10.4	19	1.0848	K.EWGDGIR.G
 8.1	32	1.0484	K.WEDVKR.-
 7.9	33	1.0484	R.QWLNSGK.I
 7.8	34	0.0134 484	P11498|PYC_HUMAN K.KVMVANR.G
 7.7	35	1.0273	R.EKSLEAR.A
 7.5	37	1.0636	M.VTEEAGAR.V
 7.0	41	1.1000	K.EGNEDIR.E
 6.7	45	1.0888	K.EGAGGGAGTR.A
 6.1	51	1.0120	K.SWGLTLR.C
 5.9	53	-0.9924	K.QEGILFK.D

Top scoring peptide matches to query 1921
File7043 Spectrum3026 scans: 2338
Score greater than 32 indicates homology
Score greater than 36 indicates identity

**Score	Expect	Delta Hit Protein	Peptide**
 31.3	0.15	1.0739	R.STFAGHGR.Y
 17.9	3.4	0.0865	R.NCIVDGR.F
 16.0	5.2	-0.9590	K.MKEPTTK.A
 15.2	6.3	0.0688	K.ENKNSNK.F
 15.2	6.3	1.0752	R.EPGSISDK.F
 10.8	17	1.0341	R.KGLQCAR.L
 9.5	23	0.0250	R.IVELMGR.E
 9.5	23	0.0389	22	O60494|CUBN_HUMAN R.LARTCGR.E
 9.5	23	0.0250	ELMIVGR
 9.5	23	0.9396	R.IKFLVGR.D

Top scoring peptide matches to query 1928
File7043 Spectrum9409 scans: 7418
Score greater than 22 indicates homology
Score greater than 35 indicates identity

**Score	Expect	Delta Hit Protein	Peptide**
 11.5	14	1.0879	R.GCSQPER.V
 9.3	23	1.0086 547	O00291|HIP1_HUMAN R.SLSEIER.K
 9.1	24	1.0814	R.EAEEAER.E
 8.6	27	-0.9390	K.SAGCRER.D
 8.6	27	-0.9277	R.SQMANER.I
 7.9	32	0.0199	R.TLRCER.H
 6.8	41	1.0086	R.EVTVTER.L
 5.9	50	1.0185	R.KNWTER.W
 5.8	52	1.0450	R.SEAEIER.E
 5.6	54	0.9610	R.KSSVSGIR.I

Top scoring peptide matches to query 1929
File7043 Spectrum10208 scans: 8081
Score greater than 33 indicates homology
Score greater than 35 indicates identity

**Score	Expect	Delta Hit Protein	Peptide**
 25.3	0.58	-0.0080 391	P12956|KU70_HUMAN K.MKAIVEK.L
 25.3	0.58	-0.0047 178	Q99623|PHB2_HUMAN R.AQFLVEK.A
 19.7	2.1	0.9760	K.MLNIAKK.I
 18.9	2.6	-0.9887	R.FAELVEK.L
 17.8	3.2	0.0284	R.KDMIAEK.L
 17.8	3.2	0.0647	K.QDMLAEK.V
 17.0	3.9	0.0059	K.MRALATR.M
 16.8	4.1	0.0317	K.YVPKAEQ.-
 16.7	4.2	-0.9557	-.MEAVIEK.E
 16.5	4.4	-0.9847	M.SSSLGKEK.D

Top scoring peptide matches to query 1938
File7043 Spectrum13519 scans: 10950
Score greater than 22 indicates homology
Score greater than 35 indicates identity

**Score	Expect	Delta Hit Protein	Peptide**
 10.4	18	-0.9849	K.SSSSSLLR.N
 9.1	24	0.0710	K.EMMPRR.L
 7.0	38	1.0307	R.ATSDKANK.L
 6.0	49	-0.9308	K.EMNISSR.D
 6.0	49	-0.9784	R.LMRASSR.S
 6.0	49	-0.9420	R.MERASSR.V
 5.9	49	-1.0227	K.RTGPHLR.F
 5.7	51	-1.0076 599	Q96BM9|ARL8A_HUMAN -.MIALFNK.L
 5.4	55	0.9757	K.MVLTRAK.A
 5.4	56	1.0233	K.ALNLEMK.R

Top scoring peptide matches to query 1939
File7043 Spectrum16423 scans: 13610
Score greater than 28 indicates homology
Score greater than 35 indicates identity

**Score	Expect	Delta Hit Protein	Peptide**
 17.4	3.5	-0.0052 391	P12956|KU70_HUMAN K.FTVPMLK.E
 14.6	6.6	0.9974	R.KFPDVTK.V
 14.4	7.1	1.0556	K.GACGLQTK.I
 14.2	7.2	0.9941	R.LDKMVTK.K
 13.4	8.9	1.0304	K.QMEVVTK.G
 12.9	9.8	0.0352	K.MTEIGIR.S
 11.5	14	0.0716	R.MTPSDLR.N
 11.2	14	1.0761	K.YWFYR.L
 10.2	18	0.0022	K.FTELVAR.R
 9.9	20	0.0199	-.MTLIWR.H

Top scoring peptide matches to query 1942
File7043 Spectrum1569 scans: 1190
Score greater than 23 indicates homology
Score greater than 35 indicates identity

**Score	Expect	Delta Hit Protein	Peptide**
 12.8	10	1.0414	M.WMTPKR.S
 12.3	11	-0.9590 746	Q96A26|F162A_HUMAN -.MGSLSGLR.L
 12.3	11	1.0389	K.SDIKESR.R
 9.7	21	0.9872	K.ASFLQLR.N
 9.6	21	0.9872	R.TPPGPPLR.L
 9.5	21	1.0380	R.ICCLLR.K
 9.5	21	1.0203	K.ISSCVLR.W
 9.5	21	1.0566	R.LACSDIR.F
 9.5	21	1.0414	R.LCWSLR.N
 9.5	21	1.0203	R.LNMATLR.M

Top scoring peptide matches to query 1944
File7043 Spectrum10220 scans: 8092
Score greater than 26 indicates homology
Score greater than 35 indicates identity

**Score	Expect	Delta Hit Protein	Peptide**
 18.8	2.5	0.9990 178	Q99623|PHB2_HUMAN R.AQFLVEK.A
 12.8	10	0.9956 391	P12956|KU70_HUMAN K.MKAIVEK.L
 12.4	11	0.0367	K.MTEIGIR.S
 10.3	18	1.0208	K.QAMKQTK.L
 10.0	19	1.0571	K.AQQMTQK.Y
 9.8	20	1.0935	R.AQAQCEK.R
 9.0	24	0.9990	R.KFPDVTK.V
 8.3	29	1.0459	R.QAMAKNR.L
 8.3	29	0.0619	R.EAMAGKGR.A
 8.2	29	0.9877	K.QAFSLLR.F

Top scoring peptide matches to query 1948
File7043 Spectrum2612 scans: 2018
Score greater than 22 indicates homology
Score greater than 35 indicates identity

**Score	Expect	Delta Hit Protein	Peptide**
 10.9	16	1.0893	K.DTTGTALR.I
 10.9	16	1.0344	K.GLMKTLR.K
 9.3	23	-0.8874	4	P98164|LRP2_HUMAN K.CAPGYLR.E
 8.9	25	1.1258	K.EGEKTDR.V
 8.8	25	-0.8722	-.MDGQTLR.K
 7.0	38	0.0867	K.MDISTLR.R
 7.0	38	0.0239	-.MLRFLR.R
 7.0	38	0.0933	-.MMERIR.K
 7.0	38	0.0933	R.MMRELR.N
 7.0	38	0.1231	R.MTEDALR.L

Top scoring peptide matches to query 1952
File7043 Spectrum6717 scans: 5252
Score greater than 24 indicates homology
Score greater than 35 indicates identity

**Score	Expect	Delta Hit Protein	Peptide**
 16.0	4.5	-0.0197	K.GKNQSMR.G
 10.1	17	0.9715	K.ALNMWGK.E
 9.7	19	-1.0116	R.DQGQHPR.A
 9.0	22	0.8334 737	Q9NZM1|MYOF_HUMAN R.LALHILR.T
 8.7	24	-1.0592	R.RSGQHPR.Y
 8.5	25	0.9286	K.IALEFDK.D
 7.2	33	0.9537	R.DDAVFIR.A
 6.8	37	0.9537	K.LAFAEER.N
 6.8	37	0.9690	R.LAGTSGSDK.G
 6.6	38	0.9537 513	Q9BS26|ERP44_HUMAN K.LAPSEYR.Y

Top scoring peptide matches to query 1965
File7043 Spectrum3801 scans: 2957
Score greater than 33 indicates homology
Score greater than 35 indicates identity

**Score	Expect	Delta Hit Protein	Peptide**
 20.2	1.6	0.9546	K.SSSSSLLR.N
 19.3	1.9	0.9393	R.QYKEIR.D
 18.4	2.4	0.9393	K.LYLGGASR.D
 18.3	2.5	-0.0447	R.FDSSIIR.I
 17.1	3.2	-0.0447	R.EYEKLR.D
 15.4	4.7	0.9393	K.QKYELR.V
 15.1	5.1	0.9723 746	Q96A26|F162A_HUMAN -.MGSLSGLR.L
 15.1	5.1	0.9393	R.YKEQLR.E
 15.1	5.1	0.9029	K.YKVTGLR.D
 12.9	8.6	0.8917	R.LQGHLLR.Q

Top scoring peptide matches to query 1966
File7043 Spectrum6580 scans: 5145
Score greater than 32 indicates homology
Score greater than 35 indicates identity

**Score	Expect	Delta Hit Protein	Peptide**
 22.5	0.92	-0.0047 715	P07305|H10_HUMAN K.GVGASGSFR.L
 17.9	2.6	1.0123	K.GKNQSMR.G
 11.0	13	-1.0186	R.AARMSFR.Q
 11.0	13	-1.0251	K.IAASSSFR.S
 11.0	13	-0.9921	K.KVSDSMR.T
 11.0	13	0.9429	R.LGRTSFR.N
 11.0	13	0.9178	R.LSKVSFR.G
 11.0	13	0.0031	R.LTVDSMR.R
 11.0	13	0.0429	R.TDPDSFR.V
 11.0	13	-1.0615	K.TKSLSFR.S

Top scoring peptide matches to query 1969
File7043 Spectrum8270 scans: 6495
Score greater than 22 indicates homology
Score greater than 35 indicates identity

**Score	Expect	Delta Hit Protein	Peptide**
 9.7	18	1.0516	K.WPSGHPR.F
 8.7	22	0.0464 715	P07305|H10_HUMAN K.GVGASGSFR.L
 8.5	23	-0.9740	R.FSLSASAR.L
 5.7	44	0.0365	K.KADSSTTK.K
 5.1	51	1.0747	K.SSGQMAQK.F
 5.1	51	1.0781	M.ESSPFNR.R
 4.7	56	1.0958	R.DPACFAR.G
 4.5	58	1.0417	R.DLVNGYR.C
 4.5	58	1.0053	K.IDKAAYR.K
 4.1	65	1.0569	R.TNSTSAQK.N

Top scoring peptide matches to query 1978
File7043 Spectrum2604 scans: 2013
Score greater than 26 indicates homology
Score greater than 34 indicates identity

**Score	Expect	Delta Hit Protein	Peptide**
 14.2	5.4	-0.8695	K.IALHTQR.Q
 13.9	5.8	-0.8219	K.IAASSSFR.S
 13.9	5.8	-0.9422	R.LALPRIR.K
 13.9	5.8	-0.9310	R.LAVLLPGR.H
 12.9	7.2	-0.8443	R.RQPGPQR.G
 9.9	14	0.0894	K.KPPLVQR.N
 8.9	18	-0.9310 497	P23229|ITA6_HUMAN R.LLLVGAPR.A
 8.0	22	0.0530	R.KKPPVIR.Q
 7.1	28	-0.8807	R.RLSAHVR.R
 6.9	29	-0.8041	R.IYCQVR.A

Top scoring peptide matches to query 1981
File7043 Spectrum30408 scans: 26909
Score greater than 17 indicates homology
Score greater than 34 indicates identity

**Score	Expect	Delta Hit Protein	Peptide**
 10.3	13	1.1101	R.LQGHLLR.Q
 4.0	56	1.1465	R.LHPAKDR.E
 3.3	65	0.2465 170	O00217|NDUS8_HUMAN R.YPSGEER.C
 3.1	69	0.2465	R.SDSFPER.R
 1.5	1e+002	1.1941	R.GPSYGLSR.E
 1.4	1e+002	0.2795	K.MQEEER.I
 1.3	1e+002	1.1942	K.IQYEQR.L
 1.1 1.1e+002	0.1513	K.NVHSKPR.T
 0.9 1.1e+002	-0.8440	K.GGHKGLNR.C

Top scoring peptide matches to query 1983
File7043 Spectrum2410 scans: 1869
Score greater than 19 indicates homology
Score greater than 34 indicates identity

**Score	Expect	Delta Hit Protein	Peptide**
 9.3	16	-0.7706	M.MAAMATAR.V
 6.0	35	1.1327	K.AGVIFTTK.K
 5.0	45	1.0487	R.IPLLPKR.W
 4.9	46	1.1909	K.VMSTVGSR.K
 4.7	48	1.1103	K.ALHNLLR.F
 3.7	60	1.1393	K.KCILFR.I
 3.5	63	1.1691	K.DSLINFK.E
 2.7	76	-0.7560	K.CYDPVGK.T
 2.6	78	0.1262	R.ITITHPR.R
 2.3	83	1.0487 267	Q12955|ANK3_HUMAN R.IIIPPRK.C

Top scoring peptide matches to query 1984
File7043 Spectrum29928 scans: 26531
Score greater than 23 indicates homology
Score greater than 34 indicates identity

**Score	Expect	Delta Hit Protein	Peptide**
 11.4	10	0.2069	R.LTVDSMR.R
 10.9	11	0.1917	R.IYPSAMR.I
 10.3	13	0.1739	R.FDSSIIR.I
 10.3	13	1.1580	R.NYASIIR.R
 10.3	13	1.1732	K.SSSSSLLR.N
 10.3	13	-0.8689	K.VNHSLLR.L
 5.5	40	-0.8074	R.AHPDSRR.A
 4.6	49	0.1991 715	P07305|H10_HUMAN K.GVGASGSFR.L
 4.0	56	0.2247	-.MEALMAR.G
 3.8	58	0.2247	K.LLECMR.K

Top scoring peptide matches to query 1995
File7043 Spectrum5888 scans: 4601
Score greater than 13 indicates homology
Score greater than 34 indicates identity

**Score	Expect	Delta Hit Protein	Peptide**
 8.5	21	0.0086	R.GMMKGGNK.Q
 8.5	21	-0.0058	80	O43451|MGA_HUMAN R.STFPSSGR.W
 7.1	30	0.0086	R.GMMKGGNK.Q
 0.1 1.5e+002	0.0054	K.TFEDAQK.S

Top scoring peptide matches to query 1997
File7043 Spectrum11358 scans: 9037
Score greater than 26 indicates homology
Score greater than 34 indicates identity

**Score	Expect	Delta Hit Protein	Peptide**
 13.8	6.4	-0.0812	R.IRPPAER.S
 12.8	8	-0.1791	K.ILKVIPR.D
 12.8	8	-0.1428	K.LIVAGLPR.F
 12.8	8	-0.1428 497	P23229|ITA6_HUMAN R.LLLVGAPR.A
 12.8	8	-0.1540	R.LLRAIPR.Q
 12.8	8	-0.1176	R.LPSRLPR.R
 12.7	8.1	-1.1129	R.GGARLPLR.R
 12.7	8.1	-0.0336	K.IAASSSFR.S
 12.7	8.1	-0.0812	K.IALHTQR.Q
 12.7	8.1	-0.1540	R.LALPRIR.K

Top scoring peptide matches to query 1998
File7043 Spectrum10186 scans: 8060
Score greater than 23 indicates homology
Score greater than 34 indicates identity

**Score	Expect	Delta Hit Protein	Peptide**
 22.9	0.78	-0.0049 434	P01857|IGHG1_HUMAN K.ALPAPIEK.T
 9.6	17	0.0090	R.ALPPRER.S
 6.6	33	0.1186	R.MACEALK.H
 6.6	33	0.0679	R.SQYEALK.E
 6.2	37	0.0342	R.HQIERR.R
 6.2	37	0.0342	R.HQLERR.K
 5.4	44	-0.0777	K.ALPVLVVK.L
 4.8	50	-0.0525	K.HKSILLK.N
 4.5	54	0.1181	R.GHSASHDK.D
 3.2	73	-0.0049	R.VLPPEVGK.L

Top scoring peptide matches to query 2000
File7043 Spectrum13785 scans: 11178
Score greater than 23 indicates homology
Score greater than 34 indicates identity

**Score	Expect	Delta Hit Protein	Peptide**
 22.6	0.82	-0.0056 497	P23229|ITA6_HUMAN R.LLLVGAPR.A
 9.0	19	1.0954	R.QSMTVKK.G
 8.1	23	0.0559	K.EGIKVHR.T
 8.0	24	-0.0168	R.LLLAPRR.G
 7.5	27	-0.9168 325	O15031|PLXB2_HUMAN K.LDIPEPR.R
 7.3	28	-0.0056	K.LLNPLIR.V
 7.1	29	1.0954	K.MAASSVKK.D
 6.2	36	-0.9896	K.LLDPLLR.I
 5.8	39	0.0559	K.NPPAGVRK.I
 5.5	42	1.0512	R.ILLGEHR.L

Top scoring peptide matches to query 2001
File7043 Spectrum4261 scans: 3330
Score greater than 19 indicates homology
Score greater than 32 indicates identity

**Score	Expect	Delta Hit Protein	Peptide**
 7.2	19	-0.6299 138	P06396|GELS_HUMAN K.MDAHPPR.L
 6.0	25	0.3983	-.MAPGMSGR.G
 4.1	39	0.3694	K.ERSMGSR.Q
 4.1	39	0.3289	R.RDFCIK.V
 3.9	41	-0.7092 325	O15031|PLXB2_HUMAN K.LDIPEPR.R
 3.7	42	0.3732	-.MAVEGGMK.C
 3.5	44	0.3806	-.MTNNSGSK.A
 3.5	44	0.3038	K.AKPYMTK.E
 3.5	45	0.2860	R.SLFLSGSK.R
 3.2	48	0.3476	K.GYASGVER.T

Top scoring peptide matches to query 2002
File7043 Spectrum17395 scans: 14522
Score greater than 27 indicates homology
Score greater than 32 indicates identity

**Score	Expect	Delta Hit Protein	Peptide**
 19.7	1.1	0.3514	R.GLVAEGHR.L
 13.7	4.2	0.3038	K.GRLSLHR.T
 10.9	8	0.3626	R.TYDGKVR.V
 8.2	15	-0.6802	M.PARTAPAR.V
 7.2	19	0.3804 644	O43175|SERA_HUMAN -.MAFANLR.K
 6.8	21	0.3402	K.GGHKGLNR.C
 5.9	26	0.3691	R.GPVLCHR.A
 5.8	26	0.4246	-.MAVEGGMK.C
 5.8	26	0.3514 268	P04003|C4BPA_HUMAN K.TPSGALHR.K
 5.7	27	0.3990	R.GLDSYQR.D

Top scoring peptide matches to query 2005
File7043 Spectrum5049 scans: 3943
Score greater than 25 indicates homology
Score greater than 34 indicates identity

**Score	Expect	Delta Hit Protein	Peptide**
 14.8	4.9	-0.0016	-.MMMVRR.G
 11.6	10	-0.0702	M.SRPPPTGK.M
 9.1	18	-0.9637	R.AGAGDFMR.Y
 6.6	32	0.9945	-.MATKESR.D
 6.1	36	0.9428 510	P00966|ASSY_HUMAN R.FIGMKSR.G
 6.0	37	0.9832	-.MRSSVSR.Q
 6.0	37	1.0374	-.MERCSR.C
 6.0	37	1.0334	R.MMQSWR.K
 5.7	40	1.0308	R.GSMGSLDR.L
 5.6	41	0.9363	K.ELGTKYK.Y

Top scoring peptide matches to query 2006
File7043 Spectrum7984 scans: 6264
Score greater than 23 indicates homology
Score greater than 34 indicates identity

**Score	Expect	Delta Hit Protein	Peptide**
 10.8	12	-1.0634	K.KPLPEEK.K
 9.7	16	-0.9729	K.FKEECK.S
 8.4	21	0.0475	K.QFGAECK.Y
 6.1	36	-1.0131	K.DVVSAHGR.E
 5.4	42	0.9951	R.IYCQVR.A
 5.4	42	0.8683	K.KPIPGAKK.L
 5.3	44	-1.1586 651	O94919|ENDD1_HUMAN K.VIRALLR.I
 5.3	44	-1.1474	K.VIVGALLR.S
 3.7	63	-1.0243	R.VSREHGR.T
 3.6	64	-0.0179	R.VGPNAEPR.F

Top scoring peptide matches to query 2008
File7043 Spectrum5275 scans: 4112
Score greater than 19 indicates homology
Score greater than 34 indicates identity

**Score	Expect	Delta Hit Protein	Peptide**
 7.1	29	-1.0326	R.GVVGPQGAR.G
 7.0	29	0.8852	K.KPIPGAKK.L
 6.9	30	-1.0802 344	Q8TCZ2|C99L2_HUMAN R.RKPGIGGR.E
 5.8	39	1.0087	K.KSMGMIR.K
 5.4	43	-1.0115 431	O43795|MYO1B_HUMAN R.RAGYAFR.Q
 5.2	45	0.9426	R.QFKIFR.E
 4.5	52	-0.9923	50	P29972|AQP1_HUMAN -.MASEFKK.K
 4.2	56	-0.9858	R.RCMVFK.E
 4.1	58	-1.0036	K.KSPPHMK.T
 3.1	72	0.9943	R.EAAYTKR.L

Top scoring peptide matches to query 2010
File7043 Spectrum2809 scans: 2163
Score greater than 22 indicates homology
Score greater than 34 indicates identity

**Score	Expect	Delta Hit Protein	Peptide**
 10.7	13	0.0695	R.DCGKGFR.Q
 9.1	18	-1.0638 344	Q8TCZ2|C99L2_HUMAN R.RKPGIGGR.E
 8.4	21	1.0470	R.TDSFVGGR.W
 6.1	36	0.9882	R.GLGGGHKGR.K
 6.0	37	0.9631	R.RLPAPER.E
 5.8	39	1.0800	R.IGMDGSSR.S
 5.8	39	0.9994	R.LYRGSSR.L
 5.1	46	1.0834 565	Q7Z7H5|TMED4_HUMAN K.EQDYQR.Y
 4.9	47	0.0332	K.KYVCGGR.F
 4.5	52	0.9379	R.AKPIPGQK.T

Top scoring peptide matches to query 2011
File7043 Spectrum4015 scans: 3135
Score greater than 18 indicates homology
Score greater than 34 indicates identity

**Score	Expect	Delta Hit Protein	Peptide**
 6.5	33	-0.1160	K.LKIPIGAK.I
 4.8	49	0.0030	R.WAHAQVK.R
 4.5	52	0.9772	R.GLEAIHAK.G
 4.5	52	-0.0069 232	P51572|BAP31_HUMAN R.LLEEHAK.L
 3.9	59	-1.0636	K.EQLLLPK.T
 3.1	72	-0.0796	R.KIIPSPGK.E
 3.0	74	0.0183	K.KASHDGPK.R
 2.6	81	-0.9037	-.MATMENK.V
 2.2	89	-0.0796	R.LQIIPGAK.H
 1.6	1e+002	-1.0174	K.RYGGFLK.R

Top scoring peptide matches to query 2012
File7043 Spectrum3993 scans: 3119
Score greater than 26 indicates homology
Score greater than 34 indicates identity

**Score	Expect	Delta Hit Protein	Peptide**
 20.5	1.3	0.9423	K.KPPLIDR.E
 12.1	9.1	-0.0781	K.LLPEVLR.D
 12.1	9.1	-0.0781	R.LLPQLQK.A
 10.4	13	1.0845	K.KDDMSAR.R
 10.2	14	0.0674	R.EPEEAHK.E
 9.3	17	-0.0053 232	P51572|BAP31_HUMAN R.LLEEHAK.L
 9.2	18	-0.1145	R.ILKPQLK.S
 9.0	18	0.1103	K.ENCGGFR.L
 8.0	23	0.0528	-.MSSGKSAR.Y
 7.5	26	-1.1210	R.ILLVRAR.E

Top scoring peptide matches to query 2013
File7043 Spectrum6488 scans: 5073
Score greater than 21 indicates homology
Score greater than 34 indicates identity

**Score	Expect	Delta Hit Protein	Peptide**
 9.7	16	-1.0786	K.VLLLGAVR.I
 7.8	25	1.0826	R.EAQTFSR.M
 7.8	25	1.0462	R.EATKYAR.W
 6.0	37	-1.0899	K.VILAIRR.A
 6.0	37	-1.0899 651	O94919|ENDD1_HUMAN K.VIRALLR.I
 6.0	37	-1.0786	K.VIVGALLR.S
 6.0	37	-1.0422 484	P11498|PYC_HUMAN	K.VLKDLPR.V
 6.0	37	-1.0899	R.VLLALRR.Y
 6.0	37	-1.0535	R.VLLRPSR.R
 6.0	37	-1.1150	R.VLLVKIR.S

Top scoring peptide matches to query 2018
File7043 Spectrum1909 scans: 1456
Score greater than 18 indicates homology
Score greater than 33 indicates identity

**Score	Expect	Delta Hit Protein	Peptide**
 6.3	29	-0.8414	R.RPVSPER.Y
 5.2	37	-0.8600	R.MPALPRR.E
 4.5	43	0.1903	R.ESPITHR.L
 2.5	70	1.1379	K.EGIKVHR.T
 2.2	75	-0.8414 153	P35749|MYH11_HUMAN K.ALDKTHR.Q
 2.2	75	0.1790	R.NSPKHTR.L
 2.1	75	1.1631	R.HAAEVRR.N
 1.4	90	1.1379	K.VNHSLLR.L
 0.9	99	0.0811	R.AAIPIAKR.C
 0.3 1.1e+002	-0.8488	-.MLLGGPPR.S

Top scoring peptide matches to query 2023
File7043 Spectrum13290 scans: 10747
Score greater than 22 indicates homology
Score greater than 32 indicates identity

**Score	Expect	Delta Hit Protein	Peptide**
 10.5	8.8	0.6601	M.PARTAPAR.V
 9.0	12	0.6826 325	O15031|PLXB2_HUMAN K.LDIPEPR.R
 7.7	17	0.7077	R.DLEAHVR.Q
 6.8	21	0.7003	K.MSFGLLR.V
 5.9	25	-0.4218	K.IVGLGLLR.V
 4.8	33	0.7077	R.HDGELLR.G
 3.5	43	-0.4218	R.GLLVGILR.C
 3.5	43	-0.4331	R.RLLGLLR.R
 2.5	55	0.7288	K.HFPAPDR.K
 2.2	59	0.6350	R.AGAAPALLR.S

Top scoring peptide matches to query 2024
File7043 Spectrum5495 scans: 4289
Score greater than 21 indicates homology
Score greater than 34 indicates identity

**Score	Expect	Delta Hit Protein	Peptide**
 25.2	0.43	-0.0055	42+ P05141|ADT2_HUMAN R.MMMQSGR.K
 7.1	28	-0.0563	K.GGFGSMLR.A
 6.9	29	-0.9969	K.MMMMGPK.E
 6.2	34	-0.0992	K.ETYKTAK.L
 6.1	35	0.9680	R.MLWMDK.E
 4.6	49	-0.0740	R.KTEYGSR.L
 4.5	50	-0.0740	K.DAPLPDGR.S
 3.8	60	-0.1104	K.EDALKHK.S
 3.0	70	-0.0311	K.EGPGHMGR.S
 2.6	78	-1.0515	R.CASKYGR.D

Top scoring peptide matches to query 2025
File7043 Spectrum5816 scans: 4543
Score greater than 22 indicates homology
Score greater than 34 indicates identity

**Score	Expect	Delta Hit Protein	Peptide**
 10.7	12	0.8518	M.ASRPRPR.T
 9.0	18	0.9331 247	P27824|CALX_HUMAN K.TGIYEEK.H
 7.9	23	-0.9815	R.CTCASSR.I
 6.7	31	0.8743	K.TGPQLAPR.K
 5.8	37	0.0138	-.MASNMDR.E
 5.4	41	-0.1210	R.SVLHQTR.S
 5.3	42	-0.1825	R.LQILTPR.A
 5.3	42	-0.2189	K.LKITIPR.G
 5.2	43	-0.2189	K.KILLTPR.R
 4.7	48	-0.0226	-.MASSGMTR.R

Top scoring peptide matches to query 2026
File7043 Spectrum5573 scans: 4352
Score greater than 21 indicates homology
Score greater than 34 indicates identity

**Score	Expect	Delta Hit Protein	Peptide**
 18.3	2.1	-0.0047	42+ P05141|ADT2_HUMAN R.MMMQSGR.K
 7.5	25	-0.0555	K.GGFGSMLR.A
 6.6	31	0.9939	R.WMNMNK.T
 4.6	49	-0.2300	K.LRLLGLR.Y
 4.3	53	-0.1136 432	Q9H9B4|SFXN1_HUMAN R.VYFNKGL.-
 4.0	57	0.9550	M.STESMIR.D
 3.4	64	0.9737	K.TASSGSSDK.D
 2.9	73	0.9688	R.MLWMDK.E
 2.1	88	-0.0667	R.VYRCSR.D
 1.9	92	0.9034	K.MLLFSGR.R

Top scoring peptide matches to query 2028
File7043 Spectrum10890 scans: 8645
Score greater than 26 indicates homology
Score greater than 34 indicates identity

**Score	Expect	Delta Hit Protein	Peptide**
 16.1	3.5	1.0809	R.EFGIMVK.D
 15.7	3.9	1.0697	R.EFMRLK.D
 15.7	3.9	1.0333 132+ P08238|HS90B_HUMAN IYRMIK
 15.7	3.9	0.0745	K.NYRMIK.K
 15.7	3.9	1.0044	K.RPSAAPIK.Q
 12.3	8.4	-1.0113	R.LKVSAPAR.V
 12.1	8.8	-0.9749	R.TGQLLPGR.Y
 11.4	10	-0.9385	R.EAGALGPAR.E
 10.8	12	-1.0001	75	Q13423|NNTM_HUMAN K.QLTVGVPK.E
 10.2	14	1.1140	R.VLAMDMK.G

Top scoring peptide matches to query 2032
File7043 Spectrum380 scans: 397
Score greater than 20 indicates homology
Score greater than 32 indicates identity

**Score	Expect	Delta Hit Protein	Peptide**
 8.8	13	0.5877	R.AYLWCK.E
 8.3	14	-0.3519	R.SCSSASSR.S
 7.0	19	0.5474 606	O00159|MYO1C_HUMAN AGFAYRR
 6.8	20	-0.3229	-.MDSSNCK.V
 6.4	22	-0.4110	-.MAMGLFR.V
 6.2	23	0.6281	K.AEFASCR.L
 4.9	31	-0.3164	-.MGCCTGR.C
 3.3	45	0.5739	R.DLPPENR.A
 3.3	45	0.4648	K.LLVPKDR.L
 3.2	46	0.5515	R.LDGHSRR.A

Top scoring peptide matches to query 2036
File7043 Spectrum6740 scans: 5271
Score greater than 21 indicates homology
Score greater than 32 indicates identity

**Score	Expect	Delta Hit Protein	Peptide**
 9.6	10	0.5387	K.VLLLGAVR.I
 8.4	14	0.6254	K.NNIPRAR.L
 6.7	21	0.6954 198	P04843|RPN1_HUMAN R.NVESYTK.L
 5.5	27	-0.3548	R.MVLEPPR.E
 4.7	33	-0.3072	K.DLAMTYK.Q
 4.7	33	0.6213	R.FKGPHVR.Y
 4.3	36	-0.3838	R.QTGPLIGR.Q
 3.3	45	-0.4453	K.IIVLDLR.-
 3.1	47	-0.2747	R.TEPHDSR.W
 2.8	51	-0.3878	K.LSLPHFK.E

Top scoring peptide matches to query 2039
File7043 Spectrum4679 scans: 3648
Score greater than 21 indicates homology
Score greater than 32 indicates identity

**Score	Expect	Delta Hit Protein	Peptide**
 20.4	0.88	0.6958 198	P04843|RPN1_HUMAN R.NVESYTK.L
 7.7	16	0.6734	K.NAQPSPAR.A
 6.3	23	0.5966	R.FPPRVPK.S
 6.2	23	0.6231	R.TLKYSTK.M
 5.6	27	0.6118	LEVRVPQ
 4.5	34	0.6846	R.ASRDYTK.Y
 2.4	56	-0.3107	K.LTEEGHR.R
 2.4	56	-0.3722	K.TLETHIK.I
 2.1	60	0.6772	K.MGKETFK.D
 2.1	60	0.6772	R.MNSLTFK.K

Top scoring peptide matches to query 2040
File7043 Spectrum6486 scans: 5072
Score greater than 18 indicates homology
Score greater than 32 indicates identity

**Score	Expect	Delta Hit Protein	Peptide**
 6.8	20	-0.2452	K.DPYGSMR.K
 4.8	32	0.5391	K.VLLLGAVR.I
 4.5	34	0.5755 484	P11498|PYC_HUMAN K.VLKDLPR.V
 4.3	36	0.6119	K.VPKNPSAK.R
 4.2	37	0.6483	R.NPNAPVTK.A
 3.9	40	-0.3721	K.DPKVPSAK.E
 3.8	41	0.5027	K.VLLVRLK.K
 3.7	41	0.5867 198	P04843|RPN1_HUMAN K.IILPEGAK.N
 3.6	42	0.6847	K.NPEEPVR.F
 2.8	51	0.5279	K.VILAIRR.A

Top scoring peptide matches to query 2042
File7043 Spectrum5813 scans: 4541
Score greater than 16 indicates homology
Score greater than 32 indicates identity

**Score	Expect	Delta Hit Protein	Peptide**
 4.9	31	0.7532	42+ P05141|ADT2_HUMAN R.MMMQSGR.K
 3.1	47	0.8082	R.MDDCGSR.S
 1.1	75	-0.2234	R.CTCASSR.I
 0.8	81	-0.3179	-.MSFQGKK.S
 0.7	81	0.7541	R.TSRTCES.-
 0.3	90	-0.3761	K.YLFTGLK.L

Top scoring peptide matches to query 2044
File7043 Spectrum7893 scans: 6192
Score greater than 22 indicates homology
Score greater than 32 indicates identity

**Score	Expect	Delta Hit Protein	Peptide**
 12.4	5.5	0.6260	R.VRSHVSR.Q
 8.3	14	0.5757 484	P11498|PYC_HUMAN K.VLKDLPR.V
 7.3	18	0.5281	K.VILAIRR.A
 7.3	18	0.5281	R.VLLALRR.Y
 7.3	18	0.5393	K.VLLLGAVR.I
 7.3	18	0.5645	R.VLLRPSR.R
 7.3	18	0.5030	R.VLLVKIR.S
 6.7	21	0.6121	K.APSSLPLR.G
 6.0	24	0.5757	K.KLVPVER.N
 5.3	29	0.6993	R.KMSVMGR.Q

Top scoring peptide matches to query 2048
File7043 Spectrum2321 scans: 1797
Score greater than 20 indicates homology
Score greater than 32 indicates identity

**Score	Expect	Delta Hit Protein	Peptide**
 8.4	14	0.6230	R.FKGPHVR.Y
 7.3	18	-0.2804	R.VQYEMR.T
 6.2	23	-0.4549	K.VKVLLNR.I
 5.8	25	0.5906 344	Q8TCZ2|C99L2_HUMAN R.RKPGIGGR.E
 5.7	26	0.5906	K.QRPGVKR.R
 5.6	27	0.6957	R.WDHQVR.R
 4.8	32	-0.4437	R.VLVAVNVK.S
 3.5	43	0.6495	K.EHIADKK.K
 3.5	44	0.6594	R.QPGVFHR.M
 3.2	46	0.6091	K.ILYYLR.Q

Top scoring peptide matches to query 2053
File7043 Spectrum2066 scans: 1589
Score greater than 19 indicates homology
Score greater than 32 indicates identity

**Score	Expect	Delta Hit Protein	Peptide**
 7.0	19	0.6235	R.FKGPHVR.Y
 5.6	26	0.6566	-.MPSPRPR.G
 5.3	29	0.7042	K.IFCASSR.I
 4.3	36	0.5409	K.VLLLGAVR.I
 3.5	43	-0.3928	R.KPRGEVR.N
 3.5	43	-0.3452	K.QPKPESR.E
 3.0	48	-0.2468	-.MMTAESR.E
 2.6	53	0.5912 344	Q8TCZ2|C99L2_HUMAN R.RKPGIGGR.E
 2.5	54	0.6963	R.WDHQVR.R
 2.0	61	0.7003	R.NNSHVNR.E

Top scoring peptide matches to query 2056
File7043 Spectrum29133 scans: 25875
Score greater than 17 indicates homology
Score greater than 32 indicates identity

**Score	Expect	Delta Hit Protein	Peptide**
 4.9	31	0.6684	K.GMPPLPGR.A
 4.8	32	0.6507	R.VPDSGIPR.D
 4.5	34	0.7049	K.KEYMNR.K
 4.5	34	0.6871	K.TRYESGK.Y
 4.5	34	0.6824	-.MQRPHR.R
 4.0	39	0.6970	K.ASSWPHR.R
 3.9	39	0.6606 431	O43795|MYO1B_HUMAN R.RAGYAFR.Q
 1.7	65	-0.3420	K.WMLVHR.T

Top scoring peptide matches to query 2057
File7043 Spectrum8749 scans: 6877
Score greater than 29 indicates homology
Score greater than 34 indicates identity

**Score	Expect	Delta Hit Protein	Peptide**
 20.6	1.3	-0.0055 206	P48047|ATPO_HUMAN K.YVDMSVK.T
 15.7	4.1	0.9785	R.YMDVKGK.K
 5.3	45	0.9785	K.YMNATLK.R
 4.4	54	0.0262	K.CRFCAK.V
 4.4	55	0.0601	K.SGTSCSSR.C
 4.3	55	0.9495	K.YRSTVSK.S
 4.2	57	-0.1073 312	P35222|CTNB1_HUMAN K.SGGIPALVK.M
 4.0	59	0.9929	K.MGKMMVK.A
 0.9 1.2e+002	0.0527	R.RMEMDK.T
 0.4 1.4e+002	-0.0246	K.FREFSR.D

Top scoring peptide matches to query 2058
File7043 Spectrum8733 scans: 6863
Score greater than 22 indicates homology
Score greater than 34 indicates identity

**Score	Expect	Delta Hit Protein	Peptide**
 11.7	10	-0.0051 206	P48047|ATPO_HUMAN K.YVDMSVK.T
 9.1	19	-0.0163	R.YVMERK.N
 7.5	27	1.0669	-.MSSEQDK.S
 6.5	33	0.0531	R.DMREMK.N
 6.5	34	-1.0334 579	Q14624|ITIH4_HUMAN K.YYLQGAK.I
 6.5	34	-0.0745	K.YVYVGLK.D
 6.1	37	-0.0051	R.VVMYEGK.K
 6.0	38	1.1098	K.CTDCER.A
 6.0	38	1.1098	R.CTDECR.T
 5.6	41	-1.0770	R.IAAANGIGR.L

Top scoring peptide matches to query 2063
File7043 Spectrum12303 scans: 9880
Score greater than 15 indicates homology
Score greater than 33 indicates identity

**Score	Expect	Delta Hit Protein	Peptide**
 7.8	19	0.3697	R.LPSGSEPR.A
 7.7	20	0.2970	R.LPKTEVR.A
 2.1	72	0.3987 185	P39023|RL3_HUMAN K.DFSSMKK.Y
 1.3	86	-0.6441	R.NPLICAR.G
 0.8	96	0.2857	LRGLLDR
 0.4 1.1e+002	0.2858 639	P21439|MDR3_HUMAN K.LLAGNAKR.D
 0.3 1.1e+002	0.3762	R.GVGHSMVR.K
 0.1 1.1e+002	0.2606	K.VVVIEKR.D
 0.1 1.1e+002	-0.7347	R.TLKVNLR.N
 0.0 1.2e+002	0.2606	K.VVREVLK.T

Top scoring peptide matches to query 2064
File7043 Spectrum5721 scans: 4471
Score greater than 16 indicates homology
Score greater than 33 indicates identity

**Score	Expect	Delta Hit Protein	Peptide**
 3.4	53	0.6008	K.TAVAPLDR.T
 3.4	54	-0.4560	97	P02679|FIBG_HUMAN K.IVNLKEK.V

Top scoring peptide matches to query 2067
File7043 Spectrum2539 scans: 1964
Score greater than 19 indicates homology
Score greater than 33 indicates identity

**Score	Expect	Delta Hit Protein	Peptide**
 9.2	14	-0.4159	97	P02679|FIBG_HUMAN K.IVNLKEK.V
 6.1	28	-0.3431	K.TPKSPGEK.T
 6.1	29	-0.4159 585	Q9Y4W6|AFG32_HUMAN R.VIGGLEKK.T
 6.0	29	-0.4522	K.LTLLQKK.I
 5.7	31	-0.3908	K.VVVVTGGGR.G
 5.1	36	0.7388	R.TGADGEHR.R
 5.0	36	0.5430	K.LVLIEKK.Q
 5.0	36	0.5641	R.VLLLAWK.F
 4.7	39	-0.3795 683	Q8TF72|SHRM3_HUMAN R.LTPEQKK.R
 4.4	42	-0.3907	K.KPSQQKK.L

Top scoring peptide matches to query 2076
File7043 Spectrum7638 scans: 5991
Score greater than 28 indicates homology
Score greater than 35 indicates identity

**Score	Expect	Delta Hit Protein	Peptide**
 21.3	1.4	-0.0410	K.VLEIKNK.A
 14.7	6.4	-0.0046 282	Q9UI12|VATH_HUMAN R.VIEQLGGK.Q
 14.0	7.4	-0.0410	K.LVLEKNK.L
 12.4	11	-0.0410 585	Q9Y4W6|AFG32_HUMAN R.VIGGLEKK.T
 11.9	12	-0.0410	R.VLELNKK.Q
 11.1	15	-0.0046	R.VLQELNK.Q
 10.5	17	1.0410	R.QKASGSHK.R
 8.3	28	0.9794	K.IANLNGLK.N
 7.9	31	0.9430	K.VLQNKLK.Q
 7.6	32	-0.0297	R.VILEELK.S

Top scoring peptide matches to query 2077
File7043 Spectrum7887 scans: 6188
Score greater than 22 indicates homology
Score greater than 35 indicates identity

**Score	Expect	Delta Hit Protein	Peptide**
 9.8	19	0.9977	K.TLKPEVR.L
 9.3	22	0.0024	R.QNKLAAAK.K
 8.8	25	-0.9816 602	Q8IYT4|KATL2_HUMAN R.ELAAVVSR.D
 8.2	29	-0.9969	K.ELIKWR.A
 7.7	32	0.1068	K.TNHCRR.V
 7.6	32	0.0752	R.SQGLPEGR.L
 7.4	34	0.0500	K.TLQGPAEK.K
 7.0	37	0.0678	K.MTIEPPR.G
 7.0	38	-0.9565	R.GQGATALAR.L
 6.8	39	-0.0227	R.KSLPASLK.V

Top scoring peptide matches to query 2078
File7043 Spectrum15464 scans: 12724
Score greater than 15 indicates homology
Score greater than 35 indicates identity

**Score	Expect	Delta Hit Protein	Peptide**
 4.7	64	0.1042	K.CMLYLK.Y
 4.7	64	0.0865	K.IPPEEMK.Q
 4.7	64	0.1736	R.MCELMK.Q
 4.7	64	0.0422 465	P35606|COPB2_HUMAN R.VAHFLEK.Q
 4.5	66	0.0786	K.HPVAEYK.E
 4.5	66	0.0462 670	Q9NZ01|TECR_HUMAN R.DLRPAGSK.T
 2.5	1e+002	0.0561	K.GQHARFK.K
 1.2 1.4e+002	1.0303	R.RGAKPEGK.A
 0.0 1.9e+002	0.0898	R.EFKEYK.C

Top scoring peptide matches to query 2082
File7043 Spectrum5037 scans: 3935
Score greater than 20 indicates homology
Score greater than 36 indicates identity

**Score	Expect	Delta Hit Protein	Peptide**
 10.6	18	-0.0061	K.RHGYGVR.Q
 6.8	43	-1.0000	R.SSVAPQTR.V
 6.1	51	-0.9789	K.RHFEEK.K
 4.6	71	-1.0728	K.RGSLTIAK.L
 4.6	72	-1.0153	R.RTWASPK.L
 3.7	88	-1.0074	98	P28331|NDUS1_HUMAN R.LTEPMVR.N
 3.6	90	0.9354	K.VLPIMVR.A
 3.5	93	-1.0768	R.GPIFVKGK.G
 3.5	94	-0.0564	K.VNIPFVR.L
 3.3	98	1.0736 409	P98187|CP4F8_HUMAN K.FAMAEMK.V

Top scoring peptide matches to query 2086
File7043 Spectrum465 scans: 451
Score greater than 24 indicates homology
Score greater than 36 indicates identity

**Score	Expect	Delta Hit Protein	Peptide**
 11.7	14	1.0178	K.IQTQTPR.K
 11.1	16	1.0390	K.LNWSAPR.D
 10.8	17	0.9676	R.LIVIEEK.D
 10.7	18	1.0608	K.LDHMRR.R
 10.7	18	-0.9726	R.EASGRLGR.F
 10.7	18	-0.9726	R.ETNRLGR.L
 9.3	24	0.9815	K.IDIQAKR.V
 9.0	26	-0.9502	R.DQTSPAVK.Q
 9.0	26	-0.9866	K.DTKSGPLK.L
 8.9	27	0.9563	97	P02679|FIBG_HUMAN K.IVNLKEK.V

Top scoring peptide matches to query 2088
File7043 Spectrum7312 scans: 5730
Score greater than 18 indicates homology
Score greater than 36 indicates identity

**Score	Expect	Delta Hit Protein	Peptide**
 6.3	49	0.0140	R.EGALVSLR.R
 5.6	58	1.0093	R.QLKALGVD.-
 5.3	62	0.9981 547	O00291|HIP1_HUMAN R.QKLGELR.K
 4.4	75	0.0617	K.EGATLEPK.Q
 1.8 1.4e+002	0.0392	K.AGLEKGNR.K
 1.4 1.5e+002	-1.0427	R.VIAVKTSK.S
 1.1 1.6e+002	1.0457	K.TPKSPGEK.T
 0.4 1.9e+002	-0.9951	R.EEITLLK.E
 0.3 1.9e+002	1.0596	R.QGQLQNR.R

Top scoring peptide matches to query 2089
File7043 Spectrum12230 scans: 9812
Score greater than 22 indicates homology
Score greater than 36 indicates identity

**Score	Expect	Delta Hit Protein	Peptide**
 15.4	6	-0.0049	R.IGAGASKLK.K
 9.0	26	0.0203	R.GLQATARK.A
 9.0	26	0.0315	R.LQQTNLK.L
 4.4	75	-0.9274	R.APTTATQR.L
 4.4	75	-0.9386	K.RITNDAR.E
 4.4	75	-1.0001	60	Q01082|SPTB2_HUMAN K.RLTVQTK.F
 4.3	77	1.1714	K.MMYDQR.Q
 4.3	78	0.0315	R.LKQQEAK.F
 3.4	94	1.1384	-.MDAGFFR.G
 3.2	1e+002	0.0567	R.LRDGVER.C

Top scoring peptide matches to query 2092
File7043 Spectrum2726 scans: 2102
Score greater than 22 indicates homology
Score greater than 34 indicates identity

**Score	Expect	Delta Hit Protein	Peptide**
 9.9	15	-0.3745	R.EAQIKEK.L
 9.8	15	-0.4109	LSQIKEK
 8.6	20	-0.2880	K.EFYQMK.Q
 8.0	23	0.7589	-.MEEYEK.F
 7.9	23	0.6095	K.EALVREK.A
 7.9	24	-0.2918	64	Q13813|SPTA2_HUMAN R.EAHDAFR.S
 7.4	26	0.6895	K.YLEYEK.K
 7.4	26	0.6782	K.QFYSTAK.K
 6.9	30	0.6347	K.TQTPNRK.E
 6.8	30	-0.3745	K.LSVDVGGAK.R

Top scoring peptide matches to query 2093
File7043 Spectrum2925 scans: 2256
Score greater than 27 indicates homology
Score greater than 34 indicates identity

**Score	Expect	Delta Hit Protein	Peptide**
 14.9	4.6	0.7589	R.EEMYEK.Y
 14.4	5.3	0.6782	K.QFYSTAK.K
 14.1	5.7	0.6558	K.QFKEHR.K
 12.8	7.5	0.6273	R.KPQACLK.A
 12.7	7.8	0.6749 300+ P08263|GSTA1_HUMAN R.KPPMDEK.S
 11.7	9.8	0.5983	K.QKIAQTR.R
 11.3	11	0.5844	K.KIVVEEK.V
 10.9	12	0.5327	K.KFLPVLK.E
 10.8	12	0.7589	-.MEEYEK.F
 9.9	15	0.6306	R.QFLPPSR.Q

Top scoring peptide matches to query 2094
File7043 Spectrum2397 scans: 1859
Score greater than 23 indicates homology
Score greater than 34 indicates identity

**Score	Expect	Delta Hit Protein	Peptide**
 11.1	11	0.5847	K.KIVVEEK.V
 10.2	14	-0.3742	R.EAQIKEK.L
 5.4	42	-0.4218	R.LSKIGATR.R
 5.3	43	0.6098 373	P10412|H14_HUMAN K.KATGAATPK.K
 5.2	43	-0.3967	R.RTALAGTR.T
 5.0	46	0.6938	R.QLDPDEK.I
 4.6	51	-0.3490	R.EKLQDGR.I
 4.3	54	-0.2763	M.AEGGSPDGR.A
 4.2	55	0.6098 740	Q06136|KDSR_HUMAN R.TKPLETR.L
 4.1	56	-0.4106	LSQIKEK

Top scoring peptide matches to query 2095
File7043 Spectrum5653 scans: 4417
Score greater than 35 indicates homology
Score greater than 36 indicates identity

**Score	Expect	Delta Hit Protein	Peptide**
 22.2	1.3	-0.0049 222	Q00325|MPCP_HUMAN R.MQVDPQK.Y
 21.1	1.7	-0.0478 674	O75865|TPC6A_HUMAN R.EELDVLK.F
 18.1	3.4	0.9175 111	P21397|AOFA_HUMAN R.LPMGAVIK.C
 16.8	4.6	-0.0842	R.EEITLLK.E
 15.2	6.7	-0.0954	K.EKNLTLK.E
 14.9	7.2	0.9250 343	P06744|G6PI_HUMAN R.KELQAAGK.S
 14.6	7.6	0.8886	K.QTKINLK.I
 14.5	7.9	-0.0478	R.EEVEVLK.E
 14.4	8	-0.0955	R.TGTVQVLK.R
 14.3	8.2	-0.0955	R.ITATQALK.M

Top scoring peptide matches to query 2096
File7043 Spectrum5576 scans: 4354
Score greater than 36 indicates identity

**Score	Expect	Delta Hit Protein	Peptide**
 37.5	0.039	-0.0477 674	O75865|TPC6A_HUMAN R.EELDVLK.F
 31.3	0.16	-0.0841	R.EEITLLK.E
 28.2	0.33	-1.0429	K.EENTLLK.Q
 25.6	0.61	-0.0953	K.EKNLTLK.E
 24.3	0.81	-1.0066	K.ELEDVNK.W
 23.7	0.94	-0.0048 222	Q00325|MPCP_HUMAN R.MQVDPQK.Y
 23.1	1.1	-0.0477	R.EEVEVLK.E
 22.6	1.2	-1.0477	K.MQLGRNK.K
 20.7	1.9	-0.0953	K.KAASDLIK.S
 20.1	2.2	-0.0953	R.AVSTNLLK.Q

Top scoring peptide matches to query 2097
File7043 Spectrum5579 scans: 4356
Score greater than 36 indicates identity

**Score	Expect	Delta Hit Protein	Peptide**
 35.1	0.068	-0.0477 674	O75865|TPC6A_HUMAN R.EELDVLK.F
 32.5	0.12	-0.0048 222	Q00325|MPCP_HUMAN R.MQVDPQK.Y
 30.7	0.19	-0.0953	R.TGTVQVLK.R
 28.0	0.35	-0.0953	K.EKNLTLK.E
 21.6	1.5	-0.0953	R.KALSDAIK.K
 21.3	1.7	-0.0225	K.EKDTPQK.L
 20.5	2	-0.0048	K.DHMSVLK.A
 20.5	2	-0.0702	R.GRADSVLK.G
 20.2	2.1	-0.0841	R.EEITLLK.E
 19.3	2.6	-0.1139	K.MQVKLVK.D

Top scoring peptide matches to query 2099
File7043 Spectrum3701 scans: 2874
Score greater than 17 indicates homology
Score greater than 36 indicates identity

**Score	Expect	Delta Hit Protein	Peptide**
 6.3	52	-0.0789	K.VSVIKGSR.S
 4.5	79	-1.0265	K.ALTEEKR.V
 4.5	79	-0.9901 433	P11277|SPTB1_HUMAN K.DVAEERK.R
 4.5	79	-0.9650	R.EREEQR.R
 2.0 1.4e+002	-1.0517	K.GDIIVTTK.S
 2.0 1.4e+002	-1.0265	K.LTALEGSR.V
 2.0 1.4e+002	-0.9902	K.VADIESGR.Q
 1.1 1.7e+002	-0.0174	R.REASVGAR.G
 0.8 1.8e+002	0.0051	K.QEEVGGVK.E
 0.2 2.1e+002	-0.0061	R.ATEQLQR.A

Top scoring peptide matches to query 2100
File7043 Spectrum7561 scans: 5929
Score greater than 26 indicates homology
Score greater than 36 indicates identity

**Score	Expect	Delta Hit Protein	Peptide**
 21.0	1.8	-0.9838 311	P01833|PIGR_HUMAN K.TENAQKR.K
 12.5	12	-0.0137	R.TGAENLLK.V
 11.7	15	-0.0435	K.LVMAQKR.G
 11.3	16	-0.0249	R.TLQSLQR.T
 10.3	21	-0.0249	K.IVSREGGK.V
 10.3	21	1.0067	K.DIAELQR.L
 9.9	23	-0.0071	37	P55084|ECHB_HUMAN R.LVMAAANR.L
 9.7	24	-0.0249	K.TLSQLQR.D
 9.1	27	0.0326	K.EVAHSFR.I
 9.1	27	0.9591	R.ILTDARR.S

Top scoring peptide matches to query 2106
File7043 Spectrum87 scans: 195
Score greater than 18 indicates homology
Score greater than 34 indicates identity

**Score	Expect	Delta Hit Protein	Peptide**
 5.9	38	0.4193	K.LLSQTQR.E
 5.7	40	0.4808	K.ENRSPSR.R
 5.6	41	0.4444	K.QDRALSR.M
 5.6	41	0.4332	R.RSRPSSR.G
 5.6	41	0.4444 343	P06744|G6PI_HUMAN K.SRGVEAAR.E
 4.7	50	0.4305	K.EGKVVDAK.T
 4.2	57	-0.4920	R.EDADIQR.E
 3.8	62	0.5098	K.EMHERK.R
 3.8	62	0.4808	K.SGSSHKSR.-
 3.8	62	0.4444	R.SGRLQER.A

Top scoring peptide matches to query 2114
File7043 Spectrum5260 scans: 4101
Score greater than 24 indicates homology
Score greater than 36 indicates identity

**Score	Expect	Delta Hit Protein	Peptide**
 18.4	3.1	-0.0050 658	Q02543|RL18A_HUMAN K.VEEIAASK.C
 10.1	21	-0.9639 357+ Q15836|VAMP3_HUMAN K.LSELDDR.A
 9.2	26	-0.9540	K.NNQWSAK.K
 8.4	31	0.9677	R.QSDVRIK.F
 8.3	32	0.9677	K.GGIIVSGSR.D
 8.0	35	0.9789	K.DTKSGPLK.L
 7.5	38	-0.0275	R.SLRNSAAK.K
 7.5	39	0.9385	R.LDFLPLK.C
 7.4	39	-0.0163	R.DLLATGTR.K
 7.1	42	0.0048	R.VEKEWR.R

Top scoring peptide matches to query 2115
File7043 Spectrum13522 scans: 10952
Score greater than 30 indicates homology
Score greater than 36 indicates identity

**Score	Expect	Delta Hit Protein	Peptide**
 24.1	0.84	-0.0051	37	P55084|ECHB_HUMAN K.LMLDLNK.A
 15.9	5.5	-0.0527	R.LMLSVRK.L
 15.7	5.9	-0.9891	K.MILDEVK.M
 15.1	6.7	-0.0051	R.LMNVIEK.E
 14.9	7	-0.9202	R.DEKDGGAR.E
 13.1	10	-1.0004	R.INNMIVK.R
 12.9	11	-0.9752	R.NMLERGK.A
 12.4	13	-0.9858	K.FPIEDVK.I
 12.3	13	-0.0164	K.LMLSVAGR.V
 12.2	13	-0.0051	K.LIMDNIK.M

Top scoring peptide matches to query 2121
File7043 Spectrum3499 scans: 2714
Score greater than 20 indicates homology
Score greater than 34 indicates identity

**Score	Expect	Delta Hit Protein	Peptide**
 8.6	20	0.6460	K.DPVRGFR.Q
 7.1	28	0.6651	K.IPTQEMK.N
 6.8	30	0.6426	K.IPGRMTR.S
 6.2	34	-0.2904	K.DPTDPFR.L
 6.1	35	0.7188	R.NYEQHR.K
 4.0	56	0.7089	R.EEAEAAAR.A
 4.0	56	0.6613	K.NKSSAPSR.V
 2.5	79	0.7556 267	Q12955|ANK3_HUMAN K.MFEMTR.S
 2.3	84	0.7089	K.DLEAENR.R
 2.0	90	0.6977	R.AEEDARR.Y

Top scoring peptide matches to query 2122
File7043 Spectrum3123 scans: 2418
Score greater than 20 indicates homology
Score greater than 34 indicates identity

**Score	Expect	Delta Hit Protein	Peptide**
 8.0	22	-0.3991	R.KTGFIGPK.G
 7.1	28	0.6981	K.NDGTVQGR.K
 6.1	35	-0.3297	R.VLPDTMR.N
 5.3	42	-0.3362 291	P26373|RL13_HUMAN R.VITEEEK.N
 4.6	50	0.6478	K.VKDVEEK.S
 3.0	72	0.7635	R.EMEEHR.Q
 2.6	78	-0.3375	R.KFQPDGR.S
 1.7	97	0.6292	-.MVTTLGPK.M
 1.3 1.1e+002	0.7206	K.EQESPEK.L
 0.7 1.2e+002	0.6479	K.EIAKEEK.K

Top scoring peptide matches to query 2123
File7043 Spectrum4950 scans: 3863
Score greater than 21 indicates homology
Score greater than 34 indicates identity

**Score	Expect	Delta Hit Protein	Peptide**
 11.6	9.9	0.6119	K.KSLLEEK.E
 7.8	24	0.6370	K.KQPSGTTK.R
 7.0	28	0.6548	K.GGTPVCKK.D
 7.0	28	0.6911	R.NTVPCQK.R
 6.4	32	0.6734	R.KSPNDTGK.V
 6.2	34	0.6006	K.KSVLSQGK.T
 6.1	35	0.5602	K.VLLVQFK.G
 5.9	37	0.6548	R.KGGTPVCK.K
 5.9	37	0.6184	16+ P35579|MYH9_HUMAN R.KLQAQMK.D
 5.9	37	0.6548	R.KTPNQMK.A

Top scoring peptide matches to query 2127
File7043 Spectrum8490 scans: 6668
Score greater than 23 indicates homology
Score greater than 36 indicates identity

**Score	Expect	Delta Hit Protein	Peptide**
 10.9	17	1.0267	K.QMDPLDK.K
 10.4	19	0.9175	R.KVIMDLK.T
 9.9	21	0.9175	R.KVMDILK.R
 9.0	26	-1.0431	K.KVVSSLES.-
 8.6	29	0.9865	K.QNKTNNK.K
 7.4	38	0.9573 227	P21283|VATC1_HUMAN K.FNIPDLK.V
 5.4	61	-0.0631	K.VYIDPLK.R
 5.0	67	1.0228	K.INQDNSR.K
 4.8	70	-0.0339	K.VGSKAGSNK.S
 4.7	72	0.9977	R.RAEDDLK.K

Top scoring peptide matches to query 2128
File7043 Spectrum6443 scans: 5037
Score greater than 17 indicates homology
Score greater than 36 indicates identity

**Score	Expect	Delta Hit Protein	Peptide**
 7.2	40	0.8745	LLKGLFR
 4.4	76	0.9439	R.LLEGMRK.W
 3.6	92	0.9989 433	P11277|SPTB1_HUMAN K.DVAEERK.R
 2.8 1.1e+002	0.9625	K.LTALEGSR.V
 2.6 1.1e+002	0.9261	K.LTSIEKR.L
 2.0 1.3e+002	0.9625	K.LAETEKR.C
 1.7 1.4e+002	0.9803	K.ITPAMAAR.I
 1.2 1.6e+002	-0.0150	K.IEMGNKR.W
 0.7 1.8e+002	0.9374	K.LTSLIDGK.V

Top scoring peptide matches to query 2130
File7043 Spectrum12138 scans: 9729
Score greater than 21 indicates homology
Score greater than 36 indicates identity

**Score	Expect	Delta Hit Protein	Peptide**
 8.7	28	-0.0040	K.SLVMELR.Y
 8.2	32	0.9436 657+ P35221|CTNA1_HUMAN R.KAVLMIR.T
 7.7	35	0.9986	R.DLLATGTR.K
 6.6	46	0.0211 391	P12956|KU70_HUMAN K.VEAMNKR.L
 6.4	48	1.0350	K.DKEGLER.M
 5.4	61	1.0164	K.MLATAPAR.G
 5.0	66	1.0714	R.DQEGELR.S
 4.6	73	1.0350	R.NSDIELR.K
 4.5	74	-1.0058	K.KVVSSLES.-
 4.0	83	-0.9079	R.ESEVGDGR.A

Top scoring peptide matches to query 2138
File7043 Spectrum4633 scans: 3612
Score greater than 29 indicates homology
Score greater than 35 indicates identity

**Score	Expect	Delta Hit Protein	Peptide**
 22.8	1	-0.0046 413	Q00765|REEP5_HUMAN K.ETADAITK.E
 15.7	5.4	-0.0020	R.NQTSRSR.V
 13.4	9	0.8992	R.LLMILTK.Q
 12.3	12	0.0317	R.DSVEGVDK.N
 11.5	14	-0.0093 301	P09210|GSTA2_HUMAN R.GRMESIR.W
 11.5	14	0.9356	R.IVVEMIK.R
 11.4	14	0.0317	R.NVEEETK.Y
 11.3	15	0.9906	K.IVEEETK.K
 11.3	15	0.9607	K.LVCLNTK.T
 10.6	17	-0.0159	R.SSLSADLR.Q

Top scoring peptide matches to query 2139
File7043 Spectrum3059 scans: 2365
Score greater than 27 indicates homology
Score greater than 35 indicates identity

**Score	Expect	Delta Hit Protein	Peptide**
 20.7	1.7	1.0014	R.VLPDTMR.N
 20.2	1.9	0.9684	R.VIEPFSR.V
 13.1	9.6	0.0062	R.DGGAILMR.L
 11.4	14	1.0412	K.DPTDPFR.L
 9.9	20	0.9321	R.KTGFIGPK.G
 9.7	21	0.9538	K.VLKQMGR.L
 8.8	26	1.0015	K.EVLPSMR.K
 8.5	28	0.9321 602	Q8IYT4|KATL2_HUMAN R.VLFELAR.Y
 8.1	30	0.9902	R.VNKEMAR.Y
 7.7	34	1.1059	K.NGGHCMR.N

Top scoring peptide matches to query 2140
File7043 Spectrum2820 scans: 2172
Score greater than 23 indicates homology
Score greater than 35 indicates identity

**Score	Expect	Delta Hit Protein	Peptide**
 12.9	10	0.9930	K.NEESLKK.C
 10.3	18	-0.0097 178	Q99623|PHB2_HUMAN K.MLGEALSK.N
 10.2	19	0.9995 391	P12956|KU70_HUMAN K.VEAMNKR.L
 9.6	21	0.9856	K.MDLLVEK.V
 9.1	24	1.0181	R.EVTTNQR.I
 9.0	25	0.9930	K.DINSTAVK.L
 8.8	26	-1.0016	R.TNPAVGYK.L
 8.5	28	0.9930	R.ELETSLR.T
 8.3	29	1.0042	K.ELTEVEK.G
 8.2	30	-1.0049	K.TEAAAMKK.Q

Top scoring peptide matches to query 2141
File7043 Spectrum9910 scans: 7829
Score greater than 24 indicates homology
Score greater than 35 indicates identity

**Score	Expect	Delta Hit Protein	Peptide**
 17.9	3.2	-1.0083	K.VFSVNGAR.G
 17.1	3.9	-0.0065	-.MIWRSR.A
 17.1	3.9	-0.0052 178	Q99623|PHB2_HUMAN K.MLGEALSK.N
 10.8	16	0.9822	R.KPNTYPK.M
 10.7	17	-0.0131	VADFGLAR
 10.1	19	1.0226	K.QDQKNSK.E
 9.9	20	0.9709	95	P35555|FBN1_HUMAN K.INGYPKR.G
 9.4	22	1.0040	K.IEMGNKR.W
 8.4	28	-0.8978	R.DEEEEAK.M
 7.5	35	0.0312	K.KDPDMVK.D

Top scoring peptide matches to query 2144
File7043 Spectrum4237 scans: 3313
Score greater than 16 indicates homology
Score greater than 35 indicates identity

**Score	Expect	Delta Hit Protein	Peptide**
 4.0	78	1.0218	R.EKCLAAR.E
 3.5	87	0.0490	R.MTISPDGK.T
 2.8	1e+002	1.0469	M.EGCAVRR.G
 2.8	1e+002	1.0768	K.ENSSGTPR.V
 2.6 1.1e+002	0.9602	K.KMVAASIK.D
 2.5 1.1e+002	1.0330	-.MPTTIER.E
 1.6 1.4e+002	1.0292	M.ERAGATSR.G
 1.6 1.4e+002	1.0469	K.ERQQMR.L
 1.6 1.4e+002	1.0152	K.DINSTAVK.L
 1.3 1.5e+002	0.0126 178	Q99623|PHB2_HUMAN K.MLGEALSK.N

Top scoring peptide matches to query 2145
File7043 Spectrum6267 scans: 4897
Score greater than 16 indicates homology
Score greater than 35 indicates identity

**Score	Expect	Delta Hit Protein	Peptide**
 3.9	81	-0.0048 347	Q9UF12|PROD2_HUMAN K.VTALTSTR.L

Top scoring peptide matches to query 2147
File7043 Spectrum10963 scans: 8705
Score greater than 18 indicates homology
Score greater than 34 indicates identity

**Score	Expect	Delta Hit Protein	Peptide**
 7.3	24	0.3124	R.LWISAMK.W
 4.9	43	-0.6425	R.SRDIACK.C
 4.8	43	0.3276	R.VAAALMEK.G
 3.9	53	0.3527	R.TLMQSPR.T
 3.3	61	0.3528	K.DRDALMK.S
 3.0	66	0.3229	81	P21281|VATB2_HUMAN -.MALRAMR.G
 2.9	67	0.3415	R.GEISRMR.E
 1.6	90	0.3528	K.KAQENMK.Q
 1.4	95	0.2721	R.LSRFGLR.T
 0.8 1.1e+002	0.3415	K.RSLEGMR.K

Top scoring peptide matches to query 2151
File7043 Spectrum3310 scans: 2565
Score greater than 23 indicates homology
Score greater than 34 indicates identity

**Score	Expect	Delta Hit Protein	Peptide**
 13.9	5.3	-0.3360	53	P20674|COX5A_HUMAN R.CAVAATTR.A
 9.5	15	-0.2884	R.MAQDAGEK.-
 8.6	18	0.6341	K.VTEQLMK.L
 8.5	19	0.6956	K.EADVVCR.Q
 7.8	22	0.6262	R.EFAVNLR.N
 7.0	26	0.6010	K.VNLVFEK.I
 6.8	28	0.7432	R.DPEACEK.L
 6.7	28	-0.3360	R.QTGREMK.V
 6.5	29	0.6228	K.VLSDMKR.H
 6.5	29	-0.3612	K.DKSLQMK.Y

Top scoring peptide matches to query 2152
File7043 Spectrum1282 scans: 1001
Score greater than 24 indicates homology
Score greater than 34 indicates identity

**Score	Expect	Delta Hit Protein	Peptide**
 11.5	9.2	0.5899	K.FAIVDKR.V
 11.5	9.2	0.6262	R.FALVDQR.V
 11.0	10	0.6627	R.VYPANER.F
 10.8	11	0.6481	R.MERLQR.E
 10.4	12	0.6415	K.TESIDKR.F
 9.1	16	0.6229 736	P17252|KPCA_HUMAN K.VMLADRK.G
 8.0	21	0.5865	K.IMTSLKR.-
 7.1	26	-0.2883	R.MAQDAGEK.-
 6.2	31	0.6779	R.VTDDKDR.R
 5.8	35	0.6262	VADFGLAR

Top scoring peptide matches to query 2153
File7043 Spectrum7079 scans: 5536
Score greater than 19 indicates homology
Score greater than 35 indicates identity

**Score	Expect	Delta Hit Protein	Peptide**
 8.9	25	-0.9999	R.RAFDGER.L
 5.6	54	-0.9920	M.LDMGDRK.E
 5.4	56	-0.9491 752	O95954|FTCD_HUMAN R.CGNLACR.S
 5.3	57	0.9443	R.DITIASTK.G
 5.2	59	1.0012	-.MARGNQR.E
 5.0	61	-0.0332	R.TGVIMANK.E
 4.2	73	-0.0332	K.VGKGTEMK.K
 3.5	87	0.9984	R.GVAVLCTE.-
 2.8	1e+002	-1.0722	R.AMKILMK.V
 2.8	1e+002	-1.0172	K.KMVEEAK.V

Top scoring peptide matches to query 2154
File7043 Spectrum6481 scans: 5068
Score greater than 28 indicates homology
Score greater than 35 indicates identity

**Score	Expect	Delta Hit Protein	Peptide**
 20.6	1.7	-0.0528	10+ Q00610|CLH1_HUMAN K.NFLKEAK.L
 14.3	7.2	-0.9488	R.DDDSVTAK.D
 12.9	9.9	-0.0164	R.QFGLQEK.S
 12.3	11	-0.9674	-.MAGELADK.K
 11.8	13	0.0054	K.EGKMAASR.R
 11.3	14	-0.0164	R.IANFQEK.L
 11.3	15	-0.0198	K.LAGDMKSK.V
 10.9	16	-0.0376 616	Q9UHQ4|BAP29_HUMAN R.KTSDALSK.A
 10.9	16	-1.0005	R.DSSAFPVK.R
 10.8	16	-0.9423	-.MAAAADER.S

Top scoring peptide matches to query 2155
File7043 Spectrum12229 scans: 9811
Score greater than 25 indicates homology
Score greater than 35 indicates identity

**Score	Expect	Delta Hit Protein	Peptide**
 12.3	11	-0.0412	K.NKSLMIK.D
 11.5	14	-0.0201	K.FAVPMLR.L
 11.2	15	-0.0048	21	P31040|DHSA_HUMAN R.SMTLEIR.E
 9.8	20	-0.0855	K.LPLHTLR.S
 8.3	29	-0.0048	K.KIENAMK.T
 7.6	33	-0.0491	R.GTFQKLR.E
 7.6	33	-0.0161	K.SAMKQLR.Q
 6.9	39	-0.0015	R.DADFLLR.V
 6.9	39	-0.0015	K.AVEDFLR.S
 6.9	39	0.0349	K.DSDPFIR.K

Top scoring peptide matches to query 2156
File7043 Spectrum12238 scans: 9819
Score greater than 30 indicates homology
Score greater than 35 indicates identity

**Score	Expect	Delta Hit Protein	Peptide**
 33.6	0.085	-0.0046	21	P31040|DHSA_HUMAN R.SMTLEIR.E
 15.1	6	-0.0046	K.AMLTELR.K
 12.2	12	-0.0158	K.SAMKQLR.Q
 11.1	15	-0.9999	-.MATLDRK.V
 10.2	19	-0.9965	K.AFTEINR.L
 9.9	20	1.0409	R.CGNTQLR.V
 9.3	23	-0.0199	K.FAVPMLR.L
 9.3	23	-1.1169	K.HILAKIR.W
 8.8	26	-0.0410	R.VMTNKLK.N
 8.6	27	-0.0012	K.AFDVLER.L

Top scoring peptide matches to query 2157
File7043 Spectrum11726 scans: 9367
Score greater than 21 indicates homology
Score greater than 35 indicates identity

**Score	Expect	Delta Hit Protein	Peptide**
 10.7	17	-0.0038	K.KIENAMK.T
 8.3	29	-0.0150 711	Q7Z4W1|DCXR_HUMAN K.AKTMLNR.I
 8.0	31	-0.0038	K.AAVEGKMK.V
 6.3	46	-0.0038	K.QISVQMK.G
 6.2	47	0.0325	R.GAPGTMTSK.R
 3.9	80	-0.0038	K.GLEKQMK.Q
 3.9	80	-0.0117	K.GNKQQFK.I
 3.9	80	-0.0368	K.VAEKQFK.E
 3.9	80	-0.0191	K.WLQKMK.E
 3.8	82	-0.0005	K.VTGSGGPFK.S

Top scoring peptide matches to query 2158
File7043 Spectrum11442 scans: 9113
Score greater than 35 indicates identity

**Score	Expect	Delta Hit Protein	Peptide**
 59.3	0.00023	-0.0043 500	P09669|COX6C_HUMAN K.AGIFQSVK.-
 36.2	0.047	-0.0407 696	P62753|RS6_HUMAN	R.KLFNLSK.E
 36.2	0.047	-0.0196 697	Q9Y2L6|FRM4B_HUMAN R.KLFQWK.Q
 25.4	0.56	-0.0771	R.KLPAPPVK.A
 24.9	0.63	-0.9454	R.MWRDVK.R
 24.3	0.72	0.0287	K.KIENAMK.T
 24.3	0.72	0.0287	K.QISVQMK.G
 22.5	1.1	-0.0155	R.AGLPLGGHK.S
 21.6	1.4	-0.0196 582	O00748|EST2_HUMAN R.LQFWKK.A
 21.6	1.4	0.0287	R.LQMINSK.W

Top scoring peptide matches to query 2159
File7043 Spectrum13986 scans: 11365
Score greater than 34 indicates homology
Score greater than 35 indicates identity

**Score	Expect	Delta Hit Protein	Peptide**
 25.3	0.58	-0.0048	R.YIEIAIK.Q
 20.4	1.8	-0.0048 680	P53990|IST1_HUMAN R.YLIEIAK.N
 15.8	5.1	-0.0161	R.YLTLAIR.M
 15.7	5.2	-0.0412	R.YLLVLTK.N
 14.4	7.1	0.0897	-.MENQLAK.S
 14.3	7.2	0.0203	R.YLIAVDR.R
 14.2	7.4	0.0050	K.YLIVWR.A
 11.1	15	0.0646	K.EMIEALK.Q
 11.1	15	0.0646	K.EMLEALK.A
 11.1	15	0.0646	K.EMLELAK.N

Top scoring peptide matches to query 2163
File7043 Spectrum4831 scans: 3769
Score greater than 19 indicates homology
Score greater than 35 indicates identity

**Score	Expect	Delta Hit Protein	Peptide**
 10.1	19	0.1267	R.YGGPGGGER.A
 5.7	52	1.0492	K.EFQAVVR.A
 5.7	53	0.0506 635	Q9HCK1|ZDBF2_HUMAN R.MGTKGSLR.V
 5.2	59	1.0669	K.YLHMLR.W
 5.2	59	0.1081	R.YNHMLR.K
 5.1	60	1.0207	R.AGSLLMLK.K
 5.1	60	1.0934	R.KSPEEMK.N
 4.7	65	0.0366	K.ETLVIMK.D
 4.6	67	1.1121	K.QSEEEVK.G
 4.0	77	0.1081	-.MAGGVWGR.S

Top scoring peptide matches to query 2175
File7043 Spectrum6071 scans: 4744
Score greater than 18 indicates homology
Score greater than 35 indicates identity

**Score	Expect	Delta Hit Protein	Peptide**
 8.9	23	-1.1519 614	P62424|RL7A_HUMAN K.VAPAPAVVK.K
 8.8	23	-0.0588	K.TADFGLAR.A
 5.2	53	0.9365	K.EVLEGFR.K
 3.7	76	0.9760	K.GNGAMMIR.I
 3.7	76	0.9219	R.AGKMTIGR.Q
 3.5	79	-0.0257	K.TQMAEVR.C
 3.4	82	0.9080	K.DLLTLMK.L
 3.2	86	-1.0349	R.DDLTIMK.R
 2.8	93	-0.0588	K.TALDAFGR.I
 2.6	99	-0.0435	K.TKESETR.C

Top scoring peptide matches to query 2178
File7043 Spectrum7299 scans: 5720
Score greater than 19 indicates homology
Score greater than 35 indicates identity

**Score	Expect	Delta Hit Protein	Peptide**
 12.5	9.9	1.0370 679	P26232|CTNA2_HUMAN R.KQGETMR.I
 12.3	10	1.0482	R.DLGGSCLK.V
 5.8	47	1.0880	R.DAFPEDR.S
 5.7	48	1.0305	K.QTLSSGEK.Y
 4.2	68	1.0582	R.NNWKCK.S
 3.8	74	0.9941 616	Q9UHQ4|BAP29_HUMAN R.KTSDALSK.A
 3.3	84	0.0279 567	O14773|TPP1_HUMAN R.VNTELMK.A
 2.9	92	1.0330	K.CNPAFLK.F
 2.4	1e+002	0.0676	K.DDLSWSK.T
 2.4	1e+002	-0.9827	R.TSFPLMR.S

Top scoring peptide matches to query 2181
File7043 Spectrum5046 scans: 3941
Score greater than 23 indicates homology
Score greater than 35 indicates identity

**Score	Expect	Delta Hit Protein	Peptide**
 10.7	15	1.0698 718	O43747|AP1G1_HUMAN KYDHMR
 10.6	15	0.0204	K.RYEVAGR.R
 10.6	15	0.0568	R.RYPGSDR.I
 9.7	19	1.0963	K.EADMQQK.K
 9.6	19	0.0317	R.QYSSLPR.Q
 8.2	27	-0.9822	R.KFCQIR.Y
 8.0	28	1.0487	K.KESACVR.L
 6.6	39	-0.9636	K.EYRQQK.K
 6.1	44	-0.9193	R.EGTMELR.T
 5.3	52	0.0316	R.SLSFSGPR.Y

Top scoring peptide matches to query 2184
File7043 Spectrum10281 scans: 8141
Score greater than 29 indicates homology
Score greater than 35 indicates identity

**Score	Expect	Delta Hit Protein	Peptide**
 25.3	0.52	-0.0052 133+ Q6FI13|H2A2A_HUMAN HLQLAIR
 14.8	5.9	-0.0415	K.HILAKIR.W
 12.3	10	0.0741	R.HLCHKR.F
 12.3	10	0.0676	R.HLEAAGPR.V
 12.3	10	0.0312	K.HLLSPQR.A
 12.1	11	0.0425	R.SYGAALLR.A
 10.1	17	0.1264	R.EAFPGDSK.S
 7.5	32	-0.0415	R.LHALKLR.S
 7.3	33	-0.0052	K.IHLQIAR.Q
 6.6	39	-0.0052	K.HIRTPVK.V

Top scoring peptide matches to query 2185
File7043 Spectrum10249 scans: 8116
Score greater than 30 indicates homology
Score greater than 35 indicates identity

**Score	Expect	Delta Hit Protein	Peptide**
 27.1	0.35	-0.0049 133+ Q6FI13|H2A2A_HUMAN HLQLAIR
 15.9	4.6	-0.0413	K.HILAKIR.W
 15.9	4.6	0.0744	R.HLCHKR.F
 15.9	4.6	0.0678	R.HLEAAGPR.V
 15.9	4.6	0.0315	K.HLLSPQR.A
 11.7	12	0.0427	R.SYGAALLR.A
 10.6	16	0.1002	R.NLDFWR.Q
 10.6	16	0.1042	R.RAFDGER.L
 8.9	23	0.0791	K.SYPNTLR.L
 8.3	26	-0.0413	R.LHALKLR.S

Top scoring peptide matches to query 2186
File7043 Spectrum2511 scans: 1942
Score greater than 24 indicates homology
Score greater than 35 indicates identity

**Score	Expect	Delta Hit Protein	Peptide**
 11.4	13	0.0747	M.SDKLPYK.V
 10.9	14	1.0917	R.ISDQKMK.D
 10.7	15	1.1467	M.AEETGQSK.L
 9.8	19	1.1029	R.SLLEMEK.R
 8.2	27	0.0601	R.SLMSLKR.K
 8.1	27	1.0475	K.KAYAQIR.V
 8.2	27	-0.0206	R.LHALKLR.S
 7.8	30	1.1103	K.SNELATSK.R
 7.7	30	1.1281	K.SLMVDER.Q
 7.4	33	1.1380 718	O43747|AP1G1_HUMAN KYDHMR

Top scoring peptide matches to query 2187
File7043 Spectrum6995 scans: 5473
Score greater than 18 indicates homology
Score greater than 33 indicates identity

**Score	Expect	Delta Hit Protein	Peptide**
 7.2	22	-0.6923	R.GGIRVGHR.I
 5.1	36	-0.6334	K.GYSQQLR.E
 4.1	45	0.3585 663	Q9NX20|RM16_HUMAN R.GTLEKMR.K
 3.5	51	0.3585	K.QSLSLMR.D
 3.4	53	0.3585	K.GTVMGTLR.S
 1.7	78	-0.6698	K.FNSSKLR.V
 1.0	92	-0.7789	K.KPIPRIK.R
 0.4 1.1e+002	-0.6810	R.SNKPIHR.A

Top scoring peptide matches to query 2193
File7043 Spectrum8636 scans: 6786
Score greater than 18 indicates homology
Score greater than 35 indicates identity

**Score	Expect	Delta Hit Protein	Peptide**
 6.0	42	1.0954	R.DPSQMEK.L
 5.9	43	1.0591	R.DMLQAEK.A
 5.5	48	1.0591	K.DMKEAEK.N
 5.3	49	0.9896	IADFGLSK
 5.2	50	1.0478	K.TGKCQEK.K
 5.0	53	1.0591	36+ P11142|HSP7C_HUMAN R.MVQEAEK.Y
 4.7	57	1.0049	K.SSISTVEK.R
 4.2	64	1.0543	-.MRAPGMR.S
 4.2	64	1.0842 176	P56199|ITA1_HUMAN K.DCGNKEK.C
 3.8	70	1.0074	R.LWMGLSK.V

Top scoring peptide matches to query 2194
File7043 Spectrum9158 scans: 7215
Score greater than 19 indicates homology
Score greater than 35 indicates identity

**Score	Expect	Delta Hit Protein	Peptide**
 9.7	18	-1.0478	K.DLAKLHR.E
 5.7	45	1.0233	R.ISELNMK.E
 5.6	46	-0.0050	58	Q16853|AOC3_HUMAN K.YTTPLTR.G
 5.6	46	0.0305	M.AMMVFPR.E
 5.5	48	-0.0202	R.IWLTYR.E
 5.5	48	-0.0202	R.LWLTYR.R
 5.3	49	1.0298	R.LSCMLAR.L
 4.7	56	0.0280	R.KVTDNMK.L
 4.7	57	1.0484	R.LSDGTMAR.K
 4.7	57	-0.9638	R.LSQDAYR.R

Top scoring peptide matches to query 2195
File7043 Spectrum8161 scans: 6404
Score greater than 22 indicates homology
Score greater than 35 indicates identity

**Score	Expect	Delta Hit Protein	Peptide**
 11.3	13	0.9907	K.VIIPSPPK.D
 9.1	21	-0.0045 614	P62424|RL7A_HUMAN K.VAPAPAVVK.K
 5.6	46	0.1926	K.DSESGGGKD.-
 5.2	50	0.0094	K.VAGRIHAK.R
 3.2	80	1.0853	-.MSGIALSR.L
 3.1	82	1.0635 707	P50053|KHK_HUMAN K.VDLTQFK.W
 3.1	83	1.1217	R.GLSMSSPR.G
 2.5	94	-0.9634	K.AVLDLHGK.S
 2.5	94	-0.9674 617	P22307|NLTP_HUMAN K.IGGIFAFK.V
 2.1	1e+002	0.9907	K.VLPPLSPK.V

Top scoring peptide matches to query 2197
File7043 Spectrum11691 scans: 9336
Score greater than 23 indicates homology
Score greater than 35 indicates identity

**Score	Expect	Delta Hit Protein	Peptide**
 10.9	13	0.1155	K.EALEAYR.R
 9.8	17	1.1438	K.QAEMEVK.R
 9.3	20	-0.8910	R.NTGGRYGK.A
 9.1	21	-0.9777	37	P55084|ECHB_HUMAN K.VGLPPLEK.F
 8.0	27	1.1437	-.MSTGPDVK.A
 5.3	49	0.1519	K.EEAPSYR.T
 5.1	52	-0.8541	-.MMAEQVK.C
 5.1	52	1.1325	K.QGGVASGMK.H
 5.1	52	-0.9637	R.AEVHLRK.S
 5.0	53	0.1294	K.RNGADYR.D

Top scoring peptide matches to query 2198
File7043 Spectrum11957 scans: 9567
Score greater than 21 indicates homology
Score greater than 35 indicates identity

**Score	Expect	Delta Hit Protein	Peptide**
 20.5	1.5	1.0921	K.QPAHLER.L
 7.6	29	0.1081	M.EPAGPAPGR.L
 5.4	48	1.1033	R.QPTFTTR.L
 5.2	51	0.1160	K.KDMLNSK.T
 5.1	51	-0.8139	K.MNTDPMK.R
 4.2	63	1.0418	K.QLTFTLK.R
 2.4	97	0.1160	R.GAATMSLGK.L
 1.3 1.2e+002	1.1211	K.KTMWER.L
 1.2 1.3e+002	1.1000	60	Q01082|SPTB2_HUMAN R.KLTGMER.D
 1.1 1.3e+002	1.1146	K.ENVYTPK.S

Top scoring peptide matches to query 2203
File7043 Spectrum2916 scans: 2248
Score greater than 22 indicates homology
Score greater than 34 indicates identity

**Score	Expect	Delta Hit Protein	Peptide**
 9.6	17	0.9755	R.EFRPFR.D
 9.6	17	1.0602	R.ERMEDR.C
 8.9	20	1.0020	K.KDGADFAK.W
 6.0	38	0.0068	K.NSFSEIR.I
 5.9	39	-1.0071	-.MAELPHR.I
 5.9	39	-1.0071	K.VCGKSFR.S
 5.5	43	-1.0210 268	P04003|C4BPA_HUMAN K.MALEVYK.L
 5.2	45	1.0238	K.EAMKSNR.-
 5.0	48	1.0160	K.ENGRYGR.R
 4.8	51	0.0510	-.MTTDEGAK.N

Top scoring peptide matches to query 2204
File7043 Spectrum7550 scans: 5919
Score greater than 28 indicates homology
Score greater than 34 indicates identity

**Score	Expect	Delta Hit Protein	Peptide**
 19.4	1.8	-0.0052 625	Q9NX63|CHCH3_HUMAN K.ILQCYR.E
 19.4	1.8	-0.0706	K.LLKEGHR.M
 14.4	5.5	-0.0634	R.ILGAFFGK.N
 14.4	5.5	-0.0668	R.LLKGYMK.I
 14.4	5.5	-0.0958	K.LLQTHLK.R
 14.2	5.7	-0.1573	K.LLLPGVIK.T
 14.2	5.7	-0.1685	R.LLLPRIK.N
 13.8	6.4	-1.0910	R.NLGALLPR.L
 12.9	7.7	-0.0706	R.LLSHINR.D
 12.5	8.6	-1.0798	K.NLLPTPAK.S

Top scoring peptide matches to query 2210
File7043 Spectrum501 scans: 476
Score greater than 16 indicates homology
Score greater than 33 indicates identity

**Score	Expect	Delta Hit Protein	Peptide**
 10.2	9.6	0.6919 569	P47914|RL29_HUMAN K.FLRNMR.F
 3.0	51	-0.2623	R.EYGSLER.S
 2.2	60	-0.2656	R.ETSMKNK.T
 2.2	61	0.7249	K.QRMMVR.A
 1.6	69	0.7110	-.MDLSMKK.F
 1.5	72	-0.3350	R.KSASFSVK.S
 1.4	73	-0.3099	K.ASKFGSTR.G
 1.2	76	-0.2479	K.MMTGELR.N
 1.2	76	-0.2479	K.MMTGELR.N
 0.4	93	-0.2987	R.TTIESFR.A

Top scoring peptide matches to query 2212
File7043 Spectrum30496 scans: 26985
Score greater than 22 indicates homology
Score greater than 33 indicates identity

**Score	Expect	Delta Hit Protein	Peptide**
 9.7	12	0.7475	R.ALKMSFR.G
 8.9	15	0.7913 622	Q9UBC0|HNF6_HUMAN K.SGRETFR.R
 8.2	17	0.6569	R.GLVVPVIR.N
 7.9	19	-0.2040	R.TRGSNYR.F
 5.0	37	0.8533	K.LAADCMR.D
 2.2	69	0.7549	R.GHLEAVAR.H
 2.2	69	-0.2035	K.MMGGKGLK.Q
 2.2	70	-0.2291	K.SPAPGAPTR.S
 2.1	71	-0.2365	K.LSLMYAR.K
 1.8	77	0.7509	R.VAFVNFR.R

Top scoring peptide matches to query 2217
File7043 Spectrum4439 scans: 3470
Score greater than 19 indicates homology
Score greater than 34 indicates identity

**Score	Expect	Delta Hit Protein	Peptide**
 7.7	25	0.0236	R.IYDGEEK.I
 5.9	38	1.0043	R.MTLSQEK.D
 5.9	38	0.9679	R.TMVTKEK.E
 5.7	40	1.0472	R.QMCQAAK.A
 4.8	48	-0.9902	R.LYAMNNI.-
 4.6	51	-0.0062	10+ Q00610|CLH1_HUMAN K.YIQAACK.T
 4.4	54	0.0084	R.YIDWEK.L
 3.6	65	-0.0274	K.KMSGTVSK.H
 3.0	74	-1.0053	R.RSSDPHR.L
 2.7	80	-1.0266	R.YLCSIAK.I

Top scoring peptide matches to query 2219
File7043 Spectrum4554 scans: 3553
Score greater than 24 indicates homology
Score greater than 34 indicates identity

**Score	Expect	Delta Hit Protein	Peptide**
 41.6	0.01	-0.0057	10+ Q00610|CLH1_HUMAN K.YIQAACK.T
 9.2	18	-0.0169	K.NRYICK.Q
 7.4	27	0.0241	R.IYDGEEK.I
 6.0	37	-0.9711 730	P22303|ACES_HUMAN R.NYTAEEK.I
 5.9	38	0.9895	R.SSMKPGFV.-
 5.4	42	0.9354	K.LKYATEK.K
 5.3	44	0.0273	K.SSVCICK.L
 4.2	56	-0.0639	K.ADFLLFK.V
 4.0	59	0.0161	-.MASTRCK.L
 3.2	70	-0.0091	-.MASKCLK.A

Top scoring peptide matches to query 2221
File7043 Spectrum7072 scans: 5529
Score greater than 30 indicates homology
Score greater than 34 indicates identity

**Score	Expect	Delta Hit Protein	Peptide**
 40.5	0.013	-0.0054	26	Q14764|MVP_HUMAN R.HADLEIR.L
 15.1	4.6	-0.0054	K.HVEAELR.S
 14.9	4.7	0.0566	K.MMTGELR.N
 14.9	4.7	0.0566	K.MMTGELR.N
 12.7	7.9	-0.0167	R.HADGKLGR.T
 11.0	12	0.0058	R.TTIESFR.A
 9.5	17	-0.9751	K.KSMGMIR.K
 9.4	17	1.0439	R.EAFGCLR.A
 8.3	22	1.0262	K.NSFSEIR.I
 8.0	23	-0.9499	-.MASRSMR.L

Top scoring peptide matches to query 2222
File7043 Spectrum6232 scans: 4870
Score greater than 21 indicates homology
Score greater than 34 indicates identity

**Score	Expect	Delta Hit Protein	Peptide**
 9.1	18	1.0673	R.SSENMIR.E
 8.1	23	0.0647	K.MMTGELR.N
 7.8	25	1.0343	K.NSFSEIR.I
 7.7	25	-0.9119	R.STTMNER.A
 7.6	26	-0.9418	-.MASRSMR.L
 7.3	28	-0.8942	K.MSSMNER.T
 7.0	29	-0.8942	K.MACTSER.C
 6.9	30	0.0065 268	P04003|C4BPA_HUMAN K.MALEVYK.L
 6.9	30	1.0860	R.ESSSSAER.Q
 6.8	31	1.0343	R.GAEYVSAR.E

Top scoring peptide matches to query 2225
File7043 Spectrum30362 scans: 26871
Score greater than 17 indicates homology
Score greater than 33 indicates identity

**Score	Expect	Delta Hit Protein	Peptide**
 7.0	22	0.2473	R.NWKDYK.Q
 3.6	49	0.2188 268	P04003|C4BPA_HUMAN K.MALEVYK.L
 3.2	54	-0.7071	K.TGGMVDMK.K
 2.9	57	0.2955	R.LSDMSER.L
 2.8	59	0.2075	K.LSLMYAR.K
 2.8	59	0.2327	-.MAELPHR.I
 2.8	59	0.2075	-.MSLLFSR.C
 2.5	63	-0.7514	K.MGYGIGTR.L
 2.5	63	-0.7691	K.STYGNGKK.Y
 2.4	64	-0.7401	K.VAMGAFDK.T

Top scoring peptide matches to query 2227
File7043 Spectrum5390 scans: 4205
Score greater than 18 indicates homology
Score greater than 32 indicates identity

**Score	Expect	Delta Hit Protein	Peptide**
 8.2	15	-0.5365	R.KSTTMDR.R
 4.8	32	0.3418 119+ P27635|RL10_HUMAN R.QKIHISK.K
 4.6	33	-0.5993	R.CGIIHVR.G
 2.9	50	0.4033	K.QQNKAHK.G
 2.3	57	0.4258	K.AGFSSGSLK.S
 2.3	57	0.3166	K.KLPPASLK.H
 2.3	57	0.3669	K.RPLAPSGR.S
 1.8	64	-0.5371	R.QGPYFDK.S
 1.3	71	0.4687	ECGKGFR
 1.3	71	0.5051	K.ECGNAFR.L

Top scoring peptide matches to query 2231
File7043 Spectrum4759 scans: 3715
Score greater than 24 indicates homology
Score greater than 34 indicates identity

**Score	Expect	Delta Hit Protein	Peptide**
 11.7	9.3	-0.0051	4	P98164|LRP2_HUMAN R.ASMDGSMR.T
 10.8	12	0.9281	R.SVPGHDNK.M
 6.3	32	0.9798	R.GGSSSESSR.V
 6.1	34	-0.0922 533	Q3LXA3|DHAK_HUMAN R.ASYISSAR.L
 5.5	39	-0.0228	R.STTMNER.A
 5.5	39	-0.1035	M.AEERPPR.L
 5.0	44	-0.1286	R.KYLTSSR.E
 4.6	48	0.8554	K.KGEPLGPR.G
 4.4	51	0.8956	K.EFIGIMK.D
 3.7	59	-1.0585	R.EFEMKR.M

Top scoring peptide matches to query 2234
File7043 Spectrum15725 scans: 12951
Score greater than 22 indicates homology
Score greater than 34 indicates identity

**Score	Expect	Delta Hit Protein	Peptide**
 24.6	0.5	-0.0061	51	P21796|VDAC1_HUMAN K.GYGFGLIK.L
 7.7	24	-0.0384	K.ESIKHLK.E
 7.1	28	-0.0067	K.CRLHLR.N
 7.1	28	0.0231	R.EAQTHIR.A
 7.1	28	0.0045	R.MAVQHLR.S
 7.1	28	0.0409	-.MGPQHLR.L
 7.1	28	-0.0067	K.RLCHIR.G
 7.1	28	-0.0132	K.TAKEHLR.K
 7.0	28	-0.0748	K.RLPDLIK.E
 6.8	29	0.0674	R.SSSMASLR.S

Top scoring peptide matches to query 2235
File7043 Spectrum15720 scans: 12947
Score greater than 26 indicates homology
Score greater than 34 indicates identity

**Score	Expect	Delta Hit Protein	Peptide**
 18.3	2.1	0.0196	K.YGGHPVPK.F
 12.3	8.5	-0.0055	51	P21796|VDAC1_HUMAN K.GYGFGLIK.L
 6.8	30	-0.9604	K.EGNLGTHK.A
 6.3	33	-0.9967	K.AVDQGLPR.R
 6.3	33	-1.0120	K.GWGLLGPR.N
 6.3	33	-1.0331	K.VNLSGLPR.D
 5.1	44	0.9098	R.LQKVIPR.H
 4.9	46	-1.0695	K.IRTIAPGK.T
 4.7	49	0.0461	R.YGETKEK.D
 4.5	50	0.0639	K.GYIDQMK.Q

Top scoring peptide matches to query 2238
File7043 Spectrum9302 scans: 7334
Score greater than 20 indicates homology
Score greater than 34 indicates identity

**Score	Expect	Delta Hit Protein	Peptide**
 7.3	27	-0.1048	K.RLANLIR.R
 7.3	27	-1.0637	R.RNNAILR.A
 6.5	33	-1.0161 101+ P19440|GGT1_HUMAN K.RAVEEPR.L
 4.7	50	-0.0473	R.RLWAGPR.V
 4.3	56	0.0445	R.TDMAFVR.G
 3.5	67	-1.0776	K.VLNILER.T
 3.3	69	0.0259	R.LCFMLR.V
 2.3	88	0.0809	K.TGDPYMR.S
 2.2	90	-1.0637	R.RVAAAAAAR.L
 2.1	91	0.0293	M.SWMFLR.D

Top scoring peptide matches to query 2240
File7043 Spectrum8593 scans: 6751
Score greater than 25 indicates homology
Score greater than 34 indicates identity

**Score	Expect	Delta Hit Protein	Peptide**
 20.6	1.3	-0.9272	K.ISSSYSGR.V
 11.0	12	0.9429 465	P35606|COPB2_HUMAN M.PLRLDIK.R
 10.2	14	-0.9247	R.VCFFAGR.E
 10.2	14	-0.9281	R.VCKMFR.Q
 9.4	17	-0.9094	K.KMDSGYR.K
 7.9	24	-0.9425	R.GYQIGYR.E
 7.8	25	-1.0840	K.VIRSILR.E
 7.8	25	-1.0112	R.VLRTDPR.Y
 7.8	25	-1.0000	R.VPDGLLSR.A
 7.8	25	-1.0364	R.VPTATLVR.V

Top scoring peptide matches to query 2242
File7043 Spectrum5990 scans: 4681
Score greater than 15 indicates homology
Score greater than 34 indicates identity

**Score	Expect	Delta Hit Protein	Peptide**
 2.9	76	0.9889	K.NLPRINK.L
 2.1	90	-0.8960 719	O00338|ST1C2_HUMAN K.VMQFMGK.K
 1.8	97	1.1197	K.NYMMPAK.G
 0.9 1.2e+002	1.0002	R.LDLLPQR.V
 0.5 1.3e+002	1.0141	R.RLGPGGAAR.L
 0.3 1.4e+002	0.0624	R.AHAGPGAFK.C
 0.2 1.4e+002	1.0001 544	P07197|NFM_HUMAN	K.VTSHAIVK.E

Top scoring peptide matches to query 2245
File7043 Spectrum6225 scans: 4865
Score greater than 27 indicates homology
Score greater than 34 indicates identity

**Score	Expect	Delta Hit Protein	Peptide**
 14.6	5.1	0.0314	KPEEKPK
 13.6	6.5	-0.9890	K.KLEEIPK.Y
 13.3	7	-0.9275	R.HSTLSSPK.T
 12.3	8.7	-0.8622 691	Q96N66|MBOA7_HUMAN K.EMASGFSK.G
 11.4	11	-1.0003	K.KNAIPVSK.S
 10.9	12	-0.0778	K.KILQLIK.S
 9.7	16	1.0769	R.DGRGPGAPK.R
 9.5	17	-1.0115	K.QLLQRAK.K
 9.2	18	-0.9825	K.QLVPLMR.L
 9.1	18	-0.9752	R.RGVPTQAK.G

Top scoring peptide matches to query 2248
File7043 Spectrum5198 scans: 4057
Score greater than 20 indicates homology
Score greater than 33 indicates identity

**Score	Expect	Delta Hit Protein	Peptide**
 8.3	15	0.6799 646	P50402|EMD_HUMAN R.AIRPENR.A
 7.4	18	0.7486	R.DYAPPHR.D
 1.3	75	0.6547	R.GLGLPVGSR.Q
 1.2	77	0.7162	R.ERPGGSPR.G
 1.0	80	0.6659	K.LLLPDER.A
 0.9	82	0.6911	R.LVQPQDR.Q
 0.9	82	-0.2970	K.KYPGTYK.R
 0.9	82	0.7903	R.SSSSSSEGK.E
 0.7	85	-0.2790	R.ARPGGADGR.V
 0.7	85	0.6322	K.ARPLSRR.G

Top scoring peptide matches to query 2249
File7043 Spectrum3632 scans: 2816
Score greater than 18 indicates homology
Score greater than 34 indicates identity

**Score	Expect	Delta Hit Protein	Peptide**
 7.7	27	0.8619 646	P50402|EMD_HUMAN	R.AIRPENR.A
 5.1	49	-1.1061	R.SSTLANHK.R
 4.8	52	0.8943	R.FGNKAYR.T
 4.7	53	0.9459	R.SGSSFSQR.A
 4.6	54	0.8691	R.SGLIFYR.K
 4.6	54	0.9095	K.EPVPNSGR.S
 4.5	56	0.9603 635	Q9HCK1|ZDBF2_HUMAN R.ERMMTR.L
 4.5	56	0.9273	K.FGTSCKR.S
 3.8	65	0.9273	K.GMAGGKYR.S
 3.7	67	0.9749	R.ECLYDR.I

Top scoring peptide matches to query 2253
File7043 Spectrum5605 scans: 4379
Score greater than 31 indicates homology
Score greater than 35 indicates identity

**Score	Expect	Delta Hit Protein	Peptide**
 34.3	0.062	-0.0051 103	P27487|DPP4_HUMAN K.AGAVNPTVK.F
 16.7	3.5	-1.0255	R.EAVLQGLK.K
 15.9	4.2	-1.0255	R.QAVELLGK.A
 15.5	4.6	-0.0415	R.AGAAAVVLGK.Q
 15.5	4.7	-1.0367	K.QAELKIR.A
 12.4	9.4	-1.0004	R.AGALEIQR.R
 12.4	9.4	-1.0367	R.KAELLQR.Q
 12.1	10	-1.0255	K.EILQAGVK.G
 11.8	11	-1.0367	R.IGQNKAVK.V
 11.4	12	-1.0619	K.EALKVIGK.K

Top scoring peptide matches to query 2254
File7043 Spectrum7820 scans: 6134
Score greater than 35 indicates identity

**Score	Expect	Delta Hit Protein	Peptide**
 35.8	0.043	-0.0047	42+ P05141|ADT2_HUMAN K.TAVAPIER.V
 20.1	1.6	0.0494	R.TMPAPAPR.A
 16.4	3.8	0.0204	K.RDAEIPR.H
 15.8	4.4	-0.0299 175	P61421|VA0D1_HUMAN R.SIAELVPK.C
 15.4	4.7	0.0819	R.GGGGGGGGGAPR.G
 10.5	15	1.0157	R.NGVPEALR.G
 9.8	17	0.0052	K.ATFKHPR.T
 9.8	17	-0.0160	R.VLRTDPR.Y
 9.7	18	-0.0047	R.VPDGLLSR.A
 8.4	24	-0.0121	M.ATMIPPVK.L

Top scoring peptide matches to query 2255
File7043 Spectrum10913 scans: 8663
Score greater than 35 indicates identity

**Score	Expect	Delta Hit Protein	Peptide**
 31.2	0.12	-0.0053	42+ P05141|ADT2_HUMAN R.GNLANVIR.Y
 26.5	0.37	0.9535	R.LGIQGVIR.G
 21.7	1.1	-0.9894	K.DGLALGVGR.D
 21.1	1.3	0.0925	R.GGGGGGGGGAPR.G
 18.8	2.2	0.0158	GNLHFIR
 15.4	4.7	0.9171	R.LGIARVVK.N
 15.1	5.1	0.9171	R.LLKVNIR.T
 15.0	5.2	1.0011	K.AVDGGIPVK.H
 14.8	5.4	0.9899	K.LGAPQTLR.S
 14.8	5.4	0.9535	K.LGGLQVLR.T

Top scoring peptide matches to query 2267
File7043 Spectrum557 scans: 512
Score greater than 24 indicates homology
Score greater than 33 indicates identity

**Score	Expect	Delta Hit Protein	Peptide**
 14.0	4.8	0.8201	42+ P05141|ADT2_HUMAN R.MMMQSGR.K
 13.9	4.9	0.8201	42+ P05141|ADT2_HUMAN R.MMMQSGR.K
 10.6	11	0.7694	R.SSCSLFR.S
 10.2	12	-0.3416	K.QVIELAGK.Q
 10.1	12	-0.3164	K.AGLVENVR.T
 9.6	13	0.6788	K.ETQILPR.Q
 8.6	17	-0.2800	R.EAPAEGKR.L
 8.2	18	-0.3528	K.GAVITQIR.L
 7.8	20	0.6676	K.SKQPGLAR.S
 7.7	21	-0.3780	K.KVVLVDGK.T

Top scoring peptide matches to query 2269
File7043 Spectrum31515 scans: 27763
Score greater than 20 indicates homology
Score greater than 33 indicates identity

**Score	Expect	Delta Hit Protein	Peptide**
 8.0	19	0.7701	R.SSCSLFR.S
 7.6	21	-0.3408	K.QVIELAGK.Q
 7.3	22	0.8209	42+ P05141|ADT2_HUMAN R.MMMQSGR.K
 7.3	22	0.8209	42+ P05141|ADT2_HUMAN R.MMMQSGR.K
 5.7	33	-0.3772	K.KVVLVDGK.T
 5.5	34	0.7411	R.QGPAGSPSR.F
 5.3	36	0.8395	K.TMNGDMR.M
 5.3	36	0.7371	K.GSFAGVYR.A
 5.0	38	0.7371	R.TWAPEPR.-
 4.7	41	-0.3408	K.QVLADIAK.Q

Top scoring peptide matches to query 2270
File7043 Spectrum13491 scans: 10922
Score greater than 23 indicates homology
Score greater than 35 indicates identity

**Score	Expect	Delta Hit Protein	Peptide**
 11.5	13	0.9002	R.VTIASLPR.N
 10.9	16	-1.0790	M.VLSELAAR.L
 9.4	22	0.8526	R.IVLIRSR.K
 9.1	24	-1.0427 510	P00966|ASSY_HUMAN R.IDIVENR.F
 8.9	24	-1.0651	R.LSARNAAR.R
 8.9	25	0.9254	R.VLRTDPR.Y
 7.1	37	0.9986	R.VMTSKMK.N
 7.1	37	0.9002	K.VLLDQLR.F
 7.1	37	0.9002 226	Q7Z406|MYH14_HUMAN R.LVLDQLR.C
 6.9	39	-1.1406	K.TLLLGTIK.M

Top scoring peptide matches to query 2271
File7043 Spectrum5124 scans: 3999
Score greater than 21 indicates homology
Score greater than 35 indicates identity

**Score	Expect	Delta Hit Protein	Peptide**
 13.3	9	1.0949	42+ P05141|ADT2_HUMAN R.MMMQSGR.K
 7.7	33	-0.9681	K.NSTAYFR.G
 7.2	36	0.9788	K.THLKDSR.L
 6.6	42	1.0949	42+ P05141|ADT2_HUMAN R.MMMQSGR.K
 6.1	47	1.0264	K.ISSSYSGR.V
 6.0	48	-0.0304	R.LPKAEGDK.S
 5.8	51	1.0516	K.QAEESHR.Q
 5.7	52	0.9350	K.QLVPLMR.L
 5.5	54	1.0151	K.HTATVDGR.T
 5.5	54	0.9675	K.HTSRSLR.L

Top scoring peptide matches to query 2272
File7043 Spectrum2959 scans: 2282
Score greater than 26 indicates homology
Score greater than 35 indicates identity

**Score	Expect	Delta Hit Protein	Peptide**
 13.6	8.4	-0.0046	R.RPEVGSGR.S
 13.4	8.7	0.1228	R.ECMVMR.D
 13.0	9.6	1.1068	42+ P05141|ADT2_HUMAN R.MMMQSGR.K
 12.7	10	1.0747	R.DFTSSGSR.K
 12.7	10	1.1618	R.MDDCGSR.S
 11.1	15	-1.0362	RIAQASGR
 10.8	16	-0.0410	R.RIADQVR.L
 10.6	17	-0.9410	K.DGPAAQDGK.I
 10.4	17	0.0356	R.FSVTTMR.D
 10.2	19	-0.0046	K.LRNPSDR.K

Top scoring peptide matches to query 2274
File7043 Spectrum7577 scans: 5942
Score greater than 20 indicates homology
Score greater than 35 indicates identity

**Score	Expect	Delta Hit Protein	Peptide**
 19.2	2.3	1.0852	R.GGGGGGGGGAPR.G
 6.1	47	0.0761	K.DGPTSQPR.L
 5.0	60	-0.9807	R.LDPGTISR.G
 4.5	68	-1.0283	R.RDVLLSR.Q
 4.4	69	0.9986	42+ P05141|ADT2_HUMAN K.TAVAPIER.V
 4.2	73	-0.8537	K.SEGYCSR.I
 3.2	92	-0.9079	M.AEGPEEAR.G
 3.0	97	-1.0170	R.KEILEAR.T
 3.0	97	-0.9806	R.QELLEAR.F
 2.7	1e+002	-1.0534	K.KTLVLER.R

Top scoring peptide matches to query 2278
File7043 Spectrum10303 scans: 8160
Score greater than 19 indicates homology
Score greater than 36 indicates identity

**Score	Expect	Delta Hit Protein	Peptide**
 8.0	34	-0.0400	K.VEPEQEK.W
 5.7	57	0.9328	K.NLPNTNGK.S
 2.7 1.1e+002	-0.0989 101+ P19440|GGT1_HUMAN K.VLREGER.L
 2.3 1.2e+002	0.9579	R.ARPDNER.Q
 1.1 1.7e+002	-0.0553	R.YTNGPPPL.-
 0.8 1.7e+002	-0.1141	K.VLRWER.A
 0.6 1.8e+002	0.8236	R.LLRLSQK.K

Top scoring peptide matches to query 2280
File7043 Spectrum8965 scans: 7058
Score greater than 20 indicates homology
Score greater than 36 indicates identity

**Score	Expect	Delta Hit Protein	Peptide**
 9.1	26	0.0048	R.INHTAFR.T
 6.6	46	-0.0051 510	P00966|ASSY_HUMAN R.IDIVENR.F
 6.5	48	0.9174	K.KVVLVDGK.T
 6.3	49	-0.0125	K.LDIICPK.V
 6.3	49	-0.0527	R.LDLSVRR.T
 6.3	49	-0.9639	K.NDLDLNR.S
 6.3	49	-0.9639	R.NDLNLDR.S
 3.5	94	-0.9388	R.SGDRDRPG.-
 3.5	95	1.0920	K.KMFESES.-
 2.6 1.1e+002	0.0340	R.GGGGRGQGGR.G

Top scoring peptide matches to query 2283
File7043 Spectrum4199 scans: 3282
Score greater than 24 indicates homology
Score greater than 36 indicates identity

**Score	Expect	Delta Hit Protein	Peptide**
 13.1	10	-0.0313	R.LLGGTLER.V
 10.5	19	-1.0378	R.RLVATGSR.F
 9.7	23	-0.0174	R.IARTQGGR.Y
 8.2	32	0.0051	K.VVEELNR.L
 7.8	35	0.0051	K.QAGLEAAAK.A
 7.8	35	1.0506	R.AGNSAQGPR.S
 7.7	36	0.9275	K.KVVLVDGK.T
 6.8	44	-0.0313	K.VVTAAAQAK.Q
 6.7	45	0.9639	K.QVLADIAK.Q
 6.7	45	0.0051 510	P00966|ASSY_HUMAN R.IDIVENR.F

Top scoring peptide matches to query 2284
File7043 Spectrum5434 scans: 4236
Score greater than 25 indicates homology
Score greater than 36 indicates identity

**Score	Expect	Delta Hit Protein	Peptide**
 20.1	2.1	-0.9467	-.MGDQRPR.D
 11.3	16	1.0915	K.EMSATFR.Q
 10.7	18	1.0261	R.GEDGKPVR.K
 10.7	18	1.0650	R.WDIMHR.L
 10.7	18	1.0796	K.WDYFAR.D
 10.6	19	1.0697	K.EGDFVYK.E
 9.8	22	1.0261	R.GEVGVPGSR.G
 9.2	26	1.0624	R.EGTPGSPGR.G
 8.9	27	-0.9177	-.MATPMHR.L
 7.7	36	-0.9354 405	P56181|NDUV3_HUMAN R.MPQPSSGR.E

Top scoring peptide matches to query 2288
File7043 Spectrum11731 scans: 9370
Score greater than 27 indicates homology
Score greater than 34 indicates identity

**Score	Expect	Delta Hit Protein	Peptide**
 19.1	1.8	-0.5735	R.KNEEVLK.R
 19.0	1.9	-0.5623	K.ELEEVLK.S
 19.0	1.9	-0.5735	K.ELQTQIK.D
 19.0	1.9	-0.5735	K.NKEEVLK.A
 19.0	1.9	-0.5847	K.NQKTQIK.L
 13.7	6.3	-0.5847	K.ISGTVNIR.T
 13.7	6.3	-0.5847 426	Q7Z3B1|NEGR1_HUMAN R.KGDTAVLR.C
 13.6	6.4	0.5197	K.VEGDPEGR.E
 12.6	8.2	-0.5596 650	Q96AG4|LRC59_HUMAN K.AGSKGGNLR.D
 11.7	10	-0.5847	R.LSKENIR.T

Top scoring peptide matches to query 2290
File7043 Spectrum12911 scans: 10396
Score greater than 19 indicates homology
Score greater than 36 indicates identity

**Score	Expect	Delta Hit Protein	Peptide**
 7.9	36	0.9205	K.MRIPVAR.V
 6.2	53	-0.0813	K.KIIGDGTR.D
 5.6	61	-0.0350	K.TPRGSWR.W
 5.6	62	0.9140	R.LSAELGLR.Q
 5.0	71	-0.0925	R.RLVATGSR.F
 4.9	72	0.9028	K.IANGTVKR.K
 4.6	76	-1.0765	R.TQKAGVTR.S
 4.3	83	0.0205	R.TMSTFTR.R
 4.0	89	0.0093 405	P56181|NDUV3_HUMAN R.MPQPSSGR.E
 3.9	91	-0.1176	R.TIKTLQR.T

Top scoring peptide matches to query 2291
File7043 Spectrum12758 scans: 10265
Score greater than 36 indicates identity

**Score	Expect	Delta Hit Protein	Peptide**
 43.0	0.011	-1.0999 622	Q9UBC0|HNF6_HUMAN R.ITTELKR.Y
 27.9	0.36	-1.0272	K.DAEEIKR.Q
 27.9	0.36	-1.0384	K.GGNSEIKR.I
 24.8	0.73	0.8793	R.LISELKR.Q
 24.8	0.73	0.9521	K.SPEELKR.I
 24.6	0.76	-1.0999	R.ITTLEKR.Y
 19.5	2.5	-0.0908	R.LSSRLQR.S
 18.0	3.5	-0.0544	K.RSSLSPGR.G
 17.0	4.4	-1.0458	K.LGCLEIR.R
 17.0	4.4	-1.0458	R.LGCLLER.L

Top scoring peptide matches to query 2293
File7043 Spectrum6633 scans: 5186
Score greater than 21 indicates homology
Score greater than 36 indicates identity

**Score	Expect	Delta Hit Protein	Peptide**
 8.6	31	0.9790	K.LDGTTPVR.Y
 8.2	34	-0.0050	26	Q14764|MVP_HUMAN K.VEVVEER.Q
 7.7	38	0.9426	R.KEQGLVGK.Y
 6.0	56	-0.0315	K.NPVFAGVR.L
 5.7	60	0.9968	K.LGGLMDPR.Q
 5.2	67	-0.0349	R.NLCLALR.G
 5.1	69	0.0704	GGGGGGGGGGGGR
 4.9	72	0.9314	R.LASNVAKR.K
 4.8	73	0.9240	K.GIGVLMIR.D
 4.5	78	1.0042	R.ERTPEAR.I

Top scoring peptide matches to query 2296
File7043 Spectrum11840 scans: 9466
Score greater than 31 indicates homology
Score greater than 36 indicates identity

**Score	Expect	Delta Hit Protein	Peptide**
 36.9	0.045	-0.0043 167	O00264|PGRC1_HUMAN R.ILMAINGK.V
 16.3	5.2	0.0144 128	P05387|RLA2_HUMAN K.VISELNGK.N
 13.0	11	0.0143	R.ILDSVQGK.S
 12.6	12	0.1010	R.DAGGGAGAAGR.D
 8.9	29	-0.9744	K.INMAGAKR.V
 8.0	35	0.0824	SRNAHMK
 6.8	46	0.0143	K.ILSDGQVK.V
 6.4	51	-0.9809	R.SDLLLSGR.D
 6.2	54	0.9871	K.TQVKNIR.Q
 6.1	54	0.9984	R.SDLLAAIR.M

Top scoring peptide matches to query 2298
File7043 Spectrum11040 scans: 8766
Score greater than 23 indicates homology
Score greater than 34 indicates identity

**Score	Expect	Delta Hit Protein	Peptide**
 16.4	3.4	-0.3317	R.ERLAEDK.L
 9.2	18	-0.3317 432	Q9H9B4|SFXN1_HUMAN R.LGESANAAK.Q
 8.6	21	0.5973	R.MRQIAIK.F
 7.6	26	0.5908	R.AESLAKIK.S
 6.8	31	0.6271	R.GITGIVGDK.G
 6.7	32	0.6635 525	P07910|HNRPC_HUMAN GDDLQAIK
 6.6	33	0.6635	R.GNLEGELK.S
 5.7	40	0.6523	K.EENARLK.N
 5.6	41	-0.4045	K.TSLVAKNK.I
 5.4	43	0.5834	R.MELIILK.A

Top scoring peptide matches to query 2300
File7043 Spectrum8023 scans: 6298
Score greater than 20 indicates homology
Score greater than 36 indicates identity

**Score	Expect	Delta Hit Protein	Peptide**
 8.0	35	0.8797	R.TLADIKAK.A
 6.8	45	0.9848	M.AEYSYVK.S
 6.5	48	0.9048 751	O15269|SPTC1_HUMAN R.SIALTQAR.Y
 5.8	58	0.9048	K.SALGDRIK.E
 5.0	68	1.0205	K.HREDMR.G
 4.8	73	0.9048	M.AAAVSSVVR.R
 4.3	81	0.9161	R.TGILQEAK.R
 4.3	81	0.9161	K.DVLNSLAK.C
 4.0	86	0.9048	R.VVSSVNVR.L
 4.0	87	0.8896	K.GAFPARLK.K

Top scoring peptide matches to query 2301
File7043 Spectrum7077 scans: 5534
Score greater than 27 indicates homology
Score greater than 36 indicates identity

**Score	Expect	Delta Hit Protein	Peptide**
 21.0	1.7	-0.0051	73	P80365|DHI2_HUMAN R.NVGQWEK.R
 12.8	11	-0.0666	R.LALTWEK.L
 9.7	23	-0.0626	R.LREVSEK.L
 6.4	50	-0.0262	R.KPSNASEK.E
 6.1	54	-0.0336	R.LMPGSVEK.F
 5.3	64	-0.0011	K.RQNEADK.T
 5.3	65	-0.0812	K.ALMIREK.Y
 5.2	66	-0.0812	K.IAIMREK.H
 4.4	80	-0.0309	R.AAAAGGMRR.D
 2.9 1.1e+002	-0.0303	K.TIPSWEK.G

Top scoring peptide matches to query 2302
File7043 Spectrum7085 scans: 5542
Score greater than 18 indicates homology
Score greater than 36 indicates identity

**Score	Expect	Delta Hit Protein	Peptide**
 8.1	34	0.9214	K.GLISGKER.A
 5.5	62	0.9140	K.VLEVMLR.N
 5.4	63	0.9426	R.GLLSGWAR.N
 5.3	65	0.0167	MHSGEKR
 5.0	69	0.8963 350+ P07355|ANXA2_HUMAN K.KELASALK.S
 4.8	72	0.9318	R.MMLVLPR.L
 4.6	76	0.9392	R.QVVNMIR.Q
 4.5	77	0.8599	K.SSKAILIK.E
 4.0	88	0.9102	R.IVASRTGR.N
 3.8	91	0.9028	R.QMIAIRK.R

Top scoring peptide matches to query 2303
File7043 Spectrum7004 scans: 5480
Score greater than 30 indicates homology
Score greater than 36 indicates identity

**Score	Expect	Delta Hit Protein	Peptide**
 17.3	4.1	-1.0575	K.DVGKTSVR.L
 16.1	5.3	-0.0305	R.AAAAGGMRR.D
 13.6	9.6	-0.0047	73	P80365|DHI2_HUMAN R.NVGQWEK.R
 11.1	17	-1.0939	R.LTSVSAKR.T
 10.8	18	0.0058	R.GRGGPGGMR.G
 10.3	21	-0.0445	K.IAGSLPMR.V
 10.2	21	-0.0371 460	O94826|TOM70_HUMAN R.GRGDASGLK.R
 10.1	21	-0.0259	K.GEKGDGGIK.G
 9.5	25	-1.0033	R.DVAACAVR.F
 9.4	25	0.0171	M.NVQPCSR.C

Top scoring peptide matches to query 2304
File7043 Spectrum7343 scans: 5753
Score greater than 25 indicates homology
Score greater than 36 indicates identity

**Score	Expect	Delta Hit Protein	Peptide**
 15.4	6.3	-0.0412	K.MVVLDAGR.Y
 15.0	6.9	0.0139	R.QDEEALR.Y
 12.2	13	-0.0490	K.GREPFVR.M
 12.2	13	-1.0252	98	P28331|NDUS1_HUMAN R.LTEPMVR.N
 11.5	16	-1.0066	R.EDELVTR.I
 10.7	19	0.9979	R.IEAGAQDR.F
 9.6	24	-0.9814	R.ENSDLQR.Q
 9.3	26	0.9979	R.LQEAENR.N
 9.1	27	0.9978	K.GGVDPTASR.V
 9.1	27	-1.0252	R.EDVVLMR.A

Top scoring peptide matches to query 2305
File7043 Spectrum13220 scans: 10677
Score greater than 22 indicates homology
Score greater than 36 indicates identity

**Score	Expect	Delta Hit Protein	Peptide**
 9.3	25	-0.0700	K.RPVLGYR.N
 8.6	30	-1.0137	K.VRDSTGAR.V
 8.0	35	-1.0541	R.QLAVFQR.N
 6.9	44	0.9405 426	Q7Z3B1|NEGR1_HUMAN R.KGDTAVLR.C
 6.6	47	0.9769	M.KSSGPVER.L
 6.3	51	-0.0370	K.RLLGDMR.F
 6.1	54	-1.0541	R.ALQQVFR.M
 5.5	62	-1.0323	R.VGRLMNR.S
 5.2	67	0.9616	K.NPVFAGVR.L
 4.6	75	-1.0653	K.VRIFGNR.E

Top scoring peptide matches to query 2309
File7043 Spectrum12498 scans: 10050
Score greater than 27 indicates homology
Score greater than 36 indicates identity

**Score	Expect	Delta Hit Protein	Peptide**
 15.8	5.7	-0.0376	K.LILYPNK.N
 15.6	6	0.0206 177	P20648|ATP4A_HUMAN R.ILAAQGCK.V
 13.8	9.1	-0.0158	K.IIVLCSR.G
 13.8	9.1	0.0569	K.LIPDSCR.R
 13.8	9.1	0.0318	R.LLPDGMSK.L
 9.6	24	0.0239	K.IPFSPGSR.V
 8.7	30	0.0206	R.IINCNVK.Y
 8.7	30	0.0280	R.ILNGSSNR.Q
 8.7	30	0.0756	R.LINDDDR.R
 8.4	32	-0.0376	K.LILNYPK.A

Top scoring peptide matches to query 2310
File7043 Spectrum14471 scans: 11813
Score greater than 34 indicates homology
Score greater than 36 indicates identity

**Score	Expect	Delta Hit Protein	Peptide**
 27.1	0.42	-0.0053	9	P25705|ATPA_HUMAN R.QMSLLLR.R
 20.4	2	-0.0747	R.FAKLLLR.L
 20.4	2	-0.0417	-.MAKIILR.H
 18.2	3.3	-0.0053	R.MQALLIR.E
 18.2	3.3	-0.0231	R.TSASIILR.G
 16.0	5.4	0.0133	R.SDLLLSGR.D
 16.0	5.5	0.0311	K.QMAELLR.S
 15.8	5.8	1.0086	R.EELSLLR.L
 14.7	7.4	0.0674	K.LIPDSCR.R
 12.0	14	-0.0747	R.KFLALLR.E

Top scoring peptide matches to query 2311
File7043 Spectrum2902 scans: 2239
Score greater than 23 indicates homology
Score greater than 36 indicates identity

**Score	Expect	Delta Hit Protein	Peptide**
 11.5	15	-0.9329	R.CINIWR.D
 10.0	22	1.0690	R.ETRSGGPR.G
 9.2	26	1.0728	K.AMGTSPAPK.S
 9.0	28	1.0173 491	O43181|NDUS4_HUMAN R.QTLWRR.R
 6.8	45	1.0979	K.QPCTTPR.A
 6.8	45	-0.9792	-.MASVLNVK.E
 6.4	50	1.0802	K.GAEGPSSVR.F
 6.3	52	-0.8813	-.MAAGGSDPR.A
 5.8	57	1.0802	R.QGAELDAR.V
 5.8	58	1.0649	K.WSDRAPK.C

Top scoring peptide matches to query 2312
File7043 Spectrum951 scans: 781
Score greater than 21 indicates homology
Score greater than 34 indicates identity

**Score	Expect	Delta Hit Protein	Peptide**
 15.8	3.8	0.3066	R.ETRDALR.E
 7.4	26	0.2927 411	O15229|KMO_HUMAN K.DLLTAAEK.Y
 5.9	37	-0.6709	K.CARIGER.K
 5.6	40	-0.6709	R.CALRDAR.V
 5.5	41	0.2992	K.EIQMLAR.D
 5.5	41	0.2629	R.EKLMLAR.R
 5.4	42	0.2563	K.ETVLSALK.E
 4.5	51	-0.6960	K.NLLMQAR.W
 3.7	62	0.2927	K.EVLDASVK.E
 3.6	63	0.3066	R.EVEATRR.A

Top scoring peptide matches to query 2313
File7043 Spectrum1361 scans: 1051
Score greater than 19 indicates homology
Score greater than 34 indicates identity

**Score	Expect	Delta Hit Protein	Peptide**
 7.5	26	0.3066	R.ETRDALR.E
 7.3	27	-0.6848	98	P28331|NDUS1_HUMAN R.LTEPMVR.N
 5.7	39	0.2914	K.GREPFVR.M
 4.1	57	-0.6670	K.VLMPMDR.E
 3.7	62	0.2914	R.DPRAVFR.S
 2.1	89	-0.6662	K.EVDKADGK.Q
 1.6	1e+002	0.3390	K.EFSGPPAR.N
 1.1 1.1e+002	0.3641	R.EQAGGGWR.A
 1.0 1.1e+002	0.2563	K.ETVLSALK.E
 0.9 1.2e+002	0.3066	R.RQGTSPSK.C

Top scoring peptide matches to query 2314
File7043 Spectrum1156 scans: 916
Score greater than 19 indicates homology
Score greater than 34 indicates identity

**Score	Expect	Delta Hit Protein	Peptide**
 7.6	25	0.2928 411	O15229|KMO_HUMAN	K.DLLTAAEK.Y
 6.2	35	0.3067	R.ETRDALR.E
 4.5	51	-0.6661	K.EVDKADGK.Q
 4.2	55	-0.6847	R.MLVAENGK.K
 3.8	61	0.2564	K.ETVLSALK.E
 3.8	61	0.3543 455	P12110|CO6A2_HUMAN R.DDDLNLR.A
 2.5	81	-0.6847	98	P28331|NDUS1_HUMAN R.LTEPMVR.N
 1.3 1.1e+002	-0.6232	-.MAAGGSDPR.A
 1.0 1.1e+002	0.3132	R.AAAAGGMRR.D
 1.0 1.2e+002	0.2815	K.ETVDKLR.E

Top scoring peptide matches to query 2315
File7043 Spectrum2376 scans: 1841
Score greater than 16 indicates homology
Score greater than 34 indicates identity

**Score	Expect	Delta Hit Protein	Peptide**
 3.9	59	-0.7389 190	P02545|LMNA_HUMAN R.KTLDSVAK.E
 3.6	63	0.2881	R.LARVMDR.F
 3.3	68	0.3026	R.GPGEVVFR.G
 3.0	73	-0.6959	K.RPADKMK.S
 2.7	77	0.3357	K.MPNGSAGVK.V
 2.7	77	0.3132	R.AAAAGGMRR.D
 2.4	83	-0.6921	R.MPLEMLK.S
 2.4	83	-0.6847	K.LLDVMDR.G
 2.3	85	0.2815	K.LGLGNSTAK.S
 1.1 1.1e+002	0.3721	R.QPPMSER.T

Top scoring peptide matches to query 2317
File7043 Spectrum2209 scans: 1706
Score greater than 18 indicates homology
Score greater than 34 indicates identity

**Score	Expect	Delta Hit Protein	Peptide**
 6.3	34	0.2930	K.KDEEVLK.K
 5.4	42	-0.7135	K.KTTTASPR.T
 5.3	42	0.3545	R.QDEEALR.Y
 4.2	56	-0.7387 190	P02545|LMNA_HUMAN R.KTLDSVAK.E
 3.3	69	0.3392	R.AFPEPGSR.V
 3.0	73	0.2518	R.RLVMGLR.E
 2.8	77	0.2817	K.EVTDLRK.I
 2.7	77	0.2930	R.EAESLIAK.K
 1.8	96	-0.5866	K.DHETMGR.N
 1.8	96	-0.7209	R.LAIMVNGK.F

Top scoring peptide matches to query 2322
File7043 Spectrum31823 scans: 27946
Score greater than 22 indicates homology
Score greater than 34 indicates identity

**Score	Expect	Delta Hit Protein	Peptide**
 12.5	8.2	-0.6869	K.VRDSTGAR.V
 8.8	19	0.2818	R.VHSPHKR.L
 7.2	27	0.3407	R.AFPEPGSR.V
 6.4	33	-0.6831	R.MANADLVK.Y
 5.5	41	-0.6831	42+ P05141|ADT2_HUMAN R.GLGDCLVK.I
 4.8	48	0.3486	-.MELQDPK.M
 4.2	55	0.3308	R.DVTPEATK.E
 4.1	57	-0.6869	K.GKGQSQTR.V
 3.9	59	-0.7736	K.LKTSTAIK.K
 3.0	73	-0.7056	K.QITGMRR.L

Top scoring peptide matches to query 2328
File7043 Spectrum4710 scans: 3677
Score greater than 19 indicates homology
Score greater than 36 indicates identity

**Score	Expect	Delta Hit Protein	Peptide**
 6.3	50	0.9059	K.EALKMLR.N
 6.3	50	0.9423	K.EALQMLR.D
 5.9	56	0.9609	R.KEALEDR.Q
 5.6	59	0.9059	K.VDKVLMR.N
 5.5	60	0.9675	R.QIQANMR.S
 5.2	65	0.9423 663	Q9NX20|RM16_HUMAN R.LVVEMGGR.C
 5.2	65	0.9423	R.VEVLMNR.Q
 5.1	66	-0.0417	R.EDVVLMR.A
 4.8	71	0.9973	K.EPSKEDR.K
 4.7	72	0.9886	R.KCWPNR.V

Top scoring peptide matches to query 2331
File7043 Spectrum3100 scans: 2399
Score greater than 33 indicates homology
Score greater than 36 indicates identity

**Score	Expect	Delta Hit Protein	Peptide**
 21.9	1.4	0.9653	R.QMLALER.E
 19.1	2.6	0.9588 411	O15229|KMO_HUMAN K.DLLTAAEK.Y
 14.9	6.9	-0.0551	R.KMVENIK.Q
 14.4	7.7	-0.1092	R.TKLTSALK.K
 13.5	9.5	-0.0001	R.ETQENIK.R
 13.4	9.9	-0.0365	K.QTETAALK.A
 13.3	10	-0.0113	K.ETQEARK.S
 13.0	11	-0.9412	-.MEDPSGAR.E
 12.5	12	0.9952	K.EEASSPLK.S
 12.4	12	-0.0663	R.KMVGTLGR.L

Top scoring peptide matches to query 2332
File7043 Spectrum11232 scans: 8933
Score greater than 36 indicates identity

**Score	Expect	Delta Hit Protein	Peptide**
 41.4	0.016	-0.0049	42+ P05141|ADT2_HUMAN R.GLGDCLVK.I
 21.1	1.7	-0.0049	R.GLEAICAK.F
 16.1	5.3	1.0267	R.TPDGMPVK.N
 15.7	5.8	-0.0591	R.TSGKELVK.Q
 15.4	6.1	-0.0413	K.VAGMLAVGK.E
 14.3	8.1	-0.0703	R.GLLRSSTK.T
 13.2	10	-0.0856	R.RAPPPVPK.K
 13.1	11	-0.1107	K.KVPVPPPK.K
 12.2	13	-0.0777	R.LGITMKAK.L
 11.5	15	0.0137	K.GVEGTDGVK.K

Top scoring peptide matches to query 2333
File7043 Spectrum11263 scans: 8960
Score greater than 34 indicates homology
Score greater than 36 indicates identity

**Score	Expect	Delta Hit Protein	Peptide**
 41.3	0.016	-0.0047	42+ P05141|ADT2_HUMAN R.GLGDCLVK.I
 19.2	2.6	-0.0411	R.MVQQLVK.K
 16.5	4.8	0.9794	R.IINCNVK.Y
 14.9	7	-0.0047	R.GLEAICAK.F
 13.5	9.5	-0.0410	K.NMEKLVK.L
 13.1	11	0.9616	K.LGLGNSTAK.S
 13.0	11	-1.0251	K.DLMEKVK.S
 12.9	11	-0.0047	R.MANADLVK.Y
 12.9	11	-0.0047	R.AVVVQMAQ.-
 12.8	11	-0.0588	R.TSGKELVK.Q

Top scoring peptide matches to query 2336
File7043 Spectrum13701 scans: 11106
Score greater than 25 indicates homology
Score greater than 36 indicates identity

**Score	Expect	Delta Hit Protein	Peptide**
 14.4	7.8	-1.0008	R.IGADFLAR.W
 14.3	8.1	-0.9314	R.LENMSPR.Q
 11.9	14	-0.0056 183	Q9Y5K8|VATD_HUMAN R.IEIFPSR.M
 11.9	14	0.0274	K.IEIMSPR.K
 11.1	17	0.9421	R.IQLFLAR.L
 11.1	17	0.8805	R.LKLFLVK.D
 11.1	17	0.9751	K.LQLMLAR.E
 9.7	23	-0.9427	R.QNEMGKR.-
 9.2	26	-0.9831	-.MAFLAGPR.L
 7.7	36	-0.9539	-.MAQRGGAR.R

Top scoring peptide matches to query 2337
File7043 Spectrum13959 scans: 11338
Score greater than 20 indicates homology
Score greater than 36 indicates identity

**Score	Expect	Delta Hit Protein	Peptide**
 9.4	24	1.0158	K.LMVTPAGR.N
 9.2	26	0.9465	R.FLLIAAGR.T
 7.3	40	0.9577	LFVGSIPK
 7.1	42	0.0869 133+ Q6FI13|H2A2A_HUMAN R.NDEELNK.L
 6.6	47	0.9795	K.LVKGMAGGK.Y
 5.3	63	1.0556	K.LHGEYKN.-
 4.7	72	0.9431	K.MLALLKR.K
 4.3	80	0.9213	R.LILFLNK.L
 4.3	80	0.9577	K.LILYPNK.N
 4.3	80	0.9907	R.LLELMNK.H

Top scoring peptide matches to query 2338
File7043 Spectrum17744 scans: 14850
Score greater than 34 indicates homology
Score greater than 36 indicates identity

**Score	Expect	Delta Hit Protein	Peptide**
 25.4	0.62	1.0675	K.EVTQVER.D
 20.1	2.1	1.0311	K.EVTVEKR.R
 12.6	12	-0.9270	K.DAPEKFR.G
 12.6	12	1.0489 663	Q9NX20|RM16_HUMAN R.LVVEMGGR.C
 11.9	14	-0.8940	R.DSEKMPR.I
 11.2	16	1.0675	K.LESIDGAR.Y
 10.8	18	-0.9205	K.HMQFKR.T
 9.1	27	1.0675	K.IDLSEQR.Q
 8.4	31	0.0722	R.DLSDAGKR.G
 8.3	32	-0.9118	R.DDELSKR.E

Top scoring peptide matches to query 2339
File7043 Spectrum7584 scans: 5948
Score greater than 29 indicates homology
Score greater than 36 indicates identity

**Score	Expect	Delta Hit Protein	Peptide**
 16.6	4.5	-0.1035	R.LTTPVFGK.G
 16.5	4.5	0.9500	98	P28331|NDUS1_HUMAN R.LTEPMVR.N
 16.5	4.6	-1.0331	R.DSATSRAR.Q
 15.5	5.7	-0.0783	R.NPIYTVR.S
 14.0	8.1	0.9929	K.ICVGCPR.D
 12.0	13	0.9387	R.TVNAMAVR.D
 11.1	16	0.9639	-.MAAERGAR.R
 10.1	20	0.9751	R.DVAACAVR.F
 9.2	25	0.9574	K.DSVSNIAR.E
 8.9	26	0.9751	K.LSTACPGR.V

Top scoring peptide matches to query 2341
File7043 Spectrum4623 scans: 3602
Score greater than 23 indicates homology
Score greater than 36 indicates identity

**Score	Expect	Delta Hit Protein	Peptide**
 33.0	0.1	-0.0051	2	P05023|AT1A1_HUMAN R.IISANGCK.V
 8.9	26	-1.0446	K.LNSAFRR.S
 8.1	32	-0.0051	K.LQSGAVCK.L
 8.1	32	-0.0666	K.LLASMLAK.A
 6.1	51	-0.0666	R.LLMAAISK.D
 5.4	59	-1.0697	R.LHIDIPR.V
 5.3	60	-1.0433	M.SQLSSTLK.R
 4.5	72	0.0612	K.SEDESAPK.E
 4.4	75	-0.9527	R.EDSPKCK.G
 4.3	76	-1.1061	R.IPHLAGKK.A

Top scoring peptide matches to query 2342
File7043 Spectrum4626 scans: 3606
Score greater than 26 indicates homology
Score greater than 36 indicates identity

**Score	Expect	Delta Hit Protein	Peptide**
 15.1	6.3	-0.0050	2	P05023|AT1A1_HUMAN R.IISANGCK.V
 12.7	11	-1.0948	R.LLSGAFKK.R
 9.5	23	-1.1060	K.ILHQPKK.S
 8.0	32	-1.0445	K.LNSAFRR.S
 7.0	41	-0.0665	K.LLASMLAK.A
 6.3	48	-1.0108	K.LLPDDYK.A
 6.3	48	-1.0366	K.IAMTQKR.L
 5.5	57	0.0249	R.LAEADSEK.A
 5.4	60	-0.0115	R.LIEGSDTK.R
 5.1	64	-0.0591	R.KPSTSSKK.T

Top scoring peptide matches to query 2343
File7043 Spectrum7837 scans: 6147
Score greater than 26 indicates homology
Score greater than 36 indicates identity

**Score	Expect	Delta Hit Protein	Peptide**
 14.7	7	0.9842	R.LSGAAVSTR.G
 14.5	7.3	-0.0039 235	P07237|PDIA1_HUMAN K.ALAPEYAK.A
 12.3	12	0.0114	R.SLDIDTAK.S
 9.1	25	-0.9111	K.DSGAAEGEK.Q
 7.5	36	-1.0467	R.LAAAAPPPR.K
 7.4	37	0.9954 206	P48047|ATPO_HUMAN K.SLNDITAK.E
 7.2	39	-0.9773	R.LAEKSCR.N
 6.6	45	-0.0151	IADFGLAR
 6.6	45	-0.0151	LADFGLAR
 5.5	57	-1.0467	R.LAVPPNPR.T

Top scoring peptide matches to query 2348
File7043 Spectrum3289 scans: 2550
Score greater than 20 indicates homology
Score greater than 35 indicates identity

**Score	Expect	Delta Hit Protein	Peptide**
 12.9	10	0.9264	R.DSLAEATR.G
 7.1	39	0.9217	-.MAGSRGAGR.T
 7.1	39	0.9442	16	P35579|MYH9_HUMAN -.MAQQAADK.Y
 6.9	40	0.9217	-.MSRQANR.G
 6.8	42	0.8999	R.QFANLNR.E
 6.7	42	1.0057	R.NNSDCPR.V
 6.7	43	0.8159	R.RLHLAPR.A
 6.1	49	0.9403	R.AGRSDSGGR.G
 6.1	49	0.9078	-.MATSAPLR.S
 5.9	51	0.8748	M.EVPPPAPR.S

Top scoring peptide matches to query 2350
File7043 Spectrum9198 scans: 7246
Score greater than 27 indicates homology
Score greater than 36 indicates identity

**Score	Expect	Delta Hit Protein	Peptide**
 14.7	6.7	-1.0298	K.ADSNVSSGK.I
 13.6	8.7	-0.9821 138	P06396|GELS_HUMAN K.DSQEEEK.T
 13.6	8.7	-1.0185	K.KDSEEEK.E
 13.6	8.7	-1.0297	K.SKDNSGEK.T
 13.2	9.5	-1.0484	R.MEEVGRK.L
 11.4	15	-1.0410	R.SSVSSGGAGR.L
 11.0	16	-1.0297	R.SSEEEKR.V
 11.0	16	-1.0297	K.SSEEREK.A
 10.9	16	-0.9756	K.CDEGVER.Y
 10.9	16	-1.0562	K.FQERER.K

Top scoring peptide matches to query 2354
File7043 Spectrum4837 scans: 3772
Score greater than 26 indicates homology
Score greater than 36 indicates identity

**Score	Expect	Delta Hit Protein	Peptide**
 14.0	8	0.0750	R.EEGSNGDR.E
 12.7	11	-0.0131	K.QEGVGAFR.E
 10.5	18	-0.0018	85	P05026|AT1B1_HUMAN K.EEGSWKK.F
 8.9	26	-0.0229	R.EVSSLNSK.L
 8.7	27	0.9425	R.IILSNMR.M
 7.1	39	-0.0018	K.QESFGPAK.L
 6.8	42	0.9095	R.LNSLLFR.W
 6.5	44	-0.0303	43	P33121|ACSL1_HUMAN R.LLMDDLK.V
 6.5	45	-0.9971	R.DLSSWTR.I
 6.4	46	0.0312	R.EEGMIER.K

Top scoring peptide matches to query 2355
File7043 Spectrum4930 scans: 3850
Score greater than 26 indicates homology
Score greater than 36 indicates identity

**Score	Expect	Delta Hit Protein	Peptide**
 16.5	4.5	-1.0948	K.KAYELIK.S
 13.0	10	-0.9704	K.KDEEDTK.F
 13.0	10	-1.0254	R.KMLEESK.T
 11.4	15	-1.0584 295	Q9UM54|MYO6_HUMAN R.KSPEYLK.A
 11.2	15	-1.0809	R.KGAAPAPPR.K
 11.1	16	-1.0122	R.AGPFGYPR.D
 10.3	19	-1.1425	R.HAVLALLK.A
 10.1	19	-1.0333	R.KSTEFPR.K
 9.8	21	-1.0632	K.KMVPPHR.A
 8.7	27	-1.0697	K.GAAYLKNK.D

Top scoring peptide matches to query 2361
File7043 Spectrum5398 scans: 4212
Score greater than 22 indicates homology
Score greater than 36 indicates identity

**Score	Expect	Delta Hit Protein	Peptide**
 12.9	10	-0.0152	K.YKFFMK.A
 12.4	12	-1.0089	K.TSSSQLNK.S
 8.6	27	0.9980	K.WVRCNK.M
 6.5	45	-1.0792	K.MGRIFLK.V
 6.2	48	1.0544	K.DAGDEDLK.K
 5.5	56	-0.0145 747	P07602|SAP_HUMAN K.MCSKLPK.S
 3.6	87	1.0378	K.WHNSYR.E
 3.4	92	0.9439	K.QGGAVKFR.D
 3.0	1e+002	-0.0323	K.MATLSVGGK.T
 2.5 1.1e+002	0.9915	R.NNPNYIK.D

Top scoring peptide matches to query 2362
File7043 Spectrum9183 scans: 7235
Score greater than 16 indicates homology
Score greater than 36 indicates identity

**Score	Expect	Delta Hit Protein	Peptide**
 4.5	72	-1.0266	33	P14625|ENPL_HUMAN K.TLDMIKK.I
 3.2	96	-0.0062	K.MATLSVGGK.T
 3.0	1e+002	-0.0253	R.RFSGLGAR.A
 3.0	1e+002	1.0254	K.CEIALEK.L
 2.5 1.1e+002	-0.9109	K.SCMNPQK.F
 2.2 1.2e+002	-1.0345	K.QFVTGGKK.L
 1.3 1.5e+002	-0.0062 530	Q13740|CD166_HUMAN K.CSLIDKK.S
 1.1 1.6e+002	1.0142	-.MNKVDQK.F
 1.0 1.6e+002	-1.0015	R.NSKMLQK.V
 0.8 1.6e+002	0.0401	K.FHTMATR.T

Top scoring peptide matches to query 2363
File7043 Spectrum3934 scans: 3067
Score greater than 36 indicates identity

**Score	Expect	Delta Hit Protein	Peptide**
 28.0	0.31	1.0688	R.EEASDRR.L
 23.0	1	-0.0066 658	Q02543|RL18A_HUMAN -.MKASGTLR.E
 17.4	3.6	0.0047	K.MKTENLK.K
 16.7	4.3	0.0080	K.AFAAGADIK.E
 16.6	4.4	0.0597	K.ENSSVEAK.D
 13.6	8.8	1.0073	R.KNESSVAK.N
 13.5	8.9	0.0960	R.ENSSDSPK.E
 13.0	9.9	1.0033	R.QLTPFEK.L
 12.7	11	1.0073	K.KDTSANVK.S
 12.3	12	-0.0317	-.MAKATTIK.E

Top scoring peptide matches to query 2368
File7043 Spectrum6541 scans: 5117
Score greater than 25 indicates homology
Score greater than 36 indicates identity

**Score	Expect	Delta Hit Protein	Peptide**
 12.7	11	1.0357	R.AHLTAHGR.A
 11.7	14	-0.9067	K.AMRALGMD.-
 9.9	20	1.0687	-.MAGSRGAGR.T
 9.3	23	1.0482	R.TLLDSEGK.Y
 9.2	24	-0.9827 279	O43707|ACTN4_HUMAN K.ALDFIASK.G
 9.1	25	0.0741	M.VFTPEDR.L
 8.6	28	1.0873	R.SGSRQDGR.R
 8.4	29	-0.9463	R.LVDDAFGK.I
 8.4	29	-0.9674	K.TTLISDSK.L
 8.3	30	-0.9860	R.MESLKLK.S

Top scoring peptide matches to query 2369
File7043 Spectrum3463 scans: 2687
Score greater than 21 indicates homology
Score greater than 36 indicates identity

**Score	Expect	Delta Hit Protein	Peptide**
 9.0	25	1.1007	16	P35579|MYH9_HUMAN -.MAQQAADK.Y
 7.9	33	1.0830	K.EQLETSR.R
 7.6	35	1.1041 484	P11498|PYC_HUMAN R.EGPEGFAR.A
 7.3	37	1.0426	K.ETPLFQK.I
 7.0	40	1.1007	K.SAPSMVDR.L
 6.5	45	1.1194	K.ENSEGAGAK.A
 6.2	48	1.1194	R.DSAPTESR.L
 6.1	49	1.0466	R.EVSSLTAR.W
 5.9	51	1.0313	M.EVPPPAPR.S
 5.9	52	1.0783	-.MAQRGGAR.R

Top scoring peptide matches to query 2372
File7043 Spectrum12086 scans: 9682
Score greater than 17 indicates homology
Score greater than 34 indicates identity

**Score	Expect	Delta Hit Protein	Peptide**
 4.3	49	0.7406	R.ALQGAMEK.L
 4.0	52	0.6599	R.LAAAAPPPR.K
 4.0	53	-0.3241	R.AIGKGSFGK.V
 3.9	54	0.6348	K.KQIYAIK.Y
 3.1	64	-0.2010	K.DGHGHEGR.A
 3.0	66	0.6851	R.KGPHPTAR.S
 1.6	91	0.6963 359	Q9NYQ3|HAOX2_HUMAN R.NLVQFSR.L
 1.4	97	0.7657	K.EMGQNLR.E
 1.4	97	-0.2183	K.MEANIDR.I
 1.4	97	-0.2369	-.MEGKPMR.R

Top scoring peptide matches to query 2373
File7043 Spectrum6262 scans: 4893
Score greater than 27 indicates homology
Score greater than 35 indicates identity

**Score	Expect	Delta Hit Protein	Peptide**
 18.3	2.7	-0.0051	9	P25705|ATPA_HUMAN R.LTDADAMK.Y
 13.9	7.6	-0.0858	K.LTARFEK.A
 13.8	7.7	-0.1109	R.LTLGGIYK.F
 12.1	11	-0.0779	K.LTMSAVVK.K
 10.3	17	-0.1221	R.LTSLFRK.V
 8.9	24	0.9558	K.WINFQR.K
 8.9	24	0.0009	R.DVSHNHR.L
 8.8	25	-1.0368	-.MVSKDTGK.C
 8.5	26	-0.1109	K.ITLDKFK.E
 8.5	26	-0.0858 156	P36542|ATPG_HUMAN K.LTLTFNR.T

Top scoring peptide matches to query 2374
File7043 Spectrum10014 scans: 7913
Score greater than 20 indicates homology
Score greater than 35 indicates identity

**Score	Expect	Delta Hit Protein	Peptide**
 8.2	28	0.9313	R.RFSGLGAR.A
 6.6	40	0.9637	K.NGLAFWR.S
 5.9	48	0.9756	K.VSMNGSIR.N
 5.2	56	0.9934	R.GMGSMPKR.G
 5.2	56	0.9677 561	P98160|PGBM_HUMAN R.SSARWTR.I
 4.8	61	0.9942	K.STGDGTLGR.A
 4.5	66	0.9677	R.WTGSRTR.G
 4.2	71	-1.0506	K.ILDTFEK.L
 4.0	74	-1.0619	R.VLTEFTR.E
 4.0	74	-1.0367	R.VQTNFTR.C

Top scoring peptide matches to query 2376
File7043 Spectrum13080 scans: 10548
Score greater than 24 indicates homology
Score greater than 35 indicates identity

**Score	Expect	Delta Hit Protein	Peptide**
 13.8	7.8	1.0511	85	P05026|AT1B1_HUMAN K.EEGSWKK.F
 10.5	16	-0.9944	K.VPPHMLR.A
 9.7	20	1.0114	R.NASLVMTK.A
 9.5	21	0.9671	K.EIHIPVR.K
 9.0	23	1.0511	R.EWSGKEK.W
 8.5	26	-0.0169	K.DVKYALR.R
 7.0	37	1.0664	K.EENSSGLK.Y
 6.9	37	-1.1101	K.VLPAPKLK.M
 6.5	41	0.9750	K.TTLIIMR.Y
 6.0	47	0.0195	R.DVQFSIR.D

Top scoring peptide matches to query 2377
File7043 Spectrum11864 scans: 9488
Score greater than 16 indicates homology
Score greater than 35 indicates identity

**Score	Expect	Delta Hit Protein	Peptide**
 4.1	71	0.9790	K.NLPTPPPK.L
 4.1	72	0.0354	K.TESSSVVR.T
 4.0	73	-0.1254	K.LLHILKK.E
 4.0	73	-1.0366	R.LLSYITR.V
 3.6	81	-0.9751	R.VQTNFTR.C
 2.5	1e+002	0.9817	R.APRNIHR.H
 2.4 1.1e+002	1.0372 391	P12956|KU70_HUMAN K.VEAMNKR.L
 2.3 1.1e+002	-1.0227	K.NLVVHQR.M
 2.2 1.1e+002	1.1099	R.DLGQCDR.F
 2.1 1.1e+002	0.0895	R.DIMDAQR.R

Top scoring peptide matches to query 2378
File7043 Spectrum9529 scans: 7516
Score greater than 29 indicates homology
Score greater than 35 indicates identity

**Score	Expect	Delta Hit Protein	Peptide**
 17.2	3.5	-0.0301	K.AYLGLSLK.Q
 16.9	3.8	0.0202	R.LFSDARR.L
 15.5	5.2	0.0202	R.HPGLADVR.N
 15.1	5.8	0.0645	R.SEMDKVR.V
 14.3	6.9	-0.0413	R.AYLKTIR.Q
 13.2	8.9	0.0315	R.LYADLNR.H
 10.9	15	-0.0049	R.IDSFVKR.Q
 10.6	16	1.0671	R.DGSSDKVR.T
 10.6	16	0.0314 711	Q7Z4W1|DCXR_HUMAN R.SFEVNLR.A
 9.9	19	0.0532	-.MESSRVR.L

Top scoring peptide matches to query 2380
File7043 Spectrum1365 scans: 1053
Score greater than 26 indicates homology
Score greater than 33 indicates identity

**Score	Expect	Delta Hit Protein	Peptide**
 21.6	0.83	-0.6974	K.KVGTGTMR.T
 12.0	7.5	-0.7304	R.GGSLKTFR.A
 12.0	7.5	0.2649	R.LLSQTFR.G
 10.3	11	0.3231	-.MSAGGSARK.S
 10.2	11	0.3013	K.EVKDAFR.R
 10.2	11	0.3377	R.EVQDAFR.C
 10.1	12	-0.6385	VEVDMEK
 9.7	13	0.3707	K.QIDDAMR.N
 9.5	13	-0.6827	K.EAELTFR.Q
 9.5	13	-0.6610 679	P26232|CTNA2_HUMAN R.KQGETMR.I

Top scoring peptide matches to query 2389
File7043 Spectrum31839 scans: 27955
Score greater than 22 indicates homology
Score greater than 33 indicates identity

**Score	Expect	Delta Hit Protein	Peptide**
 12.1	7.4	-0.7288	R.VNHTLGPK.K
 8.7	16	-0.7764	R.LPRGLPGR.A
 8.1	18	-0.7539	K.YGKVLSAK.V
 1.2	91	0.3821	R.WGQASCR.A
 0.6	1e+002	-0.6304	R.SCTPCLK.A
 0.6	1e+002	-0.6594	K.TCSVRDK.C
 0.5 1.1e+002	0.2883	K.MSSVRLR.K
 0.4 1.1e+002	-0.7063 741	P08727|K1C19_HUMAN K.EELAYLK.K
 0.4 1.1e+002	-0.6369	R.ELEAMEK.E
 0.0 1.2e+002	0.2552	R.LHVTAAPR.I

Top scoring peptide matches to query 2398
File7043 Spectrum6575 scans: 5140
Score greater than 25 indicates homology
Score greater than 35 indicates identity

**Score	Expect	Delta Hit Protein	Peptide**
 14.1	7	0.9058 711	Q7Z4W1|DCXR_HUMAN K.GALDMLTK.V
 14.1	7	0.8364	R.KIDFLTK.Q
 11.4	13	-1.0847	K.MNKTQTK.S
 10.9	14	-1.0813	K.FTLQDSR.T
 8.3	26	-0.1337	R.IRYGQTK.K
 8.0	28	-0.1476	4	P98164|LRP2_HUMAN K.IYLVETK.V
 7.3	33	0.8728	K.YLVIDNK.D
 7.2	34	-1.1217	R.LPFFTNK.T
 7.0	35	-1.1065	K.IFQTETK.Q
 7.0	35	-0.1476	K.LEYIVTK.Y

Top scoring peptide matches to query 2399
File7043 Spectrum5601 scans: 4375
Score greater than 18 indicates homology
Score greater than 35 indicates identity

**Score	Expect	Delta Hit Protein	Peptide**
 16.1	4.3	0.9982 381	P01842|LAC_HUMAN K.TVAPTECS.-
 15.8	4.7	-1.1205	K.INKQHAR.R
 4.1	69	-1.0841	R.TEPHRAR.R
 4.1	69	-1.1456	K.HKELLAR.R
 4.1	70	0.9870	K.EAASEMAR.A
 4.1	70	0.9619	R.ISAEDAMK.H
 4.0	70	-1.0616	R.AGLSYEAR.H
 4.0	70	-1.0472	K.ALMASMAR.R
 4.0	70	-1.0472	K.ALMASMAR.R
 4.0	70	-1.0439	R.CAEIFAR.G

Top scoring peptide matches to query 2409
File7043 Spectrum9153 scans: 7211
Score greater than 25 indicates homology
Score greater than 35 indicates identity

**Score	Expect	Delta Hit Protein	Peptide**
 15.7	4.4	-0.0051 106	P12821|ACE_HUMAN R.VFDGSITK.E
 15.7	4.4	-0.0237	K.VFDVMKK.G
 15.7	4.4	0.0457	K.VMDMVQK.F
 11.8	11	1.0371	-.MASAKDAR.L
 11.3	12	0.0200	R.VFNTTER.D
 7.7	28	0.0014	K.VFQGMLR.A
 6.0	41	-1.0367	R.AASKVYTK.D
 5.6	46	1.0218	R.SMKWSAR.A
 5.0	52	0.0313	K.VEESFQK.A
 3.9	67	-0.8833	K.EDDMETK.K

Top scoring peptide matches to query 2414
File7043 Spectrum9520 scans: 7511
Score greater than 18 indicates homology
Score greater than 33 indicates identity

**Score	Expect	Delta Hit Protein	Peptide**
 12.8	5.8	0.3199	K.HPDKDVR.L
 4.3	41	0.3312	K.AYELSQR.S
 4.0	44	0.3311	K.SFTDQLR.K
 3.4	50	0.3125	-.MAITQFR.L
 3.4	50	0.3204	K.MMTVLGAK.W
 3.4	50	0.3204	K.MMTVLGAK.W
 1.6	76	0.4006	R.ENMSDVR.E
 1.5	78	-0.7045	K.DAFLVFR.S
 1.3	83	0.3642	K.DSLSMAAR.V
 1.2	84	0.3199 679	P26232|CTNA2_HUMAN R.HPDVAATR.A

Top scoring peptide matches to query 2418
File7043 Spectrum9822 scans: 7756
Score greater than 25 indicates homology
Score greater than 34 indicates identity

**Score	Expect	Delta Hit Protein	Peptide**
 12.9	8.1	0.9419	K.VIDPVAPR.Y
 12.0	10	1.0477	K.MANGTAGTK.V
 10.7	13	1.0953	K.EMEQDAK.R
 8.9	20	0.9307	R.DPLRIPR.E
 8.6	22	-0.0057	R.EDPKPGPK.K
 7.9	25	0.8943	R.VLLRHTK.E
 5.4	46	1.0113	K.MNKTQTK.S
 3.3	73	0.9895 106	P12821|ACE_HUMAN R.VFDGSITK.E
 3.3	73	0.9896	R.VISEYQK.L
 3.3	74	1.0259	K.STGYDPVK.L

Top scoring peptide matches to query 2419
File7043 Spectrum3202 scans: 2480
Score greater than 23 indicates homology
Score greater than 34 indicates identity

**Score	Expect	Delta Hit Protein	Peptide**
 11.8	10	1.0567	R.EEMSKSR.H
 11.8	10	0.9873	K.EFSLSKR.K
 10.2	15	0.0792	K.SMNCLAR.K
 9.7	17	-0.0080 185	P39023|RL3_HUMAN K.GYKGVTSR.W
 7.2	30	1.0601	R.ESSGFPSR.L
 7.2	30	-0.9742	-.MSSKYPR.S
 6.7	34	0.0284	K.GLSGGFSSR.S
 5.4	45	0.0978	K.SNMQSER.A
 5.2	48	0.0978	R.SCTDSAAR.T
 5.1	49	1.0818	R.RMEGDSR.G

Top scoring peptide matches to query 2423
File7043 Spectrum5005 scans: 3909
Score greater than 29 indicates homology
Score greater than 32 indicates identity

**Score	Expect	Delta Hit Protein	Peptide**
 16.9	2	-0.5194	R.QPRAQLR.R
 15.0	3.2	-0.5194	R.RKPASPGR.T
 14.5	3.5	-0.4718	R.VQAGPGSPR.R
 11.0	7.8	0.5235	K.EPPGAQLR.T
 10.2	9.6	-0.4718	R.QLDSIHR.R
 9.2	12	-0.3920	K.MASLCMR.D
 9.2	12	-0.4428	-.MASLFSGR.I
 9.2	12	-0.4428	-.MASLYQR.F
 9.2	12	-0.3734	-.MATDMQR.K
 9.2	12	-0.4428 208	Q9GZP9|DERL2_HUMAN -.MAYQSLR.L

Top scoring peptide matches to query 2424
File7043 Spectrum12076 scans: 9674
Score greater than 19 indicates homology
Score greater than 32 indicates identity

**Score	Expect	Delta Hit Protein	Peptide**
 6.9	20	0.5719	K.HISELLR.E
 6.7	21	0.5858	K.HSARQLR.L
 6.7	21	0.5719	R.HLESLLR.V
 6.2	24	0.5858	K.HSSRKPR.L
 6.1	24	-0.2886 173	P52272|HNRPM_HUMAN R.MGQTMER.I
 6.0	25	-0.3982	K.GPAAGPRSR.A
 5.5	28	0.6083	R.HEVVLDR.H
 5.3	29	-0.4597	K.TIHKLTR.G
 4.3	37	0.5818	R.HWQKLR.E
 3.8	41	0.5719	R.HIAQTGLK.V

Top scoring peptide matches to query 2425
File7043 Spectrum11280 scans: 8973
Score greater than 16 indicates homology
Score greater than 34 indicates identity

**Score	Expect	Delta Hit Protein	Peptide**
 3.6	64	0.9614	K.DAPSPKPR.L
 3.6	64	0.9250	R.SVSPPPKR.A
 3.5	65	1.0849	R.DTVCGCR.K
 3.0	73	0.0389	K.DYSDGRR.T
 2.9	75	1.0090	K.LEGETYR.Y
 2.8	77	0.9389	K.HSSRKPR.L
 2.3	86	-1.0179	R.EGEGKPPR.G
 1.3 1.1e+002	-1.0794	96	P30101|PDIA3_HUMAN K.DPNIVIAK.M
 1.3 1.1e+002	0.9726	R.IDLSTYR.N
 1.1 1.1e+002	-1.1019	R.GAPTRIVR.K

Top scoring peptide matches to query 2427
File7043 Spectrum4151 scans: 3243
Score greater than 23 indicates homology
Score greater than 34 indicates identity

**Score	Expect	Delta Hit Protein	Peptide**
 24.6	0.5	-0.0064	25	P40939|ECHA_HUMAN K.HVAEDLGK.V
 9.0	18	-0.0469	K.VFTFGLGK.K
 8.0	23	0.0807	K.RCEEMK.Y
 7.0	29	0.0378	R.TSMSEGIK.Q
 7.0	29	1.0317	-.MANSFAAR.I
 6.9	30	0.0807	K.CEREMK.V
 5.5	41	0.0412	R.EGESYVGK.L
 5.5	41	0.0412	R.EVSYGEGK.A
 5.3	42	-1.0091	K.MYNKAVK.L
 4.9	47	0.0920	R.SVEMCDK.S

Top scoring peptide matches to query 2428
File7043 Spectrum4128 scans: 3227
Score greater than 29 indicates homology
Score greater than 34 indicates identity

**Score	Expect	Delta Hit Protein	Peptide**
 21.9	0.93	-0.0063	25	P40939|ECHA_HUMAN K.HVAEDLGK.V
 14.7	4.9	0.9955	R.SFCGKLR.S
 11.8	9.7	-0.0539	R.HVETKVR.A
 10.0	14	0.9777	K.EPPGAQLR.T
 5.7	39	0.9890	K.TYEVSLR.E
 5.0	46	0.9890	R.TTVDYLR.L
 4.9	46	0.0115	-.MANNYKK.I
 4.6	50	0.0366	R.KYCQNR.Y
 4.6	50	-0.0427	R.VHLGSVEK.S
 4.4	53	-0.8998	-.MADFDDR.V

Top scoring peptide matches to query 2431
File7043 Spectrum3932 scans: 3065
Score greater than 27 indicates homology
Score greater than 34 indicates identity

**Score	Expect	Delta Hit Protein	Peptide**
 15.3	4.2	-0.9428	K.VESESYR.K
 14.2	5.6	-0.0065	R.VQAGPGSPR.R
 12.7	7.7	-0.9905	R.LDTDHLR.Y
 11.4	11	0.1032	K.QEGMEMK.L
 10.2	14	0.9814 754	P00558|PGK1_HUMAN K.AGGFLMKK.E
 9.1	18	-0.0356	K.QVAYIFK.C
 8.8	19	1.0694	EVEMSTR
 7.8	24	0.0920	K.CEREMK.V
 7.8	24	0.0920	K.RCEEMK.Y
 7.7	25	-0.0064	R.QLDSIHR.R

Top scoring peptide matches to query 2435
File7043 Spectrum983 scans: 800
Score greater than 26 indicates homology
Score greater than 32 indicates identity

**Score	Expect	Delta Hit Protein	Peptide**
 17.0	1.9	-0.7130	K.MHAIIER.T
 12.9	4.8	-0.7560	K.ELLPEIR.E
 11.7	6.4	0.2896	R.QLDSIHR.R
 11.2	7.2	-0.7131	-.MAGAPGPLR.L
 10.4	8.8	-0.7784	43	P33121|ACSL1_HUMAN AKRPELR
 9.8	10	-0.7243	K.NMAKIHR.G
 9.7	10	0.2896	R.GPQGLPGSR.G
 9.6	10	0.1917	K.KLPIELR.D
 9.3	11	0.3372	R.NSELSYR.E
 8.0	15	-0.7560	R.LEPLLER.R

Top scoring peptide matches to query 2436
File7043 Spectrum2450 scans: 1898
Score greater than 19 indicates homology
Score greater than 32 indicates identity

**Score	Expect	Delta Hit Protein	Peptide**
 7.3	18	0.3378	R.NSELSYR.E
 7.2	18	-0.7125	-.MAGAPGPLR.L
 6.0	24	-0.7125	K.MHAIIER.T
 5.1	29	0.2537	K.VHESIKR.S
 4.3	35	-0.7554	K.ELLPEIR.E
 2.7	51	-0.8395 621	P46779|RL28_HUMAN K.GVVVVIKR.R
 2.1	59	0.2650	K.TTKLYSR.F
 1.5	68	-0.6761	K.MFSSRNK.E
 1.3	71	0.2901	R.VQAGPGSPR.R
 1.1	75	0.3303	K.EVMAQFK.E

Top scoring peptide matches to query 2449
File7043 Spectrum1931 scans: 1474
Score greater than 22 indicates homology
Score greater than 32 indicates identity

**Score	Expect	Delta Hit Protein	Peptide**
 11.4	6.9	-0.7767	43	P33121|ACSL1_HUMAN AKRPELR
 8.6	13	-0.7767	R.ARPKELR.F
 6.7	21	0.2761	K.APFHQLR.I
 6.7	21	0.3091	R.AQMPHLR.K
 6.7	21	0.2550	K.ASAITLHR.S
 6.0	24	0.3390	R.NSELSYR.E
 5.5	27	-0.6307	K.MMTGELR.N
 5.4	28	-0.7542	K.ELLPEIR.E
 5.3	28	-0.7113	K.MHAIIER.T
 5.3	28	-0.7331 249	P06756|ITAV_HUMAN R.ALFLYSR.S

Top scoring peptide matches to query 2453
File7043 Spectrum156 scans: 250
Score greater than 16 indicates homology
Score greater than 32 indicates identity

**Score	Expect	Delta Hit Protein	Peptide**
 5.1	29	0.2923	R.HSVAGELR.L
 4.9	31	0.2672	K.EPPKELR.Q
 3.3	45	0.3325	K.EVMAQFK.E
 2.0	61	0.3763 360	P16157|ANK1_HUMAN K.DHTSTPNP.-
 0.2	91	0.2923	R.THNVLER.Q

Top scoring peptide matches to query 2456
File7043 Spectrum3528 scans: 2734
Score greater than 23 indicates homology
Score greater than 34 indicates identity

**Score	Expect	Delta Hit Protein	Peptide**
 10.2	13	-0.0060	88	P01834|IGKC_HUMAN K.SFNRGEC.-

Top scoring peptide matches to query 2457
File7043 Spectrum3391 scans: 2628
Score greater than 20 indicates homology
Score greater than 34 indicates identity

**Score	Expect	Delta Hit Protein	Peptide**
 7.7	23	-0.0475	K.GATSDFSGK.A
 6.9	28	0.8525	K.ILANGGAPR.K
 5.6	38	0.8597	K.LLGQFYK.C
 5.4	40	0.7910 715	P07305|H10_HUMAN K.KPKATPVK.K
 4.1	54	-1.1631	K.QPSLRAAK.E
 3.2	66	0.8815	R.LHPGMGLK.G
 3.0	69	0.8815	K.MYGTLRK.G
 2.6	76	0.8525	K.KLTQNHK.A
 2.6	77	-0.2294	K.KPLSAIIK.E
 1.6	95	0.9808	R.ASEMASEK.E

Top scoring peptide matches to query 2459
File7043 Spectrum4344 scans: 3392
Score greater than 25 indicates homology
Score greater than 34 indicates identity

**Score	Expect	Delta Hit Protein	Peptide**
 14.5	5	1.0253	-.MATDMQR.K
 11.9	9.1	0.9018	K.TTKLYSR.F
 8.2	21	-0.0757	K.MHAIIER.T
 7.3	26	0.8906	86	P18859|ATP5J_HUMAN R.SAVSVHLR.R
 7.3	26	0.9746	R.NSELSYR.E
 6.8	30	0.8906	K.ASAITLHR.S
 6.7	30	0.9270	R.THNVLER.Q
 6.2	33	0.9157	R.TGRPGPAGR.C
 6.2	34	0.9560	-.MASLYQR.F
 6.2	34	0.9746	R.SAYPSTSR.G

Top scoring peptide matches to query 2462
File7043 Spectrum10399 scans: 8243
Score greater than 27 indicates homology
Score greater than 34 indicates identity

**Score	Expect	Delta Hit Protein	Peptide**
 17.3	2.6	-1.1120	R.KLLVAGNR.I
 13.7	6	-1.1007	87+ Q5VTE0|EF1A3_HUMAN K.QLIVGVNK.M
 8.4	20	-1.0643	R.KLPDIER.G
 8.4	20	-1.0643	K.KLPDLER.L
 7.9	23	-1.1371	R.KLLELVR.Y
 7.9	23	-1.1119	K.QILAAAKR.A
 6.7	30	0.0149	K.TETIDYK.D
 6.4	32	-1.0280	K.EQPVLER.L
 6.4	32	-1.1007	K.ILQVLER.N
 6.3	33	-1.1007	K.LQVLELR.D

Top scoring peptide matches to query 2463
File7043 Spectrum11997 scans: 9603
Score greater than 24 indicates homology
Score greater than 34 indicates identity

**Score	Expect	Delta Hit Protein	Peptide**
 12.4	8	0.9793	R.VRSYSTR.D
 10.5	13	-0.9016	K.MSSMNER.T
 10.5	13	0.0382	K.SFCGRSR.L
 10.5	13	0.0204	K.SYGSGSRR.E
 9.6	15	-0.1503 621	P46779|RL28_HUMAN K.GVVVVIKR.R
 8.5	20	0.0204	R.SAGSHPGTR.S
 8.2	21	-1.0768	R.FLLAPPGR.L
 8.2	21	-1.0153	K.FTPVHNR.T
 8.2	21	-0.1503	R.KLLVQLR.R
 7.4	26	-1.0000	R.SRTSYTR.Y

Top scoring peptide matches to query 2475
File7043 Spectrum6278 scans: 4907
Score greater than 20 indicates homology
Score greater than 34 indicates identity

**Score	Expect	Delta Hit Protein	Peptide**
 9.1	17	1.1467	R.DSEDFKK.T
 7.5	25	1.0515	R.HTKGIGQK.L
 6.7	30	1.1242	K.DPGGGGGPVR.A
 5.1	43	1.1507	K.TSSGNSTSK.G
 5.0	44	0.0628	-.MGRPRPR.A
 4.7	47	1.1611	K.DAMMKEK.H
 4.4	51	1.1862 173	P52272|HNRPM_HUMAN R.MGQTMER.I
 4.3	52	1.1420	-.MQRDFR.W
 4.0	55	1.0991	R.FQSSKSGK.I
 3.9	56	1.0726	K.NRLYFR.S

Top scoring peptide matches to query 2489
File7043 Spectrum6758 scans: 5288
Score greater than 30 indicates homology
Score greater than 35 indicates identity

**Score	Expect	Delta Hit Protein	Peptide**
 17.8	2.9	-1.1145	4	P98164|LRP2_HUMAN R.GLQVYHR.R
 16.8	3.7	-1.1145	K.AVQYVHR.V
 13.4	8	0.8436	K.APQVAKTR.N
 11.7	12	-1.1105	R.GIQERNR.G
 10.5	15	-0.1768	R.LGKVAIDR.G
 10.2	17	-0.1152	K.AGRSPEVR.S
 9.9	18	-0.1768 297	Q6UX53|MET7B_HUMAN R.KVLQEVR.R
 9.3	21	-0.0788	K.EPARAGDR.Q
 9.0	22	-1.0993	K.LGGGGDLGAR.V
 7.3	32	0.8436	R.KAQSIPAR.K

Top scoring peptide matches to query 2491
File7043 Spectrum981 scans: 799
Score greater than 23 indicates homology
Score greater than 35 indicates identity

**Score	Expect	Delta Hit Protein	Peptide**
 13.5	7.8	-0.1246	R.KEAVLLAK.M
 9.8	18	-0.0267	R.SSQLTAHK.M
 8.8	23	-1.0107	K.ESGQLPNK.K
 7.1	34	-1.0484	R.FHASVRR.L
 6.5	39	0.8846	R.KLLVAGNR.I
 6.0	44	0.9863	R.QPEMIPR.I
 5.4	51	0.8958	87+ Q5VTE0|EF1A3_HUMAN K.QLIVGVNK.M
 5.1	54	-0.1246	R.EKGLALLK.E
 4.8	57	0.9573	R.KQNLDPR.Y
 4.8	58	-0.0783	R.KIFQAHK.N

Top scoring peptide matches to query 2492
File7043 Spectrum6066 scans: 4739
Score greater than 24 indicates homology
Score greater than 35 indicates identity

**Score	Expect	Delta Hit Protein	Peptide**
 12.6	9.6	0.9898	22	O60494|CUBN_HUMAN R.GFKAEYR.Q
 10.2	17	-0.0055 108	P50440|GATM_HUMAN R.HLAPDYR.V
 8.3	26	1.0227	K.MGFGSISR.V
 7.8	29	1.0592 560	P20711|DDC_HUMAN -.MNASEFR.R
 7.3	32	0.9646	K.KFFDVSK.L
 6.3	41	1.0881	-.MGWMGEK.T
 6.1	43	1.0592	R.QMEQYR.D
 5.6	49	1.0227	K.MGYGIGTR.L
 5.1	54	1.0374	K.FGFEENK.I
 5.0	55	1.0340	K.SMSFPTGK.S

Top scoring peptide matches to query 2494
File7043 Spectrum7457 scans: 5843
Score greater than 24 indicates homology
Score greater than 35 indicates identity

**Score	Expect	Delta Hit Protein	Peptide**
 11.4	13	-1.0544	R.DLDVVRR.I
 10.7	15	-0.9816	R.NAGGEATPR.E
 10.5	16	-0.9969	R.EQHVFGR.G
 10.1	17	0.0790	R.ESMEGFR.I
 9.6	19	-0.0955 637	Q9H4M9|EHD1_HUMAN K.LNDLIKR.A
 9.6	19	-0.0591	K.LNDLIQR.L
 9.3	21	-1.0544	K.VRAELER.W
 8.4	25	0.0565	R.LNGCHDR.A
 8.3	26	-1.0180	R.GAGSQSLPR.G
 7.7	30	-1.0544	R.ARVELER.A

Top scoring peptide matches to query 2495
File7043 Spectrum4045 scans: 3162
Score greater than 27 indicates homology
Score greater than 35 indicates identity

**Score	Expect	Delta Hit Protein	Peptide**
 18.0	2.8	-1.0417	R.KVQVQDR.G
 13.8	7.3	0.1424	K.CSGCMEK.I
 12.0	11	-0.9842	R.FAVHGDAR.A
 12.0	11	0.0553	R.SSKDFCK.I
 12.0	11	0.0917	M.SSNECFK.C
 11.5	12	1.1087	4	P98164|LRP2_HUMAN R.ASMDGSMR.T
 9.8	18	0.0151	K.EPARAGDR.Q
 8.8	23	-1.0781	R.KITIQNR.A
 8.4	25	-1.0127	R.LMPDPKR.F
 7.7	30	-1.0305	R.SSAPELLR.L

Top scoring peptide matches to query 2497
File7043 Spectrum8033 scans: 6306
Score greater than 18 indicates homology
Score greater than 35 indicates identity

**Score	Expect	Delta Hit Protein	Peptide**
 5.3	52	0.0199	K.GSPRGELR.G
 5.2	53	-0.0569	R.KIVLWGR.T
 4.7	59	-0.0416	M.AAVVLAATR.L
 4.2	67	-0.0164	R.IQGRELR.T
 4.2	67	-0.0416	R.LKVNELR.E
 4.2	67	-0.0164 283	Q12846|STX4_HUMAN K.QLGREIR.L
 4.0	69	-1.0157	R.LNVKWGR.S
 3.9	71	0.0676	R.DHSDAAKK.H
 3.5	77	-1.0005	R.KVQVQDR.G
 3.3	82	0.1039	R.DHDSELR.F

Top scoring peptide matches to query 2500
File7043 Spectrum7995 scans: 6274
Score greater than 35 indicates identity

**Score	Expect	Delta Hit Protein	Peptide**
 42.5	0.0099	-0.0048 280	P05090|APOD_HUMAN K.VLNQELR.A
 27.9	0.28	-0.0300	R.VLELELR.G
 23.9	0.71	-0.0524	K.VLSRAGLR.Q
 21.2	1.3	-0.0524	K.VLAASVRR.A
 16.4	4	-0.0122	K.VLNGMLPK.S
 15.7	4.7	-0.0300	K.VLGSSALPK.E
 14.7	5.9	-0.0048	K.QIENVLR.R
 14.1	6.9	-0.0412	R.VSALNVLR.D
 13.8	7.3	-0.0524	R.VNLRTLR.G
 13.7	7.4	-0.0161	R.QEARVLR.N

Top scoring peptide matches to query 2505
File7043 Spectrum3674 scans: 2850
Score greater than 24 indicates homology
Score greater than 36 indicates identity

**Score	Expect	Delta Hit Protein	Peptide**
 12.1	12	-1.1217	R.KRPLGMR.E
 11.3	15	0.8259	K.DAKIALIK.E
 11.3	15	0.8259 300	P08263|GSTA1_HUMAN K.DAKLALIK.E
 11.3	15	-1.1282	R.ITKINER.Y
 10.4	18	-1.0918	M.AGLTDLQR.L
 10.3	19	-0.1330	K.IQTLLER.A
 10.3	19	-0.1442	R.KITIQNR.A
 9.4	23	-0.9867	R.YYAGDER.R
 9.3	23	-1.1282	K.GEKLSIAR.G
 9.3	23	-1.0820	R.KHTFGQR.C

Top scoring peptide matches to query 2506
File7043 Spectrum5034 scans: 3933
Score greater than 25 indicates homology
Score greater than 36 indicates identity

**Score	Expect	Delta Hit Protein	Peptide**
 13.4	9.1	-0.0375	R.GGRQGLER.L
 11.5	14	-1.0830	R.IGTKGLER.I
 11.0	16	0.9214	K.AVLRQER.V
 8.1	31	0.9214	R.IGVQGKNR.M
 7.4	36	-1.0467	81	P21281|VATB2_HUMAN R.EQALAVSR.N
 7.1	39	-1.0103	R.SSDPRSPK.G
 6.7	43	-0.9739	R.EGGPSAAER.R
 6.7	43	-1.0467	K.DGLREVGK.V
 6.6	44	-1.0718	R.SLAGLLGDK.K
 5.7	54	0.9690	R.LGSPSPASR.T

Top scoring peptide matches to query 2507
File7043 Spectrum6751 scans: 5281
Score greater than 36 indicates identity

**Score	Expect	Delta Hit Protein	Peptide**
 36.7	0.043	-0.0061	4	P98164|LRP2_HUMAN R.GLQVYHR.R
 23.9	0.82	-0.0061	K.AVQYVHR.V
 18.4	2.9	-0.0020	R.GIQERNR.G
 16.3	4.7	-1.0298	K.GLQVLMGR.M
 13.6	8.7	-0.0173	K.GIARHYR.I
 13.2	9.6	-0.9901	K.GIQQHYK.H
 12.7	11	1.0685	R.QMHYHR.N
 12.2	12	-1.0588	R.IVSVRSGR.E
 12.0	13	-1.0476	K.AVQVSNKK.K
 11.5	14	-1.0476	K.KIALSDAR.S

Top scoring peptide matches to query 2512
File7043 Spectrum29724 scans: 26364
Score greater than 26 indicates homology
Score greater than 36 indicates identity

**Score	Expect	Delta Hit Protein	Peptide**
 13.8	8.2	0.9839	K.QVLRSLR.L
 13.6	8.7	0.9951	R.IIKVGGER.F
 12.6	11	0.0475	K.EPLSSLAR.K
 12.1	12	0.0362	R.KVQVQDR.G
 11.9	13	0.9700	K.LNLLITGK.I
 11.7	14	-0.0001	R.EVISRLR.N
 11.7	14	0.9951 297	Q6UX53|MET7B_HUMAN R.KVLQEVR.R
 11.7	14	1.0162	K.QVLGWIR.N
 11.6	14	-0.9590	R.QTLREAR.M
 10.8	17	-0.9478	R.EVNDLKR.K

Top scoring peptide matches to query 2515
File7043 Spectrum13637 scans: 11051
Score greater than 22 indicates homology
Score greater than 34 indicates identity

**Score	Expect	Delta Hit Protein	Peptide**
 10.3	13	0.3163 439	P16662|UD2B7_HUMAN K.ADVWLIR.N
 8.6	19	0.2799 122	P19224|UD16_HUMAN K.VSVWLLR.Y
 6.4	32	0.2951	M.ADVLSVLR.Q
 6.3	32	-0.6426	K.IPSASGWR.K
 6.1	34	0.2401	K.KVMLLLR.V
 5.9	35	0.3493	K.EGGMPLLR.D
 5.8	36	0.3955	K.WTLCHR.Q
 5.5	39	-0.6790	R.QASWIIR.G
 3.9	56	0.3203	R.KVQVQDR.G
 3.6	60	-0.6637	K.LDQQTLR.N

Top scoring peptide matches to query 2518
File7043 Spectrum136 scans: 234
Score greater than 24 indicates homology
Score greater than 34 indicates identity

**Score	Expect	Delta Hit Protein	Peptide**
 14.5	4.9	0.6513	64	Q13813|SPTA2_HUMAN K.DPTNLQGK.L
 10.2	13	0.6037	K.SNLKAPSR.H
 8.8	18	0.5421	K.VITNKAVK.G
 8.0	22	-0.3956	R.FQGLVPGR.K
 6.9	28	-0.3916	R.TPGLSRSR.D
 6.2	33	0.5421	K.LVQKVASK.I
 6.1	34	0.6513	K.GGLAGPDGTK.S
 5.7	37	0.6877	K.DPAEQQGK.G
 5.7	37	0.5421	K.INVTVAKK.K
 5.6	38	0.5897	K.IVDVIGEK.I

Top scoring peptide matches to query 2519
File7043 Spectrum1467 scans: 1120
Score greater than 18 indicates homology
Score greater than 34 indicates identity

**Score	Expect	Delta Hit Protein	Peptide**
 6.5	35	0.8238	K.TMFSKDK.D
 6.1	38	0.7221	K.GVIGSLGAAK.M
 5.2	47	-0.2772	K.DIILPFR.V
 5.0	49	-0.2521	K.QNLVPFR.D
 5.0	49	-0.1317	R.YYAGDER.R
 4.9	50	0.7108	K.RLSLGAQK.G
 4.2	59	0.6857	R.ISINKAVK.C
 4.2	59	0.6857	K.VITNKAVK.G
 3.7	66	-0.2620 204	P61106|RAB14_HUMAN R.IIEVSGQK.I
 1.9	99	0.6857	K.INVTVAKK.K

Top scoring peptide matches to query 2524
File7043 Spectrum2829 scans: 2178
Score greater than 20 indicates homology
Score greater than 36 indicates identity

**Score	Expect	Delta Hit Protein	Peptide**
 9.0	27	0.9834	R.TKAGPWGR.C
 7.3	40	-0.9992	K.MLPDRSR.A
 6.2	51	-1.0144 249	P06756|ITAV_HUMAN -.MAFPPRR.R
 6.0	54	-0.9958	K.NLGGNPFR.K
 5.9	54	-0.0621	K.DIILPFR.V
 5.6	58	-0.0291	K.LLDLMPR.L
 5.5	59	-0.0370	K.QNLVPFR.D
 5.4	62	0.9623	K.ELEARVR.K
 5.4	62	0.9372	R.ESLLAALR.R
 4.7	72	-0.8797	R.MCFNMR.G

Top scoring peptide matches to query 2528
File7043 Spectrum7000 scans: 5477
Score greater than 28 indicates homology
Score greater than 36 indicates identity

**Score	Expect	Delta Hit Protein	Peptide**
 25.9	0.55	-0.0051	81	P21281|VATB2_HUMAN R.EQALAVSR.N
 13.9	8.6	1.0265	K.KDLDPER.E
 12.3	12	-0.0415	K.LAGTLRDK.L
 9.7	23	0.9637	R.SPRLPFR.G
 9.6	23	1.0331	-.MPRAEPR.A
 9.6	23	0.9538	R.IVKGGAAEK.S
 8.8	28	-0.0125	K.LLDLMPR.L
 8.6	29	-1.0003	R.VSRSNSPK.T
 8.4	31	-0.0779	K.ISKLIGSR.T
 8.3	32	1.0405	K.KGSHSNSR.K

Top scoring peptide matches to query 2531
File7043 Spectrum15998 scans: 13204
Score greater than 36 indicates identity

**Score	Expect	Delta Hit Protein	Peptide**
 31.6	0.15	0.9980 439	P16662|UD2B7_HUMAN K.ADVWLIR.N
 29.3	0.25	0.9616 122	P19224|UD16_HUMAN K.VSVWLLR.Y
 22.3	1.3	0.0028	R.QASWIIR.G
 21.7	1.4	0.9769	M.ADVLSVLR.Q
 18.2	3.2	0.9657	R.KQSQLLR.S
 18.1	3.3	0.9583	-.MVLAGLIR.K
 17.9	3.4	0.0180	R.QGDTAILR.C
 17.2	4	1.0020	K.GKTGGPSIR.I
 16.8	4.4	0.9868	R.REWLLR.K
 16.6	4.7	-1.0136	K.ERSTILR.Q

Top scoring peptide matches to query 2532
File7043 Spectrum11608 scans: 9266
Score greater than 36 indicates identity

**Score	Expect	Delta Hit Protein	Peptide**
 40.9	0.017	-0.0052 648	P10515|ODP2_HUMAN K.GIDLTQVK.G
 25.0	0.68	-0.0164	K.GLDTLARK.G
 19.6	2.3	0.9676	K.NIGLTKAR.D
 17.8	3.5	0.0200	R.GLDISNVR.H
 16.5	4.7	0.0200	R.IGEAEAKR.D
 15.6	5.9	0.0200	R.AVLDATQR.Q
 14.7	7.1	-0.0052	R.EADLAKVK.K
 14.1	8.2	0.0200	R.ERLQEAK.L
 13.3	10	-0.9753	R.NGDVSRVK.R
 12.8	11	-0.0415	K.LLSLTQAK.E

Top scoring peptide matches to query 2533
File7043 Spectrum3858 scans: 3005
Score greater than 26 indicates homology
Score greater than 36 indicates identity

**Score	Expect	Delta Hit Protein	Peptide**
 16.2	5	1.0086	K.MLSLPPAK.E
 12.5	12	0.9545	R.EEIIIKK.E
 11.9	14	0.1048	K.SSSSSQYK.A
 11.8	14	0.1008	K.FFEESSK.S
 11.5	15	0.9909	R.EEIAVALK.K
 11.0	17	0.9545	K.EELKLLK.L
 10.2	20	1.1252	K.EENESHK.W
 10.1	21	1.1178 691	Q96N66|MBOA7_HUMAN K.EMASGFSK.G
 9.9	21	1.0735	K.ESHFPQK.Y
 9.3	25	1.0524	R.KPDPSGSGK.M

Top scoring peptide matches to query 2535
File7043 Spectrum4444 scans: 3474
Score greater than 25 indicates homology
Score greater than 36 indicates identity

**Score	Expect	Delta Hit Protein	Peptide**
 12.8	11	-0.9330	R.AAAARVCR.A
 12.3	12	0.1173	R.DPASRGDR.Y
 12.1	13	-0.9507	M.RGAASASVR.E
 11.7	14	0.0921	K.DPAALGSSR.S
 11.7	14	1.0649	R.GGPATARSR.V
 10.8	18	1.0510	K.KTPSGPSAK.S
 9.4	24	0.0484	K.KPPMATTK.G
 7.0	42	-0.9031	K.KDNANANK.G
 6.4	48	-0.0244 350+ P07355|ANXA2_HUMAN R.KLMVALAK.G
 6.3	50	1.0146	R.ESLLAALR.R

Top scoring peptide matches to query 2537
File7043 Spectrum15604 scans: 12852
Score greater than 29 indicates homology
Score greater than 34 indicates identity

**Score	Expect	Delta Hit Protein	Peptide**
 24.0	0.59	-0.3709	K.NLGGNPFR.K
 14.5	5.2	0.5628	K.DIILPFR.V
 11.4	11	-0.3168	K.HSACPFR.C
 11.4	11	-0.3896 249	P06756|ITAV_HUMAN -.MAFPPRR.R
 11.4	11	0.5879	K.QNLVPFR.D
 11.0	12	0.5958	K.LLDLMPR.L
 10.9	12	-0.4284	R.KNASISVR.A
 10.5	13	0.6897	K.WTYTMR.E
 10.0	15	-0.3307	R.YIMEFR.V
 8.1	23	-0.3557	R.AREVEDR.F

Top scoring peptide matches to query 2539
File7043 Spectrum2710 scans: 2089
Score greater than 25 indicates homology
Score greater than 34 indicates identity

**Score	Expect	Delta Hit Protein	Peptide**
 13.6	6.4	0.7320	K.FVCEYR.R
 11.8	9.7	0.6455	K.NLTDAIAR.A
 11.0	12	0.6203	K.NVELLSAK.L
 10.9	12	0.6455	R.EAELKQR.V
 10.9	12	0.6819	EALEQQR
 10.9	12	0.6343	K.ESRAAALR.A
 10.9	12	0.6455	K.EVVDKQR.D
 10.6	13	0.6302	R.FQGLVPGR.K
 10.5	13	0.6632 702	O95169|NDUB8_HUMAN R.NVMPLGAR.T
 10.3	14	0.7183	R.EGEGASAPR.D

Top scoring peptide matches to query 2541
File7043 Spectrum8284 scans: 6507
Score greater than 26 indicates homology
Score greater than 36 indicates identity

**Score	Expect	Delta Hit Protein	Peptide**
 21.3	1.6	-0.0293	R.MQSPKQR.T
 12.1	13	-1.0311	R.DLSEAGKR.G
 12.1	13	0.9846	R.LEAEQQR.C
 10.8	18	0.0031	R.YGGFMKR.Y
 9.8	23	-0.0908	-.MDLVLKR.C
 9.7	23	-0.9947	K.ADEEQKR.Q
 9.7	23	-0.9947	36	P11142|HSP7C_HUMAN K.AEDEKQR.D
 9.7	23	0.9482	K.AEIEKQR.L
 9.7	23	-1.0245	AKMNQQR
 9.7	23	-0.0107	K.DEAQKQR.K

Top scoring peptide matches to query 2542
File7043 Spectrum6251 scans: 4885
Score greater than 22 indicates homology
Score greater than 36 indicates identity

**Score	Expect	Delta Hit Protein	Peptide**
 10.3	20	0.9424	K.GEKLSIAR.G
 9.3	25	-0.0140 249	P06756|ITAV_HUMAN -.MAFPPRR.R
 8.2	33	0.9536	K.NVELLSAK.L
 7.1	42	0.9172 683	Q8TF72|SHRM3_HUMAN R.KEAVSLVK.G
 6.5	48	-0.0352	K.MVVTPRR.A
 6.4	50	0.9787	K.KPIGSSER.Y
 5.3	63	-0.1046	R.FVLRALR.M
 5.3	64	0.9747	R.EWAGALVK.D
 5.0	69	-0.0304	R.SEEVAVLK.Q
 4.7	73	0.0158	R.GTAEPFPR.L

Top scoring peptide matches to query 2545
File7043 Spectrum6988 scans: 5468
Score greater than 27 indicates homology
Score greater than 36 indicates identity

**Score	Expect	Delta Hit Protein	Peptide**
 31.5	0.15	0.9984	81	P21281|VATB2_HUMAN R.EQALAVSR.N
 12.7	12	0.9621	R.ILKSAEGR.V
 12.6	12	0.9257	K.ISKLIGSR.T
 11.2	16	-1.0358 595	P08559|ODPA_HUMAN R.KMLAAVSR.V
 11.1	17	0.9984	K.EQIAGLSR.R
 10.0	22	0.9369 683	Q8TF72|SHRM3_HUMAN R.KEAVSLVK.G
 9.7	23	-0.9961	R.IDSKAWR.E
 8.8	28	1.0236	R.ENLRDAR.N
 8.6	30	0.9621	R.LLSNAISR.L
 8.1	34	0.9620	K.LLVQTGSR.D

Top scoring peptide matches to query 2546
File7043 Spectrum6872 scans: 5375
Score greater than 29 indicates homology
Score greater than 36 indicates identity

**Score	Expect	Delta Hit Protein	Peptide**
 17.5	3.9	0.9673	R.INLTIGSR.A
 17.3	4	0.9673	K.KVLTQER.V
 15.4	6.2	-1.0120	K.TQITLGSR.S
 14.2	8.2	-0.0167	R.EEVTKLR.M
 12.2	13	0.9851	K.QVLLNMR.K
 11.9	14	0.0085	K.ETAEIRR.Q
 10.9	18	-0.0280	K.VIQKGSSR.E
 10.7	18	0.0197	R.LVEVDSGR.Q
 10.1	21	-0.9942 412	Q13011|ECH1_HUMAN -.MAAGIVASR.R
 9.4	25	0.0448	K.EVQSAGQR.I

Top scoring peptide matches to query 2551
File7043 Spectrum8532 scans: 6701
Score greater than 23 indicates homology
Score greater than 34 indicates identity

**Score	Expect	Delta Hit Protein	Peptide**
 11.9	9.3	0.2556	K.LISETRR.R
 9.4	17	-0.7219	R.ARMLGATR.A
 9.1	18	-0.6921	K.DKDLQTR.R
 8.7	20	0.2945 249	P06756|ITAV_HUMAN -.MAFPPRR.R
 7.3	27	0.2920	R.ILDSRDR.N
 7.0	29	0.2807	R.TSLAGGGRR.I
 6.6	31	-0.7107	R.QVVRDMK.D
 6.6	32	0.3032	K.LLDTEQR.K
 6.5	32	0.2556	K.LLESTRR.L
 6.3	34	0.2920	R.ELEATRR.Q

Top scoring peptide matches to query 2552
File7043 Spectrum8237 scans: 6468
Score greater than 23 indicates homology
Score greater than 36 indicates identity

**Score	Expect	Delta Hit Protein	Peptide**
 11.0	17	1.0269	HTGDGPYK
 10.8	18	1.0004	82	P12236|ADT3_HUMAN K.HTQFWR.Y
 10.8	18	1.0011	R.HTRSMAR.V
 10.6	18	-0.0775	R.SLPQRFK.M
 9.7	23	0.9508	K.MLQPITR.L
 9.5	24	0.9541	K.HTGYVGLK.N
 9.4	24	-0.0119	R.QRSSQNR.Q
 9.4	25	-0.0259	K.QLTTDAAR.I
 8.5	30	-0.0259	K.KQISEDR.K
 7.9	34	0.9469	R.LQRSSQR.I

Top scoring peptide matches to query 2557
File7043 Spectrum5298 scans: 4132
Score greater than 24 indicates homology
Score greater than 34 indicates identity

**Score	Expect	Delta Hit Protein	Peptide**
 13.4	6.5	-0.2937	R.MDGRLER.T
 10.8	12	-0.2937	-.MAQKEGGR.T
 10.5	13	-0.3883	K.IITFLNR.V
 9.7	15	-0.3552	-.MSLLKER.K
 9.7	15	-0.2937	R.MTDPTRR.F
 8.9	18	0.6838	K.EAAETKAR.E
 8.7	19	0.7049	R.FQEAAGPR.E
 8.4	20	-0.3519	K.LSYIQPR.T
 8.3	21	-0.2638 611	Q16822|PCKGM_HUMAN R.LEGEDSAR.E
 7.8	23	0.6725	R.GVKGTSGNR.G

Top scoring peptide matches to query 2558
File7043 Spectrum7963 scans: 6248
Score greater than 25 indicates homology
Score greater than 34 indicates identity

**Score	Expect	Delta Hit Protein	Peptide**
 15.0	4.4	-0.2750	R.VDSEDRR.S
 11.9	9.1	0.6839	K.TITEQQR.D
 11.8	9.2	-0.2862	R.EASGSGGRR.R
 10.9	11	0.6726	R.ESLSRQR.S
 10.4	13	0.6475	R.LTEKTQR.R
 9.5	16	-0.2386	M.EGSGGGAGER.A
 9.3	16	-0.3552	K.SKEMILR.I
 8.7	19	-0.2936 311	P01833|PIGR_HUMAN K.CGLGINSR.G
 8.2	21	-0.3114	K.DSRVNASK.T
 7.9	23	0.6726	K.TSGEGRLR.A

Top scoring peptide matches to query 2560
File7043 Spectrum5958 scans: 4655
Score greater than 17 indicates homology
Score greater than 36 indicates identity

**Score	Expect	Delta Hit Protein	Peptide**
 7.4	37	0.9724	R.LLNDSTGR.I
 3.9	82	-0.9891	R.NLSGMAER.K
 3.5	90	0.9173	K.MRVVLNK.A
 3.4	92	-0.0303 663	Q9NX20|RM16_HUMAN R.LVVEMGGR.C
 3.3	94	-0.9858	K.NPYAQER.G
 2.6 1.1e+002	0.9425	K.RMLAGGVR.S
 2.6 1.1e+002	-0.9528	M.NPSEMQR.K
 2.6 1.1e+002	-0.0303	K.LADMIASR.Q
 2.5 1.1e+002	-0.0051	98	P28331|NDUS1_HUMAN R.LSVAGNCR.M
 2.0 1.3e+002	0.9537	-.MGVLAAAAR.C

Top scoring peptide matches to query 2562
File7043 Spectrum3038 scans: 2347
Score greater than 19 indicates homology
Score greater than 36 indicates identity

**Score	Expect	Delta Hit Protein	Peptide**
 7.3	38	-1.0260	K.GKLCPFR.H
 6.5	46	0.0646	K.SGGQDQER.K
 6.4	46	0.9871	R.EAQVSSVR.I
 6.4	46	0.0170	R.GSSGRQER.S
 6.0	51	1.0083	R.DVQNPFR.Y
 5.4	58	-0.9445	K.TVEEDER.I
 5.2	61	1.0413 537	P21333|FLNA_HUMAN K.CSGPGLER.A
 5.2	61	-0.9995	R.TIIMEDR.L
 5.0	63	-0.9723	R.NHAHWGR.R
 4.7	68	-0.9962	K.ELDTPFR.L

Top scoring peptide matches to query 2563
File7043 Spectrum10598 scans: 8411
Score greater than 18 indicates homology
Score greater than 36 indicates identity

**Score	Expect	Delta Hit Protein	Peptide**
 10.9	16	-0.0047	33	P14625|ENPL_HUMAN K.TFEINPR.H
 4.9	65	1.0236	R.EAAAMGPTK.F
 0.9 1.6e+002	-0.0444	K.IAIMREK.H
 0.9 1.7e+002	1.0049	R.MVLPMPR.L
 0.6 1.8e+002	0.9582	R.ISRSPSTK.A

Top scoring peptide matches to query 2564
File7043 Spectrum11478 scans: 9142
Score greater than 23 indicates homology
Score greater than 36 indicates identity

**Score	Expect	Delta Hit Protein	Peptide**
 17.4	3.6	0.0530	-.MAVQGSQR.R
 9.3	24	1.0483	-.MAAVGAEAR.G
 9.1	25	0.0239	-.MAGYLPPK.G
 8.5	28	0.0391 266	P17858|K6PL_HUMAN -.MAAVDLEK.L
 8.1	32	0.0167	-.MAAGTAARK.A
 7.6	35	0.0279	K.LPMTDKR.V
 7.4	37	0.0531	-.MAQKEGGR.T
 6.8	42	0.0279	K.AQILEMR.Y
 5.3	60	-0.9925	-.MATSNLLK.N
 5.3	60	1.0306	K.EKLENSR.S

Top scoring peptide matches to query 2565
File7043 Spectrum17829 scans: 14929
Score greater than 15 indicates homology
Score greater than 36 indicates identity

**Score	Expect	Delta Hit Protein	Peptide**
 3.5	91	-0.9776	R.SGLISTSGR.V
 2.4 1.2e+002	0.0388 633	P07858|CATB_HUMAN K.LPASFDAR.E
 2.0 1.3e+002	-0.9776	K.NISNSSKK.R

Top scoring peptide matches to query 2566
File7043 Spectrum13778 scans: 11172
Score greater than 15 indicates homology
Score greater than 36 indicates identity

**Score	Expect	Delta Hit Protein	Peptide**
 2.8 1.1e+002	-0.9854	R.VARAEFGK.N
 2.5 1.1e+002	1.0381 479	Q6ZMR3|LDH6A_HUMAN K.MPNIVSSK.D
 1.4 1.4e+002	1.0051	R.ANYVPALK.L
 1.1 1.6e+002	-0.9126	R.WRDTDGK.K
 0.7 1.7e+002	-0.0265	R.IGQVGKFK.N
 0.4 1.8e+002	1.1585	K.ASMTSTDF.-

Top scoring peptide matches to query 2572
File7043 Spectrum6490 scans: 5074
Score greater than 23 indicates homology
Score greater than 35 indicates identity

**Score	Expect	Delta Hit Protein	Peptide**
 11.4	14	-0.0420	K.DLDLVFR.V
 11.4	14	-0.0089	R.VEDIMVR.D
 11.1	15	1.0050	K.EVIDASDK.E
 10.2	18	0.9751 144	Q9NQC3|RTN4_HUMAN K.NVKDAMAK.I
 7.7	33	0.9387	R.TVMVVVGR.N
 7.5	34	1.0591	K.EDPDCIK.A
 6.6	42	1.0730	R.GGSEGGCPR.G
 5.7	52	-0.0783	K.ELITVFR.M
 4.3	72	1.0366	R.AEAACAQR.S
 4.1	75	1.0413	K.SDPESVDK.E

Top scoring peptide matches to query 2573
File7043 Spectrum7558 scans: 5927
Score greater than 27 indicates homology
Score greater than 35 indicates identity

**Score	Expect	Delta Hit Protein	Peptide**
 15.3	5.7	-0.9816	K.DLSDVAMK.V
 13.6	8.5	-1.0002	K.GIEGMIMK.R
 13.6	8.5	-1.0180	K.KTEEIMK.E
 13.6	8.5	-1.0696	R.LLNFLMK.H
 13.5	8.7	0.9673	R.FQGGGRVR.T
 13.0	9.7	-0.9928	R.DIERSMK.S
 13.0	9.7	-0.9928	R.EVERSMK.A
 12.3	11	0.0170	R.EFIPDEK.K
 12.3	11	-0.0054	81	P21281|VATB2_HUMAN R.NYLSQPR.L
 11.7	13	0.0137	R.DIEEIMK.D

Top scoring peptide matches to query 2575
File7043 Spectrum5294 scans: 4129
Score greater than 32 indicates homology
Score greater than 35 indicates identity

**Score	Expect	Delta Hit Protein	Peptide**
 22.8	1	0.9965	-.MEGAALLR.V
 17.8	3.2	0.0046	81	P21281|VATB2_HUMAN R.NYLSQPR.L
 14.4	7.1	0.9886	K.FVDPSRR.A
 14.4	7.1	0.9964	K.MVVLDAGR.Y
 12.7	10	0.9965	R.MVSPASLR.K
 11.4	14	0.9634	K.LSYIQPR.T
 10.8	16	0.9634	K.FAEQLIR.V
 10.8	16	0.8907	K.FLKSLIR.K
 10.8	16	0.9270	K.FQVTLLR.L
 10.8	16	0.9998	K.FSEIPQR.L

Top scoring peptide matches to query 2586
File7043 Spectrum15553 scans: 12805
Score greater than 19 indicates homology
Score greater than 35 indicates identity

**Score	Expect	Delta Hit Protein	Peptide**
 8.6	27	0.0532	K.KQGLEMR.I
 6.2	46	-0.8693	64	Q13813|SPTA2_HUMAN R.EQAQSCR.Q
 5.3	57	1.0807	K.LPPDCFK.N
 4.7	65	1.0544	R.HRAGGGGHK.L
 2.7	1e+002	0.0790	K.DEPLEFK.R
 2.5 1.1e+002	0.0895	K.GVQGNMQK.Q
 2.4 1.1e+002	1.0405	R.AGKPFGSGR.A
 2.3 1.1e+002	0.0161	R.AHVLYFK.N
 1.8 1.3e+002	0.0895	R.QQMDLAR.Q
 1.0 1.5e+002	1.0623	R.AAAAGGMRR.D

Top scoring peptide matches to query 2587
File7043 Spectrum12948 scans: 10431
Score greater than 25 indicates homology
Score greater than 35 indicates identity

**Score	Expect	Delta Hit Protein	Peptide**
 13.9	7.9	1.1464	R.GGSEGGCPR.G
 11.4	14	1.0307	K.SSEKNALK.L
 11.4	14	1.0446	R.SSRNSLGR.A
 10.4	18	0.0896	K.SMPNLGSR.N
 10.3	18	1.1286	M.EGSGGGAGER.A
 10.3	18	1.0922	R.GDGTGGVTGR.F
 9.8	20	0.0202	96	P30101|PDIA3_HUMAN K.LNFAVASR.K
 9.1	24	0.0710 237	Q969Z3|MOSC2_HUMAN K.KVMACPR.C
 8.9	25	-0.9097	R.MIWEQR.S
 4.4	71	1.0849	R.MPEAEKR.K

Top scoring peptide matches to query 2591
File7043 Spectrum30667 scans: 27130
Score greater than 16 indicates homology
Score greater than 34 indicates identity

**Score	Expect	Delta Hit Protein	Peptide**
 3.8	53	0.3059	R.CFLPQGR.R
 3.3	59	0.3721 185	P39023|RL3_HUMAN K.WQDEDGK.K

Top scoring peptide matches to query 2600
File7043 Spectrum7929 scans: 6221
Score greater than 30 indicates homology
Score greater than 35 indicates identity

**Score	Expect	Delta Hit Protein	Peptide**
 25.7	0.49	0.0060	K.FSEVLKR.L
 16.0	4.6	0.0423	R.FSDLLQR.L
 15.8	4.8	-0.0053 432	Q9H9B4|SFXN1_HUMAN K.HVSPLIGR.F
 14.4	6.6	0.0641	-.MSGIGNKR.A
 13.3	8.6	0.0753	R.NTISGMQK.F
 11.2	14	0.1369	-.MQANDAGR.F
 11.0	15	0.0893	-.MTRGNQR.E
 10.9	15	0.0026	K.MTITLKR.E
 9.4	21	1.0230 403	Q01813|K6PP_HUMAN K.MKTTIQR.G
 8.6	25	0.0199	R.HVKTHTR.E

Top scoring peptide matches to query 2607
File7043 Spectrum4054 scans: 3171
Score greater than 14 indicates homology
Score greater than 33 indicates identity

**Score	Expect	Delta Hit Protein	Peptide**
 4.7	42	-0.4338	K.AKYAEVAK.H
 0.8	1e+002	0.6009	K.VILMACR.E
 0.6 1.1e+002	0.6672 351	P13010|KU86_HUMAN K.VDEEQMK.Y
 0.6 1.1e+002	0.6130	K.EDVLSTSK.D
 0.5 1.1e+002	0.6625	K.NIANMCR.G
 0.3 1.1e+002	0.5614	K.YLAVPTSK.K
 0.2 1.2e+002	0.6512	R.CRQVCR.G
 0.2 1.2e+002	0.6382	K.DNSINTSK.H
 0.2 1.2e+002	0.5679	K.IFLVACR.Q

Top scoring peptide matches to query 2611
File7043 Spectrum5745 scans: 4490
Score greater than 29 indicates homology
Score greater than 35 indicates identity

**Score	Expect	Delta Hit Protein	Peptide**
 23.4	0.83	0.0314 136	P07339|CATD_HUMAN R.QVFGEATK.Q
 14.7	6.1	-0.0049	K.KVYDLNK.I
 12.9	9.2	-0.0049	R.KLSEAGFK.A
 9.4	20	-0.0386	R.QHAIRVR.I
 9.0	23	-0.9526	R.VIDFSDGK.A
 8.3	27	-0.0413	R.KYASGLLK.A
 8.2	27	-0.0083	K.KSSMVALK.R
 7.2	34	-0.9559	R.EVSMGLTK.S
 6.8	38	0.0645	K.QEEAKMK.K
 6.7	38	0.0467	R.QLSESSTK.D

Top scoring peptide matches to query 2613
File7043 Spectrum3238 scans: 2510
Score greater than 20 indicates homology
Score greater than 35 indicates identity

**Score	Expect	Delta Hit Protein	Peptide**
 8.2	27	1.0572	K.AVTAIMTR.L
 7.1	35	1.0823	K.GMATAKQR.L
 7.1	35	1.1221	R.SSHGSFTR.G
 6.2	43	-0.9048	K.NPNDKHR.S
 6.2	43	-0.9412	K.SKQPAGHR.R
 6.2	43	-1.0391	K.TLLLKHR.Q
 6.2	43	0.0752	R.VPWGQHR.A
 3.5	79	0.1048 473	Q5VT66|MOSC1_HUMAN K.RVMACSR.C
 3.3	84	-0.9486	K.KMVPPHR.A
 3.2	86	-0.9048	R.EDPRAHR.L

Top scoring peptide matches to query 2617
File7043 Spectrum3061 scans: 2366
Score greater than 29 indicates homology
Score greater than 35 indicates identity

**Score	Expect	Delta Hit Protein	Peptide**
 19.8	1.7	0.9798 110+ Q9HAW9|UD18_HUMAN R.SLFNDRK.L
 15.7	4.5	1.0161	R.DGASNIFR.C
 11.8	11	1.0492	K.SVENQMR.Y
 11.7	11	1.0373	R.DGAWGAFR.H
 10.4	15	0.0023	K.EAMARFR.K
 10.2	16	1.0240	R.GDVEMKGK.G
 9.7	18	0.9798	K.ISDNRFK.A
 9.3	19	0.9910	R.EAEKQFK.S
 9.3	19	1.0240	K.EAKEGAMK.D
 9.3	19	0.9724	K.LSCKPFK.W

Top scoring peptide matches to query 2620
File7043 Spectrum5363 scans: 4185
Score greater than 24 indicates homology
Score greater than 33 indicates identity

**Score	Expect	Delta Hit Protein	Peptide**
 11.8	6.9	0.3563 319	P50416|CPT1A_HUMAN K.IWMGMVK.I
 10.7	9	-0.6051	K.DQTDKMK.Q
 10.6	9.1	0.3563 319	P50416|CPT1A_HUMAN K.IWMGMVK.I
 9.2	13	0.3967	K.IDGKCCK.V
 9.2	13	0.3571	97	P02679|FIBG_HUMAN R.LDGSVDFK.K
 9.2	13	0.3538	R.LDGTMSIK.K
 7.6	18	0.3207	K.AVDFTTVK.F
 7.4	19	0.3095	K.IRYSDVK.K
 6.1	26	0.3902	R.ISAEDAMK.H
 5.1	33	0.3538	K.LSVMESSK.S

Top scoring peptide matches to query 2621
File7043 Spectrum3422 scans: 2655
Score greater than 18 indicates homology
Score greater than 34 indicates identity

**Score	Expect	Delta Hit Protein	Peptide**
 5.8	40	-0.9828	-.MSSEVSAR.R
 5.0	49	-1.0232	R.MASGLEFK.S
 4.7	52	1.0142	R.NTLMDMR.L
 3.6	66	0.9409	K.SKQPAGHR.R
 3.0	77	-1.0271	K.AIDAAEHR.L
 3.0	77	-0.0314	K.ITMLEMK.E
 2.9	79	-1.0635 520	P05106|ITB3_HUMAN K.THIALDGR.L
 2.6	84	-0.9907	R.HTGEEPGR.R
 2.6	84	-0.9949	K.RMAMAMR.Q
 2.5	85	0.9158	R.VPAGAVGGPR.G

Top scoring peptide matches to query 2623
File7043 Spectrum2894 scans: 2233
Score greater than 18 indicates homology
Score greater than 34 indicates identity

**Score	Expect	Delta Hit Protein	Peptide**
 8.5	21	0.9577	-.MANLFIR.K
 5.2	46	0.9539	R.LAHQQQR.Q
 4.9	49	0.9175	K.ERHGLIR.A
 4.3	57	0.9876	32	P02768|ALBU_HUMAN K.AEFAEVSK.L
 4.0	60	1.0280	K.GSSDDGKSK.I
 3.5	68	1.0271	K.TMPLSCR.K
 3.4	70	-0.0627	-.MTLLTFR.D
 1.2 1.2e+002	0.9876	K.AFVEDTAK.K
 1.1 1.2e+002	-0.9270	R.CCNVSSR.M
 1.1 1.2e+002	1.0345	K.TGNCSVSR.V

Top scoring peptide matches to query 2626
File7043 Spectrum5588 scans: 4365
Score greater than 23 indicates homology
Score greater than 34 indicates identity

**Score	Expect	Delta Hit Protein	Peptide**
 12.6	8.4	0.0027	R.FGRTTTAK.S
 12.1	9.5	1.0786	K.EVMESSAK.I
 10.1	15	0.0065	R.FMIELTK.L
 7.8	26	1.0673	K.EMSKGQGK.D
 7.5	27	1.0487 226	Q7Z406|MYH14_HUMAN R.KMQAQMK.E
 7.3	28	0.0946	K.EDTLEMK.I
 6.5	34	0.9905	K.LFVGMLGK.Q
 6.1	38	0.9575	K.VFIFNLK.L
 6.0	38	1.1150	K.DEDVQMK.R
 6.0	38	1.0231	K.EPGHQKGK.E

Top scoring peptide matches to query 2628
File7043 Spectrum9900 scans: 7820
Score greater than 25 indicates homology
Score greater than 34 indicates identity

**Score	Expect	Delta Hit Protein	Peptide**
 12.2	9.1	-0.9271	R.GSPASGPGPR.R
 11.4	11	-0.9748	R.RGVGSPPGR.K
 11.2	12	0.0820	R.AGAQAGGGHR.H
 10.7	13	-0.0046	26	Q14764|MVP_HUMAN	K.SLQPLAPR.N
 9.5	17	-0.9267	K.VTEMMKK.F
 8.1	24	-0.9635	R.TANGLPGPR.S
 7.9	25	-0.9384 182	P12109|CO6A1_HUMAN R.GGPGERGPR.G
 7.9	25	-0.9384	K.GPAGDRGPR.G
 7.3	28	0.0971	R.AQEMFQK.T
 6.3	36	-0.0199	R.KAHVIWK.K

Top scoring peptide matches to query 2629
File7043 Spectrum9897 scans: 7817
Score greater than 30 indicates homology
Score greater than 34 indicates identity

**Score	Expect	Delta Hit Protein	Peptide**
 26.8	0.32	-0.0046	26	Q14764|MVP_HUMAN	K.SLQPLAPR.N
 15.7	4.1	0.1189	R.LSQACMR.G
 14.0	6.1	0.0317	K.SLQKSYR.K
 13.8	6.3	0.0681	R.SLQNYTR.A
 9.2	18	-0.0047	R.TVAIPQPR.Q
 9.1	19	0.1045 455	P12110|CO6A2_HUMAN K.GDPGPEGPR.G
 8.8	20	0.0457	R.RGPNQGPR.G
 8.1	24	0.0052	R.RLWPGPR.E
 7.8	25	0.0205	K.DGRPLPAR.D
 7.2	29	0.0430	R.LSKNYEK.A

Top scoring peptide matches to query 2632
File7043 Spectrum13390 scans: 10833
Score greater than 29 indicates homology
Score greater than 34 indicates identity

**Score	Expect	Delta Hit Protein	Peptide**
 33.3	0.071	-0.0049 168	P30050|RL12_HUMAN K.IGPLGLSPK.K
 14.7	5.2	0.0856	K.LGCYIQK.N
 14.3	5.7	-0.0049	K.LGIPTVPGK.V
 11.4	11	-0.8919	R.LGCMFVR.C
 11.1	12	0.1979	K.MSMMGHR.V
 10.5	14	-1.0365	K.LGIVPKQK.Y
 9.6	17	-1.0365	K.GIPLKNIK.H
 9.3	18	-0.0273	R.LGLRGPLR.K
 8.9	20	1.1577	K.MSADDGIR.N
 8.5	22	0.0605	K.IGTVMTFL.-

Top scoring peptide matches to query 2637
File7043 Spectrum2289 scans: 1771
Score greater than 20 indicates homology
Score greater than 34 indicates identity

**Score	Expect	Delta Hit Protein	Peptide**
 13.1	7.2	0.0524	K.LSHIKQR.I
 6.4	34	1.1494	K.AVMEQFR.H
 5.6	40	1.1316	K.INSDYLR.D
 5.5	42	1.1091	R.LAHQQQR.Q
 5.3	43	0.1654	K.LSDMPYR.A
 5.1	45	-0.0092	R.LPLALAKR.G
 3.4	67	0.1937 740	Q06136|KDSR_HUMAN R.RCMMQR.E
 3.3	68	-0.8840 683	Q8TF72|SHRM3_HUMAN R.SSLYEKR.K
 3.1	72	1.1455	R.HPNTQQR.A
 3.1	72	-0.9065	K.HVQSQKR.E

Top scoring peptide matches to query 2639
File7043 Spectrum1226 scans: 962
Score greater than 20 indicates homology
Score greater than 34 indicates identity

**Score	Expect	Delta Hit Protein	Peptide**
 8.0	23	0.1040	K.MILTFEK.R
 6.6	32	1.1648	-.MALADSTR.G
 5.5	41	1.1350	81	P21281|VATB2_HUMAN -.MALRAMR.G
 4.9	48	1.1608	K.MLADWTK.Y
 4.5	53	0.0928	-.MTLLTFR.D
 3.9	60	0.1920	K.DIETMEK.K
 3.8	61	1.1397	R.AATTIEMK.A
 3.7	62	0.1114	K.QITASSFK.K
 3.7	63	1.1569	K.FARSDER.K
 3.5	65	0.1622	-.MAQEKMK.L

Top scoring peptide matches to query 2640
File7043 Spectrum30218 scans: 26754
Score greater than 18 indicates homology
Score greater than 34 indicates identity

**Score	Expect	Delta Hit Protein	Peptide**
 10.1	14	1.1649	K.AEKETMR.K
 4.9	48	1.1458	K.NPNDKHR.S
 4.0	59	0.0890	R.ATHGGLGLR.M
 3.0	75	0.1115	K.TQAGTYIK.E
 2.9	76	-0.9678	K.KPKPEKR.E
 2.9	76	-0.9314	K.KPPKEQR.S
 2.9	76	-0.8110	K.SAYEEQR.I
 2.9	76	-0.8838 683	Q8TF72|SHRM3_HUMAN R.SSLYEKR.K
 2.9	76	-0.9314	R.VTHIEKR.Y
 2.6	81	1.1536	K.TMRVSDR.Q

Top scoring peptide matches to query 2648
File7043 Spectrum32071 scans: 28096
Score greater than 19 indicates homology
Score greater than 34 indicates identity

**Score	Expect	Delta Hit Protein	Peptide**
 8.0	23	1.1771	R.IMQDTEK.D
 5.9	38	0.1454	R.SMTSLAQK.I
 4.3	55	1.1295	K.RMIDTTK.N
 4.2	56	1.0349	R.LEPLLAPK.F
 3.6	64	1.1957	60	Q01082|SPTB2_HUMAN K.STDEVDSK.R
 3.3	68	0.1111	K.HPTGWKR.T
 2.8	78	0.2368	K.GSSAVGSDSD.-
 2.3	87	1.1659	R.EARMTEK.L
 1.1 1.1e+002	0.1818	K.ESIETMR.T

Top scoring peptide matches to query 2650
File7043 Spectrum32374 scans: 28408
Score greater than 22 indicates homology
Score greater than 34 indicates identity

**Score	Expect	Delta Hit Protein	Peptide**
 10.7	12	-0.8649	R.DRGMLYK.D
 8.5	21	1.0854	K.ISHIPASR.W
 8.3	22	1.1508	K.IADMGFAR.L
 7.7	25	1.0378 199	P62241|RS8_HUMAN R.IHTVRVR.G
 7.7	25	1.0378	R.IHVTVRR.D
 6.9	30	1.1144	K.NMVLFTR.Q
 6.7	31	1.1694	K.IDFGDSAR.A
 5.7	40	1.0854	K.LGGVLHER.Q
 5.1	45	1.1582	K.HGPLADDR.S
 5.1	45	1.0378	R.IGRPKGPR.D

Top scoring peptide matches to query 2653
File7043 Spectrum32344 scans: 28356
Score greater than 19 indicates homology
Score greater than 34 indicates identity

**Score	Expect	Delta Hit Protein	Peptide**
 7.7	25	1.1301	R.VSQSMKGK.S
 6.1	36	1.0971	R.LNSSFKGK.M
 5.4	42	1.1512	K.IADMGFAR.L
 5.4	42	1.1438 319	P50416|CPT1A_HUMAN K.IWMGMVK.I
 3.1	72	1.1698	K.IDFGDSAR.A
 1.2 1.1e+002	1.1222	R.ATHLDPAR.L
 1.2 1.1e+002	1.1811	R.GYDSDPVK.A
 1.2 1.1e+002	1.0784	K.MRFLVAK.S
 1.2 1.1e+002	1.1148	K.NMVLFTR.Q
 1.2 1.1e+002	1.0494	K.VSVHVLAR.E

Top scoring peptide matches to query 2655
File7043 Spectrum29578 scans: 26250
Score greater than 18 indicates homology
Score greater than 33 indicates identity

**Score	Expect	Delta Hit Protein	Peptide**
 5.8	33	0.1898	M.SPEKQHR.E
 5.4	36	-0.8168	K.WKMFVR.A
 4.3	46	-0.7322	K.CNTSLCK.E
 3.1	61	-0.7434	R.GQSCMKR.H
 2.9	64	-0.9285 380	P13667|PDIA4_HUMAN K.GPVKVVVGK.T
 2.9	64	-0.8306	R.HVVDGISR.E
 2.6	69	-0.8054	K.AHRENQK.Y
 2.6	69	-0.8306	K.HAAEQAKK.A
 2.4	71	-0.7652	IGDFGMAR
 2.4	71	-0.8380	K.LFTAKMR.K

Top scoring peptide matches to query 2660
File7043 Spectrum9127 scans: 7193
Score greater than 29 indicates homology
Score greater than 34 indicates identity

**Score	Expect	Delta Hit Protein	Peptide**
 18.4	2	1.0660	K.QMMCPSK.V
 15.0	4.4	-0.0051 355	P23396|RS3_HUMAN R.FIMESGAK.G
 12.3	8.2	0.0279	R.LMMEQSK.K
 11.1	11	0.0022	K.FLDSTGSR.L
 10.2	13	0.8983	R.KAHVIWK.K
 10.1	14	-0.0051	K.EMIQFSK.D
 9.8	15	0.0279	K.MIEMQAK.I
 9.8	15	-0.0415	K.MLKEFAK.A
 9.7	15	-0.9674	-.MIMTESR.E
 9.5	16	-0.0779	K.MLKSFLK.A

Top scoring peptide matches to query 2661
File7043 Spectrum8882 scans: 6985
Score greater than 28 indicates homology
Score greater than 34 indicates identity

**Score	Expect	Delta Hit Protein	Peptide**
 20.9	1.1	-0.0053 138	P06396|GELS_HUMAN K.TASDFITK.M
 13.9	5.7	0.0311	K.GDYDTALK.L
 11.1	11	-0.9576	K.GSMGHPGPK.G
 10.0	14	0.0377	R.TAMEFQR.R
 9.3	17	-0.0641	R.VTHIEKR.Y
 8.9	18	0.0199	K.TATFTDAR.I
 8.3	21	-0.0892	R.VLPNGLAAK.A
 7.4	26	0.9563	R.ATHGGLGLR.M
 7.4	26	0.0087	K.TAERSYR.E
 6.7	30	-0.0277	R.DALTGHLR.T

Top scoring peptide matches to query 2663
File7043 Spectrum9875 scans: 7800
Score greater than 23 indicates homology
Score greater than 34 indicates identity

**Score	Expect	Delta Hit Protein	Peptide**
 24.4	0.51	0.9982	26	Q14764|MVP_HUMAN K.SLQPLAPR.N
 8.7	19	0.9982	K.QTLPVAPR.T
 8.6	19	0.9982	R.TVAIPQPR.Q
 8.3	21	1.0636	K.MPPAPSGPK.A
 6.8	30	-0.9559	M.AGPLQGGGAR.A
 6.2	34	-1.0286	R.LLPTRQR.V
 5.9	36	0.9618	R.VAIVVGAPR.T
 5.9	36	-0.8905	R.ESGFCKR.H
 4.0	55	1.0081	R.RLWPGPR.E
 4.0	56	-0.9745	-.MPAPVGRR.S

Top scoring peptide matches to query 2671
File7043 Spectrum8072 scans: 6336
Score greater than 21 indicates homology
Score greater than 34 indicates identity

**Score	Expect	Delta Hit Protein	Peptide**
 9.0	18	1.0546	R.MASGLEFK.S
 7.9	23	-0.0172 635	Q9HCK1|ZDBF2_HUMAN R.TTHRLQK.A
 7.3	27	1.0951	R.RSSEEMK.R
 7.1	28	0.1110	K.ASSQSEMK.R
 6.5	32	1.0360	R.FMACLLK.G
 6.5	32	1.0950	-.MSSEVSAR.R
 6.2	34	-0.9940	R.LYKGEFK.Q
 6.2	34	-0.8552	K.QEGMEMK.L
 6.2	34	-0.9359	R.RFASEMK.Q
 6.2	34	-0.9246	K.SNMLEFK.L

Top scoring peptide matches to query 2672
File7043 Spectrum11713 scans: 9354
Score greater than 17 indicates homology
Score greater than 34 indicates identity

**Score	Expect	Delta Hit Protein	Peptide**
 6.3	33	-0.0056 393	P24752|THIL_HUMAN R.LNVTPLAR.I
 3.7	62	0.0597	K.VKVSCYK.E
 3.5	64	0.0961	K.VMVEAYR.R
 3.4	66	0.0849 668	Q92820|GGH_HUMAN K.VMKNYGR.Y
 3.0	71	0.1875	K.DDEDSFR.R
 2.6	79	1.1165	K.DFLANMR.F
 2.6	79	1.1165	K.DMLANFR.G
 2.2	87	-0.0057	R.VVPEGVKR.E
 2.0	89	-0.9467	-.MALLHSGR.V
 2.0	91	1.0512	4	P98164|LRP2_HUMAN K.ETIINHR.L

Top scoring peptide matches to query 2675
File7043 Spectrum8343 scans: 6553
Score greater than 21 indicates homology
Score greater than 32 indicates identity

**Score	Expect	Delta Hit Protein	Peptide**
 10.6	8.3	0.4752	R.ETLNPPGR.A
 7.6	17	-0.5162	-.MSLNYIK.N
 6.8	20	0.5406	-.MGFELDR.F
 4.1	37	0.5406	K.YEAVMDR.V
 4.1	37	-0.4434	R.MSYEPNK.D
 3.4	44	0.5042	R.YMADVIR.H
 3.3	45	-0.5162	K.SMVQYLK.S
 3.0	47	0.4527 150	P12111|CO6A3_HUMAN K.GGIGNRGPR.G
 3.0	48	0.5485	K.MSEMELK.A
 3.0	48	0.4678	R.VTMFTIR.G

Top scoring peptide matches to query 2684
File7043 Spectrum6326 scans: 4944
Score greater than 19 indicates homology
Score greater than 33 indicates identity

**Score	Expect	Delta Hit Protein	Peptide**
 9.9	11	0.4463	K.QVAYIYK.C
 9.6	11	-0.5271	K.KHCEALK.D
 6.3	24	-0.6289	R.RAIAVLSR.W
 6.1	25	0.4504 451+ O94973|AP2A2_HUMAN K.AQEPPKSK.K
 5.7	28	0.4643	R.TSGRGPPGR.W
 5.4	29	-0.6289	K.RALIVASR.G
 5.1	31	0.4681	K.ACPPKPSK.W
 4.6	35	-0.5020	R.RAEPCPR.V
 3.4	47	-0.6792	K.KAILTLVK.Q
 3.0	51	-0.6428	K.KSIPLSIK.N

Top scoring peptide matches to query 2688
File7043 Spectrum7179 scans: 5621
Score greater than 20 indicates homology
Score greater than 33 indicates identity

**Score	Expect	Delta Hit Protein	Peptide**
 8.4	15	0.5854 451+ O94973|AP2A2_HUMAN K.AQEPPKSK.K
 7.5	18	-0.3920	K.KHCEALK.D
 5.4	30	-0.4462	R.VKLDPTGR.L
 4.8	34	0.6357	K.EGKGSNHR.G
 4.5	36	-0.3734	K.KHGSEEAK.R
 4.3	38	-0.4713	K.AQLLEIAK.A
 4.3	38	-0.4350	R.AQLLEPSK.S
 4.3	38	-0.5441	K.KAILTLVK.Q
 3.5	45	-0.4098	K.GLDNKNPK.I
 2.9	53	-0.4323	R.ARLNAQGR.V

Top scoring peptide matches to query 2690
File7043 Spectrum6554 scans: 5126
Score greater than 19 indicates homology
Score greater than 33 indicates identity

**Score	Expect	Delta Hit Protein	Peptide**
 6.3	24	0.6033	K.ACPPKPSK.W
 5.9	26	0.7091	R.GSGEMMTR.S
 5.5	29	0.7091	R.GSGEMMTR.S
 4.7	35	0.6172 739	Q9NQE9|HINT3_HUMAN K.KHIGNCR.T
 3.2	49	0.5631	K.GASLARPGR.G
 3.2	49	-0.3733	K.KHGSEEAK.R
 3.1	50	-0.2716	-.MADFDDR.V
 1.6	71	0.5379	R.RLGVQPSK.Y
 1.4	74	0.6761	R.DTFAAMGR.L
 1.4	75	0.5856 451+ O94973|AP2A2_HUMAN K.AQEPPKSK.K

Top scoring peptide matches to query 2692
File7043 Spectrum9370 scans: 7389
Score greater than 20 indicates homology
Score greater than 33 indicates identity

**Score	Expect	Delta Hit Protein	Peptide**
 8.5	15	-0.3839	K.AAPGEERR.R
 7.1	20	-0.4025	K.NMAKIHR.G
 5.9	27	0.6516	K.VDCSIYK.K
 4.2	39	0.5927 572	P14618|KPYM_HUMAN R.MQHLIAR.E
 4.1	40	0.6469 416	Q9UHE5|NAT8_HUMAN -.MAPCHIR.K
 4.0	41	-0.3628	K.NTPHFNR.K
 3.4	47	0.5862	K.LPEEIQR.D
 3.3	48	0.5274	R.LQALRQR.L
 3.1	50	-0.4706	K.AQLLEIAK.A
 3.1	50	-0.4342	R.AQLLEPSK.S

Top scoring peptide matches to query 2696
File7043 Spectrum30240 scans: 26771
Score greater than 18 indicates homology
Score greater than 33 indicates identity

**Score	Expect	Delta Hit Protein	Peptide**
 5.5	29	0.5721	R.TPPAGKWK.Q
 5.0	33	0.6489	K.EREHGEK.E
 4.5	37	0.6237	K.DVLSHGEK.E
 4.2	39	0.6164	R.QLYMVSK.E
 4.2	39	0.6011	K.YKMLWK.L
 4.1	40	0.6705	R.YGMCPLK.G
 3.5	46	0.5397	R.RIGGGSPIK.I
 3.2	49	0.5874 451+ O94973|AP2A2_HUMAN K.AQEPPKSK.K
 2.5	58	0.5397	R.AQLLVQGR.R
 1.6	71	0.5688	R.HKEMVLK.I

Top scoring peptide matches to query 2697
File7043 Spectrum29408 scans: 26105
Score greater than 20 indicates homology
Score greater than 33 indicates identity

**Score	Expect	Delta Hit Protein	Peptide**
 9.4	12	0.5874 451+ O94973|AP2A2_HUMAN K.AQEPPKSK.K
 7.4	19	0.5650	K.GASLARPGR.G
 7.0	20	0.5398	R.RLGVQPSK.Y
 6.3	24	0.6052	K.ACPPKPSK.W
 6.1	25	0.5034	R.IIGAAVGRK.K
 4.2	39	0.5147	R.NLNVILAK.Y
 4.0	40	0.5147	R.QALQALLK.E
 4.0	41	0.6013	R.TSGRGPPGR.W
 3.9	42	-0.4078	R.NPEKIER.M
 3.9	42	0.6126	R.SDGAKPGPR.E

Top scoring peptide matches to query 2706
File7043 Spectrum208 scans: 287
Score greater than 18 indicates homology
Score greater than 33 indicates identity

**Score	Expect	Delta Hit Protein	Peptide**
 9.9	11	0.6462	K.GDIYNFR.L
 5.0	32	-0.3701	K.KHGSEEAK.R
 3.7	44	0.6026	K.GSRPQSPR.K
 3.5	45	-0.3636	R.KHCVEGR.L
 3.2	49	0.6138	M.GTGPAQTPR.S
 3.2	49	-0.3524	K.NMYRSAK.S
 2.4	59	-0.4681	K.AQLLEIAK.A
 2.3	60	0.5887 451+ O94973|AP2A2_HUMAN K.AQEPPKSK.K
 2.3	60	-0.5408	K.KAILTLVK.Q

Top scoring peptide matches to query 2708
File7043 Spectrum32281 scans: 28274
Score greater than 16 indicates homology
Score greater than 33 indicates identity

**Score	Expect	Delta Hit Protein	Peptide**
 5.0	33	0.6066	K.ACPPKPSK.W
 2.9	53	0.5889 451+ O94973|AP2A2_HUMAN K.AQEPPKSK.K
 1.2	78	-0.4289	M.RAAAAGGGVR.T

Top scoring peptide matches to query 2710
File7043 Spectrum8894 scans: 6995
Score greater than 32 indicates homology
Score greater than 35 indicates identity

**Score	Expect	Delta Hit Protein	Peptide**
 33.9	0.068	-0.0054	10+ Q00610|CLH1_HUMAN R.AYEFAER.C
 17.8	2.8	-0.0530	R.FSEPHLR.D
 14.8	5.5	-0.0054	K.ESFFAER.F
 13.6	7.3	-0.1218	K.KAVQAVNR.K
 11.9	11	0.0276	K.YAETMDR.E
 11.7	11	-0.0377	R.APEAAAAER.E
 11.1	13	-0.1217	R.IRQLAER.S
 10.4	15	-0.0966	M.RAAAAGGGVR.T
 10.2	16	-0.0530	K.HPESFIR.M
 10.2	16	-0.0088	K.SFEVTMR.R

Top scoring peptide matches to query 2712
File7043 Spectrum8586 scans: 6746
Score greater than 29 indicates homology
Score greater than 35 indicates identity

**Score	Expect	Delta Hit Protein	Peptide**
 22.9	0.85	-0.0059	94	P30086|PEBP1_HUMAN K.VLTPTQVK.N
 14.8	5.6	-0.9284	R.NVDKDPAK.E
 12.9	8.6	-0.9549	K.VNTHLFR.H
 11.9	11	-0.0171	K.VLELQRK.L
 10.9	14	-0.0059	R.LVENAVLK.A
 9.4	19	-0.9396	R.SRSQSPPK.D
 9.3	20	-0.0324	R.VILWARK.S
 9.2	20	0.0192	R.VLEQLQR.D
 8.2	25	-0.0535	R.VIALRSVK.N
 8.2	25	0.0118	M.VLCPVIGK.L

Top scoring peptide matches to query 2713
File7043 Spectrum10149 scans: 8027
Score greater than 25 indicates homology
Score greater than 35 indicates identity

**Score	Expect	Delta Hit Protein	Peptide**
 15.3	4.9	-0.0056	K.LTVLSQPK.-
 11.6	12	0.0995	K.ITNDYIF.-
 9.5	19	0.0196 660	P30038|AL4A1_HUMAN K.AIEAALAAR.K
 9.1	21	-0.0168	R.LTVLAVNR.I
 7.3	31	0.0083	R.LAELQRR.Y
 7.3	31	0.0083	LAELRQR
 6.4	38	-0.0168	K.LTRIPSAK.K
 6.3	39	0.0195	R.LTVVSPNR.A
 6.0	42	-0.9393	R.NLSGPDKR.L
 4.8	55	0.0407	R.LAWDPRK.L

Top scoring peptide matches to query 2714
File7043 Spectrum13058 scans: 10532
Score greater than 14 indicates homology
Score greater than 33 indicates identity

**Score	Expect	Delta Hit Protein	Peptide**
 4.5	42	0.4125	R.WGDTPPGR.K
 1.6	82	0.3914	R.AELAGDPGR.Y
 0.4 1.1e+002	0.2823 267	Q12955|ANK3_HUMAN K.LLLDRGAK.I

Top scoring peptide matches to query 2719
File7043 Spectrum6792 scans: 5315
Score greater than 35 indicates identity

**Score	Expect	Delta Hit Protein	Peptide**
 42.4	0.011	-0.0061	3	P15144|AMPN_HUMAN K.IQTQLQR.D
 27.2	0.36	-0.0425	R.IKLATGQR.K
 27.1	0.37	-0.9901	K.LQTQLER.E
 24.7	0.65	-0.0425	R.IQAITGKR.G
 22.2	1.1	-0.9650	R.NQQTIGAR.K
 22.1	1.2	-1.0265	R.LKTLEQR.A
 21.4	1.4	-1.0054	R.NQLPFLR.N
 21.0	1.5	-0.9901	R.NVEELKR.K
 20.2	1.8	-0.0465	R.LQPLFLR.G
 20.2	1.8	0.0415	K.LKEEPDR.E

Top scoring peptide matches to query 2725
File7043 Spectrum631 scans: 560
Score greater than 19 indicates homology
Score greater than 34 indicates identity

**Score	Expect	Delta Hit Protein	Peptide**
 6.6	30	0.5529	R.KVSFLHR.K
 6.4	31	-0.3254	K.GAMGEPGPR.G
 6.2	32	-0.3907	K.QDNRNLK.E
 5.8	36	-0.4199	K.QLIAEWK.K
 5.7	37	-0.4410	R.KIPDSISK.F
 4.8	45	-0.4675	R.QLVFPRK.G
 4.7	46	0.5794	R.AGNSLLSPK.V
 4.0	54	0.6110	R.AGMPRQAR.D
 3.4	63	-0.3682	R.EAEPEGKK.K
 2.8	72	-0.4159 106	P12821|ACE_HUMAN K.KVQDLER.A

Top scoring peptide matches to query 2726
File7043 Spectrum12363 scans: 9932
Score greater than 36 indicates identity

**Score	Expect	Delta Hit Protein	Peptide**
 33.5	0.09	-0.0049	40	O95831|AIFM1_HUMAN K.LFNIHED.-
 25.6	0.56	-0.1252	K.LFPLRNK.E
 11.5	15	-1.0915	K.LFMPRPK.V
 10.7	17	-1.1192	K.SIISLLDK.H
 10.2	19	-1.0688	K.LASQNISR.L
 9.9	21	-0.8939	R.LMSMEMD.-
 9.8	21	0.0208	R.IFAMMDK.N
 9.8	21	-0.0817	K.IFYFLGK.Q
 9.8	21	0.0425	K.IMMMGTR.E
 9.8	21	-0.0413	R.LFLDHDK.T

Top scoring peptide matches to query 2727
File7043 Spectrum11851 scans: 9477
Score greater than 13 indicates homology
Score greater than 36 indicates identity

**Score	Expect	Delta Hit Protein	Peptide**
 1.0 1.6e+002	1.0147	K.IHADMDGK.K
 0.9 1.7e+002	0.9718	K.EEPSPISK.M
 0.4 1.9e+002	-1.0373	K.GVAGNPMVK.S
 0.4 1.9e+002	0.9130 335	Q8WW52|F151A_HUMAN K.QLALNATR.K
 0.4 1.9e+002	0.9242	R.SALGPNVTK.I
 0.4 1.9e+002	0.9857	K.TPGAVNDGR.S

Top scoring peptide matches to query 2728
File7043 Spectrum14206 scans: 11568
Score greater than 27 indicates homology
Score greater than 36 indicates identity

**Score	Expect	Delta Hit Protein	Peptide**
 40.9	0.017	-0.0058	20+ P68366|TBA4A_HUMAN FDLMYAK
 12.5	11	-0.0672	K.INAENKAK.I
 12.4	12	0.0272	K.MLDMSFK.D
 11.8	14	-0.0171	R.DFLPHMK.E
 11.4	15	-0.0746	K.QTPVIMAK.I
 9.8	22	0.8917	R.VIKGNIDK.F
 9.6	22	-0.0923	R.GTEIGVLAK.A
 9.3	24	0.9934	K.SMFEKTK.F
 9.2	24	-0.0559	R.LNEELAAK.K
 9.1	25	-1.1062	-.MVLAGLIR.K

Top scoring peptide matches to query 2729
File7043 Spectrum7781 scans: 6101
Score greater than 23 indicates homology
Score greater than 36 indicates identity

**Score	Expect	Delta Hit Protein	Peptide**
 13.6	8.9	-0.0054 134	Q8N2G8|GHDC_HUMAN R.AVGQWAGAK.L
 9.7	22	-0.0013	K.GLGKDNQR.S
 8.4	29	-0.9966	M.GIGRSEGGR.R
 7.6	35	-0.0815	R.LGGIMLRK.M
 6.5	45	0.0238	K.QNNNNRK.T
 6.0	51	-0.0781	K.QVKPLFR.H
 5.9	52	-0.0265	K.QVIEDKR.Y
 5.7	55	-1.0040	R.KGGVGMPSR.D
 5.4	59	0.0164	R.AREPGMAR.G
 5.3	60	-0.0781	R.QLVFPRK.G

Top scoring peptide matches to query 2731
File7043 Spectrum10622 scans: 8429
Score greater than 26 indicates homology
Score greater than 36 indicates identity

**Score	Expect	Delta Hit Protein	Peptide**
 13.9	8.3	-0.0053	R.WLQKQGK.Y
 13.7	8.7	0.0463	K.EAVDLANR.I
 13.4	9.4	-0.0152	R.EAVGGITIK.L
 13.1	9.9	-0.0053 253	P14927|QCR7_HUMAN K.WLDGIRK.W
 13.0	10	0.0713	K.GEIMFYK.K
 12.5	11	-0.0264	K.ISVDRGLK.W
 11.2	16	0.0100	R.EGLGVERK.S
 11.0	16	-0.0264	M.SLVDLGKR.L
 10.4	19	-0.0053	R.QPVKYPR.G
 9.8	21	0.0603	K.SGRNSNPR.Q

Top scoring peptide matches to query 2735
File7043 Spectrum7530 scans: 5901
Score greater than 16 indicates homology
Score greater than 34 indicates identity

**Score	Expect	Delta Hit Protein	Peptide**
 4.0	56	-0.4855	K.VHVPPVIK.D
 3.0	70	0.6077	K.HEAIVYR.H
 2.3	82	0.6441	K.ESHVPYR.N
 1.9	91	0.5502	R.ELTAKLGR.C
 1.9	91	0.5614 355	P23396|RS3_HUMAN R.ELTAVVQK.R
 1.9	91	0.5502	R.ELTGKLAR.C
 1.9	91	-0.3763	R.KDTPHYK.K
 1.9	91	0.5753	K.NKTGSKPR.T
 1.6	96	-0.4015	R.VPYDPGLK.A
 0.9 1.1e+002	0.5713	K.LVFPGAQR.M

Top scoring peptide matches to query 2737
File7043 Spectrum9148 scans: 7208
Score greater than 30 indicates homology
Score greater than 36 indicates identity

**Score	Expect	Delta Hit Protein	Peptide**
 18.7	2.8	-1.0989	K.GMLVGIVGK.V
 17.1	4.1	0.9606	R.QEVNELR.K
 17.0	4.2	-1.1101	-.MLRGVLGK.T
 16.1	5.1	0.8878	R.GSLLTLQR.L
 15.3	6.3	-0.0598	R.ELDDLKR.L
 15.2	6.3	0.9493	K.GLGKDNQR.S
 14.9	6.8	-0.0347 497	P23229|ITA6_HUMAN K.QIDDNRK.F
 14.8	7	-0.0711	K.TGAAGDRLK.E
 14.5	7.5	0.9453	K.GWIDLQR.L
 14.5	7.5	-1.0802	K.LDSKELGK.Y

Top scoring peptide matches to query 2739
File7043 Spectrum12714 scans: 10226
Score greater than 24 indicates homology
Score greater than 36 indicates identity

**Score	Expect	Delta Hit Protein	Peptide**
 12.3	12	0.9674	R.NQLPFLR.N
 10.5	19	1.0004	R.NLQPLMR.S
 6.9	43	-0.0167	R.DQPIFLR.L
 6.4	48	0.9826	R.ELLAASQR.L
 5.6	58	0.9640 325	O15031|PLXB2_HUMAN NPKLMLR
 4.6	73	1.0190	K.NELNELR.K
 4.2	80	1.0190 425	P01024|CO3_HUMAN	R.NEQVEIR.A
 3.7	89	-0.0200	R.DIKMPLR.K
 3.6	92	-0.1258	K.VKIIFIR.N
 3.2	1e+002	-0.9490	K.DELDELR.A

Top scoring peptide matches to query 2740
File7043 Spectrum16722 scans: 13894
Score greater than 23 indicates homology
Score greater than 36 indicates identity

**Score	Expect	Delta Hit Protein	Peptide**
 11.8	14	1.0303	K.VKEDQIR.E
 9.5	23	-0.9142	R.FFCFLR.R
 8.2	32	1.0415	K.EVELEIR.I
 8.1	32	1.0415	K.EVVEVGQK.L
 7.8	35	1.0554	R.AREQQAGK.D
 7.6	36	1.0667	R.VQEQDLR.Q
 7.0	42	1.0303	K.EVKLNER.I
 6.0	53	0.0463 466	P20073|ANXA7_HUMAN R.DLEKDIR.S
 6.0	53	0.0827	M.VEEENIR.V
 5.9	54	-0.9676	K.MVDLRGAK.V

Top scoring peptide matches to query 2743
File7043 Spectrum6694 scans: 5236
Score greater than 16 indicates homology
Score greater than 34 indicates identity

**Score	Expect	Delta Hit Protein	Peptide**
 3.1	68	0.6074	R.EGGDQRVK.S
 3.1	68	0.6187	R.LEDELNR.A
 2.9	72	-0.3402	M.ADGNEDLR.A
 2.4	80	0.6187	K.IENEEVR.K
 2.1	86	0.5922	R.HGAALYTR.K
 1.9	89	-0.3766	K.ADSEQLAR.S
 0.0 1.4e+002	-0.4316 436	P04899|GNAI2_HUMAN K.MIDKNLR.E

Top scoring peptide matches to query 2746
File7043 Spectrum547 scans: 506
Score greater than 23 indicates homology
Score greater than 34 indicates identity

**Score	Expect	Delta Hit Protein	Peptide**
 10.7	13	0.7403	R.KEGAGSLAR.F
 9.7	16	0.7879	R.ELAEEAAR.L
 7.1	30	-0.2689 490	P51153|RAB13_HUMAN R.TVDIEGKK.I
 5.1	47	0.7580	R.KGGVGMPSR.D
 4.7	51	0.7767	R.AGQEAEKR.R
 4.5	53	-0.2437	K.EGGSSLIAR.N
 3.9	61	-0.3165	R.KSLVTSVR.A
 3.6	66	0.8131	R.QAEAENAR.L
 3.5	68	0.6965	K.KVLLEMR.K
 2.9	77	-0.3066	R.ARLNLFR.H

Top scoring peptide matches to query 2747
File7043 Spectrum31330 scans: 27654
Score greater than 25 indicates homology
Score greater than 34 indicates identity

**Score	Expect	Delta Hit Protein	Peptide**
 12.6	8.3	0.7884	K.NEIDELR.T
 11.7	10	-0.2320	K.ISQDADLK.T
 11.7	10	-0.2320 319	P50416|CPT1A_HUMAN K.QSLDAVEK.A
 11.5	11	-0.2796	R.SSRIGEIK.E
 9.1	19	0.7408	K.EEVSLRR.V
 8.2	23	-0.1592	K.NSDEEAPK.T
 7.9	24	-1.1012	R.MCFNMR.G
 7.9	24	-1.1956	K.CQAEARR.L
 7.7	26	0.6891 604	Q9BWM7|SFXN3_HUMAN K.HLPPLVGR.F
 7.2	29	-0.0981	K.MMMMGPK.E

Top scoring peptide matches to query 2748
File7043 Spectrum10927 scans: 8675
Score greater than 13 indicates homology
Score greater than 36 indicates identity

**Score	Expect	Delta Hit Protein	Peptide**
 2.5 1.2e+002	0.9670 109	P24539|AT5F1_HUMAN K.KAQAQPVM.-
 0.5 1.8e+002	-1.1214	R.MLTKIIR.D
 0.1	2e+002	-1.0049	R.EEAERTR.D

Top scoring peptide matches to query 2752
File7043 Spectrum10498 scans: 8325
Score greater than 35 indicates homology
Score greater than 36 indicates identity

**Score	Expect	Delta Hit Protein	Peptide**
 30.1	0.2	0.9982 174	P01009|A1AT_HUMAN K.AVLTIDEK.G
 21.2	1.5	0.9869	R.IGLETSLR.Y
 15.9	5.2	0.9982	K.GLIDEITK.K
 15.3	6.1	1.0233	R.AVLDVTDR.L
 15.0	6.5	-1.0328	R.DGIVFVLK.C
 13.4	9.2	1.0047	R.VALPSVMR.F
 12.8	11	0.9869	R.GLKLSGGEK.Q
 12.7	11	1.0081	K.VALGNTWK.E
 11.9	13	0.9795	R.IGVDLIMK.T
 11.7	14	1.0186	R.GLLCRNR.K

Top scoring peptide matches to query 2753
File7043 Spectrum28408 scans: 25272
Score greater than 21 indicates homology
Score greater than 36 indicates identity

**Score	Expect	Delta Hit Protein	Peptide**
 12.4	12	0.1241	K.EVEPKQC.-
 7.9	33	1.0064	K.KGSTAVGIR.G
 7.3	38	-0.9113	R.AQDSRTGR.D
 6.6	44	0.0588	K.QVESGTLR.F
 6.6	44	1.0388	88	P01834|IGKC_HUMAN R.EAKVQWK.V
 6.6	44	1.0904	K.NIEVEER.E
 6.4	47	0.0700	R.NLESDIAK.I
 6.2	49	1.0428	K.DLKAQGTR.D
 6.1	50	-0.9365	4	P98164|LRP2_HUMAN R.VKTDGTNR.T
 6.1	50	0.0700	K.ISQDADLK.T

Top scoring peptide matches to query 2754
File7043 Spectrum29212 scans: 25943
Score greater than 23 indicates homology
Score greater than 35 indicates identity

**Score	Expect	Delta Hit Protein	Peptide**
 11.7	13	1.1889	K.DRGAADAGR.D
 9.9	20	0.2300	R.GSGGGGGGGGGGR.G
 9.3	24	1.1161	R.RNSSIVGR.R
 9.0	25	-0.8308	R.WKETGGGR.R
 8.9	25	1.1233	88	P01834|IGKC_HUMAN R.EAKVQWK.V
 8.1	31	1.1564	R.DLQCPKK.D
 7.8	33	0.2087	-.MQPEGAEK.G
 7.8	33	0.1169	R.RFEKPGR.K
 7.8	33	1.1638	M.AEQEQRK.I
 7.8	33	1.1638	R.AEQKEQR.H

Top scoring peptide matches to query 2760
File7043 Spectrum545 scans: 505
Score greater than 20 indicates homology
Score greater than 36 indicates identity

**Score	Expect	Delta Hit Protein	Peptide**
 8.4	29	-1.0241	K.AKETVSTR.Y
 8.1	31	1.0093	R.CISVAPSR.E
 6.9	41	-0.9779	R.EAGPAHPGR.E
 6.6	44	0.9915	K.GDTAVLSAR.V
 6.5	45	1.0530	R.AGAGSGAGGER.W
 6.1	49	0.9114	R.ILMAKSVK.Y
 5.4	58	1.0279	K.DANQGEKK.K
 5.3	59	-1.0758	K.KAIQLYR.H
 5.3	60	0.9664 490	P51153|RAB13_HUMAN R.TVDIEGKK.I
 5.0	64	-0.9038	K.DQEGGEEK.K

Top scoring peptide matches to query 2761
File7043 Spectrum28613 scans: 25450
Score greater than 17 indicates homology
Score greater than 35 indicates identity

**Score	Expect	Delta Hit Protein	Peptide**
 7.4	34	1.1565	K.NAMDVIVK.V
 4.2	71	-0.7976	-.MPAESGKR.F
 3.1	92	1.1995	-.MLPQMNR.T
 2.7	1e+002	1.1752 209	P11233|RALA_HUMAN R.QVSVEEAK.N
 1.9 1.2e+002	1.1639	R.TISLGAGDR.Q
 1.8 1.2e+002	-0.7724	R.NLNNNMR.R
 1.8 1.3e+002	-0.8591	K.LAMDLKGK.M
 1.7 1.3e+002	0.1501	K.AREVLMR.L
 1.7 1.3e+002	0.1687 547	O00291|HIP1_HUMAN K.SRAGDTGVK.L
 1.5 1.3e+002	0.1938	R.ARSASQDR.L

Top scoring peptide matches to query 2766
File7043 Spectrum16753 scans: 13922
Score greater than 23 indicates homology
Score greater than 35 indicates identity

**Score	Expect	Delta Hit Protein	Peptide**
 14.1	7.6	-0.0046 514	P51970|NDUA8_HUMAN R.FYFWTK.-
 9.2	24	-0.9853	R.YMHRNR.E
 8.5	28	0.0107 132	P08238|HS90B_HUMAN K.FYEAFSK.N
 7.7	33	-0.0733	K.YFAHIIK.E
 7.5	35	0.0106	K.YFDVYGK.V
 6.4	45	0.9953	R.AAAPVCSSK.T
 4.2	75	-0.1196	K.LIKILYE.-
 3.6	85	0.9008	R.LLVPGTYK.I
 3.6	85	0.0212	MYYSRR
 3.4	89	-0.0006	K.YFKDYR.Q

Top scoring peptide matches to query 2767
File7043 Spectrum6131 scans: 4793
Score greater than 27 indicates homology
Score greater than 35 indicates identity

**Score	Expect	Delta Hit Protein	Peptide**
 15.2	5.9	0.0271	K.KATEMGVR.L
 14.0	7.8	-0.0059 332+ Q93050|VPP1_HUMAN R.KFVNEVR.R
 12.0	12	-0.0423	K.KPKYTVR.L
 10.4	18	-0.0423	R.KVVQVYR.K
 9.7	21	0.0635	R.QMTEQVR.S
 8.1	30	-0.0310	K.KVFEAAVK.S
 7.9	32	0.0305	K.AGFDGAVVR.T
 6.7	42	-0.0059 309	P21695|GPDA_HUMAN R.KVAEAFAR.T
 6.3	46	-0.0158	K.KTVESSLK.T
 6.2	47	0.0383	R.KVVEECK.L

Top scoring peptide matches to query 2768
File7043 Spectrum8064 scans: 6330
Score greater than 22 indicates homology
Score greater than 35 indicates identity

**Score	Expect	Delta Hit Protein	Peptide**
 10.4	18	1.0429 249	P06756|ITAV_HUMAN -.MAFPPRR.R
 10.4	18	1.0581	-.MDLSRPR.W
 9.1	24	1.0462	K.FGLHNFR.T
 9.1	24	1.1776	R.MCFNMR.G
 6.5	44	1.0694	R.MDPVLSGR.S
 6.5	44	1.0330	-.MVLADLGR.K
 6.4	45	1.0152	R.ASLEGKSAK.E
 6.1	48	1.0330	K.KGCDVLAK.S
 6.0	49	-0.9389	M.SAAQVSSSR.R
 5.9	50	1.0291	R.QRAVSSSR.N

Top scoring peptide matches to query 2774
File7043 Spectrum12590 scans: 10127
Score greater than 23 indicates homology
Score greater than 35 indicates identity

**Score	Expect	Delta Hit Protein	Peptide**
 11.2	14	-1.0295	K.ARQTFDR.D
 11.2	14	-1.0911	K.KQVTVYR.L
 9.4	22	0.9286	R.SSSLRVSR.N
 8.3	28	-0.0053 655	Q9Y696|CLIC4_HUMAN K.EMTGIWR.Y
 8.0	30	0.8882	K.FLKGLASR.K
 6.4	43	0.8882	K.KPKYTVR.L
 4.3	70	-1.0547	K.LFSGSQVR.F
 3.7	81	0.9762	K.SEKDAVSR.E
 3.7	82	-0.0264	-.MASLEVSR.S
 3.4	87	0.9464	K.GVGMRLSR.A

Top scoring peptide matches to query 2775
File7043 Spectrum13508 scans: 10938
Score greater than 17 indicates homology
Score greater than 35 indicates identity

**Score	Expect	Delta Hit Protein	Peptide**
 10.3	18	-0.9787	R.GPQGQGHGR.M
 4.1	74	-0.0311	R.RHVTSHR.S
 3.9	78	-1.0178 141	Q9BSJ8|ESYT1_HUMAN K.GKSDPYVK.L
 3.2	91	-1.0013	K.RHFMFR.N
 2.6 1.1e+002	-1.0211	K.VQTAVTMK.G
 2.2 1.1e+002	-1.1018	R.LLIQPGPR.F
 1.3 1.4e+002	-1.0541	R.VGLEPPPGK.R
 1.2 1.4e+002	-0.0337 304	O75964|ATP5L_HUMAN K.RGIIGYDV.-
 1.2 1.4e+002	0.9581	K.VVGLAMGTK.D
 0.3 1.8e+002	-1.0112	R.LLNCFAR.V

Top scoring peptide matches to query 2777
File7043 Spectrum8305 scans: 6522
Score greater than 23 indicates homology
Score greater than 35 indicates identity

**Score	Expect	Delta Hit Protein	Peptide**
 28.1	0.3	-0.0052 414	P13987|CD59_HUMAN K.AGLQVYNK.C
 8.8	25	-0.9529	K.QIFQDDK.D
 8.4	28	-0.0126	R.AGIMPFLK.C
 7.9	31	-1.0256	K.KDLIYNK.V
 7.5	33	0.0026	16	P35579|MYH9_HUMAN K.KLEMDLK.D
 7.2	36	-0.9892	K.ENLLYNK.E
 7.2	36	-1.0732	R.AIAHQLIK.H
 6.4	43	-1.0442	K.KLINFMK.M
 5.8	50	-1.0369	M.AGLRYSVK.V
 5.8	50	-0.9562	R.AICSSIDK.L

Top scoring peptide matches to query 2778
File7043 Spectrum11038 scans: 8764
Score greater than 35 indicates identity

**Score	Expect	Delta Hit Protein	Peptide**
 43.2	0.0091	-0.0050	9	P25705|ATPA_HUMAN K.LELAQYR.E
 22.9	0.97	0.0280	R.IEIMGASR.R
 19.4	2.2	-0.0050	K.LELFTNR.F
 11.6	13	0.0280	K.NKLEMNK.V
 11.2	14	-1.0366	R.NLTKVYR.G
 10.9	15	0.0532	K.KNLDCSR.S
 10.2	18	0.0103	K.GGKLSSESK.T
 10.1	18	0.0280 295	Q9UM54|MYO6_HUMAN K.LEMEAKR.K
 9.2	23	0.0532	K.KNTQMDR.F
 8.9	25	0.0280	R.LEAEKMR.L

Top scoring peptide matches to query 2779
File7043 Spectrum10265 scans: 8129
Score greater than 20 indicates homology
Score greater than 35 indicates identity

**Score	Expect	Delta Hit Protein	Peptide**
 8.3	28	1.0930	K.EAMNVQTV.-
 7.1	37	1.0376	R.SYGGRPVR.R
 5.6	52	1.0818	-.MAAGGSGVGGK.R
 4.6	66	0.0978	R.EDTLCVR.G
 3.9	77	-0.9556	R.DLIDYVR.Y
 2.4 1.1e+002	-0.9305 466	P20073|ANXA7_HUMAN R.LYQAGEGR.L
 2.3 1.1e+002	0.9760	R.KVVQVYR.K
 2.1 1.2e+002	-0.9781	K.LYSRAQR.H
 1.8 1.2e+002	1.0236	K.FDPGLKSK.N
 1.8 1.3e+002	-0.9451	K.SLSRMQR.K

Top scoring peptide matches to query 2783
File7043 Spectrum12050 scans: 9650
Score greater than 25 indicates homology
Score greater than 35 indicates identity

**Score	Expect	Delta Hit Protein	Peptide**
 13.9	7.7	1.0637	R.DLIIDFR.E
 11.8	13	1.0525	K.DIKVFNR.D
 9.8	20	1.1470	R.NLNNNMR.R
 9.4	22	0.1776	R.DENPDFR.E
 6.6	42	1.0188	HRGLRPR
 5.0	59	0.1089	M.SAAQVSSSR.R
 4.1	74	0.1048	R.SSAIPDFR.D
 4.0	75	1.0161	R.KVVQVYR.K
 2.5 1.1e+002	1.1405 737	Q9NZM1|MYOF_HUMAN K.DLTGDQSR.S
 2.5 1.1e+002	0.1736	K.EYYFDR.N

Top scoring peptide matches to query 2784
File7043 Spectrum11132 scans: 8852
Score greater than 33 indicates identity

**Score	Expect	Delta Hit Protein	Peptide**
 20.7	1	0.3100	9	P25705|ATPA_HUMAN K.LELAQYR.E
 19.0	1.6	-0.7216	K.QVHTALPK.V
 11.9	8	0.2485	R.LEKVYLK.N
 10.0	12	-0.6675	R.RAQYMPK.Y
 8.5	17	-0.7104	R.KGGLDYLK.Q
 6.0	31	0.3430	R.IEIMGASR.R
 5.3	37	0.3100	K.LELFTNR.F
 5.2	38	-0.6852	R.ELNRAYK.A
 3.7	53	-0.7580	K.ELLLHIR.S
 3.7	53	-0.6740	R.LELEAYR.L

Top scoring peptide matches to query 2789
File7043 Spectrum8731 scans: 6861
Score greater than 21 indicates homology
Score greater than 35 indicates identity

**Score	Expect	Delta Hit Protein	Peptide**
 9.0	23	-1.0375 330	O95202|LETM1_HUMAN R.ICADLFR.L
 8.4	26	-1.0739	K.CLYLIGR.E
 7.9	29	-0.9859	K.GDTALDMR.G
 7.7	30	-0.0059 563	P09496|CLCA_HUMAN R.LCDFNPK.S
 6.8	37	-1.0124	R.ICAFNNR.A
 6.0	45	-0.0019	R.CLSTEQR.L
 6.0	45	-1.0297	R.LMGGMEIK.K
 5.5	51	-0.0535	K.ICGFLQR.T
 5.1	55	-1.0375	R.LCFEGLR.S
 5.0	57	-1.0665	R.TRSLDFR.E

Top scoring peptide matches to query 2790
File7043 Spectrum8799 scans: 6918
Score greater than 29 indicates homology
Score greater than 35 indicates identity

**Score	Expect	Delta Hit Protein	Peptide**
 23.5	0.8	-0.0054 563	P09496|CLCA_HUMAN R.LCDFNPK.S
 15.4	5.2	-0.9820	K.NEDFNQK.F
 12.3	11	-0.0595 653	P54289|CA2D1_HUMAN K.IDLYDVR.R
 8.2	27	-0.1296	R.LVHGGRVR.S
 7.4	32	-1.0152	K.NCVMSRK.Q
 7.1	35	0.0164	R.CACIDVR.H
 5.8	47	-1.0547	K.NDVYKQK.K
 5.8	48	-1.0291	R.LMGGMEIK.K
 5.3	53	-1.0370	K.NMPKFNK.I
 5.1	55	-0.0343	K.ATTYRGPQ.-

Top scoring peptide matches to query 2791
File7043 Spectrum23038 scans: 19952
Score greater than 23 indicates homology
Score greater than 35 indicates identity

**Score	Expect	Delta Hit Protein	Peptide**
 10.4	16	0.0062	-.MRCLAAR.V
 10.3	17	-0.0367	R.KMTSAIAR.W
 9.7	19	-0.9996	K.MPSFGLAR.G
 9.7	19	-1.0030	R.MVTMGIAR.T
 9.6	20	-0.0221	K.EIYELAR.K
 8.7	24	0.9321	-.MLLGFRR.G
 8.5	25	-0.0221	R.YIEELAR.S
 8.1	28	-1.0360	-.MLGFLSAR.Q
 7.8	30	1.0136	32	P02768|ALBU_HUMAN R.DEGKASSAK.Q
 7.7	31	-1.0142	R.VMGRIMR.E

Top scoring peptide matches to query 2796
File7043 Spectrum575 scans: 522
Score greater than 27 indicates homology
Score greater than 34 indicates identity

**Score	Expect	Delta Hit Protein	Peptide**
 15.1	4.2	0.2247	K.FGGSNQKR.Q
 13.9	5.6	1.1836	R.FINSQKR.R
 7.4	25	0.2326	R.MTTEKQR.Q
 6.4	31	0.2577	K.TCNSQKR.T
 5.2	41	-0.8936	K.KLAQIPPK.T
 4.2	52	0.2207	R.FGGAFAPAR.C
 2.3	80	1.1333	59+ O60656|UD19_HUMAN KLVEYLK
 2.1	83	1.1649	R.FIGIRMR.T
 2.1	84	0.2471	R.IVEDFDR.E
 1.8	90	0.2326	-.MESVKQR.I

Top scoring peptide matches to query 2799
File7043 Spectrum13346 scans: 10794
Score greater than 19 indicates homology
Score greater than 35 indicates identity

**Score	Expect	Delta Hit Protein	Peptide**
 7.9	27	0.9846	R.GIAPNFMK.V
 7.9	27	0.9846	R.GLAPNFMK.V
 7.5	30	0.8941	K.ILGTFKSK.K
 6.0	42	1.0064	R.GIVGGGMMR.D
 6.0	42	1.0064	R.GIVGGGMMR.D
 6.0	42	-0.9980	R.SIMEMLR.L
 6.0	42	0.9635	-.MAAATLTSK.L
 5.2	50	0.9669	R.DLIDYVR.Y
 5.2	51	0.0410	-.MSEADGLR.Q
 4.7	56	0.9669 653	P54289|CA2D1_HUMAN K.IDLYDVR.R

Top scoring peptide matches to query 2801
File7043 Spectrum5850 scans: 4570
Score greater than 27 indicates homology
Score greater than 35 indicates identity

**Score	Expect	Delta Hit Protein	Peptide**
 19.9	1.7	-0.0422	K.TLEYIKK.Q
 13.7	7	0.0272	K.TLEKMEK.H
 12.9	8.6	-0.0058	87+ Q5VTE0|EF1A3_HUMAN R.TIEKFEK.E
 9.6	18	-0.8953	R.DVGNTDMK.Y
 8.5	24	-0.0786	K.DVLPPIIK.D
 8.5	24	-0.0786	R.DVLPPLLK.T
 8.5	24	-0.0647	R.TLLHAALR.A
 7.5	29	-0.9680	K.ESKVSMSK.N
 7.4	30	-0.0170	R.QQSLPPPK.F
 7.3	31	0.0847	R.VDEWMAK.E

Top scoring peptide matches to query 2802
File7043 Spectrum5839 scans: 4561
Score greater than 20 indicates homology
Score greater than 35 indicates identity

**Score	Expect	Delta Hit Protein	Peptide**
 7.7	28	1.0288	R.ETKHGGHK.N
 6.8	34	-0.9393	K.QSDSPPHK.K
 5.7	44	-0.9830	K.KSMLWSK.M
 4.3	62	0.0850	R.VDEWMAK.E
 4.1	65	0.0275	K.TLEKMEK.H
 3.6	73	-1.0596	K.AGRIHITK.A
 3.5	74	1.0843	K.DDQEKMK.E
 3.3	78	-0.0055	87+ Q5VTE0|EF1A3_HUMAN R.TIEKFEK.E
 3.1	82	-0.8950	R.QSEEQMK.N
 2.6	92	0.9785	R.QLTLGAYK.H

Top scoring peptide matches to query 2803
File7043 Spectrum18720 scans: 15776
Score greater than 18 indicates homology
Score greater than 35 indicates identity

**Score	Expect	Delta Hit Protein	Peptide**
 7.1	32	0.0612	R.VSKGGSFGR.G
 7.0	34	-0.8832	-.MTMAGGRR.G
 5.0	53	0.0691	R.LSLATMSR.S
 4.9	53	1.1008	K.LITEMDR.S
 1.3 1.2e+002	-0.9414	K.MRAFQVK.Q
 0.9 1.4e+002	-0.0115	K.VLLEKHR.K
 0.7 1.4e+002	1.1623 205	P02748|CO9_HUMAN R.CTDAVGDR.R
 0.5 1.5e+002	1.1041	R.IVEDFDR.E
 0.5 1.5e+002	1.1623	K.NAGMETDR.I
 0.3 1.6e+002	1.1371	-.MEDEVVR.F

Top scoring peptide matches to query 2811
File7043 Spectrum6422 scans: 5020
Score greater than 22 indicates homology
Score greater than 34 indicates identity

**Score	Expect	Delta Hit Protein	Peptide**
 11.2	12	-0.0809	R.VLGVPVIAK.D
 11.2	12	0.0348	K.NVMKTFR.E
 8.7	21	1.0188	R.VIGRMFR.R
 8.7	21	1.0188	R.VLGRMFR.R
 6.7	34	1.0565	K.IVEMSTSK.T
 4.9	51	1.0599	K.IDIDYQK.L
 4.6	54	1.0664 330	O95202|LETM1_HUMAN R.ICADLFR.L
 3.3	74	1.0850	K.LTNEDFR.K
 2.5	89	1.0664	K.LVSYCPR.R
 1.8	1e+002	1.0123	R.IDLSKYR.E

Top scoring peptide matches to query 2814
File7043 Spectrum13073 scans: 10542
Score greater than 13 indicates homology
Score greater than 33 indicates identity

**Score	Expect	Delta Hit Protein	Peptide**
 1.1	79	0.6490	R.ARDQLHR.H
 1.1	79	0.6602	R.GLGDHQLR.V
 1.1	79	-0.2869 647	P13639|EF2_HUMAN K.VEDMMKK.L
 1.1	79	-0.2869 647	P13639|EF2_HUMAN K.VEDMMKK.L
 0.8	86	0.5372	R.LTPLKPVK.K
 0.8	86	0.5624	K.NLKKPAPK.S

Top scoring peptide matches to query 2817
File7043 Spectrum9031 scans: 7116
Score greater than 26 indicates homology
Score greater than 34 indicates identity

**Score	Expect	Delta Hit Protein	Peptide**
 29.6	0.16	-0.0053 111	P21397|AOFA_HUMAN K.ICELYAK.V
 29.6	0.16	-0.0053 139	P27338|AOFB_HUMAN K.LCELYAK.V
 11.5	11	-0.0166	R.LCNYKAK.Q
 11.1	12	0.0277	R.LCSEMLK.T
 7.3	28	-0.0417	K.LLTMGSFK.R
 7.3	28	0.0463	K.LSEEGMSK.A
 6.0	38	-0.0456	R.GSVHIEVR.E
 6.0	38	-0.0568	K.RHSLEVR.V
 5.1	46	-0.0893	K.ICHLLIK.G
 4.1	58	0.0277	K.ITEMCVK.M

Top scoring peptide matches to query 2819
File7043 Spectrum11952 scans: 9563
Score greater than 28 indicates homology
Score greater than 34 indicates identity

**Score	Expect	Delta Hit Protein	Peptide**
 22.8	0.79	0.9806	R.YIEELTK.E
 14.2	5.7	-0.0048	84+ P01876|IGHA1_HUMAN K.YLTWASR.Q
 14.1	5.8	0.0469	K.FEQNGSSK.N
 11.6	10	-1.0616 150	P12111|CO6A3_HUMAN K.YIAYLVR.Q
 11.6	10	-0.9922	R.YLDMISR.K
 11.5	11	0.0911	K.EMTNEEK.N
 9.7	16	-1.0285	R.YLQMKAK.R
 9.4	17	-1.0575	K.SEKPALPR.K
 9.1	19	0.9694	R.YNKELTK.E
 8.6	21	-0.9041	R.METEESR.K

Top scoring peptide matches to query 2822
File7043 Spectrum4286 scans: 3349
Score greater than 23 indicates homology
Score greater than 34 indicates identity

**Score	Expect	Delta Hit Protein	Peptide**
 13.2	7.2	-0.0074 200	P02647|APOA1_HUMAN K.LHELQEK.L
 10.0	15	0.0038	K.IETATSFK.M
 9.9	15	-1.0027	R.IHEGNISK.Q
 6.0	37	-0.9299	K.DQTGGYTR.M
 6.0	38	-1.0391	K.HLTVKGDK.E
 4.4	54	0.0797	R.QADCKMK.L
 4.0	59	-0.9815	K.NHPAPFSK.V
 3.9	62	-0.9849	R.LHKDCPK.V
 3.7	63	-0.9914	K.NEPVDPVK.L
 3.7	63	1.0130	R.SNAATLYR.C

Top scoring peptide matches to query 2826
File7043 Spectrum1169 scans: 925
Score greater than 14 indicates homology
Score greater than 32 indicates identity

**Score	Expect	Delta Hit Protein	Peptide**
 2.3	58	0.3095	R.LQPQTPGR.C
 1.1	75	-0.8088	R.QLLIGLLK.S
 0.6	85	-0.7949	4	P98164|LRP2_HUMAN R.LGAIIRVR.K

Top scoring peptide matches to query 2831
File7043 Spectrum5171 scans: 4035
Score greater than 23 indicates homology
Score greater than 34 indicates identity

**Score	Expect	Delta Hit Protein	Peptide**
 13.2	7	-0.0349	K.QLSEHQR.I
 9.6	16	-0.1328	K.LKAPAAAGAK.A
 7.7	25	1.0410	K.QDTEMTR.W
 7.0	29	-0.1216	R.GPSLIPSVK.Q
 6.5	33	-0.0713	K.IASSVHQR.V
 6.5	33	-0.0535	R.NLMVHQR.I
 6.4	34	0.9465 211	O15400|STX7_HUMAN K.ETDKYIK.E
 5.8	38	-1.0553	R.NQVGPEVR.S
 5.1	45	0.9716	K.EYNGKTGK.V
 4.8	49	-0.0059	ECGKTFR

Top scoring peptide matches to query 2837
File7043 Spectrum926 scans: 763
Score greater than 21 indicates homology
Score greater than 34 indicates identity

**Score	Expect	Delta Hit Protein	Peptide**
 9.2	18	1.1126	R.CLDMEGR.I
 8.3	21	-0.9473	K.FEGMRSR.N
 5.9	38	-0.9585	K.RPEGMHR.E
 3.6	63	-0.1266	K.LARLGLVR.C
 3.0	73	-0.0287	K.RPSPERR.E
 2.3	85	-0.0426	R.AVGEPGLVR.R
 2.0	92	1.0212	K.RALMMMK.F
 2.0	92	1.0212	K.RALMMMK.F
 1.3 1.1e+002	-0.0789	R.RLAEALPK.Q
 1.1 1.1e+002	-1.0855	16	P35579|MYH9_HUMAN K.QLVRQVR.E

Top scoring peptide matches to query 2840
File7043 Spectrum1145 scans: 908
Score greater than 19 indicates homology
Score greater than 34 indicates identity

**Score	Expect	Delta Hit Protein	Peptide**
 9.3	17	1.1162	R.CLDMEGR.I
 5.7	39	-0.9549	K.RPEGMHR.E
 4.0	59	1.0248	K.RALMMMK.F
 3.5	64	-0.0251	K.RPSPERR.E
 3.0	72	-1.0091	R.GPSPTRAGR.G
 2.5	82	-1.0230	K.EIPPSISR.V
 2.4	84	0.0226	K.RPPDEQR.L
 2.2	87	-0.9437	K.FEGMRSR.N
 1.8	96	-0.0753	R.RLAEALPK.Q
 1.6	1e+002	-1.0819	16	P35579|MYH9_HUMAN K.QLVRQVR.E

Top scoring peptide matches to query 2850
File7043 Spectrum11930 scans: 9543
Score greater than 24 indicates homology
Score greater than 34 indicates identity

**Score	Expect	Delta Hit Protein	Peptide**
 13.3	6.8	1.0152	R.YLDMISR.K
 11.2	11	0.9458 150	P12111|CO6A3_HUMAN K.YIAYLVR.Q
 9.2	18	0.9822	K.YFLELGR.I
 8.7	19	1.1033	R.METEESR.K
 8.6	20	1.0226	K.SGSLFGSSR.V
 6.8	30	0.9312	R.IVLLMHR.W
 6.2	35	-0.9528	-.MEEIYAK.F
 6.1	36	-0.0454	K.DRKPGNLV.-
 6.1	36	0.9862	R.GETLGPAPR.A
 5.2	44	-0.9640	K.YNKEMSK.R

Top scoring peptide matches to query 2856
File7043 Spectrum9404 scans: 7415
Score greater than 22 indicates homology
Score greater than 34 indicates identity

**Score	Expect	Delta Hit Protein	Peptide**
 10.2	15	0.8965	R.GPSPTRAGR.G
 9.0	19	-1.1191	M.SRPLGQSR.R
 8.8	20	0.8562	R.AAVPAWKR.E
 8.8	20	0.8714	R.QLPQKER.Q
 7.3	28	-0.1126	R.DIGLQTPR.I
 7.3	28	0.8602	R.RIVSGNPR.C
 7.2	29	-0.1126	R.ELQSAVPR.D
 6.1	37	0.7986	64	Q13813|SPTA2_HUMAN R.LKGLALQR.Q
 5.9	39	-0.0874	K.KEGAAGNPR.V
 4.2	58	-0.1602	K.EIRQVVR.M

Top scoring peptide matches to query 2857
File7043 Spectrum948 scans: 778
Score greater than 24 indicates homology
Score greater than 34 indicates identity

**Score	Expect	Delta Hit Protein	Peptide**
 13.0	7.7	-1.0698	67	P02511|CRYAB_HUMAN R.KQVSGPER.T
 12.9	7.9	-1.0698	R.QTLGTPQR.D
 11.2	12	-1.0810	R.GAQLAERR.L
 10.4	14	1.0476	R.EFDNMAR.V
 10.1	15	-1.1425	K.KAIQTAIR.K
 9.4	17	-0.9970	R.QQPDEQR.R
 9.2	18	-1.0698 150	P12111|CO6A3_HUMAN K.QDVVNAVR.Q
 8.8	20	1.0000	K.FEGMRSR.N
 8.7	21	-1.0772	R.MLVVDPAR.R
 8.6	21	-1.0698	R.KDVGPTQR.H

Top scoring peptide matches to query 2858
File7043 Spectrum732 scans: 630
Score greater than 23 indicates homology
Score greater than 34 indicates identity

**Score	Expect	Delta Hit Protein	Peptide**
 10.3	14	-1.0809	R.QQAELRR.A
 9.8	16	-1.0809	R.GAQLAERR.L
 9.4	18	1.0477	R.EFDNMAR.V
 9.3	18	-1.0810	R.QGRDAVVR.T
 9.2	18	-1.0697	R.QTLGTPQR.D
 9.0	19	-1.0697	67	P02511|CRYAB_HUMAN R.KQVSGPER.T
 8.8	20	1.0001	K.FEGMRSR.N
 8.6	21	-1.0697 150	P12111|CO6A3_HUMAN K.QDVVNAVR.Q
 8.4	22	-1.0697	R.KDVGPTQR.H
 8.4	22	-1.0486	R.QVDHVFR.E

Top scoring peptide matches to query 2859
File7043 Spectrum463 scans: 450
Score greater than 24 indicates homology
Score greater than 34 indicates identity

**Score	Expect	Delta Hit Protein	Peptide**
 14.2	5.8	-1.0654	67	P02511|CRYAB_HUMAN R.KQVSGPER.T
 13.5	6.8	-1.0655	R.QTLGTPQR.D
 10.8	13	-1.0767	R.GAQLAERR.L
 10.5	14	-1.1059	K.QKVPGFPK.R
 10.1	15	1.0520	R.EFDNMAR.V
 10.0	15	-1.0655	R.KDVGPTQR.H
 9.9	16	1.0044	K.FEGMRSR.N
 9.8	16	-1.0655 150	P12111|CO6A3_HUMAN K.QDVVNAVR.Q
 9.0	19	-1.0443	R.QVDHVFR.E
 8.8	20	-1.0906	K.AGEALSKPK.S

Top scoring peptide matches to query 2860
File7043 Spectrum166 scans: 260
Score greater than 29 indicates homology
Score greater than 34 indicates identity

**Score	Expect	Delta Hit Protein	Peptide**
 19.5	1.7	-1.0639	67	P02511|CRYAB_HUMAN R.KQVSGPER.T
 15.3	4.5	-1.0751	R.QQAELRR.A
 14.8	5.1	-1.0751	R.GAQLAERR.L
 11.9	9.9	-0.9911	R.QEDAQGPR.D
 10.6	13	-1.0580	K.KQPWWR.K
 10.6	13	-1.1003	K.QKNIIER.Q
 10.6	13	-1.0751	R.QQELRAR.Y
 10.6	13	-1.0639	K.QQIGDAIR.M
 10.6	13	-1.0639	K.QQLDGALR.Q
 10.6	13	-0.9911	R.QQPDEQR.R

Top scoring peptide matches to query 2863
File7043 Spectrum8845 scans: 6956
Score greater than 32 indicates homology
Score greater than 34 indicates identity

**Score	Expect	Delta Hit Protein	Peptide**
 21.0	1.2	-0.0054	29	P08473|NEP_HUMAN	R.AYQNYIK.K
 18.0	2.4	-0.0305	R.IYEAYLK.N
 10.9	13	0.0059	R.YLEAEFK.K
 5.1	47	0.0276	K.CSTYQIK.G
 5.1	47	-0.0305	K.SFIEYIK.S
 4.7	52	0.0389	R.YMEAELK.E
 4.6	53	0.9501	K.KMTVLYK.L
 4.5	54	0.0970 364	Q13621|S12A1_HUMAN K.EMNSGMAK.K
 4.3	57	0.9171	K.KFISYLK.G
 4.2	58	-0.0088	R.NSKMYLK.T

Top scoring peptide matches to query 2864
File7043 Spectrum8834 scans: 6946
Score greater than 21 indicates homology
Score greater than 34 indicates identity

**Score	Expect	Delta Hit Protein	Peptide**
 9.5	17	0.9833	R.DTGRGPPAK.A
 7.6	26	1.0600	K.LCEDFSK.V
 6.2	37	0.0317	R.YAQENFK.S
 6.1	37	-0.0047	K.FQGSVSFK.D
 5.6	43	0.0396	-.MEEIYAK.F
 5.5	44	1.0197	R.GGSGGGGGPPAK.R
 5.2	46	-0.0298	K.LEYIYAK.Q
 5.2	47	-0.0047	29	P08473|NEP_HUMAN R.AYQNYIK.K
 4.6	53	0.9793	K.QFKNSFK.K
 4.5	54	-0.0047	K.SFKDFQK.G

Top scoring peptide matches to query 2868
File7043 Spectrum9192 scans: 7242
Score greater than 30 indicates homology
Score greater than 34 indicates identity

**Score	Expect	Delta Hit Protein	Peptide**
 50.5	0.0014	-0.0060	1	P06576|ATPB_HUMAN K.VLDSGAPIK.I
 14.7	5.2	-0.0536	R.SRISVPIK.R
 10.7	13	-0.0423	R.VLNLEALK.Q
 10.7	13	-0.0060	R.VLTPNDLK.H
 8.6	21	-0.0172	K.EREKPLK.R
 8.6	21	-0.0610	R.MVALKPIK.I
 8.1	24	-0.0787	K.KDLLGIIK.G
 8.0	24	-0.0172	K.VQEAILAR.K
 7.3	28	-0.0536	K.QQKAALLK.N
 7.2	29	0.0668	R.EPNSDIPK.N

Top scoring peptide matches to query 2869
File7043 Spectrum9163 scans: 7220
Score greater than 21 indicates homology
Score greater than 34 indicates identity

**Score	Expect	Delta Hit Protein	Peptide**
 8.7	21	-0.0056	1	P06576|ATPB_HUMAN K.VLDSGAPIK.I
 7.5	27	-0.0419	R.VLNLEALK.Q
 7.1	30	-0.0532	K.VLKTSPVR.E
 5.0	48	1.0400	R.DGQPQVVR.V
 4.3	56	-0.0168	R.VLAALQER.L
 3.6	67	0.0447	R.DPRGIDAR.G
 3.2	73	-0.9215	K.AKEEMHR.V
 2.8	80	1.0651	R.QGGQPGAQR.M
 2.1	94	1.1053	R.DCSTFLR.T
 2.0	96	1.0288	R.DGRPQAVR.V

Top scoring peptide matches to query 2871
File7043 Spectrum7830 scans: 6141
Score greater than 27 indicates homology
Score greater than 34 indicates identity

**Score	Expect	Delta Hit Protein	Peptide**
 17.5	2.7	1.1936	K.ATEECTST.-
 13.1	7.4	1.1121	R.LGCMFVR.C
 11.9	9.9	1.0943	K.AYTGSMLR.K
 10.8	13	1.1121	R.MGAMVAFR.R
 7.9	25	-0.8849 317	Q9BYF1|ACE2_HUMAN R.SSVAYAMR.Q
 6.8	32	0.0773	K.DSVYIFR.E
 6.4	35	0.0773	R.YIGEYVR.N
 6.2	37	1.0910	R.MSTIRMK.A
 4.1	59	0.1065	K.DQSHGSLR.M
 4.0	61	1.0654	R.NNLNPAQK.L

Top scoring peptide matches to query 2872
File7043 Spectrum3977 scans: 3106
Score greater than 24 indicates homology
Score greater than 34 indicates identity

**Score	Expect	Delta Hit Protein	Peptide**
 20.6	1.3	-0.0173	R.GAILLSVAR.G
 10.1	15	-0.9220 279	O43707|ACTN4_HUMAN K.EAMLKHR.D
 8.4	22	0.0330	R.GANVNLRR.K
 8.4	22	-0.8670	K.DSGSPNPAR.G
 8.3	23	1.1334	R.AWEAEHR.D
 7.9	25	1.0031	R.GQLLAQLR.N
 7.1	30	0.9919	R.VQLINRR.N
 7.0	30	-0.8670	R.QEDAQGPR.D
 6.7	33	-0.9472	R.MLVVDPAR.R
 6.4	35	1.1777	R.EFDNMAR.V

Top scoring peptide matches to query 2873
File7043 Spectrum4408 scans: 3441
Score greater than 18 indicates homology
Score greater than 34 indicates identity

**Score	Expect	Delta Hit Protein	Peptide**
 7.6	27	-0.9870	R.VRSAAQIR.V
 5.3	45	-0.9468	R.MLVVDPAR.R
 4.8	51	0.0810	R.RPDDGALR.K
 4.1	59	0.0559	K.GKEELPAR.A
 3.7	65	0.0334	R.GANVNLRR.K
 3.7	66	1.1780	R.EFDNMAR.V
 3.5	69	0.0657	71	P36269|GGT5_HUMAN R.WRLGDPR.S
 3.5	69	0.0698	R.SSGLSRHR.R
 3.3	72	-0.9030	K.GSIEGQGPR.R
 3.3	72	1.0610	K.YFRVASR.D

Top scoring peptide matches to query 2875
File7043 Spectrum17196 scans: 14338
Score greater than 22 indicates homology
Score greater than 34 indicates identity

**Score	Expect	Delta Hit Protein	Peptide**
 10.1	15	-0.9528	R.ALALVEER.V
 9.4	18	1.0516	K.GLVVPSGGGR.F
 8.8	20	1.1533	R.NTTPMYR.T
 8.3	22	-0.8259	K.SDSCLYR.R
 7.7	26	1.0840 425	P01024|CO3_HUMAN R.AVLYNYR.Q
 5.1	47	-1.0004	R.KPKITASR.K
 3.9	62	0.1143	K.MLLGMYR.C
 1.4 1.1e+002	-0.8549	R.QQPDEQR.R

Top scoring peptide matches to query 2877
File7043 Spectrum11350 scans: 9029
Score greater than 14 indicates homology
Score greater than 34 indicates identity

**Score	Expect	Delta Hit Protein	Peptide**
 1.4 1.2e+002	-0.2675 655	Q9Y696|CLIC4_HUMAN R.GLLKTLQK.L
 1.4 1.2e+002	-1.1899	K.NGINDIKK.H

Top scoring peptide matches to query 2878
File7043 Spectrum4700 scans: 3668
Score greater than 34 indicates homology
Score greater than 35 indicates identity

**Score	Expect	Delta Hit Protein	Peptide**
 30.7	0.15	0.9424 140	O14828|SCAM3_HUMAN R.KLSPTEPK.N
 19.5	2	0.9424	R.KDPDALLK.H
 17.0	3.5	-0.0602	K.ELGMLIPK.A
 14.2	6.6	0.9061	K.DKPVLSLK.T
 13.8	7.4	0.9238	R.KALMPPVK.Q
 13.4	8	-0.9688	-.MNPQQQR.M
 13.4	8.1	0.0126	K.EDALMPPK.L
 13.4	8.1	-0.0052	R.EDDLLAPK.Q
 12.8	9.1	0.9200	K.LGNRPSKK.E
 11.6	12	0.8948	R.GAILLSVAR.G

Top scoring peptide matches to query 2879
File7043 Spectrum1347 scans: 1042
Score greater than 35 indicates identity

**Score	Expect	Delta Hit Protein	Peptide**
 22.5	0.97	-0.9907	R.EQVTATPR.I
 21.1	1.4	-0.0180	R.QGRDAVVR.T
 20.5	1.6	-1.0019	R.EQEARLR.Q
 20.5	1.6	-0.0179	R.QQAELRR.A
 16.7	3.7	-0.0067	67	P02511|CRYAB_HUMAN R.KQVSGPER.T
 14.0	7	-0.0795	K.KAIQTAIR.K
 12.9	9	0.0297	R.KADAGPEGR.L
 12.5	9.9	-0.0067	R.KDVGPTQR.H
 12.5	9.9	-0.0067	R.QTLGTPQR.D
 12.4	10	-1.0019	K.QEEALRR.Q

Top scoring peptide matches to query 2880
File7043 Spectrum1549 scans: 1177
Score greater than 31 indicates homology
Score greater than 35 indicates identity

**Score	Expect	Delta Hit Protein	Peptide**
 18.4	2.6	-1.0263	K.QKVIEER.E
 17.5	3.1	-0.9900	R.EQVTATPR.I
 17.3	3.3	-0.9899	K.QEVEQLR.R
 15.5	4.9	-0.0059	67	P02511|CRYAB_HUMAN R.KQVSGPER.T
 14.4	6.4	-1.0879	K.KVEISVVK.A
 13.7	7.4	-1.0151	K.GAESSILPK.C
 13.5	7.8	0.0152	R.QVDHVFR.E
 12.8	9.2	-0.9899	K.EQVAVAER.R
 12.8	9.2	-1.0263	R.EQVKIER.D
 12.8	9.3	0.0305	R.KADAGPEGR.L

Top scoring peptide matches to query 2881
File7043 Spectrum6612 scans: 5169
Score greater than 26 indicates homology
Score greater than 35 indicates identity

**Score	Expect	Delta Hit Protein	Peptide**
 15.2	5.3	-0.0057	40	O95831|AIFM1_HUMAN R.VNAELQAR.S
 12.7	9.4	-0.0784	R.LNSLLGRK.G
 11.2	13	-0.0308	K.QLAAELQK.V
 10.7	15	-0.1036	K.LQTAVLKK.Q
 9.6	19	-0.0421 153	P35749|MYH11_HUMAN K.VGRDVVQK.A
 9.0	22	-0.0057	R.RGSLPDQK.D
 8.7	24	-0.0825	K.VWKVLQK.C
 8.4	26	-0.0420	R.KALDNLAR.Q
 8.2	27	-0.0308	R.EVQGLQVK.V
 7.7	30	-0.0308	R.AELQALQK.T

Top scoring peptide matches to query 2882
File7043 Spectrum2684 scans: 2071
Score greater than 35 indicates identity

**Score	Expect	Delta Hit Protein	Peptide**
 34.4	0.064	-0.0051	67	P02511|CRYAB_HUMAN R.KQVSGPER.T
 31.4	0.13	-0.9891	K.EQVAVAER.R
 31.4	0.13	-1.0255	R.EQVKIER.D
 31.4	0.13	-0.9892	R.EQVTATPR.I
 24.8	0.58	-1.0255	K.QKVIEER.E
 20.5	1.5	-1.0157	K.KAVGHFSR.E
 18.7	2.4	-0.0456	K.QKVPGFPK.R
 18.6	2.4	-1.0255	R.KEEQVLR.S
 18.4	2.6	-0.0164	R.GAQLAERR.L
 18.4	2.6	0.0007	K.KQPWWR.K

Top scoring peptide matches to query 2885
File7043 Spectrum2765 scans: 2130
Score greater than 35 indicates identity

**Score	Expect	Delta Hit Protein	Peptide**
 32.4	0.1	-0.0048	67	P02511|CRYAB_HUMAN R.KQVSGPER.T
 30.7	0.15	-0.9888	K.EQVAVAER.R
 30.7	0.15	-1.0252	R.EQVKIER.D
 30.7	0.15	-0.9888	R.EQVTATPR.I
 30.5	0.16	-1.0252	K.QKVIEER.E
 18.0	2.8	-0.0452	K.QKVPGFPK.R
 17.7	3	-0.0160	R.GAQLAERR.L
 17.7	3	0.0011	K.KQPWWR.K
 17.7	3	-0.0411	K.QKNIIER.Q
 17.7	3	-0.0160	R.QQAELRR.A

Top scoring peptide matches to query 2887
File7043 Spectrum378 scans: 396
Score greater than 25 indicates homology
Score greater than 35 indicates identity

**Score	Expect	Delta Hit Protein	Peptide**
 19.4	2	-0.9868	R.EQVTATPR.I
 11.3	13	-0.0028	67	P02511|CRYAB_HUMAN R.KQVSGPER.T
 10.8	15	-0.0140	R.GAQLAERR.L
 10.6	15	-0.9980	R.EQALERR.C
 9.8	18	-0.9868	ELALGQDR
 9.8	18	-0.9505	K.EVGPTGEGR.V
 9.8	18	-0.0253	K.QRPTSRR.I
 9.8	18	-0.0432	K.QKVPGFPK.R
 9.6	19	0.9700	K.VRPERSR.R
 7.9	28	-0.9882	R.QQWQRR.S

Top scoring peptide matches to query 2888
File7043 Spectrum120 scans: 223
Score greater than 35 indicates identity

**Score	Expect	Delta Hit Protein	Peptide**
 25.7	0.47	-0.0025	67	P02511|CRYAB_HUMAN R.KQVSGPER.T
 22.4	1	-0.9865	K.EQVAVAER.R
 22.4	1	-1.0228	R.EQVKIER.D
 22.4	1	-0.9865	R.EQVTATPR.I
 17.6	3	0.0703	R.QQPDEQR.R
 17.1	3.4	-0.0137	R.GAQLAERR.L
 13.7	7.5	-0.0429	K.QKVPGFPK.R
 13.3	8.3	0.0034	K.KQPWWR.K
 13.3	8.3	-0.0388	K.QKNIIER.Q
 13.3	8.3	-0.0137	R.QQAELRR.A

Top scoring peptide matches to query 2889
File7043 Spectrum11569 scans: 9227
Score greater than 21 indicates homology
Score greater than 35 indicates identity

**Score	Expect	Delta Hit Protein	Peptide**
 9.0	22	-1.0258	56	P10809|CH60_HUMAN K.LSDGVAVLK.V
 8.3	26	-1.0258	K.VQDSVLIK.A
 5.1	54	-1.0258	R.GAVIATELK.N
 4.2	66	0.9423 230	P21912|DHSB_HUMAN M.AAVVALSLR.R
 4.2	66	0.9535	K.LINAVELK.K
 4.2	66	0.9674	K.RLAALEAR.Q
 3.7	75	-1.0258	K.QTEVLALK.L
 3.3	81	0.9899	K.INDPLVTK.V
 2.8	92	-1.0621	R.SSIAGLLLK.A
 2.6	96	1.0150	R.GSPDLAALR.G

Top scoring peptide matches to query 2891
File7043 Spectrum1899 scans: 1448
Score greater than 28 indicates homology
Score greater than 33 indicates identity

**Score	Expect	Delta Hit Protein	Peptide**
 16.8	2.6	-0.4016	R.EQVTATPR.I
 14.5	4.4	0.5824	67	P02511|CRYAB_HUMAN R.KQVSGPER.T
 10.7	11	0.5638	K.QLVKCPR.R
 10.2	12	0.5712	R.AGARLQER.E
 10.0	13	0.6002	K.KIMAHER.R
 10.0	13	0.6552	R.QQPDEQR.R
 10.0	13	0.5600	R.QRPSRTR.L
 9.8	13	0.5712	R.QGRDAVVR.T
 9.3	15	0.6036	43	P33121|ACSL1_HUMAN M.QAHELFR.Y
 8.4	18	-0.3288	K.GEAGEPGER.G

Top scoring peptide matches to query 2892
File7043 Spectrum2058 scans: 1583
Score greater than 31 indicates homology
Score greater than 33 indicates identity

**Score	Expect	Delta Hit Protein	Peptide**
 19.8	1.3	0.6556	R.QQPDEQR.R
 19.7	1.3	0.5716	R.AGARLQER.E
 17.7	2.1	-0.4012	R.EQVTATPR.I
 15.2	3.8	-0.4012	K.EQVAVAER.R
 15.2	3.8	-0.4376	R.EQVKIER.D
 15.2	3.8	0.5828	67	P02511|CRYAB_HUMAN R.KQVSGPER.T
 11.4	9.1	0.6192	R.KADAGPEGR.L
 11.0	9.9	0.5060	R.QVKLWVK.M
 10.6	11	0.5716	R.RAQLQER.L
 10.6	11	-0.4012	R.VEQLAGER.H

Top scoring peptide matches to query 2893
File7043 Spectrum2222 scans: 1717
Score greater than 26 indicates homology
Score greater than 33 indicates identity

**Score	Expect	Delta Hit Protein	Peptide**
 16.8	2.6	-0.4011	R.EQVTATPR.I
 12.2	7.6	0.5829	67	P02511|CRYAB_HUMAN R.KQVSGPER.T
 11.8	8.3	0.6557	R.QQPDEQR.R
 11.1	9.6	0.5717	R.AGARLQER.E
 10.3	12	0.5326	K.ELLLTSPK.Y
 9.9	13	0.5717	R.GAQLAERR.L
 8.9	16	-0.4374	K.EVAGLREK.I
 8.7	17	-0.4262	K.GAESSILPK.C
 8.7	17	0.6007	K.KIMAHER.R
 8.4	18	0.5425	K.QKVPGFPK.R

Top scoring peptide matches to query 2897
File7043 Spectrum3388 scans: 2626
Score greater than 25 indicates homology
Score greater than 35 indicates identity

**Score	Expect	Delta Hit Protein	Peptide**
 13.7	8.4	1.0395	R.MGGMVSFR.T
 11.6	14	0.0442	R.LCPHCSK.L
 11.3	15	-1.0633	K.KLEHFTK.L
 11.3	15	0.9741	K.REIHMAK.A
 11.1	15	1.0217 317	Q9BYF1|ACE2_HUMAN R.SSVAYAMR.Q
 9.5	22	0.0411	R.DYDVYAR.R
 8.5	28	1.0291	R.QQPDEQR.R
 6.8	42	0.8836	R.KPKITASR.K
 6.8	42	0.8836	R.KPKSATLR.A
 6.8	42	0.9622	K.KQPWWR.K

Top scoring peptide matches to query 2899
File7043 Spectrum9030 scans: 7115
Score greater than 35 indicates identity

**Score	Expect	Delta Hit Protein	Peptide**
 25.4	0.57	0.9329	R.LAQEKALK.R
 25.0	0.62	-0.0049 300+ P08263|GSTA1_HUMAN K.FLQPGSPR.K
 23.8	0.82	-0.0148	3	P15144|AMPN_HUMAN R.ALEQALEK.T
 17.7	3.3	0.9329	R.QIEAKALK.V
 17.6	3.4	-1.0100	R.SLLQAGADK.D
 16.9	4	0.9692	K.DNIVAQLK.Q
 16.0	4.9	0.0315	YHPDKNK
 15.3	5.8	-1.0325	R.GLRSVGASR.H
 15.3	5.8	-1.0828	K.KVIVTGASK.G
 15.2	5.9	0.9944	K.RDSGPAIGK.D

Top scoring peptide matches to query 2900
File7043 Spectrum8797 scans: 6917
Score greater than 25 indicates homology
Score greater than 35 indicates identity

**Score	Expect	Delta Hit Protein	Peptide**
 12.4	11	-1.0008	K.AIEEANKK.M
 11.7	13	-0.9756 223	O60716|CTND1_HUMAN R.ALEEERR.H
 11.3	15	-0.0055	3	P15144|AMPN_HUMAN R.ALEQALEK.T
 11.2	15	0.0084	R.ALEQERR.H
 10.9	16	-1.0372	R.ALESLNKK.D
 10.4	18	-0.8779	K.EFFDGCK.A
 9.6	22	-0.0419 160	O95299|NDUAA_HUMAN K.LAKEIAEK.L
 9.5	22	-1.0121	R.RLVQGDSK.L
 9.2	23	-1.0372	K.ALKSEVQK.L
 8.5	28	0.0923	K.DDQGNPQK.V

Top scoring peptide matches to query 2901
File7043 Spectrum8796 scans: 6915
Score greater than 35 indicates identity

**Score	Expect	Delta Hit Protein	Peptide**
 25.3	0.59	-0.0052	3	P15144|AMPN_HUMAN R.ALEQALEK.T
 24.8	0.66	-0.0164	K.QANKALEK.Y
 23.6	0.86	-0.0052	K.ELAAQLEK.K
 13.1	9.6	-0.0779	R.KSLLALEK.E
 12.5	11	-0.0164	R.IEAAQLTR.A
 11.9	13	-0.0052	R.LAEAAVAEK.R
 11.9	13	-0.0052	K.LAAAEGELK.I
 11.6	14	-0.0779	K.KISAIIEK.R
 11.6	14	-1.0191	R.MELKPLR.V
 11.6	14	-0.0164	R.QVREIEK.L

Top scoring peptide matches to query 2903
File7043 Spectrum4176 scans: 3263
Score greater than 28 indicates homology
Score greater than 35 indicates identity

**Score	Expect	Delta Hit Protein	Peptide**
 17.6	3.4	-0.0043 542	P02549|SPTA1_HUMAN R.RQEVLTR.Y
 14.3	7.3	-0.0294	K.GVLSEKLR.S
 14.3	7.3	0.0685	R.RAEEPGSR.G
 14.3	7.3	0.0321	K.REAEQLR.M
 14.3	7.3	0.0572	K.RGSGQSGPR.G
 14.3	7.3	-0.0043	K.RLTDLQR.D
 12.7	11	-0.9454	R.RGVPACSR.A
 12.1	12	-0.0182	K.TTLQPTLK.F
 11.6	14	-0.9130	RYGGFMR
 11.5	14	0.0685	R.RDPQETR.S

Top scoring peptide matches to query 2904
File7043 Spectrum4387 scans: 3425
Score greater than 25 indicates homology
Score greater than 35 indicates identity

**Score	Expect	Delta Hit Protein	Peptide**
 14.3	7.4	1.0060	R.QRPSRTR.L
 11.9	13	0.9557	R.QAALTKLR.Y
 11.7	13	0.1891	R.GCCFDSR.I
 11.4	14	1.0132	K.AGLAYHLR.S
 10.7	17	-0.9946	R.MELKPLR.V
 10.4	18	1.0462 185	P39023|RL3_HUMAN K.AGMTHIVR.E
 9.8	21	0.1019	R.GFSGYNTR.W
 9.5	22	1.0132	R.YLIAHQR.T
 9.4	23	1.0284	K.AGPTLAQSR.E
 8.6	27	0.0873	R.GNMGHLTR.I

Top scoring peptide matches to query 2908
File7043 Spectrum6895 scans: 5393
Score greater than 22 indicates homology
Score greater than 35 indicates identity

**Score	Expect	Delta Hit Protein	Peptide**
 13.1	9.7	1.0400	K.DGFHRLR.Q
 8.8	26	-0.9967	R.SSGLVQRR.T
 8.8	26	0.0527	-.MNPDLRR.E
 7.7	33	1.0802	R.CLFSAFR.V
 7.2	37	1.0553	R.RPQSQER.W
 6.5	44	1.0955 317	Q9BYF1|ACE2_HUMAN R.SSVAYAMR.Q
 5.9	51	-0.0266	R.EVSGLLKR.R
 5.6	54	1.0414	K.DQVNLSPK.L
 5.4	56	0.1189	K.GEAGEPGER.G
 5.3	58	-1.0470	R.QALKLTTK.C

Top scoring peptide matches to query 2912
File7043 Spectrum7497 scans: 5873
Score greater than 29 indicates homology
Score greater than 35 indicates identity

**Score	Expect	Delta Hit Protein	Peptide**
 18.0	3.1	0.0058	K.GVLSEKLR.S
 16.2	4.7	0.1149	K.GSEGEPGIR.G
 15.9	5	-0.9466	R.CQLGIRR.H
 15.9	5	0.0851	K.DPGGMLRR.N
 14.4	7.2	-0.0054 172	P84098|RL19_HUMAN K.RLASSVLR.C
 14.4	7.2	0.0673	R.RLDDNIR.T
 14.0	7.8	0.0785	K.GDLDINVR.T
 13.9	8	0.0851	K.RLEPCAR.L
 13.9	8.1	0.0997	R.GESGIWPR.H
 13.2	9.4	-0.9643	R.SSGLVQRR.T

Top scoring peptide matches to query 2914
File7043 Spectrum5296 scans: 4130
Score greater than 32 indicates homology
Score greater than 36 indicates identity

**Score	Expect	Delta Hit Protein	Peptide**
 21.1	1.6	-0.9830	R.RAESGGGGGR.C
 21.0	1.7	-0.9969	R.SPSATEVGR.I
 20.1	2	-1.0234 311	P01833|PIGR_HUMAN K.RAPAFEGR.I
 18.8	2.7	0.9922	R.GPRFDGPR.R
 17.2	3.9	-1.0016	M.CARGQVGR.G
 13.8	8.6	-1.1213	K.KVFLLAGR.K
 13.7	8.9	-0.9870	R.REAEWGR.V
 13.6	9	-0.9792	R.SCLDVPGR.L
 13.2	10	-0.9718	R.DSGANGQVR.C
 12.7	11	-0.9606	K.SSGDGPDLR.D

Top scoring peptide matches to query 2915
File7043 Spectrum7206 scans: 5641
Score greater than 19 indicates homology
Score greater than 36 indicates identity

**Score	Expect	Delta Hit Protein	Peptide**
 8.4	30	-1.0752	R.AGLRALFR.S
 6.2	50	-1.0310	K.AGLKDMLR.M
 4.7	71	0.9418 160	O95299|NDUAA_HUMAN K.LAKEIAEK.L
 4.5	75	-0.9986	R.WMKPPTK.Q
 4.3	78	0.0040	R.EFLAHSAK.V
 3.8	87	-1.0124	K.QLDSLTAR.T
 3.5	94	0.9305	K.NALQKLSK.A
 3.1	1e+002	-1.1004	R.KLINLFR.G
 3.1	1e+002	-1.1103	R.KTITITVK.S
 2.9 1.1e+002	-1.0739	K.KLVSETVK.K

Top scoring peptide matches to query 2917
File7043 Spectrum9613 scans: 7589
Score greater than 18 indicates homology
Score greater than 36 indicates identity

**Score	Expect	Delta Hit Protein	Peptide**
 8.4	30	0.0090	R.AASSPGRTR.R
 5.0	66	-0.8732	K.AEMDEHR.L
 4.8	68	-0.9824	-.MGALGLEGR.G
 4.8	68	-0.9977	R.VCLWIGR.Q
 4.5	74	-0.0162	R.KTSGLGSPR.A
 4.2	79	1.0155	R.QPLSVSDR.H
 4.1	81	-0.0161	K.VKENSAVR.V
 4.1	81	1.0002 300+ P08263|GSTA1_HUMAN K.FLQPGSPR.K
 3.7	88	1.0155	R.LDSPLSNR.Y
 3.1	1e+002	-0.9499	R.RAESGGGGGR.C

Top scoring peptide matches to query 2918
File7043 Spectrum10085 scans: 7979
Score greater than 24 indicates homology
[truncated: 4,333,405 more chars]
