# Supplementary material for: Dataset from proteomic analysis of human liver, lung, kidney and intestine microsomes
Source: Data Brief. 2018 Mar 30;18:831–4. doi: 10.1016/j.dib.2018.03.124 (PMC5996730; doi:10.1016/j.dib.2018.03.124)
Supplement: Supplementary file 5 — Supplementary material [file mmc5.doc]

Mascot Search Results

**User	: zhihong**

**Email	: peng_zhihong@hotmail.com**

**Search title	: liver**

**MS data file	: liver-2.mgf**

**Database	: SwissProt 101509 (510076 sequences; 179409349 residues)**

**Taxonomy	: Homo sapiens (human) (20335 sequences)**

**Timestamp	: 8 May 2013 at 17:10:30 GMT**

**Warning	: Invalid value (13+) specified for the "CHARGE" parameter**

**Warning	: Invalid value () specified for the "CHARGE" parameter**

**Warning	: Invalid value (17+) specified for the "CHARGE" parameter**

**Warning	: Invalid value () specified for the "CHARGE" parameter**

**Warning	: Invalid value (9+) specified for the "CHARGE" parameter**

**Warning	: Invalid value () specified for the "CHARGE" parameter**

**Warning	: Invalid value (12+) specified for the "CHARGE" parameter**

**Warning	: Invalid value () specified for the "CHARGE" parameter**

**Warning	: Invalid value (12+) specified for the "CHARGE" parameter**

**Warning	: Invalid value () specified for the "CHARGE" parameter**

**Warning	: Error 61 has been detected 38 times and only the first 10 messages have been output**

**Enzyme	: Trypsin**

**Fixed modifications	: Carbamidomethyl (C)**

**Variable modifications : Oxidation (M)**

**Mass values	: Monoisotopic**

**Protein Mass	: Unrestricted**

**Peptide Mass Tolerance : ± 1.2 Da**

**Fragment Mass Tolerance: ± 0.6 Da**

**Max Missed Cleavages	: 1**

**Instrument type	: Default**

**Number of queries	: 31987**

| **Protein hits           :** | **P31327\|CPSM_HUMAN** | Carbamoyl-phosphate synthase [ammonia], mitochondrial OS=Homo sapiens GN=CPS1 PE=1 SV=2 |
| --- | --- | --- |
|  | **P00325\|ADH1B_HUMAN** | Alcohol dehydrogenase 1B OS=Homo sapiens GN=ADH1B PE=1 SV=2 |
|  | **P35579\|MYH9_HUMAN** | Myosin-9 OS=Homo sapiens GN=MYH9 PE=1 SV=4 |
|  | **P23141\|EST1_HUMAN** | Liver carboxylesterase 1 OS=Homo sapiens GN=CES1 PE=1 SV=2 |
|  | **P08319\|ADH4_HUMAN** | Alcohol dehydrogenase 4 OS=Homo sapiens GN=ADH4 PE=1 SV=4 |
|  | **P06576\|ATPB_HUMAN** | ATP synthase subunit beta, mitochondrial OS=Homo sapiens GN=ATP5B PE=1 SV=3 |
|  | **P11021\|GRP78_HUMAN** | 78 kDa glucose-regulated protein OS=Homo sapiens GN=HSPA5 PE=1 SV=2 |
|  | **P07327\|ADH1A_HUMAN** | Alcohol dehydrogenase 1A OS=Homo sapiens GN=ADH1A PE=1 SV=2 |
|  | **P00738\|HPT_HUMAN** | Haptoglobin OS=Homo sapiens GN=HP PE=1 SV=1 |
|  | **P07099\|HYEP_HUMAN** | Epoxide hydrolase 1 OS=Homo sapiens GN=EPHX1 PE=1 SV=1 |
|  | **P60709\|ACTB_HUMAN** | Actin, cytoplasmic 1 OS=Homo sapiens GN=ACTB PE=1 SV=1 |
|  | **P00326\|ADH1G_HUMAN** | Alcohol dehydrogenase 1C OS=Homo sapiens GN=ADH1C PE=1 SV=2 |
|  | **P07437\|TBB5_HUMAN** | Tubulin beta chain OS=Homo sapiens GN=TUBB PE=1 SV=2 |
|  | **P68371\|TBB2C_HUMAN** | Tubulin beta-2C chain OS=Homo sapiens GN=TUBB2C PE=1 SV=1 |
|  | **P08684\|CP3A4_HUMAN** | Cytochrome P450 3A4 OS=Homo sapiens GN=CYP3A4 PE=1 SV=4 |
|  | **P16435\|NCPR_HUMAN** | NADPH--cytochrome P450 reductase OS=Homo sapiens GN=POR PE=1 SV=2 |
|  | **P68363\|TBA1B_HUMAN** | Tubulin alpha-1B chain OS=Homo sapiens GN=TUBA1B PE=1 SV=1 |
|  | **Q9BQE3\|TBA1C_HUMAN** | Tubulin alpha-1C chain OS=Homo sapiens GN=TUBA1C PE=1 SV=1 |
|  | **P33121\|ACSL1_HUMAN** | Long-chain-fatty-acid--CoA ligase 1 OS=Homo sapiens GN=ACSL1 PE=1 SV=1 |
|  | **P68366\|TBA4A_HUMAN** | Tubulin alpha-4A chain OS=Homo sapiens GN=TUBA4A PE=1 SV=1 |
|  | **P25705\|ATPA_HUMAN** | ATP synthase subunit alpha, mitochondrial OS=Homo sapiens GN=ATP5A1 PE=1 SV=1 |
|  | **P04350\|TBB4_HUMAN** | Tubulin beta-4 chain OS=Homo sapiens GN=TUBB4 PE=1 SV=2 |
|  | **Q71U36\|TBA1A_HUMAN** | Tubulin alpha-1A chain OS=Homo sapiens GN=TUBA1A PE=1 SV=1 |
|  | **P05181\|CP2E1_HUMAN** | Cytochrome P450 2E1 OS=Homo sapiens GN=CYP2E1 PE=1 SV=1 |
|  | **P07237\|PDIA1_HUMAN** | Protein disulfide-isomerase OS=Homo sapiens GN=P4HB PE=1 SV=3 |
|  | **Q13885\|TBB2A_HUMAN** | Tubulin beta-2A chain OS=Homo sapiens GN=TUBB2A PE=1 SV=1 |
|  | **Q9BVA1\|TBB2B_HUMAN** | Tubulin beta-2B chain OS=Homo sapiens GN=TUBB2B PE=1 SV=1 |
|  | **Q7Z4W1\|DCXR_HUMAN** | L-xylulose reductase OS=Homo sapiens GN=DCXR PE=1 SV=2 |
|  | **P05062\|ALDOB_HUMAN** | Fructose-bisphosphate aldolase B OS=Homo sapiens GN=ALDOB PE=1 SV=2 |
|  | **Q9Y2P5\|S27A5_HUMAN** | Bile acyl-CoA synthetase OS=Homo sapiens GN=SLC27A5 PE=1 SV=1 |
|  | **P11498\|PYC_HUMAN** | Pyruvate carboxylase, mitochondrial OS=Homo sapiens GN=PC PE=1 SV=2 |
|  | **P35573\|GDE_HUMAN** | Glycogen debranching enzyme OS=Homo sapiens GN=AGL PE=1 SV=3 |
|  | **Q13748\|TBA3C_HUMAN** | Tubulin alpha-3C/D chain OS=Homo sapiens GN=TUBA3C PE=1 SV=3 |
|  | **P27797\|CALR_HUMAN** | Calreticulin OS=Homo sapiens GN=CALR PE=1 SV=1 |
|  | **Q6PEY2\|TBA3E_HUMAN** | Tubulin alpha-3E chain OS=Homo sapiens GN=TUBA3E PE=1 SV=2 |
|  | **P14625\|ENPL_HUMAN** | Endoplasmin OS=Homo sapiens GN=HSP90B1 PE=1 SV=1 |
|  | **P13667\|PDIA4_HUMAN** | Protein disulfide-isomerase A4 OS=Homo sapiens GN=PDIA4 PE=1 SV=2 |
|  | **O14975\|S27A2_HUMAN** | Very long-chain acyl-CoA synthetase OS=Homo sapiens GN=SLC27A2 PE=1 SV=1 |
|  | **P21796\|VDAC1_HUMAN** | Voltage-dependent anion-selective channel protein 1 OS=Homo sapiens GN=VDAC1 PE=1 SV=2 |
|  | **P31513\|FMO3_HUMAN** | Dimethylaniline monooxygenase [N-oxide-forming] 3 OS=Homo sapiens GN=FMO3 PE=1 SV=5 |
|  | **P10620\|MGST1_HUMAN** | Microsomal glutathione S-transferase 1 OS=Homo sapiens GN=MGST1 PE=1 SV=1 |
|  | **P40939\|ECHA_HUMAN** | Trifunctional enzyme subunit alpha, mitochondrial OS=Homo sapiens GN=HADHA PE=1 SV=2 |
|  | **Q13423\|NNTM_HUMAN** | NAD(P) transhydrogenase, mitochondrial OS=Homo sapiens GN=NNT PE=1 SV=3 |
|  | **P51659\|DHB4_HUMAN** | Peroxisomal multifunctional enzyme type 2 OS=Homo sapiens GN=HSD17B4 PE=1 SV=3 |
|  | **P31040\|DHSA_HUMAN** | Succinate dehydrogenase [ubiquinone] flavoprotein subunit, mitochondrial OS=Homo sapiens GN=SDHA PE=1 SV=2 |
|  | **Q16134\|ETFD_HUMAN** | Electron transfer flavoprotein-ubiquinone oxidoreductase, mitochondrial OS=Homo sapiens GN=ETFDH PE=1 SV=1 |
|  | **P35749\|MYH11_HUMAN** | Myosin-11 OS=Homo sapiens GN=MYH11 PE=1 SV=3 |
|  | **Q9NY65\|TBA8_HUMAN** | Tubulin alpha-8 chain OS=Homo sapiens GN=TUBA8 PE=1 SV=1 |
|  | **P02671\|FIBA_HUMAN** | Fibrinogen alpha chain OS=Homo sapiens GN=FGA PE=1 SV=2 |
|  | **Q16822\|PCKGM_HUMAN** | Phosphoenolpyruvate carboxykinase [GTP], mitochondrial OS=Homo sapiens GN=PCK2 PE=1 SV=2 |
|  | **P04040\|CATA_HUMAN** | Catalase OS=Homo sapiens GN=CAT PE=1 SV=3 |
|  | **Q15084\|PDIA6_HUMAN** | Protein disulfide-isomerase A6 OS=Homo sapiens GN=PDIA6 PE=1 SV=1 |
|  | **P11509\|CP2A6_HUMAN** | Cytochrome P450 2A6 OS=Homo sapiens GN=CYP2A6 PE=1 SV=2 |
|  | **P31930\|QCR1_HUMAN** | Cytochrome b-c1 complex subunit 1, mitochondrial OS=Homo sapiens GN=UQCRC1 PE=1 SV=3 |
|  | **P06737\|PYGL_HUMAN** | Glycogen phosphorylase, liver form OS=Homo sapiens GN=PYGL PE=1 SV=4 |
|  | **Q6S8J3\|POTEE_HUMAN** | POTE ankyrin domain family member E OS=Homo sapiens GN=POTEE PE=1 SV=3 |
|  | **Q9UJS0\|CMC2_HUMAN** | Calcium-binding mitochondrial carrier protein Aralar2 OS=Homo sapiens GN=SLC25A13 PE=1 SV=2 |
|  | **P42765\|THIM_HUMAN** | 3-ketoacyl-CoA thiolase, mitochondrial OS=Homo sapiens GN=ACAA2 PE=1 SV=2 |
|  | **Q02338\|BDH_HUMAN** | D-beta-hydroxybutyrate dehydrogenase, mitochondrial OS=Homo sapiens GN=BDH1 PE=1 SV=3 |
|  | **P68032\|ACTC_HUMAN** | Actin, alpha cardiac muscle 1 OS=Homo sapiens GN=ACTC1 PE=1 SV=1 |
|  | **P30101\|PDIA3_HUMAN** | Protein disulfide-isomerase A3 OS=Homo sapiens GN=PDIA3 PE=1 SV=4 |
|  | **P21397\|AOFA_HUMAN** | Amine oxidase [flavin-containing] A OS=Homo sapiens GN=MAOA PE=1 SV=1 |
|  | **P10632\|CP2C8_HUMAN** | Cytochrome P450 2C8 OS=Homo sapiens GN=CYP2C8 PE=1 SV=2 |
|  | **P63267\|ACTH_HUMAN** | Actin, gamma-enteric smooth muscle OS=Homo sapiens GN=ACTG2 PE=1 SV=1 |
|  | **P62736\|ACTA_HUMAN** | Actin, aortic smooth muscle OS=Homo sapiens GN=ACTA2 PE=1 SV=1 |
|  | **Q02928\|CP4AB_HUMAN** | Cytochrome P450 4A11 OS=Homo sapiens GN=CYP4A11 PE=1 SV=1 |
|  | **Q13509\|TBB3_HUMAN** | Tubulin beta-3 chain OS=Homo sapiens GN=TUBB3 PE=1 SV=2 |
|  | **P22310\|UD14_HUMAN** | UDP-glucuronosyltransferase 1-4 OS=Homo sapiens GN=UGT1A4 PE=1 SV=1 |
|  | **P55084\|ECHB_HUMAN** | Trifunctional enzyme subunit beta, mitochondrial OS=Homo sapiens GN=HADHB PE=1 SV=3 |
|  | **O60656\|UD19_HUMAN** | UDP-glucuronosyltransferase 1-9 OS=Homo sapiens GN=UGT1A9 PE=1 SV=1 |
|  | **P68133\|ACTS_HUMAN** | Actin, alpha skeletal muscle OS=Homo sapiens GN=ACTA1 PE=1 SV=1 |
|  | **P22309\|UD11_HUMAN** | UDP-glucuronosyltransferase 1-1 OS=Homo sapiens GN=UGT1A1 PE=1 SV=1 |
|  | **P21549\|SPYA_HUMAN** | Serine--pyruvate aminotransferase OS=Homo sapiens GN=AGXT PE=1 SV=1 |
|  | **Q9Y4L1\|HYOU1_HUMAN** | Hypoxia up-regulated protein 1 OS=Homo sapiens GN=HYOU1 PE=1 SV=1 |
|  | **P24462\|CP3A7_HUMAN** | Cytochrome P450 3A7 OS=Homo sapiens GN=CYP3A7 PE=1 SV=2 |
|  | **P02679\|FIBG_HUMAN** | Fibrinogen gamma chain OS=Homo sapiens GN=FGG PE=1 SV=3 |
|  | **P11712\|CP2C9_HUMAN** | Cytochrome P450 2C9 OS=Homo sapiens GN=CYP2C9 PE=1 SV=3 |
|  | **P10809\|CH60_HUMAN** | 60 kDa heat shock protein, mitochondrial OS=Homo sapiens GN=HSPD1 PE=1 SV=2 |
|  | **P01024\|CO3_HUMAN** | Complement C3 OS=Homo sapiens GN=C3 PE=1 SV=2 |
|  | **O95954\|FTCD_HUMAN** | Formimidoyltransferase-cyclodeaminase OS=Homo sapiens GN=FTCD PE=1 SV=2 |
|  | **Q8NBX0\|SCPDH_HUMAN** | Probable saccharopine dehydrogenase OS=Homo sapiens GN=SCCPDH PE=1 SV=1 |
|  | **P05177\|CP1A2_HUMAN** | Cytochrome P450 1A2 OS=Homo sapiens GN=CYP1A2 PE=1 SV=3 |
|  | **P27338\|AOFB_HUMAN** | Amine oxidase [flavin-containing] B OS=Homo sapiens GN=MAOB PE=1 SV=3 |
|  | **P00367\|DHE3_HUMAN** | Glutamate dehydrogenase 1, mitochondrial OS=Homo sapiens GN=GLUD1 PE=1 SV=2 |
|  | **P35504\|UD15_HUMAN** | UDP-glucuronosyltransferase 1-5 OS=Homo sapiens GN=UGT1A5 PE=1 SV=1 |
|  | **P05091\|ALDH2_HUMAN** | Aldehyde dehydrogenase, mitochondrial OS=Homo sapiens GN=ALDH2 PE=1 SV=2 |
|  | **P11142\|HSP7C_HUMAN** | Heat shock cognate 71 kDa protein OS=Homo sapiens GN=HSPA8 PE=1 SV=1 |
|  | **P19224\|UD16_HUMAN** | UDP-glucuronosyltransferase 1-6 OS=Homo sapiens GN=UGT1A6 PE=1 SV=2 |
|  | **P54868\|HMCS2_HUMAN** | Hydroxymethylglutaryl-CoA synthase, mitochondrial OS=Homo sapiens GN=HMGCS2 PE=1 SV=1 |
|  | **P35503\|UD13_HUMAN** | UDP-glucuronosyltransferase 1-3 OS=Homo sapiens GN=UGT1A3 PE=1 SV=1 |
|  | **Q16696\|CP2AD_HUMAN** | Cytochrome P450 2A13 OS=Homo sapiens GN=CYP2A13 PE=1 SV=3 |
|  | **O14756\|H17B6_HUMAN** | Hydroxysteroid 17-beta dehydrogenase 6 OS=Homo sapiens GN=HSD17B6 PE=1 SV=1 |
|  | **P35580\|MYH10_HUMAN** | Myosin-10 OS=Homo sapiens GN=MYH10 PE=1 SV=3 |
|  | **P00966\|ASSY_HUMAN** | Argininosuccinate synthase OS=Homo sapiens GN=ASS1 PE=1 SV=2 |
|  | **Q3ZCM7\|TBB8_HUMAN** | Tubulin beta-8 chain OS=Homo sapiens GN=TUBB8 PE=1 SV=2 |
|  | **O95831\|AIFM1_HUMAN** | Apoptosis-inducing factor 1, mitochondrial OS=Homo sapiens GN=AIFM1 PE=1 SV=1 |
|  | **Q9ULC5\|ACSL5_HUMAN** | Long-chain-fatty-acid--CoA ligase 5 OS=Homo sapiens GN=ACSL5 PE=1 SV=1 |
|  | **P02675\|FIBB_HUMAN** | Fibrinogen beta chain OS=Homo sapiens GN=FGB PE=1 SV=2 |
|  | **P04406\|G3P_HUMAN** | Glyceraldehyde-3-phosphate dehydrogenase OS=Homo sapiens GN=GAPDH PE=1 SV=3 |
|  | **P30049\|ATPD_HUMAN** | ATP synthase subunit delta, mitochondrial OS=Homo sapiens GN=ATP5D PE=1 SV=2 |
|  | **P04843\|RPN1_HUMAN** | Dolichyl-diphosphooligosaccharide--protein glycosyltransferase subunit 1 OS=Homo sapiens GN=RPN1 PE=1 SV=1 |
|  | **O75452\|RDH16_HUMAN** | Retinol dehydrogenase 16 OS=Homo sapiens GN=RDH16 PE=1 SV=2 |
|  | **Q14697\|GANAB_HUMAN** | Neutral alpha-glucosidase AB OS=Homo sapiens GN=GANAB PE=1 SV=3 |
|  | **Q08426\|ECHP_HUMAN** | Peroxisomal bifunctional enzyme OS=Homo sapiens GN=EHHADH PE=1 SV=3 |
|  | **Q9UNU6\|CP8B1_HUMAN** | 7-alpha-hydroxycholest-4-en-3-one 12-alpha-hydroxylase OS=Homo sapiens GN=CYP8B1 PE=2 SV=1 |
|  | **P22695\|QCR2_HUMAN** | Cytochrome b-c1 complex subunit 2, mitochondrial OS=Homo sapiens GN=UQCRC2 PE=1 SV=3 |
|  | **P49326\|FMO5_HUMAN** | Dimethylaniline monooxygenase [N-oxide-forming] 5 OS=Homo sapiens GN=FMO5 PE=2 SV=2 |
|  | **A6NNZ2\|TBB8B_HUMAN** | Tubulin beta-8 chain B OS=Homo sapiens PE=1 SV=1 |
|  | **P02768\|ALBU_HUMAN** | Serum albumin OS=Homo sapiens GN=ALB PE=1 SV=2 |
|  | **P28332\|ADH6_HUMAN** | Alcohol dehydrogenase 6 OS=Homo sapiens GN=ADH6 PE=2 SV=2 |
|  | **P06733\|ENOA_HUMAN** | Alpha-enolase OS=Homo sapiens GN=ENO1 PE=1 SV=2 |
|  | **P0C0L4\|CO4A_HUMAN** | Complement C4-A OS=Homo sapiens GN=C4A PE=1 SV=1 |
|  | **P78329\|CP4F2_HUMAN** | Leukotriene-B(4) omega-hydroxylase 1 OS=Homo sapiens GN=CYP4F2 PE=1 SV=1 |
|  | **P12236\|ADT3_HUMAN** | ADP/ATP translocase 3 OS=Homo sapiens GN=SLC25A6 PE=1 SV=4 |
|  | **P05141\|ADT2_HUMAN** | ADP/ATP translocase 2 OS=Homo sapiens GN=SLC25A5 PE=1 SV=6 |
|  | **Q99623\|PHB2_HUMAN** | Prohibitin-2 OS=Homo sapiens GN=PHB2 PE=1 SV=2 |
|  | **P00167\|CYB5_HUMAN** | Cytochrome b5 OS=Homo sapiens GN=CYB5A PE=1 SV=2 |
|  | **P16662\|UD2B7_HUMAN** | UDP-glucuronosyltransferase 2B7 OS=Homo sapiens GN=UGT2B7 PE=1 SV=1 |
|  | **P04844\|RPN2_HUMAN** | Dolichyl-diphosphooligosaccharide--protein glycosyltransferase subunit 2 OS=Homo sapiens GN=RPN2 PE=1 SV=3 |
|  | **Q96LJ7\|DHRS1_HUMAN** | Dehydrogenase/reductase SDR family member 1 OS=Homo sapiens GN=DHRS1 PE=1 SV=1 |
|  | **Q99867\|TBB4Q_HUMAN** | Putative tubulin beta-4q chain OS=Homo sapiens GN=TUBB4Q PE=5 SV=1 |
|  | **Q9BUF5\|TBB6_HUMAN** | Tubulin beta-6 chain OS=Homo sapiens GN=TUBB6 PE=1 SV=1 |
|  | **Q9UKY3\|EST4_HUMAN** | Inactive carboxylesterase 4 OS=Homo sapiens GN=CES4 PE=2 SV=2 |
|  | **P12235\|ADT1_HUMAN** | ADP/ATP translocase 1 OS=Homo sapiens GN=SLC25A4 PE=1 SV=4 |
|  | **P24752\|THIL_HUMAN** | Acetyl-CoA acetyltransferase, mitochondrial OS=Homo sapiens GN=ACAT1 PE=1 SV=1 |
|  | **P10635\|CP2D6_HUMAN** | Cytochrome P450 2D6 OS=Homo sapiens GN=CYP2D6 PE=1 SV=2 |
|  | **P28845\|DHI1_HUMAN** | Corticosteroid 11-beta-dehydrogenase isozyme 1 OS=Homo sapiens GN=HSD11B1 PE=1 SV=3 |
|  | **Q5TCH4\|CP4AM_HUMAN** | Cytochrome P450 4A22 OS=Homo sapiens GN=CYP4A22 PE=1 SV=1 |
|  | **P02735\|SAA_HUMAN** | Serum amyloid A protein OS=Homo sapiens GN=SAA1 PE=1 SV=2 |
|  | **P05388\|RLA0_HUMAN** | 60S acidic ribosomal protein P0 OS=Homo sapiens GN=RPLP0 PE=1 SV=1 |
|  | **P30086\|PEBP1_HUMAN** | Phosphatidylethanolamine-binding protein 1 OS=Homo sapiens GN=PEBP1 PE=1 SV=3 |
|  | **P27824\|CALX_HUMAN** | Calnexin OS=Homo sapiens GN=CANX PE=1 SV=2 |
|  | **Q16851\|UGPA_HUMAN** | UTP--glucose-1-phosphate uridylyltransferase OS=Homo sapiens GN=UGP2 PE=1 SV=5 |
|  | **P28331\|NDUS1_HUMAN** | NADH-ubiquinone oxidoreductase 75 kDa subunit, mitochondrial OS=Homo sapiens GN=NDUFS1 PE=1 SV=3 |
|  | **P00352\|AL1A1_HUMAN** | Retinal dehydrogenase 1 OS=Homo sapiens GN=ALDH1A1 PE=1 SV=2 |
|  | **Q9Y5M8\|SRPRB_HUMAN** | Signal recognition particle receptor subunit beta OS=Homo sapiens GN=SRPRB PE=1 SV=3 |
|  | **A1L0T0\|ILVBL_HUMAN** | Acetolactate synthase-like protein OS=Homo sapiens GN=ILVBL PE=1 SV=2 |
|  | **P22760\|AAAD_HUMAN** | Arylacetamide deacetylase OS=Homo sapiens GN=AADAC PE=1 SV=4 |
|  | **P15144\|AMPN_HUMAN** | Aminopeptidase N OS=Homo sapiens GN=ANPEP PE=1 SV=4 |
|  | **Q9BPW8\|NIPS1_HUMAN** | Protein NipSnap homolog 1 OS=Homo sapiens GN=NIPSNAP1 PE=1 SV=1 |
|  | **P18859\|ATP5J_HUMAN** | ATP synthase-coupling factor 6, mitochondrial OS=Homo sapiens GN=ATP5J PE=1 SV=1 |
|  | **P24539\|AT5F1_HUMAN** | ATP synthase subunit b, mitochondrial OS=Homo sapiens GN=ATP5F1 PE=1 SV=2 |
|  | **Q00266\|METK1_HUMAN** | S-adenosylmethionine synthetase isoform type-1 OS=Homo sapiens GN=MAT1A PE=1 SV=2 |
|  | **P07339\|CATD_HUMAN** | Cathepsin D OS=Homo sapiens GN=CTSD PE=1 SV=1 |
|  | **P28288\|ABCD3_HUMAN** | ATP-binding cassette sub-family D member 3 OS=Homo sapiens GN=ABCD3 PE=1 SV=1 |
|  | **P20674\|COX5A_HUMAN** | Cytochrome c oxidase subunit 5A, mitochondrial OS=Homo sapiens GN=COX5A PE=1 SV=2 |
|  | **P55157\|MTP_HUMAN** | Microsomal triglyceride transfer protein large subunit OS=Homo sapiens GN=MTTP PE=1 SV=1 |
|  | **P06133\|UD2B4_HUMAN** | UDP-glucuronosyltransferase 2B4 OS=Homo sapiens GN=UGT2B4 PE=1 SV=2 |
|  | **P51648\|AL3A2_HUMAN** | Fatty aldehyde dehydrogenase OS=Homo sapiens GN=ALDH3A2 PE=1 SV=1 |
|  | **P16615\|AT2A2_HUMAN** | Sarcoplasmic/endoplasmic reticulum calcium ATPase 2 OS=Homo sapiens GN=ATP2A2 PE=1 SV=1 |
|  | **O75874\|IDHC_HUMAN** | Isocitrate dehydrogenase [NADP] cytoplasmic OS=Homo sapiens GN=IDH1 PE=1 SV=2 |
|  | **P21980\|TGM2_HUMAN** | Protein-glutamine gamma-glutamyltransferase 2 OS=Homo sapiens GN=TGM2 PE=1 SV=2 |
|  | **P09110\|THIK_HUMAN** | 3-ketoacyl-CoA thiolase, peroxisomal OS=Homo sapiens GN=ACAA1 PE=1 SV=2 |
|  | **P68871\|HBB_HUMAN** | Hemoglobin subunit beta OS=Homo sapiens GN=HBB PE=1 SV=2 |
|  | **Q08477\|CP4F3_HUMAN** | Leukotriene-B(4) omega-hydroxylase 2 OS=Homo sapiens GN=CYP4F3 PE=2 SV=2 |
|  | **A5A3E0\|POTEF_HUMAN** | POTE ankyrin domain family member F OS=Homo sapiens GN=POTEF PE=1 SV=2 |
|  | **Q5VTE0\|EF1A3_HUMAN** | Putative elongation factor 1-alpha-like 3 OS=Homo sapiens GN=EEF1AL3 PE=5 SV=1 |
|  | **P00441\|SODC_HUMAN** | Superoxide dismutase [Cu-Zn] OS=Homo sapiens GN=SOD1 PE=1 SV=2 |
|  | **P13929\|ENOB_HUMAN** | Beta-enolase OS=Homo sapiens GN=ENO3 PE=1 SV=4 |
|  | **Q00325\|MPCP_HUMAN** | Phosphate carrier protein, mitochondrial OS=Homo sapiens GN=SLC25A3 PE=1 SV=2 |
|  | **P48047\|ATPO_HUMAN** | ATP synthase subunit O, mitochondrial OS=Homo sapiens GN=ATP5O PE=1 SV=1 |
|  | **Q9H9B4\|SFXN1_HUMAN** | Sideroflexin-1 OS=Homo sapiens GN=SFXN1 PE=1 SV=4 |
|  | **P04792\|HSPB1_HUMAN** | Heat shock protein beta-1 OS=Homo sapiens GN=HSPB1 PE=1 SV=2 |
|  | **Q92928\|RAB1C_HUMAN** | Putative Ras-related protein Rab-1C OS=Homo sapiens GN=RAB1C PE=5 SV=2 |
|  | **Q9H0U4\|RAB1B_HUMAN** | Ras-related protein Rab-1B OS=Homo sapiens GN=RAB1B PE=1 SV=1 |
|  | **P60660\|MYL6_HUMAN** | Myosin light polypeptide 6 OS=Homo sapiens GN=MYL6 PE=1 SV=2 |
|  | **Q96AG4\|LRC59_HUMAN** | Leucine-rich repeat-containing protein 59 OS=Homo sapiens GN=LRRC59 PE=1 SV=1 |
|  | **P01009\|A1AT_HUMAN** | Alpha-1-antitrypsin OS=Homo sapiens GN=SERPINA1 PE=1 SV=3 |
|  | **Q9NYU2\|UGGG1_HUMAN** | UDP-glucose:glycoprotein glucosyltransferase 1 OS=Homo sapiens GN=UGCGL1 PE=1 SV=3 |
|  | **P30040\|ERP29_HUMAN** | Endoplasmic reticulum protein ERp29 OS=Homo sapiens GN=ERP29 PE=1 SV=4 |
|  | **P08133\|ANXA6_HUMAN** | Annexin A6 OS=Homo sapiens GN=ANXA6 PE=1 SV=3 |
|  | **Q00796\|DHSO_HUMAN** | Sorbitol dehydrogenase OS=Homo sapiens GN=SORD PE=1 SV=3 |
|  | **P36542\|ATPG_HUMAN** | ATP synthase subunit gamma, mitochondrial OS=Homo sapiens GN=ATP5C1 PE=1 SV=1 |
|  | **Q7Z5P4\|DHB13_HUMAN** | 17-beta hydroxysteroid dehydrogenase 13 OS=Homo sapiens GN=HSD17B13 PE=2 SV=1 |
|  | **Q6DD88\|ATLA3_HUMAN** | Atlastin-3 OS=Homo sapiens GN=ATL3 PE=1 SV=1 |
|  | **P36269\|GGT5_HUMAN** | Gamma-glutamyltransferase 5 OS=Homo sapiens GN=GGT5 PE=1 SV=2 |
|  | **Q53GQ0\|DHB12_HUMAN** | Estradiol 17-beta-dehydrogenase 12 OS=Homo sapiens GN=HSD17B12 PE=1 SV=2 |
|  | **P00387\|NB5R3_HUMAN** | NADH-cytochrome b5 reductase 3 OS=Homo sapiens GN=CYB5R3 PE=1 SV=3 |
|  | **P54819\|KAD2_HUMAN** | Adenylate kinase 2, mitochondrial OS=Homo sapiens GN=AK2 PE=1 SV=2 |
|  | **P45880\|VDAC2_HUMAN** | Voltage-dependent anion-selective channel protein 2 OS=Homo sapiens GN=VDAC2 PE=1 SV=2 |
|  | **Q562R1\|ACTBL_HUMAN** | Beta-actin-like protein 2 OS=Homo sapiens GN=ACTBL2 PE=1 SV=2 |
|  | **P60174\|TPIS_HUMAN** | Triosephosphate isomerase OS=Homo sapiens GN=TPI1 PE=1 SV=2 |
|  | **Q3LXA3\|DHAK_HUMAN** | Bifunctional ATP-dependent dihydroxyacetone kinase/FAD-AMP lyase (cyclizing) OS=Homo sapiens GN=DAK PE=1 SV=1 |
|  | **P20813\|CP2B6_HUMAN** | Cytochrome P450 2B6 OS=Homo sapiens GN=CYP2B6 PE=1 SV=1 |
|  | **Q16891\|IMMT_HUMAN** | Mitochondrial inner membrane protein OS=Homo sapiens GN=IMMT PE=1 SV=1 |
|  | **P54652\|HSP72_HUMAN** | Heat shock-related 70 kDa protein 2 OS=Homo sapiens GN=HSPA2 PE=1 SV=1 |
|  | **P62937\|PPIA_HUMAN** | Peptidyl-prolyl cis-trans isomerase A OS=Homo sapiens GN=PPIA PE=1 SV=2 |
|  | **P51570\|GALK1_HUMAN** | Galactokinase OS=Homo sapiens GN=GALK1 PE=1 SV=1 |
|  | **P34931\|HS71L_HUMAN** | Heat shock 70 kDa protein 1L OS=Homo sapiens GN=HSPA1L PE=1 SV=2 |
|  | **P30084\|ECHM_HUMAN** | Enoyl-CoA hydratase, mitochondrial OS=Homo sapiens GN=ECHS1 PE=1 SV=4 |
|  | **P17858\|K6PL_HUMAN** | 6-phosphofructokinase, liver type OS=Homo sapiens GN=PFKL PE=1 SV=6 |
|  | **P30050\|RL12_HUMAN** | 60S ribosomal protein L12 OS=Homo sapiens GN=RPL12 PE=1 SV=1 |
|  | **O43795\|MYO1B_HUMAN** | Myosin-Ib OS=Homo sapiens GN=MYO1B PE=1 SV=3 |
|  | **Q96A33\|CCD47_HUMAN** | Coiled-coil domain-containing protein 47 OS=Homo sapiens GN=CCDC47 PE=1 SV=1 |
|  | **Q9UJM8\|HAOX1_HUMAN** | Hydroxyacid oxidase 1 OS=Homo sapiens GN=HAO1 PE=1 SV=1 |
|  | **Q9UBX3\|DIC_HUMAN** | Mitochondrial dicarboxylate carrier OS=Homo sapiens GN=SLC25A10 PE=1 SV=2 |
|  | **P63244\|GBLP_HUMAN** | Guanine nucleotide-binding protein subunit beta-2-like 1 OS=Homo sapiens GN=GNB2L1 PE=1 SV=3 |
|  | **P27105\|STOM_HUMAN** | Erythrocyte band 7 integral membrane protein OS=Homo sapiens GN=STOM PE=1 SV=3 |
|  | **P23284\|PPIB_HUMAN** | Peptidyl-prolyl cis-trans isomerase B OS=Homo sapiens GN=PPIB PE=1 SV=2 |
|  | **P50225\|ST1A1_HUMAN** | Sulfotransferase 1A1 OS=Homo sapiens GN=SULT1A1 PE=1 SV=2 |
|  | **P50226\|ST1A2_HUMAN** | Sulfotransferase 1A2 OS=Homo sapiens GN=SULT1A2 PE=1 SV=1 |
|  | **O75947\|ATP5H_HUMAN** | ATP synthase subunit d, mitochondrial OS=Homo sapiens GN=ATP5H PE=1 SV=3 |
|  | **P09104\|ENOG_HUMAN** | Gamma-enolase OS=Homo sapiens GN=ENO2 PE=1 SV=3 |
|  | **P02656\|APOC3_HUMAN** | Apolipoprotein C-III OS=Homo sapiens GN=APOC3 PE=1 SV=1 |
|  | **Q00610\|CLH1_HUMAN** | Clathrin heavy chain 1 OS=Homo sapiens GN=CLTC PE=1 SV=5 |
|  | **P48735\|IDHP_HUMAN** | Isocitrate dehydrogenase [NADP], mitochondrial OS=Homo sapiens GN=IDH2 PE=1 SV=2 |
|  | **P53621\|COPA_HUMAN** | Coatomer subunit alpha OS=Homo sapiens GN=COPA PE=1 SV=2 |
|  | **P27169\|PON1_HUMAN** | Serum paraoxonase/arylesterase 1 OS=Homo sapiens GN=PON1 PE=1 SV=2 |
|  | **O14950\|ML12B_HUMAN** | Myosin regulatory light chain 12B OS=Homo sapiens GN=MYL12B PE=1 SV=2 |
|  | **P19105\|ML12A_HUMAN** | Myosin regulatory light chain 12A OS=Homo sapiens GN=MYL12A PE=1 SV=2 |
|  | **P30048\|PRDX3_HUMAN** | Thioredoxin-dependent peroxide reductase, mitochondrial OS=Homo sapiens GN=PRDX3 PE=1 SV=3 |
|  | **P08574\|CY1_HUMAN** | Cytochrome c1, heme protein, mitochondrial OS=Homo sapiens GN=CYC1 PE=1 SV=2 |
|  | **P34896\|GLYC_HUMAN** | Serine hydroxymethyltransferase, cytosolic OS=Homo sapiens GN=SHMT1 PE=1 SV=1 |
|  | **P20853\|CP2A7_HUMAN** | Cytochrome P450 2A7 OS=Homo sapiens GN=CYP2A7 PE=2 SV=2 |
|  | **P09210\|GSTA2_HUMAN** | Glutathione S-transferase A2 OS=Homo sapiens GN=GSTA2 PE=1 SV=4 |
|  | **Q8NBQ5\|DHB11_HUMAN** | Estradiol 17-beta-dehydrogenase 11 OS=Homo sapiens GN=HSD17B11 PE=1 SV=2 |
|  | **P20815\|CP3A5_HUMAN** | Cytochrome P450 3A5 OS=Homo sapiens GN=CYP3A5 PE=1 SV=1 |
|  | **P53007\|TXTP_HUMAN** | Tricarboxylate transport protein, mitochondrial OS=Homo sapiens GN=SLC25A1 PE=1 SV=2 |
|  | **Q9H8H3\|MET7A_HUMAN** | Methyltransferase-like protein 7A OS=Homo sapiens GN=METTL7A PE=1 SV=1 |
|  | **Q9BYX7\|ACTBM_HUMAN** | Beta-actin-like protein 3 OS=Homo sapiens GN=ACTBL3 PE=1 SV=1 |
|  | **P36537\|UDB10_HUMAN** | UDP-glucuronosyltransferase 2B10 OS=Homo sapiens GN=UGT2B10 PE=1 SV=1 |
|  | **P18124\|RL7_HUMAN** | 60S ribosomal protein L7 OS=Homo sapiens GN=RPL7 PE=1 SV=1 |
|  | **P54920\|SNAA_HUMAN** | Alpha-soluble NSF attachment protein OS=Homo sapiens GN=NAPA PE=1 SV=3 |
|  | **P50416\|CPT1A_HUMAN** | Carnitine O-palmitoyltransferase 1, liver isoform OS=Homo sapiens GN=CPT1A PE=1 SV=2 |
|  | **P48449\|ERG7_HUMAN** | Lanosterol synthase OS=Homo sapiens GN=LSS PE=1 SV=1 |
|  | **O75396\|SC22B_HUMAN** | Vesicle-trafficking protein SEC22b OS=Homo sapiens GN=SEC22B PE=1 SV=3 |
|  | **P08107\|HSP71_HUMAN** | Heat shock 70 kDa protein 1 OS=Homo sapiens GN=HSPA1A PE=1 SV=5 |
|  | **Q9HBI6\|CP4FB_HUMAN** | Cytochrome P450 4F11 OS=Homo sapiens GN=CYP4F11 PE=2 SV=2 |
|  | **P30041\|PRDX6_HUMAN** | Peroxiredoxin-6 OS=Homo sapiens GN=PRDX6 PE=1 SV=3 |
|  | **Q07065\|CKAP4_HUMAN** | Cytoskeleton-associated protein 4 OS=Homo sapiens GN=CKAP4 PE=1 SV=2 |
|  | **P05387\|RLA2_HUMAN** | 60S acidic ribosomal protein P2 OS=Homo sapiens GN=RPLP2 PE=1 SV=1 |
|  | **Q6UX53\|MET7B_HUMAN** | Methyltransferase-like protein 7B OS=Homo sapiens GN=METTL7B PE=2 SV=2 |
|  | **P33261\|CP2CJ_HUMAN** | Cytochrome P450 2C19 OS=Homo sapiens GN=CYP2C19 PE=1 SV=3 |
|  | **P33778\|H2B1B_HUMAN** | Histone H2B type 1-B OS=Homo sapiens GN=HIST1H2BB PE=1 SV=2 |
|  | **P23527\|H2B1O_HUMAN** | Histone H2B type 1-O OS=Homo sapiens GN=HIST1H2BO PE=1 SV=3 |
|  | **Q99879\|H2B1M_HUMAN** | Histone H2B type 1-M OS=Homo sapiens GN=HIST1H2BM PE=1 SV=3 |
|  | **Q99880\|H2B1L_HUMAN** | Histone H2B type 1-L OS=Homo sapiens GN=HIST1H2BL PE=1 SV=3 |
|  | **Q8N257\|H2B3B_HUMAN** | Histone H2B type 3-B OS=Homo sapiens GN=HIST3H2BB PE=1 SV=3 |
|  | **P57053\|H2BFS_HUMAN** | Histone H2B type F-S OS=Homo sapiens GN=H2BFS PE=1 SV=2 |
|  | **P58876\|H2B1D_HUMAN** | Histone H2B type 1-D OS=Homo sapiens GN=HIST1H2BD PE=1 SV=2 |
|  | **P49419\|AL7A1_HUMAN** | Alpha-aminoadipic semialdehyde dehydrogenase OS=Homo sapiens GN=ALDH7A1 PE=1 SV=4 |
|  | **P35232\|PHB_HUMAN** | Prohibitin OS=Homo sapiens GN=PHB PE=1 SV=1 |
|  | **Q15166\|PON3_HUMAN** | Serum paraoxonase/lactonase 3 OS=Homo sapiens GN=PON3 PE=1 SV=3 |
|  | **Q06520\|ST2A1_HUMAN** | Bile salt sulfotransferase OS=Homo sapiens GN=SULT2A1 PE=1 SV=3 |
|  | **P49755\|TMEDA_HUMAN** | Transmembrane emp24 domain-containing protein 10 OS=Homo sapiens GN=TMED10 PE=1 SV=2 |
|  | **Q68CK6\|ACS2B_HUMAN** | Acyl-coenzyme A synthetase ACSM2B, mitochondrial OS=Homo sapiens GN=ACSM2B PE=1 SV=1 |
|  | **Q08AH3\|ACS2A_HUMAN** | Acyl-coenzyme A synthetase ACSM2A, mitochondrial OS=Homo sapiens GN=ACSM2A PE=1 SV=2 |
|  | **P62424\|RL7A_HUMAN** | 60S ribosomal protein L7a OS=Homo sapiens GN=RPL7A PE=1 SV=2 |
|  | **Q9NVH1\|DJC11_HUMAN** | DnaJ homolog subfamily C member 11 OS=Homo sapiens GN=DNAJC11 PE=1 SV=2 |
|  | **Q12907\|LMAN2_HUMAN** | Vesicular integral-membrane protein VIP36 OS=Homo sapiens GN=LMAN2 PE=1 SV=1 |
|  | **P49327\|FAS_HUMAN** | Fatty acid synthase OS=Homo sapiens GN=FASN PE=1 SV=2 |
|  | **Q9Y5U8\|BR44L_HUMAN** | Brain protein 44-like protein OS=Homo sapiens GN=BRP44L PE=1 SV=1 |
|  | **Q9H853\|TBA4B_HUMAN** | Putative tubulin-like protein alpha-4B OS=Homo sapiens GN=TUBA4B PE=5 SV=2 |
|  | **P14854\|CX6B1_HUMAN** | Cytochrome c oxidase subunit 6B1 OS=Homo sapiens GN=COX6B1 PE=1 SV=2 |
|  | **P62701\|RS4X_HUMAN** | 40S ribosomal protein S4, X isoform OS=Homo sapiens GN=RPS4X PE=1 SV=2 |
|  | **P62820\|RAB1A_HUMAN** | Ras-related protein Rab-1A OS=Homo sapiens GN=RAB1A PE=1 SV=3 |
|  | **P02042\|HBD_HUMAN** | Hemoglobin subunit delta OS=Homo sapiens GN=HBD PE=1 SV=2 |
|  | **Q5K4L6\|S27A3_HUMAN** | Long-chain fatty acid transport protein 3 OS=Homo sapiens GN=SLC27A3 PE=2 SV=3 |
|  | **P62888\|RL30_HUMAN** | 60S ribosomal protein L30 OS=Homo sapiens GN=RPL30 PE=1 SV=2 |
|  | **O43678\|NDUA2_HUMAN** | NADH dehydrogenase [ubiquinone] 1 alpha subcomplex subunit 2 OS=Homo sapiens GN=NDUFA2 PE=1 SV=3 |
|  | **P43490\|NAMPT_HUMAN** | Nicotinamide phosphoribosyltransferase OS=Homo sapiens GN=NAMPT PE=1 SV=1 |
|  | **Q15043\|S39AE_HUMAN** | Zinc transporter ZIP14 OS=Homo sapiens GN=SLC39A14 PE=1 SV=2 |
|  | **P33260\|CP2CI_HUMAN** | Cytochrome P450 2C18 OS=Homo sapiens GN=CYP2C18 PE=1 SV=3 |
|  | **Q8NHW5\|RLA0L_HUMAN** | 60S acidic ribosomal protein P0-like OS=Homo sapiens PE=2 SV=1 |
|  | **P40926\|MDHM_HUMAN** | Malate dehydrogenase, mitochondrial OS=Homo sapiens GN=MDH2 PE=1 SV=3 |
|  | **P24844\|MYL9_HUMAN** | Myosin regulatory light polypeptide 9 OS=Homo sapiens GN=MYL9 PE=1 SV=4 |
|  | **Q9HCN8\|SDF2L_HUMAN** | Stromal cell-derived factor 2-like protein 1 OS=Homo sapiens GN=SDF2L1 PE=1 SV=2 |
|  | **P09467\|F16P1_HUMAN** | Fructose-1,6-bisphosphatase 1 OS=Homo sapiens GN=FBP1 PE=1 SV=4 |
|  | **Q9UJZ1\|STML2_HUMAN** | Stomatin-like protein 2 OS=Homo sapiens GN=STOML2 PE=1 SV=1 |
|  | **Q8NBS9\|TXND5_HUMAN** | Thioredoxin domain-containing protein 5 OS=Homo sapiens GN=TXNDC5 PE=1 SV=2 |
|  | **P39023\|RL3_HUMAN** | 60S ribosomal protein L3 OS=Homo sapiens GN=RPL3 PE=1 SV=2 |
|  | **P08238\|HS90B_HUMAN** | Heat shock protein HSP 90-beta OS=Homo sapiens GN=HSP90AB1 PE=1 SV=4 |
|  | **P84098\|RL19_HUMAN** | 60S ribosomal protein L19 OS=Homo sapiens GN=RPL19 PE=1 SV=1 |
|  | **P50224\|ST1A3_HUMAN** | Sulfotransferase 1A3/1A4 OS=Homo sapiens GN=SULT1A3 PE=1 SV=1 |
|  | **O00264\|PGRC1_HUMAN** | Membrane-associated progesterone receptor component 1 OS=Homo sapiens GN=PGRMC1 PE=1 SV=3 |
|  | **P61247\|RS3A_HUMAN** | 40S ribosomal protein S3a OS=Homo sapiens GN=RPS3A PE=1 SV=2 |
|  | **P52758\|UK114_HUMAN** | Ribonuclease UK114 OS=Homo sapiens GN=HRSP12 PE=1 SV=1 |
|  | **P63104\|1433Z_HUMAN** | 14-3-3 protein zeta/delta OS=Homo sapiens GN=YWHAZ PE=1 SV=1 |
|  | **P37059\|DHB2_HUMAN** | Estradiol 17-beta-dehydrogenase 2 OS=Homo sapiens GN=HSD17B2 PE=1 SV=1 |
|  | **Q03154\|ACY1_HUMAN** | Aminoacylase-1 OS=Homo sapiens GN=ACY1 PE=1 SV=1 |
|  | **P49257\|LMAN1_HUMAN** | Protein ERGIC-53 OS=Homo sapiens GN=LMAN1 PE=1 SV=2 |
|  | **O14983\|AT2A1_HUMAN** | Sarcoplasmic/endoplasmic reticulum calcium ATPase 1 OS=Homo sapiens GN=ATP2A1 PE=1 SV=1 |
|  | **Q15738\|NSDHL_HUMAN** | Sterol-4-alpha-carboxylate 3-dehydrogenase, decarboxylating OS=Homo sapiens GN=NSDHL PE=1 SV=2 |
|  | **P36578\|RL4_HUMAN** | 60S ribosomal protein L4 OS=Homo sapiens GN=RPL4 PE=1 SV=5 |
|  | **P62158\|CALM_HUMAN** | Calmodulin OS=Homo sapiens GN=CALM1 PE=1 SV=2 |
|  | **Q9UHG3\|PCYOX_HUMAN** | Prenylcysteine oxidase 1 OS=Homo sapiens GN=PCYOX1 PE=1 SV=3 |
|  | **P49411\|EFTU_HUMAN** | Elongation factor Tu, mitochondrial OS=Homo sapiens GN=TUFM PE=1 SV=2 |
|  | **Q15165\|PON2_HUMAN** | Serum paraoxonase/arylesterase 2 OS=Homo sapiens GN=PON2 PE=1 SV=2 |
|  | **Q16718\|NDUA5_HUMAN** | NADH dehydrogenase [ubiquinone] 1 alpha subcomplex subunit 5 OS=Homo sapiens GN=NDUFA5 PE=1 SV=3 |
|  | **Q9Y394\|DHRS7_HUMAN** | Dehydrogenase/reductase SDR family member 7 OS=Homo sapiens GN=DHRS7 PE=1 SV=1 |
|  | **Q92575\|UBXN4_HUMAN** | UBX domain-containing protein 4 OS=Homo sapiens GN=UBXN4 PE=1 SV=2 |
|  | **P23786\|CPT2_HUMAN** | Carnitine O-palmitoyltransferase 2, mitochondrial OS=Homo sapiens GN=CPT2 PE=1 SV=2 |
|  | **P50440\|GATM_HUMAN** | Glycine amidinotransferase, mitochondrial OS=Homo sapiens GN=GATM PE=1 SV=1 |
|  | **P16930\|FAAA_HUMAN** | Fumarylacetoacetase OS=Homo sapiens GN=FAH PE=1 SV=2 |
|  | **O00217\|NDUS8_HUMAN** | NADH dehydrogenase [ubiquinone] iron-sulfur protein 8, mitochondrial OS=Homo sapiens GN=NDUFS8 PE=1 SV=1 |
|  | **P69905\|HBA_HUMAN** | Hemoglobin subunit alpha OS=Homo sapiens GN=HBA1 PE=1 SV=2 |
|  | **P35606\|COPB2_HUMAN** | Coatomer subunit beta' OS=Homo sapiens GN=COPB2 PE=1 SV=2 |
|  | **Q9HDC9\|APMAP_HUMAN** | Adipocyte plasma membrane-associated protein OS=Homo sapiens GN=APMAP PE=1 SV=2 |
|  | **P17516\|AK1C4_HUMAN** | Aldo-keto reductase family 1 member C4 OS=Homo sapiens GN=AKR1C4 PE=1 SV=2 |
|  | **O75891\|FTHFD_HUMAN** | 10-formyltetrahydrofolate dehydrogenase OS=Homo sapiens GN=ALDH1L1 PE=1 SV=2 |
|  | **Q9UBQ7\|GRHPR_HUMAN** | Glyoxylate reductase/hydroxypyruvate reductase OS=Homo sapiens GN=GRHPR PE=1 SV=1 |
|  | **P09525\|ANXA4_HUMAN** | Annexin A4 OS=Homo sapiens GN=ANXA4 PE=1 SV=4 |
|  | **Q9Y277\|VDAC3_HUMAN** | Voltage-dependent anion-selective channel protein 3 OS=Homo sapiens GN=VDAC3 PE=1 SV=1 |
|  | **P32969\|RL9_HUMAN** | 60S ribosomal protein L9 OS=Homo sapiens GN=RPL9 PE=1 SV=1 |
|  | **P08910\|ABHD2_HUMAN** | Abhydrolase domain-containing protein 2 OS=Homo sapiens GN=ABHD2 PE=2 SV=1 |
|  | **P02787\|TRFE_HUMAN** | Serotransferrin OS=Homo sapiens GN=TF PE=1 SV=2 |
|  | **Q8N5K1\|CISD2_HUMAN** | CDGSH iron sulfur domain-containing protein 2 OS=Homo sapiens GN=CISD2 PE=1 SV=1 |
|  | **P00558\|PGK1_HUMAN** | Phosphoglycerate kinase 1 OS=Homo sapiens GN=PGK1 PE=1 SV=3 |
|  | **Q13724\|MOGS_HUMAN** | Mannosyl-oligosaccharide glucosidase OS=Homo sapiens GN=MOGS PE=1 SV=5 |
|  | **P38646\|GRP75_HUMAN** | Stress-70 protein, mitochondrial OS=Homo sapiens GN=HSPA9 PE=1 SV=2 |
|  | **Q99714\|HCD2_HUMAN** | 3-hydroxyacyl-CoA dehydrogenase type-2 OS=Homo sapiens GN=HSD17B10 PE=1 SV=3 |
|  | **Q9NQC3\|RTN4_HUMAN** | Reticulon-4 OS=Homo sapiens GN=RTN4 PE=1 SV=2 |
|  | **Q969Z3\|MOSC2_HUMAN** | MOSC domain-containing protein 2, mitochondrial OS=Homo sapiens GN=MOSC2 PE=1 SV=1 |
|  | **Q9BSE5\|SPEB_HUMAN** | Agmatinase, mitochondrial OS=Homo sapiens GN=AGMAT PE=1 SV=2 |
|  | **P17174\|AATC_HUMAN** | Aspartate aminotransferase, cytoplasmic OS=Homo sapiens GN=GOT1 PE=1 SV=3 |
|  | **O95182\|NDUA7_HUMAN** | NADH dehydrogenase [ubiquinone] 1 alpha subcomplex subunit 7 OS=Homo sapiens GN=NDUFA7 PE=1 SV=3 |
|  | **P08571\|CD14_HUMAN** | Monocyte differentiation antigen CD14 OS=Homo sapiens GN=CD14 PE=1 SV=2 |
|  | **Q9Y6C9\|MTCH2_HUMAN** | Mitochondrial carrier homolog 2 OS=Homo sapiens GN=MTCH2 PE=1 SV=1 |
|  | **P04004\|VTNC_HUMAN** | Vitronectin OS=Homo sapiens GN=VTN PE=1 SV=1 |
|  | **Q04828\|AK1C1_HUMAN** | Aldo-keto reductase family 1 member C1 OS=Homo sapiens GN=AKR1C1 PE=1 SV=1 |
|  | **P36871\|PGM1_HUMAN** | Phosphoglucomutase-1 OS=Homo sapiens GN=PGM1 PE=1 SV=3 |
|  | **O75306\|NDUS2_HUMAN** | NADH dehydrogenase [ubiquinone] iron-sulfur protein 2, mitochondrial OS=Homo sapiens GN=NDUFS2 PE=1 SV=2 |
|  | **P22307\|NLTP_HUMAN** | Non-specific lipid-transfer protein OS=Homo sapiens GN=SCP2 PE=1 SV=2 |
|  | **Q07954\|LRP1_HUMAN** | Prolow-density lipoprotein receptor-related protein 1 OS=Homo sapiens GN=LRP1 PE=1 SV=1 |
|  | **P60033\|CD81_HUMAN** | CD81 antigen OS=Homo sapiens GN=CD81 PE=1 SV=1 |
|  | **P17066\|HSP76_HUMAN** | Heat shock 70 kDa protein 6 OS=Homo sapiens GN=HSPA6 PE=1 SV=2 |
|  | **P05556\|ITB1_HUMAN** | Integrin beta-1 OS=Homo sapiens GN=ITGB1 PE=1 SV=2 |
|  | **O95479\|G6PE_HUMAN** | GDH/6PGL endoplasmic bifunctional protein OS=Homo sapiens GN=H6PD PE=1 SV=2 |
|  | **P39656\|OST48_HUMAN** | Dolichyl-diphosphooligosaccharide--protein glycosyltransferase 48 kDa subunit OS=Homo sapiens GN=DDOST PE=1 SV=4 |
|  | **Q86UE4\|LYRIC_HUMAN** | Protein LYRIC OS=Homo sapiens GN=MTDH PE=1 SV=2 |
|  | **O94826\|TOM70_HUMAN** | Mitochondrial import receptor subunit TOM70 OS=Homo sapiens GN=TOMM70A PE=1 SV=1 |
|  | **P80404\|GABT_HUMAN** | 4-aminobutyrate aminotransferase, mitochondrial OS=Homo sapiens GN=ABAT PE=1 SV=3 |
|  | **P49448\|DHE4_HUMAN** | Glutamate dehydrogenase 2, mitochondrial OS=Homo sapiens GN=GLUD2 PE=1 SV=2 |
|  | **Q9HCC0\|MCCB_HUMAN** | Methylcrotonoyl-CoA carboxylase beta chain, mitochondrial OS=Homo sapiens GN=MCCC2 PE=1 SV=1 |
|  | **Q15392\|DHC24_HUMAN** | 24-dehydrocholesterol reductase OS=Homo sapiens GN=DHCR24 PE=1 SV=2 |
|  | **O15229\|KMO_HUMAN** | Kynurenine 3-monooxygenase OS=Homo sapiens GN=KMO PE=1 SV=2 |
|  | **O94905\|ERLN2_HUMAN** | Erlin-2 OS=Homo sapiens GN=ERLIN2 PE=1 SV=1 |
|  | **P31153\|METK2_HUMAN** | S-adenosylmethionine synthetase isoform type-2 OS=Homo sapiens GN=MAT2A PE=1 SV=1 |
|  | **P51572\|BAP31_HUMAN** | B-cell receptor-associated protein 31 OS=Homo sapiens GN=BCAP31 PE=1 SV=3 |
|  | **Q6NUM9\|RETST_HUMAN** | All-trans-retinol 13,14-reductase OS=Homo sapiens GN=RETSAT PE=2 SV=2 |
|  | **O75881\|CP7B1_HUMAN** | 25-hydroxycholesterol 7-alpha-hydroxylase OS=Homo sapiens GN=CYP7B1 PE=1 SV=2 |
|  | **P32754\|HPPD_HUMAN** | 4-hydroxyphenylpyruvate dioxygenase OS=Homo sapiens GN=HPD PE=1 SV=2 |
|  | **P14927\|QCR7_HUMAN** | Cytochrome b-c1 complex subunit 7 OS=Homo sapiens GN=UQCRB PE=1 SV=2 |
|  | **Q93084\|AT2A3_HUMAN** | Sarcoplasmic/endoplasmic reticulum calcium ATPase 3 OS=Homo sapiens GN=ATP2A3 PE=1 SV=2 |
|  | **Q9P0L0\|VAPA_HUMAN** | Vesicle-associated membrane protein-associated protein A OS=Homo sapiens GN=VAPA PE=1 SV=3 |
|  | **P13073\|COX41_HUMAN** | Cytochrome c oxidase subunit 4 isoform 1, mitochondrial OS=Homo sapiens GN=COX4I1 PE=1 SV=1 |
|  | **P11216\|PYGB_HUMAN** | Glycogen phosphorylase, brain form OS=Homo sapiens GN=PYGB PE=1 SV=5 |
|  | **P07148\|FABPL_HUMAN** | Fatty acid-binding protein, liver OS=Homo sapiens GN=FABP1 PE=1 SV=1 |
|  | **P05023\|AT1A1_HUMAN** | Sodium/potassium-transporting ATPase subunit alpha-1 OS=Homo sapiens GN=ATP1A1 PE=1 SV=1 |
|  | **P04003\|C4BPA_HUMAN** | C4b-binding protein alpha chain OS=Homo sapiens GN=C4BPA PE=1 SV=2 |
|  | **P13639\|EF2_HUMAN** | Elongation factor 2 OS=Homo sapiens GN=EEF2 PE=1 SV=4 |
|  | **P37802\|TAGL2_HUMAN** | Transgelin-2 OS=Homo sapiens GN=TAGLN2 PE=1 SV=3 |
|  | **P61006\|RAB8A_HUMAN** | Ras-related protein Rab-8A OS=Homo sapiens GN=RAB8A PE=1 SV=1 |
|  | **P08865\|RSSA_HUMAN** | 40S ribosomal protein SA OS=Homo sapiens GN=RPSA PE=1 SV=4 |
|  | **P00403\|COX2_HUMAN** | Cytochrome c oxidase subunit 2 OS=Homo sapiens GN=MT-CO2 PE=1 SV=1 |
|  | **P05089\|ARGI1_HUMAN** | Arginase-1 OS=Homo sapiens GN=ARG1 PE=1 SV=2 |
|  | **P07954\|FUMH_HUMAN** | Fumarate hydratase, mitochondrial OS=Homo sapiens GN=FH PE=1 SV=3 |
|  | **P07307\|ASGR2_HUMAN** | Asialoglycoprotein receptor 2 OS=Homo sapiens GN=ASGR2 PE=1 SV=2 |
|  | **Q9UBS4\|DJB11_HUMAN** | DnaJ homolog subfamily B member 11 OS=Homo sapiens GN=DNAJB11 PE=1 SV=1 |
|  | **O75746\|CMC1_HUMAN** | Calcium-binding mitochondrial carrier protein Aralar1 OS=Homo sapiens GN=SLC25A12 PE=1 SV=2 |
|  | **P14314\|GLU2B_HUMAN** | Glucosidase 2 subunit beta OS=Homo sapiens GN=PRKCSH PE=1 SV=2 |
|  | **P55072\|TERA_HUMAN** | Transitional endoplasmic reticulum ATPase OS=Homo sapiens GN=VCP PE=1 SV=4 |
|  | **P61224\|RAP1B_HUMAN** | Ras-related protein Rap-1b OS=Homo sapiens GN=RAP1B PE=1 SV=1 |
|  | **A6NIZ1\|RP1BL_HUMAN** | Ras-related protein Rap-1b-like protein OS=Homo sapiens PE=2 SV=1 |
|  | **P07306\|ASGR1_HUMAN** | Asialoglycoprotein receptor 1 OS=Homo sapiens GN=ASGR1 PE=1 SV=2 |
|  | **Q14117\|DPYS_HUMAN** | Dihydropyrimidinase OS=Homo sapiens GN=DPYS PE=1 SV=1 |
|  | **P62280\|RS11_HUMAN** | 40S ribosomal protein S11 OS=Homo sapiens GN=RPS11 PE=1 SV=3 |
|  | **P00338\|LDHA_HUMAN** | L-lactate dehydrogenase A chain OS=Homo sapiens GN=LDHA PE=1 SV=2 |
|  | **P07900\|HS90A_HUMAN** | Heat shock protein HSP 90-alpha OS=Homo sapiens GN=HSP90AA1 PE=1 SV=5 |
|  | **P40261\|NNMT_HUMAN** | Nicotinamide N-methyltransferase OS=Homo sapiens GN=NNMT PE=1 SV=1 |
|  | **Q14165\|MLEC_HUMAN** | Malectin OS=Homo sapiens GN=MLEC PE=1 SV=1 |
|  | **P13637\|AT1A3_HUMAN** | Sodium/potassium-transporting ATPase subunit alpha-3 OS=Homo sapiens GN=ATP1A3 PE=1 SV=3 |
|  | **P11586\|C1TC_HUMAN** | C-1-tetrahydrofolate synthase, cytoplasmic OS=Homo sapiens GN=MTHFD1 PE=1 SV=3 |
|  | **Q9HCU5\|PREB_HUMAN** | Prolactin regulatory element-binding protein OS=Homo sapiens GN=PREB PE=1 SV=2 |
|  | **P52895\|AK1C2_HUMAN** | Aldo-keto reductase family 1 member C2 OS=Homo sapiens GN=AKR1C2 PE=1 SV=3 |
|  | **Q9UF12\|PROD2_HUMAN** | Probable proline dehydrogenase 2 OS=Homo sapiens GN=PRODH2 PE=2 SV=1 |
|  | **O15260\|SURF4_HUMAN** | Surfeit locus protein 4 OS=Homo sapiens GN=SURF4 PE=1 SV=3 |
|  | **P51571\|SSRD_HUMAN** | Translocon-associated protein subunit delta OS=Homo sapiens GN=SSR4 PE=1 SV=1 |
|  | **O75380\|NDUS6_HUMAN** | NADH dehydrogenase [ubiquinone] iron-sulfur protein 6, mitochondrial OS=Homo sapiens GN=NDUFS6 PE=1 SV=1 |
|  | **Q29963\|1C06_HUMAN** | HLA class I histocompatibility antigen, Cw-6 alpha chain OS=Homo sapiens GN=HLA-C PE=1 SV=2 |
|  | **P10321\|1C07_HUMAN** | HLA class I histocompatibility antigen, Cw-7 alpha chain OS=Homo sapiens GN=HLA-C PE=1 SV=3 |
|  | **O75795\|UDB17_HUMAN** | UDP-glucuronosyltransferase 2B17 OS=Homo sapiens GN=UGT2B17 PE=2 SV=1 |
|  | **P54855\|UDB15_HUMAN** | UDP-glucuronosyltransferase 2B15 OS=Homo sapiens GN=UGT2B15 PE=1 SV=2 |
|  | **Q02318\|CP27A_HUMAN** | Sterol 26-hydroxylase, mitochondrial OS=Homo sapiens GN=CYP27A1 PE=1 SV=1 |
|  | **P09382\|LEG1_HUMAN** | Galectin-1 OS=Homo sapiens GN=LGALS1 PE=1 SV=2 |
|  | **P35575\|G6PC_HUMAN** | Glucose-6-phosphatase OS=Homo sapiens GN=G6PC PE=1 SV=2 |
|  | **P62917\|RL8_HUMAN** | 60S ribosomal protein L8 OS=Homo sapiens GN=RPL8 PE=1 SV=2 |
|  | **P50993\|AT1A2_HUMAN** | Sodium/potassium-transporting ATPase subunit alpha-2 OS=Homo sapiens GN=ATP1A2 PE=1 SV=1 |
|  | **O75915\|PRAF3_HUMAN** | PRA1 family protein 3 OS=Homo sapiens GN=ARL6IP5 PE=1 SV=1 |
|  | **P61026\|RAB10_HUMAN** | Ras-related protein Rab-10 OS=Homo sapiens GN=RAB10 PE=1 SV=1 |
|  | **P07858\|CATB_HUMAN** | Cathepsin B OS=Homo sapiens GN=CTSB PE=1 SV=3 |
|  | **P01834\|IGKC_HUMAN** | Ig kappa chain C region OS=Homo sapiens GN=IGKC PE=1 SV=1 |
|  | **P62841\|RS15_HUMAN** | 40S ribosomal protein S15 OS=Homo sapiens GN=RPS15 PE=1 SV=2 |
|  | **Q14914\|PTGR1_HUMAN** | Prostaglandin reductase 1 OS=Homo sapiens GN=PTGR1 PE=1 SV=2 |
|  | **P62873\|GBB1_HUMAN** | Guanine nucleotide-binding protein G(I)/G(S)/G(T) subunit beta-1 OS=Homo sapiens GN=GNB1 PE=1 SV=3 |
|  | **Q9HCS2\|CP4FC_HUMAN** | Cytochrome P450 4F12 OS=Homo sapiens GN=CYP4F12 PE=1 SV=1 |
|  | **Q9NZ01\|TECR_HUMAN** | Trans-2,3-enoyl-CoA reductase OS=Homo sapiens GN=TECR PE=1 SV=1 |
|  | **Q96HA9\|PX11C_HUMAN** | Peroxisomal membrane protein 11C OS=Homo sapiens GN=PEX11G PE=1 SV=1 |
|  | **Q9H936\|GHC1_HUMAN** | Mitochondrial glutamate carrier 1 OS=Homo sapiens GN=SLC25A22 PE=1 SV=1 |
|  | **P29401\|TKT_HUMAN** | Transketolase OS=Homo sapiens GN=TKT PE=1 SV=3 |
|  | **Q96HR9\|REEP6_HUMAN** | Receptor expression-enhancing protein 6 OS=Homo sapiens GN=REEP6 PE=1 SV=1 |
|  | **O14773\|TPP1_HUMAN** | Tripeptidyl-peptidase 1 OS=Homo sapiens GN=TPP1 PE=1 SV=2 |
|  | **O43707\|ACTN4_HUMAN** | Alpha-actinin-4 OS=Homo sapiens GN=ACTN4 PE=1 SV=2 |
|  | **P05164\|PERM_HUMAN** | Myeloperoxidase OS=Homo sapiens GN=MPO PE=1 SV=1 |
|  | **P49189\|AL9A1_HUMAN** | 4-trimethylaminobutyraldehyde dehydrogenase OS=Homo sapiens GN=ALDH9A1 PE=1 SV=3 |
|  | **P62834\|RAP1A_HUMAN** | Ras-related protein Rap-1A OS=Homo sapiens GN=RAP1A PE=1 SV=1 |
|  | **P46782\|RS5_HUMAN** | 40S ribosomal protein S5 OS=Homo sapiens GN=RPS5 PE=1 SV=4 |
|  | **Q29865\|1C18_HUMAN** | HLA class I histocompatibility antigen, Cw-18 alpha chain OS=Homo sapiens GN=HLA-C PE=2 SV=1 |
|  | **P30499\|1C01_HUMAN** | HLA class I histocompatibility antigen, Cw-1 alpha chain OS=Homo sapiens GN=HLA-C PE=2 SV=1 |
|  | **P27635\|RL10_HUMAN** | 60S ribosomal protein L10 OS=Homo sapiens GN=RPL10 PE=1 SV=4 |
|  | **P30460\|1B08_HUMAN** | HLA class I histocompatibility antigen, B-8 alpha chain OS=Homo sapiens GN=HLA-B PE=1 SV=1 |
|  | **Q9NTJ5\|SAC1_HUMAN** | Phosphatidylinositide phosphatase SAC1 OS=Homo sapiens GN=SACM1L PE=1 SV=2 |
|  | **P56134\|ATPK_HUMAN** | ATP synthase subunit f, mitochondrial OS=Homo sapiens GN=ATP5J2 PE=1 SV=3 |
|  | **P21912\|DHSB_HUMAN** | Succinate dehydrogenase [ubiquinone] iron-sulfur subunit, mitochondrial OS=Homo sapiens GN=SDHB PE=1 SV=3 |
|  | **O14653\|GOSR2_HUMAN** | Golgi SNAP receptor complex member 2 OS=Homo sapiens GN=GOSR2 PE=1 SV=2 |
|  | **Q9H115\|SNAB_HUMAN** | Beta-soluble NSF attachment protein OS=Homo sapiens GN=NAPB PE=1 SV=2 |
|  | **P62241\|RS8_HUMAN** | 40S ribosomal protein S8 OS=Homo sapiens GN=RPS8 PE=1 SV=2 |
|  | **P35558\|PCKGC_HUMAN** | Phosphoenolpyruvate carboxykinase, cytosolic [GTP] OS=Homo sapiens GN=PCK1 PE=1 SV=3 |
|  | **Q96GC9\|TMM49_HUMAN** | Transmembrane protein 49 OS=Homo sapiens GN=TMEM49 PE=2 SV=1 |
|  | **P83111\|LACTB_HUMAN** | Serine beta-lactamase-like protein LACTB, mitochondrial OS=Homo sapiens GN=LACTB PE=1 SV=2 |
|  | **Q5VT66\|MOSC1_HUMAN** | MOSC domain-containing protein 1, mitochondrial OS=Homo sapiens GN=MOSC1 PE=1 SV=1 |
|  | **P54840\|GYS2_HUMAN** | Glycogen [starch] synthase, liver OS=Homo sapiens GN=GYS2 PE=1 SV=1 |
|  | **P21695\|GPDA_HUMAN** | Glycerol-3-phosphate dehydrogenase [NAD+], cytoplasmic OS=Homo sapiens GN=GPD1 PE=1 SV=4 |
|  | **Q13162\|PRDX4_HUMAN** | Peroxiredoxin-4 OS=Homo sapiens GN=PRDX4 PE=1 SV=1 |
|  | **O15173\|PGRC2_HUMAN** | Membrane-associated progesterone receptor component 2 OS=Homo sapiens GN=PGRMC2 PE=1 SV=1 |
|  | **Q6FI13\|H2A2A_HUMAN** | Histone H2A type 2-A OS=Homo sapiens GN=HIST2H2AA3 PE=1 SV=3 |
|  | **Q9NR31\|SAR1A_HUMAN** | GTP-binding protein SAR1a OS=Homo sapiens GN=SAR1A PE=1 SV=1 |
|  | **P49821\|NDUV1_HUMAN** | NADH dehydrogenase [ubiquinone] flavoprotein 1, mitochondrial OS=Homo sapiens GN=NDUFV1 PE=1 SV=4 |
|  | **Q86VB7\|C163A_HUMAN** | Scavenger receptor cysteine-rich type 1 protein M130 OS=Homo sapiens GN=CD163 PE=1 SV=1 |
|  | **Q9NP72\|RAB18_HUMAN** | Ras-related protein Rab-18 OS=Homo sapiens GN=RAB18 PE=1 SV=1 |
|  | **Q9H2A2\|AL8A1_HUMAN** | Aldehyde dehydrogenase family 8 member A1 OS=Homo sapiens GN=ALDH8A1 PE=1 SV=1 |
|  | **O95470\|SGPL1_HUMAN** | Sphingosine-1-phosphate lyase 1 OS=Homo sapiens GN=SGPL1 PE=1 SV=3 |
|  | **Q9BVK6\|TMED9_HUMAN** | Transmembrane emp24 domain-containing protein 9 OS=Homo sapiens GN=TMED9 PE=1 SV=2 |
|  | **Q9NX40\|OCAD1_HUMAN** | OCIA domain-containing protein 1 OS=Homo sapiens GN=OCIAD1 PE=1 SV=1 |
|  | **P61106\|RAB14_HUMAN** | Ras-related protein Rab-14 OS=Homo sapiens GN=RAB14 PE=1 SV=4 |
|  | **Q9H4B7\|TBB1_HUMAN** | Tubulin beta-1 chain OS=Homo sapiens GN=TUBB1 PE=1 SV=1 |
|  | **O43772\|MCAT_HUMAN** | Mitochondrial carnitine/acylcarnitine carrier protein OS=Homo sapiens GN=SLC25A20 PE=1 SV=1 |
|  | **Q9NR77\|PXMP2_HUMAN** | Peroxisomal membrane protein 2 OS=Homo sapiens GN=PXMP2 PE=1 SV=3 |
|  | **Q9GZP9\|DERL2_HUMAN** | Derlin-2 OS=Homo sapiens GN=DERL2 PE=1 SV=1 |
|  | **P61313\|RL15_HUMAN** | 60S ribosomal protein L15 OS=Homo sapiens GN=RPL15 PE=1 SV=2 |
|  | **P18085\|ARF4_HUMAN** | ADP-ribosylation factor 4 OS=Homo sapiens GN=ARF4 PE=1 SV=3 |
|  | **Q9Y2Q3\|GSTK1_HUMAN** | Glutathione S-transferase kappa 1 OS=Homo sapiens GN=GSTK1 PE=1 SV=3 |
|  | **P00450\|CERU_HUMAN** | Ceruloplasmin OS=Homo sapiens GN=CP PE=1 SV=1 |
|  | **Q6IAN0\|DRS7B_HUMAN** | Dehydrogenase/reductase SDR family member 7B OS=Homo sapiens GN=DHRS7B PE=1 SV=2 |
|  | **Q15800\|ERG25_HUMAN** | C-4 methylsterol oxidase OS=Homo sapiens GN=SC4MOL PE=1 SV=1 |
|  | **O00748\|EST2_HUMAN** | Carboxylesterase 2 OS=Homo sapiens GN=CES2 PE=1 SV=1 |
|  | **Q8TC12\|RDH11_HUMAN** | Retinol dehydrogenase 11 OS=Homo sapiens GN=RDH11 PE=1 SV=2 |
|  | **P61019\|RAB2A_HUMAN** | Ras-related protein Rab-2A OS=Homo sapiens GN=RAB2A PE=1 SV=1 |
|  | **P23396\|RS3_HUMAN** | 40S ribosomal protein S3 OS=Homo sapiens GN=RPS3 PE=1 SV=2 |
|  | **O15127\|SCAM2_HUMAN** | Secretory carrier-associated membrane protein 2 OS=Homo sapiens GN=SCAMP2 PE=1 SV=2 |
|  | **P47914\|RL29_HUMAN** | 60S ribosomal protein L29 OS=Homo sapiens GN=RPL29 PE=1 SV=2 |
|  | **Q6UW68\|TM205_HUMAN** | Transmembrane protein 205 OS=Homo sapiens GN=TMEM205 PE=1 SV=1 |
|  | **P52272\|HNRPM_HUMAN** | Heterogeneous nuclear ribonucleoprotein M OS=Homo sapiens GN=HNRNPM PE=1 SV=3 |
|  | **Q9Y6N5\|SQRD_HUMAN** | Sulfide:quinone oxidoreductase, mitochondrial OS=Homo sapiens GN=SQRDL PE=1 SV=1 |
|  | **P18463\|1B37_HUMAN** | HLA class I histocompatibility antigen, B-37 alpha chain OS=Homo sapiens GN=HLA-B PE=2 SV=1 |
|  | **Q29718\|1B82_HUMAN** | HLA class I histocompatibility antigen, B-82 alpha chain OS=Homo sapiens GN=HLA-B PE=2 SV=1 |
|  | **P30462\|1B14_HUMAN** | HLA class I histocompatibility antigen, B-14 alpha chain OS=Homo sapiens GN=HLA-B PE=1 SV=1 |
|  | **P30475\|1B39_HUMAN** | HLA class I histocompatibility antigen, B-39 alpha chain OS=Homo sapiens GN=HLA-B PE=2 SV=1 |
|  | **P30486\|1B48_HUMAN** | HLA class I histocompatibility antigen, B-48 alpha chain OS=Homo sapiens GN=HLA-B PE=2 SV=1 |
|  | **Q31610\|1B81_HUMAN** | HLA class I histocompatibility antigen, B-81 alpha chain OS=Homo sapiens GN=HLA-B PE=2 SV=1 |
|  | **O95497\|VNN1_HUMAN** | Pantetheinase OS=Homo sapiens GN=VNN1 PE=1 SV=2 |
|  | **P51149\|RAB7A_HUMAN** | Ras-related protein Rab-7a OS=Homo sapiens GN=RAB7A PE=1 SV=1 |
|  | **P32929\|CGL_HUMAN** | Cystathionine gamma-lyase OS=Homo sapiens GN=CTH PE=1 SV=3 |
|  | **Q14108\|SCRB2_HUMAN** | Lysosome membrane protein 2 OS=Homo sapiens GN=SCARB2 PE=1 SV=2 |
|  | **O75477\|ERLN1_HUMAN** | Erlin-1 OS=Homo sapiens GN=ERLIN1 PE=1 SV=1 |
|  | **O14880\|MGST3_HUMAN** | Microsomal glutathione S-transferase 3 OS=Homo sapiens GN=MGST3 PE=1 SV=1 |
|  | **P12814\|ACTN1_HUMAN** | Alpha-actinin-1 OS=Homo sapiens GN=ACTN1 PE=1 SV=2 |
|  | **P30038\|AL4A1_HUMAN** | Delta-1-pyrroline-5-carboxylate dehydrogenase, mitochondrial OS=Homo sapiens GN=ALDH4A1 PE=1 SV=3 |
|  | **Q7Z406\|MYH14_HUMAN** | Myosin-14 OS=Homo sapiens GN=MYH14 PE=1 SV=1 |
|  | **P16152\|CBR1_HUMAN** | Carbonyl reductase [NADPH] 1 OS=Homo sapiens GN=CBR1 PE=1 SV=3 |
|  | **Q9UN36\|NDRG2_HUMAN** | Protein NDRG2 OS=Homo sapiens GN=NDRG2 PE=1 SV=2 |
|  | **Q15005\|SPCS2_HUMAN** | Signal peptidase complex subunit 2 OS=Homo sapiens GN=SPCS2 PE=1 SV=3 |
|  | **P14406\|CX7A2_HUMAN** | Cytochrome c oxidase polypeptide 7A2, mitochondrial OS=Homo sapiens GN=COX7A2 PE=1 SV=1 |
|  | **P05090\|APOD_HUMAN** | Apolipoprotein D OS=Homo sapiens GN=APOD PE=1 SV=1 |
|  | **P18669\|PGAM1_HUMAN** | Phosphoglycerate mutase 1 OS=Homo sapiens GN=PGAM1 PE=1 SV=2 |
|  | **P43686\|PRS6B_HUMAN** | 26S protease regulatory subunit 6B OS=Homo sapiens GN=PSMC4 PE=1 SV=2 |
|  | **P12111\|CO6A3_HUMAN** | Collagen alpha-3(VI) chain OS=Homo sapiens GN=COL6A3 PE=1 SV=4 |
|  | **P16671\|CD36_HUMAN** | Platelet glycoprotein 4 OS=Homo sapiens GN=CD36 PE=1 SV=2 |
|  | **P62829\|RL23_HUMAN** | 60S ribosomal protein L23 OS=Homo sapiens GN=RPL23 PE=1 SV=1 |
|  | **P14649\|MYL6B_HUMAN** | Myosin light chain 6B OS=Homo sapiens GN=MYL6B PE=1 SV=1 |
|  | **Q7Z7H5\|TMED4_HUMAN** | Transmembrane emp24 domain-containing protein 4 OS=Homo sapiens GN=TMED4 PE=1 SV=1 |
|  | **Q16853\|AOC3_HUMAN** | Membrane primary amine oxidase OS=Homo sapiens GN=AOC3 PE=1 SV=3 |
|  | **P38435\|VKGC_HUMAN** | Vitamin K-dependent gamma-carboxylase OS=Homo sapiens GN=GGCX PE=1 SV=2 |
|  | **Q969X5\|ERGI1_HUMAN** | Endoplasmic reticulum-Golgi intermediate compartment protein 1 OS=Homo sapiens GN=ERGIC1 PE=1 SV=1 |
|  | **O75964\|ATP5L_HUMAN** | ATP synthase subunit g, mitochondrial OS=Homo sapiens GN=ATP5L PE=1 SV=3 |
|  | **P00395\|COX1_HUMAN** | Cytochrome c oxidase subunit 1 OS=Homo sapiens GN=MT-CO1 PE=1 SV=1 |
|  | **P21964\|COMT_HUMAN** | Catechol O-methyltransferase OS=Homo sapiens GN=COMT PE=1 SV=2 |
|  | **Q02252\|MMSA_HUMAN** | Methylmalonate-semialdehyde dehydrogenase [acylating], mitochondrial OS=Homo sapiens GN=ALDH6A1 PE=1 SV=2 |
|  | **P61204\|ARF3_HUMAN** | ADP-ribosylation factor 3 OS=Homo sapiens GN=ARF3 PE=1 SV=2 |
|  | **P84077\|ARF1_HUMAN** | ADP-ribosylation factor 1 OS=Homo sapiens GN=ARF1 PE=1 SV=2 |
|  | **P09417\|DHPR_HUMAN** | Dihydropteridine reductase OS=Homo sapiens GN=QDPR PE=1 SV=2 |
|  | **P30046\|DOPD_HUMAN** | D-dopachrome decarboxylase OS=Homo sapiens GN=DDT PE=1 SV=3 |
|  | **O14579\|COPE_HUMAN** | Coatomer subunit epsilon OS=Homo sapiens GN=COPE PE=1 SV=3 |
|  | **P35754\|GLRX1_HUMAN** | Glutaredoxin-1 OS=Homo sapiens GN=GLRX PE=1 SV=2 |
|  | **Q92930\|RAB8B_HUMAN** | Ras-related protein Rab-8B OS=Homo sapiens GN=RAB8B PE=1 SV=2 |
|  | **Q15286\|RAB35_HUMAN** | Ras-related protein Rab-35 OS=Homo sapiens GN=RAB35 PE=1 SV=1 |
|  | **P59190\|RAB15_HUMAN** | Ras-related protein Rab-15 OS=Homo sapiens GN=RAB15 PE=1 SV=1 |
|  | **Q14032\|BAAT_HUMAN** | Bile acid-CoA:amino acid N-acyltransferase OS=Homo sapiens GN=BAAT PE=1 SV=1 |
|  | **P02763\|A1AG1_HUMAN** | Alpha-1-acid glycoprotein 1 OS=Homo sapiens GN=ORM1 PE=1 SV=1 |
|  | **P14550\|AK1A1_HUMAN** | Alcohol dehydrogenase [NADP+] OS=Homo sapiens GN=AKR1A1 PE=1 SV=3 |
|  | **P45954\|ACDSB_HUMAN** | Short/branched chain specific acyl-CoA dehydrogenase, mitochondrial OS=Homo sapiens GN=ACADSB PE=1 SV=1 |
|  | **P61619\|S61A1_HUMAN** | Protein transport protein Sec61 subunit alpha isoform 1 OS=Homo sapiens GN=SEC61A1 PE=1 SV=2 |
|  | **Q07020\|RL18_HUMAN** | 60S ribosomal protein L18 OS=Homo sapiens GN=RPL18 PE=1 SV=2 |
|  | **Q9Y3E5\|PTH2_HUMAN** | Peptidyl-tRNA hydrolase 2, mitochondrial OS=Homo sapiens GN=PTRH2 PE=1 SV=1 |
|  | **O43175\|SERA_HUMAN** | D-3-phosphoglycerate dehydrogenase OS=Homo sapiens GN=PHGDH PE=1 SV=4 |
|  | **P11182\|ODB2_HUMAN** | Lipoamide acyltransferase component of branched-chain alpha-keto acid dehydrogenase complex, mitochondrial OS=Homo sapiens GN=DBT PE=1 SV=3 |
|  | **P30508\|1C12_HUMAN** | HLA class I histocompatibility antigen, Cw-12 alpha chain OS=Homo sapiens GN=HLA-C PE=1 SV=2 |
|  | **P04839\|CY24B_HUMAN** | Cytochrome b-245 heavy chain OS=Homo sapiens GN=CYBB PE=1 SV=2 |
|  | **P02753\|RET4_HUMAN** | Retinol-binding protein 4 OS=Homo sapiens GN=RBP4 PE=1 SV=3 |
|  | **O75489\|NDUS3_HUMAN** | NADH dehydrogenase [ubiquinone] iron-sulfur protein 3, mitochondrial OS=Homo sapiens GN=NDUFS3 PE=1 SV=1 |
|  | **P35268\|RL22_HUMAN** | 60S ribosomal protein L22 OS=Homo sapiens GN=RPL22 PE=1 SV=2 |
|  | **Q9BS26\|ERP44_HUMAN** | Endoplasmic reticulum resident protein ERp44 OS=Homo sapiens GN=ERP44 PE=1 SV=1 |
|  | **P13640\|MT1G_HUMAN** | Metallothionein-1G OS=Homo sapiens GN=MT1G PE=1 SV=2 |
|  | **P04732\|MT1E_HUMAN** | Metallothionein-1E OS=Homo sapiens GN=MT1E PE=1 SV=1 |
|  | **P26885\|FKBP2_HUMAN** | FK506-binding protein 2 OS=Homo sapiens GN=FKBP2 PE=1 SV=2 |
|  | **O75251\|NDUS7_HUMAN** | NADH dehydrogenase [ubiquinone] iron-sulfur protein 7, mitochondrial OS=Homo sapiens GN=NDUFS7 PE=1 SV=3 |
|  | **O60762\|DPM1_HUMAN** | Dolichol-phosphate mannosyltransferase OS=Homo sapiens GN=DPM1 PE=1 SV=1 |
|  | **Q5JRA6\|MIA3_HUMAN** | Melanoma inhibitory activity protein 3 OS=Homo sapiens GN=MIA3 PE=1 SV=1 |
|  | **P52597\|HNRPF_HUMAN** | Heterogeneous nuclear ribonucleoprotein F OS=Homo sapiens GN=HNRNPF PE=1 SV=3 |
|  | **P62879\|GBB2_HUMAN** | Guanine nucleotide-binding protein G(I)/G(S)/G(T) subunit beta-2 OS=Homo sapiens GN=GNB2 PE=1 SV=3 |
|  | **Q9UNL2\|SSRG_HUMAN** | Translocon-associated protein subunit gamma OS=Homo sapiens GN=SSR3 PE=1 SV=1 |
|  | **Q12797\|ASPH_HUMAN** | Aspartyl/asparaginyl beta-hydroxylase OS=Homo sapiens GN=ASPH PE=1 SV=3 |
|  | **P06396\|GELS_HUMAN** | Gelsolin OS=Homo sapiens GN=GSN PE=1 SV=1 |
|  | **O60701\|UGDH_HUMAN** | UDP-glucose 6-dehydrogenase OS=Homo sapiens GN=UGDH PE=1 SV=1 |
|  | **P11279\|LAMP1_HUMAN** | Lysosome-associated membrane glycoprotein 1 OS=Homo sapiens GN=LAMP1 PE=1 SV=3 |
|  | **P01893\|HLAH_HUMAN** | Putative HLA class I histocompatibility antigen, alpha chain H OS=Homo sapiens GN=HLA-H PE=5 SV=2 |
|  | **Q9NYQ3\|HAOX2_HUMAN** | Hydroxyacid oxidase 2 OS=Homo sapiens GN=HAO2 PE=2 SV=1 |
|  | **O60220\|TIM8A_HUMAN** | Mitochondrial import inner membrane translocase subunit Tim8 A OS=Homo sapiens GN=TIMM8A PE=1 SV=1 |
|  | **Q9Y3Z3\|SAMH1_HUMAN** | SAM domain and HD domain-containing protein 1 OS=Homo sapiens GN=SAMHD1 PE=1 SV=2 |
|  | **P01011\|AACT_HUMAN** | Alpha-1-antichymotrypsin OS=Homo sapiens GN=SERPINA3 PE=1 SV=2 |
|  | **O95169\|NDUB8_HUMAN** | NADH dehydrogenase [ubiquinone] 1 beta subcomplex subunit 8, mitochondrial OS=Homo sapiens GN=NDUFB8 PE=1 SV=1 |
|  | **P09543\|CN37_HUMAN** | 2',3'-cyclic-nucleotide 3'-phosphodiesterase OS=Homo sapiens GN=CNP PE=1 SV=2 |
|  | **P50454\|SERPH_HUMAN** | Serpin H1 OS=Homo sapiens GN=SERPINH1 PE=1 SV=2 |
|  | **P42330\|AK1C3_HUMAN** | Aldo-keto reductase family 1 member C3 OS=Homo sapiens GN=AKR1C3 PE=1 SV=3 |
|  | **Q9UL46\|PSME2_HUMAN** | Proteasome activator complex subunit 2 OS=Homo sapiens GN=PSME2 PE=1 SV=3 |
|  | **O43169\|CYB5B_HUMAN** | Cytochrome b5 type B OS=Homo sapiens GN=CYB5B PE=1 SV=2 |
|  | **Q9BY49\|PECR_HUMAN** | Peroxisomal trans-2-enoyl-CoA reductase OS=Homo sapiens GN=PECR PE=1 SV=2 |
|  | **Q02878\|RL6_HUMAN** | 60S ribosomal protein L6 OS=Homo sapiens GN=RPL6 PE=1 SV=3 |
|  | **Q9BRX8\|CJ058_HUMAN** | Uncharacterized protein C10orf58 OS=Homo sapiens GN=C10orf58 PE=1 SV=3 |
|  | **P50914\|RL14_HUMAN** | 60S ribosomal protein L14 OS=Homo sapiens GN=RPL14 PE=1 SV=4 |
|  | **P23378\|GCSP_HUMAN** | Glycine dehydrogenase [decarboxylating], mitochondrial OS=Homo sapiens GN=GLDC PE=1 SV=2 |
|  | **Q8N139\|ABCA6_HUMAN** | ATP-binding cassette sub-family A member 6 OS=Homo sapiens GN=ABCA6 PE=1 SV=2 |
|  | **P62995\|TRA2B_HUMAN** | Transformer-2 protein homolog beta OS=Homo sapiens GN=TRA2B PE=1 SV=1 |
|  | **Q14258\|TRI25_HUMAN** | Tripartite motif-containing protein 25 OS=Homo sapiens GN=TRIM25 PE=1 SV=1 |
|  | **Q07000\|1C15_HUMAN** | HLA class I histocompatibility antigen, Cw-15 alpha chain OS=Homo sapiens GN=HLA-C PE=1 SV=1 |
|  | **P30501\|1C02_HUMAN** | HLA class I histocompatibility antigen, Cw-2 alpha chain OS=Homo sapiens GN=HLA-C PE=1 SV=1 |
|  | **Q31612\|1B73_HUMAN** | HLA class I histocompatibility antigen, B-73 alpha chain OS=Homo sapiens GN=HLA-B PE=1 SV=1 |
|  | **P05026\|AT1B1_HUMAN** | Sodium/potassium-transporting ATPase subunit beta-1 OS=Homo sapiens GN=ATP1B1 PE=1 SV=1 |
|  | **P13747\|HLAE_HUMAN** | HLA class I histocompatibility antigen, alpha chain E OS=Homo sapiens GN=HLA-E PE=1 SV=3 |
|  | **P62277\|RS13_HUMAN** | 40S ribosomal protein S13 OS=Homo sapiens GN=RPS13 PE=1 SV=2 |
|  | **Q9UHQ9\|NB5R1_HUMAN** | NADH-cytochrome b5 reductase 1 OS=Homo sapiens GN=CYB5R1 PE=1 SV=1 |
|  | **O95292\|VAPB_HUMAN** | Vesicle-associated membrane protein-associated protein B/C OS=Homo sapiens GN=VAPB PE=1 SV=3 |
|  | **A6NHG4\|DDTL_HUMAN** | D-dopachrome decarboxylase-like protein OS=Homo sapiens GN=DDTL PE=2 SV=1 |
|  | **P62988\|UBIQ_HUMAN** | Ubiquitin OS=Homo sapiens GN=RPS27A PE=1 SV=1 |
|  | **P30504\|1C04_HUMAN** | HLA class I histocompatibility antigen, Cw-4 alpha chain OS=Homo sapiens GN=HLA-C PE=1 SV=1 |
|  | **O75356\|ENTP5_HUMAN** | Ectonucleoside triphosphate diphosphohydrolase 5 OS=Homo sapiens GN=ENTPD5 PE=1 SV=1 |
|  | **Q8IX05\|CD302_HUMAN** | CD302 antigen OS=Homo sapiens GN=CD302 PE=1 SV=1 |
|  | **P62899\|RL31_HUMAN** | 60S ribosomal protein L31 OS=Homo sapiens GN=RPL31 PE=1 SV=1 |
|  | **P37108\|SRP14_HUMAN** | Signal recognition particle 14 kDa protein OS=Homo sapiens GN=SRP14 PE=1 SV=2 |
|  | **Q5T8D3\|ACBD5_HUMAN** | Acyl-CoA-binding domain-containing protein 5 OS=Homo sapiens GN=ACBD5 PE=1 SV=1 |
|  | **Q9H0C2\|ADT4_HUMAN** | ADP/ATP translocase 4 OS=Homo sapiens GN=SLC25A31 PE=1 SV=1 |
|  | **Q9Y673\|ALG5_HUMAN** | Dolichyl-phosphate beta-glucosyltransferase OS=Homo sapiens GN=ALG5 PE=1 SV=1 |
|  | **P47985\|UCRI_HUMAN** | Cytochrome b-c1 complex subunit Rieske, mitochondrial OS=Homo sapiens GN=UQCRFS1 PE=1 SV=2 |
|  | **P19404\|NDUV2_HUMAN** | NADH dehydrogenase [ubiquinone] flavoprotein 2, mitochondrial OS=Homo sapiens GN=NDUFV2 PE=1 SV=2 |
|  | **P13804\|ETFA_HUMAN** | Electron transfer flavoprotein subunit alpha, mitochondrial OS=Homo sapiens GN=ETFA PE=1 SV=1 |
|  | **P50395\|GDIB_HUMAN** | Rab GDP dissociation inhibitor beta OS=Homo sapiens GN=GDI2 PE=1 SV=2 |
|  | **P31150\|GDIA_HUMAN** | Rab GDP dissociation inhibitor alpha OS=Homo sapiens GN=GDI1 PE=1 SV=2 |
|  | **Q9Y5L4\|TIM13_HUMAN** | Mitochondrial import inner membrane translocase subunit Tim13 OS=Homo sapiens GN=TIMM13 PE=1 SV=1 |
|  | **Q96G23\|LASS2_HUMAN** | LAG1 longevity assurance homolog 2 OS=Homo sapiens GN=LASS2 PE=1 SV=1 |
|  | **Q8NFQ8\|TOIP2_HUMAN** | Torsin-1A-interacting protein 2 OS=Homo sapiens GN=TOR1AIP2 PE=1 SV=1 |
|  | **Q15836\|VAMP3_HUMAN** | Vesicle-associated membrane protein 3 OS=Homo sapiens GN=VAMP3 PE=1 SV=3 |
|  | **P63027\|VAMP2_HUMAN** | Vesicle-associated membrane protein 2 OS=Homo sapiens GN=VAMP2 PE=1 SV=2 |
|  | **P11217\|PYGM_HUMAN** | Glycogen phosphorylase, muscle form OS=Homo sapiens GN=PYGM PE=1 SV=6 |
|  | **Q06278\|ADO_HUMAN** | Aldehyde oxidase OS=Homo sapiens GN=AOX1 PE=2 SV=2 |
|  | **Q10471\|GALT2_HUMAN** | Polypeptide N-acetylgalactosaminyltransferase 2 OS=Homo sapiens GN=GALNT2 PE=1 SV=1 |
|  | **P62195\|PRS8_HUMAN** | 26S protease regulatory subunit 8 OS=Homo sapiens GN=PSMC5 PE=1 SV=1 |
|  | **P30837\|AL1B1_HUMAN** | Aldehyde dehydrogenase X, mitochondrial OS=Homo sapiens GN=ALDH1B1 PE=1 SV=2 |
|  | **Q9UHE5\|NAT8_HUMAN** | Probable N-acetyltransferase 8 OS=Homo sapiens GN=NAT8 PE=2 SV=2 |
|  | **P34913\|HYES_HUMAN** | Epoxide hydrolase 2 OS=Homo sapiens GN=EPHX2 PE=1 SV=2 |
|  | **Q6IQ22\|RAB12_HUMAN** | Putative Ras-related protein Rab-12 OS=Homo sapiens GN=RAB12 PE=5 SV=3 |
|  | **Q9NRW1\|RAB6B_HUMAN** | Ras-related protein Rab-6B OS=Homo sapiens GN=RAB6B PE=1 SV=1 |
|  | **P20336\|RAB3A_HUMAN** | Ras-related protein Rab-3A OS=Homo sapiens GN=RAB3A PE=1 SV=1 |
|  | **Q96E17\|RAB3C_HUMAN** | Ras-related protein Rab-3C OS=Homo sapiens GN=RAB3C PE=2 SV=1 |
|  | **Q96AX2\|RAB37_HUMAN** | Ras-related protein Rab-37 OS=Homo sapiens GN=RAB37 PE=1 SV=3 |
|  | **P61018\|RAB4B_HUMAN** | Ras-related protein Rab-4B OS=Homo sapiens GN=RAB4B PE=1 SV=1 |
|  | **P20337\|RAB3B_HUMAN** | Ras-related protein Rab-3B OS=Homo sapiens GN=RAB3B PE=1 SV=2 |
|  | **Q14964\|RB39A_HUMAN** | Ras-related protein Rab-39A OS=Homo sapiens GN=RAB39 PE=2 SV=2 |
|  | **Q9H082\|RB33B_HUMAN** | Ras-related protein Rab-33B OS=Homo sapiens GN=RAB33B PE=1 SV=1 |
|  | **P20340\|RAB6A_HUMAN** | Ras-related protein Rab-6A OS=Homo sapiens GN=RAB6A PE=1 SV=3 |
|  | **P20338\|RAB4A_HUMAN** | Ras-related protein Rab-4A OS=Homo sapiens GN=RAB4A PE=1 SV=2 |
|  | **Q86YS6\|RAB43_HUMAN** | Ras-related protein Rab-43 OS=Homo sapiens GN=RAB43 PE=1 SV=1 |
|  | **O95716\|RAB3D_HUMAN** | Ras-related protein Rab-3D OS=Homo sapiens GN=RAB3D PE=1 SV=1 |
|  | **Q15771\|RAB30_HUMAN** | Ras-related protein Rab-30 OS=Homo sapiens GN=RAB30 PE=1 SV=2 |
|  | **P11226\|MBL2_HUMAN** | Mannose-binding protein C OS=Homo sapiens GN=MBL2 PE=1 SV=2 |
|  | **Q86VD7\|S2542_HUMAN** | Solute carrier family 25 member 42 OS=Homo sapiens GN=SLC25A42 PE=1 SV=2 |
|  | **Q9Y678\|COPG_HUMAN** | Coatomer subunit gamma OS=Homo sapiens GN=COPG PE=1 SV=1 |
|  | **O00483\|NDUA4_HUMAN** | NADH dehydrogenase [ubiquinone] 1 alpha subcomplex subunit 4 OS=Homo sapiens GN=NDUFA4 PE=1 SV=1 |
|  | **O95168\|NDUB4_HUMAN** | NADH dehydrogenase [ubiquinone] 1 beta subcomplex subunit 4 OS=Homo sapiens GN=NDUFB4 PE=1 SV=3 |
|  | **P04222\|1C03_HUMAN** | HLA class I histocompatibility antigen, Cw-3 alpha chain OS=Homo sapiens GN=HLA-C PE=1 SV=2 |
|  | **P37268\|FDFT_HUMAN** | Squalene synthetase OS=Homo sapiens GN=FDFT1 PE=1 SV=1 |
|  | **Q29960\|1C16_HUMAN** | HLA class I histocompatibility antigen, Cw-16 alpha chain OS=Homo sapiens GN=HLA-C PE=2 SV=1 |
|  | **Q95604\|1C17_HUMAN** | HLA class I histocompatibility antigen, Cw-17 alpha chain OS=Homo sapiens GN=HLA-C PE=1 SV=1 |
|  | **P02647\|APOA1_HUMAN** | Apolipoprotein A-I OS=Homo sapiens GN=APOA1 PE=1 SV=1 |
|  | **Q01581\|HMCS1_HUMAN** | Hydroxymethylglutaryl-CoA synthase, cytoplasmic OS=Homo sapiens GN=HMGCS1 PE=1 SV=2 |
|  | **P36776\|LONM_HUMAN** | Lon protease homolog, mitochondrial OS=Homo sapiens GN=LONP1 PE=1 SV=2 |
|  | **P06748\|NPM_HUMAN** | Nucleophosmin OS=Homo sapiens GN=NPM1 PE=1 SV=2 |
|  | **P62906\|RL10A_HUMAN** | 60S ribosomal protein L10a OS=Homo sapiens GN=RPL10A PE=1 SV=2 |
|  | **P01842\|LAC_HUMAN** | Ig lambda chain C regions OS=Homo sapiens GN=IGLC1 PE=1 SV=1 |
|  | **P15259\|PGAM2_HUMAN** | Phosphoglycerate mutase 2 OS=Homo sapiens GN=PGAM2 PE=1 SV=3 |
|  | **Q9BQB6\|VKOR1_HUMAN** | Vitamin K epoxide reductase complex subunit 1 OS=Homo sapiens GN=VKORC1 PE=1 SV=1 |
|  | **P02649\|APOE_HUMAN** | Apolipoprotein E OS=Homo sapiens GN=APOE PE=1 SV=1 |
|  | **P04424\|ARLY_HUMAN** | Argininosuccinate lyase OS=Homo sapiens GN=ASL PE=1 SV=4 |
|  | **P46779\|RL28_HUMAN** | 60S ribosomal protein L28 OS=Homo sapiens GN=RPL28 PE=1 SV=3 |
|  | **P05386\|RLA1_HUMAN** | 60S acidic ribosomal protein P1 OS=Homo sapiens GN=RPLP1 PE=1 SV=1 |
|  | **P14174\|MIF_HUMAN** | Macrophage migration inhibitory factor OS=Homo sapiens GN=MIF PE=1 SV=4 |
|  | **O43852\|CALU_HUMAN** | Calumenin OS=Homo sapiens GN=CALU PE=1 SV=2 |
|  | **Q13576\|IQGA2_HUMAN** | Ras GTPase-activating-like protein IQGAP2 OS=Homo sapiens GN=IQGAP2 PE=1 SV=3 |
|  | **P34897\|GLYM_HUMAN** | Serine hydroxymethyltransferase, mitochondrial OS=Homo sapiens GN=SHMT2 PE=1 SV=3 |
|  | **O75310\|UDB11_HUMAN** | UDP-glucuronosyltransferase 2B11 OS=Homo sapiens GN=UGT2B11 PE=2 SV=1 |
|  | **Q9NRP0\|OSTC_HUMAN** | Oligosaccharyltransferase complex subunit OSTC OS=Homo sapiens GN=OSTC PE=1 SV=1 |
|  | **P22626\|ROA2_HUMAN** | Heterogeneous nuclear ribonucleoproteins A2/B1 OS=Homo sapiens GN=HNRNPA2B1 PE=1 SV=2 |
|  | **O14548\|COX7R_HUMAN** | Cytochrome c oxidase subunit 7A-related protein, mitochondrial OS=Homo sapiens GN=COX7A2L PE=1 SV=2 |
|  | **P62244\|RS15A_HUMAN** | 40S ribosomal protein S15a OS=Homo sapiens GN=RPS15A PE=1 SV=2 |
|  | **P06744\|G6PI_HUMAN** | Glucose-6-phosphate isomerase OS=Homo sapiens GN=GPI PE=1 SV=4 |
|  | **Q13011\|ECH1_HUMAN** | Delta(3,5)-Delta(2,4)-dienoyl-CoA isomerase, mitochondrial OS=Homo sapiens GN=ECH1 PE=1 SV=2 |
|  | **Q6UW02\|CP20A_HUMAN** | Cytochrome P450 20A1 OS=Homo sapiens GN=CYP20A1 PE=1 SV=1 |
|  | **Q13438\|OS9_HUMAN** | Protein OS-9 OS=Homo sapiens GN=OS9 PE=1 SV=1 |
|  | **P28161\|GSTM2_HUMAN** | Glutathione S-transferase Mu 2 OS=Homo sapiens GN=GSTM2 PE=1 SV=2 |
|  | **P09488\|GSTM1_HUMAN** | Glutathione S-transferase Mu 1 OS=Homo sapiens GN=GSTM1 PE=1 SV=3 |
|  | **Q03013\|GSTM4_HUMAN** | Glutathione S-transferase Mu 4 OS=Homo sapiens GN=GSTM4 PE=1 SV=3 |
|  | **Q7Z403\|TMC6_HUMAN** | Transmembrane channel-like protein 6 OS=Homo sapiens GN=TMC6 PE=1 SV=2 |
|  | **P63010\|AP2B1_HUMAN** | AP-2 complex subunit beta OS=Homo sapiens GN=AP2B1 PE=1 SV=1 |
|  | **Q9NS69\|TOM22_HUMAN** | Mitochondrial import receptor subunit TOM22 homolog OS=Homo sapiens GN=TOMM22 PE=1 SV=3 |
|  | **Q9NRV9\|HEBP1_HUMAN** | Heme-binding protein 1 OS=Homo sapiens GN=HEBP1 PE=1 SV=1 |
|  | **Q14240\|IF4A2_HUMAN** | Eukaryotic initiation factor 4A-II OS=Homo sapiens GN=EIF4A2 PE=1 SV=2 |
|  | **P60842\|IF4A1_HUMAN** | Eukaryotic initiation factor 4A-I OS=Homo sapiens GN=EIF4A1 PE=1 SV=1 |
|  | **P98187\|CP4F8_HUMAN** | Cytochrome P450 4F8 OS=Homo sapiens GN=CYP4F8 PE=1 SV=1 |
|  | **P31946\|1433B_HUMAN** | 14-3-3 protein beta/alpha OS=Homo sapiens GN=YWHAB PE=1 SV=3 |
|  | **Q96I99\|SUCB2_HUMAN** | Succinyl-CoA ligase [GDP-forming] subunit beta, mitochondrial OS=Homo sapiens GN=SUCLG2 PE=1 SV=2 |
|  | **O75955\|FLOT1_HUMAN** | Flotillin-1 OS=Homo sapiens GN=FLOT1 PE=1 SV=3 |
|  | **O15258\|RER1_HUMAN** | Protein RER1 OS=Homo sapiens GN=RER1 PE=1 SV=1 |
|  | **Q05639\|EF1A2_HUMAN** | Elongation factor 1-alpha 2 OS=Homo sapiens GN=EEF1A2 PE=1 SV=1 |
|  | **P05109\|S10A8_HUMAN** | Protein S100-A8 OS=Homo sapiens GN=S100A8 PE=1 SV=1 |
|  | **Q02978\|M2OM_HUMAN** | Mitochondrial 2-oxoglutarate/malate carrier protein OS=Homo sapiens GN=SLC25A11 PE=1 SV=3 |
|  | **P04899\|GNAI2_HUMAN** | Guanine nucleotide-binding protein G(i), alpha-2 subunit OS=Homo sapiens GN=GNAI2 PE=1 SV=3 |
|  | **P61604\|CH10_HUMAN** | 10 kDa heat shock protein, mitochondrial OS=Homo sapiens GN=HSPE1 PE=1 SV=2 |
|  | **P60468\|SC61B_HUMAN** | Protein transport protein Sec61 subunit beta OS=Homo sapiens GN=SEC61B PE=1 SV=2 |
|  | **P26038\|MOES_HUMAN** | Moesin OS=Homo sapiens GN=MSN PE=1 SV=3 |
|  | **P46776\|RL27A_HUMAN** | 60S ribosomal protein L27a OS=Homo sapiens GN=RPL27A PE=1 SV=2 |
|  | **Q9UKU0\|ACSL6_HUMAN** | Long-chain-fatty-acid--CoA ligase 6 OS=Homo sapiens GN=ACSL6 PE=2 SV=4 |
|  | **P11310\|ACADM_HUMAN** | Medium-chain specific acyl-CoA dehydrogenase, mitochondrial OS=Homo sapiens GN=ACADM PE=1 SV=1 |
|  | **P02100\|HBE_HUMAN** | Hemoglobin subunit epsilon OS=Homo sapiens GN=HBE1 PE=1 SV=2 |
|  | **P69891\|HBG1_HUMAN** | Hemoglobin subunit gamma-1 OS=Homo sapiens GN=HBG1 PE=1 SV=2 |
|  | **P49748\|ACADV_HUMAN** | Very long-chain specific acyl-CoA dehydrogenase, mitochondrial OS=Homo sapiens GN=ACADVL PE=1 SV=1 |
|  | **P02730\|B3AT_HUMAN** | Band 3 anion transport protein OS=Homo sapiens GN=SLC4A1 PE=1 SV=3 |
|  | **Q9Y3D6\|FIS1_HUMAN** | Mitochondrial fission 1 protein OS=Homo sapiens GN=FIS1 PE=1 SV=2 |
|  | **P62857\|RS28_HUMAN** | 40S ribosomal protein S28 OS=Homo sapiens GN=RPS28 PE=1 SV=1 |
|  | **Q99497\|PARK7_HUMAN** | Protein DJ-1 OS=Homo sapiens GN=PARK7 PE=1 SV=2 |
|  | **Q9Y4P3\|TBL2_HUMAN** | Transducin beta-like protein 2 OS=Homo sapiens GN=TBL2 PE=1 SV=1 |
|  | **P40429\|RL13A_HUMAN** | 60S ribosomal protein L13a OS=Homo sapiens GN=RPL13A PE=1 SV=2 |
|  | **Q6NVV1\|R13AX_HUMAN** | Putative 60S ribosomal protein L13a-like MGC87657 OS=Homo sapiens PE=5 SV=1 |
|  | **Q16772\|GSTA3_HUMAN** | Glutathione S-transferase A3 OS=Homo sapiens GN=GSTA3 PE=1 SV=3 |
|  | **P10909\|CLUS_HUMAN** | Clusterin OS=Homo sapiens GN=CLU PE=1 SV=1 |
|  | **P38919\|IF4A3_HUMAN** | Eukaryotic initiation factor 4A-III OS=Homo sapiens GN=EIF4A3 PE=1 SV=4 |
|  | **P62851\|RS25_HUMAN** | 40S ribosomal protein S25 OS=Homo sapiens GN=RPS25 PE=1 SV=1 |
|  | **Q9UL12\|SARDH_HUMAN** | Sarcosine dehydrogenase, mitochondrial OS=Homo sapiens GN=SARDH PE=1 SV=1 |
|  | **P51687\|SUOX_HUMAN** | Sulfite oxidase, mitochondrial OS=Homo sapiens GN=SUOX PE=1 SV=2 |
|  | **P07602\|SAP_HUMAN** | Proactivator polypeptide OS=Homo sapiens GN=PSAP PE=1 SV=2 |
|  | **P67809\|YBOX1_HUMAN** | Nuclease-sensitive element-binding protein 1 OS=Homo sapiens GN=YBX1 PE=1 SV=3 |
|  | **O95299\|NDUAA_HUMAN** | NADH dehydrogenase [ubiquinone] 1 alpha subcomplex subunit 10, mitochondrial OS=Homo sapiens GN=NDUFA10 PE=1 SV=1 |
|  | **O75828\|CBR3_HUMAN** | Carbonyl reductase [NADPH] 3 OS=Homo sapiens GN=CBR3 PE=1 SV=3 |
|  | **P0C0S8\|H2A1_HUMAN** | Histone H2A type 1 OS=Homo sapiens GN=HIST1H2AG PE=1 SV=2 |
|  | **P10319\|1B58_HUMAN** | HLA class I histocompatibility antigen, B-58 alpha chain OS=Homo sapiens GN=HLA-B PE=2 SV=1 |
|  | **Q29940\|1B59_HUMAN** | HLA class I histocompatibility antigen, B-59 alpha chain OS=Homo sapiens GN=HLA-B PE=2 SV=1 |
|  | **P30464\|1B15_HUMAN** | HLA class I histocompatibility antigen, B-15 alpha chain OS=Homo sapiens GN=HLA-B PE=1 SV=2 |
|  | **P30490\|1B52_HUMAN** | HLA class I histocompatibility antigen, B-52 alpha chain OS=Homo sapiens GN=HLA-B PE=2 SV=1 |
|  | **P18465\|1B57_HUMAN** | HLA class I histocompatibility antigen, B-57 alpha chain OS=Homo sapiens GN=HLA-B PE=1 SV=1 |
|  | **Q9NQR4\|NIT2_HUMAN** | Nitrilase homolog 2 OS=Homo sapiens GN=NIT2 PE=1 SV=1 |
|  | **Q99424\|ACOX2_HUMAN** | Peroxisomal acyl-coenzyme A oxidase 2 OS=Homo sapiens GN=ACOX2 PE=1 SV=1 |
|  | **P09669\|COX6C_HUMAN** | Cytochrome c oxidase subunit 6C OS=Homo sapiens GN=COX6C PE=1 SV=2 |
|  | **P31512\|FMO4_HUMAN** | Dimethylaniline monooxygenase [N-oxide-forming] 4 OS=Homo sapiens GN=FMO4 PE=2 SV=3 |
|  | **Q9H0W9\|CK054_HUMAN** | Ester hydrolase C11orf54 OS=Homo sapiens GN=C11orf54 PE=1 SV=1 |
|  | **P19338\|NUCL_HUMAN** | Nucleolin OS=Homo sapiens GN=NCL PE=1 SV=3 |
|  | **Q8N5M9\|JAGN1_HUMAN** | Protein jagunal homolog 1 OS=Homo sapiens GN=JAGN1 PE=1 SV=1 |
|  | **P02655\|APOC2_HUMAN** | Apolipoprotein C-II OS=Homo sapiens GN=APOC2 PE=1 SV=1 |
|  | **Q15006\|TTC35_HUMAN** | Tetratricopeptide repeat protein 35 OS=Homo sapiens GN=TTC35 PE=1 SV=1 |
|  | **Q14696\|MESD_HUMAN** | LDLR chaperone MESD OS=Homo sapiens GN=MESDC2 PE=1 SV=2 |
|  | **O43181\|NDUS4_HUMAN** | NADH dehydrogenase [ubiquinone] iron-sulfur protein 4, mitochondrial OS=Homo sapiens GN=NDUFS4 PE=1 SV=1 |
|  | **P23528\|COF1_HUMAN** | Cofilin-1 OS=Homo sapiens GN=CFL1 PE=1 SV=3 |
|  | **P61421\|VA0D1_HUMAN** | V-type proton ATPase subunit d 1 OS=Homo sapiens GN=ATP6V0D1 PE=1 SV=1 |
|  | **Q06830\|PRDX1_HUMAN** | Peroxiredoxin-1 OS=Homo sapiens GN=PRDX1 PE=1 SV=1 |
|  | **O95782\|AP2A1_HUMAN** | AP-2 complex subunit alpha-1 OS=Homo sapiens GN=AP2A1 PE=1 SV=2 |
|  | **O94973\|AP2A2_HUMAN** | AP-2 complex subunit alpha-2 OS=Homo sapiens GN=AP2A2 PE=1 SV=2 |
|  | **P15880\|RS2_HUMAN** | 40S ribosomal protein S2 OS=Homo sapiens GN=RPS2 PE=1 SV=2 |
|  | **P08708\|RS17_HUMAN** | 40S ribosomal protein S17 OS=Homo sapiens GN=RPS17 PE=1 SV=2 |
|  | **Q56VL3\|OCAD2_HUMAN** | OCIA domain-containing protein 2 OS=Homo sapiens GN=OCIAD2 PE=1 SV=1 |
|  | **Q14571\|ITPR2_HUMAN** | Inositol 1,4,5-trisphosphate receptor type 2 OS=Homo sapiens GN=ITPR2 PE=1 SV=2 |
|  | **Q01082\|SPTB2_HUMAN** | Spectrin beta chain, brain 1 OS=Homo sapiens GN=SPTBN1 PE=1 SV=2 |
|  | **P12074\|CX6A1_HUMAN** | Cytochrome c oxidase subunit 6A1, mitochondrial OS=Homo sapiens GN=COX6A1 PE=1 SV=4 |
|  | **P51159\|RB27A_HUMAN** | Ras-related protein Rab-27A OS=Homo sapiens GN=RAB27A PE=1 SV=3 |
|  | **O96000\|NDUBA_HUMAN** | NADH dehydrogenase [ubiquinone] 1 beta subcomplex subunit 10 OS=Homo sapiens GN=NDUFB10 PE=1 SV=3 |
|  | **P35914\|HMGCL_HUMAN** | Hydroxymethylglutaryl-CoA lyase, mitochondrial OS=Homo sapiens GN=HMGCL PE=1 SV=2 |
|  | **P16219\|ACADS_HUMAN** | Short-chain specific acyl-CoA dehydrogenase, mitochondrial OS=Homo sapiens GN=ACADS PE=1 SV=1 |
|  | **P62263\|RS14_HUMAN** | 40S ribosomal protein S14 OS=Homo sapiens GN=RPS14 PE=1 SV=3 |
|  | **P25325\|THTM_HUMAN** | 3-mercaptopyruvate sulfurtransferase OS=Homo sapiens GN=MPST PE=1 SV=3 |
|  | **P07195\|LDHB_HUMAN** | L-lactate dehydrogenase B chain OS=Homo sapiens GN=LDHB PE=1 SV=2 |
|  | **Q6ZMR3\|LDH6A_HUMAN** | L-lactate dehydrogenase A-like 6A OS=Homo sapiens GN=LDHAL6A PE=2 SV=1 |
|  | **P07864\|LDHC_HUMAN** | L-lactate dehydrogenase C chain OS=Homo sapiens GN=LDHC PE=2 SV=4 |
|  | **Q9NZ45\|CISD1_HUMAN** | CDGSH iron sulfur domain-containing protein 1 OS=Homo sapiens GN=CISD1 PE=1 SV=1 |
|  | **Q709F0\|ACD11_HUMAN** | Acyl-CoA dehydrogenase family member 11 OS=Homo sapiens GN=ACAD11 PE=1 SV=2 |
|  | **P08590\|MYL3_HUMAN** | Myosin light chain 3 OS=Homo sapiens GN=MYL3 PE=1 SV=3 |
|  | **P05976\|MLE1_HUMAN** | Myosin light chain 1, skeletal muscle isoform OS=Homo sapiens GN=MYL1 PE=1 SV=3 |
|  | **Q9Y6B6\|SAR1B_HUMAN** | GTP-binding protein SAR1b OS=Homo sapiens GN=SAR1B PE=1 SV=1 |
|  | **P08240\|SRPR_HUMAN** | Signal recognition particle receptor subunit alpha OS=Homo sapiens GN=SRPR PE=1 SV=2 |
|  | **Q9NYL4\|FKB11_HUMAN** | FK506-binding protein 11 OS=Homo sapiens GN=FKBP11 PE=1 SV=1 |
|  | **P01876\|IGHA1_HUMAN** | Ig alpha-1 chain C region OS=Homo sapiens GN=IGHA1 PE=1 SV=2 |
|  | **P30519\|HMOX2_HUMAN** | Heme oxygenase 2 OS=Homo sapiens GN=HMOX2 PE=1 SV=2 |
|  | **Q15436\|SC23A_HUMAN** | Protein transport protein Sec23A OS=Homo sapiens GN=SEC23A PE=1 SV=2 |
|  | **Q9NNW7\|TRXR2_HUMAN** | Thioredoxin reductase 2, mitochondrial OS=Homo sapiens GN=TXNRD2 PE=1 SV=3 |
|  | **Q16881\|TRXR1_HUMAN** | Thioredoxin reductase 1, cytoplasmic OS=Homo sapiens GN=TXNRD1 PE=1 SV=3 |
|  | **P20132\|SDHL_HUMAN** | L-serine dehydratase/L-threonine deaminase OS=Homo sapiens GN=SDS PE=1 SV=2 |
|  | **Q14061\|COX17_HUMAN** | Cytochrome c oxidase copper chaperone OS=Homo sapiens GN=COX17 PE=1 SV=2 |
|  | **P10606\|COX5B_HUMAN** | Cytochrome c oxidase subunit 5B, mitochondrial OS=Homo sapiens GN=COX5B PE=1 SV=2 |
|  | **O96005\|CLPT1_HUMAN** | Cleft lip and palate transmembrane protein 1 OS=Homo sapiens GN=CLPTM1 PE=1 SV=1 |
|  | **P08758\|ANXA5_HUMAN** | Annexin A5 OS=Homo sapiens GN=ANXA5 PE=1 SV=2 |
|  | **Q9BVC6\|TM109_HUMAN** | Transmembrane protein 109 OS=Homo sapiens GN=TMEM109 PE=1 SV=1 |
|  | **P62266\|RS23_HUMAN** | 40S ribosomal protein S23 OS=Homo sapiens GN=RPS23 PE=1 SV=3 |
|  | **Q99685\|MGLL_HUMAN** | Monoglyceride lipase OS=Homo sapiens GN=MGLL PE=2 SV=2 |
|  | **P12110\|CO6A2_HUMAN** | Collagen alpha-2(VI) chain OS=Homo sapiens GN=COL6A2 PE=1 SV=4 |
|  | **P46777\|RL5_HUMAN** | 60S ribosomal protein L5 OS=Homo sapiens GN=RPL5 PE=1 SV=3 |
|  | **Q2M1Z3\|CDGAP_HUMAN** | Cdc42 GTPase-activating protein OS=Homo sapiens GN=CDGAP PE=1 SV=1 |
|  | **Q3ZCQ8\|TIM50_HUMAN** | Mitochondrial import inner membrane translocase subunit TIM50 OS=Homo sapiens GN=TIMM50 PE=1 SV=2 |
|  | **Q6NUQ4\|TM214_HUMAN** | Transmembrane protein 214 OS=Homo sapiens GN=TMEM214 PE=1 SV=2 |
|  | **P51153\|RAB13_HUMAN** | Ras-related protein Rab-13 OS=Homo sapiens GN=RAB13 PE=1 SV=1 |
|  | **P03989\|1B27_HUMAN** | HLA class I histocompatibility antigen, B-27 alpha chain OS=Homo sapiens GN=HLA-B PE=1 SV=2 |
|  | **P00480\|OTC_HUMAN** | Ornithine carbamoyltransferase, mitochondrial OS=Homo sapiens GN=OTC PE=1 SV=3 |
|  | **P62913\|RL11_HUMAN** | 60S ribosomal protein L11 OS=Homo sapiens GN=RPL11 PE=1 SV=2 |
|  | **Q16698\|DECR_HUMAN** | 2,4-dienoyl-CoA reductase, mitochondrial OS=Homo sapiens GN=DECR1 PE=1 SV=1 |
|  | **Q16836\|HCDH_HUMAN** | Hydroxyacyl-coenzyme A dehydrogenase, mitochondrial OS=Homo sapiens GN=HADH PE=1 SV=2 |
|  | **P13987\|CD59_HUMAN** | CD59 glycoprotein OS=Homo sapiens GN=CD59 PE=1 SV=1 |
|  | **P84095\|RHOG_HUMAN** | Rho-related GTP-binding protein RhoG OS=Homo sapiens GN=RHOG PE=1 SV=1 |
|  | **Q9H6S3\|ES8L2_HUMAN** | Epidermal growth factor receptor kinase substrate 8-like protein 2 OS=Homo sapiens GN=EPS8L2 PE=1 SV=2 |
|  | **Q14624\|ITIH4_HUMAN** | Inter-alpha-trypsin inhibitor heavy chain H4 OS=Homo sapiens GN=ITIH4 PE=1 SV=4 |
|  | **Q9H1K4\|GHC2_HUMAN** | Mitochondrial glutamate carrier 2 OS=Homo sapiens GN=SLC25A18 PE=2 SV=1 |
|  | **Q9NX63\|CHCH3_HUMAN** | Coiled-coil-helix-coiled-coil-helix domain-containing protein 3, mitochondrial OS=Homo sapiens GN=CHCHD3 PE=1 SV=1 |
|  | **P35241\|RADI_HUMAN** | Radixin OS=Homo sapiens GN=RDX PE=1 SV=1 |
|  | **P15311\|EZRI_HUMAN** | Ezrin OS=Homo sapiens GN=EZR PE=1 SV=4 |
|  | **P07737\|PROF1_HUMAN** | Profilin-1 OS=Homo sapiens GN=PFN1 PE=1 SV=2 |
|  | **P0C7P4\|UCRIL_HUMAN** | Cytochrome b-c1 complex subunit Rieske-like protein 1 OS=Homo sapiens GN=UQCRFSL1 PE=2 SV=1 |
|  | **P22090\|RS4Y1_HUMAN** | 40S ribosomal protein S4, Y isoform 1 OS=Homo sapiens GN=RPS4Y1 PE=2 SV=2 |
|  | **Q8TD47\|RS4Y2_HUMAN** | 40S ribosomal protein S4, Y isoform 2 OS=Homo sapiens GN=RPS4Y2 PE=1 SV=3 |
|  | **Q8NB25\|F184A_HUMAN** | Protein FAM184A OS=Homo sapiens GN=FAM184A PE=2 SV=3 |
|  | **P02749\|APOH_HUMAN** | Beta-2-glycoprotein 1 OS=Homo sapiens GN=APOH PE=1 SV=3 |
|  | **Q15437\|SC23B_HUMAN** | Protein transport protein Sec23B OS=Homo sapiens GN=SEC23B PE=1 SV=2 |
|  | **O15320\|CTGE5_HUMAN** | Cutaneous T-cell lymphoma-associated antigen 5 OS=Homo sapiens GN=CTAGE5 PE=1 SV=3 |
|  | **P67936\|TPM4_HUMAN** | Tropomyosin alpha-4 chain OS=Homo sapiens GN=TPM4 PE=1 SV=3 |
|  | **P42766\|RL35_HUMAN** | 60S ribosomal protein L35 OS=Homo sapiens GN=RPL35 PE=1 SV=2 |
|  | **P07355\|ANXA2_HUMAN** | Annexin A2 OS=Homo sapiens GN=ANXA2 PE=1 SV=2 |
|  | **Q15125\|EBP_HUMAN** | 3-beta-hydroxysteroid-Delta(8),Delta(7)-isomerase OS=Homo sapiens GN=EBP PE=1 SV=3 |
|  | **Q08830\|FGL1_HUMAN** | Fibrinogen-like protein 1 OS=Homo sapiens GN=FGL1 PE=1 SV=3 |
|  | **Q58FF8\|H90B2_HUMAN** | Putative heat shock protein HSP 90-beta 2 OS=Homo sapiens GN=HSP90AB2P PE=1 SV=2 |
|  | **Q14568\|HS902_HUMAN** | Putative heat shock protein HSP 90-alpha A2 OS=Homo sapiens GN=HSP90AA2 PE=1 SV=2 |
|  | **P18621\|RL17_HUMAN** | 60S ribosomal protein L17 OS=Homo sapiens GN=RPL17 PE=1 SV=3 |
|  | **P49458\|SRP09_HUMAN** | Signal recognition particle 9 kDa protein OS=Homo sapiens GN=SRP9 PE=1 SV=2 |
|  | **Q53H12\|AGK_HUMAN** | Acylglycerol kinase, mitochondrial OS=Homo sapiens GN=AGK PE=1 SV=2 |
|  | **Q13228\|SBP1_HUMAN** | Selenium-binding protein 1 OS=Homo sapiens GN=SELENBP1 PE=1 SV=2 |
|  | **Q86WU2\|LDHD_HUMAN** | Probable D-lactate dehydrogenase, mitochondrial OS=Homo sapiens GN=LDHD PE=1 SV=1 |
|  | **P10253\|LYAG_HUMAN** | Lysosomal alpha-glucosidase OS=Homo sapiens GN=GAA PE=1 SV=3 |
|  | **Q6ZUJ8\|BCAP_HUMAN** | Phosphoinositide 3-kinase adapter protein 1 OS=Homo sapiens GN=PIK3AP1 PE=1 SV=2 |
|  | **P26373\|RL13_HUMAN** | 60S ribosomal protein L13 OS=Homo sapiens GN=RPL13 PE=1 SV=4 |
|  | **P01019\|ANGT_HUMAN** | Angiotensinogen OS=Homo sapiens GN=AGT PE=1 SV=1 |
|  | **P05783\|K1C18_HUMAN** | Keratin, type I cytoskeletal 18 OS=Homo sapiens GN=KRT18 PE=1 SV=2 |
|  | **Q9HBH1\|DEFM_HUMAN** | Peptide deformylase, mitochondrial OS=Homo sapiens GN=PDF PE=1 SV=1 |
|  | **Q6P1J9\|CDC73_HUMAN** | Parafibromin OS=Homo sapiens GN=CDC73 PE=1 SV=1 |
|  | **O95154\|ARK73_HUMAN** | Aflatoxin B1 aldehyde reductase member 3 OS=Homo sapiens GN=AKR7A3 PE=1 SV=2 |
|  | **Q9HAV0\|GBB4_HUMAN** | Guanine nucleotide-binding protein subunit beta-4 OS=Homo sapiens GN=GNB4 PE=1 SV=3 |
|  | **P16104\|H2AX_HUMAN** | Histone H2A.x OS=Homo sapiens GN=H2AFX PE=1 SV=2 |
|  | **Q08257\|QOR_HUMAN** | Quinone oxidoreductase OS=Homo sapiens GN=CRYZ PE=1 SV=1 |
|  | **O14874\|BCKD_HUMAN** | [3-methyl-2-oxobutanoate dehydrogenase [lipoamide]] kinase, mitochondrial OS=Homo sapiens GN=BCKDK PE=1 SV=2 |
|  | **P04217\|A1BG_HUMAN** | Alpha-1B-glycoprotein OS=Homo sapiens GN=A1BG PE=1 SV=3 |
|  | **Q9BWD1\|THIC_HUMAN** | Acetyl-CoA acetyltransferase, cytosolic OS=Homo sapiens GN=ACAT2 PE=1 SV=2 |
|  | **P43307\|SSRA_HUMAN** | Translocon-associated protein subunit alpha OS=Homo sapiens GN=SSR1 PE=1 SV=3 |
|  | **P84085\|ARF5_HUMAN** | ADP-ribosylation factor 5 OS=Homo sapiens GN=ARF5 PE=1 SV=2 |
|  | **P61353\|RL27_HUMAN** | 60S ribosomal protein L27 OS=Homo sapiens GN=RPL27 PE=1 SV=2 |
|  | **Q93088\|BHMT1_HUMAN** | Betaine--homocysteine S-methyltransferase 1 OS=Homo sapiens GN=BHMT PE=1 SV=2 |
|  | **Q99536\|VAT1_HUMAN** | Synaptic vesicle membrane protein VAT-1 homolog OS=Homo sapiens GN=VAT1 PE=1 SV=2 |
|  | **P01857\|IGHG1_HUMAN** | Ig gamma-1 chain C region OS=Homo sapiens GN=IGHG1 PE=1 SV=1 |
|  | **Q9NUQ7\|UFSP2_HUMAN** | Ufm1-specific protease 2 OS=Homo sapiens GN=UFSP2 PE=2 SV=3 |
|  | **Q6UXX5\|ITH5L_HUMAN** | Inter-alpha-trypsin inhibitor heavy chain H5-like protein OS=Homo sapiens GN=ITIH5L PE=2 SV=1 |
|  | **O95298\|NDUC2_HUMAN** | NADH dehydrogenase [ubiquinone] 1 subunit C2 OS=Homo sapiens GN=NDUFC2 PE=1 SV=1 |
|  | **Q9NZS9\|BFAR_HUMAN** | Bifunctional apoptosis regulator OS=Homo sapiens GN=BFAR PE=1 SV=1 |
|  | **O43826\|G6PT1_HUMAN** | Glucose-6-phosphate translocase OS=Homo sapiens GN=SLC37A4 PE=1 SV=1 |
|  | **P80723\|BASP1_HUMAN** | Brain acid soluble protein 1 OS=Homo sapiens GN=BASP1 PE=1 SV=2 |
|  | **Q9UBR2\|CATZ_HUMAN** | Cathepsin Z OS=Homo sapiens GN=CTSZ PE=1 SV=1 |
|  | **Q7KZF4\|SND1_HUMAN** | Staphylococcal nuclease domain-containing protein 1 OS=Homo sapiens GN=SND1 PE=1 SV=1 |
|  | **P25311\|ZA2G_HUMAN** | Zinc-alpha-2-glycoprotein OS=Homo sapiens GN=AZGP1 PE=1 SV=1 |
|  | **P61981\|1433G_HUMAN** | 14-3-3 protein gamma OS=Homo sapiens GN=YWHAG PE=1 SV=2 |
|  | **P00505\|AATM_HUMAN** | Aspartate aminotransferase, mitochondrial OS=Homo sapiens GN=GOT2 PE=1 SV=2 |
|  | **Q15293\|RCN1_HUMAN** | Reticulocalbin-1 OS=Homo sapiens GN=RCN1 PE=1 SV=1 |
|  | **Q96LZ7\|RMD2_HUMAN** | Regulator of microtubule dynamics protein 2 OS=Homo sapiens GN=FAM82A1 PE=1 SV=2 |
|  | **P42126\|D3D2_HUMAN** | 3,2-trans-enoyl-CoA isomerase, mitochondrial OS=Homo sapiens GN=DCI PE=1 SV=1 |
|  | **Q15907\|RB11B_HUMAN** | Ras-related protein Rab-11B OS=Homo sapiens GN=RAB11B PE=1 SV=4 |
|  | **Q9BXJ8\|T120A_HUMAN** | Transmembrane protein 120A OS=Homo sapiens GN=TMEM120A PE=2 SV=1 |
|  | **P00533\|EGFR_HUMAN** | Epidermal growth factor receptor OS=Homo sapiens GN=EGFR PE=1 SV=2 |
|  | **Q9UFP1\|CC041_HUMAN** | Uncharacterized protein C3orf41 OS=Homo sapiens GN=C3orf41 PE=2 SV=2 |
|  | **P56385\|ATP5I_HUMAN** | ATP synthase subunit e, mitochondrial OS=Homo sapiens GN=ATP5I PE=1 SV=2 |
|  | **Q9Y2B0\|CNPY2_HUMAN** | Protein canopy homolog 2 OS=Homo sapiens GN=CNPY2 PE=1 SV=1 |
|  | **Q9Y6I9\|TX264_HUMAN** | Testis-expressed sequence 264 protein OS=Homo sapiens GN=TEX264 PE=1 SV=1 |
|  | **Q96GQ5\|CP058_HUMAN** | UPF0420 protein C16orf58 OS=Homo sapiens GN=C16orf58 PE=1 SV=2 |
|  | **Q9UHQ4\|BAP29_HUMAN** | B-cell receptor-associated protein 29 OS=Homo sapiens GN=BCAP29 PE=1 SV=2 |
|  | **Q58FG1\|HS904_HUMAN** | Putative heat shock protein HSP 90-alpha A4 OS=Homo sapiens GN=HSP90AA4P PE=5 SV=1 |
|  | **P61769\|B2MG_HUMAN** | Beta-2-microglobulin OS=Homo sapiens GN=B2M PE=1 SV=1 |
|  | **O75665\|OFD1_HUMAN** | Oral-facial-digital syndrome 1 protein OS=Homo sapiens GN=OFD1 PE=1 SV=1 |
|  | **P22413\|ENPP1_HUMAN** | Ectonucleotide pyrophosphatase/phosphodiesterase family member 1 OS=Homo sapiens GN=ENPP1 PE=1 SV=2 |
|  | **P20648\|ATP4A_HUMAN** | Potassium-transporting ATPase alpha chain 1 OS=Homo sapiens GN=ATP4A PE=2 SV=5 |
|  | **Q9BRL6\|SFR2B_HUMAN** | Splicing factor, arginine/serine-rich 2B OS=Homo sapiens GN=SFRS2B PE=1 SV=1 |
|  | **Q01130\|SFRS2_HUMAN** | Splicing factor, arginine/serine-rich 2 OS=Homo sapiens GN=SFRS2 PE=1 SV=4 |
|  | **P05534\|1A24_HUMAN** | HLA class I histocompatibility antigen, A-24 alpha chain OS=Homo sapiens GN=HLA-A PE=1 SV=2 |
|  | **P30453\|1A34_HUMAN** | HLA class I histocompatibility antigen, A-34 alpha chain OS=Homo sapiens GN=HLA-A PE=1 SV=1 |
|  | **P01892\|1A02_HUMAN** | HLA class I histocompatibility antigen, A-2 alpha chain OS=Homo sapiens GN=HLA-A PE=1 SV=1 |
|  | **P30443\|1A01_HUMAN** | HLA class I histocompatibility antigen, A-1 alpha chain OS=Homo sapiens GN=HLA-A PE=1 SV=1 |
|  | **P30447\|1A23_HUMAN** | HLA class I histocompatibility antigen, A-23 alpha chain OS=Homo sapiens GN=HLA-A PE=1 SV=1 |
|  | **P10314\|1A32_HUMAN** | HLA class I histocompatibility antigen, A-32 alpha chain OS=Homo sapiens GN=HLA-A PE=2 SV=2 |
|  | **P16188\|1A30_HUMAN** | HLA class I histocompatibility antigen, A-30 alpha chain OS=Homo sapiens GN=HLA-A PE=1 SV=2 |
|  | **P16189\|1A31_HUMAN** | HLA class I histocompatibility antigen, A-31 alpha chain OS=Homo sapiens GN=HLA-A PE=2 SV=2 |
|  | **P18462\|1A25_HUMAN** | HLA class I histocompatibility antigen, A-25 alpha chain OS=Homo sapiens GN=HLA-A PE=2 SV=1 |
|  | **O94855\|SC24D_HUMAN** | Protein transport protein Sec24D OS=Homo sapiens GN=SEC24D PE=1 SV=2 |
|  | **P07919\|QCR6_HUMAN** | Cytochrome b-c1 complex subunit 6, mitochondrial OS=Homo sapiens GN=UQCRH PE=1 SV=2 |
|  | **P51148\|RAB5C_HUMAN** | Ras-related protein Rab-5C OS=Homo sapiens GN=RAB5C PE=1 SV=2 |
|  | **P28072\|PSB6_HUMAN** | Proteasome subunit beta type-6 OS=Homo sapiens GN=PSMB6 PE=1 SV=4 |
|  | **Q8IVS8\|GLCTK_HUMAN** | Glycerate kinase OS=Homo sapiens GN=GLYCTK PE=1 SV=1 |
|  | **P37275\|ZEB1_HUMAN** | Zinc finger E-box-binding homeobox 1 OS=Homo sapiens GN=ZEB1 PE=1 SV=2 |
|  | **Q9H330\|CI005_HUMAN** | Transmembrane protein C9orf5 OS=Homo sapiens GN=C9orf5 PE=1 SV=2 |
|  | **Q8WXF1\|PSPC1_HUMAN** | Paraspeckle component 1 OS=Homo sapiens GN=PSPC1 PE=1 SV=1 |
|  | **O60313\|OPA1_HUMAN** | Dynamin-like 120 kDa protein, mitochondrial OS=Homo sapiens GN=OPA1 PE=1 SV=3 |
|  | **Q9UIJ7\|KAD3_HUMAN** | GTP:AMP phosphotransferase mitochondrial OS=Homo sapiens GN=AK3 PE=1 SV=4 |
|  | **P38117\|ETFB_HUMAN** | Electron transfer flavoprotein subunit beta OS=Homo sapiens GN=ETFB PE=1 SV=3 |
|  | **Q8N4C7\|STX19_HUMAN** | Syntaxin-19 OS=Homo sapiens GN=STX19 PE=2 SV=1 |
|  | **P35221\|CTNA1_HUMAN** | Catenin alpha-1 OS=Homo sapiens GN=CTNNA1 PE=1 SV=1 |
|  | **O14556\|G3PT_HUMAN** | Glyceraldehyde-3-phosphate dehydrogenase, testis-specific OS=Homo sapiens GN=GAPDHS PE=1 SV=2 |
|  | **Q96JI7\|SPTCS_HUMAN** | Spatacsin OS=Homo sapiens GN=SPG11 PE=1 SV=2 |
|  | **Q9Y3B3\|TMED7_HUMAN** | Transmembrane emp24 domain-containing protein 7 OS=Homo sapiens GN=TMED7 PE=1 SV=2 |
|  | **P30479\|1B41_HUMAN** | HLA class I histocompatibility antigen, B-41 alpha chain OS=Homo sapiens GN=HLA-B PE=1 SV=1 |
|  | **Q04826\|1B40_HUMAN** | HLA class I histocompatibility antigen, B-40 alpha chain OS=Homo sapiens GN=HLA-B PE=1 SV=1 |
|  | **P07108\|ACBP_HUMAN** | Acyl-CoA-binding protein OS=Homo sapiens GN=DBI PE=1 SV=2 |
|  | **Q9H3N1\|TMX1_HUMAN** | Thioredoxin-related transmembrane protein 1 OS=Homo sapiens GN=TMX1 PE=1 SV=1 |
|  | **Q71UI9\|H2AV_HUMAN** | Histone H2A.V OS=Homo sapiens GN=H2AFV PE=1 SV=3 |
|  | **O00757\|F16P2_HUMAN** | Fructose-1,6-bisphosphatase isozyme 2 OS=Homo sapiens GN=FBP2 PE=1 SV=2 |
|  | **Q9UBU6\|FA8A1_HUMAN** | Protein FAM8A1 OS=Homo sapiens GN=FAM8A1 PE=1 SV=1 |
|  | **Q00765\|REEP5_HUMAN** | Receptor expression-enhancing protein 5 OS=Homo sapiens GN=REEP5 PE=1 SV=3 |
|  | **Q9NUI1\|DECR2_HUMAN** | Peroxisomal 2,4-dienoyl-CoA reductase OS=Homo sapiens GN=DECR2 PE=1 SV=1 |
|  | **Q96IU4\|ABHEB_HUMAN** | Abhydrolase domain-containing protein 14B OS=Homo sapiens GN=ABHD14B PE=1 SV=1 |
|  | **P02774\|VTDB_HUMAN** | Vitamin D-binding protein OS=Homo sapiens GN=GC PE=1 SV=1 |
|  | **Q92947\|GCDH_HUMAN** | Glutaryl-CoA dehydrogenase, mitochondrial OS=Homo sapiens GN=GCDH PE=1 SV=1 |
|  | **P62269\|RS18_HUMAN** | 40S ribosomal protein S18 OS=Homo sapiens GN=RPS18 PE=1 SV=3 |
|  | **Q16795\|NDUA9_HUMAN** | NADH dehydrogenase [ubiquinone] 1 alpha subcomplex subunit 9, mitochondrial OS=Homo sapiens GN=NDUFA9 PE=1 SV=2 |
|  | **Q8TCT9\|HM13_HUMAN** | Minor histocompatibility antigen H13 OS=Homo sapiens GN=HM13 PE=1 SV=1 |
|  | **Q9Y617\|SERC_HUMAN** | Phosphoserine aminotransferase OS=Homo sapiens GN=PSAT1 PE=1 SV=2 |
|  | **Q99735\|MGST2_HUMAN** | Microsomal glutathione S-transferase 2 OS=Homo sapiens GN=MGST2 PE=1 SV=1 |
|  | **O75381\|PEX14_HUMAN** | Peroxisomal membrane protein PEX14 OS=Homo sapiens GN=PEX14 PE=1 SV=1 |
|  | **P54821\|PRRX1_HUMAN** | Paired mesoderm homeobox protein 1 OS=Homo sapiens GN=PRRX1 PE=2 SV=2 |
|  | **Q04446\|GLGB_HUMAN** | 1,4-alpha-glucan-branching enzyme OS=Homo sapiens GN=GBE1 PE=1 SV=2 |
|  | **O75844\|FACE1_HUMAN** | CAAX prenyl protease 1 homolog OS=Homo sapiens GN=ZMPSTE24 PE=1 SV=2 |
|  | **Q9BUP3\|HTAI2_HUMAN** | Oxidoreductase HTATIP2 OS=Homo sapiens GN=HTATIP2 PE=1 SV=1 |
|  | **P48444\|COPD_HUMAN** | Coatomer subunit delta OS=Homo sapiens GN=ARCN1 PE=1 SV=1 |
|  | **Q8N136\|WDR69_HUMAN** | WD repeat-containing protein 69 OS=Homo sapiens GN=WDR69 PE=2 SV=1 |
|  | **Q9Y2L6\|FRM4B_HUMAN** | FERM domain-containing protein 4B OS=Homo sapiens GN=FRMD4B PE=1 SV=2 |
|  | **P22830\|HEMH_HUMAN** | Ferrochelatase, mitochondrial OS=Homo sapiens GN=FECH PE=1 SV=2 |
|  | **O15121\|DEGS1_HUMAN** | Sphingolipid delta(4)-desaturase DES1 OS=Homo sapiens GN=DEGS1 PE=1 SV=1 |
|  | **O75880\|SCO1_HUMAN** | Protein SCO1 homolog, mitochondrial OS=Homo sapiens GN=SCO1 PE=1 SV=1 |
|  | **Q99518\|FMO2_HUMAN** | Dimethylaniline monooxygenase [N-oxide-forming] 2 OS=Homo sapiens GN=FMO2 PE=2 SV=3 |
|  | **P30043\|BLVRB_HUMAN** | Flavin reductase OS=Homo sapiens GN=BLVRB PE=1 SV=3 |
|  | **P61978\|HNRPK_HUMAN** | Heterogeneous nuclear ribonucleoprotein K OS=Homo sapiens GN=HNRNPK PE=1 SV=1 |
|  | **Q07955\|SFRS1_HUMAN** | Splicing factor, arginine/serine-rich 1 OS=Homo sapiens GN=SFRS1 PE=1 SV=2 |
|  | **Q9P035\|PTAD1_HUMAN** | Protein tyrosine phosphatase-like protein PTPLAD1 OS=Homo sapiens GN=PTPLAD1 PE=1 SV=2 |
|  | **Q9NY15\|STAB1_HUMAN** | Stabilin-1 OS=Homo sapiens GN=STAB1 PE=1 SV=2 |
|  | **Q9NNX6\|CD209_HUMAN** | CD209 antigen OS=Homo sapiens GN=CD209 PE=1 SV=1 |
|  | **Q9H2X3\|CLC4M_HUMAN** | C-type lectin domain family 4 member M OS=Homo sapiens GN=CLEC4M PE=1 SV=1 |
|  | **P08754\|GNAI3_HUMAN** | Guanine nucleotide-binding protein G(k) subunit alpha OS=Homo sapiens GN=GNAI3 PE=1 SV=3 |
|  | **P63096\|GNAI1_HUMAN** | Guanine nucleotide-binding protein G(i), alpha-1 subunit OS=Homo sapiens GN=GNAI1 PE=1 SV=2 |
|  | **Q8IXI2\|MIRO1_HUMAN** | Mitochondrial Rho GTPase 1 OS=Homo sapiens GN=RHOT1 PE=1 SV=2 |
|  | **P04075\|ALDOA_HUMAN** | Fructose-bisphosphate aldolase A OS=Homo sapiens GN=ALDOA PE=1 SV=2 |
|  | **P09972\|ALDOC_HUMAN** | Fructose-bisphosphate aldolase C OS=Homo sapiens GN=ALDOC PE=1 SV=2 |
|  | **P13533\|MYH6_HUMAN** | Myosin-6 OS=Homo sapiens GN=MYH6 PE=1 SV=4 |
|  | **O76081\|RGS20_HUMAN** | Regulator of G-protein signaling 20 OS=Homo sapiens GN=RGS20 PE=1 SV=4 |
|  | **Q8IYM0\|F186B_HUMAN** | Protein FAM186B OS=Homo sapiens GN=FAM186B PE=2 SV=2 |
|  | **Q7Z5H3\|RHG22_HUMAN** | Rho GTPase-activating protein 22 OS=Homo sapiens GN=ARHGAP22 PE=1 SV=1 |
|  | **P61586\|RHOA_HUMAN** | Transforming protein RhoA OS=Homo sapiens GN=RHOA PE=1 SV=1 |
|  | **Q96BM9\|ARL8A_HUMAN** | ADP-ribosylation factor-like protein 8A OS=Homo sapiens GN=ARL8A PE=1 SV=1 |
|  | **Q9NVJ2\|ARL8B_HUMAN** | ADP-ribosylation factor-like protein 8B OS=Homo sapiens GN=ARL8B PE=1 SV=1 |
|  | **P39019\|RS19_HUMAN** | 40S ribosomal protein S19 OS=Homo sapiens GN=RPS19 PE=1 SV=2 |
|  | **P01023\|A2MG_HUMAN** | Alpha-2-macroglobulin OS=Homo sapiens GN=A2M PE=1 SV=1 |
|  | **Q7Z4N2\|TRPM1_HUMAN** | Transient receptor potential cation channel subfamily M member 1 OS=Homo sapiens GN=TRPM1 PE=1 SV=2 |
|  | **Q9P2D3\|HTR5B_HUMAN** | HEAT repeat-containing protein 5B OS=Homo sapiens GN=HEATR5B PE=1 SV=2 |
|  | **Q5VSK2\|MRC1L_HUMAN** | Macrophage mannose receptor 1-like protein 1 OS=Homo sapiens GN=MRC1L1 PE=2 SV=1 |
|  | **Q02224\|CENPE_HUMAN** | Centromere-associated protein E OS=Homo sapiens GN=CENPE PE=1 SV=2 |
|  | **Q9Y490\|TLN1_HUMAN** | Talin-1 OS=Homo sapiens GN=TLN1 PE=1 SV=3 |
|  | **Q92504\|S39A7_HUMAN** | Zinc transporter SLC39A7 OS=Homo sapiens GN=SLC39A7 PE=1 SV=2 |
|  | **P12109\|CO6A1_HUMAN** | Collagen alpha-1(VI) chain OS=Homo sapiens GN=COL6A1 PE=1 SV=3 |
|  | **P55145\|MANF_HUMAN** | Mesencephalic astrocyte-derived neurotrophic factor OS=Homo sapiens GN=MANF PE=1 SV=2 |
|  | **P55957\|BID_HUMAN** | BH3-interacting domain death agonist OS=Homo sapiens GN=BID PE=1 SV=1 |
|  | **Q9BUN8\|DERL1_HUMAN** | Derlin-1 OS=Homo sapiens GN=DERL1 PE=1 SV=1 |
|  | **P41091\|IF2G_HUMAN** | Eukaryotic translation initiation factor 2 subunit 3 OS=Homo sapiens GN=EIF2S3 PE=1 SV=3 |
|  | **Q9BQE4\|SELS_HUMAN** | Selenoprotein S OS=Homo sapiens GN=SELS PE=1 SV=3 |
|  | **P55822\|SH3BG_HUMAN** | SH3 domain-binding glutamic acid-rich protein OS=Homo sapiens GN=SH3BGR PE=1 SV=3 |
|  | **O15523\|DDX3Y_HUMAN** | ATP-dependent RNA helicase DDX3Y OS=Homo sapiens GN=DDX3Y PE=1 SV=2 |
|  | **O00571\|DDX3X_HUMAN** | ATP-dependent RNA helicase DDX3X OS=Homo sapiens GN=DDX3X PE=1 SV=3 |
|  | **P10599\|THIO_HUMAN** | Thioredoxin OS=Homo sapiens GN=TXN PE=1 SV=3 |
|  | **Q96PR1\|KCNC2_HUMAN** | Potassium voltage-gated channel subfamily C member 2 OS=Homo sapiens GN=KCNC2 PE=1 SV=1 |
|  | **Q8N7J2\|F123A_HUMAN** | Protein FAM123A OS=Homo sapiens GN=FAM123A PE=2 SV=2 |
|  | **P47870\|GBRB2_HUMAN** | Gamma-aminobutyric acid receptor subunit beta-2 OS=Homo sapiens GN=GABRB2 PE=1 SV=1 |
|  | **O60488\|ACSL4_HUMAN** | Long-chain-fatty-acid--CoA ligase 4 OS=Homo sapiens GN=ACSL4 PE=1 SV=2 |
|  | **Q15041\|AR6P1_HUMAN** | ADP-ribosylation factor-like protein 6-interacting protein 1 OS=Homo sapiens GN=ARL6IP1 PE=1 SV=2 |
|  | **P21926\|CD9_HUMAN** | CD9 antigen OS=Homo sapiens GN=CD9 PE=1 SV=4 |
|  | **P62805\|H4_HUMAN** | Histone H4 OS=Homo sapiens GN=HIST1H4A PE=1 SV=2 |

Top of Form

Select Summary Report

|  | Peptide Summary Select Summary (protein hits) Select Summary (unassigned) Export Search Results |  | Help |
| --- | --- | --- | --- |
|  | Significance threshold p< | Max. number of hits |  |
|  | Standard scoring  MudPIT scoring | Ions score or expect cut-off | Show sub-sets |
|  | Show pop-ups  Suppress pop-ups | Sort unassigned Decreasing Score Increasing query / Mr Decreasing Intensity | Require bold red |

Bottom of Form

Top of Form

| **1.** | P31327\|CPSM_HUMAN    **Mass:** 165975   **Score:** 4274   **Queries matched:** 285   **emPAI:** 4.92 |
| --- | --- |
|  | Carbamoyl-phosphate synthase [ammonia], mitochondrial OS=Homo sapiens GN=CPS1 PE=1 SV=2 |

|  | **Query** | **Observed** | **Mr(expt)** | **Mr(calc)** | **Delta** | **Miss** | **Score** | **Expect** | **Rank** | **Peptide** |
| --- | --- | --- | --- | --- | --- | --- | --- | --- | --- | --- |
|  | 1317 | **615.3528** | **614.3455** | **614.3500** | **-0.0045** | **0** | **20** | **2.6** | **1** | **K.NNVIR.L** |
|  | 1332 | **622.3183** | **621.3110** | **621.3156** | **-0.0046** | **0** | **17** | **3.2** | **1** | **R.IGSSMK.S** |
|  | 1523 | **678.4135** | **677.4062** | **677.4112** | **-0.0050** | **0** | **24** | **0.81** | **2** | **R.IYAIAK.A** |
|  | 1826 | **396.1919** | **790.3692** | **790.3722** | **-0.0030** | **0** | **12** | **15** | **2** | **R.FHGTSSR.I** 1825 |
|  | 1938 | **800.4573** | **799.4500** | **798.4388** | **1.0112** | **0** | **35** | **0.068** | **1** | **K.VAQAPWK.A** |
|  | 2111 | **406.2187** | **810.4228** | **810.4276** | **-0.0048** | **0** | **19** | **1.8** | **1** | **R.SFPFVSK.T** 2110 |
|  | 2250 | **412.2154** | **822.4163** | **821.4184** | **0.9979** | **0** | **17** | **3.3** | **1** | **K.SLFHYR.Q** 2237 |
|  | 2568 | **425.0472** | **848.0799** | **848.4426** | **-0.3627** | **1** | **26** | **0.33** | **1** | **R.DKGTMLGK.I** 2575 2576 |
|  | 2627 | **427.7226** | **853.4306** | **853.4368** | **-0.0061** | **0** | **17** | **3** | **1** | **K.AMLSTGFK.I** |
|  | 2698 | **430.5539** | **859.0932** | **859.4763** | **-0.3831** | **1** | **3** | **82** | **5** | **K.ILESDRK.E** |
|  | 2703 | **430.7416** | **859.4686** | **859.4763** | **-0.0077** | **1** | **15** | **7.4** | **6** | **R.KILESDR.K** |
|  | 2801 | **433.2216** | **864.4286** | **864.4341** | **-0.0055** | **0** | **41** | **0.016** | **1** | **K.FLEEATR.V** |
|  | 3570 | **462.2598** | **922.5051** | **922.4760** | **0.0291** | **0** | **18** | **2.5** | **2** | **K.YLESNGIK.V** |
|  | 3848 | **472.7820** | **943.5495** | **943.5563** | **-0.0068** | **1** | **31** | **0.14** | **1** | **R.RTSINVVR.H** 3829 |
|  | 4074 | **480.7595** | **959.5044** | **959.5110** | **-0.0066** | **0** | **31** | **0.14** | **1** | **K.VVAVDCGIK.N** 4075 |
|  | 4308 | **489.2307** | **976.4469** | **976.4535** | **-0.0067** | **0** | **22** | **1.1** | **1** | **R.EVEMDAVGK.D** |
|  | 4541 | **497.7600** | **993.5054** | **993.5131** | **-0.0077** | **1** | **12** | **9.1** | **1** | **R.AKEIGFSDK.Q** |
|  | 4849 | **508.2366** | **1014.4586** | **1014.4658** | **-0.0072** | **0** | **21** | **1.4** | **1** | **R.TFEESFQK.A** 4854 |
|  | 4900 | **509.7680** | **1017.5215** | **1017.5277** | **-0.0062** | **0** | **52** | **0.0011** | **1** | **K.SVGEVMAIGR.T** 4883 4901 |
|  | 4927 | **511.2709** | **1020.5272** | **1020.5869** | **-0.0596** | **1** | **16** | **4.5** | **2** | **K.KNIHPWVK.Q** 4928 |
|  | 5115 | **517.7651** | **1033.5156** | **1033.5226** | **-0.0070** | **0** | **(36)** | **0.038** | **1** | **K.SVGEVMAIGR.T** |
|  | 5143 | **519.1346** | **1036.2546** | **1035.5237** | **0.7310** | **0** | **10** | **10** | **4** | **K.EIEYEVVR.D** |
|  | 5527 | **534.8080** | **1067.6015** | **1067.6087** | **-0.0072** | **1** | **38** | **0.021** | **1** | **R.LRDADPILR.C** |
|  | 6304 | **565.2817** | **1128.5489** | **1128.5564** | **-0.0074** | **0** | **24** | **0.73** | **1** | **K.EWPSNLDLR.K** 6305 |
|  | 6368 | **567.2752** | **1132.5357** | **1132.5434** | **-0.0077** | **0** | **13** | **7.9** | **1** | **K.VMIGENVDEK.H** 6354 6383 |
|  | 6371 | **567.2886** | **1132.5627** | **1132.5724** | **-0.0097** | **1** | **10** | **15** | **2** | **R.KELSEPSSTR.I** |
|  | 6551 | **574.2759** | **1146.5372** | **1146.5458** | **-0.0086** | **0** | **36** | **0.041** | **1** | **K.DYNHWLATK.S** 6552 |
|  | 6560 | **574.7857** | **1147.5569** | **1147.5656** | **-0.0087** | **0** | **30** | **0.17** | **1** | **K.CLGLTEAQTR.E** |
|  | 6745 | **581.7993** | **1161.5841** | **1161.5931** | **-0.0090** | **0** | **32** | **0.091** | **1** | **K.FVHDNYVIR.R** 6746 |
|  | 6768 | **582.3701** | **1162.7257** | **1162.7325** | **-0.0068** | **0** | **51** | **0.0012** | **1** | **K.IALGIPLPEIK.N** 6769 |
|  | 7258 | **597.3390** | **1192.6634** | **1192.6703** | **-0.0069** | **0** | **46** | **0.004** | **1** | **K.VSGLLVLDYSK.D** 7276 7278 7279 |
|  | 7754 | **609.3078** | **1216.6010** | **1216.6088** | **-0.0077** | **0** | **52** | **0.0011** | **1** | **K.SLGQWLQEEK.V** 7755 7756 |
|  | 7850 | **611.3376** | **1220.6606** | **1220.6652** | **-0.0046** | **0** | **57** | **0.00032** | **1** | **R.SIFSAVLDELK.V** 7847 7848 7871 |
|  | 7868 | **611.8412** | **1221.6678** | **1221.6757** | **-0.0079** | **0** | **20** | **1.4** | **1** | **K.ATGYPLAFIAAK.I** |
|  | 8015 | **615.8251** | **1229.6356** | **1229.6438** | **-0.0082** | **0** | **73** | **7.1e-006** | **1** | **K.IMGTSPLQIDR.A** 8014 |
|  | 8117 | **618.3307** | **1234.6469** | **1235.6874** | **-1.0404** | **0** | **46** | **0.004** | **1** | **R.VSQEHPVVLTK.F** 8134 8135 |
|  | 8331 | **623.8220** | **1245.6295** | **1245.6387** | **-0.0092** | **0** | **(15)** | **5** | **1** | **K.IMGTSPLQIDR.A** 8348 |
|  | 8541 | **629.3284** | **1256.6422** | **1256.6513** | **-0.0091** | **1** | **5** | **52** | **6** | **K.EWPSNLDLRK.E** |
|  | 8899 | **639.3052** | **1276.5958** | **1277.6867** | **-1.0909** | **0** | **56** | **0.00033** | **1** | **K.TLGVDFIDVATK.V** 8927 |
|  | 9428 | **653.3038** | **1304.5930** | **1304.6031** | **-0.0101** | **1** | **63** | **6.8e-005** | **1** | **R.EVEMDAVGKDGR.V** 9430 |
|  | 9456 | **653.8404** | **1305.6662** | **1305.6751** | **-0.0089** | **0** | **31** | **0.097** | **2** | **K.AADTIGYPVMIR.S** 9458 |
|  | 9820 | **663.5601** | **1325.1057** | **1324.7099** | **0.3958** | **0** | **25** | **0.26** | **1** | **R.GQNQPVLNITNK.Q** |
|  | 10051 | **669.3255** | **1336.6364** | **1335.7220** | **0.9144** | **1** | **1** | **1.1e+002** | **2** | **K.AMLSTGFKIPQK.G** |
|  | 10523 | **682.8613** | **1363.7080** | **1363.7169** | **-0.0090** | **0** | **77** | **3e-006** | **1** | **K.AFAMTNQILVEK.S** 10493 10514 10520 10521 10522 10537 10543 |
|  | 10796 | **690.3649** | **1378.7152** | **1379.7119** | **-0.9967** | **0** | **(14)** | **5.8** | **1** | **K.AFAMTNQILVEK.S** |
|  | 10865 | **461.5779** | **1381.7120** | **1381.7201** | **-0.0081** | **0** | **29** | **0.19** | **1** | **K.AQTAHIVLEDGTK.M** |
|  | 11509 | **710.3730** | **1418.7314** | **1418.7405** | **-0.0091** | **0** | **59** | **0.00017** | **1** | **K.AVNTLNEALEFAK.S** 11508 |
|  | 11703 | **477.8852** | **1430.6337** | **1430.6435** | **-0.0098** | **0** | **30** | **0.13** | **1** | **R.MCHPSIEGFTPR.L** |
|  | 11913 | **723.3243** | **1444.6341** | **1443.7933** | **0.8408** | **1** | **6** | **33** | **1** | **K.QNLIAEVSTKDVK.V** |
|  | 11947 | **482.9862** | **1445.9368** | **1446.6384** | **-0.7016** | **0** | **(3)** | **55** | **9** | **R.MCHPSIEGFTPR.L** |
|  | 12452 | **738.8472** | **1475.6799** | **1475.6906** | **-0.0107** | **1** | **21** | **1.1** | **1** | **R.WDLDRFHGTSSR.I** 12451 12454 |
|  | 12588 | **742.9334** | **1483.8523** | **1483.8623** | **-0.0101** | **0** | **41** | **0.0095** | **1** | **K.GILIGIQQSFRPR.F** 12587 |
|  | 12658 | **744.8912** | **1487.7678** | **1488.8017** | **-1.0339** | **1** | **55** | **0.00036** | **1** | **K.LTYIDKWFLYK.M** 12675 12676 12677 |
|  | 12713 | **746.8407** | **1491.6668** | **1491.6763** | **-0.0094** | **0** | **61** | **0.00011** | **1** | **K.AIDDNMSLDEIEK.L** |
|  | 13986 | **789.4064** | **1576.7982** | **1576.8096** | **-0.0115** | **1** | **57** | **0.00021** | **1** | **R.QLFSDKLNEINEK.I** 13988 |
|  | 14170 | **794.9040** | **1587.7934** | **1587.8045** | **-0.0111** | **0** | **18** | **2.1** | **1** | **R.FLGVAEQLHNEGFK.L** 14171 14172 14173 |
|  | 14205 | **795.4202** | **1588.8258** | **1588.8348** | **-0.0090** | **0** | **96** | **2.8e-008** | **1** | **K.IAPSFAVESIEDALK.A** 14201 14202 14203 14204 |
|  | 14259 | **796.8903** | **1591.7660** | **1591.7777** | **-0.0117** | **0** | **50** | **0.0012** | **1** | **R.SAYALGGLGSGICPNR.E** 14260 14261 |
|  | 14280 | **797.8849** | **1593.7552** | **1593.7668** | **-0.0116** | **1** | **83** | **6.4e-007** | **1** | **K.GLNSESMTEETLKR.A** 14281 14282 14284 14298 |
|  | 14461 | **804.3956** | **1606.7767** | **1606.7872** | **-0.0105** | **0** | **95** | **3.5e-008** | **1** | **K.VLGTSVESIMATEDR.Q** 14459 14460 14462 |
|  | 14768 | **543.3106** | **1626.9098** | **1627.8716** | **-0.9617** | **1** | **(4)** | **45** | **4** | **K.NGVKIMGTSPLQIDR.A** |
|  | 14950 | **819.4518** | **1636.8891** | **1636.8977** | **-0.0086** | **0** | **46** | **0.0031** | **1** | **K.AFAISGPFNVQFLVK.G** 14948 14949 14951 14960 |
|  | 15038 | **548.8052** | **1643.3939** | **1643.8665** | **-0.4726** | **1** | **6** | **20** | **4** | **K.NGVKIMGTSPLQIDR.A** |
|  | 15202 | **827.4872** | **1652.9599** | **1652.8226** | **0.1374** | **1** | **5** | **36** | **5** | **R.IGSSMKSVGEVMAIGR.T** 15176 |
|  | 15934 | **851.4277** | **1700.8409** | **1700.8515** | **-0.0106** | **1** | **15** | **3.8** | **1** | **K.IMGTSPLQIDRAEDR.S** |
|  | 16125 | **856.9481** | **1711.8817** | **1711.8715** | **0.0102** | **1** | **4** | **41** | **10** | **R.LPMNKEWPSNLDLR.K** |
|  | 16287 | **575.3214** | **1722.9422** | **1724.0196** | **-1.0774** | **0** | **30** | **0.12** | **1** | **K.ATTITSVLPKPALVASR.V** 16289 16297 16298 |
|  | 16319 | **863.9461** | **1725.8777** | **1725.8897** | **-0.0120** | **0** | **81** | **8.9e-007** | **1** | **R.DGSIDLVINLPNNNTK.F** 16320 16332 |
|  | 16638 | **874.8910** | **1747.7675** | **1747.7797** | **-0.0122** | **0** | **44** | **0.0042** | **1** | **K.TSACFEPSLDYMVTK.I** 16639 |
|  | 16657 | **875.4333** | **1748.8520** | **1748.8635** | **-0.0114** | **0** | **31** | **0.097** | **1** | **R.GAEVHLVPWNHDFTK.M** 16658 16659 |
|  | 16897 | **882.8885** | **1763.7624** | **1763.7746** | **-0.0122** | **0** | **(21)** | **0.84** | **1** | **K.TSACFEPSLDYMVTK.I** |
|  | 17401 | **902.4908** | **1802.9670** | **1802.9778** | **-0.0108** | **0** | **58** | **0.00017** | **1** | **R.TAVDSGIPLLTNFQVTK.L** 17400 17417 |
|  | 18718 | **953.4829** | **1904.9513** | **1904.9646** | **-0.0133** | **1** | **70** | **1.1e-005** | **1** | **K.RGAEVHLVPWNHDFTK.M** 18716 |
|  | 18786 | **637.6400** | **1909.8980** | **1909.1360** | **0.7620** | **1** | **6** | **22** | **2** | **K.GKATTITSVLPKPALVASR.V** |
|  | 18911 | **641.6520** | **1921.9343** | **1922.0837** | **-0.1494** | **1** | **2** | **65** | **6** | **R.LLSVKAQTAHIVLEDGTK.M** |
|  | 19014 | **965.5300** | **1929.0455** | **1929.0571** | **-0.0116** | **0** | **23** | **0.47** | **1** | **K.EPLFGISTGNLITGLAAGAK.T** |
|  | 19357 | **980.5396** | **1959.0647** | **1959.0789** | **-0.0143** | **1** | **75** | **3e-006** | **1** | **R.RTAVDSGIPLLTNFQVTK.L** |
|  | 19839 | **1001.5490** | **2001.0833** | **2001.0935** | **-0.0101** | **1** | **23** | **0.41** | **1** | **R.SIFSAVLDELKVAQAPWK.A** |
|  | 19951 | **1007.0563** | **2012.0980** | **2012.1095** | **-0.0115** | **0** | **43** | **0.0044** | **1** | **K.HLPTLDHPIIPADYVAIK.A** 19947 19948 19949 19950 |
|  | 19973 | **672.3741** | **2014.1006** | **2015.0040** | **-0.9034** | **1** | **3** | **47** | **5** | **K.MSMANRGQNQPVLNITNK.Q** |
|  | 20159 | **1014.5370** | **2027.0594** | **2027.0721** | **-0.0127** | **0** | **111** | **6.4e-010** | **1** | **K.TVLMNPNIASVQTNEVGLK.Q** 20154 20155 20156 20158 20160 20161 20172 |
|  | 20181 | **677.3309** | **2028.9708** | **2029.9779** | **-1.0071** | **1** | **1** | **79** | **2** | **K.SVGEVMAIGRTFEESFQK.A** |
|  | 20291 | **1022.5337** | **2043.0528** | **2043.0670** | **-0.0142** | **0** | **(90)** | **1e-007** | **1** | **K.TVLMNPNIASVQTNEVGLK.Q** 20290 |
|  | 20338 | **683.3365** | **2046.9876** | **2046.9939** | **-0.0062** | **1** | **(1)** | **82** | **10** | **K.MSMANRGQNQPVLNITNK.Q** |
|  | 20487 | **686.7211** | **2057.1414** | **2057.1521** | **-0.0107** | **1** | **0** | **78** | **3** | **R.KEPLFGISTGNLITGLAAGAK.T** |
|  | 21697 | **547.0261** | **2184.0754** | **2183.0780** | **0.9974** | **1** | **5** | **26** | **6** | **K.EYGVKVLGTSVESIMATEDR.Q** |
|  | 22090 | **1113.5389** | **2225.0633** | **2225.0773** | **-0.0140** | **1** | **67** | **1.5e-005** | **1** | **K.AIDDNMSLDEIEKLTYIDK.W** 22091 |
|  | 22590 | **1145.0902** | **2288.1659** | **2288.1801** | **-0.0142** | **1** | **105** | **2.6e-009** | **1** | **K.SLGQWLQEEKVPAIYGVDTR.M** 22591 22592 |
|  | 22745 | **1155.1184** | **2308.2223** | **2308.2355** | **-0.0132** | **0** | **34** | **0.033** | **1** | **K.QADTVYFLPITPQFVTEVIK.A** 22743 22744 22746 22747 |
|  | 22854 | **581.3055** | **2321.1928** | **2321.2056** | **-0.0127** | **1** | **12** | **5.2** | **1** | **K.VSGLLVLDYSKDYNHWLATK.S** |
|  | 23408 | **1197.0887** | **2392.1629** | **2392.2196** | **-0.0566** | **1** | **6** | **20** | **2** | **K.TLGVDFIDVATKVMIGENVDEK.H** 23410 |
|  | 23538 | **1208.0342** | **2414.0538** | **2414.0705** | **-0.0167** | **0** | **110** | **8e-010** | **1** | **R.CEMASTGEVACFGEGIHTAFLK.A** 23537 23539 |
|  | 24529 | **1277.1545** | **2552.2945** | **2552.3122** | **-0.0177** | **0** | **85** | **1.8e-007** | **1** | **K.VLILGSGGLSIGQAGEFDYSGSQAVK.A** 24528 24532 24533 24534 24535 24537 24540 24541 24542 24545 24547 24550 24551 24552 24553 24554 24556 24557 24558 24559 24567 24568 24570 24572 24573 24574 24575 24576 |
|  | 24888 | **1306.1381** | **2610.2616** | **2610.2782** | **-0.0166** | **1** | **66** | **1.7e-005** | **1** | **R.SAYALGGLGSGICPNRETLMDLSTK.A** 24887 |
|  | 24918 | **1309.6444** | **2617.2743** | **2617.2880** | **-0.0137** | **0** | **87** | **1.4e-007** | **1** | **K.AEQPDGLILGMGGQTALNCGVELFK.R** 24916 24917 24919 |
|  | 25395 | **1354.6995** | **2707.3844** | **2707.4003** | **-0.0159** | **0** | **90** | **5.8e-008** | **1** | **K.MEYDGILIAGGPGNPALAEPLIQNVR.K** 25391 25392 25393 25394 25396 |
|  | 25483 | **1362.6969** | **2723.3792** | **2723.3952** | **-0.0160** | **0** | **(81)** | **4.6e-007** | **1** | **K.MEYDGILIAGGPGNPALAEPLIQNVR.K** |
|  | 25737 | **925.4682** | **2773.3828** | **2773.3891** | **-0.0063** | **1** | **35** | **0.019** | **1** | **K.AEQPDGLILGMGGQTALNCGVELFKR.G** |
|  | 26006 | **1409.2105** | **2816.4063** | **2816.4232** | **-0.0169** | **1** | **69** | **8.1e-006** | **1** | **K.IEFEGQPVDFVDPNKQNLIAEVSTK.D** 26004 26005 26008 26009 26010 26011 |
|  | 26307 | **1434.2373** | **2866.4600** | **2866.4746** | **-0.0146** | **0** | **73** | **2.6e-006** | **1** | **K.GQILTMANPIIGNGGAPDTTALDELGLSK.Y** 26305 26306 26308 26313 26314 26315 26316 |
|  | 27630 | **1043.2140** | **3126.6201** | **3126.6423** | **-0.0222** | **1** | **55** | **0.00014** | **1** | **K.VMIGENVDEKHLPTLDHPIIPADYVAIK.A** 27632 |
|  | 28467 | **1124.5437** | **3370.6093** | **3370.6261** | **-0.0168** | **0** | **66** | **9.4e-006** | **1** | **R.HLGIVGECNIQFALHPTSMEYCIIEVNAR.L** |
|  | 28514 | **1129.8705** | **3386.5896** | **3386.6096** | **-0.0199** | **0** | **72** | **2.9e-006** | **1** | **K.GYSFGHPSSVAGEVVFNTGLGGYPEAITDPAYK.G** 28513 |
|  | 28592 | **1138.2443** | **3411.7110** | **3411.7319** | **-0.0210** | **0** | **40** | **0.0039** | **1** | **R.ILDIYHQEACGGCIISVGGQIPNNLAVPLYK.N** |
|  | 28613 | **1141.2144** | **3420.6213** | **3420.6404** | **-0.0191** | **1** | **57** | **8.5e-005** | **1** | **K.SVDYPCLLRPSYVLSGSAMNVVFSEDEMKK.F** 28610 28611 28612 28614 28615 |
|  | 29129 | **1203.5442** | **3607.6107** | **3607.6335** | **-0.0227** | **1** | **68** | **5e-006** | **1** | **K.TVVVNCNPETVSTDFDECDKLYFEELSLER.I** 29130 29131 |
|  | 29406 | **1247.9552** | **3740.8438** | **3740.8679** | **-0.0242** | **0** | **78** | **5.7e-007** | **1** | **R.VISHAISEHVEDAGVHSGDATLMLPTQTISQGAIEK.V** 29404 29407 29408 29409 29410 |
|  | 29452 | **940.2167** | **3756.8376** | **3756.8629** | **-0.0253** | **0** | **(16)** | **0.92** | **1** | **R.VISHAISEHVEDAGVHSGDATLMLPTQTISQGAIEK.V** |
|  | 30036 | **1323.6432** | **3967.9077** | **3967.9340** | **-0.0263** | **0** | **5** | **8.8** | **1** | **K.LFATEATSDWLNANNVPATPVAWPSQEGQNPSLSSIR.K** 30038 |
|  | 30288 | **1366.3423** | **4096.0050** | **4096.0290** | **-0.0239** | **1** | **72** | **1.5e-006** | **1** | **K.LFATEATSDWLNANNVPATPVAWPSQEGQNPSLSSIRK.L** 30287 |
|  | 30891 | **1110.8165** | **4439.2370** | **4438.2532** | **0.9838** | **1** | **17** | **0.48** | **1** | **R.GAEVHLVPWNHDFTKMEYDGILIAGGPGNPALAEPLIQNVR.K** |
|  | 31110 | **1561.0212** | **4680.0419** | **4680.0659** | **-0.0240** | **0** | **68** | **2.2e-006** | **1** | **R.DADDNCVTVCNMENVDAMGVHTGDSVVVAPAQTLSNAEFQMLR.R** |
|  | 31730 | **1425.3915** | **5697.5368** | **5697.5790** | **-0.0422** | **1** | **31** | **0.0063** | **1** | **K.EIEYEVVRDADDNCVTVCNMENVDAMGVHTGDSVVVAPAQTLSNAEFQMLR.R** 31731 31733 |
|  | 31839 | **1560.0161** | **6236.0353** | **6235.0736** | **0.9617** | **1** | **18** | **0.11** | **1** | **K.GYSFGHPSSVAGEVVFNTGLGGYPEAITDPAYKGQILTMANPIIGNGGAPDTTALDELGLSK.Y** 31840 |
|  | 31954 | **1149.2839** | **8037.9366** | **8037.9458** | **-0.0091** | **0** | **5** | **0.66** | **1** | **K.QAFITAQNHGYALDNTLPAGWKPLFVNVNDQTNEGIMHESKPFFAVQFHPEVTPGPIDTEYLFDSFFSLIK.K** |

| **2.** | P00325\|ADH1B_HUMAN    **Mass:** 40684    **Score:** 3604   **Queries matched:** 180   **emPAI:** 25.01 |
| --- | --- |
|  | Alcohol dehydrogenase 1B OS=Homo sapiens GN=ADH1B PE=1 SV=2 |

|  | **Query** | **Observed** | **Mr(expt)** | **Mr(calc)** | **Delta** | **Miss** | **Score** | **Expect** | **Rank** | **Peptide** |
| --- | --- | --- | --- | --- | --- | --- | --- | --- | --- | --- |
|  | 1134 | **564.7275** | **563.7203** | **563.3431** | **0.3772** | **1** | **4** | **57** | **2** | **K.FAKAK.E** |
|  | 1769 | **785.3839** | **784.3766** | **784.3828** | **-0.0062** | **0** | **11** | **15** | **1** | **K.NDLGNPR.G** |
|  | 1912 | **798.4093** | **797.4020** | **797.4072** | **-0.0052** | **0** | **29** | **0.17** | **1** | **K.GAVYGGFK.S** |
|  | 2553 | **847.4208** | **846.4135** | **846.4196** | **-0.0061** | **0** | **8** | **31** | **2** | **R.GTLQDGTR.R** |
|  | 3102 | **443.2709** | **884.5272** | **884.5331** | **-0.0059** | **0** | **13** | **7.6** | **7** | **R.IIAVDINK.D** |
|  | 3178 | **447.7383** | **893.4620** | **893.4681** | **-0.0061** | **0** | **39** | **0.019** | **1** | **K.LVADFMAK.K** 3177 3179 |
|  | 3337 | **453.2354** | **904.4563** | **904.4623** | **-0.0060** | **0** | **36** | **0.052** | **1** | **K.MVAVGICR.T** 3325 3327 3333 |
|  | 3404 | **455.7356** | **909.4566** | **909.4630** | **-0.0063** | **0** | **(12)** | **8.8** | **1** | **K.LVADFMAK.K** |
|  | 3471 | **458.2656** | **914.5166** | **914.5225** | **-0.0060** | **0** | **57** | **0.00037** | **1** | **K.AAVLWEVK.K** 3467 3468 3469 3486 |
|  | 3554 | **461.6588** | **921.3031** | **920.4572** | **0.8459** | **0** | **(4)** | **59** | **9** | **K.MVAVGICR.T** |
|  | 3738 | **468.5695** | **935.1245** | **935.5440** | **-0.4195** | **1** | **10** | **13** | **1** | **K.SIRTVLTF.-** |
|  | 3825 | **472.2549** | **942.4953** | **942.5022** | **-0.0069** | **0** | **33** | **0.092** | **1** | **K.IDAASPLEK.V** 3826 |
|  | 3999 | **477.7995** | **953.5845** | **953.5909** | **-0.0064** | **0** | **43** | **0.0078** | **1** | **K.KPIQEVLK.E** 3998 |
|  | 4709 | **502.7657** | **1003.5168** | **1002.5207** | **0.9962** | **1** | **6** | **48** | **8** | **R.GTLQDGTRR.F** |
|  | 5167 | **520.2565** | **1038.4984** | **1037.5579** | **0.9405** | **1** | **13** | **6.9** | **1** | **K.LVADFMAKK.F** |
|  | 6249 | **562.7519** | **1123.4893** | **1123.4968** | **-0.0074** | **0** | **42** | **0.0086** | **1** | **K.NPESNYCLK.N** 6247 6248 |
|  | 6316 | **565.3330** | **1128.6515** | **1127.6550** | **0.9965** | **1** | **23** | **0.79** | **1** | **R.IIAVDINKDK.F** 6313 |
|  | 8576 | **630.3404** | **1258.6662** | **1258.6744** | **-0.0082** | **0** | **44** | **0.0062** | **1** | **K.VIPLFTPQCGK.C** 8577 |
|  | 9913 | **665.3396** | **1328.6646** | **1328.6725** | **-0.0078** | **0** | **55** | **0.00043** | **1** | **K.INEGFDLLHSGK.S** 9908 9909 9910 9911 9912 9929 |
|  | 10841 | **461.2424** | **1380.7054** | **1381.8041** | **-1.0987** | **1** | **3** | **74** | **8** | **K.AAGAARIIAVDINK.D** |
|  | 11467 | **473.2544** | **1416.7415** | **1417.7639** | **-1.0224** | **1** | **7** | **26** | **10** | **K.EGIPKLVADFMAK.K** 11469 11484 |
|  | 12991 | **504.5672** | **1510.6798** | **1510.6908** | **-0.0110** | **1** | **35** | **0.039** | **1** | **R.VCKNPESNYCLK.N** 12990 |
|  | 13702 | **778.4170** | **1554.8194** | **1554.8293** | **-0.0099** | **0** | **52** | **0.00074** | **1** | **K.KPFSIEDVEVAPPK.A** 13701 13703 13707 |
|  | 14920 | **819.3719** | **1636.7292** | **1636.7403** | **-0.0110** | **0** | **80** | **1.2e-006** | **1** | **K.ELGATECINPQDYK.K** 14917 14918 14919 14921 14936 14938 |
|  | 16389 | **865.4758** | **1728.9370** | **1728.9451** | **-0.0081** | **0** | **86** | **3.1e-007** | **1** | **K.FSLDALITHVLPFEK.I** 16386 16388 16390 |
|  | 16809 | **879.8994** | **1757.7843** | **1757.7931** | **-0.0088** | **0** | **93** | **5.6e-008** | **1** | **K.EMTDGGVDFSFEVIGR.L** 16786 16788 16789 16790 16791 16792 16793 16794 16795 16796 16798 16799 16800 16802 16803 16804 16805 16806 16807 16808 16810 16811 16812 16813 16814 16815 16816 16819 16820 16826 16827 16828 16839 16840 |
|  | 17806 | **918.9362** | **1835.8578** | **1835.8723** | **-0.0146** | **1** | **57** | **0.00023** | **1** | **K.AKELGATECINPQDYK.K** 17807 17808 17826 |
|  | 18096 | **929.5218** | **1857.0290** | **1857.0400** | **-0.0110** | **1** | **90** | **9.9e-008** | **1** | **K.KFSLDALITHVLPFEK.I** 18094 18095 18097 18098 18100 18101 18102 18109 18111 |
|  | 18541 | **631.2869** | **1890.8390** | **1889.8690** | **0.9700** | **1** | **4** | **39** | **3** | **K.NPESNYCLKNDLGNPR.G** |
|  | 20506 | **1030.4940** | **2058.9735** | **2058.9867** | **-0.0132** | **0** | **97** | **1.7e-008** | **1** | **K.VCLIGCGFSTGYGSAVNVAK.V** 20504 20505 |
|  | 21586 | **725.0203** | **2172.0391** | **2173.1419** | **-1.1028** | **1** | **6** | **19** | **1** | **K.KPFSIEDVEVAPPKAYEVR.I** |
|  | 22865 | **1163.0658** | **2324.1170** | **2324.1328** | **-0.0157** | **0** | **106** | **1.9e-009** | **1** | **K.VTPGSTCAVFGLGGVGLSAVMGCK.A** 22866 22867 22868 22869 22870 22871 22872 22873 22874 22876 22887 |
|  | 22997 | **1171.0629** | **2340.1112** | **2340.1277** | **-0.0165** | **0** | **(96)** | **1.8e-008** | **1** | **K.VTPGSTCAVFGLGGVGLSAVMGCK.A** 22988 22998 22999 |
|  | 24695 | **858.4423** | **2572.3050** | **2572.3206** | **-0.0157** | **1** | **10** | **5.9** | **1** | **K.ELGATECINPQDYKKPIQEVLK.E** |
|  | 25290 | **898.7924** | **2693.3554** | **2693.3735** | **-0.0180** | **1** | **85** | **1.8e-007** | **1** | **K.KPIQEVLKEMTDGGVDFSFEVIGR.L** 25291 |
|  | 25412 | **903.7743** | **2708.3010** | **2709.3684** | **-1.0673** | **1** | **(2)** | **42** | **4** | **K.KPIQEVLKEMTDGGVDFSFEVIGR.L** |
|  | 26160 | **1423.7186** | **2845.4227** | **2845.4399** | **-0.0172** | **0** | **92** | **3.7e-008** | **1** | **R.GKPIHHFLGTSTFSQYTVVDENAVAK.I** 26150 26151 26153 26154 26157 26158 26159 26173 26177 |
|  | 30311 | **1369.7159** | **4106.1260** | **4106.1536** | **-0.0276** | **0** | **60** | **2.3e-005** | **1** | **R.TDDHVVSGNLVTPLPVILGHEAAGIVESVGEGVTTVKPGDK.V** 30310 30313 30314 30315 30317 30318 30319 30321 30322 30333 30334 |
|  | 30822 | **1476.3864** | **4426.1372** | **4426.1649** | **-0.0277** | **0** | **56** | **4.7e-005** | **1** | **R.LDTMMASLLCCHEACGTSVIVGVPPASQNLSINPMLLLTGR.T** 30821 30823 30824 30826 30828 30830 30831 30837 30838 30842 30844 30845 30849 30850 30851 30852 30854 30855 30858 30859 30860 |
|  | 31461 | **1070.3645** | **5346.7861** | **5346.8174** | **-0.0313** | **1** | **43** | **0.00064** | **1** | **R.TDDHVVSGNLVTPLPVILGHEAAGIVESVGEGVTTVKPGDKVIPLFTPQCGK.C** |

| **3.** | P35579\|MYH9_HUMAN    **Mass:** 227646   **Score:** 3221   **Queries matched:** 209   **emPAI:** 2.12 |
| --- | --- |
|  | Myosin-9 OS=Homo sapiens GN=MYH9 PE=1 SV=4 |

|  | **Query** | **Observed** | **Mr(expt)** | **Mr(calc)** | **Delta** | **Miss** | **Score** | **Expect** | **Rank** | **Peptide** |
| --- | --- | --- | --- | --- | --- | --- | --- | --- | --- | --- |
|  | 997 | **533.1882** | **532.1809** | **531.3017** | **0.8792** | **0** | **8** | **42** | **1** | **R.IGQSK.V** 996 1000 1001 1002 1004 1005 1008 |
|  | 1167 | **572.9950** | **571.9877** | **571.3806** | **0.6072** | **1** | **12** | **10** | **2** | **R.IKVGR.D** 1173 |
|  | 1179 | **577.0236** | **576.0163** | **575.3101** | **0.7063** | **0** | **8** | **23** | **1** | **K.NMALK.K** |
|  | 1214 | **586.2972** | **585.2900** | **585.2944** | **-0.0045** | **0** | **17** | **3.7** | **1** | **K.MNPPK.F** |
|  | 1295 | **609.3199** | **608.3127** | **608.3170** | **-0.0043** | **0** | **26** | **0.38** | **1** | **K.SFVEK.V** 1304 |
|  | 1367 | **630.3886** | **629.3813** | **629.3860** | **-0.0047** | **0** | **19** | **3.7** | **1** | **K.ALSLAR.A** |
|  | 1456 | **653.3032** | **652.2959** | **653.2843** | **-0.9884** | **0** | **13** | **7.2** | **8** | **K.GFMDGK.Q** |
|  | 1459 | **656.4042** | **655.3970** | **656.4221** | **-1.0251** | **0** | **13** | **6.4** | **3** | **K.VLLQGK.G** |
|  | 1584 | **705.3660** | **704.3588** | **704.3639** | **-0.0052** | **0** | **23** | **1.1** | **1** | **R.SMAVAAR.K** |
|  | 1631 | **719.3886** | **718.3813** | **717.4207** | **0.9606** | **1** | **18** | **3.3** | **2** | **K.MQLAKK.E** |
|  | 1635 | **721.3285** | **720.3212** | **719.4000** | **0.9213** | **1** | **14** | **7.5** | **4** | **K.NMALKK.I** |
|  | 1665 | **735.0493** | **734.0420** | **733.3792** | **0.6628** | **0** | **3** | **73** | **1** | **K.LQAQMK.D** |
|  | 1968 | **401.2853** | **800.5559** | **800.4140** | **0.1419** | **0** | **14** | **7.6** | **2** | **K.QLAAENR.L** |
|  | 2062 | **404.7288** | **807.4430** | **807.4491** | **-0.0061** | **0** | **21** | **1.2** | **1** | **R.TVGQLYK.E** |
|  | 2489 | **841.4352** | **840.4280** | **840.4341** | **-0.0061** | **0** | **35** | **0.053** | **1** | **K.SVHELEK.S** |
|  | 2530 | **423.2321** | **844.4496** | **845.4793** | **-1.0297** | **1** | **9** | **27** | **9** | **R.KLQAQMK.D** 2546 2548 |
|  | 2750 | **863.0858** | **862.0785** | **861.4742** | **0.6043** | **1** | **(0)** | **1.3e+002** | **7** | **R.KLQAQMK.D** |
|  | 2757 | **432.2277** | **862.4409** | **861.4014** | **1.0395** | **0** | **6** | **54** | **3** | **-.MAQQAADK.Y** |
|  | 3162 | **446.7122** | **891.4098** | **891.4160** | **-0.0062** | **0** | **22** | **1.2** | **2** | **K.ADEWLMK.N** |
|  | 3373 | **454.7448** | **907.4750** | **907.4803** | **-0.0053** | **0** | **19** | **2.2** | **1** | **K.FVSELWK.D** |
|  | 3506 | **459.2351** | **916.4556** | **916.4614** | **-0.0058** | **0** | **34** | **0.079** | **1** | **K.DLEGLSQR.H** 3505 |
|  | 3582 | **462.7474** | **923.4803** | **923.4865** | **-0.0062** | **0** | **44** | **0.0063** | **1** | **R.VVFQEFR.Q** |
|  | 3677 | **466.2601** | **930.5057** | **930.5134** | **-0.0077** | **1** | **7** | **42** | **8** | **R.VRTELADK.V** |
|  | 3894 | **474.2494** | **946.4843** | **946.4906** | **-0.0063** | **0** | **30** | **0.18** | **1** | **R.QQQLTAMK.V** |
|  | 4083 | **481.1506** | **960.2866** | **960.4777** | **-0.1911** | **0** | **8** | **25** | **3** | **R.NTNPNFVR.C** |
|  | 4432 | **493.7819** | **985.5493** | **985.5556** | **-0.0063** | **1** | **10** | **17** | **10** | **K.GALALEEKR.R** |
|  | 4745 | **503.7359** | **1005.4572** | **1004.5185** | **0.9387** | **1** | **4** | **75** | **9** | **K.QRSMAVAAR.K** 4731 |
|  | 4969 | **513.2107** | **1024.4068** | **1023.5237** | **0.8832** | **1** | **6** | **35** | **7** | **K.ATDKSFVEK.V** |
|  | 5107 | **517.7593** | **1033.5040** | **1033.5114** | **-0.0074** | **0** | **15** | **5.2** | **3** | **K.LQEMEGTVK.S** |
|  | 5155 | **519.2921** | **1036.5695** | **1035.5600** | **1.0095** | **1** | **14** | **6.7** | **1** | **K.VAAYDKLEK.T** |
|  | 5254 | **523.2856** | **1044.5567** | **1044.5637** | **-0.0070** | **0** | **47** | **0.0033** | **1** | **R.LEVNLQAMK.A** |
|  | 5289 | **524.7490** | **1047.4835** | **1047.4906** | **-0.0071** | **0** | **30** | **0.18** | **1** | **R.ALEEAMEQK.A** |
|  | 5571 | **536.2970** | **1070.5794** | **1070.6124** | **-0.0330** | **1** | **17** | **3.4** | **2** | **K.KLVWVPSDK.S** |
|  | 5998 | **552.2991** | **1102.5836** | **1102.5917** | **-0.0081** | **1** | **33** | **0.082** | **1** | **K.RQQQLTAMK.V** 6000 |
|  | 6171 | **559.2921** | **1116.5697** | **1115.5710** | **0.9987** | **0** | **11** | **13** | **2** | **R.DLGEELEALK.T** |
|  | 6651 | **577.7811** | **1153.5476** | **1153.5550** | **-0.0074** | **0** | **41** | **0.011** | **1** | **K.VMQEQGTHPK.F** 6650 6652 6653 6655 |
|  | 6661 | **578.0131** | **1154.0117** | **1154.6560** | **-0.6443** | **1** | **7** | **21** | **2** | **R.RGDLPFVVPR.R** |
|  | 7062 | **593.2817** | **1184.5488** | **1184.5859** | **-0.0371** | **1** | **4** | **70** | **8** | **K.DDIQKMNPPK.F** |
|  | 7254 | **597.3074** | **1192.6003** | **1192.6088** | **-0.0085** | **0** | **4** | **55** | **7** | **K.ALELDSNLYR.I** |
|  | 7274 | **597.8357** | **1193.6569** | **1193.6655** | **-0.0086** | **1** | **13** | **6.8** | **1** | **K.YKASITALEAK.I** |
|  | 7536 | **603.8130** | **1205.6115** | **1204.6339** | **0.9776** | **0** | **11** | **12** | **1** | **K.TDLLLEPYNK.Y** |
|  | 7641 | **606.7924** | **1211.5703** | **1211.5782** | **-0.0079** | **0** | **43** | **0.0071** | **1** | **K.DLEAHIDSANK.N** 7643 |
|  | 7703 | **608.3338** | **1214.6530** | **1214.6619** | **-0.0088** | **1** | **42** | **0.0094** | **1** | **R.ASREEILAQAK.E** 7702 |
|  | 7815 | **610.8254** | **1219.6363** | **1219.6448** | **-0.0085** | **1** | **56** | **0.00035** | **1** | **K.KFDQLLAEEK.T** 7814 |
|  | 7843 | **407.8885** | **1220.6437** | **1219.5754** | **1.0683** | **0** | **8** | **26** | **9** | **R.ALEQQVEEMK.T** 7789 |
|  | 8543 | **629.3329** | **1256.6513** | **1256.6221** | **0.0292** | **1** | **1** | **1.3e+002** | **4** | **K.NENARQQLER.Q** |
|  | 8564 | **420.3177** | **1257.9312** | **1257.5772** | **0.3540** | **0** | **3** | **48** | **9** | **K.THEAQIQEMR.Q** |
|  | 8640 | **631.3401** | **1260.6656** | **1260.6748** | **-0.0091** | **1** | **8** | **22** | **2** | **K.VKLQEMEGTVK.S** |
|  | 8875 | **425.8832** | **1274.6278** | **1273.6918** | **0.9360** | **0** | **1** | **1.2e+002** | **6** | **R.YEILTPNSIPK.G** |
|  | 9027 | **642.8571** | **1283.6997** | **1283.7085** | **-0.0088** | **0** | **69** | **1.5e-005** | **1** | **K.VEAQLQELQVK.F** 9006 9056 |
|  | 9435 | **653.3342** | **1304.6538** | **1304.6612** | **-0.0074** | **0** | **17** | **2.5** | **1** | **K.EQADFAIEALAK.A** |
|  | 9513 | **655.3731** | **1308.7315** | **1308.5946** | **0.1370** | **1** | **28** | **0.22** | **1** | **R.NAEQYKDQADK.A** |
|  | 9558 | **656.8732** | **1311.7318** | **1311.5733** | **0.1584** | **1** | **0** | **1.4e+002** | **10** | **K.LQAQMKDCMR.E** |
|  | 9675 | **659.8734** | **1317.7323** | **1317.7405** | **-0.0082** | **0** | **38** | **0.022** | **1** | **K.LDPHLVLDQLR.C** 9688 |
|  | 9877 | **664.3445** | **1326.6744** | **1325.5340** | **1.1404** | **1** | **1** | **1.1e+002** | **4** | **K.DCMRELDDTR.A** |
|  | 10415 | **679.3520** | **1356.6894** | **1356.6997** | **-0.0103** | **1** | **6** | **34** | **7** | **K.DVLLQVDDERR.N** |
|  | 11126 | **699.3379** | **1396.6612** | **1396.6697** | **-0.0084** | **1** | **69** | **1.6e-005** | **1** | **K.VDYKADEWLMK.N** 11124 11125 |
|  | 11248 | **703.3307** | **1404.6469** | **1403.5519** | **1.0950** | **0** | **3** | **74** | **6** | **K.DMFQETMEAMR.I** |
|  | 11393 | **706.9036** | **1411.7927** | **1411.8035** | **-0.0107** | **1** | **67** | **2.7e-005** | **1** | **K.KVEAQLQELQVK.F** 11370 11387 |
|  | 11616 | **713.3745** | **1424.7345** | **1424.7446** | **-0.0101** | **0** | **45** | **0.0045** | **1** | **K.QIATLHAQVADMK.K** 11617 |
|  | 11732 | **717.3234** | **1432.6323** | **1432.6430** | **-0.0107** | **1** | **38** | **0.02** | **1** | **R.DLQGRDEQSEEK.K** |
|  | 13031 | **505.2625** | **1512.7657** | **1511.7620** | **1.0037** | **1** | **3** | **56** | **8** | **M.AQQAADKYLYVDK.N** |
|  | 13311 | **765.8807** | **1529.7468** | **1529.7573** | **-0.0105** | **0** | **69** | **1.6e-005** | **1** | **K.IAQLEEQLDNETK.E** 13312 |
|  | 13620 | **776.3459** | **1550.6772** | **1550.6882** | **-0.0110** | **0** | **11** | **9.5** | **1** | **R.EDQSILCTGESGAGK.T** |
|  | 13663 | **777.3546** | **1552.6945** | **1551.7538** | **0.9408** | **0** | **15** | **3.6** | **1** | **K.ITDVIIGFQACCR.G** |
|  | 13670 | **777.4072** | **1552.7998** | **1552.8395** | **-0.0397** | **1** | **16** | **2.9** | **2** | **K.QIATLHAQVADMKK.K** |
|  | 13734 | **779.9018** | **1557.7890** | **1557.8515** | **-0.0624** | **1** | **6** | **29** | **3** | **R.QRYEILTPNSIPK.G** |
|  | 13834 | **783.3355** | **1564.6563** | **1564.6675** | **-0.0111** | **0** | **51** | **0.00096** | **1** | **R.ELEDATETADAMNR.E** 13835 |
|  | 13913 | **786.4249** | **1570.8353** | **1570.8468** | **-0.0114** | **0** | **35** | **0.036** | **1** | **K.VSHLLGINVTDFTR.G** |
|  | 13955 | **525.6353** | **1573.8841** | **1573.8940** | **-0.0099** | **1** | **17** | **2.4** | **1** | **K.AGKLDPHLVLDQLR.C** |
|  | 14022 | **790.4198** | **1578.8250** | **1578.8365** | **-0.0115** | **1** | **44** | **0.0047** | **1** | **R.AGVLAHLEEERDLK.I** 14024 |
|  | 14122 | **793.3798** | **1584.7450** | **1584.7566** | **-0.0116** | **1** | **34** | **0.046** | **1** | **K.NKHEAMITDLEER.L** 14123 |
|  | 14218 | **796.3485** | **1590.6825** | **1590.6944** | **-0.0119** | **0** | **74** | **5.1e-006** | **1** | **R.NTDQASMPDNTAAQK.V** 14217 |
|  | 14579 | **808.4079** | **1614.8012** | **1614.8109** | **-0.0097** | **0** | **28** | **0.18** | **1** | **R.IMGIPEEEQMGLLR.V** 14580 |
|  | 14816 | **544.2957** | **1629.8653** | **1630.8058** | **-0.9405** | **0** | **(1)** | **91** | **5** | **R.IMGIPEEEQMGLLR.V** |
|  | 15104 | **550.2948** | **1647.8626** | **1646.8008** | **1.0618** | **0** | **(6)** | **33** | **2** | **R.IMGIPEEEQMGLLR.V** |
|  | 15297 | **554.2548** | **1659.7425** | **1658.8661** | **0.8764** | **1** | **4** | **40** | **7** | **K.ALSLARALEEAMEQK.A** 15287 15288 |
|  | 15319 | **831.8976** | **1661.7806** | **1661.8671** | **-0.0865** | **1** | **0** | **1e+002** | **8** | **K.LMATLRNTNPNFVR.C** |
|  | 15464 | **837.1274** | **1672.2403** | **1671.8369** | **0.4035** | **0** | **11** | **6.6** | **1** | **K.NFINNPLAQADWAAK.K** |
|  | 15514 | **559.3157** | **1674.9254** | **1674.8610** | **0.0644** | **1** | **(4)** | **47** | **3** | **K.ALSLARALEEAMEQK.A** |
|  | 15624 | **842.8687** | **1683.7227** | **1683.7331** | **-0.0104** | **1** | **64** | **4e-005** | **1** | **R.TEMEDLMSSKDDVGK.S** 15623 15625 |
|  | 16324 | **863.9725** | **1725.9305** | **1725.9413** | **-0.0108** | **0** | **26** | **0.25** | **1** | **R.QLLQANPILEAFGNAK.T** 16312 |
|  | 16372 | **865.4339** | **1728.8532** | **1728.8642** | **-0.0109** | **1** | **47** | **0.002** | **1** | **K.QTLENERGELANEVK.V** 16370 16371 |
|  | 16684 | **876.4255** | **1750.8365** | **1750.8481** | **-0.0116** | **0** | **57** | **0.00021** | **1** | **R.LTEMETLQSQLMAEK.L** 16665 16703 |
|  | 16731 | **877.9332** | **1753.8518** | **1753.7750** | **0.0768** | **1** | **5** | **33** | **8** | **R.QEEEMMAKEEELVK.V** |
|  | 16863 | **881.4452** | **1760.8758** | **1759.8298** | **1.0460** | **0** | **33** | **0.053** | **2** | **K.LEEEQIILEDQNCK.L** 16862 |
|  | 17537 | **908.4514** | **1814.8883** | **1814.9010** | **-0.0127** | **1** | **84** | **3.7e-007** | **1** | **K.IAQLEEQLDNETKER.Q** 17535 17536 |
|  | 17632 | **912.4769** | **1822.9392** | **1823.8281** | **-0.8889** | **1** | **2** | **68** | **6** | **K.KMEDSVGCLETAEEVK.R** |
|  | 18012 | **618.2805** | **1851.8196** | **1851.8342** | **-0.0147** | **1** | **10** | **11** | **1** | **K.MEDSVGCLETAEEVKR.K** |
|  | 18264 | **934.9537** | **1867.8929** | **1867.9051** | **-0.0121** | **1** | **119** | **1.2e-010** | **1** | **R.IAEFTTNLTEEEEKSK.S** |
|  | 18283 | **935.4809** | **1868.9472** | **1868.9592** | **-0.0119** | **0** | **92** | **5.9e-008** | **1** | **K.ANLQIDQINTDLNLER.S** 18281 |
|  | 18495 | **944.9632** | **1887.9118** | **1887.9247** | **-0.0129** | **1** | **110** | **1.1e-009** | **1** | **K.KLEEEQIILEDQNCK.L** 18494 |
|  | 18839 | **639.3072** | **1914.8999** | **1913.9094** | **0.9905** | **0** | **36** | **0.025** | **1** | **R.HEMPPHIYAITDTAYR.S** |
|  | 18932 | **642.3522** | **1924.0347** | **1924.9530** | **-0.9183** | **1** | **12** | **6.4** | **1** | **K.EQADFAIEALAKATYER.M** |
|  | 19026 | **644.3679** | **1930.0819** | **1929.9043** | **0.1776** | **0** | **(4)** | **38** | **3** | **R.HEMPPHIYAITDTAYR.S** |
|  | 19196 | **973.5010** | **1944.9875** | **1945.0004** | **-0.0129** | **0** | **126** | **2.7e-011** | **1** | **K.LQVELDNVTGLLSQSDSK.S** 19206 |
|  | 19242 | **975.4949** | **1948.9752** | **1948.9854** | **-0.0102** | **0** | **98** | **1.6e-008** | **1** | **R.LQQELDDLLVDLDHQR.Q** 19241 19243 |
|  | 19380 | **981.4560** | **1960.8974** | **1960.9113** | **-0.0138** | **0** | **90** | **9.9e-008** | **1** | **K.TQLEELEDELQATEDAK.L** |
|  | 19384 | **491.2570** | **1960.9991** | **1961.9112** | **-0.9121** | **1** | **1** | **74** | **8** | **K.LQRELEDATETADAMNR.E** |
|  | 19754 | **666.0038** | **1994.9895** | **1995.0021** | **-0.0126** | **1** | **62** | **6e-005** | **1** | **K.HSQAVEELAEQLEQTKR.V** 19765 |
|  | 19755 | **499.7608** | **1995.0140** | **1995.0254** | **-0.0114** | **0** | **34** | **0.037** | **1** | **R.TFHIFYYLLSGAGEHLK.T** 19756 |
|  | 19772 | **999.5273** | **1997.0400** | **1997.0541** | **-0.0141** | **1** | **102** | **5.2e-009** | **1** | **K.KANLQIDQINTDLNLER.S** 19773 |
|  | 20007 | **1009.5289** | **2017.0432** | **2017.0554** | **-0.0122** | **0** | **69** | **1.2e-005** | **1** | **R.IIGLDQVAGMSETALPGAFK.T** 19990 |
|  | 20280 | **1021.9880** | **2041.9614** | **2041.9738** | **-0.0124** | **1** | **102** | **5.5e-009** | **1** | **K.TLEEEAKTHEAQIQEMR.Q** 20278 20279 |
|  | 20349 | **1024.9793** | **2047.9439** | **2047.9554** | **-0.0114** | **0** | **88** | **1.6e-007** | **1** | **K.SMEAEMIQLQEELAAAER.A** 20350 20352 |
|  | 20833 | **1044.9858** | **2087.9571** | **2087.9719** | **-0.0148** | **1** | **58** | **0.00015** | **1** | **R.QAQQERDELADEIANSSGK.G** 20831 20832 |
|  | 21254 | **1068.4760** | **2134.9373** | **2134.0389** | **0.8984** | **1** | **4** | **32** | **4** | **K.TELEDTLDSTAAQQELRSK.R** |
|  | 21889 | **736.7177** | **2207.1311** | **2208.2379** | **-1.1068** | **1** | **1** | **62** | **1** | **K.VSHLLGINVTDFTRGILTPR.I** |
|  | 22560 | **1143.5513** | **2285.0880** | **2285.1031** | **-0.0151** | **0** | **105** | **2.7e-009** | **1** | **K.VEDMAELTCLNEASVLHNLK.E** 22561 22562 |
|  | 22677 | **1150.4929** | **2298.9713** | **2298.9879** | **-0.0166** | **1** | **71** | **5.3e-006** | **1** | **R.SMMQDREDQSILCTGESGAGK.T** 22678 22679 |
|  | 22692 | **576.3224** | **2301.2605** | **2301.0981** | **0.1624** | **0** | **(4)** | **29** | **3** | **K.VEDMAELTCLNEASVLHNLK.E** 22696 |
|  | 22697 | **768.3776** | **2302.1110** | **2302.1288** | **-0.0178** | **1** | **42** | **0.005** | **1** | **K.IRELESQISELQEDLESER.A** |
|  | 22932 | **1167.0241** | **2332.0335** | **2332.0488** | **-0.0153** | **0** | **109** | **8.2e-010** | **1** | **K.MQQNIQELEEQLEEEESAR.Q** 22931 |
|  | 23366 | **1193.1134** | **2384.2122** | **2384.2263** | **-0.0141** | **0** | **58** | **0.00011** | **1** | **R.INFDVNGYIVGANIETYLLEK.S** |
|  | 23875 | **1231.0707** | **2460.1268** | **2460.1438** | **-0.0170** | **1** | **93** | **3.8e-008** | **1** | **K.KMQQNIQELEEQLEEEESAR.Q** 23876 |
|  | 23925 | **1234.0996** | **2466.1847** | **2466.2002** | **-0.0155** | **0** | **88** | **1.2e-007** | **1** | **K.LQQLFNHTMFILEQEEYQR.E** 23924 23932 |
|  | 23961 | **1236.5826** | **2471.1507** | **2471.1663** | **-0.0156** | **0** | **88** | **1e-007** | **1** | **R.IAQLEEELEEEQGNTELINDR.L** 23960 23962 |
|  | 24164 | **1247.0831** | **2492.1517** | **2492.1667** | **-0.0149** | **0** | **100** | **6.3e-009** | **1** | **K.DFSALESQLQDTQELLQEENR.Q** 24165 24166 24167 |
|  | 25040 | **883.4341** | **2647.2804** | **2647.2985** | **-0.0181** | **1** | **19** | **0.81** | **1** | **K.FSKVEDMAELTCLNEASVLHNLK.E** |
|  | 25593 | **917.1085** | **2748.3037** | **2748.3202** | **-0.0165** | **1** | **34** | **0.024** | **1** | **K.DFSALESQLQDTQELLQEENRQK.L** |
|  | 26102 | **945.7772** | **2834.3097** | **2834.3934** | **-0.0837** | **1** | **2** | **32** | **9** | **K.LTKDFSALESQLQDTQELLQEENR.Q** |
|  | 27088 | **1004.4548** | **3010.3427** | **3009.3783** | **0.9644** | **1** | **36** | **0.012** | **1** | **R.FLSNGHVTIPGQQDKDMFQETMEAMR.I** 27089 27090 |
|  | 27104 | **754.6237** | **3014.4655** | **3014.4807** | **-0.0152** | **1** | **14** | **2.2** | **1** | **K.NMDPLNDNIATLLHQSSDKFVSELWK.D** 27102 27103 |
|  | 27110 | **1509.2352** | **3016.4559** | **3016.4724** | **-0.0165** | **1** | **79** | **6.4e-007** | **1** | **R.DLGEELEALKTELEDTLDSTAAQQELR.S** 27111 27112 |
|  | 27702 | **1574.2394** | **3146.4642** | **3146.4812** | **-0.0170** | **1** | **76** | **1.2e-006** | **1** | **R.ALEQQVEEMKTQLEELEDELQATEDAK.L** |
|  | 30583 | **1058.2704** | **4229.0525** | **4228.0828** | **0.9696** | **1** | **0** | **24** | **3** | **R.YYSGLIYTYSGLFCVVINPYKNLPIYSEEIVEMYK.G** |

| **4.** | P23141\|EST1_HUMAN    **Mass:** 62766    **Score:** 2753   **Queries matched:** 176   **emPAI:** 10.90 |
| --- | --- |
|  | Liver carboxylesterase 1 OS=Homo sapiens GN=CES1 PE=1 SV=2 |

|  | **Query** | **Observed** | **Mr(expt)** | **Mr(calc)** | **Delta** | **Miss** | **Score** | **Expect** | **Rank** | **Peptide** |
| --- | --- | --- | --- | --- | --- | --- | --- | --- | --- | --- |
|  | 2425 | **837.1757** | **836.1684** | **835.4659** | **0.7025** | **1** | **6** | **33** | **2** | **R.LSKMVMK.F** |
|  | 3412 | **456.2266** | **910.4386** | **910.4450** | **-0.0063** | **0** | **32** | **0.084** | **1** | **K.FWANFAR.N** 3413 |
|  | 3930 | **475.2593** | **948.5041** | **948.5103** | **-0.0061** | **0** | **47** | **0.0037** | **1** | **K.TAMSLLWK.S** 3929 3931 |
|  | 5035 | **515.2733** | **1028.5321** | **1028.5390** | **-0.0069** | **0** | **19** | **2.3** | **1** | **K.ELIPEATEK.Y** |
|  | 5304 | **525.7828** | **1049.5511** | **1049.5579** | **-0.0068** | **0** | **24** | **0.71** | **1** | **K.SYPLVCIAK.E** 5303 |
|  | 5520 | **534.7601** | **1067.5056** | **1067.5135** | **-0.0079** | **0** | **29** | **0.2** | **1** | **K.YLGGTDDTVK.K** 5514 5521 5522 5523 5539 |
|  | 5582 | **536.7636** | **1071.5125** | **1071.5196** | **-0.0071** | **0** | **61** | **0.00015** | **1** | **K.TPEELQAER.N** 5575 5581 |
|  | 8600 | **630.8290** | **1259.6434** | **1259.6510** | **-0.0076** | **0** | **67** | **2.9e-005** | **1** | **K.FLSLDLQGDPR.E** 8572 |
|  | 9412 | **652.8287** | **1303.6429** | **1304.6711** | **-1.0282** | **0** | **0** | **1.3e+002** | **10** | **K.TEEELLETTLK.M** |
|  | 10227 | **674.8011** | **1347.5876** | **1346.6677** | **0.9199** | **1** | **15** | **5.2** | **2** | **K.EGASEEEIRLSK.M** 10232 |
|  | 10236 | **674.8602** | **1347.7058** | **1347.7146** | **-0.0089** | **0** | **76** | **3.7e-006** | **1** | **K.AGQLLSELFTNR.K** 10238 |
|  | 10731 | **688.3403** | **1374.6660** | **1374.6748** | **-0.0088** | **0** | **60** | **0.00015** | **1** | **K.TTTSAVMVHCLR.Q** |
|  | 11040 | **464.9056** | **1391.6951** | **1390.6697** | **1.0254** | **0** | **(5)** | **38** | **4** | **K.TTTSAVMVHCLR.Q** |
|  | 11783 | **718.8685** | **1435.7224** | **1435.7320** | **-0.0097** | **0** | **50** | **0.0012** | **1** | **R.GNWGHLDQVAALR.W** 11782 11784 11785 11793 |
|  | 12402 | **737.4247** | **1472.8349** | **1472.8450** | **-0.0101** | **0** | **101** | **9.8e-009** | **1** | **R.AISESGVALTSVLVK.K** 12403 12405 12406 12407 |
|  | 12464 | **738.9071** | **1475.7996** | **1475.8096** | **-0.0100** | **1** | **61** | **0.0001** | **1** | **K.AGQLLSELFTNRK.E** 12463 12465 12466 12476 |
|  | 12694 | **745.8910** | **1489.7674** | **1489.7776** | **-0.0102** | **0** | **32** | **0.074** | **1** | **K.AVEKPPQTEHIEL.-** |
|  | 13129 | **760.3843** | **1518.7540** | **1518.7653** | **-0.0113** | **0** | **55** | **0.00045** | **1** | **R.NFHTVPYMVGINK.Q** 13130 13159 |
|  | 13377 | **512.5906** | **1534.7501** | **1534.7602** | **-0.0101** | **0** | **(16)** | **3.2** | **1** | **R.NFHTVPYMVGINK.Q** 13378 |
|  | 13778 | **781.4141** | **1560.8137** | **1560.8246** | **-0.0109** | **1** | **48** | **0.0019** | **1** | **R.QKTEEELLETTLK.M** 13760 |
|  | 13877 | **523.6052** | **1567.7937** | **1567.8035** | **-0.0098** | **1** | **15** | **3.9** | **1** | **K.DKEVAFWTNLFAK.K** |
|  | 14224 | **796.4020** | **1590.7895** | **1590.8002** | **-0.0106** | **0** | **76** | **2.7e-006** | **1** | **K.EGYLQIGANTQAAQK.L** 14215 14225 14226 14227 14228 14229 14233 14235 14255 |
|  | 14631 | **809.9376** | **1617.8606** | **1617.8726** | **-0.0120** | **1** | **47** | **0.0022** | **1** | **K.KAVEKPPQTEHIEL.-** 14632 14634 14636 14637 |
|  | 14809 | **815.9115** | **1629.8084** | **1629.8191** | **-0.0107** | **0** | **40** | **0.011** | **1** | **R.FTPPQPAEPWSFVK.N** 14806 14807 14808 14810 14811 |
|  | 16067 | **855.4040** | **1708.7935** | **1707.8005** | **0.9930** | **0** | **69** | **1.5e-005** | **1** | **R.LGIWGFFSTGDEHSR.G** 16044 |
|  | 17646 | **913.4399** | **1824.8653** | **1826.0223** | **-1.1570** | **0** | **79** | **1.4e-006** | **1** | **R.ESQPLLGTVIDGMLLLK.T** 17664 17665 17666 17667 17668 17669 17670 17672 17673 17674 17675 17678 17679 17680 17681 17691 17692 |
|  | 17885 | **615.0427** | **1842.1063** | **1842.0172** | **0.0891** | **0** | **(4)** | **37** | **2** | **R.ESQPLLGTVIDGMLLLK.T** |
|  | 18624 | **950.0154** | **1898.0162** | **1898.0295** | **-0.0133** | **1** | **95** | **2.9e-008** | **1** | **K.KGDVKPLAEQIAITAGCK.T** 18625 |
|  | 20376 | **1025.9648** | **2049.9151** | **2049.9293** | **-0.0141** | **0** | **44** | **0.0032** | **1** | **R.NGNPNGEGLPHWPEYNQK.E** 20374 20375 20377 20393 20394 20396 |
|  | 21190 | **1065.0207** | **2128.0269** | **2128.0398** | **-0.0128** | **0** | **73** | **4e-006** | **1** | **K.LSEDCLYLNIYTPADLTK.K** 21191 21192 |
|  | 21371 | **1075.0453** | **2148.0760** | **2148.0892** | **-0.0132** | **0** | **121** | **6.7e-011** | **1** | **K.TVIGDHGDELFSVFGAPFLK.E** 21370 21372 21374 21375 21377 21378 21382 21391 |
|  | 22603 | **764.0873** | **2289.2400** | **2289.2443** | **-0.0043** | **0** | **3** | **36** | **1** | **K.DLFLDLIADVMFGVPSVIVAR.N** |
|  | 23588 | **806.7833** | **2417.3280** | **2417.3392** | **-0.0113** | **1** | **13** | **3.1** | **1** | **K.KDLFLDLIADVMFGVPSVIVAR.N** |
|  | 25996 | **939.4215** | **2815.2427** | **2815.2622** | **-0.0195** | **0** | **32** | **0.033** | **1** | **R.DAGAPTYMYEFQYRPSFSSDMKPK.T** 25994 25995 25997 25998 |
|  | 26044 | **1412.2196** | **2822.4246** | **2822.4411** | **-0.0165** | **1** | **32** | **0.037** | **1** | **K.ENIPLKLSEDCLYLNIYTPADLTK.K** 26042 26045 |
|  | 26068 | **944.7529** | **2831.2370** | **2831.2571** | **-0.0201** | **0** | **(0)** | **50** | **2** | **R.DAGAPTYMYEFQYRPSFSSDMKPK.T** |
|  | 26070 | **944.7539** | **2831.2399** | **2831.2571** | **-0.0172** | **0** | **(18)** | **0.78** | **1** | **R.DAGAPTYMYEFQYRPSFSSDMKPK.T** |
|  | 26113 | **1419.6895** | **2837.3643** | **2837.3768** | **-0.0125** | **0** | **71** | **5.2e-006** | **1** | **K.QEFGWLIPMQLMSYPLSEGQLDQK.T** 26114 26116 |
|  | 26963 | **746.1779** | **2980.6826** | **2980.6943** | **-0.0117** | **0** | **46** | **0.001** | **1** | **K.FVSLEGFAQPVAIFLGIPFAKPPLGPLR.F** 26958 26959 26960 26961 26962 26965 26966 |
|  | 27722 | **1050.5134** | **3148.5185** | **3148.5353** | **-0.0168** | **1** | **70** | **4.4e-006** | **1** | **K.TVIGDHGDELFSVFGAPFLKEGASEEEIR.L** |
|  | 28494 | **845.4953** | **3377.9521** | **3377.9632** | **-0.0111** | **1** | **41** | **0.0027** | **1** | **K.VLGKFVSLEGFAQPVAIFLGIPFAKPPLGPLR.F** |
|  | 29587 | **1895.4714** | **3788.9283** | **3788.9261** | **0.0022** | **0** | **88** | **5.6e-008** | **1** | **R.WVQDNIASFGGNPGSVTIFGESAGGESVSVLVLSPLAK.N** 29507 29509 29510 29511 29512 29524 29525 29530 29532 29533 29538 29539 29540 29545 29547 29555 29556 29557 29558 29559 29566 29570 29571 29578 29579 29584 29591 |
|  | 30328 | **822.2419** | **4106.1733** | **4107.1438** | **-0.9705** | **0** | **0** | **20** | **5** | **R.LPVMVWIHGGGLMVGAASTYDGLALAAHENVVVVTIQYR.L** |
|  | 30748 | **1447.0441** | **4338.1104** | **4338.1316** | **-0.0212** | **1** | **42** | **0.0014** | **1** | **R.NFHTVPYMVGINKQEFGWLIPMQLMSYPLSEGQLDQK.T** 30745 30746 30747 30751 30752 |
|  | 30761 | **1089.5309** | **4354.0944** | **4354.1265** | **-0.0321** | **1** | **(24)** | **0.09** | **1** | **R.NFHTVPYMVGINKQEFGWLIPMQLMSYPLSEGQLDQK.T** |
|  | 30762 | **1089.5364** | **4354.1164** | **4354.1265** | **-0.0101** | **1** | **(12)** | **1.5** | **1** | **R.NFHTVPYMVGINKQEFGWLIPMQLMSYPLSEGQLDQK.T** |

| **5.** | P08319\|ADH4_HUMAN    **Mass:** 41108    **Score:** 2629   **Queries matched:** 120   **emPAI:** 18.50 |
| --- | --- |
|  | Alcohol dehydrogenase 4 OS=Homo sapiens GN=ADH4 PE=1 SV=4 |

|  | **Query** | **Observed** | **Mr(expt)** | **Mr(calc)** | **Delta** | **Miss** | **Score** | **Expect** | **Rank** | **Peptide** |
| --- | --- | --- | --- | --- | --- | --- | --- | --- | --- | --- |
|  | 995 | **532.2833** | **531.2761** | **531.2765** | **-0.0004** | **0** | **13** | **18** | **6** | **K.AAGASR.I** 994 |
|  | 1677 | **738.3976** | **737.3904** | **737.3959** | **-0.0056** | **0** | **12** | **11** | **1** | **K.LVTDYK.N** |
|  | 5419 | **1060.4827** | **1059.4754** | **1059.4832** | **-0.0079** | **0** | **42** | **0.01** | **1** | **K.IDDDANLER.V** 5437 |
|  | 5964 | **551.3075** | **1100.6004** | **1100.6077** | **-0.0073** | **0** | **27** | **0.3** | **1** | **R.IIGIDINSEK.F** 5965 |
|  | 7109 | **594.2914** | **1186.5682** | **1186.5764** | **-0.0082** | **0** | **64** | **6.4e-005** | **1** | **K.ALGATDCLNPR.D** 7086 7098 7107 7108 7110 7111 7112 7113 |
|  | 7415 | **601.3376** | **1200.6606** | **1200.6689** | **-0.0083** | **0** | **41** | **0.012** | **1** | **K.VIPLYAPLCR.K** |
|  | 7945 | **614.3077** | **1226.6009** | **1226.6084** | **-0.0075** | **0** | **51** | **0.0012** | **1** | **R.TINGTFFGGWK.S** 7966 7967 7968 |
|  | 10228 | **674.8015** | **1347.5883** | **1347.5976** | **-0.0093** | **0** | **72** | **9.6e-006** | **1** | **K.SPASDQQLMEDK.T** 10227 |
|  | 10318 | **676.8245** | **1351.6345** | **1351.6442** | **-0.0097** | **0** | **92** | **8.8e-008** | **1** | **K.ISEAFDLMNQGK.S** 10317 10319 10320 10342 |
|  | 10485 | **682.3027** | **1362.5908** | **1363.5925** | **-1.0018** | **0** | **(32)** | **0.078** | **2** | **K.SPASDQQLMEDK.T** |
|  | 10620 | **685.2796** | **1368.5446** | **1367.6391** | **0.9055** | **0** | **(4)** | **49** | **10** | **K.ISEAFDLMNQGK.S** |
|  | 11887 | **722.4095** | **1442.8045** | **1442.8133** | **-0.0088** | **0** | **46** | **0.0034** | **1** | **K.GLTVFPEELIIGR.T** 11886 11888 11892 11893 |
|  | 16079 | **570.6428** | **1708.9066** | **1707.7734** | **1.1333** | **1** | **5** | **37** | **1** | **K.SPASDQQLMEDKTSR.F** |
|  | 16146 | **857.8682** | **1713.7218** | **1713.7338** | **-0.0121** | **0** | **94** | **4.5e-008** | **1** | **K.GGVDFALDCAGGSETMK.A** 16144 16145 16156 16157 |
|  | 16367 | **865.4163** | **1728.8180** | **1729.7288** | **-0.9108** | **0** | **(39)** | **0.015** | **2** | **K.GGVDFALDCAGGSETMK.A** 16365 |
|  | 16411 | **865.9534** | **1729.8923** | **1729.9039** | **-0.0116** | **0** | **74** | **4.7e-006** | **1** | **K.FNLDALVTHTLPFDK.I** 16371 16372 16412 16413 16414 16415 16416 16423 |
|  | 17016 | **888.5114** | **1775.0083** | **1775.0193** | **-0.0110** | **0** | **59** | **0.00015** | **1** | **R.DLHKPIQEVIIELTK.G** 17012 17013 17014 17015 17017 17019 |
|  | 18130 | **930.0013** | **1857.9880** | **1857.9989** | **-0.0109** | **1** | **85** | **3.1e-007** | **1** | **K.KFNLDALVTHTLPFDK.I** 18127 18128 18129 18145 |
|  | 18691 | **952.4685** | **1902.9225** | **1902.9357** | **-0.0132** | **1** | **91** | **8.1e-008** | **1** | **K.ISNLKSPASDQQLMEDK.T** 18690 |
|  | 18866 | **640.3029** | **1917.8868** | **1918.9306** | **-1.0438** | **1** | **(0)** | **1e+002** | **9** | **K.ISNLKSPASDQQLMEDK.T** |
|  | 20676 | **1036.9911** | **2071.9676** | **2071.9819** | **-0.0143** | **0** | **110** | **7.8e-010** | **1** | **R.VCLLGCGFSTGYGAAINNAK.V** 20647 20675 20686 20687 20688 20689 20693 |
|  | 20804 | **1043.5394** | **2085.0643** | **2085.0776** | **-0.0133** | **0** | **90** | **9.6e-008** | **1** | **R.IQIIATSLCHTDATVIDSK.F** 20803 |
|  | 22684 | **1151.0276** | **2300.0406** | **2300.0566** | **-0.0160** | **0** | **109** | **9.3e-010** | **1** | **K.AALDCTTAGWGSCTFIGVAAGSK.G** 22683 22685 22686 22687 |
|  | 22865 | 1163.0658 | 2324.1170 | 2324.1328 | -0.0157 | 0 | 106 | 1.9e-009 | 1 | K.VTPGSTCAVFGLGGVGLSAVMGCK.A 22866 22867 22868 22869 22870 22871 22872 22873 22874 22876 22887 |
|  | 22997 | 1171.0629 | 2340.1112 | 2340.1277 | -0.0165 | 0 | (96) | 1.8e-008 | 1 | K.VTPGSTCAVFGLGGVGLSAVMGCK.A 22988 22998 22999 |
|  | 23294 | **1189.6168** | **2377.2191** | **2377.2351** | **-0.0160** | **0** | **65** | **2.2e-005** | **1** | **K.AAIAWEAGKPLCIEEVEVAPPK.A** 23293 23295 23296 |
|  | 26525 | **1453.7306** | **2905.4466** | **2905.4651** | **-0.0184** | **0** | **99** | **6.6e-009** | **1** | **K.GKPVYHFFGTSTFSQYTVVSDINLAK.I** 26524 26526 26527 26529 26530 26532 |
|  | 26884 | **743.3883** | **2969.5241** | **2969.5433** | **-0.0191** | **1** | **22** | **0.32** | **1** | **K.AAIAWEAGKPLCIEEVEVAPPKAHEVR.I** |
|  | 27391 | **766.8887** | **3063.5258** | **3063.5376** | **-0.0117** | **1** | **42** | **0.0032** | **1** | **K.FNLDALVTHTLPFDKISEAFDLMNQGK.S** 27387 27388 27393 27402 27403 |
|  | 28219 | **1102.2518** | **3303.7337** | **3303.7504** | **-0.0167** | **0** | **25** | **0.11** | **1** | **K.FEGLAFPVIVGHEAAGIVESIGPGVTNVKPGDK.V** 28218 |
|  | 31474 | **896.1387** | **5370.7887** | **5370.8174** | **-0.0287** | **1** | **10** | **1.1** | **1** | **R.IQIIATSLCHTDATVIDSKFEGLAFPVIVGHEAAGIVESIGPGVTNVKPGDK.V** 31475 |

| **6.** | P06576\|ATPB_HUMAN    **Mass:** 56525    **Score:** 2357   **Queries matched:** 122   **emPAI:** 7.22 |
| --- | --- |
|  | ATP synthase subunit beta, mitochondrial OS=Homo sapiens GN=ATP5B PE=1 SV=3 |

|  | **Query** | **Observed** | **Mr(expt)** | **Mr(calc)** | **Delta** | **Miss** | **Score** | **Expect** | **Rank** | **Peptide** |
| --- | --- | --- | --- | --- | --- | --- | --- | --- | --- | --- |
|  | 1176 | **576.3048** | **575.2975** | **574.3439** | **0.9537** | **0** | **20** | **2.7** | **10** | **K.LTVSR.A** |
|  | 1764 | **779.4243** | **778.4170** | **778.4225** | **-0.0055** | **0** | **27** | **0.4** | **1** | **K.ILQDYK.S** |
|  | 4287 | **488.2816** | **974.5486** | **974.5549** | **-0.0063** | **0** | **10** | **17** | **1** | **K.IGLFGGAGVGK.T** |
|  | 5161 | **519.7969** | **1037.5793** | **1037.5869** | **-0.0076** | **0** | **19** | **1.9** | **1** | **K.IPVGPETLGR.I** |
|  | 5190 | **520.8356** | **1039.6566** | **1039.6641** | **-0.0075** | **1** | **17** | **2.7** | **1** | **K.LVPLKETIK.G** 5201 |
|  | 5757 | **543.7534** | **1085.4923** | **1085.4989** | **-0.0066** | **1** | **33** | **0.086** | **1** | **K.ADKLAEEHSS.-** |
|  | 5789 | **544.8179** | **1087.6212** | **1087.6277** | **-0.0065** | **0** | **51** | **0.0016** | **1** | **K.VVDLLAPYAK.G** |
|  | 6789 | **583.2623** | **1164.5101** | **1163.5459** | **0.9643** | **0** | **7** | **30** | **8** | **R.DYAAQTSPSPK.A** |
|  | 7739 | **609.1259** | **1216.2373** | **1216.6928** | **-0.4555** | **1** | **3** | **52** | **2** | **K.GGKIGLFGGAGVGK.T** 7741 |
|  | 8650 | **631.8196** | **1261.6247** | **1261.6336** | **-0.0089** | **0** | **43** | **0.0076** | **1** | **R.TIAMDGTEGLVR.G** 8651 8666 |
|  | 9926 | **444.2188** | **1329.6344** | **1329.7656** | **-0.1312** | **1** | **14** | **5.8** | **5** | **K.VVDLLAPYAKGGK.I** |
|  | 10909 | **693.3537** | **1384.6928** | **1384.7020** | **-0.0092** | **0** | **35** | **0.044** | **1** | **R.IMNVIGEPIDER.G** |
|  | 11269 | **703.8389** | **1405.6633** | **1405.6739** | **-0.0106** | **0** | **59** | **0.00017** | **1** | **K.AHGGYSVFAGVGER.T** 11244 |
|  | 11772 | **718.3761** | **1434.7376** | **1434.7467** | **-0.0090** | **0** | **77** | **2.7e-006** | **1** | **R.FTQAGSEVSALLGR.I** 11769 11770 |
|  | 11829 | **720.3944** | **1438.7743** | **1438.7820** | **-0.0077** | **0** | **103** | **6.6e-009** | **1** | **R.VALTGLTVAEYFR.D** |
|  | 12106 | **729.4193** | **1456.8239** | **1456.8323** | **-0.0084** | **0** | **64** | **4.7e-005** | **1** | **K.TVLIMELINNVAK.A** 12094 12108 12121 12132 |
|  | 14378 | **801.4036** | **1600.7927** | **1600.8031** | **-0.0104** | **0** | **75** | **3.4e-006** | **1** | **K.VALVYGQMNEPPGAR.A** 14377 14379 14380 14384 14385 |
|  | 15144 | **825.9568** | **1649.8990** | **1649.9101** | **-0.0110** | **0** | **60** | **9.8e-005** | **1** | **R.LVLEVAQHLGESTVR.T** 15128 15145 15148 |
|  | 15338 | **555.2988** | **1662.8747** | **1662.8227** | **0.0520** | **1** | **3** | **53** | **3** | **K.AHGGYSVFAGVGERTR.E** 15344 |
|  | 16623 | **583.2623** | **1746.7652** | **1747.8489** | **-1.0837** | **1** | **9** | **14** | **1** | **R.DYAAQTSPSPKAGAATGR.I** 16622 16624 |
|  | 17098 | **890.9792** | **1779.9438** | **1779.9553** | **-0.0114** | **1** | **58** | **0.00015** | **1** | **R.IMNVIGEPIDERGPIK.T** 17097 17099 17110 |
|  | 17532 | **908.4324** | **1814.8503** | **1814.8621** | **-0.0118** | **0** | **76** | **2.6e-006** | **1** | **R.IMDPNIVGSEHYDVAR.G** 17531 |
|  | 18875 | **960.0449** | **1918.0752** | **1918.0888** | **-0.0136** | **1** | **65** | **3.1e-005** | **1** | **K.VLDSGAPIKIPVGPETLGR.I** 18874 18876 18880 |
|  | 18898 | **961.4822** | **1920.9499** | **1920.9581** | **-0.0082** | **0** | **79** | **1.3e-006** | **1** | **R.DQEGQDVLLFIDNIFR.F** |
|  | 19670 | **994.5144** | **1987.0142** | **1987.0262** | **-0.0120** | **0** | **33** | **0.051** | **1** | **R.AIAELGIYPAVDPLDSTSR.I** |
|  | 20095 | **1012.0023** | **2021.9901** | **2022.0033** | **-0.0133** | **0** | **61** | **7.9e-005** | **1** | **R.FLSQPFQVAEVFTGHMGK.L** 20096 20097 |
|  | 20231 | **510.2764** | **2037.0764** | **2036.1014** | **0.9749** | **1** | **6** | **24** | **9** | **R.ETRLVLEVAQHLGESTVR.T** 20232 |
|  | 20516 | **1030.9957** | **2059.9769** | **2059.9884** | **-0.0115** | **0** | **65** | **3.2e-005** | **1** | **R.EGNDLYHEMIESGVINLK.D** 20517 |
|  | 21495 | **541.4925** | **2161.9409** | **2163.1073** | **-1.1664** | **1** | **3** | **37** | **8** | **R.AAPTAVHPVRDYAAQTSPSPK.A** |
|  | 22245 | **748.4654** | **2242.3743** | **2243.1005** | **-0.7261** | **1** | **1** | **49** | **6** | **R.IMDPNIVGSEHYDVARGVQK.I** |
|  | 22397 | **1133.5381** | **2265.0616** | **2265.0770** | **-0.0153** | **0** | **96** | **2.1e-008** | **1** | **R.IPSAVGYQPTLATDMGTMQER.I** 22396 22398 22399 |
|  | 22535 | **761.3592** | **2281.0557** | **2281.0719** | **-0.0161** | **0** | **(12)** | **5.1** | **1** | **R.IPSAVGYQPTLATDMGTMQER.I** |
|  | 22820 | **1159.5676** | **2317.1207** | **2317.1372** | **-0.0165** | **1** | **79** | **9.8e-007** | **1** | **R.TREGNDLYHEMIESGVINLK.D** 22819 22821 22822 |
|  | 25172 | **1338.1865** | **2674.3585** | **2674.3735** | **-0.0150** | **1** | **88** | **9.9e-008** | **1** | **K.SLQDIIAILGMDELSEEDKLTVSR.A** 25169 25170 25171 25173 25174 25175 25176 25177 25178 25179 25180 25181 25183 25185 25186 25187 25188 25189 25190 25193 25194 25195 25196 25197 25199 25200 25202 25203 25204 |
|  | 25274 | **897.7930** | **2690.3573** | **2690.3684** | **-0.0111** | **1** | **(5)** | **20** | **1** | **K.SLQDIIAILGMDELSEEDKLTVSR.A** |
|  | 27284 | **1015.5026** | **3043.4859** | **3043.5034** | **-0.0176** | **0** | **47** | **0.00089** | **1** | **K.QFAPIHAEAPEFMEMSVEQEILVTGIK.V** 27281 27282 27283 27291 |
|  | 28367 | **1114.9114** | **3341.7123** | **3341.7296** | **-0.0173** | **1** | **95** | **1.4e-008** | **1** | **R.VALTGLTVAEYFRDQEGQDVLLFIDNIFR.F** 28368 |
|  | 28401 | **1675.8159** | **3349.6173** | **3349.6329** | **-0.0156** | **0** | **69** | **5.5e-006** | **1** | **K.GFQQILAGEYDHLPEQAFYMVGPIEEAVAK.A** 28398 28399 28400 28403 28404 |
|  | 28452 | **1122.9028** | **3365.6867** | **3365.6278** | **0.0589** | **0** | **(24)** | **0.18** | **1** | **K.GFQQILAGEYDHLPEQAFYMVGPIEEAVAK.A** |
|  | 29322 | **1238.9602** | **3713.8588** | **3713.8789** | **-0.0201** | **0** | **33** | **0.019** | **1** | **K.GSITSVQAIYVPADDLTDPAPATTFAHLDATTVLSR.A** |
|  | 29776 | **1281.6559** | **3841.9458** | **3841.9738** | **-0.0280** | **1** | **80** | **3.2e-007** | **1** | **K.KGSITSVQAIYVPADDLTDPAPATTFAHLDATTVLSR.A** 29771 29779 29781 |

| **7.** | P11021\|GRP78_HUMAN    **Mass:** 72402    **Score:** 2346   **Queries matched:** 128   **emPAI:** 5.05 |
| --- | --- |
|  | 78 kDa glucose-regulated protein OS=Homo sapiens GN=HSPA5 PE=1 SV=2 |

|  | **Query** | **Observed** | **Mr(expt)** | **Mr(calc)** | **Delta** | **Miss** | **Score** | **Expect** | **Rank** | **Peptide** |
| --- | --- | --- | --- | --- | --- | --- | --- | --- | --- | --- |
|  | 1539 | **686.4146** | **685.4073** | **686.3963** | **-0.9889** | **0** | **12** | **12** | **10** | **R.LIGDAAK.N** |
|  | 1646 | **728.4612** | **727.4539** | **727.4592** | **-0.0053** | **0** | **34** | **0.074** | **1** | **K.IQQLVK.E** |
|  | 3309 | **452.2387** | **902.4629** | **902.4684** | **-0.0055** | **0** | **23** | **0.91** | **1** | **R.VMEHFIK.L** |
|  | 3513 | **459.7395** | **917.4645** | **917.4706** | **-0.0061** | **0** | **13** | **9.9** | **3** | **K.VLEDSDLK.K** |
|  | 4442 | **494.2598** | **986.5050** | **985.5080** | **0.9970** | **0** | **11** | **16** | **9** | **R.LTPEEIER.M** |
|  | 4585 | **499.2595** | **996.5045** | **996.5101** | **-0.0055** | **0** | **55** | **0.00045** | **1** | **R.ALSSQHQAR.I** 4568 4569 4581 4582 4583 4584 4586 4587 4588 4589 4590 4591 |
|  | 5246 | **523.2378** | **1044.4611** | **1045.5655** | **-1.1044** | **1** | **13** | **7.9** | **2** | **K.VLEDSDLKK.S** 5266 5273 |
|  | 5601 | **537.2804** | **1072.5462** | **1073.5465** | **-1.0003** | **0** | **20** | **2** | **1** | **K.ITITNDQNR.L** |
|  | 6071 | **555.2916** | **1108.5686** | **1108.5764** | **-0.0078** | **1** | **4** | **60** | **9** | **K.ETAEAYLGKK.V** |
|  | 7619 | **606.2839** | **1210.5532** | **1209.5778** | **0.9754** | **1** | **8** | **23** | **4** | **K.EFFNGKEPSR.G** 7616 |
|  | 7757 | **609.3150** | **1216.6154** | **1216.6234** | **-0.0080** | **0** | **63** | **8.9e-005** | **1** | **K.DAGTIAGLNVMR.I** |
|  | 7971 | **614.8130** | **1227.6115** | **1227.6207** | **-0.0092** | **0** | **24** | **0.56** | **1** | **R.VEIIANDQGNR.I** |
|  | 8217 | **620.8115** | **1239.6084** | **1239.6169** | **-0.0085** | **1** | **35** | **0.038** | **1** | **K.MKETAEAYLGK.K** 8239 |
|  | 9547 | **656.6663** | **1311.3180** | **1311.5864** | **-0.2684** | **1** | **1** | **78** | **8** | **K.LSSEDKETMEK.A** |
|  | 9632 | **658.8180** | **1315.6214** | **1315.6295** | **-0.0081** | **0** | **4** | **57** | **1** | **R.NELESYAYSLK.N** |
|  | 9636 | **658.8453** | **1315.6761** | **1315.6844** | **-0.0083** | **1** | **28** | **0.26** | **1** | **K.NKITITNDQNR.L** |
|  | 11130 | **699.3939** | **1396.7732** | **1396.7813** | **-0.0081** | **0** | **60** | **0.00013** | **1** | **K.ELEEIVQPIISK.L** |
|  | 11692 | **715.8444** | **1429.6743** | **1429.6838** | **-0.0095** | **0** | **66** | **3.5e-005** | **1** | **R.TWNDPSVQQDIK.F** 11705 |
|  | 13007 | **756.8743** | **1511.7340** | **1511.7442** | **-0.0102** | **1** | **67** | **2.6e-005** | **1** | **R.AKFEELNMDLFR.S** 13006 13008 13009 |
|  | 13236 | **509.3023** | **1524.8852** | **1524.8763** | **0.0089** | **1** | **4** | **47** | **8** | **K.KELEEIVQPIISK.L** |
|  | 13388 | **768.8980** | **1535.7813** | **1535.7905** | **-0.0092** | **0** | **69** | **1.4e-005** | **1** | **K.TFAPEEISAMVLTK.M** 13387 13389 |
|  | 13458 | **771.3984** | **1540.7823** | **1540.6715** | **0.1108** | **1** | **11** | **9.7** | **4** | **R.MVNDAEKFAEEDK.K** |
|  | 13466 | **514.7521** | **1541.2344** | **1540.9374** | **0.2970** | **0** | **1** | **58** | **8** | **K.LSLVAAMLLLLSAAR.A** |
|  | 13697 | **778.3945** | **1554.7744** | **1554.7862** | **-0.0119** | **1** | **62** | **7.6e-005** | **1** | **K.NGRVEIIANDQGNR.I** 13696 13698 13709 13710 13711 |
|  | 13853 | **522.9283** | **1565.7632** | **1565.7726** | **-0.0094** | **0** | **15** | **3.9** | **1** | **R.ITPSYVAFTPEGER.L** 13852 |
|  | 14175 | **794.9252** | **1587.8359** | **1587.8468** | **-0.0109** | **1** | **96** | **3e-008** | **1** | **K.KSDIDEIVLVGGSTR.I** 14176 14194 |
|  | 14420 | **802.9302** | **1603.8458** | **1603.8570** | **-0.0112** | **0** | **77** | **2.6e-006** | **1** | **K.TKPYIQVDIGGGQTK.T** 14417 14418 14419 |
|  | 15284 | **830.4458** | **1658.8770** | **1658.8879** | **-0.0108** | **0** | **85** | **3.9e-007** | **1** | **R.IINEPTAAAIAYGLDK.R** 15285 |
|  | 15538 | **839.4016** | **1676.7885** | **1676.8006** | **-0.0120** | **0** | **76** | **3.2e-006** | **1** | **K.NQLTSNPENTVFDAK.R** 15537 15539 15550 15551 |
|  | 16439 | **866.9770** | **1731.9394** | **1731.9519** | **-0.0125** | **1** | **89** | **1.2e-007** | **1** | **K.KTKPYIQVDIGGGQTK.T** 16438 16440 |
|  | 17375 | **901.4455** | **1800.8764** | **1800.8893** | **-0.0129** | **1** | **38** | **0.017** | **1** | **R.IDTRNELESYAYSLK.N** |
|  | 17383 | **601.3399** | **1800.9979** | **1800.0729** | **0.9250** | **1** | **17** | **2** | **1** | **-.MKLSLVAAMLLLLSAAR.A** |
|  | 17544 | **908.4955** | **1814.9765** | **1814.9890** | **-0.0125** | **1** | **86** | **2.7e-007** | **1** | **R.IINEPTAAAIAYGLDKR.E** 17545 17549 |
|  | 17767 | **917.4522** | **1832.8899** | **1832.9017** | **-0.0118** | **1** | **59** | **0.00014** | **1** | **K.NQLTSNPENTVFDAKR.L** 17766 17785 |
|  | 17814 | **918.9644** | **1835.9142** | **1835.9265** | **-0.0123** | **0** | **74** | **4.5e-006** | **1** | **K.SQIFSTASDNQPTVTIK.V** 17813 17815 |
|  | 18484 | **944.4827** | **1886.9508** | **1886.9639** | **-0.0131** | **0** | **58** | **0.00017** | **1** | **K.VTHAVVTVPAYFNDAQR.Q** 18485 18486 18487 |
|  | 19059 | **967.5049** | **1932.9953** | **1933.0058** | **-0.0104** | **0** | **46** | **0.0024** | **1** | **K.DNHLLGTFDLTGIPPAPR.G** 19051 19057 19060 |
|  | 19414 | **983.0110** | **1964.0075** | **1964.0215** | **-0.0139** | **1** | **95** | **3.2e-008** | **1** | **K.KSQIFSTASDNQPTVTIK.V** 19412 19413 |
|  | 19501 | **658.3333** | **1971.9781** | **1970.9585** | **1.0196** | **1** | **3** | **49** | **9** | **R.NELESYAYSLKNQIGDK.E** |
|  | 19525 | **987.9512** | **1973.8879** | **1973.9007** | **-0.0128** | **0** | **92** | **6e-008** | **1** | **K.IEWLESHQDADIEDFK.A** 19526 19534 |
|  | 19983 | **672.6894** | **2015.0465** | **2015.0589** | **-0.0123** | **1** | **12** | **6.3** | **1** | **K.KVTHAVVTVPAYFNDAQR.Q** |
|  | 20278 | 681.6606 | 2041.9599 | 2041.0440 | 0.9160 | 1 | 0 | 90 | 8 | K.ITITNDQNRLTPEEIER.M |
|  | 21513 | **1082.9923** | **2163.9701** | **2163.9848** | **-0.0147** | **0** | **97** | **1.8e-008** | **1** | **R.IEIESFYEGEDFSETLTR.A** 21514 21515 21525 |
|  | 21595 | **1087.5165** | **2173.0184** | **2173.0327** | **-0.0143** | **1** | **45** | **0.0027** | **1** | **K.IEWLESHQDADIEDFKAK.K** 21593 |
|  | 21608 | **1088.4933** | **2174.9720** | **2174.9855** | **-0.0135** | **1** | **100** | **8.3e-009** | **1** | **K.LYGSAGPPPTGEEDTAEKDEL.-** 21604 21606 21607 21609 |
|  | 23176 | **788.7626** | **2363.2661** | **2363.1169** | **0.1492** | **1** | **1** | **53** | **9** | **R.IEIESFYEGEDFSETLTRAK.F** |
|  | 24393 | **844.3987** | **2530.1744** | **2530.1863** | **-0.0119** | **1** | **27** | **0.13** | **1** | **K.AVEEKIEWLESHQDADIEDFK.A** |
|  | 31239 | **1225.6152** | **4898.4318** | **4898.4570** | **-0.0252** | **0** | **54** | **6.9e-005** | **1** | **R.GINPDEAVAYGAAVQAGVLSGDQDTGDLVLLDVCPLTLGIETVGGVMTK.L** 31237 31240 31241 31242 31247 31248 31252 31253 31254 31255 31257 31258 31259 31264 31265 31266 |

| **8.** | P07327\|ADH1A_HUMAN    **Mass:** 40745    **Score:** 2171   **Queries matched:** 100   **emPAI:** 5.36 |
| --- | --- |
|  | Alcohol dehydrogenase 1A OS=Homo sapiens GN=ADH1A PE=1 SV=2 |

|  | **Query** | **Observed** | **Mr(expt)** | **Mr(calc)** | **Delta** | **Miss** | **Score** | **Expect** | **Rank** | **Peptide** |
| --- | --- | --- | --- | --- | --- | --- | --- | --- | --- | --- |
|  | 1134 | 564.7275 | 563.7203 | 563.3431 | 0.3772 | 1 | 4 | 57 | 2 | K.FAKAK.E |
|  | 2619 | **853.5083** | **852.5010** | **851.4977** | **1.0033** | **1** | **6** | **34** | **8** | **K.AHEVRIK.M** |
|  | 3102 | 443.2709 | 884.5272 | 884.5331 | -0.0059 | 0 | 13 | 7.6 | 7 | R.IIAVDINK.D |
|  | 3178 | 447.7383 | 893.4620 | 893.4681 | -0.0061 | 0 | 39 | 0.019 | 1 | K.LVADFMAK.K 3177 3179 |
|  | 3404 | 455.7356 | 909.4566 | 909.4630 | -0.0063 | 0 | (12) | 8.8 | 1 | K.LVADFMAK.K |
|  | 3825 | 472.2549 | 942.4953 | 942.5022 | -0.0069 | 0 | 33 | 0.092 | 1 | K.IDAASPLEK.V 3826 |
|  | 3999 | 477.7995 | 953.5845 | 953.5909 | -0.0064 | 0 | 43 | 0.0078 | 1 | K.KPIQEVLK.E 3998 |
|  | 5167 | 520.2565 | 1038.4984 | 1037.5579 | 0.9405 | 1 | 13 | 6.9 | 1 | K.LVADFMAKK.F |
|  | 6249 | 562.7519 | 1123.4893 | 1123.4968 | -0.0074 | 0 | 42 | 0.0086 | 1 | K.NPESNYCLK.N 6247 6248 |
|  | 6316 | 565.3330 | 1128.6515 | 1127.6550 | 0.9965 | 1 | 23 | 0.79 | 1 | R.IIAVDINKDK.F 6313 |
|  | 7297 | **598.3428** | **1194.6711** | **1194.6795** | **-0.0084** | **0** | **16** | **3.4** | **1** | **K.VIPLAIPQCGK.C** 7298 |
|  | 9913 | 665.3396 | 1328.6646 | 1328.6725 | -0.0078 | 0 | 55 | 0.00043 | 1 | K.INEGFDLLHSGK.S 9908 9909 9910 9911 9912 9929 |
|  | 10841 | 461.2424 | 1380.7054 | 1381.8041 | -1.0987 | 1 | 3 | 74 | 8 | K.AAGAARIIAVDINK.D |
|  | 13227 | **763.3554** | **1524.6961** | **1524.7065** | **-0.0103** | **1** | **51** | **0.0009** | **1** | **R.ICKNPESNYCLK.N** |
|  | 14920 | 819.3719 | 1636.7292 | 1636.7403 | -0.0110 | 0 | 80 | 1.2e-006 | 1 | K.ELGATECINPQDYK.K 14917 14918 14919 14921 14936 14938 |
|  | 15686 | **845.3811** | **1688.7476** | **1687.7762** | **0.9715** | **0** | **33** | **0.05** | **1** | **K.NDVSNPQGTLQDGTSR.F** 15672 |
|  | 16389 | 865.4758 | 1728.9370 | 1728.9451 | -0.0081 | 0 | 86 | 3.1e-007 | 1 | K.FSLDALITHVLPFEK.I 16386 16388 16390 |
|  | 16809 | 879.8994 | 1757.7843 | 1757.7931 | -0.0088 | 0 | 93 | 5.6e-008 | 1 | K.EMTDGGVDFSFEVIGR.L 16786 16788 16789 16790 16791 16792 16793 16794 16795 16796 16798 16799 16800 16802 16803 16804 16805 16806 16807 16808 16810 16811 16812 16813 16814 16815 16816 16819 16820 16826 16827 16828 16839 16840 |
|  | 17806 | 918.9362 | 1835.8578 | 1835.8723 | -0.0146 | 1 | 57 | 0.00023 | 1 | K.AKELGATECINPQDYK.K 17807 17808 17826 |
|  | 18096 | 929.5218 | 1857.0290 | 1857.0400 | -0.0110 | 1 | 90 | 9.9e-008 | 1 | K.KFSLDALITHVLPFEK.I 18094 18095 18097 18098 18100 18101 18102 18109 18111 |
|  | 20506 | 1030.4940 | 2058.9735 | 2058.9867 | -0.0132 | 0 | 97 | 1.7e-008 | 1 | K.VCLIGCGFSTGYGSAVNVAK.V 20504 20505 |
|  | 22988 | 1170.5765 | 2339.1385 | 2338.1484 | 0.9901 | 0 | 50 | 0.00074 | 1 | K.VTPGSTCAVFGLGGVGLSAIMGCK.A 22985 |
|  | 24695 | 858.4423 | 2572.3050 | 2572.3206 | -0.0157 | 1 | 10 | 5.9 | 1 | K.ELGATECINPQDYKKPIQEVLK.E |
|  | 25290 | 898.7924 | 2693.3554 | 2693.3735 | -0.0180 | 1 | 85 | 1.8e-007 | 1 | K.KPIQEVLKEMTDGGVDFSFEVIGR.L 25291 |
|  | 25412 | 903.7743 | 2708.3010 | 2709.3684 | -1.0673 | 1 | (2) | 42 | 4 | K.KPIQEVLKEMTDGGVDFSFEVIGR.L |
|  | 31269 | **1225.8718** | **4899.4582** | **4898.4869** | **0.9713** | **0** | **36** | **0.0043** | **1** | **K.MVAVGICGTDDHVVSGTMVTPLPVILGHEAAGIVESVGEGVTTVKPGDK.V** |

| **9.** | P00738\|HPT_HUMAN    **Mass:** 45861    **Score:** 2130   **Queries matched:** 111   **emPAI:** 4.62 |
| --- | --- |
|  | Haptoglobin OS=Homo sapiens GN=HP PE=1 SV=1 |

|  | **Query** | **Observed** | **Mr(expt)** | **Mr(calc)** | **Delta** | **Miss** | **Score** | **Expect** | **Rank** | **Peptide** |
| --- | --- | --- | --- | --- | --- | --- | --- | --- | --- | --- |
|  | 1543 | **688.3727** | **687.3655** | **687.3704** | **-0.0049** | **0** | **14** | **10** | **5** | **K.QWINK.A** |
|  | 2161 | **408.7373** | **815.4600** | **815.4653** | **-0.0053** | **1** | **27** | **0.45** | **1** | **K.KQWINK.A** |
|  | 3191 | **448.2376** | **894.4606** | **894.4671** | **-0.0065** | **0** | **41** | **0.013** | **1** | **K.NPANPVQR.I** 3190 3192 3195 3205 3208 |
|  | 3534 | **920.4568** | **919.4495** | **919.4552** | **-0.0057** | **0** | **3** | **96** | **5** | **K.GSFPWQAK.M** |
|  | 3573 | **462.2658** | **922.5169** | **922.5236** | **-0.0067** | **0** | **43** | **0.0083** | **1** | **R.ILGGHLDAK.G** |
|  | 4358 | **490.7479** | **979.4812** | **979.4876** | **-0.0064** | **0** | **65** | **5.1e-005** | **1** | **R.VGYVSGWGR.N** 4355 4359 4372 |
|  | 4433 | **493.7940** | **985.5735** | **985.5808** | **-0.0073** | **1** | **16** | **4.4** | **1** | **K.KQLVEIEK.V** |
|  | 6532 | **573.7708** | **1145.5269** | **1145.5353** | **-0.0083** | **0** | **33** | **0.078** | **1** | **R.HYEGSTVPEK.K** 6531 6533 6535 6550 |
|  | 7490 | **602.8037** | **1203.5929** | **1202.6295** | **0.9633** | **0** | **2** | **1e+002** | **5** | **K.VTSIQDWVQK.T** |
|  | 8835 | **637.8176** | **1273.6206** | **1273.6302** | **-0.0097** | **1** | **42** | **0.0085** | **1** | **R.HYEGSTVPEKK.T** 8825 8834 8836 8837 8838 8839 8840 8841 8842 8843 8845 8846 8848 8849 8851 8884 |
|  | 9532 | **656.3021** | **1310.5897** | **1310.5990** | **-0.0093** | **0** | **67** | **3e-005** | **1** | **R.TEGDGVYTLNDK.K** |
|  | 10175 | **673.3218** | **1344.6291** | **1344.6384** | **-0.0093** | **0** | **75** | **4.4e-006** | **1** | **K.SCAVAEYGVYVK.V** 10174 10176 10177 |
|  | 11497 | **709.9114** | **1417.8083** | **1417.8181** | **-0.0097** | **1** | **33** | **0.064** | **1** | **K.DIAPTLTLYVGKK.Q** 11484 11498 |
|  | 11827 | **720.3496** | **1438.6845** | **1438.6940** | **-0.0094** | **1** | **40** | **0.012** | **1** | **R.TEGDGVYTLNDKK.Q** 11825 11826 |
|  | 14053 | **790.8948** | **1579.7751** | **1579.7842** | **-0.0091** | **1** | **87** | **2.2e-007** | **1** | **K.LRTEGDGVYTLNDK.K** 14052 |
|  | 16029 | **854.4076** | **1706.8007** | **1706.8120** | **-0.0113** | **0** | **(74)** | **4.7e-006** | **1** | **K.YVMLPVADQDQCIR.H** 16025 16028 16030 |
|  | 16047 | **854.9230** | **1707.8314** | **1707.8428** | **-0.0114** | **1** | **78** | **1.9e-006** | **1** | **K.LRTEGDGVYTLNNEK.Q** 16021 16046 16050 16051 16068 16072 16074 |
|  | 16268 | **862.4040** | **1722.7934** | **1722.8069** | **-0.0135** | **0** | **75** | **3.5e-006** | **1** | **K.YVMLPVADQDQCIR.H** |
|  | 17301 | **599.3394** | **1794.9964** | **1794.0040** | **0.9925** | **0** | **2** | **65** | **9** | **K.VVLHPNYSQVDIGLIK.L** |
|  | 17783 | **917.9572** | **1833.8999** | **1833.9117** | **-0.0118** | **1** | **43** | **0.0054** | **1** | **R.VMPICLPSKDYAEVGR.V** 17781 |
|  | 18083 | **619.9736** | **1856.8991** | **1856.9124** | **-0.0134** | **1** | **65** | **3.1e-005** | **1** | **K.AVGDKLPECEAVCGKPK.N** 18082 18084 18085 18086 |
|  | 21579 | **1086.5255** | **2171.0365** | **2171.0504** | **-0.0139** | **0** | **88** | **1.3e-007** | **1** | **K.SPVGVQPILNEHTFCAGMSK.Y** 21576 21577 21578 |
|  | 21725 | **730.0176** | **2187.0309** | **2187.0453** | **-0.0144** | **0** | **(5)** | **28** | **1** | **K.SPVGVQPILNEHTFCAGMSK.Y** |
|  | 23907 | **1233.0983** | **2464.1820** | **2464.1879** | **-0.0059** | **1** | **0** | **66** | **4** | **K.FTDHLKYVMLPVADQDQCIR.H** |
|  | 28646 | **1144.8619** | **3431.5640** | **3431.5874** | **-0.0234** | **1** | **40** | **0.0036** | **1** | **K.AVGDKLPECEADDGCPKPPEIAHGYVEHSVR.Y** 28645 28647 28649 |
|  | 29845 | **1292.2273** | **3873.6600** | **3873.6628** | **-0.0028** | **0** | **96** | **6.7e-009** | **1** | **K.YQEDTCYGDAGSAFAVHDLEEDTWYATGILSFDK.S** 29819 29820 29821 29822 29823 29824 29825 29826 29827 29828 29829 29830 29831 29832 29833 29834 29835 29836 29837 29838 29839 29840 29841 29842 29843 29844 29848 29849 |

| **10.** | P07099\|HYEP_HUMAN    **Mass:** 53143    **Score:** 2120   **Queries matched:** 160   **emPAI:** 17.45 |
| --- | --- |
|  | Epoxide hydrolase 1 OS=Homo sapiens GN=EPHX1 PE=1 SV=1 |

|  | **Query** | **Observed** | **Mr(expt)** | **Mr(calc)** | **Delta** | **Miss** | **Score** | **Expect** | **Rank** | **Peptide** |
| --- | --- | --- | --- | --- | --- | --- | --- | --- | --- | --- |
|  | 1520 | **678.3878** | **677.3805** | **677.3860** | **-0.0056** | **1** | **13** | **11** | **1** | **R.IDKFR.F** |
|  | 2254 | **412.2241** | **822.4336** | **822.4388** | **-0.0052** | **0** | **12** | **9.7** | **1** | **K.VISYWR.N** |
|  | 2404 | **418.2345** | **834.4545** | **834.4599** | **-0.0054** | **0** | **22** | **1.2** | **3** | **R.FLGLTER.D** 2417 |
|  | 2445 | **838.3671** | **837.3598** | **837.3657** | **-0.0059** | **0** | **30** | **0.16** | **1** | **R.NEFDWK.K** 2446 |
|  | 2765 | **432.2501** | **862.4857** | **862.4912** | **-0.0056** | **0** | **37** | **0.038** | **1** | **K.FLSVLER.Q** 2781 |
|  | 2902 | **436.2507** | **870.4868** | **870.4923** | **-0.0055** | **0** | **35** | **0.05** | **1** | **K.QVEILNR.Y** 2890 2900 2912 |
|  | 3468 | 458.2385 | 914.4624 | 914.4684 | -0.0060 | 0 | 30 | 0.2 | 1 | K.VFYSLMR.E 3469 |
|  | 3959 | **476.2706** | **950.5267** | **950.5338** | **-0.0071** | **1** | **15** | **5.6** | **1** | **K.KVISYWR.N** |
|  | 4156 | **483.7348** | **965.4550** | **965.4607** | **-0.0057** | **1** | **11** | **12** | **3** | **R.NEFDWKK.Q** |
|  | 4502 | **496.2789** | **990.5432** | **990.5498** | **-0.0066** | **1** | **43** | **0.0099** | **1** | **K.FLSVLERQ.-** 4503 |
|  | 4637 | **500.2970** | **998.5794** | **998.5873** | **-0.0079** | **1** | **15** | **5.3** | **4** | **K.KQVEILNR.Y** |
|  | 4781 | **505.3151** | **1008.6156** | **1008.6219** | **-0.0063** | **0** | **25** | **0.45** | **1** | **K.IIPLLTDPK.N** 4780 4782 |
|  | 5308 | **525.7847** | **1049.5548** | **1049.5618** | **-0.0070** | **1** | **69** | **2.2e-005** | **1** | **K.KGFNSVATAR.I** 5306 5307 5315 5316 |
|  | 5314 | **526.2526** | **1050.4906** | **1050.4982** | **-0.0076** | **0** | **38** | **0.027** | **1** | **R.YLEDGGLER.K** 5312 5313 |
|  | 5627 | **538.3018** | **1074.5891** | **1074.5961** | **-0.0070** | **0** | **28** | **0.25** | **1** | **R.DVELLYPVK.E** |
|  | 5710 | **542.1286** | **1082.2426** | **1082.6310** | **-0.3884** | **1** | **6** | **26** | **6** | **R.IFYKLMLR.L** |
|  | 6029 | **553.7740** | **1105.5335** | **1105.5404** | **-0.0068** | **0** | **26** | **0.42** | **1** | **R.EDDSIRPFK.V** 6044 |
|  | 6335 | **566.2930** | **1130.5715** | **1130.5794** | **-0.0079** | **0** | **37** | **0.031** | **1** | **K.LISYSYMVR.G** 6334 |
|  | 6561 | **574.7923** | **1147.5700** | **1146.5743** | **0.9957** | **0** | **(31)** | **0.14** | **1** | **K.LISYSYMVR.G** |
|  | 6957 | **590.2995** | **1178.5844** | **1178.5931** | **-0.0087** | **1** | **18** | **2.3** | **1** | **R.YLEDGGLERK.F** |
|  | 9099 | **644.7971** | **1287.5796** | **1287.5884** | **-0.0088** | **0** | **55** | **0.00042** | **1** | **K.FSTWTNTEFR.Y** 9098 9100 9118 |
|  | 9157 | **646.3040** | **1290.5935** | **1290.6027** | **-0.0092** | **0** | **(5)** | **42** | **2** | **K.ENLGQGWMTQK.H** |
|  | 9468 | **654.3021** | **1306.5897** | **1306.5976** | **-0.0079** | **0** | **17** | **2.9** | **2** | **K.ENLGQGWMTQK.H** 9496 9497 |
|  | 16022 | **854.3947** | **1706.7749** | **1706.7860** | **-0.0111** | **0** | **74** | **4.2e-006** | **1** | **K.VETSDEEIHDLHQR.I** 16023 |
|  | 16365 | 865.4156 | 1728.8166 | 1728.8294 | -0.0127 | 1 | 61 | 7.8e-005 | 1 | R.FYKENLGQGWMTQK.H 16364 16366 16367 |
|  | 16599 | **873.4130** | **1744.8115** | **1744.8243** | **-0.0128** | **1** | **(59)** | **0.00013** | **1** | **R.FYKENLGQGWMTQK.H** 16600 |
|  | 18552 | **631.3527** | **1891.0363** | **1891.0455** | **-0.0092** | **1** | **73** | **4.6e-006** | **1** | **R.FLGLTERDVELLYPVK.E** 18529 18550 18551 |
|  | 19817 | **1000.4900** | **1998.9654** | **1998.9799** | **-0.0145** | **0** | **93** | **4.7e-008** | **1** | **R.GGHFAAFEEPELLAQDIR.K** 19795 19818 19819 19820 19822 19823 |
|  | 21179 | **1064.5367** | **2127.0589** | **2127.0749** | **-0.0159** | **1** | **89** | **1.2e-007** | **1** | **R.GGHFAAFEEPELLAQDIRK.F** 21178 21180 21181 21182 21183 21184 |
|  | 22498 | **1140.0878** | **2278.1610** | **2278.1674** | **-0.0064** | **0** | **87** | **1.5e-007** | **1** | **K.VYVPTGFSAFPFELLHTPEK.W** 22476 22479 22480 22483 22484 22487 22488 22493 22496 22497 22519 22521 |
|  | 23668 | **1218.5448** | **2435.0750** | **2435.0892** | **-0.0142** | **0** | **64** | **2.6e-005** | **1** | **R.FTPPLEDSCFHYGFNSNYLK.K** 23665 23666 23667 23669 23678 23679 |
|  | 24278 | **837.8046** | **2510.3921** | **2510.4043** | **-0.0122** | **0** | **32** | **0.041** | **1** | **K.GLHLNMALVLSNFSTLTLLLGQR.F** 24275 24276 24279 24282 24283 24286 24288 24289 |
|  | 24424 | **846.7705** | **2537.2897** | **2537.3029** | **-0.0132** | **1** | **19** | **0.95** | **1** | **R.MKVYVPTGFSAFPFELLHTPEK.W** |
|  | 24577 | **852.4013** | **2554.1821** | **2553.2978** | **0.8843** | **1** | **(2)** | **39** | **4** | **R.MKVYVPTGFSAFPFELLHTPEK.W** |
|  | 24642 | **1282.5908** | **2563.1671** | **2563.1842** | **-0.0171** | **1** | **41** | **0.0052** | **1** | **R.FTPPLEDSCFHYGFNSNYLKK.V** |
|  | 25739 | **1387.7004** | **2773.3863** | **2773.3997** | **-0.0133** | **0** | **102** | **4e-009** | **1** | **K.FSLDDLLTNVMLYWTTGTIISSQR.F** 25738 25741 |
|  | 25861 | **932.4399** | **2794.2978** | **2794.3158** | **-0.0180** | **1** | **49** | **0.00083** | **1** | **R.EDDSIRPFKVETSDEEIHDLHQR.I** 25856 25857 25858 25859 25860 25863 25864 25870 25873 |
|  | 26492 | **1451.7496** | **2901.4847** | **2901.4946** | **-0.0099** | **1** | **104** | **2.4e-009** | **1** | **R.KFSLDDLLTNVMLYWTTGTIISSQR.F** 26488 26489 26491 26496 26501 |
|  | 27378 | **1532.2015** | **3062.3885** | **3062.4080** | **-0.0195** | **0** | **81** | **4e-007** | **1** | **K.NHGLSDEHVFEVICPSIPGYGFSEASSK.K** 27359 27374 27375 27376 27377 27379 27380 27381 27382 27384 |
|  | 27871 | **1064.5016** | **3190.4829** | **3190.5030** | **-0.0200** | **1** | **65** | **1.4e-005** | **1** | **K.NHGLSDEHVFEVICPSIPGYGFSEASSKK.G** 27868 27869 27870 |
|  | 28548 | **1132.5502** | **3394.6287** | **3394.6479** | **-0.0192** | **0** | **64** | **1.9e-005** | **1** | **R.LGFQEFYIQGGDWGSLICTNMAQLVPSHVK.G** 28543 28544 28545 28546 28549 28552 |
|  | 28986 | **891.9341** | **3563.7072** | **3563.7276** | **-0.0204** | **0** | **67** | **8.2e-006** | **1** | **R.ESGYMHIQCTKPDTVGSALNDSPVGLAAYILEK.F** 28985 28987 28988 28989 28990 28991 28992 28993 |
|  | 29038 | **895.9308** | **3579.6941** | **3579.7225** | **-0.0285** | **0** | **(19)** | **0.45** | **1** | **R.ESGYMHIQCTKPDTVGSALNDSPVGLAAYILEK.F** 29042 |
|  | 30174 | **1014.2557** | **4052.9939** | **4053.0194** | **-0.0255** | **1** | **3** | **12** | **2** | **K.IIPLLTDPKNHGLSDEHVFEVICPSIPGYGFSEASSK.K** |
|  | 31190 | **690.2229** | **4824.5094** | **4824.5407** | **-0.0313** | **1** | **5** | **4.4** | **2** | **K.TKIEGLDIHFIHVKPPQLPAGHTPKPLLMVHGWPGSFYEFYK.I** 31193 31195 |

| **11.** | P60709\|ACTB_HUMAN    **Mass:** 42052    **Score:** 2116   **Queries matched:** 106   **emPAI:** 14.34 |
| --- | --- |
|  | Actin, cytoplasmic 1 OS=Homo sapiens GN=ACTB PE=1 SV=1 |

|  | **Query** | **Observed** | **Mr(expt)** | **Mr(calc)** | **Delta** | **Miss** | **Score** | **Expect** | **Rank** | **Peptide** |
| --- | --- | --- | --- | --- | --- | --- | --- | --- | --- | --- |
|  | 1363 | **630.3417** | **629.3344** | **630.3701** | **-1.0357** | **0** | **4** | **99** | **2** | **K.ILTER.G** |
|  | 1421 | **644.3680** | **643.3607** | **643.3653** | **-0.0046** | **0** | **20** | **2.6** | **1** | **R.LDLAGR.D** |
|  | 1884 | **795.4671** | **794.4598** | **794.4650** | **-0.0052** | **0** | **17** | **3.4** | **2** | **K.IIAPPER.K** |
|  | 4297 | **488.7242** | **975.4339** | **975.4410** | **-0.0071** | **0** | **67** | **3.7e-005** | **1** | **K.AGFAGDDAPR.A** 4293 4296 |
|  | 4616 | **499.7438** | **997.4730** | **997.4790** | **-0.0060** | **0** | **33** | **0.076** | **1** | **R.DLTDYLMK.I** |
|  | 5140 | **518.8257** | **1035.6369** | **1035.6440** | **-0.0071** | **1** | **26** | **0.39** | **1** | **K.IKIIAPPER.K** |
|  | 6353 | **566.7636** | **1131.5125** | **1131.5197** | **-0.0071** | **0** | **40** | **0.016** | **1** | **R.GYSFTTTAER.E** 6351 6352 6354 |
|  | 6737 | **581.3089** | **1160.6032** | **1160.6111** | **-0.0078** | **0** | **48** | **0.0027** | **1** | **K.EITALAPSTMK.I** 6736 6738 |
|  | 6867 | **586.2850** | **1170.5555** | **1170.5638** | **-0.0083** | **0** | **50** | **0.0016** | **1** | **R.HQGVMVGMGQK.D** 6865 6866 |
|  | 6943 | **589.3067** | **1176.5988** | **1176.6060** | **-0.0072** | **0** | **(4)** | **67** | **3** | **K.EITALAPSTMK.I** |
|  | 7105 | **594.2822** | **1186.5499** | **1186.5587** | **-0.0088** | **0** | **(35)** | **0.046** | **1** | **R.HQGVMVGMGQK.D** |
|  | 7106 | **594.2826** | **1186.5507** | **1186.5587** | **-0.0080** | **0** | **(47)** | **0.003** | **1** | **R.HQGVMVGMGQK.D** 7093 |
|  | 7338 | **599.2899** | **1196.5652** | **1197.5150** | **-0.9498** | **0** | **29** | **0.2** | **1** | **K.DSYVGDEAQSK.R** |
|  | 7365 | **599.8527** | **1197.6908** | **1197.6982** | **-0.0075** | **0** | **58** | **0.00026** | **1** | **R.AVFPSIVGRPR.H** 7366 |
|  | 7465 | **602.2790** | **1202.5434** | **1202.5536** | **-0.0102** | **0** | **(4)** | **69** | **5** | **R.HQGVMVGMGQK.D** |
|  | 10347 | **677.8101** | **1353.6057** | **1353.6161** | **-0.0104** | **1** | **33** | **0.07** | **1** | **K.DSYVGDEAQSKR.G** 10348 10349 |
|  | 13084 | **758.8497** | **1515.6849** | **1515.6954** | **-0.0105** | **0** | **48** | **0.0021** | **1** | **K.QEYDESGPSIVHR.K** |
|  | 13087 | **758.8746** | **1515.7347** | **1514.7419** | **0.9928** | **0** | **38** | **0.022** | **1** | **K.IWHHTFYNELR.V** 13067 13068 |
|  | 14707 | **541.9487** | **1622.8244** | **1622.8338** | **-0.0094** | **1** | **10** | **12** | **4** | **R.LDLAGRDLTDYLMK.I** |
|  | 15044 | **822.8969** | **1643.7793** | **1643.7903** | **-0.0111** | **1** | **28** | **0.19** | **1** | **K.QEYDESGPSIVHRK.C** |
|  | 17255 | **895.9438** | **1789.8730** | **1789.8846** | **-0.0116** | **0** | **71** | **9.6e-006** | **1** | **K.SYELPDGQVITIGNER.F** 17256 17257 17258 17267 17268 |
|  | 18946 | **642.6774** | **1925.0103** | **1925.8169** | **-0.8066** | **0** | **(3)** | **49** | **4** | **-.MDDDIAALVVDNGSGMCK.A** |
|  | 19152 | **648.3133** | **1941.9180** | **1941.8118** | **0.1062** | **0** | **6** | **25** | **3** | **-.MDDDIAALVVDNGSGMCK.A** 19155 |
|  | 19282 | **977.5289** | **1953.0433** | **1953.0571** | **-0.0138** | **0** | **62** | **6.3e-005** | **1** | **R.VAPEEHPVLLTEAPLNPK.A** 19281 19283 19284 19285 |
|  | 21975 | **1108.0316** | **2214.0487** | **2214.0627** | **-0.0140** | **0** | **113** | **4.5e-010** | **1** | **K.DLYANTVLSGGTTMYPGIADR.M** 21969 21970 21972 21973 21974 21976 21977 21985 21986 |
|  | 22142 | **1116.0288** | **2230.0431** | **2230.0576** | **-0.0145** | **0** | **(105)** | **2.8e-009** | **1** | **K.DLYANTVLSGGTTMYPGIADR.M** 22141 |
|  | 23022 | **1172.0790** | **2342.1434** | **2342.1576** | **-0.0142** | **1** | **81** | **6.6e-007** | **1** | **R.KDLYANTVLSGGTTMYPGIADR.M** 23019 23020 23021 |
|  | 23193 | **789.4443** | **2365.3112** | **2366.2464** | **-0.9352** | **1** | **1** | **50** | **1** | **R.AVFPSIVGRPRHQGVMVGMGQK.D** |
|  | 23210 | **592.8179** | **2367.2426** | **2366.0631** | **1.1795** | **1** | **1** | **60** | **2** | **R.HQGVMVGMGQKDSYVGDEAQSK.R** |
|  | 24494 | **1275.5835** | **2549.1524** | **2549.1665** | **-0.0141** | **0** | **108** | **1.1e-009** | **1** | **K.LCYVALDFEQEMATAASSSSLEK.S** 24486 24487 24488 24490 24491 24492 24493 24495 24498 24503 24506 |
|  | 25942 | **936.4365** | **2806.2876** | **2806.3041** | **-0.0165** | **1** | **56** | **0.00014** | **1** | **K.EKLCYVALDFEQEMATAASSSSLEK.S** |
|  | 26371 | **961.7416** | **2882.2031** | **2883.2474** | **-1.0443** | **1** | **2** | **27** | **2** | **-.MDDDIAALVVDNGSGMCKAGFAGDDAPR.A** |
|  | 27842 | **1061.8693** | **3182.5860** | **3182.6071** | **-0.0211** | **0** | **64** | **2.1e-005** | **1** | **R.TTGIVMDSGDGVTHTVPIYEGYALPHAILR.L** 27841 27843 27844 27845 27847 |
|  | 27892 | **1067.2017** | **3198.5832** | **3198.6020** | **-0.0188** | **0** | **(45)** | **0.0016** | **1** | **R.TTGIVMDSGDGVTHTVPIYEGYALPHAILR.L** |
|  | 28005 | **1077.8189** | **3230.4347** | **3230.4545** | **-0.0198** | **0** | **66** | **1e-005** | **1** | **R.CPEALFQPSFLGMESCGIHETTFNSIMK.C** 28004 28007 28010 28011 28012 28013 28014 28015 |
|  | 28057 | **1083.1494** | **3246.4264** | **3246.4494** | **-0.0230** | **0** | **(16)** | **1.2** | **1** | **R.CPEALFQPSFLGMESCGIHETTFNSIMK.C** 28058 |
|  | 28895 | **1178.8742** | **3533.6006** | **3533.6240** | **-0.0234** | **1** | **66** | **7.8e-006** | **1** | **R.FRCPEALFQPSFLGMESCGIHETTFNSIMK.C** 28894 28896 28897 28898 28899 28901 |

|  | **Proteins matching a subset of these peptides:** |
| --- | --- |

|  | P63261\|ACTG_HUMAN    **Mass:** 42108    **Score:** 2116   **Queries matched:** 102 |
| --- | --- |
|  | Actin, cytoplasmic 2 OS=Homo sapiens GN=ACTG1 PE=1 SV=1 |

| **12.** | P00326\|ADH1G_HUMAN    **Mass:** 40697    **Score:** 2102   **Queries matched:** 108   **emPAI:** 5.36 |
| --- | --- |
|  | Alcohol dehydrogenase 1C OS=Homo sapiens GN=ADH1C PE=1 SV=2 |

|  | **Query** | **Observed** | **Mr(expt)** | **Mr(calc)** | **Delta** | **Miss** | **Score** | **Expect** | **Rank** | **Peptide** |
| --- | --- | --- | --- | --- | --- | --- | --- | --- | --- | --- |
|  | 1134 | 564.7275 | 563.7203 | 563.3431 | 0.3772 | 1 | 4 | 57 | 2 | K.FAKAK.E |
|  | 1769 | 785.3839 | 784.3766 | 784.3828 | -0.0062 | 0 | 11 | 15 | 1 | K.NDLGNPR.G |
|  | 2553 | 847.4208 | 846.4135 | 846.4196 | -0.0061 | 0 | 8 | 31 | 2 | R.GTLQDGTR.R |
|  | 2619 | 853.5083 | 852.5010 | 851.4977 | 1.0033 | 1 | 6 | 34 | 8 | K.AHEVRIK.M |
|  | 2959 | **438.7474** | **875.4803** | **876.4310** | **-0.9506** | **0** | **16** | **5.3** | **5** | **K.MVAAGICR.S** 2968 |
|  | 3102 | 443.2709 | 884.5272 | 884.5331 | -0.0059 | 0 | 13 | 7.6 | 7 | R.IIAVDINK.D |
|  | 3178 | 447.7383 | 893.4620 | 893.4681 | -0.0061 | 0 | 39 | 0.019 | 1 | K.LVADFMAK.K 3177 3179 |
|  | 3404 | 455.7356 | 909.4566 | 909.4630 | -0.0063 | 0 | (12) | 8.8 | 1 | K.LVADFMAK.K |
|  | 3738 | 468.5695 | 935.1245 | 935.5440 | -0.4195 | 1 | 10 | 13 | 1 | K.SIRTVLTF.- |
|  | 3825 | 472.2549 | 942.4953 | 942.5022 | -0.0069 | 0 | 33 | 0.092 | 1 | K.IDAASPLEK.V 3826 |
|  | 3999 | 477.7995 | 953.5845 | 953.5909 | -0.0064 | 0 | 43 | 0.0078 | 1 | K.KPIQEVLK.E 3998 |
|  | 4709 | 502.7657 | 1003.5168 | 1002.5207 | 0.9962 | 1 | 6 | 48 | 8 | R.GTLQDGTRR.F |
|  | 5167 | 520.2565 | 1038.4984 | 1037.5579 | 0.9405 | 1 | 13 | 6.9 | 1 | K.LVADFMAKK.F |
|  | 5627 | 538.3018 | 1074.5891 | 1075.5662 | -0.9771 | 0 | 15 | 5.6 | 3 | K.INEGFDLLR.S |
|  | 6249 | 562.7519 | 1123.4893 | 1123.4968 | -0.0074 | 0 | 42 | 0.0086 | 1 | K.NPESNYCLK.N 6247 6248 |
|  | 6316 | 565.3330 | 1128.6515 | 1127.6550 | 0.9965 | 1 | 23 | 0.79 | 1 | R.IIAVDINKDK.F 6313 |
|  | 8576 | 630.3404 | 1258.6662 | 1258.6744 | -0.0082 | 0 | 44 | 0.0062 | 1 | K.VIPLFTPQCGK.C 8577 |
|  | 10841 | 461.2424 | 1380.7054 | 1381.8041 | -1.0987 | 1 | 3 | 74 | 8 | K.AAGAARIIAVDINK.D |
|  | 12001 | **725.8683** | **1449.7220** | **1449.7537** | **-0.0317** | **1** | **10** | **15** | **4** | **K.ESVPKLVADFMAK.K** 12000 |
|  | 13227 | 763.3554 | 1524.6961 | 1524.7065 | -0.0103 | 1 | 51 | 0.0009 | 1 | R.ICKNPESNYCLK.N |
|  | 14920 | 819.3719 | 1636.7292 | 1636.7403 | -0.0110 | 0 | 80 | 1.2e-006 | 1 | K.ELGATECINPQDYK.K 14917 14918 14919 14921 14936 14938 |
|  | 16236 | **860.9760** | **1719.9373** | **1719.9447** | **-0.0074** | **0** | **35** | **0.036** | **1** | **K.FSLDALITNILPFEK.I** |
|  | 16809 | 879.8994 | 1757.7843 | 1757.7931 | -0.0088 | 0 | 93 | 5.6e-008 | 1 | K.EMTDGGVDFSFEVIGR.L 16786 16788 16789 16790 16791 16792 16793 16794 16795 16796 16798 16799 16800 16802 16803 16804 16805 16806 16807 16808 16810 16811 16812 16813 16814 16815 16816 16819 16820 16826 16827 16828 16839 16840 |
|  | 17009 | **888.4205** | **1774.8265** | **1774.8383** | **-0.0117** | **0** | **55** | **0.00032** | **1** | **K.VCLIGCGFSTGYGSAVK.V** |
|  | 17806 | 918.9362 | 1835.8578 | 1835.8723 | -0.0146 | 1 | 57 | 0.00023 | 1 | K.AKELGATECINPQDYK.K 17807 17808 17826 |
|  | 17968 | **925.5242** | **1849.0338** | **1848.0397** | **0.9941** | **1** | **109** | **1.2e-009** | **1** | **K.KFSLDALITNILPFEK.I** 17958 |
|  | 18541 | 631.2869 | 1890.8390 | 1889.8690 | 0.9700 | 1 | 4 | 39 | 3 | K.NPESNYCLKNDLGNPR.G |
|  | 20676 | 1036.9911 | 2071.9676 | 2073.0387 | -1.0711 | 1 | 49 | 0.0012 | 2 | K.VCLIGCGFSTGYGSAVKVAK.V 20675 20686 20687 20688 20689 20693 |
|  | 23081 | **1177.0835** | **2352.1524** | **2352.1641** | **-0.0116** | **0** | **75** | **2.5e-006** | **1** | **K.VTPGSTCAVFGLGGVGLSVVMGCK.A** 23080 23082 |
|  | 24695 | 858.4423 | 2572.3050 | 2572.3206 | -0.0157 | 1 | 10 | 5.9 | 1 | K.ELGATECINPQDYKKPIQEVLK.E |
|  | 25290 | 898.7924 | 2693.3554 | 2693.3735 | -0.0180 | 1 | 85 | 1.8e-007 | 1 | K.KPIQEVLKEMTDGGVDFSFEVIGR.L 25291 |
|  | 25412 | 903.7743 | 2708.3010 | 2709.3684 | -1.0673 | 1 | (2) | 42 | 4 | K.KPIQEVLKEMTDGGVDFSFEVIGR.L |
|  | 30311 | 1369.7159 | 4106.1260 | 4106.1536 | -0.0276 | 0 | 60 | 2.3e-005 | 1 | R.SDEHVVSGNLVTPLPVILGHEAAGIVESVGEGVTTVKPGDK.V 30310 30313 30314 30315 30317 30318 30319 30321 30322 30333 30334 |
|  | 31461 | 1070.3645 | 5346.7861 | 5346.8174 | -0.0313 | 1 | 43 | 0.00064 | 1 | R.SDEHVVSGNLVTPLPVILGHEAAGIVESVGEGVTTVKPGDKVIPLFTPQCGK.C |

| **13.** | P07437\|TBB5_HUMAN    **Mass:** 50095    **Score:** 2024   **Queries matched:** 113   **emPAI:** 14.38 |
| --- | --- |
|  | Tubulin beta chain OS=Homo sapiens GN=TUBB PE=1 SV=2 |

|  | **Query** | **Observed** | **Mr(expt)** | **Mr(calc)** | **Delta** | **Miss** | **Score** | **Expect** | **Rank** | **Peptide** |
| --- | --- | --- | --- | --- | --- | --- | --- | --- | --- | --- |
|  | 1603 | **709.3656** | **708.3583** | **708.3411** | **0.0172** | **1** | **14** | **6.5** | **3** | **R.GRMSMK.E** |
|  | 2066 | **404.7367** | **807.4588** | **807.3399** | **0.1190** | **0** | **7** | **33** | **7** | **R.EEYPDR.I** |
|  | 5020 | **514.7598** | **1027.5050** | **1027.5121** | **-0.0071** | **0** | **46** | **0.004** | **1** | **K.TAVCDIPPR.G** 5004 5015 5016 5017 5018 5019 5021 5022 |
|  | 5177 | **520.2972** | **1038.5798** | **1038.5862** | **-0.0064** | **0** | **53** | **0.00083** | **1** | **R.YLTVAAVFR.G** 5176 |
|  | 5494 | **533.3253** | **1064.6361** | **1064.4201** | **0.2160** | **0** | **(4)** | **60** | **6** | **K.NMMAACDPR.H** |
|  | 5638 | **539.2662** | **1076.5179** | **1076.5250** | **-0.0071** | **1** | **36** | **0.039** | **1** | **K.IREEYPDR.I** 5637 |
|  | 5678 | **541.2108** | **1080.4071** | **1080.4151** | **-0.0080** | **0** | **29** | **0.16** | **1** | **K.NMMAACDPR.H** 5679 5694 |
|  | 6327 | **565.7967** | **1129.5788** | **1129.5880** | **-0.0092** | **0** | **4** | **59** | **6** | **R.FPGQLNADLR.K** |
|  | 6499 | **572.3174** | **1142.6203** | **1142.6270** | **-0.0067** | **0** | **70** | **1.8e-005** | **1** | **K.LAVNMVPFPR.L** 6498 |
|  | 7996 | **615.2989** | **1228.5833** | **1228.5910** | **-0.0077** | **0** | **62** | **0.00011** | **1** | **R.ISEQFTAMFR.R** 7997 |
|  | 8802 | **636.3640** | **1270.7133** | **1270.7220** | **-0.0086** | **1** | **47** | **0.0027** | **1** | **R.KLAVNMVPFPR.L** |
|  | 9112 | **430.2745** | **1287.8016** | **1286.7169** | **1.0847** | **1** | **(7)** | **28** | **2** | **R.KLAVNMVPFPR.L** 9101 |
|  | 9342 | **651.3178** | **1300.6209** | **1300.6299** | **-0.0090** | **0** | **61** | **0.00011** | **1** | **R.ISVYYNEATGGK.Y** 9317 9343 |
|  | 9689 | **660.3509** | **1318.6872** | **1318.6955** | **-0.0083** | **0** | **82** | **8.3e-007** | **1** | **R.IMNTFSVVPSPK.V** 9686 9687 9696 |
|  | 10023 | **668.3477** | **1334.6809** | **1334.6904** | **-0.0095** | **0** | **(19)** | **1.8** | **1** | **R.IMNTFSVVPSPK.V** |
|  | 10908 | **693.3495** | **1384.6844** | **1384.6921** | **-0.0077** | **1** | **45** | **0.004** | **1** | **K.RISEQFTAMFR.R** |
|  | 11940 | **723.8431** | **1445.6717** | **1445.6820** | **-0.0103** | **0** | **68** | **2.2e-005** | **1** | **K.EVDEQMLNVQNK.N** 11938 11939 |
|  | 12198 | **731.8404** | **1461.6662** | **1461.6769** | **-0.0107** | **0** | **(42)** | **0.0078** | **1** | **K.EVDEQMLNVQNK.N** 12218 |
|  | 14585 | **808.4162** | **1614.8178** | **1614.8287** | **-0.0109** | **0** | **42** | **0.0069** | **1** | **R.AILVDLEPGTMDSVR.S** 14583 14586 14602 |
|  | 14658 | **810.9161** | **1619.8176** | **1619.8283** | **-0.0107** | **0** | **52** | **0.0008** | **1** | **R.LHFFMPGFAPLTSR.G** 14659 14660 14661 |
|  | 14838 | **816.4137** | **1630.8128** | **1630.8236** | **-0.0108** | **0** | **(37)** | **0.025** | **1** | **R.AILVDLEPGTMDSVR.S** |
|  | 15287 | 830.4462 | 1658.8778 | 1658.8879 | -0.0102 | 0 | 55 | 0.00035 | 1 | R.ALTVPELTQQVFDAK.N 15283 15288 |
|  | 15835 | **848.9150** | **1695.8154** | **1695.8257** | **-0.0102** | **0** | **33** | **0.051** | **1** | **K.NSSYFVEWIPNNVK.T** 15855 15856 |
|  | 17620 | **911.9583** | **1821.9019** | **1821.9156** | **-0.0136** | **0** | **67** | **2.6e-005** | **1** | **R.EIVHIQAGQCGNQIGAK.F** 17614 17621 |
|  | 18291 | **935.4880** | **1868.9615** | **1868.9706** | **-0.0091** | **0** | **91** | **7.2e-008** | **1** | **K.MAVTFIGNSTAIQELFK.R** 18288 18290 18292 18293 18302 18303 |
|  | 19345 | **979.9900** | **1957.9654** | **1957.9745** | **-0.0091** | **0** | **103** | **4.9e-009** | **1** | **K.GHYTEGAELVDSVLDVVR.K** 19346 |
|  | 20135 | **1013.5360** | **2025.0573** | **2025.0717** | **-0.0144** | **1** | **83** | **4.7e-007** | **1** | **K.MAVTFIGNSTAIQELFKR.I** 20136 20137 20138 |
|  | 20818 | **1044.0366** | **2086.0587** | **2086.0695** | **-0.0108** | **1** | **86** | **2.5e-007** | **1** | **K.GHYTEGAELVDSVLDVVRK.E** 20815 20817 20819 |
|  | 21027 | **1055.5288** | **2109.0431** | **2109.0571** | **-0.0141** | **1** | **71** | **7.2e-006** | **1** | **-.MREIVHIQAGQCGNQIGAK.F** 21028 |
|  | 23107 | **786.0563** | **2355.1470** | **2355.1583** | **-0.0113** | **1** | **2** | **44** | **7** | **R.LHFFMPGFAPLTSRGSQQYR.A** |
|  | 25368 | **902.4683** | **2704.3831** | **2705.2975** | **-0.9144** | **1** | **4** | **24** | **3** | **R.ALTVPELTQQVFDAKNMMAACDPR.H** |
|  | 25387 | **1354.6653** | **2707.3160** | **2707.3310** | **-0.0150** | **0** | **93** | **3.2e-008** | **1** | **K.LTTPTYGDLNHLVSATMSGVTTCLR.F** 25379 25385 25386 25404 25411 25412 |
|  | 25469 | **545.2800** | **2721.3638** | **2721.2924** | **0.0713** | **1** | **(0)** | **57** | **4** | **R.ALTVPELTQQVFDAKNMMAACDPR.H** |
|  | 25766 | **695.3771** | **2777.4792** | **2776.4346** | **1.0446** | **1** | **6** | **13** | **2** | **K.LAVNMVPFPRLHFFMPGFAPLTSR.G** |
|  | 25900 | **933.4471** | **2797.3194** | **2797.3361** | **-0.0167** | **0** | **97** | **1.3e-008** | **1** | **R.SGPFGQIFRPDNFVFGQSGAGNNWAK.G** 25898 25899 25903 25910 25911 25912 |
|  | 27535 | **1034.8005** | **3101.3798** | **3101.4003** | **-0.0205** | **0** | **65** | **1.5e-005** | **1** | **K.FWEVISDEHGIDPTGTYHGDSDLQLDR.I** 27536 27537 |
|  | 28244 | **1656.2616** | **3310.5086** | **3310.5268** | **-0.0182** | **0** | **(65)** | **1.3e-005** | **1** | **K.EAESCDCLQGFQLTHSLGGGTGSGMGTLLISK.I** 28241 28242 28243 28245 28246 |
|  | 28304 | **1109.8416** | **3326.5028** | **3326.5218** | **-0.0189** | **0** | **72** | **3e-006** | **1** | **K.EAESCDCLQGFQLTHSLGGGTGSGMGTLLISK.I** |
|  | 28665 | **1147.2072** | **3438.5996** | **3438.6218** | **-0.0222** | **1** | **68** | **6.1e-006** | **1** | **R.KEAESCDCLQGFQLTHSLGGGTGSGMGTLLISK.I** 28657 28663 28664 |
|  | 31036 | **1531.7026** | **4592.0861** | **4592.0999** | **-0.0139** | **0** | **50** | **0.00017** | **1** | **K.VSDTVVEPYNATLSVHQLVENTDETYCIDNEALYDICFR.T** 31032 31033 |

| **14.** | P68371\|TBB2C_HUMAN    **Mass:** 50255    **Score:** 1904   **Queries matched:** 107   **emPAI:** 11.26 |
| --- | --- |
|  | Tubulin beta-2C chain OS=Homo sapiens GN=TUBB2C PE=1 SV=1 |

|  | **Query** | **Observed** | **Mr(expt)** | **Mr(calc)** | **Delta** | **Miss** | **Score** | **Expect** | **Rank** | **Peptide** |
| --- | --- | --- | --- | --- | --- | --- | --- | --- | --- | --- |
|  | 1603 | 709.3656 | 708.3583 | 708.3411 | 0.0172 | 1 | 14 | 6.5 | 3 | R.GRMSMK.E |
|  | 2066 | 404.7367 | 807.4588 | 807.3399 | 0.1190 | 0 | 7 | 33 | 7 | R.EEYPDR.I |
|  | 5020 | 514.7598 | 1027.5050 | 1027.5121 | -0.0071 | 0 | 46 | 0.004 | 1 | K.TAVCDIPPR.G 5004 5015 5016 5017 5018 5019 5021 5022 |
|  | 5177 | 520.2972 | 1038.5798 | 1038.5862 | -0.0064 | 0 | 53 | 0.00083 | 1 | R.YLTVAAVFR.G 5176 |
|  | 5494 | 533.3253 | 1064.6361 | 1064.4201 | 0.2160 | 0 | (4) | 60 | 6 | K.NMMAACDPR.H |
|  | 5638 | 539.2662 | 1076.5179 | 1076.5250 | -0.0071 | 1 | 36 | 0.039 | 1 | K.IREEYPDR.I 5637 |
|  | 5678 | 541.2108 | 1080.4071 | 1080.4151 | -0.0080 | 0 | 29 | 0.16 | 1 | K.NMMAACDPR.H 5679 5694 |
|  | 6327 | 565.7967 | 1129.5788 | 1129.5880 | -0.0092 | 0 | 4 | 59 | 6 | R.FPGQLNADLR.K |
|  | 6499 | 572.3174 | 1142.6203 | 1142.6270 | -0.0067 | 0 | 70 | 1.8e-005 | 1 | K.LAVNMVPFPR.L 6498 |
|  | 7996 | 615.2989 | 1228.5833 | 1228.5910 | -0.0077 | 0 | 62 | 0.00011 | 1 | R.ISEQFTAMFR.R 7997 |
|  | 8802 | 636.3640 | 1270.7133 | 1270.7220 | -0.0086 | 1 | 47 | 0.0027 | 1 | R.KLAVNMVPFPR.L |
|  | 9112 | 430.2745 | 1287.8016 | 1286.7169 | 1.0847 | 1 | (7) | 28 | 2 | R.KLAVNMVPFPR.L 9101 |
|  | 9689 | 660.3509 | 1318.6872 | 1318.6955 | -0.0083 | 0 | 82 | 8.3e-007 | 1 | R.IMNTFSVVPSPK.V 9686 9687 9696 |
|  | 9887 | **664.8235** | **1327.6325** | **1327.6408** | **-0.0083** | **0** | **60** | **0.00014** | **1** | **R.INVYYNEATGGK.Y** 9886 |
|  | 10023 | 668.3477 | 1334.6809 | 1334.6904 | -0.0095 | 0 | (19) | 1.8 | 1 | R.IMNTFSVVPSPK.V |
|  | 10908 | 693.3495 | 1384.6844 | 1384.6921 | -0.0077 | 1 | 45 | 0.004 | 1 | K.RISEQFTAMFR.R |
|  | 11940 | 723.8431 | 1445.6717 | 1445.6820 | -0.0103 | 0 | 68 | 2.2e-005 | 1 | K.EVDEQMLNVQNK.N 11938 11939 |
|  | 12198 | 731.8404 | 1461.6662 | 1461.6769 | -0.0107 | 0 | (42) | 0.0078 | 1 | K.EVDEQMLNVQNK.N 12218 |
|  | 14384 | 801.4086 | 1600.8026 | 1600.8131 | -0.0105 | 0 | 67 | 2.6e-005 | 1 | R.AVLVDLEPGTMDSVR.S 14378 14379 14385 |
|  | 14658 | 810.9161 | 1619.8176 | 1619.8283 | -0.0107 | 0 | 52 | 0.0008 | 1 | R.LHFFMPGFAPLTSR.G 14659 14660 14661 |
|  | 15728 | **846.4319** | **1690.8492** | **1690.8600** | **-0.0108** | **0** | **35** | **0.032** | **1** | **R.ALTVPELTQQMFDAK.N** 15729 15730 15732 |
|  | 15835 | 848.9150 | 1695.8154 | 1695.8257 | -0.0102 | 0 | 33 | 0.051 | 1 | K.NSSYFVEWIPNNVK.T 15855 15856 |
|  | 16036 | **854.4293** | **1706.8441** | **1706.8549** | **-0.0108** | **0** | **(19)** | **1.5** | **2** | **R.ALTVPELTQQMFDAK.N** |
|  | 17620 | 911.9583 | 1821.9019 | 1821.9156 | -0.0136 | 0 | 67 | 2.6e-005 | 1 | R.EIVHLQAGQCGNQIGAK.F 17614 17621 |
|  | 18090 | **929.4694** | **1856.9242** | **1856.9342** | **-0.0101** | **0** | **88** | **1.5e-007** | **2** | **K.MSATFIGNSTAIQELFK.R** 18088 18089 18091 |
|  | 19345 | 979.9900 | 1957.9654 | 1957.9745 | -0.0091 | 0 | 103 | 4.9e-009 | 1 | K.GHYTEGAELVDSVLDVVR.K 19346 |
|  | 19959 | **1007.5189** | **2013.0232** | **2013.0353** | **-0.0122** | **1** | **75** | **2.7e-006** | **1** | **K.MSATFIGNSTAIQELFKR.I** |
|  | 20818 | 1044.0366 | 2086.0587 | 2086.0695 | -0.0108 | 1 | 86 | 2.5e-007 | 1 | K.GHYTEGAELVDSVLDVVRK.E 20815 20817 20819 |
|  | 21027 | 1055.5288 | 2109.0431 | 2109.0571 | -0.0141 | 1 | 71 | 7.2e-006 | 1 | -.MREIVHLQAGQCGNQIGAK.F 21028 |
|  | 21101 | **1059.0121** | **2116.0096** | **2116.0987** | **-0.0891** | **1** | **2** | **56** | **4** | **K.YVPRAVLVDLEPGTMDSVR.S** 21100 |
|  | 21442 | **1078.9855** | **2155.9564** | **2155.1347** | **0.8217** | **1** | **8** | **14** | **2** | **R.GLKMSATFIGNSTAIQELFK.R** 21443 |
|  | 23107 | 786.0563 | 2355.1470 | 2355.1583 | -0.0113 | 1 | 2 | 44 | 7 | R.LHFFMPGFAPLTSRGSQQYR.A |
|  | 25387 | 1354.6653 | 2707.3160 | 2707.3310 | -0.0150 | 0 | 93 | 3.2e-008 | 1 | K.LTTPTYGDLNHLVSATMSGVTTCLR.F 25379 25385 25386 25404 25411 25412 |
|  | 25766 | 695.3771 | 2777.4792 | 2776.4346 | 1.0446 | 1 | 6 | 13 | 2 | K.LAVNMVPFPRLHFFMPGFAPLTSR.G |
|  | 25900 | 933.4471 | 2797.3194 | 2797.3361 | -0.0167 | 0 | 97 | 1.3e-008 | 1 | R.SGPFGQIFRPDNFVFGQSGAGNNWAK.G 25898 25899 25903 25910 25911 25912 |
|  | 27582 | **1039.4722** | **3115.3947** | **3115.4159** | **-0.0213** | **0** | **67** | **1.1e-005** | **1** | **K.FWEVISDEHGIDPTGTYHGDSDLQLER.I** |
|  | 28244 | 1656.2616 | 3310.5086 | 3310.5268 | -0.0182 | 0 | (65) | 1.3e-005 | 1 | K.EAESCDCLQGFQLTHSLGGGTGSGMGTLLISK.I 28241 28242 28243 28245 28246 |
|  | 28304 | 1109.8416 | 3326.5028 | 3326.5218 | -0.0189 | 0 | 72 | 3e-006 | 1 | K.EAESCDCLQGFQLTHSLGGGTGSGMGTLLISK.I |
|  | 28665 | 1147.2072 | 3438.5996 | 3438.6218 | -0.0222 | 1 | 68 | 6.1e-006 | 1 | R.KEAESCDCLQGFQLTHSLGGGTGSGMGTLLISK.I 28657 28663 28664 |
|  | 31036 | 1531.7026 | 4592.0861 | 4592.0999 | -0.0139 | 0 | 50 | 0.00017 | 1 | K.VSDTVVEPYNATLSVHQLVENTDETYCIDNEALYDICFR.T 31032 31033 |

| **15.** | P08684\|CP3A4_HUMAN    **Mass:** 57705    **Score:** 1867   **Queries matched:** 107   **emPAI:** 4.41 |
| --- | --- |
|  | Cytochrome P450 3A4 OS=Homo sapiens GN=CYP3A4 PE=1 SV=4 |

|  | **Query** | **Observed** | **Mr(expt)** | **Mr(calc)** | **Delta** | **Miss** | **Score** | **Expect** | **Rank** | **Peptide** |
| --- | --- | --- | --- | --- | --- | --- | --- | --- | --- | --- |
|  | 1116 | **559.3775** | **558.3702** | **558.3741** | **-0.0039** | **0** | **24** | **1.2** | **1** | **K.TVLVK.E** |
|  | 2761 | **432.2411** | **862.4676** | **862.4735** | **-0.0059** | **0** | **17** | **4.1** | **1** | **R.LFPIAMR.L** |
|  | 3965 | **476.7212** | **951.4278** | **951.4338** | **-0.0060** | **0** | **18** | **2.7** | **1** | **K.YWTEPEK.F** |
|  | 6395 | **568.3036** | **1134.5927** | **1134.6008** | **-0.0081** | **0** | **18** | **2.5** | **1** | **R.RPFGPVGFMK.S** |
|  | 6425 | **569.3078** | **1136.6010** | **1136.6077** | **-0.0067** | **0** | **38** | **0.025** | **1** | **R.SLLSPTFTSGK.L** |
|  | 6876 | **586.3881** | **1170.7616** | **1171.6448** | **-0.8833** | **0** | **36** | **0.038** | **1** | **R.EAETGKPVTLK.D** 6886 |
|  | 8621 | **421.0562** | **1260.1466** | **1260.7012** | **-0.5546** | **1** | **2** | **69** | **9** | **R.LFPIAMRLER.V** |
|  | 8652 | **631.8217** | **1261.6289** | **1261.6376** | **-0.0088** | **0** | **50** | **0.0015** | **1** | **K.DVEINGMFIPK.G** 8653 8661 8662 |
|  | 9917 | **443.9207** | **1328.7403** | **1327.7459** | **0.9944** | **1** | **12** | **8.3** | **4** | **R.REAETGKPVTLK.D** |
|  | 10591 | **684.3566** | **1366.6986** | **1366.7067** | **-0.0082** | **0** | **28** | **0.22** | **1** | **R.VLQNFSFKPCK.E** 10590 |
|  | 10611 | **684.8675** | **1367.7204** | **1367.7296** | **-0.0092** | **0** | **11** | **10** | **2** | **K.LQEEIDAVLPNK.A** |
|  | 10772 | **689.8339** | **1377.6532** | **1376.6459** | **1.0072** | **0** | **18** | **2.3** | **1** | **K.SAISIAEDEEWK.R** |
|  | 10794 | **690.3622** | **1378.7098** | **1377.7868** | **0.9231** | **1** | **2** | **82** | **5** | **R.SLLSPTFTSGKLK.E** 10769 |
|  | 11001 | **695.8683** | **1389.7221** | **1389.7326** | **-0.0105** | **1** | **58** | **0.00023** | **1** | **K.KDVEINGMFIPK.G** 11023 |
|  | 11272 | **703.8880** | **1405.7614** | **1405.7929** | **-0.0315** | **1** | **11** | **9.4** | **4** | **R.LRSLLSPTFTSGK.L** |
|  | 11275 | **703.9634** | **1405.9123** | **1405.7275** | **0.1848** | **1** | **(3)** | **56** | **2** | **K.KDVEINGMFIPK.G** |
|  | 11771 | **479.2531** | **1434.7374** | **1435.8043** | **-1.0669** | **1** | **0** | **1.2e+002** | **4** | **R.FALMNMKLALIR.V** |
|  | 11862 | **481.2682** | **1440.7827** | **1440.7911** | **-0.0085** | **0** | **21** | **1.1** | **1** | **K.GVVVMIPSYALHR.D** |
|  | 13352 | **767.3753** | **1532.7361** | **1532.7470** | **-0.0110** | **1** | **63** | **6.6e-005** | **1** | **K.SAISIAEDEEWKR.L** 13350 13351 13353 |
|  | 14186 | **795.3166** | **1588.6187** | **1588.6295** | **-0.0108** | **1** | **58** | **0.00019** | **1** | **K.GFCMFDMECHKK.Y** 14184 14187 |
|  | 14285 | **532.2644** | **1593.7714** | **1593.7827** | **-0.0113** | **1** | **12** | **7.1** | **1** | **K.YWTEPEKFLPER.F** |
|  | 14374 | **801.3773** | **1600.7400** | **1600.7346** | **0.0054** | **1** | **(8)** | **21** | **6** | **R.NCIGMRFALMNMK.L** 14375 |
|  | 14604 | **808.9011** | **1615.7876** | **1616.7295** | **-0.9420** | **1** | **(1)** | **1e+002** | **8** | **R.NCIGMRFALMNMK.L** |
|  | 14861 | **817.3726** | **1632.7307** | **1632.7244** | **0.0062** | **1** | **8** | **19** | **3** | **R.NCIGMRFALMNMK.L** |
|  | 14924 | **819.4083** | **1636.8021** | **1636.8130** | **-0.0109** | **0** | **69** | **1.4e-005** | **1** | **R.VDFLQLMIDSQNSK.E** |
|  | 15716 | **564.3504** | **1690.0294** | **1690.0393** | **-0.0099** | **0** | **40** | **0.0087** | **1** | **K.LSLGGLLQPEKPVVLK.V** 15714 15715 |
|  | 15949 | **851.9586** | **1701.9027** | **1701.9124** | **-0.0097** | **0** | **74** | **4.9e-006** | **1** | **K.EMVPIIAQYGDVLVR.N** 15948 15951 |
|  | 17110 | 891.4844 | 1780.9542 | 1780.9658 | -0.0116 | 1 | 33 | 0.051 | 1 | K.GVVVMIPSYALHRDPK.Y 17109 17131 |
|  | 17491 | **906.4285** | **1810.8424** | **1810.8526** | **-0.0102** | **0** | **38** | **0.016** | **1** | **K.DNIDPYIYTPFGSGPR.N** |
|  | 19021 | **965.9876** | **1929.9607** | **1929.9731** | **-0.0124** | **1** | **108** | **1.8e-009** | **1** | **K.HRVDFLQLMIDSQNSK.E** 19004 19020 19022 |
|  | 19167 | **648.7004** | **1943.0795** | **1943.0914** | **-0.0119** | **1** | **42** | **0.0062** | **1** | **K.LKEMVPIIAQYGDVLVR.N** |
|  | 19374 | **654.3664** | **1960.0773** | **1959.0863** | **0.9910** | **1** | **(5)** | **27** | **1** | **K.LKEMVPIIAQYGDVLVR.N** |
|  | 20429 | **1027.4961** | **2052.9776** | **2052.9905** | **-0.0129** | **1** | **61** | **6.2e-005** | **1** | **K.NKDNIDPYIYTPFGSGPR.N** 20427 20428 20430 |
|  | 21235 | **1067.6003** | **2133.1861** | **2133.1986** | **-0.0125** | **0** | **37** | **0.018** | **1** | **K.LGIPGPTPLPFLGNILSYHK.G** |
|  | 23410 | 1197.0900 | 2392.1654 | 2392.1773 | -0.0120 | 0 | 86 | 1.9e-007 | 1 | K.VWGFYDGQQPVLAITDPDMIK.T 23403 23408 23409 |
|  | 24208 | **834.4102** | **2500.2087** | **2499.5040** | **0.7047** | **1** | **5** | **22** | **1** | **K.ETQIPLKLSLGGLLQPEKPVVLK.V** |
|  | 25309 | **1349.6505** | **2697.2865** | **2697.3030** | **-0.0165** | **0** | **63** | **3e-005** | **1** | **K.APPTYDTVLQMEYLDMVVNETLR.L** 25310 25311 25313 |
|  | 29700 | **1274.5957** | **3820.7653** | **3820.7778** | **-0.0126** | **0** | **55** | **0.0001** | **1** | **K.DVFGAYSMDVITSTSFGVNIDSLNNPQDPFVENTK.K** 29683 29684 29687 29688 29691 29694 29695 29696 29697 29698 29699 29702 29703 |
|  | 29982 | **1317.2929** | **3948.8567** | **3948.8728** | **-0.0161** | **1** | **80** | **2.8e-007** | **1** | **K.DVFGAYSMDVITSTSFGVNIDSLNNPQDPFVENTKK.L** 29971 29973 29974 29975 29978 29979 29980 29983 29984 29985 29987 29988 29994 29995 29996 |
|  | 30158 | **1350.0090** | **4047.0053** | **4047.0220** | **-0.0168** | **1** | **60** | **2.7e-005** | **1** | **K.LQEEIDAVLPNKAPPTYDTVLQMEYLDMVVNETLR.L** 30153 30154 30155 30156 30157 30159 30160 |
|  | 30198 | **1355.3381** | **4062.9926** | **4063.0170** | **-0.0244** | **1** | **(45)** | **0.00083** | **1** | **K.LQEEIDAVLPNKAPPTYDTVLQMEYLDMVVNETLR.L** |

| **16.** | P16435\|NCPR_HUMAN    **Mass:** 77097    **Score:** 1843   **Queries matched:** 103   **emPAI:** 3.49 |
| --- | --- |
|  | NADPH--cytochrome P450 reductase OS=Homo sapiens GN=POR PE=1 SV=2 |

|  | **Query** | **Observed** | **Mr(expt)** | **Mr(calc)** | **Delta** | **Miss** | **Score** | **Expect** | **Rank** | **Peptide** |
| --- | --- | --- | --- | --- | --- | --- | --- | --- | --- | --- |
|  | 2886 | **435.7370** | **869.4594** | **868.3596** | **1.0998** | **0** | **8** | **27** | **8** | **K.MASSSGEGK.E** |
|  | 3459 | **457.7532** | **913.4918** | **913.4981** | **-0.0063** | **0** | **59** | **0.0002** | **1** | **R.LEQLGAQR.I** 3458 |
|  | 3484 | **458.7509** | **915.4871** | **915.4926** | **-0.0055** | **0** | **17** | **3.8** | **1** | **K.GVATNWLR.A** 3483 |
|  | 3691 | **466.7698** | **931.5251** | **931.5313** | **-0.0063** | **0** | **10** | **19** | **1** | **R.ALVPMFVR.K** 3700 |
|  | 3815 | **471.7092** | **941.4038** | **941.4099** | **-0.0061** | **0** | **25** | **0.44** | **1** | **K.VYMGEMGR.L** 3816 |
|  | 3861 | **473.2566** | **944.4987** | **944.5039** | **-0.0052** | **1** | **29** | **0.23** | **1** | **R.KLNQGTER.H** 3851 3852 3857 3858 3863 3864 |
|  | 3991 | **477.3566** | **952.6987** | **951.5178** | **1.1809** | **0** | **4** | **44** | **3** | **K.FAVFGLGNK.T** |
|  | 4056 | **480.2086** | **958.4027** | **957.4048** | **0.9979** | **0** | **(11)** | **16** | **2** | **K.VYMGEMGR.L** |
|  | 4638 | **500.2995** | **998.5844** | **998.5913** | **-0.0069** | **0** | **51** | **0.0012** | **1** | **K.VYVQHLLK.Q** |
|  | 4716 | **502.7872** | **1003.5598** | **1003.5662** | **-0.0064** | **0** | **52** | **0.0011** | **1** | **K.IQTLTSSVR.E** 4715 4717 |
|  | 4729 | **503.2445** | **1004.4745** | **1004.4814** | **-0.0070** | **0** | **31** | **0.15** | **1** | **R.YYSIASSSK.V** |
|  | 5537 | **535.2819** | **1068.5492** | **1069.5992** | **-1.0500** | **1** | **26** | **0.36** | **1** | **K.RLEQLGAQR.I** |
|  | 5729 | **542.7820** | **1083.5495** | **1084.5261** | **-0.9766** | **1** | **6** | **43** | **3** | **R.AKEPAGENGGR.A** |
|  | 7334 | **599.2675** | **1196.5205** | **1196.5284** | **-0.0079** | **0** | **53** | **0.00066** | **1** | **K.TYEHFNAMGK.Y** 7332 7333 |
|  | 7506 | **402.2448** | **1203.7127** | **1202.6408** | **1.0719** | **0** | **8** | **23** | **3** | **K.NPFLAAVTTNR.K** |
|  | 7664 | **607.3158** | **1212.6170** | **1212.5233** | **0.0937** | **0** | **(16)** | **4** | **1** | **K.TYEHFNAMGK.Y** |
|  | 7761 | **609.3204** | **1216.6262** | **1215.5520** | **1.0742** | **1** | **15** | **5.4** | **1** | **R.RSDEDYLYR.E** 7719 |
|  | 8801 | **424.5758** | **1270.7056** | **1270.7146** | **-0.0090** | **1** | **14** | **6.3** | **1** | **R.INKGVATNWLR.A** 8800 |
|  | 10976 | **695.3357** | **1388.6569** | **1388.6659** | **-0.0090** | **1** | **13** | **7.5** | **1** | **K.KHPFPCPTSYR.T** 10975 |
|  | 11023 | 696.3632 | 1390.7119 | 1390.7205 | -0.0086 | 0 | 53 | 0.00066 | 1 | R.DGALTQLNVAFSR.E 10998 11022 11029 |
|  | 11585 | **712.3408** | **1422.6671** | **1422.6779** | **-0.0108** | **0** | **43** | **0.0069** | **1** | **K.SYENQKPPFDAK.N** 11586 |
|  | 12140 | **730.3425** | **1458.6705** | **1458.6813** | **-0.0108** | **0** | **45** | **0.0045** | **1** | **K.EVGETLLYYGCR.R** |
|  | 13400 | **769.3823** | **1536.7500** | **1536.7606** | **-0.0106** | **0** | **70** | **1.3e-005** | **1** | **R.HLMHLELDISDSK.I** 13399 13401 13402 13406 |
|  | 14374 | 801.3773 | 1600.7400 | 1600.8097 | -0.0697 | 0 | 33 | 0.057 | 2 | R.QYELVVHTDIDAAK.V 14375 14380 14383 |
|  | 14929 | **819.4252** | **1636.8359** | **1636.8461** | **-0.0101** | **0** | **65** | **3.5e-005** | **1** | **R.TALTYYLDITNPPR.T** 14927 14928 |
|  | 15353 | **555.6226** | **1663.8459** | **1663.8569** | **-0.0111** | **1** | **6** | **29** | **3** | **R.LKSYENQKPPFDAK.N** |
|  | 17543 | **605.9993** | **1814.9762** | **1814.9097** | **0.0664** | **1** | **6** | **27** | **8** | **K.EPAGENGGRALVPMFVR.K** |
|  | 20621 | **690.9833** | **2069.9282** | **2069.9442** | **-0.0161** | **1** | **43** | **0.0038** | **1** | **R.SDEDYLYREELAQFHR.D** 20622 20623 |
|  | 21101 | 1059.0121 | 2116.0096 | 2116.0225 | -0.0129 | 0 | 115 | 2.6e-010 | 1 | R.NIIVFYGSQTGTAEEFANR.L 21097 21099 21100 |
|  | 21990 | **554.7685** | **2215.0449** | **2214.0627** | **0.9822** | **1** | **1** | **67** | **8** | **K.MASSSGEGKELYLSWVVEAR.R** |
|  | 22711 | **769.4009** | **2305.1810** | **2304.1155** | **1.0655** | **0** | **5** | **23** | **3** | **K.ILGADLDVVMSLNNLDEESNK.K** |
|  | 23560 | **1209.1075** | **2416.2005** | **2416.2155** | **-0.0150** | **1** | **104** | **3e-009** | **1** | **K.ILGADLDVVMSLNNLDEESNKK.H** 23561 23562 23563 |
|  | 23743 | **815.4662** | **2443.3767** | **2444.2336** | **-0.8568** | **1** | **3** | **34** | **5** | **R.NIIVFYGSQTGTAEEFANRLSK.D** |
|  | 23758 | **1224.0874** | **2446.1602** | **2446.1765** | **-0.0162** | **0** | **57** | **0.00016** | **1** | **R.YESGDHVAVYPANDSALVNQLGK.I** 23756 23757 |
|  | 23815 | **818.4068** | **2452.1986** | **2452.2121** | **-0.0136** | **0** | **68** | **1.1e-005** | **1** | **R.TNVLYELAQYASEPSEQELLR.K** 23814 23816 23817 23818 |
|  | 23826 | **1227.5703** | **2453.1261** | **2453.1387** | **-0.0126** | **0** | **127** | **1.5e-011** | **1** | **R.IFELGLGDDDGNLEEDFITWR.E** 23824 23825 23827 23828 |
|  | 23860 | **1229.6564** | **2457.2982** | **2457.3090** | **-0.0108** | **0** | **75** | **2.4e-006** | **1** | **K.ATTPVIMVGPGTGVAPFIGFIQER.A** 23857 23858 23861 23869 |
|  | 24750 | **1291.1541** | **2580.2935** | **2580.3071** | **-0.0135** | **1** | **95** | **2e-008** | **1** | **R.TNVLYELAQYASEPSEQELLRK.M** 24746 24751 |
|  | 25116 | **889.0650** | **2664.1732** | **2664.1915** | **-0.0183** | **0** | **37** | **0.012** | **1** | **R.EQFWPAVCEHFGVEATGEESSIR.Q** |
|  | 25442 | **906.1205** | **2715.3398** | **2715.3616** | **-0.0218** | **1** | **27** | **0.13** | **1** | **K.IRYESGDHVAVYPANDSALVNQLGK.I** |
|  | 26737 | **1470.7003** | **2939.3861** | **2939.4011** | **-0.0150** | **0** | **109** | **6.8e-010** | **1** | **R.DVQNTFYDIVAELGAMEHAQAVDYIK.K** |
|  | 26924 | **744.8985** | **2975.5649** | **2975.5837** | **-0.0188** | **0** | **17** | **1.1** | **1** | **R.HILAILQDCPSLRPPIDHLCELLPR.L** 26907 26929 26931 |
|  | 27413 | **1023.5020** | **3067.4840** | **3067.4961** | **-0.0120** | **1** | **24** | **0.22** | **1** | **R.DVQNTFYDIVAELGAMEHAQAVDYIKK.L** 27412 |
|  | 27660 | **627.3407** | **3131.6671** | **3131.6848** | **-0.0177** | **1** | **22** | **0.3** | **1** | **R.RHILAILQDCPSLRPPIDHLCELLPR.L** 27661 |

| **17.** | P68363\|TBA1B_HUMAN    **Mass:** 50804    **Score:** 1795   **Queries matched:** 83   **emPAI:** 6.80 |
| --- | --- |
|  | Tubulin alpha-1B chain OS=Homo sapiens GN=TUBA1B PE=1 SV=1 |

|  | **Query** | **Observed** | **Mr(expt)** | **Mr(calc)** | **Delta** | **Miss** | **Score** | **Expect** | **Rank** | **Peptide** |
| --- | --- | --- | --- | --- | --- | --- | --- | --- | --- | --- |
|  | 1311 | **614.3460** | **613.3387** | **613.3435** | **-0.0049** | **0** | **11** | **14** | **1** | **R.GDVVPK.D** |
|  | 1757 | **775.4042** | **774.3969** | **774.4024** | **-0.0055** | **0** | **28** | **0.34** | **1** | **R.GHYTIGK.E** |
|  | 1765 | **781.4037** | **780.3964** | **780.4018** | **-0.0053** | **0** | **29** | **0.21** | **1** | **R.LSVDYGK.K** |
|  | 3389 | **455.2527** | **908.4908** | **908.4967** | **-0.0060** | **1** | **42** | **0.0094** | **1** | **R.LSVDYGKK.S** |
|  | 4864 | **508.2898** | **1014.5650** | **1014.5709** | **-0.0060** | **0** | **41** | **0.014** | **1** | **K.DVNAAIATIK.T** 4835 4861 4863 |
|  | 4948 | **512.2609** | **1022.5072** | **1022.4417** | **0.0655** | **0** | **11** | **14** | **2** | **K.EDAANNYAR.G** |
|  | 5745 | **543.3106** | **1084.6065** | **1084.6128** | **-0.0063** | **0** | **32** | **0.11** | **1** | **K.EIIDLVLDR.I** 5743 |
|  | 8386 | **625.2759** | **1248.5372** | **1248.5453** | **-0.0081** | **0** | **18** | **2.4** | **1** | **K.YMACCLLYR.G** |
|  | 10814 | **460.9011** | **1379.6815** | **1379.6907** | **-0.0092** | **1** | **31** | **0.11** | **1** | **R.LDHKFDLMYAK.R** |
|  | 11350 | **705.8859** | **1409.7573** | **1409.7667** | **-0.0094** | **0** | **6** | **31** | **1** | **R.QLFHPEQLITGK.E** 11351 |
|  | 12641 | **744.4393** | **1486.8641** | **1486.8719** | **-0.0078** | **0** | **83** | **6.7e-007** | **1** | **R.LISQIVSSITASLR.F** 12628 12639 12642 12644 12646 |
|  | 14104 | **792.8749** | **1583.7352** | **1583.7443** | **-0.0091** | **0** | **66** | **2.9e-005** | **1** | **R.SIQFVDWCPTGFK.V** 14102 14103 14123 |
|  | 15935 | **851.4510** | **1700.8874** | **1700.8985** | **-0.0111** | **0** | **60** | **0.00011** | **1** | **R.AVFVDLEPTVIDEVR.T** 15936 |
|  | 16223 | **573.9514** | **1718.8322** | **1717.8747** | **0.9575** | **0** | **4** | **47** | **7** | **R.NLDIERPTYTNLNR.L** |
|  | 16758 | **878.9794** | **1755.9442** | **1755.9559** | **-0.0117** | **0** | **73** | **4.9e-006** | **1** | **R.IHFPLATYAPVISAEK.A** 16755 16756 16757 16760 16761 16762 |
|  | 17643 | **912.9902** | **1823.9658** | **1823.9782** | **-0.0124** | **0** | **59** | **0.00015** | **1** | **K.VGINYQPPTVVPGGDLAK.V** 17640 17641 17653 |
|  | 17874 | **921.5047** | **1840.9948** | **1841.0047** | **-0.0098** | **1** | **31** | **0.077** | **1** | **R.GHYTIGKEIIDLVLDR.I** |
|  | 18197 | **932.9499** | **1863.8853** | **1863.8971** | **-0.0118** | **0** | **82** | **6.5e-007** | **1** | **R.AVCMLSNTTAIAEAWAR.L** 18198 |
|  | 19906 | **1004.4442** | **2006.8737** | **2006.8858** | **-0.0121** | **0** | **105** | **2.5e-009** | **1** | **K.TIGGGDDSFNTFFSETGAGK.H** 19902 19903 19905 |
|  | 22378 | **755.3527** | **2263.0362** | **2263.1201** | **-0.0840** | **1** | **5** | **22** | **3** | **K.VQRAVCMLSNTTAIAEAWAR.L** |
|  | 22917 | **777.6732** | **2329.9978** | **2329.0110** | **0.9869** | **0** | **37** | **0.014** | **1** | **R.AFVHWYVGEGMEEGEFSEAR.E** |
|  | 23508 | **1205.1007** | **2408.1869** | **2408.2012** | **-0.0144** | **0** | **116** | **1.9e-010** | **1** | **R.FDGALNVDLTEFQTNLVPYPR.I** 23503 23504 23505 23506 23507 23509 23510 23512 23515 |
|  | 23544 | **805.7346** | **2414.1819** | **2414.1978** | **-0.0160** | **1** | **41** | **0.0058** | **1** | **R.QLFHPEQLITGKEDAANNYAR.G** 23545 |
|  | 24192 | **833.4252** | **2497.2539** | **2496.1670** | **1.0869** | **1** | **7** | **13** | **1** | **K.TIGGGDDSFNTFFSETGAGKHVPR.A** |
|  | 25605 | **1375.6397** | **2749.2647** | **2749.2840** | **-0.0192** | **0** | **80** | **6.4e-007** | **1** | **K.AYHEQLSVAEITNACFEPANQMVK.C** 25593 25603 25604 25606 25608 |
|  | 27966 | **1073.1377** | **3216.3913** | **3216.4168** | **-0.0255** | **1** | **62** | **2.5e-005** | **1** | **R.AFVHWYVGEGMEEGEFSEAREDMAALEK.D** 27967 |
|  | 28016 | **808.8973** | **3231.5600** | **3232.4117** | **-0.8517** | **1** | **(4)** | **19** | **5** | **R.AFVHWYVGEGMEEGEFSEAREDMAALEK.D** |
|  | 28067 | **1083.8721** | **3248.5944** | **3248.4066** | **0.1877** | **1** | **(1)** | **38** | **9** | **R.AFVHWYVGEGMEEGEFSEAREDMAALEK.D** |
|  | 28528 | **1130.8743** | **3389.6010** | **3389.6173** | **-0.0164** | **0** | **68** | **7.3e-006** | **1** | **K.LADQCTGLQGFLVFHSFGGGTGSGFTSLLMER.L** 28527 28529 |
|  | 28854 | **1173.5719** | **3517.6939** | **3517.7123** | **-0.0184** | **1** | **86** | **9.8e-008** | **1** | **R.KLADQCTGLQGFLVFHSFGGGTGSGFTSLLMER.L** 28855 |
|  | 28902 | **884.7049** | **3534.7905** | **3533.7072** | **1.0833** | **1** | **(2)** | **25** | **10** | **R.KLADQCTGLQGFLVFHSFGGGTGSGFTSLLMER.L** |
|  | 30674 | **1074.9896** | **4295.9294** | **4295.9497** | **-0.0203** | **0** | **20** | **0.22** | **1** | **R.ECISIHVGQAGVQIGNACWELYCLEHGIQPDGQMPSDK.T** |
|  | 31025 | **1146.7742** | **4583.0676** | **4583.0913** | **-0.0237** | **1** | **55** | **5.3e-005** | **1** | **-.MRECISIHVGQAGVQIGNACWELYCLEHGIQPDGQMPSDK.T** 31027 |
|  | 31739 | **1434.1794** | **5732.6887** | **5731.7161** | **0.9726** | **1** | **22** | **0.053** | **1** | **K.SKLEFSIYPAPQVSTAVVEPYNSILTTHTTLEHSDCAFMVDNEAIYDICR.R** 31736 31737 |

| **18.** | Q9BQE3\|TBA1C_HUMAN    **Mass:** 50548    **Score:** 1620   **Queries matched:** 75   **emPAI:** 6.36 |
| --- | --- |
|  | Tubulin alpha-1C chain OS=Homo sapiens GN=TUBA1C PE=1 SV=1 |

|  | **Query** | **Observed** | **Mr(expt)** | **Mr(calc)** | **Delta** | **Miss** | **Score** | **Expect** | **Rank** | **Peptide** |
| --- | --- | --- | --- | --- | --- | --- | --- | --- | --- | --- |
|  | 1311 | 614.3460 | 613.3387 | 613.3435 | -0.0049 | 0 | 11 | 14 | 1 | R.GDVVPK.D |
|  | 1757 | 775.4042 | 774.3969 | 774.4024 | -0.0055 | 0 | 28 | 0.34 | 1 | R.GHYTIGK.E |
|  | 1765 | 781.4037 | 780.3964 | 780.4018 | -0.0053 | 0 | 29 | 0.21 | 1 | R.LSVDYGK.K |
|  | 3389 | 455.2527 | 908.4908 | 908.4967 | -0.0060 | 1 | 42 | 0.0094 | 1 | R.LSVDYGKK.S |
|  | 4864 | 508.2898 | 1014.5650 | 1014.5709 | -0.0060 | 0 | 41 | 0.014 | 1 | K.DVNAAIATIK.T 4835 4861 4863 |
|  | 4948 | 512.2609 | 1022.5072 | 1022.4417 | 0.0655 | 0 | 11 | 14 | 2 | K.EDAANNYAR.G |
|  | 5745 | 543.3106 | 1084.6065 | 1084.6128 | -0.0063 | 0 | 32 | 0.11 | 1 | K.EIIDLVLDR.I 5743 |
|  | 8386 | 625.2759 | 1248.5372 | 1248.5453 | -0.0081 | 0 | 18 | 2.4 | 1 | K.YMACCLLYR.G |
|  | 10814 | 460.9011 | 1379.6815 | 1379.6907 | -0.0092 | 1 | 31 | 0.11 | 1 | R.LDHKFDLMYAK.R |
|  | 11350 | 705.8859 | 1409.7573 | 1409.7667 | -0.0094 | 0 | 6 | 31 | 1 | R.QLFHPEQLITGK.E 11351 |
|  | 12641 | 744.4393 | 1486.8641 | 1486.8719 | -0.0078 | 0 | 83 | 6.7e-007 | 1 | R.LISQIVSSITASLR.F 12628 12639 12642 12644 12646 |
|  | 14334 | **799.8823** | **1597.7500** | **1597.7599** | **-0.0100** | **0** | **23** | **0.64** | **2** | **R.TIQFVDWCPTGFK.V** |
|  | 15935 | 851.4510 | 1700.8874 | 1700.8985 | -0.0111 | 0 | 60 | 0.00011 | 1 | R.AVFVDLEPTVIDEVR.T 15936 |
|  | 16223 | 573.9514 | 1718.8322 | 1717.8747 | 0.9575 | 0 | 4 | 47 | 7 | R.NLDIERPTYTNLNR.L |
|  | 16758 | 878.9794 | 1755.9442 | 1755.9559 | -0.0117 | 0 | 73 | 4.9e-006 | 1 | R.IHFPLATYAPVISAEK.A 16755 16756 16757 16760 16761 16762 |
|  | 17643 | 912.9902 | 1823.9658 | 1823.9782 | -0.0124 | 0 | 59 | 0.00015 | 1 | K.VGINYQPPTVVPGGDLAK.V 17640 17641 17653 |
|  | 17874 | 921.5047 | 1840.9948 | 1841.0047 | -0.0098 | 1 | 31 | 0.077 | 1 | R.GHYTIGKEIIDLVLDR.I |
|  | 17992 | **925.9419** | **1849.8692** | **1849.8815** | **-0.0122** | **0** | **82** | **6.1e-007** | **1** | **R.AVCMLSNTTAVAEAWAR.L** 17993 |
|  | 18241 | **933.9811** | **1865.9477** | **1865.8764** | **0.0713** | **0** | **(7)** | **19** | **4** | **R.AVCMLSNTTAVAEAWAR.L** |
|  | 19906 | 1004.4442 | 2006.8737 | 2006.8858 | -0.0121 | 0 | 105 | 2.5e-009 | 1 | K.TIGGGDDSFNTFFSETGAGK.H 19902 19903 19905 |
|  | 22917 | 777.6732 | 2329.9978 | 2329.0110 | 0.9869 | 0 | 37 | 0.014 | 1 | R.AFVHWYVGEGMEEGEFSEAR.E |
|  | 23508 | 1205.1007 | 2408.1869 | 2408.2012 | -0.0144 | 0 | 116 | 1.9e-010 | 1 | R.FDGALNVDLTEFQTNLVPYPR.I 23503 23504 23505 23506 23507 23509 23510 23512 23515 |
|  | 23544 | 805.7346 | 2414.1819 | 2414.1978 | -0.0160 | 1 | 41 | 0.0058 | 1 | R.QLFHPEQLITGKEDAANNYAR.G 23545 |
|  | 24192 | 833.4252 | 2497.2539 | 2496.1670 | 1.0869 | 1 | 7 | 13 | 1 | K.TIGGGDDSFNTFFSETGAGKHVPR.A |
|  | 25685 | **922.1008** | **2763.2805** | **2763.2996** | **-0.0191** | **0** | **22** | **0.38** | **1** | **K.AYHEQLTVAEITNACFEPANQMVK.C** |
|  | 27966 | 1073.1377 | 3216.3913 | 3216.4168 | -0.0255 | 1 | 62 | 2.5e-005 | 1 | R.AFVHWYVGEGMEEGEFSEAREDMAALEK.D 27967 |
|  | 28016 | 808.8973 | 3231.5600 | 3232.4117 | -0.8517 | 1 | (4) | 19 | 5 | R.AFVHWYVGEGMEEGEFSEAREDMAALEK.D |
|  | 28067 | 1083.8721 | 3248.5944 | 3248.4066 | 0.1877 | 1 | (1) | 38 | 9 | R.AFVHWYVGEGMEEGEFSEAREDMAALEK.D |
|  | 28528 | 1130.8743 | 3389.6010 | 3389.6173 | -0.0164 | 0 | 68 | 7.3e-006 | 1 | K.LADQCTGLQGFLVFHSFGGGTGSGFTSLLMER.L 28527 28529 |
|  | 28854 | 1173.5719 | 3517.6939 | 3517.7123 | -0.0184 | 1 | 86 | 9.8e-008 | 1 | R.KLADQCTGLQGFLVFHSFGGGTGSGFTSLLMER.L 28855 |
|  | 28902 | 884.7049 | 3534.7905 | 3533.7072 | 1.0833 | 1 | (2) | 25 | 10 | R.KLADQCTGLQGFLVFHSFGGGTGSGFTSLLMER.L |
|  | 30674 | 1074.9896 | 4295.9294 | 4295.9497 | -0.0203 | 0 | 20 | 0.22 | 1 | R.ECISIHVGQAGVQIGNACWELYCLEHGIQPDGQMPSDK.T |
|  | 31025 | 1146.7742 | 4583.0676 | 4583.0913 | -0.0237 | 1 | 55 | 5.3e-005 | 1 | -.MRECISIHVGQAGVQIGNACWELYCLEHGIQPDGQMPSDK.T 31027 |
|  | 31739 | 1434.1794 | 5732.6887 | 5731.7161 | 0.9726 | 1 | 22 | 0.053 | 1 | K.SKLEFSIYPAPQVSTAVVEPYNSILTTHTTLEHSDCAFMVDNEAIYDICR.R 31736 31737 |

| **19.** | P33121\|ACSL1_HUMAN    **Mass:** 78919    **Score:** 1610   **Queries matched:** 111   **emPAI:** 2.96 |
| --- | --- |
|  | Long-chain-fatty-acid--CoA ligase 1 OS=Homo sapiens GN=ACSL1 PE=1 SV=1 |

|  | **Query** | **Observed** | **Mr(expt)** | **Mr(calc)** | **Delta** | **Miss** | **Score** | **Expect** | **Rank** | **Peptide** |
| --- | --- | --- | --- | --- | --- | --- | --- | --- | --- | --- |
|  | 1049 | **546.2808** | **545.2735** | **544.3333** | **0.9403** | **0** | **11** | **32** | **1** | **R.SGIIR.N** |
|  | 1823 | **791.3659** | **790.3586** | **790.3643** | **-0.0057** | **0** | **28** | **0.33** | **1** | **K.AMEDLGR.A** |
|  | 2006 | **803.9178** | **802.9105** | **802.4297** | **0.4808** | **0** | **21** | **1.2** | **1** | **K.VQSSLGGR.V** 1982 1992 2002 |
|  | 2869 | **435.2664** | **868.5182** | **868.5242** | **-0.0061** | **1** | **13** | **7.7** | **3** | **K.AKRPELR.N** 2870 |
|  | 3314 | **904.4215** | **903.4142** | **903.4199** | **-0.0057** | **0** | **9** | **24** | **5** | **R.NNSLWDR.L** |
|  | 3749 | **469.2370** | **936.4594** | **937.4691** | **-1.0097** | **0** | **12** | **9.5** | **1** | **K.IENIYMR.S** |
|  | 3997 | **477.7365** | **953.4585** | **953.4640** | **-0.0056** | **0** | **(12)** | **9.8** | **1** | **K.IENIYMR.S** |
|  | 4785 | **505.7324** | **1009.4503** | **1009.4573** | **-0.0070** | **0** | **45** | **0.0044** | **1** | **R.CGVEVTSMK.A** |
|  | 4841 | **507.7917** | **1013.5689** | **1013.5757** | **-0.0067** | **0** | **12** | **11** | **1** | **K.LLLEGVENK.L** 4861 |
|  | 5327 | **526.7764** | **1051.5382** | **1051.5451** | **-0.0069** | **0** | **57** | **0.00031** | **1** | **K.IGFFQGDIR.L** 5326 |
|  | 5670 | **540.4084** | **1078.8022** | **1079.5546** | **-0.7524** | **1** | **3** | **51** | **3** | **R.LLNRMFDR.I** 5683 |
|  | 6699 | **579.7348** | **1157.4550** | **1157.5928** | **-0.1377** | **1** | **7** | **32** | **1** | **K.DPAKTAEALDK.D** |
|  | 7777 | **609.8176** | **1217.6206** | **1217.6292** | **-0.0086** | **0** | **43** | **0.0083** | **1** | **K.LAQGEYIAPEK.I** |
|  | 8199 | **620.3008** | **1238.5871** | **1238.5965** | **-0.0094** | **0** | **51** | **0.0011** | **1** | **R.NIVSDCSAFVK.A** 8198 8200 |
|  | 8367 | **624.3306** | **1246.6466** | **1246.6557** | **-0.0092** | **0** | **39** | **0.017** | **1** | **K.DSGLKPFEQVK.G** 8365 8366 8369 |
|  | 8370 | **624.3749** | **1246.7352** | **1247.6986** | **-0.9634** | **1** | **34** | **0.063** | **1** | **R.IFGQANTTLKR.W** 8402 8407 |
|  | 9923 | **665.7861** | **1329.5576** | **1329.5659** | **-0.0084** | **0** | **55** | **0.00047** | **1** | **R.GFEGSFEELCR.N** |
|  | 10312 | **676.4042** | **1350.7939** | **1350.8024** | **-0.0085** | **0** | **19** | **1.7** | **1** | **K.VLQPTVFPVVPR.L** |
|  | 10839 | **691.3597** | **1380.7049** | **1380.7137** | **-0.0087** | **1** | **72** | **8.1e-006** | **1** | **R.SQIDDLYSTIKV.-** 10820 10840 10843 |
|  | 11198 | **701.8307** | **1401.6469** | **1401.6567** | **-0.0098** | **0** | **56** | **0.00037** | **1** | **R.VVECVMLCHGAK.I** |
|  | 11475 | **473.2583** | **1416.7532** | **1417.6516** | **-0.8985** | **0** | **(9)** | **18** | **5** | **R.VVECVMLCHGAK.I** |
|  | 12097 | **729.3465** | **1456.6784** | **1457.7045** | **-1.0261** | **0** | **75** | **4.6e-006** | **1** | **R.GIQVSNNGPCLGSR.K** 12125 12127 12141 12142 12143 12144 |
|  | 12422 | **737.9061** | **1473.7976** | **1473.8079** | **-0.0103** | **0** | **41** | **0.0096** | **1** | **K.AELSLVFVDKPEK.A** 12423 |
|  | 12607 | **496.2268** | **1485.6585** | **1485.6671** | **-0.0085** | **1** | **12** | **7.8** | **1** | **K.RGFEGSFEELCR.N** |
|  | 13365 | **512.2708** | **1533.7906** | **1532.7987** | **0.9919** | **1** | **9** | **15** | **1** | **K.GPNVFQGYLKDPAK.T** |
|  | 13540 | **515.9556** | **1544.8451** | **1544.8562** | **-0.0112** | **1** | **11** | **9.6** | **1** | **R.LGKDSGLKPFEQVK.G** |
|  | 13666 | **777.3804** | **1552.7463** | **1552.7562** | **-0.0099** | **0** | **52** | **0.00074** | **1** | **R.KPDQPYEWLSYK.Q** 13664 13667 |
|  | 14894 | **545.9482** | **1634.8229** | **1634.9971** | **-0.1742** | **1** | **9** | **14** | **5** | **K.LLLEGVENKLIPGLK.I** 14897 |
|  | 15773 | **847.4399** | **1692.8652** | **1692.8756** | **-0.0104** | **0** | **81** | **9.1e-007** | **1** | **K.IIVVMDAYGSELVER.G** 15767 15768 15769 15770 15771 15772 15793 |
|  | 16605 | **873.4526** | **1744.8907** | **1744.9029** | **-0.0122** | **0** | **80** | **1e-006** | **1** | **K.QVAELSECIGSALIQK.G** 16604 |
|  | 17148 | **595.3079** | **1782.9019** | **1781.8110** | **1.0909** | **1** | **3** | **56** | **6** | **R.CGVEVTSMKAMEDLGR.A** |
|  | 17929 | **924.0204** | **1846.0262** | **1846.0387** | **-0.0124** | **0** | **75** | **3e-006** | **1** | **R.LMVTGAAPVSATVLTFLR.A** 17930 17931 17933 17935 |
|  | 18952 | **963.9750** | **1925.9355** | **1925.9483** | **-0.0128** | **1** | **78** | **1.6e-006** | **1** | **K.TAEALDKDGWLHTGDIGK.W** 18953 18970 |
|  | 21279 | **1069.5450** | **2137.0755** | **2137.0877** | **-0.0122** | **1** | **62** | **5e-005** | **1** | **K.LAQGEYIAPEKIENIYMR.S** 21276 21278 |
|  | 22654 | **1149.1067** | **2296.1988** | **2296.2137** | **-0.0149** | **0** | **73** | **3.7e-006** | **1** | **K.GITLHPELFSIDNGLLTPTMK.A** 22655 22656 22661 22662 22663 22664 |
|  | 23246 | **1186.5222** | **2371.0299** | **2371.0382** | **-0.0083** | **0** | **26** | **0.17** | **1** | **K.LVDVEEMNYMAAEGEGEVCVK.G** 23235 23248 23250 23251 23258 23261 |
|  | 25224 | **894.4677** | **2680.3811** | **2680.4007** | **-0.0195** | **1** | **78** | **9.6e-007** | **1** | **R.RKPKPPAPEDLAVICFTSGTTGNPK.G** 25225 |
|  | 27000 | **1494.7086** | **2987.4027** | **2987.4157** | **-0.0130** | **0** | **78** | **8.9e-007** | **1** | **K.ATENTVNPCPDDTLISFLPLAHMFER.V** 26996 26997 26999 27002 27003 27004 |
|  | 27323 | **1528.7172** | **3055.4198** | **3055.4339** | **-0.0141** | **0** | **76** | **1.2e-006** | **1** | **R.SALLDSDEPLVYFYDDVTTLYEGFQR.G** 27321 27322 27325 |
|  | 27940 | **1071.5151** | **3211.5236** | **3211.5350** | **-0.0114** | **1** | **93** | **2.4e-008** | **1** | **R.RSALLDSDEPLVYFYDDVTTLYEGFQR.G** 27939 |
|  | 28921 | **1181.5327** | **3541.5763** | **3540.8538** | **0.7225** | **1** | **1** | **28** | **2** | **K.DSGLKPFEQVKGITLHPELFSIDNGLLTPTMK.A** |
|  | 31154 | **1584.0071** | **4748.9994** | **4749.0348** | **-0.0354** | **0** | **61** | **1.2e-005** | **1** | **R.AALGCQFYEGYGQTECTAGCCLTMPGDWTAGHVGAPMPCNLIK.L** 31155 31156 31157 |

| **20.** | P68366\|TBA4A_HUMAN    **Mass:** 50634    **Score:** 1599   **Queries matched:** 67   **emPAI:** 3.78 |
| --- | --- |
|  | Tubulin alpha-4A chain OS=Homo sapiens GN=TUBA4A PE=1 SV=1 |

|  | **Query** | **Observed** | **Mr(expt)** | **Mr(calc)** | **Delta** | **Miss** | **Score** | **Expect** | **Rank** | **Peptide** |
| --- | --- | --- | --- | --- | --- | --- | --- | --- | --- | --- |
|  | 1311 | 614.3460 | 613.3387 | 613.3435 | -0.0049 | 0 | 11 | 14 | 1 | R.GDVVPK.D |
|  | 1757 | 775.4042 | 774.3969 | 774.4024 | -0.0055 | 0 | 28 | 0.34 | 1 | R.GHYTIGK.E |
|  | 1765 | 781.4037 | 780.3964 | 780.4018 | -0.0053 | 0 | 29 | 0.21 | 1 | R.LSVDYGK.K |
|  | 3389 | 455.2527 | 908.4908 | 908.4967 | -0.0060 | 1 | 42 | 0.0094 | 1 | R.LSVDYGKK.S |
|  | 4948 | 512.2609 | 1022.5072 | 1022.4417 | 0.0655 | 0 | 11 | 14 | 2 | K.EDAANNYAR.G |
|  | 8386 | 625.2759 | 1248.5372 | 1248.5453 | -0.0081 | 0 | 18 | 2.4 | 1 | K.YMACCLLYR.G |
|  | 10814 | 460.9011 | 1379.6815 | 1379.6907 | -0.0092 | 1 | 31 | 0.11 | 1 | R.LDHKFDLMYAK.R |
|  | 11350 | 705.8859 | 1409.7573 | 1409.7667 | -0.0094 | 0 | 6 | 31 | 1 | R.QLFHPEQLITGK.E 11351 |
|  | 12641 | 744.4393 | 1486.8641 | 1486.8719 | -0.0078 | 0 | 83 | 6.7e-007 | 1 | R.LISQIVSSITASLR.F 12628 12639 12642 12644 12646 |
|  | 14104 | 792.8749 | 1583.7352 | 1583.7443 | -0.0091 | 0 | 66 | 2.9e-005 | 1 | R.SIQFVDWCPTGFK.V 14102 14103 14123 |
|  | 16176 | **858.4595** | **1714.9044** | **1714.9142** | **-0.0098** | **0** | **74** | **4.2e-006** | **1** | **R.AVFVDLEPTVIDEIR.N** 16177 |
|  | 16223 | 573.9514 | 1718.8322 | 1717.8747 | 0.9575 | 0 | 4 | 47 | 7 | R.NLDIERPTYTNLNR.L |
|  | 16758 | 878.9794 | 1755.9442 | 1755.9559 | -0.0117 | 0 | 73 | 4.9e-006 | 1 | R.IHFPLATYAPVISAEK.A 16755 16756 16757 16760 16761 16762 |
|  | 17643 | 912.9902 | 1823.9658 | 1823.9782 | -0.0124 | 0 | 59 | 0.00015 | 1 | K.VGINYQPPTVVPGGDLAK.V 17640 17641 17653 |
|  | 18197 | 932.9499 | 1863.8853 | 1863.8971 | -0.0118 | 0 | 82 | 6.5e-007 | 1 | R.AVCMLSNTTAIAEAWAR.L 18198 |
|  | 20586 | **1034.4443** | **2066.8741** | **2066.8892** | **-0.0151** | **0** | **128** | **1.4e-011** | **1** | **K.TIGGGDDSFTTFFCETGAGK.H** 20587 |
|  | 21850 | **735.4002** | **2203.1788** | **2204.1954** | **-1.0166** | **1** | **0** | **76** | **5** | **K.HVPRAVFVDLEPTVIDEIR.N** |
|  | 22378 | 755.3527 | 2263.0362 | 2263.1201 | -0.0840 | 1 | 5 | 22 | 3 | K.VQRAVCMLSNTTAIAEAWAR.L |
|  | 22917 | 777.6732 | 2329.9978 | 2329.0110 | 0.9869 | 0 | 37 | 0.014 | 1 | R.AFVHWYVGEGMEEGEFSEAR.E |
|  | 23508 | 1205.1007 | 2408.1869 | 2408.2012 | -0.0144 | 0 | 116 | 1.9e-010 | 1 | R.FDGALNVDLTEFQTNLVPYPR.I 23503 23504 23505 23506 23507 23509 23510 23512 23515 |
|  | 23544 | 805.7346 | 2414.1819 | 2414.1978 | -0.0160 | 1 | 41 | 0.0058 | 1 | R.QLFHPEQLITGKEDAANNYAR.G 23545 |
|  | 25605 | 1375.6397 | 2749.2647 | 2749.2840 | -0.0192 | 0 | 80 | 6.4e-007 | 1 | K.AYHEQLSVAEITNACFEPANQMVK.C 25593 25603 25604 25606 25608 |
|  | 27966 | 1073.1377 | 3216.3913 | 3216.4168 | -0.0255 | 1 | 62 | 2.5e-005 | 1 | R.AFVHWYVGEGMEEGEFSEAREDMAALEK.D 27967 |
|  | 28016 | 808.8973 | 3231.5600 | 3232.4117 | -0.8517 | 1 | (4) | 19 | 5 | R.AFVHWYVGEGMEEGEFSEAREDMAALEK.D |
|  | 28067 | 1083.8721 | 3248.5944 | 3248.4066 | 0.1877 | 1 | (1) | 38 | 9 | R.AFVHWYVGEGMEEGEFSEAREDMAALEK.D |
|  | 28579 | **1136.2041** | **3405.5905** | **3405.6123** | **-0.0218** | **0** | **58** | **6.6e-005** | **1** | **K.LSDQCTGLQGFLVFHSFGGGTGSGFTSLLMER.L** |
|  | 30509 | **1043.2919** | **4169.1384** | **4168.0035** | **1.1349** | **1** | **1** | **19** | **3** | **K.LSDQCTGLQGFLVFHSFGGGTGSGFTSLLMERLSVDYGK.K** |
|  | 31739 | 1434.1794 | 5732.6887 | 5731.7161 | 0.9726 | 1 | 22 | 0.053 | 1 | K.SKLEFSIYPAPQVSTAVVEPYNSILTTHTTLEHSDCAFMVDNEAIYDICR.R 31736 31737 |

| **21.** | P25705\|ATPA_HUMAN    **Mass:** 59828    **Score:** 1596   **Queries matched:** 83   **emPAI:** 3.01 |
| --- | --- |
|  | ATP synthase subunit alpha, mitochondrial OS=Homo sapiens GN=ATP5A1 PE=1 SV=1 |

|  | **Query** | **Observed** | **Mr(expt)** | **Mr(calc)** | **Delta** | **Miss** | **Score** | **Expect** | **Rank** | **Peptide** |
| --- | --- | --- | --- | --- | --- | --- | --- | --- | --- | --- |
|  | 1790 | **395.2127** | **788.4108** | **788.4141** | **-0.0033** | **0** | **40** | **0.023** | **1** | **R.VGSAAQTR.A** 1781 1785 |
|  | 2147 | **408.2278** | **814.4411** | **814.4661** | **-0.0250** | **1** | **5** | **71** | **8** | **K.GPIGSKTR.R** |
|  | 2534 | **423.2554** | **844.4962** | **844.5018** | **-0.0056** | **0** | **40** | **0.02** | **1** | **R.STVAQLVK.R** |
|  | 2774 | **864.4076** | **863.4004** | **863.4059** | **-0.0055** | **0** | **43** | **0.01** | **1** | **R.LTDADAMK.Y** |
|  | 2963 | **439.0479** | **876.0813** | **876.4189** | **-0.3376** | **0** | **8** | **19** | **2** | **K.ISEQSDAK.L** |
|  | 3164 | **446.7449** | **891.4752** | **891.4814** | **-0.0061** | **0** | **35** | **0.06** | **1** | **K.LELAQYR.E** |
|  | 3431 | **456.7638** | **911.5130** | **910.3668** | **1.1462** | **0** | **10** | **15** | **2** | **R.FNDGSDEK.K** |
|  | 4650 | **500.7916** | **999.5686** | **999.5713** | **-0.0026** | **0** | **22** | **0.99** | **1** | **R.VLSIGDGIAR.V** |
|  | 4672 | **501.3358** | **1000.6569** | **1000.6029** | **0.0540** | **1** | **10** | **17** | **6** | **K.RSTVAQLVK.R** |
|  | 4920 | **510.7574** | **1019.5003** | **1019.5070** | **-0.0067** | **1** | **26** | **0.41** | **1** | **K.RLTDADAMK.Y** |
|  | 5009 | **514.2991** | **1026.5836** | **1025.5869** | **0.9966** | **0** | **27** | **0.33** | **1** | **K.AVDSLVPIGR.G** |
|  | 5658 | **540.2302** | **1078.4458** | **1079.5467** | **-1.1010** | **1** | **9** | **20** | **4** | **R.AMKQVAGTMK.L** |
|  | 6217 | **561.2853** | **1120.5560** | **1121.7033** | **-1.1473** | **1** | **4** | **62** | **5** | **R.VAAAVVRALPR.R** |
|  | 6574 | **575.3066** | **1148.5987** | **1148.6077** | **-0.0090** | **1** | **29** | **0.19** | **1** | **R.GYLDKLEPSK.I** |
|  | 6873 | **586.3153** | **1170.6161** | **1170.6245** | **-0.0084** | **0** | **53** | **0.00069** | **1** | **R.VVDALGNAIDGK.G** 6871 |
|  | 7991 | **615.2459** | **1228.4773** | **1228.6775** | **-0.2003** | **1** | **8** | **25** | **4** | **R.ELIIGDRQTGK.T** 7990 |
|  | 9088 | **644.3467** | **1286.6789** | **1286.6870** | **-0.0081** | **0** | **65** | **4.2e-005** | **1** | **K.HALIIYDDLSK.Q** 9078 9087 |
|  | 9638 | **658.8699** | **1315.7252** | **1315.7347** | **-0.0095** | **0** | **51** | **0.0011** | **1** | **K.TSIAIDTIINQK.R** 9639 9640 |
|  | 10441 | **680.3729** | **1358.7312** | **1357.7388** | **0.9924** | **1** | **32** | **0.089** | **1** | **R.ISVREPMQTGIK.A** |
|  | 11582 | **712.3356** | **1422.6567** | **1422.6660** | **-0.0093** | **0** | **65** | **4.5e-005** | **1** | **K.TGTAEMSSILEER.I** 11583 |
|  | 11816 | **719.9227** | **1437.8309** | **1437.8416** | **-0.0107** | **0** | **40** | **0.012** | **1** | **K.GIRPAINVGLSVSR.V** |
|  | 12072 | **728.3895** | **1454.7644** | **1454.7955** | **-0.0311** | **1** | **13** | **6.5** | **1** | **K.KLYCIYVAIGQK.R** |
|  | 12403 | 737.4248 | 1472.8350 | 1471.8358 | 0.9992 | 1 | 1 | 95 | 6 | K.TSIAIDTIINQKR.F |
|  | 13629 | **776.4201** | **1550.8256** | **1550.8344** | **-0.0088** | **1** | **69** | **1.6e-005** | **1** | **K.LKEIVTNFLAGFEA.-** 13604 13624 13628 |
|  | 13831 | **522.3104** | **1563.9094** | **1563.8555** | **0.0539** | **1** | **6** | **30** | **3** | **K.QAVAYRQMSLLLR.R** 13828 |
|  | 13963 | **788.3910** | **1574.7675** | **1574.7788** | **-0.0112** | **0** | **105** | **3.6e-009** | **1** | **R.ILGADTSVDLEETGR.V** 13962 13964 |
|  | 14724 | **812.9434** | **1623.8723** | **1623.8832** | **-0.0109** | **0** | **34** | **0.042** | **1** | **R.TGAIVDVPVGEELLGR.V** |
|  | 15390 | **834.3954** | **1666.7763** | **1666.7872** | **-0.0109** | **0** | **97** | **2.2e-008** | **1** | **R.NVQAEEMVEFSSGLK.G** 15388 15389 15401 15403 |
|  | 15689 | **563.9595** | **1688.8568** | **1688.9322** | **-0.0754** | **1** | **1** | **97** | **4** | **R.AGLVSRNALGSSFIAAR.N** |
|  | 15945 | **568.2745** | **1701.8016** | **1700.8733** | **0.9283** | **1** | **8** | **18** | **1** | **R.DNGKHALIIYDDLSK.Q** 15927 |
|  | 16090 | **855.9666** | **1709.9187** | **1709.9312** | **-0.0125** | **1** | **30** | **0.12** | **1** | **R.VVDALGNAIDGKGPIGSK.T** 16070 16091 |
|  | 17113 | **594.6657** | **1780.9751** | **1779.9843** | **0.9908** | **1** | **3** | **54** | **9** | **K.RTGAIVDVPVGEELLGR.V** |
|  | 18976 | **643.3840** | **1927.1303** | **1926.0244** | **1.1058** | **1** | **1** | **70** | **6** | **R.EPMQTGIKAVDSLVPIGR.G** |
|  | 20971 | **1052.5167** | **2103.0189** | **2103.0307** | **-0.0118** | **0** | **49** | **0.0011** | **1** | **K.GMSLNLEPDNVGVVVFGNDK.L** 20970 20972 20973 20976 20977 |
|  | 22270 | **749.3609** | **2245.0609** | **2245.1161** | **-0.0552** | **1** | **3** | **44** | **9** | **R.VHGLRNVQAEEMVEFSSGLK.G** |
|  | 22740 | **1155.0768** | **2308.1390** | **2308.1521** | **-0.0131** | **0** | **129** | **1.1e-011** | **1** | **K.QGQYSPMAIEEQVAVIYAGVR.G** 22738 22739 22742 |
|  | 22976 | **1169.5823** | **2337.1500** | **2337.1601** | **-0.0101** | **0** | **115** | **2.2e-010** | **1** | **R.EVAAFAQFGSDLDAATQQLLSR.G** 22972 22973 22975 22977 22978 22979 22980 |
|  | 23197 | **1184.1242** | **2366.2337** | **2366.2495** | **-0.0158** | **0** | **110** | **7.3e-010** | **1** | **K.FENAFLSHVVSQHQALLGTIR.A** 23196 23199 |
|  | 23854 | **820.0707** | **2457.1902** | **2457.2937** | **-0.1035** | **1** | **0** | **66** | **3** | **K.GMSLNLEPDNVGVVVFGNDKLIK.E** |
|  | 25414 | **542.6992** | **2708.4594** | **2708.4762** | **-0.0168** | **1** | **12** | **3.5** | **2** | **K.ITKFENAFLSHVVSQHQALLGTIR.A** |
|  | 28518 | **1130.1939** | **3387.5597** | **3387.5791** | **-0.0194** | **0** | **44** | **0.0018** | **1** | **K.YTIVVSATASDAAPLQYLAPYSGCSMGEYFR.D** 28516 28517 28520 |

| **22.** | P04350\|TBB4_HUMAN    **Mass:** 50010    **Score:** 1559   **Queries matched:** 92   **emPAI:** 7.05 |
| --- | --- |
|  | Tubulin beta-4 chain OS=Homo sapiens GN=TUBB4 PE=1 SV=2 |

|  | **Query** | **Observed** | **Mr(expt)** | **Mr(calc)** | **Delta** | **Miss** | **Score** | **Expect** | **Rank** | **Peptide** |
| --- | --- | --- | --- | --- | --- | --- | --- | --- | --- | --- |
|  | 1603 | 709.3656 | 708.3583 | 708.3411 | 0.0172 | 1 | 14 | 6.5 | 3 | R.GRMSMK.E |
|  | 1851 | **397.2534** | **792.4923** | **791.3450** | **1.1474** | **0** | **11** | **14** | **2** | **R.EEFPDR.I** 1840 |
|  | 5020 | 514.7598 | 1027.5050 | 1027.5121 | -0.0071 | 0 | 46 | 0.004 | 1 | K.TAVCDIPPR.G 5004 5015 5016 5017 5018 5019 5021 5022 |
|  | 5177 | 520.2972 | 1038.5798 | 1038.5862 | -0.0064 | 0 | 53 | 0.00083 | 1 | R.YLTVAAVFR.G 5176 |
|  | 5494 | 533.3253 | 1064.6361 | 1064.4201 | 0.2160 | 0 | (4) | 60 | 6 | K.NMMAACDPR.H |
|  | 5678 | 541.2108 | 1080.4071 | 1080.4151 | -0.0080 | 0 | 29 | 0.16 | 1 | K.NMMAACDPR.H 5679 5694 |
|  | 6327 | 565.7967 | 1129.5788 | 1129.5880 | -0.0092 | 0 | 4 | 59 | 6 | R.FPGQLNADLR.K |
|  | 6499 | 572.3174 | 1142.6203 | 1142.6270 | -0.0067 | 0 | 70 | 1.8e-005 | 1 | K.LAVNMVPFPR.L 6498 |
|  | 7996 | 615.2989 | 1228.5833 | 1228.5910 | -0.0077 | 0 | 62 | 0.00011 | 1 | R.ISEQFTAMFR.R 7997 |
|  | 8802 | 636.3640 | 1270.7133 | 1270.7220 | -0.0086 | 1 | 47 | 0.0027 | 1 | R.KLAVNMVPFPR.L |
|  | 9112 | 430.2745 | 1287.8016 | 1286.7169 | 1.0847 | 1 | (7) | 28 | 2 | R.KLAVNMVPFPR.L 9101 |
|  | 9689 | 660.3509 | 1318.6872 | 1318.6955 | -0.0083 | 0 | 82 | 8.3e-007 | 1 | R.IMNTFSVVPSPK.V 9686 9687 9696 |
|  | 10023 | 668.3477 | 1334.6809 | 1334.6904 | -0.0095 | 0 | (19) | 1.8 | 1 | R.IMNTFSVVPSPK.V |
|  | 10908 | 693.3495 | 1384.6844 | 1384.6921 | -0.0077 | 1 | 45 | 0.004 | 1 | K.RISEQFTAMFR.R |
|  | 14384 | 801.4086 | 1600.8026 | 1600.8131 | -0.0105 | 0 | 67 | 2.6e-005 | 1 | R.AVLVDLEPGTMDSVR.S 14378 14379 14385 |
|  | 14658 | 810.9161 | 1619.8176 | 1619.8283 | -0.0107 | 0 | 52 | 0.0008 | 1 | R.LHFFMPGFAPLTSR.G 14659 14660 14661 |
|  | 15728 | 846.4319 | 1690.8492 | 1690.8600 | -0.0108 | 0 | 35 | 0.032 | 1 | R.ALTVPELTQQMFDAK.N 15729 15730 15732 |
|  | 15835 | 848.9150 | 1695.8154 | 1695.8257 | -0.0102 | 0 | 33 | 0.051 | 1 | K.NSSYFVEWIPNNVK.T 15855 15856 |
|  | 16036 | 854.4293 | 1706.8441 | 1706.8549 | -0.0108 | 0 | (19) | 1.5 | 2 | R.ALTVPELTQQMFDAK.N |
|  | 17620 | 911.9583 | 1821.9019 | 1821.9156 | -0.0136 | 0 | 67 | 2.6e-005 | 1 | R.EIVHLQAGQCGNQIGAK.F 17614 17621 |
|  | 18090 | 929.4694 | 1856.9242 | 1856.9342 | -0.0101 | 0 | 91 | 8.2e-008 | 1 | K.MAATFIGNSTAIQELFK.R 18084 18088 18089 18091 |
|  | 19959 | 1007.5189 | 2013.0232 | 2013.0353 | -0.0122 | 1 | 75 | 2.7e-006 | 1 | K.MAATFIGNSTAIQELFKR.I |
|  | 21027 | 1055.5288 | 2109.0431 | 2109.0571 | -0.0141 | 1 | 71 | 7.2e-006 | 1 | -.MREIVHLQAGQCGNQIGAK.F 21028 |
|  | 21442 | 1078.9855 | 2155.9564 | 2155.1347 | 0.8217 | 1 | 8 | 14 | 2 | R.GLKMAATFIGNSTAIQELFK.R 21443 |
|  | 23107 | 786.0563 | 2355.1470 | 2355.1583 | -0.0113 | 1 | 2 | 44 | 7 | R.LHFFMPGFAPLTSRGSQQYR.A |
|  | 25387 | 1354.6653 | 2707.3160 | 2707.3310 | -0.0150 | 0 | 93 | 3.2e-008 | 1 | K.LTTPTYGDLNHLVSATMSGVTTCLR.F 25379 25385 25386 25404 25411 25412 |
|  | 25766 | 695.3771 | 2777.4792 | 2776.4346 | 1.0446 | 1 | 6 | 13 | 2 | K.LAVNMVPFPRLHFFMPGFAPLTSR.G |
|  | 25900 | 933.4471 | 2797.3194 | 2797.3361 | -0.0167 | 0 | 97 | 1.3e-008 | 1 | R.SGPFGQIFRPDNFVFGQSGAGNNWAK.G 25898 25899 25903 25910 25911 25912 |
|  | 27582 | 1039.4722 | 3115.3947 | 3115.4159 | -0.0213 | 0 | 67 | 1.1e-005 | 1 | K.FWEVISDEHGIDPTGTYHGDSDLQLER.I |
|  | 28244 | 1656.2616 | 3310.5086 | 3310.5268 | -0.0182 | 0 | (65) | 1.3e-005 | 1 | K.EAESCDCLQGFQLTHSLGGGTGSGMGTLLISK.I 28241 28242 28243 28245 28246 |
|  | 28304 | 1109.8416 | 3326.5028 | 3326.5218 | -0.0189 | 0 | 72 | 3e-006 | 1 | K.EAESCDCLQGFQLTHSLGGGTGSGMGTLLISK.I |
|  | 28665 | 1147.2072 | 3438.5996 | 3438.6218 | -0.0222 | 1 | 68 | 6.1e-006 | 1 | R.KEAESCDCLQGFQLTHSLGGGTGSGMGTLLISK.I 28657 28663 28664 |
|  | 31036 | 1531.7026 | 4592.0861 | 4592.0999 | -0.0139 | 0 | 50 | 0.00017 | 1 | K.VSDTVVEPYNATLSVHQLVENTDETYCIDNEALYDICFR.T 31032 31033 |

| **23.** | Q71U36\|TBA1A_HUMAN    **Mass:** 50788    **Score:** 1554   **Queries matched:** 75   **emPAI:** 5.77 |
| --- | --- |
|  | Tubulin alpha-1A chain OS=Homo sapiens GN=TUBA1A PE=1 SV=1 |

|  | **Query** | **Observed** | **Mr(expt)** | **Mr(calc)** | **Delta** | **Miss** | **Score** | **Expect** | **Rank** | **Peptide** |
| --- | --- | --- | --- | --- | --- | --- | --- | --- | --- | --- |
|  | 1311 | 614.3460 | 613.3387 | 613.3435 | -0.0049 | 0 | 11 | 14 | 1 | R.GDVVPK.D |
|  | 1757 | 775.4042 | 774.3969 | 774.4024 | -0.0055 | 0 | 28 | 0.34 | 1 | R.GHYTIGK.E |
|  | 1765 | 781.4037 | 780.3964 | 780.4018 | -0.0053 | 0 | 29 | 0.21 | 1 | R.LSVDYGK.K |
|  | 3389 | 455.2527 | 908.4908 | 908.4967 | -0.0060 | 1 | 42 | 0.0094 | 1 | R.LSVDYGKK.S |
|  | 4864 | 508.2898 | 1014.5650 | 1014.5709 | -0.0060 | 0 | 41 | 0.014 | 1 | K.DVNAAIATIK.T 4835 4861 4863 |
|  | 4948 | 512.2609 | 1022.5072 | 1022.4417 | 0.0655 | 0 | 11 | 14 | 2 | K.EDAANNYAR.G |
|  | 5745 | 543.3106 | 1084.6065 | 1084.6128 | -0.0063 | 0 | 32 | 0.11 | 1 | K.EIIDLVLDR.I 5743 |
|  | 8386 | 625.2759 | 1248.5372 | 1248.5453 | -0.0081 | 0 | 18 | 2.4 | 1 | K.YMACCLLYR.G |
|  | 10814 | 460.9011 | 1379.6815 | 1379.6907 | -0.0092 | 1 | 31 | 0.11 | 1 | R.LDHKFDLMYAK.R |
|  | 11350 | 705.8859 | 1409.7573 | 1409.7667 | -0.0094 | 0 | 6 | 31 | 1 | R.QLFHPEQLITGK.E 11351 |
|  | 12123 | **486.8886** | **1457.6440** | **1456.8613** | **0.7827** | **0** | **2** | **94** | **6** | **R.LIGQIVSSITASLR.F** |
|  | 14334 | 799.8823 | 1597.7500 | 1597.7599 | -0.0100 | 0 | 23 | 0.64 | 2 | R.TIQFVDWCPTGFK.V |
|  | 15935 | 851.4510 | 1700.8874 | 1700.8985 | -0.0111 | 0 | 60 | 0.00011 | 1 | R.AVFVDLEPTVIDEVR.T 15936 |
|  | 16223 | 573.9514 | 1718.8322 | 1717.8747 | 0.9575 | 0 | 4 | 47 | 7 | R.NLDIERPTYTNLNR.L |
|  | 16758 | 878.9794 | 1755.9442 | 1755.9559 | -0.0117 | 0 | 73 | 4.9e-006 | 1 | R.IHFPLATYAPVISAEK.A 16755 16756 16757 16760 16761 16762 |
|  | 17643 | 912.9902 | 1823.9658 | 1823.9782 | -0.0124 | 0 | 59 | 0.00015 | 1 | K.VGINYQPPTVVPGGDLAK.V 17640 17641 17653 |
|  | 17874 | 921.5047 | 1840.9948 | 1841.0047 | -0.0098 | 1 | 31 | 0.077 | 1 | R.GHYTIGKEIIDLVLDR.I |
|  | 18197 | 932.9499 | 1863.8853 | 1863.8971 | -0.0118 | 0 | 82 | 6.5e-007 | 1 | R.AVCMLSNTTAIAEAWAR.L 18198 |
|  | 19906 | 1004.4442 | 2006.8737 | 2006.8858 | -0.0121 | 0 | 105 | 2.5e-009 | 1 | K.TIGGGDDSFNTFFSETGAGK.H 19902 19903 19905 |
|  | 22378 | 755.3527 | 2263.0362 | 2263.1201 | -0.0840 | 1 | 5 | 22 | 3 | K.VQRAVCMLSNTTAIAEAWAR.L |
|  | 22917 | 777.6732 | 2329.9978 | 2329.0110 | 0.9869 | 0 | 37 | 0.014 | 1 | R.AFVHWYVGEGMEEGEFSEAR.E |
|  | 23508 | 1205.1007 | 2408.1869 | 2408.2012 | -0.0144 | 0 | 116 | 1.9e-010 | 1 | R.FDGALNVDLTEFQTNLVPYPR.I 23503 23504 23505 23506 23507 23509 23510 23512 23515 |
|  | 23544 | 805.7346 | 2414.1819 | 2414.1978 | -0.0160 | 1 | 41 | 0.0058 | 1 | R.QLFHPEQLITGKEDAANNYAR.G 23545 |
|  | 24192 | 833.4252 | 2497.2539 | 2496.1670 | 1.0869 | 1 | 7 | 13 | 1 | K.TIGGGDDSFNTFFSETGAGKHVPR.A |
|  | 25605 | 1375.6397 | 2749.2647 | 2749.2840 | -0.0192 | 0 | 80 | 6.4e-007 | 1 | K.AYHEQLSVAEITNACFEPANQMVK.C 25593 25603 25604 25606 25608 |
|  | 27966 | 1073.1377 | 3216.3913 | 3216.4168 | -0.0255 | 1 | 62 | 2.5e-005 | 1 | R.AFVHWYVGEGMEEGEFSEAREDMAALEK.D 27967 |
|  | 28016 | 808.8973 | 3231.5600 | 3232.4117 | -0.8517 | 1 | (4) | 19 | 5 | R.AFVHWYVGEGMEEGEFSEAREDMAALEK.D |
|  | 28067 | 1083.8721 | 3248.5944 | 3248.4066 | 0.1877 | 1 | (1) | 38 | 9 | R.AFVHWYVGEGMEEGEFSEAREDMAALEK.D |
|  | 28528 | 1130.8743 | 3389.6010 | 3389.6173 | -0.0164 | 0 | 68 | 7.3e-006 | 1 | K.LADQCTGLQGFLVFHSFGGGTGSGFTSLLMER.L 28527 28529 |
|  | 28854 | 1173.5719 | 3517.6939 | 3517.7123 | -0.0184 | 1 | 86 | 9.8e-008 | 1 | R.KLADQCTGLQGFLVFHSFGGGTGSGFTSLLMER.L 28855 |
|  | 28902 | 884.7049 | 3534.7905 | 3533.7072 | 1.0833 | 1 | (2) | 25 | 10 | R.KLADQCTGLQGFLVFHSFGGGTGSGFTSLLMER.L |
|  | 30674 | 1074.9896 | 4295.9294 | 4295.9497 | -0.0203 | 0 | 20 | 0.22 | 1 | R.ECISIHVGQAGVQIGNACWELYCLEHGIQPDGQMPSDK.T |
|  | 31025 | 1146.7742 | 4583.0676 | 4583.0913 | -0.0237 | 1 | 55 | 5.3e-005 | 1 | -.MRECISIHVGQAGVQIGNACWELYCLEHGIQPDGQMPSDK.T 31027 |
|  | 31739 | 1434.1794 | 5732.6887 | 5731.7161 | 0.9726 | 1 | 22 | 0.053 | 1 | K.SKLEFSIYPAPQVSTAVVEPYNSILTTHTTLEHSDCAFMVDNEAIYDICR.R 31736 31737 |

| **24.** | P05181\|CP2E1_HUMAN    **Mass:** 57269    **Score:** 1517   **Queries matched:** 84   **emPAI:** 4.48 |
| --- | --- |
|  | Cytochrome P450 2E1 OS=Homo sapiens GN=CYP2E1 PE=1 SV=1 |

|  | **Query** | **Observed** | **Mr(expt)** | **Mr(calc)** | **Delta** | **Miss** | **Score** | **Expect** | **Rank** | **Peptide** |
| --- | --- | --- | --- | --- | --- | --- | --- | --- | --- | --- |
|  | 1355 | **628.3728** | **627.3655** | **627.3704** | **-0.0049** | **0** | **12** | **9.9** | **1** | **R.VIGPSR.I** |
|  | 1447 | **650.3833** | **649.3760** | **650.3388** | **-0.9628** | **0** | **20** | **1.9** | **1** | **R.DTIFR.G** |
|  | 1474 | **659.3670** | **658.3597** | **658.3650** | **-0.0053** | **0** | **17** | **4.9** | **8** | **K.NVAEVK.E** |
|  | 1495 | **670.3840** | **669.3767** | **668.2952** | **1.0815** | **0** | **9** | **16** | **4** | **R.NYGMGK.Q** |
|  | 1608 | **711.0913** | **710.0840** | **709.3031** | **0.7809** | **0** | **7** | **17** | **2** | **K.DEFSGR.G** 1597 |
|  | 3415 | **456.2292** | **910.4439** | **910.4508** | **-0.0069** | **0** | **15** | **4.4** | **1** | **K.LHEEIDR.V** |
|  | 3942 | **475.7881** | **949.5617** | **949.5670** | **-0.0053** | **0** | **49** | **0.0023** | **1** | **R.YGLLILMK.Y** |
|  | 4130 | **482.7375** | **963.4604** | **963.4670** | **-0.0066** | **0** | **26** | **0.47** | **1** | **R.MVVMHGYK.A** 4110 4131 4145 |
|  | 4530 | **497.2375** | **992.4605** | **992.5767** | **-0.1162** | **1** | **7** | **35** | **4** | **R.RFSLTTLR.N** |
|  | 5783 | **544.7809** | **1087.5473** | **1087.5556** | **-0.0083** | **1** | **36** | **0.043** | **1** | **K.RVCAGEGLAR.M** |
|  | 6209 | **560.7824** | **1119.5503** | **1119.5574** | **-0.0071** | **0** | **43** | **0.0079** | **1** | **R.GDLPAFHAHR.D** 6208 |
|  | 8334 | **623.8287** | **1245.6429** | **1245.6506** | **-0.0077** | **0** | **42** | **0.0091** | **1** | **R.GIIFNNGPTWK.D** 8362 |
|  | 9202 | **647.7875** | **1293.5605** | **1293.6573** | **-0.0968** | **1** | **7** | **30** | **7** | **R.MVVMHGYKAVK.E** |
|  | 9873 | **664.3168** | **1326.6191** | **1325.7455** | **0.8736** | **1** | **50** | **0.0012** | **1** | **R.EAHFLLEALRK.T** 9834 9835 |
|  | 11805 | **719.8249** | **1437.6352** | **1438.6769** | **-1.0417** | **0** | **23** | **0.6** | **1** | **K.YSDYFKPFSTGK.R** 11821 11822 11824 |
|  | 12149 | **730.3655** | **1458.7164** | **1458.7255** | **-0.0091** | **0** | **38** | **0.023** | **1** | **K.FKPEHFLNENGK.F** 12147 12148 12150 |
|  | 12235 | **733.3449** | **1464.6753** | **1464.6840** | **-0.0087** | **0** | **16** | **3.1** | **1** | **R.DLTDCLLVEMEK.E** |
|  | 12662 | **497.2213** | **1488.6422** | **1488.6528** | **-0.0106** | **0** | **37** | **0.028** | **1** | **K.EHHQSLDPNCPR.D** 12661 12664 |
|  | 13474 | **771.8702** | **1541.7259** | **1541.7362** | **-0.0102** | **1** | **60** | **0.00011** | **1** | **K.EALLDYKDEFSGR.G** 13473 13475 |
|  | 14300 | **532.5961** | **1594.7666** | **1594.7780** | **-0.0114** | **1** | **29** | **0.15** | **1** | **K.YSDYFKPFSTGKR.V** 14301 14302 |
|  | 15806 | **847.9589** | **1693.9032** | **1693.9151** | **-0.0120** | **0** | **54** | **0.00044** | **1** | **R.FITLVPSNLPHEATR.D** 15807 15808 15809 |
|  | 16185 | **858.9097** | **1715.8049** | **1715.8162** | **-0.0113** | **1** | **64** | **4.8e-005** | **1** | **R.VKEHHQSLDPNCPR.D** 16180 16181 16182 16183 16184 16186 16187 16188 |
|  | 16255 | **861.9125** | **1721.8104** | **1721.8215** | **-0.0111** | **1** | **108** | **1.9e-009** | **1** | **R.DLTDCLLVEMEKEK.H** 16254 16256 16257 |
|  | 17834 | **919.9719** | **1837.9292** | **1837.9899** | **-0.0607** | **1** | **17** | **2** | **1** | **R.YGLLILMKYPEIEEK.L** |
|  | 17915 | **923.4294** | **1844.8442** | **1844.8549** | **-0.0107** | **0** | **59** | **0.00012** | **1** | **R.QEMPYMDAVVHEIQR.F** |
|  | 18738 | **636.3297** | **1905.9671** | **1905.9771** | **-0.0100** | **0** | **49** | **0.0012** | **1** | **K.DIDLSPIHIGFGCIPPR.Y** 18734 18737 18739 |
|  | 21094 | **706.3301** | **2115.9686** | **2115.9830** | **-0.0144** | **1** | **55** | **0.00027** | **1** | **K.DRQEMPYMDAVVHEIQR.F** 21093 21096 21113 |
|  | 24617 | **1281.6257** | **2561.2369** | **2561.2537** | **-0.0168** | **0** | **66** | **1.6e-005** | **1** | **K.GTVVVPTLDSVLYDNQEFPDPEK.F** 24615 24616 24619 |
|  | 26633 | **1462.7263** | **2923.4381** | **2923.4525** | **-0.0144** | **0** | **146** | **1.4e-013** | **1** | **R.LYTMDGITVTVADLFFAGTETTSTTLR.Y** 26632 26635 26638 26642 26643 26645 26652 |
|  | 26807 | **1475.7290** | **2949.4434** | **2949.4518** | **-0.0083** | **0** | **61** | **3.8e-005** | **1** | **K.TQGQPFDPTFLIGCAPCNVIADILFR.K** |
|  | 27459 | **1539.7743** | **3077.5340** | **3077.5467** | **-0.0127** | **1** | **79** | **6.1e-007** | **1** | **R.KTQGQPFDPTFLIGCAPCNVIADILFR.K** 27450 27460 27461 |

| **25.** | P07237\|PDIA1_HUMAN    **Mass:** 57480    **Score:** 1484   **Queries matched:** 108   **emPAI:** 7.99 |
| --- | --- |
|  | Protein disulfide-isomerase OS=Homo sapiens GN=P4HB PE=1 SV=3 |

|  | **Query** | **Observed** | **Mr(expt)** | **Mr(calc)** | **Delta** | **Miss** | **Score** | **Expect** | **Rank** | **Peptide** |
| --- | --- | --- | --- | --- | --- | --- | --- | --- | --- | --- |
|  | 1248 | **593.3254** | **592.3181** | **592.3221** | **-0.0039** | **0** | **22** | **0.97** | **1** | **R.FLEGK.I** |
|  | 1517 | **678.3412** | **677.3339** | **677.3748** | **-0.0409** | **0** | **7** | **38** | **1** | **R.GYPTIK.F** |
|  | 2063 | **404.7291** | **807.4437** | **807.4491** | **-0.0053** | **1** | **19** | **2.1** | **1** | **R.TLDGFKK.F** 2064 2065 |
|  | 2754 | **863.3731** | **862.3658** | **861.4596** | **0.9063** | **0** | **15** | **6.6** | **1** | **K.ALAPEYAK.A** 2738 2739 |
|  | 2882 | **870.3770** | **869.3697** | **869.3767** | **-0.0069** | **0** | **29** | **0.18** | **1** | **K.SVSDYDGK.L** |
|  | 3397 | **455.7214** | **909.4283** | **909.4345** | **-0.0061** | **0** | **27** | **0.27** | **1** | **K.FFPASADR.T** 3396 |
|  | 3628 | **464.7632** | **927.5117** | **927.5178** | **-0.0061** | **0** | **12** | **10** | **2** | **K.VHSFPTLK.F** |
|  | 3765 | **469.7466** | **937.4787** | **937.4869** | **-0.0081** | **1** | **26** | **0.41** | **1** | **K.TAAESFKGK.I** 3775 3778 |
|  | 4096 | **481.7261** | **961.4376** | **961.4440** | **-0.0063** | **0** | **36** | **0.05** | **1** | **R.ITEFCHR.F** 4095 4116 |
|  | 4163 | **483.7837** | **965.5528** | **965.5586** | **-0.0058** | **0** | **35** | **0.052** | **1** | **R.ILEFFGLK.K** 4161 |
|  | 4453 | **494.7439** | **987.4732** | **987.4807** | **-0.0075** | **1** | **23** | **0.9** | **1** | **K.KEECPAVR.L** 4456 |
|  | 4680 | **501.7790** | **1001.5434** | **1001.5505** | **-0.0071** | **1** | **28** | **0.32** | **1** | **K.LKAEGSEIR.L** 4661 4663 4664 4679 4690 4696 |
|  | 5147 | **519.2446** | **1036.4746** | **1036.4825** | **-0.0079** | **0** | **17** | **3.2** | **1** | **R.NNFEGEVTK.E** |
|  | 5480 | **533.1893** | **1064.3641** | **1065.5091** | **-1.1450** | **0** | **2** | **85** | **5** | **R.TVIDYNGER.T** |
|  | 5698 | **541.3391** | **1080.6637** | **1080.6695** | **-0.0059** | **0** | **26** | **0.35** | **1** | **K.THILLFLPK.S** 5697 5704 |
|  | 5881 | **548.3821** | **1094.7497** | **1093.6535** | **1.0962** | **1** | **0** | **1.2e+002** | **8** | **R.ILEFFGLKK.E** |
|  | 6702 | **579.7945** | **1157.5744** | **1157.5829** | **-0.0085** | **0** | **54** | **0.00066** | **1** | **K.SNFAEALAAHK.Y** 6703 |
|  | 7452 | **601.8029** | **1201.5913** | **1201.5979** | **-0.0066** | **0** | **48** | **0.0023** | **1** | **R.EADDIVNWLK.K** 7417 7451 |
|  | 7537 | **603.8140** | **1205.6135** | **1205.6213** | **-0.0078** | **0** | **44** | **0.0066** | **1** | **R.LITLEEEMTK.Y** 7536 |
|  | 7656 | **607.2684** | **1212.5222** | **1212.5299** | **-0.0077** | **0** | **23** | **0.73** | **1** | **K.NFEDVAFDEK.K** |
|  | 8213 | **414.2006** | **1239.5800** | **1238.6044** | **0.9756** | **1** | **15** | **4.1** | **1** | **K.FFRNGDTASPK.E** |
|  | 9069 | **643.8417** | **1285.6689** | **1285.6778** | **-0.0089** | **1** | **40** | **0.014** | **1** | **R.KSNFAEALAAHK.Y** 9070 |
|  | 9950 | **666.3513** | **1330.6880** | **1329.6928** | **0.9951** | **1** | **64** | **6.5e-005** | **1** | **R.EADDIVNWLKK.R** |
|  | 10101 | **671.3148** | **1340.6150** | **1340.6248** | **-0.0099** | **1** | **32** | **0.087** | **1** | **K.NFEDVAFDEKK.N** |
|  | 11595 | **712.6131** | **1423.2116** | **1423.7711** | **-0.5595** | **1** | **56** | **0.0002** | **1** | **K.YQLDKDGVVLFK.K** 11600 |
|  | 12013 | **726.3496** | **1450.6845** | **1450.6939** | **-0.0094** | **0** | **49** | **0.0015** | **1** | **K.YKPESEELTAER.I** 12009 12012 12014 12015 |
|  | 12178 | **487.5736** | **1459.6989** | **1458.6991** | **0.9998** | **1** | **7** | **27** | **1** | **K.SVSDYDGKLSNFK.T** |
|  | 12258 | **733.7855** | **1465.5565** | **1465.6797** | **-0.1232** | **1** | **1** | **84** | **8** | **R.NGDTASPKEYTAGR.E** |
|  | 14999 | **821.3726** | **1640.7307** | **1640.7431** | **-0.0124** | **1** | **33** | **0.057** | **1** | **K.FDEGRNNFEGEVTK.E** |
|  | 16377 | **865.4537** | **1728.8929** | **1728.9046** | **-0.0117** | **1** | **56** | **0.00028** | **1** | **K.LGETYKDHENIVIAK.M** 16375 16376 16378 16379 |
|  | 17092 | **890.9156** | **1779.8166** | **1779.8275** | **-0.0109** | **0** | **70** | **1e-005** | **1** | **K.VDATEESDLAQQYGVR.G** 17077 17089 17090 17091 17094 17109 17110 |
|  | 17771 | **917.4541** | **1832.8936** | **1832.9057** | **-0.0121** | **0** | **70** | **1.2e-005** | **1** | **K.ILFIFIDSDHTDNQR.I** 17768 17769 17770 |
|  | 18396 | **940.4570** | **1878.8994** | **1878.9112** | **-0.0118** | **1** | **29** | **0.14** | **1** | **K.EYTAGREADDIVNWLK.K** |
|  | 19415 | **983.0190** | **1964.0235** | **1964.0367** | **-0.0132** | **0** | **76** | **2.2e-006** | **1** | **K.HNQLPLVIEFTEQTAPK.I** 19416 19419 |
|  | 19925 | **1005.5063** | **2008.9980** | **2009.0105** | **-0.0125** | **1** | **86** | **2.5e-007** | **1** | **R.NNFEGEVTKENLLDFIK.H** 19924 19933 |
|  | 20552 | **688.7208** | **2063.1405** | **2062.9915** | **0.1490** | **1** | **11** | **7.4** | **2** | **K.EECPAVRLITLEEEMTK.Y** |
|  | 20874 | **1047.0223** | **2092.0301** | **2092.0436** | **-0.0135** | **1** | **101** | **6.4e-009** | **1** | **R.LAKVDATEESDLAQQYGVR.G** 20873 |
|  | 21839 | **735.3721** | **2203.0944** | **2202.0991** | **0.9953** | **1** | **1** | **67** | **3** | **K.MDSTANEVEAVKVHSFPTLK.F** |
|  | 23587 | **605.3140** | **2417.2267** | **2417.2413** | **-0.0146** | **1** | **46** | **0.0016** | **1** | **K.IKPHLMSQELPEDWDKQPVK.V** 23585 23586 |
|  | 23658 | **609.3121** | **2433.2194** | **2433.2362** | **-0.0168** | **1** | **(27)** | **0.14** | **1** | **K.IKPHLMSQELPEDWDKQPVK.V** |
|  | 24587 | **852.7524** | **2555.2355** | **2555.2730** | **-0.0375** | **1** | **3** | **38** | **3** | **R.FLEGKIKPHLMSQELPEDWDK.Q** |
|  | 25426 | **1357.1630** | **2712.3114** | **2712.3283** | **-0.0169** | **0** | **84** | **2.7e-007** | **1** | **K.QFLQAAEAIDDIPFGITSNSDVFSK.Y** 25427 25429 25430 |
|  | 26695 | **1468.2436** | **2934.4727** | **2934.4862** | **-0.0135** | **0** | **108** | **8.5e-010** | **1** | **R.TGPAATTLPDGAAAESLVESSEVAVIGFFK.D** 26689 26693 26694 26698 26701 26712 26716 |
|  | 29474 | **942.4659** | **3765.8344** | **3765.8473** | **-0.0128** | **1** | **48** | **0.00057** | **1** | **R.TGPAATTLPDGAAAESLVESSEVAVIGFFKDVESDSAK.Q** 29472 29475 29477 29478 |

| **26.** | Q13885\|TBB2A_HUMAN    **Mass:** 50274    **Score:** 1404   **Queries matched:** 88   **emPAI:** 6.98 |
| --- | --- |
|  | Tubulin beta-2A chain OS=Homo sapiens GN=TUBB2A PE=1 SV=1 |

|  | **Query** | **Observed** | **Mr(expt)** | **Mr(calc)** | **Delta** | **Miss** | **Score** | **Expect** | **Rank** | **Peptide** |
| --- | --- | --- | --- | --- | --- | --- | --- | --- | --- | --- |
|  | 1603 | 709.3656 | 708.3583 | 708.3411 | 0.0172 | 1 | 14 | 6.5 | 3 | R.GRMSMK.E |
|  | 2066 | 404.7367 | 807.4588 | 807.3399 | 0.1190 | 0 | 7 | 33 | 7 | R.EEYPDR.I |
|  | 5020 | 514.7598 | 1027.5050 | 1027.5121 | -0.0071 | 0 | 46 | 0.004 | 1 | K.TAVCDIPPR.G 5004 5015 5016 5017 5018 5019 5021 5022 |
|  | 5494 | 533.3253 | 1064.6361 | 1064.4201 | 0.2160 | 0 | (4) | 60 | 6 | K.NMMAACDPR.H |
|  | 5638 | 539.2662 | 1076.5179 | 1076.5250 | -0.0071 | 1 | 36 | 0.039 | 1 | K.IREEYPDR.I 5637 |
|  | 5678 | 541.2108 | 1080.4071 | 1080.4151 | -0.0080 | 0 | 29 | 0.16 | 1 | K.NMMAACDPR.H 5679 5694 |
|  | 6327 | 565.7967 | 1129.5788 | 1129.5880 | -0.0092 | 0 | 4 | 59 | 6 | R.FPGQLNADLR.K |
|  | 6499 | 572.3174 | 1142.6203 | 1142.6270 | -0.0067 | 0 | 70 | 1.8e-005 | 1 | K.LAVNMVPFPR.L 6498 |
|  | 7996 | 615.2989 | 1228.5833 | 1228.5910 | -0.0077 | 0 | 62 | 0.00011 | 1 | R.ISEQFTAMFR.R 7997 |
|  | 8802 | 636.3640 | 1270.7133 | 1270.7220 | -0.0086 | 1 | 47 | 0.0027 | 1 | R.KLAVNMVPFPR.L |
|  | 9112 | 430.2745 | 1287.8016 | 1286.7169 | 1.0847 | 1 | (7) | 28 | 2 | R.KLAVNMVPFPR.L 9101 |
|  | 10371 | **678.3285** | **1354.6424** | **1354.6517** | **-0.0093** | **0** | **68** | **2.1e-005** | **1** | **R.INVYYNEAAGNK.Y** 10370 |
|  | 10574 | **456.2892** | **1365.8456** | **1366.6625** | **-0.8169** | **0** | **4** | **45** | **10** | **R.IMNTFSVMPSPK.V** |
|  | 10873 | **692.3431** | **1382.6716** | **1382.6574** | **0.0142** | **0** | **(3)** | **67** | **5** | **R.IMNTFSVMPSPK.V** |
|  | 10908 | 693.3495 | 1384.6844 | 1384.6921 | -0.0077 | 1 | 45 | 0.004 | 1 | K.RISEQFTAMFR.R |
|  | 11940 | 723.8431 | 1445.6717 | 1445.6820 | -0.0103 | 0 | 68 | 2.2e-005 | 1 | K.EVDEQMLNVQNK.N 11938 11939 |
|  | 12198 | 731.8404 | 1461.6662 | 1461.6769 | -0.0107 | 0 | (42) | 0.0078 | 1 | K.EVDEQMLNVQNK.N 12218 |
|  | 14585 | 808.4162 | 1614.8178 | 1614.8287 | -0.0109 | 0 | 42 | 0.0069 | 1 | R.AILVDLEPGTMDSVR.S 14583 14586 14602 |
|  | 14658 | 810.9161 | 1619.8176 | 1619.8283 | -0.0107 | 0 | 52 | 0.0008 | 1 | R.LHFFMPGFAPLTSR.G 14659 14660 14661 |
|  | 14838 | 816.4137 | 1630.8128 | 1630.8236 | -0.0108 | 0 | (37) | 0.025 | 1 | R.AILVDLEPGTMDSVR.S |
|  | 15835 | 848.9150 | 1695.8154 | 1695.8257 | -0.0102 | 0 | 33 | 0.051 | 1 | K.NSSYFVEWIPNNVK.T 15855 15856 |
|  | 16036 | 854.4293 | 1706.8441 | 1706.8549 | -0.0108 | 0 | 38 | 0.019 | 1 | R.ALTVPELTQQMFDSK.N 16035 16056 |
|  | 17620 | 911.9583 | 1821.9019 | 1821.9156 | -0.0136 | 0 | 67 | 2.6e-005 | 1 | R.EIVHIQAGQCGNQIGAK.F 17614 17621 |
|  | 18090 | 929.4694 | 1856.9242 | 1856.9342 | -0.0101 | 0 | 88 | 1.5e-007 | 2 | K.MSATFIGNSTAIQELFK.R 18088 18089 18091 |
|  | 19345 | 979.9900 | 1957.9654 | 1957.9745 | -0.0091 | 0 | 103 | 4.9e-009 | 1 | K.GHYTEGAELVDSVLDVVR.K 19346 |
|  | 19959 | 1007.5189 | 2013.0232 | 2013.0353 | -0.0122 | 1 | 75 | 2.7e-006 | 1 | K.MSATFIGNSTAIQELFKR.I |
|  | 20818 | 1044.0366 | 2086.0587 | 2086.0695 | -0.0108 | 1 | 86 | 2.5e-007 | 1 | K.GHYTEGAELVDSVLDVVRK.E 20815 20817 20819 |
|  | 21027 | 1055.5288 | 2109.0431 | 2109.0571 | -0.0141 | 1 | 71 | 7.2e-006 | 1 | -.MREIVHIQAGQCGNQIGAK.F 21028 |
|  | 21442 | 1078.9855 | 2155.9564 | 2155.1347 | 0.8217 | 1 | 8 | 14 | 2 | R.GLKMSATFIGNSTAIQELFK.R 21443 |
|  | 23107 | 786.0563 | 2355.1470 | 2355.1583 | -0.0113 | 1 | 2 | 44 | 7 | R.LHFFMPGFAPLTSRGSQQYR.A |
|  | 25387 | 1354.6653 | 2707.3160 | 2707.3310 | -0.0150 | 0 | 93 | 3.2e-008 | 1 | K.LTTPTYGDLNHLVSATMSGVTTCLR.F 25379 25385 25386 25404 25411 25412 |
|  | 25766 | 695.3771 | 2777.4792 | 2776.4346 | 1.0446 | 1 | 6 | 13 | 2 | K.LAVNMVPFPRLHFFMPGFAPLTSR.G |
|  | 25900 | 933.4471 | 2797.3194 | 2797.3361 | -0.0167 | 0 | 97 | 1.3e-008 | 1 | R.SGPFGQIFRPDNFVFGQSGAGNNWAK.G 25898 25899 25903 25910 25911 25912 |
|  | 27537 | 1034.8012 | 3101.3816 | 3101.4003 | -0.0187 | 0 | 58 | 7.5e-005 | 1 | K.FWEVISDEHGIDPTGSYHGDSDLQLER.I 27535 27536 |
|  | 28303 | **1109.8408** | **3326.5006** | **3326.5218** | **-0.0211** | **0** | **50** | **0.00045** | **1** | **K.ESESCDCLQGFQLTHSLGGGTGSGMGTLLISK.I** |

| **27.** | Q9BVA1\|TBB2B_HUMAN    **Mass:** 50377    **Score:** 1384   **Queries matched:** 89   **emPAI:** 6.94 |
| --- | --- |
|  | Tubulin beta-2B chain OS=Homo sapiens GN=TUBB2B PE=1 SV=1 |

|  | **Query** | **Observed** | **Mr(expt)** | **Mr(calc)** | **Delta** | **Miss** | **Score** | **Expect** | **Rank** | **Peptide** |
| --- | --- | --- | --- | --- | --- | --- | --- | --- | --- | --- |
|  | 1603 | 709.3656 | 708.3583 | 708.3411 | 0.0172 | 1 | 14 | 6.5 | 3 | R.GRMSMK.E |
|  | 2066 | 404.7367 | 807.4588 | 807.3399 | 0.1190 | 0 | 7 | 33 | 7 | R.EEYPDR.I |
|  | 5020 | 514.7598 | 1027.5050 | 1027.5121 | -0.0071 | 0 | 46 | 0.004 | 1 | K.TAVCDIPPR.G 5004 5015 5016 5017 5018 5019 5021 5022 |
|  | 5494 | 533.3253 | 1064.6361 | 1064.4201 | 0.2160 | 0 | (4) | 60 | 6 | K.NMMAACDPR.H |
|  | 5638 | 539.2662 | 1076.5179 | 1076.5250 | -0.0071 | 1 | 36 | 0.039 | 1 | K.IREEYPDR.I 5637 |
|  | 5678 | 541.2108 | 1080.4071 | 1080.4151 | -0.0080 | 0 | 29 | 0.16 | 1 | K.NMMAACDPR.H 5679 5694 |
|  | 6327 | 565.7967 | 1129.5788 | 1129.5880 | -0.0092 | 0 | 4 | 59 | 6 | R.FPGQLNADLR.K |
|  | 6499 | 572.3174 | 1142.6203 | 1142.6270 | -0.0067 | 0 | 70 | 1.8e-005 | 1 | K.LAVNMVPFPR.L 6498 |
|  | 7996 | 615.2989 | 1228.5833 | 1228.5910 | -0.0077 | 0 | 62 | 0.00011 | 1 | R.ISEQFTAMFR.R 7997 |
|  | 8802 | 636.3640 | 1270.7133 | 1270.7220 | -0.0086 | 1 | 47 | 0.0027 | 1 | R.KLAVNMVPFPR.L |
|  | 9112 | 430.2745 | 1287.8016 | 1286.7169 | 1.0847 | 1 | (7) | 28 | 2 | R.KLAVNMVPFPR.L 9101 |
|  | 10574 | 456.2892 | 1365.8456 | 1366.6625 | -0.8169 | 0 | 4 | 45 | 10 | R.IMNTFSVMPSPK.V |
|  | 10873 | 692.3431 | 1382.6716 | 1382.6574 | 0.0142 | 0 | (3) | 67 | 5 | R.IMNTFSVMPSPK.V |
|  | 10908 | 693.3495 | 1384.6844 | 1384.6921 | -0.0077 | 1 | 45 | 0.004 | 1 | K.RISEQFTAMFR.R |
|  | 11940 | 723.8431 | 1445.6717 | 1445.6820 | -0.0103 | 0 | 68 | 2.2e-005 | 1 | K.EVDEQMLNVQNK.N 11938 11939 |
|  | 12198 | 731.8404 | 1461.6662 | 1461.6769 | -0.0107 | 0 | (42) | 0.0078 | 1 | K.EVDEQMLNVQNK.N 12218 |
|  | 14585 | 808.4162 | 1614.8178 | 1614.8287 | -0.0109 | 0 | 42 | 0.0069 | 1 | R.AILVDLEPGTMDSVR.S 14583 14586 14602 |
|  | 14658 | 810.9161 | 1619.8176 | 1619.8283 | -0.0107 | 0 | 52 | 0.0008 | 1 | R.LHFFMPGFAPLTSR.G 14659 14660 14661 |
|  | 14838 | 816.4137 | 1630.8128 | 1630.8236 | -0.0108 | 0 | (37) | 0.025 | 1 | R.AILVDLEPGTMDSVR.S |
|  | 15835 | 848.9150 | 1695.8154 | 1695.8257 | -0.0102 | 0 | 33 | 0.051 | 1 | K.NSSYFVEWIPNNVK.T 15855 15856 |
|  | 16036 | 854.4293 | 1706.8441 | 1706.8549 | -0.0108 | 0 | 38 | 0.019 | 1 | R.ALTVPELTQQMFDSK.N 16035 16056 |
|  | 17620 | 911.9583 | 1821.9019 | 1821.9156 | -0.0136 | 0 | 67 | 2.6e-005 | 1 | R.EIVHIQAGQCGNQIGAK.F 17614 17621 |
|  | 18090 | 929.4694 | 1856.9242 | 1856.9342 | -0.0101 | 0 | 88 | 1.5e-007 | 2 | K.MSATFIGNSTAIQELFK.R 18088 18089 18091 |
|  | 19345 | 979.9900 | 1957.9654 | 1957.9745 | -0.0091 | 0 | 103 | 4.9e-009 | 1 | K.GHYTEGAELVDSVLDVVR.K 19346 |
|  | 19959 | 1007.5189 | 2013.0232 | 2013.0353 | -0.0122 | 1 | 75 | 2.7e-006 | 1 | K.MSATFIGNSTAIQELFKR.I |
|  | 20818 | 1044.0366 | 2086.0587 | 2086.0695 | -0.0108 | 1 | 86 | 2.5e-007 | 1 | K.GHYTEGAELVDSVLDVVRK.E 20815 20817 20819 |
|  | 21027 | 1055.5288 | 2109.0431 | 2109.0571 | -0.0141 | 1 | 71 | 7.2e-006 | 1 | -.MREIVHIQAGQCGNQIGAK.F 21028 |
|  | 21442 | 1078.9855 | 2155.9564 | 2155.1347 | 0.8217 | 1 | 8 | 14 | 2 | R.GLKMSATFIGNSTAIQELFK.R 21443 |
|  | 23107 | 786.0563 | 2355.1470 | 2355.1583 | -0.0113 | 1 | 2 | 44 | 7 | R.LHFFMPGFAPLTSRGSQQYR.A |
|  | 25387 | 1354.6653 | 2707.3160 | 2707.3310 | -0.0150 | 0 | 93 | 3.2e-008 | 1 | K.LTTPTYGDLNHLVSATMSGVTTCLR.F 25379 25385 25386 25404 25411 25412 |
|  | 25766 | 695.3771 | 2777.4792 | 2776.4346 | 1.0446 | 1 | 6 | 13 | 2 | K.LAVNMVPFPRLHFFMPGFAPLTSR.G |
|  | 25900 | 933.4471 | 2797.3194 | 2797.3361 | -0.0167 | 0 | 97 | 1.3e-008 | 1 | R.SGPFGQIFRPDNFVFGQSGAGNNWAK.G 25898 25899 25903 25910 25911 25912 |
|  | 27537 | 1034.8012 | 3101.3816 | 3101.4003 | -0.0187 | 0 | 58 | 7.5e-005 | 1 | K.FWEVISDEHGIDPTGSYHGDSDLQLER.I 27535 27536 |
|  | 28303 | 1109.8408 | 3326.5006 | 3326.5218 | -0.0211 | 0 | 50 | 0.00045 | 1 | K.ESESCDCLQGFQLTHSLGGGTGSGMGTLLISK.I |
|  | 31036 | 1531.7026 | 4592.0861 | 4592.0999 | -0.0139 | 0 | 50 | 0.00017 | 1 | K.VSDTVVEPYNATLSVHQLVENTDETYCIDNEALYDICFR.T 31032 31033 |

| **28.** | Q7Z4W1\|DCXR_HUMAN    **Mass:** 26182    **Score:** 1355   **Queries matched:** 64   **emPAI:** 12.12 |
| --- | --- |
|  | L-xylulose reductase OS=Homo sapiens GN=DCXR PE=1 SV=2 |

|  | **Query** | **Observed** | **Mr(expt)** | **Mr(calc)** | **Delta** | **Miss** | **Score** | **Expect** | **Rank** | **Peptide** |
| --- | --- | --- | --- | --- | --- | --- | --- | --- | --- | --- |
|  | 973 | **528.2403** | **527.2330** | **526.3479** | **0.8852** | **0** | **16** | **3.4** | **2** | **R.IPLGK.F** 975 |
|  | 1366 | **630.3882** | **629.3810** | **629.3861** | **-0.0051** | **0** | **38** | **0.047** | **1** | **R.VVAVSR.T** |
|  | 1695 | **745.4605** | **744.4532** | **743.4541** | **0.9990** | **0** | **28** | **0.37** | **1** | **R.VLVTGAGK.G** |
|  | 2563 | **424.7282** | **847.4417** | **847.4473** | **-0.0056** | **0** | **23** | **0.92** | **1** | **K.GALDMLTK.V** |
|  | 2777 | **864.4514** | **863.4441** | **863.4501** | **-0.0060** | **0** | **23** | **0.97** | **1** | **R.SFEVNLR.A** |
|  | 3256 | **450.7814** | **899.5483** | **899.5553** | **-0.0069** | **1** | **53** | **0.00091** | **1** | **R.RVLVTGAGK.G** 3254 3257 3276 |
|  | 5871 | **548.2419** | **1094.4692** | **1093.5954** | **0.8738** | **0** | **12** | **9.2** | **2** | **K.VMALELGPHK.I** |
|  | 6171 | 559.2921 | 1116.5697 | 1116.5775 | -0.0078 | 0 | 54 | 0.00062 | 1 | R.TQADLDSLVR.E |
|  | 6982 | **591.3185** | **1180.6224** | **1180.6313** | **-0.0089** | **0** | **58** | **0.00023** | **1** | **R.GTVQALHATGAR.V** 6983 6984 |
|  | 7028 | **592.3578** | **1182.7011** | **1182.7085** | **-0.0073** | **0** | **70** | **1.3e-005** | **1** | **R.AVIQVSQIVAR.G** 7021 |
|  | 10560 | **683.8198** | **1365.6250** | **1365.6347** | **-0.0097** | **0** | **72** | **7.7e-006** | **1** | **R.AVTNHSVYCSTK.G** 10558 10561 10562 10563 10586 10593 |
|  | 15263 | **829.9115** | **1657.8084** | **1657.8206** | **-0.0122** | **0** | **83** | **6e-007** | **1** | **R.GVPGAIVNVSSQCSQR.A** 15249 15261 15262 15264 |
|  | 19121 | **970.9390** | **1939.8634** | **1939.0271** | **0.8363** | **1** | **0** | **86** | **5** | **K.GALDMLTKVMALELGPHK.I** |
|  | 20652 | **1036.5581** | **2071.1017** | **2071.1102** | **-0.0085** | **0** | **115** | **2.5e-010** | **1** | **K.FAEVEHVVNAILFLLSDR.S** 20650 20651 20653 20657 20666 20670 20682 20685 |
|  | 21551 | **1085.0444** | **2168.0743** | **2168.1484** | **-0.0741** | **1** | **2** | **56** | **6** | **R.GLIARGVPGAIVNVSSQCSQR.A** |
|  | 22923 | **1166.5071** | **2330.9996** | **2331.0148** | **-0.0151** | **0** | **84** | **2.7e-007** | **1** | **R.ECPGIEPVCVDLGDWEATER.A** 22921 22922 22924 |
|  | 23940 | **1235.5878** | **2469.1610** | **2469.1781** | **-0.0171** | **0** | **87** | **1.5e-007** | **1** | **R.VNAVNPTVVMTSMGQATWSDPHK.A** 23938 23941 23943 23944 23945 23951 23959 |
|  | 24086 | **829.3922** | **2485.1548** | **2485.1730** | **-0.0182** | **0** | **(15)** | **2.2** | **1** | **R.VNAVNPTVVMTSMGQATWSDPHK.A** |
|  | 26185 | **1424.8055** | **2847.5965** | **2847.6110** | **-0.0144** | **0** | **81** | **3.7e-007** | **1** | **R.ALGSVGPVDLLVNNAAVALLQPFLEVTK.E** 26182 26183 26184 26186 26189 26190 26197 26198 |
|  | 28737 | **1156.2986** | **3465.8739** | **3465.8871** | **-0.0132** | **1** | **64** | **1.5e-005** | **1** | **R.ALGSVGPVDLLVNNAAVALLQPFLEVTKEAFDR.S** 28738 |

| **29.** | P05062\|ALDOB_HUMAN    **Mass:** 39961    **Score:** 1353   **Queries matched:** 89   **emPAI:** 5.02 |
| --- | --- |
|  | Fructose-bisphosphate aldolase B OS=Homo sapiens GN=ALDOB PE=1 SV=2 |

|  | **Query** | **Observed** | **Mr(expt)** | **Mr(calc)** | **Delta** | **Miss** | **Score** | **Expect** | **Rank** | **Peptide** |
| --- | --- | --- | --- | --- | --- | --- | --- | --- | --- | --- |
|  | 1741 | **763.4657** | **762.4584** | **762.4640** | **-0.0055** | **0** | **35** | **0.058** | **1** | **K.VLAAVYK.A** |
|  | 2323 | **415.2108** | **828.4069** | **828.4130** | **-0.0060** | **0** | **9** | **24** | **1** | **K.LSFSYGR.A** 2324 |
|  | 2800 | **433.2214** | **864.4283** | **864.4341** | **-0.0059** | **1** | **6** | **43** | **10** | **K.KDGVDFGK.W** 2793 2813 |
|  | 4129 | **482.7193** | **963.4240** | **963.4266** | **-0.0025** | **0** | **39** | **0.023** | **1** | **R.AMANCQAAK.G** 4123 4124 4125 4126 4127 4128 4136 4137 4144 |
|  | 4366 | **491.1816** | **980.3487** | **979.4215** | **0.9272** | **0** | **(5)** | **50** | **10** | **R.AMANCQAAK.G** |
|  | 4491 | **495.7995** | **989.5845** | **989.4414** | **0.1431** | **0** | **8** | **30** | **9** | **K.VENTEENR.R** |
|  | 5435 | **531.2814** | **1060.5482** | **1060.5553** | **-0.0071** | **0** | **38** | **0.027** | **1** | **R.FPALTQEQK.K** 5434 |
|  | 6206 | **560.7679** | **1119.5213** | **1119.5277** | **-0.0064** | **1** | **36** | **0.043** | **1** | **K.RAMANCQAAK.G** |
|  | 8045 | **411.4189** | **1231.2349** | **1230.6204** | **0.6145** | **1** | **5** | **31** | **10** | **R.IKVENTEENR.R** |
|  | 8214 | **620.8046** | **1239.5947** | **1240.6412** | **-1.0465** | **0** | **100** | **1.4e-008** | **1** | **K.LDQGGAPLAGTNK.E** 8237 8238 8246 8259 |
|  | 8283 | **622.3394** | **1242.6642** | **1242.6720** | **-0.0079** | **0** | **81** | **1.1e-006** | **1** | **R.ALQASALAAWGGK.A** 8284 |
|  | 11493 | **709.8547** | **1417.6948** | **1417.7049** | **-0.0101** | **0** | **27** | **0.28** | **1** | **K.ETTIQGLDGLSER.C** |
|  | 11807 | **719.8480** | **1437.6814** | **1437.6922** | **-0.0108** | **1** | **52** | **0.00081** | **1** | **K.AANKEATQEAFMK.R** 11808 |
|  | 12131 | **729.8887** | **1457.7629** | **1457.7725** | **-0.0096** | **0** | **56** | **0.00036** | **1** | **K.ELSEIAQSIVANGK.G** 12156 12157 12158 |
|  | 12690 | **745.8621** | **1489.7096** | **1489.7195** | **-0.0099** | **0** | **74** | **5.8e-006** | **1** | **K.GILAADESVGTMGNR.L** 12689 |
|  | 12924 | **753.8619** | **1505.7093** | **1505.7144** | **-0.0051** | **0** | **(56)** | **0.00032** | **1** | **K.GILAADESVGTMGNR.L** 12934 |
|  | 17204 | **894.4535** | **1786.8925** | **1786.9036** | **-0.0110** | **0** | **39** | **0.014** | **1** | **K.YTPEQVAMATVTALHR.T** 17206 |
|  | 18735 | **636.3292** | **1905.9657** | **1907.0840** | **-1.1184** | **1** | **2** | **61** | **1** | **K.GIVVGIKLDQGGAPLAGTNK.E** |
|  | 18844 | **639.3355** | **1914.9847** | **1914.9986** | **-0.0138** | **1** | **50** | **0.0011** | **1** | **K.KYTPEQVAMATVTALHR.T** |
|  | 21313 | **1071.5262** | **2141.0379** | **2141.0535** | **-0.0155** | **0** | **108** | **1.3e-009** | **1** | **R.IADQCPSSLAIQENANALAR.Y** 21312 21314 21320 21321 |
|  | 25012 | **881.1128** | **2640.3167** | **2640.3355** | **-0.0188** | **1** | **40** | **0.0068** | **1** | **K.LDQGGAPLAGTNKETTIQGLDGLSER.C** |
|  | 27008 | **997.4957** | **2989.4654** | **2989.4902** | **-0.0249** | **0** | **42** | **0.0032** | **1** | **K.ALNDHHVYLEGTLLKPNMVTAGHACTK.K** 27009 27010 27020 |
|  | 27244 | **1013.1976** | **3036.5709** | **3036.5808** | **-0.0099** | **0** | **68** | **7.8e-006** | **1** | **R.EILFSVDSSINQSIGGVILFHETLYQK.D** 27223 27224 27225 27226 27227 27230 27232 27233 27234 27236 27237 27240 27242 27243 27247 27249 27250 27252 27253 |
|  | 30169 | **1351.9567** | **4052.8482** | **4051.8932** | **0.9550** | **0** | **53** | **0.00014** | **1** | **R.YASICQQNGLVPIVEPEVIPDGDHDLEHCQYVTEK.V** 30170 30171 30172 30173 |
|  | 30243 | **1361.0177** | **4080.0313** | **4080.0522** | **-0.0210** | **0** | **55** | **9.1e-005** | **1** | **R.TVPAAVPGICFLSGGMSEEDATLNLNAINLCPLPKPWK.L** 30244 30245 30247 30249 30250 30251 30252 30257 30258 |

| **30.** | Q9Y2P5\|S27A5_HUMAN    **Mass:** 76420    **Score:** 1324   **Queries matched:** 71   **emPAI:** 3.35 |
| --- | --- |
|  | Bile acyl-CoA synthetase OS=Homo sapiens GN=SLC27A5 PE=1 SV=1 |

|  | **Query** | **Observed** | **Mr(expt)** | **Mr(calc)** | **Delta** | **Miss** | **Score** | **Expect** | **Rank** | **Peptide** |
| --- | --- | --- | --- | --- | --- | --- | --- | --- | --- | --- |
|  | 1586 | **705.3914** | **704.3841** | **704.3891** | **-0.0050** | **0** | **21** | **1.6** | **1** | **R.VLQMSK.M** |
|  | 1596 | **708.3626** | **707.3553** | **707.3602** | **-0.0049** | **0** | **15** | **5.4** | **1** | **R.LGDTFR.W** |
|  | 2505 | **422.2348** | **842.4550** | **842.4610** | **-0.0060** | **0** | **19** | **2.5** | **2** | **K.LQAENIR.C** |
|  | 3665 | **466.2345** | **930.4544** | **930.4599** | **-0.0056** | **0** | **14** | **6.7** | **1** | **R.EGFLYFR.D** 3664 |
|  | 4899 | **509.7573** | **1017.5001** | **1017.5066** | **-0.0065** | **0** | **18** | **2.7** | **1** | **R.ACQAAWALK.A** |
|  | 8935 | **640.3030** | **1278.5914** | **1278.5993** | **-0.0079** | **0** | **31** | **0.1** | **1** | **R.ADVWETFQQR.F** |
|  | 8945 | **640.3390** | **1278.6634** | **1278.6721** | **-0.0087** | **0** | **44** | **0.006** | **1** | **K.VVSQQPFVGYR.G** 8946 |
|  | 10624 | **685.3318** | **1368.6490** | **1368.6595** | **-0.0105** | **0** | **38** | **0.021** | **1** | **R.IQDAMEVTSTFK.L** |
|  | 10861 | **691.8592** | **1381.7038** | **1381.7136** | **-0.0098** | **0** | **49** | **0.002** | **1** | **R.GMPLAHSVLSSGAR.V** 10863 |
|  | 10924 | **693.8102** | **1385.6058** | **1384.6544** | **0.9514** | **0** | **(2)** | **88** | **9** | **R.IQDAMEVTSTFK.L** |
|  | 11142 | **699.8567** | **1397.6988** | **1397.7085** | **-0.0097** | **0** | **(29)** | **0.17** | **1** | **R.GMPLAHSVLSSGAR.V** 11143 |
|  | 12495 | **739.8661** | **1477.7176** | **1477.7249** | **-0.0072** | **0** | **44** | **0.0055** | **1** | **K.LGCPTAWINPHGR.G** 12493 12494 |
|  | 12536 | **741.2813** | **1480.5481** | **1479.5548** | **0.9933** | **0** | **38** | **0.015** | **1** | **K.FSTSCFWDDCR.Q** |
|  | 13329 | **766.8565** | **1531.6983** | **1531.7089** | **-0.0106** | **0** | **38** | **0.021** | **1** | **R.YLCNIPQQPEDR.T** 13330 |
|  | 13481 | **514.9418** | **1541.8035** | **1541.8143** | **-0.0108** | **0** | **9** | **14** | **9** | **R.AWLPAYATPHFIR.I** 13483 |
|  | 15844 | **848.9861** | **1695.9577** | **1695.9672** | **-0.0094** | **0** | **36** | **0.027** | **1** | **R.QHGVTVILYVGELLR.Y** |
|  | 16976 | **591.6310** | **1771.8713** | **1772.8689** | **-0.9976** | **1** | **6** | **31** | **3** | **R.IQDAMEVTSTFKLMK.T** 17001 |
|  | 17575 | **909.9506** | **1817.8867** | **1817.8982** | **-0.0115** | **0** | **47** | **0.0024** | **1** | **K.VGMAAVQLAPGQTFDGEK.L** 17574 |
|  | 18685 | **952.4220** | **1902.8294** | **1902.8418** | **-0.0123** | **0** | **82** | **5.5e-007** | **1** | **R.QSGDVYYNTGDVLAMDR.E** |
|  | 19858 | **1002.0283** | **2002.0421** | **2002.0524** | **-0.0103** | **0** | **77** | **2.1e-006** | **1** | **R.EGFNVGIVVDPLFVLDNR.A** 19857 19859 19860 19861 19862 |
|  | 20551 | **1032.5768** | **2063.1390** | **2063.1514** | **-0.0124** | **1** | **35** | **0.024** | **1** | **R.VLVVDPDLRESLEEILPK.L** 20550 20552 |
|  | 21010 | **703.3563** | **2107.0470** | **2107.0269** | **0.0201** | **1** | **1** | **71** | **4** | **R.LAMGNGLRADVWETFQQR.F** |
|  | 21087 | **1058.4976** | **2114.9806** | **2116.0953** | **-1.1148** | **0** | **75** | **2.7e-006** | **1** | **R.ALLVWTGPGAGSVTFGELDAR.A** 21105 21106 21107 |
|  | 21187 | **1064.5527** | **2127.0909** | **2127.1034** | **-0.0125** | **0** | **44** | **0.0035** | **1** | **R.DNQGFCIPVGLGEPGLLLTK.V** |
|  | 22249 | **1122.5348** | **2243.0550** | **2243.0681** | **-0.0131** | **0** | **74** | **2.8e-006** | **1** | **R.IWEVYGSTEGNMGLVNYVGR.C** 22250 22254 |
|  | 24207 | **1251.0809** | **2500.1473** | **2500.1627** | **-0.0154** | **0** | **71** | **5.4e-006** | **1** | **R.AQSFRPLTAEMYQAVCEGTWR.L** 24205 |
|  | 24684 | **643.3514** | **2569.3764** | **2569.3904** | **-0.0140** | **0** | **45** | **0.0021** | **1** | **R.SPALFIYTSGTTGLPKPAILTHER.V** 24681 24682 |
|  | 26003 | **939.7665** | **2816.2778** | **2815.2912** | **0.9866** | **1** | **33** | **0.026** | **1** | **R.QSGDVYYNTGDVLAMDREGFLYFR.D** |
|  | 26146 | **949.1301** | **2844.3686** | **2845.3527** | **-0.9842** | **1** | **1** | **50** | **3** | **R.IWEVYGSTEGNMGLVNYVGRCGALGK.M** |
|  | 28377 | **1115.8867** | **3344.6383** | **3344.6612** | **-0.0229** | **0** | **52** | **0.00031** | **1** | **R.CFYLSHTSPTPGVGALGAALDAAPSHPVPADLR.A** |
|  | 28513 | 1129.8702 | 3386.5889 | 3387.7650 | -1.1761 | 1 | 3 | 21 | 4 | R.DNQGFCIPVGLGEPGLLLTKVVSQQPFVGYR.G 28514 |
|  | 29262 | **1231.9677** | **3692.8811** | **3692.8980** | **-0.0168** | **0** | **63** | **1.9e-005** | **1** | **K.AELGDPASLCAGEPTALLVLASQAVPALCMWLGLAK.L** 29263 29264 29265 |
|  | 29752 | **1278.6005** | **3832.7796** | **3832.8037** | **-0.0241** | **0** | **44** | **0.0011** | **1** | **K.GENVSTHEVEGVLSQVDFLQQVNVYGVCVPGCEGK.V** 29753 |
|  | 30425 | **1383.3247** | **4146.9523** | **4146.9780** | **-0.0257** | **1** | **109** | **3.5e-010** | **1** | **R.WKGENVSTHEVEGVLSQVDFLQQVNVYGVCVPGCEGK.V** 30426 30428 |
|  | 30702 | **1440.0509** | **4317.1309** | **4317.1524** | **-0.0215** | **1** | **64** | **8.3e-006** | **1** | **R.MLSPFELVQFDMEAAEPVRDNQGFCIPVGLGEPGLLLTK.V** 30701 30703 |
|  | 30733 | **1445.3837** | **4333.1292** | **4333.1473** | **-0.0181** | **1** | **(47)** | **0.00047** | **1** | **R.MLSPFELVQFDMEAAEPVRDNQGFCIPVGLGEPGLLLTK.V** |
|  | 30742 | **1445.7192** | **4334.1359** | **4333.1473** | **0.9886** | **1** | **(28)** | **0.034** | **1** | **R.MLSPFELVQFDMEAAEPVRDNQGFCIPVGLGEPGLLLTK.V** |

| **31.** | P11498\|PYC_HUMAN    **Mass:** 130293   **Score:** 1307   **Queries matched:** 86   **emPAI:** 1.44 |
| --- | --- |
|  | Pyruvate carboxylase, mitochondrial OS=Homo sapiens GN=PC PE=1 SV=2 |

|  | **Query** | **Observed** | **Mr(expt)** | **Mr(calc)** | **Delta** | **Miss** | **Score** | **Expect** | **Rank** | **Peptide** |
| --- | --- | --- | --- | --- | --- | --- | --- | --- | --- | --- |
|  | 1116 | 559.3775 | 558.3702 | 558.3741 | -0.0039 | 0 | 24 | 1.2 | 1 | R.SILVK.D |
|  | 1707 | **753.3551** | **752.3478** | **752.3527** | **-0.0049** | **0** | **22** | **0.92** | **1** | **K.FCEVAK.E** |
|  | 2149 | **408.2325** | **814.4504** | **813.4821** | **0.9684** | **1** | **20** | **1.8** | **1** | **R.RLQELR.E** |
|  | 2475 | **421.0561** | **840.0976** | **839.5229** | **0.5747** | **1** | **11** | **8.4** | **2** | **K.VLKDLPR.V** |
|  | 2500 | **421.7558** | **841.4970** | **841.5385** | **-0.0415** | **1** | **5** | **48** | **8** | **K.VVAGAKVAK.G** |
|  | 3326 | **453.0465** | **904.0784** | **904.4436** | **-0.3652** | **1** | **6** | **35** | **3** | **K.MGDKVEAR.A** |
|  | 3357 | **454.2142** | **906.4139** | **906.4196** | **-0.0056** | **0** | **19** | **2.5** | **1** | **R.SFQPDTGR.I** |
|  | 3520 | **460.2337** | **918.4529** | **918.4593** | **-0.0064** | **0** | **5** | **62** | **4** | **R.ACTELGIR.T** |
|  | 3996 | **477.7303** | **953.4461** | **954.4957** | **-1.0495** | **0** | **(8)** | **23** | **1** | **K.GQIGAPMPGK.V** |
|  | 4054 | **479.7663** | **957.5180** | **957.5243** | **-0.0063** | **0** | **8** | **30** | **7** | **K.ALAVSDLNR.A** |
|  | 4231 | **486.7490** | **971.4835** | **970.4906** | **0.9929** | **0** | **13** | **9.1** | **2** | **K.GQIGAPMPGK.V** |
|  | 5892 | **549.2413** | **1096.4680** | **1095.5495** | **0.9185** | **1** | **7** | **32** | **7** | **K.MSRALAEFR.V** |
|  | 6090 | **556.2931** | **1110.5717** | **1110.5782** | **-0.0064** | **0** | **7** | **25** | **6** | **R.DAHQSLLATR.V** |
|  | 6777 | **582.7896** | **1163.5647** | **1163.5723** | **-0.0077** | **0** | **38** | **0.024** | **1** | **K.HYFIEVNSR.L** |
|  | 6848 | **585.3500** | **1168.6854** | **1169.5328** | **-0.8474** | **0** | **8** | **22** | **4** | **R.FLYECPWR.R** |
|  | 6874 | **586.3209** | **1170.6272** | **1170.5993** | **0.0279** | **0** | **8** | **22** | **4** | **R.TSTAPAASPNVR.R** |
|  | 7819 | **407.5617** | **1219.6634** | **1218.6101** | **1.0533** | **0** | **4** | **56** | **10** | **K.GQPLCVLSAMK.M** |
|  | 8333 | **623.8284** | **1245.6422** | **1245.6506** | **-0.0084** | **0** | **19** | **2.1** | **1** | **K.IAPYVAHNFSK.L** |
|  | 8667 | **632.3338** | **1262.6530** | **1262.6619** | **-0.0088** | **1** | **44** | **0.0056** | **1** | **R.QKADEAYLIGR.G** |
|  | 10156 | **672.8218** | **1343.6291** | **1343.6391** | **-0.0100** | **0** | **16** | **3.2** | **1** | **R.GTPLDTEVPMER.V** |
|  | 10312 | 676.4042 | 1350.7939 | 1349.7092 | 1.0847 | 0 | 7 | 25 | 2 | R.QVFFELNGQLR.S |
|  | 10493 | 682.3362 | 1362.6579 | 1362.6667 | -0.0087 | 0 | 47 | 0.0026 | 1 | K.IAEEFEVELER.G 10520 |
|  | 10712 | **458.9192** | **1373.7359** | **1373.7455** | **-0.0096** | **1** | **15** | **4.1** | **1** | **K.KIAPYVAHNFSK.L** 10725 |
|  | 11294 | **704.8122** | **1407.6098** | **1407.6201** | **-0.0103** | **0** | **58** | **0.00021** | **1** | **R.ADFAQACQDAGVR.F** |
|  | 11927 | **723.3921** | **1444.7696** | **1444.7787** | **-0.0090** | **1** | **69** | **1.8e-005** | **1** | **K.VIAHGKDHPTAATK.M** 11914 |
|  | 13044 | **505.5944** | **1513.7613** | **1513.7711** | **-0.0098** | **0** | **28** | **0.18** | **1** | **R.NHPGLLLMDTTFR.D** 13045 |
|  | 13099 | **759.1606** | **1516.3066** | **1516.8105** | **-0.5039** | **1** | **1** | **76** | **1** | **K.VAKGQPLCVLSAMK.M** |
|  | 13165 | **761.3307** | **1520.6468** | **1520.6572** | **-0.0104** | **0** | **89** | **1.4e-007** | **1** | **R.VFDYSEYWEGAR.G** |
|  | 13638 | **518.2266** | **1551.6579** | **1551.7273** | **-0.0694** | **0** | **1** | **1e+002** | **2** | **K.METVVTSPMEGTVR.K** |
|  | 13822 | **782.9002** | **1563.7859** | **1563.7966** | **-0.0108** | **0** | **21** | **0.98** | **1** | **K.AYVEANQMLGDLIK.V** |
|  | 14783 | **814.9540** | **1627.8935** | **1627.9046** | **-0.0110** | **0** | **51** | **0.00095** | **1** | **K.VVEIAPAAHLDPQLR.T** 14784 |
|  | 15214 | **827.9017** | **1653.7888** | **1653.7999** | **-0.0111** | **0** | **56** | **0.00027** | **1** | **K.DFTATFGPLDSLNTR.L** 15216 |
|  | 16467 | **868.4019** | **1734.7892** | **1734.7995** | **-0.0104** | **0** | **90** | **1.2e-007** | **1** | **R.TVAIYSEQDTGQMHR.Q** 16465 16466 |
|  | 16628 | **874.4569** | **1746.8993** | **1746.9087** | **-0.0094** | **0** | **85** | **3.8e-007** | **1** | **K.IVGDLAQFMVQNGLSR.A** 16627 |
|  | 16632 | **874.5034** | **1746.9923** | **1747.0032** | **-0.0109** | **0** | **87** | **2.2e-007** | **1** | **R.GLAPVQAYLHIPDIIK.V** 16613 16633 |
|  | 16878 | **588.3060** | **1761.8961** | **1762.9036** | **-1.0075** | **0** | **(8)** | **19** | **1** | **K.IVGDLAQFMVQNGLSR.A** |
|  | 16920 | **884.4063** | **1766.7981** | **1766.8111** | **-0.0130** | **0** | **49** | **0.0012** | **1** | **R.VVHSYEELEENYTR.A** 16928 16929 |
|  | 17026 | **592.8820** | **1775.6241** | **1775.8439** | **-0.2198** | **1** | **2** | **48** | **3** | **R.VTTEDPARSFQPDTGR.I** |
|  | 17305 | **898.9388** | **1795.8630** | **1795.8741** | **-0.0111** | **0** | **27** | **0.22** | **1** | **K.QVGYENAGTVEFLVDR.H** |
|  | 17702 | **914.9408** | **1827.8670** | **1827.8792** | **-0.0121** | **0** | **87** | **2e-007** | **1** | **R.GANAVGYTNYPDNVVFK.F** 17703 |
|  | 17796 | **918.4498** | **1834.8850** | **1833.8971** | **0.9879** | **0** | **63** | **5.7e-005** | **1** | **K.YSLQYYMGLAEELVR.A** 17780 |
|  | 18118 | **929.9592** | **1857.9039** | **1856.9308** | **0.9731** | **0** | **15** | **3.2** | **1** | **R.AYSEALAAFGNGALFVEK.F** 18108 18119 |
|  | 18687 | **952.4439** | **1902.8731** | **1902.8860** | **-0.0129** | **0** | **47** | **0.0018** | **1** | **K.ENNVDAVHPGYGFLSER.A** |
|  | 20547 | **1032.5293** | **2063.0440** | **2063.0577** | **-0.0137** | **0** | **38** | **0.013** | **1** | **K.DMAGLLKPTACTMLVSSLR.D** |
|  | 20735 | **693.6707** | **2077.9901** | **2079.0526** | **-1.0625** | **0** | **(1)** | **80** | **5** | **K.DMAGLLKPTACTMLVSSLR.D** |
|  | 21715 | **729.4048** | **2185.1925** | **2185.2082** | **-0.0156** | **0** | **20** | **0.74** | **1** | **K.LLHYLGHVMVNGPTTPIPVK.A** |
|  | 22097 | **1113.5913** | **2225.1681** | **2225.1845** | **-0.0164** | **0** | **47** | **0.0015** | **1** | **K.ASPSPTDPVVPAVPIGPPPAGFR.D** 22088 22098 |
|  | 23030 | **1173.1027** | **2344.1908** | **2344.2063** | **-0.0155** | **0** | **78** | **1.1e-006** | **1** | **R.LDNASAFQGAVISPHYDSLLVK.V** 23033 |
|  | 23518 | **1205.6223** | **2409.2301** | **2409.2441** | **-0.0140** | **0** | **50** | **0.00068** | **1** | **R.HIEVQILGDQYGNILHLYER.D** 23517 |
|  | 23646 | **1216.6157** | **2431.2169** | **2431.2325** | **-0.0156** | **0** | **67** | **1.5e-005** | **1** | **R.SVVEFLQGYIGVPHGGFPEPFR.S** 23647 23649 |
|  | 25267 | **897.0873** | **2688.2400** | **2688.2530** | **-0.0130** | **0** | **63** | **3.4e-005** | **1** | **R.HGEEVTPEDVLSAAMYPDVFAHFK.D** |
|  | 27256 | **760.3884** | **3037.5246** | **3037.5469** | **-0.0223** | **0** | **54** | **0.00021** | **1** | **R.LQVEHTVTEEITDVDLVHAQIHVAEGR.S** |
|  | 27432 | **1025.0201** | **3072.0386** | **3072.5956** | **-0.5570** | **1** | **6** | **9.1** | **1** | **R.AGTHILCIKDMAGLLKPTACTMLVSSLR.D** |
|  | 28585 | **1137.6243** | **3409.8510** | **3408.7540** | **1.0970** | **1** | **3** | **19** | **4** | **R.ELIPNIPFQMLLRGANAVGYTNYPDNVVFK.F** 28584 |
|  | 30000 | **1317.6887** | **3950.0443** | **3950.0618** | **-0.0175** | **0** | **4** | **10** | **1** | **R.AIAIAAGVPVVPGTDAPITSLHEAHEFSNTYGFPIIFK.A** |
|  | 30177 | **1353.3163** | **4056.9270** | **4056.9449** | **-0.0179** | **0** | **28** | **0.042** | **1** | **R.VFDSLNYLPNMLLGMEAAGSAGGVVEAAISYTGDVADPSR.T** 30178 30179 30180 |
|  | 31071 | **1539.7813** | **4616.3219** | **4616.3412** | **-0.0192** | **0** | **155** | **6e-015** | **1** | **K.TNIAFLQNVLNNQQFLAGTVDTQFIDENPELFQLRPAQNR.A** 31069 31070 |

| **32.** | P35573\|GDE_HUMAN    **Mass:** 176819   **Score:** 1277   **Queries matched:** 84   **emPAI:** 0.86 |
| --- | --- |
|  | Glycogen debranching enzyme OS=Homo sapiens GN=AGL PE=1 SV=3 |

|  | **Query** | **Observed** | **Mr(expt)** | **Mr(calc)** | **Delta** | **Miss** | **Score** | **Expect** | **Rank** | **Peptide** |
| --- | --- | --- | --- | --- | --- | --- | --- | --- | --- | --- |
|  | 1230 | **589.0242** | **588.0169** | **587.3391** | **0.6778** | **0** | **1** | **2e+002** | **3** | **K.NVLSR.H** |
|  | 2317 | **414.7564** | **827.4983** | **827.4939** | **0.0044** | **0** | **6** | **42** | **6** | **K.LLGPLGMK.T** |
|  | 2392 | **417.7258** | **833.4370** | **834.4599** | **-1.0229** | **0** | **23** | **0.95** | **3** | **R.DTFIALR.G** |
|  | 3197 | **895.4921** | **894.4848** | **894.4923** | **-0.0075** | **0** | **57** | **0.00035** | **1** | **K.LHQELGAK.G** 3196 |
|  | 3980 | **477.2479** | **952.4813** | **952.4879** | **-0.0066** | **0** | **8** | **23** | **2** | **R.HYVHLER.S** |
|  | 4268 | **487.7495** | **973.4845** | **973.5444** | **-0.0599** | **1** | **18** | **3.3** | **1** | **R.LNEITKEK.E** |
|  | 4813 | **506.7202** | **1011.4258** | **1011.4332** | **-0.0073** | **0** | **27** | **0.3** | **1** | **R.FSCDVAEGK.Y** |
|  | 4870 | **508.7548** | **1015.4951** | **1015.5022** | **-0.0071** | **0** | **9** | **23** | **1** | **K.HMQGIQFR.E** 4892 |
|  | 4936 | **511.7584** | **1021.5023** | **1020.5240** | **0.9783** | **1** | **10** | **18** | **3** | **R.KIQDNFEK.L** |
|  | 5538 | **535.2839** | **1068.5532** | **1067.4672** | **1.0860** | **0** | **25** | **0.45** | **1** | **K.VSYDEWNR.K** 5519 |
|  | 5981 | **551.7995** | **1101.5844** | **1101.6103** | **-0.0259** | **0** | **6** | **43** | **5** | **R.ILLLNEMEK.L** |
|  | 6905 | **587.8151** | **1173.6156** | **1173.6241** | **-0.0085** | **0** | **11** | **12** | **1** | **R.DGSAVEIVGLSK.S** |
|  | 7141 | **396.8853** | **1187.6341** | **1187.7026** | **-0.0685** | **0** | **5** | **53** | **10** | **R.NIILAFAGTLR.H** |
|  | 8987 | **641.8204** | **1281.6263** | **1281.6353** | **-0.0090** | **0** | **17** | **2.7** | **1** | **R.LEQGYELQFR.L** |
|  | 9368 | **434.8809** | **1301.6210** | **1300.6459** | **0.9752** | **1** | **3** | **75** | **6** | **K.HMQGIQFRER.N** |
|  | 10375 | **678.3413** | **1354.6681** | **1354.6782** | **-0.0102** | **0** | **36** | **0.033** | **1** | **R.NHLTQFSPHFK.S** 10377 |
|  | 10832 | **691.3367** | **1380.6589** | **1380.6674** | **-0.0084** | **0** | **35** | **0.047** | **1** | **R.NFAEPGSEVYLR.R** 10830 |
|  | 12080 | **728.8355** | **1455.6565** | **1455.6677** | **-0.0113** | **0** | **17** | **2.8** | **1** | **K.NDLGHPFCNNLR.S** |
|  | 12512 | **740.8048** | **1479.5951** | **1479.6048** | **-0.0097** | **0** | **55** | **0.00033** | **1** | **R.EAMSAYNSHEEGR.L** 12511 |
|  | 12761 | **499.2606** | **1494.7599** | **1495.5997** | **-0.8398** | **0** | **(13)** | **6.4** | **1** | **R.EAMSAYNSHEEGR.L** 12768 |
|  | 12884 | **501.9358** | **1502.7855** | **1502.7954** | **-0.0099** | **0** | **31** | **0.11** | **1** | **R.HSPSIHQSVVAVSR.T** |
|  | 13575 | **774.8827** | **1547.7509** | **1547.7620** | **-0.0111** | **0** | **43** | **0.0066** | **1** | **K.YTEITATYFQGVR.L** |
|  | 13953 | **787.9492** | **1573.8838** | **1573.8940** | **-0.0103** | **0** | **70** | **1.2e-005** | **1** | **R.VSLDPHAQVAVGILR.N** 13954 |
|  | 14004 | **526.9388** | **1577.7947** | **1578.8042** | **-1.0095** | **1** | **3** | **58** | **1** | **R.KYTWNDVGQLVEK.L** |
|  | 14539 | **538.2963** | **1611.8670** | **1612.8283** | **-0.9613** | **1** | **7** | **27** | **1** | **K.LYFSRLMGPETTAK.T** |
|  | 14580 | 808.4084 | 1614.8022 | 1615.9219 | -1.1196 | 1 | 7 | 22 | 3 | R.LMGPETTAKTIVLVK.N |
|  | 14685 | **541.3059** | **1620.8959** | **1621.8940** | **-0.9981** | **0** | **1** | **92** | **10** | **R.HGLIPNLLGEGIYAR.Y** |
|  | 15378 | **833.9478** | **1665.8809** | **1664.8926** | **0.9883** | **1** | **36** | **0.029** | **1** | **K.LKLWEFFQVDVNK.A** |
|  | 15522 | **838.9163** | **1675.8181** | **1675.8570** | **-0.0389** | **1** | **34** | **0.044** | **1** | **K.KYTEITATYFQGVR.L** 15500 |
|  | 18943 | **963.4647** | **1924.9149** | **1924.9279** | **-0.0130** | **0** | **36** | **0.026** | **1** | **K.SDPNQHLTIIQDPEYR.R** 18942 |
|  | 19324 | **978.9794** | **1955.9443** | **1955.9589** | **-0.0146** | **0** | **46** | **0.0023** | **1** | **R.GYDELVPHQISVVSEER.F** 19325 |
|  | 19360 | **980.9514** | **1959.8883** | **1959.9948** | **-0.1066** | **1** | **2** | **54** | **7** | **K.GIPALIENDHHMNSIRK.I** |
|  | 19535 | **988.4750** | **1974.9354** | **1974.9476** | **-0.0122** | **0** | **64** | **3.7e-005** | **1** | **K.AVTVYTNYPFPGETFNR.E** 19536 |
|  | 19557 | **660.0585** | **1977.1536** | **1978.0497** | **-0.8961** | **1** | **2** | **59** | **5** | **R.HSPSIHQSVVAVSRTAFR.N** |
|  | 19913 | **502.9778** | **2007.8823** | **2007.8971** | **-0.0149** | **0** | **22** | **0.56** | **1** | **R.YGNKPEDCPYLWAHMK.K** |
|  | 21505 | **1082.5392** | **2163.0638** | **2163.0783** | **-0.0145** | **0** | **48** | **0.0014** | **1** | **K.ESGYNMIHFTPLQTLGLSR.S** 21506 21507 21508 |
|  | 21532 | **1083.5514** | **2165.0882** | **2165.1005** | **-0.0123** | **0** | **14** | **3.2** | **1** | **K.GFIQVYVDQVDEDIVAVTR.H** |
|  | 21941 | **738.0326** | **2211.0761** | **2212.1232** | **-1.0470** | **1** | **3** | **45** | **4** | **K.EVPQMCIPGKIEEVVLEAR.T** |
|  | 22200 | **560.0657** | **2236.2338** | **2235.0565** | **1.1773** | **0** | **11** | **5.7** | **1** | **K.EQCCVSLAAGLPHFSSGIFR.C** |
|  | 22373 | **1132.0070** | **2261.9994** | **2263.0909** | **-1.0916** | **0** | **47** | **0.0014** | **2** | **K.LNLQQSGSFQYYFLQGNEK.S** |
|  | 22759 | **1156.1024** | **2310.1903** | **2310.2042** | **-0.0139** | **0** | **60** | **7e-005** | **1** | **R.VGADNHVLPLDCVTLQTFLAK.C** 22760 |
|  | 22774 | **771.6920** | **2312.0541** | **2312.0678** | **-0.0137** | **0** | **36** | **0.021** | **1** | **R.LDNCHSTPLHVAEYMLDAAR.N** |
|  | 22942 | **778.7067** | **2333.0982** | **2333.1110** | **-0.0128** | **0** | **1** | **62** | **5** | **K.EWNVICITDVVYNHTAANSK.W** |
|  | 23070 | **784.7272** | **2351.1597** | **2351.1402** | **0.0194** | **1** | **3** | **38** | **9** | **R.RFGCTVDMNIALTTFIPHDK.G** |
|  | 23314 | **794.0558** | **2379.1455** | **2379.1589** | **-0.0134** | **0** | **11** | **5.3** | **1** | **K.GFNYHQGPEWLWPIGYFLR.A** |
|  | 23319 | **794.3762** | **2380.1068** | **2380.1230** | **-0.0161** | **0** | **46** | **0.0017** | **1** | **R.SCYSLANQLELNPDFSRPNR.K** |
|  | 23482 | **1203.5328** | **2405.0511** | **2405.0661** | **-0.0150** | **0** | **103** | **3.6e-009** | **1** | **R.SAYDALPSTTIVSMACCASGSTR.G** |
|  | 23955 | **1236.1099** | **2470.2052** | **2470.2241** | **-0.0189** | **0** | **80** | **7e-007** | **1** | **K.WNPEALPSNTGEVNFQSGIIAAR.C** 23956 23958 |
|  | 24260 | **628.0579** | **2508.2026** | **2508.2179** | **-0.0153** | **1** | **11** | **6.1** | **1** | **R.SCYSLANQLELNPDFSRPNRK.Y** |
|  | 24729 | **1290.6293** | **2579.2440** | **2579.2591** | **-0.0151** | **0** | **92** | **4.5e-008** | **1** | **R.LINYHQEQAVNCLLGNVFYER.L** |
|  | 25050 | **1326.1641** | **2650.3136** | **2650.3279** | **-0.0143** | **0** | **92** | **3.8e-008** | **1** | **K.GPNEYIQEIEFENLSPGSVIIFR.V** 25049 25052 |
|  | 26227 | **1426.1449** | **2850.2752** | **2850.2921** | **-0.0168** | **0** | **42** | **0.0037** | **1** | **R.YFTFPFEEIDFSMEESMIHLPNK.A** |
|  | 26820 | **1477.2440** | **2952.4735** | **2952.4869** | **-0.0134** | **0** | **78** | **9e-007** | **1** | **R.NLQPNLYVVAELFTGSEDLDNVFVTR.L** |
|  | 27125 | **1511.6492** | **3021.2838** | **3021.3008** | **-0.0170** | **0** | **90** | **4.3e-008** | **1** | **K.TLDPDDMVYCGIYDNALDNDNYNLAK.G** 27126 27127 27128 |
|  | 27795 | **1058.1904** | **3171.5495** | **3171.5712** | **-0.0218** | **0** | **49** | **0.00064** | **1** | **K.WIQEHPECAYNLVNSPHLKPAWVLDR.A** |
|  | 27910 | **1603.2672** | **3204.5199** | **3204.5359** | **-0.0160** | **0** | **82** | **3.2e-007** | **1** | **R.MYPTDDSAPLPAGTLDQPLFEVIQEAMQK.H** 27909 27911 |
|  | 29599 | **1265.2593** | **3792.7560** | **3792.7804** | **-0.0244** | **0** | **22** | **0.22** | **1** | **K.DSYGASSPWCDYQLRPNFTIAMVVAPELFTTEK.A** |
|  | 29701 | **1274.5964** | **3820.7675** | **3819.8158** | **0.9517** | **1** | **1** | **27** | **3** | **K.CPVSRMYPTDDSAPLPAGTLDQPLFEVIQEAMQK.H** |
|  | 30955 | **758.3702** | **4544.1778** | **4544.2014** | **-0.0236** | **0** | **18** | **0.33** | **1** | **R.YGGEPVGSFVQPCLRPLMPAIAHALFMDITHDNECPIVHR.S** |

| **33.** | Q13748\|TBA3C_HUMAN    **Mass:** 50612    **Score:** 1264   **Queries matched:** 61   **emPAI:** 2.60 |
| --- | --- |
|  | Tubulin alpha-3C/D chain OS=Homo sapiens GN=TUBA3C PE=1 SV=3 |

|  | **Query** | **Observed** | **Mr(expt)** | **Mr(calc)** | **Delta** | **Miss** | **Score** | **Expect** | **Rank** | **Peptide** |
| --- | --- | --- | --- | --- | --- | --- | --- | --- | --- | --- |
|  | 1311 | 614.3460 | 613.3387 | 613.3435 | -0.0049 | 0 | 11 | 14 | 1 | R.GDVVPK.D |
|  | 1757 | 775.4042 | 774.3969 | 774.4024 | -0.0055 | 0 | 28 | 0.34 | 1 | R.GHYTIGK.E |
|  | 1765 | 781.4037 | 780.3964 | 780.4018 | -0.0053 | 0 | 29 | 0.21 | 1 | R.LSVDYGK.K |
|  | 3389 | 455.2527 | 908.4908 | 908.4967 | -0.0060 | 1 | 42 | 0.0094 | 1 | R.LSVDYGKK.S |
|  | 4864 | 508.2898 | 1014.5650 | 1014.5709 | -0.0060 | 0 | 41 | 0.014 | 1 | K.DVNAAIATIK.T 4835 4861 4863 |
|  | 4948 | 512.2609 | 1022.5072 | 1022.4417 | 0.0655 | 0 | 11 | 14 | 2 | K.EDAANNYAR.G |
|  | 9326 | **434.2335** | **1299.6786** | **1298.4916** | **1.1870** | **0** | **6** | **33** | **7** | **K.YMACCMLYR.G** |
|  | 10814 | 460.9011 | 1379.6815 | 1379.6907 | -0.0092 | 1 | 31 | 0.11 | 1 | R.LDHKFDLMYAK.R |
|  | 11350 | 705.8859 | 1409.7573 | 1409.7667 | -0.0094 | 0 | 6 | 31 | 1 | R.QLFHPEQLITGK.E 11351 |
|  | 12123 | 486.8886 | 1457.6440 | 1456.8613 | 0.7827 | 0 | 2 | 94 | 6 | R.LIGQIVSSITASLR.F |
|  | 14334 | 799.8823 | 1597.7500 | 1597.7599 | -0.0100 | 0 | 23 | 0.64 | 2 | R.TIQFVDWCPTGFK.V |
|  | 16223 | 573.9514 | 1718.8322 | 1717.8747 | 0.9575 | 0 | 4 | 47 | 7 | R.NLDIERPTYTNLNR.L |
|  | 16758 | 878.9794 | 1755.9442 | 1755.9559 | -0.0117 | 0 | 73 | 4.9e-006 | 1 | R.IHFPLATYAPVISAEK.A 16755 16756 16757 16760 16761 16762 |
|  | 17643 | 912.9902 | 1823.9658 | 1823.9782 | -0.0124 | 0 | 59 | 0.00015 | 1 | K.VGINYQPPTVVPGGDLAK.V 17640 17641 17653 |
|  | 18197 | 932.9499 | 1863.8853 | 1863.8971 | -0.0118 | 0 | 82 | 6.5e-007 | 1 | R.AVCMLSNTTAIAEAWAR.L 18198 |
|  | 19906 | 1004.4442 | 2006.8737 | 2006.8858 | -0.0121 | 0 | 105 | 2.5e-009 | 1 | K.TIGGGDDSFNTFFSETGAGK.H 19902 19903 19905 |
|  | 21610 | **726.0033** | **2174.9881** | **2176.1641** | **-1.1760** | **1** | **6** | **20** | **3** | **K.HVPRAVFVDLEPTVVDEVR.T** |
|  | 22378 | 755.3527 | 2263.0362 | 2263.1201 | -0.0840 | 1 | 5 | 22 | 3 | K.VQRAVCMLSNTTAIAEAWAR.L |
|  | 22917 | 777.6732 | 2329.9978 | 2329.0110 | 0.9869 | 0 | 37 | 0.014 | 1 | R.AFVHWYVGEGMEEGEFSEAR.E |
|  | 23508 | 1205.1007 | 2408.1869 | 2408.2012 | -0.0144 | 0 | 116 | 1.9e-010 | 1 | R.FDGALNVDLTEFQTNLVPYPR.I 23503 23504 23505 23506 23507 23509 23510 23512 23515 |
|  | 23544 | 805.7346 | 2414.1819 | 2414.1978 | -0.0160 | 1 | 41 | 0.0058 | 1 | R.QLFHPEQLITGKEDAANNYAR.G 23545 |
|  | 24192 | 833.4252 | 2497.2539 | 2496.1670 | 1.0869 | 1 | 7 | 13 | 1 | K.TIGGGDDSFNTFFSETGAGKHVPR.A |
|  | 25605 | 1375.6397 | 2749.2647 | 2749.2840 | -0.0192 | 0 | 80 | 6.4e-007 | 1 | K.AYHEQLSVAEITNACFEPANQMVK.C 25593 25603 25604 25606 25608 |
|  | 30674 | 1074.9896 | 4295.9294 | 4295.9497 | -0.0203 | 0 | 20 | 0.22 | 1 | R.ECISIHVGQAGVQIGNACWELYCLEHGIQPDGQMPSDK.T |
|  | 31025 | 1146.7742 | 4583.0676 | 4583.0913 | -0.0237 | 1 | 55 | 5.3e-005 | 1 | -.MRECISIHVGQAGVQIGNACWELYCLEHGIQPDGQMPSDK.T 31027 |
|  | 31526 | **1380.1462** | **5516.5559** | **5516.5891** | **-0.0332** | **0** | **2** | **6.6** | **2** | **K.LEFAIYPAPQVSTAVVEPYNSILTTHTTLEHSDCAFMVDNEAIYDICR.R** |
|  | 31737 | 1147.3422 | 5731.6744 | 5731.7161 | -0.0416 | 1 | 11 | 0.85 | 1 | K.SKLEFAIYPAPQVSTAVVEPYNSILTTHTTLEHSDCAFMVDNEAIYDICR.R 31738 |

| **34.** | P27797\|CALR_HUMAN    **Mass:** 48283    **Score:** 1224   **Queries matched:** 77   **emPAI:** 3.44 |
| --- | --- |
|  | Calreticulin OS=Homo sapiens GN=CALR PE=1 SV=1 |

|  | **Query** | **Observed** | **Mr(expt)** | **Mr(calc)** | **Delta** | **Miss** | **Score** | **Expect** | **Rank** | **Peptide** |
| --- | --- | --- | --- | --- | --- | --- | --- | --- | --- | --- |
|  | 2178 | **409.7097** | **817.4049** | **817.4082** | **-0.0034** | **1** | **2** | **1.2e+002** | **4** | **K.HKSDFGK.F** |
|  | 4277 | **975.4777** | **974.4704** | **974.4781** | **-0.0077** | **0** | **20** | **1.9** | **1** | **K.GLQTSQDAR.F** 4279 4289 |
|  | 4914 | **510.2835** | **1018.5523** | **1018.5600** | **-0.0076** | **0** | **32** | **0.11** | **1** | **K.VHVIFNYK.G** |
|  | 4917 | **510.2887** | **1018.5629** | **1018.4818** | **0.0811** | **1** | **7** | **39** | **1** | **R.LKEEEEDK.K** |
|  | 6323 | **565.7501** | **1129.4857** | **1129.4928** | **-0.0071** | **1** | **27** | **0.33** | **1** | **K.FYGDEEKDK.G** |
|  | 7810 | **610.3519** | **1218.6893** | **1218.6972** | **-0.0079** | **0** | **21** | **1.2** | **1** | **K.GQTLVVQFTVK.H** |
|  | 11342 | **705.8130** | **1409.6115** | **1409.6212** | **-0.0096** | **0** | **16** | **2.8** | **1** | **K.EQFLDGDGWTSR.W** |
|  | 12448 | **492.8867** | **1475.6383** | **1475.6463** | **-0.0080** | **0** | **30** | **0.15** | **1** | **K.HEQNIDCGGGYVK.L** 12440 12455 12457 12477 |
|  | 14457 | **804.3854** | **1606.7562** | **1606.7667** | **-0.0105** | **0** | **42** | **0.0071** | **1** | **R.FYALSASFEPFSNK.G** |
|  | 17356 | **900.9177** | **1799.8208** | **1799.8326** | **-0.0118** | **1** | **61** | **8.5e-005** | **1** | **K.IKDPDASKPEDWDER.A** 17353 17355 |
|  | 23399 | **1196.0452** | **2390.0758** | **2390.0914** | **-0.0156** | **0** | **76** | **1.6e-006** | **1** | **K.IDNSQVESGSLEDDWDFLPPK.K** 23400 |
|  | 24331 | **1260.0914** | **2518.1683** | **2518.1864** | **-0.0180** | **1** | **68** | **1e-005** | **1** | **K.IDNSQVESGSLEDDWDFLPPKK.I** 24329 24330 24332 24333 24334 |
|  | 25667 | **920.7531** | **2759.2375** | **2759.2562** | **-0.0187** | **0** | **27** | **0.12** | **1** | **K.IDDPTDSKPEDWDKPEHIPDPDAK.K** 25668 |
|  | 26257 | **952.7943** | **2855.3609** | **2855.3800** | **-0.0191** | **1** | **38** | **0.0094** | **1** | **R.CKDDEFTHLYTLIVRPDNTYEVK.I** 26256 |
|  | 26841 | **987.1307** | **2958.3702** | **2958.3883** | **-0.0181** | **1** | **41** | **0.004** | **1** | **R.AKIDDPTDSKPEDWDKPEHIPDPDAK.K** 26839 26840 |
|  | 26849 | **1480.6412** | **2959.2679** | **2959.2858** | **-0.0179** | **0** | **62** | **2.8e-005** | **1** | **K.KPEDWDEEMDGEWEPPVIQNPEYK.G** 26846 26847 26848 |
|  | 28133 | **1634.7424** | **3267.4703** | **3267.4884** | **-0.0181** | **0** | **64** | **1.7e-005** | **1** | **K.SGTIFDNFLITNDEAYAEEFGNETWGVTK.A** 28126 28129 28130 28134 28135 28136 28137 28138 28139 28144 |
|  | 28701 | **1152.8369** | **3455.4889** | **3455.5108** | **-0.0219** | **0** | **39** | **0.004** | **1** | **K.LFPNSLDQTDMHGDSEYNIMFGPDICGPGTK.K** 28699 28700 28702 28703 28704 |
|  | 29320 | **929.1694** | **3712.6484** | **3712.6780** | **-0.0296** | **1** | **1** | **24** | **1** | **K.KPEDWDEEMDGEWEPPVIQNPEYKGEWKPR.Q** |
|  | 30433 | **1037.9980** | **4147.9631** | **4147.9844** | **-0.0213** | **0** | **74** | **1e-006** | **1** | **K.GTWIHPEIDNPEYSPDPSIYAYDNFGVLGLDLWQVK.S** 30431 30435 30436 30437 30438 30439 30440 30446 30447 30449 30450 30451 30452 30460 30462 30465 30466 |
|  | 31369 | **1281.6193** | **5122.4479** | **5121.4349** | **1.0131** | **1** | **9** | **1.9** | **1** | **R.QIDNPDYKGTWIHPEIDNPEYSPDPSIYAYDNFGVLGLDLWQVK.S** 31365 31366 31367 |

| **35.** | Q6PEY2\|TBA3E_HUMAN    **Mass:** 50568    **Score:** 1199   **Queries matched:** 55   **emPAI:** 1.52 |
| --- | --- |
|  | Tubulin alpha-3E chain OS=Homo sapiens GN=TUBA3E PE=1 SV=2 |

|  | **Query** | **Observed** | **Mr(expt)** | **Mr(calc)** | **Delta** | **Miss** | **Score** | **Expect** | **Rank** | **Peptide** |
| --- | --- | --- | --- | --- | --- | --- | --- | --- | --- | --- |
|  | 1311 | 614.3460 | 613.3387 | 613.3435 | -0.0049 | 0 | 11 | 14 | 1 | R.GDVVPK.D |
|  | 1757 | 775.4042 | 774.3969 | 774.4024 | -0.0055 | 0 | 28 | 0.34 | 1 | R.GHYTIGK.E |
|  | 4864 | 508.2898 | 1014.5650 | 1014.5709 | -0.0060 | 0 | 41 | 0.014 | 1 | K.DVNAAIATIK.T 4835 4861 4863 |
|  | 9326 | 434.2335 | 1299.6786 | 1298.4916 | 1.1870 | 0 | 6 | 33 | 7 | K.YMACCMLYR.G |
|  | 10814 | 460.9011 | 1379.6815 | 1379.7271 | -0.0456 | 1 | 7 | 27 | 5 | R.LVHKFDLMYAK.W |
|  | 11350 | 705.8859 | 1409.7573 | 1409.7667 | -0.0094 | 0 | 6 | 31 | 1 | R.QLFHPEQLITGK.E 11351 |
|  | 12123 | 486.8886 | 1457.6440 | 1456.8613 | 0.7827 | 0 | 2 | 94 | 6 | R.LIGQIVSSITASLR.F |
|  | 14334 | 799.8823 | 1597.7500 | 1597.7599 | -0.0100 | 0 | 23 | 0.64 | 2 | R.TIQFVDWCPTGFK.V |
|  | 16223 | 573.9514 | 1718.8322 | 1717.8747 | 0.9575 | 0 | 4 | 47 | 7 | R.NLDIERPTYTNLNR.L |
|  | 16758 | 878.9794 | 1755.9442 | 1755.9559 | -0.0117 | 0 | 73 | 4.9e-006 | 1 | R.IHFPLATYAPVISAEK.A 16755 16756 16757 16760 16761 16762 |
|  | 17643 | 912.9902 | 1823.9658 | 1823.9782 | -0.0124 | 0 | 59 | 0.00015 | 1 | K.VGINYQPPTVVPGGDLAK.V 17640 17641 17653 |
|  | 18197 | 932.9499 | 1863.8853 | 1863.8971 | -0.0118 | 0 | 82 | 6.5e-007 | 1 | R.AVCMLSNTTAIAEAWAR.L 18198 |
|  | 19906 | 1004.4442 | 2006.8737 | 2006.8858 | -0.0121 | 0 | 105 | 2.5e-009 | 1 | K.TIGGGDDSFNTFFSETGAGK.H 19902 19903 19905 |
|  | 21610 | 726.0033 | 2174.9881 | 2176.1641 | -1.1760 | 1 | 6 | 20 | 3 | K.HVPRAVFVDLEPTVVDEVR.T |
|  | 22378 | 755.3527 | 2263.0362 | 2263.1201 | -0.0840 | 1 | 5 | 22 | 3 | K.VQRAVCMLSNTTAIAEAWAR.L |
|  | 23508 | 1205.1007 | 2408.1869 | 2408.2012 | -0.0144 | 0 | 116 | 1.9e-010 | 1 | R.FDGALNVDLTEFQTNLVPYPR.I 23503 23504 23505 23506 23507 23509 23510 23512 23515 |
|  | 24192 | 833.4252 | 2497.2539 | 2496.1670 | 1.0869 | 1 | 7 | 13 | 1 | K.TIGGGDDSFNTFFSETGAGKHVPR.A |
|  | 25605 | 1375.6397 | 2749.2647 | 2749.2840 | -0.0192 | 0 | 80 | 6.4e-007 | 1 | K.AYHEQLSVAEITNACFEPANQMVK.C 25593 25603 25604 25606 25608 |
|  | 30674 | 1074.9896 | 4295.9294 | 4295.9497 | -0.0203 | 0 | 20 | 0.22 | 1 | R.ECISIHVGQAGVQIGNACWELYCLEHGIQPDGQMPSDK.T |
|  | 31025 | 1146.7742 | 4583.0676 | 4583.0913 | -0.0237 | 1 | 55 | 5.3e-005 | 1 | -.MRECISIHVGQAGVQIGNACWELYCLEHGIQPDGQMPSDK.T 31027 |
|  | 31526 | 1380.1462 | 5516.5559 | 5516.5891 | -0.0332 | 0 | 2 | 6.6 | 2 | K.LEFAIYPAPQVSTAVVEPYNSILTTHTTLEHSDCAFMVDNEAIYDICR.R |
|  | 31737 | 1147.3422 | 5731.6744 | 5731.7161 | -0.0416 | 1 | 11 | 0.85 | 1 | K.SKLEFAIYPAPQVSTAVVEPYNSILTTHTTLEHSDCAFMVDNEAIYDICR.R 31738 |

| **36.** | P14625\|ENPL_HUMAN    **Mass:** 92696    **Score:** 1173   **Queries matched:** 97   **emPAI:** 2.11 |
| --- | --- |
|  | Endoplasmin OS=Homo sapiens GN=HSP90B1 PE=1 SV=1 |

|  | **Query** | **Observed** | **Mr(expt)** | **Mr(calc)** | **Delta** | **Miss** | **Score** | **Expect** | **Rank** | **Peptide** |
| --- | --- | --- | --- | --- | --- | --- | --- | --- | --- | --- |
|  | 1327 | **618.3774** | **617.3701** | **616.3432** | **1.0269** | **0** | **20** | **2.9** | **3** | **R.EELVK.N** |
|  | 1386 | **634.3719** | **633.3647** | **634.3915** | **-1.0268** | **0** | **10** | **23** | **9** | **R.HPLIR.D** |
|  | 1429 | **646.4085** | **645.4012** | **645.4061** | **-0.0050** | **0** | **25** | **0.78** | **1** | **K.VIVTSK.H** 1427 1430 1431 |
|  | 1594 | **707.3770** | **706.3697** | **705.3810** | **0.9888** | **0** | **31** | **0.15** | **1** | **R.FQNVAK.E** 1588 1589 1590 |
|  | 1624 | **715.4303** | **714.4230** | **715.4228** | **-0.9998** | **0** | **11** | **17** | **1** | **K.NLGTIAK.S** |
|  | 2555 | **424.2193** | **846.4241** | **847.4837** | **-1.0596** | **1** | **8** | **30** | **10** | **K.TLDMIKK.I** |
|  | 2565 | **424.7464** | **847.4782** | **847.4837** | **-0.0055** | **1** | **18** | **3.5** | **1** | **R.KTLDMIK.K** |
|  | 2742 | **431.7447** | **861.4748** | **861.4821** | **-0.0073** | **1** | **13** | **10** | **8** | **K.RFQNVAK.E** |
|  | 2803 | **433.2307** | **864.4469** | **863.4786** | **0.9683** | **1** | **(7)** | **36** | **4** | **R.KTLDMIK.K** |
|  | 3524 | **460.2686** | **918.5226** | **918.5287** | **-0.0060** | **1** | **22** | **1.1** | **1** | **K.NKEIFLR.E** 3525 |
|  | 4117 | **482.2941** | **962.5736** | **962.5800** | **-0.0064** | **0** | **30** | **0.17** | **1** | **K.LIINSLYK.N** |
|  | 4337 | **490.2477** | **978.4807** | **978.4883** | **-0.0075** | **1** | **11** | **14** | **1** | **K.AYGDRIER.M** |
|  | 4376 | **491.7424** | **981.4702** | **981.4767** | **-0.0065** | **0** | **50** | **0.0015** | **1** | **K.SGTSEFLNK.M** 4377 |
|  | 4534 | **497.2626** | **992.5106** | **992.5179** | **-0.0072** | **0** | **14** | **7.3** | **1** | **R.SGYLLPDTK.A** |
|  | 4687 | **502.2315** | **1002.4485** | **1003.5451** | **-1.0966** | **1** | **25** | **0.61** | **1** | **K.KTFEINPR.H** 4714 |
|  | 4863 | 1015.5713 | 1014.5640 | 1014.4658 | 0.0982 | 0 | 22 | 1.1 | 2 | R.GLFDEYGSK.K |
|  | 5058 | **1031.4906** | **1030.4833** | **1030.4906** | **-0.0073** | **0** | **11** | **15** | **1** | **K.IYFMAGSSR.K** |
|  | 5685 | **541.2716** | **1080.5287** | **1080.5352** | **-0.0066** | **0** | **51** | **0.0011** | **1** | **K.FAFQAEVNR.M** 5684 |
|  | 6445 | **570.2897** | **1138.5648** | **1138.5731** | **-0.0083** | **0** | **15** | **4.7** | **1** | **K.LGVIEDHSNR.T** 6446 |
|  | 8874 | **638.3207** | **1274.6268** | **1274.6354** | **-0.0086** | **0** | **43** | **0.0078** | **1** | **R.ELISNASDALDK.I** |
|  | 8904 | **639.3301** | **1276.6457** | **1277.6252** | **-0.9794** | **1** | **24** | **0.52** | **1** | **R.KEAESSPFVER.L** |
|  | 11201 | **701.8377** | **1401.6609** | **1401.6711** | **-0.0102** | **1** | **4** | **49** | **4** | **K.QDKIYFMAGSSR.K** |
|  | 11608 | **475.7658** | **1424.2754** | **1424.7048** | **-0.4294** | **1** | **5** | **29** | **4** | **K.SEKFAFQAEVNR.M** |
|  | 12594 | **743.3755** | **1484.7365** | **1484.7471** | **-0.0106** | **0** | **77** | **2.6e-006** | **1** | **K.GVVDSDDLPLNVSR.E** 12593 |
|  | 13029 | **757.3880** | **1512.7614** | **1512.7719** | **-0.0104** | **0** | **57** | **0.00024** | **1** | **K.NLLHVTDTGVGMTR.E** 13030 |
|  | 13065 | **758.3540** | **1514.6934** | **1514.7041** | **-0.0107** | **1** | **45** | **0.0043** | **1** | **K.IADDKYNDTFWK.E** 13064 |
|  | 13229 | **763.3615** | **1524.7085** | **1524.7195** | **-0.0110** | **0** | **75** | **3.8e-006** | **1** | **K.EEASDYLELDTIK.N** |
|  | 13288 | **764.9148** | **1527.8150** | **1528.7668** | **-0.9518** | **0** | **(7)** | **28** | **3** | **K.NLLHVTDTGVGMTR.E** |
|  | 13527 | **772.9121** | **1543.8095** | **1543.8205** | **-0.0110** | **1** | **52** | **0.00085** | **1** | **R.ELISNASDALDKIR.L** 13496 13526 |
|  | 13582 | **775.3506** | **1548.6866** | **1548.6984** | **-0.0117** | **1** | **27** | **0.24** | **1** | **K.EVEEDEYKAFYK.S** |
|  | 14295 | **532.5761** | **1594.7065** | **1594.7160** | **-0.0095** | **0** | **14** | **4.5** | **1** | **R.VFITDDFHDMMPK.Y** |
|  | 14758 | **543.2665** | **1626.7778** | **1626.7058** | **0.0720** | **0** | **(6)** | **31** | **1** | **R.VFITDDFHDMMPK.Y** |
|  | 15570 | **560.6206** | **1678.8400** | **1678.8890** | **-0.0490** | **1** | **5** | **35** | **2** | **K.NLGTIAKSGTSEFLNK.M** |
|  | 16677 | **876.4097** | **1750.8049** | **1750.8171** | **-0.0122** | **1** | **48** | **0.0016** | **1** | **R.RVFITDDFHDMMPK.Y** 16678 16679 16680 |
|  | 17131 | 594.9929 | 1781.9569 | 1782.8069 | -0.8500 | 1 | (6) | 27 | 4 | R.RVFITDDFHDMMPK.Y |
|  | 18174 | **931.9658** | **1861.9171** | **1861.9284** | **-0.0113** | **1** | **53** | **0.00055** | **1** | **R.EAVEKEFEPLLNWMK.D** 18175 |
|  | 18390 | **939.9706** | **1877.9267** | **1877.9352** | **-0.0085** | **0** | **65** | **3.5e-005** | **1** | **K.YSQFINFPIYVWSSK.T** 18391 |
|  | 20309 | **1023.5272** | **2045.0399** | **2045.0528** | **-0.0129** | **0** | **115** | **2.9e-010** | **1** | **R.LISLTDENALSGNEELTVK.I** 20307 20308 20311 20312 10506 |
|  | 20357 | **513.0305** | **2048.0930** | **2047.9182** | **0.1748** | **1** | **5** | **32** | **8** | **R.ADDEVDVDGTVEEDLGKSR.E** |
|  | 21050 | **1056.5519** | **2111.0892** | **2111.1045** | **-0.0153** | **1** | **65** | **2.6e-005** | **1** | **K.NLLHVTDTGVGMTREELVK.N** 21038 21051 21059 |
|  | 21168 | **1064.0077** | **2126.0008** | **2127.0994** | **-1.0986** | **1** | **(2)** | **49** | **2** | **K.NLLHVTDTGVGMTREELVK.N** |
|  | 21266 | **713.0063** | **2135.9970** | **2136.0123** | **-0.0153** | **1** | **26** | **0.19** | **1** | **K.AQAYQTGKDISTNYYASQK.K** |
|  | 22355 | **754.0204** | **2259.0395** | **2259.0556** | **-0.0161** | **0** | **71** | **6.7e-006** | **1** | **R.FQSSHHPTDITSLDQYVER.M** 22356 22357 |
|  | 23104 | **1178.5308** | **2355.0470** | **2355.0623** | **-0.0154** | **0** | **84** | **2.9e-007** | **1** | **R.LTESPCALVASQYGWSGNMER.I** 23102 |
|  | 23804 | **818.1057** | **2451.2953** | **2451.2996** | **-0.0043** | **1** | **28** | **0.11** | **1** | **R.GTTITLVLKEEASDYLELDTIK.N** 23789 23795 23801 23805 |
|  | 24440 | **848.0972** | **2541.2699** | **2541.2838** | **-0.0140** | **0** | **35** | **0.024** | **1** | **K.TVWDWELMNDIKPIWQRPSK.E** |
|  | 27442 | **1026.1602** | **3075.4587** | **3075.4787** | **-0.0200** | **1** | **56** | **0.00014** | **1** | **K.KGYEVIYLTEPVDEYCIQALPEFDGK.R** |
|  | 27550 | **1552.7402** | **3103.4659** | **3103.4848** | **-0.0189** | **1** | **57** | **0.00011** | **1** | **K.GYEVIYLTEPVDEYCIQALPEFDGKR.F** 27549 |
|  | 28289 | **1109.1859** | **3324.5359** | **3324.5497** | **-0.0138** | **0** | **44** | **0.0018** | **1** | **K.MTEAQEDGQSTSELIGQFGVGFYSAFLVADK.V** 28286 28287 28288 28290 28291 28293 28298 |
|  | 28749 | **1157.5749** | **3469.7030** | **3470.6704** | **-0.9674** | **1** | **5** | **13** | **3** | **K.ESDDPMAYIHFTAEGEVTFKSILFVPTSAPR.G** |
|  | 30011 | **1318.6349** | **3952.8828** | **3951.9452** | **0.9376** | **1** | **25** | **0.085** | **1** | **K.MTEAQEDGQSTSELIGQFGVGFYSAFLVADKVIVTSK.H** 30007 |
|  | 31071 | 1539.7813 | 4616.3219 | 4615.7570 | 0.5649 | 0 | 2 | 13 | 4 | K.VEEEPEEEPEETAEDTTEDTEQDEDEEMDVGTDEEEETAK.E |

| **37.** | P13667\|PDIA4_HUMAN    **Mass:** 73229    **Score:** 1064   **Queries matched:** 69   **emPAI:** 2.61 |
| --- | --- |
|  | Protein disulfide-isomerase A4 OS=Homo sapiens GN=PDIA4 PE=1 SV=2 |

|  | **Query** | **Observed** | **Mr(expt)** | **Mr(calc)** | **Delta** | **Miss** | **Score** | **Expect** | **Rank** | **Peptide** |
| --- | --- | --- | --- | --- | --- | --- | --- | --- | --- | --- |
|  | 2247 | **411.7552** | **821.4959** | **821.5011** | **-0.0052** | **0** | **13** | **9.2** | **1** | **R.SPPIPLAK.V** |
|  | 2485 | **421.0580** | **840.1015** | **840.4341** | **-0.3326** | **0** | **13** | **4.9** | **5** | **R.DLEHLSK.F** |
|  | 4191 | **485.2707** | **968.5268** | **968.5331** | **-0.0063** | **1** | **10** | **16** | **1** | **R.EFVTAFKK.G** |
|  | 4266 | **487.7473** | **973.4801** | **973.4869** | **-0.0067** | **0** | **51** | **0.0016** | **1** | **K.FIEEHATK.L** |
|  | 4300 | **488.7611** | **975.5077** | **976.5229** | **-1.0152** | **1** | **8** | **32** | **6** | **K.KLAPEYEK.A** |
|  | 4334 | **489.8050** | **977.5954** | **977.6022** | **-0.0068** | **1** | **19** | **2.4** | **2** | **K.RSPPIPLAK.V** 4345 |
|  | 4786 | **505.7324** | **1009.4503** | **1010.4709** | **-1.0205** | **0** | **21** | **1.3** | **1** | **K.QFAPEYEK.I** |
|  | 4978 | **513.2945** | **1024.5744** | **1024.5818** | **-0.0074** | **0** | **27** | **0.29** | **1** | **K.YALPLVGHR.K** 4979 |
|  | 5654 | **539.7786** | **1077.5426** | **1078.6022** | **-1.0597** | **1** | **23** | **0.78** | **1** | **K.DKDPPIPVAK.I** 5668 |
|  | 6276 | **563.7889** | **1125.5633** | **1125.5706** | **-0.0073** | **0** | **50** | **0.0015** | **1** | **R.FDVSGYPTIK.I** 6275 |
|  | 6348 | **566.7506** | **1131.4867** | **1132.5612** | **-1.0745** | **0** | **34** | **0.063** | **1** | **K.VDATAETDLAK.R** 6361 |
|  | 6605 | **576.7801** | **1151.5456** | **1151.5533** | **-0.0076** | **0** | **58** | **0.00022** | **1** | **K.TFDSIVMDPK.K** 6604 |
|  | 7200 | **595.8180** | **1189.6215** | **1189.6302** | **-0.0087** | **0** | **102** | **1.1e-008** | **1** | **K.IDATSASVLASR.F** 7199 |
|  | 7267 | **597.7816** | **1193.5487** | **1193.5578** | **-0.0091** | **0** | **41** | **0.011** | **1** | **K.GRPYDYNGPR.E** 7266 7284 7286 |
|  | 7586 | **605.3022** | **1208.5898** | **1208.5786** | **0.0113** | **1** | **17** | **2.9** | **1** | **K.KGQAVDYEGSR.T** |
|  | 8597 | **630.8282** | **1259.6418** | **1260.6714** | **-1.0295** | **0** | **19** | **1.8** | **2** | **K.QLEPVYNSLAK.K** |
|  | 8969 | **641.2933** | **1280.5721** | **1281.6717** | **-1.0996** | **1** | **31** | **0.11** | **1** | **K.RFDVSGYPTLK.I** 8990 9006 |
|  | 9125 | **645.3337** | **1288.6529** | **1288.6623** | **-0.0093** | **1** | **52** | **0.00085** | **1** | **K.VDATAETDLAKR.F** |
|  | 9155 | **646.2784** | **1290.5422** | **1290.5510** | **-0.0088** | **0** | **59** | **0.00017** | **1** | **K.MDATANDVPSDR.Y** |
|  | 9234 | **648.3831** | **1294.7516** | **1295.6431** | **-0.8916** | **1** | **5** | **47** | **8** | **K.TFDSIVMDPKK.D** |
|  | 9727 | **661.4158** | **1320.8171** | **1321.6527** | **-0.8356** | **1** | **0** | **1.2e+002** | **6** | **R.KGRPYDYNGPR.E** |
|  | 11869 | **721.8820** | **1441.7494** | **1441.7599** | **-0.0105** | **0** | **75** | **4.6e-006** | **1** | **K.VSQGQLVVMQPEK.F** 11870 11884 |
|  | 12145 | **730.3554** | **1458.6961** | **1457.7548** | **0.9413** | **0** | **(5)** | **40** | **5** | **K.VSQGQLVVMQPEK.F** 12138 |
|  | 12866 | **751.8603** | **1501.7060** | **1501.7161** | **-0.0101** | **1** | **26** | **0.36** | **1** | **K.FEGGDRDLEHLSK.F** 12867 |
|  | 14896 | **545.9579** | **1634.8518** | **1633.8750** | **0.9769** | **1** | **1** | **1e+002** | **10** | **K.VVVGKTFDSIVMDPK.K** |
|  | 15945 | 568.2745 | 1701.8016 | 1701.7192 | 0.0824 | 0 | 3 | 58 | 2 | K.FAMEPEEFDSDTLR.E |
|  | 16914 | **589.6261** | **1765.8565** | **1765.7611** | **0.0954** | **0** | **1** | **85** | **4** | **R.SHMMDVQGSTQDSAIK.D** |
|  | 17140 | **892.4259** | **1782.8372** | **1782.8498** | **-0.0126** | **0** | **82** | **6.9e-007** | **1** | **K.YGIVDYMIEQSGPPSK.E** 17139 |
|  | 17927 | **616.3184** | **1845.9333** | **1846.0023** | **-0.0690** | **1** | **1** | **81** | **7** | **K.FLKVSQGQLVVMQPEK.F** |
|  | 18870 | **959.9758** | **1917.9370** | **1917.9513** | **-0.0143** | **1** | **72** | **6.8e-006** | **1** | **R.YKVEGFPTIYFAPSGDK.K** 18871 |
|  | 20091 | **674.9861** | **2021.9366** | **2021.9492** | **-0.0126** | **1** | **56** | **0.00021** | **1** | **K.KDVLIEFYAPWCGHCK.Q** 20090 |
|  | 20248 | **1020.4498** | **2038.8851** | **2038.8980** | **-0.0129** | **0** | **94** | **3e-008** | **1** | **K.GESDPAYQQYQDAANNLR.E** 20249 |
|  | 20254 | **680.9985** | **2039.9736** | **2039.9874** | **-0.0138** | **1** | **33** | **0.051** | **1** | **R.EKYGIVDYMIEQSGPPSK.E** 20253 |
|  | 21247 | **1068.0259** | **2134.0372** | **2135.1151** | **-1.0778** | **0** | **33** | **0.048** | **1** | **R.EVSQPDWTPPPEVTLVLTK.E** 21258 21259 |
|  | 22277 | **1124.0455** | **2246.0765** | **2246.0914** | **-0.0149** | **1** | **116** | **2e-010** | **1** | **K.DLGLSESGEDVNAAILDESGKK.F** 22276 22278 |
|  | 22968 | **1169.0559** | **2336.0973** | **2336.1141** | **-0.0168** | **1** | **81** | **7e-007** | **1** | **R.SHMMDVQGSTQDSAIKDFVLK.Y** 22967 22969 |
|  | 24061 | **827.7650** | **2480.2730** | **2480.2872** | **-0.0142** | **1** | **41** | **0.0053** | **1** | **K.YGIVDYMIEQSGPPSKEILTLK.Q** 24060 |
|  | 24256 | **1255.0765** | **2508.1385** | **2508.1519** | **-0.0134** | **1** | **47** | **0.0015** | **1** | **K.FAMEPEEFDSDTLREFVTAFK.K** |
|  | 24702 | **644.3463** | **2573.3559** | **2574.1258** | **-0.7699** | **1** | **0** | **60** | **8** | **K.GESDPAYQQYQDAANNLREDYK.F** |
|  | 26796 | **983.4681** | **2947.3824** | **2947.4015** | **-0.0191** | **1** | **10** | **5.8** | **1** | **K.VLEVAKDFPEYTFAIADEEDYAGEVK.D** |
|  | 27686 | **1047.4711** | **3139.3914** | **3139.4056** | **-0.0142** | **0** | **51** | **0.00038** | **1** | **K.ENFDEVVNDADIILVEFYAPWCGHCK.K** |

| **38.** | O14975\|S27A2_HUMAN    **Mass:** 71066    **Score:** 1058   **Queries matched:** 46   **emPAI:** 2.23 |
| --- | --- |
|  | Very long-chain acyl-CoA synthetase OS=Homo sapiens GN=SLC27A2 PE=1 SV=1 |

|  | **Query** | **Observed** | **Mr(expt)** | **Mr(calc)** | **Delta** | **Miss** | **Score** | **Expect** | **Rank** | **Peptide** |
| --- | --- | --- | --- | --- | --- | --- | --- | --- | --- | --- |
|  | 1314 | **615.0433** | **614.0361** | **614.3024** | **-0.2663** | **0** | **4** | **49** | **5** | **K.DEPVR.D** |
|  | 3612 | **464.2412** | **926.4679** | **926.4756** | **-0.0077** | **0** | **31** | **0.14** | **1** | **K.AAMITHQR.I** |
|  | 4547 | **497.8868** | **993.7591** | **993.5351** | **0.2240** | **1** | **6** | **31** | **6** | **R.IGMASIKMK.E** |
|  | 7722 | **608.7971** | **1215.5797** | **1215.5772** | **0.0025** | **0** | **11** | **13** | **9** | **K.DDVSIYYVSR.T** |
|  | 8091 | **617.7745** | **1233.5345** | **1233.5448** | **-0.0103** | **1** | **8** | **23** | **2** | **K.MKENHEFDGK.K** |
|  | 8790 | **636.3071** | **1270.5996** | **1270.6081** | **-0.0085** | **0** | **61** | **0.00011** | **1** | **K.DALYFLDDTAK.M** 8791 |
|  | 10158 | **448.8951** | **1343.6635** | **1343.6721** | **-0.0086** | **1** | **35** | **0.041** | **1** | **K.KDDVSIYYVSR.T** 10157 |
|  | 10541 | **683.3619** | **1364.7093** | **1364.7188** | **-0.0094** | **0** | **51** | **0.0012** | **1** | **R.IQDTIEITGTFK.H** 10553 |
|  | 12263 | **733.8600** | **1465.7054** | **1465.7161** | **-0.0107** | **1** | **17** | **2.7** | **2** | **R.DETLTYAQVDRR.S** |
|  | 12522 | **740.8882** | **1479.7619** | **1479.7722** | **-0.0102** | **0** | **44** | **0.0054** | **1** | **K.ITQLTPFNGYAGAK.A** 12521 |
|  | 13566 | **774.4054** | **1546.7962** | **1546.8065** | **-0.0103** | **0** | **72** | **7.9e-006** | **1** | **K.MTLVEEGFNPAVIK.D** 13565 |
|  | 15286 | **553.9664** | **1658.8773** | **1657.8788** | **0.9985** | **1** | **3** | **50** | **3** | **R.IQDTIEITGTFKHR.K** |
|  | 15483 | **837.9166** | **1673.8186** | **1674.9015** | **-1.0829** | **1** | **32** | **0.077** | **1** | **R.KMTLVEEGFNPAVIK.D** |
|  | 15577 | **840.9645** | **1679.9145** | **1679.9246** | **-0.0101** | **0** | **89** | **1.5e-007** | **1** | **K.YNVTVIQYIGELLR.Y** 15576 |
|  | 17474 | **905.5143** | **1809.0140** | **1808.0196** | **0.9944** | **1** | **54** | **0.00039** | **1** | **R.KYNVTVIQYIGELLR.Y** |
|  | 17918 | **923.4362** | **1844.8579** | **1844.8688** | **-0.0109** | **0** | **94** | **4.1e-008** | **1** | **K.MYVPMTEDIYNAISAK.T** 17917 17919 |
|  | 18309 | **624.6279** | **1870.8620** | **1870.8744** | **-0.0124** | **1** | **6** | **27** | **2** | **R.YLCNSPQKPNDRDHK.V** |
|  | 19207 | **649.6751** | **1946.0033** | **1946.0162** | **-0.0129** | **0** | **27** | **0.19** | **1** | **K.LFQHIADYLPSYARPR.F** |
|  | 21364 | **716.7530** | **2147.2371** | **2147.2453** | **-0.0081** | **0** | **46** | **0.0017** | **1** | **K.VLLVSPELQAAVEEILPSLK.K** |
|  | 22935 | **1167.0929** | **2332.1712** | **2332.1839** | **-0.0126** | **0** | **91** | **6.6e-008** | **1** | **R.SEVTFSTPALYIYTSGTTGLPK.A** 22934 22936 |
|  | 25584 | **916.4871** | **2746.4394** | **2747.3815** | **-0.9422** | **0** | **5** | **21** | **5** | **R.QGDCVALLMGNEPAYVWLWLGLVK.L** |
|  | 25916 | **1400.7046** | **2799.3946** | **2799.4041** | **-0.0094** | **1** | **74** | **2.2e-006** | **1** | **K.MTLVEEGFNPAVIKDALYFLDDTAK.M** 25914 25915 |
|  | 26586 | **972.7918** | **2915.3536** | **2916.3177** | **-0.9641** | **0** | **0** | **48** | **6** | **K.GDLYFNSGDLLMVDHENFIYFHDR.V** |
|  | 27220 | **1519.2101** | **3036.4056** | **3036.4200** | **-0.0144** | **1** | **71** | **3.9e-006** | **1** | **R.TSNTDGIDSFLDKVDEVSTEPIPESWR.S** 27217 27218 27219 |
|  | 27288 | **1015.8042** | **3044.3908** | **3044.4127** | **-0.0219** | **1** | **75** | **1.5e-006** | **1** | **K.KGDLYFNSGDLLMVDHENFIYFHDR.V** 27289 |
|  | 30205 | **1355.9959** | **4064.9657** | **4064.9869** | **-0.0212** | **1** | **73** | **1.5e-006** | **1** | **R.WKGENVATTEVADTVGLVDFVQEVNVYGVHVPDHEGR.I** 30202 30203 30204 30206 |

| **39.** | P21796\|VDAC1_HUMAN    **Mass:** 30868    **Score:** 1013   **Queries matched:** 39   **emPAI:** 4.66 |
| --- | --- |
|  | Voltage-dependent anion-selective channel protein 1 OS=Homo sapiens GN=VDAC1 PE=1 SV=2 |

|  | **Query** | **Observed** | **Mr(expt)** | **Mr(calc)** | **Delta** | **Miss** | **Score** | **Expect** | **Rank** | **Peptide** |
| --- | --- | --- | --- | --- | --- | --- | --- | --- | --- | --- |
|  | 1893 | **398.7224** | **795.4303** | **795.3987** | **0.0316** | **0** | **10** | **14** | **7** | **K.NVNAGGHK.L** |
|  | 2612 | **427.1963** | **852.3780** | **853.4698** | **-1.0918** | **0** | **20** | **1.4** | **1** | **K.GYGFGLIK.L** |
|  | 5056 | **515.8076** | **1029.6006** | **1029.6070** | **-0.0064** | **0** | **56** | **0.00042** | **1** | **K.LTLSALLDGK.N** |
|  | 7662 | **607.3099** | **1212.6052** | **1212.6139** | **-0.0087** | **0** | **62** | **8.9e-005** | **1** | **R.VTQSNFAVGYK.T** 7661 |
|  | 10708 | **687.8282** | **1373.6418** | **1373.6503** | **-0.0085** | **0** | **27** | **0.26** | **1** | **R.WTEYGLTFTEK.W** 10735 |
|  | 11177 | **700.8335** | **1399.6524** | **1399.6620** | **-0.0095** | **0** | **60** | **0.00014** | **1** | **K.LTFDSSFSPNTGK.K** |
|  | 12089 | **728.8760** | **1455.7375** | **1455.7470** | **-0.0095** | **1** | **40** | **0.012** | **1** | **K.SRVTQSNFAVGYK.T** 12080 |
|  | 13282 | **764.8801** | **1527.7457** | **1527.7569** | **-0.0112** | **1** | **40** | **0.011** | **1** | **K.LTFDSSFSPNTGKK.N** 13283 |
|  | 14243 | **531.3212** | **1590.9417** | **1591.8028** | **-0.8611** | **1** | **0** | **1e+002** | **9** | **-.MAVPPTYADLGKSAR.D** |
|  | 19195 | **973.5006** | **1944.9867** | **1945.0017** | **-0.0150** | **1** | **110** | **9.2e-010** | **1** | **K.KLETAVNLAWTAGNSNTR.F** |
|  | 20964 | **1052.0870** | **2102.1595** | **2102.1735** | **-0.0140** | **0** | **44** | **0.0031** | **1** | **K.VNNSSLIGLGYTQTLKPGIK.L** 20963 20965 20974 20975 |
|  | 21473 | **1080.4898** | **2158.9649** | **2158.9776** | **-0.0126** | **0** | **88** | **1.5e-007** | **1** | **R.EHINLGCDMDFDIAGPSIR.G** 21472 |
|  | 21611 | **1088.5217** | **2175.0289** | **2175.0444** | **-0.0155** | **0** | **134** | **3.4e-012** | **1** | **K.WNTDNTLGTEITVEDQLAR.G** 21612 21613 21622 |
|  | 21727 | **1095.0064** | **2187.9981** | **2188.0131** | **-0.0150** | **1** | **151** | **6.8e-014** | **1** | **K.TKSENGLEFTSSGSANTETTK.V** 21726 21728 21736 21737 21738 |
|  | 23569 | **1209.5848** | **2417.1551** | **2417.1725** | **-0.0174** | **0** | **80** | **7.6e-007** | **1** | **R.GALVLGYEGWLAGYQMNFETAK.S** 23572 23573 |
|  | 24840 | **1300.5904** | **2599.1663** | **2599.1827** | **-0.0163** | **0** | **89** | **7.3e-008** | **1** | **K.TDEFQLHTNVNDGTEFGGSIYQK.V** 24839 24841 |
|  | 25213 | **893.0765** | **2676.2078** | **2676.3006** | **-0.0928** | **1** | **1** | **42** | **4** | **R.GALVLGYEGWLAGYQMNFETAKSR.V** |
|  | 25741 | 925.4704 | 2773.3894 | 2774.3094 | -0.9200 | 1 | 4 | 25 | 2 | K.SENGLEFTSSGSANTETTKVTGSLETK.Y |

| **40.** | P31513\|FMO3_HUMAN    **Mass:** 60622    **Score:** 995    **Queries matched:** 78   **emPAI:** 3.71 |
| --- | --- |
|  | Dimethylaniline monooxygenase [N-oxide-forming] 3 OS=Homo sapiens GN=FMO3 PE=1 SV=5 |

|  | **Query** | **Observed** | **Mr(expt)** | **Mr(calc)** | **Delta** | **Miss** | **Score** | **Expect** | **Rank** | **Peptide** |
| --- | --- | --- | --- | --- | --- | --- | --- | --- | --- | --- |
|  | 1201 | **581.3245** | **580.3172** | **580.3220** | **-0.0049** | **0** | **10** | **12** | **9** | **R.ASIYK.S** 1202 |
|  | 1610 | **712.3652** | **711.3579** | **711.3956** | **-0.0377** | **0** | **9** | **18** | **4** | **R.FGTFLK.N** |
|  | 2141 | **814.4976** | **813.4903** | **814.4701** | **-0.9798** | **0** | **21** | **1.3** | **3** | **R.WAAQVIK.G** |
|  | 2636 | **428.2115** | **854.4084** | **854.4134** | **-0.0050** | **0** | **10** | **15** | **1** | **K.SVFSNSSK.E** |
|  | 4077 | **480.7651** | **959.5156** | **959.5222** | **-0.0066** | **0** | **24** | **0.7** | **1** | **R.SLKPMQTR.V** |
|  | 4301 | **488.7627** | **975.5108** | **975.5171** | **-0.0064** | **0** | **(12)** | **12** | **5** | **R.SLKPMQTR.V** |
|  | 4479 | **495.7448** | **989.4751** | **988.4978** | **0.9773** | **0** | **23** | **0.82** | **2** | **K.SNDIGGLWK.F** |
|  | 4489 | **495.7815** | **989.5485** | **989.5545** | **-0.0061** | **0** | **39** | **0.023** | **1** | **R.NNEIILFK.G** 4487 |
|  | 4636 | **500.2947** | **998.5748** | **998.5801** | **-0.0053** | **0** | **36** | **0.046** | **1** | **K.GVFPPLLEK.S** 4635 |
|  | 5279 | **524.2787** | **1046.5429** | **1046.4417** | **0.1012** | **0** | **28** | **0.26** | **1** | **K.FSDHAEEGR.A** 5284 |
|  | 6153 | **558.7893** | **1115.5639** | **1115.5723** | **-0.0084** | **0** | **45** | **0.0049** | **1** | **R.NAILTQWDR.S** |
|  | 6215 | **561.2807** | **1120.5468** | **1120.5546** | **-0.0078** | **0** | **57** | **0.00035** | **1** | **R.TAEQVMISSR.S** 6214 6216 |
|  | 6419 | **569.2773** | **1136.5401** | **1136.5496** | **-0.0094** | **0** | **(25)** | **0.48** | **1** | **R.TAEQVMISSR.S** |
|  | 6426 | **569.3085** | **1136.6024** | **1136.6091** | **-0.0067** | **0** | **56** | **0.00035** | **1** | **R.LVGPGQWPGAR.N** |
|  | 6916 | **588.2923** | **1174.5700** | **1174.5771** | **-0.0071** | **0** | **24** | **0.68** | **1** | **K.ESFPGLNHFK.G** |
|  | 7295 | **598.3372** | **1194.6599** | **1194.6648** | **-0.0049** | **0** | **38** | **0.025** | **1** | **K.IQEYIIAFAK.E** 7293 |
|  | 8071 | **617.3472** | **1232.6798** | **1232.6877** | **-0.0079** | **1** | **25** | **0.52** | **1** | **K.SRNNEIILFK.G** |
|  | 10665 | **686.3314** | **1370.6483** | **1370.7816** | **-0.1333** | **1** | **11** | **10** | **4** | **R.SLKPMQTRVVGR.L** |
|  | 10887 | **692.4154** | **1382.8163** | **1382.8245** | **-0.0083** | **0** | **59** | **0.00015** | **1** | **K.VAIIGAGVSGLASIR.S** 10886 |
|  | 14494 | **537.3440** | **1609.0101** | **1608.7532** | **0.2569** | **1** | **1** | **76** | **8** | **K.FSDHAEEGRASIYK.S** |
|  | 14883 | **817.9200** | **1633.8255** | **1632.8511** | **0.9744** | **0** | **27** | **0.21** | **1** | **K.NNLPTAISDWLYVK.Q** |
|  | 15870 | **849.8655** | **1697.7165** | **1697.7277** | **-0.0111** | **0** | **64** | **4.1e-005** | **1** | **R.SCLEEGLEPTCFEK.S** 15869 |
|  | 18156 | **930.9230** | **1859.8314** | **1859.8439** | **-0.0125** | **0** | **79** | **1.2e-006** | **1** | **K.HPDFATTGQWDVTTER.D** |
|  | 18201 | **932.9520** | **1863.8894** | **1863.8978** | **-0.0084** | **0** | **58** | **0.00016** | **1** | **R.VWDNGYPWDMLLVTR.F** 18188 18200 18202 18220 18221 18222 |
|  | 18502 | **944.9839** | **1887.9532** | **1886.9825** | **0.9707** | **1** | **17** | **2.1** | **1** | **R.FKHENYGLMPLNGVLR.K** 18501 18503 18504 18505 |
|  | 19370 | **654.3359** | **1959.9860** | **1959.0095** | **0.9765** | **0** | **6** | **21** | **1** | **R.VLVVGLGNSGCDIATELSR.T** |
|  | 19409 | **982.9488** | **1963.8831** | **1963.8961** | **-0.0129** | **0** | **64** | **3.6e-005** | **1** | **K.LAMEVYFGPCSPYQFR.L** 19410 |
|  | 20798 | **1043.4259** | **2084.8372** | **2084.8523** | **-0.0150** | **0** | **82** | **4.4e-007** | **1** | **K.GTCTLPSMEDMMNDINEK.M** 20797 20799 |
|  | 21111 | **706.3737** | **2116.0993** | **2115.1106** | **0.9887** | **1** | **44** | **0.0032** | **1** | **K.RVLVVGLGNSGCDIATELSR.T** |
|  | 21119 | **706.9099** | **2117.7079** | **2116.8421** | **0.8658** | **0** | **(0)** | **55** | **8** | **K.GTCTLPSMEDMMNDINEK.M** |
|  | 23713 | **1222.1758** | **2442.3370** | **2442.3482** | **-0.0112** | **0** | **80** | **6.9e-007** | **1** | **K.STIAVIGFVQSLGAAIPTVDLQSR.W** 23714 23717 23719 23722 23724 |
|  | 23849 | **1229.0790** | **2456.1434** | **2456.1617** | **-0.0183** | **0** | **43** | **0.0036** | **1** | **K.ESAVFDAVMVCSGHHVYPNLPK.E** 23850 23851 |
|  | 24945 | **875.7990** | **2624.3752** | **2624.3884** | **-0.0132** | **0** | **20** | **0.7** | **1** | **K.EPVFNDELPASILCGIVSVKPNVK.E** |
|  | 25471 | **908.4342** | **2722.2808** | **2722.2988** | **-0.0180** | **1** | **44** | **0.0027** | **1** | **K.TFVSSVNKHPDFATTGQWDVTTER.D** |
|  | 25602 | **1375.5570** | **2749.0995** | **2749.1110** | **-0.0115** | **0** | **30** | **0.043** | **1** | **K.EMMCFPDFPFPDDFPNFMHNSK.I** 25599 25600 25601 |
|  | 25637 | **918.4974** | **2752.4703** | **2752.4833** | **-0.0130** | **1** | **50** | **0.00065** | **1** | **R.KEPVFNDELPASILCGIVSVKPNVK.E** |
|  | 25779 | **927.7452** | **2780.2139** | **2781.1008** | **-0.8869** | **0** | **(3)** | **26** | **2** | **K.EMMCFPDFPFPDDFPNFMHNSK.I** |
|  | 28512 | **847.6532** | **3386.5837** | **3386.9749** | **-0.3912** | **1** | **0** | **42** | **4** | **R.LQKPCFFFHWLKLFAIPILLIAVFLVLT.-** |
|  | 29910 | **1311.3231** | **3930.9475** | **3930.9641** | **-0.0166** | **0** | **92** | **1.7e-008** | **1** | **K.SETIQTDYIVYMDELSSFIGAKPNIPWLFLTDPK.L** 29906 29907 29908 29909 29911 29912 29913 |

| **41.** | P10620\|MGST1_HUMAN    **Mass:** 17644    **Score:** 978    **Queries matched:** 46   **emPAI:** 9.78 |
| --- | --- |
|  | Microsomal glutathione S-transferase 1 OS=Homo sapiens GN=MGST1 PE=1 SV=1 |

|  | **Query** | **Observed** | **Mr(expt)** | **Mr(calc)** | **Delta** | **Miss** | **Score** | **Expect** | **Rank** | **Peptide** |
| --- | --- | --- | --- | --- | --- | --- | --- | --- | --- | --- |
|  | 11558 | **711.8276** | **1421.6407** | **1421.6505** | **-0.0098** | **0** | **84** | **5.3e-007** | **1** | **K.MMLMSTATAFYR.L** 11549 11550 11557 11559 11560 11561 11562 |
|  | 11806 | **719.8256** | **1437.6367** | **1437.6454** | **-0.0088** | **0** | **(73)** | **5.7e-006** | **1** | **K.MMLMSTATAFYR.L** 11805 11820 11821 11831 |
|  | 11813 | **719.8817** | **1437.7489** | **1437.6454** | **0.1034** | **0** | **(17)** | **2.5** | **1** | **K.MMLMSTATAFYR.L** |
|  | 12041 | **727.3377** | **1452.6609** | **1452.6708** | **-0.0099** | **0** | **47** | **0.0023** | **1** | **K.VFANPEDCVAFGK.G** 12042 |
|  | 14066 | **791.3850** | **1580.7553** | **1580.7657** | **-0.0104** | **1** | **44** | **0.0051** | **1** | **R.KVFANPEDCVAFGK.G** 14065 14067 |
|  | 18614 | **949.5113** | **1897.0081** | **1896.0257** | **0.9824** | **0** | **4** | **40** | **2** | **R.IYHTIAYLTPLPQPNR.A** |
|  | 19268 | **976.9555** | **1951.8965** | **1951.9098** | **-0.0133** | **1** | **104** | **3.7e-009** | **1** | **K.VFANPEDCVAFGKGENAK.K** 19262 19267 19269 |
|  | 25949 | **1404.1918** | **2806.3690** | **2806.3809** | **-0.0119** | **0** | **70** | **5.5e-006** | **1** | **M.VDLTQVMDDEVFMAFASYATIILSK.M** 25944 25945 25946 25947 25948 25950 25951 25953 25958 25959 25961 25962 25963 25964 |
|  | 26038 | **941.7958** | **2822.3657** | **2822.3758** | **-0.0101** | **0** | **(49)** | **0.00069** | **1** | **M.VDLTQVMDDEVFMAFASYATIILSK.M** 26039 |
|  | 26727 | **735.3470** | **2937.3588** | **2937.4214** | **-0.0626** | **0** | **1** | **40** | **2** | **-.MVDLTQVMDDEVFMAFASYATIILSK.M** |
|  | 29302 | **1235.3279** | **3702.9618** | **3702.9773** | **-0.0155** | **0** | **85** | **9.9e-008** | **1** | **R.AHLNDLENIIPFLGIGLLYSLSGPDPSTAILHFR.L** 29301 29303 29304 |

| **42.** | P40939\|ECHA_HUMAN    **Mass:** 83688    **Score:** 963    **Queries matched:** 79   **emPAI:** 1.71 |
| --- | --- |
|  | Trifunctional enzyme subunit alpha, mitochondrial OS=Homo sapiens GN=HADHA PE=1 SV=2 |

|  | **Query** | **Observed** | **Mr(expt)** | **Mr(calc)** | **Delta** | **Miss** | **Score** | **Expect** | **Rank** | **Peptide** |
| --- | --- | --- | --- | --- | --- | --- | --- | --- | --- | --- |
|  | 1161 | **572.3473** | **571.3400** | **571.3693** | **-0.0293** | **1** | **17** | **4.8** | **1** | **K.KISPK.R** 1158 1160 1166 |
|  | 1432 | **647.3463** | **646.3390** | **646.3439** | **-0.0048** | **0** | **20** | **2.8** | **1** | **K.FGAPQK.D** |
|  | 1481 | **661.3398** | **660.3326** | **660.3806** | **-0.0481** | **0** | **17** | **5.3** | **1** | **K.VNTLSK.E** |
|  | 1847 | **397.2216** | **792.4286** | **792.4494** | **-0.0208** | **1** | **11** | **15** | **2** | **R.QQVYKK.V** |
|  | 1943 | **801.3719** | **800.3647** | **800.3704** | **-0.0058** | **0** | **40** | **0.02** | **1** | **K.YEAAYGK.Q** |
|  | 2685 | **858.5028** | **857.4955** | **857.5011** | **-0.0055** | **0** | **25** | **0.66** | **1** | **K.GLYPAPLK.I** 2686 |
|  | 2841 | **434.7261** | **867.4377** | **867.4450** | **-0.0073** | **0** | **26** | **0.37** | **1** | **K.HVAEDLGK.V** 2834 2845 2860 |
|  | 4272 | **487.7856** | **973.5566** | **974.5033** | **-0.9467** | **0** | **25** | **0.67** | **1** | **K.DATLTALDR.G** |
|  | 4473 | **495.2710** | **988.5274** | **988.5342** | **-0.0068** | **1** | **21** | **1.7** | **1** | **K.FGAPQKDVK.H** |
|  | 4621 | **998.5454** | **997.5381** | **997.5444** | **-0.0063** | **0** | **21** | **1.3** | **1** | **R.ILQEGVDPK.K** |
|  | 4825 | **1013.4616** | **1012.4543** | **1012.4614** | **-0.0071** | **0** | **29** | **0.19** | **1** | **K.DGPGFYTTR.C** |
|  | 5201 | 521.3046 | 1040.5946 | 1039.5338 | 1.0607 | 0 | 12 | 9.1 | 1 | R.FVDLYGAQK.I |
|  | 6187 | **559.8020** | **1117.5894** | **1117.5979** | **-0.0085** | **0** | **26** | **0.39** | **1** | **K.DTSASAVAVGLK.Q** |
|  | 6790 | **583.2850** | **1164.5554** | **1165.6091** | **-1.0537** | **0** | **7** | **27** | **2** | **R.NFTGSSALLTR.T** |
|  | 7490 | 602.8037 | 1203.5929 | 1202.5972 | 0.9957 | 0 | 22 | 1.1 | 1 | K.GFYIYQEGVK.R |
|  | 9450 | **653.8149** | **1305.6153** | **1305.6243** | **-0.0090** | **0** | **15** | **4.3** | **1** | **R.CLAPMMSEVIR.I** |
|  | 9673 | **659.8641** | **1317.7136** | **1317.7214** | **-0.0078** | **0** | **63** | **6.5e-005** | **1** | **K.MQLLEIITTEK.T** 9672 |
|  | 10829 | **691.3287** | **1380.6429** | **1381.7428** | **-1.0999** | **0** | **46** | **0.0038** | **1** | **K.LTAYAMTIPFVR.Q** 10855 |
|  | 11037 | **696.8472** | **1391.6799** | **1391.6908** | **-0.0109** | **0** | **22** | **0.92** | **1** | **K.VIGMHYFSPVDK.M** 11038 11039 |
|  | 11370 | 706.3697 | 1410.7249 | 1410.6263 | 0.0987 | 0 | 13 | 6.1 | 3 | K.SEVSSDEDIQFR.L |
|  | 11753 | **717.8883** | **1433.7621** | **1433.7726** | **-0.0105** | **1** | **7** | **29** | **1** | **K.TSKDTSASAVAVGLK.Q** 11757 |
|  | 12087 | **728.8753** | **1455.7361** | **1455.7470** | **-0.0110** | **1** | **5** | **46** | **4** | **K.HVAEDLGKVFGER.F** |
|  | 12672 | **745.3817** | **1488.7489** | **1488.7581** | **-0.0092** | **0** | **43** | **0.0064** | **1** | **K.ALMGLYHGQVLCK.K** 12671 12693 |
|  | 13186 | **508.2635** | **1521.7686** | **1522.8144** | **-1.0458** | **1** | **4** | **47** | **7** | **R.FVDLYGAQKIVDR.L** |
|  | 13774 | **521.2654** | **1560.7745** | **1560.7826** | **-0.0081** | **0** | **6** | **33** | **2** | **K.MVGVPAALDMMLTGR.S** |
|  | 13983 | **789.3976** | **1576.7807** | **1576.7919** | **-0.0112** | **0** | **87** | **2.6e-007** | **1** | **R.FGGGNPELLTQMVSK.G** 13984 13985 |
|  | 13990 | **789.4236** | **1576.8327** | **1576.7775** | **0.0552** | **0** | **(0)** | **1.1e+002** | **4** | **K.MVGVPAALDMMLTGR.S** |
|  | 14271 | **797.3696** | **1592.7247** | **1592.7869** | **-0.0621** | **0** | **(20)** | **1.1** | **1** | **R.FGGGNPELLTQMVSK.G** 14264 |
|  | 14493 | **805.4768** | **1608.9389** | **1608.7674** | **0.1716** | **0** | **(4)** | **45** | **6** | **K.MVGVPAALDMMLTGR.S** |
|  | 14751 | **813.9305** | **1625.8465** | **1625.8552** | **-0.0087** | **0** | **51** | **0.00086** | **1** | **R.TIEYLEEVAITFAK.G** |
|  | 14813 | **815.9179** | **1629.8211** | **1629.8322** | **-0.0111** | **0** | **57** | **0.00026** | **1** | **K.TLQEVTQLSQEAQR.I** 14814 |
|  | 15569 | **840.4261** | **1678.8376** | **1678.8488** | **-0.0111** | **0** | **60** | **0.00012** | **1** | **K.ADMVIEAVFEDLSLK.H** |
|  | 17883 | **921.9059** | **1841.7972** | **1841.8102** | **-0.0129** | **0** | **84** | **4.1e-007** | **1** | **K.TGIEQGSDAGYLCESQK.F** 17872 17884 |
|  | 17939 | **616.6408** | **1846.9004** | **1845.9108** | **0.9896** | **1** | **39** | **0.014** | **1** | **K.LPPKSEVSSDEDIQFR.L** |
|  | 18623 | **633.6788** | **1898.0147** | **1899.0144** | **-0.9997** | **1** | **0** | **93** | **6** | **R.LPKMVGVPAALDMMLTGR.S** |
|  | 18860 | **639.9909** | **1916.9509** | **1917.9771** | **-1.0262** | **1** | **6** | **28** | **2** | **K.RPEKVIGMHYFSPVDK.M** |
|  | 18946 | 642.6774 | 1925.0103 | 1924.0492 | 0.9611 | 1 | 2 | 53 | 5 | K.GLVEKLTAYAMTIPFVR.Q |
|  | 19056 | **645.3386** | **1932.9940** | **1932.9947** | **-0.0007** | **1** | **(3)** | **48** | **2** | **K.MVGVPAALDMMLTGRSIR.A** |
|  | 19432 | **492.4990** | **1965.9670** | **1964.9846** | **0.9824** | **1** | **10** | **8.2** | **6** | **K.MVGVPAALDMMLTGRSIR.A** |
|  | 19897 | **1004.0596** | **2006.1047** | **2006.1161** | **-0.0114** | **0** | **59** | **0.00012** | **1** | **K.TVLGTPEVLLGALPGAGGTQR.L** 19895 19896 |
|  | 20226 | **679.9017** | **2036.6832** | **2036.1088** | **0.5743** | **0** | **4** | **25** | **2** | **K.HLAILGAGLMGAGIAQVSVDK.G** |
|  | 20522 | **1031.4850** | **2060.9554** | **2060.9691** | **-0.0137** | **0** | **77** | **1.8e-006** | **1** | **R.DSIFSNLTGQLDYQGFEK.A** 20523 |
|  | 20596 | **518.0077** | **2068.0019** | **2068.0160** | **-0.0141** | **1** | **1** | **66** | **8** | **K.QFTPCQLLADHANSPNKK.F** |
|  | 21248 | **1068.1058** | **2134.1971** | **2134.2110** | **-0.0139** | **1** | **90** | **8.1e-008** | **1** | **R.KTVLGTPEVLLGALPGAGGTQR.L** |
|  | 22445 | **1137.6052** | **2273.1959** | **2273.2089** | **-0.0131** | **0** | **76** | **2.1e-006** | **1** | **K.MGLVDQLVEPLGPGLKPPEER.T** 22444 |
|  | 22810 | **1159.1002** | **2316.1859** | **2316.0701** | **0.1158** | **1** | **1** | **59** | **3** | **K.DGPGFYTTRCLAPMMSEVIR.I** |
|  | 23593 | **1210.5561** | **2419.0977** | **2419.1114** | **-0.0136** | **0** | **31** | **0.058** | **1** | **K.ELHSEFSEVMNEIWASDQIR.S** |
|  | 24817 | **1297.6525** | **2593.2904** | **2593.3066** | **-0.0162** | **0** | **64** | **2.4e-005** | **1** | **R.SAVLISSKPGCFIAGADINMLAACK.T** |
|  | 25413 | **678.1221** | **2708.4592** | **2707.3965** | **1.0627** | **1** | **5** | **20** | **2** | **K.VIGMHYFSPVDKMQLLEIITTEK.T** |
|  | 26109 | **1418.7463** | **2835.4781** | **2835.4906** | **-0.0125** | **1** | **91** | **5.1e-008** | **1** | **K.KLDSLTTSFGFPVGAATLVDEVGVDVAK.H** 26105 26108 |
|  | 27417 | **1534.7753** | **3067.5360** | **3067.5536** | **-0.0176** | **0** | **52** | **0.00033** | **1** | **K.EVEAVIPDHCIFASNTSALPISEIAAVSK.R** 27416 |
|  | 28581 | **1136.9342** | **3407.7808** | **3407.8010** | **-0.0202** | **1** | **21** | **0.34** | **1** | **R.VLKEVEAVIPDHCIFASNTSALPISEIAAVSK.R** |

| **43.** | Q13423\|NNTM_HUMAN    **Mass:** 114564   **Score:** 947    **Queries matched:** 78   **emPAI:** 1.21 |
| --- | --- |
|  | NAD(P) transhydrogenase, mitochondrial OS=Homo sapiens GN=NNT PE=1 SV=3 |

|  | **Query** | **Observed** | **Mr(expt)** | **Mr(calc)** | **Delta** | **Miss** | **Score** | **Expect** | **Rank** | **Peptide** |
| --- | --- | --- | --- | --- | --- | --- | --- | --- | --- | --- |
|  | 1396 | **635.3936** | **634.3863** | **633.3520** | **1.0343** | **0** | **13** | **12** | **3** | **R.GTVVMK.D** 1382 |
|  | 1628 | **717.4290** | **716.4217** | **716.4255** | **-0.0038** | **0** | **35** | **0.07** | **1** | **K.QVIVMK.R** |
|  | 1932 | **400.2382** | **798.4618** | **799.3608** | **-0.8990** | **0** | **3** | **83** | **7** | **R.MLDMFK.R** |
|  | 2051 | **807.2560** | **806.2488** | **805.4004** | **0.8484** | **0** | **22** | **1.1** | **1** | **K.MLTEQGK.K** |
|  | 2973 | **439.2398** | **876.4651** | **876.4705** | **-0.0054** | **0** | **20** | **2** | **1** | **R.AAALEQFK.S** |
|  | 2976 | **439.2573** | **876.5000** | **875.4865** | **1.0135** | **0** | **17** | **3.5** | **4** | **K.AATITPFR.K** |
|  | 3346 | **453.7180** | **905.4214** | **905.4277** | **-0.0063** | **0** | **21** | **1.4** | **1** | **K.TCDALQAK.V** |
|  | 3489 | **458.7816** | **915.5486** | **915.5542** | **-0.0055** | **1** | **38** | **0.032** | **1** | **K.KAPVLFNK.E** |
|  | 3556 | **922.4670** | **921.4598** | **922.5236** | **-1.0638** | **0** | **38** | **0.025** | **1** | **K.NIPQGAPVK.Q** 3574 |
|  | 3721 | **467.7511** | **933.4877** | **933.4953** | **-0.0076** | **1** | **21** | **1.6** | **2** | **K.MLTEQGKK.V** 3722 |
|  | 3772 | **470.1981** | **938.3816** | **938.3882** | **-0.0067** | **0** | **25** | **0.43** | **1** | **K.FSDDHYR.V** 3787 |
|  | 3986 | **477.2664** | **952.5182** | **952.5243** | **-0.0061** | **0** | **39** | **0.02** | **1** | **R.FGIHPVAGR.M** |
|  | 4195 | **485.2889** | **968.5632** | **968.5695** | **-0.0063** | **0** | **5** | **42** | **10** | **K.VIFPAPTPK.N** |
|  | 4471 | **495.2577** | **988.5008** | **988.5077** | **-0.0068** | **0** | **14** | **7.1** | **1** | **K.TVAELEAEK.A** |
|  | 5110 | **517.7642** | **1033.5138** | **1033.5226** | **-0.0088** | **1** | **37** | **0.036** | **1** | **K.KTCDALQAK.V** 5111 5117 |
|  | 5464 | **532.2764** | **1062.5383** | **1062.5458** | **-0.0075** | **1** | **24** | **0.64** | **1** | **K.EIFQNEKR.V** |
|  | 5591 | **536.8130** | **1071.6114** | **1071.6176** | **-0.0061** | **0** | **28** | **0.31** | **1** | **K.EVLASDLVVK.V** |
|  | 5700 | **541.7607** | **1081.5069** | **1081.4676** | **0.0393** | **0** | **6** | **38** | **4** | **K.ESGEGQGGYAK.E** |
|  | 6154 | **558.7985** | **1115.5824** | **1116.6179** | **-1.0355** | **0** | **39** | **0.02** | **1** | **K.AQYPIADLVK.M** 6173 |
|  | 6465 | **570.8019** | **1139.5892** | **1139.5975** | **-0.0083** | **0** | **61** | **0.0001** | **1** | **R.FFTGQITAAGK.V** 6464 6485 |
|  | 8364 | **624.3208** | **1246.6270** | **1245.6387** | **0.9883** | **0** | **3** | **80** | **3** | **K.TTVLAMDQVPR.V** |
|  | 9235 | **648.3835** | **1294.7525** | **1294.7609** | **-0.0084** | **0** | **52** | **0.00088** | **1** | **R.VALSPAGVQNLVK.Q** 9234 |
|  | 9881 | **664.3976** | **1326.7807** | **1326.7871** | **-0.0064** | **1** | **1** | **1.1e+002** | **2** | **K.EVLASDLVVKVR.A** |
|  | 10427 | **679.8717** | **1357.7288** | **1357.7388** | **-0.0099** | **1** | **81** | **1.3e-006** | **1** | **R.KTTVLAMDQVPR.V** 10428 10429 |
|  | 11671 | **715.3706** | **1428.7267** | **1428.7361** | **-0.0095** | **0** | **53** | **0.00063** | **1** | **K.GITHIGYTDLPSR.M** |
|  | 12312 | **490.2770** | **1467.8093** | **1467.8548** | **-0.0455** | **0** | **5** | **35** | **3** | **K.EVDILISTALIPGK.K** 12328 |
|  | 12356 | **736.3394** | **1470.6642** | **1470.6734** | **-0.0092** | **1** | **41** | **0.01** | **1** | **K.EMSKEFIEAEMK.L** 12355 |
|  | 13215 | **762.9544** | **1523.8943** | **1523.9035** | **-0.0093** | **0** | **13** | **5.8** | **2** | **K.ILIVGGGVAGLASAGAAK.S** |
|  | 13938 | **525.2016** | **1572.5830** | **1572.6726** | **-0.0896** | **1** | **3** | **53** | **8** | **K.ESGEGQGGYAKEMSK.E** |
|  | 13998 | **789.8856** | **1577.7566** | **1577.7686** | **-0.0120** | **0** | **33** | **0.062** | **1** | **K.QGFNVVVESGAGEASK.F** 14027 |
|  | 14329 | **533.3090** | **1596.9051** | **1595.9498** | **0.9553** | **1** | **3** | **60** | **10** | **K.EVDILISTALIPGKK.A** |
|  | 15016 | **821.9030** | **1641.7913** | **1641.8032** | **-0.0118** | **0** | **73** | **5.7e-006** | **1** | **R.MATQASTLYSNNITK.L** 15017 15029 15030 |
|  | 15259 | **829.9005** | **1657.7865** | **1657.7981** | **-0.0116** | **0** | **(62)** | **6.6e-005** | **1** | **R.MATQASTLYSNNITK.L** |
|  | 17058 | **889.4554** | **1776.8962** | **1776.9080** | **-0.0118** | **0** | **51** | **0.00081** | **1** | **R.EANSIIITPGYGLCAAK.A** 17057 |
|  | 17416 | **602.3221** | **1803.9446** | **1803.9553** | **-0.0107** | **0** | **24** | **0.42** | **1** | **R.APMVNPTLGVHEADLLK.T** |
|  | 17591 | **607.3405** | **1818.9997** | **1819.9502** | **-0.9505** | **0** | **(4)** | **50** | **8** | **R.APMVNPTLGVHEADLLK.T** |
|  | 19999 | **673.0805** | **2016.2197** | **2016.2095** | **0.0102** | **1** | **3** | **38** | **1** | **K.VPPAKILIVGGGVAGLASAGAAK.S** |
|  | 20519 | **687.7118** | **2060.1135** | **2059.1248** | **0.9887** | **1** | **48** | **0.0014** | **1** | **K.VRAPMVNPTLGVHEADLLK.T** |
|  | 20678 | **1037.0132** | **2072.0118** | **2072.0248** | **-0.0130** | **0** | **114** | **3.1e-010** | **1** | **R.VTIAQGYDALSSMANIAGYK.A** 20636 20637 20668 20680 20681 |
|  | 21871 | **1103.5831** | **2205.1517** | **2205.1681** | **-0.0164** | **0** | **81** | **6.3e-007** | **1** | **K.TSGTLISFIYPAQNPELLNK.L** 21872 21873 21874 |
|  | 22474 | **760.3239** | **2277.9499** | **2278.1232** | **-0.1733** | **1** | **4** | **27** | **7** | **K.TVVTGCSCPLLSNLGSCKGLR.V** |
|  | 22943 | **778.7147** | **2333.1222** | **2333.1387** | **-0.0165** | **1** | **29** | **0.088** | **1** | **K.SLGAEPLEVDLKESGEGQGGYAK.E** |
|  | 25007 | **880.4028** | **2638.1865** | **2638.2951** | **-0.1086** | **1** | **0** | **61** | **6** | **-.MANLLKTVVTGCSCPLLSNLGSCK.G** |
|  | 26056 | **942.4836** | **2824.4291** | **2824.4469** | **-0.0178** | **0** | **53** | **0.0003** | **1** | **R.SLGVGYAAVDNPIFYKPNTAMLLGDAK.K** 26055 26057 |
|  | 26676 | **978.1570** | **2931.4491** | **2930.4662** | **0.9830** | **0** | **4** | **24** | **3** | **K.EGSVVVDLAAEAGGNFETTKPGELYIHK.G** |
|  | 26823 | **985.1829** | **2952.5268** | **2952.5419** | **-0.0151** | **1** | **27** | **0.11** | **1** | **R.SLGVGYAAVDNPIFYKPNTAMLLGDAKK.T** |
|  | 29494 | **945.9634** | **3779.8244** | **3778.8433** | **0.9811** | **1** | **18** | **0.5** | **1** | **K.EMIESMKEGSVVVDLAAEAGGNFETTKPGELYIHK.G** |
|  | 30348 | **1030.2621** | **4117.0192** | **4117.0460** | **-0.0268** | **0** | **1** | **21** | **3** | **R.SLANVILGGYGTTSTAGGKPMEISGTHTEINLDNAIDMIR.E** |
|  | 30800 | **1469.7411** | **4406.2014** | **4405.4151** | **0.7864** | **0** | **0** | **19** | **2** | **R.LGNALGMIGVAGGLAATLGVLKPGPELLAQMSGAMALGGTIGLTIAK.R** |

| **44.** | P51659\|DHB4_HUMAN    **Mass:** 80092    **Score:** 917    **Queries matched:** 56   **emPAI:** 1.58 |
| --- | --- |
|  | Peroxisomal multifunctional enzyme type 2 OS=Homo sapiens GN=HSD17B4 PE=1 SV=3 |

|  | **Query** | **Observed** | **Mr(expt)** | **Mr(calc)** | **Delta** | **Miss** | **Score** | **Expect** | **Rank** | **Peptide** |
| --- | --- | --- | --- | --- | --- | --- | --- | --- | --- | --- |
|  | 1733 | **762.4083** | **761.4010** | **761.4072** | **-0.0062** | **0** | **24** | **0.97** | **1** | **K.VYQGPAK.G** |
|  | 1875 | **397.8812** | **793.7479** | **793.4083** | **0.3396** | **0** | **7** | **21** | **4** | **R.GSFQVTR.A** |
|  | 3140 | **445.7385** | **889.4625** | **889.4691** | **-0.0066** | **0** | **32** | **0.13** | **1** | **R.GNIMLSQK.L** |
|  | 3280 | **451.2655** | **900.5164** | **899.5188** | **0.9975** | **1** | **5** | **59** | **7** | **K.VVEEIRR.R** |
|  | 6178 | **559.7170** | **1117.4195** | **1116.6073** | **0.8122** | **1** | **7** | **30** | **3** | **K.ARGNIMLSQK.L** |
|  | 6842 | **585.3200** | **1168.6255** | **1168.6928** | **-0.0674** | **0** | **22** | **0.95** | **1** | **R.VVLVTGAGAGLGR.A** 6829 |
|  | 8996 | **641.9227** | **1281.8308** | **1281.7405** | **0.0903** | **0** | **4** | **41** | **4** | **R.IDVVVNNAGILR.D** 9015 |
|  | 10154 | **672.8061** | **1343.5976** | **1342.7034** | **0.8943** | **0** | **4** | **54** | **7** | **K.VNAVFEWHITK.G** |
|  | 10787 | **460.5715** | **1378.6927** | **1378.7027** | **-0.0100** | **1** | **11** | **10** | **1** | **R.QKNHPMTPEAVK.A** 10792 |
|  | 10930 | **693.8782** | **1385.7419** | **1385.7514** | **-0.0095** | **1** | **9** | **17** | **1** | **K.GSLAADKVVEEIR.R** 10932 |
|  | 11019 | **696.3447** | **1390.6748** | **1390.6841** | **-0.0093** | **0** | **37** | **0.024** | **1** | **R.VLQQFADNDVSR.F** |
|  | 11217 | **702.3752** | **1402.7358** | **1402.7457** | **-0.0098** | **0** | **2** | **78** | **6** | **R.GALVVVNDLGGDFK.G** |
|  | 11343 | **705.8174** | **1409.6203** | **1409.6310** | **-0.0107** | **0** | **30** | **0.12** | **1** | **K.AVANYDSVEEGEK.V** |
|  | 11608 | 475.7658 | 1424.2754 | 1424.7300 | -0.4546 | 0 | 2 | 54 | 8 | K.LQSTFVFEEIGR.R |
|  | 11665 | **715.3320** | **1428.6494** | **1428.6593** | **-0.0099** | **0** | **77** | **2.5e-006** | **1** | **K.IDSEGGVSANHTSR.A** 11664 11691 |
|  | 12282 | **734.3701** | **1466.7257** | **1466.7365** | **-0.0108** | **0** | **13** | **6.1** | **1** | **R.ATSTATSGFAGAIGQK.L** 12264 |
|  | 12363 | **491.2701** | **1470.7885** | **1470.7983** | **-0.0098** | **1** | **34** | **0.048** | **1** | **K.KVNAVFEWHITK.G** |
|  | 13563 | **774.3942** | **1546.7739** | **1546.7852** | **-0.0113** | **1** | **44** | **0.0043** | **1** | **R.RVLQQFADNDVSR.F** 13555 |
|  | 14503 | **806.3803** | **1610.7459** | **1610.7583** | **-0.0124** | **0** | **68** | **1.9e-005** | **1** | **K.SNIHCNTIAPNAGSR.M** |
|  | 14598 | **808.8916** | **1615.7686** | **1615.9153** | **-0.1467** | **1** | **13** | **6.8** | **8** | **R.GNIMLSQKLQMILK.D** 14599 14600 |
|  | 14931 | **546.6201** | **1636.8384** | **1637.7984** | **-0.9600** | **1** | **3** | **55** | **7** | **K.NHPMTPEAVKANWK.K** |
|  | 16495 | **868.9326** | **1735.8506** | **1735.8628** | **-0.0123** | **1** | **93** | **5.1e-008** | **1** | **K.AVANYDSVEEGEKVVK.T** 16496 16497 |
|  | 16526 | **870.4283** | **1738.8420** | **1738.8533** | **-0.0113** | **1** | **78** | **1.9e-006** | **1** | **R.KSNIHCNTIAPNAGSR.M** 16524 16525 16527 |
|  | 20116 | **1012.5134** | **2023.0123** | **2023.0237** | **-0.0114** | **0** | **40** | **0.0092** | **1** | **R.FAKPVYPGQTLQTEMWK.E** 20115 |
|  | 20241 | **510.5207** | **2038.0537** | **2039.0186** | **-0.9650** | **0** | **(2)** | **53** | **10** | **R.FAKPVYPGQTLQTEMWK.E** |
|  | 23059 | **1176.0625** | **2350.1104** | **2350.1263** | **-0.0159** | **0** | **91** | **5.7e-008** | **1** | **R.IIMTSSASGIYGNFGQANYSAAK.L** 23058 |
|  | 23908 | **1233.0992** | **2464.1839** | **2464.1985** | **-0.0145** | **0** | **81** | **6e-007** | **1** | **K.FIYEGSSDFSCLPTFGVIIGQK.S** 23907 |
|  | 24419 | **1268.6337** | **2535.2528** | **2535.2704** | **-0.0176** | **0** | **79** | **8.9e-007** | **1** | **K.VQETGDIVISNAYVDLAPTSGTSAK.T** 24418 |
|  | 26300 | **1433.7660** | **2865.5174** | **2865.5348** | **-0.0174** | **0** | **24** | **0.19** | **1** | **K.VAVAIPNRPPDAVLTDTTSLNQAALYR.L** 26298 |
|  | 26361 | **960.8090** | **2879.4052** | **2879.4222** | **-0.0170** | **0** | **70** | **6.4e-006** | **1** | **K.ICDFENASKPQSIQESTGSIIEVLSK.I** |
|  | 27082 | **1003.5070** | **3007.4991** | **3007.5172** | **-0.0181** | **1** | **56** | **0.00012** | **1** | **K.KICDFENASKPQSIQESTGSIIEVLSK.I** |
|  | 27286 | **761.8944** | **3043.5483** | **3043.5616** | **-0.0133** | **1** | **79** | **5.7e-007** | **1** | **K.LPPFSYAYTELEAIMYALGVGASIKDPK.D** 27287 |
|  | 29783 | **1281.9626** | **3842.8661** | **3843.9829** | **-1.1167** | **1** | **1** | **28** | **3** | **R.IIMTSSASGIYGNFGQANYSAAKLGLLGLANSLAIEGR.K** |
|  | 30045 | **993.4931** | **3969.9433** | **3969.9625** | **-0.0192** | **0** | **53** | **0.00014** | **1** | **R.LSGDWNPLHIDPNFASLAGFDKPILHGLCTFGFSAR.R** 30047 |

| **45.** | P31040\|DHSA_HUMAN    **Mass:** 73672    **Score:** 907    **Queries matched:** 75   **emPAI:** 1.80 |
| --- | --- |
|  | Succinate dehydrogenase [ubiquinone] flavoprotein subunit, mitochondrial OS=Homo sapiens GN=SDHA PE=1 SV=2 |

|  | **Query** | **Observed** | **Mr(expt)** | **Mr(calc)** | **Delta** | **Miss** | **Score** | **Expect** | **Rank** | **Peptide** |
| --- | --- | --- | --- | --- | --- | --- | --- | --- | --- | --- |
|  | 895 | **514.2632** | **513.2559** | **514.3479** | **-1.0920** | **0** | **6** | **41** | **1** | **R.LALAK.A** |
|  | 1176 | 576.3048 | 575.2975 | 574.3075 | 0.9901 | 0 | 23 | 1.3 | 6 | R.DVVSR.S |
|  | 1436 | **649.2012** | **648.1939** | **647.3642** | **0.8297** | **0** | **17** | **5** | **4** | **R.YAPVAK.D** |
|  | 2052 | **404.2188** | **806.4229** | **806.4286** | **-0.0057** | **0** | **21** | **1.6** | **2** | **R.AFGGQSLK.F** |
|  | 3786 | **470.6719** | **939.3292** | **939.3361** | **-0.0069** | **0** | **21** | **1.2** | **1** | **R.CCCVADR.T** 3790 |
|  | 5873 | **548.2626** | **1094.5106** | **1094.5185** | **-0.0080** | **0** | **13** | **6.7** | **1** | **R.WHFYDTVK.G** |
|  | 6081 | **555.7212** | **1109.4278** | **1108.5513** | **0.8766** | **1** | **32** | **0.083** | **1** | **R.TEDGKIYQR.A** |
|  | 6186 | **559.8013** | **1117.5880** | **1116.5887** | **0.9993** | **1** | **5** | **53** | **9** | **K.DLASRDVVSR.S** |
|  | 6369 | **567.2803** | **1132.5461** | **1132.5547** | **-0.0086** | **0** | **53** | **0.00077** | **1** | **R.VGSVLQEGCGK.I** 6383 |
|  | 6721 | **580.8076** | **1159.6006** | **1159.5556** | **0.0449** | **0** | **18** | **2.4** | **1** | **K.SMQNHAAVFR.V** |
|  | 7199 | 595.8180 | 1189.6214 | 1189.6455 | -0.0241 | 1 | 11 | 14 | 4 | R.YAPVAKDLASR.D 7200 |
|  | 7238 | **398.2372** | **1191.6898** | **1191.6281** | **0.0617** | **1** | **7** | **31** | **5** | **R.TSELRLSMQK.S** |
|  | 8016 | **615.8416** | **1229.6687** | **1228.6564** | **1.0122** | **0** | **5** | **52** | **6** | **K.AWPTVLQTGTR.G** |
|  | 8872 | **638.2999** | **1274.5852** | **1275.6459** | **-1.0607** | **0** | **26** | **0.36** | **1** | **K.HTLSYVDVGTGK.V** 8892 |
|  | 9190 | **647.3101** | **1292.6057** | **1292.6837** | **-0.0780** | **1** | **37** | **0.031** | **2** | **R.AKNTVVATGGYGR.T** 9192 9195 |
|  | 9907 | **665.3379** | **1328.6613** | **1328.6684** | **-0.0071** | **0** | **38** | **0.024** | **1** | **R.GEGGILINSQGER.F** 9906 |
|  | 9965 | **666.8749** | **1331.7353** | **1331.7449** | **-0.0096** | **0** | **48** | **0.0024** | **1** | **K.VTLEYRPVIDK.T** 9966 |
|  | 10356 | **677.8601** | **1353.7055** | **1353.7153** | **-0.0098** | **0** | **53** | **0.00069** | **1** | **R.TGHSLLHTLYGR.S** 10345 10358 |
|  | 11227 | **702.8304** | **1403.6463** | **1403.7409** | **-0.0946** | **1** | **30** | **0.14** | **1** | **R.KHTLSYVDVGTGK.V** |
|  | 12399 | **737.4214** | **1472.8282** | **1472.8351** | **-0.0069** | **0** | **104** | **5.2e-009** | **1** | **R.LGANSLLDLVVFGR.A** 12397 12398 12400 12424 |
|  | 13425 | **513.8198** | **1538.4375** | **1538.7875** | **-0.3501** | **0** | **4** | **36** | **2** | **R.GVIALCIEDGSIHR.I** |
|  | 16318 | **576.2917** | **1725.8532** | **1724.9937** | **0.8595** | **1** | **3** | **58** | **3** | **R.LALAKAWPTVLQTGTR.G** |
|  | 16574 | **872.4165** | **1742.8184** | **1742.8298** | **-0.0113** | **0** | **80** | **1.1e-006** | **1** | **R.AAFGLSEAGFNTACVTK.L** 16575 |
|  | 17492 | **906.4375** | **1810.8604** | **1810.8737** | **-0.0133** | **0** | **65** | **3.2e-005** | **1** | **R.IDEYDYSKPIQGQQK.K** 17493 |
|  | 18372 | **626.3936** | **1876.1588** | **1876.8989** | **-0.7401** | **1** | **0** | **69** | **1** | **K.TLNEADCATVPPAIRSY.-** |
|  | 20583 | **1034.0219** | **2066.0291** | **2066.0432** | **-0.0141** | **1** | **53** | **0.00043** | **1** | **K.VRIDEYDYSKPIQGQQK.K** |
|  | 22040 | **741.4453** | **2221.3141** | **2222.1729** | **-0.8588** | **1** | **1** | **46** | **8** | **K.VPPIKPNAGEESVMNLDKLR.F** |
|  | 22386 | **1132.9912** | **2263.9679** | **2263.9838** | **-0.0159** | **0** | **100** | **7.6e-009** | **1** | **R.TYFSCTSAHTSTGDGTAMITR.A** 22387 22388 |
|  | 22992 | **780.7354** | **2339.1842** | **2339.1984** | **-0.0142** | **0** | **1** | **63** | **2** | **K.EPIPVLPTVHYNMGGIPTNYK.G** 22994 |
|  | 23691 | **814.3746** | **2440.1019** | **2440.1189** | **-0.0171** | **0** | **(6)** | **16** | **1** | **R.SHTVAAQGGINAALGNMEEDNWR.W** |
|  | 23843 | **819.4083** | **2455.2032** | **2456.1139** | **-0.9107** | **0** | **8** | **11** | **1** | **R.SHTVAAQGGINAALGNMEEDNWR.W** |
|  | 25871 | **699.6015** | **2794.3769** | **2794.3973** | **-0.0204** | **1** | **29** | **0.083** | **1** | **R.GCGPEKDHVYLQLHHLPPEQLATR.L** 25870 25872 25873 25874 |
|  | 26588 | **972.8173** | **2915.4301** | **2914.4461** | **0.9840** | **0** | **58** | **8.1e-005** | **1** | **K.VSDSISAQYPVVDHEFDAVVVGAGGAGLR.A** 26580 |
|  | 26810 | **984.4531** | **2950.3376** | **2950.3563** | **-0.0187** | **0** | **86** | **1.3e-007** | **1** | **R.HVNGQDQIVPGLYACGEAACASVHGANR.L** 26816 |
|  | 28450 | **1122.8556** | **3365.5449** | **3365.5632** | **-0.0182** | **0** | **18** | **0.58** | **1** | **R.AGLPCQDLEFVQFHPTGIYGAGCLITEGCR.G** 28448 |
|  | 29136 | **723.1508** | **3610.7174** | **3610.7429** | **-0.0255** | **1** | **8** | **5.6** | **1** | **R.ACALSIEESCRPGDKVPPIKPNAGEESVMNLDK.L** 29135 |
|  | 29609 | **1266.5719** | **3796.6939** | **3796.7137** | **-0.0199** | **0** | **58** | **5.4e-005** | **1** | **K.GSDWLGDQDAIHYMTEQAPAAVVELENYGMPFSR.T** 29606 29607 29610 |
|  | 30302 | **1368.6543** | **4102.9411** | **4102.9802** | **-0.0391** | **1** | **1** | **24** | **4** | **K.TFDRGMVWNTDLVETLELQNLMLCALQTIYGAEAR.K** |
|  | 30509 | 1043.2919 | 4169.1384 | 4169.1581 | -0.0197 | 1 | 57 | 4.5e-005 | 1 | R.LPGISETAMIFAGVDVTKEPIPVLPTVHYNMGGIPTNYK.G 30508 30510 30517 30518 30519 |
|  | 30533 | **1047.2921** | **4185.1393** | **4185.1530** | **-0.0137** | **1** | **(12)** | **1.6** | **1** | **R.LPGISETAMIFAGVDVTKEPIPVLPTVHYNMGGIPTNYK.G** |

| **46.** | Q16134\|ETFD_HUMAN    **Mass:** 69262    **Score:** 878    **Queries matched:** 65   **emPAI:** 1.43 |
| --- | --- |
|  | Electron transfer flavoprotein-ubiquinone oxidoreductase, mitochondrial OS=Homo sapiens GN=ETFDH PE=1 SV=1 |

|  | **Query** | **Observed** | **Mr(expt)** | **Mr(calc)** | **Delta** | **Miss** | **Score** | **Expect** | **Rank** | **Peptide** |
| --- | --- | --- | --- | --- | --- | --- | --- | --- | --- | --- |
|  | 814 | **487.0863** | **486.0790** | **486.2438** | **-0.1648** | **0** | **4** | **76** | **4** | **K.DGAPK.A** |
|  | 1099 | **557.0191** | **556.0118** | **555.3744** | **0.6374** | **0** | **18** | **1.3** | **1** | **R.LKPAK.D** 1098 1100 |
|  | 2056 | **404.2315** | **806.4485** | **806.4538** | **-0.0053** | **0** | **25** | **0.65** | **1** | **R.FGILTEK.Y** |
|  | 2191 | **819.4088** | **818.4015** | **818.4075** | **-0.0060** | **0** | **30** | **0.21** | **1** | **K.NSWVWK.E** |
|  | 5065 | **1031.5342** | **1030.5269** | **1030.5335** | **-0.0066** | **0** | **35** | **0.052** | **1** | **K.ELWVIDEK.N** |
|  | 6148 | **558.3569** | **1114.6992** | **1114.5982** | **0.1010** | **0** | **31** | **0.12** | **1** | **K.GIATNDVGIQK.D** 6141 |
|  | 6412 | **568.8393** | **1135.6640** | **1135.6713** | **-0.0073** | **1** | **49** | **0.0023** | **1** | **R.LKQLAVAHEK.D** 6411 |
|  | 8372 | **416.7287** | **1247.1644** | **1246.6856** | **0.4788** | **1** | **17** | **2** | **3** | **K.KNYLPLCAIR.W** |
|  | 8599 | **630.8287** | **1259.6429** | **1259.6510** | **-0.0081** | **0** | **52** | **0.001** | **1** | **R.ALNEGGFQSIPK.L** 8597 |
|  | 8680 | **632.8333** | **1263.6521** | **1263.6612** | **-0.0091** | **0** | **23** | **0.78** | **1** | **R.ITTHYTIYPR.D** 8689 |
|  | 8759 | **635.3206** | **1268.6266** | **1268.6361** | **-0.0095** | **0** | **37** | **0.031** | **1** | **K.GAPLNTPVTEDR.F** 8760 |
|  | 9158 | **646.3132** | **1290.6118** | **1290.6204** | **-0.0086** | **0** | **30** | **0.15** | **1** | **R.NLSIYDGPEQR.F** |
|  | 9194 | **647.3331** | **1292.6517** | **1293.6466** | **-0.9949** | **0** | **22** | **1** | **2** | **R.VDHTVGWPLDR.H** 9209 |
|  | 11996 | **725.8486** | **1449.6826** | **1449.6922** | **-0.0096** | **0** | **52** | **0.00075** | **1** | **R.ANCEPQTYGIGLK.E** 11995 |
|  | 12580 | **742.8534** | **1483.6923** | **1483.7024** | **-0.0100** | **0** | **64** | **5.2e-005** | **1** | **R.LQINAQNCVHCK.T** 12575 12581 12582 |
|  | 13253 | **764.3356** | **1526.6567** | **1525.7736** | **0.8831** | **1** | **9** | **16** | **2** | **K.EKGAPLNTPVTEDR.F** |
|  | 13429 | **513.9262** | **1538.7568** | **1538.7664** | **-0.0096** | **0** | **2** | **72** | **6** | **K.VTIFAEGCHGHLAK.Q** |
|  | 14112 | **396.9712** | **1583.8557** | **1583.8644** | **-0.0088** | **1** | **17** | **2.3** | **1** | **K.HHPSIRPTLEGGKR.I** 14115 14121 |
|  | 14501 | **806.3414** | **1610.6683** | **1609.7745** | **0.8938** | **0** | **6** | **27** | **3** | **K.LSCLAYQCFHALK.I** |
|  | 14836 | **816.4122** | **1630.8099** | **1630.8202** | **-0.0103** | **0** | **43** | **0.006** | **1** | **K.TIGLHVTEYEDNLK.N** 14837 |
|  | 19618 | **992.9920** | **1983.9694** | **1983.9836** | **-0.0142** | **0** | **84** | **3.9e-007** | **1** | **K.AAQIGAHTLSGACLDPGAFK.E** 19617 |
|  | 20937 | **1050.0537** | **2098.0929** | **2098.1059** | **-0.0130** | **0** | **87** | **1.8e-007** | **1** | **R.FAEEADVVIVGAGPAGLSAAVR.L** 20938 20940 |
|  | 20984 | **1053.0438** | **2104.0731** | **2104.0850** | **-0.0119** | **0** | **68** | **1.3e-005** | **1** | **K.LTFPGGLLIGCSPGFMNVPK.I** 20982 20983 20989 |
|  | 21589 | **1087.4979** | **2172.9813** | **2172.9939** | **-0.0126** | **0** | **89** | **1.1e-007** | **1** | **R.FCPAGVYEFVPVEQGDGFR.L** 21588 21590 21591 |
|  | 21959 | **738.7406** | **2213.2000** | **2213.2143** | **-0.0143** | **0** | **18** | **1.3** | **1** | **R.IPVPILPGLPMNNHGNYIVR.L** |
|  | 22290 | **1125.5789** | **2249.1432** | **2249.1539** | **-0.0107** | **0** | **75** | **2.4e-006** | **1** | **K.SGILAAESIFNQLTSENLQSK.T** |
|  | 23165 | **788.4485** | **2362.3236** | **2362.2363** | **0.0873** | **1** | **8** | **11** | **1** | **-.MLVPLAKLSCLAYQCFHALK.I** |
|  | 24405 | **845.4631** | **2533.3674** | **2532.3787** | **0.9887** | **1** | **28** | **0.095** | **1** | **K.YRIPVPILPGLPMNNHGNYIVR.L** |
|  | 26345 | **1438.6443** | **2875.2740** | **2875.2905** | **-0.0165** | **1** | **60** | **5.1e-005** | **1** | **K.TCDIKDPSQNINWVVPEGGGGPAYNGM.-** |
|  | 29947 | **1315.6395** | **3943.8968** | **3943.9091** | **-0.0123** | **0** | **85** | **1e-007** | **1** | **R.LGHLVSWMGEQAEALGVEVYPGYAAAEVLFHDDGSVK.G** 29929 29930 29933 29934 29935 29936 29939 29941 29944 29951 29956 |

| **47.** | P35749\|MYH11_HUMAN    **Mass:** 228054   **Score:** 876    **Queries matched:** 117   **emPAI:** 0.47 |
| --- | --- |
|  | Myosin-11 OS=Homo sapiens GN=MYH11 PE=1 SV=3 |

|  | **Query** | **Observed** | **Mr(expt)** | **Mr(calc)** | **Delta** | **Miss** | **Score** | **Expect** | **Rank** | **Peptide** |
| --- | --- | --- | --- | --- | --- | --- | --- | --- | --- | --- |
|  | 997 | 533.1882 | 532.1809 | 531.3017 | 0.8792 | 0 | 8 | 42 | 1 | R.IGQSK.I 996 1000 1001 1002 1004 1005 |
|  | 1008 | 533.3640 | 532.3567 | 531.2877 | 1.0690 | 1 | 8 | 44 | 1 | R.SGGRR.V |
|  | 1167 | 572.9950 | 571.9877 | 571.3806 | 0.6072 | 1 | 12 | 10 | 2 | R.IKVGR.D 1173 |
|  | 1214 | 586.2972 | 585.2900 | 585.2944 | -0.0045 | 0 | 17 | 3.7 | 1 | K.MNPPK.F |
|  | 1279 | **603.3430** | **602.3357** | **601.3184** | **1.0173** | **0** | **18** | **5.1** | **6** | **R.DLQAR.D** |
|  | 1295 | 609.3199 | 608.3127 | 608.3170 | -0.0043 | 0 | 26 | 0.38 | 1 | K.SFVEK.L 1304 |
|  | 1318 | **615.3780** | **614.3707** | **614.3752** | **-0.0044** | **0** | **11** | **20** | **2** | **R.VLGQAK.Q** |
|  | 1367 | 630.3886 | 629.3813 | 629.3860 | -0.0047 | 0 | 19 | 3.7 | 1 | K.ALSLAR.A |
|  | 1456 | 653.3032 | 652.2959 | 653.2843 | -0.9884 | 0 | 13 | 7.2 | 8 | K.GFMDGK.Q |
|  | 1631 | 719.3886 | 718.3813 | 717.4207 | 0.9606 | 1 | 18 | 3.3 | 2 | K.MQLAKK.E |
|  | 1665 | 735.0493 | 734.0420 | 733.3792 | 0.6628 | 0 | 3 | 73 | 1 | K.LQAQMK.D |
|  | 2062 | 404.7288 | 807.4430 | 807.4491 | -0.0061 | 0 | 21 | 1.2 | 1 | R.TVGQLYK.E |
|  | 2179 | **409.7104** | **817.4063** | **816.3977** | **1.0086** | **0** | **14** | **8** | **1** | **R.NALQDEK.R** 2175 |
|  | 2530 | 423.2321 | 844.4496 | 845.4793 | -1.0297 | 1 | 9 | 27 | 9 | R.KLQAQMK.D 2546 2548 |
|  | 2750 | 863.0858 | 862.0785 | 861.4742 | 0.6043 | 1 | (0) | 1.3e+002 | 7 | R.KLQAQMK.D |
|  | 3482 | **458.7429** | **915.4712** | **915.4774** | **-0.0062** | **1** | **16** | **4.7** | **3** | **R.ARAELNDK.V** 3509 |
|  | 3524 | 460.2686 | 918.5226 | 918.3930 | 0.1296 | 0 | 12 | 10 | 6 | R.LEEEEDR.G |
|  | 3594 | **463.2467** | **924.4788** | **923.4097** | **1.0692** | **1** | **13** | **7.8** | **1** | **K.YADERDR.A** |
|  | 3667 | **466.2373** | **930.4600** | **929.5182** | **0.9418** | **0** | **14** | **7.2** | **2** | **R.QLVSNLEK.K** 3645 3646 3660 |
|  | 3774 | **470.2359** | **938.4572** | **937.5022** | **0.9551** | **0** | **4** | **56** | **8** | **R.IVFQEFR.Q** |
|  | 3894 | 474.2494 | 946.4843 | 946.4906 | -0.0063 | 0 | 30 | 0.18 | 1 | R.QQQLTAMK.V |
|  | 4195 | 485.2889 | 968.5632 | 968.5001 | 0.0631 | 1 | 21 | 1.3 | 1 | K.IVDMYKGK.K |
|  | 4698 | **502.2659** | **1002.5172** | **1001.4890** | **1.0283** | **1** | **3** | **94** | **9** | **K.EQAEKGNAR.V** |
|  | 4969 | 513.2107 | 1024.4068 | 1023.5237 | 0.8832 | 1 | 6 | 35 | 7 | K.ATDKSFVEK.L |
|  | 5254 | 523.2856 | 1044.5567 | 1044.5637 | -0.0070 | 0 | 28 | 0.29 | 2 | R.LEVNMQALK.G |
|  | 5283 | **524.2865** | **1046.5584** | **1045.5152** | **1.0433** | **1** | **8** | **26** | **1** | **R.ELEDARASR.D** |
|  | 5382 | **529.2843** | **1056.5540** | **1057.6131** | **-1.0591** | **1** | **3** | **87** | **9** | **R.QLVSNLEKK.Q** |
|  | 5767 | **544.2890** | **1086.5634** | **1086.5305** | **0.0329** | **0** | **2** | **1.2e+002** | **4** | **K.ENADLAGELR.V** |
|  | 5998 | 552.2991 | 1102.5836 | 1102.5917 | -0.0081 | 1 | 33 | 0.082 | 1 | K.RQQQLTAMK.V 6000 |
|  | 6054 | **554.7529** | **1107.4913** | **1107.5118** | **-0.0205** | **0** | **7** | **32** | **9** | **K.AEMEDLVSSK.D** |
|  | 6171 | 559.2921 | 1116.5697 | 1115.5710 | 0.9987 | 0 | 11 | 13 | 2 | R.DLGEELEALK.T |
|  | 6647 | **577.6002** | **1153.1859** | **1152.5332** | **0.6527** | **0** | **2** | **63** | **2** | **K.MTESSLPSASK.T** |
|  | 7062 | 593.2817 | 1184.5488 | 1184.5859 | -0.0371 | 1 | 4 | 70 | 8 | K.DDIQKMNPPK.F |
|  | 7682 | **607.7783** | **1213.5421** | **1213.5509** | **-0.0089** | **0** | **46** | **0.0037** | **1** | **R.SHEAQVQEMR.Q** |
|  | 7815 | 610.8254 | 1219.6363 | 1219.6448 | -0.0085 | 1 | 56 | 0.00035 | 1 | R.KFDQLLAEEK.N 7814 |
|  | 7974 | **614.8170** | **1227.6194** | **1227.6281** | **-0.0088** | **1** | **37** | **0.033** | **1** | **R.SKLHEMEGAVK.S** 7945 |
|  | 8011 | **615.7865** | **1229.5584** | **1228.7027** | **0.8558** | **1** | **2** | **1.1e+002** | **7** | **K.LQLEKVTAEAK.I** |
|  | 8325 | **623.3716** | **1244.7286** | **1243.6230** | **1.1056** | **1** | **(3)** | **75** | **7** | **R.SKLHEMEGAVK.S** |
|  | 9152 | **431.0844** | **1290.2315** | **1290.6456** | **-0.4141** | **0** | **4** | **40** | **4** | **K.EQADFAVEALAK.A** 9153 |
|  | 9445 | **436.1963** | **1305.5670** | **1304.7122** | **0.8548** | **1** | **5** | **45** | **7** | **K.EQLGKLMTTLR.N** |
|  | 9948 | **666.3349** | **1330.6552** | **1329.6776** | **0.9777** | **1** | **6** | **39** | **9** | **K.AENELKELEQK.H** |
|  | 10522 | 682.8611 | 1363.7076 | 1364.6242 | -0.9166 | 1 | 9 | 19 | 5 | -.MAQKGQLSDDEK.F 10520 10523 |
|  | 10660 | **457.7708** | **1370.2906** | **1370.7405** | **-0.4499** | **1** | **8** | **13** | **6** | **K.EILLQVEDERK.M** |
|  | 10860 | **691.8444** | **1381.6742** | **1381.6837** | **-0.0096** | **1** | **0** | **1.3e+002** | **9** | **K.DDVGKNVHELEK.S** |
|  | 11180 | **700.8856** | **1399.7567** | **1399.7671** | **-0.0104** | **1** | **48** | **0.0023** | **1** | **K.KLEAQVQELQSK.C** |
|  | 11417 | **707.8890** | **1413.7635** | **1413.7980** | **-0.0345** | **0** | **22** | **0.91** | **2** | **K.VIQYLAVVASSHK.G** |
|  | 12582 | 495.5716 | 1483.6930 | 1483.8246 | -0.1315 | 1 | 8 | 19 | 7 | K.LKEILLQVEDER.K 12581 |
|  | 12665 | **497.2346** | **1488.6819** | **1489.7260** | **-1.0440** | **0** | **4** | **54** | **4** | **K.EELAEELASSLSGR.N** |
|  | 12878 | **501.7809** | **1502.3209** | **1502.6961** | **-0.3752** | **1** | **4** | **36** | **2** | **K.RQLEEAEEESQR.I** |
|  | 13198 | **762.3596** | **1522.7046** | **1522.7151** | **-0.0105** | **0** | **52** | **0.00086** | **1** | **K.EIENLTQQYEEK.A** |
|  | 13476 | **771.8861** | **1541.7577** | **1541.7685** | **-0.0108** | **0** | **66** | **2.8e-005** | **1** | **K.IAQLEEQVEQEAR.E** |
|  | 13478 | **771.8914** | **1541.7682** | **1541.8929** | **-0.1248** | **1** | **4** | **49** | **6** | **K.KVIQYLAVVASSHK.G** |
|  | 13620 | 776.3459 | 1550.6772 | 1550.6882 | -0.0110 | 0 | 11 | 9.5 | 1 | R.EDQSILCTGESGAGK.T |
|  | 14218 | 796.3485 | 1590.6825 | 1590.6944 | -0.0119 | 0 | 74 | 5.1e-006 | 1 | R.NTDQASMPDNTAAQK.V 14217 |
|  | 14487 | **537.2858** | **1608.8355** | **1608.8471** | **-0.0116** | **1** | **9** | **17** | **3** | **R.TGVLAHLEEERDLK.I** |
|  | 14689 | **541.5734** | **1621.6983** | **1621.7505** | **-0.0523** | **1** | **(0)** | **1e+002** | **7** | **K.AEMEDLVSSKDDVGK.N** |
|  | 14841 | **816.4767** | **1630.9388** | **1629.9818** | **0.9570** | **1** | **1** | **86** | **10** | **K.VVSSVLQLGNIVFKK.E** |
|  | 14930 | **546.6193** | **1636.8361** | **1637.7454** | **-0.9093** | **1** | **4** | **49** | **1** | **K.AEMEDLVSSKDDVGK.N** |
|  | 15037 | **548.7964** | **1643.3673** | **1642.7881** | **0.5792** | **1** | **2** | **55** | **5** | **K.GFMDGKQACILMIK.A** |
|  | 15320 | **554.9373** | **1661.7900** | **1661.8018** | **-0.0119** | **0** | **19** | **1.5** | **1** | **K.VCHLMGINVTDFTR.S** |
|  | 15325 | **831.9429** | **1661.8713** | **1661.8559** | **0.0154** | **1** | **8** | **17** | **4** | **R.LEVNMQALKGQFER.D** 15324 |
|  | 16324 | 863.9725 | 1725.9305 | 1725.9413 | -0.0108 | 0 | 26 | 0.25 | 1 | K.QLLQANPILEAFGNAK.T 16312 |
|  | 16667 | **438.4664** | **1749.8364** | **1750.8964** | **-1.0600** | **1** | **9** | **15** | **1** | **K.HLPIYSEKIVDMYK.G** |
|  | 16732 | **585.6258** | **1753.8555** | **1753.8668** | **-0.0113** | **1** | **20** | **1.2** | **1** | **K.KQELEEILHEMEAR.L** |
|  | 16892 | **588.7871** | **1763.3395** | **1763.8941** | **-0.5546** | **1** | **2** | **52** | **7** | **K.KLQDFASTVEALEEGK.K** |
|  | 16976 | 591.6310 | 1771.8713 | 1771.8840 | -0.0127 | 1 | 4 | 49 | 5 | K.EEKGDEVVVELVENGK.K 16967 |
|  | 17238 | **895.4464** | **1788.8781** | **1787.8649** | **1.0132** | **0** | **63** | **5.8e-005** | **1** | **K.ATQQAEQLSNELATER.S** |
|  | 18812 | **957.0059** | **1911.9972** | **1911.9538** | **0.0434** | **0** | **16** | **2.5** | **2** | **R.LQQELDDLVVDLDNQR.Q** 18824 |
|  | 18853 | **958.9804** | **1915.9463** | **1915.9599** | **-0.0136** | **1** | **106** | **2.5e-009** | **1** | **R.KATQQAEQLSNELATER.S** 18854 |
|  | 19215 | **974.4641** | **1946.9135** | **1946.9255** | **-0.0119** | **0** | **88** | **1.7e-007** | **1** | **K.LQNEVESVTGMLNEAEGK.A** |
|  | 19380 | 981.4560 | 1960.8974 | 1960.9113 | -0.0138 | 0 | 90 | 9.9e-008 | 1 | K.TQLEELEDELQATEDAK.L |
|  | 19842 | **1001.9401** | **2001.8656** | **2002.9881** | **-1.1225** | **1** | **3** | **41** | **3** | **K.LEDEILVMDDQNNKLSK.E** |
|  | 19845 | **1001.9858** | **2001.9570** | **2001.9677** | **-0.0107** | **0** | **77** | **1.8e-006** | **1** | **K.SLEADLMQLQEDLAAAER.A** |
|  | 20010 | **673.3554** | **2017.0444** | **2017.9626** | **-0.9182** | **0** | **(0)** | **85** | **8** | **K.SLEADLMQLQEDLAAAER.A** |
|  | 20161 | 676.6939 | 2027.0597 | 2027.9330 | -0.8733 | 1 | 6 | 21 | 7 | K.ALDEETRSHEAQVQEMR.Q 20168 |
|  | 22373 | 1132.0070 | 2261.9994 | 2262.0143 | -0.0150 | 0 | 52 | 0.00052 | 1 | K.MAQQMLDLEEQLEEEEAAR.Q |
|  | 22534 | **761.3460** | **2281.0160** | **2281.0314** | **-0.0154** | **1** | **52** | **0.00049** | **1** | **R.SMLQDREDQSILCTGESGAGK.T** |
|  | 22787 | **772.3733** | **2314.0982** | **2313.1093** | **0.9889** | **0** | **39** | **0.0088** | **1** | **K.VEDMAELTCLNEASVLHNLR.E** |
|  | 23384 | **1195.5802** | **2389.1458** | **2389.1609** | **-0.0150** | **0** | **45** | **0.0023** | **1** | **K.DVASLSSQLQDTQELLQEETR.Q** 23383 |
|  | 23402 | **797.9668** | **2390.8786** | **2390.1093** | **0.7693** | **1** | **1** | **46** | **10** | **K.KMAQQMLDLEEQLEEEEAAR.Q** |
|  | 23925 | 1234.0996 | 2466.1847 | 2466.2002 | -0.0155 | 0 | 88 | 1.2e-007 | 1 | K.LQQLFNHTMFILEQEEYQR.E 23924 23932 |
|  | 24453 | **848.6993** | **2543.0762** | **2542.1492** | **0.9269** | **1** | **0** | **60** | **3** | **K.QELEEILHEMEARLEEEEDR.G** |
|  | 26550 | **969.8068** | **2906.3986** | **2906.4120** | **-0.0134** | **1** | **23** | **0.27** | **1** | **K.NMDPLNDNVTSLLNASSDKFVADLWK.D** |
|  | 27734 | **1051.4813** | **3151.4221** | **3151.4424** | **-0.0203** | **1** | **63** | **2.3e-005** | **1** | **R.ALETQMEEMKTQLEELEDELQATEDAK.L** 27735 |

| **48.** | Q9NY65\|TBA8_HUMAN    **Mass:** 50746    **Score:** 833    **Queries matched:** 46   **emPAI:** 1.18 |
| --- | --- |
|  | Tubulin alpha-8 chain OS=Homo sapiens GN=TUBA8 PE=1 SV=1 |

|  | **Query** | **Observed** | **Mr(expt)** | **Mr(calc)** | **Delta** | **Miss** | **Score** | **Expect** | **Rank** | **Peptide** |
| --- | --- | --- | --- | --- | --- | --- | --- | --- | --- | --- |
|  | 1311 | 614.3460 | 613.3387 | 613.3435 | -0.0049 | 0 | 11 | 14 | 1 | R.GDVVPK.D |
|  | 4835 | 507.7253 | 1013.4360 | 1012.5917 | 0.8443 | 0 | 18 | 2.4 | 3 | K.DVNVAIAAIK.T |
|  | 4948 | 512.2609 | 1022.5072 | 1022.4417 | 0.0655 | 0 | 11 | 14 | 2 | K.EDAANNYAR.G |
|  | 9326 | 434.2335 | 1299.6786 | 1298.4916 | 1.1870 | 0 | 6 | 33 | 7 | K.YMACCMLYR.G |
|  | 10814 | 460.9011 | 1379.6815 | 1379.6907 | -0.0092 | 1 | 31 | 0.11 | 1 | R.LDHKFDLMYAK.R |
|  | 11350 | 705.8859 | 1409.7573 | 1409.7667 | -0.0094 | 0 | 6 | 31 | 1 | R.QLFHPEQLITGK.E 11351 |
|  | 12641 | 744.4393 | 1486.8641 | 1486.8719 | -0.0078 | 0 | 83 | 6.7e-007 | 1 | R.LISQIVSSITASLR.F 12628 12639 12642 12644 12646 |
|  | 14334 | 799.8823 | 1597.7500 | 1597.7599 | -0.0100 | 0 | 23 | 0.64 | 2 | R.TIQFVDWCPTGFK.V |
|  | 14472 | **804.8864** | **1607.7583** | **1607.9247** | **-0.1664** | **1** | **4** | **43** | **2** | **R.GDVVPKDVNVAIAAIK.T** |
|  | 15638 | **562.5958** | **1684.7657** | **1684.8706** | **-0.1049** | **0** | **(10)** | **12** | **2** | **R.AVMIDLEPTVVDEVR.A** 15636 |
|  | 15935 | 851.4510 | 1700.8874 | 1700.8655 | 0.0219 | 0 | 14 | 4 | 2 | R.AVMIDLEPTVVDEVR.A 15936 |
|  | 16223 | 573.9514 | 1718.8322 | 1717.8747 | 0.9575 | 0 | 4 | 47 | 7 | R.NLDIERPTYTNLNR.L |
|  | 17369 | **901.3199** | **1800.6253** | **1800.9370** | **-0.3117** | **1** | **0** | **71** | **4** | **R.GHYTVGKESIDLVLDR.I** |
|  | 17643 | 912.9902 | 1823.9658 | 1823.9782 | -0.0124 | 0 | 59 | 0.00015 | 1 | K.VGINYQPPTVVPGGDLAK.V 17640 17641 17653 |
|  | 18197 | 932.9499 | 1863.8853 | 1863.8971 | -0.0118 | 0 | 82 | 6.5e-007 | 1 | R.AVCMLSNTTAIAEAWAR.L 18198 |
|  | 19338 | **653.3392** | **1956.9957** | **1958.0374** | **-1.0417** | **1** | **2** | **59** | **7** | **R.AGTYRQLFHPEQLITGK.E** 19351 |
|  | 22378 | 755.3527 | 2263.0362 | 2263.1201 | -0.0840 | 1 | 5 | 22 | 3 | K.VQRAVCMLSNTTAIAEAWAR.L |
|  | 22917 | 777.6732 | 2329.9978 | 2329.0110 | 0.9869 | 0 | 37 | 0.014 | 1 | R.AFVHWYVGEGMEEGEFSEAR.E |
|  | 23508 | 1205.1007 | 2408.1869 | 2408.2012 | -0.0144 | 0 | 116 | 1.9e-010 | 1 | R.FDGALNVDLTEFQTNLVPYPR.I 23503 23504 23505 23506 23507 23509 23510 23512 23515 |
|  | 23544 | 805.7346 | 2414.1819 | 2414.1978 | -0.0160 | 1 | 41 | 0.0058 | 1 | R.QLFHPEQLITGKEDAANNYAR.G 23545 |
|  | 31526 | 1380.1462 | 5516.5559 | 5516.5891 | -0.0332 | 0 | 2 | 6.6 | 2 | K.LEFAIYPAPQVSTAVVEPYNSILTTHTTLEHSDCAFMVDNEAIYDICR.R |
|  | 31737 | 1147.3422 | 5731.6744 | 5731.7161 | -0.0416 | 1 | 11 | 0.85 | 1 | K.SKLEFAIYPAPQVSTAVVEPYNSILTTHTTLEHSDCAFMVDNEAIYDICR.R 31738 |

| **49.** | P02671\|FIBA_HUMAN    **Mass:** 95656    **Score:** 833    **Queries matched:** 65   **emPAI:** 0.98 |
| --- | --- |
|  | Fibrinogen alpha chain OS=Homo sapiens GN=FGA PE=1 SV=2 |

|  | **Query** | **Observed** | **Mr(expt)** | **Mr(calc)** | **Delta** | **Miss** | **Score** | **Expect** | **Rank** | **Peptide** |
| --- | --- | --- | --- | --- | --- | --- | --- | --- | --- | --- |
|  | 1478 | **659.4036** | **658.3964** | **657.2970** | **1.0994** | **0** | **10** | **28** | **4** | **K.SSSYSK.Q** |
|  | 2013 | **402.7151** | **803.4157** | **803.4211** | **-0.0054** | **0** | **13** | **11** | **1** | **R.AQLVDMK.R** |
|  | 3629 | **464.7739** | **927.5332** | **927.5389** | **-0.0057** | **0** | **18** | **2.8** | **1** | **K.QLEQVIAK.D** |
|  | 4169 | **484.2495** | **966.4843** | **966.4155** | **0.0688** | **0** | **8** | **26** | **1** | **R.GDFSSANNR.D** |
|  | 4303 | **976.5506** | **975.5433** | **976.4872** | **-0.9439** | **1** | **8** | **30** | **6** | **R.GSCSRALAR.E** |
|  | 4790 | **505.7563** | **1009.4980** | **1009.5040** | **-0.0060** | **0** | **50** | **0.0014** | **1** | **K.VTSGSTTTTR.R** 4772 4788 4789 4792 4793 4794 |
|  | 5013 | **514.7521** | **1027.4896** | **1027.4974** | **-0.0078** | **0** | **10** | **17** | **4** | **K.NSLFEYQK.N** |
|  | 5951 | **550.8161** | **1099.6176** | **1099.6237** | **-0.0061** | **1** | **10** | **16** | **3** | **K.RLEVDIDIK.I** |
|  | 6038 | **553.8339** | **1105.6533** | **1105.6608** | **-0.0075** | **0** | **31** | **0.14** | **1** | **K.VQHIQLLQK.N** |
|  | 7156 | **595.3039** | **1188.5932** | **1189.5364** | **-0.9431** | **0** | **3** | **82** | **9** | **K.QFTSSTSYNR.G** |
|  | 7188 | **397.4026** | **1189.1860** | **1188.6285** | **0.5576** | **1** | **1** | **88** | **8** | **K.NVRAQLVDMK.R** |
|  | 8712 | **633.9261** | **1265.8376** | **1266.6416** | **-0.8039** | **1** | **3** | **60** | **7** | **K.EKVTSGSTTTTR.R** |
|  | 8762 | **635.3411** | **1268.6676** | **1268.6765** | **-0.0089** | **1** | **34** | **0.061** | **1** | **K.LKNSLFEYQK.N** |
|  | 10815 | **690.8732** | **1379.7319** | **1379.7409** | **-0.0090** | **1** | **39** | **0.017** | **1** | **K.TVIGPDGHKEVTK.E** |
|  | 11859 | **721.3924** | **1440.7702** | **1440.7799** | **-0.0097** | **0** | **23** | **0.69** | **1** | **K.MKPVPDLVPGNFK.S** |
|  | 12844 | **751.3691** | **1500.7237** | **1500.7354** | **-0.0117** | **0** | **34** | **0.053** | **1** | **R.MELERPGGNEITR.G** 12843 12845 |
|  | 12953 | **754.8351** | **1507.6557** | **1508.6994** | **-1.0437** | **1** | **4** | **45** | **2** | **R.EVDLKDYEDQQK.Q** |
|  | 13113 | **759.8256** | **1517.6366** | **1516.7304** | **0.9062** | **0** | **(2)** | **81** | **2** | **R.MELERPGGNEITR.G** |
|  | 13919 | **786.8373** | **1571.6600** | **1571.6700** | **-0.0100** | **0** | **37** | **0.025** | **1** | **R.GGSTSYGTGSETESPR.N** |
|  | 14791 | **815.4125** | **1628.8105** | **1628.8192** | **-0.0087** | **0** | **45** | **0.0039** | **1** | **K.DSHSLTTNIMEILR.G** |
|  | 14922 | **546.5903** | **1636.7490** | **1636.7594** | **-0.0104** | **0** | **26** | **0.29** | **1** | **K.ESSSHHPGIAEFPSR.G** |
|  | 15066 | **549.3212** | **1644.9417** | **1644.8141** | **0.1276** | **0** | **(2)** | **70** | **7** | **K.DSHSLTTNIMEILR.G** |
|  | 16403 | **865.8715** | **1729.7285** | **1729.7404** | **-0.0119** | **1** | **28** | **0.15** | **1** | **R.GDFSSANNRDNTYNR.V** |
|  | 18311 | **936.4531** | **1870.8917** | **1871.7592** | **-0.8675** | **0** | **5** | **30** | **1** | **K.MADEAGSEADHEGTHSTK.R** 18310 |
|  | 18473 | **943.9355** | **1885.8564** | **1885.8707** | **-0.0143** | **1** | **70** | **1.1e-005** | **1** | **R.HRHPDEAAFFDTASTGK.T** 18474 |
|  | 18516 | **630.3777** | **1888.1114** | **1887.7541** | **0.3573** | **0** | **(2)** | **55** | **7** | **K.MADEAGSEADHEGTHSTK.R** |
|  | 19393 | **982.3688** | **1962.7230** | **1962.7367** | **-0.0137** | **0** | **11** | **6.4** | **1** | **K.DSDWPFCSDEDWNYK.C** |
|  | 19395 | **982.4216** | **1962.8287** | **1962.8416** | **-0.0129** | **0** | **112** | **6.1e-010** | **1** | **R.NPSSAGSWNSGSSGPGSTGNR.N** 19394 |
|  | 19637 | **993.5009** | **1984.9873** | **1985.0000** | **-0.0127** | **1** | **66** | **2.6e-005** | **1** | **K.NNKDSHSLTTNIMEILR.G** 19636 |
|  | 20164 | **676.9550** | **2027.8431** | **2027.8603** | **-0.0172** | **1** | **19** | **1.1** | **1** | **K.MADEAGSEADHEGTHSTKR.G** |
|  | 20262 | **1021.4513** | **2040.8880** | **2040.9025** | **-0.0145** | **1** | **66** | **2.2e-005** | **1** | **K.QFTSSTSYNRGDSTFESK.S** 20261 |
|  | 20302 | **1023.2681** | **2044.5216** | **2043.8552** | **0.6664** | **1** | **(5)** | **20** | **9** | **K.MADEAGSEADHEGTHSTKR.G** |
|  | 22390 | **1133.0243** | **2264.0340** | **2264.0460** | **-0.0120** | **0** | **42** | **0.0046** | **1** | **K.TFPGFFSPMLGEFVSETESR.G** 22389 22392 |
|  | 22530 | **1141.0216** | **2280.0287** | **2280.0409** | **-0.0122** | **0** | **(15)** | **2.7** | **1** | **K.TFPGFFSPMLGEFVSETESR.G** 22531 |
|  | 24464 | **849.3928** | **2545.1565** | **2544.2247** | **0.9318** | **1** | **3** | **34** | **2** | **K.ALTDMPQMRMELERPGGNEITR.G** |
|  | 24606 | **854.4677** | **2560.3811** | **2560.2196** | **0.1615** | **1** | **(2)** | **40** | **1** | **K.ALTDMPQMRMELERPGGNEITR.G** |
|  | 24715 | **645.0296** | **2576.0893** | **2576.2145** | **-0.1252** | **1** | **(1)** | **48** | **5** | **K.ALTDMPQMRMELERPGGNEITR.G** |
|  | 25164 | **892.3505** | **2674.0296** | **2674.0489** | **-0.0194** | **1** | **51** | **0.00032** | **1** | **R.HQSACKDSDWPFCSDEDWNYK.C** |
|  | 27430 | **768.8466** | **3071.3574** | **3070.3898** | **0.9676** | **1** | **5** | **13** | **1** | **R.MELERPGGNEITRGGSTSYGTGSETESPR.N** |
|  | 27475 | **1028.4623** | **3082.3650** | **3081.5594** | **0.8057** | **1** | **0** | **47** | **10** | **K.LPGSSKIFSVYCDQETSLGGWLLIQQR.M** |
|  | 27635 | **1564.6957** | **3127.3768** | **3127.3962** | **-0.0194** | **0** | **112** | **3.1e-010** | **1** | **K.EVVTSEDGSDCPEAMDLGTLSGIGTLDGFR.H** 27634 27637 |
|  | 28849 | **1173.1682** | **3516.4828** | **3516.5010** | **-0.0182** | **1** | **76** | **8e-007** | **1** | **R.GGSTSYGTGSETESPRNPSSAGSWNSGSSGPGSTGNR.N** |
|  | 29060 | **1195.5802** | **3583.7188** | **3584.6499** | **-0.9311** | **1** | **32** | **0.023** | **1** | **K.EVTKEVVTSEDGSDCPEAMDLGTLSGIGTLDGFR.H** |
|  | 31543 | **1384.3579** | **5533.4025** | **5533.4413** | **-0.0387** | **0** | **16** | **0.19** | **1** | **R.NPGSSGTGGTATWKPGSSGPGSTGSWNSGSSGTGSTGNQNPGSPRPGSTGTWNPGSSER.G** |
|  | 31816 | **1195.7242** | **5973.5848** | **5973.6261** | **-0.0413** | **0** | **11** | **0.55** | **1** | **R.GSAGHWTSESSVSGSTGQWHSESGSFRPDSPGSGNARPNNPDWGTFEEVSGNVSPGTR.R** 31815 31817 |

| **50.** | Q16822\|PCKGM_HUMAN    **Mass:** 71447    **Score:** 830    **Queries matched:** 54   **emPAI:** 2.05 |
| --- | --- |
|  | Phosphoenolpyruvate carboxykinase [GTP], mitochondrial OS=Homo sapiens GN=PCK2 PE=1 SV=2 |

|  | **Query** | **Observed** | **Mr(expt)** | **Mr(calc)** | **Delta** | **Miss** | **Score** | **Expect** | **Rank** | **Peptide** |
| --- | --- | --- | --- | --- | --- | --- | --- | --- | --- | --- |
|  | 1414 | **641.3686** | **640.3613** | **640.3656** | **-0.0043** | **0** | **33** | **0.058** | **1** | **K.GAQLPR.I** |
|  | 1874 | **397.8542** | **793.6939** | **793.4269** | **0.2670** | **1** | **11** | **8.7** | **1** | **K.KCFALR.I** |
|  | 3297 | **451.7477** | **901.4807** | **900.4665** | **1.0143** | **1** | **12** | **12** | **4** | **R.TDPKDVAR.V** |
|  | 4070 | **480.7267** | **959.4389** | **959.4461** | **-0.0071** | **0** | **26** | **0.45** | **1** | **R.DFVEHSAR.L** 4057 |
|  | 4649 | **500.7890** | **999.5634** | **999.5713** | **-0.0079** | **0** | **17** | **3.4** | **2** | **K.TVIVTPSQR.D** 4633 |
|  | 5056 | 515.8076 | 1029.6006 | 1029.5455 | 0.0551 | 0 | 23 | 0.78 | 3 | K.EGALDLSGLR.A 5032 |
|  | 5071 | **516.3112** | **1030.6079** | **1031.4996** | **-0.8917** | **1** | **33** | **0.082** | **1** | **R.RLEGEDSAR.E** |
|  | 5895 | **549.2511** | **1096.4876** | **1095.4920** | **0.9957** | **0** | **39** | **0.017** | **1** | **K.YNNCWLAR.T** 5893 |
|  | 6054 | 554.7529 | 1107.4913 | 1107.4985 | -0.0072 | 0 | 25 | 0.49 | 1 | K.DFWEQEVR.D |
|  | 6159 | **559.0585** | **1116.1025** | **1116.5862** | **-0.4837** | **1** | **2** | **66** | **3** | **R.VLDWICRR.L** |
|  | 6398 | **568.3110** | **1134.6074** | **1134.6146** | **-0.0072** | **0** | **24** | **0.63** | **1** | **K.TLIGHVPDQR.E** |
|  | 8798 | **636.3419** | **1270.6692** | **1270.6768** | **-0.0077** | **0** | **62** | **9.3e-005** | **1** | **K.EVLAELEALER.R** |
|  | 9991 | **667.3677** | **1332.7209** | **1332.7289** | **-0.0080** | **0** | **78** | **2.5e-006** | **1** | **R.AIDTTQLFSLPK.D** |
|  | 11631 | **713.8851** | **1425.7556** | **1424.7987** | **0.9569** | **0** | **8** | **23** | **2** | **R.VLSGDLGQLPTGIR.D** |
|  | 12005 | **725.8729** | **1449.7313** | **1449.7405** | **-0.0092** | **0** | **48** | **0.002** | **1** | **K.GVPLVYEAFNWR.H** 12006 |
|  | 13359 | **767.8866** | **1533.7586** | **1533.7675** | **-0.0088** | **0** | **14** | **5.3** | **1** | **R.SYLTEQVNQDLPK.E** 13372 |
|  | 14129 | **793.4159** | **1584.8172** | **1584.8268** | **-0.0096** | **0** | **41** | **0.011** | **1** | **K.TNLAMMRPALPGWK.V** 14128 14130 |
|  | 14442 | **803.8697** | **1605.7248** | **1605.7358** | **-0.0110** | **0** | **80** | **1.1e-006** | **1** | **R.GQLGNWMSPADFQR.A** 14443 |
|  | 14796 | **815.4407** | **1628.8669** | **1628.8774** | **-0.0105** | **0** | **79** | **1.6e-006** | **1** | **R.LGTPVLQALGDGDFVK.C** 14795 |
|  | 15876 | **849.9326** | **1697.8506** | **1697.8624** | **-0.0119** | **0** | **66** | **2.5e-005** | **1** | **R.EIISFGSGYGGNSLLGK.K** 15877 |
|  | 16054 | **854.9484** | **1707.8822** | **1708.8818** | **-0.9996** | **0** | **41** | **0.0096** | **1** | **R.IGVQLTDSAYVVASMR.I** 16055 16067 16075 16078 |
|  | 17663 | **913.9786** | **1825.9426** | **1825.9574** | **-0.0148** | **1** | **19** | **1.2** | **1** | **R.EIISFGSGYGGNSLLGKK.C** |
|  | 19105 | **969.9885** | **1937.9625** | **1937.9743** | **-0.0118** | **0** | **29** | **0.11** | **1** | **R.TMYVLPFSMGPVGSPLSR.I** |
|  | 19343 | **653.6425** | **1957.9056** | **1957.9183** | **-0.0128** | **1** | **21** | **0.75** | **1** | **R.RDEAGHFLWPGFGENAR.V** |
|  | 21277 | **1069.5448** | **2137.0750** | **2137.0878** | **-0.0127** | **0** | **99** | **1e-008** | **1** | **R.DEGWLAEHMLILGITSPAGK.K** 21280 |
|  | 21362 | **716.7014** | **2147.0824** | **2146.1456** | **0.9368** | **1** | **4** | **35** | **3** | **R.IMTRLGTPVLQALGDGDFVK.C** |
|  | 21628 | **726.3957** | **2176.1652** | **2176.0120** | **0.1533** | **1** | **2** | **54** | **8** | **R.GQLGNWMSPADFQRAVDER.F** |
|  | 23208 | **790.0819** | **2367.2237** | **2366.2343** | **0.9895** | **1** | **11** | **5.9** | **1** | **R.VLSGDLGQLPTGIRDFVEHSAR.L** |
|  | 24043 | **826.7730** | **2477.2970** | **2477.3100** | **-0.0130** | **1** | **26** | **0.18** | **1** | **R.LARDEGWLAEHMLILGITSPAGK.K** |
|  | 25555 | **913.7772** | **2738.3097** | **2738.3196** | **-0.0100** | **0** | **34** | **0.022** | **1** | **R.QCPIMDPAWEAPEGVPIDAIIFGGR.R** |
|  | 25639 | **918.7751** | **2753.3034** | **2754.3146** | **-1.0111** | **0** | **(1)** | **52** | **7** | **R.QCPIMDPAWEAPEGVPIDAIIFGGR.R** |
|  | 25806 | **1394.2156** | **2786.4166** | **2786.4337** | **-0.0171** | **1** | **74** | **2.3e-006** | **1** | **R.SYLTEQVNQDLPKEVLAELEALER.R** 25807 |
|  | 25936 | **935.7681** | **2804.2826** | **2804.3011** | **-0.0185** | **0** | **55** | **0.00017** | **1** | **K.CLHSVGQPLTGQGEPVSQWPCNPEK.T** |
|  | 26522 | **727.3661** | **2905.4353** | **2905.4223** | **0.0129** | **1** | **5** | **17** | **1** | **K.ALCAAAFPSACGKTNLAMMRPALPGWK.V** |
|  | 28775 | **1160.9005** | **3479.6797** | **3479.7025** | **-0.0228** | **0** | **58** | **5.9e-005** | **1** | **R.LCQPEGIHICDGTEAENTATLTLLEQQGLIR.K** 28774 |
|  | 29778 | **961.4943** | **3841.9482** | **3841.8108** | **0.1373** | **1** | **1** | **25** | **1** | **K.IIMHDPFAMRPFFGYNFGHYLEHWLSMEGRK.G** |

| **51.** | P04040\|CATA_HUMAN    **Mass:** 59947    **Score:** 827    **Queries matched:** 55   **emPAI:** 3.79 |
| --- | --- |
|  | Catalase OS=Homo sapiens GN=CAT PE=1 SV=3 |

|  | **Query** | **Observed** | **Mr(expt)** | **Mr(calc)** | **Delta** | **Miss** | **Score** | **Expect** | **Rank** | **Peptide** |
| --- | --- | --- | --- | --- | --- | --- | --- | --- | --- | --- |
|  | 1186 | **579.2696** | **578.2623** | **578.2482** | **0.0141** | **0** | **12** | **12** | **3** | **-.MADSR.D** 1188 |
|  | 4101 | **481.7657** | **961.5168** | **961.5233** | **-0.0065** | **0** | **28** | **0.3** | **1** | **K.DAQIFIQK.K** |
|  | 4181 | **484.7948** | **967.5751** | **967.5815** | **-0.0064** | **0** | **48** | **0.0024** | **1** | **K.LNVITVGPR.G** 4186 |
|  | 6660 | **577.7985** | **1153.5824** | **1153.5914** | **-0.0090** | **0** | **36** | **0.036** | **1** | **R.LCENIAGHLK.D** |
|  | 8929 | **427.1862** | **1278.5367** | **1277.5611** | **0.9755** | **0** | **16** | **3.4** | **1** | **R.HMNGYGSHTFK.L** 8930 8932 |
|  | 9174 | **646.8046** | **1291.5946** | **1291.6044** | **-0.0098** | **0** | **10** | **14** | **1** | **R.LSQEDPDYGIR.D** |
|  | 9205 | **647.8129** | **1293.6112** | **1293.5561** | **0.0551** | **0** | **(1)** | **1.1e+002** | **3** | **R.HMNGYGSHTFK.L** |
|  | 10051 | 669.3255 | 1336.6364 | 1336.6445 | -0.0081 | 0 | 27 | 0.26 | 1 | K.LVNANGEAVYCK.F 10069 |
|  | 11412 | **707.8574** | **1413.7002** | **1413.7100** | **-0.0098** | **0** | **59** | **0.00016** | **1** | **K.ADVLTTGAGNPVGDK.L** |
|  | 12538 | **741.3674** | **1480.7203** | **1480.7310** | **-0.0107** | **0** | **70** | **1.4e-005** | **1** | **R.AFYVNVLNEEQR.K** |
|  | 12730 | **747.3475** | **1492.6804** | **1492.6906** | **-0.0103** | **0** | **67** | **2.3e-005** | **1** | **R.FNTANDDNVTQVR.A** 12731 12739 12741 |
|  | 13287 | **510.2737** | **1527.7993** | **1527.8086** | **-0.0093** | **0** | **17** | **2.3** | **1** | **R.DPILFPSFIHSQK.R** |
|  | 15139 | **550.9353** | **1649.7841** | **1648.7917** | **0.9923** | **1** | **41** | **0.0083** | **1** | **R.RFNTANDDNVTQVR.A** 15121 |
|  | 16052 | **854.9416** | **1707.8686** | **1707.8805** | **-0.0119** | **0** | **74** | **4.8e-006** | **1** | **K.NAIHTFVQSGSHLAAR.E** |
|  | 16094 | **856.3688** | **1710.7230** | **1711.8206** | **-1.0976** | **0** | **26** | **0.27** | **1** | **K.GAGAFGYFEVTHDITK.Y** 16118 16119 |
|  | 17504 | **906.9586** | **1811.9026** | **1811.9141** | **-0.0115** | **0** | **32** | **0.061** | **1** | **R.LGPNYLHIPVNCPYR.A** 17502 17503 |
|  | 18004 | **617.9620** | **1850.8641** | **1850.8759** | **-0.0118** | **1** | **16** | **2.5** | **1** | **R.FSTVAGESGSADTVRDPR.G** 18003 18005 |
|  | 23175 | **1182.6399** | **2363.2652** | **2363.2809** | **-0.0157** | **1** | **111** | **5.2e-010** | **1** | **K.ADVLTTGAGNPVGDKLNVITVGPR.G** 23158 23176 |
|  | 23977 | **825.4047** | **2473.1922** | **2473.2060** | **-0.0139** | **1** | **36** | **0.02** | **1** | **R.GPLLVQDVVFTDEMAHFDRER.I** 23976 |
|  | 24322 | **1259.6000** | **2517.1854** | **2517.1965** | **-0.0111** | **0** | **102** | **4e-009** | **1** | **K.FYTEDGNWDLVGNNTPIFFIR.D** 24323 |
|  | 27099 | **754.3761** | **3013.4753** | **3013.4933** | **-0.0181** | **1** | **28** | **0.075** | **1** | **K.NFTEVHPDYGSHIQALLDKYNAEKPK.N** |
|  | 27812 | **1059.4955** | **3175.4646** | **3175.4808** | **-0.0162** | **0** | **61** | **3.3e-005** | **1** | **R.NPVNYFAEVEQIAFDPSNMPPGIEASPDK.M** 27813 27814 27815 |
|  | 30122 | **1007.7387** | **4026.9257** | **4026.9476** | **-0.0218** | **1** | **48** | **0.00049** | **1** | **R.NPQTHLKDPDMVWDFWSLRPESLHQVSFLFSDR.G** 30120 30121 30123 |
|  | 30124 | **1343.3326** | **4026.9761** | **4026.9945** | **-0.0184** | **1** | **49** | **0.00035** | **1** | **K.FYTEDGNWDLVGNNTPIFFIRDPILFPSFIHSQK.R** 30125 30126 30127 |
|  | 31078 | **1542.9950** | **4625.9632** | **4625.9911** | **-0.0279** | **0** | **51** | **0.00011** | **1** | **R.DGPMCMQDNQGGAPNYYPNSFGAPEQQPSALEHSIQYSGEVR.R** 31079 |
|  | 31176 | **1196.7743** | **4783.0681** | **4782.0922** | **0.9759** | **1** | **17** | **0.32** | **1** | **R.DGPMCMQDNQGGAPNYYPNSFGAPEQQPSALEHSIQYSGEVRR.F** |

| **52.** | Q15084\|PDIA6_HUMAN    **Mass:** 48490    **Score:** 827    **Queries matched:** 41   **emPAI:** 2.53 |
| --- | --- |
|  | Protein disulfide-isomerase A6 OS=Homo sapiens GN=PDIA6 PE=1 SV=1 |

|  | **Query** | **Observed** | **Mr(expt)** | **Mr(calc)** | **Delta** | **Miss** | **Score** | **Expect** | **Rank** | **Peptide** |
| --- | --- | --- | --- | --- | --- | --- | --- | --- | --- | --- |
|  | 1485 | **665.3571** | **664.3498** | **664.3544** | **-0.0046** | **0** | **33** | **0.098** | **1** | **K.IFGSNK.N** 1484 1487 |
|  | 1512 | **676.3567** | **675.3494** | **675.3551** | **-0.0057** | **0** | **14** | **9.8** | **7** | **R.SDIVSR.A** |
|  | 1676 | **737.3516** | **736.3443** | **737.3555** | **-1.0112** | **1** | **8** | **27** | **1** | **R.SDSSSKK.D** |
|  | 3297 | 451.7477 | 901.4807 | 900.5069 | 0.9739 | 1 | 8 | 32 | 8 | R.LTPEWKK.A |
|  | 3713 | **467.2553** | **932.4961** | **932.5039** | **-0.0079** | **1** | **28** | **0.27** | **1** | **R.TRSDIVSR.A** 3710 |
|  | 4847 | **508.2152** | **1014.4158** | **1014.6073** | **-0.1915** | **1** | **12** | **11** | **9** | **K.AATALKDVVK.V** |
|  | 6600 | **576.3086** | **1150.6027** | **1150.4891** | **0.1137** | **0** | **5** | **50** | **8** | **K.GESPVDYDGGR.T** |
|  | 7213 | **596.2759** | **1190.5373** | **1190.5428** | **-0.0055** | **0** | **11** | **12** | **1** | **K.NRPEDYQGGR.T** 7208 7215 |
|  | 10931 | **693.8785** | **1385.7425** | **1385.7514** | **-0.0089** | **0** | **90** | **1.4e-007** | **1** | **R.TGEAIVDAALSALR.Q** 10933 10938 |
|  | 12564 | **742.3574** | **1482.7003** | **1482.7103** | **-0.0100** | **0** | **59** | **0.00015** | **1** | **K.GSFSEQGINEFLR.E** 12565 |
|  | 13043 | **757.8726** | **1513.7306** | **1513.7412** | **-0.0107** | **0** | **67** | **2.2e-005** | **1** | **K.NLEPEWAAAASEVK.E** 13042 |
|  | 13249 | **764.1032** | **1526.1917** | **1526.8416** | **-0.6499** | **0** | **63** | **4e-005** | **1** | **K.LAAVDATVNQVLASR.Y** |
|  | 14587 | **808.4202** | **1614.8258** | **1614.8366** | **-0.0108** | **0** | **63** | **5.9e-005** | **1** | **R.GSTAPVGGGAFPTIVER.E** |
|  | 15392 | **834.3996** | **1666.7846** | **1666.7951** | **-0.0105** | **1** | **77** | **2.4e-006** | **1** | **K.IFQKGESPVDYDGGR.T** 15391 |
|  | 16746 | **878.4720** | **1754.9294** | **1754.0050** | **0.9244** | **1** | **47** | **0.0021** | **1** | **K.VKLAAVDATVNQVLASR.Y** 16745 |
|  | 19828 | **1000.9932** | **1999.9718** | **1999.9850** | **-0.0133** | **1** | **85** | **2.8e-007** | **1** | **K.NLEPEWAAAASEVKEQTK.G** 19829 |
|  | 23107 | 786.0563 | 2355.1470 | 2355.1608 | -0.0138 | 1 | 9 | 8.8 | 1 | R.GSTAPVGGGAFPTIVEREPWDGR.D |
|  | 24748 | **861.1048** | **2580.2926** | **2580.3085** | **-0.0159** | **1** | **94** | **2.6e-008** | **1** | **K.VGAVDADKHHSLGGQYGVQGFPTIK.I** 24745 24747 |
|  | 24995 | **1319.1799** | **2636.3453** | **2636.3585** | **-0.0132** | **0** | **46** | **0.0016** | **1** | **R.ALDLFSDNAPPPELLEIINEDIAK.R** 24996 24998 |
|  | 25036 | **882.7709** | **2645.2910** | **2645.3054** | **-0.0144** | **0** | **41** | **0.0054** | **1** | **R.TCEEHQLCVVAVLPHILDTGAAGR.N** 25035 |
|  | 25653 | **1379.6284** | **2757.2423** | **2757.2604** | **-0.0181** | **1** | **77** | **1.1e-006** | **1** | **R.DGELPVEDDIDLSDVELDDLGKDEL.-** 25655 |
|  | 25846 | **931.8217** | **2792.4432** | **2792.4596** | **-0.0164** | **1** | **63** | **3.1e-005** | **1** | **R.ALDLFSDNAPPPELLEIINEDIAKR.T** 25847 |

| **53.** | P11509\|CP2A6_HUMAN    **Mass:** 56652    **Score:** 822    **Queries matched:** 68   **emPAI:** 3.92 |
| --- | --- |
|  | Cytochrome P450 2A6 OS=Homo sapiens GN=CYP2A6 PE=1 SV=2 |

|  | **Query** | **Observed** | **Mr(expt)** | **Mr(calc)** | **Delta** | **Miss** | **Score** | **Expect** | **Rank** | **Peptide** |
| --- | --- | --- | --- | --- | --- | --- | --- | --- | --- | --- |
|  | 1418 | **643.3307** | **642.3234** | **641.3609** | **0.9625** | **1** | **4** | **83** | **4** | **K.NRQPK.F** |
|  | 1748 | **772.4255** | **771.4183** | **772.3967** | **-0.9784** | **0** | **7** | **41** | **9** | **K.DIDVSPK.H** |
|  | 2041 | **403.7088** | **805.4030** | **806.4650** | **-1.0620** | **0** | **3** | **99** | **7** | **R.FSIATLR.D** |
|  | 3230 | **450.2301** | **898.4456** | **898.4508** | **-0.0052** | **0** | **17** | **3.2** | **1** | **R.TLDPNSPR.D** 3229 3240 |
|  | 4132 | **482.7554** | **963.4963** | **962.5661** | **0.9302** | **1** | **14** | **7.1** | **1** | **R.RFSIATLR.D** |
|  | 4406 | **492.7800** | **983.5455** | **983.5514** | **-0.0059** | **0** | **18** | **2.5** | **2** | **R.YGFLLLMK.H** |
|  | 4593 | **499.2789** | **996.5432** | **996.5505** | **-0.0073** | **0** | **47** | **0.003** | **1** | **K.HVGFATIPR.N** 4575 4594 4611 |
|  | 6248 | 562.7517 | 1123.4889 | 1124.5502 | -1.0613 | 0 | 8 | 25 | 7 | K.NPNTEFYLK.N |
|  | 7040 | **592.7842** | **1183.5538** | **1183.5622** | **-0.0084** | **0** | **39** | **0.019** | **1** | **K.GYGVVFSNGER.A** |
|  | 7518 | **603.3176** | **1204.6206** | **1204.6274** | **-0.0068** | **0** | **57** | **0.00031** | **1** | **R.FGDVIPMSLAR.R** 7498 |
|  | 7902 | **408.7606** | **1223.2599** | **1223.6445** | **-0.3846** | **0** | **0** | **94** | **10** | **R.VVVLCGHDAVR.E** |
|  | 9219 | **647.8548** | **1293.6950** | **1293.7041** | **-0.0090** | **1** | **29** | **0.19** | **1** | **K.VHEEIDRVIGK.N** |
|  | 9232 | **648.3492** | **1294.6839** | **1294.6921** | **-0.0082** | **1** | **6** | **33** | **1** | **K.KSDAFVPFSIGK.R** |
|  | 10396 | **452.9163** | **1355.7270** | **1355.7350** | **-0.0080** | **0** | **55** | **0.00042** | **1** | **R.YGPVFTIHLGPR.R** 10395 |
|  | 10802 | **460.5898** | **1378.7475** | **1379.7456** | **-0.9981** | **1** | **33** | **0.067** | **1** | **R.RVVVLCGHDAVR.E** 10792 10819 10834 |
|  | 10874 | **692.3497** | **1382.6848** | **1382.6943** | **-0.0095** | **1** | **3** | **73** | **8** | **K.GYGVVFSNGERAK.Q** |
|  | 11346 | **470.9017** | **1409.6832** | **1409.7667** | **-0.0835** | **1** | **4** | **47** | **3** | **R.FSIATLRDFGVGK.R** |
|  | 11550 | 711.3719 | 1420.7292 | 1420.7384 | -0.0092 | 0 | 30 | 0.13 | 1 | K.GTEVYPMLGSVLR.D 11549 |
|  | 11993 | **725.8381** | **1449.6616** | **1449.6736** | **-0.0120** | **0** | **32** | **0.078** | **1** | **R.EALVDQAEEFSGR.G** |
|  | 12393 | **737.4042** | **1472.7939** | **1473.7827** | **-0.9888** | **0** | **64** | **5.7e-005** | **1** | **R.IQEEAGFLIDALR.G** 12418 |
|  | 13644 | **776.8866** | **1551.7586** | **1551.7682** | **-0.0095** | **0** | **48** | **0.0021** | **1** | **R.GTGGANIDPTFFLSR.T** 13643 |
|  | 14603 | **539.5991** | **1615.7755** | **1615.7850** | **-0.0095** | **0** | **30** | **0.11** | **1** | **K.MPYMEAVIHEIQR.F** |
|  | 15319 | 831.8976 | 1661.7806 | 1661.8234 | -0.0428 | 1 | 4 | 42 | 2 | K.VEHNQRTLDPNSPR.D |
|  | 17541 | **454.7337** | **1814.9058** | **1814.9171** | **-0.0113** | **1** | **14** | **3.8** | **1** | **R.AKMPYMEAVIHEIQR.F** |
|  | 18628 | **950.4365** | **1898.8585** | **1898.8720** | **-0.0135** | **1** | **67** | **1.8e-005** | **1** | **R.MQEEEKNPNTEFYLK.N** 18629 18630 |
|  | 23333 | **1191.1365** | **2380.2584** | **2380.2672** | **-0.0088** | **0** | **35** | **0.024** | **1** | **K.NLVMTTLNLFIGGTETVSTTLR.Y** 23326 23328 23339 23340 23343 23345 |
|  | 23496 | **803.3657** | **2407.0752** | **2407.0869** | **-0.0118** | **0** | **30** | **0.066** | **1** | **R.DPSFFSNPQDFNPQHFLNEK.G** |
|  | 24044 | **826.7730** | **2477.2972** | **2477.3067** | **-0.0095** | **0** | **77** | **1.3e-006** | **1** | **K.HLPGPQQQAFQLLQGLEDFIAK.K** 24039 24040 24042 |
|  | 24147 | **623.7916** | **2491.1371** | **2492.1761** | **-1.0390** | **1** | **5** | **21** | **3** | **R.GEQATFDWVFKGYGVVFSNGER.A** |
|  | 25661 | **1380.1421** | **2758.2696** | **2758.2875** | **-0.0178** | **1** | **47** | **0.0011** | **1** | **R.EALVDQAEEFSGRGEQATFDWVFK.G** 25660 25662 |
|  | 27633 | **626.3319** | **3126.6232** | **3125.6183** | **1.0048** | **0** | **6** | **11** | **3** | **-.MLASGMLLVALLVCLTVMVLMSVWQQR.K** |
|  | 27818 | **1588.8043** | **3175.5941** | **3175.6086** | **-0.0145** | **0** | **72** | **3e-006** | **1** | **K.LPPGPTPLPFIGNYLQLNTEQMYNSLMK.I** 27817 27819 |
|  | 28425 | **1121.2435** | **3360.7088** | **3360.7250** | **-0.0162** | **1** | **85** | **1.5e-007** | **1** | **K.GKLPPGPTPLPFIGNYLQLNTEQMYNSLMK.I** 28426 28427 28429 28430 28431 |
|  | 29636 | **1271.2738** | **3810.7996** | **3809.8148** | **0.9848** | **1** | **47** | **0.00069** | **1** | **K.GTEVYPMLGSVLRDPSFFSNPQDFNPQHFLNEK.G** 29631 |

| **54.** | P31930\|QCR1_HUMAN    **Mass:** 53297    **Score:** 821    **Queries matched:** 48   **emPAI:** 2.38 |
| --- | --- |
|  | Cytochrome b-c1 complex subunit 1, mitochondrial OS=Homo sapiens GN=UQCRC1 PE=1 SV=3 |

|  | **Query** | **Observed** | **Mr(expt)** | **Mr(calc)** | **Delta** | **Miss** | **Score** | **Expect** | **Rank** | **Peptide** |
| --- | --- | --- | --- | --- | --- | --- | --- | --- | --- | --- |
|  | 2093 | **405.7104** | **809.4062** | **808.4079** | **0.9983** | **0** | **5** | **45** | **2** | **R.FTGSEIR.H** |
|  | 3201 | **448.7233** | **895.4321** | **895.4374** | **-0.0053** | **0** | **35** | **0.048** | **2** | **R.SGMFWLR.F** |
|  | 3253 | **450.7632** | **899.5117** | **899.5188** | **-0.0071** | **1** | **12** | **11** | **8** | **K.ERDVILR.E** |
|  | 5403 | **529.6173** | **1057.2199** | **1057.5768** | **-0.3568** | **0** | **11** | **9.5** | **1** | **R.IAEVDASVVR.E** |
|  | 5570 | **536.2842** | **1070.5539** | **1069.6244** | **0.9296** | **0** | **11** | **16** | **8** | **R.AATAGAQVLLR.A** 5546 |
|  | 7627 | **606.3237** | **1210.6329** | **1209.4754** | **1.1575** | **0** | **18** | **2.3** | **1** | **R.EMQENDASMR.D** 7603 |
|  | 8523 | **628.8366** | **1255.6587** | **1255.6673** | **-0.0086** | **1** | **24** | **0.57** | **1** | **R.RIPLAEWESR.I** 8524 |
|  | 9769 | **662.3097** | **1322.6048** | **1322.6136** | **-0.0088** | **0** | **74** | **5.8e-006** | **1** | **R.LCTSATESEVAR.G** 9763 9770 9787 |
|  | 11833 | **720.8491** | **1439.6836** | **1439.6932** | **-0.0097** | **0** | **32** | **0.077** | **1** | **R.ADLTEYLSTHYK.A** 11847 |
|  | 14064 | **527.9249** | **1580.7528** | **1579.7783** | **0.9745** | **0** | **13** | **6.4** | **2** | **K.NNGAGYFLEHLAFK.G** |
|  | 15350 | **832.8832** | **1663.7519** | **1663.7624** | **-0.0105** | **0** | **25** | **0.37** | **1** | **K.EVESMGAHLNAYSTR.E** |
|  | 16943 | **885.4078** | **1768.8011** | **1768.8099** | **-0.0088** | **0** | **44** | **0.0043** | **1** | **K.IDDMMFVLQGQWMR.L** |
|  | 17375 | 901.4455 | 1800.8764 | 1800.7997 | 0.0767 | 0 | (4) | 41 | 7 | K.IDDMMFVLQGQWMR.L |
|  | 17848 | **920.5057** | **1838.9969** | **1837.8298** | **1.1671** | **1** | **4** | **37** | **1** | **R.DVILREMQENDASMR.D** |
|  | 20169 | **1014.9746** | **2027.9345** | **2027.9453** | **-0.0108** | **1** | **88** | **1.6e-007** | **1** | **R.MKIDDMMFVLQGQWMR.L** 20177 |
|  | 20426 | **1027.4955** | **2052.9764** | **2052.9899** | **-0.0135** | **0** | **63** | **4.7e-005** | **1** | **R.NALVSHLDGTTPVCEDIGR.S** 20424 20425 |
|  | 20512 | **515.7833** | **2059.1039** | **2059.9352** | **-0.8312** | **1** | **(4)** | **38** | **7** | **R.MKIDDMMFVLQGQWMR.L** |
|  | 20721 | **1039.0618** | **2076.1090** | **2075.9301** | **0.1789** | **1** | **(5)** | **31** | **4** | **R.MKIDDMMFVLQGQWMR.L** |
|  | 20865 | **1046.5577** | **2091.1009** | **2091.1146** | **-0.0137** | **0** | **98** | **1.3e-008** | **1** | **R.MVLAAAGGVEHQQLLDLAQK.H** |
|  | 21991 | **739.3556** | **2215.0451** | **2214.0745** | **0.9706** | **1** | **15** | **2.6** | **1** | **R.FETEKNNGAGYFLEHLAFK.G** 21988 |
|  | 23090 | **1177.5756** | **2353.1366** | **2353.1525** | **-0.0159** | **0** | **55** | **0.00021** | **1** | **K.HLGGIPWTYAEDAVPTLTPCR.F** 23091 23092 23093 |
|  | 23137 | **1180.5781** | **2359.1417** | **2359.1577** | **-0.0160** | **0** | **116** | **2.2e-010** | **1** | **K.AVELLGDIVQNCSLEDSQIEK.E** |
|  | 23157 | **1182.0595** | **2362.1043** | **2362.1224** | **-0.0180** | **0** | **87** | **1.3e-007** | **1** | **R.VASEQSSQPTCTVGVWIDVGSR.F** 23158 |
|  | 25032 | **1323.1495** | **2644.2845** | **2644.3014** | **-0.0168** | **1** | **77** | **1.2e-006** | **1** | **K.AVELLGDIVQNCSLEDSQIEKER.D** 25030 25031 |
|  | 25351 | **1351.6224** | **2701.2303** | **2701.2482** | **-0.0179** | **0** | **64** | **2.3e-005** | **1** | **K.YIYDQCPAVAGYGPIEQLPDYNR.I** 25349 25350 |
|  | 27605 | **1041.8565** | **3122.5475** | **3121.3977** | **1.1498** | **1** | **0** | **48** | **6** | **K.IDDMMFVLQGQWMRLCTSATESEVAR.G** |
|  | 27651 | **1044.1852** | **3129.5337** | **3129.5520** | **-0.0183** | **0** | **12** | **3** | **1** | **R.DVVFNYLHATAFQGTPLAQAVEGPSENVR.K** |
|  | 31833 | **1023.1716** | **6132.9861** | **6133.0293** | **-0.0432** | **1** | **9** | **0.86** | **1** | **R.HRDDALPFAHVAIAVEGPGWASPDNVALQVANAIIGHYDCTYGGGVHLSSPLASGAVANK.L** 31832 |

| **55.** | P06737\|PYGL_HUMAN    **Mass:** 97486    **Score:** 820    **Queries matched:** 63   **emPAI:** 1.44 |
| --- | --- |
|  | Glycogen phosphorylase, liver form OS=Homo sapiens GN=PYGL PE=1 SV=4 |

|  | **Query** | **Observed** | **Mr(expt)** | **Mr(calc)** | **Delta** | **Miss** | **Score** | **Expect** | **Rank** | **Peptide** |
| --- | --- | --- | --- | --- | --- | --- | --- | --- | --- | --- |
|  | 2018 | **804.4410** | **803.4337** | **803.4389** | **-0.0052** | **0** | **38** | **0.037** | **1** | **K.DLSQLTK.L** 2024 |
|  | 2944 | **875.4564** | **874.4491** | **874.4548** | **-0.0058** | **0** | **28** | **0.34** | **2** | **K.AWELTQK.T** |
|  | 3182 | **447.7711** | **893.5276** | **892.4079** | **1.1197** | **0** | **8** | **28** | **6** | **R.YGNPWEK.S** |
|  | 3482 | 458.7429 | 915.4712 | 915.4774 | -0.0062 | 0 | 22 | 1.3 | 2 | R.NLAENISR.V |
|  | 3854 | **473.2308** | **944.4470** | **944.4538** | **-0.0068** | **0** | **15** | **5.9** | **3** | **K.AAPGYHMAK.M** |
|  | 4497 | **496.2532** | **990.4918** | **990.4982** | **-0.0063** | **0** | **24** | **0.69** | **1** | **K.ISLSNESNK.V** |
|  | 5338 | **527.2866** | **1052.5586** | **1052.5655** | **-0.0069** | **0** | **35** | **0.05** | **1** | **K.VIFLENYR.V** |
|  | 5654 | 539.7786 | 1077.5426 | 1078.4819 | -0.9393 | 0 | 5 | 53 | 6 | K.DFSELEPDK.F |
|  | 7045 | **592.8414** | **1183.6682** | **1184.6513** | **-0.9831** | **1** | **1** | **1.1e+002** | **9** | **M.AKPLTDQEKR.R** |
|  | 8488 | **418.9050** | **1253.6931** | **1253.6179** | **0.0752** | **0** | **14** | **6.3** | **3** | **K.EYYEALPELK.L** |
|  | 8924 | **639.8160** | **1277.6175** | **1277.6285** | **-0.0110** | **1** | **33** | **0.072** | **1** | **R.MSLIEEEGSKR.I** 8942 |
|  | 9220 | **432.2447** | **1293.7122** | **1293.7445** | **-0.0322** | **1** | **10** | **13** | **6** | **K.LKVIFLENYR.V** |
|  | 9634 | **658.8289** | **1315.6432** | **1315.6918** | **-0.0486** | **1** | **13** | **7.1** | **6** | **-.MAKPLTDQEKR.R** 9604 9611 9635 9649 |
|  | 10372 | **452.5600** | **1354.6583** | **1354.6670** | **-0.0087** | **0** | **20** | **1.5** | **1** | **R.DYYFALAHTVR.D** |
|  | 10384 | **678.3940** | **1354.7735** | **1354.7820** | **-0.0085** | **1** | **36** | **0.038** | **1** | **R.GIVGVENVAELKK.S** 10383 |
|  | 10510 | **682.8431** | **1363.6717** | **1363.6806** | **-0.0089** | **0** | **62** | **8e-005** | **1** | **K.INPSSMFDVQVK.R** 10512 |
|  | 10725 | 459.1710 | 1374.4912 | 1374.6739 | -0.1827 | 1 | 5 | 39 | 10 | K.ISLSNESNKVNGN.- |
|  | 11169 | **700.3776** | **1398.7407** | **1398.7507** | **-0.0100** | **0** | **52** | **0.00081** | **1** | **R.HLEIIYEINQK.H** |
|  | 11867 | **721.8465** | **1441.6784** | **1441.6878** | **-0.0093** | **0** | **51** | **0.001** | **1** | **R.VLYPNDNFFEGK.E** 11868 |
|  | 12301 | **734.7387** | **1467.4627** | **1466.6612** | **0.8015** | **1** | **9** | **11** | **1** | **R.TQQHYYDKCPK.R** |
|  | 14233 | 796.4125 | 1590.8105 | 1590.8440 | -0.0334 | 1 | 6 | 29 | 5 | K.VKINPSSMFDVQVK.R |
|  | 14308 | **798.8752** | **1595.7359** | **1595.7467** | **-0.0108** | **1** | **42** | **0.0076** | **1** | **K.DFSELEPDKFQNK.T** |
|  | 14479 | **536.9508** | **1607.8306** | **1607.8406** | **-0.0100** | **1** | **30** | **0.12** | **1** | **K.IGEDYVKDLSQLTK.L** |
|  | 14622 | **809.8549** | **1617.6952** | **1617.7059** | **-0.0108** | **0** | **59** | **0.00013** | **1** | **R.DGWQVEEADDWLR.Y** |
|  | 14656 | **810.8405** | **1619.6665** | **1618.8501** | **0.8164** | **1** | **1** | **94** | **1** | **K.AWNTMVLKNIAASGK.F** |
|  | 14721 | **812.8982** | **1623.7819** | **1623.7893** | **-0.0073** | **0** | **33** | **0.054** | **1** | **R.DFNVGDYIQAVLDR.N** 14722 |
|  | 16037 | **427.7196** | **1706.8492** | **1706.8603** | **-0.0111** | **0** | **15** | **3.5** | **1** | **K.SRPEFMLPVHFYGK.V** 16049 |
|  | 17427 | **903.4329** | **1804.8512** | **1804.8631** | **-0.0120** | **0** | **48** | **0.0019** | **1** | **K.EYAQNIWNVEPSDLK.I** 17428 |
|  | 17856 | **921.0026** | **1839.9907** | **1838.9964** | **0.9943** | **0** | **73** | **4.8e-006** | **1** | **R.WLLLCNPGLAELIAEK.I** 17847 |
|  | 17871 | **614.6365** | **1840.8876** | **1839.9155** | **0.9721** | **1** | **7** | **21** | **3** | **R.VLYPNDNFFEGKELR.L** |
|  | 18125 | **929.9770** | **1857.9394** | **1857.9506** | **-0.0112** | **0** | **71** | **7.5e-006** | **1** | **K.LITSVADVVNNDPMVGSK.L** 18122 18124 |
|  | 18164 | **931.4695** | **1860.9244** | **1860.9370** | **-0.0126** | **0** | **98** | **1.7e-008** | **1** | **K.TFAYTNHTVLPEALER.W** 18165 |
|  | 18482 | **944.4477** | **1886.8808** | **1886.8911** | **-0.0103** | **1** | **53** | **0.00048** | **1** | **K.IRDGWQVEEADDWLR.Y** |
|  | 18893 | **641.3006** | **1920.8800** | **1920.0217** | **0.8583** | **1** | **6** | **26** | **8** | **R.HLEIIYEINQKHLDR.I** |
|  | 19910 | **1004.5635** | **2007.1124** | **2006.1200** | **0.9924** | **1** | **33** | **0.041** | **1** | **R.LKQEYFVVAATLQDIIR.R** |
|  | 20201 | **678.4135** | **2032.2185** | **2032.0279** | **0.1906** | **1** | **2** | **51** | **4** | **R.DYYFALAHTVRDHLVGR.W** |
|  | 21520 | **542.0240** | **2164.0669** | **2164.0775** | **-0.0107** | **1** | **18** | **1.5** | **1** | **K.QPDLFKDIINMLFYHDR.F** |
|  | 24034 | **1239.5978** | **2477.1810** | **2477.1955** | **-0.0145** | **0** | **122** | **5.1e-011** | **1** | **K.VIPATDLSEQISTAGTEASGTGNMK.F** 24031 24033 |
|  | 25030 | 882.4347 | 2644.2822 | 2643.3982 | 0.8841 | 1 | 5 | 21 | 2 | R.WLLLCNPGLAELIAEKIGEDYVK.D |
|  | 27924 | **1604.7860** | **3207.5575** | **3207.5733** | **-0.0158** | **0** | **40** | **0.0039** | **1** | **K.WIDTQVVLALPYDTPVPGYMNNTVNTMR.L** |
|  | 28229 | **1103.2256** | **3306.6549** | **3306.6707** | **-0.0158** | **0** | **70** | **4.4e-006** | **1** | **R.GAGTVFDAFPDQVAIQLNDTHPALAIPELMR.I** 28224 28226 28227 |
|  | 29315 | **743.1732** | **3710.8297** | **3709.6772** | **1.1525** | **0** | **1** | **28** | **1** | **K.FMLNGALTIGTMDGANVEMAEEAGEENLFIFGMR.I** |

| **56.** | Q6S8J3\|POTEE_HUMAN    **Mass:** 122882   **Score:** 786    **Queries matched:** 66   **emPAI:** 0.34 |
| --- | --- |
|  | POTE ankyrin domain family member E OS=Homo sapiens GN=POTEE PE=1 SV=3 |

|  | **Query** | **Observed** | **Mr(expt)** | **Mr(calc)** | **Delta** | **Miss** | **Score** | **Expect** | **Rank** | **Peptide** |
| --- | --- | --- | --- | --- | --- | --- | --- | --- | --- | --- |
|  | 1363 | 630.3417 | 629.3344 | 630.3701 | -1.0357 | 0 | 4 | 99 | 2 | K.ILTER.G |
|  | 1421 | 644.3680 | 643.3607 | 643.3653 | -0.0046 | 0 | 20 | 2.6 | 1 | R.LDLAGR.E |
|  | 2523 | **423.0518** | **844.0891** | **843.4702** | **0.6189** | **0** | **8** | **23** | **5** | **K.QIEVVEK.M** |
|  | 3103 | **443.2713** | **884.5281** | **885.4668** | **-0.9387** | **0** | **14** | **5.9** | **6** | **K.ANLNALDR.Y** |
|  | 3377 | **454.8929** | **907.7713** | **906.6014** | **1.1698** | **1** | **5** | **33** | **7** | **K.IRIIAPPK.R** |
|  | 4297 | 488.7242 | 975.4339 | 975.4410 | -0.0071 | 0 | 67 | 3.7e-005 | 1 | K.AGFAGDDAPR.A 4293 4296 |
|  | 7268 | **398.8572** | **1193.5497** | **1192.5831** | **0.9666** | **0** | **6** | **40** | **4** | **K.EIAALAPSMMK.I** |
|  | 7365 | 599.8527 | 1197.6908 | 1197.6982 | -0.0075 | 0 | 58 | 0.00026 | 1 | R.AVFPSIVGRPR.Q 7366 |
|  | 7902 | 408.7606 | 1223.2599 | 1222.6445 | 0.6154 | 1 | 1 | 70 | 6 | K.YLEDIESVKK.K |
|  | 8059 | **411.7251** | **1232.1536** | **1231.5260** | **0.6275** | **0** | **(6)** | **28** | **8** | **R.QQGMMGGMHQK.E** |
|  | 8380 | **624.8301** | **1247.6456** | **1247.5209** | **0.1247** | **0** | **(7)** | **28** | **3** | **R.QQGMMGGMHQK.E** 8402 |
|  | 8381 | **624.8337** | **1247.6528** | **1247.5209** | **0.1319** | **0** | **(7)** | **32** | **3** | **R.QQGMMGGMHQK.E** |
|  | 8938 | **427.2195** | **1278.6368** | **1279.5108** | **-0.8740** | **0** | **11** | **12** | **1** | **R.QQGMMGGMHQK.E** |
|  | 9688 | 660.3503 | 1318.6861 | 1318.6728 | 0.0133 | 1 | 7 | 31 | 6 | K.LTSEEESQRLK.G |
|  | 11679 | **477.2838** | **1428.8296** | **1429.7785** | **-0.9489** | **1** | **9** | **16** | **1** | **K.EIAALAPSMMKIR.I** |
|  | 11841 | **481.1506** | **1440.4300** | **1439.7368** | **0.6931** | **1** | **8** | **16** | **5** | **K.EKDVLHENSTLR.E** 11849 |
|  | 11963 | **483.2732** | **1446.7977** | **1445.7734** | **1.0243** | **1** | **(8)** | **22** | **5** | **K.EIAALAPSMMKIR.I** |
|  | 13084 | 758.8497 | 1515.6849 | 1515.6954 | -0.0105 | 0 | 48 | 0.0021 | 1 | K.QEYDESGPSIVHR.K |
|  | 13087 | 758.8746 | 1515.7347 | 1514.7419 | 0.9928 | 0 | 38 | 0.022 | 1 | K.IWHHTFYNELR.V 13067 13068 |
|  | 13561 | **516.5950** | **1546.7631** | **1546.8025** | **-0.0394** | **1** | **5** | **39** | **5** | **K.DLIVMLRDTDVNK.K** |
|  | 13578 | **775.2053** | **1548.3960** | **1548.7528** | **-0.3568** | **0** | **(1)** | **66** | **6** | **-.MVVEVDSMPAASSVK.K** |
|  | 13834 | 783.3355 | 1564.6563 | 1564.7477 | -0.0913 | 0 | (1) | 84 | 6 | -.MVVEVDSMPAASSVK.K 13818 |
|  | 13852 | 783.8886 | 1565.7625 | 1564.7477 | 1.0149 | 0 | 2 | 68 | 5 | -.MVVEVDSMPAASSVK.K |
|  | 14025 | **395.7156** | **1578.8331** | **1579.7772** | **-0.9441** | **1** | **3** | **57** | **5** | **R.MQKEIAALAPSMMK.I** |
|  | 14303 | **532.6229** | **1594.8469** | **1595.7721** | **-0.9251** | **1** | **(2)** | **81** | **8** | **R.MQKEIAALAPSMMK.I** |
|  | 14568 | **807.9148** | **1613.8150** | **1613.8195** | **-0.0045** | **1** | **12** | **6.9** | **4** | **R.LELDTMKHQSQLR.E** |
|  | 14944 | **410.2205** | **1636.8530** | **1635.8542** | **0.9988** | **1** | **9** | **16** | **1** | **R.ELPDYLMKILTER.G** |
|  | 15044 | 822.8969 | 1643.7793 | 1643.7903 | -0.0111 | 1 | 28 | 0.19 | 1 | K.QEYDESGPSIVHRK.C |
|  | 15149 | **550.9941** | **1649.9606** | **1648.8494** | **1.1112** | **1** | **0** | **1e+002** | **10** | **R.LDLAGRELPDYLMK.I** |
|  | 16276 | **862.4311** | **1722.8476** | **1722.8532** | **-0.0055** | **1** | **6** | **26** | **1** | **R.EEIAMLRLELDTMK.H** 16290 |
|  | 17255 | 895.9438 | 1789.8730 | 1789.8846 | -0.0116 | 0 | 71 | 9.6e-006 | 1 | K.SYELPDGQVITIGNER.F 17256 17257 17258 17267 17268 |
|  | 19524 | **987.9422** | **1973.8698** | **1973.9727** | **-0.1029** | **1** | **1** | **73** | **7** | **K.QMLKISSENSNPEQELK.L** |
|  | 20355 | **410.6172** | **2048.0494** | **2046.9527** | **1.0967** | **1** | **6** | **23** | **6** | **K.GSENSQPEKMSQELEINK.D** |
|  | 20543 | **1032.4503** | **2062.8861** | **2062.9477** | **-0.0616** | **1** | **(0)** | **82** | **4** | **K.GSENSQPEKMSQELEINK.D** |
|  | 21233 | **427.4260** | **2132.0936** | **2132.1300** | **-0.0364** | **1** | **7** | **18** | **6** | **M.VVEVDSMPAASSVKKPFGLR.S** |
|  | 24494 | 1275.5835 | 2549.1524 | 2549.1665 | -0.0141 | 0 | 108 | 1.1e-009 | 1 | K.LCYVALDFEQEMATAASSSSLEK.S 24486 24487 24488 24490 24491 24492 24493 24495 24498 24503 24506 |
|  | 25268 | **897.1061** | **2688.2966** | **2687.2506** | **1.0460** | **1** | **2** | **40** | **6** | **K.DLYTNTVLSGGTTMYPGMAHRMQK.E** |
|  | 25942 | 936.4365 | 2806.2876 | 2806.3041 | -0.0165 | 1 | 56 | 0.00014 | 1 | K.EKLCYVALDFEQEMATAASSSSLEK.S |
|  | 27653 | **783.3997** | **3129.5698** | **3130.5755** | **-1.0058** | **1** | **1** | **42** | **1** | **K.HESNNVGLLENLTNGVTAGNGDNGLIPQRK.S** |
|  | 29606 | 1266.5684 | 3796.6833 | 3795.7390 | 0.9443 | 1 | 1 | 26 | 4 | K.ALQLNELTMDDDTAVLVIDNGSGMCKAGFAGDDAPR.A |
|  | 29641 | **1271.3035** | **3810.8886** | **3811.7339** | **-0.8453** | **1** | **(1)** | **31** | **5** | **K.ALQLNELTMDDDTAVLVIDNGSGMCKAGFAGDDAPR.A** |

| **57.** | Q9UJS0\|CMC2_HUMAN    **Mass:** 74528    **Score:** 781    **Queries matched:** 58   **emPAI:** 1.77 |
| --- | --- |
|  | Calcium-binding mitochondrial carrier protein Aralar2 OS=Homo sapiens GN=SLC25A13 PE=1 SV=2 |

|  | **Query** | **Observed** | **Mr(expt)** | **Mr(calc)** | **Delta** | **Miss** | **Score** | **Expect** | **Rank** | **Peptide** |
| --- | --- | --- | --- | --- | --- | --- | --- | --- | --- | --- |
|  | 375 | **430.2744** | **429.2672** | **430.2288** | **-0.9617** | **0** | **1** | **1.7e+002** | **2** | **K.GAGAR.V** 374 |
|  | 992 | **531.3457** | **530.3384** | **530.3428** | **-0.0044** | **0** | **11** | **25** | **2** | **K.VALTK.R** |
|  | 1465 | **657.3996** | **656.3923** | **656.3969** | **-0.0046** | **0** | **25** | **0.56** | **1** | **R.LQVAAR.A** |
|  | 1701 | **748.4047** | **747.3974** | **747.4028** | **-0.0053** | **0** | **12** | **14** | **1** | **K.QAFVQR.D** |
|  | 3452 | **457.3125** | **912.6104** | **913.5457** | **-0.9353** | **1** | **2** | **97** | **4** | **K.TRLQVAAR.A** |
|  | 3625 | **464.7524** | **927.4903** | **926.4933** | **0.9969** | **1** | **5** | **54** | **4** | **K.RADPAELR.T** |
|  | 3631 | **465.0192** | **928.0238** | **928.5243** | **-0.5005** | **1** | **8** | **17** | **1** | **K.ALWKGAGAR.V** 3639 |
|  | 3801 | **471.2708** | **940.5269** | **940.5341** | **-0.0072** | **1** | **32** | **0.092** | **1** | **K.ILREEGPK.A** 3799 3800 |
|  | 3928 | **475.2434** | **948.4723** | **947.4746** | **0.9977** | **0** | **41** | **0.015** | **1** | **R.MTLADIER.I** |
|  | 4111 | **482.2635** | **962.5124** | **962.5185** | **-0.0062** | **0** | **39** | **0.021** | **1** | **K.LTVNDFVR.D** 4135 |
|  | 4697 | **502.2654** | **1002.5161** | **1002.5896** | **-0.0734** | **1** | **7** | **37** | **10** | **-.MAAAKVALTK.R** |
|  | 5247 | **523.2389** | **1044.4632** | **1044.4699** | **-0.0066** | **1** | **25** | **0.54** | **1** | **K.NSFDCFKK.V** |
|  | 6595 | **576.2760** | **1150.5374** | **1150.5447** | **-0.0073** | **0** | **59** | **0.00021** | **1** | **R.YEGFFGLYR.G** |
|  | 8942 | 427.2242 | 1278.6508 | 1278.6456 | 0.0052 | 1 | 19 | 1.6 | 1 | K.AGKGEVTFEDVK.Q 8924 |
|  | 9668 | **659.8169** | **1317.6192** | **1317.6275** | **-0.0083** | **0** | **11** | **12** | **1** | **R.STGSFVGELMYK.N** |
|  | 10007 | **667.9017** | **1333.7888** | **1333.7969** | **-0.0081** | **0** | **46** | **0.0034** | **1** | **R.GLLPQLLGVAPEK.A** |
|  | 10966 | **695.0616** | **1388.1086** | **1387.7559** | **0.3527** | **0** | **37** | **0.019** | **1** | **K.TVELLSGVVDQTK.D** |
|  | 12926 | **753.8777** | **1505.7408** | **1505.7514** | **-0.0106** | **0** | **71** | **9e-006** | **1** | **R.YLNIFGESQPNPK.T** 12925 12941 |
|  | 14233 | 796.4125 | 1590.8105 | 1590.8154 | -0.0049 | 0 | 35 | 0.042 | 1 | R.INLPAPNPDHVGGYK.L |
|  | 15308 | **831.3618** | **1660.7091** | **1659.7352** | **0.9739** | **0** | **21** | **0.87** | **1** | **K.NGEFFMSPNDFVTR.Y** 15296 |
|  | 15946 | **851.9084** | **1701.8022** | **1701.8145** | **-0.0122** | **1** | **63** | **5e-005** | **1** | **R.AGQTTYSGVIDCFRK.I** |
|  | 15996 | **853.4484** | **1704.8822** | **1704.8934** | **-0.0112** | **1** | **52** | **0.0007** | **1** | **K.DVEVTKEEFVLAAQK.F** |
|  | 19262 | 976.5238 | 1951.0330 | 1951.0415 | -0.0084 | 0 | 37 | 0.02 | 1 | R.SSPQFGVTLLTYELLQR.W |
|  | 19462 | **985.0174** | **1968.0202** | **1968.0316** | **-0.0114** | **0** | **62** | **5e-005** | **1** | **R.IAPLEEGTLPFNLAEAQR.Q** 19461 |
|  | 19531 | **658.9921** | **1973.9544** | **1974.9292** | **-0.9748** | **1** | **20** | **1** | **1** | **R.MQNQRSTGSFVGELMYK.N** |
|  | 19697 | **995.5155** | **1989.0164** | **1989.0279** | **-0.0115** | **0** | **39** | **0.011** | **1** | **K.ASGDSARPVLLQVAESAYR.F** 19694 19698 |
|  | 19915 | **670.3262** | **2007.9569** | **2006.9190** | **1.0379** | **1** | **(1)** | **69** | **5** | **R.MQNQRSTGSFVGELMYK.N** |
|  | 20987 | **1053.0786** | **2104.1427** | **2104.1569** | **-0.0142** | **0** | **87** | **1.7e-007** | **1** | **R.FGLGSVAGAVGATAVYPIDLVK.T** 20983 20986 20988 |
|  | 21420 | **718.6912** | **2153.0517** | **2153.0656** | **-0.0139** | **0** | **34** | **0.038** | **1** | **R.WFYIDFGGVKPMGSEPVPK.S** 21418 21419 |
|  | 22067 | **742.3616** | **2224.0631** | **2224.1851** | **-0.1221** | **1** | **4** | **31** | **1** | **R.IAPLEEGTLPFNLAEAQRQK.A** |
|  | 22263 | **749.1364** | **2244.3874** | **2245.1815** | **-0.7941** | **1** | **6** | **13** | **2** | **R.QKASGDSARPVLLQVAESAYR.F** |
|  | 23640 | **811.0894** | **2430.2463** | **2430.2583** | **-0.0121** | **0** | **65** | **2.2e-005** | **1** | **R.HLTYAEFTQFLLEIQLEHAK.Q** 23639 |
|  | 24743 | **1291.1491** | **2580.2835** | **2580.2934** | **-0.0099** | **0** | **93** | **3.3e-008** | **1** | **K.FGQVTPMEVDILFQLADLYEPR.G** 24742 |
|  | 25854 | **932.4279** | **2794.2619** | **2793.4160** | **0.8459** | **1** | **1** | **45** | **5** | **K.FGQVTPMEVDILFQLADLYEPRGR.M** |
|  | 28908 | **1769.9185** | **3537.8224** | **3537.8389** | **-0.0165** | **0** | **90** | **3.8e-008** | **1** | **K.ASFANEDGQVSPGSLLLAGAIAGMPAASLVTPADVIK.T** 28909 28911 |
|  | 29600 | **1897.9421** | **3793.8697** | **3794.9877** | **-1.1179** | **1** | **3** | **18** | **1** | **K.ASFANEDGQVSPGSLLLAGAIAGMPAASLVTPADVIKTR.L** |
|  | 29631 | 953.4554 | 3809.7924 | 3810.9826 | -1.1902 | 1 | (2) | 20 | 9 | K.ASFANEDGQVSPGSLLLAGAIAGMPAASLVTPADVIKTR.L |

| **58.** | P42765\|THIM_HUMAN    **Mass:** 42354    **Score:** 774    **Queries matched:** 47   **emPAI:** 2.88 |
| --- | --- |
|  | 3-ketoacyl-CoA thiolase, mitochondrial OS=Homo sapiens GN=ACAA2 PE=1 SV=2 |

|  | **Query** | **Observed** | **Mr(expt)** | **Mr(calc)** | **Delta** | **Miss** | **Score** | **Expect** | **Rank** | **Peptide** |
| --- | --- | --- | --- | --- | --- | --- | --- | --- | --- | --- |
|  | 1326 | **618.3608** | **617.3536** | **616.3544** | **0.9992** | **0** | **23** | **1.6** | **1** | **K.AALSAGK.V** 1325 |
|  | 3144 | **445.9981** | **889.9817** | **889.4756** | **0.5061** | **0** | **7** | **24** | **1** | **R.SLDLDISK.T** |
|  | 4008 | **478.2560** | **954.4975** | **954.5035** | **-0.0061** | **0** | **14** | **6.2** | **1** | **K.HNFTPLAR.I** |
|  | 4533 | **497.2560** | **992.4975** | **992.5039** | **-0.0064** | **0** | **21** | **1.3** | **1** | **K.YALQSQQR.W** |
|  | 4838 | **507.7788** | **1013.5429** | **1013.5505** | **-0.0076** | **0** | **23** | **0.88** | **1** | **K.ETPALTINR.L** 4853 |
|  | 5725 | **542.7360** | **1083.4574** | **1082.5985** | **0.8589** | **1** | **16** | **3.9** | **3** | **K.KHNFTPLAR.I** |
|  | 6244 | **562.3076** | **1122.6006** | **1122.6073** | **-0.0068** | **0** | **7** | **29** | **2** | **R.TPFGAYGGLLK.D** |
|  | 7142 | **594.8413** | **1187.6681** | **1187.6775** | **-0.0094** | **0** | **55** | **0.0005** | **1** | **R.ITAHLVHELR.R** 7143 |
|  | 8961 | **640.8532** | **1279.6919** | **1278.7085** | **0.9834** | **1** | **14** | **5.2** | **5** | **K.RTPFGAYGGLLK.D** |
|  | 15209 | **827.8723** | **1653.7299** | **1653.7417** | **-0.0117** | **1** | **53** | **0.00055** | **1** | **R.EECDKYALQSQQR.W** 15210 15211 |
|  | 17785 | 917.9719 | 1833.9293 | 1833.9446 | -0.0153 | 0 | 41 | 0.01 | 1 | K.TNVNGGAIALGHPLGGSGSR.I 17767 17799 17800 |
|  | 19290 | **977.9401** | **1953.8656** | **1953.8778** | **-0.0122** | **0** | **55** | **0.00029** | **1** | **K.AANDAGYFNDEMAPIEVK.T** |
|  | 21442 | 1078.9855 | 2155.9564 | 2154.9861 | 0.9703 | 0 | 20 | 0.89 | 1 | R.LCGSGFQSIVNGCQEICVK.E 21443 |
|  | 22736 | **1155.0530** | **2308.0914** | **2308.1045** | **-0.0131** | **0** | **50** | **0.00077** | **1** | **K.DMDLVEVNEAFAPQYLAVER.S** |
|  | 23013 | **1172.0162** | **2342.0179** | **2342.0341** | **-0.0162** | **0** | **89** | **9.9e-008** | **1** | **K.EAEVVLCGGTESMSQAPYCVR.N** 23010 23014 |
|  | 23321 | **1191.0891** | **2380.1637** | **2380.1805** | **-0.0168** | **0** | **36** | **0.016** | **1** | **K.QTMQVDEHARPQTTLEQLQK.L** 23322 |
|  | 24315 | **1258.6191** | **2515.2237** | **2515.2402** | **-0.0165** | **0** | **134** | **2.8e-012** | **1** | **K.DGTVTAGNASGVADGAGAVIIASEDAVK.K** 24314 |
|  | 24661 | **856.1001** | **2565.2785** | **2565.2969** | **-0.0184** | **1** | **34** | **0.028** | **1** | **K.GKQTMQVDEHARPQTTLEQLQK.L** |
|  | 25027 | **1322.6659** | **2643.3172** | **2643.3352** | **-0.0179** | **1** | **85** | **2e-007** | **1** | **K.DGTVTAGNASGVADGAGAVIIASEDAVKK.H** |
|  | 25370 | **1353.2004** | **2704.3863** | **2704.3969** | **-0.0105** | **0** | **50** | **0.0007** | **1** | **R.IVGYFVSGCDPSIMGIGPVPAISGALK.K** 25368 25369 |
|  | 25613 | **1376.1903** | **2750.3661** | **2750.3797** | **-0.0136** | **0** | **83** | **3.1e-007** | **1** | **K.VSPETVDSVIMGNVLQSSSDAIYLAR.H** 25611 25614 25623 |
|  | 26199 | **950.5349** | **2848.5827** | **2848.4867** | **0.0960** | **1** | **2** | **31** | **2** | **R.IVGYFVSGCDPSIMGIGPVPAISGALKK.A** |
|  | 27682 | **1047.1925** | **3138.5557** | **3138.5729** | **-0.0173** | **0** | **56** | **0.00013** | **1** | **K.LEDSLWVSLTDQHVQLPMAMTAENLAVK.H** 27684 |
|  | 27741 | **1052.8027** | **3155.3864** | **3154.5679** | **0.8185** | **0** | **(2)** | **31** | **9** | **K.LEDSLWVSLTDQHVQLPMAMTAENLAVK.H** |
|  | 27823 | **1060.5297** | **3178.5672** | **3179.5696** | **-1.0025** | **1** | **0** | **43** | **5** | **K.DMDLVEVNEAFAPQYLAVERSLDLDISK.T** |
|  | 28317 | **832.9116** | **3327.6174** | **3328.7085** | **-1.0912** | **1** | **0** | **43** | **3** | **K.VSPETVDSVIMGNVLQSSSDAIYLARHVGLR.V** |
|  | 29432 | **1251.6041** | **3751.7906** | **3751.9165** | **-0.1259** | **1** | **(0)** | **32** | **4** | **K.LGSDIKLEDSLWVSLTDQHVQLPMAMTAENLAVK.H** |
|  | 29504 | **1262.5776** | **3784.7111** | **3783.9063** | **0.8048** | **1** | **0** | **30** | **10** | **K.LGSDIKLEDSLWVSLTDQHVQLPMAMTAENLAVK.H** |

| **59.** | Q02338\|BDH_HUMAN    **Mass:** 38532    **Score:** 773    **Queries matched:** 53   **emPAI:** 2.35 |
| --- | --- |
|  | D-beta-hydroxybutyrate dehydrogenase, mitochondrial OS=Homo sapiens GN=BDH1 PE=1 SV=3 |

|  | **Query** | **Observed** | **Mr(expt)** | **Mr(calc)** | **Delta** | **Miss** | **Score** | **Expect** | **Rank** | **Peptide** |
| --- | --- | --- | --- | --- | --- | --- | --- | --- | --- | --- |
|  | 2631 | **427.7554** | **853.4962** | **854.4246** | **-0.9284** | **1** | **8** | **20** | **2** | **K.DKGHDGVK.E** |
|  | 2876 | **435.2684** | **868.5222** | **867.4160** | **1.1062** | **0** | **25** | **0.44** | **1** | **R.SPYCITK.F** |
|  | 5299 | **525.2739** | **1048.5333** | **1047.4832** | **1.0501** | **0** | **19** | **2.3** | **2** | **K.ELDSLNSDR.L** |
|  | 5625 | **538.2967** | **1074.5789** | **1074.5856** | **-0.0066** | **0** | **43** | **0.0089** | **1** | **R.VVNISSMLGR.M** |
|  | 5930 | **550.2747** | **1098.5349** | **1098.5419** | **-0.0071** | **0** | **32** | **0.093** | **1** | **R.YEMYPLGVK.V** |
|  | 6152 | **558.7479** | **1115.4812** | **1114.5369** | **0.9443** | **0** | **(4)** | **64** | **8** | **R.YEMYPLGVK.V** |
|  | 6293 | **564.7705** | **1127.5265** | **1128.6866** | **-1.1602** | **1** | **3** | **90** | **9** | **K.VVEIVRSSLK.D** |
|  | 7484 | **402.1782** | **1203.5128** | **1204.7002** | **-1.1873** | **1** | **15** | **5.5** | **1** | **R.MTKSFLPLIR.R** |
|  | 7813 | **610.7673** | **1219.5200** | **1220.6951** | **-1.1751** | **1** | **(7)** | **27** | **3** | **R.MTKSFLPLIR.R** 7845 |
|  | 7896 | **408.5623** | **1222.6650** | **1222.7510** | **-0.0859** | **1** | **5** | **50** | **10** | **R.LSRPLSRLPGK.T** |
|  | 8256 | **621.8190** | **1241.6235** | **1241.6301** | **-0.0066** | **0** | **50** | **0.0016** | **1** | **K.GFLVFAGCLMK.D** |
|  | 8556 | **420.2212** | **1257.6418** | **1257.6250** | **0.0168** | **0** | **(4)** | **59** | **6** | **K.GFLVFAGCLMK.D** |
|  | 8764 | **635.3471** | **1268.6797** | **1268.7677** | **-0.0880** | **1** | **4** | **53** | **6** | **M.LATRLSRPLSR.L** |
|  | 9085 | **644.3197** | **1286.6248** | **1286.6329** | **-0.0080** | **0** | **1** | **1.2e+002** | **4** | **K.MWEELPEVVR.K** |
|  | 9107 | **644.8566** | **1287.6987** | **1287.7081** | **-0.0094** | **1** | **40** | **0.014** | **1** | **K.GRVVNISSMLGR.M** 9106 |
|  | 9319 | **650.7991** | **1299.5836** | **1299.5918** | **-0.0082** | **0** | **24** | **0.59** | **2** | **K.FGVEAFSDCLR.Y** |
|  | 9647 | **659.3370** | **1316.6595** | **1316.6684** | **-0.0089** | **1** | **18** | **2.4** | **1** | **K.ELDSLNSDRLR.T** 9669 |
|  | 10047 | **669.3032** | **1336.5919** | **1335.6783** | **0.9136** | **1** | **10** | **13** | **1** | **R.RTYASAAEPVGSK.A** |
|  | 10671 | **686.6459** | **1371.2772** | **1370.7307** | **0.5465** | **0** | **42** | **0.0064** | **1** | **K.QVAEVNLWGTVR.M** |
|  | 11438 | **472.5804** | **1414.7194** | **1414.7278** | **-0.0084** | **1** | **5** | **39** | **3** | **K.KMWEELPEVVR.K** |
|  | 11449 | **708.8481** | **1415.6816** | **1414.7278** | **0.9538** | **1** | **4** | **55** | **3** | **K.MWEELPEVVRK.D** |
|  | 14670 | **811.3848** | **1620.7551** | **1620.7665** | **-0.0114** | **0** | **2** | **67** | **6** | **R.TVQLNVCSSEEVEK.V** |
|  | 16340 | **864.9281** | **1727.8416** | **1727.8553** | **-0.0136** | **0** | **71** | **8.6e-006** | **1** | **K.AVLVTGCDSGFGFSLAK.H** 16332 16339 16341 16376 16393 |
|  | 20838 | **1045.5270** | **2089.0394** | **2089.0522** | **-0.0128** | **0** | **61** | **7.1e-005** | **1** | **R.MQIMTHLPGAISDMIYIR.-** 20837 20839 20854 |
|  | 21004 | **1054.0269** | **2106.0392** | **2105.0471** | **0.9920** | **0** | **(14)** | **3.8** | **2** | **R.MQIMTHLPGAISDMIYIR.-** |
|  | 21274 | **713.3357** | **2136.9854** | **2137.0370** | **-0.0516** | **0** | **(2)** | **56** | **8** | **R.MQIMTHLPGAISDMIYIR.-** |
|  | 22811 | **1159.1002** | **2316.1859** | **2316.1995** | **-0.0136** | **1** | **89** | **9.9e-008** | **1** | **R.TVQLNVCSSEEVEKVVEIVR.S** 22809 22810 |
|  | 25275 | **1346.2070** | **2690.3995** | **2690.4167** | **-0.0172** | **0** | **63** | **2.8e-005** | **1** | **K.VSVVEPGNFIAATSLYSPESIQAIAK.K** 25276 25282 25283 |
|  | 26205 | **1425.6763** | **2849.3380** | **2849.3582** | **-0.0202** | **0** | **55** | **0.00017** | **1** | **K.GMWGLVNNAGISTFGEVEFTSLETYK.Q** 26218 26220 |
|  | 26425 | **1445.7195** | **2889.4244** | **2889.4219** | **0.0025** | **1** | **1** | **46** | **3** | **R.TYASAAEPVGSKAVLVTGCDSGFGFSLAK.H** |
|  | 28332 | **1111.8400** | **3332.4981** | **3332.5177** | **-0.0196** | **0** | **86** | **1.1e-007** | **1** | **K.METYCSSGSTDTSPVIDAVTHALTATTPYTR.Y** 28331 28333 28334 |
|  | 28386 | **1117.1718** | **3348.4934** | **3348.5126** | **-0.0192** | **0** | **(74)** | **1.5e-006** | **1** | **K.METYCSSGSTDTSPVIDAVTHALTATTPYTR.Y** |

| **60.** | P68032\|ACTC_HUMAN    **Mass:** 42334    **Score:** 746    **Queries matched:** 73   **emPAI:** 5.47 |
| --- | --- |
|  | Actin, alpha cardiac muscle 1 OS=Homo sapiens GN=ACTC1 PE=1 SV=1 |

|  | **Query** | **Observed** | **Mr(expt)** | **Mr(calc)** | **Delta** | **Miss** | **Score** | **Expect** | **Rank** | **Peptide** |
| --- | --- | --- | --- | --- | --- | --- | --- | --- | --- | --- |
|  | 1363 | 630.3417 | 629.3344 | 630.3701 | -1.0357 | 0 | 4 | 99 | 2 | K.ILTER.G |
|  | 1421 | 644.3680 | 643.3607 | 643.3653 | -0.0046 | 0 | 20 | 2.6 | 1 | R.LDLAGR.D |
|  | 1884 | 795.4671 | 794.4598 | 794.4650 | -0.0052 | 0 | 17 | 3.4 | 2 | K.IIAPPER.K |
|  | 4297 | 488.7242 | 975.4339 | 975.4410 | -0.0071 | 0 | 67 | 3.7e-005 | 1 | K.AGFAGDDAPR.A 4293 4296 |
|  | 4616 | 499.7438 | 997.4730 | 997.4790 | -0.0060 | 0 | 33 | 0.076 | 1 | R.DLTDYLMK.I |
|  | 5140 | 518.8257 | 1035.6369 | 1035.6440 | -0.0071 | 1 | 26 | 0.39 | 1 | K.IKIIAPPER.K |
|  | 6325 | **565.7733** | **1129.5321** | **1129.5404** | **-0.0083** | **0** | **46** | **0.0042** | **1** | **R.GYSFVTTAER.E** |
|  | 6737 | 581.3089 | 1160.6032 | 1160.6111 | -0.0078 | 0 | 48 | 0.0027 | 1 | K.EITALAPSTMK.I 6736 6738 |
|  | 6867 | 586.2850 | 1170.5555 | 1170.5638 | -0.0083 | 0 | 50 | 0.0016 | 1 | R.HQGVMVGMGQK.D 6865 6866 |
|  | 6943 | 589.3067 | 1176.5988 | 1176.6060 | -0.0072 | 0 | (4) | 67 | 3 | K.EITALAPSTMK.I |
|  | 7105 | 594.2822 | 1186.5499 | 1186.5587 | -0.0088 | 0 | (35) | 0.046 | 1 | R.HQGVMVGMGQK.D |
|  | 7106 | 594.2826 | 1186.5507 | 1186.5587 | -0.0080 | 0 | (47) | 0.003 | 1 | R.HQGVMVGMGQK.D 7093 |
|  | 7338 | 599.2899 | 1196.5652 | 1197.5150 | -0.9498 | 0 | 29 | 0.2 | 1 | K.DSYVGDEAQSK.R |
|  | 7365 | 599.8527 | 1197.6908 | 1197.6982 | -0.0075 | 0 | 58 | 0.00026 | 1 | R.AVFPSIVGRPR.H 7366 |
|  | 7465 | 602.2790 | 1202.5434 | 1202.5536 | -0.0102 | 0 | (4) | 69 | 5 | R.HQGVMVGMGQK.D |
|  | 10347 | 677.8101 | 1353.6057 | 1353.6161 | -0.0104 | 1 | 33 | 0.07 | 1 | K.DSYVGDEAQSKR.G 10348 10349 |
|  | 13087 | 758.8746 | 1515.7347 | 1514.7419 | 0.9928 | 0 | 38 | 0.022 | 1 | K.IWHHTFYNELR.V 13067 13068 |
|  | 14707 | 541.9487 | 1622.8244 | 1622.8338 | -0.0094 | 1 | 10 | 12 | 4 | R.LDLAGRDLTDYLMK.I |
|  | 17255 | 895.9438 | 1789.8730 | 1789.8846 | -0.0116 | 0 | 71 | 9.6e-006 | 1 | K.SYELPDGQVITIGNER.F 17256 17257 17258 17267 17268 |
|  | 19306 | **978.5182** | **1955.0219** | **1955.0364** | **-0.0144** | **0** | **54** | **0.00035** | **1** | **R.VAPEEHPTLLTEAPLNPK.A** 19307 19308 |
|  | 19360 | 980.9514 | 1959.8883 | 1959.9036 | -0.0154 | 0 | 43 | 0.0047 | 1 | K.YPIEHGIITNWDDMEK.I 19361 19362 19363 |
|  | 22116 | **1114.5293** | **2227.0440** | **2227.0579** | **-0.0139** | **0** | **60** | **7.5e-005** | **1** | **K.DLYANNVLSGGTTMYPGIADR.M** |
|  | 23105 | **786.0529** | **2355.1368** | **2355.1529** | **-0.0161** | **1** | **59** | **9.6e-005** | **1** | **R.KDLYANNVLSGGTTMYPGIADR.M** |
|  | 23193 | 789.4443 | 2365.3112 | 2366.2464 | -0.9352 | 1 | 1 | 50 | 1 | R.AVFPSIVGRPRHQGVMVGMGQK.D |
|  | 23210 | 592.8179 | 2367.2426 | 2366.0631 | 1.1795 | 1 | 1 | 60 | 2 | R.HQGVMVGMGQKDSYVGDEAQSK.R |
|  | 24542 | 1277.1577 | 2552.3009 | 2551.1458 | 1.1551 | 0 | 30 | 0.071 | 2 | K.LCYVALDFENEMATAASSSSLEK.S 24503 24506 24519 24520 24522 24531 24536 24539 24540 24541 24543 24544 24547 24548 24551 24555 24556 24558 24559 24560 |
|  | 25973 | **937.5005** | **2809.4798** | **2808.2833** | **1.1965** | **1** | **0** | **55** | **2** | **K.EKLCYVALDFENEMATAASSSSLEK.S** |
|  | 27863 | **1063.4888** | **3187.4445** | **3187.4664** | **-0.0219** | **0** | **42** | **0.0027** | **1** | **R.CPETLFQPSFIGMESAGIHETTYNSIMK.C** |
|  | 27883 | **1066.2007** | **3195.5802** | **3195.6023** | **-0.0221** | **0** | **62** | **3.3e-005** | **1** | **R.TTGIVLDSGDGVTHNVPIYEGYALPHAIMR.L** 27885 |
|  | 28706 | **865.1603** | **3456.6120** | **3456.6349** | **-0.0229** | **1** | **16** | **0.96** | **1** | **K.YPIEHGIITNWDDMEKIWHHTFYNELR.V** |

| **61.** | P30101\|PDIA3_HUMAN    **Mass:** 57146    **Score:** 744    **Queries matched:** 62   **emPAI:** 3.01 |
| --- | --- |
|  | Protein disulfide-isomerase A3 OS=Homo sapiens GN=PDIA3 PE=1 SV=4 |

|  | **Query** | **Observed** | **Mr(expt)** | **Mr(calc)** | **Delta** | **Miss** | **Score** | **Expect** | **Rank** | **Peptide** |
| --- | --- | --- | --- | --- | --- | --- | --- | --- | --- | --- |
|  | 1788 | **395.2075** | **788.4004** | **787.4188** | **0.9816** | **1** | **6** | **56** | **5** | **R.DGKALER.F** |
|  | 2810 | **866.4545** | **865.4473** | **865.4545** | **-0.0072** | **1** | **15** | **5.3** | **1** | **K.YKELGEK.L** |
|  | 2975 | **439.2452** | **876.4759** | **876.4817** | **-0.0058** | **0** | **54** | **0.0008** | **1** | **K.LNFAVASR.K** 2980 |
|  | 4559 | **498.2944** | **994.5742** | **994.5560** | **0.0183** | **0** | **9** | **19** | **8** | **K.QAGPASVPLR.T** 4558 |
|  | 5189 | **520.7869** | **1039.5593** | **1039.5662** | **-0.0069** | **0** | **28** | **0.24** | **1** | **R.TADGIVSHLK.K** |
|  | 5731 | **542.7838** | **1083.5529** | **1083.5601** | **-0.0071** | **0** | **35** | **0.055** | **1** | **K.YGVSGYPTLK.I** 5730 |
|  | 6825 | **584.8334** | **1167.6523** | **1167.6612** | **-0.0088** | **1** | **43** | **0.0069** | **1** | **R.TADGIVSHLKK.Q** 6826 |
|  | 6878 | **586.7695** | **1171.5244** | **1171.5332** | **-0.0088** | **0** | **14** | **6.7** | **1** | **K.FVMQEEFSR.D** |
|  | 7219 | **596.2999** | **1190.5853** | **1190.5931** | **-0.0078** | **0** | **46** | **0.004** | **1** | **R.LAPEYEAAATR.L** 7196 |
|  | 7345 | **599.3596** | **1196.7046** | **1196.7128** | **-0.0083** | **1** | **47** | **0.0031** | **1** | **K.LSKDPNIVIAK.M** 7318 7346 |
|  | 10105 | **671.3416** | **1340.6687** | **1340.6765** | **-0.0078** | **0** | **39** | **0.019** | **1** | **R.GFPTIYFSPANK.K** |
|  | 10212 | **674.3500** | **1346.6855** | **1346.6942** | **-0.0087** | **1** | **60** | **0.00015** | **1** | **K.RLAPEYEAAATR.L** |
|  | 10604 | **684.8312** | **1367.6479** | **1367.6569** | **-0.0089** | **0** | **16** | **3.6** | **2** | **K.SEPIPESNDGPVK.V** 10603 |
|  | 10651 | **685.8472** | **1369.6799** | **1369.6878** | **-0.0079** | **0** | **21** | **1.1** | **1** | **R.ELSDFISYLQR.E** 10645 |
|  | 11116 | **699.2885** | **1396.5623** | **1396.5711** | **-0.0088** | **0** | **59** | **0.00016** | **1** | **K.VDCTANTNTCNK.Y** 11114 11115 11118 11120 11121 11144 |
|  | 12126 | **486.9054** | **1457.6945** | **1457.7038** | **-0.0094** | **1** | **18** | **2.4** | **1** | **K.FEDKTVAYTEQK.M** |
|  | 12328 | 735.3875 | 1468.7605 | 1468.7714 | -0.0110 | 1 | 33 | 0.07 | 1 | R.GFPTIYFSPANKK.L |
|  | 12395 | **737.4144** | **1472.8142** | **1471.6766** | **1.1376** | **1** | **0** | **1.2e+002** | **9** | **K.FVMQEEFSRDGK.A** |
|  | 12749 | **747.8723** | **1493.7301** | **1494.9286** | **-1.1985** | **0** | **4** | **56** | **5** | **R.LALFPGVALLLAAAR.L** 12750 |
|  | 13072 | **758.3833** | **1514.7520** | **1514.7518** | **0.0003** | **1** | **9** | **16** | **3** | **R.FLQDYFDGNLKR.Y** |
|  | 14017 | **790.4145** | **1578.8144** | **1578.8253** | **-0.0109** | **0** | **48** | **0.002** | **1** | **R.EATNPPVIQEEKPK.K** 14018 |
|  | 15060 | **823.4092** | **1644.8038** | **1644.8736** | **-0.0698** | **1** | **13** | **5.8** | **2** | **K.FLDAGHKLNFAVASR.K** |
|  | 15175 | **826.8820** | **1651.7494** | **1651.7590** | **-0.0097** | **1** | **66** | **3e-005** | **1** | **K.IFRDGEEAGAYDGPR.T** |
|  | 15347 | **832.8768** | **1663.7390** | **1663.7512** | **-0.0122** | **0** | **75** | **3.5e-006** | **1** | **K.MDATANDVPSPYEVR.G** 15338 15348 |
|  | 16041 | **854.4677** | **1706.9207** | **1706.9203** | **0.0005** | **1** | **10** | **12** | **1** | **R.EATNPPVIQEEKPKK.K** |
|  | 23892 | **821.7364** | **2462.1875** | **2462.1206** | **0.0669** | **1** | **2** | **49** | **4** | **K.VDCTANTNTCNKYGVSGYPTLK.I** |
|  | 24707 | **1288.1471** | **2574.2796** | **2574.2966** | **-0.0170** | **0** | **104** | **2.8e-009** | **1** | **K.TFSHELSDFGLESTAGEIPVVAIR.T** 24705 24706 |
|  | 24755 | **1291.5826** | **2581.1507** | **2582.1756** | **-1.0249** | **0** | **6** | **17** | **2** | **R.ISDTGSAGLMLVEFFAPWCGHCK.R** |
|  | 24831 | **866.7221** | **2597.1445** | **2598.1706** | **-1.0261** | **0** | **(4)** | **26** | **1** | **R.ISDTGSAGLMLVEFFAPWCGHCK.R** |
|  | 25358 | **1352.1946** | **2702.3746** | **2702.3915** | **-0.0169** | **1** | **58** | **0.0001** | **1** | **R.KTFSHELSDFGLESTAGEIPVVAIR.T** 25359 |
|  | 26176 | **949.7929** | **2846.3569** | **2846.3731** | **-0.0162** | **1** | **41** | **0.0049** | **1** | **K.FIQENIFGICPHMTEDNKDLIQGK.D** |
|  | 26723 | **980.1249** | **2937.3528** | **2937.3709** | **-0.0181** | **1** | **80** | **5.8e-007** | **1** | **K.FISDKDASIVGFFDDSFSEAHSEFLK.A** 26725 |
|  | 27067 | **1001.5457** | **3001.6151** | **3002.4120** | **-0.7969** | **1** | **0** | **48** | **3** | **K.MDATANDVPSPYEVRGFPTIYFSPANK.K** |
|  | 28885 | **1177.2417** | **3528.7033** | **3528.7273** | **-0.0241** | **0** | **73** | **1.8e-006** | **1** | **R.FAHTNVESLVNEYDDNGEGIILFRPSHLTNK.F** 28884 28886 28887 |
|  | 30166 | **1013.2415** | **4048.9370** | **4047.9603** | **0.9767** | **1** | **39** | **0.0036** | **1** | **R.FAHTNVESLVNEYDDNGEGIILFRPSHLTNKFEDK.T** |
|  | 30959 | **909.8502** | **4544.2144** | **4545.0927** | **-0.8783** | **1** | **0** | **19** | **7** | **R.LAAASDVLELTDDNFESRISDTGSAGLMLVEFFAPWCGHCK.R** |

| **62.** | P21397\|AOFA_HUMAN    **Mass:** 60157    **Score:** 739    **Queries matched:** 56   **emPAI:** 1.61 |
| --- | --- |
|  | Amine oxidase [flavin-containing] A OS=Homo sapiens GN=MAOA PE=1 SV=1 |

|  | **Query** | **Observed** | **Mr(expt)** | **Mr(calc)** | **Delta** | **Miss** | **Score** | **Expect** | **Rank** | **Peptide** |
| --- | --- | --- | --- | --- | --- | --- | --- | --- | --- | --- |
|  | 1529 | **683.3465** | **682.3392** | **682.3439** | **-0.0046** | **0** | **9** | **16** | **1** | **K.TYPFR.G** |
|  | 1745 | **771.4420** | **770.4347** | **770.4399** | **-0.0051** | **0** | **35** | **0.05** | **1** | **R.NQLIQR.L** 1750 |
|  | 1882 | **398.2250** | **794.4354** | **793.3276** | **1.1078** | **0** | **13** | **7.5** | **5** | **-.MENQEK.A** |
|  | 2334 | **415.2291** | **828.4437** | **827.4939** | **0.9498** | **0** | **14** | **7.8** | **4** | **R.LPMGAVIK.C** |
|  | 2517 | **422.7490** | **843.4834** | **843.4888** | **-0.0054** | **0** | **(12)** | **12** | **1** | **R.LPMGAVIK.C** 2534 |
|  | 3202 | **448.7280** | **895.4414** | **895.4473** | **-0.0059** | **0** | **22** | **1** | **1** | **K.ICELYAK.V** 3204 |
|  | 3974 | **476.7733** | **951.5319** | **951.4913** | **0.0407** | **0** | **12** | **9.3** | **8** | **K.ELGIETYK.V** 3968 |
|  | 4965 | **512.7746** | **1023.5346** | **1023.5423** | **-0.0076** | **1** | **24** | **0.64** | **1** | **K.KICELYAK.V** |
|  | 6222 | **561.2954** | **1120.5763** | **1121.5466** | **-0.9703** | **0** | **72** | **9.3e-006** | **1** | **K.FVGGSGQVSER.I** 6233 6245 |
|  | 6338 | **566.3041** | **1130.5936** | **1130.6005** | **-0.0069** | **0** | **41** | **0.013** | **1** | **R.IMDLLGDQVK.L** |
|  | 6485 | 571.3520 | 1140.6895 | 1139.5566 | 1.1329 | 1 | 4 | 60 | 5 | K.MTMKELIDK.I |
|  | 7090 | **593.8085** | **1185.6025** | **1184.6077** | **0.9948** | **0** | **31** | **0.12** | **1** | **R.IFFAGTETATK.W** |
|  | 7567 | **403.2249** | **1206.6530** | **1206.5993** | **0.0537** | **0** | **6** | **34** | **4** | **R.IFSVTNGGQER.K** |
|  | 8418 | **625.8241** | **1249.6336** | **1249.6415** | **-0.0079** | **1** | **60** | **0.00015** | **1** | **R.KFVGGSGQVSER.I** 8417 8419 8437 |
|  | 9094 | **644.4320** | **1286.8493** | **1285.7242** | **1.1252** | **1** | **1** | **99** | **9** | **R.EVLNGLGKVTEK.D** |
|  | 10001 | **667.8383** | **1333.6621** | **1334.6943** | **-1.0322** | **1** | **7** | **26** | **9** | **R.IFSVTNGGQERK.F** 10002 |
|  | 10524 | **682.8707** | **1363.7269** | **1363.7360** | **-0.0091** | **0** | **36** | **0.037** | **1** | **K.IHFRPELPAER.N** 10525 10526 |
|  | 11181 | **700.9064** | **1399.7983** | **1399.8075** | **-0.0092** | **0** | **12** | **8.4** | **2** | **K.YVINAIPPTLTAK.I** |
|  | 13453 | **771.3329** | **1540.6512** | **1540.6616** | **-0.0104** | **0** | **89** | **1.6e-007** | **1** | **K.WSGYMEGAVEAGER.A** 13454 13472 13474 |
|  | 15659 | **844.4251** | **1686.8355** | **1686.8465** | **-0.0109** | **1** | **76** | **2.9e-006** | **1** | **K.VTEKDIWVQEPESK.D** 15658 |
|  | 15889 | **850.4199** | **1698.8252** | **1698.8366** | **-0.0114** | **0** | **70** | **1.2e-005** | **1** | **K.DVPAVEITHTFWER.N** |
|  | 16020 | **569.8419** | **1706.5038** | **1706.7900** | **-0.2862** | **0** | **5** | **23** | **1** | **K.EIPTDAPWEAQHADK.W** |
|  | 16493 | **868.9004** | **1735.7862** | **1735.8169** | **-0.0307** | **1** | **10** | **11** | **2** | **R.LPMGAVIKCMMYYK.E** |
|  | 16494 | **868.9009** | **1735.7873** | **1735.8169** | **-0.0296** | **1** | **(7)** | **22** | **5** | **R.LPMGAVIKCMMYYK.E** |
|  | 17797 | **918.4614** | **1834.9083** | **1834.9213** | **-0.0130** | **0** | **42** | **0.0078** | **1** | **K.VLGSQEALHPVHYEEK.N** |
|  | 21093 | 1058.9905 | 2115.9664 | 2116.1653 | -0.1989 | 1 | 1 | 77 | 10 | K.IHFRPELPAERNQLIQR.L |
|  | 21263 | **1068.9957** | **2135.9769** | **2135.9912** | **-0.0143** | **1** | **39** | **0.0095** | **1** | **K.EIPTDAPWEAQHADKWDK.M** 21264 |
|  | 21740 | **1095.5065** | **2188.9984** | **2189.0138** | **-0.0154** | **0** | **93** | **4.5e-008** | **1** | **R.NEHVDYVDVGGAYVGPTQNR.I** 21737 21739 |
|  | 22341 | **1129.0974** | **2256.1803** | **2256.1936** | **-0.0134** | **0** | **59** | **9e-005** | **1** | **K.ASIAGHMFDVVVIGGGISGLSAAK.L** 22340 22344 |
|  | 22443 | **758.4356** | **2272.2848** | **2272.1886** | **0.0963** | **0** | **(0)** | **67** | **1** | **K.ASIAGHMFDVVVIGGGISGLSAAK.L** |
|  | 23489 | **1203.5992** | **2405.1839** | **2405.1957** | **-0.0117** | **0** | **84** | **2.8e-007** | **1** | **R.GAFPPVWNPIAYLDYNNLWR.T** 23487 23488 23490 |
|  | 28362 | **836.3947** | **3341.5497** | **3341.5735** | **-0.0238** | **0** | **23** | **0.22** | **1** | **K.LNHPVTHVDQSSDNIIIETLNHEHYECK.Y** |

| **63.** | P10632\|CP2C8_HUMAN    **Mass:** 56587    **Score:** 739    **Queries matched:** 69   **emPAI:** 2.58 |
| --- | --- |
|  | Cytochrome P450 2C8 OS=Homo sapiens GN=CYP2C8 PE=1 SV=2 |

|  | **Query** | **Observed** | **Mr(expt)** | **Mr(calc)** | **Delta** | **Miss** | **Score** | **Expect** | **Rank** | **Peptide** |
| --- | --- | --- | --- | --- | --- | --- | --- | --- | --- | --- |
|  | 1456 | 653.3032 | 652.2959 | 652.3003 | -0.0043 | 0 | 14 | 5.1 | 6 | R.NFGMGK.R |
|  | 1766 | **781.4157** | **780.4084** | **780.4130** | **-0.0046** | **0** | **34** | **0.074** | **1** | **K.HPEVTAK.V** |
|  | 2082 | **405.2055** | **808.3964** | **808.4014** | **-0.0050** | **1** | **11** | **13** | **4** | **R.NFGMGKR.S** |
|  | 2341 | **415.7027** | **829.3909** | **829.3970** | **-0.0061** | **0** | **4** | **85** | **3** | **K.SFTNFSK.V** |
|  | 2663 | **429.1951** | **856.3756** | **857.4355** | **-1.0599** | **0** | **28** | **0.3** | **1** | **R.GNSPISQR.I** |
|  | 3694 | **466.8098** | **931.6051** | **931.6106** | **-0.0055** | **0** | **71** | **1.5e-005** | **1** | **R.YGLLLLLK.H** 3702 |
|  | 3880 | **473.7387** | **945.4629** | **945.4702** | **-0.0073** | **0** | **29** | **0.22** | **2** | **R.ICAGEGLAR.M** 3855 3891 3892 |
|  | 4093 | **481.2659** | **960.5172** | **960.5240** | **-0.0068** | **0** | **41** | **0.015** | **1** | **K.NLNTTAVTK.G** |
|  | 4378 | **491.7427** | **981.4709** | **981.4780** | **-0.0072** | **1** | **27** | **0.29** | **1** | **K.RFNENFR.I** |
|  | 4530 | 497.2375 | 992.4605 | 992.5767 | -0.1162 | 1 | 7 | 35 | 4 | R.RFSLTTLR.N |
|  | 5956 | **551.2539** | **1100.4933** | **1100.6302** | **-0.1369** | **1** | **10** | **17** | **4** | **K.GLGIISSNGKR.W** 5943 |
|  | 6069 | **555.2833** | **1108.5521** | **1108.5587** | **-0.0066** | **0** | **17** | **2.8** | **1** | **K.DQNFLTLMK.R** |
|  | 6857 | **585.7937** | **1169.5728** | **1169.5791** | **-0.0062** | **0** | **44** | **0.0063** | **1** | **R.DFIDCFLIK.M** 6856 |
|  | 7086 | 396.1775 | 1185.5106 | 1185.5131 | -0.0026 | 1 | 17 | 3 | 1 | R.HRSPCMQDR.S 7081 7085 7123 |
|  | 8411 | **625.7788** | **1249.5431** | **1248.5485** | **0.9946** | **0** | **39** | **0.018** | **1** | **K.SDYFMPFSAGK.R** |
|  | 9211 | **647.8364** | **1293.6583** | **1293.6677** | **-0.0094** | **0** | **59** | **0.00016** | **1** | **K.VQEEIDHVIGR.H** 9212 |
|  | 10745 | **688.8618** | **1375.7090** | **1376.6435** | **-0.9345** | **1** | **(4)** | **50** | **3** | **K.KSDYFMPFSAGK.R** |
|  | 10784 | **690.3321** | **1378.6496** | **1378.6589** | **-0.0093** | **0** | **57** | **0.00027** | **1** | **K.EHQASLDVNNPR.D** |
|  | 11061 | **697.3727** | **1392.7308** | **1392.6384** | **0.0924** | **1** | **12** | **8.7** | **1** | **K.KSDYFMPFSAGK.R** 11044 |
|  | 11764 | **718.3525** | **1434.6904** | **1435.6579** | **-0.9675** | **0** | **58** | **0.0002** | **1** | **K.EALIDNGEEFSGR.G** 11765 11778 |
|  | 14446 | **536.2776** | **1605.8111** | **1605.8223** | **-0.0112** | **1** | **65** | **3.9e-005** | **1** | **K.VKEHQASLDVNNPR.D** 14456 |
|  | 14506 | **806.3884** | **1610.7623** | **1610.7722** | **-0.0099** | **0** | **71** | **8.7e-006** | **1** | **R.VQEEAHCLVEELR.K** 14504 14505 |
|  | 14627 | **809.9304** | **1617.8462** | **1617.8574** | **-0.0112** | **1** | **62** | **7.7e-005** | **1** | **K.SVDDLKNLNTTAVTK.G** 14651 |
|  | 15334 | **555.2833** | **1662.8282** | **1661.8123** | **1.0158** | **1** | **9** | **15** | **2** | **R.FDYKDQNFLTLMK.R** |
|  | 15811 | **424.5080** | **1694.0029** | **1694.0130** | **-0.0101** | **1** | **25** | **0.33** | **1** | **R.YGLLLLLKHPEVTAK.V** 15802 |
|  | 16529 | **870.4347** | **1738.8548** | **1738.8672** | **-0.0124** | **1** | **61** | **9.1e-005** | **1** | **R.VQEEAHCLVEELRK.T** |
|  | 17122 | **594.9534** | **1781.8385** | **1781.8519** | **-0.0134** | **0** | **50** | **0.0012** | **1** | **R.SHMPYTDAVVHEIQR.Y** 17123 17124 17126 |
|  | 17242 | **895.4770** | **1788.9395** | **1788.9484** | **-0.0089** | **0** | **12** | **6.4** | **1** | **K.GIVSLPPSYQICFIPV.-** |
|  | 18648 | **950.9793** | **1899.9441** | **1899.9578** | **-0.0138** | **0** | **27** | **0.22** | **1** | **R.YSDLVPTGVPHAVTTDTK.F** 18649 18650 |
|  | 20790 | **1043.0721** | **2084.1297** | **2084.1380** | **-0.0083** | **0** | **6** | **20** | **1** | **R.MELFLFLTTILQNFNLK.S** |
|  | 21069 | **705.0714** | **2112.1924** | **2112.2017** | **-0.0093** | **0** | **14** | **3** | **1** | **K.LPPGPTPLPIIGNMLQIDVK.D** |
|  | 22230 | **1121.1491** | **2240.2835** | **2240.2966** | **-0.0131** | **1** | **30** | **0.075** | **1** | **R.KLPPGPTPLPIIGNMLQIDVK.D** |
|  | 22344 | 565.2560 | 2256.9948 | 2256.2915 | 0.7033 | 1 | (4) | 32 | 3 | R.KLPPGPTPLPIIGNMLQIDVK.D |
|  | 26091 | **1417.6881** | **2833.3617** | **2832.4350** | **0.9267** | **0** | **4** | **23** | **2** | **K.VYGPVFTVYFGMNPIVVFHGYEAVK.E** 26082 |
|  | 26121 | **947.4414** | **2839.3022** | **2839.3166** | **-0.0144** | **0** | **7** | **12** | **1** | **K.ASPCDPTFILGCAPCNVICSVVFQK.R** |
|  | 26986 | **1493.2478** | **2984.4810** | **2984.4979** | **-0.0168** | **0** | **128** | **8.9e-012** | **1** | **K.SEFNIENLVGTVADLFVAGTETTSTTLR.Y** 26987 26988 |
|  | 27515 | **1033.1790** | **3096.5151** | **3096.5314** | **-0.0163** | **0** | **64** | **2.2e-005** | **1** | **R.ILNSPWIQVCNNFPLLIDCFPGTHNK.V** 27517 27522 27523 |
|  | 28749 | 1157.5749 | 3469.7030 | 3469.7213 | -0.0183 | 1 | 28 | 0.066 | 1 | K.DNQKSEFNIENLVGTVADLFVAGTETTSTTLR.Y |
|  | 29046 | **1194.9296** | **3581.7669** | **3581.7865** | **-0.0196** | **1** | **37** | **0.008** | **1** | **K.GTTIMALLTSVLHDDKEFPNPNIFDPGHFLDK.N** 29049 |

| **64.** | P63267\|ACTH_HUMAN    **Mass:** 42249    **Score:** 716    **Queries matched:** 73   **emPAI:** 4.95 |
| --- | --- |
|  | Actin, gamma-enteric smooth muscle OS=Homo sapiens GN=ACTG2 PE=1 SV=1 |

|  | **Query** | **Observed** | **Mr(expt)** | **Mr(calc)** | **Delta** | **Miss** | **Score** | **Expect** | **Rank** | **Peptide** |
| --- | --- | --- | --- | --- | --- | --- | --- | --- | --- | --- |
|  | 1363 | 630.3417 | 629.3344 | 630.3701 | -1.0357 | 0 | 4 | 99 | 2 | K.ILTER.G |
|  | 1421 | 644.3680 | 643.3607 | 643.3653 | -0.0046 | 0 | 20 | 2.6 | 1 | R.LDLAGR.D |
|  | 1884 | 795.4671 | 794.4598 | 794.4650 | -0.0052 | 0 | 17 | 3.4 | 2 | K.IIAPPER.K |
|  | 4297 | 488.7242 | 975.4339 | 975.4410 | -0.0071 | 0 | 67 | 3.7e-005 | 1 | K.AGFAGDDAPR.A 4293 4296 |
|  | 4616 | 499.7438 | 997.4730 | 997.4790 | -0.0060 | 0 | 33 | 0.076 | 1 | R.DLTDYLMK.I |
|  | 5140 | 518.8257 | 1035.6369 | 1035.6440 | -0.0071 | 1 | 26 | 0.39 | 1 | K.IKIIAPPER.K |
|  | 6325 | 565.7733 | 1129.5321 | 1129.5404 | -0.0083 | 0 | 46 | 0.0042 | 1 | R.GYSFVTTAER.E |
|  | 6737 | 581.3089 | 1160.6032 | 1160.6111 | -0.0078 | 0 | 48 | 0.0027 | 1 | K.EITALAPSTMK.I 6736 6738 |
|  | 6867 | 586.2850 | 1170.5555 | 1170.5638 | -0.0083 | 0 | 50 | 0.0016 | 1 | R.HQGVMVGMGQK.D 6865 6866 |
|  | 6943 | 589.3067 | 1176.5988 | 1176.6060 | -0.0072 | 0 | (4) | 67 | 3 | K.EITALAPSTMK.I |
|  | 7105 | 594.2822 | 1186.5499 | 1186.5587 | -0.0088 | 0 | (35) | 0.046 | 1 | R.HQGVMVGMGQK.D |
|  | 7106 | 594.2826 | 1186.5507 | 1186.5587 | -0.0080 | 0 | (47) | 0.003 | 1 | R.HQGVMVGMGQK.D 7093 |
|  | 7338 | 599.2899 | 1196.5652 | 1197.5150 | -0.9498 | 0 | 29 | 0.2 | 1 | K.DSYVGDEAQSK.R |
|  | 7365 | 599.8527 | 1197.6908 | 1197.6982 | -0.0075 | 0 | 58 | 0.00026 | 1 | R.AVFPSIVGRPR.H 7366 |
|  | 7465 | 602.2790 | 1202.5434 | 1202.5536 | -0.0102 | 0 | (4) | 69 | 5 | R.HQGVMVGMGQK.D |
|  | 10347 | 677.8101 | 1353.6057 | 1353.6161 | -0.0104 | 1 | 33 | 0.07 | 1 | K.DSYVGDEAQSKR.G 10348 10349 |
|  | 12840 | **751.3654** | **1500.7163** | **1500.7262** | **-0.0099** | **0** | **28** | **0.21** | **1** | **K.IWHHSFYNELR.V** 12841 |
|  | 14707 | 541.9487 | 1622.8244 | 1622.8338 | -0.0094 | 1 | 10 | 12 | 4 | R.LDLAGRDLTDYLMK.I |
|  | 17255 | 895.9438 | 1789.8730 | 1789.8846 | -0.0116 | 0 | 71 | 9.6e-006 | 1 | K.SYELPDGQVITIGNER.F 17256 17257 17258 17267 17268 |
|  | 19306 | 978.5182 | 1955.0219 | 1955.0364 | -0.0144 | 0 | 54 | 0.00035 | 1 | R.VAPEEHPTLLTEAPLNPK.A 19307 19308 |
|  | 19360 | 980.9514 | 1959.8883 | 1959.9036 | -0.0154 | 0 | 43 | 0.0047 | 1 | K.YPIEHGIITNWDDMEK.I 19361 19362 19363 |
|  | 20278 | 681.6606 | 2041.9599 | 2041.8391 | 0.1208 | 0 | 1 | 76 | 6 | M.CEEETTALVCDNGSGLCK.A |
|  | 22116 | 1114.5293 | 2227.0440 | 2227.0579 | -0.0139 | 0 | 60 | 7.5e-005 | 1 | K.DLYANNVLSGGTTMYPGIADR.M |
|  | 23105 | 786.0529 | 2355.1368 | 2355.1529 | -0.0161 | 1 | 59 | 9.6e-005 | 1 | R.KDLYANNVLSGGTTMYPGIADR.M |
|  | 23193 | 789.4443 | 2365.3112 | 2366.2464 | -0.9352 | 1 | 1 | 50 | 1 | R.AVFPSIVGRPRHQGVMVGMGQK.D |
|  | 23210 | 592.8179 | 2367.2426 | 2366.0631 | 1.1795 | 1 | 1 | 60 | 2 | R.HQGVMVGMGQKDSYVGDEAQSK.R |
|  | 24542 | 1277.1577 | 2552.3009 | 2551.1458 | 1.1551 | 0 | 30 | 0.071 | 2 | K.LCYVALDFENEMATAASSSSLEK.S 24503 24506 24519 24520 24522 24531 24536 24539 24540 24541 24543 24544 24547 24548 24551 24555 24556 24558 24559 24560 |
|  | 25973 | 937.5005 | 2809.4798 | 2808.2833 | 1.1965 | 1 | 0 | 55 | 2 | K.EKLCYVALDFENEMATAASSSSLEK.S |
|  | 27863 | 1063.4888 | 3187.4445 | 3187.4664 | -0.0219 | 0 | 42 | 0.0027 | 1 | R.CPETLFQPSFIGMESAGIHETTYNSIMK.C |
|  | 27883 | 1066.2007 | 3195.5802 | 3195.6023 | -0.0221 | 0 | 62 | 3.3e-005 | 1 | R.TTGIVLDSGDGVTHNVPIYEGYALPHAIMR.L 27885 |
|  | 30172 | 1351.9580 | 4052.8522 | 4052.0142 | 0.8380 | 0 | 1 | 22 | 10 | K.YSVWIGGSILASLSTFQQMWISKPEYDEAGPSIVHR.K |

| **65.** | P62736\|ACTA_HUMAN    **Mass:** 42381    **Score:** 716    **Queries matched:** 72   **emPAI:** 4.92 |
| --- | --- |
|  | Actin, aortic smooth muscle OS=Homo sapiens GN=ACTA2 PE=1 SV=1 |

|  | **Query** | **Observed** | **Mr(expt)** | **Mr(calc)** | **Delta** | **Miss** | **Score** | **Expect** | **Rank** | **Peptide** |
| --- | --- | --- | --- | --- | --- | --- | --- | --- | --- | --- |
|  | 1363 | 630.3417 | 629.3344 | 630.3701 | -1.0357 | 0 | 4 | 99 | 2 | K.ILTER.G |
|  | 1421 | 644.3680 | 643.3607 | 643.3653 | -0.0046 | 0 | 20 | 2.6 | 1 | R.LDLAGR.D |
|  | 1884 | 795.4671 | 794.4598 | 794.4650 | -0.0052 | 0 | 17 | 3.4 | 2 | K.IIAPPER.K |
|  | 4297 | 488.7242 | 975.4339 | 975.4410 | -0.0071 | 0 | 67 | 3.7e-005 | 1 | K.AGFAGDDAPR.A 4293 4296 |
|  | 4616 | 499.7438 | 997.4730 | 997.4790 | -0.0060 | 0 | 33 | 0.076 | 1 | R.DLTDYLMK.I |
|  | 5140 | 518.8257 | 1035.6369 | 1035.6440 | -0.0071 | 1 | 26 | 0.39 | 1 | K.IKIIAPPER.K |
|  | 6325 | 565.7733 | 1129.5321 | 1129.5404 | -0.0083 | 0 | 46 | 0.0042 | 1 | R.GYSFVTTAER.E |
|  | 6737 | 581.3089 | 1160.6032 | 1160.6111 | -0.0078 | 0 | 48 | 0.0027 | 1 | K.EITALAPSTMK.I 6736 6738 |
|  | 6867 | 586.2850 | 1170.5555 | 1170.5638 | -0.0083 | 0 | 50 | 0.0016 | 1 | R.HQGVMVGMGQK.D 6865 6866 |
|  | 6943 | 589.3067 | 1176.5988 | 1176.6060 | -0.0072 | 0 | (4) | 67 | 3 | K.EITALAPSTMK.I |
|  | 7105 | 594.2822 | 1186.5499 | 1186.5587 | -0.0088 | 0 | (35) | 0.046 | 1 | R.HQGVMVGMGQK.D |
|  | 7106 | 594.2826 | 1186.5507 | 1186.5587 | -0.0080 | 0 | (47) | 0.003 | 1 | R.HQGVMVGMGQK.D 7093 |
|  | 7338 | 599.2899 | 1196.5652 | 1197.5150 | -0.9498 | 0 | 29 | 0.2 | 1 | K.DSYVGDEAQSK.R |
|  | 7365 | 599.8527 | 1197.6908 | 1197.6982 | -0.0075 | 0 | 58 | 0.00026 | 1 | R.AVFPSIVGRPR.H 7366 |
|  | 7465 | 602.2790 | 1202.5434 | 1202.5536 | -0.0102 | 0 | (4) | 69 | 5 | R.HQGVMVGMGQK.D |
|  | 10347 | 677.8101 | 1353.6057 | 1353.6161 | -0.0104 | 1 | 33 | 0.07 | 1 | K.DSYVGDEAQSKR.G 10348 10349 |
|  | 12840 | 751.3654 | 1500.7163 | 1500.7262 | -0.0099 | 0 | 28 | 0.21 | 1 | K.IWHHSFYNELR.V 12841 |
|  | 14707 | 541.9487 | 1622.8244 | 1622.8338 | -0.0094 | 1 | 10 | 12 | 4 | R.LDLAGRDLTDYLMK.I |
|  | 17255 | 895.9438 | 1789.8730 | 1789.8846 | -0.0116 | 0 | 71 | 9.6e-006 | 1 | K.SYELPDGQVITIGNER.F 17256 17257 17258 17267 17268 |
|  | 19306 | 978.5182 | 1955.0219 | 1955.0364 | -0.0144 | 0 | 54 | 0.00035 | 1 | R.VAPEEHPTLLTEAPLNPK.A 19307 19308 |
|  | 19360 | 980.9514 | 1959.8883 | 1959.9036 | -0.0154 | 0 | 43 | 0.0047 | 1 | K.YPIEHGIITNWDDMEK.I 19361 19362 19363 |
|  | 22116 | 1114.5293 | 2227.0440 | 2227.0579 | -0.0139 | 0 | 60 | 7.5e-005 | 1 | K.DLYANNVLSGGTTMYPGIADR.M |
|  | 22610 | **573.5440** | **2290.1467** | **2289.8858** | **0.2609** | **0** | **2** | **46** | **3** | **-.MCEEEDSTALVCDNGSGLCK.A** |
|  | 23105 | 786.0529 | 2355.1368 | 2355.1529 | -0.0161 | 1 | 59 | 9.6e-005 | 1 | R.KDLYANNVLSGGTTMYPGIADR.M |
|  | 23193 | 789.4443 | 2365.3112 | 2366.2464 | -0.9352 | 1 | 1 | 50 | 1 | R.AVFPSIVGRPRHQGVMVGMGQK.D |
|  | 23210 | 592.8179 | 2367.2426 | 2366.0631 | 1.1795 | 1 | 1 | 60 | 2 | R.HQGVMVGMGQKDSYVGDEAQSK.R |
|  | 24542 | 1277.1577 | 2552.3009 | 2551.1458 | 1.1551 | 0 | 30 | 0.071 | 2 | K.LCYVALDFENEMATAASSSSLEK.S 24503 24506 24519 24520 24522 24531 24536 24539 24540 24541 24543 24544 24547 24548 24551 24555 24556 24558 24559 24560 |
|  | 25973 | 937.5005 | 2809.4798 | 2808.2833 | 1.1965 | 1 | 0 | 55 | 2 | K.EKLCYVALDFENEMATAASSSSLEK.S |
|  | 27863 | 1063.4888 | 3187.4445 | 3187.4664 | -0.0219 | 0 | 42 | 0.0027 | 1 | R.CPETLFQPSFIGMESAGIHETTYNSIMK.C |
|  | 27883 | 1066.2007 | 3195.5802 | 3195.6023 | -0.0221 | 0 | 62 | 3.3e-005 | 1 | R.TTGIVLDSGDGVTHNVPIYEGYALPHAIMR.L 27885 |

| **66.** | Q02928\|CP4AB_HUMAN    **Mass:** 59766    **Score:** 712    **Queries matched:** 34   **emPAI:** 1.33 |
| --- | --- |
|  | Cytochrome P450 4A11 OS=Homo sapiens GN=CYP4A11 PE=1 SV=1 |

|  | **Query** | **Observed** | **Mr(expt)** | **Mr(calc)** | **Delta** | **Miss** | **Score** | **Expect** | **Rank** | **Peptide** |
| --- | --- | --- | --- | --- | --- | --- | --- | --- | --- | --- |
|  | 1101 | **557.3727** | **556.3654** | **556.3697** | **-0.0043** | **0** | **22** | **0.93** | **1** | **K.VILGR.S** |
|  | 3096 | **443.0397** | **884.0648** | **884.4035** | **-0.3387** | **1** | **1** | **80** | **3** | **K.HQERCR.E** 3083 3089 |
|  | 4355 | 490.7437 | 979.4729 | 979.4797 | -0.0068 | 0 | 41 | 0.013 | 1 | K.QFAMNELK.V |
|  | 5528 | **534.8102** | **1067.6058** | **1067.6128** | **-0.0070** | **0** | **12** | **11** | **1** | **R.LYPPVPGIGR.E** |
|  | 9081 | **429.7552** | **1286.2437** | **1286.5853** | **-0.3415** | **0** | **4** | **38** | **10** | **R.VQLYDPDYMK.V** |
|  | 11355 | **705.9176** | **1409.8206** | **1409.8282** | **-0.0076** | **0** | **66** | **2.8e-005** | **1** | **R.HLDFLDILLLAK.M** |
|  | 12489 | **739.8560** | **1477.6974** | **1476.7242** | **0.9732** | **1** | **12** | **8.9** | **1** | **K.MENGSILSDKDLR.A** 12458 |
|  | 12754 | **498.9393** | **1493.7962** | **1492.7191** | **1.0770** | **1** | **(2)** | **74** | **5** | **K.MENGSILSDKDLR.A** |
|  | 12871 | **751.8711** | **1501.7276** | **1501.7354** | **-0.0078** | **0** | **39** | **0.017** | **1** | **K.VWPNPEVFDPFR.F** |
|  | 12892 | **752.8464** | **1503.6783** | **1503.6889** | **-0.0105** | **0** | **73** | **5.6e-006** | **1** | **K.CAFSHQGSIQVDR.N** |
|  | 15397 | **834.4959** | **1666.9773** | **1666.9671** | **0.0102** | **1** | **2** | **67** | **8** | **K.AVQLYLHRQWLLK.A** |
|  | 17281 | **598.3333** | **1791.9781** | **1790.9865** | **0.9916** | **0** | **14** | **4.2** | **1** | **K.GIMVLLSIYGLHHNPK.V** |
|  | 18568 | **947.9489** | **1893.8833** | **1893.8969** | **-0.0137** | **0** | **102** | **5.8e-009** | **1** | **R.NAFHQNDTIYSLTSAGR.W** 18569 18584 |
|  | 18859 | **959.4827** | **1916.9508** | **1916.9639** | **-0.0131** | **0** | **81** | **8.2e-007** | **1** | **R.ACQLAHQHTDQVIQLR.K** 18860 |
|  | 20483 | **1029.4944** | **2056.9742** | **2056.9868** | **-0.0126** | **0** | **68** | **1.4e-005** | **1** | **R.FAPGSAQHSHAFLPFSGGSR.N** 20481 20482 |
|  | 21551 | 1085.0444 | 2168.0743 | 2168.0862 | -0.0119 | 0 | 103 | 4.5e-009 | 1 | R.NSQSYIQAISDLNNLVFSR.V 21552 21553 |
|  | 24993 | **660.0908** | **2636.3342** | **2636.3495** | **-0.0153** | **0** | **20** | **0.72** | **1** | **R.MLTPAFHYDILKPYVGLMADSVR.V** 24994 |
|  | 28639 | **1144.2223** | **3429.6450** | **3429.6677** | **-0.0226** | **0** | **26** | **0.095** | **1** | **K.ALQQFPCPPSHWLFGHIQELQQDQELQR.I** |
|  | 29235 | **919.9258** | **3675.6740** | **3675.6942** | **-0.0202** | **1** | **46** | **0.00083** | **1** | **R.CREEIHSLLGDGASITWNHLDQMPYTTMCIK.E** |
|  | 29421 | **1249.2950** | **3744.8633** | **3744.8817** | **-0.0184** | **1** | **73** | **1.8e-006** | **1** | **R.VMLDKWEELLGQDSPLEVFQHVSLMTLDTIMK.C** 29422 29423 29424 |

| **67.** | Q13509\|TBB3_HUMAN    **Mass:** 50856    **Score:** 711    **Queries matched:** 52   **emPAI:** 2.11 |
| --- | --- |
|  | Tubulin beta-3 chain OS=Homo sapiens GN=TUBB3 PE=1 SV=2 |

|  | **Query** | **Observed** | **Mr(expt)** | **Mr(calc)** | **Delta** | **Miss** | **Score** | **Expect** | **Rank** | **Peptide** |
| --- | --- | --- | --- | --- | --- | --- | --- | --- | --- | --- |
|  | 1603 | 709.3656 | 708.3583 | 708.3411 | 0.0172 | 1 | 14 | 6.5 | 3 | R.GRMSMK.E |
|  | 2066 | 404.7367 | 807.4588 | 807.3399 | 0.1190 | 0 | 7 | 33 | 7 | R.EEYPDR.I |
|  | 5004 | 514.2689 | 1026.5233 | 1025.5328 | 0.9905 | 0 | 5 | 48 | 5 | K.VAVCDIPPR.G |
|  | 5494 | 533.3253 | 1064.6361 | 1064.4201 | 0.2160 | 0 | (4) | 60 | 6 | K.NMMAACDPR.H |
|  | 5521 | 534.7601 | 1067.5057 | 1068.5968 | -1.0911 | 0 | 7 | 29 | 7 | R.YLTVATVFR.G 5522 |
|  | 5678 | 541.2108 | 1080.4071 | 1080.4151 | -0.0080 | 0 | 29 | 0.16 | 1 | K.NMMAACDPR.H 5679 5694 |
|  | 6327 | 565.7967 | 1129.5788 | 1129.5880 | -0.0092 | 0 | 4 | 59 | 6 | R.FPGQLNADLR.K |
|  | 6499 | 572.3174 | 1142.6203 | 1142.6270 | -0.0067 | 0 | 70 | 1.8e-005 | 1 | K.LAVNMVPFPR.L 6498 |
|  | 7996 | 615.2989 | 1228.5833 | 1228.5910 | -0.0077 | 0 | 62 | 0.00011 | 1 | R.ISEQFTAMFR.R 7997 |
|  | 8802 | 636.3640 | 1270.7133 | 1270.7220 | -0.0086 | 1 | 47 | 0.0027 | 1 | R.KLAVNMVPFPR.L |
|  | 9112 | 430.2745 | 1287.8016 | 1286.7169 | 1.0847 | 1 | (7) | 28 | 2 | R.KLAVNMVPFPR.L 9101 |
|  | 9689 | 660.3509 | 1318.6872 | 1318.6955 | -0.0083 | 0 | 82 | 8.3e-007 | 1 | R.IMNTFSVVPSPK.V 9686 9687 9696 |
|  | 10023 | 668.3477 | 1334.6809 | 1334.6904 | -0.0095 | 0 | (19) | 1.8 | 1 | R.IMNTFSVVPSPK.V |
|  | 10908 | 693.3495 | 1384.6844 | 1384.6921 | -0.0077 | 1 | 45 | 0.004 | 1 | K.RISEQFTAMFR.R |
|  | 11009 | **464.2834** | **1389.8284** | **1389.6810** | **0.1475** | **0** | **1** | **1.1e+002** | **9** | **K.EVDEQMLAIQSK.N** |
|  | 11137 | **466.7581** | **1397.2524** | **1396.6623** | **0.5902** | **0** | **13** | **4.1** | **2** | **R.ISVYYNEASSHK.Y** |
|  | 14585 | 808.4162 | 1614.8178 | 1614.8287 | -0.0109 | 0 | 42 | 0.0069 | 1 | R.AILVDLEPGTMDSVR.S 14583 14586 14602 |
|  | 14658 | 810.9161 | 1619.8176 | 1619.8283 | -0.0107 | 0 | 15 | 3.6 | 2 | R.LHFFMPGFAPLTAR.G 14659 |
|  | 14838 | 816.4137 | 1630.8128 | 1630.8236 | -0.0108 | 0 | (37) | 0.025 | 1 | R.AILVDLEPGTMDSVR.S |
|  | 15728 | 846.4319 | 1690.8492 | 1690.8600 | -0.0108 | 0 | 35 | 0.032 | 1 | R.ALTVPELTQQMFDAK.N 15729 15730 15732 |
|  | 15835 | 848.9150 | 1695.8154 | 1695.8257 | -0.0102 | 0 | 33 | 0.051 | 1 | K.NSSYFVEWIPNNVK.V 15855 15856 |
|  | 16036 | 854.4293 | 1706.8441 | 1706.8549 | -0.0108 | 0 | (19) | 1.5 | 2 | R.ALTVPELTQQMFDAK.N |
|  | 17620 | 911.9583 | 1821.9019 | 1821.9156 | -0.0136 | 0 | 67 | 2.6e-005 | 1 | R.EIVHIQAGQCGNQIGAK.F 17614 17621 |
|  | 19345 | 979.9900 | 1957.9654 | 1957.9745 | -0.0091 | 0 | 103 | 4.9e-009 | 1 | K.GHYTEGAELVDSVLDVVR.K 19346 |
|  | 20818 | 1044.0366 | 2086.0587 | 2086.0695 | -0.0108 | 1 | 86 | 2.5e-007 | 1 | K.GHYTEGAELVDSVLDVVRK.E 20815 20817 20819 |
|  | 21027 | 1055.5288 | 2109.0431 | 2109.0571 | -0.0141 | 1 | 71 | 7.2e-006 | 1 | -.MREIVHIQAGQCGNQIGAK.F 21028 |
|  | 29278 | **740.5325** | **3697.6260** | **3697.6957** | **-0.0698** | **1** | **4** | **12** | **8** | **K.ECENCDCLQGFQLTHSLGGGTGSGMGTLLISKVR.E** |

| **68.** | P22310\|UD14_HUMAN    **Mass:** 60613    **Score:** 711    **Queries matched:** 51   **emPAI:** 1.75 |
| --- | --- |
|  | UDP-glucuronosyltransferase 1-4 OS=Homo sapiens GN=UGT1A4 PE=1 SV=1 |

|  | **Query** | **Observed** | **Mr(expt)** | **Mr(calc)** | **Delta** | **Miss** | **Score** | **Expect** | **Rank** | **Peptide** |
| --- | --- | --- | --- | --- | --- | --- | --- | --- | --- | --- |
|  | 1474 | 659.3670 | 658.3597 | 658.3650 | -0.0053 | 0 | 23 | 1.4 | 1 | K.AVINDK.S |
|  | 4073 | **480.7594** | **959.5043** | **959.5110** | **-0.0067** | **0** | **44** | **0.0083** | **1** | **K.AMAIADALGK.I** |
|  | 4819 | **506.7970** | **1011.5795** | **1011.5865** | **-0.0070** | **0** | **28** | **0.25** | **1** | **K.IPQTVLWR.Y** 4818 |
|  | 5184 | **520.7598** | **1039.5051** | **1039.5120** | **-0.0069** | **1** | **43** | **0.0075** | **1** | **K.SYKENIMR.L** 5183 |
|  | 5366 | **528.7572** | **1055.4998** | **1055.5069** | **-0.0071** | **1** | **(12)** | **9.2** | **2** | **K.SYKENIMR.L** |
|  | 5794 | **545.2573** | **1088.5000** | **1087.6059** | **0.8940** | **1** | **11** | **15** | **2** | **K.KAMAIADALGK.I** |
|  | 5875 | **548.2790** | **1094.5434** | **1093.5992** | **0.9442** | **1** | **10** | **14** | **2** | **R.EALRELHAR.G** 5855 |
|  | 11148 | **699.8788** | **1397.7430** | **1397.7496** | **-0.0066** | **0** | **37** | **0.024** | **1** | **K.YLSIPAVFFWR.Y** 11147 |
|  | 11968 | **724.8407** | **1447.6668** | **1447.6766** | **-0.0097** | **0** | **44** | **0.0058** | **1** | **K.GTQCPNPSSYIPK.L** 11967 |
|  | 13911 | **524.6165** | **1570.8277** | **1571.7912** | **-0.9635** | **0** | **4** | **51** | **7** | **R.SMAIMNNVSLALHR.C** |
|  | 13924 | **524.9633** | **1571.8681** | **1571.7912** | **0.0769** | **0** | **(2)** | **78** | **5** | **R.SMAIMNNVSLALHR.C** |
|  | 15244 | **828.9377** | **1655.8609** | **1655.8705** | **-0.0096** | **0** | **68** | **1.7e-005** | **1** | **K.VLVVPTDGSPWLSMR.E** 15256 |
|  | 15500 | 838.3856 | 1674.7566 | 1675.8352 | -1.0786 | 0 | 53 | 0.00059 | 1 | K.LLTTNSDHMTFLQR.V 15522 15523 |
|  | 15545 | **839.4250** | **1676.8354** | **1676.8457** | **-0.0103** | **0** | **56** | **0.00028** | **1** | **K.WLPQNDLLGHPMTR.A** 15543 15544 15546 |
|  | 16989 | **887.3036** | **1772.5927** | **1771.9403** | **0.6524** | **0** | **4** | **30** | **2** | **R.GHQAVVLTPEVNMHIK.E** |
|  | 18691 | 952.4685 | 1902.9225 | 1902.9986 | -0.0761 | 1 | 2 | 67 | 6 | K.LLTTNSDHMTFLQRVK.N |
|  | 19300 | **652.3673** | **1954.0801** | **1953.0870** | **0.9931** | **1** | **18** | **1.4** | **1** | **K.AMAIADALGKIPQTVLWR.Y** |
|  | 19557 | 660.0585 | 1977.1536 | 1977.9876 | -0.8341 | 1 | 1 | 62 | 9 | R.YSRSMAIMNNVSLALHR.C |
|  | 19592 | **661.6747** | **1982.0024** | **1982.0149** | **-0.0125** | **0** | **14** | **3.5** | **1** | **R.VTLGYTQGFFETEHLLK.R** 19591 |
|  | 21465 | **720.3764** | **2158.1074** | **2158.1205** | **-0.0131** | **1** | **36** | **0.025** | **1** | **R.GHQAVVLTPEVNMHIKEEK.F** |
|  | 21832 | **1102.5536** | **2203.0926** | **2203.1042** | **-0.0116** | **0** | **109** | **1.1e-009** | **1** | **K.GAGVTLNVLEMTSEDLENALK.A** 21831 21833 21836 21838 21839 21846 |
|  | 22825 | **1159.5969** | **2317.1793** | **2317.1929** | **-0.0136** | **0** | **66** | **1.7e-005** | **1** | **K.DRPVEPLDLAVFWVEFVMR.H** 22826 |
|  | 24585 | **852.4621** | **2554.3645** | **2553.3261** | **1.0383** | **0** | **4** | **26** | **3** | **R.HLNATSFDVVLTDPVNLCGAVLAK.Y** |
|  | 26144 | **948.8229** | **2843.4468** | **2843.4586** | **-0.0118** | **1** | **54** | **0.00021** | **1** | **K.GAGVTLNVLEMTSEDLENALKAVINDK.S** |
|  | 29713 | **956.9341** | **3823.7075** | **3823.7289** | **-0.0215** | **0** | **40** | **0.0032** | **1** | **R.AFITHAGSHGVYESICNGVPMVMMPLFGDQMDNAK.R** 29712 29714 29722 29724 29725 |
|  | 30060 | **1327.6094** | **3979.8063** | **3979.8300** | **-0.0237** | **1** | **38** | **0.0046** | **1** | **R.AFITHAGSHGVYESICNGVPMVMMPLFGDQMDNAKR.M** 30062 |

| **69.** | P55084\|ECHB_HUMAN    **Mass:** 51547    **Score:** 688    **Queries matched:** 56   **emPAI:** 1.86 |
| --- | --- |
|  | Trifunctional enzyme subunit beta, mitochondrial OS=Homo sapiens GN=HADHB PE=1 SV=3 |

|  | **Query** | **Observed** | **Mr(expt)** | **Mr(calc)** | **Delta** | **Miss** | **Score** | **Expect** | **Rank** | **Peptide** |
| --- | --- | --- | --- | --- | --- | --- | --- | --- | --- | --- |
|  | 1770 | **785.4454** | **784.4381** | **784.4443** | **-0.0062** | **0** | **21** | **1.2** | **1** | **R.AAPAVQTK.T** |
|  | 2533 | **423.2327** | **844.4508** | **844.4589** | **-0.0081** | **0** | **13** | **11** | **5** | **R.LVMAAANR.L** |
|  | 2611 | **426.7602** | **851.5059** | **851.5116** | **-0.0058** | **0** | **15** | **4.7** | **1** | **K.VGLPPLEK.F** |
|  | 2726 | **431.2305** | **860.4465** | **860.4538** | **-0.0073** | **0** | **(10)** | **21** | **3** | **R.LVMAAANR.L** 2711 2716 |
|  | 3342 | **453.2611** | **904.5077** | **904.5130** | **-0.0054** | **0** | **55** | **0.00058** | **1** | **R.LAAAFAVSR.L** |
|  | 3432 | **456.7817** | **911.5489** | **911.5552** | **-0.0063** | **0** | **8** | **21** | **2** | **K.TLAKPNIR.N** |
|  | 4023 | **956.5455** | **955.5382** | **955.5451** | **-0.0069** | **0** | **46** | **0.0041** | **1** | **R.NVVVVDGVR.T** |
|  | 4272 | 487.7856 | 973.5566 | 973.5630 | -0.0064 | 1 | 20 | 1.8 | 3 | R.KLMLDLNK.A 4249 4295 |
|  | 4307 | **489.2206** | **976.4267** | **977.5368** | **-1.1101** | **0** | **40** | **0.017** | **1** | **K.ALAMGYKPK.A** 4333 |
|  | 4490 | **495.7824** | **989.5501** | **989.5579** | **-0.0078** | **1** | **(10)** | **18** | **6** | **R.KLMLDLNK.A** |
|  | 5509 | **534.2650** | **1066.5153** | **1066.5229** | **-0.0076** | **0** | **14** | **5.9** | **1** | **K.DLMPHDLAR.A** |
|  | 6325 | 565.7733 | 1129.5321 | 1128.5889 | 0.9432 | 0 | 6 | 41 | 6 | -.MTILTYPFK.N |
|  | 7669 | **607.3228** | **1212.6311** | **1212.6390** | **-0.0080** | **0** | **19** | **1.7** | **1** | **R.TPFLLSGTSYK.D** |
|  | 7805 | **610.3339** | **1218.6532** | **1218.6754** | **-0.0222** | **1** | **7** | **29** | **9** | **K.SMGQRLSLISK.F** 7804 7817 |
|  | 8533 | **419.7284** | **1256.1633** | **1255.7037** | **0.4597** | **1** | **6** | **24** | **6** | **K.NLPTASKWALR.F** |
|  | 11439 | **708.3671** | **1414.7197** | **1415.7660** | **-1.0463** | **0** | **58** | **0.00022** | **1** | **K.DQLLLGPTYATPK.V** |
|  | 12228 | **488.7791** | **1463.3156** | **1462.8620** | **0.4536** | **1** | **1** | **75** | **7** | **R.AALTGLLHRTSVPK.E** |
|  | 13534 | **773.3826** | **1544.7507** | **1544.7617** | **-0.0110** | **0** | **9** | **17** | **2** | **K.DNGIRPSSLEQMAK.L** |
|  | 15069 | **823.7813** | **1645.5481** | **1644.8723** | **0.6758** | **1** | **1** | **71** | **4** | **K.KAQDEGLLSDVVPFK.V** |
|  | 15741 | **846.8366** | **1691.6587** | **1691.6708** | **-0.0121** | **0** | **74** | **4.1e-006** | **1** | **K.AMDSDWFAENYMGR.K** 15742 |
|  | 17639 | **608.9841** | **1823.9304** | **1823.9855** | **-0.0551** | **1** | **4** | **39** | **9** | **-.MTILTYPFKNLPTASK.W** |
|  | 18022 | **927.0042** | **1851.9939** | **1851.0030** | **0.9909** | **0** | **44** | **0.0036** | **1** | **K.EVVDYIIFGTVIQEVK.T** 18008 18019 |
|  | 18624 | 950.0154 | 1898.0162 | 1898.0149 | 0.0013 | 1 | 4 | 41 | 5 | K.AQDEGLLSDVVPFKVPGK.D |
|  | 21241 | **1067.9906** | **2133.9666** | **2133.9803** | **-0.0137** | **0** | **117** | **1.9e-010** | **1** | **K.FNNWGGSLSLGHPFGATGCR.L** 21242 21243 |
|  | 22370 | **754.7201** | **2261.1386** | **2261.1514** | **-0.0128** | **1** | **37** | **0.016** | **1** | **R.TPFLLSGTSYKDLMPHDLAR.A** 22369 |
|  | 22483 | 1140.0850 | 2278.1554 | 2277.1463 | 1.0090 | 1 | (4) | 29 | 3 | R.TPFLLSGTSYKDLMPHDLAR.A 22476 22479 22480 22484 22497 |
|  | 25365 | **1353.1351** | **2704.2557** | **2704.2737** | **-0.0180** | **0** | **88** | **1.1e-007** | **1** | **K.EGGQYGLVAACAAGGQGHAMIVEAYPK.-** 25364 25366 |
|  | 26221 | **950.7908** | **2849.3507** | **2848.3636** | **0.9870** | **1** | **0** | **53** | **4** | **R.KEGGQYGLVAACAAGGQGHAMIVEAYPK.-** |
|  | 26392 | **962.7943** | **2885.3609** | **2885.3694** | **-0.0085** | **0** | **37** | **0.011** | **1** | **K.AGLTMNDIDAFEFHEAFSGQILANFK.A** 26376 26385 26393 26405 26407 |
|  | 29017 | **1191.6101** | **3571.8085** | **3571.8306** | **-0.0221** | **0** | **109** | **4.6e-010** | **1** | **K.LKPAFIKPYGTVTAANSSFLTDGASAMLIMAEEK.A** |
|  | 31037 | **1531.7166** | **4592.1278** | **4591.0195** | **1.1083** | **1** | **1** | **14** | **9** | **K.AGLTMNDIDAFEFHEAFSGQILANFKAMDSDWFAENYMGR.K** |

| **70.** | O60656\|UD19_HUMAN    **Mass:** 60757    **Score:** 685    **Queries matched:** 39   **emPAI:** 1.17 |
| --- | --- |
|  | UDP-glucuronosyltransferase 1-9 OS=Homo sapiens GN=UGT1A9 PE=1 SV=1 |

|  | **Query** | **Observed** | **Mr(expt)** | **Mr(calc)** | **Delta** | **Miss** | **Score** | **Expect** | **Rank** | **Peptide** |
| --- | --- | --- | --- | --- | --- | --- | --- | --- | --- | --- |
|  | 1474 | 659.3670 | 658.3597 | 658.3650 | -0.0053 | 0 | 23 | 1.4 | 1 | K.AVINDK.S |
|  | 4073 | 480.7594 | 959.5043 | 959.5110 | -0.0067 | 0 | 44 | 0.0083 | 1 | K.AMAIADALGK.I |
|  | 4819 | 506.7970 | 1011.5795 | 1011.5865 | -0.0070 | 0 | 28 | 0.25 | 1 | K.IPQTVLWR.Y 4818 |
|  | 5184 | 520.7598 | 1039.5051 | 1039.5120 | -0.0069 | 1 | 43 | 0.0075 | 1 | K.SYKENIMR.L 5183 |
|  | 5366 | 528.7572 | 1055.4998 | 1055.5069 | -0.0071 | 1 | (12) | 9.2 | 2 | K.SYKENIMR.L |
|  | 5794 | 545.2573 | 1088.5000 | 1087.6059 | 0.8940 | 1 | 11 | 15 | 2 | K.KAMAIADALGK.I |
|  | 15545 | 839.4250 | 1676.8354 | 1676.8457 | -0.0103 | 0 | 56 | 0.00028 | 1 | K.WLPQNDLLGHPMTR.A 15543 15544 15546 |
|  | 19300 | 652.3673 | 1954.0801 | 1953.0870 | 0.9931 | 1 | 18 | 1.4 | 1 | K.AMAIADALGKIPQTVLWR.Y |
|  | 19447 | **984.4599** | **1966.9052** | **1966.9160** | **-0.0107** | **1** | **70** | **9.2e-006** | **1** | **K.TYSTSYTLEDLDREFK.A** 19448 |
|  | 21832 | 1102.5536 | 2203.0926 | 2203.1042 | -0.0116 | 0 | 109 | 1.1e-009 | 1 | K.GAGVTLNVLEMTSEDLENALK.A 21831 21833 21836 21838 21839 21846 |
|  | 22825 | 1159.5969 | 2317.1793 | 2317.1929 | -0.0136 | 0 | 66 | 1.7e-005 | 1 | K.DRPVEPLDLAVFWVEFVMR.H 22826 |
|  | 23024 | **1172.5561** | **2343.0977** | **2343.1093** | **-0.0116** | **0** | **47** | **0.0015** | **1** | **K.ESSFDAVFLDPFDNCGLIVAK.Y** 23023 |
|  | 24384 | **844.0737** | **2529.1994** | **2529.2144** | **-0.0151** | **0** | **15** | **2.1** | **1** | **R.GILCHYLEEGAQCPAPLSYVPR.I** 24385 |
|  | 26144 | 948.8229 | 2843.4468 | 2843.4586 | -0.0118 | 1 | 54 | 0.00021 | 1 | K.GAGVTLNVLEMTSEDLENALKAVINDK.S |
|  | 27166 | **758.3851** | **3029.5112** | **3028.3989** | **1.1123** | **0** | **0** | **47** | **4** | **-.MACTGWTSPLPLCVCLLLTCGFAEAGK.L** |
|  | 28492 | **1125.9172** | **3374.7299** | **3374.7398** | **-0.0099** | **0** | **10** | **3.8** | **1** | **K.NALEIASEILQTPVTEYDLYSHTSIWLLR.T** |
|  | 29713 | 956.9341 | 3823.7075 | 3823.7289 | -0.0215 | 0 | 40 | 0.0032 | 1 | R.AFITHAGSHGVYESICNGVPMVMMPLFGDQMDNAK.R 29712 29714 29722 29724 29725 |
|  | 30060 | 1327.6094 | 3979.8063 | 3979.8300 | -0.0237 | 1 | 38 | 0.0046 | 1 | R.AFITHAGSHGVYESICNGVPMVMMPLFGDQMDNAKR.M 30062 |

| **71.** | P68133\|ACTS_HUMAN    **Mass:** 42366    **Score:** 682    **Queries matched:** 73   **emPAI:** 4.44 |
| --- | --- |
|  | Actin, alpha skeletal muscle OS=Homo sapiens GN=ACTA1 PE=1 SV=1 |

|  | **Query** | **Observed** | **Mr(expt)** | **Mr(calc)** | **Delta** | **Miss** | **Score** | **Expect** | **Rank** | **Peptide** |
| --- | --- | --- | --- | --- | --- | --- | --- | --- | --- | --- |
|  | 1363 | 630.3417 | 629.3344 | 630.3701 | -1.0357 | 0 | 4 | 99 | 2 | K.ILTER.G |
|  | 1421 | 644.3680 | 643.3607 | 643.3653 | -0.0046 | 0 | 20 | 2.6 | 1 | R.LDLAGR.D |
|  | 1884 | 795.4671 | 794.4598 | 794.4650 | -0.0052 | 0 | 17 | 3.4 | 2 | K.IIAPPER.K |
|  | 4297 | 488.7242 | 975.4339 | 975.4410 | -0.0071 | 0 | 67 | 3.7e-005 | 1 | K.AGFAGDDAPR.A 4293 4296 |
|  | 4616 | 499.7438 | 997.4730 | 997.4790 | -0.0060 | 0 | 33 | 0.076 | 1 | R.DLTDYLMK.I |
|  | 5140 | 518.8257 | 1035.6369 | 1035.6440 | -0.0071 | 1 | 26 | 0.39 | 1 | K.IKIIAPPER.K |
|  | 6325 | 565.7733 | 1129.5321 | 1129.5404 | -0.0083 | 0 | 46 | 0.0042 | 1 | R.GYSFVTTAER.E |
|  | 6737 | 581.3089 | 1160.6032 | 1160.6111 | -0.0078 | 0 | 48 | 0.0027 | 1 | K.EITALAPSTMK.I 6736 6738 |
|  | 6867 | 586.2850 | 1170.5555 | 1170.5638 | -0.0083 | 0 | 50 | 0.0016 | 1 | R.HQGVMVGMGQK.D 6865 6866 |
|  | 6943 | 589.3067 | 1176.5988 | 1176.6060 | -0.0072 | 0 | (4) | 67 | 3 | K.EITALAPSTMK.I |
|  | 7105 | 594.2822 | 1186.5499 | 1186.5587 | -0.0088 | 0 | (35) | 0.046 | 1 | R.HQGVMVGMGQK.D |
|  | 7106 | 594.2826 | 1186.5507 | 1186.5587 | -0.0080 | 0 | (47) | 0.003 | 1 | R.HQGVMVGMGQK.D 7093 |
|  | 7338 | 599.2899 | 1196.5652 | 1197.5150 | -0.9498 | 0 | 29 | 0.2 | 1 | K.DSYVGDEAQSK.R |
|  | 7365 | 599.8527 | 1197.6908 | 1197.6982 | -0.0075 | 0 | 58 | 0.00026 | 1 | R.AVFPSIVGRPR.H 7366 |
|  | 7465 | 602.2790 | 1202.5434 | 1202.5536 | -0.0102 | 0 | (4) | 69 | 5 | R.HQGVMVGMGQK.D |
|  | 10347 | 677.8101 | 1353.6057 | 1353.6161 | -0.0104 | 1 | 33 | 0.07 | 1 | K.DSYVGDEAQSKR.G 10348 10349 |
|  | 13087 | 758.8746 | 1515.7347 | 1514.7419 | 0.9928 | 0 | 38 | 0.022 | 1 | K.IWHHTFYNELR.V 13067 13068 |
|  | 14707 | 541.9487 | 1622.8244 | 1622.8338 | -0.0094 | 1 | 10 | 12 | 4 | R.LDLAGRDLTDYLMK.I |
|  | 17255 | 895.9438 | 1789.8730 | 1789.8846 | -0.0116 | 0 | 71 | 9.6e-006 | 1 | K.SYELPDGQVITIGNER.F 17256 17257 17258 17267 17268 |
|  | 19306 | 978.5182 | 1955.0219 | 1955.0364 | -0.0144 | 0 | 54 | 0.00035 | 1 | R.VAPEEHPTLLTEAPLNPK.A 19307 19308 |
|  | 19360 | 980.9514 | 1959.8883 | 1959.9036 | -0.0154 | 0 | 43 | 0.0047 | 1 | K.YPIEHGIITNWDDMEK.I 19361 19362 19363 |
|  | 22483 | 1140.0850 | 2278.1554 | 2277.0042 | 1.1512 | 0 | 2 | 56 | 8 | K.DLYANNVMSGGTTMYPGIADR.M |
|  | 23193 | 789.4443 | 2365.3112 | 2366.2464 | -0.9352 | 1 | 1 | 50 | 1 | R.AVFPSIVGRPRHQGVMVGMGQK.D |
|  | 23210 | 592.8179 | 2367.2426 | 2366.0631 | 1.1795 | 1 | 1 | 60 | 2 | R.HQGVMVGMGQKDSYVGDEAQSK.R |
|  | 24542 | 1277.1577 | 2552.3009 | 2551.1458 | 1.1551 | 0 | 30 | 0.071 | 2 | K.LCYVALDFENEMATAASSSSLEK.S 24503 24506 24519 24520 24522 24531 24536 24539 24540 24541 24543 24544 24547 24548 24551 24555 24556 24558 24559 24560 |
|  | 25115 | **889.0327** | **2664.0763** | **2664.1982** | **-0.1219** | **1** | **1** | **37** | **1** | **K.DLYANNVMSGGTTMYPGIADRMQK.E** |
|  | 25973 | 937.5005 | 2809.4798 | 2808.2833 | 1.1965 | 1 | 0 | 55 | 2 | K.EKLCYVALDFENEMATAASSSSLEK.S |
|  | 27863 | 1063.4888 | 3187.4445 | 3187.4664 | -0.0219 | 0 | 42 | 0.0027 | 1 | R.CPETLFQPSFIGMESAGIHETTYNSIMK.C |
|  | 27883 | 1066.2007 | 3195.5802 | 3195.6023 | -0.0221 | 0 | 62 | 3.3e-005 | 1 | R.TTGIVLDSGDGVTHNVPIYEGYALPHAIMR.L 27885 |
|  | 28706 | 865.1603 | 3456.6120 | 3456.6349 | -0.0229 | 1 | 16 | 0.96 | 1 | K.YPIEHGIITNWDDMEKIWHHTFYNELR.V |

| **72.** | P22309\|UD11_HUMAN    **Mass:** 60180    **Score:** 669    **Queries matched:** 39   **emPAI:** 1.61 |
| --- | --- |
|  | UDP-glucuronosyltransferase 1-1 OS=Homo sapiens GN=UGT1A1 PE=1 SV=1 |

|  | **Query** | **Observed** | **Mr(expt)** | **Mr(calc)** | **Delta** | **Miss** | **Score** | **Expect** | **Rank** | **Peptide** |
| --- | --- | --- | --- | --- | --- | --- | --- | --- | --- | --- |
|  | 1474 | 659.3670 | 658.3597 | 658.3650 | -0.0053 | 0 | 23 | 1.4 | 1 | K.AVINDK.S |
|  | 4073 | 480.7594 | 959.5043 | 959.5110 | -0.0067 | 0 | 44 | 0.0083 | 1 | K.AMAIADALGK.I |
|  | 4819 | 506.7970 | 1011.5795 | 1011.5865 | -0.0070 | 0 | 28 | 0.25 | 1 | K.IPQTVLWR.Y 4818 |
|  | 5184 | 520.7598 | 1039.5051 | 1039.5120 | -0.0069 | 1 | 43 | 0.0075 | 1 | K.SYKENIMR.L 5183 |
|  | 5366 | 528.7572 | 1055.4998 | 1055.5069 | -0.0071 | 1 | (12) | 9.2 | 2 | K.SYKENIMR.L |
|  | 5794 | 545.2573 | 1088.5000 | 1087.6059 | 0.8940 | 1 | 11 | 15 | 2 | K.KAMAIADALGK.I |
|  | 15545 | 839.4250 | 1676.8354 | 1676.8457 | -0.0103 | 0 | 56 | 0.00028 | 1 | K.WLPQNDLLGHPMTR.A 15543 15544 15546 |
|  | 16701 | **876.9801** | **1751.9456** | **1751.9570** | **-0.0114** | **0** | **53** | **0.0005** | **1** | **R.GHEIVVLAPDASLYIR.D** 16700 16702 |
|  | 19300 | 652.3673 | 1954.0801 | 1953.0870 | 0.9931 | 1 | 18 | 1.4 | 1 | K.AMAIADALGKIPQTVLWR.Y |
|  | 21832 | 1102.5536 | 2203.0926 | 2203.1042 | -0.0116 | 0 | 109 | 1.1e-009 | 1 | K.GAGVTLNVLEMTSEDLENALK.A 21831 21833 21836 21838 21839 21846 |
|  | 22096 | **742.7249** | **2225.1529** | **2224.0549** | **1.0980** | **0** | **5** | **28** | **8** | **K.ESFVSLGHNVFENDSFLQR.V** |
|  | 22825 | 1159.5969 | 2317.1793 | 2317.1929 | -0.0136 | 0 | 66 | 1.7e-005 | 1 | K.DRPVEPLDLAVFWVEFVMR.H 22826 |
|  | 25297 | **899.4313** | **2695.2722** | **2695.2878** | **-0.0156** | **1** | **51** | **0.00047** | **1** | **R.EDVKESFVSLGHNVFENDSFLQR.V** |
|  | 25447 | **1358.7461** | **2715.4776** | **2715.4894** | **-0.0118** | **0** | **61** | **5.1e-005** | **1** | **K.ILLIPVDGSHWLSMLGAIQQLQQR.G** 25445 25446 |
|  | 26144 | 948.8229 | 2843.4468 | 2843.4586 | -0.0118 | 1 | 54 | 0.00021 | 1 | K.GAGVTLNVLEMTSEDLENALKAVINDK.S |
|  | 29713 | 956.9341 | 3823.7075 | 3823.7289 | -0.0215 | 0 | 40 | 0.0032 | 1 | R.AFITHAGSHGVYESICNGVPMVMMPLFGDQMDNAK.R 29712 29714 29722 29724 29725 |
|  | 30060 | 1327.6094 | 3979.8063 | 3979.8300 | -0.0237 | 1 | 38 | 0.0046 | 1 | R.AFITHAGSHGVYESICNGVPMVMMPLFGDQMDNAKR.M 30062 |

| **73.** | P21549\|SPYA_HUMAN    **Mass:** 43325    **Score:** 658    **Queries matched:** 58   **emPAI:** 2.77 |
| --- | --- |
|  | Serine--pyruvate aminotransferase OS=Homo sapiens GN=AGXT PE=1 SV=1 |

|  | **Query** | **Observed** | **Mr(expt)** | **Mr(calc)** | **Delta** | **Miss** | **Score** | **Expect** | **Rank** | **Peptide** |
| --- | --- | --- | --- | --- | --- | --- | --- | --- | --- | --- |
|  | 1618 | **713.3796** | **712.3723** | **711.3738** | **0.9985** | **0** | **19** | **1.9** | **1** | **R.VHPMTK.D** |
|  | 3579 | **462.7387** | **923.4627** | **923.4647** | **-0.0020** | **0** | **29** | **0.21** | **1** | **R.AALQHCPK.K** 3577 3578 3586 3587 3594 |
|  | 4451 | **494.2943** | **986.5740** | **986.4933** | **0.0807** | **0** | **8** | **27** | **3** | **R.EAAAYLHGR.L** |
|  | 5612 | **537.7863** | **1073.5579** | **1073.5652** | **-0.0072** | **0** | **77** | **3.3e-006** | **1** | **R.IGLLGCNATR.E** |
|  | 8005 | **615.3809** | **1228.7472** | **1228.7543** | **-0.0072** | **0** | **60** | **0.00014** | **1** | **R.LQALGLQLFVK.D** 8002 8003 |
|  | 8218 | **620.8152** | **1239.6159** | **1239.6248** | **-0.0088** | **0** | **9** | **17** | **1** | **K.EGIQYVFQTR.N** 8225 |
|  | 9346 | **651.3392** | **1300.6639** | **1300.6735** | **-0.0096** | **1** | **28** | **0.2** | **1** | **R.ENVDRVTEALR.A** |
|  | 9353 | **651.3821** | **1300.7496** | **1300.5679** | **0.1817** | **0** | **4** | **54** | **6** | **K.DMYQIMDEIK.E** |
|  | 10426 | **453.5805** | **1357.7196** | **1357.7282** | **-0.0086** | **0** | **23** | **0.67** | **1** | **K.TKPFSFYLDIK.W** |
|  | 12750 | 747.8729 | 1493.7313 | 1493.7404 | -0.0091 | 0 | 74 | 5e-006 | 1 | R.IMAAGGLQMIGSMSK.D 12746 12749 |
|  | 12970 | **755.3989** | **1508.7832** | **1509.7353** | **-0.9522** | **0** | **(6)** | **31** | **5** | **R.IMAAGGLQMIGSMSK.D** |
|  | 12972 | **755.3999** | **1508.7852** | **1509.7353** | **-0.9501** | **0** | **(1)** | **1.1e+002** | **10** | **R.IMAAGGLQMIGSMSK.D** |
|  | 13000 | **756.4030** | **1510.7915** | **1509.7353** | **1.0562** | **0** | **(12)** | **7.2** | **1** | **R.IMAAGGLQMIGSMSK.D** |
|  | 13227 | 763.3554 | 1524.6961 | 1525.7302 | -1.0341 | 0 | (5) | 37 | 7 | R.IMAAGGLQMIGSMSK.D |
|  | 13255 | **764.3378** | **1526.6611** | **1525.7302** | **0.9309** | **0** | **(13)** | **6.4** | **3** | **R.IMAAGGLQMIGSMSK.D** |
|  | 14614 | **809.4224** | **1616.8302** | **1616.8410** | **-0.0108** | **0** | **54** | **0.00053** | **1** | **K.ALNAPPGTSLISFSDK.A** 14613 14615 14635 |
|  | 15346 | **832.8618** | **1663.7091** | **1663.7202** | **-0.0111** | **0** | **30** | **0.12** | **1** | **K.WLANFWGCDDQPR.M** |
|  | 16607 | **873.4589** | **1744.9032** | **1744.9148** | **-0.0117** | **0** | **46** | **0.0024** | **1** | **R.LPTVTTVAVPAGYDWR.D** 16606 |
|  | 17114 | **891.5300** | **1781.0454** | **1781.0563** | **-0.0109** | **1** | **63** | **5.5e-005** | **1** | **R.LQALGLQLFVKDPALR.L** |
|  | 17540 | **908.4600** | **1814.9055** | **1814.9162** | **-0.0107** | **0** | **84** | **4e-007** | **1** | **R.ESLALIAEQGLENSWR.Q** 17538 |
|  | 17541 | 454.7337 | 1814.9058 | 1815.9730 | -1.0672 | 1 | 6 | 27 | 7 | K.ALNAPPGTSLISFSDKAK.K |
|  | 17720 | **610.6587** | **1828.9542** | **1828.9658** | **-0.0115** | **0** | **36** | **0.028** | **1** | **R.MYHHTIPVISLYSLR.E** 17718 |
|  | 17919 | 615.9601 | 1844.8586 | 1844.9607 | -0.1021 | 0 | (1) | 78 | 4 | R.MYHHTIPVISLYSLR.E |
|  | 21655 | **1090.5513** | **2179.0880** | **2179.1017** | **-0.0137** | **0** | **57** | **0.00017** | **1** | **K.CLLLVDSVASLGGTPLYMDR.Q** |
|  | 22666 | **766.7419** | **2297.2038** | **2297.2168** | **-0.0130** | **1** | **5** | **24** | **1** | **K.DPALRLPTVTTVAVPAGYDWR.D** |
|  | 24231 | **835.8313** | **2504.4721** | **2504.4842** | **-0.0121** | **0** | **47** | **0.0011** | **1** | **K.ALLKPLSIPNQLLLGPGPSNLPPR.I** |
|  | 24240 | **1254.0962** | **2506.1778** | **2506.1872** | **-0.0094** | **1** | **89** | **8e-008** | **1** | **K.DMYQIMDEIKEGIQYVFQTR.N** 24239 |
|  | 24635 | **855.0931** | **2562.2576** | **2562.2676** | **-0.0100** | **0** | **30** | **0.073** | **1** | **R.DIVSYVIDHFDIEIMGGLGPSTGK.V** 24640 |
|  | 28748 | **1157.5471** | **3469.6195** | **3468.7633** | **0.8563** | **1** | **0** | **36** | **9** | **K.CLLLVDSVASLGGTPLYMDRQGIDILYSGSQK.A** |
|  | 30126 | 1007.9993 | 4027.9680 | 4028.1988 | -0.2308 | 1 | 3 | 17 | 3 | K.ALLKPLSIPNQLLLGPGPSNLPPRIMAAGGLQMIGSMSK.D |
|  | 30385 | **1378.3674** | **4132.0805** | **4131.1212** | **0.9593** | **0** | **32** | **0.017** | **1** | **R.NPLTLVISGSGHCALEAALVNVLEPGDSFLVGANGIWGQR.A** 30379 30381 30388 30390 30392 |
|  | 30664 | **1073.5450** | **4290.1511** | **4289.1719** | **0.9792** | **1** | **4** | **8** | **1** | **R.LPTVTTVAVPAGYDWRDIVSYVIDHFDIEIMGGLGPSTGK.V** |
|  | 31386 | **1034.5111** | **5167.5192** | **5167.5462** | **-0.0270** | **0** | **8** | **2** | **1** | **K.DPGGHYTLQEVEEGLAQHKPVLLFLTHGESSTGVLQPLDGFGELCHR.Y** 31385 31387 |

| **74.** | Q9Y4L1\|HYOU1_HUMAN    **Mass:** 111494   **Score:** 652    **Queries matched:** 58   **emPAI:** 0.53 |
| --- | --- |
|  | Hypoxia up-regulated protein 1 OS=Homo sapiens GN=HYOU1 PE=1 SV=1 |

|  | **Query** | **Observed** | **Mr(expt)** | **Mr(calc)** | **Delta** | **Miss** | **Score** | **Expect** | **Rank** | **Peptide** |
| --- | --- | --- | --- | --- | --- | --- | --- | --- | --- | --- |
|  | 1615 | **713.0678** | **712.0605** | **711.4252** | **0.6353** | **1** | **2** | **59** | **1** | **R.QRPRR.R** |
|  | 1980 | **401.7347** | **801.4549** | **801.4894** | **-0.0346** | **1** | **29** | **0.28** | **3** | **R.AMAKLLR.E** 1975 1986 |
|  | 2684 | **429.7533** | **857.4920** | **857.4971** | **-0.0050** | **0** | **12** | **12** | **7** | **K.LQDLTLR.D** 2669 |
|  | 3077 | **443.0380** | **884.0615** | **883.5352** | **0.5263** | **1** | **13** | **4.7** | **2** | **R.RAVLQAAR.M** |
|  | 3707 | **467.1008** | **932.1871** | **931.4545** | **0.7326** | **1** | **12** | **8.6** | **7** | **R.ENPRAMAK.L** |
|  | 4410 | **493.2287** | **984.4428** | **983.5400** | **0.9029** | **1** | **6** | **40** | **1** | **K.RPLKNDEL.-** |
|  | 4735 | **503.2759** | **1004.5373** | **1004.5443** | **-0.0071** | **0** | **33** | **0.095** | **1** | **R.YFQHLLGK.Q** |
|  | 4748 | **503.7788** | **1005.5429** | **1005.5495** | **-0.0065** | **0** | **31** | **0.13** | **1** | **R.EVQYLLNK.A** |
|  | 5029 | **515.2440** | **1028.4735** | **1028.4814** | **-0.0080** | **1** | **23** | **0.79** | **1** | **K.KYPDYESK.G** 5030 |
|  | 5280 | **524.2794** | **1046.5442** | **1046.5509** | **-0.0067** | **0** | **24** | **0.76** | **1** | **R.LAGLFNEQR.K** |
|  | 7251 | **597.2990** | **1192.5835** | **1192.6452** | **-0.0617** | **0** | **0** | **1.4e+002** | **2** | **R.VFGSQNLTTVK.L** |
|  | 8575 | **630.3404** | **1258.6662** | **1259.5856** | **-0.9194** | **0** | **4** | **58** | **8** | **R.FFGDSAASMAIK.N** |
|  | 9261 | **432.9341** | **1295.7806** | **1294.7860** | **0.9946** | **0** | **25** | **0.38** | **1** | **K.LPATEKPVLLSK.D** |
|  | 9510 | **437.2403** | **1308.6990** | **1307.6213** | **1.0776** | **1** | **4** | **53** | **6** | **K.DIEAKMMALDR.E** |
|  | 9949 | **666.3408** | **1330.6670** | **1331.6946** | **-1.0276** | **1** | **4** | **55** | **3** | **R.ERLAGLFNEQR.K** |
|  | 12026 | **484.6248** | **1450.8527** | **1449.7551** | **1.0976** | **1** | **(2)** | **83** | **2** | **R.VLFSRMGPYPQR.K** |
|  | 12294 | **734.3990** | **1466.7835** | **1465.7500** | **1.0335** | **1** | **4** | **45** | **2** | **R.VLFSRMGPYPQR.K** |
|  | 12554 | **741.8705** | **1481.7264** | **1481.7375** | **-0.0111** | **0** | **43** | **0.0066** | **1** | **K.QADNPHVALYQAR.F** |
|  | 12818 | **750.3980** | **1498.7815** | **1498.7926** | **-0.0111** | **1** | **19** | **1.6** | **1** | **K.TKEAGMQPQLQIR.G** 12819 |
|  | 13076 | **505.9344** | **1514.7813** | **1514.7875** | **-0.0062** | **1** | **(4)** | **47** | **5** | **K.TKEAGMQPQLQIR.G** 13047 |
|  | 13548 | **773.9011** | **1545.7876** | **1545.7270** | **0.0605** | **1** | **2** | **83** | **10** | **K.ENGDKSEAQKPSEK.A** |
|  | 15242 | **828.8769** | **1655.7393** | **1655.7501** | **-0.0108** | **0** | **44** | **0.0047** | **1** | **R.VEFEELCADLFER.V** |
|  | 15615 | **842.4094** | **1682.8042** | **1682.8151** | **-0.0110** | **0** | **55** | **0.00033** | **1** | **K.AANSLEAFIFETQDK.L** |
|  | 16279 | **575.2974** | **1722.8704** | **1722.8797** | **-0.0092** | **1** | **11** | **8.3** | **1** | **K.MMALDREVQYLLNK.A** |
|  | 16474 | **868.4406** | **1734.8667** | **1734.8788** | **-0.0122** | **0** | **37** | **0.02** | **1** | **K.LGNTISSLFGGGTTPDAK.E** 16462 |
|  | 17578 | **607.2062** | **1818.5969** | **1817.9345** | **0.6624** | **0** | **6** | **18** | **3** | **R.LSALDNLLNHSSMFLK.G** 17583 |
|  | 20738 | **1040.0052** | **2077.9959** | **2078.0102** | **-0.0142** | **0** | **102** | **5.3e-009** | **1** | **K.NINADEAAAMGAVYQAAALSK.A** 20736 20737 |
|  | 20884 | **698.6275** | **2092.8607** | **2094.0051** | **-1.1444** | **0** | **(6)** | **19** | **4** | **K.NINADEAAAMGAVYQAAALSK.A** |
|  | 20903 | **1048.0565** | **2094.0985** | **2094.1109** | **-0.0125** | **0** | **87** | **1.9e-007** | **1** | **K.VLQLINDNTATALSYGVFR.R** 20902 |
|  | 21649 | **1090.0442** | **2178.0738** | **2178.0878** | **-0.0140** | **0** | **111** | **7.5e-010** | **1** | **K.LSAASTWLEDEGVGATTVMLK.E** 21648 |
|  | 22404 | **1134.0563** | **2266.0980** | **2266.1113** | **-0.0133** | **0** | **31** | **0.059** | **1** | **R.LIPEMDQIFTEVEMTTLEK.V** 22402 |
|  | 24341 | **1260.5830** | **2519.1515** | **2519.1672** | **-0.0158** | **0** | **77** | **1.2e-006** | **1** | **K.TVLSANADHMAQIEGLMDDVDFK.A** 24340 24342 |
|  | 24361 | **841.7603** | **2522.2591** | **2522.1660** | **0.0931** | **0** | **1** | **57** | **9** | **K.VETGSEPGDTEPLELGGPGAEPEQK.E** |
|  | 24438 | **848.0641** | **2541.1704** | **2541.1870** | **-0.0166** | **1** | **12** | **4** | **1** | **K.LYQPEYQEVSTEEQREEISGK.L** |
|  | 24517 | **851.0950** | **2550.2631** | **2550.2709** | **-0.0079** | **1** | **1** | **54** | **2** | **K.GARLIPEMDQIFTEVEMTTLEK.V** |
|  | 25454 | **906.8283** | **2717.4629** | **2718.2993** | **-0.8364** | **1** | **4** | **21** | **2** | **K.TVLSANADHMAQIEGLMDDVDFKAK.V** |
|  | 27205 | **759.3897** | **3033.5297** | **3033.5447** | **-0.0150** | **1** | **1** | **38** | **1** | **R.SLAEDFAEQPIKDAVITVPVFFNQAER.R** |
|  | 27671 | **1568.3138** | **3134.6131** | **3134.6282** | **-0.0150** | **0** | **88** | **6.8e-008** | **1** | **R.VPGPVQQALQSAEMSLDEIEQVILVGGATR.V** 27669 27672 |
|  | 28980 | **713.5448** | **3562.6876** | **3563.6293** | **-0.9416** | **1** | **6** | **9.1** | **2** | **R.KDINTTAQNIMFYDMGSGSTVCTIVTYQMVK.T** |
|  | 30089 | **999.7325** | **3994.9011** | **3994.9171** | **-0.0161** | **1** | **6** | **7.5** | **1** | **K.AHFNLDESGVLSLDRVESVFETLVEDSAEEESTLTK.L** |
|  | 31406 | **1042.8773** | **5209.3502** | **5209.8130** | **-0.4628** | **1** | **4** | **4.7** | **1** | **R.VCWALVAVLLADLLALSDTLAVMSVDLGSESMKVAIVKPGVPMEIVLNK.E** |

| **75.** | P24462\|CP3A7_HUMAN    **Mass:** 57831    **Score:** 651    **Queries matched:** 44   **emPAI:** 1.55 |
| --- | --- |
|  | Cytochrome P450 3A7 OS=Homo sapiens GN=CYP3A7 PE=1 SV=2 |

|  | **Query** | **Observed** | **Mr(expt)** | **Mr(calc)** | **Delta** | **Miss** | **Score** | **Expect** | **Rank** | **Peptide** |
| --- | --- | --- | --- | --- | --- | --- | --- | --- | --- | --- |
|  | 1116 | 559.3775 | 558.3702 | 558.3741 | -0.0039 | 0 | 24 | 1.2 | 1 | K.TVLVK.E |
|  | 3734 | **468.2968** | **934.5790** | **934.5851** | **-0.0061** | **1** | **11** | **15** | **2** | **R.KVISFLTK.S** |
|  | 6235 | **561.8127** | **1121.6109** | **1120.6856** | **0.9253** | **1** | **3** | **72** | **4** | **K.VISFLTKSVK.Q** |
|  | 6395 | 568.3036 | 1134.5927 | 1134.6008 | -0.0081 | 0 | 18 | 2.5 | 1 | R.RPFGPVGFMK.N |
|  | 6425 | 569.3078 | 1136.6010 | 1136.6077 | -0.0067 | 0 | 38 | 0.025 | 1 | R.SLLSPTFTSGK.L |
|  | 6876 | 586.3881 | 1170.7616 | 1171.6448 | -0.8833 | 0 | 36 | 0.038 | 1 | R.EAETGKPVTLK.H 6886 |
|  | 8652 | 631.8217 | 1261.6289 | 1261.6376 | -0.0088 | 0 | 50 | 0.0015 | 1 | K.DVEINGMFIPK.G 8653 8661 8662 |
|  | 9917 | 443.9207 | 1328.7403 | 1327.7459 | 0.9944 | 1 | 12 | 8.3 | 4 | R.REAETGKPVTLK.H |
|  | 10591 | 684.3566 | 1366.6986 | 1366.7067 | -0.0082 | 0 | 28 | 0.22 | 1 | R.VLQNFSFKPCK.E 10590 |
|  | 10794 | 690.3622 | 1378.7098 | 1377.7868 | 0.9231 | 1 | 2 | 82 | 5 | R.SLLSPTFTSGKLK.E 10769 |
|  | 10855 | 691.8163 | 1381.6180 | 1382.7769 | -1.1589 | 1 | 4 | 60 | 2 | K.VQKEIDTVLPNK.A |
|  | 11001 | 695.8683 | 1389.7221 | 1389.7326 | -0.0105 | 1 | 58 | 0.00023 | 1 | K.KDVEINGMFIPK.G 11023 |
|  | 11020 | **464.5670** | **1390.6791** | **1389.8166** | **0.8624** | **1** | **11** | **11** | **1** | **R.FALVNMKLALVR.V** |
|  | 11272 | 703.8880 | 1405.7614 | 1405.7929 | -0.0315 | 1 | 11 | 9.4 | 4 | R.IRSLLSPTFTSGK.L |
|  | 11275 | 703.9634 | 1405.9123 | 1405.7275 | 0.1848 | 1 | (3) | 56 | 2 | K.KDVEINGMFIPK.G |
|  | 13752 | **780.8803** | **1559.7459** | **1559.7579** | **-0.0120** | **1** | **36** | **0.031** | **1** | **K.NAISIAEDEEWKR.I** |
|  | 14112 | 396.9712 | 1583.8557 | 1584.7575 | -0.9018 | 1 | 6 | 32 | 6 | R.NCIGMRFALVNMK.L 14115 |
|  | 14756 | **543.2561** | **1626.7465** | **1626.6847** | **0.0618** | **1** | **2** | **75** | **4** | **R.KGYWTFDMECYK.K** |
|  | 14924 | 819.4083 | 1636.8021 | 1636.8130 | -0.0109 | 0 | 69 | 1.4e-005 | 1 | R.VDFLQLMIDSQNSK.D |
|  | 15949 | 851.9586 | 1701.9027 | 1701.9124 | -0.0097 | 0 | 74 | 4.9e-006 | 1 | K.EMVPIIAQYGDVLVR.N 15948 15951 |
|  | 17491 | 906.4285 | 1810.8424 | 1810.8526 | -0.0102 | 0 | 38 | 0.016 | 1 | K.DNIDPYIYTPFGSGPR.N |
|  | 19021 | 965.9876 | 1929.9607 | 1929.9731 | -0.0124 | 1 | 108 | 1.8e-009 | 1 | K.HRVDFLQLMIDSQNSK.D 19004 19020 19022 |
|  | 19167 | 648.7004 | 1943.0795 | 1943.0914 | -0.0119 | 1 | 42 | 0.0062 | 1 | K.LKEMVPIIAQYGDVLVR.N |
|  | 19374 | 654.3664 | 1960.0773 | 1959.0863 | 0.9910 | 1 | (5) | 27 | 1 | K.LKEMVPIIAQYGDVLVR.N |
|  | 20429 | 1027.4961 | 2052.9776 | 2052.9905 | -0.0129 | 1 | 61 | 6.2e-005 | 1 | K.NKDNIDPYIYTPFGSGPR.N 20427 20428 20430 |
|  | 20908 | **699.3164** | **2094.9272** | **2094.1990** | **0.7282** | **1** | **0** | **79** | **1** | **K.KLGIPGPTPLPFLGNALSFR.K** |
|  | 23060 | **784.3809** | **2350.1208** | **2350.1111** | **0.0097** | **1** | **5** | **25** | **1** | **R.VDFLQLMIDSQNSKDSETHK.A** |
|  | 29255 | **1230.9652** | **3689.8738** | **3688.8910** | **0.9828** | **1** | **57** | **6.3e-005** | **1** | **K.EIDTVLPNKAPPTYDTVLQLEYLDMVVNETLR.L** |

| **76.** | P02679\|FIBG_HUMAN    **Mass:** 52106    **Score:** 643    **Queries matched:** 34   **emPAI:** 1.00 |
| --- | --- |
|  | Fibrinogen gamma chain OS=Homo sapiens GN=FGG PE=1 SV=3 |

|  | **Query** | **Observed** | **Mr(expt)** | **Mr(calc)** | **Delta** | **Miss** | **Score** | **Expect** | **Rank** | **Peptide** |
| --- | --- | --- | --- | --- | --- | --- | --- | --- | --- | --- |
|  | 2600 | **851.4351** | **850.4278** | **850.4337** | **-0.0059** | **0** | **31** | **0.13** | **1** | **K.NWIQYK.E** |
|  | 4927 | 511.2709 | 1020.5272 | 1020.5347 | -0.0075 | 1 | 18 | 2.5 | 1 | R.KMLEEIMK.Y 4928 4931 |
|  | 5109 | **517.7635** | **1033.5124** | **1033.5192** | **-0.0068** | **1** | **53** | **0.00086** | **1** | **K.VGPEADKYR.L** 5108 |
|  | 5136 | **518.7714** | **1035.5283** | **1035.5349** | **-0.0066** | **1** | **18** | **2.7** | **1** | **K.RLDGSVDFK.K** 5130 |
|  | 7925 | **409.4391** | **1225.2955** | **1225.6303** | **-0.3347** | **0** | **4** | **44** | **8** | **K.DTVQIHDITGK.D** |
|  | 9197 | **647.3776** | **1292.7407** | **1292.7492** | **-0.0085** | **0** | **17** | **2.8** | **1** | **K.QSGLYFIKPLK.A** 9198 |
|  | 12695 | **497.5967** | **1489.7681** | **1490.7365** | **-0.9684** | **0** | **18** | **2** | **1** | **K.YEASILTHDSSIR.Y** |
|  | 13037 | **757.4335** | **1512.8524** | **1512.7208** | **0.1316** | **0** | **48** | **0.0022** | **1** | **R.YLQEIYNSNNQK.I** 13040 |
|  | 13537 | **773.4043** | **1544.7940** | **1544.8059** | **-0.0119** | **0** | **63** | **6e-005** | **1** | **R.LTIGEGQQHHLGGAK.Q** |
|  | 15610 | **561.6544** | **1681.9413** | **1681.9515** | **-0.0103** | **0** | **32** | **0.06** | **1** | **K.IHLISTQSAIPYALR.V** |
|  | 18560 | **947.4542** | **1892.8938** | **1892.9057** | **-0.0119** | **0** | **99** | **1.2e-008** | **1** | **K.ASTPNGYDNGIIWATWK.T** |
|  | 19946 | **1006.9427** | **2011.8708** | **2010.9007** | **0.9702** | **0** | **15** | **2.8** | **1** | **K.CHAGHLNGVYYQGGTYSK.A** 19944 19945 |
|  | 21877 | **1104.0165** | **2206.0184** | **2206.0331** | **-0.0147** | **0** | **66** | **2.1e-005** | **1** | **K.EGFGHLSPTGTTEFWLGNEK.I** 21878 |
|  | 23187 | **789.3913** | **2365.1521** | **2365.1657** | **-0.0136** | **1** | **61** | **6.2e-005** | **1** | **K.MLEEIMKYEASILTHDSSIR.Y** |
|  | 23554 | **1209.0354** | **2416.0562** | **2416.0716** | **-0.0153** | **0** | **116** | **1.9e-010** | **1** | **R.FGSYCPTTCGIADFLSTYQTK.V** 23553 23555 |
|  | 24344 | **1260.6279** | **2519.2413** | **2519.2577** | **-0.0164** | **0** | **90** | **7.3e-008** | **1** | **K.AIQLTYNPDESSKPNMIDAATLK.S** 24343 24345 24352 |
|  | 25693 | **923.4439** | **2767.3097** | **2767.3269** | **-0.0172** | **1** | **46** | **0.0015** | **1** | **K.VAQLEAQCQEPCKDTVQIHDITGK.D** 25694 |
|  | 25993 | **939.4208** | **2815.2407** | **2816.3341** | **-1.0934** | **1** | **3** | **29** | **2** | **K.ANQQFLVYCEIDGSGNGWTVFQKR.L** 25998 |

| **77.** | P11712\|CP2C9_HUMAN    **Mass:** 56333    **Score:** 635    **Queries matched:** 69   **emPAI:** 3.64 |
| --- | --- |
|  | Cytochrome P450 2C9 OS=Homo sapiens GN=CYP2C9 PE=1 SV=3 |

|  | **Query** | **Observed** | **Mr(expt)** | **Mr(calc)** | **Delta** | **Miss** | **Score** | **Expect** | **Rank** | **Peptide** |
| --- | --- | --- | --- | --- | --- | --- | --- | --- | --- | --- |
|  | 1456 | 653.3032 | 652.2959 | 652.3003 | -0.0043 | 0 | 14 | 5.1 | 6 | R.NFGMGK.R |
|  | 1603 | 709.3656 | 708.3583 | 708.3629 | -0.0045 | 0 | 34 | 0.067 | 1 | K.NVAFMK.S |
|  | 1734 | **762.4299** | **761.4226** | **761.4283** | **-0.0057** | **0** | **33** | **0.11** | **1** | **K.SLTNLSK.V** |
|  | 1766 | 781.4157 | 780.4084 | 780.4130 | -0.0046 | 0 | 34 | 0.074 | 1 | K.HPEVTAK.V |
|  | 2082 | 405.2055 | 808.3964 | 808.4014 | -0.0050 | 1 | 11 | 13 | 4 | R.NFGMGKR.S |
|  | 3057 | **442.2357** | **882.4568** | **882.4633** | **-0.0065** | **0** | **20** | **1.4** | **1** | **R.FSLMTLR.N** |
|  | 3299 | **451.7559** | **901.4973** | **901.5021** | **-0.0048** | **0** | **8** | **37** | **4** | **R.GIFPLAER.A** |
|  | 3308 | **452.2098** | **902.4050** | **901.4505** | **0.9545** | **0** | **12** | **11** | **1** | **K.VQEEIER.V** |
|  | 3511 | **459.7364** | **917.4583** | **917.4640** | **-0.0057** | **0** | **34** | **0.073** | **1** | **R.CLVEELR.K** 3510 |
|  | 3888 | **473.8176** | **945.6206** | **945.6263** | **-0.0057** | **0** | **49** | **0.0022** | **1** | **R.YALLLLLK.H** |
|  | 4350 | **490.3154** | **978.6162** | **978.5750** | **0.0412** | **1** | **4** | **70** | **10** | **K.SYILEKVK.E** |
|  | 4952 | **512.2885** | **1022.5623** | **1022.5695** | **-0.0072** | **1** | **29** | **0.18** | **1** | **R.RFSLMTLR.N** 4953 |
|  | 4995 | **513.7629** | **1025.5112** | **1024.5342** | **0.9770** | **0** | **27** | **0.31** | **1** | **R.GFGIVFSNGK.K** |
|  | 5285 | **524.2869** | **1046.5593** | **1046.4895** | **0.0698** | **0** | **17** | **3.2** | **1** | **K.YFMPFSAGK.R** |
|  | 6642 | **577.3176** | **1152.6207** | **1152.6292** | **-0.0084** | **1** | **12** | **8.9** | **1** | **R.GFGIVFSNGKK.W** |
|  | 6749 | **582.2515** | **1162.4885** | **1162.4971** | **-0.0086** | **1** | **30** | **0.16** | **1** | **R.NRSPCMQDR.S** 6750 6751 6752 6753 6754 6755 6775 |
|  | 7466 | **401.8682** | **1202.5827** | **1202.5906** | **-0.0080** | **1** | **14** | **5.8** | **1** | **K.YFMPFSAGKR.I** |
|  | 8708 | **633.7903** | **1265.5661** | **1264.6122** | **0.9540** | **0** | **11** | **11** | **2** | **K.DQQFLNLMEK.L** |
|  | 9945 | **666.3270** | **1330.6394** | **1330.6477** | **-0.0083** | **1** | **37** | **0.032** | **1** | **R.SIEDRVQEEAR.C** |
|  | 11765 | 718.3525 | 1434.6904 | 1434.6991 | -0.0086 | 0 | 63 | 7.1e-005 | 1 | K.EALIDLGEEFSGR.G 11764 11778 |
|  | 15349 | **555.5905** | **1663.7497** | **1663.8529** | **-0.1032** | **1** | **7** | **22** | **3** | **K.HPEVTAKVQEEIER.V** |
|  | 16110 | **856.4823** | **1710.9500** | **1710.9516** | **-0.0016** | **0** | **1** | **88** | **4** | **K.GTTILISLTSVLHDNK.E** |
|  | 16928 | 884.9205 | 1767.8265 | 1767.8363 | -0.0098 | 0 | 54 | 0.00038 | 1 | R.SHMPYTDAVVHEVQR.Y 16920 16925 16926 16927 16929 |
|  | 17146 | **595.3039** | **1782.8898** | **1783.8312** | **-0.9413** | **0** | **(1)** | **94** | **8** | **R.SHMPYTDAVVHEVQR.Y** |
|  | 17572 | **909.9340** | **1817.8535** | **1817.8658** | **-0.0123** | **1** | **48** | **0.0016** | **1** | **R.FDYKDQQFLNLMEK.L** |
|  | 19550 | **659.8734** | **1976.5984** | **1976.0037** | **0.5947** | **1** | **(3)** | **35** | **5** | **K.DQQFLNLMEKLNENIK.I** |
|  | 19738 | **665.3569** | **1993.0490** | **1991.9986** | **1.0504** | **1** | **5** | **33** | **4** | **K.DQQFLNLMEKLNENIK.I** |
|  | 20219 | **679.5073** | **2035.5001** | **2036.2398** | **-0.7396** | **0** | **2** | **35** | **10** | **K.LPPGPTPLPVIGNILQIGIK.D** |
|  | 20460 | **1028.5363** | **2055.0579** | **2055.0711** | **-0.0131** | **0** | **59** | **9.9e-005** | **1** | **R.YIDLLPTSLPHAVTCDIK.F** 20459 20461 |
|  | 22042 | **741.4559** | **2221.3458** | **2221.3562** | **-0.0104** | **1** | **51** | **0.0005** | **1** | **R.GKLPPGPTPLPVIGNILQIGIK.D** 22041 |
|  | 26251 | **952.7480** | **2855.2221** | **2855.2387** | **-0.0165** | **1** | **64** | **2.2e-005** | **1** | **K.VKEHQESMDMNNPQDFIDCFLMK.M** 26250 26252 26253 |
|  | 26348 | **959.7856** | **2876.3351** | **2876.3482** | **-0.0131** | **0** | **27** | **0.11** | **1** | **K.ASPCDPTFILGCAPCNVICSIIFHK.R** 26349 |
|  | 26399 | **722.5901** | **2886.3315** | **2887.2285** | **-0.8970** | **1** | **(3)** | **32** | **2** | **K.VKEHQESMDMNNPQDFIDCFLMK.M** |
|  | 26749 | **981.1864** | **2940.5374** | **2940.5493** | **-0.0119** | **0** | **11** | **4.5** | **1** | **R.ICVGEALAGMELFLFLTSILQNFNLK.S** |
|  | 27351 | **1531.2551** | **3060.4957** | **3060.5168** | **-0.0210** | **0** | **60** | **5.8e-005** | **1** | **K.ILSSPWIQICNNFSPIIDYFPGTHNK.L** 27352 27355 27369 |
|  | 28732 | **1733.8259** | **3465.6373** | **3465.6536** | **-0.0163** | **0** | **67** | **7.4e-006** | **1** | **K.HNQPSEFTIESLENTAVDLFGAGTETTSTTLR.Y** 28730 28731 28733 28734 28736 28740 |
|  | 29347 | **931.6993** | **3722.7680** | **3722.7911** | **-0.0231** | **1** | **3** | **19** | **1** | **K.EKHNQPSEFTIESLENTAVDLFGAGTETTSTTLR.Y** |

| **78.** | P10809\|CH60_HUMAN    **Mass:** 61187    **Score:** 630    **Queries matched:** 59   **emPAI:** 1.15 |
| --- | --- |
|  | 60 kDa heat shock protein, mitochondrial OS=Homo sapiens GN=HSPD1 PE=1 SV=2 |

|  | **Query** | **Observed** | **Mr(expt)** | **Mr(calc)** | **Delta** | **Miss** | **Score** | **Expect** | **Rank** | **Peptide** |
| --- | --- | --- | --- | --- | --- | --- | --- | --- | --- | --- |
|  | 1399 | **636.2909** | **635.2837** | **635.3027** | **-0.0190** | **0** | **7** | **35** | **1** | **K.FGADAR.A** |
|  | 2534 | 423.2554 | 844.4962 | 843.4888 | 1.0074 | 0 | 11 | 16 | 5 | K.IPAMTIAK.N 2517 |
|  | 2642 | **428.2345** | **854.4544** | **854.4610** | **-0.0066** | **0** | **19** | **2.1** | **1** | **K.GANPVEIR.R** |
|  | 2717 | **861.0763** | **860.0690** | **859.4837** | **0.5853** | **0** | **(1)** | **1.1e+002** | **8** | **K.IPAMTIAK.N** |
|  | 3283 | **901.5299** | **900.5226** | **900.5280** | **-0.0055** | **0** | **26** | **0.49** | **1** | **K.LSDGVAVLK.V** |
|  | 4078 | **480.7816** | **959.5486** | **959.5036** | **0.0450** | **0** | **14** | **7.3** | **4** | **R.VTDALNATR.A** 4057 |
|  | 4331 | **489.7550** | **977.4954** | **977.4930** | **0.0024** | **1** | **10** | **17** | **9** | **K.DVKFGADAR.A** 4348 |
|  | 6372 | **567.2888** | **1132.5630** | **1131.6587** | **0.9043** | **1** | **3** | **82** | **5** | **-.MLRLPTVFR.Q** |
|  | 7224 | **397.8812** | **1190.6219** | **1189.6012** | **1.0206** | **0** | **11** | **12** | **3** | **K.EIGNIISDAMK.K** |
|  | 7699 | **608.3281** | **1214.6417** | **1214.6507** | **-0.0089** | **0** | **51** | **0.0013** | **1** | **K.NAGVEGSLIVEK.I** |
|  | 9685 | **660.3498** | **1318.6851** | **1317.6962** | **0.9890** | **1** | **0** | **1.3e+002** | **3** | **K.EIGNIISDAMKK.V** |
|  | 10160 | **672.8570** | **1343.6994** | **1343.7085** | **-0.0091** | **0** | **46** | **0.0031** | **1** | **R.TVIIEQSWGSPK.V** |
|  | 10982 | **695.3515** | **1388.6884** | **1388.6976** | **-0.0092** | **0** | **16** | **3.4** | **1** | **R.GYISPYFINTSK.G** 10981 |
|  | 12898 | **752.8771** | **1503.7396** | **1503.7490** | **-0.0094** | **0** | **84** | **4.9e-007** | **1** | **K.TLNDELEIIEGMK.F** |
|  | 13721 | **778.9536** | **1555.8927** | **1555.9007** | **-0.0081** | **1** | **46** | **0.0028** | **1** | **R.GVMLAVDAVIAELKK.Q** 13720 |
|  | 14818 | **815.9521** | **1629.8897** | **1629.9011** | **-0.0114** | **1** | **58** | **0.00017** | **1** | **K.VGEVIVTKDDAMLLK.G** 14813 14819 |
|  | 15628 | **842.9492** | **1683.8839** | **1683.8978** | **-0.0139** | **0** | **1** | **80** | **6** | **R.AAVEEGIVLGGGCALLR.C** |
|  | 15686 | 845.3811 | 1688.7476 | 1689.6078 | -0.8602 | 0 | (8) | 17 | 2 | K.DPGMGAMGGMGGGMGGGMF.- 15716 |
|  | 15713 | **564.3489** | **1690.0250** | **1689.6078** | **0.4172** | **0** | **(1)** | **79** | **4** | **K.DPGMGAMGGMGGGMGGGMF.-** 15697 |
|  | 15722 | **564.5905** | **1690.7497** | **1689.6078** | **1.1419** | **0** | **(2)** | **70** | **9** | **K.DPGMGAMGGMGGGMGGGMF.-** |
|  | 15995 | **569.3003** | **1704.8790** | **1705.6027** | **-0.7237** | **0** | **(5)** | **32** | **4** | **K.DPGMGAMGGMGGGMGGGMF.-** 15992 |
|  | 16002 | **569.3980** | **1705.1720** | **1705.6027** | **-0.4307** | **0** | **(1)** | **52** | **6** | **K.DPGMGAMGGMGGGMGGGMF.-** |
|  | 16005 | **569.5535** | **1705.6388** | **1705.6027** | **0.0360** | **0** | **(1)** | **76** | **5** | **K.DPGMGAMGGMGGGMGGGMF.-** |
|  | 16009 | **569.6232** | **1705.8479** | **1705.6027** | **0.2451** | **0** | **(0)** | **1e+002** | **9** | **K.DPGMGAMGGMGGGMGGGMF.-** |
|  | 16249 | **574.7977** | **1721.3712** | **1721.5977** | **-0.2265** | **0** | **12** | **4.4** | **3** | **K.DPGMGAMGGMGGGMGGGMF.-** |
|  | 16258 | **574.9487** | **1721.8244** | **1721.5977** | **0.2267** | **0** | **(5)** | **32** | **3** | **K.DPGMGAMGGMGGGMGGGMF.-** |
|  | 16517 | **580.5372** | **1738.5897** | **1737.5926** | **0.9971** | **0** | **(1)** | **67** | **3** | **K.DPGMGAMGGMGGGMGGGMF.-** |
|  | 17020 | **592.6781** | **1775.0125** | **1775.9941** | **-0.9816** | **1** | **1** | **86** | **2** | **R.QMRPVSRVLAPHLTR.A** |
|  | 17454 | **603.3775** | **1807.1107** | **1806.8941** | **0.2166** | **1** | **1** | **76** | **8** | **K.FDRGYISPYFINTSK.G** |
|  | 18879 | **640.6918** | **1919.0537** | **1918.0636** | **0.9901** | **0** | **28** | **0.17** | **1** | **K.ISSIQSIVPALEIANAHR.K** 18874 18875 |
|  | 20332 | **683.0560** | **2046.1461** | **2046.1585** | **-0.0124** | **1** | **41** | **0.0067** | **1** | **K.KISSIQSIVPALEIANAHR.K** |
|  | 20720 | **693.0377** | **2076.0913** | **2075.7879** | **0.3034** | **1** | **4** | **36** | **4** | **K.EEKDPGMGAMGGMGGGMGGGMF.-** |
|  | 20886 | **698.6591** | **2092.9555** | **2091.7829** | **1.1727** | **1** | **(0)** | **88** | **2** | **K.EEKDPGMGAMGGMGGGMGGGMF.-** |
|  | 21024 | **703.9592** | **2108.8557** | **2107.7778** | **1.0779** | **1** | **(3)** | **41** | **3** | **K.EEKDPGMGAMGGMGGGMGGGMF.-** |
|  | 21064 | **1057.0685** | **2112.1224** | **2112.1323** | **-0.0099** | **0** | **106** | **2.4e-009** | **1** | **R.ALMLQGVDLLADAVAVTMGPK.G** 21063 21065 |
|  | 21081 | **705.8641** | **2114.5706** | **2115.1059** | **-0.5353** | **1** | **1** | **46** | **5** | **K.LSDGVAVLKVGGTSDVEVNEK.K** |
|  | 24246 | **1254.5493** | **2507.0841** | **2507.1018** | **-0.0177** | **0** | **121** | **5e-011** | **1** | **K.IMQSSSEVGYDAMAGDFVNMVEK.G** 24247 24248 24255 |
|  | 24594 | **1280.6182** | **2559.2218** | **2559.2413** | **-0.0195** | **0** | **148** | **1.2e-013** | **1** | **K.LVQDVANNTNEEAGDGTTTATVLAR.S** 24599 |
|  | 28170 | **1093.8215** | **3278.4428** | **3279.4985** | **-1.0558** | **1** | **2** | **24** | **8** | **K.IMQSSSEVGYDAMAGDFVNMVEKGIIDPTK.V** 28171 |
|  | 29338 | **744.9642** | **3719.7848** | **3719.7368** | **0.0479** | **1** | **2** | **22** | **4** | **K.NAGVEGSLIVEKIMQSSSEVGYDAMAGDFVNMVEK.G** |

| **79.** | P01024\|CO3_HUMAN    **Mass:** 188569   **Score:** 630    **Queries matched:** 59   **emPAI:** 0.36 |
| --- | --- |
|  | Complement C3 OS=Homo sapiens GN=C3 PE=1 SV=2 |

|  | **Query** | **Observed** | **Mr(expt)** | **Mr(calc)** | **Delta** | **Miss** | **Score** | **Expect** | **Rank** | **Peptide** |
| --- | --- | --- | --- | --- | --- | --- | --- | --- | --- | --- |
|  | 1047 | **545.3637** | **544.3564** | **544.3333** | **0.0231** | **0** | **9** | **43** | **3** | **K.TVAVR.T** |
|  | 2002 | 402.2448 | 802.4751 | 802.4483 | 0.0268 | 1 | 8 | 35 | 8 | K.LNKLCR.D |
|  | 3222 | **449.7401** | **897.4657** | **897.4708** | **-0.0051** | **0** | **13** | **7.5** | **2** | **R.AVLYNYR.Q** |
|  | 3510 | 459.7364 | 917.4583 | 917.5117 | -0.0534 | 1 | 12 | 12 | 4 | R.MNKTVAVR.T 3530 |
|  | 4083 | 481.1506 | 960.2866 | 959.5400 | 0.7467 | 1 | 7 | 33 | 7 | R.RSVQLTEK.R |
|  | 5223 | **522.2623** | **1042.5100** | **1041.6182** | **0.8918** | **1** | **3** | **88** | **9** | **R.ISLPESLKR.I** |
|  | 7197 | **595.8107** | **1189.6069** | **1189.6132** | **-0.0063** | **0** | **10** | **15** | **1** | **K.DFDFVPPVVR.W** |
|  | 7648 | **404.8967** | **1211.6684** | **1211.6761** | **-0.0078** | **0** | **8** | **22** | **2** | **K.VTIKPAPETEK.R** 7647 |
|  | 7943 | **409.8712** | **1226.5918** | **1225.6489** | **0.9429** | **0** | **10** | **16** | **4** | **R.QPVPGQQMTLK.I** |
|  | 8214 | 620.8046 | 1239.5947 | 1239.6030 | -0.0083 | 0 | 11 | 9.8 | 2 | R.AELQCPQPAAR.R 8238 |
|  | 8380 | 624.8301 | 1247.6456 | 1248.5775 | -0.9319 | 1 | 6 | 38 | 8 | R.FYHPEKEDGK.L |
|  | 8503 | **419.2373** | **1254.6902** | **1255.6884** | **-0.9983** | **1** | **9** | **17** | **4** | **K.TVAVRTLDPER.L** 8514 |
|  | 9283 | **649.7743** | **1297.5340** | **1297.5431** | **-0.0091** | **0** | **40** | **0.014** | **1** | **R.CAEENCFIQK.S** |
|  | 9311 | **650.3571** | **1298.6996** | **1299.5805** | **-0.8810** | **0** | **13** | **6.3** | **1** | **K.ACEPGVDYVYK.T** |
|  | 9372 | **434.8811** | **1301.6214** | **1301.7707** | **-0.1493** | **1** | **2** | **97** | **10** | **K.GPLLNKFLTTAK.D** |
|  | 9504 | **437.2116** | **1308.6129** | **1307.6986** | **0.9143** | **1** | **11** | **11** | **2** | **R.QPSSAFAAFVKR.A** |
|  | 11195 | **701.4170** | **1400.8194** | **1400.8279** | **-0.0085** | **0** | **23** | **0.74** | **1** | **K.SSLSVPYVIVPLK.T** |
|  | 11798 | **479.9543** | **1436.8410** | **1436.7122** | **0.1288** | **1** | **6** | **30** | **7** | **R.YYTYLIMNKGR.L** |
|  | 12340 | **735.8308** | **1469.6471** | **1469.7726** | **-0.1255** | **0** | **8** | **19** | **2** | **R.IPIEDGSGEVVLSR.K** |
|  | 12361 | **491.2495** | **1470.7265** | **1470.7368** | **-0.0103** | **0** | **30** | **0.12** | **1** | **K.AAVYHHFISDGVR.K** |
|  | 12734 | **747.3829** | **1492.7512** | **1493.8276** | **-1.0764** | **1** | **7** | **27** | **2** | **K.NGISTKLMNIFLK.D** 12748 |
|  | 15996 | 853.4484 | 1704.8822 | 1705.8821 | -1.0000 | 1 | 6 | 26 | 3 | K.GQGTLSVVTMYHAKAK.D |
|  | 16204 | **573.2933** | **1716.8582** | **1717.9376** | **-1.0794** | **1** | **0** | **1e+002** | **9** | **K.ITHRIHWESASLLR.S** |
|  | 17195 | **596.3116** | **1785.9131** | **1786.9618** | **-1.0487** | **0** | **1** | **86** | **3** | **R.SGIPIVTSPYQIHFTK.T** |
|  | 17550 | **908.9455** | **1815.8764** | **1815.8850** | **-0.0085** | **0** | **64** | **4.1e-005** | **1** | **R.SNLDEDIIAEENIVSR.S** 17530 |
|  | 18198 | 932.9503 | 1863.8860 | 1864.9319 | -1.0460 | 0 | 4 | 47 | 8 | K.VEGTAFVIFGIQDGEQR.I |
|  | 18314 | **624.6783** | **1871.0132** | **1871.0265** | **-0.0133** | **0** | **45** | **0.0034** | **1** | **R.TELRPGETLNVNFLLR.M** |
|  | 18392 | **939.9848** | **1877.9550** | **1877.9662** | **-0.0112** | **0** | **25** | **0.32** | **1** | **K.EYVLPSFEVIVEPTEK.F** 18377 |
|  | 18537 | **473.5211** | **1890.0555** | **1890.0687** | **-0.0132** | **0** | **40** | **0.011** | **1** | **K.LSINTHPSQKPLSITVR.T** |
|  | 19634 | **993.4828** | **1984.9510** | **1984.9636** | **-0.0126** | **1** | **57** | **0.00018** | **1** | **K.SGQSEDRQPVPGQQMTLK.I** |
|  | 20727 | **520.2636** | **2077.0251** | **2077.0375** | **-0.0124** | **1** | **6** | **26** | **4** | **R.QPVPGQQMTLKIEGDHGAR.V** |
|  | 20746 | **694.0244** | **2079.0514** | **2079.0677** | **-0.0163** | **1** | **2** | **55** | **5** | **K.FYYIYNEKGLEVTITAR.F** |
|  | 21447 | **1079.0396** | **2156.0645** | **2156.0783** | **-0.0138** | **0** | **111** | **6.4e-010** | **1** | **R.ILLQGTPVAQMTEDAVDAER.L** 21448 |
|  | 21527 | **1083.4983** | **2164.9820** | **2164.9953** | **-0.0133** | **0** | **91** | **6.5e-008** | **1** | **R.AYYENSPQQVFSTEFEVK.E** 21528 |
|  | 21788 | **1099.5605** | **2197.1065** | **2197.1227** | **-0.0161** | **0** | **88** | **1.1e-007** | **1** | **R.VPVAVQGEDTVQSLTQGDGVAK.L** 21786 21787 |
|  | 22331 | **1129.0483** | **2256.0821** | **2256.0984** | **-0.0162** | **0** | **88** | **1.3e-007** | **1** | **K.VQLSNDFDEYIMAIEQTIK.S** |
|  | 23544 | 805.7346 | 2414.1819 | 2413.2523 | 0.9296 | 1 | 1 | 64 | 5 | R.ILLQGTPVAQMTEDAVDAERLK.H |
|  | 23739 | **1222.6517** | **2443.2889** | **2443.2999** | **-0.0110** | **0** | **48** | **0.0013** | **1** | **R.EPGQDLVVLPLSITTDFIPSFR.L** |
|  | 24724 | **860.1023** | **2577.2850** | **2577.3009** | **-0.0159** | **0** | **4** | **27** | **4** | **R.TMQALPYSTVGNSNNYLHLSVLR.T** |
|  | 24744 | **861.1020** | **2580.2843** | **2579.2802** | **1.0041** | **0** | **2** | **42** | **1** | **K.QKPDGVFQEDAPVIHQEMIGGLR.N** |
|  | 25592 | **917.1052** | **2748.2937** | **2748.3080** | **-0.0144** | **0** | **14** | **2.3** | **1** | **K.YFKPGMPFDLMVFVTNPDGSPAYR.V** |
|  | 27502 | **618.9213** | **3089.5699** | **3090.4984** | **-0.9284** | **1** | **2** | **30** | **7** | **K.TPKYFKPGMPFDLMVFVTNPDGSPAYR.V** |
|  | 28905 | **1179.8696** | **3536.5871** | **3536.6114** | **-0.0244** | **1** | **63** | **1.5e-005** | **1** | **K.ADIGCTPGSGKDYAGVFSDAGLTFTSSSGQQTAQR.A** |
|  | 30070 | **996.7231** | **3982.8632** | **3982.8904** | **-0.0271** | **0** | **31** | **0.022** | **1** | **K.HLIVTPSGCGEQNMIGMTPTVIAVHYLDETEQWEK.F** 30072 |
|  | 31281 | **1226.6254** | **4902.4724** | **4902.4936** | **-0.0212** | **1** | **35** | **0.0052** | **1** | **R.TVMVNIENPEGIPVKQDSLSSQNQLGVLPLSWDIPELVNMGQWK.I** 31280 31282 |

| **80.** | O95954\|FTCD_HUMAN    **Mass:** 59574    **Score:** 628    **Queries matched:** 36   **emPAI:** 0.95 |
| --- | --- |
|  | Formimidoyltransferase-cyclodeaminase OS=Homo sapiens GN=FTCD PE=1 SV=2 |

|  | **Query** | **Observed** | **Mr(expt)** | **Mr(calc)** | **Delta** | **Miss** | **Score** | **Expect** | **Rank** | **Peptide** |
| --- | --- | --- | --- | --- | --- | --- | --- | --- | --- | --- |
|  | 699 | **461.0076** | **460.0004** | **460.2645** | **-0.2642** | **0** | **2** | **1.8e+002** | **7** | **R.GLGSK.S** |
|  | 1164 | **572.7883** | **571.7811** | **572.3646** | **-0.5835** | **0** | **8** | **29** | **5** | **R.LVVSR.L** |
|  | 1502 | **673.3939** | **672.3866** | **673.3507** | **-0.9641** | **1** | **7** | **51** | **3** | **R.EQGRGK.D** |
|  | 4334 | 489.8050 | 977.5954 | 976.4865 | 1.1088 | 0 | 26 | 0.43 | 1 | R.AGEYEALPK.K |
|  | 6129 | **557.8155** | **1113.6164** | **1113.6254** | **-0.0090** | **1** | **22** | **1** | **1** | **R.TAALQEGLRR.A** |
|  | 6342 | **566.3203** | **1130.6261** | **1130.6335** | **-0.0075** | **0** | **25** | **0.53** | **1** | **R.IIEYLVPER.G** |
|  | 8252 | **414.7159** | **1241.1260** | **1241.5710** | **-0.4451** | **0** | **5** | **32** | **10** | **R.QFQSLDTTMR.R** |
|  | 9460 | **653.8490** | **1305.6834** | **1306.6557** | **-0.9723** | **0** | **1** | **99** | **7** | **K.VQGIGWYLDEK.N** |
|  | 10857 | **691.8409** | **1381.6673** | **1381.6772** | **-0.0099** | **1** | **19** | **1.8** | **1** | **R.RQFQSLDTTMR.R** |
|  | 11152 | **466.9958** | **1397.9655** | **1397.6722** | **0.2934** | **1** | **4** | **43** | **8** | **R.QFQSLDTTMRR.L** 11140 |
|  | 12429 | **492.2822** | **1473.8249** | **1473.7472** | **0.0776** | **0** | **2** | **87** | **6** | **R.MGALDVCPFIPVR.G** |
|  | 12673 | **745.3827** | **1488.7509** | **1488.7606** | **-0.0097** | **0** | **59** | **0.00018** | **1** | **K.TQAALVLDCLETR.Q** |
|  | 13901 | **785.9277** | **1569.8408** | **1569.8515** | **-0.0107** | **1** | **21** | **1.1** | **1** | **R.IIEYLVPERGPER.G** 13902 13915 |
|  | 14235 | 531.2845 | 1590.8316 | 1590.7685 | 0.0631 | 1 | 10 | 11 | 1 | R.LIDMSRHQGEHPR.M |
|  | 16299 | **575.6768** | **1724.0086** | **1723.7909** | **0.2177** | **0** | **1** | **74** | **9** | **-.MSQLVECVPNFSEGK.N** |
|  | 16613 | 873.9324 | 1745.8503 | 1745.8618 | -0.0115 | 1 | 44 | 0.0041 | 1 | K.TQAALVLDCLETRQE.- |
|  | 18581 | **948.4335** | **1894.8524** | **1894.8666** | **-0.0142** | **0** | **80** | **1.1e-006** | **1** | **R.GVSVDECVLCAQAFGQR.L** |
|  | 18758 | **954.4859** | **1906.9572** | **1906.9676** | **-0.0104** | **0** | **9** | **11** | **1** | **R.LAEELDVPVYLYGEAAR.M** 18759 |
|  | 21620 | **1088.6276** | **2175.2406** | **2175.2515** | **-0.0109** | **0** | **33** | **0.037** | **1** | **R.EAQELSLPVVGSQLVGLVPLK.A** 21599 |
|  | 21804 | **1101.0378** | **2200.0611** | **2200.0722** | **-0.0110** | **0** | **116** | **2.3e-010** | **1** | **K.LTTLVDADAEAFTAYLEAMR.L** 21805 |
|  | 22286 | **1125.5579** | **2249.1012** | **2249.1151** | **-0.0139** | **0** | **65** | **2.2e-005** | **1** | **R.TVYTFVGPPECVVEGALNAAR.V** 22287 |
|  | 24411 | **1268.2069** | **2534.3993** | **2534.4108** | **-0.0115** | **0** | **86** | **1.5e-007** | **1** | **R.AVSVPLTLAETVASLWPALQELAR.C** 24412 24413 |
|  | 25279 | **897.8411** | **2690.5014** | **2690.5119** | **-0.0106** | **1** | **6** | **14** | **1** | **R.RAVSVPLTLAETVASLWPALQELAR.C** |
|  | 25479 | **1362.6589** | **2723.3033** | **2723.3193** | **-0.0160** | **0** | **100** | **6.1e-009** | **1** | **R.SAAPGGGSVAAAAAAMGAALGSMVGLMTYGR.R** |
|  | 26799 | **983.4768** | **2947.4084** | **2947.4253** | **-0.0169** | **1** | **12** | **3.5** | **1** | **K.KLQQADWAPDFGPSSFVPSWGATATGAR.K** |
|  | 27133 | **1008.4933** | **3022.4580** | **3022.4706** | **-0.0126** | **0** | **49** | **0.00073** | **1** | **K.NLAQVSTNLLDFEVTALHTVYEETCR.E** |
|  | 29182 | **1214.5768** | **3640.7085** | **3641.7753** | **-1.0667** | **1** | **0** | **32** | **1** | **R.AFVGEVGARSAAPGGGSVAAAAAAMGAALGSMVGLMTYGR.R** |

| **81.** | Q8NBX0\|SCPDH_HUMAN    **Mass:** 47464    **Score:** 607    **Queries matched:** 31   **emPAI:** 1.48 |
| --- | --- |
|  | Probable saccharopine dehydrogenase OS=Homo sapiens GN=SCCPDH PE=1 SV=1 |

|  | **Query** | **Observed** | **Mr(expt)** | **Mr(calc)** | **Delta** | **Miss** | **Score** | **Expect** | **Rank** | **Peptide** |
| --- | --- | --- | --- | --- | --- | --- | --- | --- | --- | --- |
|  | 1700 | **748.3966** | **747.3893** | **747.3949** | **-0.0056** | **0** | **20** | **2.1** | **3** | **K.ICTQVK.G** |
|  | 6430 | **569.3249** | **1136.6353** | **1136.5549** | **0.0804** | **1** | **5** | **45** | **9** | **R.RWPISYCR.E** |
|  | 7436 | **401.2846** | **1200.8320** | **1201.5687** | **-0.7367** | **1** | **10** | **12** | **3** | **R.EQVDPERSSR.L** |
|  | 7589 | **605.3131** | **1208.6117** | **1208.6190** | **-0.0073** | **0** | **19** | **1.8** | **1** | **K.AGGVFTPGAAFSK.T** |
|  | 10981 | 695.3513 | 1388.6880 | 1389.6776 | -0.9896 | 0 | 2 | 89 | 4 | K.HGIEFSVISSSEV.- |
|  | 11640 | **714.3439** | **1426.6733** | **1426.6841** | **-0.0108** | **0** | **54** | **0.0006** | **1** | **K.SAIYGFGDQSNLR.K** 11641 |
|  | 12443 | **738.4452** | **1474.8758** | **1474.8871** | **-0.0113** | **0** | **42** | **0.0078** | **1** | **R.NVSNLKPVPLIGPK.L** 12444 |
|  | 14439 | **536.2324** | **1605.6754** | **1606.8734** | **-1.1980** | **0** | **0** | **1e+002** | **7** | **K.LMFAGLFFLFFVR.F** |
|  | 14556 | **807.3986** | **1612.7827** | **1612.7919** | **-0.0093** | **0** | **19** | **1.6** | **1** | **K.GYSIPFMGSDVSVVR.R** 14555 |
|  | 21792 | **733.6195** | **2197.8365** | **2198.0626** | **-0.2261** | **1** | **1** | **48** | **3** | **K.FPWFFSFGYFSKQGPTQK.Q** |
|  | 23455 | **1200.6206** | **2399.2267** | **2399.2373** | **-0.0106** | **0** | **90** | **8.1e-008** | **1** | **K.GVYIIGSSGFDSIPADLGVIYTR.N** 23454 |
|  | 25797 | **1393.2163** | **2784.4181** | **2784.4334** | **-0.0153** | **1** | **89** | **6.8e-008** | **1** | **K.AADKGVYIIGSSGFDSIPADLGVIYTR.N** 25802 |
|  | 26034 | **1412.1616** | **2822.3087** | **2822.3289** | **-0.0202** | **0** | **92** | **3.3e-008** | **1** | **K.ACIENGASCIDISGEPQFLELMQLK.Y** 26036 26046 26047 |
|  | 26732 | **1470.2483** | **2938.4820** | **2938.4932** | **-0.0112** | **0** | **69** | **7.4e-006** | **1** | **K.GPEAGYVATPIAMVQAAMTLLSDASHLPK.A** 26730 26731 26733 26734 |
|  | 26985 | **995.8337** | **2984.4792** | **2985.5151** | **-1.0359** | **0** | **1** | **44** | **1** | **K.LGRPTLSSEVGIIICDIANPASLDEMAK.Q** |
|  | 27529 | **775.6286** | **3098.4853** | **3097.5179** | **0.9674** | **0** | **44** | **0.0018** | **1** | **K.MNGTLTAVESFLTIHSGPEGLSIHDGTWK.S** 27526 27530 |
|  | 29488 | **1257.9551** | **3770.8434** | **3771.8198** | **-0.9764** | **1** | **0** | **33** | **9** | **R.FYGEPVIKACIENGASCIDISGEPQFLELMQLK.Y** |

| **82.** | P05177\|CP1A2_HUMAN    **Mass:** 58656    **Score:** 606    **Queries matched:** 30   **emPAI:** 1.09 |
| --- | --- |
|  | Cytochrome P450 1A2 OS=Homo sapiens GN=CYP1A2 PE=1 SV=3 |

|  | **Query** | **Observed** | **Mr(expt)** | **Mr(calc)** | **Delta** | **Miss** | **Score** | **Expect** | **Rank** | **Peptide** |
| --- | --- | --- | --- | --- | --- | --- | --- | --- | --- | --- |
|  | 3296 | **451.7475** | **901.4804** | **901.4869** | **-0.0065** | **0** | **16** | **4.9** | **1** | **K.ELDTVIGR.E** |
|  | 3423 | **456.2896** | **910.5645** | **909.4821** | **1.0825** | **1** | **13** | **7.4** | **1** | **R.FKAFNQR.F** 3408 |
|  | 3438 | **457.2337** | **912.4528** | **913.4224** | **-0.9695** | **0** | **8** | **25** | **2** | **K.MMLFGMGK.R** |
|  | 4113 | **482.2636** | **962.5127** | **962.5185** | **-0.0059** | **0** | **65** | **5.5e-005** | **1** | **R.YGDVLQIR.I** 4112 |
|  | 5369 | **528.7842** | **1055.5539** | **1055.5611** | **-0.0072** | **0** | **4** | **63** | **3** | **R.ASGNLIPQEK.I** |
|  | 5571 | 536.2970 | 1070.5794 | 1070.5873 | -0.0078 | 0 | 19 | 2.2 | 1 | R.YLPNPALQR.F |
|  | 10281 | **675.8665** | **1349.7185** | **1349.7265** | **-0.0080** | **0** | **1** | **1.2e+002** | **3** | **K.VDLTPIYGLTMK.H** |
|  | 11356 | **705.9182** | **1409.8219** | **1408.6259** | **1.1960** | **0** | **0** | **1.1e+002** | **2** | **K.TVQEHYQDFDK.N** |
|  | 15985 | **852.9561** | **1703.8977** | **1703.9094** | **-0.0117** | **0** | **82** | **7e-007** | **1** | **R.FLTADGTAINKPLSEK.M** 15986 |
|  | 16349 | **576.9643** | **1727.8710** | **1726.8791** | **0.9919** | **0** | **29** | **0.13** | **1** | **R.HSSFLPFTIPHSTTR.D** |
|  | 18216 | **933.4362** | **1864.8579** | **1864.8704** | **-0.0125** | **1** | **58** | **0.00018** | **1** | **K.TVQEHYQDFDKNSVR.D** 18217 18218 |
|  | 20785 | **695.3793** | **2083.1160** | **2083.1255** | **-0.0095** | **0** | **37** | **0.02** | **1** | **K.SPPEPWGWPLLGHVLTLGK.N** |
|  | 22730 | **1154.6167** | **2307.2188** | **2307.2263** | **-0.0074** | **0** | **64** | **3.2e-005** | **1** | **R.LSDRPQLPYLEAFILETFR.H** 22727 |
|  | 24141 | **1246.1088** | **2490.2030** | **2490.2179** | **-0.0150** | **0** | **96** | **1.9e-008** | **1** | **K.NTHEFVETASSGNPLDFFPILR.Y** 24142 24143 |
|  | 27626 | **1042.8209** | **3125.4409** | **3125.4611** | **-0.0202** | **0** | **87** | **9.9e-008** | **1** | **R.LAQNALNTFSIASDPASSSSCYLEEHVSK.E** 27624 |
|  | 29120 | **901.1586** | **3600.6052** | **3600.6303** | **-0.0251** | **1** | **30** | **0.036** | **1** | **K.KCCVFVNQWQVNHDPELWEDPSEFRPER.F** |
|  | 30110 | **1340.3004** | **4017.8794** | **4018.2297** | **-0.3503** | **1** | **3** | **14** | **8** | **K.WEIFLFLAILLQQLEFSVPPGVKVDLTPIYGLTMK.H** |
|  | 31340 | **1262.8516** | **5047.3771** | **5047.4017** | **-0.0246** | **0** | **46** | **0.00033** | **1** | **R.LQELMAGPGHFDPYNQVVVSVANVIGAMCFGQHFPESSDEMLSLVK.N** 31341 31342 |
|  | 31352 | **1274.6025** | **5094.3811** | **5095.3865** | **-1.0054** | **0** | **(1)** | **10** | **2** | **R.LQELMAGPGHFDPYNQVVVSVANVIGAMCFGQHFPESSDEMLSLVK.N** |

| **83.** | P27338\|AOFB_HUMAN    **Mass:** 59238    **Score:** 605    **Queries matched:** 64   **emPAI:** 1.82 |
| --- | --- |
|  | Amine oxidase [flavin-containing] B OS=Homo sapiens GN=MAOB PE=1 SV=3 |

|  | **Query** | **Observed** | **Mr(expt)** | **Mr(calc)** | **Delta** | **Miss** | **Score** | **Expect** | **Rank** | **Peptide** |
| --- | --- | --- | --- | --- | --- | --- | --- | --- | --- | --- |
|  | 1732 | **762.3873** | **761.3800** | **761.3854** | **-0.0054** | **0** | **34** | **0.09** | **1** | **R.NQMITR.V** |
|  | 2296 | **413.6645** | **825.3144** | **824.3157** | **0.9987** | **0** | **12** | **8.3** | **1** | **R.TMDDMGR.E** |
|  | 2468 | **420.8956** | **839.7766** | **840.3106** | **-0.5339** | **0** | **(6)** | **24** | **1** | **R.TMDDMGR.E** |
|  | 3202 | 448.7280 | 895.4414 | 895.4473 | -0.0059 | 0 | 22 | 1 | 1 | K.LCELYAK.V 3204 |
|  | 3223 | **898.4752** | **897.4679** | **897.4742** | **-0.0063** | **0** | **32** | **0.098** | **1** | **R.EILHAMGK.I** 3213 3214 3225 |
|  | 3974 | 476.7733 | 951.5319 | 951.4913 | 0.0407 | 0 | 12 | 9.3 | 8 | K.ELGLETYK.V 3968 |
|  | 4965 | 512.7746 | 1023.5346 | 1023.5423 | -0.0076 | 1 | 24 | 0.64 | 1 | K.KLCELYAK.V |
|  | 6222 | 561.2954 | 1120.5763 | 1121.5466 | -0.9703 | 0 | 72 | 9.3e-006 | 1 | K.FVGGSGQVSER.I 6233 6245 |
|  | 6908 | **587.8647** | **1173.7149** | **1174.5942** | **-0.8793** | **0** | **24** | **0.65** | **1** | **R.IISTTNGGQER.K** 6907 6918 6929 |
|  | 8418 | 625.8241 | 1249.6336 | 1249.6415 | -0.0079 | 1 | 60 | 0.00015 | 1 | R.KFVGGSGQVSER.I 8417 8419 8437 |
|  | 9419 | **652.9451** | **1303.8757** | **1302.6892** | **1.1865** | **1** | **0** | **1e+002** | **9** | **R.IISTTNGGQERK.F** |
|  | 10323 | **676.8472** | **1351.6798** | **1351.6893** | **-0.0095** | **0** | **26** | **0.38** | **1** | **K.IHFNPPLPMMR.N** |
|  | 10905 | **462.2657** | **1383.7753** | **1383.6791** | **0.0962** | **0** | **(7)** | **29** | **6** | **K.IHFNPPLPMMR.N** |
|  | 10991 | **695.3897** | **1388.7649** | **1388.7737** | **-0.0089** | **0** | **2** | **91** | **1** | **K.YVISAIPPTLGMK.I** |
|  | 12873 | **751.9145** | **1501.8145** | **1501.8253** | **-0.0107** | **0** | **48** | **0.0021** | **1** | **K.LERPVIYIDQTR.E** 12874 |
|  | 13374 | **768.3415** | **1534.6684** | **1534.6796** | **-0.0111** | **0** | **23** | **0.66** | **1** | **K.APLAEEWDNMTMK.E** |
|  | 14449 | **803.9174** | **1605.8203** | **1605.7855** | **0.0348** | **0** | **8** | **17** | **2** | **K.CDVVVVGGGISGMAAAK.L** |
|  | 14734 | **813.3951** | **1624.7757** | **1624.7845** | **-0.0088** | **0** | **37** | **0.025** | **1** | **K.YVDLGGSYVGPTQNR.I** 14733 14747 |
|  | 14888 | **817.9595** | **1633.9044** | **1633.9151** | **-0.0108** | **0** | **72** | **8.1e-006** | **1** | **K.LLHDSGLNVVVLEAR.D** 14887 |
|  | 14918 | 819.3717 | 1636.7288 | 1637.9215 | -1.1926 | 1 | 2 | 74 | 5 | R.VPLGSVIKCIVYYK.E 14919 |
|  | 15556 | **560.3003** | **1677.8790** | **1677.8573** | **0.0217** | **1** | **9** | **15** | **2** | **K.ELGLETYKVNEVER.L** 15554 15563 |
|  | 16396 | **577.3327** | **1728.9763** | **1728.9886** | **-0.0124** | **1** | **21** | **0.93** | **1** | **R.VKLERPVIYIDQTR.E** 16395 16397 |
|  | 17507 | **906.9670** | **1811.9195** | **1811.9305** | **-0.0110** | **0** | **56** | **0.00028** | **1** | **K.VLGSLEALEPVHYEEK.N** 17505 17506 |
|  | 18868 | **959.9580** | **1917.9015** | **1917.9142** | **-0.0127** | **0** | **70** | **9.7e-006** | **1** | **R.ENVLVETLNHEMYEAK.Y** 18867 18870 |
|  | 20579 | **689.3701** | **2065.0884** | **2065.9959** | **-0.9075** | **1** | **0** | **79** | **1** | **-.MSNKCDVVVVGGGISGMAAAK.L** |
|  | 21047 | **1056.4707** | **2110.9268** | **2111.0591** | **-0.1322** | **1** | **2** | **56** | **3** | **K.IHFNPPLPMMRNQMITR.V** |
|  | 21180 | 710.0269 | 2127.0589 | 2127.0540 | 0.0050 | 1 | (1) | 75 | 6 | K.IHFNPPLPMMRNQMITR.V |
|  | 21204 | **533.3133** | **2129.2241** | **2129.2361** | **-0.0120** | **1** | **26** | **0.19** | **1** | **R.LIGLTTIFSATALGFLAHKR.G** |
|  | 23954 | **824.4049** | **2470.1927** | **2469.2018** | **0.9909** | **0** | **18** | **1.1** | **1** | **R.GPFPPVWNPITYLDHNNFWR.T** |
|  | 25516 | **911.7403** | **2732.1991** | **2732.2177** | **-0.0186** | **0** | **28** | **0.085** | **1** | **R.IYFAGTETATHWSGYMEGAVEAGER.A** 25524 |
|  | 27990 | **1613.7830** | **3225.5514** | **3225.5717** | **-0.0204** | **0** | **61** | **3.5e-005** | **1** | **K.IPEDEIWQSEPESVDVPAQPITTTFLER.H** 27991 27992 |
|  | 30307 | **1027.2612** | **4105.0158** | **4105.0354** | **-0.0196** | **1** | **14** | **1.1** | **1** | **R.EILHAMGKIPEDEIWQSEPESVDVPAQPITTTFLER.H** 30310 |
|  | 30660 | **1430.6661** | **4288.9766** | **4289.0007** | **-0.0241** | **0** | **53** | **0.00011** | **1** | **K.DYCGTMIIDGEEAPVAYTLDDTKPEGNYAAIMGFILAHK.A** 30662 |

| **84.** | P00367\|DHE3_HUMAN    **Mass:** 61701    **Score:** 603    **Queries matched:** 45   **emPAI:** 1.27 |
| --- | --- |
|  | Glutamate dehydrogenase 1, mitochondrial OS=Homo sapiens GN=GLUD1 PE=1 SV=2 |

|  | **Query** | **Observed** | **Mr(expt)** | **Mr(calc)** | **Delta** | **Miss** | **Score** | **Expect** | **Rank** | **Peptide** |
| --- | --- | --- | --- | --- | --- | --- | --- | --- | --- | --- |
|  | 2299 | **413.7705** | **825.5265** | **825.5072** | **0.0193** | **1** | **15** | **4.9** | **7** | **K.AGVKINPK.N** 2304 |
|  | 2457 | **839.4550** | **838.4477** | **838.4259** | **0.0219** | **0** | **11** | **12** | **6** | **R.FTMELAK.K** 2454 |
|  | 4034 | **479.2498** | **956.4850** | **955.5127** | **0.9723** | **1** | **6** | **42** | **4** | **R.LTFKYER.D** |
|  | 4592 | **499.2697** | **996.5248** | **996.5314** | **-0.0066** | **0** | **33** | **0.074** | **1** | **K.ALASLMTYK.C** |
|  | 4830 | **507.2892** | **1012.5639** | **1013.5618** | **-0.9978** | **1** | **14** | **5.9** | **1** | **K.QLTKSNAPR.V** |
|  | 5410 | **530.2664** | **1058.5183** | **1058.5257** | **-0.0074** | **0** | **23** | **0.86** | **1** | **K.NLNHVSYGR.L** |
|  | 5662 | **540.2866** | **1078.5587** | **1078.5658** | **-0.0072** | **0** | **40** | **0.015** | **1** | **R.TAAYVNAIEK.V** |
|  | 5924 | **550.2159** | **1098.4172** | **1099.5186** | **-1.1014** | **0** | **4** | **62** | **2** | **K.VYNEAGVTFT.-** |
|  | 8231 | **621.2943** | **1240.5741** | **1240.5823** | **-0.0083** | **0** | **37** | **0.027** | **1** | **R.YSTDVSVDEVK.A** 8229 8230 |
|  | 11609 | **713.3135** | **1424.6125** | **1424.6208** | **-0.0083** | **0** | **15** | **4.5** | **1** | **R.DDGSWEVIEGYR.A** 11607 |
|  | 12923 | **502.9083** | **1505.7030** | **1506.7137** | **-1.0106** | **0** | **4** | **46** | **9** | **K.DIVHSGLAYTMER.S** |
|  | 13496 | 772.4148 | 1542.8150 | 1542.8253 | -0.0103 | 1 | 32 | 0.074 | 1 | R.GASIVEDKLVEDLR.T 13497 |
|  | 14386 | **801.4146** | **1600.8147** | **1599.8443** | **0.9704** | **1** | **1** | **85** | **5** | **-.MYRYLGEALLLSR.A** |
|  | 15041 | **822.8477** | **1643.6808** | **1643.9219** | **-0.2412** | **1** | **1** | **77** | **5** | **R.GQPAAAPQPGLALAARR.H** |
|  | 16508 | **869.4434** | **1736.8723** | **1736.8846** | **-0.0123** | **0** | **58** | **0.00017** | **1** | **K.HGGTIPIVPTAEFQDR.I** 16507 |
|  | 16644 | **874.9427** | **1747.8708** | **1747.8828** | **-0.0120** | **0** | **36** | **0.031** | **1** | **K.TFVVQGFGNVGLHSMR.Y** 16639 |
|  | 16886 | **882.3986** | **1762.7826** | **1763.8778** | **-1.0952** | **0** | **(3)** | **59** | **3** | **K.TFVVQGFGNVGLHSMR.Y** |
|  | 17339 | **600.4647** | **1798.3722** | **1798.8560** | **-0.4838** | **1** | **0** | **61** | **1** | **K.MVEGFFDRGASIVEDK.L** |
|  | 17626 | **608.3438** | **1822.0096** | **1820.8839** | **1.1257** | **1** | **8** | **18** | **3** | **K.DIVHSGLAYTMERSAR.Q** |
|  | 18565 | **632.0254** | **1893.0545** | **1893.0658** | **-0.0113** | **0** | **10** | **10** | **1** | **R.IIKPCNHVLSLSFPIR.R** |
|  | 18841 | **958.4581** | **1914.9016** | **1914.9146** | **-0.0130** | **0** | **58** | **0.00014** | **1** | **K.GFIGPGIDVPAPDMSTGER.E** 18840 |
|  | 18881 | **960.9526** | **1919.8906** | **1919.9047** | **-0.0141** | **0** | **66** | **2.2e-005** | **1** | **R.DSNYHLLMSVQESLER.K** 18882 18883 |
|  | 20287 | **1022.5059** | **2042.9972** | **2043.0095** | **-0.0124** | **1** | **58** | **0.00016** | **1** | **K.KGFIGPGIDVPAPDMSTGER.E** |
|  | 21502 | **1082.5313** | **2163.0479** | **2163.0630** | **-0.0150** | **1** | **88** | **1.4e-007** | **1** | **R.ISGASEKDIVHSGLAYTMER.S** 21501 21504 |
|  | 22235 | **1121.5808** | **2241.1471** | **2241.1641** | **-0.0170** | **1** | **78** | **1.2e-006** | **1** | **K.IIAEGANGPTTPEADKIFLER.N** 22228 22234 22242 |
|  | 24133 | **1245.6171** | **2489.2196** | **2489.2359** | **-0.0163** | **0** | **77** | **1.5e-006** | **1** | **K.AKPYEGSILEADCDILIPAASEK.Q** 24132 |
|  | 25566 | **914.8179** | **2741.4318** | **2741.4138** | **0.0179** | **0** | **17** | **1.3** | **1** | **R.NIMVIPDLYLNAGGVTVSYFEWLK.N** |
|  | 27170 | **1516.2146** | **3030.4146** | **3030.4256** | **-0.0109** | **0** | **77** | **9.4e-007** | **1** | **R.GVFHGIENFINEASYMSILGMTPGFGDK.T** |

| **85.** | P35504\|UD15_HUMAN    **Mass:** 60602    **Score:** 586    **Queries matched:** 40   **emPAI:** 1.17 |
| --- | --- |
|  | UDP-glucuronosyltransferase 1-5 OS=Homo sapiens GN=UGT1A5 PE=1 SV=1 |

|  | **Query** | **Observed** | **Mr(expt)** | **Mr(calc)** | **Delta** | **Miss** | **Score** | **Expect** | **Rank** | **Peptide** |
| --- | --- | --- | --- | --- | --- | --- | --- | --- | --- | --- |
|  | 1474 | 659.3670 | 658.3597 | 658.3650 | -0.0053 | 0 | 23 | 1.4 | 1 | K.AVINDK.S |
|  | 4073 | 480.7594 | 959.5043 | 959.5110 | -0.0067 | 0 | 44 | 0.0083 | 1 | K.AMAIADALGK.I |
|  | 4819 | 506.7970 | 1011.5795 | 1011.5865 | -0.0070 | 0 | 28 | 0.25 | 1 | K.IPQTVLWR.Y 4818 |
|  | 5184 | 520.7598 | 1039.5051 | 1039.5120 | -0.0069 | 1 | 43 | 0.0075 | 1 | K.SYKENIMR.L 5183 |
|  | 5366 | 528.7572 | 1055.4998 | 1055.5069 | -0.0071 | 1 | (12) | 9.2 | 2 | K.SYKENIMR.L |
|  | 5794 | 545.2573 | 1088.5000 | 1087.6059 | 0.8940 | 1 | 11 | 15 | 2 | K.KAMAIADALGK.I |
|  | 13485 | **514.9630** | **1541.8672** | **1542.7832** | **-0.9160** | **0** | **(1)** | **94** | **4** | **R.MAIMNNMSLIIHR.S** |
|  | 13669 | **518.6048** | **1552.7926** | **1552.8031** | **-0.0106** | **0** | **13** | **6.5** | **1** | **R.SCVELLHNEALIR.H** |
|  | 14214 | **795.9376** | **1589.8607** | **1590.7680** | **-0.9073** | **0** | **2** | **79** | **4** | **R.MAIMNNMSLIIHR.S** |
|  | 15500 | 838.3856 | 1674.7566 | 1675.8352 | -1.0786 | 0 | 53 | 0.00059 | 1 | R.LLTTNSDHMTFLQR.V 15522 15523 |
|  | 15545 | 839.4250 | 1676.8354 | 1676.8457 | -0.0103 | 0 | 56 | 0.00028 | 1 | K.WLPQNDLLGHPMTR.A 15543 15544 15546 |
|  | 16403 | 865.8715 | 1729.7285 | 1730.8742 | -1.1457 | 1 | 5 | 36 | 6 | R.RMAIMNNMSLIIHR.S |
|  | 18691 | 952.4685 | 1902.9225 | 1902.9986 | -0.0761 | 1 | 2 | 67 | 6 | R.LLTTNSDHMTFLQRVK.N |
|  | 19300 | 652.3673 | 1954.0801 | 1953.0870 | 0.9931 | 1 | 18 | 1.4 | 1 | K.AMAIADALGKIPQTVLWR.Y |
|  | 21832 | 1102.5536 | 2203.0926 | 2203.1042 | -0.0116 | 0 | 109 | 1.1e-009 | 1 | K.GAGVTLNVLEMTSEDLENALK.A 21831 21833 21836 21838 21839 21846 |
|  | 22825 | 1159.5969 | 2317.1793 | 2317.1929 | -0.0136 | 0 | 66 | 1.7e-005 | 1 | K.DRPVEPLDLAVFWVEFVMR.H 22826 |
|  | 26144 | 948.8229 | 2843.4468 | 2843.4586 | -0.0118 | 1 | 54 | 0.00021 | 1 | K.GAGVTLNVLEMTSEDLENALKAVINDK.S |
|  | 29713 | 956.9341 | 3823.7075 | 3823.7289 | -0.0215 | 0 | 40 | 0.0032 | 1 | R.AFITHAGSHGVYESICNGVPMVMMPLFGDQMDNAK.R 29712 29714 29722 29724 29725 |
|  | 30060 | 1327.6094 | 3979.8063 | 3979.8300 | -0.0237 | 1 | 38 | 0.0046 | 1 | R.AFITHAGSHGVYESICNGVPMVMMPLFGDQMDNAKR.M 30062 |
|  | 31194 | **805.0928** | **4824.5130** | **4824.6075** | **-0.0945** | **1** | **0** | **14** | **2** | **-.MATGLQVPLPQLATGLLLLLSVQPWAESGKVLVVPTDGSHWLSMR.E** |

| **86.** | P05091\|ALDH2_HUMAN    **Mass:** 56859    **Score:** 581    **Queries matched:** 41   **emPAI:** 2.14 |
| --- | --- |
|  | Aldehyde dehydrogenase, mitochondrial OS=Homo sapiens GN=ALDH2 PE=1 SV=2 |

|  | **Query** | **Observed** | **Mr(expt)** | **Mr(calc)** | **Delta** | **Miss** | **Score** | **Expect** | **Rank** | **Peptide** |
| --- | --- | --- | --- | --- | --- | --- | --- | --- | --- | --- |
|  | 2324 | 829.4149 | 828.4076 | 828.4705 | -0.0629 | 0 | 4 | 73 | 3 | R.LADLIER.D |
|  | 2928 | **437.2527** | **872.4909** | **871.4082** | **1.0827** | **1** | **10** | **19** | **4** | **R.RMDASHR.G** |
|  | 4108 | **482.2439** | **962.4732** | **961.4869** | **0.9863** | **0** | **22** | **1** | **1** | **R.VVGNPFDSK.T** |
|  | 4236 | **486.7922** | **971.5699** | **971.5764** | **-0.0064** | **1** | **26** | **0.42** | **1** | **K.RVTLELGGK.S** |
|  | 5945 | **550.8029** | **1099.5912** | **1099.5985** | **-0.0074** | **1** | **34** | **0.066** | **1** | **R.LADLIERDR.T** |
|  | 5994 | **552.2850** | **1102.5555** | **1101.5601** | **0.9954** | **0** | **55** | **0.0006** | **1** | **K.LLCGGGIAADR.G** 5993 |
|  | 6364 | **567.2476** | **1132.4806** | **1131.5825** | **0.8980** | **0** | **5** | **49** | **1** | **R.AAFQLGSPWR.R** |
|  | 7098 | 1186.6715 | 1185.6642 | 1185.6717 | -0.0075 | 0 | 49 | 0.0021 | 1 | R.VIQVAAGSSNLK.R |
|  | 9798 | **442.2561** | **1323.7466** | **1324.7826** | **-1.0361** | **1** | **8** | **21** | **3** | **R.LLNRLADLIER.D** 9794 9819 |
|  | 10133 | **671.8888** | **1341.7630** | **1341.7728** | **-0.0098** | **1** | **22** | **0.84** | **1** | **R.VIQVAAGSSNLKR.V** |
|  | 10636 | **685.3922** | **1368.7699** | **1368.7799** | **-0.0100** | **0** | **50** | **0.0013** | **1** | **K.LGPALATGNVVVMK.V** 10637 |
|  | 10907 | **693.3085** | **1384.6025** | **1384.7748** | **-0.1723** | **0** | **(10)** | **13** | **4** | **K.LGPALATGNVVVMK.V** |
|  | 12160 | **730.3827** | **1458.7509** | **1457.6728** | **1.0782** | **1** | **0** | **1.3e+002** | **6** | **R.YYAGWADKYHGK.T** |
|  | 12706 | **746.4065** | **1490.7984** | **1490.8093** | **-0.0108** | **1** | **35** | **0.038** | **1** | **K.ILGYINTGKQEGAK.L** |
|  | 12922 | **753.8522** | **1505.6898** | **1505.6998** | **-0.0100** | **0** | **39** | **0.016** | **1** | **K.TEQGPQVDETQFK.K** |
|  | 13268 | **764.3884** | **1526.7622** | **1526.7729** | **-0.0107** | **0** | **84** | **4.5e-007** | **1** | **R.ANNSTYGLAAAVFTK.D** 13267 |
|  | 13318 | **766.3705** | **1530.7265** | **1530.7355** | **-0.0089** | **0** | **4** | **50** | **5** | **K.TIPIDGDFFSYTR.H** |
|  | 14347 | **800.3929** | **1598.7712** | **1598.7828** | **-0.0116** | **0** | **64** | **4.8e-005** | **1** | **R.ELGEYGLQAYTEVK.T** 14348 |
|  | 17231 | **895.4111** | **1788.8076** | **1788.8206** | **-0.0131** | **0** | **34** | **0.043** | **1** | **R.TFVQEDIYDEFVER.S** |
|  | 21169 | **1064.0176** | **2126.0206** | **2127.1099** | **-1.0893** | **1** | **2** | **56** | **2** | **R.ELGEYGLQAYTEVKTVTVK.V** |
|  | 21596 | **725.3699** | **2173.0880** | **2174.0314** | **-0.9434** | **1** | **12** | **6.1** | **1** | **K.MSGSGRELGEYGLQAYTEVK.T** |
|  | 21721 | **1094.0360** | **2186.0575** | **2186.0718** | **-0.0144** | **0** | **97** | **1.7e-008** | **1** | **R.GYFIQPTVFGDVQDGMTIAK.E** 21722 |
|  | 21829 | **551.7786** | **2203.0851** | **2202.0667** | **1.0184** | **0** | **(6)** | **22** | **5** | **R.GYFIQPTVFGDVQDGMTIAK.E** |
|  | 23776 | **817.3944** | **2449.1612** | **2449.1762** | **-0.0149** | **1** | **14** | **3.2** | **1** | **R.VVGNPFDSKTEQGPQVDETQFK.K** |
|  | 25460 | **1360.6893** | **2719.3641** | **2719.3767** | **-0.0126** | **0** | **52** | **0.00041** | **1** | **R.HEPVGVCGQIIPWNFPLLMQAWK.L** 25461 |
|  | 27750 | **1053.2261** | **3156.6564** | **3156.6668** | **-0.0104** | **0** | **52** | **0.00029** | **1** | **R.TYLAALETLDNGKPYVISYLVDLDMVLK.C** |
|  | 29016 | **893.7082** | **3570.8037** | **3570.8143** | **-0.0106** | **1** | **6** | **9.5** | **1** | **R.GYFIQPTVFGDVQDGMTIAKEEIFGPVMQILK.F** 29015 |
|  | 29861 | **1294.9454** | **3881.8145** | **3881.8359** | **-0.0215** | **1** | **63** | **1.4e-005** | **1** | **K.DLDKANYLSQALQAGTVWVNCYDVFGAQSPFGGYK.M** 29862 |
|  | 30137 | **1345.2371** | **4032.6894** | **4032.7110** | **-0.0217** | **0** | **75** | **7.9e-007** | **1** | **K.SPNIIMSDADMDWAVEQAHFALFFNQGQCCCAGSR.T** 30136 |
|  | 30405 | **1035.2712** | **4137.0559** | **4137.0808** | **-0.0249** | **1** | **27** | **0.056** | **1** | **K.EAGFPPGVVNIVPGFGPTAGAAIASHEDVDKVAFTGSTEIGR.V** |

| **87.** | P11142\|HSP7C_HUMAN    **Mass:** 71082    **Score:** 562    **Queries matched:** 50   **emPAI:** 1.15 |
| --- | --- |
|  | Heat shock cognate 71 kDa protein OS=Homo sapiens GN=HSPA8 PE=1 SV=1 |

|  | **Query** | **Observed** | **Mr(expt)** | **Mr(calc)** | **Delta** | **Miss** | **Score** | **Expect** | **Rank** | **Peptide** |
| --- | --- | --- | --- | --- | --- | --- | --- | --- | --- | --- |
|  | 1539 | 686.4146 | 685.4073 | 686.3963 | -0.9889 | 0 | 12 | 12 | 10 | R.LIGDAAK.N |
|  | 2017 | **804.4382** | **803.4309** | **803.4389** | **-0.0080** | **0** | **23** | **1.1** | **1** | **K.ITITNDK.G** |
|  | 4472 | **495.2636** | **988.5127** | **988.5189** | **-0.0062** | **1** | **19** | **2.7** | **1** | **R.LSKEDIER.M** |
|  | 4891 | **509.2687** | **1016.5228** | **1016.5614** | **-0.0386** | **1** | **8** | **29** | **7** | **K.ITITNDKGR.L** 4890 |
|  | 7388 | **600.4399** | **1198.8652** | **1197.6717** | **1.1935** | **1** | **8** | **17** | **3** | **R.GTLDPVEKALR.D** |
|  | 7971 | 614.8130 | 1227.6115 | 1227.6207 | -0.0092 | 0 | 24 | 0.56 | 1 | K.VEIIANDQGNR.T |
|  | 8113 | **618.3115** | **1234.6084** | **1234.6169** | **-0.0085** | **0** | **33** | **0.076** | **1** | **R.MVNHFIAEFK.R** 8114 8133 |
|  | 8449 | **626.8290** | **1251.6435** | **1251.6533** | **-0.0097** | **1** | **49** | **0.0019** | **1** | **K.MKEIAEAYLGK.T** |
|  | 8456 | **418.4507** | **1252.3303** | **1252.6088** | **-0.2785** | **0** | **2** | **59** | **7** | **R.FEELNADLFR.G** |
|  | 8477 | **627.7831** | **1253.5516** | **1253.5598** | **-0.0082** | **0** | **28** | **0.22** | **1** | **R.FDDAVVQSDMK.H** 8484 |
|  | 8769 | **424.0556** | **1269.1451** | **1269.5547** | **-0.4097** | **0** | **(6)** | **22** | **4** | **R.FDDAVVQSDMK.H** 8776 8778 |
|  | 11003 | **464.2542** | **1389.7408** | **1390.7180** | **-0.9772** | **1** | **10** | **14** | **1** | **R.MVNHFIAEFKR.K** |
|  | 11345 | **705.8336** | **1409.6526** | **1409.6609** | **-0.0084** | **1** | **34** | **0.054** | **1** | **R.RFDDAVVQSDMK.H** |
|  | 12518 | **740.8753** | **1479.7361** | **1479.7470** | **-0.0109** | **1** | **36** | **0.031** | **1** | **R.ARFEELNADLFR.G** 12519 |
|  | 12631 | **744.3494** | **1486.6842** | **1486.6940** | **-0.0098** | **0** | **65** | **4.2e-005** | **1** | **R.TTPSYVAFTDTER.L** 12606 12630 |
|  | 14602 | 808.8926 | 1615.7706 | 1615.7804 | -0.0097 | 0 | 61 | 9.1e-005 | 1 | K.SFYPEEVSSMVLTK.M 14601 |
|  | 15120 | **825.3957** | **1648.7768** | **1648.7879** | **-0.0111** | **0** | **80** | **1.2e-006** | **1** | **K.NQVAMNPTNTVFDAK.R** 15119 |
|  | 15182 | **551.7995** | **1652.3767** | **1652.8246** | **-0.4479** | **0** | **18** | **1.3** | **1** | **K.HWPFMVVNDAGRPK.V** |
|  | 15284 | 830.4458 | 1658.8770 | 1658.8879 | -0.0108 | 0 | 85 | 3.9e-007 | 1 | R.IINEPTAAAIAYGLDK.K 15285 |
|  | 15356 | **555.6410** | **1663.9013** | **1664.7828** | **-0.8815** | **0** | **(9)** | **13** | **4** | **K.NQVAMNPTNTVFDAK.R** |
|  | 15720 | **846.3599** | **1690.7053** | **1690.7183** | **-0.0130** | **0** | **56** | **0.00023** | **1** | **K.STAGDTHLGGEDFDNR.M** 15721 |
|  | 15975 | **568.9254** | **1703.7544** | **1703.8188** | **-0.0644** | **1** | **2** | **65** | **5** | **K.VSSKNSLESYAFNMK.A** |
|  | 16995 | **591.9691** | **1772.8854** | **1772.9131** | **-0.0277** | **1** | **4** | **41** | **10** | **K.ILDKCNEIINWLDK.N** |
|  | 17581 | **607.2679** | **1818.7818** | **1819.0316** | **-0.2498** | **1** | **6** | **25** | **1** | **K.SQIHDIVLVGGSTRIPK.I** |
|  | 18772 | **637.0158** | **1908.0254** | **1906.9056** | **1.1198** | **1** | **3** | **54** | **7** | **K.SFYPEEVSSMVLTKMK.E** |
|  | 19581 | **991.4953** | **1980.9760** | **1980.9905** | **-0.0145** | **0** | **25** | **0.34** | **1** | **K.TVTNAVVTVPAYFNDSQR.Q** 19582 19583 |
|  | 25577 | **549.8809** | **2744.3682** | **2743.4028** | **0.9654** | **1** | **0** | **57** | **8** | **K.ELNKSINPDEAVAYGAAVQAAILSGDK.S** |
|  | 25734 | **1387.6576** | **2773.3006** | **2773.3195** | **-0.0189** | **0** | **96** | **1.5e-008** | **1** | **K.QTQTFTTYSDNQPGVLIQVYEGER.A** 25729 25733 |
|  | 28381 | **1116.1635** | **3345.4685** | **3345.4919** | **-0.0233** | **0** | **(4)** | **16** | **1** | **K.LYQSAGGMPGGMPGGFPGGGAPPSGGASSGPTIEEVD.-** |
|  | 28425 | 1121.2435 | 3360.7088 | 3361.4868 | -0.7780 | 0 | 6 | 10 | 2 | K.LYQSAGGMPGGMPGGFPGGGAPPSGGASSGPTIEEVD.- |
|  | 30636 | **611.1672** | **4271.1193** | **4270.9974** | **0.1220** | **1** | **(0)** | **22** | **2** | **K.VCNPIITKLYQSAGGMPGGMPGGFPGGGAPPSGGASSGPTIEEVD.-** |
|  | 30656 | **1072.6031** | **4286.3835** | **4286.9923** | **-0.6088** | **1** | **3** | **10** | **4** | **K.VCNPIITKLYQSAGGMPGGMPGGFPGGGAPPSGGASSGPTIEEVD.-** 30653 |

| **88.** | P19224\|UD16_HUMAN    **Mass:** 61338    **Score:** 561    **Queries matched:** 37   **emPAI:** 1.03 |
| --- | --- |
|  | UDP-glucuronosyltransferase 1-6 OS=Homo sapiens GN=UGT1A6 PE=1 SV=2 |

|  | **Query** | **Observed** | **Mr(expt)** | **Mr(calc)** | **Delta** | **Miss** | **Score** | **Expect** | **Rank** | **Peptide** |
| --- | --- | --- | --- | --- | --- | --- | --- | --- | --- | --- |
|  | 1474 | 659.3670 | 658.3597 | 658.3650 | -0.0053 | 0 | 23 | 1.4 | 1 | K.AVINDK.S |
|  | 2911 | **436.7503** | **871.4860** | **871.5280** | **-0.0420** | **0** | **42** | **0.013** | **3** | **K.VSVWLLR.Y** |
|  | 4073 | 480.7594 | 959.5043 | 959.5110 | -0.0067 | 0 | 44 | 0.0083 | 1 | K.AMAIADALGK.I |
|  | 4819 | 506.7970 | 1011.5795 | 1011.5865 | -0.0070 | 0 | 28 | 0.25 | 1 | K.IPQTVLWR.Y 4818 |
|  | 5184 | 520.7598 | 1039.5051 | 1039.5120 | -0.0069 | 1 | 43 | 0.0075 | 1 | K.SYKENIMR.L 5183 |
|  | 5366 | 528.7572 | 1055.4998 | 1055.5069 | -0.0071 | 1 | (12) | 9.2 | 2 | K.SYKENIMR.L |
|  | 5794 | 545.2573 | 1088.5000 | 1087.6059 | 0.8940 | 1 | 11 | 15 | 2 | K.KAMAIADALGK.I |
|  | 9576 | **438.5840** | **1312.7300** | **1311.6459** | **1.0841** | **0** | **2** | **87** | **5** | **R.SFLTAPQTEYR.N** |
|  | 12747 | **747.8696** | **1493.7247** | **1492.7449** | **0.9798** | **0** | **12** | **8.8** | **2** | **K.IYPVPYDQEELK.N** |
|  | 15545 | 839.4250 | 1676.8354 | 1676.8457 | -0.0103 | 0 | 56 | 0.00028 | 1 | K.WLPQNDLLGHPMTR.A 15543 15544 15546 |
|  | 16522 | **580.5969** | **1738.7689** | **1738.7924** | **-0.0235** | **1** | **0** | **1e+002** | **6** | **K.NRYQSFGNNHFAER.S** |
|  | 17633 | **608.7136** | **1823.1190** | **1822.7767** | **0.3423** | **1** | **2** | **53** | **8** | **R.CYTKFSDHMTFSQR.V** |
|  | 19300 | 652.3673 | 1954.0801 | 1953.0870 | 0.9931 | 1 | 18 | 1.4 | 1 | K.AMAIADALGKIPQTVLWR.Y |
|  | 21832 | 1102.5536 | 2203.0926 | 2203.1042 | -0.0116 | 0 | 109 | 1.1e-009 | 1 | K.GAGVTLNVLEMTSEDLENALK.A 21831 21833 21836 21838 21839 21846 |
|  | 22825 | 1159.5969 | 2317.1793 | 2317.1929 | -0.0136 | 0 | 66 | 1.7e-005 | 1 | K.DRPVEPLDLAVFWVEFVMR.H 22826 |
|  | 26144 | 948.8229 | 2843.4468 | 2843.4586 | -0.0118 | 1 | 54 | 0.00021 | 1 | K.GAGVTLNVLEMTSEDLENALKAVINDK.S |
|  | 26993 | **996.4664** | **2986.3775** | **2986.4907** | **-0.1133** | **1** | **8** | **7.9** | **3** | **R.YDFVLEYPRPVMPNMVFIGGINCKK.R** |
|  | 29713 | 956.9341 | 3823.7075 | 3823.7289 | -0.0215 | 0 | 40 | 0.0032 | 1 | R.AFITHAGSHGVYESICNGVPMVMMPLFGDQMDNAK.R 29712 29714 29722 29724 29725 |
|  | 30060 | 1327.6094 | 3979.8063 | 3979.8300 | -0.0237 | 1 | 38 | 0.0046 | 1 | R.AFITHAGSHGVYESICNGVPMVMMPLFGDQMDNAKR.M 30062 |

| **89.** | P54868\|HMCS2_HUMAN    **Mass:** 57113    **Score:** 556    **Queries matched:** 39   **emPAI:** 1.27 |
| --- | --- |
|  | Hydroxymethylglutaryl-CoA synthase, mitochondrial OS=Homo sapiens GN=HMGCS2 PE=1 SV=1 |

|  | **Query** | **Observed** | **Mr(expt)** | **Mr(calc)** | **Delta** | **Miss** | **Score** | **Expect** | **Rank** | **Peptide** |
| --- | --- | --- | --- | --- | --- | --- | --- | --- | --- | --- |
|  | 2158 | **408.7193** | **815.4240** | **815.4290** | **-0.0050** | **0** | **22** | **1.3** | **1** | **K.IQNQWK.Q** |
|  | 3165 | **446.7489** | **891.4833** | **890.4862** | **0.9971** | **0** | **25** | **0.54** | **1** | **K.GLEAFGGLK.L** 3151 |
|  | 3867 | **473.2675** | **944.5205** | **943.5239** | **0.9966** | **1** | **11** | **15** | **3** | **K.KIQNQWK.Q** |
|  | 3924 | **475.2253** | **948.4359** | **947.5222** | **0.9137** | **1** | **7** | **32** | **7** | **K.MVQKSLAR.L** |
|  | 4394 | **492.2658** | **982.5170** | **982.4607** | **0.0563** | **0** | **10** | **15** | **4** | **K.LEDTYTNK.D** |
|  | 4540 | **497.7497** | **993.4848** | **993.5244** | **-0.0396** | **0** | **17** | **3.5** | **1** | **K.YTVGLGQTR.M** |
|  | 5273 | 524.2460 | 1046.4774 | 1045.5655 | 0.9119 | 0 | 6 | 49 | 10 | K.LVSSTSDLPK.R 5259 |
|  | 5327 | 526.7764 | 1051.5382 | 1051.5484 | -0.0102 | 0 | 15 | 4.3 | 4 | K.LSIQCYLR.A |
|  | 5828 | **546.7651** | **1091.5156** | **1090.6022** | **0.9134** | **0** | **1** | **1.3e+002** | **9** | **R.FSTASAVPLAK.T** |
|  | 7456 | **601.8360** | **1201.6574** | **1201.6666** | **-0.0092** | **1** | **48** | **0.0024** | **1** | **K.LVSSTSDLPKR.L** 7455 |
|  | 7722 | 608.7971 | 1215.5797 | 1216.6551 | -1.0754 | 0 | 66 | 3.6e-005 | 1 | R.LEVGTETIIDK.S |
|  | 9057 | **643.3840** | **1284.7535** | **1283.6357** | **1.1178** | **0** | **11** | **11** | **1** | **R.VSQDAAPGSPLDK.L** 9048 |
|  | 9405 | **435.2690** | **1302.7851** | **1303.5979** | **-0.8128** | **1** | **4** | **50** | **6** | **R.ALDRCYTSYR.K** |
|  | 16519 | **870.3848** | **1738.7551** | **1738.7654** | **-0.0103** | **0** | **63** | **4.7e-005** | **1** | **K.CVSPEEFTEIMNQR.E** 16518 |
|  | 18075 | **928.9885** | **1855.9624** | **1854.9363** | **1.0260** | **1** | **5** | **35** | **9** | **K.GLEAFGGLKLEDTYTNK.D** |
|  | 18244 | **934.4308** | **1866.8471** | **1866.8604** | **-0.0132** | **1** | **87** | **1.8e-007** | **1** | **R.KCVSPEEFTEIMNQR.E** |
|  | 18453 | **628.9747** | **1883.9022** | **1882.8553** | **1.0469** | **1** | **(1)** | **87** | **2** | **R.KCVSPEEFTEIMNQR.E** |
|  | 18455 | **942.9633** | **1883.9120** | **1883.9206** | **-0.0087** | **0** | **109** | **1.3e-009** | **1** | **R.IGAFSYGSGLAASFFSFR.V** |
|  | 18613 | **633.3380** | **1896.9922** | **1896.0792** | **0.9130** | **1** | **9** | **13** | **4** | **R.ILQLTRAVQETSLTPAR.L** |
|  | 21544 | **1084.5082** | **2167.0018** | **2167.0143** | **-0.0125** | **0** | **91** | **6.3e-008** | **1** | **R.LMFNDFLSASSDTQTSLYK.G** |
|  | 22770 | **771.3993** | **2311.1760** | **2311.1907** | **-0.0147** | **1** | **12** | **5.2** | **1** | **R.VSQDAAPGSPLDKLVSSTSDLPK.R** |
|  | 23425 | **1199.0714** | **2396.1283** | **2396.1437** | **-0.0155** | **0** | **94** | **2.7e-008** | **1** | **K.VNFSPPGDTNSLFPGTWYLER.V** 23426 23427 |
|  | 24949 | **1313.6604** | **2625.3062** | **2625.3214** | **-0.0151** | **0** | **59** | **8.1e-005** | **1** | **K.DVGILALEVYFPAQYVDQTDLEK.Y** 24948 24950 |
|  | 26400 | **963.1182** | **2886.3327** | **2886.3534** | **-0.0208** | **0** | **45** | **0.0019** | **1** | **R.GTHMENVYDFYKPNLASEYPIVDGK.L** |
|  | 26402 | **722.5912** | **2886.3357** | **2886.3469** | **-0.0113** | **0** | **26** | **0.13** | **1** | **K.QAGSDRPFTLDDLQYMIFHTPFCK.M** 26401 |
|  | 28827 | **1167.9120** | **3500.7141** | **3500.7351** | **-0.0210** | **1** | **20** | **0.41** | **1** | **K.DVGILALEVYFPAQYVDQTDLEKYNNVEAGK.Y** 28829 |
|  | 30091 | **1000.2191** | **3996.8474** | **3995.8894** | **0.9580** | **0** | **34** | **0.012** | **1** | **K.ASLYLSTHNGNMYTSSLYGCLASLLSHHSAQELAGSR.I** 30090 |

| **90.** | P35503\|UD13_HUMAN    **Mass:** 60869    **Score:** 555    **Queries matched:** 37   **emPAI:** 0.92 |
| --- | --- |
|  | UDP-glucuronosyltransferase 1-3 OS=Homo sapiens GN=UGT1A3 PE=1 SV=1 |

|  | **Query** | **Observed** | **Mr(expt)** | **Mr(calc)** | **Delta** | **Miss** | **Score** | **Expect** | **Rank** | **Peptide** |
| --- | --- | --- | --- | --- | --- | --- | --- | --- | --- | --- |
|  | 1474 | 659.3670 | 658.3597 | 658.3650 | -0.0053 | 0 | 23 | 1.4 | 1 | K.AVINDK.S |
|  | 4073 | 480.7594 | 959.5043 | 959.5110 | -0.0067 | 0 | 44 | 0.0083 | 1 | K.AMAIADALGK.I |
|  | 4819 | 506.7970 | 1011.5795 | 1011.5865 | -0.0070 | 0 | 28 | 0.25 | 1 | K.IPQTVLWR.Y 4818 |
|  | 5184 | 520.7598 | 1039.5051 | 1039.5120 | -0.0069 | 1 | 43 | 0.0075 | 1 | K.SYKENIMR.L 5183 |
|  | 5366 | 528.7572 | 1055.4998 | 1055.5069 | -0.0071 | 1 | (12) | 9.2 | 2 | K.SYKENIMR.L |
|  | 5794 | 545.2573 | 1088.5000 | 1087.6059 | 0.8940 | 1 | 11 | 15 | 2 | K.KAMAIADALGK.I |
|  | 6238 | **561.8185** | **1121.6225** | **1121.6305** | **-0.0080** | **1** | **9** | **19** | **6** | **R.EVLRELHAR.G** |
|  | 13669 | 518.6048 | 1552.7926 | 1552.8031 | -0.0106 | 0 | 13 | 6.5 | 1 | R.SCVELLHNEALIR.H |
|  | 15545 | 839.4250 | 1676.8354 | 1676.8457 | -0.0103 | 0 | 56 | 0.00028 | 1 | K.WLPQNDLLGHPMTR.A 15543 15544 15546 |
|  | 15603 | **561.3240** | **1680.9501** | **1681.7738** | **-0.8237** | **0** | **2** | **67** | **7** | **R.SMAMLNNMSLVYHR.S** |
|  | 15782 | **565.3055** | **1692.8948** | **1693.7916** | **-0.8968** | **0** | **2** | **64** | **10** | **R.LLTTNSDHMTFMQR.V** |
|  | 16989 | 887.3036 | 1772.5927 | 1771.9403 | 0.6524 | 0 | 4 | 30 | 2 | R.GHQAVVLTPEVNMHIK.E |
|  | 19300 | 652.3673 | 1954.0801 | 1953.0870 | 0.9931 | 1 | 18 | 1.4 | 1 | K.AMAIADALGKIPQTVLWR.Y |
|  | 21832 | 1102.5536 | 2203.0926 | 2203.1042 | -0.0116 | 0 | 109 | 1.1e-009 | 1 | K.GAGVTLNVLEMTSEDLENALK.A 21831 21833 21836 21838 21839 21846 |
|  | 22825 | 1159.5969 | 2317.1793 | 2317.1929 | -0.0136 | 0 | 66 | 1.7e-005 | 1 | K.DRPVEPLDLAVFWVEFVMR.H 22826 |
|  | 26144 | 948.8229 | 2843.4468 | 2843.4586 | -0.0118 | 1 | 54 | 0.00021 | 1 | K.GAGVTLNVLEMTSEDLENALKAVINDK.S |
|  | 27188 | **1011.5614** | **3031.6624** | **3031.3813** | **0.2811** | **0** | **0** | **41** | **1** | **R.GDFVMDYPRPIMPNMVFIGGINCANR.K** |
|  | 29713 | 956.9341 | 3823.7075 | 3823.7289 | -0.0215 | 0 | 40 | 0.0032 | 1 | R.AFITHAGSHGVYESICNGVPMVMMPLFGDQMDNAK.R 29712 29714 29722 29724 29725 |
|  | 30060 | 1327.6094 | 3979.8063 | 3979.8300 | -0.0237 | 1 | 38 | 0.0046 | 1 | R.AFITHAGSHGVYESICNGVPMVMMPLFGDQMDNAKR.M 30062 |

| **91.** | Q16696\|CP2AD_HUMAN    **Mass:** 56822    **Score:** 548    **Queries matched:** 40   **emPAI:** 1.14 |
| --- | --- |
|  | Cytochrome P450 2A13 OS=Homo sapiens GN=CYP2A13 PE=1 SV=3 |

|  | **Query** | **Observed** | **Mr(expt)** | **Mr(calc)** | **Delta** | **Miss** | **Score** | **Expect** | **Rank** | **Peptide** |
| --- | --- | --- | --- | --- | --- | --- | --- | --- | --- | --- |
|  | 1418 | 643.3307 | 642.3234 | 641.3609 | 0.9625 | 1 | 4 | 83 | 4 | K.NRQPK.F |
|  | 1748 | 772.4255 | 771.4183 | 772.3967 | -0.9784 | 0 | 7 | 41 | 9 | K.DIDVSPK.H |
|  | 2041 | 403.7088 | 805.4030 | 806.4650 | -1.0620 | 0 | 3 | 99 | 7 | R.FSIATLR.G |
|  | 3230 | 450.2301 | 898.4456 | 898.4508 | -0.0052 | 0 | 17 | 3.2 | 1 | R.TLDPNSPR.D 3229 3240 |
|  | 4132 | 482.7554 | 963.4963 | 962.5661 | 0.9302 | 1 | 14 | 7.1 | 1 | R.RFSIATLR.G |
|  | 4406 | 492.7800 | 983.5455 | 983.5514 | -0.0059 | 0 | 18 | 2.5 | 2 | R.YGFLLLMK.H |
|  | 4593 | 499.2789 | 996.5432 | 996.5505 | -0.0073 | 0 | 47 | 0.003 | 1 | K.HVGFATIPR.N 4575 4594 4611 |
|  | 6248 | 562.7517 | 1123.4889 | 1124.5502 | -1.0613 | 0 | 8 | 25 | 7 | K.NPNTEFYLK.N |
|  | 6684 | **579.2767** | **1156.5388** | **1155.5309** | **1.0079** | **0** | **3** | **91** | **6** | **K.GYGVAFSNGER.A** |
|  | 9219 | 647.8548 | 1293.6950 | 1293.7041 | -0.0090 | 1 | 29 | 0.19 | 1 | K.VHEEIDRVIGK.N |
|  | 9232 | 648.3492 | 1294.6839 | 1294.6921 | -0.0082 | 1 | 6 | 33 | 1 | K.KSDAFVPFSIGK.R |
|  | 10396 | 452.9163 | 1355.7270 | 1355.7350 | -0.0080 | 0 | 55 | 0.00042 | 1 | R.YGPVFTIHLGPR.R 10395 |
|  | 11550 | 711.3719 | 1420.7292 | 1420.7384 | -0.0092 | 0 | 14 | 5.1 | 3 | K.GTEVFPMLGSVLR.D 11549 |
|  | 11993 | 725.8381 | 1449.6616 | 1449.6736 | -0.0120 | 0 | 32 | 0.078 | 1 | K.EALVDQAEEFSGR.G |
|  | 12393 | 737.4042 | 1472.7939 | 1473.7827 | -0.9888 | 0 | 64 | 5.7e-005 | 1 | R.IQEEAGFLIDALR.G 12418 |
|  | 15319 | 831.8976 | 1661.7806 | 1661.8234 | -0.0428 | 1 | 4 | 42 | 2 | K.VEHNQRTLDPNSPR.D |
|  | 18628 | 950.4365 | 1898.8585 | 1898.8720 | -0.0135 | 1 | 67 | 1.8e-005 | 1 | R.MQEEEKNPNTEFYLK.N 18629 18630 |
|  | 19296 | **652.3503** | **1954.0290** | **1952.9529** | **1.0761** | **0** | **0** | **93** | **8** | **R.MELFLFFTTIMQNFR.F** |
|  | 22130 | **743.8360** | **2228.4862** | **2228.1162** | **0.3699** | **1** | **2** | **34** | **7** | **R.MELFLFFTTIMQNFRFK.S** |
|  | 25188 | 892.4617 | 2674.3632 | 2674.4988 | -0.1356 | 0 | 6 | 17 | 2 | M.LASGLLLVTLLACLTVMVLMSVWR.Q |
|  | 27818 | 1588.8043 | 3175.5941 | 3175.6086 | -0.0145 | 0 | 72 | 3e-006 | 1 | K.LPPGPTPLPFIGNYLQLNTEQMYNSLMK.I 27817 27819 |
|  | 28425 | 1121.2435 | 3360.7088 | 3360.7250 | -0.0162 | 1 | 85 | 1.5e-007 | 1 | R.GKLPPGPTPLPFIGNYLQLNTEQMYNSLMK.I 28426 28427 28429 28430 28431 |
|  | 30265 | **1022.2596** | **4085.0092** | **4084.9196** | **0.0897** | **1** | **0** | **25** | **6** | **R.MMLGSFQFTATSTGQLYEMFSSVMKHLPGPQQQAFK.E** |

| **92.** | O14756\|H17B6_HUMAN    **Mass:** 36285    **Score:** 544    **Queries matched:** 33   **emPAI:** 2.98 |
| --- | --- |
|  | Hydroxysteroid 17-beta dehydrogenase 6 OS=Homo sapiens GN=HSD17B6 PE=1 SV=1 |

|  | **Query** | **Observed** | **Mr(expt)** | **Mr(calc)** | **Delta** | **Miss** | **Score** | **Expect** | **Rank** | **Peptide** |
| --- | --- | --- | --- | --- | --- | --- | --- | --- | --- | --- |
|  | 3213 | 897.4039 | 896.3967 | 896.4028 | -0.0062 | 0 | 22 | 0.84 | 1 | R.YSAGWDAK.F 3214 |
|  | 4044 | **479.2578** | **956.5011** | **956.5080** | **-0.0068** | **0** | **20** | **1.6** | **1** | **R.EIQHFGVK.I** |
|  | 4712 | **502.7725** | **1003.5304** | **1003.5372** | **-0.0068** | **0** | **51** | **0.0015** | **1** | **R.VLAACLTEK.G** 4713 |
|  | 5391 | **529.3182** | **1056.6219** | **1056.6291** | **-0.0072** | **0** | **48** | **0.0026** | **1** | **R.IVNVSSILGR.V** |
|  | 8761 | **635.3237** | **1268.6328** | **1268.6401** | **-0.0073** | **0** | **62** | **8.2e-005** | **1** | **K.YGVEAFSDILR.R** |
|  | 9984 | **667.3299** | **1332.6452** | **1332.6537** | **-0.0084** | **0** | **31** | **0.11** | **1** | **R.VAFFVGGYCVSK.Y** 9983 |
|  | 10002 | 667.8386 | 1333.6626 | 1333.6700 | -0.0074 | 0 | 65 | 4.1e-005 | 1 | K.MESIAAATQWVK.E 10001 |
|  | 10268 | **450.5861** | **1348.7364** | **1349.6649** | **-0.9286** | **0** | **(4)** | **67** | **3** | **K.MESIAAATQWVK.E** |
|  | 10599 | **684.8114** | **1367.6082** | **1367.6173** | **-0.0091** | **0** | **71** | **1e-005** | **1** | **R.TGMTNMTQSLER.M** 10598 10620 |
|  | 10894 | **692.8089** | **1383.6032** | **1383.6122** | **-0.0090** | **0** | **(53)** | **0.00071** | **1** | **R.TGMTNMTQSLER.M** |
|  | 11165 | **467.2216** | **1398.6429** | **1399.6072** | **-0.9643** | **0** | **(7)** | **28** | **10** | **R.TGMTNMTQSLER.M** |
|  | 11615 | **475.9184** | **1424.7334** | **1424.7412** | **-0.0078** | **1** | **40** | **0.012** | **1** | **K.YGVEAFSDILRR.E** |
|  | 15485 | **558.9705** | **1673.8896** | **1674.7375** | **-0.8480** | **1** | **8** | **17** | **2** | **R.TGMTNMTQSLERMK.Q** 15491 15517 |
|  | 16880 | **881.9559** | **1761.8973** | **1761.9109** | **-0.0136** | **1** | **65** | **3.7e-005** | **1** | **R.GQTSDRLETVTLDVTK.M** 16879 16881 16882 |
|  | 18521 | **945.4586** | **1888.9026** | **1888.9142** | **-0.0116** | **0** | **6** | **24** | **1** | **K.YVFITGCDSGFGNLLAR.Q** |
|  | 19603 | **992.4565** | **1982.8984** | **1982.9084** | **-0.0100** | **0** | **67** | **1.7e-005** | **1** | **K.ETYGQQYFDALYNIMK.E** 19605 |
|  | 23152 | **1181.5720** | **2361.1295** | **2361.1463** | **-0.0168** | **1** | **71** | **5.8e-006** | **1** | **K.HIKETYGQQYFDALYNIMK.E** 23153 |
|  | 23287 | **1189.1408** | **2376.2669** | **2376.2769** | **-0.0100** | **0** | **57** | **0.00015** | **1** | **K.FFFIPLSYLPTSLADYILTR.S** |
|  | 27468 | **1027.8170** | **3080.4292** | **3080.4478** | **-0.0185** | **0** | **45** | **0.0014** | **1** | **K.EGLLNCSTNLNLVTDCMEHALTSVHPR.T** 27474 |

| **93.** | P35580\|MYH10_HUMAN    **Mass:** 229827   **Score:** 536    **Queries matched:** 101   **emPAI:** 0.21 |
| --- | --- |
|  | Myosin-10 OS=Homo sapiens GN=MYH10 PE=1 SV=3 |

|  | **Query** | **Observed** | **Mr(expt)** | **Mr(calc)** | **Delta** | **Miss** | **Score** | **Expect** | **Rank** | **Peptide** |
| --- | --- | --- | --- | --- | --- | --- | --- | --- | --- | --- |
|  | 997 | 533.1882 | 532.1809 | 531.3017 | 0.8792 | 0 | 8 | 42 | 1 | R.IGQSK.I 996 1000 1001 1002 1004 1005 1008 |
|  | 1133 | **563.2704** | **562.2631** | **561.2944** | **0.9687** | **0** | **16** | **6.1** | **6** | **K.AMVNK.D** |
|  | 1167 | 572.9950 | 571.9877 | 571.3806 | 0.6072 | 1 | 12 | 10 | 2 | R.IKVGR.D 1173 |
|  | 1174 | **573.2849** | **572.2776** | **571.3693** | **0.9083** | **0** | **20** | **2.7** | **1** | **K.LQLAK.K** |
|  | 1214 | 586.2972 | 585.2900 | 585.2944 | -0.0045 | 0 | 17 | 3.7 | 1 | K.MNPPK.F |
|  | 1238 | **589.3899** | **588.3826** | **587.3503** | **1.0323** | **1** | **4** | **1.4e+002** | **6** | **K.SRAVR.Q** |
|  | 1359 | **628.7740** | **627.7668** | **628.4020** | **-0.6352** | **1** | **13** | **5.2** | **6** | **R.ARLAAK.K** |
|  | 1367 | 630.3886 | 629.3813 | 629.3860 | -0.0047 | 0 | 19 | 3.7 | 1 | K.ALSLAR.A |
|  | 1456 | 653.3032 | 652.2959 | 653.2843 | -0.9884 | 0 | 13 | 7.2 | 8 | K.GFMDGK.Q |
|  | 1665 | 735.0493 | 734.0420 | 733.3792 | 0.6628 | 0 | 3 | 73 | 1 | K.LQAQMK.D |
|  | 2062 | 404.7288 | 807.4430 | 807.4491 | -0.0061 | 0 | 21 | 1.2 | 1 | R.TVGQLYK.E |
|  | 2530 | 423.2321 | 844.4496 | 845.4793 | -1.0297 | 1 | 9 | 27 | 9 | R.KLQAQMK.D 2546 2548 |
|  | 2581 | **849.4780** | **848.4708** | **848.4756** | **-0.0048** | **1** | **9** | **24** | **9** | **R.FKANLEK.N** |
|  | 2750 | 863.0858 | 862.0785 | 861.4742 | 0.6043 | 1 | (0) | 1.3e+002 | 7 | R.KLQAQMK.D |
|  | 3162 | 446.7122 | 891.4098 | 891.4160 | -0.0062 | 0 | 22 | 1.2 | 2 | K.ADEWLMK.N |
|  | 3774 | 470.2359 | 938.4572 | 937.5022 | 0.9551 | 0 | 4 | 56 | 8 | R.IVFQEFR.Q |
|  | 3820 | **942.5307** | **941.5234** | **942.5610** | **-1.0376** | **1** | **14** | **5.5** | **2** | **K.QRALAVASK.K** |
|  | 4065 | **480.2766** | **958.5387** | **958.4872** | **0.0515** | **0** | **8** | **29** | **9** | **R.HGFEAASIK.E** |
|  | 4083 | 481.1506 | 960.2866 | 960.4777 | -0.1911 | 0 | 8 | 25 | 3 | R.NTNPNFVR.C |
|  | 4256 | **487.3202** | **972.6258** | **972.5491** | **0.0766** | **1** | **9** | **24** | **10** | **K.DEELLKVK.E** |
|  | 4437 | **494.2357** | **986.4568** | **985.5444** | **0.9124** | **0** | **5** | **60** | **3** | **K.LQELEGAVK.S** |
|  | 5158 | **519.7733** | **1037.5321** | **1037.5393** | **-0.0072** | **1** | **8** | **24** | **8** | **K.ATDKTFVEK.L** |
|  | 5232 | **522.3104** | **1042.6063** | **1042.6135** | **-0.0072** | **1** | **21** | **1.3** | **1** | **K.KQQQLSALK.V** |
|  | 5946 | **550.8050** | **1099.5954** | **1100.5826** | **-0.9871** | **1** | **0** | **1.6e+002** | **6** | **K.REQEVAELK.K** |
|  | 6157 | **558.8325** | **1115.6505** | **1114.5618** | **1.0887** | **0** | **8** | **27** | **1** | **K.EEELQGALAR.G** |
|  | 6639 | **577.3082** | **1152.6018** | **1151.6298** | **0.9720** | **1** | **14** | **6.1** | **1** | **R.KHQQLLEEK.N** |
|  | 7062 | 593.2817 | 1184.5488 | 1184.5859 | -0.0371 | 1 | 4 | 70 | 8 | K.DDIQKMNPPK.F |
|  | 7815 | 610.8254 | 1219.6363 | 1219.6448 | -0.0085 | 1 | 56 | 0.00035 | 1 | K.KFDQLLAEEK.S 7814 |
|  | 8011 | 615.7865 | 1229.5584 | 1228.7027 | 0.8558 | 1 | 2 | 1.1e+002 | 7 | K.LQLEKVTAEAK.I |
|  | 8290 | **415.2401** | **1242.6984** | **1242.6932** | **0.0053** | **1** | **5** | **42** | **3** | **K.LLKDAEALSQR.L** |
|  | 8439 | **626.3936** | **1250.7725** | **1250.6255** | **0.1471** | **1** | **8** | **20** | **2** | **R.ASRDEIFAQSK.E** |
|  | 8942 | 427.2242 | 1278.6508 | 1277.7013 | 0.9495 | 1 | 10 | 13 | 6 | K.ESLTKLMATLR.N |
|  | 9152 | 431.0844 | 1290.2315 | 1290.6456 | -0.4141 | 0 | 4 | 40 | 4 | K.EQADFAVEALAK.A 9153 |
|  | 9550 | **656.8098** | **1311.6049** | **1310.5813** | **1.0237** | **0** | **5** | **43** | **3** | **K.EIFMQVEDER.R** |
|  | 9675 | 659.8734 | 1317.7323 | 1317.7405 | -0.0082 | 0 | 38 | 0.022 | 1 | K.LDPHLVLDQLR.C 9688 |
|  | 10237 | **450.2426** | **1347.7059** | **1347.6340** | **0.0719** | **0** | **7** | **34** | **9** | **K.QEVMISDLEER.L** |
|  | 10677 | **458.2939** | **1371.8599** | **1372.6735** | **-0.8136** | **1** | **4** | **47** | **8** | **R.HGFEAASIKEER.G** |
|  | 10860 | 691.8444 | 1381.6742 | 1381.6837 | -0.0096 | 1 | 0 | 1.3e+002 | 9 | K.DDVGKNVHELEK.S |
|  | 11126 | 699.3379 | 1396.6612 | 1396.6697 | -0.0084 | 1 | 69 | 1.6e-005 | 1 | K.VDYKADEWLMK.N 11124 11125 |
|  | 11254 | **469.2529** | **1404.7370** | **1403.6827** | **1.0543** | **1** | **7** | **24** | **7** | **K.RALEQQVEEMR.T** |
|  | 11417 | 707.8890 | 1413.7635 | 1413.7827 | -0.0192 | 0 | 30 | 0.14 | 1 | K.QVLALQSQLADTK.K |
|  | 11503 | **710.3305** | **1418.6465** | **1417.6620** | **0.9845** | **1** | **1** | **1.2e+002** | **8** | **-.MAQRTGLEDPER.Y** |
|  | 11998 | **484.2477** | **1449.7211** | **1448.6631** | **1.0581** | **0** | **5** | **39** | **7** | **R.DELADEITNSASGK.S** 12008 |
|  | 12037 | **484.9332** | **1451.7777** | **1451.7885** | **-0.0108** | **0** | **6** | **33** | **4** | **K.VIQYLAHVASSHK.G** |
|  | 13351 | 767.3753 | 1532.7361 | 1533.7345 | -0.9984 | 1 | 10 | 13 | 4 | R.GDEVMVELAENGKK.A |
|  | 13376 | **768.3760** | **1534.7374** | **1535.7653** | **-1.0279** | **1** | **5** | **40** | **10** | **K.LKEIFMQVEDER.R** |
|  | 13478 | 771.8914 | 1541.7682 | 1541.8565 | -0.0884 | 1 | 9 | 16 | 2 | R.QRYEILTPNAIPK.G |
|  | 13620 | 776.3459 | 1550.6772 | 1550.6882 | -0.0110 | 0 | 11 | 9.5 | 1 | R.EDQSILCTGESGAGK.T |
|  | 13681 | **777.8880** | **1553.7614** | **1554.7018** | **-0.9403** | **1** | **3** | **57** | **9** | **K.QLRADMEDLMSSK.D** |
|  | 13955 | 525.6353 | 1573.8841 | 1573.8940 | -0.0099 | 1 | 17 | 2.4 | 1 | R.AGKLDPHLVLDQLR.C |
|  | 14022 | 790.4198 | 1578.8250 | 1578.8365 | -0.0115 | 1 | 44 | 0.0047 | 1 | R.AGVLAHLEEERDLK.I 14024 |
|  | 14170 | 794.9040 | 1587.7934 | 1588.8130 | -1.0196 | 1 | 4 | 51 | 5 | K.QEVMISDLEERLK.K |
|  | 14307 | **532.9060** | **1595.6962** | **1594.8541** | **0.8421** | **0** | **14** | **4.3** | **1** | **K.ITDIIIFFQAVCR.G** |
|  | 14587 | 808.4202 | 1614.8258 | 1614.7849 | 0.0409 | 1 | 8 | 19 | 5 | K.QVRELEAELEDER.K 14603 |
|  | 14861 | 817.3726 | 1632.7307 | 1632.7413 | -0.0106 | 0 | 43 | 0.0062 | 1 | R.NTDQASMPENTVAQK.L |
|  | 15319 | 831.8976 | 1661.7806 | 1661.8671 | -0.0865 | 1 | 0 | 1e+002 | 8 | K.LMATLRNTNPNFVR.C |
|  | 16106 | **571.3160** | **1710.9263** | **1709.8229** | **1.1034** | **1** | **6** | **30** | **2** | **R.LEVNMQAMKAQFER.D** |
|  | 16241 | **574.6148** | **1720.8226** | **1719.8259** | **0.9967** | **0** | **7** | **24** | **2** | **K.LCHLLGMNVMEFTR.A** |
|  | 16333 | **432.7242** | **1726.8679** | **1725.8178** | **1.0501** | **1** | **(3)** | **49** | **10** | **R.LEVNMQAMKAQFER.D** |
|  | 16531 | **435.7212** | **1738.8557** | **1737.8897** | **0.9660** | **1** | **11** | **8.6** | **1** | **K.KQELEEILHDLESR.V** |
|  | 17341 | **900.3734** | **1798.7323** | **1798.9061** | **-0.1738** | **1** | **0** | **88** | **1** | **K.IGQLEEQLEQEAKER.A** |
|  | 18848 | **639.3781** | **1915.1123** | **1915.9235** | **-0.8112** | **1** | **1** | **71** | **6** | **K.LQRELDDATEANEGLSR.E** |
|  | 19097 | **646.6534** | **1936.9385** | **1936.9490** | **-0.0105** | **0** | **17** | **1.8** | **1** | **R.LQQELDDLTVDLDHQR.Q** |
|  | 19380 | 981.4560 | 1960.8974 | 1960.9113 | -0.0138 | 0 | 90 | 9.9e-008 | 1 | R.TQLEELEDELQATEDAK.L |
|  | 20459 | 1028.5358 | 2055.0570 | 2055.9948 | -0.9378 | 1 | 1 | 66 | 6 | K.RHEMPPHIYAISESAYR.C |
|  | 20746 | 694.0244 | 2079.0514 | 2080.0548 | -1.0034 | 1 | 4 | 40 | 2 | R.QRHATALEELSEQLEQAK.R |
|  | 20807 | **696.3228** | **2085.9464** | **2085.9702** | **-0.0238** | **0** | **2** | **56** | **4** | **R.QLHLEGASLELSDDDTESK.T** 20811 |
|  | 20829 | **1044.5137** | **2087.0128** | **2087.0245** | **-0.0117** | **0** | **114** | **3.9e-010** | **1** | **R.IVGLDQVTGMTETAFGSAYK.T** |
|  | 21880 | **736.3812** | **2206.1219** | **2207.0931** | **-0.9712** | **1** | **4** | **36** | **2** | **K.NRLQQELDDLTVDLDHQR.Q** |
|  | 22560 | 1143.5513 | 2285.0880 | 2285.1031 | -0.0151 | 0 | 105 | 2.7e-009 | 1 | K.VEDMAELTCLNEASVLHNLK.D 22561 22562 |
|  | 22692 | 576.3224 | 2301.2605 | 2301.0981 | 0.1624 | 0 | (4) | 29 | 3 | K.VEDMAELTCLNEASVLHNLK.D 22696 |
|  | 22823 | **773.3919** | **2317.1539** | **2316.1672** | **0.9867** | **1** | **1** | **63** | **7** | **R.IVGLDQVTGMTETAFGSAYKTK.K** |
|  | 23477 | **802.1015** | **2403.2827** | **2403.1401** | **0.1426** | **0** | **0** | **72** | **9** | **K.DAASLESQLQDTQELLQEETR.Q** |
|  | 23925 | 1234.0996 | 2466.1847 | 2466.2002 | -0.0155 | 0 | 88 | 1.2e-007 | 1 | K.LQQLFNHTMFILEQEEYQR.E 23924 23932 |
|  | 25040 | 883.4341 | 2647.2804 | 2647.2985 | -0.0181 | 1 | 19 | 0.81 | 1 | K.FSKVEDMAELTCLNEASVLHNLK.D |
|  | 27156 | **1010.4925** | **3028.4556** | **3028.4713** | **-0.0156** | **1** | **1** | **44** | **1** | **K.NMDPLNDNVATLLHQSSDRFVAELWK.D** 27157 |

| **94.** | P00966\|ASSY_HUMAN    **Mass:** 46786    **Score:** 519    **Queries matched:** 41   **emPAI:** 1.16 |
| --- | --- |
|  | Argininosuccinate synthase OS=Homo sapiens GN=ASS1 PE=1 SV=2 |

|  | **Query** | **Observed** | **Mr(expt)** | **Mr(calc)** | **Delta** | **Miss** | **Score** | **Expect** | **Rank** | **Peptide** |
| --- | --- | --- | --- | --- | --- | --- | --- | --- | --- | --- |
|  | 1115 | **559.3773** | **558.3700** | **559.3330** | **-0.9630** | **0** | **14** | **9.8** | **1** | **K.VTNVK.D** 1117 |
|  | 1752 | **773.4914** | **772.4841** | **771.4854** | **0.9987** | **0** | **24** | **0.95** | **1** | **K.VQVSVLK.G** |
|  | 2659 | **429.0872** | **856.1598** | **855.5542** | **0.6056** | **1** | **11** | **11** | **5** | **K.IKQGLGLK.F** |
|  | 2682 | **429.7352** | **857.4558** | **857.4607** | **-0.0049** | **0** | **37** | **0.041** | **1** | **R.IDIVENR.F** |
|  | 4132 | 482.7554 | 963.4963 | 963.5025 | -0.0062 | 0 | 13 | 8.6 | 3 | K.VFIEDVSR.E |
|  | 4225 | **486.2816** | **970.5486** | **970.5560** | **-0.0073** | **1** | **35** | **0.052** | **1** | **R.KQVEIAQR.E** 4218 4220 4222 4224 4226 |
|  | 4386 | **492.2252** | **982.4359** | **982.4429** | **-0.0071** | **0** | **38** | **0.026** | **1** | **R.NDLMEYAK.Q** |
|  | 5793 | **545.2298** | **1088.4450** | **1087.5662** | **0.8789** | **0** | **13** | **9.6** | **1** | **K.NQAPPGLYTK.T** |
|  | 5835 | **546.8026** | **1091.5905** | **1091.5975** | **-0.0070** | **1** | **39** | **0.022** | **1** | **K.KVFIEDVSR.E** |
|  | 7069 | **593.3291** | **1184.6436** | **1184.7129** | **-0.0692** | **1** | **8** | **27** | **7** | **R.VEGKVQVSVLK.G** |
|  | 7318 | 598.8022 | 1195.5898 | 1195.5655 | 0.0243 | 1 | 16 | 3.2 | 2 | K.GRNDLMEYAK.Q 7345 |
|  | 13694 | **778.3895** | **1554.7645** | **1554.7752** | **-0.0107** | **0** | **39** | **0.014** | **1** | **R.FELSCYSLAPQIK.V** |
|  | 14162 | **529.9421** | **1586.8044** | **1587.7754** | **-0.9710** | **1** | **4** | **50** | **8** | **K.YVSHGATGKGNDQVR.F** |
|  | 14213 | **530.9607** | **1589.8602** | **1589.8712** | **-0.0109** | **0** | **13** | **6.4** | **1** | **R.YLLGTSLARPCIAR.K** 14214 |
|  | 16345 | **864.9399** | **1727.8652** | **1727.8842** | **-0.0190** | **1** | **1** | **88** | **7** | **K.NQAPPGLYTKTQDPAK.A** |
|  | 21162 | **709.3541** | **2125.0404** | **2126.0895** | **-1.0492** | **1** | **3** | **45** | **1** | **K.TQDPAKAPNTPDILEIEFK.K** |
|  | 23087 | **1177.5635** | **2353.1124** | **2353.1260** | **-0.0136** | **0** | **89** | **8.1e-008** | **1** | **K.DGTTHQTSLELFMYLNEVAGK.H** 23086 23088 |
|  | 23271 | **1187.5441** | **2373.0736** | **2373.0889** | **-0.0153** | **0** | **74** | **2.8e-006** | **1** | **K.FAELVYTGFWHSPECEFVR.H** 23270 23272 |
|  | 23628 | **1215.0802** | **2428.1458** | **2428.1587** | **-0.0128** | **0** | **71** | **5.8e-006** | **1** | **R.EFVEEFIWPAIQSSALYEDR.Y** 23629 23630 23638 |
|  | 25256 | **1344.6173** | **2687.2201** | **2687.2359** | **-0.0158** | **0** | **71** | **5e-006** | **1** | **K.NPWSMDENLMHISYEAGILENPK.N** 25253 25254 25255 |
|  | 29451 | **940.2006** | **3756.7734** | **3756.7915** | **-0.0182** | **1** | **11** | **2.4** | **1** | **K.NPWSMDENLMHISYEAGILENPKNQAPPGLYTK.T** |
|  | 29894 | **1305.9534** | **3914.8383** | **3914.8520** | **-0.0137** | **0** | **5** | **9.3** | **3** | **R.ESPLSLYNEELVSMNVQGDYEPTDATGFININSLR.L** |
|  | 30096 | **1334.3294** | **3999.9662** | **4000.0193** | **-0.0530** | **1** | **1** | **22** | **2** | **R.EFVEEFIWPAIQSSALYEDRYLLGTSLARPCIAR.K** |

| **95.** | Q3ZCM7\|TBB8_HUMAN    **Mass:** 50257    **Score:** 517    **Queries matched:** 50   **emPAI:** 1.20 |
| --- | --- |
|  | Tubulin beta-8 chain OS=Homo sapiens GN=TUBB8 PE=1 SV=2 |

|  | **Query** | **Observed** | **Mr(expt)** | **Mr(calc)** | **Delta** | **Miss** | **Score** | **Expect** | **Rank** | **Peptide** |
| --- | --- | --- | --- | --- | --- | --- | --- | --- | --- | --- |
|  | 2066 | 404.7367 | 807.4588 | 807.3399 | 0.1190 | 0 | 7 | 33 | 7 | R.EEYPDR.I |
|  | 5020 | 514.7598 | 1027.5050 | 1027.5121 | -0.0071 | 0 | 46 | 0.004 | 1 | K.TAVCDIPPR.G 5004 5015 5016 5017 5018 5019 5021 5022 |
|  | 5494 | 533.3253 | 1064.6361 | 1064.4201 | 0.2160 | 0 | (4) | 60 | 6 | K.NMMAACDPR.H |
|  | 5638 | 539.2662 | 1076.5179 | 1076.5250 | -0.0071 | 1 | 36 | 0.039 | 1 | K.IREEYPDR.I 5637 |
|  | 5678 | 541.2108 | 1080.4071 | 1080.4151 | -0.0080 | 0 | 29 | 0.16 | 1 | K.NMMAACDPR.H 5679 5694 |
|  | 6327 | 565.7967 | 1129.5788 | 1129.5880 | -0.0092 | 0 | 4 | 59 | 6 | R.FPGQLNADLR.K |
|  | 6499 | 572.3174 | 1142.6203 | 1142.6270 | -0.0067 | 0 | 70 | 1.8e-005 | 1 | K.LAVNMVPFPR.L 6498 |
|  | 8802 | 636.3640 | 1270.7133 | 1270.7220 | -0.0086 | 1 | 47 | 0.0027 | 1 | R.KLAVNMVPFPR.L |
|  | 9112 | 430.2745 | 1287.8016 | 1286.7169 | 1.0847 | 1 | (7) | 28 | 2 | R.KLAVNMVPFPR.L 9101 |
|  | 9913 | 665.3396 | 1328.6646 | 1328.7704 | -0.1057 | 0 | 11 | 11 | 3 | R.IINTFSILPSPK.V 9910 9911 |
|  | 14384 | 801.4086 | 1600.8026 | 1600.8131 | -0.0105 | 0 | 67 | 2.6e-005 | 1 | R.AVLVDLEPGTMDSVR.S 14378 14379 14385 |
|  | 14658 | 810.9161 | 1619.8176 | 1619.8283 | -0.0107 | 0 | 52 | 0.0008 | 1 | R.LHFFMPGFAPLTSR.G 14659 14660 14661 |
|  | 18121 | **620.3093** | **1857.9060** | **1856.9169** | **0.9891** | **1** | **3** | **59** | **10** | **R.INVYYNEASGGRYVPR.A** |
|  | 19931 | **503.4933** | **2009.9439** | **2009.9009** | **0.0431** | **1** | **1** | **71** | **5** | **R.MPMREVDEQMFNIQDK.N** |
|  | 21101 | 1059.0121 | 2116.0096 | 2116.0987 | -0.0891 | 1 | 2 | 56 | 4 | R.YVPRAVLVDLEPGTMDSVR.S 21100 |
|  | 23107 | 786.0563 | 2355.1470 | 2355.1583 | -0.0113 | 1 | 2 | 44 | 7 | R.LHFFMPGFAPLTSRGSQQYR.A |
|  | 25766 | 695.3771 | 2777.4792 | 2776.4346 | 1.0446 | 1 | 6 | 13 | 2 | K.LAVNMVPFPRLHFFMPGFAPLTSR.G |
|  | 28244 | 1656.2616 | 3310.5086 | 3310.5268 | -0.0182 | 0 | (65) | 1.3e-005 | 1 | K.EAESCDCLQGFQLTHSLGGGTGSGMGTLLLSK.I 28241 28242 28243 28245 28246 |
|  | 28304 | 1109.8416 | 3326.5028 | 3326.5218 | -0.0189 | 0 | 72 | 3e-006 | 1 | K.EAESCDCLQGFQLTHSLGGGTGSGMGTLLLSK.I |
|  | 28665 | 1147.2072 | 3438.5996 | 3438.6218 | -0.0222 | 1 | 68 | 6.1e-006 | 1 | R.KEAESCDCLQGFQLTHSLGGGTGSGMGTLLLSK.I 28657 28663 28664 |

| **96.** | O95831\|AIFM1_HUMAN    **Mass:** 67144    **Score:** 510    **Queries matched:** 47   **emPAI:** 1.50 |
| --- | --- |
|  | Apoptosis-inducing factor 1, mitochondrial OS=Homo sapiens GN=AIFM1 PE=1 SV=1 |

|  | **Query** | **Observed** | **Mr(expt)** | **Mr(calc)** | **Delta** | **Miss** | **Score** | **Expect** | **Rank** | **Peptide** |
| --- | --- | --- | --- | --- | --- | --- | --- | --- | --- | --- |
|  | 906 | **515.3262** | **514.3189** | **514.2500** | **0.0689** | **0** | **13** | **12** | **4** | **R.DPGAR.V** 898 903 |
|  | 1234 | **589.0247** | **588.0174** | **587.3391** | **0.6783** | **1** | **4** | **87** | **4** | **K.LKDGR.K** |
|  | 1651 | **731.1757** | **730.1684** | **730.4160** | **-0.2475** | **1** | **5** | **63** | **3** | **R.MPIARK.I** |
|  | 3112 | **444.2137** | **886.4129** | **886.4185** | **-0.0056** | **0** | **27** | **0.44** | **1** | **K.LFNIHED.-** |
|  | 3251 | **450.7455** | **899.4763** | **899.4825** | **-0.0061** | **0** | **57** | **0.00035** | **1** | **R.VNAELQAR.S** 3264 |
|  | 4358 | 490.7479 | 979.4812 | 979.4393 | 0.0419 | 0 | 5 | 45 | 8 | R.QMASSGASGGK.I |
|  | 4563 | **498.3364** | **994.6582** | **995.4342** | **-0.7760** | **0** | **(5)** | **48** | **10** | **R.QMASSGASGGK.I** |
|  | 6026 | **553.7711** | **1105.5276** | **1105.5364** | **-0.0088** | **0** | **43** | **0.0091** | **1** | **K.SATEQSGTGIR.S** 6021 6027 6028 6030 6031 |
|  | 6501 | **572.3229** | **1142.6312** | **1141.6343** | **0.9969** | **0** | **11** | **15** | **3** | **R.ISGLGLTPEQK.Q** |
|  | 11325 | **705.3572** | **1408.6998** | **1408.7285** | **-0.0287** | **0** | **1** | **99** | **6** | **R.WHVPLELQMTR.Q** |
|  | 11904 | **722.9017** | **1443.7889** | **1443.7973** | **-0.0084** | **0** | **67** | **2.5e-005** | **1** | **R.ALGTEVIQLFPEK.G** 11903 |
|  | 11994 | **725.8415** | **1449.6684** | **1449.6776** | **-0.0092** | **0** | **15** | **3.9** | **1** | **K.ELWFSDDPNVTK.T** |
|  | 12341 | **735.8422** | **1469.6698** | **1469.6787** | **-0.0089** | **0** | **48** | **0.002** | **1** | **K.TGGLEIDSDFGGFR.V** |
|  | 12566 | **742.3719** | **1482.7293** | **1482.6586** | **0.0707** | **0** | **4** | **45** | **2** | **K.DGEQHEDLNEVAK.L** |
|  | 12802 | **500.2560** | **1497.7462** | **1497.7549** | **-0.0087** | **1** | **54** | **0.00049** | **1** | **R.RVEHHDHAVVSGR.L** 12800 12801 12819 |
|  | 13306 | **765.8622** | **1529.7098** | **1529.7209** | **-0.0111** | **0** | **15** | **3.8** | **1** | **K.AALSASEGEEVPQDK.A** |
|  | 13922 | **786.9189** | **1571.8232** | **1571.8341** | **-0.0109** | **0** | **58** | **0.00019** | **1** | **K.VMPNAIVQSVGVSSGK.L** |
|  | 14704 | **812.4028** | **1622.7910** | **1622.8014** | **-0.0104** | **0** | **49** | **0.0016** | **1** | **K.ILPEYLSNWTMEK.V** |
|  | 14980 | **820.4669** | **1638.9193** | **1638.7963** | **0.1230** | **0** | **(5)** | **37** | **3** | **K.ILPEYLSNWTMEK.V** |
|  | 17712 | **915.4235** | **1828.8325** | **1828.8454** | **-0.0129** | **0** | **81** | **7.4e-007** | **1** | **R.SNIWVAGDAACFYDIK.L** 17721 |
|  | 19047 | **483.9760** | **1931.8749** | **1930.9344** | **0.9405** | **1** | **0** | **1e+002** | **10** | **K.ATAQDNPKSATEQSGTGIR.S** |
|  | 19721 | **996.5275** | **1991.0404** | **1991.0510** | **-0.0106** | **0** | **43** | **0.0045** | **1** | **K.SITIIGGGFLGSELACALGR.K** |
|  | 21679 | **1092.0845** | **2182.1544** | **2182.1707** | **-0.0164** | **0** | **54** | **0.00035** | **1** | **R.VLIVSEDPELPYMRPPLSK.E** 21680 21681 |
|  | 22154 | **1116.6079** | **2231.2013** | **2231.2161** | **-0.0149** | **1** | **63** | **4.2e-005** | **1** | **R.KVETDHIVAAVGLEPNVELAK.T** 22155 22164 |
|  | 28026 | **1078.8796** | **3233.6171** | **3233.6397** | **-0.0226** | **0** | **23** | **0.19** | **1** | **R.SIYFQPPSFYVSAQDLPHIENGGVAVLTGK.K** 28025 |
|  | 28903 | **1179.6089** | **3535.8048** | **3534.8107** | **0.9942** | **1** | **31** | **0.027** | **1** | **K.AALSASEGEEVPQDKAPSHVPFLLIGGGTAAFAAAR.S** 28902 |
|  | 31320 | **1671.4727** | **5011.3962** | **5011.4201** | **-0.0240** | **0** | **42** | **0.00085** | **1** | **R.LAGENMTGAAKPYWHQSMFWSDLGPDVGYEAIGLVDSSLPTVGVFAK.A** 31321 |

| **97.** | Q9ULC5\|ACSL5_HUMAN    **Mass:** 76855    **Score:** 503    **Queries matched:** 34   **emPAI:** 0.76 |
| --- | --- |
|  | Long-chain-fatty-acid--CoA ligase 5 OS=Homo sapiens GN=ACSL5 PE=1 SV=1 |

|  | **Query** | **Observed** | **Mr(expt)** | **Mr(calc)** | **Delta** | **Miss** | **Score** | **Expect** | **Rank** | **Peptide** |
| --- | --- | --- | --- | --- | --- | --- | --- | --- | --- | --- |
|  | 1562 | **696.3879** | **695.3807** | **695.3642** | **0.0164** | **0** | **18** | **2.2** | **1** | **R.WLSYK.Q** |
|  | 1830 | **396.2400** | **790.4654** | **791.4541** | **-0.9887** | **1** | **18** | **3.5** | **4** | **K.FKELQK.G** |
|  | 2028 | **403.2075** | **804.4004** | **804.4051** | **-0.0047** | **0** | **13** | **11** | **2** | **R.LLADDMK.T** |
|  | 2540 | **423.7273** | **845.4401** | **844.4403** | **0.9998** | **0** | **18** | **3.5** | **1** | **K.IQDSLGGR.V** |
|  | 3709 | **467.2218** | **932.4290** | **933.3981** | **-0.9691** | **0** | **9** | **24** | **2** | **R.HDSFWDK.L** |
|  | 5156 | **519.7684** | **1037.5222** | **1037.5294** | **-0.0073** | **0** | **48** | **0.0022** | **1** | **R.VGFFQGDIR.L** 5157 |
|  | 5373 | **528.8245** | **1055.6344** | **1054.6386** | **0.9957** | **0** | **45** | **0.0056** | **1** | **K.ALVLIGNVEK.G** 5351 5363 |
|  | 5596 | **537.2544** | **1072.4942** | **1072.5012** | **-0.0069** | **0** | **1** | **1.4e+002** | **3** | **K.TMYEVFQR.G** |
|  | 5966 | **551.3127** | **1100.6109** | **1099.6098** | **1.0011** | **1** | **11** | **14** | **2** | **K.IQDSLGGRVR.V** |
|  | 6037 | **553.8283** | **1105.6419** | **1104.6907** | **0.9513** | **1** | **2** | **1.2e+002** | **6** | **K.FLLKLAVSSK.F** |
|  | 7777 | 609.8176 | 1217.6206 | 1217.6292 | -0.0086 | 0 | 43 | 0.0083 | 1 | K.LAQGEYIAPEK.I |
|  | 9140 | **645.8250** | **1289.6355** | **1289.6438** | **-0.0083** | **0** | **19** | **1.6** | **2** | **R.AEYLGSCLLHK.G** |
|  | 9762 | **661.8444** | **1321.6743** | **1321.6813** | **-0.0070** | **0** | **2** | **82** | **8** | **R.IVQAVVYSCGAR.V** |
|  | 12184 | **730.8988** | **1459.7830** | **1460.7003** | **-0.9173** | **0** | **2** | **89** | **8** | **K.ADIAMVICDTPQK.A** |
|  | 12191 | **731.3417** | **1460.6689** | **1460.6783** | **-0.0094** | **0** | **40** | **0.013** | **1** | **R.TQIDSLYEHIQD.-** |
|  | 12488 | **739.8510** | **1477.6873** | **1477.6984** | **-0.0110** | **0** | **63** | **6.1e-005** | **1** | **R.GLAVSDNGPCLGYR.K** 12487 12502 12503 |
|  | 13316 | **766.3055** | **1530.5964** | **1530.6079** | **-0.0115** | **0** | **60** | **0.00011** | **1** | **K.NNDLTSCCFSDAK.T** |
|  | 13837 | **783.3674** | **1564.7203** | **1564.7304** | **-0.0101** | **0** | **32** | **0.068** | **1** | **K.GSFEELCQNQVVR.E** |
|  | 16081 | **855.4646** | **1708.9146** | **1708.9261** | **-0.0114** | **0** | **45** | **0.0031** | **1** | **R.SQPVLQIFVHGESLR.S** 16080 |
|  | 18465 | **943.4952** | **1884.9759** | **1884.9880** | **-0.0120** | **0** | **79** | **1.3e-006** | **1** | **K.GAMITHQNIVSNAAAFLK.C** 18466 |
|  | 18834 | **957.9888** | **1913.9630** | **1913.9744** | **-0.0114** | **0** | **49** | **0.0012** | **1** | **R.VIVTGAAPMSTSVMTFFR.A** |
|  | 19833 | **1001.0432** | **2000.0717** | **2000.0830** | **-0.0113** | **0** | **78** | **1.6e-006** | **1** | **R.SSLVGVVVPDTDVLPSFAAK.L** 19831 19832 |
|  | 22621 | **764.7230** | **2291.1472** | **2290.1593** | **0.9879** | **1** | **29** | **0.1** | **1** | **K.SGIEILSLYDAENLGKEHFR.K** |
|  | 22998 | 1171.0638 | 2340.1131 | 2341.2002 | -1.0870 | 1 | 1 | 68 | 4 | R.IVQAVVYSCGARVGFFQGDIR.L |

| **98.** | P02675\|FIBB_HUMAN    **Mass:** 56577    **Score:** 501    **Queries matched:** 40   **emPAI:** 1.44 |
| --- | --- |
|  | Fibrinogen beta chain OS=Homo sapiens GN=FGB PE=1 SV=2 |

|  | **Query** | **Observed** | **Mr(expt)** | **Mr(calc)** | **Delta** | **Miss** | **Score** | **Expect** | **Rank** | **Peptide** |
| --- | --- | --- | --- | --- | --- | --- | --- | --- | --- | --- |
|  | 2515 | **422.7454** | **843.4762** | **843.4814** | **-0.0052** | **0** | **26** | **0.5** | **1** | **R.SILENLR.S** |
|  | 2533 | 423.2327 | 844.4508 | 844.4555 | -0.0047 | 1 | 13 | 12 | 6 | K.DLWQKR.Q |
|  | 4359 | 980.4886 | 979.4813 | 979.4359 | 0.0454 | 0 | 7 | 33 | 5 | R.QDGSVDFGR.K |
|  | 4588 | 499.2603 | 996.5061 | 995.4355 | 1.0705 | 0 | 5 | 50 | 8 | R.CHAANPNGR.Y |
|  | 5079 | **516.7816** | **1031.5487** | **1031.5553** | **-0.0066** | **0** | **13** | **9.4** | **1** | **K.IRPFFPQQ.-** |
|  | 8194 | **620.2582** | **1238.5018** | **1238.5105** | **-0.0087** | **0** | **42** | **0.0075** | **1** | **K.EDGGGWWYNR.C** |
|  | 9487 | **654.8085** | **1307.6024** | **1307.6106** | **-0.0082** | **0** | **32** | **0.078** | **1** | **K.QGFGNVATNTDGK.N** |
|  | 13376 | 768.3760 | 1534.7374 | 1534.7164 | 0.0210 | 0 | 26 | 0.3 | 2 | K.AHYGGFTVQNEANK.Y |
|  | 13479 | **771.8937** | **1541.7728** | **1540.7530** | **1.0198** | **1** | **4** | **54** | **6** | **K.MSMKIRPFFPQQ.-** |
|  | 13515 | **772.8461** | **1543.6776** | **1543.6878** | **-0.0102** | **0** | **47** | **0.0027** | **1** | **K.HGTDDGVVWMNWK.G** |
|  | 14625 | **809.8953** | **1617.7760** | **1617.7855** | **-0.0095** | **0** | **38** | **0.021** | **1** | **R.TPCTVSCNIPVVSGK.E** |
|  | 15400 | **834.8557** | **1667.6969** | **1667.7079** | **-0.0110** | **0** | **60** | **0.00011** | **1** | **R.YYWGGQYTWDMAK.H** 15401 |
|  | 15721 | 564.5761 | 1690.7065 | 1690.7946 | -0.0881 | 0 | 3 | 57 | 2 | R.MGPTELLIEMEDWK.G |
|  | 16922 | **884.8904** | **1767.7662** | **1767.7799** | **-0.0137** | **0** | **9** | **14** | **1** | **K.DNENVVNEYSSELEK.H** |
|  | 17175 | **893.3928** | **1784.7711** | **1784.7828** | **-0.0117** | **0** | **47** | **0.0021** | **1** | **K.NYCGLPGEYWLGNDK.I** 17176 |
|  | 18754 | **954.4832** | **1906.9517** | **1907.8465** | **-0.8948** | **0** | **9** | **14** | **2** | **R.GTAGNALMDGASQLMGENR.T** |
|  | 18755 | **954.4832** | **1906.9517** | **1907.8465** | **-0.8948** | **0** | **(8)** | **16** | **2** | **R.GTAGNALMDGASQLMGENR.T** |
|  | 21012 | **703.3695** | **2107.0865** | **2106.0970** | **0.9895** | **1** | **41** | **0.0077** | **1** | **K.REEAPSLRPAPPPISGGGYR.A** 20997 |
|  | 21440 | **1078.9700** | **2155.9254** | **2155.9415** | **-0.0161** | **0** | **95** | **2.5e-008** | **1** | **R.VYCDMNTENGGWTVIQNR.Q** 21439 21452 |
|  | 22326 | **753.0265** | **2256.0576** | **2256.0732** | **-0.0156** | **0** | **54** | **0.00032** | **1** | **K.GGETSEMYLIQPDSSVKPYR.V** |
|  | 23207 | **790.0555** | **2367.1448** | **2367.1607** | **-0.0159** | **1** | **32** | **0.051** | **1** | **K.AHYGGFTVQNEANKYQISVNK.Y** |
|  | 23363 | **795.7248** | **2384.1527** | **2384.1682** | **-0.0155** | **1** | **55** | **0.00023** | **1** | **R.KGGETSEMYLIQPDSSVKPYR.V** 23364 23365 |
|  | 24480 | **850.0730** | **2547.1972** | **2547.2131** | **-0.0160** | **1** | **47** | **0.0015** | **1** | **R.TPCTVSCNIPVVSGKECEEIIR.K** 24479 |
|  | 27455 | **770.3347** | **3077.3098** | **3076.3444** | **0.9654** | **1** | **9** | **5.4** | **1** | **R.TMTIHNGMFFSTYDRDNDGWLTSDPR.K** 27454 |
|  | 27586 | **1039.8050** | **3116.3933** | **3117.3669** | **-0.9736** | **1** | **2** | **32** | **4** | **R.VYCDMNTENGGWTVIQNRQDGSVDFGR.K** |
|  | 27776 | **1056.8268** | **3167.4585** | **3166.4764** | **0.9821** | **0** | **15** | **1.3** | **1** | **R.NSVDELNNNVEAVSQTSSSSFQYMYLLK.D** |
|  | 29856 | **1292.9474** | **3875.8203** | **3875.8449** | **-0.0246** | **1** | **51** | **0.00023** | **1** | **K.DNENVVNEYSSELEKHQLYIDETVNSNIPTNLR.V** |
|  | 30016 | **989.7295** | **3954.8889** | **3954.9139** | **-0.0250** | **0** | **15** | **0.97** | **1** | **K.APDAGGCLHADPDLGVLCPTGCQLQEALLQQERPIR.N** |
|  | 30259 | **1021.7530** | **4082.9829** | **4083.0088** | **-0.0260** | **1** | **31** | **0.021** | **1** | **R.KAPDAGGCLHADPDLGVLCPTGCQLQEALLQQERPIR.N** 30260 30261 |

| **99.** | P04406\|G3P_HUMAN    **Mass:** 36201    **Score:** 481    **Queries matched:** 39   **emPAI:** 3.41 |
| --- | --- |
|  | Glyceraldehyde-3-phosphate dehydrogenase OS=Homo sapiens GN=GAPDH PE=1 SV=3 |

|  | **Query** | **Observed** | **Mr(expt)** | **Mr(calc)** | **Delta** | **Miss** | **Score** | **Expect** | **Rank** | **Peptide** |
| --- | --- | --- | --- | --- | --- | --- | --- | --- | --- | --- |
|  | 1544 | **688.3731** | **687.3658** | **687.3704** | **-0.0046** | **0** | **29** | **0.36** | **1** | **K.FHGTVK.A** |
|  | 1878 | **398.2100** | **794.4055** | **794.4109** | **-0.0054** | **0** | **38** | **0.022** | **1** | **K.LTGMAFR.V** 1877 |
|  | 2113 | **406.2468** | **810.4790** | **810.4058** | **0.0732** | **0** | **(2)** | **80** | **5** | **K.LTGMAFR.V** |
|  | 3380 | **455.0524** | **908.0902** | **908.4828** | **-0.3926** | **0** | **28** | **0.15** | **1** | **K.AGAHLQGGAK.R** 3381 3387 3389 |
|  | 5490 | **533.2960** | **1064.5774** | **1064.5839** | **-0.0065** | **1** | **37** | **0.033** | **1** | **K.AGAHLQGGAKR.V** |
|  | 7747 | **406.5326** | **1216.5759** | **1216.5944** | **-0.0185** | **0** | **3** | **79** | **6** | **R.VVDLMAHMASK.E** |
|  | 9925 | **665.8242** | **1329.6338** | **1329.6421** | **-0.0083** | **1** | **68** | **2.2e-005** | **1** | **R.VVDLMAHMASKE.-** 9914 9926 9937 9944 |
|  | 13315 | **765.8954** | **1529.7763** | **1529.7872** | **-0.0109** | **0** | **71** | **9.7e-006** | **1** | **R.VPTANVSVVDLTCR.L** 13310 13314 |
|  | 14572 | **807.9416** | **1613.8687** | **1612.8937** | **0.9751** | **0** | **24** | **0.52** | **1** | **K.LVINGNPITIFQER.D** 14532 |
|  | 16524 | 435.7175 | 1738.8409 | 1738.9326 | -0.0917 | 1 | 7 | 23 | 1 | R.DGRGALQNIIPASTGAAK.A |
|  | 16887 | **882.3990** | **1762.7834** | **1762.7951** | **-0.0116** | **0** | **65** | **3.4e-005** | **1** | **K.LISWYDNEFGYSNR.V** 16886 |
|  | 17778 | **917.9492** | **1833.8839** | **1832.9124** | **0.9715** | **0** | **32** | **0.078** | **1** | **K.IISNASCTTNCLAPLAK.V** 17772 17784 |
|  | 20258 | **681.0363** | **2040.0869** | **2040.1004** | **-0.0135** | **1** | **17** | **1.8** | **1** | **K.LVINGNPITIFQERDPSK.I** 20273 |
|  | 21947 | **738.3699** | **2212.0880** | **2212.1020** | **-0.0141** | **0** | **11** | **6.6** | **1** | **R.VIISAPSADAPMFVMGVNHEK.Y** |
|  | 22464 | **1139.0160** | **2276.0174** | **2276.0307** | **-0.0133** | **0** | **70** | **7.1e-006** | **1** | **K.WGDAGAEYVVESTGVFTTMEK.A** 22463 |
|  | 24325 | **840.0717** | **2517.1932** | **2517.2097** | **-0.0166** | **1** | **40** | **0.0069** | **1** | **K.IKWGDAGAEYVVESTGVFTTMEK.A** 24324 |
|  | 24823 | **865.7867** | **2594.3384** | **2594.3527** | **-0.0143** | **0** | **62** | **4.5e-005** | **1** | **K.VIHDNFGIVEGLMTTVHAITATQK.T** 24825 |
|  | 28230 | **1103.5217** | **3307.5434** | **3307.5570** | **-0.0136** | **0** | **51** | **0.00038** | **1** | **K.VDIVAINDPFIDLNYMVYMFQYDSTHGK.F** |
|  | 30073 | **996.7255** | **3982.8730** | **3982.8910** | **-0.0180** | **1** | **11** | **2.4** | **1** | **R.AAFNSGKVDIVAINDPFIDLNYMVYMFQYDSTHGK.F** |
|  | 30141 | **1009.9710** | **4035.8547** | **4035.8875** | **-0.0328** | **0** | **54** | **0.00011** | **1** | **K.GILGYTEHQVVSSDFNSDTHSSTFDAGAGIALNDHFVK.L** 30143 |

| **100.** | P30049\|ATPD_HUMAN    **Mass:** 17479    **Score:** 479    **Queries matched:** 24   **emPAI:** 0.83 |
| --- | --- |
|  | ATP synthase subunit delta, mitochondrial OS=Homo sapiens GN=ATP5D PE=1 SV=2 |

|  | **Query** | **Observed** | **Mr(expt)** | **Mr(calc)** | **Delta** | **Miss** | **Score** | **Expect** | **Rank** | **Peptide** |
| --- | --- | --- | --- | --- | --- | --- | --- | --- | --- | --- |
|  | 11707 | **716.3529** | **1430.6913** | **1430.7001** | **-0.0089** | **0** | **90** | **1.2e-007** | **1** | **K.AQAELVGTADEATR.A** |
|  | 13137 | **507.2887** | **1518.8444** | **1519.9133** | **-1.0689** | **1** | **7** | **24** | **2** | **-.MLPAALLRRPGLGR.L** |
|  | 21322 | **715.0376** | **2142.0910** | **2141.1076** | **0.9834** | **1** | **5** | **30** | **3** | **K.AQAELVGTADEATRAEIQIR.I** |
|  | 29091 | **1195.6028** | **3583.7865** | **3583.7967** | **-0.0102** | **0** | **62** | **2.6e-005** | **1** | **K.YFVSSGSIAVNADSSVQLLAEEAVTLDMLDLGAAK.A** 29061 29062 29063 29065 29066 29067 29068 29070 29071 29074 29076 29079 29080 29081 29084 29092 29093 29095 |
|  | 30107 | **669.8193** | **4012.8720** | **4011.9074** | **0.9645** | **1** | **2** | **19** | **4** | **R.HARAYAEAAAAPAAASGPNQMSFTFASPTQVFFNGANVR.Q** |
|  | 30330 | **1369.7521** | **4106.2344** | **4106.2529** | **-0.0185** | **0** | **38** | **0.0033** | **1** | **R.QVDVPTLTGAFGILAAHVPTLQVLRPGLVVVHAEDGTTSK.Y** |

| **101.** | P04843\|RPN1_HUMAN    **Mass:** 68641    **Score:** 477    **Queries matched:** 44   **emPAI:** 1.32 |
| --- | --- |
|  | Dolichyl-diphosphooligosaccharide--protein glycosyltransferase subunit 1 OS=Homo sapiens GN=RPN1 PE=1 SV=1 |

|  | **Query** | **Observed** | **Mr(expt)** | **Mr(calc)** | **Delta** | **Miss** | **Score** | **Expect** | **Rank** | **Peptide** |
| --- | --- | --- | --- | --- | --- | --- | --- | --- | --- | --- |
|  | 919 | **517.3309** | **516.3237** | **516.3271** | **-0.0035** | **0** | **19** | **4.2** | **1** | **K.LISGK.R** |
|  | 1271 | **601.3879** | **600.3807** | **600.3846** | **-0.0040** | **0** | **19** | **3.5** | **7** | **K.ELVLK.S** |
|  | 2463 | **840.4038** | **839.3965** | **839.4025** | **-0.0059** | **0** | **23** | **0.66** | **1** | **R.NVESYTK.L** 2479 2484 |
|  | 2465 | **420.7602** | **839.5059** | **839.5116** | **-0.0057** | **0** | **18** | **2.5** | **1** | **K.IILPEGAK.N** 2478 2485 |
|  | 2937 | **437.7622** | **873.5097** | **872.5080** | **1.0018** | **1** | **13** | **9.9** | **3** | **K.RQELVTK.I** 2918 |
|  | 4712 | 502.7725 | 1003.5304 | 1003.4756 | 0.0548 | 1 | 22 | 1.1 | 3 | K.DPAAEARMK.V 4713 |
|  | 4887 | **509.2371** | **1016.4597** | **1016.4676** | **-0.0078** | **0** | **36** | **0.05** | **1** | **R.HFDETVNR.Y** 4888 4898 |
|  | 5458 | **531.7894** | **1061.5642** | **1061.5717** | **-0.0075** | **1** | **13** | **7.9** | **1** | **R.DISTLNSGKK.S** |
|  | 5551 | **535.7917** | **1069.5688** | **1069.5768** | **-0.0079** | **0** | **29** | **0.2** | **1** | **R.TVDLSSHLAK.V** |
|  | 6468 | **570.8184** | **1139.6222** | **1138.5506** | **1.0716** | **1** | **4** | **62** | **10** | **K.KDTYIENEK.L** |
|  | 8777 | **635.7969** | **1269.5792** | **1269.5877** | **-0.0085** | **0** | **44** | **0.0058** | **1** | **R.DVPAYSQDTFK.V** |
|  | 9188 | **647.3042** | **1292.5938** | **1292.6031** | **-0.0092** | **1** | **37** | **0.03** | **1** | **R.LKTEGSDLCDR.V** 9186 9187 9190 9192 |
|  | 9352 | **651.3709** | **1300.7273** | **1300.7350** | **-0.0078** | **0** | **56** | **0.00037** | **1** | **K.ALTSEIALLQSR.L** 9319 9351 |
|  | 11763 | **718.3518** | **1434.6889** | **1434.6990** | **-0.0101** | **0** | **54** | **0.00052** | **1** | **K.NIEIDSPYEISR.A** |
|  | 14454 | **536.3151** | **1605.9234** | **1606.8930** | **-0.9697** | **1** | **10** | **11** | **2** | **R.QELVTKIDHILDAL.-** |
|  | 15199 | **827.4439** | **1652.8733** | **1652.8846** | **-0.0113** | **0** | **86** | **2.7e-007** | **1** | **K.VTAEVVLAHLGGGSTSR.A** 15201 |
|  | 15289 | **830.4466** | **1658.8786** | **1658.8879** | **-0.0093** | **0** | **64** | **4.8e-005** | **1** | **R.ATSFLLALEPELEAR.L** 15290 |
|  | 16579 | **581.9942** | **1742.9608** | **1742.9713** | **-0.0105** | **1** | **18** | **1.8** | **1** | **K.VACITEQVLTLVNKR.I** |
|  | 17167 | **892.9249** | **1783.8352** | **1783.8489** | **-0.0137** | **1** | **34** | **0.048** | **1** | **R.YDYQRQPDSGISSIR.S** 17168 |
|  | 17926 | **923.9647** | **1845.9148** | **1846.0056** | **-0.0908** | **1** | **3** | **49** | **6** | **R.MKVACITEQVLTLVNK.R** |
|  | 19710 | **996.0035** | **1989.9924** | **1990.0048** | **-0.0124** | **0** | **76** | **2.3e-006** | **1** | **R.FVDHVFDEQVIDSLTVK.I** 19711 |
|  | 19921 | **670.6594** | **2008.9564** | **2008.9677** | **-0.0112** | **0** | **17** | **1.7** | **1** | **K.VHYENNSPFLTITSMTR.V** |
|  | 27041 | **999.4997** | **2995.4772** | **2996.4596** | **-0.9823** | **0** | **68** | **9.1e-006** | **1** | **K.THYIVGYNLPSYEYLYNLGDQYALK.M** 27048 27049 27050 |

| **102.** | O75452\|RDH16_HUMAN    **Mass:** 36050    **Score:** 475    **Queries matched:** 41   **emPAI:** 3.89 |
| --- | --- |
|  | Retinol dehydrogenase 16 OS=Homo sapiens GN=RDH16 PE=1 SV=2 |

|  | **Query** | **Observed** | **Mr(expt)** | **Mr(calc)** | **Delta** | **Miss** | **Score** | **Expect** | **Rank** | **Peptide** |
| --- | --- | --- | --- | --- | --- | --- | --- | --- | --- | --- |
|  | 2886 | 435.7370 | 869.4594 | 869.4647 | -0.0053 | 1 | 11 | 13 | 2 | K.FVADYKK.S |
|  | 3213 | 897.4039 | 896.3967 | 896.4028 | -0.0062 | 0 | 22 | 0.84 | 1 | R.YSAGWDAK.L 3214 |
|  | 3818 | **471.7468** | **941.4790** | **941.4858** | **-0.0068** | **0** | **31** | **0.11** | **1** | **R.ELSYFGVK.V** 3836 |
|  | 3951 | **476.2405** | **950.4665** | **949.4175** | **1.0491** | **0** | **14** | **7.1** | **1** | **K.SAEQMEQK.C** |
|  | 4712 | 502.7725 | 1003.5304 | 1003.5372 | -0.0068 | 0 | 51 | 0.0015 | 1 | R.VLAACLTEK.G 4713 |
|  | 5281 | **524.2808** | **1046.5470** | **1046.5543** | **-0.0073** | **0** | **(28)** | **0.25** | **1** | **R.VVNVSSVMGR.V** |
|  | 5462 | **532.2699** | **1062.5252** | **1062.5492** | **-0.0239** | **0** | **32** | **0.1** | **1** | **R.VVNVSSVMGR.V** 5465 |
|  | 5484 | **533.2686** | **1064.5227** | **1064.5291** | **-0.0064** | **0** | **45** | **0.0056** | **1** | **K.SFLEIWDR.S** |
|  | 6658 | **577.7953** | **1153.5760** | **1153.5842** | **-0.0081** | **0** | **29** | **0.17** | **1** | **K.VAMIEPGYFK.T** |
|  | 8596 | **630.8281** | **1259.6417** | **1259.6510** | **-0.0093** | **0** | **54** | **0.00058** | **1** | **K.TESVAAAAQWVK.E** 8598 8636 |
|  | 8641 | **631.3424** | **1260.6701** | **1259.6768** | **0.9933** | **1** | **48** | **0.0024** | **1** | **R.GRVVNVSSVMGR.V** |
|  | 11166 | **467.2339** | **1398.6798** | **1398.6892** | **-0.0094** | **1** | **36** | **0.03** | **1** | **K.YGVEAFSDSLRR.E** |
|  | 11992 | **725.8321** | **1449.6496** | **1449.6599** | **-0.0103** | **0** | **51** | **0.0011** | **1** | **K.YVFITGCDSGFGK.L** |
|  | 15751 | **847.3918** | **1692.7691** | **1692.7818** | **-0.0126** | **1** | **66** | **3e-005** | **1** | **R.DKYVFITGCDSGFGK.L** 15753 15794 |
|  | 16754 | **586.3152** | **1755.9239** | **1756.9070** | **-0.9830** | **1** | **3** | **59** | **6** | **K.VAMIEPGYFKTAVTSK.E** |
|  | 16880 | 881.9559 | 1761.8973 | 1761.9109 | -0.0136 | 1 | 65 | 3.7e-005 | 1 | R.GQTSDRLETVTLDVTK.T 16879 16881 16882 |
|  | 18701 | **635.6240** | **1903.8501** | **1902.9662** | **0.8838** | **1** | **12** | **6.3** | **1** | **K.YVFITGCDSGFGKLLAR.Q** |
|  | 23299 | **1189.6511** | **2377.2877** | **2377.3005** | **-0.0128** | **0** | **59** | **9e-005** | **1** | **K.GLWGLVNNAGISLPTAPNELLTK.Q** 23298 23300 23305 23306 |
|  | 24933 | **1311.2095** | **2620.4044** | **2620.4224** | **-0.0180** | **1** | **62** | **4.1e-005** | **1** | **R.DKGLWGLVNNAGISLPTAPNELLTK.Q** 24934 |
|  | 24946 | **657.3016** | **2625.1772** | **2625.1920** | **-0.0148** | **0** | **27** | **0.13** | **1** | **K.CTQDLSLVTNCMEHALIACHPR.T** 24947 |
|  | 25020 | **529.2626** | **2641.2765** | **2641.1869** | **0.0895** | **0** | **(6)** | **16** | **6** | **K.CTQDLSLVTNCMEHALIACHPR.T** |
|  | 27663 | **1567.2902** | **3132.5658** | **3132.5778** | **-0.0120** | **0** | **56** | **0.00012** | **1** | **K.LLYLPMSYMPTFLVDAIMYWVSPSPAK.A** 27664 |
|  | 28392 | **1117.2294** | **3348.6663** | **3348.6888** | **-0.0225** | **1** | **0** | **42** | **6** | **K.LLYLPMSYMPTFLVDAIMYWVSPSPAKAL.-** |

| **103.** | Q14697\|GANAB_HUMAN    **Mass:** 107263   **Score:** 473    **Queries matched:** 55   **emPAI:** 0.84 |
| --- | --- |
|  | Neutral alpha-glucosidase AB OS=Homo sapiens GN=GANAB PE=1 SV=3 |

|  | **Query** | **Observed** | **Mr(expt)** | **Mr(calc)** | **Delta** | **Miss** | **Score** | **Expect** | **Rank** | **Peptide** |
| --- | --- | --- | --- | --- | --- | --- | --- | --- | --- | --- |
|  | 2284 | **413.2261** | **824.4376** | **823.4123** | **1.0253** | **1** | **8** | **20** | **7** | **K.NMTRFR.I** |
|  | 2922 | **437.2348** | **872.4550** | **872.4603** | **-0.0053** | **0** | **1** | **1.6e+002** | **9** | **R.LDLLEDR.S** |
|  | 3655 | **465.2734** | **928.5323** | **929.5116** | **-0.9794** | **0** | **11** | **15** | **1** | **-.MAAVAAVAAR.R** |
|  | 4235 | **486.7921** | **971.5697** | **971.5764** | **-0.0067** | **0** | **34** | **0.076** | **1** | **R.SLLLSVNAR.G** 4223 4246 |
|  | 4322 | **489.2749** | **976.5352** | **976.4839** | **0.0513** | **0** | **18** | **2.8** | **1** | **R.AHAHLDTGR.R** |
|  | 4750 | **504.2396** | **1006.4647** | **1006.4720** | **-0.0072** | **0** | **14** | **7.2** | **1** | **K.VTEGGEPYR.L** |
|  | 5561 | **536.2445** | **1070.4743** | **1070.4821** | **-0.0078** | **0** | **5** | **56** | **7** | **R.YFTWDPSR.F** |
|  | 6296 | **564.7894** | **1127.5643** | **1127.5723** | **-0.0080** | **0** | **25** | **0.58** | **1** | **R.GLLEFEHQR.A** |
|  | 8379 | **624.8285** | **1247.6424** | **1247.6510** | **-0.0086** | **1** | **21** | **1.3** | **1** | **R.LKVTEGGEPYR.L** |
|  | 9380 | **652.2694** | **1302.5243** | **1302.5333** | **-0.0090** | **1** | **30** | **0.16** | **1** | **K.TCEESSFCKR.Q** |
|  | 9680 | **660.3438** | **1318.6731** | **1318.6816** | **-0.0085** | **0** | **32** | **0.094** | **1** | **R.SGGMERPFVLAR.A** 9681 |
|  | 10039 | **668.8967** | **1335.7788** | **1334.6765** | **1.1023** | **0** | **(7)** | **28** | **4** | **R.SGGMERPFVLAR.A** 10021 |
|  | 10369 | **678.2925** | **1354.5705** | **1354.5803** | **-0.0098** | **0** | **27** | **0.28** | **1** | **K.DAQHYGGWEHR.D** |
|  | 13322 | **511.2709** | **1530.7909** | **1530.7162** | **0.0747** | **1** | **5** | **38** | **3** | **R.DGDKPEETQGKAEK.D** |
|  | 14270 | **797.3642** | **1592.7138** | **1592.7235** | **-0.0096** | **0** | **15** | **3.7** | **1** | **R.WYQMGAYQPFFR.A** |
|  | 15579 | **840.9698** | **1679.9250** | **1679.9359** | **-0.0109** | **1** | **10** | **10** | **1** | **R.YRVPDVLVADPPIAR.L** |
|  | 16550 | **870.9616** | **1739.9087** | **1739.9206** | **-0.0120** | **0** | **31** | **0.087** | **1** | **R.LSFQHDPETSVLVLR.K** 16521 |
|  | 18043 | **464.4870** | **1853.9189** | **1854.0761** | **-0.1572** | **1** | **6** | **24** | **4** | **K.VLLVLELQGLQKNMTR.F** |
|  | 18343 | **625.3349** | **1872.9829** | **1872.9958** | **-0.0130** | **1** | **40** | **0.01** | **1** | **R.REPWLLPSQHNDIIR.D** |
|  | 18965 | **964.4239** | **1926.8332** | **1926.8452** | **-0.0119** | **0** | **70** | **9.3e-006** | **1** | **K.MMDYLQGSGETPQTDVR.W** 18964 |
|  | 19154 | **648.3198** | **1941.9376** | **1942.8401** | **-0.9025** | **0** | **(3)** | **53** | **2** | **K.MMDYLQGSGETPQTDVR.W** 19155 |
|  | 20771 | **417.2395** | **2081.1613** | **2081.1381** | **0.0231** | **1** | **8** | **14** | **7** | **R.SLLLSVNARGLLEFEHQR.A** |
|  | 21338 | **715.6854** | **2144.0344** | **2144.0473** | **-0.0129** | **0** | **45** | **0.0031** | **1** | **R.DVHNIYGLYVHMATADGLR.Q** 21339 |
|  | 21492 | **541.2919** | **2161.1386** | **2160.0422** | **1.0964** | **0** | **(7)** | **16** | **1** | **R.DVHNIYGLYVHMATADGLR.Q** |
|  | 22535 | 761.3592 | 2281.0557 | 2281.0896 | -0.0339 | 1 | 4 | 34 | 3 | R.LSVSGRDENSVELTMAEGPYK.I |
|  | 23392 | **1195.6462** | **2389.2779** | **2389.2907** | **-0.0127** | **0** | **49** | **0.00076** | **1** | **K.HHGPQTLYLPVTLSSIPVFQR.G** |
|  | 24201 | **1250.1881** | **2498.3617** | **2498.3744** | **-0.0128** | **0** | **35** | **0.019** | **1** | **R.ALLDSLQLGPDSLTVHLIHEVTK.V** 24197 24198 24199 24200 |
|  | 24242 | **836.4148** | **2506.2225** | **2505.1516** | **1.0709** | **1** | **2** | **40** | **8** | **K.TLFGKMMDYLQGSGETPQTDVR.W** |
|  | 24416 | **846.0461** | **2535.1166** | **2534.2918** | **0.8248** | **0** | **16** | **1.6** | **2** | **R.QYASLTGTQALPPLFSLGYHQSR.W** |
|  | 26291 | **1433.5685** | **2865.1224** | **2865.1436** | **-0.0211** | **0** | **67** | **9.3e-006** | **1** | **R.DGSDYEGWCWPGSAGYPDFTNPTMR.A** 26292 |
|  | 27085 | **1004.1384** | **3009.3933** | **3009.4145** | **-0.0212** | **0** | **20** | **0.53** | **1** | **R.FSFSGNTLVSSSADPEGHFETPIWIER.V** |
|  | 27601 | **1041.7651** | **3122.2736** | **3122.2923** | **-0.0188** | **1** | **25** | **0.12** | **1** | **K.TRDGSDYEGWCWPGSAGYPDFTNPTMR.A** |
|  | 27772 | **792.3820** | **3165.4987** | **3165.5156** | **-0.0168** | **1** | **26** | **0.12** | **1** | **R.RFSFSGNTLVSSSADPEGHFETPIWIER.V** 27774 |
|  | 29189 | **1216.2842** | **3645.8307** | **3645.8442** | **-0.0135** | **0** | **105** | **1.3e-009** | **1** | **R.LYNLDVFQYELYNPMALYGSVPVLLAHNPHR.D** 29186 29187 |
|  | 30010 | **989.2139** | **3952.8266** | **3952.8513** | **-0.0246** | **0** | **29** | **0.033** | **1** | **K.THSDSKPYGPMSVGLDFSLPGMEHVYGIPEHADNLR.L** 30014 |
|  | 30046 | **794.9960** | **3969.9435** | **3968.8462** | **1.0973** | **0** | **(3)** | **16** | **6** | **K.THSDSKPYGPMSVGLDFSLPGMEHVYGIPEHADNLR.L** |
|  | 31122 | **787.3839** | **4718.2598** | **4718.0505** | **0.2093** | **0** | **1** | **14** | **9** | **R.AWWANMFSYDNYEGSAPNLFVWNDMNEPSVFNGPEVTMLK.D** |
|  | 31908 | **1449.7100** | **7243.5134** | **7243.5706** | **-0.0572** | **0** | **6** | **1.2** | **1** | **R.EGIPVMRPLWVQYPQDVTTFNIDDQYLLGDALLVHPVSDSGAHGVQVYLPGQGEVWYDIQSYQK.H** |

| **104.** | Q08426\|ECHP_HUMAN    **Mass:** 80072    **Score:** 472    **Queries matched:** 45   **emPAI:** 0.72 |
| --- | --- |
|  | Peroxisomal bifunctional enzyme OS=Homo sapiens GN=EHHADH PE=1 SV=3 |

|  | **Query** | **Observed** | **Mr(expt)** | **Mr(calc)** | **Delta** | **Miss** | **Score** | **Expect** | **Rank** | **Peptide** |
| --- | --- | --- | --- | --- | --- | --- | --- | --- | --- | --- |
|  | 2745 | **431.7579** | **861.5012** | **861.5072** | **-0.0060** | **0** | **28** | **0.36** | **1** | **R.GIVISFAR.A** |
|  | 3538 | **460.7567** | **919.4988** | **919.5048** | **-0.0060** | **0** | **12** | **12** | **1** | **K.MITSVLEK.E** |
|  | 4892 | 509.2690 | 1016.5233 | 1016.5325 | -0.0091 | 0 | 7 | 37 | 9 | K.AIVICGAEGK.F |
|  | 5022 | 514.7600 | 1027.5053 | 1027.6026 | -0.0972 | 1 | 7 | 31 | 7 | R.RILADEALK.L 5009 5052 |
|  | 6613 | **576.8157** | **1151.6168** | **1151.6662** | **-0.0494** | **1** | **16** | **3.8** | **1** | **K.KLASQGNPPLK.E** |
|  | 7470 | **602.3101** | **1202.6057** | **1202.6143** | **-0.0086** | **0** | **40** | **0.017** | **1** | **R.TISQDEILER.C** |
|  | 7818 | **610.8388** | **1219.6629** | **1219.6713** | **-0.0084** | **0** | **19** | **2.1** | **1** | **R.IHKPDPWLSK.F** 7819 |
|  | 10856 | **691.8409** | **1381.6672** | **1381.6779** | **-0.0107** | **0** | **26** | **0.36** | **1** | **K.GWYQYDKPLGR.I** |
|  | 10908 | 693.3495 | 1384.6844 | 1384.6809 | 0.0035 | 0 | 5 | 42 | 7 | R.CLYSLINEAFR.I |
|  | 11500 | **473.7387** | **1418.1943** | **1418.6942** | **-0.4999** | **1** | **5** | **27** | **7** | **K.ANKWSTPSGASWK.T** |
|  | 12030 | **484.7605** | **1451.2596** | **1450.8144** | **0.4452** | **1** | **1** | **62** | **10** | **K.EGLQKAVIDHTIK.A** |
|  | 12573 | **742.4227** | **1482.8309** | **1482.8406** | **-0.0097** | **0** | **3** | **63** | **5** | **R.LTGVPAALDLITSGR.R** |
|  | 13225 | **763.3491** | **1524.6836** | **1524.6926** | **-0.0090** | **0** | **23** | **0.61** | **1** | **R.QHPGCLAQEACVR.A** |
|  | 14258 | **531.5932** | **1591.7578** | **1591.7678** | **-0.0100** | **0** | **19** | **1.4** | **1** | **K.MQQSGHPWSGPKPR.L** |
|  | 14489 | **537.2990** | **1608.8752** | **1607.7627** | **1.1125** | **0** | **(6)** | **32** | **1** | **K.MQQSGHPWSGPKPR.L** |
|  | 14897 | 818.4349 | 1634.8553 | 1634.8668 | -0.0115 | 0 | 60 | 0.00011 | 1 | R.AVQAAVQYPYEVGIK.K |
|  | 15325 | 831.9429 | 1661.8713 | 1662.9417 | -1.0704 | 1 | 2 | 79 | 10 | K.GQGLTGPTLLPGTPARK.R |
|  | 15339 | **832.4733** | **1662.9321** | **1662.9417** | **-0.0096** | **1** | **68** | **2e-005** | **1** | **R.KGQGLTGPTLLPGTPAR.K** |
|  | 15357 | **832.9882** | **1663.9618** | **1663.9733** | **-0.0115** | **1** | **24** | **0.43** | **1** | **R.LRNPPVNAISTTLLR.D** |
|  | 15589 | **561.2679** | **1680.7820** | **1680.7937** | **-0.0117** | **1** | **25** | **0.34** | **1** | **R.RQHPGCLAQEACVR.A** |
|  | 15628 | 842.9492 | 1683.8839 | 1683.8944 | -0.0106 | 0 | 61 | 9.5e-005 | 1 | R.TFGLTLGHVVDEIQR.N 15629 |
|  | 18176 | **621.6817** | **1862.0233** | **1862.9349** | **-0.9116** | **1** | **2** | **64** | **1** | **K.MGPFRVSDLAGLDVGWK.S** |
|  | 18242 | **933.9812** | **1865.9478** | **1865.9597** | **-0.0119** | **0** | **69** | **1.2e-005** | **1** | **K.GGPMFYASTVGLPTVLEK.L** 18241 |
|  | 18649 | 950.9800 | 1899.9454 | 1901.0153 | -1.0699 | 0 | 24 | 0.38 | 1 | K.TASARPVSSVGVVGLGTMGR.G 18648 |
|  | 22041 | 1111.6786 | 2221.3426 | 2221.1552 | 0.1875 | 1 | 3 | 33 | 6 | K.ELGGVDLVIEAVFEEMSLKK.Q |
|  | 22537 | **761.3920** | **2281.1543** | **2281.1702** | **-0.0160** | **1** | **10** | **8.7** | **1** | **K.LASQGNPPLKEWQSLAGSPSSK.L** |
|  | 24155 | **1246.6630** | **2491.3114** | **2491.3244** | **-0.0130** | **0** | **80** | **6.3e-007** | **1** | **K.LLEVIPSQYSSPTTIATVMNLSK.K** 24153 |
|  | 24328 | **630.3395** | **2517.3291** | **2517.2971** | **0.0320** | **0** | **2** | **43** | **1** | **R.LCNKPIQSLPNMDSIFSEALLK.M** |
|  | 25385 | 903.4458 | 2707.3156 | 2708.4194 | -1.1038 | 1 | 2 | 35 | 4 | R.LTSSVKELGGVDLVIEAVFEEMSLK.K |
|  | 25595 | **917.1356** | **2748.3849** | **2748.4024** | **-0.0175** | **0** | **38** | **0.01** | **1** | **R.ILGEGIAASPEHIDVVYLHGYGWPR.H** 25594 25596 |
|  | 18713 | **953.4583** | **2857.3531** | **2857.4367** | **-0.0836** | **0** | **2** | **87** | **1** | **R.NEKPVVAAIQGMAFGGGLELALGCHYR.I** |
|  | 29732 | **1276.2707** | **3825.7904** | **3825.8123** | **-0.0219** | **0** | **68** | **5.4e-006** | **1** | **R.MLNPYYNQAYFLLEEGSKPEEVDQVLEEFGFK.M** 29733 29734 |
|  | 29763 | **1281.5999** | **3841.7777** | **3841.8073** | **-0.0295** | **0** | **(44)** | **0.0015** | **1** | **R.MLNPYYNQAYFLLEEGSKPEEVDQVLEEFGFK.M** 29764 |

| **105.** | Q9UNU6\|CP8B1_HUMAN    **Mass:** 58269    **Score:** 471    **Queries matched:** 34   **emPAI:** 0.64 |
| --- | --- |
|  | 7-alpha-hydroxycholest-4-en-3-one 12-alpha-hydroxylase OS=Homo sapiens GN=CYP8B1 PE=2 SV=1 |

|  | **Query** | **Observed** | **Mr(expt)** | **Mr(calc)** | **Delta** | **Miss** | **Score** | **Expect** | **Rank** | **Peptide** |
| --- | --- | --- | --- | --- | --- | --- | --- | --- | --- | --- |
|  | 1250 | **596.9539** | **595.9466** | **595.2966** | **0.6500** | **0** | **2** | **48** | **5** | **R.LHPTE.-** |
|  | 1271 | 601.3879 | 600.3807 | 599.4370 | 0.9437 | 1 | 21 | 2.2 | 1 | K.KLVLK.V |
|  | 3338 | **453.2506** | **904.4867** | **903.4563** | **1.0305** | **0** | **14** | **8** | **4** | **R.FLNPNGSR.K** 3326 |
|  | 3352 | **453.7699** | **905.5252** | **906.4963** | **-0.9711** | **0** | **10** | **18** | **3** | **K.FDLLFPR.F** |
|  | 5129 | **518.2993** | **1034.5841** | **1034.5913** | **-0.0072** | **1** | **41** | **0.014** | **1** | **R.KFDLLFPR.F** 5133 |
|  | 5519 | 534.7373 | 1067.4600 | 1068.5604 | -1.1003 | 1 | 18 | 2.6 | 1 | R.KLDFGQYAK.K 5538 |
|  | 6181 | **559.7886** | **1117.5626** | **1116.5815** | **0.9811** | **0** | **24** | **0.64** | **1** | **R.LVHEDYTLK.M** |
|  | 6542 | **573.7926** | **1145.5706** | **1144.5546** | **1.0160** | **0** | **8** | **23** | **4** | **K.MLSVSHSQEK.E** |
|  | 7148 | **595.2588** | **1188.5030** | **1188.5445** | **-0.0415** | **0** | **9** | **21** | **1** | **R.EQGVPSAMQDK.F** |
|  | 7450 | **601.8001** | **1201.5857** | **1201.5939** | **-0.0082** | **0** | **42** | **0.0098** | **1** | **R.EEATQVLGEAR.L** 7449 7480 |
|  | 9053 | **429.2404** | **1284.6994** | **1284.6938** | **0.0056** | **1** | **8** | **21** | **10** | **R.EWLEVGRLQR.L** |
|  | 11557 | 711.8276 | 1421.6406 | 1420.7575 | 0.8831 | 1 | 1 | 1.1e+002 | 4 | R.QRRPWEPPLDK.G |
|  | 13103 | **759.3461** | **1516.6777** | **1516.6882** | **-0.0104** | **0** | **15** | **4.1** | **1** | **R.WGFGTMQPSHDVR.F** 13104 |
|  | 13341 | **511.7584** | **1532.2534** | **1532.6831** | **-0.4296** | **0** | **(3)** | **41** | **9** | **R.WGFGTMQPSHDVR.F** |
|  | 13903 | **524.2944** | **1569.8613** | **1569.8238** | **0.0374** | **1** | **(0)** | **1.2e+002** | **9** | **K.GTVPWLGHAMAFRK.N** |
|  | 14150 | **793.8614** | **1585.7083** | **1585.8188** | **-0.1104** | **1** | **5** | **36** | **7** | **K.GTVPWLGHAMAFRK.N** |
|  | 14745 | **813.8752** | **1625.7359** | **1625.7468** | **-0.0108** | **0** | **63** | **6e-005** | **1** | **R.SVQGDHEMIHSASTK.H** 14744 |
|  | 20593 | **1034.9974** | **2067.9803** | **2067.9935** | **-0.0132** | **1** | **129** | **1e-011** | **1** | **K.DKEQDLLQAGELFMEFR.K** 20594 |
|  | 20721 | 1039.0618 | 2076.1090 | 2076.1215 | -0.0125 | 0 | 59 | 0.00012 | 1 | K.LGALQHTPVLDSVVEETLR.L 20722 |
|  | 26964 | **994.5689** | **2980.6847** | **2979.6918** | **0.9929** | **1** | **0** | **43** | **9** | **M.VLWGPVLGALLVVIAGYLCLPGMLRQR.R** |
|  | 27497 | **1030.1865** | **3087.5377** | **3087.5528** | **-0.0151** | **0** | **70** | **4.5e-006** | **1** | **R.HGDILALFPYLSVHMDPDIHPEPTVFK.Y** 27495 27496 |
|  | 27572 | **778.6395** | **3110.5290** | **3110.7323** | **-0.2033** | **1** | **4** | **20** | **2** | **-.MVLWGPVLGALLVVIAGYLCLPGMLRQR.R** |
|  | 28366 | **836.4333** | **3341.7041** | **3341.7159** | **-0.0119** | **1** | **13** | **2.4** | **1** | **R.TKHGDVFTVQLGGQYFTFVMDPLSFGPILK.D** |

| **106.** | P22695\|QCR2_HUMAN    **Mass:** 48584    **Score:** 470    **Queries matched:** 46   **emPAI:** 2.52 |
| --- | --- |
|  | Cytochrome b-c1 complex subunit 2, mitochondrial OS=Homo sapiens GN=UQCRC2 PE=1 SV=3 |

|  | **Query** | **Observed** | **Mr(expt)** | **Mr(calc)** | **Delta** | **Miss** | **Score** | **Expect** | **Rank** | **Peptide** |
| --- | --- | --- | --- | --- | --- | --- | --- | --- | --- | --- |
|  | 1722 | **759.4305** | **758.4232** | **758.4286** | **-0.0054** | **0** | **32** | **0.15** | **1** | **R.GGLGLSGAK.A** |
|  | 1843 | **793.4138** | **792.4065** | **792.4130** | **-0.0065** | **0** | **15** | **6** | **1** | **K.AAYNQVK.T** |
|  | 5318 | **526.2855** | **1050.5565** | **1051.6066** | **-1.0501** | **1** | **4** | **61** | **7** | **R.FYSLKVAPK.V** |
|  | 8112 | **618.3096** | **1234.6046** | **1234.6128** | **-0.0082** | **0** | **77** | **3e-006** | **1** | **K.QVAEQFLNMR.G** 8111 |
|  | 8430 | **626.2974** | **1250.5802** | **1250.6077** | **-0.0276** | **0** | **(10)** | **15** | **1** | **K.QVAEQFLNMR.G** |
|  | 9827 | **663.8519** | **1325.6893** | **1325.6979** | **-0.0086** | **0** | **41** | **0.011** | **1** | **R.WEVADLQPQLK.I** |
|  | 10907 | 693.3085 | 1384.6025 | 1384.6115 | -0.0090 | 0 | 37 | 0.025 | 1 | R.ENMAYTVECLR.G 10924 |
|  | 12000 | 725.8682 | 1449.7219 | 1449.7324 | -0.0105 | 0 | 72 | 8e-006 | 1 | R.GSNTTSHLHQAVAK.A 12001 12020 |
|  | 12168 | **487.3204** | **1458.9394** | **1457.8202** | **1.1192** | **1** | **5** | **38** | **10** | **R.GIEAVGGKLSVTATR.E** 12138 |
|  | 12556 | **741.9013** | **1481.7881** | **1481.7990** | **-0.0110** | **1** | **25** | **0.43** | **1** | **R.RWEVADLQPQLK.I** 12539 |
|  | 14449 | 803.9174 | 1605.8203 | 1605.8335 | -0.0133 | 1 | 42 | 0.008 | 1 | K.RGSNTTSHLHQAVAK.A 14448 14450 |
|  | 14799 | **815.4562** | **1628.8978** | **1629.8322** | **-0.9344** | **0** | **29** | **0.16** | **1** | **K.TIAQGNLSNTDVQAAK.N** 14841 |
|  | 15363 | **555.9424** | **1664.8053** | **1664.8158** | **-0.0105** | **0** | **52** | **0.00071** | **1** | **R.YEDFSNLGTTHLLR.L** 15379 |
|  | 16596 | **873.4056** | **1744.7967** | **1744.8090** | **-0.0123** | **0** | **28** | **0.17** | **1** | **K.SMAASGNLGHTPFVDEL.-** 16597 16598 |
|  | 18778 | **955.4798** | **1908.9450** | **1908.9581** | **-0.0131** | **0** | **5** | **28** | **3** | **K.ATAAPAGAPPQPQDLEFTK.L** |
|  | 18988 | **965.0200** | **1928.0255** | **1928.0367** | **-0.0112** | **0** | **62** | **5.9e-005** | **1** | **K.LPNGLVIASLENYSPVSR.I** |
|  | 20221 | **510.0061** | **2035.9952** | **2036.0075** | **-0.0124** | **1** | **16** | **2.4** | **1** | **K.AGSRYEDFSNLGTTHLLR.L** |
|  | 21517 | **722.3468** | **2164.0186** | **2164.0338** | **-0.0152** | **0** | **18** | **1.5** | **1** | **K.VTSEELHYFVQNHFTSAR.M** |
|  | 22509 | **760.7211** | **2279.1416** | **2279.1508** | **-0.0092** | **0** | **30** | **0.071** | **1** | **R.GDVDILMEFLLNVTTAPEFR.R** 22507 |
|  | 23054 | **1175.5975** | **2349.1805** | **2349.1978** | **-0.0173** | **0** | **87** | **1.3e-007** | **1** | **K.AVAFQNPQTHVIENLHAAAYR.N** 23055 |
|  | 29671 | **1273.9950** | **3818.9632** | **3818.9843** | **-0.0211** | **1** | **39** | **0.004** | **1** | **K.ATAAPAGAPPQPQDLEFTKLPNGLVIASLENYSPVSR.I** 29676 29680 29681 |
|  | 29758 | **1280.9517** | **3839.8332** | **3839.8530** | **-0.0199** | **0** | **40** | **0.0029** | **1** | **K.ATQQPFDVSAFNASYSDSGLFGIYTISQATAAGDVIK.A** 29760 29762 |
|  | 30265 | 1022.2596 | 4085.0092 | 4084.0403 | 0.9690 | 0 | 38 | 0.0043 | 1 | R.EQNGDSLVHAAFVAESAVAGSAEANAFSVLQHVLGAGPHVK.R 30263 |
|  | 31052 | **920.4606** | **4597.2665** | **4596.3109** | **0.9555** | **1** | **6** | **4.3** | **1** | **R.GGEIREQNGDSLVHAAFVAESAVAGSAEANAFSVLQHVLGAGPHVK.R** 31053 |
|  | 31527 | **1380.3977** | **5517.5617** | **5516.7041** | **0.8577** | **1** | **3** | **4.6** | **1** | **K.LKAGYLMSVESSECFLEEVGSQALVAGSYMPPSTVLQQIDSVANADIINAAK.K** |

| **107.** | P49326\|FMO5_HUMAN    **Mass:** 60638    **Score:** 466    **Queries matched:** 47   **emPAI:** 1.59 |
| --- | --- |
|  | Dimethylaniline monooxygenase [N-oxide-forming] 5 OS=Homo sapiens GN=FMO5 PE=2 SV=2 |

|  | **Query** | **Observed** | **Mr(expt)** | **Mr(calc)** | **Delta** | **Miss** | **Score** | **Expect** | **Rank** | **Peptide** |
| --- | --- | --- | --- | --- | --- | --- | --- | --- | --- | --- |
|  | 1201 | 581.3245 | 580.3172 | 580.3220 | -0.0049 | 0 | 10 | 12 | 9 | R.ASIYK.S 1202 |
|  | 1426 | **645.3886** | **644.3813** | **643.4017** | **0.9796** | **1** | **10** | **26** | **9** | **K.VKGNVK.E** |
|  | 1866 | **794.4014** | **793.3942** | **793.4004** | **-0.0063** | **0** | **8** | **26** | **1** | **K.TTVCSVK.K** |
|  | 2576 | 425.2252 | 848.4358 | 848.4028 | 0.0329 | 0 | 14 | 7.5 | 3 | K.NPEGFTGK.R |
|  | 2702 | **860.4659** | **859.4587** | **860.4967** | **-1.0381** | **0** | **35** | **0.067** | **1** | **K.SVIINTSK.E** |
|  | 3009 | **440.2368** | **878.4591** | **878.4650** | **-0.0060** | **0** | **17** | **3.9** | **2** | **R.WATQVFK.G** |
|  | 3313 | **452.7103** | **903.4060** | **903.4662** | **-0.0601** | **0** | **12** | **13** | **6** | **K.AILTTDDR.I** |
|  | 4483 | **495.7521** | **989.4897** | **989.4964** | **-0.0067** | **0** | **31** | **0.14** | **1** | **K.ICGQSLANK.Y** 4497 |
|  | 4732 | **503.2665** | **1004.5185** | **1004.5039** | **0.0145** | **1** | **9** | **23** | **5** | **K.NPEGFTGKR.V** |
|  | 5064 | **516.2689** | **1030.5233** | **1031.5611** | **-1.0378** | **1** | **15** | **5.9** | **5** | **R.KAILTTDDR.I** |
|  | 5078 | **516.7556** | **1031.4967** | **1031.5036** | **-0.0069** | **0** | **39** | **0.021** | **1** | **R.TDDIGGLWR.F** |
|  | 5522 | 534.7603 | 1067.5061 | 1068.5638 | -1.0577 | 1 | 3 | 74 | 6 | R.FKTTVCSVK.K |
|  | 6837 | **585.2919** | **1168.5693** | **1168.5778** | **-0.0085** | **1** | **36** | **0.034** | **1** | **K.FKGQYFHSR.D** 6836 |
|  | 6865 | 586.2844 | 1170.5542 | 1169.5942 | 0.9600 | 1 | 56 | 0.00037 | 1 | R.VQGPGKWDGAR.K |
|  | 7943 | 409.8712 | 1226.5918 | 1227.7121 | -1.1203 | 1 | 10 | 14 | 3 | R.KPLMTRVVER.S |
|  | 8021 | **411.0968** | **1230.2687** | **1229.5632** | **0.7055** | **0** | **10** | **11** | **3** | **R.SSSMTSTMTIGK.F** 8023 |
|  | 8479 | **627.7994** | **1253.5842** | **1254.5881** | **-1.0038** | **1** | **11** | **11** | **2** | **R.DYKNPEGFTGK.R** 8509 |
|  | 10231 | **450.2260** | **1347.6563** | **1347.6645** | **-0.0082** | **0** | **23** | **0.86** | **1** | **R.FDHEMFGLKPK.H** 10230 |
|  | 10395 | 678.8706 | 1355.7267 | 1356.7977 | -1.0710 | 0 | 1 | 1.1e+002 | 4 | R.IAVIGGGVSGLSSIK.C |
|  | 10527 | **455.5926** | **1363.7561** | **1363.6595** | **0.0967** | **0** | **(4)** | **55** | **3** | **R.FDHEMFGLKPK.H** |
|  | 15013 | **548.2316** | **1641.6731** | **1640.8246** | **0.8485** | **1** | **6** | **29** | **7** | **R.FDHEMFGLKPKHR.A** |
|  | 15053 | **823.3910** | **1644.7675** | **1644.7784** | **-0.0108** | **0** | **53** | **0.00061** | **1** | **R.VGDYGYPADVLFSSR.L** 15054 15064 15071 |
|  | 15705 | **845.9222** | **1689.8299** | **1689.8434** | **-0.0135** | **0** | **57** | **0.0002** | **1** | **R.ALSQHPTLNDDLPNR.I** 15707 |
|  | 17134 | **892.3702** | **1782.7258** | **1782.7375** | **-0.0117** | **0** | **75** | **3.2e-006** | **1** | **K.CCVEEGLEPVCFER.T** |
|  | 18349 | **625.3498** | **1873.0275** | **1873.0396** | **-0.0121** | **0** | **42** | **0.0064** | **1** | **K.LALHLLLGPCTPIHYR.V** 18350 18351 |
|  | 21427 | **1078.0953** | **2154.1761** | **2154.1896** | **-0.0135** | **0** | **76** | **2e-006** | **1** | **R.VIIIGIGNSGGDLAVEISQTAK.Q** 21431 21432 |
|  | 28693 | **1151.9883** | **3452.9430** | **3452.9581** | **-0.0151** | **0** | **60** | **3.2e-005** | **1** | **K.VFPPNLERPTLAIIGLIQPLGAIMPISELQGR.W** 28692 28695 |
|  | 28948 | **1184.5096** | **3550.5071** | **3550.5243** | **-0.0172** | **0** | **40** | **0.0028** | **1** | **K.EMMCFSDYPIPDHYPNFMHNAQVLEYFR.M** |
|  | 29227 | **918.7174** | **3670.8405** | **3670.8553** | **-0.0147** | **0** | **45** | **0.0011** | **1** | **R.HTIQGDYIDTMEELADLVGVRPNLLSLAFTDPK.L** 29226 |
|  | 30877 | **1109.5147** | **4434.0295** | **4433.2213** | **0.8082** | **1** | **5** | **6.4** | **3** | **R.YVESQRHTIQGDYIDTMEELADLVGVRPNLLSLAFTDPK.L** |

| **108.** | A6NNZ2\|TBB8B_HUMAN    **Mass:** 50168    **Score:** 463    **Queries matched:** 45   **emPAI:** 1.05 |
| --- | --- |
|  | Tubulin beta-8 chain B OS=Homo sapiens PE=1 SV=1 |

|  | **Query** | **Observed** | **Mr(expt)** | **Mr(calc)** | **Delta** | **Miss** | **Score** | **Expect** | **Rank** | **Peptide** |
| --- | --- | --- | --- | --- | --- | --- | --- | --- | --- | --- |
|  | 2066 | 404.7367 | 807.4588 | 807.3399 | 0.1190 | 0 | 7 | 33 | 7 | R.EEYPDR.I |
|  | 5020 | 514.7598 | 1027.5050 | 1027.5121 | -0.0071 | 0 | 46 | 0.004 | 1 | K.TAVCDIPPR.G 5004 5015 5016 5017 5018 5019 5021 5022 |
|  | 5494 | 533.3253 | 1064.6361 | 1064.4201 | 0.2160 | 0 | (4) | 60 | 6 | K.NMMAACDPR.H |
|  | 5638 | 539.2662 | 1076.5179 | 1076.5250 | -0.0071 | 1 | 36 | 0.039 | 1 | K.IREEYPDR.I 5637 |
|  | 5678 | 541.2108 | 1080.4071 | 1080.4151 | -0.0080 | 0 | 29 | 0.16 | 1 | K.NMMAACDPR.H 5679 5694 |
|  | 6327 | 565.7967 | 1129.5788 | 1129.5880 | -0.0092 | 0 | 4 | 59 | 6 | R.FPGQLNADLR.K |
|  | 6499 | 572.3174 | 1142.6203 | 1142.6270 | -0.0067 | 0 | 70 | 1.8e-005 | 1 | K.LAVNMVPFPR.L 6498 |
|  | 8802 | 636.3640 | 1270.7133 | 1270.7220 | -0.0086 | 1 | 47 | 0.0027 | 1 | R.KLAVNMVPFPR.L |
|  | 9112 | 430.2745 | 1287.8016 | 1286.7169 | 1.0847 | 1 | (7) | 28 | 2 | R.KLAVNMVPFPR.L 9101 |
|  | 9913 | 665.3396 | 1328.6646 | 1328.7704 | -0.1057 | 0 | 11 | 11 | 3 | R.IINTFSILPSPK.V 9910 9911 |
|  | 14658 | 810.9161 | 1619.8176 | 1619.8283 | -0.0107 | 0 | 52 | 0.0008 | 1 | R.LHFFMPGFAPLTSR.G 14659 14660 14661 |
|  | 17713 | **610.6197** | **1828.8372** | **1827.9241** | **0.9132** | **1** | **7** | **21** | **2** | **R.INVHHHEASGGRYVPR.A** |
|  | 19931 | 503.4933 | 2009.9439 | 2009.9009 | 0.0431 | 1 | 1 | 71 | 5 | R.MPMREVDEQMFNIQDK.N |
|  | 20170 | **676.9858** | **2027.9357** | **2026.9630** | **0.9727** | **1** | **2** | **52** | **1** | **K.GRYTEGAELTESVMDVVR.K** |
|  | 23107 | 786.0563 | 2355.1470 | 2355.1583 | -0.0113 | 1 | 2 | 44 | 7 | R.LHFFMPGFAPLTSRGSQQYR.A |
|  | 25766 | 695.3771 | 2777.4792 | 2776.4346 | 1.0446 | 1 | 6 | 13 | 2 | K.LAVNMVPFPRLHFFMPGFAPLTSR.G |
|  | 28244 | 1656.2616 | 3310.5086 | 3310.5268 | -0.0182 | 0 | (65) | 1.3e-005 | 1 | K.EAESCDCLQGFQLTHSLGGGTGSGMGTLLISK.I 28241 28242 28243 28245 28246 |
|  | 28304 | 1109.8416 | 3326.5028 | 3326.5218 | -0.0189 | 0 | 72 | 3e-006 | 1 | K.EAESCDCLQGFQLTHSLGGGTGSGMGTLLISK.I |
|  | 28665 | 1147.2072 | 3438.5996 | 3438.6218 | -0.0222 | 1 | 68 | 6.1e-006 | 1 | R.KEAESCDCLQGFQLTHSLGGGTGSGMGTLLISK.I 28657 28663 28664 |

| **109.** | P02768\|ALBU_HUMAN    **Mass:** 71317    **Score:** 462    **Queries matched:** 47   **emPAI:** 2.06 |
| --- | --- |
|  | Serum albumin OS=Homo sapiens GN=ALB PE=1 SV=2 |

|  | **Query** | **Observed** | **Mr(expt)** | **Mr(calc)** | **Delta** | **Miss** | **Score** | **Expect** | **Rank** | **Peptide** |
| --- | --- | --- | --- | --- | --- | --- | --- | --- | --- | --- |
|  | 1353 | **627.4148** | **626.4075** | **625.3296** | **1.0779** | **1** | **10** | **11** | **3** | **R.RDAHK.S** |
|  | 1749 | **772.4332** | **771.4259** | **771.4313** | **-0.0054** | **0** | **19** | **2.8** | **1** | **K.AACLLPK.L** 1750 |
|  | 1800 | **789.4664** | **788.4592** | **788.4644** | **-0.0052** | **0** | **27** | **0.51** | **1** | **K.LVTDLTK.V** |
|  | 3015 | **880.4348** | **879.4275** | **879.4338** | **-0.0062** | **0** | **28** | **0.28** | **1** | **K.AEFAEVSK.L** |
|  | 3715 | **933.5123** | **932.5051** | **932.5113** | **-0.0063** | **0** | **6** | **43** | **2** | **K.LCTVATLR.E** |
|  | 4078 | 480.7816 | 959.5486 | 959.5552 | -0.0067 | 0 | 33 | 0.093 | 1 | K.FQNALLVR.Y 4064 |
|  | 4651 | **500.8021** | **999.5896** | **999.5964** | **-0.0069** | **0** | **14** | **7.3** | **4** | **K.QTALVELVK.H** |
|  | 4843 | **1014.5956** | **1013.5883** | **1012.5917** | **0.9966** | **0** | **39** | **0.02** | **1** | **K.LVAASQAALGL.-** |
|  | 5620 | **538.2687** | **1074.5228** | **1073.5353** | **0.9875** | **1** | **12** | **11** | **7** | **K.LDELRDEGK.A** |
|  | 6431 | **569.3980** | **1136.7813** | **1137.4907** | **-0.7093** | **0** | **30** | **0.13** | **1** | **K.CCTESLVNR.R** 6427 |
|  | 7384 | **600.3666** | **1198.7187** | **1197.5336** | **1.1852** | **1** | **3** | **69** | **4** | **K.ETCFAEEGKK.L** |
|  | 7930 | **613.8018** | **1225.5890** | **1225.5979** | **-0.0089** | **1** | **30** | **0.15** | **1** | **R.FKDLGEENFK.A** 7931 |
|  | 10124 | **671.8168** | **1341.6190** | **1341.6275** | **-0.0085** | **0** | **74** | **5.2e-006** | **1** | **K.AVMDDFAAFVEK.C** 10123 |
|  | 10663 | **686.2820** | **1370.5494** | **1370.5595** | **-0.0101** | **0** | **34** | **0.054** | **1** | **K.AAFTECCQAADK.A** |
|  | 11744 | **717.7654** | **1433.5163** | **1433.5261** | **-0.0098** | **0** | **36** | **0.027** | **1** | **R.ETYGEMADCCAK.Q** |
|  | 11880 | **722.3198** | **1442.6250** | **1442.6347** | **-0.0098** | **0** | **56** | **0.00031** | **1** | **K.YICENQDSISSK.L** 11885 |
|  | 12297 | **489.9495** | **1466.8266** | **1466.8358** | **-0.0091** | **1** | **40** | **0.012** | **1** | **R.RHPDYSVVLLLR.L** |
|  | 12686 | **745.8144** | **1489.6143** | **1489.8980** | **-0.2837** | **1** | **7** | **25** | **3** | **K.QTALVELVKHKPK.A** 12673 |
|  | 12815 | **750.3333** | **1498.6521** | **1497.5712** | **1.0809** | **0** | **29** | **0.14** | **1** | **K.TCVADESAENCDK.S** |
|  | 13547 | **773.8964** | **1545.7783** | **1545.7894** | **-0.0112** | **1** | **36** | **0.03** | **1** | **K.LKECCEKPLLEK.S** 13538 13564 |
|  | 13634 | **776.7972** | **1551.5799** | **1551.5905** | **-0.0105** | **0** | **51** | **0.00091** | **1** | **K.CCAAADPHECYAK.V** 13635 |
|  | 14980 | 820.4669 | 1638.9193 | 1638.9305 | -0.0112 | 1 | 47 | 0.0021 | 1 | K.KVPQVSTPTLVEVSR.N |
|  | 15248 | **829.3740** | **1656.7335** | **1656.7453** | **-0.0119** | **0** | **24** | **0.39** | **1** | **K.QNCELFEQLGEYK.F** |
|  | 18621 | **633.6663** | **1897.9771** | **1897.9879** | **-0.0108** | **1** | **36** | **0.022** | **1** | **R.RHPYFYAPELLFFAK.R** |
|  | 18787 | **955.9631** | **1909.9116** | **1909.9244** | **-0.0128** | **0** | **39** | **0.013** | **1** | **R.RPCFSALEVDETYVPK.E** |
|  | 20298 | **682.3662** | **2044.0768** | **2044.0881** | **-0.0113** | **0** | **37** | **0.02** | **1** | **K.VFDEFKPLVEEPQNLIK.Q** 20297 20299 |
|  | 20702 | **692.3341** | **2073.9803** | **2072.8237** | **1.1566** | **1** | **0** | **82** | **6** | **R.ETYGEMADCCAKQEPER.N** |
|  | 20801 | **1043.4778** | **2084.9410** | **2085.8303** | **-0.8893** | **0** | **1** | **70** | **9** | **K.VHTECCHGDLLECADDR.A** |
|  | 24135 | **1245.6388** | **2489.2630** | **2489.2777** | **-0.0147** | **0** | **50** | **0.00069** | **1** | **K.ALVLIAFAQYLQQCPFEDHVK.L** |
|  | 24773 | **862.3711** | **2584.0916** | **2584.1105** | **-0.0188** | **1** | **45** | **0.0018** | **1** | **K.VHTECCHGDLLECADDRADLAK.Y** 24772 |
|  | 25160 | **892.1061** | **2673.2966** | **2673.3077** | **-0.0111** | **1** | **56** | **0.00014** | **1** | **K.RMPCAEDYLSVVLNQLCVLHEK.T** 25155 |
|  | 25763 | **926.7838** | **2777.3294** | **2777.3517** | **-0.0222** | **1** | **36** | **0.014** | **1** | **R.LVRPEVDVMCTAFHDNEETFLKK.Y** |
|  | 29028 | **716.5300** | **3577.6135** | **3577.6680** | **-0.0545** | **1** | **2** | **24** | **5** | **K.CCAAADPHECYAKVFDEFKPLVEEPQNLIK.Q** |

| **110.** | P28332\|ADH6_HUMAN    **Mass:** 40032    **Score:** 453    **Queries matched:** 30   **emPAI:** 2.20 |
| --- | --- |
|  | Alcohol dehydrogenase 6 OS=Homo sapiens GN=ADH6 PE=2 SV=2 |

|  | **Query** | **Observed** | **Mr(expt)** | **Mr(calc)** | **Delta** | **Miss** | **Score** | **Expect** | **Rank** | **Peptide** |
| --- | --- | --- | --- | --- | --- | --- | --- | --- | --- | --- |
|  | 1426 | 645.3886 | 644.3813 | 643.4017 | 0.9796 | 1 | 11 | 23 | 4 | K.EVRIK.V |
|  | 5167 | 520.2565 | 1038.4984 | 1038.5056 | -0.0072 | 0 | 13 | 8.6 | 2 | K.LVADYMAEK.L |
|  | 5698 | 541.3391 | 1080.6637 | 1080.5056 | 0.1581 | 0 | 5 | 47 | 4 | -.MCTTGQVIR.C |
|  | 5872 | **548.2549** | **1094.4953** | **1094.5026** | **-0.0073** | **0** | **44** | **0.0059** | **1** | **K.TQLMSDGTSR.F** |
|  | 6091 | **556.2949** | **1110.5752** | **1110.5822** | **-0.0070** | **0** | **64** | **5.5e-005** | **1** | **K.ISGQLFFSGR.S** |
|  | 6131 | **557.8229** | **1113.6313** | **1113.6394** | **-0.0080** | **1** | **4** | **65** | **5** | **R.IIGVDVNKEK.F** |
|  | 8689 | 633.3103 | 1264.6060 | 1264.6156 | -0.0095 | 0 | 27 | 0.29 | 1 | K.VVATGLCGTEMK.V |
|  | 10221 | **674.3982** | **1346.7818** | **1347.7068** | **-0.9249** | **1** | **1** | **1.2e+002** | **4** | **K.INEAVELMKTGK.W** |
|  | 12931 | **753.8991** | **1505.7837** | **1505.7946** | **-0.0109** | **1** | **60** | **0.00012** | **1** | **R.IKVVATGLCGTEMK.V** |
|  | 13187 | **761.9039** | **1521.7933** | **1521.7895** | **0.0038** | **1** | **(6)** | **34** | **4** | **R.IKVVATGLCGTEMK.V** |
|  | 17192 | **893.9298** | **1785.8449** | **1785.8567** | **-0.0117** | **0** | **73** | **5.5e-006** | **1** | **K.AQELGATECLNPQDLK.K** |
|  | 20676 | 1036.9911 | 2071.9676 | 2073.0024 | -1.0347 | 0 | 6 | 21 | 7 | K.VCLISCGFSTGFGAAINTAK.V 20688 20689 |
|  | 21543 | **723.3403** | **2166.9992** | **2166.0092** | **0.9900** | **0** | **18** | **1.2** | **1** | **K.SIYHFGNTSTFCEYTVIK.E** 21536 |
|  | 23048 | **1175.1381** | **2348.2616** | **2348.2780** | **-0.0164** | **0** | **57** | **0.00015** | **1** | **K.AAILWKPGAPFSIEEVEVAPPK.A** 23047 23050 |
|  | 23081 | 1177.0835 | 2352.1524 | 2352.1641 | -0.0116 | 0 | 75 | 2.5e-006 | 1 | K.VTPGSTCAVFGLGGVGLSVVMGCK.A 23080 23082 |
|  | 25586 | **1374.2480** | **2746.4815** | **2746.4939** | **-0.0123** | **1** | **55** | **0.00019** | **1** | **K.LNLDPLITHTLNLDKINEAVELMK.T** 25585 25587 25588 25589 |
|  | 28564 | **1134.5958** | **3400.7657** | **3400.7878** | **-0.0222** | **0** | **81** | **3.1e-007** | **1** | **K.HLDLLYPTILGHEGAGIVESIGEGVSTVKPGDK.V** 28565 28566 |

| **111.** | P06733\|ENOA_HUMAN    **Mass:** 47481    **Score:** 452    **Queries matched:** 32   **emPAI:** 0.58 |
| --- | --- |
|  | Alpha-enolase OS=Homo sapiens GN=ENO1 PE=1 SV=2 |

|  | **Query** | **Observed** | **Mr(expt)** | **Mr(calc)** | **Delta** | **Miss** | **Score** | **Expect** | **Rank** | **Peptide** |
| --- | --- | --- | --- | --- | --- | --- | --- | --- | --- | --- |
|  | 2031 | **403.2131** | **804.4117** | **805.4446** | **-1.0329** | **0** | **14** | **8.6** | **2** | **K.YNQLLR.I** |
|  | 3084 | **443.0388** | **884.0631** | **884.4426** | **-0.3795** | **1** | **4** | **41** | **9** | **R.YMGKGVSK.A** 3097 |
|  | 4751 | **504.2510** | **1006.4874** | **1006.4940** | **-0.0066** | **0** | **11** | **12** | **2** | **K.SCNCLLLK.V** |
|  | 5088 | **517.2529** | **1032.4913** | **1032.4771** | **0.0142** | **1** | **7** | **38** | **8** | **K.TGAPCRSER.L** |
|  | 5787 | **544.7971** | **1087.5797** | **1087.5873** | **-0.0076** | **1** | **26** | **0.5** | **1** | **K.KLNVTEQEK.I** 5765 |
|  | 9241 | **432.7251** | **1295.1534** | **1295.5737** | **-0.4203** | **0** | **4** | **32** | **7** | **K.LMIEMDGTENK.S** |
|  | 9278 | **649.3389** | **1296.6633** | **1295.5737** | **1.0896** | **0** | **(3)** | **66** | **2** | **K.LMIEMDGTENK.S** |
|  | 11628 | **713.8634** | **1425.7122** | **1424.7187** | **0.9935** | **0** | **48** | **0.0024** | **1** | **R.YISPDQLADLYK.S** |
|  | 13248 | **509.6260** | **1525.8562** | **1526.6956** | **-0.8394** | **1** | **8** | **18** | **5** | **K.LMIEMDGTENKSK.F** |
|  | 13429 | 513.9262 | 1538.7568 | 1539.7756 | -1.0188 | 0 | (1) | 1.1e+002 | 9 | K.VVIGMDVAASEFFR.S |
|  | 13693 | **519.2545** | **1554.7416** | **1555.7705** | **-1.0289** | **0** | **12** | **6.9** | **1** | **K.VVIGMDVAASEFFR.S** 13725 |
|  | 14931 | 546.6201 | 1636.8384 | 1635.7848 | 1.0536 | 1 | (5) | 39 | 5 | K.IDKLMIEMDGTENK.S |
|  | 15160 | **551.2899** | **1650.8478** | **1651.7797** | **-0.9319** | **1** | **5** | **36** | **10** | **K.IDKLMIEMDGTENK.S** |
|  | 15398 | **556.7568** | **1667.2487** | **1667.7746** | **-0.5259** | **1** | **(1)** | **63** | **6** | **K.IDKLMIEMDGTENK.S** |
|  | 17411 | **902.9698** | **1803.9250** | **1803.9366** | **-0.0116** | **0** | **109** | **1.3e-009** | **1** | **R.AAVPSGASTGIYEALELR.D** 17410 17412 |
|  | 18760 | **954.4908** | **1906.9670** | **1906.9797** | **-0.0127** | **0** | **32** | **0.061** | **1** | **K.LAMQEFMILPVGAANFR.E** |
|  | 19364 | **980.9598** | **1959.9050** | **1959.9174** | **-0.0124** | **0** | **98** | **1.5e-008** | **1** | **K.DATNVGDEGGFAPNILENK.E** 19365 |
|  | 22474 | 760.3239 | 2277.9499 | 2277.2117 | 0.7382 | 1 | 5 | 24 | 5 | K.GLFRAAVPSGASTGIYEALELR.D |
|  | 24267 | **1255.5525** | **2509.0904** | **2509.1074** | **-0.0170** | **0** | **58** | **9.3e-005** | **1** | **K.DYPVVSIEDPFDQDDWGAWQK.F** 24268 |
|  | 25574 | **915.1251** | **2742.3534** | **2742.3712** | **-0.0178** | **1** | **50** | **0.00057** | **1** | **K.DATNVGDEGGFAPNILENKEGLELLK.T** 25572 25573 |
|  | 27094 | **1004.8491** | **3011.5255** | **3010.5625** | **0.9631** | **0** | **17** | **1** | **1** | **R.HIADLAGNSEVILPVPAFNVINGGSHAGNK.L** |
|  | 27745 | **1053.0476** | **3156.1210** | **3155.5604** | **0.5606** | **1** | **2** | **24** | **3** | **K.VNQIGSVTESLQACKLAQANGWGVMVSHR.S** |
|  | 29857 | **969.9627** | **3875.8217** | **3874.8982** | **0.9235** | **1** | **2** | **20** | **1** | **K.LAQANGWGVMVSHRSGETEDTFIADLVVGLCTGQIK.T** |

| **112.** | P0C0L4\|CO4A_HUMAN    **Mass:** 194247   **Score:** 450    **Queries matched:** 46   **emPAI:** 0.23 |
| --- | --- |
|  | Complement C4-A OS=Homo sapiens GN=C4A PE=1 SV=1 |

|  | **Query** | **Observed** | **Mr(expt)** | **Mr(calc)** | **Delta** | **Miss** | **Score** | **Expect** | **Rank** | **Peptide** |
| --- | --- | --- | --- | --- | --- | --- | --- | --- | --- | --- |
|  | 2215 | **821.3732** | **820.3660** | **819.4491** | **0.9169** | **0** | **16** | **4.6** | **1** | **R.VFALDQK.M** |
|  | 2932 | **437.7217** | **873.4287** | **873.4854** | **-0.0567** | **1** | **6** | **53** | **4** | **R.LPMSVRR.F** |
|  | 4146 | **483.2628** | **964.5110** | **963.4960** | **1.0150** | **0** | **14** | **7.2** | **2** | **R.GQIVFMNR.E** |
|  | 4301 | 488.7627 | 975.5108 | 974.4669 | 1.0439 | 0 | 16 | 4.5 | 2 | K.VVEEQESR.V |
|  | 4385 | **492.2160** | **982.4174** | **982.4621** | **-0.0447** | **0** | **14** | **5.9** | **3** | **R.QGSFQGGFR.S** 4378 |
|  | 5173 | **520.2812** | **1038.5478** | **1037.5506** | **0.9973** | **0** | **28** | **0.23** | **1** | **K.DHAVDLIQK.G** |
|  | 5329 | **526.7801** | **1051.5456** | **1051.5523** | **-0.0066** | **0** | **23** | **0.78** | **1** | **K.SHALQLNNR.Q** 5345 |
|  | 5342 | **527.2899** | **1052.5652** | **1051.4570** | **1.1081** | **0** | **1** | **1.2e+002** | **7** | **R.GLQDEDGYR.M** |
|  | 6221 | **561.2913** | **1120.5680** | **1120.5764** | **-0.0085** | **1** | **8** | **28** | **8** | **R.FGLLDEDGKK.T** |
|  | 7676 | **405.2987** | **1212.8742** | **1212.6649** | **0.2093** | **0** | **7** | **20** | **3** | **K.GLCVATPVQLR.V** |
|  | 9777 | **441.9037** | **1322.6892** | **1322.5893** | **0.0999** | **1** | **15** | **4.2** | **1** | **R.LPMMRSCEQR.A** 9764 |
|  | 10591 | 684.3566 | 1366.6986 | 1366.7279 | -0.0293 | 1 | 4 | 53 | 4 | K.VLRTYNVLDMK.N |
|  | 11038 | 464.9009 | 1391.6810 | 1391.7885 | -0.1075 | 1 | 7 | 24 | 5 | K.VLQIEKEGAIHR.E 11026 |
|  | 12248 | **489.2749** | **1464.8028** | **1464.6667** | **0.1361** | **0** | **5** | **36** | **7** | **K.AEMADQASAWLTR.Q** |
|  | 13459 | **771.4065** | **1540.7984** | **1540.8097** | **-0.0112** | **0** | **16** | **3.1** | **1** | **K.VLSLAQEQVGGSPEK.L** |
|  | 13822 | 782.9002 | 1563.7859 | 1562.8416 | 0.9442 | 0 | 10 | 13 | 2 | R.TTNIQGINLLFSSR.R |
|  | 15215 | **552.2703** | **1653.7890** | **1654.7048** | **-0.9159** | **1** | **1** | **95** | **8** | **R.CCQDGVTRLPMMR.S** |
|  | 16909 | **589.3287** | **1764.9642** | **1763.9207** | **1.0435** | **1** | **5** | **33** | **1** | **K.VVARGSFEFPVGDAVSK.V** 16900 |
|  | 16999 | **592.2828** | **1773.8267** | **1773.8057** | **0.0210** | **0** | **2** | **73** | **6** | **R.FSDGLESNSSTQFEVK.K** |
|  | 21171 | **709.6811** | **2126.0214** | **2127.1510** | **-1.1296** | **0** | **1** | **63** | **7** | **K.VGLSGMAIADVTLLSGFHALR.A** |
|  | 21307 | **714.3697** | **2140.0874** | **2139.0974** | **0.9901** | **1** | **38** | **0.013** | **1** | **R.RGHLFLQTDQPIYNPGQR.V** |
|  | 22071 | **1113.0557** | **2224.0968** | **2224.1110** | **-0.0143** | **0** | **42** | **0.005** | **1** | **R.ALEILQEEDLIDEDDIPVR.S** 22070 |
|  | 22608 | **764.3759** | **2290.1057** | **2289.1501** | **0.9556** | **1** | **6** | **20** | **2** | **K.EGAIHREELVYELNPLDHR.G** |
|  | 23627 | **810.0364** | **2427.0875** | **2426.3104** | **0.7771** | **1** | **1** | **59** | **1** | **R.NGKVGLSGMAIADVTLLSGFHALR.A** |
|  | 24069 | **1242.1449** | **2482.2752** | **2482.2915** | **-0.0163** | **0** | **126** | **2e-011** | **1** | **R.VTASDPLDTLGSEGALSPGGVASLLR.L** 24070 |
|  | 24896 | **871.8057** | **2612.3952** | **2612.4061** | **-0.0109** | **0** | **84** | **2.5e-007** | **1** | **K.LHLETDSLALVALGALDTALYAAGSK.S** |
|  | 25216 | **894.0998** | **2679.2777** | **2679.2969** | **-0.0192** | **0** | **30** | **0.062** | **1** | **R.YVSHFETEGPHVLLYFDSVPTSR.E** |
|  | 25318 | **900.9308** | **2699.7707** | **2699.4316** | **0.3391** | **1** | **2** | **24** | **1** | **K.VGLSGMAIADVTLLSGFHALRADLEK.L** |
|  | 25699 | **923.8683** | **2768.5830** | **2768.5953** | **-0.0123** | **0** | **49** | **0.0007** | **1** | **R.LLLFSPSVVHLGVPLSVGVQLQDVPR.G** |
|  | 25940 | **702.1251** | **2804.4714** | **2804.4895** | **-0.0181** | **0** | **9** | **7.9** | **1** | **K.ITPGKPYILTVPGHLDEMQLDIQAR.Y** |
|  | 26462 | **1450.6792** | **2899.3438** | **2899.3626** | **-0.0188** | **0** | **37** | **0.0099** | **1** | **K.EVYMPSSIFQDDFVIPDISEPGTWK.I** |
|  | 27656 | **1044.7860** | **3131.3362** | **3131.4618** | **-0.1256** | **0** | **1** | **33** | **1** | **K.LQETSNWLLSQQQADGSFQDPCPVLDR.S** |
|  | 27668 | **1045.5270** | **3133.5591** | **3133.5655** | **-0.0064** | **1** | **1** | **41** | **5** | **R.VGDTLNLNLRAVGSGATFSHYYYMILSR.G** |
|  | 28300 | **832.4323** | **3325.7001** | **3325.7169** | **-0.0167** | **0** | **23** | **0.21** | **1** | **R.NPSDPMPQAPALWIETTAYALLHLLLHEGK.A** |
|  | 28819 | **875.2194** | **3496.8486** | **3496.8606** | **-0.0120** | **1** | **2** | **23** | **6** | **R.VETVDRFQILTLWLPDSLTTWEIHGLSLSK.T** |
|  | 29197 | **1219.5873** | **3655.7400** | **3655.7617** | **-0.0217** | **1** | **105** | **1e-009** | **1** | **R.ECVGFEAVQEVPVGLVQPASATLYDYYNPERR.C** |
|  | 30140 | **1009.7629** | **4035.0227** | **4035.0412** | **-0.0185** | **0** | **16** | **0.77** | **1** | **R.SMQGGLVGNDETVALTAFVTIALHHGLAVFQDEGAEPLK.Q** |
|  | 30695 | **1437.7053** | **4310.0941** | **4309.9321** | **0.1620** | **0** | **4** | **9.8** | **1** | **K.VFEAMNSYDLGCGPGGGDSALQVFQAAGLAFSDGDQWTLSR.K** |
|  | 31327 | **1254.9014** | **5015.5764** | **5014.4506** | **1.1258** | **1** | **0** | **14** | **7** | **R.TLEIPGNSDPNMIPDGDFNSYVRVTASDPLDTLGSEGALSPGGVASLLR.L** |

|  | **Proteins matching a subset of these peptides:** |
| --- | --- |

|  | P0C0L5\|CO4B_HUMAN    **Mass:** 194212   **Score:** 450    **Queries matched:** 45 |
| --- | --- |
|  | Complement C4-B OS=Homo sapiens GN=C4B PE=1 SV=1 |

| **113.** | P78329\|CP4F2_HUMAN    **Mass:** 60442    **Score:** 450    **Queries matched:** 35   **emPAI:** 0.82 |
| --- | --- |
|  | Leukotriene-B(4) omega-hydroxylase 1 OS=Homo sapiens GN=CYP4F2 PE=1 SV=1 |

|  | **Query** | **Observed** | **Mr(expt)** | **Mr(calc)** | **Delta** | **Miss** | **Score** | **Expect** | **Rank** | **Peptide** |
| --- | --- | --- | --- | --- | --- | --- | --- | --- | --- | --- |
|  | 2552 | **424.2082** | **846.4018** | **846.4083** | **-0.0065** | **0** | **12** | **15** | **1** | **K.LSDEDIR.A** |
|  | 2628 | **427.7369** | **853.4593** | **853.5385** | **-0.0792** | **0** | **5** | **49** | **1** | **R.KPELVLR.A** |
|  | 3271 | **451.2620** | **900.5095** | **900.4817** | **0.0278** | **0** | **6** | **45** | **6** | **R.AEGGLWLR.V** |
|  | 4066 | **480.2767** | **958.5388** | **957.4304** | **1.1084** | **0** | **9** | **26** | **8** | **K.HPEYQER.C** |
|  | 4431 | **493.7759** | **985.5372** | **985.5444** | **-0.0072** | **0** | **32** | **0.11** | **1** | **R.QEVQELLK.D** |
|  | 4799 | **505.8239** | **1009.6333** | **1009.6396** | **-0.0063** | **1** | **12** | **8.4** | **1** | **R.RKPELVLR.A** |
|  | 9377 | **651.8411** | **1301.6676** | **1301.6761** | **-0.0086** | **1** | **34** | **0.061** | **1** | **R.CRQEVQELLK.D** |
|  | 10264 | **450.5739** | **1348.7000** | **1348.7099** | **-0.0099** | **0** | **20** | **1.5** | **1** | **R.HVTQDIVLPDGR.V** |
|  | 10440 | **680.3709** | **1358.7271** | **1358.7347** | **-0.0075** | **0** | **36** | **0.033** | **1** | **R.SPLAFIPFSAGPR.N** |
|  | 10747 | **688.9023** | **1375.7901** | **1375.7963** | **-0.0061** | **0** | **78** | **2.3e-006** | **1** | **K.TLDFIDVLLLSK.D** |
|  | 11469 | 473.2546 | 1416.7420 | 1417.7024 | -0.9604 | 0 | 5 | 47 | 10 | K.IFNESVNIMHAK.W |
|  | 12828 | **750.8306** | **1499.6467** | **1499.6571** | **-0.0104** | **0** | **62** | **8.6e-005** | **1** | **R.NCIGQTFAMAEMK.V** |
|  | 13091 | **506.2715** | **1515.7926** | **1515.6520** | **0.1406** | **0** | **(8)** | **20** | **3** | **R.NCIGQTFAMAEMK.V** |
|  | 13117 | **759.8887** | **1517.7628** | **1517.7726** | **-0.0098** | **0** | **52** | **0.00085** | **1** | **R.TLPSQGVDDFLQAK.A** 13115 |
|  | 13479 | 771.8937 | 1541.7728 | 1541.7838 | -0.0110 | 0 | 62 | 8.4e-005 | 1 | R.LVHDFTDAVIQER.R 13480 |
|  | 13827 | **782.9360** | **1563.8575** | **1563.8661** | **-0.0086** | **0** | **70** | **1.3e-005** | **1** | **R.VLTQLVATYPQGFK.V** 13803 13825 13840 13841 |
|  | 14261 | 796.8909 | 1591.7672 | 1590.9232 | 0.8439 | 1 | 2 | 76 | 10 | K.SKTLDFIDVLLLSK.D |
|  | 18877 | **960.4878** | **1918.9610** | **1920.0092** | **-1.0481** | **1** | **11** | **7.8** | **1** | **K.TLDFIDVLLLSKDEDGK.K** |
|  | 19253 | **651.0122** | **1950.0148** | **1950.0259** | **-0.0111** | **0** | **15** | **3.3** | **2** | **R.MLTPAFHFNILKPYMK.I** |
|  | 21603 | **1088.4716** | **2174.9286** | **2174.9415** | **-0.0129** | **0** | **70** | **7.9e-006** | **1** | **R.NWFWGHQGMVNPTEEGMR.V** 21602 |
|  | 22282 | **1125.0194** | **2248.0243** | **2248.0367** | **-0.0124** | **0** | **8** | **13** | **1** | **K.EIEWDDLAHLPFLTMCMK.E** |
|  | 23176 | 788.7626 | 2363.2661 | 2363.0324 | 0.2337 | 1 | 1 | 53 | 8 | R.RNWFWGHQGMVNPTEEGMR.V |
|  | 24921 | **873.4406** | **2617.2998** | **2617.3104** | **-0.0106** | **1** | **11** | **5.7** | **1** | **K.DKFFYSFLEPWLGDGLLLSAGDK.W** |
|  | 27043 | **999.8007** | **2996.3802** | **2996.4015** | **-0.0213** | **0** | **81** | **4.6e-007** | **1** | **R.AEADTFMFEGHDTTASGLSWVLYHLAK.H** 27044 |
|  | 27983 | **1075.5250** | **3223.5532** | **3222.5399** | **1.0133** | **0** | **36** | **0.01** | **1** | **K.WQLLASEGSACLDMFEHISLMTLDSLQK.C** |
|  | 29470 | **1883.6089** | **3765.2032** | **3765.0150** | **0.1882** | **1** | **0** | **25** | **3** | **R.VLTQLVATYPQGFKVWMGPISPLLSLCHPDIIR.S** |
|  | 29726 | **957.2018** | **3824.7783** | **3824.7992** | **-0.0209** | **1** | **65** | **1e-005** | **1** | **K.LSDEDIRAEADTFMFEGHDTTASGLSWVLYHLAK.H** |

| **114.** | P12236\|ADT3_HUMAN    **Mass:** 33073    **Score:** 450    **Queries matched:** 37   **emPAI:** 1.37 |
| --- | --- |
|  | ADP/ATP translocase 3 OS=Homo sapiens GN=SLC25A6 PE=1 SV=4 |

|  | **Query** | **Observed** | **Mr(expt)** | **Mr(calc)** | **Delta** | **Miss** | **Score** | **Expect** | **Rank** | **Peptide** |
| --- | --- | --- | --- | --- | --- | --- | --- | --- | --- | --- |
|  | 1501 | **673.3835** | **672.3762** | **672.3806** | **-0.0044** | **0** | **28** | **0.4** | **1** | **R.LAADVGK.S** |
|  | 2480 | **421.0564** | **840.0982** | **839.3452** | **0.7530** | **0** | **(8)** | **15** | **3** | **R.MMMQSGR.K** |
|  | 2649 | **428.7446** | **855.4747** | **855.4814** | **-0.0067** | **0** | **24** | **0.66** | **1** | **K.TAVAPIER.V** |
|  | 2661 | **429.0876** | **856.1606** | **855.3401** | **0.8205** | **0** | **17** | **2.3** | **1** | **R.MMMQSGR.K** |
|  | 2669 | 429.2404 | 856.4663 | 855.4926 | 0.9737 | 0 | 12 | 13 | 4 | R.GNLANVIR.Y |
|  | 2725 | **431.2256** | **860.4367** | **860.4426** | **-0.0058** | **0** | **38** | **0.03** | **1** | **R.GLGDCLVK.I** |
|  | 2939 | **437.7781** | **873.5417** | **873.4246** | **0.1171** | **0** | **6** | **49** | **9** | **K.HTQFWR.Y** |
|  | 3294 | **902.4784** | **901.4712** | **901.4770** | **-0.0058** | **0** | **20** | **2.3** | **1** | **K.GAWSNVLR.G** |
|  | 4410 | 493.2287 | 984.4428 | 983.4351 | 1.0078 | 1 | (2) | 1.1e+002 | 9 | R.MMMQSGRK.G |
|  | 4551 | **498.2523** | **994.4901** | **995.4463** | **-0.9562** | **1** | **8** | **24** | **5** | **R.RMMMQSGR.K** |
|  | 4895 | **509.2857** | **1016.5569** | **1015.4249** | **1.1320** | **1** | **6** | **47** | **4** | **R.MMMQSGRK.G** |
|  | 5031 | **515.2455** | **1028.4765** | **1027.4361** | **1.0404** | **1** | **(4)** | **63** | **5** | **R.RMMMQSGR.K** |
|  | 5718 | **542.3104** | **1082.6063** | **1082.6448** | **-0.0385** | **1** | **8** | **27** | **2** | **K.TAVAPIERVK.L** 5713 5715 |
|  | 6219 | **561.2871** | **1120.5597** | **1120.5665** | **-0.0069** | **0** | **30** | **0.17** | **1** | **K.EQGVLSFWR.G** |
|  | 6397 | **568.3104** | **1134.6063** | **1135.6713** | **-1.0650** | **0** | **32** | **0.096** | **1** | **K.LLLQVQHASK.Q** 6410 |
|  | 7500 | **402.2434** | **1203.7084** | **1202.6255** | **1.0829** | **1** | **10** | **16** | **4** | **R.LAADVGKSGTER.E** |
|  | 8072 | **411.9008** | **1232.6805** | **1232.6765** | **0.0040** | **0** | **9** | **19** | **5** | **K.DFLAGGIAAAISK.T** |
|  | 10478 | **454.8929** | **1361.6569** | **1362.8347** | **-1.1778** | **1** | **7** | **25** | **2** | **R.VKLLLQVQHASK.Q** 10504 |
|  | 11941 | **723.8702** | **1445.7259** | **1445.7343** | **-0.0084** | **0** | **56** | **0.00036** | **1** | **R.YFPTQALNFAFK.D** 11955 |
|  | 15042 | **822.8551** | **1643.6956** | **1643.7072** | **-0.0116** | **0** | **45** | **0.0032** | **1** | **K.GADIMYTGTVDCWR.K** |
|  | 16970 | **886.9021** | **1771.7896** | **1771.8022** | **-0.0125** | **1** | **81** | **7.9e-007** | **1** | **R.KGADIMYTGTVDCWR.K** 16971 |
|  | 19334 | **653.3267** | **1956.9582** | **1958.0486** | **-1.0905** | **1** | **1** | **75** | **6** | **K.EQGVLSFWRGNLANVIR.Y** |
|  | 25886 | **1398.6688** | **2795.3231** | **2795.3377** | **-0.0147** | **0** | **98** | **1.1e-008** | **1** | **R.YFAGNLASGGAAGATSLCFVYPLDFAR.T** 25877 25883 25884 25885 25887 25888 |
|  | 28271 | **830.4323** | **3317.7001** | **3316.7391** | **0.9610** | **1** | **(0)** | **42** | **6** | **K.NTHIVVSWMIAQTVTAVAGVVSYPFDTVRR.R** |
|  | 28339 | **834.3652** | **3333.4318** | **3332.7340** | **0.6978** | **1** | **1** | **29** | **10** | **K.NTHIVVSWMIAQTVTAVAGVVSYPFDTVRR.R** |

| **115.** | P05141\|ADT2_HUMAN    **Mass:** 33102    **Score:** 448    **Queries matched:** 43   **emPAI:** 1.37 |
| --- | --- |
|  | ADP/ATP translocase 2 OS=Homo sapiens GN=SLC25A5 PE=1 SV=6 |

|  | **Query** | **Observed** | **Mr(expt)** | **Mr(calc)** | **Delta** | **Miss** | **Score** | **Expect** | **Rank** | **Peptide** |
| --- | --- | --- | --- | --- | --- | --- | --- | --- | --- | --- |
|  | 1501 | 673.3835 | 672.3762 | 672.3806 | -0.0044 | 0 | 28 | 0.4 | 1 | R.LAADVGK.A |
|  | 2480 | 421.0564 | 840.0982 | 839.3452 | 0.7530 | 0 | (8) | 15 | 3 | R.MMMQSGR.K |
|  | 2649 | 428.7446 | 855.4747 | 855.4814 | -0.0067 | 0 | 24 | 0.66 | 1 | K.TAVAPIER.V |
|  | 2661 | 429.0876 | 856.1606 | 855.3401 | 0.8205 | 0 | 17 | 2.3 | 1 | R.MMMQSGR.K |
|  | 2669 | 429.2404 | 856.4663 | 855.4926 | 0.9737 | 0 | 12 | 13 | 4 | R.GNLANVIR.Y |
|  | 2725 | 431.2256 | 860.4367 | 860.4426 | -0.0058 | 0 | 38 | 0.03 | 1 | R.GLGDCLVK.I |
|  | 3294 | 902.4784 | 901.4712 | 901.4770 | -0.0058 | 0 | 20 | 2.3 | 1 | K.GAWSNVLR.G |
|  | 3570 | 462.2598 | 922.5051 | 922.5124 | -0.0073 | 1 | 30 | 0.15 | 1 | K.IYKSDGIK.G |
|  | 3676 | **466.2520** | **930.4894** | **930.4957** | **-0.0063** | **0** | **20** | **1.8** | **2** | **K.GIIDCVVR.I** |
|  | 3738 | 468.5695 | 935.1245 | 934.4621 | 0.6625 | 1 | 9 | 15 | 2 | K.AGAEREFR.G 3742 |
|  | 4410 | 493.2287 | 984.4428 | 983.4351 | 1.0078 | 1 | (2) | 1.1e+002 | 9 | R.MMMQSGRK.G |
|  | 4551 | 498.2523 | 994.4901 | 995.4463 | -0.9562 | 1 | 8 | 24 | 5 | R.RMMMQSGR.K |
|  | 4895 | 509.2857 | 1016.5569 | 1015.4249 | 1.1320 | 1 | 6 | 47 | 4 | R.MMMQSGRK.G |
|  | 5031 | 515.2455 | 1028.4765 | 1027.4361 | 1.0404 | 1 | (4) | 63 | 5 | R.RMMMQSGR.K |
|  | 5718 | 542.3104 | 1082.6063 | 1082.6448 | -0.0385 | 1 | 8 | 27 | 2 | K.TAVAPIERVK.L 5713 5715 |
|  | 5874 | **548.2782** | **1094.5418** | **1093.5768** | **0.9651** | **1** | **11** | **11** | **1** | **K.QITADKQYK.G** |
|  | 6219 | 561.2871 | 1120.5597 | 1120.5665 | -0.0069 | 0 | 30 | 0.17 | 1 | K.EQGVLSFWR.G |
|  | 6362 | **566.8232** | **1131.6319** | **1131.6400** | **-0.0081** | **1** | **27** | **0.32** | **1** | **K.QIFLGGVDKR.T** |
|  | 6397 | 568.3104 | 1134.6063 | 1135.6713 | -1.0650 | 0 | 32 | 0.096 | 1 | K.LLLQVQHASK.Q 6410 |
|  | 7805 | 610.3339 | 1218.6532 | 1218.6608 | -0.0077 | 0 | 59 | 0.00018 | 1 | K.DFLAGGVAAAISK.T 7804 7806 7817 |
|  | 10278 | **450.7683** | **1349.2829** | **1349.7126** | **-0.4296** | **1** | **8** | **15** | **3** | **K.QYKGIIDCVVR.I** |
|  | 10478 | 454.8929 | 1361.6569 | 1362.8347 | -1.1778 | 1 | 7 | 25 | 2 | R.VKLLLQVQHASK.Q 10504 |
|  | 11941 | 723.8702 | 1445.7259 | 1445.7343 | -0.0084 | 0 | 56 | 0.00036 | 1 | R.YFPTQALNFAFK.D 11955 |
|  | 17534 | **908.4503** | **1814.8861** | **1815.8284** | **-0.9423** | **1** | **54** | **0.00041** | **1** | **R.KGTDIMYTGTLDCWR.K** 17564 |
|  | 18943 | 963.4647 | 1924.9149 | 1926.0363 | -1.1214 | 0 | 4 | 42 | 2 | K.GLYQGFNVSVQGIIIYR.A |
|  | 19334 | 653.3267 | 1956.9582 | 1958.0486 | -1.0905 | 1 | 1 | 75 | 6 | K.EQGVLSFWRGNLANVIR.Y |
|  | 25886 | 1398.6688 | 2795.3231 | 2795.3377 | -0.0147 | 0 | 98 | 1.1e-008 | 1 | R.YFAGNLASGGAAGATSLCFVYPLDFAR.T 25877 25883 25884 25885 25887 25888 |

| **116.** | Q99623\|PHB2_HUMAN    **Mass:** 33276    **Score:** 447    **Queries matched:** 30   **emPAI:** 1.92 |
| --- | --- |
|  | Prohibitin-2 OS=Homo sapiens GN=PHB2 PE=1 SV=2 |

|  | **Query** | **Observed** | **Mr(expt)** | **Mr(calc)** | **Delta** | **Miss** | **Score** | **Expect** | **Rank** | **Peptide** |
| --- | --- | --- | --- | --- | --- | --- | --- | --- | --- | --- |
|  | 988 | **530.2563** | **529.2490** | **530.2813** | **-1.0322** | **0** | **11** | **25** | **3** | **K.DLAGR.L** |
|  | 1516 | **677.3592** | **676.3520** | **676.3578** | **-0.0058** | **0** | **9** | **26** | **8** | **R.GMGTALK.L** 1515 |
|  | 1630 | **719.3702** | **718.3629** | **718.3861** | **-0.0232** | **0** | **24** | **0.82** | **3** | **R.GSDSLIK.G** |
|  | 1761 | **777.0341** | **776.0268** | **775.4076** | **0.6192** | **0** | **1** | **1.1e+002** | **7** | **K.ISSPTGSK.D** |
|  | 2561 | **848.4482** | **847.4409** | **847.4473** | **-0.0064** | **0** | **23** | **0.95** | **1** | **K.MLGEALSK.N** 2562 |
|  | 3341 | **453.2574** | **904.5003** | **903.5025** | **0.9978** | **1** | **19** | **2.3** | **2** | **R.KISSPTGSK.D** |
|  | 5379 | **529.2699** | **1056.5252** | **1056.5312** | **-0.0060** | **0** | **12** | **12** | **4** | **K.QVAQQEAQR.A** |
|  | 5762 | **543.8081** | **1085.6017** | **1084.5989** | **1.0028** | **1** | **0** | **1.7e+002** | **5** | **M.AQNLKDLAGR.L** |
|  | 6944 | **589.3166** | **1176.6186** | **1176.6251** | **-0.0065** | **0** | **30** | **0.17** | **1** | **K.FNASQLITQR.A** |
|  | 7135 | **594.8203** | **1187.6261** | **1187.6332** | **-0.0072** | **0** | **57** | **0.00033** | **1** | **K.DLQMVNISLR.V** 7136 |
|  | 7497 | **402.2431** | **1203.7074** | **1203.6281** | **0.0792** | **0** | **(12)** | **11** | **2** | **K.DLQMVNISLR.V** 7517 |
|  | 7701 | **405.8887** | **1214.6443** | **1215.6394** | **-0.9951** | **1** | **9** | **18** | **5** | **-.MAQNLKDLAGR.L** |
|  | 7744 | **609.2946** | **1216.5747** | **1216.5837** | **-0.0090** | **0** | **45** | **0.0047** | **1** | **R.ESVFTVEGGHR.A** |
|  | 8588 | **630.3729** | **1258.7312** | **1258.7397** | **-0.0086** | **0** | **82** | **8.3e-007** | **1** | **K.LLLGAGAVAYGVR.E** 8602 |
|  | 9036 | **643.2828** | **1284.5511** | **1283.7020** | **0.8491** | **1** | **2** | **97** | **6** | **R.LPAGPRGMGTALK.L** |
|  | 16283 | **862.4588** | **1722.9030** | **1722.9133** | **-0.0103** | **0** | **45** | **0.0033** | **1** | **R.IPWFQYPIIYDIR.A** 16282 |
|  | 18033 | **927.4915** | **1852.9685** | **1852.9796** | **-0.0111** | **0** | **74** | **3.9e-006** | **1** | **R.IGGVQQDTILAEGLHFR.I** 18032 18045 |
|  | 18500 | **944.9822** | **1887.9498** | **1887.9625** | **-0.0127** | **0** | **50** | **0.001** | **1** | **R.VLSRPNAQELPSMYQR.L** 18499 |
|  | 19181 | **649.0352** | **1944.0838** | **1945.0302** | **-0.9464** | **1** | **1** | **75** | **4** | **K.ISSPTGSKDLQMVNISLR.V** |
|  | 22080 | **1113.0696** | **2224.1246** | **2224.1375** | **-0.0129** | **0** | **107** | **1.8e-009** | **1** | **R.IYLTADNLVLNLQDESFTR.G** 22079 |

| **117.** | P00167\|CYB5_HUMAN    **Mass:** 15321    **Score:** 447    **Queries matched:** 37   **emPAI:** 8.66 |
| --- | --- |
|  | Cytochrome b5 OS=Homo sapiens GN=CYB5A PE=1 SV=2 |

|  | **Query** | **Observed** | **Mr(expt)** | **Mr(calc)** | **Delta** | **Miss** | **Score** | **Expect** | **Rank** | **Peptide** |
| --- | --- | --- | --- | --- | --- | --- | --- | --- | --- | --- |
|  | 1680 | **740.4246** | **739.4173** | **740.3051** | **-0.8877** | **0** | **9** | **17** | **5** | **R.LYMAED.-** |
|  | 6048 | **554.2637** | **1106.5128** | **1106.4914** | **0.0214** | **0** | **14** | **6.3** | **5** | **-.MAEQSDEAVK.Y** |
|  | 6256 | **562.8031** | **1123.5916** | **1122.4863** | **1.1054** | **0** | **(7)** | **28** | **9** | **-.MAEQSDEAVK.Y** |
|  | 6388 | **567.8209** | **1133.6272** | **1133.6346** | **-0.0074** | **0** | **31** | **0.14** | **1** | **K.STWLILHHK.V** 6367 6387 |
|  | 7088 | **593.7990** | **1185.5835** | **1185.5917** | **-0.0083** | **0** | **29** | **0.2** | **1** | **K.YYTLEEIQK.H** 7072 |
|  | 12994 | **756.3727** | **1510.7309** | **1510.7416** | **-0.0107** | **0** | **50** | **0.0011** | **1** | **K.FLEEHPGGEEVLR.E** 12992 12993 12995 12996 |
|  | 14935 | **819.4305** | **1636.8464** | **1636.8573** | **-0.0109** | **0** | **50** | **0.0013** | **1** | **K.TFIIGELHPDDRPK.L** 14933 14936 14937 14938 |
|  | 16508 | 869.4434 | 1736.8723 | 1736.9223 | -0.0500 | 1 | 2 | 66 | 7 | K.HNHSKSTWLILHHK.V 16492 |
|  | 21865 | **1103.4597** | **2204.9049** | **2204.9207** | **-0.0158** | **0** | **104** | **2.8e-009** | **1** | **R.EQAGGDATENFEDVGHSTDAR.E** 21863 21864 21876 |
|  | 29275 | **1233.5482** | **3697.6228** | **3697.6517** | **-0.0289** | **1** | **60** | **3e-005** | **1** | **K.FLEEHPGGEEVLREQAGGDATENFEDVGHSTDAR.E** 29276 29277 29278 29279 29280 29281 29282 29283 29284 |
|  | 30372 | **1377.7206** | **4130.1399** | **4130.1550** | **-0.0151** | **0** | **58** | **4.5e-005** | **1** | **K.LNKPPETLITTIDSSSSWWTNWVIPAISAVAVALMYR.L** 30373 30382 |

| **118.** | P16662\|UD2B7_HUMAN    **Mass:** 61168    **Score:** 446    **Queries matched:** 38   **emPAI:** 1.15 |
| --- | --- |
|  | UDP-glucuronosyltransferase 2B7 OS=Homo sapiens GN=UGT2B7 PE=1 SV=1 |

|  | **Query** | **Observed** | **Mr(expt)** | **Mr(calc)** | **Delta** | **Miss** | **Score** | **Expect** | **Rank** | **Peptide** |
| --- | --- | --- | --- | --- | --- | --- | --- | --- | --- | --- |
|  | 761 | **474.2994** | **473.2922** | **472.2758** | **1.0164** | **0** | **4** | **1.4e+002** | **7** | **R.GAAVR.V** |
|  | 2911 | 436.7503 | 871.4860 | 871.4916 | -0.0056 | 0 | 60 | 0.00019 | 1 | K.ADVWLIR.N |
|  | 4313 | **489.2631** | **976.5116** | **975.5171** | **0.9945** | **1** | **8** | **30** | **5** | **K.ENVMKLSR.I** |
|  | 5906 | **549.3357** | **1096.6569** | **1095.5383** | **1.1187** | **1** | **12** | **8.4** | **2** | **K.FCKDVVSNK.K** |
|  | 5948 | **550.8156** | **1099.6167** | **1099.6237** | **-0.0070** | **0** | **64** | **6.6e-005** | **1** | **K.TILDELIQR.G** 5950 |
|  | 6782 | **582.8255** | **1163.6364** | **1163.6438** | **-0.0073** | **0** | **27** | **0.33** | **1** | **K.IEIYPTSLTK.T** |
|  | 11471 | **473.2548** | **1416.7425** | **1416.7514** | **-0.0089** | **0** | **29** | **0.18** | **1** | **K.WIPQNDLLGHPK.T** 11466 11467 11469 |
|  | 11924 | **723.3910** | **1444.7674** | **1444.7787** | **-0.0112** | **0** | **40** | **0.013** | **1** | **R.IQHDQPVKPLDR.A** 11925 11926 |
|  | 12734 | 747.3829 | 1492.7512 | 1492.7595 | -0.0083 | 0 | 23 | 0.67 | 1 | K.TELENFIMQQIK.R |
|  | 13386 | **768.8843** | **1535.7540** | **1535.7653** | **-0.0113** | **1** | **17** | **2.3** | **1** | **R.VINDPSYKENVMK.L** |
|  | 13559 | **774.3882** | **1546.7619** | **1546.7740** | **-0.0120** | **0** | **46** | **0.0031** | **1** | **R.FDGNKPDTLGLNTR.L** 13560 13561 13562 |
|  | 16930 | **884.9373** | **1767.8601** | **1767.8713** | **-0.0112** | **0** | **74** | **3.9e-006** | **1** | **R.VDFNTMSSTDLLNALK.R** 16931 |
|  | 17167 | 892.9249 | 1783.8352 | 1783.8662 | -0.0310 | 0 | (4) | 40 | 5 | R.VDFNTMSSTDLLNALK.R 17151 |
|  | 18931 | **962.9875** | **1923.9604** | **1923.9724** | **-0.0120** | **1** | **95** | **3.2e-008** | **1** | **R.VDFNTMSSTDLLNALKR.V** 18930 |
|  | 19132 | **647.6590** | **1939.9552** | **1939.9673** | **-0.0121** | **1** | **(2)** | **58** | **3** | **R.VDFNTMSSTDLLNALKR.V** |
|  | 22041 | 1111.6786 | 2221.3426 | 2222.1365 | -0.7939 | 1 | 6 | 14 | 2 | R.GAAVRVDFNTMSSTDLLNALK.R |
|  | 23871 | **820.7310** | **2459.1711** | **2460.1631** | **-0.9921** | **0** | **19** | **0.91** | **2** | **K.WDQFYSEVLGRPTTLSETMGK.A** |
|  | 27904 | **1068.1691** | **3201.4854** | **3201.4974** | **-0.0120** | **0** | **83** | **2.5e-007** | **1** | **K.HSGGFIFPPSYVPVVMSELTDQMTFMER.V** |
|  | 28022 | **1078.4851** | **3232.4335** | **3233.4872** | **-1.0537** | **0** | **(1)** | **37** | **4** | **K.HSGGFIFPPSYVPVVMSELTDQMTFMER.V** |
|  | 30231 | **1020.5082** | **4078.0036** | **4078.0233** | **-0.0197** | **0** | **38** | **0.0039** | **1** | **R.AFITHGGANGIYEAIYHGIPMVGIPLFADQPDNIAHMK.A** 30230 30232 30233 30237 30238 |
|  | 30794 | **1465.3874** | **4393.1405** | **4393.1585** | **-0.0180** | **0** | **33** | **0.011** | **1** | **R.FDVIFADAIFPCSELLAELFNIPFVYSLSFSPGYTFEK.H** |

| **119.** | P04844\|RPN2_HUMAN    **Mass:** 69355    **Score:** 444    **Queries matched:** 28   **emPAI:** 0.97 |
| --- | --- |
|  | Dolichyl-diphosphooligosaccharide--protein glycosyltransferase subunit 2 OS=Homo sapiens GN=RPN2 PE=1 SV=3 |

|  | **Query** | **Observed** | **Mr(expt)** | **Mr(calc)** | **Delta** | **Miss** | **Score** | **Expect** | **Rank** | **Peptide** |
| --- | --- | --- | --- | --- | --- | --- | --- | --- | --- | --- |
|  | 1519 | **678.3773** | **677.3700** | **677.3748** | **-0.0048** | **0** | **21** | **1.7** | **1** | **R.VTYPAK.A** 1518 |
|  | 3326 | 453.0465 | 904.0784 | 903.4848 | 0.5936 | 0 | 4 | 53 | 6 | R.MLAQQAVK.R |
|  | 3416 | **456.2386** | **910.4626** | **910.4695** | **-0.0068** | **1** | **43** | **0.0077** | **1** | **K.KACTYIR.S** 3417 |
|  | 4554 | **498.2668** | **994.5189** | **995.4560** | **-0.9370** | **0** | **7** | **29** | **7** | **K.FELDTSER.K** 4593 |
|  | 5258 | **523.3015** | **1044.5883** | **1043.5910** | **0.9974** | **1** | **39** | **0.024** | **1** | **R.MLAQQAVKR.T** |
|  | 6150 | **558.6027** | **1115.1909** | **1115.5935** | **-0.4026** | **1** | **6** | **26** | **3** | **K.DQSIAPKTTR.V** |
|  | 12822 | **500.7648** | **1499.2727** | **1499.7256** | **-0.4529** | **1** | **2** | **51** | **10** | **K.NVYKFELDTSER.K** |
|  | 17849 | **920.5244** | **1839.0343** | **1839.0466** | **-0.0123** | **0** | **78** | **1.6e-006** | **1** | **R.LQVTNVLSQPLTQATVK.L** 17850 17851 |
|  | 20298 | 682.3662 | 2044.0768 | 2043.9976 | 0.0792 | 0 | 2 | 62 | 7 | K.TSFTPVGDVFELNFMNVK.F |
|  | 22719 | **769.7125** | **2306.1156** | **2306.1291** | **-0.0135** | **0** | **12** | **5.3** | **2** | **K.NFESLSEAFSVASAAAVLSHNR.Y** |
|  | 23662 | **812.4106** | **2434.2101** | **2434.2240** | **-0.0140** | **1** | **62** | **4.5e-005** | **1** | **K.KNFESLSEAFSVASAAAVLSHNR.Y** |
|  | 24221 | **835.0974** | **2502.2702** | **2502.2867** | **-0.0165** | **0** | **47** | **0.0014** | **1** | **R.YHVPVVVVPEGSASDTHEQAILR.L** 24220 |
|  | 24391 | **1265.6575** | **2529.3004** | **2529.3174** | **-0.0170** | **1** | **103** | **3.2e-009** | **1** | **K.ISTEVGITNVDLSTVDKDQSIAPK.T** 24390 |
|  | 26722 | **735.1424** | **2936.5405** | **2936.5567** | **-0.0162** | **1** | **54** | **0.00021** | **1** | **R.LSKEETVLATVQALQTASHLSQQADLR.S** 26721 |
|  | 28073 | **1085.2240** | **3252.6502** | **3252.6666** | **-0.0165** | **0** | **57** | **8.6e-005** | **1** | **K.ASLDRPFTNLESAFYSIVGLSSLGAQVPDAK.K** 28074 28075 |
|  | 29110 | **1199.3008** | **3594.8805** | **3594.8974** | **-0.0169** | **1** | **38** | **0.0061** | **1** | **K.NPILWNVADVVIKFPEEEAPSTVLSQNLFTPK.Q** 29111 |
|  | 29672 | **1274.2988** | **3819.8747** | **3819.9010** | **-0.0263** | **0** | **62** | **1.9e-005** | **1** | **K.GTFIADSHQNFALFFQLVDVNTGAELTPHQTFVR.L** |

| **120.** | Q96LJ7\|DHRS1_HUMAN    **Mass:** 34458    **Score:** 444    **Queries matched:** 19   **emPAI:** 1.07 |
| --- | --- |
|  | Dehydrogenase/reductase SDR family member 1 OS=Homo sapiens GN=DHRS1 PE=1 SV=1 |

|  | **Query** | **Observed** | **Mr(expt)** | **Mr(calc)** | **Delta** | **Miss** | **Score** | **Expect** | **Rank** | **Peptide** |
| --- | --- | --- | --- | --- | --- | --- | --- | --- | --- | --- |
|  | 4776 | **505.2697** | **1008.5248** | **1007.5400** | **0.9848** | **0** | **10** | **16** | **2** | **K.AGATVYITGR.H** |
|  | 6842 | 585.3200 | 1168.6255 | 1168.6339 | -0.0085 | 0 | 3 | 67 | 4 | K.EEVLQDPVLK.Q |
|  | 9535 | **656.3287** | **1310.6429** | **1310.6513** | **-0.0084** | **1** | **16** | **3.4** | **1** | **K.LAADCAHELRR.H** |
|  | 11410 | **707.8334** | **1413.6523** | **1413.6623** | **-0.0100** | **0** | **65** | **3.9e-005** | **1** | **K.SAFSSAETTELSGK.C** |
|  | 14220 | **796.3888** | **1590.7630** | **1590.7750** | **-0.0120** | **1** | **35** | **0.036** | **1** | **R.SLFEQVDREQQGR.L** |
|  | 16905 | **589.3055** | **1764.8948** | **1764.9080** | **-0.0132** | **1** | **13** | **6** | **1** | **K.EHMAKEEVLQDPVLK.Q** |
|  | 16907 | **589.3166** | **1764.9279** | **1763.8117** | **1.1162** | **0** | **1** | **90** | **10** | **-.MAAPMNGQVCVVTGASR.G** |
|  | 17097 | 890.9791 | 1779.9436 | 1780.9029 | -0.9593 | 1 | (11) | 8.1 | 3 | K.EHMAKEEVLQDPVLK.Q 17113 17114 |
|  | 20699 | **1037.5633** | **2073.1121** | **2073.1218** | **-0.0097** | **0** | **75** | **2.4e-006** | **1** | **R.LDVLVNNAYAGVQTILNTR.N** 20697 |
|  | 21147 | **1062.0631** | **2122.1117** | **2122.1245** | **-0.0128** | **0** | **90** | **7.9e-008** | **1** | **R.HGVSCVSLWPGIVQTELLK.E** 21145 |
|  | 21495 | 541.4925 | 2161.9409 | 2163.0347 | -1.0939 | 1 | 10 | 8.7 | 1 | -.MAAPMNGQVCVVTGASRGIGR.G |
|  | 22029 | **1111.5138** | **2221.0130** | **2221.0262** | **-0.0132** | **0** | **106** | **2.1e-009** | **1** | **K.AFWETPASMWDDINNVGLR.G** 22031 |
|  | 26598 | **1459.6746** | **2917.3346** | **2917.3546** | **-0.0200** | **0** | **77** | **1.2e-006** | **1** | **R.VVAQEAQSLGGQCVPVVCDSSQESEVR.S** |
|  | 27976 | **1074.5708** | **3220.6906** | **3220.7029** | **-0.0123** | **0** | **28** | **0.068** | **1** | **R.LMVPAGQGLIVVISSPGSLQYMFNVPYGVGK.A** |

| **121.** | Q99867\|TBB4Q_HUMAN    **Mass:** 48917    **Score:** 441    **Queries matched:** 37   **emPAI:** 0.80 |
| --- | --- |
|  | Putative tubulin beta-4q chain OS=Homo sapiens GN=TUBB4Q PE=5 SV=1 |

|  | **Query** | **Observed** | **Mr(expt)** | **Mr(calc)** | **Delta** | **Miss** | **Score** | **Expect** | **Rank** | **Peptide** |
| --- | --- | --- | --- | --- | --- | --- | --- | --- | --- | --- |
|  | 1602 | **709.3584** | **708.3511** | **708.3047** | **0.0464** | **0** | **8** | **25** | **2** | **K.NMMAAR.D** 1597 |
|  | 2066 | 404.7367 | 807.4588 | 807.3399 | 0.1190 | 0 | 7 | 33 | 7 | R.EEYPDR.I |
|  | 5632 | **538.7996** | **1075.5847** | **1076.4855** | **-0.9008** | **1** | **4** | **64** | **9** | **K.NMMAARDPR.H** |
|  | 5638 | 539.2662 | 1076.5179 | 1076.5250 | -0.0071 | 1 | 36 | 0.039 | 1 | K.IREEYPDR.I 5637 |
|  | 6021 | 553.2699 | 1104.5252 | 1104.4771 | 0.0482 | 0 | 16 | 4.7 | 5 | R.QCGAGNNWAK.G 6033 |
|  | 6327 | 565.7967 | 1129.5788 | 1129.5880 | -0.0092 | 0 | 4 | 59 | 6 | R.FPGQLNADLR.K |
|  | 6499 | 572.3174 | 1142.6203 | 1142.6270 | -0.0067 | 0 | 70 | 1.8e-005 | 1 | K.LAVNMVPFPR.L 6498 |
|  | 8802 | 636.3640 | 1270.7133 | 1270.7220 | -0.0086 | 1 | 47 | 0.0027 | 1 | R.KLAVNMVPFPR.L |
|  | 9112 | 430.2745 | 1287.8016 | 1286.7169 | 1.0847 | 1 | (7) | 28 | 2 | R.KLAVNMVPFPR.L 9101 |
|  | 9913 | 665.3396 | 1328.6646 | 1328.7704 | -0.1057 | 0 | 11 | 11 | 3 | R.IINTFSILPSPK.V 9910 9911 |
|  | 14384 | 801.4086 | 1600.8026 | 1600.8131 | -0.0105 | 0 | 67 | 2.6e-005 | 1 | R.AVLVDLEPGTMDSVR.S 14378 14379 14385 |
|  | 15410 | **557.0175** | **1668.0306** | **1667.7943** | **0.2362** | **0** | **4** | **38** | **6** | **K.NSSYFADWLPNNIK.T** |
|  | 17713 | 610.6197 | 1828.8372 | 1827.9241 | 0.9132 | 1 | 7 | 21 | 2 | R.INVHHHEASGGRYVPR.A |
|  | 19931 | 503.4933 | 2009.9439 | 2009.9009 | 0.0431 | 1 | 1 | 71 | 5 | R.MPMREVDEQMFNIQDK.N |
|  | 21101 | 1059.0121 | 2116.0096 | 2116.0987 | -0.0891 | 1 | 2 | 56 | 4 | R.YVPRAVLVDLEPGTMDSVR.S 21100 |
|  | 28244 | 1656.2616 | 3310.5086 | 3310.5268 | -0.0182 | 0 | (65) | 1.3e-005 | 1 | K.EAESCDCLQGFQLTHSLGGGTGSGMGTLLLSK.I 28241 28242 28243 28245 28246 |
|  | 28304 | 1109.8416 | 3326.5028 | 3326.5218 | -0.0189 | 0 | 72 | 3e-006 | 1 | K.EAESCDCLQGFQLTHSLGGGTGSGMGTLLLSK.I |
|  | 28665 | 1147.2072 | 3438.5996 | 3438.6218 | -0.0222 | 1 | 68 | 6.1e-006 | 1 | R.KEAESCDCLQGFQLTHSLGGGTGSGMGTLLLSK.I 28657 28663 28664 |

| **122.** | Q9BUF5\|TBB6_HUMAN    **Mass:** 50281    **Score:** 440    **Queries matched:** 40   **emPAI:** 0.77 |
| --- | --- |
|  | Tubulin beta-6 chain OS=Homo sapiens GN=TUBB6 PE=1 SV=1 |

|  | **Query** | **Observed** | **Mr(expt)** | **Mr(calc)** | **Delta** | **Miss** | **Score** | **Expect** | **Rank** | **Peptide** |
| --- | --- | --- | --- | --- | --- | --- | --- | --- | --- | --- |
|  | 1851 | 397.2534 | 792.4923 | 791.3450 | 1.1474 | 0 | 11 | 14 | 2 | R.EEFPDR.I 1840 |
|  | 5004 | 514.2689 | 1026.5233 | 1025.5328 | 0.9905 | 0 | 5 | 48 | 5 | K.VAVCDIPPR.G |
|  | 5494 | 533.3253 | 1064.6361 | 1064.4201 | 0.2160 | 0 | (4) | 60 | 6 | R.NMMAACDPR.H |
|  | 5521 | 534.7601 | 1067.5057 | 1068.5968 | -1.0911 | 0 | 7 | 29 | 7 | R.YLTVATVFR.G 5522 |
|  | 5678 | 541.2108 | 1080.4071 | 1080.4151 | -0.0080 | 0 | 29 | 0.16 | 1 | R.NMMAACDPR.H 5679 5694 |
|  | 6327 | 565.7967 | 1129.5788 | 1129.5880 | -0.0092 | 0 | 4 | 59 | 6 | R.FPGQLNADLR.K |
|  | 6499 | 572.3174 | 1142.6203 | 1142.6270 | -0.0067 | 0 | 70 | 1.8e-005 | 1 | K.LAVNMVPFPR.L 6498 |
|  | 8802 | 636.3640 | 1270.7133 | 1270.7220 | -0.0086 | 1 | 47 | 0.0027 | 1 | R.KLAVNMVPFPR.L |
|  | 9112 | 430.2745 | 1287.8016 | 1286.7169 | 1.0847 | 1 | (7) | 28 | 2 | R.KLAVNMVPFPR.L 9101 |
|  | 10574 | 456.2892 | 1365.8456 | 1366.6625 | -0.8169 | 0 | 4 | 45 | 10 | R.IMNTFSVMPSPK.V |
|  | 10873 | 692.3431 | 1382.6716 | 1382.6574 | 0.0142 | 0 | (3) | 67 | 5 | R.IMNTFSVMPSPK.V |
|  | 11009 | 464.2834 | 1389.8284 | 1389.6810 | 0.1475 | 0 | 1 | 1.1e+002 | 9 | K.EVDEQMLAIQSK.N |
|  | 14658 | 810.9161 | 1619.8176 | 1619.8283 | -0.0107 | 0 | 52 | 0.0008 | 1 | R.LHFFMPGFAPLTSR.G 14659 14660 14661 |
|  | 15835 | 848.9150 | 1695.8154 | 1695.8257 | -0.0102 | 0 | 33 | 0.051 | 1 | K.NSSYFVEWIPNNVK.V 15855 15856 |
|  | 16501 | **579.6563** | **1735.9471** | **1734.8611** | **1.0860** | **0** | **4** | **39** | **2** | **R.ALTVPELTQQMFDAR.N** 16500 |
|  | 18090 | 929.4694 | 1856.9242 | 1856.9342 | -0.0101 | 0 | 88 | 1.5e-007 | 2 | K.MASTFIGNSTAIQELFK.R 18088 18089 18091 |
|  | 19959 | 1007.5189 | 2013.0232 | 2013.0353 | -0.0122 | 1 | 75 | 2.7e-006 | 1 | K.MASTFIGNSTAIQELFKR.I |
|  | 21442 | 1078.9855 | 2155.9564 | 2155.1347 | 0.8217 | 1 | 4 | 30 | 4 | R.GLKMASTFIGNSTAIQELFK.R |
|  | 23107 | 786.0563 | 2355.1470 | 2355.1583 | -0.0113 | 1 | 2 | 44 | 7 | R.LHFFMPGFAPLTSRGSQQYR.A |
|  | 23836 | **410.0428** | **2454.2131** | **2454.1962** | **0.0169** | **1** | **6** | **17** | **3** | **R.GSQQYRALTVPELTQQMFDAR.N** |
|  | 25766 | 695.3771 | 2777.4792 | 2776.4346 | 1.0446 | 1 | 6 | 13 | 2 | K.LAVNMVPFPRLHFFMPGFAPLTSR.G |
|  | 29033 | **716.7436** | **3578.6816** | **3577.6422** | **1.0393** | **1** | **1** | **26** | **6** | **R.KECEHCDCLQGFQLTHSLGGGTGSGMGTLLISK.I** |
|  | 31036 | 1531.7026 | 4592.0861 | 4592.0999 | -0.0139 | 0 | 50 | 0.00017 | 1 | K.VSDTVVEPYNATLSVHQLVENTDETYCIDNEALYDICFR.T 31032 31033 |

| **123.** | Q9UKY3\|EST4_HUMAN    **Mass:** 30773    **Score:** 438    **Queries matched:** 19   **emPAI:** 1.53 |
| --- | --- |
|  | Inactive carboxylesterase 4 OS=Homo sapiens GN=CES4 PE=2 SV=2 |

|  | **Query** | **Observed** | **Mr(expt)** | **Mr(calc)** | **Delta** | **Miss** | **Score** | **Expect** | **Rank** | **Peptide** |
| --- | --- | --- | --- | --- | --- | --- | --- | --- | --- | --- |
|  | 10236 | 674.8602 | 1347.7058 | 1347.7146 | -0.0089 | 0 | 76 | 3.7e-006 | 1 | K.AGQLISELFTNR.K 10238 |
|  | 12402 | 737.4247 | 1472.8349 | 1472.8450 | -0.0101 | 0 | 101 | 9.8e-009 | 1 | R.AISESGVALTSVLVK.K 12403 12405 12406 12407 |
|  | 12464 | 738.9071 | 1475.7996 | 1475.8096 | -0.0100 | 1 | 61 | 0.0001 | 1 | K.AGQLISELFTNRK.E 12463 12465 12466 12476 |
|  | 21190 | 1065.0207 | 2128.0269 | 2128.0398 | -0.0128 | 0 | 73 | 4e-006 | 1 | K.LSEDCLYLNIYTPADLTK.K 21191 21192 |
|  | 26044 | 1412.2196 | 2822.4246 | 2822.4411 | -0.0165 | 1 | 32 | 0.037 | 1 | K.ENIPLKLSEDCLYLNIYTPADLTK.K 26042 26045 |
|  | 30328 | 822.2419 | 4106.1733 | 4107.1438 | -0.9705 | 0 | 0 | 20 | 5 | R.LPVMVWIHGGGLMVGAASTYDGLALAAHENVVVVTIQYR.L |

| **124.** | P12235\|ADT1_HUMAN    **Mass:** 33271    **Score:** 435    **Queries matched:** 36   **emPAI:** 0.90 |
| --- | --- |
|  | ADP/ATP translocase 1 OS=Homo sapiens GN=SLC25A4 PE=1 SV=4 |

|  | **Query** | **Observed** | **Mr(expt)** | **Mr(calc)** | **Delta** | **Miss** | **Score** | **Expect** | **Rank** | **Peptide** |
| --- | --- | --- | --- | --- | --- | --- | --- | --- | --- | --- |
|  | 867 | **503.1058** | **502.0985** | **501.2659** | **0.8326** | **0** | **11** | **32** | **1** | **K.GAAQR.E** |
|  | 1501 | 673.3835 | 672.3762 | 672.3806 | -0.0044 | 0 | 28 | 0.4 | 1 | R.LAADVGK.G |
|  | 2480 | 421.0564 | 840.0982 | 839.3452 | 0.7530 | 0 | (8) | 15 | 3 | R.MMMQSGR.K |
|  | 2649 | 428.7446 | 855.4747 | 855.4814 | -0.0067 | 0 | 24 | 0.66 | 1 | K.TAVAPIER.V |
|  | 2661 | 429.0876 | 856.1606 | 855.3401 | 0.8205 | 0 | 17 | 2.3 | 1 | R.MMMQSGR.K |
|  | 2669 | 429.2404 | 856.4663 | 855.4926 | 0.9737 | 0 | 12 | 13 | 4 | R.GNLANVIR.Y |
|  | 3294 | 902.4784 | 901.4712 | 901.4770 | -0.0058 | 0 | 20 | 2.3 | 1 | K.GAWSNVLR.G |
|  | 3676 | 466.2520 | 930.4894 | 930.4957 | -0.0063 | 0 | 20 | 1.8 | 2 | K.GIIDCVVR.I |
|  | 4410 | 493.2287 | 984.4428 | 983.4351 | 1.0078 | 1 | (2) | 1.1e+002 | 9 | R.MMMQSGRK.G |
|  | 4551 | 498.2523 | 994.4901 | 995.4463 | -0.9562 | 1 | 8 | 24 | 5 | R.RMMMQSGR.K |
|  | 4736 | **503.2899** | **1004.5652** | **1003.5451** | **1.0201** | **0** | **17** | **3.5** | **2** | **K.QLFLGGVDR.H** |
|  | 4895 | 509.2857 | 1016.5569 | 1015.4249 | 1.1320 | 1 | 6 | 47 | 4 | R.MMMQSGRK.G |
|  | 5031 | 515.2455 | 1028.4765 | 1027.4361 | 1.0404 | 1 | (4) | 63 | 5 | R.RMMMQSGR.K |
|  | 5718 | 542.3104 | 1082.6063 | 1082.6448 | -0.0385 | 1 | 8 | 27 | 2 | K.TAVAPIERVK.L 5713 5715 |
|  | 6397 | 568.3104 | 1134.6063 | 1135.6713 | -1.0650 | 0 | 32 | 0.096 | 1 | K.LLLQVQHASK.Q 6410 |
|  | 6852 | **585.3680** | **1168.7214** | **1168.5665** | **0.1549** | **0** | **8** | **25** | **2** | **K.EQGFLSFWR.G** |
|  | 10278 | 450.7683 | 1349.2829 | 1349.7126 | -0.4296 | 1 | 8 | 15 | 3 | K.QYKGIIDCVVR.I |
|  | 10478 | 454.8929 | 1361.6569 | 1362.8347 | -1.1778 | 1 | 7 | 25 | 2 | R.VKLLLQVQHASK.Q 10504 |
|  | 11941 | 723.8702 | 1445.7259 | 1445.7343 | -0.0084 | 0 | 56 | 0.00036 | 1 | R.YFPTQALNFAFK.D 11955 |
|  | 15042 | 822.8551 | 1643.6956 | 1643.7072 | -0.0116 | 0 | 45 | 0.0032 | 1 | K.GADIMYTGTVDCWR.K |
|  | 16970 | 886.9021 | 1771.7896 | 1771.8022 | -0.0125 | 1 | 81 | 7.9e-007 | 1 | R.KGADIMYTGTVDCWR.K 16971 |
|  | 18943 | 963.4647 | 1924.9149 | 1926.0363 | -1.1214 | 0 | 4 | 42 | 2 | R.GLYQGFNVSVQGIIIYR.A |
|  | 25886 | 1398.6688 | 2795.3231 | 2795.3377 | -0.0147 | 0 | 98 | 1.1e-008 | 1 | R.YFAGNLASGGAAGATSLCFVYPLDFAR.T 25877 25883 25884 25885 25887 25888 |
|  | 29949 | **986.9840** | **3943.9067** | **3945.0322** | **-1.1255** | **1** | **0** | **31** | **2** | **K.GMLPDPKNVHIFVSWMIAQSVTAVAGLVSYPFDTVR.R** |

| **125.** | P24752\|THIL_HUMAN    **Mass:** 45456    **Score:** 431    **Queries matched:** 38   **emPAI:** 1.38 |
| --- | --- |
|  | Acetyl-CoA acetyltransferase, mitochondrial OS=Homo sapiens GN=ACAT1 PE=1 SV=1 |

|  | **Query** | **Observed** | **Mr(expt)** | **Mr(calc)** | **Delta** | **Miss** | **Score** | **Expect** | **Rank** | **Peptide** |
| --- | --- | --- | --- | --- | --- | --- | --- | --- | --- | --- |
|  | 993 | **531.3464** | **530.3391** | **530.3064** | **0.0327** | **0** | **14** | **13** | **3** | **K.DVGLK.K** |
|  | 1706 | **752.3388** | **751.3315** | **751.3357** | **-0.0042** | **0** | **9** | **18** | **1** | **K.VCASGMK.A** |
|  | 2338 | **415.2526** | **828.4907** | **828.4957** | **-0.0050** | **0** | **23** | **1.1** | **1** | **K.LEDLIVK.D** 2337 |
|  | 2501 | **421.7633** | **841.5120** | **840.4705** | **1.0415** | **0** | **8** | **24** | **2** | **K.GQPDVVVK.E** |
|  | 2814 | **433.7496** | **865.4846** | **864.4341** | **1.0505** | **0** | **4** | **72** | **2** | **R.GSTPYGGVK.L** |
|  | 4049 | **479.2868** | **956.5590** | **956.5841** | **-0.0250** | **0** | **6** | **45** | **4** | **-.MAVLAALLR.S** |
|  | 4193 | **485.2817** | **968.5489** | **967.4247** | **1.1243** | **1** | **1** | **1e+002** | **4** | **K.EDEEYKR.V** |
|  | 4203 | **485.7785** | **969.5424** | **969.5495** | **-0.0070** | **1** | **22** | **0.91** | **1** | **K.AGIPKEEVK.E** |
|  | 4252 | **973.5606** | **972.5533** | **972.5604** | **-0.0071** | **0** | **36** | **0.045** | **1** | **K.EVVIVSATR.T** |
|  | 4256 | 487.3202 | 972.6258 | 972.4950 | 0.1308 | 0 | 11 | 14 | 3 | K.MLEIDPQK.V 4261 |
|  | 4270 | **487.7676** | **973.5205** | **972.5790** | **0.9416** | **0** | **(3)** | **99** | **7** | **-.MAVLAALLR.S** |
|  | 5180 | **520.3453** | **1038.6760** | **1038.6298** | **0.0462** | **1** | **18** | **2.3** | **2** | **K.RLNVTPLAR.I** |
|  | 5796 | **545.2714** | **1088.5283** | **1087.6502** | **0.8781** | **0** | **31** | **0.15** | **2** | **R.IVGHLTHALK.Q** |
|  | 7330 | **399.7497** | **1196.2274** | **1196.7353** | **-0.5079** | **1** | **7** | **20** | **4** | **M.AVLAALLRSGAR.S** |
|  | 9663 | **659.7926** | **1317.5706** | **1317.5805** | **-0.0099** | **0** | **48** | **0.0023** | **1** | **K.IHMGSCAENTAK.K** 9664 9665 |
|  | 11937 | **482.8955** | **1445.6648** | **1445.6755** | **-0.0107** | **1** | **30** | **0.14** | **1** | **K.IHMGSCAENTAKK.L** |
|  | 13516 | **772.8464** | **1543.6783** | **1543.6903** | **-0.0119** | **0** | **38** | **0.021** | **1** | **R.NEQDAYAINSYTR.S** |
|  | 15521 | **838.9007** | **1675.7868** | **1674.9192** | **0.8676** | **1** | **4** | **46** | **9** | **R.GSTPYGGVKLEDLIVK.D** |
|  | 15939 | **851.4893** | **1700.9641** | **1700.9713** | **-0.0072** | **0** | **87** | **2.1e-007** | **1** | **R.TPIGSFLGSLSLLPATK.L** 15955 |
|  | 18376 | **939.4374** | **1876.8603** | **1876.8737** | **-0.0134** | **0** | **68** | **1.6e-005** | **1** | **K.EAYMGNVLQGGEGQAPTR.Q** 18375 |
|  | 18757 | **636.6595** | **1906.9566** | **1905.9843** | **0.9723** | **0** | **9** | **13** | **1** | **K.VNINGGAVSLGHPIGMSGAR.I** |
|  | 19135 | **971.0211** | **1940.0276** | **1940.0401** | **-0.0125** | **0** | **75** | **3e-006** | **1** | **R.QAVLGAGLPISTPCTTINK.V** 19136 |
|  | 21099 | 1059.0116 | 2116.0086 | 2115.1364 | 0.8722 | 1 | 6 | 21 | 2 | K.AAWEAGKFGNEVIPVTVTVK.G 21100 |
|  | 23159 | **788.3762** | **2362.1067** | **2362.1223** | **-0.0156** | **1** | **31** | **0.057** | **1** | **K.EEVKEAYMGNVLQGGEGQAPTR.Q** |
|  | 26019 | **940.1713** | **2817.4922** | **2817.5027** | **-0.0105** | **0** | **55** | **0.00019** | **1** | **R.IVAFADAAVEPIDFPIAPVYAASMVLK.D** 26018 |
|  | 26281 | **716.3661** | **2861.4353** | **2860.4688** | **0.9665** | **1** | **18** | **0.79** | **1** | **K.MLEIDPQKVNINGGAVSLGHPIGMSGAR.I** |
|  | 26600 | **973.4842** | **2917.4309** | **2916.4611** | **0.9699** | **1** | **8** | **10** | **1** | **K.ENGTVTAANASTLNDGAAALVLMTADAAKR.L** |
|  | 29387 | **1244.8761** | **3731.6065** | **3731.6189** | **-0.0124** | **0** | **36** | **0.008** | **1** | **K.AIMMASQSLMCGHQDVMVAGGMESMSNVPYVMNR.G** |
|  | 30956 | **909.8430** | **4544.1787** | **4544.9186** | **-0.7399** | **1** | **3** | **9.4** | **2** | **K.VCASGMKAIMMASQSLMCGHQDVMVAGGMESMSNVPYVMNR.G** |

| **126.** | P10635\|CP2D6_HUMAN    **Mass:** 56076    **Score:** 426    **Queries matched:** 33   **emPAI:** 1.46 |
| --- | --- |
|  | Cytochrome P450 2D6 OS=Homo sapiens GN=CYP2D6 PE=1 SV=2 |

|  | **Query** | **Observed** | **Mr(expt)** | **Mr(calc)** | **Delta** | **Miss** | **Score** | **Expect** | **Rank** | **Peptide** |
| --- | --- | --- | --- | --- | --- | --- | --- | --- | --- | --- |
|  | 2093 | 405.7104 | 809.4062 | 808.4443 | 0.9619 | 0 | 11 | 13 | 1 | R.FSVSTLR.N 2094 |
|  | 2974 | **439.2449** | **876.4753** | **876.4818** | **-0.0064** | **0** | **23** | **0.96** | **1** | **R.SQGVFLAR.Y** |
|  | 4109 | **482.2452** | **962.4759** | **962.4821** | **-0.0063** | **0** | **33** | **0.087** | **1** | **R.DIEVQGFR.I** |
|  | 4151 | **483.2765** | **964.5385** | **964.5454** | **-0.0069** | **1** | **21** | **1.4** | **1** | **R.RFSVSTLR.N** |
|  | 8890 | **426.2130** | **1275.6173** | **1275.6248** | **-0.0075** | **0** | **5** | **44** | **9** | **K.DEAVWEKPFR.F** |
|  | 10214 | **674.3515** | **1346.6884** | **1346.6976** | **-0.0092** | **0** | **58** | **0.00022** | **1** | **K.AVSNVIASLTCGR.R** 10215 10221 |
|  | 11012 | **696.2872** | **1390.5598** | **1390.7932** | **-0.2335** | **1** | **8** | **20** | **1** | **R.FSVSTLRNLGLGK.K** |
|  | 12486 | **739.8237** | **1477.6329** | **1477.6433** | **-0.0104** | **0** | **56** | **0.0003** | **1** | **K.GNPESSFNDENLR.I** |
|  | 12803 | **749.8910** | **1497.7674** | **1497.7787** | **-0.0113** | **0** | **69** | **1.4e-005** | **1** | **R.VQQEIDDVIGQVR.R** |
|  | 12891 | **752.4136** | **1502.8127** | **1502.7987** | **0.0140** | **1** | **6** | **34** | **7** | **K.AVSNVIASLTCGRR.F** |
|  | 15534 | **559.9283** | **1676.7632** | **1676.7754** | **-0.0122** | **1** | **17** | **2.3** | **1** | **K.AKGNPESSFNDENLR.I** |
|  | 15643 | **843.4424** | **1684.8702** | **1684.8784** | **-0.0082** | **0** | **61** | **9e-005** | **1** | **K.AFLTQLDELLTEHR.M** 15642 |
|  | 18015 | **926.9727** | **1851.9309** | **1853.1138** | **-1.1829** | **0** | **36** | **0.026** | **1** | **R.EVLNAVPVLLHIPALAGK.V** 18036 |
|  | 18862 | **959.5116** | **1917.0086** | **1917.0207** | **-0.0121** | **1** | **78** | **1.8e-006** | **1** | **R.LLDLAQEGLKEESGFLR.E** 18861 18863 |
|  | 24660 | **856.0753** | **2565.2040** | **2565.2216** | **-0.0177** | **0** | **56** | **0.00017** | **1** | **R.RPEMGDQAHMPYTTAVIHEVQR.F** 24659 |
|  | 24713 | **859.4055** | **2575.1946** | **2575.2087** | **-0.0141** | **1** | **24** | **0.27** | **1** | **R.MTWDPAQPPRDLTEAFLAEMEK.A** |
|  | 24860 | **868.8013** | **2603.3820** | **2603.3959** | **-0.0139** | **1** | **43** | **0.003** | **1** | **K.GTTLITNLSSVLKDEAVWEKPFR.F** |
|  | 26395 | **962.8345** | **2885.4818** | **2885.5036** | **-0.0218** | **0** | **31** | **0.045** | **1** | **R.EALVTHGEDTADRPPVPITQILGFGPR.S** 26396 |
|  | 26953 | **746.1216** | **2980.4575** | **2980.4773** | **-0.0198** | **0** | **19** | **0.77** | **1** | **R.FHPEHFLDAQGHFVKPEAFLPFSAGR.R** 26955 |
|  | 28659 | **1146.9093** | **3437.7061** | **3437.7231** | **-0.0170** | **0** | **46** | **0.00094** | **1** | **R.YPPGPLPLPGLGNLLHVDFQNTPYCFDQLR.R** 28657 |
|  | 29056 | **896.9101** | **3583.6113** | **3583.9147** | **-0.3034** | **0** | **3** | **19** | **4** | **R.IVVADLFSAGMVTTSTTLAWGLLLMILHPDVQR.R** |
|  | 29106 | **899.4595** | **3593.8088** | **3593.8242** | **-0.0154** | **1** | **32** | **0.023** | **1** | **R.YPPGPLPLPGLGNLLHVDFQNTPYCFDQLRR.R** |

| **127.** | P28845\|DHI1_HUMAN    **Mass:** 32608    **Score:** 412    **Queries matched:** 35   **emPAI:** 1.68 |
| --- | --- |
|  | Corticosteroid 11-beta-dehydrogenase isozyme 1 OS=Homo sapiens GN=HSD11B1 PE=1 SV=3 |

|  | **Query** | **Observed** | **Mr(expt)** | **Mr(calc)** | **Delta** | **Miss** | **Score** | **Expect** | **Rank** | **Peptide** |
| --- | --- | --- | --- | --- | --- | --- | --- | --- | --- | --- |
|  | 755 | **473.0815** | **472.0742** | **472.2758** | **-0.2015** | **0** | **8** | **36** | **1** | **K.GGALR.Q** 753 757 |
|  | 1756 | **774.4308** | **773.4235** | **773.4647** | **-0.0412** | **0** | **28** | **0.37** | **1** | **K.VIVTGASK.G** |
|  | 3304 | **451.7828** | **901.5511** | **901.5597** | **-0.0085** | **1** | **16** | **5.8** | **2** | **K.KVIVTGASK.G** |
|  | 4097 | **481.7384** | **961.4622** | **961.4691** | **-0.0069** | **0** | **13** | **10** | **5** | **R.EMAYHLAK.M** |
|  | 4347 | **490.2976** | **978.5807** | **977.4640** | **1.1166** | **0** | **(6)** | **41** | **2** | **R.EMAYHLAK.M** |
|  | 5166 | **520.2509** | **1038.4871** | **1039.5597** | **-1.0725** | **0** | **50** | **0.0016** | **1** | **K.MGAHVVVTAR.S** 5171 5187 |
|  | 5389 | **529.3085** | **1056.6024** | **1055.5546** | **1.0478** | **0** | **(1)** | **1.4e+002** | **5** | **K.MGAHVVVTAR.S** |
|  | 9497 | 654.8543 | 1307.6941 | 1307.7020 | -0.0079 | 0 | 44 | 0.0056 | 1 | K.AVSGIVHMQAAPK.E 9468 9496 |
|  | 9794 | 662.8603 | 1323.7060 | 1323.6969 | 0.0091 | 0 | (10) | 15 | 1 | K.AVSGIVHMQAAPK.E |
|  | 10411 | **679.3399** | **1356.6652** | **1356.6747** | **-0.0095** | **0** | **60** | **0.00014** | **1** | **K.VAYPMVAAYSASK.F** |
|  | 10955 | **463.2477** | **1386.7212** | **1386.7296** | **-0.0084** | **1** | **21** | **1.1** | **2** | **K.FALDGFFSSIRK.E** 10948 |
|  | 11918 | **723.3619** | **1444.7092** | **1444.7886** | **-0.0794** | **0** | **3** | **68** | **4** | **K.QSNGSIVVVSSLAGK.V** |
|  | 16682 | **876.4138** | **1750.8131** | **1750.8236** | **-0.0105** | **0** | **69** | **1.4e-005** | **1** | **K.ILEFLYSTSYNMDR.F** 16683 |
|  | 18401 | **940.4600** | **1878.9054** | **1878.9185** | **-0.0132** | **1** | **59** | **0.00012** | **1** | **R.KILEFLYSTSYNMDR.F** 18400 |
|  | 23414 | **798.7485** | **2393.2236** | **2393.2446** | **-0.0211** | **1** | **46** | **0.0016** | **1** | **K.AVSGIVHMQAAPKEECALEIIK.G** |
|  | 23822 | **818.4507** | **2452.3302** | **2452.3473** | **-0.0171** | **1** | **(3)** | **34** | **1** | **R.KSMEVNFLSYVVLTVAALPMLK.Q** |
|  | 23939 | **823.9080** | **2468.7022** | **2468.3423** | **0.3600** | **1** | **7** | **10** | **1** | **R.KSMEVNFLSYVVLTVAALPMLK.Q** |
|  | 24077 | **828.7479** | **2483.2219** | **2484.3372** | **-1.1152** | **1** | **(1)** | **65** | **6** | **R.KSMEVNFLSYVVLTVAALPMLK.Q** |
|  | 24678 | **857.4418** | **2569.3037** | **2569.3176** | **-0.0140** | **1** | **35** | **0.02** | **1** | **K.GGALRQEEVYYDSSLWTTLLIR.N** 24676 24685 |
|  | 28895 | 1178.8742 | 3533.6006 | 3534.7243 | -1.1237 | 0 | 3 | 15 | 2 | K.YLLPILGLFMAYYYYSANEEFRPEMLQGK.K |
|  | 29399 | **1247.2506** | **3738.7300** | **3738.7480** | **-0.0180** | **0** | **84** | **1.2e-007** | **1** | **K.VVSHCLELGAASAHYIAGTMEDMTFAEQFVAQAGK.L** 29401 29402 |
|  | 29450 | **1252.6078** | **3754.8015** | **3754.7429** | **0.0586** | **0** | **(1)** | **30** | **5** | **K.VVSHCLELGAASAHYIAGTMEDMTFAEQFVAQAGK.L** |

| **128.** | Q5TCH4\|CP4AM_HUMAN    **Mass:** 59721    **Score:** 411    **Queries matched:** 26   **emPAI:** 0.53 |
| --- | --- |
|  | Cytochrome P450 4A22 OS=Homo sapiens GN=CYP4A22 PE=1 SV=1 |

|  | **Query** | **Observed** | **Mr(expt)** | **Mr(calc)** | **Delta** | **Miss** | **Score** | **Expect** | **Rank** | **Peptide** |
| --- | --- | --- | --- | --- | --- | --- | --- | --- | --- | --- |
|  | 1101 | 557.3727 | 556.3654 | 556.3697 | -0.0043 | 0 | 22 | 0.93 | 1 | K.VILGR.S |
|  | 2128 | **407.1916** | **812.3686** | **812.4578** | **-0.0893** | **0** | **1** | **1.1e+002** | **6** | **R.IPIPMAR.L** |
|  | 3096 | 443.0397 | 884.0648 | 884.4035 | -0.3387 | 1 | 1 | 80 | 3 | K.HQERCR.E 3083 3089 |
|  | 5528 | 534.8102 | 1067.6058 | 1067.6128 | -0.0070 | 0 | 12 | 11 | 1 | R.LYPPVPGIGR.E |
|  | 9081 | 429.7552 | 1286.2437 | 1286.5853 | -0.3415 | 0 | 4 | 38 | 10 | R.VQLYDPDYMK.V |
|  | 11355 | 705.9176 | 1409.8206 | 1409.8282 | -0.0076 | 0 | 66 | 2.8e-005 | 1 | R.HLDFLDILLLAK.M |
|  | 12489 | 739.8560 | 1477.6974 | 1476.7242 | 0.9732 | 1 | 12 | 8.9 | 1 | K.MENGSILSDKDLR.A 12458 |
|  | 12754 | 498.9393 | 1493.7962 | 1492.7191 | 1.0770 | 1 | (2) | 74 | 5 | K.MENGSILSDKDLR.A |
|  | 17281 | 598.3333 | 1791.9781 | 1790.9865 | 0.9916 | 0 | 14 | 4.2 | 1 | K.GIMVLLSIYGLHHNPK.V |
|  | 18569 | 947.9495 | 1893.8844 | 1894.8809 | -0.9966 | 0 | 76 | 2.6e-006 | 2 | R.NAFHENDTIYSLTSAGR.W 18568 |
|  | 18859 | 959.4827 | 1916.9508 | 1916.9639 | -0.0131 | 0 | 81 | 8.2e-007 | 1 | R.ACQLAHQHTDQVIQLR.K 18860 |
|  | 20483 | 1029.4944 | 2056.9742 | 2056.9868 | -0.0126 | 0 | 68 | 1.4e-005 | 1 | R.FAPGSAQHSHAFLPFSGGSR.N 20481 20482 |
|  | 21581 | **1086.5715** | **2171.1285** | **2171.1752** | **-0.0467** | **1** | **2** | **50** | **6** | **R.IGYGLLLLNGQTWFQHRR.M** |
|  | 28016 | 808.8973 | 3231.5600 | 3230.7063 | 0.8537 | 1 | 3 | 24 | 9 | K.GIMVLLSIYGLHHNPKVWPNLEVFDPSR.F |
|  | 29204 | **733.1699** | **3660.8129** | **3661.6786** | **-0.8657** | **1** | **6** | **8.2** | **1** | **R.CREEIHGLLGDGASITWNHLDQMPYTTMCIK.E** |
|  | 29421 | 1249.2950 | 3744.8633 | 3744.8817 | -0.0184 | 1 | 73 | 1.8e-006 | 1 | R.VMLDKWEELLGQDSPLEVFQHVSLMTLDTIMK.S 29422 29423 29424 |

| **129.** | P02735\|SAA_HUMAN    **Mass:** 13581    **Score:** 403    **Queries matched:** 23   **emPAI:** 2.58 |
| --- | --- |
|  | Serum amyloid A protein OS=Homo sapiens GN=SAA1 PE=1 SV=2 |

|  | **Query** | **Observed** | **Mr(expt)** | **Mr(calc)** | **Delta** | **Miss** | **Score** | **Expect** | **Rank** | **Peptide** |
| --- | --- | --- | --- | --- | --- | --- | --- | --- | --- | --- |
|  | 1476 | **659.3795** | **658.3722** | **658.3398** | **0.0324** | **0** | **15** | **7.7** | **6** | **R.ENIQR.F** |
|  | 4571 | **498.7321** | **995.4497** | **995.4560** | **-0.0062** | **0** | **23** | **0.67** | **1** | **R.EANYIGSDK.Y** 4595 |
|  | 13598 | **775.8637** | **1549.7127** | **1549.7202** | **-0.0074** | **0** | **60** | **0.00012** | **1** | **R.SFFSFLGEAFDGAR.D** 13597 13601 |
|  | 14533 | **806.9072** | **1611.7999** | **1611.8117** | **-0.0118** | **1** | **72** | **7.9e-006** | **1** | **K.RGPGGVWAAEAISDAR.E** 14510 14511 14512 14534 14535 14536 14537 |
|  | 14989 | **547.6075** | **1639.8008** | **1639.8107** | **-0.0099** | **1** | **13** | **6.1** | **1** | **K.DPNHFRPAGLPEKY.-** |
|  | 15425 | **557.5982** | **1669.7728** | **1669.7848** | **-0.0120** | **1** | **25** | **0.34** | **1** | **R.EANYIGSDKYFHAR.G** |
|  | 21415 | **718.6880** | **2153.0423** | **2153.9629** | **-0.9206** | **1** | **1** | **68** | **7** | **R.SFFSFLGEAFDGARDMWR.A** |
|  | 21630 | **1089.4778** | **2176.9410** | **2176.9562** | **-0.0152** | **0** | **107** | **1.6e-009** | **1** | **R.FFGHGAEDSLADQAANEWGR.S** 21629 21631 21632 21633 21634 |

| **130.** | P05388\|RLA0_HUMAN    **Mass:** 34423    **Score:** 401    **Queries matched:** 26   **emPAI:** 1.83 |
| --- | --- |
|  | 60S acidic ribosomal protein P0 OS=Homo sapiens GN=RPLP0 PE=1 SV=1 |

|  | **Query** | **Observed** | **Mr(expt)** | **Mr(calc)** | **Delta** | **Miss** | **Score** | **Expect** | **Rank** | **Peptide** |
| --- | --- | --- | --- | --- | --- | --- | --- | --- | --- | --- |
|  | 2953 | **438.6146** | **875.2147** | **874.4396** | **0.7751** | **0** | **10** | **16** | **1** | **K.EDLTEIR.D** |
|  | 7765 | **609.3382** | **1216.6618** | **1216.6703** | **-0.0085** | **0** | **38** | **0.024** | **1** | **K.IIQLLDDYPK.C** 7766 |
|  | 7837 | **611.3100** | **1220.6054** | **1220.6149** | **-0.0095** | **0** | **52** | **0.00098** | **1** | **R.GHLENNPALEK.L** 7836 |
|  | 8711 | **633.8062** | **1265.5979** | **1265.6074** | **-0.0096** | **0** | **42** | **0.0087** | **1** | **K.CFIVGADNVGSK.Q** |
|  | 9168 | **646.3835** | **1290.7524** | **1289.6696** | **1.0828** | **1** | **3** | **70** | **1** | **K.QMQQIRMSLR.G** |
|  | 9571 | **657.3549** | **1312.6952** | **1312.7027** | **-0.0075** | **0** | **22** | **0.87** | **1** | **K.TSFFQALGITTK.I** |
|  | 10541 | 683.3619 | 1364.7093 | 1365.6931 | -0.9837 | 1 | (2) | 79 | 9 | K.AVVLMGKNTMMR.K |
|  | 10546 | **455.9204** | **1364.7394** | **1365.6931** | **-0.9537** | **1** | **(2)** | **85** | **9** | **K.AVVLMGKNTMMR.K** |
|  | 10587 | **456.5440** | **1366.6102** | **1365.6931** | **0.9171** | **1** | **6** | **30** | **5** | **K.AVVLMGKNTMMR.K** |
|  | 10633 | **685.3796** | **1368.7447** | **1368.7547** | **-0.0100** | **1** | **59** | **0.00015** | **1** | **R.DMLLANKVPAAAR.A** 10634 |
|  | 11660 | **714.9153** | **1427.8160** | **1427.8235** | **-0.0075** | **0** | **7** | **30** | **1** | **R.GTIEILSDVQLIK.T** |
|  | 17638 | **912.9720** | **1823.9294** | **1823.9418** | **-0.0124** | **1** | **70** | **1e-005** | **1** | **R.GNVGFVFTKEDLTEIR.D** |
|  | 18595 | **948.4978** | **1894.9810** | **1894.9928** | **-0.0117** | **0** | **55** | **0.00029** | **1** | **R.VLALSVETDYTFPLAEK.V** 18580 |
|  | 20379 | **684.3174** | **2049.9305** | **2050.0088** | **-0.0783** | **1** | **2** | **54** | **4** | **K.CFIVGADNVGSKQMQQIR.M** |
|  | 21651 | **1090.5466** | **2179.0787** | **2179.0943** | **-0.0156** | **0** | **60** | **9.3e-005** | **1** | **R.AGAIAPCEVTVPAQNTGLGPEK.T** 21652 21654 |
|  | 25624 | **1376.7280** | **2751.4415** | **2751.4595** | **-0.0180** | **0** | **92** | **3.9e-008** | **1** | **K.AFLADPSAFVAAAPVAAATTAAPAAAAAPAK.V** 25612 25625 25626 |
|  | 27829 | **1060.5691** | **3178.6854** | **3178.7026** | **-0.0171** | **1** | **21** | **0.35** | **1** | **K.AFLADPSAFVAAAPVAAATTAAPAAAAAPAKVEAK.E** |

| **131.** | P30086\|PEBP1_HUMAN    **Mass:** 21158    **Score:** 383    **Queries matched:** 16   **emPAI:** 2.20 |
| --- | --- |
|  | Phosphatidylethanolamine-binding protein 1 OS=Homo sapiens GN=PEBP1 PE=1 SV=3 |

|  | **Query** | **Observed** | **Mr(expt)** | **Mr(calc)** | **Delta** | **Miss** | **Score** | **Expect** | **Rank** | **Peptide** |
| --- | --- | --- | --- | --- | --- | --- | --- | --- | --- | --- |
|  | 1187 | **579.3205** | **578.3132** | **578.3176** | **-0.0044** | **0** | **9** | **20** | **1** | **K.VASFR.K** |
|  | 3103 | 443.2713 | 884.5281 | 884.5331 | -0.0050 | 0 | 23 | 0.81 | 1 | K.VLTPTQVK.N |
|  | 13761 | **780.9120** | **1559.8094** | **1559.8195** | **-0.0101** | **0** | **54** | **0.00045** | **1** | **K.LYTLVLTDPDAPSR.K** 13760 |
|  | 14852 | **816.8972** | **1631.7799** | **1631.7903** | **-0.0105** | **0** | **23** | **0.67** | **1** | **K.NRPTSISWDGLDSGK.L** 14847 |
|  | 16056 | 854.9504 | 1707.8862 | 1707.8984 | -0.0122 | 0 | 46 | 0.0025 | 1 | R.YVWLVYEQDRPLK.C |
|  | 16834 | **440.4749** | **1757.8707** | **1757.8824** | **-0.0117** | **1** | **28** | **0.19** | **1** | **K.YREWHHFLVVNMK.G** |
|  | 19233 | **975.4705** | **1948.9264** | **1948.9378** | **-0.0115** | **0** | **99** | **1.1e-008** | **1** | **K.GNDISSGTVLSDYVGSGPPK.G** 19234 |
|  | 20610 | **1035.4612** | **2068.9078** | **2068.9200** | **-0.0122** | **0** | **101** | **6.6e-009** | **1** | **R.APVAGTCYQAEWDDYVPK.L** 20611 |
|  | 25842 | **931.7991** | **2792.3754** | **2792.3956** | **-0.0202** | **1** | **27** | **0.14** | **1** | **R.YVWLVYEQDRPLKCDEPILSNR.S** 25843 |
|  | 28750 | **1157.9028** | **3470.6867** | **3470.7106** | **-0.0240** | **0** | **52** | **0.00022** | **1** | **K.WSGPLSLQEVDEQPQHPLHVTYAGAAVDELGK.V** 28752 |

| **132.** | P27824\|CALX_HUMAN    **Mass:** 67982    **Score:** 382    **Queries matched:** 36   **emPAI:** 1.00 |
| --- | --- |
|  | Calnexin OS=Homo sapiens GN=CANX PE=1 SV=2 |

|  | **Query** | **Observed** | **Mr(expt)** | **Mr(calc)** | **Delta** | **Miss** | **Score** | **Expect** | **Rank** | **Peptide** |
| --- | --- | --- | --- | --- | --- | --- | --- | --- | --- | --- |
|  | 1496 | **671.2761** | **670.2688** | **669.4034** | **0.8654** | **1** | **6** | **45** | **6** | **R.NRKPR.R** |
|  | 3291 | **451.7311** | **901.4477** | **901.4545** | **-0.0068** | **1** | **29** | **0.24** | **1** | **K.TYFTDKK.T** 3292 |
|  | 4104 | **481.7777** | **961.5409** | **961.5457** | **-0.0048** | **1** | **15** | **6.6** | **1** | **R.AKHHAISAK.L** 4116 |
|  | 4147 | **483.2652** | **964.5159** | **965.4164** | **-0.9005** | **0** | **3** | **91** | **2** | **K.WEVEEMK.E** |
|  | 4688 | **502.2441** | **1002.4736** | **1001.4301** | **1.0435** | **0** | **9** | **25** | **8** | **R.EIEDPEDR.K** |
|  | 5441 | **531.2995** | **1060.5844** | **1060.5917** | **-0.0072** | **0** | **41** | **0.013** | **1** | **R.GTLSGWILSK.A** |
|  | 5800 | **545.3002** | **1088.5858** | **1087.5145** | **1.0713** | **0** | **59** | **0.00024** | **1** | **K.AEEDEILNR.S** 5782 |
|  | 6574 | 575.3066 | 1148.5987 | 1148.5197 | 0.0790 | 1 | 10 | 15 | 2 | K.KDDTDDEIAK.Y |
|  | 9203 | **647.7980** | **1293.5814** | **1293.5911** | **-0.0097** | **1** | **16** | **3.7** | **1** | **K.WEVEEMKESK.L** |
|  | 9324 | **434.2138** | **1299.6197** | **1298.6255** | **0.9942** | **0** | **12** | **8.8** | **1** | **R.KPEDWDERPK.I** |
|  | 12083 | **486.2370** | **1455.6891** | **1455.6994** | **-0.0103** | **0** | **38** | **0.02** | **1** | **K.TPELNLDQFHDK.T** |
|  | 14604 | 808.9011 | 1615.7876 | 1615.7995 | -0.0119 | 1 | 46 | 0.0033 | 1 | R.IVDDWANDGWGLKK.A 14605 |
|  | 16469 | **868.4161** | **1734.8176** | **1734.8253** | **-0.0077** | **0** | **49** | **0.0015** | **1** | **K.IPNPDFFEDLEPFR.M** |
|  | 16956 | **885.9149** | **1769.8153** | **1769.8261** | **-0.0108** | **0** | **72** | **6.3e-006** | **1** | **K.APVPTGEVYFADSFDR.G** 16955 |
|  | 18184 | **932.4631** | **1862.9116** | **1862.9203** | **-0.0087** | **1** | **50** | **0.001** | **1** | **R.KIPNPDFFEDLEPFR.M** 18182 18204 |
|  | 21950 | **738.3779** | **2212.1120** | **2212.1251** | **-0.0132** | **0** | **5** | **25** | **1** | **K.WKPPMIDNPSYQGIWKPR.K** |
|  | 22547 | **761.6836** | **2282.0289** | **2282.0451** | **-0.0161** | **1** | **13** | **4.4** | **1** | **R.EIEDPEDRKPEDWDERPK.I** |
|  | 23754 | **1224.0576** | **2446.1007** | **2446.1158** | **-0.0151** | **0** | **46** | **0.0016** | **1** | **R.CESAPGCGVWQRPVIDNPNYK.G** 23755 |
|  | 25378 | **903.1014** | **2706.2825** | **2706.3000** | **-0.0175** | **1** | **38** | **0.0097** | **1** | **K.TPELNLDQFHDKTPYTIMFGPDK.C** 25379 25380 |
|  | 25980 | **938.4715** | **2812.3927** | **2812.4072** | **-0.0145** | **1** | **31** | **0.045** | **1** | **K.APVPTGEVYFADSFDRGTLSGWILSK.A** |
|  | 29295 | **926.2267** | **3700.8776** | **3700.8963** | **-0.0187** | **0** | **16** | **0.74** | **1** | **K.LNKPFLFDTKPLIVQYEVNFQNGIECGGAYVK.L** 29296 |
|  | 30883 | **1479.7444** | **4436.2113** | **4436.2322** | **-0.0209** | **0** | **68** | **3.1e-006** | **1** | **K.THLYTLILNPDNSFEILVDQSVVNSGNLLNDMTPPVNPSR.E** 30884 30885 |
|  | 31792 | **977.4782** | **5858.8256** | **5857.7602** | **1.0654** | **0** | **1** | **7.5** | **3** | **K.WLLCMLLVLGTAIVEAHDGHDDDVIDIEDDLDDVIEEVEDSKPDTTAPPSSPK.V** |

| **133.** | Q16851\|UGPA_HUMAN    **Mass:** 57076    **Score:** 375    **Queries matched:** 28   **emPAI:** 0.46 |
| --- | --- |
|  | UTP--glucose-1-phosphate uridylyltransferase OS=Homo sapiens GN=UGP2 PE=1 SV=5 |

|  | **Query** | **Observed** | **Mr(expt)** | **Mr(calc)** | **Delta** | **Miss** | **Score** | **Expect** | **Rank** | **Peptide** |
| --- | --- | --- | --- | --- | --- | --- | --- | --- | --- | --- |
|  | 2430 | **419.2371** | **836.4596** | **835.4440** | **1.0157** | **0** | **12** | **9.7** | **5** | **R.FVQDLSK.A** |
|  | 4580 | **499.2563** | **996.4980** | **995.6015** | **0.8965** | **0** | **7** | **32** | **1** | **R.LVEIAQVPK.A** |
|  | 5016 | 514.7588 | 1027.5030 | 1027.5087 | -0.0057 | 0 | 18 | 2.7 | 2 | K.IYTFNQSR.Y 5015 |
|  | 7259 | **398.5677** | **1192.6814** | **1193.5533** | **-0.8719** | **0** | **2** | **97** | **8** | **K.LNGGLGTSMGCK.G** |
|  | 8527 | **628.8419** | **1255.6693** | **1255.6772** | **-0.0079** | **0** | **9** | **18** | **4** | **R.GLPDNISSVLNK.L** |
|  | 8704 | **422.7545** | **1265.2418** | **1264.7027** | **0.5391** | **0** | **2** | **55** | **4** | **R.IDIPPGAVLENK.I** |
|  | 12717 | **498.2749** | **1491.8029** | **1491.7174** | **0.0855** | **1** | **13** | **6.6** | **1** | **K.LNGGLGTSMGCKGPK.S** |
|  | 13033 | **757.3913** | **1512.7680** | **1512.7936** | **-0.0256** | **1** | **5** | **42** | **9** | **K.LGSSFTKVQDYLR.R** |
|  | 15372 | **833.8904** | **1665.7663** | **1665.7781** | **-0.0117** | **0** | **61** | **8.6e-005** | **1** | **K.AMSQDGASQFQEVIR.Q** 15367 15373 |
|  | 15589 | 561.2679 | 1680.7820 | 1681.7730 | -0.9910 | 0 | (4) | 45 | 3 | K.AMSQDGASQFQEVIR.Q |
|  | 15892 | **850.4297** | **1698.8448** | **1698.8577** | **-0.0128** | **0** | **46** | **0.0026** | **1** | **K.IQRPPEDSIQPYEK.I** 15893 |
|  | 16348 | **576.9643** | **1727.8710** | **1727.8842** | **-0.0132** | **1** | **21** | **0.84** | **1** | **K.ILTTASSHEFEHTKK.D** |
|  | 17449 | **452.7530** | **1806.9827** | **1806.0112** | **0.9716** | **1** | **7** | **25** | **2** | **K.NVSLKGTVIIIANHGDR.I** |
|  | 19614 | **992.5564** | **1983.0982** | **1982.1048** | **0.9935** | **0** | **56** | **0.00024** | **1** | **K.TLDGGLNVIQLETAVGAAIK.S** 19595 |
|  | 19821 | **667.3299** | **1998.9679** | **2000.0360** | **-1.0682** | **1** | **2** | **58** | **3** | **K.RLQEQNAIDMEIIVNAK.T** |
|  | 21432 | 1078.5457 | 2155.0767 | 2155.0909 | -0.0142 | 0 | 90 | 8.6e-008 | 1 | R.NENTFLDLTVQQIEHLNK.T 21431 |
|  | 24459 | **849.0704** | **2544.1895** | **2544.2054** | **-0.0159** | **1** | **18** | **1.2** | **1** | **K.TYNTDVPLVLMNSFNTDEDTKK.I** 24460 |
|  | 25258 | **1344.6704** | **2687.3263** | **2687.3397** | **-0.0135** | **0** | **105** | **1.9e-009** | **1** | **K.TTSDLLLVMSNLYSLNAGSLTMSEK.R** 25260 |
|  | 25376 | **902.9741** | **2705.9004** | **2705.3371** | **0.5633** | **1** | **9** | **4.6** | **1** | **R.RFESIPDMLELDHLTVSGDVTFGK.N** |
|  | 26345 | 1438.6443 | 2875.2740 | 2875.4307 | -0.1567 | 1 | 5 | 15 | 2 | K.TTSDLLLVMSNLYSLNAGSLTMSEKR.E |

| **134.** | P28331\|NDUS1_HUMAN    **Mass:** 80443    **Score:** 372    **Queries matched:** 46   **emPAI:** 0.57 |
| --- | --- |
|  | NADH-ubiquinone oxidoreductase 75 kDa subunit, mitochondrial OS=Homo sapiens GN=NDUFS1 PE=1 SV=3 |

|  | **Query** | **Observed** | **Mr(expt)** | **Mr(calc)** | **Delta** | **Miss** | **Score** | **Expect** | **Rank** | **Peptide** |
| --- | --- | --- | --- | --- | --- | --- | --- | --- | --- | --- |
|  | 1248 | 593.3254 | 592.3181 | 592.3221 | -0.0039 | 0 | 22 | 0.97 | 1 | R.FLEGK.R |
|  | 1263 | **600.3673** | **599.3600** | **599.3279** | **0.0321** | **0** | **21** | **1.3** | **1** | **R.QDLPK.D** |
|  | 1957 | **401.2846** | **800.5546** | **799.4374** | **1.1172** | **0** | **13** | **11** | **4** | **K.VGMQIPR.F** 1927 1940 |
|  | 2144 | **407.8894** | **813.7642** | **814.5276** | **-0.7634** | **1** | **14** | **5.4** | **9** | **R.KALVGLSK.S** |
|  | 2529 | **423.2285** | **844.4425** | **844.4477** | **-0.0051** | **0** | **9** | **26** | **1** | **R.LTEPMVR.N** |
|  | 2716 | 430.9235 | 859.8324 | 860.4426 | -0.6102 | 0 | (4) | 54 | 2 | R.LTEPMVR.N 2714 |
|  | 2957 | **438.7188** | **875.4230** | **875.4283** | **-0.0053** | **0** | **6** | **49** | **3** | **R.LSVAGNCR.M** |
|  | 6801 | **583.7955** | **1165.5764** | **1164.5961** | **0.9803** | **0** | **3** | **70** | **7** | **R.VAGMLQSFQGK.D** |
|  | 6873 | 586.3153 | 1170.6161 | 1170.6543 | -0.0382 | 1 | 7 | 34 | 8 | R.TGEVMRILPR.M |
|  | 6930 | **588.7966** | **1175.5786** | **1175.5865** | **-0.0079** | **0** | **26** | **0.38** | **1** | **K.VVAACAMPVMK.G** |
|  | 9648 | **439.8941** | **1316.6606** | **1316.6832** | **-0.0226** | **1** | **11** | **12** | **3** | **R.MCLVEIEKAPK.V** |
|  | 9996 | **667.8162** | **1333.6178** | **1332.7302** | **0.8875** | **1** | **7** | **29** | **7** | **R.FEAPLFNARIR.K** |
|  | 10351 | **677.8147** | **1353.6148** | **1353.6235** | **-0.0086** | **0** | **35** | **0.045** | **1** | **R.GNDMQVGTYIEK.M** 10350 |
|  | 10646 | **685.6694** | **1369.3243** | **1369.6184** | **-0.2941** | **0** | **(3)** | **48** | **4** | **R.GNDMQVGTYIEK.M** |
|  | 10910 | **693.3568** | **1384.6991** | **1384.7860** | **-0.0870** | **0** | **2** | **94** | **8** | **K.KPMVVLGSSALQR.N** |
|  | 11620 | **713.3890** | **1424.7635** | **1424.7735** | **-0.0100** | **1** | **45** | **0.0046** | **1** | **R.NRLEEVSPNLVR.Y** 11619 |
|  | 13289 | **764.9157** | **1527.8169** | **1527.8256** | **-0.0088** | **0** | **48** | **0.0019** | **1** | **R.NDGAAILAAVSSIAQK.I** 13290 |
|  | 13750 | **780.8460** | **1559.6775** | **1559.7137** | **-0.0363** | **0** | **0** | **1.1e+002** | **4** | **K.AVTEGAQAVEEPSIC.-** |
|  | 14474 | **804.8907** | **1607.7668** | **1607.7791** | **-0.0123** | **0** | **60** | **0.00011** | **1** | **R.FASEIAGVDDLGTTGR.G** 14475 |
|  | 14532 | 806.9012 | 1611.7878 | 1610.7940 | 0.9938 | 0 | 79 | 1.6e-006 | 1 | K.GLLTYTSWEDALSR.V |
|  | 16022 | 854.3947 | 1706.7749 | 1707.7974 | -1.0225 | 1 | 8 | 18 | 4 | K.VGMQIPRFCYHER.L |
|  | 16349 | 576.9643 | 1727.8710 | 1728.9556 | -1.0846 | 1 | 2 | 62 | 7 | K.EAKKPMVVLGSSALQR.N |
|  | 17299 | **599.3283** | **1794.9631** | **1795.0091** | **-0.0460** | **0** | **0** | **99** | **7** | **K.DVAAIAGGLVDAEALVALK.D** |
|  | 17844 | **920.4954** | **1838.9762** | **1838.9890** | **-0.0129** | **0** | **71** | **8.4e-006** | **1** | **K.ILQDIASGSHPFSQVLK.E** 17845 |
|  | 19464 | **657.0141** | **1968.0205** | **1966.8765** | **1.1440** | **1** | **10** | **8.8** | **2** | **K.DFYMTDSISRASQTMAK.C** |
|  | 20878 | **1047.0276** | **2092.0406** | **2092.0510** | **-0.0104** | **0** | **54** | **0.00035** | **1** | **R.ALSEIAGMTLPYDTLDQVR.N** 20877 |
|  | 20948 | **1050.6180** | **2099.2215** | **2099.2354** | **-0.0139** | **0** | **10** | **8.3** | **1** | **K.LVNQQLLADPLVPPQLTIK.D** 20949 |
|  | 20991 | **1053.4620** | **2104.9095** | **2103.9385** | **0.9710** | **0** | **35** | **0.03** | **1** | **R.YDDIEGANYFQQANELSK.L** 20973 20992 |
|  | 21795 | **550.5638** | **2198.2263** | **2198.2423** | **-0.0160** | **1** | **13** | **3.8** | **1** | **R.IASQVAALDLGYKPGVEAIRK.N** |
|  | 23137 | 1180.5781 | 2359.1417 | 2360.1900 | -1.0483 | 0 | 11 | 6.9 | 2 | K.VALIGSPVDLTYTYDHLGDSPK.I 23136 |
|  | 25116 | 889.0650 | 2664.1732 | 2665.3051 | -1.1320 | 1 | 2 | 34 | 4 | K.TESIDVMDAVGSNIVVSTRTGEVMR.I |
|  | 28000 | **1076.8599** | **3227.5578** | **3226.5757** | **0.9820** | **0** | **45** | **0.0015** | **1** | **K.DCFIIYQGHHGDVGAPIADVILPGAAYTEK.S** |
|  | 30682 | **616.1635** | **4306.0932** | **4305.0801** | **1.0131** | **1** | **4** | **7.9** | **1** | **K.DCFIIYQGHHGDVGAPIADVILPGAAYTEKSATYVNTEGR.A** |

| **135.** | P00352\|AL1A1_HUMAN    **Mass:** 55454    **Score:** 364    **Queries matched:** 32   **emPAI:** 1.33 |
| --- | --- |
|  | Retinal dehydrogenase 1 OS=Homo sapiens GN=ALDH1A1 PE=1 SV=2 |

|  | **Query** | **Observed** | **Mr(expt)** | **Mr(calc)** | **Delta** | **Miss** | **Score** | **Expect** | **Rank** | **Peptide** |
| --- | --- | --- | --- | --- | --- | --- | --- | --- | --- | --- |
|  | 779 | **476.1964** | **475.1892** | **474.2438** | **0.9454** | **0** | **5** | **1e+002** | **4** | **K.EAAGK.S** |
|  | 2083 | **405.2073** | **808.4001** | **808.3385** | **0.0616** | **0** | **1** | **1.3e+002** | **6** | **R.TMDASER.G** |
|  | 2324 | 829.4149 | 828.4076 | 828.4705 | -0.0629 | 0 | 4 | 73 | 3 | K.LADLIER.D |
|  | 3869 | **473.2683** | **944.5220** | **944.5291** | **-0.0071** | **1** | **19** | **2.3** | **2** | **K.SLDDVIKR.A** 3868 |
|  | 4236 | 486.7922 | 971.5699 | 971.5764 | -0.0064 | 1 | 26 | 0.42 | 1 | K.RVTLELGGK.S |
|  | 5877 | **548.2840** | **1094.5534** | **1094.5608** | **-0.0074** | **0** | **49** | **0.0018** | **1** | **K.VAFTGSTEVGK.L** |
|  | 5945 | 550.8029 | 1099.5912 | 1099.5985 | -0.0074 | 1 | 34 | 0.066 | 1 | K.LADLIERDR.L |
|  | 6145 | **558.3331** | **1114.6517** | **1114.6597** | **-0.0080** | **1** | **24** | **0.66** | **1** | **K.ILDLIESGKK.E** |
|  | 13521 | **772.8860** | **1543.7575** | **1543.7671** | **-0.0096** | **0** | **27** | **0.23** | **1** | **R.TIPIDGNFFTYTR.H** |
|  | 14190 | **795.3961** | **1588.7777** | **1588.7886** | **-0.0109** | **0** | **79** | **1.6e-006** | **1** | **R.ANNTFYGLSAGVFTK.D** |
|  | 15057 | **823.3964** | **1644.7783** | **1644.7896** | **-0.0113** | **0** | **80** | **1.1e-006** | **1** | **K.IFINNEWHDSVSGK.K** 15055 |
|  | 15058 | **823.4038** | **1644.7931** | **1644.8035** | **-0.0105** | **0** | **35** | **0.034** | **1** | **R.IFVEESIYDEFVR.R** |
|  | 15143 | **825.9385** | **1649.8624** | **1650.8069** | **-0.9445** | **1** | **13** | **5.7** | **2** | **R.DRLLLATMESMNGGK.L** 15162 15177 |
|  | 15902 | **850.8884** | **1699.7622** | **1699.7729** | **-0.0108** | **0** | **41** | **0.0091** | **1** | **R.ELGEYGFHEYTEVK.T** 15901 |
|  | 16151 | **857.9215** | **1713.8285** | **1713.8396** | **-0.0111** | **0** | **36** | **0.026** | **2** | **K.LYSNAYLNDLAGCIK.T** 16135 |
|  | 16419 | **865.9731** | **1729.9317** | **1729.9436** | **-0.0119** | **1** | **66** | **3e-005** | **1** | **R.IAKEEIFGPVQQIMK.F** 16420 |
|  | 19693 | **995.4638** | **1988.9129** | **1988.9302** | **-0.0173** | **0** | **43** | **0.0044** | **1** | **K.GYFVQPTVFSNVTDEMR.I** |
|  | 25161 | **1337.6819** | **2673.3492** | **2673.3650** | **-0.0158** | **1** | **41** | **0.0048** | **1** | **K.YILGNPLTPGVTQGPQIDKEQYDK.I** |
|  | 25584 | 916.4871 | 2746.4394 | 2746.4491 | -0.0098 | 0 | 60 | 5.8e-005 | 1 | R.HEPIGVCGQIIPWNFPLVMLIWK.I |
|  | 27407 | **1023.1713** | **3066.4920** | **3066.5121** | **-0.0201** | **0** | **13** | **2.5** | **1** | **K.EAGFPPGVVNIVPGYGPTAGAAISSHMDIDK.V** 27408 |
|  | 27853 | **796.9443** | **3183.7480** | **3183.7690** | **-0.0210** | **0** | **27** | **0.088** | **1** | **K.IGPALSCGNTVVVKPAEQTPLTALHVASLIK.E** 27854 |
|  | 30419 | **1036.7670** | **4143.0388** | **4143.0623** | **-0.0236** | **1** | **7** | **4.8** | **1** | **K.EAGFPPGVVNIVPGYGPTAGAAISSHMDIDKVAFTGSTEVGK.L** 30418 |

| **136.** | Q9Y5M8\|SRPRB_HUMAN    **Mass:** 29912    **Score:** 360    **Queries matched:** 20   **emPAI:** 1.05 |
| --- | --- |
|  | Signal recognition particle receptor subunit beta OS=Homo sapiens GN=SRPRB PE=1 SV=3 |

|  | **Query** | **Observed** | **Mr(expt)** | **Mr(calc)** | **Delta** | **Miss** | **Score** | **Expect** | **Rank** | **Peptide** |
| --- | --- | --- | --- | --- | --- | --- | --- | --- | --- | --- |
|  | 4634 | **500.2922** | **998.5698** | **998.5760** | **-0.0062** | **0** | **31** | **0.13** | **1** | **K.LIQQQLEK.E** |
|  | 8032 | **616.3355** | **1230.6563** | **1230.6642** | **-0.0079** | **0** | **41** | **0.014** | **1** | **R.AVLLVGLCDSGK.T** |
|  | 8678 | **632.8002** | **1263.5858** | **1263.6281** | **-0.0424** | **0** | **5** | **50** | **5** | **K.NTPSFLIACNK.Q** |
|  | 10197 | **673.8207** | **1345.6268** | **1345.6361** | **-0.0093** | **0** | **45** | **0.0052** | **1** | **R.GDVGSADIQDLEK.W** 10212 |
|  | 11549 | 711.3719 | 1420.7292 | 1421.7667 | -1.0375 | 0 | 32 | 0.088 | 1 | R.AIVFVVDSAAFQR.E 11550 |
|  | 14594 | **808.8586** | **1615.7026** | **1615.7148** | **-0.0122** | **0** | **6** | **29** | **2** | **R.DTQTSITDSCAVYR.V** |
|  | 14598 | 808.8916 | 1615.7686 | 1615.7802 | -0.0115 | 1 | 59 | 0.00017 | 1 | K.GGRGDVGSADIQDLEK.W 14599 14600 14604 |
|  | 16243 | **861.4746** | **1720.9345** | **1720.9108** | **0.0238** | **0** | **1** | **89** | **2** | **R.GNSLTLIDLPGHESLR.L** |
|  | 17221 | **894.9462** | **1787.8778** | **1787.8901** | **-0.0123** | **0** | **67** | **2.2e-005** | **1** | **R.SAAPSTLDSSSTAPAQLGK.K** 17222 |
|  | 20404 | **1026.5869** | **2051.1593** | **2050.0232** | **1.1360** | **1** | **0** | **77** | **9** | **R.RVADGGGAGGTFQPYLDTLR.Q** |
|  | 21324 | **715.3400** | **2142.9981** | **2144.1073** | **-1.1092** | **1** | **2** | **61** | **6** | **R.VTRSAAPSTLDSSSTAPAQLGK.K** |
|  | 22653 | **1149.1035** | **2296.1925** | **2296.2025** | **-0.0100** | **1** | **113** | **3.8e-010** | **1** | **R.EVKDVAEFLYQVLIDSMGLK.N** 22651 22652 |

| **137.** | A1L0T0\|ILVBL_HUMAN    **Mass:** 68452    **Score:** 360    **Queries matched:** 27   **emPAI:** 0.61 |
| --- | --- |
|  | Acetolactate synthase-like protein OS=Homo sapiens GN=ILVBL PE=1 SV=2 |

|  | **Query** | **Observed** | **Mr(expt)** | **Mr(calc)** | **Delta** | **Miss** | **Score** | **Expect** | **Rank** | **Peptide** |
| --- | --- | --- | --- | --- | --- | --- | --- | --- | --- | --- |
|  | 3100 | **443.2293** | **884.4441** | **885.4933** | **-1.0492** | **0** | **11** | **14** | **2** | **R.NHPLHIR.E** |
|  | 3123 | **444.7604** | **887.5063** | **887.4824** | **0.0238** | **1** | **5** | **62** | **8** | **R.ENRSAALK.K** |
|  | 3754 | **469.2529** | **936.4913** | **935.4825** | **1.0088** | **1** | **10** | **16** | **9** | **R.QKEQTFR.E** |
|  | 8032 | 616.3355 | 1230.6563 | 1230.7125 | -0.0561 | 0 | 10 | 15 | 4 | R.LGLFYQLLHK.V |
|  | 8482 | **627.7998** | **1253.5850** | **1253.5935** | **-0.0085** | **0** | **41** | **0.011** | **1** | **K.VLHDAQQQCR.D** 8480 |
|  | 10994 | **695.3917** | **1388.7689** | **1387.7936** | **0.9753** | **0** | **6** | **34** | **2** | **R.DGHPVVVNILIGR.T** |
|  | 12495 | 739.8661 | 1477.7176 | 1477.8439 | -0.1263 | 1 | (1) | 1e+002 | 10 | K.EMVPAKPPKGLVGR.V |
|  | 12748 | 747.8700 | 1493.7254 | 1493.8388 | -0.1134 | 1 | 11 | 10 | 2 | K.EMVPAKPPKGLVGR.V |
|  | 16643 | **874.9394** | **1747.8642** | **1747.8750** | **-0.0107** | **0** | **41** | **0.0085** | **1** | **R.AAVETLGVPCFLGGMAR.G** 16642 |
|  | 18988 | 965.0200 | 1928.0255 | 1926.9986 | 1.0269 | 0 | 28 | 0.17 | 2 | K.IPVMALVGNDAGWTQISR.E |
|  | 19787 | **999.5811** | **1997.1477** | **1997.0979** | **0.0498** | **0** | **16** | **1.9** | **1** | **K.RPLMVLGSQALLTPTSADK.L** |
|  | 19879 | **1003.0251** | **2004.0356** | **2004.0469** | **-0.0113** | **0** | **66** | **2.2e-005** | **1** | **R.WLDPGAFGTLGVGAGFALGAK.L** 19880 |
|  | 21687 | **1092.6079** | **2183.2013** | **2183.2162** | **-0.0149** | **0** | **53** | **0.00039** | **1** | **R.LSGTVGVAAVTAGPGLTNTVTAVK.N** 21688 |
|  | 21783 | **733.0790** | **2196.2151** | **2196.2300** | **-0.0149** | **1** | **8** | **11** | **1** | **R.AKRPLMVLGSQALLTPTSADK.L** |
|  | 23202 | **1184.1373** | **2366.2601** | **2366.2740** | **-0.0139** | **0** | **100** | **7e-009** | **1** | **K.NAQMAQSPILLLGGAASTLLQNR.G** 23201 23203 23204 |
|  | 25302 | **899.7748** | **2696.3027** | **2696.3194** | **-0.0167** | **1** | **23** | **0.3** | **1** | **K.LVEGLQGQTWAPDWVEELREADR.Q** |
|  | 28392 | 1117.2294 | 3348.6663 | 3348.6812 | -0.0150 | 1 | 54 | 0.00017 | 1 | R.NREEMLLNSDIFWKPQEAVQGDVGSFVLK.L |
|  | 30334 | 1027.5724 | 4106.2605 | 4106.1261 | 0.1344 | 1 | 1 | 17 | 6 | R.DIVPTLRAAMAAAQSGTPGPVFVELPVDVLYPYFMVQK.E |
|  | 31214 | **1215.3898** | **4857.5300** | **4857.5488** | **-0.0188** | **0** | **61** | **1.3e-005** | **1** | **K.AAMPVAQHLNPVQVLQLVEETLPDNSILVVDGGDFVGTAAHLVQPR.G** 31213 |

| **138.** | P22760\|AAAD_HUMAN    **Mass:** 45833    **Score:** 359    **Queries matched:** 18   **emPAI:** 0.37 |
| --- | --- |
|  | Arylacetamide deacetylase OS=Homo sapiens GN=AADAC PE=1 SV=4 |

|  | **Query** | **Observed** | **Mr(expt)** | **Mr(calc)** | **Delta** | **Miss** | **Score** | **Expect** | **Rank** | **Peptide** |
| --- | --- | --- | --- | --- | --- | --- | --- | --- | --- | --- |
|  | 5018 | 514.7596 | 1027.5046 | 1026.5345 | 0.9701 | 0 | 12 | 11 | 2 | R.AAPLLADDNK.L |
|  | 6507 | **572.3724** | **1142.7302** | **1142.5729** | **0.1573** | **0** | **4** | **63** | **6** | **R.MMWINAHLK.T** |
|  | 8341 | **416.2596** | **1245.7570** | **1244.6877** | **1.0693** | **1** | **5** | **42** | **10** | **K.VLAKYGVNPER.I** |
|  | 11286 | **704.3681** | **1406.7216** | **1406.7307** | **-0.0090** | **0** | **29** | **0.16** | **1** | **R.QHVPVESSHLFK.F** 11287 |
|  | 12191 | 731.3417 | 1460.6689 | 1460.8504 | -0.1814 | 1 | 2 | 87 | 5 | K.FNNILVRVYVPK.R |
|  | 16653 | **875.4070** | **1748.7995** | **1748.8118** | **-0.0123** | **0** | **74** | **4e-006** | **1** | **K.GHVYNNPNYGSSELAK.K** 16652 16654 |
|  | 18209 | **622.3441** | **1864.0104** | **1864.9432** | **-0.9328** | **1** | **1** | **74** | **6** | **R.WTADRLDAVVVSTNYR.L** |
|  | 24603 | **854.4411** | **2560.3015** | **2560.3148** | **-0.0133** | **0** | **15** | **2.4** | **1** | **K.TIQNLATFVELLGLHHFMDSFK.V** 24622 |
|  | 24951 | **1313.6654** | **2625.3163** | **2624.3293** | **0.9869** | **0** | **134** | **2.6e-012** | **1** | **R.IGISGDSAGGNLAAAVTQQLLDDPDVK.I** 24944 24952 |
|  | 25421 | **904.4449** | **2710.3128** | **2710.3252** | **-0.0124** | **0** | **22** | **0.39** | **1** | **R.NTGVQVTHNHVEDGFHGAFSFLGLK.I** |
|  | 26310 | **956.5051** | **2866.4936** | **2865.5084** | **0.9852** | **1** | **20** | **0.55** | **1** | **R.IGISGDSAGGNLAAAVTQQLLDDPDVKIK.L** |
|  | 28686 | **1726.8956** | **3451.7767** | **3451.7915** | **-0.0148** | **0** | **42** | **0.0023** | **1** | **K.IQSLIYPALQPLDVDLPSYQENSNFLFLSK.S** |

| **139.** | P15144\|AMPN_HUMAN    **Mass:** 109870   **Score:** 358    **Queries matched:** 37   **emPAI:** 0.30 |
| --- | --- |
|  | Aminopeptidase N OS=Homo sapiens GN=ANPEP PE=1 SV=4 |

|  | **Query** | **Observed** | **Mr(expt)** | **Mr(calc)** | **Delta** | **Miss** | **Score** | **Expect** | **Rank** | **Peptide** |
| --- | --- | --- | --- | --- | --- | --- | --- | --- | --- | --- |
|  | 3267 | **901.4934** | **900.4861** | **900.4916** | **-0.0055** | **0** | **16** | **4.5** | **1** | **R.ALEQALEK.T** |
|  | 5724 | **542.7359** | **1083.4572** | **1083.4655** | **-0.0082** | **0** | **17** | **3.4** | **1** | **R.SEYMEGNVR.K** |
|  | 5952 | **550.9353** | **1099.8560** | **1099.4604** | **0.3956** | **0** | **(8)** | **17** | **3** | **R.SEYMEGNVR.K** |
|  | 6857 | 585.7937 | 1169.5728 | 1168.5724 | 1.0004 | 0 | 10 | 14 | 3 | R.GVGGSQPPDIDK.T |
|  | 7903 | **612.7635** | **1223.5124** | **1223.5207** | **-0.0083** | **0** | **19** | **1.6** | **1** | **R.VNYDEENWR.K** 7894 |
|  | 9368 | 434.8809 | 1301.6210 | 1302.6932 | -1.0722 | 0 | 17 | 2.8 | 1 | R.LPNTLKPDSYR.V 9365 9367 9370 9371 9374 9390 |
|  | 10462 | **681.3412** | **1360.6679** | **1360.6769** | **-0.0089** | **0** | **72** | **9.1e-006** | **1** | **K.VVATTQMQAADAR.K** |
|  | 11096 | **466.2285** | **1395.6637** | **1396.6219** | **-0.9582** | **1** | **8** | **20** | **4** | **K.KDNEETGFGSGTR.A** 11105 |
|  | 11213 | **702.3476** | **1402.6806** | **1401.6850** | **0.9957** | **0** | **60** | **0.00015** | **1** | **R.MLSSFLSEDVFK.Q** |
|  | 11919 | **482.5790** | **1444.7150** | **1443.7068** | **1.0083** | **1** | **8** | **23** | **6** | **R.SEVYGPMKNYLK.K** |
|  | 12218 | 732.3626 | 1462.7105 | 1462.7205 | -0.0099 | 0 | 17 | 2.8 | 1 | R.QQQDYWLIDVR.A |
|  | 12261 | **489.5734** | **1465.6983** | **1466.7769** | **-1.0786** | **0** | **2** | **72** | **10** | **R.YLSYTLNPDLIR.K** |
|  | 12674 | **745.3879** | **1488.7612** | **1488.7719** | **-0.0106** | **1** | **81** | **9.2e-007** | **1** | **R.KVVATTQMQAADAR.K** 12681 |
|  | 12894 | **752.8549** | **1503.6953** | **1504.7668** | **-1.0715** | **1** | **1** | **98** | **1** | **K.VVATTQMQAADARK.S** |
|  | 14281 | 532.2592 | 1593.7558 | 1594.8719 | -1.1160 | 1 | 1 | 91 | 8 | R.YLSYTLNPDLIRK.Q |
|  | 14419 | 535.6225 | 1603.8457 | 1603.7374 | 0.1082 | 1 | 11 | 9.8 | 3 | K.LMFDRSEVYGPMK.N 14418 |
|  | 16294 | **862.9152** | **1723.8159** | **1723.8264** | **-0.0105** | **0** | **56** | **0.00025** | **1** | **R.ENSLLFDPLSSSSSNK.E** 16295 |
|  | 18892 | **641.2933** | **1920.8582** | **1919.9127** | **0.9454** | **0** | **0** | **88** | **7** | **R.QYMPWEAALSSLSYFK.L** |
|  | 19512 | **658.6471** | **1972.9194** | **1972.9326** | **-0.0131** | **0** | **24** | **0.38** | **1** | **K.QWMENPNNNPIHPNLR.S** |
|  | 22631 | **1147.0599** | **2292.1053** | **2292.1175** | **-0.0122** | **0** | **33** | **0.042** | **1** | **K.LFNDYGGGSFSFSNLIQAVTR.R** |
|  | 24567 | 1277.5983 | 2553.1820 | 2553.1958 | -0.0138 | 0 | 67 | 1.5e-005 | 1 | K.SDQIGLPDFNAGAMENWGLVTYR.E 24568 |
|  | 25499 | **910.7332** | **2729.1778** | **2728.4660** | **0.7118** | **1** | **0** | **51** | **8** | **R.LPNTLKPDSYRVTLRPYLTPNDR.G** |
|  | 26618 | **974.5085** | **2920.5036** | **2919.5230** | **0.9806** | **1** | **8** | **7.7** | **1** | **R.GVGGSQPPDIDKTELVEPTEYLVVHLK.G** |
|  | 29807 | **1287.9738** | **3860.8994** | **3859.9189** | **0.9805** | **0** | **65** | **9.1e-006** | **1** | **R.VMAVDALASSHPLSTPASEINTPAQISELFDAISYSK.G** 29806 |

| **140.** | Q9BPW8\|NIPS1_HUMAN    **Mass:** 33460    **Score:** 358    **Queries matched:** 17   **emPAI:** 1.62 |
| --- | --- |
|  | Protein NipSnap homolog 1 OS=Homo sapiens GN=NIPSNAP1 PE=1 SV=1 |

|  | **Query** | **Observed** | **Mr(expt)** | **Mr(calc)** | **Delta** | **Miss** | **Score** | **Expect** | **Rank** | **Peptide** |
| --- | --- | --- | --- | --- | --- | --- | --- | --- | --- | --- |
|  | 4380 | **491.7623** | **981.5100** | **981.5131** | **-0.0031** | **1** | **6** | **36** | **4** | **K.KETSNLYK.I** |
|  | 5830 | **546.7756** | **1091.5366** | **1091.5433** | **-0.0067** | **0** | **53** | **0.00088** | **1** | **R.MGPNIYELR.T** 5844 |
|  | 14211 | **795.8358** | **1589.6571** | **1589.6676** | **-0.0106** | **0** | **52** | **0.00073** | **1** | **R.FSGGYPALMDCMNK.L** 14210 |
|  | 16037 | 427.7196 | 1706.8492 | 1705.9223 | 0.9268 | 1 | 11 | 9.1 | 2 | R.LLGGPGPRAGDVASAAAAR.F 15995 |
|  | 16975 | **886.9424** | **1771.8702** | **1771.8828** | **-0.0126** | **0** | **41** | **0.009** | **1** | **K.LKPGTMIEWGNNWAR.A** 16974 16976 |
|  | 17956 | **617.0051** | **1847.9936** | **1846.8416** | **1.1520** | **1** | **3** | **57** | **9** | **R.FSGGYPALMDCMNKLK.N** |
|  | 18239 | **933.9724** | **1865.9301** | **1865.9424** | **-0.0123** | **1** | **65** | **3.1e-005** | **1** | **K.RGWDENVYYTVPLVR.H** |
|  | 20003 | **1009.5055** | **2016.9964** | **2017.0057** | **-0.0093** | **0** | **77** | **2.1e-006** | **1** | **R.NQLLLEFSFWNEPQPR.M** |
|  | 27723 | **788.1388** | **3148.5261** | **3148.5407** | **-0.0146** | **0** | **30** | **0.05** | **1** | **R.QENQEAVGGFFSQIGELYVVHHLWAYK.D** |
|  | 29294 | **1234.5608** | **3700.6605** | **3700.6794** | **-0.0188** | **0** | **84** | **1.4e-007** | **1** | **K.LHLDEDYPCSLVGNWNTWYGEQDQAVHLWR.F** 29292 29293 |

| **141.** | P18859\|ATP5J_HUMAN    **Mass:** 12580    **Score:** 357    **Queries matched:** 10   **emPAI:** 2.93 |
| --- | --- |
|  | ATP synthase-coupling factor 6, mitochondrial OS=Homo sapiens GN=ATP5J PE=1 SV=1 |

|  | **Query** | **Observed** | **Mr(expt)** | **Mr(calc)** | **Delta** | **Miss** | **Score** | **Expect** | **Rank** | **Peptide** |
| --- | --- | --- | --- | --- | --- | --- | --- | --- | --- | --- |
|  | 2876 | 435.2684 | 868.5222 | 867.4926 | 1.0295 | 0 | 13 | 6.5 | 10 | R.SAVSVHLR.R 2851 |
|  | 4982 | **513.3003** | **1024.5861** | **1023.5938** | **0.9924** | **1** | **1** | **1.1e+002** | **7** | **R.SAVSVHLRR.N** |
|  | 16636 | **874.8862** | **1747.7579** | **1747.7698** | **-0.0119** | **0** | **69** | **1.4e-005** | **1** | **K.QMFGNADMNTFPTFK.F** 16637 |
|  | 16896 | **882.8832** | **1763.7518** | **1763.7647** | **-0.0129** | **0** | **(26)** | **0.26** | **1** | **K.QMFGNADMNTFPTFK.F** |
|  | 20749 | **1040.9673** | **2079.9200** | **2079.9345** | **-0.0145** | **0** | **110** | **8.5e-010** | **1** | **R.QTSGGPVDASSEYQQELER.E** 20750 |
|  | 22192 | **746.3472** | **2236.0197** | **2236.0356** | **-0.0159** | **1** | **71** | **6.4e-006** | **1** | **K.RQTSGGPVDASSEYQQELER.E** 22193 |

| **142.** | P24539\|AT5F1_HUMAN    **Mass:** 28947    **Score:** 355    **Queries matched:** 31   **emPAI:** 3.94 |
| --- | --- |
|  | ATP synthase subunit b, mitochondrial OS=Homo sapiens GN=ATP5F1 PE=1 SV=2 |

|  | **Query** | **Observed** | **Mr(expt)** | **Mr(calc)** | **Delta** | **Miss** | **Score** | **Expect** | **Rank** | **Peptide** |
| --- | --- | --- | --- | --- | --- | --- | --- | --- | --- | --- |
|  | 1630 | 719.3702 | 718.3629 | 718.3683 | -0.0054 | 0 | 29 | 0.31 | 1 | K.CIADLK.L |
|  | 2916 | **437.0518** | **872.0890** | **871.4586** | **0.6304** | **1** | **3** | **63** | **6** | **K.KAQAQPVM.-** |
|  | 3266 | **451.2501** | **900.4857** | **900.4916** | **-0.0059** | **0** | **56** | **0.00051** | **1** | **K.LAQLEEAK.Q** |
|  | 3268 | **451.2552** | **900.4959** | **900.5029** | **-0.0069** | **0** | **37** | **0.04** | **1** | **K.SQQALVQK.R** 3250 3269 |
|  | 5384 | **529.3049** | **1056.5953** | **1056.6040** | **-0.0087** | **1** | **35** | **0.055** | **1** | **K.SQQALVQKR.H** |
|  | 5640 | **539.2737** | **1076.5328** | **1076.5403** | **-0.0075** | **0** | **41** | **0.015** | **1** | **R.HYLFDVQR.N** 5639 |
|  | 8100 | **412.2235** | **1233.6487** | **1232.6414** | **1.0073** | **1** | **21** | **1.3** | **1** | **K.RHYLFDVQR.N** |
|  | 9294 | **649.8523** | **1297.6900** | **1297.7605** | **-0.0705** | **0** | **4** | **50** | **4** | **R.VVLSAAATAAPSLK.N** 9279 |
|  | 10879 | **692.3604** | **1382.7061** | **1382.7154** | **-0.0092** | **0** | **70** | **1.3e-005** | **1** | **K.HVVQSISTQQEK.E** 10877 10878 |
|  | 11073 | **465.3406** | **1393.0001** | **1393.7023** | **-0.7023** | **0** | **1** | **79** | **9** | **R.NNIAMALEVTYR.E** |
|  | 12923 | 502.9083 | 1505.7030 | 1505.7119 | -0.0088 | 0 | 36 | 0.032 | 1 | R.LDYHISVQNMMR.R 12925 |
|  | 13893 | **524.2571** | **1569.7496** | **1569.7609** | **-0.0113** | **1** | **29** | **0.18** | **1** | **R.KEQEHMINWVEK.H** |
|  | 15822 | **848.4300** | **1694.8454** | **1694.8587** | **-0.0133** | **0** | **54** | **0.00041** | **1** | **K.QASIQHIQNAIDTEK.S** 15824 |
|  | 17031 | **444.9684** | **1775.8445** | **1775.8559** | **-0.0114** | **1** | **30** | **0.12** | **1** | **K.NRLDYHISVQNMMR.R** 17029 17030 |
|  | 17375 | 901.4455 | 1800.8764 | 1801.0131 | -0.1367 | 1 | 5 | 37 | 4 | -.MLSRVVLSAAATAAPSLK.N |
|  | 17468 | **603.9803** | **1808.9190** | **1807.8457** | **1.0733** | **1** | **(1)** | **94** | **7** | **K.NRLDYHISVQNMMR.R** |
|  | 17871 | 614.6365 | 1840.8876 | 1840.8996 | -0.0120 | 1 | 17 | 2.1 | 1 | K.YGPFVADFADKLNEQK.L |
|  | 18944 | **963.5115** | **1925.0084** | **1925.0218** | **-0.0134** | **1** | **103** | **4.3e-009** | **1** | **K.HVVQSISTQQEKETIAK.C** 18945 18946 |
|  | 31076 | **925.4111** | **4622.0190** | **4622.5213** | **-0.5023** | **1** | **1** | **12** | **4** | **K.TGVTGPYVLGTGLILYALSKEIYVISAETFTALSVLGVMVYGIK.K** |

| **143.** | Q00266\|METK1_HUMAN    **Mass:** 44190    **Score:** 353    **Queries matched:** 24   **emPAI:** 0.92 |
| --- | --- |
|  | S-adenosylmethionine synthetase isoform type-1 OS=Homo sapiens GN=MAT1A PE=1 SV=2 |

|  | **Query** | **Observed** | **Mr(expt)** | **Mr(calc)** | **Delta** | **Miss** | **Score** | **Expect** | **Rank** | **Peptide** |
| --- | --- | --- | --- | --- | --- | --- | --- | --- | --- | --- |
|  | 1280 | **604.4396** | **603.4324** | **604.3003** | **-0.8679** | **0** | **5** | **92** | **8** | **R.MADLR.R** |
|  | 1602 | 709.3584 | 708.3511 | 708.3555 | -0.0043 | 0 | 14 | 6.5 | 1 | R.SAAYAAR.W |
|  | 3974 | 476.7733 | 951.5319 | 951.5389 | -0.0070 | 0 | 21 | 1.2 | 1 | R.ELLDVVHK.N |
|  | 4025 | **478.7952** | **955.5758** | **955.5814** | **-0.0056** | **1** | **45** | **0.0051** | **1** | **R.ALKEQVIR.A** |
|  | 4728 | **503.2318** | **1004.4490** | **1004.4563** | **-0.0073** | **0** | **32** | **0.099** | **1** | **K.HIGYDDSAK.G** |
|  | 5524 | **534.7811** | **1067.5477** | **1067.4607** | **0.0870** | **0** | **31** | **0.12** | **1** | **K.TACYGHFGR.S** 5508 |
|  | 9055 | **643.3682** | **1284.7218** | **1284.7303** | **-0.0085** | **0** | **26** | **0.38** | **1** | **K.NFDLRPGVIVR.D** |
|  | 10454 | **680.8904** | **1359.7663** | **1359.7762** | **-0.0098** | **1** | **14** | **5.5** | **2** | **R.DLDLKKPIYQK.T** |
|  | 10613 | **456.9232** | **1367.7478** | **1367.7561** | **-0.0083** | **0** | **15** | **4.1** | **2** | **R.SGLLPWLRPDSK.T** 10614 |
|  | 11694 | **715.8674** | **1429.7202** | **1429.7314** | **-0.0112** | **0** | **70** | **1.3e-005** | **1** | **R.FVIGGPQGDAGVTGR.K** 11695 |
|  | 13214 | **508.9561** | **1523.8466** | **1523.8572** | **-0.0106** | **1** | **2** | **77** | **4** | **R.RSGLLPWLRPDSK.T** |
|  | 15862 | **566.6187** | **1696.8341** | **1696.8454** | **-0.0113** | **0** | **2** | **67** | **3** | **K.ICDQISDAVLDAHLK.Q** |
|  | 19235 | **975.4714** | **1948.9283** | **1948.9432** | **-0.0149** | **0** | **87** | **1.9e-007** | **1** | **K.IIVDTYGGWGAHGGGAFSGK.D** 19236 |
|  | 20022 | **1010.0143** | **2018.0141** | **2018.0255** | **-0.0114** | **0** | **69** | **1.2e-005** | **1** | **K.TQVTVQYMQDNGAVIPVR.I** 20023 |
|  | 20727 | 520.2636 | 2077.0251 | 2077.0381 | -0.0131 | 1 | 11 | 7.9 | 2 | R.KIIVDTYGGWGAHGGGAFSGK.D |
|  | 29443 | **1252.2285** | **3753.6637** | **3753.6848** | **-0.0211** | **0** | **67** | **5.5e-006** | **1** | **R.NEEDVGAGDQGLMFGYATDETEECMPLTIILAHK.L** |
|  | 29488 | 1257.9551 | 3770.8434 | 3769.6797 | 1.1637 | 0 | (6) | 7.7 | 1 | R.NEEDVGAGDQGLMFGYATDETEECMPLTIILAHK.L |
|  | 31859 | **1281.4011** | **6401.9692** | **6400.9695** | **0.9997** | **1** | **12** | **0.44** | **1** | **K.TCNVLVALEQQSPDIAQCVHLDRNEEDVGAGDQGLMFGYATDETEECMPLTIILAHK.L** 31858 |

| **144.** | P07339\|CATD_HUMAN    **Mass:** 45037    **Score:** 353    **Queries matched:** 17   **emPAI:** 0.61 |
| --- | --- |
|  | Cathepsin D OS=Homo sapiens GN=CTSD PE=1 SV=1 |

|  | **Query** | **Observed** | **Mr(expt)** | **Mr(calc)** | **Delta** | **Miss** | **Score** | **Expect** | **Rank** | **Peptide** |
| --- | --- | --- | --- | --- | --- | --- | --- | --- | --- | --- |
|  | 1530 | **683.8199** | **682.8126** | **683.3490** | **-0.5364** | **0** | **2** | **63** | **9** | **K.SSTYVK.N** |
|  | 2202 | **820.3386** | **819.3313** | **819.4239** | **-0.0926** | **0** | **25** | **0.59** | **1** | **R.VGFAEAAR.L** 2210 |
|  | 2983 | **878.3532** | **877.3459** | **878.4498** | **-1.1039** | **0** | **6** | **45** | **8** | **R.QVFGEATK.Q** |
|  | 8203 | **620.3098** | **1238.6051** | **1238.6118** | **-0.0067** | **0** | **60** | **0.00014** | **1** | **K.FDGILGMAYPR.I** |
|  | 12197 | **731.8336** | **1461.6527** | **1461.6637** | **-0.0110** | **1** | **19** | **1.9** | **1** | **R.YYTVFDRDNNR.V** |
|  | 14386 | 801.4146 | 1600.8147 | 1600.8249 | -0.0103 | 0 | 74 | 4.6e-006 | 1 | K.LVDQNIFSFYLSR.D 14387 |
|  | 17200 | **894.4031** | **1786.7916** | **1786.8044** | **-0.0127** | **0** | **73** | **5.8e-006** | **1** | **R.DPDAQPGGELMLGGTDSK.Y** 17201 |
|  | 19680 | **995.0067** | **1987.9989** | **1988.0111** | **-0.0122** | **0** | **21** | **0.77** | **1** | **K.AIGAVPLIQGEYMIPCEK.V** 19679 |
|  | 19878 | **1003.0043** | **2003.9940** | **2004.0060** | **-0.0120** | **0** | **(0)** | **91** | **3** | **K.AIGAVPLIQGEYMIPCEK.V** |
|  | 22941 | **1167.5441** | **2333.0736** | **2333.0879** | **-0.0143** | **0** | **92** | **4.2e-008** | **1** | **K.EGCEAIVDTGTSLMVGPVDEVR.E** 22940 |
|  | 28465 | **1124.2072** | **3369.5996** | **3369.6187** | **-0.0191** | **1** | **58** | **7.2e-005** | **1** | **K.LVDQNIFSFYLSRDPDAQPGGELMLGGTDSK.Y** |
|  | 30310 | 1369.7154 | 4106.1245 | 4106.0145 | 0.1101 | 1 | 0 | 22 | 5 | K.TLCLSGFMGMDIPPPSGPLWILGDVFIGRYYTVFDR.D |

| **145.** | P28288\|ABCD3_HUMAN    **Mass:** 75941    **Score:** 353    **Queries matched:** 37   **emPAI:** 0.61 |
| --- | --- |
|  | ATP-binding cassette sub-family D member 3 OS=Homo sapiens GN=ABCD3 PE=1 SV=1 |

|  | **Query** | **Observed** | **Mr(expt)** | **Mr(calc)** | **Delta** | **Miss** | **Score** | **Expect** | **Rank** | **Peptide** |
| --- | --- | --- | --- | --- | --- | --- | --- | --- | --- | --- |
|  | 1186 | 579.2696 | 578.2623 | 578.2668 | -0.0045 | 0 | 23 | 0.78 | 1 | R.MAMAR.L |
|  | 1357 | **628.4094** | **627.4021** | **627.4068** | **-0.0047** | **0** | **5** | **50** | **8** | **R.IVLAGR.E** |
|  | 2163 | **408.7423** | **815.4700** | **815.4501** | **0.0199** | **1** | **5** | **61** | **5** | **K.ERAVVDK.V** |
|  | 2754 | 863.3731 | 862.3658 | 862.4265 | -0.0607 | 1 | 1 | 1.6e+002 | 10 | K.QRMAMAR.L |
|  | 4239 | **486.8050** | **971.5954** | **971.6015** | **-0.0061** | **1** | **27** | **0.37** | **1** | **R.KGISDLVLK.E** |
|  | 5453 | **531.7604** | **1061.5062** | **1061.5142** | **-0.0080** | **0** | **30** | **0.19** | **1** | **R.DQVIYPDGR.E** |
|  | 5613 | **537.8120** | **1073.6095** | **1073.6154** | **-0.0060** | **0** | **33** | **0.09** | **1** | **R.ITELMQVLK.D** |
|  | 6354 | 566.7642 | 1131.5138 | 1130.5468 | 0.9669 | 1 | 8 | 24 | 2 | K.DLNHGKYER.T |
|  | 8035 | **616.3616** | **1230.7087** | **1229.6517** | **1.0570** | **1** | **1** | **1.2e+002** | **3** | **R.EKQTVHSVFR.K** |
|  | 8555 | **420.2175** | **1257.6306** | **1257.6539** | **-0.0234** | **1** | **1** | **1.1e+002** | **8** | **-.MAAFSKYLTAR.N** |
|  | 9172 | **646.7981** | **1291.5816** | **1290.6900** | **0.8916** | **1** | **8** | **23** | **2** | **R.MLLRMSQALGR.I** |
|  | 9872 | **664.3152** | **1326.6159** | **1327.6846** | **-1.0687** | **0** | **(4)** | **59** | **5** | **R.FSMGFIDSIIAK.Y** |
|  | 10140 | **672.3738** | **1342.7330** | **1342.7398** | **-0.0068** | **0** | **46** | **0.0033** | **1** | **R.VLGELWPLFGGR.L** |
|  | 10195 | **449.2771** | **1344.8096** | **1343.6795** | **1.1300** | **0** | **18** | **2.4** | **2** | **R.FSMGFIDSIIAK.Y** |
|  | 13791 | **521.5831** | **1561.7274** | **1561.7372** | **-0.0099** | **1** | **13** | **6.8** | **1** | **R.DQVIYPDGREDQK.R** |
|  | 14094 | **792.4119** | **1582.8092** | **1582.8202** | **-0.0110** | **0** | **71** | **9.7e-006** | **1** | **R.IANPDQLLTQDVEK.F** 14095 |
|  | 15081 | **824.3817** | **1646.7489** | **1646.7610** | **-0.0121** | **1** | **53** | **0.00061** | **1** | **K.MTITEQKYEGEYR.Y** |
|  | 15583 | **841.0504** | **1680.0863** | **1678.9328** | **1.1535** | **0** | **2** | **55** | **1** | **K.ETGYLVLIAVMLVSR.T** |
|  | 15878 | **849.9387** | **1697.8629** | **1697.8737** | **-0.0108** | **0** | **60** | **0.0001** | **1** | **K.EYLDNVQLGHILER.E** |
|  | 16887 | 882.3990 | 1762.7834 | 1762.9366 | -0.1532 | 0 | 1 | 82 | 4 | K.FDHVPLATPNGDVLIR.D |
|  | 17165 | **892.9017** | **1783.7889** | **1783.8013** | **-0.0124** | **0** | **61** | **7.9e-005** | **1** | **K.STHSELLEDYYQSGR.M** |
|  | 17426 | **602.6239** | **1804.8499** | **1806.0073** | **-1.1575** | **1** | **10** | **10** | **3** | **R.LAGFTARITELMQVLK.D** |
|  | 17706 | **914.9697** | **1827.9248** | **1827.0076** | **0.9171** | **0** | **3** | **53** | **7** | **R.NSSLAGAAFLLLCLLHK.R** |
|  | 18266 | **623.6511** | **1867.9314** | **1867.9428** | **-0.0114** | **1** | **6** | **27** | **6** | **R.LITNSEEIAFYNGNKR.E** |
|  | 20895 | **1047.9513** | **2093.8880** | **2093.9001** | **-0.0120** | **0** | **108** | **1.3e-009** | **1** | **R.EGGWDSVQDWMDVLSGGEK.Q** |
|  | 22745 | 1155.1184 | 2308.2223 | 2309.2857 | -1.0634 | 0 | 1 | 62 | 2 | R.YLLNFIAAMPLISLVNNFLK.Y 22744 22746 |
|  | 22925 | **583.7639** | **2331.0263** | **2331.1495** | **-0.1232** | **1** | **0** | **67** | **10** | **R.YVNSRLITNSEEIAFYNGNK.R** |
|  | 23696 | **814.4010** | **2440.1812** | **2441.1719** | **-0.9907** | **0** | **1** | **57** | **2** | **R.TYCDVWMIQNGTLIESGIIGR.S** |
|  | 24365 | **842.1262** | **2523.3567** | **2523.3697** | **-0.0130** | **1** | **21** | **0.51** | **1** | **K.GISDLVLKEYLDNVQLGHILER.E** |
|  | 24445 | **848.1625** | **2541.4658** | **2541.4781** | **-0.0124** | **0** | **54** | **0.00021** | **1** | **K.GIEGVQVIPLIPGAGEIIIADNIIK.F** 24444 24446 |
|  | 26627 | **487.9054** | **2921.3885** | **2922.5823** | **-1.1938** | **1** | **5** | **16** | **1** | **K.LTSAIGAQGPASMMAYLVVSGLFLTRLR.R** |
|  | 30656 | 1072.6031 | 4286.3835 | 4286.4042 | -0.0207 | 1 | 23 | 0.09 | 1 | K.GIEGVQVIPLIPGAGEIIIADNIIKFDHVPLATPNGDVLIR.D |

| **146.** | P20674\|COX5A_HUMAN    **Mass:** 16923    **Score:** 352    **Queries matched:** 20   **emPAI:** 3.26 |
| --- | --- |
|  | Cytochrome c oxidase subunit 5A, mitochondrial OS=Homo sapiens GN=COX5A PE=1 SV=2 |

|  | **Query** | **Observed** | **Mr(expt)** | **Mr(calc)** | **Delta** | **Miss** | **Score** | **Expect** | **Rank** | **Peptide** |
| --- | --- | --- | --- | --- | --- | --- | --- | --- | --- | --- |
|  | 1746 | **771.4666** | **770.4593** | **770.4650** | **-0.0057** | **0** | **24** | **0.67** | **1** | **K.IIDAALR.A** 1744 |
|  | 3293 | **451.7372** | **901.4599** | **902.5120** | **-1.0520** | **1** | **10** | **19** | **6** | **-.MLGAALRR.C** |
|  | 4937 | **511.7632** | **1021.5119** | **1021.5193** | **-0.0073** | **0** | **63** | **7.4e-005** | **1** | **R.LNDFASTVR.I** |
|  | 6694 | **579.3210** | **1156.6274** | **1157.6339** | **-1.0065** | **1** | **1** | **1.2e+002** | **8** | **K.IIDAALRACR.R** |
|  | 6959 | **590.3060** | **1178.5974** | **1177.6204** | **0.9770** | **1** | **6** | **44** | **2** | **R.RLNDFASTVR.I** |
|  | 15524 | **838.9262** | **1675.8377** | **1675.8491** | **-0.0114** | **0** | **94** | **4.9e-008** | **1** | **K.GINTLVTYDMVPEPK.I** 15525 |
|  | 20414 | **1027.0064** | **2051.9981** | **2052.0105** | **-0.0124** | **0** | **67** | **1.9e-005** | **1** | **R.WVTYFNKPDIDAWELR.K** 20415 20430 |
|  | 21661 | **727.7046** | **2180.0919** | **2180.1055** | **-0.0135** | **1** | **40** | **0.0083** | **1** | **R.WVTYFNKPDIDAWELRK.G** 21662 21663 |
|  | 28629 | **1143.6049** | **3427.7928** | **3427.8126** | **-0.0199** | **1** | **52** | **0.00022** | **1** | **K.EIYPYVIQELRPTLNELGISTPEELGLDKV.-** 28626 28627 28634 28635 28637 |

| **147.** | P55157\|MTP_HUMAN    **Mass:** 99916    **Score:** 346    **Queries matched:** 42   **emPAI:** 0.34 |
| --- | --- |
|  | Microsomal triglyceride transfer protein large subunit OS=Homo sapiens GN=MTTP PE=1 SV=1 |

|  | **Query** | **Observed** | **Mr(expt)** | **Mr(calc)** | **Delta** | **Miss** | **Score** | **Expect** | **Rank** | **Peptide** |
| --- | --- | --- | --- | --- | --- | --- | --- | --- | --- | --- |
|  | 1047 | 545.3637 | 544.3564 | 544.2969 | 0.0595 | 0 | 10 | 32 | 2 | K.AEAVR.N |
|  | 1074 | **550.3789** | **549.3716** | **550.2785** | **-0.9068** | **0** | **2** | **1.5e+002** | **2** | **R.LMSGK.Q** |
|  | 1620 | **714.4461** | **713.4388** | **713.4435** | **-0.0047** | **0** | **11** | **14** | **2** | **K.QAAAIIK.A** |
|  | 3725 | **468.1685** | **934.3225** | **933.4953** | **0.8272** | **1** | **9** | **23** | **1** | **K.IKALDSCK.I** |
|  | 3749 | 469.2370 | 936.4594 | 936.4665 | -0.0070 | 0 | 9 | 18 | 4 | K.LQDSVGYR.I |
|  | 4206 | **485.8155** | **969.6164** | **969.6222** | **-0.0059** | **1** | **24** | **0.6** | **3** | **K.KLILGGLEK.A** 4205 |
|  | 7521 | **603.3259** | **1204.6373** | **1204.6452** | **-0.0078** | **1** | **8** | **27** | **2** | **R.KYLQPDNLSK.A** |
|  | 8552 | **420.2086** | **1257.6039** | **1257.6982** | **-0.0944** | **0** | **5** | **49** | **9** | **R.NFLAFIQHLR.T** |
|  | 8665 | **632.3276** | **1262.6407** | **1262.6289** | **0.0119** | **1** | **3** | **77** | **4** | **K.TTEAGPRLMSGK.Q** |
|  | 9058 | **643.4095** | **1284.8044** | **1283.6218** | **1.1826** | **0** | **15** | **3.8** | **1** | **K.GHTTGLSLNNDR.L** 9041 |
|  | 9508 | **655.3494** | **1308.6842** | **1308.6925** | **-0.0083** | **0** | **50** | **0.0015** | **1** | **K.LTYSTEVLLDR.G** |
|  | 9587 | **657.8312** | **1313.6478** | **1313.6324** | **0.0154** | **0** | **7** | **29** | **5** | **K.DVNVENVNQQR.G** |
|  | 9973 | **445.0338** | **1332.0797** | **1331.7483** | **0.3314** | **0** | **2** | **59** | **8** | **R.ETVMIITGTLVR.K** |
|  | 10004 | **667.8753** | **1333.7361** | **1333.7428** | **-0.0067** | **0** | **(9)** | **19** | **4** | **K.YMLAIVQDILR.F** |
|  | 10304 | **451.2620** | **1350.7642** | **1349.7377** | **1.0265** | **0** | **10** | **13** | **1** | **K.YMLAIVQDILR.F** |
|  | 10529 | **682.9069** | **1363.7992** | **1363.8075** | **-0.0083** | **0** | **16** | **3.2** | **1** | **K.NALLPEGIPSLLK.Y** |
|  | 10667 | **686.3614** | **1370.7083** | **1371.7510** | **-1.0427** | **0** | **1** | **1.1e+002** | **2** | **R.ISSNVDVALLWR.N** |
|  | 10756 | **689.3129** | **1376.6113** | **1376.6208** | **-0.0095** | **0** | **60** | **0.00015** | **1** | **R.SGSSSAYTGYIER.S** 10755 |
|  | 12288 | **734.3853** | **1466.7559** | **1466.7657** | **-0.0097** | **1** | **27** | **0.26** | **1** | **R.YDLPFITDEVKK.T** |
|  | 13172 | **761.3946** | **1520.7746** | **1520.7835** | **-0.0089** | **0** | **51** | **0.0011** | **1** | **R.SGFTTPNQVLGVSSK.A** |
|  | 14719 | **812.8805** | **1623.7465** | **1623.7570** | **-0.0104** | **0** | **6** | **27** | **1** | **K.VVFAPQPDSTSSGWF.-** |
|  | 15880 | **849.9533** | **1697.8921** | **1697.9022** | **-0.0101** | **0** | **61** | **7.9e-005** | **1** | **K.NILLSIGELPQEMNK.Y** 15881 |
|  | 15940 | **851.5000** | **1700.9854** | **1701.7880** | **-0.8025** | **0** | **23** | **0.57** | **1** | **R.NPDGDDDQLIQITMK.D** |
|  | 16184 | 429.9585 | 1715.8048 | 1716.8067 | -1.0019 | 1 | 9 | 12 | 5 | R.SGSSSAYTGYIERSPR.S |
|  | 18719 | **953.4838** | **1904.9531** | **1904.9666** | **-0.0134** | **0** | **68** | **1.6e-005** | **1** | **R.TAAAAIILNNNPSYMDVK.N** 18715 18720 |
|  | 19619 | **993.0017** | **1983.9887** | **1984.0014** | **-0.0126** | **0** | **104** | **4e-009** | **1** | **K.YAEAGEGPISHLATTALQR.Y** |
|  | 20112 | **675.3239** | **2022.9499** | **2022.9622** | **-0.0123** | **0** | **21** | **0.71** | **1** | **R.FLYACGFASHPNEELLR.A** |
|  | 20775 | **695.0616** | **2082.1629** | **2081.1745** | **0.9884** | **0** | **23** | **0.42** | **1** | **K.ENLEALQRPTLLHLIHGK.V** 20769 |
|  | 21345 | **716.0308** | **2145.0705** | **2144.0789** | **0.9915** | **1** | **12** | **5.1** | **1** | **K.VKEFYSYQNEAVAIENIK.R** |
|  | 22370 | 754.7201 | 2261.1386 | 2261.1838 | -0.0451 | 1 | 1 | 72 | 2 | K.TVRTAAAAIILNNNPSYMDVK.N |
|  | 22493 | 760.3937 | 2278.1592 | 2277.2153 | 0.9439 | 0 | 2 | 45 | 6 | -.MILLAVLFLCFISSYSASVK.G |
|  | 23180 | **789.0082** | **2364.0027** | **2364.0184** | **-0.0157** | **0** | **1** | **52** | **3** | **K.ESVLAGCEFPLHQENSEMCK.V** |
|  | 23311 | **794.0342** | **2379.0807** | **2380.0134** | **-0.9326** | **0** | **(1)** | **60** | **6** | **K.ESVLAGCEFPLHQENSEMCK.V** |
|  | 26792 | **1474.6952** | **2947.3758** | **2947.5331** | **-0.1573** | **0** | **32** | **0.031** | **1** | **K.IEDSFVIAVLAEETHNFGLNFLQTIK.G** |
|  | 27766 | **1055.5418** | **3163.6034** | **3163.6176** | **-0.0142** | **0** | **5** | **15** | **2** | **K.EVLPQLVDAVTSAQTSDSLEAILDFLDFK.S** |

| **148.** | P06133\|UD2B4_HUMAN    **Mass:** 60872    **Score:** 344    **Queries matched:** 43   **emPAI:** 0.92 |
| --- | --- |
|  | UDP-glucuronosyltransferase 2B4 OS=Homo sapiens GN=UGT2B4 PE=1 SV=2 |

|  | **Query** | **Observed** | **Mr(expt)** | **Mr(calc)** | **Delta** | **Miss** | **Score** | **Expect** | **Rank** | **Peptide** |
| --- | --- | --- | --- | --- | --- | --- | --- | --- | --- | --- |
|  | 3918 | **474.9767** | **947.9387** | **947.4858** | **0.4529** | **1** | **2** | **82** | **9** | **K.ENAMKLSR.I** |
|  | 4049 | 479.2868 | 956.5590 | 956.5654 | -0.0064 | 0 | 8 | 28 | 2 | R.ANVIASALAK.I |
|  | 4543 | **497.7863** | **993.5581** | **993.5647** | **-0.0067** | **0** | **17** | **2.9** | **1** | **K.IPFVYSLR.F** |
|  | 4808 | **506.2576** | **1010.5006** | **1010.5073** | **-0.0067** | **0** | **26** | **0.38** | **1** | **R.FSPGYAIEK.H** |
|  | 5161 | 519.7969 | 1037.5793 | 1038.6338 | -1.0545 | 1 | 8 | 24 | 4 | K.IPQKVLWR.F |
|  | 5459 | **531.7919** | **1061.5692** | **1061.5757** | **-0.0065** | **0** | **16** | **4.1** | **1** | **K.TVINDPLYK.E** |
|  | 11471 | 473.2548 | 1416.7425 | 1416.7514 | -0.0089 | 0 | 29 | 0.18 | 1 | K.WIPQNDLLGHPK.T 11466 11467 11469 |
|  | 12076 | **728.4362** | **1454.8578** | **1453.7790** | **1.0788** | **0** | **21** | **0.92** | **1** | **R.IHHDQPVKPLDR.A** 12065 12066 |
|  | 13559 | 774.3882 | 1546.7619 | 1546.7740 | -0.0120 | 0 | 46 | 0.0031 | 1 | R.FDGNKPDTLGLNTR.L 13560 13561 13562 |
|  | 14893 | **818.4185** | **1634.8224** | **1634.8338** | **-0.0114** | **1** | **33** | **0.058** | **1** | **K.TVINDPLYKENAMK.L** 14894 14895 |
|  | 15156 | **826.4159** | **1650.8173** | **1650.8287** | **-0.0113** | **1** | **(21)** | **0.97** | **2** | **K.TVINDPLYKENAMK.L** |
|  | 18678 | **634.9871** | **1901.9394** | **1901.9498** | **-0.0105** | **0** | **8** | **16** | **2** | **K.VLVWPTEFSHWMNIK.T** |
|  | 21746 | **1096.0491** | **2190.0836** | **2190.0991** | **-0.0155** | **0** | **82** | **5.3e-007** | **1** | **K.GAAVSLDFHTMSSTDLLNALK.T** 21747 21748 |
|  | 22715 | **1153.6328** | **2305.2511** | **2305.2610** | **-0.0099** | **0** | **77** | **1.4e-006** | **1** | **R.FDVVLADAVFPFGELLAELLK.I** 22717 22718 |
|  | 23399 | 1196.0452 | 2390.0758 | 2389.2311 | 0.8447 | 1 | 5 | 21 | 2 | K.AKGAAVSLDFHTMSSTDLLNALK.T |
|  | 23871 | 820.7310 | 2459.1711 | 2458.1839 | 0.9872 | 0 | 30 | 0.076 | 1 | K.WDQFYSEVLGRPTTLSETMAK.A |
|  | 24776 | **862.7462** | **2585.2168** | **2586.2788** | **-1.0620** | **1** | **0** | **64** | **2** | **K.KWDQFYSEVLGRPTTLSETMAK.A** |
|  | 24816 | **865.4241** | **2593.2504** | **2593.2426** | **0.0078** | **1** | **0** | **66** | **2** | **K.NMIYVLYFEFWFQIFDMKK.W** |
|  | 26252 | 714.8129 | 2855.2224 | 2854.3526 | 0.8698 | 1 | 3 | 26 | 3 | -.MSMKWTSALLLIQLSCYFSSGSCGK.V |
|  | 27677 | **1046.1826** | **3135.5260** | **3135.5409** | **-0.0149** | **0** | **21** | **0.39** | **1** | **K.HSGGLLFPPSYVPVVMSELSDQMTFIER.V** |
|  | 30210 | **1017.2502** | **4064.9716** | **4064.0077** | **0.9639** | **0** | **27** | **0.053** | **1** | **R.AFITHGGANGIYEAIYHGIPMVGVPLFADQPDNIAHMK.A** 30200 30209 30212 30213 30214 |
|  | 30233 | 1020.7534 | 4078.9843 | 4080.0026 | -1.0183 | 0 | (22) | 0.16 | 1 | R.AFITHGGANGIYEAIYHGIPMVGVPLFADQPDNIAHMK.A 30237 30238 |

| **149.** | P51648\|AL3A2_HUMAN    **Mass:** 55269    **Score:** 342    **Queries matched:** 29   **emPAI:** 1.34 |
| --- | --- |
|  | Fatty aldehyde dehydrogenase OS=Homo sapiens GN=ALDH3A2 PE=1 SV=1 |

|  | **Query** | **Observed** | **Mr(expt)** | **Mr(calc)** | **Delta** | **Miss** | **Score** | **Expect** | **Rank** | **Peptide** |
| --- | --- | --- | --- | --- | --- | --- | --- | --- | --- | --- |
|  | 1828 | **396.2166** | **790.4186** | **791.3847** | **-0.9661** | **0** | **1** | **1.7e+002** | **6** | **-.MELEVR.R** |
|  | 3541 | **461.0080** | **920.0014** | **919.4399** | **0.5615** | **0** | **4** | **45** | **2** | **R.YPPNSQSK.V** |
|  | 4206 | 485.8155 | 969.6164 | 969.5607 | 0.0557 | 0 | 18 | 2.4 | 10 | R.LQQLEALR.R 4205 |
|  | 6683 | **579.2626** | **1156.5106** | **1155.6975** | **0.8131** | **1** | **24** | **0.68** | **1** | **K.RILSLLEGQK.I** |
|  | 8709 | **633.7970** | **1265.5794** | **1265.5888** | **-0.0093** | **0** | **75** | **3.9e-006** | **1** | **K.IAFGGETDEATR.Y** |
|  | 11710 | **716.3852** | **1430.7558** | **1430.7657** | **-0.0099** | **0** | **59** | **0.00018** | **1** | **R.YIAPTVLTDVDPK.T** 11711 |
|  | 13764 | **780.9145** | **1559.8145** | **1559.8229** | **-0.0083** | **1** | **58** | **0.00022** | **1** | **R.EKDILTAIAADLCK.S** 13762 |
|  | 13884 | **785.3832** | **1568.7519** | **1568.7624** | **-0.0104** | **0** | **17** | **2.7** | **1** | **R.FDHIFYTGNTAVGK.I** |
|  | 15608 | **561.6345** | **1681.8817** | **1681.8940** | **-0.0123** | **0** | **17** | **2.3** | **1** | **R.EKPLALYVFSHNHK.L** |
|  | 18031 | **618.6438** | **1852.9096** | **1852.9220** | **-0.0125** | **1** | **26** | **0.27** | **1** | **K.QRFDHIFYTGNTAVGK.I** |
|  | 18318 | **468.9817** | **1871.8975** | **1871.9101** | **-0.0126** | **0** | **17** | **2.2** | **1** | **K.HSFDTFSHQRPCLLK.S** |
|  | 18361 | **938.4172** | **1874.8198** | **1874.8322** | **-0.0124** | **1** | **52** | **0.00055** | **1** | **K.EFYGENIKESPDYER.I** 18362 |
|  | 18774 | **955.0406** | **1908.0666** | **1908.0794** | **-0.0128** | **0** | **43** | **0.005** | **1** | **K.VMQEEIFGPILPIVPVK.N** 18775 |
|  | 20166 | **676.9603** | **2027.8592** | **2027.8751** | **-0.0159** | **1** | **28** | **0.12** | **1** | **K.SPCYIDKDCDLDIVCR.R** |
|  | 20892 | **1047.5394** | **2093.0643** | **2093.1554** | **-0.0911** | **1** | **9** | **13** | **2** | **K.IVMEAAAKHLTPVTLELGGK.S** |
|  | 26942 | **993.1962** | **2976.5669** | **2975.5743** | **0.9926** | **0** | **47** | **0.00095** | **1** | **K.LLPQYLDQDLYIVINGGVEETTELLK.Q** 26920 26925 26938 |
|  | 28163 | **1092.4959** | **3274.4657** | **3274.4919** | **-0.0262** | **0** | **20** | **0.37** | **1** | **K.YMNCGQTCIAPDYILCEASLQNQIVWK.I** 28164 |
|  | 30192 | **1354.6126** | **4060.8158** | **4060.8394** | **-0.0236** | **0** | **57** | **5.2e-005** | **1** | **R.MIDETSSGGVTGNDVIMHFTLNSFPFGGVGSSGMGAYHGK.H** 30193 30194 |

| **150.** | P16615\|AT2A2_HUMAN    **Mass:** 116336   **Score:** 335    **Queries matched:** 36   **emPAI:** 0.50 |
| --- | --- |
|  | Sarcoplasmic/endoplasmic reticulum calcium ATPase 2 OS=Homo sapiens GN=ATP2A2 PE=1 SV=1 |

|  | **Query** | **Observed** | **Mr(expt)** | **Mr(calc)** | **Delta** | **Miss** | **Score** | **Expect** | **Rank** | **Peptide** |
| --- | --- | --- | --- | --- | --- | --- | --- | --- | --- | --- |
|  | 1044 | **545.2759** | **544.2686** | **543.3129** | **0.9557** | **0** | **6** | **88** | **9** | **R.QAGIR.V** |
|  | 1122 | **561.3561** | **560.3489** | **560.3533** | **-0.0045** | **0** | **19** | **4** | **1** | **R.LTSIK.S** 1126 |
|  | 1159 | **572.3469** | **571.3396** | **571.3442** | **-0.0046** | **0** | **16** | **5.3** | **1** | **K.NAIVR.S** |
|  | 1852 | **397.4022** | **792.7899** | **793.4156** | **-0.6257** | **0** | **10** | **12** | **5** | **R.MFILDR.V** |
|  | 5090 | **517.2530** | **1032.4914** | **1032.4989** | **-0.0074** | **0** | **13** | **8.1** | **3** | **K.HTDPVPDPR.A** |
|  | 5886 | **548.7673** | **1095.5201** | **1095.5270** | **-0.0069** | **0** | **25** | **0.5** | **1** | **K.MNVFDTELK.G** |
|  | 6681 | **578.9090** | **1155.8034** | **1155.5924** | **0.2110** | **1** | **7** | **25** | **1** | **K.KEFTLEFSR.D** |
|  | 9149 | **430.9051** | **1289.6935** | **1289.7013** | **-0.0078** | **1** | **10** | **15** | **3** | **R.VGSTKVPMTSGVK.Q** 9155 |
|  | 9450 | 653.8149 | 1305.6153 | 1305.6963 | -0.0809 | 1 | (6) | 31 | 10 | R.VGSTKVPMTSGVK.Q |
|  | 9773 | **662.3356** | **1322.6566** | **1322.6653** | **-0.0087** | **0** | **4** | **56** | **2** | **K.NMLFSGTNIAAGK.A** |
|  | 11296 | **704.8463** | **1407.6779** | **1407.6882** | **-0.0102** | **0** | **50** | **0.0014** | **1** | **R.IGIFGQDEDVTSK.A** |
|  | 11996 | 725.8486 | 1449.6826 | 1450.7602 | -1.0776 | 1 | 13 | 6.9 | 2 | K.KNMLFSGTNIAAGK.A 11995 |
|  | 12359 | **491.2355** | **1470.6846** | **1470.8367** | **-0.1521** | **0** | **9** | **18** | **2** | **K.ISLPVILMDETLK.F** |
|  | 12458 | 738.8539 | 1475.6933 | 1475.7038 | -0.0105 | 1 | 57 | 0.00025 | 1 | K.IRDEMVATEQER.T |
|  | 12885 | **752.4078** | **1502.8011** | **1502.8126** | **-0.0115** | **1** | **85** | **4.3e-007** | **1** | **K.KAEIGIAMGSGTAVAK.T** 12886 |
|  | 13243 | **763.8527** | **1525.6909** | **1525.7017** | **-0.0108** | **0** | **47** | **0.0026** | **1** | **K.SMSVYCTPNKPSR.T** 13242 |
|  | 13546 | **516.2562** | **1545.7467** | **1544.8232** | **0.9234** | **0** | **5** | **40** | **7** | **K.AMGVVVATGVNTEIGK.I** |
|  | 13790 | **781.8561** | **1561.6977** | **1561.7082** | **-0.0105** | **0** | **38** | **0.019** | **1** | **R.EEMHLEDSANFIK.Y** |
|  | 13820 | **782.8967** | **1563.7788** | **1563.7893** | **-0.0105** | **1** | **56** | **0.0003** | **1** | **R.RIGIFGQDEDVTSK.A** 13821 |
|  | 13952 | **787.9310** | **1573.8474** | **1573.8563** | **-0.0089** | **0** | **64** | **5.2e-005** | **1** | **R.VDQSILTGESVSVIK.H** |
|  | 14653 | **540.6265** | **1618.8578** | **1619.8076** | **-0.9499** | **0** | **1** | **90** | **10** | **K.VGEATETALTCLVEK.M** |
|  | 16524 | 435.7175 | 1738.8409 | 1737.9513 | 0.8896 | 1 | 6 | 30 | 4 | K.AKDIVPGDIVEIAVGDK.V |
|  | 19270 | **976.9625** | **1951.9105** | **1951.9231** | **-0.0126** | **0** | **52** | **0.00056** | **1** | **R.SLPSVETLGCTSVICSDK.T** 19271 |
|  | 23942 | **618.2977** | **2469.1618** | **2468.1866** | **0.9752** | **1** | **2** | **49** | **3** | **R.EWGSGSDTLRCLALATHDNPLR.R** |
|  | 23964 | **824.7540** | **2471.2401** | **2471.2544** | **-0.0143** | **0** | **25** | **0.22** | **1** | **K.TVEEVLGHFGVNESTGLSLEQVK.K** |
|  | 24849 | **867.7862** | **2600.3367** | **2599.3494** | **0.9874** | **1** | **47** | **0.0013** | **1** | **K.TVEEVLGHFGVNESTGLSLEQVKK.L** |
|  | 26563 | **970.4830** | **2908.4273** | **2908.4528** | **-0.0256** | **1** | **9** | **6.7** | **1** | **K.IVEFLQSFDEITAMTGDGVNDAPALKK.A** |
|  | 28162 | **1092.2274** | **3273.6604** | **3274.5486** | **-0.8881** | **1** | **2** | **32** | **6** | **K.TASEMVLADDNFSTIVAAVEEGRAIYNNMK.Q** |
|  | 29669 | **954.4909** | **3813.9345** | **3812.8448** | **1.0897** | **1** | **0** | **31** | **1** | **K.AEIGIAMGSGTAVAKTASEMVLADDNFSTIVAAVEEGR.A** |

| **151.** | O75874\|IDHC_HUMAN    **Mass:** 46915    **Score:** 332    **Queries matched:** 27   **emPAI:** 0.85 |
| --- | --- |
|  | Isocitrate dehydrogenase [NADP] cytoplasmic OS=Homo sapiens GN=IDH1 PE=1 SV=2 |

|  | **Query** | **Observed** | **Mr(expt)** | **Mr(calc)** | **Delta** | **Miss** | **Score** | **Expect** | **Rank** | **Peptide** |
| --- | --- | --- | --- | --- | --- | --- | --- | --- | --- | --- |
|  | 1228 | **588.3668** | **587.3595** | **587.3642** | **-0.0047** | **0** | **33** | **0.15** | **1** | **K.NTILK.K** |
|  | 1768 | **783.4412** | **782.4339** | **782.4399** | **-0.0060** | **0** | **14** | **6** | **1** | **K.GLPNVQR.S** |
|  | 1815 | **790.4069** | **789.3996** | **789.4055** | **-0.0059** | **0** | **31** | **0.17** | **1** | **K.DLAACIK.G** |
|  | 3313 | 452.7103 | 903.4060 | 902.4399 | 0.9662 | 0 | 9 | 23 | 10 | K.IWYEHR.L |
|  | 4303 | 976.5506 | 975.5433 | 975.5502 | -0.0068 | 0 | 25 | 0.64 | 1 | R.NILGGTVFR.E 4304 4305 |
|  | 4792 | 505.7566 | 1009.4986 | 1008.4413 | 1.0573 | 0 | 8 | 25 | 1 | R.HAYGDQYR.A |
|  | 6856 | 585.7933 | 1169.5721 | 1169.5604 | 0.0117 | 0 | 7 | 28 | 7 | K.DIFQEIYDK.Q |
|  | 7185 | **397.4022** | **1189.1849** | **1189.5761** | **-0.3912** | **1** | **9** | **13** | **7** | **K.CATITPDEKR.V** |
|  | 10103 | **671.3367** | **1340.6588** | **1340.6684** | **-0.0097** | **0** | **28** | **0.23** | **1** | **K.TVEAEAAHGTVTR.H** |
|  | 11799 | **719.4462** | **1436.8779** | **1436.8867** | **-0.0088** | **0** | **40** | **0.011** | **1** | **R.LVSGWVKPIIIGR.H** |
|  | 11809 | **719.8518** | **1437.6891** | **1437.6987** | **-0.0097** | **0** | **10** | **13** | **1** | **K.VEITYTPSDGTQK.V** |
|  | 11919 | 482.5790 | 1444.7150 | 1444.7238 | -0.0088 | 1 | 24 | 0.52 | 1 | R.FKDIFQEIYDK.Q 11920 |
|  | 14883 | 817.9200 | 1633.8255 | 1632.9239 | 0.9016 | 1 | 1 | 89 | 10 | K.GWPLYLSTKNTILK.K |
|  | 15816 | **848.3818** | **1694.7491** | **1694.7604** | **-0.0112** | **0** | **104** | **4.4e-009** | **1** | **K.ISGGSVVEMQGDEMTR.I** 15815 |
|  | 17316 | **899.4220** | **1796.8294** | **1796.8403** | **-0.0109** | **0** | **69** | **1.3e-005** | **1** | **K.SIEDFAHSSFQMALSK.G** 17314 |
|  | 18050 | **619.0256** | **1854.0549** | **1854.8452** | **-0.7903** | **1** | **4** | **39** | **2** | **K.KISGGSVVEMQGDEMTR.I** 18058 |
|  | 18388 | **939.9648** | **1877.9150** | **1877.9272** | **-0.0122** | **0** | **71** | **7.6e-006** | **1** | **K.GQETSTNPIASIFAWTR.G** |
|  | 23493 | **1203.6137** | **2405.2127** | **2405.2267** | **-0.0139** | **0** | **48** | **0.0012** | **1** | **K.LIFPYVELDLHSYDLGIENR.D** 23491 23492 |
|  | 24243 | **836.4213** | **2506.2420** | **2506.2592** | **-0.0172** | **1** | **5** | **24** | **1** | **R.ATDFVVPGPGKVEITYTPSDGTQK.V** |

| **152.** | P21980\|TGM2_HUMAN    **Mass:** 78420    **Score:** 330    **Queries matched:** 30   **emPAI:** 0.52 |
| --- | --- |
|  | Protein-glutamine gamma-glutamyltransferase 2 OS=Homo sapiens GN=TGM2 PE=1 SV=2 |

|  | **Query** | **Observed** | **Mr(expt)** | **Mr(calc)** | **Delta** | **Miss** | **Score** | **Expect** | **Rank** | **Peptide** |
| --- | --- | --- | --- | --- | --- | --- | --- | --- | --- | --- |
|  | 2038 | **403.2422** | **804.4698** | **804.4606** | **0.0092** | **1** | **23** | **0.95** | **9** | **K.FLKNAGR.D** |
|  | 3743 | **468.7552** | **935.4958** | **934.4039** | **1.0919** | **1** | **8** | **29** | **8** | **K.NAGRDCSR.R** |
|  | 4763 | **504.3182** | **1006.6218** | **1005.4767** | **1.1451** | **0** | **3** | **93** | **7** | **R.EDITHTYK.Y** |
|  | 4925 | **511.2589** | **1020.5033** | **1019.5512** | **0.9520** | **1** | **17** | **3.6** | **2** | **R.RSSPVYVGR.V** |
|  | 7608 | **404.2189** | **1209.6348** | **1208.5641** | **1.0706** | **1** | **12** | **7.9** | **1** | **K.EETGMAMRIR.V** 7610 |
|  | 10535 | **683.3235** | **1364.6325** | **1364.6428** | **-0.0102** | **1** | **42** | **0.0089** | **1** | **K.LAEKEETGMAMR.I** |
|  | 10825 | **691.3071** | **1380.5996** | **1380.6204** | **-0.0209** | **1** | **7** | **26** | **4** | **R.DHHTADLCREK.L** |
|  | 11230 | **468.9183** | **1403.7331** | **1403.7417** | **-0.0087** | **0** | **(7)** | **24** | **2** | **R.MDLLPLHMGLHK.L** |
|  | 11526 | **474.2863** | **1419.8371** | **1419.7366** | **0.1005** | **0** | **8** | **20** | **2** | **R.MDLLPLHMGLHK.L** |
|  | 12537 | **741.3239** | **1480.6333** | **1480.6439** | **-0.0106** | **0** | **45** | **0.0037** | **1** | **K.SEGTYCCGPVPVR.A** |
|  | 12997 | **756.3743** | **1510.7340** | **1510.7449** | **-0.0110** | **1** | **38** | **0.02** | **1** | **K.YRDCLTESNLIK.V** 12998 |
|  | 13440 | **514.2628** | **1539.7665** | **1539.8119** | **-0.0455** | **1** | **2** | **87** | **3** | **K.SVPLCILYEKYR.D** |
|  | 14021 | **790.4177** | **1578.8208** | **1578.8293** | **-0.0085** | **0** | **81** | **9e-007** | **1** | **K.YLLNLNLEPFSEK.S** 14020 |
|  | 15724 | **846.3940** | **1690.7734** | **1690.9011** | **-0.1277** | **1** | **3** | **52** | **6** | **K.VRMDLLPLHMGLHK.L** 15723 15735 |
|  | 17350 | **900.5121** | **1799.0096** | **1799.0192** | **-0.0096** | **0** | **46** | **0.0025** | **1** | **R.ALLVEPVINSYLLAER.D** 17329 |
|  | 19048 | **966.9603** | **1931.9061** | **1931.9194** | **-0.0132** | **0** | **52** | **0.00066** | **1** | **R.VVSGMVNCNDDQGVLLGR.W** |
|  | 24058 | **1241.0718** | **2480.1290** | **2480.1431** | **-0.0141** | **0** | **85** | **2.3e-007** | **1** | **R.WDNNYGDGVSPMSWIGSVDILR.R** 24059 |
|  | 24181 | **624.8339** | **2495.3063** | **2496.1380** | **-0.8316** | **0** | **(3)** | **31** | **5** | **R.WDNNYGDGVSPMSWIGSVDILR.R** |
|  | 24186 | **833.0690** | **2496.1853** | **2496.2033** | **-0.0181** | **0** | **44** | **0.0026** | **1** | **R.VVTNYNSAHDQNSNLLIEYFR.N** |
|  | 25001 | **879.8990** | **2636.6751** | **2636.2442** | **0.4310** | **1** | **2** | **32** | **1** | **R.WDNNYGDGVSPMSWIGSVDILRR.W** |
|  | 25067 | **1328.1346** | **2654.2547** | **2654.2712** | **-0.0164** | **0** | **18** | **0.93** | **1** | **R.NYEASVDSLTFSVVTGPAPSQEAGTK.A** |
|  | 25865 | **559.8686** | **2794.3066** | **2793.3538** | **0.9527** | **1** | **7** | **14** | **4** | **R.SSPVYVGRVVSGMVNCNDDQGVLLGR.W** 25848 |

| **153.** | P09110\|THIK_HUMAN    **Mass:** 44834    **Score:** 325    **Queries matched:** 21   **emPAI:** 0.90 |
| --- | --- |
|  | 3-ketoacyl-CoA thiolase, peroxisomal OS=Homo sapiens GN=ACAA1 PE=1 SV=2 |

|  | **Query** | **Observed** | **Mr(expt)** | **Mr(calc)** | **Delta** | **Miss** | **Score** | **Expect** | **Rank** | **Peptide** |
| --- | --- | --- | --- | --- | --- | --- | --- | --- | --- | --- |
|  | 3478 | **458.3055** | **914.5964** | **914.4570** | **0.1394** | **0** | **38** | **0.028** | **1** | **R.GNPGNITSR.L** 3488 |
|  | 6863 | **585.8546** | **1169.6945** | **1169.7019** | **-0.0074** | **0** | **46** | **0.0035** | **1** | **R.QVITLLNELK.R** |
|  | 9851 | **443.0382** | **1326.0929** | **1325.8031** | **0.2898** | **1** | **7** | **19** | **4** | **R.QVITLLNELKR.R** 9852 9869 |
|  | 11452 | **708.8597** | **1415.7048** | **1415.7191** | **-0.0143** | **1** | **1** | **99** | **9** | **R.GNPGNITSRLMEK.E** |
|  | 12272 | **489.6164** | **1465.8273** | **1464.8347** | **0.9926** | **1** | **3** | **64** | **10** | **-.MQRLQVVLGHLR.G** |
|  | 12733 | **747.3780** | **1492.7414** | **1492.7522** | **-0.0107** | **1** | **60** | **0.00012** | **1** | **R.EKQDTFALASQQK.A** |
|  | 14290 | **532.3193** | **1593.9360** | **1593.9454** | **-0.0094** | **1** | **30** | **0.11** | **1** | **R.SKAEELGLPILGVLR.S** |
|  | 18855 | **959.0036** | **1915.9926** | **1916.0051** | **-0.0124** | **0** | **68** | **1.7e-005** | **1** | **K.VNPLGGAVALGHPLGCTGAR.Q** |
|  | 21368 | **1074.9462** | **2147.8778** | **2147.8922** | **-0.0144** | **0** | **6** | **21** | **4** | **R.NGSYDIGMACGVESMSLADR.G** |
|  | 21488 | **1081.5309** | **2161.0472** | **2161.0611** | **-0.0139** | **0** | **127** | **1.7e-011** | **1** | **K.DGSTTAGNSSQVSDGAAAILLAR.R** |
|  | 22171 | **1117.5626** | **2233.1107** | **2233.1260** | **-0.0153** | **0** | **32** | **0.053** | **1** | **R.SITVTQDEGIRPSTTMEGLAK.L** 22172 |
|  | 22606 | **764.3409** | **2290.0010** | **2289.1561** | **0.8449** | **1** | **6** | **20** | **1** | **K.KDGSTTAGNSSQVSDGAAAILLAR.R** 22608 |
|  | 22696 | 768.3770 | 2302.1092 | 2302.1264 | -0.0172 | 1 | 15 | 2.3 | 1 | K.GCFQAEIVPVTTTVHDDKGTK.R |
|  | 24953 | **1313.7118** | **2625.4090** | **2625.4240** | **-0.0150** | **0** | **56** | **0.00014** | **1** | **R.SYAVVGVPPDIMGIGPAYAIPVALQK.A** 24954 24956 |

| **154.** | P68871\|HBB_HUMAN    **Mass:** 16102    **Score:** 324    **Queries matched:** 21   **emPAI:** 3.55 |
| --- | --- |
|  | Hemoglobin subunit beta OS=Homo sapiens GN=HBB PE=1 SV=2 |

|  | **Query** | **Observed** | **Mr(expt)** | **Mr(calc)** | **Delta** | **Miss** | **Score** | **Expect** | **Rank** | **Peptide** |
| --- | --- | --- | --- | --- | --- | --- | --- | --- | --- | --- |
|  | 3971 | **476.7556** | **951.4965** | **951.5025** | **-0.0060** | **0** | **2** | **1e+002** | **9** | **M.VHLTPEEK.S** |
|  | 6273 | **563.7812** | **1125.5478** | **1125.5567** | **-0.0089** | **0** | **9** | **17** | **2** | **K.LHVDPENFR.L** |
|  | 6579 | **575.3358** | **1148.6571** | **1148.6666** | **-0.0095** | **0** | **48** | **0.0024** | **1** | **K.VVAGVANALAHK.Y** 6559 6560 |
|  | 8859 | **637.8631** | **1273.7116** | **1273.7183** | **-0.0066** | **0** | **56** | **0.00035** | **1** | **R.LLVVYPWTQR.F** 8858 8877 |
|  | 9587 | 657.8312 | 1313.6478 | 1313.6575 | -0.0097 | 0 | 47 | 0.0026 | 1 | K.VNVDEVGGEALGR.L 9569 |
|  | 11984 | **725.3973** | **1448.7801** | **1448.7888** | **-0.0087** | **1** | **15** | **3.8** | **1** | **K.VVAGVANALAHKYH.-** 11983 11985 |
|  | 15418 | **835.4432** | **1668.8718** | **1668.8835** | **-0.0117** | **0** | **84** | **4.1e-007** | **1** | **K.VLGAFSDGLAHLDNLK.G** |
|  | 17322 | **899.4909** | **1796.9673** | **1796.9785** | **-0.0112** | **1** | **55** | **0.00034** | **1** | **K.KVLGAFSDGLAHLDNLK.G** 17323 |
|  | 20490 | **1029.9700** | **2057.9254** | **2057.9405** | **-0.0151** | **0** | **69** | **1.1e-005** | **1** | **R.FFESFGDLSTPDAVMGNPK.V** 20491 20492 |
|  | 24776 | 862.7462 | 2585.2168 | 2585.2333 | -0.0164 | 1 | 47 | 0.0012 | 1 | K.GTFATLSELHCDKLHVDPENFR.L 24778 |

| **155.** | Q08477\|CP4F3_HUMAN    **Mass:** 60663    **Score:** 322    **Queries matched:** 28   **emPAI:** 0.61 |
| --- | --- |
|  | Leukotriene-B(4) omega-hydroxylase 2 OS=Homo sapiens GN=CYP4F3 PE=2 SV=2 |

|  | **Query** | **Observed** | **Mr(expt)** | **Mr(calc)** | **Delta** | **Miss** | **Score** | **Expect** | **Rank** | **Peptide** |
| --- | --- | --- | --- | --- | --- | --- | --- | --- | --- | --- |
|  | 2552 | 424.2082 | 846.4018 | 846.4083 | -0.0065 | 0 | 12 | 15 | 1 | K.LSDEDIR.A |
|  | 2628 | 427.7369 | 853.4593 | 853.5385 | -0.0792 | 0 | 5 | 49 | 1 | R.KPELVLR.A |
|  | 3271 | 451.2620 | 900.5095 | 900.4817 | 0.0278 | 0 | 6 | 45 | 6 | R.AEGGLWLR.V |
|  | 4066 | 480.2767 | 958.5388 | 957.4304 | 1.1084 | 0 | 9 | 26 | 8 | K.HPEYQER.C |
|  | 4403 | **492.3312** | **982.6479** | **982.6539** | **-0.0060** | **0** | **23** | **0.76** | **1** | **K.VVLGLTLLR.F** |
|  | 4431 | 493.7759 | 985.5372 | 985.5444 | -0.0072 | 0 | 32 | 0.11 | 1 | R.QEVQELLK.D |
|  | 4799 | 505.8239 | 1009.6333 | 1009.6396 | -0.0063 | 1 | 12 | 8.4 | 1 | R.RKPELVLR.A |
|  | 5045 | **515.3271** | **1028.6396** | **1028.5226** | **0.1171** | **1** | **5** | **50** | **10** | **R.CFPQPPKR.N** |
|  | 5592 | **536.8134** | **1071.6121** | **1071.6189** | **-0.0068** | **0** | **1** | **1.5e+002** | **1** | **R.LHPPVPAVSR.C** |
|  | 9377 | 651.8411 | 1301.6676 | 1301.6761 | -0.0086 | 1 | 34 | 0.061 | 1 | R.CRQEVQELLK.D |
|  | 10440 | 680.3709 | 1358.7271 | 1358.7347 | -0.0075 | 0 | 36 | 0.033 | 1 | R.SPLAFIPFSAGPR.N |
|  | 10747 | 688.9023 | 1375.7901 | 1375.7963 | -0.0061 | 0 | 78 | 2.3e-006 | 1 | K.TLDFIDVLLLSK.D |
|  | 11469 | 473.2546 | 1416.7420 | 1417.7024 | -0.9604 | 0 | 5 | 47 | 10 | K.IFNESVNIMHAK.W |
|  | 11748 | **717.8292** | **1433.6439** | **1432.6439** | **1.0000** | **0** | **8** | **20** | **4** | **R.CCTQDIVLPDGR.V** |
|  | 12338 | **735.8257** | **1469.6368** | **1469.6465** | **-0.0097** | **0** | **58** | **0.0002** | **1** | **R.NCIGQAFAMAEMK.V** |
|  | 13117 | 759.8887 | 1517.7628 | 1517.7726 | -0.0098 | 0 | 52 | 0.00085 | 1 | R.TLPSQGVDDFLQAK.A 13115 |
|  | 13479 | 771.8937 | 1541.7728 | 1541.7838 | -0.0110 | 0 | 62 | 8.4e-005 | 1 | R.LVHDFTDAVIQER.R 13480 |
|  | 14261 | 796.8909 | 1591.7672 | 1590.9232 | 0.8439 | 1 | 2 | 76 | 10 | K.SKTLDFIDVLLLSK.D |
|  | 18293 | 623.9947 | 1868.9622 | 1869.9441 | -0.9819 | 1 | 6 | 27 | 9 | R.CCTQDIVLPDGRVIPK.G |
|  | 18877 | 960.4878 | 1918.9610 | 1920.0092 | -1.0481 | 1 | 11 | 7.8 | 1 | K.TLDFIDVLLLSKDEDGK.K |
|  | 19253 | 651.0122 | 1950.0148 | 1950.0259 | -0.0111 | 0 | 15 | 3.3 | 2 | R.MLTPAFHFNILKPYMK.I |
|  | 20046 | **1011.0021** | **2019.9896** | **2020.0009** | **-0.0113** | **0** | **13** | **4.3** | **1** | **R.LDMFEHISLMTLDSLQK.C** |
|  | 24765 | **861.7956** | **2582.3649** | **2582.3784** | **-0.0135** | **1** | **45** | **0.002** | **1** | **K.DKVFYSFLKPWLGDGLLLSAGEK.W** |
|  | 27043 | 999.8007 | 2996.3802 | 2996.4015 | -0.0213 | 0 | 81 | 4.6e-007 | 1 | R.AEADTFMFEGHDTTASGLSWVLYHLAK.H 27044 |
|  | 29726 | 957.2018 | 3824.7783 | 3824.7992 | -0.0209 | 1 | 65 | 1e-005 | 1 | K.LSDEDIRAEADTFMFEGHDTTASGLSWVLYHLAK.H |

| **156.** | A5A3E0\|POTEF_HUMAN    **Mass:** 123020   **Score:** 321    **Queries matched:** 50   **emPAI:** 0.23 |
| --- | --- |
|  | POTE ankyrin domain family member F OS=Homo sapiens GN=POTEF PE=1 SV=2 |

|  | **Query** | **Observed** | **Mr(expt)** | **Mr(calc)** | **Delta** | **Miss** | **Score** | **Expect** | **Rank** | **Peptide** |
| --- | --- | --- | --- | --- | --- | --- | --- | --- | --- | --- |
|  | 1421 | 644.3680 | 643.3607 | 643.3653 | -0.0046 | 0 | 20 | 2.6 | 1 | R.LDLAGR.E |
|  | 2523 | 423.0518 | 844.0891 | 843.4702 | 0.6189 | 0 | 8 | 23 | 5 | K.QIEVVEK.M |
|  | 3103 | 443.2713 | 884.5281 | 885.4668 | -0.9387 | 0 | 14 | 5.9 | 6 | K.ANLNALDR.Y |
|  | 3377 | 454.8929 | 907.7713 | 906.6014 | 1.1698 | 1 | 5 | 33 | 7 | K.IRIIAPPK.R |
|  | 4297 | 488.7242 | 975.4339 | 975.4410 | -0.0071 | 0 | 67 | 3.7e-005 | 1 | K.AGFAGDDAPR.A 4293 4296 |
|  | 7268 | 398.8572 | 1193.5497 | 1192.5831 | 0.9666 | 0 | 6 | 40 | 4 | K.EIAALAPSMMK.I |
|  | 7365 | 599.8527 | 1197.6908 | 1197.6982 | -0.0075 | 0 | 58 | 0.00026 | 1 | R.AVFPSIVGRPR.Q 7366 |
|  | 7902 | 408.7606 | 1223.2599 | 1222.6445 | 0.6154 | 1 | 1 | 70 | 6 | K.YLEDIESVKK.R |
|  | 8059 | 411.7251 | 1232.1536 | 1231.5260 | 0.6275 | 0 | (6) | 28 | 8 | R.QQGMMGGMHQK.E |
|  | 8380 | 624.8301 | 1247.6456 | 1247.5209 | 0.1247 | 0 | (7) | 28 | 3 | R.QQGMMGGMHQK.E 8402 |
|  | 8381 | 624.8337 | 1247.6528 | 1247.5209 | 0.1319 | 0 | (7) | 32 | 3 | R.QQGMMGGMHQK.E |
|  | 8938 | 427.2195 | 1278.6368 | 1279.5108 | -0.8740 | 0 | 11 | 12 | 1 | R.QQGMMGGMHQK.E |
|  | 9688 | 660.3503 | 1318.6861 | 1318.6728 | 0.0133 | 1 | 7 | 31 | 6 | K.LTSEEESQRLK.G |
|  | 11679 | 477.2838 | 1428.8296 | 1429.7785 | -0.9489 | 1 | 9 | 16 | 1 | K.EIAALAPSMMKIR.I |
|  | 11963 | 483.2732 | 1446.7977 | 1445.7734 | 1.0243 | 1 | (8) | 22 | 5 | K.EIAALAPSMMKIR.I |
|  | 13084 | 758.8497 | 1515.6849 | 1515.6954 | -0.0105 | 0 | 48 | 0.0021 | 1 | K.QEYDESGPSIVHR.K |
|  | 13087 | 758.8746 | 1515.7347 | 1514.7419 | 0.9928 | 0 | 38 | 0.022 | 1 | K.IWHHTFYNELR.V 13067 13068 |
|  | 13561 | 516.5950 | 1546.7631 | 1546.8025 | -0.0394 | 1 | 5 | 39 | 5 | K.DLIVMLRDTDVNK.Q |
|  | 13578 | 775.2053 | 1548.3960 | 1548.7528 | -0.3568 | 0 | (1) | 66 | 6 | -.MVVEVDSMPAASSVK.K |
|  | 13834 | 783.3355 | 1564.6563 | 1564.7477 | -0.0913 | 0 | (1) | 84 | 6 | -.MVVEVDSMPAASSVK.K 13818 |
|  | 13852 | 783.8886 | 1565.7625 | 1564.7477 | 1.0149 | 0 | 2 | 68 | 5 | -.MVVEVDSMPAASSVK.K |
|  | 14025 | 395.7156 | 1578.8331 | 1579.7772 | -0.9441 | 1 | 3 | 57 | 5 | R.MQKEIAALAPSMMK.I |
|  | 14303 | 532.6229 | 1594.8469 | 1595.7721 | -0.9251 | 1 | (2) | 81 | 8 | R.MQKEIAALAPSMMK.I |
|  | 14568 | 807.9148 | 1613.8150 | 1613.8195 | -0.0045 | 1 | 12 | 6.9 | 4 | R.LELDTMKHQSQLR.E |
|  | 15044 | 822.8969 | 1643.7793 | 1643.7903 | -0.0111 | 1 | 28 | 0.19 | 1 | K.QEYDESGPSIVHRK.C |
|  | 15149 | 550.9941 | 1649.9606 | 1648.8494 | 1.1112 | 1 | 0 | 1e+002 | 10 | R.LDLAGRELPDYLMK.I |
|  | 16276 | 862.4311 | 1722.8476 | 1722.8532 | -0.0055 | 1 | 6 | 26 | 1 | R.EEIAMLRLELDTMK.H 16290 |
|  | 17255 | 895.9438 | 1789.8730 | 1789.8846 | -0.0116 | 0 | 71 | 9.6e-006 | 1 | K.SYELPDGQVITIGNER.F 17256 17257 17258 17267 17268 |
|  | 19364 | 980.9598 | 1959.9050 | 1959.9571 | -0.0521 | 1 | 7 | 21 | 4 | K.QMLKISSENSNPEQDLK.L |
|  | 20355 | 410.6172 | 2048.0494 | 2046.9164 | 1.1331 | 1 | 6 | 23 | 6 | K.GSENSQPEKMSQEPEINK.D |
|  | 21233 | 427.4260 | 2132.0936 | 2132.1300 | -0.0364 | 1 | 7 | 18 | 6 | M.VVEVDSMPAASSVKKPFGLR.S |
|  | 25268 | 897.1061 | 2688.2966 | 2687.2506 | 1.0460 | 1 | 2 | 40 | 6 | K.DLYTNTVLSGGTTMYPGMAHRMQK.E |
|  | 25401 | **677.8561** | **2707.3955** | **2706.2998** | **1.0957** | **0** | **3** | **33** | **8** | **R.STHVGFPENLTNGATAGNGDDGLIPPR.K** |
|  | 27653 | 783.3997 | 3129.5698 | 3130.5755 | -1.0058 | 1 | 1 | 42 | 1 | K.HESNNVGLLENLTNGVTAGNGDNGLIPQRK.S |
|  | 28259 | **1106.1185** | **3315.3338** | **3314.6025** | **0.7312** | **0** | **0** | **32** | **9** | **K.MTQIMFETFNTPAMYVAIQAVLSLYTSGR.T** |
|  | 29606 | 1266.5684 | 3796.6833 | 3795.7390 | 0.9443 | 1 | 1 | 26 | 4 | K.ALQLNELTMDDDTAVLVIDNGSGMCKAGFAGDDAPR.A |
|  | 29641 | 1271.3035 | 3810.8886 | 3811.7339 | -0.8453 | 1 | (1) | 31 | 5 | K.ALQLNELTMDDDTAVLVIDNGSGMCKAGFAGDDAPR.A |

| **157.** | Q5VTE0\|EF1A3_HUMAN    **Mass:** 50495    **Score:** 320    **Queries matched:** 37   **emPAI:** 1.19 |
| --- | --- |
|  | Putative elongation factor 1-alpha-like 3 OS=Homo sapiens GN=EEF1AL3 PE=5 SV=1 |

|  | **Query** | **Observed** | **Mr(expt)** | **Mr(calc)** | **Delta** | **Miss** | **Score** | **Expect** | **Rank** | **Peptide** |
| --- | --- | --- | --- | --- | --- | --- | --- | --- | --- | --- |
|  | 2456 | **839.4452** | **838.4379** | **838.4436** | **-0.0057** | **0** | **21** | **1.1** | **1** | **K.EVSTYIK.K** |
|  | 2881 | **435.6920** | **869.3694** | **869.5334** | **-0.1640** | **0** | **14** | **6.1** | **6** | **K.QLIVGVNK.M** 2896 2903 |
|  | 4172 | **484.2731** | **966.5316** | **966.5386** | **-0.0070** | **1** | **31** | **0.12** | **1** | **K.EVSTYIKK.I** |
|  | 4983 | **513.3048** | **1024.5951** | **1024.6030** | **-0.0079** | **0** | **52** | **0.001** | **1** | **K.IGGIGTVPVGR.V** 4984 |
|  | 6657 | **577.7952** | **1153.5759** | **1154.5277** | **-0.9518** | **1** | **4** | **54** | **1** | **K.FEKEAAEMGK.G** |
|  | 6852 | 585.3680 | 1168.7214 | 1169.5387 | -0.8172 | 1 | 3 | 69 | 8 | K.EAAEMGKGSFK.Y |
|  | 9596 | **657.8701** | **1313.7257** | **1313.7343** | **-0.0086** | **0** | **26** | **0.32** | **1** | **R.EHALLAYTLGVK.Q** |
|  | 9893 | **443.5826** | **1327.7261** | **1326.7871** | **0.9390** | **1** | **3** | **79** | **9** | **R.QTVAVGVIKAVDK.K** |
|  | 11229 | **702.8627** | **1403.7109** | **1403.7197** | **-0.0088** | **0** | **22** | **0.84** | **1** | **K.YYVTIIDAPGHR.D** |
|  | 14186 | 795.3166 | 1588.6187 | 1587.8733 | 0.7454 | 0 | 23 | 0.62 | 2 | K.THINIVVIGHVDSGK.S 14184 14187 |
|  | 18740 | **636.3303** | **1905.9690** | **1906.9941** | **-1.0252** | **1** | **3** | **45** | **5** | **K.YYVTIIDAPGHRDFIK.N** |
|  | 24309 | **1258.1871** | **2514.3597** | **2514.3768** | **-0.0171** | **0** | **61** | **5.3e-005** | **1** | **R.VETGVLKPGMVVTFAPVNVTTEVK.S** 24310 24312 |
|  | 27030 | **999.1321** | **2994.3744** | **2994.3926** | **-0.0182** | **0** | **23** | **0.25** | **1** | **K.SGDAAIVDMVPGKPMCVESFSDYPPLGR.F** 27031 |
|  | 27123 | **756.1439** | **3020.5463** | **3020.5601** | **-0.0137** | **0** | **8** | **7.2** | **1** | **K.DGNASGTTLLEALDCILPPTRPTDKPLR.L** |
|  | 27726 | **1050.5525** | **3148.6356** | **3148.6550** | **-0.0194** | **1** | **10** | **4.5** | **1** | **R.KDGNASGTTLLEALDCILPPTRPTDKPLR.L** |
|  | 29008 | **1784.8325** | **3567.6505** | **3566.6639** | **0.9866** | **0** | **55** | **0.00012** | **1** | **K.IGYNPDTVAFVPISGWNGDNMLEPSANMPWFK.G** 28997 28999 29000 29002 29005 29007 |
|  | 29057 | **1195.5636** | **3583.6690** | **3582.6588** | **1.0102** | **0** | **(8)** | **6.5** | **1** | **K.IGYNPDTVAFVPISGWNGDNMLEPSANMPWFK.G** 29058 29059 |
|  | 29269 | **1232.5873** | **3694.7400** | **3694.7589** | **-0.0188** | **1** | **50** | **0.00039** | **1** | **K.KIGYNPDTVAFVPISGWNGDNMLEPSANMPWFK.G** 29268 29273 |
|  | 30054 | **1326.6968** | **3977.0685** | **3977.0932** | **-0.0247** | **1** | **9** | **3.5** | **1** | **K.DGNASGTTLLEALDCILPPTRPTDKPLRLPLQDVYK.I** |

|  | **Proteins matching the same set of peptides:** |
| --- | --- |

|  | P68104\|EF1A1_HUMAN    **Mass:** 50451    **Score:** 320    **Queries matched:** 37 |
| --- | --- |
|  | Elongation factor 1-alpha 1 OS=Homo sapiens GN=EEF1A1 PE=1 SV=1 |

| **158.** | P00441\|SODC_HUMAN    **Mass:** 16154    **Score:** 318    **Queries matched:** 17   **emPAI:** 3.50 |
| --- | --- |
|  | Superoxide dismutase [Cu-Zn] OS=Homo sapiens GN=SOD1 PE=1 SV=2 |

|  | **Query** | **Observed** | **Mr(expt)** | **Mr(calc)** | **Delta** | **Miss** | **Score** | **Expect** | **Rank** | **Peptide** |
| --- | --- | --- | --- | --- | --- | --- | --- | --- | --- | --- |
|  | 1345 | **624.3776** | **623.3703** | **622.3551** | **1.0153** | **1** | **8** | **19** | **1** | **R.KHGGPK.D** |
|  | 1552 | **689.3929** | **688.3857** | **688.3908** | **-0.0051** | **0** | **22** | **1.8** | **1** | **K.VWGSIK.G** |
|  | 2280 | **413.2139** | **824.4132** | **824.4756** | **-0.0624** | **0** | **8** | **22** | **5** | **R.TLVVHEK.A** |
|  | 6209 | 560.7824 | 1119.5503 | 1119.6144 | -0.0641 | 1 | 12 | 11 | 2 | -.MATKAVCVLK.G |
|  | 12846 | **751.3803** | **1500.7459** | **1500.7573** | **-0.0113** | **0** | **60** | **0.00014** | **1** | **K.GDGPVQGIINFEQK.E** 12847 |
|  | 21581 | 1086.5715 | 2171.1285 | 2171.1409 | -0.0124 | 1 | 52 | 0.00055 | 1 | K.AVCVLKGDGPVQGIINFEQK.E 21579 |
|  | 28860 | **1173.8722** | **3518.5947** | **3518.6175** | **-0.0227** | **0** | **63** | **2.1e-005** | **1** | **K.GLTEGLHGFHVHEFGDNTAGCTSAGPHFNPLSR.K** 28852 28857 28858 28859 28861 |
|  | 29340 | **1241.2692** | **3720.7857** | **3719.8061** | **0.9796** | **1** | **71** | **2.7e-006** | **1** | **R.HVGDLGNVTADKDGVADVSIEDSVISLSGDHCIIGR.T** 29337 29338 |

| **159.** | P13929\|ENOB_HUMAN    **Mass:** 47244    **Score:** 316    **Queries matched:** 28   **emPAI:** 0.46 |
| --- | --- |
|  | Beta-enolase OS=Homo sapiens GN=ENO3 PE=1 SV=4 |

|  | **Query** | **Observed** | **Mr(expt)** | **Mr(calc)** | **Delta** | **Miss** | **Score** | **Expect** | **Rank** | **Peptide** |
| --- | --- | --- | --- | --- | --- | --- | --- | --- | --- | --- |
|  | 1099 | 557.0191 | 556.0118 | 556.3333 | -0.3214 | 1 | 11 | 7.1 | 6 | R.NPKAK.- |
|  | 1385 | **634.3629** | **633.3556** | **633.3598** | **-0.0042** | **0** | **23** | **1.3** | **1** | **K.AIFAGR.K** |
|  | 4124 | 482.7173 | 963.4201 | 963.5324 | -0.1123 | 1 | 15 | 5 | 2 | M.AMQKIFAR.E 4123 4125 4128 4136 4137 4144 |
|  | 5088 | 517.2529 | 1032.4913 | 1032.4771 | 0.0142 | 1 | 7 | 38 | 8 | K.TGAPCRSER.L |
|  | 6596 | **576.3020** | **1150.5894** | **1151.6121** | **-1.0226** | **1** | **4** | **59** | **8** | **R.LAKYNQLMR.I** 6646 |
|  | 6807 | **583.8150** | **1165.6154** | **1165.6244** | **-0.0089** | **0** | **25** | **0.42** | **1** | **R.IGAEVYHHLK.G** |
|  | 12684 | **497.4863** | **1489.4370** | **1488.7936** | **0.6434** | **1** | **4** | **34** | **2** | **R.IEEALGDKAIFAGR.K** |
|  | 13446 | **514.2965** | **1539.8675** | **1540.7569** | **-0.8894** | **0** | **3** | **60** | **7** | **K.LAQSNGWGVMVSHR.S** |
|  | 14254 | **796.8760** | **1591.7374** | **1592.8271** | **-1.0897** | **1** | **4** | **46** | **8** | **R.GNPTVEVDLHTAKGR.F** 14255 |
|  | 14951 | 819.4556 | 1636.8967 | 1637.7971 | -0.9003 | 1 | 3 | 61 | 8 | K.VDKFMIELDGTENK.S |
|  | 15818 | **848.3873** | **1694.7601** | **1694.8297** | **-0.0696** | **1** | **2** | **71** | **7** | **K.YNQLMRIEEALGDK.A** |
|  | 17411 | 902.9698 | 1803.9250 | 1803.9366 | -0.0116 | 0 | 109 | 1.3e-009 | 1 | R.AAVPSGASTGIYEALELR.D 17410 17412 |
|  | 18258 | **934.7076** | **1867.4007** | **1867.9576** | **-0.5569** | **0** | **2** | **40** | **1** | **K.LAMQEFMILPVGASSFK.E** |
|  | 20336 | **1024.4697** | **2046.9249** | **2046.0932** | **0.8317** | **1** | **0** | **84** | **2** | **K.FGANAILGVSLAVCKAGAAEK.G** |
|  | 25153 | **891.7810** | **2672.3212** | **2671.3316** | **0.9896** | **1** | **39** | **0.0082** | **1** | **K.TAIQAAGYPDKVVIGMDVAASEFYR.N** |
|  | 25572 | 1372.1642 | 2742.3138 | 2742.3348 | -0.0210 | 0 | 51 | 0.00052 | 1 | K.DATNVGDEGGFAPNILENNEALELLK.T 25573 25574 |

| **160.** | Q00325\|MPCP_HUMAN    **Mass:** 40525    **Score:** 315    **Queries matched:** 33   **emPAI:** 0.70 |
| --- | --- |
|  | Phosphate carrier protein, mitochondrial OS=Homo sapiens GN=SLC25A3 PE=1 SV=2 |

|  | **Query** | **Observed** | **Mr(expt)** | **Mr(calc)** | **Delta** | **Miss** | **Score** | **Expect** | **Rank** | **Peptide** |
| --- | --- | --- | --- | --- | --- | --- | --- | --- | --- | --- |
|  | 1477 | **659.4034** | **658.3961** | **658.4014** | **-0.0053** | **1** | **25** | **0.76** | **2** | **K.KLGLTQ.-** |
|  | 2256 | **412.2291** | **822.4436** | **822.4487** | **-0.0051** | **0** | **28** | **0.23** | **1** | **R.TVEALYK.F** 2261 |
|  | 2501 | 421.7633 | 841.5120 | 841.5174 | -0.0054 | 0 | 10 | 16 | 1 | K.FVVPKPR.S |
|  | 2525 | **423.2103** | **844.4060** | **844.4113** | **-0.0053** | **0** | **22** | **1.4** | **1** | **R.MQVDPQK.Y** 2537 |
|  | 2744 | **431.7545** | **861.4944** | **860.4967** | **0.9977** | **0** | **10** | **19** | **2** | **K.GSSASLVLK.R** |
|  | 3648 | **465.2520** | **928.4895** | **928.4953** | **-0.0058** | **0** | **26** | **0.51** | **1** | **K.GVAPLWMR.Q** 3647 |
|  | 4612 | **499.7310** | **997.4475** | **996.4950** | **0.9525** | **1** | **18** | **2.3** | **1** | **K.MYKEEGLK.A** 4611 |
|  | 4871 | **508.7593** | **1015.5039** | **1016.5978** | **-1.0939** | **1** | **44** | **0.0078** | **1** | **K.GSSASLVLKR.L** 4872 4896 |
|  | 6190 | **559.8201** | **1117.6257** | **1117.6343** | **-0.0086** | **1** | **16** | **4.7** | **1** | **K.EKGSSASLVLK.R** |
|  | 6619 | **577.1246** | **1152.2346** | **1151.5645** | **0.6701** | **1** | **1** | **82** | **8** | **R.MQVDPQKYK.G** 6616 |
|  | 10463 | **681.3574** | **1360.7002** | **1360.7099** | **-0.0097** | **0** | **32** | **0.1** | **1** | **R.IQTQPGYANTLR.D** 10480 |
|  | 12419 | **492.2652** | **1473.7738** | **1472.8028** | **0.9710** | **1** | **5** | **39** | **5** | **K.YKGIFNGFSVTLK.E** |
|  | 16535 | **870.4460** | **1738.8774** | **1737.9050** | **0.9724** | **1** | **37** | **0.019** | **1** | **K.GIFNGFSVTLKEDGVR.G** |
|  | 17530 | 908.4257 | 1814.8368 | 1814.8484 | -0.0116 | 0 | 78 | 1.8e-006 | 1 | K.GWAPTFLGYSMQGLCK.F 17529 |
|  | 17822 | **613.3159** | **1836.9259** | **1836.8361** | **0.0898** | **1** | **1** | **82** | **9** | **R.QIPYTMMKFACFER.T** |
|  | 19660 | **994.4766** | **1986.9387** | **1986.9509** | **-0.0122** | **0** | **87** | **1.9e-007** | **1** | **K.VLYSNMLGEENTYLWR.T** 19659 |
|  | 25086 | **1330.1652** | **2658.3158** | **2658.3250** | **-0.0093** | **0** | **62** | **4.1e-005** | **1** | **R.TSLYLAASASAEFFADIALAPMEAAK.V** 25079 25081 25093 25097 25100 |
|  | 25843 | 699.1020 | 2792.3791 | 2792.4975 | -0.1184 | 1 | 2 | 44 | 7 | R.IIMIGTLTALQWFIYDSVKVYFR.L |

| **161.** | P48047\|ATPO_HUMAN    **Mass:** 23377    **Score:** 315    **Queries matched:** 25   **emPAI:** 1.88 |
| --- | --- |
|  | ATP synthase subunit O, mitochondrial OS=Homo sapiens GN=ATP5O PE=1 SV=1 |

|  | **Query** | **Observed** | **Mr(expt)** | **Mr(calc)** | **Delta** | **Miss** | **Score** | **Expect** | **Rank** | **Peptide** |
| --- | --- | --- | --- | --- | --- | --- | --- | --- | --- | --- |
|  | 2486 | **421.2073** | **840.4001** | **840.4051** | **-0.0051** | **0** | **14** | **6** | **1** | **K.YVDMSVK.T** |
|  | 4986 | **1025.6289** | **1024.6216** | **1024.6281** | **-0.0064** | **1** | **37** | **0.031** | **1** | **R.VAQILKEPK.V** 5001 |
|  | 5616 | **538.2483** | **1074.4821** | **1074.5492** | **-0.0670** | **0** | **2** | **1e+002** | **2** | **-.MAAPAVSGLSR.Q** |
|  | 6035 | **553.8103** | **1105.6060** | **1105.6132** | **-0.0071** | **0** | **65** | **5.6e-005** | **1** | **K.SFLSQGQVLK.L** 6032 6036 |
|  | 6518 | **573.2919** | **1144.5693** | **1144.5764** | **-0.0071** | **0** | **0** | **1.5e+002** | **9** | **R.YATALYSAASK.Q** |
|  | 8558 | **629.8411** | **1257.6677** | **1257.6751** | **-0.0074** | **0** | **40** | **0.013** | **1** | **K.TDPSILGGMIVR.I** 8557 |
|  | 9027 | 642.8571 | 1283.6997 | 1283.6431 | 0.0566 | 1 | 8 | 22 | 7 | R.IGEKYVDMSVK.T |
|  | 13075 | **505.9299** | **1514.7680** | **1513.8504** | **0.9176** | **1** | **5** | **44** | **1** | **K.EPKVAASVLNPYVK.R** |
|  | 13566 | 774.4054 | 1546.7962 | 1546.9083 | -0.1120 | 1 | 5 | 36 | 5 | K.TVLKSFLSQGQVLK.L |
|  | 14081 | **528.3035** | **1581.8886** | **1581.8991** | **-0.0105** | **0** | **12** | **7.8** | **1** | **K.LVRPPVQVYGIEGR.Y** 14063 |
|  | 18328 | **937.0079** | **1872.0012** | **1872.0105** | **-0.0093** | **0** | **92** | **5.9e-008** | **1** | **R.FSPLTTNLINLLAENGR.L** 18327 18347 18348 18351 |
|  | 20397 | **684.6672** | **2050.9799** | **2050.9928** | **-0.0130** | **0** | **48** | **0.0015** | **1** | **R.LSNTQGVVSAFSTMMSVHR.G** |
|  | 20590 | **690.3077** | **2067.9012** | **2066.9878** | **0.9134** | **0** | **(4)** | **38** | **3** | **R.LSNTQGVVSAFSTMMSVHR.G** |
|  | 20777 | **1042.3483** | **2082.6820** | **2082.9827** | **-0.3007** | **0** | **(7)** | **12** | **1** | **R.LSNTQGVVSAFSTMMSVHR.G** |
|  | 22818 | **1159.5671** | **2317.1197** | **2317.1359** | **-0.0162** | **0** | **63** | **3.3e-005** | **1** | **R.GEVPCTVTSASPLEEATLSELK.T** 22817 |

| **162.** | Q9H9B4\|SFXN1_HUMAN    **Mass:** 35881    **Score:** 315    **Queries matched:** 28   **emPAI:** 1.22 |
| --- | --- |
|  | Sideroflexin-1 OS=Homo sapiens GN=SFXN1 PE=1 SV=4 |

|  | **Query** | **Observed** | **Mr(expt)** | **Mr(calc)** | **Delta** | **Miss** | **Score** | **Expect** | **Rank** | **Peptide** |
| --- | --- | --- | --- | --- | --- | --- | --- | --- | --- | --- |
|  | 1576 | **703.0165** | **702.0092** | **701.4258** | **0.5834** | **0** | **0** | **1.7e+002** | **10** | **K.MILIGR.M** |
|  | 1974 | **802.4143** | **801.4070** | **801.4133** | **-0.0064** | **0** | **14** | **9.5** | **2** | **K.IVHDYR.Q** |
|  | 2722 | **431.0848** | **860.1551** | **859.4399** | **0.7152** | **0** | **10** | **15** | **4** | **R.LGESANAAK.Q** |
|  | 2989 | **439.7609** | **877.5073** | **877.5134** | **-0.0061** | **0** | **14** | **7.4** | **1** | **K.HVSPLIGR.F** |
|  | 3660 | 465.7576 | 929.5006 | 929.5083 | -0.0076 | 1 | 24 | 0.79 | 1 | R.KIVHDYR.Q 3651 3666 3667 |
|  | 6059 | **554.7874** | **1107.5603** | **1107.5672** | **-0.0070** | **0** | **50** | **0.0014** | **1** | **K.IQESHPELR.R** |
|  | 8779 | **635.8186** | **1269.6226** | **1269.6313** | **-0.0087** | **0** | **14** | **5.7** | **1** | **K.VGIPVTDENGNR.L** |
|  | 12831 | **750.8994** | **1499.7841** | **1499.7943** | **-0.0102** | **0** | **69** | **1.6e-005** | **1** | **R.NILLTNEQLESAR.K** 12832 |
|  | 14046 | **790.8896** | **1579.7647** | **1579.7763** | **-0.0116** | **0** | **58** | **0.0002** | **1** | **K.SSMSVTSLEAELQAK.I** 14045 |
|  | 15014 | **821.8668** | **1641.7190** | **1641.7311** | **-0.0121** | **0** | **54** | **0.0004** | **1** | **K.YIYDSAFHPDTGEK.M** 15026 |
|  | 16055 | 854.9485 | 1707.8824 | 1707.8944 | -0.0120 | 0 | 43 | 0.0052 | 1 | R.QGIVPPGLTENELWR.A 16054 |
|  | 16102 | **571.2980** | **1710.8721** | **1709.8770** | **0.9951** | **1** | **3** | **62** | **7** | **-.MSGELPPNINIKEPR.W** |
|  | 21069 | 705.0714 | 2112.1924 | 2111.0607 | 1.1317 | 1 | 5 | 29 | 4 | K.VGIPVTDENGNRLGESANAAK.Q |
|  | 21456 | **1079.5725** | **2157.1305** | **2157.1400** | **-0.0095** | **0** | **25** | **0.27** | **1** | **R.ILMAAPGMAIPPFIMNTLEK.K** 21455 |
|  | 21881 | **552.5380** | **2206.1230** | **2205.1247** | **0.9983** | **0** | **(3)** | **44** | **6** | **R.ILMAAPGMAIPPFIMNTLEK.K** |
|  | 22952 | **778.7880** | **2333.3422** | **2333.2197** | **0.1226** | **1** | **2** | **43** | **2** | **R.ILMAAPGMAIPPFIMNTLEKK.A** |
|  | 23654 | **609.0833** | **2432.3041** | **2432.9966** | **-0.6924** | **0** | **4** | **25** | **3** | **R.MSAQVPMNMTITGCMMTFYR.T** |
|  | 25248 | **672.6267** | **2686.4775** | **2685.3279** | **1.1496** | **1** | **1** | **49** | **2** | **K.SSMSVTSLEAELQAKIQESHPELR.R** |
|  | 28061 | **1624.3497** | **3246.6849** | **3246.6983** | **-0.0134** | **0** | **101** | **3.4e-009** | **1** | **R.SGDAPLTVNELGTAYVSATTGAVATALGLNALTK.H** 28060 |

| **163.** | P04792\|HSPB1_HUMAN    **Mass:** 22826    **Score:** 313    **Queries matched:** 15   **emPAI:** 1.95 |
| --- | --- |
|  | Heat shock protein beta-1 OS=Homo sapiens GN=HSPB1 PE=1 SV=2 |

|  | **Query** | **Observed** | **Mr(expt)** | **Mr(calc)** | **Delta** | **Miss** | **Score** | **Expect** | **Rank** | **Peptide** |
| --- | --- | --- | --- | --- | --- | --- | --- | --- | --- | --- |
|  | 2368 | **416.5444** | **831.0742** | **830.5014** | **0.5728** | **0** | **10** | **16** | **8** | **R.VPFSLLR.G** |
|  | 5623 | **538.2871** | **1074.5595** | **1074.5669** | **-0.0074** | **0** | **16** | **4.5** | **2** | **R.QLSSGVSEIR.H** |
|  | 6004 | **552.5848** | **1103.1550** | **1103.4996** | **-0.3446** | **0** | **11** | **9.7** | **1** | **R.QDEHGYISR.C** |
|  | 6546 | **573.8174** | **1145.6202** | **1145.6292** | **-0.0090** | **1** | **33** | **0.085** | **1** | **K.TKDGVVEITGK.H** |
|  | 6765 | **582.3104** | **1162.6063** | **1162.6135** | **-0.0072** | **0** | **58** | **0.00027** | **1** | **R.LFDQAFGLPR.L** |
|  | 15231 | **552.5848** | **1654.7325** | **1654.7448** | **-0.0123** | **1** | **36** | **0.026** | **1** | **K.HEERQDEHGYISR.C** |
|  | 17150 | **892.4595** | **1782.9044** | **1782.9152** | **-0.0108** | **0** | **65** | **3.4e-005** | **1** | **R.VSLDVNHFAPDELTVK.T** 17149 |
|  | 18721 | **953.4933** | **1904.9721** | **1904.9843** | **-0.0122** | **0** | **33** | **0.048** | **1** | **K.LATQSNEITIPVTFESR.A** 18724 |
|  | 27996 | **1076.2170** | **3225.6293** | **3225.6479** | **-0.0186** | **1** | **55** | **0.00013** | **1** | **R.KYTLPPGVDPTQVSSSLSPEGTLTVEAPMPK.L** 27994 27995 |
|  | 30281 | **1365.0172** | **4092.0298** | **4092.0533** | **-0.0235** | **0** | **79** | **3.6e-007** | **1** | **R.LPEEWSQWLGGSSWPGYVRPLPPAAIESPAVAAPAYSR.A** 30283 |

| **164.** | Q92928\|RAB1C_HUMAN    **Mass:** 22231    **Score:** 312    **Queries matched:** 26   **emPAI:** 0.89 |
| --- | --- |
|  | Putative Ras-related protein Rab-1C OS=Homo sapiens GN=RAB1C PE=5 SV=2 |

|  | **Query** | **Observed** | **Mr(expt)** | **Mr(calc)** | **Delta** | **Miss** | **Score** | **Expect** | **Rank** | **Peptide** |
| --- | --- | --- | --- | --- | --- | --- | --- | --- | --- | --- |
|  | 4286 | **488.2803** | **974.5461** | **974.5397** | **0.0065** | **1** | **8** | **28** | **6** | **K.KVVDNTTAK.E** |
|  | 5578 | **536.3207** | **1070.6269** | **1070.6336** | **-0.0066** | **0** | **53** | **0.00096** | **1** | **K.LLLIGDSGVGK.S** 5577 |
|  | 8595 | **630.8000** | **1259.5854** | **1259.5315** | **0.0539** | **0** | **8** | **21** | **6** | **-.MNPGYDCLFK.L** |
|  | 9082 | **644.2776** | **1286.5406** | **1287.7398** | **-1.1992** | **1** | **7** | **29** | **2** | **K.LLVGNKSDLTTK.K** |
|  | 9635 | 658.8289 | 1315.6433 | 1315.6521 | -0.0088 | 0 | 60 | 0.00015 | 1 | K.LQIWDTAGQER.F 9612 9633 9634 |
|  | 10441 | 680.3729 | 1358.7312 | 1357.7817 | 0.9495 | 1 | 16 | 3.9 | 2 | K.IQTIELDGKTIK.L 10442 |
|  | 14114 | **792.9352** | **1583.8559** | **1584.7678** | **-0.9119** | **0** | **13** | **5.5** | **2** | **K.QMGPGAASGGERPNLK.I** 14129 14138 |
|  | 16119 | 571.6105 | 1711.8096 | 1712.8628 | -1.0532 | 1 | 6 | 32 | 2 | K.KQMGPGAASGGERPNLK.I 16118 |
|  | 17502 | 604.9745 | 1811.9016 | 1813.0495 | -1.1479 | 1 | 7 | 20 | 3 | K.LLLIGDSGVGKSCLLLR.F |
|  | 18276 | **935.4440** | **1868.8735** | **1867.8808** | **0.9927** | **0** | **111** | **8.5e-010** | **1** | **K.NATNVEQAFMTMAAEIK.K** 18261 |
|  | 18451 | **942.9408** | **1883.8670** | **1883.8757** | **-0.0087** | **0** | **(66)** | **2.4e-005** | **2** | **K.NATNVEQAFMTMAAEIK.K** 18449 18450 |
|  | 18654 | **951.0151** | **1900.0157** | **1899.8706** | **0.1451** | **0** | **(7)** | **21** | **4** | **K.NATNVEQAFMTMAAEIK.K** |
|  | 19774 | **666.6886** | **1997.0440** | **1995.9757** | **1.0682** | **1** | **(2)** | **64** | **7** | **K.NATNVEQAFMTMAAEIKK.Q** |
|  | 19954 | **671.8677** | **2012.5814** | **2011.9707** | **0.6107** | **1** | **8** | **11** | **2** | **K.NATNVEQAFMTMAAEIKK.Q** |
|  | 26979 | **746.8492** | **2983.3676** | **2984.3903** | **-1.0227** | **1** | **0** | **47** | **7** | **K.QMGPGAASGGERPNLKIDSTPVKPAGGGCC.-** |

| **165.** | Q9H0U4\|RAB1B_HUMAN    **Mass:** 22328    **Score:** 312    **Queries matched:** 23   **emPAI:** 0.88 |
| --- | --- |
|  | Ras-related protein Rab-1B OS=Homo sapiens GN=RAB1B PE=1 SV=1 |

|  | **Query** | **Observed** | **Mr(expt)** | **Mr(calc)** | **Delta** | **Miss** | **Score** | **Expect** | **Rank** | **Peptide** |
| --- | --- | --- | --- | --- | --- | --- | --- | --- | --- | --- |
|  | 3586 | 463.0037 | 923.9928 | 923.4348 | 0.5579 | 0 | 8 | 15 | 6 | R.YASENVNK.L 3579 |
|  | 4286 | 488.2803 | 974.5461 | 974.5397 | 0.0065 | 1 | 8 | 28 | 6 | K.KVVDNTTAK.E |
|  | 5578 | 536.3207 | 1070.6269 | 1070.6336 | -0.0066 | 0 | 53 | 0.00096 | 1 | K.LLLIGDSGVGK.S 5577 |
|  | 7161 | **595.3152** | **1188.6158** | **1187.5499** | **1.0660** | **0** | **5** | **56** | **6** | **M.NPEYDYLFK.L** |
|  | 9082 | 644.2776 | 1286.5406 | 1287.7398 | -1.1992 | 1 | 7 | 29 | 2 | K.LLVGNKSDLTTK.K |
|  | 9635 | 658.8289 | 1315.6433 | 1315.6521 | -0.0088 | 0 | 60 | 0.00015 | 1 | K.LQIWDTAGQER.F 9612 9633 9634 |
|  | 11841 | 481.1506 | 1440.4300 | 1440.7143 | -0.2844 | 0 | 10 | 8.6 | 3 | R.MGPGAASGGERPNLK.I 11851 11852 |
|  | 17502 | 604.9745 | 1811.9016 | 1813.0495 | -1.1479 | 1 | 7 | 20 | 3 | K.LLLIGDSGVGKSCLLLR.F |
|  | 18276 | 935.4440 | 1868.8735 | 1867.8808 | 0.9927 | 0 | 111 | 8.5e-010 | 1 | K.NATNVEQAFMTMAAEIK.K 18261 |
|  | 18451 | 942.9408 | 1883.8670 | 1883.8757 | -0.0087 | 0 | (66) | 2.4e-005 | 2 | K.NATNVEQAFMTMAAEIK.K 18449 18450 |
|  | 18654 | 951.0151 | 1900.0157 | 1899.8706 | 0.1451 | 0 | (7) | 21 | 4 | K.NATNVEQAFMTMAAEIK.K |
|  | 19774 | 666.6886 | 1997.0440 | 1995.9757 | 1.0682 | 1 | (2) | 64 | 7 | K.NATNVEQAFMTMAAEIKK.R |
|  | 19954 | 671.8677 | 2012.5814 | 2011.9707 | 0.6107 | 1 | 8 | 11 | 2 | K.NATNVEQAFMTMAAEIKK.R |

| **166.** | P60660\|MYL6_HUMAN    **Mass:** 17090    **Score:** 310    **Queries matched:** 24   **emPAI:** 3.18 |
| --- | --- |
|  | Myosin light polypeptide 6 OS=Homo sapiens GN=MYL6 PE=1 SV=2 |

|  | **Query** | **Observed** | **Mr(expt)** | **Mr(calc)** | **Delta** | **Miss** | **Score** | **Expect** | **Rank** | **Peptide** |
| --- | --- | --- | --- | --- | --- | --- | --- | --- | --- | --- |
|  | 971 | **527.0981** | **526.0909** | **525.2911** | **0.7998** | **0** | **7** | **17** | **1** | **R.HILSG.-** |
|  | 2235 | **822.3596** | **821.3523** | **821.3589** | **-0.0066** | **0** | **30** | **0.15** | **1** | **K.SDEMNVK.V** |
|  | 4559 | 498.2944 | 994.5742 | 994.5811 | -0.0069 | 0 | 36 | 0.041 | 1 | R.HVLVTLGEK.M 4558 |
|  | 10100 | **671.3138** | **1340.6131** | **1340.6217** | **-0.0086** | **0** | **56** | **0.00035** | **1** | **K.ILYSQCGDVMR.A** |
|  | 10359 | **677.8650** | **1353.7154** | **1353.7252** | **-0.0098** | **0** | **44** | **0.0058** | **1** | **R.ALGQNPTNAEVLK.V** 10346 10360 10373 10376 |
|  | 16259 | **861.9221** | **1721.8296** | **1721.8407** | **-0.0111** | **1** | **51** | **0.00082** | **1** | **R.VFDKEGNGTVMGAEIR.H** 16249 16262 16270 16271 16272 |
|  | 16536 | **580.6337** | **1738.8794** | **1737.8356** | **1.0438** | **1** | **(4)** | **49** | **5** | **R.VFDKEGNGTVMGAEIR.H** 16532 |
|  | 17188 | **893.9096** | **1785.8047** | **1785.8169** | **-0.0123** | **1** | **72** | **7.3e-006** | **1** | **K.NKDQGTYEDYVEGLR.V** 17186 17187 |
|  | 18490 | **944.5005** | **1886.9865** | **1886.9964** | **-0.0099** | **0** | **69** | **1.1e-005** | **1** | **K.VLDFEHFLPMLQTVAK.N** 18488 18489 |

| **167.** | Q96AG4\|LRC59_HUMAN    **Mass:** 35308    **Score:** 307    **Queries matched:** 23   **emPAI:** 1.25 |
| --- | --- |
|  | Leucine-rich repeat-containing protein 59 OS=Homo sapiens GN=LRRC59 PE=1 SV=1 |

|  | **Query** | **Observed** | **Mr(expt)** | **Mr(calc)** | **Delta** | **Miss** | **Score** | **Expect** | **Rank** | **Peptide** |
| --- | --- | --- | --- | --- | --- | --- | --- | --- | --- | --- |
|  | 1580 | **703.4049** | **702.3976** | **702.4064** | **-0.0089** | **0** | **15** | **7.9** | **4** | **R.SWAVLK.L** |
|  | 2170 | **817.4359** | **816.4286** | **816.4705** | **-0.0419** | **1** | **10** | **23** | **8** | **K.LDLSKNK.L** |
|  | 2701 | **430.7366** | **859.4586** | **858.4671** | **0.9915** | **1** | **9** | **25** | **7** | **K.AGSKGGNLR.D** |
|  | 2817 | **866.5030** | **865.4957** | **865.4545** | **0.0412** | **1** | **9** | **19** | **9** | **R.KEYDALK.A** |
|  | 2932 | 437.7217 | 873.4287 | 873.4780 | -0.0493 | 1 | 6 | 60 | 5 | K.SKSGSRPR.K |
|  | 6384 | **567.7916** | **1133.5686** | **1133.5750** | **-0.0065** | **0** | **7** | **31** | **1** | **K.ATILDLSCNK.L** |
|  | 9620 | **658.3996** | **1314.7846** | **1314.7911** | **-0.0065** | **0** | **33** | **0.075** | **1** | **K.LVTLPVSFAQLK.N** |
|  | 10910 | 693.3568 | 1384.6991 | 1385.7415 | -1.0425 | 1 | 2 | 90 | 7 | K.NKLQQLPADFGR.L |
|  | 13355 | **767.4358** | **1532.8571** | **1532.8674** | **-0.0103** | **0** | **69** | **1.4e-005** | **1** | **R.LVNLQHLDLLNNK.L** 13356 |
|  | 16500 | 579.6545 | 1735.9416 | 1735.9509 | -0.0092 | 1 | 36 | 0.027 | 1 | K.WLDLKDNPLDPVLAK.V 16483 16498 16499 |
|  | 17379 | **901.4740** | **1800.9334** | **1800.9444** | **-0.0110** | **0** | **51** | **0.00093** | **1** | **K.LTTLPSDFCGLTHLVK.L** 17378 |
|  | 18415 | **940.9782** | **1879.9419** | **1879.9541** | **-0.0122** | **1** | **90** | **9.6e-008** | **1** | **R.RHEILQWVLQTDSQQ.-** 18414 18422 |
|  | 20614 | **690.6556** | **2068.9449** | **2069.0528** | **-0.1079** | **0** | **5** | **27** | **1** | **K.LDGNELDLSLSDLNEVPVK.E** |
|  | 22778 | **771.7275** | **2312.1606** | **2312.1747** | **-0.0141** | **1** | **47** | **0.0014** | **1** | **R.DKLDGNELDLSLSDLNEVPVK.E** 22770 |
|  | 25841 | **931.7723** | **2792.2952** | **2791.4855** | **0.8097** | **1** | **17** | **1.3** | **1** | **K.LDGNELDLSLSDLNEVPVKELAALPK.A** |

| **168.** | P01009\|A1AT_HUMAN    **Mass:** 46878    **Score:** 307    **Queries matched:** 28   **emPAI:** 1.51 |
| --- | --- |
|  | Alpha-1-antitrypsin OS=Homo sapiens GN=SERPINA1 PE=1 SV=3 |

|  | **Query** | **Observed** | **Mr(expt)** | **Mr(calc)** | **Delta** | **Miss** | **Score** | **Expect** | **Rank** | **Peptide** |
| --- | --- | --- | --- | --- | --- | --- | --- | --- | --- | --- |
|  | 1769 | 785.3839 | 784.3766 | 784.4443 | -0.0677 | 0 | 7 | 37 | 5 | K.VVNPTQK.- |
|  | 2610 | **426.7479** | **851.4812** | **851.4865** | **-0.0053** | **0** | **31** | **0.13** | **1** | **R.SASLHLPK.L** |
|  | 3122 | **444.7526** | **887.4906** | **887.4964** | **-0.0058** | **0** | **21** | **1.6** | **1** | **K.AVLTIDEK.G** |
|  | 3560 | **462.0099** | **922.0052** | **921.4192** | **0.5860** | **0** | **19** | **1.3** | **2** | **K.FLENEDR.R** |
|  | 4866 | **508.3076** | **1014.6006** | **1014.6073** | **-0.0067** | **0** | **23** | **0.89** | **1** | **K.SVLGQLGITK.V** |
|  | 5652 | **539.7639** | **1077.5131** | **1077.5203** | **-0.0071** | **1** | **17** | **3** | **1** | **K.FLENEDRR.S** |
|  | 6093 | **556.3039** | **1110.5932** | **1109.5968** | **0.9964** | **0** | **51** | **0.0012** | **1** | **K.LSITGTYDLK.S** 6096 |
|  | 6412 | 568.8393 | 1135.6640 | 1135.5873 | 0.0767 | 1 | 15 | 5.3 | 5 | K.KQINDYVEK.G 6411 |
|  | 8331 | 623.8220 | 1245.6295 | 1246.5951 | -0.9656 | 0 | 7 | 28 | 3 | R.LGMFNIQHCK.K |
|  | 12513 | **494.2357** | **1479.6852** | **1478.7365** | **0.9487** | **1** | **9** | **16** | **4** | **K.QINDYVEKGTQGK.I** |
|  | 15005 | **821.4294** | **1640.8443** | **1640.8562** | **-0.0119** | **0** | **67** | **2.5e-005** | **1** | **K.ITPNLAEFAFSLYR.Q** 15007 15013 |
|  | 17076 | **593.9232** | **1778.7478** | **1778.7609** | **-0.0130** | **0** | **27** | **0.2** | **1** | **K.TDTSHHDQDHPTFNK.I** |
|  | 17774 | **917.4585** | **1832.9024** | **1832.9156** | **-0.0132** | **0** | **69** | **1.4e-005** | **1** | **K.VFSNGADLSGVTEEAPLK.L** |
|  | 18075 | 928.9885 | 1855.9624 | 1854.9702 | 0.9922 | 0 | 64 | 4e-005 | 1 | K.FNKPFVFLMIEQNTK.S 18062 |
|  | 20843 | **697.3679** | **2089.0819** | **2089.0884** | **-0.0065** | **1** | **47** | **0.0017** | **1** | **K.ELDRDTVFALVNYIFFK.G** 20842 |
|  | 21490 | **721.3832** | **2161.1277** | **2161.1266** | **0.0011** | **1** | **2** | **58** | **5** | **K.VFSNGADLSGVTEEAPLKLSK.A** |
|  | 21700 | **729.3464** | **2185.0175** | **2185.0327** | **-0.0153** | **1** | **32** | **0.046** | **1** | **K.LYHSEAFTVNFGDTEEAKK.Q** |
|  | 24699 | **1287.6659** | **2573.3172** | **2573.3337** | **-0.0165** | **0** | **95** | **2.3e-008** | **1** | **R.TLNQPDSQLQLTTGNGLFLSEGLK.L** 24701 24708 24709 24710 |

| **169.** | Q9NYU2\|UGGG1_HUMAN    **Mass:** 177819   **Score:** 303    **Queries matched:** 36   **emPAI:** 0.18 |
| --- | --- |
|  | UDP-glucose:glycoprotein glucosyltransferase 1 OS=Homo sapiens GN=UGCGL1 PE=1 SV=3 |

|  | **Query** | **Observed** | **Mr(expt)** | **Mr(calc)** | **Delta** | **Miss** | **Score** | **Expect** | **Rank** | **Peptide** |
| --- | --- | --- | --- | --- | --- | --- | --- | --- | --- | --- |
|  | 779 | 476.1964 | 475.1892 | 475.2390 | -0.0499 | 0 | 6 | 71 | 1 | K.SNAGK.I |
|  | 1477 | 659.4034 | 658.3961 | 658.4126 | -0.0165 | 1 | 25 | 0.86 | 5 | K.ARAITK.T |
|  | 1764 | 779.4243 | 778.4170 | 778.4377 | -0.0208 | 0 | 16 | 4.8 | 4 | R.IIWGYK.I |
|  | 3897 | **474.2790** | **946.5434** | **945.4821** | **1.0613** | **0** | **12** | **11** | **1** | **R.HYIFNPR.K** |
|  | 3953 | **476.2551** | **950.4956** | **951.5211** | **-1.0255** | **1** | **9** | **22** | **4** | **K.NFITKMAK.E** |
|  | 4114 | **482.2759** | **962.5373** | **962.5437** | **-0.0064** | **0** | **12** | **10** | **6** | **R.QLLYDAIK.H** |
|  | 5158 | 519.7733 | 1037.5321 | 1037.5393 | -0.0072 | 1 | 7 | 30 | 10 | K.EKETGALYK.E |
|  | 5185 | **520.7623** | **1039.5101** | **1040.5866** | **-1.0765** | **0** | **5** | **48** | **2** | **K.VDALLSAQPK.G** |
|  | 5534 | **535.2702** | **1068.5258** | **1068.5352** | **-0.0094** | **0** | **8** | **26** | **2** | **R.WLHQQTEK.Q** |
|  | 10028 | **668.4043** | **1334.7940** | **1333.6435** | **1.1505** | **0** | **3** | **74** | **6** | **R.VEEDVASDLVMK.V** |
|  | 10570 | **683.8863** | **1365.7581** | **1364.8028** | **0.9554** | **1** | **7** | **28** | **6** | **K.VKVEHVVSVLEK.K** 10521 |
|  | 10577 | **456.3150** | **1365.9232** | **1365.7876** | **0.1356** | **1** | **10** | **11** | **2** | **R.FLRIMMLSVLK.N** |
|  | 11430 | **708.2770** | **1414.5394** | **1413.6776** | **0.8618** | **1** | **4** | **48** | **4** | **R.TEVEENQKYFK.G** |
|  | 11530 | **710.9467** | **1419.8789** | **1419.7762** | **0.1027** | **0** | **4** | **45** | **1** | **K.YHISALYVVDLK.K** |
|  | 12258 | 733.7855 | 1465.5565 | 1465.7889 | -0.2324 | 1 | 2 | 74 | 6 | K.VDALLSAQPKGDPR.I |
|  | 12925 | 753.8776 | 1505.7406 | 1504.7378 | 1.0028 | 1 | 1 | 1.1e+002 | 8 | R.AKTIDLCNNPMTK.E |
|  | 13588 | **775.4293** | **1548.8440** | **1547.8711** | **0.9728** | **1** | **4** | **46** | **2** | **R.KYHISALYVVDLK.K** |
|  | 13941 | **787.4152** | **1572.8158** | **1572.8260** | **-0.0102** | **0** | **26** | **0.34** | **1** | **R.AIWAALQTQTSNAAK.N** |
|  | 14907 | **818.9556** | **1635.8967** | **1636.8712** | **-0.9745** | **0** | **6** | **27** | **8** | **K.EPVYLSGYGVELAIK.S** |
|  | 15322 | **831.9276** | **1661.8407** | **1661.8526** | **-0.0119** | **0** | **70** | **1.3e-005** | **1** | **K.VWQLQDLSFQTAAR.I** 15323 |
|  | 15976 | **568.9396** | **1703.7971** | **1702.8526** | **0.9445** | **0** | **4** | **42** | **10** | **K.LNIQPSEADYAVDIR.S** |
|  | 17071 | **593.8140** | **1778.4201** | **1778.9567** | **-0.5366** | **1** | **1** | **53** | **1** | **K.FLFVDADQIVRTDLK.E** |
|  | 17335 | **600.3666** | **1798.0781** | **1797.8455** | **0.2326** | **0** | **5** | **29** | **3** | **R.EQLDPDELETITMHK.I** |
|  | 25029 | **882.4231** | **2644.2475** | **2644.2657** | **-0.0182** | **0** | **31** | **0.047** | **1** | **R.IYSHDGTDSPPDADEVVIVLNNFK.S** |
|  | 25515 | **683.8650** | **2731.4309** | **2731.4466** | **-0.0157** | **0** | **37** | **0.013** | **1** | **K.TCESDTLEALLLTASERPKPLLFK.G** |
|  | 26034 | 1412.1616 | 2822.3087 | 2821.3559 | 0.9528 | 1 | 2 | 35 | 3 | R.ILTAERDYLDLTASNNFFVDDYAR.F |
|  | 26603 | **973.8007** | **2918.3802** | **2917.3923** | **0.9878** | **0** | **4** | **22** | **1** | **K.EIYDDSFIRPVTFWIVGDFDSPSGR.Q** |
|  | 26661 | **975.8422** | **2924.5047** | **2924.5171** | **-0.0125** | **0** | **26** | **0.15** | **1** | **R.IIGPLEDSELFNQDDFHLLENIILK.T** |
|  | 27308 | **1526.2451** | **3050.4757** | **3050.4882** | **-0.0125** | **0** | **83** | **2.4e-007** | **1** | **K.FLDMPQSPLFTLNLNTPESWMVESVR.T** 27310 |
|  | 27406 | **614.0967** | **3065.4470** | **3066.4831** | **-1.0361** | **0** | **(2)** | **32** | **2** | **K.FLDMPQSPLFTLNLNTPESWMVESVR.T** |
|  | 28723 | **1155.2284** | **3462.6633** | **3462.6838** | **-0.0204** | **0** | **83** | **2.2e-007** | **1** | **R.GQYQGLSQDPNSLSNLDQDLPNNMIHQVPIK.S** 28722 |
|  | 29887 | **781.9854** | **3904.8907** | **3904.9062** | **-0.0156** | **0** | **10** | **3** | **3** | **K.NLHNMVFIVDPAHETTAELMNTAEMFLSNHIPLR.I** |

| **170.** | P30040\|ERP29_HUMAN    **Mass:** 29032    **Score:** 303    **Queries matched:** 19   **emPAI:** 0.84 |
| --- | --- |
|  | Endoplasmic reticulum protein ERp29 OS=Homo sapiens GN=ERP29 PE=1 SV=4 |

|  | **Query** | **Observed** | **Mr(expt)** | **Mr(calc)** | **Delta** | **Miss** | **Score** | **Expect** | **Rank** | **Peptide** |
| --- | --- | --- | --- | --- | --- | --- | --- | --- | --- | --- |
|  | 1657 | **732.3197** | **731.3124** | **730.3796** | **0.9328** | **0** | **9** | **29** | **10** | **-.MAAAVPR.A** |
|  | 2213 | **411.1689** | **820.3231** | **819.5218** | **0.8013** | **1** | **3** | **94** | **5** | **K.SKFVLVK.F** |
|  | 3750 | **469.2394** | **936.4642** | **936.4705** | **-0.0063** | **0** | **12** | **11** | **1** | **K.WAEQYLK.I** |
|  | 5620 | 538.2687 | 1074.5228 | 1074.5305 | -0.0077 | 0 | 15 | 6.1 | 1 | K.QGQDNLSSVK.E |
|  | 8259 | 621.8343 | 1241.6540 | 1241.7092 | -0.0551 | 1 | 12 | 9.2 | 5 | R.ASGVEARQALLK.Q |
|  | 8355 | **624.2782** | **1246.5418** | **1246.5506** | **-0.0088** | **0** | **26** | **0.36** | **1** | **K.FDTQYPYGEK.Q** |
|  | 8806 | **636.4210** | **1270.8274** | **1269.6274** | **1.1999** | **1** | **0** | **1.2e+002** | **7** | **K.LNMELSEKYK.L** |
|  | 9704 | **660.8311** | **1319.6477** | **1319.6550** | **-0.0073** | **0** | **23** | **0.65** | **1** | **K.ESYPVFYLFR.D** |
|  | 14470 | **804.8751** | **1607.7357** | **1607.7468** | **-0.0111** | **0** | **36** | **0.03** | **1** | **R.DGDFENPVPYTGAVK.V** |
|  | 14794 | **543.9487** | **1628.8242** | **1627.8893** | **0.9349** | **1** | **1** | **96** | **10** | **R.QALLKQGQDNLSSVK.E** |
|  | 16043 | **854.8905** | **1707.7664** | **1707.7774** | **-0.0109** | **0** | **74** | **4.7e-006** | **1** | **K.ILDQGEDFPASEMTR.I** |
|  | 20571 | **689.0545** | **2064.1417** | **2063.9946** | **0.1471** | **1** | **0** | **77** | **2** | **K.ILDQGEDFPASEMTRIAR.L** 20550 |
|  | 21422 | **718.7399** | **2153.1980** | **2153.0133** | **0.1847** | **1** | **6** | **18** | **6** | **K.IMGKILDQGEDFPASEMTR.I** |
|  | 24763 | **1292.1325** | **2582.2503** | **2582.2662** | **-0.0158** | **0** | **79** | **8.7e-007** | **1** | **K.GQGVYLGMPGCLPVYDALAGEFIR.A** 24762 |
|  | 28588 | **1138.2082** | **3411.6029** | **3411.6239** | **-0.0209** | **1** | **87** | **7.9e-008** | **1** | **R.LAENSASSDDLLVAEVGISDYGDKLNMELSEK.Y** 28589 28590 |

| **171.** | P08133\|ANXA6_HUMAN    **Mass:** 76168    **Score:** 302    **Queries matched:** 33   **emPAI:** 0.69 |
| --- | --- |
|  | Annexin A6 OS=Homo sapiens GN=ANXA6 PE=1 SV=3 |

|  | **Query** | **Observed** | **Mr(expt)** | **Mr(calc)** | **Delta** | **Miss** | **Score** | **Expect** | **Rank** | **Peptide** |
| --- | --- | --- | --- | --- | --- | --- | --- | --- | --- | --- |
|  | 1854 | **397.4025** | **792.7904** | **792.3436** | **0.4469** | **0** | **6** | **32** | **2** | **K.EMSGDVR.D** 1861 |
|  | 3438 | 457.2337 | 912.4528 | 912.3825 | 0.0704 | 0 | 1 | 1.3e+002 | 5 | K.NDTSGEYK.K |
|  | 3483 | 916.4941 | 915.4869 | 916.4800 | -0.9931 | 0 | 0 | 1.9e+002 | 8 | -.MAKPAQGAK.Y |
|  | 4640 | **500.3105** | **998.6064** | **998.6124** | **-0.0060** | **0** | **36** | **0.045** | **1** | **K.ALIEILATR.T** |
|  | 5193 | **521.2276** | **1040.4406** | **1040.5147** | **-0.0741** | **0** | **10** | **16** | **6** | **R.FMTILCTR.S** |
|  | 5566 | **536.2778** | **1070.5410** | **1071.5924** | **-1.0514** | **0** | **12** | **10** | **3** | **R.SEIDLLNIR.R** 5567 |
|  | 5571 | 536.2970 | 1070.5794 | 1070.5284 | 0.0511 | 1 | 8 | 28 | 6 | R.EFIEKYDK.S |
|  | 5670 | 540.4084 | 1078.8022 | 1079.6451 | -0.8429 | 0 | 6 | 29 | 1 | R.ILISLATGHR.E |
|  | 6524 | **573.4052** | **1144.7957** | **1144.6638** | **0.1319** | **0** | **6** | **35** | **1** | **K.MLVVLLQGTR.E** 6543 |
|  | 10276 | **450.7346** | **1349.1819** | **1349.6285** | **-0.4466** | **0** | **5** | **30** | **5** | **K.MTNYDVEHTIK.K** |
|  | 11759 | **718.3008** | **1434.5871** | **1434.6926** | **-0.1054** | **1** | **1** | **1e+002** | **7** | **K.SHFGRDLMTDLK.S** |
|  | 12492 | **739.8641** | **1477.7136** | **1477.7235** | **-0.0099** | **1** | **30** | **0.14** | **1** | **K.MTNYDVEHTIKK.E** 12491 |
|  | 13336 | **766.8749** | **1531.7352** | **1531.7453** | **-0.0101** | **0** | **47** | **0.0024** | **1** | **R.TNEQMHQLVAAYK.D** |
|  | 14666 | **811.3701** | **1620.7257** | **1620.7380** | **-0.0123** | **0** | **70** | **1.2e-005** | **1** | **K.SLEDALSSDTSGHFR.R** 14667 |
|  | 15672 | 844.8889 | 1687.7633 | 1686.7962 | 0.9671 | 0 | 8 | 20 | 2 | K.GTVRPANDFNPDADAK.A |
|  | 15963 | **568.6333** | **1702.8781** | **1702.8898** | **-0.0118** | **0** | **27** | **0.21** | **1** | **R.LIVGLMRPPAYCDAK.E** 15949 |
|  | 16202 | **859.4169** | **1716.8192** | **1716.8319** | **-0.0127** | **0** | **59** | **0.00015** | **1** | **K.SLHQAIEGDTSGDFLK.A** |
|  | 16888 | **882.4398** | **1762.8651** | **1762.8771** | **-0.0120** | **1** | **69** | **1.4e-005** | **1** | **R.DLMTDLKSEISGDLAR.L** |
|  | 16935 | **884.9531** | **1767.8916** | **1767.9003** | **-0.0087** | **0** | **60** | **9.8e-005** | **1** | **K.GLGTDEDTIIDIITHR.S** 16934 |
|  | 21491 | **721.3865** | **2161.1376** | **2160.1096** | **1.0280** | **1** | **3** | **41** | **4** | **K.DAISGIGTDEKCLIEILASR.T** |
|  | 22494 | **760.3939** | **2278.1599** | **2278.1665** | **-0.0066** | **1** | **3** | **42** | **2** | **R.ILISLATGHREEGGENLDQAR.E** |
|  | 23311 | 794.0342 | 2379.0807 | 2379.0979 | -0.0172 | 1 | 35 | 0.022 | 1 | K.DAYERDLEADIIGDTSGHFQK.M |
|  | 24363 | **842.0489** | **2523.1248** | **2523.1376** | **-0.0128** | **0** | **33** | **0.035** | **1** | **R.GSIHDFPGFDPNQDAEALYTAMK.G** |
|  | 26129 | **948.4357** | **2842.2854** | **2842.3021** | **-0.0167** | **1** | **67** | **1.1e-005** | **1** | **K.YRGSIHDFPGFDPNQDAEALYTAMK.G** |
|  | 28266 | **829.9030** | **3315.5827** | **3315.5143** | **0.0684** | **0** | **0** | **42** | **3** | **K.LSGGDDDAAGQFFPEAAQVAYQMWELSAVAR.V** |
|  | 30048 | **1325.9890** | **3974.9452** | **3974.9671** | **-0.0219** | **1** | **24** | **0.13** | **1** | **K.MLVVLLQGTREEDDVVSEDLVQQDVQDLYEAGELK.W** |

| **172.** | Q00796\|DHSO_HUMAN    **Mass:** 38899    **Score:** 297    **Queries matched:** 24   **emPAI:** 1.09 |
| --- | --- |
|  | Sorbitol dehydrogenase OS=Homo sapiens GN=SORD PE=1 SV=3 |

|  | **Query** | **Observed** | **Mr(expt)** | **Mr(calc)** | **Delta** | **Miss** | **Score** | **Expect** | **Rank** | **Peptide** |
| --- | --- | --- | --- | --- | --- | --- | --- | --- | --- | --- |
|  | 818 | **487.3205** | **486.3132** | **486.3166** | **-0.0034** | **0** | **19** | **3.8** | **1** | **K.GLGLK.I** |
|  | 3391 | **455.2582** | **908.5019** | **908.5080** | **-0.0061** | **0** | **34** | **0.064** | **1** | **R.VAIEPGAPR.E** 3384 |
|  | 4260 | **487.3208** | **972.6270** | **971.6201** | **1.0069** | **1** | **24** | **0.82** | **1** | **K.GLGLKIMLK.C** |
|  | 4801 | **506.2277** | **1010.4408** | **1009.4440** | **0.9968** | **0** | **18** | **2.2** | **1** | **K.HNAAFCYK.L** |
|  | 5404 | **529.7252** | **1057.4359** | **1056.5563** | **0.8796** | **1** | **6** | **44** | **6** | **K.ESPQEIARK.V** |
|  | 7017 | **592.3174** | **1182.6203** | **1182.6284** | **-0.0081** | **1** | **31** | **0.11** | **1** | **K.ALEAFETFKK.G** 7018 |
|  | 14075 | **791.9109** | **1581.8072** | **1581.8185** | **-0.0112** | **0** | **52** | **0.00066** | **1** | **K.KPMVLGHEASGTVEK.V** |
|  | 14200 | **795.4146** | **1588.8147** | **1588.8243** | **-0.0096** | **0** | **94** | **5.6e-008** | **1** | **K.AMGAAQVVVTDLSATR.L** |
|  | 14333 | **799.8712** | **1597.7279** | **1597.8134** | **-0.0855** | **0** | **(4)** | **51** | **7** | **K.KPMVLGHEASGTVEK.V** |
|  | 14444 | **536.2662** | **1605.7767** | **1604.8192** | **0.9575** | **0** | **(5)** | **38** | **2** | **K.AMGAAQVVVTDLSATR.L** |
|  | 16546 | **870.9205** | **1739.8265** | **1739.8375** | **-0.0110** | **0** | **45** | **0.0036** | **1** | **R.YCNTWPVAISMLASK.S** |
|  | 17324 | **899.5150** | **1797.0155** | **1797.0257** | **-0.0102** | **0** | **42** | **0.0063** | **1** | **K.VLVCGAGPIGMVTLLVAK.A** |
|  | 19244 | **975.5138** | **1949.0130** | **1949.0258** | **-0.0128** | **0** | **54** | **0.00041** | **1** | **R.LENYPIPEPGPNEVLLR.M** |
|  | 21409 | **538.9853** | **2151.9121** | **2151.9255** | **-0.0134** | **0** | **11** | **6.8** | **1** | **R.MHSVGICGSDVHYWEYGR.I** |
|  | 23734 | **1222.5472** | **2443.0799** | **2443.0937** | **-0.0137** | **0** | **103** | **3.9e-009** | **1** | **R.YNLSPSIFFCATPPDDGNLCR.F** 23707 23733 |
|  | 24156 | **831.4450** | **2491.3132** | **2491.3832** | **-0.0700** | **0** | **2** | **43** | **1** | **R.SGGTLVLVGLGSEMTTVPLLHAAIR.E** |
|  | 28511 | **847.1534** | **3384.5847** | **3383.8343** | **0.7504** | **1** | **9** | **5.3** | **2** | **K.VLVCGAGPIGMVTLLVAKAMGAAQVVVTDLSATR.L** |
|  | 29909 | 1311.3231 | 3930.9475 | 3930.1115 | 0.8360 | 1 | 5 | 8.4 | 4 | M.AAAAKPNNLSLVVHGPGDLRLENYPIPEPGPNEVLLR.M 29907 |
|  | 30293 | **1025.7588** | **4099.0061** | **4098.9356** | **0.0704** | **1** | **1** | **20** | **1** | **R.LENYPIPEPGPNEVLLRMHSVGICGSDVHYWEYGR.I** |

| **173.** | P36542\|ATPG_HUMAN    **Mass:** 33032    **Score:** 296    **Queries matched:** 28   **emPAI:** 1.37 |
| --- | --- |
|  | ATP synthase subunit gamma, mitochondrial OS=Homo sapiens GN=ATP5C1 PE=1 SV=1 |

|  | **Query** | **Observed** | **Mr(expt)** | **Mr(calc)** | **Delta** | **Miss** | **Score** | **Expect** | **Rank** | **Peptide** |
| --- | --- | --- | --- | --- | --- | --- | --- | --- | --- | --- |
|  | 1244 | **591.2767** | **590.2694** | **589.3257** | **0.9436** | **0** | **34** | **0.089** | **1** | **K.MVAAAK.Y** |
|  | 1477 | 659.4034 | 658.3961 | 658.4014 | -0.0053 | 0 | 25 | 0.76 | 1 | R.QAVITK.E |
|  | 1562 | 696.3879 | 695.3807 | 695.3854 | -0.0047 | 0 | 18 | 2.2 | 1 | R.SVISYK.T |
|  | 2779 | **432.7479** | **863.4812** | **863.4865** | **-0.0053** | **0** | **20** | **1.8** | **2** | **K.LTLTFNR.T** |
|  | 3947 | **476.1950** | **950.3754** | **951.4881** | **-1.1127** | **1** | **4** | **67** | **3** | **K.SMKMVAAAK.Y** |
|  | 4176 | **484.7089** | **967.4033** | **967.4103** | **-0.0070** | **0** | **40** | **0.016** | **1** | **R.MTAMDNASK.N** |
|  | 4231 | 486.7490 | 971.4835 | 971.4924 | -0.0089 | 1 | 18 | 3.2 | 1 | K.ADIKGPEDK.K 4212 |
|  | 5291 | **524.7856** | **1047.5566** | **1046.5608** | **0.9958** | **0** | **41** | **0.012** | **1** | **K.SEVATLTAAGK.E** |
|  | 5540 | **535.2873** | **1068.5600** | **1068.6039** | **-0.0439** | **1** | **1** | **1.2e+002** | **9** | **R.AERELKPAR.I** |
|  | 5883 | **548.7441** | **1095.4736** | **1094.4840** | **0.9896** | **0** | **10** | **15** | **4** | **K.ESTTSEQSAR.M** 5885 |
|  | 7638 | **606.7701** | **1211.5256** | **1212.6285** | **-1.1029** | **0** | **68** | **2.3e-005** | **1** | **R.GLCGAIHSSIAK.Q** 7666 |
|  | 7922 | **409.2528** | **1224.7366** | **1223.6986** | **1.0380** | **1** | **5** | **48** | **8** | **K.KHLLIGVSSDR.G** |
|  | 9177 | **431.5562** | **1291.6469** | **1291.6561** | **-0.0092** | **0** | **26** | **0.41** | **1** | **R.THSDQFLVAFK.E** |
|  | 9830 | **663.8646** | **1325.7147** | **1325.7231** | **-0.0084** | **0** | **65** | **4.7e-005** | **1** | **R.IYGLGSLALYEK.A** |
|  | 10165 | **448.9991** | **1343.9756** | **1344.7435** | **-0.7679** | **1** | **8** | **17** | **2** | **K.EVMLVGIGDKIR.G** |
|  | 12022 | **726.3957** | **1450.7768** | **1449.7497** | **1.0271** | **1** | **3** | **65** | **8** | **K.QMKSEVATLTAAGK.E** |
|  | 16697 | **876.9451** | **1751.8756** | **1751.8876** | **-0.0120** | **1** | **97** | **1.9e-008** | **1** | **K.NASEMIDKLTLTFNR.T** 16713 |
|  | 18342 | **625.3335** | **1872.9787** | **1871.8063** | **1.1724** | **1** | **1** | **83** | **8** | **R.MTAMDNASKNASEMIDK.L** |
|  | 28473 | **843.6780** | **3370.6831** | **3370.6973** | **-0.0142** | **0** | **24** | **0.15** | **1** | **R.KPPTFGDASVIALELLNSGYEFDEGSIIFNK.F** 28469 28474 28476 28483 |
|  | 29665 | **1271.9418** | **3812.8035** | **3811.9308** | **0.8726** | **1** | **1** | **26** | **5** | **K.EVGRKPPTFGDASVIALELLNSGYEFDEGSIIFNK.F** |

| **174.** | Q7Z5P4\|DHB13_HUMAN    **Mass:** 33976    **Score:** 294    **Queries matched:** 28   **emPAI:** 1.86 |
| --- | --- |
|  | 17-beta hydroxysteroid dehydrogenase 13 OS=Homo sapiens GN=HSD17B13 PE=2 SV=1 |

|  | **Query** | **Observed** | **Mr(expt)** | **Mr(calc)** | **Delta** | **Miss** | **Score** | **Expect** | **Rank** | **Peptide** |
| --- | --- | --- | --- | --- | --- | --- | --- | --- | --- | --- |
|  | 3339 | **453.2552** | **904.4958** | **903.4715** | **1.0243** | **0** | **10** | **20** | **2** | **K.FAAVGFHR.G** |
|  | 5605 | **537.3102** | **1072.6059** | **1072.6128** | **-0.0069** | **0** | **47** | **0.0043** | **1** | **R.SLIDGILTNK.K** |
|  | 5665 | **540.2974** | **1078.5802** | **1078.5151** | **0.0651** | **0** | **9** | **21** | **2** | **K.ALLPSMMER.N** |
|  | 6155 | **558.8128** | **1115.6111** | **1115.6186** | **-0.0076** | **0** | **58** | **0.00028** | **1** | **R.GLTSELQALGK.T** 6156 |
|  | 7421 | **601.3569** | **1200.6993** | **1200.7078** | **-0.0085** | **1** | **35** | **0.05** | **1** | **R.SLIDGILTNKK.M** 7395 7422 |
|  | 8389 | **625.2908** | **1248.5670** | **1248.5768** | **-0.0098** | **1** | **16** | **3.6** | **1** | **R.GVEETAAECRK.L** |
|  | 12074 | **728.3914** | **1454.7683** | **1454.7769** | **-0.0086** | **0** | **76** | **3.6e-006** | **1** | **R.LWPVLETDEVVR.S** 12073 |
|  | 12586 | **495.6213** | **1483.8421** | **1483.8511** | **-0.0090** | **1** | **38** | **0.019** | **1** | **R.QSILVLWDINKR.G** |
|  | 12830 | **750.8793** | **1499.7441** | **1499.7555** | **-0.0114** | **0** | **57** | **0.00025** | **1** | **R.MQNIQFEAVVGHK.I** 12826 |
|  | 14542 | **806.9464** | **1611.8781** | **1611.8847** | **-0.0065** | **0** | **3** | **54** | **1** | **K.MIFVPSYINIFLR.L** |
|  | 15970 | **852.4830** | **1702.9515** | **1703.9035** | **-0.9520** | **0** | **14** | **4.4** | **1** | **K.TFEVNILGHFWITK.A** 15968 |
|  | 16013 | **853.9753** | **1705.9360** | **1705.9475** | **-0.0115** | **0** | **77** | **2e-006** | **1** | **K.SVAGEIVLITGAGHGIGR.Q** |
|  | 16407 | **865.9103** | **1729.8060** | **1729.8168** | **-0.0108** | **0** | **41** | **0.0092** | **1** | **K.TSCLCPVFVNTGFTK.N** 16408 |
|  | 16754 | 586.3152 | 1755.9239 | 1756.9294 | -1.0055 | 1 | 4 | 48 | 4 | R.MQNIQFEAVVGHKIK.M 16748 |
|  | 17782 | **612.3071** | **1833.8996** | **1834.0425** | **-0.1429** | **1** | **33** | **0.054** | **1** | **R.KSVAGEIVLITGAGHGIGR.Q** |
|  | 19767 | **666.3513** | **1996.0320** | **1997.1172** | **-1.0853** | **1** | **3** | **47** | **7** | **K.MIFVPSYINIFLRLQK.F** 19788 |
|  | 27032 | **999.1506** | **2994.4301** | **2994.4480** | **-0.0180** | **0** | **19** | **0.62** | **1** | **R.NHGHIVTVASVCGHEGIPYLIPYCSSK.F** |
|  | 27866 | **1064.2109** | **3189.6110** | **3189.6293** | **-0.0183** | **1** | **34** | **0.019** | **1** | **K.EVGDVTIVVNNAGTVYPADLLSTKDEEITK.T** 27872 |

| **175.** | Q6DD88\|ATLA3_HUMAN    **Mass:** 60960    **Score:** 293    **Queries matched:** 16   **emPAI:** 0.61 |
| --- | --- |
|  | Atlastin-3 OS=Homo sapiens GN=ATL3 PE=1 SV=1 |

|  | **Query** | **Observed** | **Mr(expt)** | **Mr(calc)** | **Delta** | **Miss** | **Score** | **Expect** | **Rank** | **Peptide** |
| --- | --- | --- | --- | --- | --- | --- | --- | --- | --- | --- |
|  | 4223 | 486.2704 | 970.5262 | 970.5236 | 0.0026 | 0 | 17 | 3.3 | 4 | K.QLALDHFK.K |
|  | 7940 | **409.7307** | **1226.1703** | **1225.6891** | **0.4812** | **1** | **9** | **12** | **5** | **M.LSPQRVAAAASR.G** |
|  | 9376 | **651.8408** | **1301.6670** | **1301.6762** | **-0.0092** | **1** | **24** | **0.56** | **1** | **R.DAVVGRPSMDKK.A** |
|  | 10283 | **675.8987** | **1349.7829** | **1348.7827** | **1.0003** | **0** | **4** | **61** | **3** | **K.ALASILLQDHIR.D** |
|  | 11490 | **709.8373** | **1417.6600** | **1417.6698** | **-0.0098** | **0** | **24** | **0.5** | **1** | **K.EHQHEEIQNVR.N** |
|  | 16120 | **856.9122** | **1711.8098** | **1711.9508** | **-0.1410** | **0** | **22** | **0.65** | **1** | **K.EQLQALIPYVLNPSK.L** 16098 |
|  | 17750 | **916.9562** | **1831.8979** | **1831.9097** | **-0.0118** | **0** | **115** | **3.6e-010** | **1** | **K.SMLQATAEANNLAAAASAK.D** 17749 |
|  | 21426 | **1078.0496** | **2154.0846** | **2154.0991** | **-0.0145** | **0** | **63** | **4.3e-005** | **1** | **R.GADDAMESSKPGPVQVVLVQK.D** 21423 21434 |
|  | 22903 | **776.4109** | **2326.2110** | **2326.2218** | **-0.0107** | **0** | **27** | **0.16** | **1** | **R.LAMDEIFQKPFQTLMFLVR.D** |
|  | 23404 | **798.0347** | **2391.0824** | **2391.0940** | **-0.0116** | **1** | **9** | **9.1** | **1** | **R.YQQELEEEIKELYENFCK.H** |
|  | 24924 | **873.7646** | **2618.2719** | **2618.2864** | **-0.0145** | **0** | **34** | **0.023** | **1** | **R.GGSDPETTGIQIWSEVFTVEKPGGK.K** |
|  | 27555 | **1035.8001** | **3104.3783** | **3104.3994** | **-0.0211** | **0** | **42** | **0.0026** | **1** | **K.DIYYNNMEEVCGGEKPYLSPDILEEK.H** |

| **176.** | P36269\|GGT5_HUMAN    **Mass:** 62736    **Score:** 292    **Queries matched:** 20   **emPAI:** 0.41 |
| --- | --- |
|  | Gamma-glutamyltransferase 5 OS=Homo sapiens GN=GGT5 PE=1 SV=2 |

|  | **Query** | **Observed** | **Mr(expt)** | **Mr(calc)** | **Delta** | **Miss** | **Score** | **Expect** | **Rank** | **Peptide** |
| --- | --- | --- | --- | --- | --- | --- | --- | --- | --- | --- |
|  | 7761 | 609.3204 | 1216.6262 | 1215.6533 | 0.9729 | 0 | 2 | 91 | 10 | R.LGQMLVEDIAK.E |
|  | 10116 | **671.3873** | **1340.7600** | **1340.7663** | **-0.0064** | **0** | **56** | **0.00031** | **1** | **R.DLLGETLAQLIR.Q** 10115 |
|  | 10693 | **687.3467** | **1372.6789** | **1371.7432** | **0.9358** | **0** | **8** | **24** | **1** | **R.SPSSMVPSILINK.A** |
|  | 14816 | 544.2957 | 1629.8653 | 1629.7529 | 0.1124 | 1 | 2 | 76 | 3 | R.CPRGSGTTPSPVSGDR.V |
|  | 16906 | **883.4690** | **1764.9234** | **1766.0243** | **-1.1009** | **0** | **54** | **0.00042** | **1** | **R.LPWAQLFQPTIALLR.G** |
|  | 16979 | **886.9590** | **1771.9034** | **1771.9138** | **-0.0104** | **0** | **53** | **0.0006** | **1** | **R.TGIILNNELLDLCER.C** 16980 |
|  | 17869 | **921.4036** | **1840.7926** | **1840.8050** | **-0.0124** | **0** | **66** | **2.7e-005** | **1** | **K.GCVEYEPNFSQEVQR.G** 17870 |
|  | 18129 | 465.5042 | 1857.9879 | 1858.9822 | -0.9944 | 1 | 8 | 18 | 7 | R.SPSSMVPSILINKAQGSK.L 18156 |
|  | 19275 | **489.0277** | **1952.0816** | **1952.0956** | **-0.0139** | **0** | **15** | **2.7** | **1** | **R.FLHNSILRPSLQASTLR.Q** 19274 |
|  | 20241 | 510.5207 | 2038.0537 | 2038.1171 | -0.0634 | 1 | 4 | 34 | 7 | R.DLLGETLAQLIRQQIDGR.G |
|  | 29175 | **1213.9503** | **3638.8291** | **3638.8515** | **-0.0224** | **0** | **50** | **0.00035** | **1** | **R.ETVPASHAPSLLDQCAQALPLGTGAQWIGVPGELR.G** 29177 |
|  | 30222 | **1356.7245** | **4067.1516** | **4067.1660** | **-0.0143** | **0** | **61** | **2.1e-005** | **1** | **K.FQPEVVDALEVPLGDYTLYSPPPPAGGAILSFILNVLR.G** |
|  | 31411 | **1045.8756** | **5224.3417** | **5225.4538** | **-1.1121** | **0** | **1** | **7.7** | **10** | **R.GDHQLSHYSLAEAWGHGTGTSHVSVLGEDGSAVAATSTINTPFGAMVYSPR.T** |
|  | 31715 | **1892.3554** | **5674.0442** | **5673.9069** | **0.1373** | **1** | **4** | **3.2** | **5** | **R.QLFFNGTEPLRPQDPLPWPALATTLETVATEGVEVFYTGRLGQMLVEDIAK.E** |

| **177.** | Q53GQ0\|DHB12_HUMAN    **Mass:** 34416    **Score:** 288    **Queries matched:** 23   **emPAI:** 0.87 |
| --- | --- |
|  | Estradiol 17-beta-dehydrogenase 12 OS=Homo sapiens GN=HSD17B12 PE=1 SV=2 |

|  | **Query** | **Observed** | **Mr(expt)** | **Mr(calc)** | **Delta** | **Miss** | **Score** | **Expect** | **Rank** | **Peptide** |
| --- | --- | --- | --- | --- | --- | --- | --- | --- | --- | --- |
|  | 1372 | **631.3518** | **630.3445** | **630.3489** | **-0.0044** | **0** | **24** | **1.3** | **1** | **R.AHYLK.K** |
|  | 3033 | **441.2954** | **880.5762** | **880.4146** | **0.1616** | **0** | **3** | **72** | **7** | **K.IVMNMNK.S** |
|  | 3401 | **910.4453** | **909.4380** | **909.4443** | **-0.0063** | **0** | **18** | **2.2** | **1** | **K.SYAEELAK.H** 3402 |
|  | 9394 | **652.3503** | **1302.6860** | **1303.6992** | **-1.0132** | **0** | **56** | **0.00033** | **1** | **K.MININILSVCK.M** |
|  | 10697 | **687.3638** | **1372.7130** | **1372.7207** | **-0.0077** | **0** | **44** | **0.0059** | **1** | **K.MTQLVLPGMVER.S** 10696 |
|  | 11006 | **464.2585** | **1389.7537** | **1388.7156** | **1.0381** | **0** | **(8)** | **24** | **1** | **K.MTQLVLPGMVER.S** |
|  | 11248 | 703.3307 | 1404.6469 | 1404.7105 | -0.0636 | 0 | (3) | 69 | 5 | K.MTQLVLPGMVER.S 11228 |
|  | 14156 | **793.9229** | **1585.8313** | **1585.8716** | **-0.0403** | **0** | **9** | **15** | **2** | **R.KPTLDKPSPETFVK.S** |
|  | 14678 | **541.2791** | **1620.8155** | **1619.8375** | **0.9780** | **1** | **0** | **1.1e+002** | **9** | **K.MTQLVLPGMVERSK.G** |
|  | 17687 | **914.4661** | **1826.9177** | **1826.9302** | **-0.0125** | **1** | **71** | **7.6e-006** | **1** | **R.TIAVDFASEDIYDKIK.T** 17676 |
|  | 19551 | **659.9611** | **1976.8614** | **1976.8727** | **-0.0114** | **0** | **39** | **0.011** | **1** | **K.TFVDFFSQCLHEEYR.S** |
|  | 19630 | **662.6324** | **1984.8755** | **1985.1197** | **-0.2442** | **1** | **4** | **37** | **1** | **R.KPTLDKPSPETFVKSAIK.T** |
|  | 24222 | **1252.1875** | **2502.3604** | **2502.3767** | **-0.0163** | **0** | **106** | **1.5e-009** | **1** | **K.GAILNISSGSGMLPVPLLTIYSATK.T** 24223 24224 |
|  | 25456 | **906.8383** | **2717.4931** | **2717.5037** | **-0.0106** | **1** | **15** | **1.8** | **1** | **R.SKGAILNISSGSGMLPVPLLTIYSATK.T** 25449 |
|  | 30077 | **797.9835** | **3984.8809** | **3985.9911** | **-1.1102** | **0** | **0** | **28** | **2** | **K.TGLAGLEIGILVNNVGMSYEYPEYFLDVPDLDNVIK.K** |
|  | 30291 | **1367.0315** | **4098.0726** | **4098.0911** | **-0.0185** | **1** | **83** | **1.2e-007** | **1** | **K.TGLAGLEIGILVNNVGMSYEYPEYFLDVPDLDNVIKK.M** |

| **178.** | P00387\|NB5R3_HUMAN    **Mass:** 34441    **Score:** 288    **Queries matched:** 34   **emPAI:** 2.14 |
| --- | --- |
|  | NADH-cytochrome b5 reductase 3 OS=Homo sapiens GN=CYB5R3 PE=1 SV=3 |

|  | **Query** | **Observed** | **Mr(expt)** | **Mr(calc)** | **Delta** | **Miss** | **Score** | **Expect** | **Rank** | **Peptide** |
| --- | --- | --- | --- | --- | --- | --- | --- | --- | --- | --- |
|  | 6189 | **559.8101** | **1117.6056** | **1117.6132** | **-0.0076** | **0** | **34** | **0.066** | **1** | **R.GPSGLLVYQGK.G** 6165 6176 6188 |
|  | 6278 | **563.7973** | **1125.5800** | **1125.5891** | **-0.0090** | **1** | **24** | **0.61** | **1** | **R.EIISHDTRR.F** 6277 6288 |
|  | 6393 | **568.2579** | **1134.5012** | **1133.5869** | **0.9142** | **1** | **15** | **5** | **1** | **K.VYFKDTHPK.F** 6385 |
|  | 8242 | **621.3331** | **1240.6516** | **1240.6604** | **-0.0088** | **1** | **23** | **0.65** | **1** | **R.FKLWYTLDR.A** |
|  | 12292 | **489.9317** | **1466.7732** | **1466.7841** | **-0.0109** | **1** | **28** | **0.2** | **1** | **R.LIDREIISHDTR.R** 12291 12293 |
|  | 12765 | **748.4228** | **1494.8310** | **1494.8405** | **-0.0095** | **0** | **38** | **0.019** | **1** | **K.DILLRPELEELR.N** 12766 |
|  | 18654 | 951.0151 | 1900.0157 | 1900.0274 | -0.0117 | 0 | 65 | 2.9e-005 | 1 | K.SVGMIAGGTGITPMLQVIR.A 18651 18655 18660 18661 |
|  | 18656 | **951.0170** | **1900.0195** | **1900.0305** | **-0.0110** | **1** | **61** | **7e-005** | **1** | **R.STPAITLESPDIKYPLR.L** |
|  | 20335 | **1024.4697** | **2046.9249** | **2046.9390** | **-0.0141** | **0** | **58** | **0.00013** | **1** | **K.MSQYLESMQIGDTIEFR.G** 20336 |
|  | 21047 | 1056.4707 | 2110.9268 | 2110.9418 | -0.0150 | 0 | 83 | 4.3e-007 | 1 | R.APEAWDYGQGFVNEEMIR.D |
|  | 22136 | **558.2913** | **2229.1359** | **2228.2385** | **0.8974** | **1** | **7** | **16** | **9** | **R.TVKSVGMIAGGTGITPMLQVIR.A** |
|  | 24303 | **838.8000** | **2513.3781** | **2513.3907** | **-0.0125** | **0** | **26** | **0.19** | **1** | **R.FALPSPQHILGLPVGQHIYLSAR.I** |
|  | 24760 | **861.7430** | **2582.2071** | **2582.2257** | **-0.0186** | **1** | **41** | **0.0049** | **1** | **R.AIMKDPDDHTVCHLLFANQTEK.D** |
|  | 26909 | **992.5226** | **2974.5461** | **2974.5652** | **-0.0191** | **1** | **44** | **0.0019** | **1** | **R.IDGNLVVRPYTPISSDDDKGFVDLVIK.V** 26912 26913 |
|  | 28622 | **857.4556** | **3425.7934** | **3425.8219** | **-0.0284** | **1** | **1** | **35** | **5** | **-.MGAQLSTLGHMVLFPVWFLYSLLMKLFQR.S** |
|  | 30763 | **1089.7666** | **4355.0373** | **4355.0636** | **-0.0263** | **0** | **22** | **0.13** | **1** | **R.DHLPPPEEEPLVLMCGPPPMIQYACLPNLDHVGHPTER.C** 30764 |
|  | 31864 | **1290.7998** | **6448.9626** | **6447.9948** | **0.9678** | **1** | **5** | **1.7** | **1** | **R.APEAWDYGQGFVNEEMIRDHLPPPEEEPLVLMCGPPPMIQYACLPNLDHVGHPTER.C** |

| **179.** | P54819\|KAD2_HUMAN    **Mass:** 26689    **Score:** 286    **Queries matched:** 27   **emPAI:** 1.90 |
| --- | --- |
|  | Adenylate kinase 2, mitochondrial OS=Homo sapiens GN=AK2 PE=1 SV=2 |

|  | **Query** | **Observed** | **Mr(expt)** | **Mr(calc)** | **Delta** | **Miss** | **Score** | **Expect** | **Rank** | **Peptide** |
| --- | --- | --- | --- | --- | --- | --- | --- | --- | --- | --- |
|  | 4268 | 487.7495 | 973.4845 | 973.4902 | -0.0058 | 0 | 9 | 26 | 6 | K.NLETPLCK.N |
|  | 4347 | 490.2976 | 978.5807 | 978.5862 | -0.0055 | 0 | 12 | 9.7 | 1 | R.AVLLGPPGAGK.G |
|  | 5489 | **533.2899** | **1064.5652** | **1064.5172** | **0.0480** | **0** | **4** | **65** | **8** | **R.AMVASGSELGK.K** |
|  | 6407 | **568.7871** | **1135.5597** | **1134.5822** | **0.9775** | **0** | **15** | **5.4** | **1** | **K.NGFLLDGFPR.T** |
|  | 6940 | **589.2897** | **1176.5649** | **1176.6172** | **-0.0523** | **1** | **22** | **0.93** | **1** | **R.AMVASGSELGKK.L** |
|  | 9756 | **661.7976** | **1321.5807** | **1321.5894** | **-0.0087** | **0** | **42** | **0.0091** | **1** | **R.QAEMLDDLMEK.R** 9743 |
|  | 10068 | **669.8117** | **1337.6089** | **1337.5843** | **0.0246** | **0** | **(3)** | **62** | **4** | **R.QAEMLDDLMEK.R** |
|  | 10373 | 678.3408 | 1354.6671 | 1354.6768 | -0.0098 | 0 | 31 | 0.11 | 1 | M.APSVPAAEPEYPK.G 10376 |
|  | 12620 | **496.2844** | **1485.8313** | **1485.7173** | **0.1139** | **0** | **9** | **15** | **1** | **-.MAPSVPAAEPEYPK.G** |
|  | 12842 | **501.2470** | **1500.7191** | **1501.7123** | **-0.9931** | **0** | **(5)** | **38** | **5** | **-.MAPSVPAAEPEYPK.G** |
|  | 12882 | **752.3976** | **1502.7806** | **1502.7902** | **-0.0096** | **0** | **90** | **1.3e-007** | **1** | **K.LVSDEMVVELIEK.N** |
|  | 15594 | **561.2807** | **1680.8203** | **1680.8835** | **-0.0632** | **1** | **6** | **30** | **3** | **M.APSVPAAEPEYPKGIR.A** |
|  | 15764 | **424.2193** | **1692.8482** | **1693.8015** | **-0.9533** | **1** | **8** | **19** | **2** | **R.TVRQAEMLDDLMEK.R** |
|  | 17556 | **909.0022** | **1815.9898** | **1815.9982** | **-0.0084** | **0** | **3** | **51** | **1** | **K.LDSVIEFSIPDSLLIR.R** 17537 |
|  | 19766 | **999.0035** | **1995.9925** | **1996.0054** | **-0.0129** | **0** | **52** | **0.00053** | **1** | **R.LQAYHTQTTPLIEYYR.K** 19750 |
|  | 19892 | **1003.9587** | **2005.9028** | **2005.9172** | **-0.0144** | **0** | **72** | **5.6e-006** | **1** | **R.LAENFCVCHLATGDMLR.A** 19907 19908 |
|  | 20700 | **1037.5685** | **2073.1224** | **2073.1357** | **-0.0133** | **1** | **30** | **0.078** | **1** | **K.EKLDSVIEFSIPDSLLIR.R** |
|  | 21633 | 726.6548 | 2176.9425 | 2177.0959 | -0.1534 | 1 | 3 | 38 | 6 | K.ATMDAGKLVSDEMVVELIEK.N |
|  | 24462 | **1273.1630** | **2544.3114** | **2544.3224** | **-0.0110** | **0** | **83** | **3.4e-007** | **1** | **R.GIHSAIDASQTPDVVFASILAAFSK.A** 24461 24463 |

| **180.** | P45880\|VDAC2_HUMAN    **Mass:** 32060    **Score:** 285    **Queries matched:** 19   **emPAI:** 1.44 |
| --- | --- |
|  | Voltage-dependent anion-selective channel protein 2 OS=Homo sapiens GN=VDAC2 PE=1 SV=2 |

|  | **Query** | **Observed** | **Mr(expt)** | **Mr(calc)** | **Delta** | **Miss** | **Score** | **Expect** | **Rank** | **Peptide** |
| --- | --- | --- | --- | --- | --- | --- | --- | --- | --- | --- |
|  | 2569 | **425.0476** | **848.0807** | **847.4651** | **0.6156** | **0** | **17** | **2.3** | **1** | **K.VTGTLETK.Y** |
|  | 3788 | **470.7319** | **939.4493** | **939.4563** | **-0.0069** | **0** | **32** | **0.085** | **1** | **R.NNFAVGYR.T** 3789 |
|  | 4876 | **508.7996** | **1015.5847** | **1015.5914** | **-0.0066** | **0** | **43** | **0.0098** | **1** | **K.LTLSALVDGK.S** 4866 |
|  | 9194 | 647.3331 | 1292.6517 | 1292.6612 | -0.0095 | 0 | 29 | 0.2 | 1 | K.YQLDPTASISAK.V |
|  | 11656 | **714.8486** | **1427.6826** | **1427.6933** | **-0.0107** | **0** | **61** | **0.0001** | **1** | **K.LTFDTTFSPNTGK.K** |
|  | 11731 | **717.3196** | **1432.6246** | **1432.6333** | **-0.0087** | **0** | **7** | **25** | **1** | **K.WCEYGLTFTEK.W** |
|  | 13712 | **778.8962** | **1555.7778** | **1555.7882** | **-0.0104** | **1** | **36** | **0.034** | **1** | **K.LTFDTTFSPNTGKK.S** |
|  | 18414 | 940.9780 | 1879.9415 | 1880.0043 | -0.0628 | 1 | 4 | 42 | 6 | R.FGIAAKYQLDPTASISAK.V |
|  | 18748 | **954.3934** | **1906.7722** | **1906.7851** | **-0.0129** | **0** | **64** | **3.2e-005** | **1** | **K.SCSGVEFSTSGSSNTDTGK.V** |
|  | 20963 | 1052.0741 | 2102.1336 | 2102.1484 | -0.0148 | 0 | 73 | 4e-006 | 1 | K.VNNSSLIGVGYTQTLRPGVK.L 20964 20965 20975 |
|  | 24336 | **1260.0995** | **2518.1844** | **2518.2010** | **-0.0165** | **0** | **126** | **1.5e-011** | **1** | **K.WNTDNTLGTEIAIEDQICQGLK.L** 24337 |
|  | 25500 | **910.7561** | **2729.2465** | **2728.2996** | **0.9469** | **1** | **0** | **60** | **4** | **M.ATHGQTCARPMCIPPSYADLGKAAR.D** |
|  | 25543 | **1368.6543** | **2735.2940** | **2736.2396** | **-0.9456** | **1** | **1** | **55** | **3** | **K.SCSGVEFSTSGSSNTDTGKVTGTLETK.Y** |

| **181.** | Q562R1\|ACTBL_HUMAN    **Mass:** 42318    **Score:** 285    **Queries matched:** 31   **emPAI:** 0.66 |
| --- | --- |
|  | Beta-actin-like protein 2 OS=Homo sapiens GN=ACTBL2 PE=1 SV=2 |

|  | **Query** | **Observed** | **Mr(expt)** | **Mr(calc)** | **Delta** | **Miss** | **Score** | **Expect** | **Rank** | **Peptide** |
| --- | --- | --- | --- | --- | --- | --- | --- | --- | --- | --- |
|  | 1363 | 630.3417 | 629.3344 | 630.3701 | -1.0357 | 0 | 4 | 99 | 2 | K.ILTER.G |
|  | 1421 | 644.3680 | 643.3607 | 643.3653 | -0.0046 | 0 | 20 | 2.6 | 1 | R.LDLAGR.D |
|  | 1884 | 795.4671 | 794.4598 | 794.4650 | -0.0052 | 0 | 17 | 3.4 | 2 | K.IIAPPER.K |
|  | 4616 | 499.7438 | 997.4730 | 997.4790 | -0.0060 | 0 | 33 | 0.076 | 1 | R.DLTDYLMK.I |
|  | 5140 | 518.8257 | 1035.6369 | 1035.6440 | -0.0071 | 1 | 26 | 0.39 | 1 | K.IKIIAPPER.K |
|  | 6867 | 586.2850 | 1170.5555 | 1170.5638 | -0.0083 | 0 | 50 | 0.0016 | 1 | R.HQGVMVGMGQK.D 6865 6866 |
|  | 7105 | 594.2822 | 1186.5499 | 1186.5587 | -0.0088 | 0 | (35) | 0.046 | 1 | R.HQGVMVGMGQK.D |
|  | 7106 | 594.2826 | 1186.5507 | 1186.5587 | -0.0080 | 0 | (47) | 0.003 | 1 | R.HQGVMVGMGQK.D 7093 |
|  | 7465 | 602.2790 | 1202.5434 | 1202.5536 | -0.0102 | 0 | (4) | 69 | 5 | R.HQGVMVGMGQK.D |
|  | 14707 | 541.9487 | 1622.8244 | 1622.8338 | -0.0094 | 1 | 10 | 12 | 4 | R.LDLAGRDLTDYLMK.I |
|  | 16973 | **886.9161** | **1771.8177** | **1771.8273** | **-0.0096** | **0** | **2** | **71** | **4** | **K.LCYVALDFEQEMVR.A** |
|  | 17255 | 895.9438 | 1789.8730 | 1789.8846 | -0.0116 | 0 | 71 | 9.6e-006 | 1 | R.SYELPDGQVITIGNER.F 17256 17257 17258 17267 17268 |
|  | 18772 | 637.0158 | 1908.0254 | 1908.8557 | -0.8303 | 0 | 3 | 50 | 6 | M.TDNELSALVVDNGSGMCK.A |
|  | 19228 | **650.3574** | **1948.0504** | **1947.8673** | **0.1832** | **0** | **9** | **12** | **1** | **K.YPIEHGVVTNWDDMEK.I** |
|  | 19285 | 652.0222 | 1953.0448 | 1953.0571 | -0.0123 | 0 | 27 | 0.17 | 2 | R.VAPDEHPILLTEAPLNPK.I 19281 19282 19283 19284 |
|  | 21986 | 1108.5231 | 2215.0316 | 2216.0419 | -1.0104 | 0 | 8 | 12 | 2 | K.DLYANTVLSGGSTMYPGIADR.M 21985 |
|  | 26237 | **951.5194** | **2851.5362** | **2852.2705** | **-0.7343** | **1** | **0** | **47** | **5** | **M.TDNELSALVVDNGSGMCKAGFGGDDAPR.A** |
|  | 28089 | **1086.1782** | **3255.5128** | **3254.5814** | **0.9314** | **0** | **2** | **34** | **1** | **K.MTQIMFEAFNTPAMYVAIQAVLSLYASGR.T** |

| **182.** | P60174\|TPIS_HUMAN    **Mass:** 26938    **Score:** 284    **Queries matched:** 19   **emPAI:** 2.27 |
| --- | --- |
|  | Triosephosphate isomerase OS=Homo sapiens GN=TPI1 PE=1 SV=2 |

|  | **Query** | **Observed** | **Mr(expt)** | **Mr(calc)** | **Delta** | **Miss** | **Score** | **Expect** | **Rank** | **Peptide** |
| --- | --- | --- | --- | --- | --- | --- | --- | --- | --- | --- |
|  | 1720 | **758.4352** | **757.4280** | **757.4334** | **-0.0054** | **0** | **25** | **0.76** | **1** | **K.VIADNVK.D** |
|  | 8854 | **637.8362** | **1273.6578** | **1273.6666** | **-0.0088** | **1** | **24** | **0.63** | **1** | **K.VIADNVKDWSK.V** |
|  | 9823 | **663.8351** | **1325.6556** | **1325.6649** | **-0.0093** | **0** | **44** | **0.0051** | **1** | **R.IIYGGSVTGATCK.E** 9830 |
|  | 11418 | **707.8937** | **1413.7729** | **1413.7827** | **-0.0098** | **0** | **30** | **0.13** | **1** | **K.QSLGELIGTLNAAK.V** 11401 |
|  | 12128 | **729.8602** | **1457.7058** | **1457.7151** | **-0.0093** | **0** | **41** | **0.011** | **1** | **R.HVFGESDELIGQK.V** |
|  | 12264 | 733.8604 | 1465.7063 | 1465.7161 | -0.0098 | 0 | 54 | 0.00053 | 1 | K.TATPQQAQEVHEK.L 12262 |
|  | 13503 | **515.3265** | **1542.9576** | **1541.8777** | **1.0799** | **1** | **5** | **36** | **8** | **R.KQSLGELIGTLNAAK.V** |
|  | 14151 | **793.8674** | **1585.7202** | **1585.7307** | **-0.0106** | **0** | **66** | **3.1e-005** | **1** | **K.DCGATWVVLGHSER.R** 14161 |
|  | 14403 | **802.4448** | **1602.8750** | **1601.8817** | **0.9932** | **0** | **56** | **0.00029** | **1** | **K.VVLAYEPVWAIGTGK.T** 14377 |
|  | 14691 | **811.9040** | **1621.7934** | **1620.8181** | **0.9753** | **0** | **16** | **3.1** | **1** | **K.VTNGAFTGEISPGMIK.D** |
|  | 21751 | **1096.5309** | **2191.0472** | **2191.0620** | **-0.0148** | **0** | **66** | **2e-005** | **1** | **K.VPADTEVVCAPPTAYIDFAR.Q** 21753 |
|  | 27163 | **1010.5277** | **3028.5613** | **3028.5757** | **-0.0144** | **0** | **46** | **0.0014** | **1** | **K.ELASQPDVDGFLVGGASLKPEFVDIINAK.Q** 27162 |

| **183.** | Q3LXA3\|DHAK_HUMAN    **Mass:** 59282    **Score:** 282    **Queries matched:** 31   **emPAI:** 0.63 |
| --- | --- |
|  | Bifunctional ATP-dependent dihydroxyacetone kinase/FAD-AMP lyase (cyclizing) OS=Homo sapiens GN=DAK PE=1 SV=1 |

|  | **Query** | **Observed** | **Mr(expt)** | **Mr(calc)** | **Delta** | **Miss** | **Score** | **Expect** | **Rank** | **Peptide** |
| --- | --- | --- | --- | --- | --- | --- | --- | --- | --- | --- |
|  | 2625 | **854.4318** | **853.4245** | **853.4294** | **-0.0048** | **0** | **24** | **0.56** | **1** | **R.ASYISSAR.L** |
|  | 3465 | **457.7969** | **913.5793** | **913.5848** | **-0.0055** | **0** | **16** | **4** | **1** | **K.LSVLLLEK.M** |
|  | 4501 | **496.2788** | **990.5430** | **989.5142** | **1.0288** | **1** | **8** | **28** | **3** | **R.SDLDSLKGR.V** |
|  | 4699 | **502.2663** | **1002.5180** | **1002.5644** | **-0.0464** | **1** | **10** | **20** | **10** | **K.RMALVLER.V** 4709 |
|  | 7079 | **593.7472** | **1185.4798** | **1184.6665** | **0.8133** | **1** | **5** | **44** | **2** | **R.AARAIQEWLK.E** |
|  | 7777 | 609.8176 | 1217.6206 | 1217.6689 | -0.0483 | 1 | 4 | 55 | 10 | R.IKMATADEIVK.L |
|  | 8468 | **418.5738** | **1252.6997** | **1252.7074** | **-0.0077** | **1** | **21** | **1.2** | **1** | **R.RGLCGTVLIHK.V** |
|  | 11037 | 696.8472 | 1391.6799 | 1390.7456 | 0.9343 | 0 | 8 | 22 | 2 | K.EGPPPASPAQLLSK.L |
|  | 11842 | **481.1506** | **1440.4301** | **1439.8712** | **0.5589** | **0** | **7** | **17** | **1** | **R.AVAQAGTVGTLLIVK.N** 11843 |
|  | 14241 | **796.4383** | **1590.8620** | **1590.8729** | **-0.0109** | **0** | **30** | **0.11** | **1** | **R.LEQPDPGAVAAAAILR.A** |
|  | 14983 | **547.3412** | **1639.0019** | **1639.7736** | **-0.7717** | **1** | **(2)** | **58** | **7** | **K.NMEAGAGRASYISSAR.L** |
|  | 15231 | 552.5848 | 1654.7325 | 1655.7685 | -1.0360 | 1 | 3 | 60 | 5 | K.NMEAGAGRASYISSAR.L |
|  | 19118 | **647.3311** | **1938.9715** | **1938.9833** | **-0.0118** | **0** | **21** | **0.7** | **1** | **R.VCSTLLGLEEHLNALDR.A** |
|  | 19124 | **970.9572** | **1939.8999** | **1939.9123** | **-0.0124** | **0** | **77** | **2e-006** | **1** | **R.VAPAEPQEAPDSTAAGGSASK.R** 19125 |
|  | 19216 | **974.4799** | **1946.9453** | **1946.9560** | **-0.0107** | **0** | **28** | **0.17** | **1** | **R.TMLDSLWAAGQELQAWK.S** |
|  | 19384 | 491.2570 | 1960.9991 | 1961.0119 | -0.0128 | 0 | 5 | 34 | 4 | R.VALLSGGGSGHEPAHAGFIGK.G |
|  | 21360 | **716.6879** | **2147.0418** | **2146.1746** | **0.8672** | **1** | **0** | **84** | **10** | **R.AVAQAGTVGTLLIVKNYTGDR.L** |
|  | 21749 | **1096.0685** | **2190.1224** | **2189.1290** | **0.9934** | **0** | **49** | **0.001** | **1** | **R.AEGIPVEMVVIGDDSAFTVLK.K** |
|  | 21903 | **1105.0386** | **2208.0626** | **2208.0554** | **0.0071** | **1** | **2** | **49** | **2** | **K.AKTSLPAWSAAMDAGLEAMQK.Y** |
|  | 22113 | **1114.0828** | **2226.1510** | **2226.1644** | **-0.0135** | **0** | **78** | **1.4e-006** | **1** | **K.LIDAETTAAAWPNVAAVSITGR.K** 22112 |
|  | 23229 | **791.0843** | **2370.2312** | **2371.3297** | **-1.0985** | **0** | **5** | **23** | **3** | **K.GMLTGVIAGAVFTSPAVGSILAAIR.A** |
|  | 23745 | **612.0809** | **2444.2944** | **2444.3638** | **-0.0695** | **1** | **1** | **64** | **6** | **R.LEQPDPGAVAAAAILRAILEVLQS.-** |
|  | 25183 | 892.4611 | 2674.3614 | 2673.3684 | 0.9930 | 1 | 4 | 24 | 4 | R.EQARAEGIPVEMVVIGDDSAFTVLK.K 25181 25195 25199 |
|  | 27700 | **1049.5269** | **3145.5588** | **3145.5761** | **-0.0173** | **0** | **59** | **5.8e-005** | **1** | **K.LVNSVAGCADDALAGLVACNPNLQLLQGHR.V** |

| **184.** | P20813\|CP2B6_HUMAN    **Mass:** 56527    **Score:** 273    **Queries matched:** 22   **emPAI:** 0.67 |
| --- | --- |
|  | Cytochrome P450 2B6 OS=Homo sapiens GN=CYP2B6 PE=1 SV=1 |

|  | **Query** | **Observed** | **Mr(expt)** | **Mr(calc)** | **Delta** | **Miss** | **Score** | **Expect** | **Rank** | **Peptide** |
| --- | --- | --- | --- | --- | --- | --- | --- | --- | --- | --- |
|  | 1456 | 653.3032 | 652.2959 | 653.2843 | -0.9884 | 0 | 24 | 0.58 | 2 | R.DFGMGK.R |
|  | 2082 | 405.2055 | 808.3964 | 809.3854 | -0.9890 | 1 | 9 | 20 | 8 | R.DFGMGKR.S |
|  | 4406 | 492.7800 | 983.5455 | 983.5514 | -0.0059 | 0 | 24 | 0.68 | 1 | R.YGFLLMLK.Y |
|  | 4446 | **494.2813** | **986.5481** | **986.5549** | **-0.0068** | **0** | **11** | **14** | **1** | **K.IPPTYQIR.F** |
|  | 4456 | 494.7625 | 987.5105 | 987.5171 | -0.0066 | 0 | 27 | 0.36 | 1 | R.ICLGEGIAR.A 4445 |
|  | 4569 | 498.7056 | 995.3966 | 996.5175 | -1.1208 | 1 | 8 | 25 | 7 | R.RFSVTTMR.D |
|  | 8420 | **417.5520** | **1249.6341** | **1249.6415** | **-0.0074** | **1** | **39** | **0.019** | **1** | **K.HRETLDPSAPK.D** 8416 8421 |
|  | 10057 | **669.3723** | **1336.7299** | **1336.7391** | **-0.0091** | **1** | **43** | **0.0069** | **1** | **K.KTEAFIPFSLGK.R** |
|  | 11041 | **696.8552** | **1391.6959** | **1391.7045** | **-0.0086** | **1** | **63** | **6.7e-005** | **1** | **R.EALVDKAEAFSGR.G** |
|  | 12691 | **745.8769** | **1489.7393** | **1489.7486** | **-0.0093** | **0** | **71** | **9.6e-006** | **1** | **K.DLIDTYLLHMEK.E** |
|  | 14531 | **806.8999** | **1611.7852** | **1611.7966** | **-0.0114** | **0** | **87** | **2.7e-007** | **1** | **K.MPYTEAVIYEIQR.F** |
|  | 16155 | **857.9358** | **1713.8570** | **1713.8686** | **-0.0115** | **0** | **68** | **1.9e-005** | **1** | **K.NLQEINAYIGHSVEK.H** 16159 |
|  | 16835 | **879.9505** | **1757.8864** | **1757.8981** | **-0.0117** | **1** | **65** | **3.3e-005** | **1** | **R.IQEEAQCLIEELRK.S** |
|  | 22158 | **744.8709** | **2231.5909** | **2232.1538** | **-0.5629** | **1** | **3** | **27** | **2** | **R.QVYKNLQEINAYIGHSVEK.H** |
|  | 22538 | **571.2962** | **2281.1557** | **2281.1678** | **-0.0121** | **0** | **24** | **0.3** | **1** | **R.FSDLLPMGVPHIVTQHTSFR.G** |
|  | 22651 | 766.4046 | 2296.1920 | 2297.1627 | -0.9707 | 0 | (11) | 6 | 3 | R.FSDLLPMGVPHIVTQHTSFR.G |
|  | 25240 | **895.8000** | **2684.3781** | **2684.3931** | **-0.0150** | **0** | **9** | **7.9** | **1** | **K.YGDVFTVHLGPRPVVMLCGVEAIR.E** |
|  | 26586 | 972.7918 | 2915.3536 | 2914.5085 | 0.8451 | 1 | 3 | 29 | 2 | K.GALMDPTFLFQSITANIICSIVFGKR.F |

| **185.** | Q16891\|IMMT_HUMAN    **Mass:** 84026    **Score:** 273    **Queries matched:** 41   **emPAI:** 0.54 |
| --- | --- |
|  | Mitochondrial inner membrane protein OS=Homo sapiens GN=IMMT PE=1 SV=1 |

|  | **Query** | **Observed** | **Mr(expt)** | **Mr(calc)** | **Delta** | **Miss** | **Score** | **Expect** | **Rank** | **Peptide** |
| --- | --- | --- | --- | --- | --- | --- | --- | --- | --- | --- |
|  | 1726 | **760.4142** | **759.4069** | **759.4126** | **-0.0057** | **0** | **11** | **19** | **1** | **K.SVIENAK.K** |
|  | 2052 | 404.2188 | 806.4229 | 807.4127 | -0.9897 | 0 | 10 | 20 | 9 | R.AFDSAVAK.A |
|  | 3121 | **444.7401** | **887.4656** | **887.5076** | **-0.0420** | **1** | **11** | **18** | **2** | **K.SVIENAKK.K** |
|  | 4117 | 482.2941 | 962.5736 | 963.5138 | -0.9402 | 1 | 7 | 33 | 8 | K.RAFDSAVAK.A |
|  | 4349 | **490.3099** | **978.6053** | **978.5056** | **0.0998** | **0** | **5** | **47** | **7** | **K.ISSVSEVMK.E** 4338 |
|  | 4681 | **501.7790** | **1001.5435** | **1001.5505** | **-0.0070** | **1** | **7** | **36** | **2** | **R.TVEGALKER.R** |
|  | 5103 | **517.7377** | **1033.4609** | **1034.5430** | **-1.0821** | **1** | **8** | **25** | **5** | **K.MKSVIENAK.K** 5125 |
|  | 5415 | **530.2906** | **1058.5666** | **1058.5720** | **-0.0054** | **1** | **31** | **0.16** | **1** | **K.KVQAAQSEAK.V** 5425 |
|  | 6636 | **577.2990** | **1152.5835** | **1152.5887** | **-0.0052** | **0** | **10** | **14** | **4** | **R.ERPPEEVAAR.L** |
|  | 7619 | 606.2839 | 1210.5532 | 1209.5513 | 1.0019 | 0 | 28 | 0.24 | 1 | K.SEFEQNLSEK.L |
|  | 8288 | **622.3441** | **1242.6736** | **1242.6819** | **-0.0084** | **1** | **23** | **0.73** | **1** | **R.KAVDEAADALLK.A** 8257 |
|  | 9588 | **657.8317** | **1313.6489** | **1312.7550** | **0.8939** | **0** | **0** | **1.4e+002** | **7** | **K.FVLRPLRPCR.R** |
|  | 10485 | 682.3027 | 1362.5908 | 1362.6449 | -0.0541 | 1 | 44 | 0.006 | 1 | K.AAMDNSEIAGEKK.S 10516 |
|  | 12973 | **503.9379** | **1508.7920** | **1508.8021** | **-0.0101** | **0** | **12** | **8.4** | **1** | **K.LHNMIVDLDNVVK.K** |
|  | 13194 | **508.2928** | **1521.8567** | **1521.8667** | **-0.0100** | **1** | **14** | **4.6** | **1** | **R.KAHQLWLSVEALK.Y** |
|  | 13269 | **764.4123** | **1526.8100** | **1526.8205** | **-0.0105** | **0** | **70** | **1.3e-005** | **1** | **K.VVSQYHELVVQAR.D** 13270 13271 |
|  | 14096 | **792.4150** | **1582.8154** | **1582.8243** | **-0.0089** | **0** | **31** | **0.096** | **1** | **R.ELDSITPEVLPGWK.G** |
|  | 14670 | 811.3848 | 1620.7551 | 1620.7235 | 0.0316 | 1 | 5 | 39 | 4 | K.IEEVRDAMENEMR.T 14687 |
|  | 15221 | **552.2891** | **1653.8455** | **1652.8920** | **0.9536** | **1** | **4** | **43** | **4** | **K.LHNMIVDLDNVVKK.V** |
|  | 15341 | **555.3461** | **1663.0164** | **1661.8485** | **1.1679** | **0** | **8** | **17** | **1** | **K.LSTDDLNSLIAHAHR.R** |
|  | 17994 | **617.6386** | **1849.8938** | **1849.9646** | **-0.0707** | **1** | **1** | **83** | **3** | **R.ERPPEEVAARLAQQEK.Q** |
|  | 18313 | **936.4954** | **1870.9763** | **1870.9888** | **-0.0125** | **0** | **54** | **0.00037** | **1** | **K.TSSAETPTIPLGSAVEAIK.A** 18326 |
|  | 20559 | **1033.0005** | **2063.9864** | **2064.0011** | **-0.0147** | **1** | **70** | **9.3e-006** | **1** | **R.VQEQELKSEFEQNLSEK.L** 20560 20575 |
|  | 22655 | 1149.1067 | 2296.1988 | 2297.0749 | -0.8760 | 1 | 2 | 45 | 4 | -.MLRACQLSGVTAAAQSCLCGK.F |
|  | 23094 | **785.3992** | **2353.1757** | **2353.1662** | **0.0095** | **1** | **12** | **4.8** | **1** | **R.RLSQEQVDNFTLDINTAYAR.L** |
|  | 24238 | **836.3945** | **2506.1616** | **2505.1805** | **0.9810** | **0** | **2** | **48** | **7** | **K.ANCSDNEFTQALTAAIPPESLTR.G** |
|  | 25112 | **888.8068** | **2663.3986** | **2662.4080** | **0.9906** | **1** | **36** | **0.016** | **1** | **K.TIPYSDKLFEMVLGPAAYNVPLPK.K** |
|  | 25116 | 889.0650 | 2664.1732 | 2665.3130 | -1.1398 | 1 | 2 | 38 | 7 | K.GMSVSDLADKLSTDDLNSLIAHAHR.R |
|  | 26073 | **944.8431** | **2831.5074** | **2831.5253** | **-0.0179** | **0** | **66** | **1.5e-005** | **1** | **R.QTASVTLQAIAAQNAAVQAVNAHSNILK.A** 26074 |

| **186.** | P54652\|HSP72_HUMAN    **Mass:** 70263    **Score:** 273    **Queries matched:** 24   **emPAI:** 0.36 |
| --- | --- |
|  | Heat shock-related 70 kDa protein 2 OS=Homo sapiens GN=HSPA2 PE=1 SV=1 |

|  | **Query** | **Observed** | **Mr(expt)** | **Mr(calc)** | **Delta** | **Miss** | **Score** | **Expect** | **Rank** | **Peptide** |
| --- | --- | --- | --- | --- | --- | --- | --- | --- | --- | --- |
|  | 1518 | 678.3768 | 677.3695 | 677.2803 | 0.0892 | 0 | 10 | 18 | 3 | K.GCAGGEK.N |
|  | 1539 | 686.4146 | 685.4073 | 686.3963 | -0.9889 | 0 | 12 | 12 | 10 | R.LIGDAAK.N |
|  | 1654 | **731.1765** | **730.1692** | **729.4385** | **0.7307** | **1** | **5** | **55** | **3** | **K.NKILDK.C** |
|  | 2017 | 804.4382 | 803.4309 | 803.4389 | -0.0080 | 0 | 23 | 1.1 | 1 | K.ITITNDK.G |
|  | 2565 | 424.7464 | 847.4782 | 847.3923 | 0.0859 | 0 | 16 | 4.4 | 2 | K.QTVEDEK.L |
|  | 4098 | **481.7402** | **961.4658** | **960.4876** | **0.9783** | **1** | **14** | **7.5** | **7** | **K.ISEQDKNK.I** |
|  | 4891 | 509.2687 | 1016.5228 | 1016.5614 | -0.0386 | 1 | 8 | 29 | 7 | K.ITITNDKGR.L 4890 |
|  | 7672 | **607.3405** | **1212.6665** | **1211.6874** | **0.9791** | **1** | **6** | **34** | **2** | **R.GTLEPVEKALR.D** |
|  | 7971 | 614.8130 | 1227.6115 | 1227.6207 | -0.0092 | 0 | 24 | 0.56 | 1 | K.VEIIANDQGNR.T |
|  | 8456 | 418.4507 | 1252.3303 | 1252.6088 | -0.2785 | 0 | 2 | 59 | 7 | R.FEELNADLFR.G |
|  | 9829 | **663.8644** | **1325.7143** | **1324.6696** | **1.0447** | **1** | **2** | **91** | **1** | **K.MKEIAEAYLGGK.V** |
|  | 12096 | **486.3361** | **1455.9866** | **1455.8046** | **0.1821** | **0** | **3** | **47** | **4** | **K.GQIQEIVLVGGSTR.I** |
|  | 12518 | 740.8753 | 1479.7361 | 1479.7470 | -0.0109 | 1 | 36 | 0.031 | 1 | R.ARFEELNADLFR.G 12519 |
|  | 12631 | 744.3494 | 1486.6842 | 1486.6940 | -0.0098 | 0 | 65 | 4.2e-005 | 1 | R.TTPSYVAFTDTER.L 12606 12630 |
|  | 15042 | 822.8551 | 1643.6956 | 1643.8117 | -0.1160 | 0 | 3 | 50 | 4 | K.TFFPEEISSMVLTK.M |
|  | 15284 | 830.4458 | 1658.8770 | 1658.8879 | -0.0108 | 0 | 85 | 3.9e-007 | 1 | R.IINEPTAAAIAYGLDK.K 15285 |
|  | 15720 | 846.3599 | 1690.7053 | 1690.7183 | -0.0130 | 0 | 56 | 0.00023 | 1 | K.STAGDTHLGGEDFDNR.M 15721 |
|  | 19096 | **646.6487** | **1936.9242** | **1937.0370** | **-0.1128** | **1** | **2** | **55** | **5** | **K.DNNLLGKFDLTGIPPAPR.G** |

| **187.** | P62937\|PPIA_HUMAN    **Mass:** 18229    **Score:** 272    **Queries matched:** 18   **emPAI:** 3.66 |
| --- | --- |
|  | Peptidyl-prolyl cis-trans isomerase A OS=Homo sapiens GN=PPIA PE=1 SV=2 |

|  | **Query** | **Observed** | **Mr(expt)** | **Mr(calc)** | **Delta** | **Miss** | **Score** | **Expect** | **Rank** | **Peptide** |
| --- | --- | --- | --- | --- | --- | --- | --- | --- | --- | --- |
|  | 1541 | **687.3981** | **686.3908** | **685.3912** | **0.9996** | **0** | **25** | **0.8** | **1** | **K.HVVFGK.V** |
|  | 8361 | **624.3140** | **1246.6135** | **1246.6227** | **-0.0092** | **1** | **50** | **0.0014** | **1** | **K.KITIADCGQLE.-** |
|  | 9220 | 432.2447 | 1293.7122 | 1293.5693 | 0.1430 | 0 | 11 | 12 | 4 | K.EGMNIVEAMER.F |
|  | 9526 | **437.7396** | **1310.1971** | **1309.5642** | **0.6329** | **0** | **(3)** | **43** | **7** | **K.EGMNIVEAMER.F** |
|  | 10799 | **690.3773** | **1378.7401** | **1378.7496** | **-0.0096** | **1** | **62** | **9.9e-005** | **1** | **R.VSFELFADKVPK.T** 10800 |
|  | 12912 | **753.3715** | **1504.7284** | **1504.7377** | **-0.0094** | **1** | **58** | **0.0002** | **1** | **K.VKEGMNIVEAMER.F** 12913 |
|  | 13162 | **507.7917** | **1520.3534** | **1520.7327** | **-0.3792** | **1** | **(2)** | **59** | **8** | **K.VKEGMNIVEAMER.F** |
|  | 13387 | 512.9344 | 1535.7813 | 1536.7276 | -0.9462 | 1 | (3) | 60 | 2 | K.VKEGMNIVEAMER.F |
|  | 14333 | 799.8712 | 1597.7279 | 1597.7381 | -0.0103 | 0 | 45 | 0.0038 | 1 | R.IIPGFMCQGGDFTR.H |
|  | 16780 | **586.7935** | **1757.3587** | **1756.7872** | **0.5715** | **1** | **0** | **64** | **9** | **K.EGMNIVEAMERFGSR.N** |
|  | 17736 | **916.4537** | **1830.8929** | **1830.9039** | **-0.0110** | **1** | **65** | **3e-005** | **1** | **K.SIYGEKFEDENFILK.H** 17737 |
|  | 19191 | **973.4997** | **1944.9848** | **1944.9946** | **-0.0097** | **0** | **31** | **0.068** | **1** | **M.VNPTVFFDIAVDGEPLGR.V** |
|  | 25833 | **931.1084** | **2790.3034** | **2790.3218** | **-0.0184** | **0** | **69** | **7.1e-006** | **1** | **K.HTGPGILSMANAGPNTNGSQFFICTAK.T** 25836 25837 |

| **188.** | P51570\|GALK1_HUMAN    **Mass:** 42702    **Score:** 271    **Queries matched:** 26   **emPAI:** 1.13 |
| --- | --- |
|  | Galactokinase OS=Homo sapiens GN=GALK1 PE=1 SV=1 |

|  | **Query** | **Observed** | **Mr(expt)** | **Mr(calc)** | **Delta** | **Miss** | **Score** | **Expect** | **Rank** | **Peptide** |
| --- | --- | --- | --- | --- | --- | --- | --- | --- | --- | --- |
|  | 2085 | **405.2325** | **808.4505** | **808.4555** | **-0.0050** | **0** | **31** | **0.12** | **1** | **R.HVVGEIR.R** 2084 |
|  | 2648 | **428.7270** | **855.4394** | **855.4450** | **-0.0056** | **0** | **18** | **2.4** | **4** | **R.SLEPGTPR.W** |
|  | 2897 | **436.2220** | **870.4294** | **871.4875** | **-1.0581** | **0** | **19** | **2.2** | **1** | **R.TAQAAAALR.R** |
|  | 3112 | 444.2137 | 886.4129 | 886.4331 | -0.0202 | 0 | 12 | 13 | 5 | R.LMVESHR.S 3107 3116 |
|  | 5027 | **514.9418** | **1027.8690** | **1027.5886** | **0.2803** | **1** | **13** | **5.4** | **1** | **R.TAQAAAALRR.G** |
|  | 5305 | **525.7829** | **1049.5512** | **1049.5506** | **0.0007** | **1** | **3** | **78** | **8** | **R.DLVSKEGFR.R** |
|  | 7382 | **600.3544** | **1198.6943** | **1198.7034** | **-0.0090** | **0** | **56** | **0.00038** | **1** | **K.LAVLITNSNVR.H** |
|  | 8315 | **623.3108** | **1244.6071** | **1244.6149** | **-0.0078** | **0** | **40** | **0.015** | **1** | **R.HSLASSEYPVR.R** |
|  | 8618 | **421.0560** | **1260.1463** | **1259.6292** | **0.5171** | **1** | **9** | **12** | **10** | **R.QCEEVARALGK.E** |
|  | 9795 | **662.8607** | **1323.7068** | **1323.7146** | **-0.0079** | **0** | **37** | **0.028** | **1** | **R.QPQVAELLAEAR.R** 9816 |
|  | 10912 | **693.3751** | **1384.7357** | **1384.7449** | **-0.0093** | **0** | **43** | **0.0072** | **1** | **R.SLETSLVPLSDPK.L** 10913 |
|  | 11192 | **467.9094** | **1400.7064** | **1400.7160** | **-0.0096** | **1** | **14** | **5.1** | **1** | **R.HSLASSEYPVRR.R** |
|  | 19978 | **1008.4417** | **2014.8687** | **2015.0171** | **-0.1484** | **1** | **71** | **6.7e-006** | **1** | **R.KDGLVSLLTTSEGADEPQR.L** 19996 |
|  | 23182 | **789.0400** | **2364.0981** | **2364.1135** | **-0.0154** | **0** | **50** | **0.00066** | **1** | **R.HIQEHYGGTATFYLSQAADGAK.V** |
|  | 28153 | **1090.4891** | **3268.4456** | **3268.4596** | **-0.0140** | **0** | **82** | **2.9e-007** | **1** | **R.AQVCQQAEHSFAGMPCGIMDQFISLMGQK.G** 28154 |
|  | 28237 | **1103.5370** | **3307.5891** | **3307.6031** | **-0.0139** | **1** | **25** | **0.15** | **1** | **R.SLRDDYEVSCPELDQLVEAALAVPGVYGSR.M** 28236 |
|  | 28499 | **1127.2585** | **3378.7538** | **3378.7680** | **-0.0142** | **0** | **10** | **4** | **1** | **R.VNLIGEHTDYNQGLVLPMALELMTVLVGSPR.K** 28498 |

| **189.** | P34931\|HS71L_HUMAN    **Mass:** 70730    **Score:** 270    **Queries matched:** 36   **emPAI:** 0.29 |
| --- | --- |
|  | Heat shock 70 kDa protein 1L OS=Homo sapiens GN=HSPA1L PE=1 SV=2 |

|  | **Query** | **Observed** | **Mr(expt)** | **Mr(calc)** | **Delta** | **Miss** | **Score** | **Expect** | **Rank** | **Peptide** |
| --- | --- | --- | --- | --- | --- | --- | --- | --- | --- | --- |
|  | 1539 | 686.4146 | 685.4073 | 686.3963 | -0.9889 | 0 | 12 | 12 | 10 | R.LIGDAAK.N |
|  | 1654 | 731.1765 | 730.1692 | 729.4385 | 0.7307 | 1 | 5 | 55 | 3 | K.NKILDK.C |
|  | 2017 | 804.4382 | 803.4309 | 803.4389 | -0.0080 | 0 | 23 | 1.1 | 1 | K.ITITNDK.G |
|  | 4891 | 509.2687 | 1016.5228 | 1016.5614 | -0.0386 | 1 | 8 | 29 | 7 | K.ITITNDKGR.L 4890 |
|  | 6105 | **556.7713** | **1111.5280** | **1111.5583** | **-0.0303** | **1** | **12** | **9.5** | **4** | **R.MVLDAEKYK.A** |
|  | 7672 | 607.3405 | 1212.6665 | 1211.6874 | 0.9791 | 1 | 6 | 34 | 2 | R.GTLEPVEKALR.D |
|  | 7971 | 614.8130 | 1227.6115 | 1227.6207 | -0.0092 | 0 | 24 | 0.56 | 1 | K.VEIIANDQGNR.T |
|  | 8103 | **412.2278** | **1233.6617** | **1233.6394** | **0.0223** | **0** | **4** | **59** | **5** | **R.LVSHFVEEFK.R** |
|  | 8366 | 416.5560 | 1246.6462 | 1247.6292 | -0.9830 | 1 | 12 | 9.3 | 4 | R.AMTKDNNLLGR.F |
|  | 8691 | **422.5445** | **1264.6116** | **1265.7092** | **-1.0976** | **0** | **6** | **35** | **7** | **K.IHDIVLVGGSTR.I** |
|  | 9084 | **644.3015** | **1286.5885** | **1286.5965** | **-0.0080** | **0** | **30** | **0.16** | **1** | **K.NALESYAFNMK.S** 9083 |
|  | 9307 | **650.2986** | **1298.5826** | **1298.5965** | **-0.0139** | **0** | **8** | **20** | **7** | **R.FEELCADLFR.G** |
|  | 12177 | **487.5734** | **1459.6983** | **1460.7180** | **-1.0197** | **1** | **10** | **14** | **3** | **K.EEIERMVLDAEK.Y** 12197 |
|  | 12261 | 489.5734 | 1465.6983 | 1464.8413 | 0.8571 | 1 | 5 | 41 | 4 | K.AKIHDIVLVGGSTR.I |
|  | 12461 | **492.9316** | **1475.7730** | **1474.7160** | **1.0570** | **0** | **4** | **51** | **3** | **K.ELEQMCNPIITK.L** |
|  | 12490 | **493.5731** | **1477.6974** | **1476.7130** | **0.9845** | **1** | **(5)** | **38** | **8** | **K.EEIERMVLDAEK.Y** |
|  | 12631 | 744.3494 | 1486.6842 | 1486.6940 | -0.0098 | 0 | 65 | 4.2e-005 | 1 | R.TTPSYVAFTDTER.L 12606 12630 |
|  | 14661 | 540.9471 | 1619.8196 | 1618.8058 | 1.0138 | 1 | 7 | 24 | 9 | R.KELEQMCNPIITK.L 14633 |
|  | 14734 | 813.3951 | 1624.7757 | 1624.9260 | -0.1503 | 1 | 13 | 6.1 | 2 | R.QATKDAGVIAGLNVLR.I 14733 14747 |
|  | 14809 | 815.9115 | 1629.8084 | 1629.7960 | 0.0125 | 0 | 5 | 41 | 9 | K.AFYPEEISSMVLTK.L |
|  | 15284 | 830.4458 | 1658.8770 | 1658.8879 | -0.0108 | 0 | 85 | 3.9e-007 | 1 | R.IINEPTAAAIAYGLDK.G 15285 |
|  | 15498 | **838.3627** | **1674.7108** | **1674.7234** | **-0.0126** | **0** | **65** | **3.5e-005** | **1** | **K.ATAGDTHLGGEDFDNR.L** |
|  | 15517 | 559.3773 | 1675.1101 | 1675.7988 | -0.6887 | 0 | (3) | 46 | 2 | K.NQVAMNPQNTVFDAK.R |
|  | 15732 | 846.4323 | 1690.8501 | 1691.7937 | -0.9436 | 0 | 4 | 40 | 3 | K.NQVAMNPQNTVFDAK.R |
|  | 18321 | **624.9905** | **1871.9498** | **1870.9750** | **0.9748** | **1** | **2** | **67** | **4** | **K.AFYPEEISSMVLTKLK.E** |
|  | 23136 | 787.3878 | 2359.1415 | 2360.1794 | -1.0380 | 1 | 1 | 71 | 6 | R.LIGDAAKNQVAMNPQNTVFDAK.R |
|  | 25890 | **932.7847** | **2795.3324** | **2794.4146** | **0.9177** | **1** | **3** | **28** | **1** | **-.MATAKGIAIGIDLGTTYSCVGVFQHGK.V** |

| **190.** | P30084\|ECHM_HUMAN    **Mass:** 31823    **Score:** 269    **Queries matched:** 24   **emPAI:** 1.19 |
| --- | --- |
|  | Enoyl-CoA hydratase, mitochondrial OS=Homo sapiens GN=ECHS1 PE=1 SV=4 |

|  | **Query** | **Observed** | **Mr(expt)** | **Mr(calc)** | **Delta** | **Miss** | **Score** | **Expect** | **Rank** | **Peptide** |
| --- | --- | --- | --- | --- | --- | --- | --- | --- | --- | --- |
|  | 3160 | **446.6842** | **891.3538** | **890.5450** | **0.8088** | **0** | **6** | **52** | **7** | **R.GPLRPPVR.C** |
|  | 6764 | **582.2971** | **1162.5796** | **1162.5883** | **-0.0088** | **0** | **14** | **6.5** | **1** | **K.HWDHLTQVK.K** |
|  | 7042 | **395.5463** | **1183.6171** | **1182.5703** | **1.0468** | **1** | **9** | **20** | **3** | **K.EGMTAFVEKR.K** 7034 |
|  | 10346 | 677.7925 | 1353.5704 | 1353.6268 | -0.0564 | 0 | 8 | 24 | 7 | K.SLAMEMVLTGDR.I 10376 |
|  | 12217 | **488.5739** | **1462.6998** | **1462.7092** | **-0.0094** | **1** | **22** | **0.84** | **1** | **K.LFYSTFATDDRK.E** |
|  | 12270 | **733.9203** | **1465.8261** | **1465.8365** | **-0.0103** | **0** | **56** | **0.00027** | **1** | **K.NNTVGLIQLNRPK.A** 12271 |
|  | 15797 | **565.5638** | **1693.6695** | **1692.8539** | **0.8156** | **1** | **5** | **29** | **6** | **R.AVGKSLAMEMVLTGDR.I** |
|  | 16406 | **433.4524** | **1729.7803** | **1728.7876** | **0.9927** | **0** | **9** | **15** | **1** | **K.ESVNAAFEMTLTEGSK.L** 16346 |
|  | 16490 | **868.8608** | **1735.7070** | **1735.7182** | **-0.0111** | **0** | **67** | **2e-005** | **1** | **K.EMQNLSFQDCYSSK.F** 16489 |
|  | 17576 | **909.9530** | **1817.8914** | **1817.9047** | **-0.0133** | **0** | **26** | **0.25** | **1** | **K.TFEEDPAVGAIVLTGGDK.A** |
|  | 19488 | **986.0092** | **1970.0038** | **1970.0142** | **-0.0105** | **0** | **29** | **0.12** | **1** | **K.ALNALCDGLIDELNQALK.T** |
|  | 19677 | **663.6569** | **1987.9489** | **1987.9594** | **-0.0105** | **0** | **35** | **0.03** | **1** | **K.ICPVETLVEEAIQCAEK.I** |
|  | 20781 | **1042.5104** | **2083.0062** | **2083.0143** | **-0.0081** | **1** | **(2)** | **53** | **1** | **K.ESVNAAFEMTLTEGSKLEK.K** |
|  | 20936 | **700.3280** | **2097.9622** | **2099.0092** | **-1.0470** | **1** | **3** | **40** | **1** | **K.ESVNAAFEMTLTEGSKLEK.K** |
|  | 21155 | **1063.0664** | **2124.1183** | **2124.1328** | **-0.0145** | **0** | **92** | **6e-008** | **1** | **K.AQFAQPEILIGTIPGAGGTQR.L** 21156 21157 21164 |
|  | 30785 | **1461.3203** | **4380.9391** | **4381.0792** | **-0.1401** | **1** | **0** | **17** | **2** | **K.HWDHLTQVKKPVIAAVNGYAFGGGCELAMMCDIIYAGEK.A** |

| **191.** | P17858\|K6PL_HUMAN    **Mass:** 85762    **Score:** 267    **Queries matched:** 32   **emPAI:** 0.53 |
| --- | --- |
|  | 6-phosphofructokinase, liver type OS=Homo sapiens GN=PFKL PE=1 SV=6 |

|  | **Query** | **Observed** | **Mr(expt)** | **Mr(calc)** | **Delta** | **Miss** | **Score** | **Expect** | **Rank** | **Peptide** |
| --- | --- | --- | --- | --- | --- | --- | --- | --- | --- | --- |
|  | 1899 | **399.2322** | **796.4498** | **796.3901** | **0.0597** | **0** | **8** | **24** | **6** | **K.MLAQYR.I** |
|  | 2613 | **427.2116** | **852.4087** | **853.4368** | **-1.0280** | **0** | **5** | **43** | **4** | **R.MGIYVGAK.V** |
|  | 2825 | **434.2242** | **866.4339** | **865.4327** | **1.0012** | **1** | **2** | **93** | **7** | **R.RTLSMDK.G** |
|  | 2959 | 438.7474 | 875.4803 | 875.4422 | 0.0381 | 0 | 21 | 1.5 | 2 | -.MAAVDLEK.L |
|  | 3162 | 446.7122 | 891.4098 | 890.4644 | 0.9455 | 1 | 8 | 28 | 9 | K.MKTDIQR.G |
|  | 4311 | **489.2566** | **976.4987** | **976.5052** | **-0.0065** | **0** | **36** | **0.049** | **1** | **K.AMLWLSEK.L** |
|  | 5072 | **516.3173** | **1030.6201** | **1030.6287** | **-0.0086** | **0** | **11** | **14** | **2** | **K.LLAHQKPPK.E** |
|  | 5317 | **526.2808** | **1050.5470** | **1050.5532** | **-0.0062** | **0** | **30** | **0.15** | **1** | **R.TFVLEVMGR.H** |
|  | 5524 | 534.7811 | 1067.5477 | 1066.5481 | 0.9996 | 0 | (10) | 16 | 5 | R.TFVLEVMGR.H |
|  | 5758 | **543.7620** | **1085.5094** | **1085.5175** | **-0.0082** | **0** | **27** | **0.37** | **1** | **K.VNVEHMTEK.M** 5759 |
|  | 8527 | 628.8419 | 1255.6693 | 1256.6724 | -1.0032 | 0 | 54 | 0.00063 | 1 | K.GQLESIVENIR.I |
|  | 8990 | 428.2288 | 1281.6645 | 1281.6937 | -0.0292 | 1 | 5 | 43 | 9 | R.LMLKMLAQYR.I |
|  | 9140 | 645.8250 | 1289.6355 | 1288.7391 | 0.8964 | 1 | 20 | 1.5 | 1 | K.AVAFSPVTELKK.D |
|  | 10267 | **675.3663** | **1348.7181** | **1348.6553** | **0.0628** | **0** | **3** | **85** | **7** | **R.LPLMECVQMTK.E** |
|  | 11463 | **709.3539** | **1416.6932** | **1417.7678** | **-1.0746** | **1** | **6** | **38** | **2** | **K.DLVVQRLGFDTR.V** |
|  | 13777 | **781.3997** | **1560.7849** | **1560.7970** | **-0.0121** | **0** | **49** | **0.0016** | **1** | **R.VFANAPDSACVIGLK.K** |
|  | 15073 | **823.9119** | **1645.8093** | **1645.8199** | **-0.0106** | **0** | **40** | **0.013** | **1** | **R.SEWGSLLEELVAEGK.I** 15074 |
|  | 17203 | **894.4505** | **1786.8864** | **1786.8996** | **-0.0131** | **0** | **32** | **0.062** | **1** | **K.AIGVLTSGGDAQGMNAAVR.A** 17192 |
|  | 18754 | 954.4832 | 1906.9517 | 1906.9650 | -0.0132 | 0 | 73 | 5.4e-006 | 1 | R.TNVLGHLQQGGAPTPFDR.N 18755 |
|  | 18820 | **638.6586** | **1912.9541** | **1912.9677** | **-0.0136** | **0** | **12** | **6** | **1** | **R.IMEVIDAITTTAQSHQR.T** |
|  | 21326 | **1072.5604** | **2143.1063** | **2143.1208** | **-0.0145** | **0** | **56** | **0.00024** | **1** | **K.SNFSLAILNVGAPAAGMNAAVR.S** 21327 |
|  | 22055 | **742.0381** | **2223.0924** | **2223.1073** | **-0.0149** | **0** | **33** | **0.041** | **1** | **R.TGISHGHTVYVVHDGFEGLAK.G** 22054 |
|  | 22133 | **744.0394** | **2229.0963** | **2230.1488** | **-1.0525** | **1** | **3** | **47** | **9** | **K.AIGVLTSGGDAQGMNAAVRAVTR.M** |
|  | 28950 | **888.6641** | **3550.6274** | **3550.6457** | **-0.0183** | **0** | **10** | **3.3** | **1** | **R.TYSHLNIAGLVGSIDNDFCGTDMTIGTDSALHR.I** |
|  | 30745 | 1447.0414 | 4338.1023 | 4339.0858 | -0.9835 | 0 | 1 | 19 | 4 | R.VFIVETMGGYCGYLATVTGIAVGADAAYVFEDPFNIHDLK.V |
|  | 30949 | **908.4207** | **4537.0672** | **4538.0056** | **-0.9384** | **0** | **2** | **10** | **2** | **R.YEELCIVMCVIPATISNNVPGTDFSLGSDTAVNAAMESCDR.I** |

| **192.** | P30050\|RL12_HUMAN    **Mass:** 17979    **Score:** 263    **Queries matched:** 12   **emPAI:** 1.18 |
| --- | --- |
|  | 60S ribosomal protein L12 OS=Homo sapiens GN=RPL12 PE=1 SV=1 |

|  | **Query** | **Observed** | **Mr(expt)** | **Mr(calc)** | **Delta** | **Miss** | **Score** | **Expect** | **Rank** | **Peptide** |
| --- | --- | --- | --- | --- | --- | --- | --- | --- | --- | --- |
|  | 1696 | **747.4200** | **746.4127** | **746.4174** | **-0.0047** | **0** | **16** | **5.6** | **1** | **R.ELSGTIK.E** |
|  | 3032 | **441.2739** | **880.5332** | **880.5382** | **-0.0050** | **0** | **27** | **0.31** | **1** | **K.IGPLGLSPK.K** |
|  | 4687 | 502.2315 | 1002.4485 | 1002.5247 | -0.0762 | 1 | 13 | 8.5 | 8 | K.ATGDWKGLR.I |
|  | 11492 | **709.8462** | **1417.6778** | **1417.6871** | **-0.0093** | **0** | **46** | **0.0036** | **1** | **R.CTGGEVGATSALAPK.I** |
|  | 15383 | **833.9844** | **1665.9542** | **1665.9665** | **-0.0123** | **0** | **31** | **0.084** | **1** | **R.QAQIEVVPSASALIIK.A** |
|  | 15503 | **838.4016** | **1674.7887** | **1674.7995** | **-0.0109** | **0** | **65** | **3.3e-005** | **1** | **K.EILGTAQSVGCNVDGR.H** 15504 |
|  | 15641 | **843.4288** | **1684.8431** | **1684.8533** | **-0.0102** | **0** | **73** | **5.6e-006** | **1** | **K.HSGNITFDEIVNIAR.Q** |
|  | 20324 | **1023.9560** | **2045.8974** | **2045.9113** | **-0.0138** | **0** | **70** | **8e-006** | **1** | **R.HPHDIIDDINSGAVECPAS.-** 20322 |
|  | 23476 | **802.0729** | **2403.1970** | **2403.2064** | **-0.0094** | **1** | **10** | **7.4** | **1** | **R.ELSGTIKEILGTAQSVGCNVDGR.H** |
|  | 29302 | 1235.3279 | 3702.9618 | 3702.7003 | 0.2616 | 1 | 1 | 23 | 9 | K.EILGTAQSVGCNVDGRHPHDIIDDINSGAVECPAS.- |

| **193.** | O43795\|MYO1B_HUMAN    **Mass:** 132928   **Score:** 263    **Queries matched:** 38   **emPAI:** 0.18 |
| --- | --- |
|  | Myosin-Ib OS=Homo sapiens GN=MYO1B PE=1 SV=3 |

|  | **Query** | **Observed** | **Mr(expt)** | **Mr(calc)** | **Delta** | **Miss** | **Score** | **Expect** | **Rank** | **Peptide** |
| --- | --- | --- | --- | --- | --- | --- | --- | --- | --- | --- |
|  | 697 | **460.2629** | **459.2556** | **459.2441** | **0.0115** | **0** | **7** | **73** | **1** | **R.ANAGK.K** 696 |
|  | 1238 | 589.3899 | 588.3826 | 587.3755 | 1.0071 | 1 | 4 | 1.4e+002 | 6 | K.SRVVK.Q |
|  | 3397 | 455.7214 | 909.4283 | 909.4821 | -0.0537 | 1 | 10 | 16 | 5 | K.FFRANAGK.K |
|  | 5407 | **529.9421** | **1057.8696** | **1058.5396** | **-0.6700** | **0** | **2** | **73** | **5** | **K.SLFPEGNPAK.I** |
|  | 5561 | 536.2445 | 1070.4743 | 1071.5423 | -1.0679 | 1 | 7 | 37 | 2 | K.YFLEMKNK.M |
|  | 5786 | **544.7919** | **1087.5693** | **1087.5774** | **-0.0081** | **0** | **23** | **0.88** | **1** | **K.RPPTAGSQFK.A** 5812 |
|  | 6254 | **562.7897** | **1123.5648** | **1123.6349** | **-0.0701** | **1** | **7** | **30** | **7** | **K.NNRLLEVAVP.-** |
|  | 6878 | 586.7695 | 1171.5244 | 1172.5713 | -1.0469 | 0 | 3 | 85 | 3 | R.YNYLSLDSAK.V |
|  | 7539 | **603.8287** | **1205.6428** | **1205.6557** | **-0.0129** | **0** | **12** | **10** | **2** | **K.SQIVIAAWYR.R** 7538 |
|  | 9129 | **645.3524** | **1288.6903** | **1288.6775** | **0.0128** | **0** | **1** | **1.3e+002** | **10** | **K.LGNIEFKPESR.V** |
|  | 9270 | **649.3165** | **1296.6184** | **1297.7104** | **-1.0920** | **1** | **5** | **47** | **10** | **R.IVQKYFLEMK.N** |
|  | 11252 | **469.2502** | **1404.7288** | **1403.7806** | **0.9482** | **1** | **19** | **1.7** | **1** | **K.ASVATLMKNLQTK.N** |
|  | 12089 | 728.8760 | 1455.7375 | 1455.6816 | 0.0559 | 1 | 2 | 80 | 5 | R.QAYEPCLERYK.M |
|  | 13281 | **764.8696** | **1527.7246** | **1526.8456** | **0.8789** | **1** | **6** | **35** | **5** | **K.SLFPEGNPAKINLK.R** |
|  | 13413 | **513.3104** | **1536.9093** | **1536.8664** | **0.0429** | **1** | **1** | **85** | **5** | **K.IYEFTLQRIVQK.Y** |
|  | 16314 | **576.2695** | **1725.7866** | **1725.8005** | **-0.0140** | **0** | **17** | **2.1** | **1** | **K.LNQVCATHQHFESR.M** |
|  | 16392 | **865.4905** | **1728.9665** | **1728.9774** | **-0.0109** | **0** | **79** | **1.3e-006** | **1** | **R.IFLLTNNNLLLADQK.S** 16393 16415 |
|  | 16871 | **881.9031** | **1761.7917** | **1760.8615** | **0.9303** | **0** | **5** | **39** | **3** | **K.EICELTGIDQSVLER.A** |
|  | 18460 | **629.2795** | **1884.8166** | **1884.0105** | **0.8061** | **1** | **0** | **94** | **7** | **R.YQQTKSSALVIQSYIR.G** |
|  | 18740 | 636.3303 | 1905.9690 | 1905.9995 | -0.0306 | 1 | 10 | 9.1 | 1 | K.KAAHIFNEALVCHQIR.Y |
|  | 21585 | **725.0143** | **2172.0212** | **2171.0323** | **0.9889** | **0** | **20** | **0.88** | **1** | **R.NFYELSPHIFALSDEAYR.S** |
|  | 21905 | **1105.0397** | **2208.0648** | **2208.1215** | **-0.0567** | **0** | **72** | **5.2e-006** | **1** | **R.NFHVFYQLLSGASEELLNK.L** 21904 |
|  | 21961 | **738.8424** | **2213.5054** | **2213.1804** | **0.3250** | **1** | **0** | **52** | **3** | **K.EQLLQSNPVLEAFGNAKTVR.N** |
|  | 23530 | **1207.0927** | **2412.1707** | **2412.1849** | **-0.0141** | **0** | **3** | **40** | **1** | **R.SGVEVLFNELEIPVEEYSFGR.S** 23531 |
|  | 24353 | **841.1019** | **2520.2840** | **2520.3012** | **-0.0173** | **1** | **16** | **2** | **1** | **K.ALYPSSVGQPFQGAYLEINKNPK.Y** |
|  | 25021 | **1321.6820** | **2641.3495** | **2641.3608** | **-0.0113** | **0** | **73** | **3.3e-006** | **1** | **R.NAMQIVGFMDHEAESVLAVVAAVLK.L** 25023 |
|  | 25681 | **921.7879** | **2762.3419** | **2762.3513** | **-0.0094** | **1** | **26** | **0.16** | **1** | **K.YMDIEFDFKGDPLGGVISNYLLEK.S** |
|  | 25763 | 926.7838 | 2777.3294 | 2778.3462 | -1.0168 | 1 | (0) | 58 | 5 | K.YMDIEFDFKGDPLGGVISNYLLEK.S |
|  | 27047 | **750.1040** | **2996.3869** | **2997.5780** | **-1.1911** | **1** | **3** | **30** | **1** | **R.TVRNAMQIVGFMDHEAESVLAVVAAVLK.L** |
|  | 27103 | 1005.8288 | 3014.4646 | 3013.5729 | 0.8917 | 1 | (1) | 42 | 4 | R.TVRNAMQIVGFMDHEAESVLAVVAAVLK.L |

| **194.** | Q96A33\|CCD47_HUMAN    **Mass:** 56123    **Score:** 259    **Queries matched:** 25   **emPAI:** 0.67 |
| --- | --- |
|  | Coiled-coil domain-containing protein 47 OS=Homo sapiens GN=CCDC47 PE=1 SV=1 |

|  | **Query** | **Observed** | **Mr(expt)** | **Mr(calc)** | **Delta** | **Miss** | **Score** | **Expect** | **Rank** | **Peptide** |
| --- | --- | --- | --- | --- | --- | --- | --- | --- | --- | --- |
|  | 4046 | **479.2739** | **956.5332** | **956.5403** | **-0.0070** | **1** | **16** | **4.3** | **2** | **R.RLEEAALR.R** |
|  | 7119 | **594.3103** | **1186.6060** | **1187.5856** | **-0.9796** | **0** | **3** | **85** | **3** | **K.IMQEEGQPLK.L** |
|  | 8283 | 622.3394 | 1242.6642 | 1242.6258 | 0.0384 | 0 | 8 | 24 | 4 | R.LAQAWFNTHR.E 8284 |
|  | 10771 | **689.8309** | **1377.6473** | **1377.6567** | **-0.0094** | **0** | **5** | **40** | **4** | **R.VCCEGMLIQLR.F** |
|  | 11708 | **716.3718** | **1430.7290** | **1430.7374** | **-0.0084** | **0** | **34** | **0.054** | **1** | **R.MMRPVSDQVQIK.V** |
|  | 13993 | **526.7540** | **1577.2401** | **1576.7522** | **0.4879** | **0** | **3** | **36** | **5** | **K.IESVHFSDQFSGPK.I** |
|  | 16134 | **857.3707** | **1712.7268** | **1712.7386** | **-0.0118** | **0** | **53** | **0.00049** | **1** | **K.EMQDLSEFCSDKPK.S** |
|  | 17143 | **595.2961** | **1782.8666** | **1781.8990** | **0.9675** | **1** | **8** | **15** | **2** | **R.VCCEGMLIQLRFLK.R** |
|  | 18060 | **928.4774** | **1854.9402** | **1854.9516** | **-0.0114** | **0** | **16** | **2.9** | **1** | **R.TLLFTFNVPGSGNTYPK.D** |
|  | 20197 | **678.3099** | **2031.9080** | **2031.9221** | **-0.0141** | **0** | **34** | **0.037** | **1** | **K.LNQENEHIYNLWCSGR.V** |
|  | 20543 | 1032.4503 | 2062.8861 | 2062.8976 | -0.0115 | 0 | 48 | 0.0013 | 1 | K.VTMNDEDMDTYVFAVGTR.K 20544 |
|  | 20910 | **1048.5225** | **2095.0304** | **2095.0403** | **-0.0099** | **0** | **32** | **0.053** | **1** | **K.DMEALLPLMNMVIYSIDK.A** 20913 |
|  | 20938 | 1050.0537 | 2098.0929 | 2097.9711 | 0.1218 | 1 | 5 | 27 | 3 | R.LQKEMQDLSEFCSDKPK.S |
|  | 22896 | **776.3309** | **2325.9710** | **2326.1622** | **-0.1913** | **1** | **5** | **22** | **1** | **K.DMEALLPLMNMVIYSIDKAK.K** |
|  | 24092 | **829.4807** | **2485.4203** | **2485.3145** | **0.1058** | **1** | **12** | **4** | **1** | **R.QDLLNVLARMMRPVSDQVQIK.V** 24081 24086 |
|  | 24690 | **1287.0879** | **2572.1612** | **2572.1747** | **-0.0134** | **0** | **98** | **9.1e-009** | **1** | **K.YGLPDSLAILSEMGEVTDGMMDTK.M** 24691 24692 |
|  | 26609 | **584.8841** | **2919.3841** | **2919.4014** | **-0.0173** | **1** | **2** | **32** | **2** | **K.MVHFLTHYADKIESVHFSDQFSGPK.I** |
|  | 30062 | 996.2048 | 3980.7900 | 3979.9661 | 0.8239 | 1 | 2 | 16 | 2 | R.TLLFTFNVPGSGNTYPKDMEALLPLMNMVIYSIDK.A |

| **195.** | Q9UJM8\|HAOX1_HUMAN    **Mass:** 41127    **Score:** 259    **Queries matched:** 22   **emPAI:** 1.01 |
| --- | --- |
|  | Hydroxyacid oxidase 1 OS=Homo sapiens GN=HAO1 PE=1 SV=1 |

|  | **Query** | **Observed** | **Mr(expt)** | **Mr(calc)** | **Delta** | **Miss** | **Score** | **Expect** | **Rank** | **Peptide** |
| --- | --- | --- | --- | --- | --- | --- | --- | --- | --- | --- |
|  | 1423 | **644.4134** | **643.4061** | **642.4064** | **0.9997** | **0** | **26** | **0.67** | **1** | **K.ALALGAK.A** |
|  | 2652 | **428.7634** | **855.5122** | **855.5178** | **-0.0056** | **1** | **20** | **1.8** | **1** | **R.KNPLAVSK.I** |
|  | 5918 | **549.8152** | **1097.6158** | **1096.6968** | **0.9190** | **1** | **15** | **4.6** | **1** | **R.RLTSLPIVAK.G** |
|  | 9484 | **436.7547** | **1307.2423** | **1306.6373** | **0.6049** | **0** | **6** | **24** | **8** | **R.LAMALSGCQNVK.V** |
|  | 9566 | **657.2576** | **1312.5007** | **1313.6398** | **-1.1391** | **0** | **1** | **90** | **3** | **R.MAHVDGELATVR.A** 9600 |
|  | 10814 | 460.9011 | 1379.6815 | 1379.8864 | -0.2049 | 1 | 12 | 9.9 | 2 | R.LTSLPIVAKGILR.G |
|  | 11921 | **482.5882** | **1444.7428** | **1443.7695** | **0.9733** | **0** | **33** | **0.071** | **1** | **K.HGLNGILVSNHGAR.Q** |
|  | 13156 | **760.8673** | **1519.7201** | **1519.7309** | **-0.0109** | **0** | **30** | **0.13** | **1** | **R.VSMPICVGATAMQR.M** |
|  | 13836 | **783.3656** | **1564.7166** | **1564.8250** | **-0.1083** | **0** | **8** | **18** | **3** | **K.AIFVTVDTPYLGNR.L** |
|  | 14194 | 795.4045 | 1588.7945 | 1588.8057 | -0.0111 | 0 | 67 | 2.7e-005 | 1 | R.NVAETDLSTSVLGQR.V |
|  | 15486 | **837.9525** | **1673.8903** | **1673.8988** | **-0.0085** | **1** | **89** | **1.6e-007** | **1** | **K.GVQDVLEILKEEFR.L** |
|  | 17706 | 914.9697 | 1827.9248 | 1827.9519 | -0.0271 | 1 | 4 | 39 | 3 | K.AIDPSISWEDIKWLR.R |
|  | 18412 | **940.9283** | **1879.8421** | **1879.8548** | **-0.0126** | **0** | **85** | **3e-007** | **1** | **R.SGANDEETLADNIAAFSR.W** 18420 |
|  | 19518 | **658.7001** | **1973.0786** | **1973.0887** | **-0.0102** | **0** | **41** | **0.0068** | **1** | **K.AVFVGRPIVWGLAFQGEK.G** |
|  | 22636 | **765.0808** | **2292.2206** | **2291.2260** | **0.9946** | **0** | **12** | **4.3** | **1** | **R.QLDGVPATIDVLPEIVEAVEGK.V** |
|  | 26446 | **1448.6520** | **2895.2894** | **2895.3087** | **-0.0192** | **0** | **63** | **2.7e-005** | **1** | **K.NFETSTLSFSPEENFGDDSGLAAYVAK.A** |
|  | 27409 | **1023.4606** | **3067.3601** | **3067.7223** | **-0.3622** | **1** | **0** | **42** | **7** | **K.AVFVGRPIVWGLAFQGEKGVQDVLEILK.E** |
|  | 27743 | **1578.7894** | **3155.5643** | **3154.4441** | **1.1202** | **1** | **0** | **48** | **6** | **R.MKNFETSTLSFSPEENFGDDSGLAAYVAK.A** |
|  | 28435 | **1121.9351** | **3362.7834** | **3362.7974** | **-0.0140** | **1** | **37** | **0.0072** | **1** | **R.QLDGVPATIDVLPEIVEAVEGKVEVFLDGGVR.K** 28434 |

| **196.** | Q9UBX3\|DIC_HUMAN    **Mass:** 31718    **Score:** 256    **Queries matched:** 17   **emPAI:** 0.57 |
| --- | --- |
|  | Mitochondrial dicarboxylate carrier OS=Homo sapiens GN=SLC25A10 PE=1 SV=2 |

|  | **Query** | **Observed** | **Mr(expt)** | **Mr(calc)** | **Delta** | **Miss** | **Score** | **Expect** | **Rank** | **Peptide** |
| --- | --- | --- | --- | --- | --- | --- | --- | --- | --- | --- |
|  | 1871 | **397.8527** | **793.6908** | **794.3779** | **-0.6870** | **0** | **8** | **16** | **8** | **R.MTGMALR.V** |
|  | 3374 | **454.7623** | **907.5100** | **907.5167** | **-0.0067** | **0** | **21** | **1.2** | **1** | **K.LGPLAFYK.G** |
|  | 7318 | 598.8022 | 1195.5898 | 1195.5986 | -0.0087 | 0 | 17 | 2.7 | 1 | K.GSQGPLPFHEK.V |
|  | 7590 | **605.3289** | **1208.6432** | **1208.6513** | **-0.0082** | **0** | **33** | **0.075** | **1** | **K.VHLQTQQEVK.L** 7591 |
|  | 9009 | **642.3244** | **1282.6342** | **1282.6452** | **-0.0109** | **1** | **6** | **34** | **3** | **R.RLFSGATMASSR.G** |
|  | 9315 | **434.0128** | **1299.0166** | **1298.6401** | **0.3765** | **1** | **(4)** | **35** | **1** | **R.RLFSGATMASSR.G** 9295 9297 |
|  | 15444 | **836.4599** | **1670.9052** | **1670.9872** | **-0.0819** | **1** | **3** | **59** | **5** | **K.LGPLAFYKGLVPAGIR.L** |
|  | 15819 | **848.3873** | **1694.7601** | **1694.7723** | **-0.0122** | **0** | **57** | **0.00022** | **1** | **K.GEYQGVFHCAVETAK.L** 15818 |
|  | 16069 | **855.4243** | **1708.8341** | **1708.8454** | **-0.0113** | **0** | **28** | **0.17** | **2** | **R.GALVTVGQLSCYDQAK.Q** 16070 |
|  | 22670 | **1149.6382** | **2297.2618** | **2297.2744** | **-0.0126** | **0** | **102** | **4.4e-009** | **1** | **K.VLLGSVSGLAGGFVGTPADLVNVR.M** 22668 |
|  | 24184 | **833.0485** | **2496.1237** | **2496.1389** | **-0.0151** | **0** | **25** | **0.21** | **1** | **R.WYFGGLASCGAACCTHPLDLLK.V** |

| **197.** | P63244\|GBLP_HUMAN    **Mass:** 35511    **Score:** 255    **Queries matched:** 18   **emPAI:** 0.65 |
| --- | --- |
|  | Guanine nucleotide-binding protein subunit beta-2-like 1 OS=Homo sapiens GN=GNB2L1 PE=1 SV=3 |

|  | **Query** | **Observed** | **Mr(expt)** | **Mr(calc)** | **Delta** | **Miss** | **Score** | **Expect** | **Rank** | **Peptide** |
| --- | --- | --- | --- | --- | --- | --- | --- | --- | --- | --- |
|  | 2317 | 414.7564 | 827.4983 | 828.4957 | -0.9973 | 0 | 5 | 55 | 9 | K.IIVDELK.Q |
|  | 4993 | **513.7482** | **1025.4819** | **1024.4681** | **1.0138** | **0** | **0** | **1.5e+002** | **7** | **-.MTEQMTLR.G** |
|  | 8694 | **633.3361** | **1264.6577** | **1263.6459** | **1.0118** | **0** | **38** | **0.023** | **1** | **R.LWDLTTGTTTR.R** |
|  | 9518 | **655.8199** | **1309.6252** | **1308.6310** | **0.9942** | **0** | **18** | **2.3** | **1** | **K.DVLSVAFSSDNR.Q** |
|  | 11321 | **705.3192** | **1408.6239** | **1407.7214** | **0.9025** | **1** | **(1)** | **91** | **8** | **-.MTEQMTLRGTLK.G** |
|  | 11584 | **475.2288** | **1422.6645** | **1423.7163** | **-1.0518** | **1** | **6** | **36** | **6** | **-.MTEQMTLRGTLK.G** |
|  | 12449 | **738.8387** | **1475.6628** | **1475.6715** | **-0.0087** | **0** | **30** | **0.14** | **1** | **K.DGQAMLWDLNEGK.H** |
|  | 17226 | **894.9957** | **1787.9768** | **1787.9880** | **-0.0112** | **1** | **85** | **3.6e-007** | **1** | **K.IIVDELKQEVISTSSK.A** 17224 17225 |
|  | 18750 | **954.4449** | **1906.8753** | **1906.8884** | **-0.0130** | **0** | **57** | **0.0002** | **1** | **R.FSPNSSNPIIVSCGWDK.L** 18751 |
|  | 19589 | **661.6251** | **1981.8534** | **1980.8636** | **0.9898** | **0** | **2** | **56** | **4** | **K.YTVQDESHSEWVSCVR.F** |
|  | 24960 | **876.7626** | **2627.2659** | **2626.2962** | **0.9697** | **0** | **10** | **6.6** | **1** | **K.GHNGWVTQIATTPQFPDMILSASR.D** |
|  | 25571 | **915.1024** | **2742.2853** | **2742.3032** | **-0.0179** | **0** | **58** | **9.3e-005** | **1** | **K.TNHIGHTGYLNTVTVSPDGSLCASGGK.D** 25569 25570 |
|  | 29387 | 1244.8761 | 3731.6065 | 3732.7664 | -1.1600 | 1 | 2 | 21 | 4 | K.DGQAMLWDLNEGKHLYTLDGGDIINALCFSPNR.Y |

| **198.** | P27105\|STOM_HUMAN    **Mass:** 31882    **Score:** 253    **Queries matched:** 17   **emPAI:** 0.57 |
| --- | --- |
|  | Erythrocyte band 7 integral membrane protein OS=Homo sapiens GN=STOM PE=1 SV=3 |

|  | **Query** | **Observed** | **Mr(expt)** | **Mr(calc)** | **Delta** | **Miss** | **Score** | **Expect** | **Rank** | **Peptide** |
| --- | --- | --- | --- | --- | --- | --- | --- | --- | --- | --- |
|  | 1538 | **686.4136** | **685.4064** | **685.4123** | **-0.0059** | **0** | **28** | **0.36** | **1** | **R.ILQGGAK.G** |
|  | 3473 | **915.5566** | **914.5493** | **914.5549** | **-0.0056** | **0** | **18** | **3** | **1** | **R.LLAQTTLR.N** 3480 |
|  | 5938 | **550.3113** | **1098.6080** | **1098.5166** | **0.0914** | **1** | **8** | **26** | **9** | **R.HTRDSEAQR.L** 5934 |
|  | 7839 | **407.8856** | **1220.6349** | **1219.6085** | **1.0264** | **1** | **6** | **37** | **10** | **R.LPDSFKDSPSK.G** |
|  | 8357 | **624.3015** | **1246.5883** | **1246.5975** | **-0.0092** | **0** | **46** | **0.0038** | **1** | **K.VIAAEGEMNASR.A** |
|  | 8663 | **632.3147** | **1262.6148** | **1262.5925** | **0.0224** | **0** | **(3)** | **80** | **4** | **K.VIAAEGEMNASR.A** |
|  | 10321 | **676.8428** | **1351.6711** | **1350.7395** | **0.9317** | **0** | **1** | **1.2e+002** | **8** | **R.YLQTLTTIAAEK.N** |
|  | 19010 | **965.4967** | **1928.9788** | **1928.9915** | **-0.0127** | **0** | **121** | **8.4e-011** | **1** | **R.VQNATLAVANITNADSATR.L** 19011 |
|  | 20158 | 1014.5367 | 2027.0589 | 2027.1085 | -0.0495 | 1 | 1 | 68 | 3 | R.ALKEASMVITESPAALQLR.Y 20160 |
|  | 21172 | **1064.0931** | **2126.1717** | **2126.1809** | **-0.0092** | **0** | **56** | **0.0002** | **1** | **K.NSTIVFPLPIDMLQGIIGAK.H** 21173 |
|  | 25162 | **669.3532** | **2673.3837** | **2673.4312** | **-0.0475** | **1** | **0** | **54** | **5** | **K.NSTIVFPLPIDMLQGIIGAKHSHLG.-** |
|  | 28466 | **1124.5388** | **3370.5946** | **3370.6099** | **-0.0153** | **1** | **55** | **0.00012** | **1** | **K.NLSQILSDREEIAHNMQSTLDDATDAWGIK.V** |

| **199.** | P23284\|PPIB_HUMAN    **Mass:** 23785    **Score:** 252    **Queries matched:** 25   **emPAI:** 3.43 |
| --- | --- |
|  | Peptidyl-prolyl cis-trans isomerase B OS=Homo sapiens GN=PPIB PE=1 SV=2 |

|  | **Query** | **Observed** | **Mr(expt)** | **Mr(calc)** | **Delta** | **Miss** | **Score** | **Expect** | **Rank** | **Peptide** |
| --- | --- | --- | --- | --- | --- | --- | --- | --- | --- | --- |
|  | 1541 | 687.3981 | 686.3908 | 685.3912 | 0.9996 | 0 | 25 | 0.8 | 1 | K.HVVFGK.V |
|  | 1813 | **395.7058** | **789.3971** | **789.4021** | **-0.0050** | **0** | **12** | **13** | **3** | **K.TAWLDGK.H** |
|  | 2110 | 406.2186 | 810.4226 | 811.4228 | -1.0002 | 0 | 11 | 9.9 | 3 | K.VYFDLR.I |
|  | 5066 | **516.2780** | **1030.5413** | **1030.5481** | **-0.0068** | **0** | **66** | **5e-005** | **1** | **K.VLEGMEVVR.K** |
|  | 8302 | **622.8616** | **1243.7086** | **1243.7176** | **-0.0090** | **0** | **13** | **7.9** | **1** | **K.IEVEKPFAIAK.E** |
|  | 10514 | 682.8517 | 1363.6888 | 1363.6983 | -0.0095 | 0 | 68 | 2.4e-005 | 1 | K.TVDNFVALATGEK.G 10542 10543 |
|  | 12124 | **729.8535** | **1457.6925** | **1456.7198** | **0.9727** | **0** | **12** | **8.3** | **1** | **K.DTNGSQFFITTVK.T** |
|  | 12410 | **737.8428** | **1473.6710** | **1473.6823** | **-0.0113** | **0** | **59** | **0.00015** | **1** | **K.HYGPGWVSMANAGK.D** 12409 12415 |
|  | 13912 | **786.4212** | **1570.8278** | **1570.8389** | **-0.0110** | **1** | **44** | **0.0047** | **1** | **R.DKPLKDVIIADCGK.I** 13911 |
|  | 14375 | 801.3773 | 1600.7400 | 1600.7522 | -0.0122 | 1 | 45 | 0.0039 | 1 | K.SIYGERFPDENFK.L 14372 14374 |
|  | 14749 | **542.9451** | **1625.8136** | **1625.8236** | **-0.0100** | **1** | **45** | **0.004** | **1** | **R.VIKDFMIQGGDFTR.G** 14748 14750 |
|  | 16171 | **572.6233** | **1714.8480** | **1714.8613** | **-0.0133** | **1** | **43** | **0.0059** | **1** | **K.LKHYGPGWVSMANAGK.D** |
|  | 16266 | **862.0104** | **1722.0063** | **1720.9148** | **1.0915** | **1** | **38** | **0.017** | **1** | **R.IGDEDVGRVIFGLFGK.T** 16265 |
|  | 18097 | 465.2646 | 1857.0294 | 1857.8316 | -0.8022 | 1 | 10 | 10 | 5 | K.DFMIQGGDFTRGDGTGGK.S 18106 |

| **200.** | P50225\|ST1A1_HUMAN    **Mass:** 34289    **Score:** 250    **Queries matched:** 15   **emPAI:** 0.68 |
| --- | --- |
|  | Sulfotransferase 1A1 OS=Homo sapiens GN=SULT1A1 PE=1 SV=2 |

|  | **Query** | **Observed** | **Mr(expt)** | **Mr(calc)** | **Delta** | **Miss** | **Score** | **Expect** | **Rank** | **Peptide** |
| --- | --- | --- | --- | --- | --- | --- | --- | --- | --- | --- |
|  | 1592 | **707.2511** | **706.2438** | **705.4174** | **0.8265** | **0** | **15** | **5.8** | **3** | **K.VVYVAR.N** |
|  | 3357 | 454.2142 | 906.4139 | 907.4222 | -1.0083 | 1 | 12 | 12 | 3 | R.KGMAGDWK.T 3387 |
|  | 3693 | **466.7707** | **931.5268** | **932.5807** | **-1.0539** | **1** | **16** | **4.4** | **2** | **K.VKVVYVAR.N** |
|  | 6817 | **584.3174** | **1166.6203** | **1165.5728** | **1.0476** | **0** | **3** | **68** | **7** | **K.TTFTVAQNER.F** |
|  | 7083 | **396.1761** | **1185.5065** | **1186.5852** | **-1.0786** | **1** | **2** | **91** | **10** | **K.CHRAPIFMR.V** |
|  | 15004 | **821.3939** | **1640.7733** | **1640.7835** | **-0.0102** | **0** | **19** | **1.3** | **1** | **K.VHPEPGTWDSFLEK.F** |
|  | 16405 | **865.8974** | **1729.7802** | **1729.7923** | **-0.0120** | **0** | **50** | **0.001** | **1** | **K.DVAVSYYHFYHMAK.V** 16404 |
|  | 17366 | **901.0276** | **1800.0406** | **1800.0509** | **-0.0103** | **0** | **53** | **0.0005** | **1** | **K.THLPLALLPQTLLDQK.V** |
|  | 19060 | 645.3392 | 1932.9959 | 1933.9819 | -0.9860 | 0 | 1 | 74 | 9 | -.MELIQDTSRPPLEYVK.G |
|  | 23001 | **1171.0669** | **2340.1192** | **2340.1308** | **-0.0115** | **0** | **102** | **4.3e-009** | **1** | **K.SGTTWVSQILDMIYQGGDLEK.C** 23000 23002 |
|  | 26741 | **980.8369** | **2939.4889** | **2939.5069** | **-0.0180** | **0** | **18** | **0.78** | **1** | **K.YFAEALGPLQSFQARPDDLLISTYPK.S** |

| **201.** | P50226\|ST1A2_HUMAN    **Mass:** 34377    **Score:** 250    **Queries matched:** 17   **emPAI:** 0.68 |
| --- | --- |
|  | Sulfotransferase 1A2 OS=Homo sapiens GN=SULT1A2 PE=1 SV=1 |

|  | **Query** | **Observed** | **Mr(expt)** | **Mr(calc)** | **Delta** | **Miss** | **Score** | **Expect** | **Rank** | **Peptide** |
| --- | --- | --- | --- | --- | --- | --- | --- | --- | --- | --- |
|  | 1592 | 707.2511 | 706.2438 | 705.4174 | 0.8265 | 0 | 15 | 5.8 | 3 | K.VVYVAR.N |
|  | 3357 | 454.2142 | 906.4139 | 907.4222 | -1.0083 | 1 | 12 | 12 | 3 | R.KGMAGDWK.T 3387 |
|  | 3693 | 466.7707 | 931.5268 | 932.5807 | -1.0539 | 1 | 16 | 4.4 | 2 | K.VKVVYVAR.N |
|  | 6817 | 584.3174 | 1166.6203 | 1165.5728 | 1.0476 | 0 | 3 | 68 | 7 | K.TTFTVAQNER.F |
|  | 7083 | 396.1761 | 1185.5065 | 1186.5852 | -1.0786 | 1 | 2 | 91 | 10 | K.CHRAPIFMR.V |
|  | 13023 | **505.2591** | **1512.7554** | **1511.6537** | **1.1017** | **0** | **6** | **32** | **4** | **R.EFMDHSISPFMR.K** |
|  | 13024 | **757.3860** | **1512.7575** | **1511.6537** | **1.1038** | **0** | **(2)** | **79** | **1** | **R.EFMDHSISPFMR.K** |
|  | 16405 | 865.8974 | 1729.7802 | 1729.7923 | -0.0120 | 0 | 50 | 0.001 | 1 | K.DVAVSYYHFYHMAK.V 16404 |
|  | 17366 | 901.0276 | 1800.0406 | 1800.0509 | -0.0103 | 0 | 53 | 0.0005 | 1 | K.THLPLALLPQTLLDQK.V |
|  | 19060 | 645.3392 | 1932.9959 | 1933.9819 | -0.9860 | 0 | 1 | 74 | 9 | -.MELIQDTSRPPLEYVK.G |
|  | 20983 | 1053.0438 | 2104.0731 | 2104.1279 | -0.0548 | 1 | 7 | 17 | 3 | R.VPFLEFKVPGIPSGMETLK.N |
|  | 23001 | 1171.0669 | 2340.1192 | 2340.1308 | -0.0115 | 0 | 102 | 4.3e-009 | 1 | K.SGTTWVSQILDMIYQGGDLEK.C 23000 23002 |
|  | 26741 | 980.8369 | 2939.4889 | 2939.5069 | -0.0180 | 0 | 18 | 0.78 | 1 | K.YFAEALGPLQSFQARPDDLLISTYPK.S |

| **202.** | O75947\|ATP5H_HUMAN    **Mass:** 18537    **Score:** 249    **Queries matched:** 19   **emPAI:** 2.11 |
| --- | --- |
|  | ATP synthase subunit d, mitochondrial OS=Homo sapiens GN=ATP5H PE=1 SV=3 |

|  | **Query** | **Observed** | **Mr(expt)** | **Mr(calc)** | **Delta** | **Miss** | **Score** | **Expect** | **Rank** | **Peptide** |
| --- | --- | --- | --- | --- | --- | --- | --- | --- | --- | --- |
|  | 1163 | **572.4088** | **571.4015** | **571.4057** | **-0.0042** | **1** | **26** | **0.52** | **1** | **R.KLALK.T** |
|  | 1554 | **689.4148** | **688.4075** | **688.4119** | **-0.0044** | **0** | **20** | **2.4** | **1** | **K.AIASSLK.S** |
|  | 1635 | 721.3285 | 720.3212 | 719.4330 | 0.8882 | 1 | 15 | 5.4 | 3 | K.KFNALK.V 1638 |
|  | 5842 | **547.2637** | **1092.5128** | **1092.5200** | **-0.0072** | **0** | **21** | **1.2** | **1** | **K.SWNETLTSR.L** |
|  | 6221 | 561.2913 | 1120.5680 | 1120.5764 | -0.0085 | 1 | 35 | 0.052 | 1 | K.AGLVDDFEKK.F |
|  | 9270 | 649.3165 | 1296.6184 | 1296.6271 | -0.0087 | 1 | 38 | 0.024 | 1 | R.IVEYEKEMEK.M |
|  | 13694 | 778.3895 | 1554.7645 | 1555.7459 | -0.9814 | 0 | 19 | 1.5 | 2 | K.YPYWPHQPIENL.- |
|  | 18323 | **936.9913** | **1871.9680** | **1871.9781** | **-0.0101** | **0** | **54** | **0.00039** | **1** | **K.TIDWVAFAEIIPQNQK.A** 18324 |
|  | 19038 | **966.4924** | **1930.9702** | **1930.9828** | **-0.0127** | **0** | **75** | **3e-006** | **1** | **R.LAALPENPPAIDWAYYK.A** 19039 19040 19050 19051 |
|  | 23904 | **1233.0925** | **2464.1705** | **2464.1832** | **-0.0127** | **0** | **81** | **5.8e-007** | **1** | **K.NLIPFDQMTIEDLNEAFPETK.L** 23903 23905 23906 |

| **203.** | P09104\|ENOG_HUMAN    **Mass:** 47581    **Score:** 248    **Queries matched:** 10   **emPAI:** 0.08 |
| --- | --- |
|  | Gamma-enolase OS=Homo sapiens GN=ENO2 PE=1 SV=3 |

|  | **Query** | **Observed** | **Mr(expt)** | **Mr(calc)** | **Delta** | **Miss** | **Score** | **Expect** | **Rank** | **Peptide** |
| --- | --- | --- | --- | --- | --- | --- | --- | --- | --- | --- |
|  | 4251 | **487.2736** | **972.5326** | **972.5240** | **0.0087** | **1** | **6** | **47** | **7** | **R.IERAVEEK.A** |
|  | 5088 | 517.2529 | 1032.4913 | 1032.4771 | 0.0142 | 1 | 7 | 38 | 8 | K.TGAPCRSER.L |
|  | 6596 | 576.3020 | 1150.5894 | 1151.6121 | -1.0226 | 1 | 4 | 59 | 8 | R.LAKYNQLMR.I 6646 |
|  | 10436 | **680.3564** | **1358.6982** | **1357.6891** | **1.0091** | **1** | **2** | **95** | **3** | **R.FAGHNFRNPSVL.-** |
|  | 17411 | 902.9698 | 1803.9250 | 1803.9366 | -0.0116 | 0 | 109 | 1.3e-009 | 1 | R.AAVPSGASTGIYEALELR.D 17410 17412 |
|  | 19472 | **657.2788** | **1968.8146** | **1969.9641** | **-1.1495** | **0** | **2** | **52** | **1** | **K.LAMQEFMILPVGAESFR.D** |
|  | 22474 | 760.3239 | 2277.9499 | 2277.2117 | 0.7382 | 1 | 5 | 24 | 5 | K.GLFRAAVPSGASTGIYEALELR.D |

| **204.** | P02656\|APOC3_HUMAN    **Mass:** 10846    **Score:** 247    **Queries matched:** 8   **emPAI:** 0.87 |
| --- | --- |
|  | Apolipoprotein C-III OS=Homo sapiens GN=APOC3 PE=1 SV=1 |

|  | **Query** | **Observed** | **Mr(expt)** | **Mr(calc)** | **Delta** | **Miss** | **Score** | **Expect** | **Rank** | **Peptide** |
| --- | --- | --- | --- | --- | --- | --- | --- | --- | --- | --- |
|  | 7317 | **598.7977** | **1195.5809** | **1195.5874** | **-0.0064** | **0** | **5** | **49** | **6** | **R.GWVTDGFSSLK.D** |
|  | 16170 | **858.4113** | **1714.8081** | **1715.8438** | **-1.0357** | **0** | **93** | **5.4e-008** | **1** | **K.DALSSVQESQVAQQAR.G** 16189 16190 |
|  | 19912 | **670.2897** | **2007.8472** | **2008.8758** | **-1.0286** | **0** | **5** | **28** | **1** | **R.ASEAEDASLLSFMQGYMK.H** |
|  | 20716 | **692.6700** | **2074.9881** | **2075.0000** | **-0.0119** | **1** | **1** | **65** | **2** | **R.GWVTDGFSSLKDYWSTVK.D** |
|  | 23313 | **1190.5698** | **2379.1251** | **2379.1383** | **-0.0132** | **1** | **56** | **0.00018** | **1** | **K.DKFSEFWDLDPEVRPTSAVAA.-** |
|  | 28820 | **875.2194** | **3496.8486** | **3495.8720** | **0.9765** | **1** | **2** | **22** | **2** | **R.VLLVVALLALLASARASEAEDASLLSFMQGYMK.H** |

| **205.** | Q00610\|CLH1_HUMAN    **Mass:** 193260   **Score:** 247    **Queries matched:** 42   **emPAI:** 0.21 |
| --- | --- |
|  | Clathrin heavy chain 1 OS=Homo sapiens GN=CLTC PE=1 SV=5 |

|  | **Query** | **Observed** | **Mr(expt)** | **Mr(calc)** | **Delta** | **Miss** | **Score** | **Expect** | **Rank** | **Peptide** |
| --- | --- | --- | --- | --- | --- | --- | --- | --- | --- | --- |
|  | 2179 | 409.7104 | 817.4063 | 817.4334 | -0.0271 | 1 | 6 | 50 | 6 | K.WLKEDK.L |
|  | 2402 | **418.2182** | **834.4218** | **833.5374** | **0.8843** | **1** | **4** | **81** | **10** | **K.IVLYAKK.V** |
|  | 3584 | **462.8864** | **923.7582** | **923.4535** | **0.3047** | **0** | **10** | **9.6** | **3** | **R.VMEYINR.L** |
|  | 4682 | **501.7809** | **1001.5473** | **1001.5692** | **-0.0219** | **1** | **19** | **2.1** | **2** | **K.AQLQKGMVK.E** |
|  | 5215 | **522.2344** | **1042.4542** | **1042.4356** | **0.0186** | **0** | **4** | **74** | **5** | **R.ENPYYDSR.V** |
|  | 5535 | **535.2725** | **1068.5305** | **1068.5386** | **-0.0081** | **0** | **19** | **2** | **1** | **R.AHIAQLCEK.A** 5536 |
|  | 8061 | **617.2866** | **1232.5586** | **1231.6408** | **0.9178** | **1** | **0** | **1.5e+002** | **9** | **K.VDKLDASESLR.K** |
|  | 8693 | **422.5577** | **1264.6511** | **1265.6880** | **-1.0369** | **1** | **4** | **53** | **7** | **R.IAAYLFKGNNR.W** |
|  | 9392 | **435.2326** | **1302.6759** | **1303.6520** | **-0.9761** | **0** | **6** | **36** | **5** | **R.NNLAGAEELFAR.K** |
|  | 9996 | 667.8162 | 1333.6178 | 1333.6262 | -0.0084 | 0 | 29 | 0.18 | 1 | K.IYIDSNNNPER.F |
|  | 10306 | **451.2627** | **1350.7662** | **1350.8387** | **-0.0725** | **1** | **7** | **31** | **5** | **R.LKLLLPWLEAR.I** |
|  | 10607 | **684.8478** | **1367.6810** | **1367.6906** | **-0.0095** | **0** | **34** | **0.049** | **1** | **K.NNRPSEGPLQTR.L** |
|  | 10648 | **685.8314** | **1369.6483** | **1368.6959** | **0.9524** | **1** | **5** | **39** | **2** | **K.GMVKEAIDSYIK.A** 10649 |
|  | 12315 | **490.2948** | **1467.8625** | **1466.7803** | **1.0822** | **1** | **7** | **22** | **3** | **K.TLQIFNIEMKSK.M** 12290 |
|  | 12935 | **503.1055** | **1506.2948** | **1506.7501** | **-0.4553** | **0** | **2** | **52** | **9** | **K.VIQCFAETGQVQK.I** |
|  | 13941 | 787.4152 | 1572.8158 | 1572.7930 | 0.0228 | 0 | 6 | 35 | 3 | R.RPISADSAIMNPASK.V |
|  | 14465 | **536.6331** | **1606.8774** | **1606.8871** | **-0.0098** | **1** | **28** | **0.17** | **1** | **K.KVGYTPDWIFLLR.N** |
|  | 14489 | 537.2990 | 1608.8752 | 1607.8671 | 1.0081 | 1 | 1 | 91 | 9 | K.YIEIYVQKVNPSR.L |
|  | 14662 | **540.9473** | **1619.8202** | **1619.8307** | **-0.0106** | **1** | **36** | **0.029** | **1** | **R.ALEHFTDLYDIKR.A** |
|  | 16008 | **853.9128** | **1705.8111** | **1706.8475** | **-1.0364** | **1** | **5** | **35** | **8** | **R.GQFSTDELVAEVEKR.N** |
|  | 16833 | **879.9381** | **1757.8617** | **1757.8736** | **-0.0120** | **1** | **63** | **5.7e-005** | **1** | **R.KFNALFAQGNYSEAAK.V** |
|  | 17028 | **592.9452** | **1775.8139** | **1775.8260** | **-0.0121** | **0** | **27** | **0.22** | **1** | **R.IHEGCEEPATHNALAK.I** |
|  | 17907 | **615.6343** | **1843.8812** | **1843.9581** | **-0.0769** | **1** | **6** | **24** | **2** | **R.KVSQPIEGHAASFAQFK.M** |
|  | 18377 | 939.4597 | 1876.9049 | 1875.8131 | 1.0918 | 0 | 2 | 59 | 2 | K.MEGNAEESTLFCFAVR.G |
|  | 19499 | **658.0116** | **1971.0130** | **1970.0221** | **0.9908** | **0** | **30** | **0.1** | **1** | **R.LASTLVHLGEYQAAVDGAR.K** |
|  | 19635 | **993.4941** | **1984.9737** | **1983.9409** | **1.0328** | **0** | **38** | **0.014** | **1** | **R.HNIMDFAMPYFIQVMK.E** |
|  | 20928 | **1049.5436** | **2097.0726** | **2097.1616** | **-0.0890** | **1** | **1** | **74** | **5** | **R.RPISADSAIMNPASKVIALK.A** |
|  | 22466 | **759.7147** | **2276.1223** | **2276.1324** | **-0.0101** | **1** | **13** | **4.3** | **1** | **K.DTELAEELLQWFLQEEKR.E** |
|  | 23084 | **785.0837** | **2352.2292** | **2352.2438** | **-0.0145** | **0** | **12** | **4.1** | **1** | **R.ISGETIFVTAPHEATAGIIGVNR.K** |
|  | 23098 | **1178.0696** | **2354.1246** | **2354.1390** | **-0.0144** | **0** | **110** | **7.3e-010** | **1** | **K.SVNESLNNLFITEEDYQALR.T** |
|  | 23209 | **790.0872** | **2367.2398** | **2367.2547** | **-0.0148** | **1** | **74** | **3.2e-006** | **1** | **R.KFDVNTSAVQVLIEHIGNLDR.A** |
|  | 23708 | **815.0696** | **2442.1871** | **2442.3020** | **-0.1149** | **1** | **0** | **70** | **7** | **R.GQAGGKLHIIEVGTPPTGNQPFPK.K** |
|  | 25376 | 902.9741 | 2705.9004 | 2706.3363 | -0.4360 | 1 | 3 | 23 | 2 | K.KAVDVFFPPEAQNDFPVAMQISEK.H |
|  | 27700 | 1049.5269 | 3145.5588 | 3144.5008 | 1.0579 | 0 | 4 | 19 | 2 | R.LAQMCGLHIVVHADELEELINYYQDR.G |
|  | 27804 | **1058.8519** | **3173.5340** | **3173.5492** | **-0.0152** | **0** | **29** | **0.056** | **1** | **K.WISLNTVALVTDNAVYHWSMEGESQPVK.M** |
|  | 27874 | **1064.5548** | **3190.6426** | **3189.5441** | **1.0985** | **0** | **(0)** | **41** | **2** | **K.WISLNTVALVTDNAVYHWSMEGESQPVK.M** |
|  | 28861 | 880.6561 | 3518.5952 | 3517.6129 | 0.9822 | 1 | 1 | 28 | 2 | K.ADDPSSYMEVVQAANTSGNWEELVKYLQMAR.K |
|  | 29357 | **931.9614** | **3723.8163** | **3722.7861** | **1.0302** | **1** | **1** | **29** | **8** | **K.WISLNTVALVTDNAVYHWSMEGESQPVKMFDR.H** |
|  | 30842 | 1107.5447 | 4426.1496 | 4426.0714 | 0.0782 | 1 | 0 | 18 | 6 | R.AAEQAHLWAELVFLYDKYEEYDNAIITMMNHPTDAWK.E |

| **206.** | P48735\|IDHP_HUMAN    **Mass:** 51333    **Score:** 247    **Queries matched:** 40   **emPAI:** 0.64 |
| --- | --- |
|  | Isocitrate dehydrogenase [NADP], mitochondrial OS=Homo sapiens GN=IDH2 PE=1 SV=2 |

|  | **Query** | **Observed** | **Mr(expt)** | **Mr(calc)** | **Delta** | **Miss** | **Score** | **Expect** | **Rank** | **Peptide** |
| --- | --- | --- | --- | --- | --- | --- | --- | --- | --- | --- |
|  | 1195 | **580.3173** | **579.3100** | **578.3176** | **0.9924** | **0** | **12** | **12** | **1** | **M.AGYLR.V** |
|  | 1228 | 588.3668 | 587.3595 | 587.3642 | -0.0047 | 0 | 33 | 0.15 | 1 | K.NTILK.A |
|  | 2815 | **866.4924** | **865.4851** | **865.4368** | **0.0483** | **0** | **15** | **5.3** | **3** | **R.FAQMLEK.V** |
|  | 3173 | **447.7162** | **893.4178** | **893.4243** | **-0.0065** | **0** | **53** | **0.00085** | **1** | **K.ATDFVADR.A** |
|  | 3313 | 452.7103 | 903.4060 | 902.4399 | 0.9662 | 0 | 9 | 23 | 10 | K.IWYEHR.L |
|  | 4303 | 976.5506 | 975.5433 | 975.5502 | -0.0068 | 0 | 25 | 0.64 | 1 | R.NILGGTVFR.E 4304 4305 |
|  | 4670 | **501.3112** | **1000.6078** | **1000.5414** | **0.0665** | **1** | **5** | **62** | **2** | **K.SNLDRALGR.Q** |
|  | 4973 | **513.2572** | **1024.4998** | **1024.5052** | **-0.0053** | **0** | **7** | **30** | **3** | **K.WPLYMSTK.N** |
|  | 6642 | 577.3176 | 1152.6207 | 1153.5655 | -0.9448 | 0 | 4 | 61 | 6 | K.DIFQEIFDK.H |
|  | 7932 | **613.8235** | **1225.6324** | **1225.6529** | **-0.0205** | **1** | **5** | **41** | **8** | **R.AGTFKMVFTPK.D** |
|  | 7976 | **614.8311** | **1227.6475** | **1227.6571** | **-0.0096** | **1** | **21** | **1.3** | **1** | **R.GKLDGNQDLIR.F** 7946 7961 |
|  | 8220 | **620.8170** | **1239.6195** | **1238.6805** | **0.9390** | **1** | **3** | **62** | **9** | **R.EPIICKNIPR.L** |
|  | 8300 | **622.8454** | **1243.6762** | **1243.6846** | **-0.0084** | **0** | **57** | **0.00027** | **1** | **R.LIDDMVAQVLK.S** 8299 |
|  | 8569 | **630.2997** | **1258.5848** | **1259.6795** | **-1.0947** | **0** | **(8)** | **23** | **2** | **R.LIDDMVAQVLK.S** |
|  | 10875 | **692.3502** | **1382.6858** | **1382.6976** | **-0.0119** | **0** | **59** | **0.00019** | **1** | **K.DLAGCIHGLSNVK.L** |
|  | 11359 | **706.2960** | **1410.5775** | **1409.6531** | **0.9244** | **0** | **50** | **0.0013** | **1** | **K.VCVETVESGAMTK.D** 11340 11345 |
|  | 11671 | 715.3706 | 1428.7267 | 1428.7289 | -0.0022 | 1 | 6 | 33 | 9 | R.FKDIFQEIFDK.H |
|  | 11787 | **479.7668** | **1436.2785** | **1436.8504** | **-0.5719** | **0** | **5** | **28** | **3** | **R.LVPGWTKPITIGR.H** |
|  | 13521 | 772.8860 | 1543.7575 | 1544.9290 | -1.1714 | 1 | 3 | 62 | 6 | K.EKLILPHVDIQLK.Y |
|  | 15518 | **838.8968** | **1675.7790** | **1675.7909** | **-0.0119** | **0** | **42** | **0.0068** | **1** | **K.VAKPVVEMDGDEMTR.I** 15519 15520 |
|  | 16054 | 854.9484 | 1707.8822 | 1707.7808 | 0.1014 | 0 | (6) | 29 | 4 | K.VAKPVVEMDGDEMTR.I 16055 |
|  | 16193 | **858.9717** | **1715.9289** | **1715.9393** | **-0.0103** | **1** | **17** | **2.4** | **2** | **R.NILGGTVFREPIICK.N** |
|  | 18364 | **938.4933** | **1874.9721** | **1873.9799** | **0.9923** | **0** | **24** | **0.41** | **1** | **K.GRPTSTNPIASIFAWTR.G** 18357 |
|  | 18886 | **960.9828** | **1919.9511** | **1919.9629** | **-0.0117** | **0** | **69** | **1.3e-005** | **1** | **K.LNEHFLNTTDFLDTIK.S** 18900 |
|  | 19251 | **650.9836** | **1949.9291** | **1948.9598** | **0.9693** | **1** | **6** | **24** | **1** | **R.IKVAKPVVEMDGDEMTR.I** 19243 |
|  | 23001 | 1171.0669 | 2340.1192 | 2339.1654 | 0.9538 | 1 | 0 | 71 | 7 | R.LIDDMVAQVLKSSGGFVWACK.N |
|  | 24995 | 1319.1799 | 2636.3453 | 2636.3342 | 0.0111 | 1 | 1 | 55 | 4 | K.VAKPVVEMDGDEMTRIIWQFIK.E |

| **207.** | P53621\|COPA_HUMAN    **Mass:** 139797   **Score:** 246    **Queries matched:** 41   **emPAI:** 0.26 |
| --- | --- |
|  | Coatomer subunit alpha OS=Homo sapiens GN=COPA PE=1 SV=2 |

|  | **Query** | **Observed** | **Mr(expt)** | **Mr(calc)** | **Delta** | **Miss** | **Score** | **Expect** | **Rank** | **Peptide** |
| --- | --- | --- | --- | --- | --- | --- | --- | --- | --- | --- |
|  | 2069 | **405.0413** | **808.0680** | **807.4239** | **0.6441** | **0** | **9** | **14** | **1** | **R.TGVQTFR.R** |
|  | 2207 | **820.4719** | **819.4646** | **819.4160** | **0.0486** | **0** | **6** | **45** | **3** | **K.ILSACEK.N** |
|  | 2718 | **861.1348** | **860.1275** | **859.4916** | **0.6359** | **0** | **6** | **41** | **1** | **R.ISPLQFR.-** |
|  | 3885 | **473.7682** | **945.5219** | **945.5859** | **-0.0640** | **1** | **11** | **14** | **3** | **R.TLASVKISK.V** |
|  | 4064 | 480.2765 | 958.5384 | 958.5447 | -0.0063 | 1 | 30 | 0.19 | 1 | K.NLKNEITK.K 4063 |
|  | 4099 | **481.7411** | **961.4676** | **961.4730** | **-0.0054** | **0** | **20** | **1.9** | **1** | **K.HVLEGHDR.G** |
|  | 4276 | **488.2400** | **974.4654** | **974.4722** | **-0.0069** | **0** | **19** | **2.3** | **1** | **R.VWNWQSR.T** |
|  | 4815 | **506.7713** | **1011.5281** | **1012.5263** | **-0.9982** | **1** | **14** | **5.5** | **4** | **-.MLTKFETK.S** 4816 |
|  | 4834 | **507.3274** | **1012.6402** | **1011.5865** | **1.0537** | **1** | **1** | **1.2e+002** | **2** | **R.VKGLSFHPK.R** |
|  | 7624 | **606.3003** | **1210.5861** | **1211.7060** | **-1.1198** | **1** | **2** | **80** | **9** | **R.MHSLLIKNLK.N** |
|  | 8214 | 620.8046 | 1239.5947 | 1240.6452 | -1.0505 | 1 | 2 | 82 | 10 | K.GFFEGTIASKGK.G |
|  | 8922 | **639.7882** | **1277.5619** | **1277.5710** | **-0.0091** | **1** | **13** | **6.6** | **1** | **K.ALDDKNCWEK.L** |
|  | 10070 | **669.8324** | **1337.6502** | **1336.6558** | **0.9945** | **1** | **42** | **0.0088** | **1** | **R.VKGNNVYCLDR.E** 10052 |
|  | 10072 | **446.9020** | **1337.6841** | **1337.7667** | **-0.0826** | **1** | **3** | **69** | **9** | **K.DAGLKNGVPAVGLK.L** |
|  | 10311 | **451.2696** | **1350.7869** | **1351.7361** | **-0.9491** | **1** | **6** | **34** | **3** | **K.QLFLQTYARGR.T** |
|  | 12829 | **500.9127** | **1499.7162** | **1498.6648** | **1.0514** | **1** | **5** | **44** | **1** | **K.DADSQNPDAPEGKR.S** |
|  | 13023 | 505.2591 | 1512.7554 | 1512.8776 | -0.1222 | 1 | 8 | 22 | 2 | K.DVIGLRISPLQFR.- |
|  | 14368 | **534.3104** | **1599.9094** | **1599.9196** | **-0.0102** | **1** | **9** | **13** | **2** | **R.VTTVTEIGKDVIGLR.I** |
|  | 15250 | **553.2562** | **1656.7469** | **1656.7579** | **-0.0111** | **0** | **25** | **0.32** | **1** | **R.GHYNNVSCAVFHPR.Q** |
|  | 15558 | **560.3170** | **1677.9292** | **1677.9413** | **-0.0121** | **0** | **50** | **0.0012** | **1** | **R.LLELGPKPEVAQQTR.K** |
|  | 16003 | **853.7794** | **1705.5442** | **1705.9839** | **-0.4397** | **1** | **3** | **35** | **1** | **K.NGVPAVGLKLNDLIQR.L** |
|  | 17438 | **903.9670** | **1805.9195** | **1806.0363** | **-0.1168** | **1** | **3** | **52** | **3** | **R.LLELGPKPEVAQQTRK.I** |
|  | 17501 | **906.9549** | **1811.8952** | **1812.0873** | **-0.1920** | **1** | **4** | **45** | **4** | **K.FRSILLSVPLLVVDNK.Q** |
|  | 17704 | **914.9460** | **1827.8774** | **1827.8890** | **-0.0116** | **0** | **91** | **7.4e-008** | **1** | **R.ASNLENSTYDLYTIPK.D** 17685 |
|  | 18248 | **934.4620** | **1866.9095** | **1865.9305** | **0.9790** | **1** | **6** | **25** | **6** | **R.QLDFNSSKDVAVMQLR.S** |
|  | 18461 | **629.2855** | **1884.8347** | **1883.9464** | **0.8883** | **0** | **21** | **0.83** | **1** | **R.ERPAYAVHGNMLHYVK.D** |
|  | 19229 | **650.4381** | **1948.2925** | **1947.8819** | **0.4106** | **1** | **5** | **22** | **2** | **R.MCTLIDKFDEHDGPVR.G** |
|  | 19973 | 672.3741 | 2014.1006 | 2014.0775 | 0.0231 | 1 | 11 | 6.8 | 1 | K.NFDKLSFLYLITGNLEK.L |
|  | 21048 | **704.6677** | **2110.9813** | **2110.9960** | **-0.0147** | **0** | **33** | **0.048** | **1** | **K.SGAWDESGVFIYTTSNHIK.Y** |
|  | 22313 | **1128.0526** | **2254.0907** | **2253.1001** | **0.9906** | **0** | **52** | **0.00048** | **1** | **R.GVNWAAFHPTMPLIVSGADDR.Q** 22310 |
|  | 22475 | **1140.0706** | **2278.1266** | **2278.1416** | **-0.0150** | **0** | **69** | **1.1e-005** | **1** | **K.VQVPNCDEIFYAGTGNLLLR.D** |
|  | 23746 | **1223.1803** | **2444.3460** | **2443.3549** | **0.9912** | **0** | **8** | **11** | **1** | **K.LLQPPAPIMPLDTNWPLLTVSK.G** |
|  | 24947 | 657.3016 | 2625.1775 | 2624.3170 | 0.8605 | 1 | 1 | 50 | 5 | R.GVNWAAFHPTMPLIVSGADDRQVK.I |
|  | 25357 | **901.7689** | **2702.2849** | **2701.2992** | **0.9857** | **1** | **8** | **11** | **1** | **R.SGSKFPVFNMSYNPAENAVLLCTR.A** |
|  | 27662 | **522.9522** | **3131.6696** | **3132.6774** | **-1.0078** | **1** | **3** | **21** | **8** | **K.GLSFHPKRPWILTSLHNGVIQLWDYR.M** |
|  | 30559 | **842.1891** | **4205.9091** | **4205.9397** | **-0.0306** | **1** | **12** | **1.4** | **1** | **K.ILSACEKNPTDAYQLNYDMHNPFDICAASYRPIYR.G** |

| **208.** | P27169\|PON1_HUMAN    **Mass:** 39895    **Score:** 246    **Queries matched:** 8   **emPAI:** 0.43 |
| --- | --- |
|  | Serum paraoxonase/arylesterase 1 OS=Homo sapiens GN=PON1 PE=1 SV=2 |

|  | **Query** | **Observed** | **Mr(expt)** | **Mr(calc)** | **Delta** | **Miss** | **Score** | **Expect** | **Rank** | **Peptide** |
| --- | --- | --- | --- | --- | --- | --- | --- | --- | --- | --- |
|  | 2257 | **823.4509** | **822.4437** | **822.4487** | **-0.0051** | **0** | **26** | **0.43** | **1** | **K.STVELFK.F** |
|  | 14958 | **819.9239** | **1637.8332** | **1637.8447** | **-0.0115** | **0** | **48** | **0.0017** | **1** | **R.EVQPVELPNCNLVK.G** 14957 |
|  | 18442 | **942.4564** | **1882.8983** | **1882.9101** | **-0.0118** | **0** | **65** | **2.9e-005** | **1** | **K.IFFYDSENPPASEVLR.I** |
|  | 23459 | **1200.6255** | **2399.2364** | **2399.2505** | **-0.0141** | **0** | **78** | **1.1e-006** | **1** | **K.ILLMDLNEEDPTVLELGITGSK.F** 23458 |
|  | 28323 | **1110.8464** | **3329.5175** | **3329.5333** | **-0.0158** | **0** | **53** | **0.00019** | **1** | **K.SLDFNTLVDNISVDPETGDLWVGCHPNGMK.I** |
|  | 29035 | **895.9163** | **3579.6362** | **3579.6617** | **-0.0255** | **0** | **8** | **5** | **1** | **K.FDVSSFNPHGISTFTDEDNAMYLLVVNHPDAK.S** |

| **209.** | O14950\|ML12B_HUMAN    **Mass:** 19824    **Score:** 242    **Queries matched:** 16   **emPAI:** 1.03 |
| --- | --- |
|  | Myosin regulatory light chain 12B OS=Homo sapiens GN=MYL12B PE=1 SV=2 |

|  | **Query** | **Observed** | **Mr(expt)** | **Mr(calc)** | **Delta** | **Miss** | **Score** | **Expect** | **Rank** | **Peptide** |
| --- | --- | --- | --- | --- | --- | --- | --- | --- | --- | --- |
|  | 5112 | **517.7648** | **1033.5151** | **1034.5066** | **-0.9915** | **0** | **6** | **38** | **1** | **R.ELLTTMGDR.F** |
|  | 7970 | **614.8079** | **1227.6012** | **1227.6095** | **-0.0084** | **0** | **25** | **0.49** | **1** | **K.LNGTDPEDVIR.N** |
|  | 8474 | **418.7264** | **1253.1572** | **1252.5506** | **0.6066** | **0** | **6** | **24** | **8** | **K.EAFNMIDQNR.D** |
|  | 8595 | 630.8000 | 1259.5854 | 1259.5935 | -0.0080 | 0 | 66 | 3.4e-005 | 1 | K.GNFNYIEFTR.I |
|  | 10985 | **463.9178** | **1388.7315** | **1387.6884** | **1.0430** | **1** | **3** | **70** | **4** | **K.KGNFNYIEFTR.I** |
|  | 11433 | **708.3155** | **1414.6164** | **1414.6252** | **-0.0088** | **0** | **8** | **22** | **2** | **R.FTDEEVDELYR.E** |
|  | 19868 | **1002.4852** | **2002.9558** | **2002.9670** | **-0.0112** | **1** | **92** | **6.4e-008** | **1** | **R.DGFIDKEDLHDMLASLGK.N** 19869 19871 |
|  | 20599 | **690.3575** | **2068.0508** | **2067.9637** | **0.0871** | **1** | **1** | **74** | **3** | **R.FTDEEVDELYREAPIDK.K** |
|  | 20850 | **1045.9896** | **2089.9647** | **2089.9779** | **-0.0132** | **0** | **114** | **3.8e-010** | **1** | **R.ATSNVFAMFDQSQIQEFK.E** 20839 20851 |
|  | 23643 | **1216.5591** | **2431.1036** | **2431.1213** | **-0.0177** | **1** | **41** | **0.0056** | **1** | **R.ELLTTMGDRFTDEEVDELYR.E** 23642 |
|  | 28447 | **1122.8392** | **3365.4959** | **3365.5117** | **-0.0158** | **0** | **4** | **13** | **1** | **K.NPTDAYLDAMMNEAPGPINFTMFLTMFGEK.L** |

| **210.** | P19105\|ML12A_HUMAN    **Mass:** 19839    **Score:** 242    **Queries matched:** 16   **emPAI:** 1.03 |
| --- | --- |
|  | Myosin regulatory light chain 12A OS=Homo sapiens GN=MYL12A PE=1 SV=2 |

|  | **Query** | **Observed** | **Mr(expt)** | **Mr(calc)** | **Delta** | **Miss** | **Score** | **Expect** | **Rank** | **Peptide** |
| --- | --- | --- | --- | --- | --- | --- | --- | --- | --- | --- |
|  | 5112 | 517.7648 | 1033.5151 | 1034.5066 | -0.9915 | 0 | 6 | 38 | 1 | R.ELLTTMGDR.F |
|  | 7970 | 614.8079 | 1227.6012 | 1227.6095 | -0.0084 | 0 | 25 | 0.49 | 1 | K.LNGTDPEDVIR.N |
|  | 8474 | 418.7264 | 1253.1572 | 1252.5506 | 0.6066 | 0 | 6 | 24 | 8 | K.EAFNMIDQNR.D |
|  | 8595 | 630.8000 | 1259.5854 | 1259.5935 | -0.0080 | 0 | 66 | 3.4e-005 | 1 | K.GNFNYIEFTR.I |
|  | 10985 | 463.9178 | 1388.7315 | 1387.6884 | 1.0430 | 1 | 3 | 70 | 4 | K.KGNFNYIEFTR.I |
|  | 11433 | 708.3155 | 1414.6164 | 1414.6252 | -0.0088 | 0 | 8 | 22 | 2 | R.FTDEEVDELYR.E |
|  | 19868 | 1002.4852 | 2002.9558 | 2002.9670 | -0.0112 | 1 | 92 | 6.4e-008 | 1 | R.DGFIDKEDLHDMLASLGK.N 19869 19871 |
|  | 20599 | 690.3575 | 2068.0508 | 2067.9637 | 0.0871 | 1 | 1 | 74 | 3 | R.FTDEEVDELYREAPIDK.K |
|  | 20850 | 1045.9896 | 2089.9647 | 2089.9779 | -0.0132 | 0 | 114 | 3.8e-010 | 1 | R.ATSNVFAMFDQSQIQEFK.E 20839 20851 |
|  | 23643 | 1216.5591 | 2431.1036 | 2431.1213 | -0.0177 | 1 | 41 | 0.0056 | 1 | R.ELLTTMGDRFTDEEVDELYR.E 23642 |
|  | 31173 | **1196.0378** | **4780.1223** | **4781.1355** | **-1.0133** | **1** | **2** | **11** | **3** | **K.EDLHDMLASLGKNPTDEYLDAMMNEAPGPINFTMFLTMFGEK.L** |

| **211.** | P30048\|PRDX3_HUMAN    **Mass:** 28017    **Score:** 240    **Queries matched:** 14   **emPAI:** 0.89 |
| --- | --- |
|  | Thioredoxin-dependent peroxide reductase, mitochondrial OS=Homo sapiens GN=PRDX3 PE=1 SV=3 |

|  | **Query** | **Observed** | **Mr(expt)** | **Mr(calc)** | **Delta** | **Miss** | **Score** | **Expect** | **Rank** | **Peptide** |
| --- | --- | --- | --- | --- | --- | --- | --- | --- | --- | --- |
|  | 1623 | **715.4052** | **714.3979** | **713.3384** | **1.0594** | **0** | **13** | **9.4** | **1** | **K.EYFQK.V** |
|  | 2376 | **833.3874** | **832.3801** | **832.4290** | **-0.0489** | **0** | **8** | **36** | **4** | **R.SVEETLR.L** |
|  | 5376 | **529.2334** | **1056.4522** | **1056.6226** | **-0.1703** | **1** | **9** | **21** | **8** | **-.MAAAVGRLLR.A** 5388 5398 |
|  | 7539 | 603.8287 | 1205.6428 | 1205.6517 | -0.0089 | 0 | 42 | 0.0094 | 1 | K.HLSVNDLPVGR.S 7538 |
|  | 12207 | **731.8946** | **1461.7747** | **1461.7827** | **-0.0080** | **0** | **98** | **2.1e-008** | **1** | **R.DYGVLLEGSGLALR.G** |
|  | 16714 | **877.4606** | **1752.9067** | **1752.9192** | **-0.0125** | **0** | **64** | **4e-005** | **1** | **K.NGGLGHMNIALLSDLTK.Q** 16733 |
|  | 18423 | **628.0079** | **1881.0020** | **1881.0142** | **-0.0122** | **1** | **62** | **6.5e-005** | **1** | **R.KNGGLGHMNIALLSDLTK.Q** 18432 |
|  | 28511 | 847.1534 | 3384.5847 | 3384.6085 | -0.0238 | 0 | 35 | 0.012 | 1 | K.AFQYVETHGEVCPANWTPDSPTIKPSPAASK.E 28510 |

| **212.** | P08574\|CY1_HUMAN    **Mass:** 35709    **Score:** 240    **Queries matched:** 16   **emPAI:** 0.82 |
| --- | --- |
|  | Cytochrome c1, heme protein, mitochondrial OS=Homo sapiens GN=CYC1 PE=1 SV=2 |

|  | **Query** | **Observed** | **Mr(expt)** | **Mr(calc)** | **Delta** | **Miss** | **Score** | **Expect** | **Rank** | **Peptide** |
| --- | --- | --- | --- | --- | --- | --- | --- | --- | --- | --- |
|  | 4237 | **486.7995** | **971.5845** | **971.5916** | **-0.0071** | **1** | **19** | **2.4** | **1** | **R.KLAYRPPK.-** 4232 4260 |
|  | 9279 | 649.3450 | 1296.6755 | 1297.6990 | -1.0235 | 0 | 39 | 0.016 | 1 | R.GLLSSLDHTSIR.R 9294 |
|  | 13177 | **761.4283** | **1520.8421** | **1519.6977** | **1.1444** | **0** | **20** | **1.2** | **1** | **R.HLVGVCYTEDEAK.E** 13167 |
|  | 15441 | **836.4335** | **1670.8524** | **1669.8787** | **0.9737** | **0** | **1** | **1e+002** | **4** | **R.AANNGALPPDLSYIVR.A** |
|  | 18605 | **633.1576** | **1896.4509** | **1897.0170** | **-0.5660** | **1** | **0** | **61** | **5** | **R.AANNGALPPDLSYIVRAR.H** |
|  | 18830 | **957.9663** | **1913.9181** | **1913.9312** | **-0.0131** | **0** | **35** | **0.029** | **1** | **K.LFDYFPKPYPNSEAAR.A** |
|  | 25060 | **1327.6238** | **2653.2330** | **2653.2483** | **-0.0153** | **0** | **82** | **4.2e-007** | **1** | **R.HGGEDYVFSLLTGYCEPPTGVSLR.E** 25061 25062 |
|  | 25222 | **894.4631** | **2680.3674** | **2679.1792** | **1.1882** | **0** | **1** | **52** | **6** | **K.ELAAEVEVQDGPNEDGEMFMRPGK.L** |
|  | 30458 | **1038.2194** | **4148.8483** | **4148.8765** | **-0.0282** | **1** | **85** | **8e-008** | **1** | **R.HLVGVCYTEDEAKELAAEVEVQDGPNEDGEMFMRPGK.L** |
|  | 30778 | **1460.3624** | **4378.0655** | **4378.0854** | **-0.0199** | **0** | **9** | **3.2** | **1** | **R.EGLYFNPYFPGQAIAMAPPIYTDVLEFDDGTPATMSQIAK.D** |

| **213.** | P34896\|GLYC_HUMAN    **Mass:** 53619    **Score:** 239    **Queries matched:** 26   **emPAI:** 0.40 |
| --- | --- |
|  | Serine hydroxymethyltransferase, cytosolic OS=Homo sapiens GN=SHMT1 PE=1 SV=1 |

|  | **Query** | **Observed** | **Mr(expt)** | **Mr(calc)** | **Delta** | **Miss** | **Score** | **Expect** | **Rank** | **Peptide** |
| --- | --- | --- | --- | --- | --- | --- | --- | --- | --- | --- |
|  | 1558 | **693.3877** | **692.3804** | **692.3857** | **-0.0053** | **0** | **11** | **17** | **1** | **R.ALQAYK.L** |
|  | 2666 | **429.2180** | **856.4214** | **856.4265** | **-0.0052** | **0** | **12** | **13** | **9** | **R.AGMIFYR.K** |
|  | 3478 | 458.3055 | 914.5964 | 914.5185 | 0.0779 | 0 | 14 | 6.5 | 2 | R.LGTPALTSR.G |
|  | 4211 | **486.2480** | **970.4815** | **969.4702** | **1.0113** | **0** | **0** | **1.5e+002** | **6** | **M.TMPVNGAHK.D** |
|  | 5646 | **539.2965** | **1076.5785** | **1076.5866** | **-0.0081** | **1** | **16** | **3.9** | **1** | **R.GLLEKDFQK.V** |
|  | 7989 | **615.2289** | **1228.4432** | **1229.5798** | **-1.1365** | **1** | **4** | **53** | **8** | **R.GCRAGMIFYR.K** |
|  | 8221 | **620.8171** | **1239.6196** | **1239.6281** | **-0.0085** | **0** | **31** | **0.11** | **1** | **K.LIIAGTSCYSR.N** 8220 8241 |
|  | 8678 | 632.8002 | 1263.5858 | 1263.5951 | -0.0093 | 0 | 47 | 0.0027 | 1 | K.VLEACSIACNK.N |
|  | 9215 | **647.8442** | **1293.6739** | **1293.6816** | **-0.0077** | **0** | **76** | **3.4e-006** | **1** | **R.ALSEALTELGYK.I** 9214 |
|  | 10690 | **687.3361** | **1372.6576** | **1372.6670** | **-0.0094** | **0** | **39** | **0.02** | **1** | **K.VYQHQVVANCR.A** 10678 10691 |
|  | 12869 | **751.8697** | **1501.7249** | **1502.8205** | **-1.0955** | **1** | **4** | **50** | **4** | **R.LAGDKYQAAVQALR.E** |
|  | 14648 | **540.6003** | **1618.7792** | **1619.7905** | **-1.0113** | **0** | **1** | **88** | **4** | **K.ISATSIFFESMPYK.V** |
|  | 16263 | **861.9764** | **1721.9382** | **1720.9472** | **0.9910** | **0** | **7** | **21** | **2** | **K.IVTGGSDNHLILVDLR.S** |
|  | 21040 | **1055.9810** | **2109.9474** | **2109.9603** | **-0.0129** | **0** | **88** | **1.3e-007** | **1** | **K.VNPDTGYINYDQLEENAR.L** 21039 |
|  | 21425 | **539.5095** | **2154.0090** | **2154.0238** | **-0.0148** | **0** | **7** | **17** | **1** | **R.IMGLDLPDGGHLTHGFMTDK.K** |
|  | 22332 | **753.0365** | **2256.0877** | **2255.0099** | **1.0777** | **1** | **8** | **13** | **1** | **-.MTMPVNGAHKDADLWSSHDK.M** |
|  | 22536 | **761.3882** | **2281.1429** | **2282.1188** | **-0.9759** | **1** | **2** | **55** | **4** | **R.IMGLDLPDGGHLTHGFMTDKK.K** |
|  | 22663 | 766.7357 | 2297.1853 | 2298.1137 | -0.9284 | 1 | (1) | 63 | 5 | R.IMGLDLPDGGHLTHGFMTDKK.K |
|  | 28883 | **1177.2297** | **3528.6674** | **3528.6885** | **-0.0211** | **0** | **21** | **0.27** | **1** | **K.LDPQCWGVNVQPYSGSPANFAVYTALVEPHGR.I** 28888 |

| **214.** | P20853\|CP2A7_HUMAN    **Mass:** 56674    **Score:** 237    **Queries matched:** 27   **emPAI:** 0.77 |
| --- | --- |
|  | Cytochrome P450 2A7 OS=Homo sapiens GN=CYP2A7 PE=2 SV=2 |

|  | **Query** | **Observed** | **Mr(expt)** | **Mr(calc)** | **Delta** | **Miss** | **Score** | **Expect** | **Rank** | **Peptide** |
| --- | --- | --- | --- | --- | --- | --- | --- | --- | --- | --- |
|  | 1418 | 643.3307 | 642.3234 | 641.3609 | 0.9625 | 1 | 4 | 83 | 4 | K.NRQPK.F |
|  | 1748 | 772.4255 | 771.4183 | 772.3967 | -0.9784 | 0 | 7 | 41 | 9 | K.DIDVSPK.H |
|  | 4406 | 492.7800 | 983.5455 | 983.5514 | -0.0059 | 0 | 18 | 2.5 | 2 | R.YGFLLLMK.H |
|  | 6248 | 562.7517 | 1123.4889 | 1124.5502 | -1.0613 | 0 | 8 | 25 | 7 | K.NPNTEFYLK.N |
|  | 6684 | 579.2767 | 1156.5388 | 1155.5309 | 1.0079 | 0 | 3 | 91 | 6 | K.GYGVAFSNGER.A |
|  | 7518 | 603.3176 | 1204.6206 | 1204.6274 | -0.0068 | 0 | 57 | 0.00031 | 1 | R.FGDVIPMSLAR.R 7498 |
|  | 7902 | 408.7606 | 1223.2599 | 1223.6445 | -0.3846 | 0 | 0 | 94 | 10 | R.VVVLCGHDAVR.E |
|  | 9219 | 647.8548 | 1293.6950 | 1293.7041 | -0.0090 | 1 | 29 | 0.19 | 1 | K.VHEEIDRVIGK.N |
|  | 9232 | 648.3492 | 1294.6839 | 1294.6921 | -0.0082 | 1 | 6 | 33 | 1 | K.KSDAFVPFSIGK.R |
|  | 10802 | 460.5898 | 1378.7475 | 1379.7456 | -0.9981 | 1 | 33 | 0.067 | 1 | R.RVVVLCGHDAVR.E 10792 10819 10834 |
|  | 11550 | 711.3719 | 1420.7292 | 1420.7384 | -0.0092 | 0 | 14 | 5.1 | 3 | K.GTEVFPMLGSVLR.D 11549 |
|  | 11993 | 725.8381 | 1449.6616 | 1449.6736 | -0.0120 | 0 | 32 | 0.078 | 1 | R.EALVDQAEEFSGR.G |
|  | 12916 | **753.4093** | **1504.8040** | **1503.7933** | **1.0108** | **0** | **1** | **92** | **6** | **R.IQEESGFLIEAIR.S** |
|  | 14603 | 539.5991 | 1615.7755 | 1615.7850 | -0.0095 | 0 | 30 | 0.11 | 1 | K.MPYMEAVIHEIQR.F |
|  | 17915 | 923.4294 | 1844.8442 | 1844.9277 | -0.0835 | 1 | 13 | 5.5 | 2 | R.TKMPYMEAVIHEIQR.F |
|  | 24044 | 826.7730 | 2477.2972 | 2477.3431 | -0.0459 | 1 | 77 | 1.3e-006 | 1 | K.HLPGPQQQAFKLLQGLEDFIAK.K 24039 24040 24042 |
|  | 25661 | 1380.1421 | 2758.2696 | 2758.2875 | -0.0178 | 1 | 47 | 0.0011 | 1 | R.EALVDQAEEFSGRGEQATFDWVFK.G 25660 25662 |

| **215.** | P09210\|GSTA2_HUMAN    **Mass:** 25648    **Score:** 236    **Queries matched:** 25   **emPAI:** 1.63 |
| --- | --- |
|  | Glutathione S-transferase A2 OS=Homo sapiens GN=GSTA2 PE=1 SV=4 |

|  | **Query** | **Observed** | **Mr(expt)** | **Mr(calc)** | **Delta** | **Miss** | **Score** | **Expect** | **Rank** | **Peptide** |
| --- | --- | --- | --- | --- | --- | --- | --- | --- | --- | --- |
|  | 2903 | 436.2636 | 870.5127 | 870.5175 | -0.0048 | 0 | 18 | 2.8 | 4 | R.ISNLPTVK.K |
|  | 3265 | **451.2452** | **900.4759** | **900.4818** | **-0.0059** | **0** | **20** | **2.1** | **1** | **K.FLQPGSPR.K** 3280 3288 3302 3303 |
|  | 4519 | **992.5702** | **991.5629** | **991.5702** | **-0.0073** | **0** | **27** | **0.34** | **1** | **R.AILNYIASK.Y** 4518 |
|  | 4613 | **499.7349** | **997.4552** | **998.6124** | **-1.1572** | **1** | **16** | **3.4** | **4** | **R.ISNLPTVKK.F** 4620 |
|  | 5037 | **515.2913** | **1028.5681** | **1028.5767** | **-0.0086** | **1** | **32** | **0.11** | **1** | **K.KFLQPGSPR.K** 5029 5038 |
|  | 5268 | **523.8002** | **1045.5858** | **1045.5404** | **0.0454** | **1** | **15** | **6.1** | **10** | **K.SAEDLDKLR.N** |
|  | 7745 | **609.2951** | **1216.5756** | **1216.5836** | **-0.0080** | **0** | **40** | **0.014** | **1** | **K.SHGQDYLVGNK.L** 7743 7746 7772 |
|  | 12205 | **731.8782** | **1461.7419** | **1461.7504** | **-0.0084** | **0** | **56** | **0.00032** | **1** | **R.WLLAAAGVEFEEK.F** |
|  | 14127 | **793.4149** | **1584.8152** | **1585.8398** | **-1.0247** | **1** | **2** | **78** | **2** | **-.MAEKPKLHYSNIR.G** |
|  | 16559 | **581.2822** | **1740.8247** | **1741.8821** | **-1.0574** | **1** | **2** | **61** | **2** | **K.FLQPGSPRKPPMDEK.S** |
|  | 21967 | **1108.0089** | **2214.0033** | **2214.0159** | **-0.0127** | **0** | **100** | **8.6e-009** | **1** | **R.NDGYLMFQQVPMVEIDGMK.L** 21965 21966 |
|  | 27560 | **1036.2203** | **3105.6392** | **3105.6525** | **-0.0133** | **0** | **25** | **0.14** | **1** | **R.ADIHLVELLYYVEELDSSLISSFPLLK.A** |

|  | **Proteins matching a subset of these peptides:** |
| --- | --- |

|  | P08263\|GSTA1_HUMAN    **Mass:** 25672    **Score:** 236    **Queries matched:** 24 |
| --- | --- |
|  | Glutathione S-transferase A1 OS=Homo sapiens GN=GSTA1 PE=1 SV=3 |

| **216.** | Q8NBQ5\|DHB11_HUMAN    **Mass:** 33285    **Score:** 231    **Queries matched:** 20   **emPAI:** 1.12 |
| --- | --- |
|  | Estradiol 17-beta-dehydrogenase 11 OS=Homo sapiens GN=HSD17B11 PE=1 SV=2 |

|  | **Query** | **Observed** | **Mr(expt)** | **Mr(calc)** | **Delta** | **Miss** | **Score** | **Expect** | **Rank** | **Peptide** |
| --- | --- | --- | --- | --- | --- | --- | --- | --- | --- | --- |
|  | 1554 | 689.4148 | 688.4075 | 689.4476 | -1.0401 | 0 | 5 | 76 | 8 | R.FLAVLK.R |
|  | 3010 | **440.2384** | **878.4623** | **877.4731** | **0.9892** | **0** | **12** | **10** | **2** | **K.AFLPAMTK.N** |
|  | 3185 | **447.8937** | **893.7729** | **893.4681** | **0.3048** | **0** | **(5)** | **32** | **8** | **K.AFLPAMTK.N** |
|  | 3430 | **456.7422** | **911.4698** | **911.4753** | **-0.0054** | **0** | **35** | **0.047** | **1** | **K.FDAVIGYK.M** |
|  | 7103 | **594.2815** | **1186.5484** | **1186.6056** | **-0.0572** | **1** | **1** | **1.4e+002** | **5** | **K.FDAVIGYKMK.A** |
|  | 10072 | 446.9020 | 1337.6841 | 1338.7547 | -1.0706 | 1 | 4 | 60 | 8 | K.ISVKFDAVIGYK.M |
|  | 13925 | **786.9415** | **1571.8685** | **1571.8770** | **-0.0085** | **0** | **76** | **3.5e-006** | **1** | **K.TLTDELAALQITGVK.T** 13923 |
|  | 15745 | **564.9602** | **1691.8588** | **1691.8672** | **-0.0084** | **0** | **14** | **4.6** | **1** | **K.TFEVNVLAHFWTTK.A** |
|  | 16476 | **868.4452** | **1734.8758** | **1735.9581** | **-1.0823** | **0** | **44** | **0.0042** | **1** | **K.SVTGEIVLITGAGHGIGR.L** 16501 16504 |
|  | 16964 | **886.4234** | **1770.8322** | **1770.8433** | **-0.0111** | **0** | **42** | **0.0077** | **1** | **K.TTCLCPNFVNTGFIK.N** 16963 |
|  | 19086 | **646.3502** | **1936.0288** | **1936.0418** | **-0.0129** | **1** | **31** | **0.075** | **1** | **K.LVLWDINKHGLEETAAK.C** |
|  | 19103 | **969.9854** | **1937.9563** | **1937.9694** | **-0.0132** | **0** | **72** | **5.5e-006** | **1** | **K.NPSTSLGPTLEPEEVVNR.L** 19102 19110 |
|  | 22102 | **743.0142** | **2226.0208** | **2226.0375** | **-0.0167** | **1** | **16** | **2.2** | **1** | **K.VHTFVVDCSNREDIYSSAK.K** |
|  | 29261 | **1231.6344** | **3691.8814** | **3690.8022** | **1.0792** | **1** | **0** | **31** | **8** | **K.TTCLCPNFVNTGFIKNPSTSLGPTLEPEEVVNR.L** |

| **217.** | P20815\|CP3A5_HUMAN    **Mass:** 57357    **Score:** 228    **Queries matched:** 27   **emPAI:** 0.65 |
| --- | --- |
|  | Cytochrome P450 3A5 OS=Homo sapiens GN=CYP3A5 PE=1 SV=1 |

|  | **Query** | **Observed** | **Mr(expt)** | **Mr(calc)** | **Delta** | **Miss** | **Score** | **Expect** | **Rank** | **Peptide** |
| --- | --- | --- | --- | --- | --- | --- | --- | --- | --- | --- |
|  | 1116 | 559.3775 | 558.3702 | 558.3741 | -0.0039 | 0 | 24 | 1.2 | 1 | R.TVLVK.E |
|  | 6425 | 569.3078 | 1136.6010 | 1136.6077 | -0.0067 | 0 | 38 | 0.025 | 1 | R.SLLSPTFTSGK.L |
|  | 7660 | **405.2073** | **1212.6002** | **1212.7343** | **-0.1341** | **1** | **8** | **21** | **2** | **R.LFPVAIRLER.T** |
|  | 10591 | 684.3566 | 1366.6986 | 1366.7067 | -0.0082 | 0 | 28 | 0.22 | 1 | R.VLQNFSFKPCK.E 10590 |
|  | 10772 | 689.8339 | 1377.6532 | 1376.6459 | 1.0072 | 0 | 18 | 2.3 | 1 | K.SAISLAEDEEWK.R |
|  | 10794 | 690.3622 | 1378.7098 | 1377.7868 | 0.9231 | 1 | 2 | 82 | 5 | R.SLLSPTFTSGKLK.E 10769 |
|  | 11272 | 703.8880 | 1405.7614 | 1405.7929 | -0.0315 | 1 | 11 | 9.4 | 4 | R.IRSLLSPTFTSGK.L |
|  | 11771 | 479.2531 | 1434.7374 | 1435.8043 | -1.0669 | 1 | 0 | 1.2e+002 | 4 | R.FALMNMKLALIR.V |
|  | 13352 | 767.3753 | 1532.7361 | 1532.7470 | -0.0110 | 1 | 63 | 6.6e-005 | 1 | K.SAISLAEDEEWKR.I 13350 13351 13353 |
|  | 14374 | 801.3773 | 1600.7400 | 1600.7346 | 0.0054 | 1 | (8) | 21 | 6 | R.NCIGMRFALMNMK.L 14375 |
|  | 14604 | 808.9011 | 1615.7876 | 1616.7295 | -0.9420 | 1 | (1) | 1e+002 | 8 | R.NCIGMRFALMNMK.L |
|  | 14861 | 817.3726 | 1632.7307 | 1632.7244 | 0.0062 | 1 | 8 | 19 | 3 | R.NCIGMRFALMNMK.L |
|  | 19547 | **989.0742** | **1976.1338** | **1976.1459** | **-0.0121** | **0** | **67** | **1.7e-005** | **1** | **R.LGIPGPTPLPLLGNVLSYR.Q** 19556 |
|  | 19723 | **664.7009** | **1991.0810** | **1991.0914** | **-0.0104** | **1** | **25** | **0.3** | **1** | **K.LKEMFPIIAQYGDVLVR.N** |
|  | 23278 | **792.4051** | **2374.1934** | **2373.2039** | **0.9896** | **0** | **19** | **0.99** | **1** | **K.MWGTYEGQLPVLAITDPDVIR.T** |
|  | 24852 | **867.8410** | **2600.5012** | **2600.5153** | **-0.0141** | **1** | **58** | **8.5e-005** | **1** | **K.ETQIPLKLDTQGLLQPEKPIVLK.V** |
|  | 27913 | **802.3577** | **3205.4018** | **3204.7872** | **0.6146** | **0** | **0** | **40** | **3** | **-.MDLIPNLAVETWLLLAVSLVLLYLYGTR.T** |
|  | 29169 | **1211.9391** | **3632.7954** | **3632.8106** | **-0.0152** | **1** | **97** | **7.5e-009** | **1** | **K.EIDAVLPNKAPPTYDAVVQMEYLDMVVNETLR.L** 29170 29172 |

| **218.** | P53007\|TXTP_HUMAN    **Mass:** 34333    **Score:** 228    **Queries matched:** 29   **emPAI:** 1.30 |
| --- | --- |
|  | Tricarboxylate transport protein, mitochondrial OS=Homo sapiens GN=SLC25A1 PE=1 SV=2 |

|  | **Query** | **Observed** | **Mr(expt)** | **Mr(calc)** | **Delta** | **Miss** | **Score** | **Expect** | **Rank** | **Peptide** |
| --- | --- | --- | --- | --- | --- | --- | --- | --- | --- | --- |
|  | 987 | **530.1989** | **529.1916** | **528.3020** | **0.8896** | **0** | **15** | **10** | **1** | **K.GTVPR.L** |
|  | 1759 | **776.3669** | **775.3597** | **775.3647** | **-0.0050** | **0** | **14** | **9.5** | **1** | **R.GIGDCVR.Q** |
|  | 2600 | 851.4351 | 850.4278 | 850.5025 | -0.0746 | 1 | 23 | 0.79 | 2 | K.AKLTHPGK.A |
|  | 2635 | **428.2109** | **854.4073** | **854.5086** | **-0.1014** | **1** | **2** | **95** | **8** | **K.GTVPRLGR.V** |
|  | 3454 | **457.7306** | **913.4466** | **912.4487** | **0.9979** | **0** | **20** | **1.9** | **1** | **R.MQGLEAHK.Y** |
|  | 3644 | **465.2362** | **928.4578** | **928.4436** | **0.0142** | **0** | **(3)** | **93** | **5** | **R.MQGLEAHK.Y** |
|  | 4647 | **500.7648** | **999.5151** | **999.5212** | **-0.0061** | **0** | **31** | **0.16** | **1** | **R.FFVMTSLR.N** 4652 |
|  | 4677 | **501.7608** | **1001.5071** | **1001.5142** | **-0.0071** | **0** | **23** | **0.97** | **1** | **K.TQLQLDER.S** 4678 |
|  | 8034 | **411.2401** | **1230.6983** | **1231.6131** | **-0.9148** | **1** | **2** | **92** | **6** | **R.MQGLEAHKYR.N** |
|  | 8105 | **617.8519** | **1233.6892** | **1233.6969** | **-0.0077** | **0** | **41** | **0.013** | **1** | **R.GLSSLLYGSIPK.A** 8121 |
|  | 8436 | **626.3467** | **1250.6789** | **1250.6871** | **-0.0081** | **0** | **37** | **0.029** | **1** | **K.GTYQGLTATVLK.Q** |
|  | 9007 | **642.3179** | **1282.6213** | **1282.6306** | **-0.0093** | **0** | **38** | **0.021** | **1** | **K.FIHDQTSPNPK.Y** 9002 9008 |
|  | 10210 | **674.3353** | **1346.6561** | **1346.6653** | **-0.0092** | **0** | **38** | **0.026** | **1** | **R.NTWDCGLQILK.K** 10211 |
|  | 12427 | **492.2818** | **1473.8236** | **1474.7602** | **-0.9367** | **1** | **8** | **18** | **3** | **R.NTWDCGLQILKK.E** |
|  | 12979 | **504.2680** | **1509.7823** | **1509.7940** | **-0.0117** | **1** | **14** | **5** | **1** | **K.VKFIHDQTSPNPK.Y** |
|  | 13061 | **505.8976** | **1514.6711** | **1514.6799** | **-0.0088** | **0** | **39** | **0.017** | **1** | **R.FGMFEFLSNHMR.D** 13062 |
|  | 18993 | **965.2244** | **1928.4342** | **1927.9185** | **0.5157** | **1** | **1** | **58** | **2** | **K.AAVRFGMFEFLSNHMR.D** |
|  | 23738 | **1222.6372** | **2443.2599** | **2442.2685** | **0.9914** | **0** | **84** | **2.7e-007** | **1** | **R.GLLCGLGAGVAEAVVVVCPMETIK.V** 23709 |
|  | 28804 | **1165.9421** | **3494.8046** | **3494.8232** | **-0.0186** | **0** | **87** | **6.6e-008** | **1** | **R.GDNPNKPMNPLITGVFGAIAGAASVFGNTPLDVIK.T** |
|  | 30379 | 1033.7836 | 4131.1052 | 4130.1048 | 1.0004 | 1 | 12 | 1.7 | 1 | R.NWYRGDNPNKPMNPLITGVFGAIAGAASVFGNTPLDVIK.T 30368 |

| **219.** | Q9H8H3\|MET7A_HUMAN    **Mass:** 28814    **Score:** 228    **Queries matched:** 17   **emPAI:** 0.64 |
| --- | --- |
|  | Methyltransferase-like protein 7A OS=Homo sapiens GN=METTL7A PE=1 SV=1 |

|  | **Query** | **Observed** | **Mr(expt)** | **Mr(calc)** | **Delta** | **Miss** | **Score** | **Expect** | **Rank** | **Peptide** |
| --- | --- | --- | --- | --- | --- | --- | --- | --- | --- | --- |
|  | 2325 | **415.2162** | **828.4179** | **828.4242** | **-0.0063** | **0** | **32** | **0.13** | **1** | **R.HLQFER.F** 2327 2336 |
|  | 3195 | 448.2382 | 894.4619 | 895.4011 | -0.9392 | 0 | 9 | 21 | 5 | K.FYPPGCR.V |
|  | 11706 | **716.3497** | **1430.6849** | **1429.6911** | **0.9938** | **0** | **51** | **0.0012** | **1** | **R.FTVIYNEQMASK.K** 11693 |
|  | 11733 | **717.3353** | **1432.6560** | **1432.6657** | **-0.0097** | **0** | **40** | **0.013** | **1** | **R.VTCIDPNPNFEK.F** 11734 |
|  | 13840 | 783.3976 | 1564.7806 | 1564.7919 | -0.0113 | 0 | 92 | 7.7e-008 | 1 | K.LSLLEVGCGTGANFK.F 13841 13842 |
|  | 25455 | **680.3731** | **2717.4631** | **2717.4805** | **-0.0174** | **0** | **11** | **4.7** | **1** | **K.LQHIQAPLSWELVRPHIYGYAVK.-** 25449 25454 25456 |
|  | 26844 | **592.7352** | **2958.6398** | **2958.6596** | **-0.0198** | **1** | **37** | **0.01** | **1** | **K.LKLQHIQAPLSWELVRPHIYGYAVK.-** |
|  | 27787 | **1057.4955** | **3169.4646** | **3169.5754** | **-0.1108** | **1** | **0** | **47** | **5** | **R.ELFSNLQEFAGPSGKLSLLEVGCGTGANFK.F** |

| **220.** | Q9BYX7\|ACTBM_HUMAN    **Mass:** 42331    **Score:** 227    **Queries matched:** 20   **emPAI:** 0.53 |
| --- | --- |
|  | Beta-actin-like protein 3 OS=Homo sapiens GN=ACTBL3 PE=1 SV=1 |

|  | **Query** | **Observed** | **Mr(expt)** | **Mr(calc)** | **Delta** | **Miss** | **Score** | **Expect** | **Rank** | **Peptide** |
| --- | --- | --- | --- | --- | --- | --- | --- | --- | --- | --- |
|  | 1363 | 630.3417 | 629.3344 | 630.3701 | -1.0357 | 0 | 4 | 99 | 2 | K.ILTER.G |
|  | 1421 | 644.3680 | 643.3607 | 643.3653 | -0.0046 | 0 | 20 | 2.6 | 1 | R.LDLAGR.E |
|  | 2167 | **409.2121** | **816.4097** | **817.4844** | **-1.0747** | **1** | **4** | **82** | **2** | **K.RGMLTLK.Y** |
|  | 9919 | **443.9266** | **1328.7580** | **1328.5424** | **0.2156** | **0** | **6** | **35** | **3** | **R.HQGMMEGMHQK.E** |
|  | 13084 | 758.8497 | 1515.6849 | 1515.6954 | -0.0105 | 0 | 48 | 0.0021 | 1 | K.QEYDESGPSIVHR.K |
|  | 13087 | 758.8746 | 1515.7347 | 1514.7419 | 0.9928 | 0 | 38 | 0.022 | 1 | K.IWHHTFYNELR.V 13067 13068 |
|  | 13969 | **788.8369** | **1575.6591** | **1575.8364** | **-0.1772** | **1** | **1** | **92** | **5** | **R.MQKEITALAPSIMK.I** |
|  | 15044 | 822.8969 | 1643.7793 | 1643.7903 | -0.0111 | 1 | 28 | 0.19 | 1 | K.QEYDESGPSIVHRK.C |
|  | 15177 | 413.9481 | 1651.7631 | 1652.8443 | -1.0812 | 1 | 10 | 13 | 8 | R.LDLAGRELTDYLMK.I |
|  | 17255 | 895.9438 | 1789.8730 | 1789.8846 | -0.0116 | 0 | 71 | 9.6e-006 | 1 | K.SYELPDGQVITIGNER.F 17256 17257 17258 17267 17268 |
|  | 19327 | **490.0043** | **1955.9881** | **1955.8275** | **0.1606** | **0** | **5** | **30** | **4** | **-.MDDDTAVLVIDNGSGMCK.A** |
|  | 24422 | **635.3286** | **2537.2853** | **2537.0971** | **0.1882** | **0** | **3** | **35** | **8** | **K.LCYVALDSEQEMAMAASSSSVEK.S** |
|  | 28259 | 1106.1185 | 3315.3338 | 3314.6025 | 0.7312 | 0 | 0 | 32 | 9 | K.MTQIMFETFNTPAMYVAIQAVLSLYTSGR.T |

| **221.** | P36537\|UDB10_HUMAN    **Mass:** 61190    **Score:** 226    **Queries matched:** 34   **emPAI:** 0.43 |
| --- | --- |
|  | UDP-glucuronosyltransferase 2B10 OS=Homo sapiens GN=UGT2B10 PE=1 SV=1 |

|  | **Query** | **Observed** | **Mr(expt)** | **Mr(calc)** | **Delta** | **Miss** | **Score** | **Expect** | **Rank** | **Peptide** |
| --- | --- | --- | --- | --- | --- | --- | --- | --- | --- | --- |
|  | 761 | 474.2994 | 473.2922 | 472.2758 | 1.0164 | 0 | 4 | 1.4e+002 | 7 | K.GAAVR.V |
|  | 1382 | 634.3110 | 633.3037 | 633.3156 | -0.0119 | 0 | 12 | 15 | 6 | K.ENIMK.L |
|  | 4227 | **486.2945** | **970.5744** | **970.5811** | **-0.0067** | **0** | **13** | **8** | **1** | **R.ANVIATALAK.I** 4217 |
|  | 4477 | **495.7325** | **989.4504** | **989.5328** | **-0.0824** | **1** | **8** | **27** | **7** | **K.ENIMKLSR.I** |
|  | 5081 | **516.7835** | **1031.5523** | **1031.5586** | **-0.0062** | **1** | **59** | **0.00023** | **1** | **R.KADIWLMR.N** 5080 |
|  | 5161 | 519.7969 | 1037.5793 | 1038.6338 | -1.0545 | 1 | 8 | 24 | 4 | K.IPQKVLWR.F |
|  | 5950 | 550.8157 | 1099.6169 | 1098.6761 | 0.9408 | 1 | 23 | 0.9 | 2 | K.TILKELVQR.G |
|  | 7616 | 404.4434 | 1210.3083 | 1209.5812 | 0.7270 | 1 | 13 | 4.9 | 1 | R.NFCKDVVSNK.K |
|  | 11471 | 473.2548 | 1416.7425 | 1416.7514 | -0.0089 | 0 | 29 | 0.18 | 1 | K.WIPQNDLLGHPK.T 11466 11467 11469 |
|  | 11924 | 723.3910 | 1444.7674 | 1444.7787 | -0.0112 | 0 | 40 | 0.013 | 1 | R.IQHDQPVKPLDR.A 11925 11926 |
|  | 12256 | **489.3186** | **1464.9339** | **1463.7694** | **1.1645** | **0** | **15** | **3.5** | **2** | **K.TEFENIIMQLVK.R** 12227 |
|  | 15156 | 826.4159 | 1650.8173 | 1650.8287 | -0.0113 | 1 | 72 | 7.2e-006 | 1 | K.TVINDPSYKENIMK.L |
|  | 16930 | 884.9373 | 1767.8601 | 1767.8713 | -0.0112 | 0 | 74 | 3.9e-006 | 1 | R.VDFNTMSSTDLLNALK.T 16931 |
|  | 17167 | 892.9249 | 1783.8352 | 1783.8662 | -0.0310 | 0 | (4) | 40 | 5 | R.VDFNTMSSTDLLNALK.T 17151 |
|  | 18092 | **929.4810** | **1856.9475** | **1855.9001** | **1.0474** | **0** | **1** | **82** | **3** | **K.VLVWAAEYSLWMNMK.T** |
|  | 19241 | 975.4944 | 1948.9742 | 1947.9917 | 0.9825 | 0 | 4 | 41 | 4 | R.HSGGFIFPPSYVPVVMSK.L |
|  | 22041 | 1111.6786 | 2221.3426 | 2222.1365 | -0.7939 | 1 | 6 | 14 | 2 | K.GAAVRVDFNTMSSTDLLNALK.T |
|  | 23557 | **806.3754** | **2416.1043** | **2415.1529** | **0.9514** | **0** | **1** | **52** | **3** | **K.WDQFYSEVLGRPTTLSETMR.K** |
|  | 24892 | **1306.6445** | **2611.2745** | **2611.3819** | **-0.1074** | **1** | **3** | **34** | **2** | **K.LEVYPTSLTKTEFENIIMQLVK.R** |
|  | 25977 | **469.5885** | **2811.4875** | **2811.5047** | **-0.0172** | **0** | **1** | **43** | **6** | **K.FPHPFLPNVDFVGGLHCKPAKPLPK.E** |
|  | 27904 | 1068.1691 | 3201.4854 | 3202.5290 | -1.0436 | 1 | 46 | 0.0012 | 2 | R.HSGGFIFPPSYVPVVMSKLSDQMTFMER.V |
|  | 30490 | **1039.5150** | **4154.0309** | **4154.0546** | **-0.0237** | **0** | **49** | **0.00033** | **1** | **R.AFITHGGANGIYEAIYHGIPMVGIPLFFDQPDNIAHMK.A** 30489 30492 |

| **222.** | P18124\|RL7_HUMAN    **Mass:** 29264    **Score:** 226    **Queries matched:** 22   **emPAI:** 2.37 |
| --- | --- |
|  | 60S ribosomal protein L7 OS=Homo sapiens GN=RPL7 PE=1 SV=1 |

|  | **Query** | **Observed** | **Mr(expt)** | **Mr(calc)** | **Delta** | **Miss** | **Score** | **Expect** | **Rank** | **Peptide** |
| --- | --- | --- | --- | --- | --- | --- | --- | --- | --- | --- |
|  | 1636 | **721.3830** | **720.3757** | **720.3806** | **-0.0049** | **0** | **33** | **0.085** | **1** | **R.NFAELK.I** |
|  | 1748 | 772.4255 | 771.4183 | 770.4286 | 0.9896 | 0 | 7 | 37 | 5 | R.GINGVSPK.V |
|  | 2016 | **402.7211** | **803.4277** | **803.4323** | **-0.0046** | **0** | **44** | **0.0083** | **1** | **K.ASINMLR.I** 2014 |
|  | 4148 | **483.2656** | **964.5166** | **964.5229** | **-0.0064** | **0** | **15** | **5.4** | **1** | **K.SVNELIYK.R** |
|  | 5335 | **527.2828** | **1052.5510** | **1052.5655** | **-0.0145** | **0** | **1** | **1.2e+002** | **3** | **R.QIFNGTFVK.L** |
|  | 6227 | **561.3153** | **1120.6161** | **1120.6240** | **-0.0080** | **1** | **30** | **0.18** | **1** | **K.SVNELIYKR.G** |
|  | 6861 | **585.8418** | **1169.6690** | **1169.6768** | **-0.0077** | **0** | **57** | **0.00033** | **1** | **R.IALTDNALIAR.S** 6860 |
|  | 7622 | **606.2948** | **1210.5750** | **1209.6969** | **0.8782** | **1** | **4** | **57** | **8** | **K.KEVPAVPETLK.K** 7623 |
|  | 9709 | **660.8461** | **1319.6777** | **1319.6874** | **-0.0097** | **1** | **47** | **0.0027** | **1** | **R.KAGNFYVPAEPK.L** 9686 9708 |
|  | 13442 | **770.8964** | **1539.7782** | **1539.7874** | **-0.0092** | **1** | **54** | **0.00056** | **1** | **R.FKEANNFLWPFK.L** 13458 |
|  | 15325 | 831.9429 | 1661.8713 | 1661.8817 | -0.0104 | 0 | 55 | 0.00034 | 1 | R.IVEPYIAWGYPNLK.S 15324 |
|  | 21085 | **1058.4869** | **2114.9593** | **2114.9730** | **-0.0136** | **1** | **22** | **0.5** | **1** | **K.TTHFVEGGDAGNREDQINR.L** 21086 |
|  | 21416 | **1077.5289** | **2153.0433** | **2153.0537** | **-0.0104** | **0** | **47** | **0.0017** | **1** | **K.YGIICMEDLIHEIYTVGK.R** |
|  | 22753 | **770.7218** | **2309.1436** | **2309.1548** | **-0.0112** | **1** | **17** | **1.5** | **1** | **K.YGIICMEDLIHEIYTVGKR.F** |

| **223.** | P54920\|SNAA_HUMAN    **Mass:** 33667    **Score:** 226    **Queries matched:** 23   **emPAI:** 1.34 |
| --- | --- |
|  | Alpha-soluble NSF attachment protein OS=Homo sapiens GN=NAPA PE=1 SV=3 |

|  | **Query** | **Observed** | **Mr(expt)** | **Mr(calc)** | **Delta** | **Miss** | **Score** | **Expect** | **Rank** | **Peptide** |
| --- | --- | --- | --- | --- | --- | --- | --- | --- | --- | --- |
|  | 1527 | **681.3342** | **680.3269** | **680.3316** | **-0.0047** | **0** | **25** | **0.47** | **1** | **R.AANMFK.M** |
|  | 6844 | **585.3311** | **1168.6475** | **1167.5600** | **1.0875** | **1** | **8** | **22** | **1** | **K.YSAKDYFFK.A** |
|  | 7018 | 395.2144 | 1182.6213 | 1183.5543 | -0.9330 | 0 | 4 | 51 | 9 | R.AIEIYTDMGR.F |
|  | 8459 | **627.2908** | **1252.5670** | **1252.5757** | **-0.0088** | **0** | **44** | **0.0062** | **1** | **K.IEEACEIYAR.A** |
|  | 10988 | **695.3777** | **1388.7408** | **1388.7486** | **-0.0078** | **0** | **49** | **0.0016** | **1** | **R.LDQWLTTMLLR.I** 10989 |
|  | 13583 | **775.3629** | **1548.7113** | **1548.7209** | **-0.0096** | **0** | **60** | **0.00011** | **1** | **K.NSQSFFSGLFGGSSK.I** |
|  | 14702 | **812.3594** | **1622.7042** | **1622.7148** | **-0.0105** | **0** | **54** | **0.00043** | **1** | **K.HDAATCFVDAGNAFK.K** |
|  | 17552 | **606.3003** | **1815.8792** | **1814.9236** | **0.9556** | **1** | **2** | **72** | **6** | **R.AIEIYTDMGRFTIAAK.H** |
|  | 20382 | **1026.0132** | **2050.0118** | **2050.9299** | **-0.9181** | **1** | **(4)** | **36** | **2** | **-.MDNSGKEAEAMALLAEAER.K** |
|  | 20384 | **513.5104** | **2050.0127** | **2050.9299** | **-0.9172** | **1** | **5** | **27** | **1** | **-.MDNSGKEAEAMALLAEAER.K** 20385 |
|  | 20736 | 1040.0050 | 2077.9954 | 2077.0401 | 0.9553 | 0 | 21 | 0.67 | 2 | K.AIDIYEQVGTNAMDSPLLK.Y 20727 20738 |
|  | 21274 | 713.3357 | 2136.9854 | 2138.0895 | -1.1041 | 0 | 1 | 62 | 10 | K.HHISIAEIYETELVDIEK.A |
|  | 22326 | 753.0265 | 2256.0576 | 2255.1256 | 0.9320 | 1 | 0 | 73 | 10 | K.EYDSISRLDQWLTTMLLR.I |
|  | 23736 | **815.3986** | **2443.1740** | **2442.1862** | **0.9878** | **0** | **12** | **4.9** | **1** | **K.NWSAAGNAFCQAAQLHLQLQSK.H** |
|  | 24457 | **509.6844** | **2543.3856** | **2542.2625** | **1.1231** | **1** | **4** | **29** | **8** | **K.AIDIYEQVGTNAMDSPLLKYSAK.D** |
|  | 24609 | **854.7112** | **2561.1117** | **2561.1306** | **-0.0189** | **1** | **66** | **1.4e-005** | **1** | **K.AIAHYEQSADYYKGEESNSSANK.C** 24608 |
|  | 26744 | **981.1209** | **2940.3409** | **2940.3624** | **-0.0215** | **1** | **58** | **7.4e-005** | **1** | **K.LLEAHEEQNVDSYTESVKEYDSISR.L** 26745 |

| **224.** | P50416\|CPT1A_HUMAN    **Mass:** 88995    **Score:** 224    **Queries matched:** 41   **emPAI:** 0.33 |
| --- | --- |
|  | Carnitine O-palmitoyltransferase 1, liver isoform OS=Homo sapiens GN=CPT1A PE=1 SV=2 |

|  | **Query** | **Observed** | **Mr(expt)** | **Mr(calc)** | **Delta** | **Miss** | **Score** | **Expect** | **Rank** | **Peptide** |
| --- | --- | --- | --- | --- | --- | --- | --- | --- | --- | --- |
|  | 1639 | **723.4462** | **722.4389** | **722.4691** | **-0.0302** | **0** | **18** | **2.2** | **4** | **R.LPVPAVK.D** |
|  | 2259 | **412.2585** | **822.5024** | **822.3872** | **0.1152** | **1** | **15** | **4.8** | **2** | **K.EEDFKR.M** |
|  | 2910 | **436.7482** | **871.4818** | **870.3919** | **1.0899** | **0** | **7** | **44** | **8** | **K.HQHMYR.L** 2897 |
|  | 3118 | **444.5535** | **887.0925** | **886.3868** | **0.7057** | **0** | **(1)** | **1.1e+002** | **5** | **K.HQHMYR.L** |
|  | 4271 | **487.7680** | **973.5214** | **973.5273** | **-0.0059** | **0** | **12** | **12** | **3** | **K.SFTFVVFK.N** |
|  | 5149 | **519.2545** | **1036.4944** | **1035.5019** | **0.9925** | **0** | **10** | **15** | **5** | **R.LAMTGSGIDR.H** |
|  | 5370 | **528.7870** | **1055.5594** | **1056.4923** | **-0.9329** | **1** | **4** | **58** | **2** | **R.CRQAYFGR.G** |
|  | 5658 | 540.2302 | 1078.4458 | 1078.4535 | -0.0078 | 0 | 28 | 0.26 | 1 | R.EMEQQMQR.I |
|  | 5890 | **548.8052** | **1095.5959** | **1094.4485** | **1.1475** | **0** | **(4)** | **58** | **4** | **R.EMEQQMQR.I** |
|  | 6092 | **556.2981** | **1110.5816** | **1110.4434** | **0.1383** | **0** | **(9)** | **19** | **7** | **R.EMEQQMQR.I** |
|  | 6336 | **566.2996** | **1130.5847** | **1130.5931** | **-0.0084** | **1** | **22** | **1.2** | **1** | **K.NKQSLDAVEK.A** |
|  | 6777 | 582.7896 | 1163.5647 | 1163.6195 | -0.0548 | 1 | 6 | 37 | 8 | R.ATKIWMGMVK.I |
|  | 6810 | **583.9658** | **1165.9170** | **1165.6383** | **0.2787** | **0** | **0** | **90** | **2** | **K.YLAVESPFLK.E** |
|  | 8695 | **633.3431** | **1264.6717** | **1264.6638** | **0.0079** | **0** | **0** | **1.3e+002** | **4** | **R.HLFCLYVVSK.Y** |
|  | 9541 | **437.9199** | **1310.7380** | **1310.7459** | **-0.0078** | **0** | **12** | **8.8** | **1** | **R.AGNAIHAILLYR.R** |
|  | 11847 | 481.2363 | 1440.6870 | 1439.7417 | 0.9452 | 1 | 11 | 11 | 3 | K.IWMGMVKIFSGR.K |
|  | 11905 | **482.2759** | **1443.8059** | **1444.5664** | **-0.7605** | **0** | **15** | **4.2** | **1** | **R.SEDPDTSMDSYAK.S** |
|  | 12260 | **489.5662** | **1465.6767** | **1466.8470** | **-1.1702** | **1** | **6** | **33** | **9** | **R.AGNAIHAILLYRR.K** |
|  | 12339 | **735.8267** | **1469.6389** | **1469.6490** | **-0.0101** | **0** | **66** | **3.2e-005** | **1** | **R.TLETANCMSSQTK.N** |
|  | 13245 | **509.5768** | **1525.7084** | **1525.7195** | **-0.0111** | **0** | **17** | **2.3** | **1** | **R.IPGEETDTIQHMR.D** |
|  | 13246 | **763.8704** | **1525.7263** | **1525.7372** | **-0.0109** | **0** | **6** | **33** | **3** | **R.ILDNTSEPQPGEAR.L** |
|  | 13548 | 773.9011 | 1545.7876 | 1545.7973 | -0.0098 | 0 | 57 | 0.00022 | 1 | R.MTALAQDFAVGLGPR.L |
|  | 13777 | 781.3997 | 1560.7849 | 1561.7923 | -1.0074 | 0 | (7) | 26 | 3 | R.MTALAQDFAVGLGPR.L 13769 |
|  | 14157 | **793.9615** | **1585.9085** | **1584.8294** | **1.0792** | **1** | **1** | **91** | **7** | **R.AMVDPAQTVEQRLK.L** |
|  | 16409 | **865.9426** | **1729.8707** | **1729.8821** | **-0.0114** | **0** | **39** | **0.015** | **1** | **R.LLGSTIPLCSAQWER.M** 16410 |
|  | 17639 | 608.9841 | 1823.9304 | 1824.7845 | -0.8541 | 1 | 6 | 29 | 4 | K.DMGKFCLTYEASMTR.L |
|  | 17686 | **914.4610** | **1826.9074** | **1825.9131** | **0.9943** | **0** | **53** | **0.00054** | **1** | **K.EAMTDIITLFGLSSNSK.K** |
|  | 22041 | 1111.6786 | 2221.3426 | 2220.1460 | 1.1966 | 1 | 3 | 32 | 5 | R.HLKEAMTDIITLFGLSSNSK.K |
|  | 22375 | **755.1022** | **2262.2847** | **2262.0521** | **0.2326** | **1** | **14** | **2.8** | **1** | **R.MFNTSRIPGEETDTIQHMR.D** |
|  | 24856 | **651.5742** | **2602.2675** | **2602.1751** | **0.0924** | **1** | **2** | **43** | **8** | **R.EMEQQMQRILDNTSEPQPGEAR.L** |
|  | 24922 | **873.5001** | **2617.4784** | **2617.2548** | **0.2236** | **1** | **2** | **35** | **4** | **K.QSLDAVEKAAFFVTLDETEEGYR.S** |
|  | 27803 | **1058.7900** | **3173.3483** | **3173.3659** | **-0.0176** | **1** | **74** | **1.5e-006** | **1** | **K.AAFFVTLDETEEGYRSEDPDTSMDSYAK.S** |
|  | 29328 | **1239.5742** | **3715.7008** | **3714.8784** | **0.8225** | **1** | **1** | **28** | **2** | **R.TLETANCMSSQTKNVVSGVLFGTGLWVALIVTMR.Y** |
|  | 30190 | **677.6803** | **4060.0381** | **4061.1985** | **-1.1604** | **1** | **(0)** | **26** | **7** | **K.NGIITGVYPASPSSWLIVVVGVMTTMYAKIDPSLGIIAK.I** |
|  | 30231 | 1020.5082 | 4078.0036 | 4077.1934 | 0.8102 | 1 | (1) | 21 | 6 | K.NGIITGVYPASPSSWLIVVVGVMTTMYAKIDPSLGIIAK.I |
|  | 30281 | 1365.0172 | 4092.0298 | 4093.1884 | -1.1586 | 1 | 5 | 8.9 | 4 | K.NGIITGVYPASPSSWLIVVVGVMTTMYAKIDPSLGIIAK.I |
|  | 30815 | **1106.2847** | **4421.1096** | **4421.1315** | **-0.0219** | **0** | **22** | **0.12** | **1** | **R.LQWDIPGECQEVIETSLNTANLLANDVDFHSFPFVAFGK.G** |
|  | 31752 | **1151.1376** | **5750.6515** | **5750.7021** | **-0.0506** | **1** | **34** | **0.0032** | **1** | **K.MGLNAEHSWADAPIVAHLWEYVMSIDSLQLGYAEDGHCKGDINPNIPYPTR.L** |

| **225.** | P48449\|ERG7_HUMAN    **Mass:** 84453    **Score:** 224    **Queries matched:** 25   **emPAI:** 0.29 |
| --- | --- |
|  | Lanosterol synthase OS=Homo sapiens GN=LSS PE=1 SV=1 |

|  | **Query** | **Observed** | **Mr(expt)** | **Mr(calc)** | **Delta** | **Miss** | **Score** | **Expect** | **Rank** | **Peptide** |
| --- | --- | --- | --- | --- | --- | --- | --- | --- | --- | --- |
|  | 1139 | **565.3046** | **564.2974** | **564.3020** | **-0.0046** | **0** | **5** | **52** | **5** | **R.ALAGHP.-** |
|  | 1347 | **625.0812** | **624.0740** | **623.3755** | **0.6985** | **0** | **7** | **17** | **5** | **R.NILHK.K** |
|  | 4369 | **491.2412** | **980.4678** | **980.4749** | **-0.0071** | **0** | **11** | **12** | **1** | **R.ACDFLLSR.Q** |
|  | 4520 | **496.9103** | **991.8060** | **991.4869** | **0.3191** | **1** | **8** | **18** | **6** | **M.TEGTCLRR.R** |
|  | 5185 | 520.7623 | 1039.5101 | 1038.5134 | 0.9967 | 0 | 10 | 15 | 1 | R.FSQLYPER.A |
|  | 9306 | **650.2911** | **1298.5677** | **1298.5779** | **-0.0102** | **0** | **55** | **0.00045** | **1** | **R.NPDGGFATYETK.R** |
|  | 10081 | **670.3129** | **1338.6112** | **1338.6204** | **-0.0092** | **0** | **55** | **0.00045** | **1** | **R.QTWTYLQDER.A** |
|  | 10337 | **677.3225** | **1352.6303** | **1352.6394** | **-0.0091** | **0** | **8** | **23** | **1** | **R.ETLTQGLEFCR.R** |
|  | 10542 | 683.3671 | 1364.7197 | 1364.7300 | -0.0103 | 0 | 34 | 0.053 | 1 | R.ILGVGPDDPDLVR.A 10543 |
|  | 10710 | **687.8619** | **1373.7093** | **1373.7159** | **-0.0066** | **0** | **86** | **3.3e-007** | **1** | **R.LCDAVAVLLNMR.N** |
|  | 11146 | **699.8783** | **1397.7420** | **1397.7514** | **-0.0094** | **0** | **6** | **34** | **1** | **R.LSAAEDPLVQSLR.Q** |
|  | 12067 | **485.6782** | **1454.0127** | **1454.6790** | **-0.6663** | **1** | **5** | **29** | **7** | **R.NPDGGFATYETKR.G** |
|  | 12843 | 751.3687 | 1500.7227 | 1499.7256 | 0.9971 | 0 | 20 | 1.3 | 1 | R.LSQVPDNPPDYQK.Y |
|  | 13416 | **769.8686** | **1537.7226** | **1537.7309** | **-0.0083** | **0** | **20** | **1.2** | **1** | **R.IPDYLWMGLDGMK.M** 13417 13430 |
|  | 13625 | **517.9241** | **1550.7506** | **1551.7430** | **-0.9924** | **1** | **9** | **15** | **2** | **R.GRQTWTYLQDER.A** |
|  | 14463 | **804.4116** | **1606.8086** | **1605.8151** | **0.9935** | **1** | **18** | **2** | **2** | **K.LYEHIVADDRFTK.S** |
|  | 14477 | **804.9041** | **1607.7937** | **1607.8042** | **-0.0106** | **0** | **81** | **1.1e-006** | **1** | **R.EQTGLEAYALGLDTK.N** |
|  | 19235 | 975.4714 | 1948.9283 | 1948.9068 | 0.0215 | 0 | 12 | 6 | 2 | R.WYVDGPASTAFQEHVSR.I |
|  | 19843 | **1001.9403** | **2001.8659** | **2001.7469** | **0.1190** | **0** | **5** | **30** | **3** | **R.QMADGGWGEDFESCEER.R** |
|  | 20129 | **1013.0066** | **2023.9986** | **2024.0116** | **-0.0129** | **0** | **46** | **0.0025** | **1** | **R.NNVAPDELYTPHSWLLR.V** |
|  | 21601 | **725.9066** | **2174.6979** | **2173.8429** | **0.8549** | **1** | **4** | **22** | **1** | **R.QMADGGWGEDFESCEERR.Y** |
|  | 28749 | 1157.5749 | 3469.7030 | 3468.6271 | 1.0759 | 1 | 0 | 38 | 6 | R.WYVDGPASTAFQEHVSRIPDYLWMGLDGMK.M |

| **226.** | O75396\|SC22B_HUMAN    **Mass:** 24896    **Score:** 224    **Queries matched:** 17   **emPAI:** 1.35 |
| --- | --- |
|  | Vesicle-trafficking protein SEC22b OS=Homo sapiens GN=SEC22B PE=1 SV=3 |

|  | **Query** | **Observed** | **Mr(expt)** | **Mr(calc)** | **Delta** | **Miss** | **Score** | **Expect** | **Rank** | **Peptide** |
| --- | --- | --- | --- | --- | --- | --- | --- | --- | --- | --- |
|  | 3198 | **448.2736** | **894.5326** | **893.4970** | **1.0355** | **1** | **6** | **38** | **3** | **K.KLYIDSR.A** |
|  | 3711 | **467.2505** | **932.4864** | **932.4927** | **-0.0062** | **0** | **19** | **2.3** | **1** | **K.ANNLSSLSK.K** 3712 3719 |
|  | 5265 | **523.7759** | **1045.5372** | **1046.5980** | **-1.0608** | **0** | **7** | **34** | **8** | **-.MVLLTMIAR.V** |
|  | 5431 | **531.2659** | **1060.5173** | **1060.5876** | **-0.0703** | **1** | **10** | **18** | **5** | **K.ANNLSSLSKK.Y** |
|  | 5586 | **536.7870** | **1071.5594** | **1071.5672** | **-0.0078** | **1** | **47** | **0.0034** | **1** | **R.KLNEQSPTR.C** 5587 5588 |
|  | 7573 | **604.7944** | **1207.5742** | **1207.5833** | **-0.0091** | **0** | **35** | **0.042** | **1** | **R.DLQQYQSQAK.Q** |
|  | 11415 | **707.8851** | **1413.7556** | **1413.7650** | **-0.0094** | **0** | **81** | **1.1e-006** | **1** | **R.IMVANIEEVLQR.G** |
|  | 14153 | **793.9045** | **1585.7945** | **1585.8060** | **-0.0115** | **0** | **62** | **7.9e-005** | **1** | **R.NLGSINTELQDVQR.I** |
|  | 19510 | **987.4581** | **1972.9016** | **1972.9160** | **-0.0144** | **0** | **74** | **3.5e-006** | **1** | **R.VADGLPLAASMQEDEQSGR.D** 19511 |
|  | 23371 | **796.0889** | **2385.2448** | **2385.2573** | **-0.0125** | **1** | **25** | **0.22** | **1** | **R.IMVANIEEVLQRGEALSALDSK.A** |
|  | 23468 | **801.4251** | **2401.2533** | **2401.2682** | **-0.0149** | **1** | **14** | **2.7** | **1** | **K.KVPTVSRPYSFIEFDTFIQK.T** |
|  | 26512 | **968.5031** | **2902.4875** | **2902.4528** | **0.0347** | **1** | **1** | **41** | **1** | **M.VLLTMIARVADGLPLAASMQEDEQSGR.D** |

| **227.** | P08107\|HSP71_HUMAN    **Mass:** 70294    **Score:** 221    **Queries matched:** 30   **emPAI:** 0.36 |
| --- | --- |
|  | Heat shock 70 kDa protein 1 OS=Homo sapiens GN=HSPA1A PE=1 SV=5 |

|  | **Query** | **Observed** | **Mr(expt)** | **Mr(calc)** | **Delta** | **Miss** | **Score** | **Expect** | **Rank** | **Peptide** |
| --- | --- | --- | --- | --- | --- | --- | --- | --- | --- | --- |
|  | 1539 | 686.4146 | 685.4073 | 686.3963 | -0.9889 | 0 | 12 | 12 | 10 | R.LIGDAAK.N |
|  | 1555 | **690.0817** | **689.0744** | **688.3868** | **0.6876** | **1** | **21** | **1.4** | **1** | **R.ERVSAK.N** |
|  | 2017 | 804.4382 | 803.4309 | 803.4389 | -0.0080 | 0 | 23 | 1.1 | 1 | K.ITITNDK.G |
|  | 4891 | 509.2687 | 1016.5228 | 1016.5614 | -0.0386 | 1 | 8 | 29 | 7 | K.ITITNDKGR.L 4890 |
|  | 6085 | **555.8871** | **1109.7596** | **1108.5665** | **1.1931** | **0** | **3** | **64** | **7** | **K.LLQDFFNGR.D** |
|  | 7856 | **611.7879** | **1221.5612** | **1221.5700** | **-0.0087** | **0** | **46** | **0.0041** | **1** | **K.FGDPVVQSDMK.H** |
|  | 7971 | 614.8130 | 1227.6115 | 1227.6207 | -0.0092 | 0 | 24 | 0.56 | 1 | K.VEIIANDQGNR.T |
|  | 8366 | 416.5560 | 1246.6462 | 1247.6292 | -0.9830 | 1 | 12 | 9.3 | 4 | R.AMTKDNNLLGR.F |
|  | 8613 | **630.9919** | **1259.9693** | **1260.6503** | **-0.6809** | **0** | **8** | **15** | **1** | **R.LVNHFVEEFK.R** |
|  | 9084 | 644.3015 | 1286.5885 | 1286.5965 | -0.0080 | 0 | 30 | 0.16 | 1 | K.NALESYAFNMK.S 9083 |
|  | 10304 | 451.2620 | 1350.7642 | 1349.6649 | 1.0993 | 1 | (7) | 29 | 6 | R.KFGDPVVQSDMK.H |
|  | 10564 | **456.2163** | **1365.6269** | **1365.6599** | **-0.0329** | **1** | **7** | **26** | **4** | **R.KFGDPVVQSDMK.H** |
|  | 12261 | 489.5734 | 1465.6983 | 1464.8049 | 0.8935 | 0 | 4 | 52 | 8 | K.AQIHDLVLVGGSTR.I |
|  | 12631 | 744.3494 | 1486.6842 | 1486.6940 | -0.0098 | 0 | 65 | 4.2e-005 | 1 | R.TTPSYVAFTDTER.L 12606 12630 |
|  | 14734 | 813.3951 | 1624.7757 | 1624.9260 | -0.1503 | 1 | 13 | 6.1 | 2 | R.QATKDAGVIAGLNVLR.I 14733 14747 |
|  | 14809 | 815.9115 | 1629.8084 | 1629.7960 | 0.0125 | 0 | 5 | 41 | 9 | K.AFYPEEISSMVLTK.M |
|  | 15266 | **829.9232** | **1657.8319** | **1657.8424** | **-0.0105** | **0** | **68** | **1.6e-005** | **1** | **K.NQVALNPQNTVFDAK.R** 15259 |
|  | 15498 | 838.3627 | 1674.7108 | 1674.7234 | -0.0126 | 0 | 65 | 3.5e-005 | 1 | K.ATAGDTHLGGEDFDNR.L |
|  | 15684 | **563.8006** | **1688.3800** | **1687.8239** | **0.5561** | **1** | **1** | **54** | **4** | **R.VSAKNALESYAFNMK.S** 15676 |
|  | 18521 | 945.4586 | 1888.9026 | 1888.9314 | -0.0289 | 1 | 0 | 97 | 5 | K.AFYPEEISSMVLTKMK.E |
|  | 20184 | **677.3671** | **2029.0794** | **2029.0078** | **0.0716** | **1** | **11** | **8** | **1** | **K.GETKAFYPEEISSMVLTK.M** |
|  | 28111 | **1087.5413** | **3259.6020** | **3259.6223** | **-0.0204** | **1** | **24** | **0.2** | **1** | **K.MKEIAEAYLGYPVTNAVITVPAYFNDSQR.Q** |

| **228.** | Q9HBI6\|CP4FB_HUMAN    **Mass:** 60843    **Score:** 221    **Queries matched:** 19   **emPAI:** 0.61 |
| --- | --- |
|  | Cytochrome P450 4F11 OS=Homo sapiens GN=CYP4F11 PE=2 SV=2 |

|  | **Query** | **Observed** | **Mr(expt)** | **Mr(calc)** | **Delta** | **Miss** | **Score** | **Expect** | **Rank** | **Peptide** |
| --- | --- | --- | --- | --- | --- | --- | --- | --- | --- | --- |
|  | 2878 | **435.2685** | **868.5224** | **867.5542** | **0.9683** | **0** | **7** | **28** | **7** | **R.KPELILR.A** |
|  | 3271 | 451.2620 | 900.5095 | 900.4817 | 0.0278 | 0 | 6 | 45 | 6 | R.AEGGLWLR.V |
|  | 3460 | **457.7575** | **913.5005** | **913.4505** | **0.0500** | **0** | **10** | **18** | **2** | **R.VEPLGANSQ.-** |
|  | 4431 | 493.7759 | 985.5372 | 985.5444 | -0.0072 | 0 | 32 | 0.11 | 1 | R.QEVQELLK.D |
|  | 6141 | 558.3129 | 1114.6112 | 1113.5641 | 1.0471 | 0 | 9 | 20 | 1 | R.LQCFPQPPK.Q |
|  | 10440 | 680.3709 | 1358.7271 | 1358.7347 | -0.0075 | 0 | 36 | 0.033 | 1 | R.SPLAFIPFSAGPR.N |
|  | 10747 | 688.9023 | 1375.7901 | 1375.7963 | -0.0061 | 0 | 78 | 2.3e-006 | 1 | K.TLDFIDVLLLSK.D |
|  | 12338 | 735.8257 | 1469.6368 | 1469.6465 | -0.0097 | 0 | 58 | 0.0002 | 1 | R.NCIGQAFAMAEMK.V |
|  | 14261 | 796.8909 | 1591.7672 | 1590.9232 | 0.8439 | 1 | 2 | 76 | 10 | K.SKTLDFIDVLLLSK.D |
|  | 14311 | **798.9299** | **1595.8453** | **1595.8559** | **-0.0106** | **0** | **44** | **0.0044** | **1** | **K.TLTQLVTTYPQGFK.L** |
|  | 18786 | 637.6400 | 1909.8980 | 1909.9105 | -0.0125 | 0 | 34 | 0.039 | 1 | R.ACHLVHDFTDAVIQER.R |
|  | 18877 | 960.4878 | 1918.9610 | 1920.0092 | -1.0481 | 1 | 11 | 7.8 | 1 | K.TLDFIDVLLLSKDEDGK.E |
|  | 19253 | 651.0122 | 1950.0148 | 1950.0259 | -0.0111 | 0 | 15 | 3.3 | 2 | R.MLTPAFHFNILKPYMK.I |
|  | 20046 | 1011.0021 | 2019.9896 | 2020.0009 | -0.0113 | 0 | 13 | 4.3 | 1 | R.LDMFEHISLMTLDSLQK.C |
|  | 23663 | **812.4113** | **2434.2119** | **2434.2281** | **-0.0162** | **0** | **25** | **0.23** | **1** | **R.NQQILLHTDFLYYLTPDGQR.F** |
|  | 26889 | **743.6314** | **2970.4963** | **2970.5102** | **-0.0139** | **1** | **19** | **0.73** | **1** | **K.DMIFYGFLKPWLGDGLLLSGGDKWSR.H** 26890 |
|  | 27043 | 999.8007 | 2996.3802 | 2996.4015 | -0.0213 | 0 | 81 | 4.6e-007 | 1 | R.AEADTFMFEGHDTTASGLSWVLYHLAK.H 27044 |

| **229.** | P30041\|PRDX6_HUMAN    **Mass:** 25133    **Score:** 220    **Queries matched:** 20   **emPAI:** 0.76 |
| --- | --- |
|  | Peroxiredoxin-6 OS=Homo sapiens GN=PRDX6 PE=1 SV=3 |

|  | **Query** | **Observed** | **Mr(expt)** | **Mr(calc)** | **Delta** | **Miss** | **Score** | **Expect** | **Rank** | **Peptide** |
| --- | --- | --- | --- | --- | --- | --- | --- | --- | --- | --- |
|  | 5761 | **543.8081** | **1085.6017** | **1084.5917** | **1.0100** | **0** | **26** | **0.42** | **1** | **K.LPFPIIDDR.N** |
|  | 6185 | **559.7977** | **1117.5809** | **1118.5390** | **-0.9581** | **1** | **12** | **9.9** | **1** | **K.DEKGMPVTAR.V** 6182 6184 6207 |
|  | 11084 | **698.3276** | **1394.6406** | **1394.6500** | **-0.0094** | **0** | **43** | **0.0072** | **1** | **R.DFTPVCTTELGR.A** |
|  | 16221 | **573.7926** | **1718.3560** | **1718.9178** | **-0.5619** | **1** | **3** | **39** | **8** | **K.GMPVTARVVFVFGPDK.K** |
|  | 18458 | **942.9973** | **1883.9799** | **1883.9914** | **-0.0114** | **1** | **50** | **0.0011** | **1** | **R.ELAILLGMLDPAEKDEK.G** 18467 |
|  | 18601 | **949.0009** | **1895.9873** | **1895.9993** | **-0.0120** | **0** | **56** | **0.00023** | **1** | **K.LIALSIDSVEDHLAWSK.D** 18600 18613 |
|  | 18646 | **634.3189** | **1899.9347** | **1899.9863** | **-0.0516** | **1** | **(1)** | **83** | **2** | **R.ELAILLGMLDPAEKDEK.G** |
|  | 20191 | **508.4995** | **2029.9688** | **2029.9799** | **-0.0111** | **0** | **14** | **3.7** | **1** | **R.FHDFLGDSWGILFSHPR.D** 20190 |
|  | 20928 | 1049.5436 | 2097.0726 | 2097.0855 | -0.0129 | 0 | 80 | 9.8e-007 | 1 | M.PGGLLLGDVAPNFEANTTVGR.I 20929 |
|  | 22129 | **558.0503** | **2228.1723** | **2228.1260** | **0.0463** | **0** | **12** | **4.7** | **1** | **-.MPGGLLLGDVAPNFEANTTVGR.I** |
|  | 22256 | **748.8586** | **2243.5539** | **2244.1209** | **-0.5670** | **0** | **(4)** | **23** | **1** | **-.MPGGLLLGDVAPNFEANTTVGR.I** |
|  | 25490 | **909.4526** | **2725.3361** | **2725.3521** | **-0.0160** | **1** | **2** | **37** | **1** | **R.VATPVDWKDGDSVMVLPTIPEEEAK.K** |

| **230.** | Q07065\|CKAP4_HUMAN    **Mass:** 66097    **Score:** 218    **Queries matched:** 22   **emPAI:** 0.47 |
| --- | --- |
|  | Cytoskeleton-associated protein 4 OS=Homo sapiens GN=CKAP4 PE=1 SV=2 |

|  | **Query** | **Observed** | **Mr(expt)** | **Mr(calc)** | **Delta** | **Miss** | **Score** | **Expect** | **Rank** | **Peptide** |
| --- | --- | --- | --- | --- | --- | --- | --- | --- | --- | --- |
|  | 1582 | **705.2689** | **704.2616** | **703.4116** | **0.8500** | **0** | **14** | **8.9** | **1** | **R.LTELTK.S** |
|  | 1717 | **758.3886** | **757.3813** | **757.4698** | **-0.0884** | **1** | **17** | **4.6** | **5** | **K.ALKEAVK.E** |
|  | 2670 | **429.2551** | **856.4956** | **856.5018** | **-0.0061** | **0** | **40** | **0.02** | **1** | **K.LQNEILK.D** |
|  | 3162 | 446.7122 | 891.4098 | 890.4094 | 1.0005 | 0 | 10 | 21 | 7 | K.QGESEVSR.I |
|  | 3846 | **472.7582** | **943.5019** | **943.5087** | **-0.0067** | **1** | **31** | **0.14** | **1** | **R.RLEEELR.Q** |
|  | 5069 | **516.2939** | **1030.5732** | **1030.5659** | **0.0074** | **1** | **9** | **21** | **2** | **K.QDLTEKAVK.Q** |
|  | 5929 | **550.2580** | **1098.5014** | **1099.5662** | **-1.0648** | **0** | **6** | **42** | **6** | **R.LPPQDFLDR.L** |
|  | 6543 | 573.7993 | 1145.5840 | 1145.5928 | -0.0088 | 0 | 12 | 11 | 1 | K.ASVSQVEADLK.M |
|  | 11415 | 707.8851 | 1413.7556 | 1413.7100 | 0.0456 | 0 | 10 | 13 | 7 | R.EELGQGLQGVEQK.V 11410 |
|  | 12391 | **737.3881** | **1472.7617** | **1472.7722** | **-0.0105** | **0** | **33** | **0.061** | **1** | **R.SVGELPSTVESLQK.V** |
|  | 15069 | 823.7813 | 1645.5481 | 1645.8159 | -0.2678 | 1 | 17 | 1.6 | 1 | K.VASLEESEGNKQDLK.A |
|  | 16292 | **862.9018** | **1723.7890** | **1723.8013** | **-0.0123** | **1** | **5** | **34** | **1** | **R.ERDFTSLENTVEER.L** |
|  | 16629 | **583.3120** | **1746.9140** | **1746.9264** | **-0.0124** | **1** | **5** | **32** | **7** | **K.SINDNIAIFTEVQKR.S** |
|  | 16973 | 886.9161 | 1771.8177 | 1771.8298 | -0.0121 | 0 | 46 | 0.0026 | 1 | R.STLQTMESDIYTEVR.E |
|  | 17307 | **898.9750** | **1795.9354** | **1795.9469** | **-0.0114** | **0** | **53** | **0.00057** | **1** | **K.VQSLQATFGTFESILR.S** |
|  | 17853 | **614.3029** | **1839.8868** | **1838.9309** | **0.9559** | **0** | **5** | **36** | **3** | **R.LQHVEDGVLSMQVASAR.Q** |
|  | 18888 | **481.0213** | **1920.0562** | **1920.0680** | **-0.0118** | **1** | **44** | **0.0038** | **1** | **K.LQNEILKDLSDGIHVVK.D** |
|  | 20071 | **1011.5144** | **2021.0142** | **2021.0290** | **-0.0148** | **0** | **97** | **2e-008** | **1** | **K.VQEQVHTLLSQDQAQAAR.L** 20070 |
|  | 21463 | **720.3755** | **2158.1046** | **2158.9992** | **-0.8945** | **1** | **1** | **69** | **3** | **K.GGHGAASPSEKGAHPSGGADDVAK.K** |
|  | 28801 | **874.4338** | **3493.7060** | **3492.7664** | **0.9396** | **0** | **3** | **19** | **4** | **K.KPPPAPQQPPPPPAPHPQQHPQQHPQNQAHGK.G** |

| **231.** | P05387\|RLA2_HUMAN    **Mass:** 11658    **Score:** 218    **Queries matched:** 11   **emPAI:** 4.81 |
| --- | --- |
|  | 60S acidic ribosomal protein P2 OS=Homo sapiens GN=RPLP2 PE=1 SV=1 |

|  | **Query** | **Observed** | **Mr(expt)** | **Mr(calc)** | **Delta** | **Miss** | **Score** | **Expect** | **Rank** | **Peptide** |
| --- | --- | --- | --- | --- | --- | --- | --- | --- | --- | --- |
|  | 8526 | **628.8417** | **1255.6688** | **1255.6772** | **-0.0084** | **0** | **39** | **0.019** | **1** | **K.NIEDVIAQGIGK.L** |
|  | 16994 | **887.4269** | **1772.8392** | **1771.8952** | **0.9440** | **1** | **27** | **0.22** | **1** | **K.ILDSVGIEADDDRLNK.V** 16995 |
|  | 18270 | **934.9863** | **1867.9581** | **1867.9679** | **-0.0098** | **0** | **80** | **9.8e-007** | **1** | **R.YVASYLLAALGGNSSPSAK.D** 18267 18268 18279 |
|  | 20932 | **1049.5673** | **2097.1200** | **2096.1477** | **0.9723** | **1** | **42** | **0.0049** | **1** | **K.VISELNGKNIEDVIAQGIGK.L** |
|  | 25743 | **1387.7095** | **2773.4044** | **2773.4246** | **-0.0202** | **0** | **63** | **2.7e-005** | **1** | **K.LASVPAGGAVAVSAAPGSAAPAAGSAPAAAEEK.K** 25742 25744 |

| **232.** | Q6UX53\|MET7B_HUMAN    **Mass:** 28269    **Score:** 217    **Queries matched:** 13   **emPAI:** 0.65 |
| --- | --- |
|  | Methyltransferase-like protein 7B OS=Homo sapiens GN=METTL7B PE=2 SV=2 |

|  | **Query** | **Observed** | **Mr(expt)** | **Mr(calc)** | **Delta** | **Miss** | **Score** | **Expect** | **Rank** | **Peptide** |
| --- | --- | --- | --- | --- | --- | --- | --- | --- | --- | --- |
|  | 2526 | **845.4195** | **844.4122** | **844.4191** | **-0.0069** | **0** | **20** | **2.3** | **1** | **R.HLQYER.F** |
|  | 7150 | **595.2787** | **1188.5429** | **1187.5176** | **1.0254** | **0** | **8** | **27** | **1** | **K.HIGDGCCLTR.E** |
|  | 8102 | **412.2278** | **1233.6615** | **1233.6692** | **-0.0077** | **0** | **19** | **1.7** | **1** | **K.WLPVGPHIMGK.A** 8103 |
|  | 12082 | **728.8436** | **1455.6726** | **1455.6817** | **-0.0091** | **0** | **37** | **0.028** | **1** | **R.VTCLDPNPHFEK.F** 12081 |
|  | 22308 | **1127.5186** | **2253.0225** | **2253.0372** | **-0.0146** | **1** | **90** | **8e-008** | **1** | **R.ETWKDLENAQFSEIQMER.Q** 22307 22309 |
|  | 22423 | **757.3860** | **2269.1363** | **2269.0321** | **0.1042** | **1** | **(2)** | **55** | **8** | **R.ETWKDLENAQFSEIQMER.Q** |
|  | 23621 | **1214.0735** | **2426.1324** | **2426.1511** | **-0.0187** | **0** | **60** | **7.7e-005** | **1** | **K.VALLELGCGTGANFQFYPPGCR.V** 23622 |
|  | 29211 | **1222.8959** | **3665.6658** | **3666.7515** | **-1.0857** | **1** | **1** | **25** | **3** | **R.FVVAPGEDMRQLADGSMDVVVCTLVLCSVQSPR.K** |

| **233.** | P33261\|CP2CJ_HUMAN    **Mass:** 56636    **Score:** 217    **Queries matched:** 38   **emPAI:** 1.02 |
| --- | --- |
|  | Cytochrome P450 2C19 OS=Homo sapiens GN=CYP2C19 PE=1 SV=3 |

|  | **Query** | **Observed** | **Mr(expt)** | **Mr(calc)** | **Delta** | **Miss** | **Score** | **Expect** | **Rank** | **Peptide** |
| --- | --- | --- | --- | --- | --- | --- | --- | --- | --- | --- |
|  | 1456 | 653.3032 | 652.2959 | 652.3003 | -0.0043 | 0 | 14 | 5.1 | 6 | R.NFGMGK.R |
|  | 1734 | 762.4299 | 761.4226 | 761.4283 | -0.0057 | 0 | 33 | 0.11 | 1 | K.SLTNLSK.I |
|  | 1766 | 781.4157 | 780.4084 | 780.4130 | -0.0046 | 0 | 34 | 0.074 | 1 | K.HPEVTAK.V |
|  | 2082 | 405.2055 | 808.3964 | 808.4014 | -0.0050 | 1 | 11 | 13 | 4 | R.NFGMGKR.S |
|  | 3057 | 442.2357 | 882.4568 | 882.4633 | -0.0065 | 0 | 20 | 1.4 | 1 | R.FSLMTLR.N |
|  | 3308 | 452.2098 | 902.4050 | 901.4505 | 0.9545 | 0 | 12 | 11 | 1 | K.VQEEIER.V |
|  | 3511 | 459.7364 | 917.4583 | 917.4640 | -0.0057 | 0 | 34 | 0.073 | 1 | R.CLVEELR.K 3510 |
|  | 3888 | 473.8176 | 945.6206 | 945.6263 | -0.0057 | 0 | 49 | 0.0022 | 1 | R.YALLLLLK.H |
|  | 4275 | **487.9090** | **973.8034** | **973.5015** | **0.3020** | **0** | **7** | **26** | **1** | **R.ICVGEGLAR.M** |
|  | 4952 | 512.2885 | 1022.5623 | 1022.5695 | -0.0072 | 1 | 29 | 0.18 | 1 | R.RFSLMTLR.N 4953 |
|  | 4995 | 513.7629 | 1025.5112 | 1024.5342 | 0.9770 | 0 | 27 | 0.31 | 1 | R.GFGIVFSNGK.R |
|  | 6749 | 582.2515 | 1162.4885 | 1162.4971 | -0.0086 | 1 | 30 | 0.16 | 1 | R.NRSPCMQDR.G 6750 6751 6752 6753 6754 6755 6775 |
|  | 6857 | 585.7937 | 1169.5728 | 1169.5791 | -0.0062 | 0 | 44 | 0.0063 | 1 | R.DFIDCFLIK.M 6856 |
|  | 6970 | **590.8045** | **1179.5944** | **1180.6353** | **-1.0409** | **1** | **2** | **1.1e+002** | **3** | **R.GFGIVFSNGKR.W** |
|  | 8708 | 633.7903 | 1265.5661 | 1264.6122 | 0.9540 | 0 | 11 | 11 | 2 | K.DQQFLNLMEK.L |
|  | 9160 | **431.2163** | **1290.6269** | **1290.6357** | **-0.0088** | **1** | **24** | **0.55** | **1** | **R.HFLDEGGNFKK.S** |
|  | 9945 | 666.3270 | 1330.6394 | 1330.6477 | -0.0083 | 1 | 37 | 0.032 | 1 | R.SIEDRVQEEAR.C |
|  | 10745 | 688.8618 | 1375.7090 | 1375.6594 | 0.0495 | 1 | 3 | 65 | 4 | K.KSNYFMPFSAGK.R |
|  | 11598 | **712.8061** | **1423.5976** | **1424.6857** | **-1.0881** | **0** | **6** | **29** | **4** | **K.NLAFMESDILEK.V** |
|  | 11765 | 718.3525 | 1434.6904 | 1434.6991 | -0.0086 | 0 | 63 | 7.1e-005 | 1 | K.EALIDLGEEFSGR.G 11764 11778 |
|  | 15349 | 555.5905 | 1663.7497 | 1663.8529 | -0.1032 | 1 | 7 | 22 | 3 | K.HPEVTAKVQEEIER.V |
|  | 15830 | **848.9013** | **1695.7879** | **1695.7998** | **-0.0119** | **1** | **57** | **0.00022** | **1** | **K.VKEHQESMDINNPR.D** 15831 |
|  | 17572 | 909.9340 | 1817.8535 | 1817.8658 | -0.0123 | 1 | 48 | 0.0016 | 1 | R.FDYKDQQFLNLMEK.L |
|  | 20272 | **681.3538** | **2041.0395** | **2041.0554** | **-0.0160** | **0** | **1** | **67** | **2** | **R.YIDLIPTSLPHAVTCDVK.F** |
|  | 27438 | **1025.8450** | **3074.5131** | **3074.5324** | **-0.0193** | **0** | **6** | **13** | **1** | **R.IVSTPWIQICNNFPTIIDYFPGTHNK.L** |

| **234.** | P33778\|H2B1B_HUMAN    **Mass:** 13942    **Score:** 217    **Queries matched:** 14   **emPAI:** 0.28 |
| --- | --- |
|  | Histone H2B type 1-B OS=Homo sapiens GN=HIST1H2BB PE=1 SV=2 |

|  | **Query** | **Observed** | **Mr(expt)** | **Mr(calc)** | **Delta** | **Miss** | **Score** | **Expect** | **Rank** | **Peptide** |
| --- | --- | --- | --- | --- | --- | --- | --- | --- | --- | --- |
|  | 2160 | **816.4524** | **815.4451** | **815.4501** | **-0.0050** | **0** | **17** | **4.1** | **1** | **R.EIQTAVR.L** |
|  | 2202 | 820.3386 | 819.3313 | 819.4563 | -0.1250 | 1 | 12 | 12 | 3 | K.RSTITSR.E |
|  | 2333 | **415.2289** | **828.4431** | **827.4137** | **1.0294** | **0** | **9** | **29** | **5** | **K.HAVSEGTK.A** 2320 2321 |
|  | 3990 | **477.3019** | **952.5892** | **952.5957** | **-0.0065** | **0** | **18** | **2.4** | **2** | **R.LLLPGELAK.H** 3986 3994 |
|  | 8524 | 628.8367 | 1255.6588 | 1254.6278 | 1.0310 | 1 | 9 | 19 | 2 | -.MPEPSKSAPAPK.K |
|  | 8940 | **640.3275** | **1278.6405** | **1278.6496** | **-0.0091** | **1** | **11** | **11** | **1** | **R.KESYSIYVYK.V** |
|  | 16572 | **872.4073** | **1742.8000** | **1742.8120** | **-0.0120** | **0** | **120** | **1.1e-010** | **1** | **K.AMGIMNSFVNDIFER.I** 16571 16573 16585 |

|  | **Proteins matching a subset of these peptides:** |
| --- | --- |

|  | P06899\|H2B1J_HUMAN    **Mass:** 13896    **Score:** 217    **Queries matched:** 13 |
| --- | --- |
|  | Histone H2B type 1-J OS=Homo sapiens GN=HIST1H2BJ PE=1 SV=3 |

|  | Q16778\|H2B2E_HUMAN    **Mass:** 13912    **Score:** 217    **Queries matched:** 13 |
| --- | --- |
|  | Histone H2B type 2-E OS=Homo sapiens GN=HIST2H2BE PE=1 SV=3 |

|  | Q99877\|H2B1N_HUMAN    **Mass:** 13914    **Score:** 217    **Queries matched:** 13 |
| --- | --- |
|  | Histone H2B type 1-N OS=Homo sapiens GN=HIST1H2BN PE=1 SV=3 |

|  | O60814\|H2B1K_HUMAN    **Mass:** 13882    **Score:** 217    **Queries matched:** 12 |
| --- | --- |
|  | Histone H2B type 1-K OS=Homo sapiens GN=HIST1H2BK PE=1 SV=3 |

|  | P62807\|H2B1C_HUMAN    **Mass:** 13811    **Score:** 217    **Queries matched:** 12 |
| --- | --- |
|  | Histone H2B type 1-C/E/F/G/I OS=Homo sapiens GN=HIST1H2BC PE=1 SV=3 |

| **235.** | P23527\|H2B1O_HUMAN    **Mass:** 13898    **Score:** 217    **Queries matched:** 15   **emPAI:** 0.28 |
| --- | --- |
|  | Histone H2B type 1-O OS=Homo sapiens GN=HIST1H2BO PE=1 SV=3 |

|  | **Query** | **Observed** | **Mr(expt)** | **Mr(calc)** | **Delta** | **Miss** | **Score** | **Expect** | **Rank** | **Peptide** |
| --- | --- | --- | --- | --- | --- | --- | --- | --- | --- | --- |
|  | 2160 | 816.4524 | 815.4451 | 815.4501 | -0.0050 | 0 | 17 | 4.1 | 1 | R.EIQTAVR.L |
|  | 2202 | 820.3386 | 819.3313 | 819.4563 | -0.1250 | 1 | 12 | 12 | 3 | K.RSTITSR.E |
|  | 2333 | 415.2289 | 828.4431 | 827.4137 | 1.0294 | 0 | 9 | 29 | 5 | K.HAVSEGTK.A 2320 2321 |
|  | 3990 | 477.3019 | 952.5892 | 952.5957 | -0.0065 | 0 | 18 | 2.4 | 2 | R.LLLPGELAK.H 3986 3994 |
|  | 5660 | **540.2666** | **1078.5186** | **1077.5818** | **0.9368** | **1** | **2** | **99** | **3** | **M.PDPAKSAPAPK.K** |
|  | 7577 | **604.8005** | **1207.5865** | **1208.6223** | **-1.0358** | **1** | **3** | **81** | **7** | **-.MPDPAKSAPAPK.K** |
|  | 8940 | 640.3275 | 1278.6405 | 1278.6496 | -0.0091 | 1 | 11 | 11 | 1 | R.KESYSIYVYK.V |
|  | 16572 | 872.4073 | 1742.8000 | 1742.8120 | -0.0120 | 0 | 120 | 1.1e-010 | 1 | K.AMGIMNSFVNDIFER.I 16571 16573 16585 |

|  | **Proteins matching a subset of these peptides:** |
| --- | --- |

|  | Q5QNW6\|H2B2F_HUMAN    **Mass:** 13912    **Score:** 217    **Queries matched:** 14 |
| --- | --- |
|  | Histone H2B type 2-F OS=Homo sapiens GN=HIST2H2BF PE=1 SV=3 |

|  | Q93079\|H2B1H_HUMAN    **Mass:** 13884    **Score:** 217    **Queries matched:** 14 |
| --- | --- |
|  | Histone H2B type 1-H OS=Homo sapiens GN=HIST1H2BH PE=1 SV=3 |

| **236.** | Q99879\|H2B1M_HUMAN    **Mass:** 13981    **Score:** 217    **Queries matched:** 13   **emPAI:** 0.28 |
| --- | --- |
|  | Histone H2B type 1-M OS=Homo sapiens GN=HIST1H2BM PE=1 SV=3 |

|  | **Query** | **Observed** | **Mr(expt)** | **Mr(calc)** | **Delta** | **Miss** | **Score** | **Expect** | **Rank** | **Peptide** |
| --- | --- | --- | --- | --- | --- | --- | --- | --- | --- | --- |
|  | 2160 | 816.4524 | 815.4451 | 815.4501 | -0.0050 | 0 | 17 | 4.1 | 1 | R.EIQTAVR.L |
|  | 2202 | 820.3386 | 819.3313 | 819.4563 | -0.1250 | 1 | 12 | 12 | 3 | K.RSTITSR.E |
|  | 2333 | 415.2289 | 828.4431 | 827.4137 | 1.0294 | 0 | 9 | 29 | 5 | K.HAVSEGTK.A 2320 2321 |
|  | 3990 | 477.3019 | 952.5892 | 952.5957 | -0.0065 | 0 | 18 | 2.4 | 2 | R.LLLPGELAK.H 3986 3994 |
|  | 9235 | 648.3835 | 1294.7525 | 1294.6955 | 0.0570 | 1 | 8 | 22 | 5 | -.MPEPVKSAPVPK.K |
|  | 16572 | 872.4073 | 1742.8000 | 1742.8120 | -0.0120 | 0 | 120 | 1.1e-010 | 1 | K.AMGIMNSFVNDIFER.I 16571 16573 16585 |

| **237.** | Q99880\|H2B1L_HUMAN    **Mass:** 13944    **Score:** 217    **Queries matched:** 13   **emPAI:** 0.28 |
| --- | --- |
|  | Histone H2B type 1-L OS=Homo sapiens GN=HIST1H2BL PE=1 SV=3 |

|  | **Query** | **Observed** | **Mr(expt)** | **Mr(calc)** | **Delta** | **Miss** | **Score** | **Expect** | **Rank** | **Peptide** |
| --- | --- | --- | --- | --- | --- | --- | --- | --- | --- | --- |
|  | 2160 | 816.4524 | 815.4451 | 815.4501 | -0.0050 | 0 | 17 | 4.1 | 1 | R.EIQTAVR.L |
|  | 2202 | 820.3386 | 819.3313 | 819.4563 | -0.1250 | 1 | 12 | 12 | 3 | K.RSTITSR.E |
|  | 2333 | 415.2289 | 828.4431 | 827.4137 | 1.0294 | 0 | 9 | 29 | 5 | K.HAVSEGTK.A 2320 2321 |
|  | 3990 | 477.3019 | 952.5892 | 952.5957 | -0.0065 | 0 | 18 | 2.4 | 2 | R.LLLPGELAK.H 3986 3994 |
|  | 8524 | 628.8367 | 1255.6588 | 1254.6642 | 0.9946 | 1 | 6 | 42 | 4 | -.MPELAKSAPAPK.K |
|  | 16572 | 872.4073 | 1742.8000 | 1742.8120 | -0.0120 | 0 | 120 | 1.1e-010 | 1 | K.AMGIMNSFVNDIFER.I 16571 16573 16585 |

| **238.** | Q8N257\|H2B3B_HUMAN    **Mass:** 13900    **Score:** 217    **Queries matched:** 15   **emPAI:** 0.28 |
| --- | --- |
|  | Histone H2B type 3-B OS=Homo sapiens GN=HIST3H2BB PE=1 SV=3 |

|  | **Query** | **Observed** | **Mr(expt)** | **Mr(calc)** | **Delta** | **Miss** | **Score** | **Expect** | **Rank** | **Peptide** |
| --- | --- | --- | --- | --- | --- | --- | --- | --- | --- | --- |
|  | 2202 | 820.3386 | 819.3313 | 819.4563 | -0.1250 | 1 | 12 | 12 | 3 | K.RSTITSR.E |
|  | 2333 | 415.2289 | 828.4431 | 827.4137 | 1.0294 | 0 | 9 | 29 | 5 | K.HAVSEGTK.A 2320 2321 |
|  | 3990 | 477.3019 | 952.5892 | 952.5957 | -0.0065 | 0 | 18 | 2.4 | 2 | R.LLLPGELAK.H 3986 3994 |
|  | 7919 | **613.3374** | **1224.6602** | **1224.6172** | **0.0430** | **1** | **(4)** | **54** | **1** | **-.MPDPSKSAPAPK.K** |
|  | 8212 | **620.7972** | **1239.5799** | **1240.6122** | **-1.0322** | **1** | **5** | **39** | **5** | **-.MPDPSKSAPAPK.K** |
|  | 8940 | 640.3275 | 1278.6405 | 1278.6496 | -0.0091 | 1 | 11 | 11 | 1 | R.KESYSIYVYK.V |
|  | 16483 | 579.3234 | 1734.9483 | 1736.0196 | -1.0713 | 1 | 5 | 32 | 3 | R.EVQTAVRLLLPGELAK.H |
|  | 16572 | 872.4073 | 1742.8000 | 1742.8120 | -0.0120 | 0 | 120 | 1.1e-010 | 1 | K.AMGIMNSFVNDIFER.I 16571 16573 16585 |

| **239.** | P57053\|H2BFS_HUMAN    **Mass:** 13936    **Score:** 217    **Queries matched:** 13   **emPAI:** 0.28 |
| --- | --- |
|  | Histone H2B type F-S OS=Homo sapiens GN=H2BFS PE=1 SV=2 |

|  | **Query** | **Observed** | **Mr(expt)** | **Mr(calc)** | **Delta** | **Miss** | **Score** | **Expect** | **Rank** | **Peptide** |
| --- | --- | --- | --- | --- | --- | --- | --- | --- | --- | --- |
|  | 2160 | 816.4524 | 815.4451 | 815.4501 | -0.0050 | 0 | 17 | 4.1 | 1 | R.EIQTAVR.L |
|  | 2202 | 820.3386 | 819.3313 | 819.4563 | -0.1250 | 1 | 12 | 12 | 3 | K.RSTITSR.E |
|  | 2333 | 415.2289 | 828.4431 | 827.4137 | 1.0294 | 0 | 9 | 29 | 5 | K.HAVSEGTK.A 2320 2321 |
|  | 3990 | 477.3019 | 952.5892 | 952.5957 | -0.0065 | 0 | 18 | 2.4 | 2 | R.LLLPGELAK.H 3986 3994 |
|  | 12062 | **727.8803** | **1453.7461** | **1454.7630** | **-1.0169** | **1** | **4** | **48** | **6** | **R.IAGEASRLPHYNK.R** |
|  | 16572 | 872.4073 | 1742.8000 | 1742.8120 | -0.0120 | 0 | 120 | 1.1e-010 | 1 | K.AMGIMNSFVNDIFER.I 16571 16573 16585 |

| **240.** | P58876\|H2B1D_HUMAN    **Mass:** 13928    **Score:** 217    **Queries matched:** 13   **emPAI:** 0.28 |
| --- | --- |
|  | Histone H2B type 1-D OS=Homo sapiens GN=HIST1H2BD PE=1 SV=2 |

|  | **Query** | **Observed** | **Mr(expt)** | **Mr(calc)** | **Delta** | **Miss** | **Score** | **Expect** | **Rank** | **Peptide** |
| --- | --- | --- | --- | --- | --- | --- | --- | --- | --- | --- |
|  | 2160 | 816.4524 | 815.4451 | 815.4501 | -0.0050 | 0 | 17 | 4.1 | 1 | R.EIQTAVR.L |
|  | 2202 | 820.3386 | 819.3313 | 819.4563 | -0.1250 | 1 | 12 | 12 | 3 | K.RSTITSR.E |
|  | 2333 | 415.2289 | 828.4431 | 827.4137 | 1.0294 | 0 | 9 | 29 | 5 | K.HAVSEGTK.A 2320 2321 |
|  | 3990 | 477.3019 | 952.5892 | 952.5957 | -0.0065 | 0 | 18 | 2.4 | 2 | R.LLLPGELAK.H 3986 3994 |
|  | 8776 | 635.6240 | 1269.2334 | 1268.6434 | 0.5899 | 1 | 1 | 68 | 2 | -.MPEPTKSAPAPK.K |
|  | 16572 | 872.4073 | 1742.8000 | 1742.8120 | -0.0120 | 0 | 120 | 1.1e-010 | 1 | K.AMGIMNSFVNDIFER.I 16571 16573 16585 |

| **241.** | P49419\|AL7A1_HUMAN    **Mass:** 55845    **Score:** 216    **Queries matched:** 14   **emPAI:** 0.29 |
| --- | --- |
|  | Alpha-aminoadipic semialdehyde dehydrogenase OS=Homo sapiens GN=ALDH7A1 PE=1 SV=4 |

|  | **Query** | **Observed** | **Mr(expt)** | **Mr(calc)** | **Delta** | **Miss** | **Score** | **Expect** | **Rank** | **Peptide** |
| --- | --- | --- | --- | --- | --- | --- | --- | --- | --- | --- |
|  | 5415 | 530.2906 | 1058.5666 | 1058.5543 | 0.0124 | 0 | (7) | 37 | 9 | K.QVGLMVQER.F |
|  | 5612 | 537.7863 | 1073.5579 | 1074.5492 | -0.9912 | 0 | 7 | 34 | 9 | K.QVGLMVQER.F |
|  | 6488 | **571.7501** | **1141.4856** | **1141.6706** | **-0.1851** | **1** | **7** | **33** | **4** | **K.IIAKVLEDNK.L** |
|  | 6840 | **585.2986** | **1168.5826** | **1168.6274** | **-0.0448** | **0** | **5** | **49** | **1** | **R.MIGGPILPSER.S** |
|  | 12022 | 726.3957 | 1450.7768 | 1449.7828 | 0.9941 | 0 | 41 | 0.011 | 1 | R.VNLLSFTGSTQVGK.Q |
|  | 14708 | **812.4274** | **1622.8403** | **1622.8338** | **0.0065** | **1** | **4** | **43** | **6** | **K.QAVSMFLGAVEEAKK.E** |
|  | 16610 | **582.8255** | **1745.4547** | **1745.9597** | **-0.5050** | **1** | **2** | **50** | **6** | **R.EKIQVLGSLVSLEMGK.I** |
|  | 18703 | **952.9554** | **1903.8963** | **1903.9098** | **-0.0135** | **0** | **74** | **4.2e-006** | **1** | **R.GEVITTYCPANNEPIAR.V** 18702 |
|  | 19228 | 650.3574 | 1948.0504 | 1948.0530 | -0.0026 | 1 | 9 | 13 | 2 | R.LFIHESIHDEVVNRLK.K |
|  | 20536 | **688.3534** | **2062.0383** | **2062.9850** | **-0.9466** | **0** | **2** | **58** | **4** | **K.LPGAICSLTCGGADIGTAMAK.D** |
|  | 24234 | **1253.5891** | **2505.1637** | **2505.1806** | **-0.0169** | **0** | **83** | **3.5e-007** | **1** | **K.GSDCGIVNVNIPTSGAEIGGAFGGEK.H** |
|  | 24238 | 836.3945 | 2506.1616 | 2506.3214 | -0.1598 | 1 | 3 | 38 | 3 | R.VNLLSFTGSTQVGKQVGLMVQER.F |
|  | 25677 | **921.4501** | **2761.3286** | **2761.3449** | **-0.0163** | **1** | **45** | **0.002** | **1** | **K.VLEDNKLPGAICSLTCGGADIGTAMAK.D** |

| **242.** | P35232\|PHB_HUMAN    **Mass:** 29843    **Score:** 216    **Queries matched:** 21   **emPAI:** 1.30 |
| --- | --- |
|  | Prohibitin OS=Homo sapiens GN=PHB PE=1 SV=1 |

|  | **Query** | **Observed** | **Mr(expt)** | **Mr(calc)** | **Delta** | **Miss** | **Score** | **Expect** | **Rank** | **Peptide** |
| --- | --- | --- | --- | --- | --- | --- | --- | --- | --- | --- |
|  | 1330 | **621.3561** | **620.3489** | **620.3534** | **-0.0045** | **0** | **20** | **2.1** | **1** | **R.FVVEK.A** |
|  | 7069 | 593.3291 | 1184.6436 | 1184.6513 | -0.0077 | 0 | 45 | 0.0049 | 1 | K.DLQNVNITLR.I |
|  | 11108 | **698.9196** | **1395.8246** | **1395.8350** | **-0.0105** | **0** | **20** | **1.2** | **1** | **R.ILFRPVASQLPR.I** |
|  | 14430 | **803.4283** | **1604.8421** | **1605.8362** | **-0.9941** | **1** | **80** | **1.1e-006** | **1** | **R.KLEAAEDIAYQLSR.S** 14451 14459 |
|  | 15310 | **554.6144** | **1660.8213** | **1660.8897** | **-0.0684** | **1** | **6** | **32** | **2** | **K.SVVARFDAGELITQR.E** |
|  | 18046 | **928.0143** | **1854.0140** | **1854.0251** | **-0.0111** | **0** | **22** | **0.6** | **1** | **R.NITYLPAGQSVLLQLPQ.-** |
|  | 19776 | **999.5413** | **1997.0680** | **1997.0793** | **-0.0113** | **0** | **27** | **0.17** | **1** | **K.AAELIANSLATAGDGLIELR.K** 19775 19795 |
|  | 21121 | **1060.0686** | **2118.1226** | **2118.1361** | **-0.0135** | **0** | **68** | **1.3e-005** | **1** | **R.AATFGLILDDVSLTHLTFGK.E** 21128 |
|  | 23229 | 791.0843 | 2370.2312 | 2370.2444 | -0.0132 | 0 | 27 | 0.13 | 1 | K.FGLALAVAGGVVNSALYNVDAGHR.A 23230 23238 23240 23248 |
|  | 27610 | **1041.8805** | **3122.6196** | **3122.6376** | **-0.0179** | **0** | **52** | **0.00032** | **1** | **R.GVQDIVVGEGTHFLIPWVQKPIIFDCR.S** 27609 |
|  | 28622 | 857.4556 | 3425.7934 | 3425.8071 | -0.0137 | 1 | 38 | 0.0064 | 1 | R.FRGVQDIVVGEGTHFLIPWVQKPIIFDCR.S |

| **243.** | Q15166\|PON3_HUMAN    **Mass:** 39810    **Score:** 215    **Queries matched:** 17   **emPAI:** 0.88 |
| --- | --- |
|  | Serum paraoxonase/lactonase 3 OS=Homo sapiens GN=PON3 PE=1 SV=3 |

|  | **Query** | **Observed** | **Mr(expt)** | **Mr(calc)** | **Delta** | **Miss** | **Score** | **Expect** | **Rank** | **Peptide** |
| --- | --- | --- | --- | --- | --- | --- | --- | --- | --- | --- |
|  | 2257 | 823.4509 | 822.4437 | 822.4487 | -0.0051 | 0 | 26 | 0.43 | 1 | K.STVEIFK.F |
|  | 5916 | **549.7913** | **1097.5680** | **1097.5757** | **-0.0077** | **0** | **30** | **0.15** | **1** | **K.YVYVADVAAK.N** |
|  | 8755 | **635.3105** | **1268.6064** | **1268.6149** | **-0.0085** | **0** | **18** | **2** | **1** | **K.HDNWDLTQLK.V** |
|  | 9924 | **665.7930** | **1329.5715** | **1330.6710** | **-1.0995** | **0** | **2** | **84** | **6** | **R.WTYVLFYSPR.E** |
|  | 12667 | **745.3723** | **1488.7301** | **1488.7395** | **-0.0094** | **0** | **59** | **0.00015** | **1** | **K.IFLMDLNEQNPR.A** 12664 12668 |
|  | 13125 | **760.3425** | **1518.6705** | **1518.6813** | **-0.0108** | **0** | **62** | **7.5e-005** | **1** | **K.YPGMPNFAPDEPGK.I** |
|  | 13673 | **518.7709** | **1553.2908** | **1553.7144** | **-0.4236** | **0** | **3** | **44** | **3** | **K.GFCSANGITVSADQK.Y** |
|  | 17500 | **906.9531** | **1811.8916** | **1811.9054** | **-0.0138** | **0** | **34** | **0.038** | **1** | **K.LLNYNPEDPPGSEVLR.I** 17501 |
|  | 17709 | **610.3425** | **1828.0056** | **1828.8930** | **-0.8875** | **0** | **2** | **68** | **1** | **K.DNTVYLYVVNHPHMK.S** |
|  | 18761 | **954.4909** | **1906.9673** | **1906.9789** | **-0.0116** | **0** | **41** | **0.0082** | **1** | **K.SVNDIVVLGPEQFYATR.D** |
|  | 27018 | **997.8060** | **2990.3963** | **2989.4102** | **0.9860** | **1** | **63** | **2.7e-005** | **1** | **K.YPGMPNFAPDEPGKIFLMDLNEQNPR.A** |
|  | 30485 | **1384.6721** | **4150.9945** | **4151.0144** | **-0.0198** | **0** | **29** | **0.033** | **1** | **R.EVEPVEPENCHLIEELESGSEDIDILPSGLAFISSGLK.Y** 30486 |
|  | 31708 | **1885.2018** | **5652.5835** | **5651.6851** | **0.8984** | **1** | **2** | **6.3** | **4** | **R.EVEPVEPENCHLIEELESGSEDIDILPSGLAFISSGLKYPGMPNFAPDEPGK.I** |

| **244.** | Q06520\|ST2A1_HUMAN    **Mass:** 33929    **Score:** 213    **Queries matched:** 18   **emPAI:** 0.37 |
| --- | --- |
|  | Bile salt sulfotransferase OS=Homo sapiens GN=SULT2A1 PE=1 SV=3 |

|  | **Query** | **Observed** | **Mr(expt)** | **Mr(calc)** | **Delta** | **Miss** | **Score** | **Expect** | **Rank** | **Peptide** |
| --- | --- | --- | --- | --- | --- | --- | --- | --- | --- | --- |
|  | 2191 | 819.4088 | 818.4015 | 819.3803 | -0.9788 | 0 | 7 | 45 | 10 | R.ELFPWE.- |
|  | 2776 | **432.7251** | **863.4356** | **864.4528** | **-1.0172** | **0** | **13** | **9.2** | **2** | **K.ICQFLGK.T** |
|  | 4792 | 505.7566 | 1009.4986 | 1008.5790 | 0.9196 | 1 | 1 | 1.2e+002 | 10 | K.AKVIYLMR.N |
|  | 9290 | **649.8434** | **1297.6723** | **1298.5925** | **-0.9201** | **1** | **2** | **93** | **1** | **K.NSSFQSMKENK.M** |
|  | 9570 | **657.3490** | **1312.6834** | **1312.6928** | **-0.0093** | **0** | **17** | **3** | **1** | **K.WIQSVPIWER.S** |
|  | 10612 | **684.8698** | **1367.7251** | **1367.7336** | **-0.0086** | **0** | **31** | **0.12** | **1** | **K.NFLLLSYEELK.Q** 10608 |
|  | 11374 | **706.4014** | **1410.7882** | **1410.7970** | **-0.0088** | **0** | **23** | **0.64** | **1** | **K.TLEPEELNLILK.N** 11348 11361 11363 |
|  | 13248 | 509.6260 | 1525.8562 | 1525.8657 | -0.0095 | 0 | 27 | 0.26 | 1 | R.LFSSHLPIQLFPK.S |
|  | 16653 | 875.4070 | 1748.7995 | 1748.8596 | -0.0601 | 1 | 1 | 93 | 7 | R.DVLVSGYFFWKNMK.F |
|  | 21537 | **723.0146** | **2166.0221** | **2166.0382** | **-0.0161** | **1** | **39** | **0.0099** | **1** | **K.NHFTVAQAEDFDKLFQEK.M** 21531 |
|  | 22207 | **1119.5242** | **2237.0338** | **2237.0488** | **-0.0150** | **0** | **128** | **1.2e-011** | **1** | **R.SPWVESEIGYTALSETESPR.L** 22206 |
|  | 25468 | **1361.6481** | **2721.2816** | **2721.2897** | **-0.0081** | **1** | **0** | **56** | **2** | **M.SDDFLWFEGIAFPTMGFRSETLR.K** |

| **245.** | P49755\|TMEDA_HUMAN    **Mass:** 25131    **Score:** 212    **Queries matched:** 8   **emPAI:** 0.53 |
| --- | --- |
|  | Transmembrane emp24 domain-containing protein 10 OS=Homo sapiens GN=TMED10 PE=1 SV=2 |

|  | **Query** | **Observed** | **Mr(expt)** | **Mr(calc)** | **Delta** | **Miss** | **Score** | **Expect** | **Rank** | **Peptide** |
| --- | --- | --- | --- | --- | --- | --- | --- | --- | --- | --- |
|  | 7857 | **611.8149** | **1221.6152** | **1221.6241** | **-0.0089** | **1** | **27** | **0.33** | **1** | **K.NYEEIAKVEK.L** 7873 |
|  | 9415 | **652.8415** | **1303.6684** | **1303.6772** | **-0.0088** | **0** | **73** | **7.1e-006** | **1** | **K.ITDSAGHILYSK.E** |
|  | 17950 | **616.9780** | **1847.9121** | **1847.9265** | **-0.0144** | **1** | **17** | **2.2** | **1** | **K.ITDSAGHILYSKEDATK.G** 17951 |
|  | 20182 | **1015.4935** | **2028.9724** | **2028.9826** | **-0.0102** | **1** | **96** | **2.4e-008** | **1** | **R.RLEDLSESIVNDFAYMK.K** 20180 20181 |

| **246.** | Q68CK6\|ACS2B_HUMAN    **Mass:** 64786    **Score:** 212    **Queries matched:** 15   **emPAI:** 0.18 |
| --- | --- |
|  | Acyl-coenzyme A synthetase ACSM2B, mitochondrial OS=Homo sapiens GN=ACSM2B PE=1 SV=1 |

|  | **Query** | **Observed** | **Mr(expt)** | **Mr(calc)** | **Delta** | **Miss** | **Score** | **Expect** | **Rank** | **Peptide** |
| --- | --- | --- | --- | --- | --- | --- | --- | --- | --- | --- |
|  | 1967 | **401.2850** | **800.5555** | **800.4578** | **0.0977** | **0** | **12** | **12** | **1** | **R.VAVMLPR.V** 1972 |
|  | 3033 | 441.2954 | 880.5762 | 880.4113 | 0.1649 | 1 | 2 | 1e+002 | 10 | K.EWKMSGK.A |
|  | 7597 | **404.0300** | **1209.0681** | **1209.5626** | **-0.4945** | **0** | **11** | **7.8** | **1** | **R.ADDIINSSGYR.I** |
|  | 9759 | **661.8194** | **1321.6242** | **1321.6336** | **-0.0094** | **0** | **39** | **0.018** | **1** | **K.MAEHSYSSLGLK.A** 9760 |
|  | 11280 | **704.3071** | **1406.5997** | **1405.7462** | **0.8536** | **0** | **7** | **24** | **1** | **R.AGLIFMPGTIQMK.S** |
|  | 11816 | 719.9227 | 1437.8309 | 1437.7360 | 0.0949 | 0 | (4) | 49 | 5 | R.AGLIFMPGTIQMK.S |
|  | 22150 | **1116.5298** | **2231.0450** | **2231.0600** | **-0.0150** | **0** | **112** | **5.1e-010** | **1** | **R.ELSENSQQAANVLSGACGLQR.G** 22149 22151 |
|  | 22245 | 748.4654 | 2242.3743 | 2241.9956 | 0.3787 | 1 | 0 | 56 | 9 | R.EFYGQTETGLTCMVSKTMK.I |
|  | 23855 | **1229.6025** | **2457.1905** | **2457.2057** | **-0.0152** | **0** | **20** | **0.73** | **1** | **K.AIVAGDEVIQEVDTVASECPSLR.I** |
|  | 23886 | **821.4198** | **2461.2376** | **2462.2007** | **-0.9632** | **1** | **1** | **59** | **3** | **K.SMMGAPIVYRMLLQQDLSSYK.F** |
|  | 31501 | **1811.6638** | **5431.9696** | **5432.7288** | **-0.7591** | **1** | **0** | **9.9** | **6** | **K.AKMDAGWTGLQASDIMWTISDTGWILNILGSLLESWTLGACTFVHLLPK.F** |

| **247.** | Q08AH3\|ACS2A_HUMAN    **Mass:** 64809    **Score:** 212    **Queries matched:** 12   **emPAI:** 0.18 |
| --- | --- |
|  | Acyl-coenzyme A synthetase ACSM2A, mitochondrial OS=Homo sapiens GN=ACSM2A PE=1 SV=2 |

|  | **Query** | **Observed** | **Mr(expt)** | **Mr(calc)** | **Delta** | **Miss** | **Score** | **Expect** | **Rank** | **Peptide** |
| --- | --- | --- | --- | --- | --- | --- | --- | --- | --- | --- |
|  | 3033 | 441.2954 | 880.5762 | 880.4113 | 0.1649 | 1 | 2 | 1e+002 | 10 | K.EWKMSGK.A |
|  | 9759 | 661.8194 | 1321.6242 | 1321.6336 | -0.0094 | 0 | 39 | 0.018 | 1 | K.MAEHSYSSLGLK.A 9760 |
|  | 11280 | 704.3071 | 1406.5997 | 1405.7462 | 0.8536 | 0 | 7 | 24 | 1 | R.AGLIFMPGTIQMK.S |
|  | 11816 | 719.9227 | 1437.8309 | 1437.7360 | 0.0949 | 0 | (4) | 49 | 5 | R.AGLIFMPGTIQMK.S |
|  | 22150 | 1116.5298 | 2231.0450 | 2231.0600 | -0.0150 | 0 | 112 | 5.1e-010 | 1 | R.ELSENSQQAANVLSGACGLQR.G 22149 22151 |
|  | 23855 | 1229.6025 | 2457.1905 | 2457.2057 | -0.0152 | 0 | 20 | 0.73 | 1 | K.AIVAGDEVIQEVDTVASECPSLR.I |
|  | 23886 | 821.4198 | 2461.2376 | 2462.2007 | -0.9632 | 1 | 1 | 59 | 3 | K.SMMGAPIVYRMLLQQDLSSYK.F |
|  | 25371 | **902.4908** | **2704.4507** | **2704.2910** | **0.1597** | **1** | **2** | **42** | **3** | **K.TMKIKPGYMGTAASCYDVQIIDDK.G** |
|  | 31468 | **1789.6014** | **5365.7825** | **5366.5623** | **-0.7798** | **0** | **1** | **7.3** | **5** | **K.MDAGWTGLQASDIMWTISDTGWILNILCSLMEPWALGACTFVHLLPK.F** |

| **248.** | P62424\|RL7A_HUMAN    **Mass:** 30148    **Score:** 210    **Queries matched:** 21   **emPAI:** 1.03 |
| --- | --- |
|  | 60S ribosomal protein L7a OS=Homo sapiens GN=RPL7A PE=1 SV=2 |

|  | **Query** | **Observed** | **Mr(expt)** | **Mr(calc)** | **Delta** | **Miss** | **Score** | **Expect** | **Rank** | **Peptide** |
| --- | --- | --- | --- | --- | --- | --- | --- | --- | --- | --- |
|  | 1869 | **794.4352** | **793.4279** | **792.4130** | **1.0149** | **0** | **6** | **46** | **7** | **K.YRPETK.Q** |
|  | 2604 | **426.2686** | **850.5226** | **850.5276** | **-0.0050** | **0** | **6** | **44** | **7** | **K.VAPAPAVVK.K** |
|  | 4350 | 490.3154 | 978.6162 | 978.6226 | -0.0064 | 1 | 24 | 0.65 | 1 | K.KVAPAPAVVK.K |
|  | 5476 | **532.7817** | **1063.5488** | **1063.5563** | **-0.0075** | **0** | **21** | **1.3** | **1** | **R.HWGGNVLGPK.S** |
|  | 5606 | **537.3176** | **1072.6207** | **1072.6281** | **-0.0074** | **1** | **18** | **2.8** | **1** | **K.KVVNPLFEK.R** |
|  | 6466 | **570.8022** | **1139.5899** | **1139.7026** | **-0.1127** | **1** | **7** | **26** | **2** | **K.GALAKLVEAIR.T** |
|  | 10005 | **445.5987** | **1333.7742** | **1333.7830** | **-0.0089** | **1** | **3** | **64** | **9** | **K.GDVPTKRPPVLR.A** |
|  | 10189 | **673.3649** | **1344.7152** | **1344.7249** | **-0.0097** | **0** | **68** | **2.1e-005** | **1** | **R.AGVNTVTTLVENK.K** 10174 10190 10192 |
|  | 12122 | **729.8290** | **1457.6434** | **1457.6535** | **-0.0101** | **1** | **20** | **1.2** | **1** | **R.TNYNDRYDEIR.R** |
|  | 12394 | **737.4125** | **1472.8104** | **1472.8199** | **-0.0094** | **1** | **24** | **0.53** | **1** | **R.AGVNTVTTLVENKK.A** |
|  | 13886 | **785.4175** | **1568.8205** | **1568.8311** | **-0.0106** | **0** | **60** | **0.00014** | **1** | **K.VPPAINQFTQALDR.Q** |
|  | 17489 | **604.3411** | **1810.0014** | **1810.0101** | **-0.0087** | **1** | **26** | **0.26** | **1** | **R.LKVPPAINQFTQALDR.Q** 17491 |
|  | 21302 | **1071.0226** | **2140.0306** | **2140.0470** | **-0.0164** | **1** | **92** | **5.2e-008** | **1** | **K.TCTTVAFTQVNSEDKGALAK.L** 21311 |
|  | 25263 | **896.8342** | **2687.4809** | **2686.4880** | **0.9928** | **0** | **15** | **1.8** | **1** | **K.AQLVVIAHDVDPIELVVFLPALCR.K** |
|  | 25993 | 939.4208 | 2815.2407 | 2814.5830 | 0.6577 | 1 | 3 | 27 | 1 | K.AQLVVIAHDVDPIELVVFLPALCRK.M 25996 |

| **249.** | Q9NVH1\|DJC11_HUMAN    **Mass:** 63524    **Score:** 209    **Queries matched:** 15   **emPAI:** 0.12 |
| --- | --- |
|  | DnaJ homolog subfamily C member 11 OS=Homo sapiens GN=DNAJC11 PE=1 SV=2 |

|  | **Query** | **Observed** | **Mr(expt)** | **Mr(calc)** | **Delta** | **Miss** | **Score** | **Expect** | **Rank** | **Peptide** |
| --- | --- | --- | --- | --- | --- | --- | --- | --- | --- | --- |
|  | 2004 | **402.2881** | **802.5616** | **801.4344** | **1.1272** | **1** | **13** | **11** | **1** | **K.ELEKQR.E** |
|  | 2740 | **431.7357** | **861.4569** | **861.4378** | **0.0191** | **0** | **9** | **26** | **7** | **R.LMQESVR.R** |
|  | 4985 | **513.3104** | **1024.6062** | **1023.4258** | **1.1804** | **0** | **5** | **42** | **5** | **K.FQDDDQTR.V** 4967 |
|  | 5932 | **550.2844** | **1098.5542** | **1097.5869** | **0.9673** | **1** | **9** | **20** | **1** | **R.AIYDIYGKR.G** |
|  | 10197 | 673.8207 | 1345.6268 | 1344.6997 | 0.9271 | 1 | 7 | 29 | 4 | K.QRESAATDVLQK.K |
|  | 10806 | **690.4505** | **1378.8864** | **1379.7271** | **-0.8407** | **0** | **3** | **56** | **8** | **R.MGLIIVNAWYGK.F** |
|  | 12567 | **495.2525** | **1482.7356** | **1482.8480** | **-0.1124** | **0** | **2** | **84** | **1** | **K.VIDVTVPLQCLVK.D** |
|  | 14205 | 795.4202 | 1588.8258 | 1588.7668 | 0.0590 | 1 | 6 | 29 | 9 | R.GLEMEGWEVVERR.R 14202 |
|  | 15604 | **561.3361** | **1680.9864** | **1681.8821** | **-0.8958** | **0** | **5** | **30** | **4** | **R.GVLHQVMVLDSEALR.I** |
|  | 16448 | **434.2342** | **1732.9075** | **1731.8573** | **1.0502** | **1** | **1** | **79** | **9** | **K.QEAESAVRLMQESVR.R** |
|  | 20853 | **1046.0259** | **2090.0372** | **2090.0473** | **-0.0101** | **0** | **118** | **1.4e-010** | **1** | **K.GWGELEFGAGDLQGPLFGLK.L** 20852 |
|  | 29875 | **1299.2626** | **3894.7659** | **3894.7960** | **-0.0301** | **1** | **26** | **0.065** | **1** | **K.GTISVGVDATDLFDRYDEEYEDVSGSSFPQIEINK.M** |

| **250.** | Q12907\|LMAN2_HUMAN    **Mass:** 40545    **Score:** 209    **Queries matched:** 13   **emPAI:** 1.03 |
| --- | --- |
|  | Vesicular integral-membrane protein VIP36 OS=Homo sapiens GN=LMAN2 PE=1 SV=1 |

|  | **Query** | **Observed** | **Mr(expt)** | **Mr(calc)** | **Delta** | **Miss** | **Score** | **Expect** | **Rank** | **Peptide** |
| --- | --- | --- | --- | --- | --- | --- | --- | --- | --- | --- |
|  | 1409 | **639.2965** | **638.2892** | **637.3296** | **0.9596** | **1** | **5** | **38** | **6** | **R.YSRGR.L** |
|  | 1510 | **675.3979** | **674.3906** | **673.3507** | **1.0399** | **1** | **8** | **37** | **10** | **R.QERNK.R** |
|  | 8811 | **424.7282** | **1271.1626** | **1270.7034** | **0.4592** | **1** | **17** | **2.1** | **1** | **R.DRLVPGPVFGSK.D** |
|  | 11627 | **713.8196** | **1425.6246** | **1425.6347** | **-0.0101** | **0** | **34** | **0.054** | **1** | **R.WTELAGCTADFR.N** |
|  | 13072 | 758.3833 | 1514.7520 | 1514.7630 | -0.0110 | 0 | 33 | 0.058 | 1 | K.NLHGDGIALWYTR.D |
|  | 16712 | **585.2989** | **1752.8748** | **1753.7842** | **-0.9094** | **1** | **3** | **61** | **5** | **K.DGRWTELAGCTADFR.N** |
|  | 16854 | **587.8179** | **1760.4320** | **1760.8358** | **-0.4038** | **1** | **5** | **24** | **5** | **-.MAAEGWIWRWGWGR.R** |
|  | 22812 | **773.3663** | **2317.0772** | **2317.0936** | **-0.0165** | **0** | **29** | **0.092** | **1** | **K.LFQLMVEHTPDEESIDWTK.I** |
|  | 23927 | **1234.5643** | **2467.1141** | **2467.1292** | **-0.0151** | **0** | **72** | **4.2e-006** | **1** | **K.DNFHGLAIFLDTYPNDETTER.V** |
|  | 25500 | 910.7561 | 2729.2465 | 2729.2643 | -0.0178 | 0 | 84 | 2.2e-007 | 1 | R.LPTGYYFGASAGTGDLSDNHDIISMK.L |
|  | 28379 | **1115.8881** | **3344.6424** | **3344.6639** | **-0.0215** | **1** | **43** | **0.0022** | **1** | **K.LFQLMVEHTPDEESIDWTKIEPSVNFLK.S** |
|  | 29382 | **1243.9345** | **3728.7815** | **3728.7967** | **-0.0152** | **0** | **31** | **0.025** | **1** | **R.EHSLIKPYQGVGSSSMPLWDFQGSTMLTSQYVR.L** 29381 |

| **251.** | P49327\|FAS_HUMAN    **Mass:** 275850   **Score:** 208    **Queries matched:** 46   **emPAI:** 0.16 |
| --- | --- |
|  | Fatty acid synthase OS=Homo sapiens GN=FASN PE=1 SV=2 |

|  | **Query** | **Observed** | **Mr(expt)** | **Mr(calc)** | **Delta** | **Miss** | **Score** | **Expect** | **Rank** | **Peptide** |
| --- | --- | --- | --- | --- | --- | --- | --- | --- | --- | --- |
|  | 1377 | **633.1577** | **632.1504** | **632.3242** | **-0.1737** | **1** | **5** | **77** | **2** | **R.DASGKR.V** |
|  | 2998 | **440.0299** | **878.0452** | **878.4035** | **-0.3583** | **0** | **2** | **72** | **7** | **R.DGAWGAFR.H** |
|  | 3040 | **441.2964** | **880.5782** | **879.4306** | **1.1476** | **0** | **7** | **31** | **10** | **R.GMGLSLMR.L** |
|  | 3669 | **466.2374** | **930.4602** | **931.5338** | **-1.0736** | **1** | **38** | **0.031** | **1** | **R.KLQELSSK.A** 3692 |
|  | 4022 | **478.7659** | **955.5172** | **955.5239** | **-0.0068** | **0** | **29** | **0.22** | **1** | **K.HGLYLPTR.V** |
|  | 4855 | **508.2635** | **1014.5124** | **1013.5982** | **0.9142** | **1** | **8** | **27** | **3** | **R.EVRQLTLR.K** |
|  | 5235 | **522.7451** | **1043.4757** | **1042.5771** | **0.8986** | **0** | **0** | **1.6e+002** | **5** | **K.AQVADVVVSR.W** |
|  | 6723 | **580.8228** | **1159.6309** | **1159.6502** | **-0.0192** | **1** | **14** | **6.3** | **1** | **R.WKAGLYGLPR.R** |
|  | 7340 | **599.3149** | **1196.6153** | **1195.5768** | **1.0385** | **0** | **8** | **24** | **2** | **K.QAHTMDPQLR.L** |
|  | 8031 | **616.3311** | **1230.6477** | **1230.5088** | **0.1389** | **0** | **6** | **40** | **2** | **K.AFDTAGNGYCR.S** |
|  | 8287 | **622.3401** | **1242.6656** | **1242.7547** | **-0.0891** | **1** | **1** | **1.2e+002** | **5** | **R.SEGVVAVLLTKK.S** |
|  | 8328 | **623.8091** | **1245.6036** | **1246.6418** | **-1.0382** | **0** | **4** | **58** | **4** | **K.VSVHVIEGDHR.T** |
|  | 8342 | **416.2596** | **1245.7571** | **1245.7115** | **0.0456** | **1** | **11** | **13** | **4** | **K.GAKPKLMSAISK.T** |
|  | 8973 | **641.3152** | **1280.6159** | **1279.6377** | **0.9783** | **1** | **2** | **90** | **6** | **R.GMGLSLMRLDR.F** 8979 |
|  | 9010 | **428.5670** | **1282.6791** | **1281.5945** | **1.0847** | **0** | **1** | **97** | **7** | **-.MEEVVIAGMSGK.L** |
|  | 10323 | 676.8472 | 1351.6798 | 1351.7282 | -0.0484 | 0 | 22 | 0.76 | 2 | K.MVVPGLDGAQIPR.D |
|  | 10944 | **694.3340** | **1386.6534** | **1385.7402** | **0.9132** | **0** | **6** | **35** | **9** | **K.GVDLVLNSLAEEK.L** |
|  | 11259 | **703.4090** | **1404.8034** | **1404.8089** | **-0.0054** | **0** | **23** | **0.69** | **1** | **R.DLVEAVAHILGIR.D** |
|  | 11631 | 713.8851 | 1425.7556 | 1425.7650 | -0.0094 | 0 | 22 | 0.95 | 1 | R.SLLVNPEGPTLMR.L |
|  | 11879 | **481.8479** | **1442.5220** | **1441.7599** | **0.7621** | **0** | **(9)** | **14** | **2** | **R.SLLVNPEGPTLMR.L** |
|  | 14278 | **797.4515** | **1592.8884** | **1592.8967** | **-0.0082** | **0** | **10** | **11** | **1** | **R.VLFPATGYLSIVWK.T** |
|  | 14558 | **807.4105** | **1612.8065** | **1612.8169** | **-0.0104** | **0** | **30** | **0.11** | **1** | **K.EDGLAQQQTQLNLR.S** |
|  | 14938 | 819.4307 | 1636.8468 | 1635.7464 | 1.1004 | 0 | 3 | 52 | 3 | K.CTVFHGAQVEDAFR.Y |
|  | 15393 | **834.4288** | **1666.8431** | **1665.8144** | **1.0287** | **0** | **8** | **16** | **3** | **K.SNMGHPEPASGLAALAK.V** |
|  | 15640 | **562.6213** | **1684.8420** | **1684.8784** | **-0.0364** | **0** | **1** | **99** | **9** | **K.GILADEDSSRPVWLK.A** |
|  | 16135 | 857.3936 | 1712.7725 | 1712.7828 | -0.0103 | 0 | 86 | 2.6e-007 | 1 | K.WTSQDSLLGMEFSGR.D 16151 |
|  | 16163 | **572.3264** | **1713.9574** | **1713.8547** | **0.1028** | **1** | **2** | **62** | **10** | **K.SHQGLDRQELSFAAR.S** |
|  | 16384 | **577.3178** | **1728.9316** | **1728.7777** | **0.1539** | **0** | **(8)** | **17** | **1** | **K.WTSQDSLLGMEFSGR.D** |
|  | 16704 | **585.2407** | **1752.7002** | **1751.8276** | **0.8726** | **1** | **6** | **25** | **1** | **R.AMMANRLSFFFDFR.G** |
|  | 16991 | **887.3782** | **1772.7418** | **1772.7536** | **-0.0118** | **0** | **35** | **0.031** | **1** | **R.GNAGQSNYGFANSAMER.I** |
|  | 17060 | **889.4958** | **1776.9770** | **1776.9873** | **-0.0103** | **0** | **42** | **0.0067** | **1** | **K.LPEDPLLSGLLDSPALK.A** |
|  | 18863 | 640.0103 | 1917.0089 | 1915.9640 | 1.0450 | 0 | 3 | 45 | 7 | K.EQGVTFPSGDIQEQLIR.S |
|  | 23608 | **808.1082** | **2421.3028** | **2421.3115** | **-0.0086** | **0** | **35** | **0.023** | **1** | **R.TLLEGSGLESIISIIHSSLAEPR.V** |
|  | 23965 | **824.7668** | **2471.2787** | **2471.2961** | **-0.0174** | **0** | **17** | **1.5** | **1** | **R.LHLSGIDANPNALFPPVEFPAPR.G** |
|  | 24439 | **848.0690** | **2541.1851** | **2541.2023** | **-0.0173** | **0** | **10** | **6.6** | **1** | **R.SLYQSAGVAPESFEYIEAHGTGTK.V** 24445 |
|  | 25118 | **534.0581** | **2665.2542** | **2666.3221** | **-1.0680** | **0** | **7** | **14** | **2** | **R.CVLLSNLSSTSHVPEVDPGSAELQK.V** |
|  | 25586 | 1374.2480 | 2746.4815 | 2747.3411 | -0.8595 | 0 | 0 | 51 | 6 | R.HSQDLAFLSMLNDIAAVPATAMPFR.G |
|  | 25798 | **929.1973** | **2784.5702** | **2784.5790** | **-0.0088** | **0** | **32** | **0.034** | **1** | **R.ALGLGVEQLPVVFEDVVLHQATILPK.T** |
|  | 26124 | **947.4697** | **2839.3874** | **2839.4068** | **-0.0195** | **0** | **58** | **0.00011** | **1** | **R.LFDHPESPTPNPTEPLFLAQAEVYK.E** |
|  | 26550 | 969.8068 | 2906.3986 | 2907.4191 | -1.0205 | 1 | 1 | 41 | 2 | R.GYDYGPHFQGILEASLEGDSGRLLWK.D |
|  | 27989 | **1076.1602** | **3225.4587** | **3226.5613** | **-1.1027** | **1** | **(0)** | **38** | **3** | **K.DNWVSFMDTMLQMSILGSAKHGLYLPTR.V** |
|  | 28110 | **1087.1256** | **3258.3550** | **3258.5512** | **-0.1962** | **1** | **1** | **28** | **4** | **K.DNWVSFMDTMLQMSILGSAKHGLYLPTR.V** |

| **252.** | Q9Y5U8\|BR44L_HUMAN    **Mass:** 12510    **Score:** 207    **Queries matched:** 13   **emPAI:** 2.93 |
| --- | --- |
|  | Brain protein 44-like protein OS=Homo sapiens GN=BRP44L PE=1 SV=1 |

|  | **Query** | **Observed** | **Mr(expt)** | **Mr(calc)** | **Delta** | **Miss** | **Score** | **Expect** | **Rank** | **Peptide** |
| --- | --- | --- | --- | --- | --- | --- | --- | --- | --- | --- |
|  | 2245 | **411.7251** | **821.4357** | **821.4395** | **-0.0038** | **1** | **22** | **0.96** | **1** | **R.KAADYVR.S** 2243 2244 2258 |
|  | 4432 | 493.7819 | 985.5493 | 985.5556 | -0.0063 | 1 | 46 | 0.0042 | 1 | K.KSPEIISGR.M |
|  | 4634 | 500.2922 | 998.5698 | 998.5583 | 0.0116 | 1 | 22 | 1.1 | 3 | R.LIKHEMTK.T |
|  | 17382 | **901.4796** | **1800.9447** | **1799.7867** | **1.1579** | **0** | **1** | **90** | **6** | **R.MTFALCCYSLTFMR.F** |
|  | 23443 | **1199.6033** | **2397.1920** | **2397.2012** | **-0.0092** | **0** | **52** | **0.00045** | **1** | **R.NWLLFACHATNEVAQLIQGGR.L** |
|  | 26782 | **983.1426** | **2946.4059** | **2946.4197** | **-0.0138** | **0** | **82** | **3.4e-007** | **1** | **R.DYLMSTHFWGPVANWGLPIAAINDMK.K** 26780 26781 26783 |
|  | 27437 | **769.6326** | **3074.5012** | **3074.5146** | **-0.0135** | **1** | **18** | **0.74** | **1** | **R.DYLMSTHFWGPVANWGLPIAAINDMKK.S** |

| **253.** | Q9H853\|TBA4B_HUMAN    **Mass:** 27819    **Score:** 207    **Queries matched:** 9   **emPAI:** 0.29 |
| --- | --- |
|  | Putative tubulin-like protein alpha-4B OS=Homo sapiens GN=TUBA4B PE=5 SV=2 |

|  | **Query** | **Observed** | **Mr(expt)** | **Mr(calc)** | **Delta** | **Miss** | **Score** | **Expect** | **Rank** | **Peptide** |
| --- | --- | --- | --- | --- | --- | --- | --- | --- | --- | --- |
|  | 1911 | **399.6883** | **797.3620** | **797.3780** | **-0.0160** | **0** | **11** | **13** | **8** | **R.HQQTER.Q** |
|  | 11350 | 705.8859 | 1409.7573 | 1409.7667 | -0.0094 | 0 | 6 | 31 | 1 | R.QIFHPEQLITGK.E 11351 |
|  | 12641 | 744.4393 | 1486.8641 | 1486.8719 | -0.0078 | 0 | 83 | 6.7e-007 | 1 | R.LISQIVSSITASLR.F 12628 12639 12642 12644 12646 |

| **254.** | P14854\|CX6B1_HUMAN    **Mass:** 10414    **Score:** 205    **Queries matched:** 9   **emPAI:** 2.67 |
| --- | --- |
|  | Cytochrome c oxidase subunit 6B1 OS=Homo sapiens GN=COX6B1 PE=1 SV=2 |

|  | **Query** | **Observed** | **Mr(expt)** | **Mr(calc)** | **Delta** | **Miss** | **Score** | **Expect** | **Rank** | **Peptide** |
| --- | --- | --- | --- | --- | --- | --- | --- | --- | --- | --- |
|  | 2051 | 807.2560 | 806.2488 | 805.3970 | 0.8517 | 0 | 7 | 36 | 9 | R.AEGTFPGK.I |
|  | 4707 | **502.7459** | **1003.4771** | **1003.4835** | **-0.0064** | **0** | **36** | **0.041** | **1** | **R.FPNQNQTR.N** 4705 4706 |
|  | 7635 | **404.7168** | **1211.1287** | **1210.5573** | **0.5713** | **1** | **7** | **19** | **4** | **-.MAEDMETKIK.N** |
|  | 12325 | **735.3222** | **1468.6298** | **1468.6405** | **-0.0107** | **0** | **56** | **0.00033** | **1** | **K.GGDISVCEWYQR.V** |
|  | 22417 | **1135.5057** | **2268.9969** | **2269.0110** | **-0.0141** | **0** | **101** | **5.7e-009** | **1** | **R.VYQSLCPTSWVTDWDEQR.A** 22418 |
|  | 27332 | **1019.7995** | **3056.3767** | **3056.3975** | **-0.0208** | **1** | **47** | **0.0009** | **1** | **R.VYQSLCPTSWVTDWDEQRAEGTFPGK.I** |

| **255.** | P62701\|RS4X_HUMAN    **Mass:** 29807    **Score:** 205    **Queries matched:** 30   **emPAI:** 0.82 |
| --- | --- |
|  | 40S ribosomal protein S4, X isoform OS=Homo sapiens GN=RPS4X PE=1 SV=2 |

|  | **Query** | **Observed** | **Mr(expt)** | **Mr(calc)** | **Delta** | **Miss** | **Score** | **Expect** | **Rank** | **Peptide** |
| --- | --- | --- | --- | --- | --- | --- | --- | --- | --- | --- |
|  | 1593 | **707.3278** | **706.3205** | **706.3254** | **-0.0049** | **0** | **6** | **44** | **6** | **K.ICMQR.F** |
|  | 1832 | **396.5331** | **791.0517** | **791.4905** | **-0.4389** | **1** | **10** | **15** | **5** | **R.KIFVGTK.G** 1831 |
|  | 2322 | **415.2021** | **828.3897** | **828.3952** | **-0.0055** | **0** | **23** | **1.1** | **1** | **K.HWMLDK.L** |
|  | 2352 | **416.2294** | **830.4442** | **830.4498** | **-0.0055** | **0** | **7** | **40** | **1** | **R.LTIAEER.D** |
|  | 2418 | **418.7376** | **835.4607** | **834.4204** | **1.0403** | **1** | **18** | **2.9** | **1** | **K.KICMQR.F** |
|  | 2542 | **423.7382** | **845.4619** | **844.4694** | **0.9925** | **0** | **11** | **18** | **5** | **R.YPDPLIK.V** |
|  | 4491 | 495.7995 | 989.5845 | 989.5910 | -0.0065 | 0 | 55 | 0.0006 | 1 | R.LSNIFVIGK.G |
|  | 4559 | 498.2944 | 994.5742 | 994.4971 | 0.0771 | 0 | 9 | 18 | 7 | K.YALTGDEVK.K 4553 |
|  | 5323 | **526.7377** | **1051.4608** | **1051.4683** | **-0.0075** | **0** | **46** | **0.0036** | **1** | **K.DANGNSFATR.L** 5329 |
|  | 5601 | 537.2804 | 1072.5462 | 1073.5717 | -1.0254 | 1 | 5 | 69 | 7 | R.LTIAEERDK.R |
|  | 6247 | 562.7515 | 1123.4885 | 1122.5921 | 0.8964 | 1 | 13 | 7 | 4 | K.YALTGDEVKK.I |
|  | 7700 | **405.8887** | **1214.6442** | **1214.6520** | **-0.0078** | **0** | **17** | **3.5** | **1** | **K.GIPHLVTHDAR.T** |
|  | 11923 | **723.3862** | **1444.7579** | **1444.7409** | **0.0170** | **0** | **6** | **36** | **4** | **K.VNDTIQIDLETGK.I** |
|  | 13492 | **772.3727** | **1542.7308** | **1541.9116** | **0.8192** | **1** | **65** | **4.2e-005** | **1** | **K.LRECLPLIIFLR.N** 13485 13486 13505 |
|  | 14107 | **792.8931** | **1583.7716** | **1584.8849** | **-1.1133** | **1** | **9** | **14** | **4** | **K.GIPHLVTHDARTIR.Y** 14109 14114 |
|  | 17120 | **891.9114** | **1781.8082** | **1781.8189** | **-0.0107** | **0** | **75** | **3.4e-006** | **1** | **K.FDTGNLCMVTGGANLGR.I** 17121 17153 |
|  | 21428 | **719.3137** | **2154.9192** | **2156.0824** | **-1.1632** | **1** | **0** | **73** | **1** | **K.VRTDITYPAGFMDVISIDK.T** |
|  | 22317 | **752.4130** | **2254.2172** | **2254.0880** | **0.1293** | **1** | **4** | **34** | **2** | **R.HPGSFDVVHVKDANGNSFATR.L** |
|  | 24552 | 851.7751 | 2552.3034 | 2551.2636 | 1.0399 | 1 | 4 | 28 | 3 | K.FDTGNLCMVTGGANLGRIGVITNR.E |
|  | 24864 | **869.3984** | **2605.1735** | **2605.2370** | **-0.0635** | **1** | **1** | **55** | **8** | **R.TDITYPAGFMDVISIDKTGENFR.L** |

| **256.** | P62820\|RAB1A_HUMAN    **Mass:** 22891    **Score:** 203    **Queries matched:** 24   **emPAI:** 0.86 |
| --- | --- |
|  | Ras-related protein Rab-1A OS=Homo sapiens GN=RAB1A PE=1 SV=3 |

|  | **Query** | **Observed** | **Mr(expt)** | **Mr(calc)** | **Delta** | **Miss** | **Score** | **Expect** | **Rank** | **Peptide** |
| --- | --- | --- | --- | --- | --- | --- | --- | --- | --- | --- |
|  | 3209 | **448.9991** | **895.9837** | **895.4651** | **0.5186** | **0** | **1** | **82** | **9** | **K.VVDYTTAK.E** |
|  | 3586 | 463.0037 | 923.9928 | 923.4348 | 0.5579 | 0 | 8 | 15 | 6 | R.YASENVNK.L 3579 |
|  | 4950 | **512.2786** | **1022.5427** | **1023.5601** | **-1.0174** | **1** | **8** | **26** | **1** | **K.KVVDYTTAK.E** 4985 |
|  | 5262 | **1046.4866** | **1045.4793** | **1045.4862** | **-0.0069** | **0** | **30** | **0.17** | **1** | **R.MGPGATAGGAEK.S** 5271 |
|  | 5434 | 531.2813 | 1060.5481 | 1061.4812 | -0.9331 | 0 | (16) | 4.9 | 4 | R.MGPGATAGGAEK.S |
|  | 5578 | 536.3207 | 1070.6269 | 1070.6336 | -0.0066 | 0 | 53 | 0.00096 | 1 | K.LLLIGDSGVGK.S 5577 |
|  | 7419 | **601.3399** | **1200.6652** | **1201.5873** | **-0.9221** | **1** | **11** | **12** | **8** | **K.RMGPGATAGGAEK.S** |
|  | 9635 | 658.8289 | 1315.6433 | 1315.6521 | -0.0088 | 0 | 60 | 0.00015 | 1 | K.LQIWDTAGQER.F 9612 9633 9634 |
|  | 12739 | 747.4353 | 1492.8560 | 1492.6544 | 0.2016 | 0 | 5 | 44 | 6 | M.SSMNPEYDYLFK.L |
|  | 15251 | **553.2769** | **1656.8088** | **1655.6847** | **1.1240** | **0** | **2** | **69** | **9** | **-.MSSMNPEYDYLFK.L** |
|  | 17502 | 604.9745 | 1811.9016 | 1813.0495 | -1.1479 | 1 | 7 | 20 | 3 | K.LLLIGDSGVGKSCLLLR.F |
|  | 18450 | 942.9408 | 1883.8670 | 1883.8757 | -0.0087 | 0 | 90 | 9.2e-008 | 1 | K.NATNVEQSFMTMAAEIK.K 18449 18451 |
|  | 19954 | 671.8677 | 2012.5814 | 2011.9707 | 0.6107 | 1 | 6 | 16 | 4 | K.NATNVEQSFMTMAAEIKK.R |
|  | 24618 | **854.7532** | **2561.2379** | **2561.2723** | **-0.0345** | **1** | **11** | **4.9** | **1** | **M.SSMNPEYDYLFKLLLIGDSGVGK.S** |
|  | 29104 | **898.6563** | **3590.5961** | **3589.7320** | **0.8641** | **1** | **3** | **16** | **8** | **K.EFADSLGIPFLETSAKNATNVEQSFMTMAAEIK.K** |

| **257.** | P02042\|HBD_HUMAN    **Mass:** 16159    **Score:** 203    **Queries matched:** 15   **emPAI:** 1.36 |
| --- | --- |
|  | Hemoglobin subunit delta OS=Homo sapiens GN=HBD PE=1 SV=2 |

|  | **Query** | **Observed** | **Mr(expt)** | **Mr(calc)** | **Delta** | **Miss** | **Score** | **Expect** | **Rank** | **Peptide** |
| --- | --- | --- | --- | --- | --- | --- | --- | --- | --- | --- |
|  | 3971 | 476.7556 | 951.4965 | 951.5025 | -0.0060 | 0 | 2 | 1e+002 | 9 | M.VHLTPEEK.T |
|  | 6273 | 563.7812 | 1125.5478 | 1125.5567 | -0.0089 | 0 | 9 | 17 | 2 | K.LHVDPENFR.L |
|  | 6579 | 575.3358 | 1148.6571 | 1148.6666 | -0.0095 | 0 | 48 | 0.0024 | 1 | K.VVAGVANALAHK.Y 6559 6560 |
|  | 8859 | 637.8631 | 1273.7116 | 1273.7183 | -0.0066 | 0 | 56 | 0.00035 | 1 | R.LLVVYPWTQR.F 8858 8877 |
|  | 11984 | 725.3973 | 1448.7801 | 1448.7888 | -0.0087 | 1 | 15 | 3.8 | 1 | K.VVAGVANALAHKYH.- 11983 11985 |
|  | 12126 | 486.9054 | 1457.6945 | 1456.6656 | 1.0288 | 0 | 2 | 92 | 8 | K.EFTPQMQAAYQK.V |
|  | 15418 | 835.4432 | 1668.8718 | 1668.8835 | -0.0117 | 0 | 84 | 4.1e-007 | 1 | K.VLGAFSDGLAHLDNLK.G |
|  | 17322 | 899.4909 | 1796.9673 | 1796.9785 | -0.0112 | 1 | 55 | 0.00034 | 1 | K.KVLGAFSDGLAHLDNLK.G 17323 |

| **258.** | Q5K4L6\|S27A3_HUMAN    **Mass:** 79393    **Score:** 203    **Queries matched:** 16   **emPAI:** 0.05 |
| --- | --- |
|  | Long-chain fatty acid transport protein 3 OS=Homo sapiens GN=SLC27A3 PE=2 SV=3 |

|  | **Query** | **Observed** | **Mr(expt)** | **Mr(calc)** | **Delta** | **Miss** | **Score** | **Expect** | **Rank** | **Peptide** |
| --- | --- | --- | --- | --- | --- | --- | --- | --- | --- | --- |
|  | 674 | **459.0117** | **458.0044** | **458.2601** | **-0.2557** | **0** | **4** | **84** | **1** | **R.GAVGR.A** 671 676 677 681 683 |
|  | 5444 | **531.3214** | **1060.6281** | **1061.6134** | **-0.9853** | **0** | **16** | **4.7** | **9** | **K.LHLWPQLR.W** 5445 |
|  | 7205 | **397.7031** | **1190.0874** | **1189.6819** | **0.4056** | **1** | **9** | **14** | **5** | **R.YSALLAGNLRI.-** 7191 |
|  | 7537 | 603.8140 | 1205.6135 | 1206.6509 | -1.0374 | 1 | 9 | 20 | 4 | R.GAVGRASWLYK.H 7568 |
|  | 19523 | **987.9416** | **1973.8686** | **1973.8827** | **-0.0141** | **0** | **126** | **2.3e-011** | **1** | **R.AAPGAGDAAAGSGAEFAGGDGAAR.G** 19524 |
|  | 30164 | **1013.0076** | **4048.0014** | **4047.9459** | **0.0555** | **0** | **4** | **12** | **2** | **R.DPQGHCMATSPGEPGLLVAPVSQQSPFLGYAGGPELAQGK.L** |
|  | 31395 | **1295.9298** | **5179.6901** | **5179.5020** | **0.1882** | **1** | **2** | **6.9** | **5** | **R.YDVTTGEPIRDPQGHCMATSPGEPGLLVAPVSQQSPFLGYAGGPELAQGK.L** |

| **259.** | P62888\|RL30_HUMAN    **Mass:** 12947    **Score:** 203    **Queries matched:** 7   **emPAI:** 1.22 |
| --- | --- |
|  | 60S ribosomal protein L30 OS=Homo sapiens GN=RPL30 PE=1 SV=2 |

|  | **Query** | **Observed** | **Mr(expt)** | **Mr(calc)** | **Delta** | **Miss** | **Score** | **Expect** | **Rank** | **Peptide** |
| --- | --- | --- | --- | --- | --- | --- | --- | --- | --- | --- |
|  | 10342 | 677.3821 | 1352.7497 | 1352.7598 | -0.0101 | 0 | 19 | 1.8 | 2 | K.LVILANNCPALR.K |
|  | 12523 | **494.2690** | **1479.7850** | **1480.8548** | **-1.0697** | **1** | **6** | **29** | **6** | **K.LVILANNCPALRK.S** |
|  | 16775 | **879.4529** | **1756.8912** | **1756.9029** | **-0.0117** | **0** | **82** | **6.1e-007** | **1** | **R.VCTLAIIDPGDSDIIR.S** |
|  | 19963 | **1007.9669** | **2013.9193** | **2013.9327** | **-0.0134** | **0** | **90** | **1e-007** | **1** | **K.TGVHHYSGNNIELGTACGK.Y** 19961 19962 |
|  | 25669 | **921.0026** | **2759.9860** | **2760.3310** | **-0.3450** | **1** | **0** | **39** | **9** | **R.VCTLAIIDPGDSDIIRSMPEQTGEK.-** |

| **260.** | O43678\|NDUA2_HUMAN    **Mass:** 11029    **Score:** 202    **Queries matched:** 5   **emPAI:** 0.36 |
| --- | --- |
|  | NADH dehydrogenase [ubiquinone] 1 alpha subcomplex subunit 2 OS=Homo sapiens GN=NDUFA2 PE=1 SV=3 |

|  | **Query** | **Observed** | **Mr(expt)** | **Mr(calc)** | **Delta** | **Miss** | **Score** | **Expect** | **Rank** | **Peptide** |
| --- | --- | --- | --- | --- | --- | --- | --- | --- | --- | --- |
|  | 2296 | 413.6645 | 825.3144 | 825.4279 | -0.1136 | 0 | 4 | 56 | 6 | R.IHLCQR.S |
|  | 2669 | 429.2404 | 856.4663 | 855.5290 | 0.9373 | 1 | 10 | 17 | 5 | K.LGLREIR.I |
|  | 20555 | **1032.8850** | **2063.7555** | **2064.0674** | **-0.3119** | **1** | **5** | **23** | **2** | **K.ANPDLPILIRECSDVQPK.L** |
|  | 23228 | **1186.0626** | **2370.1107** | **2370.1240** | **-0.0134** | **0** | **137** | **1.5e-012** | **1** | **R.YAFGQETNVPLNNFSADQVTR.A** 23227 |

| **261.** | P43490\|NAMPT_HUMAN    **Mass:** 55772    **Score:** 201    **Queries matched:** 11   **emPAI:** 0.30 |
| --- | --- |
|  | Nicotinamide phosphoribosyltransferase OS=Homo sapiens GN=NAMPT PE=1 SV=1 |

|  | **Query** | **Observed** | **Mr(expt)** | **Mr(calc)** | **Delta** | **Miss** | **Score** | **Expect** | **Rank** | **Peptide** |
| --- | --- | --- | --- | --- | --- | --- | --- | --- | --- | --- |
|  | 1158 | 572.3192 | 571.3119 | 571.4057 | -0.0938 | 1 | 15 | 6.9 | 4 | K.KILAK.Y 1161 |
|  | 2907 | **436.5798** | **871.1450** | **870.5327** | **0.6123** | **0** | **11** | **10** | **9** | **K.LLPPYLR.V** |
|  | 4939 | **511.7659** | **1021.5172** | **1021.5233** | **-0.0060** | **0** | **12** | **11** | **2** | **K.GWNYILEK.Y** |
|  | 6708 | **579.8361** | **1157.6576** | **1157.6656** | **-0.0080** | **0** | **15** | **5.5** | **1** | **K.GTDTVAGLALIK.K** |
|  | 18800 | **638.2904** | **1911.8494** | **1911.8639** | **-0.0145** | **1** | **30** | **0.096** | **1** | **K.DVYKEHFQDDVFNEK.G** |
|  | 21453 | **1079.5497** | **2157.0848** | **2157.0987** | **-0.0139** | **0** | **126** | **2e-011** | **1** | **R.VIQGDGVDINTLQEIVEGMK.Q** 21465 |
|  | 22183 | **1118.1133** | **2234.2120** | **2234.2270** | **-0.0150** | **0** | **71** | **6.6e-006** | **1** | **R.STQAPLIIRPDSGNPLDTVLK.V** |
|  | 27918 | **802.6474** | **3206.5605** | **3206.5772** | **-0.0167** | **1** | **34** | **0.018** | **1** | **R.TPAGNFVTLEEGKGDLEEYGQDLLHTVFK.N** 27920 |

| **262.** | Q15043\|S39AE_HUMAN    **Mass:** 54902    **Score:** 200    **Queries matched:** 6   **emPAI:** 0.22 |
| --- | --- |
|  | Zinc transporter ZIP14 OS=Homo sapiens GN=SLC39A14 PE=1 SV=2 |

|  | **Query** | **Observed** | **Mr(expt)** | **Mr(calc)** | **Delta** | **Miss** | **Score** | **Expect** | **Rank** | **Peptide** |
| --- | --- | --- | --- | --- | --- | --- | --- | --- | --- | --- |
|  | 8671 | **421.9079** | **1262.7018** | **1262.7095** | **-0.0077** | **0** | **33** | **0.073** | **1** | **K.ALLNHLDVGVGR.G** |
|  | 12020 | 726.3673 | 1450.7201 | 1450.7304 | -0.0103 | 0 | 68 | 1.8e-005 | 1 | R.YGEGDSLTLQQLK.A |
|  | 22839 | **1161.0682** | **2320.1219** | **2320.1369** | **-0.0150** | **0** | **78** | **1.1e-006** | **1** | **R.IGSSELQEFCPTILQQLDSR.A** 22838 22840 22850 |

| **263.** | P33260\|CP2CI_HUMAN    **Mass:** 56530    **Score:** 199    **Queries matched:** 37   **emPAI:** 0.67 |
| --- | --- |
|  | Cytochrome P450 2C18 OS=Homo sapiens GN=CYP2C18 PE=1 SV=3 |

|  | **Query** | **Observed** | **Mr(expt)** | **Mr(calc)** | **Delta** | **Miss** | **Score** | **Expect** | **Rank** | **Peptide** |
| --- | --- | --- | --- | --- | --- | --- | --- | --- | --- | --- |
|  | 1456 | 653.3032 | 652.2959 | 652.3003 | -0.0043 | 0 | 14 | 5.1 | 6 | R.NFGMGK.R |
|  | 2082 | 405.2055 | 808.3964 | 808.4014 | -0.0050 | 1 | 11 | 13 | 4 | R.NFGMGKR.S |
|  | 3511 | 459.7364 | 917.4583 | 917.4640 | -0.0057 | 0 | 34 | 0.073 | 1 | R.CLVEELR.K 3510 |
|  | 3694 | 466.8098 | 931.6051 | 931.6106 | -0.0055 | 0 | 71 | 1.5e-005 | 1 | R.YGLLLLLK.Y 3702 |
|  | 4022 | 478.7659 | 955.5172 | 955.4619 | 0.0552 | 0 | 7 | 35 | 6 | R.FCLMTLR.N |
|  | 4234 | **486.7869** | **971.5592** | **970.4508** | **1.1084** | **1** | **8** | **28** | **3** | **R.FDYKDQR.F** |
|  | 4752 | **504.2585** | **1006.5025** | **1006.5811** | **-0.0786** | **1** | **12** | **12** | **10** | **K.SYVLERIK.E** |
|  | 4948 | 512.2609 | 1022.5072 | 1023.4300 | -0.9228 | 0 | 6 | 41 | 6 | R.MCMGEGLAR.M |
|  | 5166 | 520.2509 | 1038.4871 | 1039.4249 | -0.9377 | 0 | (5) | 47 | 7 | R.MCMGEGLAR.M |
|  | 6749 | 582.2515 | 1162.4885 | 1162.4971 | -0.0086 | 1 | 30 | 0.16 | 1 | R.NRSPCMQDR.S 6750 6751 6752 6753 6754 6755 6775 |
|  | 6857 | 585.7937 | 1169.5728 | 1169.5791 | -0.0062 | 0 | 44 | 0.0063 | 1 | R.DFIDCFLIK.M 6856 |
|  | 7327 | **399.7487** | **1196.2242** | **1195.5260** | **0.6982** | **1** | **10** | **9.3** | **3** | **K.RMCMGEGLAR.M** |
|  | 8411 | 625.7788 | 1249.5431 | 1248.5485 | 0.9946 | 0 | 39 | 0.018 | 1 | K.SDYFMPFSAGK.R |
|  | 8818 | **425.0466** | **1272.1181** | **1272.6020** | **-0.4839** | **1** | **1** | **82** | **8** | **K.DMSKSLTNFSK.V** |
|  | 9945 | 666.3270 | 1330.6394 | 1330.6477 | -0.0083 | 1 | 37 | 0.032 | 1 | R.SIEDRVQEEAR.C |
|  | 10745 | 688.8618 | 1375.7090 | 1376.6435 | -0.9345 | 1 | (4) | 50 | 3 | K.KSDYFMPFSAGK.R |
|  | 11061 | 697.3727 | 1392.7308 | 1392.6384 | 0.0924 | 1 | 12 | 8.7 | 1 | K.KSDYFMPFSAGK.R 11044 |
|  | 14153 | 793.9045 | 1585.7945 | 1586.7835 | -0.9890 | 1 | 1 | 1e+002 | 5 | K.VQEEIECVVGRNR.S |
|  | 15626 | **562.2491** | **1683.7256** | **1682.8450** | **0.8806** | **1** | **9** | **13** | **1** | **R.FLNLMEKFNENLR.I** |
|  | 17122 | 594.9534 | 1781.8385 | 1781.8519 | -0.0134 | 0 | 50 | 0.0012 | 1 | R.SHMPYTDAVVHEIQR.Y 17123 17124 17126 |
|  | 20790 | 1043.0721 | 2084.1297 | 2084.1380 | -0.0083 | 0 | 6 | 20 | 1 | R.MELFLFLTTILQNFNLK.S |
|  | 20991 | 1053.4620 | 2104.9095 | 2105.0463 | -0.1368 | 1 | 6 | 24 | 2 | K.YPEVTAKVQEEIECVVGR.N 20992 |

| **264.** | Q8NHW5\|RLA0L_HUMAN    **Mass:** 34514    **Score:** 197    **Queries matched:** 17   **emPAI:** 0.86 |
| --- | --- |
|  | 60S acidic ribosomal protein P0-like OS=Homo sapiens PE=2 SV=1 |

|  | **Query** | **Observed** | **Mr(expt)** | **Mr(calc)** | **Delta** | **Miss** | **Score** | **Expect** | **Rank** | **Peptide** |
| --- | --- | --- | --- | --- | --- | --- | --- | --- | --- | --- |
|  | 2953 | 438.6146 | 875.2147 | 874.4396 | 0.7751 | 0 | 10 | 16 | 1 | K.EDLTEIR.D |
|  | 7765 | 609.3382 | 1216.6618 | 1216.6703 | -0.0085 | 0 | 38 | 0.024 | 1 | K.IIQLLDDYPK.C 7766 |
|  | 7837 | 611.3100 | 1220.6054 | 1220.6149 | -0.0095 | 0 | 52 | 0.00098 | 1 | R.GHLENNPALEK.L 7836 |
|  | 8711 | 633.8062 | 1265.5979 | 1265.6074 | -0.0096 | 0 | 42 | 0.0087 | 1 | K.CFIVGADNVGSK.Q |
|  | 9168 | 646.3835 | 1290.7524 | 1289.6696 | 1.0828 | 1 | 3 | 70 | 1 | K.QMQQIRMSLR.G |
|  | 9571 | 657.3549 | 1312.6952 | 1312.7027 | -0.0075 | 0 | 22 | 0.87 | 1 | K.TSFFQALGITTK.I |
|  | 10633 | 685.3796 | 1368.7447 | 1368.7547 | -0.0100 | 1 | 59 | 0.00015 | 1 | R.DMLLANKVPAAAR.A 10634 |
|  | 11660 | 714.9153 | 1427.8160 | 1427.8235 | -0.0075 | 0 | 7 | 30 | 1 | R.GTIEILSDVQLIK.T |
|  | 20379 | 684.3174 | 2049.9305 | 2050.0088 | -0.0783 | 1 | 2 | 54 | 4 | K.CFIVGADNVGSKQMQQIR.M |
|  | 21651 | 1090.5466 | 2179.0787 | 2179.0943 | -0.0156 | 0 | 60 | 9.3e-005 | 1 | R.AGAIAPCEVTVPAQNTGLGPEK.T 21652 21654 |
|  | 30745 | 1447.0414 | 4338.1023 | 4339.2086 | -1.1063 | 0 | (1) | 19 | 5 | K.VGASEATLLNMLNISPFSFGLVIQQVFDNGSIYNPEVLDK.T |
|  | 30761 | 1089.5309 | 4354.0944 | 4355.2035 | -1.1091 | 0 | 1 | 16 | 5 | K.VGASEATLLNMLNISPFSFGLVIQQVFDNGSIYNPEVLDK.T |

| **265.** | P40926\|MDHM_HUMAN    **Mass:** 35937    **Score:** 197    **Queries matched:** 22   **emPAI:** 0.64 |
| --- | --- |
|  | Malate dehydrogenase, mitochondrial OS=Homo sapiens GN=MDH2 PE=1 SV=3 |

|  | **Query** | **Observed** | **Mr(expt)** | **Mr(calc)** | **Delta** | **Miss** | **Score** | **Expect** | **Rank** | **Peptide** |
| --- | --- | --- | --- | --- | --- | --- | --- | --- | --- | --- |
|  | 1748 | 772.4255 | 771.4183 | 771.4239 | -0.0056 | 0 | 18 | 3.3 | 1 | K.NSPLVSR.L |
|  | 2222 | **411.2328** | **820.4510** | **821.4283** | **-0.9773** | **1** | **10** | **18** | **6** | **K.KGEDFVK.T** 2219 2227 |
|  | 3654 | **465.2707** | **928.5268** | **927.4563** | **1.0706** | **0** | **14** | **6.7** | **2** | **K.HGVYNPNK.I** 3614 3624 |
|  | 4514 | **496.7526** | **991.4906** | **991.5338** | **-0.0432** | **0** | **16** | **4.6** | **1** | **R.ANTFVAELK.G** |
|  | 6134 | **558.2662** | **1114.5179** | **1115.5896** | **-1.0717** | **0** | **14** | **6.9** | **1** | **K.MISDAIPELK.A** 6154 |
|  | 8070 | **617.3467** | **1232.6788** | **1232.7129** | **-0.0341** | **0** | **4** | **66** | **7** | **K.IFGVTTLDIVR.A** |
|  | 10074 | **669.8587** | **1337.7028** | **1337.7126** | **-0.0097** | **0** | **48** | **0.0021** | **1** | **K.GCDVVVIPAGVPR.K** |
|  | 10154 | 672.8061 | 1343.5976 | 1342.6591 | 0.9385 | 0 | 5 | 41 | 6 | R.FVFSLVDAMNGK.E |
|  | 12339 | 735.8267 | 1469.6389 | 1469.6933 | -0.0544 | 0 | 0 | 1.2e+002 | 6 | K.AGAGSATLSMAYAGAR.F |
|  | 13328 | **511.5582** | **1531.6527** | **1530.8327** | **0.8200** | **1** | **1** | **87** | **5** | **K.MISDAIPELKASIK.K** |
|  | 13757 | **780.8991** | **1559.7837** | **1559.7944** | **-0.0107** | **0** | **82** | **7.3e-007** | **1** | **K.VDFPQDQLTALTGR.I** |
|  | 17285 | **897.0440** | **1792.0733** | **1792.0822** | **-0.0089** | **0** | **101** | **7.2e-009** | **1** | **K.VAVLGASGGIGQPLSLLLK.N** |
|  | 19114 | **970.2714** | **1938.5283** | **1937.9656** | **0.5627** | **1** | **13** | **3.3** | **1** | **K.VSSFEEKMISDAIPELK.A** |
|  | 23184 | **789.0804** | **2364.2195** | **2364.2325** | **-0.0130** | **0** | **32** | **0.049** | **1** | **R.LTLYDIAHTPGVAADLSHIETK.A** 23191 |
|  | 24196 | **833.7841** | **2498.3304** | **2498.4043** | **-0.0740** | **1** | **1** | **52** | **9** | **R.VNVPVIGGHAGKTIIPLISQCTPK.V** |
|  | 25875 | **932.5081** | **2794.5025** | **2794.3418** | **0.1607** | **1** | **1** | **49** | **3** | **K.AGAGSATLSMAYAGARFVFSLVDAMNGK.E** |

| **266.** | P24844\|MYL9_HUMAN    **Mass:** 19871    **Score:** 196    **Queries matched:** 10   **emPAI:** 0.70 |
| --- | --- |
|  | Myosin regulatory light polypeptide 9 OS=Homo sapiens GN=MYL9 PE=1 SV=4 |

|  | **Query** | **Observed** | **Mr(expt)** | **Mr(calc)** | **Delta** | **Miss** | **Score** | **Expect** | **Rank** | **Peptide** |
| --- | --- | --- | --- | --- | --- | --- | --- | --- | --- | --- |
|  | 5112 | 517.7648 | 1033.5151 | 1034.5066 | -0.9915 | 0 | 6 | 38 | 1 | R.ELLTTMGDR.F |
|  | 7970 | 614.8079 | 1227.6012 | 1227.6095 | -0.0084 | 0 | 25 | 0.49 | 1 | K.LNGTDPEDVIR.N |
|  | 8327 | **623.7921** | **1245.5695** | **1245.5778** | **-0.0083** | **0** | **39** | **0.02** | **1** | **K.GNFNYVEFTR.I** |
|  | 8474 | 418.7264 | 1253.1572 | 1252.5506 | 0.6066 | 0 | 6 | 24 | 8 | K.EAFNMIDQNR.D |
|  | 19868 | 1002.4852 | 2002.9558 | 2002.9670 | -0.0112 | 1 | 92 | 6.4e-008 | 1 | R.DGFIDKEDLHDMLASLGK.N 19869 19871 |
|  | 20850 | 1045.9896 | 2089.9647 | 2089.9779 | -0.0132 | 0 | 114 | 3.8e-010 | 1 | R.ATSNVFAMFDQSQIQEFK.E 20839 20851 |

| **267.** | Q9HCN8\|SDF2L_HUMAN    **Mass:** 23812    **Score:** 195    **Queries matched:** 13   **emPAI:** 0.35 |
| --- | --- |
|  | Stromal cell-derived factor 2-like protein 1 OS=Homo sapiens GN=SDF2L1 PE=1 SV=2 |

|  | **Query** | **Observed** | **Mr(expt)** | **Mr(calc)** | **Delta** | **Miss** | **Score** | **Expect** | **Rank** | **Peptide** |
| --- | --- | --- | --- | --- | --- | --- | --- | --- | --- | --- |
|  | 905 | **515.3257** | **514.3185** | **514.2863** | **0.0321** | **0** | **16** | **5** | **5** | **R.GSPVR.C** |
|  | 1047 | 545.3637 | 544.3564 | 544.2969 | 0.0595 | 0 | 9 | 43 | 3 | R.EAAVR.F |
|  | 2969 | **439.2309** | **876.4472** | **875.3556** | **1.0917** | **0** | **4** | **75** | **2** | **R.GGSEGGCPR.G** |
|  | 6517 | **572.9967** | **1143.9788** | **1144.5407** | **-0.5619** | **1** | **4** | **48** | **4** | **R.IRGGSEGGCPR.G** 6528 |
|  | 20186 | **677.6421** | **2029.9044** | **2029.9177** | **-0.0133** | **0** | **12** | **5.4** | **1** | **R.GQHEVHGMPSANTHNTWK.A** |
|  | 21169 | 1064.0176 | 2126.0206 | 2126.0354 | -0.0148 | 0 | 57 | 0.00018 | 1 | K.AMEGIFIKPSVEPSAGHDEL.- 21170 21171 |
|  | 23516 | **803.7399** | **2408.1980** | **2408.2125** | **-0.0145** | **0** | **8** | **11** | **1** | **R.FQHVGTSVFLSVTGEQYGSPIR.G** |
|  | 24348 | **1261.0380** | **2520.0614** | **2520.0789** | **-0.0176** | **0** | **126** | **1.6e-011** | **1** | **K.YGSGSGQQSVTGVEASDDANSYWR.I** |
|  | 26502 | **968.1705** | **2901.4896** | **2901.6415** | **-0.1519** | **1** | **0** | **54** | **2** | **-.MWSAGRGGAAWPVLLGLLLALLVPGGGAAK.T** |
|  | 30885 | 1480.0720 | 4437.1942 | 4436.1145 | 1.0797 | 1 | 3 | 12 | 2 | R.FQHVGTSVFLSVTGEQYGSPIRGQHEVHGMPSANTHNTWK.A |

| **268.** | P09467\|F16P1_HUMAN    **Mass:** 37190    **Score:** 194    **Queries matched:** 18   **emPAI:** 0.62 |
| --- | --- |
|  | Fructose-1,6-bisphosphatase 1 OS=Homo sapiens GN=FBP1 PE=1 SV=4 |

|  | **Query** | **Observed** | **Mr(expt)** | **Mr(calc)** | **Delta** | **Miss** | **Score** | **Expect** | **Rank** | **Peptide** |
| --- | --- | --- | --- | --- | --- | --- | --- | --- | --- | --- |
|  | 866 | **503.1058** | **502.0985** | **501.2547** | **0.8438** | **0** | **5** | **1.2e+002** | **1** | **K.SPNGK.L** |
|  | 1580 | 703.4049 | 702.3976 | 702.4024 | -0.0048 | 0 | 17 | 4.9 | 3 | K.AISSAVR.K |
|  | 1860 | **397.4034** | **792.7922** | **792.3800** | **0.4122** | **0** | **13** | **6.2** | **3** | **K.AGGMATTGK.E** 1850 |
|  | 4541 | 497.7600 | 993.5054 | 994.4906 | -0.9852 | 1 | 7 | 31 | 9 | R.FVMEEGRK.A |
|  | 6687 | **579.2913** | **1156.5681** | **1156.5764** | **-0.0083** | **0** | **38** | **0.026** | **1** | **K.IYSLNEGYAK.D** |
|  | 9167 | **431.2581** | **1290.7524** | **1290.5993** | **0.1531** | **0** | **5** | **42** | **9** | **K.FPPDNSAPYGAR.Y** 9142 |
|  | 10104 | **447.8937** | **1340.6593** | **1341.6928** | **-1.0335** | **1** | **5** | **39** | **6** | **K.GKIYSLNEGYAK.D** |
|  | 14423 | **803.3351** | **1604.6557** | **1604.8522** | **-0.1965** | **0** | **3** | **52** | **2** | **K.EAVLDVIPTDIHQR.A** |
|  | 16127 | **856.9727** | **1711.9309** | **1711.9396** | **-0.0087** | **0** | **50** | **0.0011** | **1** | **R.APVILGSPDDVLEFLK.V** |
|  | 16435 | **866.9653** | **1731.9161** | **1731.9263** | **-0.0102** | **1** | **70** | **1.1e-005** | **1** | **K.KLDVLSNDLVMNMLK.S** |
|  | 16632 | 874.5034 | 1746.9923 | 1747.9212 | -0.9289 | 1 | (5) | 33 | 6 | K.KLDVLSNDLVMNMLK.S |
|  | 16846 | **880.9010** | **1759.7874** | **1759.7983** | **-0.0108** | **0** | **55** | **0.00032** | **1** | **R.LLYECNPMAYVMEK.A** 16845 |
|  | 17430 | **903.4734** | **1804.9323** | **1803.9400** | **0.9923** | **0** | **7** | **20** | **1** | **R.GTGELTQLLNSLCTAVK.A** |
|  | 20643 | **691.3638** | **2071.0695** | **2071.0698** | **-0.0003** | **0** | **1** | **68** | **4** | **K.AGIAHLYGIAGSTNVTGDQVK.K** |
|  | 21800 | **734.0573** | **2199.1501** | **2199.1648** | **-0.0147** | **1** | **51** | **0.00069** | **1** | **R.KAGIAHLYGIAGSTNVTGDQVK.K** |

| **269.** | Q9UJZ1\|STML2_HUMAN    **Mass:** 38624    **Score:** 194    **Queries matched:** 18   **emPAI:** 0.32 |
| --- | --- |
|  | Stomatin-like protein 2 OS=Homo sapiens GN=STOML2 PE=1 SV=1 |

|  | **Query** | **Observed** | **Mr(expt)** | **Mr(calc)** | **Delta** | **Miss** | **Score** | **Expect** | **Rank** | **Peptide** |
| --- | --- | --- | --- | --- | --- | --- | --- | --- | --- | --- |
|  | 1962 | **401.2848** | **800.5550** | **799.4916** | **1.0634** | **0** | **12** | **15** | **3** | **R.GTGALLLR.G** 1965 |
|  | 2024 | 403.0464 | 804.0782 | 803.4436 | 0.6347 | 1 | 5 | 41 | 6 | -.MLARAAR.G |
|  | 4883 | 509.2279 | 1016.4413 | 1016.5138 | -0.0725 | 0 | 7 | 39 | 3 | R.ESAINVAEGK.K |
|  | 5472 | **532.3206** | **1062.6266** | **1061.5353** | **1.0913** | **0** | **3** | **82** | **6** | **R.ATVLESEGTR.E** |
|  | 5923 | **550.2157** | **1098.4168** | **1097.6669** | **0.7499** | **1** | **0** | **1.4e+002** | **5** | **R.AARGTGALLLR.G** |
|  | 6520 | **573.3074** | **1144.6002** | **1144.6088** | **-0.0086** | **1** | **28** | **0.29** | **1** | **R.ESAINVAEGKK.Q** |
|  | 9608 | **658.3292** | **1314.6438** | **1314.7143** | **-0.0705** | **1** | **6** | **39** | **5** | **K.KQAQILASEAEK.A** |
|  | 12979 | 504.2680 | 1509.7823 | 1508.6711 | 1.1112 | 1 | 10 | 14 | 6 | K.ESMQMQVEAERR.K |
|  | 14307 | 532.9060 | 1595.6962 | 1595.7283 | -0.0321 | 1 | 1 | 96 | 9 | R.VKESMQMQVEAER.R |
|  | 15511 | **838.4520** | **1674.8894** | **1674.0080** | **0.8814** | **0** | **4** | **40** | **1** | **R.ILEPGLNILIPVLDR.I** |
|  | 22693 | **1152.0523** | **2302.0899** | **2302.1012** | **-0.0112** | **0** | **25** | **0.24** | **1** | **R.ESLNASIVDAINQAADCWGIR.C** |
|  | 22913 | **1165.5438** | **2329.0731** | **2329.0896** | **-0.0165** | **0** | **94** | **2.9e-008** | **1** | **K.ASYGVEDPEYAVTQLAQTTMR.S** 22914 |
|  | 25220 | **894.4169** | **2680.2290** | **2681.4289** | **-1.2000** | **1** | **4** | **22** | **1** | **R.ASSGLPRNTVVLFVPQQEAWVVER.M** |
|  | 25511 | **911.4207** | **2731.2403** | **2731.3170** | **-0.0767** | **1** | **0** | **57** | **1** | **R.ESLNASIVDAINQAADCWGIRCLR.Y** |
|  | 27404 | **1022.5638** | **3064.6695** | **3065.5049** | **-0.8354** | **0** | **5** | **14** | **2** | **K.DSNTILLPSNPGDVTSMVAQAMGVYGALTK.A** |
|  | 27449 | **1026.4845** | **3076.4317** | **3076.4521** | **-0.0205** | **1** | **42** | **0.0029** | **1** | **R.IMDPYKASYGVEDPEYAVTQLAQTTMR.S** |

| **270.** | Q8NBS9\|TXND5_HUMAN    **Mass:** 48283    **Score:** 192    **Queries matched:** 13   **emPAI:** 0.35 |
| --- | --- |
|  | Thioredoxin domain-containing protein 5 OS=Homo sapiens GN=TXNDC5 PE=1 SV=2 |

|  | **Query** | **Observed** | **Mr(expt)** | **Mr(calc)** | **Delta** | **Miss** | **Score** | **Expect** | **Rank** | **Peptide** |
| --- | --- | --- | --- | --- | --- | --- | --- | --- | --- | --- |
|  | 1189 | **579.3454** | **578.3381** | **578.3428** | **-0.0047** | **0** | **13** | **8.1** | **1** | **K.VYVAK.V** |
|  | 1454 | **652.6113** | **651.6040** | **651.3228** | **0.2812** | **0** | **13** | **5** | **2** | **K.VDQYK.G** |
|  | 1517 | 678.3412 | 677.3339 | 677.3748 | -0.0409 | 0 | 7 | 38 | 1 | R.GYPTLK.L |
|  | 5669 | **540.3129** | **1078.6113** | **1078.6175** | **-0.0062** | **0** | **3** | **77** | **1** | **R.GYPTLLLFR.G** |
|  | 14138 | 529.3085 | 1584.9036 | 1583.8824 | 1.0212 | 1 | 3 | 65 | 4 | K.YSVRGYPTLLLFR.G 14141 |
|  | 16856 | **881.3796** | **1760.7446** | **1760.7570** | **-0.0124** | **0** | **77** | **1.9e-006** | **1** | **K.VDCTAHSDVCSAQGVR.G** 16857 |
|  | 18204 | 932.9689 | 1863.9233 | 1863.9326 | -0.0093 | 1 | 3 | 51 | 5 | R.DLESLREYVESQLQR.T 18184 |
|  | 19424 | **983.4236** | **1964.8327** | **1964.8469** | **-0.0142** | **0** | **70** | **8.8e-006** | **1** | **K.VDCTQHYELCSGNQVR.G** |
|  | 29621 | **1269.9425** | **3806.8057** | **3806.8237** | **-0.0180** | **0** | **40** | **0.0032** | **1** | **R.DFQTLENWMLQTLNEEPVTPEPEVEPPSAPELK.Q** |
|  | 31306 | **1245.1138** | **4976.4260** | **4976.4554** | **-0.0294** | **1** | **31** | **0.012** | **1** | **R.TETGATETVTPSEAPVLAAEPEADKGTVLALTENNFDDTIAEGITFIK.F** |

| **271.** | P39023\|RL3_HUMAN    **Mass:** 46365    **Score:** 192    **Queries matched:** 26   **emPAI:** 0.59 |
| --- | --- |
|  | 60S ribosomal protein L3 OS=Homo sapiens GN=RPL3 PE=1 SV=2 |

|  | **Query** | **Observed** | **Mr(expt)** | **Mr(calc)** | **Delta** | **Miss** | **Score** | **Expect** | **Rank** | **Peptide** |
| --- | --- | --- | --- | --- | --- | --- | --- | --- | --- | --- |
|  | 1632 | **720.3648** | **719.3575** | **719.3636** | **-0.0061** | **0** | **14** | **9.1** | **1** | **K.GCVVGTK.K** |
|  | 1774 | **788.4827** | **787.4755** | **787.4803** | **-0.0049** | **0** | **30** | **0.25** | **1** | **K.SLLVQTK.R** 1775 |
|  | 3151 | 446.2657 | 890.5168 | 890.5225 | -0.0057 | 0 | 59 | 0.00023 | 1 | K.IGQGYLIK.D |
|  | 3608 | **464.2262** | **926.4378** | **927.4008** | **-0.9629** | **0** | **2** | **95** | **5** | **R.FQTMEEK.K** |
|  | 4019 | **478.7313** | **955.4481** | **954.5069** | **0.9412** | **0** | **(8)** | **28** | **4** | **R.VIAHTQMR.L** |
|  | 4214 | **486.2554** | **970.4963** | **970.5018** | **-0.0055** | **0** | **16** | **3.8** | **1** | **R.VIAHTQMR.L** |
|  | 7196 | 595.7852 | 1189.5559 | 1190.6659 | -1.1100 | 1 | 35 | 0.051 | 1 | K.IGQGYLIKDGK.L |
|  | 10120 | **671.7870** | **1341.5594** | **1341.5684** | **-0.0090** | **0** | **43** | **0.0067** | **1** | **K.NNASTDYDLSDK.S** 10142 |
|  | 10536 | **683.3274** | **1364.6402** | **1364.6837** | **-0.0435** | **1** | **6** | **38** | **7** | **K.FIDTTSKFGHGR.F** |
|  | 11760 | **718.3250** | **1434.6355** | **1434.6449** | **-0.0095** | **0** | **55** | **0.00038** | **1** | **K.TVFAEHISDECK.R** 11761 |
|  | 13466 | 514.7521 | 1541.2344 | 1540.8263 | 0.4081 | 1 | 4 | 35 | 4 | K.FSAPRHGSLGFLPR.K |
|  | 14219 | **796.3749** | **1590.7353** | **1590.7460** | **-0.0107** | **1** | **54** | **0.00044** | **1** | **K.TVFAEHISDECKR.R** |
|  | 15861 | **849.4241** | **1696.8337** | **1695.8614** | **0.9723** | **0** | **16** | **2.7** | **1** | **K.AHLMEIQVNGGTVAEK.L** |
|  | 16707 | **585.2807** | **1752.8203** | **1751.9101** | **0.9102** | **1** | **3** | **58** | **8** | **K.AGMTHIVREVDRPGSK.V** |
|  | 17649 | **609.3163** | **1824.9270** | **1823.9563** | **0.9707** | **1** | **20** | **1.1** | **1** | **K.KAHLMEIQVNGGTVAEK.L** |
|  | 22279 | **562.5466** | **2246.1572** | **2246.1736** | **-0.0164** | **1** | **9** | **11** | **1** | **K.SFPKDDPSKPVHLTAFLGYK.A** |
|  | 23682 | **610.2855** | **2437.1127** | **2436.2148** | **0.8980** | **0** | **2** | **42** | **4** | **K.SINPLGGFVHYGEVTNDFVMLK.G** |
|  | 25241 | **1343.6831** | **2685.3517** | **2685.3684** | **-0.0167** | **0** | **73** | **3.7e-006** | **1** | **R.LEQQVPVNQVFGQDEMIDVIGVTK.G** |
|  | 25329 | **1351.5815** | **2701.1485** | **2701.3633** | **-0.2148** | **0** | **(2)** | **35** | **3** | **R.LEQQVPVNQVFGQDEMIDVIGVTK.G** 25323 25326 |
|  | 26887 | **991.1719** | **2970.4940** | **2970.5121** | **-0.0181** | **1** | **28** | **0.083** | **1** | **R.ERLEQQVPVNQVFGQDEMIDVIGVTK.G** |
|  | 27090 | 1004.4562 | 3010.3469 | 3009.6461 | 0.7008 | 1 | 1 | 44 | 3 | K.KEVVEAVTIVETPPMVVVGIVGYVETPR.G |

| **272.** | P08238\|HS90B_HUMAN    **Mass:** 83554    **Score:** 192    **Queries matched:** 23   **emPAI:** 0.48 |
| --- | --- |
|  | Heat shock protein HSP 90-beta OS=Homo sapiens GN=HSP90AB1 PE=1 SV=4 |

|  | **Query** | **Observed** | **Mr(expt)** | **Mr(calc)** | **Delta** | **Miss** | **Score** | **Expect** | **Rank** | **Peptide** |
| --- | --- | --- | --- | --- | --- | --- | --- | --- | --- | --- |
|  | 996 | 533.1879 | 532.1807 | 532.2493 | -0.0686 | 0 | 7 | 57 | 8 | K.AEADK.N |
|  | 2756 | **432.2167** | **862.4189** | **862.4218** | **-0.0029** | **0** | **13** | **11** | **4** | **R.EMLQQSK.I** |
|  | 3147 | **446.2207** | **890.4268** | **890.4174** | **0.0094** | **0** | **6** | **52** | **1** | **K.FYEAFSK.N** |
|  | 8142 | **618.9986** | **1235.9826** | **1235.6299** | **0.3528** | **1** | **2** | **64** | **2** | **R.RAPFDLFENK.K** |
|  | 8264 | **621.8519** | **1241.6893** | **1241.6979** | **-0.0086** | **0** | **45** | **0.0042** | **1** | **K.ADLINNLGTIAK.S** 8293 |
|  | 8391 | **625.2974** | **1248.5802** | **1247.4984** | **1.0817** | **0** | **24** | **0.53** | **1** | **R.DNSTMGYMMAK.K** |
|  | 8874 | 638.3207 | 1274.6268 | 1274.6354 | -0.0086 | 0 | 43 | 0.0078 | 1 | R.ELISNASDALDK.I |
|  | 9295 | 433.5708 | 1297.6907 | 1296.6534 | 1.0372 | 1 | 7 | 30 | 1 | K.LGIHEDSTNRR.R |
|  | 10267 | 675.3663 | 1348.7181 | 1348.7272 | -0.0091 | 0 | 40 | 0.015 | 1 | R.TLTLVDTGIGMTK.A |
|  | 11581 | **475.2253** | **1422.6539** | **1423.5782** | **-0.9242** | **1** | **12** | **7.8** | **1** | **R.DNSTMGYMMAKK.H** |
|  | 13034 | **757.3917** | **1512.7688** | **1512.7784** | **-0.0096** | **0** | **22** | **0.77** | **1** | **R.GVVDSEDLPLNISR.E** |
|  | 13263 | **764.3709** | **1526.7271** | **1526.7365** | **-0.0094** | **0** | **46** | **0.0028** | **1** | **K.SLTNDWEDHLAVK.H** 13262 |
|  | 13527 | 772.9121 | 1543.8095 | 1543.8205 | -0.0110 | 1 | 52 | 0.00085 | 1 | R.ELISNASDALDKIR.Y 13496 13526 |
|  | 15060 | 823.4092 | 1644.8038 | 1645.8199 | -1.0161 | 1 | 5 | 38 | 7 | K.ETQKSIYYITGESK.E |
|  | 17184 | **596.2705** | **1785.7897** | **1786.8164** | **-1.0267** | **1** | **4** | **47** | **5** | **K.AQALRDNSTMGYMMAK.K** |
|  | 17937 | **924.3958** | **1846.7771** | **1846.7897** | **-0.0127** | **0** | **71** | **7.8e-006** | **1** | **R.NPDDITQEEYGEFYK.S** |
|  | 20200 | **678.3444** | **2032.0113** | **2033.0582** | **-1.0469** | **1** | **1** | **76** | **6** | **K.YIDQEELNKTKPIWTR.N** |
|  | 23765 | **1224.5689** | **2447.1231** | **2447.1389** | **-0.0157** | **0** | **59** | **9.8e-005** | **1** | **R.GFEVVYMTEPIDEYCVQQLK.E** |
|  | 29230 | **1225.1710** | **3672.4912** | **3672.7844** | **-0.2932** | **1** | **1** | **20** | **7** | **K.SGTKAFMEALQAGADISMIGQFGVGFYSAYLVAEK.V** |

| **273.** | P84098\|RL19_HUMAN    **Mass:** 23565    **Score:** 191    **Queries matched:** 10   **emPAI:** 0.35 |
| --- | --- |
|  | 60S ribosomal protein L19 OS=Homo sapiens GN=RPL19 PE=1 SV=1 |

|  | **Query** | **Observed** | **Mr(expt)** | **Mr(calc)** | **Delta** | **Miss** | **Score** | **Expect** | **Rank** | **Peptide** |
| --- | --- | --- | --- | --- | --- | --- | --- | --- | --- | --- |
|  | 1375 | **632.3568** | **631.3495** | **630.3588** | **0.9907** | **0** | **12** | **18** | **3** | **K.EEIIK.T** 1371 |
|  | 1417 | **643.0626** | **642.0553** | **641.3319** | **0.7234** | **0** | **1** | **85** | **3** | **R.HMGIGK.R** |
|  | 3252 | **450.7582** | **899.5018** | **900.5505** | **-1.0487** | **1** | **14** | **7.1** | **1** | **K.RLASSVLR.C** |
|  | 5173 | 520.2812 | 1038.5478 | 1037.5361 | 1.0117 | 1 | 11 | 12 | 2 | -.MSMLRLQK.R |
|  | 7232 | **596.8000** | **1191.5854** | **1190.5906** | **0.9948** | **0** | **22** | **0.9** | **1** | **R.HMYHSLYLK.V** 7226 |
|  | 7551 | **604.2512** | **1206.4879** | **1206.5855** | **-0.0977** | **0** | **(8)** | **23** | **1** | **R.HMYHSLYLK.V** |
|  | 19151 | **971.9595** | **1941.9044** | **1941.9180** | **-0.0136** | **0** | **115** | **3e-010** | **1** | **K.VWLDPNETNEIANANSR.Q** 19150 |

| **274.** | P50224\|ST1A3_HUMAN    **Mass:** 34288    **Score:** 191    **Queries matched:** 14   **emPAI:** 0.23 |
| --- | --- |
|  | Sulfotransferase 1A3/1A4 OS=Homo sapiens GN=SULT1A3 PE=1 SV=1 |

|  | **Query** | **Observed** | **Mr(expt)** | **Mr(calc)** | **Delta** | **Miss** | **Score** | **Expect** | **Rank** | **Peptide** |
| --- | --- | --- | --- | --- | --- | --- | --- | --- | --- | --- |
|  | 1592 | 707.2511 | 706.2438 | 705.4174 | 0.8265 | 0 | 15 | 5.8 | 3 | K.VVYVAR.N |
|  | 3357 | 454.2142 | 906.4139 | 907.4222 | -1.0083 | 1 | 12 | 12 | 3 | R.KGMAGDWK.T 3387 |
|  | 3693 | 466.7707 | 931.5268 | 932.5807 | -1.0539 | 1 | 16 | 4.4 | 2 | K.VKVVYVAR.N |
|  | 6817 | 584.3174 | 1166.6203 | 1165.5728 | 1.0476 | 0 | 3 | 68 | 7 | K.TTFTVAQNER.F |
|  | 19060 | 645.3392 | 1932.9959 | 1933.9819 | -0.9860 | 0 | 1 | 74 | 9 | -.MELIQDTSRPPLEYVK.G |
|  | 20509 | **687.3582** | **2059.0526** | **2057.9074** | **1.1452** | **0** | **2** | **65** | **6** | **R.SLPEETMDFMVQHTSFK.E** |
|  | 23001 | 1171.0669 | 2340.1192 | 2340.1308 | -0.0115 | 0 | 102 | 4.3e-009 | 1 | K.SGTTWVSQILDMIYQGGDLEK.C 23000 23002 |
|  | 23649 | 1216.6175 | 2431.2205 | 2430.0905 | 1.1300 | 1 | (1) | 56 | 4 | R.SLPEETMDFMVQHTSFKEMK.K 23646 |
|  | 23762 | **816.4137** | **2446.2193** | **2446.0855** | **0.1338** | **1** | **5** | **23** | **3** | **R.SLPEETMDFMVQHTSFKEMK.K** |
|  | 23894 | **821.7593** | **2462.2560** | **2462.0804** | **0.1756** | **1** | **(1)** | **55** | **4** | **R.SLPEETMDFMVQHTSFKEMK.K** |

| **275.** | O00264\|PGRC1_HUMAN    **Mass:** 21772    **Score:** 189    **Queries matched:** 11   **emPAI:** 1.65 |
| --- | --- |
|  | Membrane-associated progesterone receptor component 1 OS=Homo sapiens GN=PGRMC1 PE=1 SV=3 |

|  | **Query** | **Observed** | **Mr(expt)** | **Mr(calc)** | **Delta** | **Miss** | **Score** | **Expect** | **Rank** | **Peptide** |
| --- | --- | --- | --- | --- | --- | --- | --- | --- | --- | --- |
|  | 2691 | **430.2545** | **858.4944** | **858.4997** | **-0.0053** | **0** | **17** | **4.7** | **1** | **R.ILMAINGK.V** 2705 |
|  | 3709 | 467.2218 | 932.4290 | 932.4352 | -0.0062 | 0 | 24 | 0.7 | 1 | R.FDGVQDPR.I |
|  | 6010 | **552.7895** | **1103.5644** | **1103.5723** | **-0.0079** | **1** | **9** | **24** | **3** | **R.DFTPAELRR.F** |
|  | 6011 | **552.7897** | **1103.5648** | **1103.5723** | **-0.0076** | **1** | **36** | **0.047** | **1** | **R.RDFTPAELR.R** |
|  | 13086 | **758.8596** | **1515.7047** | **1515.7147** | **-0.0100** | **0** | **39** | **0.015** | **1** | **K.FYGPEGPYGVFAGR.D** 13085 |
|  | 15045 | **822.9066** | **1643.7986** | **1643.8096** | **-0.0111** | **1** | **59** | **0.00014** | **1** | **R.KFYGPEGPYGVFAGR.D** 15041 15059 |
|  | 29156 | **1208.5498** | **3622.6276** | **3622.6475** | **-0.0199** | **1** | **110** | **3.2e-010** | **1** | **K.EALKDEYDDLSDLTAAQQETLSDWESQFTFK.Y** |

| **276.** | P61247\|RS3A_HUMAN    **Mass:** 30154    **Score:** 189    **Queries matched:** 27   **emPAI:** 1.03 |
| --- | --- |
|  | 40S ribosomal protein S3a OS=Homo sapiens GN=RPS3A PE=1 SV=2 |

|  | **Query** | **Observed** | **Mr(expt)** | **Mr(calc)** | **Delta** | **Miss** | **Score** | **Expect** | **Rank** | **Peptide** |
| --- | --- | --- | --- | --- | --- | --- | --- | --- | --- | --- |
|  | 1237 | **589.3621** | **588.3548** | **588.3595** | **-0.0047** | **0** | **15** | **9.4** | **1** | **K.TLVTR.T** |
|  | 3403 | **455.7319** | **909.4493** | **910.4074** | **-0.9581** | **0** | **8** | **26** | **5** | **K.MMEIMTR.E** |
|  | 3628 | 464.7632 | 927.5117 | 926.4024 | 1.1094 | 0 | (5) | 48 | 7 | K.MMEIMTR.E |
|  | 3738 | 468.5695 | 935.1245 | 934.4695 | 0.6551 | 0 | 7 | 24 | 3 | K.APAMFNIR.N |
|  | 4826 | **507.2556** | **1012.4966** | **1013.4344** | **-0.9378** | **1** | **16** | **4.4** | **1** | **R.DKMCSMVK.K** |
|  | 5554 | **536.1620** | **1070.3094** | **1070.4922** | **-0.1828** | **1** | **(5)** | **37** | **1** | **K.KMMEIMTR.E** |
|  | 5769 | **544.2924** | **1086.5703** | **1086.4872** | **0.0831** | **1** | **16** | **4.2** | **3** | **K.KMMEIMTR.E** 5770 |
|  | 7748 | **609.2996** | **1216.5846** | **1216.5949** | **-0.0103** | **0** | **57** | **0.00029** | **1** | **K.TSYAQHQQVR.Q** 7749 |
|  | 8098 | **412.2165** | **1233.6278** | **1232.5997** | **1.0281** | **1** | **7** | **27** | **1** | **K.ATGDETGAKVER.A** |
|  | 9146 | **430.8954** | **1289.6645** | **1289.5776** | **0.0870** | **0** | **5** | **44** | **8** | **R.ADGYEPPVQESV.-** |
|  | 9880 | **664.3723** | **1326.7301** | **1326.7395** | **-0.0094** | **1** | **18** | **2.2** | **1** | **K.LIPDSIGKDIEK.A** 9830 9882 |
|  | 9939 | **666.3111** | **1330.6076** | **1330.6187** | **-0.0111** | **0** | **52** | **0.00098** | **1** | **K.LMELHGEGSSSGK.A** 9940 |
|  | 10180 | **673.3481** | **1344.6817** | **1344.6898** | **-0.0081** | **1** | **42** | **0.0098** | **1** | **R.KTSYAQHQQVR.Q** 10171 |
|  | 13077 | **758.3982** | **1514.7819** | **1514.7940** | **-0.0121** | **1** | **76** | **3e-006** | **1** | **R.EVQTNDLKEVVNK.L** 13042 13043 |
|  | 13997 | **526.9062** | **1577.6967** | **1577.7079** | **-0.0111** | **0** | **20** | **1.1** | **2** | **K.NCLTNFHGMDLTR.D** |
|  | 15978 | **852.9241** | **1703.8337** | **1703.8454** | **-0.0117** | **0** | **48** | **0.0017** | **1** | **K.ACQSIYPLHDVFVR.K** 15979 |
|  | 17823 | **613.3262** | **1836.9569** | **1836.8247** | **0.1322** | **1** | **8** | **18** | **3** | **K.NCLTNFHGMDLTRDK.M** |
|  | 20746 | 694.0244 | 2079.0514 | 2079.0637 | -0.0123 | 1 | 11 | 7.3 | 1 | R.VFEVSLADLQNDEVAFRK.F |

| **277.** | P52758\|UK114_HUMAN    **Mass:** 14542    **Score:** 187    **Queries matched:** 6   **emPAI:** 0.61 |
| --- | --- |
|  | Ribonuclease UK114 OS=Homo sapiens GN=HRSP12 PE=1 SV=1 |

|  | **Query** | **Observed** | **Mr(expt)** | **Mr(calc)** | **Delta** | **Miss** | **Score** | **Expect** | **Rank** | **Peptide** |
| --- | --- | --- | --- | --- | --- | --- | --- | --- | --- | --- |
|  | 5069 | 516.2939 | 1030.5732 | 1030.5811 | -0.0079 | 0 | 39 | 0.025 | 1 | R.AAYQVAALPK.G |
|  | 9941 | **444.5436** | **1330.6090** | **1330.7357** | **-0.1267** | **1** | **7** | **32** | **6** | **R.AAYQVAALPKGSR.I** |
|  | 21676 | **1092.0590** | **2182.1034** | **2182.1158** | **-0.0124** | **0** | **95** | **2.3e-008** | **1** | **K.TTVLLADINDFNTVNEIYK.Q** 21675 21677 |
|  | 30657 | **1072.8075** | **4287.2009** | **4288.1685** | **-0.9677** | **1** | **1** | **17** | **2** | **K.APGAIGPYSQAVLVDRTIYISGQIGMDPSSGQLVSGGVAEEAK.Q** |

| **278.** | P63104\|1433Z_HUMAN    **Mass:** 27899    **Score:** 186    **Queries matched:** 13   **emPAI:** 0.47 |
| --- | --- |
|  | 14-3-3 protein zeta/delta OS=Homo sapiens GN=YWHAZ PE=1 SV=1 |

|  | **Query** | **Observed** | **Mr(expt)** | **Mr(calc)** | **Delta** | **Miss** | **Score** | **Expect** | **Rank** | **Peptide** |
| --- | --- | --- | --- | --- | --- | --- | --- | --- | --- | --- |
|  | 1446 | **650.2773** | **649.2701** | **648.3265** | **0.9436** | **0** | **3** | **1e+002** | **7** | **K.ACSLAK.T** |
|  | 3143 | **445.7517** | **889.4888** | **888.4916** | **0.9972** | **0** | **5** | **59** | **7** | **R.VVSSIEQK.T** |
|  | 3688 | **466.7581** | **931.5016** | **931.4222** | **0.0795** | **1** | **12** | **13** | **10** | **K.MKGDYYR.Y** |
|  | 3849 | **472.7915** | **943.5684** | **943.5201** | **0.0484** | **1** | **5** | **58** | **3** | **K.VFYLKMK.G** |
|  | 6057 | **554.7784** | **1107.5423** | **1107.5495** | **-0.0072** | **0** | **22** | **0.99** | **1** | **K.EMQPTHPIR.L** |
|  | 7502 | **402.2437** | **1203.7093** | **1204.6485** | **-0.9392** | **0** | **12** | **9.5** | **8** | **K.DSTLIMQLLR.D** |
|  | 8936 | **640.3250** | **1278.6355** | **1278.6456** | **-0.0101** | **1** | **10** | **15** | **1** | **R.YLAEVAAGDDKK.G** |
|  | 9427 | **653.3024** | **1304.5903** | **1303.6772** | **0.9131** | **0** | **3** | **68** | **9** | **K.FLIPNASQAESK.V** |
|  | 11503 | 710.3305 | 1418.6465 | 1417.7123 | 0.9342 | 0 | 7 | 25 | 3 | R.DICNDVLSLLEK.F |
|  | 12913 | 502.5836 | 1504.7290 | 1503.7780 | 0.9510 | 1 | 5 | 38 | 7 | R.VVSSIEQKTEGAEK.K |
|  | 13573 | **774.8550** | **1547.6954** | **1547.7063** | **-0.0109** | **0** | **44** | **0.005** | **1** | **K.SVTEQGAELSNEER.N** |
|  | 20253 | 1020.9900 | 2039.9654 | 2039.9800 | -0.0146 | 0 | 122 | 5.3e-011 | 1 | K.GIVDQSQQAYQEAFEISK.K |
|  | 28214 | **1101.5457** | **3301.6151** | **3301.6275** | **-0.0124** | **1** | **71** | **4e-006** | **1** | **K.TAFDEAIAELDTLSEESYKDSTLIMQLLR.D** |

| **279.** | P37059\|DHB2_HUMAN    **Mass:** 43441    **Score:** 185    **Queries matched:** 22   **emPAI:** 0.64 |
| --- | --- |
|  | Estradiol 17-beta-dehydrogenase 2 OS=Homo sapiens GN=HSD17B2 PE=1 SV=1 |

|  | **Query** | **Observed** | **Mr(expt)** | **Mr(calc)** | **Delta** | **Miss** | **Score** | **Expect** | **Rank** | **Peptide** |
| --- | --- | --- | --- | --- | --- | --- | --- | --- | --- | --- |
|  | 2117 | **812.4085** | **811.4012** | **811.4076** | **-0.0064** | **0** | **33** | **0.071** | **1** | **R.LASYGSSK.A** |
|  | 2693 | **430.2710** | **858.5275** | **858.5327** | **-0.0052** | **0** | **11** | **16** | **1** | **K.TFLPLLR.K** |
|  | 4751 | 504.2510 | 1006.4874 | 1007.5764 | -1.0889 | 0 | 36 | 0.042 | 1 | R.DIQHAILAK.S 4750 4771 |
|  | 7702 | 608.3337 | 1214.6529 | 1214.5788 | 0.0742 | 0 | 8 | 24 | 2 | K.AAVTMFSSVMR.L |
|  | 7946 | 409.8872 | 1226.6396 | 1227.5819 | -0.9423 | 0 | 8 | 23 | 1 | R.HFGQDKPMPR.A |
|  | 9965 | 666.8749 | 1331.7353 | 1331.7813 | -0.0459 | 0 | 8 | 22 | 5 | R.NFLLLINSLASK.D |
|  | 10620 | 685.2796 | 1368.5446 | 1367.6881 | 0.8566 | 1 | 11 | 11 | 1 | K.RHFGQDKPMPR.A |
|  | 10903 | **462.2638** | **1383.7697** | **1383.6830** | **0.0867** | **1** | **(8)** | **22** | **1** | **K.RHFGQDKPMPR.A** |
|  | 12560 | **495.0600** | **1482.1582** | **1482.7137** | **-0.5555** | **1** | **4** | **29** | **4** | **K.DAYSKVAAMLQDR.G** |
|  | 12895 | **752.8604** | **1503.7061** | **1503.7174** | **-0.0112** | **0** | **71** | **9.1e-006** | **1** | **R.LVNVSSMGGGAPMER.L** 12896 12907 |
|  | 13162 | 507.7917 | 1520.3534 | 1519.7123 | 0.6411 | 0 | (7) | 19 | 4 | R.LVNVSSMGGGAPMER.L |
|  | 16128 | **856.9968** | **1711.9790** | **1711.9906** | **-0.0116** | **0** | **39** | **0.014** | **1** | **R.LSVLQMDITKPVQIK.D** |
|  | 16330 | **864.4258** | **1726.8370** | **1726.8495** | **-0.0125** | **0** | **77** | **2.3e-006** | **1** | **K.AVLVTGGDCGLGHALCK.Y** |
|  | 16441 | **578.3623** | **1732.0651** | **1732.8349** | **-0.7698** | **1** | **4** | **33** | **6** | **K.GRLVNVSSMGGGAPMER.L** |
|  | 16925 | 442.9635 | 1767.8248 | 1768.9215 | -1.0968 | 1 | 13 | 5.3 | 2 | K.AAVTMFSSVMRLELSK.W |
|  | 22833 | **773.7321** | **2318.1744** | **2318.1907** | **-0.0163** | **1** | **6** | **18** | **1** | **K.VASIQPGGFLTNIAGTSDKWEK.L** |
|  | 25353 | **901.4373** | **2701.2900** | **2700.3031** | **0.9869** | **0** | **8** | **10** | **1** | **K.DILDHLPAEVQEDYGQDYILAQR.N** |
|  | 26296 | **956.1458** | **2865.4154** | **2865.4297** | **-0.0143** | **1** | **37** | **0.011** | **1** | **K.YLDELGFTVFAGVLNENGPGAEELRR.T** |

| **280.** | Q03154\|ACY1_HUMAN    **Mass:** 46084    **Score:** 182    **Queries matched:** 13   **emPAI:** 0.17 |
| --- | --- |
|  | Aminoacylase-1 OS=Homo sapiens GN=ACY1 PE=1 SV=1 |

|  | **Query** | **Observed** | **Mr(expt)** | **Mr(calc)** | **Delta** | **Miss** | **Score** | **Expect** | **Rank** | **Peptide** |
| --- | --- | --- | --- | --- | --- | --- | --- | --- | --- | --- |
|  | 4904 | **509.8024** | **1017.5902** | **1017.5971** | **-0.0069** | **0** | **17** | **3.7** | **1** | **K.VVNSILAFR.E** |
|  | 5193 | 521.2276 | 1040.4406 | 1040.4484 | -0.0078 | 0 | 12 | 10 | 2 | R.FMEDTAAEK.L |
|  | 8879 | **638.3388** | **1274.6629** | **1274.7347** | **-0.0717** | **1** | **3** | **73** | **8** | **K.VVNSILAFREK.E** |
|  | 10050 | **446.5502** | **1336.6288** | **1336.6809** | **-0.0521** | **0** | **10** | **12** | **1** | **K.CVSIQYLEAVR.R** |
|  | 11700 | **477.7992** | **1430.3757** | **1430.7340** | **-0.3583** | **0** | **3** | **46** | **10** | **R.AVGVPALGFSPMNR.T** |
|  | 12732 | **747.3543** | **1492.6941** | **1492.7820** | **-0.0879** | **1** | **0** | **1.2e+002** | **6** | **K.CVSIQYLEAVRR.L** |
|  | 19432 | 492.4990 | 1965.9670 | 1966.9604 | -0.9935 | 1 | 19 | 1.1 | 1 | R.GAQDMKCVSIQYLEAVR.R |
|  | 20188 | **1015.9731** | **2029.9316** | **2029.9448** | **-0.0132** | **0** | **39** | **0.011** | **1** | **K.DMNLTLEPEIMPAATDNR.Y** 20191 |
|  | 21789 | **550.2888** | **2197.1259** | **2196.0851** | **1.0408** | **0** | **3** | **43** | **5** | **R.TVQPKPDYGAAVAFFEETAR.Q** |
|  | 24139 | **1246.0862** | **2490.1578** | **2490.1703** | **-0.0125** | **0** | **103** | **3.7e-009** | **1** | **R.AGFALDEGIANPTDAFTVFYSER.S** 24140 |
|  | 29990 | **1317.3750** | **3949.1032** | **3949.1241** | **-0.0210** | **0** | **7** | **5.1** | **1** | **K.VEVAPGYVVTVLTWPGTNPTLSSILLNSHTDVVPVFK.E** |

| **281.** | P49257\|LMAN1_HUMAN    **Mass:** 57798    **Score:** 182    **Queries matched:** 24   **emPAI:** 0.45 |
| --- | --- |
|  | Protein ERGIC-53 OS=Homo sapiens GN=LMAN1 PE=1 SV=2 |

|  | **Query** | **Observed** | **Mr(expt)** | **Mr(calc)** | **Delta** | **Miss** | **Score** | **Expect** | **Rank** | **Peptide** |
| --- | --- | --- | --- | --- | --- | --- | --- | --- | --- | --- |
|  | 1312 | **614.3827** | **613.3754** | **613.3799** | **-0.0045** | **0** | **22** | **1.1** | **1** | **R.VAPSLK.S** |
|  | 2043 | **403.7328** | **805.4511** | **804.4024** | **1.0487** | **1** | **14** | **7.7** | **6** | **-.MAGSRQR.G** |
|  | 2089 | **405.2992** | **808.5838** | **807.4126** | **1.1711** | **1** | **6** | **33** | **3** | **K.KEEFQK.G** |
|  | 2924 | **437.2471** | **872.4797** | **872.4868** | **-0.0071** | **0** | **15** | **6.6** | **3** | **R.NKPYPVR.A** |
|  | 3207 | **448.7478** | **895.4810** | **895.4875** | **-0.0065** | **0** | **9** | **18** | **3** | **K.TQHEILR.Q** 3208 |
|  | 5236 | **522.7575** | **1043.5005** | **1043.5069** | **-0.0065** | **0** | **19** | **2.1** | **1** | **R.NMPSNEKPK.C** |
|  | 8522 | **628.8093** | **1255.6040** | **1254.6343** | **0.9697** | **0** | **6** | **40** | **2** | **R.YVSSLTEEISK.R** 8517 |
|  | 10708 | 687.8282 | 1373.6418 | 1374.7004 | -1.0586 | 1 | 8 | 24 | 6 | R.ELRQVFEGQNR.I |
|  | 11370 | 706.3697 | 1410.7249 | 1410.7354 | -0.0105 | 1 | 21 | 1 | 2 | R.YVSSLTEEISKR.G 11371 |
|  | 11453 | **708.8616** | **1415.7086** | **1415.7191** | **-0.0105** | **1** | **11** | **12** | **7** | **R.QLDMILDEQRR.Y** 11457 |
|  | 14334 | 799.8823 | 1597.7500 | 1596.7572 | 0.9927 | 0 | 23 | 0.54 | 1 | K.AAFENWEVEVTFR.V |
|  | 15189 | **551.9274** | **1652.7605** | **1651.7658** | **0.9947** | **1** | **20** | **1.2** | **1** | **R.QVNEMKNSMSETVR.L** 15188 |
|  | 21915 | **1105.5394** | **2209.0643** | **2209.0798** | **-0.0155** | **0** | **37** | **0.017** | **1** | **R.GAGMPGQHGQITQQELDTVVK.T** 21925 |
|  | 23189 | **789.3951** | **2365.1636** | **2365.1809** | **-0.0173** | **1** | **54** | **0.00033** | **1** | **K.RGAGMPGQHGQITQQELDTVVK.T** |
|  | 25142 | **891.4367** | **2671.2883** | **2671.3065** | **-0.0182** | **0** | **30** | **0.065** | **1** | **R.LVSGMQHPGSAGGVYETTQHFIDIK.E** |
|  | 26834 | **986.4862** | **2956.4368** | **2956.4580** | **-0.0212** | **0** | **54** | **0.00023** | **1** | **K.GPHLVQSDGTVPFWAHAGNAIPSSDQIR.V** 26833 |
|  | 28767 | **1159.2134** | **3474.6183** | **3474.6447** | **-0.0264** | **0** | **61** | **2.7e-005** | **1** | **K.NNPAIVIIGNNGQIHYDHQNDGASQALASCQR.D** |

| **282.** | O14983\|AT2A1_HUMAN    **Mass:** 111550   **Score:** 181    **Queries matched:** 29   **emPAI:** 0.14 |
| --- | --- |
|  | Sarcoplasmic/endoplasmic reticulum calcium ATPase 1 OS=Homo sapiens GN=ATP2A1 PE=1 SV=1 |

|  | **Query** | **Observed** | **Mr(expt)** | **Mr(calc)** | **Delta** | **Miss** | **Score** | **Expect** | **Rank** | **Peptide** |
| --- | --- | --- | --- | --- | --- | --- | --- | --- | --- | --- |
|  | 994 | 532.2831 | 531.2759 | 530.2813 | 0.9946 | 0 | 12 | 24 | 5 | R.DAGIR.V 993 |
|  | 1009 | **534.1858** | **533.1786** | **532.2969** | **0.8817** | **0** | **6** | **78** | **3** | **R.VGTTR.V** |
|  | 1159 | 572.3469 | 571.3396 | 571.3442 | -0.0046 | 0 | 16 | 5.3 | 1 | K.NAIVR.S |
|  | 3123 | 444.7604 | 887.5063 | 888.4777 | -0.9714 | 1 | 7 | 38 | 3 | K.SSRAAVGNK.M |
|  | 4872 | 508.7678 | 1015.5211 | 1016.5549 | -1.0338 | 1 | 12 | 11 | 4 | K.GTAIAICRR.I |
|  | 6149 | **558.3571** | **1114.6996** | **1114.7074** | **-0.0078** | **1** | **15** | **4.6** | **1** | **R.ILAIKSTTLR.V** 6135 |
|  | 6607 | **576.7817** | **1151.5488** | **1151.4765** | **0.0724** | **0** | **12** | **9.9** | **4** | **R.DQMAATEQDK.T** |
|  | 6681 | 578.9090 | 1155.8034 | 1155.5924 | 0.2110 | 1 | 7 | 25 | 1 | K.KEFTLEFSR.D |
|  | 9664 | 659.7933 | 1317.5721 | 1318.6405 | -1.0683 | 0 | 1 | 1.1e+002 | 3 | K.YGLNELPAEEGK.T |
|  | 9773 | 662.3356 | 1322.6566 | 1322.6653 | -0.0087 | 0 | 4 | 56 | 2 | K.NMLFSGTNIAAGK.A |
|  | 9963 | **444.9135** | **1331.7186** | **1331.6357** | **0.0829** | **0** | **4** | **65** | **7** | **R.EFDDLPLAEQR.E** |
|  | 11996 | 725.8486 | 1449.6826 | 1450.7602 | -1.0776 | 1 | 13 | 6.9 | 2 | K.KNMLFSGTNIAAGK.A 11995 |
|  | 12885 | 752.4078 | 1502.8011 | 1502.8126 | -0.0115 | 1 | 85 | 4.3e-007 | 1 | K.KAEIGIAMGSGTAVAK.T 12886 |
|  | 13271 | 509.9443 | 1526.8112 | 1525.7559 | 1.0553 | 1 | 4 | 43 | 4 | K.MNVFNTDVRSLSK.V |
|  | 13772 | **521.2606** | **1560.7599** | **1560.8247** | **-0.0648** | **0** | **7** | **26** | **2** | **K.VGEATETALTTLVEK.M** |
|  | 13952 | 787.9310 | 1573.8474 | 1573.8563 | -0.0089 | 0 | 64 | 5.2e-005 | 1 | R.VDQSILTGESVSVIK.H |
|  | 16168 | **572.3726** | **1714.0959** | **1714.9804** | **-0.8845** | **1** | **4** | **37** | **4** | **K.LRALDLTQWLMVLK.I** |
|  | 18399 | **627.3087** | **1878.9043** | **1879.9064** | **-1.0021** | **1** | **2** | **60** | **1** | **R.AYTGREFDDLPLAEQR.E** |
|  | 19270 | 976.9625 | 1951.9105 | 1951.9231 | -0.0126 | 0 | 52 | 0.00056 | 1 | R.SLPSVETLGCTSVICSDK.T 19271 |
|  | 24859 | **651.8524** | **2603.3806** | **2602.3603** | **1.0203** | **1** | **1** | **53** | **6** | **R.VDQSILTGESVSVIKHTEPVPDPR.A** |
|  | 26756 | **981.5026** | **2941.4859** | **2940.4426** | **1.0432** | **1** | **3** | **26** | **1** | **K.IVEYLQSYDEITAMTGDGVNDAPALKK.A** |
|  | 27920 | 802.6482 | 3206.5637 | 3205.6648 | 0.8989 | 0 | 2 | 28 | 4 | K.VISLICVAVWLINIGHFNDPVHGGSWFR.G |
|  | 28162 | 1092.2274 | 3273.6604 | 3274.5486 | -0.8881 | 1 | 2 | 32 | 6 | K.TASEMVLADDNFSTIVAAVEEGRAIYNNMK.Q |
|  | 29669 | 954.4909 | 3813.9345 | 3812.8448 | 1.0897 | 1 | 0 | 31 | 1 | K.AEIGIAMGSGTAVAKTASEMVLADDNFSTIVAAVEEGR.A |

| **283.** | Q15738\|NSDHL_HUMAN    **Mass:** 42159    **Score:** 181    **Queries matched:** 14   **emPAI:** 0.29 |
| --- | --- |
|  | Sterol-4-alpha-carboxylate 3-dehydrogenase, decarboxylating OS=Homo sapiens GN=NSDHL PE=1 SV=2 |

|  | **Query** | **Observed** | **Mr(expt)** | **Mr(calc)** | **Delta** | **Miss** | **Score** | **Expect** | **Rank** | **Peptide** |
| --- | --- | --- | --- | --- | --- | --- | --- | --- | --- | --- |
|  | 1869 | 794.4352 | 793.4279 | 793.4334 | -0.0055 | 0 | 31 | 0.15 | 1 | R.VNYIGTK.N |
|  | 4791 | **505.7563** | **1009.4981** | **1008.5426** | **0.9555** | **1** | **2** | **92** | **4** | **K.MKFVIGNGK.N** |
|  | 5195 | **521.2606** | **1040.5066** | **1040.5138** | **-0.0073** | **0** | **13** | **8.7** | **1** | **R.THLTEDTPK.V** |
|  | 13903 | 524.2944 | 1569.8613 | 1569.8111 | 0.0502 | 1 | 9 | 15 | 2 | K.VNADIEKVNQNQAK.R |
|  | 14134 | **529.3055** | **1584.8946** | **1583.7726** | **1.1220** | **1** | **5** | **42** | **8** | **M.EPAVSEPMRDQVAR.T** |
|  | 14495 | **537.5945** | **1609.7616** | **1609.8060** | **-0.0444** | **1** | **1** | **99** | **10** | **R.DQVARTHLTEDTPK.V** |
|  | 14948 | 819.4517 | 1636.8888 | 1637.8447 | -0.9559 | 1 | 7 | 22 | 8 | R.VNYIGTKNVIETCK.E |
|  | 18124 | 929.9770 | 1857.9394 | 1858.7899 | -0.8505 | 0 | (0) | 95 | 4 | K.AMGYQPLVTMDDAMER.T |
|  | 18360 | **625.9470** | **1874.8192** | **1874.7849** | **0.0344** | **0** | **1** | **78** | **6** | **K.AMGYQPLVTMDDAMER.T** |
|  | 21539 | **1084.0244** | **2166.0343** | **2166.0494** | **-0.0152** | **0** | **90** | **7.6e-008** | **1** | **R.GYAVNVFDIQQGFDNPQVR.F** 21538 21540 |
|  | 25403 | **903.4734** | **2707.3983** | **2707.4082** | **-0.0099** | **0** | **36** | **0.014** | **1** | **K.NLVDFTFVENVVHGHILAAEQLSR.D** 25401 |

| **284.** | P36578\|RL4_HUMAN    **Mass:** 47953    **Score:** 180    **Queries matched:** 23   **emPAI:** 0.25 |
| --- | --- |
|  | 60S ribosomal protein L4 OS=Homo sapiens GN=RPL4 PE=1 SV=5 |

|  | **Query** | **Observed** | **Mr(expt)** | **Mr(calc)** | **Delta** | **Miss** | **Score** | **Expect** | **Rank** | **Peptide** |
| --- | --- | --- | --- | --- | --- | --- | --- | --- | --- | --- |
|  | 1262 | **599.3834** | **598.3761** | **598.3802** | **-0.0041** | **1** | **8** | **22** | **10** | **K.KNPLK.N** |
|  | 1605 | **710.2909** | **709.2837** | **709.3469** | **-0.0632** | **0** | **4** | **57** | **5** | **R.MFAPTK.T** |
|  | 2763 | **432.2428** | **862.4710** | **862.4773** | **-0.0064** | **0** | **17** | **4** | **2** | **K.LAPGGHVGR.F** 2751 |
|  | 3935 | **475.2847** | **948.5549** | **948.4698** | **0.0851** | **0** | **4** | **65** | **8** | **K.MINTDLSR.I** |
|  | 5547 | **535.7466** | **1069.4787** | **1068.6040** | **0.8748** | **1** | **9** | **20** | **3** | **K.SPEIQRALR.A** |
|  | 6083 | **555.8129** | **1109.6112** | **1108.5699** | **1.0413** | **1** | **2** | **92** | **6** | **K.LNPYAKTMR.R** |
|  | 6623 | **577.2717** | **1152.5289** | **1152.5750** | **-0.0461** | **1** | **4** | **55** | **8** | **R.MFAPTKTWR.R** |
|  | 7378 | **600.3311** | **1198.6477** | **1198.6557** | **-0.0080** | **1** | **5** | **54** | **2** | **K.KPTTEEKKPAA.-** |
|  | 7394 | **600.7434** | **1199.4723** | **1199.4812** | **-0.0089** | **0** | **52** | **0.001** | **1** | **R.SGQGAFGNMCR.G** 7393 |
|  | 8745 | **634.8783** | **1267.7420** | **1267.7500** | **-0.0079** | **0** | **12** | **8.2** | **2** | **R.NIPGITLLNVSK.L** |
|  | 9673 | 659.8641 | 1317.7136 | 1318.7278 | -1.0143 | 1 | 20 | 1.5 | 2 | K.MINTDLSRILK.S |
|  | 11431 | **708.2933** | **1414.5720** | **1414.7415** | **-0.1696** | **1** | **1** | **1e+002** | **5** | **K.AAAAAAALQAKSDEK.A** |
|  | 12739 | 747.4353 | 1492.8560 | 1492.7708 | 0.0853 | 0 | 5 | 42 | 5 | M.ACARPLISVYSEK.G |
|  | 13349 | **511.9192** | **1532.7359** | **1532.8199** | **-0.0839** | **1** | **0** | **1.2e+002** | **9** | **K.GESSGKNVTLPAVFK.A** |
|  | 14429 | **803.4278** | **1604.8410** | **1604.7868** | **0.0542** | **0** | **2** | **80** | **10** | **R.GPCIIYNEDNGIIK.A** |
|  | 14471 | **804.8763** | **1607.7380** | **1607.8658** | **-0.1278** | **0** | **6** | **30** | **3** | **R.IEEVPELPLVVEDK.V** |
|  | 16863 | 881.4452 | 1760.8758 | 1760.8879 | -0.0121 | 1 | 39 | 0.015 | 1 | R.RGPCIIYNEDNGIIK.A 16862 16875 |
|  | 19084 | **969.0095** | **1936.0044** | **1936.0161** | **-0.0118** | **0** | **86** | **2.2e-007** | **1** | **R.YAICSALAASALPALVMSK.G** 19083 |

| **285.** | P62158\|CALM_HUMAN    **Mass:** 16827    **Score:** 179    **Queries matched:** 19   **emPAI:** 0.86 |
| --- | --- |
|  | Calmodulin OS=Homo sapiens GN=CALM1 PE=1 SV=2 |

|  | **Query** | **Observed** | **Mr(expt)** | **Mr(calc)** | **Delta** | **Miss** | **Score** | **Expect** | **Rank** | **Peptide** |
| --- | --- | --- | --- | --- | --- | --- | --- | --- | --- | --- |
|  | 2030 | **805.4183** | **804.4111** | **804.4164** | **-0.0053** | **0** | **17** | **4** | **1** | **K.ELGTVMR.S** 2031 |
|  | 2227 | 411.2446 | 820.4746 | 820.4113 | 0.0634 | 0 | (8) | 29 | 9 | K.ELGTVMR.S 2220 |
|  | 5864 | **547.8026** | **1093.5905** | **1092.4571** | **1.1335** | **0** | **8** | **23** | **9** | **K.DTDSEEEIR.E** |
|  | 10315 | **676.7671** | **1351.5196** | **1351.5925** | **-0.0729** | **1** | **19** | **1.7** | **1** | **K.MKDTDSEEEIR.E** |
|  | 14307 | 532.9060 | 1595.6962 | 1595.7063 | -0.0101 | 1 | 14 | 5 | 2 | K.DTDSEEEIREAFR.V |
|  | 16731 | 877.9332 | 1753.8518 | 1753.8635 | -0.0117 | 1 | 92 | 6.3e-008 | 1 | R.VFDKDGNGYISAAELR.H 16705 16726 16729 16737 16741 16742 |
|  | 17906 | **922.9460** | **1843.8775** | **1843.8840** | **-0.0065** | **1** | **36** | **0.024** | **1** | **K.EAFSLFDKDGDGTITTK.E** |
|  | 23279 | **792.4559** | **2374.3458** | **2374.1144** | **0.2313** | **1** | **12** | **3.9** | **1** | **R.HVMTNLGEKLTDEEVDEMIR.E** |
|  | 24131 | **1245.5365** | **2489.0584** | **2489.0726** | **-0.0142** | **0** | **93** | **2.9e-008** | **1** | **R.EADIDGDGQVNYEEFVQMMTAK.-** |
|  | 30226 | **1357.6107** | **4069.8103** | **4068.8391** | **0.9713** | **0** | **16** | **0.63** | **1** | **R.SLGQNPTEAELQDMINEVDADGNGTIDFPEFLTMMAR.K** |
|  | 30569 | **602.7260** | **4212.0307** | **4212.9290** | **-0.8982** | **1** | **3** | **12** | **1** | **R.SLGQNPTEAELQDMINEVDADGNGTIDFPEFLTMMARK.M** |

| **286.** | Q9UHG3\|PCYOX_HUMAN    **Mass:** 57003    **Score:** 178    **Queries matched:** 23   **emPAI:** 0.66 |
| --- | --- |
|  | Prenylcysteine oxidase 1 OS=Homo sapiens GN=PCYOX1 PE=1 SV=3 |

|  | **Query** | **Observed** | **Mr(expt)** | **Mr(calc)** | **Delta** | **Miss** | **Score** | **Expect** | **Rank** | **Peptide** |
| --- | --- | --- | --- | --- | --- | --- | --- | --- | --- | --- |
|  | 2873 | **435.2678** | **868.5210** | **869.4395** | **-0.9185** | **0** | **7** | **31** | **3** | **R.YGFQSLR.M** |
|  | 6921 | **588.3220** | **1174.6295** | **1174.6380** | **-0.0085** | **0** | **13** | **7.9** | **1** | **K.LVCSGLLQASK.S** |
|  | 11217 | 702.3752 | 1402.7358 | 1401.6421 | 1.0937 | 0 | 26 | 0.38 | 1 | R.MHMWVEDVLDK.F |
|  | 11507 | **473.9092** | **1418.7057** | **1418.7154** | **-0.0097** | **1** | **18** | **2.3** | **1** | **K.IDLFEREEVGGR.L** |
|  | 11589 | **475.2776** | **1422.8111** | **1423.7724** | **-0.9613** | **1** | **6** | **32** | **3** | **K.LVWRYGFQSLR.M** |
|  | 11733 | 717.3353 | 1432.6560 | 1433.6319 | -0.9759 | 0 | (5) | 40 | 6 | R.MHMWVEDVLDK.F |
|  | 13750 | 780.8460 | 1559.6775 | 1559.6892 | -0.0118 | 0 | 44 | 0.0047 | 1 | R.YQSHDYAFSSVEK.L 13751 |
|  | 14168 | **794.8825** | **1587.7505** | **1587.7603** | **-0.0098** | **0** | **76** | **3.1e-006** | **1** | **K.MYEVVYQIGTETR.S** |
|  | 14413 | **802.9076** | **1603.8006** | **1603.7552** | **0.0454** | **0** | **(5)** | **37** | **5** | **K.MYEVVYQIGTETR.S** |
|  | 14652 | **810.4352** | **1618.8559** | **1618.9294** | **-0.0735** | **1** | **2** | **84** | **6** | **K.IFSQETLTKAQILK.L** |
|  | 16261 | **861.9513** | **1721.8880** | **1721.8988** | **-0.0108** | **0** | **22** | **0.63** | **1** | **R.SDFYDIVLVATPLNR.K** 16262 |
|  | 16916 | **883.9878** | **1765.9610** | **1765.9726** | **-0.0116** | **0** | **31** | **0.075** | **1** | **K.IAIIGAGIGGTSAAYYLR.Q** 16906 |
|  | 17325 | **600.2760** | **1797.8062** | **1797.9182** | **-0.1120** | **1** | **2** | **63** | **5** | **K.SNLISGSVMYIEEKTK.T** |
|  | 17970 | **617.3573** | **1849.0501** | **1849.9938** | **-0.9437** | **1** | **1** | **89** | **9** | **R.SDFYDIVLVATPLNRK.M** |
|  | 18909 | **641.6141** | **1921.8204** | **1920.8312** | **0.9892** | **0** | **20** | **0.86** | **1** | **R.WNGHTDMIDQDGLYEK.L** |
|  | 25840 | **1396.7380** | **2791.4615** | **2791.4756** | **-0.0141** | **0** | **74** | **2.4e-006** | **1** | **K.FGLNTVLTTDNSDLFINSIGIVPSVR.E** 25839 |
|  | 26510 | **968.4843** | **2902.4311** | **2901.3823** | **1.0488** | **0** | **7** | **12** | **1** | **R.LATMMVQGQEYEAGGSVIHPLNLHMK.R** |
|  | 28540 | **849.6591** | **3394.6071** | **3394.6260** | **-0.0188** | **0** | **10** | **3.9** | **1** | **R.LYYLNGIECAASAMEMSAIAAHNAALLAYHR.W** 28551 |

| **287.** | P49411\|EFTU_HUMAN    **Mass:** 49852    **Score:** 178    **Queries matched:** 18   **emPAI:** 0.66 |
| --- | --- |
|  | Elongation factor Tu, mitochondrial OS=Homo sapiens GN=TUFM PE=1 SV=2 |

|  | **Query** | **Observed** | **Mr(expt)** | **Mr(calc)** | **Delta** | **Miss** | **Score** | **Expect** | **Rank** | **Peptide** |
| --- | --- | --- | --- | --- | --- | --- | --- | --- | --- | --- |
|  | 5082 | **516.7842** | **1031.5539** | **1031.5611** | **-0.0072** | **0** | **32** | **0.11** | **1** | **R.GTVVTGTLER.G** |
|  | 6584 | **575.8169** | **1149.6192** | **1148.6441** | **0.9751** | **0** | **23** | **0.7** | **1** | **K.VEAQVYILSK.E** |
|  | 8598 | 630.8287 | 1259.6428 | 1260.6537 | -1.0109 | 0 | 8 | 23 | 3 | R.TVVTGIEMFHK.S |
|  | 11872 | **721.9005** | **1441.7865** | **1442.8457** | **-1.0592** | **1** | **2** | **80** | **8** | **R.GTVVTGTLERGILK.K** |
|  | 13483 | 771.9256 | 1541.8366 | 1541.8453 | -0.0087 | 0 | 55 | 0.00037 | 1 | K.LLDAVDTYIPVPAR.D |
|  | 15474 | **837.4283** | **1672.8420** | **1672.8533** | **-0.0112** | **0** | **58** | **0.0002** | **1** | **R.GITINAAHVEYSTAAR.H** |
|  | 15556 | 560.3003 | 1677.8790 | 1676.8733 | 1.0057 | 1 | 5 | 39 | 5 | K.VEAQVYILSKEEGGR.H 15544 |
|  | 16953 | **590.9226** | **1769.7460** | **1769.7580** | **-0.0120** | **0** | **32** | **0.061** | **1** | **R.HYAHTDCPGHADYVK.N** 16952 16954 |
|  | 17467 | **603.9802** | **1808.9187** | **1808.9282** | **-0.0096** | **0** | **27** | **0.2** | **1** | **R.DKPHVNVGTIGHVDHGK.T** 17468 |
|  | 18753 | **954.4779** | **1906.9413** | **1906.9558** | **-0.0145** | **0** | **44** | **0.0036** | **1** | **R.TIGTGLVTNTLAMTEEEK.N** 18752 |
|  | 18914 | **481.5253** | **1922.0720** | **1922.9507** | **-0.8787** | **0** | **(5)** | **29** | **2** | **R.TIGTGLVTNTLAMTEEEK.N** |
|  | 22105 | **557.5154** | **2226.0327** | **2225.2433** | **0.7894** | **1** | **2** | **52** | **3** | **R.ATPHFSGLAAGRTFLLQGLLR.L** |
|  | 31006 | **1145.5597** | **4578.2097** | **4579.1141** | **-0.9045** | **1** | **0** | **17** | **1** | **R.HYAHTDCPGHADYVKNMITGTAPLDGCILVVAANDGPMPQTR.E** |

| **288.** | Q15165\|PON2_HUMAN    **Mass:** 39658    **Score:** 178    **Queries matched:** 8   **emPAI:** 0.31 |
| --- | --- |
|  | Serum paraoxonase/arylesterase 2 OS=Homo sapiens GN=PON2 PE=1 SV=2 |

|  | **Query** | **Observed** | **Mr(expt)** | **Mr(calc)** | **Delta** | **Miss** | **Score** | **Expect** | **Rank** | **Peptide** |
| --- | --- | --- | --- | --- | --- | --- | --- | --- | --- | --- |
|  | 5748 | **543.3283** | **1084.6421** | **1084.6393** | **0.0028** | **0** | **13** | **8** | **4** | **K.LLIGTLYHR.A** |
|  | 9618 | **658.3839** | **1314.7531** | **1314.7772** | **-0.0240** | **1** | **10** | **16** | **4** | **R.AGFLGERLLALR.N** |
|  | 15506 | **559.2744** | **1674.8012** | **1674.8399** | **-0.0387** | **0** | **4** | **48** | **4** | **R.EVESVDLPHCHLIK.G** |
|  | 17926 | 923.9647 | 1845.9148 | 1845.9261 | -0.0113 | 0 | 67 | 2.2e-005 | 1 | K.LFVYDPNNPPSSEVLR.I |
|  | 20748 | **694.0386** | **2079.0939** | **2078.9779** | **0.1160** | **1** | **6** | **23** | **2** | **-.MGAWVGCGLAGDRAGFLGER.L** |
|  | 23889 | **1231.6393** | **2461.2640** | **2461.2740** | **-0.0100** | **0** | **67** | **1.5e-005** | **1** | **K.GIEAGSEDIDILPNGLAFFSVGLK.F** |
|  | 26569 | **485.9197** | **2909.4744** | **2909.4932** | **-0.0187** | **1** | **12** | **3.7** | **1** | **K.FPGLHSFAPDKPGGILMMDLKEEKPR.A** |
|  | 28168 | **1093.5325** | **3277.5756** | **3277.5925** | **-0.0169** | **0** | **91** | **4e-008** | **1** | **K.VLELDTLVDNLSIDPSSGDIWVGCHPNGQK.L** |

| **289.** | Q16718\|NDUA5_HUMAN    **Mass:** 13507    **Score:** 177    **Queries matched:** 7   **emPAI:** 1.15 |
| --- | --- |
|  | NADH dehydrogenase [ubiquinone] 1 alpha subcomplex subunit 5 OS=Homo sapiens GN=NDUFA5 PE=1 SV=3 |

|  | **Query** | **Observed** | **Mr(expt)** | **Mr(calc)** | **Delta** | **Miss** | **Score** | **Expect** | **Rank** | **Peptide** |
| --- | --- | --- | --- | --- | --- | --- | --- | --- | --- | --- |
|  | 818 | 487.3205 | 486.3132 | 486.3166 | -0.0034 | 0 | 19 | 3.8 | 1 | M.AGVLK.K |
|  | 17810 | **918.9557** | **1835.8968** | **1835.9094** | **-0.0126** | **0** | **55** | **0.00038** | **1** | **K.LWEPLVEEPPADQWK.W** 17811 |
|  | 18035 | **618.6720** | **1852.9942** | **1851.9625** | **1.0316** | **1** | **2** | **71** | **4** | **K.KTTGLVGLAVCNTPHER.L** |
|  | 22163 | **1117.0646** | **2232.1146** | **2232.1255** | **-0.0109** | **1** | **7** | **15** | **1** | **K.LWEPLVEEPPADQWKWPI.-** |
|  | 26147 | **949.1569** | **2844.4488** | **2844.4617** | **-0.0129** | **0** | **36** | **0.014** | **1** | **K.LEDQLQGGQLEEVILQAEHELNLAR.K** |
|  | 26895 | **991.8538** | **2972.5395** | **2972.5567** | **-0.0172** | **1** | **105** | **1.5e-009** | **1** | **K.KLEDQLQGGQLEEVILQAEHELNLAR.K** |

| **290.** | Q9Y394\|DHRS7_HUMAN    **Mass:** 38673    **Score:** 176    **Queries matched:** 22   **emPAI:** 0.74 |
| --- | --- |
|  | Dehydrogenase/reductase SDR family member 7 OS=Homo sapiens GN=DHRS7 PE=1 SV=1 |

|  | **Query** | **Observed** | **Mr(expt)** | **Mr(calc)** | **Delta** | **Miss** | **Score** | **Expect** | **Rank** | **Peptide** |
| --- | --- | --- | --- | --- | --- | --- | --- | --- | --- | --- |
|  | 6913 | **588.2606** | **1174.5067** | **1174.5142** | **-0.0075** | **0** | **44** | **0.007** | **1** | **K.SGVDADSSYFK.I** 6912 |
|  | 7469 | **602.3079** | **1202.6012** | **1203.5917** | **-0.9906** | **1** | **6** | **41** | **3** | **R.CLENGNLKEK.D** |
|  | 9087 | 644.3466 | 1286.6787 | 1286.6996 | -0.0209 | 1 | 6 | 41 | 3 | K.HALRGFFNGLR.T 9088 |
|  | 11454 | **708.8621** | **1415.7096** | **1415.7191** | **-0.0095** | **0** | **46** | **0.0032** | **1** | **R.IDILVNNGGMSQR.S** 11463 |
|  | 12423 | 492.2734 | 1473.7983 | 1474.6432 | -0.8449 | 0 | 6 | 30 | 4 | R.SLCMDTSLDVYR.K 12415 |
|  | 13809 | **782.4487** | **1562.8828** | **1562.8919** | **-0.0091** | **0** | **57** | **0.00025** | **1** | **K.LIELNYLGTVSLTK.C** |
|  | 14161 | 794.3728 | 1586.7310 | 1586.7433 | -0.0122 | 1 | 17 | 2.5 | 2 | R.SLCMDTSLDVYRK.L 14151 |
|  | 15718 | **846.0917** | **1690.1689** | **1690.9869** | **-0.8180** | **1** | **13** | **3.9** | **1** | **R.KLIELNYLGTVSLTK.C** |
|  | 21009 | **1054.5298** | **2107.0450** | **2108.1001** | **-1.0551** | **0** | **29** | **0.11** | **1** | **K.DILVLPLDLTDTGSHEAATK.A** 21020 21021 |
|  | 21345 | 716.0308 | 2145.0705 | 2146.0960 | -1.0255 | 1 | 1 | 75 | 9 | R.FLRADGDLTLLWAEWQGR.R |
|  | 22935 | 1167.0929 | 2332.1712 | 2332.1958 | -0.0245 | 1 | 5 | 26 | 2 | K.AVLQEFGRIDILVNNGGMSQR.S |
|  | 23478 | **802.1190** | **2403.3351** | **2403.3447** | **-0.0097** | **0** | **22** | **0.45** | **1** | **K.IVTVNSILGIISVPLSIGYCASK.H** |
|  | 29010 | **1785.9109** | **3569.8072** | **3569.8287** | **-0.0215** | **0** | **70** | **3.8e-006** | **1** | **R.TELATYPGIIVSNICPGPVQSNIVENSLAGEVTK.T** 29013 29014 |

| **291.** | Q92575\|UBXN4_HUMAN    **Mass:** 57028    **Score:** 173    **Queries matched:** 18   **emPAI:** 0.21 |
| --- | --- |
|  | UBX domain-containing protein 4 OS=Homo sapiens GN=UBXN4 PE=1 SV=2 |

|  | **Query** | **Observed** | **Mr(expt)** | **Mr(calc)** | **Delta** | **Miss** | **Score** | **Expect** | **Rank** | **Peptide** |
| --- | --- | --- | --- | --- | --- | --- | --- | --- | --- | --- |
|  | 4467 | **495.2524** | **988.4903** | **989.4964** | **-1.0061** | **1** | **13** | **11** | **4** | **K.QAEMEVKR.E** |
|  | 4750 | 504.2396 | 1006.4647 | 1005.4913 | 0.9734 | 1 | (4) | 72 | 5 | K.QAEMEVKR.E |
|  | 5723 | **542.7288** | **1083.4430** | **1083.6400** | **-0.1970** | **1** | **29** | **0.2** | **1** | **R.IKQQIALDR.A** 5745 |
|  | 10317 | 676.8245 | 1351.6345 | 1351.6983 | -0.0638 | 0 | 23 | 0.67 | 2 | K.VTEASSNSFVAIK.I 10318 10319 10320 10342 |
|  | 10944 | 694.3340 | 1386.6534 | 1386.6627 | -0.0093 | 0 | 30 | 0.15 | 1 | R.VTSSEPPNPASSSK.S 10943 10957 |
|  | 12675 | 497.2714 | 1488.7923 | 1487.8017 | 0.9906 | 1 | 6 | 33 | 6 | K.AAALLAKQAEMEVK.R |
|  | 13967 | **788.4277** | **1574.8408** | **1573.7294** | **1.1114** | **0** | **3** | **55** | **4** | **R.NAELCEIPPTSDTK.S** |
|  | 22471 | **1139.5272** | **2277.0399** | **2277.0549** | **-0.0151** | **0** | **100** | **7.5e-009** | **1** | **R.LPDGSSFTNQFPSDAPLEEAR.Q** 22470 22472 |
|  | 23685 | **813.3947** | **2437.1623** | **2436.1532** | **1.0091** | **0** | **1** | **60** | **9** | **R.QFAAQTVGNTYGNFSLATMFPR.R** |

| **292.** | P23786\|CPT2_HUMAN    **Mass:** 74244    **Score:** 173    **Queries matched:** 24   **emPAI:** 0.41 |
| --- | --- |
|  | Carnitine O-palmitoyltransferase 2, mitochondrial OS=Homo sapiens GN=CPT2 PE=1 SV=2 |

|  | **Query** | **Observed** | **Mr(expt)** | **Mr(calc)** | **Delta** | **Miss** | **Score** | **Expect** | **Rank** | **Peptide** |
| --- | --- | --- | --- | --- | --- | --- | --- | --- | --- | --- |
|  | 1483 | **661.3824** | **660.3752** | **660.3806** | **-0.0055** | **0** | **18** | **3.8** | **1** | **K.TGITAAK.E** |
|  | 3330 | **453.2174** | **904.4203** | **904.5382** | **-0.1179** | **0** | **16** | **5.4** | **3** | **K.SFNLIIAK.D** 3347 |
|  | 3812 | **471.3251** | **940.6356** | **939.4484** | **1.1873** | **1** | **8** | **21** | **2** | **R.KTEQFCK.S** |
|  | 5324 | **526.7540** | **1051.4934** | **1051.5008** | **-0.0074** | **0** | **8** | **26** | **5** | **R.EFLQCVEK.A** |
|  | 5680 | **541.2364** | **1080.4582** | **1080.4658** | **-0.0076** | **0** | **38** | **0.022** | **1** | **K.EAAMGQGFDR.H** |
|  | 7580 | **605.0494** | **1208.0842** | **1208.5673** | **-0.4831** | **1** | **3** | **47** | **10** | **R.DELFTDDKAR.H** |
|  | 7875 | **612.2873** | **1222.5600** | **1221.5659** | **0.9941** | **0** | **36** | **0.04** | **1** | **K.LMSSGNEESLR.K** |
|  | 10544 | **683.3679** | **1364.7213** | **1364.7300** | **-0.0087** | **0** | **4** | **56** | **3** | **R.TETIRPASVYTK.R** |
|  | 10567 | **456.2251** | **1365.6535** | **1365.6558** | **-0.0023** | **1** | **5** | **40** | **7** | **K.LMSSGNEESLRK.V** |
|  | 10813 | **690.8459** | **1379.6772** | **1379.6868** | **-0.0095** | **0** | **18** | **2.1** | **1** | **K.TLTIDCVQFQR.G** |
|  | 12476 | 493.2690 | 1476.7853 | 1477.7195 | -0.9342 | 1 | 8 | 20 | 5 | R.QKLMSSGNEESLR.K |
|  | 15031 | **548.6082** | **1642.8028** | **1642.8137** | **-0.0109** | **0** | **8** | **19** | **2** | **R.SIVPTMHYQDSLPR.L** |
|  | 16661 | **875.4614** | **1748.9082** | **1748.9209** | **-0.0128** | **0** | **42** | **0.0069** | **1** | **R.YLSAQKPLLNDGQFR.K** |
|  | 18381 | **939.4972** | **1876.9798** | **1876.9869** | **-0.0071** | **0** | **16** | **2.6** | **1** | **K.LSPDAVAQLAFQMAFLR.Q** |
|  | 21287 | **535.5019** | **2137.9787** | **2139.0134** | **-1.0347** | **0** | **6** | **24** | **2** | **K.DGSTAVHFEHSWGDGVAVLR.F** |
|  | 22179 | **745.7120** | **2234.1143** | **2234.9718** | **-0.8575** | **1** | **3** | **42** | **3** | **R.EPSRHSAGELQQMMVECSK.Y** |
|  | 23947 | **1235.6043** | **2469.1939** | **2469.2063** | **-0.0124** | **0** | **72** | **4.9e-006** | **1** | **K.YILSDSSPAPEFPLAYLTSENR.D** |
|  | 26605 | **973.8173** | **2918.4300** | **2918.4450** | **-0.0151** | **0** | **64** | **2.5e-005** | **1** | **K.GNFYIFDVLDQDGNIVSPSEIQAHLK.Y** 26604 |
|  | 27296 | **762.6382** | **3046.5239** | **3046.5400** | **-0.0161** | **1** | **11** | **4** | **1** | **R.KGNFYIFDVLDQDGNIVSPSEIQAHLK.Y** |
|  | 28824 | **1167.2377** | **3498.6912** | **3498.7155** | **-0.0243** | **1** | **54** | **0.00015** | **1** | **R.FFNEVFKDSTQTPAVTPQSQPATTDSTVTVQK.L** |
|  | 30287 | 1366.3417 | 4096.0032 | 4094.8972 | 1.1060 | 1 | 0 | 25 | 9 | K.HTSYISGPWFDMYLSARDSVVLNFNPFMAFNPDPK.S |
|  | 31894 | **1708.8446** | **6831.3493** | **6831.3708** | **-0.0215** | **0** | **5** | **1.3** | **1** | **K.GIILPELYLDPAYGQINHNVLSTSTLSSPAVNLGGFAPVVSDGFGVGYAVHDNWIGCNVSSYPGR.N** |

| **293.** | P50440\|GATM_HUMAN    **Mass:** 48938    **Score:** 172    **Queries matched:** 19   **emPAI:** 0.25 |
| --- | --- |
|  | Glycine amidinotransferase, mitochondrial OS=Homo sapiens GN=GATM PE=1 SV=1 |

|  | **Query** | **Observed** | **Mr(expt)** | **Mr(calc)** | **Delta** | **Miss** | **Score** | **Expect** | **Rank** | **Peptide** |
| --- | --- | --- | --- | --- | --- | --- | --- | --- | --- | --- |
|  | 2910 | 436.7482 | 871.4818 | 870.4348 | 1.0470 | 0 | 15 | 5.9 | 1 | R.HLAPDYR.V 2897 |
|  | 7970 | 614.8079 | 1227.6012 | 1226.7710 | 0.8301 | 1 | 8 | 26 | 8 | K.LGITTIKVNIR.N |
|  | 8950 | **640.3573** | **1278.7000** | **1279.7210** | **-1.0209** | **1** | **2** | **94** | **9** | **K.MFEKLGITTIK.V** 8973 |
|  | 10114 | **671.3673** | **1340.7201** | **1341.6962** | **-0.9762** | **0** | **12** | **9.1** | **6** | **R.VMVDANEVPIQK.M** |
|  | 12739 | 747.4353 | 1492.8560 | 1493.7258 | -0.8698 | 0 | 8 | 18 | 2 | K.WLSMNVLMLDEK.R |
|  | 13376 | 768.3760 | 1534.7374 | 1534.7371 | 0.0003 | 0 | 8 | 19 | 5 | K.AVAEIEEMCNILK.T |
|  | 14597 | **539.5869** | **1615.7387** | **1615.7512** | **-0.0124** | **1** | **8** | **19** | **1** | **R.NSCAADDKATEPLPK.D** |
|  | 15126 | **550.6244** | **1648.8513** | **1649.8269** | **-0.9756** | **1** | **1** | **82** | **2** | **K.WLSMNVLMLDEKR.V** |
|  | 15679 | **563.6414** | **1687.9024** | **1686.7559** | **1.1465** | **0** | **6** | **25** | **1** | **K.TPDFESTGLYSAMPR.D** |
|  | 18574 | **474.5073** | **1894.0003** | **1892.9376** | **1.0627** | **1** | **5** | **33** | **10** | **R.VMVDANEVPIQKMFEK.L** |
|  | 20251 | **1020.5505** | **2039.0865** | **2038.0921** | **0.9944** | **0** | **17** | **1.8** | **1** | **R.DILIVVGNEIIEAPMAWR.S** 20243 |
|  | 20557 | **1032.9664** | **2063.9183** | **2063.9299** | **-0.0116** | **0** | **49** | **0.0012** | **1** | **K.FVTTEFEPCFDAADFIR.A** |
|  | 23171 | **1182.5377** | **2363.0609** | **2363.0740** | **-0.0131** | **0** | **58** | **0.00012** | **1** | **K.DCPVSSYNEWDPLEEVIVGR.A** |
|  | 27448 | **1026.4749** | **3076.4027** | **3076.4236** | **-0.0209** | **0** | **67** | **1e-005** | **1** | **K.WTTAPKPTMADELYNQDYPIHSVEDR.H** 27447 |
|  | 31098 | **934.0808** | **4665.3677** | **4665.3988** | **-0.0312** | **1** | **0** | **15** | **5** | **R.VHIISFKDPNPMHIDATFNIIGPGIVLSNPDRPCHQIDLFK.K** |

| **294.** | P16930\|FAAA_HUMAN    **Mass:** 46743    **Score:** 172    **Queries matched:** 11   **emPAI:** 0.26 |
| --- | --- |
|  | Fumarylacetoacetase OS=Homo sapiens GN=FAH PE=1 SV=2 |

|  | **Query** | **Observed** | **Mr(expt)** | **Mr(calc)** | **Delta** | **Miss** | **Score** | **Expect** | **Rank** | **Peptide** |
| --- | --- | --- | --- | --- | --- | --- | --- | --- | --- | --- |
|  | 5918 | 549.8152 | 1097.6158 | 1097.6233 | -0.0075 | 0 | 3 | 68 | 3 | K.HLFTGPVLSK.H |
|  | 8440 | **626.7822** | **1251.5498** | **1251.5587** | **-0.0090** | **0** | **50** | **0.0012** | **1** | **K.GEGMSQAATICK.S** |
|  | 12405 | 737.4250 | 1472.8354 | 1473.8304 | -0.9949 | 0 | 13 | 6.3 | 4 | R.VFLQNLLSVSQAR.L 12402 12406 |
|  | 15397 | 834.4959 | 1666.9773 | 1666.9869 | -0.0096 | 0 | 85 | 3.2e-007 | 1 | R.IGVAIGDQILDLSIIK.H 15396 15398 |
|  | 25261 | **1344.6757** | **2687.3368** | **2687.3492** | **-0.0124** | **0** | **36** | **0.015** | **1** | **K.SFGTTVSPWVVPMDALMPFAVPNPK.Q** 25262 |
|  | 30548 | **840.6094** | **4198.0108** | **4196.9978** | **1.0130** | **1** | **0** | **22** | **1** | **R.RPMGQMKPDDSKPPVYGACKLLDMELEMAFFVGPGNR.L** |

| **295.** | O00217\|NDUS8_HUMAN    **Mass:** 24203    **Score:** 169    **Queries matched:** 7   **emPAI:** 0.34 |
| --- | --- |
|  | NADH dehydrogenase [ubiquinone] iron-sulfur protein 8, mitochondrial OS=Homo sapiens GN=NDUFS8 PE=1 SV=1 |

|  | **Query** | **Observed** | **Mr(expt)** | **Mr(calc)** | **Delta** | **Miss** | **Score** | **Expect** | **Rank** | **Peptide** |
| --- | --- | --- | --- | --- | --- | --- | --- | --- | --- | --- |
|  | 4530 | 497.2375 | 992.4605 | 992.4675 | -0.0070 | 1 | 7 | 32 | 3 | R.RYPSGEER.C |
|  | 8557 | 629.8407 | 1257.6668 | 1258.5863 | -0.9195 | 1 | 4 | 54 | 10 | R.TTRYDIDMTK.C |
|  | 8587 | **630.3648** | **1258.7151** | **1259.6842** | **-0.9692** | **1** | **9** | **19** | **6** | **M.RCLTTPMLLR.A** |
|  | 12827 | **750.8065** | **1499.5985** | **1499.6095** | **-0.0110** | **0** | **56** | **0.00032** | **1** | **K.YVNMQDPEMDMK.S** |
|  | 20268 | **1021.4985** | **2040.9824** | **2040.9972** | **-0.0148** | **0** | **118** | **1.6e-010** | **1** | **K.LCEAICPAQAITIEAEPR.A** 20269 |
|  | 24474 | **849.7639** | **2546.2697** | **2546.2879** | **-0.0182** | **1** | **7** | **13** | **1** | **R.GLGMTLSYLFREPATINYPFEK.G** |

| **296.** | P69905\|HBA_HUMAN    **Mass:** 15305    **Score:** 169    **Queries matched:** 18   **emPAI:** 2.90 |
| --- | --- |
|  | Hemoglobin subunit alpha OS=Homo sapiens GN=HBA1 PE=1 SV=2 |

|  | **Query** | **Observed** | **Mr(expt)** | **Mr(calc)** | **Delta** | **Miss** | **Score** | **Expect** | **Rank** | **Peptide** |
| --- | --- | --- | --- | --- | --- | --- | --- | --- | --- | --- |
|  | 995 | 532.2833 | 531.2761 | 531.2805 | -0.0045 | 0 | 15 | 11 | 1 | K.AAWGK.V 994 |
|  | 2977 | **439.2582** | **876.5017** | **875.4422** | **1.0595** | **0** | **7** | **36** | **8** | **-.MVLSPADK.T** |
|  | 5566 | 536.2778 | 1070.5410 | 1070.5471 | -0.0061 | 0 | 28 | 0.29 | 1 | R.MFLSFPTTK.T 5555 5567 |
|  | 13296 | **765.3655** | **1528.7164** | **1528.7270** | **-0.0106** | **0** | **76** | **3e-006** | **1** | **K.VGAHAGEYGAEALER.M** 13297 13299 13308 |
|  | 17761 | **917.4437** | **1832.8729** | **1832.8846** | **-0.0117** | **0** | **48** | **0.0015** | **1** | **K.TYFPHFDLSHGSAQVK.G** 17762 |
|  | 27036 | **999.4951** | **2995.4635** | **2995.4821** | **-0.0186** | **0** | **82** | **3.2e-007** | **1** | **K.VADALTNAVAHVDDMPNALSALSDLHAHK.L** 27039 |
|  | 27615 | **521.6004** | **3123.5587** | **3123.5771** | **-0.0184** | **1** | **9** | **6.7** | **1** | **K.KVADALTNAVAHVDDMPNALSALSDLHAHK.L** 27614 |
|  | 30619 | **710.5583** | **4257.3061** | **4257.3235** | **-0.0174** | **1** | **14** | **0.72** | **1** | **K.LLSHCLLVTLAAHLPAEFTPAVHASLDKFLASVSTVLTSK.Y** 30618 |

| **297.** | P35606\|COPB2_HUMAN    **Mass:** 103278   **Score:** 168    **Queries matched:** 29   **emPAI:** 0.23 |
| --- | --- |
|  | Coatomer subunit beta' OS=Homo sapiens GN=COPB2 PE=1 SV=2 |

|  | **Query** | **Observed** | **Mr(expt)** | **Mr(calc)** | **Delta** | **Miss** | **Score** | **Expect** | **Rank** | **Peptide** |
| --- | --- | --- | --- | --- | --- | --- | --- | --- | --- | --- |
|  | 1233 | **589.0245** | **588.0173** | **587.3755** | **0.6418** | **1** | **7** | **51** | **1** | **R.KLTAR.S** |
|  | 1597 | 709.1922 | 708.1849 | 707.3854 | 0.7995 | 0 | 11 | 10 | 1 | R.LYLGDK.E |
|  | 1934 | **400.5084** | **799.0023** | **799.5068** | **-0.5045** | **1** | **7** | **23** | **7** | **R.VVKLWR.E** |
|  | 2136 | **407.6923** | **813.3701** | **812.3374** | **1.0327** | **0** | **8** | **26** | **1** | **R.DFSMADK.V** |
|  | 4238 | **486.8001** | **971.5857** | **971.5651** | **0.0206** | **0** | **18** | **2.8** | **1** | **K.QLAELAISK.C** |
|  | 4445 | 494.2808 | 986.5470 | 986.5549 | -0.0079 | 0 | 13 | 8.6 | 2 | R.LPEAAFLAR.T |
|  | 4484 | **495.7550** | **989.4954** | **989.5117** | **-0.0162** | **0** | **6** | **46** | **7** | **R.VWCVASLR.G** |
|  | 5900 | **549.3059** | **1096.5973** | **1096.6240** | **-0.0268** | **1** | **3** | **77** | **5** | **K.DGERLPLAVK.D** |
|  | 6632 | **577.2976** | **1152.5805** | **1152.5887** | **-0.0082** | **0** | **53** | **0.00084** | **1** | **K.HSEVQQANLK.A** 6630 6631 6652 6653 6655 |
|  | 7458 | **401.5686** | **1201.6840** | **1200.6536** | **1.0303** | **0** | **10** | **17** | **3** | **K.VDACLELLIR.T** |
|  | 8092 | **412.1908** | **1233.5506** | **1234.6234** | **-1.0728** | **0** | **8** | **21** | **3** | **K.EAFVVEEWVK.E** |
|  | 9550 | 656.8098 | 1311.6049 | 1311.6129 | -0.0079 | 0 | 12 | 8 | 1 | R.LESTLNYGMER.V |
|  | 10046 | **669.3027** | **1336.5908** | **1336.6007** | **-0.0100** | **0** | **58** | **0.00023** | **1** | **K.DNNQFASASLDR.T** |
|  | 12893 | **502.2346** | **1503.6821** | **1503.6929** | **-0.0107** | **0** | **14** | **4.7** | **1** | **R.VHMFEAHSDYIR.C** |
|  | 13161 | **507.7253** | **1520.1540** | **1519.7089** | **0.4451** | **0** | **6** | **25** | **4** | **K.NWVVTGADDMQIR.V** |
|  | 13785 | **521.3046** | **1560.8921** | **1560.8222** | **0.0699** | **1** | **9** | **16** | **1** | **R.DFSMADKVLPTIPK.E** |
|  | 16475 | **868.4411** | **1734.8676** | **1734.8788** | **-0.0112** | **0** | **27** | **0.24** | **1** | **R.GSNNVALGYDEGSIIVK.L** |
|  | 18912 | **641.6625** | **1921.9658** | **1921.8913** | **0.0745** | **1** | **9** | **12** | **5** | **R.EEPAMSMDANGKIIWAK.H** |
|  | 19957 | **1007.4750** | **2012.9355** | **2011.9639** | **0.9716** | **0** | **41** | **0.0074** | **1** | **R.SVNGLAFYDWDNTELIR.R** |
|  | 20562 | **1033.0034** | **2063.9923** | **2064.0051** | **-0.0128** | **0** | **44** | **0.0032** | **1** | **K.AAESLADPTEYENLFPGLK.E** 20561 |
|  | 21755 | **731.3996** | **2191.1770** | **2192.0646** | **-0.8876** | **0** | **0** | **82** | **9** | **R.FVVVCGDGEYIIYTAMALR.N** |
|  | 22350 | **753.6699** | **2257.9879** | **2258.0028** | **-0.0149** | **0** | **28** | **0.13** | **1** | **K.SFGSAQEFAWAHDSSEYAIR.E** |
|  | 27186 | **1011.5076** | **3031.5009** | **3032.5414** | **-1.0405** | **1** | **2** | **32** | **2** | **R.STAQQELDGKPASPTPVIVASHTANKEEK.S** |

| **298.** | Q9HDC9\|APMAP_HUMAN    **Mass:** 46622    **Score:** 167    **Queries matched:** 29   **emPAI:** 0.85 |
| --- | --- |
|  | Adipocyte plasma membrane-associated protein OS=Homo sapiens GN=APMAP PE=1 SV=2 |

|  | **Query** | **Observed** | **Mr(expt)** | **Mr(calc)** | **Delta** | **Miss** | **Score** | **Expect** | **Rank** | **Peptide** |
| --- | --- | --- | --- | --- | --- | --- | --- | --- | --- | --- |
|  | 2640 | **428.2288** | **854.4430** | **855.5178** | **-1.0748** | **0** | **13** | **6.9** | **1** | **K.VLLDQLR.F** |
|  | 3173 | 447.7162 | 893.4178 | 893.3913 | 0.0265 | 0 | 8 | 26 | 9 | -.MSEADGLR.Q |
|  | 5705 | **541.7775** | **1081.5404** | **1081.5478** | **-0.0074** | **0** | **26** | **0.41** | **1** | **K.LFSQETVMK.F** |
|  | 5798 | **545.2871** | **1088.5597** | **1088.5655** | **-0.0058** | **0** | **27** | **0.37** | **1** | **K.GLFEVNPWK.R** |
|  | 6070 | **555.2914** | **1108.5682** | **1108.5764** | **-0.0082** | **0** | **19** | **1.7** | **1** | **R.LLEYDTVTR.E** |
|  | 6759 | **582.2786** | **1162.5426** | **1161.5560** | **0.9865** | **1** | **(1)** | **1.3e+002** | **7** | **-.MSEADGLRQR.R** |
|  | 6950 | **589.7913** | **1177.5680** | **1177.5509** | **0.0170** | **1** | **6** | **36** | **5** | **-.MSEADGLRQR.R** |
|  | 8317 | **623.3152** | **1244.6158** | **1243.6408** | **0.9750** | **0** | **22** | **1** | **1** | **K.LENGEIETIAR.F** |
|  | 9074 | **643.8595** | **1285.7044** | **1285.7129** | **-0.0085** | **0** | **41** | **0.011** | **1** | **K.LLLSSETPIEGK.N** 9073 |
|  | 12554 | 741.8705 | 1481.7264 | 1482.7249 | -0.9985 | 0 | 8 | 22 | 6 | R.DDEPVCGRPLGIR.A |
|  | 13252 | **509.7802** | **1526.3189** | **1526.8820** | **-0.5632** | **0** | **1** | **70** | **3** | **K.EPPLLLGVLHPNTK.L** |
|  | 13903 | 524.2944 | 1569.8613 | 1569.8726 | -0.0113 | 1 | 15 | 4.1 | 1 | R.VVKLENGEIETIAR.F 13888 13915 |
|  | 16557 | **581.2698** | **1740.7877** | **1739.8737** | **0.9140** | **1** | **4** | **36** | **4** | **K.TRDDEPVCGRPLGIR.A** 16563 |
|  | 20459 | 1028.5358 | 2055.0570 | 2055.0789 | -0.0219 | 1 | 1 | 65 | 5 | K.FVPRYSLVLELSDSGAFR.R |
|  | 20863 | **698.0263** | **2091.0571** | **2091.0709** | **-0.0138** | **0** | **25** | **0.31** | **1** | **R.RPLRPQVVTDDDGQAPEAK.D** 20864 20880 |
|  | 23280 | **792.7456** | **2375.2150** | **2375.2306** | **-0.0156** | **1** | **18** | **1** | **1** | **R.QRRPLRPQVVTDDDGQAPEAK.D** 23281 |
|  | 24149 | **1246.6223** | **2491.2301** | **2491.2417** | **-0.0116** | **0** | **61** | **5.3e-005** | **1** | **R.FPNGVQLSPAEDFVLVAETTMAR.I** 24148 24151 |
|  | 26336 | **1437.6993** | **2873.3841** | **2873.4018** | **-0.0177** | **0** | **76** | **1.5e-006** | **1** | **R.LFENQLVGPESIAHIGDVMFTGTADGR.V** 26338 |
|  | 26950 | **745.8728** | **2979.4621** | **2979.4859** | **-0.0238** | **1** | **3** | **26** | **3** | **K.LLLSSETPIEGKNMSFVNDLTVTQDGR.K** |

| **299.** | P17516\|AK1C4_HUMAN    **Mass:** 37470    **Score:** 165    **Queries matched:** 14   **emPAI:** 0.77 |
| --- | --- |
|  | Aldo-keto reductase family 1 member C4 OS=Homo sapiens GN=AKR1C4 PE=1 SV=2 |

|  | **Query** | **Observed** | **Mr(expt)** | **Mr(calc)** | **Delta** | **Miss** | **Score** | **Expect** | **Rank** | **Peptide** |
| --- | --- | --- | --- | --- | --- | --- | --- | --- | --- | --- |
|  | 1536 | **685.4556** | **684.4483** | **684.4534** | **-0.0051** | **0** | **21** | **1.3** | **4** | **R.GVVVLAK.S** |
|  | 1550 | **689.3528** | **688.3455** | **688.3755** | **-0.0300** | **0** | **23** | **1.4** | **1** | **K.IADGSVK.R** |
|  | 3501 | **459.1707** | **916.3269** | **915.5138** | **0.8131** | **1** | **0** | **1.8e+002** | **8** | **R.NRAVEVTK.L** |
|  | 4799 | 505.8239 | 1009.6333 | 1009.6396 | -0.0063 | 1 | 11 | 11 | 3 | K.RTPALIALR.Y |
|  | 6701 | **579.7891** | **1157.5637** | **1157.5717** | **-0.0080** | **1** | **32** | **0.11** | **1** | **K.REDIFYTSK.L** 6700 |
|  | 10883 | **461.9362** | **1382.7867** | **1382.7955** | **-0.0088** | **0** | **26** | **0.32** | **1** | **R.QLEMILNKPGLK.Y** |
|  | 15810 | **565.6526** | **1693.9359** | **1693.9475** | **-0.0116** | **1** | **36** | **0.024** | **1** | **K.SKDIVLVAHSALGTQR.H** |
|  | 22378 | 755.3527 | 2263.0362 | 2263.0514 | -0.0152 | 0 | 25 | 0.23 | 1 | K.YKPVCNQVECHPYLNQSK.L |
|  | 23045 | **1175.1240** | **2348.2335** | **2348.2450** | **-0.0115** | **0** | **52** | **0.00052** | **1** | **K.LWVDPNSPVLLEDPVLCALAK.K** |
|  | 23352 | **1191.9943** | **2381.9740** | **2381.9861** | **-0.0121** | **0** | **58** | **0.00011** | **1** | **R.YVVMDFLMDHPDYPFSDEY.-** 23351 23353 |
|  | 25024 | **881.7703** | **2642.2891** | **2641.2999** | **0.9892** | **0** | **28** | **0.1** | **1** | **R.VELNDGHFMPVLGFGTYAPPEVPR.N** |

| **300.** | O75891\|FTHFD_HUMAN    **Mass:** 99622    **Score:** 165    **Queries matched:** 37   **emPAI:** 0.39 |
| --- | --- |
|  | 10-formyltetrahydrofolate dehydrogenase OS=Homo sapiens GN=ALDH1L1 PE=1 SV=2 |

|  | **Query** | **Observed** | **Mr(expt)** | **Mr(calc)** | **Delta** | **Miss** | **Score** | **Expect** | **Rank** | **Peptide** |
| --- | --- | --- | --- | --- | --- | --- | --- | --- | --- | --- |
|  | 1628 | 717.4290 | 716.4217 | 716.4181 | 0.0036 | 0 | 10 | 22 | 10 | R.NLTLTR.K |
|  | 1725 | **760.4075** | **759.4003** | **759.4061** | **-0.0059** | **0** | **13** | **14** | **3** | **K.GMVQAVR.L** |
|  | 2511 | **422.7269** | **843.4392** | **844.5130** | **-1.0738** | **1** | **10** | **23** | **2** | **R.NLTLTRK.E** 2529 |
|  | 3482 | 458.7429 | 915.4712 | 915.4661 | 0.0050 | 0 | 11 | 15 | 8 | K.NIQLEDGK.M |
|  | 3762 | **469.7347** | **937.4548** | **937.4617** | **-0.0069** | **0** | **9** | **20** | **6** | **R.LSDHPDVR.K** |
|  | 5542 | **535.3193** | **1068.6240** | **1069.5630** | **-0.9390** | **0** | **7** | **30** | **2** | **K.MILASNFFK.G** |
|  | 5877 | 548.2840 | 1094.5534 | 1094.5608 | -0.0074 | 0 | 40 | 0.015 | 2 | K.IGFTGSTEVGK.H |
|  | 7340 | 599.3149 | 1196.6153 | 1195.6925 | 0.9229 | 1 | 26 | 0.39 | 1 | R.AKGQALPDVVAK.Y 7304 7324 |
|  | 8675 | **632.7897** | **1263.5648** | **1263.5740** | **-0.0092** | **0** | **5** | **49** | **3** | **K.LMEYCQHGVK.E** |
|  | 10511 | **682.8431** | **1363.6717** | **1362.6779** | **0.9938** | **0** | **4** | **60** | **4** | **K.DLGEAALNEYLR.V** |
|  | 11655 | **714.8450** | **1427.6755** | **1427.6820** | **-0.0065** | **0** | **10** | **15** | **1** | **K.VLEVEDSTDFFK.S** |
|  | 12062 | 727.8803 | 1453.7461 | 1453.7565 | -0.0105 | 0 | 39 | 0.018 | 1 | K.LQAGTVFVNTYNK.T |
|  | 12678 | **497.2775** | **1488.8107** | **1488.8201** | **-0.0094** | **0** | **32** | **0.083** | **1** | **R.HGSIIYHPSLLPR.H** |
|  | 12919 | **502.7684** | **1505.2835** | **1504.7596** | **0.5239** | **0** | **1** | **63** | **8** | **K.SPLIIFADCDLNK.A** |
|  | 13102 | **759.2203** | **1516.4260** | **1515.8330** | **0.5930** | **1** | **1** | **69** | **8** | **K.MLLVKNIQLEDGK.M** |
|  | 13419 | **769.9305** | **1537.8464** | **1537.8577** | **-0.0113** | **0** | **13** | **5.5** | **1** | **K.GVVNVLPGSGSLVGQR.L** 13418 |
|  | 13438 | **770.8858** | **1539.7570** | **1539.7682** | **-0.0111** | **0** | **78** | **1.9e-006** | **1** | **R.ANATEFGLASGVFTR.D** 13439 |
|  | 14425 | **535.9318** | **1604.7735** | **1604.7835** | **-0.0100** | **0** | **3** | **53** | **5** | **R.LFVEDSIHDEFVR.R** |
|  | 15356 | 555.6410 | 1663.9013 | 1663.9118 | -0.0105 | 0 | 20 | 1.1 | 1 | K.IQGSTIPINQARPNR.N |
|  | 16019 | **569.8015** | **1706.3827** | **1706.9178** | **-0.5351** | **1** | **6** | **19** | **10** | **R.FLFPEGIKGMVQAVR.L** |
|  | 16611 | **582.8311** | **1745.4715** | **1745.9750** | **-0.5034** | **1** | **3** | **35** | **2** | **K.AGLILFGNDDKMLLVK.N** |
|  | 17223 | **596.9735** | **1787.8987** | **1786.9101** | **0.9886** | **1** | **10** | **11** | **1** | **R.LPQPEEGATYEGIQKK.E** |
|  | 18172 | **621.6446** | **1861.9121** | **1861.9223** | **-0.0102** | **0** | **6** | **23** | **1** | **K.INWDQPAEAIHNWIR.G** |
|  | 18783 | **955.9374** | **1909.8602** | **1909.8728** | **-0.0125** | **0** | **44** | **0.0038** | **1** | **K.ECEVLPDDTVSTLYNR.F** 18784 |
|  | 19596 | **661.7914** | **1982.3523** | **1983.0135** | **-0.6612** | **1** | **2** | **40** | **1** | **K.NIQLEDGKMILASNFFK.G** |
|  | 23861 | 1229.6566 | 2457.2987 | 2457.3089 | -0.0102 | 1 | 4 | 31 | 2 | K.GVVNVLPGSGSLVGQRLSDHPDVR.K |
|  | 24251 | **836.8071** | **2507.3996** | **2507.4145** | **-0.0150** | **0** | **19** | **0.77** | **1** | **K.TAACLAAGNTVVIKPAQVTPLTALK.F** |
|  | 27395 | **1532.7788** | **3063.5431** | **3063.5562** | **-0.0131** | **0** | **51** | **0.00036** | **1** | **K.YQALGAELNVLPFCSQFIPMEIISAPR.H** 27396 |
|  | 28185 | **1096.8859** | **3287.6358** | **3287.6555** | **-0.0198** | **1** | **28** | **0.067** | **1** | **K.TSETINPTDGSVICQVSLAQVTDVDKAVAAAK.D** |
|  | 30499 | **1387.6582** | **4159.9528** | **4158.9732** | **0.9795** | **1** | **9** | **3.4** | **1** | **K.GGFSIFWADDGLDTGDLLLQKECEVLPDDTVSTLYNR.F** |

| **301.** | Q9UBQ7\|GRHPR_HUMAN    **Mass:** 36045    **Score:** 164    **Queries matched:** 13   **emPAI:** 0.49 |
| --- | --- |
|  | Glyoxylate reductase/hydroxypyruvate reductase OS=Homo sapiens GN=GRHPR PE=1 SV=1 |

|  | **Query** | **Observed** | **Mr(expt)** | **Mr(calc)** | **Delta** | **Miss** | **Score** | **Expect** | **Rank** | **Peptide** |
| --- | --- | --- | --- | --- | --- | --- | --- | --- | --- | --- |
|  | 1330 | 621.3561 | 620.3489 | 620.3646 | -0.0157 | 0 | 16 | 4.7 | 9 | K.VFVTR.R |
|  | 1921 | **399.7502** | **797.4859** | **797.4508** | **0.0351** | **1** | **8** | **21** | **4** | **R.RIPAEGR.V** |
|  | 3832 | **472.2844** | **942.5543** | **943.5603** | **-1.0060** | **0** | **3** | **95** | **2** | **R.LKPFGVQR.F** |
|  | 4418 | **493.2837** | **984.5528** | **984.5604** | **-0.0075** | **0** | **45** | **0.0048** | **1** | **R.ILDAAGANLK.V** 4419 |
|  | 7023 | **592.3333** | **1182.6521** | **1182.6608** | **-0.0087** | **1** | **21** | **1.2** | **1** | **R.RLPEAIEEVK.N** |
|  | 15443 | **836.4592** | **1670.9038** | **1670.9137** | **-0.0099** | **0** | **38** | **0.018** | **1** | **R.NTMSLLAANNLLAGLR.G** 15444 |
|  | 17278 | **896.9334** | **1791.8521** | **1791.8639** | **-0.0118** | **0** | **81** | **8.1e-007** | **1** | **R.GDVVNQDDLYQALASGK.I** |
|  | 18985 | **483.0032** | **1927.9836** | **1928.0625** | **-0.0789** | **1** | **4** | **40** | **7** | **R.TRNTMSLLAANNLLAGLR.G** |
|  | 23934 | **1234.6827** | **2467.3509** | **2467.3686** | **-0.0177** | **0** | **30** | **0.071** | **1** | **K.IAAAGLDVTSPEPLPTNHPLLTLK.N** |
|  | 24783 | **863.0532** | **2586.1377** | **2586.1544** | **-0.0167** | **1** | **66** | **1.6e-005** | **1** | **R.AADCEVEQWDSDEPIPAKELER.G** |
|  | 26626 | **974.7787** | **2921.3142** | **2922.4723** | **-1.1581** | **1** | **2** | **37** | **3** | **K.ETAVFINISRGDVVNQDDLYQALASGK.I** |

| **302.** | P09525\|ANXA4_HUMAN    **Mass:** 36088    **Score:** 164    **Queries matched:** 15   **emPAI:** 0.64 |
| --- | --- |
|  | Annexin A4 OS=Homo sapiens GN=ANXA4 PE=1 SV=4 |

|  | **Query** | **Observed** | **Mr(expt)** | **Mr(calc)** | **Delta** | **Miss** | **Score** | **Expect** | **Rank** | **Peptide** |
| --- | --- | --- | --- | --- | --- | --- | --- | --- | --- | --- |
|  | 699 | 461.0076 | 460.0004 | 460.2646 | -0.2642 | 0 | 6 | 73 | 1 | K.GGTVK.A |
|  | 3280 | 451.2655 | 900.5164 | 899.4825 | 1.0339 | 1 | 5 | 59 | 7 | R.TPEEIRR.I |
|  | 3373 | 454.7448 | 907.4750 | 907.4797 | -0.0047 | 1 | 14 | 7.5 | 3 | -.MATKGGTVK.A 3392 |
|  | 6151 | **558.6251** | **1115.2357** | **1114.5843** | **0.6514** | **1** | **8** | **17** | **4** | **R.NTAQRQEIR.T** |
|  | 10665 | 686.3314 | 1370.6483 | 1370.6579 | -0.0096 | 0 | 44 | 0.0056 | 1 | R.ISQTYQQQYGR.S |
|  | 13177 | 761.4283 | 1520.8421 | 1519.7664 | 1.0757 | 1 | 1 | 94 | 7 | K.SMKGLGTDDNTLIR.V |
|  | 14063 | 791.3648 | 1580.7151 | 1580.7253 | -0.0102 | 0 | 39 | 0.015 | 1 | K.AASGFNAMEDAQTLR.K 14047 |
|  | 15309 | **831.4064** | **1660.7982** | **1660.8090** | **-0.0108** | **0** | **36** | **0.026** | **1** | **K.GAGTDEGCLIEILASR.T** |
|  | 15393 | 834.4288 | 1666.8431 | 1665.8461 | 0.9970 | 0 | 65 | 3.7e-005 | 1 | K.SETSGSFEDALLAIVK.C |
|  | 15746 | **846.9379** | **1691.8612** | **1691.8730** | **-0.0118** | **0** | **79** | **1.4e-006** | **1** | **K.GLGTDEDAIISVLAYR.N** 15777 |
|  | 16884 | **588.3673** | **1762.0801** | **1761.9043** | **0.1758** | **1** | **1** | **67** | **9** | **K.GLGTDDNTLIRVMVSR.A** |
|  | 19387 | **654.8266** | **1961.4580** | **1961.8789** | **-0.4209** | **1** | **3** | **31** | **1** | **R.SLEDDIRSDTSFMFQR.V** |

| **303.** | Q9Y277\|VDAC3_HUMAN    **Mass:** 30981    **Score:** 164    **Queries matched:** 11   **emPAI:** 1.00 |
| --- | --- |
|  | Voltage-dependent anion-selective channel protein 3 OS=Homo sapiens GN=VDAC3 PE=1 SV=1 |

|  | **Query** | **Observed** | **Mr(expt)** | **Mr(calc)** | **Delta** | **Miss** | **Score** | **Expect** | **Rank** | **Peptide** |
| --- | --- | --- | --- | --- | --- | --- | --- | --- | --- | --- |
|  | 2678 | **429.7097** | **857.4049** | **857.4106** | **-0.0057** | **0** | **8** | **37** | **3** | **K.GYGFGMVK.I** |
|  | 5056 | 515.8076 | 1029.6006 | 1029.6070 | -0.0064 | 0 | 56 | 0.00042 | 1 | K.LTLSALIDGK.N |
|  | 8486 | **627.8227** | **1253.6308** | **1253.6404** | **-0.0096** | **0** | **54** | **0.00061** | **1** | **K.LSQNNFALGYK.A** |
|  | 9868 | **443.1396** | **1326.3970** | **1326.7006** | **-0.3036** | **1** | **7** | **20** | **2** | **K.GYGFGMVKIDLK.T** 9869 |
|  | 13567 | **516.6305** | **1546.8698** | **1545.8766** | **0.9932** | **1** | **26** | **0.28** | **1** | **K.LTLDTIFVPNTGKK.S** 13538 |
|  | 14671 | **541.2696** | **1620.7871** | **1620.7970** | **-0.0099** | **1** | **9** | **14** | **1** | **K.YKVCNYGLTFTQK.W** |
|  | 19705 | **995.9185** | **1989.8224** | **1989.8375** | **-0.0151** | **0** | **51** | **0.00065** | **1** | **K.SCSGVEFSTSGHAYTDTGK.A** |
|  | 20955 | **1051.0846** | **2100.1546** | **2100.1691** | **-0.0145** | **0** | **77** | **1.6e-006** | **1** | **K.VNNASLIGLGYTQTLRPGVK.L** |
|  | 24336 | 1260.0995 | 2518.1844 | 2518.2339 | -0.0495 | 1 | 45 | 0.0021 | 2 | K.WNTDNTLGTEISWENKLAEGLK.L |

| **304.** | P32969\|RL9_HUMAN    **Mass:** 21964    **Score:** 164    **Queries matched:** 13   **emPAI:** 0.90 |
| --- | --- |
|  | 60S ribosomal protein L9 OS=Homo sapiens GN=RPL9 PE=1 SV=1 |

|  | **Query** | **Observed** | **Mr(expt)** | **Mr(calc)** | **Delta** | **Miss** | **Score** | **Expect** | **Rank** | **Peptide** |
| --- | --- | --- | --- | --- | --- | --- | --- | --- | --- | --- |
|  | 1116 | 559.3775 | 558.3702 | 558.3741 | -0.0039 | 0 | 24 | 1.2 | 1 | R.TVIVK.G |
|  | 6858 | **585.8016** | **1169.5887** | **1169.5968** | **-0.0081** | **0** | **48** | **0.0022** | **1** | **K.FLDGIYVSEK.G** |
|  | 9291 | **649.8483** | **1297.6821** | **1297.6918** | **-0.0097** | **1** | **81** | **1.1e-006** | **1** | **R.KFLDGIYVSEK.G** |
|  | 13121 | **760.3239** | **1518.6333** | **1517.7443** | **0.8890** | **0** | **9** | **15** | **5** | **R.MRPGVACSVSQAQK.D** |
|  | 14337 | **799.9254** | **1597.8363** | **1597.8464** | **-0.0101** | **0** | **20** | **1.1** | **1** | **R.DFNHINVELSLLGK.K** |
|  | 16716 | **439.2384** | **1752.9244** | **1753.9475** | **-1.0231** | **1** | **2** | **75** | **7** | **R.RDFNHINVELSLLGK.K** |
|  | 19995 | **505.0176** | **2016.0413** | **2015.0292** | **1.0121** | **1** | **1** | **73** | **8** | **K.ELATVRTICSHVQNMIK.G** |
|  | 21062 | **1057.0667** | **2112.1187** | **2112.1314** | **-0.0127** | **0** | **72** | **5.5e-006** | **1** | **K.TILSNQTVDIPENVDITLK.G** 21060 |
|  | 23229 | 791.0843 | 2370.2312 | 2371.2668 | -1.0356 | 1 | 2 | 51 | 5 | -.MKTILSNQTVDIPENVDITLK.G |
|  | 24083 | **1243.1584** | **2484.3023** | **2483.3172** | **0.9851** | **0** | **8** | **10** | **1** | **R.SVYAHFPINVVIQENGSLVEIR.N** 24081 24082 |

| **305.** | P08910\|ABHD2_HUMAN    **Mass:** 49081    **Score:** 163    **Queries matched:** 13   **emPAI:** 0.16 |
| --- | --- |
|  | Abhydrolase domain-containing protein 2 OS=Homo sapiens GN=ABHD2 PE=2 SV=1 |

|  | **Query** | **Observed** | **Mr(expt)** | **Mr(calc)** | **Delta** | **Miss** | **Score** | **Expect** | **Rank** | **Peptide** |
| --- | --- | --- | --- | --- | --- | --- | --- | --- | --- | --- |
|  | 11336 | **470.6585** | **1408.9537** | **1408.6155** | **0.3382** | **0** | **8** | **16** | **1** | **R.FYNFLMADNMK.K** |
|  | 13159 | 507.5949 | 1519.7628 | 1520.7156 | -0.9527 | 1 | (2) | 77 | 3 | R.FYNFLMADNMKK.I |
|  | 13651 | **776.9350** | **1551.8554** | **1552.7054** | **-0.8500** | **1** | **11** | **11** | **4** | **R.FYNFLMADNMKK.I** 13650 |
|  | 18299 | **935.9494** | **1869.8842** | **1869.8965** | **-0.0122** | **0** | **111** | **8e-010** | **1** | **R.LYTATSLMQIDDNVMR.K** 18300 |
|  | 19563 | **660.3462** | **1978.0167** | **1976.9587** | **1.0580** | **0** | **2** | **57** | **6** | **-.MNAMLETPELPAVFDGVK.L** |
|  | 19634 | 993.4828 | 1984.9510 | 1985.1859 | -0.2349 | 1 | 2 | 56 | 7 | K.LAAVAAVLYVIVRCLNLK.S |
|  | 19982 | **672.6893** | **2015.0460** | **2013.9863** | **1.0597** | **1** | **7** | **17** | **1** | **R.LYTATSLMQIDDNVMRK.F** |
|  | 20708 | **692.3674** | **2074.0803** | **2074.0993** | **-0.0190** | **0** | **3** | **47** | **4** | **R.CAVLNHLGALPNIELTSPR.M** |
|  | 22166 | **1117.4187** | **2232.8228** | **2232.9683** | **-0.1454** | **0** | **3** | **32** | **1** | **R.MFTYGCTWEFGAMVNYIK.K** |
|  | 23224 | **790.7240** | **2369.1502** | **2370.2736** | **-1.1234** | **1** | **0** | **70** | **2** | **K.EYIPPLIWGKSGHIQTALYGK.M** |
|  | 25676 | **921.1736** | **2760.4991** | **2760.5136** | **-0.0144** | **0** | **49** | **0.00064** | **1** | **R.IYVPLMLVNAADDPLVHESLLTIPK.S** |

| **306.** | P02787\|TRFE_HUMAN    **Mass:** 79280    **Score:** 163    **Queries matched:** 18   **emPAI:** 0.20 |
| --- | --- |
|  | Serotransferrin OS=Homo sapiens GN=TF PE=1 SV=2 |

|  | **Query** | **Observed** | **Mr(expt)** | **Mr(calc)** | **Delta** | **Miss** | **Score** | **Expect** | **Rank** | **Peptide** |
| --- | --- | --- | --- | --- | --- | --- | --- | --- | --- | --- |
|  | 2942 | **438.2243** | **874.4340** | **873.4345** | **0.9996** | **0** | **13** | **11** | **1** | **K.DSAHGFLK.V** |
|  | 6804 | **583.7983** | **1165.5820** | **1165.5840** | **-0.0020** | **0** | **9** | **17** | **1** | **K.HQTVPQNTGGK.N** 6803 |
|  | 8897 | **426.2686** | **1275.7839** | **1275.6248** | **0.1592** | **0** | **7** | **27** | **5** | **K.EFQLFSSPHGK.D** |
|  | 9003 | **642.2839** | **1282.5532** | **1282.5618** | **-0.0087** | **0** | **12** | **7.4** | **1** | **K.EGYYGYTGAFR.C** |
|  | 11436 | **708.3588** | **1414.7030** | **1414.7126** | **-0.0097** | **0** | **42** | **0.0085** | **1** | **K.SVIPSDGPSVACVK.K** |
|  | 11733 | 717.3353 | 1432.6560 | 1433.7150 | -1.0591 | 1 | 3 | 72 | 10 | K.SASDLTWDNLKGK.K |
|  | 12471 | **739.3558** | **1476.6971** | **1475.7442** | **0.9529** | **0** | **2** | **79** | **4** | **K.CGLVPVLAENYNK.S** |
|  | 12691 | 745.8769 | 1489.7393 | 1489.7929 | -0.0536 | 1 | 9 | 17 | 4 | K.DLLFKDSAHGFLK.V |
|  | 13170 | **507.9199** | **1520.7378** | **1520.7293** | **0.0084** | **1** | **7** | **24** | **8** | **R.LKCDEWSVNSVGK.I** |
|  | 13987 | **789.4066** | **1576.7986** | **1576.8072** | **-0.0086** | **0** | **5** | **37** | **1** | **R.TAGWNIPMGLLYNK.I** |
|  | 14806 | 815.9112 | 1629.8078 | 1628.8086 | 0.9992 | 0 | 8 | 21 | 2 | K.EDPQTFYYAVAVVK.K |
|  | 21469 | **720.3986** | **2158.1740** | **2158.0075** | **0.1666** | **0** | **2** | **57** | **2** | **K.IMNGEADAMSLDGGFVYIAGK.C** |
|  | 21564 | **1086.0447** | **2170.0748** | **2170.0881** | **-0.0133** | **0** | **98** | **1.2e-008** | **1** | **R.SAGWNIPIGLLYCDLPEPR.K** |
|  | 23890 | **821.4314** | **2461.2724** | **2461.1995** | **0.0729** | **1** | **3** | **39** | **3** | **K.SCHTAVGRTAGWNIPMGLLYNK.I** |
|  | 24386 | **844.0803** | **2529.2191** | **2529.2322** | **-0.0130** | **1** | **36** | **0.015** | **1** | **R.SMGGKEDLIWELLNQAQEHFGK.D** 24387 |
|  | 31311 | **1247.7707** | **4987.0539** | **4987.0904** | **-0.0365** | **0** | **39** | **0.0013** | **1** | **K.AVANFFSGSCAPCADGTDFPQLCQLCPGCGCSTLNQYFGYSGAFK.C** |

| **307.** | Q8N5K1\|CISD2_HUMAN    **Mass:** 15497    **Score:** 162    **Queries matched:** 7   **emPAI:** 0.56 |
| --- | --- |
|  | CDGSH iron sulfur domain-containing protein 2 OS=Homo sapiens GN=CISD2 PE=1 SV=1 |

|  | **Query** | **Observed** | **Mr(expt)** | **Mr(calc)** | **Delta** | **Miss** | **Score** | **Expect** | **Rank** | **Peptide** |
| --- | --- | --- | --- | --- | --- | --- | --- | --- | --- | --- |
|  | 8298 | **622.8114** | **1243.6082** | **1243.7322** | **-0.1240** | **1** | **7** | **31** | **8** | **-.MVLESVARIVK.V** |
|  | 11961 | **483.2628** | **1446.7665** | **1447.6514** | **-0.8849** | **1** | **12** | **8.3** | **1** | **R.SKTFPACDGSHNK.H** 11952 11965 |
|  | 14652 | 810.4352 | 1618.8559 | 1618.8679 | -0.0119 | 0 | 69 | 1.5e-005 | 1 | K.HNELTGDNVGPLILK.K |
|  | 15280 | **830.4291** | **1658.8437** | **1658.8549** | **-0.0112** | **0** | **82** | **6.3e-007** | **1** | **K.VVNEINIEDLCLTK.A** 15281 |

| **308.** | P00558\|PGK1_HUMAN    **Mass:** 44985    **Score:** 161    **Queries matched:** 22   **emPAI:** 0.38 |
| --- | --- |
|  | Phosphoglycerate kinase 1 OS=Homo sapiens GN=PGK1 PE=1 SV=3 |

|  | **Query** | **Observed** | **Mr(expt)** | **Mr(calc)** | **Delta** | **Miss** | **Score** | **Expect** | **Rank** | **Peptide** |
| --- | --- | --- | --- | --- | --- | --- | --- | --- | --- | --- |
|  | 3548 | **461.2475** | **920.4804** | **919.4685** | **1.0119** | **0** | **0** | **1.6e+002** | **9** | **K.ALMDEVVK.A** |
|  | 5961 | **551.2899** | **1100.5652** | **1100.5323** | **0.0329** | **0** | **33** | **0.076** | **1** | **K.NNQITNNQR.I** |
|  | 6194 | **560.1840** | **1118.3535** | **1117.6343** | **0.7192** | **1** | **9** | **16** | **2** | **M.SLSNKLTLDK.L** |
|  | 7765 | 609.3382 | 1216.6618 | 1216.6485 | 0.0133 | 0 | 13 | 7.3 | 3 | K.IQLINNMLDK.V |
|  | 10040 | **446.2689** | **1335.7848** | **1334.6864** | **1.0984** | **1** | **13** | **6** | **1** | **K.ALMDEVVKATSR.G** |
|  | 10588 | **684.3542** | **1366.6938** | **1366.7027** | **-0.0089** | **0** | **42** | **0.008** | **1** | **R.AHSSMVGVNLPQK.A** |
|  | 11995 | 725.8484 | 1449.6823 | 1449.7472 | -0.0649 | 1 | (0) | 1.2e+002 | 8 | R.VVMRVDFNVPMK.N |
|  | 12290 | 734.3900 | 1466.7655 | 1465.7422 | 1.0233 | 1 | 9 | 14 | 1 | R.VVMRVDFNVPMK.N 12251 |
|  | 16546 | 870.9205 | 1739.8265 | 1739.9054 | -0.0789 | 0 | 15 | 2.9 | 2 | K.VSHVSTGGGASLELLEGK.V |
|  | 16727 | **877.8914** | **1753.7682** | **1753.7798** | **-0.0116** | **0** | **37** | **0.022** | **1** | **R.GCITIIGGGDTATCCAK.W** |
|  | 16937 | **884.9664** | **1767.9183** | **1767.9301** | **-0.0118** | **0** | **92** | **7.1e-008** | **1** | **K.ACANPAAGSVILLENLR.F** |
|  | 19430 | **983.9695** | **1965.9244** | **1965.9353** | **-0.0109** | **0** | **67** | **1.8e-005** | **1** | **K.VLNNMEIGTSLFDEEGAK.I** 19427 |
|  | 20100 | **675.0133** | **2022.0181** | **2022.0310** | **-0.0129** | **1** | **5** | **32** | **3** | **K.ITLPVDFVTADKFDENAK.T** |
|  | 20358 | **683.7055** | **2048.0947** | **2046.9905** | **1.1042** | **1** | **4** | **37** | **2** | **R.VDFNVPMKNNQITNNQR.I** |
|  | 20364 | **513.2955** | **2049.1530** | **2050.0340** | **-0.8810** | **0** | **1** | **65** | **5** | **K.SVVLMSHLGRPDGVPMPDK.Y** |
|  | 22855 | **1161.9507** | **2321.8868** | **2322.1777** | **-0.2909** | **1** | **4** | **24** | **5** | **K.VLNNMEIGTSLFDEEGAKIVK.D** |
|  | 24861 | **869.0779** | **2604.2118** | **2604.2312** | **-0.0194** | **1** | **7** | **13** | **1** | **K.TGQATVASGIPAGWMGLDCGPESSKK.Y** |
|  | 28200 | **1099.5588** | **3295.6547** | **3294.6992** | **0.9555** | **1** | **7** | **8.3** | **1** | **K.SVVLMSHLGRPDGVPMPDKYSLEPVAVELK.S** |
|  | 29341 | **931.4318** | **3721.6979** | **3722.7882** | **-1.0902** | **1** | **1** | **25** | **10** | **K.VNEMIIGGGMAFTFLKVLNNMEIGTSLFDEEGAK.I** 29342 |

| **309.** | Q13724\|MOGS_HUMAN    **Mass:** 92032    **Score:** 159    **Queries matched:** 17   **emPAI:** 0.13 |
| --- | --- |
|  | Mannosyl-oligosaccharide glucosidase OS=Homo sapiens GN=MOGS PE=1 SV=5 |

|  | **Query** | **Observed** | **Mr(expt)** | **Mr(calc)** | **Delta** | **Miss** | **Score** | **Expect** | **Rank** | **Peptide** |
| --- | --- | --- | --- | --- | --- | --- | --- | --- | --- | --- |
|  | 522 | **443.0396** | **442.0323** | **442.2288** | **-0.1966** | **0** | **3** | **21** | **1** | **R.GGPGR.R** 529 |
|  | 1917 | **399.7487** | **797.4828** | **797.4395** | **0.0433** | **0** | **14** | **6.5** | **8** | **R.AVPAEGVR.T** |
|  | 4011 | **478.2628** | **954.5110** | **953.5406** | **0.9703** | **1** | **4** | **58** | **4** | **R.RAVPAEGVR.T** |
|  | 5062 | **516.2640** | **1030.5134** | **1029.5091** | **1.0043** | **0** | **5** | **51** | **7** | **R.EQILGDEAR.A** |
|  | 11006 | 464.2585 | 1389.7537 | 1390.7932 | -1.0395 | 1 | 6 | 34 | 8 | R.GRDPALPTLLNPK.T |
|  | 13466 | 514.7521 | 1541.2344 | 1540.8137 | 0.4207 | 0 | 5 | 23 | 2 | R.FTLLPPTSPGDTAPK.Y |
|  | 13731 | **520.2633** | **1557.7681** | **1557.9467** | **-0.1786** | **0** | **3** | **65** | **2** | **K.AVQLKPRPPQGLVR.V** |
|  | 18638 | **950.5687** | **1899.1228** | **1898.9850** | **0.1378** | **0** | **21** | **0.61** | **1** | **R.LAGSLLTQALESHAEGFR.E** |
|  | 21686 | **728.7410** | **2183.2013** | **2184.1287** | **-0.9274** | **1** | **6** | **21** | **1** | **R.LAGSLLTQALESHAEGFRER.F** |
|  | 22893 | **776.0891** | **2325.2455** | **2324.2199** | **1.0256** | **0** | **1** | **61** | **3** | **R.SPKPLLTGLMWAQQGTTPGTPK.L** |
|  | 24317 | **1258.6227** | **2515.2308** | **2515.2457** | **-0.0149** | **0** | **83** | **3.4e-007** | **1** | **K.YGSYNVFWTSNPGLPLLTEMVK.S** |
|  | 25382 | **903.3859** | **2707.1358** | **2707.1510** | **-0.0153** | **0** | **49** | **0.00072** | **1** | **R.HTCEQGDGVGPYGWEFHDGLSFGR.Q** 25408 |
|  | 29885 | **1302.3595** | **3904.0567** | **3904.0762** | **-0.0195** | **1** | **57** | **6.3e-005** | **1** | **R.VTVEPQDSGTSALPLVSLFFYVVTDGKEVLLPEVGAK.G** 29884 |
|  | 31491 | **1349.6838** | **5394.7063** | **5393.7208** | **0.9855** | **1** | **1** | **8.3** | **2** | **R.VLTRLAEHLGEAEVAAELGPLAASLEAAESLDELHWAPELGVFADFGNHTK.A** |

| **310.** | P38646\|GRP75_HUMAN    **Mass:** 73920    **Score:** 159    **Queries matched:** 41   **emPAI:** 0.48 |
| --- | --- |
|  | Stress-70 protein, mitochondrial OS=Homo sapiens GN=HSPA9 PE=1 SV=2 |

|  | **Query** | **Observed** | **Mr(expt)** | **Mr(calc)** | **Delta** | **Miss** | **Score** | **Expect** | **Rank** | **Peptide** |
| --- | --- | --- | --- | --- | --- | --- | --- | --- | --- | --- |
|  | 672 | **459.0113** | **458.0040** | **458.2601** | **-0.2561** | **0** | **12** | **13** | **1** | **R.AAAAR.L** |
|  | 1526 | **680.4208** | **679.4136** | **679.3323** | **0.0813** | **0** | **12** | **8.8** | **3** | **-.MISASR.A** |
|  | 2751 | 863.0864 | 862.0791 | 862.3967 | -0.3176 | 0 | 5 | 43 | 6 | K.DNMALQR.V |
|  | 4041 | **479.2562** | **956.4979** | **957.4879** | **-0.9900** | **0** | **0** | **1.6e+002** | **6** | **K.VLENAEGAR.T** |
|  | 4071 | **480.7268** | **959.4391** | **959.5440** | **-0.1049** | **1** | **14** | **7.1** | **5** | **K.DIKNVPFK.I** |
|  | 4241 | **487.0873** | **972.1601** | **972.5175** | **-0.3574** | **1** | **7** | **28** | **5** | **R.RTIAPCQK.A** |
|  | 4532 | **993.5043** | **992.4970** | **993.4437** | **-0.9467** | **0** | **2** | **1.2e+002** | **10** | **K.AMQDAEVSK.S** |
|  | 5990 | **552.2572** | **1102.4998** | **1103.5869** | **-1.0871** | **1** | **4** | **70** | **6** | **-.MISASRAAAAR.L** |
|  | 6564 | **574.9908** | **1147.9671** | **1147.5469** | **0.4202** | **1** | **3** | **51** | **9** | **R.KDSETGENIR.Q** |
|  | 6569 | **575.2764** | **1148.5382** | **1148.5462** | **-0.0080** | **1** | **18** | **2.5** | **1** | **R.RYDDPEVQK.D** |
|  | 9145 | **645.8393** | **1289.6640** | **1289.6728** | **-0.0088** | **0** | **24** | **0.56** | **1** | **K.VQQTVQDLFGR.A** |
|  | 9270 | 649.3165 | 1296.6184 | 1296.7262 | -0.1078 | 1 | 7 | 25 | 5 | R.LVGAAASRGPTAAR.H |
|  | 9962 | **666.8666** | **1331.7186** | **1332.6310** | **-0.9124** | **0** | **2** | **91** | **8** | **K.ETAENYLGHTAK.N** |
|  | 11997 | **725.8570** | **1449.6994** | **1449.7100** | **-0.0106** | **0** | **8** | **22** | **3** | **R.TTPSVVAFTADGER.L** 12005 |
|  | 12202 | **488.2468** | **1461.7187** | **1461.7497** | **-0.0311** | **0** | **9** | **17** | **2** | **K.SDIGEVILVGGMTR.M** 12190 12204 12213 |
|  | 12376 | **491.5977** | **1471.7713** | **1472.7835** | **-1.0121** | **0** | **4** | **50** | **10** | **R.EQQIVIQSSGGLSK.D** |
|  | 12953 | 754.8351 | 1507.6557 | 1508.6348 | -0.9790 | 1 | 2 | 76 | 5 | K.VCQGEREMAGDNK.L |
|  | 13671 | **777.4197** | **1552.8249** | **1552.8323** | **-0.0074** | **0** | **37** | **0.025** | **1** | **K.LYSPSQIGAFVLMK.M** |
|  | 13876 | **784.8833** | **1567.7520** | **1567.7631** | **-0.0110** | **0** | **27** | **0.28** | **1** | **R.QAVTNPNNTFYATK.R** |
|  | 14257 | **796.8852** | **1591.7558** | **1591.7664** | **-0.0106** | **1** | **23** | **0.59** | **1** | **K.MKETAENYLGHTAK.N** 14256 |
|  | 15062 | **823.4380** | **1644.8615** | **1644.8723** | **-0.0107** | **0** | **69** | **1.3e-005** | **1** | **R.VINEPTAAALAYGLDK.S** |
|  | 15804 | **847.9230** | **1693.8315** | **1693.8424** | **-0.0109** | **0** | **39** | **0.013** | **1** | **K.NAVITVPAYFNDSQR.Q** 15760 15786 15789 15803 |
|  | 15827 | **566.2650** | **1695.7732** | **1696.7322** | **-0.9590** | **1** | **12** | **7.3** | **2** | **K.MASEREGSGSSGTGEQK.E** |
|  | 16066 | **855.3986** | **1708.7827** | **1708.8645** | **-0.0818** | **1** | **7** | **24** | **3** | **K.IVRASNGDAWVEAHGK.L** |
|  | 16148 | **572.2499** | **1713.7280** | **1712.7271** | **1.0009** | **1** | **(3)** | **51** | **10** | **K.MASEREGSGSSGTGEQK.E** |
|  | 18308 | **936.4182** | **1870.8219** | **1871.8935** | **-1.0716** | **0** | **4** | **40** | **1** | **R.VEAVNMAEGIIHDTETK.M** 18342 |
|  | 20463 | **1028.9705** | **2055.9264** | **2054.9545** | **0.9718** | **0** | **58** | **0.00013** | **1** | **K.STNGDTFLGGEDFDQALLR.H** 20447 20448 |
|  | 21056 | **705.0170** | **2112.0291** | **2113.0877** | **-1.0586** | **1** | **0** | **83** | **6** | **R.QAASSLQQASLKLFEMAYK.K** |
|  | 23581 | **806.7388** | **2417.1945** | **2417.2108** | **-0.0163** | **1** | **18** | **1.2** | **1** | **R.EQQIVIQSSGGLSKDDIENMVK.N** |

| **311.** | Q99714\|HCD2_HUMAN    **Mass:** 27134    **Score:** 159    **Queries matched:** 18   **emPAI:** 0.93 |
| --- | --- |
|  | 3-hydroxyacyl-CoA dehydrogenase type-2 OS=Homo sapiens GN=HSD17B10 PE=1 SV=3 |

|  | **Query** | **Observed** | **Mr(expt)** | **Mr(calc)** | **Delta** | **Miss** | **Score** | **Expect** | **Rank** | **Peptide** |
| --- | --- | --- | --- | --- | --- | --- | --- | --- | --- | --- |
|  | 1421 | 644.3680 | 643.3607 | 643.3653 | -0.0046 | 0 | 19 | 3.4 | 5 | R.LDGAIR.M |
|  | 7044 | **592.8411** | **1183.6677** | **1183.6747** | **-0.0070** | **0** | **58** | **0.00022** | **1** | **K.GGIVGMTLPIAR.D** |
|  | 7426 | **601.3876** | **1200.7607** | **1199.6696** | **1.0911** | **0** | **(6)** | **42** | **2** | **K.GGIVGMTLPIAR.D** 7377 |
|  | 12419 | 492.2652 | 1473.7738 | 1472.7657 | 1.0081 | 0 | 5 | 44 | 7 | R.VDVAVNCAGIAVASK.T 12368 |
|  | 14203 | 795.4202 | 1588.8258 | 1589.8600 | -1.0342 | 0 | 16 | 3 | 2 | R.VLDVNLMGTFNVIR.L |
|  | 14675 | **811.4057** | **1620.7968** | **1620.8083** | **-0.0114** | **0** | **51** | **0.00092** | **1** | **K.VCNFLASQVPFPSR.L** |
|  | 17178 | **893.4069** | **1784.7993** | **1784.8112** | **-0.0119** | **0** | **58** | **0.00016** | **1** | **R.LVAGEMGQNEPDQGGQR.G** |
|  | 17380 | **601.3185** | **1800.9338** | **1800.8061** | **0.1277** | **0** | **(1)** | **83** | **5** | **R.LVAGEMGQNEPDQGGQR.G** |
|  | 17509 | **907.0070** | **1811.9995** | **1812.0105** | **-0.0110** | **0** | **54** | **0.0004** | **1** | **K.GLVAVITGGASGLGLATAER.L** |
|  | 20784 | **695.3790** | **2083.1153** | **2084.1591** | **-1.0439** | **0** | **3** | **49** | **1** | **R.VMTIAPGLFGTPLLTSLPEK.V** |
|  | 20876 | **698.3537** | **2092.0393** | **2092.0987** | **-0.0594** | **1** | **6** | **24** | **1** | **R.VDVAVNCAGIAVASKTYNLK.K** |
|  | 20950 | **700.7487** | **2099.2243** | **2100.1541** | **-0.9297** | **0** | **(2)** | **53** | **4** | **R.VMTIAPGLFGTPLLTSLPEK.V** |
|  | 21768 | **1098.0794** | **2194.1441** | **2194.1593** | **-0.0152** | **0** | **37** | **0.015** | **1** | **R.LVGQGASAVLLDLPNSGGEAQAK.K** |
|  | 25519 | **911.7977** | **2732.3714** | **2731.3851** | **0.9863** | **1** | **2** | **36** | **2** | **K.LGNNCVFAPADVTSEKDVQTALALAK.G** |
|  | 26609 | 584.8841 | 2919.3841 | 2919.6507 | -0.2667 | 1 | 2 | 37 | 3 | R.DLAPIGIRVMTIAPGLFGTPLLTSLPEK.V |
|  | 26722 | 735.1424 | 2936.5405 | 2935.6456 | 0.8949 | 1 | (1) | 37 | 2 | R.DLAPIGIRVMTIAPGLFGTPLLTSLPEK.V |

| **312.** | Q9NQC3\|RTN4_HUMAN    **Mass:** 130250   **Score:** 159    **Queries matched:** 18   **emPAI:** 0.09 |
| --- | --- |
|  | Reticulon-4 OS=Homo sapiens GN=RTN4 PE=1 SV=2 |

|  | **Query** | **Observed** | **Mr(expt)** | **Mr(calc)** | **Delta** | **Miss** | **Score** | **Expect** | **Rank** | **Peptide** |
| --- | --- | --- | --- | --- | --- | --- | --- | --- | --- | --- |
|  | 1262 | 599.3834 | 598.3761 | 598.3438 | 0.0323 | 0 | 9 | 19 | 7 | K.NIQPK.V |
|  | 1374 | **632.3099** | **631.3026** | **630.4064** | **0.8962** | **1** | **12** | **18** | **8** | **K.KVSGIK.E** |
|  | 1776 | **395.0780** | **788.1414** | **787.4440** | **0.6975** | **0** | **9** | **19** | **9** | **K.AQIVTEK.N** |
|  | 3113 | **444.2188** | **886.4229** | **887.4535** | **-1.0305** | **0** | **5** | **69** | **2** | **R.VAVEAPMR.E** |
|  | 3840 | **472.3205** | **942.6265** | **943.4148** | **-0.7883** | **0** | **20** | **1.7** | **1** | **K.SDEGHPFR.A** |
|  | 4259 | **487.3206** | **972.6267** | **971.5651** | **1.0616** | **1** | **10** | **19** | **3** | **R.EEIIVKNK.D** |
|  | 4469 | **495.2530** | **988.4914** | **989.5182** | **-1.0268** | **1** | **2** | **1.3e+002** | **1** | **R.VWEVKDSK.E** |
|  | 4550 | **498.2463** | **994.4780** | **993.4437** | **1.0343** | **0** | **6** | **35** | **5** | **R.SSAENMDLK.E** |
|  | 9701 | **660.8060** | **1319.5975** | **1318.7092** | **0.8883** | **1** | **7** | **27** | **1** | **K.AQIVTEKNTSTK.T** 9679 |
|  | 13320 | **511.2535** | **1530.7388** | **1531.8610** | **-1.1222** | **1** | **2** | **75** | **10** | **R.RLFLVDDLVDSLK.F** |
|  | 13804 | **782.3774** | **1562.7403** | **1562.7511** | **-0.0108** | **0** | **55** | **0.00037** | **1** | **K.YSNSALGHVNCTIK.E** 13805 13818 |
|  | 14464 | **804.4122** | **1606.8098** | **1606.8216** | **-0.0118** | **0** | **91** | **1.1e-007** | **1** | **R.HQAQIDHYLGLANK.N** 14460 14463 |
|  | 17741 | **611.3269** | **1830.9589** | **1831.9720** | **-1.0131** | **0** | **1** | **90** | **3** | **K.LSALPPEGGKPYLESFK.L** |

| **313.** | Q969Z3\|MOSC2_HUMAN    **Mass:** 38569    **Score:** 157    **Queries matched:** 32   **emPAI:** 0.92 |
| --- | --- |
|  | MOSC domain-containing protein 2, mitochondrial OS=Homo sapiens GN=MOSC2 PE=1 SV=1 |

|  | **Query** | **Observed** | **Mr(expt)** | **Mr(calc)** | **Delta** | **Miss** | **Score** | **Expect** | **Rank** | **Peptide** |
| --- | --- | --- | --- | --- | --- | --- | --- | --- | --- | --- |
|  | 1662 | **733.3431** | **732.3359** | **732.3411** | **-0.0052** | **0** | **12** | **13** | **2** | **K.VMACPR.C** |
|  | 2029 | **403.2109** | **804.4072** | **804.4130** | **-0.0058** | **0** | **22** | **1.4** | **1** | **R.VGDPVYR.M** |
|  | 2038 | 403.2422 | 804.4698 | 804.4745 | -0.0048 | 0 | 49 | 0.0029 | 1 | R.IFGLDIK.G 2037 |
|  | 2955 | **438.6946** | **875.3747** | **875.3807** | **-0.0060** | **0** | **15** | **6.9** | **6** | **R.LCDPSER.E** |
|  | 2975 | 439.2452 | 876.4759 | 876.4310 | 0.0450 | 1 | 17 | 4.3 | 8 | K.KVMACPR.C 2986 |
|  | 3516 | **459.7731** | **917.5317** | **917.5375** | **-0.0058** | **0** | **4** | **82** | **7** | **K.LWIYPVK.S** |
|  | 3823 | **471.7816** | **941.5487** | **941.5546** | **-0.0059** | **1** | **20** | **1.4** | **1** | **R.KQPLDTLK.S** 3822 |
|  | 3828 | **472.2786** | **942.5426** | **942.5498** | **-0.0072** | **0** | **35** | **0.048** | **1** | **R.LQQVGTVAK.L** |
|  | 4007 | **478.2526** | **954.4906** | **954.4963** | **-0.0058** | **0** | **21** | **1.2** | **2** | **K.WFTNFLK.T** |
|  | 5145 | **519.1361** | **1036.2577** | **1036.4971** | **-0.2394** | **0** | **(6)** | **25** | **1** | **-.MGASSSSALAR.L** |
|  | 5345 | 527.3506 | 1052.6867 | 1052.4920 | 0.1947 | 0 | 9 | 17 | 5 | -.MGASSSSALAR.L 5325 5330 5334 5339 |
|  | 5939 | **550.3290** | **1098.6435** | **1098.6509** | **-0.0074** | **1** | **24** | **0.61** | **1** | **R.RLQQVGTVAK.L** |
|  | 6068 | **555.2828** | **1108.5511** | **1108.5587** | **-0.0075** | **0** | **40** | **0.016** | **1** | **R.LVQFETNMK.G** |
|  | 6249 | 562.7519 | 1123.4893 | 1124.5536 | -1.0642 | 0 | (7) | 28 | 9 | R.LVQFETNMK.G |
|  | 7843 | 407.8885 | 1220.6437 | 1219.6561 | 0.9877 | 1 | 13 | 8 | 2 | K.QPLDTLKSYR.L |
|  | 9561 | **656.8914** | **1311.7682** | **1312.6697** | **-0.9015** | **0** | **8** | **21** | **2** | **R.APDMDQLVLPSK.Q** |
|  | 11339 | **470.7319** | **1409.1740** | **1408.6656** | **0.5083** | **1** | **1** | **61** | **7** | **R.LCDPSERELYK.L** |
|  | 13079 | **758.4039** | **1514.7933** | **1514.8021** | **-0.0088** | **0** | **10** | **13** | **1** | **K.LSPLFGIYYSVEK.I** |
|  | 13972 | **788.8712** | **1575.7279** | **1575.7385** | **-0.0106** | **0** | **66** | **3.4e-005** | **1** | **K.GVPVSEAECTAMGLR.S** |
|  | 15483 | 837.9166 | 1673.8186 | 1673.8295 | -0.0109 | 0 | 10 | 11 | 2 | R.CILTTVDPDTGVIDR.K |
|  | 17377 | **451.2384** | **1800.9246** | **1800.9345** | **-0.0099** | **1** | **35** | **0.03** | **1** | **R.FWLVIKEDGHMVTAR.Q** |
|  | 20357 | 513.0305 | 2048.0930 | 2049.1232 | -1.0302 | 1 | 3 | 47 | 9 | M.GASSSSALARLGLPARPWPR.W |
|  | 29918 | **1313.3330** | **3936.9772** | **3937.0005** | **-0.0233** | **1** | **64** | **1.2e-005** | **1** | **R.KLLPTLDQNFQVAYPDYCPLLIMTDASLVDLNTR.M** 29920 29921 |

| **314.** | Q9BSE5\|SPEB_HUMAN    **Mass:** 38206    **Score:** 156    **Queries matched:** 23   **emPAI:** 0.60 |
| --- | --- |
|  | Agmatinase, mitochondrial OS=Homo sapiens GN=AGMAT PE=1 SV=2 |

|  | **Query** | **Observed** | **Mr(expt)** | **Mr(calc)** | **Delta** | **Miss** | **Score** | **Expect** | **Rank** | **Peptide** |
| --- | --- | --- | --- | --- | --- | --- | --- | --- | --- | --- |
|  | 2539 | **423.7259** | **845.4373** | **846.4382** | **-1.0009** | **0** | **2** | **1.4e+002** | **6** | **R.LLASGCAR.G** |
|  | 5134 | **518.7705** | **1035.5265** | **1035.5349** | **-0.0084** | **1** | **13** | **7.6** | **2** | **R.RIQEAYEK.I** 5135 |
|  | 6146 | **558.3336** | **1114.6527** | **1114.5479** | **0.1048** | **1** | **3** | **85** | **5** | **R.QSRQASDAPR.N** |
|  | 8422 | **625.8278** | **1249.6411** | **1249.5835** | **0.0576** | **0** | **11** | **13** | **2** | **R.VVLAEDCWMK.S** |
|  | 10516 | 455.5753 | 1363.7040 | 1363.6224 | 0.0815 | 1 | 8 | 24 | 4 | R.CVDEGLLDCKR.V |
|  | 17878 | **614.8130** | **1841.4173** | **1840.8600** | **0.5573** | **1** | **2** | **39** | **5** | **R.SQGFRVVLAEDCWMK.S** |
|  | 18803 | **638.3170** | **1911.9291** | **1911.9439** | **-0.0149** | **0** | **24** | **0.36** | **1** | **K.HGPVGLLHVDAHTDTTDK.A** 18804 18818 |
|  | 22346 | **753.3798** | **2257.1175** | **2258.0759** | **-0.9584** | **0** | **5** | **26** | **4** | **R.NQPPSPEFVARPVGVCSMMR.L** 22351 |
|  | 23050 | 783.7623 | 2348.2652 | 2347.1738 | 1.0913 | 1 | 1 | 59 | 10 | R.VVLAEDCWMKSLVPLMGEVR.Q |
|  | 23521 | **603.5595** | **2410.2089** | **2410.2241** | **-0.0152** | **1** | **16** | **1.9** | **1** | **K.HGPVGLLHVDAHTDTTDKALGEK.L** |
|  | 26051 | **942.1690** | **2823.4852** | **2823.5027** | **-0.0175** | **0** | **21** | **0.4** | **1** | **K.IVAAGCIPLTLGGDHTITYPILQAMAK.K** |
|  | 26823 | 985.1829 | 2952.5268 | 2951.5976 | 0.9291 | 1 | 0 | 49 | 6 | K.IVAAGCIPLTLGGDHTITYPILQAMAKK.H |
|  | 27214 | **1012.8572** | **3035.5499** | **3035.5676** | **-0.0177** | **0** | **68** | **7.6e-006** | **1** | **R.LPVQTSPEGLDAAFIGVPLDTGTSNRPGAR.F** |
|  | 30937 | **1129.5776** | **4514.2815** | **4514.3043** | **-0.0228** | **0** | **44** | **0.00086** | **1** | **R.QQMGGKPIYISFDIDALDPAYAPGTGTPEIAGLTPSQALEIIR.G** 30934 30935 30936 30938 30939 |

| **315.** | P17174\|AATC_HUMAN    **Mass:** 46447    **Score:** 156    **Queries matched:** 19   **emPAI:** 0.47 |
| --- | --- |
|  | Aspartate aminotransferase, cytoplasmic OS=Homo sapiens GN=GOT1 PE=1 SV=3 |

|  | **Query** | **Observed** | **Mr(expt)** | **Mr(calc)** | **Delta** | **Miss** | **Score** | **Expect** | **Rank** | **Peptide** |
| --- | --- | --- | --- | --- | --- | --- | --- | --- | --- | --- |
|  | 3693 | 466.7707 | 931.5268 | 931.5338 | -0.0070 | 0 | 21 | 1.5 | 1 | R.INVSGLTTK.N 3687 |
|  | 5361 | **528.2995** | **1054.5844** | **1054.5923** | **-0.0079** | **0** | **10** | **17** | **1** | **K.HIYLLPSGR.I** |
|  | 5623 | 538.2871 | 1074.5595 | 1075.6138 | -1.0543 | 1 | 20 | 1.6 | 1 | R.KVNLGVGAYR.T |
|  | 6378 | **567.5941** | **1133.1736** | **1133.6227** | **-0.4491** | **1** | **10** | **11** | **1** | **R.ILTMRSELR.A** |
|  | 7893 | **408.5614** | **1222.6624** | **1222.6162** | **0.0462** | **1** | **12** | **9.3** | **1** | **K.TMADRILTMR.S** 7866 |
|  | 8222 | **414.2684** | **1239.7835** | **1238.6111** | **1.1723** | **1** | **(3)** | **69** | **8** | **K.TMADRILTMR.S** |
|  | 8800 | 636.3522 | 1270.6899 | 1270.6994 | -0.0095 | 0 | 33 | 0.064 | 1 | R.VGGVQSLGGTGALR.I |
|  | 9275 | **649.3314** | **1296.6482** | **1296.6575** | **-0.0093** | **0** | **40** | **0.014** | **1** | **R.ITWSNPPAQGAR.I** 9274 |
|  | 13827 | 782.9360 | 1563.8575 | 1564.7708 | -0.9133 | 0 | 1 | 92 | 5 | R.TDDCHPWVLPVVK.K |
|  | 14799 | 815.4562 | 1628.8978 | 1629.8283 | -0.9305 | 0 | 12 | 6.8 | 2 | K.EPESILQVLSQMEK.I |
|  | 19772 | 999.5273 | 1997.0400 | 1998.0819 | -1.0419 | 1 | 3 | 51 | 2 | K.EPESILQVLSQMEKIVR.I |
|  | 22173 | **1117.5647** | **2233.1148** | **2233.1266** | **-0.0118** | **0** | **82** | **5.3e-007** | **1** | **R.IVASTLSNPELFEEWTGNVK.T** 22174 |
|  | 22775 | **1157.0447** | **2312.0748** | **2312.0902** | **-0.0154** | **0** | **38** | **0.012** | **1** | **R.FLFPFFDSAYQGFASGNLER.D** |
|  | 23450 | **800.4123** | **2398.2150** | **2398.2281** | **-0.0130** | **0** | **6** | **20** | **1** | **K.IANDNSLNHEYLPILGLAEFR.S** |
|  | 24196 | 833.7841 | 2498.3304 | 2497.3462 | 0.9842 | 1 | 30 | 0.059 | 1 | R.VGNLTVVGKEPESILQVLSQMEK.I |

| **316.** | O95182\|NDUA7_HUMAN    **Mass:** 12601    **Score:** 155    **Queries matched:** 8   **emPAI:** 1.96 |
| --- | --- |
|  | NADH dehydrogenase [ubiquinone] 1 alpha subcomplex subunit 7 OS=Homo sapiens GN=NDUFA7 PE=1 SV=3 |

|  | **Query** | **Observed** | **Mr(expt)** | **Mr(calc)** | **Delta** | **Miss** | **Score** | **Expect** | **Rank** | **Peptide** |
| --- | --- | --- | --- | --- | --- | --- | --- | --- | --- | --- |
|  | 2260 | **412.5435** | **823.0724** | **822.4712** | **0.6012** | **1** | **3** | **49** | **4** | **K.RTQPPPK.L** |
|  | 3566 | **462.2471** | **922.4797** | **922.4872** | **-0.0075** | **1** | **7** | **32** | **5** | **R.YQEISKR.T** |
|  | 5302 | **525.3234** | **1048.6323** | **1048.6393** | **-0.0070** | **1** | **40** | **0.017** | **1** | **K.AVTPAPPIKR.W** |
|  | 7195 | **595.7621** | **1189.5096** | **1189.5186** | **-0.0090** | **0** | **40** | **0.014** | **1** | **K.LSNNYYCTR.D** |
|  | 7631 | **606.3565** | **1210.6983** | **1211.5683** | **-0.8700** | **0** | **12** | **9.5** | **2** | **R.NWASGHDLQGK.L** |
|  | 16174 | **858.4562** | **1714.8979** | **1714.9101** | **-0.0122** | **0** | **90** | **1.2e-007** | **1** | **K.ALVSGKPAESSAVAATEK.K** 16172 16173 |

| **317.** | P08571\|CD14_HUMAN    **Mass:** 40678    **Score:** 154    **Queries matched:** 12   **emPAI:** 0.30 |
| --- | --- |
|  | Monocyte differentiation antigen CD14 OS=Homo sapiens GN=CD14 PE=1 SV=2 |

|  | **Query** | **Observed** | **Mr(expt)** | **Mr(calc)** | **Delta** | **Miss** | **Score** | **Expect** | **Rank** | **Peptide** |
| --- | --- | --- | --- | --- | --- | --- | --- | --- | --- | --- |
|  | 3428 | **456.7218** | **911.4290** | **911.4825** | **-0.0534** | **0** | **11** | **11** | **1** | **R.ATVNPSAPR.C** |
|  | 5901 | **549.3089** | **1096.6032** | **1096.5521** | **0.0511** | **0** | **3** | **79** | **10** | **R.GLMAALCPHK.F** |
|  | 17064 | **889.5337** | **1777.0528** | **1776.0621** | **0.9907** | **0** | **33** | **0.052** | **1** | **R.LTVGAAQVPAQLLVGALR.V** 17043 17057 17058 17061 |
|  | 19502 | **986.9968** | **1971.9791** | **1971.9902** | **-0.0111** | **0** | **100** | **9.1e-009** | **1** | **R.AFPALTSLDLSDNPGLGER.G** 19503 |
|  | 19824 | **667.3336** | **1998.9790** | **1998.9945** | **-0.0155** | **0** | **32** | **0.06** | **1** | **K.VLSIAQAHSPAFSCEQVR.A** |
|  | 21282 | **1069.5917** | **2137.1688** | **2137.1817** | **-0.0129** | **0** | **5** | **28** | **4** | **K.ITGTMPPLPLEATGLALSSLR.L** |
|  | 23610 | **808.4147** | **2422.2222** | **2422.3617** | **-0.1396** | **1** | **2** | **38** | **2** | **K.ITGTMPPLPLEATGLALSSLRLR.N** |

| **318.** | Q9Y6C9\|MTCH2_HUMAN    **Mass:** 33936    **Score:** 154    **Queries matched:** 15   **emPAI:** 0.69 |
| --- | --- |
|  | Mitochondrial carrier homolog 2 OS=Homo sapiens GN=MTCH2 PE=1 SV=1 |

|  | **Query** | **Observed** | **Mr(expt)** | **Mr(calc)** | **Delta** | **Miss** | **Score** | **Expect** | **Rank** | **Peptide** |
| --- | --- | --- | --- | --- | --- | --- | --- | --- | --- | --- |
|  | 2488 | **421.2211** | **840.4276** | **839.4290** | **0.9986** | **0** | **10** | **16** | **2** | **R.GNSLFFR.K** |
|  | 6153 | 558.7893 | 1115.5639 | 1116.6404 | -1.0764 | 1 | 19 | 2.2 | 5 | R.RGLFTGLTPR.L |
|  | 8358 | **624.3126** | **1246.6106** | **1246.6194** | **-0.0088** | **0** | **20** | **1.5** | **1** | **K.EVSSSFDHVIK.E** |
|  | 9828 | **663.8586** | **1325.7027** | **1325.7126** | **-0.0099** | **0** | **52** | **0.00079** | **1** | **R.LCSGVLGTVVHGK.V** |
|  | 16689 | **876.5007** | **1750.9869** | **1750.9982** | **-0.0112** | **0** | **25** | **0.35** | **1** | **K.VLIQVGYEPLPPTIGR.N** |
|  | 17039 | **889.0030** | **1775.9914** | **1776.0046** | **-0.0132** | **0** | **34** | **0.046** | **1** | **R.SAATLITHPFHVITLR.S** 17062 |
|  | 23101 | **1178.0740** | **2354.1334** | **2354.1502** | **-0.0169** | **1** | **60** | **7.1e-005** | **1** | **K.VLQHYQESDKGEELGPGNVQK.E** 23099 23100 |
|  | 23141 | **787.3951** | **2359.1634** | **2359.1743** | **-0.0109** | **0** | **25** | **0.28** | **1** | **R.QVCQLPGLFSYAQHIASIDGR.R** |
|  | 24318 | **629.8225** | **2515.2609** | **2515.2754** | **-0.0145** | **1** | **2** | **38** | **2** | **R.QVCQLPGLFSYAQHIASIDGRR.G** |
|  | 24864 | 869.3984 | 2605.1735 | 2605.3859 | -0.2124 | 0 | (0) | 59 | 9 | -.MADAASQVLLGSGLTILSQPLMYVK.V |
|  | 24935 | **874.9484** | **2621.8233** | **2621.3808** | **0.4424** | **0** | **3** | **23** | **1** | **-.MADAASQVLLGSGLTILSQPLMYVK.V** |
|  | 31844 | **1048.8406** | **6286.9998** | **6287.8773** | **-0.8774** | **0** | **0** | **6.7** | **1** | **K.SYSQAVTGFFASMLTYPFVLVSNLMAVNNCGLAGGCPPYSPIYTSWIDCWCMLQK.E** |

| **319.** | P04004\|VTNC_HUMAN    **Mass:** 55069    **Score:** 154    **Queries matched:** 9   **emPAI:** 0.22 |
| --- | --- |
|  | Vitronectin OS=Homo sapiens GN=VTN PE=1 SV=1 |

|  | **Query** | **Observed** | **Mr(expt)** | **Mr(calc)** | **Delta** | **Miss** | **Score** | **Expect** | **Rank** | **Peptide** |
| --- | --- | --- | --- | --- | --- | --- | --- | --- | --- | --- |
|  | 7352 | **599.7885** | **1197.5623** | **1196.5496** | **1.0128** | **1** | **9** | **21** | **5** | **R.CTEGFNVDKK.C** |
|  | 9410 | **652.7720** | **1303.5294** | **1303.5390** | **-0.0097** | **0** | **20** | **1.5** | **1** | **R.GQYCYELDEK.A** |
|  | 14824 | **544.3207** | **1629.9404** | **1630.9228** | **-0.9824** | **1** | **1** | **90** | **7** | **R.IYISGMAPRPSLAKK.Q** |
|  | 15072 | **823.9080** | **1645.8015** | **1645.8100** | **-0.0085** | **0** | **50** | **0.0011** | **1** | **R.DVWGIEGPIDAAFTR.I** |
|  | 15371 | **833.8862** | **1665.7579** | **1665.7682** | **-0.0103** | **0** | **28** | **0.19** | **1** | **R.DWHGVPGQVDAAMAGR.I** |
|  | 23311 | 794.0342 | 2379.0807 | 2380.1619 | -1.0812 | 0 | 0 | 70 | 9 | K.NNATVHEQVGGPSLTSDLQAQSK.G |
|  | 24697 | **1287.5995** | **2573.1844** | **2573.1971** | **-0.0127** | **0** | **57** | **0.00013** | **1** | **R.MDWLVPATCEPIQSVFFFSGDK.Y** |
|  | 28779 | **1161.4471** | **3481.3196** | **3481.3448** | **-0.0252** | **0** | **92** | **1.9e-008** | **1** | **K.CQCDELCSYYQSCCTDYTAECKPQVTR.G** |
|  | 30285 | **1024.4370** | **4093.7189** | **4093.7458** | **-0.0269** | **0** | **12** | **1.2** | **1** | **K.QYWEYQFQHQPSQEECEGSSLSAVFEHFAMMQR.D** |

| **320.** | Q04828\|AK1C1_HUMAN    **Mass:** 37221    **Score:** 154    **Queries matched:** 23   **emPAI:** 0.78 |
| --- | --- |
|  | Aldo-keto reductase family 1 member C1 OS=Homo sapiens GN=AKR1C1 PE=1 SV=1 |

|  | **Query** | **Observed** | **Mr(expt)** | **Mr(calc)** | **Delta** | **Miss** | **Score** | **Expect** | **Rank** | **Peptide** |
| --- | --- | --- | --- | --- | --- | --- | --- | --- | --- | --- |
|  | 1536 | 685.4556 | 684.4483 | 684.4534 | -0.0051 | 0 | 21 | 1.3 | 4 | R.GVVVLAK.S |
|  | 1550 | 689.3528 | 688.3455 | 688.3755 | -0.0300 | 0 | 23 | 1.4 | 1 | K.IADGSVK.R |
|  | 1719 | **758.4101** | **757.4028** | **757.4082** | **-0.0054** | **0** | **12** | **15** | **1** | **K.AIDGLNR.N** |
|  | 3583 | **462.7530** | **923.4915** | **922.4946** | **0.9969** | **1** | **25** | **0.44** | **1** | **R.KLLDFCK.S** |
|  | 4532 | 993.5043 | 992.4970 | 992.5039 | -0.0069 | 0 | 15 | 5.3 | 1 | K.SIGVSNFNR.R |
|  | 4799 | 505.8239 | 1009.6333 | 1009.6396 | -0.0063 | 1 | 11 | 11 | 3 | K.RTPALIALR.Y |
|  | 6573 | **575.3054** | **1148.5963** | **1148.6050** | **-0.0088** | **1** | **9** | **21** | **2** | **K.SIGVSNFNRR.Q** 6581 |
|  | 6701 | 579.7891 | 1157.5637 | 1157.5717 | -0.0080 | 1 | 32 | 0.11 | 1 | K.REDIFYTSK.L 6700 |
|  | 10883 | 461.9362 | 1382.7867 | 1382.7955 | -0.0088 | 0 | 26 | 0.32 | 1 | R.QLEMILNKPGLK.Y |
|  | 12660 | **745.1659** | **1488.3172** | **1488.8300** | **-0.5128** | **1** | **3** | **42** | **2** | **K.ALEATKLAIEAGFR.H** |
|  | 19320 | **978.9654** | **1955.9162** | **1955.9299** | **-0.0136** | **0** | **58** | **0.00014** | **1** | **R.QNVQVFEFQLTSEEMK.A** |
|  | 21647 | **727.0293** | **2178.0661** | **2178.0817** | **-0.0157** | **0** | **68** | **1.5e-005** | **1** | **R.HIDSAHLYNNEEQVGLAIR.S** |
|  | 22093 | **742.7079** | **2225.1018** | **2225.1150** | **-0.0132** | **1** | **7** | **16** | **1** | **R.IRQNVQVFEFQLTSEEMK.A** |
|  | 22212 | **560.5055** | **2237.9931** | **2238.0099** | **-0.0168** | **0** | **40** | **0.0086** | **1** | **K.YKPVCNQVECHPYFNQR.K** 22213 22214 |
|  | 23138 | **1180.5829** | **2359.1512** | **2359.1671** | **-0.0159** | **0** | **39** | **0.0095** | **1** | **K.LNDGHFMPVLGFGTYAPAEVPK.S** 23139 23140 |
|  | 27622 | **1042.5630** | **3124.6671** | **3124.6849** | **-0.0177** | **0** | **13** | **2.2** | **1** | **K.NLQLDYVDLYLIHFPVSVKPGEEVIPK.D** 27623 |

| **321.** | P36871\|PGM1_HUMAN    **Mass:** 61696    **Score:** 153    **Queries matched:** 30   **emPAI:** 0.60 |
| --- | --- |
|  | Phosphoglucomutase-1 OS=Homo sapiens GN=PGM1 PE=1 SV=3 |

|  | **Query** | **Observed** | **Mr(expt)** | **Mr(calc)** | **Delta** | **Miss** | **Score** | **Expect** | **Rank** | **Peptide** |
| --- | --- | --- | --- | --- | --- | --- | --- | --- | --- | --- |
|  | 3081 | **443.0382** | **884.0619** | **884.4716** | **-0.4096** | **0** | **3** | **49** | **5** | **K.ELLSGPNR.L** |
|  | 3715 | 933.5123 | 932.5051 | 932.5729 | -0.0678 | 1 | 7 | 41 | 1 | -.MVKIVTVK.T 3702 |
|  | 5037 | 515.2913 | 1028.5681 | 1029.5018 | -0.9338 | 0 | 13 | 8 | 4 | R.LYIDSYEK.D 5038 5059 |
|  | 5105 | **517.7471** | **1033.4797** | **1033.4862** | **-0.0065** | **0** | **20** | **1.9** | **1** | **R.SMPTSGALDR.V** |
|  | 5815 | **545.7919** | **1089.5692** | **1089.5778** | **-0.0086** | **0** | **48** | **0.0026** | **1** | **R.LSGTGSAGATIR.L** 5796 |
|  | 6305 | 565.2817 | 1128.5489 | 1128.5274 | 0.0216 | 0 | 3 | 88 | 2 | K.FFGNLMDASK.L |
|  | 7413 | **401.2128** | **1200.6166** | **1200.6099** | **0.0067** | **0** | **10** | **17** | **6** | **R.QEATLVVGGDGR.F** 7446 7450 |
|  | 10930 | 693.8782 | 1385.7419 | 1384.7173 | 1.0246 | 0 | 4 | 50 | 3 | R.IDAMHGVVGPYVK.K |
|  | 11789 | **719.3450** | **1436.6755** | **1436.6857** | **-0.0102** | **0** | **37** | **0.024** | **1** | **K.TIEEYAVCPDLK.V** |
|  | 11883 | **722.3673** | **1442.7199** | **1442.7293** | **-0.0093** | **1** | **38** | **0.021** | **1** | **R.LYIDSYEKDVAK.I** |
|  | 13091 | 506.2715 | 1515.7926 | 1514.6931 | 1.0995 | 1 | 11 | 10 | 2 | K.MMKDLEALMFDR.S |
|  | 14834 | **816.4016** | **1630.7887** | **1629.7998** | **0.9888** | **0** | **30** | **0.12** | **1** | **K.FNISNGGPAPEAITDK.I** 14832 14833 |
|  | 15122 | **825.4094** | **1648.8043** | **1648.8169** | **-0.0126** | **0** | **59** | **0.00015** | **1** | **K.TQAYQDQKPGTSGLR.K** 15142 |
|  | 16962 | **886.3858** | **1770.7570** | **1770.7697** | **-0.0126** | **0** | **75** | **3.2e-006** | **1** | **K.ADNFEYSDPVDGSISR.N** |
|  | 18215 | **622.6182** | **1864.8327** | **1863.8421** | **0.9905** | **0** | **13** | **5.8** | **1** | **K.LSLCGEESFGTGSDHIR.E** |
|  | 22802 | **772.7337** | **2315.1793** | **2315.1871** | **-0.0079** | **0** | **18** | **1.1** | **1** | **K.FKPFTVEIVDSVEAYATMLR.S** |
|  | 23759 | **816.3958** | **2446.1654** | **2446.3332** | **-0.1678** | **1** | **0** | **69** | **8** | **K.IKAIGGIILTASHNPGGPNGDFGIK.F** |
|  | 25906 | **933.4670** | **2797.3793** | **2798.3875** | **-1.0082** | **0** | **9** | **6.8** | **1** | **K.VFQSSANYAENFIQSIISTVEPAQR.Q** |
|  | 28175 | **1094.5580** | **3280.6521** | **3280.6670** | **-0.0149** | **0** | **19** | **0.53** | **1** | **K.HGFFVNPSDSVAVIAANIFSIPYFQQTGVR.G** |
|  | 30014 | 791.7742 | 3953.8345 | 3953.0298 | 0.8046 | 1 | 1 | 22 | 7 | R.NMILGKHGFFVNPSDSVAVIAANIFSIPYFQQTGVR.G |
|  | 30972 | **1142.0380** | **4564.1227** | **4564.1349** | **-0.0121** | **0** | **5** | **5.6** | **2** | **K.ILCEELGAPANSAVNCVPLEDFGGHHPDPNLTYAADLVETMK.S** |

| **322.** | O75306\|NDUS2_HUMAN    **Mass:** 52911    **Score:** 153    **Queries matched:** 24   **emPAI:** 0.23 |
| --- | --- |
|  | NADH dehydrogenase [ubiquinone] iron-sulfur protein 2, mitochondrial OS=Homo sapiens GN=NDUFS2 PE=1 SV=2 |

|  | **Query** | **Observed** | **Mr(expt)** | **Mr(calc)** | **Delta** | **Miss** | **Score** | **Expect** | **Rank** | **Peptide** |
| --- | --- | --- | --- | --- | --- | --- | --- | --- | --- | --- |
|  | 4062 | **480.2675** | **958.5204** | **958.5270** | **-0.0065** | **0** | **28** | **0.29** | **1** | **R.IIAQCLNK.M** |
|  | 5612 | 537.7863 | 1073.5579 | 1074.6662 | -1.1082 | 0 | 8 | 32 | 8 | K.LLNIRPPPR.A |
|  | 6775 | 582.7824 | 1163.5503 | 1162.5764 | 0.9738 | 1 | 7 | 34 | 5 | R.VEEMRQSLR.I |
|  | 7291 | **598.3256** | **1194.6367** | **1195.6349** | **-0.9983** | **0** | **4** | **65** | **4** | **K.APGFAHLAGLDK.M** |
|  | 8968 | **427.7138** | **1280.1196** | **1280.6482** | **-0.5286** | **1** | **0** | **86** | **4** | **-.MAALRALCGFR.G** |
|  | 9325 | **650.8257** | **1299.6369** | **1298.6540** | **0.9829** | **1** | **3** | **69** | **9** | **K.MPPGEIKVDDAK.V** |
|  | 9628 | **658.8040** | **1315.5935** | **1314.6489** | **0.9445** | **1** | **(2)** | **91** | **10** | **K.MPPGEIKVDDAK.V** |
|  | 9903 | **665.3302** | **1328.6458** | **1328.6547** | **-0.0088** | **0** | **12** | **10** | **3** | **K.TSMESLIHHFK.L** |
|  | 11268 | **469.5463** | **1405.6172** | **1406.7261** | **-1.1090** | **1** | **4** | **56** | **2** | **R.LVMELSGEMVRK.C** |
|  | 11818 | **480.2849** | **1437.8328** | **1436.8140** | **1.0188** | **1** | **6** | **33** | **1** | **K.IKAPGFAHLAGLDK.M** 11817 |
|  | 16113 | **571.3885** | **1711.1436** | **1710.9160** | **0.2276** | **1** | **3** | **42** | **2** | **R.IIAQCLNKMPPGEIK.V** |
|  | 16380 | **577.3050** | **1728.8931** | **1729.0263** | **-0.1332** | **1** | **7** | **24** | **2** | **K.LLNIRPPPRAQWIR.V** 16348 |
|  | 18493 | **944.9567** | **1887.8988** | **1887.9115** | **-0.0127** | **0** | **26** | **0.23** | **1** | **K.GEFGVYLVSDGSSRPYR.C** |
|  | 19156 | **648.3475** | **1942.0208** | **1941.0333** | **0.9875** | **0** | **14** | **3.9** | **1** | **K.NITLNFGPQHPAAHGVLR.L** |
|  | 22420 | **757.3405** | **2268.9997** | **2268.1314** | **0.8683** | **0** | **1** | **63** | **2** | **K.LYTEGYQVPPGATYTAIEAPK.G** |
|  | 23785 | **1226.5658** | **2451.1170** | **2451.1318** | **-0.0147** | **0** | **101** | **5e-009** | **1** | **R.QWQPDVEWAQQFGGAVMYPSK.E** 23786 |
|  | 23945 | 824.0619 | 2469.1638 | 2470.2526 | -1.0888 | 0 | 3 | 39 | 5 | K.GHMLADVVAIIGTQDIVFGEVDR.- |
|  | 26002 | **705.0673** | **2816.2399** | **2816.4201** | **-0.1802** | **1** | **2** | **31** | **1** | **K.MSKGHMLADVVAIIGTQDIVFGEVDR.-** 26010 |
|  | 26017 | **940.1540** | **2817.4401** | **2816.4201** | **1.0200** | **1** | **(1)** | **47** | **4** | **K.MSKGHMLADVVAIIGTQDIVFGEVDR.-** |
|  | 27793 | **793.8944** | **3171.5483** | **3171.5634** | **-0.0151** | **0** | **25** | **0.15** | **1** | **R.MHAAYIRPGGVHQDLPLGLMDDIYQFSK.N** |

| **323.** | P22307\|NLTP_HUMAN    **Mass:** 59640    **Score:** 153    **Queries matched:** 22   **emPAI:** 0.44 |
| --- | --- |
|  | Non-specific lipid-transfer protein OS=Homo sapiens GN=SCP2 PE=1 SV=2 |

|  | **Query** | **Observed** | **Mr(expt)** | **Mr(calc)** | **Delta** | **Miss** | **Score** | **Expect** | **Rank** | **Peptide** |
| --- | --- | --- | --- | --- | --- | --- | --- | --- | --- | --- |
|  | 3032 | 441.2739 | 880.5332 | 881.4872 | -0.9540 | 1 | 12 | 10 | 4 | K.IGWKNHK.H |
|  | 4356 | **490.7437** | **979.4729** | **979.5855** | **-0.1126** | **1** | **2** | **90** | **8** | **K.KIGGIFAFK.V** |
|  | 5139 | **518.7932** | **1035.5719** | **1035.5787** | **-0.0069** | **0** | **64** | **6.7e-005** | **1** | **R.VFVVGVGMTK.F** |
|  | 7395 | 600.7769 | 1199.5393 | 1198.5441 | 0.9952 | 0 | 29 | 0.19 | 1 | K.MGFPEAASSFR.T |
|  | 7713 | **406.0261** | **1215.0564** | **1214.5390** | **0.5174** | **0** | **(7)** | **20** | **4** | **K.MGFPEAASSFR.T** |
|  | 8478 | **627.7957** | **1253.5767** | **1253.5863** | **-0.0095** | **0** | **39** | **0.019** | **1** | **K.MNPQSAFFQGK.L** |
|  | 8784 | **635.8572** | **1269.6999** | **1269.7081** | **-0.0082** | **0** | **20** | **1.4** | **1** | **K.WVINPSGGLISK.G** 8785 |
|  | 9176 | **646.8249** | **1291.6352** | **1291.6992** | **-0.0640** | **1** | **5** | **43** | **6** | **K.LKITGNMGLAMK.L** |
|  | 9765 | **661.9819** | **1321.9492** | **1322.7306** | **-0.7814** | **0** | **11** | **7.8** | **1** | **K.LQNLQLQPGNAK.L** |
|  | 10020 | **668.3384** | **1334.6622** | **1334.6718** | **-0.0096** | **1** | **72** | **8.5e-006** | **1** | **K.KLEEEGEQFVK.K** 10018 |
|  | 10937 | **693.8869** | **1385.7592** | **1385.7051** | **0.0541** | **1** | **10** | **15** | **4** | **M.SSSPWEPATLRR.V** 10938 |
|  | 12997 | 756.3743 | 1510.7340 | 1510.7602 | -0.0262 | 1 | 16 | 2.8 | 3 | K.MNPQSAFFQGKLK.I |
|  | 14466 | **536.6413** | **1606.9021** | **1605.9090** | **0.9931** | **1** | **3** | **62** | **10** | **R.TIPTDKHVDLLINK.Y** |
|  | 17018 | **592.6769** | **1775.0088** | **1773.8533** | **1.1555** | **0** | **4** | **45** | **2** | **R.THQIEAVPTSSASDGFK.A** |
|  | 22211 | **746.6964** | **2237.0674** | **2237.0834** | **-0.0160** | **0** | **48** | **0.0013** | **1** | **K.GHPLGATGLAQCAELCWQLR.G** |
|  | 24403 | **507.4676** | **2532.3014** | **2531.4588** | **0.8426** | **1** | **2** | **44** | **4** | **R.QVPGAKVALQHNLGIGGAVVVTLYK.M** |
|  | 24746 | 861.1044 | 2580.2915 | 2579.1937 | 1.0978 | 1 | 1 | 46 | 4 | K.YGLSAHPVAPQMFGYAGKEHMEK.Y |
|  | 28223 | **1653.7659** | **3305.5172** | **3306.6311** | **-1.1139** | **0** | **0** | **40** | **8** | **R.AIYHSLGMTGIPIINVNNNCATGSTALFMAR.Q** |
|  | 31499 | **1357.1364** | **5424.5163** | **5424.5402** | **-0.0239** | **1** | **35** | **0.0028** | **1** | **K.SGLTPNDIDVIELHDCFSTNELLTYEALGLCPEGQGATLVDRGDNTYGGK.W** |

| **324.** | Q07954\|LRP1_HUMAN    **Mass:** 523119   **Score:** 152    **Queries matched:** 78   **emPAI:** 0.05 |
| --- | --- |
|  | Prolow-density lipoprotein receptor-related protein 1 OS=Homo sapiens GN=LRP1 PE=1 SV=1 |

|  | **Query** | **Observed** | **Mr(expt)** | **Mr(calc)** | **Delta** | **Miss** | **Score** | **Expect** | **Rank** | **Peptide** |
| --- | --- | --- | --- | --- | --- | --- | --- | --- | --- | --- |
|  | 1840 | 397.0906 | 792.1667 | 791.3861 | 0.7806 | 0 | 5 | 49 | 7 | R.CRPGFR.L |
|  | 2309 | **414.4647** | **826.9148** | **826.4297** | **0.4851** | **0** | **7** | **21** | **5** | **R.THANGSIK.R** 2305 |
|  | 3115 | **444.2681** | **886.5216** | **885.5072** | **1.0144** | **0** | **10** | **19** | **5** | **R.ILWIDAR.S** |
|  | 3777 | **470.2417** | **938.4688** | **938.4756** | **-0.0068** | **0** | **7** | **33** | **7** | **K.SGHCIPLR.W** |
|  | 4030 | **479.2378** | **956.4611** | **956.4676** | **-0.0065** | **0** | **20** | **1.5** | **1** | **R.AGTSPGTPNR.I** |
|  | 4092 | **481.2539** | **960.4933** | **959.5036** | **0.9897** | **0** | **3** | **1e+002** | **3** | **K.ADGSGSVVLR.N** |
|  | 5296 | **525.2524** | **1048.4903** | **1048.4971** | **-0.0068** | **0** | **57** | **0.00037** | **1** | **R.IEAASMSGAGR.R** |
|  | 5512 | **534.2997** | **1066.5849** | **1065.5567** | **1.0282** | **1** | **6** | **35** | **6** | **R.SGSVYRLER.G** |
|  | 6568 | **575.2723** | **1148.5301** | **1148.5318** | **-0.0017** | **0** | **20** | **1.3** | **1** | **R.CLEGACVVNK.Q** |
|  | 6943 | 589.3067 | 1176.5988 | 1176.5809 | 0.0180 | 0 | 1 | 1.2e+002 | 9 | K.IETAAMDGTLR.E |
|  | 7273 | **597.8288** | **1193.6430** | **1192.5580** | **1.0850** | **0** | **5** | **42** | **6** | **R.NSTTLVMHMK.V** |
|  | 7913 | **613.3136** | **1224.6126** | **1223.5023** | **1.1103** | **1** | **7** | **29** | **2** | **R.CDMDGQNRTK.L** |
|  | 8029 | **616.3152** | **1230.6159** | **1230.4468** | **0.1692** | **0** | **14** | **5.9** | **2** | **R.CDMDQFQCK.S** |
|  | 8148 | **619.2838** | **1236.5531** | **1236.7190** | **-0.1660** | **0** | **4** | **53** | **4** | **R.GVGGAPPTVTLLR.S** |
|  | 8832 | **637.5642** | **1273.1137** | **1273.6878** | **-0.5740** | **1** | **5** | **31** | **2** | **K.IDGTERETILK.D** |
|  | 8975 | **641.3323** | **1280.6501** | **1279.5802** | **1.0700** | **1** | **0** | **1.2e+002** | **9** | **R.NFMKTHNTCK.A** |
|  | 9309 | **650.3002** | **1298.5858** | **1297.6812** | **0.9045** | **1** | **6** | **33** | **2** | **K.TLIEGKMTHPR.A** |
|  | 9347 | **434.5623** | **1300.6650** | **1299.6670** | **0.9979** | **0** | **14** | **5.8** | **1** | **R.LNGTDPIVAADSK.R** |
|  | 9432 | **653.3267** | **1304.6388** | **1304.5707** | **0.0681** | **1** | **7** | **31** | **8** | **K.DNTTCYEFKK.F** |
|  | 9526 | 437.7396 | 1310.1971 | 1310.6765 | -0.4794 | 1 | (5) | 28 | 5 | R.IDAMDVHVKAGR.V |
|  | 9859 | **443.0394** | **1326.0963** | **1326.6714** | **-0.5751** | **1** | **6** | **25** | **3** | **R.IDAMDVHVKAGR.V** 9845 9849 9851 9862 |
|  | 10161 | **448.9229** | **1343.7469** | **1342.6517** | **1.0952** | **0** | **3** | **74** | **7** | **R.FNSTEYQVVTR.V** |
|  | 10714 | **687.8857** | **1373.7569** | **1373.7667** | **-0.0098** | **0** | **17** | **3** | **1** | **R.YVVISQGLDKPR.A** |
|  | 11172 | **700.3874** | **1398.7603** | **1398.8082** | **-0.0478** | **0** | **36** | **0.034** | **1** | **R.AALSGANVLTLIEK.D** |
|  | 12344 | **735.8612** | **1469.7079** | **1468.5671** | **1.1407** | **0** | **2** | **88** | **2** | **K.QSGDVTCNCTDGR.V** |
|  | 12417 | **492.2640** | **1473.7700** | **1472.6136** | **1.1564** | **0** | **5** | **45** | **9** | **K.CNASSQFLCSSGR.C** |
|  | 12971 | **755.3989** | **1508.7833** | **1509.6882** | **-0.9049** | **0** | **76** | **2.9e-006** | **1** | **R.MYDAQQQQVGTNK.C** |
|  | 13145 | **760.7273** | **1519.4400** | **1518.8076** | **0.6325** | **1** | **1** | **79** | **3** | **R.SQLGKATALAIMGDK.L** |
|  | 13231 | **509.2690** | **1524.7850** | **1525.6831** | **-0.8981** | **0** | **(2)** | **78** | **9** | **R.MYDAQQQQVGTNK.C** 13230 |
|  | 13363 | **767.8970** | **1533.7795** | **1533.6453** | **0.1342** | **1** | **5** | **40** | **5** | **R.QCRCTAYFEGSR.C** |
|  | 14050 | **790.8912** | **1579.7679** | **1579.5151** | **0.2528** | **0** | **16** | **3.2** | **1** | **K.CDGDHDCADGSDEK.D** 14051 |
|  | 14118 | **793.3271** | **1584.6397** | **1584.6838** | **-0.0441** | **1** | **16** | **2.6** | **1** | **K.DKSDEKPSYCNSR.R** 14145 |
|  | 15727 | **423.7151** | **1690.8313** | **1690.9406** | **-0.1094** | **1** | **8** | **19** | **7** | **R.LYWADAKLSVIGSIR.L** 15711 |
|  | 15959 | **852.4013** | **1702.7881** | **1701.7940** | **0.9941** | **0** | **48** | **0.0017** | **1** | **K.GYLFWTEWGQYPR.I** |
|  | 16541 | **870.4983** | **1738.9820** | **1738.8018** | **0.1802** | **0** | **4** | **43** | **2** | **R.MTNGAMNVEIGNPTYK.M** |
|  | 17028 | 592.9452 | 1775.8139 | 1776.9046 | -1.0907 | 0 | 2 | 74 | 9 | K.ITWPNGLTLDYVTER.I |
|  | 17266 | **896.4337** | **1790.8529** | **1790.8628** | **-0.0099** | **0** | **29** | **0.15** | **1** | **R.DGILFWTDWDASLPR.I** |
|  | 17805 | **612.9088** | **1835.7046** | **1835.7171** | **-0.0125** | **0** | **11** | **7.2** | **1** | **R.ACACAHGMLAEDGASCR.E** |
|  | 17893 | **615.2911** | **1842.8516** | **1841.8149** | **1.0367** | **1** | **6** | **26** | **1** | **R.MYDAQQQQVGTNKCR.V** |
|  | 17918 | 923.4362 | 1844.8579 | 1843.7296 | 1.1283 | 0 | 5 | 37 | 5 | K.MMPECQCPPHMTGPR.C 17896 17917 |
|  | 18059 | **619.3190** | **1854.9353** | **1855.9792** | **-1.0439** | **0** | **9** | **14** | **1** | **R.ITIVENVGSVEGLAYHR.G** |
|  | 19477 | **657.3563** | **1969.0470** | **1968.7102** | **0.3367** | **1** | **2** | **62** | **4** | **R.WKCDGEDDCGDGSDEPK.E** |
|  | 19718 | **664.6498** | **1990.9277** | **1991.6932** | **-0.7655** | **1** | **1** | **70** | **8** | **R.WRCDGDTDCMDSSDEK.S** |
|  | 20718 | **692.7061** | **2075.0965** | **2075.1085** | **-0.0120** | **0** | **24** | **0.34** | **1** | **K.TLISGMIDEPHAIVVDPLR.G** |
|  | 21406 | **538.7613** | **2151.0163** | **2150.9118** | **0.1045** | **1** | **2** | **59** | **3** | **R.CDMDQFQCKSGHCIPLR.W** |
|  | 21610 | 726.0033 | 2174.9881 | 2174.8997 | 0.0884 | 0 | 2 | 50 | 7 | K.LYWTDGDNISMANMDGSNR.T |
|  | 22432 | **758.0077** | **2271.0012** | **2271.0161** | **-0.0149** | **0** | **31** | **0.061** | **1** | **R.SGFHTVPGQPGCQDINECLR.F** |
|  | 22545 | **761.6798** | **2282.0176** | **2283.1610** | **-1.1433** | **1** | **0** | **71** | **9** | **K.GIALDPAMGKVFFTDYGQIPK.V** |
|  | 25076 | **887.0467** | **2658.1182** | **2658.1910** | **-0.0728** | **1** | **1** | **38** | **1** | **R.GMDMGAKVPDEHMIPIENLMNPR.A** |
|  | 25621 | **918.1060** | **2751.2962** | **2752.2374** | **-0.9411** | **1** | **2** | **41** | **8** | **R.GTMYWSDWGNHPKIETAAMDGTLR.E** |
|  | 26625 | **974.7783** | **2921.3131** | **2921.3330** | **-0.0199** | **1** | **1** | **40** | **1** | **R.AIVVDPLNGWMYWTDWEEDPKDSR.R** |
|  | 26667 | **732.8791** | **2927.4873** | **2926.4825** | **1.0048** | **1** | **0** | **52** | **1** | **K.LNLDGSNYTLLKQGLNNAVALDFDYR.E** |
|  | 27240 | 1013.1973 | 3036.5702 | 3035.5029 | 1.0673 | 0 | 4 | 21 | 3 | R.VEGIAVDWIAGNIYWTDQGFDVIEVAR.L 27226 27227 27232 27242 27243 |
|  | 27408 | 1023.1721 | 3066.4944 | 3065.3859 | 1.1085 | 1 | 3 | 26 | 2 | K.LYWTDGDNISMANMDGSNRTLLFSGQK.G 27407 |
|  | 27428 | **1024.3173** | **3069.9300** | **3070.3266** | **-0.3966** | **0** | **3** | **20** | **6** | **R.QPMAPNPCEANGGQGPCSHLCLINYNR.T** |
|  | 27700 | 1049.5269 | 3145.5588 | 3144.3924 | 1.1664 | 1 | 1 | 40 | 10 | R.SHACENDQYGKPGGCSDICLLANSHKAR.T |
|  | 29705 | **1274.8874** | **3821.6405** | **3820.9312** | **0.7093** | **1** | **9** | **3.3** | **2** | **K.GDYSVLVPGLRNTIALDFHLSQSALYWTDVVEDK.I** 29672 |
|  | 30045 | 993.4931 | 3969.9433 | 3969.6479 | 0.2954 | 1 | 4 | 13 | 3 | K.GTNPCSVNNGDCSQLCLPTSETTRSCMCTAGYSLR.S |
|  | 30708 | **1082.0306** | **4324.0935** | **4324.7260** | **-0.6326** | **1** | **0** | **24** | **4** | **R.VAPSCLTCVGHCSNGGSCTMNSKMMPECQCPPHMTGPR.C** |
|  | 31712 | **1418.6508** | **5670.5739** | **5670.5832** | **-0.0092** | **1** | **4** | **3.7** | **1** | **K.MYEGGEPDDVGGLLDADFALDPDKPTNFTNPVYATLYMGGHGSRHSLASTDEK.R** |

| **325.** | P60033\|CD81_HUMAN    **Mass:** 26476    **Score:** 151    **Queries matched:** 8   **emPAI:** 0.49 |
| --- | --- |
|  | CD81 antigen OS=Homo sapiens GN=CD81 PE=1 SV=1 |

|  | **Query** | **Observed** | **Mr(expt)** | **Mr(calc)** | **Delta** | **Miss** | **Score** | **Expect** | **Rank** | **Peptide** |
| --- | --- | --- | --- | --- | --- | --- | --- | --- | --- | --- |
|  | 3497 | **459.0119** | **916.0092** | **915.5025** | **0.5067** | **1** | **15** | **4.5** | **1** | **K.DQIAKDVK.Q** 3493 |
|  | 17055 | **889.4422** | **1776.8698** | **1776.8828** | **-0.0130** | **0** | **61** | **8.5e-005** | **1** | **K.NNLCPSGSNIISNLFK.E** 17046 17056 |
|  | 22301 | **1127.0169** | **2252.0191** | **2252.0345** | **-0.0154** | **0** | **58** | **0.00013** | **1** | **K.QFYDQALQQAVVDDDANNAK.A** 22300 22302 |

| **326.** | P17066\|HSP76_HUMAN    **Mass:** 71440    **Score:** 151    **Queries matched:** 21   **emPAI:** 0.16 |
| --- | --- |
|  | Heat shock 70 kDa protein 6 OS=Homo sapiens GN=HSPA6 PE=1 SV=2 |

|  | **Query** | **Observed** | **Mr(expt)** | **Mr(calc)** | **Delta** | **Miss** | **Score** | **Expect** | **Rank** | **Peptide** |
| --- | --- | --- | --- | --- | --- | --- | --- | --- | --- | --- |
|  | 1328 | **618.3778** | **617.3705** | **617.2955** | **0.0750** | **0** | **5** | **90** | **2** | **-.MQAPR.E** |
|  | 2017 | 804.4382 | 803.4309 | 803.4389 | -0.0080 | 0 | 23 | 1.1 | 1 | K.ITITNDK.G |
|  | 2187 | **410.0945** | **818.1744** | **817.3566** | **0.8178** | **0** | **9** | **20** | **3** | **K.AEDEAQR.D** |
|  | 4472 | 495.2636 | 988.5127 | 988.5189 | -0.0062 | 1 | 12 | 13 | 3 | R.LSKEEVER.M |
|  | 4891 | 509.2687 | 1016.5228 | 1016.5614 | -0.0386 | 1 | 8 | 29 | 7 | K.ITITNDKGR.L 4890 |
|  | 4918 | **510.2898** | **1018.5650** | **1017.5091** | **1.0560** | **0** | **3** | **1e+002** | **7** | **K.GSLQEESLR.D** |
|  | 7971 | 614.8130 | 1227.6115 | 1227.6207 | -0.0092 | 0 | 24 | 0.56 | 1 | R.VEILANDQGNR.T |
|  | 8366 | 416.5560 | 1246.6462 | 1247.6292 | -0.9830 | 1 | 12 | 9.3 | 4 | R.AMTKDNNLLGR.F |
|  | 8556 | 420.2212 | 1257.6418 | 1257.5547 | 0.0871 | 0 | 5 | 44 | 2 | K.FADTTVQSDMK.H 8541 |
|  | 9458 | 653.8409 | 1305.6672 | 1304.6612 | 1.0060 | 0 | 6 | 32 | 6 | K.ETAEAYLGQPVK.H |
|  | 12631 | 744.3494 | 1486.6842 | 1486.6940 | -0.0098 | 0 | 65 | 4.2e-005 | 1 | R.TTPSYVAFTDTER.L 12606 12630 |
|  | 12721 | **498.3363** | **1491.9872** | **1492.7245** | **-0.7373** | **1** | **12** | **6.5** | **1** | **R.LVNHFMEEFRR.K** |
|  | 15136 | **550.8161** | **1649.4265** | **1648.9049** | **0.5216** | **1** | **2** | **47** | **7** | **R.VAAKNSLEAHVFHVK.G** |
|  | 15498 | 838.3627 | 1674.7108 | 1674.7234 | -0.0126 | 0 | 65 | 3.5e-005 | 1 | K.ATAGDTHLGGEDFDNR.L |
|  | 17908 | **615.6389** | **1843.8949** | **1842.9951** | **0.8998** | **1** | **5** | **35** | **1** | **R.IINEPTAAAIAYGLDRR.G** |
|  | 21313 | 1071.5262 | 2141.0379 | 2141.0641 | -0.0261 | 1 | 1 | 73 | 6 | R.TTPSYVAFTDTERLVGDAAK.S |
|  | 26966 | 994.5695 | 2980.6866 | 2980.4553 | 0.2312 | 0 | 2 | 25 | 3 | R.TLSSSTQATLEIDSLFEGVDFYTSITR.A |

|  | **Proteins matching a subset of these peptides:** |
| --- | --- |

|  | P48741\|HSP77_HUMAN    **Mass:** 40448    **Score:** 151    **Queries matched:** 11 |
| --- | --- |
|  | Putative heat shock 70 kDa protein 7 OS=Homo sapiens GN=HSPA7 PE=5 SV=2 |

| **327.** | P05556\|ITB1_HUMAN    **Mass:** 91664    **Score:** 151    **Queries matched:** 27   **emPAI:** 0.17 |
| --- | --- |
|  | Integrin beta-1 OS=Homo sapiens GN=ITGB1 PE=1 SV=2 |

|  | **Query** | **Observed** | **Mr(expt)** | **Mr(calc)** | **Delta** | **Miss** | **Score** | **Expect** | **Rank** | **Peptide** |
| --- | --- | --- | --- | --- | --- | --- | --- | --- | --- | --- |
|  | 1211 | **584.3717** | **583.3644** | **583.3693** | **-0.0049** | **0** | **15** | **3.3** | **3** | **K.NLIPK.S** |
|  | 3175 | **447.7307** | **893.4469** | **893.4719** | **-0.0250** | **1** | **5** | **50** | **9** | **R.RITSDFR.I** |
|  | 3997 | 477.7365 | 953.4585 | 953.5909 | -0.1325 | 1 | 5 | 43 | 5 | K.ELKNLIPK.S |
|  | 4390 | **492.2602** | **982.5059** | **982.5124** | **-0.0064** | **0** | **17** | **3.2** | **1** | **R.IGFGSFVEK.T** |
|  | 8708 | 633.7903 | 1265.5661 | 1265.5744 | -0.0083 | 0 | 25 | 0.4 | 1 | K.SLGTDLMNEMR.R |
|  | 8998 | **641.9232** | **1281.8318** | **1281.5693** | **0.2625** | **0** | **(8)** | **17** | **1** | **K.SLGTDLMNEMR.R** |
|  | 11132 | **699.3991** | **1396.7837** | **1396.6405** | **0.1432** | **1** | **10** | **13** | **1** | **K.KGCPPDDIENPR.G** |
|  | 11824 | 480.5630 | 1438.6672 | 1437.6704 | 0.9968 | 1 | 2 | 75 | 5 | K.SLGTDLMNEMRR.I |
|  | 13382 | **768.7628** | **1535.5110** | **1535.5228** | **-0.0118** | **0** | **17** | **1.7** | **1** | **K.FCECDNFNCDR.S** |
|  | 14538 | **538.2941** | **1611.8606** | **1611.7060** | **0.1546** | **1** | **1** | **98** | **1** | **K.SYCKNGVNGTGENGR.K** |
|  | 15608 | 561.6345 | 1681.8817 | 1681.7770 | 0.1048 | 1 | 4 | 47 | 7 | K.MNAKWDTGENPIYK.S |
|  | 15610 | 561.6544 | 1681.9413 | 1682.6885 | -0.7472 | 1 | 14 | 4 | 2 | R.GICECGVCKCTDPK.F 15611 |
|  | 15941 | **851.7749** | **1701.5352** | **1701.8726** | **-0.3374** | **1** | **1** | **61** | **1** | **R.ITSDFRIGFGSFVEK.T** |
|  | 17910 | **615.6447** | **1843.9123** | **1843.9251** | **-0.0128** | **1** | **12** | **6.8** | **1** | **R.DKLPQPVQPDPVSHCK.E** |
|  | 18562 | **631.9792** | **1892.9157** | **1893.8237** | **-0.9080** | **1** | **1** | **88** | **5** | **K.KDTCTQECSYFNITK.V** |
|  | 18875 | 960.0449 | 1918.0752 | 1918.7826 | -0.7074 | 0 | 2 | 55 | 6 | R.NPCTSEQNCTSPFSYK.N |
|  | 20026 | **1010.0778** | **2018.1410** | **2018.1524** | **-0.0114** | **0** | **54** | **0.00033** | **1** | **K.LKPEDITQIQPQQLVLR.L** 20024 20025 |
|  | 23742 | **815.4562** | **2443.3467** | **2442.9848** | **0.3620** | **0** | **1** | **51** | **7** | **K.FQGQTCEMCQTCLGVCAEHK.E** |
|  | 23949 | **1235.6223** | **2469.2301** | **2469.2427** | **-0.0127** | **0** | **85** | **2.3e-007** | **1** | **K.LSENNIQTIFAVTEEFQPVYK.E** 23936 23950 |
|  | 27291 | 1015.8762 | 3044.6067 | 3044.2378 | 0.3689 | 1 | 4 | 21 | 6 | K.CTDPKFQGQTCEMCQTCLGVCAEHK.E |
|  | 29454 | **752.5183** | **3757.5552** | **3756.4274** | **1.1278** | **0** | **0** | **23** | **2** | **R.VCECNPNYTGSACDCSLDTSTCEASNGQICNGR.G** |
|  | 30525 | **836.3922** | **4176.9247** | **4176.9496** | **-0.0249** | **0** | **2** | **14** | **2** | **K.LGGIVLPNDGQCHLENNMYTMSHYYDYPSIAHLVQK.L** |

| **328.** | O95479\|G6PE_HUMAN    **Mass:** 89407    **Score:** 150    **Queries matched:** 23   **emPAI:** 0.08 |
| --- | --- |
|  | GDH/6PGL endoplasmic bifunctional protein OS=Homo sapiens GN=H6PD PE=1 SV=2 |

|  | **Query** | **Observed** | **Mr(expt)** | **Mr(calc)** | **Delta** | **Miss** | **Score** | **Expect** | **Rank** | **Peptide** |
| --- | --- | --- | --- | --- | --- | --- | --- | --- | --- | --- |
|  | 3552 | **461.2880** | **920.5614** | **919.4433** | **1.1181** | **0** | **4** | **73** | **9** | **K.QGQELMAK.A** |
|  | 4411 | **493.2389** | **984.4633** | **984.4698** | **-0.0065** | **0** | **18** | **2.4** | **1** | **K.EMEGPPGLR.L** |
|  | 4468 | **495.2525** | **988.4904** | **987.5899** | **0.9005** | **1** | **3** | **1e+002** | **9** | **K.KVAVLVMGR.M** |
|  | 5605 | 537.3102 | 1072.6059 | 1071.5825 | 1.0234 | 1 | 9 | 23 | 2 | R.KALDGLWNR.H |
|  | 10296 | **676.3514** | **1350.6882** | **1349.7051** | **0.9831** | **0** | **5** | **48** | **2** | **K.DIEAQLQHAGLR.E** |
|  | 10437 | **680.3566** | **1358.6986** | **1357.7751** | **0.9235** | **1** | **11** | **12** | **1** | **R.MSLSLPLINRAK.K** |
|  | 10776 | **689.8489** | **1377.6833** | **1378.6955** | **-1.0122** | **0** | **4** | **53** | **7** | **R.WEGVPFILMSGK.A** |
|  | 13266 | **509.9245** | **1526.7517** | **1526.7630** | **-0.0113** | **0** | **15** | **4.1** | **1** | **R.GHSFSFHGAALTAPK.Q** |
|  | 14201 | 530.6158 | 1588.8257 | 1588.8130 | 0.0127 | 1 | 9 | 16 | 8 | R.VEIIMKETVDAEGR.T 14181 14209 |
|  | 16772 | **879.4202** | **1756.8258** | **1756.8380** | **-0.0122** | **0** | **71** | **8e-006** | **1** | **R.GSAVVGQYQSYSEQVR.R** 16771 |
|  | 18343 | 625.3349 | 1872.9829 | 1872.9707 | 0.0122 | 1 | 8 | 15 | 5 | R.ERDAHSVLLSHIFHGR.K |
|  | 18715 | 635.9896 | 1904.9470 | 1904.9335 | 0.0135 | 1 | 4 | 36 | 4 | K.QGQELMAKALESLSCPK.D |
|  | 19077 | **484.7615** | **1935.0171** | **1933.9614** | **1.0556** | **0** | **10** | **10** | **1** | **R.IFYFSVPPFAYEDIAR.N** 19074 |
|  | 20072 | **506.2646** | **2021.0294** | **2021.0305** | **-0.0011** | **0** | **2** | **54** | **4** | **R.IPYYNIHPMPVHLQQR.L** |
|  | 23542 | **805.7256** | **2414.1549** | **2413.2212** | **0.9337** | **1** | **2** | **43** | **7** | **R.NLFRPSLPSSWKEMEGPPGLR.L** |
|  | 24077 | 828.7479 | 2483.2219 | 2483.2404 | -0.0185 | 1 | 71 | 6e-006 | 1 | K.TAEDYQALNKDIEAQLQHAGLR.E |
|  | 26802 | **737.8922** | **2947.5397** | **2947.5556** | **-0.0159** | **0** | **6** | **12** | **1** | **R.ELQKPDSFHSLTPTFAAVLVHIDNLR.W** |
|  | 30806 | **1471.5951** | **4411.7634** | **4411.2962** | **0.4673** | **1** | **(0)** | **14** | **5** | **-.MWNMLIVAMCLALLGCLQAQELQGHVSIILLGATGDLAKK.Y** |
|  | 30826 | 1476.3877 | 4426.1413 | 4427.2911 | -1.1498 | 1 | 2 | 14 | 3 | -.MWNMLIVAMCLALLGCLQAQELQGHVSIILLGATGDLAKK.Y |

| **329.** | P39656\|OST48_HUMAN    **Mass:** 50940    **Score:** 149    **Queries matched:** 15   **emPAI:** 0.53 |
| --- | --- |
|  | Dolichyl-diphosphooligosaccharide--protein glycosyltransferase 48 kDa subunit OS=Homo sapiens GN=DDOST PE=1 SV=4 |

|  | **Query** | **Observed** | **Mr(expt)** | **Mr(calc)** | **Delta** | **Miss** | **Score** | **Expect** | **Rank** | **Peptide** |
| --- | --- | --- | --- | --- | --- | --- | --- | --- | --- | --- |
|  | 4480 | **495.7450** | **989.4754** | **989.4964** | **-0.0209** | **1** | **3** | **90** | **8** | **R.KMEPSTAAR.A** |
|  | 5138 | **518.7929** | **1035.5713** | **1035.5138** | **0.0575** | **0** | **6** | **38** | **1** | **R.ETHSLFFR.S** |
|  | 5268 | 523.8002 | 1045.5858 | 1045.5920 | -0.0062 | 0 | 48 | 0.0031 | 1 | K.SSLNPILFR.G |
|  | 6847 | **585.3500** | **1168.6854** | **1168.6928** | **-0.0074** | **0** | **43** | **0.0073** | **1** | **K.NTLLIAGLQAR.N** 6848 |
|  | 8666 | 632.3288 | 1262.6430 | 1261.6819 | 0.9611 | 1 | 53 | 0.00071 | 1 | R.WVFKEEGVLR.V 8650 8651 |
|  | 8765 | **635.3762** | **1268.7378** | **1268.7452** | **-0.0075** | **0** | **23** | **0.64** | **1** | **R.TLVLLDNLNVR.E** |
|  | 15439 | **836.4148** | **1670.8150** | **1670.8264** | **-0.0113** | **0** | **28** | **0.2** | **1** | **R.YSQTGNYELAVALSR.W** |
|  | 29468 | **942.2444** | **3764.9484** | **3763.8896** | **1.0588** | **1** | **0** | **32** | **2** | **R.FIPSAYPYYASAFSMMLGLFIFSIVFLHMKEK.E** |
|  | 30736 | **1445.3857** | **4333.1354** | **4333.1617** | **-0.0263** | **0** | **(49)** | **0.00028** | **1** | **R.GVGMVADPDNPLVLDILTGSSTSYSFFPDKPITQYPHAVGK.N** 30737 30739 |
|  | 30758 | **1088.2922** | **4349.1399** | **4349.1566** | **-0.0168** | **0** | **60** | **1.9e-005** | **1** | **R.GVGMVADPDNPLVLDILTGSSTSYSFFPDKPITQYPHAVGK.N** |

| **330.** | Q86UE4\|LYRIC_HUMAN    **Mass:** 63856    **Score:** 149    **Queries matched:** 12   **emPAI:** 0.06 |
| --- | --- |
|  | Protein LYRIC OS=Homo sapiens GN=MTDH PE=1 SV=2 |

|  | **Query** | **Observed** | **Mr(expt)** | **Mr(calc)** | **Delta** | **Miss** | **Score** | **Expect** | **Rank** | **Peptide** |
| --- | --- | --- | --- | --- | --- | --- | --- | --- | --- | --- |
|  | 5760 | **543.8024** | **1085.5903** | **1084.5513** | **1.0390** | **0** | **4** | **76** | **3** | **K.QNSVPPSQTK.S** |
|  | 6270 | **563.5970** | **1125.1795** | **1124.6302** | **0.5494** | **0** | **1** | **83** | **9** | **K.LSEKPKPNGR.T** |
|  | 6759 | 582.2786 | 1162.5426 | 1161.5738 | 0.9688 | 1 | 2 | 1e+002 | 5 | K.SDAKAVQNSSR.H |
|  | 8131 | **412.8777** | **1235.6114** | **1236.6536** | **-1.0422** | **0** | **6** | **39** | **2** | **R.EMLSVGLGFLR.T** |
|  | 10442 | 453.9352 | 1358.7836 | 1358.7293 | 0.0543 | 0 | 5 | 43 | 4 | K.TISTSDPAEVLVK.N |
|  | 12930 | **502.9343** | **1505.7811** | **1505.8388** | **-0.0577** | **1** | **5** | **35** | **4** | **R.LREMLSVGLGFLR.T** 12949 |
|  | 17406 | **902.9022** | **1803.7899** | **1803.8023** | **-0.0124** | **0** | **91** | **8.2e-008** | **1** | **R.SWQDELAQQAEEGSAR.L** 17405 |
|  | 18884 | **960.9570** | **1919.8994** | **1920.0316** | **-0.1322** | **1** | **5** | **32** | **2** | **K.REEAAAVPAAAPDDLALLK.N** |
|  | 24095 | **829.7194** | **2486.1363** | **2486.3454** | **-0.2092** | **1** | **3** | **33** | **3** | **R.EMLSVGLGFLRTELGLDLGLEPK.R** |
|  | 26176 | 949.7929 | 2846.3569 | 2847.3635 | -1.0066 | 1 | 1 | 44 | 9 | K.SGKGDSTLQVSSGLNENLTVNGGGWNEK.S |

| **331.** | O94826\|TOM70_HUMAN    **Mass:** 68096    **Score:** 149    **Queries matched:** 30   **emPAI:** 0.38 |
| --- | --- |
|  | Mitochondrial import receptor subunit TOM70 OS=Homo sapiens GN=TOMM70A PE=1 SV=1 |

|  | **Query** | **Observed** | **Mr(expt)** | **Mr(calc)** | **Delta** | **Miss** | **Score** | **Expect** | **Rank** | **Peptide** |
| --- | --- | --- | --- | --- | --- | --- | --- | --- | --- | --- |
|  | 1115 | 559.3773 | 558.3700 | 559.2966 | -0.9266 | 0 | 14 | 9.8 | 1 | K.EANVK.L 1117 |
|  | 1360 | **629.3362** | **628.3289** | **628.3908** | **-0.0619** | **0** | **22** | **1.5** | **3** | **R.ANALIK.R** |
|  | 1978 | **401.7296** | **801.4445** | **802.4297** | **-0.9852** | **1** | **8** | **36** | **7** | **R.GDASGLKR.N** |
|  | 2340 | **415.5761** | **829.1376** | **828.4817** | **0.6559** | **1** | **3** | **79** | **7** | **K.EANVKLR.A** |
|  | 2640 | 428.2288 | 854.4430 | 853.4004 | 1.0426 | 0 | 5 | 42 | 8 | K.AIDMFNK.A |
|  | 4423 | **493.2924** | **984.5702** | **984.5756** | **-0.0055** | **0** | **30** | **0.18** | **1** | **K.GLLQLQWK.Q** |
|  | 4733 | **503.2684** | **1004.5223** | **1004.5291** | **-0.0068** | **0** | **29** | **0.24** | **1** | **R.AAAFEQLQK.W** |
|  | 5664 | **540.2960** | **1078.5774** | **1078.5845** | **-0.0071** | **0** | **45** | **0.0046** | **1** | **K.YMAEALLLR.A** |
|  | 6154 | 558.7985 | 1115.5824 | 1116.5662 | -0.9839 | 1 | 5 | 49 | 8 | K.DKEGEALEVK.E |
|  | 9073 | 643.8594 | 1285.7043 | 1285.6740 | 0.0303 | 0 | 4 | 61 | 10 | R.EPLMPSPQFIK.S 9041 |
|  | 9540 | **656.3680** | **1310.7214** | **1310.7306** | **-0.0092** | **0** | **52** | **0.00082** | **1** | **R.LRPESALAQAQK.C** 9539 |
|  | 13500 | **515.3255** | **1542.9547** | **1541.8664** | **1.0882** | **1** | **7** | **22** | **9** | **R.GLELISKAIEIDNK.C** |
|  | 16096 | **856.4004** | **1710.7862** | **1710.7995** | **-0.0133** | **0** | **58** | **0.00018** | **1** | **R.QAYTGNNSSQIQAAMK.G** |
|  | 19569 | **990.4722** | **1978.9298** | **1977.9394** | **0.9904** | **0** | **48** | **0.0017** | **1** | **K.SYFSSFTDDIISQPMLK.G** |
|  | 21244 | **1068.0140** | **2134.0135** | **2134.0252** | **-0.0117** | **0** | **7** | **16** | **1** | **K.ILLDQVEEAVADFDECIR.L** |
|  | 21524 | **722.6452** | **2164.9138** | **2164.9292** | **-0.0155** | **1** | **11** | **6** | **1** | **K.QYMEEENYDKIISECSK.E** |
|  | 22368 | **1131.5198** | **2261.0250** | **2261.0423** | **-0.0173** | **0** | **35** | **0.025** | **1** | **R.CAEGYALYAQALTDQQQFGK.A** |
|  | 22637 | **765.3706** | **2293.0900** | **2292.0984** | **0.9916** | **1** | **15** | **2.5** | **1** | **K.SYFSSFTDDIISQPMLKGEK.S** |
|  | 24705 | 859.0994 | 2574.2763 | 2574.4421 | -0.1658 | 1 | 5 | 21 | 2 | R.ATFYLLIGNANAAKPDLDKVISLK.E |
|  | 24924 | 873.7646 | 2618.2719 | 2618.1992 | 0.0727 | 1 | 2 | 38 | 5 | K.AIEIDNKCDFAYETMGTIEVQR.G |
|  | 26664 | **976.1304** | **2925.3695** | **2925.3424** | **0.0271** | **1** | **1** | **40** | **4** | **K.TPEGRASPAPGSGHPEGPGAHLDMNSLDR.A** 26663 |
|  | 28408 | **1118.2262** | **3351.6568** | **3350.6560** | **1.0008** | **1** | **0** | **42** | **9** | **K.ECLEDVTAVCILEGFQNQQSMLLADKVLK.L** |
|  | 29968 | **988.1913** | **3948.7360** | **3948.7618** | **-0.0258** | **0** | **36** | **0.0069** | **1** | **R.GSMYMQQQQPLLSTQDFNMAADIDPQNADVYHHR.G** 29967 |
|  | 30306 | **821.9752** | **4104.8397** | **4104.8629** | **-0.0232** | **1** | **7** | **4.7** | **1** | **K.RGSMYMQQQQPLLSTQDFNMAADIDPQNADVYHHR.G** |
|  | 30407 | **1035.2720** | **4137.0588** | **4136.8527** | **0.2061** | **1** | **(0)** | **24** | **6** | **K.RGSMYMQQQQPLLSTQDFNMAADIDPQNADVYHHR.G** |

| **332.** | P80404\|GABT_HUMAN    **Mass:** 57087    **Score:** 148    **Queries matched:** 35   **emPAI:** 0.37 |
| --- | --- |
|  | 4-aminobutyrate aminotransferase, mitochondrial OS=Homo sapiens GN=ABAT PE=1 SV=3 |

|  | **Query** | **Observed** | **Mr(expt)** | **Mr(calc)** | **Delta** | **Miss** | **Score** | **Expect** | **Rank** | **Peptide** |
| --- | --- | --- | --- | --- | --- | --- | --- | --- | --- | --- |
|  | 3798 | **471.2526** | **940.4907** | **939.6229** | **0.8678** | **1** | **5** | **48** | **6** | **R.NKLILIAR.N** |
|  | 3811 | **471.2975** | **940.5803** | **939.6229** | **0.9575** | **1** | **4** | **59** | **10** | **K.LILIARNK.G** |
|  | 4869 | **508.7516** | **1015.4887** | **1015.4949** | **-0.0062** | **0** | **12** | **13** | **4** | **K.TIFMWYR.S** |
|  | 4971 | **513.2523** | **1024.4901** | **1023.5277** | **0.9624** | **0** | **2** | **85** | **5** | **K.YPLEEFVK.E** |
|  | 5555 | 536.1636 | 1070.3126 | 1070.4678 | -0.1552 | 0 | 3 | 56 | 1 | K.MMTGGFFHK.E |
|  | 7398 | **600.8228** | **1199.6309** | **1198.5627** | **1.0682** | **1** | **(1)** | **1.3e+002** | **3** | **K.KMMTGGFFHK.E** |
|  | 7468 | **602.3072** | **1202.5999** | **1202.6078** | **-0.0078** | **1** | **25** | **0.48** | **1** | **R.NKGVVLGGCGDK.S** 7493 |
|  | 7534 | **603.7800** | **1205.5454** | **1205.5533** | **-0.0079** | **0** | **16** | **3.7** | **1** | **R.TMGCLATTHSK.A** |
|  | 7715 | **406.0262** | **1215.0568** | **1214.5576** | **0.4992** | **1** | **7** | **23** | **3** | **K.KMMTGGFFHK.E** 7686 7714 |
|  | 8687 | **633.2604** | **1264.5063** | **1264.7067** | **-0.2004** | **1** | **2** | **76** | **7** | **R.LKYPLEEFVK.E** |
|  | 8758 | **635.3182** | **1268.6219** | **1267.5768** | **1.0451** | **0** | **5** | **46** | **10** | **R.LACSFQHSYR.L** |
|  | 9017 | **642.3839** | **1282.7531** | **1282.7609** | **-0.0077** | **0** | **1** | **1.1e+002** | **1** | **K.ALLTGLLDLQAR.Y** |
|  | 11298 | **704.8489** | **1407.6833** | **1407.7218** | **-0.0385** | **1** | **12** | **8.3** | **2** | **K.REDLLNNAAHAGK.A** |
|  | 13259 | **764.3504** | **1526.6862** | **1526.6963** | **-0.0101** | **0** | **64** | **4.4e-005** | **1** | **K.VDVEFDYDGPLMK.T** |
|  | 14610 | **809.3408** | **1616.6670** | **1616.6777** | **-0.0108** | **0** | **11** | **9.5** | **1** | **R.GTFCSFDTPDDSIR.N** |
|  | 15445 | **418.7357** | **1670.9135** | **1670.8232** | **0.0903** | **1** | **5** | **39** | **9** | **R.TMGCLATTHSKAIHK.I** |
|  | 16960 | **885.9601** | **1769.9057** | **1769.9141** | **-0.0084** | **0** | **18** | **1.7** | **1** | **K.IDIPSFDWPIAPFPR.L** |
|  | 18152 | **620.8046** | **1859.3921** | **1858.8156** | **0.5765** | **1** | **1** | **59** | **4** | **R.GTFCSFDTPDDSIRNK.L** |
|  | 21724 | **729.8875** | **2186.6407** | **2186.9428** | **-0.3021** | **0** | **(1)** | **47** | **4** | **K.GMSQLITMACGSCSNENALK.T** |
|  | 21836 | 735.3718 | 2203.0935 | 2202.9377 | 0.1557 | 0 | 8 | 15 | 2 | K.GMSQLITMACGSCSNENALK.T |
|  | 23652 | **811.4298** | **2431.2676** | **2431.2821** | **-0.0145** | **0** | **48** | **0.0011** | **1** | **R.MLDLYSQISSVPIGYSHPALLK.L** 23651 |
|  | 25155 | 892.0908 | 2673.2505 | 2673.2671 | -0.0166 | 0 | 22 | 0.41 | 1 | K.TVAGIIVEPIQSEGGDNHASDDFFR.K |
|  | 27637 | 1564.6960 | 3127.3775 | 3126.4817 | 0.8958 | 1 | 2 | 29 | 5 | R.QSLLSVAPKGMSQLITMACGSCSNENALK.T 27640 |
|  | 27730 | **1050.8909** | **3149.6508** | **3149.6695** | **-0.0188** | **0** | **22** | **0.28** | **1** | **K.LIQQPQNASMFVNRPALGILPPENFVEK.L** |
|  | 29429 | **1250.5491** | **3748.6254** | **3748.6419** | **-0.0165** | **0** | **64** | **1.3e-005** | **1** | **R.GFSQEELETCMINQAPGCPDYSILSFMGAFHGR.T** |
|  | 30332 | **822.2545** | **4106.2362** | **4105.8179** | **0.4183** | **1** | **5** | **6.5** | **3** | **R.GQRGFSQEELETCMINQAPGCPDYSILSFMGAFHGR.T** 30321 30322 |
|  | 30333 | 1027.5669 | 4106.2385 | 4105.8179 | 0.4205 | 1 | (2) | 12 | 3 | R.GQRGFSQEELETCMINQAPGCPDYSILSFMGAFHGR.T 30311 |

| **333.** | P49448\|DHE4_HUMAN    **Mass:** 61738    **Score:** 148    **Queries matched:** 27   **emPAI:** 0.34 |
| --- | --- |
|  | Glutamate dehydrogenase 2, mitochondrial OS=Homo sapiens GN=GLUD2 PE=1 SV=2 |

|  | **Query** | **Observed** | **Mr(expt)** | **Mr(calc)** | **Delta** | **Miss** | **Score** | **Expect** | **Rank** | **Peptide** |
| --- | --- | --- | --- | --- | --- | --- | --- | --- | --- | --- |
|  | 2299 | 413.7705 | 825.5265 | 825.5072 | 0.0193 | 1 | 15 | 4.9 | 7 | K.AGVKINPK.N 2304 |
|  | 2457 | 839.4550 | 838.4477 | 838.4259 | 0.0219 | 0 | 11 | 12 | 6 | R.FTMELAK.K 2454 |
|  | 4034 | 479.2498 | 956.4850 | 955.5127 | 0.9723 | 1 | 6 | 42 | 4 | R.LTFKYER.D |
|  | 4592 | 499.2697 | 996.5248 | 996.5314 | -0.0066 | 0 | 33 | 0.074 | 1 | K.ALASLMTYK.C |
|  | 4830 | 507.2892 | 1012.5639 | 1013.5618 | -0.9978 | 1 | 14 | 5.9 | 1 | K.QLTKSNAPR.V |
|  | 5410 | 530.2664 | 1058.5183 | 1058.5257 | -0.0074 | 0 | 23 | 0.86 | 1 | K.NLNHVSYGR.L |
|  | 5662 | 540.2866 | 1078.5587 | 1078.5658 | -0.0072 | 0 | 40 | 0.015 | 1 | R.TAAYVNAIEK.V |
|  | 8231 | 621.2943 | 1240.5741 | 1240.5823 | -0.0083 | 0 | 37 | 0.027 | 1 | R.YSTDVSVDEVK.A 8229 8230 |
|  | 11609 | 713.3135 | 1424.6125 | 1424.6208 | -0.0083 | 0 | 15 | 4.5 | 1 | R.DDGSWEVIEGYR.A 11607 |
|  | 14877 | **545.3369** | **1632.9887** | **1633.9012** | **-0.9125** | **1** | **3** | **52** | **8** | **R.GQPAAASQPGLALAARR.H** |
|  | 16644 | 874.9427 | 1747.8708 | 1747.8828 | -0.0120 | 0 | 36 | 0.031 | 1 | K.TFVVQGFGNVGLHSMR.Y 16639 |
|  | 16886 | 882.3986 | 1762.7826 | 1763.8778 | -1.0952 | 0 | (3) | 59 | 3 | K.TFVVQGFGNVGLHSMR.Y |
|  | 17339 | 600.4647 | 1798.3722 | 1798.8560 | -0.4838 | 1 | 0 | 61 | 1 | K.MVEGFFDRGASIVEDK.L |
|  | 18565 | 632.0254 | 1893.0545 | 1893.0658 | -0.0113 | 0 | 10 | 10 | 1 | R.IIKPCNHVLSLSFPIR.R |
|  | 18666 | **634.5947** | **1900.7622** | **1901.9483** | **-1.1861** | **0** | **1** | **73** | **4** | **R.DSNYHLLLSVQESLER.K** |
|  | 18970 | 643.3198 | 1926.9376 | 1927.9098 | -0.9722 | 0 | 6 | 26 | 6 | K.GFIGPGVDVPAPDMNTGER.E 19000 |
|  | 22235 | 1121.5808 | 2241.1471 | 2241.1641 | -0.0170 | 1 | 78 | 1.2e-006 | 1 | K.IIAEGANGPTTPEADKIFLER.N 22228 22234 22242 |

| **334.** | Q9HCC0\|MCCB_HUMAN    **Mass:** 61808    **Score:** 147    **Queries matched:** 32   **emPAI:** 0.26 |
| --- | --- |
|  | Methylcrotonoyl-CoA carboxylase beta chain, mitochondrial OS=Homo sapiens GN=MCCC2 PE=1 SV=1 |

|  | **Query** | **Observed** | **Mr(expt)** | **Mr(calc)** | **Delta** | **Miss** | **Score** | **Expect** | **Rank** | **Peptide** |
| --- | --- | --- | --- | --- | --- | --- | --- | --- | --- | --- |
|  | 5444 | 531.3214 | 1060.6281 | 1059.5131 | 1.1151 | 0 | 21 | 1.5 | 5 | R.AQEIAMQNR.L 5436 5439 5442 5445 5448 |
|  | 5665 | 540.2974 | 1078.5802 | 1078.5883 | -0.0081 | 0 | 46 | 0.0045 | 1 | K.ALVNQLHER.V 5666 |
|  | 6383 | 567.7763 | 1133.5381 | 1132.6353 | 0.9028 | 1 | 9 | 21 | 4 | K.VVRNLNYQK.K |
|  | 6805 | **583.8091** | **1165.6036** | **1165.5615** | **0.0421** | **0** | **8** | **26** | **3** | **K.QFSSADEAALK.E** 6813 |
|  | 7235 | **596.8209** | **1191.6272** | **1190.6408** | **0.9864** | **1** | **6** | **36** | **4** | **R.SFDVREVIAR.I** |
|  | 8283 | 622.3394 | 1242.6642 | 1243.6417 | -0.9775 | 0 | (6) | 41 | 6 | K.MVAAVACAQVPK.I 8284 8302 |
|  | 8596 | 630.8281 | 1259.6417 | 1259.6366 | 0.0051 | 0 | 8 | 25 | 7 | K.MVAAVACAQVPK.I |
|  | 11464 | **709.3541** | **1416.6936** | **1416.7038** | **-0.0102** | **0** | **34** | **0.06** | **1** | **K.AFYGDTLVTGFAR.I** |
|  | 13347 | **511.9151** | **1532.7236** | **1532.7341** | **-0.0105** | **1** | **4** | **53** | **3** | **K.KGTHFVQLCCQR.N** |
|  | 13564 | 516.6005 | 1546.7796 | 1547.6528 | -0.8733 | 0 | 4 | 48 | 5 | K.FEEEGNPYYSSAR.V |
|  | 14825 | **544.3312** | **1629.9717** | **1630.8171** | **-0.8454** | **1** | **5** | **34** | **2** | **K.DGAKMVAAVACAQVPK.I** 14844 |
|  | 15989 | **853.3809** | **1704.7473** | **1705.8379** | **-1.0906** | **0** | **8** | **15** | **3** | **R.VSGVECMIIANDATVK.G** 16032 |
|  | 16255 | 861.9125 | 1721.8104 | 1721.8328 | -0.0224 | 0 | (8) | 17 | 3 | R.VSGVECMIIANDATVK.G |
|  | 16334 | **864.4436** | **1726.8726** | **1727.9439** | **-1.0713** | **1** | **3** | **54** | **1** | **-.MWAVLRLALRPCAR.A** |
|  | 18866 | 640.3029 | 1917.8868 | 1916.8839 | 1.0028 | 1 | 2 | 64 | 4 | R.DHFGRTFYNQAIMSSK.N |
|  | 20475 | **686.6398** | **2056.8977** | **2056.9120** | **-0.0143** | **0** | **41** | **0.007** | **1** | **K.AATGEEVSAEDLGGADLHCR.K** |
|  | 21555 | **1085.0587** | **2168.1029** | **2167.1314** | **0.9715** | **0** | **23** | **0.38** | **1** | **R.IFGYPVGIVGNNGVLFSESAK.K** |
|  | 22647 | **765.8823** | **2294.6250** | **2294.1305** | **0.4945** | **1** | **4** | **19** | **1** | **R.KSGVSDHWALDDHHALHLTR.K** |
|  | 27898 | **800.9351** | **3199.7114** | **3200.5603** | **-0.8489** | **1** | **5** | **15** | **1** | **K.MVAAVACAQVPKITLIIGGSYGAGNYGMCGR.A** |
|  | 30899 | **1484.0690** | **4449.1851** | **4449.2128** | **-0.0278** | **1** | **54** | **8e-005** | **1** | **R.ERIDNLIDPGSPFLELSQFAGYQLYDNEEVPGGGIITGIGR.V** 30900 |

| **335.** | Q15392\|DHC24_HUMAN    **Mass:** 60803    **Score:** 147    **Queries matched:** 27   **emPAI:** 0.43 |
| --- | --- |
|  | 24-dehydrocholesterol reductase OS=Homo sapiens GN=DHCR24 PE=1 SV=2 |

|  | **Query** | **Observed** | **Mr(expt)** | **Mr(calc)** | **Delta** | **Miss** | **Score** | **Expect** | **Rank** | **Peptide** |
| --- | --- | --- | --- | --- | --- | --- | --- | --- | --- | --- |
|  | 2725 | 431.2256 | 860.4367 | 860.4426 | -0.0058 | 0 | 11 | 15 | 3 | R.GLEAICAK.F |
|  | 3293 | 451.7372 | 901.4599 | 901.4657 | -0.0058 | 0 | 23 | 0.93 | 1 | K.HVENYLK.T |
|  | 3315 | **452.7146** | **903.4146** | **903.4199** | **-0.0053** | **0** | **13** | **11** | **1** | **K.FTHESQR.Q** |
|  | 3487 | **458.7688** | **915.5230** | **915.5290** | **-0.0060** | **1** | **22** | **1.1** | **1** | **K.LRFEPVR.G** 3488 |
|  | 3507 | **459.2531** | **916.4917** | **916.4978** | **-0.0061** | **0** | **27** | **0.35** | **1** | **K.LTQGETLR.K** |
|  | 5256 | **523.2999** | **1044.5853** | **1044.5927** | **-0.0074** | **1** | **24** | **0.71** | **1** | **K.LTQGETLRK.L** 5238 5257 |
|  | 5324 | 526.7540 | 1051.4934 | 1050.4950 | 0.9984 | 1 | 15 | 5.1 | 1 | R.SCMRQLEK.F |
|  | 8466 | **627.3451** | **1252.6757** | **1252.6315** | **0.0443** | **0** | **1** | **1.1e+002** | **5** | **R.YLFGWMVPPK.I** |
|  | 12183 | **730.8911** | **1459.7677** | **1459.7783** | **-0.0106** | **1** | **25** | **0.41** | **1** | **K.TNREGLEYIPLR.H** |
|  | 13454 | 771.3452 | 1540.6759 | 1540.6868 | -0.0109 | 0 | 83 | 6.5e-007 | 1 | K.LGCQDAFPEVYDK.I 13455 |
|  | 17280 | **598.3256** | **1791.9550** | **1792.8951** | **-0.9400** | **0** | **2** | **75** | **5** | **K.NIMINLMDILEVDTK.K** |
|  | 18420 | 941.4715 | 1880.9284 | 1880.9390 | -0.0105 | 0 | 14 | 3.9 | 1 | K.TFMCTGRPGWLTVSLR.V |
|  | 18722 | **635.9983** | **1904.9732** | **1904.9951** | **-0.0219** | **1** | **1** | **84** | **4** | **K.NIMINLMDILEVDTKK.Q** |
|  | 19449 | **656.6633** | **1966.9682** | **1965.9805** | **0.9877** | **0** | **33** | **0.043** | **1** | **K.LYEQHHVVQDMLVPMK.C** 19432 |
|  | 19908 | 1004.4809 | 2006.9472 | 2006.8437 | 0.1035 | 0 | 0 | 82 | 5 | R.SVHGFQMLYADCYMNR.E |
|  | 20901 | **699.0272** | **2094.0597** | **2094.0754** | **-0.0158** | **1** | **30** | **0.087** | **1** | **R.KLYEQHHVVQDMLVPMK.C** 20892 |
|  | 21172 | 1064.0931 | 2126.1717 | 2126.0653 | 0.1065 | 1 | (4) | 29 | 5 | R.KLYEQHHVVQDMLVPMK.C |
|  | 22711 | 769.4009 | 2305.1810 | 2305.1895 | -0.0085 | 0 | 17 | 1.6 | 1 | R.SIFWELQDIIPFGNNPIFR.Y 22710 |
|  | 30503 | **1042.5397** | **4166.1296** | **4166.1387** | **-0.0091** | **0** | **34** | **0.01** | **1** | **K.CLQQALHTFQNDIHVYPIWLCPFILPSQPGLVHPK.G** |
|  | 31487 | **1347.8447** | **5387.3498** | **5386.8772** | **0.4726** | **1** | **0** | **10** | **3** | **K.QIVRVEPLVTMGQVTALLTSIGWTLPVLPELDDLTVGGLIMGTGIESSSHK.Y** |

| **336.** | O15229\|KMO_HUMAN    **Mass:** 56344    **Score:** 146    **Queries matched:** 22   **emPAI:** 0.47 |
| --- | --- |
|  | Kynurenine 3-monooxygenase OS=Homo sapiens GN=KMO PE=1 SV=2 |

|  | **Query** | **Observed** | **Mr(expt)** | **Mr(calc)** | **Delta** | **Miss** | **Score** | **Expect** | **Rank** | **Peptide** |
| --- | --- | --- | --- | --- | --- | --- | --- | --- | --- | --- |
|  | 1273 | **602.3574** | **601.3501** | **600.3959** | **0.9543** | **1** | **18** | **4.3** | **2** | **K.KVINK.G** |
|  | 2494 | **421.2491** | **840.4837** | **840.3813** | **0.1024** | **0** | **1** | **1.2e+002** | **9** | **K.MHFNHR.L** |
|  | 4135 | 482.7736 | 963.5327 | 963.5389 | -0.0062 | 1 | 25 | 0.54 | 1 | K.KSAIPYGTK.S |
|  | 4798 | **505.7874** | **1009.5601** | **1009.5668** | **-0.0067** | **0** | **33** | **0.079** | **1** | **R.SINLALSHR.G** 4788 4797 4806 |
|  | 7890 | **408.5605** | **1222.6596** | **1222.6894** | **-0.0299** | **1** | **7** | **27** | **7** | **R.GRSINLALSHR.G** |
|  | 13211 | **762.8677** | **1523.7209** | **1523.7298** | **-0.0089** | **0** | **29** | **0.14** | **1** | **R.NTFMMIALPNMNK.S** |
|  | 13230 | 763.3944 | 1524.7741 | 1524.7824 | -0.0083 | 0 | 66 | 3.1e-005 | 1 | K.LLTSNDVVDFFQK.Y |
|  | 13459 | 771.4065 | 1540.7984 | 1539.7247 | 1.0737 | 0 | (5) | 37 | 2 | R.NTFMMIALPNMNK.S |
|  | 13528 | **772.9129** | **1543.8112** | **1543.8206** | **-0.0093** | **0** | **76** | **3.4e-006** | **1** | **K.AVDSLEQISNLISR.-** |
|  | 16125 | 856.9481 | 1711.8817 | 1711.8927 | -0.0110 | 0 | 43 | 0.0058 | 1 | K.AVGLEDQIVSQGIPMR.A |
|  | 16333 | 432.7242 | 1726.8679 | 1727.8876 | -1.0198 | 0 | (4) | 43 | 9 | K.AVGLEDQIVSQGIPMR.A |
|  | 18136 | **620.4863** | **1858.4372** | **1859.0339** | **-0.5967** | **0** | **1** | **53** | **1** | **K.VAVIGGGLVGSLQACFLAK.R** |
|  | 18934 | **642.3773** | **1924.1100** | **1923.0036** | **1.1063** | **1** | **3** | **47** | **9** | **K.FSNDLSLCLPVFSRLR.I** |
|  | 19327 | 490.0043 | 1955.9881 | 1955.0258 | 0.9623 | 1 | 8 | 13 | 1 | K.AVGLEDQIVSQGIPMRAR.M |
|  | 19827 | **1000.9493** | **1999.8840** | **1999.8980** | **-0.0140** | **0** | **33** | **0.043** | **1** | **K.DVTCDLIVGCDGAYSTVR.S** |
|  | 19846 | **1001.9858** | **2001.9570** | **2003.0067** | **-1.0497** | **1** | **2** | **57** | **1** | **R.LLKCNPEEGMITVLGSDK.V** |
|  | 20703 | **692.3431** | **2074.0074** | **2074.1537** | **-0.1463** | **0** | **2** | **52** | **5** | **K.LLVQDFFLLPAQPMISVK.C** |
|  | 24126 | **830.3985** | **2488.1737** | **2487.2695** | **0.9042** | **0** | **1** | **56** | **7** | **R.FLHAIMPSTFIPLYTMVTFSR.I** |
|  | 28739 | **1734.0352** | **3466.0558** | **3465.5463** | **0.5094** | **1** | **2** | **19** | **3** | **R.NTFMMIALPNMNKSFTCTLFMPFEEFEK.L** |

| **337.** | O94905\|ERLN2_HUMAN    **Mass:** 38044    **Score:** 146    **Queries matched:** 18   **emPAI:** 0.33 |
| --- | --- |
|  | Erlin-2 OS=Homo sapiens GN=ERLIN2 PE=1 SV=1 |

|  | **Query** | **Observed** | **Mr(expt)** | **Mr(calc)** | **Delta** | **Miss** | **Score** | **Expect** | **Rank** | **Peptide** |
| --- | --- | --- | --- | --- | --- | --- | --- | --- | --- | --- |
|  | 1422 | **644.3934** | **643.3861** | **644.3493** | **-0.9632** | **0** | **14** | **9** | **1** | **K.IAEANK.L** |
|  | 3266 | 451.2501 | 900.4857 | 900.5280 | -0.0423 | 1 | 22 | 1.1 | 5 | K.KALIEAEK.V 3270 |
|  | 6709 | **580.2095** | **1158.4045** | **1158.4685** | **-0.0640** | **0** | **6** | **33** | **5** | **K.ADAECYTAMK.I** 6712 |
|  | 7187 | **397.4025** | **1189.1857** | **1188.5986** | **0.5871** | **1** | **2** | **67** | **8** | **K.VVEKEAETER.K** 7188 |
|  | 9460 | 653.8490 | 1305.6834 | 1305.6929 | -0.0094 | 0 | 67 | 2.7e-005 | 1 | K.VAQVAEITYGQK.V |
|  | 9589 | **657.8319** | **1313.6491** | **1313.5921** | **0.0570** | **1** | **13** | **6.7** | **4** | **R.RNYELMESEK.T** |
|  | 10234 | **674.8464** | **1347.6782** | **1347.6882** | **-0.0100** | **0** | **61** | **0.00012** | **1** | **K.SVQTTLQTDEVK.N** 10208 |
|  | 10963 | **694.7695** | **1387.5245** | **1386.6337** | **0.8909** | **1** | **3** | **66** | **7** | **R.NYELMESEKTK.L** |
|  | 12222 | **732.3987** | **1462.7828** | **1461.7827** | **1.0001** | **1** | **44** | **0.0054** | **1** | **K.KISEIEDAAFLAR.E** |
|  | 13456 | **514.5894** | **1540.7463** | **1541.8163** | **-1.0701** | **1** | **3** | **64** | **7** | **K.LTPEYLQLMKYK.A** |
|  | 16967 | 591.3234 | 1770.9485 | 1771.8022 | -0.8537 | 0 | 3 | 56 | 4 | K.NVPCGTSGGVMIYFDR.I |
|  | 17487 | **604.3333** | **1809.9781** | **1808.9342** | **1.0439** | **1** | **0** | **91** | **2** | **K.VAQVAEITYGQKVMEK.E** |
|  | 23922 | **617.3566** | **2465.3972** | **2466.2730** | **-0.8758** | **0** | **6** | **15** | **3** | **M.AQLGAVVAVASSFFCASLFSAVHK.I** |
|  | 29894 | 1305.9534 | 3914.8383 | 3913.9648 | 0.8735 | 1 | 0 | 27 | 4 | -.MAQLGAVVAVASSFFCASLFSAVHKIEEGHIGVYYR.G |

| **338.** | P31153\|METK2_HUMAN    **Mass:** 43975    **Score:** 145    **Queries matched:** 9   **emPAI:** 0.18 |
| --- | --- |
|  | S-adenosylmethionine synthetase isoform type-2 OS=Homo sapiens GN=MAT2A PE=1 SV=1 |

|  | **Query** | **Observed** | **Mr(expt)** | **Mr(calc)** | **Delta** | **Miss** | **Score** | **Expect** | **Rank** | **Peptide** |
| --- | --- | --- | --- | --- | --- | --- | --- | --- | --- | --- |
|  | 1323 | **616.3982** | **615.3910** | **615.3955** | **-0.0046** | **1** | **13** | **16** | **3** | **K.EKVIK.A** 1321 |
|  | 1602 | 709.3584 | 708.3511 | 708.3555 | -0.0043 | 0 | 14 | 6.5 | 1 | R.SAAYAAR.W |
|  | 9055 | 643.3682 | 1284.7218 | 1284.7303 | -0.0085 | 0 | 26 | 0.38 | 1 | K.NFDLRPGVIVR.D |
|  | 10466 | **681.5141** | **1361.0136** | **1360.7384** | **0.2752** | **0** | **1** | **84** | **1** | **K.TGMILLAGEITSR.A** |
|  | 10890 | **462.0124** | **1383.0153** | **1383.6453** | **-0.6299** | **0** | **4** | **37** | **5** | **K.TQVTVQYMQDR.G** |
|  | 19235 | 975.4714 | 1948.9283 | 1948.9432 | -0.0149 | 0 | 87 | 1.9e-007 | 1 | K.IIVDTYGGWGAHGGGAFSGK.D 19236 |
|  | 20727 | 520.2636 | 2077.0251 | 2077.0381 | -0.0131 | 1 | 11 | 7.9 | 2 | R.KIIVDTYGGWGAHGGGAFSGK.D |

| **339.** | P51572\|BAP31_HUMAN    **Mass:** 28031    **Score:** 145    **Queries matched:** 13   **emPAI:** 0.13 |
| --- | --- |
|  | B-cell receptor-associated protein 31 OS=Homo sapiens GN=BCAP31 PE=1 SV=3 |

|  | **Query** | **Observed** | **Mr(expt)** | **Mr(calc)** | **Delta** | **Miss** | **Score** | **Expect** | **Rank** | **Peptide** |
| --- | --- | --- | --- | --- | --- | --- | --- | --- | --- | --- |
|  | 2457 | 839.4550 | 838.4477 | 838.4548 | -0.0071 | 0 | 21 | 1.3 | 1 | R.LLEEHAK.L 2461 |
|  | 2569 | 425.0476 | 848.0807 | 848.4908 | -0.4102 | 1 | 6 | 31 | 8 | R.WQKIFK.S |
|  | 3873 | **473.2927** | **944.5708** | **943.4975** | **1.0733** | **0** | **7** | **41** | **3** | **K.LDVGNAEVK.L** |
|  | 4736 | 503.2899 | 1004.5652 | 1003.5549 | 1.0102 | 1 | 13 | 9.2 | 3 | K.LKDELASTK.Q |
|  | 5276 | **524.2654** | **1046.5163** | **1046.5179** | **-0.0015** | **0** | **9** | **22** | **5** | **K.AENQVLAMR.K** |
|  | 6504 | **572.3357** | **1142.6569** | **1143.5594** | **-0.9025** | **0** | **16** | **4.4** | **3** | **K.LQAAVDGPMDK.K** |
|  | 6825 | 584.8334 | 1167.6523 | 1168.5070 | -0.8547 | 0 | 7 | 32 | 7 | K.YMEENDQLK.K |
|  | 9268 | **649.3043** | **1296.5941** | **1296.6020** | **-0.0079** | **1** | **13** | **7.4** | **1** | **K.KYMEENDQLK.K** |
|  | 9578 | **657.4031** | **1312.7916** | **1312.5969** | **0.1947** | **1** | **1** | **1.1e+002** | **4** | **K.YMEENDQLKK.G** |
|  | 11205 | **468.2654** | **1401.7745** | **1400.7445** | **1.0300** | **1** | **2** | **94** | **10** | **K.LEKAENQVLAMR.K** |
|  | 16153 | **857.9280** | **1713.8415** | **1713.8533** | **-0.0118** | **1** | **89** | **1.5e-007** | **1** | **K.LDVGNAEVKLEEENR.S** 16154 |

| **340.** | Q6NUM9\|RETST_HUMAN    **Mass:** 67461    **Score:** 145    **Queries matched:** 29   **emPAI:** 0.38 |
| --- | --- |
|  | All-trans-retinol 13,14-reductase OS=Homo sapiens GN=RETSAT PE=2 SV=2 |

|  | **Query** | **Observed** | **Mr(expt)** | **Mr(calc)** | **Delta** | **Miss** | **Score** | **Expect** | **Rank** | **Peptide** |
| --- | --- | --- | --- | --- | --- | --- | --- | --- | --- | --- |
|  | 1625 | **716.4249** | **715.4176** | **715.4228** | **-0.0052** | **0** | **24** | **0.95** | **2** | **R.AGGAVLTK.A** 1627 |
|  | 3011 | **440.2387** | **878.4629** | **877.3963** | **1.0665** | **0** | **9** | **21** | **4** | **R.MEEGSIGR.F** |
|  | 5859 | **547.7241** | **1093.4336** | **1093.4434** | **-0.0098** | **0** | **28** | **0.24** | **1** | **K.AGGCCHTFGK.N** 5871 |
|  | 5976 | **551.7580** | **1101.5014** | **1101.5091** | **-0.0076** | **1** | **26** | **0.41** | **1** | **K.RGSDYETFK.N** 5999 |
|  | 8011 | 615.7865 | 1229.5584 | 1230.5590 | -1.0006 | 1 | 3 | 78 | 6 | R.KEYPMYSGEK.A 8017 |
|  | 9154 | **646.2728** | **1290.5310** | **1290.5411** | **-0.0101** | **0** | **54** | **0.0006** | **1** | **R.GACYGADHDLGR.L** 9163 |
|  | 9169 | **431.2597** | **1290.7572** | **1290.7660** | **-0.0088** | **0** | **17** | **3** | **1** | **K.VVSSGAPHAILLK.F** |
|  | 10729 | **459.2146** | **1374.6219** | **1374.6317** | **-0.0098** | **1** | **14** | **5.9** | **1** | **K.DPTWEDRFPGR.S** 10738 |
|  | 11357 | **471.0709** | **1410.1910** | **1409.7224** | **0.4686** | **0** | **4** | **32** | **5** | **K.NSFVEASMSVVLK.L** |
|  | 11987 | **483.9404** | **1448.7995** | **1448.8099** | **-0.0105** | **1** | **58** | **0.00021** | **1** | **K.RPPAPLVTDKEAR.K** 11979 11986 |
|  | 14221 | **531.2633** | **1590.7681** | **1590.7791** | **-0.0110** | **0** | **13** | **5.6** | **1** | **K.NGLEFDTGIHYIGR.M** |
|  | 19681 | **498.0073** | **1988.0001** | **1989.0565** | **-1.0564** | **1** | **1** | **74** | **10** | **K.ATVQSVLLDSAGKACGVSVK.K** |
|  | 20004 | **1009.5195** | **2017.0245** | **2016.0356** | **0.9889** | **0** | **34** | **0.037** | **1** | **R.EEAAEHIPLLFFAFPSAK.D** |
|  | 21028 | 704.0217 | 2109.0432 | 2109.2133 | -0.1701 | 1 | 1 | 79 | 7 | K.FLPLPVVQLLDRCGLLTR.F |
|  | 21134 | **1061.0446** | **2120.0746** | **2120.0902** | **-0.0156** | **0** | **75** | **3e-006** | **1** | **K.VESVTAGSPLTNQFYLAAPR.G** |
|  | 21770 | **1098.2628** | **2194.5111** | **2193.3397** | **1.1714** | **0** | **3** | **26** | **7** | **-.MWLPLVLLLAVLLLAVLCK.V** |
|  | 23077 | **589.0385** | **2352.1249** | **2353.1148** | **-0.9898** | **1** | **0** | **70** | **2** | **R.GSDYETFKNSFVEASMSVVLK.L** |
|  | 25440 | **1358.6492** | **2715.2838** | **2715.2964** | **-0.0126** | **0** | **20** | **0.6** | **1** | **R.STMIMLIPTAYEWFEEWQAELK.G** |
|  | 28759 | **1158.7736** | **3473.2989** | **3473.7177** | **-0.4189** | **0** | **1** | **24** | **4** | **R.FILDQITEGQLDWAPLSSPFDIMVLEGPNGR.K** 28762 |
|  | 29542 | **1263.9775** | **3788.9108** | **3788.9845** | **-0.0737** | **0** | **2** | **22** | **2** | **R.AQSPIPNLYLTGQDIFTCGLVGALQGALLCSSAILK.R** |

| **341.** | O75881\|CP7B1_HUMAN    **Mass:** 58732    **Score:** 144    **Queries matched:** 16   **emPAI:** 0.13 |
| --- | --- |
|  | 25-hydroxycholesterol 7-alpha-hydroxylase OS=Homo sapiens GN=CYP7B1 PE=1 SV=2 |

|  | **Query** | **Observed** | **Mr(expt)** | **Mr(calc)** | **Delta** | **Miss** | **Score** | **Expect** | **Rank** | **Peptide** |
| --- | --- | --- | --- | --- | --- | --- | --- | --- | --- | --- |
|  | 3197 | 895.4921 | 894.4848 | 894.4997 | -0.0149 | 1 | 16 | 4.4 | 8 | R.FMKTLQK.Q |
|  | 3395 | **455.7211** | **909.4277** | **910.4946** | **-1.0669** | **1** | **(5)** | **43** | **4** | **R.FMKTLQK.Q** |
|  | 3559 | **461.9362** | **921.8578** | **921.4742** | **0.3836** | **1** | **5** | **31** | **6** | **K.DPLRFMK.T** |
|  | 4582 | 499.2589 | 996.5033 | 996.4811 | 0.0222 | 0 | 8 | 23 | 7 | R.HPEAMAAVR.D 4588 |
|  | 5500 | **533.7944** | **1065.5742** | **1064.4921** | **1.0821** | **0** | **7** | **32** | **2** | **-.MAGEVSAATGR.F** 5486 |
|  | 12822 | 500.7648 | 1499.2727 | 1498.8660 | 0.4066 | 0 | 3 | 39 | 6 | K.GWLPYLGVVLNLR.K |
|  | 12912 | 753.3715 | 1504.7284 | 1503.8660 | 0.8623 | 1 | 2 | 81 | 8 | K.LLEKAFSISQLQK.N |
|  | 14721 | 812.8982 | 1623.7819 | 1624.7627 | -0.9808 | 1 | 6 | 32 | 6 | R.HPEAMAAVRDEIDR.L |
|  | 14884 | **817.9249** | **1633.8352** | **1633.8419** | **-0.0067** | **0** | **53** | **0.0006** | **1** | **K.SLDILLESMMQNLK.Q** 14898 |
|  | 15878 | 849.9387 | 1697.8629 | 1696.8203 | 1.0426 | 1 | 16 | 2.6 | 2 | -.MAGEVSAATGRFSLER.L 15844 |
|  | 17129 | **891.9698** | **1781.9250** | **1781.9352** | **-0.0102** | **0** | **65** | **3.4e-005** | **1** | **R.LLFGIQYPDSDVLFR.Y** 17128 |

| **342.** | P32754\|HPPD_HUMAN    **Mass:** 45077    **Score:** 143    **Queries matched:** 13   **emPAI:** 0.17 |
| --- | --- |
|  | 4-hydroxyphenylpyruvate dioxygenase OS=Homo sapiens GN=HPD PE=1 SV=2 |

|  | **Query** | **Observed** | **Mr(expt)** | **Mr(calc)** | **Delta** | **Miss** | **Score** | **Expect** | **Rank** | **Peptide** |
| --- | --- | --- | --- | --- | --- | --- | --- | --- | --- | --- |
|  | 10314 | **676.6600** | **1351.3054** | **1350.7031** | **0.6023** | **0** | **9** | **11** | **1** | **R.SIVVANYEESIK.M** |
|  | 12817 | **750.3912** | **1498.7678** | **1498.7086** | **0.0592** | **1** | **3** | **64** | **9** | **-.MTTYSDKGAKPER.G** |
|  | 13829 | **522.3003** | **1563.8790** | **1564.6862** | **-0.8071** | **0** | **0** | **1.1e+002** | **10** | **R.GNLTNMETNGVVPGM.-** |
|  | 14166 | **794.4437** | **1586.8729** | **1586.8820** | **-0.0091** | **0** | **57** | **0.00026** | **1** | **K.IVFVLSSALNPWNK.E** 14165 |
|  | 14502 | **806.3420** | **1610.6694** | **1609.8788** | **0.7906** | **1** | **10** | **10** | **2** | **R.GLETGSREVVSHVIK.Q** 14501 |
|  | 18912 | 641.6625 | 1921.9658 | 1921.9786 | -0.0128 | 0 | 15 | 2.8 | 2 | K.FAVLQTYGDTTHTLVEK.M |
|  | 20612 | **690.6522** | **2068.9348** | **2068.9491** | **-0.0142** | **0** | **11** | **6.5** | **1** | **R.FWSVDDTQVHTEYSSLR.S** |
|  | 24891 | **1306.6445** | **2611.2745** | **2611.2855** | **-0.0110** | **0** | **45** | **0.002** | **1** | **K.MNYIGQFLPGYEAPAFMDPLLPK.L** 24890 24892 |
|  | 28570 | **1134.9523** | **3401.8350** | **3401.6416** | **0.1934** | **1** | **5** | **11** | **1** | **R.FWSVDDTQVHTEYSSLRSIVVANYEESIK.M** |

| **343.** | P14927\|QCR7_HUMAN    **Mass:** 13522    **Score:** 143    **Queries matched:** 12   **emPAI:** 1.15 |
| --- | --- |
|  | Cytochrome b-c1 complex subunit 7 OS=Homo sapiens GN=UQCRB PE=1 SV=2 |

|  | **Query** | **Observed** | **Mr(expt)** | **Mr(calc)** | **Delta** | **Miss** | **Score** | **Expect** | **Rank** | **Peptide** |
| --- | --- | --- | --- | --- | --- | --- | --- | --- | --- | --- |
|  | 1672 | **735.3829** | **734.3756** | **734.4439** | **-0.0683** | **0** | **3** | **97** | **1** | **K.HQILPK.E** 1674 |
|  | 3122 | 444.7526 | 887.4906 | 886.5025 | 0.9881 | 1 | 20 | 2 | 2 | K.WLDGIRK.W |
|  | 3817 | **471.7464** | **941.4783** | **941.5658** | **-0.0875** | **1** | **11** | **13** | **7** | **K.RALDLNLK.H** |
|  | 9123 | **645.3288** | **1288.6430** | **1288.6524** | **-0.0093** | **1** | **33** | **0.065** | **1** | **R.RLPENLYNDR.M** 9144 |
|  | 10460 | **681.3301** | **1360.6456** | **1360.6564** | **-0.0108** | **1** | **19** | **1.7** | **1** | **R.KWYYNAAGFNK.L** |
|  | 16494 | 868.9009 | 1735.7873 | 1735.7981 | -0.0107 | 0 | 42 | 0.0067 | 1 | K.YEEENFYLEPYLK.E 16493 |
|  | 18795 | **956.4786** | **1910.9426** | **1910.8931** | **0.0495** | **1** | **2** | **61** | **1** | **K.LGLMRDDTIYEDEDVK.E** |
|  | 23501 | **1205.0598** | **2408.1051** | **2408.1212** | **-0.0161** | **1** | **69** | **9.1e-006** | **1** | **K.EQWTKYEEENFYLEPYLK.E** 23502 |

| **344.** | Q93084\|AT2A3_HUMAN    **Mass:** 115444   **Score:** 143    **Queries matched:** 23   **emPAI:** 0.10 |
| --- | --- |
|  | Sarcoplasmic/endoplasmic reticulum calcium ATPase 3 OS=Homo sapiens GN=ATP2A3 PE=1 SV=2 |

|  | **Query** | **Observed** | **Mr(expt)** | **Mr(calc)** | **Delta** | **Miss** | **Score** | **Expect** | **Rank** | **Peptide** |
| --- | --- | --- | --- | --- | --- | --- | --- | --- | --- | --- |
|  | 1094 | **556.3608** | **555.3536** | **554.2813** | **1.0723** | **0** | **16** | **3.5** | **9** | **R.DAPPR.K** 1086 |
|  | 1159 | 572.3469 | 571.3396 | 571.3442 | -0.0046 | 0 | 16 | 5.3 | 1 | K.NAIVR.S |
|  | 1930 | **400.2259** | **798.4373** | **798.3984** | **0.0389** | **0** | **6** | **43** | **10** | **R.QGDQPVR.C** |
|  | 6681 | 578.9090 | 1155.8034 | 1155.5924 | 0.2110 | 1 | 7 | 25 | 1 | R.KEFTLEFSR.D |
|  | 8692 | **633.3309** | **1264.6472** | **1265.7707** | **-1.1235** | **1** | **1** | **1.1e+002** | **7** | **K.VPADLRLIEIK.S** |
|  | 10501 | **682.3831** | **1362.7517** | **1361.6688** | **1.0829** | **1** | **5** | **40** | **4** | **K.IRDWGSGSDTLR.C** |
|  | 10599 | 684.8114 | 1367.6082 | 1368.6707 | -1.0625 | 0 | 13 | 7 | 3 | K.NMLFSGTNITSGK.A 10598 |
|  | 12885 | 752.4078 | 1502.8011 | 1502.8126 | -0.0115 | 1 | 85 | 4.3e-007 | 1 | K.KAEIGIAMGSGTAVAK.S 12886 |
|  | 13019 | **505.2354** | **1512.6844** | **1512.7606** | **-0.0762** | **1** | **3** | **65** | **3** | **K.KNMLFSGTNITSGK.A** |
|  | 13783 | **521.3043** | **1560.8910** | **1561.8199** | **-0.9290** | **0** | **1** | **1e+002** | **9** | **R.VDQSILTGESVSVTK.H** |
|  | 14320 | **533.1893** | **1596.5462** | **1596.7653** | **-0.2192** | **0** | **2** | **60** | **5** | **R.NHMHACLYPGLLR.T** 14319 |
|  | 14653 | 540.6265 | 1618.8578 | 1619.8076 | -0.9499 | 0 | 1 | 90 | 10 | K.VGEATETALTCLVEK.M |
|  | 17405 | 902.9022 | 1803.7899 | 1803.9335 | -0.1436 | 1 | 0 | 95 | 7 | R.VVMITGDNKGTAVAICR.R |
|  | 19270 | 976.9625 | 1951.9105 | 1951.9231 | -0.0126 | 0 | 52 | 0.00056 | 1 | R.SLPSVETLGCTSVICSDK.T 19271 |
|  | 26337 | **719.3538** | **2873.3859** | **2874.4433** | **-1.0574** | **1** | **1** | **39** | **1** | **R.IVENLQSFNEITAMTGDGVNDAPALKK.A** |
|  | 27703 | **1049.8289** | **3146.4648** | **3146.3995** | **0.0653** | **0** | **11** | **4.2** | **1** | **R.CGQFDGLVELATICALCNDSALDYNEAK.G** |
|  | 29444 | **1252.2725** | **3753.7956** | **3752.8237** | **0.9719** | **1** | **2** | **22** | **4** | **K.AEIGIAMGSGTAVAKSAAEMVLSDDNFASIVAAVEEGR.A** |
|  | 31671 | **1860.5464** | **5578.6173** | **5579.6027** | **-0.9853** | **1** | **1** | **7.2** | **2** | **K.CSEDNPLFAGIDCEVFESRFPTTMALSVLVTIEMCNALNSVSENQSLLR.M** |

| **345.** | Q9P0L0\|VAPA_HUMAN    **Mass:** 28103    **Score:** 141    **Queries matched:** 22   **emPAI:** 1.75 |
| --- | --- |
|  | Vesicle-associated membrane protein-associated protein A OS=Homo sapiens GN=VAPA PE=1 SV=3 |

|  | **Query** | **Observed** | **Mr(expt)** | **Mr(calc)** | **Delta** | **Miss** | **Score** | **Expect** | **Rank** | **Peptide** |
| --- | --- | --- | --- | --- | --- | --- | --- | --- | --- | --- |
|  | 2414 | **836.4061** | **835.3988** | **835.3932** | **0.0056** | **0** | **10** | **19** | **1** | **R.LQGEMMK.L** 2425 |
|  | 2843 | **434.7275** | **867.4405** | **867.3830** | **0.0575** | **0** | **(4)** | **56** | **7** | **R.LQGEMMK.L** |
|  | 4034 | 479.2498 | 956.4850 | 955.4103 | 1.0747 | 0 | 10 | 17 | 2 | -.MASASGAMAK.H |
|  | 4155 | **483.7193** | **965.4240** | **964.4470** | **0.9770** | **1** | **6** | **43** | **3** | **K.LMEECKR.L** |
|  | 4544 | **497.7891** | **993.5637** | **994.5308** | **-0.9671** | **1** | **11** | **13** | **4** | **R.HLRDEGLR.L** |
|  | 8648 | **631.7955** | **1261.5765** | **1261.5860** | **-0.0095** | **0** | **24** | **0.54** | **1** | **K.EAKPDELMDSK.L** |
|  | 13326 | **766.5288** | **1531.0431** | **1530.7711** | **0.2719** | **1** | **0** | **84** | **2** | **K.EAKPDELMDSKLR.C** |
|  | 13857 | **783.9247** | **1565.8348** | **1565.8454** | **-0.0106** | **1** | **31** | **0.1** | **1** | **K.FKGPFTDVVTTNLK.L** 13866 |
|  | 14616 | **809.4408** | **1616.8670** | **1616.8774** | **-0.0103** | **0** | **49** | **0.0014** | **1** | **K.HEQILVLDPPTDLK.F** 14617 |
|  | 17010 | **592.6351** | **1774.8834** | **1774.8962** | **-0.0128** | **1** | **35** | **0.034** | **1** | **R.KVAHSDKPGSTSTASFR.D** |
|  | 21017 | **703.6674** | **2107.9803** | **2107.9957** | **-0.0154** | **0** | **36** | **0.024** | **1** | **K.QDGPMPKPHSVSLNDTETR.K** 21016 |
|  | 22649 | **766.3291** | **2295.9655** | **2295.9810** | **-0.0155** | **1** | **3** | **39** | **1** | **R.CVFEMPNENDKLNDMEPSK.A** |
|  | 22765 | **1156.5520** | **2311.0894** | **2311.1017** | **-0.0123** | **0** | **29** | **0.089** | **1** | **K.FMVQTIFAPPNTSDMEAVWK.E** 22764 |
|  | 29114 | **1200.2369** | **3597.6890** | **3597.7120** | **-0.0230** | **0** | **41** | **0.0027** | **1** | **R.YCVRPNSGIIDPGSTVTVSVMLQPFDYDPNEK.S** |
|  | 29444 | 1252.2725 | 3753.7956 | 3753.8131 | -0.0175 | 1 | 27 | 0.074 | 1 | R.RYCVRPNSGIIDPGSTVTVSVMLQPFDYDPNEK.S 29445 29446 |

| **346.** | P13073\|COX41_HUMAN    **Mass:** 19621    **Score:** 140    **Queries matched:** 22   **emPAI:** 1.45 |
| --- | --- |
|  | Cytochrome c oxidase subunit 4 isoform 1, mitochondrial OS=Homo sapiens GN=COX4I1 PE=1 SV=1 |

|  | **Query** | **Observed** | **Mr(expt)** | **Mr(calc)** | **Delta** | **Miss** | **Score** | **Expect** | **Rank** | **Peptide** |
| --- | --- | --- | --- | --- | --- | --- | --- | --- | --- | --- |
|  | 1406 | **637.2998** | **636.2925** | **636.2975** | **-0.0050** | **0** | **12** | **12** | **1** | **R.MLDMK.V** |
|  | 1743 | **768.6448** | **767.6376** | **768.4130** | **-0.7754** | **0** | **19** | **1.4** | **1** | **R.AHESVVK.S** |
|  | 4376 | 491.7424 | 981.4702 | 982.4178 | -0.9476 | 0 | 15 | 4.8 | 8 | K.ESFAEMNR.G |
|  | 4999 | **513.7972** | **1025.5798** | **1025.5869** | **-0.0071** | **0** | **42** | **0.0084** | **1** | **K.VNPIQGLASK.W** 4998 5000 |
|  | 5719 | **542.3107** | **1082.6069** | **1081.6244** | **0.9825** | **1** | **2** | **89** | **6** | **K.HLSASQKALK.E** |
|  | 6564 | 574.9908 | 1147.9671 | 1147.6132 | 0.3540 | 1 | 4 | 40 | 5 | K.RAISTSVCVR.A |
|  | 8200 | 1239.5953 | 1238.5881 | 1239.5441 | -0.9561 | 0 | 27 | 0.25 | 2 | K.ASWSSLSMDEK.V |
|  | 8240 | **621.3311** | **1240.6477** | **1240.6564** | **-0.0087** | **0** | **30** | **0.15** | **1** | **R.DHPLPEVAHVK.H** 8241 |
|  | 8540 | **629.3204** | **1256.6263** | **1257.5812** | **-0.9549** | **1** | **7** | **32** | **5** | **K.FKESFAEMNR.G** |
|  | 9266 | **649.2916** | **1296.5687** | **1296.5775** | **-0.0088** | **1** | **27** | **0.25** | **1** | **K.WDYEKNEWK.K** |
|  | 10063 | **446.7122** | **1337.1147** | **1336.7537** | **0.3610** | **1** | **2** | **52** | **9** | **-.MLATRVFSLVGK.R** 10034 |
|  | 11686 | **715.8118** | **1429.6090** | **1429.6184** | **-0.0094** | **0** | **58** | **0.00024** | **1** | **K.SEDFSLPAYMDR.R** 11685 |
|  | 13023 | 505.2591 | 1512.7554 | 1512.6766 | 0.0789 | 1 | 7 | 26 | 3 | K.EKASWSSLSMDEK.V 13025 13032 |
|  | 14150 | 793.8614 | 1585.7083 | 1585.7195 | -0.0111 | 1 | 20 | 1.1 | 1 | K.SEDFSLPAYMDRR.D |
|  | 21784 | **733.1590** | **2196.4552** | **2196.0157** | **0.4395** | **1** | **0** | **50** | **4** | **R.AHESVVKSEDFSLPAYMDR.R** |

| **347.** | P11216\|PYGB_HUMAN    **Mass:** 97319    **Score:** 138    **Queries matched:** 28   **emPAI:** 0.16 |
| --- | --- |
|  | Glycogen phosphorylase, brain form OS=Homo sapiens GN=PYGB PE=1 SV=5 |

|  | **Query** | **Observed** | **Mr(expt)** | **Mr(calc)** | **Delta** | **Miss** | **Score** | **Expect** | **Rank** | **Peptide** |
| --- | --- | --- | --- | --- | --- | --- | --- | --- | --- | --- |
|  | 1595 | **707.3774** | **706.3702** | **707.3714** | **-1.0013** | **1** | **2** | **1.2e+002** | **10** | **R.KGYNAR.E** |
|  | 1650 | **730.4197** | **729.4125** | **729.4497** | **-0.0372** | **1** | **8** | **41** | **7** | **R.KQISVR.G** |
|  | 1884 | 795.4671 | 794.4598 | 794.3269 | 0.1329 | 0 | 6 | 38 | 10 | K.EPDCFK.D |
|  | 2489 | 841.4352 | 840.4280 | 840.4381 | -0.0102 | 0 | 21 | 1.2 | 2 | K.FSAFLEK.E |
|  | 2944 | 875.4564 | 874.4491 | 874.4912 | -0.0421 | 1 | 28 | 0.34 | 2 | K.AWEITKK.T |
|  | 3182 | 447.7711 | 893.5276 | 892.4079 | 1.1197 | 0 | 8 | 28 | 6 | R.YGNPWEK.A |
|  | 3482 | 458.7429 | 915.4712 | 915.4774 | -0.0062 | 0 | 22 | 1.3 | 2 | R.NLAENISR.V |
|  | 3614 | 464.2542 | 926.4939 | 927.4886 | -0.9947 | 1 | 3 | 86 | 3 | K.DRNVATPR.D |
|  | 3854 | 473.2308 | 944.4470 | 944.4538 | -0.0068 | 0 | 15 | 5.9 | 3 | K.AAPGYHMAK.L |
|  | 4368 | **491.2355** | **980.4564** | **980.4927** | **-0.0363** | **0** | **16** | **4.1** | **1** | **R.VEHTPDGVK.W** |
|  | 4737 | **503.2904** | **1004.5663** | **1005.4814** | **-0.9152** | **1** | **6** | **46** | **6** | **K.FGCRDPVR.T** |
|  | 5338 | 527.2866 | 1052.5586 | 1052.5655 | -0.0069 | 0 | 35 | 0.05 | 1 | K.VIFLENYR.V |
|  | 9163 | 431.2373 | 1290.6901 | 1290.7118 | -0.0218 | 1 | 6 | 33 | 10 | K.AFVPRTVMIGGK.A |
|  | 9167 | 431.2581 | 1290.7524 | 1290.6602 | 0.0922 | 1 | 7 | 29 | 4 | -.MAKPLTDSEKR.K |
|  | 9220 | 432.2447 | 1293.7122 | 1293.7445 | -0.0322 | 1 | 10 | 13 | 6 | R.LKVIFLENYR.V |
|  | 10601 | **456.8828** | **1367.6265** | **1368.7513** | **-1.1248** | **0** | **7** | **27** | **5** | **R.HLEIIYAINQR.H** 10602 |
|  | 11867 | 721.8465 | 1441.6784 | 1441.6878 | -0.0093 | 0 | 51 | 0.001 | 1 | R.VLYPNDNFFEGK.E 11868 |
|  | 15321 | **554.9412** | **1661.8017** | **1662.8222** | **-1.0205** | **1** | **3** | **53** | **5** | **R.TVMIGGKAAPGYHMAK.L** |
|  | 16527 | 580.6215 | 1738.8427 | 1738.9690 | -0.1262 | 1 | 3 | 51 | 3 | K.QISVRGLAGLGDVAEVR.K |
|  | 16577 | **581.9571** | **1742.8494** | **1741.9297** | **0.9197** | **0** | **3** | **51** | **5** | **R.QLLNCLHVVTLYNR.I** |
|  | 17722 | **915.4901** | **1828.9657** | **1828.8744** | **0.0913** | **0** | **6** | **28** | **5** | **K.IVNGWQVEEADDWLR.Y** |
|  | 17856 | 921.0026 | 1839.9907 | 1840.9757 | -0.9850 | 0 | 9 | 14 | 2 | R.WLLLCNPGLADTIVEK.I |
|  | 17871 | 614.6365 | 1840.8876 | 1839.9155 | 0.9721 | 1 | 7 | 21 | 3 | R.VLYPNDNFFEGKELR.L |
|  | 18248 | 934.4620 | 1866.9095 | 1865.9159 | 0.9936 | 0 | 97 | 1.9e-008 | 1 | K.LQDFNVGDYIEAVLDR.N |
|  | 19910 | 1004.5635 | 2007.1124 | 2006.1200 | 0.9924 | 1 | 33 | 0.041 | 1 | R.LKQEYFVVAATLQDIIR.R |
|  | 25684 | **921.8369** | **2762.4887** | **2763.3827** | **-0.8940** | **1** | **0** | **54** | **7** | **K.LQDFNVGDYIEAVLDRNLAENISR.V** |

| **348.** | P07148\|FABPL_HUMAN    **Mass:** 14256    **Score:** 137    **Queries matched:** 10   **emPAI:** 2.38 |
| --- | --- |
|  | Fatty acid-binding protein, liver OS=Homo sapiens GN=FABP1 PE=1 SV=1 |

|  | **Query** | **Observed** | **Mr(expt)** | **Mr(calc)** | **Delta** | **Miss** | **Score** | **Expect** | **Rank** | **Peptide** |
| --- | --- | --- | --- | --- | --- | --- | --- | --- | --- | --- |
|  | 2262 | **824.4464** | **823.4391** | **823.4440** | **-0.0049** | **0** | **39** | **0.018** | **1** | **K.FTITAGSK.V** |
|  | 5063 | **516.2681** | **1030.5216** | **1029.5455** | **0.9761** | **0** | **22** | **1.2** | **1** | **K.GVSEIVQNGK.H** |
|  | 7612 | **605.8520** | **1209.6894** | **1209.6969** | **-0.0074** | **0** | **36** | **0.039** | **1** | **K.AIGLPEELIQK.G** |
|  | 9911 | 443.8952 | 1328.6638 | 1328.7300 | -0.0662 | 1 | 6 | 39 | 8 | K.VKTVVQLEGDNK.L |
|  | 16871 | 881.9031 | 1761.7917 | 1761.8032 | -0.0115 | 0 | 47 | 0.0024 | 1 | K.YQLQSQENFEAFMK.A 16870 |
|  | 23348 | **1191.6029** | **2381.1913** | **2380.2196** | **0.9717** | **0** | **81** | **6.2e-007** | **1** | **K.SVTELNGDIITNTMTLGDIVFK.R** 23323 |
|  | 23545 | 604.5530 | 2414.1831 | 2415.0875 | -0.9044 | 1 | 0 | 68 | 5 | -.MSFSGKYQLQSQENFEAFMK.A |
|  | 24421 | **846.4429** | **2536.3068** | **2536.3207** | **-0.0139** | **1** | **23** | **0.36** | **1** | **K.SVTELNGDIITNTMTLGDIVFKR.I** |

| **349.** | P05023\|AT1A1_HUMAN    **Mass:** 114135   **Score:** 137    **Queries matched:** 36   **emPAI:** 0.21 |
| --- | --- |
|  | Sodium/potassium-transporting ATPase subunit alpha-1 OS=Homo sapiens GN=ATP1A1 PE=1 SV=1 |

|  | **Query** | **Observed** | **Mr(expt)** | **Mr(calc)** | **Delta** | **Miss** | **Score** | **Expect** | **Rank** | **Peptide** |
| --- | --- | --- | --- | --- | --- | --- | --- | --- | --- | --- |
|  | 1381 | **633.3794** | **632.3721** | **632.3316** | **0.0405** | **0** | **13** | **14** | **3** | **K.NCLVK.N** |
|  | 1638 | 721.4193 | 720.4120 | 719.3748 | 1.0371 | 1 | 9 | 24 | 3 | -.MGKGVGR.D |
|  | 2741 | **862.4644** | **861.4571** | **861.4378** | **0.0193** | **0** | **3** | **1.1e+002** | **8** | **R.IISANGCK.V** |
|  | 3121 | 444.7401 | 887.4656 | 887.4865 | -0.0209 | 0 | 8 | 30 | 4 | K.YQLSIHK.N |
|  | 4306 | **488.9782** | **975.9417** | **975.3968** | **0.5450** | **0** | **6** | **35** | **4** | **K.EVSMDDHK.L** |
|  | 4338 | 490.2615 | 978.5085 | 978.5135 | -0.0050 | 0 | 13 | 7.8 | 1 | R.GIVVYTGDR.T |
|  | 5527 | 534.8080 | 1067.6015 | 1068.5676 | -0.9661 | 1 | 23 | 0.79 | 2 | K.GGDRIPADLR.I |
|  | 5751 | **543.3576** | **1084.7006** | **1084.5335** | **0.1671** | **0** | **18** | **2.7** | **1** | **K.ACVVHGSDLK.D** |
|  | 6024 | **553.2999** | **1104.5852** | **1104.5927** | **-0.0076** | **0** | **41** | **0.013** | **1** | **K.TSATWLALSR.I** |
|  | 6093 | 556.3039 | 1110.5932 | 1109.6193 | 0.9739 | 1 | 12 | 9.5 | 9 | K.LSLDELHRK.Y |
|  | 7590 | 605.3289 | 1208.6432 | 1209.5924 | -0.9493 | 1 | 4 | 62 | 7 | R.RNSVFQQGMK.N |
|  | 8139 | **618.8524** | **1235.6902** | **1235.6986** | **-0.0084** | **0** | **30** | **0.13** | **1** | **R.LNIPVSQVNPR.D** |
|  | 8611 | **630.9324** | **1259.8502** | **1260.6714** | **-0.8212** | **0** | **0** | **1.1e+002** | **1** | **K.IVEIPFNSTNK.Y** |
|  | 8743 | **634.8563** | **1267.6981** | **1267.7071** | **-0.0090** | **0** | **8** | **22** | **3** | **K.NMVPQQALVIR.N** |
|  | 9047 | **643.3311** | **1284.6475** | **1283.7020** | **0.9456** | **0** | **(3)** | **71** | **4** | **K.NMVPQQALVIR.N** |
|  | 11097 | **466.2328** | **1395.6766** | **1396.7384** | **-1.0618** | **0** | **1** | **1.1e+002** | **9** | **K.VIMVTGDHPITAK.A** |
|  | 13398 | **513.2558** | **1536.7456** | **1535.7103** | **1.0352** | **1** | **17** | **2.2** | **1** | **R.DKYEPAAVSEQGDK.K** 13375 |
|  | 13424 | **513.7847** | **1538.3322** | **1538.7875** | **-0.4554** | **1** | **13** | **4.5** | **1** | **R.GIVVYTGDRTVMGR.I** 13447 |
|  | 13614 | **517.7669** | **1550.2789** | **1549.8576** | **0.4213** | **1** | **2** | **51** | **6** | **R.LNIPVSQVNPRDAK.A** 13615 |
|  | 14114 | 792.9352 | 1583.8559 | 1583.8671 | -0.0112 | 0 | 33 | 0.064 | 1 | R.AVFQANQENLPILK.R |
|  | 14459 | 804.3953 | 1606.7761 | 1606.8137 | -0.0376 | 0 | 3 | 61 | 3 | K.NPNTSEPQHLLVMK.G |
|  | 14645 | **810.3738** | **1618.7331** | **1618.7435** | **-0.0103** | **0** | **40** | **0.012** | **1** | **K.VDNSSLTGESEPQTR.S** |
|  | 14892 | **818.4097** | **1634.8049** | **1633.8345** | **0.9704** | **1** | **1** | **90** | **4** | **K.KADIGVAMGIAGSDVSK.Q** |
|  | 16547 | **580.9738** | **1739.8994** | **1739.9682** | **-0.0688** | **1** | **4** | **45** | **4** | **R.AVFQANQENLPILKR.A** 16536 |
|  | 17090 | 594.2792 | 1779.8159 | 1779.9917 | -0.1758 | 1 | 7 | 20 | 2 | K.VIMVTGDHPITAKAIAK.G 17091 |
|  | 17716 | **915.4601** | **1828.9056** | **1828.9167** | **-0.0110** | **0** | **103** | **5.4e-009** | **1** | **K.GVGIISEGNETVEDIAAR.L** |
|  | 18015 | 926.9727 | 1851.9309 | 1853.0081 | -1.0772 | 1 | 4 | 44 | 2 | R.SAGIKVIMVTGDHPITAK.A |
|  | 18928 | **642.3124** | **1923.9153** | **1922.9156** | **0.9997** | **1** | **5** | **30** | **5** | **K.EVSMDDHKLSLDELHR.K** |
|  | 22454 | **759.1606** | **2274.4599** | **2274.1413** | **0.3186** | **1** | **4** | **20** | **1** | **R.NGEKMSINAEEVVVGDLVEVK.G** |
|  | 24059 | 1241.0720 | 2480.1295 | 2479.1901 | 0.9394 | 0 | 2 | 45 | 2 | K.QAADMILLDDNFASIVTGVEEGR.L |
|  | 26091 | 1417.6881 | 2833.3617 | 2833.3770 | -0.0153 | 1 | 18 | 0.93 | 1 | K.EQPLDEELKDAFQNAYLELGGLGER.V |

| **350.** | P04003\|C4BPA_HUMAN    **Mass:** 69042    **Score:** 137    **Queries matched:** 11   **emPAI:** 0.23 |
| --- | --- |
|  | C4b-binding protein alpha chain OS=Homo sapiens GN=C4BPA PE=1 SV=2 |

|  | **Query** | **Observed** | **Mr(expt)** | **Mr(calc)** | **Delta** | **Miss** | **Score** | **Expect** | **Rank** | **Peptide** |
| --- | --- | --- | --- | --- | --- | --- | --- | --- | --- | --- |
|  | 4366 | 491.1816 | 980.3487 | 980.4538 | -0.1051 | 0 | 5 | 48 | 9 | K.MAAWPFSR.L |
|  | 4962 | **512.7551** | **1023.4957** | **1023.5032** | **-0.0075** | **1** | **15** | **5.3** | **3** | **R.CRHPGELR.N** |
|  | 8863 | **637.9789** | **1273.9433** | **1274.5561** | **-0.6128** | **0** | **1** | **74** | **6** | **R.GSSVIHCDADSK.W** |
|  | 9297 | 433.5841 | 1297.7305 | 1296.7051 | 1.0254 | 1 | 10 | 13 | 1 | M.HPPKTPSGALHR.K |
|  | 11636 | **714.3367** | **1426.6589** | **1427.7456** | **-1.0867** | **1** | **1** | **1.1e+002** | **2** | **-.MHPPKTPSGALHR.K** |
|  | 12366 | **736.4088** | **1470.8031** | **1469.8089** | **0.9942** | **0** | **81** | **9.4e-007** | **1** | **K.LSLEIEQLELQR.D** |
|  | 14062 | **791.3540** | **1580.6934** | **1580.7042** | **-0.0107** | **0** | **35** | **0.032** | **1** | **R.FSAICQGDGTWSPR.T** |
|  | 16024 | **854.4014** | **1706.7883** | **1705.8610** | **0.9273** | **0** | **4** | **46** | **5** | **R.GVGWSHPLPQCEIVK.C** |
|  | 19750 | 998.4173 | 1994.8200 | 1994.8325 | -0.0124 | 0 | 60 | 9.5e-005 | 1 | R.WTPYQGCEALCCPEPK.L 19766 |
|  | 26628 | **975.1074** | **2922.3004** | **2922.3204** | **-0.0200** | **1** | **35** | **0.015** | **1** | **R.TWYPEVPKCEWETPEGCEQVLTGK.R** |

| **351.** | P13639\|EF2_HUMAN    **Mass:** 96246    **Score:** 137    **Queries matched:** 22   **emPAI:** 0.25 |
| --- | --- |
|  | Elongation factor 2 OS=Homo sapiens GN=EEF2 PE=1 SV=4 |

|  | **Query** | **Observed** | **Mr(expt)** | **Mr(calc)** | **Delta** | **Miss** | **Score** | **Expect** | **Rank** | **Peptide** |
| --- | --- | --- | --- | --- | --- | --- | --- | --- | --- | --- |
|  | 1473 | **659.3287** | **658.3215** | **657.4061** | **0.9154** | **0** | **14** | **12** | **3** | **K.LVEGLK.R** |
|  | 4192 | **485.2742** | **968.5339** | **968.5403** | **-0.0064** | **0** | **21** | **1.2** | **2** | **R.GGGQIIPTAR.R** |
|  | 5639 | 539.2736 | 1076.5326 | 1076.5325 | 0.0001 | 0 | 5 | 49 | 10 | R.IMGPNYTPGK.K |
|  | 5727 | **542.7750** | **1083.5354** | **1083.5423** | **-0.0069** | **0** | **20** | **1.7** | **1** | **K.VFDAIMNFK.K** |
|  | 7673 | **607.3628** | **1212.7111** | **1211.6258** | **1.0853** | **1** | **7** | **29** | **1** | **K.GEGQLGPAERAK.K** |
|  | 8248 | **414.5639** | **1240.6700** | **1240.7325** | **-0.0626** | **1** | **8** | **24** | **4** | **K.EGKPLLKAVMR.R** |
|  | 8542 | **419.8887** | **1256.6443** | **1256.7274** | **-0.0832** | **1** | **(5)** | **45** | **4** | **K.EGKPLLKAVMR.R** |
|  | 9493 | **654.8266** | **1307.6386** | **1306.6452** | **0.9934** | **0** | **50** | **0.0014** | **1** | **R.NMSVIAHVDHGK.S** |
|  | 10314 | 676.6600 | 1351.3054 | 1350.7507 | 0.5547 | 1 | 2 | 56 | 3 | R.VAVEAKNPADLPK.L |
|  | 11831 | 720.4023 | 1438.7901 | 1438.6762 | 0.1139 | 0 | 1 | 93 | 3 | K.DLEEDHACIPIK.K |
|  | 12885 | 752.4078 | 1502.8011 | 1502.7591 | 0.0420 | 1 | 3 | 71 | 8 | K.QFAEMYVAKFAAK.G |
|  | 12923 | 502.9083 | 1505.7030 | 1506.7720 | -1.0690 | 1 | 17 | 2.3 | 2 | R.IKPVLMMNKMDR.A |
|  | 14577 | **539.2560** | **1614.7461** | **1614.7573** | **-0.0112** | **0** | **12** | **7.1** | **1** | **K.TGTITTFEHAHNMR.V** 14596 |
|  | 17345 | **900.4466** | **1798.8786** | **1798.8890** | **-0.0104** | **0** | **69** | **1.3e-005** | **1** | **K.AYLPVNESFGFTADLR.S** 17344 |
|  | 21322 | 715.0376 | 2142.0910 | 2142.0705 | 0.0204 | 1 | 24 | 0.35 | 1 | K.ARPFPDGLAEDIDKGEVSAR.Q |
|  | 22014 | **1110.5748** | **2219.1351** | **2219.1474** | **-0.0122** | **0** | **43** | **0.004** | **1** | **R.ALLELQLEPEELYQTFQR.I** 22015 |
|  | 24846 | **867.4767** | **2599.4082** | **2599.4196** | **-0.0114** | **0** | **24** | **0.23** | **1** | **R.WLPAGDALLQMITIHLPSPVTAQK.Y** |
|  | 27718 | **630.6960** | **3148.4435** | **3148.5903** | **-0.1468** | **1** | **0** | **40** | **4** | **K.IWCFGPDGTGPNILTDITKGVQYLNEIK.D** |
|  | 28435 | 1121.9351 | 3362.7834 | 3363.6149 | -0.8315 | 0 | 4 | 16 | 4 | K.SDPMVQCIIEESGEHIIAGAGELHLEICLK.D |

| **352.** | P37802\|TAGL2_HUMAN    **Mass:** 22548    **Score:** 135    **Queries matched:** 15   **emPAI:** 0.17 |
| --- | --- |
|  | Transgelin-2 OS=Homo sapiens GN=TAGLN2 PE=1 SV=3 |

|  | **Query** | **Observed** | **Mr(expt)** | **Mr(calc)** | **Delta** | **Miss** | **Score** | **Expect** | **Rank** | **Peptide** |
| --- | --- | --- | --- | --- | --- | --- | --- | --- | --- | --- |
|  | 4572 | **498.7588** | **995.5031** | **995.5110** | **-0.0079** | **0** | **7** | **28** | **3** | **K.IQASTMAFK.Q** |
|  | 6080 | **555.7108** | **1109.4071** | **1108.6101** | **0.7970** | **1** | **4** | **56** | **6** | **R.KDVGRPQPGR.E** 6075 |
|  | 7703 | 608.3338 | 1214.6530 | 1214.6805 | -0.0275 | 0 | 13 | 7.9 | 6 | R.TLMNLGGLAVAR.D |
|  | 9184 | **431.7291** | **1292.1654** | **1291.6455** | **0.5198** | **1** | **6** | **27** | **5** | **-.MANRGPAYGLSR.E** |
|  | 10873 | 692.3431 | 1382.6716 | 1382.6071 | 0.0645 | 0 | 8 | 20 | 2 | R.GASQAGMTGYGMPR.Q |
|  | 11177 | 700.8335 | 1399.6524 | 1398.6020 | 1.0504 | 0 | (0) | 1.3e+002 | 8 | R.GASQAGMTGYGMPR.Q |
|  | 11409 | **707.8140** | **1413.6134** | **1414.5969** | **-0.9836** | **0** | **(3)** | **60** | **2** | **R.GASQAGMTGYGMPR.Q** |
|  | 11721 | **716.8539** | **1431.6933** | **1431.7470** | **-0.0537** | **1** | **9** | **18** | **5** | **R.GPAYGLSREVQQK.I** |
|  | 15808 | 847.9595 | 1693.9044 | 1693.8093 | 0.0950 | 0 | 6 | 30 | 3 | K.QMEQISQFLQAAER.Y |
|  | 16941 | **590.3463** | **1768.0171** | **1768.8236** | **-0.8065** | **1** | **9** | **12** | **1** | **R.GASQAGMTGYGMPRQIL.-** |
|  | 20254 | 680.9985 | 2039.9736 | 2040.0289 | -0.0554 | 1 | 1 | 73 | 7 | K.DVGRPQPGRENFQNWLK.D |
|  | 20841 | **697.3626** | **2089.0660** | **2090.0547** | **-0.9887** | **1** | **0** | **83** | **8** | **K.NMACVQRTLMNLGGLAVAR.D** |
|  | 20945 | **1050.5134** | **2099.0123** | **2099.0211** | **-0.0088** | **0** | **135** | **2.6e-012** | **1** | **R.YGINTTDIFQTVDLWEGK.N** |
|  | 24044 | 826.7730 | 2477.2972 | 2478.1921 | -0.8949 | 1 | 6 | 18 | 5 | R.NFSDNQLQEGKNVIGLQMGTNR.G |

| **353.** | P61006\|RAB8A_HUMAN    **Mass:** 23824    **Score:** 134    **Queries matched:** 17   **emPAI:** 0.81 |
| --- | --- |
|  | Ras-related protein Rab-8A OS=Homo sapiens GN=RAB8A PE=1 SV=1 |

|  | **Query** | **Observed** | **Mr(expt)** | **Mr(calc)** | **Delta** | **Miss** | **Score** | **Expect** | **Rank** | **Peptide** |
| --- | --- | --- | --- | --- | --- | --- | --- | --- | --- | --- |
|  | 1925 | **799.3966** | **798.3893** | **798.4137** | **-0.0244** | **1** | **5** | **51** | **4** | **K.RSSFFR.C** |
|  | 5578 | 536.3207 | 1070.6269 | 1070.6336 | -0.0066 | 0 | 53 | 0.00096 | 1 | K.LLLIGDSGVGK.T 5577 |
|  | 9225 | **432.2579** | **1293.7519** | **1294.6267** | **-0.8749** | **1** | **11** | **11** | **1** | **-.MAKTYDYLFK.L** |
|  | 9635 | 658.8289 | 1315.6433 | 1315.6521 | -0.0088 | 0 | 60 | 0.00015 | 1 | K.LQIWDTAGQER.F 9612 9633 9634 |
|  | 11442 | **708.3881** | **1414.7617** | **1413.6848** | **1.0769** | **0** | **3** | **61** | **4** | **K.LEGNSPQGSNQGVK.I** 11409 |
|  | 11558 | 711.8276 | 1421.6407 | 1421.6643 | -0.0235 | 1 | 4 | 45 | 7 | K.MILGNKCDVNDK.R |
|  | 13494 | **772.3932** | **1542.7718** | **1541.7798** | **0.9921** | **1** | **51** | **0.00091** | **1** | **K.KLEGNSPQGSNQGVK.I** 13477 13478 13480 |
|  | 14016 | **790.4100** | **1578.8055** | **1578.8154** | **-0.0099** | **0** | **22** | **0.81** | **1** | **K.ANINVENAFFTLAR.D** 14007 |

| **354.** | P08865\|RSSA_HUMAN    **Mass:** 32947    **Score:** 133    **Queries matched:** 11   **emPAI:** 0.38 |
| --- | --- |
|  | 40S ribosomal protein SA OS=Homo sapiens GN=RPSA PE=1 SV=4 |

|  | **Query** | **Observed** | **Mr(expt)** | **Mr(calc)** | **Delta** | **Miss** | **Score** | **Expect** | **Rank** | **Peptide** |
| --- | --- | --- | --- | --- | --- | --- | --- | --- | --- | --- |
|  | 2442 | **419.3141** | **836.6137** | **835.4334** | **1.1803** | **1** | **5** | **42** | **10** | **R.MRGTISR.E** |
|  | 7594 | **605.3978** | **1208.7810** | **1207.5941** | **1.1869** | **0** | **(3)** | **63** | **3** | **-.MSGALDVLQMK.E** |
|  | 7921 | **613.3446** | **1224.6746** | **1223.5890** | **1.0857** | **0** | **4** | **58** | **3** | **-.MSGALDVLQMK.E** |
|  | 8671 | 421.9079 | 1262.7018 | 1262.7234 | -0.0216 | 1 | 9 | 19 | 6 | R.KSDGIYIINLK.R |
|  | 9452 | **653.8219** | **1305.6292** | **1305.6387** | **-0.0095** | **0** | **25** | **0.47** | **1** | **R.YVDIAIPCNNK.G** |
|  | 15875 | **849.9283** | **1697.8420** | **1697.8526** | **-0.0106** | **0** | **56** | **0.0003** | **1** | **R.FTPGTFTNQIQAAFR.E** |
|  | 16552 | **870.9731** | **1739.9317** | **1739.9417** | **-0.0100** | **0** | **81** | **8.1e-007** | **1** | **R.AIVAIENPADVSVISSR.N** |
|  | 18902 | **641.3329** | **1920.9768** | **1920.9536** | **0.0232** | **1** | **2** | **57** | **8** | **-.MSGALDVLQMKEEDVLK.F** 18913 |
|  | 27034 | **999.4902** | **2995.4489** | **2995.4709** | **-0.0221** | **0** | **26** | **0.12** | **1** | **R.ADHQPLTEASYVNLPTIALCNTDSPLR.Y** 27035 |

| **355.** | P00403\|COX2_HUMAN    **Mass:** 25719    **Score:** 131    **Queries matched:** 15   **emPAI:** 0.73 |
| --- | --- |
|  | Cytochrome c oxidase subunit 2 OS=Homo sapiens GN=MT-CO2 PE=1 SV=1 |

|  | **Query** | **Observed** | **Mr(expt)** | **Mr(calc)** | **Delta** | **Miss** | **Score** | **Expect** | **Rank** | **Peptide** |
| --- | --- | --- | --- | --- | --- | --- | --- | --- | --- | --- |
|  | 1647 | **729.3837** | **728.3765** | **728.3817** | **-0.0052** | **0** | **13** | **11** | **1** | **K.TDAIPGR.L** 1644 |
|  | 2511 | 422.7269 | 843.4392 | 843.4450 | -0.0058 | 0 | 22 | 1.3 | 1 | R.LLDVDNR.V 2519 |
|  | 6040 | **553.8470** | **1105.6795** | **1105.6859** | **-0.0064** | **0** | **22** | **1.1** | **1** | **R.VVLPIEAPIR.M** |
|  | 18012 | 618.2805 | 1851.8196 | 1850.9335 | 0.8860 | 0 | 3 | 54 | 7 | R.ILYMTDEVNDPSLTIK.S |
|  | 22111 | **1114.0779** | **2226.1412** | **2226.1541** | **-0.0129** | **0** | **68** | **1.2e-005** | **1** | **R.MMITSQDVLHSWAVPTLGLK.T** 22092 22110 |
|  | 30819 | **1107.3015** | **4425.1769** | **4425.1960** | **-0.0190** | **0** | **24** | **0.08** | **1** | **R.LNQTTFTATRPGVYYGQCSEICGANHSFMPIVLELIPLK.I** 30816 30817 30818 30820 |
|  | 31687 | **1119.5593** | **5592.7603** | **5591.8985** | **0.8618** | **0** | **3** | **4.8** | **1** | **-.MAHAAQVGLQDATSPIMEELITFHDHALMIIFLICFLVLYALFLTLTTK.L** |

| **356.** | P05089\|ARGI1_HUMAN    **Mass:** 34884    **Score:** 131    **Queries matched:** 18   **emPAI:** 0.85 |
| --- | --- |
|  | Arginase-1 OS=Homo sapiens GN=ARG1 PE=1 SV=2 |

|  | **Query** | **Observed** | **Mr(expt)** | **Mr(calc)** | **Delta** | **Miss** | **Score** | **Expect** | **Rank** | **Peptide** |
| --- | --- | --- | --- | --- | --- | --- | --- | --- | --- | --- |
|  | 3913 | **474.7967** | **947.5789** | **947.5440** | **0.0349** | **0** | **9** | **23** | **2** | **K.DIVYIGLR.D** |
|  | 6549 | **574.2548** | **1146.4950** | **1146.5016** | **-0.0066** | **0** | **10** | **16** | **2** | **K.YFSMTEVDR.L** |
|  | 8246 | 621.3418 | 1240.6690 | 1240.6776 | -0.0085 | 1 | 43 | 0.0064 | 1 | R.GGVEEGPTVLRK.A 8254 |
|  | 9044 | **643.3201** | **1284.6256** | **1284.6350** | **-0.0094** | **0** | **25** | **0.49** | **1** | **R.DVDPGEHYILK.T** 9051 |
|  | 10395 | 678.8706 | 1355.7267 | 1356.6813 | -0.9546 | 0 | 1 | 1.2e+002 | 5 | R.EGLYITEEIYK.T |
|  | 11348 | 705.8641 | 1409.7137 | 1409.7224 | -0.0087 | 0 | 32 | 0.082 | 1 | K.VMEETLSYLLGR.K 11374 |
|  | 11624 | **476.1948** | **1425.5625** | **1425.7173** | **-0.1548** | **0** | **(10)** | **13** | **3** | **K.VMEETLSYLLGR.K** 11629 11632 |
|  | 17043 | 889.0327 | 1776.0509 | 1776.9556 | -0.9048 | 0 | 0 | 94 | 9 | R.TVNTAVAITLACFGLAR.E |
|  | 18114 | **620.2764** | **1857.8073** | **1858.9710** | **-1.1637** | **0** | **5** | **33** | **7** | **K.TGLLSGLDIMEVNPSLGK.T** |
|  | 18338 | **937.4738** | **1872.9331** | **1872.9444** | **-0.0113** | **0** | **69** | **1.3e-005** | **1** | **K.IPDVPGFSWVTPCISAK.D** |
|  | 20509 | 687.3582 | 2059.0526 | 2058.0609 | 0.9918 | 1 | 25 | 0.29 | 1 | K.GKIPDVPGFSWVTPCISAK.D |
|  | 22297 | **1126.0440** | **2250.0733** | **2250.0845** | **-0.0111** | **0** | **54** | **0.00034** | **1** | **K.DYGDLPFADIPNDSPFQIVK.N** |
|  | 28181 | **822.4247** | **3285.6696** | **3284.6830** | **0.9866** | **0** | **9** | **6.2** | **1** | **K.RPIHLSFDVDGLDPSFTPATGTPVVGGLTYR.E** |

| **357.** | P07954\|FUMH_HUMAN    **Mass:** 54773    **Score:** 131    **Queries matched:** 13   **emPAI:** 0.14 |
| --- | --- |
|  | Fumarate hydratase, mitochondrial OS=Homo sapiens GN=FH PE=1 SV=3 |

|  | **Query** | **Observed** | **Mr(expt)** | **Mr(calc)** | **Delta** | **Miss** | **Score** | **Expect** | **Rank** | **Peptide** |
| --- | --- | --- | --- | --- | --- | --- | --- | --- | --- | --- |
|  | 1893 | 398.7224 | 795.4303 | 795.4351 | -0.0048 | 0 | 16 | 3.6 | 1 | K.NVLHSAR.L |
|  | 2088 | **405.2987** | **808.5828** | **808.4377** | **0.1451** | **1** | **8** | **20** | **6** | **-.MYRALR.L** |
|  | 2117 | 812.4085 | 811.4012 | 811.5392 | -0.1380 | 1 | 2 | 91 | 9 | R.ALRLLAR.S |
|  | 7520 | **603.3205** | **1204.6264** | **1203.6169** | **1.0095** | **0** | **12** | **9.9** | **2** | **R.AIEMLGGELGSK.I** |
|  | 12686 | 745.8144 | 1489.6143 | 1489.7049 | -0.0905 | 0 | 8 | 18 | 2 | R.AAAEVNQDYGLDPK.I |
|  | 15073 | 823.9119 | 1645.8093 | 1645.8345 | -0.0252 | 1 | 4 | 47 | 4 | K.IANAIMKAADEVAEGK.L |
|  | 21154 | **1063.0393** | **2124.0641** | **2124.0772** | **-0.0132** | **0** | **9** | **12** | **1** | **R.SGLGELILPENEPGSSIMPGK.V** |
|  | 21307 | 714.3697 | 2140.0874 | 2140.0722 | 0.0153 | 0 | (7) | 19 | 3 | R.SGLGELILPENEPGSSIMPGK.V |
|  | 22353 | **753.7178** | **2258.1315** | **2258.1439** | **-0.0124** | **0** | **83** | **4.2e-007** | **1** | **K.LMNESLMLVTALNPHIGYDK.A** |
|  | 22711 | 769.4009 | 2305.1810 | 2305.1848 | -0.0039 | 1 | 2 | 48 | 6 | K.AAMPRIYELAAGGTAVGTGLNTR.I |
|  | 23926 | **823.0803** | **2466.2191** | **2466.2318** | **-0.0127** | **0** | **10** | **6.6** | **1** | **K.ETAIELGYLTAEQFDEWVKPK.D** |
|  | 28158 | **1090.8641** | **3269.5706** | **3269.5888** | **-0.0182** | **0** | **63** | **2.4e-005** | **1** | **K.LNDHFPLVVWQTGSGTQTNMNVNEVISNR.A** 28157 |

| **358.** | P07307\|ASGR2_HUMAN    **Mass:** 35697    **Score:** 130    **Queries matched:** 5   **emPAI:** 0.35 |
| --- | --- |
|  | Asialoglycoprotein receptor 2 OS=Homo sapiens GN=ASGR2 PE=1 SV=2 |

|  | **Query** | **Observed** | **Mr(expt)** | **Mr(calc)** | **Delta** | **Miss** | **Score** | **Expect** | **Rank** | **Peptide** |
| --- | --- | --- | --- | --- | --- | --- | --- | --- | --- | --- |
|  | 1553 | **689.4140** | **688.4067** | **688.4119** | **-0.0052** | **0** | **22** | **1.7** | **1** | **K.ITSLGAK.L** |
|  | 6329 | **565.8377** | **1129.6609** | **1130.6196** | **-0.9588** | **0** | **1** | **1.5e+002** | **9** | **K.GPPPAQPLAQR.L** |
|  | 13082 | **758.8344** | **1515.6543** | **1514.6613** | **0.9930** | **0** | **69** | **1.6e-005** | **1** | **R.WNDDFCLQVYR.W** |
|  | 25108 | **888.4288** | **2662.2647** | **2662.2816** | **-0.0170** | **0** | **52** | **0.00039** | **1** | **K.FIVQHTNPFNTWIGLTDSDGSWK.W** |
|  | 28188 | **1097.1423** | **3288.4052** | **3288.4279** | **-0.0228** | **0** | **48** | **0.00061** | **1** | **K.NWAVTQPDNWHGHELGGSEDCVEVQPDGR.W** |

| **359.** | Q9UBS4\|DJB11_HUMAN    **Mass:** 40774    **Score:** 130    **Queries matched:** 16   **emPAI:** 0.09 |
| --- | --- |
|  | DnaJ homolog subfamily B member 11 OS=Homo sapiens GN=DNAJB11 PE=1 SV=1 |

|  | **Query** | **Observed** | **Mr(expt)** | **Mr(calc)** | **Delta** | **Miss** | **Score** | **Expect** | **Rank** | **Peptide** |
| --- | --- | --- | --- | --- | --- | --- | --- | --- | --- | --- |
|  | 2331 | **829.4466** | **828.4393** | **828.4454** | **-0.0061** | **0** | **14** | **8.4** | **3** | **R.TTQLGPGR.F** |
|  | 4006 | **478.2525** | **954.4905** | **953.5195** | **0.9710** | **1** | **4** | **54** | **9** | **K.HPIFERR.G** |
|  | 4295 | 488.3156 | 974.6167 | 974.4669 | 0.1498 | 0 | 8 | 32 | 6 | K.EQLTEEAR.E |
|  | 4422 | **493.2907** | **984.5668** | **984.5716** | **-0.0048** | **1** | **4** | **62** | **10** | **R.DKITRPGAK.L** |
|  | 4992 | **513.7482** | **1025.4819** | **1025.5366** | **-0.0547** | **1** | **5** | **45** | **2** | **R.QQDRNIPR.G** 5007 |
|  | 7185 | 397.4022 | 1189.1849 | 1189.6931 | -0.5082 | 1 | 12 | 6.7 | 2 | R.KLALQLHPDR.N |
|  | 7548 | **403.0460** | **1206.1161** | **1206.6761** | **-0.5600** | **1** | **6** | **24** | **4** | **R.DFYKILGVPR.S** |
|  | 8254 | 621.8157 | 1241.6168 | 1240.6663 | 0.9505 | 0 | 17 | 2.6 | 2 | R.TLEVEIEPGVR.D |
|  | 9358 | **434.7275** | **1301.1608** | **1301.5775** | **-0.4168** | **0** | **4** | **34** | **3** | **K.QYDTYGEEGLK.D** 9359 |
|  | 18974 | **643.3422** | **1927.0047** | **1926.9323** | **0.0724** | **1** | **17** | **1.8** | **1** | **K.FQDLGAAYEVLSDSEKR.K** |
|  | 19601 | **992.4269** | **1982.8392** | **1982.8536** | **-0.0144** | **0** | **91** | **6.6e-008** | **1** | **R.FQMTQEVVCDECPNVK.L** 19602 |
|  | 24217 | **834.9982** | **2501.9729** | **2502.4032** | **-0.4304** | **0** | **3** | **29** | **1** | **M.APQNLSTFCLLLLYLIGAVIAGR.D** |
|  | 25859 | 699.5815 | 2794.2968 | 2793.2885 | 1.0084 | 1 | 1 | 53 | 2 | R.TTQLGPGRFQMTQEVVCDECPNVK.L |

| **360.** | O75746\|CMC1_HUMAN    **Mass:** 75114    **Score:** 129    **Queries matched:** 24   **emPAI:** 0.21 |
| --- | --- |
|  | Calcium-binding mitochondrial carrier protein Aralar1 OS=Homo sapiens GN=SLC25A12 PE=1 SV=2 |

|  | **Query** | **Observed** | **Mr(expt)** | **Mr(calc)** | **Delta** | **Miss** | **Score** | **Expect** | **Rank** | **Peptide** |
| --- | --- | --- | --- | --- | --- | --- | --- | --- | --- | --- |
|  | 763 | **474.9745** | **473.9672** | **474.2550** | **-0.2878** | **0** | **2** | **1.7e+002** | **2** | **K.GTAAR.V** 768 777 |
|  | 1465 | 657.3996 | 656.3923 | 656.3969 | -0.0046 | 0 | 25 | 0.56 | 1 | R.LQVAAR.A |
|  | 2257 | 823.4509 | 822.4437 | 822.3984 | 0.0452 | 0 | 8 | 25 | 7 | R.GDPHELR.N |
|  | 2969 | 439.2309 | 876.4472 | 876.4930 | -0.0457 | 1 | 3 | 92 | 7 | K.GTAARVFR.S |
|  | 3452 | 457.3125 | 912.6104 | 913.5457 | -0.9353 | 1 | 2 | 97 | 4 | K.TRLQVAAR.A |
|  | 4111 | 482.2635 | 962.5124 | 962.5185 | -0.0062 | 0 | 39 | 0.021 | 1 | K.LTVNDFVR.D 4135 |
|  | 4337 | 490.2477 | 978.4807 | 978.4995 | -0.0188 | 1 | 7 | 30 | 2 | K.RGDPHELR.N |
|  | 5247 | 523.2389 | 1044.4632 | 1044.4699 | -0.0066 | 1 | 25 | 0.54 | 1 | K.NSFDCFKK.V |
|  | 6595 | 576.2760 | 1150.5374 | 1150.5447 | -0.0073 | 0 | 59 | 0.00021 | 1 | R.YEGFFGLYR.G |
|  | 8120 | **618.3489** | **1234.6832** | **1234.6557** | **0.0275** | **0** | **6** | **33** | **5** | **R.LATATFAGIENK.F** |
|  | 8273 | **415.2021** | **1242.5845** | **1241.5962** | **0.9883** | **0** | **7** | **33** | **6** | **R.GSGSVVGELMYK.N** |
|  | 10007 | 667.9017 | 1333.7888 | 1333.7969 | -0.0081 | 0 | 46 | 0.0034 | 1 | R.GLIPQLIGVAPEK.A |
|  | 15946 | 851.9084 | 1701.8022 | 1701.8145 | -0.0122 | 1 | 63 | 5e-005 | 1 | R.AGQTTYSGVIDCFRK.I |
|  | 18852 | **639.6557** | **1915.9453** | **1914.8928** | **1.0525** | **1** | **0** | **95** | **4** | **R.MQNQRGSGSVVGELMYK.N** |
|  | 20666 | 691.3754 | 2071.1043 | 2072.0282 | -0.9239 | 1 | 15 | 2.5 | 4 | K.SKSGMISGLDFSDIMVTIR.S 20650 20652 20682 |
|  | 28908 | 1769.9185 | 3537.8224 | 3537.9229 | -0.1005 | 0 | 26 | 0.084 | 2 | K.LLLADENGHVGGLNLLAAGAMAGVPAASLVTPADVIK.T 28909 28911 |

| **361.** | P14314\|GLU2B_HUMAN    **Mass:** 60357    **Score:** 128    **Queries matched:** 19   **emPAI:** 0.61 |
| --- | --- |
|  | Glucosidase 2 subunit beta OS=Homo sapiens GN=PRKCSH PE=1 SV=2 |

|  | **Query** | **Observed** | **Mr(expt)** | **Mr(calc)** | **Delta** | **Miss** | **Score** | **Expect** | **Rank** | **Peptide** |
| --- | --- | --- | --- | --- | --- | --- | --- | --- | --- | --- |
|  | 4619 | **499.7659** | **997.5172** | **998.6124** | **-1.0952** | **1** | **4** | **63** | **4** | **K.LIELQAGKK.S** |
|  | 6705 | **579.8075** | **1157.6004** | **1157.6080** | **-0.0076** | **0** | **59** | **0.00021** | **1** | **K.LWEEQLAAAK.A** 6704 |
|  | 7794 | **610.2992** | **1218.5838** | **1218.5914** | **-0.0076** | **0** | **4** | **68** | **4** | **K.SLEDQVEMLR.T** |
|  | 8118 | **618.3360** | **1234.6574** | **1234.5863** | **0.0711** | **0** | **(2)** | **95** | **5** | **K.SLEDQVEMLR.T** |
|  | 9159 | **646.3148** | **1290.6151** | **1290.6238** | **-0.0087** | **0** | **40** | **0.014** | **1** | **R.ESLQQMAEVTR.E** |
|  | 9610 | **658.3412** | **1314.6679** | **1314.6779** | **-0.0100** | **1** | **34** | **0.062** | **1** | **R.TVKEEAEKPER.E** |
|  | 10494 | **682.3422** | **1362.6698** | **1362.6813** | **-0.0115** | **1** | **3** | **75** | **3** | **K.KSLEDQVEMLR.T** |
|  | 11888 | 722.4097 | 1442.8048 | 1443.8272 | -1.0224 | 1 | 8 | 22 | 4 | R.LCPFKLVSQKPK.L 11887 |
|  | 13636 | **776.8281** | **1551.6416** | **1551.6525** | **-0.0109** | **0** | **47** | **0.0026** | **1** | **K.YEQGTGCWQGPNR.S** |
|  | 13800 | **521.8254** | **1562.4545** | **1562.7974** | **-0.3429** | **1** | **6** | **21** | **2** | **K.SLEDQVEMLRTVK.E** |
|  | 23562 | 806.4080 | 2416.2021 | 2416.4214 | -0.2194 | 1 | 1 | 57 | 8 | M.LLPLLLLLPMCWAVEVKRPR.G |
|  | 25513 | **911.4369** | **2731.2888** | **2731.3065** | **-0.0176** | **1** | **23** | **0.33** | **1** | **K.LGGSPTSLGTWGSWIGPDHDKFSAMK.Y** |
|  | 31104 | **1559.6619** | **4675.9638** | **4675.9896** | **-0.0259** | **1** | **33** | **0.0082** | **1** | **K.ETMVTSTTEPSRCEYLMELMTPAACPEPPPEAPTEDDHDEL.-** 31103 31105 |
|  | 31625 | **1112.9515** | **5559.7213** | **5560.4232** | **-0.7018** | **0** | **2** | **7.7** | **1** | **K.ELDDDMDGTVSVTELQTHPELDTDGDGALSEAEAQALLSGDTQTDATSFYDR.V** |
|  | 31899 | **1396.0225** | **6975.0759** | **6974.1120** | **0.9640** | **1** | **1** | **3.4** | **1** | **K.AQQEQELAADAFKELDDDMDGTVSVTELQTHPELDTDGDGALSEAEAQALLSGDTQTDATSFYDR.V** |

| **362.** | P55072\|TERA_HUMAN    **Mass:** 89950    **Score:** 128    **Queries matched:** 27   **emPAI:** 0.04 |
| --- | --- |
|  | Transitional endoplasmic reticulum ATPase OS=Homo sapiens GN=VCP PE=1 SV=4 |

|  | **Query** | **Observed** | **Mr(expt)** | **Mr(calc)** | **Delta** | **Miss** | **Score** | **Expect** | **Rank** | **Peptide** |
| --- | --- | --- | --- | --- | --- | --- | --- | --- | --- | --- |
|  | 3667 | 466.2373 | 930.4600 | 929.5546 | 0.9054 | 1 | 10 | 18 | 8 | R.GDTVLLKGK.K |
|  | 3899 | **474.2844** | **946.5543** | **946.5600** | **-0.0057** | **1** | **5** | **64** | **5** | **R.KGDIFLVR.G** |
|  | 6169 | **559.2820** | **1116.5495** | **1115.4956** | **1.0540** | **0** | **2** | **1.1e+002** | **4** | **R.GGNIGDGGGAADR.V** |
|  | 6181 | 559.7886 | 1117.5626 | 1117.5727 | -0.0101 | 1 | 8 | 26 | 7 | R.ESIESEIRR.E 6178 |
|  | 6854 | **585.7550** | **1169.4954** | **1170.6107** | **-1.1153** | **1** | **1** | **1.2e+002** | **9** | **K.FLKFGMTPSK.G** |
|  | 7468 | 602.3072 | 1202.5999 | 1202.6255 | -0.0255 | 1 | 11 | 12 | 6 | K.LAGESESNLRK.A |
|  | 8276 | **622.3262** | **1242.6379** | **1241.6252** | **1.0127** | **0** | **24** | **0.54** | **1** | **R.EVDIGIPDATGR.L** 8254 8277 |
|  | 9913 | 665.3396 | 1328.6646 | 1328.6837 | -0.0190 | 0 | 14 | 5.6 | 2 | R.WALSQSNPSALR.E |
|  | 13310 | 765.8718 | 1529.7290 | 1530.8293 | -1.1004 | 1 | 2 | 73 | 5 | K.SPVAKDVDLEFLAK.M |
|  | 13717 | **778.9274** | **1555.8402** | **1555.8497** | **-0.0095** | **0** | **3** | **55** | **2** | **R.LDQLIYIPLPDEK.S** |
|  | 14823 | **544.3179** | **1629.9318** | **1628.7981** | **1.1337** | **1** | **15** | **3.5** | **1** | **R.KYEMFAQTLQQSR.G** |
|  | 17048 | **593.1561** | **1776.4464** | **1776.9444** | **-0.4980** | **1** | **2** | **44** | **5** | **K.MDELQLFRGDTVLLK.G** |
|  | 17345 | 900.4466 | 1798.8786 | 1798.7978 | 0.0808 | 0 | 8 | 17 | 5 | K.MTNGFSGADLTEICQR.A 17344 |
|  | 17488 | **905.9968** | **1809.9790** | **1809.9876** | **-0.0086** | **0** | **3** | **46** | **2** | **K.NAPAIIFIDELDAIAPK.R** |
|  | 21559 | **1085.5581** | **2169.1017** | **2169.1165** | **-0.0148** | **0** | **86** | **2.2e-007** | **1** | **R.LIVDEAINEDNSVVSLSQPK.M** 21560 |
|  | 22781 | **771.8914** | **2312.6523** | **2312.9703** | **-0.3180** | **1** | **2** | **37** | **5** | **R.EDEEESLNEVGYDDIGGCRK.Q** 22788 |
|  | 23844 | **819.4202** | **2455.2387** | **2456.1125** | **-0.8738** | **1** | **0** | **70** | **8** | **R.ERQTNPSAMEVEEDDPVPEIR.R** |
|  | 24192 | 833.4252 | 2497.2539 | 2497.2701 | -0.0162 | 1 | 3 | 38 | 8 | R.ETVVEVPQVTWEDIGGLEDVKR.E |
|  | 25844 | **699.1020** | **2792.3791** | **2792.3069** | **0.0722** | **1** | **0** | **57** | **10** | **R.GGNIGDGGGAADRVINQILTEMDGMSTK.K** |
|  | 27581 | **779.8359** | **3115.3144** | **3116.3876** | **-1.0732** | **0** | **3** | **23** | **3** | **K.MDLIDLEDETIDAEVMNSLAVTMDDFR.W** |
|  | 30808 | **1471.5956** | **4411.7649** | **4411.0658** | **0.6991** | **1** | **1** | **11** | **4** | **K.MDLIDLEDETIDAEVMNSLAVTMDDFRWALSQSNPSALR.E** |

| **363.** | P61224\|RAP1B_HUMAN    **Mass:** 21040    **Score:** 127    **Queries matched:** 13   **emPAI:** 0.65 |
| --- | --- |
|  | Ras-related protein Rap-1b OS=Homo sapiens GN=RAP1B PE=1 SV=1 |

|  | **Query** | **Observed** | **Mr(expt)** | **Mr(calc)** | **Delta** | **Miss** | **Score** | **Expect** | **Rank** | **Peptide** |
| --- | --- | --- | --- | --- | --- | --- | --- | --- | --- | --- |
|  | 2293 | **413.2655** | **824.5164** | **824.4868** | **0.0296** | **1** | **5** | **40** | **4** | **K.TPVPGKAR.K** |
|  | 3747 | **469.2074** | **936.4002** | **935.3654** | **1.0348** | **0** | **5** | **46** | **2** | **K.CDLEDER.V** |
|  | 4425 | **493.3024** | **984.5903** | **984.5968** | **-0.0065** | **0** | **4** | **64** | **7** | **K.LVVLGSGGVGK.S** 4409 |
|  | 12751 | **747.8981** | **1493.7816** | **1493.7878** | **-0.0062** | **0** | **77** | **2.7e-006** | **1** | **K.INVNEIFYDLVR.Q** |
|  | 13680 | **777.8486** | **1553.6827** | **1553.6933** | **-0.0105** | **0** | **42** | **0.0075** | **1** | **R.QWNNCAFLESSAK.S** |
|  | 15015 | **548.2626** | **1641.7659** | **1642.8600** | **-1.0941** | **1** | **5** | **37** | **5** | **R.VKDTDDVPMILVGNK.C** 15014 |
|  | 15378 | 833.9478 | 1665.8809 | 1664.9138 | 0.9672 | 0 | 13 | 6.1 | 2 | K.SALTVQFVQGIFVEK.Y 15347 |
|  | 16075 | 855.4589 | 1708.9033 | 1708.9148 | -0.0115 | 1 | 49 | 0.0015 | 1 | K.SKINVNEIFYDLVR.Q 16078 |
|  | 19887 | **669.3632** | **2005.0677** | **2005.0745** | **-0.0068** | **1** | **4** | **33** | **4** | **K.INVNEIFYDLVRQINR.K** |

| **364.** | A6NIZ1\|RP1BL_HUMAN    **Mass:** 21140    **Score:** 127    **Queries matched:** 12   **emPAI:** 0.65 |
| --- | --- |
|  | Ras-related protein Rap-1b-like protein OS=Homo sapiens PE=2 SV=1 |

|  | **Query** | **Observed** | **Mr(expt)** | **Mr(calc)** | **Delta** | **Miss** | **Score** | **Expect** | **Rank** | **Peptide** |
| --- | --- | --- | --- | --- | --- | --- | --- | --- | --- | --- |
|  | 2293 | 413.2655 | 824.5164 | 824.4868 | 0.0296 | 1 | 5 | 40 | 4 | K.TPVPGKAR.K |
|  | 3747 | 469.2074 | 936.4002 | 935.3654 | 1.0348 | 0 | 5 | 46 | 2 | K.CDLEDER.V |
|  | 12751 | 747.8981 | 1493.7816 | 1493.7878 | -0.0062 | 0 | 77 | 2.7e-006 | 1 | K.INVNEIFYDLVR.Q |
|  | 13680 | 777.8486 | 1553.6827 | 1553.6933 | -0.0105 | 0 | 42 | 0.0075 | 1 | R.QWNNCAFLESSAK.S |
|  | 15015 | 548.2626 | 1641.7659 | 1642.8600 | -1.0941 | 1 | 5 | 37 | 5 | R.VKDTDDVPMILVGNK.C 15014 |
|  | 15378 | 833.9478 | 1665.8809 | 1664.9138 | 0.9672 | 0 | 13 | 6.1 | 2 | K.SALTVQFVQGIFVEK.Y 15347 |
|  | 16075 | 855.4589 | 1708.9033 | 1708.9148 | -0.0115 | 1 | 49 | 0.0015 | 1 | K.SKINVNEIFYDLVR.Q 16078 |
|  | 19887 | 669.3632 | 2005.0677 | 2005.0745 | -0.0068 | 1 | 4 | 33 | 4 | K.INVNEIFYDLVRQINR.K |
|  | 27699 | **787.3259** | **3145.2746** | **3144.4050** | **0.8696** | **0** | **2** | **27** | **1** | **R.EQVEVDAQQCMLEILDTAGTEQFTAMR.D** |

| **365.** | P07306\|ASGR1_HUMAN    **Mass:** 33735    **Score:** 127    **Queries matched:** 12   **emPAI:** 1.33 |
| --- | --- |
|  | Asialoglycoprotein receptor 1 OS=Homo sapiens GN=ASGR1 PE=1 SV=2 |

|  | **Query** | **Observed** | **Mr(expt)** | **Mr(calc)** | **Delta** | **Miss** | **Score** | **Expect** | **Rank** | **Peptide** |
| --- | --- | --- | --- | --- | --- | --- | --- | --- | --- | --- |
|  | 6906 | **587.8179** | **1173.6213** | **1172.6262** | **0.9951** | **1** | **34** | **0.065** | **1** | **K.GLSTQGGNVGRK.M** |
|  | 8227 | **621.2494** | **1240.4842** | **1240.4931** | **-0.0089** | **0** | **36** | **0.035** | **1** | **K.AWADADNYCR.L** |
|  | 11461 | **709.3120** | **1416.6093** | **1416.6198** | **-0.0104** | **0** | **68** | **2.1e-005** | **1** | **K.WVDGTDYETGFK.N** 11493 |
|  | 12951 | **754.8336** | **1507.6527** | **1507.6626** | **-0.0100** | **0** | **10** | **13** | **2** | **R.WNDDVCQRPYR.W** |
|  | 14160 | **794.3414** | **1586.6683** | **1585.6766** | **0.9917** | **0** | **33** | **0.065** | **1** | **R.TCCPVNWVEHER.S** 14149 |
|  | 14264 | 531.6107 | 1591.8102 | 1591.8206 | -0.0104 | 0 | 30 | 0.12 | 1 | K.DLSEDHSSLLLHVK.Q |
|  | 18428 | **628.3185** | **1881.9338** | **1881.9472** | **-0.0134** | **0** | **36** | **0.026** | **1** | **R.LEDAHLVVVTSWEEQK.F** |
|  | 19977 | **1008.3380** | **2014.6615** | **2013.9717** | **0.6897** | **1** | **22** | **0.41** | **1** | **R.WVCETELDKASQEPPLL.-** |
|  | 24981 | **879.0695** | **2634.1866** | **2634.2058** | **-0.0193** | **1** | **37** | **0.012** | **1** | **M.TKEYQDLQHLDNEESDHHQLR.K** |
|  | 30979 | **1145.5360** | **4578.1149** | **4577.2339** | **0.8810** | **1** | **1** | **13** | **6** | **R.LEDAHLVVVTSWEEQKFVQHHIGPVNTWMGLHDQNGPWK.W** |

| **366.** | Q14117\|DPYS_HUMAN    **Mass:** 57107    **Score:** 126    **Queries matched:** 14   **emPAI:** 0.07 |
| --- | --- |
|  | Dihydropyrimidinase OS=Homo sapiens GN=DPYS PE=1 SV=1 |

|  | **Query** | **Observed** | **Mr(expt)** | **Mr(calc)** | **Delta** | **Miss** | **Score** | **Expect** | **Rank** | **Peptide** |
| --- | --- | --- | --- | --- | --- | --- | --- | --- | --- | --- |
|  | 850 | **500.2753** | **499.2681** | **500.2707** | **-1.0026** | **0** | **8** | **30** | **5** | **M.AAPSR.L** |
|  | 1500 | **673.3824** | **672.3752** | **672.3806** | **-0.0055** | **0** | **34** | **0.092** | **1** | **R.VLDAAGK.L** 1501 |
|  | 3356 | **454.2112** | **906.4079** | **907.4473** | **-1.0395** | **0** | **10** | **20** | **1** | **R.MSVIWEK.G** |
|  | 5444 | 531.3214 | 1060.6281 | 1059.5019 | 1.1262 | 0 | 16 | 4.7 | 8 | R.TCTPTPVER.A |
|  | 12376 | 491.5977 | 1471.7713 | 1472.7446 | -0.9733 | 1 | 6 | 34 | 7 | R.MSVIWEKGVHSGK.M |
|  | 14498 | **806.0705** | **1610.1264** | **1610.8893** | **-0.7628** | **0** | **3** | **37** | **1** | **R.ALGHDLLPPGGAPAGLR.V** |
|  | 20982 | 1053.0436 | 2104.0726 | 2105.0753 | -1.0026 | 0 | 4 | 33 | 2 | K.EIGAIAQVHAENGDLIAEGAK.K 20990 |
|  | 21616 | **1088.5363** | **2175.0579** | **2175.0696** | **-0.0116** | **0** | **83** | **4.5e-007** | **1** | **R.VVNDDFSEVADVLVEDGVVR.A** 21598 21615 |
|  | 25608 | 917.7570 | 2750.2491 | 2751.2634 | -1.0143 | 1 | 4 | 23 | 2 | K.MFMAYKDLYMVTDLELYEAFSR.C |
|  | 26856 | **988.4751** | **2962.4035** | **2962.4695** | **-0.0660** | **0** | **1** | **40** | **1** | **K.THHQAVNFNIFEGMVCHGVPLVTISR.G** |

| **367.** | P62280\|RS11_HUMAN    **Mass:** 18590    **Score:** 126    **Queries matched:** 8   **emPAI:** 0.21 |
| --- | --- |
|  | 40S ribosomal protein S11 OS=Homo sapiens GN=RPS11 PE=1 SV=3 |

|  | **Query** | **Observed** | **Mr(expt)** | **Mr(calc)** | **Delta** | **Miss** | **Score** | **Expect** | **Rank** | **Peptide** |
| --- | --- | --- | --- | --- | --- | --- | --- | --- | --- | --- |
|  | 5589 | **536.7995** | **1071.5844** | **1072.6128** | **-1.0284** | **1** | **18** | **3.3** | **1** | **R.VLLGETGKEK.L** |
|  | 10242 | **450.2783** | **1347.8132** | **1346.6224** | **1.1909** | **0** | **2** | **85** | **10** | **K.NMSVHLSPCFR.D** |
|  | 12312 | 490.2770 | 1467.8093 | 1468.6980 | -0.8887 | 1 | 5 | 43 | 4 | -.MADIQTERAYQK.Q |
|  | 14539 | 538.2963 | 1611.8670 | 1611.7762 | 0.0907 | 1 | 2 | 78 | 3 | R.HKNMSVHLSPCFR.D |
|  | 19640 | **993.5136** | **1985.0125** | **1985.0252** | **-0.0126** | **0** | **78** | **1.3e-006** | **1** | **R.DVQIGDIVTVGECRPLSK.T** 19639 19649 |
|  | 28324 | **666.9549** | **3329.7381** | **3329.6319** | **0.1062** | **1** | **0** | **39** | **4** | **K.NMSVHLSPCFRDVQIGDIVTVGECRPLSK.T** |

| **368.** | P00338\|LDHA_HUMAN    **Mass:** 36950    **Score:** 126    **Queries matched:** 21   **emPAI:** 0.62 |
| --- | --- |
|  | L-lactate dehydrogenase A chain OS=Homo sapiens GN=LDHA PE=1 SV=2 |

|  | **Query** | **Observed** | **Mr(expt)** | **Mr(calc)** | **Delta** | **Miss** | **Score** | **Expect** | **Rank** | **Peptide** |
| --- | --- | --- | --- | --- | --- | --- | --- | --- | --- | --- |
|  | 1663 | **734.4153** | **733.4080** | **733.4123** | **-0.0043** | **0** | **37** | **0.044** | **1** | **R.NVNIFK.F** |
|  | 2378 | **417.2429** | **832.4712** | **832.3675** | **0.1037** | **0** | **12** | **14** | **3** | **R.QQEGESR.L** |
|  | 3435 | **456.8829** | **911.7512** | **912.5756** | **-0.8245** | **0** | **11** | **8.3** | **1** | **K.LVIITAGAR.Q** 3434 |
|  | 4237 | 486.7995 | 971.5845 | 972.4512 | -0.8667 | 0 | 14 | 7.4 | 4 | K.EEQTPQNK.I |
|  | 4803 | **506.2372** | **1010.4599** | **1010.4669** | **-0.0070** | **0** | **39** | **0.02** | **1** | **K.DYNVTANSK.L** 4804 |
|  | 6820 | **584.3329** | **1166.6513** | **1166.6594** | **-0.0080** | **1** | **15** | **4.8** | **1** | **R.RVHPVSTMIK.G** |
|  | 8375 | **624.7991** | **1247.5836** | **1247.5928** | **-0.0092** | **0** | **52** | **0.00096** | **1** | **R.VIGSGCNLDSAR.F** |
|  | 18356 | **469.4928** | **1873.9420** | **1873.9542** | **-0.0123** | **1** | **5** | **36** | **2** | **K.LKGEMMDLQHGSLFLR.T** |
|  | 18634 | **634.0134** | **1899.0185** | **1898.0248** | **0.9937** | **1** | **14** | **3.6** | **1** | **K.DLADELALVDVIEDKLK.G** 18613 |
|  | 19170 | **972.5596** | **1943.1046** | **1943.1132** | **-0.0086** | **0** | **16** | **2.5** | **1** | **K.LLIVSNPVDILTYVAWK.I** |
|  | 19889 | **669.3984** | **2005.1733** | **2005.0774** | **0.0959** | **0** | **5** | **26** | **1** | **K.ITVVGVGAVGMACAISILMK.D** |
|  | 20693 | 1037.5375 | 2073.0604 | 2073.0742 | -0.0138 | 1 | 80 | 8.3e-007 | 1 | K.DQLIYNLLKEEQTPQNK.I |
|  | 21065 | 1057.0685 | 2112.1224 | 2111.0609 | 1.0615 | 0 | 36 | 0.024 | 1 | K.GYTSWAIGLSVADLAESIMK.N 21061 21064 |
|  | 23085 | **785.0841** | **2352.2303** | **2352.2399** | **-0.0096** | **1** | **14** | **3** | **1** | **K.LKGYTSWAIGLSVADLAESIMK.N** 23078 |
|  | 26620 | **1461.2632** | **2920.5118** | **2919.5416** | **0.9702** | **1** | **12** | **3.7** | **1** | **K.GLYGIKDDVFLSVPCILGQNGISDLVK.V** |

| **369.** | P07900\|HS90A_HUMAN    **Mass:** 85006    **Score:** 125    **Queries matched:** 24   **emPAI:** 0.29 |
| --- | --- |
|  | Heat shock protein HSP 90-alpha OS=Homo sapiens GN=HSP90AA1 PE=1 SV=5 |

|  | **Query** | **Observed** | **Mr(expt)** | **Mr(calc)** | **Delta** | **Miss** | **Score** | **Expect** | **Rank** | **Peptide** |
| --- | --- | --- | --- | --- | --- | --- | --- | --- | --- | --- |
|  | 996 | 533.1879 | 532.1807 | 532.2493 | -0.0686 | 0 | 7 | 57 | 8 | K.AEADK.N |
|  | 2756 | 432.2167 | 862.4189 | 862.4218 | -0.0029 | 0 | 13 | 11 | 4 | R.EMLQQSK.I |
|  | 3906 | **474.7233** | **947.4320** | **947.4389** | **-0.0068** | **0** | **22** | **1.3** | **1** | **K.FYEQFSK.N** 3924 |
|  | 7131 | **594.7509** | **1187.4871** | **1187.4951** | **-0.0079** | **0** | **28** | **0.24** | **1** | **R.DNSTMGYMAAK.K** |
|  | 7217 | **596.2830** | **1190.5515** | **1189.6819** | **0.8696** | **0** | **3** | **73** | **6** | **K.ELHINLIPNK.Q** |
|  | 7799 | **407.2164** | **1218.6273** | **1219.4849** | **-0.8576** | **0** | **(5)** | **47** | **9** | **R.DNSTMGYMAAK.K** |
|  | 8264 | 621.8519 | 1241.6893 | 1241.6979 | -0.0086 | 0 | 45 | 0.0042 | 1 | K.ADLINNLGTIAK.S 8293 |
|  | 8682 | **422.2445** | **1263.7117** | **1264.5718** | **-0.8601** | **1** | **10** | **15** | **4** | **R.TDTGEPMGRGTK.V** |
|  | 10236 | 674.8602 | 1347.7058 | 1347.5799 | 0.1259 | 1 | 8 | 24 | 7 | R.DNSTMGYMAAKK.H |
|  | 10267 | 675.3663 | 1348.7181 | 1348.7272 | -0.0091 | 0 | 40 | 0.015 | 1 | R.TLTIVDTGIGMTK.A |
|  | 11047 | **465.0192** | **1392.0357** | **1392.5892** | **-0.5535** | **1** | **4** | **32** | **4** | **R.DKEVSDDEAEEK.E** |
|  | 13034 | 757.3917 | 1512.7688 | 1512.7784 | -0.0096 | 0 | 22 | 0.77 | 1 | R.GVVDSEDLPLNISR.E |
|  | 13185 | **508.2631** | **1521.7674** | **1522.7852** | **-1.0177** | **1** | **4** | **54** | **2** | **K.NIKLGIHEDSQNR.K** |
|  | 13263 | 764.3709 | 1526.7271 | 1526.7365 | -0.0094 | 0 | 46 | 0.0028 | 1 | K.SLTNDWEDHLAVK.H 13262 |
|  | 17067 | **593.6499** | **1777.9279** | **1777.9403** | **-0.0124** | **0** | **10** | **10** | **1** | **K.HSQFIGYPITLFVEK.E** |
|  | 17760 | **917.3878** | **1832.7611** | **1832.7741** | **-0.0130** | **0** | **71** | **7.1e-006** | **1** | **R.NPDDITNEEYGEFYK.S** |
|  | 18743 | **636.3640** | **1906.0700** | **1906.0353** | **0.0348** | **1** | **4** | **39** | **4** | **K.KHSQFIGYPITLFVEK.E** |
|  | 20200 | 678.3444 | 2032.0113 | 2033.0582 | -1.0469 | 1 | 1 | 76 | 6 | K.YIDQEELNKTKPIWTR.N |
|  | 20556 | **1032.8854** | **2063.7562** | **2063.0840** | **0.6722** | **1** | **2** | **41** | **4** | **K.HSQFIGYPITLFVEKER.D** |
|  | 24716 | **859.7559** | **2576.2458** | **2576.2655** | **-0.0197** | **0** | **16** | **1.7** | **1** | **K.HGLEVIYMIEPIDEYCVQQLK.E** |
|  | 29230 | 1225.1710 | 3672.4912 | 3672.7844 | -0.2932 | 1 | 1 | 20 | 7 | K.SGTKAFMEALQAGADISMIGQFGVGFYSAYLVAEK.V |

| **370.** | P40261\|NNMT_HUMAN    **Mass:** 30011    **Score:** 125    **Queries matched:** 15   **emPAI:** 0.61 |
| --- | --- |
|  | Nicotinamide N-methyltransferase OS=Homo sapiens GN=NNMT PE=1 SV=1 |

|  | **Query** | **Observed** | **Mr(expt)** | **Mr(calc)** | **Delta** | **Miss** | **Score** | **Expect** | **Rank** | **Peptide** |
| --- | --- | --- | --- | --- | --- | --- | --- | --- | --- | --- |
|  | 2160 | 816.4524 | 815.4451 | 815.4389 | 0.0063 | 0 | 10 | 21 | 6 | R.EAVEAAVK.E |
|  | 4815 | 506.7713 | 1011.5281 | 1011.5349 | -0.0068 | 0 | 46 | 0.0036 | 1 | R.HSAESQILK.H 4816 |
|  | 7513 | **603.2740** | **1204.5335** | **1204.5434** | **-0.0098** | **0** | **38** | **0.024** | **1** | **K.SSYYMIGEQK.F** 7530 |
|  | 8423 | **625.8359** | **1249.6572** | **1248.5887** | **1.0685** | **0** | **21** | **1.2** | **2** | **K.DTYLSHFNPR.D** |
|  | 19650 | **663.0244** | **1986.0514** | **1984.9279** | **1.1235** | **1** | **2** | **63** | **2** | **M.ESGFTSKDTYLSHFNPR.D** |
|  | 19653 | **994.0715** | **1986.1285** | **1985.1383** | **0.9902** | **0** | **6** | **20** | **1** | **R.NLGSLLKPGGFLVIMDALK.S** 19634 19644 |
|  | 20093 | **1011.9906** | **2021.9666** | **2021.9793** | **-0.0127** | **0** | **61** | **6.8e-005** | **1** | **K.EIVVTDYSDQNLQELEK.W** |
|  | 21230 | **534.0159** | **2132.0344** | **2131.9633** | **0.0711** | **1** | **5** | **32** | **5** | **-.MESGFTSKDTYLSHFNPR.D** |
|  | 24068 | **1242.0557** | **2482.0968** | **2482.1111** | **-0.0143** | **0** | **39** | **0.0087** | **1** | **K.EPEAFDWSPVVTYVCDLEGNR.V** 24075 |
|  | 30808 | 1471.5956 | 4411.7649 | 4412.1158 | -0.3509 | 1 | 5 | 5 | 1 | R.EAVEAAVKEAGYTIEWFEVISQSYSSTMANNEGLFSLVAR.K |

| **371.** | Q14165\|MLEC_HUMAN    **Mass:** 32385    **Score:** 125    **Queries matched:** 9   **emPAI:** 0.39 |
| --- | --- |
|  | Malectin OS=Homo sapiens GN=MLEC PE=1 SV=1 |

|  | **Query** | **Observed** | **Mr(expt)** | **Mr(calc)** | **Delta** | **Miss** | **Score** | **Expect** | **Rank** | **Peptide** |
| --- | --- | --- | --- | --- | --- | --- | --- | --- | --- | --- |
|  | 2123 | **407.1330** | **812.2514** | **813.4344** | **-1.1830** | **1** | **2** | **84** | **7** | **R.KDPLEGR.V** |
|  | 11916 | **723.3515** | **1444.6884** | **1444.6987** | **-0.0102** | **0** | **44** | **0.0049** | **1** | **K.FAEVYFAQSQQK.V** |
|  | 13384 | **512.9053** | **1535.6942** | **1536.8148** | **-1.1206** | **1** | **8** | **18** | **2** | **K.GKLSVQGEVSTFTGK.L** |
|  | 14254 | 796.8760 | 1591.7374 | 1591.7478 | -0.0104 | 0 | 58 | 0.00018 | 1 | R.SNPEDQILYQTER.Y 14255 |
|  | 15250 | 553.2562 | 1656.7469 | 1656.9021 | -0.1553 | 0 | 7 | 20 | 3 | -.MLGAWAVEGTAVALLR.L |
|  | 15470 | **837.3868** | **1672.7590** | **1672.8970** | **-0.1380** | **0** | **(1)** | **97** | **7** | **-.MLGAWAVEGTAVALLR.L** |
|  | 25533 | **912.1156** | **2733.3250** | **2733.3425** | **-0.0175** | **1** | **52** | **0.00035** | **1** | **R.YNEETFGYEVPIKEEGDYVLVLK.F** 25534 |

| **372.** | P13637\|AT1A3_HUMAN    **Mass:** 113102   **Score:** 124    **Queries matched:** 21   **emPAI:** 0.10 |
| --- | --- |
|  | Sodium/potassium-transporting ATPase subunit alpha-3 OS=Homo sapiens GN=ATP1A3 PE=1 SV=3 |

|  | **Query** | **Observed** | **Mr(expt)** | **Mr(calc)** | **Delta** | **Miss** | **Score** | **Expect** | **Rank** | **Peptide** |
| --- | --- | --- | --- | --- | --- | --- | --- | --- | --- | --- |
|  | 1381 | 633.3794 | 632.3721 | 632.3316 | 0.0405 | 0 | 13 | 14 | 3 | K.NCLVK.N |
|  | 1654 | 731.1765 | 730.1692 | 729.3769 | 0.7922 | 1 | 5 | 55 | 2 | R.NPRTDK.L |
|  | 3101 | **443.2707** | **884.5269** | **884.4538** | **0.0731** | **0** | **6** | **38** | **8** | **R.IISAHGCK.V** |
|  | 5673 | **540.6237** | **1079.2328** | **1078.5329** | **0.6999** | **0** | **5** | **36** | **1** | **K.CIELSSGSVK.L** |
|  | 6382 | **567.7724** | **1133.5302** | **1133.4910** | **0.0392** | **0** | **1** | **1.4e+002** | **3** | **K.EQPLDEEMK.E** |
|  | 7295 | 598.3372 | 1194.6599 | 1195.6673 | -1.0074 | 1 | 8 | 22 | 6 | K.GGQDNIPVLKR.D |
|  | 7590 | 605.3289 | 1208.6432 | 1209.5924 | -0.9493 | 1 | 4 | 62 | 7 | R.RNSVFQQGMK.N |
|  | 8139 | 618.8524 | 1235.6902 | 1235.6986 | -0.0084 | 0 | 30 | 0.13 | 1 | R.LNIPVSQVNPR.D |
|  | 8743 | 634.8563 | 1267.6981 | 1267.7071 | -0.0090 | 0 | 8 | 22 | 3 | K.NMVPQQALVIR.E |
|  | 9047 | 643.3311 | 1284.6475 | 1283.7020 | 0.9456 | 0 | (3) | 71 | 4 | K.NMVPQQALVIR.E |
|  | 11097 | 466.2328 | 1395.6766 | 1396.7384 | -1.0618 | 0 | 1 | 1.1e+002 | 9 | K.VIMVTGDHPITAK.A |
|  | 13614 | 517.7669 | 1550.2789 | 1549.8576 | 0.4213 | 1 | 2 | 51 | 6 | R.LNIPVSQVNPRDAK.A 13615 |
|  | 14645 | 810.3738 | 1618.7331 | 1618.7435 | -0.0103 | 0 | 40 | 0.012 | 1 | K.VDNSSLTGESEPQTR.S |
|  | 14892 | 818.4097 | 1634.8049 | 1633.8345 | 0.9704 | 1 | 1 | 90 | 4 | K.KADIGVAMGIAGSDVSK.Q |
|  | 17090 | 594.2792 | 1779.8159 | 1779.9917 | -0.1758 | 1 | 7 | 20 | 2 | K.VIMVTGDHPITAKAIAK.G 17091 |
|  | 17716 | 915.4601 | 1828.9056 | 1828.9167 | -0.0110 | 0 | 103 | 5.4e-009 | 1 | K.GVGIISEGNETVEDIAAR.L |
|  | 18015 | 926.9727 | 1851.9309 | 1853.0081 | -1.0772 | 1 | 4 | 44 | 2 | R.SAGIKVIMVTGDHPITAK.A |
|  | 19426 | **492.2580** | **1965.0030** | **1965.8594** | **-0.8564** | **1** | **5** | **30** | **1** | **K.EVAMTEHKMSVEEVCR.K** |
|  | 24059 | 1241.0720 | 2480.1295 | 2479.1901 | 0.9394 | 0 | 2 | 45 | 2 | K.QAADMILLDDNFASIVTGVEEGR.L |

| **373.** | P11586\|C1TC_HUMAN    **Mass:** 102180   **Score:** 124    **Queries matched:** 35   **emPAI:** 0.15 |
| --- | --- |
|  | C-1-tetrahydrofolate synthase, cytoplasmic OS=Homo sapiens GN=MTHFD1 PE=1 SV=3 |

|  | **Query** | **Observed** | **Mr(expt)** | **Mr(calc)** | **Delta** | **Miss** | **Score** | **Expect** | **Rank** | **Peptide** |
| --- | --- | --- | --- | --- | --- | --- | --- | --- | --- | --- |
|  | 2347 | **415.7680** | **829.5214** | **829.4545** | **0.0669** | **0** | **16** | **5.1** | **10** | **K.AAEEIGIK.A** 2351 |
|  | 2360 | **416.2596** | **830.5047** | **831.4086** | **-0.9040** | **0** | **8** | **35** | **2** | **R.VLDTNDR.F** |
|  | 3231 | **450.2426** | **898.4706** | **898.4872** | **-0.0166** | **0** | **8** | **26** | **7** | **K.ETGVPIAGR.H** |
|  | 3729 | **468.2708** | **934.5270** | **934.5712** | **-0.0442** | **1** | **6** | **50** | **4** | **K.ATHIKLPR.T** |
|  | 5120 | **517.7871** | **1033.5597** | **1033.5669** | **-0.0072** | **1** | **8** | **29** | **1** | **R.RFSDIQIR.R** |
|  | 5569 | **536.2841** | **1070.5536** | **1070.6448** | **-0.0912** | **1** | **4** | **74** | **9** | **R.LKNQVTQLK.E** |
|  | 5904 | **549.3212** | **1096.6278** | **1096.6353** | **-0.0075** | **0** | **8** | **22** | **2** | **K.GALALAQAVQR.A** |
|  | 7068 | **593.3290** | **1184.6434** | **1184.6765** | **-0.0331** | **1** | **10** | **15** | **8** | **R.KITIGQAPTEK.G** |
|  | 8029 | 616.3152 | 1230.6159 | 1230.6245 | -0.0085 | 0 | 18 | 2.7 | 1 | R.IFHELTQTDK.A |
|  | 10805 | **690.4140** | **1378.8134** | **1377.6623** | **1.1511** | **0** | **6** | **36** | **3** | **K.TDTESELDLISR.L** |
|  | 10817 | **461.0072** | **1379.9999** | **1379.7772** | **0.2226** | **1** | **5** | **33** | **4** | **K.AAEEIGIKATHIK.L** |
|  | 12704 | **746.3586** | **1490.7026** | **1490.8320** | **-0.1293** | **0** | **4** | **50** | **1** | **R.MFGIPVVVAVNAFK.T** |
|  | 14609 | **539.7786** | **1616.3139** | **1616.9111** | **-0.5972** | **1** | **2** | **50** | **10** | **K.ETGVPIAGRHAVVVGR.S** |
|  | 14817 | **815.9509** | **1629.8872** | **1629.8978** | **-0.0106** | **0** | **65** | **3.9e-005** | **1** | **K.YVVVTGITPTPLGEGK.S** |
|  | 16925 | 442.9635 | 1767.8248 | 1768.9658 | -1.1411 | 0 | 3 | 52 | 5 | R.YSGLCPHVVVLVATVR.A |
|  | 17447 | **603.3262** | **1806.9569** | **1807.9091** | **-0.9522** | **0** | **2** | **63** | **4** | **R.EIGLLSEEVELYGETK.A** |
|  | 17501 | 906.9549 | 1811.8952 | 1810.9101 | 0.9852 | 0 | 8 | 14 | 2 | K.IYGADDIELLPEAQHK.A |
|  | 19677 | 663.6569 | 1987.9489 | 1988.0136 | -0.0647 | 0 | 12 | 6.3 | 2 | K.GEPVSAEDLGVSGALTVLMK.D |
|  | 20960 | **526.2903** | **2101.1320** | **2100.1136** | **1.0184** | **1** | **9** | **12** | **2** | **K.KGEPVSAEDLGVSGALTVLMK.D** |
|  | 24723 | **645.3238** | **2577.2663** | **2577.2832** | **-0.0169** | **0** | **3** | **35** | **7** | **K.IVGAPMHDLLLWNNATVTTCHSK.T** |
|  | 25394 | 1354.6995 | 2707.3844 | 2706.3370 | 1.0474 | 1 | 0 | 57 | 2 | K.QGFGNLPICMAKTHLSLSHNPEQK.G |
|  | 25483 | 1362.6969 | 2723.3792 | 2722.3319 | 1.0473 | 1 | (0) | 60 | 3 | K.QGFGNLPICMAKTHLSLSHNPEQK.G |
|  | 26143 | **711.8656** | **2843.4333** | **2843.3933** | **0.0400** | **0** | **3** | **26** | **5** | **R.TAQFDISVASEIMAVLALTTSLEDMR.E** |
|  | 27025 | **998.5160** | **2992.5263** | **2993.5025** | **-0.9761** | **1** | **2** | **29** | **2** | **R.ASFITPVPGGVGPMTVAMLMQSTVESAKR.F** |
|  | 27093 | **1004.5635** | **3010.6686** | **3010.5335** | **0.1351** | **1** | **2** | **29** | **1** | **K.GEWIKPGAIVIDCGINYVPDDKKPNGR.K** |
|  | 27600 | **1041.5548** | **3121.6426** | **3122.5450** | **-0.9024** | **1** | **(0)** | **47** | **6** | **K.ERASFITPVPGGVGPMTVAMLMQSTVESAK.R** |
|  | 27602 | **1041.8154** | **3122.4245** | **3122.5450** | **-0.1206** | **1** | **1** | **37** | **1** | **K.ERASFITPVPGGVGPMTVAMLMQSTVESAK.R** |
|  | 30558 | **1052.2760** | **4205.0749** | **4205.0964** | **-0.0215** | **0** | **40** | **0.0028** | **1** | **K.GGAAGGGYSQVIPMEEFNLHLTGDIHAITAANNLVAAAIDAR.I** 30557 30561 30562 |
|  | 31503 | **1359.6748** | **5434.6701** | **5433.7052** | **0.9649** | **1** | **5** | **3.9** | **2** | **K.GGAAGGGYSQVIPMEEFNLHLTGDIHAITAANNLVAAAIDARIFHELTQTDK.A** |
|  | 31515 | **1375.4354** | **5497.7126** | **5497.7376** | **-0.0250** | **1** | **40** | **0.0011** | **1** | **K.YITSLNEDSTVHGFLVQLPLDSENSINTEEVINAIAPEKDVDGLTSINAGR.L** 31516 |

| **374.** | Q9HCU5\|PREB_HUMAN    **Mass:** 46010    **Score:** 124    **Queries matched:** 8   **emPAI:** 0.17 |
| --- | --- |
|  | Prolactin regulatory element-binding protein OS=Homo sapiens GN=PREB PE=1 SV=2 |

|  | **Query** | **Observed** | **Mr(expt)** | **Mr(calc)** | **Delta** | **Miss** | **Score** | **Expect** | **Rank** | **Peptide** |
| --- | --- | --- | --- | --- | --- | --- | --- | --- | --- | --- |
|  | 9056 | 429.2551 | 1284.7435 | 1283.6622 | 1.0812 | 0 | 7 | 29 | 10 | R.FGQVPDQPAGLR.L |
|  | 9104 | **644.8182** | **1287.6218** | **1287.7438** | **-0.1220** | **1** | **4** | **57** | **5** | **K.VPSLEKVLEFK.A** |
|  | 12816 | **750.3354** | **1498.6562** | **1498.6834** | **-0.0272** | **0** | **2** | **76** | **9** | **K.CGAETQHEGLELR.V** |
|  | 13854 | **522.9300** | **1565.7681** | **1565.7798** | **-0.0116** | **0** | **28** | **0.17** | **1** | **R.LSASLLHSHDTETR.A** |
|  | 14757 | **814.3832** | **1626.7518** | **1626.7784** | **-0.0266** | **1** | **7** | **22** | **2** | **K.KCGAETQHEGLELR.V** |
|  | 20021 | **1009.9988** | **2017.9830** | **2017.9957** | **-0.0126** | **0** | **80** | **9.6e-007** | **1** | **R.VENLQAVQTDFSSDPLQK.V** 20020 |
|  | 24361 | 841.7603 | 2522.2591 | 2522.2733 | -0.0142 | 0 | 13 | 3.3 | 2 | R.ATMNLALAGDILAAGQDAHCQLLR.F |

| **375.** | P52895\|AK1C2_HUMAN    **Mass:** 37111    **Score:** 124    **Queries matched:** 21   **emPAI:** 0.79 |
| --- | --- |
|  | Aldo-keto reductase family 1 member C2 OS=Homo sapiens GN=AKR1C2 PE=1 SV=3 |

|  | **Query** | **Observed** | **Mr(expt)** | **Mr(calc)** | **Delta** | **Miss** | **Score** | **Expect** | **Rank** | **Peptide** |
| --- | --- | --- | --- | --- | --- | --- | --- | --- | --- | --- |
|  | 1536 | 685.4556 | 684.4483 | 684.4534 | -0.0051 | 0 | 21 | 1.3 | 4 | R.GVVVLAK.S |
|  | 1550 | 689.3528 | 688.3455 | 688.3755 | -0.0300 | 0 | 23 | 1.4 | 1 | K.IADGSVK.R |
|  | 1719 | 758.4101 | 757.4028 | 757.4082 | -0.0054 | 0 | 12 | 15 | 1 | K.AIDGLNR.N |
|  | 3583 | 462.7530 | 923.4915 | 922.4946 | 0.9969 | 1 | 25 | 0.44 | 1 | R.KLLDFCK.S |
|  | 4799 | 505.8239 | 1009.6333 | 1009.6396 | -0.0063 | 1 | 11 | 11 | 3 | K.RTPALIALR.Y |
|  | 6701 | 579.7891 | 1157.5637 | 1157.5717 | -0.0080 | 1 | 32 | 0.11 | 1 | K.REDIFYTSK.L 6700 |
|  | 10639 | **685.4150** | **1368.8155** | **1367.8210** | **0.9945** | **0** | **44** | **0.0047** | **1** | **R.LLEMILNKPGLK.Y** |
|  | 10883 | 461.9362 | 1382.7867 | 1383.8159 | -1.0292 | 0 | (13) | 6.5 | 3 | R.LLEMILNKPGLK.Y |
|  | 19320 | 978.9654 | 1955.9162 | 1955.9299 | -0.0136 | 0 | 58 | 0.00014 | 1 | R.QNVQVFEFQLTSEEMK.A |
|  | 20466 | **1028.9874** | **2055.9603** | **2056.9486** | **-0.9883** | **0** | **1** | **73** | **4** | **K.ILFDTVDLCATWEAMEK.C** |
|  | 22093 | 742.7079 | 2225.1018 | 2225.1150 | -0.0132 | 1 | 7 | 16 | 1 | R.IRQNVQVFEFQLTSEEMK.A |
|  | 22212 | 560.5055 | 2237.9931 | 2238.0099 | -0.0168 | 0 | 40 | 0.0086 | 1 | K.YKPVCNQVECHPYFNQR.K 22213 22214 |
|  | 23138 | 1180.5829 | 2359.1512 | 2359.1671 | -0.0159 | 0 | 39 | 0.0095 | 1 | K.LNDGHFMPVLGFGTYAPAEVPK.S 23139 23140 |
|  | 27069 | **751.6271** | **3002.4792** | **3002.4998** | **-0.0206** | **0** | **9** | **7.4** | **1** | **K.LAIEAGFHHIDSAHVYNNEEQVGLAIR.S** |
|  | 27622 | 1042.5630 | 3124.6671 | 3124.6849 | -0.0177 | 0 | 13 | 2.2 | 1 | K.NLQLDYVDLYLIHFPVSVKPGEEVIPK.D 27623 |

| **376.** | Q9UF12\|PROD2_HUMAN    **Mass:** 59689    **Score:** 123    **Queries matched:** 9   **emPAI:** 0.20 |
| --- | --- |
|  | Probable proline dehydrogenase 2 OS=Homo sapiens GN=PRODH2 PE=2 SV=1 |

|  | **Query** | **Observed** | **Mr(expt)** | **Mr(calc)** | **Delta** | **Miss** | **Score** | **Expect** | **Rank** | **Peptide** |
| --- | --- | --- | --- | --- | --- | --- | --- | --- | --- | --- |
|  | 1046 | **545.3609** | **544.3536** | **544.3333** | **0.0203** | **0** | **9** | **41** | **7** | **R.LLGSR.L** |
|  | 2564 | **848.4782** | **847.4709** | **847.4763** | **-0.0055** | **0** | **6** | **55** | **1** | **K.VTALTSTR.L** |
|  | 9111 | **430.2685** | **1287.7837** | **1288.7615** | **-0.9778** | **1** | **7** | **31** | **6** | **R.LLGSRLSGAFLR.A** |
|  | 9900 | **443.7560** | **1328.2461** | **1327.6956** | **0.5505** | **1** | **6** | **24** | **5** | **R.AQENRSVLQGAR.R** |
|  | 24297 | **838.1482** | **2511.4229** | **2511.4312** | **-0.0083** | **0** | **31** | **0.051** | **1** | **R.LLVDAEYTSLNPALSLLVAALAVR.W** |
|  | 24445 | 848.1625 | 2541.4658 | 2542.3135 | -0.8477 | 1 | 5 | 17 | 2 | R.CVDLSRGLLEPPSLAEASLMQLK.V |
|  | 25779 | 927.7452 | 2780.2139 | 2780.2348 | -0.0209 | 0 | 42 | 0.0037 | 1 | R.AVAQLHGMEDPTQPDYEATSQSYSR.C |
|  | 26062 | **942.7727** | **2825.2963** | **2825.3184** | **-0.0221** | **0** | **91** | **4.5e-008** | **1** | **R.LAEAMDSGQNLQVSCLNAEQNQHLR.A** |
|  | 30348 | 1030.2621 | 4117.0192 | 4118.0021 | -0.9829 | 1 | 1 | 23 | 4 | R.RPGASLELSPERLAEAMDSGQNLQVSCLNAEQNQHLR.A |

| **377.** | O15260\|SURF4_HUMAN    **Mass:** 30602    **Score:** 123    **Queries matched:** 13   **emPAI:** 0.12 |
| --- | --- |
|  | Surfeit locus protein 4 OS=Homo sapiens GN=SURF4 PE=1 SV=3 |

|  | **Query** | **Observed** | **Mr(expt)** | **Mr(calc)** | **Delta** | **Miss** | **Score** | **Expect** | **Rank** | **Peptide** |
| --- | --- | --- | --- | --- | --- | --- | --- | --- | --- | --- |
|  | 4400 | **492.2891** | **982.5636** | **982.5348** | **0.0288** | **0** | **13** | **6.9** | **2** | **K.QYLPHVAR.L** 4379 4393 |
|  | 5896 | **549.2746** | **1096.5346** | **1095.5205** | **1.0141** | **0** | **5** | **49** | **3** | **K.SMFAGVPTMR.E** |
|  | 9281 | **649.3554** | **1296.6961** | **1296.5710** | **0.1252** | **0** | **6** | **33** | **3** | **R.MWFQWSEQR.D** |
|  | 13026 | **505.2599** | **1512.7579** | **1512.7065** | **0.0515** | **1** | **5** | **39** | **4** | **R.SEGKSMFAGVPTMR.E** |
|  | 13392 | **768.9043** | **1535.7940** | **1535.8018** | **-0.0077** | **0** | **8** | **18** | **1** | **R.LCLISTFLEDGIR.M** |
|  | 14727 | **542.3107** | **1623.9104** | **1623.7749** | **0.1355** | **1** | **6** | **30** | **3** | **K.SMFAGVPTMRESSPK.Q** |
|  | 19943 | **671.4405** | **2011.2996** | **2010.1473** | **1.1523** | **1** | **0** | **62** | **8** | **R.NLALGGGLLLLLAESRSEGK.S** |
|  | 20151 | **1014.4550** | **2026.8954** | **2026.9055** | **-0.0101** | **0** | **73** | **4e-006** | **1** | **M.GQNDLMGTAEDFADQFLR.V** 20152 20168 |
|  | 24087 | **829.4171** | **2485.2293** | **2486.1570** | **-0.9277** | **1** | **5** | **24** | **1** | **-.MGQNDLMGTAEDFADQFLRVTK.Q** |

| **378.** | P51571\|SSRD_HUMAN    **Mass:** 19158    **Score:** 123    **Queries matched:** 11   **emPAI:** 1.08 |
| --- | --- |
|  | Translocon-associated protein subunit delta OS=Homo sapiens GN=SSR4 PE=1 SV=1 |

|  | **Query** | **Observed** | **Mr(expt)** | **Mr(calc)** | **Delta** | **Miss** | **Score** | **Expect** | **Rank** | **Peptide** |
| --- | --- | --- | --- | --- | --- | --- | --- | --- | --- | --- |
|  | 5814 | **545.7637** | **1089.5128** | **1089.5203** | **-0.0075** | **0** | **42** | **0.012** | **1** | **K.SAHAGTYEVR.F** |
|  | 10539 | **683.3406** | **1364.6667** | **1364.6758** | **-0.0091** | **0** | **65** | **4.7e-005** | **1** | **R.VQNMALYADVGGK.Q** 10538 |
|  | 11248 | 703.3307 | 1404.6469 | 1404.6561 | -0.0092 | 0 | 26 | 0.32 | 1 | R.FFDEESYSLLR.K |
|  | 15143 | 825.9385 | 1649.8624 | 1650.8148 | -0.9524 | 1 | 8 | 18 | 3 | K.NRVQNMALYADVGGK.Q |
|  | 18098 | 465.2647 | 1857.0296 | 1857.0757 | -0.0461 | 0 | 13 | 5.2 | 4 | M.AAMASLGALALLLLSSLSR.C 18097 |
|  | 18356 | 469.4928 | 1873.9420 | 1873.0706 | 0.8714 | 0 | (3) | 49 | 5 | M.AAMASLGALALLLLSSLSR.C |
|  | 19887 | 669.3632 | 2005.0677 | 2004.1111 | 0.9566 | 0 | 2 | 58 | 9 | -.MAAMASLGALALLLLSSLSR.C |
|  | 20884 | 698.6275 | 2092.8607 | 2093.0728 | -0.2121 | 1 | 7 | 18 | 3 | R.VQNMALYADVGGKQFPVTR.G |
|  | 21536 | 722.7073 | 2165.1002 | 2165.1117 | -0.0115 | 0 | 27 | 0.18 | 1 | R.NNEDISIIPPLFTVSVDHR.G |

| **379.** | O75380\|NDUS6_HUMAN    **Mass:** 14045    **Score:** 123    **Queries matched:** 4   **emPAI:** 1.09 |
| --- | --- |
|  | NADH dehydrogenase [ubiquinone] iron-sulfur protein 6, mitochondrial OS=Homo sapiens GN=NDUFS6 PE=1 SV=1 |

|  | **Query** | **Observed** | **Mr(expt)** | **Mr(calc)** | **Delta** | **Miss** | **Score** | **Expect** | **Rank** | **Peptide** |
| --- | --- | --- | --- | --- | --- | --- | --- | --- | --- | --- |
|  | 11298 | 704.8489 | 1407.6833 | 1407.6929 | -0.0096 | 0 | 64 | 5e-005 | 1 | R.VIACDGGGGALGHPK.V |
|  | 11502 | **710.3060** | **1418.5974** | **1418.6071** | **-0.0097** | **0** | **51** | **0.00094** | **1** | **K.TGTCGYCGLQFR.Q** |
|  | 15828 | **424.9509** | **1695.7746** | **1695.7853** | **-0.0107** | **1** | **5** | **31** | **1** | **K.VTHTGQVYDDKDYR.R** |
|  | 25656 | **920.1290** | **2757.3651** | **2757.3821** | **-0.0170** | **1** | **58** | **8.8e-005** | **1** | **R.QKEVNENFAIDLIAEQPVSEVETR.V** |

| **380.** | Q29963\|1C06_HUMAN    **Mass:** 41399    **Score:** 122    **Queries matched:** 10   **emPAI:** 0.41 |
| --- | --- |
|  | HLA class I histocompatibility antigen, Cw-6 alpha chain OS=Homo sapiens GN=HLA-C PE=1 SV=2 |

|  | **Query** | **Observed** | **Mr(expt)** | **Mr(calc)** | **Delta** | **Miss** | **Score** | **Expect** | **Rank** | **Peptide** |
| --- | --- | --- | --- | --- | --- | --- | --- | --- | --- | --- |
|  | 4142 | **483.2169** | **964.4193** | **964.4250** | **-0.0057** | **0** | **53** | **0.00075** | **1** | **R.FDSDAASPR.G** |
|  | 7831 | **611.2879** | **1220.5612** | **1220.6037** | **-0.0424** | **0** | **2** | **89** | **3** | **K.DYIALNEDLR.S** |
|  | 10294 | **676.3427** | **1350.6709** | **1349.6939** | **0.9770** | **1** | **8** | **24** | **1** | **R.YLENGKETLQR.A** |
|  | 11642 | **714.3621** | **1426.7097** | **1426.7205** | **-0.0108** | **0** | **11** | **10** | **1** | **K.WAAVVVPSGEEQR.Y** |
|  | 12774 | **748.8586** | **1495.7026** | **1495.7129** | **-0.0103** | **0** | **11** | **9.6** | **2** | **R.AYLEGTCVEWLR.R** |
|  | 13126 | **760.3734** | **1518.7323** | **1518.7427** | **-0.0104** | **0** | **41** | **0.011** | **1** | **R.SWTAADTAAQITQR.K** |
|  | 15060 | 823.4092 | 1644.8038 | 1644.8148 | -0.0110 | 0 | 75 | 3.4e-006 | 1 | R.FISVGYVDDTQFVR.F |
|  | 17823 | 613.3262 | 1836.9569 | 1835.9027 | 1.0542 | 0 | 9 | 14 | 2 | K.THVTHHPVSDHEATLR.C |
|  | 24990 | **879.7289** | **2636.1650** | **2636.1838** | **-0.0188** | **0** | **27** | **0.14** | **1** | **R.DGEDQTQDTELVETRPAGDGTFQK.W** 24989 |

| **381.** | P10321\|1C07_HUMAN    **Mass:** 41136    **Score:** 122    **Queries matched:** 9   **emPAI:** 0.42 |
| --- | --- |
|  | HLA class I histocompatibility antigen, Cw-7 alpha chain OS=Homo sapiens GN=HLA-C PE=1 SV=3 |

|  | **Query** | **Observed** | **Mr(expt)** | **Mr(calc)** | **Delta** | **Miss** | **Score** | **Expect** | **Rank** | **Peptide** |
| --- | --- | --- | --- | --- | --- | --- | --- | --- | --- | --- |
|  | 4142 | 483.2169 | 964.4193 | 964.4250 | -0.0057 | 0 | 53 | 0.00075 | 1 | R.FDSDAASPR.G |
|  | 7831 | 611.2879 | 1220.5612 | 1220.6037 | -0.0424 | 0 | 2 | 89 | 3 | K.DYIALNEDLR.S |
|  | 10294 | 676.3427 | 1350.6709 | 1349.6939 | 0.9770 | 1 | 8 | 24 | 1 | R.YLENGKETLQR.A |
|  | 12774 | 748.8586 | 1495.7026 | 1495.7129 | -0.0103 | 0 | 11 | 9.6 | 2 | R.AYLEGTCVEWLR.R |
|  | 13126 | 760.3734 | 1518.7323 | 1518.7427 | -0.0104 | 0 | 41 | 0.011 | 1 | R.SWTAADTAAQITQR.K |
|  | 15060 | 823.4092 | 1644.8038 | 1644.8148 | -0.0110 | 0 | 75 | 3.4e-006 | 1 | R.FISVGYVDDTQFVR.F |
|  | 24990 | 879.7289 | 2636.1650 | 2636.1838 | -0.0188 | 0 | 27 | 0.14 | 1 | R.DGEDQTQDTELVETRPAGDGTFQK.W 24989 |
|  | 27428 | 1024.3173 | 3069.9300 | 3069.5926 | 0.3374 | 1 | 4 | 13 | 2 | R.VMAPRALLLLLSGGLALTETWACSHSMR.Y |

| **382.** | O75795\|UDB17_HUMAN    **Mass:** 61625    **Score:** 122    **Queries matched:** 37   **emPAI:** 0.42 |
| --- | --- |
|  | UDP-glucuronosyltransferase 2B17 OS=Homo sapiens GN=UGT2B17 PE=2 SV=1 |

|  | **Query** | **Observed** | **Mr(expt)** | **Mr(calc)** | **Delta** | **Miss** | **Score** | **Expect** | **Rank** | **Peptide** |
| --- | --- | --- | --- | --- | --- | --- | --- | --- | --- | --- |
|  | 1382 | 634.3110 | 633.3037 | 633.3156 | -0.0119 | 0 | 12 | 15 | 6 | K.ENIMK.L |
|  | 3270 | 451.2557 | 900.4968 | 900.5029 | -0.0061 | 0 | 46 | 0.0053 | 1 | K.GAALSVDIR.T 3275 |
|  | 4477 | 495.7325 | 989.4504 | 989.5328 | -0.0824 | 1 | 8 | 27 | 7 | K.ENIMKLSR.I |
|  | 5769 | 544.2924 | 1086.5703 | 1086.5782 | -0.0079 | 0 | 37 | 0.039 | 1 | K.KPNTLGSNTR.L 5756 5768 5770 |
|  | 5950 | 550.8157 | 1099.6169 | 1099.6237 | -0.0068 | 0 | 60 | 0.00015 | 1 | K.TILEELVQR.G 5948 |
|  | 5957 | **551.2843** | **1100.5540** | **1099.6349** | **0.9191** | **1** | **14** | **6.4** | **2** | **K.AKGAALSVDIR.T** |
|  | 6714 | **580.3182** | **1158.6219** | **1157.5063** | **1.1156** | **0** | **15** | **5.1** | **1** | **K.NDLEDFFMK.M** 6696 |
|  | 11471 | 473.2548 | 1416.7425 | 1416.7514 | -0.0089 | 0 | 29 | 0.18 | 1 | K.WLPQNDLLGHPK.T 11466 11467 11469 |
|  | 12076 | 728.4362 | 1454.8578 | 1453.7790 | 1.0788 | 0 | 21 | 0.92 | 1 | R.IHHDQPVKPLDR.A 12065 12066 |
|  | 13363 | 767.8970 | 1533.7795 | 1533.7899 | -0.0104 | 1 | 33 | 0.069 | 1 | R.FDGKKPNTLGSNTR.L 13357 13362 13364 |
|  | 14907 | 818.9556 | 1635.8967 | 1635.9083 | -0.0116 | 1 | 49 | 0.0016 | 1 | K.SSAIKLEVYPTSLTK.N 14906 14908 |
|  | 15075 | **549.6106** | **1645.8100** | **1645.8940** | **-0.0840** | **1** | **2** | **68** | **4** | **K.WLPQNDLLGHPKTK.A** |
|  | 18678 | 634.9871 | 1901.9394 | 1901.9498 | -0.0105 | 0 | 8 | 16 | 2 | K.VLVWPTEYSHWINMK.T |
|  | 24235 | **836.0715** | **2505.1926** | **2504.2046** | **0.9880** | **0** | **33** | **0.036** | **1** | **K.WDQFYSEVLGRPTTLFETMGK.A** |
|  | 25046 | **663.3171** | **2649.2392** | **2648.2945** | **0.9447** | **1** | **0** | **60** | **5** | **K.KWDQFYSEVLGRPTTLFETMGK.A** |
|  | 30473 | **692.5077** | **4149.0025** | **4148.0401** | **0.9624** | **0** | **3** | **12** | **3** | **K.AFITHGGTNGIYEAIYHGIPMVGIPLFADQHDNIAHMK.A** 30468 30469 30472 30476 |
|  | 31117 | **1177.0631** | **4704.2233** | **4705.1907** | **-0.9674** | **0** | **1** | **14** | **7** | **K.EMEEFVQSSGENGIVVFSLGSMISNMSEESANMIASALAQIPQK.V** |

| **383.** | P54855\|UDB15_HUMAN    **Mass:** 61461    **Score:** 122    **Queries matched:** 39   **emPAI:** 0.42 |
| --- | --- |
|  | UDP-glucuronosyltransferase 2B15 OS=Homo sapiens GN=UGT2B15 PE=1 SV=2 |

|  | **Query** | **Observed** | **Mr(expt)** | **Mr(calc)** | **Delta** | **Miss** | **Score** | **Expect** | **Rank** | **Peptide** |
| --- | --- | --- | --- | --- | --- | --- | --- | --- | --- | --- |
|  | 3270 | 451.2557 | 900.4968 | 900.5029 | -0.0061 | 0 | 46 | 0.0053 | 1 | K.GAALSVDIR.T 3275 |
|  | 4313 | 489.2631 | 976.5116 | 975.5171 | 0.9945 | 1 | 8 | 30 | 5 | K.ENVMKLSR.I |
|  | 5119 | **517.7761** | **1033.5376** | **1033.5444** | **-0.0068** | **0** | **9** | **20** | **2** | **K.SVINDPVYK.E** |
|  | 5636 | **539.2628** | **1076.5110** | **1076.5179** | **-0.0069** | **0** | **6** | **41** | **3** | **R.FSVGYTFEK.N** |
|  | 5769 | 544.2924 | 1086.5703 | 1086.5782 | -0.0079 | 0 | 37 | 0.039 | 1 | K.KPNTLGSNTR.L 5756 5768 5770 |
|  | 5950 | 550.8157 | 1099.6169 | 1099.6237 | -0.0068 | 0 | 60 | 0.00015 | 1 | K.TILEELVQR.G 5948 |
|  | 5957 | 551.2843 | 1100.5540 | 1099.6349 | 0.9191 | 1 | 14 | 6.4 | 2 | K.AKGAALSVDIR.T |
|  | 11471 | 473.2548 | 1416.7425 | 1416.7514 | -0.0089 | 0 | 29 | 0.18 | 1 | K.WLPQNDLLGHPK.T 11466 11467 11469 |
|  | 12863 | **501.3359** | **1500.9860** | **1501.7460** | **-0.7600** | **0** | **3** | **54** | **6** | **R.IHHDQPMKPLDR.A** |
|  | 13363 | 767.8970 | 1533.7795 | 1533.7899 | -0.0104 | 1 | 33 | 0.069 | 1 | R.FDGKKPNTLGSNTR.L 13357 13362 13364 |
|  | 14893 | 818.4185 | 1634.8224 | 1634.8338 | -0.0114 | 1 | 12 | 7.3 | 3 | K.SVINDPVYKENVMK.L |
|  | 14907 | 818.9556 | 1635.8967 | 1635.9083 | -0.0116 | 1 | 49 | 0.0016 | 1 | K.SSAIKLEVYPTSLTK.N 14906 14908 |
|  | 15075 | 549.6106 | 1645.8100 | 1645.8940 | -0.0840 | 1 | 2 | 68 | 4 | K.WLPQNDLLGHPKTK.A |
|  | 18121 | 620.3093 | 1857.9060 | 1857.9632 | -0.0572 | 1 | 4 | 39 | 4 | K.LSRIHHDQPMKPLDR.A |
|  | 18678 | 634.9871 | 1901.9394 | 1901.9498 | -0.0105 | 0 | 8 | 16 | 2 | K.VLVWPTEYSHWINMK.T |
|  | 24235 | 836.0715 | 2505.1926 | 2504.2046 | 0.9880 | 0 | 33 | 0.036 | 1 | K.WDQFYSEVLGRPTTLFETMGK.A |
|  | 25046 | 663.3171 | 2649.2392 | 2648.2945 | 0.9447 | 1 | 0 | 60 | 5 | K.KWDQFYSEVLGRPTTLFETMGK.A |
|  | 25899 | 1399.6666 | 2797.3187 | 2796.3992 | 0.9195 | 1 | 5 | 20 | 2 | R.IHHDQPMKPLDRAVFWIEFVMR.H 25898 25900 |
|  | 30473 | 692.5077 | 4149.0025 | 4148.0401 | 0.9624 | 0 | 3 | 12 | 3 | K.AFITHGGTNGIYEAIYHGIPMVGIPLFADQHDNIAHMK.A 30468 30469 30472 30476 |
|  | 31117 | 1177.0631 | 4704.2233 | 4705.1907 | -0.9674 | 0 | 1 | 14 | 7 | K.EMEEFVQSSGENGIVVFSLGSMISNMSEESANMIASALAQIPQK.V |

| **384.** | Q02318\|CP27A_HUMAN    **Mass:** 60595    **Score:** 122    **Queries matched:** 24   **emPAI:** 0.27 |
| --- | --- |
|  | Sterol 26-hydroxylase, mitochondrial OS=Homo sapiens GN=CYP27A1 PE=1 SV=1 |

|  | **Query** | **Observed** | **Mr(expt)** | **Mr(calc)** | **Delta** | **Miss** | **Score** | **Expect** | **Rank** | **Peptide** |
| --- | --- | --- | --- | --- | --- | --- | --- | --- | --- | --- |
|  | 2571 | **425.0489** | **848.0833** | **848.3997** | **-0.3163** | **0** | **4** | **55** | **9** | **-.MAALGCAR.L** 2567 |
|  | 2837 | **434.5623** | **867.1100** | **866.4181** | **0.6919** | **0** | **5** | **29** | **3** | **R.GLCPHGAR.A** |
|  | 4079 | **480.7817** | **959.5489** | **959.5553** | **-0.0064** | **0** | **53** | **0.00097** | **1** | **K.VGLQFLQR.Q** |
|  | 4804 | 506.2373 | 1010.4600 | 1009.5305 | 0.9295 | 0 | 4 | 55 | 9 | K.ATGAPGAGPGVR.R |
|  | 5795 | **545.2665** | **1088.5184** | **1087.6502** | **0.8682** | **1** | **14** | **7.8** | **1** | **K.KVGLQFLQR.Q** 5794 5801 |
|  | 6203 | **560.3560** | **1118.6974** | **1117.5848** | **1.1125** | **1** | **6** | **38** | **3** | **-.MAALGCARLR.W** |
|  | 10711 | **458.9104** | **1373.7094** | **1372.6194** | **1.0900** | **1** | **9** | **18** | **1** | **R.NDMELWKEHR.D** |
|  | 11175 | **700.4553** | **1398.8961** | **1398.7904** | **0.1057** | **0** | **(6)** | **26** | **5** | **R.IAELEMQLLLAR.L** |
|  | 11427 | **472.3203** | **1413.9390** | **1414.7853** | **-0.8463** | **0** | **9** | **12** | **10** | **R.IAELEMQLLLAR.L** 11421 |
|  | 12005 | 725.8729 | 1449.7313 | 1449.7074 | 0.0238 | 1 | (4) | 56 | 5 | K.YPVRNDMELWK.E |
|  | 12254 | **489.2996** | **1464.8771** | **1465.7024** | **-0.8252** | **1** | **7** | **22** | **4** | **K.YPVRNDMELWK.E** |
|  | 12586 | 495.6213 | 1483.8421 | 1484.8405 | -0.9984 | 1 | 7 | 23 | 5 | K.WTRPVLPFWKR.Y |
|  | 15303 | **830.9283** | **1659.8420** | **1659.8522** | **-0.0102** | **0** | **34** | **0.047** | **1** | **R.IQHPFGSVPFGYGVR.A** |
|  | 19023 | **966.0077** | **1930.0008** | **1929.0070** | **0.9938** | **0** | **72** | **6.9e-006** | **1** | **R.SIGLMFQNSLYATFLPK.W** |
|  | 27972 | **1073.8539** | **3218.5398** | **3218.5537** | **-0.0139** | **0** | **46** | **0.0011** | **1** | **K.YGPMWMSYLGPQMHVNLASAPLLEQVMR.Q** |
|  | 28026 | 1078.8796 | 3233.6171 | 3234.5486 | -0.9316 | 0 | (1) | 32 | 9 | K.YGPMWMSYLGPQMHVNLASAPLLEQVMR.Q |
|  | 28067 | 1083.8721 | 3248.5944 | 3248.6097 | -0.0153 | 0 | 17 | 0.98 | 1 | R.EAMGSLPELLMAGVDTTSNTLTWALYHLSK.D |
|  | 28143 | **1090.1656** | **3267.4751** | **3266.5385** | **0.9366** | **0** | **(0)** | **43** | **1** | **K.YGPMWMSYLGPQMHVNLASAPLLEQVMR.Q** |
|  | 29107 | **1199.1448** | **3594.4125** | **3593.6734** | **0.7391** | **0** | **0** | **27** | **6** | **R.AESASGNQVSDMAQLFYYFALEAICYILFEK.R** |
|  | 29158 | **907.6617** | **3626.6179** | **3626.8039** | **-0.1860** | **0** | **5** | **9.7** | **2** | **K.LEDMEAQLQAAGPDGIQVSGYLHFLLASGQLSPR.E** |

| **385.** | P09382\|LEG1_HUMAN    **Mass:** 15048    **Score:** 121    **Queries matched:** 8   **emPAI:** 0.58 |
| --- | --- |
|  | Galectin-1 OS=Homo sapiens GN=LGALS1 PE=1 SV=2 |

|  | **Query** | **Observed** | **Mr(expt)** | **Mr(calc)** | **Delta** | **Miss** | **Score** | **Expect** | **Rank** | **Peptide** |
| --- | --- | --- | --- | --- | --- | --- | --- | --- | --- | --- |
|  | 2978 | **439.2582** | **876.5019** | **876.5069** | **-0.0050** | **0** | **31** | **0.14** | **1** | **K.SFVLNLGK.D** 2977 |
|  | 4195 | 485.2889 | 968.5632 | 967.4651 | 1.0981 | 0 | 6 | 40 | 9 | K.LPDGYEFK.F |
|  | 15080 | **824.3758** | **1646.7370** | **1646.7471** | **-0.0101** | **0** | **33** | **0.06** | **1** | **R.FNAHGDANTIVCNSK.D** |
|  | 17166 | **892.9239** | **1783.8333** | **1783.8450** | **-0.0117** | **0** | **114** | **4.4e-010** | **1** | **R.LNLEAINYMAADGDFK.I** 17154 17182 |
|  | 23032 | **782.7084** | **2345.1035** | **2344.1746** | **0.9289** | **1** | **4** | **29** | **1** | **K.SFVLNLGKDSNNLCLHFNPR.F** |

| **386.** | P35575\|G6PC_HUMAN    **Mass:** 40856    **Score:** 121    **Queries matched:** 2   **emPAI:** 0.09 |
| --- | --- |
|  | Glucose-6-phosphatase OS=Homo sapiens GN=G6PC PE=1 SV=2 |

|  | **Query** | **Observed** | **Mr(expt)** | **Mr(calc)** | **Delta** | **Miss** | **Score** | **Expect** | **Rank** | **Peptide** |
| --- | --- | --- | --- | --- | --- | --- | --- | --- | --- | --- |
|  | 5276 | 524.2654 | 1046.5163 | 1045.6073 | 0.9091 | 1 | 7 | 35 | 7 | K.LSKWLPFR.L |
|  | 18973 | **964.5053** | **1926.9961** | **1926.0033** | **0.9928** | **0** | **121** | **7.5e-011** | **1** | **K.NLGTLFGLGLALNSSMYR.E** |

| **387.** | P62917\|RL8_HUMAN    **Mass:** 28235    **Score:** 120    **Queries matched:** 18   **emPAI:** 0.66 |
| --- | --- |
|  | 60S ribosomal protein L8 OS=Homo sapiens GN=RPL8 PE=1 SV=2 |

|  | **Query** | **Observed** | **Mr(expt)** | **Mr(calc)** | **Delta** | **Miss** | **Score** | **Expect** | **Rank** | **Peptide** |
| --- | --- | --- | --- | --- | --- | --- | --- | --- | --- | --- |
|  | 867 | 503.1058 | 502.0985 | 501.3023 | 0.7962 | 1 | 11 | 32 | 1 | R.KGAAR.L |
|  | 1093 | **556.3412** | **555.3340** | **555.3380** | **-0.0041** | **0** | **20** | **1.2** | **1** | **R.GAPLAK.V** |
|  | 1657 | 732.3197 | 731.3124 | 731.3173 | -0.0049 | 0 | 12 | 15 | 4 | R.NCWPR.V |
|  | 2058 | **404.4434** | **806.8722** | **806.3923** | **0.4799** | **0** | **25** | **0.36** | **1** | **R.AVDFAER.H** |
|  | 2222 | 411.2328 | 820.4510 | 820.4555 | -0.0045 | 1 | 19 | 2.1 | 1 | R.KGAGSVFR.A 2210 2234 |
|  | 2299 | 413.7705 | 825.5265 | 825.5324 | -0.0059 | 0 | 22 | 0.83 | 1 | R.IDKPILK.A |
|  | 2316 | **414.7517** | **827.4888** | **826.5388** | **0.9500** | **1** | **5** | **55** | **8** | **R.KVGLIAAR.R** |
|  | 3554 | 461.6588 | 921.3031 | 921.4668 | -0.1637 | 0 | 12 | 9.3 | 1 | K.DIIHDPGR.G 3549 |
|  | 3803 | **471.2769** | **940.5393** | **940.5454** | **-0.0062** | **0** | **52** | **0.00089** | **1** | **R.AVVGVVAGGGR.I** |
|  | 5385 | **529.3055** | **1056.5964** | **1056.6444** | **-0.0480** | **1** | **13** | **9.7** | **2** | **R.GAPLAKVVFR.D** |
|  | 15676 | 844.9099 | 1687.8051 | 1687.8165 | -0.0114 | 0 | 81 | 1e-006 | 1 | R.ASGNYATVISHNPETK.K |
|  | 17553 | **606.3073** | **1815.9001** | **1815.9115** | **-0.0114** | **1** | **3** | **54** | **1** | **R.ASGNYATVISHNPETKK.T** |
|  | 27020 | 599.0985 | 2990.4562 | 2991.5362 | -1.0801 | 1 | 9 | 6.7 | 2 | R.VRGVAMNPVEHPFGGGNHQHIGKPSTIR.R |
|  | 28125 | **1089.8715** | **3266.5926** | **3266.6098** | **-0.0172** | **0** | **45** | **0.0015** | **1** | **K.AQLNIGNVLPVGTMPEGTIVCCLEEKPGDR.G** |
|  | 28585 | 1137.6243 | 3409.8510 | 3410.6996 | -0.8486 | 1 | 1 | 34 | 8 | K.KAQLNIGNVLPVGTMPEGTIVCCLEEKPGDR.G |

| **388.** | P50993\|AT1A2_HUMAN    **Mass:** 113505   **Score:** 119    **Queries matched:** 30   **emPAI:** 0.07 |
| --- | --- |
|  | Sodium/potassium-transporting ATPase subunit alpha-2 OS=Homo sapiens GN=ATP1A2 PE=1 SV=1 |

|  | **Query** | **Observed** | **Mr(expt)** | **Mr(calc)** | **Delta** | **Miss** | **Score** | **Expect** | **Rank** | **Peptide** |
| --- | --- | --- | --- | --- | --- | --- | --- | --- | --- | --- |
|  | 1381 | 633.3794 | 632.3721 | 632.3316 | 0.0405 | 0 | 13 | 14 | 3 | K.NCLVK.N |
|  | 5036 | **515.2866** | **1028.5587** | **1029.5818** | **-1.0231** | **1** | **9** | **23** | **4** | **K.LSLDELGRK.Y** |
|  | 5751 | 543.3576 | 1084.7006 | 1084.5335 | 0.1671 | 0 | 18 | 2.7 | 1 | K.ACVVHGSDLK.D |
|  | 7590 | 605.3289 | 1208.6432 | 1209.5924 | -0.9493 | 1 | 4 | 62 | 7 | R.RNSVFQQGMK.N |
|  | 8743 | 634.8563 | 1267.6981 | 1267.7071 | -0.0090 | 0 | 8 | 22 | 3 | K.NMVPQQALVIR.E |
|  | 9047 | 643.3311 | 1284.6475 | 1283.7020 | 0.9456 | 0 | (3) | 71 | 4 | K.NMVPQQALVIR.E |
|  | 9368 | 434.8809 | 1301.6210 | 1302.6528 | -1.0317 | 1 | 10 | 15 | 2 | R.NSQTDKLVNER.L |
|  | 10628 | **457.2417** | **1368.7034** | **1368.6707** | **0.0326** | **0** | **8** | **20** | **3** | **R.EDSPQSHVLVMK.G** 10637 |
|  | 11097 | 466.2328 | 1395.6766 | 1396.7384 | -1.0618 | 0 | 1 | 1.1e+002 | 9 | K.VIMVTGDHPITAK.A |
|  | 11530 | 710.9467 | 1419.8789 | 1420.6755 | -0.7966 | 0 | 0 | 1.1e+002 | 3 | K.DMTSEQLDEILK.N |
|  | 13155 | **507.5795** | **1519.7168** | **1519.7552** | **-0.0384** | **0** | **8** | **20** | **2** | **K.ADIGIAMGISGSDVSK.Q** |
|  | 13156 | 760.8673 | 1519.7201 | 1520.7947 | -1.0746 | 1 | 7 | 29 | 6 | K.YQVDLSKGLTNQR.A |
|  | 14645 | 810.3738 | 1618.7331 | 1618.7435 | -0.0103 | 0 | 40 | 0.012 | 1 | K.VDNSSLTGESEPQTR.S |
|  | 15381 | **556.3156** | **1665.9248** | **1666.7881** | **-0.8633** | **0** | **1** | **87** | **6** | **K.LCFVGLMSMIDPPR.A** |
|  | 16375 | 577.3046 | 1728.8919 | 1729.9509 | -1.0590 | 1 | (4) | 49 | 7 | R.TVMGRIATLASGLEVGR.T |
|  | 16635 | **583.3386** | **1746.9940** | **1745.9458** | **1.0482** | **1** | **7** | **23** | **1** | **R.TVMGRIATLASGLEVGR.T** 16622 |
|  | 17090 | 594.2792 | 1779.8159 | 1779.9917 | -0.1758 | 1 | 7 | 20 | 2 | K.VIMVTGDHPITAKAIAK.G 17091 |
|  | 17664 | 914.0130 | 1826.0114 | 1826.8832 | -0.8718 | 1 | 2 | 69 | 4 | K.EVAMDDHKLSLDELGR.K 17668 |
|  | 17716 | 915.4601 | 1828.9056 | 1828.9167 | -0.0110 | 0 | 103 | 5.4e-009 | 1 | K.GVGIISEGNETVEDIAAR.L |
|  | 18015 | 926.9727 | 1851.9309 | 1853.0081 | -1.0772 | 1 | 4 | 44 | 2 | R.SAGIKVIMVTGDHPITAK.A |
|  | 21609 | 1088.4933 | 2174.9720 | 2173.9408 | 1.0312 | 0 | 2 | 57 | 6 | K.EMQDAFQNAYMELGGLGER.V |
|  | 24059 | 1241.0720 | 2480.1295 | 2479.1901 | 0.9394 | 0 | 2 | 45 | 2 | K.QAADMILLDDNFASIVTGVEEGR.L |
|  | 24134 | **830.7574** | **2489.2503** | **2488.2268** | **1.0236** | **1** | **1** | **53** | **2** | **K.DMTSEQLDEILKNHTEIVFAR.T** |
|  | 27746 | **790.1342** | **3156.5075** | **3156.4596** | **0.0480** | **1** | **1** | **37** | **7** | **K.VDNSSLTGESEPQTRSPEFTHENPLETR.N** |
|  | 28483 | 1124.9066 | 3371.6980 | 3372.6756 | -0.9775 | 1 | 0 | 38 | 4 | R.MYPLKVTWWFCAFPYSLLIFIYDEVR.K |
|  | 29405 | **624.4810** | **3740.8425** | **3739.9146** | **0.9278** | **0** | **1** | **30** | **3** | **R.LISMAYGQIGMIQALGGFFTYFVILAENGFLPSR.L** |

| **389.** | O75915\|PRAF3_HUMAN    **Mass:** 21600    **Score:** 119    **Queries matched:** 6   **emPAI:** 0.18 |
| --- | --- |
|  | PRA1 family protein 3 OS=Homo sapiens GN=ARL6IP5 PE=1 SV=1 |

|  | **Query** | **Observed** | **Mr(expt)** | **Mr(calc)** | **Delta** | **Miss** | **Score** | **Expect** | **Rank** | **Peptide** |
| --- | --- | --- | --- | --- | --- | --- | --- | --- | --- | --- |
|  | 3225 | 449.7418 | 897.4690 | 896.5080 | 0.9610 | 0 | 16 | 3.7 | 3 | M.DVNIAPLR.A |
|  | 3303 | 451.7661 | 901.5177 | 902.5007 | -0.9830 | 1 | 11 | 15 | 8 | K.MEGIGLKR.T |
|  | 8370 | 624.3749 | 1246.7352 | 1246.6591 | 0.0761 | 1 | 4 | 55 | 6 | K.LENKMEGIGLK.R |
|  | 21057 | **1057.0277** | **2112.0409** | **2112.0521** | **-0.0112** | **0** | **119** | **1.2e-010** | **1** | **R.TPMGIVLDALEQQEEGINR.L** |
|  | 22415 | **757.0542** | **2268.1408** | **2268.1532** | **-0.0124** | **1** | **22** | **0.45** | **1** | **K.RTPMGIVLDALEQQEEGINR.L** |
|  | 31398 | **1299.6226** | **5194.4611** | **5193.7199** | **0.7413** | **1** | **6** | **3.4** | **1** | **K.RYPTTFVMVVMLASYFLISMFGGVMVFVFGITFPLLLMFIHASLR.L** |

| **390.** | P61026\|RAB10_HUMAN    **Mass:** 22755    **Score:** 118    **Queries matched:** 12   **emPAI:** 0.59 |
| --- | --- |
|  | Ras-related protein Rab-10 OS=Homo sapiens GN=RAB10 PE=1 SV=1 |

|  | **Query** | **Observed** | **Mr(expt)** | **Mr(calc)** | **Delta** | **Miss** | **Score** | **Expect** | **Rank** | **Peptide** |
| --- | --- | --- | --- | --- | --- | --- | --- | --- | --- | --- |
|  | 5578 | 536.3207 | 1070.6269 | 1070.6336 | -0.0066 | 0 | 53 | 0.00096 | 1 | K.LLLIGDSGVGK.T 5577 |
|  | 8643 | **631.3579** | **1260.7013** | **1260.7078** | **-0.0065** | **0** | **52** | **0.00088** | **1** | **K.AFLTLAEDILR.K** 8642 |
|  | 9635 | 658.8289 | 1315.6433 | 1315.6521 | -0.0088 | 0 | 60 | 0.00015 | 1 | K.LQIWDTAGQER.F 9612 9633 9634 |
|  | 14066 | 791.3850 | 1580.7553 | 1581.7895 | -1.0341 | 0 | 12 | 6.6 | 2 | R.GAMGIMLVYDITNGK.S 14065 |
|  | 14563 | **538.7695** | **1613.2868** | **1613.7793** | **-0.4925** | **0** | **(0)** | **77** | **7** | **R.GAMGIMLVYDITNGK.S** |
|  | 21390 | **717.0351** | **2148.0835** | **2147.0131** | **1.0704** | **1** | **2** | **58** | **2** | **K.EPNSENVDISSGGGVTGWKSK.C** |

| **391.** | P07858\|CATB_HUMAN    **Mass:** 38766    **Score:** 118    **Queries matched:** 15   **emPAI:** 0.59 |
| --- | --- |
|  | Cathepsin B OS=Homo sapiens GN=CTSB PE=1 SV=3 |

|  | **Query** | **Observed** | **Mr(expt)** | **Mr(calc)** | **Delta** | **Miss** | **Score** | **Expect** | **Rank** | **Peptide** |
| --- | --- | --- | --- | --- | --- | --- | --- | --- | --- | --- |
|  | 9585 | **657.8006** | **1313.5866** | **1313.5962** | **-0.0095** | **0** | **37** | **0.031** | **1** | **K.ICEPGYSPTYK.Q** |
|  | 15636 | 843.3439 | 1684.6732 | 1684.7767 | -0.1035 | 1 | 21 | 0.78 | 1 | K.ICEPGYSPTYKQDK.H 15637 15638 |
|  | 15644 | **843.4706** | **1684.9267** | **1683.8403** | **1.0865** | **1** | **5** | **36** | **3** | **R.EQWPQCPTIKEIR.D** |
|  | 19026 | 644.3679 | 1930.0819 | 1928.8985 | 1.1834 | 0 | 20 | 0.95 | 1 | K.SGVYQHVTGEMMGGHAIR.I 19003 |
|  | 19884 | **1003.4935** | **2004.9724** | **2004.9833** | **-0.0109** | **0** | **91** | **6.5e-008** | **1** | **K.NGPVEGAFSVYSDFLLYK.S** |
|  | 21589 | 1087.4979 | 2172.9813 | 2171.9001 | 1.0812 | 0 | 12 | 4.8 | 2 | R.DQGSCGSCWAFGAVEAISDR.I 21590 |
|  | 24832 | **866.7264** | **2597.1575** | **2597.1744** | **-0.0169** | **1** | **24** | **0.23** | **1** | **K.HYGYNSYSVSNSEKDIMAEIYK.N** |
|  | 29987 | 1317.2955 | 3948.8648 | 3947.8611 | 1.0036 | 1 | 8 | 4.8 | 2 | K.NGPVEGAFSVYSDFLLYKSGVYQHVTGEMMGGHAIR.I 29965 29975 29982 |

| **392.** | P01834\|IGKC_HUMAN    **Mass:** 11773    **Score:** 118    **Queries matched:** 5   **emPAI:** 0.79 |
| --- | --- |
|  | Ig kappa chain C region OS=Homo sapiens GN=IGKC PE=1 SV=1 |

|  | **Query** | **Observed** | **Mr(expt)** | **Mr(calc)** | **Delta** | **Miss** | **Score** | **Expect** | **Rank** | **Peptide** |
| --- | --- | --- | --- | --- | --- | --- | --- | --- | --- | --- |
|  | 3131 | **445.0338** | **888.0531** | **887.4865** | **0.5666** | **1** | **4** | **49** | **1** | **R.EAKVQWK.V** |
|  | 12866 | 751.8603 | 1501.7060 | 1501.7512 | -0.0451 | 0 | 12 | 7.5 | 3 | K.DSTYSLSSTLTLSK.A |
|  | 18363 | **938.4601** | **1874.9057** | **1874.9197** | **-0.0139** | **0** | **61** | **7.8e-005** | **1** | **K.VYACEVTHQGLSSPVTK.S** |
|  | 21016 | 528.0020 | 2107.9789 | 2108.0161 | -0.0371 | 1 | 3 | 44 | 6 | K.DSTYSLSSTLTLSKADYEK.H |
|  | 29153 | **1207.2335** | **3618.6787** | **3618.7020** | **-0.0233** | **1** | **79** | **4.8e-007** | **1** | **K.VDNALQSGNSQESVTEQDSKDSTYSLSSTLTLSK.A** |

| **393.** | P62841\|RS15_HUMAN    **Mass:** 17029    **Score:** 118    **Queries matched:** 8   **emPAI:** 1.80 |
| --- | --- |
|  | 40S ribosomal protein S15 OS=Homo sapiens GN=RPS15 PE=1 SV=2 |

|  | **Query** | **Observed** | **Mr(expt)** | **Mr(calc)** | **Delta** | **Miss** | **Score** | **Expect** | **Rank** | **Peptide** |
| --- | --- | --- | --- | --- | --- | --- | --- | --- | --- | --- |
|  | 9959 | **666.8437** | **1331.6728** | **1331.6807** | **-0.0079** | **0** | **21** | **1.2** | **1** | **K.HGRPGIGATHSSR.F** |
|  | 10339 | **677.3527** | **1352.6909** | **1352.7010** | **-0.0101** | **0** | **34** | **0.049** | **1** | **K.EAPPMEKPEVVK.T** |
|  | 20618 | **1035.5745** | **2069.1344** | **2068.0043** | **1.1301** | **0** | **2** | **59** | **4** | **R.DMIILPEMVGSMVGVYNGK.T** |
|  | 24795 | **1294.6163** | **2587.2181** | **2587.2298** | **-0.0117** | **0** | **81** | **5.6e-007** | **1** | **R.GVDLDQLLDMSYEQLMQLYSAR.Q** 24798 |
|  | 27780 | **792.9122** | **3167.6196** | **3167.6365** | **-0.0169** | **0** | **17** | **0.96** | **1** | **K.TFNQVEIKPEMIGHYLGEFSITYKPVK.H** 27778 27779 |

| **394.** | Q14914\|PTGR1_HUMAN    **Mass:** 36075    **Score:** 118    **Queries matched:** 16   **emPAI:** 0.49 |
| --- | --- |
|  | Prostaglandin reductase 1 OS=Homo sapiens GN=PTGR1 PE=1 SV=2 |

|  | **Query** | **Observed** | **Mr(expt)** | **Mr(calc)** | **Delta** | **Miss** | **Score** | **Expect** | **Rank** | **Peptide** |
| --- | --- | --- | --- | --- | --- | --- | --- | --- | --- | --- |
|  | 993 | 531.3464 | 530.3391 | 530.3428 | -0.0037 | 1 | 17 | 6.2 | 2 | K.TIVKA.- |
|  | 4085 | **481.1511** | **960.2876** | **959.4924** | **0.7952** | **0** | **8** | **24** | **4** | **K.VVGAVGSDEK.V** 4075 |
|  | 6997 | **395.0784** | **1182.2133** | **1182.6972** | **-0.4839** | **1** | **7** | **20** | **10** | **K.VVESKNVALPK.G** |
|  | 9202 | 647.7875 | 1293.5605 | 1293.5693 | -0.0088 | 0 | 33 | 0.065 | 1 | K.EGDTMMGQQVAK.V |
|  | 13376 | 768.3760 | 1534.7374 | 1534.7483 | -0.0109 | 1 | 55 | 0.00042 | 1 | R.LKEGDTMMGQQVAK.V |
|  | 13616 | **517.7761** | **1550.3064** | **1550.7433** | **-0.4369** | **1** | **(2)** | **59** | **7** | **R.LKEGDTMMGQQVAK.V** |
|  | 15193 | **827.3932** | **1652.7719** | **1652.7835** | **-0.0115** | **0** | **8** | **18** | **1** | **K.HFVGYPTNSDFELK.T** |
|  | 15339 | 832.4733 | 1662.9321 | 1661.8988 | 1.0333 | 1 | 2 | 67 | 8 | K.VVGAVGSDEKVAYLQK.L |
|  | 20684 | **1037.0663** | **2072.1180** | **2072.1306** | **-0.0126** | **0** | **50** | **0.00076** | **1** | **R.TGPLPPGPPPEIVIYQELR.M** |
|  | 21744 | **1096.0124** | **2190.0103** | **2190.0199** | **-0.0096** | **0** | **56** | **0.00023** | **1** | **K.EYIIEGFENMPAAFMGMLK.G** 21743 |
|  | 23527 | **804.7460** | **2411.2161** | **2411.2333** | **-0.0172** | **1** | **6** | **18** | **1** | **K.GTIVLASPGWTTHSISDGKDLEK.L** |
|  | 23918 | **494.0823** | **2465.3753** | **2464.3036** | **1.0718** | **1** | **6** | **16** | **4** | **K.NGEVLLEALFLTVDPYMRVAAK.R** 23921 |
|  | 26399 | 722.5901 | 2886.3315 | 2886.2113 | 0.1202 | 0 | 3 | 32 | 1 | K.ASPDGYDCYFDNVGGEFSNTVIGQMK.K |

| **395.** | P62873\|GBB1_HUMAN    **Mass:** 38151    **Score:** 117    **Queries matched:** 21   **emPAI:** 0.60 |
| --- | --- |
|  | Guanine nucleotide-binding protein G(I)/G(S)/G(T) subunit beta-1 OS=Homo sapiens GN=GNB1 PE=1 SV=3 |

|  | **Query** | **Observed** | **Mr(expt)** | **Mr(calc)** | **Delta** | **Miss** | **Score** | **Expect** | **Rank** | **Peptide** |
| --- | --- | --- | --- | --- | --- | --- | --- | --- | --- | --- |
|  | 2033 | **403.2249** | **804.4353** | **804.4970** | **-0.0616** | **0** | **4** | **82** | **10** | **K.VHAIPLR.S** 2013 |
|  | 4485 | **495.7585** | **989.5025** | **990.4804** | **-0.9779** | **0** | **14** | **6.5** | **4** | **-.MSELDQLR.Q** |
|  | 4752 | 504.2585 | 1006.5025 | 1006.4753 | 0.0271 | 0 | (12) | 11 | 9 | -.MSELDQLR.Q |
|  | 4773 | **505.2582** | **1008.5019** | **1008.5101** | **-0.0082** | **0** | **44** | **0.0056** | **1** | **R.AGVLAGHDNR.V** 4778 4788 4789 4791 4792 4796 |
|  | 4893 | **509.2791** | **1016.5437** | **1016.5502** | **-0.0065** | **0** | **28** | **0.31** | **1** | **R.LLVSASQDGK.L** 4903 |
|  | 10032 | **668.8043** | **1335.5941** | **1335.6030** | **-0.0089** | **0** | **23** | **0.67** | **1** | **K.IYAMHWGTDSR.L** |
|  | 21168 | 1064.0077 | 2126.0008 | 2126.0143 | -0.0134 | 0 | 66 | 2.3e-005 | 1 | R.LLLAGYDDFNCNVWDALK.A 21187 |
|  | 21294 | **713.8634** | **2138.5684** | **2139.1840** | **-0.6157** | **1** | **1** | **41** | **8** | **K.LIIWDSYTTNKVHAIPLR.S** |
|  | 23546 | **1208.5550** | **2415.0955** | **2415.1087** | **-0.0131** | **0** | **57** | **0.00015** | **1** | **R.VSCLGVTDDGMAVATGSWDSFLK.I** |
|  | 27104 | 754.6237 | 3014.4655 | 3015.4066 | -0.9411 | 1 | 5 | 16 | 3 | R.ADQELMTYSHDNIICGITSVSFSKSGR.L |
|  | 28430 | 1121.2448 | 3360.7124 | 3359.6166 | 1.0958 | 1 | 2 | 27 | 5 | R.LFDLRADQELMTYSHDNIICGITSVSFSK.S |
|  | 30513 | **1043.4902** | **4169.9318** | **4169.7361** | **0.1957** | **1** | **2** | **15** | **1** | **R.EGMCRQTFTGHESDINAICFFPNGNAFATGSDDATCR.L** |

| **396.** | Q9HCS2\|CP4FC_HUMAN    **Mass:** 60955    **Score:** 117    **Queries matched:** 19   **emPAI:** 0.19 |
| --- | --- |
|  | Cytochrome P450 4F12 OS=Homo sapiens GN=CYP4F12 PE=1 SV=1 |

|  | **Query** | **Observed** | **Mr(expt)** | **Mr(calc)** | **Delta** | **Miss** | **Score** | **Expect** | **Rank** | **Peptide** |
| --- | --- | --- | --- | --- | --- | --- | --- | --- | --- | --- |
|  | 3271 | 451.2620 | 900.5095 | 900.4817 | 0.0278 | 0 | 6 | 45 | 6 | R.AEGGLWLR.V |
|  | 4066 | 480.2767 | 958.5388 | 957.4304 | 1.1084 | 0 | 9 | 26 | 8 | R.HPEYQER.C |
|  | 4431 | 493.7759 | 985.5372 | 985.5444 | -0.0072 | 0 | 32 | 0.11 | 1 | R.QEVQELLK.D |
|  | 5296 | 525.2524 | 1048.4903 | 1048.4938 | -0.0034 | 1 | 5 | 52 | 6 | R.FDPENSKGR.S |
|  | 6141 | 558.3129 | 1114.6112 | 1113.5641 | 1.0471 | 0 | 9 | 20 | 1 | R.LQCFPQPPK.R |
|  | 6400 | **568.3234** | **1134.6323** | **1133.6346** | **0.9978** | **0** | **0** | **1.5e+002** | **9** | **R.LHPPAPFISR.C** |
|  | 9377 | 651.8411 | 1301.6676 | 1301.6761 | -0.0086 | 1 | 34 | 0.061 | 1 | R.CRQEVQELLK.D |
|  | 10440 | 680.3709 | 1358.7271 | 1358.7347 | -0.0075 | 0 | 36 | 0.033 | 1 | R.SPLAFIPFSAGPR.N |
|  | 10747 | 688.9023 | 1375.7901 | 1375.7963 | -0.0061 | 0 | 78 | 2.3e-006 | 1 | K.TLDFIDVLLLSK.D |
|  | 11748 | 717.8292 | 1433.6439 | 1432.6439 | 1.0000 | 0 | 8 | 20 | 4 | R.CCTQDIVLPDGR.V |
|  | 12338 | 735.8257 | 1469.6368 | 1469.6465 | -0.0097 | 0 | 58 | 0.0002 | 1 | R.NCIGQAFAMAEMK.V |
|  | 14261 | 796.8909 | 1591.7672 | 1590.9232 | 0.8439 | 1 | 2 | 76 | 10 | K.SKTLDFIDVLLLSK.D |
|  | 14637 | 540.2949 | 1617.8628 | 1618.8943 | -1.0316 | 1 | 9 | 15 | 2 | K.ESLRLHPPAPFISR.C 14638 |
|  | 15478 | **558.6542** | **1672.9409** | **1671.9130** | **1.0279** | **1** | **1** | **94** | **7** | **K.LELIMRAEGGLWLR.V** |
|  | 15700 | **564.2685** | **1689.7836** | **1690.7926** | **-1.0089** | **0** | **1** | **82** | **7** | **R.ILAWTYAFYNNCR.R** |
|  | 18293 | 623.9947 | 1868.9622 | 1869.9441 | -0.9819 | 1 | 6 | 27 | 9 | R.CCTQDIVLPDGRVIPK.G |
|  | 18877 | 960.4878 | 1918.9610 | 1920.0092 | -1.0481 | 1 | 11 | 7.8 | 1 | K.TLDFIDVLLLSKDEDGK.A |
|  | 20046 | 1011.0021 | 2019.9896 | 2020.0009 | -0.0113 | 0 | 13 | 4.3 | 1 | R.LDMFEHISLMTLDSLQK.C |

| **397.** | Q9NZ01\|TECR_HUMAN    **Mass:** 36410    **Score:** 117    **Queries matched:** 10   **emPAI:** 0.34 |
| --- | --- |
|  | Trans-2,3-enoyl-CoA reductase OS=Homo sapiens GN=TECR PE=1 SV=1 |

|  | **Query** | **Observed** | **Mr(expt)** | **Mr(calc)** | **Delta** | **Miss** | **Score** | **Expect** | **Rank** | **Peptide** |
| --- | --- | --- | --- | --- | --- | --- | --- | --- | --- | --- |
|  | 1887 | **398.2430** | **794.4715** | **794.3997** | **0.0718** | **0** | **6** | **42** | **9** | **K.LCFLDK.V** |
|  | 6679 | **578.8097** | **1155.6048** | **1154.5621** | **1.0427** | **0** | **8** | **22** | **2** | **K.THPQWYPAR.Q** |
|  | 7244 | **596.9735** | **1191.9325** | **1191.6036** | **0.3288** | **1** | **8** | **15** | **1** | **K.EFRDYPPLR.M** |
|  | 7725 | **608.8099** | **1215.6053** | **1215.6135** | **-0.0082** | **0** | **59** | **0.00021** | **1** | **K.HYEVEILDAK.T** |
|  | 10089 | **670.3602** | **1338.7059** | **1337.7343** | **0.9716** | **0** | **1** | **1.1e+002** | **4** | **K.LPVGTTATLYFR.D** |
|  | 12431 | **492.2895** | **1473.8466** | **1472.7623** | **1.0843** | **1** | **3** | **70** | **8** | **K.HYEVEILDAKTR.E** |
|  | 12438 | **738.3767** | **1474.7389** | **1474.7490** | **-0.0101** | **1** | **51** | **0.0012** | **1** | **-.MKHYEVEILDAK.T** |
|  | 13793 | **521.5997** | **1561.7774** | **1562.8028** | **-1.0254** | **1** | **5** | **43** | **5** | **R.FSHGTMPLRNIFK.N** |
|  | 19610 | **992.5258** | **1983.0371** | **1983.0499** | **-0.0128** | **1** | **68** | **1.4e-005** | **1** | **K.LCFLDKVEPHATIAEIK.N** 19611 |

| **398.** | Q96HA9\|PX11C_HUMAN    **Mass:** 26961    **Score:** 116    **Queries matched:** 11   **emPAI:** 0.14 |
| --- | --- |
|  | Peroxisomal membrane protein 11C OS=Homo sapiens GN=PEX11G PE=1 SV=1 |

|  | **Query** | **Observed** | **Mr(expt)** | **Mr(calc)** | **Delta** | **Miss** | **Score** | **Expect** | **Rank** | **Peptide** |
| --- | --- | --- | --- | --- | --- | --- | --- | --- | --- | --- |
|  | 12492 | 739.8641 | 1477.7136 | 1477.7163 | -0.0027 | 0 | 0 | 1.2e+002 | 5 | R.LFDDLAMFVYTK.Q |
|  | 13443 | **514.2945** | **1539.8616** | **1538.8569** | **1.0047** | **1** | **6** | **32** | **6** | **R.LRSPTAPFTSPLPR.G** |
|  | 13689 | **519.1361** | **1554.3865** | **1554.7711** | **-0.3846** | **0** | **7** | **15** | **2** | **-.MASLSGLASALESYR.G** |
|  | 14090 | **792.3605** | **1582.7065** | **1581.7423** | **0.9642** | **0** | **5** | **34** | **2** | **K.QYGLGAQEEDAFVR.C** |
|  | 20280 | 1021.9880 | 2041.9614 | 2041.0739 | 0.8875 | 1 | 9 | 11 | 2 | R.SEVGTRLLVVSTQLSHCR.T 20279 |
|  | 24565 | **851.9597** | **2552.8572** | **2553.3124** | **-0.4552** | **0** | **1** | **40** | **4** | **R.FPPWLVGLMGTISSILSMYQAAR.A** |
|  | 27334 | **1019.8740** | **3056.6002** | **3057.7242** | **-1.1239** | **1** | **1** | **44** | **8** | **R.WWTLSTTLWALSLLLGVARSLWMLLK.L** |
|  | 27824 | **1060.5320** | **3178.5741** | **3178.5903** | **-0.0162** | **0** | **97** | **9.6e-009** | **1** | **R.AMEAQMQSEALSLLSNLADLANAVHWLPR.G** 27825 |
|  | 28455 | **1122.9362** | **3365.7867** | **3366.6812** | **-0.8946** | **1** | **6** | **9.8** | **1** | **R.RAMEAQMQSEALSLLSNLADLANAVHWLPR.G** |

| **399.** | Q9H936\|GHC1_HUMAN    **Mass:** 34904    **Score:** 116    **Queries matched:** 7   **emPAI:** 0.11 |
| --- | --- |
|  | Mitochondrial glutamate carrier 1 OS=Homo sapiens GN=SLC25A22 PE=1 SV=1 |

|  | **Query** | **Observed** | **Mr(expt)** | **Mr(calc)** | **Delta** | **Miss** | **Score** | **Expect** | **Rank** | **Peptide** |
| --- | --- | --- | --- | --- | --- | --- | --- | --- | --- | --- |
|  | 7753 | **406.5383** | **1216.5932** | **1216.6485** | **-0.0554** | **1** | **5** | **56** | **7** | **-.MADKQISLPAK.L** 7743 |
|  | 9539 | 437.9142 | 1310.7207 | 1311.7398 | -1.0191 | 0 | 9 | 18 | 8 | R.GAAVNLTLVTPEK.A |
|  | 12869 | 751.8697 | 1501.7249 | 1501.8980 | -0.1731 | 1 | 3 | 69 | 7 | R.GIAGLYKGLGATLLR.D |
|  | 15415 | **835.3727** | **1668.7308** | **1668.7413** | **-0.0105** | **0** | **12** | **7.4** | **1** | **R.GVNEDTYSGILDCAR.K** |
|  | 28093 | **1086.2434** | **3255.7084** | **3255.7324** | **-0.0240** | **0** | **116** | **1.1e-010** | **1** | **K.ILAAQGQLSAQGGAQPSVEAPAAPRPTATQLTR.D** 28106 |

| **400.** | P29401\|TKT_HUMAN    **Mass:** 68519    **Score:** 116    **Queries matched:** 17   **emPAI:** 0.17 |
| --- | --- |
|  | Transketolase OS=Homo sapiens GN=TKT PE=1 SV=3 |

|  | **Query** | **Observed** | **Mr(expt)** | **Mr(calc)** | **Delta** | **Miss** | **Score** | **Expect** | **Rank** | **Peptide** |
| --- | --- | --- | --- | --- | --- | --- | --- | --- | --- | --- |
|  | 1182 | **577.3180** | **576.3108** | **575.2663** | **1.0444** | **0** | **3** | **1.1e+002** | **6** | **K.DTANR.L** |
|  | 2169 | **409.2168** | **816.4191** | **817.4182** | **-0.9990** | **0** | **3** | **1e+002** | **7** | **R.GITGVEDK.E** |
|  | 3468 | 458.2385 | 914.4624 | 914.5549 | -0.0925 | 1 | 10 | 17 | 5 | R.KLILDSAR.A 3469 3471 |
|  | 3470 | **458.2636** | **914.5126** | **915.5025** | **-0.9899** | **0** | **5** | **54** | **9** | **K.AVELAANTK.G** |
|  | 3766 | **469.7582** | **937.5019** | **937.4579** | **0.0440** | **0** | **11** | **11** | **3** | **R.MPSLPSYK.V** |
|  | 10069 | 669.8172 | 1337.6198 | 1336.6697 | 0.9502 | 1 | 2 | 94 | 8 | R.MPSLPSYKVGDK.I |
|  | 13797 | **521.6049** | **1561.7929** | **1561.8035** | **-0.0106** | **1** | **8** | **21** | **2** | **K.MFGIDRDAIAQAVR.G** |
|  | 18454 | **942.9587** | **1883.9028** | **1883.9153** | **-0.0125** | **0** | **24** | **0.42** | **1** | **R.SVPTSTVFYPSDGVATEK.A** |
|  | 19367 | **654.3216** | **1959.9429** | **1958.9884** | **0.9546** | **1** | **3** | **44** | **3** | **-.MESYHKPDQQKLQALK.D** |
|  | 20042 | **1010.5324** | **2019.0501** | **2019.0636** | **-0.0135** | **0** | **63** | **4.9e-005** | **1** | **K.ILATPPQEDAPSVDIANIR.M** 20043 |
|  | 21368 | 1074.9462 | 2147.8778 | 2147.1586 | 0.7192 | 1 | 3 | 38 | 7 | K.KILATPPQEDAPSVDIANIR.M |
|  | 24249 | **836.7366** | **2507.1879** | **2507.2040** | **-0.0162** | **0** | **33** | **0.037** | **1** | **R.TSRPENAIIYNNNEDFQVGQAK.V** |
|  | 30038 | 1323.6443 | 3967.9110 | 3966.7678 | 1.1432 | 0 | (0) | 28 | 5 | R.MAAISESNINLCGSHCGVSIGEDGPSQMALEDLAMFR.S |
|  | 30075 | **996.9512** | **3983.7758** | **3982.7628** | **1.0131** | **0** | **1** | **20** | **1** | **R.MAAISESNINLCGSHCGVSIGEDGPSQMALEDLAMFR.S** |

| **401.** | Q96HR9\|REEP6_HUMAN    **Mass:** 20891    **Score:** 115    **Queries matched:** 21   **emPAI:** 0.96 |
| --- | --- |
|  | Receptor expression-enhancing protein 6 OS=Homo sapiens GN=REEP6 PE=1 SV=1 |

|  | **Query** | **Observed** | **Mr(expt)** | **Mr(calc)** | **Delta** | **Miss** | **Score** | **Expect** | **Rank** | **Peptide** |
| --- | --- | --- | --- | --- | --- | --- | --- | --- | --- | --- |
|  | 3334 | **453.2262** | **904.4379** | **904.4436** | **-0.0057** | **0** | **10** | **19** | **1** | **R.IMNDLSGR.A** |
|  | 4054 | 479.7663 | 957.5180 | 957.5243 | -0.0063 | 0 | 78 | 2.9e-006 | 1 | R.ALDAAAGITR.N |
|  | 7881 | **612.3359** | **1222.6573** | **1222.6670** | **-0.0096** | **0** | **43** | **0.007** | **1** | **R.NVKPSQTPQPK.D** 7853 7880 7883 7884 7885 7886 7887 7888 7894 |
|  | 10110 | **671.3516** | **1340.6886** | **1340.6949** | **-0.0063** | **1** | **33** | **0.076** | **1** | **R.QRVEHFLEQR.N** 10109 |
|  | 10397 | **678.8868** | **1355.7591** | **1355.7660** | **-0.0069** | **0** | **4** | **63** | **1** | **R.NLVTEVLGALEAK.T** 10370 10371 |
|  | 15783 | **565.3058** | **1692.8957** | **1692.8114** | **0.0843** | **1** | **4** | **44** | **1** | **R.HHGAVDRIMNDLSGR.A** |
|  | 18283 | 935.4809 | 1868.9472 | 1870.0411 | -1.0939 | 1 | 2 | 56 | 7 | R.NLVTEVLGALEAKTGVEK.R 18281 18306 |

| **402.** | O14773\|TPP1_HUMAN    **Mass:** 61723    **Score:** 115    **Queries matched:** 11   **emPAI:** 0.19 |
| --- | --- |
|  | Tripeptidyl-peptidase 1 OS=Homo sapiens GN=TPP1 PE=1 SV=2 |

|  | **Query** | **Observed** | **Mr(expt)** | **Mr(calc)** | **Delta** | **Miss** | **Score** | **Expect** | **Rank** | **Peptide** |
| --- | --- | --- | --- | --- | --- | --- | --- | --- | --- | --- |
|  | 1098 | 557.0184 | 556.0111 | 556.3221 | -0.3110 | 0 | 3 | 45 | 5 | K.TLLNP.- |
|  | 2390 | **834.4332** | **833.4259** | **833.4317** | **-0.0058** | **0** | **19** | **2.6** | **1** | **R.VNTELMK.A** |
|  | 7010 | **395.2037** | **1182.5893** | **1181.6557** | **0.9336** | **0** | **11** | **11** | **2** | **R.TLPPGWVSLGR.A** |
|  | 12414 | **492.2530** | **1473.7372** | **1473.7477** | **-0.0105** | **0** | **4** | **58** | **3** | **R.LFGGNFAHQASVAR.V** |
|  | 13380 | **512.7622** | **1535.2646** | **1535.8936** | **-0.6290** | **0** | **6** | **19** | **4** | **R.ILSGRPPLGFLNPR.L** |
|  | 14413 | 802.9076 | 1603.8006 | 1603.8107 | -0.0101 | 0 | 60 | 0.00012 | 1 | R.LYQQHGAGLFDVTR.G 14412 14414 |
|  | 19590 | **991.9782** | **1981.9417** | **1981.9534** | **-0.0116** | **0** | **64** | **4e-005** | **1** | **R.AYPDVAALSDGYWVVSNR.V** |
|  | 23927 | 1234.5643 | 2467.1141 | 2466.2074 | 0.9067 | 1 | 0 | 67 | 4 | K.AAARGLTLLFASGDSGAGCWSVSGR.H |
|  | 31182 | **1200.3644** | **4797.4284** | **4797.4555** | **-0.0271** | **1** | **21** | **0.13** | **1** | **R.AYPDVAALSDGYWVVSNRVPIPWVSGTSASTPVFGGILSLINEHR.I** |

| **403.** | O43707\|ACTN4_HUMAN    **Mass:** 105245   **Score:** 115    **Queries matched:** 27   **emPAI:** 0.15 |
| --- | --- |
|  | Alpha-actinin-4 OS=Homo sapiens GN=ACTN4 PE=1 SV=2 |

|  | **Query** | **Observed** | **Mr(expt)** | **Mr(calc)** | **Delta** | **Miss** | **Score** | **Expect** | **Rank** | **Peptide** |
| --- | --- | --- | --- | --- | --- | --- | --- | --- | --- | --- |
|  | 3226 | **450.1733** | **898.3319** | **899.4647** | **-1.1327** | **1** | **5** | **47** | **3** | **K.EAMLKHR.D** |
|  | 3422 | **456.2892** | **910.5637** | **910.4872** | **0.0766** | **0** | **10** | **12** | **5** | **R.IAESNHIK.L** |
|  | 9359 | 434.7278 | 1301.1616 | 1300.5982 | 0.5634 | 0 | 6 | 26 | 5 | K.HTNYTMEHIR.V 9360 9368 9374 |
|  | 10316 | **676.8120** | **1351.6095** | **1351.6190** | **-0.0096** | **0** | **25** | **0.41** | **1** | **K.GISQEQMQEFR.A** |
|  | 10937 | 693.8869 | 1385.7592 | 1385.7667 | -0.0074 | 0 | 74 | 5.8e-006 | 1 | R.VGWEQLLTTIAR.T 10938 |
|  | 11673 | **715.3812** | **1428.7478** | **1428.7572** | **-0.0095** | **0** | **41** | **0.0099** | **1** | **R.TINEVENQILTR.D** |
|  | 13136 | **507.2686** | **1518.7841** | **1519.7354** | **-0.9513** | **1** | **3** | **72** | **5** | **K.TFTAWCNSHLRK.A** |
|  | 13398 | 513.2558 | 1536.7456 | 1536.7671 | -0.0216 | 0 | 12 | 8 | 5 | R.FAIQDISVEETSAK.E |
|  | 13565 | 774.4050 | 1546.7954 | 1547.7580 | -0.9626 | 1 | 11 | 9 | 2 | K.HRDYETATLSDIK.A |
|  | 14717 | **542.1301** | **1623.3686** | **1623.7563** | **-0.3877** | **0** | **1** | **69** | **8** | **K.ACLISLGYDVENDR.Q** |
|  | 16060 | **570.3534** | **1708.0383** | **1707.9043** | **0.1341** | **1** | **2** | **59** | **9** | **R.DYETATLSDIKALIR.K** |
|  | 17169 | **595.6369** | **1783.8889** | **1785.0070** | **-1.1181** | **1** | **0** | **99** | **7** | **R.DGLKLMLLLEVISGER.L** |
|  | 17176 | 893.3929 | 1784.7713 | 1785.8719 | -1.1006 | 1 | 1 | 88 | 6 | K.ASIHEAWTDGKEAMLK.H |
|  | 17271 | **896.4455** | **1790.8764** | **1791.8502** | **-0.9737** | **0** | **3** | **51** | **2** | **R.MAPYQGPDAVPGALDYK.S** |
|  | 17351 | **600.6778** | **1799.0116** | **1797.9836** | **1.0280** | **1** | **2** | **57** | **7** | **K.GVKLVSIGAEEIVDGNAK.M** |
|  | 17750 | 916.9562 | 1831.8979 | 1831.8622 | 0.0358 | 0 | 3 | 57 | 9 | K.MLDAEDIVNTARPDEK.A |
|  | 18878 | **960.5005** | **1918.9865** | **1919.0000** | **-0.0135** | **0** | **40** | **0.01** | **1** | **K.LSGSNPYTTVTPQIINSK.W** |
|  | 20090 | 674.9855 | 2021.9346 | 2022.9509 | -1.0164 | 0 | 4 | 36 | 5 | K.AIMTYVSSFYHAFSGAQK.A |
|  | 25548 | **685.0705** | **2736.2529** | **2736.2966** | **-0.0437** | **1** | **1** | **54** | **4** | **K.AIMTYVSSFYHAFSGAQKAETAANR.I** |
|  | 29230 | 1225.1710 | 3672.4912 | 3671.7501 | 0.7411 | 1 | (0) | 24 | 10 | R.QGEAEFNRIMSLVDPNHSGLVTFQAFIDFMSR.E |
|  | 29252 | **922.9605** | **3687.8129** | **3687.7450** | **0.0679** | **1** | **2** | **23** | **2** | **R.QGEAEFNRIMSLVDPNHSGLVTFQAFIDFMSR.E** |
|  | 30584 | **1058.4697** | **4229.8498** | **4230.6651** | **-0.8152** | **0** | **0** | **20** | **7** | **-.MVDYHAANQSYQYGPSSAGNGAGGGGSMGDYMAQEDDWDR.D** |
|  | 30888 | **888.6514** | **4438.2205** | **4438.0232** | **0.1973** | **0** | **3** | **11** | **1** | **R.AAPFNNWMESAMEDLQDMFIVHTIEEIEGLISAHDQFK.S** |

| **404.** | P05164\|PERM_HUMAN    **Mass:** 84784    **Score:** 114    **Queries matched:** 30   **emPAI:** 0.14 |
| --- | --- |
|  | Myeloperoxidase OS=Homo sapiens GN=MPO PE=1 SV=1 |

|  | **Query** | **Observed** | **Mr(expt)** | **Mr(calc)** | **Delta** | **Miss** | **Score** | **Expect** | **Rank** | **Peptide** |
| --- | --- | --- | --- | --- | --- | --- | --- | --- | --- | --- |
|  | 2086 | **405.2504** | **808.4862** | **807.4239** | **1.0623** | **0** | **5** | **51** | **5** | **K.IPPNDPR.I** |
|  | 3555 | **461.7164** | **921.4182** | **921.4742** | **-0.0560** | **0** | **(10)** | **15** | **4** | **R.LFEQVMR.I** |
|  | 3783 | **470.3303** | **938.6460** | **937.4691** | **1.1769** | **0** | **28** | **0.21** | **1** | **R.LFEQVMR.I** |
|  | 3851 | 473.0812 | 944.1479 | 943.5239 | 0.6240 | 0 | 10 | 13 | 4 | R.NGFPVALAR.A |
|  | 4776 | 505.2697 | 1008.5248 | 1008.5393 | -0.0145 | 0 | 13 | 8.3 | 1 | M.GVPFFSSLR.C 4799 |
|  | 5405 | **529.7844** | **1057.5543** | **1057.5628** | **-0.0085** | **1** | **24** | **0.7** | **1** | **R.RSPTLGASNR.A** 5411 |
|  | 5711 | **542.1288** | **1082.2430** | **1081.4645** | **0.7785** | **0** | **12** | **5.8** | **1** | **R.TITGMCNNR.R** 5692 |
|  | 7869 | **408.2323** | **1221.6750** | **1222.6128** | **-0.9378** | **1** | **9** | **17** | **5** | **R.ERLFEQVMR.I** |
|  | 10109 | 447.9027 | 1340.6862 | 1339.7282 | 0.9580 | 0 | 10 | 15 | 3 | R.IGLDLPALNMQR.S |
|  | 10387 | **452.7500** | **1355.2281** | **1355.7231** | **-0.4950** | **0** | **(1)** | **79** | **9** | **R.IGLDLPALNMQR.S** |
|  | 10407 | **679.3081** | **1356.6017** | **1357.6085** | **-1.0068** | **0** | **5** | **44** | **7** | **K.NNIFMSNSYPR.D** 10422 |
|  | 10508 | **682.8130** | **1363.6115** | **1362.7693** | **0.8422** | **0** | **1** | **1.2e+002** | **6** | **K.IVGAMVQIITYR.D** |
|  | 10713 | **458.9209** | **1373.7409** | **1374.7368** | **-0.9959** | **1** | **8** | **24** | **7** | **R.SPTLGASNRAFVR.W** |
|  | 12939 | **503.1064** | **1506.2974** | **1506.8592** | **-0.5618** | **1** | **4** | **34** | **9** | **R.KIVGAMVQIITYR.D** |
|  | 12965 | **755.3055** | **1508.5964** | **1508.7598** | **-0.1634** | **0** | **2** | **78** | **6** | **R.YGHTLIQPFMFR.L** |
|  | 13532 | **772.9301** | **1543.8455** | **1543.8545** | **-0.0089** | **0** | **74** | **4.9e-006** | **1** | **R.VGPLLACIIGTQFR.K** 13531 |
|  | 13938 | 525.2016 | 1572.5830 | 1572.8042 | -0.2212 | 0 | 2 | 61 | 9 | R.NMSNQLGLLAVNQR.F |
|  | 16323 | **576.3142** | **1725.9208** | **1725.9413** | **-0.0205** | **1** | **7** | **22** | **3** | **R.AADYLHVALDLLERK.L** |
|  | 17509 | 907.0070 | 1811.9995 | 1812.0006 | -0.0011 | 1 | 3 | 52 | 3 | R.NGFPVALARAVSNEIVR.F |
|  | 18120 | **929.9597** | **1857.9048** | **1858.8097** | **-0.9049** | **0** | **6** | **30** | **2** | **R.FWWENEGVFSMQQR.Q** 18113 |
|  | 19938 | **671.3077** | **2010.9012** | **2010.8672** | **0.0340** | **0** | **0** | **79** | **8** | **R.CMVDLGPCWAGGLTAEMK.L** |
|  | 21151 | **531.7961** | **2123.1555** | **2123.0470** | **0.1085** | **0** | **2** | **49** | **3** | **R.ALLPFDNLHDDPCLLTNR.S** |
|  | 23526 | **1206.5742** | **2411.1339** | **2411.1474** | **-0.0135** | **0** | **59** | **9.3e-005** | **1** | **R.WLPAEYEDGFSLPYGWTPGVK.R** |
|  | 26014 | **940.1110** | **2817.3112** | **2817.3255** | **-0.0142** | **0** | **19** | **0.67** | **1** | **R.SLMFMQWGQLLDHDLDFTPEPAAR.A** |

| **405.** | P49189\|AL9A1_HUMAN    **Mass:** 54679    **Score:** 114    **Queries matched:** 12   **emPAI:** 0.14 |
| --- | --- |
|  | 4-trimethylaminobutyraldehyde dehydrogenase OS=Homo sapiens GN=ALDH9A1 PE=1 SV=3 |

|  | **Query** | **Observed** | **Mr(expt)** | **Mr(calc)** | **Delta** | **Miss** | **Score** | **Expect** | **Rank** | **Peptide** |
| --- | --- | --- | --- | --- | --- | --- | --- | --- | --- | --- |
|  | 7605 | **605.8065** | **1209.5984** | **1209.6064** | **-0.0080** | **0** | **9** | **17** | **2** | **K.VSFTGSVPTGMK.I** |
|  | 7658 | **405.1864** | **1212.5375** | **1211.5856** | **0.9518** | **0** | **11** | **12** | **2** | **R.VIATFTCSGEK.E** |
|  | 8152 | **619.3116** | **1236.6087** | **1236.5921** | **0.0167** | **1** | **1** | **1.2e+002** | **1** | **K.IWSQKSGMER.C** |
|  | 11724 | **478.2618** | **1431.7634** | **1431.7769** | **-0.0134** | **0** | **6** | **33** | **2** | **R.MGPLINRPHLER.V** |
|  | 13438 | 770.8858 | 1539.7570 | 1539.7682 | -0.0111 | 0 | 10 | 14 | 2 | R.ANDTTFGLAAGVFTR.D |
|  | 17605 | **911.3976** | **1820.7806** | **1819.7804** | **1.0002** | **0** | **1** | **98** | **9** | **K.DGYYMRPCVLTNCR.D** |
|  | 17629 | **912.4311** | **1822.8476** | **1822.8593** | **-0.0117** | **0** | **70** | **1.1e-005** | **1** | **K.SPLIIFSDCDMNNAVK.G** |
|  | 18933 | **642.3680** | **1924.0821** | **1923.8302** | **0.2519** | **1** | **3** | **46** | **3** | **R.EREDEIATMECINNGK.S** |
|  | 19045 | **966.8864** | **1931.7583** | **1931.9225** | **-0.1642** | **1** | **1** | **67** | **4** | **R.VEPADASGTEKAFEPATGR.V** |
|  | 20201 | 678.4135 | 2032.2185 | 2033.0901 | -0.8715 | 1 | 0 | 73 | 7 | K.IMEMSAKGIKPVTLELGGK.S |
|  | 20259 | **681.1426** | **2040.4061** | **2040.9939** | **-0.5878** | **1** | **0** | **53** | **3** | **K.AFEPATGRVIATFTCSGEK.E** |
|  | 25041 | **883.4476** | **2647.3209** | **2647.3369** | **-0.0161** | **1** | **66** | **1.6e-005** | **1** | **R.REPLGVCVGIGAWNYPFQIASWK.S** |

| **406.** | P62834\|RAP1A_HUMAN    **Mass:** 21316    **Score:** 114    **Queries matched:** 10   **emPAI:** 0.39 |
| --- | --- |
|  | Ras-related protein Rap-1A OS=Homo sapiens GN=RAP1A PE=1 SV=1 |

|  | **Query** | **Observed** | **Mr(expt)** | **Mr(calc)** | **Delta** | **Miss** | **Score** | **Expect** | **Rank** | **Peptide** |
| --- | --- | --- | --- | --- | --- | --- | --- | --- | --- | --- |
|  | 3747 | 469.2074 | 936.4002 | 935.3654 | 1.0348 | 0 | 5 | 46 | 2 | K.CDLEDER.V |
|  | 4425 | 493.3024 | 984.5903 | 984.5968 | -0.0065 | 0 | 4 | 64 | 7 | K.LVVLGSGGVGK.S 4409 |
|  | 12751 | 747.8981 | 1493.7816 | 1493.7878 | -0.0062 | 0 | 77 | 2.7e-006 | 1 | K.INVNEIFYDLVR.Q |
|  | 15378 | 833.9478 | 1665.8809 | 1664.9138 | 0.9672 | 0 | 13 | 6.1 | 2 | K.SALTVQFVQGIFVEK.Y 15347 |
|  | 16075 | 855.4589 | 1708.9033 | 1708.9148 | -0.0115 | 1 | 49 | 0.0015 | 1 | K.SKINVNEIFYDLVR.Q 16078 |
|  | 19887 | 669.3632 | 2005.0677 | 2005.0745 | -0.0068 | 1 | 4 | 33 | 4 | K.INVNEIFYDLVRQINR.K |
|  | 29447 | **1252.3075** | **3753.9007** | **3754.6657** | **-0.7650** | **1** | **1** | **27** | **9** | **K.QVEVDCQQCMLEILDTAGTEQFTAMRDLYMK.N** |

| **407.** | P46782\|RS5_HUMAN    **Mass:** 23033    **Score:** 113    **Queries matched:** 23   **emPAI:** 0.36 |
| --- | --- |
|  | 40S ribosomal protein S5 OS=Homo sapiens GN=RPS5 PE=1 SV=4 |

|  | **Query** | **Observed** | **Mr(expt)** | **Mr(calc)** | **Delta** | **Miss** | **Score** | **Expect** | **Rank** | **Peptide** |
| --- | --- | --- | --- | --- | --- | --- | --- | --- | --- | --- |
|  | 1118 | **561.0730** | **560.0657** | **559.3078** | **0.7579** | **1** | **1** | **2.1e+002** | **4** | **R.NNGKK.L** |
|  | 3600 | **463.7293** | **925.4441** | **925.4505** | **-0.0064** | **0** | **11** | **13** | **1** | **K.GSSNSYAIK.K** |
|  | 3721 | 467.7511 | 933.4877 | 932.4675 | 1.0202 | 1 | 22 | 1.3 | 1 | R.EDSTRIGR.A 3684 3722 |
|  | 4230 | **486.7468** | **971.4790** | **971.4858** | **-0.0068** | **0** | **8** | **29** | **3** | **K.AQCPIVER.L** 4244 |
|  | 7233 | **398.2046** | **1191.5920** | **1192.5151** | **-0.9231** | **0** | **2** | **86** | **8** | **R.LTNSMMMHGR.N** |
|  | 8641 | 631.3424 | 1260.6701 | 1261.6567 | -0.9866 | 1 | 4 | 61 | 7 | K.YAKYLPHSAGR.Y |
|  | 12849 | **751.3952** | **1500.7758** | **1500.7871** | **-0.0113** | **0** | **17** | **2.7** | **1** | **R.VNQAIWLLCTGAR.E** |
|  | 14185 | **398.1619** | **1588.6185** | **1589.7225** | **-1.1040** | **1** | **(6)** | **26** | **7** | **R.LTNSMMMHGRNNGK.K** |
|  | 14466 | 536.6413 | 1606.9021 | 1605.7174 | 1.1847 | 1 | (4) | 42 | 5 | R.LTNSMMMHGRNNGK.K 14423 |
|  | 14682 | **541.3048** | **1620.8926** | **1621.7123** | **-0.8197** | **1** | **(5)** | **40** | **3** | **R.LTNSMMMHGRNNGK.K** |
|  | 14684 | **541.3055** | **1620.8946** | **1621.7123** | **-0.8177** | **1** | **(4)** | **51** | **3** | **R.LTNSMMMHGRNNGK.K** |
|  | 14839 | **816.4140** | **1630.8134** | **1630.8236** | **-0.0101** | **0** | **49** | **0.0016** | **2** | **K.TIAECLADELINAAK.G** 14855 |
|  | 14977 | **547.2993** | **1638.8760** | **1637.7072** | **1.1687** | **1** | **8** | **17** | **2** | **R.LTNSMMMHGRNNGK.K** 14952 |
|  | 19639 | 993.5136 | 1985.0125 | 1986.0455 | -1.0330 | 1 | 1 | 73 | 5 | R.NIKTIAECLADELINAAK.G |
|  | 20716 | 692.6700 | 2074.9881 | 2075.0734 | -0.0853 | 1 | 0 | 85 | 7 | R.VNQAIWLLCTGAREAAFR.N |
|  | 22850 | 1161.5693 | 2321.1241 | 2322.1380 | -1.0138 | 0 | 17 | 1.4 | 2 | K.WSTDDVQINDISLQDYIAVK.E |
|  | 27404 | 1022.5638 | 3064.6695 | 3064.6822 | -0.0126 | 0 | 89 | 5.4e-008 | 1 | K.HAFEIIHLLTGENPLQVLVNAIINSGPR.E |

| **408.** | Q29865\|1C18_HUMAN    **Mass:** 41363    **Score:** 113    **Queries matched:** 10   **emPAI:** 0.30 |
| --- | --- |
|  | HLA class I histocompatibility antigen, Cw-18 alpha chain OS=Homo sapiens GN=HLA-C PE=2 SV=1 |

|  | **Query** | **Observed** | **Mr(expt)** | **Mr(calc)** | **Delta** | **Miss** | **Score** | **Expect** | **Rank** | **Peptide** |
| --- | --- | --- | --- | --- | --- | --- | --- | --- | --- | --- |
|  | 4142 | 483.2169 | 964.4193 | 964.4250 | -0.0057 | 0 | 53 | 0.00075 | 1 | R.FDSDAASPR.G |
|  | 7831 | 611.2879 | 1220.5612 | 1220.6037 | -0.0424 | 0 | 2 | 89 | 3 | K.DYIALNEDLR.S |
|  | 8239 | 621.3292 | 1240.6439 | 1239.5013 | 1.1426 | 0 | 16 | 3.5 | 1 | R.MFGCDLGPDGR.L |
|  | 10294 | 676.3427 | 1350.6709 | 1349.6939 | 0.9770 | 1 | 8 | 24 | 1 | R.YLENGKETLQR.A |
|  | 11642 | 714.3621 | 1426.7097 | 1426.7205 | -0.0108 | 0 | 11 | 10 | 1 | K.WAAVVVPSGEEQR.Y |
|  | 12774 | 748.8586 | 1495.7026 | 1495.7129 | -0.0103 | 0 | 11 | 9.6 | 2 | R.AYLEGTCVEWLR.R |
|  | 13126 | 760.3734 | 1518.7323 | 1518.7427 | -0.0104 | 0 | 41 | 0.011 | 1 | R.SWTAADTAAQITQR.K |
|  | 15060 | 823.4092 | 1644.8038 | 1644.8148 | -0.0110 | 0 | 75 | 3.4e-006 | 1 | R.FISVGYVDDTQFVR.F |
|  | 17823 | 613.3262 | 1836.9569 | 1835.9027 | 1.0542 | 0 | 9 | 14 | 2 | K.THVTHHPVSDHEATLR.C |
|  | 27428 | 1024.3173 | 3069.9300 | 3069.5926 | 0.3374 | 1 | 4 | 13 | 2 | R.VMAPRALLLLLSGGLALTETWACSHSMR.Y |

| **409.** | P30499\|1C01_HUMAN    **Mass:** 41395    **Score:** 113    **Queries matched:** 12   **emPAI:** 0.30 |
| --- | --- |
|  | HLA class I histocompatibility antigen, Cw-1 alpha chain OS=Homo sapiens GN=HLA-C PE=2 SV=1 |

|  | **Query** | **Observed** | **Mr(expt)** | **Mr(calc)** | **Delta** | **Miss** | **Score** | **Expect** | **Rank** | **Peptide** |
| --- | --- | --- | --- | --- | --- | --- | --- | --- | --- | --- |
|  | 4142 | 483.2169 | 964.4193 | 964.4250 | -0.0057 | 0 | 53 | 0.00075 | 1 | R.FDSDAASPR.G |
|  | 7831 | 611.2879 | 1220.5612 | 1220.6037 | -0.0424 | 0 | 2 | 89 | 3 | K.DYIALNEDLR.S |
|  | 9645 | **439.8373** | **1316.4902** | **1315.6673** | **0.8229** | **0** | **5** | **38** | **8** | **K.YFFTSVSRPGR.G** 9609 9648 |
|  | 12426 | **492.2814** | **1473.8225** | **1474.6875** | **-0.8650** | **0** | **7** | **23** | **1** | **K.WAAVMVPSGEEQR.Y** 12428 |
|  | 12774 | 748.8586 | 1495.7026 | 1495.7129 | -0.0103 | 0 | 11 | 9.6 | 2 | R.AYLEGTCVEWLR.R |
|  | 13126 | 760.3734 | 1518.7323 | 1518.7427 | -0.0104 | 0 | 41 | 0.011 | 1 | R.SWTAADTAAQITQR.K |
|  | 15060 | 823.4092 | 1644.8038 | 1644.8148 | -0.0110 | 0 | 75 | 3.4e-006 | 1 | R.FISVGYVDDTQFVR.F |
|  | 17823 | 613.3262 | 1836.9569 | 1835.9027 | 1.0542 | 0 | 9 | 14 | 2 | K.THVTHHPVSDHEATLR.C |
|  | 27754 | **790.6561** | **3158.5954** | **3159.6130** | **-1.0176** | **1** | **1** | **35** | **3** | **R.VMEPRTLILLLSGALALTETWACSHSMK.Y** |

| **410.** | P27635\|RL10_HUMAN    **Mass:** 25044    **Score:** 113    **Queries matched:** 13   **emPAI:** 0.33 |
| --- | --- |
|  | 60S ribosomal protein L10 OS=Homo sapiens GN=RPL10 PE=1 SV=4 |

|  | **Query** | **Observed** | **Mr(expt)** | **Mr(calc)** | **Delta** | **Miss** | **Score** | **Expect** | **Rank** | **Peptide** |
| --- | --- | --- | --- | --- | --- | --- | --- | --- | --- | --- |
|  | 1256 | **597.3668** | **596.3595** | **596.3646** | **-0.0050** | **0** | **25** | **0.37** | **1** | **K.IHISK.K** |
|  | 4171 | **484.2707** | **966.5269** | **965.5294** | **0.9975** | **0** | **10** | **14** | **4** | **K.EHVIEALR.R** |
|  | 4362 | **490.7750** | **979.5354** | **979.4215** | **0.1138** | **0** | **12** | **9.9** | **2** | **K.MLSCAGADR.L** 4342 4351 |
|  | 4563 | 498.3364 | 994.6582 | 995.4164 | -0.7583 | 0 | (6) | 34 | 4 | K.MLSCAGADR.L |
|  | 6238 | 561.8185 | 1121.6225 | 1121.6305 | -0.0080 | 1 | 25 | 0.51 | 1 | K.EHVIEALRR.A 6237 |
|  | 7139 | **594.8235** | **1187.6325** | **1187.6411** | **-0.0086** | **0** | **26** | **0.42** | **1** | **R.GAFGKPQGTVAR.V** |
|  | 8462 | **627.3160** | **1252.6174** | **1251.7122** | **0.9053** | **0** | **3** | **68** | **2** | **R.VHIGQVIMSIR.T** |
|  | 18531 | **945.9826** | **1889.9506** | **1889.9894** | **-0.0388** | **1** | **0** | **1e+002** | **2** | **R.LQTGMRGAFGKPQGTVAR.V** |
|  | 27473 | **1028.1243** | **3081.3510** | **3081.3695** | **-0.0186** | **0** | **45** | **0.0016** | **1** | **K.VDEFPLCGHMVSDEYEQLSSEALEAAR.I** |
|  | 28174 | **1094.5016** | **3280.4829** | **3280.5016** | **-0.0187** | **1** | **92** | **2.9e-008** | **1** | **K.AKVDEFPLCGHMVSDEYEQLSSEALEAAR.I** |

| **411.** | P30460\|1B08_HUMAN    **Mass:** 40648    **Score:** 112    **Queries matched:** 11   **emPAI:** 0.42 |
| --- | --- |
|  | HLA class I histocompatibility antigen, B-8 alpha chain OS=Homo sapiens GN=HLA-B PE=1 SV=1 |

|  | **Query** | **Observed** | **Mr(expt)** | **Mr(calc)** | **Delta** | **Miss** | **Score** | **Expect** | **Rank** | **Peptide** |
| --- | --- | --- | --- | --- | --- | --- | --- | --- | --- | --- |
|  | 4142 | 483.2169 | 964.4193 | 964.4250 | -0.0057 | 0 | 53 | 0.00075 | 1 | R.FDSDAASPR.E |
|  | 7831 | 611.2879 | 1220.5612 | 1220.6037 | -0.0424 | 0 | 2 | 89 | 3 | K.DYIALNEDLR.S |
|  | 9648 | 439.8941 | 1316.6606 | 1315.5979 | 1.0626 | 0 | 3 | 74 | 8 | R.YFDTAMSRPGR.G |
|  | 11642 | 714.3621 | 1426.7097 | 1426.7205 | -0.0108 | 0 | 11 | 10 | 1 | K.WAAVVVPSGEEQR.Y |
|  | 12774 | 748.8586 | 1495.7026 | 1495.7129 | -0.0103 | 0 | 11 | 9.6 | 2 | R.AYLEGTCVEWLR.R |
|  | 13126 | 760.3734 | 1518.7323 | 1518.7427 | -0.0104 | 0 | 41 | 0.011 | 1 | R.SWTAADTAAQITQR.K |
|  | 15060 | 823.4092 | 1644.8038 | 1644.8148 | -0.0110 | 0 | 75 | 3.4e-006 | 1 | R.FISVGYVDDTQFVR.F |
|  | 22148 | **744.6662** | **2230.9768** | **2230.9938** | **-0.0170** | **0** | **17** | **1.6** | **1** | **R.DGEDQTQDTELVETRPAGDR.T** |
|  | 29581 | **948.2367** | **3788.9177** | **3790.1002** | **-1.1825** | **1** | **4** | **13** | **1** | **R.WEPSSQSTVPIVGIVAGLAVLAVVVIGAVVAAVMCRR.K** 29529 29557 |

|  | **Proteins matching a subset of these peptides:** |
| --- | --- |

|  | P30480\|1B42_HUMAN    **Mass:** 40650    **Score:** 112    **Queries matched:** 10 |
| --- | --- |
|  | HLA class I histocompatibility antigen, B-42 alpha chain OS=Homo sapiens GN=HLA-B PE=1 SV=1 |

|  | P01889\|1B07_HUMAN    **Mass:** 40777    **Score:** 112    **Queries matched:** 9 |
| --- | --- |
|  | HLA class I histocompatibility antigen, B-7 alpha chain OS=Homo sapiens GN=HLA-B PE=1 SV=3 |

| **412.** | Q9NTJ5\|SAC1_HUMAN    **Mass:** 67437    **Score:** 112    **Queries matched:** 19   **emPAI:** 0.17 |
| --- | --- |
|  | Phosphatidylinositide phosphatase SAC1 OS=Homo sapiens GN=SACM1L PE=1 SV=2 |

|  | **Query** | **Observed** | **Mr(expt)** | **Mr(calc)** | **Delta** | **Miss** | **Score** | **Expect** | **Rank** | **Peptide** |
| --- | --- | --- | --- | --- | --- | --- | --- | --- | --- | --- |
|  | 2080 | **405.1943** | **808.3741** | **807.4239** | **0.9502** | **0** | **4** | **63** | **10** | **K.ASFVQTR.G** |
|  | 6902 | **587.7706** | **1173.5267** | **1173.5349** | **-0.0082** | **0** | **27** | **0.31** | **1** | **K.VANHMDGFQR.H** |
|  | 7100 | **396.2336** | **1185.6791** | **1185.4213** | **0.2578** | **0** | **10** | **18** | **2** | **R.SNCMDCLDR.T** |
|  | 7185 | 397.4022 | 1189.1849 | 1189.5298 | -0.3449 | 0 | (9) | 15 | 9 | K.VANHMDGFQR.H |
|  | 8765 | 635.3762 | 1268.7378 | 1269.7517 | -1.0139 | 1 | 9 | 17 | 5 | R.TNVIQSLLARR.S |
|  | 9451 | **436.2145** | **1305.6215** | **1304.5567** | **1.0648** | **0** | **4** | **60** | **7** | **K.NAWADNANACAK.Q** |
|  | 9454 | **653.8384** | **1305.6622** | **1305.6717** | **-0.0095** | **0** | **61** | **0.00011** | **1** | **R.HFDSQVIIYGK.Q** 9455 |
|  | 13948 | **787.8614** | **1573.7082** | **1574.8609** | **-1.1527** | **1** | **1** | **1e+002** | **5** | **K.IKVGEFFSHVVWK.A** |
|  | 16056 | 854.9504 | 1707.8862 | 1708.7991 | -0.9129 | 1 | 3 | 55 | 6 | K.IYKNAWADNANACAK.Q |
|  | 16582 | **582.2522** | **1743.7348** | **1742.8596** | **0.8752** | **0** | **3** | **53** | **9** | **R.THLGLIMDGWNSMIR.Y** |
|  | 17325 | 600.2760 | 1797.8062 | 1796.9454 | 0.8607 | 1 | 5 | 31 | 1 | K.KTMLHLTDIQLQDNK.T |
|  | 18413 | **940.9491** | **1879.8836** | **1879.8985** | **-0.0149** | **0** | **62** | **5.7e-005** | **1** | **R.LSNTSPEFQEMSLLER.A** |
|  | 18847 | **639.3488** | **1915.0244** | **1914.9556** | **0.0688** | **1** | **3** | **45** | **4** | **K.RTHLGLIMDGWNSMIR.Y** |
|  | 19031 | **644.4296** | **1930.2669** | **1930.9506** | **-0.6837** | **1** | **(3)** | **36** | **2** | **K.RTHLGLIMDGWNSMIR.Y** |
|  | 19162 | **972.4651** | **1942.9157** | **1943.0186** | **-0.1028** | **1** | **0** | **90** | **1** | **-.MATAAYEQLKLHITPEK.F** |
|  | 23735 | **815.3861** | **2443.1365** | **2443.1545** | **-0.0180** | **0** | **4** | **32** | **1** | **K.GSEKPLEQTFATMVSSLGSGMMR.Y** |
|  | 23864 | **820.3821** | **2458.1244** | **2459.1495** | **-1.0250** | **0** | **(0)** | **65** | **10** | **K.GSEKPLEQTFATMVSSLGSGMMR.Y** |
|  | 24143 | 831.0752 | 2490.2038 | 2491.1393 | -0.9355 | 0 | (1) | 60 | 6 | K.GSEKPLEQTFATMVSSLGSGMMR.Y |

| **413.** | P56134\|ATPK_HUMAN    **Mass:** 11025    **Score:** 112    **Queries matched:** 4   **emPAI:** 0.86 |
| --- | --- |
|  | ATP synthase subunit f, mitochondrial OS=Homo sapiens GN=ATP5J2 PE=1 SV=3 |

|  | **Query** | **Observed** | **Mr(expt)** | **Mr(calc)** | **Delta** | **Miss** | **Score** | **Expect** | **Rank** | **Peptide** |
| --- | --- | --- | --- | --- | --- | --- | --- | --- | --- | --- |
|  | 9593 | **657.8622** | **1313.7099** | **1313.7166** | **-0.0066** | **0** | **66** | **3.5e-005** | **1** | **K.LGELPSWILMR.D** 9592 |
|  | 11653 | **714.8445** | **1427.6745** | **1427.6834** | **-0.0089** | **0** | **52** | **0.00093** | **1** | **R.DFSPSGIFGAFQR.G** 11657 |

| **414.** | P21912\|DHSB_HUMAN    **Mass:** 32407    **Score:** 112    **Queries matched:** 27   **emPAI:** 1.16 |
| --- | --- |
|  | Succinate dehydrogenase [ubiquinone] iron-sulfur subunit, mitochondrial OS=Homo sapiens GN=SDHB PE=1 SV=3 |

|  | **Query** | **Observed** | **Mr(expt)** | **Mr(calc)** | **Delta** | **Miss** | **Score** | **Expect** | **Rank** | **Peptide** |
| --- | --- | --- | --- | --- | --- | --- | --- | --- | --- | --- |
|  | 1494 | **669.3680** | **668.3607** | **668.3646** | **-0.0039** | **0** | **20** | **1.2** | **1** | **K.FAIYR.W** |
|  | 1693 | **744.3367** | **743.3295** | **743.3346** | **-0.0051** | **0** | **19** | **3.2** | **1** | **K.MMATYK.E** |
|  | 2170 | 817.4359 | 816.4286 | 816.4341 | -0.0055 | 0 | 27 | 0.41 | 1 | R.IDTNLNK.V |
|  | 4250 | **487.2716** | **972.5287** | **972.5352** | **-0.0065** | **1** | **27** | **0.42** | **1** | **R.RIDTNLNK.V** |
|  | 4860 | **508.2861** | **1014.5576** | **1013.5254** | **1.0322** | **0** | **9** | **22** | **2** | **R.GAQTAAATAPR.I** 4845 |
|  | 6432 | **569.7947** | **1137.5749** | **1137.5818** | **-0.0069** | **0** | **39** | **0.021** | **1** | **K.LQDPFSLYR.C** 6448 |
|  | 7246 | **597.2522** | **1192.4898** | **1191.4947** | **0.9951** | **0** | **27** | **0.32** | **1** | **R.CHTIMNCTR.T** 7229 |
|  | 7465 | 602.2790 | 1202.5434 | 1201.6965 | 0.8469 | 1 | 2 | 1.1e+002 | 7 | -.MAAVVALSLRR.R |
|  | 7616 | 404.4434 | 1210.3083 | 1209.7081 | 0.6002 | 1 | 4 | 42 | 7 | K.GLNPGKAIAEIK.K |
|  | 10843 | 691.3638 | 1380.7130 | 1380.7224 | -0.0094 | 0 | 53 | 0.00072 | 1 | K.YLGPAVLMQAYR.W |
|  | 11566 | **474.9212** | **1421.7417** | **1421.7514** | **-0.0098** | **1** | **28** | **0.2** | **1** | **K.IKNEVDSTLTFR.R** |
|  | 12108 | 729.4199 | 1456.8252 | 1456.8146 | 0.0106 | 1 | 18 | 1.8 | 4 | K.CGPMVLDALIKIK.N 12106 |
|  | 15424 | **418.4498** | **1669.7701** | **1670.8886** | **-1.1185** | **1** | **10** | **11** | **1** | **R.RLPATTLGGACLQASR.G** |
|  | 15449 | **836.9095** | **1671.8045** | **1671.8144** | **-0.0099** | **0** | **40** | **0.012** | **1** | **K.DLVPDLSNFYAQYK.S** |
|  | 17916 | **615.9598** | **1844.8577** | **1844.8727** | **-0.0150** | **0** | **39** | **0.013** | **1** | **K.AGDKPHMQTYEVDLNK.C** 17920 |
|  | 22323 | **752.9817** | **2255.9232** | **2254.9551** | **0.9681** | **0** | **15** | **2.1** | **1** | **R.EGICGSCAMNINGGNTLACTR.R** 22324 22325 |
|  | 22427 | **568.5441** | **2270.1474** | **2270.9501** | **-0.8026** | **0** | **(2)** | **54** | **2** | **R.EGICGSCAMNINGGNTLACTR.R** |
|  | 22758 | **578.5485** | **2310.1647** | **2311.0660** | **-0.9013** | **1** | **8** | **11** | **2** | **K.LQDPFSLYRCHTIMNCTR.T** |
|  | 24095 | 829.7194 | 2486.1363 | 2486.1536 | -0.0174 | 1 | 34 | 0.029 | 1 | R.WDPDKAGDKPHMQTYEVDLNK.C 24096 |

| **415.** | O14653\|GOSR2_HUMAN    **Mass:** 24874    **Score:** 112    **Queries matched:** 8   **emPAI:** 0.33 |
| --- | --- |
|  | Golgi SNAP receptor complex member 2 OS=Homo sapiens GN=GOSR2 PE=1 SV=2 |

|  | **Query** | **Observed** | **Mr(expt)** | **Mr(calc)** | **Delta** | **Miss** | **Score** | **Expect** | **Rank** | **Peptide** |
| --- | --- | --- | --- | --- | --- | --- | --- | --- | --- | --- |
|  | 8607 | **630.8399** | **1259.6652** | **1259.5969** | **0.0684** | **0** | **0** | **1.4e+002** | **5** | **-.MDPLFQQTHK.Q** |
|  | 10137 | **448.5706** | **1342.6900** | **1342.6993** | **-0.0093** | **0** | **22** | **0.84** | **1** | **K.YDVQHLQTALR.N** |
|  | 16848 | **880.9691** | **1759.9237** | **1759.9324** | **-0.0087** | **0** | **74** | **4.2e-006** | **1** | **K.ILDIANMLGLSNTVMR.L** 16849 |
|  | 18696 | **635.3411** | **1903.0014** | **1904.0223** | **-1.0210** | **1** | **2** | **68** | **5** | **K.KILDIANMLGLSNTVMR.L** |
|  | 21994 | **739.3661** | **2215.0764** | **2216.1008** | **-1.0244** | **0** | **18** | **1.3** | **1** | **K.VHNGMDDLILDGHNILDGLR.T** |
|  | 22245 | 748.4654 | 2242.3743 | 2243.2381 | -0.8638 | 1 | 2 | 34 | 1 | K.ILDIANMLGLSNTVMRLIEK.R |
|  | 26885 | **594.9125** | **2969.5260** | **2969.5094** | **0.0166** | **1** | **0** | **49** | **5** | **R.LETADKQSVHIVENEIQASIDQIFSR.L** |

| **416.** | Q9H115\|SNAB_HUMAN    **Mass:** 33878    **Score:** 112    **Queries matched:** 12   **emPAI:** 0.53 |
| --- | --- |
|  | Beta-soluble NSF attachment protein OS=Homo sapiens GN=NAPB PE=1 SV=2 |

|  | **Query** | **Observed** | **Mr(expt)** | **Mr(calc)** | **Delta** | **Miss** | **Score** | **Expect** | **Rank** | **Peptide** |
| --- | --- | --- | --- | --- | --- | --- | --- | --- | --- | --- |
|  | 1527 | 681.3342 | 680.3269 | 680.3316 | -0.0047 | 0 | 25 | 0.47 | 1 | R.AANMFK.M |
|  | 2180 | **409.7213** | **817.4281** | **816.4242** | **1.0038** | **0** | **5** | **61** | **7** | **K.ASHSFLR.G** |
|  | 2230 | **411.4184** | **820.8222** | **820.4192** | **0.4031** | **0** | **2** | **74** | **2** | **R.GLFGGNTR.I** |
|  | 6844 | 585.3311 | 1168.6475 | 1167.5600 | 1.0875 | 1 | 8 | 22 | 1 | K.YSAKDYFFK.A |
|  | 9122 | **430.5539** | **1288.6398** | **1289.5735** | **-0.9337** | **0** | **6** | **33** | **7** | **K.SIQGDGEGDGDLK.-** |
|  | 10988 | 695.3777 | 1388.7408 | 1388.7486 | -0.0078 | 0 | 49 | 0.0016 | 1 | R.LDQWLTTMLLR.I 10989 |
|  | 19560 | **660.3160** | **1977.9263** | **1978.8587** | **-0.9324** | **1** | **2** | **57** | **8** | **R.IEEACEMYTRAANMFK.M** |
|  | 24609 | 854.7112 | 2561.1117 | 2561.1306 | -0.0189 | 1 | 66 | 1.4e-005 | 1 | K.AIAHYEQSADYYKGEESNSSANK.C 24608 |
|  | 24648 | **855.4311** | **2563.2714** | **2563.2125** | **0.0589** | **1** | **1** | **52** | **7** | **K.LHMQLQSKHDSATSFVDAGNAYK.K** |
|  | 24766 | **861.9764** | **2582.9073** | **2583.2890** | **-0.3817** | **1** | **6** | **11** | **3** | **K.AIEIYEQVGANTMDNPLLKYSAK.D** |

| **417.** | P62241\|RS8_HUMAN    **Mass:** 24475    **Score:** 111    **Queries matched:** 12   **emPAI:** 1.06 |
| --- | --- |
|  | 40S ribosomal protein S8 OS=Homo sapiens GN=RPS8 PE=1 SV=2 |

|  | **Query** | **Observed** | **Mr(expt)** | **Mr(calc)** | **Delta** | **Miss** | **Score** | **Expect** | **Rank** | **Peptide** |
| --- | --- | --- | --- | --- | --- | --- | --- | --- | --- | --- |
|  | 7798 | **610.3208** | **1218.6270** | **1218.6357** | **-0.0086** | **0** | **26** | **0.38** | **1** | **K.YELGRPAANTK.I** 7780 7781 7813 |
|  | 8566 | **630.2906** | **1258.5666** | **1258.5877** | **-0.0210** | **1** | **5** | **47** | **1** | **-.MGISRDNWHK.R** |
|  | 9590 | **657.8387** | **1313.6628** | **1313.6714** | **-0.0086** | **0** | **45** | **0.0041** | **1** | **K.LTPEEEEILNK.K** |
|  | 12928 | **753.8884** | **1505.7623** | **1505.7726** | **-0.0102** | **0** | **57** | **0.00024** | **1** | **K.ISSLLEEQFQQGK.L** |
|  | 13732 | **779.8951** | **1557.7756** | **1557.7868** | **-0.0112** | **0** | **7** | **26** | **2** | **K.LLACIASRPGQCGR.A** |
|  | 14648 | 540.6003 | 1618.7792 | 1618.7892 | -0.0100 | 0 | 14 | 4.3 | 1 | R.QWYESHYALPLGR.K |
|  | 16212 | **859.9521** | **1717.8896** | **1717.8999** | **-0.0102** | **0** | **22** | **0.75** | **1** | **R.IIDVVYNASNNELVR.T** |
|  | 17199 | **894.3715** | **1786.7284** | **1786.7403** | **-0.0120** | **0** | **54** | **0.00042** | **1** | **R.LDVGNFSWGSECCTR.K** |
|  | 27311 | **1018.1350** | **3051.3832** | **3050.5072** | **0.8760** | **1** | **0** | **43** | **10** | **K.NCIVLIDSTPYRQWYESHYALPLGR.K** |

| **418.** | P35558\|PCKGC_HUMAN    **Mass:** 69948    **Score:** 111    **Queries matched:** 13   **emPAI:** 0.11 |
| --- | --- |
|  | Phosphoenolpyruvate carboxykinase, cytosolic [GTP] OS=Homo sapiens GN=PCK1 PE=1 SV=3 |

|  | **Query** | **Observed** | **Mr(expt)** | **Mr(calc)** | **Delta** | **Miss** | **Score** | **Expect** | **Rank** | **Peptide** |
| --- | --- | --- | --- | --- | --- | --- | --- | --- | --- | --- |
|  | 1874 | 397.8542 | 793.6939 | 793.4269 | 0.2670 | 1 | 11 | 8.7 | 1 | K.KCFALR.M |
|  | 2352 | 416.2294 | 830.4442 | 830.4610 | -0.0168 | 0 | 6 | 61 | 3 | K.TGLSQLGR.W |
|  | 6102 | **556.7568** | **1111.4991** | **1110.5426** | **0.9565** | **1** | **10** | **16** | **4** | **K.CFALRMASR.L** |
|  | 11222 | **468.7523** | **1403.2350** | **1402.7126** | **0.5224** | **0** | **6** | **23** | **4** | **R.LLGQMEEEGILR.R** |
|  | 14263 | **796.9043** | **1591.7940** | **1590.7534** | **1.0406** | **0** | **1** | **87** | **9** | **K.TNLAMMNPSLPGWK.V** |
|  | 15876 | 849.9326 | 1697.8506 | 1697.8624 | -0.0119 | 0 | 66 | 2.5e-005 | 1 | R.EIISFGSGYGGNSLLGK.K 15877 |
|  | 17663 | 913.9786 | 1825.9426 | 1825.9574 | -0.0148 | 1 | 19 | 1.2 | 1 | R.EIISFGSGYGGNSLLGKK.C |
|  | 20184 | 677.3671 | 2029.0794 | 2027.9258 | 1.1535 | 1 | 1 | 82 | 10 | K.TGLSQLGRWMSEEDFEK.A |
|  | 21520 | 542.0240 | 2164.0669 | 2165.0861 | -1.0192 | 1 | 2 | 55 | 3 | R.IMTRMGTPVLEAVGDGEFVK.C |
|  | 24173 | **832.0679** | **2493.1820** | **2493.2573** | **-0.0753** | **1** | **0** | **66** | **5** | **K.EEGWLAEHMLILGITNPEGEKK.Y** |
|  | 25977 | 469.5885 | 2811.4875 | 2811.3546 | 0.1329 | 1 | 1 | 50 | 10 | K.YLAAAFPSACGKTNLAMMNPSLPGWK.V |
|  | 31042 | **766.8731** | **4595.1946** | **4596.0591** | **-0.8645** | **1** | **3** | **8.6** | **1** | **R.EFLENNAELCQPDHIHICDGSEEENGRLLGQMEEEGILR.R** |

| **419.** | Q96GC9\|TMM49_HUMAN    **Mass:** 46607    **Score:** 110    **Queries matched:** 8   **emPAI:** 0.08 |
| --- | --- |
|  | Transmembrane protein 49 OS=Homo sapiens GN=TMEM49 PE=2 SV=1 |

|  | **Query** | **Observed** | **Mr(expt)** | **Mr(calc)** | **Delta** | **Miss** | **Score** | **Expect** | **Rank** | **Peptide** |
| --- | --- | --- | --- | --- | --- | --- | --- | --- | --- | --- |
|  | 3651 | 465.2532 | 928.4918 | 927.5290 | 0.9628 | 0 | 0 | 1.8e+002 | 10 | R.QNIVLWR.Q |
|  | 7195 | 595.7621 | 1189.5096 | 1190.5098 | -1.0002 | 1 | 8 | 28 | 7 | M.AENGKNCDQR.R |
|  | 8313 | **623.3099** | **1244.6053** | **1243.6520** | **0.9533** | **1** | **8** | **26** | **2** | **R.IQQRLNSEEK.T** |
|  | 25112 | 888.8068 | 2663.3986 | 2664.3729 | -0.9743 | 0 | 1 | 53 | 9 | K.LVVVMVCYFILSIINSMAQSYAK.R |
|  | 25896 | **700.3294** | **2797.2885** | **2796.3549** | **0.9336** | **1** | **1** | **43** | **3** | **R.IEACMWGIGTAIGELPPYFMARAAR.L** |
|  | 28713 | **1153.8143** | **3458.4212** | **3458.4368** | **-0.0156** | **0** | **81** | **2.5e-007** | **1** | **R.LSGAEPDDEEYQEFEEMLEHAESAQDFASR.A** 28712 |
|  | 29719 | **1275.8897** | **3824.6471** | **3824.0083** | **0.6388** | **1** | **0** | **28** | **7** | **K.HIVEQMVAFIGAVPGIGPSLQKPFQEYLEAQRQK.L** |

| **420.** | P83111\|LACTB_HUMAN    **Mass:** 61111    **Score:** 110    **Queries matched:** 14   **emPAI:** 0.06 |
| --- | --- |
|  | Serine beta-lactamase-like protein LACTB, mitochondrial OS=Homo sapiens GN=LACTB PE=1 SV=2 |

|  | **Query** | **Observed** | **Mr(expt)** | **Mr(calc)** | **Delta** | **Miss** | **Score** | **Expect** | **Rank** | **Peptide** |
| --- | --- | --- | --- | --- | --- | --- | --- | --- | --- | --- |
|  | 1558 | 693.3877 | 692.3804 | 692.4221 | -0.0416 | 1 | 2 | 1.1e+002 | 7 | K.AYKALK.M |
|  | 2761 | 432.2411 | 862.4676 | 863.4535 | -0.9858 | 0 | 9 | 28 | 6 | R.LMSAVTAR.A 2760 |
|  | 6052 | **554.5258** | **1107.0371** | **1107.6764** | **-0.6393** | **0** | **0** | **97** | **3** | **R.LLISHLSGIR.H** |
|  | 9272 | **433.2214** | **1296.6424** | **1297.6635** | **-1.0211** | **1** | **5** | **41** | **3** | **-.MYRLMSAVTAR.A** |
|  | 9821 | **442.7261** | **1325.1564** | **1324.6598** | **0.4966** | **1** | **7** | **20** | **1** | **K.YAMAWGVVERK.Q** |
|  | 11277 | **469.7203** | **1406.1389** | **1406.6677** | **-0.5288** | **1** | **7** | **17** | **4** | **K.ENVAFEQEKEGK.S** |
|  | 11391 | **706.8514** | **1411.6883** | **1411.6983** | **-0.0100** | **0** | **13** | **7** | **1** | **R.LVNTPYVDNSYK.W** |
|  | 13005 | **504.8952** | **1511.6637** | **1510.7416** | **0.9221** | **1** | **3** | **56** | **5** | **K.KNDFEQGELYLR.E** |
|  | 13324 | **766.4080** | **1530.8015** | **1531.9007** | **-1.0992** | **1** | **0** | **1.2e+002** | **8** | **R.IASISKSLTMVALAK.L** |
|  | 16033 | **854.4268** | **1706.8391** | **1706.8490** | **-0.0100** | **0** | **70** | **1.1e-005** | **1** | **K.FGNAMLYGYQVGLFK.N** 16034 |
|  | 26399 | 722.5901 | 2886.3315 | 2885.6505 | 0.6810 | 1 | 1 | 43 | 7 | R.GVHQRAGLPPLGHGWVGGLGLGLGLALGVK.L |
|  | 29432 | 1251.6041 | 3751.7906 | 3751.7394 | 0.0512 | 0 | 3 | 15 | 1 | K.NSNENLLPGYLKPETMVMMWTPVPNTEMSWDK.E |

| **421.** | Q5VT66\|MOSC1_HUMAN    **Mass:** 37989    **Score:** 109    **Queries matched:** 13   **emPAI:** 0.33 |
| --- | --- |
|  | MOSC domain-containing protein 1, mitochondrial OS=Homo sapiens GN=MOSC1 PE=1 SV=1 |

|  | **Query** | **Observed** | **Mr(expt)** | **Mr(calc)** | **Delta** | **Miss** | **Score** | **Expect** | **Rank** | **Peptide** |
| --- | --- | --- | --- | --- | --- | --- | --- | --- | --- | --- |
|  | 2709 | **430.7521** | **859.4897** | **859.4512** | **0.0386** | **0** | **16** | **5.3** | **1** | **M.GAAGSSALAR.F** |
|  | 3161 | **892.3679** | **891.3606** | **890.3552** | **1.0054** | **0** | **6** | **46** | **3** | **R.QCDPSER.K** |
|  | 4026 | **478.7983** | **955.5819** | **956.5542** | **-0.9723** | **1** | **3** | **74** | **6** | **R.KEPLETLK.S** |
|  | 4184 | **485.2281** | **968.4417** | **967.5087** | **0.9330** | **0** | **37** | **0.029** | **1** | **K.TPTTNAVHK.C** 4177 |
|  | 11470 | **709.3783** | **1416.7420** | **1417.7401** | **-0.9980** | **0** | **(3)** | **69** | **2** | **R.LVHFEPHMRPR.R** |
|  | 11745 | **478.8848** | **1433.6326** | **1433.7350** | **-0.1024** | **0** | **4** | **54** | **7** | **R.LVHFEPHMRPR.R** |
|  | 13972 | 788.8712 | 1575.7279 | 1575.7385 | -0.0106 | 0 | 66 | 3.4e-005 | 1 | K.GVPVSEAECTAMGLR.S |
|  | 16309 | **575.9414** | **1724.8024** | **1725.8032** | **-1.0008** | **0** | **16** | **2.7** | **1** | **R.DCGEATAQWITSFLK.S** |
|  | 17460 | **603.6776** | **1808.0108** | **1807.8808** | **0.1300** | **1** | **3** | **49** | **6** | **R.CILTTVDPDTGVMSRK.E** |
|  | 18780 | **955.5009** | **1908.9871** | **1908.9986** | **-0.0114** | **0** | **7** | **21** | **1** | **K.SPLFGQYFVLENPGTIK.V** |
|  | 20565 | **689.0305** | **2064.0697** | **2065.0428** | **-0.9731** | **1** | **2** | **54** | **3** | **K.SQPYRLVHFEPHMRPR.R** |
|  | 25488 | **1363.6679** | **2725.3211** | **2725.3446** | **-0.0235** | **0** | **63** | **2.8e-005** | **1** | **K.DQIAYSDTSPFLILSEASLADLNSR.L** |

| **422.** | P54840\|GYS2_HUMAN    **Mass:** 81533    **Score:** 109    **Queries matched:** 30   **emPAI:** 0.49 |
| --- | --- |
|  | Glycogen [starch] synthase, liver OS=Homo sapiens GN=GYS2 PE=1 SV=1 |

|  | **Query** | **Observed** | **Mr(expt)** | **Mr(calc)** | **Delta** | **Miss** | **Score** | **Expect** | **Rank** | **Peptide** |
| --- | --- | --- | --- | --- | --- | --- | --- | --- | --- | --- |
|  | 1699 | **748.3602** | **747.3529** | **747.3585** | **-0.0056** | **0** | **27** | **0.4** | **1** | **R.AVDAMNK.H** |
|  | 2247 | 411.7552 | 821.4959 | 821.4395 | 0.0563 | 0 | 7 | 31 | 4 | R.AIFSTQR.Q |
|  | 2780 | **432.7521** | **863.4896** | **862.4912** | **0.9984** | **0** | **28** | **0.28** | **1** | **K.LYDALLR.G** |
|  | 3542 | **461.0086** | **920.0026** | **919.4545** | **0.5481** | **1** | **4** | **49** | **3** | **R.RAVDAMNK.H** |
|  | 3716 | **467.7252** | **933.4359** | **933.4419** | **-0.0060** | **0** | **4** | **78** | **3** | **K.FLYGFCK.Q** |
|  | 4869 | 508.7516 | 1015.4887 | 1016.5291 | -1.0403 | 0 | 40 | 0.019 | 1 | R.LSDLLDWR.Y 4891 |
|  | 6944 | 589.3166 | 1176.6186 | 1177.6707 | -1.0521 | 0 | 27 | 0.33 | 2 | K.VGGIYTVIQTK.A |
|  | 6956 | **590.2994** | **1178.5842** | **1178.5931** | **-0.0089** | **0** | **27** | **0.35** | **1** | **K.TNNFNVETLK.G** |
|  | 7097 | **396.2287** | **1185.6642** | **1186.6645** | **-1.0003** | **1** | **14** | **6** | **3** | **R.LNFLLRMHK.S** 7096 |
|  | 7572 | **604.7726** | **1207.5306** | **1206.6244** | **0.9062** | **0** | **5** | **44** | **10** | **K.GADIFLESLSR.L** |
|  | 8694 | 633.3361 | 1264.6577 | 1263.5401 | 1.1176 | 0 | 11 | 11 | 4 | R.SPDDSCNQLTK.F |
|  | 9522 | **655.8597** | **1309.7049** | **1309.7143** | **-0.0093** | **1** | **13** | **6.4** | **1** | **R.KQLWDVAHSVK.E** |
|  | 13380 | 512.7622 | 1535.2646 | 1535.8784 | -0.6138 | 1 | 3 | 46 | 8 | K.RKPDVVTPNGLNVK.K |
|  | 13528 | 772.9129 | 1543.8112 | 1543.8008 | 0.0105 | 1 | 5 | 37 | 8 | R.YYQHARHLTLSR.A |
|  | 15043 | **822.8799** | **1643.7452** | **1643.7573** | **-0.0121** | **0** | **57** | **0.00022** | **1** | **K.TQVEQCEPVNDAVR.R** |
|  | 16299 | 575.6768 | 1724.0086 | 1723.9985 | 0.0101 | 0 | 2 | 63 | 5 | K.LPIATIFTTHATLLGR.Y |
|  | 17372 | **601.2908** | **1800.8505** | **1799.8584** | **0.9920** | **1** | **6** | **30** | **4** | **K.TQVEQCEPVNDAVRR.A** 17357 |
|  | 17618 | **608.2931** | **1821.8574** | **1820.8668** | **0.9906** | **0** | **44** | **0.0045** | **1** | **K.FSAVHEFQNLHAMYK.A** 17606 |
|  | 17826 | 919.4928 | 1836.9710 | 1836.8617 | 0.1093 | 0 | (0) | 1e+002 | 5 | K.FSAVHEFQNLHAMYK.A |
|  | 18027 | **618.3676** | **1852.0810** | **1852.0934** | **-0.0124** | **1** | **18** | **1.4** | **1** | **R.KLPIATIFTTHATLLGR.Y** |
|  | 19238 | **488.2445** | **1948.9491** | **1948.9618** | **-0.0127** | **1** | **17** | **1.9** | **1** | **K.KFSAVHEFQNLHAMYK.A** |
|  | 19553 | **989.5019** | **1976.9892** | **1978.0420** | **-1.0528** | **1** | **1** | **79** | **8** | **R.MHKSDITVVVFFIMPAK.T** |
|  | 21711 | **1093.5474** | **2185.0802** | **2185.0936** | **-0.0135** | **1** | **33** | **0.037** | **1** | **R.GEIPDLNDILDRDDLTIMK.R** |
|  | 25106 | **888.0638** | **2661.1695** | **2661.1846** | **-0.0151** | **0** | **34** | **0.023** | **1** | **K.TTADEWGENYFLIGPYFEHNMK.T** |
|  | 26034 | 1412.1616 | 2822.3087 | 2822.4233 | -0.1146 | 1 | 2 | 41 | 8 | R.QSLPPVTTHNMIDDSTDPILSTIRR.I |
|  | 31862 | **1605.2261** | **6416.8752** | **6416.9091** | **-0.0339** | **0** | **6** | **1.6** | **1** | **R.GCHLGVFPSYYEPWGYTPAECTVMGIPSVTTNLSGFGCFMQEHVADPTAYGIYIVDR.R** |

| **423.** | P21695\|GPDA_HUMAN    **Mass:** 38171    **Score:** 109    **Queries matched:** 10   **emPAI:** 0.33 |
| --- | --- |
|  | Glycerol-3-phosphate dehydrogenase [NAD+], cytoplasmic OS=Homo sapiens GN=GPD1 PE=1 SV=4 |

|  | **Query** | **Observed** | **Mr(expt)** | **Mr(calc)** | **Delta** | **Miss** | **Score** | **Expect** | **Rank** | **Peptide** |
| --- | --- | --- | --- | --- | --- | --- | --- | --- | --- | --- |
|  | 2898 | **436.2325** | **870.4505** | **870.4559** | **-0.0054** | **0** | **15** | **5** | **8** | **K.LQGPETAR.E** |
|  | 3163 | **446.7181** | **891.4217** | **890.4974** | **0.9244** | **1** | **18** | **3.1** | **1** | **R.KVAEAFAR.T** |
|  | 13702 | 778.4170 | 1554.8194 | 1555.8107 | -0.9913 | 0 | 38 | 0.021 | 2 | K.IVGGNAAQLAQFDPR.V 13728 |
|  | 15395 | **834.4662** | **1666.9178** | **1665.9050** | **1.0129** | **1** | **38** | **0.018** | **1** | **K.KLTEIINTQHENVK.Y** 15378 15394 |
|  | 27615 | 521.6004 | 3123.5587 | 3124.4304 | -0.8717 | 0 | 2 | 34 | 6 | K.LFCSGPVSSATFLESCGVADLITTCYGGR.N |
|  | 28584 | 1137.6237 | 3409.8492 | 3409.8650 | -0.0158 | 0 | 60 | 3.6e-005 | 1 | K.LPPNVVAVPDVVQAAEDADILIFVVPHQFIGK.I 28585 |

| **424.** | Q13162\|PRDX4_HUMAN    **Mass:** 30749    **Score:** 108    **Queries matched:** 9   **emPAI:** 0.42 |
| --- | --- |
|  | Peroxiredoxin-4 OS=Homo sapiens GN=PRDX4 PE=1 SV=1 |

|  | **Query** | **Observed** | **Mr(expt)** | **Mr(calc)** | **Delta** | **Miss** | **Score** | **Expect** | **Rank** | **Peptide** |
| --- | --- | --- | --- | --- | --- | --- | --- | --- | --- | --- |
|  | 1915 | **399.7486** | **797.4826** | **796.4555** | **1.0271** | **0** | **10** | **17** | **7** | **R.QGGLGPIR.I** |
|  | 3537 | **460.7552** | **919.4958** | **919.5015** | **-0.0057** | **0** | **39** | **0.023** | **1** | **R.GLFIIDDK.G** |
|  | 3987 | **477.2836** | **952.5527** | **952.5566** | **-0.0039** | **1** | **5** | **44** | **9** | **R.RQGGLGPIR.I** |
|  | 21719 | **729.4571** | **2185.3494** | **2184.9171** | **0.4324** | **0** | **0** | **58** | **3** | **R.EEECHFYAGGQVYPGEASR.V** |
|  | 23472 | **801.7243** | **2402.1511** | **2402.1689** | **-0.0178** | **0** | **14** | **2.7** | **1** | **K.HGEVCPAGWKPGSETIIPDPAGK.L** |
|  | 23704 | **815.0233** | **2442.0481** | **2442.0659** | **-0.0177** | **1** | **61** | **5.1e-005** | **1** | **R.TREEECHFYAGGQVYPGEASR.V** 23705 23706 |
|  | 24019 | **620.0754** | **2476.2727** | **2477.2842** | **-1.0115** | **1** | **0** | **70** | **5** | **K.ISKPAPYWEGTAVIDGEFKELK.L** |

| **425.** | O15173\|PGRC2_HUMAN    **Mass:** 23861    **Score:** 108    **Queries matched:** 6   **emPAI:** 0.35 |
| --- | --- |
|  | Membrane-associated progesterone receptor component 2 OS=Homo sapiens GN=PGRMC2 PE=1 SV=1 |

|  | **Query** | **Observed** | **Mr(expt)** | **Mr(calc)** | **Delta** | **Miss** | **Score** | **Expect** | **Rank** | **Peptide** |
| --- | --- | --- | --- | --- | --- | --- | --- | --- | --- | --- |
|  | 2776 | 432.7251 | 863.4356 | 862.3855 | 1.0501 | 0 | 11 | 16 | 6 | -.MAAGDGDVK.L |
|  | 4752 | 504.2585 | 1006.5025 | 1006.5083 | -0.0059 | 0 | 40 | 0.016 | 1 | R.DFSLEQLR.Q |
|  | 12369 | **736.8644** | **1471.7143** | **1471.7248** | **-0.0105** | **0** | **69** | **1.5e-005** | **1** | **K.FYGPAGPYGIFAGR.D** 12370 |
|  | 16577 | 581.9571 | 1742.8494 | 1743.8733 | -1.0238 | 1 | 3 | 52 | 6 | K.GSKFYGPAGPYGIFAGR.D |
|  | 18045 | 618.9986 | 1853.9740 | 1852.9391 | 1.0349 | 1 | 3 | 58 | 10 | R.RGLGAGAGAGEESPATSLPR.M |

| **426.** | Q6FI13\|H2A2A_HUMAN    **Mass:** 14087    **Score:** 106    **Queries matched:** 7   **emPAI:** 1.09 |
| --- | --- |
|  | Histone H2A type 2-A OS=Homo sapiens GN=HIST2H2AA3 PE=1 SV=3 |

|  | **Query** | **Observed** | **Mr(expt)** | **Mr(calc)** | **Delta** | **Miss** | **Score** | **Expect** | **Rank** | **Peptide** |
| --- | --- | --- | --- | --- | --- | --- | --- | --- | --- | --- |
|  | 2590 | **425.7635** | **849.5125** | **849.5184** | **-0.0060** | **0** | **25** | **0.55** | **1** | **R.HLQLAIR.N** |
|  | 3867 | 473.2675 | 944.5205 | 943.5240 | 0.9965 | 0 | 38 | 0.031 | 1 | R.AGLQFPVGR.V 3847 |
|  | 8814 | **636.8391** | **1271.6635** | **1271.6721** | **-0.0085** | **1** | **29** | **0.17** | **1** | **R.NDEELNKLLGK.V** |
|  | 15722 | 564.5905 | 1690.7497 | 1691.8954 | -1.1457 | 1 | 11 | 9.3 | 3 | R.HLQLAIRNDEELNK.L |
|  | 19030 | **966.0826** | **1930.1506** | **1930.1615** | **-0.0109** | **0** | **33** | **0.041** | **1** | **K.VTIAQGGVLPNIQAVLLPK.K** |
|  | 26684 | **1467.2698** | **2932.5250** | **2932.5368** | **-0.0118** | **0** | **88** | **7.7e-008** | **1** | **R.VGAGAPVYMAAVLEYLTAEILELAGNAAR.D** |

|  | **Proteins matching the same set of peptides:** |
| --- | --- |

|  | Q16777\|H2A2C_HUMAN    **Mass:** 13980    **Score:** 106    **Queries matched:** 7 |
| --- | --- |
|  | Histone H2A type 2-C OS=Homo sapiens GN=HIST2H2AC PE=1 SV=4 |

| **427.** | Q9NR31\|SAR1A_HUMAN    **Mass:** 22467    **Score:** 106    **Queries matched:** 6   **emPAI:** 0.60 |
| --- | --- |
|  | GTP-binding protein SAR1a OS=Homo sapiens GN=SAR1A PE=1 SV=1 |

|  | **Query** | **Observed** | **Mr(expt)** | **Mr(calc)** | **Delta** | **Miss** | **Score** | **Expect** | **Rank** | **Peptide** |
| --- | --- | --- | --- | --- | --- | --- | --- | --- | --- | --- |
|  | 1374 | 632.3099 | 631.3026 | 630.3701 | 0.9325 | 0 | 12 | 17 | 4 | K.GNVTLK.E |
|  | 3437 | **457.2085** | **912.4025** | **912.4090** | **-0.0065** | **0** | **21** | **1.1** | **1** | **R.QGYGEGFR.W** |
|  | 6547 | **573.8263** | **1145.6380** | **1145.6445** | **-0.0064** | **0** | **51** | **0.0012** | **1** | **K.LVFLGLDNAGK.T** |
|  | 10131 | **448.2402** | **1341.6989** | **1341.7075** | **-0.0086** | **1** | **12** | **8.8** | **2** | **K.TTLLHMLKDDR.L** |
|  | 14089 | **792.3560** | **1582.6974** | **1581.8515** | **0.8459** | **1** | **38** | **0.019** | **1** | **K.LREIFGLYGQTTGK.G** |
|  | 29257 | **923.7051** | **3690.7912** | **3690.8101** | **-0.0189** | **0** | **66** | **7.9e-006** | **1** | **R.LGQHVPTLHPTSEELTIAGMTFTTFDLGGHEQAR.R** |

| **428.** | P49821\|NDUV1_HUMAN    **Mass:** 51469    **Score:** 106    **Queries matched:** 22   **emPAI:** 0.15 |
| --- | --- |
|  | NADH dehydrogenase [ubiquinone] flavoprotein 1, mitochondrial OS=Homo sapiens GN=NDUFV1 PE=1 SV=4 |

|  | **Query** | **Observed** | **Mr(expt)** | **Mr(calc)** | **Delta** | **Miss** | **Score** | **Expect** | **Rank** | **Peptide** |
| --- | --- | --- | --- | --- | --- | --- | --- | --- | --- | --- |
|  | 938 | **520.1362** | **519.1289** | **520.2428** | **-1.1139** | **0** | **2** | **1.6e+002** | **4** | **R.AMGAR.A** |
|  | 1316 | **615.3417** | **614.3344** | **614.4003** | **-0.0659** | **0** | **19** | **3.3** | **1** | **K.EILLK.G** |
|  | 2870 | 435.2665 | 868.5185 | 868.4807 | 0.0379 | 0 | 10 | 15 | 7 | R.AAYIYIR.G |
|  | 4394 | 492.2658 | 982.5170 | 982.5236 | -0.0066 | 0 | 24 | 0.67 | 1 | R.IFTNLYGR.H |
|  | 6838 | **585.2948** | **1168.5750** | **1167.6876** | **0.8874** | **1** | **5** | **43** | **4** | **R.RLLGWSLPAR.V** |
|  | 7645 | **404.8730** | **1211.5971** | **1211.6047** | **-0.0075** | **0** | **26** | **0.32** | **1** | **R.HFRPELEER.M** 7644 |
|  | 10652 | **685.8686** | **1369.7226** | **1370.7128** | **-0.9902** | **1** | **3** | **65** | **4** | **R.AMGARAAYIYIR.G** |
|  | 12035 | **484.8939** | **1451.6599** | **1450.6697** | **0.9902** | **1** | **6** | **29** | **4** | **R.EGVDWMNKVMAR.F** 12014 |
|  | 12278 | **734.3075** | **1466.6004** | **1466.6646** | **-0.0642** | **1** | **(1)** | **96** | **7** | **R.EGVDWMNKVMAR.F** |
|  | 13595 | **775.7067** | **1549.3989** | **1548.7507** | **0.6482** | **0** | **2** | **53** | **4** | **K.WSFMNKPSDGRPK.Y** |
|  | 15988 | **853.3803** | **1704.7459** | **1704.7566** | **-0.0107** | **0** | **75** | **3.3e-006** | **1** | **K.NACGSGYDFDVFVVR.G** |
|  | 16088 | **855.9202** | **1709.8258** | **1709.8373** | **-0.0115** | **0** | **9** | **13** | **1** | **R.GEFYNEASNLQVAIR.E** |
|  | 17181 | **595.9831** | **1784.9274** | **1785.8897** | **-0.9623** | **0** | **14** | **3.9** | **1** | **R.GDARPAEIDSLWEISK.Q** 17193 |
|  | 18910 | **641.6306** | **1921.8700** | **1921.8840** | **-0.0140** | **1** | **1** | **71** | **4** | **K.YLVVNADEGEPGTCKDR.E** |
|  | 23887 | **821.4199** | **2461.2379** | **2461.2536** | **-0.0157** | **0** | **6** | **19** | **1** | **K.QIEGHTICALGDGAAWPVQGLIR.H** |
|  | 24900 | **1307.6940** | **2613.3734** | **2613.3915** | **-0.0181** | **0** | **53** | **0.0003** | **1** | **K.HAGGVTGGWDNLLAVIPGGSSTPLIPK.S** |
|  | 27997 | **807.6464** | **3226.5563** | **3225.5720** | **0.9843** | **0** | **2** | **27** | **1** | **K.SVCETVLMDFDALVQAQTGLGTAAVIVMDR.S** |
|  | 28068 | **650.7280** | **3248.6035** | **3248.6209** | **-0.0175** | **1** | **8** | **6.6** | **2** | **K.LFNISGHVNHPCTVEEEMSVPLKELIEK.H** |
|  | 28562 | **1133.9126** | **3398.7160** | **3398.7367** | **-0.0208** | **0** | **11** | **3.2** | **1** | **R.LKPPFPADVGVFGCPTTVANVETVAVSPTICR.R** |

| **429.** | Q86VB7\|C163A_HUMAN    **Mass:** 129575   **Score:** 106    **Queries matched:** 15   **emPAI:** 0.12 |
| --- | --- |
|  | Scavenger receptor cysteine-rich type 1 protein M130 OS=Homo sapiens GN=CD163 PE=1 SV=1 |

|  | **Query** | **Observed** | **Mr(expt)** | **Mr(calc)** | **Delta** | **Miss** | **Score** | **Expect** | **Rank** | **Peptide** |
| --- | --- | --- | --- | --- | --- | --- | --- | --- | --- | --- |
|  | 1574 | **702.4092** | **701.4020** | **701.3933** | **0.0087** | **1** | **10** | **23** | **1** | **R.LRGGGSR.C** |
|  | 2569 | 425.0476 | 848.0807 | 847.3858 | 0.6949 | 0 | 6 | 30 | 7 | K.EADVVCR.Q |
|  | 3474 | **458.2939** | **914.5733** | **915.5250** | **-0.9517** | **1** | **12** | **11** | **2** | **R.QRLAVSSR.G** |
|  | 7207 | **596.2705** | **1190.5265** | **1190.5350** | **-0.0085** | **0** | **29** | **0.21** | **1** | **R.LQEGPTSCSGR.V** |
|  | 11387 | 706.8238 | 1411.6330 | 1411.6436 | -0.0105 | 0 | 33 | 0.065 | 1 | K.SSMSETTVGVVCR.Q 11390 |
|  | 12162 | **487.2723** | **1458.7950** | **1459.7202** | **-0.9251** | **1** | **1** | **98** | **6** | **K.IRLQEGPTSCSGR.V** |
|  | 16053 | **427.9747** | **1707.8697** | **1707.8614** | **0.0083** | **1** | **5** | **35** | **3** | **K.LRMVLLEDSGSADFR.R** |
|  | 17008 | **888.4115** | **1774.8084** | **1774.8205** | **-0.0120** | **0** | **37** | **0.021** | **1** | **K.AMSIPMWVDNVQCPK.G** |
|  | 18123 | **620.3204** | **1857.9393** | **1858.8057** | **-0.8664** | **1** | **1** | **75** | **4** | **R.GNESALWDCKHDGWGK.H** |
|  | 18355 | **937.9617** | **1873.9088** | **1873.9210** | **-0.0122** | **0** | **68** | **1.6e-005** | **1** | **K.EAEFGQGTGPIWLNEVK.C** |
|  | 20294 | **682.3295** | **2043.9668** | **2045.0503** | **-1.0835** | **1** | **1** | **71** | **6** | **K.FLPISGMEKEAILSHTEK.E** |
|  | 21809 | **734.3990** | **2200.1753** | **2199.9923** | **0.1831** | **1** | **2** | **48** | **5** | **K.SSMSETTVGVVCRQLGCADK.G** |
|  | 24966 | **877.0447** | **2628.1122** | **2629.2702** | **-1.1580** | **1** | **0** | **52** | **2** | **K.INPASLDKAMSIPMWVDNVQCPK.G** |
|  | 30184 | **1353.9375** | **4058.7907** | **4057.8034** | **0.9873** | **0** | **34** | **0.01** | **1** | **R.VEIWHGGSWGTVCDDSWDLDDAQVVCQQLGCGPALK.A** |

| **430.** | Q9NP72\|RAB18_HUMAN    **Mass:** 23248    **Score:** 105    **Queries matched:** 4   **emPAI:** 0.16 |
| --- | --- |
|  | Ras-related protein Rab-18 OS=Homo sapiens GN=RAB18 PE=1 SV=1 |

|  | **Query** | **Observed** | **Mr(expt)** | **Mr(calc)** | **Delta** | **Miss** | **Score** | **Expect** | **Rank** | **Peptide** |
| --- | --- | --- | --- | --- | --- | --- | --- | --- | --- | --- |
|  | 3719 | 467.7421 | 933.4696 | 933.5032 | -0.0335 | 1 | 8 | 34 | 5 | R.NEGLKFAR.K |
|  | 5748 | 543.3283 | 1084.6421 | 1084.6492 | -0.0071 | 0 | 31 | 0.13 | 1 | K.ILIIGESGVGK.S |
|  | 16750 | **878.9417** | **1755.8689** | **1755.8791** | **-0.0103** | **0** | **81** | **9.7e-007** | **1** | **K.IIQTPGLWESENQNK.G** 16749 |

| **431.** | Q9H2A2\|AL8A1_HUMAN    **Mass:** 54165    **Score:** 105    **Queries matched:** 11   **emPAI:** 0.22 |
| --- | --- |
|  | Aldehyde dehydrogenase family 8 member A1 OS=Homo sapiens GN=ALDH8A1 PE=1 SV=1 |

|  | **Query** | **Observed** | **Mr(expt)** | **Mr(calc)** | **Delta** | **Miss** | **Score** | **Expect** | **Rank** | **Peptide** |
| --- | --- | --- | --- | --- | --- | --- | --- | --- | --- | --- |
|  | 1394 | **635.3340** | **634.3267** | **633.3850** | **0.9417** | **0** | **11** | **18** | **1** | **R.IFVQK.S** |
|  | 1460 | **657.1157** | **656.1084** | **655.4017** | **0.7067** | **0** | **1** | **88** | **1** | **K.LSLPAR.N** |
|  | 5558 | **536.2247** | **1070.4348** | **1071.6036** | **-1.1688** | **1** | **5** | **52** | **4** | **K.DQGKTLALAR.T** |
|  | 6635 | **577.2986** | **1152.5826** | **1153.5914** | **-1.0088** | **0** | **6** | **42** | **8** | **R.ITQLSAPHCK.K** |
|  | 15368 | **833.4889** | **1664.9632** | **1664.9713** | **-0.0080** | **0** | **21** | **0.86** | **1** | **K.VGIPSDPLVSIGALISK.A** 15351 |
|  | 17815 | 918.9644 | 1835.9142 | 1836.9114 | -0.9972 | 0 | 6 | 28 | 2 | -.MAGTNALLMLENFIDGK.F 17813 |
|  | 23878 | **821.0831** | **2460.2274** | **2460.2384** | **-0.0110** | **0** | **49** | **0.001** | **1** | **R.VLNQVADLLEQSLEEFAQAESK.D** |
|  | 24313 | **1258.5320** | **2515.0494** | **2515.0672** | **-0.0178** | **0** | **67** | **1.1e-005** | **1** | **K.FLPCSSYIDSYDPSTGEVYCR.V** |
|  | 30078 | **797.9847** | **3984.8870** | **3985.7505** | **-0.8635** | **1** | **1** | **21** | **3** | **R.SVQNFRFFASSSLHHTSECTQMDHLGCMHYTVR.A** |

| **432.** | O95470\|SGPL1_HUMAN    **Mass:** 64053    **Score:** 105    **Queries matched:** 21   **emPAI:** 0.18 |
| --- | --- |
|  | Sphingosine-1-phosphate lyase 1 OS=Homo sapiens GN=SGPL1 PE=1 SV=3 |

|  | **Query** | **Observed** | **Mr(expt)** | **Mr(calc)** | **Delta** | **Miss** | **Score** | **Expect** | **Rank** | **Peptide** |
| --- | --- | --- | --- | --- | --- | --- | --- | --- | --- | --- |
|  | 1099 | 557.0191 | 556.0118 | 556.3333 | -0.3214 | 1 | 11 | 7.1 | 6 | K.NPKAK.T |
|  | 1565 | **698.4256** | **697.4183** | **697.3435** | **0.0748** | **0** | **17** | **2.8** | **1** | **K.YGYAPK.G** |
|  | 2337 | 415.2519 | 828.4893 | 828.4705 | 0.0188 | 0 | 9 | 25 | 7 | K.IEAEIVR.I |
|  | 3013 | **879.9007** | **878.8934** | **878.3990** | **0.4944** | **0** | **9** | **16** | **1** | **K.MMEVDVR.A** 3018 |
|  | 3166 | **446.9020** | **891.7894** | **892.4688** | **-0.6794** | **0** | **3** | **56** | **6** | **R.LSNLMTAK.G** |
|  | 4047 | **479.2865** | **956.5584** | **956.5654** | **-0.0070** | **1** | **15** | **5.6** | **1** | **R.KIEAEIVR.I** |
|  | 4894 | **509.2828** | **1016.5509** | **1016.5576** | **-0.0067** | **0** | **18** | **2.8** | **1** | **M.PSTDLLMLK.A** |
|  | 8564 | 420.3177 | 1257.9312 | 1257.6540 | 0.2772 | 1 | 4 | 39 | 8 | K.AASYFGMKIVR.V 8548 |
|  | 9146 | 430.8954 | 1289.6645 | 1289.6649 | -0.0004 | 1 | 10 | 14 | 2 | R.ESVTQIMKNPK.A |
|  | 11950 | **483.2127** | **1446.6163** | **1447.6884** | **-1.0721** | **0** | **3** | **69** | **8** | **K.AGYPLEHPFDFR.V** |
|  | 12618 | **743.9042** | **1485.7939** | **1485.8038** | **-0.0099** | **0** | **75** | **4.5e-006** | **1** | **K.ALPSQGLSSSAVLEK.L** |
|  | 17406 | 902.9022 | 1803.7899 | 1803.8132 | -0.0233 | 0 | 2 | 67 | 5 | K.TTGMGAIYGMAQTTVDR.N |
|  | 18366 | **938.5228** | **1875.0311** | **1874.0414** | **0.9897** | **0** | **57** | **0.00019** | **1** | **K.GIFVFGNPQLSVIALGSR.D** 18358 |
|  | 18923 | **481.7660** | **1923.0350** | **1923.0201** | **0.0149** | **1** | **4** | **41** | **6** | **R.ASGTVYSGEEKLTELLVK.A** |
|  | 19681 | 498.0073 | 1988.0001 | 1986.9503 | 1.0498 | 1 | 1 | 73 | 9 | K.AKTTGMGAIYGMAQTTVDR.N |
|  | 22441 | **758.3718** | **2272.0935** | **2272.1065** | **-0.0131** | **0** | **24** | **0.35** | **1** | **K.AYGDFAWSNPLHPDIFPGLR.K** |
|  | 25372 | **902.7997** | **2705.3772** | **2706.3510** | **-0.9738** | **0** | **2** | **42** | **2** | **R.NTAMLVCSTPQFPHGVIDPVPEVAK.L** |
|  | 27413 | 1023.5020 | 3067.4840 | 3068.6124 | -1.1283 | 1 | 1 | 43 | 7 | K.LTELLVKAYGDFAWSNPLHPDIFPGLR.K |

| **433.** | Q9BVK6\|TMED9_HUMAN    **Mass:** 27374    **Score:** 105    **Queries matched:** 11   **emPAI:** 0.30 |
| --- | --- |
|  | Transmembrane emp24 domain-containing protein 9 OS=Homo sapiens GN=TMED9 PE=1 SV=2 |

|  | **Query** | **Observed** | **Mr(expt)** | **Mr(calc)** | **Delta** | **Miss** | **Score** | **Expect** | **Rank** | **Peptide** |
| --- | --- | --- | --- | --- | --- | --- | --- | --- | --- | --- |
|  | 1899 | 399.2322 | 796.4498 | 795.3511 | 1.0987 | 0 | 15 | 4.2 | 1 | R.QYGSEGR.F |
|  | 5307 | 525.7843 | 1049.5540 | 1049.4738 | 0.0803 | 0 | 17 | 3 | 6 | R.QTSESTNQR.V 5308 |
|  | 5966 | 551.3127 | 1100.6109 | 1100.6189 | -0.0080 | 1 | 12 | 11 | 1 | K.DKLSELQLR.V |
|  | 10114 | 671.3673 | 1340.7201 | 1340.7300 | -0.0099 | 0 | 57 | 0.00031 | 1 | R.QLVEQVEQIQK.E |
|  | 15623 | 562.2477 | 1683.7214 | 1684.0222 | -0.3008 | 1 | (3) | 59 | 10 | R.VMRTLLLVLWLATR.G |
|  | 15930 | **567.8754** | **1700.6045** | **1700.0171** | **0.5874** | **1** | **6** | **22** | **2** | **R.VMRTLLLVLWLATR.G** |
|  | 18573 | **632.3401** | **1893.9984** | **1893.9182** | **0.0802** | **0** | **2** | **56** | **10** | **R.EEYQPATPGLGMFVEVK.D** |
|  | 20800 | **1043.4778** | **2084.9410** | **2084.9547** | **-0.0137** | **0** | **52** | **0.00056** | **1** | **K.CFIEEIPDETMVIGNYR.T** 20801 |
|  | 25507 | **1365.6897** | **2729.3648** | **2730.5108** | **-1.1460** | **1** | **3** | **35** | **5** | **R.TLLLVLWLATRGSALYFHIGETEK.K** |

| **434.** | Q9NX40\|OCAD1_HUMAN    **Mass:** 27780    **Score:** 104    **Queries matched:** 10   **emPAI:** 0.47 |
| --- | --- |
|  | OCIA domain-containing protein 1 OS=Homo sapiens GN=OCIAD1 PE=1 SV=1 |

|  | **Query** | **Observed** | **Mr(expt)** | **Mr(calc)** | **Delta** | **Miss** | **Score** | **Expect** | **Rank** | **Peptide** |
| --- | --- | --- | --- | --- | --- | --- | --- | --- | --- | --- |
|  | 6750 | 582.2515 | 1162.4885 | 1162.5407 | -0.0522 | 0 | 10 | 15 | 2 | R.SSPPGHYYQK.S |
|  | 7383 | **600.3544** | **1198.6943** | **1197.6353** | **1.0590** | **0** | **36** | **0.039** | **1** | **K.LENSPLGEALR.S** |
|  | 9699 | **440.7391** | **1319.1954** | **1318.6418** | **0.5536** | **1** | **7** | **21** | **4** | **R.RSSPPGHYYQK.S** 9687 |
|  | 9832 | **663.8680** | **1325.7214** | **1325.7303** | **-0.0089** | **1** | **76** | **3.8e-006** | **1** | **K.KLENSPLGEALR.S** 9831 9841 |
|  | 12044 | **727.3624** | **1452.7102** | **1452.7208** | **-0.0107** | **1** | **38** | **0.022** | **1** | **K.NRESYEVSLTQK.T** |
|  | 28896 | 884.4081 | 3533.6032 | 3532.7371 | 0.8662 | 1 | 5 | 11 | 4 | R.VFAECNDESFWFRSVPLAATSMLITQGLISK.G 28897 |

| **435.** | P61106\|RAB14_HUMAN    **Mass:** 24110    **Score:** 104    **Queries matched:** 12   **emPAI:** 0.55 |
| --- | --- |
|  | Ras-related protein Rab-14 OS=Homo sapiens GN=RAB14 PE=1 SV=4 |

|  | **Query** | **Observed** | **Mr(expt)** | **Mr(calc)** | **Delta** | **Miss** | **Score** | **Expect** | **Rank** | **Peptide** |
| --- | --- | --- | --- | --- | --- | --- | --- | --- | --- | --- |
|  | 9635 | 658.8289 | 1315.6433 | 1315.6521 | -0.0088 | 0 | 60 | 0.00015 | 1 | K.LQIWDTAGQER.F 9612 9633 9634 |
|  | 12732 | 747.3543 | 1492.6941 | 1492.7045 | -0.0105 | 0 | 35 | 0.04 | 1 | K.TGENVEDAFLEAAK.K |
|  | 14710 | **812.4701** | **1622.9256** | **1622.9355** | **-0.0099** | **0** | **7** | **23** | **1** | **R.NLTNPNTVIILIGNK.A** |
|  | 17833 | **613.6134** | **1837.8184** | **1836.8288** | **0.9896** | **0** | **12** | **6.8** | **1** | **K.FMADCPHTIGVEFGTR.I** |
|  | 18756 | **954.4832** | **1906.9517** | **1905.9407** | **1.0111** | **1** | **2** | **66** | **2** | **R.SYYRGAAGALMVYDITR.R** |
|  | 23492 | 802.7446 | 2405.2121 | 2406.3230 | -1.1110 | 1 | 5 | 26 | 4 | R.NLTNPNTVIILIGNKADLEAQR.D |
|  | 25323 | 1351.5801 | 2701.1456 | 2701.3122 | -0.1666 | 1 | 3 | 30 | 4 | R.DVTYEEAKQFAEENGLLFLEASAK.T |
|  | 25507 | 1365.6897 | 2729.3648 | 2730.3437 | -0.9789 | 1 | 2 | 44 | 9 | -.MATAPYNYSYIFKYIIIGDMGVGK.S |
|  | 27727 | **1050.8495** | **3149.5266** | **3149.5490** | **-0.0223** | **0** | **49** | **0.00052** | **1** | **K.IYQNIQDGSLDLNAAESGVQHKPSAPQGGR.L** |

| **436.** | Q9H4B7\|TBB1_HUMAN    **Mass:** 50865    **Score:** 103    **Queries matched:** 19   **emPAI:** 0.24 |
| --- | --- |
|  | Tubulin beta-1 chain OS=Homo sapiens GN=TUBB1 PE=1 SV=1 |

|  | **Query** | **Observed** | **Mr(expt)** | **Mr(calc)** | **Delta** | **Miss** | **Score** | **Expect** | **Rank** | **Peptide** |
| --- | --- | --- | --- | --- | --- | --- | --- | --- | --- | --- |
|  | 2066 | 404.7367 | 807.4588 | 807.3399 | 0.1190 | 0 | 7 | 33 | 7 | R.EEYPDR.I |
|  | 5004 | 514.2689 | 1026.5233 | 1025.5328 | 0.9905 | 0 | 5 | 48 | 5 | K.VAVCDIPPR.G |
|  | 5638 | 539.2662 | 1076.5179 | 1076.5250 | -0.0071 | 1 | 36 | 0.039 | 1 | K.IREEYPDR.I 5637 |
|  | 6327 | 565.7967 | 1129.5788 | 1129.5880 | -0.0092 | 0 | 4 | 59 | 6 | R.FPGQLNADLR.K |
|  | 6499 | 572.3174 | 1142.6203 | 1142.6270 | -0.0067 | 0 | 70 | 1.8e-005 | 1 | K.LAVNMVPFPR.L 6498 |
|  | 6985 | **591.3234** | **1180.6323** | **1181.5539** | **-0.9216** | **0** | **1** | **1.2e+002** | **9** | **R.VSEHFSAMFK.R** |
|  | 8802 | 636.3640 | 1270.7133 | 1270.7220 | -0.0086 | 1 | 47 | 0.0027 | 1 | R.KLAVNMVPFPR.L |
|  | 9112 | 430.2745 | 1287.8016 | 1286.7169 | 1.0847 | 1 | (7) | 28 | 2 | R.KLAVNMVPFPR.L 9101 |
|  | 10005 | 445.5987 | 1333.7742 | 1333.6302 | 0.1439 | 0 | 3 | 64 | 8 | R.ISVYYNEAYGR.K |
|  | 10374 | **678.3412** | **1354.6679** | **1353.6499** | **1.0180** | **1** | **3** | **66** | **2** | **R.VSEHFSAMFKR.K** |
|  | 10381 | **452.5670** | **1354.6791** | **1354.7180** | **-0.0389** | **1** | **4** | **50** | **3** | **R.GRYLTVACIFR.G** |
|  | 11411 | **472.2335** | **1413.6787** | **1414.7416** | **-1.0629** | **0** | **6** | **34** | **8** | **K.EVDQQLLSVQTR.N** |
|  | 15774 | **847.4401** | **1692.8657** | **1692.7930** | **0.0727** | **0** | **4** | **44** | **1** | **R.NSSCFVEWIPNNVK.V** |
|  | 18857 | **639.6808** | **1916.0207** | **1916.9877** | **-0.9670** | **1** | **1** | **73** | **5** | **R.AVLVDLEPGTMDSIRSSK.L** |
|  | 23373 | **796.3355** | **2385.9847** | **2386.1454** | **-0.1607** | **0** | **3** | **32** | **6** | **K.LGALFQPDSFVHGNSGAGNNWAK.G** |
|  | 24399 | **844.5095** | **2530.5066** | **2530.0540** | **0.4525** | **1** | **2** | **34** | **4** | **K.AVLEEDEEVTEEAEMEPEDKGH.-** |

| **437.** | O43772\|MCAT_HUMAN    **Mass:** 33264    **Score:** 103    **Queries matched:** 12   **emPAI:** 0.54 |
| --- | --- |
|  | Mitochondrial carnitine/acylcarnitine carrier protein OS=Homo sapiens GN=SLC25A20 PE=1 SV=1 |

|  | **Query** | **Observed** | **Mr(expt)** | **Mr(calc)** | **Delta** | **Miss** | **Score** | **Expect** | **Rank** | **Peptide** |
| --- | --- | --- | --- | --- | --- | --- | --- | --- | --- | --- |
|  | 2512 | **422.7346** | **843.4546** | **844.4443** | **-0.9897** | **0** | **3** | **93** | **1** | **R.FQTAPPGK.Y** |
|  | 4996 | **513.7725** | **1025.5304** | **1024.5342** | **0.9962** | **0** | **28** | **0.24** | **1** | **K.LYQEFGIR.G** 4961 |
|  | 5422 | **530.7808** | **1059.5470** | **1060.5665** | **-1.0196** | **1** | **21** | **1.4** | **1** | **K.NIFTPEGKR.V** |
|  | 5810 | **545.3362** | **1088.6579** | **1087.5774** | **1.0805** | **1** | **5** | **56** | **4** | **K.SRFQTAPPGK.Y** |
|  | 6643 | **577.3178** | **1152.6211** | **1152.6291** | **-0.0081** | **1** | **40** | **0.015** | **1** | **K.KLYQEFGIR.G** |
|  | 13187 | 761.9039 | 1521.7933 | 1521.8038 | -0.0105 | 1 | 31 | 0.091 | 1 | R.ELIRDEGVTSLYK.G |
|  | 14051 | 790.8912 | 1579.7679 | 1578.7943 | 0.9736 | 1 | 3 | 56 | 9 | R.FQTAPPGKYPNGFR.D |
|  | 24940 | **1312.6054** | **2623.1961** | **2623.2200** | **-0.0238** | **0** | **29** | **0.073** | **1** | **R.LQTQPPSLPGQPPMYSGTFDCFR.K** |
|  | 25620 | **918.1060** | **2751.2962** | **2751.3149** | **-0.0187** | **1** | **42** | **0.0039** | **1** | **R.LQTQPPSLPGQPPMYSGTFDCFRK.T** 25621 |
|  | 26119 | **710.8165** | **2839.2370** | **2840.3587** | **-1.1217** | **1** | **4** | **20** | **4** | **K.GTVLTLMRDVPASGMYFMTYEWLK.N** |

| **438.** | Q9NR77\|PXMP2_HUMAN    **Mass:** 22238    **Score:** 102    **Queries matched:** 4   **emPAI:** 0.37 |
| --- | --- |
|  | Peroxisomal membrane protein 2 OS=Homo sapiens GN=PXMP2 PE=1 SV=3 |

|  | **Query** | **Observed** | **Mr(expt)** | **Mr(calc)** | **Delta** | **Miss** | **Score** | **Expect** | **Rank** | **Peptide** |
| --- | --- | --- | --- | --- | --- | --- | --- | --- | --- | --- |
|  | 11143 | 699.8581 | 1397.7016 | 1398.7215 | -1.0199 | 1 | 7 | 28 | 7 | K.ENSRSLDVGGPLR.Y |
|  | 20208 | **1018.0433** | **2034.0720** | **2034.0819** | **-0.0099** | **0** | **89** | **1.2e-007** | **1** | **K.AATSGILSALGNFLAQMIEK.K** 20209 |
|  | 28073 | 1085.2240 | 3252.6502 | 3253.6960 | -1.0458 | 1 | 3 | 23 | 7 | R.LVFAPAFLMLFFLIMNFLEGKDASAFAAK.M |

| **439.** | Q9GZP9\|DERL2_HUMAN    **Mass:** 27663    **Score:** 102    **Queries matched:** 6   **emPAI:** 0.14 |
| --- | --- |
|  | Derlin-2 OS=Homo sapiens GN=DERL2 PE=1 SV=1 |

|  | **Query** | **Observed** | **Mr(expt)** | **Mr(calc)** | **Delta** | **Miss** | **Score** | **Expect** | **Rank** | **Peptide** |
| --- | --- | --- | --- | --- | --- | --- | --- | --- | --- | --- |
|  | 2035 | **403.2292** | **804.4439** | **803.4402** | **1.0037** | **1** | **7** | **44** | **10** | **R.RNPYVR.M** |
|  | 2873 | 435.2678 | 868.5210 | 867.4272 | 1.0938 | 0 | 6 | 38 | 5 | -.MAYQSLR.L |
|  | 4168 | **484.2484** | **966.4823** | **967.4433** | **-0.9610** | **0** | **6** | **34** | **1** | **R.MLEEGSFR.G** 4166 |
|  | 28487 | **1125.5092** | **3373.5057** | **3373.5276** | **-0.0219** | **0** | **64** | **1.7e-005** | **1** | **K.AIFDTPDEDPNYNPLPEERPGGFAWGEGQR.L** 28488 |

| **440.** | P61313\|RL15_HUMAN    **Mass:** 24245    **Score:** 102    **Queries matched:** 16   **emPAI:** 1.41 |
| --- | --- |
|  | 60S ribosomal protein L15 OS=Homo sapiens GN=RPL15 PE=1 SV=2 |

|  | **Query** | **Observed** | **Mr(expt)** | **Mr(calc)** | **Delta** | **Miss** | **Score** | **Expect** | **Rank** | **Peptide** |
| --- | --- | --- | --- | --- | --- | --- | --- | --- | --- | --- |
|  | 3006 | **440.1898** | **878.3650** | **878.4280** | **-0.0630** | **1** | **13** | **9.7** | **5** | **K.KQSDVMR.F** 3005 |
|  | 3030 | **441.2484** | **880.4822** | **880.4879** | **-0.0057** | **0** | **15** | **5.1** | **2** | **R.NTLQLHR.Y** |
|  | 3221 | **449.7396** | **897.4647** | **897.4708** | **-0.0061** | **0** | **15** | **4.1** | **4** | **K.QGYVIYR.I** |
|  | 4606 | **998.2851** | **997.2778** | **996.5036** | **0.7743** | **1** | **3** | **57** | **4** | **R.AGRHCGALR.V** |
|  | 4754 | **504.2660** | **1006.5175** | **1006.5236** | **-0.0061** | **0** | **14** | **7.3** | **1** | **K.YIQELWR.K** |
|  | 4805 | **506.2552** | **1010.4959** | **1010.5046** | **-0.0087** | **0** | **12** | **8.8** | **2** | **K.FHHTIGGSR.R** |
|  | 12903 | **502.2754** | **1503.8044** | **1503.8126** | **-0.0082** | **0** | **33** | **0.057** | **1** | **K.FFEVILIDPFHK.A** 12902 |
|  | 15299 | **830.8908** | **1659.7671** | **1659.7780** | **-0.0110** | **0** | **57** | **0.00022** | **1** | **R.VLNSYWVGEDSTYK.F** 15298 |
|  | 16000 | **853.4557** | **1704.8969** | **1704.9060** | **-0.0090** | **0** | **46** | **0.0025** | **1** | **K.GATYGKPVHHGVNQLK.F** 15998 15999 |
|  | 16226 | **573.9767** | **1718.9082** | **1718.9216** | **-0.0134** | **1** | **23** | **0.64** | **1** | **R.RNPDTQWITKPVHK.H** |
|  | 22549 | **761.7567** | **2282.2483** | **2282.2760** | **-0.0277** | **1** | **1** | **65** | **6** | **K.RPVPKGATYGKPVHHGVNQLK.F** |

| **441.** | P18085\|ARF4_HUMAN    **Mass:** 20612    **Score:** 100    **Queries matched:** 14   **emPAI:** 0.98 |
| --- | --- |
|  | ADP-ribosylation factor 4 OS=Homo sapiens GN=ARF4 PE=1 SV=3 |

|  | **Query** | **Observed** | **Mr(expt)** | **Mr(calc)** | **Delta** | **Miss** | **Score** | **Expect** | **Rank** | **Peptide** |
| --- | --- | --- | --- | --- | --- | --- | --- | --- | --- | --- |
|  | 5695 | **541.3063** | **1080.5981** | **1079.5975** | **1.0006** | **0** | **7** | **31** | **3** | **M.GLTISSLFSR.L** |
|  | 5774 | **544.3091** | **1086.6036** | **1086.6107** | **-0.0071** | **0** | **28** | **0.26** | **1** | **R.ILMVGLDAAGK.T** 5773 |
|  | 6884 | **586.8079** | **1171.6012** | **1171.6084** | **-0.0073** | **0** | **21** | **1.4** | **1** | **R.IQEVADELQK.M** |
|  | 13375 | 512.5765 | 1534.7076 | 1533.8007 | 0.9069 | 1 | 6 | 32 | 2 | K.QMRILMVGLDAAGK.T 13370 |
|  | 15331 | **832.3811** | **1662.7476** | **1662.7593** | **-0.0116** | **0** | **81** | **9.7e-007** | **1** | **K.QDLPNAMAISEMTDK.L** |
|  | 15550 | 839.8936 | 1677.7727 | 1678.7542 | -0.9815 | 0 | (3) | 64 | 6 | K.QDLPNAMAISEMTDK.L |
|  | 15800 | **847.8946** | **1693.7747** | **1694.7491** | **-0.9744** | **0** | **(6)** | **29** | **5** | **K.QDLPNAMAISEMTDK.L** |
|  | 17448 | **603.3307** | **1806.9704** | **1806.0325** | **0.9380** | **1** | **3** | **57** | **9** | **R.ILMVGLDAAGKTTILYK.L** |
|  | 22859 | **1162.1183** | **2322.2220** | **2322.2359** | **-0.0139** | **0** | **3** | **41** | **1** | **K.LGEIVTTIPTIGFNVETVEYK.N** |
|  | 23685 | 813.3947 | 2437.1623 | 2437.1775 | -0.0152 | 1 | 25 | 0.24 | 1 | K.HYFQNTQGLIFVVDSNDRER.I |
|  | 24652 | **855.4747** | **2563.4024** | **2563.4149** | **-0.0125** | **1** | **33** | **0.03** | **1** | **K.LKLGEIVTTIPTIGFNVETVEYK.N** |
|  | 30043 | **794.9757** | **3969.8422** | **3969.9823** | **-0.1401** | **1** | **0** | **27** | **1** | **K.LGEIVTTIPTIGFNVETVEYKNICFTVWDVGGQDR.I** |

| **442.** | Q9Y2Q3\|GSTK1_HUMAN    **Mass:** 25594    **Score:** 100    **Queries matched:** 13   **emPAI:** 0.32 |
| --- | --- |
|  | Glutathione S-transferase kappa 1 OS=Homo sapiens GN=GSTK1 PE=1 SV=3 |

|  | **Query** | **Observed** | **Mr(expt)** | **Mr(calc)** | **Delta** | **Miss** | **Score** | **Expect** | **Rank** | **Peptide** |
| --- | --- | --- | --- | --- | --- | --- | --- | --- | --- | --- |
|  | 5175 | **520.2958** | **1038.5771** | **1039.5008** | **-0.9237** | **0** | **4** | **66** | **4** | **K.GLYMANDLK.L** |
|  | 6639 | 577.3082 | 1152.6018 | 1151.6008 | 1.0009 | 1 | 7 | 27 | 10 | R.KGLYMANDLK.L |
|  | 11721 | 716.8539 | 1431.6933 | 1431.7027 | -0.0094 | 0 | 75 | 4.4e-006 | 1 | K.AGMSAEQAQGLLEK.I 11710 |
|  | 11988 | **725.4255** | **1448.8364** | **1447.6977** | **1.1387** | **0** | **(23)** | **0.61** | **1** | **K.AGMSAEQAQGLLEK.I** 11983 |
|  | 16342 | **864.9309** | **1727.8473** | **1727.8577** | **-0.0104** | **0** | **22** | **0.67** | **1** | **R.NEDITEPQSILAAAEK.A** |
|  | 17185 | **596.2740** | **1785.8001** | **1785.8971** | **-0.0970** | **0** | **1** | **91** | **9** | **R.FLTAVNLEHPEMLEK.A** |
|  | 17330 | **600.3097** | **1797.9072** | **1798.8957** | **-0.9885** | **1** | **3** | **48** | **2** | **K.DFLSVMLEKGSLSAMR.F** |
|  | 19350 | **653.6811** | **1958.0214** | **1958.0142** | **0.0072** | **1** | **5** | **28** | **1** | **K.AGMSAEQAQGLLEKIATPK.V** |
|  | 24698 | **644.3190** | **2573.2470** | **2574.3450** | **-1.0980** | **1** | **1** | **55** | **10** | **R.MELLAHLLGEKWMGPIPPAVNAR.L** |
|  | 25268 | 897.1061 | 2688.2966 | 2688.3119 | -0.0153 | 0 | 50 | 0.00065 | 1 | R.YGAFGLPITVAHVDGQTHMLFGSDR.M 25269 |

| **443.** | P00450\|CERU_HUMAN    **Mass:** 122983   **Score:** 99     **Queries matched:** 24   **emPAI:** 0.09 |
| --- | --- |
|  | Ceruloplasmin OS=Homo sapiens GN=CP PE=1 SV=1 |

|  | **Query** | **Observed** | **Mr(expt)** | **Mr(calc)** | **Delta** | **Miss** | **Score** | **Expect** | **Rank** | **Peptide** |
| --- | --- | --- | --- | --- | --- | --- | --- | --- | --- | --- |
|  | 3787 | 470.7157 | 939.4168 | 939.4232 | -0.0065 | 0 | 25 | 0.46 | 1 | K.YTVNQCR.R |
|  | 7568 | 403.2286 | 1206.6641 | 1206.6319 | 0.0322 | 0 | 5 | 52 | 6 | K.DIFTGLIGPMK.I |
|  | 8752 | **423.7388** | **1268.1947** | **1267.5755** | **0.6192** | **0** | **3** | **44** | **7** | **R.MFTTAPDQVDK.E** |
|  | 9101 | 430.2092 | 1287.6058 | 1288.7404 | -1.1346 | 1 | 7 | 30 | 2 | R.RPYLKVFNPR.R 9135 |
|  | 9948 | 666.3349 | 1330.6552 | 1330.6153 | 0.0399 | 1 | 12 | 9 | 1 | R.EYTDASFTNRK.E |
|  | 10668 | **686.3808** | **1370.7470** | **1370.7558** | **-0.0087** | **0** | **27** | **0.28** | **1** | **K.GAYPLSIEPIGVR.F** |
|  | 12329 | **735.4042** | **1468.7939** | **1468.8323** | **-0.0384** | **0** | **11** | **9.8** | **3** | **K.DIASGLIGPLIICK.K** |
|  | 12344 | 735.8612 | 1469.7079 | 1469.7184 | -0.0106 | 0 | 15 | 4.4 | 1 | K.EVGPTNADPVCLAK.M |
|  | 13118 | **506.9369** | **1517.7889** | **1518.7354** | **-0.9465** | **0** | **9** | **16** | **3** | **K.ALYLQYTDETFR.T** |
|  | 14153 | 793.9045 | 1585.7945 | 1586.7325 | -0.9380 | 1 | 1 | 91 | 4 | R.RQSEDSTFYLGER.T |
|  | 20171 | **1014.9905** | **2027.9664** | **2027.9735** | **-0.0071** | **1** | **0** | **82** | **9** | **K.AGLQAFFQVQECNKSSSK.D** |
|  | 21164 | 709.3928 | 2125.1566 | 2125.9916 | -0.8350 | 0 | 1 | 68 | 3 | K.ENLTAPGSDSAVFFEQGTTR.I |
|  | 22844 | **774.4382** | **2320.2927** | **2321.0196** | **-0.7269** | **1** | **2** | **40** | **5** | **K.EHEGAIYPDNTTDFQRADDK.V** |
|  | 23036 | **1174.0574** | **2346.1002** | **2346.1640** | **-0.0638** | **1** | **19** | **0.87** | **1** | **K.MYYSAVDPTKDIFTGLIGPMK.I** |
|  | 23943 | 824.0615 | 2469.1626 | 2470.0603 | -0.8978 | 0 | 2 | 51 | 6 | R.MYSVNGYTFGSLPGLSMCAEDR.V 23945 |
|  | 25127 | **1335.1262** | **2668.2379** | **2668.2520** | **-0.0141** | **0** | **60** | **6.4e-005** | **1** | **R.GVYSSDVFDIFPGTYQTLEMFPR.T** 25128 |
|  | 25316 | **900.4498** | **2698.3275** | **2697.2237** | **1.1038** | **1** | **2** | **37** | **1** | **R.MYSVNGYTFGSLPGLSMCAEDRVK.W** |
|  | 27939 | 1606.7673 | 3211.5201 | 3212.6619 | -1.1417 | 1 | 1 | 40 | 3 | R.GPEEEHLGILGPVIWAEVGDTIRVTFHNK.G |
|  | 29916 | **1313.2432** | **3936.7077** | **3935.8815** | **0.8261** | **1** | **1** | **20** | **4** | **K.EFYLFPTVFDENESLLLEDNIRMFTTAPDQVDK.E** |
|  | 31402 | **1300.8508** | **5199.3742** | **5198.3949** | **0.9793** | **1** | **0** | **10** | **1** | **R.MFGNLQGLTMHVGDEVNWYLMGMGNEIDLHTVHFHGHSFQYKHR.G** |
|  | 31734 | **951.0901** | **5700.4969** | **5700.7302** | **-0.2333** | **1** | **1** | **5.9** | **7** | **R.IDTINLFPATLFDAYMVAQNPGEWMLSCQNLNHLKAGLQAFFQVQECNK.S** |

| **444.** | Q6IAN0\|DRS7B_HUMAN    **Mass:** 35382    **Score:** 99     **Queries matched:** 5   **emPAI:** 0.22 |
| --- | --- |
|  | Dehydrogenase/reductase SDR family member 7B OS=Homo sapiens GN=DHRS7B PE=1 SV=2 |

|  | **Query** | **Observed** | **Mr(expt)** | **Mr(calc)** | **Delta** | **Miss** | **Score** | **Expect** | **Rank** | **Peptide** |
| --- | --- | --- | --- | --- | --- | --- | --- | --- | --- | --- |
|  | 9308 | **650.2987** | **1298.5829** | **1298.5925** | **-0.0096** | **0** | **68** | **2.2e-005** | **1** | **R.YGVMDTTTAQGR.S** |
|  | 9527 | **437.7397** | **1310.1973** | **1309.6072** | **0.5902** | **0** | **7** | **17** | **6** | **R.GTIMDTTVDVDK.R** |
|  | 10527 | 455.5926 | 1363.7561 | 1364.6296 | -0.8734 | 0 | 1 | 1.1e+002 | 6 | K.HATQAFFDCLR.A |
|  | 13249 | 764.1032 | 1526.1917 | 1525.7963 | 0.3955 | 0 | 0 | 71 | 7 | R.TLAPGLFFSLMASR.A |
|  | 13885 | **785.3992** | **1568.7838** | **1568.7909** | **-0.0071** | **0** | **54** | **0.00047** | **1** | **R.VMETNYFGPVALTK.A** |

| **445.** | Q15800\|ERG25_HUMAN    **Mass:** 35592    **Score:** 99     **Queries matched:** 2   **emPAI:** 0.11 |
| --- | --- |
|  | C-4 methylsterol oxidase OS=Homo sapiens GN=SC4MOL PE=1 SV=1 |

|  | **Query** | **Observed** | **Mr(expt)** | **Mr(calc)** | **Delta** | **Miss** | **Score** | **Expect** | **Rank** | **Peptide** |
| --- | --- | --- | --- | --- | --- | --- | --- | --- | --- | --- |
|  | 13576 | **516.9402** | **1547.7989** | **1546.6874** | **1.1115** | **0** | **7** | **25** | **3** | **K.NAWNYMLNNYTK.F** |
|  | 15117 | **825.3701** | **1648.7256** | **1648.7369** | **-0.0113** | **0** | **99** | **1.5e-008** | **1** | **R.IFGTDSQYNAYNEK.R** |

| **446.** | O00748\|EST2_HUMAN    **Mass:** 62109    **Score:** 98     **Queries matched:** 14   **emPAI:** 0.19 |
| --- | --- |
|  | Carboxylesterase 2 OS=Homo sapiens GN=CES2 PE=1 SV=1 |

|  | **Query** | **Observed** | **Mr(expt)** | **Mr(calc)** | **Delta** | **Miss** | **Score** | **Expect** | **Rank** | **Peptide** |
| --- | --- | --- | --- | --- | --- | --- | --- | --- | --- | --- |
|  | 2579 | **425.2419** | **848.4691** | **848.4908** | **-0.0217** | **1** | **19** | **2.4** | **6** | **R.LQFWKK.A** |
|  | 9377 | 651.8411 | 1301.6676 | 1300.7390 | 0.9285 | 0 | 24 | 0.58 | 3 | K.EEILAINKPFK.M |
|  | 11172 | 700.3874 | 1398.7603 | 1398.7694 | -0.0090 | 0 | 24 | 0.57 | 2 | K.MIPGVVDGVFLPR.H |
|  | 13096 | **758.9350** | **1515.8554** | **1515.8660** | **-0.0106** | **1** | **63** | **6.7e-005** | **1** | **K.SKEEILAINKPFK.M** |
|  | 13978 | **526.2944** | **1575.8615** | **1575.8733** | **-0.0119** | **0** | **17** | **2.5** | **1** | **R.TTHTGQVLGSLVHVK.G** |
|  | 16511 | **580.2813** | **1737.8219** | **1737.8336** | **-0.0116** | **0** | **22** | **0.74** | **1** | **R.WVQQNIAHFGGNPDR.V** |
|  | 22844 | 774.4382 | 2320.2927 | 2320.3056 | -0.0129 | 0 | 44 | 0.0027 | 1 | K.GANAGVQTFLGIPFAKPPLGPLR.F |
|  | 23557 | 806.3754 | 2416.1043 | 2415.1648 | 0.9395 | 1 | 0 | 68 | 6 | K.ADHGDELPFVFRSFFGGNYIK.F |
|  | 24815 | **865.4103** | **2593.2092** | **2593.4374** | **-0.2282** | **1** | **3** | **35** | **1** | **R.LSAVACGLLLLLVRGQGQDSASPIR.T** |
|  | 25656 | 920.1290 | 2757.3651 | 2756.4968 | 0.8682 | 1 | 9 | 7.4 | 2 | R.EASQAALQKMLTLLMLPPTFGDLLR.E |
|  | 27164 | **1010.8386** | **3029.4939** | **3028.5170** | **0.9768** | **1** | **0** | **45** | **1** | **R.APVYFYEFQHQPSWLKNIRPPHMK.A** |
|  | 28601 | **1139.5853** | **3415.7342** | **3415.7565** | **-0.0223** | **0** | **27** | **0.08** | **1** | **R.HPQELLASADFQPVPSIVGVNNNEFGWLIPK.V** 28599 28602 |

| **447.** | Q8TC12\|RDH11_HUMAN    **Mass:** 35763    **Score:** 98     **Queries matched:** 10   **emPAI:** 0.49 |
| --- | --- |
|  | Retinol dehydrogenase 11 OS=Homo sapiens GN=RDH11 PE=1 SV=2 |

|  | **Query** | **Observed** | **Mr(expt)** | **Mr(calc)** | **Delta** | **Miss** | **Score** | **Expect** | **Rank** | **Peptide** |
| --- | --- | --- | --- | --- | --- | --- | --- | --- | --- | --- |
|  | 3251 | 450.7455 | 899.4763 | 899.4937 | -0.0173 | 1 | 11 | 13 | 6 | K.ELAQRGAR.V |
|  | 3523 | **460.2552** | **918.4958** | **918.5022** | **-0.0064** | **1** | **14** | **6.8** | **1** | **R.KLDLSDTK.S** |
|  | 5772 | **544.2991** | **1086.5837** | **1086.5921** | **-0.0084** | **1** | **40** | **0.018** | **1** | **R.DVEKGELVAK.E** |
|  | 7600 | **404.0301** | **1209.0686** | **1209.6393** | **-0.5707** | **1** | **0** | **87** | **4** | **R.AFAKGFLAEEK.H** |
|  | 11689 | **715.8251** | **1429.6356** | **1429.6449** | **-0.0093** | **0** | **15** | **4** | **1** | **K.FYNAGLAYCHSK.L** 11669 |
|  | 14155 | **793.9228** | **1585.8310** | **1585.8424** | **-0.0114** | **0** | **41** | **0.01** | **1** | **K.EIQTTTGNQQVLVR.K** |
|  | 15352 | **832.9150** | **1663.8155** | **1663.8273** | **-0.0118** | **0** | **5** | **36** | **1** | **K.MLSSGVCTSTVQLPGK.V** |
|  | 16978 | **886.9536** | **1771.8927** | **1770.8975** | **0.9952** | **1** | **24** | **0.45** | **1** | **R.RLWDVSCDLLGLPID.-** |
|  | 20692 | **1037.5250** | **2073.0355** | **2073.0491** | **-0.0136** | **0** | **62** | **5.8e-005** | **1** | **K.GSGVTTYSVHPGTVQSELVR.H** |

| **448.** | P61019\|RAB2A_HUMAN    **Mass:** 23702    **Score:** 98     **Queries matched:** 10   **emPAI:** 0.56 |
| --- | --- |
|  | Ras-related protein Rab-2A OS=Homo sapiens GN=RAB2A PE=1 SV=1 |

|  | **Query** | **Observed** | **Mr(expt)** | **Mr(calc)** | **Delta** | **Miss** | **Score** | **Expect** | **Rank** | **Peptide** |
| --- | --- | --- | --- | --- | --- | --- | --- | --- | --- | --- |
|  | 7913 | 613.3136 | 1224.6126 | 1223.6220 | 0.9906 | 0 | 1 | 1.2e+002 | 9 | K.SCLLLQFTDK.R |
|  | 9691 | **660.3655** | **1318.7164** | **1318.7245** | **-0.0081** | **0** | **24** | **0.52** | **1** | **R.GAAGALLVYDITR.R** |
|  | 10478 | 454.8929 | 1361.6569 | 1361.6649 | -0.0080 | 0 | 7 | 25 | 3 | R.EHGLIFMETSAK.T |
|  | 12748 | 747.8700 | 1493.7254 | 1493.7362 | -0.0107 | 0 | 60 | 0.00011 | 1 | K.TASNVEEAFINTAK.E 12747 |
|  | 13602 | **775.8786** | **1549.7426** | **1549.7525** | **-0.0099** | **0** | **28** | **0.22** | **1** | **K.LQIWDTAGQESFR.S** 13609 |
|  | 16210 | **859.9045** | **1717.7945** | **1717.8060** | **-0.0115** | **0** | **61** | **9.7e-005** | **1** | **R.DTFNHLTTWLEDAR.Q** |
|  | 16858 | **881.4307** | **1760.8469** | **1759.8741** | **0.9729** | **0** | **12** | **7.7** | **1** | **K.IQEGVFDINNEANGIK.I** |
|  | 18485 | 629.9911 | 1886.9514 | 1887.9843 | -1.0328 | 1 | 1 | 72 | 7 | R.SYYRGAAGALLVYDITR.R |

| **449.** | P23396\|RS3_HUMAN    **Mass:** 26842    **Score:** 98     **Queries matched:** 18   **emPAI:** 0.49 |
| --- | --- |
|  | 40S ribosomal protein S3 OS=Homo sapiens GN=RPS3 PE=1 SV=2 |

|  | **Query** | **Observed** | **Mr(expt)** | **Mr(calc)** | **Delta** | **Miss** | **Score** | **Expect** | **Rank** | **Peptide** |
| --- | --- | --- | --- | --- | --- | --- | --- | --- | --- | --- |
|  | 1407 | **637.4100** | **636.4028** | **636.4071** | **-0.0044** | **0** | **15** | **5.1** | **1** | **R.HVLLR.Q** |
|  | 4562 | **498.3363** | **994.6581** | **993.5178** | **1.1403** | **1** | **5** | **41** | **10** | **R.RACYGVLR.F** |
|  | 4967 | 512.7916 | 1023.5687 | 1023.5753 | -0.0066 | 1 | 10 | 17 | 2 | R.KFVADGIFK.A 4979 |
|  | 5039 | **515.3151** | **1028.6157** | **1028.6230** | **-0.0073** | **0** | **31** | **0.13** | **1** | **R.TEIIILATR.T** |
|  | 5834 | **546.7844** | **1091.5543** | **1091.5611** | **-0.0068** | **0** | **49** | **0.0021** | **1** | **K.AELNEFLTR.E** |
|  | 5959 | **551.2859** | **1100.5572** | **1100.5938** | **-0.0366** | **1** | **7** | **37** | **10** | **R.TQNVLGEKGR.R** |
|  | 6680 | **578.8154** | **1155.6162** | **1155.6975** | **-0.0814** | **1** | **9** | **19** | **10** | **R.IRELTAVVQK.R** |
|  | 9098 | 644.7968 | 1287.5791 | 1287.6605 | -0.0814 | 0 | 15 | 4.7 | 1 | R.GLCAIAQAESLR.Y |
|  | 9971 | **445.0337** | **1332.0793** | **1331.8401** | **0.2392** | **1** | **4** | **41** | **5** | **R.HVLLRQGVLGIK.V** |
|  | 12135 | **486.9455** | **1457.8146** | **1457.8242** | **-0.0096** | **0** | **7** | **26** | **2** | **K.KPLPDHVSIVEPK.D** 12127 |
|  | 13940 | **787.4002** | **1572.7857** | **1572.7970** | **-0.0113** | **0** | **4** | **50** | **2** | **K.GGKPEPPAMPQPVPTA.-** |
|  | 18620 | **475.5015** | **1897.9769** | **1898.0836** | **-0.1068** | **1** | **4** | **43** | **6** | **R.TEIIILATRTQNVLGEK.G** |
|  | 19549 | **659.8049** | **1976.3928** | **1976.9803** | **-0.5875** | **1** | **5** | **20** | **5** | **R.ELAEDGYSGVEVRVTPTR.T** |
|  | 23930 | **1234.5898** | **2467.1651** | **2467.1842** | **-0.0191** | **0** | **76** | **2e-006** | **1** | **K.FVDGLMIHSGDPVNYYVDTAVR.H** |
|  | 26571 | **728.3960** | **2909.5549** | **2909.5750** | **-0.0201** | **1** | **9** | **7** | **2** | **K.KPLPDHVSIVEPKDEILPTTPISEQK.G** |
|  | 27536 | 776.3525 | 3101.3811 | 3101.5757 | -0.1946 | 1 | 2 | 28 | 5 | K.FVDGLMIHSGDPVNYYVDTAVRHVLLR.Q |

| **450.** | O15127\|SCAM2_HUMAN    **Mass:** 37082    **Score:** 98     **Queries matched:** 3   **emPAI:** 0.21 |
| --- | --- |
|  | Secretory carrier-associated membrane protein 2 OS=Homo sapiens GN=SCAMP2 PE=1 SV=2 |

|  | **Query** | **Observed** | **Mr(expt)** | **Mr(calc)** | **Delta** | **Miss** | **Score** | **Expect** | **Rank** | **Peptide** |
| --- | --- | --- | --- | --- | --- | --- | --- | --- | --- | --- |
|  | 11063 | **465.2532** | **1392.7378** | **1392.7473** | **-0.0096** | **0** | **27** | **0.3** | **1** | **R.ELQNTVANLHVR.Q** |
|  | 16234 | **574.2903** | **1719.8492** | **1719.8077** | **0.0415** | **1** | **6** | **30** | **4** | **R.TFHRAASSAAQGAFQGN.-** |
|  | 21854 | **1103.0071** | **2203.9996** | **2204.0134** | **-0.0138** | **0** | **93** | **4.2e-008** | **1** | **R.TGASFQQAQEEFSQGIFSSR.T** |

| **451.** | P47914\|RL29_HUMAN    **Mass:** 17798    **Score:** 97     **Queries matched:** 10   **emPAI:** 0.22 |
| --- | --- |
|  | 60S ribosomal protein L29 OS=Homo sapiens GN=RPL29 PE=1 SV=2 |

|  | **Query** | **Observed** | **Mr(expt)** | **Mr(calc)** | **Delta** | **Miss** | **Score** | **Expect** | **Rank** | **Peptide** |
| --- | --- | --- | --- | --- | --- | --- | --- | --- | --- | --- |
|  | 991 | **531.2306** | **530.2233** | **530.3064** | **-0.0831** | **0** | **10** | **34** | **5** | **R.AEAIK.A** |
|  | 3422 | 456.2892 | 910.5637 | 911.5229 | -0.9591 | 0 | 17 | 3 | 1 | R.LAYIAHPK.L 3439 |
|  | 6402 | **568.3411** | **1134.6677** | **1134.5921** | **0.0756** | **1** | **4** | **59** | **4** | **R.YESLKGVDPK.F** |
|  | 9271 | **649.3265** | **1296.6384** | **1295.7350** | **0.9034** | **1** | **2** | **95** | **8** | **K.LDRLAYIAHPK.L** |
|  | 9490 | **654.8177** | **1307.6208** | **1307.6074** | **0.0134** | **1** | **11** | **11** | **2** | **K.MQANNAKAMSAR.A** 9486 |
|  | 10763 | **689.3737** | **1376.7329** | **1376.7412** | **-0.0083** | **0** | **72** | **8.1e-006** | **1** | **K.AQAAAPASVPAQAPK.R** 10764 |
|  | 17981 | **925.8027** | **1849.5909** | **1848.9693** | **0.6216** | **1** | **1** | **54** | **2** | **K.DQTKAQAAAPASVPAQAPK.R** |

| **452.** | Q6UW68\|TM205_HUMAN    **Mass:** 21469    **Score:** 96     **Queries matched:** 11   **emPAI:** 0.39 |
| --- | --- |
|  | Transmembrane protein 205 OS=Homo sapiens GN=TMEM205 PE=1 SV=1 |

|  | **Query** | **Observed** | **Mr(expt)** | **Mr(calc)** | **Delta** | **Miss** | **Score** | **Expect** | **Rank** | **Peptide** |
| --- | --- | --- | --- | --- | --- | --- | --- | --- | --- | --- |
|  | 5749 | **543.3465** | **1084.6784** | **1085.5717** | **-0.8932** | **0** | **1** | **1.1e+002** | **7** | **M.EEGGNLGGLIK.M** |
|  | 7740 | **609.2121** | **1216.4096** | **1216.6122** | **-0.2025** | **0** | **(3)** | **68** | **4** | **-.MEEGGNLGGLIK.M** |
|  | 8088 | **617.6018** | **1233.1891** | **1232.6071** | **0.5820** | **0** | **5** | **34** | **3** | **-.MEEGGNLGGLIK.M** |
|  | 11982 | **725.3699** | **1448.7253** | **1448.7333** | **-0.0080** | **0** | **32** | **0.092** | **1** | **R.TTAAMWALQTVEK.E** |
|  | 16253 | **861.9080** | **1721.8014** | **1721.8122** | **-0.0108** | **0** | **68** | **1.8e-005** | **1** | **R.GLGGEVPGSHQGPDPYR.Q** 16250 16252 |
|  | 21220 | **711.0925** | **2130.2556** | **2130.0932** | **0.1624** | **1** | **1** | **59** | **4** | **R.WLEPRTTAAMWALQTVEK.E** |
|  | 27625 | **782.3672** | **3125.4397** | **3124.6217** | **0.8179** | **0** | **5** | **15** | **4** | **K.MVHLLVLSGAWGMQMWVTFVSGFLLFR.S** |
|  | 30708 | 1082.0306 | 4324.0935 | 4323.2233 | 0.8701 | 1 | (0) | 24 | 10 | -.MEEGGNLGGLIKMVHLLVLSGAWGMQMWVTFVSGFLLFR.S |
|  | 30765 | **1089.7775** | **4355.0808** | **4355.2132** | **-0.1324** | **1** | **2** | **14** | **2** | **-.MEEGGNLGGLIKMVHLLVLSGAWGMQMWVTFVSGFLLFR.S** |

| **453.** | P52272\|HNRPM_HUMAN    **Mass:** 77749    **Score:** 96     **Queries matched:** 71   **emPAI:** 0.20 |
| --- | --- |
|  | Heterogeneous nuclear ribonucleoprotein M OS=Homo sapiens GN=HNRNPM PE=1 SV=3 |

|  | **Query** | **Observed** | **Mr(expt)** | **Mr(calc)** | **Delta** | **Miss** | **Score** | **Expect** | **Rank** | **Peptide** |
| --- | --- | --- | --- | --- | --- | --- | --- | --- | --- | --- |
|  | 1692 | **744.3249** | **743.3177** | **742.2812** | **1.0365** | **0** | **4** | **83** | **5** | **R.MMNGMK.L** |
|  | 1786 | **395.1966** | **788.3786** | **788.3851** | **-0.0064** | **0** | **7** | **50** | **3** | **R.MGPAIER.M** |
|  | 1790 | 395.2127 | 788.4108 | 788.4028 | 0.0080 | 0 | 23 | 1.1 | 2 | R.VGSEIER.M 1781 1785 |
|  | 1797 | **395.2286** | **788.4427** | **788.3851** | **0.0576** | **0** | **4** | **1e+002** | **9** | **R.MAAPIDR.V** |
|  | 2003 | **402.2798** | **802.5451** | **801.4345** | **1.1106** | **0** | **25** | **0.66** | **1** | **R.VGQTIER.M** |
|  | 2247 | 411.7552 | 821.4959 | 820.3935 | 1.1024 | 0 | 9 | 20 | 3 | R.MGLVMDR.M |
|  | 2436 | **419.2425** | **836.4704** | **836.3521** | **0.1183** | **0** | **8** | **25** | **4** | **R.MGPVMDR.M** 2428 |
|  | 2606 | **852.3635** | **851.3562** | **851.3629** | **-0.0068** | **0** | **17** | **3.3** | **1** | **R.MGQTMER.I** |
|  | 2841 | 434.7261 | 867.4377 | 867.3579 | 0.0799 | 0 | (11) | 11 | 5 | R.MGQTMER.I 2835 2840 2857 |
|  | 3213 | 897.4039 | 896.3967 | 897.3936 | -0.9969 | 1 | 8 | 25 | 7 | K.MEESMKK.A |
|  | 3398 | **455.7219** | **909.4293** | **908.4426** | **0.9868** | **0** | **4** | **64** | **1** | **R.GCAVVEFK.M** |
|  | 4218 | 486.2632 | 970.5119 | 971.3953 | -0.8834 | 0 | 11 | 14 | 8 | R.FGSGMNMGR.I |
|  | 4544 | 497.7891 | 993.5637 | 993.5719 | -0.0083 | 0 | 15 | 4.8 | 2 | K.HSLSGRPLK.V |
|  | 5963 | **551.3073** | **1100.6001** | **1100.4855** | **0.1145** | **0** | **(4)** | **64** | **5** | **R.MGAGLGHGMDR.V** |
|  | 5999 | 552.2991 | 1102.5837 | 1102.5506 | 0.0331 | 1 | 9 | 22 | 4 | R.ADILEDKDGK.S |
|  | 6280 | **563.8108** | **1125.6071** | **1124.5107** | **1.0965** | **0** | **(4)** | **54** | **5** | **R.MGAGMGFGLER.M** |
|  | 6381 | **567.7571** | **1133.4997** | **1132.4754** | **1.0244** | **0** | **7** | **35** | **1** | **R.MGAGLGHGMDR.V** 6373 |
|  | 6471 | **571.0996** | **1140.1847** | **1140.5056** | **-0.3209** | **0** | **(4)** | **38** | **3** | **R.MGAGMGFGLER.M** |
|  | 6675 | **578.7849** | **1155.5553** | **1155.5199** | **0.0354** | **1** | **7** | **28** | **2** | **R.MMNGMKLSGR.E** |
|  | 6683 | 579.2626 | 1156.5106 | 1156.5005 | 0.0101 | 0 | 7 | 34 | 10 | R.MGAGMGFGLER.M |
|  | 7507 | **402.2798** | **1203.8176** | **1203.5740** | **0.2436** | **0** | **21** | **1.1** | **1** | **R.MVPAGMGAGLER.M** |
|  | 7827 | **611.2742** | **1220.5339** | **1219.5689** | **0.9650** | **0** | **(7)** | **33** | **2** | **R.MVPAGMGAGLER.M** 7818 |
|  | 8792 | **636.3075** | **1270.6004** | **1269.7404** | **0.8600** | **1** | **1** | **1.2e+002** | **1** | **R.INEILSNALKR.G** |
|  | 9023 | **642.8141** | **1283.6136** | **1283.6219** | **-0.0082** | **0** | **33** | **0.066** | **2** | **K.QGGGGGGGSVPGIER.M** |
|  | 11153 | **466.9959** | **1397.9659** | **1398.6020** | **-0.6361** | **0** | **5** | **33** | **3** | **R.MGLAMGGGGGASFDR.A** |
|  | 11652 | **714.8368** | **1427.6590** | **1426.7061** | **0.9530** | **0** | **(3)** | **68** | **10** | **R.MGPAMGPALGAGIER.M** |
|  | 11775 | **479.2562** | **1434.7467** | **1433.8065** | **0.9402** | **1** | **8** | **20** | **4** | **K.LKEVFSMAGVVVR.A** |
|  | 12184 | 730.8988 | 1459.7830 | 1458.6959 | 1.0871 | 0 | 5 | 43 | 4 | R.MGPAMGPALGAGIER.M 12152 |
|  | 12680 | **497.2992** | **1488.8757** | **1488.7177** | **0.1580** | **1** | **8** | **20** | **2** | **R.MGSGVERMGPAIER.M** |
|  | 12775 | **499.5901** | **1495.7486** | **1495.6945** | **0.0541** | **1** | **10** | **12** | **3** | **R.MGLVMDRMGSVER.M** |
|  | 12927 | **502.9263** | **1505.7572** | **1505.7078** | **0.0493** | **1** | **6** | **34** | **7** | **R.MGANNLERMGLER.M** |
|  | 12933 | **754.1440** | **1506.2734** | **1505.7078** | **0.5656** | **1** | **(1)** | **58** | **6** | **R.MGANNLERMGLER.M** |
|  | 13178 | **508.2139** | **1521.6200** | **1520.7075** | **0.9124** | **1** | **(7)** | **24** | **2** | **R.MGSGVERMGPAIER.M** 13169 |
|  | 13590 | **517.3057** | **1548.8952** | **1547.7184** | **1.1767** | **1** | **5** | **35** | **3** | **R.MGPGIDRLGGAGMER.M** |
|  | 13613 | **517.7650** | **1550.2732** | **1549.7341** | **0.5391** | **1** | **1** | **71** | **10** | **R.MGQTMERIGSGVER.M** |
|  | 14280 | 797.8849 | 1593.7552 | 1594.7266 | -0.9713 | 1 | 12 | 7.8 | 2 | R.MGPVMDRMATGLER.M 14298 |
|  | 14306 | **532.7896** | **1595.3470** | **1594.7266** | **0.6204** | **1** | **(2)** | **46** | **9** | **R.MGPVMDRMATGLER.M** |
|  | 14551 | **538.5942** | **1612.7609** | **1612.7701** | **-0.0092** | **0** | **30** | **0.11** | **1** | **R.MGPLGLDHMASSIER.M** |
|  | 14746 | **407.4417** | **1625.7376** | **1624.7371** | **1.0005** | **1** | **8** | **21** | **4** | **R.MGPAIERMGLSMER.M** |
|  | 15061 | **549.2779** | **1644.8118** | **1644.7600** | **0.0519** | **0** | **(3)** | **57** | **2** | **R.MGPLGLDHMASSIER.M** |
|  | 16238 | **861.0748** | **1720.1351** | **1718.9791** | **1.1560** | **1** | **2** | **57** | **7** | **K.AAEVLNKHSLSGRPLK.V** |
|  | 16621 | **583.2387** | **1746.6941** | **1745.6630** | **1.0311** | **0** | **2** | **69** | **7** | **K.MGGMEGPFGGGMENMGR.F** |
|  | 18070 | **619.5248** | **1855.5527** | **1854.8717** | **0.6810** | **1** | **2** | **49** | **2** | **R.IGSGVERMGAGMGFGLER.M** |
|  | 18569 | 947.9495 | 1893.8844 | 1894.8852 | -1.0008 | 1 | (2) | 58 | 8 | R.MGAGMGFGLERMAAPIDR.V |
|  | 18571 | **632.3174** | **1893.9305** | **1894.8852** | **-0.9547** | **1** | **6** | **26** | **2** | **R.MGAGMGFGLERMAAPIDR.V** |
|  | 19737 | **665.3490** | **1993.0252** | **1993.0190** | **0.0061** | **1** | **8** | **15** | **3** | **K.EVFSMAGVVVRADILEDK.D** |
|  | 19886 | **502.2631** | **2005.0231** | **2005.9206** | **-0.8975** | **1** | **4** | **38** | **8** | **R.MVPAGMGAGLERMGPVMDR.M** |
|  | 20132 | **675.9953** | **2024.9641** | **2024.9976** | **-0.0335** | **1** | **1** | **80** | **9** | **K.EKVGEVTYVELLMDAEGK.S** |
|  | 21483 | **541.0169** | **2160.0385** | **2159.9398** | **0.0987** | **1** | **4** | **36** | **5** | **R.MGLAMGGGGGASFDRAIEMER.G** |
|  | 21638 | **1089.5223** | **2177.0301** | **2177.0432** | **-0.0130** | **0** | **48** | **0.0015** | **1** | **K.GIGMGNIGPAGMGMEGIGFGINK.M** 21637 |
|  | 21919 | **737.3778** | **2209.1116** | **2209.0330** | **0.0786** | **0** | **(1)** | **60** | **6** | **K.GIGMGNIGPAGMGMEGIGFGINK.M** |
|  | 22821 | 773.3809 | 2317.1209 | 2317.0977 | 0.0233 | 1 | 6 | 17 | 5 | R.MGANSLERMGPAMGPALGAGIER.M 22824 |
|  | 23762 | 816.4137 | 2446.2193 | 2446.1225 | 0.0968 | 1 | 6 | 20 | 2 | R.MGPLGLDHMASSIERMGQTMER.I |
|  | 25444 | **906.1396** | **2715.3969** | **2715.0427** | **0.3543** | **1** | **(0)** | **62** | **4** | **K.MGGMEGPFGGGMENMGRFGSGMNMGR.I** |
|  | 25516 | 911.7403 | 2732.1991 | 2731.0376 | 1.1615 | 1 | 2 | 38 | 2 | K.MGGMEGPFGGGMENMGRFGSGMNMGR.I |
|  | 26262 | **953.0914** | **2856.2523** | **2855.2823** | **0.9700** | **1** | **5** | **17** | **1** | **R.MGPAMGPALGAGIERMGLAMGGGGGASFDR.A** |
|  | 29207 | **916.7186** | **3662.8454** | **3662.8589** | **-0.0135** | **0** | **41** | **0.0028** | **1** | **R.GIGTVTFEQSIEAVQAISMFNGQLLFDRPMHVK.M** |
|  | 29884 | 1302.3594 | 3904.0563 | 3904.6956 | -0.6393 | 1 | 1 | 23 | 7 | K.GIGMGNIGPAGMGMEGIGFGINKMGGMEGPFGGGMENMGR.F |

| **454.** | Q9Y6N5\|SQRD_HUMAN    **Mass:** 50214    **Score:** 96     **Queries matched:** 24   **emPAI:** 0.54 |
| --- | --- |
|  | Sulfide:quinone oxidoreductase, mitochondrial OS=Homo sapiens GN=SQRDL PE=1 SV=1 |

|  | **Query** | **Observed** | **Mr(expt)** | **Mr(calc)** | **Delta** | **Miss** | **Score** | **Expect** | **Rank** | **Peptide** |
| --- | --- | --- | --- | --- | --- | --- | --- | --- | --- | --- |
|  | 2601 | **851.4569** | **850.4496** | **850.4548** | **-0.0053** | **0** | **18** | **2.9** | **1** | **R.NLTVNYK.K** |
|  | 2846 | **434.7310** | **867.4475** | **866.4498** | **0.9978** | **0** | **10** | **14** | **1** | **K.IGSNYSVK.T** |
|  | 5834 | 546.7844 | 1091.5543 | 1090.5957 | 0.9586 | 0 | 10 | 18 | 5 | R.AQLFACLLR.L |
|  | 7428 | **401.2617** | **1200.7633** | **1201.5835** | **-0.8201** | **1** | **12** | **9** | **2** | **K.ERLSMYLMK.A** 7432 |
|  | 7892 | **408.5612** | **1222.6617** | **1223.7060** | **-1.0444** | **0** | **9** | **19** | **5** | **-.MVPLVAVVSGPR.A** |
|  | 8200 | 1239.5953 | 1238.5881 | 1239.7009 | -1.1129 | 0 | (6) | 33 | 8 | -.MVPLVAVVSGPR.A |
|  | 9175 | **646.8167** | **1291.6189** | **1291.6271** | **-0.0082** | **0** | **10** | **14** | **1** | **K.IMYLSEAYFR.K** |
|  | 9192 | 647.3314 | 1292.6482 | 1292.7241 | -0.0759 | 1 | 6 | 36 | 4 | K.IKGLPEGFAHPK.I 9185 |
|  | 12326 | **735.3785** | **1468.7424** | **1468.7522** | **-0.0097** | **0** | **42** | **0.0085** | **1** | **K.VGAENVAIVEPSER.H** |
|  | 13361 | **767.8937** | **1533.7729** | **1533.7827** | **-0.0098** | **0** | **25** | **0.41** | **1** | **K.EGNAIFTFPNTPVK.C** |
|  | 13973 | **526.2789** | **1575.8148** | **1575.8256** | **-0.0108** | **1** | **27** | **0.23** | **1** | **K.KYADALQEIIQER.N** |
|  | 14335 | **400.4581** | **1597.8032** | **1596.8471** | **0.9561** | **1** | **11** | **8.5** | **3** | **R.KVGAENVAIVEPSER.H** |
|  | 15655 | **844.4131** | **1686.8116** | **1686.8882** | **-0.0766** | **0** | **9** | **14** | **1** | **R.HFYQPIWTLVGAGAK.Q** |
|  | 16902 | **883.3890** | **1764.7634** | **1764.7777** | **-0.0143** | **0** | **10** | **12** | **1** | **K.YDGYTSCPLVTGYNR.V** |
|  | 17359 | **900.9321** | **1799.8496** | **1799.8561** | **-0.0065** | **0** | **36** | **0.025** | **1** | **K.ADLMPFLYWNMMLR.G** 17360 |
|  | 17756 | **611.7879** | **1832.3419** | **1831.8459** | **0.4959** | **0** | **(2)** | **47** | **4** | **K.ADLMPFLYWNMMLR.G** |
|  | 24791 | **863.0983** | **2586.2732** | **2586.2894** | **-0.0162** | **1** | **24** | **0.3** | **1** | **R.VILAEFDYKAEPLETFPFDQSK.E** |
|  | 25231 | **894.8305** | **2681.4697** | **2682.2904** | **-0.8207** | **1** | **(1)** | **51** | **4** | **R.LSMYLMKADLMPFLYWNMMLR.G** |
|  | 25440 | 1358.6492 | 2715.2838 | 2714.2802 | 1.0035 | 1 | 1 | 45 | 3 | R.LSMYLMKADLMPFLYWNMMLR.G |
|  | 30601 | **1064.0266** | **4252.0773** | **4252.1072** | **-0.0298** | **1** | **34** | **0.0085** | **1** | **R.ADKQEAVFENLDKPGETQVISYEMLHVTPPMSPPDVLK.T** 30602 |

| **455.** | P18463\|1B37_HUMAN    **Mass:** 40716    **Score:** 96     **Queries matched:** 10   **emPAI:** 0.30 |
| --- | --- |
|  | HLA class I histocompatibility antigen, B-37 alpha chain OS=Homo sapiens GN=HLA-B PE=2 SV=1 |

|  | **Query** | **Observed** | **Mr(expt)** | **Mr(calc)** | **Delta** | **Miss** | **Score** | **Expect** | **Rank** | **Peptide** |
| --- | --- | --- | --- | --- | --- | --- | --- | --- | --- | --- |
|  | 1586 | 705.3914 | 704.3841 | 704.3705 | 0.0136 | 0 | 8 | 31 | 8 | R.ETQISK.T |
|  | 4142 | 483.2169 | 964.4193 | 964.4250 | -0.0057 | 0 | 53 | 0.00075 | 1 | R.FDSDAASPR.T |
|  | 6587 | **575.8830** | **1149.7514** | **1149.4543** | **0.2971** | **0** | **3** | **68** | **7** | **R.MSGCDVGPDGR.L** |
|  | 10294 | 676.3427 | 1350.6709 | 1349.6939 | 0.9770 | 1 | 8 | 24 | 1 | R.YLENGKETLQR.A |
|  | 11642 | 714.3621 | 1426.7097 | 1426.7205 | -0.0108 | 0 | 11 | 10 | 1 | K.WAAVVVPSGEEQR.Y |
|  | 12774 | 748.8586 | 1495.7026 | 1495.7129 | -0.0103 | 0 | 11 | 9.6 | 2 | R.AYLEGTCVEWLR.R |
|  | 13333 | **511.5789** | **1531.7149** | **1531.7235** | **-0.0086** | **1** | **5** | **44** | **4** | **R.MSGCDVGPDGRLLR.G** |
|  | 15060 | 823.4092 | 1644.8038 | 1644.8148 | -0.0110 | 0 | 75 | 3.4e-006 | 1 | R.FISVGYVDDTQFVR.F |
|  | 22148 | 744.6662 | 2230.9768 | 2230.9938 | -0.0170 | 0 | 17 | 1.6 | 1 | R.DGEDQTQDTELVETRPAGDR.T |
|  | 25923 | **701.3510** | **2801.3747** | **2800.1929** | **1.1817** | **1** | **0** | **57** | **3** | **R.YYNQSEAGSHTIQRMSGCDVGPDGR.L** |

| **456.** | Q29718\|1B82_HUMAN    **Mass:** 40681    **Score:** 96     **Queries matched:** 6   **emPAI:** 0.30 |
| --- | --- |
|  | HLA class I histocompatibility antigen, B-82 alpha chain OS=Homo sapiens GN=HLA-B PE=2 SV=1 |

|  | **Query** | **Observed** | **Mr(expt)** | **Mr(calc)** | **Delta** | **Miss** | **Score** | **Expect** | **Rank** | **Peptide** |
| --- | --- | --- | --- | --- | --- | --- | --- | --- | --- | --- |
|  | 4142 | 483.2169 | 964.4193 | 964.4250 | -0.0057 | 0 | 53 | 0.00075 | 1 | R.FDSDAASPR.E |
|  | 8239 | 621.3292 | 1240.6439 | 1239.5013 | 1.1426 | 0 | 16 | 3.5 | 1 | R.MFGCDLGPDGR.L |
|  | 10294 | 676.3427 | 1350.6709 | 1349.6939 | 0.9770 | 1 | 8 | 24 | 1 | R.YLENGKETLQR.A |
|  | 11642 | 714.3621 | 1426.7097 | 1426.7205 | -0.0108 | 0 | 11 | 10 | 1 | K.WAAVVVPSGEEQR.Y |
|  | 15060 | 823.4092 | 1644.8038 | 1644.8148 | -0.0110 | 0 | 75 | 3.4e-006 | 1 | R.FISVGYVDDTQFVR.F |
|  | 22148 | 744.6662 | 2230.9768 | 2230.9938 | -0.0170 | 0 | 17 | 1.6 | 1 | R.DGEDQTQDTELVETRPAGDR.T |

| **457.** | P30462\|1B14_HUMAN    **Mass:** 40675    **Score:** 96     **Queries matched:** 9   **emPAI:** 0.30 |
| --- | --- |
|  | HLA class I histocompatibility antigen, B-14 alpha chain OS=Homo sapiens GN=HLA-B PE=1 SV=1 |

|  | **Query** | **Observed** | **Mr(expt)** | **Mr(calc)** | **Delta** | **Miss** | **Score** | **Expect** | **Rank** | **Peptide** |
| --- | --- | --- | --- | --- | --- | --- | --- | --- | --- | --- |
|  | 1736 | **763.1153** | **762.1080** | **762.3694** | **-0.2614** | **0** | **7** | **31** | **7** | **R.NTQICK.T** |
|  | 4142 | 483.2169 | 964.4193 | 964.4250 | -0.0057 | 0 | 53 | 0.00075 | 1 | R.FDSDAASPR.E |
|  | 11642 | 714.3621 | 1426.7097 | 1426.7205 | -0.0108 | 0 | 11 | 10 | 1 | K.WAAVVVPSGEEQR.Y |
|  | 12774 | 748.8586 | 1495.7026 | 1495.7129 | -0.0103 | 0 | 11 | 9.6 | 2 | R.AYLEGTCVEWLR.R |
|  | 15060 | 823.4092 | 1644.8038 | 1644.8148 | -0.0110 | 0 | 75 | 3.4e-006 | 1 | R.FISVGYVDDTQFVR.F |
|  | 22148 | 744.6662 | 2230.9768 | 2230.9938 | -0.0170 | 0 | 17 | 1.6 | 1 | R.DGEDQTQDTELVETRPAGDR.T |
|  | 29581 | 948.2367 | 3788.9177 | 3790.1002 | -1.1825 | 1 | 4 | 13 | 1 | R.WEPSSQSTVPIVGIVAGLAVLAVVVIGAVVAAVMCRR.K 29529 29557 |

| **458.** | P30475\|1B39_HUMAN    **Mass:** 40645    **Score:** 96     **Queries matched:** 9   **emPAI:** 0.30 |
| --- | --- |
|  | HLA class I histocompatibility antigen, B-39 alpha chain OS=Homo sapiens GN=HLA-B PE=2 SV=1 |

|  | **Query** | **Observed** | **Mr(expt)** | **Mr(calc)** | **Delta** | **Miss** | **Score** | **Expect** | **Rank** | **Peptide** |
| --- | --- | --- | --- | --- | --- | --- | --- | --- | --- | --- |
|  | 1736 | 763.1153 | 762.1080 | 762.3694 | -0.2614 | 0 | 7 | 31 | 7 | R.NTQICK.T |
|  | 4142 | 483.2169 | 964.4193 | 964.4250 | -0.0057 | 0 | 53 | 0.00075 | 1 | R.FDSDAASPR.E |
|  | 10294 | 676.3427 | 1350.6709 | 1349.6939 | 0.9770 | 1 | 8 | 24 | 1 | R.YLENGKETLQR.A |
|  | 11642 | 714.3621 | 1426.7097 | 1426.7205 | -0.0108 | 0 | 11 | 10 | 1 | K.WAAVVVPSGEEQR.Y |
|  | 15060 | 823.4092 | 1644.8038 | 1644.8148 | -0.0110 | 0 | 75 | 3.4e-006 | 1 | R.FISVGYVDDTQFVR.F |
|  | 22148 | 744.6662 | 2230.9768 | 2230.9938 | -0.0170 | 0 | 17 | 1.6 | 1 | R.DGEDQTQDTELVETRPAGDR.T |
|  | 29581 | 948.2367 | 3788.9177 | 3790.1002 | -1.1825 | 1 | 4 | 13 | 1 | R.WEPSSQSTVPIVGIVAGLAVLAVVVIGAVVAAVMCRR.K 29529 29557 |

|  | **Proteins matching the same set of peptides:** |
| --- | --- |

|  | Q95365\|1B38_HUMAN    **Mass:** 40733    **Score:** 96     **Queries matched:** 9 |
| --- | --- |
|  | HLA class I histocompatibility antigen, B-38 alpha chain OS=Homo sapiens GN=HLA-B PE=2 SV=1 |

|  | **Proteins matching a subset of these peptides:** |
| --- | --- |

|  | Q29836\|1B67_HUMAN    **Mass:** 40602    **Score:** 96     **Queries matched:** 8 |
| --- | --- |
|  | HLA class I histocompatibility antigen, B-67 alpha chain OS=Homo sapiens GN=HLA-B PE=1 SV=1 |

| **459.** | P30486\|1B48_HUMAN    **Mass:** 40679    **Score:** 96     **Queries matched:** 8   **emPAI:** 0.30 |
| --- | --- |
|  | HLA class I histocompatibility antigen, B-48 alpha chain OS=Homo sapiens GN=HLA-B PE=2 SV=1 |

|  | **Query** | **Observed** | **Mr(expt)** | **Mr(calc)** | **Delta** | **Miss** | **Score** | **Expect** | **Rank** | **Peptide** |
| --- | --- | --- | --- | --- | --- | --- | --- | --- | --- | --- |
|  | 1586 | 705.3914 | 704.3841 | 704.3705 | 0.0136 | 0 | 8 | 31 | 8 | R.ETQISK.T |
|  | 4142 | 483.2169 | 964.4193 | 964.4250 | -0.0057 | 0 | 53 | 0.00075 | 1 | R.FDSDAASPR.E |
|  | 7831 | 611.2879 | 1220.5612 | 1220.6037 | -0.0424 | 0 | 2 | 89 | 3 | K.DYIALNEDLR.S |
|  | 15060 | 823.4092 | 1644.8038 | 1644.8148 | -0.0110 | 0 | 75 | 3.4e-006 | 1 | R.FISVGYVDDTQFVR.F |
|  | 22148 | 744.6662 | 2230.9768 | 2230.9938 | -0.0170 | 0 | 17 | 1.6 | 1 | R.DGEDQTQDTELVETRPAGDR.T |
|  | 29581 | 948.2367 | 3788.9177 | 3790.1002 | -1.1825 | 1 | 4 | 13 | 1 | R.WEPSSQSTVPIVGIVAGLAVLAVVVIGAVVAAVMCRR.K 29529 29557 |

| **460.** | Q31610\|1B81_HUMAN    **Mass:** 40717    **Score:** 96     **Queries matched:** 8   **emPAI:** 0.30 |
| --- | --- |
|  | HLA class I histocompatibility antigen, B-81 alpha chain OS=Homo sapiens GN=HLA-B PE=2 SV=1 |

|  | **Query** | **Observed** | **Mr(expt)** | **Mr(calc)** | **Delta** | **Miss** | **Score** | **Expect** | **Rank** | **Peptide** |
| --- | --- | --- | --- | --- | --- | --- | --- | --- | --- | --- |
|  | 4142 | 483.2169 | 964.4193 | 964.4250 | -0.0057 | 0 | 53 | 0.00075 | 1 | R.FDSDAASPR.E |
|  | 7831 | 611.2879 | 1220.5612 | 1220.6037 | -0.0424 | 0 | 2 | 89 | 3 | K.DYIALNEDLR.S |
|  | 15060 | 823.4092 | 1644.8038 | 1644.8148 | -0.0110 | 0 | 75 | 3.4e-006 | 1 | R.FISVGYVDDTQFVR.F |
|  | 22148 | 744.6662 | 2230.9768 | 2230.9938 | -0.0170 | 0 | 17 | 1.6 | 1 | R.DGEDQTQDTELVETRPAGDR.T |
|  | 27938 | **1071.5135** | **3211.5188** | **3210.7046** | **0.8142** | **1** | **0** | **42** | **8** | **M.LVMAPRTVLLLLWGAVALTETWAGSHSMR.Y** |
|  | 29581 | 948.2367 | 3788.9177 | 3790.1002 | -1.1825 | 1 | 4 | 13 | 1 | R.WEPSSQSTVPIVGIVAGLAVLAVVVIGAVVAAVMCRR.K 29529 29557 |

| **461.** | O95497\|VNN1_HUMAN    **Mass:** 57716    **Score:** 96     **Queries matched:** 4   **emPAI:** 0.06 |
| --- | --- |
|  | Pantetheinase OS=Homo sapiens GN=VNN1 PE=1 SV=2 |

|  | **Query** | **Observed** | **Mr(expt)** | **Mr(calc)** | **Delta** | **Miss** | **Score** | **Expect** | **Rank** | **Peptide** |
| --- | --- | --- | --- | --- | --- | --- | --- | --- | --- | --- |
|  | 12324 | **490.4837** | **1468.4292** | **1467.7140** | **0.7152** | **1** | **3** | **40** | **9** | **K.KMTGSGIYAPNSSR.A** |
|  | 20194 | **508.7739** | **2031.0665** | **2032.1721** | **-1.1056** | **0** | **4** | **34** | **5** | **R.LFSLKPTSGPVLTVTLFGR.L** |
|  | 29242 | **1228.9414** | **3683.8024** | **3683.8220** | **-0.0196** | **0** | **63** | **1.7e-005** | **1** | **R.NLDILEGAITSAADQGAHIIVTPEDAIYGWNFNR.D** 29243 |

| **462.** | P51149\|RAB7A_HUMAN    **Mass:** 23760    **Score:** 95     **Queries matched:** 7   **emPAI:** 0.56 |
| --- | --- |
|  | Ras-related protein Rab-7a OS=Homo sapiens GN=RAB7A PE=1 SV=1 |

|  | **Query** | **Observed** | **Mr(expt)** | **Mr(calc)** | **Delta** | **Miss** | **Score** | **Expect** | **Rank** | **Peptide** |
| --- | --- | --- | --- | --- | --- | --- | --- | --- | --- | --- |
|  | 9006 | 642.3124 | 1282.6102 | 1282.6193 | -0.0091 | 0 | 10 | 14 | 2 | K.NNIPYFETSAK.E |
|  | 9825 | **442.8994** | **1325.6764** | **1324.6809** | **0.9955** | **1** | **8** | **21** | **1** | **K.TSLMNQYVNKK.F** |
|  | 12418 | 737.8937 | 1473.7729 | 1474.7456 | -0.9727 | 0 | 38 | 0.022 | 2 | R.DPENFPFVVLGNK.I |
|  | 14196 | **795.4127** | **1588.8109** | **1588.8209** | **-0.0100** | **0** | **41** | **0.011** | **1** | **K.EAINVEQAFQTIAR.N** |
|  | 15084 | **824.4061** | **1646.7976** | **1646.8086** | **-0.0111** | **0** | **74** | **4.7e-006** | **1** | **R.LVTMQIWDTAGQER.F** |
|  | 19063 | **645.5743** | **1933.7010** | **1932.9694** | **0.7317** | **1** | **4** | **33** | **4** | **K.TLDSWRDEFLIQASPR.D** |
|  | 19985 | **672.8003** | **2015.3792** | **2015.0799** | **0.2993** | **1** | **0** | **55** | **4** | **K.EAINVEQAFQTIARNALK.Q** |

| **463.** | P32929\|CGL_HUMAN    **Mass:** 45050    **Score:** 95     **Queries matched:** 7   **emPAI:** 0.08 |
| --- | --- |
|  | Cystathionine gamma-lyase OS=Homo sapiens GN=CTH PE=1 SV=3 |

|  | **Query** | **Observed** | **Mr(expt)** | **Mr(calc)** | **Delta** | **Miss** | **Score** | **Expect** | **Rank** | **Peptide** |
| --- | --- | --- | --- | --- | --- | --- | --- | --- | --- | --- |
|  | 7062 | 593.2817 | 1184.5488 | 1184.6652 | -0.1164 | 0 | 6 | 42 | 3 | K.LLEAAITPETK.L 7056 |
|  | 18633 | **633.9984** | **1898.9734** | **1899.8091** | **-0.8357** | **0** | **3** | **48** | **2** | **K.AGDQIICMDDVYGGTNR.Y** |
|  | 22253 | **1122.5536** | **2243.0926** | **2243.1056** | **-0.0130** | **0** | **95** | **2.5e-008** | **1** | **R.LSVGLEDEEDLLEDLDQALK.A** |
|  | 24915 | **655.0780** | **2616.2829** | **2617.1836** | **-0.9007** | **0** | **4** | **25** | **1** | **K.YMNGHSDVVMGLVSVNCESLHNR.L** |
|  | 30135 | **1008.7304** | **4030.8923** | **4030.9591** | **-0.0668** | **1** | **3** | **13** | **2** | **K.YCLAFASGLAATVTITHLLKAGDQIICMDDVYGGTNR.Y** |
|  | 31862 | 1605.2261 | 6416.8752 | 6417.0425 | -0.1674 | 1 | 0 | 5.7 | 3 | K.HGDIILVVDNTFMSPYFQRPLALGADISMYSATKYMNGHSDVVMGLVSVNCESLHNR.L |

| **464.** | Q14108\|SCRB2_HUMAN    **Mass:** 54712    **Score:** 95     **Queries matched:** 16   **emPAI:** 0.22 |
| --- | --- |
|  | Lysosome membrane protein 2 OS=Homo sapiens GN=SCARB2 PE=1 SV=2 |

|  | **Query** | **Observed** | **Mr(expt)** | **Mr(calc)** | **Delta** | **Miss** | **Score** | **Expect** | **Rank** | **Peptide** |
| --- | --- | --- | --- | --- | --- | --- | --- | --- | --- | --- |
|  | 2514 | **844.4830** | **843.4758** | **844.4443** | **-0.9685** | **0** | **8** | **37** | **8** | **K.IVEWNGK.T** |
|  | 3861 | 473.2566 | 944.4987 | 945.5205 | -1.0218 | 0 | 16 | 5.2 | 9 | R.EIIEAMLK.A |
|  | 7643 | 606.7943 | 1211.5739 | 1211.5822 | -0.0083 | 0 | 25 | 0.47 | 1 | R.VEEVGPYTYR.E 7642 |
|  | 10592 | **456.5736** | **1366.6990** | **1367.5259** | **-0.8269** | **0** | **6** | **32** | **3** | **K.GQGSMDEGTADER.A** 10618 |
|  | 11279 | **469.7582** | **1406.2529** | **1406.7042** | **-0.4513** | **1** | **12** | **5.3** | **1** | **K.KLDDFVETGDIR.T** |
|  | 14492 | **537.3176** | **1608.9311** | **1609.7736** | **-0.8426** | **1** | **17** | **2.3** | **1** | **K.AYVFERDQSVGDPK.I** 14495 |
|  | 20242 | **680.3596** | **2038.0569** | **2037.9904** | **0.0665** | **0** | **3** | **43** | **1** | **R.TMVFPVMYLNESVHIDK.E** |
|  | 20885 | **1047.4849** | **2092.9552** | **2091.9836** | **0.9715** | **0** | **53** | **0.00049** | **1** | **K.NGAPIIMSFPHFYQADER.F** |
|  | 21032 | **528.2933** | **2109.1442** | **2107.9785** | **1.1657** | **0** | **(0)** | **84** | **10** | **K.NGAPIIMSFPHFYQADER.F** |
|  | 24667 | **856.6914** | **2567.0524** | **2566.2560** | **0.7964** | **1** | **1** | **42** | **3** | **R.TMVFPVMYLNESVHIDKETASR.L** |
|  | 27171 | **758.6362** | **3030.5156** | **3030.6069** | **-0.0913** | **1** | **0** | **45** | **8** | **-.MGRCCFYTAGTLSLLLLVTSVTLLVAR.V** |
|  | 28436 | **1122.2274** | **3363.6604** | **3363.6809** | **-0.0205** | **0** | **57** | **8.3e-005** | **1** | **R.FVSAIEGMHPNQEDHETFVDINPLTGIILK.A** 28443 |

| **465.** | O75477\|ERLN1_HUMAN    **Mass:** 39072    **Score:** 94     **Queries matched:** 14   **emPAI:** 0.20 |
| --- | --- |
|  | Erlin-1 OS=Homo sapiens GN=ERLIN1 PE=1 SV=1 |

|  | **Query** | **Observed** | **Mr(expt)** | **Mr(calc)** | **Delta** | **Miss** | **Score** | **Expect** | **Rank** | **Peptide** |
| --- | --- | --- | --- | --- | --- | --- | --- | --- | --- | --- |
|  | 5441 | 531.2995 | 1060.5844 | 1060.5513 | 0.0332 | 1 | 11 | 13 | 3 | R.TGRESSLPSK.E |
|  | 7187 | 397.4025 | 1189.1857 | 1188.5986 | 0.5871 | 1 | 2 | 67 | 8 | K.VVEKEAETER.K 7188 |
|  | 10072 | 446.9020 | 1337.6841 | 1336.6411 | 1.0430 | 1 | 8 | 20 | 3 | K.AKADAEYYAAHK.Y |
|  | 10234 | 674.8464 | 1347.6782 | 1347.6882 | -0.0100 | 0 | 61 | 0.00012 | 1 | R.SVQTTLQTDEVK.N 10208 |
|  | 10633 | 685.3796 | 1368.7447 | 1368.7976 | -0.0529 | 1 | 9 | 15 | 3 | K.AVIEAEKIAQVAK.I 10634 |
|  | 13259 | 764.3504 | 1526.6862 | 1526.7576 | -0.0714 | 0 | 3 | 57 | 4 | K.EALEPSGENVIQNK.E |
|  | 16720 | **585.3440** | **1753.0101** | **1753.8128** | **-0.8026** | **0** | **4** | **38** | **2** | **K.NVPCGTSGGVMIYIDR.I** 16723 |
|  | 21496 | **1082.0098** | **2162.0050** | **2162.0176** | **-0.0127** | **0** | **30** | **0.084** | **1** | **K.IYFGSNIPNMFVDSSCALK.Y** 21509 |
|  | 27612 | **1042.1760** | **3123.5063** | **3124.4998** | **-0.9935** | **1** | **0** | **47** | **2** | **K.YQAIASNSKIYFGSNIPNMFVDSSCALK.Y** |

| **466.** | O14880\|MGST3_HUMAN    **Mass:** 16734    **Score:** 94     **Queries matched:** 9   **emPAI:** 0.52 |
| --- | --- |
|  | Microsomal glutathione S-transferase 3 OS=Homo sapiens GN=MGST3 PE=1 SV=1 |

|  | **Query** | **Observed** | **Mr(expt)** | **Mr(calc)** | **Delta** | **Miss** | **Score** | **Expect** | **Rank** | **Peptide** |
| --- | --- | --- | --- | --- | --- | --- | --- | --- | --- | --- |
|  | 9560 | **656.8871** | **1311.7596** | **1311.7663** | **-0.0067** | **0** | **8** | **24** | **1** | **R.IASGLGLAWIVGR.V** |
|  | 25646 | **919.1660** | **2754.4762** | **2754.4891** | **-0.0129** | **0** | **63** | **2.9e-005** | **1** | **R.GALGSIALLGLVGTTVCSAFQHLGWVK.S** |
|  | 26905 | **992.4620** | **2974.3641** | **2973.3789** | **0.9852** | **1** | **52** | **0.00032** | **1** | **K.YKVEYPIMYSTDPENGHIFNCIQR.A** 26899 26900 26901 26902 26904 |
|  | 27723 | 788.1388 | 3148.5261 | 3148.7358 | -0.2098 | 1 | 1 | 37 | 3 | M.AVLSKEYGFVLLTGAASFIMVAHLAINVSK.A |

| **467.** | P12814\|ACTN1_HUMAN    **Mass:** 103563   **Score:** 93     **Queries matched:** 16   **emPAI:** 0.07 |
| --- | --- |
|  | Alpha-actinin-1 OS=Homo sapiens GN=ACTN1 PE=1 SV=2 |

|  | **Query** | **Observed** | **Mr(expt)** | **Mr(calc)** | **Delta** | **Miss** | **Score** | **Expect** | **Rank** | **Peptide** |
| --- | --- | --- | --- | --- | --- | --- | --- | --- | --- | --- |
|  | 6838 | 585.2948 | 1168.5750 | 1169.5387 | -0.9636 | 0 | 2 | 90 | 9 | K.NYITMDELR.R |
|  | 9359 | 434.7278 | 1301.1616 | 1300.5982 | 0.5634 | 0 | 6 | 26 | 5 | K.HTNYTMEHIR.V 9360 9368 9374 |
|  | 10937 | 693.8869 | 1385.7592 | 1385.7667 | -0.0074 | 0 | 74 | 5.8e-006 | 1 | R.VGWEQLLTTIAR.T 10938 |
|  | 11673 | 715.3812 | 1428.7478 | 1428.7572 | -0.0095 | 0 | 41 | 0.0099 | 1 | R.TINEVENQILTR.D |
|  | 13136 | 507.2686 | 1518.7841 | 1519.7354 | -0.9513 | 1 | 3 | 72 | 5 | K.TFTAWCNSHLRK.A |
|  | 13398 | 513.2558 | 1536.7456 | 1536.7671 | -0.0216 | 0 | 12 | 8 | 5 | R.FAIQDISVEETSAK.E |
|  | 15762 | **847.4311** | **1692.8476** | **1693.9138** | **-1.0661** | **1** | **6** | **29** | **8** | **K.DYETATLSEIKALLK.K** 15761 |
|  | 17169 | 595.6369 | 1783.8889 | 1785.0070 | -1.1181 | 1 | 0 | 99 | 7 | R.DGLKLMLLLEVISGER.L |
|  | 20090 | 674.9855 | 2021.9346 | 2022.9509 | -1.0164 | 0 | 4 | 36 | 5 | K.AIMTYVSSFYHAFSGAQK.A |
|  | 25548 | 685.0705 | 2736.2529 | 2736.2966 | -0.0437 | 1 | 1 | 54 | 4 | K.AIMTYVSSFYHAFSGAQKAETAANR.I |
|  | 29059 | 1195.5638 | 3583.6697 | 3582.7678 | 0.9019 | 1 | 1 | 30 | 2 | K.IVQTYHVNMAGTNPYTTITPQEINGKWDHVR.Q |

| **468.** | P30038\|AL4A1_HUMAN    **Mass:** 62137    **Score:** 93     **Queries matched:** 20   **emPAI:** 0.42 |
| --- | --- |
|  | Delta-1-pyrroline-5-carboxylate dehydrogenase, mitochondrial OS=Homo sapiens GN=ALDH4A1 PE=1 SV=3 |

|  | **Query** | **Observed** | **Mr(expt)** | **Mr(calc)** | **Delta** | **Miss** | **Score** | **Expect** | **Rank** | **Peptide** |
| --- | --- | --- | --- | --- | --- | --- | --- | --- | --- | --- |
|  | 1939 | **400.7363** | **799.4580** | **799.4915** | **-0.0335** | **1** | **10** | **18** | **7** | **R.RAEILAK.T** |
|  | 3498 | **459.0119** | **916.0092** | **916.4436** | **-0.4344** | **0** | **4** | **49** | **10** | **K.AADMLSGPR.R** |
|  | 3827 | **472.2683** | **942.5220** | **942.4883** | **0.0337** | **0** | **10** | **16** | **2** | **K.QVAQNLDR.F** |
|  | 4032 | **479.2453** | **956.4760** | **955.4777** | **0.9984** | **0** | **18** | **2.9** | **2** | **K.NFHFVHR.S** |
|  | 4427 | **493.7292** | **985.4438** | **985.4505** | **-0.0067** | **0** | **25** | **0.5** | **1** | **R.SAFEYGGQK.C** |
|  | 9688 | 660.3503 | 1318.6861 | 1318.6729 | 0.0133 | 0 | 7 | 30 | 5 | R.SADVESVVSGTLR.S |
|  | 10199 | **673.8596** | **1345.7046** | **1344.7435** | **0.9611** | **1** | **10** | **14** | **1** | **R.AEILAKTMVGQGK.T** |
|  | 10653 | **457.5825** | **1369.7256** | **1369.7354** | **-0.0097** | **1** | **12** | **7.9** | **2** | **R.KEWDLKPIADR.A** |
|  | 12195 | **731.3710** | **1460.7274** | **1460.7372** | **-0.0098** | **0** | **37** | **0.028** | **1** | **K.STGSIVGQQPFGGAR.A** |
|  | 12524 | **494.2790** | **1479.8151** | **1479.8238** | **-0.0087** | **0** | **3** | **66** | **4** | **R.LYVPHSLWPQIK.G** 12544 |
|  | 15591 | **841.4068** | **1680.7990** | **1681.8536** | **-1.0546** | **0** | **19** | **1.5** | **1** | **R.ASGTNDKPGGPHYILR.W** |
|  | 16157 | 857.9358 | 1713.8571 | 1713.8686 | -0.0114 | 0 | 49 | 0.0013 | 1 | K.VANEPVLAFTQGSPER.D 16156 |
|  | 20101 | **1012.0272** | **2022.0398** | **2021.0469** | **0.9928** | **0** | **1** | **74** | **1** | **K.TVIQAEIDAAAELIDFFR.F** |
|  | 22109 | **743.0419** | **2226.1040** | **2226.1209** | **-0.0169** | **1** | **40** | **0.0081** | **1** | **R.IKVGDPAEDFGTFFSAVIDAK.S** 22106 |
|  | 22424 | **757.3923** | **2269.1550** | **2269.1702** | **-0.0153** | **1** | **21** | **0.57** | **1** | **K.VANEPVLAFTQGSPERDALQK.A** |
|  | 28708 | **865.3883** | **3457.5241** | **3457.5708** | **-0.0466** | **0** | **18** | **0.57** | **1** | **R.MEAIPCVVGDEEVWTSDVQYQVSPFNHGHK.V** |
|  | 30868 | **1477.3965** | **4429.1676** | **4429.1941** | **-0.0265** | **0** | **9** | **2.7** | **1** | **R.EAGLPPNIIQFVPADGPLFGDTVTSSEHLCGINFTGSVPTFK.H** |

| **469.** | Q7Z406\|MYH14_HUMAN    **Mass:** 228889   **Score:** 93     **Queries matched:** 69   **emPAI:** 0.08 |
| --- | --- |
|  | Myosin-14 OS=Homo sapiens GN=MYH14 PE=1 SV=1 |

|  | **Query** | **Observed** | **Mr(expt)** | **Mr(calc)** | **Delta** | **Miss** | **Score** | **Expect** | **Rank** | **Peptide** |
| --- | --- | --- | --- | --- | --- | --- | --- | --- | --- | --- |
|  | 919 | 517.3309 | 516.3237 | 516.3271 | -0.0035 | 0 | 19 | 4.2 | 1 | R.ILSGK.L |
|  | 1167 | 572.9950 | 571.9877 | 571.3806 | 0.6072 | 1 | 12 | 10 | 2 | R.IKVGR.D 1173 |
|  | 1214 | 586.2972 | 585.2900 | 585.2944 | -0.0045 | 0 | 17 | 3.7 | 1 | R.MNPPK.F |
|  | 1295 | 609.3199 | 608.3127 | 608.3170 | -0.0043 | 0 | 26 | 0.38 | 1 | K.SFVEK.V 1304 |
|  | 1456 | 653.3032 | 652.2959 | 653.2843 | -0.9884 | 0 | 13 | 7.2 | 8 | K.GFMDGK.Q |
|  | 1984 | **402.1784** | **802.3422** | **803.3409** | **-0.9987** | **0** | **4** | **87** | **8** | **R.AEDEGGAR.A** |
|  | 2062 | 404.7288 | 807.4430 | 807.4491 | -0.0061 | 0 | 21 | 1.2 | 1 | R.TVGQLYK.E |
|  | 2362 | **416.2596** | **830.5047** | **830.3995** | **0.1053** | **1** | **6** | **55** | **4** | **R.AGDGERAR.A** |
|  | 2640 | 428.2288 | 854.4430 | 855.5178 | -1.0748 | 0 | 8 | 26 | 4 | R.LVLDQLR.C |
|  | 2710 | **430.8546** | **859.6946** | **859.4803** | **0.2143** | **0** | **8** | **24** | **6** | **R.LTAEIWK.D** |
|  | 2875 | **435.2683** | **868.5221** | **868.4403** | **0.0819** | **0** | **8** | **20** | **10** | **K.SVHELER.A** 2852 |
|  | 3029 | **441.2481** | **880.4816** | **879.4306** | **1.0510** | **1** | **2** | **1e+002** | **9** | **R.KMQAQMK.E** |
|  | 3101 | 443.2707 | 884.5269 | 884.5807 | -0.0538 | 1 | 7 | 35 | 7 | R.ILSGKLVR.R |
|  | 3162 | 446.7122 | 891.4098 | 890.4320 | 0.9778 | 0 | 8 | 31 | 10 | K.ANEWLMK.N |
|  | 3357 | 454.2142 | 906.4139 | 907.4433 | -1.0294 | 1 | 11 | 13 | 6 | R.QMQTEKK.R |
|  | 3489 | 458.7816 | 915.5486 | 916.4614 | -0.9128 | 0 | 16 | 5.4 | 7 | R.EAEALTQR.L |
|  | 3507 | 459.2531 | 916.4917 | 916.4250 | 0.0667 | 0 | 9 | 25 | 5 | R.LGEEDAGAR.A |
|  | 3633 | **465.0195** | **928.0244** | **928.5341** | **-0.5097** | **1** | **4** | **52** | **9** | **K.AAILEEKR.Q** |
|  | 4335 | **489.9246** | **977.8347** | **978.5056** | **-0.6709** | **0** | **5** | **35** | **1** | **K.MTIAALESK.L** |
|  | 4715 | 502.7871 | 1003.5597 | 1003.5121 | 0.0476 | 0 | 19 | 2.2 | 3 | M.AAVTMSVPGR.K 4716 4717 |
|  | 4958 | **512.3156** | **1022.6167** | **1021.4828** | **1.1338** | **0** | **4** | **64** | **2** | **R.EEIFSQNR.E** |
|  | 4969 | 513.2107 | 1024.4068 | 1023.5237 | 0.8832 | 1 | 6 | 35 | 7 | K.ATDKSFVEK.V |
|  | 5622 | **538.2795** | **1074.5444** | **1074.5669** | **-0.0225** | **0** | **3** | **96** | **2** | **R.AELSSLQTAR.Q** |
|  | 6295 | **564.7856** | **1127.5567** | **1126.6710** | **0.8858** | **1** | **9** | **23** | **3** | **R.LKGLEAEVLR.L** |
|  | 6302 | **565.2700** | **1128.5255** | **1129.5476** | **-1.0221** | **1** | **7** | **31** | **8** | **R.GRLGEEDAGAR.A** |
|  | 6537 | **573.7791** | **1145.5437** | **1144.5360** | **1.0077** | **0** | **8** | **27** | **3** | **R.EQLEEEAAAR.E** |
|  | 6608 | **576.7850** | **1151.5555** | **1150.5475** | **1.0080** | **0** | **6** | **42** | **10** | **-.MAAVTMSVPGR.K** |
|  | 6798 | **583.7540** | **1165.4935** | **1164.6251** | **0.8684** | **0** | **9** | **17** | **2** | **R.IHEAAVQELR.Q** |
|  | 7125 | **594.3285** | **1186.6425** | **1185.6102** | **1.0324** | **0** | **25** | **0.55** | **1** | **K.VQELQQQSAR.E** |
|  | 7604 | **605.8060** | **1209.5974** | **1209.6353** | **-0.0379** | **1** | **2** | **82** | **4** | **R.KEPGVPGELER.Q** |
|  | 7706 | **608.3438** | **1214.6731** | **1213.6302** | **1.0428** | **0** | **4** | **62** | **2** | **R.AGVLAQLEEER.D** |
|  | 7815 | 610.8254 | 1219.6363 | 1219.6448 | -0.0085 | 1 | 56 | 0.00035 | 1 | R.KFDQLLAEEK.A 7814 |
|  | 9435 | 653.3342 | 1304.6538 | 1304.6612 | -0.0074 | 0 | 17 | 2.5 | 1 | K.EQADFALEALAK.A |
|  | 10304 | 451.2620 | 1350.7642 | 1350.7983 | -0.0341 | 1 | 10 | 13 | 1 | K.LEPRLVLDQLR.C |
|  | 11194 | **701.4042** | **1400.7939** | **1399.8034** | **0.9905** | **1** | **3** | **66** | **10** | **R.ALRAELEALLSSK.D** |
|  | 11844 | **481.1515** | **1440.4327** | **1439.7844** | **0.6483** | **1** | **5** | **29** | **3** | **R.QQLERQIQELR.G** 11845 |
|  | 12458 | 738.8539 | 1475.6933 | 1475.7005 | -0.0071 | 0 | 12 | 9.8 | 3 | R.FLTNGPSSSPGQER.E |
|  | 13035 | **505.2672** | **1512.7796** | **1513.7988** | **-1.0191** | **0** | **2** | **76** | **8** | **K.QELELVVSELEAR.V** 13047 |
|  | 13478 | 771.8914 | 1541.7682 | 1541.8565 | -0.0884 | 1 | 9 | 16 | 2 | R.QRYEILTPNAIPK.G |
|  | 13613 | 517.7650 | 1550.2732 | 1549.7923 | 0.4809 | 0 | 2 | 49 | 4 | R.LMATLSNTNPSFVR.C |
|  | 13620 | 776.3459 | 1550.6772 | 1550.6882 | -0.0110 | 0 | 11 | 9.5 | 1 | R.EDQSILCTGESGAGK.T |
|  | 14223 | **531.2675** | **1590.7806** | **1590.8075** | **-0.0270** | **0** | **4** | **51** | **3** | **K.MIQALELDPNLYR.V** |
|  | 14723 | **542.2895** | **1623.8466** | **1624.6886** | **-0.8420** | **0** | **3** | **67** | **7** | **R.ELEDVTESAESMNR.E** 14738 |
|  | 16324 | 863.9725 | 1725.9305 | 1725.9413 | -0.0108 | 0 | 26 | 0.25 | 1 | R.QLLQANPILEAFGNAK.T 16312 |
|  | 16975 | 886.9424 | 1771.8702 | 1772.8289 | -0.9586 | 1 | 1 | 89 | 9 | R.QLEEAEEEASRAQAGR.R |
|  | 18540 | **631.1890** | **1890.5451** | **1889.8537** | **0.6913** | **1** | **0** | **66** | **7** | **R.ERNTDQATMPDNTAAQK.L** |
|  | 18932 | 642.3522 | 1924.0347 | 1924.9530 | -0.9183 | 1 | 12 | 6.4 | 1 | K.EQADFALEALAKATYER.L |
|  | 19246 | **975.5556** | **1949.0966** | **1950.0687** | **-0.9720** | **1** | **2** | **64** | **6** | **K.VTDIIVSFQAAARGYLAR.R** 19247 |
|  | 20385 | 513.5104 | 2050.0127 | 2049.9208 | 0.0919 | 1 | 2 | 63 | 7 | R.NTDQATMPDNTAAQKLCR.L |
|  | 21155 | 1063.0664 | 2124.1183 | 2123.0065 | 1.1117 | 0 | 2 | 60 | 7 | K.NMDPLNDNVAALLHQSTDR.L 21156 |
|  | 21244 | 1068.0140 | 2134.0135 | 2132.9644 | 1.0491 | 0 | 1 | 71 | 2 | R.LDGESSELQEQMVEQQQR.A |
|  | 22534 | 761.3460 | 2281.0160 | 2281.0314 | -0.0154 | 1 | 52 | 0.00049 | 1 | R.SMLQDREDQSILCTGESGAGK.T |
|  | 22854 | 581.3055 | 2321.1928 | 2321.2478 | -0.0550 | 1 | 7 | 16 | 2 | K.LLLQVESLTTELSAERSFSAK.A |
|  | 23932 | 823.4158 | 2467.2257 | 2468.1794 | -0.9538 | 0 | 3 | 40 | 5 | K.LQQLFNHTMFVLEQEEYQR.E |
|  | 24468 | **849.4653** | **2545.3740** | **2546.3201** | **-0.9461** | **1** | **2** | **40** | **2** | **K.DVEGIVGLEQVSSLGDGPPGGRPRR.G** |
|  | 26966 | 994.5695 | 2980.6866 | 2980.4712 | 0.2153 | 1 | 1 | 32 | 5 | K.NMDPLNDNVAALLHQSTDRLTAEIWK.D |
|  | 27097 | **1005.1762** | **3012.5068** | **3011.4229** | **1.0839** | **1** | **1** | **40** | **1** | **K.ANEWLMKNMDPLNDNVAALLHQSTDR.L** |

| **470.** | P16152\|CBR1_HUMAN    **Mass:** 30641    **Score:** 93     **Queries matched:** 12   **emPAI:** 0.59 |
| --- | --- |
|  | Carbonyl reductase [NADPH] 1 OS=Homo sapiens GN=CBR1 PE=1 SV=3 |

|  | **Query** | **Observed** | **Mr(expt)** | **Mr(calc)** | **Delta** | **Miss** | **Score** | **Expect** | **Rank** | **Peptide** |
| --- | --- | --- | --- | --- | --- | --- | --- | --- | --- | --- |
|  | 2829 | **434.2376** | **866.4607** | **865.4545** | **1.0061** | **1** | **6** | **35** | **1** | **K.FVEDTKK.G** |
|  | 6945 | **589.3285** | **1176.6425** | **1176.6503** | **-0.0077** | **0** | **18** | **2.5** | **1** | **R.LFSGDVVLTAR.D** 6950 |
|  | 7175 | **595.3795** | **1188.7444** | **1189.6489** | **-0.9045** | **0** | **(3)** | **69** | **3** | **R.VVNVSSIMSVR.A** |
|  | 7569 | **403.2292** | **1206.6658** | **1205.6438** | **1.0220** | **0** | **6** | **34** | **7** | **R.VVNVSSIMSVR.A** |
|  | 12129 | **729.8654** | **1457.7163** | **1457.7272** | **-0.0109** | **0** | **55** | **0.00046** | **1** | **K.ILLNACCPGWVR.T** |
|  | 13107 | **759.7457** | **1517.4768** | **1517.8599** | **-0.3832** | **1** | **3** | **45** | **7** | **R.VVNVSSIMSVRALK.S** |
|  | 14349 | **800.4047** | **1598.7949** | **1598.8053** | **-0.0104** | **0** | **42** | **0.0069** | **1** | **R.FHQLDIDDLQSIR.A** 14350 |
|  | 15178 | **826.9336** | **1651.8527** | **1651.8642** | **-0.0114** | **0** | **29** | **0.14** | **1** | **R.GQAAVQQLQAEGLSPR.F** |
|  | 15771 | 847.4395 | 1692.8645 | 1691.8287 | 1.0357 | 0 | 6 | 27 | 3 | R.SETITEEELVGLMNK.F |
|  | 27764 | **1055.5110** | **3163.5111** | **3163.5350** | **-0.0238** | **0** | **40** | **0.0052** | **1** | **K.SPEEGAETPVYLALLPPDAEGPHGQFVSEK.R** |

| **471.** | Q9UN36\|NDRG2_HUMAN    **Mass:** 41114    **Score:** 93     **Queries matched:** 7   **emPAI:** 0.19 |
| --- | --- |
|  | Protein NDRG2 OS=Homo sapiens GN=NDRG2 PE=1 SV=2 |

|  | **Query** | **Observed** | **Mr(expt)** | **Mr(calc)** | **Delta** | **Miss** | **Score** | **Expect** | **Rank** | **Peptide** |
| --- | --- | --- | --- | --- | --- | --- | --- | --- | --- | --- |
|  | 10269 | **675.3820** | **1348.7494** | **1348.6583** | **0.0911** | **0** | **65** | **5.3e-005** | **1** | **R.TASLTSAASVDGNR.S** |
|  | 13041 | **757.8619** | **1513.7093** | **1513.7195** | **-0.0102** | **0** | **51** | **0.00093** | **1** | **K.MADSGGQPQLTQPGK.L** |
|  | 14228 | 796.4023 | 1590.7901 | 1591.7914 | -1.0013 | 1 | 5 | 35 | 9 | R.TASLTSAASVDGNRSR.S |
|  | 18871 | 640.3200 | 1917.9382 | 1917.7882 | 0.1500 | 0 | (1) | 76 | 2 | K.YFLQGMGYMASSCMTR.L |
|  | 19074 | 968.4858 | 1934.9570 | 1933.7831 | 1.1739 | 0 | 2 | 58 | 5 | K.YFLQGMGYMASSCMTR.L |
|  | 27760 | **791.6581** | **3162.6032** | **3162.6238** | **-0.0205** | **0** | **4** | **20** | **5** | **R.ILLDQGQTHSVETPYGSVTFTVYGTPKPK.R** |
|  | 28479 | **1124.5807** | **3370.7202** | **3370.7330** | **-0.0127** | **0** | **9** | **4.7** | **1** | **K.LTGLTSSIPEMILGHLFSQEELSGNSELIQK.Y** |

| **472.** | Q15005\|SPCS2_HUMAN    **Mass:** 25272    **Score:** 93     **Queries matched:** 11   **emPAI:** 0.52 |
| --- | --- |
|  | Signal peptidase complex subunit 2 OS=Homo sapiens GN=SPCS2 PE=1 SV=3 |

|  | **Query** | **Observed** | **Mr(expt)** | **Mr(calc)** | **Delta** | **Miss** | **Score** | **Expect** | **Rank** | **Peptide** |
| --- | --- | --- | --- | --- | --- | --- | --- | --- | --- | --- |
|  | 1640 | **724.3823** | **723.3750** | **723.3439** | **0.0311** | **0** | **8** | **20** | **5** | **R.EAEFTK.S** |
|  | 1931 | **400.2280** | **798.4414** | **799.4300** | **-0.9886** | **0** | **10** | **18** | **7** | **M.AAAAVQGGR.S** 1950 |
|  | 3884 | **473.7680** | **945.5215** | **945.5284** | **-0.0068** | **1** | **23** | **0.98** | **1** | **R.SGLLDKWK.I** |
|  | 5336 | **527.2845** | **1052.5544** | **1052.5614** | **-0.0070** | **0** | **32** | **0.09** | **1** | **R.LHDSLAIER.K** |
|  | 6182 | 559.7914 | 1117.5683 | 1117.5768 | -0.0084 | 1 | 12 | 12 | 2 | K.IDKWDGSAVK.N |
|  | 6306 | **565.2908** | **1128.5671** | **1127.6339** | **0.9332** | **1** | **11** | **14** | **1** | **K.WKIDDKPVK.I** |
|  | 8970 | **641.3006** | **1280.5866** | **1281.6353** | **-1.0487** | **0** | **7** | **25** | **4** | **K.YVENFGLIDGR.L** |
|  | 18079 | **929.3583** | **1856.7020** | **1856.7126** | **-0.0106** | **0** | **81** | **6.5e-007** | **1** | **R.SGGSGGCSGAGGASNCGTGSGR.S** |
|  | 21482 | **721.0098** | **2160.0075** | **2160.0157** | **-0.0083** | **1** | **2** | **48** | **3** | **K.DPTGMDPDDIWQLSSSLKR.F** |
|  | 21956 | **738.6727** | **2212.9964** | **2213.0099** | **-0.0136** | **0** | **20** | **0.76** | **1** | **K.FFDHSGTLVMDAYEPEISR.L** |

| **473.** | P14406\|CX7A2_HUMAN    **Mass:** 9390     **Score:** 93     **Queries matched:** 3   **emPAI:** 1.04 |
| --- | --- |
|  | Cytochrome c oxidase polypeptide 7A2, mitochondrial OS=Homo sapiens GN=COX7A2 PE=1 SV=1 |

|  | **Query** | **Observed** | **Mr(expt)** | **Mr(calc)** | **Delta** | **Miss** | **Score** | **Expect** | **Rank** | **Peptide** |
| --- | --- | --- | --- | --- | --- | --- | --- | --- | --- | --- |
|  | 5124 | **518.2835** | **1034.5523** | **1033.5556** | **0.9967** | **0** | **43** | **0.0087** | **1** | **K.GGVADALLYR.A** |
|  | 14692 | **811.9139** | **1621.8133** | **1621.8239** | **-0.0106** | **0** | **72** | **6.8e-006** | **1** | **K.LFQEDDEIPLYLK.G** 14693 |

| **474.** | P05090\|APOD_HUMAN    **Mass:** 21547    **Score:** 92     **Queries matched:** 7   **emPAI:** 0.39 |
| --- | --- |
|  | Apolipoprotein D OS=Homo sapiens GN=APOD PE=1 SV=1 |

|  | **Query** | **Observed** | **Mr(expt)** | **Mr(calc)** | **Delta** | **Miss** | **Score** | **Expect** | **Rank** | **Peptide** |
| --- | --- | --- | --- | --- | --- | --- | --- | --- | --- | --- |
|  | 2913 | **436.7547** | **871.4948** | **870.4923** | **1.0026** | **0** | **3** | **95** | **10** | **K.VLNQELR.A** |
|  | 9172 | 646.7981 | 1291.5816 | 1291.5901 | -0.0084 | 0 | 46 | 0.004 | 1 | K.MTVTDQVNCPK.L |
|  | 11516 | **710.8444** | **1419.6742** | **1419.6850** | **-0.0109** | **1** | **59** | **0.00018** | **1** | **K.KMTVTDQVNCPK.L** 11517 |
|  | 15261 | 829.9113 | 1657.8079 | 1656.7566 | 1.0513 | 0 | 28 | 0.18 | 2 | K.CPNPPVQENFDVNK.Y 15249 |
|  | 17168 | 892.9249 | 1783.8352 | 1783.8597 | -0.0245 | 1 | 2 | 70 | 4 | R.CIQANYSLMENGKIK.V |

| **475.** | P18669\|PGAM1_HUMAN    **Mass:** 28900    **Score:** 92     **Queries matched:** 14   **emPAI:** 0.64 |
| --- | --- |
|  | Phosphoglycerate mutase 1 OS=Homo sapiens GN=PGAM1 PE=1 SV=2 |

|  | **Query** | **Observed** | **Mr(expt)** | **Mr(calc)** | **Delta** | **Miss** | **Score** | **Expect** | **Rank** | **Peptide** |
| --- | --- | --- | --- | --- | --- | --- | --- | --- | --- | --- |
|  | 1244 | 591.2767 | 590.2694 | 589.3071 | 0.9623 | 0 | 17 | 5 | 2 | K.AETAAK.H |
|  | 4280 | **488.2468** | **974.4791** | **974.4855** | **-0.0064** | **0** | **35** | **0.056** | **1** | **K.AMEAVAAQGK.A** |
|  | 5422 | 530.7808 | 1059.5470 | 1058.5509 | 0.9961 | 0 | 12 | 12 | 2 | R.HYGGLTGLNK.A 5414 |
|  | 5995 | **552.2934** | **1102.5722** | **1102.5804** | **-0.0082** | **1** | **61** | **0.00012** | **1** | **R.KAMEAVAAQGK.A** |
|  | 9548 | **656.8008** | **1311.5870** | **1311.5956** | **-0.0086** | **0** | **48** | **0.0024** | **1** | **R.HGESAWNLENR.F** |
|  | 13902 | 524.2879 | 1569.8419 | 1570.8216 | -0.9797 | 1 | 8 | 21 | 5 | R.LNERHYGGLTGLNK.A |
|  | 15617 | **842.4536** | **1682.8927** | **1682.9032** | **-0.0105** | **0** | **28** | **0.17** | **1** | **R.ALPFWNEEIVPQIK.E** |
|  | 18767 | **636.6825** | **1907.0257** | **1906.0173** | **1.0084** | **1** | **0** | **89** | **9** | **K.LVLIRHGESAWNLENR.F** |
|  | 19063 | 645.5743 | 1933.7010 | 1934.8833 | -1.1823 | 1 | 2 | 51 | 6 | R.DAGYEFDICFTSVQKR.A |
|  | 21079 | **529.5341** | **2114.1071** | **2114.1194** | **-0.0122** | **0** | **8** | **15** | **1** | **K.NLKPIKPMQFLGDEETVR.K** |
|  | 21207 | **710.9467** | **2129.8183** | **2130.1143** | **-0.2960** | **0** | **(0)** | **61** | **9** | **K.NLKPIKPMQFLGDEETVR.K** |
|  | 21569 | **724.3777** | **2170.1112** | **2171.1449** | **-1.0337** | **0** | **1** | **70** | **2** | **R.TLWTVLDAIDQMWLPVVR.T** |
|  | 23615 | **809.0513** | **2424.1320** | **2424.1478** | **-0.0159** | **1** | **15** | **2.1** | **1** | **R.YADLTEDQLPSCESLKDTIAR.A** |

| **476.** | P43686\|PRS6B_HUMAN    **Mass:** 47451    **Score:** 92     **Queries matched:** 14   **emPAI:** 0.08 |
| --- | --- |
|  | 26S protease regulatory subunit 6B OS=Homo sapiens GN=PSMC4 PE=1 SV=2 |

|  | **Query** | **Observed** | **Mr(expt)** | **Mr(calc)** | **Delta** | **Miss** | **Score** | **Expect** | **Rank** | **Peptide** |
| --- | --- | --- | --- | --- | --- | --- | --- | --- | --- | --- |
|  | 2015 | **402.7210** | **803.4275** | **803.4323** | **-0.0048** | **0** | **14** | **8.3** | **1** | **K.VIMATNR.A** |
|  | 2193 | **410.2116** | **818.4087** | **819.4273** | **-1.0185** | **0** | **(4)** | **89** | **6** | **K.VIMATNR.A** |
|  | 3247 | **450.5861** | **899.1576** | **899.4389** | **-0.2813** | **1** | **3** | **52** | **1** | **K.DFEKAYK.T** |
|  | 4442 | 494.2598 | 986.5050 | 985.5233 | 0.9817 | 0 | 15 | 5.9 | 2 | K.IEFPLPDR.R 4434 4444 |
|  | 6027 | 553.7716 | 1105.5285 | 1104.6291 | 0.8994 | 1 | 10 | 18 | 5 | R.ENRYIVLAK.D |
|  | 6127 | **557.7852** | **1113.5559** | **1113.6182** | **-0.0623** | **1** | **0** | **1.5e+002** | **4** | **R.KIEFPLPDR.R** |
|  | 6421 | **569.2889** | **1136.5632** | **1135.5370** | **1.0262** | **1** | **10** | **14** | **2** | **K.RFDAQTGADR.E** |
|  | 9218 | **432.2358** | **1293.6855** | **1293.6942** | **-0.0087** | **0** | **9** | **20** | **1** | **K.AVAHHTTAAFIR.V** |
|  | 21199 | **710.4152** | **2128.2237** | **2127.0605** | **1.1631** | **1** | **1** | **54** | **2** | **K.QIGIDPPRGVLMYGPPGCGK.T** |
|  | 21524 | 722.6452 | 2164.9138 | 2165.1255 | -0.2118 | 1 | 4 | 34 | 3 | K.KLQQELEFLEVQEEYIK.D |
|  | 21823 | **1102.0677** | **2202.1209** | **2202.1354** | **-0.0145** | **0** | **87** | **1.7e-007** | **1** | **R.ILLELLNQMDGFDQNVNVK.V** 21822 |

| **477.** | P12111\|CO6A3_HUMAN    **Mass:** 345163   **Score:** 91     **Queries matched:** 68   **emPAI:** 0.03 |
| --- | --- |
|  | Collagen alpha-3(VI) chain OS=Homo sapiens GN=COL6A3 PE=1 SV=4 |

|  | **Query** | **Observed** | **Mr(expt)** | **Mr(calc)** | **Delta** | **Miss** | **Score** | **Expect** | **Rank** | **Peptide** |
| --- | --- | --- | --- | --- | --- | --- | --- | --- | --- | --- |
|  | 639 | **448.3617** | **447.3544** | **446.2237** | **1.1307** | **0** | **7** | **43** | **1** | **R.AGGSR.V** 618 |
|  | 720 | **462.1442** | **461.1370** | **460.2394** | **0.8976** | **0** | **4** | **1.2e+002** | **6** | **K.AAGSR.A** 721 722 |
|  | 854 | **501.0456** | **500.0383** | **499.2503** | **0.7881** | **0** | **1** | **1.2e+002** | **6** | **K.GNPGR.R** |
|  | 1171 | **572.9959** | **571.9886** | **572.3031** | **-0.3144** | **0** | **9** | **23** | **5** | **K.GGIGNR.G** |
|  | 1236 | **589.2908** | **588.2835** | **587.3027** | **0.9808** | **0** | **10** | **34** | **2** | **R.QEGVR.A** |
|  | 2041 | 403.7088 | 805.4030 | 805.3566 | 0.0464 | 0 | 4 | 80 | 3 | K.SQDDVSR.F |
|  | 2118 | **406.7217** | **811.4287** | **812.4617** | **-1.0329** | **1** | **7** | **27** | **2** | **R.GRTGPLGR.K** |
|  | 2286 | **413.2638** | **824.5130** | **824.4756** | **0.0374** | **0** | **7** | **30** | **2** | **R.GPIGSIGPK.G** 2291 |
|  | 3065 | **884.2894** | **883.2822** | **882.4784** | **0.8038** | **1** | **2** | **87** | **6** | **K.GGIGNRGPR.G** |
|  | 3220 | **449.7159** | **897.4172** | **896.5120** | **0.9052** | **0** | **4** | **56** | **10** | **K.YIAYLVR.Q** |
|  | 3233 | **450.2479** | **898.4812** | **899.4712** | **-0.9901** | **0** | **2** | **1e+002** | **10** | **R.VDGPASNLK.Q** |
|  | 3460 | 457.7575 | 913.5005 | 912.6008 | 0.8997 | 0 | 7 | 36 | 6 | K.LLVLITGGK.S |
|  | 3952 | **476.2448** | **950.4749** | **950.3772** | **0.0977** | **0** | **8** | **25** | **6** | **K.ACCGVPCK.C** |
|  | 4404 | **492.6126** | **983.2105** | **982.6175** | **0.5931** | **0** | **5** | **32** | **7** | **R.ALILVGLER.V** |
|  | 5155 | 519.2921 | 1036.5695 | 1035.5349 | 1.0346 | 0 | 6 | 37 | 7 | R.VAVVQYSDR.T |
|  | 5553 | **535.9318** | **1069.8490** | **1069.6244** | **0.2246** | **1** | **1** | **79** | **1** | **K.RQIIDAINK.V** |
|  | 5557 | **536.1643** | **1070.3141** | **1069.5040** | **0.8100** | **0** | **4** | **47** | **2** | **R.DGVPGGPGETGK.N** |
|  | 5921 | **550.2150** | **1098.4155** | **1098.6145** | **-0.1990** | **1** | **3** | **80** | **3** | **R.LRGGSPLNTGK.A** |
|  | 6549 | 574.2548 | 1146.4950 | 1145.6041 | 0.8909 | 0 | 14 | 6.8 | 1 | R.SSGIVSLGVGDR.N |
|  | 6664 | **578.2703** | **1154.5261** | **1153.5801** | **0.9460** | **0** | **0** | **1.5e+002** | **6** | **R.SSIMAFAIGNK.G** |
|  | 7712 | **406.0258** | **1215.0556** | **1215.6095** | **-0.5539** | **0** | **3** | **49** | **10** | **R.VVESLDVGQDR.V** |
|  | 7916 | **613.3262** | **1224.6379** | **1224.6938** | **-0.0559** | **1** | **8** | **21** | **1** | **R.SQAPVLDAIRR.L** |
|  | 8049 | **616.8104** | **1231.6062** | **1231.6197** | **-0.0136** | **0** | **4** | **69** | **7** | **R.VGVVQFSDEPR.T** |
|  | 8293 | 415.2526 | 1242.7360 | 1243.6561 | -0.9200 | 0 | 9 | 17 | 4 | K.VEFLLNAHSSK.D |
|  | 8729 | **423.2327** | **1266.6762** | **1265.6840** | **0.9921** | **1** | **5** | **49** | **7** | **R.RGNSGPPGIVGQK.G** 8730 |
|  | 10155 | **448.8735** | **1343.5987** | **1344.6633** | **-1.0647** | **1** | **3** | **71** | **7** | **R.SSDRVDGPASNLK.Q** |
|  | 10410 | **679.3384** | **1356.6622** | **1357.6296** | **-0.9674** | **0** | **2** | **82** | **9** | **K.GETGDLGPMGVPGR.D** |
|  | 10665 | 686.3314 | 1370.6483 | 1370.7154 | -0.0671 | 1 | 6 | 36 | 7 | R.SGDDVRNPSVVVK.R |
|  | 11260 | **469.2756** | **1404.8049** | **1404.7249** | **0.0800** | **0** | **6** | **35** | **9** | **K.DVVFLLDGSEGVR.S** |
|  | 11540 | **711.0920** | **1420.1694** | **1420.7059** | **-0.5365** | **1** | **1** | **65** | **3** | **K.GEPGEPGPKGGIGNR.G** |
|  | 12556 | 741.9013 | 1481.7881 | 1482.7137 | -0.9256 | 0 | 2 | 81 | 6 | K.QQSLETAMSFVAR.N |
|  | 12887 | **752.4104** | **1502.8062** | **1501.7300** | **1.0762** | **1** | **1** | **1.1e+002** | **6** | **K.VEFSLTDYGSKEK.L** |
|  | 13565 | 774.4050 | 1546.7954 | 1546.7661 | 0.0293 | 0 | 6 | 28 | 10 | R.GDSIDQCALIQSIK.D |
|  | 13623 | **776.3807** | **1550.7468** | **1551.8304** | **-1.0835** | **1** | **0** | **1.1e+002** | **7** | **K.VDAILNRISQMHR.V** |
|  | 14157 | 793.9615 | 1585.9085 | 1585.9192 | -0.0106 | 0 | 21 | 1 | 1 | R.LQPVLQPLPSPGVGGK.R |
|  | 14611 | **809.3613** | **1616.7081** | **1616.8886** | **-0.1805** | **1** | **3** | **60** | **1** | **K.RDIIFLLDGSANVGK.T** |
|  | 14835 | **816.4114** | **1630.8083** | **1630.8162** | **-0.0079** | **0** | **9** | **15** | **5** | **K.SVEDAQDVSLALTQR.G** |
|  | 14952 | 546.7651 | 1637.2734 | 1637.7394 | -0.4660 | 1 | 7 | 16 | 8 | R.GDPGNPGQDSQERGPK.G |
|  | 15251 | 553.2769 | 1656.8088 | 1657.7809 | -0.9721 | 1 | 3 | 56 | 5 | R.DGVPGGPGETGKNGGFGR.R |
|  | 15574 | **560.9559** | **1679.8458** | **1678.8526** | **0.9932** | **1** | **17** | **2.4** | **1** | **R.IAVAQYSDDVKVESR.F** |
|  | 15645 | **843.4825** | **1684.9504** | **1684.9611** | **-0.0107** | **0** | **11** | **8.2** | **1** | **K.LLTPITTLTSEQIQK.L** |
|  | 16212 | 859.9521 | 1717.8896 | 1717.8999 | -0.0103 | 1 | 1 | 87 | 8 | K.VFAVGVRNIDSEEVGK.I |
|  | 16510 | **869.5038** | **1736.9930** | **1737.0036** | **-0.0106** | **0** | **25** | **0.3** | **1** | **R.ITEGVPQLLIVLTADR.S** |
|  | 17281 | 598.3333 | 1791.9781 | 1790.9527 | 1.0254 | 0 | 14 | 4.7 | 2 | R.AGDGVPQVIVVLTDGHSK.D |
|  | 18371 | **626.3822** | **1876.1248** | **1874.9487** | **1.1761** | **1** | **2** | **48** | **2** | **K.EFVQRVVESLDVGQDR.V** |
|  | 19213 | **649.8643** | **1946.5710** | **1946.9520** | **-0.3810** | **1** | **1** | **55** | **6** | **R.NMRSQHPYVLTEDTLK.V** |
|  | 19667 | **994.5062** | **1986.9979** | **1987.0091** | **-0.0112** | **0** | **87** | **1.7e-007** | **1** | **R.VGVVQFSNDVFPEFYLK.T** |
|  | 20458 | **1028.5199** | **2055.0252** | **2056.0299** | **-1.0047** | **0** | **0** | **80** | **4** | **K.MKPLDGSALYTGSALDFVR.N** |
|  | 21481 | **721.0084** | **2160.0033** | **2161.0838** | **-1.0805** | **0** | **(3)** | **40** | **3** | **R.VAVVQHAPSESVDNASMPPVK.V** |
|  | 21631 | 726.6544 | 2176.9413 | 2177.0787 | -0.1374 | 0 | 7 | 17 | 2 | R.VAVVQHAPSESVDNASMPPVK.V |
|  | 21886 | **1104.5348** | **2207.0550** | **2206.1787** | **0.8764** | **0** | **9** | **9.7** | **1** | **R.DILFLFDGSANLVGQFPVVR.D** |
|  | 22239 | **561.5051** | **2241.9912** | **2242.1787** | **-0.1875** | **1** | **6** | **19** | **2** | **K.VRVGVVQFSNDVFPEFYLK.T** |
|  | 22364 | **566.0474** | **2260.1606** | **2260.1266** | **0.0340** | **1** | **5** | **24** | **1** | **K.ECEKVCAPVLAKPGVISVMGT.-** |
|  | 22930 | **778.0649** | **2331.1728** | **2331.1845** | **-0.0117** | **0** | **17** | **1.4** | **1** | **R.LNLLDLDYELAEQLDNIAEK.A** 22925 |
|  | 23682 | 610.2855 | 2437.1127 | 2437.2635 | -0.1508 | 1 | 2 | 45 | 6 | K.NLQVALTSKQQSLETAMSFVAR.N |
|  | 25102 | **887.5317** | **2659.5732** | **2658.4428** | **1.1304** | **1** | **2** | **28** | **2** | **R.VIGGLLAGQTYHVAVVCYLRSQVR.A** |
|  | 26412 | **578.4647** | **2887.2872** | **2887.4505** | **-0.1632** | **0** | **0** | **49** | **3** | **K.NGAAADIIFLVDSSWTIGEEHFQLVR.E** |
|  | 27844 | 796.6539 | 3182.5864 | 3182.5381 | 0.0483 | 0 | 2 | 27 | 3 | R.QLQLQGGSGLNTGSALSYVYANHFTEAGGSR.I |
|  | 28116 | **653.7164** | **3263.5458** | **3263.5512** | **-0.0054** | **1** | **6** | **11** | **3** | **R.LNLLDLDYELAEQLDNIAEKACCGVPCK.C** |
|  | 28285 | **831.9325** | **3323.7009** | **3323.5578** | **0.1431** | **1** | **1** | **34** | **1** | **R.AAGSDVDIDMAFILDSAETTTLFQFNEMKK.Y** |
|  | 29357 | 931.9614 | 3723.8163 | 3722.8495 | 0.9668 | 1 | 1 | 26 | 7 | K.SQPPPPQPARSASSSTINLMVSTEPLALTETDICK.L |
|  | 29528 | **948.2344** | **3788.9084** | **3788.8607** | **0.0477** | **1** | **1** | **25** | **1** | **R.SSIMAFAIGNKGADQAELEEIAFDSSLVFIPAEFR.A** |

| **478.** | P16671\|CD36_HUMAN    **Mass:** 53589    **Score:** 91     **Queries matched:** 7   **emPAI:** 0.07 |
| --- | --- |
|  | Platelet glycoprotein 4 OS=Homo sapiens GN=CD36 PE=1 SV=2 |

|  | **Query** | **Observed** | **Mr(expt)** | **Mr(calc)** | **Delta** | **Miss** | **Score** | **Expect** | **Rank** | **Peptide** |
| --- | --- | --- | --- | --- | --- | --- | --- | --- | --- | --- |
|  | 8471 | **627.3779** | **1252.7412** | **1253.6187** | **-0.8775** | **1** | **3** | **77** | **7** | **K.ANMFRSQVTGK.I** |
|  | 13197 | **508.5592** | **1522.6559** | **1522.9195** | **-0.2636** | **1** | **3** | **57** | **9** | **K.RLQVNLLVKPSEK.I** |
|  | 21368 | 1074.9462 | 2147.8778 | 2147.8928 | -0.0150 | 0 | 91 | 6.3e-008 | 1 | K.AFASPVENPDNYCFCTEK.I |
|  | 29091 | 1195.6028 | 3583.7865 | 3582.9089 | 0.8777 | 1 | 7 | 8.3 | 2 | M.GCDRNCGLIAGAVIGAVLAVFGGILMPVGDLLIQK.T |
|  | 29205 | **733.3638** | **3661.7825** | **3662.9134** | **-1.1310** | **1** | **2** | **20** | **2** | **K.INLLGLIEMILLSVGVVMFVAFMISYCACRSK.T** |
|  | 30157 | 1350.0075 | 4047.0005 | 4048.1096 | -1.1091 | 1 | 1 | 22 | 2 | R.SQVTGKINLLGLIEMILLSVGVVMFVAFMISYCACR.S |
|  | 31024 | **1528.0610** | **4581.1613** | **4582.0668** | **-0.9055** | **1** | **5** | **5.7** | **2** | **R.NLSYWESHCDMINGTDAASFPPFVEKSQVLQFFSSDICR.S** |

| **479.** | P62829\|RL23_HUMAN    **Mass:** 14970    **Score:** 90     **Queries matched:** 7   **emPAI:** 0.26 |
| --- | --- |
|  | 60S ribosomal protein L23 OS=Homo sapiens GN=RPL23 PE=1 SV=1 |

|  | **Query** | **Observed** | **Mr(expt)** | **Mr(calc)** | **Delta** | **Miss** | **Score** | **Expect** | **Rank** | **Peptide** |
| --- | --- | --- | --- | --- | --- | --- | --- | --- | --- | --- |
|  | 3142 | **445.7449** | **889.4752** | **889.5498** | **-0.0745** | **0** | **14** | **9** | **1** | **K.VHPAVVIR.Q** |
|  | 3286 | **451.2795** | **900.5445** | **899.5076** | **1.0368** | **0** | **21** | **1.7** | **2** | **K.GSAITGPVAK.E** |
|  | 3930 | 475.2593 | 948.5041 | 948.5644 | -0.0603 | 0 | 12 | 12 | 4 | K.NLYIISVK.G |
|  | 5280 | 524.2794 | 1046.5442 | 1045.4651 | 1.0791 | 0 | 22 | 1.1 | 2 | K.ECADLWPR.I |
|  | 17893 | 615.2911 | 1842.8516 | 1841.9856 | 0.8660 | 1 | (2) | 74 | 6 | R.LNRLPAAGVGDMVMATVK.K |
|  | 18099 | **465.2648** | **1857.0300** | **1857.9805** | **-0.9505** | **1** | **3** | **50** | **10** | **R.LNRLPAAGVGDMVMATVK.K** |
|  | 19476 | **985.5164** | **1969.0183** | **1969.0303** | **-0.0120** | **0** | **90** | **8.3e-008** | **1** | **R.ISLGLPVGAVINCADNTGAK.N** |

| **480.** | P14649\|MYL6B_HUMAN    **Mass:** 22864    **Score:** 90     **Queries matched:** 10   **emPAI:** 0.36 |
| --- | --- |
|  | Myosin light chain 6B OS=Homo sapiens GN=MYL6B PE=1 SV=1 |

|  | **Query** | **Observed** | **Mr(expt)** | **Mr(calc)** | **Delta** | **Miss** | **Score** | **Expect** | **Rank** | **Peptide** |
| --- | --- | --- | --- | --- | --- | --- | --- | --- | --- | --- |
|  | 4593 | 499.2789 | 996.5432 | 996.5604 | -0.0172 | 0 | 4 | 64 | 6 | R.HVLTTLGEK.M |
|  | 7402 | **600.9226** | **1199.8305** | **1198.6557** | **1.1748** | **1** | **1** | **97** | **4** | **K.VLGNPKSDELK.S** |
|  | 8872 | 638.2999 | 1274.5852 | 1275.6241 | -1.0389 | 1 | 16 | 3.4 | 3 | K.EGNGKVMGAELR.H 8892 |
|  | 10100 | 671.3138 | 1340.6131 | 1340.6217 | -0.0086 | 0 | 56 | 0.00035 | 1 | K.ILYSQCGDVMR.A |
|  | 10359 | 677.8650 | 1353.7154 | 1353.7252 | -0.0098 | 0 | 44 | 0.0058 | 1 | R.ALGQNPTNAEVLK.V 10346 10360 10373 10376 |

| **481.** | Q7Z7H5\|TMED4_HUMAN    **Mass:** 26097    **Score:** 90     **Queries matched:** 11   **emPAI:** 0.31 |
| --- | --- |
|  | Transmembrane emp24 domain-containing protein 4 OS=Homo sapiens GN=TMED4 PE=1 SV=1 |

|  | **Query** | **Observed** | **Mr(expt)** | **Mr(calc)** | **Delta** | **Miss** | **Score** | **Expect** | **Rank** | **Peptide** |
| --- | --- | --- | --- | --- | --- | --- | --- | --- | --- | --- |
|  | 1899 | 399.2322 | 796.4498 | 795.3511 | 1.0987 | 0 | 15 | 4.2 | 1 | R.QYGSEGR.F |
|  | 5472 | 532.3206 | 1062.6266 | 1062.6008 | 0.0258 | 1 | (2) | 1e+002 | 10 | R.MALFAGGKLR.V |
|  | 5657 | **540.0258** | **1078.0371** | **1078.5957** | **-0.5586** | **1** | **10** | **9.8** | **1** | **R.MALFAGGKLR.V** |
|  | 5750 | **543.3572** | **1084.6998** | **1084.5877** | **0.1121** | **1** | **8** | **22** | **5** | **K.DPDGKVVLSR.Q** |
|  | 7970 | 614.8079 | 1227.6012 | 1227.6506 | -0.0495 | 1 | 8 | 27 | 9 | M.AGVGAGPLRAMGR.Q 7942 |
|  | 10114 | 671.3673 | 1340.7201 | 1340.7300 | -0.0099 | 0 | 42 | 0.0094 | 2 | R.QLLDQVEQIQK.E |
|  | 10717 | **459.0118** | **1374.0134** | **1374.6860** | **-0.6726** | **1** | **8** | **14** | **1** | **-.MAGVGAGPLRAMGR.Q** 10716 |
|  | 20800 | 1043.4778 | 2084.9410 | 2084.9547 | -0.0137 | 0 | 52 | 0.00056 | 1 | R.CFIEEIPDETMVIGNYR.T 20801 |

| **482.** | Q16853\|AOC3_HUMAN    **Mass:** 85138    **Score:** 90     **Queries matched:** 10   **emPAI:** 0.09 |
| --- | --- |
|  | Membrane primary amine oxidase OS=Homo sapiens GN=AOC3 PE=1 SV=3 |

|  | **Query** | **Observed** | **Mr(expt)** | **Mr(calc)** | **Delta** | **Miss** | **Score** | **Expect** | **Rank** | **Peptide** |
| --- | --- | --- | --- | --- | --- | --- | --- | --- | --- | --- |
|  | 1322 | **616.3732** | **615.3660** | **615.3704** | **-0.0045** | **0** | **11** | **22** | **2** | **R.LQVTR.K** |
|  | 17154 | 595.3156 | 1782.9250 | 1783.9733 | -1.0483 | 1 | 4 | 45 | 3 | R.GSPPPAREALAIVFFGR.Q |
|  | 18187 | **621.9915** | **1862.9526** | **1861.9033** | **1.0493** | **1** | **5** | **30** | **10** | **R.YVDGGFGMGKYTTPLTR.G** |
|  | 18391 | 939.9706 | 1877.9267 | 1877.8982 | 0.0285 | 1 | (3) | 52 | 2 | R.YVDGGFGMGKYTTPLTR.G |
|  | 19837 | **1001.5457** | **2001.0767** | **2001.0472** | **0.0295** | **0** | **16** | **2.3** | **1** | **K.SPVPPGPAPPLQFYPQGPR.F** |
|  | 20993 | **527.2666** | **2105.0373** | **2105.0827** | **-0.0454** | **1** | **3** | **43** | **7** | **R.KLLEMEEQAAFLVGSATPR.Y** |
|  | 22650 | **766.3926** | **2296.1559** | **2296.1674** | **-0.0115** | **0** | **48** | **0.0014** | **1** | **R.RPVLFQEYLDIDQMIFNR.E** |
|  | 25404 | 903.4734 | 2707.3985 | 2708.2945 | -0.8960 | 0 | 7 | 14 | 1 | R.GVDCPYLATYVDWHFLLESQAPK.T |
|  | 26947 | **596.6978** | **2978.4527** | **2977.5331** | **0.9195** | **0** | **1** | **45** | **7** | **R.LGPGLVDAAQARPSDNCVFSVELQLPPK.A** |
|  | 29705 | 1274.8874 | 3821.6405 | 3821.6621 | -0.0216 | 0 | 65 | 9.5e-006 | 1 | R.GDQDAGACEVNPLACLPQAAACAPDLPAFSHGGFSHN.- |

| **483.** | P38435\|VKGC_HUMAN    **Mass:** 88075    **Score:** 89     **Queries matched:** 12   **emPAI:** 0.13 |
| --- | --- |
|  | Vitamin K-dependent gamma-carboxylase OS=Homo sapiens GN=GGCX PE=1 SV=2 |

|  | **Query** | **Observed** | **Mr(expt)** | **Mr(calc)** | **Delta** | **Miss** | **Score** | **Expect** | **Rank** | **Peptide** |
| --- | --- | --- | --- | --- | --- | --- | --- | --- | --- | --- |
|  | 2941 | **438.2208** | **874.4270** | **874.4872** | **-0.0602** | **1** | **15** | **6.8** | **6** | **R.GLSSLDRK.Y** |
|  | 3179 | 447.7383 | 893.4620 | 893.4429 | 0.0191 | 0 | 4 | 60 | 9 | K.LVSYCPR.R |
|  | 3281 | **451.2683** | **900.5221** | **899.4250** | **1.0971** | **0** | **2** | **1.2e+002** | **8** | **R.NTPFHER.F** |
|  | 4959 | **512.3536** | **1022.6927** | **1021.5379** | **1.1549** | **1** | **8** | **22** | **1** | **R.KLVSYCPR.R** |
|  | 5630 | **538.7800** | **1075.5454** | **1075.5509** | **-0.0056** | **1** | **10** | **17** | **2** | **R.TSPSSDKVQK.D** |
|  | 7561 | **403.2109** | **1206.6108** | **1205.6305** | **0.9803** | **1** | **9** | **18** | **1** | **R.FQQRIFDPR.V** |
|  | 8752 | 423.7388 | 1268.1947 | 1267.6785 | 0.5161 | 1 | 3 | 50 | 10 | R.SHQHVKITYR.D |
|  | 14484 | **805.3986** | **1608.7826** | **1608.7936** | **-0.0111** | **0** | **50** | **0.0012** | **1** | **K.LLGFEWTDLSSWR.R** |
|  | 14596 | 539.5859 | 1615.7360 | 1615.7664 | -0.0304 | 1 | 3 | 54 | 4 | R.EGEKMQLPAGEYHK.V |
|  | 16513 | **869.9354** | **1737.8563** | **1737.8686** | **-0.0123** | **0** | **31** | **0.079** | **1** | **R.TGELGYLNPGVFTQSR.R** 16514 |
|  | 17706 | 914.9697 | 1827.9248 | 1827.9342 | -0.0094 | 0 | 54 | 0.00037 | 1 | R.TSWVQPLLMDLSPWR.A |

| **484.** | Q969X5\|ERGI1_HUMAN    **Mass:** 32971    **Score:** 89     **Queries matched:** 10   **emPAI:** 0.11 |
| --- | --- |
|  | Endoplasmic reticulum-Golgi intermediate compartment protein 1 OS=Homo sapiens GN=ERGIC1 PE=1 SV=1 |

|  | **Query** | **Observed** | **Mr(expt)** | **Mr(calc)** | **Delta** | **Miss** | **Score** | **Expect** | **Rank** | **Peptide** |
| --- | --- | --- | --- | --- | --- | --- | --- | --- | --- | --- |
|  | 2427 | **419.2008** | **836.3871** | **836.4293** | **-0.0422** | **1** | **26** | **0.44** | **1** | **M.PFDFRR.F** |
|  | 2863 | **435.2321** | **868.4496** | **868.4555** | **-0.0059** | **1** | **20** | **1.6** | **1** | **R.RFDIYR.K** |
|  | 4198 | **485.3045** | **968.5945** | **967.4698** | **1.1247** | **1** | **1** | **1.2e+002** | **6** | **-.MPFDFRR.F** |
|  | 5532 | **535.2657** | **1068.5168** | **1068.5240** | **-0.0072** | **0** | **8** | **27** | **2** | **R.FEGQFSINK.V** |
|  | 5579 | **536.6000** | **1071.1854** | **1070.5291** | **0.6563** | **0** | **5** | **45** | **2** | **K.IPLNNGAGCR.F** |
|  | 6988 | **591.7761** | **1181.5377** | **1181.5465** | **-0.0088** | **0** | **20** | **1.4** | **1** | **K.EYVAYSHTGR.I** |
|  | 8149 | **619.2957** | **1236.5767** | **1235.5822** | **0.9945** | **0** | **3** | **66** | **4** | **R.YSYQYTVANK.E** |
|  | 24209 | **834.4177** | **2500.2312** | **2500.2459** | **-0.0147** | **0** | **61** | **5.6e-005** | **1** | **K.LSFGDTLQVQNIHGAFNALGGADR.L** 24210 |
|  | 28841 | **1170.5366** | **3508.5880** | **3509.7130** | **-1.1250** | **1** | **3** | **19** | **3** | **K.DSGGKIDVSLNISLPNLHCELVGLDIQDEMGR.H** |

| **485.** | O75964\|ATP5L_HUMAN    **Mass:** 11421    **Score:** 89     **Queries matched:** 9   **emPAI:** 2.30 |
| --- | --- |
|  | ATP synthase subunit g, mitochondrial OS=Homo sapiens GN=ATP5L PE=1 SV=3 |

|  | **Query** | **Observed** | **Mr(expt)** | **Mr(calc)** | **Delta** | **Miss** | **Score** | **Expect** | **Rank** | **Peptide** |
| --- | --- | --- | --- | --- | --- | --- | --- | --- | --- | --- |
|  | 1480 | **660.4082** | **659.4009** | **658.4014** | **0.9996** | **0** | **15** | **7.5** | **2** | **R.AIQSLK.K** |
|  | 6597 | **576.3020** | **1150.5894** | **1150.5982** | **-0.0088** | **0** | **32** | **0.092** | **1** | **K.IVNSAQTGSFK.Q** |
|  | 8948 | **640.3496** | **1278.6845** | **1278.6932** | **-0.0086** | **1** | **63** | **7.1e-005** | **1** | **K.KIVNSAQTGSFK.Q** 8949 |
|  | 11471 | 473.2548 | 1416.7425 | 1416.7977 | -0.0552 | 0 | 10 | 12 | 4 | K.VELVPPTPAEIPR.A 11454 11463 |
|  | 14163 | **794.4409** | **1586.8673** | **1586.8780** | **-0.0107** | **0** | **33** | **0.066** | **1** | **K.TPALVNAAVTYSKPR.L** 14164 |

| **486.** | P00395\|COX1_HUMAN    **Mass:** 57060    **Score:** 89     **Queries matched:** 8   **emPAI:** 0.13 |
| --- | --- |
|  | Cytochrome c oxidase subunit 1 OS=Homo sapiens GN=MT-CO1 PE=1 SV=1 |

|  | **Query** | **Observed** | **Mr(expt)** | **Mr(calc)** | **Delta** | **Miss** | **Score** | **Expect** | **Rank** | **Peptide** |
| --- | --- | --- | --- | --- | --- | --- | --- | --- | --- | --- |
|  | 17138 | **595.2831** | **1782.8274** | **1782.9590** | **-0.1316** | **0** | **1** | **81** | **6** | **R.AYFTSATMIIAIPTGVK.V** |
|  | 27670 | **1045.8779** | **3134.6120** | **3135.6191** | **-1.0071** | **0** | **0** | **43** | **4** | **K.IHFTIMFIGVNLTFFPQHFLGLSGMPR.R** |
|  | 29503 | **1262.5774** | **3784.7103** | **3784.7325** | **-0.0222** | **0** | **68** | **5.8e-006** | **1** | **K.VLMVEEPSMNLEWLYGCPPPYHTFEEPVYMK.S** 29504 |
|  | 29817 | **1291.5863** | **3871.7371** | **3871.7646** | **-0.0275** | **1** | **18** | **0.52** | **1** | **K.VLMVEEPSMNLEWLYGCPPPYHTFEEPVYMKS.-** |
|  | 29885 | 1302.3595 | 3904.0567 | 3903.7544 | 0.3023 | 1 | (2) | 20 | 6 | K.VLMVEEPSMNLEWLYGCPPPYHTFEEPVYMKS.- |
|  | 30820 | 1107.3015 | 4425.1769 | 4426.0369 | -0.8599 | 1 | 1 | 17 | 5 | K.KEPFGYMGMVWAMMSIGFLGFIVWAHHMFTVGMDVDTR.A |
|  | 30853 | **1107.5509** | **4426.1745** | **4426.0369** | **0.1376** | **1** | **(0)** | **20** | **1** | **K.KEPFGYMGMVWAMMSIGFLGFIVWAHHMFTVGMDVDTR.A** |

| **487.** | P21964\|COMT_HUMAN    **Mass:** 30474    **Score:** 88     **Queries matched:** 10   **emPAI:** 0.60 |
| --- | --- |
|  | Catechol O-methyltransferase OS=Homo sapiens GN=COMT PE=1 SV=2 |

|  | **Query** | **Observed** | **Mr(expt)** | **Mr(calc)** | **Delta** | **Miss** | **Score** | **Expect** | **Rank** | **Peptide** |
| --- | --- | --- | --- | --- | --- | --- | --- | --- | --- | --- |
|  | 6081 | 555.7212 | 1109.4278 | 1108.5587 | 0.8691 | 1 | (9) | 17 | 2 | R.MVDFAGVKDK.V 6075 |
|  | 6262 | **563.2819** | **1124.5492** | **1124.5536** | **-0.0044** | **1** | **18** | **2.3** | **1** | **R.MVDFAGVKDK.V** |
|  | 6533 | 573.7732 | 1145.5319 | 1145.5717 | -0.0397 | 1 | 5 | 55 | 9 | K.AIYKGPGSEAGP.- |
|  | 17413 | **902.9741** | **1803.9336** | **1803.9440** | **-0.0105** | **0** | **45** | **0.0034** | **1** | **R.YLPDTLLLEECGLLR.K** |
|  | 18920 | **641.9815** | **1922.9225** | **1923.1041** | **-0.1816** | **1** | **0** | **96** | **7** | **K.DKVTLVVGASQDIIPQLK.K** |
|  | 20132 | 675.9953 | 2024.9641 | 2023.9714 | 0.9927 | 1 | 56 | 0.00026 | 1 | K.KYDVDTLDMVFLDHWK.D |
|  | 26994 | **996.5082** | **2986.5027** | **2986.5222** | **-0.0195** | **0** | **26** | **0.15** | **1** | **K.IVDAVIQEHQPSVLLELGAYCGYSAVR.M** |
|  | 27820 | **795.1411** | **3176.5353** | **3176.5560** | **-0.0207** | **0** | **17** | **1** | **1** | **R.ILNHVLQHAEPGNAQSVLEAIDTYCEQK.E** |
|  | 29104 | 898.6563 | 3590.5961 | 3589.7583 | 0.8378 | 1 | 5 | 9.4 | 2 | K.EQRILNHVLQHAEPGNAQSVLEAIDTYCEQK.E |

| **488.** | Q02252\|MMSA_HUMAN    **Mass:** 58259    **Score:** 88     **Queries matched:** 18   **emPAI:** 0.13 |
| --- | --- |
|  | Methylmalonate-semialdehyde dehydrogenase [acylating], mitochondrial OS=Homo sapiens GN=ALDH6A1 PE=1 SV=2 |

|  | **Query** | **Observed** | **Mr(expt)** | **Mr(calc)** | **Delta** | **Miss** | **Score** | **Expect** | **Rank** | **Peptide** |
| --- | --- | --- | --- | --- | --- | --- | --- | --- | --- | --- |
|  | 4256 | 487.3202 | 972.6258 | 973.5127 | -0.8869 | 1 | 13 | 9.8 | 1 | K.RVQANMGAK.N |
|  | 4672 | 501.3358 | 1000.6569 | 1000.6281 | 0.0289 | 1 | 10 | 16 | 5 | R.ILQVSSKVK.S |
|  | 5039 | 515.3151 | 1028.6157 | 1029.5455 | -0.9298 | 0 | 9 | 20 | 8 | K.EGASILLDGR.K |
|  | 5401 | **529.3802** | **1056.7458** | **1056.6113** | **0.1345** | **0** | **3** | **74** | **8** | **-.MAALLAAAAVR.A** |
|  | 6032 | 553.7753 | 1105.5360 | 1105.5438 | -0.0078 | 0 | 10 | 19 | 1 | R.VCNLIDSGTK.E |
|  | 6705 | 579.8075 | 1157.6004 | 1157.6404 | -0.0400 | 1 | 12 | 11 | 7 | K.EGASILLDGRK.I |
|  | 7248 | **597.2896** | **1192.5647** | **1192.5724** | **-0.0077** | **0** | **25** | **0.44** | **1** | **K.TLADAEGDVFR.G** |
|  | 11518 | **710.8613** | **1419.7081** | **1419.7147** | **-0.0066** | **0** | **19** | **1.5** | **1** | **R.AFPAWADTSVLSR.Q** |
|  | 12244 | **733.3970** | **1464.7794** | **1464.7316** | **0.0478** | **0** | **4** | **54** | **4** | **R.CMALSTAVLVGEAK.K** 12252 |
|  | 19385 | **981.5117** | **1961.0088** | **1961.0218** | **-0.0130** | **0** | **47** | **0.0018** | **1** | **R.VNAGDQPGADLGPLITPQAK.E** 19362 19386 |
|  | 26951 | **994.1620** | **2979.4641** | **2978.4781** | **0.9861** | **1** | **51** | **0.00046** | **1** | **K.NHGVVMPDANKENTLNQLVGAAFGAAGQR.C** 26950 |
|  | 27984 | **806.9346** | **3223.7092** | **3223.7216** | **-0.0125** | **1** | **3** | **19** | **2** | **R.KYAHLVDVGQVGVNVPIPVPLPMFSFTGSR.S** |
|  | 31534 | **1105.7715** | **5523.8210** | **5524.7601** | **-0.9391** | **1** | **5** | **3.3** | **1** | **R.LPLGVCAGIAPFNFPAMIPLWMFPMAMVCGNTFLMKPSERVPGATMLLAK.L** |
|  | 31564 | **1112.3754** | **5556.8405** | **5556.7499** | **0.0906** | **1** | **(0)** | **8.6** | **2** | **R.LPLGVCAGIAPFNFPAMIPLWMFPMAMVCGNTFLMKPSERVPGATMLLAK.L** |

| **489.** | P61204\|ARF3_HUMAN    **Mass:** 20645    **Score:** 88     **Queries matched:** 20   **emPAI:** 1.35 |
| --- | --- |
|  | ADP-ribosylation factor 3 OS=Homo sapiens GN=ARF3 PE=1 SV=2 |

|  | **Query** | **Observed** | **Mr(expt)** | **Mr(calc)** | **Delta** | **Miss** | **Score** | **Expect** | **Rank** | **Peptide** |
| --- | --- | --- | --- | --- | --- | --- | --- | --- | --- | --- |
|  | 1887 | 398.2430 | 794.4715 | 794.4763 | -0.0048 | 0 | 17 | 3 | 1 | K.LGLHSLR.H 1886 |
|  | 2466 | **420.7607** | **839.5067** | **839.5130** | **-0.0062** | **0** | **6** | **33** | **1** | **K.IRPLWR.H** |
|  | 5774 | 544.3091 | 1086.6036 | 1086.6107 | -0.0071 | 0 | 28 | 0.26 | 1 | R.ILMVGLDAAGK.T 5773 |
|  | 5807 | **545.3157** | **1088.6169** | **1088.6230** | **-0.0061** | **0** | **7** | **34** | **2** | **R.DAVLLVFANK.Q** 5789 |
|  | 6035 | 553.8103 | 1105.6060 | 1105.5954 | 0.0107 | 0 | 21 | 1.4 | 2 | -.MGNIFGNLLK.S 6032 6036 |
|  | 8328 | 623.8091 | 1245.6036 | 1245.6135 | -0.0099 | 1 | 24 | 0.65 | 1 | R.VNEAREELMR.M |
|  | 13370 | 512.2930 | 1533.8573 | 1534.7847 | -0.9275 | 1 | 3 | 55 | 8 | K.EMRILMVGLDAAGK.T |
|  | 14803 | **815.8852** | **1629.7558** | **1629.7668** | **-0.0110** | **0** | **6** | **30** | **1** | **K.QDLPNAMNAAEITDK.L** |
|  | 14870 | **545.3008** | **1632.8805** | **1633.7803** | **-0.8998** | **1** | **9** | **14** | **3** | **R.EELMRMLAEDELR.D** |
|  | 17448 | 603.3307 | 1806.9704 | 1806.0325 | 0.9380 | 1 | 3 | 57 | 9 | R.ILMVGLDAAGKTTILYK.L |
|  | 20330 | **683.0316** | **2046.0729** | **2046.0819** | **-0.0091** | **1** | **46** | **0.0025** | **1** | **R.MLAEDELRDAVLLVFANK.Q** 20329 |
|  | 22859 | 1162.1183 | 2322.2220 | 2322.2359 | -0.0139 | 0 | 3 | 41 | 1 | K.LGEIVTTIPTIGFNVETVEYK.N |
|  | 23685 | 813.3947 | 2437.1623 | 2437.1775 | -0.0152 | 1 | 25 | 0.24 | 1 | R.HYFQNTQGLIFVVDSNDRER.V |
|  | 24652 | 855.4747 | 2563.4024 | 2563.4149 | -0.0125 | 1 | 33 | 0.03 | 1 | K.LKLGEIVTTIPTIGFNVETVEYK.N |

| **490.** | P84077\|ARF1_HUMAN    **Mass:** 20741    **Score:** 88     **Queries matched:** 18   **emPAI:** 1.34 |
| --- | --- |
|  | ADP-ribosylation factor 1 OS=Homo sapiens GN=ARF1 PE=1 SV=2 |

|  | **Query** | **Observed** | **Mr(expt)** | **Mr(calc)** | **Delta** | **Miss** | **Score** | **Expect** | **Rank** | **Peptide** |
| --- | --- | --- | --- | --- | --- | --- | --- | --- | --- | --- |
|  | 1887 | 398.2430 | 794.4715 | 794.4763 | -0.0048 | 0 | 17 | 3 | 1 | K.LGLHSLR.H 1886 |
|  | 2466 | 420.7607 | 839.5067 | 839.5130 | -0.0062 | 0 | 6 | 33 | 1 | K.IRPLWR.H |
|  | 5774 | 544.3091 | 1086.6036 | 1086.6107 | -0.0071 | 0 | 28 | 0.26 | 1 | R.ILMVGLDAAGK.T 5773 |
|  | 5807 | 545.3157 | 1088.6169 | 1088.6230 | -0.0061 | 0 | 7 | 34 | 2 | R.DAVLLVFANK.Q 5789 |
|  | 8328 | 623.8091 | 1245.6036 | 1245.6135 | -0.0099 | 1 | 24 | 0.65 | 1 | R.VNEAREELMR.M |
|  | 13246 | 763.8704 | 1525.7263 | 1524.8453 | 0.8810 | 1 | 1 | 89 | 8 | M.GNIFANLFKGLFGK.K |
|  | 13370 | 512.2930 | 1533.8573 | 1534.7847 | -0.9275 | 1 | 3 | 55 | 8 | K.EMRILMVGLDAAGK.T |
|  | 14803 | 815.8852 | 1629.7558 | 1629.7668 | -0.0110 | 0 | 6 | 30 | 1 | K.QDLPNAMNAAEITDK.L |
|  | 14870 | 545.3008 | 1632.8805 | 1633.7803 | -0.8998 | 1 | 9 | 14 | 3 | R.EELMRMLAEDELR.D |
|  | 17448 | 603.3307 | 1806.9704 | 1806.0325 | 0.9380 | 1 | 3 | 57 | 9 | R.ILMVGLDAAGKTTILYK.L |
|  | 20330 | 683.0316 | 2046.0729 | 2046.0819 | -0.0091 | 1 | 46 | 0.0025 | 1 | R.MLAEDELRDAVLLVFANK.Q 20329 |
|  | 22859 | 1162.1183 | 2322.2220 | 2322.2359 | -0.0139 | 0 | 3 | 41 | 1 | K.LGEIVTTIPTIGFNVETVEYK.N |
|  | 23685 | 813.3947 | 2437.1623 | 2437.1775 | -0.0152 | 1 | 25 | 0.24 | 1 | R.HYFQNTQGLIFVVDSNDRER.V |
|  | 24652 | 855.4747 | 2563.4024 | 2563.4149 | -0.0125 | 1 | 33 | 0.03 | 1 | K.LKLGEIVTTIPTIGFNVETVEYK.N |

| **491.** | P09417\|DHPR_HUMAN    **Mass:** 26001    **Score:** 88     **Queries matched:** 9   **emPAI:** 0.31 |
| --- | --- |
|  | Dihydropteridine reductase OS=Homo sapiens GN=QDPR PE=1 SV=2 |

|  | **Query** | **Observed** | **Mr(expt)** | **Mr(calc)** | **Delta** | **Miss** | **Score** | **Expect** | **Rank** | **Peptide** |
| --- | --- | --- | --- | --- | --- | --- | --- | --- | --- | --- |
|  | 4485 | 495.7585 | 989.5025 | 988.4760 | 1.0265 | 0 | 13 | 9.1 | 7 | -.MAAAAAAGEAR.R 4468 |
|  | 10604 | 684.8312 | 1367.6479 | 1367.6980 | -0.0500 | 0 | 38 | 0.019 | 1 | K.GAVHQLCQSLAGK.N 10603 |
|  | 11286 | 704.3681 | 1406.7216 | 1406.8245 | -0.1029 | 1 | 5 | 39 | 8 | K.HLKEGGLLTLAGAK.A |
|  | 18356 | 469.4928 | 1873.9420 | 1872.9516 | 0.9904 | 1 | 3 | 54 | 7 | K.VDAILCVAGGWAGGNAKSK.S |
|  | 19314 | **978.9398** | **1955.8650** | **1955.8783** | **-0.0133** | **0** | **73** | **4.2e-006** | **1** | **K.MTDSFTEQADQVTAEVGK.L** 19315 |
|  | 24565 | 851.9597 | 2552.8572 | 2552.3091 | 0.5481 | 1 | 2 | 27 | 2 | K.NSGMPPGAAAIAVLPVTLDTPMNRK.S |

| **492.** | P30046\|DOPD_HUMAN    **Mass:** 12818    **Score:** 87     **Queries matched:** 11   **emPAI:** 1.24 |
| --- | --- |
|  | D-dopachrome decarboxylase OS=Homo sapiens GN=DDT PE=1 SV=3 |

|  | **Query** | **Observed** | **Mr(expt)** | **Mr(calc)** | **Delta** | **Miss** | **Score** | **Expect** | **Rank** | **Peptide** |
| --- | --- | --- | --- | --- | --- | --- | --- | --- | --- | --- |
|  | 10297 | **676.3519** | **1350.6893** | **1350.6972** | **-0.0079** | **0** | **41** | **0.011** | **1** | **R.FFPLESWQIGK.I** 10296 |
|  | 12817 | 750.3912 | 1498.7678 | 1498.7780 | -0.0102 | 0 | 64 | 4.5e-005 | 1 | M.PFLELDTNLPANR.V |
|  | 13028 | **757.3880** | **1512.7614** | **1512.8082** | **-0.0468** | **0** | **10** | **11** | **3** | **R.LCAAAASILGKPADR.V** 13036 |
|  | 15064 | 549.2968 | 1644.8686 | 1645.8134 | -0.9448 | 0 | 10 | 12 | 2 | -.MPFLELDTNLPANR.V |
|  | 15426 | **557.6211** | **1669.8416** | **1668.9093** | **0.9323** | **1** | **4** | **42** | **1** | **K.RLCAAAASILGKPADR.V** |
|  | 17925 | **616.3118** | **1845.9135** | **1846.0505** | **-0.1370** | **1** | **3** | **52** | **4** | **R.ILIRFFPLESWQIGK.I** |
|  | 21764 | **732.0623** | **2193.1650** | **2193.1793** | **-0.0144** | **1** | **32** | **0.053** | **1** | **M.PFLELDTNLPANRVPAGLEK.R** |
|  | 23004 | **781.3394** | **2340.9963** | **2340.2147** | **0.7815** | **1** | **0** | **64** | **8** | **-.MPFLELDTNLPANRVPAGLEK.R** |
|  | 31468 | 1789.6014 | 5365.7825 | 5366.7385 | -0.9560 | 1 | 1 | 6.8 | 2 | R.LCAAAASILGKPADRVNVTVRPGLAMALSGSTEPCAQLSISSIGVVGTAEDNR.S |

| **493.** | O14579\|COPE_HUMAN    **Mass:** 34688    **Score:** 87     **Queries matched:** 6   **emPAI:** 0.23 |
| --- | --- |
|  | Coatomer subunit epsilon OS=Homo sapiens GN=COPE PE=1 SV=3 |

|  | **Query** | **Observed** | **Mr(expt)** | **Mr(calc)** | **Delta** | **Miss** | **Score** | **Expect** | **Rank** | **Peptide** |
| --- | --- | --- | --- | --- | --- | --- | --- | --- | --- | --- |
|  | 16457 | **867.9008** | **1733.7871** | **1733.7970** | **-0.0100** | **0** | **11** | **9** | **1** | **K.LQDAYYIFQEMADK.C** |
|  | 20520 | **1031.4691** | **2060.9237** | **2060.9374** | **-0.0137** | **0** | **74** | **3.5e-006** | **1** | **K.NAFYIGSYQQCINEAQR.V** |
|  | 22549 | 761.7567 | 2282.2483 | 2282.2634 | -0.0151 | 1 | 35 | 0.026 | 1 | R.KFGVVLDEIKPSSAPELQAVR.M |
|  | 30656 | 1072.6031 | 4286.3835 | 4285.9685 | 0.4150 | 1 | 1 | 17 | 8 | -.MAPPAPGPASGGSGEVDELFDVKNAFYIGSYQQCINEAQR.V |
|  | 30945 | **906.8723** | **4529.3252** | **4529.3442** | **-0.0190** | **1** | **18** | **0.27** | **1** | **R.WEAAEGLLQEALDKDSGYPETLVNLIVLSQHLGKPPEVTNR.Y** 30947 |

| **494.** | P35754\|GLRX1_HUMAN    **Mass:** 12053    **Score:** 87     **Queries matched:** 3   **emPAI:** 0.33 |
| --- | --- |
|  | Glutaredoxin-1 OS=Homo sapiens GN=GLRX PE=1 SV=2 |

|  | **Query** | **Observed** | **Mr(expt)** | **Mr(calc)** | **Delta** | **Miss** | **Score** | **Expect** | **Rank** | **Peptide** |
| --- | --- | --- | --- | --- | --- | --- | --- | --- | --- | --- |
|  | 4541 | 497.7600 | 993.5054 | 994.4542 | -0.9488 | 0 | 6 | 36 | 10 | M.AQEFVNCK.I |
|  | 15134 | **550.8157** | **1649.4252** | **1648.8065** | **0.6187** | **1** | **11** | **6.1** | **1** | **-.MAQEFVNCKIQPGK.V** |
|  | 28183 | **1096.8823** | **3287.6251** | **3287.6422** | **-0.0171** | **0** | **87** | **1e-007** | **1** | **K.QGLLEFVDITATNHTNEIQDYLQQLTGAR.T** |

| **495.** | Q92930\|RAB8B_HUMAN    **Mass:** 23740    **Score:** 86     **Queries matched:** 14   **emPAI:** 0.35 |
| --- | --- |
|  | Ras-related protein Rab-8B OS=Homo sapiens GN=RAB8B PE=1 SV=2 |

|  | **Query** | **Observed** | **Mr(expt)** | **Mr(calc)** | **Delta** | **Miss** | **Score** | **Expect** | **Rank** | **Peptide** |
| --- | --- | --- | --- | --- | --- | --- | --- | --- | --- | --- |
|  | 1888 | **398.6815** | **795.3485** | **794.4174** | **0.9311** | **0** | **10** | **16** | **2** | **K.FLETSAK.S** |
|  | 5578 | 536.3207 | 1070.6269 | 1070.6336 | -0.0066 | 0 | 53 | 0.00096 | 1 | K.LLLIGDSGVGK.T 5577 |
|  | 9177 | 431.5562 | 1291.6469 | 1291.6673 | -0.0204 | 1 | 11 | 11 | 6 | K.SFDNIKNWIR.N |
|  | 9225 | 432.2579 | 1293.7519 | 1294.6267 | -0.8749 | 1 | 11 | 11 | 1 | -.MAKTYDYLFK.L |
|  | 9422 | **435.7346** | **1304.1819** | **1303.5827** | **0.5992** | **0** | **3** | **45** | **6** | **K.MNDSNSAGAGGPVK.I** |
|  | 9635 | 658.8289 | 1315.6433 | 1315.6521 | -0.0088 | 0 | 60 | 0.00015 | 1 | K.LQIWDTAGQER.F 9612 9633 9634 |
|  | 10908 | 693.3495 | 1384.6844 | 1384.6219 | 0.0626 | 0 | 10 | 14 | 2 | R.NIEEHASSDVER.M |
|  | 11963 | 483.2732 | 1446.7977 | 1447.6725 | -0.8749 | 1 | 11 | 10 | 1 | R.KMNDSNSAGAGGPVK.I 11971 |
|  | 26759 | **589.3096** | **2941.5118** | **2942.4525** | **-0.9407** | **1** | **2** | **35** | **3** | **K.TCLLFRFSEDAFNTTFISTIGIDFK.I** |

| **496.** | Q15286\|RAB35_HUMAN    **Mass:** 23296    **Score:** 86     **Queries matched:** 8   **emPAI:** 0.35 |
| --- | --- |
|  | Ras-related protein Rab-35 OS=Homo sapiens GN=RAB35 PE=1 SV=1 |

|  | **Query** | **Observed** | **Mr(expt)** | **Mr(calc)** | **Delta** | **Miss** | **Score** | **Expect** | **Rank** | **Peptide** |
| --- | --- | --- | --- | --- | --- | --- | --- | --- | --- | --- |
|  | 5346 | **527.3516** | **1052.6887** | **1052.5026** | **0.1861** | **0** | **1** | **1.1e+002** | **4** | **K.VVETEDAYK.F** |
|  | 5578 | 536.3207 | 1070.6269 | 1070.6336 | -0.0066 | 0 | 53 | 0.00096 | 1 | K.LLIIGDSGVGK.S 5577 |
|  | 9635 | 658.8289 | 1315.6433 | 1315.6521 | -0.0088 | 0 | 60 | 0.00015 | 1 | K.LQIWDTAGQER.F 9612 9633 9634 |
|  | 19962 | 672.3132 | 2013.9179 | 2013.8898 | 0.0281 | 1 | 3 | 48 | 6 | K.RWLHEINQNCDDVCR.I |

| **497.** | P59190\|RAB15_HUMAN    **Mass:** 24660    **Score:** 86     **Queries matched:** 7   **emPAI:** 0.33 |
| --- | --- |
|  | Ras-related protein Rab-15 OS=Homo sapiens GN=RAB15 PE=1 SV=1 |

|  | **Query** | **Observed** | **Mr(expt)** | **Mr(calc)** | **Delta** | **Miss** | **Score** | **Expect** | **Rank** | **Peptide** |
| --- | --- | --- | --- | --- | --- | --- | --- | --- | --- | --- |
|  | 5578 | 536.3207 | 1070.6269 | 1070.6336 | -0.0066 | 0 | 53 | 0.00096 | 1 | R.LLLIGDSGVGK.T 5577 |
|  | 9635 | 658.8289 | 1315.6433 | 1315.6521 | -0.0088 | 0 | 60 | 0.00015 | 1 | R.IQIWDTAGQER.Y 9612 9633 9634 |
|  | 26402 | 722.5912 | 2886.3357 | 2885.3712 | 0.9645 | 1 | 1 | 50 | 8 | R.MRASNELALAELEEEEGKPEGPANSSK.T |

| **498.** | Q14032\|BAAT_HUMAN    **Mass:** 46441    **Score:** 86     **Queries matched:** 19   **emPAI:** 0.47 |
| --- | --- |
|  | Bile acid-CoA:amino acid N-acyltransferase OS=Homo sapiens GN=BAAT PE=1 SV=1 |

|  | **Query** | **Observed** | **Mr(expt)** | **Mr(calc)** | **Delta** | **Miss** | **Score** | **Expect** | **Rank** | **Peptide** |
| --- | --- | --- | --- | --- | --- | --- | --- | --- | --- | --- |
|  | 1626 | **717.3734** | **716.3661** | **716.4181** | **-0.0519** | **0** | **5** | **80** | **4** | **R.ASLLASR.G** |
|  | 5290 | **524.7789** | **1047.5432** | **1047.5502** | **-0.0070** | **0** | **10** | **15** | **1** | **R.WYVAPGVTR.I** |
|  | 6241 | **562.2491** | **1122.4837** | **1121.6081** | **0.8757** | **0** | **4** | **64** | **3** | **K.HLIPDVTSQL.-** |
|  | 9137 | **430.8546** | **1289.5419** | **1288.7292** | **0.8127** | **1** | **8** | **21** | **8** | **R.WYVAPGVTRIK.V** 9126 9132 9134 9143 |
|  | 9722 | **661.3647** | **1320.7149** | **1320.7262** | **-0.0112** | **1** | **46** | **0.0033** | **1** | **K.AHAEQAIGQLKR.H** 9764 |
|  | 12181 | **730.8827** | **1459.7509** | **1459.7606** | **-0.0096** | **0** | **31** | **0.1** | **1** | **R.DVMNRPFQVQVK.L** |
|  | 20509 | 687.3582 | 2059.0526 | 2058.1473 | 0.9053 | 0 | 1 | 66 | 7 | M.IQLTATPVSALVDEPVHIR.A |
|  | 21265 | **535.0018** | **2135.9782** | **2136.0653** | **-0.0871** | **0** | **9** | **10** | **2** | **R.LHWGGEVIPHAAAQEHAWK.E** |
|  | 21872 | 736.0584 | 2205.1532 | 2205.1827 | -0.0295 | 0 | 1 | 62 | 3 | -.MIQLTATPVSALVDEPVHIR.A |
|  | 22553 | **762.0513** | **2283.1322** | **2283.1423** | **-0.0101** | **0** | **8** | **11** | **2** | **R.KPEVTDLEYFEEAANFLLR.H** |
|  | 27164 | 1010.8386 | 3029.4939 | 3029.6195 | -0.1256 | 1 | 0 | 48 | 2 | R.HPKVFGSGVGVVSVCQGVQIGLSMAIYLK.Q |
|  | 29748 | **958.9522** | **3831.7797** | **3831.8025** | **-0.0228** | **0** | **19** | **0.42** | **1** | **R.ANEFGEVDLNHASSLGGDYMGVHPMGLFWSLKPEK.L** 29749 |
|  | 30278 | **1364.6681** | **4090.9824** | **4092.0368** | **-1.0544** | **1** | **51** | **0.00023** | **1** | **R.TFETTQVGASQYLFPIEEAQGQFLFIVGEGDKTINSK.A** |

| **499.** | P02763\|A1AG1_HUMAN    **Mass:** 23725    **Score:** 86     **Queries matched:** 10   **emPAI:** 0.81 |
| --- | --- |
|  | Alpha-1-acid glycoprotein 1 OS=Homo sapiens GN=ORM1 PE=1 SV=1 |

|  | **Query** | **Observed** | **Mr(expt)** | **Mr(calc)** | **Delta** | **Miss** | **Score** | **Expect** | **Rank** | **Peptide** |
| --- | --- | --- | --- | --- | --- | --- | --- | --- | --- | --- |
|  | 6103 | **556.7628** | **1111.5111** | **1111.5186** | **-0.0075** | **0** | **14** | **5.8** | **1** | **K.SDVVYTDWK.K** |
|  | 6720 | **580.7950** | **1159.5754** | **1159.5815** | **-0.0060** | **0** | **15** | **5.5** | **1** | **K.WFYIASAFR.N** |
|  | 8215 | **620.8090** | **1239.6034** | **1239.6136** | **-0.0102** | **1** | **45** | **0.0043** | **1** | **K.SDVVYTDWKK.D** 8216 |
|  | 11920 | 723.3652 | 1444.7159 | 1444.6544 | 0.0615 | 0 | 5 | 44 | 8 | K.TYMLAFDVNDEK.N |
|  | 16568 | **871.9016** | **1741.7887** | **1741.7981** | **-0.0094** | **0** | **29** | **0.13** | **1** | **K.EQLGEFYEALDCLR.I** |
|  | 16698 | **876.9752** | **1751.9359** | **1751.9471** | **-0.0112** | **0** | **42** | **0.0072** | **1** | **R.YVGGQEHFAHLLILR.D** 16699 |
|  | 27675 | **1046.1648** | **3135.4725** | **3134.4906** | **0.9819** | **1** | **35** | **0.015** | **1** | **K.TYMLAFDVNDEKNWGLSVYADKPETTK.E** 27674 |

| **500.** | P14550\|AK1A1_HUMAN    **Mass:** 36892    **Score:** 85     **Queries matched:** 10   **emPAI:** 0.21 |
| --- | --- |
|  | Alcohol dehydrogenase [NADP+] OS=Homo sapiens GN=AKR1A1 PE=1 SV=3 |

|  | **Query** | **Observed** | **Mr(expt)** | **Mr(calc)** | **Delta** | **Miss** | **Score** | **Expect** | **Rank** | **Peptide** |
| --- | --- | --- | --- | --- | --- | --- | --- | --- | --- | --- |
|  | 1571 | **701.7840** | **700.7767** | **700.3392** | **0.4376** | **0** | **6** | **37** | **4** | **K.EDVGPGK.A** |
|  | 2652 | 428.7634 | 855.5122 | 856.5204 | -1.0082 | 1 | 12 | 10 | 8 | R.KVICIPK.S 2679 2687 |
|  | 11033 | **464.7524** | **1391.2354** | **1390.7643** | **0.4712** | **1** | **10** | **9.2** | **2** | **R.YIVPMLTVDGKR.V** |
|  | 12413 | **492.2529** | **1473.7368** | **1474.7892** | **-1.0524** | **0** | **5** | **45** | **6** | **K.GLVQALGLSNFNSR.Q** |
|  | 13623 | 776.3807 | 1550.7468 | 1550.7576 | -0.0108 | 0 | 56 | 0.00033 | 1 | R.GLEVTAYSPLGSSDR.A 13622 |
|  | 14280 | 797.8849 | 1593.7552 | 1593.8402 | -0.0850 | 1 | 8 | 19 | 5 | R.EELFVTSKLWNTK.H |
|  | 24136 | **830.7789** | **2489.3150** | **2488.3213** | **0.9937** | **1** | **33** | **0.034** | **1** | **R.AWRDPDEPVLLEEPVVLALAEK.Y** |

| **501.** | P45954\|ACDSB_HUMAN    **Mass:** 47797    **Score:** 85     **Queries matched:** 12   **emPAI:** 0.46 |
| --- | --- |
|  | Short/branched chain specific acyl-CoA dehydrogenase, mitochondrial OS=Homo sapiens GN=ACADSB PE=1 SV=1 |

|  | **Query** | **Observed** | **Mr(expt)** | **Mr(calc)** | **Delta** | **Miss** | **Score** | **Expect** | **Rank** | **Peptide** |
| --- | --- | --- | --- | --- | --- | --- | --- | --- | --- | --- |
|  | 1821 | **395.8958** | **789.7771** | **790.4007** | **-0.6236** | **0** | **14** | **6.1** | **2** | **-.MEGLAVR.L** |
|  | 3551 | **461.2583** | **920.5021** | **920.5079** | **-0.0058** | **0** | **43** | **0.008** | **1** | **R.LLTYNAAR.L** |
|  | 11224 | **468.8766** | **1403.6079** | **1404.7361** | **-1.1283** | **0** | **2** | **74** | **6** | **R.DTPGLHIGKPENK.L** |
|  | 11491 | **709.8385** | **1417.6624** | **1417.6725** | **-0.0101** | **0** | **50** | **0.0014** | **1** | **K.YYASEIAGQTTSK.C** |
|  | 19907 | 1004.4809 | 2006.9472 | 2006.9619 | -0.0146 | 0 | 40 | 0.0092 | 1 | K.FAQEQIAPLVSTMDENSK.M 19908 |
|  | 20094 | **675.0039** | **2021.9897** | **2022.9568** | **-0.9671** | **0** | **(3)** | **50** | **2** | **K.FAQEQIAPLVSTMDENSK.M** |
|  | 23414 | 798.7485 | 2393.2236 | 2393.2703 | -0.0467 | 1 | 10 | 7.3 | 2 | K.GITSFLVDRDTPGLHIGKPENK.L |
|  | 24049 | **827.0946** | **2478.2620** | **2478.2768** | **-0.0148** | **0** | **21** | **0.61** | **1** | **R.LFDFQGLQHQVAHVATQLEAAR.L** |
|  | 25781 | **557.0758** | **2780.3427** | **2781.3506** | **-1.0079** | **0** | **3** | **29** | **1** | **K.MWISSAEHAGLFLVMANVDPTIGYK.G** |
|  | 27165 | **1010.8435** | **3029.5087** | **3029.5280** | **-0.0193** | **1** | **33** | **0.027** | **1** | **R.ASSTCPLTFENVKVPEANILGQIGHGYK.Y** |
|  | 30934 | 1129.5757 | 4514.2736 | 4515.2805 | -1.0069 | 1 | 0 | 20 | 3 | K.MEKSVIQGLFQQGLMGIEVDPEYGGTGASFLSTVLVIEELAK.V |

| **502.** | P61619\|S61A1_HUMAN    **Mass:** 52687    **Score:** 85     **Queries matched:** 13   **emPAI:** 0.15 |
| --- | --- |
|  | Protein transport protein Sec61 subunit alpha isoform 1 OS=Homo sapiens GN=SEC61A1 PE=1 SV=2 |

|  | **Query** | **Observed** | **Mr(expt)** | **Mr(calc)** | **Delta** | **Miss** | **Score** | **Expect** | **Rank** | **Peptide** |
| --- | --- | --- | --- | --- | --- | --- | --- | --- | --- | --- |
|  | 1951 | **801.4768** | **800.4695** | **800.4756** | **-0.0061** | **1** | **10** | **22** | **6** | **K.DVAKQLK.E** |
|  | 3555 | 461.7164 | 921.4182 | 920.4208 | 0.9974 | 0 | 13 | 9.1 | 2 | K.EQQMVMR.G |
|  | 5721 | **542.3458** | **1082.6770** | **1082.5396** | **0.1373** | **0** | **6** | **36** | **3** | **R.GQYNTYPIK.L** |
|  | 8258 | **621.8341** | **1241.6535** | **1241.6616** | **-0.0080** | **1** | **33** | **0.071** | **1** | **K.IIEVGDTPKDR.A** 8259 8272 |
|  | 10575 | **456.2896** | **1365.8468** | **1366.6439** | **-0.7970** | **0** | **4** | **44** | **9** | **K.EQSEVGSMGALLF.-** |
|  | 14067 | 527.9258 | 1580.7555 | 1580.7478 | 0.0078 | 1 | 4 | 50 | 3 | R.GHRETSMVHELNR.Y |
|  | 19682 | **663.6894** | **1988.0463** | **1988.0553** | **-0.0090** | **0** | **10** | **9.2** | **1** | **R.GMEFEGAIIALFHLLATR.T** |
|  | 22852 | **1161.5840** | **2321.1534** | **2321.1652** | **-0.0118** | **0** | **58** | **0.00011** | **1** | **R.FSGNLLVSLLGTWSDTSSGGPAR.A** |
|  | 23840 | **819.1263** | **2454.3570** | **2454.3708** | **-0.0138** | **0** | **46** | **0.0014** | **1** | **K.FLEVIKPFCVILPEIQKPER.K** |
|  | 24297 | 838.1482 | 2511.4229 | 2512.4008 | -0.9779 | 0 | 7 | 10 | 2 | R.GTLMELGISPIVTSGLIMQLLAGAK.I |
|  | 24388 | **633.3130** | **2529.2229** | **2528.3958** | **0.8271** | **0** | **(2)** | **45** | **7** | **R.GTLMELGISPIVTSGLIMQLLAGAK.I** |

| **503.** | Q07020\|RL18_HUMAN    **Mass:** 21735    **Score:** 85     **Queries matched:** 12   **emPAI:** 0.91 |
| --- | --- |
|  | 60S ribosomal protein L18 OS=Homo sapiens GN=RPL18 PE=1 SV=2 |

|  | **Query** | **Observed** | **Mr(expt)** | **Mr(calc)** | **Delta** | **Miss** | **Score** | **Expect** | **Rank** | **Peptide** |
| --- | --- | --- | --- | --- | --- | --- | --- | --- | --- | --- |
|  | 1456 | 653.3032 | 652.2959 | 652.3367 | -0.0407 | 0 | 14 | 4.9 | 4 | R.LFMSR.T 1457 |
|  | 1569 | **700.4302** | **699.4229** | **698.3963** | **1.0266** | **0** | **13** | **9.9** | **8** | **R.VQEVPK.L** |
|  | 1627 | 717.4199 | 716.4126 | 716.4003 | 0.0123 | 1 | 6 | 59 | 4 | K.MKLPGR.E |
|  | 2684 | 429.7533 | 857.4920 | 858.5109 | -1.0189 | 1 | 17 | 4.2 | 2 | K.LKVCALR.V |
|  | 6469 | **570.8239** | **1139.6333** | **1139.6411** | **-0.0078** | **0** | **24** | **0.61** | **1** | **R.TNRPPLSLSR.M** |
|  | 8423 | 625.8359 | 1249.6572 | 1249.6667 | -0.0094 | 0 | 33 | 0.081 | 1 | R.TNSTFNQVVLK.R |
|  | 10191 | **673.3651** | **1344.7155** | **1344.7249** | **-0.0094** | **0** | **70** | **1.6e-005** | **1** | **K.TAVVVGTITDDVR.V** 10193 |
|  | 11271 | **703.8860** | **1405.7575** | **1405.7678** | **-0.0102** | **1** | **44** | **0.0048** | **1** | **R.TNSTFNQVVLKR.L** |
|  | 11287 | 469.9146 | 1406.7218 | 1405.7678 | 0.9541 | 1 | 6 | 36 | 8 | R.RTNSTFNQVVLK.R |
|  | 12184 | 730.8988 | 1459.7830 | 1459.7922 | -0.0092 | 0 | 23 | 0.62 | 1 | K.ILTFDQLALDSPK.G |

| **504.** | Q9Y3E5\|PTH2_HUMAN    **Mass:** 19466    **Score:** 85     **Queries matched:** 8   **emPAI:** 0.44 |
| --- | --- |
|  | Peptidyl-tRNA hydrolase 2, mitochondrial OS=Homo sapiens GN=PTRH2 PE=1 SV=1 |

|  | **Query** | **Observed** | **Mr(expt)** | **Mr(calc)** | **Delta** | **Miss** | **Score** | **Expect** | **Rank** | **Peptide** |
| --- | --- | --- | --- | --- | --- | --- | --- | --- | --- | --- |
|  | 2036 | **805.4545** | **804.4473** | **804.4164** | **0.0309** | **1** | **12** | **14** | **5** | **R.NDLKMGK.G** |
|  | 12199 | **731.8538** | **1461.6930** | **1461.7034** | **-0.0105** | **0** | **59** | **0.00016** | **1** | **K.VAAQCSHAAVSAYK.Q** |
|  | 12393 | 737.4042 | 1472.7939 | 1472.8021 | -0.0082 | 0 | 31 | 0.094 | 3 | K.MLGLTVSLIQDAGR.T |
|  | 12688 | **745.8578** | **1489.7010** | **1488.7970** | **0.9040** | **0** | **(2)** | **92** | **3** | **K.MLGLTVSLIQDAGR.T** |
|  | 14239 | **531.2913** | **1590.8520** | **1590.8617** | **-0.0097** | **0** | **50** | **0.001** | **1** | **K.APDEETLIALLAHAK.M** |
|  | 18930 | 962.9875 | 1923.9604 | 1922.8655 | 1.0949 | 1 | 0 | 88 | 4 | R.NPEMLKQWEYCGQPK.V |
|  | 19646 | **662.9503** | **1985.8290** | **1987.0058** | **-1.1768** | **1** | **5** | **27** | **2** | **K.VAAQCSHAAVSAYKQIQR.R** |
|  | 29395 | **1246.9877** | **3737.9412** | **3738.8064** | **-0.8652** | **1** | **2** | **22** | **9** | **-.MPSKSLVMEYLAHPSTLGLAVGVACGMCLGWSLR.V** |

| **505.** | O43175\|SERA_HUMAN    **Mass:** 57356    **Score:** 85     **Queries matched:** 14   **emPAI:** 0.13 |
| --- | --- |
|  | D-3-phosphoglycerate dehydrogenase OS=Homo sapiens GN=PHGDH PE=1 SV=4 |

|  | **Query** | **Observed** | **Mr(expt)** | **Mr(calc)** | **Delta** | **Miss** | **Score** | **Expect** | **Rank** | **Peptide** |
| --- | --- | --- | --- | --- | --- | --- | --- | --- | --- | --- |
|  | 2231 | **411.4184** | **820.8222** | **821.4218** | **-0.5995** | **0** | **8** | **20** | **3** | **-.MAFANLR.K** |
|  | 3244 | **450.2844** | **898.5542** | **898.5600** | **-0.0058** | **0** | **35** | **0.05** | **1** | **K.TLGILGLGR.I** |
|  | 4576 | **498.8540** | **995.6935** | **995.4746** | **0.2189** | **0** | **8** | **19** | **1** | **K.FMGTELNGK.T** |
|  | 5626 | **538.3018** | **1074.5890** | **1073.5539** | **1.0351** | **0** | **2** | **1.1e+002** | **1** | **R.QIPQATASMK.D** |
|  | 6481 | **571.3156** | **1140.6167** | **1139.5645** | **1.0522** | **1** | **5** | **45** | **7** | **K.KFMGTELNGK.T** |
|  | 10698 | **687.3739** | **1372.7332** | **1373.6973** | **-0.9640** | **1** | **(1)** | **1.2e+002** | **5** | **R.QIPQATASMKDGK.W** |
|  | 11007 | **464.2632** | **1389.7676** | **1389.6922** | **0.0754** | **1** | **4** | **55** | **10** | **R.QIPQATASMKDGK.W** |
|  | 12593 | 743.3754 | 1484.7363 | 1485.7787 | -1.0424 | 1 | 9 | 17 | 3 | K.EASKQADVNLVNAK.L 12594 |
|  | 12656 | **744.8632** | **1487.7118** | **1487.7216** | **-0.0098** | **0** | **84** | **5.5e-007** | **1** | **R.AGTGVDNVDLEAATR.K** |
|  | 17109 | 891.4844 | 1780.9542 | 1781.9999 | -1.0457 | 1 | 2 | 63 | 8 | K.VTADVINAAEKLQVVGR.A |
|  | 17324 | 899.5150 | 1797.0155 | 1797.9519 | -0.9365 | 1 | 2 | 67 | 5 | R.VVNCARGGIVDEGALLR.A |
|  | 18253 | **934.4705** | **1866.9264** | **1868.0367** | **-1.1103** | **1** | **8** | **16** | **2** | **K.ILQDGGLQVVEKQNLSK.E** |
|  | 30251 | 1361.0203 | 4080.0390 | 4080.9597 | -0.9207 | 1 | 1 | 20 | 7 | K.GILVMNTPNGNSLSAAELTCGMIMCLARQIPQATASMK.D |

| **506.** | P11182\|ODB2_HUMAN    **Mass:** 53852    **Score:** 84     **Queries matched:** 15   **emPAI:** 0.14 |
| --- | --- |
|  | Lipoamide acyltransferase component of branched-chain alpha-keto acid dehydrogenase complex, mitochondrial OS=Homo sapiens GN=DBT PE=1 SV=3 |

|  | **Query** | **Observed** | **Mr(expt)** | **Mr(calc)** | **Delta** | **Miss** | **Score** | **Expect** | **Rank** | **Peptide** |
| --- | --- | --- | --- | --- | --- | --- | --- | --- | --- | --- |
|  | 2952 | **438.5862** | **875.1579** | **874.4760** | **0.6819** | **0** | **20** | **1.4** | **2** | **K.LSEVVGSGK.D** |
|  | 3936 | **475.2847** | **948.5549** | **948.4963** | **0.0586** | **1** | **6** | **44** | **9** | **R.MLRTWSR.N** |
|  | 4132 | 482.7554 | 963.4963 | 962.5154 | 0.9810 | 1 | 6 | 41 | 8 | -.MAAVRMLR.T 4103 4122 |
|  | 6071 | 555.2916 | 1108.5686 | 1107.6288 | 0.9398 | 0 | 7 | 31 | 3 | K.QTGAILPPSPK.V |
|  | 6118 | **557.3370** | **1112.6595** | **1111.5662** | **1.0933** | **1** | **26** | **0.37** | **1** | **R.FNQKGEVYK.A** |
|  | 6807 | 583.8150 | 1165.6154 | 1165.6199 | -0.0044 | 1 | (1) | 1.1e+002 | 7 | K.AMVKTMSAALK.I |
|  | 7004 | **592.2828** | **1182.5511** | **1181.6148** | **0.9363** | **1** | **2** | **82** | **3** | **K.AMVKTMSAALK.I** |
|  | 12697 | **497.6175** | **1489.8306** | **1489.8391** | **-0.0086** | **1** | **33** | **0.072** | **1** | **R.ILKEDILNYLEK.Q** |
|  | 13301 | **765.3982** | **1528.7819** | **1529.7045** | **-0.9225** | **0** | **5** | **38** | **7** | **K.AQIMNVSWSADHR.V** |
|  | 15196 | **827.4280** | **1652.8414** | **1652.8483** | **-0.0069** | **0** | **86** | **2.8e-007** | **1** | **K.SYLENPAFMLLDLK.-** 15197 |
|  | 16556 | **436.1608** | **1740.6140** | **1739.8665** | **0.7475** | **1** | **6** | **18** | **5** | **R.VIDGATMSRFSNLWK.S** |
|  | 17912 | **923.0162** | **1844.0178** | **1844.0118** | **0.0060** | **0** | **7** | **19** | **2** | **K.DMTVPILVSKPPVFTGK.D** |

| **507.** | P30508\|1C12_HUMAN    **Mass:** 41316    **Score:** 84     **Queries matched:** 13   **emPAI:** 0.42 |
| --- | --- |
|  | HLA class I histocompatibility antigen, Cw-12 alpha chain OS=Homo sapiens GN=HLA-C PE=1 SV=2 |

|  | **Query** | **Observed** | **Mr(expt)** | **Mr(calc)** | **Delta** | **Miss** | **Score** | **Expect** | **Rank** | **Peptide** |
| --- | --- | --- | --- | --- | --- | --- | --- | --- | --- | --- |
|  | 4142 | 483.2169 | 964.4193 | 964.4250 | -0.0057 | 0 | 53 | 0.00075 | 1 | R.FDSDAASPR.G |
|  | 7831 | 611.2879 | 1220.5612 | 1220.6037 | -0.0424 | 0 | 2 | 89 | 3 | K.DYIALNEDLR.S |
|  | 8239 | 621.3292 | 1240.6439 | 1239.5013 | 1.1426 | 0 | 11 | 12 | 3 | R.MYGCDLGPDGR.L |
|  | 8528 | **628.9625** | **1255.9104** | **1255.4962** | **0.4142** | **0** | **(1)** | **78** | **5** | **R.MYGCDLGPDGR.L** |
|  | 9648 | 439.8941 | 1316.6606 | 1315.6673 | 0.9932 | 0 | 8 | 21 | 4 | R.YFYTAVSRPGR.G |
|  | 10294 | 676.3427 | 1350.6709 | 1349.6939 | 0.9770 | 1 | 8 | 24 | 1 | R.YLENGKETLQR.A |
|  | 11642 | 714.3621 | 1426.7097 | 1426.7205 | -0.0108 | 0 | 11 | 10 | 1 | K.WAAVVVPSGEEQR.Y |
|  | 12774 | 748.8586 | 1495.7026 | 1495.7129 | -0.0103 | 0 | 11 | 9.6 | 2 | R.AYLEGTCVEWLR.R |
|  | 13126 | 760.3734 | 1518.7323 | 1518.7427 | -0.0104 | 0 | 41 | 0.011 | 1 | R.SWTAADTAAQITQR.K |
|  | 14790 | **815.4116** | **1628.8086** | **1628.8199** | **-0.0113** | **0** | **27** | **0.26** | **1** | **R.FIAVGYVDDTQFVR.F** |
|  | 17823 | 613.3262 | 1836.9569 | 1835.9027 | 1.0542 | 0 | 9 | 14 | 2 | K.THVTHHPVSDHEATLR.C |
|  | 24990 | 879.7289 | 2636.1650 | 2636.1838 | -0.0188 | 0 | 27 | 0.14 | 1 | R.DGEDQTQDTELVETRPAGDGTFQK.W 24989 |

|  | **Proteins matching a subset of these peptides:** |
| --- | --- |

|  | P30510\|1C14_HUMAN    **Mass:** 41268    **Score:** 73     **Queries matched:** 8 |
| --- | --- |
|  | HLA class I histocompatibility antigen, Cw-14 alpha chain OS=Homo sapiens GN=HLA-C PE=2 SV=2 |

| **508.** | P04839\|CY24B_HUMAN    **Mass:** 66206    **Score:** 83     **Queries matched:** 11   **emPAI:** 0.18 |
| --- | --- |
|  | Cytochrome b-245 heavy chain OS=Homo sapiens GN=CYBB PE=1 SV=2 |

|  | **Query** | **Observed** | **Mr(expt)** | **Mr(calc)** | **Delta** | **Miss** | **Score** | **Expect** | **Rank** | **Peptide** |
| --- | --- | --- | --- | --- | --- | --- | --- | --- | --- | --- |
|  | 2304 | 414.2562 | 826.4978 | 826.4701 | 0.0277 | 0 | 13 | 7.2 | 1 | K.VVTHPFK.T |
|  | 2981 | **439.5322** | **877.0498** | **877.4579** | **-0.4080** | **0** | **3** | **58** | **10** | **K.TIELQMK.K** 2973 |
|  | 4410 | 493.2287 | 984.4428 | 983.6127 | 0.8301 | 0 | 2 | 1e+002 | 8 | K.LLGSALALAR.A |
|  | 6254 | 562.7897 | 1123.5648 | 1123.5734 | -0.0086 | 0 | 50 | 0.0013 | 1 | K.TIASQHPNTR.I |
|  | 6729 | **581.2743** | **1160.5340** | **1160.5422** | **-0.0081** | **0** | **42** | **0.011** | **1** | **K.QSISNSESGPR.G** |
|  | 8003 | 615.3630 | 1228.7114 | 1228.6162 | 0.0952 | 0 | 3 | 80 | 3 | K.MEVGQYIFVK.C |
|  | 15874 | **849.9272** | **1697.8398** | **1698.8262** | **-0.9864** | **0** | **2** | **66** | **1** | **K.WIVGPMFLYLCER.L** |
|  | 17800 | 612.6461 | 1834.9165 | 1834.8697 | 0.0468 | 0 | 5 | 40 | 5 | R.VNNSDPYSVALSELGDR.Q |
|  | 18582 | **948.4368** | **1894.8591** | **1894.9326** | **-0.0735** | **1** | **6** | **22** | **3** | **K.QKTLYGRPNWDNEFK.T** |
|  | 26897 | **992.1028** | **2973.2865** | **2973.3062** | **-0.0197** | **1** | **42** | **0.003** | **1** | **R.IVGDWTEGLFNACGCDKQEFQDAWK.L** |

| **509.** | P02753\|RET4_HUMAN    **Mass:** 23337    **Score:** 83     **Queries matched:** 7   **emPAI:** 0.35 |
| --- | --- |
|  | Retinol-binding protein 4 OS=Homo sapiens GN=RBP4 PE=1 SV=3 |

|  | **Query** | **Observed** | **Mr(expt)** | **Mr(calc)** | **Delta** | **Miss** | **Score** | **Expect** | **Rank** | **Peptide** |
| --- | --- | --- | --- | --- | --- | --- | --- | --- | --- | --- |
|  | 3004 | **879.0607** | **878.0534** | **878.4498** | **-0.3964** | **1** | **11** | **9.3** | **1** | **R.VKENFDK.A** 2983 |
|  | 4918 | 510.2898 | 1018.5650 | 1017.4913 | 1.0737 | 0 | 4 | 69 | 2 | R.QEELCLAR.Q |
|  | 6934 | **588.8674** | **1175.7202** | **1176.5274** | **-0.8072** | **0** | **5** | **54** | **8** | **R.FSGTWYAMAK.K** |
|  | 9386 | **652.3099** | **1302.6053** | **1302.6139** | **-0.0086** | **0** | **30** | **0.16** | **1** | **R.LIVHNGYCDGR.S** |
|  | 20558 | **1032.9818** | **2063.9491** | **2063.9623** | **-0.0132** | **0** | **80** | **9.5e-007** | **1** | **R.LLNLDGTCADSYSFVFSR.D** |
|  | 25285 | **898.3722** | **2692.0947** | **2692.1136** | **-0.0189** | **0** | **22** | **0.31** | **1** | **K.GNDDHWIVDTDYDTYAVQYSCR.L** |

| **510.** | O75489\|NDUS3_HUMAN    **Mass:** 30337    **Score:** 82     **Queries matched:** 10   **emPAI:** 0.42 |
| --- | --- |
|  | NADH dehydrogenase [ubiquinone] iron-sulfur protein 3, mitochondrial OS=Homo sapiens GN=NDUFS3 PE=1 SV=1 |

|  | **Query** | **Observed** | **Mr(expt)** | **Mr(calc)** | **Delta** | **Miss** | **Score** | **Expect** | **Rank** | **Peptide** |
| --- | --- | --- | --- | --- | --- | --- | --- | --- | --- | --- |
|  | 1535 | **684.2018** | **683.1946** | **682.3398** | **0.8547** | **0** | **12** | **7.5** | **2** | **R.NDVAHK.Q** |
|  | 1897 | **399.2266** | **796.4386** | **797.4283** | **-0.9897** | **0** | **5** | **45** | **7** | **R.QPPESLK.L** |
|  | 10534 | **455.8789** | **1364.6148** | **1363.8300** | **0.7848** | **0** | **7** | **30** | **1** | **R.GTGRPSVLLLPVR.R** |
|  | 10570 | 683.8863 | 1365.7581 | 1365.7656 | -0.0075 | 0 | 44 | 0.0057 | 1 | R.FEIVYNLLSLR.F |
|  | 10928 | **693.8723** | **1385.7301** | **1384.7562** | **0.9739** | **0** | **19** | **1.5** | **1** | **K.SLVDLTAVDVPTR.Q** |
|  | 12617 | **743.8933** | **1485.7721** | **1485.7827** | **-0.0107** | **0** | **63** | **6.4e-005** | **1** | **R.VVAEPVELAQEFR.K** 12640 |
|  | 14571 | **538.9629** | **1613.8670** | **1613.8777** | **-0.0107** | **1** | **2** | **69** | **3** | **R.VVAEPVELAQEFRK.F** |
|  | 15343 | **555.3527** | **1663.0363** | **1663.8821** | **-0.8458** | **0** | **4** | **41** | **8** | **K.QLSAFGEYVAEILPK.Y** |
|  | 20873 | 698.3504 | 2092.0294 | 2092.9829 | -0.9536 | 0 | 3 | 46 | 9 | R.EIWDMFGVFFANHPDLR.R |

| **511.** | P35268\|RL22_HUMAN    **Mass:** 14835    **Score:** 82     **Queries matched:** 10   **emPAI:** 1.01 |
| --- | --- |
|  | 60S ribosomal protein L22 OS=Homo sapiens GN=RPL22 PE=1 SV=2 |

|  | **Query** | **Observed** | **Mr(expt)** | **Mr(calc)** | **Delta** | **Miss** | **Score** | **Expect** | **Rank** | **Peptide** |
| --- | --- | --- | --- | --- | --- | --- | --- | --- | --- | --- |
|  | 1318 | 615.3780 | 614.3707 | 614.4115 | -0.0408 | 1 | 6 | 67 | 7 | K.KQVLK.F |
|  | 1555 | 690.0817 | 689.0744 | 688.3942 | 0.6802 | 1 | 14 | 7 | 3 | -.MAPVKK.L |
|  | 1892 | **398.7188** | **795.4231** | **795.3763** | **0.0469** | **0** | **8** | **25** | **5** | **K.ESYELR.Y** 1879 |
|  | 3631 | 465.0192 | 928.0238 | 927.5429 | 0.4809 | 1 | 4 | 44 | 4 | R.YLKYLTK.K |
|  | 7564 | **604.3279** | **1206.6412** | **1206.6496** | **-0.0084** | **0** | **39** | **0.02** | **1** | **K.ITVTSEVPFSK.R** |
|  | 8261 | **621.8393** | **1241.6640** | **1241.6728** | **-0.0088** | **0** | **39** | **0.017** | **1** | **K.AGNLGGGVVTIER.S** 8252 |
|  | 10500 | **455.2544** | **1362.7413** | **1362.7507** | **-0.0094** | **1** | **13** | **7.1** | **1** | **K.ITVTSEVPFSKR.Y** |
|  | 27475 | 1028.4623 | 3082.3650 | 3082.3801 | -0.0151 | 0 | 56 | 0.00013 | 1 | K.FTLDCTHPVEDGIMDAANFEQFLQER.I |

| **512.** | Q9BS26\|ERP44_HUMAN    **Mass:** 47341    **Score:** 82     **Queries matched:** 11   **emPAI:** 0.16 |
| --- | --- |
|  | Endoplasmic reticulum resident protein ERp44 OS=Homo sapiens GN=ERP44 PE=1 SV=1 |

|  | **Query** | **Observed** | **Mr(expt)** | **Mr(calc)** | **Delta** | **Miss** | **Score** | **Expect** | **Rank** | **Peptide** |
| --- | --- | --- | --- | --- | --- | --- | --- | --- | --- | --- |
|  | 1692 | 744.3249 | 743.3177 | 742.2812 | 1.0365 | 0 | 4 | 83 | 5 | R.NGMMMK.R |
|  | 6495 | **572.2659** | **1142.5173** | **1142.5399** | **-0.0225** | **1** | **3** | **77** | **2** | **K.LFRNGMMMK.R** |
|  | 12514 | **494.2370** | **1479.6892** | **1480.8038** | **-1.1146** | **1** | **4** | **48** | **4** | **K.LAPSEYRYTLLR.D** |
|  | 12760 | **499.2603** | **1494.7591** | **1494.8017** | **-0.0426** | **0** | **4** | **46** | **5** | **-.MHPAVFLSLPDLR.C** |
|  | 24597 | **854.4152** | **2560.2237** | **2560.2380** | **-0.0144** | **0** | **47** | **0.0013** | **1** | **R.VANILHDDCAFLSAFGDVSKPER.Y** |
|  | 26104 | **946.1374** | **2835.3903** | **2835.4041** | **-0.0137** | **0** | **22** | **0.36** | **1** | **R.EITFENGEELTEEGLPFLILFHMK.E** |
|  | 28944 | **1184.2422** | **3549.7047** | **3549.7238** | **-0.0191** | **1** | **54** | **0.00015** | **1** | **R.FSQMLHPIFEEASDVIKEEFPNENQVVFAR.V** 28945 28946 28947 |
|  | 30575 | **1055.9929** | **4219.9426** | **4219.9659** | **-0.0234** | **0** | **12** | **1.6** | **1** | **R.YSGDNIIYKPPGHSAPDMVYLGAMTNFDVTYNWIQDK.C** |

| **513.** | P13640\|MT1G_HUMAN    **Mass:** 7277     **Score:** 81     **Queries matched:** 9   **emPAI:** 1.46 |
| --- | --- |
|  | Metallothionein-1G OS=Homo sapiens GN=MT1G PE=1 SV=2 |

|  | **Query** | **Observed** | **Mr(expt)** | **Mr(calc)** | **Delta** | **Miss** | **Score** | **Expect** | **Rank** | **Peptide** |
| --- | --- | --- | --- | --- | --- | --- | --- | --- | --- | --- |
|  | 4567 | **498.7025** | **995.3905** | **995.3987** | **-0.0082** | **0** | **29** | **0.2** | **1** | **K.CAQGCICK.G** 4568 4569 4589 4590 |
|  | 11910 | **723.2540** | **1444.4934** | **1444.5026** | **-0.0092** | **0** | **56** | **0.00029** | **1** | **K.SCCSCCPVGCAK.C** 11909 |
|  | 12289 | **734.3900** | **1466.7653** | **1467.6268** | **-0.8615** | **1** | **2** | **75** | **6** | **K.CAQGCICKGASEK.C** |
|  | 13939 | **787.3003** | **1572.5860** | **1572.5976** | **-0.0115** | **1** | **44** | **0.0038** | **1** | **K.KSCCSCCPVGCAK.C** |

|  | **Proteins matching a subset of these peptides:** |
| --- | --- |

|  | P02795\|MT2_HUMAN    **Mass:** 7178     **Score:** 81     **Queries matched:** 8 |
| --- | --- |
|  | Metallothionein-2 OS=Homo sapiens GN=MT2A PE=1 SV=1 |

|  | P80297\|MT1X_HUMAN    **Mass:** 7204     **Score:** 81     **Queries matched:** 8 |
| --- | --- |
|  | Metallothionein-1X OS=Homo sapiens GN=MT1X PE=1 SV=1 |

|  | Q8N339\|MT1M_HUMAN    **Mass:** 7246     **Score:** 81     **Queries matched:** 3 |
| --- | --- |
|  | Metallothionein-1M OS=Homo sapiens GN=MT1M PE=2 SV=2 |

| **514.** | P04732\|MT1E_HUMAN    **Mass:** 7150     **Score:** 81     **Queries matched:** 4   **emPAI:** 1.52 |
| --- | --- |
|  | Metallothionein-1E OS=Homo sapiens GN=MT1E PE=1 SV=1 |

|  | **Query** | **Observed** | **Mr(expt)** | **Mr(calc)** | **Delta** | **Miss** | **Score** | **Expect** | **Rank** | **Peptide** |
| --- | --- | --- | --- | --- | --- | --- | --- | --- | --- | --- |
|  | 4399 | **492.2822** | **982.5499** | **981.3831** | **1.1669** | **0** | **9** | **18** | **2** | **K.CAQGCVCK.G** |
|  | 11910 | 723.2540 | 1444.4934 | 1444.5026 | -0.0092 | 0 | 56 | 0.00029 | 1 | K.SCCSCCPVGCAK.C 11909 |
|  | 13939 | 787.3003 | 1572.5860 | 1572.5976 | -0.0115 | 1 | 44 | 0.0038 | 1 | K.KSCCSCCPVGCAK.C |

| **515.** | P26885\|FKBP2_HUMAN    **Mass:** 15810    **Score:** 81     **Queries matched:** 5   **emPAI:** 0.25 |
| --- | --- |
|  | FK506-binding protein 2 OS=Homo sapiens GN=FKBP2 PE=1 SV=2 |

|  | **Query** | **Observed** | **Mr(expt)** | **Mr(calc)** | **Delta** | **Miss** | **Score** | **Expect** | **Rank** | **Peptide** |
| --- | --- | --- | --- | --- | --- | --- | --- | --- | --- | --- |
|  | 1465 | 657.3996 | 656.3923 | 656.4221 | -0.0298 | 0 | 18 | 2.8 | 4 | K.LQIGVK.K |
|  | 12185 | **487.6053** | **1459.7940** | **1459.8035** | **-0.0094** | **1** | **13** | **6.2** | **1** | **R.KLVIPSELGYGER.G** |
|  | 14010 | **790.3424** | **1578.6703** | **1578.6807** | **-0.0104** | **0** | **9** | **13** | **1** | **K.GWDQGLLGMCEGEK.R** |
|  | 14137 | **793.4591** | **1584.9035** | **1584.9127** | **-0.0091** | **0** | **81** | **9.2e-007** | **1** | **K.IPGGATLVFEVELLK.I** |
|  | 20216 | **679.3939** | **2035.1599** | **2035.1718** | **-0.0118** | **1** | **12** | **5.6** | **1** | **R.GAPPKIPGGATLVFEVELLK.I** |

| **516.** | O75251\|NDUS7_HUMAN    **Mass:** 23833    **Score:** 81     **Queries matched:** 16   **emPAI:** 0.16 |
| --- | --- |
|  | NADH dehydrogenase [ubiquinone] iron-sulfur protein 7, mitochondrial OS=Homo sapiens GN=NDUFS7 PE=1 SV=3 |

|  | **Query** | **Observed** | **Mr(expt)** | **Mr(calc)** | **Delta** | **Miss** | **Score** | **Expect** | **Rank** | **Peptide** |
| --- | --- | --- | --- | --- | --- | --- | --- | --- | --- | --- |
|  | 2988 | **439.7450** | **877.4755** | **877.4810** | **-0.0055** | **0** | **16** | **4.9** | **1** | **R.LQIWYR.R** |
|  | 3431 | 456.7638 | 911.5130 | 911.5188 | -0.0058 | 0 | 18 | 2.1 | 1 | R.AVAPKPSSR.G 3418 3441 |
|  | 5041 | **515.3264** | **1028.6383** | **1029.5641** | **-0.9258** | **0** | **2** | **97** | **10** | **-.MAVLSAPGLR.G** |
|  | 6385 | 567.7972 | 1133.5799 | 1133.5175 | 0.0624 | 0 | 9 | 19 | 2 | K.VYDQMPEPR.Y 6393 |
|  | 12460 | **738.8847** | **1475.7548** | **1475.7654** | **-0.0105** | **0** | **81** | **1.2e-006** | **1** | **R.QSDVMIVAGTLTNK.M** 12456 12461 12462 12471 |
|  | 13217 | **508.9805** | **1523.9197** | **1522.8943** | **1.0253** | **1** | **5** | **36** | **10** | **R.ILGLRSSVGPAVQAR.G** |
|  | 21362 | 716.7014 | 2147.0824 | 2147.1078 | -0.0254 | 1 | 3 | 44 | 4 | R.QSDVMIVAGTLTNKMAPALR.K |
|  | 27084 | **1003.8834** | **3008.6284** | **3008.6409** | **-0.0125** | **0** | **9** | **5.4** | **1** | **R.IVPVDIYIPGCPPTAEALLYGILQLQR.K** |
|  | 28780 | **1161.5131** | **3481.5174** | **3481.4514** | **0.0659** | **1** | **0** | **35** | **4** | **R.SSLWPMTFGLACCAVEMMHMAAPRYDMDR.F** |

| **517.** | O60762\|DPM1_HUMAN    **Mass:** 29673    **Score:** 81     **Queries matched:** 9   **emPAI:** 0.27 |
| --- | --- |
|  | Dolichol-phosphate mannosyltransferase OS=Homo sapiens GN=DPM1 PE=1 SV=1 |

|  | **Query** | **Observed** | **Mr(expt)** | **Mr(calc)** | **Delta** | **Miss** | **Score** | **Expect** | **Rank** | **Peptide** |
| --- | --- | --- | --- | --- | --- | --- | --- | --- | --- | --- |
|  | 7270 | **597.7869** | **1193.5592** | **1193.5677** | **-0.0085** | **0** | **39** | **0.018** | **1** | **K.EGNFDIVSGTR.Y** 7269 |
|  | 8371 | **416.7264** | **1247.1574** | **1247.6292** | **-0.4718** | **1** | **3** | **52** | **9** | **-.MASLEVSRSPR.R** |
|  | 8607 | 630.8399 | 1259.6652 | 1259.6696 | -0.0044 | 0 | 15 | 5 | 1 | K.LGLGTAYIHGMK.H |
|  | 9722 | 661.3647 | 1320.7149 | 1320.6575 | 0.0575 | 1 | 6 | 38 | 7 | K.GNGGVYGWDLKR.K |
|  | 11211 | **468.5647** | **1402.6723** | **1403.7595** | **-1.0872** | **1** | **1** | **1e+002** | **10** | **K.KLGLGTAYIHGMK.H** |
|  | 17996 | **925.9804** | **1849.9461** | **1849.9574** | **-0.0113** | **0** | **56** | **0.00024** | **1** | **R.QLNYTIGEVPISFVDR.V** |
|  | 18405 | **627.3451** | **1879.0136** | **1877.8838** | **1.1298** | **1** | **1** | **79** | **7** | **K.CVSKGYVFQMEMIVR.A** |
|  | 21230 | 534.0159 | 2132.0344 | 2132.0473 | -0.0129 | 0 | 11 | 7.5 | 2 | K.HATGNYIIIMDADLSHHPK.F |

| **518.** | Q5JRA6\|MIA3_HUMAN    **Mass:** 214255   **Score:** 80     **Queries matched:** 32   **emPAI:** 0.05 |
| --- | --- |
|  | Melanoma inhibitory activity protein 3 OS=Homo sapiens GN=MIA3 PE=1 SV=1 |

|  | **Query** | **Observed** | **Mr(expt)** | **Mr(calc)** | **Delta** | **Miss** | **Score** | **Expect** | **Rank** | **Peptide** |
| --- | --- | --- | --- | --- | --- | --- | --- | --- | --- | --- |
|  | 1511 | **676.2846** | **675.2773** | **674.3421** | **0.9352** | **0** | **21** | **2** | **3** | **K.VNMAPK.G** |
|  | 3017 | **440.7229** | **879.4313** | **879.4008** | **0.0305** | **0** | **14** | **7.2** | **2** | **K.TLQDMEK.N** |
|  | 3034 | **441.2958** | **880.5769** | **881.3735** | **-0.7966** | **0** | **(1)** | **1.2e+002** | **6** | **K.QMMDVSR.T** |
|  | 3045 | **441.2966** | **880.5787** | **880.4290** | **0.1497** | **0** | **9** | **18** | **10** | **K.LSNYEQK.I** |
|  | 3054 | **442.2165** | **882.4184** | **881.3735** | **1.0449** | **0** | **2** | **87** | **6** | **K.QMMDVSR.T** |
|  | 4687 | 502.2315 | 1002.4485 | 1002.5723 | -0.1238 | 1 | 17 | 3.3 | 4 | R.RDLPLHPR.G |
|  | 6817 | 584.3174 | 1166.6203 | 1166.6295 | -0.0092 | 0 | 26 | 0.34 | 1 | K.LHAELSEQIK.S |
|  | 8618 | 421.0560 | 1260.1463 | 1260.6384 | -0.4921 | 0 | 16 | 2.4 | 3 | K.QNMILSDEAIK.Y 8652 |
|  | 8757 | **635.3174** | **1268.6202** | **1268.6612** | **-0.0410** | **0** | **1** | **1e+002** | **3** | **R.ELEDEVPILGR.N** |
|  | 8914 | **639.4456** | **1276.8766** | **1276.6333** | **0.2433** | **0** | **(3)** | **59** | **3** | **K.QNMILSDEAIK.Y** |
|  | 9620 | 658.3996 | 1314.7846 | 1315.6806 | -0.8959 | 1 | 3 | 69 | 6 | R.VLDEGKVNMAPK.G |
|  | 9915 | **665.3490** | **1328.6834** | **1329.6677** | **-0.9843** | **0** | **2** | **88** | **8** | **R.QFDVNLQVPDR.A** |
|  | 10984 | **695.3679** | **1388.7212** | **1388.7333** | **-0.0121** | **1** | **69** | **1.6e-005** | **1** | **R.KQNMILSDEAIK.Y** |
|  | 11255 | **703.3832** | **1404.7519** | **1404.7282** | **0.0237** | **1** | **(2)** | **78** | **5** | **R.KQNMILSDEAIK.Y** |
|  | 11989 | **484.0125** | **1449.0156** | **1448.7545** | **0.2612** | **1** | **2** | **62** | **3** | **K.TIMKENTELVQK.L** |
|  | 12194 | **731.3673** | **1460.7201** | **1460.7293** | **-0.0092** | **1** | **45** | **0.0046** | **1** | **R.VMLESEREQNVK.N** |
|  | 12291 | 734.3935 | 1466.7724 | 1466.8133 | -0.0409 | 1 | 5 | 34 | 10 | K.REDLLIISSFFK.E 12292 |
|  | 14961 | **819.9421** | **1637.8697** | **1637.9691** | **-0.0994** | **0** | **7** | **25** | **8** | **-.MAAAPGLLVWLLVLR.L** |
|  | 15261 | 829.9113 | 1657.8079 | 1657.8271 | -0.0191 | 1 | 10 | 12 | 3 | R.EQNVKNQDLISENK.K |
|  | 15548 | **559.9768** | **1676.9086** | **1677.8951** | **-0.9865** | **1** | **1** | **1e+002** | **7** | **R.LPWRVPGQLDPSTGR.R** |
|  | 17543 | 605.9993 | 1814.9762 | 1815.8275 | -0.8513 | 1 | 6 | 24 | 7 | K.DSDYLKNDNPEEHLK.T |
|  | 20719 | **692.8544** | **2075.5413** | **2075.0283** | **0.5129** | **0** | **10** | **5.8** | **1** | **K.TAELNVQVPEEPTHLDQR.V** |
|  | 21713 | **729.3682** | **2185.0829** | **2184.1255** | **0.9573** | **1** | **2** | **50** | **2** | **R.TFGYFPKDLIQVVHEYTK.E** |
|  | 21958 | **738.7232** | **2213.1478** | **2213.9998** | **-0.8520** | **0** | **0** | **73** | **2** | **K.DLDPGPVTTEDTPMDAIDANK.Q** |
|  | 23064 | **588.5705** | **2350.2529** | **2350.1335** | **0.1194** | **1** | **6** | **17** | **4** | **R.KIQQESLGSAPLMGDDHPNASR.D** |
|  | 24564 | **851.8245** | **2552.4516** | **2551.2839** | **1.1676** | **1** | **1** | **47** | **1** | **K.LKDVISMNASEFSEVQIALNEAK.L** |
|  | 26017 | 940.1540 | 2817.4401 | 2817.4339 | 0.0062 | 0 | 0 | 56 | 5 | K.MAMLQEEPVIVKPMPGKPNTQNPPR.R |
|  | 27412 | 767.8776 | 3067.4811 | 3066.5857 | 0.8954 | 1 | (1) | 44 | 8 | K.VNMAPKGPPPFPGVPLMSTPMGGPVPPPIR.Y |
|  | 27413 | 1023.5020 | 3067.4840 | 3066.5857 | 0.8983 | 1 | 8 | 8.5 | 2 | K.VNMAPKGPPPFPGVPLMSTPMGGPVPPPIR.Y |
|  | 31488 | **1797.8424** | **5390.5054** | **5390.3395** | **0.1659** | **1** | **6** | **2.6** | **1** | **R.LVTSLEDDFDEELDTEYYAVGKEDEENQEDFDELPLLTFTDGEDMK.T** |

| **519.** | P52597\|HNRPF_HUMAN    **Mass:** 45985    **Score:** 80     **Queries matched:** 13   **emPAI:** 0.08 |
| --- | --- |
|  | Heterogeneous nuclear ribonucleoprotein F OS=Homo sapiens GN=HNRNPF PE=1 SV=3 |

|  | **Query** | **Observed** | **Mr(expt)** | **Mr(calc)** | **Delta** | **Miss** | **Score** | **Expect** | **Rank** | **Peptide** |
| --- | --- | --- | --- | --- | --- | --- | --- | --- | --- | --- |
|  | 2393 | **417.7293** | **833.4441** | **833.3879** | **0.0562** | **0** | **8** | **29** | **6** | **K.SSQEEVR.S** |
|  | 3365 | **454.2528** | **906.4911** | **905.4494** | **1.0417** | **0** | **8** | **28** | **3** | **R.SYSDPPLK.F** |
|  | 5210 | **521.8254** | **1041.6363** | **1042.4726** | **-0.8363** | **1** | **7** | **33** | **1** | **K.DRANMQHR.Y** |
|  | 5835 | 546.8026 | 1091.5905 | 1091.5724 | 0.0182 | 0 | 28 | 0.26 | 2 | R.VHIEIGPDGR.V |
|  | 8639 | **421.2237** | **1260.6492** | **1260.6979** | **-0.0486** | **1** | **1** | **1.2e+002** | **4** | **R.IGHRYIEVFK.S** |
|  | 10017 | **668.3253** | **1334.6360** | **1334.6541** | **-0.0181** | **0** | **3** | **70** | **7** | **M.MLGPEGGEGFVVK.L** 10018 |
|  | 16080 | 570.6453 | 1708.9140 | 1708.7792 | 0.1348 | 0 | 6 | 31 | 1 | R.QSGEAFVELGSEDDVK.M 16049 |
|  | 18253 | 934.4705 | 1866.9264 | 1866.9363 | -0.0100 | 0 | 80 | 8.9e-007 | 1 | K.ITGEAFVQFASQELAEK.A 18259 |
|  | 18644 | **634.2933** | **1899.8582** | **1898.8983** | **0.9598** | **1** | **1** | **74** | **3** | **K.HSGPNSADSANDGFVRLR.G** |
|  | 21757 | **549.0289** | **2192.0866** | **2191.0103** | **1.0763** | **0** | **7** | **18** | **2** | **R.VTGEADVEFATHEEAVAAMSK.D** |

| **520.** | P62879\|GBB2_HUMAN    **Mass:** 38048    **Score:** 80     **Queries matched:** 22   **emPAI:** 0.60 |
| --- | --- |
|  | Guanine nucleotide-binding protein G(I)/G(S)/G(T) subunit beta-2 OS=Homo sapiens GN=GNB2 PE=1 SV=3 |

|  | **Query** | **Observed** | **Mr(expt)** | **Mr(calc)** | **Delta** | **Miss** | **Score** | **Expect** | **Rank** | **Peptide** |
| --- | --- | --- | --- | --- | --- | --- | --- | --- | --- | --- |
|  | 1492 | **668.3134** | **667.3061** | **667.2418** | **0.0644** | **0** | **5** | **34** | **2** | **R.DSMCR.Q** |
|  | 2033 | 403.2249 | 804.4353 | 804.4970 | -0.0616 | 0 | 4 | 82 | 10 | K.VHAIPLR.S 2013 |
|  | 4773 | 505.2582 | 1008.5019 | 1008.5101 | -0.0082 | 0 | 44 | 0.0056 | 1 | R.AGVLAGHDNR.V 4778 4788 4789 4791 4792 4796 |
|  | 4893 | 509.2791 | 1016.5437 | 1016.5502 | -0.0065 | 0 | 28 | 0.31 | 1 | R.LLVSASQDGK.L 4903 |
|  | 8521 | **628.7856** | **1255.5566** | **1254.5914** | **0.9652** | **0** | **19** | **1.9** | **1** | **R.TFVSGACDASIK.L** |
|  | 10032 | 668.8043 | 1335.5941 | 1335.6030 | -0.0089 | 0 | 23 | 0.67 | 1 | K.IYAMHWGTDSR.L |
|  | 10050 | 446.5502 | 1336.6288 | 1336.6596 | -0.0308 | 1 | 9 | 15 | 3 | K.GDRAGVLAGHDNR.V |
|  | 16385 | **577.3186** | **1728.9340** | **1727.8802** | **1.0538** | **1** | **2** | **71** | **8** | **M.SELEQLRQEAEQLR.N** |
|  | 21294 | 713.8634 | 2138.5684 | 2139.1840 | -0.6157 | 1 | 1 | 41 | 8 | K.LIIWDSYTTNKVHAIPLR.S |
|  | 21462 | **1079.9940** | **2157.9735** | **2157.9863** | **-0.0128** | **0** | **27** | **0.16** | **1** | **R.LLLAGYDDFNCNIWDAMK.G** 21461 |
|  | 23546 | 1208.5550 | 2415.0955 | 2415.1087 | -0.0131 | 0 | 57 | 0.00015 | 1 | R.VSCLGVTDDGMAVATGSWDSFLK.I |
|  | 24094 | **829.7151** | **2486.1236** | **2486.1358** | **-0.0122** | **1** | **30** | **0.068** | **1** | **R.LLLAGYDDFNCNIWDAMKGDR.A** |
|  | 28840 | **877.9720** | **3507.8591** | **3508.5630** | **-0.7039** | **0** | **4** | **16** | **2** | **R.QTFIGHESDINAVAFFPNGYAFTTGSDDATCR.L** |

| **521.** | Q9UNL2\|SSRG_HUMAN    **Mass:** 21067    **Score:** 80     **Queries matched:** 7   **emPAI:** 0.18 |
| --- | --- |
|  | Translocon-associated protein subunit gamma OS=Homo sapiens GN=SSR3 PE=1 SV=1 |

|  | **Query** | **Observed** | **Mr(expt)** | **Mr(calc)** | **Delta** | **Miss** | **Score** | **Expect** | **Rank** | **Peptide** |
| --- | --- | --- | --- | --- | --- | --- | --- | --- | --- | --- |
|  | 1985 | **402.1786** | **802.3426** | **803.3773** | **-1.0347** | **0** | **5** | **69** | **10** | **K.LSEADNR.K** |
|  | 3671 | **466.2379** | **930.4613** | **931.4723** | **-1.0110** | **1** | **20** | **2** | **2** | **R.KLSEADNR.K** 3707 |
|  | 6385 | 567.7972 | 1133.5799 | 1132.5724 | 1.0075 | 1 | 7 | 30 | 3 | R.EDAVSKEVTR.K |
|  | 16027 | **854.4073** | **1706.8000** | **1706.8111** | **-0.0111** | **0** | **56** | **0.0003** | **1** | **K.QQSEEDLLLQDFSR.N** 16026 |
|  | 28494 | 845.4953 | 3377.9521 | 3377.7444 | 0.2077 | 0 | 2 | 21 | 10 | R.IWHMDLIQSAVLYSVMTLVSTYLVAFAYK.N |

| **522.** | Q12797\|ASPH_HUMAN    **Mass:** 86266    **Score:** 80     **Queries matched:** 15   **emPAI:** 0.18 |
| --- | --- |
|  | Aspartyl/asparaginyl beta-hydroxylase OS=Homo sapiens GN=ASPH PE=1 SV=3 |

|  | **Query** | **Observed** | **Mr(expt)** | **Mr(calc)** | **Delta** | **Miss** | **Score** | **Expect** | **Rank** | **Peptide** |
| --- | --- | --- | --- | --- | --- | --- | --- | --- | --- | --- |
|  | 5797 | **545.2860** | **1088.5575** | **1087.5080** | **1.0495** | **1** | **4** | **73** | **8** | **R.NENACKGAPK.T** |
|  | 9186 | 431.8718 | 1292.5937 | 1291.7798 | 0.8138 | 1 | 2 | 1e+002 | 8 | R.LRMHLGLVIPK.E |
|  | 10897 | **692.8544** | **1383.6942** | **1383.6394** | **0.0548** | **0** | **10** | **15** | **1** | **R.FYFHLGDAMQR.V** 10910 |
|  | 12419 | 492.2652 | 1473.7738 | 1473.8191 | -0.0453 | 1 | 7 | 24 | 2 | K.AQNKIAESIPYLK.E |
|  | 12862 | **501.3359** | **1500.9860** | **1500.7936** | **0.1924** | **1** | **0** | **96** | **10** | **K.AKGLFLPEDENLR.E** |
|  | 13214 | 508.9561 | 1523.8466 | 1523.7831 | 0.0635 | 1 | 2 | 83 | 5 | K.ETGYTELVKSLER.N 13238 |
|  | 14471 | 804.8763 | 1607.7380 | 1607.7481 | -0.0101 | 0 | 36 | 0.027 | 1 | K.GDWSQFTLWQQGR.R |
|  | 18668 | **951.4450** | **1900.8755** | **1899.8011** | **1.0744** | **0** | **54** | **0.00043** | **1** | **K.LGIYDADGDGDFDVDDAK.V** 18643 |
|  | 22119 | **1114.5745** | **2227.1344** | **2228.1576** | **-1.0232** | **0** | **17** | **1.6** | **1** | **R.GAIETYQEVASLPDVPADLLK.L** |
|  | 23545 | 604.5530 | 2414.1831 | 2415.0494 | -0.8664 | 1 | 1 | 57 | 4 | K.NAKSSGNSSSSGSGSGSTSAGSSSPGAR.R 23556 |
|  | 24364 | **842.0852** | **2523.2338** | **2523.2381** | **-0.0043** | **1** | **24** | **0.27** | **1** | **K.LGIYDADGDGDFDVDDAKVLLGLK.E** |

| **523.** | P06396\|GELS_HUMAN    **Mass:** 86043    **Score:** 80     **Queries matched:** 17   **emPAI:** 0.18 |
| --- | --- |
|  | Gelsolin OS=Homo sapiens GN=GSN PE=1 SV=1 |

|  | **Query** | **Observed** | **Mr(expt)** | **Mr(calc)** | **Delta** | **Miss** | **Score** | **Expect** | **Rank** | **Peptide** |
| --- | --- | --- | --- | --- | --- | --- | --- | --- | --- | --- |
|  | 2454 | 420.2175 | 838.4204 | 838.3756 | 0.0448 | 0 | 8 | 21 | 8 | K.MDAHPPR.L |
|  | 3451 | **457.2922** | **912.5699** | **912.5757** | **-0.0058** | **1** | **20** | **1.7** | **2** | **R.RTPITVVK.Q** |
|  | 5615 | **538.1589** | **1074.3032** | **1073.5101** | **0.7930** | **0** | **8** | **19** | **2** | **R.EGGQTAPASTR.L** |
|  | 8882 | **638.3577** | **1274.7009** | **1274.7095** | **-0.0086** | **0** | **20** | **1.5** | **1** | **K.HVVPNEVVVQR.L** |
|  | 9684 | **660.3470** | **1318.6794** | **1318.6881** | **-0.0087** | **0** | **66** | **4e-005** | **1** | **K.AGALNSNDAFVLK.T** |
|  | 10363 | **677.8839** | **1353.7533** | **1353.7405** | **0.0128** | **1** | **5** | **48** | **4** | **K.EPGLQIWRVEK.F** |
|  | 13318 | 766.3705 | 1530.7265 | 1531.7339 | -1.0074 | 1 | 11 | 9.2 | 1 | K.GGTSREGGQTAPASTR.L |
|  | 16257 | 574.9445 | 1721.8117 | 1721.8301 | -0.0184 | 0 | 3 | 59 | 7 | R.EVQGFESATFLGYFK.S |
|  | 17717 | **915.4793** | **1828.9441** | **1828.9571** | **-0.0130** | **0** | **37** | **0.021** | **1** | **K.QTQVSVLPEGGETPLFK.Q** |
|  | 20354 | **683.6877** | **2048.0414** | **2047.0634** | **0.9779** | **0** | **3** | **46** | **1** | **K.EPAHLMSLFGGKPMIIYK.G** 20338 20340 |
|  | 24456 | **509.6843** | **2543.3853** | **2542.3804** | **1.0049** | **1** | **3** | **31** | **4** | **R.VVQGKEPAHLMSLFGGKPMIIYK.G** |
|  | 25361 | **1352.6957** | **2703.3768** | **2703.3836** | **-0.0068** | **0** | **21** | **0.52** | **1** | **K.FDLVPVPTNLYGDFFTGDAYVILK.T** |
|  | 27345 | **1020.8643** | **3059.5710** | **3059.5896** | **-0.0186** | **1** | **21** | **0.41** | **1** | **R.VEKFDLVPVPTNLYGDFFTGDAYVILK.T** |
|  | 27435 | **1025.4938** | **3073.4595** | **3074.5356** | **-1.0761** | **1** | **1** | **38** | **2** | **R.GASQAGAPQGRVPEARPNSMVVEHPEFLK.A** |
|  | 28560 | **1133.8812** | **3398.6219** | **3398.6459** | **-0.0240** | **1** | **16** | **0.99** | **1** | **R.IEGSNKVPVDPATYGQFYGGDSYIILYNYR.H** |

| **524.** | O60701\|UGDH_HUMAN    **Mass:** 55674    **Score:** 80     **Queries matched:** 18   **emPAI:** 0.14 |
| --- | --- |
|  | UDP-glucose 6-dehydrogenase OS=Homo sapiens GN=UGDH PE=1 SV=1 |

|  | **Query** | **Observed** | **Mr(expt)** | **Mr(calc)** | **Delta** | **Miss** | **Score** | **Expect** | **Rank** | **Peptide** |
| --- | --- | --- | --- | --- | --- | --- | --- | --- | --- | --- |
|  | 1481 | 661.3398 | 660.3326 | 659.4218 | 0.9108 | 0 | 8 | 38 | 4 | R.LVTISK.D |
|  | 1840 | 397.0906 | 792.1667 | 791.3773 | 0.7893 | 1 | 4 | 58 | 8 | K.KDTGDTR.E |
|  | 2856 | **434.8811** | **867.7476** | **868.4225** | **-0.6749** | **1** | **5** | **32** | **6** | **K.TYGMGKGR.A** 2855 |
|  | 4356 | 490.7437 | 979.4729 | 978.5902 | 0.8827 | 0 | 7 | 34 | 4 | K.IAILGFAFK.K |
|  | 5597 | **537.2598** | **1072.5050** | **1073.5981** | **-1.0932** | **0** | **13** | **10** | **6** | **K.LAANAFLAQR.I** 5598 |
|  | 9189 | **647.3099** | **1292.6053** | **1293.6904** | **-1.0850** | **0** | **4** | **62** | **7** | **K.MLKPAFIFDGR.R** |
|  | 11130 | 699.3939 | 1396.7732 | 1397.7667 | -0.9935 | 1 | 15 | 4.4 | 3 | K.FSLQDPPNKKPK.V 11171 |
|  | 13360 | **512.2609** | **1533.7608** | **1532.8086** | **0.9521** | **0** | **5** | **36** | **3** | **K.EADLVFISVNTPTK.T** |
|  | 15592 | **841.4112** | **1680.8078** | **1681.8675** | **-1.0597** | **1** | **0** | **97** | **4** | **R.GKNLFFSTNIDDAIK.E** |
|  | 16783 | **586.9141** | **1757.7205** | **1757.7831** | **-0.0626** | **0** | **2** | **64** | **7** | **R.YWQQVIDMNDYQR.R** |
|  | 18812 | 957.0059 | 1911.9972 | 1912.0094 | -0.0122 | 0 | 28 | 0.14 | 1 | R.INAWNSPTLPIYEPGLK.E |
|  | 21897 | **737.0240** | **2208.0501** | **2208.0659** | **-0.0157** | **0** | **32** | **0.055** | **1** | **R.EQIVVDLSHPGVSEDDQVSR.L** |
|  | 25759 | **556.2656** | **2776.2914** | **2777.2580** | **-0.9666** | **0** | **2** | **38** | **2** | **K.ICCIGAGYVGGPTCSVIAHMCPEIR.V** |
|  | 27094 | 1004.8491 | 3011.5255 | 3011.5492 | -0.0236 | 1 | 1 | 42 | 8 | K.NLFFSTNIDDAIKEADLVFISVNTPTK.T |
|  | 27334 | 1019.8740 | 3056.6002 | 3056.6182 | -0.0180 | 0 | 72 | 3e-006 | 1 | R.IFDANTKPNLNLQVLSNPEFLAEGTAIK.D |

| **525.** | P11279\|LAMP1_HUMAN    **Mass:** 45367    **Score:** 80     **Queries matched:** 13   **emPAI:** 0.27 |
| --- | --- |
|  | Lysosome-associated membrane glycoprotein 1 OS=Homo sapiens GN=LAMP1 PE=1 SV=3 |

|  | **Query** | **Observed** | **Mr(expt)** | **Mr(calc)** | **Delta** | **Miss** | **Score** | **Expect** | **Rank** | **Peptide** |
| --- | --- | --- | --- | --- | --- | --- | --- | --- | --- | --- |
|  | 3602 | **463.7416** | **925.4686** | **924.5392** | **0.9294** | **0** | **7** | **31** | **3** | **R.LLNINPNK.T** 3603 |
|  | 5095 | **517.2763** | **1032.5379** | **1032.5451** | **-0.0072** | **0** | **46** | **0.004** | **1** | **K.TVESITDIR.A** |
|  | 6599 | **576.3026** | **1150.5907** | **1150.5982** | **-0.0076** | **0** | **53** | **0.00074** | **1** | **R.ALQATVGNSYK.C** 6596 6609 |
|  | 8323 | **623.3568** | **1244.6991** | **1245.6466** | **-0.9475** | **1** | **10** | **15** | **5** | **R.DPAFKAANGSLR.A** 8332 8338 8359 |
|  | 8452 | **418.2400** | **1251.6982** | **1252.7180** | **-1.0197** | **1** | **10** | **15** | **3** | **R.VTKAFSVNIFK.V** |
|  | 23477 | 802.1015 | 2403.2827 | 2403.2951 | -0.0124 | 1 | 26 | 0.18 | 1 | R.FFLQGIQLNTILPDARDPAFK.A |
|  | 29380 | **1243.2783** | **3726.8131** | **3726.0407** | **0.7725** | **1** | **2** | **22** | **3** | **-.MAAPGSARRPLLLLLLLLLLGLMHCASAAMFMVK.N** |

| **526.** | P01893\|HLAH_HUMAN    **Mass:** 41052    **Score:** 80     **Queries matched:** 12   **emPAI:** 0.19 |
| --- | --- |
|  | Putative HLA class I histocompatibility antigen, alpha chain H OS=Homo sapiens GN=HLA-H PE=5 SV=2 |

|  | **Query** | **Observed** | **Mr(expt)** | **Mr(calc)** | **Delta** | **Miss** | **Score** | **Expect** | **Rank** | **Peptide** |
| --- | --- | --- | --- | --- | --- | --- | --- | --- | --- | --- |
|  | 1736 | 763.1153 | 762.1080 | 762.3694 | -0.2614 | 0 | 7 | 31 | 7 | R.NTQICK.A |
|  | 7831 | 611.2879 | 1220.5612 | 1220.6037 | -0.0424 | 0 | 2 | 89 | 3 | K.DYIALNEDLR.S |
|  | 10294 | 676.3427 | 1350.6709 | 1349.6939 | 0.9770 | 1 | 8 | 24 | 1 | R.YLENGKETLQR.A |
|  | 11047 | 465.0192 | 1392.0357 | 1392.6707 | -0.6350 | 0 | 3 | 48 | 9 | R.SWTAADMAAQITK.R |
|  | 11642 | 714.3621 | 1426.7097 | 1426.7205 | -0.0108 | 0 | 11 | 10 | 1 | K.WAAVVVPSGEEQR.Y |
|  | 13579 | **517.1576** | **1548.4509** | **1548.7718** | **-0.3209** | **1** | **1** | **69** | **9** | **R.SWTAADMAAQITKR.K** |
|  | 15060 | 823.4092 | 1644.8038 | 1644.8148 | -0.0110 | 0 | 75 | 3.4e-006 | 1 | R.FISVGYVDDTQFVR.F |
|  | 16649 | **583.8150** | **1748.4232** | **1747.7988** | **0.6244** | **0** | **2** | **42** | **4** | **R.YFYTTMSRPGAGEPR.F** |
|  | 19138 | **647.7875** | **1940.3408** | **1940.1459** | **0.1949** | **0** | **5** | **20** | **3** | **R.TLLLLLSGALALTQTWAR.S** |
|  | 24990 | 879.7289 | 2636.1650 | 2636.1838 | -0.0188 | 0 | 27 | 0.14 | 1 | R.DGEDQTQDTELVETRPAGDGTFQK.W 24989 |
|  | 25425 | **905.0901** | **2712.2484** | **2712.5183** | **-0.2698** | **1** | **3** | **28** | **1** | **-.MAVMAPRTLLLLLSGALALTQTWAR.S** |

| **527.** | Q9NYQ3\|HAOX2_HUMAN    **Mass:** 39384    **Score:** 80     **Queries matched:** 10   **emPAI:** 0.20 |
| --- | --- |
|  | Hydroxyacid oxidase 2 OS=Homo sapiens GN=HAO2 PE=2 SV=1 |

|  | **Query** | **Observed** | **Mr(expt)** | **Mr(calc)** | **Delta** | **Miss** | **Score** | **Expect** | **Rank** | **Peptide** |
| --- | --- | --- | --- | --- | --- | --- | --- | --- | --- | --- |
|  | 1423 | 644.4134 | 643.4061 | 642.4064 | 0.9997 | 0 | 26 | 0.67 | 1 | K.ALALGAK.C |
|  | 3536 | **460.7551** | **919.4956** | **919.4247** | **0.0709** | **0** | **13** | **9.6** | **7** | **R.DVSEVDTR.T** |
|  | 10639 | 685.4150 | 1368.8155 | 1369.7929 | -0.9774 | 1 | 9 | 16 | 5 | R.TGNDVLKALALGAK.C |
|  | 11482 | **473.3045** | **1416.8918** | **1416.8089** | **0.0829** | **1** | **7** | **22** | **7** | **K.QLIQRVESLGFK.A** 11480 |
|  | 12635 | **496.5956** | **1486.7649** | **1486.7753** | **-0.0104** | **0** | **68** | **2.2e-005** | **1** | **K.HNVQGIIVSNHGGR.Q** |
|  | 13266 | 509.9245 | 1526.7517 | 1527.8079 | -1.0562 | 0 | 0 | 1.2e+002 | 6 | K.ALVITLDTPVCGNR.R |
|  | 22854 | 581.3055 | 2321.1928 | 2321.1144 | 0.0784 | 0 | 5 | 22 | 3 | K.EVLNILTNEFHTSMALTGCR.S |
|  | 29183 | **1215.5707** | **3643.6902** | **3643.7109** | **-0.0207** | **0** | **33** | **0.019** | **1** | **R.TTIQGEEISAPICIAPTGFHCLVWPDGEMSTAR.A** |
|  | 30343 | **1370.6786** | **4109.0139** | **4110.1111** | **-1.0971** | **1** | **1** | **21** | **9** | **K.GNAIPYFQMTPISTSLCWNDLSWFQSITRLPIILK.G** |

| **528.** | O60220\|TIM8A_HUMAN    **Mass:** 11219    **Score:** 79     **Queries matched:** 4   **emPAI:** 0.84 |
| --- | --- |
|  | Mitochondrial import inner membrane translocase subunit Tim8 A OS=Homo sapiens GN=TIMM8A PE=1 SV=1 |

|  | **Query** | **Observed** | **Mr(expt)** | **Mr(calc)** | **Delta** | **Miss** | **Score** | **Expect** | **Rank** | **Peptide** |
| --- | --- | --- | --- | --- | --- | --- | --- | --- | --- | --- |
|  | 7287 | **598.2915** | **1194.5684** | **1194.5768** | **-0.0084** | **0** | **10** | **16** | **1** | **K.SKPVFSESLSD.-** |
|  | 10346 | 677.7925 | 1353.5704 | 1353.5806 | -0.0101 | 0 | 30 | 0.14 | 1 | R.AEACFVNCVER.F 10376 |
|  | 19544 | **988.9651** | **1975.9156** | **1975.9284** | **-0.0128** | **0** | **73** | **5.2e-006** | **1** | **R.FQQLVHQMTELCWEK.C** |

| **529.** | Q9Y3Z3\|SAMH1_HUMAN    **Mass:** 72896    **Score:** 79     **Queries matched:** 20   **emPAI:** 0.05 |
| --- | --- |
|  | SAM domain and HD domain-containing protein 1 OS=Homo sapiens GN=SAMHD1 PE=1 SV=2 |

|  | **Query** | **Observed** | **Mr(expt)** | **Mr(calc)** | **Delta** | **Miss** | **Score** | **Expect** | **Rank** | **Peptide** |
| --- | --- | --- | --- | --- | --- | --- | --- | --- | --- | --- |
|  | 3496 | **459.0118** | **916.0089** | **915.4774** | **0.5316** | **1** | **7** | **27** | **7** | **K.RNGIDVDK.W** 3498 |
|  | 6860 | 585.8415 | 1169.6684 | 1168.6968 | 0.9716 | 0 | 7 | 33 | 10 | R.FIPLARPEVK.W 6855 |
|  | 7040 | 592.7842 | 1183.5538 | 1182.6431 | 0.9107 | 0 | 17 | 3.1 | 2 | R.LVQIHVDTMK.V |
|  | 7630 | **606.3276** | **1210.6407** | **1209.6506** | **0.9902** | **1** | **4** | **51** | **5** | **K.QIEYRNLFK.Y** |
|  | 9457 | **653.8406** | **1305.6667** | **1306.6517** | **-0.9850** | **0** | **1** | **98** | **3** | **R.FENLGVSSLGER.K** |
|  | 10794 | 690.3622 | 1378.7098 | 1378.6340 | 0.0758 | 0 | 4 | 61 | 3 | K.NPIDHVSFYCK.T |
|  | 12391 | 737.3881 | 1472.7617 | 1472.6705 | 0.0912 | 0 | 9 | 17 | 5 | R.ISTAIDDMEAYTK.L 12426 12428 12429 |
|  | 12774 | 748.8586 | 1495.7026 | 1495.8974 | -0.1947 | 1 | 1 | 1.1e+002 | 6 | K.EVASAKPKVLLDVK.L |
|  | 15162 | 826.4394 | 1650.8643 | 1649.8698 | 0.9945 | 0 | 60 | 0.00013 | 1 | K.VGNIIDTMITDAFLK.A 15143 |
|  | 17252 | **895.9266** | **1789.8386** | **1789.9097** | **-0.0712** | **1** | **1** | **96** | **5** | **R.EDYESLPKEVASAKPK.V** |
|  | 17400 | 902.4902 | 1802.9658 | 1801.8707 | 1.0951 | 1 | 1 | 92 | 6 | K.EWNDSTSVQNPTRLR.E |
|  | 21720 | **729.4814** | **2185.4223** | **2185.0943** | **0.3280** | **0** | **0** | **54** | **4** | **K.EQIVGPLESPVEDSLWPYK.G** |
|  | 23266 | **792.0201** | **2373.0384** | **2373.1232** | **-0.0848** | **1** | **4** | **26** | **1** | **K.LKAEDFIVDVINMDYGMQEK.N** |
|  | 23386 | **797.4069** | **2389.1989** | **2389.1181** | **0.0808** | **1** | **(1)** | **57** | **3** | **K.LKAEDFIVDVINMDYGMQEK.N** |

| **530.** | P01011\|AACT_HUMAN    **Mass:** 47792    **Score:** 79     **Queries matched:** 10   **emPAI:** 0.08 |
| --- | --- |
|  | Alpha-1-antichymotrypsin OS=Homo sapiens GN=SERPINA3 PE=1 SV=2 |

|  | **Query** | **Observed** | **Mr(expt)** | **Mr(calc)** | **Delta** | **Miss** | **Score** | **Expect** | **Rank** | **Peptide** |
| --- | --- | --- | --- | --- | --- | --- | --- | --- | --- | --- |
|  | 1271 | 601.3879 | 600.3807 | 599.4006 | 0.9800 | 0 | 21 | 2.2 | 1 | K.QLVLK.A |
|  | 1377 | 633.1577 | 632.1504 | 631.3402 | 0.8103 | 1 | 1 | 2e+002 | 6 | K.NGTRGK.I |
|  | 1605 | 710.2909 | 709.2837 | 709.3283 | -0.0446 | 0 | 2 | 93 | 6 | R.FTEDAK.R |
|  | 5858 | **547.6174** | **1093.2203** | **1093.6244** | **-0.4041** | **0** | **0** | **92** | **4** | **R.NLAVSQVVHK.A** |
|  | 6056 | **554.7761** | **1107.5377** | **1107.5461** | **-0.0084** | **1** | **10** | **16** | **1** | **R.WRDSLEFR.E** |
|  | 15117 | 825.3701 | 1648.7256 | 1647.8099 | 0.9157 | 0 | 10 | 11 | 6 | K.MEEVEAMLLPETLK.R |
|  | 18542 | **946.4332** | **1890.8519** | **1890.8636** | **-0.0116** | **0** | **79** | **1.2e-006** | **1** | **R.LYGSEAFATDFQDSAAAK.K** |
|  | 22513 | **760.7282** | **2279.1628** | **2278.1378** | **1.0250** | **1** | **2** | **54** | **3** | **K.DLDSQTMMVLVNYIFFKAK.W** |
|  | 27844 | 796.6539 | 3182.5864 | 3182.5587 | 0.0276 | 1 | (2) | 31 | 4 | R.TLNQSSDELQLSMGNAMFVKEQLSLLDR.F |
|  | 27896 | **1067.2223** | **3198.6450** | **3198.5536** | **0.0914** | **1** | **3** | **23** | **1** | **R.TLNQSSDELQLSMGNAMFVKEQLSLLDR.F** |

| **531.** | O95169\|NDUB8_HUMAN    **Mass:** 21865    **Score:** 78     **Queries matched:** 14   **emPAI:** 0.38 |
| --- | --- |
|  | NADH dehydrogenase [ubiquinone] 1 beta subcomplex subunit 8, mitochondrial OS=Homo sapiens GN=NDUFB8 PE=1 SV=1 |

|  | **Query** | **Observed** | **Mr(expt)** | **Mr(calc)** | **Delta** | **Miss** | **Score** | **Expect** | **Rank** | **Peptide** |
| --- | --- | --- | --- | --- | --- | --- | --- | --- | --- | --- |
|  | 388 | **430.8870** | **429.8798** | **430.2540** | **-0.3742** | **0** | **1** | **1.6e+002** | **2** | **R.AAAAK.K** 372 |
|  | 14810 | 815.9117 | 1629.8089 | 1628.8127 | 0.9963 | 1 | 10 | 12 | 2 | R.NVMPLGARTASHMTK.D 14808 |
|  | 16029 | 854.4076 | 1706.8007 | 1706.7722 | 0.0285 | 1 | 8 | 17 | 4 | K.DMFPGPYPRTPEER.A 16025 |
|  | 16953 | 590.9226 | 1769.7460 | 1769.9723 | -0.2263 | 1 | 0 | 95 | 5 | -.MAVARAGVLGVQWLQR.A |
|  | 18004 | 617.9620 | 1850.8641 | 1850.8444 | 0.0197 | 1 | 3 | 46 | 2 | R.TASHMTKDMFPGPYPR.T 18003 |
|  | 21444 | **719.6628** | **2155.9667** | **2155.9824** | **-0.0157** | **1** | **25** | **0.29** | **1** | **R.SQHERDPWYSWDQPGLR.L** |
|  | 21911 | **1105.4407** | **2208.8668** | **2208.8834** | **-0.0166** | **0** | **48** | **0.0011** | **1** | **R.VEDYEPYPDDGMGYGDYPK.L** 21912 |
|  | 25273 | **897.7172** | **2690.1298** | **2690.1483** | **-0.0184** | **1** | **14** | **2.2** | **1** | **R.VEDYEPYPDDGMGYGDYPKLPDR.S** |
|  | 31477 | **1791.5358** | **5371.5855** | **5370.4973** | **1.0882** | **1** | **2** | **7.6** | **1** | **R.NRVDTSPTPVSWHVMCMQLFGFLAFMIFMCWVGDVYPVYQPVGPK.Q** |

| **532.** | P09543\|CN37_HUMAN    **Mass:** 47948    **Score:** 78     **Queries matched:** 8   **emPAI:** 0.08 |
| --- | --- |
|  | 2',3'-cyclic-nucleotide 3'-phosphodiesterase OS=Homo sapiens GN=CNP PE=1 SV=2 |

|  | **Query** | **Observed** | **Mr(expt)** | **Mr(calc)** | **Delta** | **Miss** | **Score** | **Expect** | **Rank** | **Peptide** |
| --- | --- | --- | --- | --- | --- | --- | --- | --- | --- | --- |
|  | 1313 | **615.0427** | **614.0354** | **613.3548** | **0.6807** | **0** | **6** | **31** | **6** | **K.ITPGAR.G** |
|  | 2119 | **406.7275** | **811.4405** | **810.3905** | **1.0500** | **1** | **6** | **34** | **2** | **R.KMSSSGAK.D** |
|  | 3247 | 450.5861 | 899.1576 | 899.4059 | -0.2483 | 0 | 3 | 52 | 1 | K.MVSADAYK.I |
|  | 3431 | 456.7638 | 911.5130 | 911.5804 | -0.0673 | 1 | 8 | 23 | 6 | K.KLKPGLEK.D 3445 |
|  | 5469 | **532.2893** | **1062.5639** | **1063.5774** | **-1.0135** | **1** | **1** | **1.3e+002** | **9** | **R.GKLYSLGNGR.W** |
|  | 11917 | **723.3612** | **1444.7079** | **1444.7310** | **-0.0232** | **1** | **4** | **58** | **3** | **K.NQWQLSADDLKK.L** |
|  | 27078 | **1003.1772** | **3006.5097** | **3006.5298** | **-0.0201** | **1** | **78** | **7.8e-007** | **1** | **R.VELSEQQLQLWPSDVDKLSPTDNLPR.G** |

| **533.** | P50454\|SERPH_HUMAN    **Mass:** 46525    **Score:** 78     **Queries matched:** 11   **emPAI:** 0.08 |
| --- | --- |
|  | Serpin H1 OS=Homo sapiens GN=SERPINH1 PE=1 SV=2 |

|  | **Query** | **Observed** | **Mr(expt)** | **Mr(calc)** | **Delta** | **Miss** | **Score** | **Expect** | **Rank** | **Peptide** |
| --- | --- | --- | --- | --- | --- | --- | --- | --- | --- | --- |
|  | 6998 | **395.0784** | **1182.2135** | **1182.5716** | **-0.3582** | **1** | **5** | **29** | **9** | **K.FHHKMVDNR.G** |
|  | 7448 | **601.7806** | **1201.5467** | **1201.4934** | **0.0533** | **0** | **4** | **56** | **5** | **K.QHYNCEHSK.I** |
|  | 9815 | **442.5643** | **1324.6711** | **1324.6380** | **0.0331** | **1** | **2** | **78** | **4** | **K.MVDNRGFMVTR.S** |
|  | 10054 | **669.3341** | **1336.6537** | **1336.7350** | **-0.0814** | **0** | **4** | **52** | **4** | **K.HLAGLGLTEAIDK.N** |
|  | 10125 | **448.2224** | **1341.6455** | **1342.5832** | **-0.9377** | **0** | **2** | **84** | **5** | **R.SYTVGVMMMHR.T** |
|  | 15300 | **830.9004** | **1659.7862** | **1658.7941** | **0.9922** | **0** | **59** | **0.00015** | **1** | **R.LYGPSSVSFADDFVR.S** 15278 |
|  | 19613 | **496.7784** | **1983.0844** | **1983.0975** | **-0.0131** | **0** | **18** | **1.2** | **1** | **K.LSSLIILMPHHVEPLER.L** |
|  | 19831 | 1001.0423 | 2000.0700 | 1999.0924 | 0.9776 | 0 | (1) | 80 | 6 | K.LSSLIILMPHHVEPLER.L |
|  | 24097 | **829.7574** | **2486.2505** | **2486.2653** | **-0.0147** | **1** | **18** | **1** | **1** | **R.SALQSINEWAAQTTDGKLPEVTK.D** |
|  | 24458 | **424.9049** | **2543.3858** | **2542.3755** | **1.0103** | **1** | **5** | **21** | **5** | **K.GVVEVTHDLQKHLAGLGLTEAIDK.N** |

| **534.** | P42330\|AK1C3_HUMAN    **Mass:** 37220    **Score:** 77     **Queries matched:** 15   **emPAI:** 0.33 |
| --- | --- |
|  | Aldo-keto reductase family 1 member C3 OS=Homo sapiens GN=AKR1C3 PE=1 SV=3 |

|  | **Query** | **Observed** | **Mr(expt)** | **Mr(calc)** | **Delta** | **Miss** | **Score** | **Expect** | **Rank** | **Peptide** |
| --- | --- | --- | --- | --- | --- | --- | --- | --- | --- | --- |
|  | 1536 | 685.4556 | 684.4483 | 684.4534 | -0.0051 | 0 | 21 | 1.3 | 4 | R.GVVVLAK.S |
|  | 1550 | 689.3528 | 688.3455 | 688.3755 | -0.0300 | 0 | 23 | 1.4 | 1 | K.IADGSVK.R |
|  | 1719 | 758.4101 | 757.4028 | 758.3923 | -0.9894 | 0 | 6 | 58 | 9 | K.AIDGLDR.N |
|  | 4532 | 993.5043 | 992.4970 | 992.5039 | -0.0069 | 0 | 15 | 5.3 | 1 | K.SIGVSNFNR.R |
|  | 4799 | 505.8239 | 1009.6333 | 1009.6396 | -0.0063 | 1 | 11 | 11 | 3 | K.RTPALIALR.Y |
|  | 6573 | 575.3054 | 1148.5963 | 1148.6050 | -0.0088 | 1 | 9 | 21 | 2 | K.SIGVSNFNRR.Q 6581 |
|  | 6701 | 579.7891 | 1157.5637 | 1157.5717 | -0.0080 | 1 | 32 | 0.11 | 1 | K.REDIFYTSK.L 6700 |
|  | 10883 | 461.9362 | 1382.7867 | 1382.7955 | -0.0088 | 0 | 26 | 0.32 | 1 | R.QLEMILNKPGLK.Y |
|  | 16008 | 853.9128 | 1705.8111 | 1705.9363 | -0.1251 | 1 | 5 | 37 | 9 | K.SKDIVLVAYSALGSQR.D |
|  | 21647 | 727.0293 | 2178.0661 | 2178.0817 | -0.0157 | 0 | 68 | 1.5e-005 | 1 | R.HIDSAHLYNNEEQVGLAIR.S |
|  | 21777 | **732.7041** | **2195.0905** | **2195.1045** | **-0.0140** | **1** | **24** | **0.34** | **1** | **R.IRQNVQVFEFQLTAEDMK.A** 21776 |
|  | 22190 | **1118.5815** | **2235.1485** | **2235.1609** | **-0.0124** | **0** | **25** | **0.26** | **1** | **R.WVDPNSPVLLEDPVLCALAK.K** |

| **535.** | Q9UL46\|PSME2_HUMAN    **Mass:** 27515    **Score:** 77     **Queries matched:** 8   **emPAI:** 0.14 |
| --- | --- |
|  | Proteasome activator complex subunit 2 OS=Homo sapiens GN=PSME2 PE=1 SV=3 |

|  | **Query** | **Observed** | **Mr(expt)** | **Mr(calc)** | **Delta** | **Miss** | **Score** | **Expect** | **Rank** | **Peptide** |
| --- | --- | --- | --- | --- | --- | --- | --- | --- | --- | --- |
|  | 1156 | **571.3159** | **570.3086** | **569.3537** | **0.9549** | **0** | **21** | **1.3** | **1** | **K.IVNPK.G** 1151 |
|  | 4619 | 499.7659 | 997.5172 | 997.3546 | 0.1626 | 0 | 10 | 17 | 2 | K.DDEMETDK.Q |
|  | 10327 | **676.8695** | **1351.7245** | **1351.7347** | **-0.0103** | **1** | **25** | **0.4** | **1** | **K.TKVEAFQTTISK.Y** |
|  | 12718 | **498.2805** | **1491.8197** | **1490.7439** | **1.0759** | **1** | **2** | **82** | **5** | **K.IVNPKGEEKPSMY.-** |
|  | 16368 | **577.2817** | **1728.8234** | **1727.8591** | **0.9643** | **1** | **5** | **37** | **3** | **R.ALVHERDEAAYGELR.A** |
|  | 19700 | **664.0292** | **1989.0657** | **1990.1438** | **-1.0781** | **1** | **3** | **44** | **1** | **K.EKCILVITWIQHLIPK.I** |
|  | 24397 | **1266.1826** | **2530.3507** | **2530.3642** | **-0.0135** | **0** | **77** | **1.3e-006** | **1** | **K.IIYLNQLLQEDSLNVADLTSLR.A** |

| **536.** | O43169\|CYB5B_HUMAN    **Mass:** 16436    **Score:** 77     **Queries matched:** 9   **emPAI:** 0.89 |
| --- | --- |
|  | Cytochrome b5 type B OS=Homo sapiens GN=CYB5B PE=1 SV=2 |

|  | **Query** | **Observed** | **Mr(expt)** | **Mr(calc)** | **Delta** | **Miss** | **Score** | **Expect** | **Rank** | **Peptide** |
| --- | --- | --- | --- | --- | --- | --- | --- | --- | --- | --- |
|  | 1398 | **636.1176** | **635.1103** | **636.2537** | **-1.1434** | **0** | **1** | **1.3e+002** | **1** | **K.NDTCK.S** |
|  | 6442 | **570.2750** | **1138.5354** | **1139.4765** | **-0.9411** | **0** | **35** | **0.05** | **2** | **-.MATAEASGSDGK.G** 6441 6465 |
|  | 13829 | 522.3003 | 1563.8790 | 1563.8885 | -0.0095 | 1 | 38 | 0.019 | 1 | R.NSLKELWLVIHGR.V |
|  | 23493 | 1203.6137 | 2405.2127 | 2405.0983 | 0.1145 | 1 | 4 | 27 | 2 | M.ATAEASGSDGKGQEVETSVTYYR.L |
|  | 24621 | **854.7594** | **2561.2564** | **2560.2445** | **1.0118** | **1** | **4** | **30** | **7** | **K.QYYIGDIHPSDLKPESGSKDPSK.N** 24600 |
|  | 29158 | 907.6617 | 3626.6179 | 3626.6397 | -0.0219 | 0 | 54 | 0.00014 | 1 | R.FLNEHPGGEEVLLEQAGVDASESFEDVGHSSDAR.E |

| **537.** | Q9BY49\|PECR_HUMAN    **Mass:** 32809    **Score:** 77     **Queries matched:** 6   **emPAI:** 0.11 |
| --- | --- |
|  | Peroxisomal trans-2-enoyl-CoA reductase OS=Homo sapiens GN=PECR PE=1 SV=2 |

|  | **Query** | **Observed** | **Mr(expt)** | **Mr(calc)** | **Delta** | **Miss** | **Score** | **Expect** | **Rank** | **Peptide** |
| --- | --- | --- | --- | --- | --- | --- | --- | --- | --- | --- |
|  | 1212 | **585.0895** | **584.0823** | **583.3806** | **0.7017** | **1** | **6** | **17** | **1** | **K.IPAKR.I** |
|  | 10460 | 681.3301 | 1360.6456 | 1361.6761 | -1.0305 | 0 | 10 | 14 | 3 | K.SLALEWACSGIR.I |
|  | 21907 | **737.0353** | **2208.0840** | **2208.1361** | **-0.0521** | **1** | **4** | **32** | **2** | **R.AGVYNLTKSLALEWACSGIR.I** 21885 21899 |
|  | 22428 | **1136.1328** | **2270.2511** | **2270.2634** | **-0.0124** | **0** | **77** | **1.6e-006** | **1** | **R.SYLAPGLLQGQVAIVTGGATGIGK.A** |

| **538.** | Q02878\|RL6_HUMAN    **Mass:** 32765    **Score:** 77     **Queries matched:** 21   **emPAI:** 0.24 |
| --- | --- |
|  | 60S ribosomal protein L6 OS=Homo sapiens GN=RPL6 PE=1 SV=3 |

|  | **Query** | **Observed** | **Mr(expt)** | **Mr(calc)** | **Delta** | **Miss** | **Score** | **Expect** | **Rank** | **Peptide** |
| --- | --- | --- | --- | --- | --- | --- | --- | --- | --- | --- |
|  | 871 | **504.6874** | **503.6801** | **503.2452** | **0.4349** | **0** | **4** | **93** | **2** | **K.NGGTR.V** 862 872 |
|  | 1773 | **788.4456** | **787.4383** | **787.4440** | **-0.0056** | **0** | **16** | **5.9** | **1** | **K.IDISNVK.I** |
|  | 2815 | 866.4924 | 865.4851 | 865.4909 | -0.0058 | 0 | 25 | 0.5 | 1 | K.FVIATSTK.I |
|  | 3781 | **470.2942** | **938.5739** | **937.4505** | **1.1235** | **0** | **19** | **2.1** | **1** | **K.YEITEQR.K** |
|  | 4204 | **970.5505** | **969.5433** | **969.5495** | **-0.0062** | **0** | **25** | **0.43** | **1** | **K.AVDSQILPK.I** 4208 |
|  | 4540 | 497.7497 | 993.4848 | 993.4920 | -0.0072 | 0 | 7 | 32 | 7 | K.HLTDAYFK.K |
|  | 6289 | **564.3104** | **1126.6063** | **1125.6757** | **0.9306** | **1** | **4** | **60** | **7** | **K.IDISNVKIPK.H** |
|  | 6440 | **570.2679** | **1138.5213** | **1138.5295** | **-0.0082** | **0** | **1** | **1.2e+002** | **1** | **R.YYPTEDVPR.K** |
|  | 7413 | 401.2128 | 1200.6166 | 1200.6350 | -0.0184 | 1 | 9 | 18 | 8 | M.AGEKVEKPDTK.E |
|  | 8719 | **634.3151** | **1266.6156** | **1266.6244** | **-0.0089** | **1** | **10** | **13** | **1** | **R.YYPTEDVPRK.L** |
|  | 9023 | 642.8141 | 1283.6136 | 1283.7449 | -0.1313 | 0 | 35 | 0.047 | 1 | K.VLATVTKPVGGDK.N 9047 |
|  | 11960 | **483.2547** | **1446.7424** | **1445.7667** | **0.9757** | **0** | **10** | **14** | **5** | **R.SVFALTNGIYPHK.L** 11957 11958 |
|  | 13192 | **508.2861** | **1521.8364** | **1522.7238** | **-0.8874** | **1** | **6** | **32** | **2** | **K.MPRYYPTEDVPR.K** |
|  | 13449 | **770.9509** | **1539.8873** | **1540.8825** | **-0.9952** | **1** | **7** | **27** | **6** | **K.EKVLATVTKPVGGDK.N** |
|  | 16891 | **882.5361** | **1763.0576** | **1763.0669** | **-0.0093** | **0** | **67** | **2.2e-005** | **1** | **K.QLASGLLLVTGPLVLNR.V** |

| **539.** | Q9BRX8\|CJ058_HUMAN    **Mass:** 25861    **Score:** 77     **Queries matched:** 13   **emPAI:** 0.32 |
| --- | --- |
|  | Uncharacterized protein C10orf58 OS=Homo sapiens GN=C10orf58 PE=1 SV=3 |

|  | **Query** | **Observed** | **Mr(expt)** | **Mr(calc)** | **Delta** | **Miss** | **Score** | **Expect** | **Rank** | **Peptide** |
| --- | --- | --- | --- | --- | --- | --- | --- | --- | --- | --- |
|  | 3661 | **465.7599** | **929.5053** | **929.5117** | **-0.0064** | **0** | **37** | **0.035** | **1** | **K.NGAVIMAVR.R** 3676 |
|  | 3877 | **473.5766** | **945.1386** | **945.5066** | **-0.3680** | **0** | **(1)** | **95** | **9** | **K.NGAVIMAVR.R** |
|  | 7403 | **600.9746** | **1199.9347** | **1200.6080** | **-0.6733** | **0** | **4** | **43** | **3** | **R.LGVWYNFFR.A** |
|  | 8322 | **623.3429** | **1244.6712** | **1244.6798** | **-0.0086** | **0** | **10** | **14** | **2** | **K.MIKPQTLASEK.K** |
|  | 8648 | 631.7955 | 1261.5765 | 1260.6747 | 0.9018 | 0 | (9) | 17 | 5 | K.MIKPQTLASEK.K |
|  | 11201 | 701.8377 | 1401.6609 | 1400.6976 | 0.9633 | 1 | 1 | 1.2e+002 | 7 | R.TEVKDFQPYFK.G |
|  | 14835 | 816.4114 | 1630.8083 | 1630.8501 | -0.0418 | 1 | 8 | 18 | 6 | K.ELWEKNGAVIMAVR.R |
|  | 14839 | 816.4140 | 1630.8134 | 1631.8957 | -1.0822 | 0 | 51 | 0.001 | 1 | K.SMLDQLGVPLYAVVK.E 14854 14855 |
|  | 15122 | 825.4094 | 1648.8043 | 1647.8906 | 0.9137 | 0 | (7) | 24 | 5 | K.SMLDQLGVPLYAVVK.E |
|  | 30971 | **1142.0355** | **4564.1130** | **4563.2892** | **0.8238** | **0** | **1** | **14** | **2** | **-.MSFLQDPSFFTMGMWSIGAGALGAAALALLLANTDVFLSKPQK.A** |

| **540.** | P50914\|RL14_HUMAN    **Mass:** 23531    **Score:** 76     **Queries matched:** 14   **emPAI:** 0.57 |
| --- | --- |
|  | 60S ribosomal protein L14 OS=Homo sapiens GN=RPL14 PE=1 SV=4 |

|  | **Query** | **Observed** | **Mr(expt)** | **Mr(calc)** | **Delta** | **Miss** | **Score** | **Expect** | **Rank** | **Peptide** |
| --- | --- | --- | --- | --- | --- | --- | --- | --- | --- | --- |
|  | 1090 | **555.2567** | **554.2494** | **553.3224** | **0.9270** | **0** | **15** | **3.8** | **1** | **K.AAPAPK.A** 1087 1091 |
|  | 1587 | **706.3829** | **705.3757** | **705.3810** | **-0.0053** | **0** | **27** | **0.38** | **1** | **R.FVEVGR.V** |
|  | 2971 | **439.2359** | **876.4572** | **876.4640** | **-0.0068** | **1** | **17** | **3.8** | **5** | **R.RQAMPFK.C** |
|  | 3176 | **447.7347** | **893.4549** | **892.4589** | **0.9960** | **1** | **(4)** | **67** | **3** | **R.RQAMPFK.C** 3168 |
|  | 3951 | 476.2405 | 950.4665 | 950.4722 | -0.0057 | 0 | 11 | 13 | 2 | K.FPHSAHQK.Y |
|  | 5407 | 529.9421 | 1057.8696 | 1058.4855 | -0.6159 | 1 | 5 | 41 | 2 | K.MTDFDRFK.V |
|  | 7697 | **608.3072** | **1214.5999** | **1214.6078** | **-0.0078** | **0** | **28** | **0.23** | **1** | **R.ALVDGPCTQVR.R** |
|  | 8740 | **634.8190** | **1267.6234** | **1267.6305** | **-0.0071** | **0** | **41** | **0.01** | **1** | **K.CMQLTDFILK.F** |
|  | 10363 | 677.8839 | 1353.7533 | 1353.7616 | -0.0083 | 0 | 59 | 0.00019 | 1 | K.LVAIVDVIDQNR.A |
|  | 11351 | 470.9265 | 1409.7577 | 1409.7990 | -0.0413 | 1 | 5 | 40 | 1 | K.GTAAAAAAAAAAKVPAK.K |
|  | 12340 | 735.8308 | 1469.6471 | 1468.7997 | 0.8473 | 1 | 4 | 47 | 7 | K.APGTKGTAAAAAAAAAAK.V |

| **541.** | P23378\|GCSP_HUMAN    **Mass:** 114254   **Score:** 76     **Queries matched:** 26   **emPAI:** 0.10 |
| --- | --- |
|  | Glycine dehydrogenase [decarboxylating], mitochondrial OS=Homo sapiens GN=GLDC PE=1 SV=2 |

|  | **Query** | **Observed** | **Mr(expt)** | **Mr(calc)** | **Delta** | **Miss** | **Score** | **Expect** | **Rank** | **Peptide** |
| --- | --- | --- | --- | --- | --- | --- | --- | --- | --- | --- |
|  | 2211 | **411.0969** | **820.1792** | **820.4299** | **-0.2507** | **1** | **4** | **57** | **9** | **K.MMGGKGLK.Q** |
|  | 3504 | **459.2291** | **916.4437** | **915.4311** | **1.0126** | **1** | **16** | **5.2** | **1** | **R.HDDFARR.H** |
|  | 7388 | 600.4399 | 1198.8652 | 1198.4998 | 0.3654 | 0 | 3 | 60 | 7 | K.LPCEMDFSGK.D |
|  | 7550 | **604.1199** | **1206.2252** | **1206.5849** | **-0.3597** | **1** | **9** | **12** | **1** | **R.ESLVRMMPGR.M** |
|  | 7870 | **408.2465** | **1221.7176** | **1221.6969** | **0.0208** | **1** | **5** | **48** | **8** | **R.AKYTGVLTELK.L** |
|  | 8129 | **412.8690** | **1235.5852** | **1235.6034** | **-0.0181** | **0** | **5** | **41** | **7** | **K.VEDFTELVER.A** |
|  | 8417 | 625.8238 | 1249.6331 | 1249.6094 | 0.0238 | 1 | 12 | 9.2 | 10 | R.MMPGRMVGVTR.D |
|  | 10245 | **450.2793** | **1347.8162** | **1346.7459** | **1.0703** | **1** | **6** | **36** | **5** | **R.LETHYRILFR.G** |
|  | 10558 | 683.8196 | 1365.6247 | 1365.7187 | -0.0940 | 1 | 5 | 38 | 3 | K.GEGHRTVCLIPK.S 10562 |
|  | 13861 | **523.2378** | **1566.6917** | **1566.7031** | **-0.0114** | **0** | **15** | **3.9** | **2** | **K.SAHGTNPASAHMAGMK.I** |
|  | 15207 | **552.0527** | **1653.1362** | **1653.7886** | **-0.6525** | **0** | **4** | **34** | **4** | **K.DVSGVLFQYPDTEGK.V** 15226 |
|  | 15612 | **561.6593** | **1681.9561** | **1681.9668** | **-0.0107** | **0** | **28** | **0.18** | **1** | **K.HLAPFLPNHPVISLK.R** |
|  | 16946 | **590.6335** | **1768.8786** | **1768.8897** | **-0.0111** | **0** | **16** | **3** | **1** | **R.AGHQLQHDLFFDTLK.I** |
|  | 17478 | **905.9102** | **1809.8059** | **1809.9043** | **-0.0984** | **1** | **3** | **53** | **4** | **K.QATETAILNANYMAKR.L** |
|  | 18042 | **927.9347** | **1853.8548** | **1853.8917** | **-0.0369** | **0** | **8** | **16** | **1** | **K.TFCIPHGGGGPGMGPIGVK.K** |
|  | 18187 | 621.9915 | 1862.9526 | 1861.9628 | 0.9898 | 0 | 10 | 9.9 | 1 | R.FGVPLGYGGPHAAFFAVR.E |
|  | 18682 | **952.0206** | **1902.0267** | **1902.0019** | **0.0248** | **0** | **5** | **29** | **3** | **R.EMLQTLGLASIDELIEK.T** |
|  | 22812 | 773.3663 | 2317.0772 | 2317.1835 | -0.1063 | 1 | 2 | 49 | 4 | K.DQREMLQTLGLASIDELIEK.T |
|  | 24017 | **826.4061** | **2476.1964** | **2476.2135** | **-0.0171** | **0** | **14** | **2.9** | **1** | **R.TSPFLTHQVFNSYHSETNIVR.Y** |
|  | 24920 | **873.4360** | **2617.2861** | **2617.3078** | **-0.0217** | **0** | **12** | **4.7** | **1** | **K.EFANIHPFVPLDQAQGYQQLFR.E** |
|  | 25214 | **1340.6445** | **2679.2745** | **2679.2929** | **-0.0184** | **0** | **68** | **9.9e-006** | **1** | **R.NLLENSGWITQYTPYQPEVSQGR.L** |
|  | 26087 | **945.4586** | **2833.3539** | **2834.2779** | **-0.9241** | **1** | **13** | **3.1** | **1** | **K.LPCEMDFSGKDVSGVLFQYPDTEGK.V** |
|  | 27698 | **1049.4321** | **3145.2746** | **3146.3519** | **-1.0773** | **0** | **1** | **34** | **5** | **K.DLDDLLWIFGCESSAELVAESMGEECR.G** |
|  | 30265 | 1022.2596 | 4085.0092 | 4085.9880 | -0.9788 | 1 | 2 | 17 | 3 | K.MSPHSLTCVTSSHWDRPYSREVAAFPLPFVKPENK.F |

| **542.** | Q8N139\|ABCA6_HUMAN    **Mass:** 185706   **Score:** 76     **Queries matched:** 15   **emPAI:** 0.02 |
| --- | --- |
|  | ATP-binding cassette sub-family A member 6 OS=Homo sapiens GN=ABCA6 PE=1 SV=2 |

|  | **Query** | **Observed** | **Mr(expt)** | **Mr(calc)** | **Delta** | **Miss** | **Score** | **Expect** | **Rank** | **Peptide** |
| --- | --- | --- | --- | --- | --- | --- | --- | --- | --- | --- |
|  | 1836 | **396.7194** | **791.4242** | **791.3960** | **0.0283** | **0** | **11** | **15** | **3** | **K.VIMSNGR.L** |
|  | 2566 | **424.8895** | **847.7644** | **847.4586** | **0.3059** | **0** | **2** | **78** | **3** | **R.VAIMVSGR.L** |
|  | 3324 | **453.0464** | **904.0782** | **903.4960** | **0.5822** | **1** | **13** | **6.7** | **2** | **R.KVIMSNGR.L** |
|  | 4408 | **492.9002** | **983.7858** | **984.4521** | **-0.6663** | **0** | **4** | **46** | **2** | **R.MQVFAMAR.L** |
|  | 6086 | **555.9462** | **1109.8779** | **1109.5652** | **0.3127** | **1** | **2** | **59** | **10** | **R.FSVVCNTKR.L** |
|  | 6192 | **559.8683** | **1117.7221** | **1116.6437** | **1.0784** | **1** | **8** | **27** | **1** | **R.VAIMVSGRLR.C** |
|  | 6731 | **581.2779** | **1160.5412** | **1161.6757** | **-1.1345** | **1** | **3** | **86** | **5** | **K.ENLSLFAKIK.G** |
|  | 7232 | 596.8000 | 1191.5854 | 1190.6910 | 0.8944 | 1 | 6 | 39 | 4 | K.LGKDYILELK.V |
|  | 9278 | 649.3389 | 1296.6633 | 1296.6972 | -0.0339 | 1 | 1 | 1.2e+002 | 4 | R.CIGSIQHLKNK.L |
|  | 9352 | 651.3709 | 1300.7273 | 1300.6888 | 0.0385 | 1 | 18 | 2.4 | 4 | K.DPVFRISPQSR.D |
|  | 18305 | **935.9951** | **1869.9757** | **1869.9870** | **-0.0113** | **0** | **76** | **2.6e-006** | **1** | **R.ILLELDMQNIQDNLAK.H** |
|  | 18884 | 960.9570 | 1919.8994 | 1920.9905 | -1.0911 | 1 | 2 | 60 | 4 | R.NGTDGLSYNGAIIVSGKQK.D |
|  | 24881 | **870.4460** | **2608.3161** | **2608.2252** | **0.0909** | **1** | **1** | **54** | **5** | **R.DAKPNPEEPIDEDEDIQTERIR.T** |
|  | 26893 | **743.9164** | **2971.6367** | **2972.4987** | **-0.8621** | **1** | **4** | **18** | **2** | **R.VDKFNSSSLMVVYTPISNLTQQIMNK.T** |
|  | 31062 | **768.3682** | **4604.1653** | **4603.4355** | **0.7298** | **1** | **3** | **9.2** | **1** | **R.GFVALQTAINTAIIEITTNHPVMEELMSVTAITMKTLPFITK.N** |

| **543.** | P62995\|TRA2B_HUMAN    **Mass:** 33760    **Score:** 76     **Queries matched:** 4   **emPAI:** 0.11 |
| --- | --- |
|  | Transformer-2 protein homolog beta OS=Homo sapiens GN=TRA2B PE=1 SV=1 |

|  | **Query** | **Observed** | **Mr(expt)** | **Mr(calc)** | **Delta** | **Miss** | **Score** | **Expect** | **Rank** | **Peptide** |
| --- | --- | --- | --- | --- | --- | --- | --- | --- | --- | --- |
|  | 8384 | **416.9041** | **1247.6905** | **1246.5724** | **1.1181** | **1** | **10** | **16** | **4** | **K.ERANGMELDGR.R** 8351 |
|  | 14548 | **807.3763** | **1612.7381** | **1612.6713** | **0.0668** | **1** | **1** | **93** | **4** | **M.SDSGEQNYGERESR.S** |
|  | 17482 | **905.9463** | **1809.8780** | **1809.8897** | **-0.0117** | **0** | **76** | **2.7e-006** | **1** | **K.YGPIADVSIVYDQQSR.R** |

| **544.** | Q14258\|TRI25_HUMAN    **Mass:** 72597    **Score:** 76     **Queries matched:** 18   **emPAI:** 0.05 |
| --- | --- |
|  | Tripartite motif-containing protein 25 OS=Homo sapiens GN=TRIM25 PE=1 SV=1 |

|  | **Query** | **Observed** | **Mr(expt)** | **Mr(calc)** | **Delta** | **Miss** | **Score** | **Expect** | **Rank** | **Peptide** |
| --- | --- | --- | --- | --- | --- | --- | --- | --- | --- | --- |
|  | 4054 | 479.7663 | 957.5180 | 957.4992 | 0.0188 | 1 | 7 | 34 | 9 | R.ALDDVRNR.Q |
|  | 4626 | **500.2230** | **998.4314** | **998.5257** | **-0.0944** | **1** | **7** | **33** | **2** | **R.QGPESRLGR.N** |
|  | 6170 | **559.2893** | **1116.5641** | **1116.5710** | **-0.0069** | **1** | **6** | **38** | **7** | **K.NELKQCIGR.L** 6184 |
|  | 6188 | 559.8020 | 1117.5894 | 1118.6699 | -1.0805 | 1 | 7 | 34 | 4 | K.AKVLETFLAK.S 6183 |
|  | 8050 | **616.8106** | **1231.6067** | **1231.6408** | **-0.0341** | **1** | **8** | **23** | **8** | **K.EEIEQSLTKR.D** |
|  | 8065 | **617.3104** | **1232.6062** | **1233.5884** | **-0.9822** | **1** | **11** | **13** | **4** | **R.QQDVRMTANR.K** 8108 |
|  | 8671 | 421.9079 | 1262.7018 | 1263.6306 | -0.9289 | 0 | 9 | 17 | 5 | K.ALLDASETTSTR.K |
|  | 11062 | **465.2520** | **1392.7342** | **1391.7256** | **1.0086** | **1** | **8** | **23** | **6** | **K.ALLDASETTSTRK.I** 11064 |
|  | 11373 | **706.3940** | **1410.7735** | **1409.7554** | **1.0181** | **0** | **1** | **90** | **10** | **K.SRPELLEYYIK.V** |
|  | 15195 | **827.4053** | **1652.7961** | **1652.8079** | **-0.0118** | **1** | **76** | **3.1e-006** | **1** | **R.KVEQLQQEYTEMK.A** |
|  | 22473 | **760.0496** | **2277.1269** | **2277.3096** | **-0.1828** | **1** | **0** | **75** | **5** | **R.GISTKPVYIPEVELNHKLIK.G** |
|  | 26019 | 940.1713 | 2817.4922 | 2816.3148 | 1.1774 | 1 | 1 | 42 | 7 | K.QCIGRLQELTPSSGDPGEHDPASTHK.S |
|  | 27698 | 1049.4321 | 3145.2746 | 3146.4161 | -1.1415 | 1 | 0 | 35 | 7 | K.GIHYWEVELQKNNFCGVGICYGSMNR.Q |
|  | 30534 | **1396.2933** | **4185.8582** | **4187.0316** | **-1.1735** | **1** | **2** | **14** | **1** | **K.VILDYNTAHNKVALSECYTVASVAEMPQNYRPHPQR.F** |

| **545.** | Q07000\|1C15_HUMAN    **Mass:** 41293    **Score:** 75     **Queries matched:** 14   **emPAI:** 0.30 |
| --- | --- |
|  | HLA class I histocompatibility antigen, Cw-15 alpha chain OS=Homo sapiens GN=HLA-C PE=1 SV=1 |

|  | **Query** | **Observed** | **Mr(expt)** | **Mr(calc)** | **Delta** | **Miss** | **Score** | **Expect** | **Rank** | **Peptide** |
| --- | --- | --- | --- | --- | --- | --- | --- | --- | --- | --- |
|  | 4142 | 483.2169 | 964.4193 | 964.4250 | -0.0057 | 0 | 53 | 0.00075 | 1 | R.FDSDAASPR.G |
|  | 7393 | 600.7434 | 1199.4723 | 1199.6371 | -0.1648 | 1 | 8 | 22 | 4 | R.QAQTDRVNLR.K 7421 |
|  | 7831 | 611.2879 | 1220.5612 | 1220.6037 | -0.0424 | 0 | 2 | 89 | 3 | K.DYIALNEDLR.S |
|  | 8239 | 621.3292 | 1240.6439 | 1239.5013 | 1.1426 | 0 | 11 | 12 | 3 | R.MYGCDLGPDGR.L |
|  | 8528 | 628.9625 | 1255.9104 | 1255.4962 | 0.4142 | 0 | (1) | 78 | 5 | R.MYGCDLGPDGR.L |
|  | 9648 | 439.8941 | 1316.6606 | 1315.6673 | 0.9932 | 0 | 8 | 21 | 4 | R.YFYTAVSRPGR.G |
|  | 10294 | 676.3427 | 1350.6709 | 1349.6939 | 0.9770 | 1 | 8 | 24 | 1 | R.YLENGKETLQR.A |
|  | 11642 | 714.3621 | 1426.7097 | 1426.7205 | -0.0108 | 0 | 11 | 10 | 1 | K.WAAVVVPSGEEQR.Y |
|  | 12774 | 748.8586 | 1495.7026 | 1495.7129 | -0.0103 | 0 | 11 | 9.6 | 2 | R.AYLEGTCVEWLR.R |
|  | 13126 | 760.3734 | 1518.7323 | 1518.7427 | -0.0104 | 0 | 41 | 0.011 | 1 | R.SWTAADTAAQITQR.K |
|  | 17823 | 613.3262 | 1836.9569 | 1835.9027 | 1.0542 | 0 | 9 | 14 | 2 | K.THVTHHPVSDHEATLR.C |
|  | 24990 | 879.7289 | 2636.1650 | 2636.1838 | -0.0188 | 0 | 27 | 0.14 | 1 | R.DGEDQTQDTELVETRPAGDGTFQK.W 24989 |

| **546.** | P30501\|1C02_HUMAN    **Mass:** 41468    **Score:** 75     **Queries matched:** 12   **emPAI:** 0.30 |
| --- | --- |
|  | HLA class I histocompatibility antigen, Cw-2 alpha chain OS=Homo sapiens GN=HLA-C PE=1 SV=1 |

|  | **Query** | **Observed** | **Mr(expt)** | **Mr(calc)** | **Delta** | **Miss** | **Score** | **Expect** | **Rank** | **Peptide** |
| --- | --- | --- | --- | --- | --- | --- | --- | --- | --- | --- |
|  | 4142 | 483.2169 | 964.4193 | 964.4250 | -0.0057 | 0 | 53 | 0.00075 | 1 | R.FDSDAASPR.G |
|  | 4355 | 490.7437 | 979.4729 | 979.4974 | -0.0246 | 1 | 12 | 9.2 | 9 | R.YLENGKEK.L |
|  | 7393 | 600.7434 | 1199.4723 | 1199.6371 | -0.1648 | 1 | 8 | 22 | 4 | R.QAQTDRVNLR.K 7421 |
|  | 7831 | 611.2879 | 1220.5612 | 1220.6037 | -0.0424 | 0 | 2 | 89 | 3 | K.DYIALNEDLR.S |
|  | 8239 | 621.3292 | 1240.6439 | 1239.5013 | 1.1426 | 0 | 11 | 12 | 3 | R.MYGCDLGPDGR.L |
|  | 8528 | 628.9625 | 1255.9104 | 1255.4962 | 0.4142 | 0 | (1) | 78 | 5 | R.MYGCDLGPDGR.L |
|  | 11642 | 714.3621 | 1426.7097 | 1426.7205 | -0.0108 | 0 | 11 | 10 | 1 | K.WAAVVVPSGEEQR.Y |
|  | 13126 | 760.3734 | 1518.7323 | 1518.7427 | -0.0104 | 0 | 41 | 0.011 | 1 | R.SWTAADTAAQITQR.K |
|  | 17823 | 613.3262 | 1836.9569 | 1835.9027 | 1.0542 | 0 | 9 | 14 | 2 | K.THVTHHPVSDHEATLR.C |
|  | 24990 | 879.7289 | 2636.1650 | 2636.1838 | -0.0188 | 0 | 27 | 0.14 | 1 | R.DGEDQTQDTELVETRPAGDGTFQK.W 24989 |

| **547.** | Q31612\|1B73_HUMAN    **Mass:** 40809    **Score:** 75     **Queries matched:** 7   **emPAI:** 0.30 |
| --- | --- |
|  | HLA class I histocompatibility antigen, B-73 alpha chain OS=Homo sapiens GN=HLA-B PE=1 SV=1 |

|  | **Query** | **Observed** | **Mr(expt)** | **Mr(calc)** | **Delta** | **Miss** | **Score** | **Expect** | **Rank** | **Peptide** |
| --- | --- | --- | --- | --- | --- | --- | --- | --- | --- | --- |
|  | 1736 | 763.1153 | 762.1080 | 762.3694 | -0.2614 | 0 | 7 | 31 | 7 | R.NTQICK.A |
|  | 4098 | 481.7402 | 961.4658 | 961.5015 | -0.0356 | 1 | 14 | 7.8 | 8 | R.NTQICKAK.A |
|  | 4142 | 483.2169 | 964.4193 | 964.4250 | -0.0057 | 0 | 53 | 0.00075 | 1 | R.FDSDAASPR.E |
|  | 7831 | 611.2879 | 1220.5612 | 1220.6037 | -0.0424 | 0 | 2 | 89 | 3 | K.DYIALNEDLR.S |
|  | 13126 | 760.3734 | 1518.7323 | 1518.7427 | -0.0104 | 0 | 41 | 0.011 | 1 | R.SWTAADTAAQITQR.K |
|  | 24990 | 879.7289 | 2636.1650 | 2636.1838 | -0.0188 | 0 | 27 | 0.14 | 1 | R.DGEDQTQDTELVETRPAGDGTFQK.W 24989 |

| **548.** | P05026\|AT1B1_HUMAN    **Mass:** 35438    **Score:** 75     **Queries matched:** 9   **emPAI:** 0.22 |
| --- | --- |
|  | Sodium/potassium-transporting ATPase subunit beta-1 OS=Homo sapiens GN=ATP1B1 PE=1 SV=1 |

|  | **Query** | **Observed** | **Mr(expt)** | **Mr(calc)** | **Delta** | **Miss** | **Score** | **Expect** | **Rank** | **Peptide** |
| --- | --- | --- | --- | --- | --- | --- | --- | --- | --- | --- |
|  | 5324 | 526.7540 | 1051.4934 | 1050.5498 | 0.9436 | 1 | 8 | 24 | 4 | K.KFIWNSEK.K |
|  | 6084 | **555.8339** | **1109.6533** | **1109.6961** | **-0.0428** | **0** | **0** | **1.3e+002** | **6** | **R.VLGFKPKPPK.N** |
|  | 9387 | **652.3311** | **1302.6477** | **1302.6568** | **-0.0091** | **0** | **5** | **46** | **7** | **K.TEISFRPNDPK.S** |
|  | 9508 | 655.3494 | 1308.6842 | 1309.6224 | -0.9382 | 0 | 3 | 70 | 3 | K.NESLETYPVMK.Y |
|  | 11847 | 481.2363 | 1440.6870 | 1439.8282 | 0.8588 | 1 | 10 | 13 | 5 | K.EGKPCIIIKLNR.V |
|  | 12679 | **745.4357** | **1488.8568** | **1488.8664** | **-0.0096** | **0** | **41** | **0.01** | **1** | **R.VAPPGLTQIPQIQK.T** 12696 12698 |
|  | 26064 | **1415.1724** | **2828.3302** | **2828.3486** | **-0.0185** | **0** | **56** | **0.00014** | **1** | **K.VGNVEYFGLGNSPGFPLQYYPYYGK.L** |

| **549.** | P13747\|HLAE_HUMAN    **Mass:** 40360    **Score:** 75     **Queries matched:** 7   **emPAI:** 0.09 |
| --- | --- |
|  | HLA class I histocompatibility antigen, alpha chain E OS=Homo sapiens GN=HLA-E PE=1 SV=3 |

|  | **Query** | **Observed** | **Mr(expt)** | **Mr(calc)** | **Delta** | **Miss** | **Score** | **Expect** | **Rank** | **Peptide** |
| --- | --- | --- | --- | --- | --- | --- | --- | --- | --- | --- |
|  | 2585 | **425.7215** | **849.4284** | **849.4345** | **-0.0061** | **0** | **10** | **20** | **6** | **R.DTAQIFR.V** |
|  | 4834 | 507.3274 | 1012.6402 | 1013.4778 | -0.8376 | 1 | 4 | 60 | 1 | K.SSGGKGGSYSK.A |
|  | 8443 | **418.2032** | **1251.5877** | **1250.6143** | **0.9735** | **0** | **4** | **57** | **7** | **K.DYLTLNEDLR.S** |
|  | 9968 | **666.8950** | **1331.7754** | **1331.7310** | **0.0444** | **1** | **8** | **22** | **4** | **R.DTAQIFRVNLR.T** |
|  | 11642 | 714.3621 | 1426.7097 | 1426.7205 | -0.0108 | 0 | 11 | 10 | 1 | K.WAAVVVPSGEEQR.Y |
|  | 15060 | 823.4092 | 1644.8038 | 1644.8148 | -0.0110 | 0 | 75 | 3.4e-006 | 1 | R.FISVGYVDDTQFVR.F |
|  | 17123 | 594.9537 | 1781.8392 | 1782.7308 | -0.8916 | 0 | 2 | 76 | 10 | R.APWMEQEGSEYWDR.E |

| **550.** | P62277\|RS13_HUMAN    **Mass:** 17212    **Score:** 75     **Queries matched:** 19   **emPAI:** 0.23 |
| --- | --- |
|  | 40S ribosomal protein S13 OS=Homo sapiens GN=RPS13 PE=1 SV=2 |

|  | **Query** | **Observed** | **Mr(expt)** | **Mr(calc)** | **Delta** | **Miss** | **Score** | **Expect** | **Rank** | **Peptide** |
| --- | --- | --- | --- | --- | --- | --- | --- | --- | --- | --- |
|  | 904 | **515.3256** | **514.3183** | **514.3227** | **-0.0044** | **0** | **13** | **12** | **5** | **K.AVAVR.K** 901 905 906 |
|  | 1484 | 665.3566 | 664.3493 | 664.3544 | -0.0051 | 0 | 25 | 0.59 | 1 | R.FVTGNK.I 1487 |
|  | 2608 | **426.7241** | **851.4336** | **852.4858** | **-1.0522** | **0** | **6** | **35** | **4** | **R.VLPPNWK.Y** |
|  | 4179 | **484.7451** | **967.4756** | **967.4836** | **-0.0079** | **0** | **11** | **10** | **2** | **R.DSHGVAQVR.F** 4182 |
|  | 4895 | 509.2857 | 1016.5569 | 1015.4691 | 1.0878 | 1 | 8 | 31 | 2 | -.MGRMHAPGK.G |
|  | 5920 | **549.9511** | **1097.8877** | **1097.5240** | **0.3636** | **0** | **6** | **28** | **1** | **K.YESSTASALVA.-** |
|  | 8450 | **418.2348** | **1251.6825** | **1252.7503** | **-1.0678** | **0** | **10** | **14** | **9** | **K.GLTPSQIGVILR.D** 8489 |
|  | 10853 | **461.2860** | **1380.8362** | **1380.8453** | **-0.0091** | **1** | **7** | **27** | **1** | **K.KGLTPSQIGVILR.D** 10813 |
|  | 11811 | **719.8697** | **1437.7249** | **1437.7351** | **-0.0102** | **1** | **75** | **4.4e-006** | **1** | **K.LTSDDVKEQIYK.L** |
|  | 15777 | 847.4429 | 1692.8713 | 1692.9086 | -0.0373 | 0 | 30 | 0.097 | 2 | K.GLAPDLPEDLYHLIK.K 15746 |
|  | 16133 | **857.3063** | **1712.5981** | **1711.8828** | **0.7153** | **1** | **5** | **25** | **7** | **R.MHAPGKGLSQSALPYR.R** |

| **551.** | Q9UHQ9\|NB5R1_HUMAN    **Mass:** 34244    **Score:** 74     **Queries matched:** 15   **emPAI:** 0.23 |
| --- | --- |
|  | NADH-cytochrome b5 reductase 1 OS=Homo sapiens GN=CYB5R1 PE=1 SV=1 |

|  | **Query** | **Observed** | **Mr(expt)** | **Mr(calc)** | **Delta** | **Miss** | **Score** | **Expect** | **Rank** | **Peptide** |
| --- | --- | --- | --- | --- | --- | --- | --- | --- | --- | --- |
|  | 1383 | **634.3151** | **633.3078** | **633.3122** | **-0.0044** | **0** | **9** | **28** | **9** | **K.FPEGGK.M** |
|  | 1561 | **695.3671** | **694.3598** | **694.3650** | **-0.0052** | **0** | **15** | **4.5** | **1** | **K.LGYSQK.M** |
|  | 5202 | **521.3046** | **1040.5947** | **1039.6178** | **0.9769** | **1** | **7** | **31** | **3** | **K.VYLKGVHPK.F** |
|  | 5951 | 550.8161 | 1099.6176 | 1099.5219 | 0.0957 | 0 | 10 | 18 | 5 | K.MSQYLDSLK.V |
|  | 5996 | **552.2936** | **1102.5727** | **1101.5302** | **1.0425** | **0** | **1** | **1.3e+002** | **7** | **R.EDLEELQAR.Y** |
|  | 7005 | **592.2874** | **1182.5603** | **1181.6305** | **0.9297** | **1** | **19** | **2** | **1** | **K.GHFNIQPNKK.S** |
|  | 14847 | 816.8810 | 1631.7475 | 1631.7903 | -0.0428 | 1 | 1 | 96 | 3 | R.EDLEELQARYPNR.F |
|  | 15271 | **415.4872** | **1657.9196** | **1657.9304** | **-0.0108** | **0** | **16** | **2.5** | **1** | **R.FALPTAHHTLGLPVGK.H** |
|  | 17877 | **921.5158** | **1841.0169** | **1841.0267** | **-0.0097** | **0** | **69** | **1.4e-005** | **1** | **K.LGMIAGGTGITPMLQLIR.A** |
|  | 18094 | 929.5218 | 1857.0290 | 1857.0216 | 0.0074 | 0 | (1) | 83 | 9 | K.LGMIAGGTGITPMLQLIR.A |
|  | 19616 | **662.3244** | **1983.9514** | **1985.1166** | **-1.1652** | **1** | **(1)** | **71** | **2** | **K.KLGMIAGGTGITPMLQLIR.A** |
|  | 19637 | 993.5009 | 1984.9873 | 1985.1166 | -0.1293 | 1 | 2 | 60 | 6 | K.KLGMIAGGTGITPMLQLIR.A |
|  | 22547 | 761.6836 | 2282.0289 | 2282.3218 | -0.2929 | 1 | 4 | 34 | 2 | K.LGMIAGGTGITPMLQLIRAILK.V |
|  | 27025 | 998.5160 | 2992.5263 | 2991.5441 | 0.9822 | 0 | 27 | 0.11 | 1 | R.IDGSLVIRPYTPVTSDEDQGYVDLVIK.V |
|  | 28569 | **851.4258** | **3401.6740** | **3401.6934** | **-0.0194** | **0** | **0** | **41** | **8** | **R.EHLPAPGDDVLVLLCGPPPMVQLACHPNLDK.L** |

| **552.** | O95292\|VAPB_HUMAN    **Mass:** 27439    **Score:** 74     **Queries matched:** 10   **emPAI:** 0.30 |
| --- | --- |
|  | Vesicle-associated membrane protein-associated protein B/C OS=Homo sapiens GN=VAPB PE=1 SV=3 |

|  | **Query** | **Observed** | **Mr(expt)** | **Mr(calc)** | **Delta** | **Miss** | **Score** | **Expect** | **Rank** | **Peptide** |
| --- | --- | --- | --- | --- | --- | --- | --- | --- | --- | --- |
|  | 2207 | 820.4719 | 819.4646 | 819.4702 | -0.0055 | 0 | 20 | 2.1 | 1 | K.IISTTASK.T |
|  | 4160 | **483.7708** | **965.5271** | **966.4263** | **-0.8991** | **1** | **10** | **15** | **2** | **K.VMEECKR.L** |
|  | 8648 | 631.7955 | 1261.5765 | 1261.5860 | -0.0095 | 0 | 14 | 5.5 | 3 | K.EAKPEDLMDSK.L |
|  | 13326 | 766.5288 | 1531.0431 | 1530.7711 | 0.2719 | 1 | 1 | 71 | 1 | K.EAKPEDLMDSKLR.C |
|  | 15106 | **824.9434** | **1647.8722** | **1647.8832** | **-0.0110** | **0** | **40** | **0.012** | **1** | **K.VEQVLSLEPQHELK.F** |
|  | 20302 | 1023.2681 | 2044.5216 | 2044.0589 | 0.4626 | 1 | 5 | 20 | 8 | R.GPFTDVVTTNLKLGNPTDR.N 10506 |
|  | 22849 | **1161.5131** | **2321.0116** | **2320.0214** | **0.9901** | **0** | **54** | **0.00026** | **1** | **K.FMVQSMFAPTDTSDMEAVWK.E** 22837 |
|  | 29135 | 903.6863 | 3610.7163 | 3611.5816 | -0.8653 | 1 | 2 | 25 | 3 | K.FMVQSMFAPTDTSDMEAVWKEAKPEDLMDSK.L |

| **553.** | A6NHG4\|DDTL_HUMAN    **Mass:** 14414    **Score:** 74     **Queries matched:** 9   **emPAI:** 0.62 |
| --- | --- |
|  | D-dopachrome decarboxylase-like protein OS=Homo sapiens GN=DDTL PE=2 SV=1 |

|  | **Query** | **Observed** | **Mr(expt)** | **Mr(calc)** | **Delta** | **Miss** | **Score** | **Expect** | **Rank** | **Peptide** |
| --- | --- | --- | --- | --- | --- | --- | --- | --- | --- | --- |
|  | 12817 | 750.3912 | 1498.7678 | 1498.7780 | -0.0102 | 0 | 64 | 4.5e-005 | 1 | M.PFLELDTNLPANR.V |
|  | 13028 | 757.3880 | 1512.7614 | 1512.8082 | -0.0468 | 0 | 10 | 11 | 3 | R.LCAAAASILGKPADR.V 13036 |
|  | 14287 | **532.2822** | **1593.8249** | **1593.8264** | **-0.0015** | **0** | **2** | **82** | **2** | **R.FPTVLSTSPAAHGGPR.C** |
|  | 15064 | 549.2968 | 1644.8686 | 1645.8134 | -0.9448 | 0 | 10 | 12 | 2 | -.MPFLELDTNLPANR.V |
|  | 15426 | 557.6211 | 1669.8416 | 1668.9093 | 0.9323 | 1 | 4 | 42 | 1 | K.RLCAAAASILGKPADR.V |
|  | 21764 | 732.0623 | 2193.1650 | 2193.1793 | -0.0144 | 1 | 32 | 0.053 | 1 | M.PFLELDTNLPANRVPAGLEK.R |
|  | 23004 | 781.3394 | 2340.9963 | 2340.2147 | 0.7815 | 1 | 0 | 64 | 8 | -.MPFLELDTNLPANRVPAGLEK.R |
|  | 31468 | 1789.6014 | 5365.7825 | 5366.7385 | -0.9560 | 1 | 1 | 6.8 | 2 | R.LCAAAASILGKPADRVNVTVRPGLAMALSGSTEPCAQLSISSIGVVGTAEDNR.S |

| **554.** | P62988\|UBIQ_HUMAN    **Mass:** 8560     **Score:** 73     **Queries matched:** 7   **emPAI:** 1.18 |
| --- | --- |
|  | Ubiquitin OS=Homo sapiens GN=RPS27A PE=1 SV=1 |

|  | **Query** | **Observed** | **Mr(expt)** | **Mr(calc)** | **Delta** | **Miss** | **Score** | **Expect** | **Rank** | **Peptide** |
| --- | --- | --- | --- | --- | --- | --- | --- | --- | --- | --- |
|  | 5686 | **541.2755** | **1080.5365** | **1080.5451** | **-0.0087** | **0** | **29** | **0.2** | **1** | **R.TLSDYNIQK.E** |
|  | 6387 | 567.8202 | 1133.6258 | 1133.6808 | -0.0550 | 1 | 18 | 2.9 | 4 | M.QIFVKTLTGK.T |
|  | 8698 | **633.3673** | **1264.7201** | **1264.7213** | **-0.0013** | **1** | **7** | **25** | **2** | **-.MQIFVKTLTGK.T** 8711 |
|  | 17209 | **894.4614** | **1786.9082** | **1786.9200** | **-0.0118** | **0** | **47** | **0.0021** | **1** | **K.TITLEVEPSDTIENVK.A** 17208 |
|  | 21218 | **711.0919** | **2130.2539** | **2129.1480** | **1.1059** | **1** | **1** | **56** | **7** | **R.TLSDYNIQKESTLHLVLR.L** |

| **555.** | P30504\|1C04_HUMAN    **Mass:** 41368    **Score:** 73     **Queries matched:** 10   **emPAI:** 0.30 |
| --- | --- |
|  | HLA class I histocompatibility antigen, Cw-4 alpha chain OS=Homo sapiens GN=HLA-C PE=1 SV=1 |

|  | **Query** | **Observed** | **Mr(expt)** | **Mr(calc)** | **Delta** | **Miss** | **Score** | **Expect** | **Rank** | **Peptide** |
| --- | --- | --- | --- | --- | --- | --- | --- | --- | --- | --- |
|  | 4142 | 483.2169 | 964.4193 | 964.4250 | -0.0057 | 0 | 53 | 0.00075 | 1 | R.FDSDAASPR.G |
|  | 7831 | 611.2879 | 1220.5612 | 1220.6037 | -0.0424 | 0 | 2 | 89 | 3 | K.DYIALNEDLR.S |
|  | 8239 | 621.3292 | 1240.6439 | 1239.5013 | 1.1426 | 0 | 16 | 3.5 | 1 | R.MFGCDLGPDGR.L |
|  | 10294 | 676.3427 | 1350.6709 | 1349.6939 | 0.9770 | 1 | 8 | 24 | 1 | R.YLENGKETLQR.A |
|  | 11642 | 714.3621 | 1426.7097 | 1426.7205 | -0.0108 | 0 | 11 | 10 | 1 | K.WAAVVVPSGEEQR.Y |
|  | 12774 | 748.8586 | 1495.7026 | 1495.7129 | -0.0103 | 0 | 11 | 9.6 | 2 | R.AYLEGTCVEWLR.R |
|  | 13126 | 760.3734 | 1518.7323 | 1518.7427 | -0.0104 | 0 | 41 | 0.011 | 1 | R.SWTAADTAAQITQR.K |
|  | 14790 | 815.4116 | 1628.8086 | 1628.8199 | -0.0113 | 0 | 27 | 0.26 | 1 | R.FIAVGYVDDTQFVR.F |
|  | 17823 | 613.3262 | 1836.9569 | 1835.9027 | 1.0542 | 0 | 9 | 14 | 2 | K.THVTHHPVSDHEATLR.C |
|  | 23860 | 1229.6564 | 2457.2982 | 2456.3097 | 0.9885 | 0 | 2 | 44 | 2 | R.TLILLLSGALALTETWAGSHSMR.Y |

| **556.** | O75356\|ENTP5_HUMAN    **Mass:** 47886    **Score:** 73     **Queries matched:** 12   **emPAI:** 0.46 |
| --- | --- |
|  | Ectonucleoside triphosphate diphosphohydrolase 5 OS=Homo sapiens GN=ENTPD5 PE=1 SV=1 |

|  | **Query** | **Observed** | **Mr(expt)** | **Mr(calc)** | **Delta** | **Miss** | **Score** | **Expect** | **Rank** | **Peptide** |
| --- | --- | --- | --- | --- | --- | --- | --- | --- | --- | --- |
|  | 818 | 487.3205 | 486.3132 | 486.3166 | -0.0034 | 0 | 19 | 3.8 | 1 | K.GGILK.V 816 |
|  | 9305 | **650.2881** | **1298.5617** | **1298.5700** | **-0.0083** | **0** | **28** | **0.22** | **1** | **R.AVDTDMIDYEK.G** |
|  | 9719 | **441.2367** | **1320.6882** | **1319.6946** | **0.9937** | **1** | **35** | **0.04** | **1** | **R.GKLHQPEEVQR.G** |
|  | 11752 | **717.8705** | **1433.7264** | **1433.7343** | **-0.0079** | **0** | **24** | **0.5** | **1** | **R.WLEAEWIFGGVK.Y** 11751 |
|  | 13415 | **769.8278** | **1537.6410** | **1537.6514** | **-0.0104** | **0** | **20** | **1.2** | **1** | **R.GSFYAFSYYYDR.A** |
|  | 16331 | **864.4264** | **1726.8382** | **1725.8938** | **0.9445** | **1** | **0** | **1.1e+002** | **5** | **K.DGFGFADSTVLQLTKK.V** |
|  | 25373 | **902.8126** | **2705.4159** | **2705.4289** | **-0.0131** | **1** | **25** | **0.19** | **1** | **K.KVNNIETGWALGATFHLLQSLGISH.-** |
|  | 27881 | **799.8988** | **3195.5661** | **3195.5772** | **-0.0111** | **0** | **42** | **0.0029** | **1** | **K.GSVSIMDGSDEGILAWVTVNFLTGQLHGHR.Q** 27882 |
|  | 28775 | 1160.9005 | 3479.6797 | 3479.6411 | 0.0386 | 1 | 0 | 36 | 9 | K.AREVCDNLENFTSGSPFLCMDLSYITALLK.D |

| **557.** | Q8IX05\|CD302_HUMAN    **Mass:** 26565    **Score:** 73     **Queries matched:** 3   **emPAI:** 0.14 |
| --- | --- |
|  | CD302 antigen OS=Homo sapiens GN=CD302 PE=1 SV=1 |

|  | **Query** | **Observed** | **Mr(expt)** | **Mr(calc)** | **Delta** | **Miss** | **Score** | **Expect** | **Rank** | **Peptide** |
| --- | --- | --- | --- | --- | --- | --- | --- | --- | --- | --- |
|  | 3880 | 473.7387 | 945.4629 | 945.4767 | -0.0138 | 0 | 14 | 7.7 | 7 | K.VESIEDVR.N |
|  | 21128 | 1060.4736 | 2118.9327 | 2118.9456 | -0.0129 | 0 | 73 | 4.3e-006 | 1 | K.GPDDILLGMFYDTDDASFK.W 21129 |

| **558.** | P62899\|RL31_HUMAN    **Mass:** 14454    **Score:** 73     **Queries matched:** 5   **emPAI:** 0.62 |
| --- | --- |
|  | 60S ribosomal protein L31 OS=Homo sapiens GN=RPL31 PE=1 SV=1 |

|  | **Query** | **Observed** | **Mr(expt)** | **Mr(calc)** | **Delta** | **Miss** | **Score** | **Expect** | **Rank** | **Peptide** |
| --- | --- | --- | --- | --- | --- | --- | --- | --- | --- | --- |
|  | 933 | **519.1368** | **518.1295** | **517.2860** | **0.8435** | **1** | **8** | **41** | **4** | **K.KGGEK.K** |
|  | 1434 | **648.3411** | **647.3339** | **647.3391** | **-0.0052** | **0** | **8** | **36** | **5** | **R.NVPYR.I** |
|  | 4459 | **494.7714** | **987.5283** | **987.5349** | **-0.0066** | **0** | **55** | **0.00056** | **1** | **R.SAINEVVTR.E** |
|  | 4892 | 509.2690 | 1016.5233 | 1016.5291 | -0.0057 | 0 | 13 | 10 | 1 | R.EYTINIHK.R |
|  | 15046 | **822.9611** | **1643.9076** | **1643.9175** | **-0.0099** | **0** | **48** | **0.0019** | **1** | **K.LYTLVTYVPVTTFK.N** |

| **559.** | P37108\|SRP14_HUMAN    **Mass:** 14675    **Score:** 73     **Queries matched:** 3   **emPAI:** 0.27 |
| --- | --- |
|  | Signal recognition particle 14 kDa protein OS=Homo sapiens GN=SRP14 PE=1 SV=2 |

|  | **Query** | **Observed** | **Mr(expt)** | **Mr(calc)** | **Delta** | **Miss** | **Score** | **Expect** | **Rank** | **Peptide** |
| --- | --- | --- | --- | --- | --- | --- | --- | --- | --- | --- |
|  | 8571 | **630.3212** | **1258.6278** | **1257.6176** | **1.0102** | **0** | **3** | **73** | **6** | **K.FQMAYSNLLR.A** |
|  | 15547 | **839.4514** | **1676.8881** | **1676.8985** | **-0.0103** | **0** | **73** | **6.4e-006** | **1** | **M.VLLESEQFLTELTR.L** |
|  | 17409 | **602.3155** | **1803.9246** | **1804.8778** | **-0.9531** | **1** | **4** | **38** | **2** | **K.GTVEGFEPADNKCLLR.A** |

| **560.** | Q5T8D3\|ACBD5_HUMAN    **Mass:** 60510    **Score:** 72     **Queries matched:** 12   **emPAI:** 0.06 |
| --- | --- |
|  | Acyl-CoA-binding domain-containing protein 5 OS=Homo sapiens GN=ACBD5 PE=1 SV=1 |

|  | **Query** | **Observed** | **Mr(expt)** | **Mr(calc)** | **Delta** | **Miss** | **Score** | **Expect** | **Rank** | **Peptide** |
| --- | --- | --- | --- | --- | --- | --- | --- | --- | --- | --- |
|  | 6777 | 582.7896 | 1163.5647 | 1163.5895 | -0.0248 | 1 | 6 | 39 | 9 | K.SGRSSDITSVR.L |
|  | 10510 | 682.8431 | 1363.6717 | 1363.6983 | -0.0266 | 0 | 10 | 14 | 3 | K.NLEVIVTNGYDK.D 10512 |
|  | 10692 | **687.3406** | **1372.6667** | **1372.6769** | **-0.0102** | **0** | **72** | **9.6e-006** | **1** | **R.LQEDMQNVLQR.L** |
|  | 11003 | 464.2542 | 1389.7408 | 1388.6718 | 1.0690 | 0 | (4) | 60 | 4 | R.LQEDMQNVLQR.L |
|  | 11089 | **465.9329** | **1394.7768** | **1395.6677** | **-0.8909** | **1** | **5** | **45** | **6** | **R.GHRMQHLSEGTK.G** |
|  | 11903 | 722.9013 | 1443.7879 | 1442.7915 | 0.9964 | 0 | 4 | 55 | 10 | R.GSLNEQIALVLMR.L |
|  | 12140 | 730.3425 | 1458.6705 | 1458.7864 | -0.1159 | 0 | (1) | 1.1e+002 | 6 | R.GSLNEQIALVLMR.L |
|  | 16835 | 879.9505 | 1757.8864 | 1757.9094 | -0.0229 | 1 | 1 | 80 | 9 | R.LQEDMQNVLQRLQK.L 16780 |
|  | 22136 | 558.2913 | 2229.1359 | 2230.1263 | -0.9904 | 1 | 9 | 12 | 5 | K.CLEDLGNVLTSTPNAKTVNGK.A |
|  | 23339 | 794.4270 | 2380.2592 | 2380.1693 | 0.0899 | 0 | 1 | 58 | 3 | R.EDIQVPPGNGNIGNMQVVAVEGK.G |

| **561.** | Q9H0C2\|ADT4_HUMAN    **Mass:** 35285    **Score:** 72     **Queries matched:** 21   **emPAI:** 0.11 |
| --- | --- |
|  | ADP/ATP translocase 4 OS=Homo sapiens GN=SLC25A31 PE=1 SV=1 |

|  | **Query** | **Observed** | **Mr(expt)** | **Mr(calc)** | **Delta** | **Miss** | **Score** | **Expect** | **Rank** | **Peptide** |
| --- | --- | --- | --- | --- | --- | --- | --- | --- | --- | --- |
|  | 2649 | 428.7446 | 855.4747 | 855.4814 | -0.0067 | 0 | 24 | 0.66 | 1 | K.TAVAPIER.V |
|  | 2669 | 429.2404 | 856.4663 | 855.4926 | 0.9737 | 0 | 12 | 13 | 4 | R.GNLANVIR.Y |
|  | 3083 | 443.0387 | 884.0628 | 883.4334 | 0.6295 | 1 | 3 | 50 | 3 | -.MHREPAK.K |
|  | 5718 | 542.3104 | 1082.6063 | 1082.6448 | -0.0385 | 1 | 8 | 27 | 2 | K.TAVAPIERVK.L 5713 5715 |
|  | 7043 | **592.8251** | **1183.6357** | **1183.5148** | **0.1210** | **1** | **(1)** | **1.3e+002** | **4** | **R.MMMQSGEAKR.Q** |
|  | 7061 | **593.2764** | **1184.5383** | **1183.5148** | **1.0236** | **1** | **3** | **84** | **9** | **R.RMMMQSGEAK.R** |
|  | 7388 | 600.4399 | 1198.8652 | 1199.5097 | -0.6445 | 1 | (1) | 84 | 10 | R.RMMMQSGEAK.R 7398 |
|  | 7392 | **400.7382** | **1199.1928** | **1199.5097** | **-0.3168** | **1** | **10** | **10** | **1** | **R.MMMQSGEAKR.Q** |
|  | 7414 | **601.3185** | **1200.6225** | **1199.5097** | **1.1129** | **1** | **(2)** | **95** | **7** | **R.RMMMQSGEAK.R** |
|  | 7721 | **608.7913** | **1215.5680** | **1215.5046** | **0.0634** | **1** | **(2)** | **96** | **9** | **R.RMMMQSGEAK.R** |
|  | 7812 | **407.4906** | **1219.4500** | **1220.6257** | **-1.1757** | **1** | **12** | **9.3** | **4** | **K.GLGDCIMKIAK.S** |
|  | 9579 | **438.6146** | **1312.8220** | **1312.8078** | **0.0142** | **1** | **10** | **13** | **1** | **R.VKLLLQVQASSK.Q** |
|  | 11692 | 715.8444 | 1429.6743 | 1430.7670 | -1.0927 | 1 | 19 | 1.7 | 2 | K.IKEFFHIDIGGR.- 11705 |
|  | 11941 | 723.8702 | 1445.7259 | 1445.7343 | -0.0084 | 0 | 56 | 0.00036 | 1 | R.YFPTQALNFAFK.D 11955 |
|  | 13568 | **774.4537** | **1546.8928** | **1545.9130** | **0.9798** | **1** | **1** | **93** | **9** | **R.GTGGALVLVLYDKIK.E** |
|  | 23474 | **801.7374** | **2402.1903** | **2403.1682** | **-0.9779** | **1** | **4** | **29** | **4** | **K.GTLDCFVKIYQHEGISSFFR.G** |

| **562.** | Q9Y673\|ALG5_HUMAN    **Mass:** 37150    **Score:** 72     **Queries matched:** 19   **emPAI:** 0.10 |
| --- | --- |
|  | Dolichyl-phosphate beta-glucosyltransferase OS=Homo sapiens GN=ALG5 PE=1 SV=1 |

|  | **Query** | **Observed** | **Mr(expt)** | **Mr(calc)** | **Delta** | **Miss** | **Score** | **Expect** | **Rank** | **Peptide** |
| --- | --- | --- | --- | --- | --- | --- | --- | --- | --- | --- |
|  | 755 | 473.0815 | 472.0742 | 472.2758 | -0.2015 | 0 | 8 | 36 | 1 | K.GGAIR.M 753 757 |
|  | 1581 | **704.3157** | **703.3084** | **702.3660** | **0.9424** | **0** | **12** | **14** | **5** | **K.ESIAQR.S** |
|  | 2253 | **412.2235** | **822.4325** | **823.4188** | **-0.9863** | **1** | **10** | **14** | **4** | **K.YGSDKVR.V** 2252 |
|  | 3612 | 464.2412 | 926.4679 | 926.6277 | -0.1598 | 1 | 19 | 1.9 | 2 | K.VRVITLVK.N |
|  | 5294 | **525.2016** | **1048.3886** | **1049.5618** | **-1.1731** | **1** | **10** | **17** | **6** | **K.LFTREAASR.T** |
|  | 5614 | **537.9465** | **1073.8785** | **1074.5458** | **-0.6673** | **0** | **3** | **54** | **4** | **R.TFSSLHVER.W** |
|  | 6027 | 553.7716 | 1105.5285 | 1104.5485 | 0.9801 | 0 | 15 | 5.9 | 1 | K.ILMADADGATK.F |
|  | 8739 | **634.7248** | **1267.4350** | **1266.6503** | **0.7847** | **1** | **2** | **76** | **4** | **K.GGAIRMGIFSSR.G** |
|  | 11025 | **696.3668** | **1390.7190** | **1390.6881** | **0.0309** | **1** | **12** | **8.1** | **1** | **R.HEEEKFFLNAK.G** |
|  | 11513 | **473.9670** | **1418.8793** | **1418.7075** | **0.1718** | **1** | **7** | **25** | **3** | **R.GEKILMADADGATK.F** |
|  | 12693 | 745.8827 | 1489.7508 | 1490.7617 | -1.0108 | 0 | 12 | 8.1 | 2 | K.QLSVVVPSYNEEK.R |
|  | 17649 | 609.3163 | 1824.9270 | 1825.8954 | -0.9684 | 1 | 1 | 79 | 9 | K.RLPVMMDEALSYLEK.R |
|  | 17825 | **613.3305** | **1836.9697** | **1835.8975** | **1.0722** | **1** | **1** | **82** | **4** | **K.ILMADADGATKFPDVEK.L** |
|  | 21177 | **1064.5002** | **2126.9859** | **2126.9990** | **-0.0130** | **0** | **54** | **0.00032** | **1** | **K.GLNDLQPWPNQMAIACGSR.A** 21176 |
|  | 22785 | **772.3688** | **2314.0847** | **2313.1013** | **0.9834** | **1** | **3** | **34** | **3** | **R.DPAFTYEVIVVDDGSKDQTSK.V** |

| **563.** | P47985\|UCRI_HUMAN    **Mass:** 29934    **Score:** 71     **Queries matched:** 16   **emPAI:** 0.61 |
| --- | --- |
|  | Cytochrome b-c1 complex subunit Rieske, mitochondrial OS=Homo sapiens GN=UQCRFS1 PE=1 SV=2 |

|  | **Query** | **Observed** | **Mr(expt)** | **Mr(calc)** | **Delta** | **Miss** | **Score** | **Expect** | **Rank** | **Peptide** |
| --- | --- | --- | --- | --- | --- | --- | --- | --- | --- | --- |
|  | 1296 | **609.3201** | **608.3129** | **609.2944** | **-0.9816** | **0** | **14** | **6** | **1** | **K.NMAFK.W** |
|  | 3881 | **946.4887** | **945.4814** | **945.4879** | **-0.0066** | **0** | **32** | **0.13** | **1** | **R.ESLSGQAVR.R** 3855 3880 3891 3898 |
|  | 4814 | **506.7482** | **1011.4819** | **1011.4662** | **0.0158** | **0** | **8** | **22** | **3** | **K.VPDFSEYR.R** |
|  | 5428 | **530.7996** | **1059.5847** | **1059.5924** | **-0.0077** | **1** | **34** | **0.075** | **1** | **R.RLEVLDSTK.S** 5429 |
|  | 7885 | 612.3364 | 1222.6582 | 1221.6102 | 1.0480 | 1 | 5 | 50 | 9 | R.DPQHDLDRVK.K |
|  | 14568 | 807.9148 | 1613.8150 | 1613.8260 | -0.0110 | 0 | 62 | 7.5e-005 | 1 | K.EIEQEAAVELSQLR.D |
|  | 21031 | **704.0299** | **2109.0677** | **2109.0776** | **-0.0099** | **0** | **22** | **0.6** | **1** | **K.NAVTQFVSSMSASADVLALAK.I** |
|  | 21172 | 1064.0931 | 2126.1717 | 2125.0725 | 1.0992 | 0 | (7) | 17 | 2 | K.NAVTQFVSSMSASADVLALAK.I 21173 |
|  | 24719 | **1289.1370** | **2576.2594** | **2576.2720** | **-0.0127** | **0** | **6** | **18** | **1** | **R.LGPAPLNLEVPTYEFTSDDMVIVG.-** |
|  | 24808 | **864.7576** | **2591.2511** | **2590.2623** | **0.9888** | **1** | **26** | **0.18** | **1** | **K.EIEQEAAVELSQLRDPQHDLDR.V** |

| **564.** | P19404\|NDUV2_HUMAN    **Mass:** 27659    **Score:** 71     **Queries matched:** 8   **emPAI:** 0.47 |
| --- | --- |
|  | NADH dehydrogenase [ubiquinone] flavoprotein 2, mitochondrial OS=Homo sapiens GN=NDUFV2 PE=1 SV=2 |

|  | **Query** | **Observed** | **Mr(expt)** | **Mr(calc)** | **Delta** | **Miss** | **Score** | **Expect** | **Rank** | **Peptide** |
| --- | --- | --- | --- | --- | --- | --- | --- | --- | --- | --- |
|  | 4053 | **479.5818** | **957.1490** | **957.4742** | **-0.3252** | **0** | **4** | **44** | **4** | **-.MFFSAALR.A** |
|  | 10059 | **669.3984** | **1336.7822** | **1335.7874** | **0.9948** | **0** | **34** | **0.048** | **1** | **K.AAAVLPVLDLAQR.Q** |
|  | 10736 | **459.2550** | **1374.7432** | **1373.6761** | **1.0670** | **0** | **6** | **35** | **7** | **R.QNGWLPISAMNK.V** |
|  | 13711 | 778.8874 | 1555.7603 | 1556.7882 | -1.0278 | 0 | 8 | 22 | 2 | K.TVMQNGAGGALFVHR.D |
|  | 17093 | **890.9156** | **1779.8166** | **1778.8266** | **0.9900** | **0** | **33** | **0.051** | **1** | **K.YHIQVCTTTPCMLR.N** |
|  | 20359 | **683.7118** | **2048.1135** | **2049.0690** | **-0.9555** | **1** | **9** | **12** | **1** | **R.NLHKTVMQNGAGGALFVHR.D** |
|  | 25156 | **1337.6541** | **2673.2935** | **2673.3109** | **-0.0173** | **1** | **46** | **0.0014** | **1** | **R.FSCEPAGGLTSLTEPPKGPGFGVQAGL.-** 25157 |

| **565.** | P13804\|ETFA_HUMAN    **Mass:** 35400    **Score:** 71     **Queries matched:** 14   **emPAI:** 0.50 |
| --- | --- |
|  | Electron transfer flavoprotein subunit alpha, mitochondrial OS=Homo sapiens GN=ETFA PE=1 SV=1 |

|  | **Query** | **Observed** | **Mr(expt)** | **Mr(calc)** | **Delta** | **Miss** | **Score** | **Expect** | **Rank** | **Peptide** |
| --- | --- | --- | --- | --- | --- | --- | --- | --- | --- | --- |
|  | 1527 | 681.3342 | 680.3269 | 680.3129 | 0.0140 | 0 | 9 | 20 | 2 | K.SGENFK.L |
|  | 6875 | **586.3232** | **1170.6319** | **1170.6397** | **-0.0078** | **0** | **25** | **0.49** | **1** | **K.VLVAQHDVYK.G** |
|  | 6921 | 588.3220 | 1174.6295 | 1173.6315 | 0.9980 | 0 | 4 | 66 | 3 | K.VVPEMTEILK.K |
|  | 7728 | **608.8178** | **1215.6211** | **1216.6499** | **-1.0288** | **1** | **3** | **76** | **2** | **-.MFRAAAPGQLR.R** |
|  | 9079 | **429.7198** | **1286.1375** | **1285.7315** | **0.4059** | **1** | **4** | **42** | **8** | **K.VVPEMTEILKK.K** |
|  | 9370 | 434.8810 | 1301.6212 | 1301.7265 | -0.1053 | 1 | (1) | 1.2e+002 | 10 | K.VVPEMTEILKK.K |
|  | 10852 | **691.4144** | **1380.8142** | **1380.8228** | **-0.0086** | **0** | **1** | **1e+002** | **2** | **K.LEVAPISDIIAIK.S** |
|  | 14802 | **815.8580** | **1629.7015** | **1629.7118** | **-0.0103** | **0** | **18** | **1.9** | **1** | **R.GTSFDAAATSGGSASSEK.A** |
|  | 17032 | **888.9301** | **1775.8457** | **1775.8577** | **-0.0121** | **0** | **51** | **0.00091** | **1** | **K.ASSTSPVEISEWLDQK.L** |
|  | 18705 | **952.9561** | **1903.8975** | **1903.9098** | **-0.0123** | **0** | **40** | **0.0092** | **1** | **R.AAVDAGFVPNDMQVGQTGK.I** 18696 18704 |
|  | 21683 | **728.4362** | **2182.2867** | **2183.2202** | **-0.9335** | **1** | **3** | **35** | **1** | **K.LEVAPISDIIAIKSPDTFVR.T** |
|  | 29032 | **597.3324** | **3577.9507** | **3578.9420** | **-0.9913** | **1** | **3** | **15** | **5** | **R.AASLLRFQSTLVIAEHANDSLAPITLNTITAATR.L** |

| **566.** | P50395\|GDIB_HUMAN    **Mass:** 51087    **Score:** 71     **Queries matched:** 10   **emPAI:** 0.07 |
| --- | --- |
|  | Rab GDP dissociation inhibitor beta OS=Homo sapiens GN=GDI2 PE=1 SV=2 |

|  | **Query** | **Observed** | **Mr(expt)** | **Mr(calc)** | **Delta** | **Miss** | **Score** | **Expect** | **Rank** | **Peptide** |
| --- | --- | --- | --- | --- | --- | --- | --- | --- | --- | --- |
|  | 1498 | **672.3263** | **671.3190** | **670.4126** | **0.9064** | **0** | **20** | **2.3** | **1** | **K.VGQVIR.V** |
|  | 5041 | 515.3264 | 1028.6383 | 1028.4961 | 0.1422 | 1 | 4 | 68 | 5 | K.TTMRDVYK.K |
|  | 9796 | **442.2455** | **1323.7146** | **1323.7286** | **-0.0140** | **0** | **11** | **9.6** | **1** | **K.YIAIVSTTVETK.E** |
|  | 10377 | 452.5637 | 1354.6692 | 1355.6616 | -0.9924 | 1 | 5 | 42 | 9 | K.IPGSPPESMGRGR.D |
|  | 14698 | **541.7374** | **1622.1905** | **1621.6752** | **0.5152** | **1** | **(2)** | **49** | **3** | **K.RMTGSEFDFEEMK.R** |
|  | 14962 | **547.0682** | **1638.1829** | **1637.6702** | **0.5127** | **1** | **6** | **19** | **3** | **K.RMTGSEFDFEEMK.R** |
|  | 18869 | **640.3082** | **1917.9029** | **1916.8098** | **1.0930** | **0** | **1** | **89** | **9** | **R.TYDATTHFETTCDDIK.N** |
|  | 21306 | **1071.0501** | **2140.0855** | **2140.0993** | **-0.0138** | **0** | **71** | **7.3e-006** | **1** | **K.SPYLYPLYGLGELPQGFAR.L** |
|  | 21411 | **539.0202** | **2152.0517** | **2151.0881** | **0.9636** | **1** | **6** | **24** | **3** | **K.VPSTEAEALASSLMGLFEKR.R** |
|  | 25054 | **884.7972** | **2651.3697** | **2650.4040** | **0.9657** | **0** | **3** | **34** | **2** | **R.LSAIYGGTYMLNKPIEEIIVQNGK.V** |

| **567.** | P31150\|GDIA_HUMAN    **Mass:** 51177    **Score:** 71     **Queries matched:** 8   **emPAI:** 0.07 |
| --- | --- |
|  | Rab GDP dissociation inhibitor alpha OS=Homo sapiens GN=GDI1 PE=1 SV=2 |

|  | **Query** | **Observed** | **Mr(expt)** | **Mr(calc)** | **Delta** | **Miss** | **Score** | **Expect** | **Rank** | **Peptide** |
| --- | --- | --- | --- | --- | --- | --- | --- | --- | --- | --- |
|  | 6421 | 569.2889 | 1136.5632 | 1135.4782 | 1.0850 | 0 | 9 | 19 | 4 | K.QNDVFGEAEQ.- |
|  | 12179 | **730.8570** | **1459.6994** | **1459.7129** | **-0.0135** | **0** | **(9)** | **17** | **7** | **R.FQLLEGPPESMGR.G** 12147 |
|  | 12480 | **493.2907** | **1476.8502** | **1475.7079** | **1.1424** | **0** | **9** | **15** | **2** | **R.FQLLEGPPESMGR.G** |
|  | 15681 | **563.7836** | **1688.3291** | **1688.8304** | **-0.5014** | **1** | **5** | **23** | **5** | **R.FQLLEGPPESMGRGR.D** 15689 |
|  | 17560 | **606.3826** | **1816.1261** | **1817.0709** | **-0.9449** | **1** | **0** | **78** | **3** | **K.AGQVIRIICILSHPIK.N** |
|  | 21306 | 1071.0501 | 2140.0855 | 2140.0993 | -0.0138 | 0 | 71 | 7.3e-006 | 1 | K.SPYLYPLYGLGELPQGFAR.L |

| **568.** | Q9Y5L4\|TIM13_HUMAN    **Mass:** 10721    **Score:** 70     **Queries matched:** 5   **emPAI:** 0.37 |
| --- | --- |
|  | Mitochondrial import inner membrane translocase subunit Tim13 OS=Homo sapiens GN=TIMM13 PE=1 SV=1 |

|  | **Query** | **Observed** | **Mr(expt)** | **Mr(calc)** | **Delta** | **Miss** | **Score** | **Expect** | **Rank** | **Peptide** |
| --- | --- | --- | --- | --- | --- | --- | --- | --- | --- | --- |
|  | 8588 | 630.3729 | 1258.7312 | 1257.6639 | 1.0673 | 0 | 8 | 21 | 7 | K.LDPGLIMEQVK.V |
|  | 13652 | **776.9385** | **1551.8625** | **1551.8733** | **-0.0107** | **0** | **70** | **1.1e-005** | **1** | **K.VQIAVANAQELLQR.M** |
|  | 14188 | **530.5870** | **1588.7393** | **1588.7515** | **-0.0122** | **0** | **17** | **2.7** | **1** | **K.CIGKPGGSLDNSEQK.C** |
|  | 22524 | **760.8773** | **2279.6101** | **2278.9414** | **0.6687** | **1** | **2** | **33** | **5** | **K.CIAMCMDRYMDAWNTVSR.A** |
|  | 25439 | **679.8214** | **2715.2565** | **2715.2520** | **0.0045** | **1** | **5** | **21** | **1** | **-.MEGGFGSDFGGSGSGKLDPGLIMEQVK.V** |

| **569.** | Q96G23\|LASS2_HUMAN    **Mass:** 44961    **Score:** 70     **Queries matched:** 9   **emPAI:** 0.17 |
| --- | --- |
|  | LAG1 longevity assurance homolog 2 OS=Homo sapiens GN=LASS2 PE=1 SV=1 |

|  | **Query** | **Observed** | **Mr(expt)** | **Mr(calc)** | **Delta** | **Miss** | **Score** | **Expect** | **Rank** | **Peptide** |
| --- | --- | --- | --- | --- | --- | --- | --- | --- | --- | --- |
|  | 5062 | 516.2640 | 1030.5134 | 1031.4535 | -0.9401 | 0 | 5 | 61 | 9 | K.MFNYAGWK.N |
|  | 5538 | 535.2839 | 1068.5532 | 1069.5880 | -1.0348 | 0 | 2 | 93 | 10 | R.NQDRPSLLK.K |
|  | 7374 | **600.3082** | **1198.6019** | **1197.6829** | **0.9190** | **1** | **2** | **96** | **9** | **R.NQDRPSLLKK.F** |
|  | 16223 | 573.9514 | 1718.8322 | 1718.8093 | 0.0229 | 0 | 11 | 8.7 | 4 | M.LQTLYDYFWWER.L |
|  | 16602 | **873.4406** | **1744.8665** | **1744.8784** | **-0.0119** | **0** | **43** | **0.005** | **1** | **R.APPNATLEHFYLTSGK.Q** 16603 |
|  | 17990 | **925.9285** | **1849.8424** | **1849.8498** | **-0.0074** | **0** | **51** | **0.00075** | **1** | **-.MLQTLYDYFWWER.L** 17989 |
|  | 19970 | **504.5199** | **2014.0505** | **2014.0636** | **-0.0131** | **1** | **15** | **3.1** | **1** | **R.LRAPPNATLEHFYLTSGK.Q** |

| **570.** | Q8NFQ8\|TOIP2_HUMAN    **Mass:** 51460    **Score:** 70     **Queries matched:** 6   **emPAI:** 0.07 |
| --- | --- |
|  | Torsin-1A-interacting protein 2 OS=Homo sapiens GN=TOR1AIP2 PE=1 SV=1 |

|  | **Query** | **Observed** | **Mr(expt)** | **Mr(calc)** | **Delta** | **Miss** | **Score** | **Expect** | **Rank** | **Peptide** |
| --- | --- | --- | --- | --- | --- | --- | --- | --- | --- | --- |
|  | 4371 | **491.2681** | **980.5217** | **979.4029** | **1.1188** | **0** | **5** | **44** | **8** | **K.ENAQDTMR.Q** |
|  | 12769 | **499.2952** | **1494.8639** | **1493.7110** | **1.1528** | **1** | **9** | **14** | **3** | **K.SESPDEANVGKHPK.D** 12749 12750 |
|  | 13854 | 522.9300 | 1565.7681 | 1566.7162 | -0.9480 | 1 | 3 | 64 | 9 | K.TEDENKQSFLDGGK.G |
|  | 14415 | **802.9174** | **1603.8203** | **1603.8318** | **-0.0115** | **0** | **70** | **1.2e-005** | **1** | **R.LLAPEAGSHPQQTQK.L** |

| **571.** | Q15836\|VAMP3_HUMAN    **Mass:** 11359    **Score:** 70     **Queries matched:** 6   **emPAI:** 0.83 |
| --- | --- |
|  | Vesicle-associated membrane protein 3 OS=Homo sapiens GN=VAMP3 PE=1 SV=3 |

|  | **Query** | **Observed** | **Mr(expt)** | **Mr(calc)** | **Delta** | **Miss** | **Score** | **Expect** | **Rank** | **Peptide** |
| --- | --- | --- | --- | --- | --- | --- | --- | --- | --- | --- |
|  | 8857 | **425.5718** | **1273.6935** | **1274.6327** | **-0.9392** | **1** | **2** | **95** | **7** | **M.STGPTAATGSNRR.L** |
|  | 11572 | **474.9771** | **1421.9094** | **1421.6681** | **0.2413** | **1** | **4** | **45** | **5** | **-.MSTGPTAATGSNRR.L** |
|  | 15348 | 832.8774 | 1663.7403 | 1664.8006 | -1.0602 | 0 | 33 | 0.058 | 1 | R.ADALQAGASQFETSAAK.L |
|  | 24173 | 832.0679 | 2493.1820 | 2493.1983 | -0.0163 | 1 | 60 | 7e-005 | 1 | K.LSELDDRADALQAGASQFETSAAK.L 24161 24162 |

| **572.** | P63027\|VAMP2_HUMAN    **Mass:** 12698    **Score:** 70     **Queries matched:** 5   **emPAI:** 0.72 |
| --- | --- |
|  | Vesicle-associated membrane protein 2 OS=Homo sapiens GN=VAMP2 PE=1 SV=2 |

|  | **Query** | **Observed** | **Mr(expt)** | **Mr(calc)** | **Delta** | **Miss** | **Score** | **Expect** | **Rank** | **Peptide** |
| --- | --- | --- | --- | --- | --- | --- | --- | --- | --- | --- |
|  | 15348 | 832.8774 | 1663.7403 | 1664.8006 | -1.0602 | 0 | 33 | 0.058 | 1 | R.ADALQAGASQFETSAAK.L |
|  | 20153 | **676.6600** | **2026.9581** | **2028.0422** | **-1.0841** | **1** | **0** | **87** | **5** | **R.RLQQTQAQVDEVVDIMR.V** |
|  | 24173 | 832.0679 | 2493.1820 | 2493.1983 | -0.0163 | 1 | 60 | 7e-005 | 1 | K.LSELDDRADALQAGASQFETSAAK.L 24161 24162 |

| **573.** | P11217\|PYGM_HUMAN    **Mass:** 97487    **Score:** 70     **Queries matched:** 32   **emPAI:** 0.16 |
| --- | --- |
|  | Glycogen phosphorylase, muscle form OS=Homo sapiens GN=PYGM PE=1 SV=6 |

|  | **Query** | **Observed** | **Mr(expt)** | **Mr(calc)** | **Delta** | **Miss** | **Score** | **Expect** | **Rank** | **Peptide** |
| --- | --- | --- | --- | --- | --- | --- | --- | --- | --- | --- |
|  | 1650 | 730.4197 | 729.4125 | 729.4497 | -0.0372 | 1 | 8 | 41 | 7 | R.KQISVR.G |
|  | 2857 | 434.8811 | 867.7477 | 868.4443 | -0.6966 | 0 | 2 | 59 | 4 | K.FAAYLER.E |
|  | 3182 | 447.7711 | 893.5276 | 892.4079 | 1.1197 | 0 | 8 | 28 | 6 | R.YGNPWEK.A |
|  | 3482 | 458.7429 | 915.4712 | 915.4774 | -0.0062 | 0 | 22 | 1.3 | 2 | R.NLAENISR.V |
|  | 3614 | 464.2542 | 926.4939 | 927.4886 | -0.9947 | 1 | 3 | 86 | 3 | K.DRNVATPR.D |
|  | 3854 | 473.2308 | 944.4470 | 944.4538 | -0.0068 | 0 | 15 | 5.9 | 3 | K.AAPGYHMAK.M |
|  | 3912 | **474.7637** | **947.5128** | **946.4654** | **1.0473** | **0** | **14** | **7.8** | **8** | **R.NNVVNTMR.L** |
|  | 4737 | 503.2904 | 1004.5663 | 1005.4814 | -0.9152 | 1 | 6 | 46 | 6 | K.FGCRDPVR.T |
|  | 5338 | 527.2866 | 1052.5586 | 1052.5655 | -0.0069 | 0 | 35 | 0.05 | 1 | R.VIFLENYR.V |
|  | 7712 | 406.0258 | 1215.0556 | 1215.6095 | -0.5539 | 1 | 13 | 5.8 | 1 | R.VEDVDKLDQR.G 7702 |
|  | 7771 | **406.7275** | **1217.1608** | **1217.6438** | **-0.4830** | **1** | **(7)** | **21** | **8** | **R.MSLVEEGAVKR.I** |
|  | 8002 | 615.3541 | 1228.6937 | 1228.6663 | 0.0274 | 0 | 6 | 41 | 7 | R.GLAGVENVTELK.K |
|  | 8100 | 412.2235 | 1233.6487 | 1233.6387 | 0.0100 | 1 | 9 | 19 | 9 | R.MSLVEEGAVKR.I |
|  | 8993 | **428.2838** | **1281.8295** | **1281.6313** | **0.1982** | **1** | **3** | **61** | **9** | **R.NIATSGKFSSDR.T** |
|  | 10297 | 676.3519 | 1350.6893 | 1350.7482 | -0.0589 | 1 | 10 | 13 | 4 | K.FFVPRTVMIGGK.A |
|  | 10372 | 452.5600 | 1354.6583 | 1354.6670 | -0.0087 | 0 | 20 | 1.5 | 1 | R.DYYFALAHTVR.D |
|  | 11867 | 721.8465 | 1441.6784 | 1441.6878 | -0.0093 | 0 | 51 | 0.001 | 1 | R.VLYPNDNFFEGK.E 11868 |
|  | 12376 | 491.5977 | 1471.7713 | 1472.8504 | -1.0790 | 0 | 8 | 23 | 5 | R.WPVHLLETLLPR.H |
|  | 12387 | **737.3273** | **1472.6400** | **1473.7584** | **-1.1184** | **1** | **1** | **1e+002** | **10** | **K.AAPGYHMAKMIIR.L** |
|  | 13135 | **760.3940** | **1518.7735** | **1519.7518** | **-0.9783** | **0** | **6** | **36** | **1** | **R.IGEDFISDLDQLR.K** |
|  | 13228 | **509.2401** | **1524.6984** | **1524.8314** | **-0.1329** | **1** | **3** | **65** | **4** | **K.NFNRHLHFTLVK.D** |
|  | 14729 | **542.3458** | **1624.0155** | **1622.8780** | **1.1374** | **0** | **4** | **37** | **8** | **K.VHINPNSLFDIQVK.R** |
|  | 15321 | 554.9412 | 1661.8017 | 1662.8222 | -1.0205 | 1 | 3 | 53 | 5 | R.TVMIGGKAAPGYHMAK.M |
|  | 17816 | **918.9670** | **1835.9194** | **1836.9622** | **-1.0428** | **1** | **4** | **42** | **2** | **K.LLSFVDDEAFIRDVAK.V** |
|  | 17871 | 614.6365 | 1840.8876 | 1839.9155 | 0.9721 | 1 | 7 | 21 | 3 | R.VLYPNDNFFEGKELR.L |
|  | 19708 | **664.3371** | **1989.9895** | **1989.9989** | **-0.0094** | **0** | **1** | **70** | **7** | **R.INMAHLCIAGSHAVNGVAR.I** |
|  | 19910 | 1004.5635 | 2007.1124 | 2006.1200 | 0.9924 | 1 | 33 | 0.041 | 1 | R.LKQEYFVVAATLQDIIR.R |
|  | 20201 | 678.4135 | 2032.2185 | 2032.0279 | 0.1906 | 1 | 2 | 51 | 4 | R.DYYFALAHTVRDHLVGR.W |
|  | 25357 | 901.7689 | 2702.2849 | 2701.3282 | 0.9567 | 1 | 2 | 47 | 10 | K.AWDVTVRTCAYTNHTVLPEALER.W |
|  | 29455 | **1253.9088** | **3758.7046** | **3759.6565** | **-0.9519** | **0** | **1** | **22** | **7** | **K.FMLNGALTIGTMDGANVEMAEEAGEENFFIFGMR.V** |

| **574.** | Q06278\|ADO_HUMAN    **Mass:** 150217   **Score:** 70     **Queries matched:** 34   **emPAI:** 0.08 |
| --- | --- |
|  | Aldehyde oxidase OS=Homo sapiens GN=AOX1 PE=2 SV=2 |

|  | **Query** | **Observed** | **Mr(expt)** | **Mr(calc)** | **Delta** | **Miss** | **Score** | **Expect** | **Rank** | **Peptide** |
| --- | --- | --- | --- | --- | --- | --- | --- | --- | --- | --- |
|  | 1904 | **399.3455** | **796.6764** | **796.4153** | **0.2611** | **0** | **3** | **44** | **6** | **R.IINMYK.E** |
|  | 2994 | **440.0289** | **878.0433** | **877.4770** | **0.5663** | **0** | **8** | **17** | **2** | **R.IHPVQER.I** |
|  | 3908 | **474.7412** | **947.4679** | **947.4746** | **-0.0067** | **0** | **10** | **17** | **1** | **K.DACQTLLK.R** |
|  | 4015 | **478.2975** | **954.5803** | **954.5862** | **-0.0058** | **1** | **10** | **16** | **1** | **K.RLEPIISK.N** |
|  | 4107 | **482.2421** | **962.4697** | **961.4916** | **0.9781** | **1** | **8** | **30** | **10** | **K.FPNLRCR.G** |
|  | 4239 | 486.8050 | 971.5954 | 971.5838 | 0.0117 | 1 | 23 | 0.82 | 2 | K.KGLAMVPLK.F |
|  | 5269 | **524.1737** | **1046.3329** | **1047.5019** | **-1.1690** | **0** | **(2)** | **91** | **5** | **R.GEDMLITGGR.H** |
|  | 5478 | **533.1871** | **1064.3597** | **1063.4968** | **0.8629** | **0** | **6** | **38** | **7** | **R.GEDMLITGGR.H** 5473 5488 |
|  | 5954 | **551.2392** | **1100.4638** | **1099.5267** | **0.9372** | **0** | **4** | **62** | **7** | **R.MPMSNVHLR.G** |
|  | 6434 | **569.8019** | **1137.5892** | **1137.6757** | **-0.0865** | **1** | **9** | **20** | **9** | **R.LEPIISKNPK.G** |
|  | 8213 | 414.2006 | 1239.5800 | 1238.6911 | 0.8890 | 0 | 7 | 24 | 6 | K.FYLEVSQILK.K |
|  | 8484 | 627.8117 | 1253.6089 | 1254.6503 | -1.0414 | 0 | 28 | 0.22 | 1 | R.NMASLGGHIISR.H 8477 8495 |
|  | 10218 | **674.3817** | **1346.7487** | **1346.7558** | **-0.0070** | **0** | **22** | **1.1** | **1** | **K.TGIIAAVTAFAANK.H** |
|  | 12849 | 751.3952 | 1500.7758 | 1499.7442 | 1.0316 | 1 | 3 | 67 | 10 | K.KMDPVHYPSLADK.Y |
|  | 13044 | 505.5944 | 1513.7613 | 1513.7493 | 0.0120 | 1 | 7 | 25 | 2 | R.ELRMPMSNVHLR.G |
|  | 13758 | **780.9036** | **1559.7927** | **1559.8017** | **-0.0090** | **0** | **11** | **10** | **1** | **K.NVDPETMLLPYLR.K** |
|  | 13864 | **523.2699** | **1566.7879** | **1566.8981** | **-0.1102** | **0** | **3** | **58** | **5** | **R.LILNEVSLLGSAPGGK.V** |
|  | 15673 | **563.5970** | **1687.7693** | **1687.8967** | **-0.1274** | **1** | **2** | **67** | **3** | **K.NVDPETMLLPYLRK.K** 15653 |
|  | 16804 | 879.8990 | 1757.7834 | 1758.8723 | -1.0889 | 1 | 3 | 55 | 2 | R.GEDMLITGGRHPYLGK.Y |
|  | 16869 | **881.8534** | **1761.6923** | **1761.7056** | **-0.0132** | **0** | **42** | **0.0069** | **1** | **K.YGCGGGGCGACTVMISR.Y** |
|  | 23787 | **818.0853** | **2451.2342** | **2451.0682** | **0.1659** | **0** | **1** | **54** | **6** | **K.ENGVCCLDQGINGLPEFEEGSK.T** |
|  | 24807 | **648.7894** | **2591.1284** | **2592.3118** | **-1.1835** | **1** | **2** | **43** | **4** | **R.QENALAIVNSGMRVFFGEGDGIIR.E** |
|  | 26991 | **995.9036** | **2984.6889** | **2985.4729** | **-0.7840** | **1** | **1** | **37** | **5** | **R.VFFGEGDGIIRELCISYGGVGPATICAK.N** |
|  | 27823 | 1060.5297 | 3178.5672 | 3178.5930 | -0.0258 | 0 | 43 | 0.0026 | 1 | R.ILALDMEHYSNAGASLDESLFVIEMGLLK.M |
|  | 28193 | **1097.5549** | **3289.6430** | **3289.6542** | **-0.0112** | **0** | **18** | **0.82** | **1** | **K.LFAEEEFLPLDPTQELIFPPELMIMAEK.Q** |
|  | 28621 | **857.2080** | **3424.8029** | **3424.8242** | **-0.0212** | **1** | **26** | **0.091** | **1** | **R.VKIVYQDLEPLILTIEESIQHNSSFKPER.K** |
|  | 29315 | 743.1732 | 3710.8297 | 3709.9501 | 0.8796 | 1 | 0 | 29 | 3 | R.QIPLNEQFLSKCPNADLKPQEILVSVNIPYSR.K |
|  | 29899 | **980.5237** | **3918.0656** | **3916.8936** | **1.1720** | **1** | **2** | **18** | **2** | **R.ILALDMEHYSNAGASLDESLFVIEMGLLKMDNAYK.F** |
|  | 31440 | **1063.7498** | **5313.7124** | **5313.4655** | **0.2469** | **1** | **0** | **12** | **6** | **K.VVDQILEGEIHMGGQEHFYMETQSMLVVPKGEDQEMDVYVSTQFPK.Y** |

| **575.** | Q10471\|GALT2_HUMAN    **Mass:** 65433    **Score:** 69     **Queries matched:** 10   **emPAI:** 0.06 |
| --- | --- |
|  | Polypeptide N-acetylgalactosaminyltransferase 2 OS=Homo sapiens GN=GALNT2 PE=1 SV=1 |

|  | **Query** | **Observed** | **Mr(expt)** | **Mr(calc)** | **Delta** | **Miss** | **Score** | **Expect** | **Rank** | **Peptide** |
| --- | --- | --- | --- | --- | --- | --- | --- | --- | --- | --- |
|  | 2569 | 425.0476 | 848.0807 | 847.4334 | 0.6473 | 1 | 6 | 31 | 9 | R.EGLMRSR.V |
|  | 6547 | 573.8263 | 1145.6380 | 1146.5782 | -0.9401 | 0 | 12 | 11 | 2 | R.NVPYGNIQSR.L |
|  | 10450 | **454.2416** | **1359.7029** | **1358.6942** | **1.0087** | **1** | **4** | **62** | **9** | **K.WEQIEGNSKLR.H** |
|  | 11105 | 466.2520 | 1395.7341 | 1396.6194 | -0.8853 | 1 | 9 | 17 | 2 | K.WDYMTPEQRR.S |
|  | 11670 | **477.2479** | **1428.7219** | **1428.7395** | **-0.0176** | **1** | **6** | **32** | **4** | **K.AQSMETLPPGKVR.W** |
|  | 15666 | **563.3164** | **1686.9272** | **1687.8386** | **-0.9114** | **1** | **2** | **66** | **4** | **K.SVKHMDLCLTVVDR.A** |
|  | 16248 | **574.7923** | **1721.3551** | **1720.8322** | **0.5229** | **0** | **3** | **38** | **5** | **K.QHPYTFPGGSGTVFAR.N** 16238 |
|  | 21613 | 1088.5291 | 2175.0435 | 2175.0583 | -0.0147 | 0 | 69 | 9.8e-006 | 1 | K.EIILVDDYSNDPEDGALLGK.I |
|  | 22230 | 1121.1491 | 2240.2835 | 2239.9224 | 0.3611 | 0 | 6 | 17 | 4 | K.YDMMMDVWGGENLEISFR.V |

| **576.** | P62195\|PRS8_HUMAN    **Mass:** 45768    **Score:** 69     **Queries matched:** 28   **emPAI:** 0.08 |
| --- | --- |
|  | 26S protease regulatory subunit 8 OS=Homo sapiens GN=PSMC5 PE=1 SV=1 |

|  | **Query** | **Observed** | **Mr(expt)** | **Mr(calc)** | **Delta** | **Miss** | **Score** | **Expect** | **Rank** | **Peptide** |
| --- | --- | --- | --- | --- | --- | --- | --- | --- | --- | --- |
|  | 1213 | **585.0898** | **584.0825** | **583.3693** | **0.7132** | **0** | **7** | **15** | **1** | **K.ILPNK.V** |
|  | 1732 | 762.3873 | 761.3800 | 761.4218 | -0.0418 | 1 | 14 | 10 | 7 | R.KMNLTR.G |
|  | 2015 | 402.7210 | 803.4275 | 803.4323 | -0.0048 | 0 | 14 | 8.3 | 1 | K.VIMATNR.I |
|  | 2193 | 410.2116 | 818.4087 | 819.4273 | -1.0185 | 0 | (4) | 89 | 6 | K.VIMATNR.I |
|  | 3618 | **464.2834** | **926.5523** | **925.5484** | **1.0039** | **0** | **4** | **59** | **4** | **K.EVIELPVK.H** |
|  | 5872 | 548.2549 | 1094.4953 | 1093.6608 | 0.8346 | 1 | 6 | 40 | 10 | R.LDILKIHSR.K |
|  | 6593 | **576.2676** | **1150.5207** | **1150.5917** | **-0.0710** | **1** | **6** | **40** | **2** | **K.FIGEGARMVR.E** |
|  | 7129 | **396.7319** | **1187.1740** | **1186.6604** | **0.5135** | **1** | **6** | **26** | **3** | **K.MNLTRGINLR.K** |
|  | 8733 | **634.3881** | **1266.7616** | **1266.6577** | **0.1039** | **1** | **8** | **20** | **7** | **R.MVRELFVMAR.E** |
|  | 9150 | **645.8647** | **1289.7148** | **1289.5848** | **0.1301** | **0** | **2** | **94** | **4** | **R.LEGGSGGDSEVQR.T** |
|  | 9290 | 649.8434 | 1297.6723 | 1297.6302 | 0.0421 | 0 | 2 | 96 | 2 | K.IEFPPPNEEAR.L |
|  | 9888 | **664.8283** | **1327.6421** | **1326.6060** | **1.0360** | **0** | **4** | **56** | **3** | **K.GVCTEAGMYALR.E** |
|  | 10767 | **689.4222** | **1376.8299** | **1375.7091** | **1.1208** | **0** | **0** | **1.3e+002** | **1** | **K.VDPLVSLMMVEK.V** |
|  | 11641 | 714.3443 | 1426.6740 | 1426.6776 | -0.0035 | 0 | 6 | 37 | 5 | R.AVAHHTDCTFIR.V |
|  | 13479 | 771.8937 | 1541.7728 | 1541.8202 | -0.0474 | 1 | 4 | 49 | 5 | R.NDSYTLHKILPNK.V |
|  | 13778 | 781.4141 | 1560.8137 | 1560.6977 | 0.1160 | 0 | 5 | 39 | 7 | M.ALDGPEQMELEEGK.A |
|  | 14762 | **543.2842** | **1626.8307** | **1627.7446** | **-0.9139** | **1** | **6** | **29** | **1** | **K.GVCTEAGMYALRER.R** |
|  | 15757 | **565.2817** | **1692.8234** | **1691.7382** | **1.0852** | **0** | **1** | **77** | **8** | **-.MALDGPEQMELEEGK.A** |
|  | 16137 | **571.9377** | **1712.7914** | **1711.9985** | **0.7929** | **1** | **2** | **67** | **9** | **K.GVLLYGPPGTGKTLLAR.A** |
|  | 17622 | **911.9605** | **1821.9065** | **1821.9182** | **-0.0118** | **0** | **69** | **1.5e-005** | **1** | **R.TMLELLNQLDGFEATK.N** 17623 |
|  | 19344 | **653.6617** | **1957.9634** | **1957.0628** | **0.9006** | **1** | **2** | **51** | **1** | **K.ILPNKVDPLVSLMMVEK.V** |
|  | 20901 | 699.0272 | 2094.0597 | 2095.1201 | -1.0604 | 1 | 1 | 79 | 9 | R.QYYLSKIEELQLIVNDK.S |
|  | 21637 | 1089.4989 | 2176.9832 | 2177.1402 | -0.1569 | 1 | 2 | 52 | 3 | R.TMLELLNQLDGFEATKNIK.V |
|  | 23852 | **492.2451** | **2456.1891** | **2457.3227** | **-1.1336** | **1** | **3** | **33** | **4** | **R.LLREELQLLQEQGSYVGEVVR.A** |
|  | 25298 | **1348.6503** | **2695.2860** | **2696.2972** | **-1.0112** | **1** | **3** | **33** | **1** | **K.IAELMPGASGAEVKGVCTEAGMYALR.E** |
|  | 26460 | **580.7275** | **2898.6013** | **2897.4112** | **1.1901** | **1** | **1** | **41** | **4** | **K.VDPLVSLMMVEKVPDSTYEMIGGLDK.Q** |
|  | 28489 | **1125.5872** | **3373.7397** | **3374.5685** | **-0.8288** | **1** | **3** | **22** | **2** | **R.EHAPSIIFMDEIDSIGSSRLEGGSGGDSEVQR.T** |

| **577.** | P30837\|AL1B1_HUMAN    **Mass:** 57658    **Score:** 69     **Queries matched:** 11   **emPAI:** 0.06 |
| --- | --- |
|  | Aldehyde dehydrogenase X, mitochondrial OS=Homo sapiens GN=ALDH1B1 PE=1 SV=2 |

|  | **Query** | **Observed** | **Mr(expt)** | **Mr(calc)** | **Delta** | **Miss** | **Score** | **Expect** | **Rank** | **Peptide** |
| --- | --- | --- | --- | --- | --- | --- | --- | --- | --- | --- |
|  | 2198 | **410.2498** | **818.4850** | **819.4351** | **-0.9501** | **1** | **6** | **57** | **2** | **K.AAREAFR.L** |
|  | 4236 | 486.7922 | 971.5699 | 971.5764 | -0.0064 | 1 | 26 | 0.42 | 1 | K.RVTLELGGK.S |
|  | 4906 | **509.8446** | **1017.6746** | **1018.5746** | **-0.9000** | **1** | **9** | **23** | **1** | **-.MLRFLAPR.L** |
|  | 5514 | 534.3357 | 1066.6569 | 1067.5764 | -0.9194 | 0 | 7 | 27 | 6 | R.YGLAAAVFTR.D |
|  | 10633 | 685.3796 | 1368.7447 | 1369.8194 | -1.0747 | 1 | 5 | 42 | 10 | R.FLAPRLLSLQGR.T |
|  | 13433 | **513.9638** | **1538.8694** | **1538.8780** | **-0.0086** | **1** | **2** | **74** | **2** | **R.LLNLLADLVERDR.V** |
|  | 16178 | **572.6589** | **1714.9548** | **1714.9658** | **-0.0110** | **1** | **1** | **89** | **8** | **R.IAKEEIFGPVQPLFK.F** |
|  | 17004 | **592.3410** | **1774.0012** | **1774.8414** | **-0.8402** | **0** | **1** | **94** | **9** | **R.TFVEESIYNEFLER.T** |
|  | 17557 | **909.0215** | **1816.0284** | **1816.0346** | **-0.0062** | **0** | **69** | **1.3e-005** | **1** | **K.VAEQTPLSALYLASLIK.E** 17542 |
|  | 22170 | **745.3655** | **2233.0748** | **2232.0950** | **0.9798** | **1** | **4** | **34** | **1** | **R.TFVEESIYNEFLERTVEK.A** |

| **578.** | Q9UHE5\|NAT8_HUMAN    **Mass:** 25831    **Score:** 69     **Queries matched:** 3   **emPAI:** 0.15 |
| --- | --- |
|  | Probable N-acetyltransferase 8 OS=Homo sapiens GN=NAT8 PE=2 SV=2 |

|  | **Query** | **Observed** | **Mr(expt)** | **Mr(calc)** | **Delta** | **Miss** | **Score** | **Expect** | **Rank** | **Peptide** |
| --- | --- | --- | --- | --- | --- | --- | --- | --- | --- | --- |
|  | 3069 | **442.8994** | **883.7843** | **883.4156** | **0.3686** | **0** | **4** | **45** | **10** | **-.MAPCHIR.K** |
|  | 14961 | 819.9421 | 1637.8697 | 1637.8811 | -0.0114 | 0 | 69 | 1.6e-005 | 1 | K.VVGMVGALPVDDPTLR.E |
|  | 27853 | 796.9443 | 3183.7480 | 3184.4403 | -0.6923 | 1 | 2 | 27 | 5 | K.KPWTEYVDMTLCTDMSDITKSYLSER.G |

| **579.** | P34913\|HYES_HUMAN    **Mass:** 63316    **Score:** 69     **Queries matched:** 20   **emPAI:** 0.06 |
| --- | --- |
|  | Epoxide hydrolase 2 OS=Homo sapiens GN=EPHX2 PE=1 SV=2 |

|  | **Query** | **Observed** | **Mr(expt)** | **Mr(calc)** | **Delta** | **Miss** | **Score** | **Expect** | **Rank** | **Peptide** |
| --- | --- | --- | --- | --- | --- | --- | --- | --- | --- | --- |
|  | 2242 | **411.7245** | **821.4344** | **822.3905** | **-0.9561** | **1** | **4** | **63** | **9** | **R.KCSETAK.V** |
|  | 2254 | 412.2241 | 822.4336 | 822.3979 | 0.0356 | 0 | 9 | 21 | 7 | R.VLAMDMK.G |
|  | 2754 | 863.3731 | 862.3658 | 861.3981 | 0.9678 | 0 | 6 | 56 | 4 | K.WLDSDAR.N |
|  | 8071 | 617.3472 | 1232.6798 | 1232.6183 | 0.0615 | 1 | 11 | 12 | 9 | K.GGPEGATTRLMK.G |
|  | 9197 | 647.3776 | 1292.7407 | 1292.6104 | 0.1302 | 0 | 7 | 28 | 2 | R.DGLAQLMCELK.M |
|  | 9314 | **650.4381** | **1298.8617** | **1297.7679** | **1.0938** | **0** | **1** | **82** | **8** | **K.ILIPALMVTAEK.D** |
|  | 11618 | **475.9284** | **1424.7634** | **1425.8629** | **-1.0995** | **1** | **5** | **38** | **2** | **R.KILIPALMVTAEK.D** |
|  | 13486 | 514.9746 | 1541.9018 | 1541.8534 | 0.0484 | 0 | 8 | 18 | 10 | K.INRPMLQAALMLR.K 13492 13505 |
|  | 15136 | 550.8161 | 1649.4265 | 1648.7912 | 0.6352 | 1 | 1 | 53 | 9 | R.AERDGLAQLMCELK.M |
|  | 15670 | **563.3618** | **1687.0636** | **1685.9433** | **1.1204** | **1** | **4** | **36** | **4** | **R.KINRPMLQAALMLR.K** |
|  | 16289 | 575.3466 | 1723.0180 | 1721.8407 | 1.1774 | 1 | 4 | 36 | 4 | K.SLFRASDESVLSMHK.V |
|  | 20356 | **683.7039** | **2048.0899** | **2046.9680** | **1.1219** | **0** | **1** | **79** | **6** | **K.VCEAGGLFVNSPEEPSLSR.M** |
|  | 20754 | **694.3375** | **2079.9906** | **2079.0385** | **0.9520** | **1** | **4** | **41** | **1** | **K.GFTTAILTNTWLDDRAER.D** |
|  | 23049 | **783.7623** | **2348.2652** | **2347.2192** | **1.0459** | **1** | **0** | **70** | **7** | **R.DLGMVTILVQDTDTALKELEK.V** |
|  | 23522 | **1206.1274** | **2410.2403** | **2410.2566** | **-0.0163** | **0** | **53** | **0.00036** | **1** | **R.AVASLNTPFIPANPNMSPLESIK.A** 23523 |
|  | 28680 | **863.4374** | **3449.7204** | **3449.7436** | **-0.0231** | **0** | **11** | **3.2** | **1** | **K.VTGIQLLNTPAPLPTSCNPSDMSHGYVTVKPR.V** |
|  | 29383 | **746.7948** | **3728.9376** | **3728.9496** | **-0.0120** | **1** | **11** | **2.7** | **1** | **K.ILIPALMVTAEKDFVLVPQMSQHMEDWIPHLK.R** |

| **580.** | Q6IQ22\|RAB12_HUMAN    **Mass:** 27573    **Score:** 68     **Queries matched:** 11   **emPAI:** 0.14 |
| --- | --- |
|  | Putative Ras-related protein Rab-12 OS=Homo sapiens GN=RAB12 PE=5 SV=3 |

|  | **Query** | **Observed** | **Mr(expt)** | **Mr(calc)** | **Delta** | **Miss** | **Score** | **Expect** | **Rank** | **Peptide** |
| --- | --- | --- | --- | --- | --- | --- | --- | --- | --- | --- |
|  | 2669 | 429.2404 | 856.4663 | 857.5334 | -1.0671 | 1 | 14 | 7 | 1 | K.IKTVELR.G |
|  | 4131 | 482.7376 | 963.4606 | 963.4549 | 0.0057 | 0 | 10 | 19 | 4 | K.ETFDDLPK.W 4130 |
|  | 4285 | **488.2803** | **974.5460** | **973.4651** | **1.0809** | **0** | **4** | **71** | **9** | **-.MDPGAALQR.R** |
|  | 9635 | 658.8289 | 1315.6433 | 1315.6521 | -0.0088 | 0 | 60 | 0.00015 | 1 | R.LQIWDTAGQER.F 9612 9633 9634 |
|  | 14953 | **546.7839** | **1637.3300** | **1636.7991** | **0.5309** | **1** | **6** | **21** | **3** | **R.QQGEKFAQQITGMR.F** |
|  | 16529 | 870.4347 | 1738.8548 | 1738.8823 | -0.0275 | 1 | 4 | 43 | 4 | R.RAGGGGGLGAGSPALSGGQGR.R |
|  | 21362 | 716.7014 | 2147.0824 | 2146.0041 | 1.0783 | 1 | 4 | 31 | 2 | R.FCEASAKDNFNVDEIFLK.L |

| **581.** | Q9NRW1\|RAB6B_HUMAN    **Score:** 68     **Queries matched:** 9 |
| --- | --- |
|  | Ras-related protein Rab-6B OS=Homo sapiens GN=RAB6B PE=1 SV=1 |

|  | **Query** | **Observed** | **Mr(expt)** | **Mr(calc)** | **Delta** | **Miss** | **Score** | **Expect** | **Rank** | **Peptide** |
| --- | --- | --- | --- | --- | --- | --- | --- | --- | --- | --- |
|  | 2205 | **410.7166** | **819.4187** | **820.4000** | **-0.9813** | **0** | **8** | **33** | **9** | **K.EGMIDIK.L** |
|  | 5822 | **546.2997** | **1090.5849** | **1089.5203** | **1.0646** | **0** | **6** | **41** | **6** | **M.SAGGDFGNPLR.K** |
|  | 7481 | **602.3917** | **1202.7688** | **1201.5939** | **1.1749** | **0** | **3** | **74** | **6** | **R.QITIEEGEQR.A** |
|  | 7934 | **409.5521** | **1225.6344** | **1224.6615** | **0.9729** | **1** | **1** | **1.1e+002** | **8** | **K.TGYNVKQLFR.R** |
|  | 8175 | **619.5247** | **1237.0348** | **1236.5557** | **0.4790** | **0** | **5** | **32** | **2** | **-.MSAGGDFGNPLR.K** |
|  | 9635 | 658.8289 | 1315.6433 | 1315.6521 | -0.0088 | 0 | 60 | 0.00015 | 1 | R.LQLWDTAGQER.F 9612 9633 9634 |

| **582.** | P20336\|RAB3A_HUMAN    **Score:** 68     **Queries matched:** 8 |
| --- | --- |
|  | Ras-related protein Rab-3A OS=Homo sapiens GN=RAB3A PE=1 SV=1 |

|  | **Query** | **Observed** | **Mr(expt)** | **Mr(calc)** | **Delta** | **Miss** | **Score** | **Expect** | **Rank** | **Peptide** |
| --- | --- | --- | --- | --- | --- | --- | --- | --- | --- | --- |
|  | 1510 | 675.3979 | 674.3906 | 675.3551 | -0.9645 | 0 | 15 | 7.6 | 1 | R.VVSSER.G |
|  | 4199 | **485.3456** | **968.6765** | **969.3168** | **-0.6402** | **0** | **2** | **70** | **9** | **K.CDMEDER.V** |
|  | 5974 | **551.3936** | **1100.7725** | **1099.6601** | **1.1125** | **0** | **3** | **65** | **9** | **K.ILIIGNSSVGK.T** |
|  | 9635 | 658.8289 | 1315.6433 | 1315.6521 | -0.0088 | 0 | 60 | 0.00015 | 1 | K.LQIWDTAGQER.Y 9612 9633 9634 |
|  | 16013 | 853.9753 | 1705.9360 | 1706.8628 | -0.9268 | 0 | 0 | 1e+002 | 9 | K.TYSWDNAQVLLVGNK.C |

| **583.** | Q96E17\|RAB3C_HUMAN    **Score:** 68     **Queries matched:** 9 |
| --- | --- |
|  | Ras-related protein Rab-3C OS=Homo sapiens GN=RAB3C PE=2 SV=1 |

|  | **Query** | **Observed** | **Mr(expt)** | **Mr(calc)** | **Delta** | **Miss** | **Score** | **Expect** | **Rank** | **Peptide** |
| --- | --- | --- | --- | --- | --- | --- | --- | --- | --- | --- |
|  | 4199 | 485.3456 | 968.6765 | 969.3168 | -0.6402 | 0 | 2 | 70 | 9 | K.CDMEDER.V |
|  | 5974 | 551.3936 | 1100.7725 | 1099.6601 | 1.1125 | 0 | 3 | 65 | 9 | K.LLIIGNSSVGK.T |
|  | 9635 | 658.8289 | 1315.6433 | 1315.6521 | -0.0088 | 0 | 60 | 0.00015 | 1 | K.LQIWDTAGQER.Y 9612 9633 9634 |
|  | 16013 | 853.9753 | 1705.9360 | 1706.8628 | -0.9268 | 0 | 0 | 1e+002 | 9 | K.TYSWDNAQVILVGNK.C |
|  | 16372 | 865.4339 | 1728.8532 | 1729.7624 | -0.9092 | 1 | 9 | 14 | 7 | M.RHEAPMQMASAQDAR.Y |
|  | 20366 | **684.1990** | **2049.5753** | **2049.8996** | **-0.3243** | **1** | **1** | **52** | **5** | **R.HEAPMQMASAQDARYGQK.D** |

| **584.** | Q96AX2\|RAB37_HUMAN    **Mass:** 25028    **Score:** 68     **Queries matched:** 9   **emPAI:** 0.15 |
| --- | --- |
|  | Ras-related protein Rab-37 OS=Homo sapiens GN=RAB37 PE=1 SV=3 |

|  | **Query** | **Observed** | **Mr(expt)** | **Mr(calc)** | **Delta** | **Miss** | **Score** | **Expect** | **Rank** | **Peptide** |
| --- | --- | --- | --- | --- | --- | --- | --- | --- | --- | --- |
|  | 5946 | 550.8050 | 1099.5954 | 1100.6264 | -1.0309 | 0 | 1 | 1.4e+002 | 3 | R.DVVIMLLGNK.A |
|  | 6746 | 1162.5927 | 1161.5854 | 1162.5764 | -0.9911 | 1 | 5 | 57 | 4 | K.ADMSSERVIR.S |
|  | 9635 | 658.8289 | 1315.6433 | 1315.6521 | -0.0088 | 0 | 60 | 0.00015 | 1 | K.LQIWDTAGQER.F 9612 9633 9634 |
|  | 17742 | **611.3271** | **1830.9594** | **1830.8530** | **0.1064** | **1** | **2** | **73** | **5** | **-.MTGTPGAVATRDGEAPER.S** |
|  | 18485 | 629.9911 | 1886.9514 | 1885.9574 | 0.9940 | 0 | 7 | 19 | 1 | K.DGAFLSGTFIATVGIDFR.N |
|  | 18566 | **947.6312** | **1893.2479** | **1892.9336** | **0.3143** | **1** | **1** | **58** | **7** | **R.DVVIMLLGNKADMSSER.V** |

| **585.** | P61018\|RAB4B_HUMAN    **Score:** 68     **Queries matched:** 6 |
| --- | --- |
|  | Ras-related protein Rab-4B OS=Homo sapiens GN=RAB4B PE=1 SV=1 |

|  | **Query** | **Observed** | **Mr(expt)** | **Mr(calc)** | **Delta** | **Miss** | **Score** | **Expect** | **Rank** | **Peptide** |
| --- | --- | --- | --- | --- | --- | --- | --- | --- | --- | --- |
|  | 2926 | **437.2476** | **872.4806** | **871.4399** | **1.0407** | **1** | **10** | **20** | **9** | **K.KDLDPER.E** |
|  | 9635 | 658.8289 | 1315.6433 | 1315.6521 | -0.0088 | 0 | 60 | 0.00015 | 1 | K.LQIWDTAGQER.F 9612 9633 9634 |
|  | 17049 | **593.2672** | **1776.7796** | **1775.8690** | **0.9106** | **1** | **2** | **70** | **7** | **K.DLDPEREVTFLEASR.F** |

| **586.** | P20337\|RAB3B_HUMAN    **Mass:** 24970    **Score:** 68     **Queries matched:** 9   **emPAI:** 0.15 |
| --- | --- |
|  | Ras-related protein Rab-3B OS=Homo sapiens GN=RAB3B PE=1 SV=2 |

|  | **Query** | **Observed** | **Mr(expt)** | **Mr(calc)** | **Delta** | **Miss** | **Score** | **Expect** | **Rank** | **Peptide** |
| --- | --- | --- | --- | --- | --- | --- | --- | --- | --- | --- |
|  | 5974 | 551.3936 | 1100.7725 | 1099.6601 | 1.1125 | 0 | 3 | 65 | 9 | K.LLIIGNSSVGK.T |
|  | 7591 | 605.3390 | 1208.6634 | 1208.6071 | 0.0563 | 1 | 2 | 97 | 7 | -.MASVTDGKTGVK.D |
|  | 9635 | 658.8289 | 1315.6433 | 1315.6521 | -0.0088 | 0 | 60 | 0.00015 | 1 | K.LQIWDTAGQER.Y 9612 9633 9634 |
|  | 14691 | 811.9040 | 1621.7934 | 1620.7123 | 1.0811 | 1 | 2 | 73 | 5 | K.CDMEEERVVPTEK.G |
|  | 16013 | 853.9753 | 1705.9360 | 1706.8628 | -0.9268 | 0 | 0 | 1e+002 | 9 | K.TYSWDNAQVILVGNK.C |
|  | 18227 | **622.6552** | **1864.9438** | **1864.8302** | **0.1136** | **1** | **4** | **42** | **1** | **K.TGVKDASDQNFDYMFK.L** |

| **587.** | Q14964\|RB39A_HUMAN    **Score:** 68     **Queries matched:** 9 |
| --- | --- |
|  | Ras-related protein Rab-39A OS=Homo sapiens GN=RAB39 PE=2 SV=2 |

|  | **Query** | **Observed** | **Mr(expt)** | **Mr(calc)** | **Delta** | **Miss** | **Score** | **Expect** | **Rank** | **Peptide** |
| --- | --- | --- | --- | --- | --- | --- | --- | --- | --- | --- |
|  | 3213 | 897.4039 | 896.3967 | 897.5171 | -1.1205 | 0 | 7 | 27 | 10 | R.LLEIEPGK.R |
|  | 9635 | 658.8289 | 1315.6433 | 1315.6521 | -0.0088 | 0 | 60 | 0.00015 | 1 | K.LQLWDTAGQER.F 9612 9633 9634 |
|  | 9997 | **667.8171** | **1333.6196** | **1332.6568** | **0.9628** | **1** | **2** | **82** | **8** | **K.CDLASQRQVTR.E** |
|  | 15697 | 564.2532 | 1689.7377 | 1688.7749 | 0.9627 | 1 | 0 | 98 | 8 | K.LSADCGMKYIETSAK.D |
|  | 17690 | **609.9840** | **1826.9302** | **1826.8661** | **0.0641** | **1** | **2** | **58** | **2** | **K.DWLEEAKMYVQPFR.I** |
|  | 19206 | 974.0029 | 1945.9913 | 1946.0964 | -0.1051 | 1 | 0 | 96 | 3 | K.MYVQPFRIVFLLVGHK.C |

| **588.** | Q9H082\|RB33B_HUMAN    **Score:** 68     **Queries matched:** 9 |
| --- | --- |
|  | Ras-related protein Rab-33B OS=Homo sapiens GN=RAB33B PE=1 SV=1 |

|  | **Query** | **Observed** | **Mr(expt)** | **Mr(calc)** | **Delta** | **Miss** | **Score** | **Expect** | **Rank** | **Peptide** |
| --- | --- | --- | --- | --- | --- | --- | --- | --- | --- | --- |
|  | 9635 | 658.8289 | 1315.6433 | 1315.6521 | -0.0088 | 0 | 60 | 0.00015 | 1 | K.IQLWDTAGQER.F 9612 9633 9634 |
|  | 12844 | 751.3691 | 1500.7237 | 1501.8868 | -1.1631 | 1 | 7 | 25 | 6 | R.IFKIIVIGDSNVGK.T 12843 |
|  | 15874 | 849.9272 | 1697.8398 | 1696.7767 | 1.0632 | 0 | 0 | 1e+002 | 2 | K.FADTHSMPLFETSAK.N |
|  | 29123 | **1201.8561** | **3602.5464** | **3601.8281** | **0.7183** | **1** | **3** | **15** | **3** | **K.LKSHKPLMLSQPPDNGIILKPEPKPAMTCWC.-** |
|  | 29460 | **1254.5916** | **3760.7528** | **3759.7297** | **1.0231** | **1** | **0** | **33** | **2** | **K.FADTHSMPLFETSAKNPNDNDHVEAIFMTLAHK.L** |

| **589.** | P20340\|RAB6A_HUMAN    **Score:** 68     **Queries matched:** 10 |
| --- | --- |
|  | Ras-related protein Rab-6A OS=Homo sapiens GN=RAB6A PE=1 SV=3 |

|  | **Query** | **Observed** | **Mr(expt)** | **Mr(calc)** | **Delta** | **Miss** | **Score** | **Expect** | **Rank** | **Peptide** |
| --- | --- | --- | --- | --- | --- | --- | --- | --- | --- | --- |
|  | 9635 | 658.8289 | 1315.6433 | 1315.6521 | -0.0088 | 0 | 60 | 0.00015 | 1 | R.LQLWDTAGQER.F 9612 9633 9634 |
|  | 11936 | **482.7554** | **1445.2445** | **1444.6980** | **0.5464** | **0** | **0** | **82** | **9** | **R.VAAALPGMESTQDR.S** |
|  | 14046 | 790.8896 | 1579.7647 | 1579.8279 | -0.0632 | 1 | (2) | 71 | 9 | K.AKELNVMFIETSAK.A |
|  | 14309 | **798.8769** | **1595.7392** | **1595.8229** | **-0.0836** | **1** | **2** | **68** | **3** | **K.AKELNVMFIETSAK.A** 14305 |
|  | 15993 | **569.2889** | **1704.8448** | **1703.8261** | **1.0187** | **1** | **7** | **25** | **6** | **R.VAAALPGMESTQDRSR.E** |
|  | 25041 | 883.4476 | 2647.3209 | 2648.1768 | -0.8559 | 1 | 1 | 52 | 5 | R.EDMIDIKLEKPQEQPVSEGGCSC.- |

| **590.** | P20338\|RAB4A_HUMAN    **Score:** 68     **Queries matched:** 7 |
| --- | --- |
|  | Ras-related protein Rab-4A OS=Homo sapiens GN=RAB4A PE=1 SV=2 |

|  | **Query** | **Observed** | **Mr(expt)** | **Mr(calc)** | **Delta** | **Miss** | **Score** | **Expect** | **Rank** | **Peptide** |
| --- | --- | --- | --- | --- | --- | --- | --- | --- | --- | --- |
|  | 9635 | 658.8289 | 1315.6433 | 1315.6521 | -0.0088 | 0 | 60 | 0.00015 | 1 | K.LQIWDTAGQER.F 9612 9633 9634 |
|  | 16019 | 569.8015 | 1706.3827 | 1705.8788 | 0.5039 | 1 | 6 | 17 | 9 | K.YVKLQIWDTAGQER.F |
|  | 17366 | 901.0276 | 1800.0406 | 1800.9954 | -0.9547 | 1 | 2 | 62 | 4 | R.MLASQNIVIILCGNKK.D |
|  | 28770 | **870.1892** | **3476.7275** | **3476.6228** | **0.1047** | **1** | **1** | **27** | **6** | **R.FAQENELMFLETSALTGENVEEAFVQCARK.I** |

| **591.** | Q86YS6\|RAB43_HUMAN    **Score:** 68     **Queries matched:** 5 |
| --- | --- |
|  | Ras-related protein Rab-43 OS=Homo sapiens GN=RAB43 PE=1 SV=1 |

|  | **Query** | **Observed** | **Mr(expt)** | **Mr(calc)** | **Delta** | **Miss** | **Score** | **Expect** | **Rank** | **Peptide** |
| --- | --- | --- | --- | --- | --- | --- | --- | --- | --- | --- |
|  | 5391 | 529.3182 | 1056.6219 | 1056.6179 | 0.0040 | 0 | 6 | 40 | 8 | K.LVLVGDASVGK.T |
|  | 9635 | 658.8289 | 1315.6433 | 1315.6521 | -0.0088 | 0 | 60 | 0.00015 | 1 | K.LQIWDTAGQER.F 9612 9633 9634 |

| **592.** | O95716\|RAB3D_HUMAN    **Score:** 68     **Queries matched:** 7 |
| --- | --- |
|  | Ras-related protein Rab-3D OS=Homo sapiens GN=RAB3D PE=1 SV=1 |

|  | **Query** | **Observed** | **Mr(expt)** | **Mr(calc)** | **Delta** | **Miss** | **Score** | **Expect** | **Rank** | **Peptide** |
| --- | --- | --- | --- | --- | --- | --- | --- | --- | --- | --- |
|  | 3747 | 469.2074 | 936.4002 | 935.3654 | 1.0348 | 0 | 5 | 46 | 2 | K.CDLEDER.V |
|  | 9635 | 658.8289 | 1315.6433 | 1315.6521 | -0.0088 | 0 | 60 | 0.00015 | 1 | K.LQIWDTAGQER.Y 9612 9633 9634 |
|  | 16013 | 853.9753 | 1705.9360 | 1706.8628 | -0.9268 | 0 | 0 | 1e+002 | 9 | K.TYSWDNAQVILVGNK.C |
|  | 24155 | 1246.6630 | 2491.3114 | 2491.0710 | 0.2404 | 1 | 0 | 60 | 9 | M.ASAGDTQAGPRDAADQNFDYMFK.L |

| **593.** | Q15771\|RAB30_HUMAN    **Mass:** 23386    **Score:** 68     **Queries matched:** 6   **emPAI:** 0.16 |
| --- | --- |
|  | Ras-related protein Rab-30 OS=Homo sapiens GN=RAB30 PE=1 SV=2 |

|  | **Query** | **Observed** | **Mr(expt)** | **Mr(calc)** | **Delta** | **Miss** | **Score** | **Expect** | **Rank** | **Peptide** |
| --- | --- | --- | --- | --- | --- | --- | --- | --- | --- | --- |
|  | 9635 | 658.8289 | 1315.6433 | 1315.6521 | -0.0088 | 0 | 60 | 0.00015 | 1 | K.LQIWDTAGQER.F 9612 9633 9634 |
|  | 21049 | **1056.5039** | **2110.9933** | **2110.9041** | **0.0892** | **0** | **5** | **28** | **1** | **R.AEEFSEAQDMYYLETSAK.E** |
|  | 23761 | **816.4114** | **2446.2125** | **2446.2276** | **-0.0151** | **1** | **1** | **64** | **7** | **-.MSMEDYDFLFKIVLIGNAGVGK.T** |

| **594.** | P11226\|MBL2_HUMAN    **Mass:** 26526    **Score:** 68     **Queries matched:** 9   **emPAI:** 0.14 |
| --- | --- |
|  | Mannose-binding protein C OS=Homo sapiens GN=MBL2 PE=1 SV=2 |

|  | **Query** | **Observed** | **Mr(expt)** | **Mr(calc)** | **Delta** | **Miss** | **Score** | **Expect** | **Rank** | **Peptide** |
| --- | --- | --- | --- | --- | --- | --- | --- | --- | --- | --- |
|  | 2136 | 407.6923 | 813.3701 | 812.4141 | 0.9561 | 0 | 7 | 35 | 3 | K.GEPGQGLR.G |
|  | 3955 | **476.2574** | **950.5002** | **950.5226** | **-0.0223** | **0** | **9** | **21** | **4** | **K.WLTFSLGK.Q** |
|  | 4300 | 488.7611 | 975.5077 | 975.5138 | -0.0061 | 0 | 24 | 0.71 | 1 | K.FQASVATPR.N 4296 |
|  | 11452 | 708.8597 | 1415.7048 | 1414.7205 | 0.9843 | 0 | 3 | 63 | 7 | K.LGPPGNPGPSGSPGPK.G 11449 |
|  | 14278 | 797.4515 | 1592.8884 | 1591.7592 | 1.1292 | 0 | 8 | 17 | 2 | K.FFLTNGEIMTFEK.V |
|  | 26235 | **1426.6460** | **2851.2774** | **2851.2970** | **-0.0196** | **0** | **68** | **8e-006** | **1** | **R.LTYTNWNEGEPNNAGSDEDCVLLLK.N** |
|  | 27787 | 1057.4955 | 3169.4646 | 3169.5927 | -0.1281 | 0 | 8 | 7.5 | 1 | M.SLFPSLPLLLLSMVAASYSETVTCEDAQK.T |

| **595.** | Q86VD7\|S2542_HUMAN    **Mass:** 35444    **Score:** 68     **Queries matched:** 13   **emPAI:** 0.11 |
| --- | --- |
|  | Solute carrier family 25 member 42 OS=Homo sapiens GN=SLC25A42 PE=1 SV=2 |

|  | **Query** | **Observed** | **Mr(expt)** | **Mr(calc)** | **Delta** | **Miss** | **Score** | **Expect** | **Rank** | **Peptide** |
| --- | --- | --- | --- | --- | --- | --- | --- | --- | --- | --- |
|  | 777 | 475.2385 | 474.2313 | 473.2598 | 0.9715 | 0 | 1 | 2.8e+002 | 2 | M.GNGVK.E |
|  | 2504 | **422.1958** | **842.3770** | **841.4658** | **0.9112** | **0** | **3** | **86** | **4** | **K.TAVAPLDR.T** |
|  | 3759 | **469.3275** | **936.6405** | **935.4501** | **1.1904** | **0** | **3** | **72** | **4** | **R.QPYPFER.M** |
|  | 4821 | **507.2206** | **1012.4267** | **1011.5461** | **0.8805** | **1** | **2** | **1e+002** | **10** | **M.GNGVKEGPVR.L** |
|  | 6721 | 580.8076 | 1159.6006 | 1158.5815 | 1.0190 | 1 | 3 | 76 | 10 | -.MGNGVKEGPVR.L |
|  | 6969 | **590.7877** | **1179.5609** | **1179.5706** | **-0.0098** | **0** | **17** | **3.1** | **1** | **R.MQTAGVTGYPR.A** |
|  | 14353 | **800.4752** | **1598.9359** | **1597.9403** | **0.9956** | **0** | **60** | **0.00011** | **1** | **R.QVLSSLLSGALAGALAK.T** 14339 14341 |
|  | 15563 | 560.4137 | 1678.2193 | 1677.8621 | 0.3572 | 1 | 10 | 7.9 | 1 | R.MQTAGVTGYPRASIAR.T |
|  | 15785 | **565.3075** | **1692.9007** | **1693.8570** | **-0.9563** | **1** | **(3)** | **59** | **7** | **R.MQTAGVTGYPRASIAR.T** |
|  | 21799 | **734.0381** | **2199.0924** | **2198.1016** | **0.9908** | **1** | **0** | **79** | **5** | **R.MAVTPKEMYSNIFHVFIR.I** |
|  | 22873 | 1163.0665 | 2324.1185 | 2324.1657 | -0.0472 | 0 | 1 | 68 | 3 | R.MIFGACAGLIGQSASYPLDVVR.R |

| **596.** | Q9Y678\|COPG_HUMAN    **Mass:** 98967    **Score:** 68     **Queries matched:** 19   **emPAI:** 0.04 |
| --- | --- |
|  | Coatomer subunit gamma OS=Homo sapiens GN=COPG PE=1 SV=1 |

|  | **Query** | **Observed** | **Mr(expt)** | **Mr(calc)** | **Delta** | **Miss** | **Score** | **Expect** | **Rank** | **Peptide** |
| --- | --- | --- | --- | --- | --- | --- | --- | --- | --- | --- |
|  | 847 | **498.6939** | **497.6867** | **498.2914** | **-0.6048** | **0** | **6** | **15** | **3** | **R.GPAVR.A** 848 |
|  | 3544 | **461.1678** | **920.3211** | **919.5161** | **0.8050** | **1** | **7** | **32** | **2** | **R.TLNKVAMK.H** |
|  | 3631 | 465.0192 | 928.0238 | 927.4558 | 0.5680 | 0 | 4 | 47 | 9 | R.MCYLTIK.E |
|  | 4634 | 500.2922 | 998.5698 | 998.5509 | 0.0189 | 1 | 13 | 7.6 | 9 | K.QPEKVAATR.Q |
|  | 6085 | 555.8871 | 1109.7596 | 1108.5877 | 1.1719 | 0 | 3 | 64 | 6 | R.VVLEHEEVR.A |
|  | 7520 | 603.3205 | 1204.6264 | 1203.6975 | 0.9289 | 0 | 6 | 40 | 5 | R.ILHLLGQEGPK.T |
|  | 8755 | 635.3105 | 1268.6064 | 1267.4809 | 1.1255 | 0 | 1 | 1.1e+002 | 4 | R.CVMDDDNEVR.D |
|  | 10129 | **448.2361** | **1341.6865** | **1340.7565** | **0.9300** | **0** | **4** | **50** | **6** | **K.NTHTLLLAGVFR.G** |
|  | 14174 | **397.9613** | **1587.8162** | **1588.8858** | **-1.0696** | **0** | **5** | **37** | **2** | **R.LLLLDTVTMQVTAR.S** 14183 |
|  | 18673 | **951.5194** | **1901.0241** | **1901.0332** | **-0.0090** | **0** | **48** | **0.0016** | **1** | **K.FGAQNEEMLPSILVLLK.R** 18682 |
|  | 19759 | **666.1277** | **1995.3612** | **1994.9904** | **0.3708** | **0** | **2** | **37** | **2** | **K.EMSCIAEDVIIVTSSLTK.D** |
|  | 20208 | 1018.0433 | 2034.0720 | 2034.9642 | -0.8922 | 1 | 1 | 72 | 7 | R.LMKQISSFMSEISDEFK.V |
|  | 22364 | 566.0474 | 2260.1606 | 2261.0820 | -0.9214 | 0 | 1 | 67 | 10 | K.HEMVVYEAASAIVNLPGCSAK.E |
|  | 24473 | **510.2529** | **2546.2281** | **2547.2290** | **-1.0009** | **1** | **3** | **38** | **10** | **K.HAVLMNFLFTMLREEGGFEYK.R** |
|  | 24601 | **641.0823** | **2560.3003** | **2559.2118** | **1.0885** | **1** | **1** | **60** | **4** | **K.EMSCIAEDVIIVTSSLTKDMTGK.E** |
|  | 27298 | **1016.8114** | **3047.4124** | **3047.4288** | **-0.0164** | **1** | **4** | **17** | **1** | **K.LNFEAAWDEVGDEFEKEETFTLSTIK.T** |

| **597.** | O00483\|NDUA4_HUMAN    **Mass:** 9421     **Score:** 67     **Queries matched:** 4   **emPAI:** 1.04 |
| --- | --- |
|  | NADH dehydrogenase [ubiquinone] 1 alpha subcomplex subunit 4 OS=Homo sapiens GN=NDUFA4 PE=1 SV=1 |

|  | **Query** | **Observed** | **Mr(expt)** | **Mr(calc)** | **Delta** | **Miss** | **Score** | **Expect** | **Rank** | **Peptide** |
| --- | --- | --- | --- | --- | --- | --- | --- | --- | --- | --- |
|  | 3717 | **467.7315** | **933.4483** | **933.4556** | **-0.0072** | **0** | **47** | **0.0041** | **1** | **K.LGPNDQYK.F** |
|  | 4613 | 499.7349 | 997.4552 | 997.4617 | -0.0065 | 0 | 19 | 1.8 | 2 | R.NNPEPWNK.L 4630 |
|  | 7833 | **611.2891** | **1220.5636** | **1220.5713** | **-0.0078** | **0** | **53** | **0.00084** | **1** | **K.FYSVNVDYSK.L** |

| **598.** | O95168\|NDUB4_HUMAN    **Mass:** 15256    **Score:** 67     **Queries matched:** 9   **emPAI:** 0.26 |
| --- | --- |
|  | NADH dehydrogenase [ubiquinone] 1 beta subcomplex subunit 4 OS=Homo sapiens GN=NDUFB4 PE=1 SV=3 |

|  | **Query** | **Observed** | **Mr(expt)** | **Mr(calc)** | **Delta** | **Miss** | **Score** | **Expect** | **Rank** | **Peptide** |
| --- | --- | --- | --- | --- | --- | --- | --- | --- | --- | --- |
|  | 5880 | **548.3256** | **1094.6367** | **1094.6448** | **-0.0081** | **0** | **18** | **2.4** | **1** | **R.GLIENPALLR.W** |
|  | 8420 | 417.5520 | 1249.6341 | 1250.7459 | -1.1118 | 1 | 15 | 4.5 | 4 | R.RGLIENPALLR.W 8421 8455 |
|  | 12087 | 728.8753 | 1455.7361 | 1455.7544 | -0.0183 | 1 | 9 | 16 | 2 | -.MSFPKYKPSSLR.T 12086 |
|  | 13538 | 515.9390 | 1544.7951 | 1545.8304 | -1.0353 | 0 | 9 | 17 | 2 | R.TINVYPNFRPTPK.N |
|  | 14044 | **527.5951** | **1579.7636** | **1579.7743** | **-0.0106** | **1** | **7** | **23** | **1** | **R.EYLLQYNDPNRR.G** |
|  | 20305 | **1023.4968** | **2044.9791** | **2044.9953** | **-0.0162** | **0** | **67** | **1.8e-005** | **1** | **R.TLPETLDPAEYNISPETR.R** |

| **599.** | P04222\|1C03_HUMAN    **Mass:** 41234    **Score:** 67     **Queries matched:** 7   **emPAI:** 0.19 |
| --- | --- |
|  | HLA class I histocompatibility antigen, Cw-3 alpha chain OS=Homo sapiens GN=HLA-C PE=1 SV=2 |

|  | **Query** | **Observed** | **Mr(expt)** | **Mr(calc)** | **Delta** | **Miss** | **Score** | **Expect** | **Rank** | **Peptide** |
| --- | --- | --- | --- | --- | --- | --- | --- | --- | --- | --- |
|  | 4142 | 483.2169 | 964.4193 | 964.4250 | -0.0057 | 0 | 53 | 0.00075 | 1 | R.FDSDAASPR.G |
|  | 7831 | 611.2879 | 1220.5612 | 1220.6037 | -0.0424 | 0 | 2 | 89 | 3 | K.DYIALNEDLR.S |
|  | 9648 | 439.8941 | 1316.6606 | 1315.6673 | 0.9932 | 0 | 8 | 21 | 4 | R.YFYTAVSRPGR.G |
|  | 11642 | 714.3621 | 1426.7097 | 1426.7205 | -0.0108 | 0 | 11 | 10 | 1 | K.WAAVVVPSGEEQR.Y |
|  | 13126 | 760.3734 | 1518.7323 | 1518.7427 | -0.0104 | 0 | 41 | 0.011 | 1 | R.SWTAADTAAQITQR.K |
|  | 17823 | 613.3262 | 1836.9569 | 1835.9027 | 1.0542 | 0 | 9 | 14 | 2 | K.THVTHHPVSDHEATLR.C |
|  | 23860 | 1229.6564 | 2457.2982 | 2456.3097 | 0.9885 | 0 | 2 | 44 | 2 | R.TLILLLSGALALTETWAGSHSMR.Y |

| **600.** | P37268\|FDFT_HUMAN    **Mass:** 48597    **Score:** 67     **Queries matched:** 13   **emPAI:** 0.16 |
| --- | --- |
|  | Squalene synthetase OS=Homo sapiens GN=FDFT1 PE=1 SV=1 |

|  | **Query** | **Observed** | **Mr(expt)** | **Mr(calc)** | **Delta** | **Miss** | **Score** | **Expect** | **Rank** | **Peptide** |
| --- | --- | --- | --- | --- | --- | --- | --- | --- | --- | --- |
|  | 2329 | **415.2221** | **828.4296** | **829.5021** | **-1.0725** | **0** | **7** | **45** | **2** | **R.QIISTIR.T** |
|  | 6970 | 590.8045 | 1179.5944 | 1179.5156 | 0.0788 | 0 | 1 | 1.1e+002 | 5 | R.DYLEDQQGGR.E |
|  | 9139 | **430.8857** | **1289.6354** | **1288.6259** | **1.0095** | **1** | **3** | **73** | **6** | **R.IPDSDPSSSKTR.Q** |
|  | 10887 | 692.4154 | 1382.8163 | 1382.6686 | 0.1476 | 1 | 1 | 1e+002 | 5 | R.RMGIGMAEFLDK.H |
|  | 11884 | 722.3673 | 1442.7201 | 1442.7300 | -0.0099 | 0 | 18 | 2.3 | 1 | R.TQNLPNCQLISR.S |
|  | 12956 | **754.8430** | **1507.6715** | **1506.7500** | **0.9214** | **0** | **40** | **0.013** | **1** | **R.SFAAVIQALDGEMR.N** |
|  | 13179 | **508.2215** | **1521.6428** | **1522.7450** | **-1.1022** | **0** | **(3)** | **65** | **4** | **R.SFAAVIQALDGEMR.N** |
|  | 15330 | **555.2322** | **1662.6749** | **1663.8504** | **-1.1756** | **0** | **12** | **7** | **1** | **K.YCHYVAGLVGIGLSR.L** |
|  | 15767 | 847.4387 | 1692.8628 | 1692.8723 | -0.0095 | 0 | 54 | 0.00039 | 1 | R.QVLEDFPTISLEFR.N |
|  | 18890 | **641.2822** | **1920.8249** | **1920.9471** | **-0.1223** | **1** | **2** | **62** | **5** | **R.KGQAVTLMMDATNMPAVK.A** |
|  | 20264 | **1021.4685** | **2040.9225** | **2039.9324** | **0.9901** | **0** | **2** | **59** | **1** | **R.LFSASEFEDPLVGEDTER.A** |
|  | 28851 | **704.5158** | **3517.5424** | **3518.6706** | **-1.1282** | **1** | **0** | **35** | **4** | **K.GQAVTLMMDATNMPAVKAIIYQYMEEIYHR.I** |
|  | 29618 | **951.5098** | **3802.0102** | **3801.8793** | **0.1309** | **1** | **0** | **27** | **4** | **R.NQSVFNFCAIPQVMAIATLAACYNNQQVFKGAVK.I** |

| **601.** | Q29960\|1C16_HUMAN    **Mass:** 41240    **Score:** 67     **Queries matched:** 12   **emPAI:** 0.30 |
| --- | --- |
|  | HLA class I histocompatibility antigen, Cw-16 alpha chain OS=Homo sapiens GN=HLA-C PE=2 SV=1 |

|  | **Query** | **Observed** | **Mr(expt)** | **Mr(calc)** | **Delta** | **Miss** | **Score** | **Expect** | **Rank** | **Peptide** |
| --- | --- | --- | --- | --- | --- | --- | --- | --- | --- | --- |
|  | 4142 | 483.2169 | 964.4193 | 964.4250 | -0.0057 | 0 | 53 | 0.00075 | 1 | R.FDSDAASPR.G |
|  | 8290 | 415.2401 | 1242.6984 | 1242.6357 | 0.0628 | 0 | 3 | 70 | 7 | K.DYIALNEHLR.S |
|  | 9648 | 439.8941 | 1316.6606 | 1315.6673 | 0.9932 | 0 | 8 | 21 | 4 | R.YFYTAVSRPGR.G |
|  | 10294 | 676.3427 | 1350.6709 | 1349.6939 | 0.9770 | 1 | 8 | 24 | 1 | R.YLENGKETLQR.A |
|  | 11642 | 714.3621 | 1426.7097 | 1426.7205 | -0.0108 | 0 | 11 | 10 | 1 | K.WAAVVVPSGEEQR.Y |
|  | 12720 | **498.3357** | **1491.9853** | **1492.6940** | **-0.7087** | **0** | **6** | **25** | **9** | **R.SCTAADTAAQITQR.K** 12754 |
|  | 12774 | 748.8586 | 1495.7026 | 1495.7129 | -0.0103 | 0 | 11 | 9.6 | 2 | R.AYLEGTCVEWLR.R |
|  | 14685 | 541.3059 | 1620.8959 | 1620.7890 | 0.1069 | 1 | 3 | 58 | 5 | R.SCTAADTAAQITQRK.W |
|  | 14790 | 815.4116 | 1628.8086 | 1628.8199 | -0.0113 | 0 | 27 | 0.26 | 1 | R.FIAVGYVDDTQFVR.F |
|  | 24990 | 879.7289 | 2636.1650 | 2636.1838 | -0.0188 | 0 | 27 | 0.14 | 1 | R.DGEDQTQDTELVETRPAGDGTFQK.W 24989 |

| **602.** | Q95604\|1C17_HUMAN    **Mass:** 41612    **Score:** 67     **Queries matched:** 12   **emPAI:** 0.30 |
| --- | --- |
|  | HLA class I histocompatibility antigen, Cw-17 alpha chain OS=Homo sapiens GN=HLA-C PE=1 SV=1 |

|  | **Query** | **Observed** | **Mr(expt)** | **Mr(calc)** | **Delta** | **Miss** | **Score** | **Expect** | **Rank** | **Peptide** |
| --- | --- | --- | --- | --- | --- | --- | --- | --- | --- | --- |
|  | 4142 | 483.2169 | 964.4193 | 964.4250 | -0.0057 | 0 | 53 | 0.00075 | 1 | R.FDSDAASPR.G |
|  | 7831 | 611.2879 | 1220.5612 | 1220.6037 | -0.0424 | 0 | 2 | 89 | 3 | K.DYIALNEDLR.S |
|  | 8239 | 621.3292 | 1240.6439 | 1239.5013 | 1.1426 | 0 | 11 | 12 | 3 | R.MYGCDLGPDGR.L |
|  | 8528 | 628.9625 | 1255.9104 | 1255.4962 | 0.4142 | 0 | (1) | 78 | 5 | R.MYGCDLGPDGR.L |
|  | 9648 | 439.8941 | 1316.6606 | 1315.6673 | 0.9932 | 0 | 8 | 21 | 4 | R.YFYTAVSRPGR.G |
|  | 11308 | **470.2942** | **1407.8609** | **1406.7154** | **1.1456** | **1** | **16** | **3.2** | **1** | **R.GYLENGKETLQR.A** |
|  | 14790 | 815.4116 | 1628.8086 | 1628.8199 | -0.0113 | 0 | 27 | 0.26 | 1 | R.FIAVGYVDDTQFVR.F |
|  | 17823 | 613.3262 | 1836.9569 | 1835.9027 | 1.0542 | 0 | 9 | 14 | 2 | K.THVTHHPVSDHEATLR.C |
|  | 24990 | 879.7289 | 2636.1650 | 2636.1838 | -0.0188 | 0 | 27 | 0.14 | 1 | R.DGEDQTQDTELVETRPAGDGTFQK.W 24989 |
|  | 28154 | 1090.4904 | 3268.4493 | 3267.7294 | 0.7199 | 1 | 2 | 28 | 7 | -.MRVMAPQALLLLLSGALALIETWAGSHSMR.Y 28153 |

| **603.** | P02647\|APOA1_HUMAN    **Mass:** 30759    **Score:** 67     **Queries matched:** 7   **emPAI:** 0.12 |
| --- | --- |
|  | Apolipoprotein A-I OS=Homo sapiens GN=APOA1 PE=1 SV=1 |

|  | **Query** | **Observed** | **Mr(expt)** | **Mr(calc)** | **Delta** | **Miss** | **Score** | **Expect** | **Rank** | **Peptide** |
| --- | --- | --- | --- | --- | --- | --- | --- | --- | --- | --- |
|  | 1279 | 603.3430 | 602.3357 | 602.2772 | 0.0585 | 0 | 22 | 1.9 | 3 | K.ENGGAR.L |
|  | 2869 | 435.2664 | 868.5182 | 868.5130 | 0.0051 | 1 | 12 | 8.1 | 6 | R.QKVEPLR.A |
|  | 9346 | 651.3392 | 1300.6639 | 1300.6411 | 0.0228 | 0 | 15 | 4.3 | 3 | R.THLAPYSDELR.Q |
|  | 10014 | **668.2917** | **1334.5689** | **1334.6387** | **-0.0698** | **1** | **6** | **32** | **8** | **R.QEMSKDLEEVK.A** |
|  | 11448 | **708.8113** | **1415.6080** | **1414.6953** | **0.9127** | **1** | **3** | **66** | **1** | **K.ENGGARLAEYHAK.A** |
|  | 14530 | **806.8920** | **1611.7695** | **1611.7781** | **-0.0086** | **0** | **67** | **2.6e-005** | **1** | **K.LLDNWDSVTSTFSK.L** |
|  | 19701 | **498.2739** | **1989.0666** | **1989.1445** | **-0.0779** | **1** | **3** | **46** | **10** | **-.MKAAVLTLAVLFLTGSQAR.H** |

| **604.** | Q01581\|HMCS1_HUMAN    **Mass:** 57828    **Score:** 66     **Queries matched:** 12   **emPAI:** 0.06 |
| --- | --- |
|  | Hydroxymethylglutaryl-CoA synthase, cytoplasmic OS=Homo sapiens GN=HMGCS1 PE=1 SV=2 |

|  | **Query** | **Observed** | **Mr(expt)** | **Mr(calc)** | **Delta** | **Miss** | **Score** | **Expect** | **Rank** | **Peptide** |
| --- | --- | --- | --- | --- | --- | --- | --- | --- | --- | --- |
|  | 1062 | **549.1952** | **548.1880** | **547.3329** | **0.8550** | **1** | **7** | **41** | **8** | **K.SKSVK.T** |
|  | 3402 | 455.7266 | 909.4386 | 909.4821 | -0.0434 | 0 | 11 | 12 | 3 | K.IHAQWQK.E |
|  | 7722 | 608.7971 | 1215.5797 | 1216.6551 | -1.0754 | 0 | 66 | 3.6e-005 | 1 | R.LEVGTETIIDK.S |
|  | 8650 | 631.8196 | 1261.6247 | 1262.6653 | -1.0405 | 1 | 8 | 23 | 8 | K.ITASLCDLKSR.L |
|  | 12431 | 492.2895 | 1473.8466 | 1472.7333 | 1.1133 | 1 | 4 | 50 | 3 | K.AFMKASSELFSQK.T |
|  | 15087 | **549.9511** | **1646.8315** | **1646.8450** | **-0.0135** | **1** | **1** | **1.1e+002** | **7** | **R.TGVAPDVFAENMKLR.E** |
|  | 15439 | 836.4148 | 1670.8150 | 1669.7956 | 1.0194 | 0 | 1 | 96 | 7 | -.MPGSLPLNAEACWPK.D |
|  | 17781 | 917.9569 | 1833.8993 | 1832.8549 | 1.0443 | 1 | 2 | 66 | 10 | K.YTIGLGQAKMGFCTDR.E |
|  | 19872 | **501.7530** | **2002.9829** | **2002.0887** | **0.8941** | **1** | **3** | **46** | **9** | **K.RIGVFSYGSGLAATLYSLK.V** |
|  | 20775 | 695.0616 | 2082.1629 | 2082.9384 | -0.7755 | 0 | 1 | 67 | 10 | R.EDINSLCMTVVQNLMER.N |
|  | 24949 | 1313.6604 | 2625.3062 | 2625.3214 | -0.0151 | 0 | 8 | 10 | 2 | K.DVGIVALEIYFPSQYVDQAELEK.Y 24950 |

| **605.** | P36776\|LONM_HUMAN    **Mass:** 106936   **Score:** 66     **Queries matched:** 34   **emPAI:** 0.07 |
| --- | --- |
|  | Lon protease homolog, mitochondrial OS=Homo sapiens GN=LONP1 PE=1 SV=2 |

|  | **Query** | **Observed** | **Mr(expt)** | **Mr(calc)** | **Delta** | **Miss** | **Score** | **Expect** | **Rank** | **Peptide** |
| --- | --- | --- | --- | --- | --- | --- | --- | --- | --- | --- |
|  | 1052 | **547.1418** | **546.1346** | **546.2762** | **-0.1416** | **0** | **11** | **25** | **1** | **R.ESGVR.N** |
|  | 1883 | **398.2371** | **794.4596** | **795.5218** | **-1.0622** | **0** | **7** | **29** | **4** | **K.ILPVGGIK.E** |
|  | 2902 | 436.2507 | 870.4868 | 870.5287 | -0.0419 | 1 | 15 | 6.1 | 10 | K.QVEKVLR.K 2912 |
|  | 3808 | **471.2903** | **940.5661** | **940.4879** | **0.0782** | **0** | **7** | **28** | **2** | **R.GPAIGGQWR.G** |
|  | 4431 | 493.7759 | 985.5372 | 985.5556 | -0.0184 | 1 | 16 | 4.2 | 5 | R.NLQKQVEK.V 4414 |
|  | 4838 | 507.7788 | 1013.5429 | 1014.5393 | -0.9963 | 0 | 6 | 39 | 9 | R.RPMLAAAGGR.V |
|  | 5879 | **548.3201** | **1094.6257** | **1094.5543** | **0.0714** | **1** | **2** | **88** | **3** | **R.RTYVGAMPGK.I** |
|  | 6667 | **578.3188** | **1154.6230** | **1153.5298** | **1.0932** | **1** | **5** | **48** | **8** | **K.QYCRESGVR.N** |
|  | 7102 | **396.2433** | **1185.7080** | **1186.6710** | **-0.9630** | **0** | **4** | **59** | **5** | **R.DIIALNPLYR.E** |
|  | 7274 | 597.8357 | 1193.6569 | 1193.5676 | 0.0893 | 0 | 2 | 91 | 9 | K.YSNENLDLAR.A |
|  | 10353 | **677.8376** | **1353.6606** | **1352.6646** | **0.9960** | **0** | **30** | **0.14** | **1** | **R.FSVGGMTDVAEIK.G** |
|  | 11443 | **708.3961** | **1414.7776** | **1415.6602** | **-0.8827** | **0** | **1** | **1e+002** | **6** | **K.HVMDVVDEELSK.L** |
|  | 11978 | **725.2089** | **1448.4032** | **1447.7341** | **0.6691** | **0** | **3** | **50** | **6** | **R.QNLAMTGEVSLTGK.I** |
|  | 12681 | 745.4511 | 1488.8875 | 1488.8817 | 0.0059 | 1 | 6 | 31 | 1 | K.VRLAQPYVGVFLK.R |
|  | 14273 | **797.4052** | **1592.7959** | **1591.8035** | **0.9924** | **0** | **45** | **0.0041** | **1** | **R.NYLDWLTSIPWGK.Y** 14263 |
|  | 14485 | **537.2751** | **1608.8034** | **1608.8195** | **-0.0160** | **1** | **(1)** | **1.1e+002** | **10** | **-.MAASTGYVRLWGAAR.C** |
|  | 14721 | 812.8982 | 1623.7819 | 1624.8144 | -1.0324 | 1 | 4 | 45 | 8 | -.MAASTGYVRLWGAAR.C |
|  | 15537 | 839.4009 | 1676.7873 | 1677.8946 | -1.1073 | 1 | (2) | 79 | 8 | R.TYVGAMPGKIIQCLK.K |
|  | 15797 | 565.5638 | 1693.6695 | 1693.8895 | -0.2200 | 1 | 13 | 4.6 | 1 | R.TYVGAMPGKIIQCLK.K 15796 |
|  | 16358 | **577.1247** | **1728.3522** | **1728.9028** | **-0.5506** | **1** | **5** | **22** | **1** | **R.CWVLRRPMLAAAGGR.V** |
|  | 17448 | 603.3307 | 1806.9704 | 1807.9866 | -1.0162 | 1 | 3 | 51 | 8 | K.LSSDVLTLLIKQYCR.E |
|  | 17775 | **612.2873** | **1833.8400** | **1832.8251** | **1.0150** | **0** | **1** | **1e+002** | **8** | **R.AQAVLEEDHYGMEDVK.K** |
|  | 18302 | 935.9789 | 1869.9433 | 1868.8760 | 1.0673 | 1 | 6 | 27 | 5 | R.DRMEMINVSGYVAQEK.L 18291 18303 |
|  | 20055 | **1011.1191** | **2020.2237** | **2020.9735** | **-0.7498** | **1** | **2** | **49** | **6** | **K.DGSLEVTGQLGEVMKESAR.I** 20071 |
|  | 20490 | 1029.9700 | 2057.9254 | 2058.0820 | -0.1566 | 0 | 1 | 74 | 6 | K.VLFICTANVTDTIPEPLR.D |
|  | 22092 | 742.7077 | 2225.1013 | 2225.2453 | -0.1441 | 1 | 4 | 30 | 6 | R.QNLAMTGEVSLTGKILPVGGIK.E |
|  | 29650 | **1271.3053** | **3810.8941** | **3809.8943** | **0.9997** | **1** | **0** | **32** | **6** | **R.MYDVTPPGVVMGLAWTAMGGSTLFVETSLRRPQDK.D** |

| **606.** | P06748\|NPM_HUMAN    **Mass:** 32726    **Score:** 66     **Queries matched:** 10   **emPAI:** 0.12 |
| --- | --- |
|  | Nucleophosmin OS=Homo sapiens GN=NPM1 PE=1 SV=2 |

|  | **Query** | **Observed** | **Mr(expt)** | **Mr(calc)** | **Delta** | **Miss** | **Score** | **Expect** | **Rank** | **Peptide** |
| --- | --- | --- | --- | --- | --- | --- | --- | --- | --- | --- |
|  | 1105 | **558.3566** | **557.3494** | **557.3173** | **0.0320** | **0** | **10** | **21** | **1** | **K.GGSLPK.V** 1109 |
|  | 6116 | **557.2696** | **1112.5247** | **1111.5986** | **0.9262** | **1** | **7** | **29** | **4** | **R.SAPGGGSKVPQK.K** |
|  | 6303 | **565.2811** | **1128.5476** | **1129.5979** | **-1.0503** | **1** | **8** | **27** | **3** | **K.GPSSVEDIKAK.M** 6332 |
|  | 10159 | **672.8516** | **1343.6887** | **1344.7071** | **-1.0184** | **1** | **1** | **1.2e+002** | **2** | **K.MQASIEKGGSLPK.V** 10151 |
|  | 17789 | **612.3781** | **1834.1125** | **1834.8308** | **-0.7183** | **0** | **4** | **41** | **2** | **R.MTDQEAIQDLWQWR.K** |
|  | 21485 | **721.1577** | **2160.4513** | **2160.0045** | **0.4468** | **0** | **0** | **55** | **5** | **K.DELHIVEAEAMNYEGSPIK.V** |
|  | 22114 | **1114.1050** | **2226.1954** | **2226.2083** | **-0.0128** | **0** | **66** | **1.8e-005** | **1** | **K.MSVQPTVSLGGFEITPPVVLR.L** |

| **607.** | P62906\|RL10A_HUMAN    **Mass:** 24987    **Score:** 66     **Queries matched:** 18   **emPAI:** 0.53 |
| --- | --- |
|  | 60S ribosomal protein L10a OS=Homo sapiens GN=RPL10A PE=1 SV=2 |

|  | **Query** | **Observed** | **Mr(expt)** | **Mr(calc)** | **Delta** | **Miss** | **Score** | **Expect** | **Rank** | **Peptide** |
| --- | --- | --- | --- | --- | --- | --- | --- | --- | --- | --- |
|  | 2096 | **405.8691** | **809.7237** | **809.4065** | **0.3171** | **1** | **9** | **13** | **4** | **-.MSSKVSR.D** |
|  | 3959 | 476.2706 | 950.5267 | 951.4886 | -0.9619 | 0 | 14 | 6.6 | 2 | R.EVLHGNQR.K |
|  | 4153 | **483.7191** | **965.4237** | **965.4818** | **-0.0581** | **0** | **12** | **11** | **8** | **R.DTLYEAVR.E** 4164 4167 |
|  | 11049 | **465.0195** | **1392.0366** | **1392.8275** | **-0.7909** | **1** | **13** | **4.5** | **1** | **K.KVLCLAVAVGHVK.M** |
|  | 11216 | **702.3623** | **1402.7100** | **1403.7673** | **-1.0573** | **1** | **3** | **73** | **2** | **K.NWQNVRALYIK.S** |
|  | 11533 | **710.9608** | **1419.9071** | **1418.8021** | **1.1050** | **0** | **6** | **26** | **1** | **K.FLETVELQISLK.N** |
|  | 12021 | **484.5871** | **1450.7395** | **1450.7490** | **-0.0095** | **0** | **32** | **0.089** | **1** | **K.AVDIPHMDIEALK.K** |
|  | 12286 | **489.9253** | **1466.7540** | **1466.7439** | **0.0101** | **0** | **(5)** | **36** | **4** | **K.AVDIPHMDIEALK.K** 12258 |
|  | 13547 | 773.8964 | 1545.7783 | 1546.8970 | -1.1187 | 1 | 6 | 27 | 10 | R.KFLETVELQISLK.N |
|  | 14026 | **527.2853** | **1578.8340** | **1578.8439** | **-0.0099** | **1** | **9** | **16** | **3** | **K.AVDIPHMDIEALKK.L** |
|  | 14363 | **534.2727** | **1599.7963** | **1599.8079** | **-0.0116** | **0** | **12** | **7.3** | **1** | **K.FPSLLTHNENMVAK.V** |
|  | 18074 | **619.6563** | **1855.9471** | **1855.9614** | **-0.0143** | **1** | **19** | **1.2** | **1** | **K.AGKFPSLLTHNENMVAK.V** 18093 |
|  | 18318 | 468.9817 | 1871.8975 | 1871.9563 | -0.0588 | 1 | (3) | 46 | 3 | K.AGKFPSLLTHNENMVAK.V |
|  | 18556 | **946.9107** | **1891.8069** | **1891.8193** | **-0.0124** | **0** | **58** | **0.00016** | **1** | **K.FSVCVLGDQQHCDEAK.A** |

| **608.** | P01842\|LAC_HUMAN    **Mass:** 11401    **Score:** 66     **Queries matched:** 4   **emPAI:** 0.82 |
| --- | --- |
|  | Ig lambda chain C regions OS=Homo sapiens GN=IGLC1 PE=1 SV=1 |

|  | **Query** | **Observed** | **Mr(expt)** | **Mr(calc)** | **Delta** | **Miss** | **Score** | **Expect** | **Rank** | **Peptide** |
| --- | --- | --- | --- | --- | --- | --- | --- | --- | --- | --- |
|  | 4467 | 495.2524 | 988.4903 | 989.5029 | -1.0126 | 0 | 47 | 0.0037 | 1 | K.AGVETTTPSK.Q |
|  | 13782 | **521.3038** | **1560.8895** | **1560.7744** | **0.1151** | **1** | **2** | **67** | **10** | **K.AGVETTTPSKQSNNK.Y** |
|  | 16110 | 856.4823 | 1710.9500 | 1710.7519 | 0.1981 | 0 | 46 | 0.0026 | 1 | R.SYSCQVTHEGSTVEK.T |
|  | 16576 | **872.4272** | **1742.8399** | **1742.8515** | **-0.0116** | **0** | **2** | **64** | **1** | **K.YAASSYLSLTPEQWK.S** |

| **609.** | P15259\|PGAM2_HUMAN    **Mass:** 28919    **Score:** 66     **Queries matched:** 13   **emPAI:** 0.45 |
| --- | --- |
|  | Phosphoglycerate mutase 2 OS=Homo sapiens GN=PGAM2 PE=1 SV=3 |

|  | **Query** | **Observed** | **Mr(expt)** | **Mr(calc)** | **Delta** | **Miss** | **Score** | **Expect** | **Rank** | **Peptide** |
| --- | --- | --- | --- | --- | --- | --- | --- | --- | --- | --- |
|  | 1244 | 591.2767 | 590.2694 | 589.3071 | 0.9623 | 0 | 17 | 5 | 2 | K.AETAAK.H |
|  | 4280 | 488.2468 | 974.4791 | 974.4855 | -0.0064 | 0 | 35 | 0.056 | 1 | K.AMEAVAAQGK.A |
|  | 5422 | 530.7808 | 1059.5470 | 1058.5509 | 0.9961 | 0 | 12 | 12 | 2 | R.HYGGLTGLNK.A 5414 |
|  | 5911 | **549.7219** | **1097.4293** | **1097.6128** | **-0.1835** | **1** | **9** | **19** | **3** | **M.ATHRLVMVR.H** |
|  | 5995 | 552.2934 | 1102.5722 | 1102.5804 | -0.0082 | 1 | 61 | 0.00012 | 1 | R.KAMEAVAAQGK.A |
|  | 8003 | 615.3630 | 1228.7114 | 1228.6533 | 0.0581 | 1 | 3 | 90 | 5 | -.MATHRLVMVR.H |
|  | 12930 | 502.9343 | 1505.7811 | 1504.6942 | 1.0869 | 0 | 2 | 73 | 8 | K.MEFDICYTSVLK.R |
|  | 13501 | **515.3264** | **1542.9574** | **1543.7341** | **-0.7766** | **1** | **5** | **39** | **5** | **R.RSFDIPPPPMDEK.H** |
|  | 13902 | 524.2879 | 1569.8419 | 1570.8216 | -0.9797 | 1 | 8 | 21 | 5 | R.LNERHYGGLTGLNK.A |
|  | 15617 | 842.4536 | 1682.8927 | 1682.9032 | -0.0105 | 0 | 28 | 0.17 | 1 | R.ALPFWNEEIVPQIK.A |
|  | 16871 | 881.9031 | 1761.7917 | 1761.8971 | -0.1054 | 0 | 7 | 23 | 2 | R.YAGLKPGELPTCESLK.D |
|  | 24702 | 644.3463 | 2573.3559 | 2572.3261 | 1.0298 | 1 | 5 | 20 | 3 | R.TLWAILDGTDQMWLPVVRTWR.L |

| **610.** | Q9BQB6\|VKOR1_HUMAN    **Mass:** 18622    **Score:** 66     **Queries matched:** 3   **emPAI:** 0.21 |
| --- | --- |
|  | Vitamin K epoxide reductase complex subunit 1 OS=Homo sapiens GN=VKORC1 PE=1 SV=1 |

|  | **Query** | **Observed** | **Mr(expt)** | **Mr(calc)** | **Delta** | **Miss** | **Score** | **Expect** | **Rank** | **Peptide** |
| --- | --- | --- | --- | --- | --- | --- | --- | --- | --- | --- |
|  | 11323 | **705.3247** | **1408.6347** | **1408.6439** | **-0.0091** | **0** | **66** | **3.2e-005** | **1** | **R.ALCDVGTAISCSR.V** 11322 |
|  | 17894 | **615.2966** | **1842.8679** | **1842.8353** | **0.0326** | **1** | **3** | **54** | **3** | **R.DYRALCDVGTAISCSR.V** |

| **611.** | P02649\|APOE_HUMAN    **Mass:** 36246    **Score:** 66     **Queries matched:** 14   **emPAI:** 0.22 |
| --- | --- |
|  | Apolipoprotein E OS=Homo sapiens GN=APOE PE=1 SV=1 |

|  | **Query** | **Observed** | **Mr(expt)** | **Mr(calc)** | **Delta** | **Miss** | **Score** | **Expect** | **Rank** | **Peptide** |
| --- | --- | --- | --- | --- | --- | --- | --- | --- | --- | --- |
|  | 2654 | **428.7636** | **855.5127** | **854.3262** | **1.1865** | **0** | **2** | **1.1e+002** | **5** | **R.MEEMGSR.T** |
|  | 3286 | 451.2795 | 900.5445 | 900.5141 | 0.0304 | 1 | 12 | 13 | 9 | R.GLSAIRER.L |
|  | 3926 | **475.2384** | **948.4622** | **947.5188** | **0.9434** | **0** | **6** | **46** | **7** | **R.LAVYQAGAR.E** |
|  | 5698 | 541.3391 | 1080.6637 | 1081.4644 | -0.8008 | 1 | 1 | 1.1e+002 | 10 | R.ARMEEMGSR.T |
|  | 7096 | 396.2286 | 1185.6641 | 1185.6353 | 0.0288 | 1 | 15 | 5 | 3 | R.LLRDADDLQK.R 7072 7097 |
|  | 7700 | 405.8887 | 1214.6442 | 1213.6778 | 0.9663 | 1 | 5 | 49 | 10 | R.LSKELQAAQAR.L |
|  | 8356 | **624.2883** | **1246.5621** | **1246.5691** | **-0.0070** | **0** | **30** | **0.16** | **1** | **R.QQTEWQSGQR.W** |
|  | 9574 | **438.5738** | **1312.6995** | **1312.7099** | **-0.0103** | **1** | **25** | **0.45** | **1** | **R.AKLEEQAQQIR.L** |
|  | 10081 | 670.3129 | 1338.6112 | 1339.6363 | -1.0251 | 1 | 5 | 45 | 10 | R.ALMDETMKELK.A |
|  | 12788 | **749.3997** | **1496.7848** | **1496.7947** | **-0.0099** | **0** | **62** | **7.7e-005** | **1** | **R.AATVGSLAGQPLQER.A** |
|  | 16385 | 577.3186 | 1728.9340 | 1729.8370 | -0.9030 | 0 | 3 | 58 | 6 | K.SELEEQLTPVAEETR.A |
|  | 21241 | 1067.9906 | 2133.9666 | 2135.1635 | -1.1969 | 1 | 0 | 80 | 7 | -.MKVLWAALLVTFLAGCQAK.V |

| **612.** | P04424\|ARLY_HUMAN    **Mass:** 51910    **Score:** 66     **Queries matched:** 13   **emPAI:** 0.07 |
| --- | --- |
|  | Argininosuccinate lyase OS=Homo sapiens GN=ASL PE=1 SV=4 |

|  | **Query** | **Observed** | **Mr(expt)** | **Mr(calc)** | **Delta** | **Miss** | **Score** | **Expect** | **Rank** | **Peptide** |
| --- | --- | --- | --- | --- | --- | --- | --- | --- | --- | --- |
|  | 4733 | 503.2684 | 1004.5223 | 1005.5351 | -1.0128 | 0 | 10 | 16 | 4 | R.CAGLLMTLK.G |
|  | 7866 | 408.2278 | 1221.6617 | 1220.6111 | 1.0506 | 0 | 6 | 40 | 7 | R.FVGAVDPIMEK.F |
|  | 7925 | 409.4391 | 1225.2955 | 1224.6938 | 0.6017 | 1 | 11 | 8.6 | 1 | R.QVRALLQAQQA.- |
|  | 9271 | 649.3265 | 1296.6384 | 1296.6463 | -0.0078 | 0 | 18 | 2.4 | 1 | R.HLWEVDVQGSK.A |
|  | 10391 | **678.8362** | **1355.6579** | **1355.6465** | **0.0114** | **0** | **5** | **43** | **2** | **R.MAEDLILYCTK.E** |
|  | 10923 | **462.7530** | **1385.2373** | **1384.6656** | **0.5717** | **0** | **14** | **3.8** | **1** | **K.AEMDQILHGLDK.V** 10911 10924 |
|  | 23743 | 815.4662 | 2443.3767 | 2443.3911 | -0.0143 | 1 | 14 | 2.4 | 1 | R.INVLPLGSGAIAGNPLGVDRELLR.A 23730 23731 23742 |
|  | 24909 | **872.7592** | **2615.2557** | **2615.2690** | **-0.0133** | **1** | **66** | **1.8e-005** | **1** | **K.AEMDQILHGLDKVAEEWAQGTFK.L** |

| **613.** | P46779\|RL28_HUMAN    **Mass:** 15795    **Score:** 66     **Queries matched:** 8   **emPAI:** 0.55 |
| --- | --- |
|  | 60S ribosomal protein L28 OS=Homo sapiens GN=RPL28 PE=1 SV=3 |

|  | **Query** | **Observed** | **Mr(expt)** | **Mr(calc)** | **Delta** | **Miss** | **Score** | **Expect** | **Rank** | **Peptide** |
| --- | --- | --- | --- | --- | --- | --- | --- | --- | --- | --- |
|  | 2848 | **434.7632** | **867.5119** | **868.5858** | **-1.0740** | **1** | **3** | **65** | **5** | **K.GVVVVIKR.R** |
|  | 2928 | 437.2527 | 872.4909 | 871.4664 | 1.0245 | 0 | 8 | 37 | 10 | R.YNGLIHR.K |
|  | 4178 | **484.7440** | **967.4734** | **967.4797** | **-0.0063** | **0** | **22** | **1** | **1** | **R.NCSSFLIK.R** |
|  | 5802 | **545.3008** | **1088.5870** | **1087.6098** | **0.9772** | **1** | **13** | **9.8** | **2** | **K.NARATLSSIR.H** |
|  | 6872 | **586.3152** | **1170.6159** | **1170.6245** | **-0.0085** | **1** | **47** | **0.0031** | **1** | **R.KTVGVEPAADGK.G** |
|  | 10690 | 687.3361 | 1372.6576 | 1372.6744 | -0.0168 | 0 | 4 | 54 | 10 | -.MSAHLQWMVVR.N |
|  | 13385 | **768.8799** | **1535.7452** | **1535.7579** | **-0.0127** | **1** | **50** | **0.0011** | **1** | **R.NKQTYSTEPNNLK.A** |
|  | 21759 | **549.0420** | **2192.1389** | **2191.1030** | **1.0358** | **1** | **7** | **16** | **4** | **M.SAHLQWMVVRNCSSFLIK.R** |

| **614.** | P05386\|RLA1_HUMAN    **Mass:** 11621    **Score:** 65     **Queries matched:** 3   **emPAI:** 0.80 |
| --- | --- |
|  | 60S acidic ribosomal protein P1 OS=Homo sapiens GN=RPLP1 PE=1 SV=1 |

|  | **Query** | **Observed** | **Mr(expt)** | **Mr(calc)** | **Delta** | **Miss** | **Score** | **Expect** | **Rank** | **Peptide** |
| --- | --- | --- | --- | --- | --- | --- | --- | --- | --- | --- |
|  | 15947 | **851.9461** | **1701.8777** | **1701.8879** | **-0.0102** | **0** | **59** | **0.00016** | **1** | **K.AAGVNVEPFWPGLFAK.A** |
|  | 29623 | **952.9823** | **3807.9001** | **3807.9213** | **-0.0212** | **0** | **30** | **0.029** | **1** | **K.ALANVNIGSLICNVGAGGPAPAAGAAPAGGPAPSTAAAPAEEK.K** 29625 |

| **615.** | P14174\|MIF_HUMAN    **Mass:** 12639    **Score:** 65     **Queries matched:** 8   **emPAI:** 0.31 |
| --- | --- |
|  | Macrophage migration inhibitory factor OS=Homo sapiens GN=MIF PE=1 SV=4 |

|  | **Query** | **Observed** | **Mr(expt)** | **Mr(calc)** | **Delta** | **Miss** | **Score** | **Expect** | **Rank** | **Peptide** |
| --- | --- | --- | --- | --- | --- | --- | --- | --- | --- | --- |
|  | 9086 | **644.3434** | **1286.6722** | **1286.6805** | **-0.0083** | **0** | **65** | **4.5e-005** | **1** | **M.PMFIVNTNVPR.A** 9072 |
|  | 9413 | **652.8328** | **1303.6511** | **1302.6754** | **0.9756** | **0** | **(1)** | **1.1e+002** | **4** | **M.PMFIVNTNVPR.A** |
|  | 11746 | **478.8855** | **1433.6348** | **1433.7159** | **-0.0812** | **0** | **8** | **21** | **3** | **-.MPMFIVNTNVPR.A** 11774 |
|  | 31767 | **1162.3777** | **5806.8521** | **5806.8797** | **-0.0277** | **0** | **11** | **0.53** | **1** | **R.ASVPDGFLSELTQQLAQATGKPPQYIAVHVVPDQLMAFGGSSEPCALCSLHSIGK.I** 31768 31769 |

| **616.** | O43852\|CALU_HUMAN    **Mass:** 37198    **Score:** 65     **Queries matched:** 7   **emPAI:** 0.21 |
| --- | --- |
|  | Calumenin OS=Homo sapiens GN=CALU PE=1 SV=2 |

|  | **Query** | **Observed** | **Mr(expt)** | **Mr(calc)** | **Delta** | **Miss** | **Score** | **Expect** | **Rank** | **Peptide** |
| --- | --- | --- | --- | --- | --- | --- | --- | --- | --- | --- |
|  | 5468 | **532.2890** | **1062.5634** | **1063.4903** | **-0.9268** | **1** | **6** | **46** | **4** | **K.QMMVRDER.R** |
|  | 5613 | 537.8120 | 1073.6095 | 1073.5968 | 0.0127 | 1 | 12 | 13 | 3 | K.LTKEEIVDK.Y |
|  | 12172 | **487.3210** | **1458.9413** | **1459.7168** | **-0.7755** | **1** | **8** | **19** | **1** | **K.DRVHHEPQLSDK.V** 12159 |
|  | 13334 | **766.8649** | **1531.7152** | **1531.7267** | **-0.0115** | **1** | **52** | **0.00085** | **1** | **R.HLVYESDQNKDGK.L** 13333 13344 |

| **617.** | Q13576\|IQGA2_HUMAN    **Mass:** 181071   **Score:** 64     **Queries matched:** 44   **emPAI:** 0.02 |
| --- | --- |
|  | Ras GTPase-activating-like protein IQGAP2 OS=Homo sapiens GN=IQGAP2 PE=1 SV=3 |

|  | **Query** | **Observed** | **Mr(expt)** | **Mr(calc)** | **Delta** | **Miss** | **Score** | **Expect** | **Rank** | **Peptide** |
| --- | --- | --- | --- | --- | --- | --- | --- | --- | --- | --- |
|  | 1347 | 625.0812 | 624.0740 | 623.3755 | 0.6985 | 0 | 8 | 14 | 1 | K.LNLHK.K 1345 |
|  | 1456 | 653.3032 | 652.2959 | 653.2843 | -0.9883 | 0 | 14 | 5.9 | 7 | K.FNMDK.Y |
|  | 1521 | **678.3880** | **677.3807** | **676.3690** | **1.0117** | **1** | **11** | **14** | **2** | **R.MATARK.S** |
|  | 1647 | 729.3837 | 728.3765 | 728.4545 | -0.0780 | 0 | 9 | 27 | 9 | K.IQSLLR.A |
|  | 2522 | **845.0936** | **844.0863** | **843.4926** | **0.5937** | **1** | **6** | **39** | **6** | **R.RNLGSVAK.V** 2513 |
|  | 3004 | 879.0607 | 878.0534 | 878.4068 | -0.3535 | 1 | 8 | 21 | 2 | R.KEYMHR.R |
|  | 4242 | **487.0874** | **972.1602** | **971.5261** | **0.6342** | **1** | **6** | **35** | **3** | **R.GARGQNTLR.Q** |
|  | 4254 | **487.3200** | **972.6255** | **972.5604** | **0.0651** | **1** | **9** | **23** | **2** | **R.EEVVTKIR.A** |
|  | 4757 | **504.2680** | **1006.5215** | **1007.5651** | **-1.0436** | **0** | **8** | **25** | **3** | **R.YANTLLSVK.L** |
|  | 5234 | **522.4341** | **1042.8536** | **1043.5611** | **-0.7075** | **0** | **2** | **76** | **5** | **K.LEASIENLR.R** |
|  | 5889 | **548.7964** | **1095.5782** | **1094.4550** | **1.1233** | **0** | **3** | **79** | **10** | **R.LSAEEMDER.R** |
|  | 6390 | **567.8754** | **1133.7363** | **1133.5903** | **0.1460** | **0** | **5** | **53** | **10** | **K.LIFQMPQNK.S** |
|  | 6488 | 571.7501 | 1141.4856 | 1141.6455 | -0.1599 | 0 | 3 | 75 | 7 | K.LQQTLNALNK.K |
|  | 7351 | **599.7808** | **1197.5471** | **1198.6056** | **-1.0585** | **1** | **16** | **3.6** | **1** | **K.FFAPKMVSEK.K** |
|  | 8662 | 632.3145 | 1262.6145 | 1263.6306 | -1.0162 | 1 | 7 | 31 | 5 | K.SQKLGDSESVSK.V 8661 8672 |
|  | 9096 | **429.9584** | **1286.8534** | **1285.6990** | **1.1544** | **1** | **7** | **25** | **4** | **K.NKLEASIENLR.R** |
|  | 11596 | **712.6133** | **1423.2120** | **1422.6483** | **0.5637** | **1** | **7** | **15** | **2** | **R.AMIDSRTPEEMK.H** |
|  | 11656 | 714.8486 | 1427.6826 | 1428.6845 | -1.0019 | 0 | 5 | 41 | 3 | R.TLEQTGHVSSENK.Y |
|  | 12324 | 490.4837 | 1468.4292 | 1468.6504 | -0.2212 | 0 | 5 | 27 | 3 | K.VDFTEEEISNMR.K |
|  | 12673 | 745.3827 | 1488.7509 | 1488.8188 | -0.0678 | 0 | 5 | 46 | 6 | K.SLIINTNPVEVYK.A |
|  | 13169 | 507.9174 | 1520.7305 | 1521.7787 | -1.0482 | 1 | 1 | 1.1e+002 | 8 | R.RQTFIDNTDSVVK.I |
|  | 14094 | 792.4119 | 1582.8092 | 1582.8467 | -0.0375 | 1 | 2 | 83 | 9 | R.VSSDGSWLKLNLHK.K |
|  | 14326 | **533.2822** | **1596.8249** | **1596.7453** | **0.0795** | **1** | **2** | **81** | **7** | **K.VDFTEEEISNMRK.E** |
|  | 14368 | 534.3104 | 1599.9094 | 1599.9269 | -0.0175 | 1 | 7 | 24 | 6 | K.DLNLMDIKIGLLVK.N |
|  | 14428 | **803.4275** | **1604.8404** | **1604.8093** | **0.0311** | **0** | **7** | **25** | **9** | **-.MPHEELPSLQRPR.Y** 14430 |
|  | 14905 | **546.2997** | **1635.8774** | **1635.9056** | **-0.0282** | **1** | **3** | **54** | **7** | **R.NLGSVAKVLQHAASNK.L** |
|  | 16199 | **859.3953** | **1716.7761** | **1717.8887** | **-1.1126** | **0** | **2** | **63** | **2** | **K.GVLLDIDDLQTNQFK.N** |
|  | 16250 | 574.9403 | 1721.7991 | 1720.7574 | 1.0417 | 0 | 0 | 1e+002 | 9 | K.SSTSNANDIIPECADK.Y |
|  | 16649 | 583.8150 | 1748.4232 | 1747.8047 | 0.6185 | 0 | 2 | 43 | 6 | K.GGEMEILNNTDNQGIK.S |
|  | 18039 | **927.8383** | **1853.6620** | **1852.8989** | **0.7631** | **0** | **(1)** | **68** | **8** | **K.HSQSMIEDAQLPLEQK.K** |
|  | 18301 | **624.3208** | **1869.9406** | **1868.8938** | **1.0468** | **0** | **3** | **56** | **7** | **K.HSQSMIEDAQLPLEQK.K** |
|  | 19632 | **993.4546** | **1984.8946** | **1984.9742** | **-0.0795** | **1** | **0** | **87** | **6** | **K.NSIHEKFPDATEDELLK.I** |
|  | 23107 | 786.0563 | 2355.1470 | 2356.1183 | -0.9713 | 0 | 4 | 33 | 4 | K.VLWLDEIQQAVDEANVDEDR.A |
|  | 24587 | 852.7524 | 2555.2355 | 2555.2503 | -0.0148 | 1 | 13 | 3.4 | 1 | K.VLWLDEIQQAVDEANVDEDRAK.Q |
|  | 25268 | 897.1061 | 2688.2966 | 2687.4574 | 0.8392 | 1 | 2 | 45 | 10 | R.KTLETYQQLFYLLQTNPLYLAK.L |
|  | 26258 | **714.8481** | **2855.3632** | **2856.4224** | **-1.0592** | **1** | **10** | **5.8** | **1** | **K.VQLNIQDLLQMQYEGVAVMKMFDK.V** |
|  | 27884 | **799.9025** | **3195.5808** | **3196.4917** | **-0.9110** | **1** | **1** | **41** | **8** | **R.YMNPAIVAPDGFDIIDMTAGGQINSDQRR.N** |
|  | 29132 | **1203.9293** | **3608.7661** | **3608.7886** | **-0.0225** | **1** | **64** | **1.4e-005** | **1** | **K.AWVNQLETQTGEASKLPYDVTTEQALTYPEVK.N** |
|  | 30222 | 1356.7245 | 4067.1516 | 4068.1241 | -0.9725 | 1 | 1 | 19 | 9 | K.YGIQMPSFSKIGGILANELSVDEAALHAAVIAINEAVEK.G |

| **618.** | P34897\|GLYM_HUMAN    **Mass:** 56414    **Score:** 64     **Queries matched:** 19   **emPAI:** 0.14 |
| --- | --- |
|  | Serine hydroxymethyltransferase, mitochondrial OS=Homo sapiens GN=SHMT2 PE=1 SV=3 |

|  | **Query** | **Observed** | **Mr(expt)** | **Mr(calc)** | **Delta** | **Miss** | **Score** | **Expect** | **Rank** | **Peptide** |
| --- | --- | --- | --- | --- | --- | --- | --- | --- | --- | --- |
|  | 2427 | 419.2008 | 836.3871 | 836.3777 | 0.0095 | 1 | 19 | 2.3 | 4 | R.EDDFRR.V |
|  | 3106 | **443.7500** | **885.4854** | **884.5080** | **0.9775** | **0** | **4** | **76** | **10** | **R.LGAPALTSR.Q** |
|  | 4470 | **495.2544** | **988.4942** | **988.5011** | **-0.0070** | **0** | **19** | **2.5** | **2** | **R.AMADALLER.G** |
|  | 6400 | 568.3234 | 1134.6323 | 1134.6397 | -0.0074 | 0 | 21 | 1.4 | 1 | R.LIIAGTSAYAR.L 6399 |
|  | 6772 | **582.7186** | **1163.4226** | **1162.4754** | **0.9472** | **0** | **3** | **78** | **3** | **R.AFPMPGFDEH.-** |
|  | 6851 | **585.3679** | **1168.7212** | **1168.5400** | **0.1811** | **0** | **2** | **90** | **9** | **R.EIPYTFEDR.I** |
|  | 7903 | 612.7635 | 1223.5124 | 1222.6306 | 0.8818 | 0 | 6 | 38 | 3 | K.HADIVTTTTHK.T |
|  | 10200 | **674.0129** | **1346.0113** | **1345.6772** | **0.3341** | **1** | **1** | **73** | **7** | **K.NARAMADALLER.G** |
|  | 11341 | **470.7605** | **1409.2596** | **1409.7150** | **-0.4555** | **1** | **10** | **8.4** | **2** | **K.SFLLKDSETSQR.L** 11344 |
|  | 14648 | 540.6003 | 1618.7792 | 1619.7905 | -1.0113 | 0 | 1 | 88 | 4 | R.ISATSIFFESMPYK.L |
|  | 15444 | 836.4599 | 1670.9052 | 1670.8159 | 0.0894 | 1 | 0 | 1.1e+002 | 10 | K.NTCPGDRSAITPGGLR.L |
|  | 18666 | 634.5947 | 1900.7622 | 1901.0258 | -0.2637 | 1 | 0 | 85 | 7 | R.RVVDFIDEGVNIGLEVK.S |
|  | 19649 | 663.0123 | 1986.0152 | 1986.0204 | -0.0053 | 1 | 5 | 33 | 8 | R.VLELVSITANKNTCPGDR.S |
|  | 20838 | 1045.5270 | 2089.0394 | 2088.0601 | 0.9793 | 1 | 1 | 78 | 10 | R.ISATSIFFESMPYKLNPK.T |
|  | 25023 | 881.4580 | 2641.3522 | 2641.4493 | -0.0971 | 0 | 4 | 24 | 2 | R.INFAVFPSLQGGPHNHAIAAVAVALK.Q |
|  | 30361 | **1375.6846** | **4124.0319** | **4123.0439** | **0.9880** | **0** | **63** | **1.4e-005** | **1** | **R.ALEAFDLDPAQWGVNVQPYSGSPANLAVYTALLQPHDR.I** |
|  | 30415 | **1036.7639** | **4143.0266** | **4143.8577** | **-0.8311** | **1** | **3** | **14** | **3** | **R.AQHSNAAQTQTGEANRGWTGQESLSDSDPEMWELLQR.E** |

| **619.** | O75310\|UDB11_HUMAN    **Mass:** 61455    **Score:** 64     **Queries matched:** 23   **emPAI:** 0.19 |
| --- | --- |
|  | UDP-glucuronosyltransferase 2B11 OS=Homo sapiens GN=UGT2B11 PE=2 SV=1 |

|  | **Query** | **Observed** | **Mr(expt)** | **Mr(calc)** | **Delta** | **Miss** | **Score** | **Expect** | **Rank** | **Peptide** |
| --- | --- | --- | --- | --- | --- | --- | --- | --- | --- | --- |
|  | 761 | 474.2994 | 473.2922 | 472.2758 | 1.0164 | 0 | 4 | 1.4e+002 | 7 | K.GAAVR.L |
|  | 1382 | 634.3110 | 633.3037 | 633.3156 | -0.0119 | 0 | 12 | 15 | 6 | K.ENIMK.L |
|  | 4227 | 486.2945 | 970.5744 | 970.5811 | -0.0067 | 0 | 13 | 8 | 1 | R.ANVIATALAK.I 4217 |
|  | 4477 | 495.7325 | 989.4504 | 989.5328 | -0.0824 | 1 | 8 | 27 | 7 | K.ENIMKLSR.I |
|  | 5161 | 519.7969 | 1037.5793 | 1038.6338 | -1.0545 | 1 | 8 | 24 | 4 | K.IPQKVLWR.F |
|  | 5459 | 531.7919 | 1061.5692 | 1061.5757 | -0.0065 | 0 | 16 | 4.1 | 1 | K.TVINDPLYK.E |
|  | 5950 | 550.8157 | 1099.6169 | 1098.6761 | 0.9408 | 1 | 23 | 0.9 | 2 | K.TILKELVQR.G |
|  | 7544 | **402.9453** | **1205.8140** | **1206.5355** | **-0.7214** | **0** | **3** | **61** | **6** | **K.FCLFCFWK.F** |
|  | 7616 | 404.4434 | 1210.3083 | 1209.5812 | 0.7270 | 1 | 13 | 4.9 | 1 | R.NFCKDVVSNK.K |
|  | 11471 | 473.2548 | 1416.7425 | 1416.7514 | -0.0089 | 0 | 29 | 0.18 | 1 | K.WIPQNDLLGHPK.T 11466 11467 11469 |
|  | 11924 | 723.3910 | 1444.7674 | 1444.7787 | -0.0112 | 0 | 40 | 0.013 | 1 | R.IQHDQPVKPLDR.A 11925 11926 |
|  | 17945 | **616.8151** | **1847.4234** | **1847.9570** | **-0.5337** | **1** | **4** | **26** | **1** | **R.FVYSLRFTPGYTIER.H** |
|  | 24235 | 836.0715 | 2505.1926 | 2504.2046 | 0.9880 | 0 | 33 | 0.036 | 1 | K.WDQFYSEVLGRPTTLFETMGK.A |
|  | 25046 | 663.3171 | 2649.2392 | 2648.2945 | 0.9447 | 1 | 0 | 60 | 5 | K.KWDQFYSEVLGRPTTLFETMGK.A |
|  | 30490 | 1039.5150 | 4154.0309 | 4154.0546 | -0.0237 | 0 | 49 | 0.00033 | 1 | R.AFITHGGANGIYEAIYHGIPMVGIPLFFDQPDNIAHMK.A 30489 30492 |

| **620.** | Q9NRP0\|OSTC_HUMAN    **Mass:** 16932    **Score:** 64     **Queries matched:** 5   **emPAI:** 0.23 |
| --- | --- |
|  | Oligosaccharyltransferase complex subunit OSTC OS=Homo sapiens GN=OSTC PE=1 SV=1 |

|  | **Query** | **Observed** | **Mr(expt)** | **Mr(calc)** | **Delta** | **Miss** | **Score** | **Expect** | **Rank** | **Peptide** |
| --- | --- | --- | --- | --- | --- | --- | --- | --- | --- | --- |
|  | 2521 | **422.7637** | **843.5128** | **842.4143** | **1.0985** | **1** | **3** | **1.1e+002** | **10** | **R.VFMRMK.L** |
|  | 4978 | 513.2945 | 1024.5744 | 1024.5085 | 0.0659 | 1 | 5 | 45 | 10 | R.MKLPGYLMG.- |
|  | 4982 | 513.3003 | 1024.5861 | 1024.5085 | 0.0776 | 1 | (4) | 53 | 3 | R.MKLPGYLMG.- |
|  | 11658 | **714.8950** | **1427.7755** | **1427.7847** | **-0.0092** | **0** | **64** | **5.4e-005** | **1** | **R.VPFLVLECPNLK.L** |
|  | 20856 | **697.8166** | **2090.4279** | **2090.1234** | **0.3045** | **1** | **2** | **39** | **1** | **M.ETLYRVPFLVLECPNLK.L** |

| **621.** | P22626\|ROA2_HUMAN    **Mass:** 37464    **Score:** 64     **Queries matched:** 9   **emPAI:** 0.21 |
| --- | --- |
|  | Heterogeneous nuclear ribonucleoproteins A2/B1 OS=Homo sapiens GN=HNRNPA2B1 PE=1 SV=2 |

|  | **Query** | **Observed** | **Mr(expt)** | **Mr(calc)** | **Delta** | **Miss** | **Score** | **Expect** | **Rank** | **Peptide** |
| --- | --- | --- | --- | --- | --- | --- | --- | --- | --- | --- |
|  | 3442 | **457.2427** | **912.4707** | **911.3733** | **1.0974** | **0** | **5** | **47** | **4** | **R.GGSDGYGSGR.G** |
|  | 5396 | **529.3671** | **1056.7196** | **1056.5815** | **0.1381** | **0** | **2** | **93** | **6** | **K.TLETVPLER.K** |
|  | 10071 | **669.8484** | **1337.6823** | **1337.6939** | **-0.0116** | **0** | **38** | **0.02** | **1** | **R.EESGKPGAHVTVK.K** |
|  | 15332 | **555.2735** | **1662.7987** | **1663.7948** | **-0.9961** | **1** | **4** | **51** | **3** | **K.ALSRQEMQEVQSSR.S** |
|  | 17329 | 899.9589 | 1797.9033 | 1797.9149 | -0.0116 | 0 | 49 | 0.0012 | 1 | K.LFIGGLSFETTEESLR.N 17349 |
|  | 20534 | **688.3160** | **2061.9261** | **2060.9448** | **0.9813** | **1** | **1** | **81** | **2** | **R.NYYEQWGKLTDCVVMR.D** |
|  | 23647 | 811.4135 | 2431.2185 | 2432.0313 | -0.8127 | 1 | 1 | 60 | 6 | R.NMGGPYGGGNYGPGGSGGSGGYGGRSR.Y |
|  | 26878 | **990.4435** | **2968.3088** | **2967.2339** | **1.0748** | **1** | **6** | **14** | **1** | **K.SGNFGGSRNMGGPYGGGNYGPGGSGGSGGYGGR.S** |

| **622.** | O14548\|COX7R_HUMAN    **Mass:** 12664    **Score:** 64     **Queries matched:** 7   **emPAI:** 0.72 |
| --- | --- |
|  | Cytochrome c oxidase subunit 7A-related protein, mitochondrial OS=Homo sapiens GN=COX7A2L PE=1 SV=2 |

|  | **Query** | **Observed** | **Mr(expt)** | **Mr(calc)** | **Delta** | **Miss** | **Score** | **Expect** | **Rank** | **Peptide** |
| --- | --- | --- | --- | --- | --- | --- | --- | --- | --- | --- |
|  | 4014 | **478.2784** | **954.5423** | **954.5498** | **-0.0075** | **1** | **10** | **15** | **3** | **K.NKVPELQK.F** |
|  | 4094 | **481.2686** | **960.5226** | **960.5280** | **-0.0054** | **0** | **8** | **27** | **1** | **K.ADGVPVYLK.R** |
|  | 8395 | **625.3149** | **1248.6153** | **1247.6445** | **0.9709** | **1** | **3** | **69** | **7** | **K.RGLPDQMLYR.T** |
|  | 11503 | 710.3305 | 1418.6465 | 1418.6565 | -0.0101 | 0 | 41 | 0.012 | 1 | K.LTSDSTVYDYAGK.N 11504 |
|  | 28553 | **1133.2660** | **3396.7761** | **3396.7969** | **-0.0208** | **0** | **44** | **0.0017** | **1** | **K.LAGAWASEAYSPQGLKPVVSTEAPPIIFATPTK.L** |
|  | 29265 | 1847.4479 | 3692.8812 | 3691.8486 | 1.0326 | 1 | 2 | 24 | 2 | R.GLPDQMLYRTTMALTVGGTIYCLIALYMASQPK.N |

| **623.** | P62244\|RS15A_HUMAN    **Mass:** 14944    **Score:** 64     **Queries matched:** 16   **emPAI:** 0.59 |
| --- | --- |
|  | 40S ribosomal protein S15a OS=Homo sapiens GN=RPS15A PE=1 SV=2 |

|  | **Query** | **Observed** | **Mr(expt)** | **Mr(calc)** | **Delta** | **Miss** | **Score** | **Expect** | **Rank** | **Peptide** |
| --- | --- | --- | --- | --- | --- | --- | --- | --- | --- | --- |
|  | 1784 | **395.1884** | **788.3621** | **787.4011** | **0.9611** | **0** | **37** | **0.045** | **1** | **K.CGVISPR.F** |
|  | 2905 | **436.2688** | **870.5231** | **870.5287** | **-0.0056** | **0** | **13** | **8.4** | **1** | **K.IVVNLTGR.L** 2891 |
|  | 3105 | **443.7456** | **885.4767** | **884.4500** | **1.0267** | **0** | **14** | **7.2** | **3** | **R.FLTVMMK.H** |
|  | 4269 | **487.7675** | **973.5205** | **973.5266** | **-0.0061** | **0** | **24** | **0.74** | **1** | **R.MNVLADALK.S** 4247 4294 |
|  | 4500 | **496.2734** | **990.5323** | **989.5215** | **1.0108** | **0** | **(6)** | **43** | **1** | **R.MNVLADALK.S** |
|  | 6499 | 572.3174 | 1142.6203 | 1142.6230 | -0.0027 | 1 | 8 | 26 | 4 | R.LNKCGVISPR.F |
|  | 10730 | **688.3331** | **1374.6516** | **1375.7316** | **-1.0800** | **1** | **(0)** | **1.3e+002** | **8** | **-.MVRMNVLADALK.S** |
|  | 10737 | **459.2621** | **1374.7645** | **1375.7316** | **-0.9670** | **1** | **6** | **32** | **4** | **-.MVRMNVLADALK.S** |
|  | 11034 | **464.7576** | **1391.2510** | **1391.7265** | **-0.4755** | **1** | **(6)** | **21** | **1** | **-.MVRMNVLADALK.S** |
|  | 14559 | **807.4301** | **1612.8455** | **1611.8369** | **1.0087** | **1** | **6** | **30** | **2** | **K.DLEKWQNNLLPSR.Q** |
|  | 15907 | **567.6022** | **1699.7849** | **1699.7954** | **-0.0106** | **0** | **51** | **0.00084** | **1** | **K.HGYIGEFEIIDDHR.A** 15906 |
|  | 16392 | 865.4905 | 1728.9665 | 1729.9032 | -0.9367 | 1 | 12 | 6.3 | 2 | R.MNVLADALKSINNAEK.R |

| **624.** | P06744\|G6PI_HUMAN    **Mass:** 63335    **Score:** 64     **Queries matched:** 13   **emPAI:** 0.19 |
| --- | --- |
|  | Glucose-6-phosphate isomerase OS=Homo sapiens GN=GPI PE=1 SV=4 |

|  | **Query** | **Observed** | **Mr(expt)** | **Mr(calc)** | **Delta** | **Miss** | **Score** | **Expect** | **Rank** | **Peptide** |
| --- | --- | --- | --- | --- | --- | --- | --- | --- | --- | --- |
|  | 3000 | **440.0307** | **878.0468** | **877.5498** | **0.4971** | **0** | **2** | **78** | **5** | **R.AVLHVALR.N** |
|  | 3968 | 476.7493 | 951.4841 | 951.4709 | 0.0132 | 1 | 1 | 1.4e+002 | 7 | K.SFCQRVR.S |
|  | 4340 | **490.2632** | **978.5118** | **977.4930** | **1.0188** | **1** | **3** | **77** | **10** | **R.LFDANKDR.F** |
|  | 5645 | **539.2960** | **1076.5775** | **1075.5332** | **1.0443** | **0** | **4** | **66** | **9** | **K.NLVTEDVMR.M** |
|  | 6578 | **575.3214** | **1148.6281** | **1147.6938** | **0.9344** | **1** | **3** | **72** | **3** | **R.AVLHVALRNR.S** |
|  | 11909 | 723.2535 | 1444.4925 | 1444.8038 | -0.3113 | 1 | 1 | 81 | 5 | K.HFVALSTNTTKVK.E |
|  | 13487 | **515.0294** | **1542.0663** | **1541.7685** | **0.2977** | **1** | **7** | **16** | **1** | **K.ELQAAGKSPEDLER.L** 13501 |
|  | 15967 | **852.4758** | **1702.9370** | **1702.9440** | **-0.0070** | **0** | **53** | **0.00053** | **1** | **K.ILLANFLAQTEALMR.G** 15965 |
|  | 16088 | 855.9202 | 1709.8258 | 1710.9529 | -1.1271 | 1 | 5 | 36 | 2 | K.INYTEGRAVLHVALR.N |
|  | 17744 | **916.5064** | **1830.9983** | **1831.0091** | **-0.0108** | **0** | **37** | **0.02** | **1** | **K.TLAQLNPESSLFIIASK.T** |
|  | 28319 | **1110.2487** | **3327.7242** | **3327.7384** | **-0.0143** | **0** | **21** | **0.35** | **1** | **K.TITDVINIGIGGSDLGPLMVTEALKPYSSGGPR.V** |

| **625.** | Q13011\|ECH1_HUMAN    **Mass:** 36136    **Score:** 63     **Queries matched:** 12   **emPAI:** 0.35 |
| --- | --- |
|  | Delta(3,5)-Delta(2,4)-dienoyl-CoA isomerase, mitochondrial OS=Homo sapiens GN=ECH1 PE=1 SV=2 |

|  | **Query** | **Observed** | **Mr(expt)** | **Mr(calc)** | **Delta** | **Miss** | **Score** | **Expect** | **Rank** | **Peptide** |
| --- | --- | --- | --- | --- | --- | --- | --- | --- | --- | --- |
|  | 1373 | **631.7742** | **630.7670** | **631.2926** | **-0.5256** | **0** | **8** | **38** | **1** | **K.GDDVAR.I** |
|  | 3273 | **451.2627** | **900.5108** | **899.5301** | **0.9807** | **1** | **5** | **65** | **8** | **M.AAGIVASRR.L** |
|  | 5274 | **524.2623** | **1046.5101** | **1046.5655** | **-0.0554** | **1** | **9** | **24** | **9** | **-.MAAGIVASRR.L** |
|  | 5371 | **528.7884** | **1055.5622** | **1055.4416** | **0.1206** | **0** | **3** | **83** | **8** | **R.EMVECFNK.I** |
|  | 9288 | **649.8181** | **1297.6217** | **1297.6302** | **-0.0086** | **0** | **42** | **0.0087** | **1** | **R.YQETFNVIER.C** 9287 |
|  | 12494 | 739.8661 | 1477.7176 | 1476.7936 | 0.9240 | 0 | 0 | 1.2e+002 | 10 | R.LTGSNYPGLSISLR.L |
|  | 13489 | **772.2360** | **1542.4574** | **1541.8049** | **0.6524** | **0** | **1** | **69** | **6** | **K.EVDVGLAADVGTLQR.L** |
|  | 14030 | **527.2899** | **1578.8478** | **1579.7698** | **-0.9220** | **1** | **6** | **32** | **3** | **R.KMMADEALGSGLVSR.V** |
|  | 16425 | **866.4674** | **1730.9203** | **1730.9315** | **-0.0113** | **0** | **28** | **0.16** | **1** | **K.VIGNQSLVNELAFTAR.K** |
|  | 20921 | **1049.0020** | **2095.9893** | **2095.9992** | **-0.0099** | **0** | **28** | **0.15** | **1** | **K.MFTAGIDLMDMASDILQPK.G** |
|  | 26447 | **966.1044** | **2895.2913** | **2894.4592** | **0.8321** | **1** | **1** | **39** | **7** | **R.AVVISGAGKMFTAGIDLMDMASDILQPK.G** |

| **626.** | Q6UW02\|CP20A_HUMAN    **Mass:** 52741    **Score:** 63     **Queries matched:** 10   **emPAI:** 0.07 |
| --- | --- |
|  | Cytochrome P450 20A1 OS=Homo sapiens GN=CYP20A1 PE=1 SV=1 |

|  | **Query** | **Observed** | **Mr(expt)** | **Mr(calc)** | **Delta** | **Miss** | **Score** | **Expect** | **Rank** | **Peptide** |
| --- | --- | --- | --- | --- | --- | --- | --- | --- | --- | --- |
|  | 2591 | **425.7639** | **849.5132** | **849.4457** | **0.0675** | **0** | **11** | **14** | **1** | **K.QHINPNK.T** |
|  | 5048 | **515.3279** | **1028.6413** | **1028.6131** | **0.0282** | **1** | **8** | **26** | **9** | **K.IDRFIIPR.E** 5043 |
|  | 6826 | 584.8338 | 1167.6530 | 1167.5482 | 0.1049 | 0 | 7 | 30 | 5 | K.TSDPFETMLK.S |
|  | 12239 | **489.2478** | **1464.7215** | **1463.6650** | **1.0565** | **0** | **10** | **11** | **1** | **R.YCQHVLCETVR.T** |
|  | 12348 | **490.9290** | **1469.7651** | **1468.6980** | **1.0670** | **1** | **3** | **70** | **3** | **K.GFLDGSLDKNMTR.K** |
|  | 12950 | **754.8255** | **1507.6364** | **1507.6474** | **-0.0109** | **0** | **63** | **6.1e-005** | **1** | **R.YQSGGGSVSENHMR.K** |
|  | 15169 | **551.3922** | **1651.1548** | **1651.7373** | **-0.5825** | **1** | **4** | **29** | **5** | **R.YQSGGGSVSENHMRK.K** |
|  | 16236 | 860.9760 | 1719.9373 | 1720.9836 | -1.0462 | 1 | 1 | 92 | 7 | K.RLHLLSVEGQVIETK.Y |
|  | 17835 | **613.6672** | **1837.9799** | **1837.9244** | **0.0555** | **1** | **2** | **71** | **6** | **K.KQYEDALMQLESVLR.N** |

| **627.** | Q13438\|OS9_HUMAN    **Mass:** 75971    **Score:** 63     **Queries matched:** 8   **emPAI:** 0.05 |
| --- | --- |
|  | Protein OS-9 OS=Homo sapiens GN=OS9 PE=1 SV=1 |

|  | **Query** | **Observed** | **Mr(expt)** | **Mr(calc)** | **Delta** | **Miss** | **Score** | **Expect** | **Rank** | **Peptide** |
| --- | --- | --- | --- | --- | --- | --- | --- | --- | --- | --- |
|  | 1195 | 580.3173 | 579.3100 | 578.2482 | 1.0618 | 0 | 12 | 12 | 1 | K.AGMER.E |
|  | 1903 | **399.3453** | **796.6761** | **796.3392** | **0.3369** | **0** | **2** | **62** | **3** | **K.DSDFWK.M** |
|  | 6821 | **584.3718** | **1166.7291** | **1165.5727** | **1.1564** | **1** | **0** | **1.2e+002** | **10** | **R.QKELESNYR.R** |
|  | 11910 | 723.2540 | 1444.4934 | 1444.7344 | -0.2410 | 1 | 2 | 59 | 4 | K.SGVAPQKMAGASPTK.D |
|  | 17365 | **600.9752** | **1799.9038** | **1798.9611** | **0.9427** | **1** | **1** | **88** | **8** | **K.LRLGGPNQDLTVLEMK.R** |
|  | 17491 | 906.4285 | 1810.8424 | 1810.7773 | 0.0650 | 1 | 5 | 29 | 3 | K.TKDWWTYEFCYGR.H |
|  | 21936 | **737.9969** | **2210.9688** | **2212.1110** | **-1.1422** | **1** | **3** | **40** | **3** | **R.ELENIIQETEKELDPDGLK.K** |
|  | 28606 | **1140.8675** | **3419.5808** | **3419.6014** | **-0.0205** | **1** | **63** | **1.9e-005** | **1** | **R.FLCDEGAGISGDYIDRVDEPLSCSYVLTIR.T** |

| **628.** | P28161\|GSTM2_HUMAN    **Mass:** 25899    **Score:** 63     **Queries matched:** 10   **emPAI:** 0.15 |
| --- | --- |
|  | Glutathione S-transferase Mu 2 OS=Homo sapiens GN=GSTM2 PE=1 SV=2 |

|  | **Query** | **Observed** | **Mr(expt)** | **Mr(calc)** | **Delta** | **Miss** | **Score** | **Expect** | **Rank** | **Peptide** |
| --- | --- | --- | --- | --- | --- | --- | --- | --- | --- | --- |
|  | 1610 | 712.3652 | 711.3579 | 711.3625 | -0.0046 | 0 | 16 | 3.5 | 1 | K.ISAYMK.S 1617 |
|  | 2043 | 403.7328 | 805.4511 | 804.3952 | 1.0559 | 0 | (15) | 6.6 | 4 | K.MAVWGNK.- |
|  | 2215 | 821.3732 | 820.3660 | 820.3902 | -0.0242 | 0 | 15 | 5.6 | 2 | K.MAVWGNK.- |
|  | 5610 | **537.7662** | **1073.5179** | **1072.4607** | **1.0572** | **0** | **8** | **28** | **5** | **K.HNLCGESEK.E** |
|  | 8392 | **417.2010** | **1248.5812** | **1249.6277** | **-1.0465** | **0** | **7** | **29** | **10** | **M.PMTLGYWNIR.G** |
|  | 9378 | **434.8966** | **1301.6679** | **1302.5187** | **-0.8507** | **0** | **3** | **74** | **8** | **K.YTMGDAPDYDR.S** |
|  | 11702 | **716.3094** | **1430.6043** | **1430.6136** | **-0.0093** | **1** | **63** | **6.6e-005** | **1** | **K.KYTMGDAPDYDR.S** |
|  | 11956 | **483.2511** | **1446.7314** | **1446.6085** | **0.1228** | **1** | **(2)** | **88** | **7** | **K.KYTMGDAPDYDR.S** |
|  | 20775 | 695.0616 | 2082.1629 | 2082.9714 | -0.8085 | 1 | 4 | 33 | 4 | R.EDILENQFMDSRMQLAK.L |

| **629.** | P09488\|GSTM1_HUMAN    **Mass:** 25923    **Score:** 63     **Queries matched:** 9   **emPAI:** 0.15 |
| --- | --- |
|  | Glutathione S-transferase Mu 1 OS=Homo sapiens GN=GSTM1 PE=1 SV=3 |

|  | **Query** | **Observed** | **Mr(expt)** | **Mr(calc)** | **Delta** | **Miss** | **Score** | **Expect** | **Rank** | **Peptide** |
| --- | --- | --- | --- | --- | --- | --- | --- | --- | --- | --- |
|  | 1610 | 712.3652 | 711.3579 | 711.3625 | -0.0046 | 0 | 16 | 3.5 | 1 | K.ISAYMK.S 1617 |
|  | 2043 | 403.7328 | 805.4511 | 804.3952 | 1.0559 | 0 | (15) | 6.6 | 4 | K.MAVWGNK.- |
|  | 2215 | 821.3732 | 820.3660 | 820.3902 | -0.0242 | 0 | 15 | 5.6 | 2 | K.MAVWGNK.- |
|  | 9378 | 434.8966 | 1301.6679 | 1302.5187 | -0.8507 | 0 | 3 | 74 | 8 | K.YTMGDAPDYDR.S |
|  | 11702 | 716.3094 | 1430.6043 | 1430.6136 | -0.0093 | 1 | 63 | 6.6e-005 | 1 | K.KYTMGDAPDYDR.S |
|  | 11956 | 483.2511 | 1446.7314 | 1446.6085 | 0.1228 | 1 | (2) | 88 | 7 | K.KYTMGDAPDYDR.S |
|  | 14576 | **539.2501** | **1614.7285** | **1613.7467** | **0.9818** | **1** | **1** | **97** | **7** | **K.HNLCGETEEEKIR.V** |
|  | 19771 | **666.6787** | **1997.0143** | **1997.0669** | **-0.0526** | **1** | **4** | **36** | **5** | **M.PMILGYWDIRGLAHAIR.L** |

| **630.** | Q03013\|GSTM4_HUMAN    **Mass:** 25773    **Score:** 63     **Queries matched:** 8   **emPAI:** 0.15 |
| --- | --- |
|  | Glutathione S-transferase Mu 4 OS=Homo sapiens GN=GSTM4 PE=1 SV=3 |

|  | **Query** | **Observed** | **Mr(expt)** | **Mr(calc)** | **Delta** | **Miss** | **Score** | **Expect** | **Rank** | **Peptide** |
| --- | --- | --- | --- | --- | --- | --- | --- | --- | --- | --- |
|  | 1610 | 712.3652 | 711.3579 | 711.3625 | -0.0046 | 0 | 16 | 3.5 | 1 | K.ISAYMK.S 1617 |
|  | 9378 | 434.8966 | 1301.6679 | 1302.5187 | -0.8507 | 0 | 3 | 74 | 8 | K.YTMGDAPDYDR.S |
|  | 10682 | **458.3306** | **1371.9699** | **1371.6315** | **0.3384** | **0** | **7** | **21** | **9** | **-.MSMTLGYWDIR.G** |
|  | 11702 | 716.3094 | 1430.6043 | 1430.6136 | -0.0093 | 1 | 63 | 6.6e-005 | 1 | K.KYTMGDAPDYDR.S |
|  | 11956 | 483.2511 | 1446.7314 | 1446.6085 | 0.1228 | 1 | (2) | 88 | 7 | K.KYTMGDAPDYDR.S |
|  | 14576 | 539.2501 | 1614.7285 | 1613.7467 | 0.9818 | 1 | 1 | 97 | 7 | K.HNLCGETEEEKIR.V |
|  | 29215 | **1223.1803** | **3666.5191** | **3666.8367** | **-0.3176** | **1** | **0** | **26** | **4** | **K.LKPEYLEELPTMMQHFSQFLGKRPWFVGDK.I** |

| **631.** | Q7Z403\|TMC6_HUMAN    **Mass:** 91356    **Score:** 63     **Queries matched:** 16   **emPAI:** 0.04 |
| --- | --- |
|  | Transmembrane channel-like protein 6 OS=Homo sapiens GN=TMC6 PE=1 SV=2 |

|  | **Query** | **Observed** | **Mr(expt)** | **Mr(calc)** | **Delta** | **Miss** | **Score** | **Expect** | **Rank** | **Peptide** |
| --- | --- | --- | --- | --- | --- | --- | --- | --- | --- | --- |
|  | 1629 | **718.3861** | **717.3788** | **716.4432** | **0.9356** | **1** | **7** | **51** | **3** | **R.IISEKK.L** |
|  | 2590 | 425.7635 | 849.5125 | 849.4457 | 0.0668 | 0 | 8 | 29 | 5 | R.HLEAAGPR.V |
|  | 4280 | 488.2468 | 974.4791 | 974.5185 | -0.0394 | 0 | 12 | 12 | 4 | R.KPEFDIAR.N |
|  | 9658 | **440.0299** | **1317.0678** | **1316.7234** | **0.3444** | **1** | **6** | **27** | **10** | **R.ILASMPSRTIGR.S** |
|  | 10428 | 679.8718 | 1357.7290 | 1357.7136 | 0.0154 | 1 | 52 | 0.00093 | 2 | K.KTSLLANCQAPR.R 10427 |
|  | 11161 | **467.1008** | **1398.2807** | **1397.8030** | **0.4776** | **1** | **4** | **39** | **5** | **R.LKELLAEWQLR.H** |
|  | 15055 | 823.3960 | 1644.7774 | 1644.9351 | -0.1577 | 1 | 13 | 5.9 | 3 | K.IFLINKLHSIYER.K 15057 |
|  | 17086 | **594.2617** | **1779.7632** | **1779.7352** | **0.0279** | **1** | **2** | **66** | **3** | **K.WRGQPGSGGVCSCCGR.L** |
|  | 17095 | **594.2992** | **1779.8757** | **1780.9584** | **-1.0826** | **1** | **13** | **5.4** | **1** | **R.GAIISQYYNRTVQLR.C** 17109 17110 |
|  | 18187 | 621.9915 | 1862.9526 | 1861.9978 | 0.9547 | 0 | 6 | 28 | 7 | R.IGGQFGSSVLSYFLFLK.T |
|  | 19786 | **666.7023** | **1997.0852** | **1997.9429** | **-0.8578** | **0** | **2** | **55** | **6** | **R.VGTTEEAAAPPALLTDEQDA.-** |
|  | 25790 | **696.5931** | **2782.3432** | **2782.5026** | **-0.1594** | **0** | **2** | **41** | **4** | **R.YACVLALHSLGLALLSALQALMPWR.Y** |

| **632.** | P63010\|AP2B1_HUMAN    **Mass:** 105398   **Score:** 63     **Queries matched:** 15   **emPAI:** 0.04 |
| --- | --- |
|  | AP-2 complex subunit beta OS=Homo sapiens GN=AP2B1 PE=1 SV=1 |

|  | **Query** | **Observed** | **Mr(expt)** | **Mr(calc)** | **Delta** | **Miss** | **Score** | **Expect** | **Rank** | **Peptide** |
| --- | --- | --- | --- | --- | --- | --- | --- | --- | --- | --- |
|  | 1756 | 774.4308 | 773.4235 | 772.3755 | 1.0480 | 0 | 9 | 32 | 7 | K.YFTTNK.K |
|  | 1762 | **777.0349** | **776.0276** | **775.4262** | **0.6014** | **0** | **7** | **29** | **7** | **K.LDIMIR.L** |
|  | 4904 | 509.8024 | 1017.5902 | 1016.6052 | 0.9850 | 1 | 4 | 70 | 4 | K.KVIAAMTVGK.D |
|  | 8332 | 416.2213 | 1245.6421 | 1246.6638 | -1.0217 | 1 | 5 | 46 | 5 | R.ALAVRTMGCIR.V |
|  | 8463 | **627.3185** | **1252.6224** | **1252.7503** | **-0.1279** | **1** | **2** | **85** | **1** | **K.RPEILKQEIK.V** 8470 |
|  | 11896 | **482.2421** | **1443.7046** | **1444.7092** | **-1.0047** | **1** | **8** | **22** | **1** | **R.EAQSICERVTPR.L** |
|  | 13373 | **512.4129** | **1534.2169** | **1533.8265** | **0.3904** | **1** | **0** | **78** | **7** | **K.KLVYLYLMNYAK.S** |
|  | 14854 | 816.9507 | 1631.8868 | 1632.8358 | -0.9490 | 1 | 4 | 50 | 6 | K.GEIFELKAELNNEK.K |
|  | 15411 | **557.0179** | **1668.0320** | **1668.7851** | **-0.7531** | **0** | **0** | **83** | **7** | **K.SQPDMAIMAVNSFVK.D** |
|  | 15839 | **566.3079** | **1695.9018** | **1695.8250** | **0.0768** | **1** | **3** | **50** | **6** | **K.RNVEGQDMLYQSLK.L** |
|  | 29882 | **1300.6992** | **3899.0758** | **3898.0737** | **1.0022** | **0** | **50** | **0.00025** | **1** | **K.NSFGVIPSTPLAIHTPLMPNQSIDVSLPLNTLGPVMK.M** 29881 |
|  | 29893 | **1305.5310** | **3913.5712** | **3914.0686** | **-0.4974** | **0** | **(2)** | **13** | **5** | **K.NSFGVIPSTPLAIHTPLMPNQSIDVSLPLNTLGPVMK.M** |
|  | 31602 | **1112.7665** | **5558.7960** | **5558.8768** | **-0.0808** | **1** | **2** | **6.2** | **1** | **K.ALQHMTDFAIQFNKNSFGVIPSTPLAIHTPLMPNQSIDVSLPLNTLGPVMK.M** |

| **633.** | Q9NS69\|TOM22_HUMAN    **Mass:** 15512    **Score:** 62     **Queries matched:** 4   **emPAI:** 0.25 |
| --- | --- |
|  | Mitochondrial import receptor subunit TOM22 homolog OS=Homo sapiens GN=TOMM22 PE=1 SV=3 |

|  | **Query** | **Observed** | **Mr(expt)** | **Mr(calc)** | **Delta** | **Miss** | **Score** | **Expect** | **Rank** | **Peptide** |
| --- | --- | --- | --- | --- | --- | --- | --- | --- | --- | --- |
|  | 11080 | **465.7571** | **1394.2495** | **1393.6700** | **0.5795** | **0** | **4** | **33** | **9** | **R.LWGLTEMFPER.V** |
|  | 13727 | **779.3882** | **1556.7618** | **1556.7729** | **-0.0111** | **0** | **62** | **7.9e-005** | **1** | **K.LQMEQQQQLQQR.Q** |
|  | 15135 | **550.8157** | **1649.4254** | **1648.8395** | **0.5858** | **1** | **5** | **25** | **5** | **R.LWGLTEMFPERVR.S** |
|  | 24735 | **860.9411** | **2579.8015** | **2580.3913** | **-0.5899** | **0** | **11** | **3.6** | **1** | **R.AALWIGTTSFMILVLPVVFETEK.L** |

| **634.** | Q9NRV9\|HEBP1_HUMAN    **Mass:** 21198    **Score:** 62     **Queries matched:** 7   **emPAI:** 0.39 |
| --- | --- |
|  | Heme-binding protein 1 OS=Homo sapiens GN=HEBP1 PE=1 SV=1 |

|  | **Query** | **Observed** | **Mr(expt)** | **Mr(calc)** | **Delta** | **Miss** | **Score** | **Expect** | **Rank** | **Peptide** |
| --- | --- | --- | --- | --- | --- | --- | --- | --- | --- | --- |
|  | 2938 | **437.7630** | **873.5114** | **872.4789** | **1.0324** | **1** | **15** | **7** | **6** | **R.EAMPKVAK.Y** |
|  | 3478 | 458.3055 | 914.5964 | 914.5225 | 0.0739 | 0 | 11 | 15 | 6 | R.NEIWLLK.T |
|  | 9726 | **661.3767** | **1320.7389** | **1320.7150** | **0.0239** | **1** | **1** | **1.1e+002** | **3** | **R.LRAALEGTATYR.G** |
|  | 14987 | **820.8933** | **1639.7721** | **1639.7842** | **-0.0121** | **0** | **19** | **1.5** | **1** | **R.IPNQFQSDPPAPSDK.S** |
|  | 17260 | **597.6463** | **1789.9171** | **1788.9258** | **0.9913** | **0** | **19** | **1.3** | **1** | **K.FATVEVTDKPVDEALR.E** |
|  | 19553 | 989.5019 | 1976.9892 | 1976.9996 | -0.0104 | 0 | 62 | 5.9e-005 | 1 | K.NSLFGSVETWPWQVLSK.G |
|  | 25710 | **693.6342** | **2770.5075** | **2770.2908** | **0.2167** | **1** | **0** | **50** | **2** | **R.EGITVYSMQFGGYAKEADYVAQATR.L** |

| **635.** | Q14240\|IF4A2_HUMAN    **Mass:** 46601    **Score:** 62     **Queries matched:** 14   **emPAI:** 0.08 |
| --- | --- |
|  | Eukaryotic initiation factor 4A-II OS=Homo sapiens GN=EIF4A2 PE=1 SV=2 |

|  | **Query** | **Observed** | **Mr(expt)** | **Mr(calc)** | **Delta** | **Miss** | **Score** | **Expect** | **Rank** | **Peptide** |
| --- | --- | --- | --- | --- | --- | --- | --- | --- | --- | --- |
|  | 2350 | **416.2238** | **830.4330** | **830.4035** | **0.0296** | **0** | **8** | **38** | **1** | **R.ENYIHR.I** |
|  | 3606 | **463.9204** | **925.8263** | **925.3890** | **0.4374** | **0** | **7** | **18** | **2** | **M.SGGSADYNR.E** |
|  | 5378 | **529.2686** | **1056.5225** | **1056.4294** | **0.0931** | **0** | **6** | **45** | **9** | **-.MSGGSADYNR.E** |
|  | 5539 | 535.2840 | 1068.5534 | 1067.5400 | 1.0135 | 0 | 9 | 18 | 3 | K.QFYINVER.E |
|  | 6712 | 580.3049 | 1158.5953 | 1158.6496 | -0.0543 | 1 | 6 | 47 | 10 | K.KEELTLEGIK.Q |
|  | 7384 | 600.3666 | 1198.7187 | 1197.6717 | 1.0470 | 0 | 2 | 94 | 9 | K.ETQALVLAPTR.E |
|  | 11992 | 725.8321 | 1449.6496 | 1448.7511 | 0.8985 | 1 | 1 | 1.1e+002 | 9 | R.KGVAINFVTEEDK.R |
|  | 13708 | **778.8576** | **1555.7006** | **1554.7058** | **0.9949** | **0** | **62** | **8.5e-005** | **1** | **K.MFVLDEADEMLSR.G** |
|  | 13804 | 782.3774 | 1562.7403 | 1562.7035 | 0.0368 | 0 | 7 | 26 | 5 | R.DFTVSALHGDMDQK.E |
|  | 13914 | **524.6194** | **1570.8363** | **1570.7007** | **0.1356** | **0** | **(3)** | **62** | **2** | **K.MFVLDEADEMLSR.G** |
|  | 19788 | 666.8950 | 1997.6631 | 1997.9590 | -0.2960 | 1 | 8 | 12 | 2 | K.WIKMFVLDEADEMLSR.G |
|  | 21339 | 537.0162 | 2144.0359 | 2143.1273 | 0.9085 | 0 | 5 | 28 | 3 | R.GIDVQQVSLVINYDLPTNR.E |
|  | 24910 | **654.8215** | **2615.2568** | **2615.4456** | **-0.1888** | **1** | **1** | **60** | **4** | **K.LNTSIQVVLLSATMPTDVLEVTKK.F** |
|  | 30784 | **1461.3203** | **4380.9391** | **4379.8608** | **1.0783** | **1** | **1** | **13** | **6** | **M.SGGSADYNREHGGPEGMDPDGVIESNWNEIVDNFDDMNLK.E** |

| **636.** | P60842\|IF4A1_HUMAN    **Mass:** 46353    **Score:** 62     **Queries matched:** 16   **emPAI:** 0.08 |
| --- | --- |
|  | Eukaryotic initiation factor 4A-I OS=Homo sapiens GN=EIF4A1 PE=1 SV=1 |

|  | **Query** | **Observed** | **Mr(expt)** | **Mr(calc)** | **Delta** | **Miss** | **Score** | **Expect** | **Rank** | **Peptide** |
| --- | --- | --- | --- | --- | --- | --- | --- | --- | --- | --- |
|  | 2350 | 416.2238 | 830.4330 | 830.4035 | 0.0296 | 0 | 8 | 38 | 1 | R.ENYIHR.I |
|  | 5539 | 535.2840 | 1068.5534 | 1067.5400 | 1.0135 | 0 | 9 | 18 | 3 | R.QFYINVER.E |
|  | 6261 | **563.2725** | **1124.5304** | **1123.5040** | **1.0264** | **1** | **2** | **98** | **7** | **-.MSASQDSRSR.D** |
|  | 6859 | **585.8135** | **1169.6125** | **1168.5764** | **1.0361** | **0** | **5** | **46** | **3** | **K.DQIYDIFQK.L** |
|  | 11992 | 725.8321 | 1449.6496 | 1448.7181 | 0.9315 | 1 | 1 | 1.1e+002 | 9 | R.KGVAINMVTEEDK.R |
|  | 13708 | 778.8576 | 1555.7006 | 1554.7058 | 0.9949 | 0 | 62 | 8.5e-005 | 1 | K.MFVLDEADEMLSR.G |
|  | 13914 | 524.6194 | 1570.8363 | 1570.7007 | 0.1356 | 0 | (3) | 62 | 2 | K.MFVLDEADEMLSR.G |
|  | 14072 | **528.2517** | **1581.7333** | **1580.6599** | **1.0734** | **0** | **6** | **31** | **5** | **R.DFTVSAMHGDMDQK.E** |
|  | 14308 | 798.8752 | 1595.7359 | 1596.6549 | -0.9189 | 0 | (3) | 53 | 4 | R.DFTVSAMHGDMDQK.E |
|  | 14632 | 540.2943 | 1617.8609 | 1617.8661 | -0.0052 | 0 | 4 | 52 | 9 | K.LQMEAPHIIVGTPGR.V 14652 |
|  | 19353 | **654.0047** | **1958.9923** | **1958.9481** | **0.0441** | **1** | **(3)** | **53** | **5** | **K.YIKMFVLDEADEMLSR.G** |
|  | 19525 | 987.9512 | 1973.8879 | 1974.9431 | -1.0551 | 1 | 4 | 39 | 4 | K.YIKMFVLDEADEMLSR.G |
|  | 21339 | 537.0162 | 2144.0359 | 2143.1273 | 0.9085 | 0 | 5 | 28 | 3 | R.GIDVQQVSLVINYDLPTNR.E |
|  | 24796 | **863.4545** | **2587.3416** | **2586.3938** | **0.9477** | **1** | **5** | **19** | **1** | **K.LNSNTQVVLLSATMPSDVLEVTKK.F** |
|  | 30503 | 1042.5397 | 4166.1296 | 4165.8481 | 0.2815 | 1 | 1 | 20 | 6 | R.SRDNGPDGMEPEGVIESNWNEIVDSFDDMNLSESLLR.G |

| **637.** | P98187\|CP4F8_HUMAN    **Mass:** 60640    **Score:** 62     **Queries matched:** 15   **emPAI:** 0.06 |
| --- | --- |
|  | Cytochrome P450 4F8 OS=Homo sapiens GN=CYP4F8 PE=1 SV=1 |

|  | **Query** | **Observed** | **Mr(expt)** | **Mr(calc)** | **Delta** | **Miss** | **Score** | **Expect** | **Rank** | **Peptide** |
| --- | --- | --- | --- | --- | --- | --- | --- | --- | --- | --- |
|  | 4066 | 480.2767 | 958.5388 | 957.4304 | 1.1084 | 0 | 9 | 26 | 8 | R.HPEYQER.C |
|  | 4431 | 493.7759 | 985.5372 | 985.5444 | -0.0072 | 0 | 32 | 0.11 | 1 | R.QEVQELLK.D |
|  | 6350 | **566.7624** | **1131.5102** | **1131.6149** | **-0.1046** | **1** | **5** | **53** | **4** | **R.ILPDHREPR.R** |
|  | 8862 | **425.5828** | **1273.7266** | **1274.6102** | **-0.8836** | **1** | **4** | **54** | **6** | **K.NGKELSDEDIR.A** |
|  | 9377 | 651.8411 | 1301.6676 | 1301.6761 | -0.0086 | 1 | 34 | 0.061 | 1 | R.CRQEVQELLK.D |
|  | 10469 | **454.7425** | **1361.2057** | **1361.7125** | **-0.5068** | **1** | **4** | **36** | **2** | **K.IFSKSANIMHAK.W** |
|  | 13352 | 767.3753 | 1532.7361 | 1532.7922 | -0.0561 | 1 | 12 | 8 | 3 | K.RSPMAFIPFSAGPR.N 13350 |
|  | 13479 | 771.8937 | 1541.7728 | 1541.7838 | -0.0110 | 0 | 62 | 8.4e-005 | 1 | R.LVHDFTDAVIQER.R 13480 |
|  | 13755 | **780.8932** | **1559.7718** | **1558.6942** | **1.0776** | **1** | **12** | **8.5** | **10** | **R.NCIGQKFAMAEMK.V** 13757 13761 |
|  | 17828 | **613.3374** | **1836.9904** | **1837.0205** | **-0.0302** | **1** | **0** | **94** | **4** | **K.FAMAEMKVVLALTLLR.F** |
|  | 28232 | **827.8987** | **3307.5656** | **3306.5669** | **0.9987** | **0** | **1** | **32** | **9** | **K.GNVCNINIFAIHHNPSVWPDPEVYDPFR.F** |

| **638.** | P31946\|1433B_HUMAN    **Mass:** 28179    **Score:** 62     **Queries matched:** 9   **emPAI:** 0.13 |
| --- | --- |
|  | 14-3-3 protein beta/alpha OS=Homo sapiens GN=YWHAB PE=1 SV=3 |

|  | **Query** | **Observed** | **Mr(expt)** | **Mr(calc)** | **Delta** | **Miss** | **Score** | **Expect** | **Rank** | **Peptide** |
| --- | --- | --- | --- | --- | --- | --- | --- | --- | --- | --- |
|  | 1446 | 650.2773 | 649.2701 | 648.3265 | 0.9436 | 0 | 3 | 1e+002 | 7 | K.ACSLAK.T |
|  | 1581 | 704.3157 | 703.3084 | 702.3912 | 0.9172 | 0 | 17 | 5.3 | 1 | K.SELVQK.A |
|  | 3664 | 466.2344 | 930.4543 | 931.4222 | -0.9679 | 1 | 7 | 40 | 7 | K.MKGDYFR.Y |
|  | 3849 | 472.7915 | 943.5684 | 943.5201 | 0.0484 | 1 | 5 | 58 | 3 | K.VFYLKMK.G |
|  | 6057 | 554.7784 | 1107.5423 | 1107.5495 | -0.0072 | 0 | 22 | 0.99 | 1 | K.EMQPTHPIR.L |
|  | 7502 | 402.2437 | 1203.7093 | 1204.6485 | -0.9392 | 0 | 12 | 9.5 | 8 | K.DSTLIMQLLR.D |
|  | 11490 | 709.8373 | 1417.6600 | 1418.7518 | -1.0917 | 1 | 5 | 41 | 9 | R.SSWRVISSIEQK.T |
|  | 14331 | **533.5817** | **1597.7234** | **1597.7332** | **-0.0098** | **0** | **16** | **3.2** | **1** | **K.AVTEQGHELSNEER.N** |
|  | 28320 | **1110.5483** | **3328.6232** | **3328.6384** | **-0.0152** | **1** | **62** | **3e-005** | **1** | **K.TAFDEAIAELDTLNEESYKDSTLIMQLLR.D** |

| **639.** | Q96I99\|SUCB2_HUMAN    **Mass:** 46824    **Score:** 61     **Queries matched:** 19   **emPAI:** 0.08 |
| --- | --- |
|  | Succinyl-CoA ligase [GDP-forming] subunit beta, mitochondrial OS=Homo sapiens GN=SUCLG2 PE=1 SV=2 |

|  | **Query** | **Observed** | **Mr(expt)** | **Mr(calc)** | **Delta** | **Miss** | **Score** | **Expect** | **Rank** | **Peptide** |
| --- | --- | --- | --- | --- | --- | --- | --- | --- | --- | --- |
|  | 1271 | 601.3879 | 600.3807 | 600.3846 | -0.0040 | 0 | 19 | 3.5 | 7 | K.EIVLK.A |
|  | 1425 | **645.3883** | **644.3810** | **644.3857** | **-0.0047** | **0** | **21** | **2.3** | **1** | **K.AVASVAK.K** |
|  | 2227 | 411.2446 | 820.4746 | 820.4443 | 0.0303 | 0 | 16 | 4.2 | 1 | K.GVFNSGLK.G |
|  | 3995 | **477.7174** | **953.4203** | **953.4164** | **0.0039** | **0** | **8** | **23** | **6** | **K.DIFAMDDK.S** |
|  | 4899 | 509.7573 | 1017.5001 | 1017.5277 | -0.0276 | 1 | 3 | 93 | 10 | K.ACRELELK.V |
|  | 5194 | **521.2349** | **1040.4552** | **1039.5662** | **0.8890** | **0** | **7** | **28** | **1** | **K.DPNVVGQLAK.Q** |
|  | 7587 | **605.3064** | **1208.5982** | **1209.5700** | **-0.9717** | **1** | **2** | **83** | **3** | **R.QKDIFAMDDK.S** |
|  | 8224 | **621.0601** | **1240.1057** | **1239.5520** | **0.5537** | **0** | **1** | **73** | **6** | **K.INFDDNAEFR.Q** |
|  | 8867 | **425.7217** | **1274.1431** | **1274.6693** | **-0.5262** | **0** | **4** | **40** | **6** | **R.MAENLGFVGPLK.S** |
|  | 11369 | **471.2470** | **1410.7192** | **1411.7969** | **-1.0777** | **1** | **5** | **40** | **2** | **-.MASPVAAQAGKLLR.A** |
|  | 13774 | 521.2654 | 1560.7745 | 1559.8341 | 0.9404 | 1 | 4 | 44 | 5 | K.VNKVMVAEALDISR.E |
|  | 19563 | 660.3462 | 1978.0167 | 1979.0112 | -0.9945 | 1 | 6 | 24 | 1 | R.VQRFFVADTANEALEAAK.R |
|  | 20447 | 1028.0455 | 2054.0765 | 2054.0895 | -0.0130 | 0 | 52 | 0.00055 | 1 | K.ILNNSGLPITSAIDLEDAAK.K 20448 |
|  | 21685 | **728.7315** | **2183.1725** | **2182.1845** | **0.9881** | **1** | **15** | **2.6** | **1** | **K.ILNNSGLPITSAIDLEDAAKK.A** |
|  | 22092 | 742.7077 | 2225.1013 | 2225.2756 | -0.1744 | 1 | 8 | 14 | 3 | R.ALALRPRFLAAGSQAVQLTSR.R 22110 |
|  | 22408 | **756.4030** | **2266.1872** | **2265.0107** | **1.1766** | **1** | **6** | **19** | **1** | **K.DIFAMDDKSENEPIENEAAK.Y** |
|  | 22540 | **761.3988** | **2281.1746** | **2281.0056** | **0.1690** | **1** | **(2)** | **56** | **3** | **K.DIFAMDDKSENEPIENEAAK.Y** |

| **640.** | O75955\|FLOT1_HUMAN    **Mass:** 47554    **Score:** 61     **Queries matched:** 12   **emPAI:** 0.08 |
| --- | --- |
|  | Flotillin-1 OS=Homo sapiens GN=FLOT1 PE=1 SV=3 |

|  | **Query** | **Observed** | **Mr(expt)** | **Mr(calc)** | **Delta** | **Miss** | **Score** | **Expect** | **Rank** | **Peptide** |
| --- | --- | --- | --- | --- | --- | --- | --- | --- | --- | --- |
|  | 994 | 532.2831 | 531.2759 | 530.2813 | 0.9946 | 0 | 12 | 24 | 5 | R.DAGIR.E 993 |
|  | 2898 | 436.2325 | 870.4505 | 871.4875 | -1.0370 | 1 | 21 | 1.2 | 2 | K.ELEARVR.K |
|  | 3167 | **446.9060** | **891.7975** | **892.3960** | **-0.5985** | **0** | **6** | **29** | **6** | **R.AEAEQMAK.K** 3183 |
|  | 6706 | **579.8215** | **1157.6285** | **1158.5993** | **-0.9708** | **1** | **8** | **25** | **1** | **K.TKQQIEEQR.V** 6702 6703 |
|  | 9670 | **440.2358** | **1317.6856** | **1317.6789** | **0.0067** | **1** | **3** | **65** | **9** | **R.GEAEAFAIGARAR.A** |
|  | 10781 | **460.3097** | **1377.9072** | **1377.6823** | **0.2250** | **1** | **8** | **20** | **3** | **R.MRGEAEAFAIGAR.A** |
|  | 12327 | **735.3831** | **1468.7517** | **1468.7634** | **-0.0117** | **0** | **61** | **0.00011** | **1** | **R.AQQVAVQEQEIAR.R** |
|  | 14648 | 540.6003 | 1618.7792 | 1618.7984 | -0.0192 | 0 | 0 | 1.1e+002 | 6 | K.SQLIMQAEAEAASVR.M |

| **641.** | O15258\|RER1_HUMAN    **Mass:** 23057    **Score:** 61     **Queries matched:** 5   **emPAI:** 0.17 |
| --- | --- |
|  | Protein RER1 OS=Homo sapiens GN=RER1 PE=1 SV=1 |

|  | **Query** | **Observed** | **Mr(expt)** | **Mr(calc)** | **Delta** | **Miss** | **Score** | **Expect** | **Rank** | **Peptide** |
| --- | --- | --- | --- | --- | --- | --- | --- | --- | --- | --- |
|  | 10288 | **676.2972** | **1350.5798** | **1349.6979** | **0.8819** | **0** | **6** | **34** | **1** | **R.LGQIYQSWLDK.S** |
|  | 18676 | **951.9289** | **1901.8432** | **1901.8564** | **-0.0132** | **0** | **61** | **8.3e-005** | **1** | **K.VDPSLMEDSDDGPSLPTK.Q** 18675 |
|  | 21270 | **428.2178** | **2136.0528** | **2134.9953** | **1.0574** | **0** | **5** | **29** | **6** | **-.MSEGDSVGESVHGKPSVVYR.F** |
|  | 30784 | 1461.3203 | 4380.9391 | 4381.1637 | -0.2245 | 0 | 5 | 5.3 | 1 | K.GILVAMVCTFFDAFNVPVFWPILVMYFIMLFCITMK.R |

| **642.** | Q05639\|EF1A2_HUMAN    **Mass:** 50780    **Score:** 61     **Queries matched:** 16   **emPAI:** 0.24 |
| --- | --- |
|  | Elongation factor 1-alpha 2 OS=Homo sapiens GN=EEF1A2 PE=1 SV=1 |

|  | **Query** | **Observed** | **Mr(expt)** | **Mr(calc)** | **Delta** | **Miss** | **Score** | **Expect** | **Rank** | **Peptide** |
| --- | --- | --- | --- | --- | --- | --- | --- | --- | --- | --- |
|  | 779 | 476.1964 | 475.1892 | 475.2391 | -0.0499 | 0 | 5 | 87 | 3 | K.SGGAGK.V |
|  | 2881 | 435.6920 | 869.3694 | 869.5334 | -0.1640 | 0 | 14 | 6.1 | 6 | K.QLIVGVNK.M 2896 2903 |
|  | 4983 | 513.3048 | 1024.5951 | 1024.6030 | -0.0079 | 0 | 52 | 0.001 | 1 | K.IGGIGTVPVGR.V 4984 |
|  | 6657 | 577.7952 | 1153.5759 | 1154.5277 | -0.9518 | 1 | 4 | 54 | 1 | K.FEKEAAEMGK.G |
|  | 6852 | 585.3680 | 1168.7214 | 1169.5387 | -0.8172 | 1 | 3 | 69 | 8 | K.EAAEMGKGSFK.Y |
|  | 8852 | **637.8256** | **1273.6366** | **1272.5180** | **1.1186** | **0** | **3** | **80** | **1** | **K.MDSTEPAYSEK.R** |
|  | 9596 | 657.8701 | 1313.7257 | 1313.7343 | -0.0086 | 0 | 26 | 0.32 | 1 | R.EHALLAYTLGVK.Q |
|  | 11653 | 714.8445 | 1427.6745 | 1428.6191 | -0.9446 | 1 | 10 | 14 | 5 | K.MDSTEPAYSEKR.Y |
|  | 14186 | 795.3166 | 1588.6187 | 1587.8733 | 0.7454 | 0 | 23 | 0.62 | 2 | K.THINIVVIGHVDSGK.S 14184 14187 |
|  | 21023 | **703.8881** | **2108.6425** | **2108.0459** | **0.5966** | **1** | **1** | **53** | **4** | **K.QLIVGVNKMDSTEPAYSEK.R** |
|  | 31340 | 1262.8516 | 5047.3771 | 5047.5787 | -0.2016 | 1 | 2 | 7.9 | 3 | R.VETGILRPGMVVTFAPVNITTEVKSVEMHHEALSEALPGDNVGFNVK.N |

| **643.** | P05109\|S10A8_HUMAN    **Mass:** 10885    **Score:** 60     **Queries matched:** 3   **emPAI:** 0.37 |
| --- | --- |
|  | Protein S100-A8 OS=Homo sapiens GN=S100A8 PE=1 SV=1 |

|  | **Query** | **Observed** | **Mr(expt)** | **Mr(calc)** | **Delta** | **Miss** | **Score** | **Expect** | **Rank** | **Peptide** |
| --- | --- | --- | --- | --- | --- | --- | --- | --- | --- | --- |
|  | 11548 | **711.3538** | **1420.6930** | **1420.7020** | **-0.0091** | **0** | **60** | **0.00011** | **1** | **K.LLETECPQYIR.K** |
|  | 13582 | 775.3506 | 1548.6866 | 1548.7970 | -0.1104 | 1 | 4 | 45 | 7 | K.KLLETECPQYIR.K |
|  | 13876 | 784.8833 | 1567.7520 | 1566.8307 | 0.9214 | 1 | 2 | 87 | 5 | K.YSLIKGNFHAVYR.D |

| **644.** | Q02978\|M2OM_HUMAN    **Mass:** 34211    **Score:** 60     **Queries matched:** 17   **emPAI:** 0.11 |
| --- | --- |
|  | Mitochondrial 2-oxoglutarate/malate carrier protein OS=Homo sapiens GN=SLC25A11 PE=1 SV=3 |

|  | **Query** | **Observed** | **Mr(expt)** | **Mr(calc)** | **Delta** | **Miss** | **Score** | **Expect** | **Rank** | **Peptide** |
| --- | --- | --- | --- | --- | --- | --- | --- | --- | --- | --- |
|  | 3533 | **460.7256** | **919.4367** | **919.4433** | **-0.0066** | **0** | **22** | **1.2** | **1** | **R.MQLSGEGAK.T** |
|  | 3538 | 460.7567 | 919.4988 | 920.4208 | -0.9220 | 0 | 9 | 25 | 4 | R.GCIPTMAR.A |
|  | 3801 | 471.2708 | 940.5269 | 940.4614 | 0.0655 | 0 | 14 | 6.6 | 3 | R.QATYTTTR.L |
|  | 4265 | **487.7367** | **973.4588** | **973.4658** | **-0.0069** | **0** | **12** | **11** | **10** | **K.GFTPYYAR.L** |
|  | 6949 | **589.7624** | **1177.5102** | **1176.5921** | **0.9181** | **1** | **6** | **36** | **4** | **R.MQLSGEGAKTR.E** |
|  | 8589 | **630.3777** | **1258.7409** | **1258.7398** | **0.0012** | **1** | **11** | **11** | **3** | **K.NGLDVLFKVVR.Y** |
|  | 9944 | 444.5535 | 1330.6388 | 1329.6459 | 0.9928 | 1 | (17) | 2.7 | 2 | R.MTADGRLPADQR.R 9927 |
|  | 10175 | 673.3218 | 1344.6291 | 1345.6408 | -1.0117 | 1 | 18 | 2.6 | 2 | R.MTADGRLPADQR.R |
|  | 10913 | 693.3755 | 1384.7364 | 1385.7555 | -1.0191 | 0 | 16 | 3.2 | 2 | R.LTGADGTPPGFLLK.A |
|  | 11172 | 700.3874 | 1398.7603 | 1398.7215 | 0.0388 | 0 | 5 | 45 | 7 | M.AATASAGAGGIDGKPR.T 11183 |
|  | 12377 | **491.6091** | **1471.8056** | **1471.8147** | **-0.0091** | **1** | **7** | **25** | **2** | **R.ITREEGVLTLWR.G** |
|  | 13558 | **774.2408** | **1546.4671** | **1545.7569** | **0.7102** | **0** | **1** | **68** | **3** | **-.MAATASAGAGGIDGKPR.T** |
|  | 16613 | 873.9324 | 1745.8503 | 1745.9788 | -0.1285 | 1 | 1 | 94 | 7 | K.AEGLRGIYTGLSAGLLR.Q |
|  | 22523 | **1140.6217** | **2279.2288** | **2279.2388** | **-0.0100** | **0** | **60** | **6.8e-005** | **1** | **K.FLFGGLAGMGATVFVQPLDLVK.N** 22507 |

| **645.** | P04899\|GNAI2_HUMAN    **Mass:** 40995    **Score:** 60     **Queries matched:** 16   **emPAI:** 0.19 |
| --- | --- |
|  | Guanine nucleotide-binding protein G(i), alpha-2 subunit OS=Homo sapiens GN=GNAI2 PE=1 SV=3 |

|  | **Query** | **Observed** | **Mr(expt)** | **Mr(calc)** | **Delta** | **Miss** | **Score** | **Expect** | **Rank** | **Peptide** |
| --- | --- | --- | --- | --- | --- | --- | --- | --- | --- | --- |
|  | 2953 | 438.6146 | 875.2147 | 874.4144 | 0.8003 | 1 | 6 | 37 | 4 | R.EDGEKAAR.E |
|  | 3383 | **455.2129** | **908.4113** | **908.4175** | **-0.0062** | **0** | **16** | **3.9** | **1** | **K.MFDVGGQR.S** |
|  | 5380 | **529.2714** | **1056.5283** | **1056.6179** | **-0.0896** | **0** | **4** | **70** | **1** | **K.LLLLGAGESGK.S** |
|  | 6082 | **555.7621** | **1109.5096** | **1109.5175** | **-0.0079** | **0** | **36** | **0.038** | **1** | **K.LFDSICNNK.W** |
|  | 8872 | 638.2999 | 1274.5852 | 1273.5826 | 1.0026 | 0 | 19 | 2 | 2 | K.YDEAASYIQSK.F |
|  | 13370 | 512.2930 | 1533.8573 | 1533.7246 | 0.1327 | 0 | 8 | 17 | 2 | K.AMGNLQIDFADPSR.A |
|  | 13612 | **517.7648** | **1550.2727** | **1549.7195** | **0.5532** | **0** | **(3)** | **42** | **8** | **K.AMGNLQIDFADPSR.A** |
|  | 14156 | 793.9229 | 1585.8313 | 1585.7083 | 0.1230 | 0 | 2 | 87 | 8 | K.EIYTHFTCATDTK.N |
|  | 16495 | 868.9326 | 1735.8506 | 1735.9542 | -0.1037 | 0 | 4 | 48 | 3 | R.AVVYSNTIQSIMAIVK.A 16497 |
|  | 16615 | **873.9488** | **1745.8831** | **1745.8948** | **-0.0117** | **0** | **51** | **0.00086** | **1** | **R.IAQSDYIPTQQDVLR.T** |
|  | 18462 | **629.2999** | **1884.8780** | **1884.8168** | **0.0611** | **1** | **3** | **48** | **1** | **R.MHESMKLFDSICNNK.W** |
|  | 21801 | **550.7955** | **2199.1530** | **2199.1721** | **-0.0191** | **1** | **5** | **28** | **3** | **R.QYRAVVYSNTIQSIMAIVK.A** |
|  | 22834 | **774.0281** | **2319.0624** | **2319.0767** | **-0.0143** | **1** | **16** | **1.9** | **1** | **R.SREYQLNDSAAYYLNDLER.I** |
|  | 28216 | **826.8944** | **3303.5485** | **3303.5758** | **-0.0272** | **1** | **14** | **1.5** | **1** | **K.ITHSPLTICFPEYTGANKYDEAASYIQSK.F** |
|  | 30944 | **906.8626** | **4529.2767** | **4530.0826** | **-0.8060** | **1** | **0** | **18** | **6** | **K.WIHCFEGVTAIIFCVALSAYDLVLAEDEEMNRMHESMK.L** |

| **646.** | P61604\|CH10_HUMAN    **Mass:** 10925    **Score:** 60     **Queries matched:** 3   **emPAI:** 0.37 |
| --- | --- |
|  | 10 kDa heat shock protein, mitochondrial OS=Homo sapiens GN=HSPE1 PE=1 SV=2 |

|  | **Query** | **Observed** | **Mr(expt)** | **Mr(calc)** | **Delta** | **Miss** | **Score** | **Expect** | **Rank** | **Peptide** |
| --- | --- | --- | --- | --- | --- | --- | --- | --- | --- | --- |
|  | 5632 | 538.7996 | 1075.5847 | 1075.5914 | -0.0067 | 0 | 15 | 5.4 | 2 | K.VLLPEYGGTK.V |
|  | 13301 | 765.3982 | 1528.7819 | 1528.7926 | -0.0106 | 1 | 60 | 0.00013 | 1 | K.VVLDDKDYFLFR.D 13302 |

| **647.** | P60468\|SC61B_HUMAN    **Mass:** 10025    **Score:** 60     **Queries matched:** 8   **emPAI:** 0.97 |
| --- | --- |
|  | Protein transport protein Sec61 subunit beta OS=Homo sapiens GN=SEC61B PE=1 SV=2 |

|  | **Query** | **Observed** | **Mr(expt)** | **Mr(calc)** | **Delta** | **Miss** | **Score** | **Expect** | **Rank** | **Peptide** |
| --- | --- | --- | --- | --- | --- | --- | --- | --- | --- | --- |
|  | 6250 | **562.7573** | **1123.5001** | **1123.5081** | **-0.0080** | **0** | **46** | **0.0036** | **1** | **R.TTSAGTGGMWR.F** 6251 6252 |
|  | 6472 | **571.2563** | **1140.4980** | **1139.5030** | **0.9951** | **0** | **(24)** | **0.57** | **1** | **R.TTSAGTGGMWR.F** |
|  | 10648 | 685.8314 | 1369.6483 | 1369.6586 | -0.0103 | 0 | 31 | 0.11 | 1 | M.PGPTPSGTNVGSSGR.S 10649 |
|  | 13110 | **506.8806** | **1517.6200** | **1516.6940** | **0.9260** | **0** | **0** | **1.1e+002** | **7** | **-.MPGPTPSGTNVGSSGR.S** |
|  | 22493 | 760.3937 | 2278.1592 | 2277.0372 | 1.1220 | 1 | 4 | 29 | 2 | R.TTSAGTGGMWRFYTEDSPGLK.V |

| **648.** | P26038\|MOES_HUMAN    **Mass:** 67892    **Score:** 60     **Queries matched:** 21   **emPAI:** 0.17 |
| --- | --- |
|  | Moesin OS=Homo sapiens GN=MSN PE=1 SV=3 |

|  | **Query** | **Observed** | **Mr(expt)** | **Mr(calc)** | **Delta** | **Miss** | **Score** | **Expect** | **Rank** | **Peptide** |
| --- | --- | --- | --- | --- | --- | --- | --- | --- | --- | --- |
|  | 3116 | 444.2788 | 886.5430 | 886.4872 | 0.0558 | 0 | 8 | 29 | 2 | K.EALLQASR.D |
|  | 3484 | 458.7509 | 915.4871 | 915.5138 | -0.0266 | 1 | 9 | 23 | 8 | K.KVTAQDVR.K |
|  | 3689 | **466.7586** | **931.5027** | **930.5321** | **0.9707** | **1** | **14** | **8.2** | **2** | **-.MPKTISVR.V** |
|  | 4314 | **489.2655** | **976.5164** | **977.4851** | **-0.9687** | **1** | **10** | **19** | **7** | **R.AMLENEKK.K** |
|  | 6174 | **559.3156** | **1116.6167** | **1115.6186** | **0.9981** | **1** | **22** | **1** | **1** | **R.LKQIEEQTK.K** |
|  | 7196 | 595.7852 | 1189.5559 | 1189.5649 | -0.0090 | 0 | 18 | 2.5 | 2 | K.AQMVQEDLEK.T 7212 |
|  | 8061 | 617.2866 | 1232.5586 | 1232.5673 | -0.0087 | 0 | 4 | 57 | 3 | K.ESEAVEWQQK.A |
|  | 8068 | **411.8849** | **1232.6328** | **1232.6050** | **0.0278** | **0** | **1** | **1.2e+002** | **9** | **R.IQVWHEEHR.G** |
|  | 8128 | **618.7987** | **1235.5829** | **1235.5935** | **-0.0106** | **1** | **23** | **0.71** | **1** | **K.YGDFNKEVHK.S** |
|  | 9399 | **435.2670** | **1302.7791** | **1302.6568** | **0.1223** | **1** | **4** | **54** | **6** | **K.EVHKSGYLAGDK.L** |
|  | 9490 | 654.8177 | 1307.6208 | 1307.5841 | 0.0368 | 1 | 10 | 13 | 3 | R.SEEERTTEAEK.N |
|  | 10949 | **463.2325** | **1386.6757** | **1386.6851** | **-0.0094** | **1** | **7** | **29** | **2** | **K.AQQELEEQTRR.A** |
|  | 11750 | **717.8565** | **1433.6985** | **1433.6027** | **0.0957** | **0** | **4** | **49** | **1** | **K.TANDMIHAENMR.L** |
|  | 12375 | **736.8928** | **1471.7710** | **1471.7817** | **-0.0107** | **1** | **47** | **0.0029** | **1** | **R.RKPDTIEVQQMK.A** 12376 |
|  | 13391 | **512.9383** | **1535.7930** | **1535.8017** | **-0.0087** | **1** | **41** | **0.0095** | **1** | **R.GMLREDAVLEYLK.I** |
|  | 16384 | 577.3178 | 1728.9316 | 1727.8195 | 1.1121 | 1 | 4 | 47 | 7 | K.TANDMIHAENMRLGR.D |
|  | 17736 | 916.4537 | 1830.8929 | 1830.9509 | -0.0580 | 1 | 14 | 4.3 | 2 | K.KTQEQLALEMAELTAR.I |
|  | 23149 | **1181.5272** | **2361.0399** | **2361.1601** | **-0.1202** | **0** | **11** | **6** | **1** | **K.GSELWLGVDALGLNIYEQNDR.L** |
|  | 29445 | 939.4576 | 3753.8015 | 3752.6707 | 1.1308 | 1 | 4 | 12 | 2 | R.AELKTAMSTPHVAEPAENEQDEQDENGAEASADLR.A |

| **649.** | P46776\|RL27A_HUMAN    **Mass:** 16665    **Score:** 59     **Queries matched:** 7   **emPAI:** 0.87 |
| --- | --- |
|  | 60S ribosomal protein L27a OS=Homo sapiens GN=RPL27A PE=1 SV=2 |

|  | **Query** | **Observed** | **Mr(expt)** | **Mr(calc)** | **Delta** | **Miss** | **Score** | **Expect** | **Rank** | **Peptide** |
| --- | --- | --- | --- | --- | --- | --- | --- | --- | --- | --- |
|  | 6096 | 556.3235 | 1110.6325 | 1110.6397 | -0.0072 | 0 | 3 | 65 | 8 | K.TGAAPIIDVVR.S |
|  | 8053 | **616.8312** | **1231.6478** | **1231.6561** | **-0.0083** | **0** | **46** | **0.0042** | **1** | **K.LWTLVSEQTR.V** 8038 |
|  | 10350 | 677.8143 | 1353.6140 | 1352.7776 | 0.8364 | 1 | 21 | 1.1 | 2 | K.NKTGAAPIIDVVR.S 10351 |
|  | 11564 | **711.8330** | **1421.6515** | **1421.6609** | **-0.0095** | **0** | **44** | **0.0049** | **1** | **R.NQSFCPTVNLDK.L** |
|  | 24988 | **879.4388** | **2635.2945** | **2635.3064** | **-0.0119** | **1** | **18** | **0.99** | **1** | **R.NQSFCPTVNLDKLWTLVSEQTR.V** |

| **650.** | Q9UKU0\|ACSL6_HUMAN    **Mass:** 78728    **Score:** 59     **Queries matched:** 20   **emPAI:** 0.05 |
| --- | --- |
|  | Long-chain-fatty-acid--CoA ligase 6 OS=Homo sapiens GN=ACSL6 PE=2 SV=4 |

|  | **Query** | **Observed** | **Mr(expt)** | **Mr(calc)** | **Delta** | **Miss** | **Score** | **Expect** | **Rank** | **Peptide** |
| --- | --- | --- | --- | --- | --- | --- | --- | --- | --- | --- |
|  | 1049 | 546.2808 | 545.2735 | 544.3333 | 0.9403 | 0 | 11 | 32 | 1 | R.SGIIR.N |
|  | 1623 | 715.4052 | 714.3979 | 713.3748 | 1.0231 | 1 | 13 | 9.4 | 1 | R.EYFKK.Q |
|  | 2212 | **411.0970** | **820.1795** | **820.4000** | **-0.2206** | **0** | **1** | **1.1e+002** | **6** | **R.LLSDDMK.A** |
|  | 2869 | 435.2664 | 868.5182 | 868.5242 | -0.0061 | 1 | 13 | 7.7 | 3 | K.AKRPELR.E 2870 |
|  | 3527 | **460.2926** | **918.5706** | **919.5127** | **-0.9421** | **0** | **4** | **67** | **1** | **K.IENIYIR.S** |
|  | 3858 | 473.2564 | 944.4982 | 943.4586 | 1.0396 | 0 | 11 | 17 | 3 | R.TMYQVFR.R |
|  | 5156 | 519.7684 | 1037.5222 | 1037.5294 | -0.0073 | 0 | 48 | 0.0022 | 1 | R.VGFFQGDIR.L 5157 |
|  | 5800 | 545.3002 | 1088.5858 | 1087.6390 | 0.9468 | 1 | 17 | 3.9 | 7 | K.VTEKVIFPR.Q 5782 |
|  | 6161 | **559.0963** | **1116.1781** | **1115.5546** | **0.6235** | **1** | **1** | **85** | **9** | **R.TMYQVFRR.G** 6162 |
|  | 6369 | 567.2803 | 1132.5461 | 1132.6393 | -0.0932 | 1 | 10 | 17 | 4 | K.RWLLEFAAK.R |
|  | 8582 | **630.3417** | **1258.6689** | **1259.6146** | **-0.9457** | **0** | **2** | **86** | **9** | **K.ESGLHSFEQVK.A** 8631 |
|  | 13549 | **516.2705** | **1545.7897** | **1546.8316** | **-1.0419** | **0** | **1** | **93** | **7** | **K.LIILMDPFEEALK.E** |
|  | 21441 | **719.6494** | **2155.9264** | **2156.1802** | **-0.2538** | **1** | **4** | **36** | **1** | **K.ETPGLKLIILMDPFEEALK.E** |
|  | 29681 | 1274.3230 | 3819.9472 | 3820.9862 | -1.0391 | 1 | 2 | 21 | 5 | R.SQPVAQIYVHGDSLKAFLVGIVVPDPEVMPSWAQK.R 29680 |

| **651.** | P11310\|ACADM_HUMAN    **Mass:** 47015    **Score:** 59     **Queries matched:** 15   **emPAI:** 0.17 |
| --- | --- |
|  | Medium-chain specific acyl-CoA dehydrogenase, mitochondrial OS=Homo sapiens GN=ACADM PE=1 SV=1 |

|  | **Query** | **Observed** | **Mr(expt)** | **Mr(calc)** | **Delta** | **Miss** | **Score** | **Expect** | **Rank** | **Peptide** |
| --- | --- | --- | --- | --- | --- | --- | --- | --- | --- | --- |
|  | 2770 | **432.2535** | **862.4925** | **862.3967** | **0.0958** | **0** | **14** | **7.3** | **5** | **K.ELNMGQR.C** |
|  | 8753 | **635.1327** | **1268.2509** | **1267.6343** | **0.6167** | **1** | **2** | **55** | **4** | **K.VELARMSYQR.A** |
|  | 12251 | 733.4284 | 1464.8423 | 1464.8525 | -0.0102 | 0 | 40 | 0.011 | 1 | K.TRPVVAAGAVGLAQR.A 12259 |
|  | 12548 | **741.7836** | **1481.5526** | **1481.6351** | **-0.0825** | **1** | **5** | **31** | **3** | **K.ELNMGQRCSDTR.G** |
|  | 14351 | **800.4085** | **1598.8023** | **1597.8100** | **0.9923** | **0** | **44** | **0.0044** | **1** | **K.IYQIYEGTSQIQR.L** 14355 |
|  | 20096 | 675.0041 | 2021.9906 | 2022.9833 | -0.9927 | 1 | 10 | 8.9 | 2 | K.GDEYIINGQKMWITNGGK.A 20094 20112 |
|  | 20258 | 681.0363 | 2040.0869 | 2038.9782 | 1.1087 | 1 | (8) | 13 | 6 | K.GDEYIINGQKMWITNGGK.A |
|  | 21038 | 704.3099 | 2109.9080 | 2109.0308 | 0.8772 | 0 | 1 | 69 | 9 | K.LLVEHQAISFMLAEMAMK.V |
|  | 25106 | 888.0638 | 2661.1695 | 2661.3692 | -0.1997 | 1 | 3 | 31 | 5 | K.LLVEHQAISFMLAEMAMKVELAR.M |
|  | 29175 | 1213.9503 | 3638.8291 | 3638.8039 | 0.0253 | 1 | 8 | 6.4 | 5 | K.AFAGDIANQLATDAVQILGGNGFNTEYPVEKLMR.D 29177 |

| **652.** | P02100\|HBE_HUMAN    **Mass:** 16249    **Score:** 59     **Queries matched:** 5   **emPAI:** 0.24 |
| --- | --- |
|  | Hemoglobin subunit epsilon OS=Homo sapiens GN=HBE1 PE=1 SV=2 |

|  | **Query** | **Observed** | **Mr(expt)** | **Mr(calc)** | **Delta** | **Miss** | **Score** | **Expect** | **Rank** | **Peptide** |
| --- | --- | --- | --- | --- | --- | --- | --- | --- | --- | --- |
|  | 5304 | 525.7828 | 1049.5511 | 1049.5757 | -0.0246 | 0 | 7 | 34 | 10 | K.VLTSFGDAIK.N |
|  | 8859 | 637.8631 | 1273.7116 | 1273.7183 | -0.0066 | 0 | 56 | 0.00035 | 1 | R.LLVVYPWTQR.F 8858 8877 |
|  | 9948 | 666.3349 | 1330.6552 | 1331.6139 | -0.9587 | 0 | 5 | 46 | 10 | K.MNVEEAGGEALGR.L |

| **653.** | P69891\|HBG1_HUMAN    **Mass:** 16187    **Score:** 59     **Queries matched:** 6   **emPAI:** 0.24 |
| --- | --- |
|  | Hemoglobin subunit gamma-1 OS=Homo sapiens GN=HBG1 PE=1 SV=2 |

|  | **Query** | **Observed** | **Mr(expt)** | **Mr(calc)** | **Delta** | **Miss** | **Score** | **Expect** | **Rank** | **Peptide** |
| --- | --- | --- | --- | --- | --- | --- | --- | --- | --- | --- |
|  | 7244 | 596.9735 | 1191.9325 | 1191.6281 | 0.3043 | 0 | 3 | 53 | 10 | K.MVTAVASALSSR.Y |
|  | 8859 | 637.8631 | 1273.7116 | 1273.7183 | -0.0066 | 0 | 56 | 0.00035 | 1 | R.LLVVYPWTQR.F 8858 8877 |
|  | 22447 | **758.8746** | **2273.6021** | **2273.1652** | **0.4369** | **1** | **1** | **48** | **8** | **K.ATITSLWGKVNVEDAGGETLGR.L** |
|  | 27614 | 625.7190 | 3123.5586 | 3123.7121 | -0.1535 | 1 | 2 | 28 | 3 | K.LLGNVLVTVLAIHFGKEFTPEVQASWQK.M |

|  | **Proteins matching a subset of these peptides:** |
| --- | --- |

|  | P69892\|HBG2_HUMAN    **Mass:** 16173    **Score:** 59     **Queries matched:** 5 |
| --- | --- |
|  | Hemoglobin subunit gamma-2 OS=Homo sapiens GN=HBG2 PE=1 SV=2 |

| **654.** | P49748\|ACADV_HUMAN    **Mass:** 70745    **Score:** 59     **Queries matched:** 34   **emPAI:** 0.17 |
| --- | --- |
|  | Very long-chain specific acyl-CoA dehydrogenase, mitochondrial OS=Homo sapiens GN=ACADVL PE=1 SV=1 |

|  | **Query** | **Observed** | **Mr(expt)** | **Mr(calc)** | **Delta** | **Miss** | **Score** | **Expect** | **Rank** | **Peptide** |
| --- | --- | --- | --- | --- | --- | --- | --- | --- | --- | --- |
|  | 1182 | 577.3180 | 576.3108 | 575.3465 | 0.9643 | 1 | 6 | 56 | 2 | K.KMGIK.A |
|  | 2672 | **429.2557** | **856.4967** | **857.4970** | **-1.0003** | **1** | **1** | **1.5e+002** | **5** | **K.KPAKAESK.S** |
|  | 4696 | 502.2646 | 1002.5147 | 1001.5869 | 0.9278 | 1 | 10 | 16 | 6 | K.SISKALVER.G 4662 4664 4667 4690 |
|  | 6326 | **565.7844** | **1129.5543** | **1130.5978** | **-1.0435** | **1** | **1** | **1.2e+002** | **5** | **M.QAARMAASLGR.Q** |
|  | 6679 | 578.8097 | 1155.6048 | 1155.6136 | -0.0087 | 0 | 11 | 11 | 1 | K.TPVTDPATGAVK.E 6688 |
|  | 8942 | 427.2242 | 1278.6508 | 1277.6332 | 1.0176 | 1 | 8 | 22 | 8 | -.MQAARMAASLGR.Q 8903 |
|  | 9197 | 647.3776 | 1292.7407 | 1293.6281 | -0.8875 | 1 | (7) | 30 | 3 | -.MQAARMAASLGR.Q 9237 |
|  | 9350 | **434.5829** | **1300.7270** | **1300.6485** | **0.0785** | **1** | **11** | **9.8** | **2** | **K.AESKSFAVGMFK.G** |
|  | 9607 | **658.3292** | **1314.6438** | **1315.7248** | **-1.0811** | **0** | **4** | **61** | **8** | **K.GIVNEQFLLQR.L** |
|  | 12130 | **729.8875** | **1457.7605** | **1456.7674** | **0.9931** | **0** | **39** | **0.016** | **1** | **K.NPFGNAGLLLGEAGK.Q** 12099 |
|  | 12268 | **733.8986** | **1465.7826** | **1465.7421** | **0.0404** | **1** | **7** | **23** | **6** | **R.LFVALQGCMDKGK.E** |
|  | 12548 | 741.7836 | 1481.5526 | 1481.7371 | -0.1845 | 1 | (4) | 37 | 4 | R.LFVALQGCMDKGK.E |
|  | 13114 | **759.8881** | **1517.7616** | **1517.7726** | **-0.0110** | **0** | **13** | **6.5** | **1** | **R.VPSENVLGEVGSGFK.V** |
|  | 15279 | **553.9428** | **1658.8066** | **1658.9607** | **-0.1541** | **1** | **6** | **31** | **5** | **R.ALEQFATVVEAKLIK.H** |
|  | 18006 | **926.5008** | **1850.9870** | **1850.9931** | **-0.0060** | **0** | **8** | **17** | **1** | **K.LWISNGGLADIFTVFAK.T** |
|  | 21121 | 1060.0686 | 2118.1226 | 2119.1459 | -1.0233 | 1 | 2 | 59 | 8 | R.LADGAIDLYAMVVVLSRASR.S |
|  | 22805 | **773.0399** | **2316.0979** | **2317.1606** | **-1.0626** | **1** | **2** | **47** | **1** | **K.VAMHILNNGRFGMAAALAGTMR.G** 22812 |
|  | 23841 | **614.5966** | **2454.3571** | **2455.2240** | **-0.8668** | **1** | **2** | **36** | **8** | **R.IFEGTNDILRLFVALQGCMDK.G** |
|  | 25766 | 695.3771 | 2777.4792 | 2777.4428 | 0.0364 | 1 | 1 | 50 | 10 | K.YYTLNGSKLWISNGGLADIFTVFAK.T |
|  | 25785 | **1391.6913** | **2781.3680** | **2781.3861** | **-0.0181** | **0** | **42** | **0.0045** | **1** | **K.GQLTTDQVFPYPSVLNEEQTQFLK.E** |
|  | 25937 | **702.0927** | **2804.3415** | **2805.2998** | **-0.9583** | **1** | **2** | **35** | **7** | **K.IFGSEAAWKVTDECIQIMGGMGFMK.E** |
|  | 27263 | **1014.1407** | **3039.4002** | **3039.4171** | **-0.0169** | **1** | **18** | **0.89** | **1** | **R.FFEEVNDPAKNDALEMVEETTWQGLK.E** |
|  | 29568 | **1263.9788** | **3788.9145** | **3788.7115** | **0.2029** | **0** | **1** | **27** | **7** | **R.MVMLQYVTESMAYMVSANMDQGATDFQIEAAISK.I** |
|  | 29676 | 1274.3215 | 3819.9428 | 3820.7014 | -0.7586 | 0 | (0) | 31 | 9 | R.MVMLQYVTESMAYMVSANMDQGATDFQIEAAISK.I |
|  | 29709 | **956.4813** | **3821.8962** | **3820.7014** | **1.1948** | **0** | **(1)** | **25** | **4** | **R.MVMLQYVTESMAYMVSANMDQGATDFQIEAAISK.I** |

| **655.** | P02730\|B3AT_HUMAN    **Mass:** 102013   **Score:** 59     **Queries matched:** 18   **emPAI:** 0.07 |
| --- | --- |
|  | Band 3 anion transport protein OS=Homo sapiens GN=SLC4A1 PE=1 SV=3 |

|  | **Query** | **Observed** | **Mr(expt)** | **Mr(calc)** | **Delta** | **Miss** | **Score** | **Expect** | **Rank** | **Peptide** |
| --- | --- | --- | --- | --- | --- | --- | --- | --- | --- | --- |
|  | 3919 | **474.9767** | **947.9389** | **948.4698** | **-0.5309** | **0** | **2** | **76** | **5** | **R.AAATLMSER.V** |
|  | 5349 | **527.7490** | **1053.4834** | **1053.4913** | **-0.0079** | **0** | **58** | **0.00022** | **1** | **R.IDAYMAQSR.G** |
|  | 6268 | **563.3618** | **1124.7091** | **1125.7386** | **-1.0295** | **1** | **6** | **34** | **9** | **R.RVLLPLIFR.N** |
|  | 6554 | **574.3258** | **1146.6371** | **1146.6662** | **-0.0291** | **0** | **9** | **22** | **3** | **R.GWVIHPLGLR.S** 6548 |
|  | 7336 | **599.2886** | **1196.5626** | **1196.7492** | **-0.1866** | **1** | **8** | **26** | **6** | **R.EELLRALLLK.H** |
|  | 10141 | **672.3741** | **1342.7337** | **1343.6867** | **-0.9530** | **0** | **10** | **14** | **1** | **R.SVTHANALTVMGK.A** 10155 |
|  | 10291 | **451.2260** | **1350.6563** | **1350.7078** | **-0.0515** | **1** | **6** | **36** | **9** | **R.AAATLMSERVFR.I** |
|  | 10666 | **686.3497** | **1370.6848** | **1369.7201** | **0.9647** | **0** | **1** | **1e+002** | **7** | **K.ASTPGAAAQIQEVK.E** |
|  | 12243 | **489.2631** | **1464.7674** | **1465.8140** | **-1.0466** | **0** | **8** | **21** | **4** | **K.IPPDSEATLVLVGR.A** |
|  | 12527 | **494.2932** | **1479.8578** | **1478.8609** | **0.9969** | **1** | **9** | **17** | **2** | **K.LIKIFQDHPLQK.T** |
|  | 22563 | **762.7438** | **2285.2097** | **2285.2208** | **-0.0111** | **0** | **23** | **0.36** | **1** | **R.FLFVLLGPEAPHIDYTQLGR.A** |
|  | 22637 | 765.3706 | 2293.0900 | 2292.3854 | 0.7046 | 0 | 1 | 69 | 9 | R.ISGLLVAVLVGLSILMEPILSR.I |
|  | 23915 | **494.0820** | **2465.3735** | **2466.2503** | **-0.8769** | **0** | **1** | **53** | **8** | **K.GLDLNGGPDDPLQQTGQLFGGLVR.D** |
|  | 28182 | **658.3409** | **3286.6680** | **3287.7199** | **-1.0519** | **0** | **5** | **14** | **3** | **K.GSGFHLDLLLVVGMGGVAALFGMPWLSATTVR.S** |
|  | 29208 | **916.7277** | **3662.8815** | **3661.9187** | **0.9628** | **1** | **1** | **26** | **3** | **K.MVKGSGFHLDLLLVVGMGGVAALFGMPWLSATTVR.S** |
|  | 31057 | **657.9187** | **4598.3800** | **4597.4012** | **0.9788** | **1** | **3** | **8.4** | **3** | **K.GSGFHLDLLLVVGMGGVAALFGMPWLSATTVRSVTHANALTVMGK.A** |

| **656.** | Q9Y3D6\|FIS1_HUMAN    **Mass:** 16984    **Score:** 59     **Queries matched:** 8   **emPAI:** 0.23 |
| --- | --- |
|  | Mitochondrial fission 1 protein OS=Homo sapiens GN=FIS1 PE=1 SV=2 |

|  | **Query** | **Observed** | **Mr(expt)** | **Mr(calc)** | **Delta** | **Miss** | **Score** | **Expect** | **Rank** | **Peptide** |
| --- | --- | --- | --- | --- | --- | --- | --- | --- | --- | --- |
|  | 7899 | **612.3804** | **1222.7462** | **1222.7536** | **-0.0075** | **0** | **27** | **0.27** | **1** | **K.GIVLLEELLPK.G** |
|  | 11836 | **720.8705** | **1439.7265** | **1439.7368** | **-0.0103** | **0** | **47** | **0.0028** | **1** | **R.GLLQTEPQNNQAK.E** 11835 11846 |
|  | 17375 | 901.4455 | 1800.8764 | 1800.9543 | -0.0778 | 0 | 4 | 38 | 6 | -.MEAVLNELVSVEDLLK.F |
|  | 21878 | 1104.0171 | 2206.0196 | 2205.1602 | 0.8594 | 1 | 1 | 59 | 2 | -.MEAVLNELVSVEDLLKFEK.K |
|  | 24958 | **876.4289** | **2626.2648** | **2625.4598** | **0.8051** | **0** | **6** | **17** | **2** | **K.DGLVGMAIVGGMALGVAGLAGLIGLAVSK.S** |
|  | 26056 | 942.4836 | 2824.4291 | 2824.5918 | -0.1628 | 1 | 0 | 52 | 7 | K.DGLVGMAIVGGMALGVAGLAGLIGLAVSKSK.S |

| **657.** | P62857\|RS28_HUMAN    **Mass:** 7893     **Score:** 59     **Queries matched:** 5   **emPAI:** 0.52 |
| --- | --- |
|  | 40S ribosomal protein S28 OS=Homo sapiens GN=RPS28 PE=1 SV=1 |

|  | **Query** | **Observed** | **Mr(expt)** | **Mr(calc)** | **Delta** | **Miss** | **Score** | **Expect** | **Rank** | **Peptide** |
| --- | --- | --- | --- | --- | --- | --- | --- | --- | --- | --- |
|  | 7156 | 595.3039 | 1188.5932 | 1189.6125 | -1.0193 | 1 | 3 | 77 | 7 | -.MDTSRVQPIK.L |
|  | 7829 | **611.2815** | **1220.5484** | **1220.5568** | **-0.0084** | **0** | **59** | **0.00022** | **1** | **R.TGSQGQCTQVR.V** 7826 7853 |
|  | 15063 | **549.2945** | **1644.8616** | **1645.8319** | **-0.9702** | **1** | **2** | **80** | **6** | **K.VLGRTGSQGQCTQVR.V** |

| **658.** | Q99497\|PARK7_HUMAN    **Mass:** 20050    **Score:** 58     **Queries matched:** 17   **emPAI:** 0.69 |
| --- | --- |
|  | Protein DJ-1 OS=Homo sapiens GN=PARK7 PE=1 SV=2 |

|  | **Query** | **Observed** | **Mr(expt)** | **Mr(calc)** | **Delta** | **Miss** | **Score** | **Expect** | **Rank** | **Peptide** |
| --- | --- | --- | --- | --- | --- | --- | --- | --- | --- | --- |
|  | 1413 | **641.2459** | **640.2386** | **639.4319** | **0.8067** | **0** | **10** | **13** | **9** | **K.APLVLK.D** |
|  | 2732 | **431.5371** | **861.0597** | **860.3811** | **0.6786** | **0** | **12** | **9.6** | **8** | **K.DPVQCSR.D** |
|  | 2817 | 866.5030 | 865.4957 | 865.5022 | -0.0064 | 0 | 23 | 0.92 | 1 | K.VTTHPLAK.D |
|  | 2945 | **438.2335** | **874.4525** | **873.4920** | **0.9605** | **0** | **7** | **44** | **3** | **K.DGLILTSR.G** |
|  | 7053 | **593.1553** | **1184.2961** | **1183.7289** | **0.5673** | **1** | **2** | **72** | **2** | **R.AGIKVTVAGLAGK.D** |
|  | 8012 | **615.8220** | **1229.6295** | **1229.6979** | **-0.0684** | **1** | **3** | **75** | **3** | **R.VEKDGLILTSR.G** |
|  | 10556 | **683.8017** | **1365.5888** | **1364.8391** | **0.7497** | **1** | **3** | **70** | **8** | **K.EVAAQVKAPLVLK.D** |
|  | 14242 | **531.3207** | **1590.9404** | **1590.6191** | **0.3213** | **0** | **5** | **36** | **3** | **K.MMNGGHYTYSENR.V** |
|  | 15255 | **829.4326** | **1656.8507** | **1656.8618** | **-0.0111** | **1** | **33** | **0.053** | **1** | **K.VTVAGLAGKDPVQCSR.D** |
|  | 15279 | 553.9428 | 1658.8066 | 1658.8185 | -0.0119 | 1 | 9 | 13 | 2 | R.DVVICPDASLEDAKK.E |
|  | 15521 | 838.9007 | 1675.7868 | 1674.7957 | 0.9912 | 0 | 22 | 0.76 | 1 | K.GAEEMETVIPVDVMR.R |
|  | 18203 | **622.3038** | **1863.8895** | **1862.8866** | **1.0029** | **1** | **5** | **36** | **1** | **K.GAEEMETVIPVDVMRR.A** |
|  | 18901 | **641.3323** | **1920.9752** | **1919.9993** | **0.9759** | **0** | **3** | **47** | **3** | **R.GPGTSFEFALAIVEALNGK.E** |
|  | 19217 | **649.9894** | **1946.9465** | **1946.8251** | **0.1214** | **1** | **2** | **57** | **2** | **K.MMNGGHYTYSENRVEK.D** 19216 |
|  | 24768 | **1292.6575** | **2583.3004** | **2583.3180** | **-0.0177** | **0** | **46** | **0.0017** | **1** | **K.EGPYDVVVLPGGNLGAQNLSESAAVK.E** 24769 |

| **659.** | Q9Y4P3\|TBL2_HUMAN    **Mass:** 50393    **Score:** 58     **Queries matched:** 18   **emPAI:** 0.24 |
| --- | --- |
|  | Transducin beta-like protein 2 OS=Homo sapiens GN=TBL2 PE=1 SV=1 |

|  | **Query** | **Observed** | **Mr(expt)** | **Mr(calc)** | **Delta** | **Miss** | **Score** | **Expect** | **Rank** | **Peptide** |
| --- | --- | --- | --- | --- | --- | --- | --- | --- | --- | --- |
|  | 3599 | **463.7119** | **925.4091** | **924.4124** | **0.9968** | **0** | **21** | **1.3** | **1** | **R.FLASCGDR.A** |
|  | 5653 | **539.7770** | **1077.5394** | **1077.5468** | **-0.0074** | **0** | **11** | **13** | **7** | **R.LFHNTPGHR.A** |
|  | 5725 | 542.7360 | 1083.4574 | 1083.4655 | -0.0081 | 0 | 39 | 0.017 | 1 | K.YLATCADDR.T |
|  | 6949 | 589.7624 | 1177.5102 | 1177.5186 | -0.0084 | 0 | 19 | 1.8 | 1 | R.FEEAAGAAPCR.L |
|  | 8811 | 424.7282 | 1271.1626 | 1271.6002 | -0.4376 | 0 | (3) | 52 | 8 | R.AMVEEMQGHLK.R |
|  | 9112 | 430.2745 | 1287.8016 | 1287.5951 | 0.2065 | 0 | 7 | 30 | 4 | R.AMVEEMQGHLK.R |
|  | 9434 | **435.8872** | **1304.6399** | **1303.5900** | **1.0498** | **0** | **(6)** | **35** | **10** | **R.AMVEEMQGHLK.R** |
|  | 12714 | **746.8469** | **1491.6793** | **1491.6888** | **-0.0096** | **1** | **16** | **3.6** | **1** | **K.TGRFEEAAGAAPCR.L** |
|  | 13288 | 764.9148 | 1527.8150 | 1527.8257 | -0.0106 | 0 | 46 | 0.0035 | 1 | R.LQQQLTQAQETLK.S |
|  | 13339 | **511.7320** | **1532.1743** | **1532.8675** | **-0.6932** | **1** | **5** | **24** | **2** | **K.HKAPVIDIGIANTGK.F** |
|  | 19008 | **965.4833** | **1928.9520** | **1929.8745** | **-0.9225** | **1** | **11** | **7.6** | **1** | **K.REDGGYTFTATPEDFPK.K** 19007 |
|  | 20963 | 1052.0741 | 2102.1336 | 2101.1544 | 0.9792 | 1 | 0 | 78 | 8 | K.EKPQQHNFTHRLLAAALK.S |
|  | 23162 | **788.4178** | **2362.2315** | **2363.1263** | **-0.8948** | **1** | **1** | **59** | **6** | **R.LFHNTPGHRAMVEEMQGHLK.R** |
|  | 24188 | **833.1202** | **2496.3387** | **2495.3496** | **0.9891** | **0** | **26** | **0.16** | **1** | **R.LALSPNAQVLALASGSSIHLYNTR.R** |
|  | 24683 | **643.3511** | **2569.3752** | **2569.2126** | **0.1626** | **0** | **0** | **58** | **6** | **K.GQVLSTINTNQMNNTHAAVSPCGR.F** |
|  | 26422 | **723.1423** | **2888.5402** | **2887.5584** | **0.9818** | **0** | **2** | **33** | **6** | **M.ELSQMSELMGLSVLLGLLALMATAAVAR.G** |
|  | 30912 | **1488.7050** | **4463.0931** | **4463.2036** | **-0.1105** | **1** | **1** | **16** | **5** | **K.FIMTASSDTTVLIWSLKGQVLSTINTNQMNNTHAAVSPCGR.F** |

| **660.** | P40429\|RL13A_HUMAN    **Mass:** 23619    **Score:** 58     **Queries matched:** 12   **emPAI:** 0.16 |
| --- | --- |
|  | 60S ribosomal protein L13a OS=Homo sapiens GN=RPL13A PE=1 SV=2 |

|  | **Query** | **Observed** | **Mr(expt)** | **Mr(calc)** | **Delta** | **Miss** | **Score** | **Expect** | **Rank** | **Peptide** |
| --- | --- | --- | --- | --- | --- | --- | --- | --- | --- | --- |
|  | 1536 | 685.4556 | 684.4483 | 684.4534 | -0.0051 | 0 | 23 | 0.81 | 3 | R.LAAIVAK.Q |
|  | 1568 | **699.4821** | **698.4748** | **698.4803** | **-0.0055** | **1** | **23** | **0.85** | **1** | **R.KVVVVR.C** |
|  | 2315 | **414.7516** | **827.4887** | **827.4939** | **-0.0051** | **0** | **11** | **14** | **3** | **R.MVVPAALK.V** |
|  | 3103 | 443.2713 | 884.5281 | 885.4780 | -0.9499 | 1 | 15 | 4.9 | 5 | K.RGQAALDR.L |
|  | 4636 | 500.2947 | 998.5748 | 999.5899 | -1.0151 | 1 | 16 | 4.2 | 2 | K.RMVVPAALK.V 4635 |
|  | 8446 | **626.8206** | **1251.6266** | **1251.6347** | **-0.0081** | **0** | **42** | **0.009** | **1** | **K.YQAVTATLEEK.R** 8445 |
|  | 9666 | **440.1981** | **1317.5724** | **1317.8245** | **-0.2521** | **1** | **6** | **38** | **9** | **R.GHLLGRLAAIVAK.Q** |
|  | 9905 | **443.8894** | **1328.6465** | **1328.7122** | **-0.0658** | **0** | **5** | **45** | **1** | **-.MAEVQVLVLDGR.G** |
|  | 11322 | 705.3240 | 1408.6334 | 1407.7358 | 0.8976 | 1 | 2 | 84 | 3 | K.YQAVTATLEEKR.K |
|  | 11693 | 715.8480 | 1429.6815 | 1428.6456 | 1.0359 | 0 | 7 | 26 | 2 | R.CEGINISGNFYR.N |

| **661.** | Q6NVV1\|R13AX_HUMAN    **Mass:** 12184    **Score:** 58     **Queries matched:** 7   **emPAI:** 0.32 |
| --- | --- |
|  | Putative 60S ribosomal protein L13a-like MGC87657 OS=Homo sapiens PE=5 SV=1 |

|  | **Query** | **Observed** | **Mr(expt)** | **Mr(calc)** | **Delta** | **Miss** | **Score** | **Expect** | **Rank** | **Peptide** |
| --- | --- | --- | --- | --- | --- | --- | --- | --- | --- | --- |
|  | 1994 | **402.2433** | **802.4720** | **803.5017** | **-1.0298** | **1** | **1** | **1.7e+002** | **7** | **R.KFALLGR.Q** |
|  | 2315 | 414.7516 | 827.4887 | 827.4939 | -0.0051 | 0 | 11 | 14 | 3 | R.MVVPAALK.V |
|  | 4636 | 500.2947 | 998.5748 | 999.5899 | -1.0151 | 1 | 16 | 4.2 | 2 | K.RMVVPAALK.V 4635 |
|  | 8446 | 626.8206 | 1251.6266 | 1251.6347 | -0.0081 | 0 | 42 | 0.009 | 1 | K.YQAVTATLEEK.R 8445 |
|  | 11322 | 705.3240 | 1408.6334 | 1407.7358 | 0.8976 | 1 | 2 | 84 | 3 | K.YQAVTATLEEKR.K |

| **662.** | Q16772\|GSTA3_HUMAN    **Mass:** 25342    **Score:** 58     **Queries matched:** 21   **emPAI:** 0.75 |
| --- | --- |
|  | Glutathione S-transferase A3 OS=Homo sapiens GN=GSTA3 PE=1 SV=3 |

|  | **Query** | **Observed** | **Mr(expt)** | **Mr(calc)** | **Delta** | **Miss** | **Score** | **Expect** | **Rank** | **Peptide** |
| --- | --- | --- | --- | --- | --- | --- | --- | --- | --- | --- |
|  | 2903 | 436.2636 | 870.5127 | 870.5175 | -0.0048 | 0 | 18 | 2.8 | 4 | R.ISNLPTVK.K |
|  | 3265 | 451.2452 | 900.4759 | 900.4818 | -0.0059 | 0 | 20 | 2.1 | 1 | K.FLQPGSPR.K 3280 3288 3302 3303 |
|  | 4519 | 992.5702 | 991.5629 | 991.5702 | -0.0073 | 0 | 27 | 0.34 | 1 | R.AILNYIASK.Y 4518 |
|  | 4613 | 499.7349 | 997.4552 | 998.6124 | -1.1572 | 1 | 16 | 3.4 | 4 | R.ISNLPTVKK.F 4620 |
|  | 5037 | 515.2913 | 1028.5681 | 1028.5767 | -0.0086 | 1 | 32 | 0.11 | 1 | K.KFLQPGSPR.K 5029 5038 |
|  | 9437 | **435.8976** | **1304.6711** | **1304.7088** | **-0.0377** | **1** | **7** | **27** | **5** | **K.FIGSAEDLGKLR.N** |
|  | 12205 | 731.8782 | 1461.7419 | 1461.7504 | -0.0084 | 0 | 56 | 0.00032 | 1 | R.WLLAAAGVEFEEK.F |
|  | 13358 | **512.2555** | **1533.7446** | **1533.7874** | **-0.0428** | **1** | **7** | **25** | **7** | **-.MAGKPKLHYFNGR.G** |
|  | 20976 | 1053.0071 | 2103.9996 | 2104.0663 | -0.0667 | 1 | 1 | 71 | 8 | R.MEPIRWLLAAAGVEFEEK.F |
|  | 21282 | 1069.5917 | 2137.1688 | 2137.9846 | -0.8158 | 0 | 12 | 4.8 | 1 | R.NDGSLMFQQVPMVEIDGMK.L |
|  | 21431 | 1078.5454 | 2155.0763 | 2153.9796 | 1.0967 | 0 | (2) | 50 | 4 | R.NDGSLMFQQVPMVEIDGMK.L |
|  | 25621 | 918.1060 | 2751.2962 | 2751.3394 | -0.0432 | 1 | 3 | 31 | 3 | R.NDGSLMFQQVPMVEIDGMKLVQTR.A |
|  | 28173 | **1093.8606** | **3278.5600** | **3277.6106** | **0.9494** | **0** | **0** | **45** | **7** | **R.ALIDMYTEGMADLNEMILLLPLCRPEEK.D** |

| **663.** | P10909\|CLUS_HUMAN    **Mass:** 53031    **Score:** 58     **Queries matched:** 12   **emPAI:** 0.07 |
| --- | --- |
|  | Clusterin OS=Homo sapiens GN=CLU PE=1 SV=1 |

|  | **Query** | **Observed** | **Mr(expt)** | **Mr(calc)** | **Delta** | **Miss** | **Score** | **Expect** | **Rank** | **Peptide** |
| --- | --- | --- | --- | --- | --- | --- | --- | --- | --- | --- |
|  | 3996 | 477.7303 | 953.4461 | 953.4528 | -0.0067 | 0 | 4 | 63 | 5 | K.FMETVAEK.A |
|  | 7487 | **402.1791** | **1203.5153** | **1203.5529** | **-0.0376** | **1** | **11** | **12** | **3** | **K.QTCMKFYAR.V** 7485 |
|  | 7774 | **406.8670** | **1217.5791** | **1218.5598** | **-0.9807** | **1** | **3** | **86** | **8** | **R.HNSTGCLRMK.D** |
|  | 15617 | 842.4536 | 1682.8927 | 1682.8264 | 0.0663 | 0 | 2 | 74 | 6 | R.LANLTQGEDQYYLR.V |
|  | 18341 | **937.4936** | **1872.9726** | **1872.9833** | **-0.0107** | **0** | **58** | **0.00017** | **1** | **K.LFDSDPITVTVPVEVSR.K** |
|  | 19828 | 1000.9932 | 1999.9718 | 1998.9588 | 1.0130 | 0 | 1 | 70 | 6 | R.EPQDTYHYLPFSLPHR.R 19829 |
|  | 26848 | 987.4299 | 2959.2678 | 2959.3553 | -0.0875 | 1 | 3 | 23 | 2 | R.IDSLLENDRQQTHMLDVMQDHFSR.A 26846 |
|  | 30206 | 1017.2488 | 4064.9660 | 4065.0253 | -0.0592 | 0 | 0 | 27 | 8 | K.TLLLFVGLLLTWESGQVLGDQTVSDNELQEMSNQGSK.Y |
|  | 30713 | **618.7368** | **4324.1064** | **4324.1607** | **-0.0543** | **1** | **0** | **22** | **4** | **M.MKTLLLFVGLLLTWESGQVLGDQTVSDNELQEMSNQGSK.Y** |

| **664.** | P38919\|IF4A3_HUMAN    **Mass:** 47126    **Score:** 57     **Queries matched:** 13   **emPAI:** 0.08 |
| --- | --- |
|  | Eukaryotic initiation factor 4A-III OS=Homo sapiens GN=EIF4A3 PE=1 SV=4 |

|  | **Query** | **Observed** | **Mr(expt)** | **Mr(calc)** | **Delta** | **Miss** | **Score** | **Expect** | **Rank** | **Peptide** |
| --- | --- | --- | --- | --- | --- | --- | --- | --- | --- | --- |
|  | 2556 | **424.2438** | **846.4730** | **846.4963** | **-0.0233** | **0** | **6** | **56** | **2** | **K.GVAINFVK.N** |
|  | 4273 | **487.8317** | **973.6488** | **974.5913** | **-0.9424** | **1** | **3** | **91** | **10** | **R.KGVAINFVK.N** |
|  | 7248 | 597.2896 | 1192.5647 | 1192.5910 | -0.0264 | 1 | 6 | 37 | 5 | K.VDWLTEKMR.E |
|  | 7670 | **607.3265** | **1212.6385** | **1211.6874** | **0.9512** | **0** | **6** | **41** | **10** | **R.ETQALILAPTR.E** |
|  | 10339 | 677.3527 | 1352.6909 | 1352.6718 | 0.0191 | 1 | 7 | 28 | 6 | M.ATTATMATSGSARK.R |
|  | 10677 | 458.2939 | 1371.8599 | 1371.6122 | 0.2477 | 0 | (6) | 31 | 4 | -.MATTATMATSGSAR.K |
|  | 10955 | 463.2477 | 1386.7212 | 1387.6072 | -0.8860 | 0 | 8 | 21 | 6 | -.MATTATMATSGSAR.K 10968 |
|  | 11215 | **702.3603** | **1402.7060** | **1402.7165** | **-0.0104** | **1** | **57** | **0.00026** | **1** | **K.GRDVIAQSQSGTGK.T** |
|  | 17299 | 599.3283 | 1794.9631 | 1793.7713 | 1.1918 | 0 | 2 | 71 | 4 | R.EANFTVSSMHGDMPQK.E |
|  | 17479 | **905.9102** | **1809.8059** | **1809.7662** | **0.0397** | **0** | **(1)** | **87** | **4** | **R.EANFTVSSMHGDMPQK.E** |
|  | 20933 | **700.0475** | **2097.1206** | **2096.9078** | **0.2129** | **1** | **1** | **71** | **7** | **K.MREANFTVSSMHGDMPQK.E** |
|  | 25864 | 559.8669 | 2794.2983 | 2793.4986 | 0.7997 | 0 | 1 | 49 | 7 | R.YLPPATQVVLISATLPHEILEMTNK.F |

| **665.** | P62851\|RS25_HUMAN    **Mass:** 13791    **Score:** 57     **Queries matched:** 5   **emPAI:** 0.65 |
| --- | --- |
|  | 40S ribosomal protein S25 OS=Homo sapiens GN=RPS25 PE=1 SV=1 |

|  | **Query** | **Observed** | **Mr(expt)** | **Mr(calc)** | **Delta** | **Miss** | **Score** | **Expect** | **Rank** | **Peptide** |
| --- | --- | --- | --- | --- | --- | --- | --- | --- | --- | --- |
|  | 2542 | 423.7382 | 845.4619 | 845.3953 | 0.0666 | 1 | 11 | 18 | 4 | -.MPPKDDK.K |
|  | 4216 | **486.2623** | **970.5101** | **971.5651** | **-1.0550** | **0** | **47** | **0.0033** | **1** | **R.AALQELLSK.G** 4233 |
|  | 5729 | 542.7820 | 1083.5495 | 1083.6288 | -0.0793 | 0 | 2 | 91 | 5 | K.LITPAVVSER.L |
|  | 12092 | **486.2623** | **1455.7652** | **1455.8409** | **-0.0757** | **1** | **31** | **0.1** | **1** | **R.GSLARAALQELLSK.G** |

| **666.** | Q9UL12\|SARDH_HUMAN    **Mass:** 101942   **Score:** 57     **Queries matched:** 19   **emPAI:** 0.07 |
| --- | --- |
|  | Sarcosine dehydrogenase, mitochondrial OS=Homo sapiens GN=SARDH PE=1 SV=1 |

|  | **Query** | **Observed** | **Mr(expt)** | **Mr(calc)** | **Delta** | **Miss** | **Score** | **Expect** | **Rank** | **Peptide** |
| --- | --- | --- | --- | --- | --- | --- | --- | --- | --- | --- |
|  | 763 | 474.9745 | 473.9672 | 474.2550 | -0.2878 | 0 | 2 | 1.7e+002 | 2 | R.AASAR.G |
|  | 943 | **521.3040** | **520.2967** | **520.2679** | **0.0288** | **0** | **12** | **15** | **1** | **R.MAGVK.V** |
|  | 2217 | **411.2015** | **820.3885** | **819.4636** | **0.9248** | **1** | **3** | **88** | **4** | **K.RLMSLGK.A** |
|  | 2934 | **437.7396** | **873.4647** | **872.4352** | **1.0296** | **0** | **20** | **2.1** | **2** | **R.EALEQQR.A** |
|  | 4146 | 483.2628 | 964.5110 | 964.5203 | -0.0093 | 0 | 17 | 3.4 | 1 | R.NGQVVGHVR.R 4150 |
|  | 4655 | **501.1824** | **1000.3503** | **999.5250** | **0.8253** | **0** | **14** | **6.1** | **1** | **K.HGLINAGYR.A** |
|  | 5334 | 527.2737 | 1052.5329 | 1052.5655 | -0.0325 | 0 | 7 | 27 | 5 | K.FYLVGLDAR.K |
|  | 5618 | **538.2584** | **1074.5023** | **1074.5094** | **-0.0071** | **0** | **6** | **45** | **2** | **R.DHDASVYLR.L** |
|  | 6959 | 590.3060 | 1178.5974 | 1177.5298 | 1.0676 | 1 | 4 | 62 | 10 | K.DMHGYDIRR.F |
|  | 13028 | 757.3880 | 1512.7614 | 1512.7719 | -0.0104 | 0 | 36 | 0.028 | 1 | R.GAQVIENCPVTGIR.V |
|  | 22083 | **742.4227** | **2224.2464** | **2223.1656** | **1.0807** | **1** | **1** | **60** | **9** | **R.MAGVKVPLVAMHHAYVVTER.I** |
|  | 23744 | **815.7435** | **2444.2086** | **2444.2223** | **-0.0138** | **0** | **5** | **25** | **1** | **R.AILQEVLDADLSNEAFPFSTHK.L** |
|  | 24149 | 1246.6223 | 2491.2301 | 2491.1683 | 0.0618 | 0 | 14 | 2.8 | 2 | K.SQCQLIDSSEDLGMISIQGPASR.A 24148 |
|  | 26997 | 996.8077 | 2987.4012 | 2988.5426 | -1.1414 | 1 | 2 | 29 | 2 | K.VPLVAMHHAYVVTERIEGIQNMPNVR.D 27004 |
|  | 29486 | **1257.9207** | **3770.7401** | **3770.7630** | **-0.0229** | **0** | **37** | **0.0072** | **1** | **K.TLYPLMNVDDLYGTLYVPHDGTMDPAGTCTTLAR.A** 29485 |

| **667.** | P51687\|SUOX_HUMAN    **Mass:** 60759    **Score:** 57     **Queries matched:** 16   **emPAI:** 0.06 |
| --- | --- |
|  | Sulfite oxidase, mitochondrial OS=Homo sapiens GN=SUOX PE=1 SV=2 |

|  | **Query** | **Observed** | **Mr(expt)** | **Mr(calc)** | **Delta** | **Miss** | **Score** | **Expect** | **Rank** | **Peptide** |
| --- | --- | --- | --- | --- | --- | --- | --- | --- | --- | --- |
|  | 2314 | **414.7159** | **827.4173** | **826.4086** | **1.0087** | **0** | **8** | **29** | **6** | **R.DHGFPVR.V** 2303 |
|  | 4886 | **509.2366** | **1016.4586** | **1015.5451** | **0.9135** | **1** | **6** | **44** | **3** | **K.EVKGLEWR.T** 4850 |
|  | 5180 | 520.3453 | 1038.6760 | 1038.5359 | 0.1401 | 0 | 17 | 2.8 | 3 | R.GVLSNAWHR.V |
|  | 5942 | **550.7808** | **1099.5470** | **1098.5418** | **1.0052** | **0** | **6** | **39** | **1** | **K.LDGEEQRPR.K** 5928 |
|  | 7936 | **409.5537** | **1225.6394** | **1226.6367** | **-0.9973** | **1** | **8** | **22** | **6** | **K.LDGEEQRPRK.A** |
|  | 10280 | **450.7895** | **1349.3467** | **1348.7000** | **0.6467** | **1** | **7** | **21** | **8** | **K.GYAWSGGGRAVIR.V** |
|  | 16430 | **578.2703** | **1731.7891** | **1730.8012** | **0.9879** | **0** | **4** | **46** | **8** | **R.VSVQPEESYSHWQR.R** |
|  | 17742 | 611.3271 | 1830.9594 | 1830.8636 | 0.0959 | 0 | 1 | 87 | 8 | K.VAPTVETSDPYADDPVR.H |
|  | 18483 | **629.9891** | **1886.9456** | **1886.9023** | **0.0432** | **1** | **2** | **61** | **8** | **R.VSVQPEESYSHWQRR.D** |
|  | 18819 | **638.6534** | **1912.9383** | **1913.0193** | **-0.0810** | **0** | **1** | **74** | **4** | **R.VMGTLLGLGAVLAYQDHR.C** |
|  | 22254 | 1122.5541 | 2243.0936 | 2242.1019 | 0.9918 | 0 | 25 | 0.28 | 2 | K.AVDDGYNVQPDTVAPIWNLR.G |
|  | 24384 | 844.0737 | 2529.1994 | 2528.3136 | 0.8858 | 0 | 3 | 32 | 5 | R.LHVVGAPGGQSLSLSLDDLHNFPR.Y |
|  | 28828 | **1167.9243** | **3500.7511** | **3500.7728** | **-0.0217** | **0** | **57** | **7.5e-005** | **1** | **K.VNSQRPFNAEPPPELLTENYITPNPIFFTR.N** |

| **668.** | P07602\|SAP_HUMAN    **Mass:** 59899    **Score:** 57     **Queries matched:** 11   **emPAI:** 0.13 |
| --- | --- |
|  | Proactivator polypeptide OS=Homo sapiens GN=PSAP PE=1 SV=2 |

|  | **Query** | **Observed** | **Mr(expt)** | **Mr(calc)** | **Delta** | **Miss** | **Score** | **Expect** | **Rank** | **Peptide** |
| --- | --- | --- | --- | --- | --- | --- | --- | --- | --- | --- |
|  | 4121 | **482.5882** | **963.1618** | **962.5549** | **0.6069** | **1** | **22** | **0.73** | **1** | **K.KLVGYLDR.N** 4105 |
|  | 5430 | **531.2557** | **1060.4969** | **1060.5045** | **-0.0076** | **0** | **35** | **0.059** | **1** | **R.LGPGMADICK.N** |
|  | 6403 | **568.5942** | **1135.1739** | **1134.5591** | **0.6148** | **0** | **1** | **81** | **4** | **K.DVVTAAGDMLK.D** |
|  | 8635 | **421.2211** | **1260.6414** | **1259.6254** | **1.0160** | **0** | **17** | **2.9** | **1** | **K.EMPMQTLVPAK.V** |
|  | 13923 | 786.9410 | 1571.8674 | 1571.8308 | 0.0366 | 0 | 3 | 62 | 6 | R.TNSTFVQALVEHVK.E |
|  | 16399 | **865.4995** | **1728.9843** | **1728.9913** | **-0.0070** | **0** | **44** | **0.0038** | **1** | **K.EIVDSYLPVILDIIK.G** 16400 |
|  | 23707 | 815.0430 | 2442.1073 | 2442.1204 | -0.0131 | 0 | 10 | 8 | 1 | K.NYISQYSEIAIQMMMHMQPK.E |
|  | 23870 | **820.4546** | **2458.3419** | **2458.1153** | **0.2267** | **0** | **(6)** | **17** | **1** | **K.NYISQYSEIAIQMMMHMQPK.E** |
|  | 24147 | 623.7916 | 2491.1371 | 2490.1051 | 1.0320 | 0 | (5) | 22 | 5 | K.NYISQYSEIAIQMMMHMQPK.E |

| **669.** | P67809\|YBOX1_HUMAN    **Mass:** 35903    **Score:** 57     **Queries matched:** 5   **emPAI:** 0.10 |
| --- | --- |
|  | Nuclease-sensitive element-binding protein 1 OS=Homo sapiens GN=YBX1 PE=1 SV=3 |

|  | **Query** | **Observed** | **Mr(expt)** | **Mr(calc)** | **Delta** | **Miss** | **Score** | **Expect** | **Rank** | **Peptide** |
| --- | --- | --- | --- | --- | --- | --- | --- | --- | --- | --- |
|  | 4465 | **495.0623** | **988.1101** | **988.4477** | **-0.3375** | **0** | **9** | **16** | **5** | **R.FPPYYMR.R** 4464 |
|  | 11147 | 699.8785 | 1397.7425 | 1397.6688 | 0.0738 | 1 | 4 | 57 | 4 | R.NGYGFINRNDTK.E |
|  | 15823 | **848.4303** | **1694.8460** | **1694.8588** | **-0.0127** | **0** | **57** | **0.00024** | **1** | **K.GAEAANVTGPGGVPVQGSK.Y** |
|  | 28046 | **648.9231** | **3239.5791** | **3238.5174** | **1.0617** | **0** | **0** | **45** | **4** | **R.RPQYSNPPVQGEVMEGADNQGAGEQGRPVR.Q** |

| **670.** | O95299\|NDUAA_HUMAN    **Mass:** 41067    **Score:** 56     **Queries matched:** 14   **emPAI:** 0.55 |
| --- | --- |
|  | NADH dehydrogenase [ubiquinone] 1 alpha subcomplex subunit 10, mitochondrial OS=Homo sapiens GN=NDUFA10 PE=1 SV=1 |

|  | **Query** | **Observed** | **Mr(expt)** | **Mr(calc)** | **Delta** | **Miss** | **Score** | **Expect** | **Rank** | **Peptide** |
| --- | --- | --- | --- | --- | --- | --- | --- | --- | --- | --- |
|  | 1926 | **400.2024** | **798.3903** | **798.4501** | **-0.0598** | **0** | **6** | **40** | **3** | **R.VLHQFR.E** |
|  | 2340 | 415.5761 | 829.1376 | 828.3436 | 0.7940 | 0 | 4 | 65 | 5 | K.GDPHEMK.I |
|  | 3266 | 451.2501 | 900.4857 | 900.5280 | -0.0423 | 1 | 44 | 0.0071 | 2 | K.LAKEIAEK.L 3287 |
|  | 3420 | **456.2672** | **910.5197** | **911.3733** | **-0.8536** | **0** | **4** | **59** | **8** | **R.SNDGNSYR.L** |
|  | 5008 | **514.2965** | **1026.5783** | **1025.6094** | **0.9689** | **1** | **7** | **33** | **1** | **R.VVAAGAQRVR.G** |
|  | 5682 | **541.2696** | **1080.5247** | **1080.5161** | **0.0086** | **0** | **3** | **73** | **7** | **K.TFLPEMSEK.C** |
|  | 8119 | **618.3433** | **1234.6720** | **1234.6809** | **-0.0089** | **1** | **31** | **0.11** | **1** | **K.KVVEDIEYLK.F** 8094 |
|  | 8893 | **638.8270** | **1275.6394** | **1275.6493** | **-0.0099** | **0** | **29** | **0.18** | **1** | **R.VITVDGNICTGK.G** |
|  | 9901 | **665.2972** | **1328.5798** | **1328.5885** | **-0.0086** | **0** | **25** | **0.44** | **1** | **K.YSPGYNTEVGDK.W** |
|  | 12807 | **500.3025** | **1497.8857** | **1497.8667** | **0.0190** | **1** | **2** | **77** | **9** | **R.TLYHLRLLVQDK.F** |
|  | 16751 | **586.3049** | **1755.8928** | **1755.9043** | **-0.0115** | **1** | **3** | **51** | **4** | **K.ITSAYLQDIENAYKK.T** |
|  | 28964 | **890.2282** | **3556.8837** | **3555.8977** | **0.9860** | **1** | **23** | **0.16** | **1** | **R.LLVQDKFEVLNYTSIPIFLPEVTIGAHQTDR.V** |

| **671.** | O75828\|CBR3_HUMAN    **Mass:** 31230    **Score:** 56     **Queries matched:** 10   **emPAI:** 0.26 |
| --- | --- |
|  | Carbonyl reductase [NADPH] 3 OS=Homo sapiens GN=CBR3 PE=1 SV=3 |

|  | **Query** | **Observed** | **Mr(expt)** | **Mr(calc)** | **Delta** | **Miss** | **Score** | **Expect** | **Rank** | **Peptide** |
| --- | --- | --- | --- | --- | --- | --- | --- | --- | --- | --- |
|  | 14349 | 800.4047 | 1598.7949 | 1598.8053 | -0.0104 | 0 | 42 | 0.0069 | 1 | R.FHQLDIDDLQSIR.A 14350 |
|  | 14486 | **537.2804** | **1608.8194** | **1607.7872** | **1.0322** | **1** | **4** | **50** | **5** | **-.MSSCSRVALVTGANR.G** |
|  | 15158 | **551.2843** | **1650.8311** | **1650.9893** | **-0.1582** | **1** | **6** | **26** | **8** | **R.VALVTGANRGIGLAIAR.E** |
|  | 15178 | 826.9336 | 1651.8527 | 1651.8642 | -0.0114 | 0 | 29 | 0.14 | 1 | R.GQAAVQQLQAEGLSPR.F |
|  | 17078 | **890.4622** | **1778.9099** | **1777.9363** | **0.9736** | **0** | **4** | **48** | **1** | **K.EYGGLNVLVNNAAVAFK.S** |
|  | 17787 | **612.3455** | **1834.0147** | **1833.8819** | **0.1329** | **0** | **1** | **91** | **3** | **R.FHSETLTEGDLVDLMK.K** |
|  | 19556 | 989.5762 | 1977.1378 | 1977.9717 | -0.8340 | 1 | 1 | 75 | 3 | R.FHSETLTEGDLVDLMKK.F |
|  | 19706 | **664.3152** | **1989.9239** | **1989.9322** | **-0.0083** | **1** | **3** | **45** | **2** | **R.ILVNACCPGPVKTDMDGK.D** |
|  | 25107 | **888.4115** | **2662.2127** | **2662.2930** | **-0.0804** | **1** | **1** | **44** | **8** | **K.TNFFATRNMCNELLPIMKPHGR.V** |

| **672.** | P0C0S8\|H2A1_HUMAN    **Mass:** 14083    **Score:** 56     **Queries matched:** 7   **emPAI:** 1.09 |
| --- | --- |
|  | Histone H2A type 1 OS=Homo sapiens GN=HIST1H2AG PE=1 SV=2 |

|  | **Query** | **Observed** | **Mr(expt)** | **Mr(calc)** | **Delta** | **Miss** | **Score** | **Expect** | **Rank** | **Peptide** |
| --- | --- | --- | --- | --- | --- | --- | --- | --- | --- | --- |
|  | 2590 | 425.7635 | 849.5125 | 849.5184 | -0.0060 | 0 | 25 | 0.55 | 1 | R.HLQLAIR.N |
|  | 3867 | 473.2675 | 944.5205 | 943.5240 | 0.9965 | 0 | 38 | 0.031 | 1 | R.AGLQFPVGR.V 3847 |
|  | 8814 | 636.8391 | 1271.6635 | 1271.6721 | -0.0085 | 1 | 29 | 0.17 | 1 | R.NDEELNKLLGK.V |
|  | 15722 | 564.5905 | 1690.7497 | 1691.8954 | -1.1457 | 1 | 11 | 9.3 | 3 | R.HLQLAIRNDEELNK.L |
|  | 19030 | 966.0826 | 1930.1506 | 1930.1615 | -0.0109 | 0 | 33 | 0.041 | 1 | K.VTIAQGGVLPNIQAVLLPK.K |
|  | 26583 | **972.5317** | **2914.5732** | **2914.5804** | **-0.0071** | **0** | **27** | **0.09** | **1** | **R.VGAGAPVYLAAVLEYLTAEILELAGNAAR.D** |

|  | **Proteins matching the same set of peptides:** |
| --- | --- |

|  | Q9BTM1\|H2AJ_HUMAN    **Mass:** 14011    **Score:** 56     **Queries matched:** 7 |
| --- | --- |
|  | Histone H2A.J OS=Homo sapiens GN=H2AFJ PE=1 SV=1 |

|  | Q96KK5\|H2A1H_HUMAN    **Mass:** 13898    **Score:** 56     **Queries matched:** 7 |
| --- | --- |
|  | Histone H2A type 1-H OS=Homo sapiens GN=HIST1H2AH PE=1 SV=3 |

|  | P20671\|H2A1D_HUMAN    **Mass:** 14099    **Score:** 56     **Queries matched:** 7 |
| --- | --- |
|  | Histone H2A type 1-D OS=Homo sapiens GN=HIST1H2AD PE=1 SV=2 |

|  | Q99878\|H2A1J_HUMAN    **Mass:** 13928    **Score:** 56     **Queries matched:** 7 |
| --- | --- |
|  | Histone H2A type 1-J OS=Homo sapiens GN=HIST1H2AJ PE=1 SV=3 |

|  | **Proteins matching a subset of these peptides:** |
| --- | --- |

|  | Q7L7L0\|H2A3_HUMAN    **Mass:** 14113    **Score:** 56     **Queries matched:** 6 |
| --- | --- |
|  | Histone H2A type 3 OS=Homo sapiens GN=HIST3H2A PE=1 SV=3 |

|  | P04908\|H2A1B_HUMAN    **Mass:** 14127    **Score:** 56     **Queries matched:** 6 |
| --- | --- |
|  | Histone H2A type 1-B/E OS=Homo sapiens GN=HIST1H2AB PE=1 SV=2 |

|  | Q93077\|H2A1C_HUMAN    **Mass:** 14097    **Score:** 56     **Queries matched:** 6 |
| --- | --- |
|  | Histone H2A type 1-C OS=Homo sapiens GN=HIST1H2AC PE=1 SV=3 |

| **673.** | P10319\|1B58_HUMAN    **Mass:** 40597    **Score:** 56     **Queries matched:** 8   **emPAI:** 0.30 |
| --- | --- |
|  | HLA class I histocompatibility antigen, B-58 alpha chain OS=Homo sapiens GN=HLA-B PE=2 SV=1 |

|  | **Query** | **Observed** | **Mr(expt)** | **Mr(calc)** | **Delta** | **Miss** | **Score** | **Expect** | **Rank** | **Peptide** |
| --- | --- | --- | --- | --- | --- | --- | --- | --- | --- | --- |
|  | 4142 | 483.2169 | 964.4193 | 964.4250 | -0.0057 | 0 | 53 | 0.00075 | 1 | R.FDSDAASPR.T |
|  | 8239 | 621.3292 | 1240.6439 | 1239.5013 | 1.1426 | 0 | 11 | 12 | 3 | R.MYGCDLGPDGR.L |
|  | 8528 | 628.9625 | 1255.9104 | 1255.4962 | 0.4142 | 0 | (1) | 78 | 5 | R.MYGCDLGPDGR.L |
|  | 10294 | 676.3427 | 1350.6709 | 1349.6939 | 0.9770 | 1 | 8 | 24 | 1 | R.YLENGKETLQR.A |
|  | 11642 | 714.3621 | 1426.7097 | 1426.7205 | -0.0108 | 0 | 11 | 10 | 1 | K.WAAVVVPSGEEQR.Y |
|  | 14790 | 815.4116 | 1628.8086 | 1628.8199 | -0.0113 | 0 | 27 | 0.26 | 1 | R.FIAVGYVDDTQFVR.F |
|  | 17823 | 613.3262 | 1836.9569 | 1835.9027 | 1.0542 | 0 | 9 | 14 | 2 | K.THVTHHPVSDHEATLR.C |
|  | 22148 | 744.6662 | 2230.9768 | 2230.9938 | -0.0170 | 0 | 17 | 1.6 | 1 | R.DGEDQTQDTELVETRPAGDR.T |

|  | **Proteins matching the same set of peptides:** |
| --- | --- |

|  | P30491\|1B53_HUMAN    **Mass:** 40755    **Score:** 56     **Queries matched:** 8 |
| --- | --- |
|  | HLA class I histocompatibility antigen, B-53 alpha chain OS=Homo sapiens GN=HLA-B PE=1 SV=1 |

|  | P30685\|1B35_HUMAN    **Mass:** 40715    **Score:** 56     **Queries matched:** 8 |
| --- | --- |
|  | HLA class I histocompatibility antigen, B-35 alpha chain OS=Homo sapiens GN=HLA-B PE=1 SV=1 |

|  | **Proteins matching a subset of these peptides:** |
| --- | --- |

|  | P18464\|1B51_HUMAN    **Mass:** 40826    **Score:** 56     **Queries matched:** 5 |
| --- | --- |
|  | HLA class I histocompatibility antigen, B-51 alpha chain OS=Homo sapiens GN=HLA-B PE=1 SV=1 |

|  | P30498\|1B78_HUMAN    **Mass:** 40738    **Score:** 56     **Queries matched:** 5 |
| --- | --- |
|  | HLA class I histocompatibility antigen, B-78 alpha chain OS=Homo sapiens GN=HLA-B PE=2 SV=1 |

|  | P30495\|1B56_HUMAN    **Mass:** 40738    **Score:** 56     **Queries matched:** 5 |
| --- | --- |
|  | HLA class I histocompatibility antigen, B-56 alpha chain OS=Homo sapiens GN=HLA-B PE=2 SV=1 |

| **674.** | Q29940\|1B59_HUMAN    **Mass:** 40844    **Score:** 56     **Queries matched:** 6   **emPAI:** 0.30 |
| --- | --- |
|  | HLA class I histocompatibility antigen, B-59 alpha chain OS=Homo sapiens GN=HLA-B PE=2 SV=1 |

|  | **Query** | **Observed** | **Mr(expt)** | **Mr(calc)** | **Delta** | **Miss** | **Score** | **Expect** | **Rank** | **Peptide** |
| --- | --- | --- | --- | --- | --- | --- | --- | --- | --- | --- |
|  | 4142 | 483.2169 | 964.4193 | 964.4250 | -0.0057 | 0 | 53 | 0.00075 | 1 | R.FDSDAASPR.E |
|  | 10294 | 676.3427 | 1350.6709 | 1349.6939 | 0.9770 | 1 | 8 | 24 | 1 | R.YLENGKETLQR.A |
|  | 11642 | 714.3621 | 1426.7097 | 1426.7205 | -0.0108 | 0 | 11 | 10 | 1 | K.WAAVVVPSGEEQR.Y |
|  | 12774 | 748.8586 | 1495.7026 | 1495.7129 | -0.0103 | 0 | 11 | 9.6 | 2 | R.AYLEGTCVEWLR.R |
|  | 14790 | 815.4116 | 1628.8086 | 1628.8199 | -0.0113 | 0 | 27 | 0.26 | 1 | R.FIAVGYVDDTQFVR.F |
|  | 22148 | 744.6662 | 2230.9768 | 2230.9938 | -0.0170 | 0 | 17 | 1.6 | 1 | R.DGEDQTQDTELVETRPAGDR.T |

|  | **Proteins matching the same set of peptides:** |
| --- | --- |

|  | P30492\|1B54_HUMAN    **Mass:** 40640    **Score:** 56     **Queries matched:** 6 |
| --- | --- |
|  | HLA class I histocompatibility antigen, B-54 alpha chain OS=Homo sapiens GN=HLA-B PE=2 SV=1 |

|  | P30493\|1B55_HUMAN    **Mass:** 40756    **Score:** 56     **Queries matched:** 6 |
| --- | --- |
|  | HLA class I histocompatibility antigen, B-55 alpha chain OS=Homo sapiens GN=HLA-B PE=1 SV=1 |

| **675.** | P30464\|1B15_HUMAN    **Mass:** 40648    **Score:** 56     **Queries matched:** 7   **emPAI:** 0.30 |
| --- | --- |
|  | HLA class I histocompatibility antigen, B-15 alpha chain OS=Homo sapiens GN=HLA-B PE=1 SV=2 |

|  | **Query** | **Observed** | **Mr(expt)** | **Mr(calc)** | **Delta** | **Miss** | **Score** | **Expect** | **Rank** | **Peptide** |
| --- | --- | --- | --- | --- | --- | --- | --- | --- | --- | --- |
|  | 1586 | 705.3914 | 704.3841 | 704.3705 | 0.0136 | 0 | 8 | 31 | 8 | R.ETQISK.T |
|  | 4142 | 483.2169 | 964.4193 | 964.4250 | -0.0057 | 0 | 53 | 0.00075 | 1 | R.FDSDAASPR.M |
|  | 10294 | 676.3427 | 1350.6709 | 1349.6939 | 0.9770 | 1 | 8 | 24 | 1 | R.YLENGKETLQR.A |
|  | 11642 | 714.3621 | 1426.7097 | 1426.7205 | -0.0108 | 0 | 11 | 10 | 1 | K.WAAVVVPSGEEQR.Y |
|  | 14790 | 815.4116 | 1628.8086 | 1628.8199 | -0.0113 | 0 | 27 | 0.26 | 1 | R.FIAVGYVDDTQFVR.F |
|  | 22148 | 744.6662 | 2230.9768 | 2230.9938 | -0.0170 | 0 | 17 | 1.6 | 1 | R.DGEDQTQDTELVETRPAGDR.T |
|  | 29523 | **1263.9766** | **3788.9079** | **3787.9178** | **0.9901** | **1** | **4** | **15** | **1** | **R.TVLLLLSGALALTETWAGSHSMRYFYTAMSRPGR.G** |

|  | **Proteins matching a subset of these peptides:** |
| --- | --- |

|  | P30484\|1B46_HUMAN    **Mass:** 40700    **Score:** 56     **Queries matched:** 6 |
| --- | --- |
|  | HLA class I histocompatibility antigen, B-46 alpha chain OS=Homo sapiens GN=HLA-B PE=2 SV=1 |

| **676.** | P30490\|1B52_HUMAN    **Mass:** 40781    **Score:** 56     **Queries matched:** 6   **emPAI:** 0.30 |
| --- | --- |
|  | HLA class I histocompatibility antigen, B-52 alpha chain OS=Homo sapiens GN=HLA-B PE=2 SV=1 |

|  | **Query** | **Observed** | **Mr(expt)** | **Mr(calc)** | **Delta** | **Miss** | **Score** | **Expect** | **Rank** | **Peptide** |
| --- | --- | --- | --- | --- | --- | --- | --- | --- | --- | --- |
|  | 1586 | 705.3914 | 704.3841 | 704.3705 | 0.0136 | 0 | 8 | 31 | 8 | R.ETQISK.T |
|  | 4142 | 483.2169 | 964.4193 | 964.4250 | -0.0057 | 0 | 53 | 0.00075 | 1 | R.FDSDAASPR.T |
|  | 11642 | 714.3621 | 1426.7097 | 1426.7205 | -0.0108 | 0 | 11 | 10 | 1 | K.WAAVVVPSGEEQR.Y |
|  | 14790 | 815.4116 | 1628.8086 | 1628.8199 | -0.0113 | 0 | 27 | 0.26 | 1 | R.FIAVGYVDDTQFVR.F |
|  | 17823 | 613.3262 | 1836.9569 | 1835.9027 | 1.0542 | 0 | 9 | 14 | 2 | K.THVTHHPVSDHEATLR.C |
|  | 22148 | 744.6662 | 2230.9768 | 2230.9938 | -0.0170 | 0 | 17 | 1.6 | 1 | R.DGEDQTQDTELVETRPAGDR.T |

| **677.** | P18465\|1B57_HUMAN    **Mass:** 40541    **Score:** 56     **Queries matched:** 8   **emPAI:** 0.30 |
| --- | --- |
|  | HLA class I histocompatibility antigen, B-57 alpha chain OS=Homo sapiens GN=HLA-B PE=1 SV=1 |

|  | **Query** | **Observed** | **Mr(expt)** | **Mr(calc)** | **Delta** | **Miss** | **Score** | **Expect** | **Rank** | **Peptide** |
| --- | --- | --- | --- | --- | --- | --- | --- | --- | --- | --- |
|  | 4142 | 483.2169 | 964.4193 | 964.4250 | -0.0057 | 0 | 53 | 0.00075 | 1 | R.FDSDAASPR.M |
|  | 10294 | 676.3427 | 1350.6709 | 1349.6939 | 0.9770 | 1 | 8 | 24 | 1 | R.YLENGKETLQR.A |
|  | 11642 | 714.3621 | 1426.7097 | 1426.7205 | -0.0108 | 0 | 11 | 10 | 1 | K.WAAVVVPSGEEQR.Y |
|  | 14790 | 815.4116 | 1628.8086 | 1628.8199 | -0.0113 | 0 | 27 | 0.26 | 1 | R.FIAVGYVDDTQFVR.F |
|  | 22148 | 744.6662 | 2230.9768 | 2230.9938 | -0.0170 | 0 | 17 | 1.6 | 1 | R.DGEDQTQDTELVETRPAGDR.T |
|  | 29581 | 948.2367 | 3788.9177 | 3790.1002 | -1.1825 | 1 | 4 | 13 | 1 | R.WEPSSQSTVPIVGIVAGLAVLAVVVIGAVVAAVMCRR.K 29529 29557 |

| **678.** | Q9NQR4\|NIT2_HUMAN    **Mass:** 30988    **Score:** 56     **Queries matched:** 7   **emPAI:** 0.26 |
| --- | --- |
|  | Nitrilase homolog 2 OS=Homo sapiens GN=NIT2 PE=1 SV=1 |

|  | **Query** | **Observed** | **Mr(expt)** | **Mr(calc)** | **Delta** | **Miss** | **Score** | **Expect** | **Rank** | **Peptide** |
| --- | --- | --- | --- | --- | --- | --- | --- | --- | --- | --- |
|  | 1430 | 647.2818 | 646.2745 | 645.3697 | 0.9048 | 0 | 13 | 12 | 3 | K.LSEVAK.E |
|  | 7950 | **614.3510** | **1226.6875** | **1226.5965** | **0.0910** | **1** | **8** | **27** | **9** | **K.RSDLYAVEMK.K** |
|  | 13776 | **781.3962** | **1560.7778** | **1560.7896** | **-0.0118** | **0** | **47** | **0.0024** | **1** | **R.AVDNQVYVATASPAR.D** |
|  | 14711 | **542.1284** | **1623.3633** | **1622.8039** | **0.5593** | **0** | **0** | **71** | **6** | **K.AGTEEAIVYSDIDLK.K** |
|  | 15592 | 841.4112 | 1680.8078 | 1680.8181 | -0.0103 | 0 | 26 | 0.3 | 1 | K.IVSLPECFNSPYGAK.Y |
|  | 18472 | **629.6025** | **1885.7858** | **1885.9098** | **-0.1240** | **1** | **4** | **40** | **1** | **K.YFPEYAEKIPGESTQK.L** |
|  | 19404 | **655.3774** | **1963.1103** | **1964.1128** | **-1.0025** | **1** | **4** | **36** | **1** | **-.MTSFRLALIQLQISSIK.S** |

| **679.** | Q99424\|ACOX2_HUMAN    **Mass:** 77576    **Score:** 56     **Queries matched:** 24   **emPAI:** 0.05 |
| --- | --- |
|  | Peroxisomal acyl-coenzyme A oxidase 2 OS=Homo sapiens GN=ACOX2 PE=1 SV=1 |

|  | **Query** | **Observed** | **Mr(expt)** | **Mr(calc)** | **Delta** | **Miss** | **Score** | **Expect** | **Rank** | **Peptide** |
| --- | --- | --- | --- | --- | --- | --- | --- | --- | --- | --- |
|  | 2897 | 436.2220 | 870.4294 | 870.4235 | 0.0059 | 0 | 5 | 60 | 3 | R.ELGYAYR.A |
|  | 4844 | **507.8031** | **1013.5916** | **1013.6121** | **-0.0205** | **0** | **7** | **31** | **4** | **K.LSGLPSLVTK.L** |
|  | 5901 | 549.3089 | 1096.6032 | 1095.6665 | 0.9367 | 1 | 5 | 49 | 8 | R.AFHIRLIAR.R |
|  | 8559 | **420.2322** | **1257.6747** | **1256.5455** | **1.1292** | **0** | **10** | **15** | **4** | **R.QMHPDIESER.Y** |
|  | 8687 | 633.2604 | 1264.5063 | 1264.6888 | -0.1824 | 0 | 5 | 44 | 2 | R.ALSGDVALNIHR.V |
|  | 11186 | **467.7521** | **1400.2344** | **1400.7623** | **-0.5279** | **1** | **9** | **12** | **5** | **R.ALRSLGSEEQIAK.W** 11187 |
|  | 13714 | **778.8967** | **1555.7788** | **1555.8318** | **-0.0530** | **0** | **18** | **2.1** | **1** | **R.LTNILDGGAQNTALR.R** 13693 13710 |
|  | 15313 | **831.4450** | **1660.8755** | **1659.8832** | **0.9923** | **0** | **56** | **0.00032** | **1** | **R.SLSPSVAYLTAPDLAR.C** |
|  | 15325 | 831.9429 | 1661.8713 | 1662.8763 | -1.0050 | 0 | 11 | 9.3 | 3 | K.LGTAQSNYLPMVVVR.V 15324 15349 |
|  | 16137 | 571.9377 | 1712.7914 | 1711.9329 | 0.8585 | 1 | 2 | 67 | 9 | R.LTNILDGGAQNTALRR.K |
|  | 17154 | 595.3156 | 1782.9250 | 1783.8788 | -0.9538 | 1 | 9 | 13 | 1 | -.MGSPVHRVSLGDTWSR.Q 17153 |
|  | 17626 | 608.3438 | 1822.0096 | 1822.8923 | -0.8827 | 1 | 10 | 11 | 2 | R.KVESIIHSYPEFSCK.D |
|  | 17637 | **608.8743** | **1823.6012** | **1823.6769** | **-0.0757** | **0** | **6** | **19** | **3** | **K.AMMSEFCTQGAEMCR.R** |
|  | 18068 | **619.5245** | **1855.5516** | **1855.6667** | **-0.1151** | **0** | **(5)** | **24** | **1** | **K.AMMSEFCTQGAEMCR.R** 18070 |
|  | 19769 | **666.3779** | **1996.1118** | **1995.7729** | **0.3388** | **1** | **2** | **54** | **3** | **K.AMMSEFCTQGAEMCRR.A** |
|  | 23096 | **785.5078** | **2353.5016** | **2352.3273** | **1.1743** | **1** | **7** | **11** | **2** | **R.VELLSGEILPILQKACVIAMR.Y** |
|  | 31319 | **1253.1266** | **5008.4773** | **5008.4982** | **-0.0209** | **0** | **5** | **4.1** | **1** | **K.NIQIIATYAQTELGHGTYLQGLETEATYDAATQEFVIHSPTLTATK.W** |

| **680.** | P09669\|COX6C_HUMAN    **Mass:** 8776     **Score:** 55     **Queries matched:** 7   **emPAI:** 0.46 |
| --- | --- |
|  | Cytochrome c oxidase subunit 6C OS=Homo sapiens GN=COX6C PE=1 SV=2 |

|  | **Query** | **Observed** | **Mr(expt)** | **Mr(calc)** | **Delta** | **Miss** | **Score** | **Expect** | **Rank** | **Peptide** |
| --- | --- | --- | --- | --- | --- | --- | --- | --- | --- | --- |
|  | 2579 | 425.2419 | 848.4691 | 848.4756 | -0.0065 | 0 | 55 | 0.00055 | 1 | K.AGIFQSVK.- 2581 |
|  | 3329 | **905.4087** | **904.4014** | **904.4079** | **-0.0065** | **0** | **12** | **13** | **1** | **K.AYADFYR.N** |
|  | 3995 | 477.7174 | 953.4203 | 953.4276 | -0.0073 | 1 | 7 | 29 | 9 | K.DFEEMRK.A |
|  | 4308 | 489.2307 | 976.4469 | 976.5706 | -0.1237 | 1 | 11 | 14 | 4 | R.KAGIFQSVK.- |
|  | 13969 | 788.8369 | 1575.6591 | 1575.6697 | -0.0106 | 1 | 23 | 0.53 | 1 | R.NYDVMKDFEEMR.K |
|  | 22057 | **556.7893** | **2223.1279** | **2222.2034** | **0.9245** | **1** | **1** | **64** | **8** | **R.NHMAVAFVLSLGVAALYKFR.V** |

| **681.** | P31512\|FMO4_HUMAN    **Mass:** 64214    **Score:** 55     **Queries matched:** 13   **emPAI:** 0.12 |
| --- | --- |
|  | Dimethylaniline monooxygenase [N-oxide-forming] 4 OS=Homo sapiens GN=FMO4 PE=2 SV=3 |

|  | **Query** | **Observed** | **Mr(expt)** | **Mr(calc)** | **Delta** | **Miss** | **Score** | **Expect** | **Rank** | **Peptide** |
| --- | --- | --- | --- | --- | --- | --- | --- | --- | --- | --- |
|  | 3038 | **441.2963** | **880.5780** | **880.4654** | **0.1125** | **1** | **3** | **73** | **10** | **R.GVFKDTSK.D** |
|  | 3126 | **445.0336** | **888.0526** | **888.4818** | **-0.4291** | **0** | **2** | **92** | **9** | **R.TGTWVLGR.S** |
|  | 4175 | **484.6528** | **967.2911** | **968.4749** | **-1.1838** | **1** | **0** | **1.2e+002** | **4** | **K.DGMTRVYK.S** |
|  | 4479 | 495.7448 | 989.4751 | 989.4818 | -0.0067 | 0 | 45 | 0.0056 | 1 | R.SDDIGGLWK.F |
|  | 6153 | 558.7893 | 1115.5639 | 1115.5723 | -0.0084 | 0 | 45 | 0.0049 | 1 | R.NAILTQWDR.T |
|  | 6297 | **564.7914** | **1127.5683** | **1126.6169** | **0.9515** | **1** | **3** | **89** | **6** | **K.GLCKIPPSQK.L** |
|  | 11817 | 480.2843 | 1437.8311 | 1436.6904 | 1.1406 | 0 | 1 | 1.1e+002 | 10 | R.CCSFIAQVLPSR.F |
|  | 12598 | **743.4355** | **1484.8564** | **1484.8926** | **-0.0362** | **1** | **6** | **34** | **3** | **K.KVAVIGAGVSGLSSIK.C** |
|  | 14277 | **531.9625** | **1592.8656** | **1592.7915** | **0.0740** | **1** | **12** | **7.3** | **3** | **R.RCCSFIAQVLPSR.F** |
|  | 16801 | **879.8987** | **1757.7828** | **1758.8974** | **-1.1146** | **0** | **2** | **75** | **3** | **R.IVPDSSKPASMSHYLK.A** |
|  | 16976 | 591.6310 | 1771.8713 | 1772.9533 | -1.0820 | 1 | 1 | 85 | 10 | K.GSILSGTELQARWVTR.V |
|  | 29406 | 1247.9552 | 3740.8438 | 3740.5680 | 0.2757 | 1 | 4 | 14 | 3 | K.SLVTNVCKEMSCYSDFPFHEDYPNFMNHEK.F 29408 |

| **682.** | Q9H0W9\|CK054_HUMAN    **Mass:** 35608    **Score:** 55     **Queries matched:** 7   **emPAI:** 0.11 |
| --- | --- |
|  | Ester hydrolase C11orf54 OS=Homo sapiens GN=C11orf54 PE=1 SV=1 |

|  | **Query** | **Observed** | **Mr(expt)** | **Mr(calc)** | **Delta** | **Miss** | **Score** | **Expect** | **Rank** | **Peptide** |
| --- | --- | --- | --- | --- | --- | --- | --- | --- | --- | --- |
|  | 2983 | 878.3532 | 877.3459 | 878.4861 | -1.1403 | 1 | 6 | 45 | 7 | K.KVYDLNK.I 3011 |
|  | 17475 | **905.5218** | **1809.0291** | **1809.0400** | **-0.0109** | **0** | **55** | **0.0003** | **1** | **R.IAEVGGVPYLLPLVNQK.K** |
|  | 18807 | **478.9937** | **1911.9456** | **1910.9230** | **1.0226** | **1** | **6** | **27** | **1** | **R.TGPLNFVTCMRETLEK.H** |
|  | 24019 | 620.0754 | 2476.2727 | 2476.2784 | -0.0058 | 1 | 1 | 64 | 2 | R.ETLEKHYGNKPIGMGGTFIIQK.G |
|  | 28920 | **1181.5272** | **3541.5598** | **3540.5788** | **0.9810** | **1** | **1** | **31** | **2** | **K.SHIMPAEFSSCPLNSDEEVNKWLHFYEMK.A** |
|  | 31808 | **1183.7769** | **5913.8479** | **5912.8971** | **0.9508** | **0** | **10** | **0.66** | **1** | **K.LPGAFILGAGAGPFQTLGFNSEFMPVIQTESEHKPPVNGSYFAHVNPADGGCLLEK.Y** |

| **683.** | P19338\|NUCL_HUMAN    **Mass:** 76625    **Score:** 55     **Queries matched:** 21   **emPAI:** 0.10 |
| --- | --- |
|  | Nucleolin OS=Homo sapiens GN=NCL PE=1 SV=3 |

|  | **Query** | **Observed** | **Mr(expt)** | **Mr(calc)** | **Delta** | **Miss** | **Score** | **Expect** | **Rank** | **Peptide** |
| --- | --- | --- | --- | --- | --- | --- | --- | --- | --- | --- |
|  | 1065 | **550.2139** | **549.2066** | **549.2660** | **-0.0594** | **0** | **4** | **89** | **4** | **R.GGFGGR.G** 1077 |
|  | 1354 | **628.3714** | **627.3641** | **626.3751** | **0.9890** | **0** | **7** | **33** | **8** | **K.GAAIPAK.G** |
|  | 1361 | **629.3574** | **628.3501** | **628.3544** | **-0.0043** | **0** | **14** | **9.5** | **5** | **K.AAATPAK.K** |
|  | 3095 | **443.0396** | **884.0647** | **883.5491** | **0.5156** | **1** | **6** | **27** | **1** | **K.KVAVATPAK.K** |
|  | 3443 | **457.2428** | **912.4711** | **913.4617** | **-0.9906** | **1** | **10** | **15** | **7** | **K.AGKNQGDPK.K** 3447 |
|  | 3842 | **472.7366** | **943.4587** | **942.5246** | **0.9341** | **1** | **6** | **51** | **5** | **R.EIEGRAIR.L** |
|  | 4651 | 500.8021 | 999.5896 | 999.5349 | 0.0547 | 0 | 13 | 8.2 | 6 | K.NDLAVVDVR.I |
|  | 5399 | **529.3799** | **1056.7453** | **1056.5968** | **0.1486** | **0** | **3** | **80** | **10** | **K.VTLDWAKPK.G** |
|  | 6287 | **564.2753** | **1126.5361** | **1127.5683** | **-1.0322** | **1** | **1** | **1.3e+002** | **6** | **R.GSPNARSQPSK.T** |
|  | 11057 | **465.2382** | **1392.6927** | **1393.7426** | **-1.0499** | **1** | **7** | **26** | **2** | **R.LELQGPRGSPNAR.S** |
|  | 11938 | 723.8431 | 1445.6716 | 1446.7718 | -1.1002 | 1 | 17 | 2.9 | 4 | K.NLPYKVTQDELK.E |
|  | 13768 | **781.3394** | **1560.6642** | **1560.6733** | **-0.0091** | **0** | **45** | **0.0041** | **1** | **K.GFGFVDFNSEEDAK.A** |
|  | 13800 | 521.8254 | 1562.4545 | 1561.8352 | 0.6193 | 1 | 2 | 53 | 6 | K.SAPELKTGISDVFAK.N |
|  | 14264 | 531.6107 | 1591.8102 | 1592.6988 | -0.8886 | 1 | 6 | 33 | 6 | K.AAKEAMEDGEIDGNK.V |
|  | 14846 | **544.8287** | **1631.4644** | **1630.9294** | **0.5350** | **1** | **2** | **49** | **9** | **K.ALELTGLKVFGNEIK.L** 14841 |
|  | 17741 | 611.3269 | 1830.9589 | 1830.8424 | 0.1164 | 1 | 2 | 73 | 2 | K.GFGFVDFNSEEDAKAAK.E |
|  | 21798 | **734.0083** | **2199.0031** | **2199.0179** | **-0.0148** | **1** | **30** | **0.086** | **1** | **K.GLSEDTTEETLKESFDGSVR.A** |
|  | 29973 | 988.2196 | 3948.8493 | 3949.6252 | -0.7759 | 1 | 1 | 26 | 2 | K.MAPPPKEVEEDSEDEEMSEDEEDDSSGEEVVIPQK.K |

| **684.** | Q8N5M9\|JAGN1_HUMAN    **Mass:** 21111    **Score:** 55     **Queries matched:** 6   **emPAI:** 0.18 |
| --- | --- |
|  | Protein jagunal homolog 1 OS=Homo sapiens GN=JAGN1 PE=1 SV=1 |

|  | **Query** | **Observed** | **Mr(expt)** | **Mr(calc)** | **Delta** | **Miss** | **Score** | **Expect** | **Rank** | **Peptide** |
| --- | --- | --- | --- | --- | --- | --- | --- | --- | --- | --- |
|  | 2157 | **408.7115** | **815.4085** | **814.4120** | **0.9966** | **0** | **7** | **40** | **2** | **K.MSVGHLR.L** |
|  | 8632 | **631.2766** | **1260.5387** | **1260.5483** | **-0.0097** | **0** | **55** | **0.00048** | **1** | **R.AAGTDGSDFQHR.E** |
|  | 11591 | **475.2841** | **1422.8304** | **1422.6999** | **0.1305** | **0** | **10** | **13** | **2** | **R.VAMHYQMSVTLK.Y** |
|  | 16036 | 854.4293 | 1706.8441 | 1707.8436 | -0.9995 | 1 | 4 | 48 | 4 | R.ERVAMHYQMSVTLK.Y |
|  | 16277 | **575.2900** | **1722.8483** | **1723.8385** | **-0.9903** | **1** | **(2)** | **72** | **7** | **R.ERVAMHYQMSVTLK.Y** |
|  | 19510 | 987.4581 | 1972.9016 | 1971.9798 | 0.9218 | 1 | 4 | 37 | 5 | R.VAMHYQMSVTLKYEIK.K |

| **685.** | P02655\|APOC2_HUMAN    **Mass:** 11277    **Score:** 55     **Queries matched:** 4   **emPAI:** 0.83 |
| --- | --- |
|  | Apolipoprotein C-II OS=Homo sapiens GN=APOC2 PE=1 SV=1 |

|  | **Query** | **Observed** | **Mr(expt)** | **Mr(calc)** | **Delta** | **Miss** | **Score** | **Expect** | **Rank** | **Peptide** |
| --- | --- | --- | --- | --- | --- | --- | --- | --- | --- | --- |
|  | 5152 | **519.2628** | **1036.5110** | **1036.5189** | **-0.0079** | **0** | **30** | **0.15** | **1** | **K.TAAQNLYEK.T** |
|  | 9402 | **435.2679** | **1302.7818** | **1303.7136** | **-0.9318** | **1** | **13** | **7** | **2** | **K.TYLPAVDEKLR.D** |
|  | 24481 | **1274.6215** | **2547.2284** | **2547.2414** | **-0.0131** | **1** | **52** | **0.00041** | **1** | **K.STAAMSTYTGIFTDQVLSVLKGEE.-** 24482 |

| **686.** | Q15006\|TTC35_HUMAN    **Mass:** 34982    **Score:** 55     **Queries matched:** 5   **emPAI:** 0.11 |
| --- | --- |
|  | Tetratricopeptide repeat protein 35 OS=Homo sapiens GN=TTC35 PE=1 SV=1 |

|  | **Query** | **Observed** | **Mr(expt)** | **Mr(calc)** | **Delta** | **Miss** | **Score** | **Expect** | **Rank** | **Peptide** |
| --- | --- | --- | --- | --- | --- | --- | --- | --- | --- | --- |
|  | 8729 | 423.2327 | 1266.6762 | 1265.6153 | 1.0609 | 0 | 4 | 53 | 8 | K.YASWAASQINR.A |
|  | 9889 | **664.8391** | **1327.6635** | **1327.6732** | **-0.0096** | **0** | **55** | **0.00047** | **1** | **R.ILQEDPTNTAAR.K** 9908 9910 9911 |

| **687.** | Q14696\|MESD_HUMAN    **Mass:** 26231    **Score:** 55     **Queries matched:** 6   **emPAI:** 0.15 |
| --- | --- |
|  | LDLR chaperone MESD OS=Homo sapiens GN=MESDC2 PE=1 SV=2 |

|  | **Query** | **Observed** | **Mr(expt)** | **Mr(calc)** | **Delta** | **Miss** | **Score** | **Expect** | **Rank** | **Peptide** |
| --- | --- | --- | --- | --- | --- | --- | --- | --- | --- | --- |
|  | 1552 | 689.3929 | 688.3857 | 688.3755 | 0.0101 | 1 | 14 | 11 | 3 | K.KEGDLK.S |
|  | 3916 | **474.9752** | **947.9358** | **947.4759** | **0.4599** | **1** | **8** | **21** | **4** | **-.MAASRWAR.K** |
|  | 13399 | 513.2572 | 1536.7498 | 1535.7290 | 1.0208 | 0 | 9 | 14 | 2 | R.CADVTLEGQVYPGK.G 13400 |
|  | 14156 | 793.9229 | 1585.8313 | 1585.8749 | -0.0436 | 1 | 7 | 23 | 4 | K.IDPSKPESILKMTK.K |
|  | 30565 | **1403.9980** | **4208.9723** | **4208.0082** | **0.9641** | **1** | **55** | **7.5e-005** | **1** | **K.TLMMFVTVSGSPTEKETEEITSLWQGSLFNANYDVQR.F** |

| **688.** | O43181\|NDUS4_HUMAN    **Mass:** 20095    **Score:** 55     **Queries matched:** 8   **emPAI:** 0.19 |
| --- | --- |
|  | NADH dehydrogenase [ubiquinone] iron-sulfur protein 4, mitochondrial OS=Homo sapiens GN=NDUFS4 PE=1 SV=1 |

|  | **Query** | **Observed** | **Mr(expt)** | **Mr(calc)** | **Delta** | **Miss** | **Score** | **Expect** | **Rank** | **Peptide** |
| --- | --- | --- | --- | --- | --- | --- | --- | --- | --- | --- |
|  | 7363 | **400.2373** | **1197.6900** | **1198.7146** | **-1.0246** | **1** | **7** | **29** | **6** | **R.RAVAVAALSVSR.V** |
|  | 7555 | **604.2795** | **1206.5444** | **1205.5459** | **0.9985** | **0** | **17** | **3** | **2** | **R.NNMQSGVNNTK.K** |
|  | 9901 | 665.2972 | 1328.5798 | 1328.6262 | -0.0464 | 1 | 2 | 89 | 5 | K.SYGANFSWNKR.T |
|  | 9998 | **667.8237** | **1333.6328** | **1333.6408** | **-0.0080** | **1** | **27** | **0.27** | **1** | **R.NNMQSGVNNTKK.W** |
|  | 10965 | **694.8488** | **1387.6829** | **1387.6520** | **0.0309** | **1** | **0** | **1.4e+002** | **8** | **K.SKSYGANFSWNK.R** |
|  | 18734 | 636.3291 | 1905.9655 | 1904.9527 | 1.0128 | 1 | 1 | 73 | 10 | R.IFVPARNNMQSGVNNTK.K |
|  | 25891 | **1398.6941** | **2795.3736** | **2795.3299** | **0.0438** | **0** | **55** | **0.00021** | **1** | **R.WENPLMGWASTADPLSNMVLTFSTK.E** |
|  | 29518 | **1263.9762** | **3788.9068** | **3787.7749** | **1.1318** | **1** | **4** | **12** | **1** | **R.WENPLMGWASTADPLSNMVLTFSTKEDAVSFAEK.N** |

| **689.** | P23528\|COF1_HUMAN    **Mass:** 18719    **Score:** 55     **Queries matched:** 15   **emPAI:** 0.45 |
| --- | --- |
|  | Cofilin-1 OS=Homo sapiens GN=CFL1 PE=1 SV=3 |

|  | **Query** | **Observed** | **Mr(expt)** | **Mr(calc)** | **Delta** | **Miss** | **Score** | **Expect** | **Rank** | **Peptide** |
| --- | --- | --- | --- | --- | --- | --- | --- | --- | --- | --- |
|  | 1925 | 799.3966 | 798.3893 | 798.3945 | -0.0053 | 0 | 26 | 0.44 | 1 | K.MIYASSK.D 1926 |
|  | 5103 | 517.7377 | 1033.4609 | 1033.4685 | -0.0076 | 1 | 16 | 4.5 | 1 | K.MLPDKDCR.Y |
|  | 5240 | **522.8067** | **1043.5988** | **1042.6022** | **0.9966** | **1** | **6** | **42** | **7** | **K.KNIILEEGK.E** |
|  | 7926 | **409.4392** | **1225.2958** | **1225.6376** | **-0.3418** | **1** | **3** | **44** | **4** | **K.MIYASSKDAIK.K** |
|  | 8106 | **412.2469** | **1233.7188** | **1232.6435** | **1.0753** | **0** | **8** | **24** | **7** | **-.MASGVAVSDGVIK.V** 8052 |
|  | 8384 | 416.9041 | 1247.6905 | 1248.6384 | -0.9479 | 0 | (5) | 42 | 10 | -.MASGVAVSDGVIK.V 8391 |
|  | 10048 | **669.3123** | **1336.6101** | **1336.6187** | **-0.0086** | **0** | **25** | **0.44** | **1** | **R.YALYDATYETK.E** |
|  | 17251 | **597.6054** | **1789.7944** | **1789.8053** | **-0.0109** | **1** | **50** | **0.001** | **1** | **K.HELQANCYEEVKDR.C** |
|  | 17799 | 612.6461 | 1834.9164 | 1835.9451 | -1.0288 | 1 | 5 | 38 | 8 | M.ASGVAVSDGVIKVFNDMK.V 17800 |
|  | 19598 | **661.7976** | **1982.3710** | **1982.9805** | **-0.6095** | **1** | **1** | **48** | **2** | **-.MASGVAVSDGVIKVFNDMK.V** |
|  | 20287 | 1022.5059 | 2042.9972 | 2042.1082 | 0.8890 | 1 | 1 | 75 | 2 | R.CTLAEKLGGSAVISLEGKPL.- |

| **690.** | P61421\|VA0D1_HUMAN    **Mass:** 40759    **Score:** 54     **Queries matched:** 10   **emPAI:** 0.19 |
| --- | --- |
|  | V-type proton ATPase subunit d 1 OS=Homo sapiens GN=ATP6V0D1 PE=1 SV=1 |

|  | **Query** | **Observed** | **Mr(expt)** | **Mr(calc)** | **Delta** | **Miss** | **Score** | **Expect** | **Rank** | **Peptide** |
| --- | --- | --- | --- | --- | --- | --- | --- | --- | --- | --- |
|  | 2649 | 428.7446 | 855.4747 | 855.5065 | -0.0318 | 0 | 17 | 3.6 | 4 | R.SIAELVPK.C |
|  | 5348 | **527.6161** | **1053.2176** | **1052.5325** | **0.6852** | **1** | **7** | **19** | **1** | **K.EKMVVEFR.H** |
|  | 8130 | **412.8777** | **1235.6113** | **1235.5903** | **0.0210** | **1** | **10** | **15** | **3** | **K.MVVEFRHMR.N** |
|  | 9424 | **653.2681** | **1304.5216** | **1303.6408** | **0.8808** | **0** | **26** | **0.33** | **1** | **K.LLFEGAGSNPGDK.T** 9427 |
|  | 21608 | 1088.4933 | 2174.9720 | 2174.0361 | 0.9360 | 1 | 0 | 83 | 7 | K.EQECRNIVWIAECIAQR.H |
|  | 26051 | 942.1690 | 2823.4852 | 2823.3942 | 0.0910 | 1 | 3 | 29 | 3 | -.MSFFPELYFNVDNGYLEGLVRGLK.A |
|  | 27963 | **804.8501** | **3215.3713** | **3215.6462** | **-0.2749** | **1** | **1** | **33** | **5** | **K.LHLQSTDYGNFLANEASPLTVSVIDDRLK.E** |
|  | 31503 | 1359.6748 | 5434.6701 | 5434.6879 | -0.0178 | 1 | 47 | 0.00023 | 1 | K.AGVLSQADYLNLVQCETLEDLKLHLQSTDYGNFLANEASPLTVSVIDDR.L 31502 |

| **691.** | Q06830\|PRDX1_HUMAN    **Mass:** 22324    **Score:** 54     **Queries matched:** 16   **emPAI:** 1.21 |
| --- | --- |
|  | Peroxiredoxin-1 OS=Homo sapiens GN=PRDX1 PE=1 SV=1 |

|  | **Query** | **Observed** | **Mr(expt)** | **Mr(calc)** | **Delta** | **Miss** | **Score** | **Expect** | **Rank** | **Peptide** |
| --- | --- | --- | --- | --- | --- | --- | --- | --- | --- | --- |
|  | 3537 | 460.7552 | 919.4958 | 919.5015 | -0.0057 | 0 | 39 | 0.023 | 1 | R.GLFIIDDK.G |
|  | 4361 | **490.7658** | **979.5171** | **979.5239** | **-0.0068** | **0** | **18** | **2.2** | **1** | **K.IGHPAPNFK.A** |
|  | 6776 | **582.7853** | **1163.5560** | **1163.5645** | **-0.0085** | **0** | **37** | **0.028** | **1** | **K.ATAVMPDGQFK.D** |
|  | 7287 | 598.2915 | 1194.5684 | 1195.6237 | -1.0553 | 0 | 6 | 37 | 2 | R.LVQAFQFTDK.H |
|  | 8659 | **632.3124** | **1262.6103** | **1262.6983** | **-0.0879** | **1** | **29** | **0.19** | **1** | **K.RTIAQDYGVLK.A** |
|  | 15135 | 550.8157 | 1649.4254 | 1649.9577 | -0.5323 | 1 | 2 | 42 | 7 | K.GILRQITVNDLPVGR.S 15142 |
|  | 15424 | 418.4498 | 1669.7701 | 1670.8199 | -1.0498 | 1 | 5 | 38 | 7 | -.MSSGNAKIGHPAPNFK.A |
|  | 16656 | **438.2201** | **1748.8515** | **1749.9447** | **-1.0932** | **1** | **6** | **26** | **5** | **K.KQGGLGPMNIPLVSDPK.R** |
|  | 19581 | 991.4953 | 1980.9760 | 1982.0109 | -1.0348 | 1 | 1 | 73 | 6 | R.TIAQDYGVLKADEGISFR.G |
|  | 19940 | **671.3418** | **2011.0036** | **2011.0698** | **-0.0663** | **1** | **4** | **34** | **2** | **R.QITVNDLPVGRSVDETLR.L** 19931 |
|  | 20823 | **696.3986** | **2086.1739** | **2085.0089** | **1.1650** | **1** | **1** | **64** | **6** | **K.ATAVMPDGQFKDISLSDYK.G** |
|  | 23483 | **802.7274** | **2405.1604** | **2405.1798** | **-0.0194** | **0** | **25** | **0.25** | **1** | **K.HGEVCPAGWKPGSDTIKPDVQK.S** 23484 23485 |

| **692.** | O95782\|AP2A1_HUMAN    **Mass:** 108570   **Score:** 54     **Queries matched:** 19   **emPAI:** 0.03 |
| --- | --- |
|  | AP-2 complex subunit alpha-1 OS=Homo sapiens GN=AP2A1 PE=1 SV=2 |

|  | **Query** | **Observed** | **Mr(expt)** | **Mr(calc)** | **Delta** | **Miss** | **Score** | **Expect** | **Rank** | **Peptide** |
| --- | --- | --- | --- | --- | --- | --- | --- | --- | --- | --- |
|  | 3079 | **443.0381** | **884.0616** | **883.4763** | **0.5853** | **1** | **4** | **40** | **3** | **K.AQEPPKSK.K** 3072 |
|  | 4502 | 496.2789 | 990.5432 | 991.5121 | -0.9689 | 0 | 16 | 4.5 | 9 | K.TCVSLAVSR.L |
|  | 5013 | 514.7521 | 1027.4896 | 1028.5801 | -1.0905 | 0 | 11 | 12 | 2 | K.ALQVGCLLR.L |
|  | 7657 | **607.2720** | **1212.5295** | **1211.5605** | **0.9691** | **0** | **11** | **11** | **1** | **K.ANHPMDAEVTK.A** |
|  | 8762 | 635.3411 | 1268.6676 | 1268.6724 | -0.0049 | 0 | 8 | 21 | 4 | K.QLSLPQQEAQK.I |
|  | 11619 | 713.3890 | 1424.7634 | 1425.7616 | -0.9982 | 0 | 4 | 48 | 4 | R.SSPPVQFSLLHSK.F |
|  | 12740 | **498.6356** | **1492.8849** | **1491.7868** | **1.0981** | **1** | **1** | **97** | **9** | **R.GLAVFISDIRNCK.S** |
|  | 14329 | 533.3090 | 1596.9051 | 1597.7341 | -0.8290 | 1 | 3 | 55 | 9 | R.QRAADLLYAMCDR.S |
|  | 14630 | **540.2941** | **1617.8606** | **1618.7926** | **-0.9320** | **1** | **1** | **87** | **10** | **R.QNLGRMYLFYGNK.T** 14633 |
|  | 15852 | **566.6041** | **1696.7904** | **1697.7865** | **-0.9961** | **1** | **6** | **27** | **6** | **R.AADLLYAMCDRSNAK.Q** |
|  | 17395 | **601.7806** | **1802.3201** | **1802.9461** | **-0.6260** | **1** | **2** | **49** | **3** | **R.LEPNAQAQMYRLTLR.T** |
|  | 17954 | **924.9832** | **1847.9519** | **1846.9577** | **0.9941** | **0** | **54** | **0.00044** | **1** | **K.VGGYILGEFGNLIAGDPR.S** |
|  | 29571 | 1263.9790 | 3788.9152 | 3788.8654 | 0.0497 | 1 | 6 | 8 | 2 | K.TVFEALQAPACHENMVKVGGYILGEFGNLIAGDPR.S 29510 29530 29559 29566 |

| **693.** | O94973\|AP2A2_HUMAN    **Mass:** 104807   **Score:** 54     **Queries matched:** 8   **emPAI:** 0.04 |
| --- | --- |
|  | AP-2 complex subunit alpha-2 OS=Homo sapiens GN=AP2A2 PE=1 SV=2 |

|  | **Query** | **Observed** | **Mr(expt)** | **Mr(calc)** | **Delta** | **Miss** | **Score** | **Expect** | **Rank** | **Peptide** |
| --- | --- | --- | --- | --- | --- | --- | --- | --- | --- | --- |
|  | 3079 | 443.0381 | 884.0616 | 883.4763 | 0.5853 | 1 | 4 | 40 | 3 | K.AQEPPKSK.K 3072 |
|  | 9472 | **654.3311** | **1306.6477** | **1307.6067** | **-0.9590** | **0** | **1** | **1e+002** | **7** | **R.EMAEAFAGEIPK.V** |
|  | 12740 | 498.6356 | 1492.8849 | 1491.7868 | 1.0981 | 1 | 1 | 97 | 9 | R.GLAVFISDIRNCK.S |
|  | 13553 | **516.3071** | **1545.8994** | **1545.8185** | **0.0809** | **1** | **8** | **20** | **7** | **R.GRLTECLETILNK.A** |
|  | 15330 | 555.2322 | 1662.6749 | 1662.8577 | -0.1828 | 1 | 8 | 16 | 5 | K.NPEEFKTSVSLAVSR.L |
|  | 17954 | 924.9832 | 1847.9519 | 1846.9577 | 0.9941 | 0 | 54 | 0.00044 | 1 | K.VGGYILGEFGNLIAGDPR.S |
|  | 18971 | **643.3201** | **1926.9384** | **1925.9669** | **0.9715** | **0** | **2** | **61** | **8** | **K.TVFEALQAPACHENLVK.V** |

| **694.** | P15880\|RS2_HUMAN    **Mass:** 31590    **Score:** 54     **Queries matched:** 31   **emPAI:** 0.40 |
| --- | --- |
|  | 40S ribosomal protein S2 OS=Homo sapiens GN=RPS2 PE=1 SV=2 |

|  | **Query** | **Observed** | **Mr(expt)** | **Mr(calc)** | **Delta** | **Miss** | **Score** | **Expect** | **Rank** | **Peptide** |
| --- | --- | --- | --- | --- | --- | --- | --- | --- | --- | --- |
|  | 1379 | **633.2982** | **632.2909** | **633.3520** | **-1.0610** | **1** | **10** | **26** | **9** | **K.DMKIK.S** |
|  | 1583 | **705.3657** | **704.3584** | **703.3501** | **1.0084** | **1** | **9** | **30** | **8** | **R.GGKAEDK.E** |
|  | 1640 | 724.3823 | 723.3750 | 723.3803 | -0.0053 | 0 | 16 | 3.5 | 1 | K.ETVFTK.S |
|  | 1649 | **730.4042** | **729.3969** | **730.4047** | **-1.0078** | **0** | **13** | **12** | **4** | **K.IMPVQK.Q** |
|  | 1723 | **759.4307** | **758.4234** | **758.4286** | **-0.0053** | **0** | **19** | **3.2** | **1** | **K.EVATAIR.G** |
|  | 4110 | 482.2459 | 962.4773 | 962.5046 | -0.0273 | 1 | 3 | 79 | 3 | R.GGFGSGIRGR.G |
|  | 4980 | **513.2994** | **1024.5843** | **1024.5917** | **-0.0074** | **0** | **27** | **0.29** | **1** | **R.GTGIVSAPVPK.K** 4975 |
|  | 5968 | **551.3146** | **1100.6147** | **1099.6172** | **0.9975** | **1** | **6** | **43** | **7** | **K.IMPVQKQTR.A** |
|  | 6408 | **568.8118** | **1135.6091** | **1135.6172** | **-0.0081** | **0** | **9** | **21** | **2** | **K.IGKPHTVPCK.V** |
|  | 6442 | 570.2750 | 1138.5354 | 1138.5441 | -0.0087 | 0 | 42 | 0.0091 | 1 | R.GCTATLGNFAK.A 6441 |
|  | 6609 | 576.7943 | 1151.5739 | 1152.6867 | -1.1127 | 1 | 6 | 35 | 4 | R.GTGIVSAPVPKK.L |
|  | 9991 | 667.3677 | 1332.7209 | 1332.6384 | 0.0826 | 1 | 7 | 27 | 9 | K.AEDKEWMPVTK.L |
|  | 10927 | **693.8464** | **1385.6782** | **1385.6867** | **-0.0085** | **0** | **39** | **0.016** | **1** | **K.TYSYLTPDLWK.E** |
|  | 13630 | **776.4312** | **1550.8477** | **1550.8596** | **-0.0118** | **0** | **25** | **0.4** | **1** | **K.SLEEIYLFSLPIK.E** 13631 |
|  | 13887 | **785.4250** | **1568.8354** | **1567.8134** | **1.0221** | **0** | **2** | **85** | **1** | **K.ESEIIDFFLGASLK.D** |
|  | 16189 | 858.9230 | 1715.8314 | 1715.8995 | -0.0681 | 0 | 6 | 26 | 9 | K.AFVAIGDYNGHVGLGVK.C |
|  | 16193 | 858.9717 | 1715.9289 | 1715.7681 | 0.1608 | 0 | 17 | 2.2 | 1 | K.LLMMAGIDDCYTSAR.G |
|  | 16738 | **439.7071** | **1754.7994** | **1753.7438** | **1.0555** | **0** | **7** | **22** | **3** | **M.ADDAGAAGGPGGPGGPGMGNR.G** 16717 |
|  | 17906 | 922.9460 | 1843.8775 | 1843.8630 | 0.0145 | 1 | 31 | 0.082 | 2 | K.KLLMMAGIDDCYTSAR.G 17905 |
|  | 18293 | 623.9947 | 1868.9622 | 1868.7894 | 0.1728 | 0 | 8 | 16 | 6 | -.MADDAGAAGGPGGPGGPGMGNR.G 18265 |
|  | 18459 | **629.0234** | **1884.0483** | **1884.7843** | **-0.7360** | **0** | **(1)** | **84** | **6** | **-.MADDAGAAGGPGGPGGPGMGNR.G** 18467 |
|  | 18461 | 629.2855 | 1884.8347 | 1884.7843 | 0.0504 | 0 | (1) | 71 | 5 | -.MADDAGAAGGPGGPGGPGMGNR.G |
|  | 21416 | 1077.5289 | 2153.0433 | 2152.1303 | 0.9130 | 1 | 17 | 1.5 | 2 | K.ESEIIDFFLGASLKDEVLK.I |
|  | 22826 | 773.4011 | 2317.1815 | 2317.9917 | -0.8102 | 1 | 2 | 48 | 2 | -.MADDAGAAGGPGGPGGPGMGNRGGFR.G |

| **695.** | P08708\|RS17_HUMAN    **Mass:** 15597    **Score:** 54     **Queries matched:** 5   **emPAI:** 0.56 |
| --- | --- |
|  | 40S ribosomal protein S17 OS=Homo sapiens GN=RPS17 PE=1 SV=2 |

|  | **Query** | **Observed** | **Mr(expt)** | **Mr(calc)** | **Delta** | **Miss** | **Score** | **Expect** | **Rank** | **Peptide** |
| --- | --- | --- | --- | --- | --- | --- | --- | --- | --- | --- |
|  | 7409 | **601.3031** | **1200.5916** | **1200.6000** | **-0.0083** | **1** | **53** | **0.00079** | **1** | **R.LGNDFHTNKR.V** 7400 7453 |
|  | 10935 | **693.8818** | **1385.7491** | **1385.7588** | **-0.0097** | **1** | **26** | **0.32** | **1** | **R.VCEEIAIIPSKK.L** 10936 |

| **696.** | Q56VL3\|OCAD2_HUMAN    **Mass:** 17285    **Score:** 54     **Queries matched:** 10   **emPAI:** 0.83 |
| --- | --- |
|  | OCIA domain-containing protein 2 OS=Homo sapiens GN=OCIAD2 PE=1 SV=1 |

|  | **Query** | **Observed** | **Mr(expt)** | **Mr(calc)** | **Delta** | **Miss** | **Score** | **Expect** | **Rank** | **Peptide** |
| --- | --- | --- | --- | --- | --- | --- | --- | --- | --- | --- |
|  | 4515 | **496.7622** | **991.5098** | **991.5161** | **-0.0063** | **0** | **6** | **39** | **6** | **K.QSLLFCPK.S** |
|  | 5182 | **520.7505** | **1039.4864** | **1039.4948** | **-0.0084** | **0** | **40** | **0.014** | **1** | **R.GAGFGPQHNR.H** 5174 5198 |
|  | 8106 | 412.2469 | 1233.7188 | 1234.5724 | -0.8537 | 1 | 8 | 23 | 4 | -.MASASARGNQDK.D 8115 |
|  | 10256 | **675.2809** | **1348.5472** | **1348.5574** | **-0.0102** | **0** | **41** | **0.012** | **1** | **R.HCLLTCEECK.I** |
|  | 11163 | **700.3212** | **1398.6278** | **1398.6375** | **-0.0097** | **1** | **24** | **0.57** | **1** | **K.HGLSEKGDSQPSAS.-** |
|  | 13397 | **513.2480** | **1536.7221** | **1536.7321** | **-0.0100** | **1** | **18** | **1.9** | **1** | **R.GNQDKDAHFPPPSK.Q** 13410 |

| **697.** | Q14571\|ITPR2_HUMAN    **Mass:** 311060   **Score:** 53     **Queries matched:** 54   **emPAI:** 0.04 |
| --- | --- |
|  | Inositol 1,4,5-trisphosphate receptor type 2 OS=Homo sapiens GN=ITPR2 PE=1 SV=2 |

|  | **Query** | **Observed** | **Mr(expt)** | **Mr(calc)** | **Delta** | **Miss** | **Score** | **Expect** | **Rank** | **Peptide** |
| --- | --- | --- | --- | --- | --- | --- | --- | --- | --- | --- |
|  | 996 | 533.1879 | 532.1807 | 532.2605 | -0.0798 | 0 | 8 | 50 | 7 | K.NNASK.L |
|  | 1276 | **602.9719** | **601.9646** | **601.3184** | **0.6462** | **0** | **7** | **38** | **5** | **K.GGDVVR.L** |
|  | 1375 | 632.3568 | 631.3495 | 630.3588 | 0.9907 | 0 | 12 | 18 | 3 | K.EEILK.T 1371 |
|  | 2125 | **407.1337** | **812.2529** | **812.4392** | **-0.1863** | **0** | **5** | **37** | **3** | **R.DSTLHLK.E** |
|  | 2400 | **418.2168** | **834.4190** | **835.3858** | **-0.9667** | **0** | **10** | **19** | **5** | **K.AGEGSMLR.L** |
|  | 2695 | **430.2993** | **858.5840** | **858.5538** | **0.0302** | **1** | **11** | **17** | **1** | **K.VLATTVKK.L** |
|  | 3364 | **454.2508** | **906.4870** | **906.5035** | **-0.0166** | **1** | **9** | **25** | **6** | **R.NSYVRLR.H** |
|  | 3524 | 460.2686 | 918.5226 | 918.4923 | 0.0303 | 0 | 19 | 2.3 | 2 | R.ELQNFLR.N 3525 |
|  | 4546 | **497.8451** | **993.6757** | **992.5403** | **1.1355** | **1** | **7** | **26** | **1** | **K.SNNYRIVK.E** |
|  | 5456 | **531.7698** | **1061.5250** | **1061.5804** | **-0.0554** | **0** | **11** | **13** | **4** | **R.ILFNMRPR.E** 5465 |
|  | 7005 | 592.2874 | 1182.5603 | 1183.6713 | -1.1111 | 0 | 7 | 28 | 7 | K.LTFEVVHLAR.N |
|  | 7532 | **402.7210** | **1205.1413** | **1205.6656** | **-0.5243** | **0** | **10** | **10** | **4** | **K.EIETFVSLLR.R** 7553 |
|  | 8086 | **617.4323** | **1232.8501** | **1233.5778** | **-0.7278** | **0** | **1** | **88** | **8** | **K.EALGGPAWDYR.N** |
|  | 8522 | 628.8093 | 1255.6040 | 1254.7118 | 0.8922 | 1 | 2 | 1.1e+002 | 6 | R.LPALLEKNAMR.V |
|  | 8941 | **427.2234** | **1278.6483** | **1277.7166** | **0.9317** | **1** | **2** | **94** | **6** | **K.YRMDLVLQLK.N** |
|  | 9196 | **647.3767** | **1292.7387** | **1293.7041** | **-0.9653** | **1** | **8** | **22** | **2** | **K.KLQHAAELEQK.Q** |
|  | 9370 | 434.8810 | 1301.6212 | 1300.6636 | 0.9576 | 1 | 1 | 1.2e+002 | 9 | R.VLRHSQQDYR.K |
|  | 10355 | **677.8409** | **1353.6673** | **1353.6751** | **-0.0078** | **0** | **21** | **1.1** | **1** | **K.LFENFLVDMAR.V** |
|  | 11082 | **698.3128** | **1394.6111** | **1393.6837** | **0.9273** | **1** | **1** | **1.1e+002** | **4** | **K.DSFVEEGNTLRK.I** |
|  | 11191 | **701.3321** | **1400.6496** | **1400.7260** | **-0.0763** | **1** | **10** | **12** | **2** | **K.LRSEGDNIVVGDK.V** 11196 |
|  | 11366 | **706.3458** | **1410.6771** | **1410.6813** | **-0.0042** | **0** | **1** | **1e+002** | **3** | **R.VVTHDDCIPEVK.I** |
|  | 11394 | **471.6052** | **1411.7937** | **1410.7692** | **1.0246** | **1** | **6** | **31** | **3** | **R.NGGGVGDVLRRPSK.D** |
|  | 11639 | **476.5640** | **1426.6701** | **1425.8092** | **0.8608** | **1** | **8** | **22** | **10** | **K.NKLTFEVVHLAR.N** |
|  | 11900 | **482.2629** | **1443.7668** | **1442.8861** | **0.8807** | **0** | **1** | **1.1e+002** | **9** | **K.IIEILQFILSVR.L** |
|  | 12147 | 487.2458 | 1458.7155 | 1459.7922 | -1.0768 | 1 | 12 | 8.3 | 2 | R.LTVEKSELWVEK.S |
|  | 12379 | **736.9230** | **1471.8315** | **1471.8398** | **-0.0083** | **0** | **35** | **0.038** | **1** | **R.EQNILAQVFGILK.A** |
|  | 12566 | 742.3719 | 1482.7293 | 1482.6587 | 0.0707 | 0 | 10 | 13 | 1 | K.TAQVGGSFSGQDSDK.M |
|  | 13177 | 761.4283 | 1520.8421 | 1520.6525 | 0.1896 | 1 | 1 | 93 | 6 | K.RDDDNELMTSGPR.M |
|  | 13189 | **508.2732** | **1521.7977** | **1521.7432** | **0.0545** | **1** | **2** | **85** | **9** | **R.CGAFMSKLINHTK.K** |
|  | 14765 | **543.2987** | **1626.8743** | **1625.7463** | **1.1280** | **0** | **1** | **91** | **4** | **K.MGISMSDIQCLLDK.E** |
|  | 16835 | 879.9505 | 1757.8864 | 1758.9476 | -1.0611 | 1 | 6 | 24 | 4 | K.EIRSTVTVNTIDLGNK.K |
|  | 17739 | **916.4638** | **1830.9131** | **1830.9264** | **-0.0133** | **1** | **1** | **81** | **2** | **K.EALGGPAWDYRNIIEK.L** |
|  | 17776 | **612.2956** | **1833.8649** | **1833.9472** | **-0.0823** | **1** | **1** | **89** | **8** | **R.DLDFANDANKVLATTVK.K** |
|  | 19904 | **669.9651** | **2006.8736** | **2006.9534** | **-0.0797** | **0** | **4** | **34** | **3** | **R.LGFLGSNTPHVNHHMPPH.-** |
|  | 20684 | 1037.0663 | 2072.1180 | 2072.0646 | 0.0534 | 0 | 5 | 27 | 5 | K.SAEEVTMSPAIAIMQPILR.F |
|  | 20708 | 692.3674 | 2074.0803 | 2074.1344 | -0.0541 | 1 | 6 | 24 | 2 | K.LESTMSLVKQLSGQLAELK.E |
|  | 21007 | **703.3307** | **2106.9704** | **2106.0636** | **0.9069** | **0** | **0** | **87** | **3** | **R.TCDTLLMCIVTVLNQGLR.N** |
|  | 21301 | **714.3404** | **2139.9993** | **2139.9652** | **0.0341** | **0** | **2** | **50** | **3** | **K.MNEVMNLAHTFLQNFCR.G** |
|  | 21792 | 733.6195 | 2197.8365 | 2196.9739 | 0.8626 | 1 | 0 | 60 | 6 | R.MRAMSLVSNEGDSEQNEIR.S |
|  | 21809 | 734.3990 | 2200.1753 | 2199.0729 | 1.1024 | 0 | 2 | 54 | 8 | K.LLQQMLKPGSDPDEGDEALK.Y |
|  | 23314 | 794.0558 | 2379.1455 | 2380.3188 | -1.1733 | 1 | 1 | 57 | 5 | R.LLKNMGAHSVVLDLLQIPYEK.N |
|  | 24679 | **857.4477** | **2569.3212** | **2569.2556** | **0.0656** | **1** | **1** | **49** | **2** | **R.TMEQIVFPVPNICEYLTRESK.C** |
|  | 25017 | **661.3247** | **2641.2697** | **2641.2999** | **-0.0301** | **1** | **3** | **28** | **7** | **K.EIYTSNHIWKLFENFLVDMAR.V** |
|  | 25801 | **929.4769** | **2785.4088** | **2785.4255** | **-0.0167** | **0** | **33** | **0.032** | **1** | **K.VVLMPVNAGQPLHASNIELLDNPGCK.E** |
|  | 29732 | 1276.2707 | 3825.7904 | 3825.8562 | -0.0658 | 1 | 1 | 24 | 9 | R.TIHGVGEMMTQMVLSRGSIFPMSVPDVPPSIHPSK.Q |
|  | 29749 | 767.3642 | 3831.7846 | 3830.9335 | 0.8511 | 1 | 1 | 27 | 10 | K.IMYTLVSVPHGNDIASLFELDATTLQRADCLVPR.N |
|  | 30815 | 1106.2847 | 4421.1096 | 4420.0128 | 1.0967 | 1 | 0 | 19 | 4 | K.CQDMVMTELINGGEDVLIFYNDRASFPILLHMMCSER.D |
|  | 30914 | **1117.7584** | **4467.0046** | **4467.0184** | **-0.0138** | **0** | **1** | **13** | **2** | **K.EFGEDNDNAETSASGSPDTLLPSAIVPDIDEIAAQAETMFAGR.K** |
|  | 30981 | **1527.0512** | **4578.1316** | **4579.1184** | **-0.9868** | **1** | **1** | **15** | **6** | **K.KEFGEDNDNAETSASGSPDTLLPSAIVPDIDEIAAQAETMFAGR.K** |

| **698.** | Q01082\|SPTB2_HUMAN    **Mass:** 275237   **Score:** 53     **Queries matched:** 48   **emPAI:** 0.03 |
| --- | --- |
|  | Spectrin beta chain, brain 1 OS=Homo sapiens GN=SPTBN1 PE=1 SV=2 |

|  | **Query** | **Observed** | **Mr(expt)** | **Mr(calc)** | **Delta** | **Miss** | **Score** | **Expect** | **Rank** | **Peptide** |
| --- | --- | --- | --- | --- | --- | --- | --- | --- | --- | --- |
|  | 2009 | **402.7041** | **803.3936** | **803.4654** | **-0.0717** | **0** | **13** | **10** | **3** | **K.LQQFLR.D** |
|  | 4040 | **479.2562** | **956.4978** | **957.5243** | **-1.0266** | **0** | **5** | **56** | **8** | **K.INAVVETGR.R** |
|  | 4100 | **481.7500** | **961.4855** | **961.5266** | **-0.0411** | **1** | **7** | **40** | **3** | **K.MKALAVEGK.R** |
|  | 4620 | 499.7713 | 997.5280 | 996.4764 | 1.0517 | 0 | 1 | 1.1e+002 | 6 | K.ILSSDDYGK.D |
|  | 5029 | 515.2440 | 1028.4735 | 1028.5727 | -0.0992 | 1 | 5 | 59 | 7 | R.VRGVNASAQK.F |
|  | 5609 | **537.7659** | **1073.5173** | **1074.4941** | **-0.9768** | **1** | **4** | **75** | **4** | **R.QQEEEERK.R** |
|  | 6376 | **567.4081** | **1132.8017** | **1131.6400** | **1.1617** | **1** | **1** | **91** | **9** | **K.ALQFLKEQR.V** |
|  | 7514 | **603.2759** | **1204.5372** | **1205.5822** | **-1.0450** | **1** | **10** | **14** | **2** | **K.SLLDACESRR.V** |
|  | 7537 | 603.8140 | 1205.6135 | 1206.6258 | -1.0123 | 1 | 9 | 18 | 2 | K.LNTRWSQFR.E |
|  | 7799 | 407.2164 | 1218.6273 | 1219.6131 | -0.9859 | 1 | 7 | 34 | 4 | R.ANNQKVYMPR.E |
|  | 8179 | **619.5478** | **1237.0810** | **1236.5445** | **0.5366** | **1** | **3** | **46** | **1** | **K.EMIDKWEDR.W** |
|  | 8690 | **633.3127** | **1264.6109** | **1263.7187** | **0.8923** | **0** | **4** | **54** | **4** | **K.HLLGVEDLLQK.H** |
|  | 9210 | **647.8338** | **1293.6530** | **1292.6257** | **1.0273** | **0** | **2** | **97** | **6** | **K.DALLLWCQMK.T** |
|  | 9481 | **654.4253** | **1306.8360** | **1305.6598** | **1.1762** | **1** | **21** | **0.91** | **1** | **K.AKDEQSAVSMLK.K** |
|  | 10040 | 446.2689 | 1335.7848 | 1334.6612 | 1.1235 | 1 | 6 | 35 | 5 | K.NRETASELLMR.L 10017 |
|  | 11437 | **708.3632** | **1414.7118** | **1413.6857** | **1.0261** | **1** | **5** | **42** | **1** | **R.MRIHCLENVDK.A** |
|  | 11596 | 712.6133 | 1423.2120 | 1423.7017 | -0.4897 | 1 | 3 | 43 | 4 | K.EAVCEVALDYKK.K |
|  | 13104 | 506.5666 | 1516.6780 | 1515.8694 | 0.8086 | 1 | 12 | 8.5 | 2 | R.MLIKLLEVLSGER.L |
|  | 13231 | 509.2690 | 1524.7850 | 1523.7732 | 1.0118 | 0 | 2 | 72 | 3 | R.LQALDTGWNELHK.M |
|  | 13330 | 766.8569 | 1531.6992 | 1532.7439 | -1.0447 | 1 | (2) | 72 | 4 | K.KQQMLENQMEVR.K |
|  | 13579 | 517.1576 | 1548.4509 | 1548.7388 | -0.2879 | 1 | (1) | 69 | 8 | K.KQQMLENQMEVR.K |
|  | 13837 | 783.3674 | 1564.7203 | 1564.7337 | -0.0134 | 1 | 15 | 4 | 2 | K.KQQMLENQMEVR.K |
|  | 13957 | **525.7826** | **1574.3259** | **1573.8980** | **0.4279** | **1** | **6** | **19** | **2** | **R.LWEYLLELLRAR.R** |
|  | 14250 | **531.3467** | **1591.0184** | **1590.7572** | **0.2612** | **1** | **12** | **6** | **2** | **K.AAMRETWLSENQR.L** |
|  | 14931 | 546.6201 | 1636.8384 | 1635.8039 | 1.0345 | 1 | 6 | 30 | 4 | R.QLMHSGHPSEKEIK.A |
|  | 15574 | 560.9559 | 1679.8458 | 1678.9981 | 0.8477 | 1 | 2 | 78 | 8 | K.LLEVLSGERLPKPTK.G |
|  | 15986 | 852.9562 | 1703.8979 | 1703.6655 | 0.2325 | 0 | 18 | 1.7 | 2 | K.QEDFMTTMDANEEK.I 15985 15994 |
|  | 16069 | 855.4243 | 1708.8341 | 1707.7886 | 1.0455 | 0 | 55 | 0.00037 | 1 | R.TQETPSAQMEGFLNR.K 16070 |
|  | 16308 | **575.8837** | **1724.6292** | **1723.7835** | **0.8456** | **0** | **(2)** | **57** | **3** | **R.TQETPSAQMEGFLNR.K** |
|  | 16727 | 877.8914 | 1753.7682 | 1752.8391 | 0.9291 | 0 | 2 | 67 | 5 | K.GEQVSQNGLPAEQGSPR.M |
|  | 17326 | **600.2834** | **1797.8283** | **1798.8771** | **-1.0488** | **1** | **0** | **93** | **9** | **K.ILSSDDYGKDLTSVMR.L** |
|  | 17958 | 925.0223 | 1848.0301 | 1847.7553 | 0.2748 | 1 | 2 | 60 | 10 | K.KQEDFMTTMDANEEK.I |
|  | 18130 | 930.0013 | 1857.9880 | 1858.7753 | -0.7873 | 0 | 3 | 51 | 9 | K.MFSEADACELWIDEK.E |
|  | 18357 | 625.6643 | 1873.9711 | 1874.8799 | -0.9088 | 0 | 1 | 83 | 4 | K.WLHGLESQIQSDDYGK.D |
|  | 19151 | 971.9595 | 1941.9044 | 1942.9636 | -1.0592 | 0 | 2 | 65 | 6 | K.DGLNEAWADLLELIDTR.T |
|  | 20099 | **675.0074** | **2022.0005** | **2021.0106** | **0.9899** | **0** | **2** | **61** | **2** | **R.LVSQDNFGFDLPAVEAATK.K** |
|  | 20357 | 513.0305 | 2048.0930 | 2048.9572 | -0.8642 | 0 | 6 | 23 | 5 | R.TQTAIASEDMPNTLTEAEK.L |
|  | 22425 | **757.4335** | **2269.2786** | **2269.9918** | **-0.7132** | **0** | **1** | **61** | **4** | **R.VAHMEFCYQELCQLAAER.R** |
|  | 23208 | 790.0819 | 2367.2237 | 2366.3209 | 0.9028 | 1 | 0 | 71 | 6 | K.VLVLSQDYGKHLLGVEDLLQK.H |
|  | 24347 | **840.7941** | **2519.3606** | **2520.1196** | **-0.7590** | **1** | **1** | **52** | **3** | **R.DMGEMVTQGQTDAQYMFLRQR.L** |
|  | 24577 | 852.4013 | 2554.1821 | 2555.0913 | -0.9092 | 1 | 0 | 63 | 5 | K.MRDMGEMVTQGQTDAQYMFLR.Q |
|  | 26463 | **967.4616** | **2899.3630** | **2899.3770** | **-0.0140** | **1** | **2** | **40** | **3** | **K.LEDLEVIQHRFESLEPEMNNQASR.V** |
|  | 28088 | **1086.1615** | **3255.4627** | **3256.5204** | **-1.0578** | **1** | **1** | **34** | **5** | **K.IFQEMLYIMDWMDEMKVLVLSQDYGK.H** |
|  | 28356 | **668.7042** | **3338.4847** | **3337.7717** | **0.7130** | **1** | **0** | **39** | **10** | **R.VHLENMGSHDIVDGNHRLTLGLIWTIILR.F** |

| **699.** | P12074\|CX6A1_HUMAN    **Mass:** 12147    **Score:** 53     **Queries matched:** 5   **emPAI:** 0.76 |
| --- | --- |
|  | Cytochrome c oxidase subunit 6A1, mitochondrial OS=Homo sapiens GN=COX6A1 PE=1 SV=4 |

|  | **Query** | **Observed** | **Mr(expt)** | **Mr(calc)** | **Delta** | **Miss** | **Score** | **Expect** | **Rank** | **Peptide** |
| --- | --- | --- | --- | --- | --- | --- | --- | --- | --- | --- |
|  | 5814 | 545.7637 | 1089.5128 | 1090.5805 | -1.0677 | 0 | 6 | 42 | 5 | -.MAVVGVSSVSR.L |
|  | 22551 | **1142.1390** | **2282.2635** | **2282.2749** | **-0.0113** | **0** | **42** | **0.0043** | **1** | **K.TLTFFVALPGVAVSMLNVYLK.S** |
|  | 28261 | **1106.1796** | **3315.5169** | **3315.5374** | **-0.0205** | **0** | **27** | **0.091** | **1** | **R.TKPFPWGDGNHTLFHNPHVNPLPTGYEDE.-** 28260 28265 |

| **700.** | P51159\|RB27A_HUMAN    **Mass:** 25137    **Score:** 53     **Queries matched:** 9   **emPAI:** 0.33 |
| --- | --- |
|  | Ras-related protein Rab-27A OS=Homo sapiens GN=RAB27A PE=1 SV=3 |

|  | **Query** | **Observed** | **Mr(expt)** | **Mr(calc)** | **Delta** | **Miss** | **Score** | **Expect** | **Rank** | **Peptide** |
| --- | --- | --- | --- | --- | --- | --- | --- | --- | --- | --- |
|  | 8826 | **637.3187** | **1272.6228** | **1273.6190** | **-0.9963** | **0** | **10** | **16** | **1** | **K.TSVLYQYTDGK.F** |
|  | 9673 | 659.8641 | 1317.7136 | 1318.5751 | -0.8615 | 0 | 14 | 5.7 | 6 | -.MSDGDYDYLIK.F |
|  | 10000 | **667.8362** | **1333.6579** | **1334.5700** | **-0.9121** | **0** | **(2)** | **96** | **2** | **-.MSDGDYDYLIK.F** |
|  | 11225 | **702.8262** | **1403.6378** | **1404.7110** | **-1.0732** | **1** | **8** | **22** | **1** | **R.VVYRASGPDGATGR.G** |
|  | 11659 | **714.8951** | **1427.7757** | **1427.7871** | **-0.0114** | **1** | **54** | **0.00061** | **1** | **R.VVKEEEAIALAEK.Y** |
|  | 13849 | **783.8121** | **1565.6096** | **1565.7950** | **-0.1854** | **0** | **19** | **1.2** | **1** | **R.IHLQLWDTAGQER.F** 13838 13855 |
|  | 25823 | **930.4849** | **2788.4328** | **2789.2724** | **-0.8396** | **0** | **3** | **27** | **1** | **R.NWISQLQMHAYCENPDIVLCGNK.S** |

| **701.** | O96000\|NDUBA_HUMAN    **Mass:** 21048    **Score:** 53     **Queries matched:** 14   **emPAI:** 0.65 |
| --- | --- |
|  | NADH dehydrogenase [ubiquinone] 1 beta subcomplex subunit 10 OS=Homo sapiens GN=NDUFB10 PE=1 SV=3 |

|  | **Query** | **Observed** | **Mr(expt)** | **Mr(calc)** | **Delta** | **Miss** | **Score** | **Expect** | **Rank** | **Peptide** |
| --- | --- | --- | --- | --- | --- | --- | --- | --- | --- | --- |
|  | 6951 | **589.8021** | **1177.5896** | **1177.5979** | **-0.0083** | **0** | **40** | **0.016** | **1** | **K.EVEQFTQVAK.A** 6958 |
|  | 8011 | 615.7865 | 1229.5584 | 1229.5676 | -0.0092 | 0 | 16 | 3.8 | 1 | R.YQDLGAYSSAR.K |
|  | 10825 | 691.3071 | 1380.5996 | 1380.6092 | -0.0096 | 0 | 35 | 0.044 | 1 | R.EGQNYQQNCIK.E |
|  | 12695 | 497.5967 | 1489.7681 | 1488.7242 | 1.0439 | 0 | 6 | 36 | 6 | K.VDQEIINIMQDR.L |
|  | 14562 | **538.6476** | **1612.9209** | **1612.9301** | **-0.0092** | **0** | **20** | **1.3** | **1** | **K.AFDLIVDRPVTLVR.E** 14561 |
|  | 15698 | **564.2629** | **1689.7670** | **1688.8742** | **0.8928** | **1** | **9** | **14** | **1** | **R.RTPVQPNPIVYMMK.A** 15683 |
|  | 15903 | **567.5984** | **1699.7735** | **1699.7842** | **-0.0107** | **1** | **20** | **1.2** | **1** | **M.PDSWDKDVYPEPPR.R** 15934 |
|  | 16409 | 865.9426 | 1729.8707 | 1729.9032 | -0.0325 | 1 | 2 | 65 | 6 | K.VDQEIINIMQDRLK.A 16410 |
|  | 21660 | **727.6729** | **2179.9969** | **2179.8682** | **0.1287** | **1** | **0** | **80** | **6** | **K.EEDIMCMYEAEMQWKR.D** |

| **702.** | P35914\|HMGCL_HUMAN    **Mass:** 34794    **Score:** 52     **Queries matched:** 10   **emPAI:** 0.11 |
| --- | --- |
|  | Hydroxymethylglutaryl-CoA lyase, mitochondrial OS=Homo sapiens GN=HMGCL PE=1 SV=2 |

|  | **Query** | **Observed** | **Mr(expt)** | **Mr(calc)** | **Delta** | **Miss** | **Score** | **Expect** | **Rank** | **Peptide** |
| --- | --- | --- | --- | --- | --- | --- | --- | --- | --- | --- |
|  | 1080 | **551.1834** | **550.1761** | **549.3122** | **0.8639** | **1** | **2** | **1.1e+002** | **5** | **R.KTSSK.V** |
|  | 1186 | 579.2696 | 578.2623 | 578.2668 | -0.0045 | 0 | 18 | 2.7 | 2 | -.MAAMR.K |
|  | 4873 | **508.7741** | **1015.5336** | **1015.5410** | **-0.0074** | **0** | **52** | **0.0011** | **1** | **K.AAQSANISVR.G** |
|  | 7298 | 598.3433 | 1194.6721 | 1193.5962 | 1.0759 | 0 | 5 | 47 | 10 | R.AVSTSSMGTLPK.R 7273 |
|  | 10279 | **450.7818** | **1349.3236** | **1349.6973** | **-0.3737** | **1** | **3** | **55** | **6** | **R.AVSTSSMGTLPKR.V** |
|  | 12772 | **499.3396** | **1494.9971** | **1495.6725** | **-0.6755** | **0** | **9** | **11** | **1** | **K.NINCSIEESFQR.F** |
|  | 13702 | 778.4170 | 1554.8194 | 1554.7501 | 0.0694 | 0 | 4 | 53 | 9 | K.WVPQMGDHTEVLK.G |
|  | 20482 | 1029.4944 | 2056.9742 | 2055.9758 | 0.9984 | 1 | 4 | 32 | 2 | R.GYVSCALGCPYEGKISPAK.V |
|  | 23076 | **589.0226** | **2352.0612** | **2352.2134** | **-0.1522** | **0** | **2** | **43** | **6** | **K.LIDMLSEAGLSVIETTSFVSPK.W** |

| **703.** | P16219\|ACADS_HUMAN    **Mass:** 44611    **Score:** 52     **Queries matched:** 13   **emPAI:** 0.17 |
| --- | --- |
|  | Short-chain specific acyl-CoA dehydrogenase, mitochondrial OS=Homo sapiens GN=ACADS PE=1 SV=1 |

|  | **Query** | **Observed** | **Mr(expt)** | **Mr(calc)** | **Delta** | **Miss** | **Score** | **Expect** | **Rank** | **Peptide** |
| --- | --- | --- | --- | --- | --- | --- | --- | --- | --- | --- |
|  | 3720 | **467.7505** | **933.4864** | **934.4946** | **-1.0082** | **0** | **(3)** | **1e+002** | **9** | **R.MAFGAPLTK.L** |
|  | 3943 | **475.8324** | **949.6502** | **950.4895** | **-0.8393** | **0** | **12** | **11** | **3** | **R.MAFGAPLTK.L** 3941 |
|  | 6405 | **568.7603** | **1135.5059** | **1134.5525** | **0.9534** | **0** | **8** | **28** | **8** | **K.IAMQTLDMGR.I** |
|  | 7888 | 612.3370 | 1222.6594 | 1223.7098 | -1.0504 | 1 | 6 | 36 | 7 | M.AAALLARASGPAR.R 7886 7894 |
|  | 8891 | **426.2178** | **1275.6316** | **1275.6492** | **-0.0177** | **0** | **10** | **13** | **2** | **K.LADMALALESAR.L** |
|  | 10661 | **457.7969** | **1370.3690** | **1370.7452** | **-0.3762** | **1** | **5** | **27** | **8** | **-.MAAALLARASGPAR.R** |
|  | 13417 | 769.8833 | 1537.7520 | 1537.7624 | -0.0103 | 0 | 48 | 0.0019 | 1 | R.ITEIYEGTSEIQR.L |
|  | 17332 | **900.0054** | **1797.9963** | **1798.0063** | **-0.0100** | **0** | **(6)** | **22** | **1** | **K.GISAFLVPMPTPGLTLGK.K** |
|  | 17526 | **605.7410** | **1814.2011** | **1814.0012** | **0.1999** | **0** | **7** | **14** | **1** | **K.GISAFLVPMPTPGLTLGK.K** |
|  | 27859 | **797.3989** | **3185.5666** | **3185.5849** | **-0.0183** | **0** | **25** | **0.15** | **1** | **K.LAASEAATAISHQAIQILGGMGYVTEMPAER.H** |

| **704.** | P62263\|RS14_HUMAN    **Mass:** 16434    **Score:** 52     **Queries matched:** 6   **emPAI:** 0.53 |
| --- | --- |
|  | 40S ribosomal protein S14 OS=Homo sapiens GN=RPS14 PE=1 SV=3 |

|  | **Query** | **Observed** | **Mr(expt)** | **Mr(calc)** | **Delta** | **Miss** | **Score** | **Expect** | **Rank** | **Peptide** |
| --- | --- | --- | --- | --- | --- | --- | --- | --- | --- | --- |
|  | 1247 | **593.1558** | **592.1485** | **591.3050** | **0.8435** | **0** | **1** | **1.3e+002** | **1** | **R.VTGGMK.V** |
|  | 2399 | **418.2058** | **834.3969** | **834.4633** | **-0.0664** | **1** | **1** | **1.6e+002** | **9** | **R.VTGGMKVK.A** |
|  | 5352 | **527.7816** | **1053.5486** | **1053.5567** | **-0.0081** | **0** | **21** | **1.2** | **1** | **K.TPGPGAQSALR.A** |
|  | 9026 | **642.8535** | **1283.6925** | **1282.6993** | **0.9931** | **1** | **21** | **1.2** | **1** | **R.TKTPGPGAQSALR.A** |
|  | 18901 | 641.3323 | 1920.9752 | 1921.8840 | -0.9088 | 0 | 0 | 87 | 9 | R.DESSPYAAMLAAQDVAQR.C |
|  | 22395 | **756.0196** | **2265.0371** | **2264.0491** | **0.9880** | **1** | **54** | **0.0003** | **1** | **K.ADRDESSPYAAMLAAQDVAQR.C** |

| **705.** | P25325\|THTM_HUMAN    **Mass:** 33443    **Score:** 52     **Queries matched:** 5   **emPAI:** 0.11 |
| --- | --- |
|  | 3-mercaptopyruvate sulfurtransferase OS=Homo sapiens GN=MPST PE=1 SV=3 |

|  | **Query** | **Observed** | **Mr(expt)** | **Mr(calc)** | **Delta** | **Miss** | **Score** | **Expect** | **Rank** | **Peptide** |
| --- | --- | --- | --- | --- | --- | --- | --- | --- | --- | --- |
|  | 1947 | **401.2126** | **800.4107** | **800.4181** | **-0.0074** | **0** | **19** | **2.9** | **1** | **R.HLFQEK.K** |
|  | 4682 | 501.7809 | 1001.5473 | 1001.5546 | -0.0073 | 0 | 33 | 0.089 | 1 | R.AQLDPAFIK.T |
|  | 17347 | **900.4829** | **1798.9513** | **1798.9617** | **-0.0105** | **0** | **52** | **0.00065** | **1** | **R.AGQPLQLLDASWYLPK.L** |
|  | 18962 | **643.2828** | **1926.8267** | **1925.9959** | **0.8308** | **1** | **2** | **51** | **6** | **R.QNLPLSSGKSQPAPAEFR.A** |
|  | 23102 | 1178.5304 | 2355.0462 | 2356.2144 | -1.1681 | 1 | 2 | 47 | 4 | -.MASPQLCRALVSAQWVAEALR.A |

| **706.** | P07195\|LDHB_HUMAN    **Mass:** 36900    **Score:** 52     **Queries matched:** 15   **emPAI:** 0.10 |
| --- | --- |
|  | L-lactate dehydrogenase B chain OS=Homo sapiens GN=LDHB PE=1 SV=2 |

|  | **Query** | **Observed** | **Mr(expt)** | **Mr(calc)** | **Delta** | **Miss** | **Score** | **Expect** | **Rank** | **Peptide** |
| --- | --- | --- | --- | --- | --- | --- | --- | --- | --- | --- |
|  | 1552 | 689.3929 | 688.3857 | 688.4119 | -0.0262 | 1 | 12 | 15 | 9 | M.ATLKEK.L |
|  | 2378 | 417.2429 | 832.4712 | 832.3675 | 0.1037 | 0 | 12 | 14 | 3 | R.QQEGESR.L |
|  | 5237 | **522.7789** | **1043.5433** | **1044.5451** | **-1.0018** | **1** | **3** | **90** | **10** | **K.DDEVAQLKK.S** |
|  | 8375 | 624.7991 | 1247.5836 | 1247.5928 | -0.0092 | 0 | 52 | 0.00096 | 1 | R.VIGSGCNLDSAR.F |
|  | 12989 | **756.0746** | **1510.1346** | **1509.7675** | **0.3671** | **1** | **5** | **23** | **1** | **K.IVADKDYSVTANSK.I** |
|  | 15786 | 424.2403 | 1692.9322 | 1693.8886 | -0.9564 | 0 | 6 | 28 | 8 | K.LIAPVAEEEATVPNNK.I 15814 |
|  | 17064 | 889.5337 | 1777.0528 | 1775.9128 | 1.1401 | 1 | 6 | 23 | 2 | K.EVHKMVVESAYEVIK.L 17012 17013 17014 17015 17016 17043 |
|  | 19255 | **651.1257** | **1950.3554** | **1951.0262** | **-0.6708** | **1** | **1** | **52** | **8** | **K.EKLIAPVAEEEATVPNNK.I** |

| **707.** | Q6ZMR3\|LDH6A_HUMAN    **Mass:** 36826    **Score:** 52     **Queries matched:** 5   **emPAI:** 0.10 |
| --- | --- |
|  | L-lactate dehydrogenase A-like 6A OS=Homo sapiens GN=LDHAL6A PE=2 SV=1 |

|  | **Query** | **Observed** | **Mr(expt)** | **Mr(calc)** | **Delta** | **Miss** | **Score** | **Expect** | **Rank** | **Peptide** |
| --- | --- | --- | --- | --- | --- | --- | --- | --- | --- | --- |
|  | 6246 | **562.6179** | **1123.2213** | **1122.6509** | **0.5703** | **1** | **7** | **19** | **1** | **R.RVHPVSTLSK.G** |
|  | 6297 | 564.7914 | 1127.5683 | 1127.5533 | 0.0151 | 0 | 8 | 23 | 2 | K.VISSGYEMVK.M |
|  | 8375 | 624.7991 | 1247.5836 | 1247.5928 | -0.0092 | 0 | 52 | 0.00096 | 1 | R.VIGSGCNLDSAR.F |
|  | 8530 | **629.0234** | **1256.0322** | **1255.6482** | **0.3840** | **1** | **2** | **59** | **2** | **K.KVISSGYEMVK.M** |
|  | 11687 | **715.8238** | **1429.6331** | **1430.8245** | **-1.1914** | **1** | **2** | **77** | **9** | **R.LDLVQRNVSIFK.L** |

| **708.** | P07864\|LDHC_HUMAN    **Mass:** 36630    **Score:** 52     **Queries matched:** 8   **emPAI:** 0.10 |
| --- | --- |
|  | L-lactate dehydrogenase C chain OS=Homo sapiens GN=LDHC PE=2 SV=4 |

|  | **Query** | **Observed** | **Mr(expt)** | **Mr(calc)** | **Delta** | **Miss** | **Score** | **Expect** | **Rank** | **Peptide** |
| --- | --- | --- | --- | --- | --- | --- | --- | --- | --- | --- |
|  | 8375 | 624.7991 | 1247.5836 | 1247.5928 | -0.0092 | 0 | 52 | 0.00096 | 1 | R.VIGSGCNLDSAR.F |
|  | 9447 | **653.7921** | **1305.5697** | **1304.7010** | **0.8687** | **1** | **2** | **88** | **8** | **-.MSTVKEQLIEK.L** |
|  | 15035 | **548.7871** | **1643.3393** | **1643.9069** | **-0.5676** | **1** | **3** | **43** | **4** | **R.VHPVSTMVKGLYGIK.E** 15036 |
|  | 17125 | **594.9539** | **1781.8399** | **1782.9992** | **-1.1592** | **1** | **1** | **81** | **10** | **K.NIHKQVIQSAYEIIK.L** |
|  | 21585 | 725.0143 | 2172.0212 | 2171.0391 | 0.9821 | 1 | 3 | 47 | 3 | K.LKGEMMDLQHGSLFFSTSK.I |
|  | 23188 | **592.2960** | **2365.1547** | **2366.2919** | **-1.1372** | **1** | **2** | **45** | **4** | **K.LKGYTSWAIGLSVMDLVGSILK.N** |
|  | 29099 | **1195.8932** | **3584.6577** | **3583.9456** | **0.7121** | **1** | **4** | **14** | **1** | **K.ITIVGTGAVGMACAISILLKDLADELALVDVALDK.L** |

| **709.** | Q9NZ45\|CISD1_HUMAN    **Mass:** 12362    **Score:** 52     **Queries matched:** 9   **emPAI:** 2.05 |
| --- | --- |
|  | CDGSH iron sulfur domain-containing protein 1 OS=Homo sapiens GN=CISD1 PE=1 SV=1 |

|  | **Query** | **Observed** | **Mr(expt)** | **Mr(calc)** | **Delta** | **Miss** | **Score** | **Expect** | **Rank** | **Peptide** |
| --- | --- | --- | --- | --- | --- | --- | --- | --- | --- | --- |
|  | 1492 | 668.3134 | 667.3061 | 667.3112 | -0.0050 | 0 | 1 | 1.1e+002 | 8 | K.AVYCR.C |
|  | 5526 | **534.8030** | **1067.5914** | **1066.5957** | **0.9957** | **0** | **31** | **0.11** | **1** | **K.AMINLHIQK.D** |
|  | 9487 | 654.8085 | 1307.6024 | 1306.6129 | 0.9895 | 1 | 18 | 2 | 2 | K.KFPFCDGAHTK.H |
|  | 12665 | 497.2346 | 1488.6819 | 1488.6919 | -0.0099 | 0 | 28 | 0.22 | 1 | K.IVHAFDMEDLGDK.A |
|  | 14893 | 818.4185 | 1634.8224 | 1634.8264 | -0.0040 | 0 | 12 | 6.9 | 2 | K.HNEETGDNVGPLIIK.K 14894 |
|  | 16891 | 882.5361 | 1763.0576 | 1762.9213 | 0.1362 | 1 | 8 | 14 | 3 | K.HNEETGDNVGPLIIKK.K |
|  | 21286 | **713.6666** | **2137.9779** | **2137.9925** | **-0.0146** | **1** | **34** | **0.037** | **1** | **K.IVHAFDMEDLGDKAVYCR.C** 21287 |

| **710.** | Q709F0\|ACD11_HUMAN    **Mass:** 88026    **Score:** 52     **Queries matched:** 12   **emPAI:** 0.04 |
| --- | --- |
|  | Acyl-CoA dehydrogenase family member 11 OS=Homo sapiens GN=ACAD11 PE=1 SV=2 |

|  | **Query** | **Observed** | **Mr(expt)** | **Mr(calc)** | **Delta** | **Miss** | **Score** | **Expect** | **Rank** | **Peptide** |
| --- | --- | --- | --- | --- | --- | --- | --- | --- | --- | --- |
|  | 850 | 500.2753 | 499.2681 | 498.2914 | 0.9766 | 0 | 6 | 39 | 10 | R.LGPGR.I |
|  | 897 | **514.2946** | **513.2873** | **512.3071** | **0.9803** | **0** | **22** | **1** | **1** | **K.VAAPR.A** 894 |
|  | 6850 | **585.3678** | **1168.7210** | **1167.5706** | **1.1504** | **0** | **4** | **51** | **10** | **K.MAGIAQGVYSR.Y** |
|  | 7341 | **599.3283** | **1196.6421** | **1197.6903** | **-1.0483** | **1** | **5** | **48** | **6** | **K.EIAMIKVAAPR.A** |
|  | 14899 | **545.9764** | **1634.9073** | **1633.9039** | **1.0034** | **0** | **5** | **33** | **1** | **R.SAIYVATVETLAQLR.S** |
|  | 19056 | 645.3386 | 1932.9940 | 1932.0074 | 0.9867 | 1 | (0) | 1e+002 | 9 | R.HKQHSMILVPMNTPGVK.I |
|  | 19232 | **650.6420** | **1948.9041** | **1948.0023** | **0.9018** | **1** | **2** | **63** | **6** | **R.HKQHSMILVPMNTPGVK.I** |
|  | 24855 | **868.4161** | **2602.2264** | **2602.2308** | **-0.0044** | **0** | **0** | **60** | **5** | **K.QYQAAAHQDIPAMQQLSEWLMK.N** |
|  | 27643 | **1043.5440** | **3127.6100** | **3127.6230** | **-0.0130** | **0** | **52** | **0.00031** | **1** | **K.VEGLWNLFLPAVSGLSHVDYALIAEETGK.C** 27642 |
|  | 30813 | **1105.5342** | **4418.1076** | **4417.2264** | **0.8813** | **1** | **0** | **20** | **2** | **K.MAGIAQGVYSRYLLGNNSSEDSFLFANIVQPLAETGLQLSK.R** |

| **711.** | P08590\|MYL3_HUMAN    **Score:** 51     **Queries matched:** 18 |
| --- | --- |
|  | Myosin light chain 3 OS=Homo sapiens GN=MYL3 PE=1 SV=3 |

|  | **Query** | **Observed** | **Mr(expt)** | **Mr(calc)** | **Delta** | **Miss** | **Score** | **Expect** | **Rank** | **Peptide** |
| --- | --- | --- | --- | --- | --- | --- | --- | --- | --- | --- |
|  | 4096 | 481.7261 | 961.4376 | 961.4604 | -0.0227 | 0 | 22 | 1.2 | 2 | R.LTEDEVEK.L 4095 |
|  | 4559 | 498.2944 | 994.5742 | 994.5560 | 0.0183 | 0 | 16 | 4 | 3 | R.HVLATLGER.L 4558 |
|  | 4986 | 1025.6289 | 1024.6216 | 1024.5739 | 0.0477 | 1 | 11 | 11 | 4 | -.MAPKKPEPK.K 4987 4995 |
|  | 10343 | **677.4230** | **1352.8314** | **1353.6751** | **-0.8437** | **1** | **4** | **47** | **6** | **K.EAFMLFDRTPK.C** |
|  | 16259 | 861.9221 | 1721.8296 | 1721.8407 | -0.0111 | 1 | 51 | 0.00082 | 1 | R.VFDKEGNGTVMGAELR.H 16249 16262 16270 16271 16272 |
|  | 16536 | 580.6337 | 1738.8794 | 1737.8356 | 1.0438 | 1 | (4) | 49 | 5 | R.VFDKEGNGTVMGAELR.H |
|  | 17721 | 915.4901 | 1828.9657 | 1828.8270 | 0.1387 | 1 | 5 | 30 | 5 | K.CEMKITYGQCGDVLR.A 17722 |
|  | 19824 | 667.3336 | 1998.9790 | 1998.9253 | 0.0537 | 0 | 0 | 87 | 9 | K.MMDFETFLPMLQHISK.N |

| **712.** | P05976\|MLE1_HUMAN    **Mass:** 21189    **Score:** 51     **Queries matched:** 13   **emPAI:** 0.18 |
| --- | --- |
|  | Myosin light chain 1, skeletal muscle isoform OS=Homo sapiens GN=MYL1 PE=1 SV=3 |

|  | **Query** | **Observed** | **Mr(expt)** | **Mr(calc)** | **Delta** | **Miss** | **Score** | **Expect** | **Rank** | **Peptide** |
| --- | --- | --- | --- | --- | --- | --- | --- | --- | --- | --- |
|  | 1336 | **624.2328** | **623.2256** | **622.3326** | **0.8930** | **0** | **2** | **92** | **2** | **K.IEFSK.E** |
|  | 7419 | 601.3399 | 1200.6652 | 1199.6874 | 0.9779 | 0 | 17 | 3.1 | 1 | K.ITLSQVGDVLR.A |
|  | 8236 | **621.3127** | **1240.6109** | **1241.6364** | **-1.0255** | **0** | **2** | **90** | **7** | **R.ALGTNPTNAEVR.K** |
|  | 10900 | **462.2472** | **1383.7198** | **1383.6994** | **0.0205** | **0** | **4** | **57** | **4** | **K.VLGNPSNEELNAK.K** |
|  | 16259 | 861.9221 | 1721.8296 | 1721.8407 | -0.0111 | 1 | 51 | 0.00082 | 1 | R.VFDKEGNGTVMGAELR.H 16249 16262 16270 16271 16272 |
|  | 16536 | 580.6337 | 1738.8794 | 1737.8356 | 1.0438 | 1 | (4) | 49 | 5 | R.VFDKEGNGTVMGAELR.H |
|  | 20652 | 1036.5581 | 2071.1017 | 2070.9754 | 0.1263 | 0 | 0 | 76 | 9 | K.IEFEQFLPMMQAISNNK.D |
|  | 21685 | 728.7315 | 2183.1725 | 2183.0755 | 0.0971 | 1 | 0 | 74 | 8 | K.KIEFEQFLPMMQAISNNK.D |

|  | **Proteins matching a subset of these peptides:** |
| --- | --- |

|  | P06741\|MLE3_HUMAN    **Mass:** 16730    **Score:** 51     **Queries matched:** 12 |
| --- | --- |
|  | Myosin light chain 3, skeletal muscle isoform OS=Homo sapiens GN=MYL1 PE=2 SV=2 |

| **713.** | Q9Y6B6\|SAR1B_HUMAN    **Mass:** 22510    **Score:** 51     **Queries matched:** 12   **emPAI:** 0.17 |
| --- | --- |
|  | GTP-binding protein SAR1b OS=Homo sapiens GN=SAR1B PE=1 SV=1 |

|  | **Query** | **Observed** | **Mr(expt)** | **Mr(calc)** | **Delta** | **Miss** | **Score** | **Expect** | **Rank** | **Peptide** |
| --- | --- | --- | --- | --- | --- | --- | --- | --- | --- | --- |
|  | 3437 | 457.2085 | 912.4025 | 912.4090 | -0.0065 | 0 | 21 | 1.1 | 1 | R.QGYGEGFR.W |
|  | 6547 | 573.8263 | 1145.6380 | 1145.6445 | -0.0064 | 0 | 51 | 0.0012 | 1 | K.LVFLGLDNAGK.T |
|  | 9589 | 657.8319 | 1313.6491 | 1313.6575 | -0.0084 | 0 | 22 | 0.85 | 1 | K.IDRPEAISEER.L 9606 |
|  | 9942 | **666.3144** | **1330.6143** | **1330.6227** | **-0.0084** | **0** | **5** | **43** | **1** | **R.EMFGLYGQTTGK.G** 9944 |
|  | 10131 | 448.2402 | 1341.6989 | 1341.7075 | -0.0086 | 1 | 12 | 8.8 | 2 | K.TTLLHMLKDDR.L |
|  | 10202 | **449.7159** | **1346.1258** | **1346.6177** | **-0.4918** | **0** | **(4)** | **40** | **9** | **R.EMFGLYGQTTGK.G** |
|  | 14379 | 801.4037 | 1600.7929 | 1599.8079 | 0.9850 | 1 | 24 | 0.51 | 2 | R.LREMFGLYGQTTGK.G 14382 |
|  | 20728 | **693.3568** | **2077.0486** | **2077.0812** | **-0.0326** | **1** | **5** | **32** | **3** | **K.ELNARPLEVFMCSVLKR.Q** |
|  | 24913 | **524.2574** | **2616.2509** | **2616.3305** | **-0.0796** | **0** | **1** | **54** | **10** | **M.SFIFDWIYSGFSSVLQFLGLYK.K** |

| **714.** | P08240\|SRPR_HUMAN    **Mass:** 70223    **Score:** 51     **Queries matched:** 24   **emPAI:** 0.05 |
| --- | --- |
|  | Signal recognition particle receptor subunit alpha OS=Homo sapiens GN=SRPR PE=1 SV=2 |

|  | **Query** | **Observed** | **Mr(expt)** | **Mr(calc)** | **Delta** | **Miss** | **Score** | **Expect** | **Rank** | **Peptide** |
| --- | --- | --- | --- | --- | --- | --- | --- | --- | --- | --- |
|  | 2514 | 844.4830 | 843.4758 | 843.4814 | -0.0056 | 0 | 8 | 30 | 3 | R.SVLLQER.G |
|  | 2577 | **425.2257** | **848.4369** | **847.3858** | **1.0511** | **0** | **7** | **42** | **8** | **R.DIMDAQR.R** |
|  | 4497 | 496.2532 | 990.4918 | 990.5498 | -0.0580 | 0 | 10 | 19 | 9 | R.LSALHPPEK.H |
|  | 4547 | 497.8868 | 993.7591 | 994.5157 | -0.7566 | 0 | 10 | 11 | 3 | R.TMVQLFEK.G |
|  | 8376 | **624.8169** | **1247.6192** | **1247.6260** | **-0.0068** | **0** | **51** | **0.0012** | **1** | **-.MLDFFTIFSK.G** |
|  | 10058 | **446.5841** | **1336.7304** | **1337.7164** | **-0.9860** | **1** | **4** | **50** | **9** | **R.AGAVEQLRTHTR.R** 10077 |
|  | 10313 | **451.2720** | **1350.7943** | **1351.6112** | **-0.8169** | **1** | **5** | **41** | **9** | **R.EDMESVLDKMR.D** |
|  | 11529 | **710.9467** | **1419.8789** | **1418.6897** | **1.1891** | **0** | **6** | **27** | **1** | **R.MQDNAPLMTALAK.L** |
|  | 11565 | **474.9099** | **1421.7079** | **1421.6973** | **0.0106** | **0** | **6** | **30** | **8** | **K.DAAGIAMEAIAFAR.N** 11567 11592 |
|  | 12657 | **744.8709** | **1487.7273** | **1487.7368** | **-0.0096** | **0** | **27** | **0.28** | **1** | **R.GGNNSFTHEALTLK.Y** |
|  | 12673 | 745.3827 | 1488.7509 | 1489.7525 | -1.0016 | 0 | 5 | 40 | 4 | R.NQGFDVVLVDTAGR.M |
|  | 15175 | 826.8820 | 1651.7494 | 1651.8790 | -0.1296 | 1 | 0 | 1.1e+002 | 7 | K.GTLGGMFGMLKGLVGSK.S |
|  | 15598 | **561.2873** | **1680.8400** | **1681.8523** | **-1.0122** | **0** | **3** | **49** | **3** | **K.EGSDGPLATSKPVPAEK.S** |
|  | 16372 | 865.4339 | 1728.8532 | 1729.8318 | -0.9786 | 1 | 8 | 16 | 10 | K.FNRALADHSMAQTPR.L 16393 |
|  | 17359 | 900.9321 | 1799.8496 | 1800.9040 | -1.0544 | 0 | 2 | 75 | 8 | K.NVAADIAVQLCESVANK.L |
|  | 17696 | **610.1815** | **1827.5226** | **1826.8985** | **0.6241** | **1** | **1** | **58** | **8** | **K.GYGKDAAGIAMEAIAFAR.N** |
|  | 19079 | **646.2784** | **1935.8133** | **1936.0102** | **-0.1969** | **1** | **6** | **21** | **2** | **R.RQRPYVVTFCGVNGVGK.S** |
|  | 26532 | 727.3695 | 2905.4487 | 2906.4266 | -0.9779 | 1 | 6 | 14 | 3 | R.NQGFDVVLVDTAGRMQDNAPLMTALAK.L 26524 |
|  | 29030 | **895.4115** | **3577.6169** | **3576.8320** | **0.7849** | **1** | **0** | **35** | **8** | **K.VGAAISMTYITSKPIVFVGTGQTYCDLRSLNAK.A** |

| **715.** | Q9NYL4\|FKB11_HUMAN    **Mass:** 22337    **Score:** 51     **Queries matched:** 2   **emPAI:** 0.17 |
| --- | --- |
|  | FK506-binding protein 11 OS=Homo sapiens GN=FKBP11 PE=1 SV=1 |

|  | **Query** | **Observed** | **Mr(expt)** | **Mr(calc)** | **Delta** | **Miss** | **Score** | **Expect** | **Rank** | **Peptide** |
| --- | --- | --- | --- | --- | --- | --- | --- | --- | --- | --- |
|  | 19979 | **1008.5045** | **2014.9945** | **2015.0067** | **-0.0123** | **0** | **51** | **0.00076** | **1** | **K.QVIPGLEQSLLDMCVGEK.R** |
|  | 24758 | **861.5770** | **2581.7093** | **2582.4481** | **-0.7388** | **0** | **3** | **24** | **1** | **K.GILPLVGMAMVPALLGLIGYHLYR.K** |

| **716.** | P01876\|IGHA1_HUMAN    **Mass:** 38486    **Score:** 51     **Queries matched:** 4   **emPAI:** 0.20 |
| --- | --- |
|  | Ig alpha-1 chain C region OS=Homo sapiens GN=IGHA1 PE=1 SV=2 |

|  | **Query** | **Observed** | **Mr(expt)** | **Mr(calc)** | **Delta** | **Miss** | **Score** | **Expect** | **Rank** | **Peptide** |
| --- | --- | --- | --- | --- | --- | --- | --- | --- | --- | --- |
|  | 3678 | **466.2731** | **930.5317** | **930.5386** | **-0.0069** | **0** | **35** | **0.065** | **1** | **K.TPLTATLSK.S** |
|  | 10727 | **688.3088** | **1374.6030** | **1374.6126** | **-0.0096** | **0** | **39** | **0.017** | **1** | **K.TFTCTAAYPESK.T** |
|  | 23074 | **784.8773** | **2351.6100** | **2352.1243** | **-0.5143** | **1** | **0** | **47** | **3** | **K.KGDTFSCMVGHEALPLAFTQK.T** |
|  | 29978 | 1317.2920 | 3948.8541 | 3948.7216 | 0.1326 | 1 | 0 | 27 | 7 | R.DLCGCYSVSSVLPGCAEPWNHGKTFTCTAAYPESK.T |

| **717.** | P30519\|HMOX2_HUMAN    **Mass:** 36181    **Score:** 50     **Queries matched:** 16   **emPAI:** 0.10 |
| --- | --- |
|  | Heme oxygenase 2 OS=Homo sapiens GN=HMOX2 PE=1 SV=2 |

|  | **Query** | **Observed** | **Mr(expt)** | **Mr(calc)** | **Delta** | **Miss** | **Score** | **Expect** | **Rank** | **Peptide** |
| --- | --- | --- | --- | --- | --- | --- | --- | --- | --- | --- |
|  | 4913 | **510.2723** | **1018.5301** | **1018.5369** | **-0.0067** | **0** | **31** | **0.16** | **1** | **R.MADLSELLK.E** |
|  | 5110 | 517.7642 | 1033.5138 | 1034.5318 | -1.0180 | 0 | (9) | 23 | 9 | R.MADLSELLK.E |
|  | 5303 | 525.7826 | 1049.5506 | 1048.5045 | 1.0461 | 0 | 17 | 3.4 | 1 | R.MNALDLNMK.T |
|  | 9013 | **642.3680** | **1282.7214** | **1282.6227** | **0.0987** | **0** | **2** | **76** | **2** | **R.YMGDLSGGQVLK.K** |
|  | 9226 | **648.2980** | **1294.5814** | **1293.6421** | **0.9393** | **1** | **6** | **33** | **2** | **R.MNALDLNMKTK.E** |
|  | 10743 | **688.8281** | **1375.6417** | **1375.6514** | **-0.0097** | **1** | **27** | **0.28** | **1** | **K.NSGALEKENQMR.M** |
|  | 18293 | 623.9947 | 1868.9622 | 1869.8149 | -0.8527 | 1 | 6 | 27 | 10 | -.MSAEVETSEGVDESEKK.N 18288 18290 18292 |
|  | 21674 | **546.5235** | **2182.0649** | **2182.0782** | **-0.0133** | **1** | **8** | **12** | **1** | **R.NKDHPAFAPLYFPMELHR.K** |
|  | 22082 | **742.3843** | **2224.1312** | **2223.1436** | **0.9876** | **0** | **11** | **6.8** | **1** | **R.IHYIGQNEPELLVAHAYTR.Y** 22057 |
|  | 24666 | **856.6909** | **2567.0509** | **2566.4021** | **0.6488** | **0** | **1** | **47** | **3** | **R.KPSLQFILAAGVALAAGLLAWYYM.-** |
|  | 26773 | **982.4894** | **2944.4463** | **2944.4607** | **-0.0144** | **1** | **7** | **10** | **1** | **R.ALKLPSTGEGTQFYLFENVDNAQQFK.Q** |
|  | 27901 | **1067.8496** | **3200.5270** | **3200.5448** | **-0.0178** | **1** | **50** | **0.00045** | **1** | **R.IVEEANKAFEYNMQIFNELDQAGSTLAR.E** |

| **718.** | Q15436\|SC23A_HUMAN    **Mass:** 87018    **Score:** 50     **Queries matched:** 12   **emPAI:** 0.13 |
| --- | --- |
|  | Protein transport protein Sec23A OS=Homo sapiens GN=SEC23A PE=1 SV=2 |

|  | **Query** | **Observed** | **Mr(expt)** | **Mr(calc)** | **Delta** | **Miss** | **Score** | **Expect** | **Rank** | **Peptide** |
| --- | --- | --- | --- | --- | --- | --- | --- | --- | --- | --- |
|  | 4019 | 478.7313 | 955.4481 | 956.5080 | -1.0599 | 1 | 9 | 20 | 1 | K.SYVFRGTK.D |
|  | 11204 | **468.2486** | **1401.7241** | **1400.8102** | **0.9139** | **0** | **9** | **17** | **1** | **R.MVVPVAALFTPLK.E** |
|  | 11384 | **471.4767** | **1411.4084** | **1411.7130** | **-0.3045** | **1** | **2** | **52** | **7** | **K.MGFGGTLEIKTSR.E** |
|  | 14152 | **529.5895** | **1585.7466** | **1585.7592** | **-0.0126** | **0** | **25** | **0.44** | **1** | **R.MVQVHELGCEGISK.S** |
|  | 14835 | 816.4114 | 1630.8083 | 1629.8396 | 0.9687 | 0 | 46 | 0.0032 | 1 | K.IDMNLTDLLGELQR.D |
|  | 15513 | **559.3156** | **1674.9250** | **1675.8814** | **-0.9564** | **1** | **4** | **45** | **5** | **K.DLSAKQLQEMLGLSK.V** |
|  | 20184 | 677.3671 | 2029.0794 | 2028.1037 | 0.9756 | 1 | 4 | 41 | 3 | K.QLQEMLGLSKVPLTQATR.G |
|  | 22503 | **760.4780** | **2278.4123** | **2279.1000** | **-0.6878** | **0** | **1** | **49** | **5** | **R.IMMFIGGPATQGPGMVVGDELK.T** |
|  | 24984 | **879.1182** | **2634.3329** | **2634.3476** | **-0.0147** | **1** | **19** | **0.85** | **1** | **K.IDMNLTDLLGELQRDPWPVPQGK.R** 24983 |
|  | 25507 | 1365.6897 | 2729.3648 | 2730.3907 | -1.0259 | 1 | 3 | 33 | 4 | R.IMMFIGGPATQGPGMVVGDELKTPIR.S |
|  | 26757 | **589.3093** | **2941.5103** | **2940.3959** | **1.1144** | **0** | **6** | **12** | **2** | **R.GPQMPLIFLYVVDTCMEDEDLQALK.E** |

| **719.** | Q9NNW7\|TRXR2_HUMAN    **Mass:** 57156    **Score:** 50     **Queries matched:** 28   **emPAI:** 0.07 |
| --- | --- |
|  | Thioredoxin reductase 2, mitochondrial OS=Homo sapiens GN=TXNRD2 PE=1 SV=3 |

|  | **Query** | **Observed** | **Mr(expt)** | **Mr(calc)** | **Delta** | **Miss** | **Score** | **Expect** | **Rank** | **Peptide** |
| --- | --- | --- | --- | --- | --- | --- | --- | --- | --- | --- |
|  | 682 | **459.0128** | **458.0055** | **458.2601** | **-0.2546** | **0** | **3** | **1e+002** | **1** | **R.GLGGR.F** 678 680 683 |
|  | 1990 | **402.2177** | **802.4208** | **801.4531** | **0.9677** | **0** | **7** | **48** | **3** | **M.AAMAVALR.G** |
|  | 2864 | **435.2323** | **868.4500** | **868.4304** | **0.0197** | **0** | **12** | **10** | **8** | **K.SLNWGHR.V** |
|  | 3927 | **475.2395** | **948.4645** | **948.4885** | **-0.0240** | **0** | **11** | **13** | **4** | **-.MAAMAVALR.G** 3915 3916 3933 3936 |
|  | 4417 | **493.2778** | **984.5411** | **984.5213** | **0.0198** | **1** | **8** | **25** | **5** | **R.GAARGAAAGQR.D** |
|  | 7440 | **401.2848** | **1200.8326** | **1199.6523** | **1.1803** | **1** | **8** | **18** | **3** | **R.WRTQAVAGGVR.G** |
|  | 8567 | **630.2971** | **1258.5797** | **1257.6976** | **0.8821** | **1** | **5** | **52** | **5** | **M.AAMAVALRGLGGR.F** 8545 |
|  | 8804 | **636.3667** | **1270.7188** | **1269.6499** | **1.0689** | **1** | **5** | **44** | **7** | **R.KMAEAVQNHVK.S** |
|  | 10626 | **457.2408** | **1368.7006** | **1369.5199** | **-0.8193** | **1** | **7** | **25** | **3** | **K.RSGLDPTVTGCUG.-** |
|  | 10683 | **458.3307** | **1371.9702** | **1372.7431** | **-0.7730** | **1** | **(5)** | **32** | **6** | **-.MAAMAVALRGLGGR.F** |
|  | 11004 | **464.2578** | **1389.7517** | **1388.7380** | **1.0137** | **1** | **5** | **43** | **8** | **-.MAAMAVALRGLGGR.F** |
|  | 11263 | **469.3273** | **1404.9601** | **1404.7330** | **0.2271** | **1** | **(0)** | **92** | **8** | **-.MAAMAVALRGLGGR.F** |
|  | 14615 | 809.4224 | 1616.8303 | 1616.7804 | 0.0499 | 0 | 50 | 0.0011 | 1 | R.WGLGGTCVNVGCIPK.K |
|  | 16692 | **584.8420** | **1751.5041** | **1750.8560** | **0.6481** | **0** | **5** | **23** | **1** | **R.DYDLLVVGGGSGGLACAK.E** 16674 16684 16695 |
|  | 21594 | **544.2617** | **2173.0178** | **2173.2331** | **-0.2153** | **1** | **2** | **56** | **9** | **K.GGKEILLSADHIIIATGGRPR.Y** |
|  | 22518 | **760.7290** | **2279.1652** | **2279.9722** | **-0.8070** | **0** | **2** | **46** | **4** | **R.GFDQQMSSMVIEHMASHGTR.F** |
|  | 28962 | **889.9440** | **3555.7468** | **3556.7980** | **-1.0512** | **1** | **2** | **22** | **7** | **K.ESPGKTLVVGASYVALECAGFLTGIGLDTTIMMR.S** |

| **720.** | Q16881\|TRXR1_HUMAN    **Mass:** 71832    **Score:** 50     **Queries matched:** 11   **emPAI:** 0.05 |
| --- | --- |
|  | Thioredoxin reductase 1, cytoplasmic OS=Homo sapiens GN=TXNRD1 PE=1 SV=3 |

|  | **Query** | **Observed** | **Mr(expt)** | **Mr(calc)** | **Delta** | **Miss** | **Score** | **Expect** | **Rank** | **Peptide** |
| --- | --- | --- | --- | --- | --- | --- | --- | --- | --- | --- |
|  | 922 | **519.1350** | **518.1277** | **517.2245** | **0.9032** | **0** | **6** | **67** | **8** | **K.NGDGR.R** |
|  | 4834 | 507.3274 | 1012.6402 | 1011.4444 | 1.1959 | 1 | 1 | 1.2e+002 | 3 | R.DNNKCYAK.I |
|  | 6708 | 579.8361 | 1157.6576 | 1158.6509 | -0.9934 | 0 | 13 | 8.1 | 2 | R.FLIATGERPR.Y |
|  | 12062 | 727.8803 | 1453.7461 | 1454.8093 | -1.0632 | 1 | 9 | 16 | 3 | K.AVAAAAPTELQTKGK.N |
|  | 14464 | 804.4122 | 1606.8098 | 1606.8137 | -0.0039 | 1 | 5 | 35 | 6 | R.SILLRGFDQDMANK.I 14463 |
|  | 14615 | 809.4224 | 1616.8303 | 1616.7804 | 0.0499 | 0 | 50 | 0.0011 | 1 | R.WGLGGTCVNVGCIPK.K |
|  | 16667 | 438.4664 | 1749.8364 | 1748.8634 | 0.9729 | 0 | 4 | 43 | 7 | K.VVYENAYGQFIGPHR.I |
|  | 18260 | **623.6072** | **1867.7999** | **1868.0156** | **-0.2157** | **1** | **4** | **41** | **1** | **R.QFVPIKVEQIEAGTPGR.L** |
|  | 18486 | 629.9914 | 1886.9523 | 1888.0207 | -1.0683 | 0 | 1 | 82 | 9 | R.ALLQAYIDGHSVVIFSR.S |
|  | 24234 | 1253.5891 | 2505.1637 | 2506.1837 | -1.0200 | 1 | 1 | 55 | 4 | K.DHHPGKTLPENPAGFTSTATADSR.A |

| **721.** | P20132\|SDHL_HUMAN    **Mass:** 34945    **Score:** 50     **Queries matched:** 17   **emPAI:** 0.36 |
| --- | --- |
|  | L-serine dehydratase/L-threonine deaminase OS=Homo sapiens GN=SDS PE=1 SV=2 |

|  | **Query** | **Observed** | **Mr(expt)** | **Mr(calc)** | **Delta** | **Miss** | **Score** | **Expect** | **Rank** | **Peptide** |
| --- | --- | --- | --- | --- | --- | --- | --- | --- | --- | --- |
|  | 2186 | **410.0944** | **818.1743** | **817.3905** | **0.7838** | **0** | **1** | **1.3e+002** | **7** | **R.GIGHFCK.R** |
|  | 4150 | 483.2754 | 964.5363 | 963.4444 | 1.0920 | 0 | 3 | 92 | 6 | K.EQLGMTNR.L |
|  | 4824 | **507.2303** | **1012.4460** | **1012.5012** | **-0.0551** | **0** | **4** | **68** | **3** | **M.MSGEPLHVK.T** |
|  | 7787 | **407.1332** | **1218.3779** | **1217.6438** | **0.7341** | **1** | **4** | **42** | **8** | **K.TPIRDSMALSK.M** |
|  | 8604 | **630.8359** | **1259.6572** | **1259.6656** | **-0.0084** | **1** | **25** | **0.52** | **1** | **R.ALKEQLGMTNR.L** 8586 |
|  | 9050 | **429.2314** | **1284.6722** | **1285.6812** | **-1.0090** | **1** | **10** | **13** | **4** | **K.EQLGMTNRLPK.-** 9090 |
|  | 13335 | **766.8729** | **1531.7313** | **1531.8134** | **-0.0821** | **0** | **3** | **65** | **8** | **K.VVGELLDEAFELAK.A** |
|  | 14281 | 532.2592 | 1593.7558 | 1594.8323 | -1.0765 | 1 | 2 | 76 | 4 | -.MMSGEPLHVKTPIR.D |
|  | 16209 | **573.3868** | **1717.1385** | **1716.8426** | **0.2959** | **1** | **(2)** | **47** | **2** | **R.DSMALSKMAGTSVYLK.M** 16212 |
|  | 16450 | **578.6440** | **1732.9103** | **1732.8375** | **0.0728** | **1** | **3** | **56** | **3** | **R.DSMALSKMAGTSVYLK.M** |
|  | 21499 | **1082.1162** | **2162.2179** | **2162.2311** | **-0.0132** | **0** | **22** | **0.51** | **1** | **R.QLGVPATIVVPSTTPALTIER.L** |
|  | 22155 | 1116.6083 | 2231.2020 | 2231.1685 | 0.0335 | 1 | 4 | 33 | 3 | K.NEGATVKVVGELLDEAFELAK.A |
|  | 24844 | **867.4402** | **2599.2989** | **2599.3170** | **-0.0180** | **0** | **47** | **0.0012** | **1** | **K.LFQEHPIFSEVISDQEAVAAIEK.F** |
|  | 25494 | **910.4357** | **2728.2852** | **2728.2057** | **0.0795** | **1** | **0** | **59** | **5** | **R.WAKQGCAHFVCSSAGNAGMAAAYAAR.Q** |

| **722.** | Q14061\|COX17_HUMAN    **Mass:** 7253     **Score:** 50     **Queries matched:** 2   **emPAI:** 0.58 |
| --- | --- |
|  | Cytochrome c oxidase copper chaperone OS=Homo sapiens GN=COX17 PE=1 SV=2 |

|  | **Query** | **Observed** | **Mr(expt)** | **Mr(calc)** | **Delta** | **Miss** | **Score** | **Expect** | **Rank** | **Peptide** |
| --- | --- | --- | --- | --- | --- | --- | --- | --- | --- | --- |
|  | 15351 | 832.9050 | 1663.7954 | 1663.8053 | -0.0099 | 0 | 50 | 0.0011 | 1 | M.PGLVDSNPAPPESQEK.K |
|  | 17491 | 906.4285 | 1810.8424 | 1810.8407 | 0.0017 | 0 | 1 | 77 | 9 | -.MPGLVDSNPAPPESQEK.K |

| **723.** | P10606\|COX5B_HUMAN    **Mass:** 13915    **Score:** 50     **Queries matched:** 12   **emPAI:** 0.28 |
| --- | --- |
|  | Cytochrome c oxidase subunit 5B, mitochondrial OS=Homo sapiens GN=COX5B PE=1 SV=2 |

|  | **Query** | **Observed** | **Mr(expt)** | **Mr(calc)** | **Delta** | **Miss** | **Score** | **Expect** | **Rank** | **Peptide** |
| --- | --- | --- | --- | --- | --- | --- | --- | --- | --- | --- |
|  | 1758 | **775.4328** | **774.4255** | **774.4309** | **-0.0054** | **0** | **31** | **0.17** | **1** | **R.EIMLAAK.K** |
|  | 2182 | **409.7230** | **817.4314** | **816.3912** | **1.0402** | **0** | **17** | **4.1** | **1** | **R.GPSGAAAMR.S** |
|  | 5024 | **514.7983** | **1027.5820** | **1027.5774** | **0.0046** | **0** | **9** | **22** | **6** | **R.GAGTLAAQALR.A** |
|  | 9594 | **657.8696** | **1313.7247** | **1313.7343** | **-0.0096** | **1** | **50** | **0.0015** | **1** | **K.KGLDPYNVLAPK.G** 9622 |
|  | 12305 | **734.8860** | **1467.7574** | **1467.7681** | **-0.0107** | **1** | **14** | **4.8** | **1** | **R.EDPNLVPSISNKR.I** 12282 12306 12316 |
|  | 17872 | 614.6456 | 1840.9149 | 1840.9279 | -0.0130 | 1 | 7 | 21 | 3 | K.GASGTREDPNLVPSISNK.R |
|  | 24172 | **831.7647** | **2492.2723** | **2491.1665** | **1.1058** | **0** | **2** | **48** | **9** | **R.IVGCICEEDNTSVVWFWLHK.G** |
|  | 25781 | 557.0758 | 2780.3427 | 2779.3004 | 1.0423 | 1 | 1 | 51 | 7 | R.SMASGGGVPTDEEQATGLEREIMLAAK.K |

| **724.** | O96005\|CLPT1_HUMAN    **Mass:** 76277    **Score:** 50     **Queries matched:** 17   **emPAI:** 0.05 |
| --- | --- |
|  | Cleft lip and palate transmembrane protein 1 OS=Homo sapiens GN=CLPTM1 PE=1 SV=1 |

|  | **Query** | **Observed** | **Mr(expt)** | **Mr(calc)** | **Delta** | **Miss** | **Score** | **Expect** | **Rank** | **Peptide** |
| --- | --- | --- | --- | --- | --- | --- | --- | --- | --- | --- |
|  | 4065 | 480.2766 | 958.5387 | 958.4468 | 0.0919 | 0 | 13 | 8.9 | 1 | M.AAAQEADGAR.S 4058 4068 |
|  | 4098 | 481.7402 | 961.4658 | 960.5426 | 0.9232 | 1 | 21 | 1.5 | 1 | K.ITKVMDVR.L |
|  | 5065 | 1031.5342 | 1030.5269 | 1030.5923 | -0.0654 | 1 | 13 | 9.7 | 5 | R.VASRNLFPK.D |
|  | 5800 | 545.3002 | 1088.5858 | 1089.4873 | -0.9015 | 0 | 20 | 2 | 3 | -.MAAAQEADGAR.S |
|  | 6049 | **554.2979** | **1106.5811** | **1105.4822** | **1.0990** | **0** | **(9)** | **22** | **1** | **-.MAAAQEADGAR.S** |
|  | 11460 | **709.3086** | **1416.6026** | **1415.7377** | **0.8649** | **1** | **0** | **1.3e+002** | **6** | **R.LATVHMSRMINK.Y** |
|  | 14616 | 809.4408 | 1616.8670 | 1616.8497 | 0.0173 | 1 | 4 | 53 | 3 | K.SVAHLPWRMLTYK.A 14617 |
|  | 17544 | 908.4955 | 1814.9765 | 1815.9036 | -0.9271 | 1 | 5 | 33 | 3 | K.NLLTGETEADPEMIKR.A |
|  | 18738 | 636.3297 | 1905.9671 | 1904.9401 | 1.0270 | 1 | 7 | 20 | 5 | K.TKNLLTGETEADPEMIK.R 18732 |
|  | 25503 | **683.3329** | **2729.3025** | **2730.2642** | **-0.9617** | **0** | **0** | **62** | **10** | **K.FDAVSGDYYPIIYFNDYWNLQK.D** |
|  | 27030 | 999.1321 | 2994.3744 | 2995.5266 | -1.1522 | 1 | 2 | 36 | 2 | K.DYYPINESLASLPLRVSFCPLSLWR.W |
|  | 28120 | **1089.1876** | **3264.5410** | **3264.5633** | **-0.0223** | **0** | **50** | **0.00045** | **1** | **R.EEASTSLPTKPTQGASSASEPQEAPPKPAEDK.K** 28121 |

| **725.** | P08758\|ANXA5_HUMAN    **Mass:** 35971    **Score:** 50     **Queries matched:** 6   **emPAI:** 0.10 |
| --- | --- |
|  | Annexin A5 OS=Homo sapiens GN=ANXA5 PE=1 SV=2 |

|  | **Query** | **Observed** | **Mr(expt)** | **Mr(calc)** | **Delta** | **Miss** | **Score** | **Expect** | **Rank** | **Peptide** |
| --- | --- | --- | --- | --- | --- | --- | --- | --- | --- | --- |
|  | 3316 | **452.7336** | **903.4526** | **902.4821** | **0.9705** | **1** | **27** | **0.35** | **1** | **R.ADAETLRK.A** |
|  | 4650 | 500.7916 | 999.5686 | 1000.5917 | -1.0230 | 0 | 9 | 23 | 10 | K.VLTEIIASR.T |
|  | 8348 | 416.2844 | 1245.8315 | 1245.6275 | 0.2041 | 1 | 4 | 54 | 10 | K.KALLLLCGEDD.- |
|  | 16459 | **867.9431** | **1733.8717** | **1732.8745** | **0.9971** | **0** | **16** | **2.9** | **1** | **R.SIPAYLAETLYYAMK.G** |
|  | 29605 | **1265.9733** | **3794.8980** | **3794.9149** | **-0.0169** | **1** | **50** | **0.00035** | **1** | **R.MLVVLLQANRDPDAGIDEAQVEQDAQALFQAGELK.W** |
|  | 29631 | 953.4554 | 3809.7924 | 3810.9098 | -1.1174 | 1 | (2) | 21 | 10 | R.MLVVLLQANRDPDAGIDEAQVEQDAQALFQAGELK.W |

| **726.** | Q9BVC6\|TM109_HUMAN    **Mass:** 26194    **Score:** 50     **Queries matched:** 3   **emPAI:** 0.15 |
| --- | --- |
|  | Transmembrane protein 109 OS=Homo sapiens GN=TMEM109 PE=1 SV=1 |

|  | **Query** | **Observed** | **Mr(expt)** | **Mr(calc)** | **Delta** | **Miss** | **Score** | **Expect** | **Rank** | **Peptide** |
| --- | --- | --- | --- | --- | --- | --- | --- | --- | --- | --- |
|  | 1003 | **533.1887** | **532.1814** | **532.2969** | **-0.1155** | **0** | **3** | **1.6e+002** | **4** | **R.LTGSR.A** |
|  | 9281 | 649.3554 | 1296.6961 | 1296.7038 | -0.0076 | 0 | 50 | 0.0014 | 1 | R.EAPVDVLTQIGR.S |
|  | 12038 | **484.9404** | **1451.7995** | **1452.8049** | **-1.0054** | **1** | **2** | **79** | **9** | **K.REAPVDVLTQIGR.S** |

| **727.** | P62266\|RS23_HUMAN    **Mass:** 15969    **Score:** 49     **Queries matched:** 10   **emPAI:** 0.24 |
| --- | --- |
|  | 40S ribosomal protein S23 OS=Homo sapiens GN=RPS23 PE=1 SV=3 |

|  | **Query** | **Observed** | **Mr(expt)** | **Mr(calc)** | **Delta** | **Miss** | **Score** | **Expect** | **Rank** | **Peptide** |
| --- | --- | --- | --- | --- | --- | --- | --- | --- | --- | --- |
|  | 2079 | **809.3738** | **808.3665** | **809.4759** | **-1.1094** | **0** | **19** | **2** | **1** | **K.AHLGTALK.A** 2095 |
|  | 7524 | **603.3369** | **1204.6593** | **1204.6677** | **-0.0084** | **1** | **49** | **0.0018** | **1** | **R.KGHAVGDIPGVR.F** 7498 7503 7542 |
|  | 8242 | 621.3331 | 1240.6516 | 1240.7391 | -0.0875 | 1 | 5 | 42 | 9 | K.GIVLEKVGVEAK.Q |
|  | 10607 | 684.8478 | 1367.6810 | 1367.7521 | -0.0711 | 1 | 6 | 37 | 6 | K.VGVEAKQPNSAIR.K |
|  | 15832 | **566.2735** | **1695.7987** | **1694.9104** | **0.8883** | **1** | **9** | **15** | **2** | **K.ANPFGGASHAKGIVLEK.V** 15802 |

| **728.** | Q99685\|MGLL_HUMAN    **Mass:** 33468    **Score:** 49     **Queries matched:** 5   **emPAI:** 0.11 |
| --- | --- |
|  | Monoglyceride lipase OS=Homo sapiens GN=MGLL PE=2 SV=2 |

|  | **Query** | **Observed** | **Mr(expt)** | **Mr(calc)** | **Delta** | **Miss** | **Score** | **Expect** | **Rank** | **Peptide** |
| --- | --- | --- | --- | --- | --- | --- | --- | --- | --- | --- |
|  | 3934 | **475.2843** | **948.5540** | **947.4018** | **1.1522** | **0** | **9** | **21** | **4** | **-.MPEESSPR.R** |
|  | 9627 | **439.5322** | **1315.5748** | **1314.6238** | **0.9510** | **0** | **9** | **19** | **2** | **R.DVLQHVDSMQK.D** |
|  | 13304 | **765.4336** | **1528.8527** | **1528.8613** | **-0.0086** | **0** | **49** | **0.0014** | **1** | **K.LTVPFLLLQGSADR.L** |
|  | 14090 | 792.3605 | 1582.7065 | 1581.8072 | 0.8993 | 1 | 2 | 83 | 4 | K.GAYLLMELAKSQDK.T |
|  | 25039 | **530.4571** | **2647.2494** | **2647.2635** | **-0.0142** | **0** | **15** | **1.9** | **1** | **R.MLMGLDLLVFAHDHVGHGQSEGER.M** |

| **729.** | P12110\|CO6A2_HUMAN    **Mass:** 109709   **Score:** 49     **Queries matched:** 25   **emPAI:** 0.03 |
| --- | --- |
|  | Collagen alpha-2(VI) chain OS=Homo sapiens GN=COL6A2 PE=1 SV=4 |

|  | **Query** | **Observed** | **Mr(expt)** | **Mr(calc)** | **Delta** | **Miss** | **Score** | **Expect** | **Rank** | **Peptide** |
| --- | --- | --- | --- | --- | --- | --- | --- | --- | --- | --- |
|  | 1031 | **540.9760** | **539.9687** | **539.2816** | **0.6871** | **0** | **7** | **15** | **1** | **K.GGPGPR.G** |
|  | 1102 | **558.2877** | **557.2804** | **557.2809** | **-0.0005** | **0** | **11** | **19** | **1** | **R.GAPGEK.G** |
|  | 1174 | 573.2849 | 572.2776 | 571.3693 | 0.9083 | 0 | 20 | 2.7 | 1 | R.LGAIAK.D 1166 |
|  | 2505 | 422.2348 | 842.4550 | 842.4722 | -0.0172 | 1 | 13 | 9 | 7 | K.LQAERAR.E |
|  | 3043 | **441.2965** | **880.5784** | **880.4039** | **0.1745** | **0** | **3** | **81** | **2** | **K.GDPGPEGPR.G** |
|  | 3431 | 456.7638 | 911.5130 | 911.5440 | -0.0310 | 1 | 10 | 15 | 3 | R.LGAIAKDPK.S |
|  | 4687 | 502.2315 | 1002.4485 | 1003.5563 | -1.1078 | 1 | 17 | 3.3 | 3 | R.RFVEQVAR.R |
|  | 4789 | 505.7560 | 1009.4975 | 1009.5193 | -0.0217 | 0 | 0 | 1.3e+002 | 8 | R.GPQGALGEPGK.Q |
|  | 7315 | **598.7735** | **1195.5324** | **1194.5629** | **0.9695** | **1** | **2** | **82** | **6** | **K.GEKGDPGPEGPR.G** |
|  | 9775 | **662.3470** | **1322.6795** | **1322.7082** | **-0.0286** | **1** | **1** | **96** | **2** | **R.IDSLSSFKEAVK.N** |
|  | 10077 | 670.2701 | 1338.5257 | 1339.6806 | -1.1549 | 0 | 3 | 65 | 4 | K.VSCLEIPGPSGPK.G |
|  | 10659 | **457.7530** | **1370.2371** | **1369.6950** | **0.5422** | **1** | **3** | **47** | **7** | **K.GAPGLAGKNGTDGQK.G** |
|  | 10741 | **688.8140** | **1375.6135** | **1374.6528** | **0.9607** | **0** | **4** | **52** | **2** | **K.GYQGNSGAPGSPGVK.G** |
|  | 11407 | **471.9575** | **1412.8506** | **1412.6467** | **0.2039** | **1** | **2** | **72** | **8** | **K.GNMGEPGEPGQKGR.Q** |
|  | 13028 | 757.3880 | 1512.7614 | 1513.6693 | -0.9079 | 1 | 3 | 63 | 8 | K.VMKHEAYGECYK.V |
|  | 16517 | 580.5372 | 1738.5897 | 1738.8387 | -0.2490 | 1 | 0 | 72 | 5 | K.GEPADPGPPGEPGPRGPR.G |
|  | 16590 | **872.9533** | **1743.8919** | **1742.8992** | **0.9928** | **0** | **49** | **0.0013** | **1** | **K.NLEWIAGGTWTPSALK.F** |
|  | 17322 | 899.4909 | 1796.9673 | 1797.9737 | -1.0064 | 1 | 5 | 31 | 5 | R.EEGIRLFAVAPNQNLK.E |
|  | 19647 | **662.9724** | **1985.8954** | **1986.8668** | **-0.9714** | **1** | **3** | **48** | **9** | **R.GPDGYPGEAGSPGERGDQGGK.G** |
|  | 21405 | **718.0077** | **2151.0014** | **2150.1161** | **0.8853** | **1** | **1** | **71** | **5** | **R.QGDPGIEGPIGFPGPKGVPGFK.G** |
|  | 26528 | **727.3691** | **2905.4472** | **2905.4869** | **-0.0397** | **1** | **0** | **48** | **3** | **K.GTVHFAVVITDGHVTGSPCGGIKLQAER.A** |
|  | 26891 | **991.4827** | **2971.4264** | **2971.5536** | **-0.1273** | **1** | **1** | **42** | **1** | **R.KQNVVPTVLALGSDVDMDVLTTLSLGDR.A** |
|  | 27539 | **1551.7245** | **3101.4344** | **3100.4673** | **0.9672** | **1** | **0** | **51** | **3** | **R.HAELSFVFLTDGVTGNDSLHESAHSMRK.Q** |
|  | 29133 | **722.9615** | **3609.7711** | **3609.8713** | **-0.1002** | **1** | **1** | **27** | **6** | **K.QNVVPTVLALGSDVDMDVLTTLSLGDRAAVFHEK.D** |

| **730.** | P46777\|RL5_HUMAN    **Mass:** 34569    **Score:** 49     **Queries matched:** 23   **emPAI:** 0.36 |
| --- | --- |
|  | 60S ribosomal protein L5 OS=Homo sapiens GN=RPL5 PE=1 SV=3 |

|  | **Query** | **Observed** | **Mr(expt)** | **Mr(calc)** | **Delta** | **Miss** | **Score** | **Expect** | **Rank** | **Peptide** |
| --- | --- | --- | --- | --- | --- | --- | --- | --- | --- | --- |
|  | 1410 | **639.3292** | **638.3219** | **637.3660** | **0.9559** | **0** | **5** | **36** | **2** | **K.AHAAIR.E** |
|  | 1624 | 715.4303 | 714.4230 | 714.4276 | -0.0046 | 0 | 5 | 64 | 4 | R.LVIQDK.N |
|  | 3001 | **440.0308** | **878.0470** | **877.4181** | **0.6288** | **0** | **1** | **1e+002** | **4** | **R.ENPVYEK.K** |
|  | 7060 | **593.2761** | **1184.5377** | **1184.5462** | **-0.0085** | **1** | **40** | **0.015** | **1** | **K.RFPGYDSESK.E** 7059 7064 7078 |
|  | 10046 | 669.3027 | 1336.5908 | 1337.6939 | -1.1032 | 0 | 4 | 60 | 9 | K.GAVDGGLSIPHSTK.R |
|  | 11748 | 717.8292 | 1433.6439 | 1433.6544 | -0.0105 | 0 | 21 | 1.1 | 1 | K.HIMGQNVADYMR.Y 11741 11743 11747 |
|  | 12009 | 484.2707 | 1449.7903 | 1449.6493 | 0.1410 | 0 | (7) | 27 | 8 | K.HIMGQNVADYMR.Y |
|  | 12751 | 747.8981 | 1493.7816 | 1493.7950 | -0.0135 | 1 | 8 | 19 | 6 | K.GAVDGGLSIPHSTKR.F |
|  | 13346 | **767.3373** | **1532.6601** | **1532.6704** | **-0.0103** | **1** | **46** | **0.0028** | **1** | **R.YLMEEDEDAYKK.Q** 13345 |
|  | 13806 | **521.9212** | **1562.7418** | **1561.7493** | **0.9924** | **1** | **(3)** | **62** | **2** | **R.KHIMGQNVADYMR.Y** |
|  | 14023 | **395.7139** | **1578.8265** | **1577.7442** | **1.0822** | **1** | **9** | **16** | **2** | **R.KHIMGQNVADYMR.Y** 14001 |
|  | 14190 | 795.3961 | 1588.7777 | 1589.6411 | -0.8635 | 0 | 2 | 77 | 6 | K.NSVTPDMMEEMYK.K |
|  | 15722 | 564.5905 | 1690.7497 | 1691.8777 | -1.1280 | 1 | 13 | 6 | 1 | R.VTNRDIICQIAYAR.I |
|  | 15944 | **568.2509** | **1701.7309** | **1701.7412** | **-0.0102** | **1** | **9** | **13** | **1** | **K.NSVTPDMMEEMYKK.A** |
|  | 16673 | **584.3236** | **1749.9490** | **1749.7259** | **0.2231** | **1** | **(4)** | **46** | **6** | **K.NSVTPDMMEEMYKK.A** |

| **731.** | Q2M1Z3\|CDGAP_HUMAN    **Mass:** 158286   **Score:** 49     **Queries matched:** 19   **emPAI:** 0.02 |
| --- | --- |
|  | Cdc42 GTPase-activating protein OS=Homo sapiens GN=CDGAP PE=1 SV=1 |

|  | **Query** | **Observed** | **Mr(expt)** | **Mr(calc)** | **Delta** | **Miss** | **Score** | **Expect** | **Rank** | **Peptide** |
| --- | --- | --- | --- | --- | --- | --- | --- | --- | --- | --- |
|  | 920 | **517.7639** | **516.7566** | **516.3020** | **0.4546** | **1** | **4** | **1.1e+002** | **2** | **K.NKGAK.Q** |
|  | 3419 | **456.2539** | **910.4932** | **910.4508** | **0.0424** | **0** | **4** | **55** | **8** | **K.GAEAPPNQK.G** |
|  | 6360 | **566.8109** | **1131.6073** | **1132.5472** | **-0.9400** | **1** | **4** | **64** | **4** | **K.QNSKNAASEGK.G** |
|  | 9577 | **438.5862** | **1312.7368** | **1311.7874** | **0.9494** | **1** | **49** | **0.0018** | **1** | **R.LSVEKATIRPAK.S** |
|  | 12257 | **733.6195** | **1465.2243** | **1465.7897** | **-0.5654** | **1** | **(0)** | **76** | **2** | **K.MCQARAVPVIPPK.I** |
|  | 12308 | **490.2633** | **1467.7681** | **1466.7742** | **0.9939** | **0** | **2** | **70** | **4** | **K.LERPSGGSKPFHR.S** |
|  | 12554 | 741.8705 | 1481.7264 | 1481.7847 | -0.0583 | 1 | 8 | 19 | 4 | K.MCQARAVPVIPPK.I |
|  | 13912 | 786.4212 | 1570.8278 | 1571.7943 | -0.9665 | 1 | 4 | 56 | 7 | R.EFSGLKGAEAPPNQK.G |
|  | 13983 | 789.3976 | 1576.7807 | 1576.6902 | 0.0906 | 0 | 11 | 9.7 | 3 | K.MLGMFYTSNDSPSK.S 14001 |
|  | 14543 | **538.3001** | **1611.8785** | **1611.8984** | **-0.0199** | **0** | **6** | **32** | **3** | **K.ALNISEPFAVSVPLR.V** |
|  | 14899 | 545.9764 | 1634.9073 | 1634.7471 | 0.1602 | 1 | 2 | 79 | 8 | K.NAASEGKGCGFPSPTR.E |
|  | 16526 | 870.4283 | 1738.8420 | 1739.9934 | -1.1514 | 1 | 2 | 65 | 6 | R.KALNISEPFAVSVPLR.V 16525 |
|  | 18475 | **472.4715** | **1885.8570** | **1886.8444** | **-0.9874** | **1** | **3** | **46** | **7** | **K.VTWMTSSYCKADPWR.V** |
|  | 18575 | **632.3467** | **1894.0184** | **1892.8534** | **1.1650** | **1** | **2** | **59** | **2** | **R.EAGEMESSTLQESPRAR.A** |
|  | 24746 | 861.1044 | 2580.2915 | 2581.4016 | -1.1101 | 1 | 0 | 59 | 10 | R.IQNVIQELPPSHYRTLEYLIR.H |
|  | 29002 | 1784.3303 | 3566.6461 | 3566.9097 | -0.2636 | 1 | 0 | 35 | 4 | R.AVPVIPPKIQYTQIPQPLPSQSSGENGVQPLER.S |
|  | 30088 | **999.2725** | **3993.0610** | **3993.0054** | **0.0555** | **0** | **2** | **18** | **9** | **R.VQQVVIEFILNHVDQIFNNGAPGSLENDENRPIMK.S** |

| **732.** | Q3ZCQ8\|TIM50_HUMAN    **Mass:** 39850    **Score:** 48     **Queries matched:** 8   **emPAI:** 0.20 |
| --- | --- |
|  | Mitochondrial import inner membrane translocase subunit TIM50 OS=Homo sapiens GN=TIMM50 PE=1 SV=2 |

|  | **Query** | **Observed** | **Mr(expt)** | **Mr(calc)** | **Delta** | **Miss** | **Score** | **Expect** | **Rank** | **Peptide** |
| --- | --- | --- | --- | --- | --- | --- | --- | --- | --- | --- |
|  | 2186 | 410.0944 | 818.1743 | 817.4368 | 0.7376 | 0 | 1 | 1.2e+002 | 3 | R.VVVVDCK.K |
|  | 4777 | **505.2731** | **1008.5317** | **1009.5015** | **-0.9698** | **0** | **7** | **28** | **8** | **-.MAASAAVFSR.L** |
|  | 7070 | **395.8886** | **1184.6439** | **1185.6353** | **-0.9914** | **0** | **7** | **32** | **4** | **K.TIALNGVEDVR.T** |
|  | 8780 | **635.8240** | **1269.6334** | **1269.6425** | **-0.0091** | **1** | **41** | **0.011** | **1** | **R.RAPDQAAEIGSR.G** |
|  | 9241 | 432.7251 | 1295.1534 | 1294.6816 | 0.4718 | 1 | 6 | 22 | 2 | -.MAASAAVFSRLR.S |
|  | 16362 | **865.4044** | **1728.7942** | **1728.8067** | **-0.0125** | **0** | **27** | **0.2** | **1** | **K.AQGPQQQPGSEGPSYAK.K** |
|  | 18133 | **620.3724** | **1858.0953** | **1856.9017** | **1.1936** | **1** | **5** | **32** | **4** | **K.AQGPQQQPGSEGPSYAKK.V** |
|  | 31513 | **1826.3176** | **5475.9311** | **5476.7767** | **-0.8457** | **0** | **0** | **8.8** | **8** | **R.QMIIEPTSPCLLPDPLQEPYYQPPYTLVLELTGVLLHPEWSLATGWR.F** |

| **733.** | Q6NUQ4\|TM214_HUMAN    **Mass:** 77957    **Score:** 48     **Queries matched:** 12   **emPAI:** 0.05 |
| --- | --- |
|  | Transmembrane protein 214 OS=Homo sapiens GN=TMEM214 PE=1 SV=2 |

|  | **Query** | **Observed** | **Mr(expt)** | **Mr(calc)** | **Delta** | **Miss** | **Score** | **Expect** | **Rank** | **Peptide** |
| --- | --- | --- | --- | --- | --- | --- | --- | --- | --- | --- |
|  | 3981 | **477.2523** | **952.4901** | **953.5155** | **-1.0254** | **0** | **0** | **1.4e+002** | **5** | **R.RPGVGAGAGGR.G** |
|  | 4244 | 487.2479 | 972.4812 | 971.5287 | 0.9525 | 0 | 11 | 16 | 1 | K.ALDVADLQK.E |
|  | 6955 | **590.2640** | **1178.5134** | **1178.6845** | **-0.1711** | **0** | **6** | **37** | **4** | **R.LLLMHPNLTK.G** |
|  | 7183 | **595.4194** | **1188.8243** | **1189.6343** | **-0.8100** | **0** | **1** | **1e+002** | **7** | **K.EQVPPPAVEPK.K** |
|  | 7418 | **401.2283** | **1200.6632** | **1199.6411** | **1.0221** | **1** | **11** | **13** | **4** | **R.RALGEANGVWK.Y** |
|  | 8330 | **623.8210** | **1245.6274** | **1244.5893** | **1.0381** | **1** | **0** | **1.5e+002** | **2** | **R.ATPSCPPEMKK.E** |
|  | 19792 | **1000.0216** | **1998.0285** | **1998.0795** | **-0.0509** | **1** | **1** | **73** | **3** | **R.LLLMHPNLTKGFGMIGPK.D** |
|  | 22639 | **765.4336** | **2293.2791** | **2293.1599** | **0.1192** | **0** | **1** | **52** | **9** | **K.CLTIMWALGQAGFANLTEGLK.V** |
|  | 23752 | **816.1063** | **2445.2971** | **2444.3063** | **0.9908** | **0** | **48** | **0.0011** | **1** | **K.HLSQSSLLLEHLLSSWEQIPK.K** |
|  | 24440 | 848.0972 | 2541.2699 | 2542.3981 | -1.1283 | 1 | 0 | 69 | 9 | K.SLSPFAITYLDRLLLMHPNLTK.G |
|  | 27854 | 796.9449 | 3183.7505 | 3184.6274 | -0.8769 | 1 | 2 | 24 | 5 | R.SHQSRPAKCLTIMWALGQAGFANLTEGLK.V |
|  | 29383 | 746.7948 | 3728.9376 | 3729.0224 | -0.0848 | 1 | 1 | 27 | 6 | K.CLTIMWALGQAGFANLTEGLKVWLGIMLPVLGIK.S |

| **734.** | P51153\|RAB13_HUMAN    **Mass:** 22988    **Score:** 48     **Queries matched:** 11   **emPAI:** 0.17 |
| --- | --- |
|  | Ras-related protein Rab-13 OS=Homo sapiens GN=RAB13 PE=1 SV=1 |

|  | **Query** | **Observed** | **Mr(expt)** | **Mr(calc)** | **Delta** | **Miss** | **Score** | **Expect** | **Rank** | **Peptide** |
| --- | --- | --- | --- | --- | --- | --- | --- | --- | --- | --- |
|  | 1008 | 533.3640 | 532.3567 | 531.2877 | 1.0690 | 1 | 8 | 44 | 1 | K.SGGRR.S |
|  | 3143 | 445.7517 | 889.4888 | 888.4916 | 0.9972 | 1 | 7 | 41 | 1 | R.TVDIEGKK.I 3134 |
|  | 3172 | **447.2613** | **892.5080** | **892.4443** | **0.0637** | **0** | **9** | **22** | **5** | **K.AYDHLFK.L** |
|  | 3960 | **476.2712** | **950.5279** | **950.5410** | **-0.0130** | **1** | **11** | **13** | **1** | **K.LAREHGIR.F** 3966 |
|  | 5578 | 536.3207 | 1070.6269 | 1070.6336 | -0.0066 | 0 | 53 | 0.00096 | 1 | K.LLLIGDSGVGK.T 5577 |
|  | 9596 | 657.8701 | 1313.7257 | 1313.6575 | 0.0682 | 0 | 1 | 1.1e+002 | 9 | R.SGNGNKPPSTDLK.T |
|  | 13890 | **785.8439** | **1569.6733** | **1569.8474** | **-0.1741** | **1** | **3** | **58** | **4** | **K.ENASAGVERLLLGNK.C** |
|  | 19207 | 649.6751 | 1946.0033 | 1945.0673 | 0.9360 | 1 | 4 | 42 | 5 | K.AYDHLFKLLLIGDSGVGK.T |

| **735.** | P03989\|1B27_HUMAN    **Mass:** 40802    **Score:** 48     **Queries matched:** 8   **emPAI:** 0.19 |
| --- | --- |
|  | HLA class I histocompatibility antigen, B-27 alpha chain OS=Homo sapiens GN=HLA-B PE=1 SV=2 |

|  | **Query** | **Observed** | **Mr(expt)** | **Mr(calc)** | **Delta** | **Miss** | **Score** | **Expect** | **Rank** | **Peptide** |
| --- | --- | --- | --- | --- | --- | --- | --- | --- | --- | --- |
|  | 4142 | 483.2169 | 964.4193 | 964.4250 | -0.0057 | 0 | 53 | 0.00075 | 1 | R.FDSDAASPR.E |
|  | 10294 | 676.3427 | 1350.6709 | 1349.6939 | 0.9770 | 1 | 8 | 24 | 1 | R.YLENGKETLQR.A |
|  | 11642 | 714.3621 | 1426.7097 | 1426.7205 | -0.0108 | 0 | 11 | 10 | 1 | K.WAAVVVPSGEEQR.Y |
|  | 22148 | 744.6662 | 2230.9768 | 2230.9938 | -0.0170 | 0 | 17 | 1.6 | 1 | R.DGEDQTQDTELVETRPAGDR.T |
|  | 28271 | 830.4323 | 3317.7001 | 3317.4830 | 0.2172 | 1 | 0 | 43 | 8 | R.TLLRYYNQSEAGSHTLQNMYGCDVGPDGR.L |
|  | 29581 | 948.2367 | 3788.9177 | 3790.1002 | -1.1825 | 1 | 4 | 13 | 1 | R.WEPSSQSTVPIVGIVAGLAVLAVVVIGAVVAAVMCRR.K 29529 29557 |

| **736.** | P00480\|OTC_HUMAN    **Mass:** 40081    **Score:** 48     **Queries matched:** 10   **emPAI:** 0.20 |
| --- | --- |
|  | Ornithine carbamoyltransferase, mitochondrial OS=Homo sapiens GN=OTC PE=1 SV=3 |

|  | **Query** | **Observed** | **Mr(expt)** | **Mr(calc)** | **Delta** | **Miss** | **Score** | **Expect** | **Rank** | **Peptide** |
| --- | --- | --- | --- | --- | --- | --- | --- | --- | --- | --- |
|  | 4467 | 495.2524 | 988.4903 | 989.5029 | -1.0126 | 0 | 22 | 1.2 | 2 | K.QSDLDTLAK.E |
|  | 7691 | **608.2880** | **1214.5615** | **1215.6070** | **-1.0455** | **0** | **8** | **22** | **7** | **K.FGMHLQAATPK.G** |
|  | 14940 | **410.2190** | **1636.8469** | **1637.8811** | **-1.0342** | **1** | **7** | **23** | **5** | **R.VLSSMADAVLARVYK.Q** |
|  | 16066 | 855.3986 | 1708.7827 | 1708.7944 | -0.0117 | 0 | 40 | 0.011 | 1 | R.KPEEVDDEVFYSPR.S |
|  | 16418 | **577.6512** | **1729.9317** | **1728.8869** | **1.0448** | **1** | **4** | **46** | **4** | **R.LQAFQGYQVTMKTAK.V** |
|  | 17425 | **602.6156** | **1804.8250** | **1805.0134** | **-0.1884** | **1** | **(1)** | **81** | **9** | **-.MLFNLRILLNNAAFR.N** |
|  | 17627 | **608.3752** | **1822.1039** | **1821.0083** | **1.0956** | **1** | **2** | **59** | **1** | **-.MLFNLRILLNNAAFR.N** |
|  | 23633 | **1215.1112** | **2428.2079** | **2428.2243** | **-0.0164** | **0** | **18** | **1.1** | **1** | **K.GLTLSWIGDGNNILHSIMMSAAK.F** 23634 |
|  | 28538 | **1132.2269** | **3393.6590** | **3393.6762** | **-0.0172** | **0** | **27** | **0.092** | **1** | **K.LLLTNDPLEAAHGGNVLITDTWISMGQEEEK.K** |

| **737.** | P62913\|RL11_HUMAN    **Mass:** 20468    **Score:** 47     **Queries matched:** 9   **emPAI:** 0.68 |
| --- | --- |
|  | 60S ribosomal protein L11 OS=Homo sapiens GN=RPL11 PE=1 SV=2 |

|  | **Query** | **Observed** | **Mr(expt)** | **Mr(calc)** | **Delta** | **Miss** | **Score** | **Expect** | **Rank** | **Peptide** |
| --- | --- | --- | --- | --- | --- | --- | --- | --- | --- | --- |
|  | 1395 | **635.3934** | **634.3861** | **634.2744** | **0.1117** | **0** | **11** | **20** | **5** | **K.EEAMR.W** |
|  | 2748 | **432.0402** | **862.0658** | **861.4491** | **0.6167** | **1** | **10** | **13** | **5** | **R.RTGCIGAK.H** |
|  | 4281 | **488.2760** | **974.5375** | **974.5437** | **-0.0062** | **0** | **34** | **0.072** | **1** | **K.YDGIILPGK.-** |
|  | 5446 | **531.3231** | **1060.6316** | **1059.5131** | **1.1185** | **1** | **11** | **15** | **5** | **K.ENPMRELR.I** 5442 |
|  | 13551 | **773.9222** | **1545.8298** | **1545.8403** | **-0.0105** | **0** | **45** | **0.0036** | **1** | **K.VLEQLTGQTPVFSK.A** |
|  | 13585 | **517.2680** | **1548.7822** | **1548.6660** | **0.1162** | **1** | **(4)** | **53** | **2** | **-.MAQDQGEKENPMR.E** |
|  | 13832 | **522.3244** | **1563.9514** | **1564.6610** | **-0.7096** | **1** | **5** | **35** | **2** | **-.MAQDQGEKENPMR.E** |
|  | 27015 | **748.3927** | **2989.5417** | **2989.5589** | **-0.0172** | **1** | **23** | **0.28** | **1** | **K.YDPSIGIYGLDFYVVLGRPGFSIADKK.R** |

| **738.** | Q16698\|DECR_HUMAN    **Mass:** 36330    **Score:** 47     **Queries matched:** 13   **emPAI:** 0.34 |
| --- | --- |
|  | 2,4-dienoyl-CoA reductase, mitochondrial OS=Homo sapiens GN=DECR1 PE=1 SV=1 |

|  | **Query** | **Observed** | **Mr(expt)** | **Mr(calc)** | **Delta** | **Miss** | **Score** | **Expect** | **Rank** | **Peptide** |
| --- | --- | --- | --- | --- | --- | --- | --- | --- | --- | --- |
|  | 2812 | **433.7394** | **865.4643** | **865.4698** | **-0.0055** | **0** | **20** | **1.7** | **1** | **K.FFSPLQK.A** |
|  | 3061 | **442.2504** | **882.4862** | **882.4745** | **0.0117** | **0** | **14** | **5.6** | **2** | **R.LPCGLAPR.R** |
|  | 6099 | **556.3797** | **1110.7448** | **1111.6390** | **-0.8941** | **0** | **5** | **39** | **1** | **R.FNVIQPGPIK.T** |
|  | 6953 | **589.8306** | **1177.6467** | **1176.6503** | **0.9964** | **0** | **45** | **0.0054** | **1** | **K.VAFITGGGTGLGK.G** 6934 |
|  | 7512 | **402.4630** | **1204.3671** | **1203.5852** | **0.7819** | **1** | **5** | **37** | **9** | **K.EMIGRIPCGR.L** |
|  | 9995 | **667.7211** | **1333.4277** | **1333.6474** | **-0.2196** | **0** | **23** | **0.52** | **1** | **K.ATAEQISSQTGNK.V** |
|  | 10112 | **671.3596** | **1340.7047** | **1340.7816** | **-0.0769** | **1** | **13** | **7.6** | **7** | **R.FNVIQPGPIKTK.G** |
|  | 20418 | **685.1995** | **2052.5767** | **2052.0503** | **0.5265** | **1** | **2** | **38** | **6** | **K.FFSPLQKAMLPPNSFQGK.V** |
|  | 22626 | **1146.6659** | **2291.3172** | **2290.1818** | **1.1354** | **0** | **1** | **51** | **10** | **K.VAGHPNIVINNAAGNFISPTER.L** |
|  | 23526 | 1206.5742 | 2411.1339 | 2412.1816 | -1.0477 | 1 | 3 | 31 | 2 | K.ATAEQISSQTGNKVHAIQCDVR.D |
|  | 24090 | **829.4391** | **2485.2954** | **2486.4360** | **-1.1405** | **1** | **3** | **33** | **4** | **K.TITDIVLNGTAFVTLEIGKQLIK.A** |
|  | 25123 | **889.7741** | **2666.3005** | **2666.3156** | **-0.0151** | **1** | **28** | **0.1** | **1** | **K.VHAIQCDVRDPDMVQNTVSELIK.V** |

| **739.** | Q16836\|HCDH_HUMAN    **Mass:** 34313    **Score:** 47     **Queries matched:** 15   **emPAI:** 0.37 |
| --- | --- |
|  | Hydroxyacyl-coenzyme A dehydrogenase, mitochondrial OS=Homo sapiens GN=HADH PE=1 SV=2 |

|  | **Query** | **Observed** | **Mr(expt)** | **Mr(calc)** | **Delta** | **Miss** | **Score** | **Expect** | **Rank** | **Peptide** |
| --- | --- | --- | --- | --- | --- | --- | --- | --- | --- | --- |
|  | 785 | **478.3275** | **477.3203** | **476.2231** | **1.0972** | **0** | **8** | **31** | **1** | **R.GDASK.E** |
|  | 1264 | **600.4396** | **599.4324** | **599.4370** | **-0.0046** | **1** | **11** | **13** | **5** | **K.KIIVK.H** |
|  | 4898 | 509.7343 | 1017.4541 | 1017.5244 | -0.0702 | 0 | 19 | 2.6 | 1 | K.DTPGFIVNR.L |
|  | 5889 | 548.7964 | 1095.5782 | 1095.5859 | -0.0077 | 1 | 4 | 57 | 4 | K.ALGKHPVSCK.D |
|  | 6879 | **586.7921** | **1171.5697** | **1171.5761** | **-0.0064** | **0** | **25** | **0.54** | **1** | **K.TFESLVDFSK.A** |
|  | 9356 | **434.7215** | **1301.1427** | **1301.6373** | **-0.4946** | **1** | **(0)** | **85** | **6** | **-.MAFVTRQFMR.S** |
|  | 9389 | **435.2319** | **1302.6740** | **1301.6373** | **1.0367** | **1** | **5** | **47** | **6** | **-.MAFVTRQFMR.S** |
|  | 9653 | **659.3798** | **1316.7450** | **1316.7526** | **-0.0076** | **0** | **9** | **19** | **1** | **R.LLVPYLMEAIR.L** |
|  | 10837 | **691.3555** | **1380.6965** | **1379.6238** | **1.0727** | **1** | **28** | **0.21** | **1** | **R.GDASKEDIDTAMK.L** 10836 |
|  | 14400 | **535.2835** | **1602.8287** | **1602.8381** | **-0.0094** | **0** | **22** | **0.86** | **1** | **R.FAGLHFFNPVPVMK.L** |
|  | 23116 | **786.3927** | **2356.1563** | **2356.1661** | **-0.0098** | **0** | **13** | **3.4** | **1** | **K.LGAGYPMGPFELLDYVGLDTTK.F** 23115 |
|  | 27477 | **1028.8772** | **3083.6098** | **3083.6238** | **-0.0140** | **0** | **31** | **0.039** | **1** | **K.TLSTIATSTDAASVVHSTDLVVEAIVENLK.V** |
|  | 29142 | **903.9866** | **3611.9174** | **3611.9346** | **-0.0171** | **0** | **2** | **24** | **2** | **K.HVTVIGGGLMGAGIAQVAAATGHTVVLVDQTEDILAK.S** |

| **740.** | P13987\|CD59_HUMAN    **Mass:** 14795    **Score:** 47     **Queries matched:** 2   **emPAI:** 0.60 |
| --- | --- |
|  | CD59 glycoprotein OS=Homo sapiens GN=CD59 PE=1 SV=1 |

|  | **Query** | **Observed** | **Mr(expt)** | **Mr(calc)** | **Delta** | **Miss** | **Score** | **Expect** | **Rank** | **Peptide** |
| --- | --- | --- | --- | --- | --- | --- | --- | --- | --- | --- |
|  | 13426 | **770.3301** | **1538.6457** | **1538.6572** | **-0.0115** | **0** | **30** | **0.12** | **1** | **K.FEHCNFNDVTTR.L** |
|  | 15095 | **824.8707** | **1647.7268** | **1647.7385** | **-0.0117** | **1** | **35** | **0.038** | **1** | **R.LRENELTYYCCK.K** |

| **741.** | P84095\|RHOG_HUMAN    **Mass:** 21751    **Score:** 47     **Queries matched:** 4   **emPAI:** 0.18 |
| --- | --- |
|  | Rho-related GTP-binding protein RhoG OS=Homo sapiens GN=RHOG PE=1 SV=1 |

|  | **Query** | **Observed** | **Mr(expt)** | **Mr(calc)** | **Delta** | **Miss** | **Score** | **Expect** | **Rank** | **Peptide** |
| --- | --- | --- | --- | --- | --- | --- | --- | --- | --- | --- |
|  | 4013 | **478.2662** | **954.5178** | **955.5199** | **-1.0021** | **1** | **9** | **21** | **2** | **R.AQPDTLRR.L** |
|  | 12967 | **755.3450** | **1508.6755** | **1509.7133** | **-1.0378** | **0** | **47** | **0.0026** | **1** | **R.YLECSALQQDGVK.E** |
|  | 19888 | **669.3682** | **2005.0829** | **2005.0956** | **-0.0127** | **1** | **20** | **0.82** | **1** | **R.LKEQGQAPITPQQGQALAK.Q** |
|  | 21216 | **711.0782** | **2130.2127** | **2130.9858** | **-0.7731** | **0** | **1** | **57** | **2** | **K.EYIPTVFDNYSAQSAVDGR.T** |

| **742.** | Q9H6S3\|ES8L2_HUMAN    **Mass:** 81197    **Score:** 47     **Queries matched:** 20   **emPAI:** 0.05 |
| --- | --- |
|  | Epidermal growth factor receptor kinase substrate 8-like protein 2 OS=Homo sapiens GN=EPS8L2 PE=1 SV=2 |

|  | **Query** | **Observed** | **Mr(expt)** | **Mr(calc)** | **Delta** | **Miss** | **Score** | **Expect** | **Rank** | **Peptide** |
| --- | --- | --- | --- | --- | --- | --- | --- | --- | --- | --- |
|  | 1277 | **602.9723** | **601.9650** | **601.3547** | **0.6103** | **0** | **1** | **1.7e+002** | **6** | **K.ISNIR.A** |
|  | 2807 | **433.7195** | **865.4244** | **866.4722** | **-1.0478** | **1** | **10** | **16** | **6** | **K.GHQEKIR.Q** |
|  | 4901 | 509.7680 | 1017.5215 | 1017.5277 | -0.0062 | 0 | 8 | 32 | 10 | R.SVSCPLLSR.D |
|  | 4908 | **510.2452** | **1018.4758** | **1018.5117** | **-0.0359** | **1** | **3** | **88** | **10** | **R.SDGVAKMSPK.D** |
|  | 5074 | **516.3889** | **1030.7631** | **1030.6022** | **0.1609** | **1** | **2** | **83** | **10** | **K.LVQLSSKEK.I** |
|  | 5084 | **516.9224** | **1031.8302** | **1032.5022** | **-0.6721** | **1** | **6** | **30** | **5** | **K.KVCGEEGVR.V** |
|  | 5132 | **518.3225** | **1034.6303** | **1034.5066** | **0.1237** | **1** | **(3)** | **87** | **2** | **R.SDGVAKMSPK.D** |
|  | 5878 | **548.3153** | **1094.6161** | **1095.7379** | **-1.1218** | **1** | **5** | **50** | **8** | **K.IKLAINLLAK.L** |
|  | 8847 | **425.5498** | **1273.6276** | **1274.6771** | **-1.0496** | **1** | **3** | **75** | **6** | **R.AWLEAKAFSPR.I** |
|  | 10846 | **461.2514** | **1380.7325** | **1381.7354** | **-1.0030** | **0** | **7** | **30** | **4** | **R.VGPQVPLSEPGFR.R** 10847 |
|  | 12033 | **726.7959** | **1451.5772** | **1452.7435** | **-1.1663** | **1** | **4** | **42** | **4** | **R.GYQPTPAMAKYVK.I** |
|  | 12736 | **747.4018** | **1492.7890** | **1491.6875** | **1.1015** | **0** | **5** | **40** | **6** | **K.QQSGSELEELMNK.F** |
|  | 19636 | 497.2541 | 1984.9873 | 1985.8822 | -0.8950 | 0 | 5 | 28 | 6 | K.DELMQHMDEVNDELIR.K |
|  | 20096 | 675.0041 | 2021.9906 | 2021.0040 | 0.9866 | 1 | 6 | 24 | 9 | R.ARPPSEGEFIDCFQKIK.L |
|  | 20137 | 676.0272 | 2025.0597 | 2024.9084 | 0.1513 | 0 | 4 | 33 | 4 | K.EMSLWESLGESWMRPR.S |
|  | 24685 | 857.7756 | 2570.3049 | 2569.1319 | 1.1729 | 1 | 0 | 62 | 4 | -.MSQSGAVSCCPGATNGSLGRSDGVAK.M |
|  | 25466 | **907.5043** | **2719.4910** | **2720.4034** | **-0.9125** | **1** | **0** | **52** | **4** | **R.QSILPPPQGPAPIPFQHRGGDSPEAK.N** |
|  | 26836 | **592.3143** | **2956.5350** | **2957.4380** | **-0.9030** | **1** | **3** | **26** | **4** | **R.NSQKHSPTSEPTPPGDALPPVSSPHTHR.G** |
|  | 29783 | 1281.9626 | 3842.8661 | 3842.8792 | -0.0130 | 0 | 47 | 0.00073 | 1 | K.FHSGWEPPVDVLQEAPWEVEGLASAPIEEVSPVSR.Q |

| **743.** | Q14624\|ITIH4_HUMAN    **Mass:** 103521   **Score:** 47     **Queries matched:** 12   **emPAI:** 0.04 |
| --- | --- |
|  | Inter-alpha-trypsin inhibitor heavy chain H4 OS=Homo sapiens GN=ITIH4 PE=1 SV=4 |

|  | **Query** | **Observed** | **Mr(expt)** | **Mr(calc)** | **Delta** | **Miss** | **Score** | **Expect** | **Rank** | **Peptide** |
| --- | --- | --- | --- | --- | --- | --- | --- | --- | --- | --- |
|  | 3724 | **467.8350** | **933.6555** | **932.5331** | **1.1224** | **0** | **2** | **1.1e+002** | **7** | **K.NVVFVIDK.S** |
|  | 4564 | **498.3364** | **994.6583** | **995.5698** | **-0.9115** | **0** | **10** | **14** | **2** | **-.MKPPRPVR.T** |
|  | 5005 | **514.2945** | **1026.5744** | **1025.6345** | **0.9399** | **1** | **8** | **26** | **3** | **R.GEGLRLLLR.D** |
|  | 8893 | 638.8270 | 1275.6394 | 1275.6976 | -0.0582 | 0 | 12 | 9.4 | 2 | K.VTIGLLFWDGR.G |
|  | 8966 | **427.5756** | **1279.7050** | **1278.6503** | **1.0547** | **0** | **4** | **54** | **6** | **R.MNFRPGVLSSR.Q** |
|  | 11062 | 465.2520 | 1392.7342 | 1392.6899 | 0.0444 | 1 | 9 | 18 | 4 | R.NRNVHSGSTFFK.Y 11040 |
|  | 18359 | **625.7788** | **1874.3146** | **1874.8828** | **-0.5682** | **1** | **3** | **33** | **8** | **K.ETLFSVMPGLKMTMDK.T** |
|  | 21922 | **737.4144** | **2209.2213** | **2209.1869** | **0.0344** | **1** | **1** | **57** | **10** | **K.NPLVWVHASPEHVVVTRNR.R** |
|  | 22279 | 562.5466 | 2246.1572 | 2247.1319 | -0.9748 | 0 | 3 | 44 | 4 | K.AFITNFSMIIDGMTYPGIIK.E |
|  | 28699 | 1152.8350 | 3455.4831 | 3454.5815 | 0.9016 | 1 | 0 | 35 | 5 | R.DTDRFSSHVGGTLGQFYQEVLWGSPAASDDGR.R |
|  | 31399 | **1300.3700** | **5197.4509** | **5197.4905** | **-0.0396** | **1** | **47** | **0.00026** | **1** | **R.RIHEDSDSALQLQDFYQEVANPLLTAVTFEYPSNAVEEVTQNNFR.L** |

| **744.** | Q9H1K4\|GHC2_HUMAN    **Mass:** 34340    **Score:** 47     **Queries matched:** 14   **emPAI:** 0.11 |
| --- | --- |
|  | Mitochondrial glutamate carrier 2 OS=Homo sapiens GN=SLC25A18 PE=2 SV=1 |

|  | **Query** | **Observed** | **Mr(expt)** | **Mr(calc)** | **Delta** | **Miss** | **Score** | **Expect** | **Rank** | **Peptide** |
| --- | --- | --- | --- | --- | --- | --- | --- | --- | --- | --- |
|  | 4332 | **489.7689** | **977.5232** | **977.5294** | **-0.0063** | **0** | **20** | **1.9** | **1** | **R.TQGLAGLYR.G** |
|  | 8393 | **625.3005** | **1248.5865** | **1247.6114** | **0.9751** | **1** | **11** | **12** | **1** | **R.RLLMEDGMQR.N** |
|  | 8939 | **427.2204** | **1278.6395** | **1279.6013** | **-0.9618** | **1** | **(8)** | **23** | **5** | **R.RLLMEDGMQR.N** |
|  | 9539 | 437.9142 | 1310.7207 | 1311.7398 | -1.0191 | 0 | 9 | 18 | 8 | R.GAAVNLTLVTPEK.A |
|  | 9654 | **659.4034** | **1316.7922** | **1316.6659** | **0.1263** | **1** | **1** | **1.2e+002** | **3** | **R.LQNQHGKAMYK.G** |
|  | 12505 | **493.9228** | **1478.7465** | **1478.7221** | **0.0244** | **1** | **10** | **12** | **10** | **R.LLMEDGMQRNLK.M** |
|  | 13214 | 508.9561 | 1523.8466 | 1524.8776 | -1.0310 | 0 | 2 | 83 | 6 | R.RPSATLIAWELLR.T |
|  | 14426 | **535.9371** | **1604.7894** | **1604.8019** | **-0.0125** | **0** | **47** | **0.0026** | **1** | **R.LAVHHQGSASAPSTSR.S** 14436 |
|  | 15041 | 822.8477 | 1643.6808 | 1643.6920 | -0.0112 | 0 | 23 | 0.53 | 2 | K.GLGEDMYSGITDCAR.K 15042 |
|  | 15290 | 830.4468 | 1658.8790 | 1659.6869 | -0.8079 | 0 | (4) | 49 | 7 | K.GLGEDMYSGITDCAR.K |
|  | 16970 | 886.9021 | 1771.7896 | 1771.7869 | 0.0027 | 1 | 10 | 10 | 3 | K.KGLGEDMYSGITDCAR.K |
|  | 25457 | **907.0897** | **2718.2472** | **2718.1510** | **0.0961** | **0** | **3** | **27** | **1** | **K.MEMLAGCGAGMCQVVVTCPMEMLK.I** |

| **745.** | Q9NX63\|CHCH3_HUMAN    **Mass:** 26421    **Score:** 46     **Queries matched:** 5   **emPAI:** 0.50 |
| --- | --- |
|  | Coiled-coil-helix-coiled-coil-helix domain-containing protein 3, mitochondrial OS=Homo sapiens GN=CHCHD3 PE=1 SV=1 |

|  | **Query** | **Observed** | **Mr(expt)** | **Mr(calc)** | **Delta** | **Miss** | **Score** | **Expect** | **Rank** | **Peptide** |
| --- | --- | --- | --- | --- | --- | --- | --- | --- | --- | --- |
|  | 2113 | 406.2468 | 810.4790 | 809.3702 | 1.1089 | 0 | 5 | 41 | 2 | -.MGGTTSTR.R |
|  | 4207 | **485.9092** | **969.8039** | **969.4879** | **0.3160** | **0** | **5** | **36** | **7** | **R.ENTHQTLK.C** |
|  | 9892 | **664.8699** | **1327.7253** | **1327.7347** | **-0.0094** | **1** | **46** | **0.0036** | **1** | **R.VAEELALEQAKK.E** |
|  | 13959 | **788.3567** | **1574.6988** | **1574.7100** | **-0.0112** | **0** | **21** | **0.9** | **1** | **R.YSGAYGASVSDEELK.R** |
|  | 15471 | **558.6027** | **1672.7863** | **1672.7991** | **-0.0128** | **1** | **26** | **0.31** | **1** | **K.RYESHPVCADLQAK.I** |

| **746.** | P35241\|RADI_HUMAN    **Mass:** 68635    **Score:** 46     **Queries matched:** 14   **emPAI:** 0.11 |
| --- | --- |
|  | Radixin OS=Homo sapiens GN=RDX PE=1 SV=1 |

|  | **Query** | **Observed** | **Mr(expt)** | **Mr(calc)** | **Delta** | **Miss** | **Score** | **Expect** | **Rank** | **Peptide** |
| --- | --- | --- | --- | --- | --- | --- | --- | --- | --- | --- |
|  | 1829 | **396.2169** | **790.4193** | **790.3643** | **0.0550** | **0** | **4** | **86** | **7** | **K.QAADQMK.N** |
|  | 2612 | 427.1963 | 852.3780 | 853.4334 | -1.0554 | 0 | 8 | 22 | 4 | K.GYSTWLK.L |
|  | 4458 | **494.7711** | **987.5277** | **987.5237** | **0.0041** | **0** | **14** | **7.9** | **5** | **K.QIEEQTIK.A** |
|  | 5079 | 516.7816 | 1031.5487 | 1031.5247 | 0.0240 | 1 | 8 | 29 | 3 | K.ELEEQTRK.A |
|  | 8002 | 615.3541 | 1228.6937 | 1228.7027 | -0.0090 | 1 | 20 | 1.6 | 1 | R.LKQIEEQTIK.A 8005 |
|  | 8389 | 625.2908 | 1248.5670 | 1247.5795 | 0.9874 | 0 | 10 | 15 | 3 | R.IQNWHEEHR.G |
|  | 11417 | 707.8890 | 1413.7635 | 1413.7576 | 0.0060 | 0 | 10 | 13 | 4 | K.QLQALSSELAQAR.D |
|  | 12375 | 736.8928 | 1471.7710 | 1471.7817 | -0.0107 | 1 | 47 | 0.0029 | 1 | R.RKPDTIEVQQMK.A 12376 |
|  | 12768 | 499.2949 | 1494.8628 | 1494.7790 | 0.0837 | 1 | 13 | 6.3 | 1 | K.KTQNDVLHAENVK.A |
|  | 13510 | **515.3512** | **1543.0318** | **1541.8525** | **1.1793** | **1** | **4** | **40** | **6** | **K.KQLQALSSELAQAR.D** 13487 |
|  | 27507 | **619.3166** | **3091.5469** | **3090.3912** | **1.1557** | **1** | **3** | **28** | **2** | **R.EDSMMEYLKIAQDLEMYGVNYFEIK.N** |

| **747.** | P15311\|EZRI_HUMAN    **Mass:** 69484    **Score:** 46     **Queries matched:** 17   **emPAI:** 0.11 |
| --- | --- |
|  | Ezrin OS=Homo sapiens GN=EZR PE=1 SV=4 |

|  | **Query** | **Observed** | **Mr(expt)** | **Mr(calc)** | **Delta** | **Miss** | **Score** | **Expect** | **Rank** | **Peptide** |
| --- | --- | --- | --- | --- | --- | --- | --- | --- | --- | --- |
|  | 1760 | **776.8281** | **775.8208** | **775.4374** | **0.3834** | **1** | **11** | **12** | **3** | **R.MAALRAK.E** |
|  | 1785 | 395.1953 | 788.3761 | 787.4188 | 0.9573 | 0 | 8 | 35 | 6 | K.VSAQEVR.K |
|  | 2045 | **404.0298** | **806.0451** | **805.4004** | **0.6447** | **0** | **12** | **8.3** | **2** | **K.EELMLR.L** |
|  | 3489 | 458.7816 | 915.5486 | 916.4502 | -0.9015 | 0 | 16 | 5.5 | 10 | K.EAQDDLVK.T |
|  | 8128 | 618.7987 | 1235.5829 | 1235.5935 | -0.0106 | 1 | 9 | 19 | 4 | K.FGDYNKEVHK.S |
|  | 10368 | **452.5304** | **1354.5694** | **1355.5742** | **-1.0048** | **0** | **5** | **44** | **5** | **K.EDEVEEWQHR.A** |
|  | 10510 | 682.8431 | 1363.6717 | 1363.7394 | -0.0677 | 1 | 6 | 37 | 8 | R.LIPQRVMDQHK.L |
|  | 11044 | 464.9230 | 1391.7473 | 1390.6841 | 1.0632 | 1 | 9 | 18 | 1 | K.EVHKSGYLSSER.L |
|  | 11457 | 472.9987 | 1415.9744 | 1415.6640 | 0.3104 | 1 | 4 | 41 | 6 | R.AQEEAERLEADR.M |
|  | 12375 | 736.8928 | 1471.7710 | 1471.7817 | -0.0107 | 1 | 47 | 0.0029 | 1 | R.RKPDTIEVQQMK.A 12376 |
|  | 13703 | 519.2805 | 1554.8196 | 1554.7063 | 0.1133 | 1 | 5 | 35 | 5 | K.EDEVEEWQHRAK.E |
|  | 13735 | **520.2724** | **1557.7954** | **1556.7578** | **1.0376** | **1** | **0** | **1.1e+002** | **10** | **R.GMLKDNAMLEYLK.I** |
|  | 15125 | **550.6242** | **1648.8508** | **1648.7852** | **0.0656** | **1** | **1** | **84** | **6** | **K.RTHNDIIHNENMR.Q** |
|  | 17290 | **598.7735** | **1793.2987** | **1792.8422** | **0.4564** | **0** | **(1)** | **59** | **5** | **R.ILQLCMGNHELYMR.R** |
|  | 17467 | 603.9802 | 1808.9187 | 1808.8372 | 0.0815 | 0 | 1 | 83 | 4 | R.ILQLCMGNHELYMR.R |
|  | 31613 | **927.4786** | **5558.8282** | **5557.6875** | **1.1407** | **1** | **2** | **6.8** | **1** | **K.TKEELHLVMTAPPPPPPPVYEPVSYHVQESLQDEGAEPTGYSAELSSEGIR.D** |

| **748.** | P07737\|PROF1_HUMAN    **Mass:** 15216    **Score:** 46     **Queries matched:** 11   **emPAI:** 0.58 |
| --- | --- |
|  | Profilin-1 OS=Homo sapiens GN=PFN1 PE=1 SV=2 |

|  | **Query** | **Observed** | **Mr(expt)** | **Mr(calc)** | **Delta** | **Miss** | **Score** | **Expect** | **Rank** | **Peptide** |
| --- | --- | --- | --- | --- | --- | --- | --- | --- | --- | --- |
|  | 2950 | **438.2739** | **874.5333** | **873.5358** | **0.9976** | **0** | **26** | **0.5** | **1** | **K.TLVLLMGK.E** |
|  | 6798 | 583.7540 | 1165.4935 | 1165.5008 | -0.0073 | 0 | 43 | 0.0079 | 1 | K.CYEMASHLR.R |
|  | 6991 | **591.8205** | **1181.6264** | **1181.4957** | **0.1307** | **0** | **(9)** | **20** | **10** | **K.CYEMASHLR.R** 6985 |
|  | 9223 | **432.2534** | **1293.7383** | **1293.5958** | **0.1425** | **1** | **(5)** | **42** | **5** | **K.KCYEMASHLR.R** |
|  | 9525 | **437.7217** | **1310.1431** | **1309.5907** | **0.5524** | **1** | **8** | **14** | **2** | **K.KCYEMASHLR.R** |
|  | 12360 | **736.3701** | **1470.7256** | **1469.7515** | **0.9741** | **0** | **27** | **0.28** | **1** | **R.SSFYVNGLTLGGQK.C** 12338 |
|  | 16562 | **581.3129** | **1740.9170** | **1740.8795** | **0.0375** | **1** | **1** | **82** | **6** | **K.DRSSFYVNGLTLGGQK.C** |
|  | 18827 | **638.8313** | **1913.4721** | **1914.0575** | **-0.5854** | **1** | **6** | **16** | **2** | **K.TFVNITPAEVGVLVGKDR.S** 18823 |

| **749.** | P0C7P4\|UCRIL_HUMAN    **Mass:** 31081    **Score:** 46     **Queries matched:** 15   **emPAI:** 0.41 |
| --- | --- |
|  | Cytochrome b-c1 complex subunit Rieske-like protein 1 OS=Homo sapiens GN=UQCRFSL1 PE=2 SV=1 |

|  | **Query** | **Observed** | **Mr(expt)** | **Mr(calc)** | **Delta** | **Miss** | **Score** | **Expect** | **Rank** | **Peptide** |
| --- | --- | --- | --- | --- | --- | --- | --- | --- | --- | --- |
|  | 1296 | 609.3201 | 608.3129 | 609.2944 | -0.9816 | 0 | 14 | 6 | 1 | K.NMAFK.W |
|  | 3304 | 451.7828 | 901.5511 | 900.5141 | 1.0370 | 1 | 13 | 11 | 9 | K.EIRSVAAR.S |
|  | 3881 | 946.4887 | 945.4814 | 945.4879 | -0.0066 | 0 | 32 | 0.13 | 1 | R.ESLSGQAVR.R 3855 3880 3891 3898 |
|  | 4814 | 506.7482 | 1011.4819 | 1011.4662 | 0.0158 | 0 | 8 | 22 | 3 | K.VPDFSEYR.R |
|  | 5428 | 530.7996 | 1059.5847 | 1059.5924 | -0.0077 | 1 | 34 | 0.075 | 1 | R.RLEVLDSTK.S 5429 |
|  | 7885 | 612.3364 | 1222.6582 | 1221.6102 | 1.0480 | 1 | 5 | 50 | 9 | R.DPQHDLDRVK.K |
|  | 14568 | 807.9148 | 1613.8150 | 1612.8784 | 0.9366 | 1 | 39 | 0.015 | 2 | K.EIKQEAAVELSQLR.D |
|  | 21031 | 704.0299 | 2109.0677 | 2109.0776 | -0.0099 | 0 | 22 | 0.6 | 1 | K.NAVTQFVSSMSASADVLALAK.I |
|  | 21172 | 1064.0931 | 2126.1717 | 2125.0725 | 1.0992 | 0 | (7) | 17 | 2 | K.NAVTQFVSSMSASADVLALAK.I 21173 |

| **750.** | P22090\|RS4Y1_HUMAN    **Mass:** 29665    **Score:** 46     **Queries matched:** 16   **emPAI:** 0.13 |
| --- | --- |
|  | 40S ribosomal protein S4, Y isoform 1 OS=Homo sapiens GN=RPS4Y1 PE=2 SV=2 |

|  | **Query** | **Observed** | **Mr(expt)** | **Mr(calc)** | **Delta** | **Miss** | **Score** | **Expect** | **Rank** | **Peptide** |
| --- | --- | --- | --- | --- | --- | --- | --- | --- | --- | --- |
|  | 1593 | 707.3278 | 706.3205 | 706.3254 | -0.0049 | 0 | 6 | 44 | 6 | K.ICMQR.F |
|  | 2322 | 415.2021 | 828.3897 | 828.3952 | -0.0055 | 0 | 23 | 1.1 | 1 | K.HWMLDK.L |
|  | 2418 | 418.7376 | 835.4607 | 834.4204 | 1.0403 | 1 | 18 | 2.9 | 1 | K.KICMQR.F |
|  | 3962 | **476.2816** | **950.5487** | **950.5185** | **0.0302** | **1** | **3** | **75** | **5** | **R.LVYDTKGR.F** |
|  | 4559 | 498.2944 | 994.5742 | 994.4971 | 0.0771 | 0 | 9 | 18 | 7 | K.YALTGDEVK.K 4553 |
|  | 5323 | 526.7377 | 1051.4608 | 1051.4683 | -0.0075 | 0 | 46 | 0.0036 | 1 | K.DANGNSFATR.L 5329 |
|  | 6247 | 562.7515 | 1123.4885 | 1122.5921 | 0.8964 | 1 | 13 | 7 | 4 | K.YALTGDEVKK.I |
|  | 7700 | 405.8887 | 1214.6442 | 1214.6520 | -0.0078 | 0 | 17 | 3.5 | 1 | K.GIPHLVTHDAR.T |
|  | 14107 | 792.8931 | 1583.7716 | 1584.8849 | -1.1133 | 1 | 9 | 14 | 4 | K.GIPHLVTHDARTIR.Y 14109 14114 |
|  | 22317 | 752.4130 | 2254.2172 | 2254.0880 | 0.1293 | 1 | 4 | 34 | 2 | R.HPGSFDVVHVKDANGNSFATR.L |
|  | 24953 | 1313.7118 | 2625.4090 | 2626.2738 | -0.8647 | 1 | 2 | 41 | 4 | R.VDVTYPAGFMDVISIEKTGEHFR.L 24954 |

| **751.** | Q8TD47\|RS4Y2_HUMAN    **Mass:** 29619    **Score:** 46     **Queries matched:** 14   **emPAI:** 0.13 |
| --- | --- |
|  | 40S ribosomal protein S4, Y isoform 2 OS=Homo sapiens GN=RPS4Y2 PE=1 SV=3 |

|  | **Query** | **Observed** | **Mr(expt)** | **Mr(calc)** | **Delta** | **Miss** | **Score** | **Expect** | **Rank** | **Peptide** |
| --- | --- | --- | --- | --- | --- | --- | --- | --- | --- | --- |
|  | 2322 | 415.2021 | 828.3897 | 828.3952 | -0.0055 | 0 | 23 | 1.1 | 1 | K.HWMLDK.L |
|  | 2352 | 416.2294 | 830.4442 | 830.4498 | -0.0055 | 0 | 7 | 40 | 1 | R.LTIAEER.D |
|  | 2542 | 423.7382 | 845.4619 | 844.4694 | 0.9925 | 0 | 11 | 18 | 5 | R.YPDPLIK.V |
|  | 4559 | 498.2944 | 994.5742 | 994.4971 | 0.0771 | 0 | 9 | 18 | 7 | K.YALTGDEVK.K 4553 |
|  | 5323 | 526.7377 | 1051.4608 | 1051.4683 | -0.0075 | 0 | 46 | 0.0036 | 1 | K.DANGNSFATR.I 5329 |
|  | 5601 | 537.2804 | 1072.5462 | 1073.5717 | -1.0254 | 1 | 5 | 69 | 7 | R.LTIAEERDK.R |
|  | 6247 | 562.7515 | 1123.4885 | 1122.5921 | 0.8964 | 1 | 13 | 7 | 4 | K.YALTGDEVKK.I |
|  | 7700 | 405.8887 | 1214.6442 | 1214.6520 | -0.0078 | 0 | 17 | 3.5 | 1 | K.GIPHLVTHDAR.T |
|  | 14107 | 792.8931 | 1583.7716 | 1584.8849 | -1.1133 | 1 | 9 | 14 | 4 | K.GIPHLVTHDARTIR.Y 14109 14114 |
|  | 17301 | 599.3394 | 1794.9964 | 1793.8553 | 1.1411 | 0 | 2 | 65 | 10 | K.FDTGNVCMVIAGANLGR.V |

| **752.** | Q8NB25\|F184A_HUMAN    **Mass:** 133111   **Score:** 46     **Queries matched:** 34   **emPAI:** 0.03 |
| --- | --- |
|  | Protein FAM184A OS=Homo sapiens GN=FAM184A PE=2 SV=3 |

|  | **Query** | **Observed** | **Mr(expt)** | **Mr(calc)** | **Delta** | **Miss** | **Score** | **Expect** | **Rank** | **Peptide** |
| --- | --- | --- | --- | --- | --- | --- | --- | --- | --- | --- |
|  | 1272 | **602.2965** | **601.2892** | **602.3024** | **-1.0131** | **0** | **18** | **4.2** | **8** | **K.EADLR.K** 1280 |
|  | 1309 | **613.3210** | **612.3138** | **611.3755** | **0.9383** | **1** | **1** | **98** | **8** | **R.HKSLK.E** |
|  | 2629 | **427.7369** | **853.4593** | **854.3592** | **-0.9000** | **0** | **(2)** | **1e+002** | **4** | **R.ESMEGFR.I** 2628 |
|  | 2719 | **861.1348** | **860.1276** | **859.4287** | **0.6989** | **0** | **7** | **31** | **4** | **K.LNLEEDK.N** 2720 |
|  | 2910 | 436.7482 | 871.4818 | 870.3542 | 1.1276 | 0 | 7 | 37 | 6 | R.ESMEGFR.I |
|  | 2948 | **438.2555** | **874.4964** | **873.4920** | **1.0045** | **1** | **12** | **15** | **7** | **R.ELDTLKR.S** |
|  | 4163 | 483.7837 | 965.5528 | 965.4706 | 0.0823 | 0 | 15 | 5.2 | 2 | K.LIEDYEGK.L 4161 |
|  | 4281 | 488.2760 | 974.5375 | 973.5080 | 1.0295 | 0 | 8 | 31 | 4 | K.VTEELDLR.R |
|  | 4815 | 506.7713 | 1011.5281 | 1011.5349 | -0.0068 | 0 | 46 | 0.0036 | 1 | K.HSAELQSLK.D 4816 |
|  | 5384 | 529.3049 | 1056.5953 | 1056.5134 | 0.0819 | 1 | 10 | 20 | 3 | R.QSKEHICR.I |
|  | 7526 | **603.3427** | **1204.6707** | **1203.6281** | **1.0426** | **1** | **1** | **1.2e+002** | **10** | **K.MLEELRLER.K** |
|  | 8647 | **631.7922** | **1261.5699** | **1261.7064** | **-0.1364** | **1** | **8** | **25** | **3** | **K.KSAMSQLLQLK.D** 8658 |
|  | 9322 | **434.2129** | **1299.6168** | **1298.6289** | **0.9879** | **0** | **8** | **22** | **7** | **K.ELDVMTADHLR.E** |
|  | 11030 | **464.7313** | **1391.1721** | **1391.7633** | **-0.5912** | **1** | **11** | **7.3** | **2** | **K.RLALEDLQAAHR.R** 11020 |
|  | 13019 | 505.2354 | 1512.6844 | 1511.7402 | 0.9442 | 1 | 1 | 95 | 8 | K.KLQMDLEEQHNK.D |
|  | 14357 | **800.8240** | **1599.6335** | **1600.7892** | **-1.1557** | **0** | **1** | **80** | **1** | **K.HHMAIEAVHSNAIR.D** |
|  | 15621 | **842.8539** | **1683.6933** | **1684.8454** | **-1.1521** | **1** | **0** | **92** | **6** | **R.LEKELDVMTADHLR.E** |
|  | 16956 | 885.9149 | 1769.8153 | 1770.8723 | -1.0570 | 1 | 3 | 62 | 5 | K.MAHDLEIKWTENLR.Q |
|  | 17970 | 617.3573 | 1849.0501 | 1847.8822 | 1.1679 | 0 | 1 | 74 | 6 | K.EINAALQVSLEEMEEK.Y |
|  | 19440 | **656.4290** | **1966.2651** | **1966.0054** | **0.2596** | **1** | **5** | **20** | **1** | **K.LNTANQEIGHLQDMVRK.S** |
|  | 19676 | **994.9358** | **1987.8571** | **1988.9877** | **-1.1305** | **1** | **1** | **63** | **3** | **K.SLKEAHVLAFQTMEEEK.E** |
|  | 19990 | 1008.9483 | 2015.8820 | 2017.0401 | -1.1581 | 1 | (1) | 73 | 5 | K.LKTELQMVQDEAGSLLDK.C |
|  | 20195 | **1016.9312** | **2031.8479** | **2033.0350** | **-1.1872** | **1** | **7** | **17** | **1** | **K.LKTELQMVQDEAGSLLDK.C** |
|  | 22378 | 755.3527 | 2263.0362 | 2264.1801 | -1.1439 | 1 | 3 | 43 | 9 | K.EVQHLHENISALTKELEFK.G |
|  | 23760 | **816.3959** | **2446.1658** | **2445.2910** | **0.8748** | **1** | **3** | **36** | **3** | **K.AMLASLRSELNHQHAAAIDLLR.H** |
|  | 31113 | **1567.9402** | **4700.7987** | **4700.0981** | **0.7006** | **1** | **2** | **8.2** | **1** | **-.MATPGMSWQQHYYGGSAAKFAPSPATAQLAGHSMDYSQEMHLK.M** 31114 |

| **753.** | P02749\|APOH_HUMAN    **Mass:** 39584    **Score:** 46     **Queries matched:** 7   **emPAI:** 0.09 |
| --- | --- |
|  | Beta-2-glycoprotein 1 OS=Homo sapiens GN=APOH PE=1 SV=3 |

|  | **Query** | **Observed** | **Mr(expt)** | **Mr(calc)** | **Delta** | **Miss** | **Score** | **Expect** | **Rank** | **Peptide** |
| --- | --- | --- | --- | --- | --- | --- | --- | --- | --- | --- |
|  | 1061 | **549.1950** | **548.1877** | **547.2900** | **0.8977** | **1** | **7** | **44** | **6** | **R.GGMRK.F** |
|  | 3111 | **887.0453** | **886.0380** | **886.3967** | **-0.3587** | **0** | **5** | **43** | **1** | **K.NGMLHGDK.V** |
|  | 4520 | 496.9103 | 991.8060 | 991.4281 | 0.3779 | 0 | 9 | 16 | 4 | K.TDASDVKPC.- |
|  | 12881 | **752.3790** | **1502.7434** | **1501.7711** | **0.9723** | **0** | **25** | **0.44** | **1** | **R.VCPFAGILENGAVR.Y** |
|  | 16982 | **886.9883** | **1771.9620** | **1771.9695** | **-0.0075** | **0** | **46** | **0.0028** | **1** | **K.FICPLTGLWPINTLK.C** |
|  | 26023 | **940.4866** | **2818.4381** | **2818.4550** | **-0.0170** | **0** | **3** | **33** | **1** | **K.WSPELPVCAPIICPPPSIPTFATLR.V** |
|  | 28876 | **1175.2441** | **3522.7106** | **3522.7350** | **-0.0244** | **1** | **2** | **25** | **1** | **K.CTEEGKWSPELPVCAPIICPPPSIPTFATLR.V** |

| **754.** | Q15437\|SC23B_HUMAN    **Mass:** 87393    **Score:** 46     **Queries matched:** 19   **emPAI:** 0.04 |
| --- | --- |
|  | Protein transport protein Sec23B OS=Homo sapiens GN=SEC23B PE=1 SV=2 |

|  | **Query** | **Observed** | **Mr(expt)** | **Mr(calc)** | **Delta** | **Miss** | **Score** | **Expect** | **Rank** | **Peptide** |
| --- | --- | --- | --- | --- | --- | --- | --- | --- | --- | --- |
|  | 3207 | 448.7478 | 895.4810 | 896.4174 | -0.9364 | 1 | 11 | 12 | 1 | K.DNARFMK.K |
|  | 4019 | 478.7313 | 955.4481 | 956.5080 | -1.0599 | 1 | 9 | 20 | 1 | K.SYVFRGTK.D |
|  | 7457 | **401.5600** | **1201.6581** | **1200.5622** | **1.0959** | **0** | **3** | **74** | **8** | **R.AESEEGPDVLR.W** |
|  | 7709 | **608.3733** | **1214.7321** | **1215.5996** | **-0.8675** | **0** | **3** | **75** | **4** | **R.NWADVQSQLR.H** |
|  | 12341 | 735.8422 | 1469.6698 | 1469.8350 | -0.1652 | 0 | 9 | 15 | 7 | R.MVVPLACLLTPLK.E 12353 |
|  | 14835 | 816.4114 | 1630.8083 | 1629.8396 | 0.9687 | 0 | 46 | 0.0032 | 1 | K.IDMNLTDLLGELQR.D |
|  | 16479 | **579.3199** | **1734.9378** | **1734.7559** | **0.1819** | **1** | **1** | **88** | **6** | **R.KAGYQDMPEYENFK.H** |
|  | 19657 | **663.2908** | **1986.8507** | **1986.9662** | **-0.1155** | **0** | **2** | **61** | **10** | **R.LSDSFSLYPQFMFHLR.R** |
|  | 22454 | 759.1606 | 2274.4599 | 2275.1229 | -0.6630 | 0 | 4 | 24 | 2 | R.IMLFTGGPPTQGPGMVVGDELK.I |
|  | 24225 | **1252.6078** | **2503.2010** | **2504.0481** | **-0.8470** | **0** | **1** | **59** | **5** | **K.CCANLTGGYMVMGDSFNTSLFK.Q** |
|  | 25131 | **890.7371** | **2669.1894** | **2669.4291** | **-0.2398** | **0** | **0** | **60** | **2** | **R.ILLMDTFFQIVIYLGETIAQWR.K** |
|  | 25480 | **908.7764** | **2723.3075** | **2722.4550** | **0.8524** | **1** | **(1)** | **50** | **2** | **R.IMLFTGGPPTQGPGMVVGDELKIPIR.S** |
|  | 25647 | **689.8313** | **2755.2961** | **2754.4449** | **0.8512** | **1** | **5** | **18** | **3** | **R.IMLFTGGPPTQGPGMVVGDELKIPIR.S** |
|  | 25900 | 933.4471 | 2797.3194 | 2797.5241 | -0.2047 | 1 | (4) | 25 | 2 | R.ILLMDTFFQIVIYLGETIAQWRK.A |
|  | 25990 | **704.5852** | **2814.3117** | **2813.5190** | **0.7928** | **1** | **5** | **20** | **1** | **R.ILLMDTFFQIVIYLGETIAQWRK.A** 25980 |
|  | 30468 | 830.8068 | 4148.9974 | 4149.0558 | -0.0583 | 1 | (1) | 22 | 8 | K.DLTAKQIQDMLGLTKPAMPMQQARPAQPQEHPFASSR.F |
|  | 30479 | **1038.4993** | **4149.9680** | **4149.0558** | **0.9122** | **1** | **2** | **18** | **3** | **K.DLTAKQIQDMLGLTKPAMPMQQARPAQPQEHPFASSR.F** |

| **755.** | O15320\|CTGE5_HUMAN    **Mass:** 91197    **Score:** 46     **Queries matched:** 23   **emPAI:** 0.13 |
| --- | --- |
|  | Cutaneous T-cell lymphoma-associated antigen 5 OS=Homo sapiens GN=CTAGE5 PE=1 SV=3 |

|  | **Query** | **Observed** | **Mr(expt)** | **Mr(calc)** | **Delta** | **Miss** | **Score** | **Expect** | **Rank** | **Peptide** |
| --- | --- | --- | --- | --- | --- | --- | --- | --- | --- | --- |
|  | 1453 | **652.4096** | **651.4023** | **651.4068** | **-0.0044** | **0** | **24** | **0.53** | **2** | **K.LIHAAK.L** |
|  | 1582 | 705.2689 | 704.2616 | 703.3501 | 0.9115 | 0 | 5 | 68 | 9 | K.TLEGER.N |
|  | 2494 | 421.2491 | 840.4837 | 841.4657 | -0.9820 | 0 | 1 | 1.1e+002 | 8 | R.LSGPAELR.S |
|  | 2847 | **434.7496** | **867.4846** | **867.4311** | **0.0536** | **1** | **8** | **23** | **4** | **R.KENAHNR.Q** |
|  | 5294 | 525.2016 | 1048.3886 | 1047.5084 | 0.8803 | 0 | 10 | 15 | 4 | R.IQSLEDESK.S |
|  | 8277 | 622.3262 | 1242.6379 | 1242.6608 | -0.0229 | 0 | 8 | 22 | 3 | K.QLLQEAEVWK.E |
|  | 11917 | 723.3612 | 1444.7079 | 1443.8370 | 0.8708 | 1 | 1 | 1.2e+002 | 5 | K.KLALMLSGLIEEK.S |
|  | 11954 | **724.3716** | **1446.7287** | **1447.6943** | **-0.9656** | **0** | **17** | **2.5** | **1** | **K.ISHATEELETYR.K** |
|  | 12430 | **492.2891** | **1473.8454** | **1474.5374** | **-0.6920** | **0** | **8** | **22** | **2** | **K.MDGSMPSEMESSR.N** |
|  | 13028 | 757.3880 | 1512.7614 | 1513.6792 | -0.9178 | 0 | 20 | 1.1 | 2 | K.VMTELYQENEMK.L |
|  | 14991 | **547.6140** | **1639.8202** | **1639.8318** | **-0.0116** | **0** | **40** | **0.011** | **1** | **R.TIHSYQGQIISHEK.K** |
|  | 15264 | 829.9116 | 1657.8087 | 1657.7729 | 0.0357 | 1 | 2 | 66 | 8 | K.HSEQDELMADISKR.I |
|  | 15514 | 559.3157 | 1674.9254 | 1673.7679 | 1.1575 | 1 | (2) | 76 | 10 | K.HSEQDELMADISKR.I |
|  | 15537 | 839.4009 | 1676.7873 | 1677.8937 | -1.1064 | 1 | 7 | 25 | 5 | R.NQIYIQLSEVDKTK.E |
|  | 15840 | **566.3195** | **1695.9366** | **1694.8239** | **1.1126** | **0** | **4** | **47** | **8** | **R.GPPFPPPPPGAMFGASR.D** |
|  | 16617 | **582.9714** | **1745.8923** | **1745.9060** | **-0.0137** | **1** | **30** | **0.11** | **1** | **K.VHAEQVLNDKESHIK.T** |
|  | 18867 | 959.9576 | 1917.9007 | 1916.7550 | 1.1457 | 1 | 1 | 88 | 8 | K.MDGSMPSEMESSRNDTK.D |
|  | 21023 | 703.8881 | 2108.6425 | 2108.9426 | -0.3000 | 1 | 4 | 22 | 2 | K.EYEGYEVESSLKDASFEK.E |
|  | 21155 | 1063.0664 | 2124.1183 | 2124.1102 | 0.0080 | 0 | 2 | 51 | 6 | M.EEPGATPQPYLGLLLEELR.R |
|  | 24040 | 1239.6555 | 2477.2965 | 2478.0171 | -0.7206 | 1 | (3) | 39 | 3 | R.SFNMPSLDKMDGSMPSEMESSR.N |
|  | 24042 | 1239.6558 | 2477.2970 | 2478.0171 | -0.7201 | 1 | (4) | 30 | 3 | R.SFNMPSLDKMDGSMPSEMESSR.N |
|  | 24044 | 826.7730 | 2477.2972 | 2478.0171 | -0.7199 | 1 | 7 | 15 | 4 | R.SFNMPSLDKMDGSMPSEMESSR.N |
|  | 24794 | **863.3983** | **2587.1731** | **2588.1739** | **-1.0007** | **0** | **4** | **28** | **1** | **K.NLQTEQASLQSENTHFENENQK.L** |

| **756.** | P67936\|TPM4_HUMAN    **Mass:** 28619    **Score:** 46     **Queries matched:** 10   **emPAI:** 0.13 |
| --- | --- |
|  | Tropomyosin alpha-4 chain OS=Homo sapiens GN=TPM4 PE=1 SV=3 |

|  | **Query** | **Observed** | **Mr(expt)** | **Mr(calc)** | **Delta** | **Miss** | **Score** | **Expect** | **Rank** | **Peptide** |
| --- | --- | --- | --- | --- | --- | --- | --- | --- | --- | --- |
|  | 3805 | **471.2897** | **940.5648** | **939.4410** | **1.1238** | **0** | **15** | **5.3** | **1** | **K.HIAEEADR.K** 3806 3810 |
|  | 6361 | 566.8173 | 1131.6200 | 1131.5958 | 0.0242 | 0 | 4 | 69 | 3 | -.MAGLNSLEAVK.R |
|  | 6576 | **575.3076** | **1148.6007** | **1147.5907** | **1.0100** | **0** | **(4)** | **66** | **2** | **-.MAGLNSLEAVK.R** |
|  | 8276 | 622.3262 | 1242.6379 | 1242.6456 | -0.0076 | 0 | 18 | 2.2 | 2 | R.IQLVEEELDR.A 8277 |
|  | 8742 | **634.8281** | **1267.6416** | **1267.6156** | **0.0259** | **1** | **3** | **74** | **6** | **K.EAKHIAEEADR.K** |
|  | 10103 | 671.3367 | 1340.6588 | 1340.6459 | 0.0129 | 1 | 12 | 8.9 | 2 | K.SLEAASEKYSEK.E |
|  | 14565 | **807.8842** | **1613.7539** | **1613.7645** | **-0.0106** | **0** | **46** | **0.0033** | **1** | **K.IQALQQQADEAEDR.A** |

| **757.** | P42766\|RL35_HUMAN    **Mass:** 14543    **Score:** 45     **Queries matched:** 7   **emPAI:** 0.61 |
| --- | --- |
|  | 60S ribosomal protein L35 OS=Homo sapiens GN=RPL35 PE=1 SV=2 |

|  | **Query** | **Observed** | **Mr(expt)** | **Mr(calc)** | **Delta** | **Miss** | **Score** | **Expect** | **Rank** | **Peptide** |
| --- | --- | --- | --- | --- | --- | --- | --- | --- | --- | --- |
|  | 1375 | 632.3568 | 631.3495 | 630.3588 | 0.9907 | 0 | 12 | 18 | 3 | K.EELLK.Q 1371 |
|  | 1476 | 659.3795 | 658.3722 | 658.3762 | -0.0039 | 1 | 15 | 7.7 | 6 | K.ENLRK.F |
|  | 6504 | 572.3357 | 1142.6569 | 1142.6659 | -0.0090 | 0 | 39 | 0.021 | 1 | R.VLTVINQTQK.E |
|  | 10148 | **448.7443** | **1343.2111** | **1342.7344** | **0.4768** | **1** | **8** | **13** | **1** | **K.EELLKQLDDLK.V** |
|  | 13719 | **519.6231** | **1555.8475** | **1555.8569** | **-0.0094** | **1** | **33** | **0.058** | **1** | **K.QLDDLKVELSQLR.V** 13718 |

| **758.** | P07355\|ANXA2_HUMAN    **Mass:** 38808    **Score:** 45     **Queries matched:** 11   **emPAI:** 0.10 |
| --- | --- |
|  | Annexin A2 OS=Homo sapiens GN=ANXA2 PE=1 SV=2 |

|  | **Query** | **Observed** | **Mr(expt)** | **Mr(calc)** | **Delta** | **Miss** | **Score** | **Expect** | **Rank** | **Peptide** |
| --- | --- | --- | --- | --- | --- | --- | --- | --- | --- | --- |
|  | 4043 | **479.2576** | **956.5006** | **957.5793** | **-1.0787** | **1** | **(13)** | **8.3** | **1** | **K.LMVALAKGR.R** |
|  | 4281 | 488.2760 | 974.5375 | 973.5742 | 0.9633 | 1 | 20 | 2 | 2 | K.LMVALAKGR.R |
|  | 6002 | **552.3230** | **1102.6314** | **1101.6692** | **0.9622** | **1** | **2** | **1.1e+002** | **1** | **K.VLIRIMVSR.S** |
|  | 6092 | 556.2981 | 1110.5816 | 1110.5458 | 0.0359 | 0 | 10 | 16 | 6 | R.QDIAFAYQR.R |
|  | 7934 | 409.5521 | 1225.6344 | 1224.5623 | 1.0722 | 0 | 5 | 44 | 2 | K.DIISDTSGDFR.K |
|  | 8298 | 622.8114 | 1243.6082 | 1243.6156 | -0.0074 | 0 | 45 | 0.0043 | 1 | R.TNQELQEINR.V |
|  | 13464 | **514.7161** | **1541.1264** | **1541.8413** | **-0.7150** | **0** | **3** | **37** | **4** | **K.GVDEVTIVNILTNR.S** |
|  | 14865 | **545.2860** | **1632.8362** | **1633.8423** | **-1.0061** | **1** | **1** | **94** | **9** | **R.TNQELQEINRVYK.E** |
|  | 17858 | **614.3466** | **1840.0180** | **1839.9665** | **0.0515** | **1** | **7** | **19** | **3** | **K.WISIMTERSVPHLQK.V** |
|  | 17907 | 615.6343 | 1843.8812 | 1843.8952 | -0.0140 | 0 | 7 | 21 | 1 | K.LSLEGDHSTPPSAYGSVK.A |
|  | 23159 | 788.3762 | 2362.1067 | 2362.3835 | -0.2768 | 1 | 1 | 52 | 4 | K.ELASALKSALSGHLETVILGLLK.T |

|  | **Proteins matching a subset of these peptides:** |
| --- | --- |

|  | A6NMY6\|AXA2L_HUMAN    **Mass:** 38806    **Score:** 45     **Queries matched:** 9 |
| --- | --- |
|  | Putative annexin A2-like protein OS=Homo sapiens GN=ANXA2P2 PE=5 SV=2 |

| **759.** | Q15125\|EBP_HUMAN    **Mass:** 26564    **Score:** 45     **Queries matched:** 1   **emPAI:** 0.14 |
| --- | --- |
|  | 3-beta-hydroxysteroid-Delta(8),Delta(7)-isomerase OS=Homo sapiens GN=EBP PE=1 SV=3 |

|  | **Query** | **Observed** | **Mr(expt)** | **Mr(calc)** | **Delta** | **Miss** | **Score** | **Expect** | **Rank** | **Peptide** |
| --- | --- | --- | --- | --- | --- | --- | --- | --- | --- | --- |
|  | 9717 | **441.2308** | **1320.6706** | **1320.6786** | **-0.0080** | **0** | **45** | **0.0041** | **1** | **K.HLTHAQSTLDAK.A** |

| **760.** | Q08830\|FGL1_HUMAN    **Mass:** 36642    **Score:** 45     **Queries matched:** 4   **emPAI:** 0.10 |
| --- | --- |
|  | Fibrinogen-like protein 1 OS=Homo sapiens GN=FGL1 PE=1 SV=3 |

|  | **Query** | **Observed** | **Mr(expt)** | **Mr(calc)** | **Delta** | **Miss** | **Score** | **Expect** | **Rank** | **Peptide** |
| --- | --- | --- | --- | --- | --- | --- | --- | --- | --- | --- |
|  | 11118 | 699.2886 | 1396.5626 | 1395.6167 | 0.9459 | 1 | 2 | 72 | 10 | R.SDGSENFNRGWK.D |
|  | 27266 | **1014.4814** | **3040.4223** | **3040.4423** | **-0.0200** | **0** | **45** | **0.0015** | **1** | **K.IKPLQSPAEFSVYCDMSDGGGWTVIQR.R** |
|  | 27334 | 1019.8740 | 3056.6002 | 3056.4372 | 0.1630 | 0 | (2) | 29 | 5 | K.IKPLQSPAEFSVYCDMSDGGGWTVIQR.R |
|  | 27938 | 1071.5135 | 3211.5188 | 3212.5383 | -1.0195 | 1 | 0 | 42 | 9 | K.IKPLQSPAEFSVYCDMSDGGGWTVIQRR.S |

| **761.** | Q58FF8\|H90B2_HUMAN    **Mass:** 44492    **Score:** 45     **Queries matched:** 8   **emPAI:** 0.08 |
| --- | --- |
|  | Putative heat shock protein HSP 90-beta 2 OS=Homo sapiens GN=HSP90AB2P PE=1 SV=2 |

|  | **Query** | **Observed** | **Mr(expt)** | **Mr(calc)** | **Delta** | **Miss** | **Score** | **Expect** | **Rank** | **Peptide** |
| --- | --- | --- | --- | --- | --- | --- | --- | --- | --- | --- |
|  | 1830 | 396.2400 | 790.4654 | 791.4211 | -0.9557 | 1 | 18 | 3.5 | 4 | R.MKEIQK.S |
|  | 7058 | **593.2739** | **1184.5333** | **1183.5543** | **0.9790** | **0** | **6** | **42** | **3** | **-.MPEEVHLGEK.E** 7065 |
|  | 8264 | 621.8519 | 1241.6893 | 1241.6979 | -0.0086 | 0 | 45 | 0.0042 | 1 | K.ADLINNLGTIAK.F 8293 |
|  | 15426 | 557.6211 | 1669.8416 | 1670.8226 | -0.9809 | 0 | 4 | 43 | 2 | R.VVYMTEPIDGYQLK.E |
|  | 20200 | 678.3444 | 2032.0113 | 2033.0582 | -1.0469 | 1 | 1 | 76 | 6 | K.YIDQEELNKTKPIWTR.N |
|  | 30117 | **1342.8850** | **4025.6332** | **4025.0132** | **0.6200** | **1** | **0** | **19** | **3** | **M.PEEVHLGEKEVETFAFQAEIAQLMSLIINTFYSNK.E** |

| **762.** | Q14568\|HS902_HUMAN    **Mass:** 39454    **Score:** 45     **Queries matched:** 5   **emPAI:** 0.10 |
| --- | --- |
|  | Putative heat shock protein HSP 90-alpha A2 OS=Homo sapiens GN=HSP90AA2 PE=1 SV=2 |

|  | **Query** | **Observed** | **Mr(expt)** | **Mr(calc)** | **Delta** | **Miss** | **Score** | **Expect** | **Rank** | **Peptide** |
| --- | --- | --- | --- | --- | --- | --- | --- | --- | --- | --- |
|  | 7217 | 596.2830 | 1190.5515 | 1189.6819 | 0.8696 | 0 | 3 | 73 | 6 | K.ELHINLIPNK.Q |
|  | 8264 | 621.8519 | 1241.6893 | 1241.6979 | -0.0086 | 0 | 45 | 0.0042 | 1 | K.ADLINNLGTIAK.S 8293 |
|  | 16598 | 873.4059 | 1744.7972 | 1743.9559 | 0.8413 | 0 | 2 | 71 | 8 | K.HSQLIGYPITLFVEK.E |
|  | 20200 | 678.3444 | 2032.0113 | 2033.0582 | -1.0469 | 1 | 1 | 76 | 6 | K.YIDQEELNKTKPIWTR.N |

| **763.** | P18621\|RL17_HUMAN    **Mass:** 21611    **Score:** 45     **Queries matched:** 9   **emPAI:** 0.18 |
| --- | --- |
|  | 60S ribosomal protein L17 OS=Homo sapiens GN=RPL17 PE=1 SV=3 |

|  | **Query** | **Observed** | **Mr(expt)** | **Mr(calc)** | **Delta** | **Miss** | **Score** | **Expect** | **Rank** | **Peptide** |
| --- | --- | --- | --- | --- | --- | --- | --- | --- | --- | --- |
|  | 1308 | **613.3187** | **612.3115** | **612.3166** | **-0.0051** | **0** | **9** | **16** | **4** | **K.GMHIR.K** |
|  | 1780 | **395.0786** | **788.1427** | **788.4253** | **-0.2826** | **1** | **6** | **40** | **10** | **K.SRGSNLR.V** |
|  | 6057 | 554.7784 | 1107.5423 | 1106.6335 | 0.9088 | 1 | 15 | 5.5 | 3 | K.YLKDVTLQK.Q |
|  | 6772 | 582.7186 | 1163.4226 | 1162.5506 | 0.8720 | 0 | 1 | 1.1e+002 | 4 | R.YSLDPENPTK.S |
|  | 8932 | 427.1866 | 1278.5378 | 1279.6091 | -1.0713 | 1 | 4 | 61 | 3 | R.YNGGVGRCAQAK.Q |
|  | 10658 | **457.7391** | **1370.1956** | **1369.7136** | **0.4820** | **1** | **10** | **9.7** | **3** | **R.ETAQAIKGMHIR.K** |
|  | 14708 | 812.4274 | 1622.8403 | 1622.8515 | -0.0112 | 0 | 41 | 0.0082 | 1 | K.EQIVPKPEEEVAQK.K 14709 |
|  | 21500 | **722.0104** | **2163.0093** | **2162.0210** | **0.9883** | **0** | **9** | **9.9** | **1** | **R.INPYMSSPCHIEMILTEK.E** |

| **764.** | P49458\|SRP09_HUMAN    **Mass:** 10219    **Score:** 45     **Queries matched:** 4   **emPAI:** 0.39 |
| --- | --- |
|  | Signal recognition particle 9 kDa protein OS=Homo sapiens GN=SRP9 PE=1 SV=2 |

|  | **Query** | **Observed** | **Mr(expt)** | **Mr(calc)** | **Delta** | **Miss** | **Score** | **Expect** | **Rank** | **Peptide** |
| --- | --- | --- | --- | --- | --- | --- | --- | --- | --- | --- |
|  | 2454 | 420.2175 | 838.4204 | 838.3378 | 0.0825 | 0 | 14 | 5.6 | 1 | R.NVTMETE.- |
|  | 3714 | **467.2596** | **932.5047** | **932.5113** | **-0.0066** | **1** | **5** | **63** | **7** | **R.LMVAKEAR.N** |
|  | 6957 | 590.2995 | 1178.5844 | 1178.5237 | 0.0607 | 1 | 6 | 39 | 7 | K.EARNVTMETE.- |
|  | 12340 | 735.8308 | 1469.6471 | 1469.6575 | -0.0105 | 0 | 45 | 0.0041 | 1 | M.PQYQTWEEFSR.A |

| **765.** | Q53H12\|AGK_HUMAN    **Mass:** 47564    **Score:** 45     **Queries matched:** 8   **emPAI:** 0.08 |
| --- | --- |
|  | Acylglycerol kinase, mitochondrial OS=Homo sapiens GN=AGK PE=1 SV=2 |

|  | **Query** | **Observed** | **Mr(expt)** | **Mr(calc)** | **Delta** | **Miss** | **Score** | **Expect** | **Rank** | **Peptide** |
| --- | --- | --- | --- | --- | --- | --- | --- | --- | --- | --- |
|  | 5710 | 542.1286 | 1082.2426 | 1082.5403 | -0.2977 | 1 | 6 | 26 | 7 | K.HCDNLLRR.A |
|  | 6223 | **561.2996** | **1120.5846** | **1121.5618** | **-0.9772** | **1** | **1** | **1.2e+002** | **10** | **R.WGSFRDAGVK.V** |
|  | 6480 | **571.3072** | **1140.5999** | **1141.6318** | **-1.0318** | **1** | **10** | **14** | **5** | **-.MTVFFKTLR.N** |
|  | 7179 | **595.4186** | **1188.8227** | **1189.5761** | **-0.7534** | **1** | **5** | **38** | **1** | **K.REQMLTSPTQ.-** |
|  | 11284 | **469.9056** | **1406.6950** | **1407.8086** | **-1.1136** | **0** | **1** | **1e+002** | **9** | **K.VQHITDATLAIVK.G** |
|  | 22536 | 761.3882 | 2281.1429 | 2282.1477 | -1.0048 | 0 | 0 | 77 | 7 | R.AACQEAQVFGNQLIPPNAQVK.K |
|  | 24903 | **872.4576** | **2614.3511** | **2614.3643** | **-0.0132** | **0** | **45** | **0.0021** | **1** | **K.IPIGFIPLGETSSLSHTLFAESGNK.V** |
|  | 25835 | **1396.2933** | **2790.5721** | **2790.3382** | **0.2339** | **1** | **2** | **35** | **1** | **R.NNQLDPTSKEDFLNICIEPDTISK.G** |

| **766.** | Q13228\|SBP1_HUMAN    **Mass:** 52928    **Score:** 45     **Queries matched:** 18   **emPAI:** 0.07 |
| --- | --- |
|  | Selenium-binding protein 1 OS=Homo sapiens GN=SELENBP1 PE=1 SV=2 |

|  | **Query** | **Observed** | **Mr(expt)** | **Mr(calc)** | **Delta** | **Miss** | **Score** | **Expect** | **Rank** | **Peptide** |
| --- | --- | --- | --- | --- | --- | --- | --- | --- | --- | --- |
|  | 2311 | **414.5568** | **827.0991** | **827.4575** | **-0.3584** | **0** | **2** | **62** | **10** | **R.LPMPNLK.D** |
|  | 5318 | 526.2855 | 1050.5565 | 1050.5498 | 0.0067 | 0 | 2 | 90 | 10 | K.QFYPDLIR.E |
|  | 6559 | 574.7856 | 1147.5566 | 1148.6553 | -1.0987 | 1 | 2 | 90 | 5 | K.VIEPKDIHAK.C 6579 |
|  | 9403 | **435.2683** | **1302.7832** | **1301.7092** | **1.0740** | **0** | **9** | **20** | **2** | **K.EPLGPALAHELR.Y** 9385 |
|  | 10691 | 458.5601 | 1372.6584 | 1373.6688 | -1.0103 | 0 | 9 | 19 | 7 | R.QYDISDPQRPR.L |
|  | 13045 | 757.8880 | 1513.7614 | 1512.7970 | 0.9644 | 0 | 4 | 55 | 2 | R.VAGGPQMIQLSLDGK.R |
|  | 13134 | **760.3932** | **1518.7718** | **1518.7600** | **0.0118** | **0** | **2** | **76** | **7** | **R.EGSVMLQVDVDTVK.G** 13135 |
|  | 18712 | **953.4504** | **1904.8862** | **1904.9004** | **-0.0142** | **0** | **16** | **2.7** | **1** | **R.NTGTEAPDYLATVDVDPK.S** 18711 18724 |
|  | 23391 | **797.4275** | **2389.2606** | **2389.2740** | **-0.0134** | **1** | **3** | **36** | **2** | **K.GGPVQVLEDEELKSQPEPLVVK.G** |
|  | 24473 | 510.2529 | 2546.2281 | 2546.3645 | -0.1364 | 1 | 3 | 37 | 9 | K.LNPNFLVDFGKEPLGPALAHELR.Y |
|  | 25268 | 897.1061 | 2688.2966 | 2687.4395 | 0.8571 | 1 | 2 | 42 | 8 | R.QYDISDPQRPRLTGQLFLGGSIVK.G |
|  | 25854 | 932.4279 | 2794.2619 | 2794.2776 | -0.0157 | 0 | 45 | 0.002 | 1 | R.DGFNPADVEAGLYGSHLYVWDWQR.H |
|  | 26349 | 959.7856 | 2876.3351 | 2876.3507 | -0.0156 | 0 | 2 | 31 | 8 | K.CELAFLHTSHCLASGEVMISSLGDVK.G |

| **767.** | Q86WU2\|LDHD_HUMAN    **Mass:** 55691    **Score:** 45     **Queries matched:** 6   **emPAI:** 0.07 |
| --- | --- |
|  | Probable D-lactate dehydrogenase, mitochondrial OS=Homo sapiens GN=LDHD PE=1 SV=1 |

|  | **Query** | **Observed** | **Mr(expt)** | **Mr(calc)** | **Delta** | **Miss** | **Score** | **Expect** | **Rank** | **Peptide** |
| --- | --- | --- | --- | --- | --- | --- | --- | --- | --- | --- |
|  | 4359 | 980.4886 | 979.4813 | 979.5675 | -0.0862 | 1 | 7 | 33 | 5 | R.LLHTAGRGR.H |
|  | 11328 | **705.3915** | **1408.7685** | **1408.7787** | **-0.0102** | **0** | **45** | **0.0042** | **1** | **K.AVVGGSHVSTAAVVR.E** |
|  | 16191 | **572.9514** | **1715.8324** | **1714.8362** | **0.9963** | **0** | **8** | **17** | **3** | **R.HNAWYAALATRPGCK.G** |
|  | 16998 | **887.5317** | **1773.0488** | **1773.8931** | **-0.8443** | **0** | **2** | **58** | **4** | **R.QLLQEEVGAVGVETMR.Q** |
|  | 27266 | 1014.4814 | 3040.4223 | 3040.5440 | -0.1217 | 0 | 2 | 35 | 2 | K.LNCLVAPTLFLEFHGSQQALEEQLQR.T |
|  | 29468 | 942.2444 | 3764.9484 | 3764.9672 | -0.0188 | 0 | 9 | 4.6 | 1 | R.LHPAPEATVAATCAFPSVQAAVDSTVHILQAAVPVAR.I |

| **768.** | P10253\|LYAG_HUMAN    **Mass:** 106107   **Score:** 45     **Queries matched:** 15   **emPAI:** 0.03 |
| --- | --- |
|  | Lysosomal alpha-glucosidase OS=Homo sapiens GN=GAA PE=1 SV=3 |

|  | **Query** | **Observed** | **Mr(expt)** | **Mr(calc)** | **Delta** | **Miss** | **Score** | **Expect** | **Rank** | **Peptide** |
| --- | --- | --- | --- | --- | --- | --- | --- | --- | --- | --- |
|  | 819 | **488.9783** | **487.9710** | **488.2343** | **-0.2633** | **0** | **1** | **2.3e+002** | **7** | **K.GGEAR.G** 820 |
|  | 1234 | 589.0247 | 588.0174 | 587.3027 | 0.7147 | 0 | 6 | 57 | 1 | R.QLDGR.V |
|  | 2363 | **416.2600** | **830.5054** | **831.3988** | **-0.8933** | **0** | **8** | **35** | **3** | **R.STFAGHGR.Y** |
|  | 2615 | **427.2234** | **852.4322** | **851.3484** | **1.0838** | **0** | **5** | **44** | **2** | **R.FDCAPDK.A** |
|  | 4293 | 488.3043 | 974.5941 | 975.4808 | -0.8867 | 0 | 7 | 35 | 6 | R.QVVENMTR.A 4306 |
|  | 7515 | **603.2780** | **1204.5415** | **1204.5506** | **-0.0091** | **0** | **45** | **0.0055** | **1** | **K.AITQEQCEAR.G** |
|  | 8750 | **423.7259** | **1268.1559** | **1268.5377** | **-0.3817** | **0** | **11** | **7.6** | **1** | **R.LDVMMETENR.L** |
|  | 13333 | 511.5789 | 1531.7149 | 1531.7282 | -0.0133 | 0 | 11 | 11 | 2 | R.WTQLGAFYPFMR.N |
|  | 16227 | **430.7348** | **1718.9100** | **1718.8852** | **0.0247** | **1** | **5** | **35** | **6** | **R.RYEVPLETPHVHSR.A** |
|  | 18312 | **624.6382** | **1870.8927** | **1871.8935** | **-1.0008** | **0** | **2** | **57** | **1** | **K.LENLSSSEMGYTATLTR.T** |
|  | 18326 | 936.9974 | 1871.9802 | 1872.9945 | -1.0143 | 0 | 4 | 42 | 6 | R.AGYIIPLQGPGLTTTESR.Q |
|  | 18493 | 944.9567 | 1887.8988 | 1887.8884 | 0.0104 | 0 | (0) | 97 | 7 | K.LENLSSSEMGYTATLTR.T |
|  | 30954 | **1515.3376** | **4542.9911** | **4543.0923** | **-0.1012** | **1** | **1** | **15** | **7** | **K.QGLQGAQMGQPWCFFPPSYPSYKLENLSSSEMGYTATLTR.T** |

| **769.** | Q6ZUJ8\|BCAP_HUMAN    **Mass:** 91139    **Score:** 44     **Queries matched:** 18   **emPAI:** 0.04 |
| --- | --- |
|  | Phosphoinositide 3-kinase adapter protein 1 OS=Homo sapiens GN=PIK3AP1 PE=1 SV=2 |

|  | **Query** | **Observed** | **Mr(expt)** | **Mr(calc)** | **Delta** | **Miss** | **Score** | **Expect** | **Rank** | **Peptide** |
| --- | --- | --- | --- | --- | --- | --- | --- | --- | --- | --- |
|  | 1902 | **399.2333** | **796.4521** | **795.4967** | **0.9554** | **1** | **5** | **40** | **6** | **R.KSVIPPR.T** |
|  | 2714 | 430.8871 | 859.7596 | 860.4406 | -0.6809 | 0 | 2 | 83 | 7 | R.AFHPPHR.V |
|  | 3355 | **454.2097** | **906.4048** | **905.4607** | **0.9442** | **0** | **13** | **9.5** | **6** | **R.FQQENLK.R** |
|  | 10630 | **457.2427** | **1368.7061** | **1369.7143** | **-1.0082** | **0** | **5** | **43** | **9** | **R.ETFHPPPPVPPR.G** |
|  | 11716 | **478.0165** | **1431.0275** | **1431.8059** | **-0.7783** | **1** | **4** | **37** | **3** | **R.VPPRAASQRPPTR.E** |
|  | 13662 | **777.3544** | **1552.6942** | **1552.8613** | **-0.1671** | **0** | **3** | **59** | **3** | **K.LGIVNVDEAVLHFK.E** |
|  | 14562 | 538.6476 | 1612.9209 | 1613.7178 | -0.7968 | 0 | (3) | 58 | 7 | K.FLEGNSMGMTNLER.D |
|  | 14573 | **538.9999** | **1613.9778** | **1613.7178** | **0.2601** | **0** | **(4)** | **41** | **7** | **K.FLEGNSMGMTNLER.D** |
|  | 14823 | 544.3179 | 1629.9318 | 1629.7127 | 0.2191 | 0 | 7 | 26 | 7 | K.FLEGNSMGMTNLER.D 14805 |
|  | 16321 | **576.3021** | **1725.8845** | **1726.7985** | **-0.9139** | **0** | **17** | **2.2** | **1** | **R.DRPQSSIYDPFAGMK.T** |
|  | 18071 | **619.5253** | **1855.5540** | **1854.8860** | **0.6680** | **0** | **6** | **16** | **1** | **K.SQERPGNFYVSSESIR.K** 18068 |
|  | 20238 | **680.3564** | **2038.0473** | **2039.0211** | **-0.9738** | **0** | **3** | **45** | **4** | **R.LGPEASFSAEDLSLFLSTR.C** |
|  | 22294 | **751.0151** | **2250.0236** | **2250.0851** | **-0.0616** | **1** | **0** | **69** | **4** | **R.DRPQSSIYDPFAGMKTPGQR.Q** |
|  | 22763 | **771.3329** | **2310.9768** | **2310.9645** | **0.0123** | **1** | **6** | **19** | **1** | **K.KAISEDSGCDSVTDTEPEDEK.V** |
|  | 23901 | **822.3923** | **2464.1550** | **2463.1071** | **1.0479** | **0** | **4** | **25** | **1** | **R.SLLSVSSGMEGDNEDNEVPEVTR.S** |
|  | 29868 | **1297.2284** | **3888.6633** | **3888.6778** | **-0.0144** | **0** | **44** | **0.00096** | **1** | **R.DSEEFLDFFPDWAHWQELTCDDEPETYVAAVK.K** |

| **770.** | P26373\|RL13_HUMAN    **Mass:** 24304    **Score:** 44     **Queries matched:** 15   **emPAI:** 0.34 |
| --- | --- |
|  | 60S ribosomal protein L13 OS=Homo sapiens GN=RPL13 PE=1 SV=4 |

|  | **Query** | **Observed** | **Mr(expt)** | **Mr(calc)** | **Delta** | **Miss** | **Score** | **Expect** | **Rank** | **Peptide** |
| --- | --- | --- | --- | --- | --- | --- | --- | --- | --- | --- |
|  | 1354 | 628.3714 | 627.3641 | 626.3751 | 0.9890 | 0 | 7 | 31 | 5 | R.KPSAPK.K |
|  | 2550 | **424.0564** | **846.0982** | **846.4334** | **-0.3353** | **0** | **5** | **43** | **6** | **R.VITEEEK.N** 2545 |
|  | 4048 | **479.2867** | **956.5589** | **956.5291** | **0.0298** | **0** | **13** | **9.2** | **2** | **R.TIGISVDPR.R** 4038 4042 |
|  | 8050 | 616.8106 | 1231.6067 | 1231.6157 | -0.0090 | 0 | 40 | 0.018 | 1 | K.STESLQANVQR.L 8049 |
|  | 9001 | **642.0647** | **1282.1148** | **1282.7357** | **-0.6209** | **1** | **5** | **29** | **1** | **K.VARTIGISVDPR.R** |
|  | 10183 | **673.3542** | **1344.6939** | **1344.7051** | **-0.0111** | **1** | **4** | **62** | **1** | **R.RVATWFNQPAR.K** |
|  | 10867 | **691.8901** | **1381.7657** | **1381.7752** | **-0.0094** | **0** | **35** | **0.049** | **1** | **K.LATQLTGPVMPVR.N** |
|  | 14358 | **534.2495** | **1599.7265** | **1598.9733** | **0.7533** | **0** | **3** | **59** | **3** | **R.IAPRPASGPIRPIVR.C** |
|  | 16462 | 868.0608 | 1734.1071 | 1733.9148 | 0.1924 | 1 | 1 | 69 | 6 | M.APSRNGMVLKPHFHK.D |
|  | 18487 | 944.4837 | 1886.9528 | 1886.0448 | 0.9081 | 1 | 7 | 21 | 3 | K.LATQLTGPVMPVRNVYK.K 18484 |

| **771.** | P01019\|ANGT_HUMAN    **Mass:** 53406    **Score:** 44     **Queries matched:** 10   **emPAI:** 0.14 |
| --- | --- |
|  | Angiotensinogen OS=Homo sapiens GN=AGT PE=1 SV=1 |

|  | **Query** | **Observed** | **Mr(expt)** | **Mr(calc)** | **Delta** | **Miss** | **Score** | **Expect** | **Rank** | **Peptide** |
| --- | --- | --- | --- | --- | --- | --- | --- | --- | --- | --- |
|  | 1264 | 600.4396 | 599.4324 | 599.3755 | 0.0569 | 1 | 13 | 8.2 | 1 | K.KLSPR.T |
|  | 4485 | 495.7585 | 989.5025 | 988.5189 | 0.9836 | 1 | 15 | 5.6 | 3 | K.LDTEDKLR.A |
|  | 5515 | **534.3454** | **1066.6762** | **1066.5270** | **0.1493** | **0** | **8** | **24** | **2** | **R.FMQAVTGWK.T** 5518 |
|  | 10588 | 684.3542 | 1366.6938 | 1366.7973 | -0.1034 | 1 | 5 | 42 | 8 | R.LQAILGVPWKDK.N |
|  | 11791 | **719.3534** | **1436.6923** | **1436.7035** | **-0.0111** | **0** | **21** | **1.1** | **1** | **R.SLDFTELDVAAEK.I** |
|  | 12797 | **500.2230** | **1497.6471** | **1496.7632** | **0.8839** | **0** | **5** | **37** | **3** | **R.AAMVGMLANFLGFR.I** |
|  | 16265 | 575.0091 | 1722.0054 | 1722.0152 | -0.0098 | 0 | 6 | 23 | 4 | K.VLSALQAVQGLLVAQGR.A |
|  | 23914 | **822.7944** | **2465.3615** | **2464.3690** | **0.9925** | **0** | **44** | **0.0027** | **1** | **R.ADSQAQLLLSTVVGVFTAPGLHLK.Q** |
|  | 27840 | **796.6532** | **3182.5837** | **3181.6376** | **0.9461** | **1** | **2** | **27** | **2** | **R.APQSEMAPAGVSLRATILCLLAWAGLAAGDR.V** |

| **772.** | P05783\|K1C18_HUMAN    **Mass:** 48029    **Score:** 44     **Queries matched:** 8   **emPAI:** 0.08 |
| --- | --- |
|  | Keratin, type I cytoskeletal 18 OS=Homo sapiens GN=KRT18 PE=1 SV=2 |

|  | **Query** | **Observed** | **Mr(expt)** | **Mr(calc)** | **Delta** | **Miss** | **Score** | **Expect** | **Rank** | **Peptide** |
| --- | --- | --- | --- | --- | --- | --- | --- | --- | --- | --- |
|  | 9181 | **647.0010** | **1291.9875** | **1291.7136** | **0.2740** | **1** | **1** | **80** | **1** | **K.VKLEAEIATYR.R** |
|  | 15566 | **560.6182** | **1678.8327** | **1679.8301** | **-0.9974** | **1** | **7** | **23** | **3** | **K.IMADIRAQYDELAR.K** |
|  | 18963 | **643.2833** | **1926.8280** | **1926.9105** | **-0.0826** | **1** | **0** | **88** | **2** | **K.ETMQSLNDRLASYLDR.V** |
|  | 21627 | **1089.0876** | **2176.1607** | **2176.1700** | **-0.0093** | **1** | **12** | **5.9** | **1** | **R.LQLETEIEALKEELLFMK.K** |
|  | 25133 | **890.7993** | **2669.3760** | **2669.3846** | **-0.0087** | **0** | **34** | **0.028** | **1** | **R.YALQMEQLNGILLHLESELAQTR.A** 25138 |
|  | 26318 | **957.4047** | **2869.1923** | **2870.3651** | **-1.1728** | **1** | **2** | **28** | **1** | **R.STSFRGGMGSGGLATGIAGGLAGMGGIQNEK.E** |
|  | 27995 | 1076.2162 | 3225.6267 | 3226.6404 | -1.0137 | 1 | 0 | 40 | 6 | R.YALQMEQLNGILLHLESELAQTRAEGQR.Q |

| **773.** | Q9HBH1\|DEFM_HUMAN    **Mass:** 27452    **Score:** 44     **Queries matched:** 2   **emPAI:** 0.14 |
| --- | --- |
|  | Peptide deformylase, mitochondrial OS=Homo sapiens GN=PDF PE=1 SV=1 |

|  | **Query** | **Observed** | **Mr(expt)** | **Mr(calc)** | **Delta** | **Miss** | **Score** | **Expect** | **Rank** | **Peptide** |
| --- | --- | --- | --- | --- | --- | --- | --- | --- | --- | --- |
|  | 5510 | **534.2740** | **1066.5334** | **1067.5723** | **-1.0389** | **0** | **5** | **51** | **7** | **R.AQLGGPELQR.L** |
|  | 21918 | **1105.5569** | **2209.0992** | **2208.0708** | **1.0284** | **0** | **44** | **0.0033** | **1** | **R.LVTFPEGCESVAGFLACVPR.F** |

| **774.** | Q6P1J9\|CDC73_HUMAN    **Mass:** 60653    **Score:** 44     **Queries matched:** 16   **emPAI:** 0.06 |
| --- | --- |
|  | Parafibromin OS=Homo sapiens GN=CDC73 PE=1 SV=1 |

|  | **Query** | **Observed** | **Mr(expt)** | **Mr(calc)** | **Delta** | **Miss** | **Score** | **Expect** | **Rank** | **Peptide** |
| --- | --- | --- | --- | --- | --- | --- | --- | --- | --- | --- |
|  | 1519 | 678.3773 | 677.3700 | 676.3941 | 0.9758 | 1 | 9 | 25 | 7 | K.AKIMAK.K |
|  | 2911 | 436.7503 | 871.4860 | 871.5127 | -0.0267 | 0 | 44 | 0.0084 | 2 | M.ADVLSVLR.Q |
|  | 4694 | **502.2642** | **1002.5139** | **1002.5532** | **-0.0393** | **0** | **11** | **16** | **2** | **-.MADVLSVLR.Q** 4695 |
|  | 4964 | **512.7622** | **1023.5097** | **1023.4873** | **0.0225** | **1** | **9** | **18** | **2** | **K.GKEETEGFK.I** |
|  | 7544 | 402.9453 | 1205.8140 | 1204.6776 | 1.1365 | 1 | 4 | 50 | 5 | R.TRTTILQSTGK.N |
|  | 8547 | **419.9061** | **1256.6966** | **1257.6829** | **-0.9864** | **1** | **8** | **25** | **8** | **R.TKQPIPAAYNR.Y** 8586 |
|  | 12823 | **500.7801** | **1499.3186** | **1498.6796** | **0.6390** | **0** | **5** | **28** | **5** | **K.IDTMGTYHGMTLK.S** |
|  | 14265 | **531.7535** | **1592.2388** | **1591.8491** | **0.3897** | **1** | **3** | **44** | **6** | **R.SLSEAMSVEKIAAIK.A** |
|  | 19223 | **650.3002** | **1947.8787** | **1946.9884** | **0.8903** | **1** | **3** | **54** | **1** | **R.KDQMQPGGTAISVTVPYR.V** |
|  | 23921 | 494.0825 | 2465.3759 | 2464.3299 | 1.0460 | 0 | 10 | 6.9 | 1 | K.TQTPAAQPVPRPVSQARPPPNQK.K 23915 23916 23918 23920 |

| **775.** | O95154\|ARK73_HUMAN    **Mass:** 37582    **Score:** 44     **Queries matched:** 8   **emPAI:** 0.10 |
| --- | --- |
|  | Aflatoxin B1 aldehyde reductase member 3 OS=Homo sapiens GN=AKR7A3 PE=1 SV=2 |

|  | **Query** | **Observed** | **Mr(expt)** | **Mr(calc)** | **Delta** | **Miss** | **Score** | **Expect** | **Rank** | **Peptide** |
| --- | --- | --- | --- | --- | --- | --- | --- | --- | --- | --- |
|  | 2975 | 439.2452 | 876.4759 | 876.4599 | 0.0160 | 1 | 17 | 3.5 | 5 | -.MSRQLSR.A |
|  | 11022 | 696.3630 | 1390.7114 | 1390.6915 | 0.0199 | 0 | 5 | 47 | 7 | R.QVETELFPCLR.H |
|  | 12718 | 498.2805 | 1491.8197 | 1490.7334 | 1.0864 | 0 | 1 | 1e+002 | 10 | R.ARPATVLGAMEMGR.R |
|  | 14822 | **544.3162** | **1629.9268** | **1630.8395** | **-0.9127** | **1** | **6** | **32** | **6** | **R.ARPATVLGAMEMGRR.M** 14844 |
|  | 18238 | **933.9655** | **1865.9163** | **1865.9305** | **-0.0142** | **0** | **44** | **0.0043** | **1** | **K.ALQAAYGASAPSMTSATLR.W** 18237 |
|  | 28799 | **874.3942** | **3493.5478** | **3494.6736** | **-1.1258** | **1** | **2** | **23** | **1** | **R.GHTEIDTAFVYSEGQSETILGGLGLRLGGSDCR.V** |

| **776.** | Q9HAV0\|GBB4_HUMAN    **Mass:** 38284    **Score:** 44     **Queries matched:** 13   **emPAI:** 0.21 |
| --- | --- |
|  | Guanine nucleotide-binding protein subunit beta-4 OS=Homo sapiens GN=GNB4 PE=1 SV=3 |

|  | **Query** | **Observed** | **Mr(expt)** | **Mr(calc)** | **Delta** | **Miss** | **Score** | **Expect** | **Rank** | **Peptide** |
| --- | --- | --- | --- | --- | --- | --- | --- | --- | --- | --- |
|  | 4773 | 505.2582 | 1008.5019 | 1008.5101 | -0.0082 | 0 | 44 | 0.0056 | 1 | R.AGVLAGHDNR.V 4778 4788 4789 4791 4792 4796 |
|  | 4893 | 509.2791 | 1016.5437 | 1016.5502 | -0.0065 | 0 | 28 | 0.31 | 1 | R.LLVSASQDGK.L 4903 |
|  | 10050 | 446.5502 | 1336.6288 | 1336.6596 | -0.0308 | 1 | 9 | 15 | 3 | K.GDRAGVLAGHDNR.V |
|  | 16385 | 577.3186 | 1728.9340 | 1727.8802 | 1.0538 | 1 | 2 | 71 | 8 | M.SELEQLRQEAEQLR.N |
|  | 21582 | **724.7347** | **2171.1824** | **2171.1561** | **0.0263** | **1** | **7** | **16** | **1** | **K.LIIWDSYTTNKMHAIPLR.S** 21563 |

| **777.** | P16104\|H2AX_HUMAN    **Mass:** 15135    **Score:** 44     **Queries matched:** 6   **emPAI:** 0.58 |
| --- | --- |
|  | Histone H2A.x OS=Homo sapiens GN=H2AFX PE=1 SV=2 |

|  | **Query** | **Observed** | **Mr(expt)** | **Mr(calc)** | **Delta** | **Miss** | **Score** | **Expect** | **Rank** | **Peptide** |
| --- | --- | --- | --- | --- | --- | --- | --- | --- | --- | --- |
|  | 917 | **517.1575** | **516.1503** | **515.2704** | **0.8799** | **0** | **5** | **1.2e+002** | **2** | **K.APSGGK.K** |
|  | 2590 | 425.7635 | 849.5125 | 849.5184 | -0.0060 | 0 | 25 | 0.55 | 1 | R.HLQLAIR.N |
|  | 3867 | 473.2675 | 944.5205 | 943.5240 | 0.9965 | 0 | 38 | 0.031 | 1 | R.AGLQFPVGR.V 3847 |
|  | 15722 | 564.5905 | 1690.7497 | 1691.8954 | -1.1457 | 1 | 11 | 9.3 | 3 | R.HLQLAIRNDEELNK.L |
|  | 26583 | 972.5317 | 2914.5732 | 2914.5804 | -0.0071 | 0 | 27 | 0.09 | 1 | R.VGAGAPVYLAAVLEYLTAEILELAGNAAR.D |

| **778.** | Q08257\|QOR_HUMAN    **Mass:** 35356    **Score:** 43     **Queries matched:** 7   **emPAI:** 0.11 |
| --- | --- |
|  | Quinone oxidoreductase OS=Homo sapiens GN=CRYZ PE=1 SV=1 |

|  | **Query** | **Observed** | **Mr(expt)** | **Mr(calc)** | **Delta** | **Miss** | **Score** | **Expect** | **Rank** | **Peptide** |
| --- | --- | --- | --- | --- | --- | --- | --- | --- | --- | --- |
|  | 4591 | 499.2617 | 996.5088 | 997.5379 | -1.0291 | 0 | 5 | 48 | 6 | R.ALIHSACVK.A |
|  | 7572 | 604.7726 | 1207.5306 | 1207.7288 | -0.1982 | 1 | 7 | 26 | 5 | K.LRSDIAVPIPK.D |
|  | 14482 | **537.2544** | **1608.7413** | **1608.9311** | **-0.1898** | **1** | **6** | **29** | **2** | **R.VIVVGSRGTIEINPR.D** |
|  | 14582 | **539.2789** | **1614.8150** | **1613.7984** | **1.0165** | **0** | **2** | **68** | **5** | **K.VHACGVNPVETYIR.S** |
|  | 15708 | **564.2841** | **1689.8305** | **1689.8434** | **-0.0129** | **0** | **43** | **0.005** | **1** | **K.VAEAHENIIHGSGATGK.M** 15706 |
|  | 25444 | 906.1396 | 2715.3969 | 2715.4119 | -0.0150 | 0 | 10 | 6.2 | 1 | R.KPLLPYTPGSDVAGVIEAVGDNASAFK.K |

| **779.** | O14874\|BCKD_HUMAN    **Mass:** 46616    **Score:** 43     **Queries matched:** 8   **emPAI:** 0.08 |
| --- | --- |
|  | [3-methyl-2-oxobutanoate dehydrogenase [lipoamide]] kinase, mitochondrial OS=Homo sapiens GN=BCKDK PE=1 SV=2 |

|  | **Query** | **Observed** | **Mr(expt)** | **Mr(calc)** | **Delta** | **Miss** | **Score** | **Expect** | **Rank** | **Peptide** |
| --- | --- | --- | --- | --- | --- | --- | --- | --- | --- | --- |
|  | 1184 | **578.2668** | **577.2596** | **576.3231** | **0.9365** | **0** | **9** | **28** | **7** | **K.TLTSR.L** |
|  | 6093 | 556.3039 | 1110.5932 | 1109.5941 | 0.9991 | 1 | 11 | 12 | 10 | R.ISDRGGGIAHK.D |
|  | 7101 | **593.8520** | **1185.6894** | **1184.6765** | **1.0129** | **0** | **1** | **1.2e+002** | **4** | **K.DVVTLLAEGLR.E** |
|  | 9275 | 649.3314 | 1296.6482 | 1297.6738 | -1.0257 | 1 | 7 | 29 | 5 | R.SQDGSHLLKSAR.Y |
|  | 13741 | **520.3453** | **1558.0140** | **1557.6842** | **0.3298** | **0** | **3** | **56** | **6** | **R.STSATDTHHVEMAR.E** |
|  | 20317 | **1023.5665** | **2045.1184** | **2045.1311** | **-0.0127** | **0** | **43** | **0.0043** | **1** | **R.FPFIPMPLDYILPELLK.N** |
|  | 22285 | **563.2759** | **2249.0744** | **2250.1137** | **-1.0392** | **1** | **3** | **37** | **2** | **R.LTPTMMLYAGRSQDGSHLLK.S** |
|  | 25198 | **892.4621** | **2674.3645** | **2673.3519** | **1.0125** | **0** | **1** | **49** | **1** | **R.MLATHHLALHEDKPDFVGIICTR.L** |

| **780.** | P04217\|A1BG_HUMAN    **Mass:** 54809    **Score:** 43     **Queries matched:** 4   **emPAI:** 0.07 |
| --- | --- |
|  | Alpha-1B-glycoprotein OS=Homo sapiens GN=A1BG PE=1 SV=3 |

|  | **Query** | **Observed** | **Mr(expt)** | **Mr(calc)** | **Delta** | **Miss** | **Score** | **Expect** | **Rank** | **Peptide** |
| --- | --- | --- | --- | --- | --- | --- | --- | --- | --- | --- |
|  | 2008 | **402.6964** | **803.3783** | **804.4858** | **-1.1075** | **0** | **6** | **51** | **10** | **R.GVTFLLR.R** |
|  | 8024 | **411.1689** | **1230.4847** | **1229.7608** | **0.7239** | **1** | **8** | **23** | **2** | **R.GVLRGVTFLLR.R** |
|  | 15029 | 822.3950 | 1642.7754 | 1641.8951 | 0.8803 | 1 | 4 | 49 | 8 | R.ATWSGAVLAGRDAVLR.C |
|  | 18365 | **938.4983** | **1874.9820** | **1874.9924** | **-0.0104** | **0** | **43** | **0.0052** | **1** | **K.VTLTCVAPLSGVDFQLR.R** |

| **781.** | Q9BWD1\|THIC_HUMAN    **Mass:** 41838    **Score:** 43     **Queries matched:** 14   **emPAI:** 0.19 |
| --- | --- |
|  | Acetyl-CoA acetyltransferase, cytosolic OS=Homo sapiens GN=ACAT2 PE=1 SV=2 |

|  | **Query** | **Observed** | **Mr(expt)** | **Mr(calc)** | **Delta** | **Miss** | **Score** | **Expect** | **Rank** | **Peptide** |
| --- | --- | --- | --- | --- | --- | --- | --- | --- | --- | --- |
|  | 1288 | **606.7614** | **605.7541** | **605.3067** | **0.4474** | **1** | **6** | **36** | **9** | **R.MGRSR.G** |
|  | 2012 | **804.4195** | **803.4122** | **802.4450** | **0.9673** | **1** | **10** | **22** | **6** | **K.KWQVSR.E** |
|  | 3049 | **441.7244** | **881.4343** | **882.5399** | **-1.1056** | **1** | **7** | **29** | **9** | **K.RGLTPLAR.I** |
|  | 5610 | 537.7662 | 1073.5179 | 1072.4971 | 1.0208 | 0 | 6 | 42 | 10 | R.HGSNIEAMSK.L |
|  | 12868 | **751.8695** | **1501.7243** | **1501.7559** | **-0.0315** | **0** | **10** | **12** | **1** | **-.MNAGSDPVVIVSAAR.T** |
|  | 19246 | 975.5556 | 1949.0966 | 1947.9949 | 1.1018 | 0 | 1 | 80 | 8 | K.VNIEGGAIALGHPLGASGCR.I |
|  | 20366 | 684.1990 | 2049.5753 | 2048.9628 | 0.6125 | 1 | 1 | 47 | 3 | R.GVAALCIGGGMGIAMCVQRE.- |
|  | 21775 | **549.7789** | **2195.0864** | **2195.0432** | **0.0432** | **1** | **3** | **40** | **2** | **R.SRGVAALCIGGGMGIAMCVQR.E** |
|  | 23302 | **1189.6536** | **2377.2926** | **2377.3079** | **-0.0154** | **0** | **31** | **0.055** | **1** | **R.IVSWSQVGVEPSIMGIGPIPAIK.Q** |
|  | 27872 | 1064.5432 | 3190.6078 | 3191.6537 | -1.0459 | 0 | 35 | 0.014 | 1 | K.LKPYFLTDGTGTVTPANASGINDGAAAVVLMK.K |
|  | 27923 | **1070.1930** | **3207.5571** | **3207.6486** | **-0.0914** | **0** | **(0)** | **38** | **1** | **K.LKPYFLTDGTGTVTPANASGINDGAAAVVLMK.K** |
|  | 29161 | **908.4169** | **3629.6384** | **3629.6623** | **-0.0239** | **0** | **8** | **4.8** | **1** | **K.IGEMPLTDSILCDGLTDAFHNCHMGITAENVAK.K** |
|  | 29162 | **908.4169** | **3629.6384** | **3629.8368** | **-0.1984** | **1** | **1** | **26** | **9** | **K.AVCLAVQSIGIGDSSIVVAGGMENMSKAPHLAYLR.T** |
|  | 29564 | **948.2357** | **3788.9138** | **3789.7471** | **-0.8333** | **1** | **0** | **34** | **10** | **K.IGEMPLTDSILCDGLTDAFHNCHMGITAENVAKK.W** |

| **782.** | P43307\|SSRA_HUMAN    **Mass:** 32215    **Score:** 43     **Queries matched:** 6   **emPAI:** 0.12 |
| --- | --- |
|  | Translocon-associated protein subunit alpha OS=Homo sapiens GN=SSR1 PE=1 SV=3 |

|  | **Query** | **Observed** | **Mr(expt)** | **Mr(calc)** | **Delta** | **Miss** | **Score** | **Expect** | **Rank** | **Peptide** |
| --- | --- | --- | --- | --- | --- | --- | --- | --- | --- | --- |
|  | 3595 | **463.2574** | **924.5003** | **924.5069** | **-0.0066** | **0** | **43** | **0.0078** | **1** | **K.FLVGFTNK.G** 3596 3599 |
|  | 7476 | **602.3468** | **1202.6790** | **1202.5931** | **0.0859** | **0** | **2** | **1.1e+002** | **6** | **K.GEDFPANNIVK.F** |
|  | 27051 | **600.2974** | **2996.4507** | **2995.3539** | **1.0968** | **0** | **2** | **37** | **4** | **K.VEMGTSSQNDVDMSWIPQETLNQINK.A** |
|  | 27751 | **1053.8651** | **3158.5735** | **3158.5899** | **-0.0164** | **0** | **3** | **26** | **1** | **R.QATFEYSFIPAEPMGGRPFGLVINLNYK.D** |

| **783.** | P84085\|ARF5_HUMAN    **Mass:** 20631    **Score:** 43     **Queries matched:** 12   **emPAI:** 0.67 |
| --- | --- |
|  | ADP-ribosylation factor 5 OS=Homo sapiens GN=ARF5 PE=1 SV=2 |

|  | **Query** | **Observed** | **Mr(expt)** | **Mr(calc)** | **Delta** | **Miss** | **Score** | **Expect** | **Rank** | **Peptide** |
| --- | --- | --- | --- | --- | --- | --- | --- | --- | --- | --- |
|  | 2466 | 420.7607 | 839.5067 | 839.5130 | -0.0062 | 0 | 6 | 33 | 1 | K.IRPLWR.H |
|  | 5774 | 544.3091 | 1086.6036 | 1086.6107 | -0.0071 | 0 | 28 | 0.26 | 1 | R.ILMVGLDAAGK.T 5773 |
|  | 5807 | 545.3157 | 1088.6169 | 1088.6230 | -0.0061 | 0 | 7 | 34 | 2 | R.DAVLLVFANK.Q 5789 |
|  | 13375 | 512.5765 | 1534.7076 | 1533.8007 | 0.9069 | 1 | 6 | 32 | 2 | K.QMRILMVGLDAAGK.T 13370 |
|  | 15740 | **564.8259** | **1691.4558** | **1690.7542** | **0.7016** | **0** | **1** | **55** | **3** | **K.QDMPNAMPVSELTDK.L** |
|  | 17448 | 603.3307 | 1806.9704 | 1806.0325 | 0.9380 | 1 | 3 | 57 | 9 | R.ILMVGLDAAGKTTILYK.L |
|  | 22859 | 1162.1183 | 2322.2220 | 2322.2359 | -0.0139 | 0 | 3 | 41 | 1 | K.LGEIVTTIPTIGFNVETVEYK.N |
|  | 23685 | 813.3947 | 2437.1623 | 2437.1775 | -0.0152 | 1 | 25 | 0.24 | 1 | R.HYFQNTQGLIFVVDSNDRER.V |
|  | 24652 | 855.4747 | 2563.4024 | 2563.4149 | -0.0125 | 1 | 33 | 0.03 | 1 | K.LKLGEIVTTIPTIGFNVETVEYK.N |

| **784.** | P61353\|RL27_HUMAN    **Mass:** 15788    **Score:** 43     **Queries matched:** 9   **emPAI:** 0.25 |
| --- | --- |
|  | 60S ribosomal protein L27 OS=Homo sapiens GN=RPL27 PE=1 SV=2 |

|  | **Query** | **Observed** | **Mr(expt)** | **Mr(calc)** | **Delta** | **Miss** | **Score** | **Expect** | **Rank** | **Peptide** |
| --- | --- | --- | --- | --- | --- | --- | --- | --- | --- | --- |
|  | 1466 | **657.4606** | **656.4533** | **656.4585** | **-0.0052** | **1** | **24** | **0.72** | **1** | **R.KAVIVK.N** |
|  | 2036 | 805.4545 | 804.4473 | 804.4527 | -0.0055 | 1 | 43 | 0.012 | 1 | R.KVTAAMGK.K |
|  | 2240 | **411.7189** | **821.4232** | **820.4477** | **0.9755** | **1** | **(7)** | **30** | **7** | **R.KVTAAMGK.K** |
|  | 3172 | 447.2613 | 892.5080 | 891.5000 | 1.0079 | 1 | 12 | 12 | 1 | M.GKFMKPGK.V |
|  | 5440 | **531.2911** | **1060.5676** | **1059.5713** | **0.9963** | **1** | **10** | **19** | **5** | **K.DVFRDPALK.R** 5426 |
|  | 11283 | **704.3406** | **1406.6667** | **1406.6765** | **-0.0098** | **0** | **7** | **27** | **1** | **K.VYNYNHLMPTR.Y** 11269 |
|  | 25263 | 896.8342 | 2687.4809 | 2687.3052 | 0.1757 | 1 | 6 | 15 | 2 | K.NIDDGTSDRPYSHALVAGIDRYPR.K |

| **785.** | Q93088\|BHMT1_HUMAN    **Mass:** 45426    **Score:** 43     **Queries matched:** 21   **emPAI:** 0.37 |
| --- | --- |
|  | Betaine--homocysteine S-methyltransferase 1 OS=Homo sapiens GN=BHMT PE=1 SV=2 |

|  | **Query** | **Observed** | **Mr(expt)** | **Mr(calc)** | **Delta** | **Miss** | **Score** | **Expect** | **Rank** | **Peptide** |
| --- | --- | --- | --- | --- | --- | --- | --- | --- | --- | --- |
|  | 1571 | 701.7840 | 700.7767 | 700.3578 | 0.4189 | 0 | 8 | 29 | 2 | -.MPPVGGK.K |
|  | 2123 | 407.1330 | 812.2514 | 812.4578 | -0.2064 | 1 | 4 | 46 | 4 | -.MPPVGGKK.A |
|  | 2215 | 821.3732 | 820.3660 | 819.4338 | 0.9322 | 1 | 6 | 49 | 9 | K.SETEVKK.V |
|  | 3557 | **461.7502** | **921.4859** | **920.4716** | **1.0143** | **0** | **3** | **83** | **10** | **R.EAYNLGVR.Y** |
|  | 4446 | 494.2813 | 986.5481 | 985.5556 | 0.9925 | 1 | 7 | 38 | 10 | K.EGLEAARLK.A |
|  | 4730 | **503.2520** | **1004.4895** | **1004.4961** | **-0.0066** | **0** | **34** | **0.068** | **1** | **K.GTAELMQQK.E** |
|  | 5276 | 524.2654 | 1046.5163 | 1046.5244 | -0.0081 | 0 | 18 | 2.7 | 1 | K.EATTEQQLK.E |
|  | 5914 | **549.7890** | **1097.5634** | **1097.5716** | **-0.0082** | **0** | **31** | **0.11** | **1** | **R.AIAEELAPER.G** 5915 |
|  | 6185 | 559.7977 | 1117.5809 | 1116.5961 | 0.9848 | 1 | 8 | 27 | 2 | K.LMKEGLEAAR.L 6182 |
|  | 10023 | 668.3477 | 1334.6809 | 1333.6990 | 0.9819 | 1 | 4 | 61 | 4 | K.LENRGNYVLEK.I |
|  | 13230 | 763.3944 | 1524.7741 | 1524.8374 | -0.0633 | 1 | 11 | 9 | 2 | K.VFLQQLEVFMKK.N |
|  | 14847 | 816.8810 | 1631.7475 | 1631.7573 | -0.0098 | 0 | 27 | 0.26 | 1 | K.ISGQEVNEAACDIAR.Q |
|  | 18796 | **637.9886** | **1910.9439** | **1910.8356** | **0.1083** | **0** | **2** | **69** | **3** | **R.AGSNVMQTFTFYASEDK.L** |
|  | 19246 | 975.5556 | 1949.0966 | 1949.9319 | -0.8353 | 0 | 0 | 88 | 9 | K.HGSWGSGLDMHTKPWVR.A |
|  | 19795 | 667.0303 | 1998.0692 | 1999.0197 | -0.9505 | 0 | 3 | 43 | 2 | K.AGASIIGVNCHFDPTISLK.T |
|  | 20387 | **684.3566** | **2050.0479** | **2049.0048** | **1.0431** | **1** | **2** | **54** | **5** | **K.GTAELMQQKEATTEQQLK.E** |
|  | 21640 | **726.7959** | **2177.3659** | **2177.0701** | **0.2958** | **1** | **0** | **55** | **7** | **K.HGSWGSGLDMHTKPWVRAR.A** |
|  | 22280 | **750.0387** | **2247.0943** | **2247.1106** | **-0.0164** | **0** | **25** | **0.24** | **1** | **R.IASGRPYNPSMSKPDGWGVTK.G** |
|  | 23497 | **803.3726** | **2407.0959** | **2407.1114** | **-0.0156** | **1** | **13** | **3.2** | **1** | **R.AGSNVMQTFTFYASEDKLENR.G** |

| **786.** | Q99536\|VAT1_HUMAN    **Mass:** 42122    **Score:** 42     **Queries matched:** 14   **emPAI:** 0.19 |
| --- | --- |
|  | Synaptic vesicle membrane protein VAT-1 homolog OS=Homo sapiens GN=VAT1 PE=1 SV=2 |

|  | **Query** | **Observed** | **Mr(expt)** | **Mr(calc)** | **Delta** | **Miss** | **Score** | **Expect** | **Rank** | **Peptide** |
| --- | --- | --- | --- | --- | --- | --- | --- | --- | --- | --- |
|  | 1161 | 572.3473 | 571.3400 | 571.3693 | -0.0293 | 1 | 17 | 4.8 | 1 | K.KISPK.G 1158 1160 1166 |
|  | 3838 | **472.3203** | **942.6260** | **943.5385** | **-0.9125** | **1** | **13** | **8.2** | **10** | **K.RNLMALAR.T** |
|  | 7272 | **597.8106** | **1193.6067** | **1193.6656** | **-0.0589** | **1** | **8** | **21** | **6** | **K.VLLVPGPEKEN.-** |
|  | 12221 | **732.3948** | **1462.7750** | **1462.7854** | **-0.0104** | **0** | **24** | **0.48** | **1** | **K.VVTYGMANLLTGPK.R** |
|  | 13950 | **787.8846** | **1573.7547** | **1573.7658** | **-0.0111** | **0** | **40** | **0.012** | **1** | **K.GVDIVMDPLGGSDTAK.G** 13951 |
|  | 19964 | **504.5068** | **2013.9980** | **2015.0245** | **-1.0265** | **1** | **7** | **19** | **7** | **K.ISPKGVDIVMDPLGGSDTAK.G** |
|  | 22608 | 764.3759 | 2290.1057 | 2289.0067 | 1.0991 | 1 | 0 | 77 | 6 | -.MSDEREVAEAATGEDASSPPPK.T |
|  | 24403 | 507.4676 | 2532.3014 | 2532.3258 | -0.0244 | 0 | 5 | 20 | 1 | R.LPPLPVTPGMEGAGVVIAVGEGVSDR.K |
|  | 26628 | 975.1074 | 2922.3004 | 2923.3988 | -1.0984 | 1 | 1 | 46 | 5 | K.HEALKENGVTHPIDYHTTDYVDEIK.K |
|  | 28115 | **544.9312** | **3263.5436** | **3264.6813** | **-1.1377** | **1** | **2** | **29** | **4** | **R.QGLYDRLPPLPVTPGMEGAGVVIAVGEGVSDR.K** |

| **787.** | P01857\|IGHG1_HUMAN    **Mass:** 36596    **Score:** 42     **Queries matched:** 16   **emPAI:** 0.10 |
| --- | --- |
|  | Ig gamma-1 chain C region OS=Homo sapiens GN=IGHG1 PE=1 SV=1 |

|  | **Query** | **Observed** | **Mr(expt)** | **Mr(calc)** | **Delta** | **Miss** | **Score** | **Expect** | **Rank** | **Peptide** |
| --- | --- | --- | --- | --- | --- | --- | --- | --- | --- | --- |
|  | 1776 | 395.0780 | 788.1414 | 787.4440 | 0.6975 | 0 | 15 | 4.6 | 1 | K.SLSLSPGK.- 1775 1777 1780 1789 1791 |
|  | 2415 | **418.7136** | **835.4127** | **834.4269** | **0.9858** | **0** | **14** | **7.4** | **3** | **K.DTLMISR.T** 2401 |
|  | 2602 | **426.2323** | **850.4501** | **850.4218** | **0.0283** | **0** | **(4)** | **71** | **7** | **K.DTLMISR.T** |
|  | 7089 | **593.7994** | **1185.5842** | **1185.6394** | **-0.0552** | **0** | **1** | **1.2e+002** | **4** | **K.GPSVFPLAPSSK.S** |
|  | 8723 | **634.3342** | **1266.6538** | **1266.7547** | **-0.1009** | **1** | **2** | **88** | **1** | **K.ALPAPIEKTISK.A** |
|  | 15536 | **839.3986** | **1676.7827** | **1676.7947** | **-0.0120** | **0** | **42** | **0.0066** | **1** | **K.FNWYVDGVEVHNAK.T** |
|  | 21288 | **713.6758** | **2138.0055** | **2138.0202** | **-0.0147** | **0** | **3** | **40** | **2** | **R.TPEVTCVVVDVSHEDPEVK.F** |
|  | 24123 | **622.8305** | **2487.2929** | **2488.2996** | **-1.0066** | **1** | **3** | **36** | **9** | **K.GPSVFPLAPSSKSTSGGTAALGCLVK.D** 24121 |
|  | 31887 | **1343.4603** | **6712.2653** | **6712.3072** | **-0.0420** | **0** | **12** | **0.31** | **1** | **K.DYFPEPVTVSWNSGALTSGVHTFPAVLQSSGLYSLSSVVTVPSSSLGTQTYICNVNHKPSNTK.V** |

| **788.** | Q9NUQ7\|UFSP2_HUMAN    **Mass:** 53626    **Score:** 42     **Queries matched:** 11   **emPAI:** 0.14 |
| --- | --- |
|  | Ufm1-specific protease 2 OS=Homo sapiens GN=UFSP2 PE=2 SV=3 |

|  | **Query** | **Observed** | **Mr(expt)** | **Mr(calc)** | **Delta** | **Miss** | **Score** | **Expect** | **Rank** | **Peptide** |
| --- | --- | --- | --- | --- | --- | --- | --- | --- | --- | --- |
|  | 4630 | 500.2732 | 998.5318 | 998.5397 | -0.0078 | 0 | 10 | 15 | 2 | K.HVLSDLSTK.L |
|  | 11090 | **698.3964** | **1394.7782** | **1393.6765** | **1.1016** | **0** | **24** | **0.49** | **1** | **R.FIQFEPEEDIK.R** 11091 |
|  | 13612 | 517.7648 | 1550.2727 | 1549.7776 | 0.4950 | 1 | 5 | 26 | 2 | R.FIQFEPEEDIKR.K |
|  | 14260 | 796.8904 | 1591.7662 | 1590.8366 | 0.9296 | 0 | 46 | 0.0031 | 1 | K.ILFVSQGSEIASQGR.E |
|  | 16058 | **570.3041** | **1707.8904** | **1708.9004** | **-1.0100** | **1** | **4** | **40** | **6** | **-.MVISESMDILFRIR.G** |
|  | 19026 | 644.3679 | 1930.0819 | 1931.1244 | -1.0425 | 1 | 2 | 68 | 5 | K.GTSIVVPEPLHFLLPGKK.N |
|  | 19190 | **649.3317** | **1944.9734** | **1946.0513** | **-1.0779** | **0** | **10** | **9.2** | **1** | **R.GGLDLAFQLATPNEIFLK.K** |
|  | 20631 | **691.3174** | **2070.9303** | **2070.9435** | **-0.0132** | **1** | **20** | **0.76** | **1** | **R.SNAYHFPDEPYKDGYIR.N** |
|  | 22105 | 557.5154 | 2226.0327 | 2225.2282 | 0.8044 | 1 | 2 | 55 | 4 | K.YMKGTSIVVPEPLHFLLPGK.K |
|  | 27256 | 760.3884 | 3037.5246 | 3038.4444 | -0.9198 | 0 | 1 | 40 | 7 | R.ESGGHHYVNMTLPVDAVISVAPEETWGK.V |

| **789.** | Q6UXX5\|ITH5L_HUMAN    **Mass:** 143611   **Score:** 42     **Queries matched:** 8   **emPAI:** 0.03 |
| --- | --- |
|  | Inter-alpha-trypsin inhibitor heavy chain H5-like protein OS=Homo sapiens GN=ITIH5L PE=2 SV=1 |

|  | **Query** | **Observed** | **Mr(expt)** | **Mr(calc)** | **Delta** | **Miss** | **Score** | **Expect** | **Rank** | **Peptide** |
| --- | --- | --- | --- | --- | --- | --- | --- | --- | --- | --- |
|  | 1453 | 652.4096 | 651.4023 | 651.4068 | -0.0044 | 0 | 42 | 0.0086 | 1 | R.HLLAAK.V |
|  | 5180 | 520.3453 | 1038.6760 | 1038.5723 | 0.1037 | 0 | 15 | 4.9 | 4 | K.YPLHTRPR.V |
|  | 7935 | **409.5523** | **1225.6349** | **1225.6779** | **-0.0430** | **1** | **6** | **34** | **4** | **R.DTTTRHLLAAK.V** |
|  | 10993 | **695.3911** | **1388.7677** | **1388.7888** | **-0.0211** | **1** | **2** | **93** | **3** | **K.QELGIHLAARGPK.D** |
|  | 13049 | **505.7306** | **1514.1700** | **1514.8821** | **-0.7120** | **0** | **6** | **23** | **5** | **R.TQVPPVKPGIPASPK.A** |
|  | 17763 | **459.2256** | **1832.8731** | **1831.9978** | **0.8753** | **1** | **6** | **26** | **5** | **R.NNMPHLGPGILLSKTPK.I** |
|  | 18546 | **631.3308** | **1890.9706** | **1891.0904** | **-0.1198** | **1** | **3** | **56** | **5** | **K.AGLHVSGKLLGAPPRPGHK.D** |
|  | 30718 | **721.8620** | **4325.1283** | **4324.2449** | **0.8835** | **1** | **5** | **8.1** | **1** | **R.YLICVSFLLTILLELTYQGPPVPASSSTKLLMTSYSMR.S** |

| **790.** | O95298\|NDUC2_HUMAN    **Mass:** 14235    **Score:** 42     **Queries matched:** 5   **emPAI:** 0.28 |
| --- | --- |
|  | NADH dehydrogenase [ubiquinone] 1 subunit C2 OS=Homo sapiens GN=NDUFC2 PE=1 SV=1 |

|  | **Query** | **Observed** | **Mr(expt)** | **Mr(calc)** | **Delta** | **Miss** | **Score** | **Expect** | **Rank** | **Peptide** |
| --- | --- | --- | --- | --- | --- | --- | --- | --- | --- | --- |
|  | 1408 | **637.8211** | **636.8138** | **637.3799** | **-0.5661** | **0** | **10** | **10** | **8** | **R.SLPPPK.L** |
|  | 3028 | **441.2480** | **880.4814** | **880.4879** | **-0.0065** | **1** | **42** | **0.0097** | **1** | **R.RNPEPLR.F** |
|  | 7842 | **611.3290** | **1220.6434** | **1219.6925** | **0.9509** | **1** | **6** | **38** | **3** | **R.SLPPPKLTDPR.L** 7798 |
|  | 27643 | 1043.5440 | 3127.6100 | 3128.7327 | -1.1227 | 1 | 1 | 34 | 8 | R.RPIATAGLHRQLLYITAFFFAGYYLVK.R |

| **791.** | Q9NZS9\|BFAR_HUMAN    **Mass:** 53331    **Score:** 42     **Queries matched:** 11   **emPAI:** 0.07 |
| --- | --- |
|  | Bifunctional apoptosis regulator OS=Homo sapiens GN=BFAR PE=1 SV=1 |

|  | **Query** | **Observed** | **Mr(expt)** | **Mr(calc)** | **Delta** | **Miss** | **Score** | **Expect** | **Rank** | **Peptide** |
| --- | --- | --- | --- | --- | --- | --- | --- | --- | --- | --- |
|  | 1269 | **601.1239** | **600.1166** | **600.3119** | **-0.1953** | **0** | **10** | **15** | **3** | **R.DEPLK.S** 1265 |
|  | 4470 | 495.2544 | 988.4942 | 989.5215 | -1.0274 | 0 | 42 | 0.012 | 1 | R.AILMELER.V |
|  | 8258 | 621.8341 | 1241.6535 | 1241.7019 | -0.0484 | 1 | 16 | 3.4 | 6 | K.LLDLKEPTWK.Q 8235 8259 |
|  | 8300 | 622.8454 | 1243.6762 | 1242.5550 | 1.1212 | 0 | 7 | 31 | 3 | K.SYVNTMDLER.D 8266 |
|  | 11903 | 722.9013 | 1443.7879 | 1443.8238 | -0.0359 | 1 | 4 | 52 | 9 | K.WEGFPKVSILLR.D |
|  | 21885 | 736.6923 | 2207.0550 | 2207.0858 | -0.0309 | 0 | 3 | 42 | 10 | R.FEDIQQNNDIVQSLAAFQK.Y 21887 |

| **792.** | O43826\|G6PT1_HUMAN    **Mass:** 46729    **Score:** 42     **Queries matched:** 5   **emPAI:** 0.08 |
| --- | --- |
|  | Glucose-6-phosphate translocase OS=Homo sapiens GN=SLC37A4 PE=1 SV=1 |

|  | **Query** | **Observed** | **Mr(expt)** | **Mr(calc)** | **Delta** | **Miss** | **Score** | **Expect** | **Rank** | **Peptide** |
| --- | --- | --- | --- | --- | --- | --- | --- | --- | --- | --- |
|  | 7696 | **405.8700** | **1214.5881** | **1214.5965** | **-0.0084** | **1** | **10** | **15** | **2** | **R.NLDPMPSEGKK.G** |
|  | 19045 | 966.8864 | 1931.7583 | 1931.8546 | -0.0964 | 0 | 2 | 63 | 3 | K.TCCTDWGQFFLIQEK.G |
|  | 22160 | **745.0141** | **2232.0205** | **2233.0920** | **-1.0715** | **0** | **0** | **74** | **6** | **R.HGLLLFMMAGMTVSMYLFR.V** |
|  | 28125 | 1089.8715 | 3266.5926 | 3266.5765 | 0.0160 | 1 | 2 | 28 | 7 | K.DDLGFITSSQSAAYAISKFVSGVLSDQMSAR.W |
|  | 29620 | **1269.6361** | **3805.8865** | **3805.9012** | **-0.0147** | **1** | **42** | **0.002** | **1** | **K.TFSFVMPSLVEEIPLDKDDLGFITSSQSAAYAISK.F** |

| **793.** | P80723\|BASP1_HUMAN    **Mass:** 22680    **Score:** 42     **Queries matched:** 7   **emPAI:** 0.17 |
| --- | --- |
|  | Brain acid soluble protein 1 OS=Homo sapiens GN=BASP1 PE=1 SV=2 |

|  | **Query** | **Observed** | **Mr(expt)** | **Mr(calc)** | **Delta** | **Miss** | **Score** | **Expect** | **Rank** | **Peptide** |
| --- | --- | --- | --- | --- | --- | --- | --- | --- | --- | --- |
|  | 9039 | **643.2899** | **1284.5653** | **1283.6357** | **0.9296** | **0** | **1** | **1.2e+002** | **5** | **K.TEAPAAPAAQETK.S** |
|  | 9265 | **433.1967** | **1296.5682** | **1297.6514** | **-1.0832** | **1** | **3** | **63** | **4** | **K.TEGAAEAKAEPPK.A** |
|  | 11404 | **707.3790** | **1412.7434** | **1411.7307** | **1.0127** | **1** | **42** | **0.0089** | **1** | **K.KTEAPAAPAAQETK.S** |
|  | 17000 | **592.3044** | **1773.8915** | **1774.8486** | **-0.9571** | **0** | **3** | **51** | **2** | **K.APEQEQAAPGPAAGGEAPK.A** 17028 |
|  | 19046 | **644.9592** | **1931.8559** | **1931.8708** | **-0.0150** | **0** | **10** | **11** | **1** | **K.SDGAPASDSKPGSSEAAPSSK.E** |
|  | 19437 | **656.3623** | **1966.0651** | **1965.9279** | **0.1372** | **0** | **2** | **55** | **5** | **K.AAEAAAAPAESAAPAAGEEPSK.E** |

| **794.** | Q9UBR2\|CATZ_HUMAN    **Mass:** 34530    **Score:** 42     **Queries matched:** 4   **emPAI:** 0.11 |
| --- | --- |
|  | Cathepsin Z OS=Homo sapiens GN=CTSZ PE=1 SV=1 |

|  | **Query** | **Observed** | **Mr(expt)** | **Mr(calc)** | **Delta** | **Miss** | **Score** | **Expect** | **Rank** | **Peptide** |
| --- | --- | --- | --- | --- | --- | --- | --- | --- | --- | --- |
|  | 2616 | **427.2242** | **852.4339** | **851.4290** | **1.0049** | **0** | **4** | **60** | **8** | **R.NYTLWR.V** |
|  | 4787 | **505.7454** | **1009.4762** | **1009.4829** | **-0.0067** | **0** | **42** | **0.01** | **1** | **R.VGDYGSLSGR.E** |
|  | 17906 | 922.9460 | 1843.8775 | 1842.9013 | 0.9762 | 1 | 4 | 46 | 4 | R.NYTLWRVGDYGSLSGR.E |
|  | 22131 | **743.9390** | **2228.7951** | **2229.9890** | **-1.1940** | **0** | **1** | **44** | **2** | **K.MMAEIYANGPISCGIMATER.L** |

| **795.** | Q7KZF4\|SND1_HUMAN    **Mass:** 102618   **Score:** 41     **Queries matched:** 26   **emPAI:** 0.04 |
| --- | --- |
|  | Staphylococcal nuclease domain-containing protein 1 OS=Homo sapiens GN=SND1 PE=1 SV=1 |

|  | **Query** | **Observed** | **Mr(expt)** | **Mr(calc)** | **Delta** | **Miss** | **Score** | **Expect** | **Rank** | **Peptide** |
| --- | --- | --- | --- | --- | --- | --- | --- | --- | --- | --- |
|  | 2901 | **436.2506** | **870.4867** | **871.4399** | **-0.9533** | **0** | **3** | **79** | **9** | **R.AAATQPDAK.D** |
|  | 3002 | **879.0601** | **878.0528** | **877.5134** | **0.5395** | **1** | **6** | **28** | **3** | **K.KEVPIHR.V** |
|  | 4047 | 479.2865 | 956.5584 | 956.5403 | 0.0181 | 0 | 10 | 16 | 6 | R.QINLSNIR.A |
|  | 5096 | **517.3057** | **1032.5968** | **1032.5088** | **0.0880** | **0** | **22** | **1.1** | **1** | **R.VADISGDTQK.A** |
|  | 8052 | 411.5496 | 1231.6270 | 1231.6408 | -0.0138 | 1 | 7 | 34 | 2 | R.VADISGDTQKAK.Q |
|  | 8086 | 617.4323 | 1232.8501 | 1233.6573 | -0.8073 | 0 | 2 | 83 | 6 | K.MVLSGCAIIVR.G |
|  | 8914 | 639.4456 | 1276.8766 | 1275.7411 | 1.1354 | 0 | 0 | 1.1e+002 | 9 | K.TIHLSSIRPPR.L |
|  | 9295 | 433.5708 | 1297.6907 | 1297.6204 | 0.0703 | 1 | 4 | 61 | 6 | K.FVDGEWYRAR.V |
|  | 9956 | **444.7295** | **1331.1666** | **1331.7462** | **-0.5796** | **1** | **10** | **11** | **2** | **K.QFLPFLQRAGR.S** |
|  | 10493 | 682.3362 | 1362.6579 | 1362.6449 | 0.0130 | 1 | 3 | 70 | 7 | R.LSECEEQAKAAK.K |
|  | 11182 | **700.9070** | **1399.7995** | **1398.7905** | **1.0091** | **0** | **41** | **0.0094** | **1** | **K.VMQVLNADAIVVK.L** 11139 11158 11160 |
|  | 11694 | 715.8674 | 1429.7202 | 1430.6320 | -0.9118 | 1 | 7 | 25 | 7 | R.EGMRANNPEQNR.L 11726 |
|  | 13738 | **520.2949** | **1557.8628** | **1558.7310** | **-0.8683** | **1** | **4** | **47** | **1** | **K.KGMWSEGNGSHTIR.D** |
|  | 13995 | **526.7801** | **1577.3184** | **1576.7733** | **0.5452** | **1** | **4** | **34** | **3** | **R.DYVAPTANLDQKDK.Q** |
|  | 14822 | 544.3162 | 1629.9268 | 1628.9470 | 0.9799 | 1 | 6 | 28 | 3 | R.GIIKMVLSGCAIIVR.G |
|  | 16161 | **572.3191** | **1713.9354** | **1712.8482** | **1.0873** | **1** | **2** | **75** | **4** | **R.AGRSEAVVEYVFSGSR.L** |
|  | 17066 | **593.6387** | **1777.8942** | **1778.9162** | **-1.0220** | **1** | **1** | **94** | **6** | **K.VITEYLNAQESAKSAR.L** |
|  | 18438 | **628.4099** | **1882.2077** | **1882.7758** | **-0.5681** | **1** | **3** | **34** | **2** | **R.YGDFRADDADEFGYSR.-** |
|  | 18873 | **640.3613** | **1918.0620** | **1917.0241** | **1.0379** | **0** | **2** | **64** | **4** | **R.TCATVTIGGINIAEALVSK.G** |
|  | 19225 | **650.3460** | **1948.0160** | **1946.9116** | **1.1044** | **0** | **7** | **18** | **1** | **-.MASSAQSGGSSGGPAVPTVQR.G** |
|  | 19346 | 979.9904 | 1957.9663 | 1958.9545 | -0.9882 | 0 | 3 | 50 | 8 | K.DTNGENIAESLVAEGLATR.R |
|  | 28974 | **890.6881** | **3558.7233** | **3558.7406** | **-0.0173** | **0** | **3** | **18** | **2** | **R.SASYKPVFVTEITDDLHFYVQDVETGTQLEK.L** |

| **796.** | P25311\|ZA2G_HUMAN    **Mass:** 34079    **Score:** 41     **Queries matched:** 6   **emPAI:** 0.11 |
| --- | --- |
|  | Zinc-alpha-2-glycoprotein OS=Homo sapiens GN=AZGP1 PE=1 SV=1 |

|  | **Query** | **Observed** | **Mr(expt)** | **Mr(calc)** | **Delta** | **Miss** | **Score** | **Expect** | **Rank** | **Peptide** |
| --- | --- | --- | --- | --- | --- | --- | --- | --- | --- | --- |
|  | 5603 | **537.2996** | **1072.5846** | **1072.5625** | **0.0221** | **1** | **4** | **71** | **7** | **K.QDSQLQKAR.E** |
|  | 6420 | **569.2781** | **1136.5417** | **1136.4808** | **0.0609** | **0** | **5** | **48** | **8** | **R.QVEGMEDWK.Q** |
|  | 11309 | **470.3303** | **1407.9690** | **1407.7286** | **0.2405** | **0** | **3** | **45** | **5** | **R.YSLTYIYTGLSK.H** |
|  | 16202 | 859.4169 | 1716.8192 | 1716.7671 | 0.0521 | 1 | 1 | 94 | 4 | K.YYYDGKDYIEFNK.E |
|  | 17010 | 592.6351 | 1774.8834 | 1774.8850 | -0.0016 | 0 | 5 | 37 | 2 | R.QDPPSVVVTSHQAPGEK.K |
|  | 23474 | 801.7374 | 2402.1903 | 2402.2019 | -0.0116 | 0 | 41 | 0.0054 | 1 | K.HVEDVPAFQALGSLNDLQFFR.Y |

| **797.** | P61981\|1433G_HUMAN    **Mass:** 28456    **Score:** 41     **Queries matched:** 7   **emPAI:** 0.13 |
| --- | --- |
|  | 14-3-3 protein gamma OS=Homo sapiens GN=YWHAG PE=1 SV=2 |

|  | **Query** | **Observed** | **Mr(expt)** | **Mr(calc)** | **Delta** | **Miss** | **Score** | **Expect** | **Rank** | **Peptide** |
| --- | --- | --- | --- | --- | --- | --- | --- | --- | --- | --- |
|  | 3688 | 466.7581 | 931.5016 | 931.4222 | 0.0795 | 1 | 12 | 13 | 10 | K.MKGDYYR.Y |
|  | 3849 | 472.7915 | 943.5684 | 943.5201 | 0.0484 | 1 | 5 | 58 | 3 | K.VFYLKMK.G |
|  | 5218 | **522.2351** | **1042.4557** | **1042.5519** | **-0.0962** | **1** | **3** | **88** | **7** | **K.ARLAEQAER.Y** |
|  | 7502 | 402.2437 | 1203.7093 | 1204.6485 | -0.9392 | 0 | 12 | 9.5 | 8 | K.DSTLIMQLLR.D |
|  | 8307 | **415.7375** | **1244.1907** | **1244.6084** | **-0.4177** | **0** | **7** | **21** | **10** | **K.EHMQPTHPIR.L** |
|  | 11490 | 709.8373 | 1417.6600 | 1418.7518 | -1.0917 | 1 | 5 | 41 | 9 | R.SSWRVISSIEQK.T |
|  | 28209 | **1101.2051** | **3300.5934** | **3300.6071** | **-0.0137** | **1** | **41** | **0.0035** | **1** | **K.TAFDDAIAELDTLNEDSYKDSTLIMQLLR.D** |

| **798.** | P00505\|AATM_HUMAN    **Mass:** 47844    **Score:** 41     **Queries matched:** 15   **emPAI:** 0.16 |
| --- | --- |
|  | Aspartate aminotransferase, mitochondrial OS=Homo sapiens GN=GOT2 PE=1 SV=2 |

|  | **Query** | **Observed** | **Mr(expt)** | **Mr(calc)** | **Delta** | **Miss** | **Score** | **Expect** | **Rank** | **Peptide** |
| --- | --- | --- | --- | --- | --- | --- | --- | --- | --- | --- |
|  | 3162 | 446.7122 | 891.4098 | 890.4974 | 0.9124 | 0 | 37 | 0.036 | 1 | R.IGASFLQR.F |
|  | 3294 | 902.4784 | 901.4712 | 901.5233 | -0.0521 | 0 | 6 | 56 | 9 | R.TQLVSNLK.K |
|  | 3654 | 465.2707 | 928.5268 | 928.5341 | -0.0073 | 1 | 23 | 0.91 | 1 | R.KAEAQIAAK.N |
|  | 3773 | **470.2338** | **938.4531** | **938.4280** | **0.0251** | **0** | **9** | **18** | **3** | **K.NMGLYGER.V** 3771 |
|  | 4814 | 506.7482 | 1011.4819 | 1011.4882 | -0.0063 | 0 | 39 | 0.021 | 1 | R.VGAFTMVCK.D |
|  | 5406 | **529.7995** | **1057.5844** | **1057.5920** | **-0.0076** | **1** | **10** | **19** | **1** | **R.KQWLQEVK.G** |
|  | 8789 | **636.3046** | **1270.5947** | **1270.6881** | **-0.0934** | **1** | **3** | **71** | **4** | **K.AEAQIAAKNLDK.E** |
|  | 13324 | 766.4080 | 1530.8015 | 1529.7937 | 1.0078 | 0 | 3 | 57 | 4 | K.ASAELALGENSEVLK.S |
|  | 15058 | 823.4038 | 1644.7931 | 1645.7294 | -0.9363 | 0 | 2 | 80 | 10 | K.TCGFDFTGAVEDISK.I |
|  | 22846 | **774.5551** | **2320.6433** | **2321.2856** | **-0.6423** | **1** | **1** | **44** | **2** | **R.FVTVQTISGTGALRIGASFLQR.F** |
|  | 25492 | **909.7705** | **2726.2897** | **2725.4962** | **0.7935** | **1** | **3** | **31** | **1** | **-.MALLHSGRVLPGIAAAFHPGLAAAASAR.A** |
|  | 26999 | 996.8079 | 2987.4019 | 2986.3888 | 1.0132 | 1 | 3 | 28 | 5 | R.HFIEQGINVCLCQSYAKNMGLYGER.V |
|  | 27755 | **1054.1610** | **3159.4612** | **3159.5713** | **-0.1101** | **1** | **4** | **19** | **4** | **R.DVFLPKPTWGNHTPIFRDAGMQLQGYR.Y** |
|  | 31122 | 787.3839 | 4718.2598 | 4719.2850 | -1.0252 | 1 | 2 | 10 | 3 | K.TCGFDFTGAVEDISKIPEQSVLLLHACAHNPTGVDPRPEQWK.E |

| **799.** | Q15293\|RCN1_HUMAN    **Mass:** 38866    **Score:** 41     **Queries matched:** 4   **emPAI:** 0.20 |
| --- | --- |
|  | Reticulocalbin-1 OS=Homo sapiens GN=RCN1 PE=1 SV=1 |

|  | **Query** | **Observed** | **Mr(expt)** | **Mr(calc)** | **Delta** | **Miss** | **Score** | **Expect** | **Rank** | **Peptide** |
| --- | --- | --- | --- | --- | --- | --- | --- | --- | --- | --- |
|  | 8978 | **641.3572** | **1280.6998** | **1279.5932** | **1.1066** | **0** | **16** | **3.5** | **1** | **K.TFDQLTPDESK.E** |
|  | 13838 | 783.3696 | 1564.7247 | 1564.7369 | -0.0122 | 1 | 34 | 0.05 | 1 | K.TFDQLTPDESKER.L |
|  | 19232 | 650.6420 | 1948.9041 | 1948.9180 | -0.0139 | 0 | 31 | 0.077 | 1 | R.HWILPQDYDHAQAEAR.H |
|  | 27648 | **1044.1669** | **3129.4788** | **3129.4964** | **-0.0177** | **1** | **4** | **17** | **1** | **K.LTKEEILENWNMFVGSQATNYGEDLTK.N** |

| **800.** | Q96LZ7\|RMD2_HUMAN    **Mass:** 47711    **Score:** 41     **Queries matched:** 7   **emPAI:** 0.08 |
| --- | --- |
|  | Regulator of microtubule dynamics protein 2 OS=Homo sapiens GN=FAM82A1 PE=1 SV=2 |

|  | **Query** | **Observed** | **Mr(expt)** | **Mr(calc)** | **Delta** | **Miss** | **Score** | **Expect** | **Rank** | **Peptide** |
| --- | --- | --- | --- | --- | --- | --- | --- | --- | --- | --- |
|  | 1993 | **402.2431** | **802.4716** | **801.4133** | **1.0583** | **0** | **14** | **8.5** | **3** | **K.HYANIGK.T** |
|  | 2431 | **419.2373** | **836.4601** | **837.4418** | **-0.9817** | **0** | **6** | **44** | **8** | **K.MAATLFGK.I** |
|  | 2577 | 425.2257 | 848.4369 | 847.4949 | 0.9420 | 1 | (9) | 25 | 3 | K.IMTSLKR.- |
|  | 2761 | 432.2411 | 862.4676 | 863.4899 | -1.0222 | 1 | 10 | 19 | 3 | K.IMTSLKR.- 2760 |
|  | 11053 | **697.3408** | **1392.6671** | **1392.6772** | **-0.0102** | **0** | **41** | **0.01** | **1** | **K.LEEYIQDELGGK.I** |
|  | 12817 | 750.3912 | 1498.7678 | 1498.7027 | 0.0651 | 1 | 4 | 51 | 4 | R.DEIEFMWRFAR.A |

| **801.** | P42126\|D3D2_HUMAN    **Mass:** 33080    **Score:** 41     **Queries matched:** 3   **emPAI:** 0.11 |
| --- | --- |
|  | 3,2-trans-enoyl-CoA isomerase, mitochondrial OS=Homo sapiens GN=DCI PE=1 SV=1 |

|  | **Query** | **Observed** | **Mr(expt)** | **Mr(calc)** | **Delta** | **Miss** | **Score** | **Expect** | **Rank** | **Peptide** |
| --- | --- | --- | --- | --- | --- | --- | --- | --- | --- | --- |
|  | 6171 | 559.2921 | 1116.5697 | 1116.5272 | 0.0425 | 1 | 7 | 32 | 6 | R.TERAAGGGDGAR.R |
|  | 12364 | **491.2727** | **1470.7963** | **1470.7752** | **0.0211** | **0** | **5** | **39** | **5** | **R.VLVEPDAGAGVAVMK.F** |
|  | 24514 | **851.0877** | **2550.2413** | **2550.2571** | **-0.0158** | **0** | **41** | **0.0051** | **1** | **R.GVILTSDRPGVFSAGLDLTEMCGR.S** |

| **802.** | Q15907\|RB11B_HUMAN    **Mass:** 24588    **Score:** 41     **Queries matched:** 8   **emPAI:** 0.15 |
| --- | --- |
|  | Ras-related protein Rab-11B OS=Homo sapiens GN=RAB11B PE=1 SV=4 |

|  | **Query** | **Observed** | **Mr(expt)** | **Mr(calc)** | **Delta** | **Miss** | **Score** | **Expect** | **Rank** | **Peptide** |
| --- | --- | --- | --- | --- | --- | --- | --- | --- | --- | --- |
|  | 5240 | 522.8067 | 1043.5988 | 1042.6023 | 0.9966 | 0 | 23 | 0.84 | 1 | K.VVLIGDSGVGK.S |
|  | 5834 | 546.7844 | 1091.5543 | 1092.6040 | -1.0497 | 1 | 10 | 17 | 4 | K.SNLLSRFTR.N |
|  | 6705 | 579.8075 | 1157.6004 | 1156.6564 | 0.9440 | 1 | 11 | 12 | 8 | R.IVSQKQIADR.A |
|  | 8833 | **637.8056** | **1273.5966** | **1273.6051** | **-0.0085** | **0** | **41** | **0.012** | **1** | **K.AQIWDTAGQER.Y** |
|  | 15015 | 548.2626 | 1641.7659 | 1640.8192 | 0.9467 | 0 | 5 | 38 | 6 | R.DHADSNIVIMLVGNK.S |
|  | 17460 | 603.6776 | 1808.0108 | 1806.9476 | 1.0632 | 1 | 11 | 8 | 1 | K.STIGVEFATRSIQVDGK.T |
|  | 20094 | 675.0039 | 2021.9897 | 2023.0520 | -1.0623 | 1 | 1 | 71 | 4 | K.ELRDHADSNIVIMLVGNK.S |
|  | 28471 | **1124.5679** | **3370.6818** | **3369.6549** | **1.0269** | **1** | **2** | **29** | **1** | **K.QIADRAAHDESPGNNVVDISVPPTTDGQKPNK.L** |

|  | **Proteins matching a subset of these peptides:** |
| --- | --- |

|  | P62491\|RB11A_HUMAN    **Mass:** 24492    **Score:** 41     **Queries matched:** 6 |
| --- | --- |
|  | Ras-related protein Rab-11A OS=Homo sapiens GN=RAB11A PE=1 SV=3 |

| **803.** | Q9BXJ8\|T120A_HUMAN    **Mass:** 41040    **Score:** 41     **Queries matched:** 4   **emPAI:** 0.09 |
| --- | --- |
|  | Transmembrane protein 120A OS=Homo sapiens GN=TMEM120A PE=2 SV=1 |

|  | **Query** | **Observed** | **Mr(expt)** | **Mr(calc)** | **Delta** | **Miss** | **Score** | **Expect** | **Rank** | **Peptide** |
| --- | --- | --- | --- | --- | --- | --- | --- | --- | --- | --- |
|  | 9190 | 647.3101 | 1292.6057 | 1292.6143 | -0.0086 | 0 | 41 | 0.013 | 1 | K.LQNNCTSSITR.Q 9188 9192 9193 |

| **804.** | P00533\|EGFR_HUMAN    **Mass:** 137612   **Score:** 41     **Queries matched:** 25   **emPAI:** 0.03 |
| --- | --- |
|  | Epidermal growth factor receptor OS=Homo sapiens GN=EGFR PE=1 SV=2 |

|  | **Query** | **Observed** | **Mr(expt)** | **Mr(calc)** | **Delta** | **Miss** | **Score** | **Expect** | **Rank** | **Peptide** |
| --- | --- | --- | --- | --- | --- | --- | --- | --- | --- | --- |
|  | 2779 | 432.7479 | 863.4812 | 863.4753 | 0.0060 | 0 | 9 | 24 | 8 | K.ITDFGLAK.L |
|  | 3186 | **447.9027** | **893.7908** | **893.4243** | **0.3665** | **0** | **6** | **29** | **6** | **K.EDSFLQR.Y** 3188 |
|  | 3879 | **946.3978** | **945.3906** | **944.4927** | **0.8979** | **1** | **2** | **1.3e+002** | **8** | **R.EATSPKANK.E** |
|  | 4317 | **489.2712** | **976.5278** | **977.5433** | **-1.0155** | **0** | **2** | **1.1e+002** | **8** | **R.ELIIEFSK.M** |
|  | 5071 | 516.3112 | 1030.6079 | 1029.5454 | 1.0624 | 1 | 7 | 37 | 7 | K.ELREATSPK.A 5069 |
|  | 5161 | 519.7969 | 1037.5793 | 1037.6597 | -0.0804 | 1 | 9 | 21 | 2 | K.IPVAIKELR.E |
|  | 5162 | **519.8286** | **1037.6427** | **1037.4270** | **0.2157** | **0** | **0** | **1.5e+002** | **7** | **R.ESDCLVCR.K** |
|  | 5842 | 547.2637 | 1092.5128 | 1091.5611 | 0.9517 | 0 | 5 | 47 | 4 | R.YLVIQGDER.M |
|  | 10020 | 668.3384 | 1334.6622 | 1335.7220 | -1.0598 | 1 | 7 | 31 | 9 | R.ELIIEFSKMAR.D |
|  | 10334 | **451.7828** | **1352.3267** | **1351.7169** | **0.6098** | **1** | **(5)** | **30** | **5** | **R.ELIIEFSKMAR.D** |
|  | 10782 | **460.3098** | **1377.9074** | **1377.6169** | **0.2905** | **0** | **6** | **31** | **6** | **K.CWMIDADSRPK.F** |
|  | 11811 | 719.8697 | 1437.7249 | 1438.8184 | -1.0935 | 1 | 2 | 88 | 10 | K.IKVLGSGAFGTVYK.G |
|  | 14176 | 530.2860 | 1587.8362 | 1587.8005 | 0.0357 | 1 | 8 | 19 | 6 | R.DPQRYLVIQGDER.M |
|  | 14182 | **530.3457** | **1588.0153** | **1586.8416** | **1.1736** | **1** | **4** | **42** | **5** | **K.DSLSINATNIKHFK.N** |
|  | 14802 | 815.8580 | 1629.7015 | 1629.7998 | -0.0983 | 1 | 1 | 86 | 6 | K.LLGAEEKEYHAEGGK.V |
|  | 15777 | 847.4429 | 1692.8713 | 1691.9545 | 0.9168 | 1 | 3 | 54 | 6 | K.VPIKWMALESILHR.I |
|  | 15973 | **568.7806** | **1703.3201** | **1703.6403** | **-0.3202** | **0** | **1** | **60** | **7** | **R.ACGADSYEMEEDGVR.K** |
|  | 17749 | 916.9562 | 1831.8979 | 1831.7353 | 0.1627 | 1 | 9 | 13 | 3 | R.ACGADSYEMEEDGVRK.C |
|  | 22011 | **740.6263** | **2218.8570** | **2218.8725** | **-0.0155** | **1** | **41** | **0.0059** | **1** | **R.GKSPSDCCHNQCAAGCTGPR.E** |
|  | 22650 | 766.3926 | 2296.1559 | 2296.0971 | 0.0588 | 1 | 4 | 32 | 2 | K.GSTAENAEYLRVAPQSSEFIGA.- |
|  | 23145 | **787.6379** | **2359.8920** | **2359.2102** | **0.6818** | **1** | **1** | **45** | **1** | **K.GERLPQPPICTIDVYMIMVK.C** |
|  | 28513 | 1129.8702 | 3386.5889 | 3387.4767 | -0.8879 | 1 | 7 | 8.1 | 2 | R.DEATCKDTCPPLMLYNPTTYQMDVNPEGK.Y 28514 |

| **805.** | Q9UFP1\|CC041_HUMAN    **Mass:** 64264    **Score:** 41     **Queries matched:** 11   **emPAI:** 0.06 |
| --- | --- |
|  | Uncharacterized protein C3orf41 OS=Homo sapiens GN=C3orf41 PE=2 SV=2 |

|  | **Query** | **Observed** | **Mr(expt)** | **Mr(calc)** | **Delta** | **Miss** | **Score** | **Expect** | **Rank** | **Peptide** |
| --- | --- | --- | --- | --- | --- | --- | --- | --- | --- | --- |
|  | 1101 | 557.3727 | 556.3654 | 556.3697 | -0.0043 | 0 | 11 | 11 | 8 | R.VLGLR.R |
|  | 2864 | 435.2323 | 868.4500 | 868.5243 | -0.0742 | 1 | 41 | 0.012 | 1 | R.SLPAVARR.F |
|  | 2871 | **435.2670** | **868.5194** | **868.5243** | **-0.0049** | **1** | **11** | **12** | **1** | **R.RSLPAVAR.R** 2880 |
|  | 5332 | **527.2499** | **1052.4852** | **1052.4974** | **-0.0122** | **1** | **8** | **24** | **10** | **R.NMGFWRSR.A** |
|  | 5550 | **535.7869** | **1069.5592** | **1070.5972** | **-1.0380** | **0** | **8** | **28** | **2** | **R.LLAQGEVVDK.A** |
|  | 10375 | 678.3413 | 1354.6681 | 1355.6470 | -0.9789 | 1 | 1 | 1.1e+002 | 5 | K.HNLTLFRDEDP.- |
|  | 11951 | **483.2195** | **1446.6366** | **1445.7734** | **0.8632** | **0** | **12** | **9.1** | **1** | **K.VLASGCLQNMLLK.S** 11933 |
|  | 12207 | 731.8946 | 1461.7747 | 1461.7683 | 0.0064 | 0 | (9) | 18 | 4 | K.VLASGCLQNMLLK.S |
|  | 20722 | 693.0439 | 2076.1098 | 2076.9939 | -0.8841 | 0 | 2 | 62 | 8 | K.SLQMDPVFWESQGGAQGLK.Q |

| **806.** | P56385\|ATP5I_HUMAN    **Mass:** 7928     **Score:** 41     **Queries matched:** 7   **emPAI:** 0.52 |
| --- | --- |
|  | ATP synthase subunit e, mitochondrial OS=Homo sapiens GN=ATP5I PE=1 SV=2 |

|  | **Query** | **Observed** | **Mr(expt)** | **Mr(calc)** | **Delta** | **Miss** | **Score** | **Expect** | **Rank** | **Peptide** |
| --- | --- | --- | --- | --- | --- | --- | --- | --- | --- | --- |
|  | 3983 | **477.2606** | **952.5066** | **952.5130** | **-0.0064** | **0** | **22** | **1** | **3** | **R.YNYLKPR.A** |
|  | 6934 | 588.8674 | 1175.7202 | 1175.7278 | -0.0076 | 0 | 41 | 0.014 | 1 | M.VPPVQVSPLIK.L |
|  | 9759 | 661.8194 | 1321.6242 | 1322.7632 | -1.1390 | 0 | 8 | 20 | 4 | -.MVPPVQVSPLIK.L |
|  | 12658 | 744.8912 | 1487.7678 | 1487.7773 | -0.0095 | 0 | 26 | 0.31 | 2 | R.YSALFLGVAYGATR.Y 12675 12677 |
|  | 12862 | 501.3359 | 1500.9860 | 1501.9344 | -0.9484 | 1 | 4 | 45 | 5 | M.VPPVQVSPLIKLGR.Y |

| **807.** | Q9Y2B0\|CNPY2_HUMAN    **Mass:** 20981    **Score:** 40     **Queries matched:** 3   **emPAI:** 0.18 |
| --- | --- |
|  | Protein canopy homolog 2 OS=Homo sapiens GN=CNPY2 PE=1 SV=1 |

|  | **Query** | **Observed** | **Mr(expt)** | **Mr(calc)** | **Delta** | **Miss** | **Score** | **Expect** | **Rank** | **Peptide** |
| --- | --- | --- | --- | --- | --- | --- | --- | --- | --- | --- |
|  | 4090 | **481.2429** | **960.4712** | **961.5457** | **-1.0745** | **1** | **10** | **19** | **2** | **K.NYVRVVGR.N** |
|  | 5292 | **525.1029** | **1048.1913** | **1047.5448** | **0.6465** | **0** | **0** | **1e+002** | **6** | **R.IDSDISGTLK.F** |
|  | 7463 | **602.2488** | **1202.4831** | **1202.4921** | **-0.0090** | **0** | **40** | **0.014** | **1** | **R.SQDLHCGACR.A** |

| **808.** | Q9Y6I9\|TX264_HUMAN    **Mass:** 34452    **Score:** 40     **Queries matched:** 4   **emPAI:** 0.11 |
| --- | --- |
|  | Testis-expressed sequence 264 protein OS=Homo sapiens GN=TEX264 PE=1 SV=1 |

|  | **Query** | **Observed** | **Mr(expt)** | **Mr(calc)** | **Delta** | **Miss** | **Score** | **Expect** | **Rank** | **Peptide** |
| --- | --- | --- | --- | --- | --- | --- | --- | --- | --- | --- |
|  | 9999 | **667.8269** | **1333.6392** | **1333.6474** | **-0.0081** | **0** | **40** | **0.013** | **1** | **R.ETSAATLSPGASSR.G** |
|  | 21625 | **1089.0514** | **2176.0882** | **2177.0034** | **-0.9152** | **1** | **1** | **75** | **1** | **R.SIAVYYDNPHMVPPDKCR.C** |
|  | 24318 | 629.8225 | 2515.2609 | 2516.1828 | -0.9219 | 1 | 0 | 62 | 7 | K.FHMGLYGETGRLFTESCSISPK.L |
|  | 28167 | **820.3492** | **3277.3679** | **3277.7652** | **-0.3974** | **1** | **1** | **28** | **5** | **K.VFSFPAPSHVVTATFPYTTILSIWLATRR.V** |

| **809.** | Q96GQ5\|CP058_HUMAN    **Mass:** 51441    **Score:** 40     **Queries matched:** 10   **emPAI:** 0.07 |
| --- | --- |
|  | UPF0420 protein C16orf58 OS=Homo sapiens GN=C16orf58 PE=1 SV=2 |

|  | **Query** | **Observed** | **Mr(expt)** | **Mr(calc)** | **Delta** | **Miss** | **Score** | **Expect** | **Rank** | **Peptide** |
| --- | --- | --- | --- | --- | --- | --- | --- | --- | --- | --- |
|  | 2430 | 419.2371 | 836.4596 | 835.3858 | 1.0738 | 0 | 8 | 26 | 8 | K.DSTGMLGR.I 2421 |
|  | 2642 | 428.2345 | 854.4544 | 854.5338 | -0.0793 | 1 | 12 | 8.7 | 4 | K.AGPKTILR.A |
|  | 4255 | **487.3201** | **972.6257** | **973.5597** | **-0.9340** | **1** | **10** | **20** | **3** | **K.KESWVVVK.E** |
|  | 6021 | 553.2699 | 1104.5252 | 1104.5346 | -0.0093 | 1 | 40 | 0.017 | 1 | R.RNNMADVSAK.D |
|  | 8363 | **624.3205** | **1246.6264** | **1247.6180** | **-0.9915** | **0** | **1** | **1.1e+002** | **10** | **R.ALVMETLNEGR.L** |
|  | 12863 | 501.3359 | 1500.9860 | 1500.8082 | 0.1778 | 1 | 2 | 69 | 8 | R.ALVMETLNEGRLR.L |
|  | 24156 | 831.4450 | 2491.3132 | 2490.2882 | 1.0250 | 0 | 1 | 58 | 3 | R.MEPLWTGFWPAPSLSLGVPLHR.L |
|  | 26159 | 712.3629 | 2845.4226 | 2844.4552 | 0.9674 | 1 | 4 | 20 | 3 | R.AATHGLMLGALQGDGPLPAELEELRNR.V 26154 |

| **810.** | Q9UHQ4\|BAP29_HUMAN    **Mass:** 28416    **Score:** 40     **Queries matched:** 4   **emPAI:** 0.13 |
| --- | --- |
|  | B-cell receptor-associated protein 29 OS=Homo sapiens GN=BCAP29 PE=1 SV=2 |

|  | **Query** | **Observed** | **Mr(expt)** | **Mr(calc)** | **Delta** | **Miss** | **Score** | **Expect** | **Rank** | **Peptide** |
| --- | --- | --- | --- | --- | --- | --- | --- | --- | --- | --- |
|  | 2592 | **425.7639** | **849.5132** | **848.4603** | **1.0529** | **1** | **13** | **8.1** | **2** | **R.KTSDALSK.A** |
|  | 8125 | **412.7686** | **1235.2840** | **1235.6761** | **-0.3921** | **1** | **5** | **28** | **6** | **R.LSKEYDQLLK.E** |
|  | 16505 | **579.9294** | **1736.7663** | **1736.7788** | **-0.0125** | **0** | **40** | **0.011** | **1** | **K.SSTSRPDAYEHTQMK.L** |
|  | 21562 | **543.5208** | **2170.0539** | **2169.0273** | **1.0266** | **1** | **1** | **64** | **4** | **K.SSTSRPDAYEHTQMKLFR.S** |

| **811.** | Q58FG1\|HS904_HUMAN    **Mass:** 47796    **Score:** 40     **Queries matched:** 12   **emPAI:** 0.08 |
| --- | --- |
|  | Putative heat shock protein HSP 90-alpha A4 OS=Homo sapiens GN=HSP90AA4P PE=5 SV=1 |

|  | **Query** | **Observed** | **Mr(expt)** | **Mr(calc)** | **Delta** | **Miss** | **Score** | **Expect** | **Rank** | **Peptide** |
| --- | --- | --- | --- | --- | --- | --- | --- | --- | --- | --- |
|  | 4757 | 504.2680 | 1006.5215 | 1006.4641 | 0.0574 | 0 | 3 | 83 | 5 | -.MESLTDPSK.L |
|  | 6561 | 574.7923 | 1147.5700 | 1146.6397 | 0.9304 | 0 | 12 | 9.3 | 2 | K.EPHISLIPNK.Q |
|  | 9043 | **643.3198** | **1284.6251** | **1285.5972** | **-0.9721** | **1** | **(2)** | **84** | **6** | **R.DNSTTGYMAAKK.H** 9087 |
|  | 9360 | 434.7288 | 1301.1645 | 1301.5921 | -0.4277 | 1 | 12 | 6.2 | 1 | R.DNSTTGYMAAKK.H 9366 9400 |
|  | 10267 | 675.3663 | 1348.7181 | 1348.7272 | -0.0091 | 0 | 40 | 0.015 | 1 | R.TLTIVDTGIGMTK.A |
|  | 12955 | **754.8429** | **1507.6712** | **1506.7236** | **0.9477** | **1** | **6** | **34** | **9** | **-.MESLTDPSKLDSGK.E** |
|  | 14194 | 795.4045 | 1588.7945 | 1587.8216 | 0.9729 | 1 | 3 | 64 | 5 | R.ETKDQVANSTIVQR.L |
|  | 24924 | 873.7646 | 2618.2719 | 2618.4201 | -0.1481 | 1 | 6 | 17 | 2 | R.TLTIVDTGIGMTKADLINNLGTITK.S |
|  | 30078 | 797.9847 | 3984.8870 | 3985.0257 | -0.1387 | 1 | 5 | 8.7 | 1 | K.VFMEVLQAGADISMIGQFSVGFYSAYSVAEKVTVITK.H |

| **812.** | P61769\|B2MG_HUMAN    **Mass:** 13820    **Score:** 40     **Queries matched:** 4   **emPAI:** 1.11 |
| --- | --- |
|  | Beta-2-microglobulin OS=Homo sapiens GN=B2M PE=1 SV=1 |

|  | **Query** | **Observed** | **Mr(expt)** | **Mr(calc)** | **Delta** | **Miss** | **Score** | **Expect** | **Rank** | **Peptide** |
| --- | --- | --- | --- | --- | --- | --- | --- | --- | --- | --- |
|  | 6235 | 561.8127 | 1121.6109 | 1121.6193 | -0.0084 | 0 | 24 | 0.64 | 1 | R.VNHVTLSQPK.I |
|  | 6557 | **574.7788** | **1147.5431** | **1147.5509** | **-0.0079** | **0** | **37** | **0.031** | **1** | **K.VEHSDLSFSK.D** |
|  | 24569 | **852.0759** | **2553.2058** | **2553.2210** | **-0.0152** | **0** | **27** | **0.14** | **1** | **K.SNFLNCYVSGFHPSDIEVDLLK.N** 24570 |

| **813.** | O75665\|OFD1_HUMAN    **Mass:** 117055   **Score:** 40     **Queries matched:** 31   **emPAI:** 0.03 |
| --- | --- |
|  | Oral-facial-digital syndrome 1 protein OS=Homo sapiens GN=OFD1 PE=1 SV=1 |

|  | **Query** | **Observed** | **Mr(expt)** | **Mr(calc)** | **Delta** | **Miss** | **Score** | **Expect** | **Rank** | **Peptide** |
| --- | --- | --- | --- | --- | --- | --- | --- | --- | --- | --- |
|  | 1138 | **564.9678** | **563.9605** | **563.2663** | **0.6942** | **0** | **6** | **37** | **2** | **R.GGTSSR.R** 1141 1142 |
|  | 1839 | **396.8853** | **791.7561** | **791.4025** | **0.3536** | **0** | **6** | **36** | **9** | **K.SLVSGSDK.E** |
|  | 2670 | 429.2551 | 856.4956 | 856.5382 | -0.0425 | 1 | 40 | 0.02 | 1 | K.LKNELLK.Y |
|  | 2801 | 433.2216 | 864.4286 | 864.4262 | 0.0023 | 0 | 34 | 0.066 | 3 | K.MIEESLK.I |
|  | 2934 | 437.7396 | 873.4647 | 872.4715 | 0.9932 | 1 | 20 | 2.1 | 2 | R.EAELKQR.V |
|  | 4501 | 496.2788 | 990.5430 | 989.4778 | 1.0652 | 0 | 6 | 48 | 8 | K.NITSSSPER.H |
|  | 5793 | 545.2298 | 1088.4450 | 1089.6103 | -1.1653 | 1 | 5 | 59 | 6 | K.MIEESLKIK.I |
|  | 6323 | 565.7501 | 1129.4857 | 1129.5437 | -0.0581 | 0 | 13 | 9 | 2 | R.MPLPSPTESR.H 6339 |
|  | 6930 | 588.7966 | 1175.5786 | 1176.6866 | -1.1080 | 1 | 4 | 58 | 6 | K.AQILGYKASVK.S |
|  | 7139 | 594.8235 | 1187.6325 | 1187.6285 | 0.0041 | 0 | 11 | 12 | 5 | K.ELELELESVK.A |
|  | 7715 | 406.0262 | 1215.0568 | 1215.6207 | -0.5639 | 1 | 7 | 23 | 2 | K.KEELNQSVNR.V 7733 7736 7751 |
|  | 8012 | 615.8220 | 1229.6295 | 1228.6411 | 0.9884 | 1 | 0 | 1.5e+002 | 5 | R.VKELQQEAER.L |
|  | 8996 | 641.9227 | 1281.8308 | 1281.8020 | 0.0288 | 1 | 3 | 57 | 6 | K.LELLAQNKLLK.Q |
|  | 9549 | **656.8029** | **1311.5912** | **1311.6744** | **-0.0832** | **1** | **5** | **42** | **4** | **K.VKEMSDYSLLK.E** |
|  | 10992 | **695.3900** | **1388.7653** | **1388.7194** | **0.0459** | **1** | **0** | **1.4e+002** | **5** | **K.QENVLARMVASR.I** |
|  | 10993 | 695.3911 | 1388.7677 | 1388.6896 | 0.0781 | 0 | 2 | 97 | 5 | R.NDVVEALTGSAASR.L |
|  | 11421 | 472.2844 | 1413.8314 | 1414.7919 | -0.9604 | 1 | 6 | 32 | 2 | R.VKELELELESVK.A |
|  | 11976 | **483.7348** | **1448.1825** | **1447.8109** | **0.3716** | **0** | **2** | **53** | **6** | **K.VFTMQDLLQLIK.I** |
|  | 13820 | 782.8967 | 1563.7788 | 1563.8620 | -0.0832 | 0 | 6 | 28 | 3 | K.AVHLQEELIAINSK.K |
|  | 16942 | **590.4245** | **1768.2517** | **1767.8163** | **0.4354** | **1** | **2** | **49** | **5** | **R.QTELQDKSEFSDVDK.L** |
|  | 21434 | 719.3697 | 2155.0872 | 2154.0085 | 1.0787 | 0 | 0 | 78 | 9 | M.MAQSNMFTVADVLSQDELR.K |
|  | 22074 | **742.3752** | **2224.1037** | **2223.9875** | **0.1163** | **1** | **4** | **31** | **1** | **K.ELEMENELEMSNQEIKDK.S** |
|  | 22793 | **1158.5148** | **2315.0150** | **2314.0933** | **0.9217** | **1** | **0** | **69** | **3** | **M.MAQSNMFTVADVLSQDELRK.K** |
|  | 23761 | 816.4114 | 2446.2125 | 2445.1338 | 1.0787 | 1 | 5 | 24 | 2 | -.MMAQSNMFTVADVLSQDELRK.K |
|  | 29246 | **922.2486** | **3684.9653** | **3684.8318** | **0.1335** | **1** | **3** | **16** | **1** | **R.SVNGLINGNVVPCNGEISGDFLNNPFKQENVLAR.M** |

| **814.** | P22413\|ENPP1_HUMAN    **Mass:** 107024   **Score:** 40     **Queries matched:** 29   **emPAI:** 0.03 |
| --- | --- |
|  | Ectonucleotide pyrophosphatase/phosphodiesterase family member 1 OS=Homo sapiens GN=ENPP1 PE=1 SV=2 |

|  | **Query** | **Observed** | **Mr(expt)** | **Mr(calc)** | **Delta** | **Miss** | **Score** | **Expect** | **Rank** | **Peptide** |
| --- | --- | --- | --- | --- | --- | --- | --- | --- | --- | --- |
|  | 2404 | 418.2345 | 834.4545 | 835.3243 | -0.8698 | 0 | 23 | 0.9 | 2 | R.DGCAGGGSR.G 2420 |
|  | 2555 | 424.2193 | 846.4241 | 845.4759 | 0.9482 | 0 | 10 | 21 | 5 | K.VIYGPAAR.L 2527 |
|  | 3218 | **449.2449** | **896.4753** | **896.4426** | **0.0327** | **0** | **10** | **14** | **9** | **K.MNASFSLK.S** |
|  | 5031 | 515.2455 | 1028.4765 | 1027.4795 | 0.9970 | 1 | 9 | 21 | 1 | R.EGPAGNGRDR.G |
|  | 7212 | 397.8529 | 1190.5369 | 1191.6400 | -1.1031 | 1 | 13 | 8.2 | 2 | R.YFHDTLLRK.Y 7225 |
|  | 7411 | **401.2119** | **1200.6137** | **1200.6979** | **-0.0841** | **1** | **3** | **74** | **9** | **K.NIKVIYGPAAR.L** |
|  | 10161 | 448.9229 | 1343.7469 | 1343.5816 | 0.1653 | 0 | 3 | 67 | 6 | K.MYNGSVPFEER.I |
|  | 10882 | **692.3929** | **1382.7713** | **1381.6302** | **1.1411** | **0** | **6** | **39** | **2** | **K.YYSFNYEGIAR.N** |
|  | 11125 | 466.5610 | 1396.6611 | 1396.6401 | 0.0210 | 0 | (2) | 96 | 9 | R.VDGMVGMLMDGLK.E |
|  | 11126 | 699.3379 | 1396.6612 | 1396.6401 | 0.0212 | 0 | 7 | 27 | 5 | R.VDGMVGMLMDGLK.E |
|  | 11456 | **708.8787** | **1415.7429** | **1414.7092** | **1.0336** | **1** | **0** | **1.3e+002** | **9** | **K.LKTHLPTFSQED.-** |
|  | 13544 | **773.8652** | **1545.7158** | **1546.7160** | **-1.0002** | **1** | **(0)** | **1.1e+002** | **5** | **K.MYDPKMNASFSLK.S** |
|  | 13790 | 781.8561 | 1561.6977 | 1562.7109 | -1.0131 | 1 | 4 | 48 | 4 | K.MYDPKMNASFSLK.S |
|  | 16940 | **590.3450** | **1768.0131** | **1766.8410** | **1.1721** | **0** | **1** | **82** | **7** | **K.HDSSWVEELLMLHR.A** |
|  | 17726 | **610.9774** | **1829.9104** | **1830.8757** | **-0.9652** | **1** | **8** | **15** | **1** | **K.CGTYTKNMRPVYPTK.T** |
|  | 18210 | **622.3512** | **1864.0318** | **1864.9209** | **-0.8891** | **1** | **7** | **21** | **2** | **K.ALQRVDGMVGMLMDGLK.E** 18209 |
|  | 18214 | **622.5424** | **1864.6054** | **1864.9209** | **-0.3155** | **1** | **(1)** | **63** | **6** | **K.ALQRVDGMVGMLMDGLK.E** 18205 |
|  | 21460 | **1079.9877** | **2157.9608** | **2159.1449** | **-1.1841** | **0** | **3** | **48** | **3** | **R.NQEILIPTHFFIVLTSCK.D** |
|  | 24134 | 830.7574 | 2489.2503 | 2488.1951 | 1.0552 | 0 | 0 | 66 | 3 | K.SGTFFWPGSDVEINGIFPDIYK.M |
|  | 26462 | 1450.6792 | 2899.3438 | 2900.3862 | -1.0423 | 0 | 2 | 39 | 4 | R.SHAAEAPGDPQAAASLLAPMDVGEEPLEK.A |
|  | 26953 | 746.1216 | 2980.4575 | 2981.3396 | -0.8821 | 1 | 2 | 37 | 10 | R.TDNSESCVHGKHDSSWVEELLMLHR.A |
|  | 27664 | 1567.2902 | 3132.5658 | 3132.5590 | 0.0068 | 0 | 6 | 13 | 2 | K.NSSGIYSEALLTTNIVPMYQSFQVIWR.Y 27663 |
|  | 29628 | **1270.5776** | **3808.7111** | **3808.7389** | **-0.0278** | **0** | **40** | **0.0032** | **1** | **K.SWVEEPCESINEPQCPAGFETPPTLLFSLDGFR.A** |

| **815.** | P20648\|ATP4A_HUMAN    **Mass:** 115756   **Score:** 40     **Queries matched:** 18   **emPAI:** 0.03 |
| --- | --- |
|  | Potassium-transporting ATPase alpha chain 1 OS=Homo sapiens GN=ATP4A PE=2 SV=5 |

|  | **Query** | **Observed** | **Mr(expt)** | **Mr(calc)** | **Delta** | **Miss** | **Score** | **Expect** | **Rank** | **Peptide** |
| --- | --- | --- | --- | --- | --- | --- | --- | --- | --- | --- |
|  | 2388 | **417.2572** | **832.4999** | **832.4337** | **0.0662** | **1** | **15** | **7.3** | **10** | **K.CRTAGIR.V** 2389 |
|  | 3145 | **446.1880** | **890.3615** | **890.4531** | **-0.0916** | **1** | **12** | **11** | **2** | **K.EKLENMK.K** |
|  | 5992 | **552.2737** | **1102.5329** | **1102.5805** | **-0.0475** | **0** | **8** | **26** | **4** | **K.LVIVESCQR.L** 6009 |
|  | 6248 | 562.7517 | 1123.4889 | 1123.6172 | -0.1283 | 1 | 7 | 32 | 10 | R.DPRHLLVMK.G |
|  | 7412 | **401.2126** | **1200.6160** | **1199.6087** | **1.0073** | **0** | **9** | **19** | **5** | **R.LSAFQQGFFR.N** |
|  | 8434 | **417.8882** | **1250.6428** | **1249.6965** | **0.9463** | **1** | **11** | **12** | **2** | **R.HLLVMKGAPER.V** |
|  | 9903 | 665.3302 | 1328.6458 | 1328.7551 | -0.1093 | 0 | 4 | 62 | 4 | R.IVIGDASETALLK.F |
|  | 10105 | 671.3416 | 1340.6687 | 1339.6666 | 1.0020 | 0 | 8 | 23 | 2 | K.AESDIMHLRPR.N |
|  | 11097 | 466.2328 | 1395.6766 | 1396.7384 | -1.0618 | 0 | 1 | 1.1e+002 | 9 | R.VIMVTGDHPITAK.A |
|  | 12082 | 728.8436 | 1455.6726 | 1454.7518 | 0.9208 | 0 | 10 | 13 | 3 | K.FQLSIHTLEDPR.D |
|  | 12583 | **742.8563** | **1483.6981** | **1484.8562** | **-1.1581** | **1** | **10** | **13** | **2** | **K.RIVIGDASETALLK.F** |
|  | 14645 | 810.3738 | 1618.7331 | 1618.7435 | -0.0103 | 0 | 40 | 0.012 | 1 | K.VDNSSLTGESEPQTR.S |
|  | 23056 | **588.3093** | **2349.2080** | **2349.1522** | **0.0557** | **1** | **8** | **12** | **2** | **R.DGDKFQINADQLVVGDLVEMK.G** |
|  | 24915 | 655.0780 | 2616.2829 | 2615.1883 | 1.0946 | 1 | 1 | 58 | 5 | -.MGKAENYELYSVELGPGPGGDMAAK.M |
|  | 28240 | **662.5501** | **3307.7142** | **3307.6758** | **0.0383** | **1** | **1** | **30** | **1** | **K.NAADMILLDDNFASIVTGVEQGRLIFDNLK.K** |
|  | 31806 | **844.1106** | **5901.7233** | **5902.1735** | **-0.4502** | **1** | **1** | **6.5** | **1** | **K.SIAYTLTKNIPELTPYLIYITVSVPLPLGCITILFIELCTDIFPSVSLAYEK.A** |

| **816.** | Q9BRL6\|SFR2B_HUMAN    **Mass:** 32382    **Score:** 40     **Queries matched:** 8   **emPAI:** 0.12 |
| --- | --- |
|  | Splicing factor, arginine/serine-rich 2B OS=Homo sapiens GN=SFRS2B PE=1 SV=1 |

|  | **Query** | **Observed** | **Mr(expt)** | **Mr(calc)** | **Delta** | **Miss** | **Score** | **Expect** | **Rank** | **Peptide** |
| --- | --- | --- | --- | --- | --- | --- | --- | --- | --- | --- |
|  | 1140 | **565.3046** | **564.2974** | **565.2609** | **-0.9635** | **0** | **5** | **56** | **2** | **R.GGGYGR.R** |
|  | 1217 | **586.9151** | **585.9078** | **585.2871** | **0.6208** | **0** | **14** | **5.1** | **1** | **R.QGEPR.G** |
|  | 1921 | 399.7502 | 797.4859 | 798.4096 | -0.9238 | 1 | 8 | 25 | 5 | R.QGEPRGR.S |
|  | 2433 | **419.2408** | **836.4671** | **836.4352** | **0.0319** | **1** | **11** | **13** | **7** | **R.SKSSSVSR.S** |
|  | 2818 | **433.7618** | **865.5089** | **864.4413** | **1.0676** | **1** | **10** | **17** | **4** | **K.SSSVSRSR.S** |
|  | 3515 | **459.7525** | **917.4905** | **917.4971** | **-0.0066** | **0** | **40** | **0.02** | **1** | **R.VGDVYIPR.E** |
|  | 18507 | **630.3295** | **1887.9668** | **1888.8845** | **-0.9178** | **0** | **0** | **98** | **9** | **-.MSCGRPPPDVDGMITLK.V** |
|  | 18537 | 473.5211 | 1890.0555 | 1889.8173 | 0.2382 | 1 | 10 | 9.6 | 3 | R.RDAQDAEAAMDGAELDGR.E |

| **817.** | Q01130\|SFRS2_HUMAN    **Mass:** 25461    **Score:** 40     **Queries matched:** 7   **emPAI:** 0.15 |
| --- | --- |
|  | Splicing factor, arginine/serine-rich 2 OS=Homo sapiens GN=SFRS2 PE=1 SV=4 |

|  | **Query** | **Observed** | **Mr(expt)** | **Mr(calc)** | **Delta** | **Miss** | **Score** | **Expect** | **Rank** | **Peptide** |
| --- | --- | --- | --- | --- | --- | --- | --- | --- | --- | --- |
|  | 2433 | 419.2408 | 836.4671 | 836.4352 | 0.0319 | 1 | 11 | 13 | 7 | K.SKSSSVSR.S |
|  | 2818 | 433.7618 | 865.5089 | 864.4413 | 1.0676 | 1 | 10 | 17 | 4 | K.SSSVSRSR.S |
|  | 3515 | 459.7525 | 917.4905 | 917.4971 | -0.0066 | 0 | 40 | 0.02 | 1 | R.VGDVYIPR.D |
|  | 3807 | **471.2901** | **940.5656** | **941.4468** | **-0.8811** | **1** | **1** | **1.2e+002** | **10** | **R.RYGGGGYGR.R** |
|  | 9511 | **437.2474** | **1308.7205** | **1307.6119** | **1.1086** | **0** | **8** | **21** | **7** | **R.YGRPPDSHHSR.R** |
|  | 17128 | 891.9698 | 1781.9250 | 1782.7036 | -0.7786 | 0 | 1 | 86 | 7 | R.DAEDAMDAMDGAVLDGR.E 17142 |

| **818.** | P05534\|1A24_HUMAN    **Mass:** 40948    **Score:** 40     **Queries matched:** 11   **emPAI:** 0.19 |
| --- | --- |
|  | HLA class I histocompatibility antigen, A-24 alpha chain OS=Homo sapiens GN=HLA-A PE=1 SV=2 |

|  | **Query** | **Observed** | **Mr(expt)** | **Mr(calc)** | **Delta** | **Miss** | **Score** | **Expect** | **Rank** | **Peptide** |
| --- | --- | --- | --- | --- | --- | --- | --- | --- | --- | --- |
|  | 1186 | 579.2696 | 578.2623 | 577.2456 | 1.0167 | 0 | 10 | 17 | 10 | R.NSSDR.K |
|  | 7459 | **601.8498** | **1201.6851** | **1200.5200** | **1.1652** | **0** | **12** | **9** | **1** | **R.GYHQYAYDGK.D** |
|  | 9800 | **442.2748** | **1323.8025** | **1323.6320** | **0.1705** | **0** | **6** | **34** | **9** | **K.WEAAHVAEQQR.A** |
|  | 10294 | 676.3427 | 1350.6709 | 1349.6939 | 0.9770 | 1 | 8 | 24 | 1 | R.YLENGKETLQR.T |
|  | 11047 | 465.0192 | 1392.0357 | 1392.6707 | -0.6350 | 0 | 3 | 48 | 9 | R.SWTAADMAAQITK.R |
|  | 11642 | 714.3621 | 1426.7097 | 1426.7205 | -0.0108 | 0 | 11 | 10 | 1 | K.WAAVVVPSGEEQR.Y |
|  | 13579 | 517.1576 | 1548.4509 | 1548.7718 | -0.3209 | 1 | 1 | 69 | 9 | R.SWTAADMAAQITKR.K |
|  | 14790 | 815.4116 | 1628.8086 | 1628.8199 | -0.0113 | 0 | 27 | 0.26 | 1 | R.FIAVGYVDDTQFVR.F |
|  | 24990 | 879.7289 | 2636.1650 | 2636.1838 | -0.0188 | 0 | 27 | 0.14 | 1 | R.DGEDQTQDTELVETRPAGDGTFQK.W 24989 |
|  | 27415 | **1023.5189** | **3067.5349** | **3066.6470** | **0.8879** | **1** | **1** | **36** | **3** | **M.AVMAPRTLVLLLSGALALTQTWAGSHSMR.Y** |

| **819.** | P30453\|1A34_HUMAN    **Mass:** 41314    **Score:** 40     **Queries matched:** 13   **emPAI:** 0.19 |
| --- | --- |
|  | HLA class I histocompatibility antigen, A-34 alpha chain OS=Homo sapiens GN=HLA-A PE=1 SV=1 |

|  | **Query** | **Observed** | **Mr(expt)** | **Mr(calc)** | **Delta** | **Miss** | **Score** | **Expect** | **Rank** | **Peptide** |
| --- | --- | --- | --- | --- | --- | --- | --- | --- | --- | --- |
|  | 2043 | 403.7328 | 805.4511 | 804.3986 | 1.0526 | 0 | 17 | 4.2 | 2 | -.MAIMAPR.T |
|  | 7831 | 611.2879 | 1220.5612 | 1220.6037 | -0.0424 | 0 | 2 | 89 | 3 | K.DYIALNEDLR.S |
|  | 10294 | 676.3427 | 1350.6709 | 1349.6939 | 0.9770 | 1 | 8 | 24 | 1 | R.YLENGKETLQR.T |
|  | 11883 | 722.3673 | 1442.7199 | 1441.7314 | 0.9886 | 0 | 8 | 22 | 3 | K.WASVVVPSGQEQR.Y 11865 |
|  | 12774 | 748.8586 | 1495.7026 | 1495.7129 | -0.0103 | 0 | 11 | 9.6 | 2 | R.AYLEGTCVEWLR.R |
|  | 13579 | 517.1576 | 1548.4509 | 1548.7355 | -0.2845 | 0 | 1 | 69 | 9 | R.SWTAADMAAQITQR.K |
|  | 14790 | 815.4116 | 1628.8086 | 1628.8199 | -0.0113 | 0 | 27 | 0.26 | 1 | R.FIAVGYVDDTQFVR.F |
|  | 15291 | **830.4475** | **1658.8805** | **1657.7341** | **1.1464** | **1** | **3** | **52** | **1** | **R.MYGCDVGPDGRFLR.G** |
|  | 15532 | **559.8101** | **1676.4084** | **1676.8304** | **-0.4221** | **1** | **1** | **69** | **8** | **R.SWTAADMAAQITQRK.W** |
|  | 24990 | 879.7289 | 2636.1650 | 2636.1838 | -0.0188 | 0 | 27 | 0.14 | 1 | R.DGEDQTQDTELVETRPAGDGTFQK.W 24989 |
|  | 27474 | 771.3614 | 3081.4167 | 3080.6627 | 0.7540 | 1 | 4 | 20 | 3 | M.AIMAPRTLVLLLSGALALTQTWAGSHSMR.Y |

| **820.** | P01892\|1A02_HUMAN    **Mass:** 41181    **Score:** 40     **Queries matched:** 13   **emPAI:** 0.19 |
| --- | --- |
|  | HLA class I histocompatibility antigen, A-2 alpha chain OS=Homo sapiens GN=HLA-A PE=1 SV=1 |

|  | **Query** | **Observed** | **Mr(expt)** | **Mr(calc)** | **Delta** | **Miss** | **Score** | **Expect** | **Rank** | **Peptide** |
| --- | --- | --- | --- | --- | --- | --- | --- | --- | --- | --- |
|  | 5462 | 532.2699 | 1062.5252 | 1062.5683 | -0.0430 | 1 | 3 | 89 | 7 | K.VKAHSQTHR.V |
|  | 7459 | 601.8498 | 1201.6851 | 1200.5200 | 1.1652 | 0 | 12 | 9 | 1 | R.GYHQYAYDGK.D |
|  | 9645 | 439.8373 | 1316.4902 | 1315.6673 | 0.8229 | 0 | 5 | 38 | 8 | R.YFFTSVSRPGR.G 9609 9648 |
|  | 10294 | 676.3427 | 1350.6709 | 1349.6939 | 0.9770 | 1 | 8 | 24 | 1 | R.YLENGKETLQR.T |
|  | 10828 | **691.3192** | **1380.6237** | **1380.6344** | **-0.0106** | **0** | **7** | **30** | **1** | **R.SWTAADMAAQTTK.H** |
|  | 12774 | 748.8586 | 1495.7026 | 1495.7129 | -0.0103 | 0 | 11 | 9.6 | 2 | R.AYLEGTCVEWLR.R |
|  | 14790 | 815.4116 | 1628.8086 | 1628.8199 | -0.0113 | 0 | 27 | 0.26 | 1 | R.FIAVGYVDDTQFVR.F |
|  | 15070 | **823.8478** | **1645.6810** | **1645.7882** | **-0.1072** | **1** | **3** | **63** | **1** | **R.SWTAADMAAQTTKHK.W** |
|  | 24990 | 879.7289 | 2636.1650 | 2636.1838 | -0.0188 | 0 | 27 | 0.14 | 1 | R.DGEDQTQDTELVETRPAGDGTFQK.W 24989 |
|  | 27415 | 1023.5189 | 3067.5349 | 3066.6470 | 0.8879 | 1 | 1 | 36 | 3 | M.AVMAPRTLVLLLSGALALTQTWAGSHSMR.Y |

|  | **Proteins matching a subset of these peptides:** |
| --- | --- |

|  | P10316\|1A69_HUMAN    **Mass:** 41236    **Score:** 40     **Queries matched:** 9 |
| --- | --- |
|  | HLA class I histocompatibility antigen, A-69 alpha chain OS=Homo sapiens GN=HLA-A PE=1 SV=2 |

|  | P01891\|1A68_HUMAN    **Mass:** 41168    **Score:** 40     **Queries matched:** 8 |
| --- | --- |
|  | HLA class I histocompatibility antigen, A-68 alpha chain OS=Homo sapiens GN=HLA-A PE=1 SV=4 |

| **821.** | P30443\|1A01_HUMAN    **Mass:** 41105    **Score:** 40     **Queries matched:** 14   **emPAI:** 0.19 |
| --- | --- |
|  | HLA class I histocompatibility antigen, A-1 alpha chain OS=Homo sapiens GN=HLA-A PE=1 SV=1 |

|  | **Query** | **Observed** | **Mr(expt)** | **Mr(calc)** | **Delta** | **Miss** | **Score** | **Expect** | **Rank** | **Peptide** |
| --- | --- | --- | --- | --- | --- | --- | --- | --- | --- | --- |
|  | 4178 | 484.7440 | 967.4734 | 967.4247 | 0.0487 | 0 | 11 | 12 | 7 | R.FDSDAASQK.M |
|  | 7831 | 611.2879 | 1220.5612 | 1220.6037 | -0.0424 | 0 | 2 | 89 | 3 | K.DYIALNEDLR.S |
|  | 9645 | 439.8373 | 1316.4902 | 1315.6673 | 0.8229 | 0 | 5 | 38 | 8 | R.YFFTSVSRPGR.G 9609 9648 |
|  | 10294 | 676.3427 | 1350.6709 | 1349.6939 | 0.9770 | 1 | 8 | 24 | 1 | R.YLENGKETLQR.T |
|  | 10313 | 451.2720 | 1350.7943 | 1351.6745 | -0.8802 | 1 | 11 | 10 | 1 | K.WEAVHAAEQRR.V 10331 |
|  | 11047 | 465.0192 | 1392.0357 | 1392.6707 | -0.6350 | 0 | 3 | 48 | 9 | R.SWTAADMAAQITK.R |
|  | 11642 | 714.3621 | 1426.7097 | 1426.7205 | -0.0108 | 0 | 11 | 10 | 1 | K.WAAVVVPSGEEQR.Y |
|  | 13579 | 517.1576 | 1548.4509 | 1548.7718 | -0.3209 | 1 | 1 | 69 | 9 | R.SWTAADMAAQITKR.K |
|  | 14790 | 815.4116 | 1628.8086 | 1628.8199 | -0.0113 | 0 | 27 | 0.26 | 1 | R.FIAVGYVDDTQFVR.F |
|  | 24990 | 879.7289 | 2636.1650 | 2636.1838 | -0.0188 | 0 | 27 | 0.14 | 1 | R.DGEDQTQDTELVETRPAGDGTFQK.W 24989 |

|  | **Proteins matching the same set of peptides:** |
| --- | --- |

|  | P30455\|1A36_HUMAN    **Mass:** 41193    **Score:** 40     **Queries matched:** 14 |
| --- | --- |
|  | HLA class I histocompatibility antigen, A-36 alpha chain OS=Homo sapiens GN=HLA-A PE=1 SV=1 |

|  | **Proteins matching a subset of these peptides:** |
| --- | --- |

|  | P04439\|1A03_HUMAN    **Mass:** 41100    **Score:** 40     **Queries matched:** 11 |
| --- | --- |
|  | HLA class I histocompatibility antigen, A-3 alpha chain OS=Homo sapiens GN=HLA-A PE=1 SV=2 |

|  | P13746\|1A11_HUMAN    **Mass:** 41196    **Score:** 40     **Queries matched:** 8 |
| --- | --- |
|  | HLA class I histocompatibility antigen, A-11 alpha chain OS=Homo sapiens GN=HLA-A PE=1 SV=1 |

| **822.** | P30447\|1A23_HUMAN    **Mass:** 40992    **Score:** 40     **Queries matched:** 11   **emPAI:** 0.19 |
| --- | --- |
|  | HLA class I histocompatibility antigen, A-23 alpha chain OS=Homo sapiens GN=HLA-A PE=1 SV=1 |

|  | **Query** | **Observed** | **Mr(expt)** | **Mr(calc)** | **Delta** | **Miss** | **Score** | **Expect** | **Rank** | **Peptide** |
| --- | --- | --- | --- | --- | --- | --- | --- | --- | --- | --- |
|  | 1186 | 579.2696 | 578.2623 | 577.2456 | 1.0167 | 0 | 10 | 17 | 10 | R.NSSDR.K |
|  | 7459 | 601.8498 | 1201.6851 | 1200.5200 | 1.1652 | 0 | 12 | 9 | 1 | R.GYHQYAYDGK.D |
|  | 10294 | 676.3427 | 1350.6709 | 1349.6939 | 0.9770 | 1 | 8 | 24 | 1 | R.YLENGKETLQR.T |
|  | 11642 | 714.3621 | 1426.7097 | 1426.7205 | -0.0108 | 0 | 11 | 10 | 1 | K.WAAVVVPSGEEQR.Y |
|  | 13579 | 517.1576 | 1548.4509 | 1548.7355 | -0.2845 | 0 | 1 | 69 | 9 | R.SWTAADMAAQITQR.K |
|  | 14790 | 815.4116 | 1628.8086 | 1628.8199 | -0.0113 | 0 | 27 | 0.26 | 1 | R.FIAVGYVDDTQFVR.F |
|  | 15532 | 559.8101 | 1676.4084 | 1676.8304 | -0.4221 | 1 | 1 | 69 | 8 | R.SWTAADMAAQITQRK.W |
|  | 20747 | **694.0385** | **2079.0935** | **2077.9851** | **1.1084** | **1** | **1** | **71** | **7** | **K.EDLRSWTAADMAAQITQR.K** |
|  | 24990 | 879.7289 | 2636.1650 | 2636.1838 | -0.0188 | 0 | 27 | 0.14 | 1 | R.DGEDQTQDTELVETRPAGDGTFQK.W 24989 |
|  | 27415 | 1023.5189 | 3067.5349 | 3066.6470 | 0.8879 | 1 | 1 | 36 | 3 | M.AVMAPRTLVLLLSGALALTQTWAGSHSMR.Y |

| **823.** | P10314\|1A32_HUMAN    **Mass:** 41307    **Score:** 40     **Queries matched:** 16   **emPAI:** 0.19 |
| --- | --- |
|  | HLA class I histocompatibility antigen, A-32 alpha chain OS=Homo sapiens GN=HLA-A PE=2 SV=2 |

|  | **Query** | **Observed** | **Mr(expt)** | **Mr(calc)** | **Delta** | **Miss** | **Score** | **Expect** | **Rank** | **Peptide** |
| --- | --- | --- | --- | --- | --- | --- | --- | --- | --- | --- |
|  | 7831 | 611.2879 | 1220.5612 | 1220.6037 | -0.0424 | 0 | 2 | 89 | 3 | K.DYIALNEDLR.S |
|  | 9321 | **650.8076** | **1299.6007** | **1298.6327** | **0.9680** | **1** | **2** | **86** | **2** | **K.AHSQTDRESLR.I** |
|  | 9645 | 439.8373 | 1316.4902 | 1315.6673 | 0.8229 | 0 | 5 | 38 | 8 | R.YFFTSVSRPGR.G 9609 9648 |
|  | 10294 | 676.3427 | 1350.6709 | 1349.6939 | 0.9770 | 1 | 8 | 24 | 1 | R.YLENGKETLQR.T |
|  | 11883 | 722.3673 | 1442.7199 | 1441.7314 | 0.9886 | 0 | 8 | 22 | 3 | K.WASVVVPSGQEQR.Y 11865 |
|  | 12774 | 748.8586 | 1495.7026 | 1495.7129 | -0.0103 | 0 | 11 | 9.6 | 2 | R.AYLEGTCVEWLR.R |
|  | 13579 | 517.1576 | 1548.4509 | 1548.7355 | -0.2845 | 0 | 1 | 69 | 9 | R.SWTAADMAAQITQR.K |
|  | 14790 | 815.4116 | 1628.8086 | 1628.8199 | -0.0113 | 0 | 27 | 0.26 | 1 | R.FIAVGYVDDTQFVR.F |
|  | 15532 | 559.8101 | 1676.4084 | 1676.8304 | -0.4221 | 1 | 1 | 69 | 8 | R.SWTAADMAAQITQRK.W |
|  | 23923 | **822.8395** | **2465.4968** | **2465.3828** | **0.1140** | **0** | **(0)** | **53** | **10** | **R.TLLLLLLGALALTQTWAGSHSMR.Y** |
|  | 24063 | **828.0273** | **2481.0602** | **2481.3777** | **-0.3175** | **0** | **8** | **11** | **1** | **R.TLLLLLLGALALTQTWAGSHSMR.Y** |
|  | 24990 | 879.7289 | 2636.1650 | 2636.1838 | -0.0188 | 0 | 27 | 0.14 | 1 | R.DGEDQTQDTELVETRPAGDGTFQK.W 24989 |

|  | **Proteins matching a subset of these peptides:** |
| --- | --- |

|  | P30512\|1A29_HUMAN    **Mass:** 41122    **Score:** 40     **Queries matched:** 12 |
| --- | --- |
|  | HLA class I histocompatibility antigen, A-29 alpha chain OS=Homo sapiens GN=HLA-A PE=2 SV=2 |

|  | P30459\|1A74_HUMAN    **Mass:** 41150    **Score:** 40     **Queries matched:** 13 |
| --- | --- |
|  | HLA class I histocompatibility antigen, A-74 alpha chain OS=Homo sapiens GN=HLA-A PE=1 SV=1 |

|  | P16190\|1A33_HUMAN    **Mass:** 41151    **Score:** 40     **Queries matched:** 11 |
| --- | --- |
|  | HLA class I histocompatibility antigen, A-33 alpha chain OS=Homo sapiens GN=HLA-A PE=1 SV=3 |

| **824.** | P16188\|1A30_HUMAN    **Mass:** 41164    **Score:** 40     **Queries matched:** 11   **emPAI:** 0.19 |
| --- | --- |
|  | HLA class I histocompatibility antigen, A-30 alpha chain OS=Homo sapiens GN=HLA-A PE=1 SV=2 |

|  | **Query** | **Observed** | **Mr(expt)** | **Mr(calc)** | **Delta** | **Miss** | **Score** | **Expect** | **Rank** | **Peptide** |
| --- | --- | --- | --- | --- | --- | --- | --- | --- | --- | --- |
|  | 7831 | 611.2879 | 1220.5612 | 1220.6037 | -0.0424 | 0 | 2 | 89 | 3 | K.DYIALNEDLR.S |
|  | 10294 | 676.3427 | 1350.6709 | 1349.6939 | 0.9770 | 1 | 8 | 24 | 1 | R.YLENGKETLQR.T |
|  | 11642 | 714.3621 | 1426.7097 | 1426.7205 | -0.0108 | 0 | 11 | 10 | 1 | K.WAAVVVPSGEEQR.Y |
|  | 12774 | 748.8586 | 1495.7026 | 1495.7129 | -0.0103 | 0 | 11 | 9.6 | 2 | R.AYLEGTCVEWLR.R |
|  | 13579 | 517.1576 | 1548.4509 | 1548.7355 | -0.2845 | 0 | 1 | 69 | 9 | R.SWTAADMAAQITQR.K |
|  | 14790 | 815.4116 | 1628.8086 | 1628.8199 | -0.0113 | 0 | 27 | 0.26 | 1 | R.FIAVGYVDDTQFVR.F |
|  | 15532 | 559.8101 | 1676.4084 | 1676.8304 | -0.4221 | 1 | 1 | 69 | 8 | R.SWTAADMAAQITQRK.W |
|  | 24697 | 1287.5995 | 2573.1844 | 2573.2299 | -0.0454 | 1 | 0 | 63 | 5 | R.APWIEQERPEYWDQETRNVK.A |
|  | 24990 | 879.7289 | 2636.1650 | 2636.1838 | -0.0188 | 0 | 27 | 0.14 | 1 | R.DGEDQTQDTELVETRPAGDGTFQK.W 24989 |
|  | 27912 | **1069.1811** | **3204.5216** | **3204.7086** | **-0.1870** | **1** | **3** | **25** | **1** | **-.MAVMAPRTLLLLLSGALALTHTWAGSHSMR.Y** |

| **825.** | P16189\|1A31_HUMAN    **Mass:** 41263    **Score:** 40     **Queries matched:** 13   **emPAI:** 0.19 |
| --- | --- |
|  | HLA class I histocompatibility antigen, A-31 alpha chain OS=Homo sapiens GN=HLA-A PE=2 SV=2 |

|  | **Query** | **Observed** | **Mr(expt)** | **Mr(calc)** | **Delta** | **Miss** | **Score** | **Expect** | **Rank** | **Peptide** |
| --- | --- | --- | --- | --- | --- | --- | --- | --- | --- | --- |
|  | 7831 | 611.2879 | 1220.5612 | 1220.6037 | -0.0424 | 0 | 2 | 89 | 3 | K.DYIALNEDLR.S |
|  | 10294 | 676.3427 | 1350.6709 | 1349.6939 | 0.9770 | 1 | 8 | 24 | 1 | R.YLENGKETLQR.T |
|  | 11883 | 722.3673 | 1442.7199 | 1441.7314 | 0.9886 | 0 | 8 | 22 | 3 | K.WASVVVPSGQEQR.Y 11865 |
|  | 12774 | 748.8586 | 1495.7026 | 1495.7129 | -0.0103 | 0 | 11 | 9.6 | 2 | R.AYLEGTCVEWLR.R |
|  | 13579 | 517.1576 | 1548.4509 | 1548.7355 | -0.2845 | 0 | 1 | 69 | 9 | R.SWTAADMAAQITQR.K |
|  | 14790 | 815.4116 | 1628.8086 | 1628.8199 | -0.0113 | 0 | 27 | 0.26 | 1 | R.FIAVGYVDDTQFVR.F |
|  | 15532 | 559.8101 | 1676.4084 | 1676.8304 | -0.4221 | 1 | 1 | 69 | 8 | R.SWTAADMAAQITQRK.W |
|  | 23923 | 822.8395 | 2465.4968 | 2465.3828 | 0.1140 | 0 | (0) | 53 | 10 | R.TLLLLLLGALALTQTWAGSHSMR.Y |
|  | 24063 | 828.0273 | 2481.0602 | 2481.3777 | -0.3175 | 0 | 8 | 11 | 1 | R.TLLLLLLGALALTQTWAGSHSMR.Y |
|  | 24697 | 1287.5995 | 2573.1844 | 2573.2299 | -0.0454 | 1 | 0 | 63 | 5 | R.APWIEQERPEYWDQETRNVK.A |
|  | 24990 | 879.7289 | 2636.1650 | 2636.1838 | -0.0188 | 0 | 27 | 0.14 | 1 | R.DGEDQTQDTELVETRPAGDGTFQK.W 24989 |

| **826.** | P18462\|1A25_HUMAN    **Mass:** 41477    **Score:** 40     **Queries matched:** 12   **emPAI:** 0.19 |
| --- | --- |
|  | HLA class I histocompatibility antigen, A-25 alpha chain OS=Homo sapiens GN=HLA-A PE=2 SV=1 |

|  | **Query** | **Observed** | **Mr(expt)** | **Mr(calc)** | **Delta** | **Miss** | **Score** | **Expect** | **Rank** | **Peptide** |
| --- | --- | --- | --- | --- | --- | --- | --- | --- | --- | --- |
|  | 7831 | 611.2879 | 1220.5612 | 1220.6037 | -0.0424 | 0 | 2 | 89 | 3 | K.DYIALNEDLR.S |
|  | 9321 | 650.8076 | 1299.6007 | 1298.6327 | 0.9680 | 1 | 2 | 86 | 2 | K.AHSQTDRESLR.I |
|  | 10294 | 676.3427 | 1350.6709 | 1349.6939 | 0.9770 | 1 | 8 | 24 | 1 | R.YLENGKETLQR.T |
|  | 11883 | 722.3673 | 1442.7199 | 1441.7314 | 0.9886 | 0 | 8 | 22 | 3 | K.WASVVVPSGQEQR.Y 11865 |
[truncated: 6,965,356 more chars]
